# COVID-19 treatment of hospital patients worldwide at the onset of the pandemic in 2020: a systematic review

**DOI:** 10.1186/s12879-025-12368-2

**Published:** 2025-12-17

**Authors:** Antoine Bosquet, Comlan Affo, Florent Happe, Hélène Helfer, Edouard Versini, Isabelle Mahé

**Affiliations:** 1 Assistance Publique-Hôpitaux de Paris (AP-HP), DMU ESPRIT, Service de Médecine Interne, Hôpital Louis-Mourier, Université Paris Cité, 178 rue des Renouillers, Colombes, 92700 France; 2 Université Paris Cité, Assistance Publique-Hôpitaux de Paris (AP-HP), DMU ESPRIT, Service de Médecine Interne, Hôpital Louis-Mourier, 178 rue des Renouillers, Colombes, 92700 France; 3 INSERM Unité Mixte de Recherche S970, Paris Cardiovascular Research Center, Team “Endotheliopathy and Hemostasis Disorders”, Paris, France

**Keywords:** Covid-19, Pandemic, Treatment, Hospital, Repurposed drugs

## Abstract

In early 2020, no drug had proven efficacy to treat COVID-19 in-patients. This work aimed to describe COVID-19 treatment for in-patients worldwide until June, 2020. A PubMed search was performed with the terms “retrospective observational study”, “hospital”, “treatment” and “COVID” to identify English-written studies describing treatments given to adult in-patients before June 30, 2020. The identified reports were analyzed, with data extracted regarding patient characteristics and treatments across continents and countries. Overall, 178 studies involving 181,510 patients in 28 countries were analyzed, including 484 patients from Africa, 36,840 from Asia, 69,088 from Europa and 68,524 from North America. The most prescribed drugs were hydroxychloroquine (64.3%, i.e. 41.9% of all patients), corticosteroids (31.0%, i.e. 21.0%) and lopinavir/ritonavir (30.8%, i.e. 12.0%). Corticosteroids was used worldwide with similar rates in Asia, Europe and North America. Hydroxychloroquine dominated prescriptions in Africa, Europe and North America. Asia exhibited unique features: less frequent hydroxychloroquine use, higher oseltamivir use and exclusive use of umifenovir. In Europe, repurposed drugs prescription was the highest in Spain and Italy. Our study is not an exhaustive review of the literature on COVID-19 treatment of in-patients early in 2020 but is the first one to provide a comprehensive overview of COVID-19 treatment modalities in such a broad range of countries worldwide. Our work reveals high prescription rates but also heterogeneity across continents and countries in the treatment of COVID-19 in-patients worldwide. These findings emphasize the critical role of international collaboration in generating and disseminating reliable information for managing emerging diseases.

**Supplementary Information:**

The online version contains supplementary material available at 10.1186/s12879-025-12368-2.

## Graphical Abstract

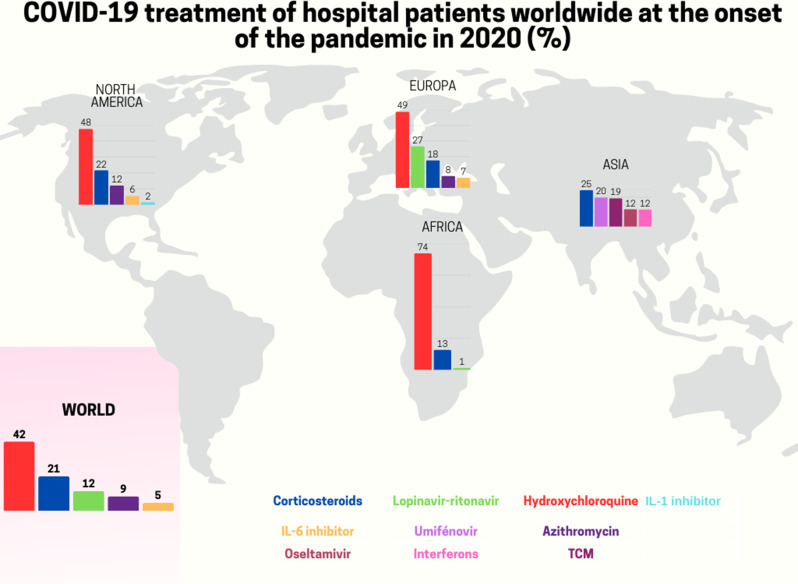

## Introduction

By the end of June 2020, the coronavirus-disease 2019 (COVID-19) pandemic had impacted more than 10 million of persons and caused over 500,000 deaths worldwide [1]. At that time, no treatment with proven efficacy was available for patients suffering from severe acute respiratory syndrome–coronavirus-2 (SARS-CoV-2). However, several molecules were proposed as potential treatments for COVID-19 patients, based on their in vitro activity against SARS-Cov2 or their immunomodulatory effect [2]. As of June 2020, more robust evidence on the efficacy or ineffectiveness of various treatments had emerged, such as hydroxychloroquine (HCQ) [3, 4], corticosteroids (CS) [5, 6] and IL-6 inhibitors (IL-6i) [7]. Surveys have reported the percentage of physicians who prescribed treatments with unproven efficacy at the onset of the pandemic as well as prescribing criteria [8]. Repurposed treatments have been widely administered to hospitalized COVID-19 patients [8, 9]. However, there is limited data on the real-life treatments administered to COVID-19 in-patients worldwide [9]. A multinational study indicates in particular that HCQ, the combination of lopinavir and ritonavir, umifenovir and azithromycin (AZM) were widely prescribed to in-patients but with significant variations across countries [9]. However, this study only includes data of patients from four countries (USA, South Korea, Spain and China) with the majority (> 95%) being treated in the USA. In this systematic review, our objective was to describe real life anti-COVID-19 treatments in PubMed-published series of adult patients in non-intensive care unit (ICU) wards hospitalized worldwide until June 30, 2020.

## Methods

The research question was, “what treatment against COVID-19 did in non-ICU wards hospitalized adult patients receive worldwide until June 30, 2020, regardless of whether they were participating to a therapeutic trial ?”

### Identifying relevant studies

Studies were identified through a search on the PubMed website performed on March 28, 2022. The search aimed to retrieve observational data on specific SARS-Cov2 treatment administered in non-ICU wards to hospitalized adult COVID-19 patients in real-life settings. Search terms used were: “retrospective observational study” AND “hospital” AND “treatment” AND “COVID”.

### Study selection

One author (AB) reviewed each search result obtained from the PubMed site. First, duplicate records were removed. Second, all remaining records, including an abstract, were screened to exclude those that did not meet the inclusion and exclusion criteria. The inclusion criteria were: any English-language articles or letters providing information about treatments given for adult COVID-19 to in-patients in real-life settings and hospitalized in non-ICU wards until to June 30, 2020. The exclusion criteria were: studies exclusively focused on ICU patients or patients younger than 18 years old, studies with an inclusion period extending after June 30, 2020, and without separate analysis of patients hospitalized before June 30, studies including in and out-patients without information on treatments administered exclusively to hospitalized in-patients, studies lacking information on treatments administered throughout the entire duration of hospitalization and studies that from an initial population of patients hospitalized and treated for COVID-19 selected a subpopulation of patients who received one or more COVID-19 treatments and did not provide information on the initial population size (see Supplementary Material 1 for details). Third, full articles were assessed to determine their eligibility for inclusion based on the mentioned inclusion and exclusion criteria.

### Drugs of interest

Medications used to treat in-patients with COVID-19, as described in the included reports, were considered treatments of interest, regardless of their mechanism of action or whether they were evaluated in a therapeutic trial. The analysis aimed to include all treatments for COVID-19 received by patients in real-life settings, without limiting the study to a pre-defined list of treatments. The exceptions include anticoagulant treatments as well as purely symptomatic treatments such as antipyretics and analgesics.

### Data extraction and risk of bias assessment

For each included report, we collected following data: ID number of report, name of first author, inclusion period, place (town, region, country, continent) where patients were hospitalized, primary objective and outcome of the study, inclusion and exclusion criteria of the study, number of hospital or centre of the study, patient characteristics (age, sex, number of deceased patients, number of patients included in a therapeutic trial), treatments given to in-patients for COVID-19 (see Supplementary Material 1 for details). If data regarding the treatments administered to patients were not provided in the article for a study, we intended to explore whether this information was available in supplementary files and, if so, extract the relevant data. We conducted a structured narrative assessment of biases in the included studies, from a standardized data extraction process. We focused on patient selection bias and information (classification) bias related to COVID-19 treatment ascertainment. We considered several subtypes of selection bias, including studies restricted to specific COVID-19 subpopulations, studies with unclear patient sampling (i.e., not reporting consecutive inclusion or complete capture of all eligible patients), inclusion of some ICU patients and inclusion of minors. We also accounted for potential duplication bias, where some patients could have been included in more than one study due to overlapping hospitals, units, or enrollment periods. An information bias related to treatment was deemed present when the study did not clearly specify how treatment data were obtained.

### Statistical methods

We present the number of studies, patients and hospitals, and the geographical scope of the studies (hospital, city, region, country, continent, etc.) as their sum per country and continent.

For variables such as sex, positive SARS-Cov2 PCR, hospitalization in an ICU, death and treatments administered, we summarize the results as proportions (the sum of patients in a category divided by the total number of patients for whom the information is available within each country and continent).

Specifically for the analysis of treatment administration, we have also chosen to present the results in terms of proportions, using the total number of patients for each country and continent as a denominator: i.e. patients with known treatment status alone (available-data denominator) or with those whose treatment status are unknown (overall denominator). In our work, we refer to the proportion of patients treated with a treatment as the reported prescription rate (RPR) using the available-data denominator. In addition, we also summarize treatment administration by ranking in descending order the absolute number of patients who received the 10 most frequently administered treatments within each continent and country. For the presentation by country, we have made the choice of presenting data on the most commonly prescribed drugs only for countries with information on at least 1000 patients.

### Patients and public involvement

No patients were involved in the formulation of the research question, data collection, or analysis of results for this review.

## Results

**Fig. 1:**
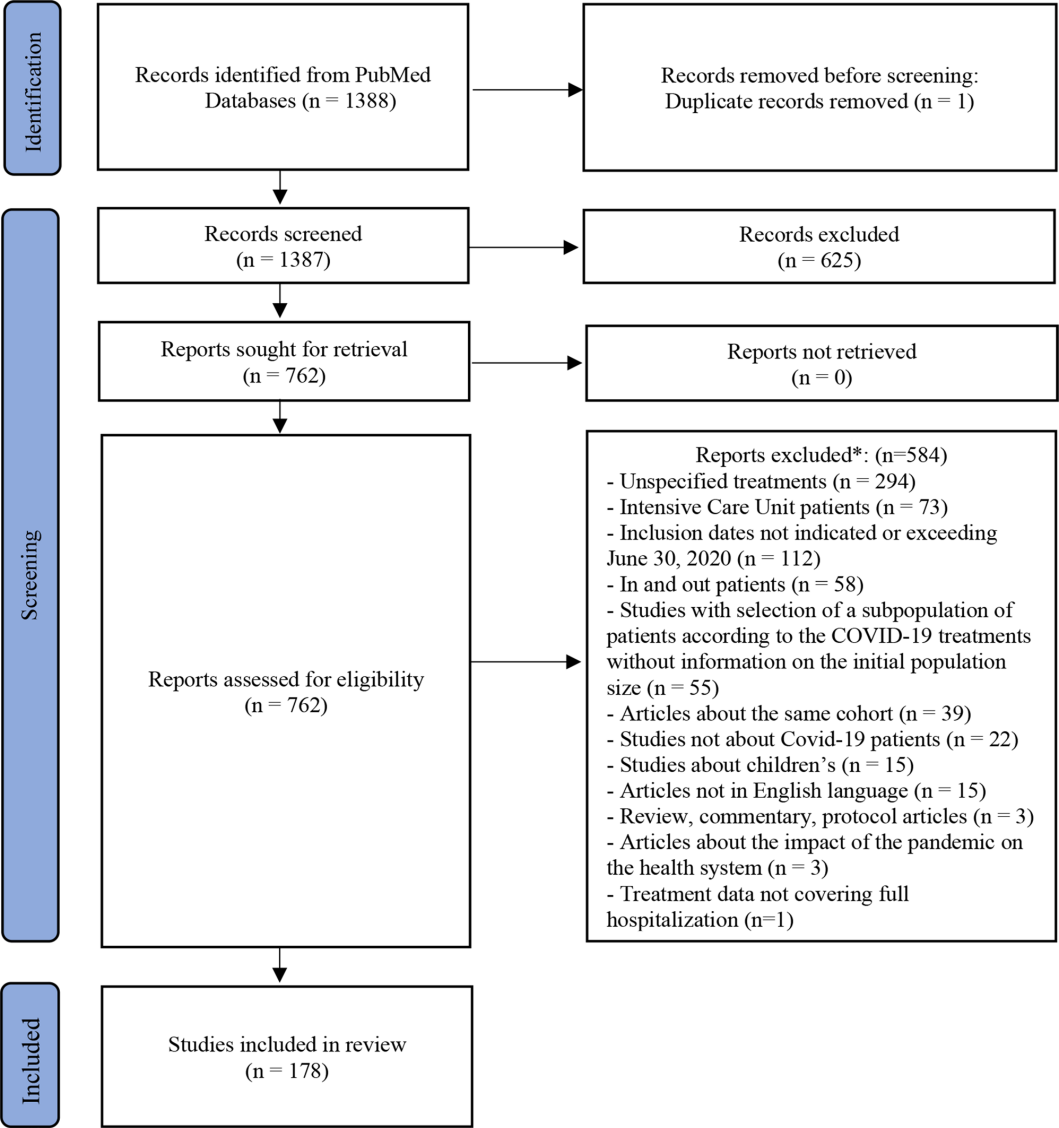
PRISMA 2020 flow diagram. * Several reasons are possible. *From*: Page MJ, McKenzie JE, Bossuyt PM, Boutron I, Hoffmann TC, Mulrow CD, et al. The PRISMA 2020 statement: an updated guideline for reporting systematic reviews. BMJ 2021;372:n71. 10.1136/bmj.n71

The Preferred Report Items for Systematic and Meta-Analysis (PRISMA) flow diagram adapted for this systematic review summarizes the study selection process (Fig. 1). This work was conduct, and the manuscript was written using the PRISMA checklist (Supplementary Materials 1 and 2). Our search retrieved 1388 reports from the PubMed database (Supplementary Materials 3 and 4). After screening titles and abstracts, we excluded one duplicate article and 625 studies, based on the inclusion and exclusion criteria (Fig. 1). The remaining 762 articles underwent full-text analysis. Ultimately, 178 studies met the pre-defined criteria and were included in this systematic review.

Risk of bias assessment is presented in Table 1 and Supplementary Material 5. The most frequent source of bias was selection bias, present in 81.5% of studies. This was mainly driven by the partial inclusion of ICU patients (117 studies); among the 89 reporting proportions, ICU patients represented on average 15.3% of participants. Subpopulation selection occurred in 26.4% of studies, although the variety of subgroups largely reflects the heterogeneity of hospitalized COVID-19 patients. Furthermore, 21% of studies did not specify whether the sample consisted of consecutive or all eligible patients, indicating unclear sampling. A duplication bias—reflecting the inability to fully exclude overlapping study populations—was identified in 39% of studies. An information bias related to treatment exposure was also observed in 22% of studies.

**Table 1:** Risk of bias assessment in included studies

Biases	Duplication Biass	Bias selection*					Information bias
		Subpopulation bias	Unclear sampling	Partial inclusion of ICU patients	Partial inclusion of minors		Treatment
**Studies (%)**	**39.2%**	26.4%	20.8%	65.7%	3.4%	**81.5%**	**22.0%**

*Selection bias was considered present if at least one of the following was identified: subpopulation bias, unclear sampling, partial inclusion of ICU patients, or partial inclusion of minors

The selected studies are presented in Table 2, Supplementary Materials 6, 7 and 8. A total of 178 studies involving 181,510 in-patients from 1986 hospitals across 28 countries on 5 continents were included in this analysis. Patients are distributed in Africa (484 patients from 2 countries), Asia (36840 patients from 9 countries, including 29169 patients from China), Europe (69088 patients from 11 countries), North America (68524 patients from 3 countries, including 66549 patients from USA), and South America (1 patient from Bolivia) (Table 2). Moreover, two multicontinental studies included 6573 patients from South and North America, Asia and Europa (Table 2). The inclusion and exclusion criteria for each study are outlined in the supplementary raw data file. Patient characteristics are detailed in Table 2. The proportion of women was 45.0% (68812/152823, missing data 28687). The percentage of patients with a SARS-Cov2 positive PCR was 97.8% (97104/100037, missing data 45402), and of patients – at least in part - hospitalized in an ICU 15.3% (17830/116554, missing data 64956), or deceased 17.6% (24461/138779, missing data 42731) (Table 2). Three-quarters of the patients analyzed in this work were included in studies carried out in more than one single hospital, at the scale of a city, a region, or even one or more states (Supplementary Material 7).

**Table 2:** Studies included in the systematic review

	Studies	Hospitals	Patients				
**Location (continent/**country**)**	**(n)**	**(n)**	**(n)**	**Sex female** **n/total (%)**	**Patients PCR+** **n/total** ^‡^ **(%)**	**ICU** ^µ^ **patients** **n/total (%)**	**Deceased patients** **n/total (%)**
**Africa**	**2**	**2**	**484**	**130/484 (26.9)**	**202/202 (100.0)**	**144/484 (29.8)**	**11/202 (5.4)**
Cameroon	1	1	282	90/282 (31.9)		91/282 (32.3)	
Egypt	1	1	202	40/202 (19.8)	202/202 (100.0)	53/202 (26.2)	11/202 (5.4)
**Asia**	**65**	327*	**36,840**	**15,890/31,879 (49.8)**	**19,890/21,124 (94.2)**	**1980/12,496 (15.8)**	**3053/27,491 (11.1)**
China	51	184	29,169	14,544/28,665 (50.7)	16,116/16,433 (98.1)	1223/9079 (13.5)	2534/24,348 (10.4)
India	1	1	108	38/108 (35.2)	108/108 (100.0)		
Iran	1	1	60	35/60 (58.3)	60/60 (100.0)	0/60 (0.0)	1/60 (1.7)
Malaysia	1	1	247	75/247 (30.4)	247/247 (100.0)	6/247 (2.4)	1/247 (0.4)
Pakistan	2	5	1461	2/23 (8.7)	1461/1461 (100.0)	1/23 (4.3)	1/23 (4.3)
Saudi Arabia	1	1	401	80/401 (20.0)		0/401 (0.0)	
South Korea	3	3	405	214/405 (52.8)	174/267 (65.2)	27/138 (19.6)	22/265 (8.3)
Thailand	1	1	193	80/193 (41.5)	193/193 (100.0)	32/193 (16.6)	4/193 (2.1)
Turkey	4	129^*^	4796	822/1777 (46.3)	1531/2355 (65.0)	691/2355 (29.3)	490/2355 (20.8)
**Europa**	**71**	**724** ^†^	**69,088**	**26,201/59,439 (44.1)**	**37,714/38,528 (97.9)**	**5349/55,801 (9.6)**	**11,807/64,042 (18.4)**
Belgium	2	110	8991	4060/8900 (45.6)		495/8991 (5.5)	1948/8991 (21.7)
France	11	85†	19,243	9429/18,963 (49.7)	18,461/18,668 (98.9)	1545/18,805 (8.2)	1894/18,917 (10.0)
Germany	1	3	10			6/10 (60.0)	
Greece	2	7	272	100/272 (36.8)	272/272 (100.0)	42/272 (15.4)	17/272 (6.3)
Italy	26	184†	13,966	2434/7193 (33.8)	10,824/10,824 (100.0)	738/4643 (15.9)	2783 (22.9)
Malta	1	1	93	33/93 (35.5)	93/93 (100.0)	7/93 (7.5)	0/93 (0.0)
Poland	1	1	70	44/70 (62.9)	70/70 (100.0)	4/70 (5.7)	21/70 (30.0)
Romania	1	1	37	18/37 (48.6)	37/37 (100.0)		7/37 (18.9)
Spain	20	315	23,283	9170/21,718 (42.2)	6444/7051 (91.4)	2329/21,800 (10.7)	4629/21,302 (21.7)
Swiss	3	9	1367	153/437 (35.0)	437/437 (100.0)	97/437 (22.2)	82/437 (18.8)
UK	3	8†	1756	760/1756 (43.3)	1076/1076 (100.0)	86/680 (12.6)	426/1756 (24.3)
**North America**	**37**	**866**	**68,524**	**23,990/54,447 (44.1)**	**38,563/39,355 (98.0)**	**9694/41,199 (23.5)**	**8203/40,470 (20.3)**
International^δ^	1	36	1790			786/1790 (43.9)	334/1790 (18.7)
Mexico	1	1	185	127/185 (68.6)	185/185 (100.0)		185/185 (100.0)
USA	35	829	66,549	23,863/54,262 (44.0)	38,378/39,170 (98.0)	8908/39,409 (22.6)	7684/38,495 (20.0)
**South America**	**1**	**1**	**1**	**1/1 (100.0)**	**1/1** (100.0)	**0/1 (0.0)**	**0/1 (0.0)**
Bolivia	1	1	1	1/1 (100.0)	1/1 (100.0)	0/1 (0.0)	0/1 (100.0)
**Multicontinental**	**2**	**66**	**6573**	**2600/6573 (39.6)**	**734/827 (88.8)**	**663/6573 (10.1)**	**1387/6573 (21.1)**
International^δ^	2	66	6573	2600/6573 (39.6)	734/827 (88.8)	663/6573 (10.1)	1387/6573 (21.1)
**Total**	**178**	1986*	**181,510**	**68,812/152,823 (45.0)**	**97,104/100,037(97.8)**	**17,830/116,554 (15.6)**	**24,461/138,779 (17.6)**

NB: In case of missing data, the denominator used to calculate percentages does not correspond to the total number of patients in the country or continent. To avoid overloading the table, only percentages based on available data are shown

* Amoung the four Turkish studies, two were conducted under the aegis of the Turkish Nephrology Society and share identical characteristics: country wide study, each involving 47 centres. These are studies number 567 and 1210

† For three studies, the number of involved centers or hospitals remains unspecified. These included studies 297 from Crossette-Thambiah C. et al. (UK, country wide study, 72 patients), 928 from Caillard S. et al. (France, country wide study, 243 patients) and 964 from Wu MA. et al. (Italy, region wide study, 48 patients)

‡ “Total” is the sum of the number of patients for whom the SARS-Cov2 PCR result is specified in each retrieved study. The difference between the overall number of patients studied in this review and this number corresponds to the sum of Covid-19 patients included in studies for which a positive SARS-Cov2 PCR is not part of the inclusion criteria and those for which it is part but without information about the number of patients with positive PCR in the article

δ International studies include a North America study from Yang J.Y. et al. (Canada, USA) and 2 multicontinental studies (study by Perez-Nieto O.R. et al. with patients from Mexico and Ecuator and study by Pepe M. et al.’ from Spain, Italy, Germany, Canada, Ecuator, Cuba and China). For all of these studies, patient distribution by country is not known

μ ICU: intensive care unit

Table 3, Supplementary Materials 9 and 10 reports information on the drugs administered to patients worldwide, categorized by countries and continents. Table 3 and Supplementary Material 10 display the 10 most commonly prescribed drugs by continent (Table 3) and by countries where treatments of at least 1,000 patients are analyzed (Supplementary Material 10). Over 32 different treatments were administered to hospitalized patients globally (Table 3). HCQ (76092/118288, 64.3%), CS (38174/123002, 31.0%) and the combination of ritonavir and lopinavir (21828/70964, 30.8%) were the most used drugs, prescribed to more than 10% of all patients (respectively, 41.9%, 21.0% and 12.0%). Azithromycin, IL-6i, umifenovir, interferon, traditional chinese medicine (TCM), oseltamivir, ribavirin and intravenous immunoglobulins (IVIg) were given to a percentage of patients ranging from 6.4% to 55.0% (i.e., from 1.0% to 8.8% of the total population) (Table 3, Supplementary Material 9). The type of IL-6i was indicated for 85.0% of patients, most often tocilizumab (99.9% of cases, 7639/7648). Other therapeutic interventions, including darunavir, convalescent plasma, and JaK2 inhibitors, were employed in less than 1% of the total patient population (Supplementary Material 9).

**Table 3:** Top ten most commonly prescribed treatments for COVID-19 during the first wave of the pandemic according the continent

Continent	Rank	Treatment	Treated Patients (*n*)	Patients without MD‡ (*n*)	Treated patients (%)	Treated patients (overall*) (%)
**World**	**1**	**Hydroxychloroquine**	**76,092**	**118,288**	**64,3**	**41,9**
*n* = 181,510	**2**	**Corticosteroids**	**38,174**	**123,002**	**31**,**0**	**21**,**0**
**32 drugs†**	**3**	**Lopinavir-ritonavir**	**21,828**	**70,964**	**30**,**8**	**12**,**0**
	**4**	**Azithromycin**	**15,921**	**28,961**	**55**,**0**	**8** **8**
	**5**	**IL-6 inhibitors**	**8966**	**91,377**	**9**,**8**	**4**,**9**
	**6**	**Interferons**	**7739**	**43,406**	**17**,**8**	**4**,**3**
	**7**	**Umifénovir**	**7382**	**13,125**	**56**,**2**	**4**,**1**
	**8**	**TCM‡**	**7192**	**14,589**	**49**,**3**	**4**,**0**
	**9**	**Oseltamivir**	**4671**	**36,353**	**12**,**8**	**2**,**6**
	**10**	**Ribavirin**	**2348**	**32,521**	**7**,**2**	**1**,**3**
**Africa**	1	Hydroxychloroquine	358	484	74,0	74,0
*n* = 484	2	Corticosteroids	62	282	22,0	12,8
3 drugs^†^	3	Lopinavir-ritonavir	6	282	2,1	1,2
**Asia**	1	Corticosteroids	9233	31,606	29.2	24.9
*n* = 36,840	2	Umifénovir	7382	13,124	56.2	19.9
23 drugs^†^	3	TCM‡	7192	14,589	49.3	19.4
	4	Oseltamivir	4398	16,473	26.7	11.9
	5	Interferons	4364	12,988	33.6	11.8
	6	Hydroxychloroquine	3834	8490	45.2	10.3
	7	Lopinavir-ritonavir	2751	16,069	17.1	7.4
	8	Ribavirin	2347	11,586	20.3	6.3
	9	IVIg‡	1778	6376	27.9	4.8
	10	Azithromycin	1619	2223	72.8	4.4
**Europa**	1	Hydroxychloroquine	33,683	47,015	71.6	48.8
*n* = 69,088	2	Lopinavir-ritonavir	18,430	41,523	44.4	26.7
18 drugs^†^	3	Corticosteroids	12,296	37,575	32.7	17.8
	4	Azithromycin	5369	10,724	50.1	7.8
	5	IL-6 inhibitors	4389	43,057	10.4	6.5
	6	Interferons	2639	20,061	13.2	3.8
	7	Darunavir	1181	8373	14.1	1.7
	8	Remdesivir	382	33,672	1.1	0.6
	9	Colchicine	162	14,064	1.2	0.2
	10	IL-1 inhibitors	162	15,016	1.1	0.2
**North America**	1	Hydroxychloroquine	33,085	55,831	59.3	47.7
*n* = 69,524	2	Corticosteroids	15,014	47,148	31.8	21.7
13 drugs^†^	3	Azithromycin	8384	15,187	55.2	12.1
	4	IL-6 inhibitors	3910	37,762	10.4	5.6
	5	IL-1 inhibitors	1142	10,630	10.7	1.6
	6	Remdesivir	714	40,328	1.8	1.0
	7	Lopinavir-ritonavir	560	12,262	4.6	0.8
	8	Zinc	411	1647	2.0	0.6
	9	Atazanavir	140	2154	6.5	0.2
	10	Convalescent Plasma	41	1896	2.2	0.1

* Percentage of the number of patients treated out of the total number of patients in the country including patients whose information on drug use is not known

† The number indicated corresponds to the total number of different treatments on a continent

‡ TCM: Traditional Chinese Medicine, IVIg: intravenous immunoglobulins, MD: Missing Data

Detailed information on the number of treatments received by each patient is only provided in 14 studies (22,259 patients): 26.8% (*n* = 5976), 46.3% (*n* = 10298), 15.4% (*n* = 3440), 1.2% (*n* = 263), 0.1% (*n* = 25), 0.0% (*n* = 1), 0.0% (*n* = 2) received none, 1, 2, 3, 4, 5, 6 or 7 treatments, respectively. The percentage of patients treated with the standard of care was 58.3% (18840/32338) based on 26 studies, primarily conducted in Europe (*n* = 14), but involving only 17.7% of the entire population (Supplementary Material 9). Similarly, only 1.0% (82/8343) of patients were included in therapeutic trials, according to the nine studies that provided the only available data on trial participation and representing 4.6% of all patients (8343/182541).

As a preamble, we have not detailed the results for South America, because in this continent treatment data are only available for a single in-patient.

The 3 most commonly used treatments globally (HCQ, CS and lopinavir and ritonavir combination) have been prescribed on the 4 continents: Africa, Asia, Europe and North America. The molecules prescribed vary depending across continents and countries.

For the most commonly prescribed drugs, the RPR varies by continent (Table 3, Supplementary Material 9). HCQ is the most prescribed drug in Africa, Europa (with a similar RPR from 74.0% to 71.6% respectively) and in North America (59,3% RPR) but not in Asia, only ranking 6th with a 45.2% RPR (Table 3, Supplementary Material 9). CS have been used at a comparable percentage worldwide, although the RPR was lower in Africa (22.0%) compared to the rest of the world (32.7% in Europe, 31.8% in North America and 29.8% in Asia). Lopinavir and ritonavir combination is primarily used in Europe (RPR 44.4%, 2nd rank) versus in Asia, in North America and in Africa with RPRs at 17.1% (7th rank), 4.6% (7th rank) and 2.1% (3rd rank) respectively (Table 3).

Comparing prescriptions in Europe and North America reveals commonalities: HCQ, CS, AZM and IL-6i are among the top five most prescribed drugs (in more than 5% of all patients), while remdesivir has been less frequently (in less than 2% of patients) (Table 3, Supplementary Material 9). In contrast, lopinavir and ritonavir combination has been used more in Europe compared than in North America (Table 3, Supplementary Material 9). In Asia, the prescription profile differs from other continents. Indeed, HCQ is less commonly prescribed than elsewhere, and some treatments are exclusively (umifenovir, TCM, ribavirin, favipavir) or significantly more prescribed (oseltamivir) (Table 3, Supplementary Material 9). Additionally, the number of different treatments prescribed to more than 10% or 5% of patients is higher in this continent (Table 3, Supplementary Material 9). In Asia, 23 different treatments were prescribed to patients, while this numbers were 18, 13 and 3 in Europe, North America and Africa, respectively (Supplementary Material 9). Furthermore, the number of treatments prescribed to more than 10% of patients varies across continents (2 in Africa and North America, 3 in Europe, and 6 in Asia) with HCQ and CS consistently included (Table 3). In Africa, few drugs appear to have been used, namely HCQ, CS and lopinavir/ritonavir combination, possibly due to the small number of patients included in the studies (*n* = 484 in 2 countries) (Table 3).

Moreover, prescription pattern is not consistent within the same continent and may differ between countries. For instance, in Asia, China differs from others countries in term of drug utilization: there was limited use of HCQ (11th rank, prescribed to less than 0.4% of all patients with information on the possible administration of HCQ being available in only 3% of them), no mention of AZM prescription, use of treatments that have not been used elsewhere such as umifenovir, TCM, ribavirin, ganciclovir, thymosin and thymalfasin and use of a greater number of different treatments prescribed in all or more than 10 or 5% of in-patients (Supplementary Materials 9 and 10). In Asia, countries like Pakistan, Turkey and South Korea differed from China in their higher use of HCQ (first rank in Turkey with 62.8% and in South Korea with 37.3%, 3th rank in Pakistan with 14.4%) and of AZM (first rank in Pakistan with 87.8%) (Supplementary Material 9).

The USA share similarities with Europe: HCQ is the most prescribed drug, as observed in Europe, particularly in Belgium, Italy, Spain, Switzerland and France, CS and AZM are widely used, as in Europe with close RPR, especially in Italy and Spain (Supplementary Material 9). Similarly, prescription of IL-6i appears comparable in the USA (10.6%, 5.6% of all patients) and in Europe (10.4%, 6.5%) while it is low in China (1.5%, 0.1%) (Table 3, Supplementary Material 10). In Europe, prescription predominates in Spain (11.6%, 11.1%) and Italy (18.4%, 12.4%) (Supplementary Materials 9 and 10).

In all European countries, except for the UK and Germany, HCQ is the most prescribed treatment, although the RPR varies across countries: 21.4%, 35.9%, 77.8%, and 85.1% (Supplementary Materials 9 and 10), in France, Switzerland, Italy and Spain, respectively. The ranks and RPR of CS, IL-6i and lopinavir and ritonavir combination also differ among European countries (Supplementary Material 9). Italy and Spain have the highest RPR for the five most commonly used drugs (HCQ, lopinavir/ritonavir, CS, AZM and IL-6i) from 17.8 to 77.8% and 11.6 to 85.1% respectively (Supplementary Materials 9 and 10). The number of treatments prescribed to more than 10% of patients is higher in Italy (*n* = 4, HCQ, CS, lopinavir/ritonavir and IL-6i) and in Spain (*n* = 6, HCQ, lopinavir/ritonavir, CS, AZM, IFN and IL-6i) compared to other European countries where only HCQ exceeds this threshold, except in France (Supplementary Materials 9 and 10). In Europe, France is distinctive in that even HCQ, the most prescribed treatment, is administered to less than 5% of all patients (Supplementary Material 9). This may be attributable to the fact that the majority of patients (78.5%) were included into a single study that did not provide details on the treatments administered to patients [10]. It only revealed the proportion of those treated by the standard of care and, therefore, did not receive any of the following agents: HCQ, AZM, remdesivir, tocilizumab, sarilumab, or dexamethasone.

## Discussion

This systematic review reports treatments administered to more than 180,000 in-patients with COVID-19 across 28 countries on 4 continents during the initial phase of the pandemic. We observed a high frequency of use of repurposed drugs until June 30 2020, in particular for the three most prescribed worldwide, HCQ (64.3%, i.e., 41.9% of all patients), CS (31.0%, i.e., 21.0%) and lopinavir/ritonavir (30.8%, i.e., 12.0%). The comparison of treatments prescribed on the different continents shows common points (CS among the most prescribed drugs on all continents and with similar RPR in Asia, Europe and North America) and differences (HCQ most prescribed treatment in Africa, Europe and North America but only 6th in Asia, use of more different molecules and more frequent use of oseltamivir in Asia than on other continents, exclusive use of umifenovir and TCM in China). Analyzing prescriptions within each continent highlight’s significant heterogeneity in the type of drug and the percentage of patients receiving repurposed drugs. For instance, in Italy and Spain for example, HCQ, CS, lopinavir/ritonavir or IL-6i were more frequently prescribed than in other European countries. Similarly, in Asia, HCQ is not one of the 10 most prescribed treatments, while it is ranked first in Turkey and third in Pakistan.

The limited dataset concerning patients treated in Africa (484 cases across 2 hospitals and 2 countries) does allow us only to present exploratory findings about COVID-19 treatment modalities in this continent: HCQ is clearly the most prescribed treatment (74%; i.e., 74% of all patients), ahead of CS (22%; i.e., 12.8%) and lopinavir and ritonavir combination (2.1%, i.e., 1.2%) with no mention of other treatments. The rest of the discussion will therefore focus on treatments used on the continents where there are more data available: Asia, Europe and North America. However, it is important to point that the treatments used in North America are predominantly administered in the USA, accounting for 97% of the patients analyzed within that region. This observation also holds true for Asia and China, albeit to a lesser degree: 79% of Asia in-patients were treated in China.

In the PubMed database, we identified only one study that compare the different treatments administered around the world to hospitalized COVID-19 patients during the first wave of the pandemic [9]. The study analyzed treatments administered from January to December 2020, mainly in the USA (*n* = 290131 versus 66524 in our review) but also in South Korea (*n* = 7599 versus 405), Spain (*n* = 5230 versus 23285) and China (*n* = 304 versus 29169), using electronic health records. The number of patients in the USA and in Spain in this study and in our review is sufficient to further investigate the prescriptions. The limited number of patients analyzed in China in Prats-Uribe study and in South Korea in ours makes the comparison less meaningful for these countries.

As in Prats-Uribe study [9], our work confirms that HCQ has been widely used worldwide in early 2020, with the notable exceptions of Asia, particularly China and the UK in Europe, where there is no mention of prescribing HCQ. Nearly 50% of all patients analyzed in this review received HCQ in Europe and North America, mainly in the USA. The relation between the publication of studies of insufficient quality, their media impact, and prescription of HCQ has been previously discussed in France [11] and the USA [12, 13]. The factors that may have prompted physicians to prescribe HCQ include its relatively low cost, widespread availability, an estimated safety profile considered as favourable, and the existing legal framework that authorizes its prescription [8]. In the Prats-Uribe work [9], HCQ use was lower. This may be linked to the period of analysis, which ended in June 2020 in this review but extends to December 2020 in this study. Indeed, most HCQ prescriptions occurred between March and April 2020 [9, 14, 15] following by a significant decline, likely due to the May 2020 publication of retrospective studies that concluded HCQ was ineffective [16–18]. Additionally, the preliminary results of the Solidarity and Recovery trials [3, 19], released in May and June 2020, further confirmed the ineffectiveness of HCQ in treating COVID-19 and led the WHO to announce the suspension of HCQ evaluation in Solidarity trial. No rebound in HCQ prescription was observed afterwards [9].

CS were widely used worldwide during the first half of 2020, in around 30% of in-patients (28.9% in Asia, 31.8% in North America and up to 32.7% in Europe). However, there were initially discussions regarding their interest to treat COVID-19 (considered potentially harmful by analogy to other viral conditions like influenza) [20] and most national guidelines did not recommend their use [9, 21]. Possible explanations for this extensive use include their low cost, ease of use, availability, the large prior experience of their use in other indications by hospitalists and the potential severity of Covid-19 infection [8]. CS prescription significantly increased after the release of preliminary results of the RECOVERY trial in June 2020 [5]. The observation of higher prescription rates in the USA in Prats-Uribe study (from 34% to 67% versus 31.0% noted in our work) may be attributed to the extended analysis period.

AZM has been widely used in Europe (Spain, Italy, Greece and France), the USA but not in Asia, except in Pakistan and Saudi Arabia. Prats-Uribe study also found high prescription rates in the USA (from 8.7 to 47.4%) and Spain (from 7.9 to 57.9%). However, not all national guidelines have recommended its use [21]. Its alleged efficacy has not been confirmed [22] and its association with HCQ has been considered potentially toxic [23].

The use of lopinavir/ritonavir is less homogeneous worldwide. It is higher in Europe (overall 44.4%), especially in Spain (63.8%), Italy (45.9%) and Switzerland (29.3%), than in China (18.5%) and in the USA (4.4%). The percentage of patients treated with lopinavir/ritonavir appears higher in our study than in the Prats-Uribe study in Spain (4.4% and 50.5%) and the USA (< 1%) [9]. Prats-Uribe et al. mention that one explanation of the broad prescription of the lopinavir and ritonavir combination in Spain and in South Korea may be related its recommendation in the national guidelines [9]. We found evidence in support of this hypothesis: the observation of high RPRs in countries where lopinavir/ritonavir also appears in clinical recommendations according to severity (in Italy and China) [21, 24] and, on the contrary, lower RPRs in countries where lopinavir/ritonavir is not recommended as first-line treatment in (0.3% in Belgium) [24] or outside any clinical trials (4.4% in the USA, 6.9% in France) [25, 26].

The RPR of IL-6i in Spain and in the USA in our work (11.6%, i.e. 11.1% of all patients, almost exclusively tocilizumab and 10.6%, i.e. 5.6%) are comparable to the rates in the Prats-Uribe study [9]: from 0 to 17% and from 0 to 9% respectively.

Remdesivir has consistently been prescribed to a small number of patients worldwide, although been recommended for use in countries like Italy and Spain [21]. Factors such as its intravenous administration, its tolerability profile, and its price may contribute to its lower RPR [27].

We have highlighted both convergences and significant heterogeneity in prescriptions between continents and countries. It is particularly complex to analyze the reasons for these differences, in particular given the multitude of factors that can influence prescribing practices. Factors such as treatment specifications (price, availability, ease of use, administration route), the country’s health system (organization, financial resources, number of ICU for example), differences in national health policies, local recommendations, and local prescribing habits can all play a role. It is plausible, if not probable, that media pressure affected prescriptions early in 2020, either directly through prescribers or indirectly through patients, their families, or policymakers. Further analysis is needed to understand the various factors that influence prescriptions in this pandemic context. The WHO proposes an emergency decision-making framework, called “MEURI” (“monitored emergency use of unregistered and experimental interventions”), for introducing a non-validated treatment in an emergency context when no effective treatment is available and it is not possible to initiate therapeutic trials immediately [28–30]. Our work points that the prescription of non-validated treatments was widespread during the first wave, particularly in the USA, Europe and Asia. Among the 32 different treatments administered to in-patients for the treatment of Covid-19 infection in our review, only 4 are finally recommended for the treatment of COVID-19, strongly for CS, IL-6i and Jak2i, conditionally for remdesivir [31]. This observation, which confirms experience from previous epidemics [32], suggests caution when prescribing treatments of uncertain benefit. The contrasting examples of HCQ (unconfirmed efficacy) and CS (initially discussed and then confirmed efficacy) illustrate the difficulty of this reflection. In the event of a sudden outbreak of a new pandemic, it remains crucial to conduct therapeutic trials as promptly as possible to obtain reliable data on the treatments of an emerging infectious disease [33, 34]. Furthermore, the dissemination of this robust, up-to-date and easily accessible information, as the WHO has done with its “early who guidance” guides, is also essential/important [35].

Our work has some limitations. Ideally, our systematic review should include searches of multiples databases but PubMed is the most comprehensive and widely used database of peer-reviewed biomedical journal literature, particularly during the COVID-19 pandemic.

Although partial ICU inclusion contributed substantially to the high rate of selection bias, the proportion of ICU patients was relatively small, and most treatments were administered across both ICU and non-ICU wards, which may mitigate the impact of this bias. Possible duplication bias may have led to overrepresentation of some populations because overlapping hospitals or recruitment periods could not be excluded. Frequent use of specific subgroups reflects real clinical heterogeneity. Unclear sampling in 21% of studies, together with insufficient reporting on how treatment data were collected, also point to the occasionally suboptimal quality of some early-pandemic studies [36].

Our review allows to analyze the treatments administered to only a minority of patients hospitalized for COVID-19, even in the most represented countries. Moreover, therapeutic modalities could not be studied in many countries in South America, Africa or Europe and in any countries of Oceania. Furthermore, patients included in published series may not have been managed in the same manner as those whose treatments were not disclosed in publications accessible on PubMed website. Consequently, this work offers a fragmentary reflection of the treatment practices of COVID-19 patient worldwide. However, the prescription rates observed in the USA and Spain, especially for HCQ, CS, AZM, in our work and in Prats-Uribe study [9] are close despite a different methodology. This reinforces the results of the Prats-Uribe study as well as ours.

Another limitation is that our study did not analyze variations in RPR during the inclusion period. Most retrieved studies did not provide data to study variations in prescriptions over time.

The inclusion of a large number of heterogeneous observational studies, many of which were not primarily designed to analyze patients’ treatments, resulted in the occurrence of significant missing data for some of the treatments examined in this review.

Our work did not cover the entire range of COVID-19 treatments. We specifically focused on treatments prescribed for patients with expected direct antiviral or adjuvant targets. We excluded others treatments, like anticoagulants that are usually recommended for hospitalized patients with acute infectious conditions and combine various risk factors, which are often present in patients hospitalized for COVID-19.

However, to the best of our knowledge, this systematic review is the only one about the treatment of COVID-19 in-patients during the first half of 2020. Apart from the Prats-Uribe study [9], our review analyzed the largest number of in-patients treated for Covid-19 worldwide during this period. The analysis of data from several countries in Europe (such as Spain, France) and Asia (such as China, Turkey and Pakistan) allows the analysis of prescriptions across these different countries.

## Conclusion

The onset of the SARS-Cov2 pandemic in 2020 placed the healthcare systems of numerous countries in an unprecedented situation. They faced challenges such as difficulty of providing care to all patients, a lack of proven effective treatment apart from supportive measures, shortages of materials and drugs, media hype surrounding COVID-19 but also international collaboration to swiftly generate reliable data on the therapeutic modalities of COVID-19 [37]. Initially, most publications on COVID-19 focused on assessing the benefit/risk ratio of different molecules, the reasons that led to invalidated treatment prescriptions, and determining how to evaluate potential treatments during a pandemic. Limited data has been published on real-life treatment when no specific treatment was available. Our work has described the treatment modalities of COVID-19 in-patients worldwide, highlighting both the commonalities of a high RPR across most countries and the significant heterogeneity in prescriptions. This data underscore that it is essential to anticipate rigorous research tant can be rapidly implemented during a pandemic within early international collaborations, enabling the quick and robust identification of the best benefit/risk ratio treatments.

## Supplementary Information

Below is the link to the electronic supplementary material.

Supplementary Material 1

Supplementary Material 2

Supplementary Material 3

Supplementary Material 4

Supplementary Material 5

Supplementary Material 6

Supplementary Material 7

Supplementary Material 8

Supplementary Material 9

Supplementary Material 10

## Data Availability

Raw data are available in the supplementary files.

## Abbreviations

AZM: Azithromycin
CS: Corticosteroids
COVID-19: Coronavirus Disease 2019
HCQ: Hydroxychloroquine
ICU: Intensive Care Unit
IL-6i: IL-6 Inhibitors
Jak2i: Janus Kinase 2 Inhibitors
MEURI: Monitored Emergency Use of Unregistered and Experimental Interventions
PRISMA: Preferred Report Items for Systematic and Meta-Analysis
RPR: Reported Prescription Rate
SARS-CoV-2: Severe Acute Respiratory Syndrome–Coronavirus-2
TCM: Traditional Chinese Medicine
UK: United Kingdom
WHO: World Health Organization

## References

[1] WHO Coronavirus (COVID-19.) Dashboard [Internet]. [cité 11 mars 2021]. Disponible sur: https://covid19.who.int

[2] Sanders JM, Monogue ML, Jodlowski TZ, Cutrell JB. Pharmacologic treatments for coronavirus disease 2019 (COVID-19): a review. JAMA. 2020;323(18):1824–36. 10.1001/jama.2020.6019 32282022

[3] RECOVERY Collaborative Group, Horby P, Mafham M, Linsell L, Bell JL, Staplin N, et al. Effect of hydroxychloroquine in hospitalized patients with Covid-19. N Engl J Med. 2020;19(21):2030–40. 10.1056/NEJMoa2022926 PMC7556338 33031652

[4] Repurposed Antiviral Drugs for Covid-19 — Interim WHO Solidarity Trial Results. N Engl J Med. 2021;384(6):497–511. 10.1056/NEJMoa2023184 PMC7727327 33264556

[5] Horby P, Lim WS, Emberson J, Mafham M, Bell J, Linsell L, et al. Effect of Dexamethasone in Hospitalized Patients with COVID-19 – Preliminary Report [Internet]. medRxiv; 2020 [cité 11 août 2023]. p. 2020.06.22.20137273. Disponible sur: https://www.medrxiv.org/content/10.1101/2020.06.22.20137273v1

[6] The WHO Rapid Evidence Appraisal for COVID-19 Therapies (REACT) Working Group. Association between administration of systemic corticosteroids and mortality among critically ill patients with COVID-19: a meta-analysis. JAMA. 2020;6(13):1330–41. 10.1001/jama.2020.17023 PMC7489434 32876694

[7] Wise J. Covid-19: arthritis drug Tocilizumab reduces deaths in hospitalised patients, study shows. BMJ. 2021;372:n433. 10.1136/bmj.n433 33574097

[8] Bosquet A, Affo C, Plaisance L, Poenou G, Mortier E, Mahé I. Outside any therapeutic trial prescription of hydroxychloroquine for hospitalized patients with covid-19 during the first wave of the pandemic: A national inquiry of prescription patterns among French hospitalists. PLOS ONE. 2022;17(1):e0261843. 10.1371/journal.pone.0261843 PMC8782345 35061735

[9] Prats-Uribe A, Sena AG, Lai LYH, Ahmed WUR, Alghoul H, Alser O, et al. Use of repurposed and adjuvant drugs in hospital patients with Covid-19: multinational network cohort study. BMJ. 2021;n1038. 10.1136/bmj.n1038 PMC8111167 33975825

[10] Sánchez-Rico M, Limosin F, Vernet R, Beeker N, Neuraz A, Blanco C, et al. Hydroxyzine use and mortality in patients hospitalized for COVID-19: A multicenter observational study. J Clin Med. 2021;10(24):5891. 10.3390/jcm10245891 PMC8707307 34945186

[11] EPI-PHARE [Internet]. 2021 [cité 29 sept 2023]. Covid-19: usage des médicaments - rapport 6. Disponible sur: https://www.epi-phare.fr/rapports-detudes-et-publications/covid-19-usage-des-medicaments-rapport-6/

[12] Vaduganathan M, van Meijgaard J, Mehra MR, Joseph J, O’Donnell CJ, Warraich HJ. Prescription Fill Patterns for Commonly Used Drugs During the COVID-19 Pandemic in the United States. JAMA. 2020;323(24):2524–6. 10.1001/jama.2020.9184 PMC7256862 32463459

[13] Bull-Otterson L, Gray EB, Budnitz DS, Strosnider HM, Schieber LZ, Courtney J, et al. Hydroxychloroquine and chloroquine prescribing patterns by provider specialty following initial reports of potential benefit for COVID-19 Treatment - United States, January-June 2020. MMWR Morb Mortal Wkly Rep. 2020;69(35):1210–5. 10.15585/mmwr.mm6935a4 PMC7470458 32881845

[14] Lin KJ, Schneeweiss S, Tesfaye H, D’Andrea E, Liu J, Lii J, et al. Pharmacotherapy for hospitalized patients with COVID-19: treatment patterns by disease severity. Drugs. 2020;80(18):1961–72. 10.1007/s40265-020-01424-7 PMC7643089 33151482

[15] Gourieux B, Reisz F, Belmas AS, Danion F, Fourtage M, Nai T, et al. nov. Prescribing practices of lopinavir/ritonavir, hydroxychloroquine and Azithromycin during the COVID-19 epidemic crisis and pharmaceutical interventions in a French teaching hospital. Eur J Hosp Pharm Sci Pract. 2020;25. 10.1136/ejhpharm-2020-002449 PMC7689541 33239282

[16] Geleris J, Sun Y, Platt J, Zucker J, Baldwin M, Hripcsak G, et al. Observational Study of Hydroxychloroquine in Hospitalized Patients with Covid-19. N Engl J Med. 2020;382(25):2411–8. 10.1056/NEJMoa2012410 PMC7224609 32379955

[17] Rosenberg ES, Dufort EM, Udo T, Wilberschied LA, Kumar J, Tesoriero J, et al. Association of Treatment With Hydroxychloroquine or Azithromycin With In-Hospital Mortality in Patients With COVID-19 in New York State. JAMA. 2020;323(24):2493–502. 10.1001/jama.2020.8630 PMC7215635 32392282

[18] Mahévas M, Tran VT, Roumier M, Chabrol A, Paule R, Guillaud C, et al. Clinical efficacy of hydroxychloroquine in patients with covid-19 pneumonia who require oxygen: observational comparative study using routine care data. BMJ. 2020;369:m1844. 10.1136/bmj.m1844 PMC7221472 32409486

[19] Torjesen I. Covid-19: hydroxychloroquine does not benefit hospitalised patients, UK trial finds. BMJ. 2020;369:m2263. 10.1136/bmj.m2263 32513810

[20] Russell CD, Millar JE, Baillie JK. Clinical evidence does not support corticosteroid treatment for 2019-nCoV lung injury. Lancet. 2020;395(10223):473–5. 10.1016/S0140-6736(20)30317-2 PMC7134694 32043983

[21] Dagens A, Sigfrid L, Cai E, Lipworth S, Cheng V, Harris E, et al. Scope, quality, and inclusivity of clinical guidelines produced early in the covid-19 pandemic: rapid review. BMJ. 2020;369:m1936. 10.1136/bmj.m1936 PMC7249097 32457027

[22] Azithromycin in patients admitted. To hospital with COVID-19 (RECOVERY): a randomised, controlled, open-label, platform trial. Lancet. 2021;397(10274):605–12. 10.1016/S0140-6736(21)00149-5 PMC7884931 33545096

[23] Fiolet T, Guihur A, Rebeaud ME, Mulot M, Peiffer-Smadja N, Mahamat-Saleh Y. Effect of hydroxychloroquine with or without Azithromycin on the mortality of coronavirus disease 2019 (COVID-19) patients: a systematic review and meta-analysis. Clin Microbiol Infect. 2021;27(1):19–27. 10.1016/j.cmi.2020.08.022 PMC7449662 32860962

[24] Xu X, Ong YK, Wang DY. Role of adjunctive treatment strategies in COVID-19 and a review of international and National clinical guidelines. Mil Med Res. 2020;7(1):22. 10.1186/s40779-020-00251-x PMC7199873 32370766

[25] Subramanian K, Nalli A, Senthil V, Jain S, Nayak A, Bhat A. Perspectives on the early quality of evidence guiding the therapeutic management of SARS-CoV-2: A systematic literature review. Adv Ther. 2020;37(10):4107–31. 10.1007/s12325-020-01460-5 PMC7433267 32809210

[26] HCSP. SARS-CoV-2: therapeutic recommendations [Internet]. Rapport de l’HCSP. Paris: Haut Conseil de la Santé Publique; 2020 mars [cité 11 mars 2021]. Disponible sur: https://www.hcsp.fr/Explore.cgi/avisrapportsdomaine?clefr=801

[27] Schumock GT, Walton SM, Park HY, Nutescu EA, Blackburn JC, Finley JM, et al. Factors that influence prescribing decisions. Ann Pharmacother. 2004;38(4):557–62. 10.1345/aph.1D390 14966259

[28] Emergency use of unproven interventions outside of research. Ethics guidance for the COVID-19 pandemic, 25 June 2020 - PAHO/WHO | Pan American Health Organization [Internet]. [cité 11 août 2023]. Disponible sur: https://www.paho.org/en/documents/emergency-use-unproven-interventions-outside-research-ethics-guidance-covid-19-pandemic-0

[29] World Health Organization. Guidance for managing ethical issues in infectious disease outbreaks [Internet]. World Health Organization. 2016 [cité 11 août 2023]. 68 p. Disponible sur: https://apps.who.int/iris/handle/10665/250580

[30] Emergency use of. unproven clinical interventions outside clinical trials: ethical considerations [Internet]. [cité 20 juin 2023]. Disponible sur: https://www.who.int/publications-detail-redirect/9789240041745

[31] Lamontagne F, Agarwal A, Rochwerg B, Siemieniuk RA, Agoritsas T, Askie L, et al. A living WHO guideline on drugs for Covid-19. BMJ. 2020;370:m3379. 10.1136/bmj.m3379 32887691

[32] Stolbach AI, Mazer-Amirshahi M, Marino R, Nelson LS, Sugarman J. ACMT position statement: Off-Label prescribing during COVID-19 pandemic. J Med Toxicol. 2020;16(3):342–5. 10.1007/s13181-020-00784-6 PMC7272106 32500283

[33] Kalil AC. Treating COVID-19-Off-Label Drug Use, Compassionate Use, and Randomized Clinical Trials During Pandemics. JAMA. 2020;323(19):1897–8. 10.1001/jama.2020.4742 32208486

[34] Caplan AL, Waldstreicher J, Childers K, Maree A. Drugs of unproven benefit for COVID-19: a pharma perspective on ethical allocation of available therapies. J Clin Invest. 2020;130(11):5622–3. 10.1172/JCI144186 PMC7598031 32931482

[35] Clinical management of COVID-19 [Internet]. [cité 28 nov 2025]. Disponible sur: https://www.who.int/publications/i/item/clinical-management-of-covid-19

[36] Ziemann S, Paetzolt I, Grüßer L, Coburn M, Rossaint R, Kowark A. Poor reporting quality of observational clinical studies comparing treatments of COVID-19 – a retrospective cross-sectional study. BMC Med Res Methodol. 2022;22(1):23. 10.1186/s12874-021-01501-9 PMC8771183 35057739

[37] Solidarity clinical trial for COVID. -19 treatments [Internet]. [cité 11 août 2023]. Disponible sur: https://www.who.int/emergencies/diseases/novel-coronavirus-2019/global-research-on-novel-coronavirus-2019-ncov/solidarity-clinical-trial-for-covid-19-treatments

