# Supplementary material for: COVID-19 treatment of hospital patients worldwide at the onset of the pandemic in 2020: a systematic review
Source: BMC Infect Dis. 2025 Dec 17;26:107. doi: 10.1186/s12879-025-12368-2 (PMC12822144; doi:10.1186/s12879-025-12368-2)
Supplement: Supplementary file 1 — Supplementary Material 1 [file 12879_2025_12368_MOESM1_ESM.docx]

**Supplementary Material** **1. Checklist for Reporting Results of Systematic Reviews***

| **Section and Topic** | **Item #** | **Checklist item** | **Location where item is reported** |
| --- | --- | --- | --- |
| **TITLE** | | |  |
| Title | 1 | Identify the report as a systematic review. | Title Page |
| **ABSTRACT** | | |  |
| Abstract | 2 | See the PRISMA 2020 for Abstracts checklist. | Page 8 (S2 Appendix) |
| **INTRODUCTION** | | |  |
| Rationale | 3 | Describe the rationale for the review in the context of existing knowledge. | Page 1 |
| Objectives | 4 | Provide an explicit statement of the objective(s) or question(s) the review addresses. | Page 1 |
| **METHODS** | | |  |
| Eligibility criteria | 5 | Specify the inclusion and exclusion criteria for the review and how studies were grouped for the syntheses.  Details  Inclusion criteria: article or letter written in English retrieving information about treatment given to adult inpatients hospitalized in non-ICU wards for Covid-19 until June 30, 2020.  Exclusion criteria:  - study exclusively on ICU patients (for example, studies 9, 15, 17) or patients younger than 18 years old (for example, studies 34, 83, 85),  - studies with an inclusion period extending after June 30, 2020, and without separate analysis of patients hospitalized before June 30  - studies including in and out-patients and do not allow the treatment of hospitalized patients to be analysed separately (for example, study 61).  - studies lacking information on treatments administered throughout the entire duration of hospitalization  - studies that from an initial population of patients hospitalized and treated for COVID-19 selected a subpopulation of patients who received one or more COVID-19 treatments and did not provide information on the initial population size (for example, studies 53, 54),  - Study without information about treatments given to inpatients (for example, studies 2, 3, and 5). For some studies, the therapeutic protocol is indicated in the "materials and methods" chapter or the introduction, but the number of patients who actually received a particular treatment is not indicated in the results. These studies are excluded from the analysis for this same reason.  - Studies that do not detail the treatments received during the entire duration of hospitalization  NB: in the case of the same cohort presented in several publications, we keep for the analysis only the one that includes the largest number of patients (for example, due to the longest inclusion period, a less selected population of patients) excluding the others. For example, study 1140 whose patients correspond to some of the patients analyzed in study 335 (same hospital, Seventh Hospital of Wuhan, but less extended inclusion period) was not kept for the analysis. | Page 2 |
| Information sources | 6 | Specify all databases, registers, websites, organisations, reference lists and other sources searched or consulted to identify studies. Specify the date when each source was last searched or consulted. | Page 2 |
| Search strategy | 7 | Present the full search strategies for all databases, registers and websites, including any filters and limits used. | Page 2 |
| Selection process | 8 | Specify the methods used to decide whether a study met the inclusion criteria of the review, including how many reviewers screened each record and each report retrieved, whether they worked independently, and if applicable, details of automation tools used in the process. | Page 2 |
| Data collection process | 9 | Specify the methods used to collect data from reports, including how many reviewers collected data from each report, whether they worked independently, any processes for obtaining or confirming data from study investigators, and if applicable, details of automation tools used in the process. | Page 2 |
| Data items | 10a | List and define all outcomes for which data were sought. Specify whether all results that were compatible with each outcome domain in each study were sought (e.g. for all measures, time points, analyses), and if not, the methods used to decide which results to collect.  We plan to collect the following data:   - ID number of each pubmed research article, name of first author, year of publication, - inclusion period, country, continent, department-wide study (yes/no), department name (if applicable), hospital or center-wide study (yes/no), hospital or center name (if applicable), city-wide study (yes/no), city name (if applicable), region or state (if multistates country)-wide study, region or state name (if applicable), country-wide study (yes/no), county name (if applicable), multinational study (yes/no), countries name (if applicable), primary objective, primary outcome, inclusion and exclusion criteria, study number of hospital or center for each study, - number of patients, number of patients admitted in intensive care unit during hospitalization, consecutive patient (yes, no), belonging of patients to a particular population (for example, patients with haematological malignancies or haemodialysis patients), study whose inclusion criteria include the performance of a SARS-Cov2 PCR (yes/no), - age of patients (median and IQR, mean and ET), sex of patients (male, female), number of deceased patients, number of patients with a PCR SARS-Cov2 positive, number of patients included in therapeutic clinical trial, number of patients treated only with standard of care (SOC), number of patients treated by hydroxychloroquine and/or chloroquine, corticosteroid, IL1-inhibitors, IL6-inhibitors, infliximab, rituximab, Jak2 inhibitor, remdesivir, convaslescent plasma, and any other treatment against Covid-19 if applicable, number of repurposed treatment received, DOI or web-article link. | Page 3 |
|  | 10b | List and define all other variables for which data were sought (e.g. participant and intervention characteristics, funding sources). Describe any assumptions made about any missing or unclear information. | Page 3-4 |
| Study risk of bias assessment | 11 | Specify the methods used to assess risk of bias in the included studies, including details of the tool(s) used, how many reviewers assessed each study and whether they worked independently, and if applicable, details of automation tools used in the process. | N.A. |
| Effect measures | 12 | Specify for each outcome the effect measure(s) (e.g. risk ratio, mean difference) used in the synthesis or presentation of results. | N.A. |
| Synthesis methods | 13a | Describe the processes used to decide which studies were eligible for each synthesis (e.g. tabulating the study intervention characteristics and comparing against the planned groups for each synthesis (item #5)). | N.A. |
|  | 13b | Describe any methods required to prepare the data for presentation or synthesis, such as handling of missing summary statistics, or data conversions. | Page 3-4 |
|  | 13c | Describe any methods used to tabulate or visually display results of individual studies and syntheses. | Page 3-4 |
|  | 13d | Describe any methods used to synthesize results and provide a rationale for the choice(s). If meta-analysis was performed, describe the model(s), method(s) to identify the presence and extent of statistical heterogeneity, and software package(s) used. | N.A. |
|  | 13e | Describe any methods used to explore possible causes of heterogeneity among study results (e.g. subgroup analysis, meta-regression). | N.A. |
|  | 13f | Describe any sensitivity analyses conducted to assess robustness of the synthesized results. | N.A. |
| Reporting bias assessment | 14 | Describe any methods used to assess risk of bias due to missing results in a synthesis (arising from reporting biases). | N.A. |
| Certainty assessment | 15 | Describe any methods used to assess certainty (or confidence) in the body of evidence for an outcome. | N.A. |
| **RESULTS** | | |  |
| Study selection | 16a | Describe the results of the search and selection process, from the number of records identified in the search to the number of studies included in the review, ideally using a flow diagram. | Page 4, Page 30 (Figure 1), supplementary files page 10-52 (Supplementary file Table A.1) |
|  | 16b | Cite studies that might appear to meet the inclusion criteria, but which were excluded, and explain why they were excluded. | Page 34 (figure 1), supplementary files page 10-52 (Supplementary file Table A.1) |
| Study characteristics | 17 | Cite each included study and present its characteristics. | Supplementary files (Appendix A: Table A.2. Page 52-68, Table A.2, A.3, Page 69-71), excel file with raw data |
| Risk of bias in studies | 18 | Present assessments of risk of bias for each included study. | N.A. |
| Results of individual studies | 19 | For all outcomes, present, for each study: (a) summary statistics for each group (where appropriate) and (b) an effect estimate and its precision (e.g. confidence/credible interval), ideally using structured tables or plots. | N.A. |
| Results of syntheses | 20a | For each synthesis, briefly summarise the characteristics and risk of bias among contributing studies. | N.A. |
|  | 20b | Present results of all statistical syntheses conducted. If meta-analysis was done, present for each the summary estimate and its precision (e.g. confidence/credible interval) and measures of statistical heterogeneity. If comparing groups, describe the direction of the effect. | N.A. |
|  | 20c | Present results of all investigations of possible causes of heterogeneity among study results. | N.A. |
|  | 20d | Present results of all sensitivity analyses conducted to assess the robustness of the synthesized results. | N.A. |
| Reporting biases | 21 | Present assessments of risk of bias due to missing results (arising from reporting biases) for each synthesis assessed. | N.A. |
| Certainty of evidence | 22 | Present assessments of certainty (or confidence) in the body of evidence for each outcome assessed. | N.A. |
| **DISCUSSION** | | |  |
| Discussion | 23a | Provide a general interpretation of the results in the context of other evidence. | Page 9-13 |
|  | 23b | Discuss any limitations of the evidence included in the review. | Page 13-14 |
|  | 23c | Discuss any limitations of the review processes used. | Page 13-14 |
|  | 23d | Discuss implications of the results for practice, policy, and future research. | Page 14-15 |
| **OTHER INFORMATION** | | |  |
| Registration and protocol | 24a | Provide registration information for the review, including register name and registration number, or state that the review was not registered. | N.A. |
|  | 24b | Indicate where the review protocol can be accessed, or state that a protocol was not prepared. | N.A. |
|  | 24c | Describe and explain any amendments to information provided at registration or in the protocol. | N.A. |
| Support | 25 | Describe sources of financial or non-financial support for the review, and the role of the funders or sponsors in the review. | N.A. |
| Competing interests | 26 | Declare any competing interests of review authors. | Page 1-2 (title page), submission platform |
| Availability of data, code and other materials | 27 | Report which of the following are publicly available and where they can be found: template data collection forms; data extracted from included studies; data used for all analyses; analytic code; any other materials used in the review. | Supplementary files (Appendix A: Table A.1, Page 10-51, Table A.2., Page 52-68), excel file with raw data |

**From:* Page MJ, McKenzie JE, Bossuyt PM, Boutron I, Hoffmann TC, Mulrow CD, et al. The PRISMA 2020 statement: an updated guideline for reporting systematic reviews. BMJ 2021;372:n71. doi: 10.1136/bmj.n71.
