# Supplementary material for: COVID-19 treatment of hospital patients worldwide at the onset of the pandemic in 2020: a systematic review
Source: BMC Infect Dis. 2025 Dec 17;26:107. doi: 10.1186/s12879-025-12368-2 (PMC12822144; doi:10.1186/s12879-025-12368-2)
Supplement: Supplementary file 3 — Supplementary Material 3 [file 12879_2025_12368_MOESM3_ESM.docx]

**Supplementary Material** **3. Selection process and list of studies identified by PubMed research (n=1388)**

| **Publication** | | | | **Study selection** | | | |
| --- | --- | --- | --- | --- | --- | --- | --- |
| Study number | First author | Year of publication | doi (*or PMID if doi not available in PubMed search result) | Removed before screening (n=1) | Records excluded after screening (n=625) | Reports assessed for eligibility and excluded (n=584) | Studies included for analysis (n=178) |
| 111 | CAROSI G | 2021 | 10.1210/clinem/dgaa793 | 1 | 0 | 0 | 0 |
| 15 | HUANG E | 2021 | 10.1016/j.ijid.2021.02.057 | 0 | 1 | 0 | 0 |
| 16 | FAZIO S | 2021 | 10.12659/MSM.935379 | 0 | 1 | 0 | 0 |
| 17 | BASKARAN V | 2021 | 10.1099/jmm.0.001350 | 0 | 1 | 0 | 0 |
| 23 | BLONZ G | 2021 | 10.1186/s13054-021-03493-w | 0 | 1 | 0 | 0 |
| 24 | JIANDANI MP | 2020 | *32978920 | 0 | 1 | 0 | 0 |
| 27 | ANGEL Y | 2021 | 10.1001/jama.2021.7152 | 0 | 1 | 0 | 0 |
| 28 | SEMENZATO L | 2021 | 10.1161/HYPERTENSIONAHA.120.16314 | 0 | 1 | 0 | 0 |
| 32 | DONG Y | 2021 | 10.1097/MD.0000000000027685 | 0 | 1 | 0 | 0 |
| 33 | INGUALE A | 2021 | *34227770 | 0 | 1 | 0 | 0 |
| 34 | RAMCHARAN T | 2020 | 10.1007/s00246-020-02391-2 | 0 | 1 | 0 | 0 |
| 37 | SONTI R | 2021 | 10.1177/0885066620976525 | 0 | 1 | 0 | 0 |
| 38 | GANESH R | 2021 | 10.1093/infdis/jiab377 | 0 | 1 | 0 | 0 |
| 44 | BHATTACHARYA B | 2021 | 10.1093/femspd/ftaa064 | 0 | 1 | 0 | 0 |
| 49 | BEZUIDENHOUT MC | 2022 | 10.1177/0004563220972539 | 0 | 1 | 0 | 0 |
| 52 | ALAA A | 2020 | 10.1136/bmjopen-2020-042712 | 0 | 1 | 0 | 0 |
| 55 | MARGUS C | 2021 | 10.2196/28615 | 0 | 1 | 0 | 0 |
| 56 | KENDORI K | 2021 | 10.1038/s41598-021-92415-5 | 0 | 1 | 0 | 0 |
| 58 | CECCARELLI G | 2021 | 10.1002/jmv.26925 | 0 | 1 | 0 | 0 |
| 61 | SANTOS CS | 2021 | 10.1136/rmdopen-2020-001439 | 0 | 1 | 0 | 0 |
| 65 | IP A | 2021 | 10.1186/s12879-021-05773-w | 0 | 1 | 0 | 0 |
| 68 | BOTTI C | 2021 | 10.1177/0145561320965196 | 0 | 1 | 0 | 0 |
| 69 | ITO J | 2021 | 10.1007/s00540-021-02897-w | 0 | 1 | 0 | 0 |
| 70 | HO KMA | 2020 | 10.7861/clinmed.2020-0483 | 0 | 1 | 0 | 0 |
| 72 | WALTER E | 2021 | 10.3390/ijerph18157963 | 0 | 1 | 0 | 0 |
| 73 | MEHTA N | 2020 | 10.1001/jamacardio.2020.1855 | 0 | 1 | 0 | 0 |
| 74 | NASER MN | 2021 | 10.1371/journal.pone.0257253 | 0 | 1 | 0 | 0 |
| 79 | PROVENZANO BC | 2021 | 10.1590/1806-9282.20210433 | 0 | 1 | 0 | 0 |
| 80 | BABAR I | 2021 | 10.14423/SMJ.0000000000001245 | 0 | 1 | 0 | 0 |
| 81 | HERMANN ML | 2021 | 10.1371/journal.pone.0253154 | 0 | 1 | 0 | 0 |
| 82 | DIEBOLD M | 2021 | 10.4414/smw.2021.20482 | 0 | 1 | 0 | 0 |
| 83 | MOUROUVAYE M | 2021 | 10.1136/archdischild-2020-320628 | 0 | 1 | 0 | 0 |
| 84 | RAMIREZ P | 2021 | 10.1016/j.medin.2020.06.015 | 0 | 1 | 0 | 0 |
| 85 | WEI Y | 2020 | 10.1186/s13052-020-00915-3 | 0 | 1 | 0 | 0 |
| 86 | VERSCHELDEN G | 2021 | 10.1016/j.intimp.2021.108163 | 0 | 1 | 0 | 0 |
| 87 | DALE CR | 2021 | 10.1186/s13054-021-03504-w | 0 | 1 | 0 | 0 |
| 90 | ALANLI R | 2021 | 10.1590/1806-9282.20210675 | 0 | 1 | 0 | 0 |
| 95 | VAN MARCKE C | 2021 | 10.1186/s12885-021-08349-8 | 0 | 1 | 0 | 0 |
| 97 | BRAWNER CA | 2021 | 10.1016/j.mayocp.2020.10.003 | 0 | 1 | 0 | 0 |
| 98 | LOPEZ A | 2021 | 10.1016/j.jcrc.2021.06.014 | 0 | 1 | 0 | 0 |
| 99 | BODNAR W | 2021 | *34919079 | 0 | 1 | 0 | 0 |
| 103 | SHIONOYA Y | 2021 | 10.1371/journal.pone.0256977 | 0 | 1 | 0 | 0 |
| 104 | MIYAGAMI T | 2021 | 10.1515/dx-2020-0114 | 0 | 1 | 0 | 0 |
| 105 | CAI Y | 2021 | 10.7189/jogh.11.05023 | 0 | 1 | 0 | 0 |
| 107 | RYAN R | 2021 | 10.23736/S0375-9393.21.15595-6 | 0 | 1 | 0 | 0 |
| 113 | SHAKESPEARE C | 2021 | 10.1186/s12884-021-03884-5 | 0 | 1 | 0 | 0 |
| 116 | CHOI DH | 2021 | 10.3346/jkms.2021.36.e44 | 0 | 1 | 0 | 0 |
| 120 | NAJATI N | 2021 | 10.1016/j.psychres.2021.113999 | 0 | 1 | 0 | 0 |
| 126 | BECKER G | 2021 | 10.26355/eurrev_202101_24686 | 0 | 1 | 0 | 0 |
| 127 | LI S | 2021 | 10.1016/j.rmed.2020.106271 | 0 | 1 | 0 | 0 |
| 128 | HASSELI R | 2021 | 10.1136/rmdopen-2020-001464 | 0 | 1 | 0 | 0 |
| 136 | MUSHTAQ MZ | 2022 | 10.1016/j.intimp.2021.108384 | 0 | 1 | 0 | 0 |
| 137 | ZINI A | 2020 | 10.1007/s10072-020-04754-2 | 0 | 1 | 0 | 0 |
| 139 | PORTZ JD | 2020 | 10.2196/21385 | 0 | 1 | 0 | 0 |
| 140 | WINDERS HR | 2021 | 10.1016/j.ijantimicag.2021.106453 | 0 | 1 | 0 | 0 |
| 141 | LINSSEN J | 2021 | 10.7554/eLife.63195 | 0 | 1 | 0 | 0 |
| 144 | BANNO A | 2021 | 10.1186/s12879-021-05840-2 | 0 | 1 | 0 | 0 |
| 147 | LIU D | 2020 | 10.2196/20108 | 0 | 1 | 0 | 0 |
| 150 | KORKMAZ MF | 2020 | 10.3346/jkms.2020.35.e236 | 0 | 1 | 0 | 0 |
| 153 | VACANTI G | 2020 | 10.1007/s00059-020-04991-3 | 0 | 1 | 0 | 0 |
| 154 | ZIEMANN S | 2022 | 10.1186/s12874-021-01501-9 | 0 | 1 | 0 | 0 |
| 155 | SUGAND K | 2020 | 10.1080/17453674.2020.1807092 | 0 | 1 | 0 | 0 |
| 157 | AKIYAMA Y | 2020 | 10.2169/internalmedicine.5614-20 | 0 | 1 | 0 | 0 |
| 159 | POUWELS S | 2021 | 10.3390/medicina57070674 | 0 | 1 | 0 | 0 |
| 161 | CERNIGLIARO A | 2020 | 10.19191/EP20.5-6.S2.132 | 0 | 1 | 0 | 0 |
| 162 | SHI J | 2020 | 10.18632/aging.202223 | 0 | 1 | 0 | 0 |
| 163 | SCHEEN AJ | 2021 | 10.1016/j.diabet.2020.101220 | 0 | 1 | 0 | 0 |
| 166 | SAWCZUNSKA K | 2021 | 10.5603/PJNNS.a2021.0037 | 0 | 1 | 0 | 0 |
| 174 | SCHULER PJ | 2021 | 10.1007/s00106-021-01021-4 | 0 | 1 | 0 | 0 |
| 178 | MALDONADO A | 2020 | 10.1007/s11606-020-05906-y | 0 | 1 | 0 | 0 |
| 180 | CARBONI BISSO I | 2021 | *34453793 | 0 | 1 | 0 | 0 |
| 182 | NENE RV | 2021 | 10.1016/j.ajem.2021.04.070 | 0 | 1 | 0 | 0 |
| 183 | ORTONOBES ROIG S | 2021 | 10.37201/req/130.2020 | 0 | 1 | 0 | 0 |
| 191 | XIONG X | 2022 | 10.1111/irv.12919 | 0 | 1 | 0 | 0 |
| 194 | EMAN A | 2021 | 10.3346/jkms.2021.36.e309 | 0 | 1 | 0 | 0 |
| 196 | GINE SERVEN E | 2021 | 10.1080/08039488.2021.1885061 | 0 | 1 | 0 | 0 |
| 197 | BALETTO AA | 2021 | *34137700 | 0 | 1 | 0 | 0 |
| 199 | MOUSTAKIS J | 2020 | 10.7196/SAMJ.2020.v110i9.15025 | 0 | 1 | 0 | 0 |
| 201 | CAN TOPCU A | 2021 | 10.1016/j.avsg.2021.03.003 | 0 | 1 | 0 | 0 |
| 203 | DONGIOO LEE D | 2021 | 10.5811/westjem.2021.1.50123 | 0 | 1 | 0 | 0 |
| 206 | BUDHIRAJA S | 2021 | 10.1016/j.bcmd.2021.102548 | 0 | 1 | 0 | 0 |
| 207 | MORADPOUR G | 2022 | 10.1007/s11695-021-05761-8 | 0 | 1 | 0 | 0 |
| 209 | SOVIK S | 2021 | 10.1111/aas.13726 | 0 | 1 | 0 | 0 |
| 210 | ANTAKIA R | 2021 | 10.1016/j.ijsu.2020.12.009 | 0 | 1 | 0 | 0 |
| 211 | PUZO M | 2021 | 10.1186/s12886-021-02169-x | 0 | 1 | 0 | 0 |
| 213 | SHANTHA JG | 2021 | 10.1080/09273948.2021.1952278 | 0 | 1 | 0 | 0 |
| 214 | BIANCARI F | 2021 | 10.1053/j.jvca.2021.01.027 | 0 | 1 | 0 | 0 |
| 216 | THEOFANOPOULOS A | 2021 | 10.1007/s10072-021-05190-6 | 0 | 1 | 0 | 0 |
| 217 | WENDEL-GARCIA PD | 2022 | 10.1186/s13054-022-03905-5 | 0 | 1 | 0 | 0 |
| 220 | DAVIES P | 2021 | 10.1136/emermed-2021-211220 | 0 | 1 | 0 | 0 |
| 223 | SCHUH A | 2021 | 10.1055/a-1529-6726 | 0 | 1 | 0 | 0 |
| 226 | ALHOSANI FI | 2022 | 10.1016/j.vaccine.2022.02.039 | 0 | 1 | 0 | 0 |
| 228 | BIARNES-SUNE A | 2021 | 10.1016/j.redar.2020.10.003 | 0 | 1 | 0 | 0 |
| 229 | TEJADA MEZA H | 2020 | 10.1177/1747493020938301 | 0 | 1 | 0 | 0 |
| 230 | KARAALI R | 2021 | 10.1016/j.ajem.2021.06.045 | 0 | 1 | 0 | 0 |
| 232 | CHUN-ERN NG D | 2021 | 10.1016/j.ijid.2021.05.073 | 0 | 1 | 0 | 0 |
| 236 | SANTI L | 2021 | 10.1371/journal.pone.0248995 | 0 | 1 | 0 | 0 |
| 244 | KROL P | 2021 | 10.1111/tmi.13542 | 0 | 1 | 0 | 0 |
| 246 | REYES C | 2021 | 10.1136/bmjopen-2021-057632 | 0 | 1 | 0 | 0 |
| 247 | FISTERA D | 2021 | 10.1186/s12879-021-06663-x | 0 | 1 | 0 | 0 |
| 248 | LEE SY | 2020 | 10.3346/jkms.2020.35.e367 | 0 | 1 | 0 | 0 |
| 250 | STRINGER H | 2021 | 10.1016/j.foot.2020.101772 | 0 | 1 | 0 | 0 |
| 251 | SANDERS J | 2021 | 10.1186/s13019-021-01424-y | 0 | 1 | 0 | 0 |
| 252 | LIBRUDER C | 2021 | 10.1159/000516753 | 0 | 1 | 0 | 0 |
| 257 | PAREEK A | 2021 | 10.1016/j.dsx.2021.05.004 | 0 | 1 | 0 | 0 |
| 263 | TRAMUNT B | 2021 | 10.1530/EJE-21-0068 | 0 | 1 | 0 | 0 |
| 265 | DOLCI G | 2022 | 10.1016/j.bjid.2021.101702 | 0 | 1 | 0 | 0 |
| 266 | TSAI Y | 2021 | 10.7326/M21-1102 | 0 | 1 | 0 | 0 |
| 268 | LI S | 2021 | 10.1097/ANA.0000000000000748 | 0 | 1 | 0 | 0 |
| 272 | GRIFFITH AM | 2021 | 10.1007/s10140-021-01907-4 | 0 | 1 | 0 | 0 |
| 274 | JAMJOOM RS | 2021 | 10.15537/smj.2021.1.25572 | 0 | 1 | 0 | 0 |
| 277 | CHAN J | 2021 | 10.1177/0033354921999385 | 0 | 1 | 0 | 0 |
| 281 | CALAGNAN E | 2020 | 10.19191/EP20.5-6.S2.133 | 0 | 1 | 0 | 0 |
| 282 | MUKHTAR A | 2021 | 10.1016/j.ajem.2021.07.049 | 0 | 1 | 0 | 0 |
| 286 | VON RENESSE J | 2021 | 10.1016/j.clnesp.2021.06.016 | 0 | 1 | 0 | 0 |
| 288 | RIPOLL B | 2022 | 10.1177/0391398821989065 | 0 | 1 | 0 | 0 |
| 289 | PEPPER MP | 2021 | 10.1097/MD.0000000000026583 | 0 | 1 | 0 | 0 |
| 290 | PERRIN N | 2020 | 10.4414/smw.2020.20448 | 0 | 1 | 0 | 0 |
| 292 | RUSSO V | 2020 | 10.3390/ijerph18010102 | 0 | 1 | 0 | 0 |
| 293 | COSTAMAGNA G | 2021 | 10.7429/pi.2021.741021 | 0 | 1 | 0 | 0 |
| 294 | MALIK-TABASSUM K | 2021 | 10.1308/rcsann.2020.7071 | 0 | 1 | 0 | 0 |
| 295 | LASKAR NS | 2021 | 10.1016/j.surge.2020.08.015 | 0 | 1 | 0 | 0 |
| 298 | MERIC S | 2021 | 10.14744/tjtes.2020.46487 | 0 | 1 | 0 | 0 |
| 299 | AOUN M | 2021 | 10.1186/s12882-021-02270-9 | 0 | 1 | 0 | 0 |
| 301 | TIGNANELLI CJ | 2021 | 10.1016/j.soard.2021.05.029 | 0 | 1 | 0 | 0 |
| 302 | JANZ DR | 2021 | 10.1016/j.chest.2020.08.2114 | 0 | 1 | 0 | 0 |
| 305 | HARO PEREZ AM | 2022 | *35194012 | 0 | 1 | 0 | 0 |
| 306 | ARDIZZONE S | 2021 | 10.1111/jgh.15591 | 0 | 1 | 0 | 0 |
| 307 | MAMIDANNA R | 2021 | 10.1308/rcsann.2021.0053 | 0 | 1 | 0 | 0 |
| 308 | TRENTINI F | 2022 | 10.1093/aje/kwab252 | 0 | 1 | 0 | 0 |
| 309 | MALDONADO M | 2021 | 10.1016/j.nefro.2020.09.002 | 0 | 1 | 0 | 0 |
| 311 | MARENCO-HILLEMBRAND L | 2021 | 10.1016/j.wneu.2021.06.147 | 0 | 1 | 0 | 0 |
| 315 | PESCATORE JM | 2021 | 10.1515/jom-2021-0182 | 0 | 1 | 0 | 0 |
| 316 | MARINO L | 2021 | 10.1111/ijcp.14426 | 0 | 1 | 0 | 0 |
| 317 | PERK O | 2021 | 10.1093/tropej/fmab102 | 0 | 1 | 0 | 0 |
| 321 | RYU BH | 2021 | 10.3346/jkms.2021.36.e341 | 0 | 1 | 0 | 0 |
| 322 | GOKSOY B | 2020 | 10.14744/etd.2020.67927 | 0 | 1 | 0 | 0 |
| 323 | PADMANABHAN N | 23021 | 10.1007/s10072-020-04775-x | 0 | 1 | 0 | 0 |
| 324 | SHEPPARD JP | 2021 | 10.1161/HYPERTENSIONAHA.120.16472 | 0 | 1 | 0 | 0 |
| 328 | QURESHI SS | 2021 | 10.1097/MD.0000000000026752 | 0 | 1 | 0 | 0 |
| 329 | ADEBAYO PB | 2020 | 10.11604/pamj.supp.2020.35.2.24977 | 0 | 1 | 0 | 0 |
| 332 | PARISIAN MILITARY ANTI-COVID-19 GROUP | 2021 | 10.37765/ajmc.2021.88623 | 0 | 1 | 0 | 0 |
| 333 | AMENDOLA A | 2021 | *34312333 | 0 | 1 | 0 | 0 |
| 336 | MCKAY B | 2021 | 10.1016/j.ajem.2021.08.077 | 0 | 1 | 0 | 0 |
| 338 | DOOLUB G | 2021 | 10.1002/ehf2.13158 | 0 | 1 | 0 | 0 |
| 340 | LAMBRACOS S | 2021 | *34239150 | 0 | 1 | 0 | 0 |
| 341 | DE MAIO NASCIMENTO M | 2021 | 10.1016/j.archger.2021.104462 | 0 | 1 | 0 | 0 |
| 343 | GODAERT L | 2021 | 10.4269/ajtmh.21-0480 | 0 | 1 | 0 | 0 |
| 346 | YAN W | 2021 | 10.1097/MD.0000000000024720 | 0 | 1 | 0 | 0 |
| 348 | VAISHYA R | 2021 | 10.1016/j.dsx.2021.102306 | 0 | 1 | 0 | 0 |
| 351 | ALFADDA AA | 2021 | 10.1016/j.jiph.2021.09.014 | 0 | 1 | 0 | 0 |
| 352 | HARRIES M | 2021 | 10.1136/bmjopen-2020-045718 | 0 | 1 | 0 | 0 |
| 359 | GROAH SL | 2022 | 10.1002/pmrj.12645 | 0 | 1 | 0 | 0 |
| 363 | RUSSO V | 2020 | 10.1080/17434440.2020.1841632 | 0 | 1 | 0 | 0 |
| 364 | MOYER JD | 2021 | 10.1186/s13049-021-00864-8 | 0 | 1 | 0 | 0 |
| 365 | BERGMAN ZR | 2021 | 10.1097/MAT.0000000000001403 | 0 | 1 | 0 | 0 |
| 366 | SZARFER JL | 2021 | 10.1093/intqhc/mzab029 | 0 | 1 | 0 | 0 |
| 367 | HANDLEY SC | 2022 | 10.1542/peds.2021-053498 | 0 | 1 | 0 | 0 |
| 369 | JI C | 2022 | 10.1016/j.vaccine.2022.02.008 | 0 | 1 | 0 | 0 |
| 372 | HWANG C | 2021 | 10.1002/cnr2.1388 | 0 | 1 | 0 | 0 |
| 378 | AHMED S | 2021 | *34958058 | 0 | 1 | 0 | 0 |
| 380 | LOPEZ-OTERO D | 2020 | 10.1016/j.rec.2020.05.018 | 0 | 1 | 0 | 0 |
| 381 | ROVIRA A | 2021 | 10.1007/s00405-020-06597-1 | 0 | 1 | 0 | 0 |
| 385 | PULIDO L | 2020 | *33048785 | 0 | 1 | 0 | 0 |
| 386 | HENRY N | 2021 | 10.1177/0091217420982100 | 0 | 1 | 0 | 0 |
| 387 | SHARMA S | 2021 | 10.33314/jnhrc.v19i2.3623 | 0 | 1 | 0 | 0 |
| 391 | FULGENCIO-BARBARIN J | 2020 | 10.1111/ijd.15157 | 0 | 1 | 0 | 0 |
| 392 | BARBHAYA D | 2021 | 10.1177/21501327211017016 | 0 | 1 | 0 | 0 |
| 393 | SHIMIZU M | 2021 | 10.1007/s00540-021-02986-w | 0 | 1 | 0 | 0 |
| 394 | MARIET AS | 2021 | 10.1161/STROKEAHA.120.032312 | 0 | 1 | 0 | 0 |
| 397 | KODDE C | 2021 | 10.1016/j.cmi.2021.09.002 | 0 | 1 | 0 | 0 |
| 398 | ALBERTO EC | 2021 | 10.1016/j.annemergmed.2021.05.022 | 0 | 1 | 0 | 0 |
| 399 | PIMLOTT N | 2021 | 10.1093/fampra/cmaa130 | 0 | 1 | 0 | 0 |
| 401 | KOKOSKA-BARGIEL I | 2020 | 10.12659/MSM.926974 | 0 | 1 | 0 | 0 |
| 403 | LEULSEGED TW | 2021 | 10.4314/ejhs.v31i4.3 | 0 | 1 | 0 | 0 |
| 404 | CARPAGNANO G | 2021 | 10.1186/s12931-021-01613-2 | 0 | 1 | 0 | 0 |
| 406 | CEM BULUT O | 2022 | 10.1007/s00106-021-01121-1 | 0 | 1 | 0 | 0 |
| 408 | DESAI A | 2021 | 10.1016/j.ijid.2021.10.032 | 0 | 1 | 0 | 0 |
| 409 | HERNIGOU J | 2020 | 10.1007/s00264-020-04619-5 | 0 | 1 | 0 | 0 |
| 410 | RABHA AC | 2020 | 10.1590/1984-0462/2021/39/2020305 | 0 | 1 | 0 | 0 |
| 413 | TSIOUFIS K | 2020 | 10.1007/s00392-020-01682-1 | 0 | 1 | 0 | 0 |
| 414 | ABDELAZIZ HK | 2020 | 10.1016/j.ahj.2020.04.022 | 0 | 1 | 0 | 0 |
| 417 | PIERI M | 2022 | 10.1177/03913988211052572 | 0 | 1 | 0 | 0 |
| 419 | ZENG M | 2020 | 10.1016/j.wneu.2020.08.007 | 0 | 1 | 0 | 0 |
| 424 | SOYSAL A | 2021 | 10.7883/yoken.JJID.2020.781 | 0 | 1 | 0 | 0 |
| 425 | ANTEBY R | 2020 | 10.1089/lap.2020.0465 | 0 | 1 | 0 | 0 |
| 427 | RUIZ DOMINGUEZ JA | 2021 | *33978342 | 0 | 1 | 0 | 0 |
| 429 | SILVESTRI C | 2020 | *33295704 | 0 | 1 | 0 | 0 |
| 430 | WU Z | 2021 | 10.1536/ihj.20-522 | 0 | 1 | 0 | 0 |
| 432 | VAQUERO-RONCERO LM | 2021 | 10.1016/j.redare.2020.11.008 | 0 | 1 | 0 | 0 |
| 433 | AGUILA-GORDO D | 2021 | 10.1016/j.regg.2020.09.006 | 0 | 1 | 0 | 0 |
| 436 | PINZON-ESPITIA OL | 2022 | 10.20960/nh.03738 | 0 | 1 | 0 | 0 |
| 438 | BUN S | 2021 | 10.1016/j.alit.2021.06.001 | 0 | 1 | 0 | 0 |
| 441 | KANG Y | 2020 | 10.1097/MD.0000000000023797 | 0 | 1 | 0 | 0 |
| 442 | ALBERT A | 2022 | *35103442 | 0 | 1 | 0 | 0 |
| 443 | MADANELO M | 2020 | 10.1111/bju.15109 | 0 | 1 | 0 | 0 |
| 444 | GUIDAVAL F | 2020 | 10.5578/tt.70128 | 0 | 1 | 0 | 0 |
| 447 | ORLANDI D | 2021 | 10.1016/j.ultrasmedbio.2021.07.014 | 0 | 1 | 0 | 0 |
| 448 | OLIVINI N | 2020 | 10.1186/s13052-020-00947-9 | 0 | 1 | 0 | 0 |
| 449 | PAPAFAKLIS MI | 2020 | 10.1002/clc.23424 | 0 | 1 | 0 | 0 |
| 450 | GONZALEZ-DIAZ A | 2020 | 10.1016/j.acuro.2020.09.007 | 0 | 1 | 0 | 0 |
| 451 | MAATAOUI N | 2021 | 10.1007/s10096-021-04213-6 | 0 | 1 | 0 | 0 |
| 454 | LOPEZ-BARBEITO B | 2020 | *33275358 | 0 | 1 | 0 | 0 |
| 455 | PABLO-MARCOS D | 2020 | 10.37201/req/077.2020 | 0 | 1 | 0 | 0 |
| 456 | YANG SS | 2020 | 10.9778/cmajo.20200159 | 0 | 1 | 0 | 0 |
| 457 | BLAIR JE | 2021 | 10.1097/MD.0000000000026371 | 0 | 1 | 0 | 0 |
| 458 | ALTERI C | 2021 | 10.1038/s41467-020-20688-x | 0 | 1 | 0 | 0 |
| 459 | BODY A | 2022 | 10.1186/s12879-021-07019-1 | 0 | 1 | 0 | 0 |
| 460 | DE LA MATTA M | 2021 | 10.1016/j.redare.2020.10.002 | 0 | 1 | 0 | 0 |
| 463 | MISRADRAEE S | 2021 | 10.1097/CCM.0000000000004890 | 0 | 1 | 0 | 0 |
| 466 | TSAI TC | 2022 | 10.1016/j.hjdsi.2021.100611 | 0 | 1 | 0 | 0 |
| 467 | AGETA K | 2020 | 10.18926/AMO/61210 | 0 | 1 | 0 | 0 |
| 468 | GANESH SK | 2022 | 10.4103/ijo.IJO_2735_21 | 0 | 1 | 0 | 0 |
| 469 | GATTA M | 2022 | 10.1186/s13052-022-01213-w | 0 | 1 | 0 | 0 |
| 475 | UNGAR SP | 2022 | 10.1177/00099228211065898 | 0 | 1 | 0 | 0 |
| 477 | WONG B | 2020 | *32994610 | 0 | 1 | 0 | 0 |
| 478 | MORTON S | 2022 | 10.1016/j.amj.2021.10.012 | 0 | 1 | 0 | 0 |
| 480 | ELMORE JG | 2020 | 10.2196/21562 | 0 | 1 | 0 | 0 |
| 481 | APRIL MD | 2022 | 10.14423/SMJ.0000000000001368 | 0 | 1 | 0 | 0 |
| 482 | LEFRANT JY | 2020 | 10.1016/j.accpm.2020.09.007 | 0 | 1 | 0 | 0 |
| 485 | RAI N | 2022 | 10.1002/ppul.25752 | 0 | 1 | 0 | 0 |
| 489 | STONE R | 2022 | 10.4140/TCP.n.2022.62 | 0 | 1 | 0 | 0 |
| 490 | NYMAN MA | 2022 | 10.1177/21501319211069748 | 0 | 1 | 0 | 0 |
| 492 | ZUGLIAN G | 2022 | 10.1186/s12879-022-07176-x | 0 | 1 | 0 | 0 |
| 493 | HOLMBERG V | 2022 | 10.1016/j.cmi.2021.08.022 | 0 | 1 | 0 | 0 |
| 495 | PLECKO D | 2022 | 10.1111/aas.13991 | 0 | 1 | 0 | 0 |
| 496 | PAETZOLD J | 2022 | 10.1038/s41467-022-28233-8 | 0 | 1 | 0 | 0 |
| 499 | NARAYANAN P | 2022 | 10.1016/j.ymgme.2021.08.004 | 0 | 1 | 0 | 0 |
| 502 | KONDILIS E | 2021 | 10.1016/j.puhe.2021.06.025 | 0 | 1 | 0 | 0 |
| 506 | SAKAMOTO D | 2021 | 10.1371/journal.pone.0260743 | 0 | 1 | 0 | 0 |
| 513 | SARTORI A | 2021 | 10.1007/s13304-021-01126-z | 0 | 1 | 0 | 0 |
| 515 | CORREIA M | 2020 | 10.1016/j.avsg.2020.08.002 | 0 | 1 | 0 | 0 |
| 518 | ALBUALI WH | 202 | 10.1136/bmjopen-2021-053722 | 0 | 1 | 0 | 0 |
| 520 | LIU Y | 2021 | 10.1186/s12879-021-06502-z | 0 | 1 | 0 | 0 |
| 524 | D'ONOFRIO L | 2021 | 10.1111/dom.14380 | 0 | 1 | 0 | 0 |
| 528 | HAY D | 2021 | 10.1308/rcsann.2020.7026 | 0 | 1 | 0 | 0 |
| 530 | CHIMA M | 2021 | 10.1542/hpeds.2021-005866 | 0 | 1 | 0 | 0 |
| 532 | KAHN MR | 2021 | 10.1177/0885066621989959 | 0 | 1 | 0 | 0 |
| 534 | ZHANG P | 2021 | 10.1016/j.clnu.2020.05.051 | 0 | 1 | 0 | 0 |
| 535 | SEWELL M | 2021 | 10.1016/j.wneu.2021.06.046 | 0 | 1 | 0 | 0 |
| 540 | KEITA H | 2021 | 10.1016/j.accpm.2021.100937 | 0 | 1 | 0 | 0 |
| 545 | BHATTACHARYA B | 2021 | 10.1093/femspd/ftaa064 | 0 | 1 | 0 | 0 |
| 546 | KUMARI J | 2021 | 10.1007/s12098-020-03590-8 | 0 | 1 | 0 | 0 |
| 547 | VASUDEV R | 2020 | 10.1111/echo.14825 | 0 | 1 | 0 | 0 |
| 550 | LAWTON MT | 2021 | 10.1186/s12883-021-02109-8 | 0 | 1 | 0 | 0 |
| 556 | PATEL VM | 2021 | 10.1016/j.amjoto.2021.103123 | 0 | 1 | 0 | 0 |
| 557 | RODRIGUES M | 2021 | 10.33588/rn.7303.2020445 | 0 | 1 | 0 | 0 |
| 559 | STRAW S | 2021 | 10.1186/s12904-021-00711-8 | 0 | 1 | 0 | 0 |
| 560 | ROMANI S | 2021 | 10.1111/cts.12883 | 0 | 1 | 0 | 0 |
| 561 | MANCILLA-GALINDO J | 2021 | 10.1017/S095026882100234X | 0 | 1 | 0 | 0 |
| 564 | GIMENO-MIGUEL A | 2021 | 10.1371/journal.pone.0259822 | 0 | 1 | 0 | 0 |
| 565 | PISCITANI L | 2022 | 10.1007/s40292-021-00502-5 | 0 | 1 | 0 | 0 |
| 566 | PAN Z | 2021 | 10.25011/cim.v44i2.36355 | 0 | 1 | 0 | 0 |
| 568 | BAGHERI SR | 2021 | 10.1016/j.jocn.2021.09.029 | 0 | 1 | 0 | 0 |
| 570 | TRONCOSO JR RD | 2021 | 10.1016/j.amj.2021.04.001 | 0 | 1 | 0 | 0 |
| 577 | TANAKA K | 2021 | 10.1111/ggi.14207 | 0 | 1 | 0 | 0 |
| 578 | RABIE AA | 2021 | 10.1007/s00134-021-06451-w | 0 | 1 | 0 | 0 |
| 579 | DOMNICH A | 2022 | 10.1016/j.vaccine.2022.02.013 | 0 | 1 | 0 | 0 |
| 580 | BOZZANI A | 2021 | 10.1016/j.avsg.2020.12.001 | 0 | 1 | 0 | 0 |
| 581 | BENFANTE A | 2021 | 10.1016/j.pupt.2021.102038 | 0 | 1 | 0 | 0 |
| 584 | PONZO V | 2021 | 10.3390/nu13051721 | 0 | 1 | 0 | 0 |
| 585 | OJETTI V | 2020 | 10.3390/medicina56100512 | 0 | 1 | 0 | 0 |
| 588 | FAULDS ER | 2021 | 10.1210/clinem/dgab409 | 0 | 1 | 0 | 0 |
| 589 | MOLINA GUTIERREZ MA | 2020 | 10.1016/j.anpedi.2020.06.021 | 0 | 1 | 0 | 0 |
| 590 | ZAYAT R | 2021 | 10.1111/aor.13873 | 0 | 1 | 0 | 0 |
| 591 | HAMEED S | 2021 | 10.1016/j.clinph.2021.10.001 | 0 | 1 | 0 | 0 |
| 593 | JEFFERY MM | 2020 | 10.1001/jamainternmed.2020.3288 | 0 | 1 | 0 | 0 |
| 594 | WASSER LM | 2021 | 10.1097/PEC.0000000000002468 | 0 | 1 | 0 | 0 |
| 600 | MAZEDA C | 2021 | *34628458 | 0 | 1 | 0 | 0 |
| 601 | VALENT A | 2020 | 10.1016/j.accpm.2020.10.001 | 0 | 1 | 0 | 0 |
| 602 | TEO KYC | 2021 | 10.1016/j.oret.2021.02.005 | 0 | 1 | 0 | 0 |
| 603 | IQBAL HOSSAIN M | 2021 | 10.1097/MD.0000000000027281 | 0 | 1 | 0 | 0 |
| 604 | BARRICK L | 2021 | 10.1016/j.ajem.2021.05.066 | 0 | 1 | 0 | 0 |
| 605 | VEGA ML | 2022 | 10.1016/j.pulmoe.2021.04.003 | 0 | 1 | 0 | 0 |
| 606 | ARGUELLES-ARIAS F | 2022 | 10.17235/reed.2021.8320/2021 | 0 | 1 | 0 | 0 |
| 607 | PRASAD NK | 2022 | 10.1097/SLA.0000000000005176 | 0 | 1 | 0 | 0 |
| 609 | XIONG X | 2020 | 10.1016/j.jpeds.2020.06.041 | 0 | 1 | 0 | 0 |
| 610 | NAFAKHI H | 2021 | 10.1016/j.dsx.2020.12.014 | 0 | 1 | 0 | 0 |
| 613 | APARICIO T | 2022 | 10.1016/j.dld.2021.09.017 | 0 | 1 | 0 | 0 |
| 615 | CHAUDHARY S | 2021 | 10.4187/respcare.08319 | 0 | 1 | 0 | 0 |
| 617 | YU JH | 2021 | 10.1136/emermed-2020-210409 | 0 | 1 | 0 | 0 |
| 618 | FABRIS E | 2021 | 10.2459/JCM.0000000000001153 | 0 | 1 | 0 | 0 |
| 619 | CHEN Z | 2020 | 10.1007/s10439-020-02648-0 | 0 | 1 | 0 | 0 |
| 620 | GARG A | 2021 | 10.2196/21327 | 0 | 1 | 0 | 0 |
| 621 | NAKAMURA S | 2021 | 10.1007/s10147-020-01837-0 | 0 | 1 | 0 | 0 |
| 623 | MASUR J | 2020 | 10.3174/ajnr.A6728 | 0 | 1 | 0 | 0 |
| 624 | WOLF M | 2021 | 10.1002/oby.23142 | 0 | 1 | 0 | 0 |
| 631 | GANTER J | 2021 | 10.1097/MD.0000000000026526 | 0 | 1 | 0 | 0 |
| 633 | SAMAAN F | 2022 | 10.1371/journal.pone.0261958 | 0 | 1 | 0 | 0 |
| 634 | JALADHAR P | 2021 | *34472811 | 0 | 1 | 0 | 0 |
| 637 | MUKHTAR A | 2020 | 10.1016/j.accpm.2020.07.012 | 0 | 1 | 0 | 0 |
| 638 | SAYNHALATH R | 2021 | 10.1213/ANE.0000000000005606 | 0 | 1 | 0 | 0 |
| 640 | HERRAIZ I | 2020 | 10.1515/jpm-2020-0236 | 0 | 1 | 0 | 0 |
| 641 | CORSO MCM | 2021 | 10.1002/pbc.29223 | 0 | 1 | 0 | 0 |
| 643 | FERREIRA JC | 2020 | 10.6061/clinics/2020/e2294 | 0 | 1 | 0 | 0 |
| 645 | NOGUEIRA RG | 2021 | 10.1177/1747493021991652 | 0 | 1 | 0 | 0 |
| 646 | BRUNETTI V | 2021 | 10.1007/s10072-021-05045-0 | 0 | 1 | 0 | 0 |
| 651 | MERINO-MATEO L | 2020 | 10.1016/j.acuro.2020.09.003 | 0 | 1 | 0 | 0 |
| 652 | BRENES SANCHEZ JM | 2021 | 10.1016/j.clbc.2020.10.006 | 0 | 1 | 0 | 0 |
| 653 | MILLEN GC | 2021 | 10.1038/s41416-020-01181-0 | 0 | 1 | 0 | 0 |
| 655 | WANG X | 2021 | 10.1007/s00467-020-04715-z | 0 | 1 | 0 | 0 |
| 656 | KANG S | 2021 | 10.1097/MD.0000000000026847 | 0 | 1 | 0 | 0 |
| 658 | BRUYNEEL A | 2021 | 10.1016/j.iccn.2020.102967 | 0 | 1 | 0 | 0 |
| 659 | RZEPINSKI L | 2022 | 10.5603/PJNNS.a2021.0054 | 0 | 1 | 0 | 0 |
| 660 | GARCIA-PACHON E | 2020 | 10.1016/j.rmed.2020.106084 | 0 | 1 | 0 | 0 |
| 663 | AMADOUCCI AM | 2021 | 10.1016/j.ajem.2021.07.058 | 0 | 1 | 0 | 0 |
| 664 | CEYLAN S | 2022 | 10.1177/08258597211036579 | 0 | 1 | 0 | 0 |
| 665 | COSTA VDO | 2021 | 10.1371/journal.pone.0256331 | 0 | 1 | 0 | 0 |
| 667 | COROMINAS H | 2021 | 10.1097/MD.0000000000025923 | 0 | 1 | 0 | 0 |
| 670 | SIEGLER JE | 2020 | 10.1016/j.jstrokecerebrovasdis.2020.104953 | 0 | 1 | 0 | 0 |
| 675 | VELASCO F | 2021 | 10.12788/jhm.3717 | 0 | 1 | 0 | 0 |
| 676 | SIBBEL S | 2022 | 10.1681/ASN.2021060778 | 0 | 1 | 0 | 0 |
| 677 | TREVISAN C | 2021 | 10.1016/j.ejim.2021.01.017 | 0 | 1 | 0 | 0 |
| 679 | MACIAS J | 2021 | 10.1371/journal.pone.0249036 | 0 | 1 | 0 | 0 |
| 680 | SABANOVIC ADILOVIC A | 2021 | 10.17392/1394-21 | 0 | 1 | 0 | 0 |
| 681 | DERESPINA KR | 2020 | 10.1016/j.jpeds.2020.07.039 | 0 | 1 | 0 | 0 |
| 682 | BHASKARA RAJASEKARAN R | 2021 | 10.1097/CORR.0000000000001568 | 0 | 1 | 0 | 0 |
| 683 | DURSUN P | 2020 | 10.1002/ijgo.13296 | 0 | 1 | 0 | 0 |
| 685 | BAYRAK V | 2021 | 10.3906/sag-2101-89 | 0 | 1 | 0 | 0 |
| 687 | TOY S | 2021 | 10.1097/MD.0000000000027166 | 0 | 1 | 0 | 0 |
| 688 | NOURAZARI S | 2021 | 10.1016/j.ajem.2020.11.029 | 0 | 1 | 0 | 0 |
| 690 | TAJADA MEZA H | 2020 | 10.1111/ene.14467 | 0 | 1 | 0 | 0 |
| 691 | LOPEZ A | 2020 | 10.1016/j.ijantimicag.2020.106136 | 0 | 1 | 0 | 0 |
| 693 | PIERI M | 2022 | 10.1053/j.jvca.2021.04.037 | 0 | 1 | 0 | 0 |
| 694 | NASREEN S | 2021 | 10.1136/bmjopen-2021-052019 | 0 | 1 | 0 | 0 |
| 696 | WATANABE Y | 2021 | 10.2169/internalmedicine.8220-21 | 0 | 1 | 0 | 0 |
| 698 | DONG CT | 2021 | 10.1097/TA.0000000000003202 | 0 | 1 | 0 | 0 |
| 699 | STORCH-DE-GRACIA P | 2020 | 10.1016/j.anpedi.2020.07.025 | 0 | 1 | 0 | 0 |
| 705 | MURETT J | 2021 | 10.5811/westjem.2021.5.49968 | 0 | 1 | 0 | 0 |
| 706 | GOLINELLI D | 2021 | 10.1016/j.ajem.2021.07.010 | 0 | 1 | 0 | 0 |
| 707 | SECCO GG | 2020 | 10.1016/j.cjca.2020.05.023 | 0 | 1 | 0 | 0 |
| 709 | OIKONOMOU E | 2020 | 10.1016/j.puhe.2020.08.007 | 0 | 1 | 0 | 0 |
| 710 | AVATEF FAZELI M | 2021 | 10.1111/myc.13351 | 0 | 1 | 0 | 0 |
| 711 | MA HH | 2021 | 10.1097/JCMA.0000000000000503 | 0 | 1 | 0 | 0 |
| 715 | IYENGAR KP | 2021 | 10.1177/17504589211026067 | 0 | 1 | 0 | 0 |
| 717 | HESSHEIMER AJ | 2021 | 10.1371/journal.pone.0252919 | 0 | 1 | 0 | 0 |
| 720 | SIVERRI GA | 2021 | 10.1016/j.numecd.2020.09.028 | 0 | 1 | 0 | 0 |
| 722 | BHATTARAM S | 2022 | 10.1016/j.ajem.2021.11.008 | 0 | 1 | 0 | 0 |
| 723 | PROSSO I | 2021 | *34392620 | 0 | 1 | 0 | 0 |
| 725 | RUBIO-RIVAS M | 2021 | 10.1002/phar.2627 | 0 | 1 | 0 | 0 |
| 726 | RUSSO V | 2021 | 10.2459/JCM.0000000000001156 | 0 | 1 | 0 | 0 |
| 727 | VELAYOS M | 2020 | 10.1016/j.anpedi.2020.04.022 | 0 | 1 | 0 | 0 |
| 729 | RIEG S | 2020 | 10.1371/journal.pone.0242127 | 0 | 1 | 0 | 0 |
| 731 | NUGROHO CW | 2021 | 10.12688/f1000research.45046.1 | 0 | 1 | 0 | 0 |
| 733 | KATAYAMA Y | 2021 | 10.3390/ijerph18115911 | 0 | 1 | 0 | 0 |
| 734 | GARRIDO P | 2021 | 10.1016/j.ajem.2020.11.011 | 0 | 1 | 0 | 0 |
| 735 | ZHANG S | 2020 | 10.1016/j.sleep.2020.08.010 | 0 | 1 | 0 | 0 |
| 736 | PAN C | 2020 | 10.1164/rccm.202003-0527LE | 0 | 1 | 0 | 0 |
| 737 | MARTINEZ-PIAS E | 2021 | 10.33588/rn.7306.2021028 | 0 | 1 | 0 | 0 |
| 738 | DEZMAN ZDW | 2021 | 10.1016/j.amjmed.2021.06.008 | 0 | 1 | 0 | 0 |
| 739 | GARCIA-MARTINEZ A | 2021 | *34251140 | 0 | 1 | 0 | 0 |
| 740 | SANCHEZ JB | 2021 | 10.1016/j.avsg.2020.12.005 | 0 | 1 | 0 | 0 |
| 744 | QIAN H | 2021 | 10.1053/j.jvca.2020.10.019 | 0 | 1 | 0 | 0 |
| 749 | TRUNFIO R | 2021 | 10.1016/j.avsg.2021.07.006 | 0 | 1 | 0 | 0 |
| 750 | KIT DELGADO M | 2022 | 10.7326/M21-2019 | 0 | 1 | 0 | 0 |
| 751 | AZUL FREITAS A | 2021 | 10.1016/j.repce.2021.07.014 | 0 | 1 | 0 | 0 |
| 752 | DI LIBERTO IA | 2020 | 10.2459/JCM.0000000000001053 | 0 | 1 | 0 | 0 |
| 753 | SOBOLEWSKI P | 2022 | 10.1111/ane.13520 | 0 | 1 | 0 | 0 |
| 754 | METTIAS B | 2021 | 10.1002/lary.29667 | 0 | 1 | 0 | 0 |
| 755 | HOOGENBOOM WS | 2022 | 10.1371/journal.pone.0262811 | 0 | 1 | 0 | 0 |
| 757 | MURPHY P | 2021 | 10.1016/j.jss.2020.10.013 | 0 | 1 | 0 | 0 |
| 758 | VENTER A | 2020 | 10.7196/SAMJ.2021.v111i1.15289 | 0 | 1 | 0 | 0 |
| 759 | MCLEAN RC | 2020 | 10.1016/j.ijsu.2020.09.011 | 0 | 1 | 0 | 0 |
| 760 | KANG EK | 2021 | 10.3346/jkms.2021.36.e33 | 0 | 1 | 0 | 0 |
| 762 | GARCIA-GRIMSHAW M | 2021 | 10.1016/j.clim.2021.108818 | 0 | 1 | 0 | 0 |
| 764 | BEZERRA R | 2021 | 10.1080/0886022X.2021.1933530 | 0 | 1 | 0 | 0 |
| 767 | SALMARON JIMENEZ M | 2020 | 10.1093/ejcts/ezaa287 | 0 | 1 | 0 | 0 |
| 770 | PICETTI E | 2020 | 10.1371/journal.pone.0240014 | 0 | 1 | 0 | 0 |
| 771 | SANGALLI D | 2021 | 10.1016/j.jns.2021.117479 | 0 | 1 | 0 | 0 |
| 773 | WILDE H | 2021 | 10.1186/s12916-021-02096-0 | 0 | 1 | 0 | 0 |
| 774 | BROWN J | 2022 | 10.1016/j.apmr.2021.10.018 | 0 | 1 | 0 | 0 |
| 777 | VILA-CORCOLES A | 2020 | 10.1111/jch.13948 | 0 | 1 | 0 | 0 |
| 778 | CHIRIAC U | 2021 | 10.1097/MD.0000000000026253 | 0 | 1 | 0 | 0 |
| 779 | OSAIKHUWUOMWAN J | 2021 | 10.11604/pamj.2021.39.134.27627 | 0 | 1 | 0 | 0 |
| 781 | RAJ KUMAR B | 2020 | 10.1002/jso.26193 | 0 | 1 | 0 | 0 |
| 782 | BASU S | 2022 | 10.1097/PCC.0000000000002850 | 0 | 1 | 0 | 0 |
| 783 | LAU LHS | 2020 | 10.1053/j.gastro.2020.07.042 | 0 | 1 | 0 | 0 |
| 784 | ELKHALIFA AME | 2022 | 10.1155/2022/4620037 | 0 | 1 | 0 | 0 |
| 786 | NSEIR S | 2021 | 10.1186/s13054-021-03588-4 | 0 | 1 | 0 | 0 |
| 787 | CHEONG RCT | 2021 | 10.1001/jamaoto.2020.5698 | 0 | 1 | 0 | 0 |
| 788 | BANSAL R | 2021 | 10.4103/ijo.IJO_2243_21 | 0 | 1 | 0 | 0 |
| 789 | RENAUD-PICARD B | 2020 | 10.1111/ctr.14119 | 0 | 1 | 0 | 0 |
| 790 | SATO Y | 2021 | 10.1016/j.ccell.2021.01.002 | 0 | 1 | 0 | 0 |
| 792 | NAKAI T | 2021 | 10.1016/j.jos.2020.10.009 | 0 | 1 | 0 | 0 |
| 793 | CHEN W | 2021 | 10.1016/j.kint.2021.04.017 | 0 | 1 | 0 | 0 |
| 795 | NAVARATNAM AV | 2021 | 10.1016/S2213-2600(20)30579-8 | 0 | 1 | 0 | 0 |
| 798 | NORDERFELDT J | 2021 | 10.1111/aas.13819 | 0 | 1 | 0 | 0 |
| 799 | SINDAL MD | 2021 | 10.4103/ijo.IJO_2807_20 | 0 | 1 | 0 | 0 |
| 802 | THOMAS B | 2021 | 10.1136/emermed-2020-210783 | 0 | 1 | 0 | 0 |
| 807 | RESENBAUM J | 2021 | 10.1016/j.ajem.2020.12.047 | 0 | 1 | 0 | 0 |
| 808 | ORTIZ PEREZ S | 2021 | *34803159 | 0 | 1 | 0 | 0 |
| 811 | FERRARESE C | 2020 | 10.1007/s10072-020-04450-1 | 0 | 1 | 0 | 0 |
| 813 | ACEHAN S | 2021 | 10.1016/j.clnesp.2021.08.030 | 0 | 1 | 0 | 0 |
| 814 | DU PLESSIS EM | 2021 | *34382564 | 0 | 1 | 0 | 0 |
| 815 | HERTH FJF | 2020 | 10.1159/000511376 | 0 | 1 | 0 | 0 |
| 816 | NANAVATI R | 2021 | 10.1007/s00431-021-03967-7 | 0 | 1 | 0 | 0 |
| 817 | GISONDI P | 2020 | 10.1111/bjd.19158 | 0 | 1 | 0 | 0 |
| 819 | GUALDI G | 2021 | 10.1016/j.clindermatol.2021.05.015 | 0 | 1 | 0 | 0 |
| 820 | GHERMANDI R | 2020 | 10.26355/eurrev_202007_21926 | 0 | 1 | 0 | 0 |
| 822 | MADRAZO Z | 2021 | 10.1097/MD.0000000000024409 | 0 | 1 | 0 | 0 |
| 823 | POLLY M | 2022 | 10.1016/j.ajic.2021.09.018 | 0 | 1 | 0 | 0 |
| 824 | STAUB MB | 2021 | 10.1017/ice.2020.1291 | 0 | 1 | 0 | 0 |
| 825 | SATO K | 2021 | 10.5582/bst.2021.01194 | 0 | 1 | 0 | 0 |
| 827 | LUGON JR | 2022 | 10.1007/s11255-021-02920-9 | 0 | 1 | 0 | 0 |
| 828 | HUYUT MT | 2022 | 10.4103/2045-9912.326002 | 0 | 1 | 0 | 0 |
| 831 | LIU Y | 2021 | 10.1016/S2589-7500(21)00059-5 | 0 | 1 | 0 | 0 |
| 832 | PAOLUCCI M | 2021 | 10.1007/s10072-020-04914-4 | 0 | 1 | 0 | 0 |
| 834 | KUMARA FONSECA M | 2020 | 10.1177/0003134820972098 | 0 | 1 | 0 | 0 |
| 835 | MATSUO K | 2020 | 10.1016/j.ygyno.2020.05.019 | 0 | 1 | 0 | 0 |
| 837 | BROAD J | 2021 | 10.1136/archdischild-2020-320388 | 0 | 1 | 0 | 0 |
| 838 | QUIROS-ROLDAN E | 2020 | 10.1186/s12981-020-00314-y | 0 | 1 | 0 | 0 |
| 844 | THAU L | 2021 | 10.1016/j.jstrokecerebrovasdis.2021.105857 | 0 | 1 | 0 | 0 |
| 845 | SMITH ME | 2022 | 10.1111/coa.13869 | 0 | 1 | 0 | 0 |
| 846 | DICKER B | 2020 | 10.1136/bmjopen-2020-044726 | 0 | 1 | 0 | 0 |
| 847 | BACHILLER-CORRAL J | 2021 | 10.3899/jrheum.200755 | 0 | 1 | 0 | 0 |
| 849 | FLAMENT J | 2021 | 10.1016/j.ajem.2021.05.014 | 0 | 1 | 0 | 0 |
| 850 | SHAH A | 2020 | 10.1186/s13054-020-03260-3 | 0 | 1 | 0 | 0 |
| 852 | SO M | 2021 | 10.1007/s11239-021-02477-5 | 0 | 1 | 0 | 0 |
| 853 | CHOI A | 2021 | 10.1371/journal.pone.0256116 | 0 | 1 | 0 | 0 |
| 857 | DARVALL JN | 2020 | 10.1016/j.bja.2020.07.049 | 0 | 1 | 0 | 0 |
| 858 | PRABHU NAVIS J | 2021 | 10.1007/s00592-020-01614-5 | 0 | 1 | 0 | 0 |
| 859 | AUSTRIA B | 2021 | 10.1371/journal.pone.0258916 | 0 | 1 | 0 | 0 |
| 861 | GRASSELI G | 2020 | 10.1001/jamainternmed.2020.3539 | 0 | 1 | 0 | 0 |
| 862 | BERGUIGUA H | 2021 | 10.1097/MD.0000000000027881 | 0 | 1 | 0 | 0 |
| 865 | BEGHI M | 2020 | 10.1080/13651501.2020.1859120 | 0 | 1 | 0 | 0 |
| 866 | JESENSEK PAPEZ B | 2021 | 10.23736/S1973-9087.21.06678-8 | 0 | 1 | 0 | 0 |
| 868 | ZHANG L | 2020 | 10.1007/s00540-020-02778-8 | 0 | 1 | 0 | 0 |
| 877 | CAREY SA | 2020 | 10.1111/ctr.14105 | 0 | 1 | 0 | 0 |
| 878 | GARDINER FW | 2020 | 10.1111/imj.15091 | 0 | 1 | 0 | 0 |
| 881 | JIVELLA DV | 2022 | 10.1007/s00415-021-10734-z | 0 | 1 | 0 | 0 |
| 883 | SPAGNOLELLO O | 2021 | 10.1002/jmv.26980 | 0 | 1 | 0 | 0 |
| 884 | LI S | 2020 | 10.1111/echo.14849 | 0 | 1 | 0 | 0 |
| 885 | BONAZZETTI C | 2021 | 10.1097/CCM.0000000000004748 | 0 | 1 | 0 | 0 |
| 889 | MAILLARD A | 2022 | 10.1182/blood.2021014232 | 0 | 1 | 0 | 0 |
| 890 | PIZZI R | 2020 | 10.1016/j.thromres.2020.09.004 | 0 | 1 | 0 | 0 |
| 892 | GUEMES M | 2020 | 10.1515/jpem-2020-0481 | 0 | 1 | 0 | 0 |
| 893 | GUO Z | 2021 | 10.1053/j.jvca.2020.08.067 | 0 | 1 | 0 | 0 |
| 894 | SCHMITT J | 2021 | 10.1016/j.idnow.2021.06.303 | 0 | 1 | 0 | 0 |
| 898 | WALKER MJ | 2021 | 10.1016/j.ypmed.2021.106586 | 0 | 1 | 0 | 0 |
| 900 | HERREROS B | 2022 | 10.1186/s13010-022-00117-1 | 0 | 1 | 0 | 0 |
| 904 | CHEN S | 2021 | 10.1007/s11239-020-02181-w | 0 | 1 | 0 | 0 |
| 906 | WEISS TT | 2021 | 10.1016/j.bja.2020.09.042 | 0 | 1 | 0 | 0 |
| 907 | ABENZA6ABILDUA MJ | 2020 | 10.1016/j.nrl.2020.07.014 | 0 | 1 | 0 | 0 |
| 909 | LEVI-SETTI PE | 2021 | 10.1038/s41598-021-96134-9 | 0 | 1 | 0 | 0 |
| 910 | MUSAJEE M | 2022 | 10.1016/j.avsg.2021.10.021 | 0 | 1 | 0 | 0 |
| 911 | DOVLE AJ | 2021 | 10.1097/CCM.0000000000004971 | 0 | 1 | 0 | 0 |
| 912 | SHI G | 2021 | 10.1016/j.jtcvs.2020.11.074 | 0 | 1 | 0 | 0 |
| 914 | PENA-GALO E | 2021 | 10.1016/j.semerg.2021.01.004 | 0 | 1 | 0 | 0 |
| 915 | SUNDER A | 2022 | 10.15537/smj.2022.43.1.20210694 | 0 | 1 | 0 | 0 |
| 916 | MEDRANDA GA | 2022 | 10.1016/j.carrev.2021.01.026 | 0 | 1 | 0 | 0 |
| 917 | BANCHINI F | 2021 | 10.1186/s13017-021-00354-3 | 0 | 1 | 0 | 0 |
| 918 | TU Y | 2021 | 10.7150/ijms.50039 | 0 | 1 | 0 | 0 |
| 919 | CHIBA H | 2021 | 10.1097/TA.0000000000003061 | 0 | 1 | 0 | 0 |
| 925 | MAHIDA RY | 2020 | 10.1136/bmjresp-2020-000731 | 0 | 1 | 0 | 0 |
| 926 | GOUEL-CHERON A | 2021 | 10.1016/j.bja.2021.04.001 | 0 | 1 | 0 | 0 |
| 927 | EL MOUSSAOUI M | 2021 | *34308800 | 0 | 1 | 0 | 0 |
| 929 | BECKER A | 2021 | 10.1177/0885066621995386 | 0 | 1 | 0 | 0 |
| 931 | WANG Y | 2020 | 10.1097/MD.0000000000023257 | 0 | 1 | 0 | 0 |
| 932 | SHAH A | 2021 | 10.1177/0885066621989920 | 0 | 1 | 0 | 0 |
| 933 | MAES M | 2021 | 10.1186/s13054-021-03460-5 | 0 | 1 | 0 | 0 |
| 935 | NG BH | 2020 | *32918430 | 0 | 1 | 0 | 0 |
| 937 | JAMOUS F | 2020 | *32805781 | 0 | 1 | 0 | 0 |
| 938 | DI LORENZO R | 2021 | 10.1007/s11126-021-09907-w | 0 | 1 | 0 | 0 |
| 940 | SOLER E | 2021 | 10.1186/s13018-021-02217-8 | 0 | 1 | 0 | 0 |
| 941 | VERDONI L | 2020 | 10.1016/S0140-6736(20)31103-X | 0 | 1 | 0 | 0 |
| 943 | CARUSO I | 2021 | 10.1002/dmrr.3404 | 0 | 1 | 0 | 0 |
| 944 | VAN ACKERBROECK S | 2021 | 10.1186/s13054-021-03728-w | 0 | 1 | 0 | 0 |
| 945 | SETIA R | 2021 | 10.1016/j.transci.2021.103160 | 0 | 1 | 0 | 0 |
| 946 | PARSQUINI Z | 2020 | 10.1093/jac/dkaa321 | 0 | 1 | 0 | 0 |
| 948 | MATSUSHITA K | 2021 | 10.1007/s11239-020-02340-z | 0 | 1 | 0 | 0 |
| 949 | WILK M | 2021 | 10.1155/2021/5515902 | 0 | 1 | 0 | 0 |
| 950 | CAPLAN M | 2021 | 10.1097/CCM.0000000000004711 | 0 | 1 | 0 | 0 |
| 951 | BOTTA M | 2021 | 10.1016/S2213-2600(20)30459-8 | 0 | 1 | 0 | 0 |
| 953 | PAYAN-PERNIA S | 2021 | 10.1093/labmed/lmaa105 | 0 | 1 | 0 | 0 |
| 956 | BOSCH X | 2021 | 10.1016/j.ajem.2021.03.087 | 0 | 1 | 0 | 0 |
| 959 | DE LA MATTA M | 2021 | 10.1016/j.redar.2020.10.006 | 0 | 1 | 0 | 0 |
| 963 | TAN SC | 2022 | 10.1111/1742-6723.13835 | 0 | 1 | 0 | 0 |
| 965 | QIU H | 2020 | 10.1016/S1473-3099(20)30198-5 | 0 | 1 | 0 | 0 |
| 968 | SLESS RT | 2021 | 10.1136/emermed-2020-209992 | 0 | 1 | 0 | 0 |
| 970 | HUESPE IA | 2021 | 10.5935/0103-507X.20210006 | 0 | 1 | 0 | 0 |
| 974 | WANG R | 2021 | 10.1016/j.sapharm.2020.06.005 | 0 | 1 | 0 | 0 |
| 975 | MURGELLA-SANCHO A | 2021 | *34806585 | 0 | 1 | 0 | 0 |
| 984 | GOMEZ S | 2021 | 10.5543/tkda.2021.87750 | 0 | 1 | 0 | 0 |
| 985 | DEMIR OM | 2022 | 10.1002/ccd.30056 | 0 | 1 | 0 | 0 |
| 987 | MI B | 2020 | 10.1080/17453674.2020.1816617 | 0 | 1 | 0 | 0 |
| 992 | COVINO M | 2021 | 10.1111/jgs.16956 | 0 | 1 | 0 | 0 |
| 993 | CLEMENT ND | 2021 | 10.1302/0301-620X.103B.BJJ-2020-1776.R1 | 0 | 1 | 0 | 0 |
| 994 | SOLANO-LOPEZ J | 2020 | 10.1016/j.rec.2020.07.009 | 0 | 1 | 0 | 0 |
| 1000 | CSEREP G | 2022 | 10.1007/s10157-021-02118-4 | 0 | 1 | 0 | 0 |
| 1004 | LAZZERONI P | 2021 | 10.23750/abm.v92i5.11911 | 0 | 1 | 0 | 0 |
| 1009 | RUBBI I | 2020 | 10.23750/abm.v91i12-S.10814 | 0 | 1 | 0 | 0 |
| 1011 | SHAH H | 2021 | 10.1177/11206721211001315 | 0 | 1 | 0 | 0 |
| 1014 | MESA A | 2020 | 10.1016/j.diabres.2020.108354 | 0 | 1 | 0 | 0 |
| 1015 | YANG K | 2021 | 10.1007/s11596-021-2315-4 | 0 | 1 | 0 | 0 |
| 1018 | NAB M | 2021 | 10.1186/s12873-021-00449-9 | 0 | 1 | 0 | 0 |
| 1020 | DUAN J | 2020 | 10.1177/1753466620963019 | 0 | 1 | 0 | 0 |
| 1022 | KITAHARA S | 2021 | 10.1136/openhrt-2020-001497 | 0 | 1 | 0 | 0 |
| 1024 | KANE AD | 2020 | 10.1111/anae.15281 | 0 | 1 | 0 | 0 |
| 1025 | ZHAO N | 2020 | 10.18632/aging.103839 | 0 | 1 | 0 | 0 |
| 1026 | PERO G | 2021 | 10.1016/j.jstrokecerebrovasdis.2021.106028 | 0 | 1 | 0 | 0 |
| 1038 | FAZIO N | 2021 | 10.1016/j.ejca.2021.06.029 | 0 | 1 | 0 | 0 |
| 1039 | XIANG NG O | 2021 | 10.1111/1742-6723.13668 | 0 | 1 | 0 | 0 |
| 1042 | MAYER MA | 2021 | 10.2196/25452 | 0 | 1 | 0 | 0 |
| 1046 | ORTEGA-GUTIERREZ S | 2021 | 10.1016/j.clineuro.2020.106436 | 0 | 1 | 0 | 0 |
| 1047 | HERRANZ-LARRANETA J | 2021 | 10.1016/j.amjoto.2020.102865 | 0 | 1 | 0 | 0 |
| 1058 | RODRIGUEZ-LEOR O | 2020 | 10.1016/j.rec.2020.08.002 | 0 | 1 | 0 | 0 |
| 1064 | EL HAJRA I | 2021 | 10.17235/reed.2020.7543/2020 | 0 | 1 | 0 | 0 |
| 1065 | DINUZZI VP | 2021 | *33448938 | 0 | 1 | 0 | 0 |
| 1067 | TUECH JJ | 2021 | 10.1007/s00384-021-03847-4 | 0 | 1 | 0 | 0 |
| 1068 | COMPAGNUCCI P | 2020 | 10.1161/CIRCEP.120.008774 | 0 | 1 | 0 | 0 |
| 1074 | NISHIZAWA H | 2021 | 10.1002/mus.27348 | 0 | 1 | 0 | 0 |
| 1078 | CASTILLO C | 2021 | 10.4103/jcrt.JCRT_1689_20 | 0 | 1 | 0 | 0 |
| 1082 | HAJDU SD | 2020 | 10.1161/STROKEAHA.120.030794 | 0 | 1 | 0 | 0 |
| 1083 | VAN-DE-L'ISLE Y | 2021 | 10.1111/1471-0528.16482 | 0 | 1 | 0 | 0 |
| 1084 | ZHANG YF | 2021 | 10.1111/jgh.15205 | 0 | 1 | 0 | 0 |
| 1087 | NGUYEN TN | 2021 | 10.1136/svn-2020-000695 | 0 | 1 | 0 | 0 |
| 1090 | KANG JM | 2021 | 10.1161/CIRCULATIONAHA.121.054785 | 0 | 1 | 0 | 0 |
| 1098 | BONILLA L | 2021 | 10.1016/j.anpedi.2020.12.003 | 0 | 1 | 0 | 0 |
| 1101 | LOSURDO P | 2020 | 10.1007/s13304-020-00884-6 | 0 | 1 | 0 | 0 |
| 1103 | BALLESTA-MARTINEZ S | 2020 | *33275371 | 0 | 1 | 0 | 0 |
| 1106 | AGARWAL MA | 2020 | 10.1016/j.mayocp.2020.09.022 | 0 | 1 | 0 | 0 |
| 1110 | GARNER O | 2021 | 10.1016/j.rmed.2021.106667 | 0 | 1 | 0 | 0 |
| 1111 | LUBANSU A | 2020 | 10.1016/j.wneu.2020.08.168 | 0 | 1 | 0 | 0 |
| 1114 | MORALES X | 2020 | *33275368 | 0 | 1 | 0 | 0 |
| 1115 | MAJIDI S | 2020 | 10.1161/STROKEAHA.120.030397 | 0 | 1 | 0 | 0 |
| 1116 | TOALE C | 2020 | 10.1002/bjs.12058 | 0 | 1 | 0 | 0 |
| 1119 | LI Y | 2020 | 10.1136/svn-2020-000431 | 0 | 1 | 0 | 0 |
| 1125 | BOSCHETTI GA | 2021 | 10.1016/j.avsg.2021.01.072 | 0 | 1 | 0 | 0 |
| 1127 | BONATO F | 2021 | 10.1016/j.clindermatol.2020.11.006 | 0 | 1 | 0 | 0 |
| 1128 | TEJERA-VAQUERIZO A | 2020 | 10.1016/j.ad.2020.05.001 | 0 | 1 | 0 | 0 |
| 1132 | MCMAHON SR | 2020 | 10.1016/j.echo.2020.05.005 | 0 | 1 | 0 | 0 |
| 1133 | BARRIO P | 2021 | 10.1111/acer.14555 | 0 | 1 | 0 | 0 |
| 1135 | VAN ZELST CM | 2021 | 10.1186/s12931-021-01685-0 | 0 | 1 | 0 | 0 |
| 1138 | SCHIANCHI A | 2021 | 10.26355/eurrev_202112_27414 | 0 | 1 | 0 | 0 |
| 1141 | KAVANAGH FG | 2021 | 10.1017/S002221512100061X | 0 | 1 | 0 | 0 |
| 1143 | JOHNSON SA | 2020 | 10.12788/jhm.3475 | 0 | 1 | 0 | 0 |
| 1144 | PELLEGRINI M | 2020 | 10.3390/nu12072016 | 0 | 1 | 0 | 0 |
| 1149 | BYRNE L | 2021 | 10.1136/openhrt-2021-001716 | 0 | 1 | 0 | 0 |
| 1150 | KATSUMATA M | 2021 | 10.1016/j.jstrokecerebrovasdis.2021.106051 | 0 | 1 | 0 | 0 |
| 1151 | KRUGER EC | 2020 | 10.7196/SAMJ.2020.v110i12.15294 | 0 | 1 | 0 | 0 |
| 1154 | MEEK CL | 2021 | 10.1111/dme.14380 | 0 | 1 | 0 | 0 |
| 1155 | HUANG B | 2020 | 10.1097/FJC.0000000000000909 | 0 | 1 | 0 | 0 |
| 1156 | CAI M | 2020 | 10.1002/bjs.11643 | 0 | 1 | 0 | 0 |
| 1158 | DUZENLI T | 2021 | 10.1007/s10620-021-06940-4 | 0 | 1 | 0 | 0 |
| 1159 | NAVARRRO-CORREAL E | 2021 | 10.1016/j.gastrohep.2020.11.018 | 0 | 1 | 0 | 0 |
| 1160 | RUPARELIA N | 2020 | 10.1177/1753944720977732 | 0 | 1 | 0 | 0 |
| 1163 | BALLOSTA R | 2021 | 10.1016/j.ejvs.2021.01.037 | 0 | 1 | 0 | 0 |
| 1165 | LIM A | 2020 | 10.1071/AH20180 | 0 | 1 | 0 | 0 |
| 1168 | PARK MJ | 2021 | 10.3346/jkms.2021.36.e196 | 0 | 1 | 0 | 0 |
| 1169 | LAVINIO A | 2021 | 10.1186/s13054-021-03543-3 | 0 | 1 | 0 | 0 |
| 1170 | THAKRAR A | 2020 | 10.1097/BOT.0000000000001889 | 0 | 1 | 0 | 0 |
| 1171 | LI D | 2021 | 10.1093/infdis/jiaa626 | 0 | 1 | 0 | 0 |
| 1176 | RIDOUT KK | 2021 | 10.4088/JCP.20m13685 | 0 | 1 | 0 | 0 |
| 1178 | SEN M | 2021 | 10.4103/ijo.IJO_1565_21 | 0 | 1 | 0 | 0 |
| 1179 | VINALS C | 2021 | 10.1007/s00592-020-01625-2 | 0 | 1 | 0 | 0 |
| 1180 | PARZY G | 2020 | 10.1097/CCM.0000000000004504 | 0 | 1 | 0 | 0 |
| 1185 | JANKE AT | 2021 | 10.1111/jgs.17227 | 0 | 1 | 0 | 0 |
| 1186 | LITTLE CD | 2020 | 10.1136/openhrt-2020-001432 | 0 | 1 | 0 | 0 |
| 1193 | MCGINLAY M | 2021 | 10.31083/j.rcm2202034 | 0 | 1 | 0 | 0 |
| 1197 | CLEMENT ND | 2021 | 10.1302/0301-620X.103B.BJJ-2021-0104.R1 | 0 | 1 | 0 | 0 |
| 1198 | ZEPPIERI M | 2021 | 10.52586/E883 | 0 | 1 | 0 | 0 |
| 1200 | MITCHELL RD | 2020 | 10.1111/1742-6723.13606 | 0 | 1 | 0 | 0 |
| 1204 | BOURDON H | 2021 | 10.1016/j.jfo.2020.12.002 | 0 | 1 | 0 | 0 |
| 1206 | ROMERO J | 2020 | 10.1016/j.jacr.2020.06.002 | 0 | 1 | 0 | 0 |
| 1209 | AREVALOS V | 2021 | 10.1371/journal.pone.0255263 | 0 | 1 | 0 | 0 |
| 1215 | ROLFES L | 2021 | 10.1212/NXI.0000000000001035 | 0 | 1 | 0 | 0 |
| 1221 | NADIMI AE | 2022 | 10.1097/DSS.0000000000003291 | 0 | 1 | 0 | 0 |
| 1222 | PUGLIESE L | 2020 | 10.21873/invivo.12223 | 0 | 1 | 0 | 0 |
| 1223 | LU X | 2020 | 10.1371/journal.pone.0238679 | 0 | 1 | 0 | 0 |
| 1224 | GARSTANG J | 2020 | 10.1136/bmjopen-2020-042867 | 0 | 1 | 0 | 0 |
| 1226 | O'BRIEN CM | 2020 | 10.1016/j.jacr.2020.08.010 | 0 | 1 | 0 | 0 |
| 1227 | ISLAM M | 2021 | 10.1136/bmjhci-2020-100310 | 0 | 1 | 0 | 0 |
| 1230 | MONDONI M | 2020 | 10.1183/13993003.02767-2020 | 0 | 1 | 0 | 0 |
| 1231 | SUTER F | 2021 | 10.1016/j.eclinm.2021.100941 | 0 | 1 | 0 | 0 |
| 1234 | BERLANDA G | 2021 | 10.20945/2359-3997000000332 | 0 | 1 | 0 | 0 |
| 1235 | NILI A | 2020 | 10.1016/j.intimp.2020.107093 | 0 | 1 | 0 | 0 |
| 1237 | CHEN L | 2021 | 10.1038/s41598-021-97595-8 | 0 | 1 | 0 | 0 |
| 1245 | CADEGIANI FA | 2021 | 10.1016/j.nmni.2021.100915 | 0 | 1 | 0 | 0 |
| 1248 | VOHRA LM | 2021 | 10.1016/j.amsu.2021.01.099 | 0 | 1 | 0 | 0 |
| 1249 | FERNANDEZ-DIAZ E | 2021 | 10.1002/acn3.51282 | 0 | 1 | 0 | 0 |
| 1255 | GILISSEN LPL | 2021 | 10.1007/s10238-021-00755-3 | 0 | 1 | 0 | 0 |
| 1256 | VELAYOS M | 2020 | 10.1016/j.anpede.2020.04.010 | 0 | 1 | 0 | 0 |
| 1263 | THOMPSON LA | 2021 | 10.1016/j.eclinm.2021.100936 | 0 | 1 | 0 | 0 |
| 1281 | TAN Q | 2021 | 10.3389/fneur.2021.673703 | 0 | 1 | 0 | 0 |
| 1283 | LEE H | 2021 | 10.1016/j.lanwpc.2020.100088 | 0 | 1 | 0 | 0 |
| 1284 | VAN CHI MAI D | 2021 | 10.1016/j.amsu.2021.02.006 | 0 | 1 | 0 | 0 |
| 1292 | ACHARYA R | 2021 | 10.14740/jocmr4507 | 0 | 1 | 0 | 0 |
| 1293 | XU G | 2020 | 10.3389/fmed.2020.593133 | 0 | 1 | 0 | 0 |
| 1295 | CHEN N | 2022 | 10.3389/fneur.2021.822342 | 0 | 1 | 0 | 0 |
| 1297 | BITKER L | 2020 | 10.1186/s13613-020-00783-4 | 0 | 1 | 0 | 0 |
| 1299 | MUMTAZ S | 2022 | 10.1177/19433875211007008 | 0 | 1 | 0 | 0 |
| 1300 | PETROVIC M | 2021 | 10.1007/s12471-021-01554-x | 0 | 1 | 0 | 0 |
| 1301 | PADHAN S | 2021 | 10.7759/cureus.19723 | 0 | 1 | 0 | 0 |
| 1308 | LAM C | 2021 | 10.3390/jcm10225314 | 0 | 1 | 0 | 0 |
| 1314 | FITZGERALD MJ | 2021 | 10.1302/2633-1462.24.BJO-2021-0005.R1 | 0 | 1 | 0 | 0 |
| 1315 | SINGH B | 2021 | 10.7759/cureus.20571 | 0 | 1 | 0 | 0 |
| 1317 | BRAQUEHAIS MD | 2022 | 10.3390/ijerph19063665 | 0 | 1 | 0 | 0 |
| 1320 | ARRUABARRENA C | 2021 | 10.1016/j.oftal.2021.02.012 | 0 | 1 | 0 | 0 |
| 1329 | BALGHITH MA | 2020 | 10.4103/HEARTVIEWS.HEARTVIEWS_128_20 | 0 | 1 | 0 | 0 |
| 1333 | PARK C | 2021 | 10.1007/s11845-021-02687-z | 0 | 1 | 0 | 0 |
| 1334 | YAPP LZ | 2021 | 10.1302/2633-1462.23.BJO-2020-0193.R1 | 0 | 1 | 0 | 0 |
| 1336 | TERAKAWA A | 2022 | 10.1111/jdi.13758 | 0 | 1 | 0 | 0 |
| 1338 | HUEMER J | 2020 | 10.1136/bmjophth-2020-000560 | 0 | 1 | 0 | 0 |
| 1342 | PARK HA | 2022 | 10.3390/jcm11040906 | 0 | 1 | 0 | 0 |
| 1344 | MOHAN K | 2021 | 10.7759/cureus.15833 | 0 | 1 | 0 | 0 |
| 1345 | YING X | 2021 | 10.3389/fonc.2021.754838 | 0 | 1 | 0 | 0 |
| 1349 | IBRAHIM Y | 2021 | 10.1302/2633-1462.22.BJO-2020-0152.R1 | 0 | 1 | 0 | 0 |
| 1353 | SALEH OA | 2020 | 10.2147/OPTH.S269179 | 0 | 1 | 0 | 0 |
| 1355 | RAFFALDI I | 2021 | 10.1016/j.lanepe.2021.100081 | 0 | 1 | 0 | 0 |
| 1364 | WORRALL AP | 2021 | 10.2196/21817 | 0 | 1 | 0 | 0 |
| 1365 | TROMANS S | 2020 | 10.1192/bjo.2020.104 | 0 | 1 | 0 | 0 |
| 1371 | HOFFMEISTER B | 2021 | 10.3390/microorganisms9091941 | 0 | 1 | 0 | 0 |
| 1372 | RAMOS OLIVEIRA SILVA A | 2021 | 10.3389/fphar.2021.778386 | 0 | 1 | 0 | 0 |
| 1373 | MEHTA Y | 2022 | 10.4103/ijabmr.ijabmr_602_21 | 0 | 1 | 0 | 0 |
| 1375 | KLOSIEWICZ T | 2021 | 10.3390/healthcare10010018 | 0 | 1 | 0 | 0 |
| 1376 | RYDWIK E | 2021 | 10.1186/s13102-021-00299-9 | 0 | 1 | 0 | 0 |
| 1377 | SCORCIA V | 2021 | 10.1007/s40123-021-00395-6 | 0 | 1 | 0 | 0 |
| 1378 | AINSLIE M | 2022 | 10.2196/33092 | 0 | 1 | 0 | 0 |
| 1379 | MIKACENIC C | 2022 | 10.1097/CCM.0000000000005333 | 0 | 1 | 0 | 0 |
| 1380 | ELFALAH M | 2021 | 10.2147/OPTH.S289068 | 0 | 1 | 0 | 0 |
| 1381 | COSTA VO | 2021 | 10.1155/2021/6689669 | 0 | 1 | 0 | 0 |
| 1382 | ZHANG J | 2020 | 10.1007/s10389-020-01291-2 | 0 | 1 | 0 | 0 |
| 1383 | KATLAN B | 2022 | 10.1002/jca.21971 | 0 | 1 | 0 | 0 |
| 1385 | ZIGARELLI A | 2022 | 10.2196/29967 | 0 | 1 | 0 | 0 |
| 1386 | BRAQUEHAIS MD | 2022 | 10.1111/jan.15189 | 0 | 1 | 0 | 0 |
| 2 | AYOUBKHANI D | 2021 | 10.1136/bmj.n693 | 0 | 0 | 1 | 0 |
| 3 | SOLERTE SB | 2020 | 10.2337/dc20-1521 | 0 | 0 | 1 | 0 |
| 4 | MORAN BLANCO JI | 2021 | 10.1016/j.pupt.2021.101989 | 0 | 0 | 1 | 0 |
| 5 | HUNDT MA | 2020 | 10.1002/hep.31487 | 0 | 0 | 1 | 0 |
| 9 | CHANDEL A | 2021 | 10.4187/respcare.08631 | 0 | 0 | 1 | 0 |
| 10 | LI G | 2021 | 10.1016/j.clnu.2020.09.040 | 0 | 0 | 1 | 0 |
| 11 | MAO L | 2021 | 10.1001/jamaneurol.2020.1127 | 0 | 0 | 1 | 0 |
| 12 | WARGNY | 2021 | 10.1007/s00125-020-05351-w | 0 | 0 | 1 | 0 |
| 13 | NG JH | 2021 | 10.1053/j.ajkd.2020.09.002 | 0 | 0 | 1 | 0 |
| 18 | HOERTEL | 2021 | 10.1038/s41380-021-01021-4 | 0 | 0 | 1 | 0 |
| 20 | LIU L | 2021 | 10.1016/S2589-7500(20)30316-2 | 0 | 0 | 1 | 0 |
| 21 | KARAMI | 2021 | 10.1080/23744235.2020.1839672 | 0 | 0 | 1 | 0 |
| 22 | UCAN A | 2021 | 10.1186/s12985-021-01577-1 | 0 | 0 | 1 | 0 |
| 25 | BHARGAVA A | 2021 | 10.1093/cid/ciaa674 | 0 | 0 | 1 | 0 |
| 29 | HOERTEL | 2021 | 10.1007/s40261-021-01001-0 | 0 | 0 | 1 | 0 |
| 30 | HAGHAGHI-MORAD M | 2021 | 10.1186/s12879-020-05617-z | 0 | 0 | 1 | 0 |
| 35 | BERTOLUCCI F | 2021 | 10.23736/S1973-9087.21.06674-0 | 0 | 0 | 1 | 0 |
| 36 | FISHER M | 2021 | 10.1681/ASN.2020040509 | 0 | 0 | 1 | 0 |
| 39 | AL-SALAMEH A | 2021 | 10.1002/dmrr.3388 | 0 | 0 | 1 | 0 |
| 41 | BODE B | 2021 | 10.1177/1932296820924469 | 0 | 0 | 1 | 0 |
| 43 | BOWLES KH | 2021 | 10.7326/M20-5206 | 0 | 0 | 1 | 0 |
| 45 | KHEDR EM | 2021 | 10.1016/j.eplepsyres.2021.106650 | 0 | 0 | 1 | 0 |
| 48 | WANG Y | 2021 | 10.12809/hkmj208725 | 0 | 0 | 1 | 0 |
| 50 | JENSEN HI | 2021 | 10.1186/s13049-021-00984-1 | 0 | 0 | 1 | 0 |
| 51 | CARPAGNANO GE | 2021 | 10.1007/s40618-020-01370-x | 0 | 0 | 1 | 0 |
| 53 | MANENTI L | 2021 | 10.1371/journal.pone.0248276 | 0 | 0 | 1 | 0 |
| 54 | CARLUCCI PM | 2021 | *32930657 | 0 | 0 | 1 | 0 |
| 59 | RAMOS RINCON JM | 2021 | 10.1093/gerona/glaa243 | 0 | 0 | 1 | 0 |
| 60 | ROMERO-DUARTE A | 2021 | 10.1186/s12916-021-02003-7 | 0 | 0 | 1 | 0 |
| 63 | MUELLER T | 2021 | 10.1136/bmjopen-2021-054861 | 0 | 0 | 1 | 0 |
| 64 | SUN M | 2021 | 10.1016/j.ijid.2020.09.1475 | 0 | 0 | 1 | 0 |
| 66 | OWEN RK | 2021 | 10.1093/ageing/afaa167 | 0 | 0 | 1 | 0 |
| 67 | SHIWANI HA | 2021 | 10.1016/j.rmed.2021.106314 | 0 | 0 | 1 | 0 |
| 71 | LU Y | 2021 | 10.1186/s12941-020-00412-9 | 0 | 0 | 1 | 0 |
| 75 | LAN X | 2021 | 10.5414/CP203861 | 0 | 0 | 1 | 0 |
| 78 | GERVASONI C | 2020 | 10.1093/cid/ciaa579 | 0 | 0 | 1 | 0 |
| 88 | FENG J | 2021 | 10.4014/jmb.2009.09029 | 0 | 0 | 1 | 0 |
| 89 | JOHN S | 2020 | 10.1016/j.clineuro.2020.106227 | 0 | 0 | 1 | 0 |
| 94 | FAKIH MG | 2022 | 10.1136/bmjqs-2021-013721 | 0 | 0 | 1 | 0 |
| 100 | BIAGI A | 2020 | 10.1002/jmv.26147 | 0 | 0 | 1 | 0 |
| 102 | SIEBER P | 2021 | 10.1186/s12879-021-05957-4 | 0 | 0 | 1 | 0 |
| 106 | BELTRAME A | 2022 | 10.3389/fimmu.2022.834851 | 0 | 0 | 1 | 0 |
| 109 | GALIERO R | 2020 | 10.1371/journal.pone.0243700 | 0 | 0 | 1 | 0 |
| 110 | CAROSI G | 2021 | 10.1210/clinem/dgaa793 | 0 | 0 | 1 | 0 |
| 112 | MARINESCU AR | 2021 | 10.3390/medicina57101099 | 0 | 0 | 1 | 0 |
| 115 | HOERTEL N | 2021 | 10.1111/bcp.14784 | 0 | 0 | 1 | 0 |
| 117 | SOUSA FM | 2021 | 10.4414/smw.2021.20547 | 0 | 0 | 1 | 0 |
| 119 | FU W | 2021 | 10.1002/jmv.26794 | 0 | 0 | 1 | 0 |
| 122 | RUSSO V | 2021 | 10.1097/FJC.0000000000001041 | 0 | 0 | 1 | 0 |
| 123 | CHEN Y | 2021 | 10.1002/jmv.26617 | 0 | 0 | 1 | 0 |
| 124 | LI BASSI G | 2020 | 10.1136/bmjopen-2020-041417 | 0 | 0 | 1 | 0 |
| 125 | LANDOAS A | 2021 | 10.1186/s13756-021-00984-x | 0 | 0 | 1 | 0 |
| 130 | BOLOURANI S | 2021 | 10.2196/24246 | 0 | 0 | 1 | 0 |
| 131 | GOODACRE S | 2020 | 10.1371/journal.pone.0240206 | 0 | 0 | 1 | 0 |
| 132 | VILLAMANAN F | 2021 | 10.1016/j.pupt.2021.102007 | 0 | 0 | 1 | 0 |
| 133 | BASU A | 2021 | 10.1136/bmjopen-2020-047561 | 0 | 0 | 1 | 0 |
| 134 | HUANG S | 2020 | 10.12659/MSM.928755 | 0 | 0 | 1 | 0 |
| 135 | GOODALL JW | 2020 | 10.1017/S0950268820002472 | 0 | 0 | 1 | 0 |
| 138 | LJUNGQUIST O | 2022 | 10.1080/23744235.2021.2013528 | 0 | 0 | 1 | 0 |
| 142 | ZHONG Y | 2020 | 10.1177/0300060520979151 | 0 | 0 | 1 | 0 |
| 143 | RIVERA-IZQUIERDO M | 2020 | 10.1371/journal.pone.0235107 | 0 | 0 | 1 | 0 |
| 145 | ABDELA SG | 2020 | 10.4269/ajtmh.20-1356 | 0 | 0 | 1 | 0 |
| 146 | OZDEMIR IH | 2021 | 10.1111/ijcp.13896 | 0 | 0 | 1 | 0 |
| 148 | GUPTA N | 2021 | 10.5582/ddt.2020.03068 | 0 | 0 | 1 | 0 |
| 149 | SHASTRI M | 2022 | *35062798 | 0 | 0 | 1 | 0 |
| 151 | SHAO S | 2020 | 10.1097/MD.0000000000022971 | 0 | 0 | 0 | 1 |
| 152 | GOMEZ-BELDA AB | 2021 | 10.1111/ggi.14102 | 0 | 0 | 1 | 0 |
| 156 | WALKER J | 2020 | 10.1371/journal.pone.0244857 | 0 | 0 | 1 | 0 |
| 160 | SCHREVER KE | 2021 | 10.5811/westjem.2020.12.49206 | 0 | 0 | 1 | 0 |
| 165 | DI DOMENICO SL | 2021 | 10.1007/s11739-020-02548-0 | 0 | 0 | 1 | 0 |
| 168 | HAYIROGLU MI | 2021 | 10.1590/1806-9282.20200896 | 0 | 0 | 1 | 0 |
| 169 | FARRUGIA Y | 2021 | 10.1155/2021/5533123 | 0 | 0 | 1 | 0 |
| 170 | COPPOCK D | 2021 | 10.1371/journal.pone.0252591 | 0 | 0 | 1 | 0 |
| 171 | KRISHNAMOORTHY G | 2021 | 10.1016/j.puhe.2020.11.021 | 0 | 0 | 0 | 1 |
| 172 | VU CA |  | 10.1186/s12879-020-05701-4 | 0 | 0 | 1 | 0 |
| 173 | HERMEL DJ | 2022 | 10.2217/fon-2021-1116 | 0 | 0 | 1 | 0 |
| 175 | REES CA | 2021 | 10.1073/pnas.2101708118 | 0 | 0 | 1 | 0 |
| 176 | DEMIR A | 2020 | 10.3906/sag-2009-140 | 0 | 0 | 1 | 0 |
| 177 | RAMOS MARTINEZ A | 2021 | 10.1038/s41598-021-93076-0 | 0 | 0 | 1 | 0 |
| 179 | SAMPEDRO-NUNEZ M | 2021 | 10.1007/s12020-021-02881-0 | 0 | 0 | 1 | 0 |
| 181 | IEZZI R | 2020 | 10.1007/s00330-020-07041-y | 0 | 0 | 1 | 0 |
| 185 | ALVAREZ-ARROVO L | 2021 | 10.24875/GMM.M21000525 | 0 | 0 | 1 | 0 |
| 186 | JHA PK | 2021 | 10.1111/ctr.14423 | 0 | 0 | 1 | 0 |
| 187 | PASCUAL PAREJA JF | 2021 | 10.1016/j.medcli.2020.11.004 | 0 | 0 | 1 | 0 |
| 188 | SHUKLA AP | 2021 | 10.1016/j.jdiacomp.2021.107967 | 0 | 0 | 1 | 0 |
| 189 | NEJAB JH | 2021 | 10.1016/j.clineuro.2021.106985 | 0 | 0 | 1 | 0 |
| 190 | OZCAN S | 2021 | 10.1093/tropej/fmab052 | 0 | 0 | 1 | 0 |
| 192 | DIEDISHEIM M | 2021 | 10.1210/clinem/dgab393 | 0 | 0 | 1 | 0 |
| 193 | RONDA VE | 2021 | 10.1016/j.medcli.2021.01.002 | 0 | 0 | 1 | 0 |
| 195 | NACHTIGALL I | 2020 | 10.1016/j.cmi.2020.08.011 | 0 | 0 | 1 | 0 |
| 198 | TAN R | 2022 | 10.1093/qjmed/hcab184 | 0 | 0 | 1 | 0 |
| 204 | BARRITEAU CM | 2020 | 10.1111/trf.15947 | 0 | 0 | 1 | 0 |
| 205 | VIDAL-CORTES P | 2021 | 10.37201/req/091.2020 | 0 | 0 | 1 | 0 |
| 208 | BROSNAHAN SB | 2021 | 10.1016/j.jvsv.2020.11.004 | 0 | 0 | 1 | 0 |
| 212 | JEYARAMAN P | 2021 | 10.1016/j.transci.2021.103075 | 0 | 0 | 1 | 0 |
| 215 | LAGANA N | 2021 | 10.1097/MD.0000000000024552 | 0 | 0 | 1 | 0 |
| 218 | LI Y | 2021 | 10.1097/MD.0000000000025287 | 0 | 0 | 1 | 0 |
| 219 | ANTUNEZ MUINOS PJ | 2021 | 10.1038/s41598-021-88679-6 | 0 | 0 | 1 | 0 |
| 224 | BALDON DOS SANTOS V | 2022 | 10.1590/S1678-9946202264020 | 0 | 0 | 1 | 0 |
| 225 | ALHATEM A | 2021 | 10.1016/j.clindermatol.2020.11.013 | 0 | 0 | 1 | 0 |
| 227 | TURKKAN S | 2021 | 10.1111/tid.13700 | 0 | 0 | 1 | 0 |
| 233 | SALVATI L | 2020 | 10.1016/j.imlet.2020.10.009 | 0 | 0 | 1 | 0 |
| 234 | EBINGER JE | 2020 | 10.1371/journal.pone.0236240 | 0 | 0 | 1 | 0 |
| 235 | ZAHR N | 2021 | 10.1016/j.therap.2021.01.056 | 0 | 0 | 1 | 0 |
| 237 | TALAVERA B | 2020 | 10.1016/j.jns.2020.117163 | 0 | 0 | 1 | 0 |
| 238 | MESSIN L | 2021 | 10.3390/v13112151 | 0 | 0 | 1 | 0 |
| 239 | WILLIAMS S | 2021 | 10.1111/resp.13985 | 0 | 0 | 1 | 0 |
| 240 | GONZALEZ-GANCEDO J | 2021 | 10.26355/eurrev_202104_25750 | 0 | 0 | 1 | 0 |
| 241 | RUIZ-BASTIAN M | 2021 | 10.1016/j.diagmicrobio.2021.115477 | 0 | 0 | 1 | 0 |
| 243 | ZHANG Y | 2021 | 10.1536/ihj.20-323 | 0 | 0 | 1 | 0 |
| 245 | AHMED Y | 2021 | 10.1002/lary.29391 | 0 | 0 | 1 | 0 |
| 255 | BORGES DO NASCIMENTO IJ | 2020 | 10.1590/1516-3180.2020.00365.R1.08092020 | 0 | 0 | 1 | 0 |
| 256 | EL-HAMSHARI Y | 2021 | 10.3855/jidc.14709 | 0 | 0 | 1 | 0 |
| 258 | MORELL-GARCIA D | 2021 | 10.1038/s41598-021-90610-y | 0 | 0 | 1 | 0 |
| 259 | BENGELLOUN A | 2021 | 10.1097/CM9.0000000000001798 | 0 | 0 | 1 | 0 |
| 260 | HUNT C | 2021 | 10.7861/clinmed.2021-0308 | 0 | 0 | 1 | 0 |
| 261 | MARTINOT M | 2021 | 10.1016/j.idnow.2021.07.002 | 0 | 0 | 1 | 0 |
| 264 | TUYGUN N | 2021 | 10.1016/j.arcped.2021.09.014 | 0 | 0 | 1 | 0 |
| 267 | BICKEL A | 2022 | 10.1186/s12876-021-02024-9 | 0 | 0 | 1 | 0 |
| 269 | IQBAL A | 2021 | 10.1016/j.diabres.2021.108955 | 0 | 0 | 1 | 0 |
| 276 | LARA OD | 2021 | 10.1002/cncr.33335 | 0 | 0 | 1 | 0 |
| 279 | O'MALLEY G | 2021 | 10.1210/clinem/dgaa825 | 0 | 0 | 1 | 0 |
| 280 | HILL JA | 2021 | 10.1002/jmv.26674 | 0 | 0 | 1 | 0 |
| 283 | SUTTER W | 2021 | 10.1016/j.diabet.2020.101222 | 0 | 0 | 1 | 0 |
| 285 | MASLENNIKOV R | 2021 | 10.1097/MD.0000000000027528 | 0 | 0 | 1 | 0 |
| 287 | BREHM TT | 2021 | 10.1038/s41598-021-85081-0 | 0 | 0 | 1 | 0 |
| 291 | WAYNE MT | 2021 | 10.1097/MD.0000000000027265 | 0 | 0 | 1 | 0 |
| 296 | TATLOW C | 2022 | 10.1016/j.physio.2021.09.001 | 0 | 0 | 1 | 0 |
| 303 | THONDAPU V | 2021 | 10.1016/j.jvsv.2020.11.006 | 0 | 0 | 1 | 0 |
| 304 | DEEB A | 2021 | 10.1155/2021/5822259 | 0 | 0 | 1 | 0 |
| 310 | GUNSTER C | 2021 | 10.1371/journal.pone.0255427 | 0 | 0 | 1 | 0 |
| 312 | GORMEZ S | 2021 | 10.4149/BLL_2021_093 | 0 | 0 | 1 | 0 |
| 313 | DEGLI ESPOSTI L | 2021 | 10.1002/jbmr.4419 | 0 | 0 | 1 | 0 |
| 314 | ALBAHHRANI S | 2021 | 10.2991/jegh.k.210112.001 | 0 | 0 | 1 | 0 |
| 318 | TERAN REDONDO M | 2022 | 10.1016/j.transproceed.2021.09.067 | 0 | 0 | 1 | 0 |
| 325 | BERNI A | 2021 | 10.1530/EJE-20-1447 | 0 | 0 | 1 | 0 |
| 326 | GARCIA-PACHON E | 2021 | 10.1016/j.rmed.2021.106495 | 0 | 0 | 1 | 0 |
| 327 | NASREDDINE R | 2021 | 10.1002/jmv.26828 | 0 | 0 | 1 | 0 |
| 330 | YENDRAPALLI U | 2021 | 10.1016/j.jiph.2021.09.013 | 0 | 0 | 1 | 0 |
| 339 | AGENO W | 2021 | 10.1007/s11739-020-02617-4 | 0 | 0 | 1 | 0 |
| 344 | BAKHSHALIYEV N | 2021 | 10.1111/anec.12846 | 0 | 0 | 1 | 0 |
| 345 | DIAZ DE TERAN T | 2021 | 10.23736/S0026-4806.21.07134-2 | 0 | 0 | 1 | 0 |
| 347 | CHOUDHURI J | 2020 | 10.1371/journal.pone.0244777 | 0 | 0 | 1 | 0 |
| 349 | TURCOTTE JJ | 2020 | 10.1371/journal.pone.0237558 | 0 | 0 | 1 | 0 |
| 350 | PADILLA R | 2021 | 10.18433/jpps31969 | 0 | 0 | 1 | 0 |
| 353 | CUI N | 2021 | 10.4269/ajtmh.21-0234 | 0 | 0 | 1 | 0 |
| 354 | BESUTTI G | 2021 | 10.1371/journal.pone.0251768 | 0 | 0 | 1 | 0 |
| 356 | GROVER S | 2021 | 10.1097/MCG.0000000000001462 | 0 | 0 | 1 | 0 |
| 358 | AOMAR-MILLAN IF | 2021 | 10.1016/j.medcli.2021.01.006 | 0 | 0 | 1 | 0 |
| 360 | YUE T | 2021 | 10.1097/MD.0000000000025083 | 0 | 0 | 1 | 0 |
| 361 | CHOW DS | 2020 | 10.1371/journal.pone.0242953 | 0 | 0 | 1 | 0 |
| 362 | RECHTMAN E | 2020 | 10.1038/s41598-020-78392-1 | 0 | 0 | 1 | 0 |
| 368 | ALTINBILEK A | 2020 | 10.23750/abm.v91i4.10227 | 0 | 0 | 1 | 0 |
| 371 | FIGUEIREDO DALMAZZO LF | 2021 | 10.1111/vox.13087 | 0 | 0 | 1 | 0 |
| 373 | FIORENTINO M | 2020 | 10.1177/0269216320940566 | 0 | 0 | 1 | 0 |
| 374 | BERRY DA | 2021 | 10.1371/journal.pone.0255228 | 0 | 0 | 1 | 0 |
| 375 | BARTIER S | 2021 | 10.1016/j.anorl.2021.03.002 | 0 | 0 | 1 | 0 |
| 377 | MELTON JD | 2021 | 10.1016/j.ajem.2021.08.032 | 0 | 0 | 1 | 0 |
| 379 | ASGHAR MS | 2021 | 10.4103/1319-2442.335450 | 0 | 0 | 1 | 0 |
| 383 | SALBACH C | 2021 | 10.1080/1354750X.2021.1921031 | 0 | 0 | 1 | 0 |
| 384 | WU Y | 2020 | 10.1007/s12250-020-00281-8 | 0 | 0 | 1 | 0 |
| 388 | SUBRAMANIAN K | 2021 | 10.1016/j.clnesp.2021.10.007 | 0 | 0 | 1 | 0 |
| 389 | FISMAN DN | 2021 | 10.1503/cmaj.211248 | 0 | 0 | 1 | 0 |
| 390 | SHARIFPOUR M | 2020 | 10.1371/journal.pone.0242400 | 0 | 0 | 1 | 0 |
| 400 | DIEZ-MANGLANO J | 2021 | 10.1371/journal.pone.0247422 | 0 | 0 | 1 | 0 |
| 402 | DAMANTI S | 2021 | 10.1111/imj.15345 | 0 | 0 | 1 | 0 |
| 405 | MOWLA A | 2020 | 10.1016/j.jns.2020.117183 | 0 | 0 | 1 | 0 |
| 407 | COLANERI M | 2021 | 10.1093/pubmed/fdaa195 | 0 | 0 | 1 | 0 |
| 411 | CARRASQUER A | 2020 | 10.4269/ajtmh.20-1427 | 0 | 0 | 1 | 0 |
| 412 | FAVERIO P | 2021 | 10.1371/journal.pone.0258754 | 0 | 0 | 1 | 0 |
| 415 | RODRIGUEZ-SERRANO DA | 2021 | 10.1038/s41598-021-92497-1 | 0 | 0 | 1 | 0 |
| 416 | CABEZON-VILLALBA G | 2021 | 10.5603/CJ.a2021.0034 | 0 | 0 | 1 | 0 |
| 418 | DOEHN JM | 2021 | 10.1007/s15010-021-01606-9 | 0 | 0 | 1 | 0 |
| 420 | BUONAMICO E | 2021 | 10.1016/j.resinv.2021.05.002 | 0 | 0 | 1 | 0 |
| 422 | XIA QI | 2021 | 10.1002/jmv.26798 | 0 | 0 | 1 | 0 |
| 423 | SARMIENTO-MONROY JC | 2021 | 10.1016/j.jaut.2020.102580 | 0 | 0 | 1 | 0 |
| 426 | LUCAR J | 2021 | 10.14423/SMJ.0000000000001222 | 0 | 0 | 1 | 0 |
| 431 | JACKSON BR | 2021 | 10.1093/cid/ciaa1459 | 0 | 0 | 1 | 0 |
| 435 | MEDETALIBEYOGLU A | 2020 | 10.1007/s12603-020-1477-2 | 0 | 0 | 1 | 0 |
| 437 | PRAKASH S | 2020 | 10.4081/monaldi.2020.1357 | 0 | 0 | 1 | 0 |
| 439 | CARPAGNANO GE | 2021 | 10.1080/17476348.2021.1866546 | 0 | 0 | 1 | 0 |
| 440 | MAJURE DT | 2021 | 10.1016/j.amjcard.2020.09.060 | 0 | 0 | 1 | 0 |
| 445 | KAR S | 2021 | 10.1038/s41598-021-92146-7 | 0 | 0 | 1 | 0 |
| 446 | MAHAJAN NN | 2021 | 10.1097/AOG.0000000000004529 | 0 | 0 | 1 | 0 |
| 452 | GOMEZ RI | 2022 | 10.1136/ejhpharm-2021-002741 | 0 | 0 | 1 | 0 |
| 461 | KNOX DB | 2022 | 10.1016/j.dsx.2022.102407 | 0 | 0 | 1 | 0 |
| 464 | IZQUIERDO JL | 2022 | 10.1177/00368504221074574 | 0 | 0 | 1 | 0 |
| 465 | REN L | 2020 | 10.1097/MD.0000000000022899 | 0 | 0 | 1 | 0 |
| 470 | VOIOSU A | 2022 | 10.2478/rjim-2021-0027 | 0 | 0 | 1 | 0 |
| 472 | INAMDAR S | 2020 | 10.1053/j.gastro.2020.08.044 | 0 | 0 | 1 | 0 |
| 473 | STOECKLE K | 2022 | 10.1002/jmv.27280 | 0 | 0 | 1 | 0 |
| 474 | FRANSVEA P | 2022 | 10.26355/eurrev_202202_28135 | 0 | 0 | 1 | 0 |
| 476 | CHILIMURI S | 2022 | 10.1016/j.jnma.2021.08.036 | 0 | 0 | 1 | 0 |
| 479 | CORROCHANO M | 2022 | 10.1007/s11239-021-02507-2 | 0 | 0 | 1 | 0 |
| 483 | GUNAY S | 2022 | 10.7754/Clin.Lab.2021.210745 | 0 | 0 | 1 | 0 |
| 484 | RYSZ S | 2022 | 10.1111/aas.13982 | 0 | 0 | 1 | 0 |
| 486 | MELILLO F | 2022 | 10.1111/eci.13703 | 0 | 0 | 1 | 0 |
| 487 | BLANCHARD C | 2022 | 10.1002/oby.23314 | 0 | 0 | 1 | 0 |
| 488 | SINDHU C | 2022 | 10.1007/s10157-021-02123-7 | 0 | 0 | 1 | 0 |
| 494 | WILES MD | 2022 | 10.1111/anae.15581 | 0 | 0 | 1 | 0 |
| 497 | LOINAZ C | 2020 | 10.1111/tid.13372 | 0 | 0 | 1 | 0 |
| 500 | SARGIN ALTUNOK E | 2022 | 10.1002/jmv.27319 | 0 | 0 | 1 | 0 |
| 501 | PANDA S | 2022 | 10.1002/jmv.27315 | 0 | 0 | 1 | 0 |
| 504 | WONGVIBULSIN S | 2021 | 10.7326/M20-6754 | 0 | 0 | 1 | 0 |
| 505 | ROCHA DE MACEDO B | 2021 | 10.36416/1806-3756/e20200545 | 0 | 0 | 1 | 0 |
| 507 | SINGH S | 2021 | 10.1161/HYPERTENSIONAHA.121.17328 | 0 | 0 | 1 | 0 |
| 508 | FILLMORE N | 2021 | 10.1371/journal.pone.0259061 | 0 | 0 | 1 | 0 |
| 509 | LARA OD | 2020 | 10.1002/cncr.33084 | 0 | 0 | 1 | 0 |
| 511 | MENDEZ SR | 2021 | 10.1016/j.chest.2021.01.073 | 0 | 0 | 1 | 0 |
| 512 | BLIEK-BUENO K | 2021 | 10.3390/ijerph182211786 | 0 | 0 | 1 | 0 |
| 516 | BIRTAY T | 2021 | 10.5144/0256-4947.2021.327 | 0 | 0 | 1 | 0 |
| 517 | PEERAPOMRATANA S | 2022 | 10.1159/000515628 | 0 | 0 | 1 | 0 |
| 519 | MEHTA M | 2021 | 10.1136/bmjopen-2021-053810 | 0 | 0 | 1 | 0 |
| 521 | SADEGHI A | 2020 | 10.23750/abm.v91i4.10175 | 0 | 0 | 1 | 0 |
| 522 | VALK CMA | 2021 | 10.1097/EJA.0000000000001565 | 0 | 0 | 1 | 0 |
| 523 | TRUJILLO H | 2020 | 10.1111/ctr.14072 | 0 | 0 | 1 | 0 |
| 525 | MONFARDINI L | 2020 | 10.1259/bjr.20200407 | 0 | 0 | 1 | 0 |
| 531 | MENA G | 2021 | 10.1016/j.medcli.2020.11.002 | 0 | 0 | 1 | 0 |
| 533 | PRICE-HAYWOOD EG | 2020 | 10.1056/NEJMsa2011686 | 0 | 0 | 1 | 0 |
| 536 | KO RE | 2022 | 10.1177/17534666221081035 | 0 | 0 | 1 | 0 |
| 537 | DANIELS LB | 2020 | 10.1016/j.amjcard.2020.09.012 | 0 | 0 | 1 | 0 |
| 538 | AKDOGAN D | 2021 | 10.3855/jidc.14072 | 0 | 0 | 1 | 0 |
| 541 | DING J | 2021 | 10.1097/MD.0000000000026538 | 0 | 0 | 1 | 0 |
| 543 | CASSONE G | 2022 | 10.1371/journal.pone.0262908 | 0 | 0 | 1 | 0 |
| 544 | CHEN M | 2021 | 10.1089/vbz.2021.0023 | 0 | 0 | 1 | 0 |
| 549 | RAMASWAMI A | 2021 | 10.1093/qjmed/hcab190 | 0 | 0 | 1 | 0 |
| 551 | CHAN L | 2021 | 10.1681/ASN.2020050615 | 0 | 0 | 1 | 0 |
| 552 | ROEDL K | 2021 | 10.1016/j.aucc.2020.10.009 | 0 | 0 | 1 | 0 |
| 553 | MARTIN-VILLARES C | 2021 | 10.1007/s00405-020-06220-3 | 0 | 0 | 1 | 0 |
| 558 | APPELBAUM B | 2022 | 10.1038/s41598-022-06276-7 | 0 | 0 | 1 | 0 |
| 562 | MORICONI D | 2020 | 10.1016/j.orcp.2020.05.009 | 0 | 0 | 1 | 0 |
| 563 | XU J | 2021 | 10.1177/0885066620970858 | 0 | 0 | 1 | 0 |
| 567 | PAN M | 2021 | 10.1161/JAHA.121.023535 | 0 | 0 | 1 | 0 |
| 569 | FIGUEIRA GONCALVES JM | 2020 | 10.1186/s13104-020-05402-w | 0 | 0 | 1 | 0 |
| 571 | CHOW JH | 2021 | 10.1111/jth.15517 | 0 | 0 | 1 | 0 |
| 573 | ELEMAM O | 2021 | 10.32471/exp-oncology.2312-8852.vol-43-no-1.15964 | 0 | 0 | 1 | 0 |
| 574 | ROSSI L | 2021 | 10.1007/s15010-020-01550-0 | 0 | 0 | 1 | 0 |
| 582 | ORTIZ MOLINA E | 2020 | 10.5603/GP.a2020.0130 | 0 | 0 | 1 | 0 |
| 586 | LIABEUF S | 2021 | 10.1093/ehjcvp/pvaa062 | 0 | 0 | 1 | 0 |
| 587 | KIM R | 2020 | 10.1371/journal.pone.0241956 | 0 | 0 | 1 | 0 |
| 592 | GALLUZZI F | 2021 | 10.1159/000514888 | 0 | 0 | 1 | 0 |
| 595 | MILLION M | 2021 | 10.31083/j.rcm2203116 | 0 | 0 | 1 | 0 |
| 596 | SOLEIMANI A | 2020 | 10.1093/ajh/hpaa149 | 0 | 0 | 1 | 0 |
| 597 | WANG Z | 2020 | 10.1136/bmjopen-2020-040441 | 0 | 0 | 1 | 0 |
| 599 | JANG JG | 2020 | 10.3346/jkms.2020.35.e209 | 0 | 0 | 0 | 1 |
| 608 | GUERSON-GIL A | 2021 | 10.1007/s10096-021-04260-z | 0 | 0 | 1 | 0 |
| 611 | LOPEZ-ESCOBAR A | 2021 | 10.1136/jim-2021-001810 | 0 | 0 | 1 | 0 |
| 612 | TRACARICHI E | 2020 | 10.1038/s41598-020-77641-7 | 0 | 0 | 1 | 0 |
| 614 | GISONDI P | 2020 | 10.1016/j.jaad.2020.04.085 | 0 | 0 | 1 | 0 |
| 616 | YUAN N | 2021 | 10.1016/j.echo.2021.03.010 | 0 | 0 | 1 | 0 |
| 622 | WANG F | 2021 | 10.1093/ije/dyaa180 | 0 | 0 | 1 | 0 |
| 625 | DUAN J | 2020 | 10.1002/jmv.26082 | 0 | 0 | 1 | 0 |
| 626 | GULER T | 2021 | 10.1007/s00296-021-05003-1 | 0 | 0 | 1 | 0 |
| 627 | TANG L | 2021 | 10.1111/cns.13687 | 0 | 0 | 1 | 0 |
| 629 | FAUVEL C | 2020 | 10.1093/eurheartj/ehaa500 | 0 | 0 | 1 | 0 |
| 632 | GORMEZ S | 2021 | 10.1038/s41371-020-00405-3 | 0 | 0 | 1 | 0 |
| 635 | FILOSTO M | 2021 | 10.1136/jnnp-2020-324837 | 0 | 0 | 1 | 0 |
| 636 | BIOH G | 2021 | 10.1136/openhrt-2021-001833 | 0 | 0 | 1 | 0 |
| 639 | DE CACERES C | 2020 | 10.1007/s43440-020-00186-z | 0 | 0 | 1 | 0 |
| 642 | RAHEJA H | 2021 | 10.1093/qjmed/hcab029 | 0 | 0 | 1 | 0 |
| 644 | DUAN J | 2021 | 10.1016/j.ajem.2020.07.071 | 0 | 0 | 1 | 0 |
| 648 | MATLI K | 2021 | 10.1136/openhrt-2021-001785 | 0 | 0 | 1 | 0 |
| 657 | HOERTEL N | 2021 | 10.1002/cpt.2317 | 0 | 0 | 1 | 0 |
| 662 | FRANZETTI M | 2021 | 10.4049/jimmunol.2001126 | 0 | 0 | 1 | 0 |
| 666 | GOKHALE Y | 2021 | 10.1186/s12879-021-05912-3 | 0 | 0 | 1 | 0 |
| 668 | ROMERO J | 2020 | 10.1007/s10840-020-00896-7 | 0 | 0 | 1 | 0 |
| 669 | ALLOU N | 2021 | 10.1097/MD.0000000000024524 | 0 | 0 | 1 | 0 |
| 671 | RUSSO V | 2020 | 10.1111/eci.13387 | 0 | 0 | 1 | 0 |
| 672 | GOEL N | 2020 | 10.4081/monaldi.2020.1568 | 0 | 0 | 1 | 0 |
| 686 | LI S | 2021 | 10.1172/JCI151418 | 0 | 0 | 1 | 0 |
| 689 | CHOW JH | 2021 | 10.1213/ANE.0000000000005292 | 0 | 0 | 1 | 0 |
| 692 | LOFFI M | 2021 | 10.1136/openhrt-2020-001428 | 0 | 0 | 1 | 0 |
| 695 | MASTROIANNI A | 2021 | 10.1177/20587384211059675 | 0 | 0 | 1 | 0 |
| 697 | SAKAI T | 2020 | 10.2340/16501977-2731 | 0 | 0 | 1 | 0 |
| 700 | BIRLUTIU V | 2021 | 10.1097/MD.0000000000025832 | 0 | 0 | 1 | 0 |
| 701 | BIGNAMI E | 2021 | 10.23750/abm.v92i5.11417 | 0 | 0 | 1 | 0 |
| 703 | DENG Q | 2020 | 10.1016/j.ijcard.2020.03.087 | 0 | 0 | 1 | 0 |
| 708 | OZTURK B | 2021 | 10.1016/j.jen.2021.03.013 | 0 | 0 | 1 | 0 |
| 712 | PALAZZUOLI A | 2020 | 10.1161/JAHA.120.017364 | 0 | 0 | 1 | 0 |
| 714 | PERAYRA D | 2021 | 10.1093/cvr/cvab308 | 0 | 0 | 1 | 0 |
| 716 | CORRAO G | 2021 | 10.1136/bmjopen-2021-053281 | 0 | 0 | 1 | 0 |
| 718 | TURNER J | 2020 | 10.1016/j.jpainsymman.2020.04.031 | 0 | 0 | 1 | 0 |
| 719 | LANG CN | 2021 | 10.1007/s12028-021-01202-7 | 0 | 0 | 1 | 0 |
| 721 | KARAHAN S | 2021 | 10.1007/s12603-020-1479-0 | 0 | 0 | 1 | 0 |
| 730 | QJAN F | 2020 | 10.1186/s12879-020-05637-9 | 0 | 0 | 1 | 0 |
| 732 | MONTREAL E | 2021 | 10.1007/s10096-020-04078-1 | 0 | 0 | 1 | 0 |
| 742 | SCHIAFFINO S | 2021 | 10.1097/MD.0000000000024002 | 0 | 0 | 1 | 0 |
| 743 | DEEB A | 2021 | 10.1155/2021/6695707 | 0 | 0 | 1 | 0 |
| 745 | CHEN APL | 2021 | 10.1111/irv.12858 | 0 | 0 | 1 | 0 |
| 746 | FRANCHIN G | 2021 | 10.12659/MSM.934267 | 0 | 0 | 1 | 0 |
| 747 | BENIAS PC | 2021 | 10.1097/MPA.0000000000001922 | 0 | 0 | 1 | 0 |
| 748 | BAKHSHALIYEV N | 2020 | 10.1016/j.jelectrocard.2020.08.008 | 0 | 0 | 1 | 0 |
| 756 | LING SF | 2020 | 10.3390/nu12123799 | 0 | 0 | 1 | 0 |
| 761 | PALLOTTO C | 2021 | 10.1002/jmv.26898 | 0 | 0 | 1 | 0 |
| 763 | GOZALBO-ROVIRA R | 2020 | 10.1016/j.jcv.2020.104611 | 0 | 0 | 1 | 0 |
| 765 | SATLIN MJ | 2020 | 10.1371/journal.pone.0236778 | 0 | 0 | 1 | 0 |
| 766 | FUMAGALLI S | 2022 | 10.1055/a-1503-3875 | 0 | 0 | 1 | 0 |
| 768 | WANG W | 2021 | 10.3389/fendo.2021.791476 | 0 | 0 | 1 | 0 |
| 772 | BARNATO AD | 2022 | 10.1111/jgs.17463 | 0 | 0 | 1 | 0 |
| 776 | CHOCRON R | 2021 | 10.1016/j.acvd.2021.02.003 | 0 | 0 | 1 | 0 |
| 780 | NG JH | 2020 | 10.1016/j.kint.2020.07.030 | 0 | 0 | 1 | 0 |
| 785 | KRISHNAN A | 2022 | 10.3748/wjg.v28.i5.570 | 0 | 0 | 1 | 0 |
| 791 | MELAZZINI F | 2021 | 10.1007/s11739-020-02550-6 | 0 | 0 | 1 | 0 |
| 796 | LUM E | 2020 | 10.1016/j.transproceed.2020.09.005 | 0 | 0 | 1 | 0 |
| 800 | PEIRO OM | 2021 | 10.1080/1354750X.2021.1874052 | 0 | 0 | 1 | 0 |
| 803 | LANG S | 2021 | 10.1080/07435800.2021.1924770 | 0 | 0 | 1 | 0 |
| 805 | LOUAPRE C | 2021 | 10.1111/ene.14612 | 0 | 0 | 1 | 0 |
| 806 | PANIGADA M | 2021 | 10.1097/MAT.0000000000001380 | 0 | 0 | 1 | 0 |
| 809 | PARRA A | 2020 | 10.1016/j.psychres.2020.113254 | 0 | 0 | 1 | 0 |
| 810 | BIANCALANA E | 2021 | 10.2147/CIA.S313028 | 0 | 0 | 1 | 0 |
| 812 | WANG P | 2021 | 10.1097/MD.0000000000024604 | 0 | 0 | 1 | 0 |
| 818 | SUN R | 2021 | 10.1016/j.ijrobp.2021.02.022 | 0 | 0 | 1 | 0 |
| 821 | GIANNIS D | 2021 | 10.1007/s11239-021-02413-7 | 0 | 0 | 1 | 0 |
| 826 | MEIZLISH ML | 2021 | 10.1002/ajh.26102 | 0 | 0 | 1 | 0 |
| 829 | PORTACCI A | 2021 | 10.1016/j.rmed.2021.106556 | 0 | 0 | 1 | 0 |
| 830 | ZHANG X | 2020 | 10.1002/hed.26261 | 0 | 0 | 1 | 0 |
| 839 | PATEL A | 2020 | 10.1371/journal.pone.0240960 | 0 | 0 | 1 | 0 |
| 842 | KAMMAR-GARCIA A | 2020 | 10.24875/RIC.20000207 | 0 | 0 | 1 | 0 |
| 843 | SONG F | 2021 | 10.1186/s12937-021-00702-8 | 0 | 0 | 1 | 0 |
| 851 | NAEHRLICH L | 2021 | 10.1016/j.jcf.2021.03.017 | 0 | 0 | 1 | 0 |
| 854 | RODILLA E | 2021 | 10.1161/HYPERTENSIONAHA.120.16563 | 0 | 0 | 1 | 0 |
| 855 | EL-MALKY AM | 2021 | 10.4103/1319-2442.335459 | 0 | 0 | 1 | 0 |
| 856 | OMMA A | 2021 | 10.1016/j.intimp.2021.107891 | 0 | 0 | 1 | 0 |
| 860 | OGAMBA I | 2020 | 10.1515/jpm-2020-0446 | 0 | 0 | 1 | 0 |
| 863 | GAO C | 2020 | 10.1093/eurheartj/ehaa433 | 0 | 0 | 1 | 0 |
| 867 | ALOISIO E | 2020 | 10.5858/arpa.2020-0389-SA | 0 | 0 | 1 | 0 |
| 869 | MASSANA L | 2022 | 10.1093/ehjcvp/pvaa128 | 0 | 0 | 1 | 0 |
| 870 | COVINO M | 2020 | 10.1016/j.resuscitation.2020.08.124 | 0 | 0 | 1 | 0 |
| 872 | HUANG S | 2020 | 10.1038/s41440-020-0485-2 | 0 | 0 | 1 | 0 |
| 874 | HUGHES S | 2020 | 10.1016/j.cmi.2020.06.025 | 0 | 0 | 1 | 0 |
| 875 | LIVINGSTON G | 2020 | 10.1016/S2215-0366(20)30434-X | 0 | 0 | 1 | 0 |
| 876 | GOYAL P | 2020 | 10.7326/M20-2730 | 0 | 0 | 1 | 0 |
| 879 | PANAGIDES V | 2021 | 10.1016/j.acvd.2021.04.003 | 0 | 0 | 1 | 0 |
| 880 | ZHANG W | 2021 | 10.1155/2021/8812304 | 0 | 0 | 1 | 0 |
| 882 | WANG L | 2021 | 10.1093/jac/dkaa475 | 0 | 0 | 1 | 0 |
| 886 | CAMMALLERI V | 2020 | 10.1161/JAHA.120.017126 | 0 | 0 | 1 | 0 |
| 888 | CUI X | 2020 | 10.1159/000509517 | 0 | 0 | 1 | 0 |
| 891 | LUIGETTI M | 2020 | 10.1111/ene.14444 | 0 | 0 | 1 | 0 |
| 896 | TIAN J | 2021 | 10.4049/jimmunol.2000981 | 0 | 0 | 1 | 0 |
| 897 | BORAH P | 2021 | 10.1016/j.bcmd.2020.102525 | 0 | 0 | 1 | 0 |
| 899 | GENET B | 2020 | 10.1016/j.jamda.2020.09.004 | 0 | 0 | 1 | 0 |
| 901 | ALI J | 2021 | 10.1016/j.hemonc.2020.12.001 | 0 | 0 | 1 | 0 |
| 902 | DEBIASI RL | 2020 | 10.1016/j.jpeds.2020.05.007 | 0 | 0 | 1 | 0 |
| 903 | PIZARRO-SANCHEZ MS | 2021 | 10.1159/000512535 | 0 | 0 | 1 | 0 |
| 913 | KIM I | 2021 | 10.3390/medicina57090931 | 0 | 0 | 1 | 0 |
| 920 | KERR AD | 2020 | 10.7861/clinmed.2020-0423 | 0 | 0 | 1 | 0 |
| 921 | AL-SHAMALI Y | 2021 | 10.1371/journal.pone.0254379 | 0 | 0 | 1 | 0 |
| 930 | LIU Y | 2020 | 10.18632/aging.103745 | 0 | 0 | 1 | 0 |
| 934 | ARENAS-JIMENEZ MD | 2021 | 10.3390/nu13082559 | 0 | 0 | 1 | 0 |
| 936 | GASPARINI M | 2021 | 10.1111/anae.15293 | 0 | 0 | 1 | 0 |
| 939 | ALFANO G | 2021 | 10.1007/s10157-020-01996-4 | 0 | 0 | 1 | 0 |
| 942 | KLOPFENSTEIN T | 2020 | 10.1016/j.medmal.2020.04.006 | 0 | 0 | 1 | 0 |
| 947 | MANOCHA KK | 2021 | 10.1161/JAHA.120.018477 | 0 | 0 | 1 | 0 |
| 954 | CHEN N | 2020 | 10.1097/MD.0000000000022635 | 0 | 0 | 1 | 0 |
| 958 | LUI X | 2021 | 10.1016/j.medcli.2020.11.036 | 0 | 0 | 1 | 0 |
| 962 | DESAI A | 2021 | 10.1016/j.ijcard.2020.09.062 | 0 | 0 | 1 | 0 |
| 966 | ROUSSEAU MC | 2021 | 10.1016/j.arcped.2021.04.004 | 0 | 0 | 1 | 0 |
| 969 | JIMENO S | 2021 | 10.1111/eci.13404 | 0 | 0 | 1 | 0 |
| 971 | DA BL | 2021 | 10.1111/joim.13292 | 0 | 0 | 1 | 0 |
| 972 | OTTOLINA D | 2022 | 10.1007/s40620-021-01100-3 | 0 | 0 | 1 | 0 |
| 973 | RIAHI S | 2021 | 10.1080/07435800.2020.1856865 | 0 | 0 | 1 | 0 |
| 976 | MA X | 2020 | 10.1017/S0950268820001727 | 0 | 0 | 1 | 0 |
| 977 | SHAO F | 2020 | 10.1016/j.resuscitation.2020.04.005 | 0 | 0 | 1 | 0 |
| 978 | TREMBLAY D | 2020 | 10.1182/blood.2020006941 | 0 | 0 | 1 | 0 |
| 979 | CERUTI S | 2021 | 10.1371/journal.pone.0260318 | 0 | 0 | 1 | 0 |
| 980 | KUMAR S | 2021 | 10.1093/jbcr/iraa217 | 0 | 0 | 1 | 0 |
| 981 | DENNIS JM | 2021 | 10.1097/CCM.0000000000004747 | 0 | 0 | 1 | 0 |
| 982 | GAO J | 2020 | 10.1002/jcla.23562 | 0 | 0 | 1 | 0 |
| 983 | CHAN KH | 2020 | 10.1016/j.diabres.2020.108279 | 0 | 0 | 1 | 0 |
| 988 | BURCU UGURLU I | 2021 | 10.14744/AnatolJCardiol.2020.79138 | 0 | 0 | 1 | 0 |
| 989 | HAIMOVICH AD | 2020 | 10.1016/j.annemergmed.2020.07.022 | 0 | 0 | 1 | 0 |
| 991 | ZOU X | 2020 | 10.1097/CCM.0000000000004411 | 0 | 0 | 1 | 0 |
| 995 | LI L | 2021 | 10.1016/j.psychres.2021.113776 | 0 | 0 | 1 | 0 |
| 996 | HE L | 2021 | 10.1186/s12964-021-00754-7 | 0 | 0 | 1 | 0 |
| 997 | YAO W | 2020 | 10.1016/j.bja.2020.03.026 | 0 | 0 | 1 | 0 |
| 998 | CHOI JJ | 2020 | 10.1016/j.thromres.2020.09.022 | 0 | 0 | 1 | 0 |
| 1003 | VULTAGGIO A | 2020 | 10.1016/j.jaip.2020.06.013 | 0 | 0 | 1 | 0 |
| 1006 | ZENG OL | 2020 | 10.1093/infdis/jiaa228 | 0 | 0 | 1 | 0 |
| 1008 | MORALES-QUINTEROS L | 2021 | 10.1186/s13054-021-03570-0 | 0 | 0 | 1 | 0 |
| 1013 | KATAGIRI D | 2021 | 10.1002/jca.21861 | 0 | 0 | 1 | 0 |
| 1016 | IERARDI AM | 2021 | 10.14309/ajg.0000000000000978 | 0 | 0 | 1 | 0 |
| 1017 | POGGIALI E | 2020 | 10.1016/j.cca.2020.06.012 | 0 | 0 | 1 | 0 |
| 1019 | BARROSO LOPEZ KR | 2021 | 10.1016/j.aprim.2020.10.005 | 0 | 0 | 1 | 0 |
| 1021 | KIM SW | 2021 | 10.3803/EnM.2021.1040 | 0 | 0 | 1 | 0 |
| 1023 | ALBALADEJO P | 2021 | 10.1016/j.chest.2021.01.017 | 0 | 0 | 1 | 0 |
| 1028 | MATSUZAWA Y | 2020 | 10.1038/s41440-020-00535-8 | 0 | 0 | 1 | 0 |
| 1029 | CHEN Y | 2020 | 10.21037/apm-20-1273 | 0 | 0 | 1 | 0 |
| 1031 | OULDALI N | 2021 | 10.1001/jama.2021.0694 | 0 | 0 | 1 | 0 |
| 1033 | GOURIEUX B | 2021 | 0.1136/ejhpharm-2020-002449 | 0 | 0 | 1 | 0 |
| 1035 | GUNDOGAN K | 2021 | 10.5152/balkanmedj.2021.21188 | 0 | 0 | 1 | 0 |
| 1036 | JIMENEZ HERNANDEZ S | 2020 | *32692002 | 0 | 0 | 1 | 0 |
| 1037 | CHEN S | 2020 | 10.1080/03009734.2020.1822960 | 0 | 0 | 1 | 0 |
| 1040 | LANZANI C | 2021 | 10.1007/s40620-021-00997-0 | 0 | 0 | 1 | 0 |
| 1041 | DUTCHER L | 2022 | 10.1542/peds.2021-053079 | 0 | 0 | 1 | 0 |
| 1043 | TABACOF L | 2022 | 10.1097/PHM.0000000000001910 | 0 | 0 | 1 | 0 |
| 1044 | SCHIAFFINO S | 2021 | 10.1148/radiol.2021204141 | 0 | 0 | 1 | 0 |
| 1045 | DHAMOON MS | 2021 | 10.1161/STROKEAHA.120.031668 | 0 | 0 | 1 | 0 |
| 1048 | COVID-19 RISK AND TREATMENTS (CORIST) COLLABORATION | 2020 | 10.1016/j.ejim.2020.08.019 | 0 | 0 | 1 | 0 |
| 1049 | GANDOLFO C | 2021 | 10.23736/S0026-4806.21.07585-6 | 0 | 0 | 1 | 0 |
| 1050 | CALLEJAS RUBIO JL | 2020 | 10.1016/j.medcli.2020.04.018 | 0 | 0 | 1 | 0 |
| 1051 | OUALBA M | 2020 | 10.1016/j.arcped.2020.05.010 | 0 | 0 | 1 | 0 |
| 1052 | LUI J | 2020 | 10.1097/MD.0000000000021012 | 0 | 0 | 1 | 0 |
| 1054 | IHLE-HANSEN H | 2020 | 10.4045/tidsskr.20.0301 | 0 | 0 | 1 | 0 |
| 1055 | LENTI MV | 2020 | 10.1007/s11739-020-02425-w | 0 | 0 | 1 | 0 |
| 1056 | BRAVI F | 2020 | 10.1371/journal.pone.0235248 | 0 | 0 | 1 | 0 |
| 1059 | LOUAPRE C | 2020 | 10.1001/jamaneurol.2020.2581 | 0 | 0 | 1 | 0 |
| 1060 | FRONTERA JA | 2020 | 10.1097/CCM.0000000000004605 | 0 | 0 | 1 | 0 |
| 1061 | BORGEL D | 2021 | 10.1097/CCM.0000000000005093 | 0 | 0 | 1 | 0 |
| 1062 | SJOSTROM A | 2021 | 10.1055/a-1477-3829 | 0 | 0 | 1 | 0 |
| 1063 | FLINSPACH AN | 2021 | 10.1371/journal.pone.0253778 | 0 | 0 | 1 | 0 |
| 1066 | JANG JG | 2020 | 10.3346/jkms.2020.35.e234 | 0 | 0 | 1 | 0 |
| 1069 | KHRIHNAN S | 2020 | 10.1016/j.jclinane.2020.110005 | 0 | 0 | 1 | 0 |
| 1070 | IRIE K | 2021 | 10.1002/psp4.12685 | 0 | 0 | 1 | 0 |
| 1071 | DE OLIVEIRA VALLE E | 2021 | 10.1186/s13054-021-03729-9 | 0 | 0 | 1 | 0 |
| 1072 | ABDALKADER M | 2021 | 10.1016/j.jstrokecerebrovasdis.2021.105733 | 0 | 0 | 1 | 0 |
| 1073 | TEJADA CIFUENTES F | 2021 | 10.1016/j.medcli.2020.10.006 | 0 | 0 | 1 | 0 |
| 1075 | ABIZANDA P | 2021 | 10.1111/jgs.17357 | 0 | 0 | 1 | 0 |
| 1076 | COLOMBO C | 2022 | 10.7748/en.2021.e2091 | 0 | 0 | 1 | 0 |
| 1077 | FATTORUTTO M | 2022 | 10.1007/s11239-021-02514-3 | 0 | 0 | 1 | 0 |
| 1079 | HOILAND RL | 2020 | 10.1182/bloodadvances.2020002623 | 0 | 0 | 1 | 0 |
| 1081 | SINHA P | 2021 | 10.1164/rccm.202105-1302OC | 0 | 0 | 1 | 0 |
| 1086 | HAMEED S | 2021 | 10.1159/000516641 | 0 | 0 | 1 | 0 |
| 1088 | GIRAUD R | 2021 | 10.14814/phy2.14715 | 0 | 0 | 1 | 0 |
| 1089 | TORRES-PENA JD | 2021 | 10.1007/s40265-021-01498-x | 0 | 0 | 1 | 0 |
| 1091 | DOPFER C | 2020 | 10.1186/s12887-020-02303-6 | 0 | 0 | 1 | 0 |
| 1092 | BRETAGNE S | 2021 | 10.1128/Spectrum.01138-21 | 0 | 0 | 1 | 0 |
| 1093 | HALVATSIOTIS P | 2020 | 10.1016/j.diabres.2020.108331 | 0 | 0 | 1 | 0 |
| 1094 | MONTERO F | 2020 | 10.1007/s00296-020-04676-4 | 0 | 0 | 1 | 0 |
| 1095 | LA TORRE G | 2022 | 10.26355/eurrev_202202_28017 | 0 | 0 | 1 | 0 |
| 1096 | LI BASSI G | 2021 | 10.1186/s13054-021-03518-4 | 0 | 0 | 1 | 0 |
| 1097 | MATHEW T | 2021 | 10.1177/1747493020968236 | 0 | 0 | 1 | 0 |
| 1099 | PAGAN ME | 2022 | 10.1055/s-0041-1739292 | 0 | 0 | 1 | 0 |
| 1102 | ZHANG C | 2020 | 10.1371/journal.pmed.1003130 | 0 | 0 | 1 | 0 |
| 1104 | MISHRA S | 2021 | 10.1016/j.jstrokecerebrovasdis.2021.105603 | 0 | 0 | 1 | 0 |
| 1105 | DALAN R | 2021 | 10.1093/ehjcvp/pvaa098 | 0 | 0 | 1 | 0 |
| 1108 | FAN X | 2021 | 10.1186/s12985-021-01538-8 | 0 | 0 | 1 | 0 |
| 1112 | YANG X | 2020 | 10.1016/S2213-2600(20)30079-5 | 0 | 0 | 1 | 0 |
| 1113 | RICO ESPINEIRA C | 2021 | *33507637 | 0 | 0 | 1 | 0 |
| 1117 | TANG H | 2020 | 10.1053/j.ajkd.2020.06.008 | 0 | 0 | 1 | 0 |
| 1120 | GUPTA R | 2021 | 10.1016/j.dsx.2021.102322 | 0 | 0 | 1 | 0 |
| 1121 | YAGHI S | 2020 | 10.1161/STROKEAHA.120.030335 | 0 | 0 | 1 | 0 |
| 1122 | WIGNALL A | 2021 | 10.1186/s13018-021-02301-z | 0 | 0 | 1 | 0 |
| 1123 | COVID-19 RISK AND TREATMENTS (CORIST) COLLABORATION | 2020 | 10.1016/j.vph.2020.106805 | 0 | 0 | 1 | 0 |
| 1124 | GUNAY S | 2021 | 10.1684/mrh.2021.0485 | 0 | 0 | 1 | 0 |
| 1126 | NOLL E | 2021 | 10.1016/j.accpm.2020.10.014 | 0 | 0 | 1 | 0 |
| 1130 | GARCIA-OLIVE I | 2020 | 10.1016/j.rmed.2020.106023 | 0 | 0 | 1 | 0 |
| 1131 | TORRES A | 2021 | 10.1186/s13054-021-03727-x | 0 | 0 | 1 | 0 |
| 1134 | SIEGLER J | 2021 | 10.1177/1747493020959216 | 0 | 0 | 1 | 0 |
| 1139 | SEE YP | 2021 | 10.1159/000514064 | 0 | 0 | 1 | 0 |
| 1142 | DI CASTELNUOVO A | 2020 | 10.1016/j.numecd.2020.07.031 | 0 | 0 | 1 | 0 |
| 1145 | LEE J | 2021 | 10.3904/kjim.2020.390 | 0 | 0 | 1 | 0 |
| 1146 | KNORR JP | 2020 | 10.1002/jmv.26191 | 0 | 0 | 1 | 0 |
| 1147 | CAO J | 2020 | 10.7150/thno.47980 | 0 | 0 | 1 | 0 |
| 1148 | PENG PP | 2020 | 10.7150/thno.46833 | 0 | 0 | 1 | 0 |
| 1153 | ROKOHL AC | 2020 | 10.1016/j.cmi.2020.08.018 | 0 | 0 | 1 | 0 |
| 1157 | ALI ALMADHI M | 2021 | 10.1038/s41598-021-84810-9 | 0 | 0 | 1 | 0 |
| 1161 | RUIZ S | 2021 | 10.1016/j.ijantimicag.2020.106247 | 0 | 0 | 1 | 0 |
| 1162 | CAMPOROTA L | 2020 | 10.1016/j.bja.2020.08.047 | 0 | 0 | 1 | 0 |
| 1166 | DI CASTELNUOVO A | 2021 | 10.1055/a-1347-6070 | 0 | 0 | 1 | 0 |
| 1172 | CHOUGAR L | 2020 | 10.1148/radiol.2020202422 | 0 | 0 | 1 | 0 |
| 1173 | MONDEJAR-LOPEZ P | 2020 | 10.1016/j.rmed.2020.106062 | 0 | 0 | 1 | 0 |
| 1174 | SAMUEL S | 2020 | 10.1016/j.hrthm.2020.06.033 | 0 | 0 | 1 | 0 |
| 1175 | LANI-LOUZADA R | 2020 | 10.1371/journal.pone.0243346 | 0 | 0 | 1 | 0 |
| 1177 | CALLEJAS RUBIO JL | 2020 | 10.1016/j.regg.2020.05.004 | 0 | 0 | 1 | 0 |
| 1182 | KREMER S | 2020 | 10.1148/radiol.2020202222 | 0 | 0 | 1 | 0 |
| 1183 | KAUSHIK S | 2020 | 10.1016/j.jpeds.2020.06.045 | 0 | 0 | 1 | 0 |
| 1184 | FERNANDEZ RUIZ M | 2020 | 10.1111/ajt.15929 | 0 | 0 | 1 | 0 |
| 1187 | MORRISON AR | 2020 | 10.1016/j.jaut.2020.102512 | 0 | 0 | 1 | 0 |
| 1188 | NIGHTINGALE R | 2020 | 10.1136/bmjresp-2020-000639 | 0 | 0 | 1 | 0 |
| 1189 | VROTSOU K | 2021 | 10.1136/bmjopen-2021-049066 | 0 | 0 | 1 | 0 |
| 1190 | LIN YH | 2020 | 10.1097/MD.0000000000023064 | 0 | 0 | 1 | 0 |
| 1191 | LIU Q | 2020 | 10.1017/S0950268820001442 | 0 | 0 | 1 | 0 |
| 1192 | KIM MK | 2020 | 10.4093/dmj.2020.0146 | 0 | 0 | 1 | 0 |
| 1194 | YOUNG BE | 2020 | 10.1016/S0140-6736(20)31757-8 | 0 | 0 | 1 | 0 |
| 1195 | THIEL SL | 2020 | 10.4414/smw.2020.20361 | 0 | 0 | 1 | 0 |
| 1199 | MAO Y | 2021 | 10.3389/fendo.2020.593179 | 0 | 0 | 1 | 0 |
| 1201 | SCHIAFFINI R | 2020 | 10.1016/j.diabres.2020.108302 | 0 | 0 | 1 | 0 |
| 1202 | MOLINA-ITURRITZA E | 2020 | 10.1097/QAD.0000000000002608 | 0 | 0 | 1 | 0 |
| 1203 | MOTTA JC | 2020 | 10.7705/biomedica.5764 | 0 | 0 | 1 | 0 |
| 1205 | GOICOECHEA M | 2020 | 10.1016/j.kint.2020.04.031 | 0 | 0 | 1 | 0 |
| 1208 | HIRSCH J | 2020 | 10.1016/j.kint.2020.05.006 | 0 | 0 | 1 | 0 |
| 1210 | YASUKAWA K | 2020 | 10.4269/ajtmh.20-0280 | 0 | 0 | 1 | 0 |
| 1211 | DAI M | 2020 | 10.7150/ijms.51159 | 0 | 0 | 1 | 0 |
| 1212 | NOTZ Q | 2020 | 10.3389/fimmu.2020.581338 | 0 | 0 | 1 | 0 |
| 1213 | PIZZINI A | 2020 | 10.3390/nu12092775 | 0 | 0 | 1 | 0 |
| 1214 | ARRAMBIDE G | 2021 | 10.1212/NXI.0000000000001024 | 0 | 0 | 1 | 0 |
| 1216 | BELLAN M | 2021 | 10.1155/2021/8863053 | 0 | 0 | 1 | 0 |
| 1218 | WANG J | 2020 | 10.1177/0300060520955037 | 0 | 0 | 1 | 0 |
| 1220 | COJUTTI PG | 2020 | 10.1007/s40262-020-00933-8 | 0 | 0 | 1 | 0 |
| 1225 | DE LORENZO R | 2020 | 10.1371/journal.pone.0239570 | 0 | 0 | 1 | 0 |
| 1229 | TRIFAN G | 2020 | 10.1016/j.jstrokecerebrovasdis.2020.105314 | 0 | 0 | 1 | 0 |
| 1232 | LUZ ROMERO RM | 2021 | 10.1016/j.medcli.2021.11.016 | 0 | 0 | 1 | 0 |
| 1233 | SIVAYOHAM N | 2021 | 10.1080/07853890.2021.1992495 | 0 | 0 | 1 | 0 |
| 1236 | PARANJAPE N | 2021 | 10.1097/IPC.0000000000001023 | 0 | 0 | 1 | 0 |
| 1238 | RAUCHMAN SH | 2021 | 10.3390/jcm11010070 | 0 | 0 | 1 | 0 |
| 1241 | LUZ ROMERO RM | 2022 | 10.1016/j.medcle.2021.11.004 | 0 | 0 | 1 | 0 |
| 1242 | SCHIAROLI E | 2021 | 10.4084/MJHID.2021.061 | 0 | 0 | 1 | 0 |
| 1243 | SANCHEZ-RICO M | 2022 | 10.1093/jtm/taab195 | 0 | 0 | 1 | 0 |
| 1244 | REY JR | 2021 | 10.1016/j.medcli.2021.07.003 | 0 | 0 | 1 | 0 |
| 1246 | HAROUN MW | 2021 | 10.7759/cureus.12552 | 0 | 0 | 1 | 0 |
| 1247 | LIU X | 2021 | 10.1016/j.medcle.2020.11.016 | 0 | 0 | 1 | 0 |
| 1251 | RUSSO V | 2020 | 10.3389/fmed.2020.569567 | 0 | 0 | 1 | 0 |
| 1253 | SINKELER FS | 2020 | 10.1007/s12471-020-01462-6 | 0 | 0 | 1 | 0 |
| 1254 | DE WATEVILLE A | 2021 | 10.1016/j.clnu.2021.05.024 | 0 | 0 | 1 | 0 |
| 1257 | BARBIERI L | 2021 | 10.3389/fcvm.2021.792804 | 0 | 0 | 1 | 0 |
| 1258 | MARIMUTHU A | 2021 | 10.4103/lungindia.lungindia_935_20 | 0 | 0 | 1 | 0 |
| 1259 | DESAI A | 2021 | 10.3390/jcm10040686 | 0 | 0 | 1 | 0 |
| 1260 | XU Y | 2020 | 10.3389/fmed.2020.576457 | 0 | 0 | 1 | 0 |
| 1261 | FABRE V | 2021 | 10.1017/ice.2021.175 | 0 | 0 | 1 | 0 |
| 1262 | IGARASHI Y | 2021 | 10.1272/jnms.JNMS.2022_89-210 | 0 | 0 | 1 | 0 |
| 1265 | JOSE J | 2021 | 10.1186/s12879-021-05771-y | 0 | 0 | 1 | 0 |
| 1266 | YAMANAKA S | 2022 | 10.3389/fmed.2022.846525 | 0 | 0 | 1 | 0 |
| 1267 | CERINO P | 2021 | 10.2144/fsoa-2021-0064 | 0 | 0 | 1 | 0 |
| 1269 | PARK HY | 2020 | 10.1016/j.lanwpc.2020.100061 | 0 | 0 | 1 | 0 |
| 1270 | FINK G | 2020 | 10.1136/bmjebm-2020-111549 | 0 | 0 | 1 | 0 |
| 1271 | BURABEE AS | 2021 | 10.1051/sicotj/2021001 | 0 | 0 | 1 | 0 |
| 1272 | MYSZENSKI A | 2022 | 10.1097/JAT.0000000000000163 | 0 | 0 | 1 | 0 |
| 1273 | MANSOUR A | 2020 | 10.17179/excli2020-2988 | 0 | 0 | 1 | 0 |
| 1274 | HAYEK ME | 2021 | 10.1016/j.mayocpiqo.2021.03.007 | 0 | 0 | 1 | 0 |
| 1275 | SILVA MG | 2022 | 10.1016/j.lfs.2022.120324 | 0 | 0 | 1 | 0 |
| 1276 | KUMAR N | 2021 | 10.4103/ija.ija_474_21 | 0 | 0 | 1 | 0 |
| 1277 | JAMIR L | 2021 | 10.7759/cureus.20394 | 0 | 0 | 1 | 0 |
| 1278 | GAYOSO CANTERO D | 2021 | 10.1016/j.nrl.2021.06.008 | 0 | 0 | 1 | 0 |
| 1279 | GOMEZ LLUCH MT | 2022 | 10.37201/req/122.2021 | 0 | 0 | 1 | 0 |
| 1280 | DECAVEL P | 2021 | 10.1159/000519226 | 0 | 0 | 1 | 0 |
| 1282 | HAFEZ W | 2022 | 10.1016/j.ijid.2022.02.019 | 0 | 0 | 1 | 0 |
| 1285 | BOLOGNA C | 2021 | 10.3390/jcm10245857 | 0 | 0 | 1 | 0 |
| 1286 | ALJUHANI O | 2022 | 10.1016/j.jsps.2022.01.022 | 0 | 0 | 1 | 0 |
| 1287 | GUISADO-VASCO P | 2020 | 10.1016/j.eclinm.2020.100591 | 0 | 0 | 1 | 0 |
| 1288 | OGUZHAN KUCUK A | 2022 | 10.4103/2452-2473.336106 | 0 | 0 | 1 | 0 |
| 1289 | BOUILLON R | 2021 | 10.1002/jbm4.10576 | 0 | 0 | 1 | 0 |
| 1290 | GUR E | 2022 | 10.1007/s10157-022-02180-6 | 0 | 0 | 1 | 0 |
| 1291 | XU Y | 2021 | 10.2147/IDR.S330743 | 0 | 0 | 1 | 0 |
| 1294 | LEE JY | 2021 | 10.1186/s40545-021-00370-3 | 0 | 0 | 1 | 0 |
| 1298 | PAWAR N | 2021 | 10.4103/ijn.IJN_460_20 | 0 | 0 | 0 | 1 |
| 1302 | RICH C | 2021 | 10.1093/cei/uxab024 | 0 | 0 | 1 | 0 |
| 1303 | CACHO J | 2021 | 10.1016/j.nefro.2021.05.005 | 0 | 0 | 1 | 0 |
| 1304 | RAJYALAKSHMI B | 2021 | 10.5005/jp-journals-10071-23765 | 0 | 0 | 1 | 0 |
| 1305 | KIM T | 2022 | 10.3390/jcm11051412 | 0 | 0 | 1 | 0 |
| 1307 | PANADERO C | 2020 | 10.4081/mrm.2020.693 | 0 | 0 | 1 | 0 |
| 1311 | BARMAN ROY D | 2021 | 10.7759/cureus.20072 | 0 | 0 | 1 | 0 |
| 1313 | POSTERARO B | 2021 | 10.3390/jcm10081752 | 0 | 0 | 1 | 0 |
| 1316 | LASBLEIZ A | 2020 | 10.3390/jcm9113726 | 0 | 0 | 1 | 0 |
| 1318 | MURALIDHAR REDDY Y | 2022 | 10.1016/j.mycmed.2022.101252 | 0 | 0 | 1 | 0 |
| 1319 | LUMLERTGUL N | 2021 | 10.1186/s13613-021-00914-5 | 0 | 0 | 1 | 0 |
| 1321 | ALAM MM | 2020 | 10.7759/cureus.9658 | 0 | 0 | 1 | 0 |
| 1322 | MASTROIANI A | 2020 | 10.1016/j.eclinm.2020.100410 | 0 | 0 | 1 | 0 |
| 1323 | TANG Y | 2020 | 10.3389/fmed.2020.615845 | 0 | 0 | 1 | 0 |
| 1324 | KUMAR R | 2020 | 10.4103/jfmpc.jfmpc_1198_20 | 0 | 0 | 1 | 0 |
| 1325 | BASTOLA A | 2021 | 10.3390/tropicalmed6030137 | 0 | 0 | 1 | 0 |
| 1326 | DE OLIVEIRA SM | 2022 | 10.1016/j.semarthrit.2022.151987 | 0 | 0 | 1 | 0 |
| 1327 | KANDINATA N | 2021 | *34901517 | 0 | 0 | 1 | 0 |
| 1328 | SOHAIB ASGHAR M | 2021 | 10.1080/20009666.2020.1835214 | 0 | 0 | 1 | 0 |
| 1330 | DI CASTELNUOVO A | 2021 | 10.3389/fmed.2021.639970 | 0 | 0 | 1 | 0 |
| 1331 | YAKUSHIJI Y | 2022 | 10.1111/jdi.13784 | 0 | 0 | 1 | 0 |
| 1332 | CHENG Y | 2021 | 10.1159/000512270 | 0 | 0 | 1 | 0 |
| 1335 | ZHOU H | 2020 | 10.21037/atm-20-2119a | 0 | 0 | 1 | 0 |
| 1337 | HERMANN J | 2020 | 10.3389/fmed.2020.599533 | 0 | 0 | 1 | 0 |
| 1339 | SUNDARAMURTHY R | 2021 | 10.7759/cureus.19791 | 0 | 0 | 1 | 0 |
| 1340 | BHANDARI S | 2021 | 10.5005/jp-journals-10071-23747 | 0 | 0 | 1 | 0 |
| 1341 | AL-QAANEH AM | 2022 | 10.3390/pharmaceutics14030624 | 0 | 0 | 1 | 0 |
| 1343 | WU F | 2020 | 10.21037/jtd-20-1914 | 0 | 0 | 1 | 0 |
| 1346 | BIRAN N | 2020 | 10.1016/S2665-9913(20)30277-0 | 0 | 0 | 1 | 0 |
| 1347 | SURANA PD | 2022 | 10.4103/jfmpc.jfmpc_817_21 | 0 | 0 | 1 | 0 |
| 1348 | PASCALE R | 2021 | 10.1093/jacamr/dlab174 | 0 | 0 | 1 | 0 |
| 1350 | ALSHEHAIL B | 2022 | 10.1016/j.jsps.2022.01.021 | 0 | 0 | 1 | 0 |
| 1351 | GUO H | 2020 | 10.3389/fmed.2020.572989 | 0 | 0 | 1 | 0 |
| 1352 | CHOTALIA M | 2022 | 10.1111/anae.15700 | 0 | 0 | 1 | 0 |
| 1356 | SANTOS JLF | 2021 | 10.3390/jcm10235599 | 0 | 0 | 1 | 0 |
| 1357 | LIU Z | 2021 | 10.1155/2021/4303380 | 0 | 0 | 1 | 0 |
| 1358 | SZAKMANY T | 2021 | 10.3390/jcm10153290 | 0 | 0 | 1 | 0 |
| 1359 | USSAID A | 2020 | 10.7759/cureus.12039 | 0 | 0 | 1 | 0 |
| 1360 | WU F | 2021 | 10.3389/fmed.2021.762740 | 0 | 0 | 1 | 0 |
| 1361 | TOMACRUZ ID | 2021 | 10.2147/IJNRD.S287455 | 0 | 0 | 1 | 0 |
| 1362 | BALAZ D | 2021 | 10.3390/jcm10194610 | 0 | 0 | 1 | 0 |
| 1363 | KASUGAI D | 2021 | 10.3390/jcm10112513 | 0 | 0 | 1 | 0 |
| 1366 | JOHNSON SW | 2022 | 10.1097/CCE.0000000000000638 | 0 | 0 | 1 | 0 |
| 1367 | KOCHNEVA OL | 2022 | 10.1080/10428194.2022.2034157 | 0 | 0 | 1 | 0 |
| 1368 | AREVALOS V | 2021 | 10.3390/jcm10102096 | 0 | 0 | 1 | 0 |
| 1369 | CITU C | 2022 | 10.3390/diagnostics12030703 | 0 | 0 | 1 | 0 |
| 1370 | RAI DK | 2021 | 10.5005/jp-journals-10071-24048 | 0 | 0 | 1 | 0 |
| 1374 | GERI G | 2021 | 10.1186/s13613-021-00875-9 | 0 | 0 | 1 | 0 |
| 1384 | PINANA JL | 2020 | 10.1186/s40164-020-00177-z | 0 | 0 | 1 | 0 |
| 1387 | MCPADDEN J | 2020 | 10.1101/2020.07.19.20157305 | 0 | 0 | 1 | 0 |
| 1388 | BENETT TD | 2021 | 10.1101/2021.01.12.21249511 | 0 | 0 | 1 | 0 |
| 1 | GARCIA-VIDAL C | 2021 | 10.1016/j.cmi.2020.07.041 | 0 | 0 | 0 | 1 |
| 6 | DE SMET R | 2020 | 10.1016/j.jamda.2020.06.008 | 0 | 0 | 0 | 1 |
| 7 | XIONG S | 2020 | 10.1186/s12879-020-05452-2 | 0 | 0 | 0 | 1 |
| 8 | BARTOLETTI M | 2021 | 10.1016/j.cmi.2020.09.014 | 0 | 0 | 0 | 1 |
| 14 | YAO JS | 2021 | 10.1016/j.chest.2020.06.082 | 0 | 0 | 0 | 1 |
| 19 | ARSHAD S | 2020 | 10.1016/j.ijid.2020.06.099 | 0 | 0 | 0 | 1 |
| 26 | GUGLIELMETTI L | 2021 | 10.1038/s41598-021-00243-4 | 0 | 0 | 0 | 1 |
| 31 | DUBERNET A | 2020 | 10.1016/j.jgar.2020.08.001 | 0 | 0 | 0 | 1 |
| 40 | OZTURK S | 2020 | 10.1093/ndt/gfaa271 | 0 | 0 | 0 | 1 |
| 42 | CHOPRA V | 2021 | 10.1136/bmjopen-2020-044921 | 0 | 0 | 0 | 1 |
| 46 | KIM J | 2021 | 10.1186/s12879-021-06588-5 | 0 | 0 | 0 | 1 |
| 47 | ZHAO X | 2021 | 10.1002/jpen.1953 | 0 | 0 | 0 | 1 |
| 57 | RUIZ-QUINONEZ JA | 2021 | 10.1371/journal.pone.0245394 | 0 | 0 | 0 | 1 |
| 62 | ANGELIDI AM | 2021 | 10.1016/j.mayocp.2021.01.001 | 0 | 0 | 0 | 1 |
| 76 | CHEN K | 2021 | 10.1038/s41598-021-94570-1 | 0 | 0 | 0 | 1 |
| 77 | XU K | 2020 | 10.1093/cid/ciaa351 | 0 | 0 | 0 | 1 |
| 91 | BETTI M | 2021 | 10.1371/journal.pone.0248829 | 0 | 0 | 0 | 1 |
| 92 | Wen XS | 2021 | 10.1186/s12879-020-05741-w | 0 | 0 | 0 | 1 |
| 93 | Liu Q | 2021 | 10.1097/MD.0000000000024544 | 0 | 0 | 0 | 1 |
| 96 | WANG Y | 2021 | 10.12659/MSM.926751 | 0 | 0 | 0 | 1 |
| 101 | FRIED MW | 2021 | 10.1093/cid/ciaa1268 | 0 | 0 | 0 | 1 |
| 108 | HE XL | 2021 | 10.1007/s11596-021-2434-y | 0 | 0 | 0 | 1 |
| 114 | TEJPAL A | 2021 | 10.1089/jwh.2020.8974 | 0 | 0 | 0 | 1 |
| 118 | XIA G | 2021 | 10.18632/aging.203503 | 0 | 0 | 0 | 1 |
| 121 | SAIB A | 2021 | 10.1371/journal.pone.0252388 | 0 | 0 | 0 | 1 |
| 129 | REGINA J | 2020 | 10.1371/journal.pone.0240781 | 0 | 0 | 0 | 1 |
| 158 | LOHIA P | 2021 | 10.1186/s12933-021-01336-0 | 0 | 0 | 0 | 1 |
| 164 | BEST JH | 2021 | 10.1002/jmv.27049 | 0 | 0 | 0 | 1 |
| 167 | SUARDI LR | 2020 | 10.1016/j.ijid.2020.09.012 | 0 | 0 | 0 | 1 |
| 184 | OKOH A | 2021 | 10.1002/jmv.26471 | 0 | 0 | 0 | 1 |
| 200 | FRONTERA JA | 2021 | 10.1007/s12028-021-01220-5 | 0 | 0 | 0 | 1 |
| 202 | ZHENG Y | 2021 | 10.1097/MD.0000000000024771 | 0 | 0 | 0 | 1 |
| 221 | VERNAZ N | 2020 | 10.4414/smw.2020.20446 | 0 | 0 | 0 | 1 |
| 222 | KUNAL S | 2020 | 10.1016/j.ihj.2020.10.005 | 0 | 0 | 0 | 1 |
| 231 | DU HW | 2021 | 10.1186/s12879-021-06970-3 | 0 | 0 | 0 | 1 |
| 242 | LOZANO-MONTOYA I | 2021 | 10.1007/s41999-021-00541-0 | 0 | 0 | 0 | 1 |
| 249 | YANG JY | 2021 | 10.1001/jamanetworkopen.2020.35699 | 0 | 0 | 0 | 1 |
| 253 | ZIELINSKA-TUREK J | 2021 | 10.5603/PJNNS.a2021.0011 | 0 | 0 | 0 | 1 |
| 254 | YAN Q | 2021 | 10.1093/gerona/glaa181 | 0 | 0 | 0 | 1 |
| 262 | PATEL AJ | 2021 | 10.1016/j.eprac.2021.07.008 | 0 | 0 | 0 | 1 |
| 270 | FOX T | 2021 | 10.1007/s00592-020-01592-8 | 0 | 0 | 0 | 1 |
| 271 | ELENI M | 2021 | 10.1080/20477724.2021.1893485 | 0 | 0 | 0 | 1 |
| 273 | TURGUTALP K | 2021 | 10.1186/s12882-021-02233-0 | 0 | 0 | 0 | 1 |
| 275 | PACCOUD O | 2021 | 10.1093/cid/ciaa791 | 0 | 0 | 0 | 1 |
| 278 | BARDAJI A | 2021 | 10.1016/j.rec.2020.08.027 | 0 | 0 | 0 | 1 |
| 284 | AWAD N | 2021 | 10.1093/ajhp/zxab056 | 0 | 0 | 0 | 1 |
| 297 | CROSSETTE-THAMBIAH C | 2021 | 10.1111/bjh.17579 | 0 | 0 | 0 | 1 |
| 300 | LEE HW | 2021 | 10.3904/kjim.2020.329 | 0 | 0 | 0 | 1 |
| 319 | ARIKAN H | 2021 | 10.1371/journal.pone.0256023 | 0 | 0 | 0 | 1 |
| 320 | PORTACCI A | 2021 | 10.1080/17476348.2021.1960824 | 0 | 0 | 0 | 1 |
| 331 | YEO I | 2021 | 10.1111/joim.13241 | 0 | 0 | 0 | 1 |
| 334 | MEKOLO D | 2021 | 10.11604/pamj.2021.38.246.28169 | 0 | 0 | 0 | 1 |
| 335 | GONG X | 2021 | 10.1186/s12879-021-06282-6 | 0 | 0 | 0 | 1 |
| 337 | SNIPELISKY D | 2020 | 10.14423/SMJ.0000000000001182 | 0 | 0 | 0 | 1 |
| 342 | LAURIOLA M | 2020 | 10.1111/cts.12860 | 0 | 0 | 0 | 1 |
| 355 | MUSSINI C | 2021 | 10.1016/j.cmi.2020.12.010 | 0 | 0 | 0 | 1 |
| 357 | SHU Z | 2020 | 10.1007/s11684-020-0803-8 | 0 | 0 | 0 | 1 |
| 370 | PIETRI L | 2021 | 10.1016/j.metabol.2021.154703 | 0 | 0 | 0 | 1 |
| 376 | CHANG MC | 2021 | 10.1097/MD.0000000000025917 | 0 | 0 | 0 | 1 |
| 382 | MONTREAL E | 2021 | 10.1002/jmv.26656 | 0 | 0 | 0 | 1 |
| 395 | RAMIREZ GA | 2021 | 10.1016/j.cmi.2021.05.023 | 0 | 0 | 0 | 1 |
| 396 | LANZA E | 2020 | 10.1007/s00330-020-07013-2 | 0 | 0 | 0 | 1 |
| 421 | CATTELAN AM | 2020 | 10.1186/s12879-020-05647-7 | 0 | 0 | 0 | 1 |
| 428 | STEFAN G | 2021 | 10.1080/0886022X.2020.1853571 | 0 | 0 | 0 | 1 |
| 434 | SOH TV | 2020 | *32918413 | 0 | 0 | 0 | 1 |
| 453 | KUNO T | 2022 | 10.1016/j.jjcc.2021.12.012 | 0 | 0 | 0 | 1 |
| 462 | LEI C | 2020 | 10.1016/j.jcv.2020.104661 | 0 | 0 | 0 | 1 |
| 471 | FREEMAN A | 2022 | 10.1016/j.jcv.2021.105031 | 0 | 0 | 0 | 1 |
| 491 | LLANERA DK | 2022 | 10.3389/fendo.2021.777130 | 0 | 0 | 0 | 1 |
| 498 | DUARTE-MILLAN MA | 2022 | 10.1002/jmv.27488 | 0 | 0 | 0 | 1 |
| 503 | BAHL A | 2021 | 10.1007/s11739-021-02655-6 | 0 | 0 | 0 | 1 |
| 510 | HUANG J | 2021 | 10.1186/s12890-021-01487-6 | 0 | 0 | 0 | 1 |
| 514 | CARDINAL-FERNANDEZ P | 2021 | 10.37201/req/050.2021 | 0 | 0 | 0 | 1 |
| 526 | AL MUTAIR A | 2020 | 10.1186/s40001-020-00462-x | 0 | 0 | 0 | 1 |
| 527 | CAPDEVILA-RENIU A | 2021 | 10.1097/MD.0000000000024750 | 0 | 0 | 0 | 1 |
| 529 | PEREZ-NIETO OR | 2022 | 10.1183/13993003.00265-2021 | 0 | 0 | 0 | 1 |
| 539 | PEREZ-DE-LLANO L | 2021 | 10.1371/journal.pone.0253465 | 0 | 0 | 0 | 1 |
| 542 | PEPE M | 2021 | 10.1007/s10238-021-00684-1 | 0 | 0 | 0 | 1 |
| 548 | FUSINA F | 2021 | 10.1002/cpt.2245 | 0 | 0 | 0 | 1 |
| 554 | AKHTAR H | 2021 | 10.2196/28594 | 0 | 0 | 0 | 1 |
| 555 | LI Y | 2020 | 10.1097/MD.0000000000023547 | 0 | 0 | 0 | 1 |
| 572 | LARA OD | 2022 | 10.1016/j.ygyno.2021.12.004 | 0 | 0 | 0 | 1 |
| 575 | WANG Z | 2020 | 10.1155/2020/2138387 | 0 | 0 | 0 | 1 |
| 576 | SONG J | 2020 | 10.12659/MSM.925047 | 0 | 0 | 0 | 1 |
| 583 | KUMAR G | 2022 | 10.1002/jmv.27357 | 0 | 0 | 0 | 1 |
| 598 | ZHANG Q | 2021 | 10.1016/j.jdiacomp.2020.107666 | 0 | 0 | 0 | 1 |
| 628 | HUR K | 2020 | 10.1177/0194599820929640 | 0 | 0 | 0 | 1 |
| 630 | ZHANG Q | 2021 | 10.1097/MD.0000000000025913 | 0 | 0 | 0 | 1 |
| 647 | BOTTIO T | 2021 | 10.1016/j.jchf.2020.10.009 | 0 | 0 | 0 | 1 |
| 649 | MILIC J | 2021 | 10.1089/AID.2020.0305 | 0 | 0 | 0 | 1 |
| 650 | VILLA L | 2021 | 10.1097/MD.0000000000024893 | 0 | 0 | 0 | 1 |
| 654 | YANG D | 2021 | 10.1002/clc.23628 | 0 | 0 | 0 | 1 |
| 661 | SHEN L | 2021 | 10.1007/s10557-020-07133-3 | 0 | 0 | 0 | 1 |
| 673 | RHODES NJ | 2021 | 10.1093/ajhp/zxaa426 | 0 | 0 | 0 | 1 |
| 674 | CHEN Q | 2020 | 10.1007/s15010-020-01432-5 | 0 | 0 | 0 | 1 |
| 678 | SUN L | 2020 | 10.1002/jmv.25966 | 0 | 0 | 0 | 1 |
| 684 | KEVORKIAN JP | 2021 | 10.1016/j.jinf.2020.08.045 | 0 | 0 | 0 | 1 |
| 702 | CHEN Q | 2021 | 10.1161/JAHA.120.018451 | 0 | 0 | 0 | 1 |
| 704 | PAFUNDI PC | 2021 | 10.1371/journal.pone.0256903 | 0 | 0 | 0 | 1 |
| 713 | RIVA G | 2021 | 10.1038/s41598-021-92236-6 | 0 | 0 | 0 | 1 |
| 724 | KHANUM I | 2021 | 10.4081/monaldi.2021.1561 | 0 | 0 | 0 | 1 |
| 728 | VAHEDI E | 2020 | 10.1007/s40199-020-00353-w | 0 | 0 | 0 | 1 |
| 741 | CUI J | 2021 | 10.1097/MD.0000000000027400 | 0 | 0 | 0 | 1 |
| 769 | ROSENBERG ES | 2020 | 10.1001/jama.2020.8630 | 0 | 0 | 0 | 1 |
| 775 | MATHER JF | 2020 | 10.14309/ajg.0000000000000832 | 0 | 0 | 0 | 1 |
| 794 | CHANGAL K | 2021 | 10.1186/s12872-021-01963-1 | 0 | 0 | 0 | 1 |
| 797 | DOUVILLE NJ | 2021 | 10.1016/j.bja.2020.11.034 | 0 | 0 | 0 | 1 |
| 801 | PONGPIRUL WA | 2020 | 10.1371/journal.pntd.0008806 | 0 | 0 | 0 | 1 |
| 804 | IP A | 2020 | 10.1371/journal.pone.0237693 | 0 | 0 | 0 | 1 |
| 833 | PELTZER B | 2020 | 10.1111/jce.14770 | 0 | 0 | 0 | 1 |
| 836 | ZHANG L | 2021 | 10.1016/j.phymed.2021.153531 | 0 | 0 | 0 | 1 |
| 840 | FAVA A | 2020 | 10.1111/ajt.16246 | 0 | 0 | 0 | 1 |
| 841 | WU C | 2020 | 10.1186/s13054-020-03340-4 | 0 | 0 | 0 | 1 |
| 848 | BROSETA JJ | 2021 | 10.1159/000510557 | 0 | 0 | 0 | 1 |
| 864 | LI M | 2021 | 10.1016/j.amjms.2020.11.005 | 0 | 0 | 0 | 1 |
| 871 | RUSSO E | 2021 | 10.1007/s40620-020-00875-1 | 0 | 0 | 0 | 1 |
| 873 | DI CASTELNUOVO A | 2021 | 10.1155/2021/5556207 | 0 | 0 | 0 | 1 |
| 887 | THOREAU B | 2021 | 10.3390/v13050758 | 0 | 0 | 0 | 1 |
| 895 | GUNER R | 2021 | 10.1016/j.jiph.2020.12.017 | 0 | 0 | 0 | 1 |
| 905 | ZHANG Y | 2020 | 10.1111/dom.14086 | 0 | 0 | 0 | 1 |
| 908 | FERGUSON J | 2020 | 10.3201/eid2608.201776 | 0 | 0 | 0 | 1 |
| 922 | GARCIA-CABRERA L | 2021 | 10.1186/s12877-021-02565-4 | 0 | 0 | 0 | 1 |
| 923 | MOUSSEAUX E | 2021 | 10.1016/j.diii.2021.06.007 | 0 | 0 | 0 | 1 |
| 924 | YAN Y | 2020 | 10.1136/bmjdrc-2020-001343 | 0 | 0 | 0 | 1 |
| 928 | CAILLARD S | 2020 | 10.1016/j.kint.2020.08.005 | 0 | 0 | 0 | 1 |
| 952 | SISO-ALMIRALL A | 2020 | 10.1371/journal.pone.0237960 | 0 | 0 | 0 | 1 |
| 955 | FALDINI GP | 2020 | 10.1016/j.diabres.2020.108374 | 0 | 0 | 0 | 1 |
| 957 | BERENGUER J | 2020 | 10.1016/j.cmi.2020.07.024 | 0 | 0 | 0 | 1 |
| 960 | XU B | 2020 | 10.1016/j.jinf.2020.04.012 | 0 | 0 | 0 | 1 |
| 961 | LIU J | 2020 | 10.1172/JCI140617 | 0 | 0 | 0 | 1 |
| 964 | WU MA | 2021 | 10.1186/s13054-021-03846-5 | 0 | 0 | 0 | 1 |
| 967 | MONTEIRO AC | 2020 | 10.1371/journal.pone.0238552 | 0 | 0 | 0 | 1 |
| 986 | LORE NI | 2021 | 10.1186/s10020-021-00390-4 | 0 | 0 | 0 | 1 |
| 990 | ADAMI G | 2021 | 10.4081/reumatismo.2020.1333 | 0 | 0 | 0 | 1 |
| 999 | WANG N | 2020 | 10.1016/j.chom.2020.07.005 | 0 | 0 | 0 | 1 |
| 1001 | LI HY | 2020 | 10.1097/MD.0000000000022847 | 0 | 0 | 0 | 1 |
| 1002 | CATTEAU L | 2020 | 10.1016/j.ijantimicag.2020.106144 | 0 | 0 | 0 | 1 |
| 1005 | SCUDIERO F | 2021 | 10.1016/j.thromres.2020.11.017 | 0 | 0 | 0 | 1 |
| 1007 | AOMAR-MILLAN IF | 2021 | 10.1007/s11739-020-02600-z | 0 | 0 | 0 | 1 |
| 1010 | GALVAN-ROMAN JM | 2021 | 10.1016/j.jaci.2020.09.018 | 0 | 0 | 0 | 1 |
| 1012 | PIAZZA G | 2020 | 10.1016/j.jacc.2020.08.070 | 0 | 0 | 0 | 1 |
| 1027 | LOARCE-MARTOS J | 2020 | 10.1007/s00296-020-04699-x | 0 | 0 | 0 | 1 |
| 1030 | MO Y | 2021 | 10.1002/jcph.1787 | 0 | 0 | 0 | 1 |
| 1032 | TZOUVELEKIS A | 2021 | 10.5603/ARM.a2021.0087 | 0 | 0 | 0 | 1 |
| 1034 | SAEED O | 2020 | 10.1161/JAHA.120.018475 | 0 | 0 | 0 | 1 |
| 1053 | LIU Z | 2020 | 10.3389/fendo.2020.00478 | 0 | 0 | 0 | 1 |
| 1057 | DU Y | 2020 | 10.1164/rccm.202003-0543OC | 0 | 0 | 0 | 1 |
| 1080 | WEIZMAN O | 2021 | 10.1016/j.acvd.2021.04.002 | 0 | 0 | 0 | 1 |
| 1085 | ESCALERA-ANTEZANAN JP | 2020 | 10.1016/j.tmaid.2020.101653 | 0 | 0 | 0 | 1 |
| 1100 | FAN L | 2021 | 10.1097/MD.0000000000023923 | 0 | 0 | 0 | 1 |
| 1107 | FERNANDINO BRUNO P | 2021 | 10.1159/000515128 | 0 | 0 | 0 | 1 |
| 1109 | MOEY MYY | 2020 | 10.1161/CIRCEP.120.009023 | 0 | 0 | 0 | 1 |
| 1118 | RAO X | 2020 | 10.1097/MD.0000000000022766 | 0 | 0 | 0 | 1 |
| 1129 | MILLACEF S | 2021 | 10.1371/journal.pone.0239389 | 0 | 0 | 0 | 1 |
| 1136 | PASSAMONTI F | 2020 | 10.1016/S2352-3026(20)30251-9 | 0 | 0 | 0 | 1 |
| 1137 | DU H | 2020 | 10.1186/s12931-020-01510-0 | 0 | 0 | 0 | 1 |
| 1140 | GUO T | 2020 | 10.1001/jamacardio.2020.1017 | 0 | 0 | 0 | 1 |
| 1152 | CHENG Y | 2020 | 10.2215/CJN.04650420 | 0 | 0 | 0 | 1 |
| 1164 | ZHANG H | 2020 | 10.1002/cncr.33042 | 0 | 0 | 0 | 1 |
| 1167 | MCPADDEN J | 2021 | 10.1371/journal.pone.0243291 | 0 | 0 | 0 | 1 |
| 1181 | ALVISET S | 2020 | 10.1371/journal.pone.0240645 | 0 | 0 | 0 | 1 |
| 1196 | HUANG L | 2020 | 10.1016/j.jcmg.2020.05.004 | 0 | 0 | 0 | 1 |
| 1207 | ZHAO Y | 2020 | 10.1186/s40249-020-00723-1 | 0 | 0 | 0 | 1 |
| 1217 | RUIZ-IRASTORZA G | 2020 | 10.1371/journal.pone.0239401 | 0 | 0 | 0 | 1 |
| 1219 | XIONG F | 2020 | 10.1681/ASN.2020030354 | 0 | 0 | 0 | 1 |
| 1228 | ZHENG J | 2021 | 10.1186/s12902-021-00896-2 | 0 | 0 | 0 | 1 |
| 1239 | PASCUAL PAREJA JF | 2020 | 10.1016/j.medcle.2020.11.006 | 0 | 0 | 0 | 1 |
| 1240 | CHEN SL | 2020 | 10.1093/ofid/ofaa432 | 0 | 0 | 0 | 1 |
| 1250 | LEO M | 2021 | 10.1016/j.dld.2021.12.014 | 0 | 0 | 0 | 1 |
| 1252 | BERTUZZI AF | 2020 | 10.3390/cancers12092352 | 0 | 0 | 0 | 1 |
| 1264 | CARDONA-PASCUAL I | 2021 | 10.1016/j.medcli.2021.03.005 | 0 | 0 | 0 | 1 |
| 1268 | SANCHEZ-RICO M | 2021 | 10.3390/jcm10245891 | 0 | 0 | 0 | 1 |
| 1296 | LOFTY SM | 2021 | 10.5152/TurkThoracJ.2021.20180 | 0 | 0 | 0 | 1 |
| 1306 | PAOLI D | 2022 | 10.1007/s11739-021-02891-w | 0 | 0 | 0 | 1 |
| 1309 | JIANG S | 2020 | 10.3389/fmed.2020.00347 | 0 | 0 | 0 | 1 |
| 1310 | JOSA-LAORDEN C | 2021 | 10.3390/jcm10050899 | 0 | 0 | 0 | 1 |
| 1312 | HUANG F | 2021 | 10.21037/atm-21-1561 | 0 | 0 | 0 | 1 |
| 1354 | ZHAO X | 2021 | 10.2147/IDR.S335868 | 0 | 0 | 0 | 1 |
