# Supplementary material for: COVID-19 treatment of hospital patients worldwide at the onset of the pandemic in 2020: a systematic review
Source: BMC Infect Dis. 2025 Dec 17;26:107. doi: 10.1186/s12879-025-12368-2 (PMC12822144; doi:10.1186/s12879-025-12368-2)
Supplement: Supplementary file 4 — Supplementary Material 4 [file 12879_2025_12368_MOESM4_ESM.zip › 12879_2025_12368_MOESM4_ESM/Search Pubmed 2022 03 28 retrospective observational study hospital treatment covid 1001-1200.pdf]

Skip to main page content

COVID-19 Information

Public health information (CDC)

Research information (NIH)

SARS-CoV-2 data (NCBI)

Prevention and treatment information (HHS)

Español

Close

Account

Logged in as:
username

- Dashboard
- Publications
- Account settings
- Log out

Access keys NCBI Homepage MyNCBI Homepage Main Content Main Navigation

Search Page

pubmed

Search: retrospective observation Search
Advanced Create alert Create RSS Clipboard
User Guide
Filters 0 Timeline
Sorted by: Best match
Sorted by: Best match Display options
Save Email
Send to

Save citations to file

Selection: All results on this page

Format: Summary (text)
Create file Cancel

# Email citations

Subject: retrospective observational study hospital treatm - PubMed
To: antoine.bosquet@lmr.apl
Selection: All results on this page
Format: Summary
MeSH and other data
Send email Cancel

Send citations to clipboard

Selection: All results on this page
Send Cancel

Add to Collections

Selection: All results on this page

- Create a new collection
- Add to an existing collection

Name your collection:
Name must be less than 100 characters
Choose a collection:
Unable to load your collection due to an error
Please try again
Add Cancel

Add to My Bibliography

Selection: All results on this page

- My Bibliography

Unable to load your delegates due to an error
Please try again
Add Cancel

Create a file for external citation management software

Selection: All results on this page
Create file Cancel

Your saved search

Name of saved search: retrospective observation

Search terms: retrospective observational study

Test search terms

Would you like email updates of new search results?

Saved Search Alert Radio Buttons

- Yes
- No

Email: antoine.bosquet@lmr.aphp.fr (change)

Frequency: Monthly

Which day? The first Sunday

Which day? Sunday

Report format: Summary

Send at most: 5 items

Send even when there aren't any new results

Optional text in email:

Save Cancel

Your RSS Feed

Name of RSS Feed: retrospective observation

Number of items displayed: 15

Create RSS Cancel

RSS Link Your RSS Feed Link Copy

My NCBI Filters

- All (1,388)
- Assistance Publique Hopitaux de Paris (0)
- clinical trial (17)
- Review (1)

Show Fewer

Results by year Expand/collapse timeline

Reset

Table representation of search results timeline featuring number of search results per year.

Year	Number of Results
2020	548
2021	893
2022	147

Text availability

- Abstract
- Free full text
- Full text

Article attribute

- Associated data

Article type

- Books and Documents
- Clinical Trial
- Meta-Analysis
- Randomized Controlled Trial
- Review
- Systematic Review

Publication date

- 1 year
- 5 years
- 10 years
- Custom Range

Additional filters Reset all filters

Search Results

clear all

1,388 results

first first previous previous

Page
6
of 7

next next last last

Use COVID-19 filters from PubMed Clinical Queries to refine your search

- Treatment
- Mechanism
- Transmission
- More filters

See more SARS-CoV-2 literature, sequence, and clinical content from NCBI

Results by year Expand/collapse timeline

Reset

Filters applied: . *Clear all* Select search result to email or save

Page 6

☐ 1,001

Observational Study

Medicine (Baltimore)

. 2020 Oct 30;99(44):e22847.

doi: 10.1097/MD.0000000000022847.

Clinical analysis of 132 cases COVID-19 from Wuhan

Hai-Yan Li [1], Jin-Wei Wang [2], Li-Wei Xu [3], Xu-Ling Zhao [1], Jia-Xi Feng [1], You-Zu Xu [1]

Affiliations Expand

Affiliations

- [1] Department of Respiratory Medicine, TaiZhou Hospital of Zhejiang Province, Shaoxing University, Linhai, Zhejiang, PR China.
- [2] Functional Inspection Section, Taizhou Hospital of Zhejiang Province affiliated to Wenzhou Medical University.
- [3] Endocrine Department, Taizhou Hospital of Zhejiang Province affiliated to Wenzhou Medical University.

- PMID: **33126325**
- PMCID: PMC7598820
- DOI: 10.1097/MD.0000000000022847

Free PMC article

Observational Study

Clinical analysis of 132 cases COVID-19 from Wuhan

Hai-Yan Li et al. Medicine (Baltimore). 2020.
Free PMC article
Show details
Medicine (Baltimore)
. 2020 Oct 30;99(44):e22847.
doi: 10.1097/MD.0000000000022847.

Authors

Hai-Yan Li [1], Jin-Wei Wang [2], Li-Wei Xu [3], Xu-Ling Zhao [1], Jia-Xi Feng [1], You-Zu Xu [1]

Affiliations

- [1] Department of Respiratory Medicine, TaiZhou Hospital of Zhejiang Province, Shaoxing University, Linhai, Zhejiang, PR China.
- [2] Functional Inspection Section, Taizhou Hospital of Zhejiang Province affiliated to Wenzhou Medical University.
- [3] Endocrine Department, Taizhou Hospital of Zhejiang Province affiliated to Wenzhou Medical University.

- PMID: **33126325**
- PMCID: PMC7598820
- DOI: 10.1097/MD.0000000000022847

Abstract

Numerous cases of pneumonia from a novel coronavirus (SARS-CoV-2) emerged in Wuhan, China during December 2019.We determined the correlations of patient parameters with disease severity in patients with COVID-19.A total of 132 patients from Wuhan Fourth Hospital who had COVID-19 from February 1 to February 29 in 2020 were retrospectively analyzed.Ninety patients had mild disease, 32 had severe disease, and 10 had critical disease. The severe/critical group was older ($P < .05$), had a higher proportion of males ($P < .05$), and had a greater mortality rate (0% vs 61.9%, $P < .05$). The main symptoms were fever (n = 112, 84.8%) and cough (n = 96, 72.7%). Patients were treated with antiviral agents (n = 94, 71.2%), antibiotics (n = 92, 69.7%), glucocorticoids (n = 46, 34.8%), intravenous immunoglobulin (n = 38, 27.3%), and/or traditional Chinese medicine (n = 40, 30.3%). Patients in the severe/critical group received mechanical ventilation (n = 22, 16.7%) or high-flow nasal can-nula oxygen therapy (n = 6, 4.5%). Chest computed tomography (CT) indicated bilateral pneumonia in all patients. Relative to the mild group, the severe/critical group had higher levels of leukocytes, C-reactive protein (CRP), procalcitonin (PCT), D-dimer, B-type natriuretic peptide (BNP), liver enzymes, and myocardial enzymes ($P < .05$), and decreased levels of lymphocytes and blood oxygen partial pressure ($P < .05$).The main clinical symptoms of patients from Wuhan who had COVID-19 were fever and cough. Patients with severe/critical disease were more likely to be male and elderly. Disease severity correlated with increased leukocytes, CRP, PCT, BNP, D-dimer, liver enzymes, and myocardial enzymes, and with decreased lymphocytes and blood oxygen partial pressure.

Conflict of interest statement

The authors declare that they have no conflict of interest.

- 16 references

Supplementary info

Publication types, MeSH terms Expand

Publication types

- Observational Study

MeSH terms

- Adult
- Aged
- Betacoronavirus / isolation & purification
- COVID-19
- China / epidemiology
- Coronavirus Infections / blood
- Coronavirus Infections / epidemiology*
- Coronavirus Infections / therapy
- Female
- Humans
- Male
- Middle Aged
- Pandemics
- Pneumonia, Viral / blood
- Pneumonia, Viral / epidemiology*
- Pneumonia, Viral / therapy
- Retrospective Studies
- SARS-CoV-2

Full text links

Wolters Kluwer Wolters Kluwer Free PMC article

Proceed to details

Cite

Share

☐ 1,002

Observational Study

Int J Antimicrob Agents

. 2020 Oct;56(4):106144.
doi: 10.1016/j.ijantimicag.2020.106144. Epub 2020 Aug 24.

Low-dose hydroxychloroquine therapy and mortality in hospitalised patients with COVID-19: a nationwide observational study of 8075 participants

Lucy Catteau [1], Nicolas Dauby [2], Marion Montourcy [1], Emmanuel Bottieau [3], Joris Hautekiet [4], Els Goetghebeur [5], Sabrina van Ierssel [6], Els Duysburgh [1], Herman Van Oyen [7], Chloé Wyndham-Thomas [1], Dominique Van Beckhoven [1], Belgian Collaborative Group on COVID-19 Hospital Surveillance
Collaborators, Affiliations Expand

Collaborators

- **Belgian Collaborative Group on COVID-19 Hospital Surveillance**: Kristof Bafort, Leïla Belkhir, Nathalie Bossuyt, Philippe Caprasse, Vincent Colombie, Paul De Munter, Jessika Deblonde, Didier Delmarcelle, Mélanie Delvallee, Rémy Demeester, Thierry Dugernier, Xavier Holemans, Benjamin Kerzmann, Pierre Yves Machurot, Philippe Minette, Jean-Marc Minon, Saphia Mokrane, Catherine Nachtergal, Séverine Noirhomme, Denis Piérard, Camelia Rossi, Carole Schirvel, Erica Sermijn, Frank Staelens, Filip Triest, Nina Van Goethem, Jens Van Praet, Anke Vanhoenacker, Roeland Verstraete, Elise Willems

Affiliations

- [1] Department of Epidemiology and public health, Sciensano, Brussels, Belgium.
- [2] Department of Infectious Diseases, CHU Saint-Pierre, Brussels, Belgium; Institute for Medical Immunology, Université Libre de Bruxelles (ULB), Brussels, Belgium; Environmental Health Research Centre, Public Health School, Université Libre de Bruxelles (ULB), Brussels, Belgium. Electronic address: Nicolas.dauby@ulb.ac.be.
- [3] Department of Clinical Sciences, Institute of Tropical Medicine, Antwerp, Belgium.
- [4] Department of Epidemiology and public health, Sciensano, Brussels, Belgium; Department of Applied Mathematics, Computer Science and Statistics, Ghent University, Ghent, Belgium.
- [5] Department of Applied Mathematics, Computer Science and Statistics, Ghent University, Ghent, Belgium.
- [6] Department of General Internal Medicine, Infectious Diseases and Tropical Medicine, University Hospital Antwerp (UZA), Edegem, Belgium.
- [7] Department of Epidemiology and public health, Sciensano, Brussels, Belgium; Public Health and Primary Care, Gent University, Gent, Belgium.

- PMID: **32853673**
- PMCID: PMC7444610
- DOI: 10.1016/j.ijantimicag.2020.106144

Free PMC article
Observational Study

Low-dose hydroxychloroquine therapy and mortality in hospitalised patients with COVID-19: a nationwide observational study of 8075 participants

Lucy Catteau et al. Int J Antimicrob Agents. 2020 Oct.
Free PMC article
Show details
Int J Antimicrob Agents
. 2020 Oct;56(4):106144.
doi: 10.1016/j.ijantimicag.2020.106144. Epub 2020 Aug 24.

Authors

Lucy Catteau [1], Nicolas Dauby [2], Marion Montourcy [1], Emmanuel Bottieau [3], Joris Hautekiet [4], Els Goetghebeur [5], Sabrina van Ierssel [6], Els Duysburgh [1], Herman Van Oyen [7], Chloé Wyndham-Thomas [1], Dominique Van Beckhoven [1], Belgian Collaborative Group on COVID-19 Hospital Surveillance

Collaborators

- **Belgian Collaborative Group on COVID-19 Hospital Surveillance**: Kristof Bafort, Leïla Belkhir, Nathalie Bossuyt, Philippe Caprasse, Vincent Colombie, Paul De Munter, Jessika Deblonde, Didier Delmarcelle, Mélanie Delvallee, Rémy Demeester, Thierry Dugernier, Xavier Holemans, Benjamin Kerzmann, Pierre Yves Machurot, Philippe Minette, Jean-Marc Minon, Saphia Mokrane, Catherine Nachtergal, Séverine Noirhomme, Denis Piérard, Camelia Rossi, Carole Schirvel, Erica Sermijn, Frank Staelens, Filip Triest, Nina Van Goethem, Jens Van Praet, Anke Vanhoenacker, Roeland Verstraete, Elise Willems

Affiliations

- [1] Department of Epidemiology and public health, Sciensano, Brussels, Belgium.
- [2] Department of Infectious Diseases, CHU Saint-Pierre, Brussels, Belgium; Institute for Medical Immunology, Université Libre de Bruxelles (ULB), Brussels, Belgium; Environmental Health Research Centre, Public Health School, Université Libre de Bruxelles (ULB), Brussels, Belgium. Electronic address: Nicolas.dauby@ulb.ac.be.
- [3] Department of Clinical Sciences, Institute of Tropical Medicine, Antwerp, Belgium.
- [4] Department of Epidemiology and public health, Sciensano, Brussels, Belgium; Department of Applied Mathematics, Computer Science and Statistics, Ghent University, Ghent, Belgium.
- [5] Department of Applied Mathematics, Computer Science and Statistics, Ghent University, Ghent, Belgium.
- [6] Department of General Internal Medicine, Infectious Diseases and Tropical Medicine, University Hospital Antwerp (UZA), Edegem, Belgium.

- [7] Department of Epidemiology and public health, Sciensano, Brussels, Belgium; Public Health and Primary Care, Gent University, Gent, Belgium.

- PMID: **32853673**
- PMCID: PMC7444610
- DOI: 10.1016/j.ijantimicag.2020.106144

Abstract

Hydroxychloroquine (HCQ) has been largely used and investigated as therapy for COVID-19 across various settings at a total dose usually ranging from 2400 mg to 9600 mg. In Belgium, off-label use of low-dose HCQ (total 2400 mg over 5 days) was recommended for hospitalised patients with COVID-19. We conducted a retrospective analysis of in-hospital mortality in the Belgian national COVID-19 hospital surveillance data. Patients treated either with HCQ monotherapy and supportive care (HCQ group) were compared with patients treated with supportive care only (no-HCQ group) using a competing risks proportional hazards regression with discharge alive as competing risk, adjusted for demographic and clinical features with robust standard errors. Of 8075 patients with complete discharge data on 24 May 2020 and diagnosed before 1 May 2020, 4542 received HCQ in monotherapy and 3533 were in the no-HCQ group. Death was reported in 804/4542 (17.7%) and 957/3533 (27.1%), respectively. In the multivariable analysis, mortality was lower in the HCQ group compared with the no-HCQ group [adjusted hazard ratio (aHR) = 0.684, 95% confidence interval (CI) 0.617-0.758]. Compared with the no-HCQ group, mortality in the HCQ group was reduced both in patients diagnosed ≤5 days (n = 3975) and >5 days (n = 3487) after symptom onset [aHR = 0.701 (95% CI 0.617-0.796) and aHR = 0.647 (95% CI 0.525-0.797), respectively]. Compared with supportive care only, low-dose HCQ monotherapy was independently associated with lower mortality in hospitalised patients with COVID-19 diagnosed and treated early or later after symptom onset.

Keywords: COVID-19; Hydroxychloroquine; Mortality; Observational study; SARS-CoV-2.

Copyright © 2020 Elsevier Ltd and International Society of Antimicrobial Chemotherapy. All rights reserved.

Comment in

- Perceived efficacy of hydroxychloroquine in observational studies: Results of the confounding effect of "goals of care".
  Tleyjeh PIM, Tlayjeh H. Tleyjeh PIM, et al. Int J Antimicrob Agents. 2021 Apr;57(4):106308. doi: 10.1016/j.ijantimicag.2021.106308. Epub 2021 Feb 17. Int J Antimicrob Agents. 2021. PMID: 33609717 Free PMC article. No abstract available.

- 45 references
- 3 figures

Supplementary info

Publication types, MeSH terms, Substances Expand

Publication types

- Observational Study

MeSH terms

- Adolescent
- Adult
- Aged
- Aged, 80 and over
- Antimalarials / therapeutic use*
- Betacoronavirus / drug effects*
- Betacoronavirus / pathogenicity
- C-Reactive Protein / metabolism
- COVID-19
- Coronavirus Infections / diagnostic imaging
- Coronavirus Infections / drug therapy*
- Coronavirus Infections / mortality
- Coronavirus Infections / pathology
- Disease Progression
- Drug Dosage Calculations
- Drug Repositioning
- Female
- Hospital Mortality
- Humans
- Hydroxychloroquine / therapeutic use*
- Intensive Care Units
- Male
- Middle Aged
- Pandemics
- Patient Safety
- Pneumonia, Viral / diagnostic imaging
- Pneumonia, Viral / drug therapy*
- Pneumonia, Viral / mortality
- Pneumonia, Viral / pathology
- Prognosis
- Proportional Hazards Models
- Retrospective Studies
- SARS-CoV-2
- T-Lymphocytes / pathology
- T-Lymphocytes / virology
- Tomography, X-Ray Computed
- Treatment Outcome

Substances

- Antimalarials
- Hydroxychloroquine
- C-Reactive Protein

Full text links

ELSEVIER FULL-TEXT ARTICLE Elsevier Science Free PMC article
Proceed to details
Cite
Share
1,003
Observational Study
J Allergy Clin Immunol Pract
. 2020 Sep;8(8):2575-2581.e2.
doi: 10.1016/j.jaip.2020.06.013. Epub 2020 Jun 19.

Prompt Predicting of Early Clinical Deterioration of Moderate-to-Severe COVID-19 Patients: Usefulness of a Combined Score Using IL-6 in a Preliminary Study

Alessandra Vultaggio [1], Emanuele Vivarelli [1], Gianni Virgili [2], Ersilia Lucenteforte [3], Alessandro Bartoloni [4], Carlo Nozzoli [5], Alessandro Morettini [6], Andrea Berni [7], Danilo Malandrino [7], Oliviero Rossi [1], Francesca Nencini [1], Filippo Pieralli [8], Adriano Peris [9], Filippo Lagi [4], Giulia Scocchera [6], Michele Spinicci [4], Michele Trotta [4], Marcello Mazzetti [4], Paola Parronchi [10], Lorenzo Cosmi [10], Francesco Liotta [10], Paolo Fontanari [11], Alessio Mazzoni [12], Lorenzo Salvati [10], Enrico Maggi [13], Francesco Annunziato [12], Fabio Almerigogna [1], Andrea Matucci [14]
Affiliations Expand

Affiliations

- [1] Immunoallergology Unit, Careggi University Hospital, Florence, Italy.
- [2] Department of Ophthalmology, Careggi University Hospital, Florence, Italy.
- [3] Department of Clinical and Experimental Medicine, University of Pisa, Pisa, Italy.
- [4] Department of Experimental and Clinical Medicine, Infectious and Tropical Diseases Unit, Careggi University Hospital, Florence, Italy.
- [5] Internal Medicine Unit 1, Careggi University Hospital, Florence, Italy.
- [6] Internal Medicine Unit 2, Careggi University Hospital, Florence, Italy.
- [7] Internal Medicine Unit 3, Careggi University Hospital, Florence, Italy.
- [8] Intermediate Care Unit, Careggi University Hospital, Florence, Italy.
- [9] Intensive Care Unit and Regional ECMO Referral Centre, Careggi University Hospital, Florence, Italy.

- [10] Department of Experimental and Clinical Medicine, Immunology and Cell Therapy Unit, Careggi University Hospital, Florence, Italy.
- [11] Cardiac Anesthesia and Intensive Care Unit, Careggi University Hospital, Florence, Italy.
- [12] Department of Experimental and Clinical Medicine, Flow Cytometric Diagnostic Centre and Immunotherapy (CDCI), Careggi University Hospital, Florence, Italy.
- [13] Translational Immunology Unit, Immunology Area, Pediatric Hospital Bambino Gesù, IRCCS, Rome, Italy.
- [14] Immunoallergology Unit, Careggi University Hospital, Florence, Italy. Electronic address: andrea.matucci@unifi.it.

- PMID: **32565226**
- PMCID: PMC7303032
- DOI: 10.1016/j.jaip.2020.06.013

Free PMC article
Observational Study

Prompt Predicting of Early Clinical Deterioration of Moderate-to-Severe COVID-19 Patients: Usefulness of a Combined Score Using IL-6 in a Preliminary Study

Alessandra Vultaggio et al. J Allergy Clin Immunol Pract. 2020 Sep.
Free PMC article
Show details
J Allergy Clin Immunol Pract
. 2020 Sep;8(8):2575-2581.e2.
doi: 10.1016/j.jaip.2020.06.013. Epub 2020 Jun 19.

Authors

Alessandra Vultaggio [1], Emanuele Vivarelli [1], Gianni Virgili [2], Ersilia Lucenteforte [3], Alessandro Bartoloni [4], Carlo Nozzoli [5], Alessandro Morettini [6], Andrea Berni [7], Danilo Malandrino [7], Oliviero Rossi [1], Francesca Nencini [1], Filippo Pieralli [8], Adriano Peris [9], Filippo Lagi [4], Giulia Scocchera [6], Michele Spinicci [4], Michele Trotta [4], Marcello Mazzetti [4], Paola Parronchi [10], Lorenzo Cosmi [10], Francesco Liotta [10], Paolo Fontanari [11], Alessio Mazzoni [12], Lorenzo Salvati [10], Enrico Maggi [13], Francesco Annunziato [12], Fabio Almerigogna [1], Andrea Matucci [14]

Affiliations

- [1] Immunoallergology Unit, Careggi University Hospital, Florence, Italy.
- [2] Department of Ophthalmology, Careggi University Hospital, Florence, Italy.
- [3] Department of Clinical and Experimental Medicine, University of Pisa, Pisa, Italy.

- [4] Department of Experimental and Clinical Medicine, Infectious and Tropical Diseases Unit, Careggi University Hospital, Florence, Italy.
- [5] Internal Medicine Unit 1, Careggi University Hospital, Florence, Italy.
- [6] Internal Medicine Unit 2, Careggi University Hospital, Florence, Italy.
- [7] Internal Medicine Unit 3, Careggi University Hospital, Florence, Italy.
- [8] Intermediate Care Unit, Careggi University Hospital, Florence, Italy.
- [9] Intensive Care Unit and Regional ECMO Referral Centre, Careggi University Hospital, Florence, Italy.
- [10] Department of Experimental and Clinical Medicine, Immunology and Cell Therapy Unit, Careggi University Hospital, Florence, Italy.
- [11] Cardiac Anesthesia and Intensive Care Unit, Careggi University Hospital, Florence, Italy.
- [12] Department of Experimental and Clinical Medicine, Flow Cytometric Diagnostic Centre and Immunotherapy (CDCI), Careggi University Hospital, Florence, Italy.
- [13] Translational Immunology Unit, Immunology Area, Pediatric Hospital Bambino Gesù, IRCCS, Rome, Italy.
- [14] Immunoallergology Unit, Careggi University Hospital, Florence, Italy. Electronic address: andrea.matucci@unifi.it.

- PMID: **32565226**
- PMCID: PMC7303032
- DOI: 10.1016/j.jaip.2020.06.013

Abstract

Background: The early identification of patients at risk of clinical deterioration is of interest considering the timeline of COVID-19 after the onset of symptoms.

Objective: The aim of our study was to evaluate the usefulness of testing serum IL-6 and other serological and clinical biomarkers, to predict a short-term negative clinical course of patients with noncritical COVID-19.

Methods: A total of 208 patients with noncritical COVID-19 pneumonia at admission were consecutively enrolled. Clinical and laboratory findings obtained on admission were analyzed by using survival analysis and stepwise logistic regression for variable selection. Three-day worsening as outcome in a logistic model to generate a prognostic score was used.

Results: Clinical worsening occurred in 63 patients (16 = died; 39 = transferred to intensive care unit; 8 worsening of respiratory failure). Forty-five of them worsened within 3 days after admission. The risk of clinical worsening was progressively enhanced along with increasing quartiles of IL-6 levels. Multivariate analysis showed that IL-6 ($P = .005$), C-reactive protein (CRP) ($P = .003$), and SaO_2/FiO_2 ($P = .014$) were the best predictors for clinical deterioration in the first 3 days after admission. The combined score yielded an area under the curve = 0.88 (95% confidence interval: 0.83-0.93). A nomogram predicting the probability of 3-day worsening was generated. The score also showed good performance for 7-day and 14- or 21-day worsening and in predicting death occurring during all the follow-up.

Conclusions: Combining IL-6, CRP, and SaO_2/FiO_2 in a score may help clinicians to identify on admission those patients with COVID-19 who are at high risk for a further 3-day clinical deterioration.

Keywords: C-reactive protein; COVID-19; IL-6; Risk factors; SARS-CoV-2.

Copyright © 2020 American Academy of Allergy, Asthma & Immunology. Published by Elsevier Inc. All rights reserved.

Comment in

- Predicting Severe Outcomes in COVID-19.
  Lipworth B, Chan R, Kuo CR. Lipworth B, et al. J Allergy Clin Immunol Pract. 2020 Sep;8 (8):2582-2584. doi: 10.1016/j.jaip.2020.06.039. Epub 2020 Jun 29. J Allergy Clin Immunol Pract. 2020. PMID: 32615256 Free PMC article. No abstract available.

- 27 references
- 4 figures

Supplementary info

Publication types, MeSH terms, Substances Expand

Publication types

- Observational Study

MeSH terms

- Adult
- Aged
- Aged, 80 and over
- Betacoronavirus
- Biomarkers
- C-Reactive Protein / analysis
- COVID-19
- Clinical Deterioration*
- Comorbidity
- Coronavirus Infections / blood
- Coronavirus Infections / epidemiology*
- Coronavirus Infections / mortality
- Coronavirus Infections / physiopathology*
- Female
- Humans
- Interleukin-6 / blood*
- Kaplan-Meier Estimate
- Length of Stay
- Male
- Middle Aged
- Oxygen / blood
- Pandemics

- Pneumonia, Viral / blood
- Pneumonia, Viral / epidemiology*
- Pneumonia, Viral / mortality
- Pneumonia, Viral / physiopathology*
- ROC Curve
- Retrospective Studies
- SARS-CoV-2
- Time Factors
- Young Adult

Substances

- Biomarkers
- Interleukin-6
- C-Reactive Protein
- Oxygen

Full text links

ELSEVIER FULL-TEXT ARTICLE Elsevier Science Free PMC article
Proceed to details
Cite
Share
1,004
Observational Study
Acta Biomed
. 2021 Nov 3;92(5):e2021399.
doi: 10.23750/abm.v92i5.11911.

Improvement in glycaemic control in paediatric and young adult type 1 diabetes patients during COVID-19 pandemic: role of telemedicine and lifestyle changes

Pietro Lazzeroni [1], Matteo Motta [2], Sara Monaco [3], Serena Rosa Laudisio [4], Daria Furoncoli [5], Valentina Maffini [6], Monica Rubini [7], Bertrand Tchana [8], Claudio Ruberto [9], Icilio Dodi [10], Brunella Iovane [11]
Affiliations Expand

Affiliations

- [1] Centre for Diabetes in Children and Adolescents, Department of Woman and Child Health, Parma University Hospital, Parma, Italy. plazzeroni@ao.pr.it.
- [2] Post-graduate School of Pediatrics, University of Parma, Parma, Italy. matteo.motta92@gmail.com.
- [3] Post-graduate School of Pediatrics, University of Parma, Parma, Italy. s.monaco1410@gmail.com.
- [4] Post-graduate School of Pediatrics, University of Parma, Parma, Italy. serenalaudisio@gmail.com.
- [5] Centre for Diabetes in Children and Adolescents, Department of Woman and Child Health, Parma University Hospital, Parma, Italy. dfuroncoli@ao.pr.it.
- [6] Department of Woman and Child Health, Parma University Hospital, Parma, Italy. vmaffini@ao.pr.it.
- [7] Department of Woman and Child Health, Parma University Hospital, Parma, Italy. mrubini@ao.pr.it.
- [8] Department of Woman and Child Health, Parma University Hospital, Parma, Italy. btchana@ao.pr.it.
- [9] Department of Woman and Child Health, Parma University Hospital, Parma, Italy. cruberto@ao.pr.it.
- [10] Department of Woman and Child Health, Parma University Hospital, Parma, Italy. idodi@ao.pr.it.
- [11] Centre for Diabetes in Children and Adolescents, Department of Woman and Child Health, Parma University Hospital, Parma, Italy. biovane@ao.pr.it.

- PMID: **34738562**
- PMCID: PMC8689301
- DOI: 10.23750/abm.v92i5.11911

Free PMC article
Observational Study

Improvement in glycaemic control in paediatric and young adult type 1 diabetes patients during COVID-19 pandemic: role of telemedicine and lifestyle changes

Pietro Lazzeroni et al. Acta Biomed. 2021.
Free PMC article

Show details

Acta Biomed
. 2021 Nov 3;92(5):e2021399.
doi: 10.23750/abm.v92i5.11911.

Authors

Pietro Lazzeroni [1], Matteo Motta [2], Sara Monaco [3], Serena Rosa Laudisio [4], Daria Furoncoli [5], Valentina Maffini [6], Monica Rubini [7], Bertrand Tchana [8], Claudio Ruberto [9], Icilio Dodi [10], Brunella Iovane [11]

Affiliations

- [1] Centre for Diabetes in Children and Adolescents, Department of Woman and Child Health, Parma University Hospital, Parma, Italy. plazzeroni@ao.pr.it.
- [2] Post-graduate School of Pediatrics, University of Parma, Parma, Italy. matteo.motta92@gmail.com.
- [3] Post-graduate School of Pediatrics, University of Parma, Parma, Italy. s.monaco1410@gmail.com.
- [4] Post-graduate School of Pediatrics, University of Parma, Parma, Italy. serenalaudisio@gmail.com.
- [5] Centre for Diabetes in Children and Adolescents, Department of Woman and Child Health, Parma University Hospital, Parma, Italy. dfuroncoli@ao.pr.it.
- [6] Department of Woman and Child Health, Parma University Hospital, Parma, Italy. vmaffini@ao.pr.it.
- [7] Department of Woman and Child Health, Parma University Hospital, Parma, Italy. mrubini@ao.pr.it.
- [8] Department of Woman and Child Health, Parma University Hospital, Parma, Italy. btchana@ao.pr.it.
- [9] Department of Woman and Child Health, Parma University Hospital, Parma, Italy. cruberto@ao.pr.it.
- [10] Department of Woman and Child Health, Parma University Hospital, Parma, Italy. idodi@ao.pr.it.
- [11] Centre for Diabetes in Children and Adolescents, Department of Woman and Child Health, Parma University Hospital, Parma, Italy. biovane@ao.pr.it.

- PMID: **34738562**
- PMCID: PMC8689301
- DOI: 10.23750/abm.v92i5.11911

Abstract

Background and aim: COVID-19 pandemic determined a profound impact in everyday life and in routine follow-up of patients with type 1 diabetes (T1D). In this context, telemedicine represented an important tool to guarantee a regular care for these patients. Aim of our work was to assess metabolic control before and after lockdown in the cohort of T1D patients followed-up by our Service, to evaluate the impact of restrictive measures and of disease management through telemedicine.

Methods: This is a retrospective observational study. Subjects were enrolled among children, adolescents and young adults affected by T1D and followed at the Regional Paediatric Diabetology Centre of the University-Hospital of Parma, Italy. We collected data about age, gender, ethnicity, anthropometric measurements, duration of disease, type of blood glucose monitoring used, type of insulin administration, daily insulin requirement and metabolic control, assessed using capillary HbA1c.

Results: We enrolled 139 patients, mean age 13.9 years. During lockdown, we reported significantly more contacts through telemedicine between patients and medical team. Global glycol-metabolic control significantly improved, without differences in daily insulin requirement. Patients with a previous poor-controlled diabetes showed a greater improvement. Finally, mean weekly hours of physical activity decreased significantly, without worsening in BMI z-score.

Conclusions: Our results show a global improvement in mean HbA1c, with a stronger result for patients with a previous non satisfactory control. In our setting, despite regulatory rules and physical and logistic limitations related to pandemic, no worsening of metabolic control has been shown for patients with type 1 diabetes.

Conflict of interest statement

Each author declares that he or she has no commercial associations (e.g. consultancies, stock ownership, equity interest, patent/licensing arrangement etc.) that might pose a conflict of interest in connection with the submitted article

- 42 references

Supplementary info

Publication types, MeSH terms, Substances Expand

Publication types

- Observational Study

MeSH terms

- Adolescent
- Blood Glucose
- Blood Glucose Self-Monitoring
- COVID-19*
- Communicable Disease Control
- Diabetes Mellitus, Type 1* / epidemiology
- Glycemic Control
- Humans
- Life Style
- Pandemics
- SARS-CoV-2
- Telemedicine*
- Young Adult

Substances

- Blood Glucose

Full text links

Free PMC article
Proceed to details
Cite
Share
1,005
Observational Study
Thromb Res
. 2021 Feb;198:34-39.
doi: 10.1016/j.thromres.2020.11.017. Epub 2020 Nov 17.

Pulmonary embolism in COVID-19 patients: prevalence, predictors and clinical outcome

Fernando Scudiero [1], Angelo Silverio [2], Marco Di Maio [3], Vincenzo Russo [4], Rodolfo Citro [2], Davide Personeni [1], Andrea Cafro [1], Antonello D'Andrea [5], Emilio Attena [6], Salvatore Pezzullo [7], Mario Enrico Canonico [8], Gennaro Galasso [2], Antonino Pitì [1], Guido Parodi [9], Cov-IT Network
Affiliations Expand

Affiliations

- [1] Division of Cardiology, "Bolognini" Hospital, ASST Bergamo est, Seriate, Italy.
- [2] Division of Cardiology, Cardiovascolar and Thoracic Department, San Giovanni di Dio e Ruggi d'Aragona University Hospital, Salerno, Italy.
- [3] Division of Cardiology, Maria SS. Addolorata Hospital, Eboli, Salerno, Italy.
- [4] Chair of Cardiology, Department of Translational Medical Sciences, University of Campania "Luigi Vanvitelli" - Monaldi and Cotugno Hospital, Naples, Italy.
- [5] Department of Cardiology and Intensive Care Unit, Umberto I Hospital, Nocera Inferiore, Italy.
- [6] Division of Cardiology, San Giuliano Hospital, Naples, Italy.
- [7] Division of Cardiology, Villa dei Fiori Hospital, Acerra, Naples, Italy.
- [8] Clinical and Interventional Cardiology, Sassari University Hospital, Sassari, Italy.
- [9] Clinical and Interventional Cardiology, Sassari University Hospital, Sassari, Italy. Electronic address: gparodi@uniss.it.

- PMID: **33271421**
- PMCID: PMC7669475
- DOI: 10.1016/j.thromres.2020.11.017

Free PMC article
Observational Study

Pulmonary embolism in COVID-19 patients: prevalence, predictors and clinical outcome

Fernando Scudiero et al. Thromb Res. 2021 Feb.
Free PMC article
Show details
Thromb Res
. 2021 Feb;198:34-39.
doi: 10.1016/j.thromres.2020.11.017. Epub 2020 Nov 17.

Authors

Fernando Scudiero [1], Angelo Silverio [2], Marco Di Maio [3], Vincenzo Russo [4], Rodolfo Citro [2], Davide Personeni [1], Andrea Cafro [1], Antonello D'Andrea [5], Emilio Attena [6], Salvatore Pezzullo [7], Mario Enrico Canonico [8], Gennaro Galasso [2], Antonino Pitì [1], Guido Parodi [9], Cov-IT Network

Affiliations

- [1] Division of Cardiology, "Bolognini" Hospital, ASST Bergamo est, Seriate, Italy.
- [2] Division of Cardiology, Cardiovascolar and Thoracic Department, San Giovanni di Dio e Ruggi d'Aragona University Hospital, Salerno, Italy.
- [3] Division of Cardiology, Maria SS. Addolorata Hospital, Eboli, Salerno, Italy.
- [4] Chair of Cardiology, Department of Translational Medical Sciences, University of Campania "Luigi Vanvitelli" - Monaldi and Cotugno Hospital, Naples, Italy.
- [5] Department of Cardiology and Intensive Care Unit, Umberto I Hospital, Nocera Inferiore, Italy.
- [6] Division of Cardiology, San Giuliano Hospital, Naples, Italy.
- [7] Division of Cardiology, Villa dei Fiori Hospital, Acerra, Naples, Italy.
- [8] Clinical and Interventional Cardiology, Sassari University Hospital, Sassari, Italy.
- [9] Clinical and Interventional Cardiology, Sassari University Hospital, Sassari, Italy. Electronic address: gparodi@uniss.it.

- PMID: **33271421**
- PMCID: PMC7669475
- DOI: 10.1016/j.thromres.2020.11.017

Abstract

Introduction: The incidence, characteristics, and prognosis of pulmonary embolism (PE) in Coronavirus disease 2019 (COVID-19) have been poorly investigated. We aimed to investigate the prevalence and the correlates with the occurrence of PE as well as the association between PE and the risk of mortality in COVID-19.

Methods: Retrospective multicenter study on consecutive COVID-19 patients hospitalized at 7 Italian Hospitals. At admission, all patients underwent medical history, laboratory and echocardiographic evaluation.

Results: The study population consisted of 224 patients (mean age 69 ± 14, male sex 62%); PE was diagnosed in 32 cases (14%). Patients with PE were hospitalized after a longer time since symptoms onset (7 IQR 3-11 days, 3 IQR 1-6 days; p = 0.001) and showed higher D-dimers level (1819 IQR 568-5017 ng/ml vs 555 IQR 13-1530 ng/ml; p < 0.001) and higher prevalence of myocardial injury (47% vs 28%, p = 0.033). At multivariable analysis, tricuspid annular plane systolic excursion (TAPSE; HR = 0.84; 95% CI 0.66-0.98; p = 0.046) and systolic pulmonary arterial pressure (sPAP; HR = 1.12; 95% CI 1.03-1.23; p = 0.008) resulted the only parameters independently associated with PE occurrence. Mortality rates (50% vs 27%; p = 0.010) and cardiogenic shock (37% vs 14%; p = 0.001) were significantly higher in PE as compared with non-PE patients. At multivariate analysis PE was significant associated with mortality.

Conclusion: PE is relatively common complication in COVID-19 and is associated with increased mortality risk. TAPSE and sPAP resulted the only parameters independently associated with PE occurrence in COVID-19 patients.

Keywords: COVID-19; Echocardiography; Pulmonary embolism.

Copyright © 2020 Elsevier Ltd. All rights reserved.

Conflict of interest statement

Prof. Parodi reported receiving consulting or lecture fees from AstraZeneca, Bayer, Chiesi, Daiichi Sankyo/Eli Lilly, and Merck Sharp Dohme. The remaining Authors have no conflict of interest to disclose.

- 24 references
- 1 figure

Supplementary info

Publication types, MeSH terms Expand

Publication types

- Letter
- Multicenter Study
- Observational Study

MeSH terms

- Aged
- Aged, 80 and over
- COVID-19 / diagnosis
- COVID-19 / epidemiology*
- COVID-19 / mortality
- COVID-19 / therapy
- Female
- Humans

- Hypertension, Pulmonary / epidemiology
- Italy / epidemiology
- Male
- Middle Aged
- Prevalence
- Prognosis
- Pulmonary Embolism / diagnosis
- Pulmonary Embolism / epidemiology*
- Pulmonary Embolism / mortality
- Pulmonary Embolism / therapy
- Registries
- Retrospective Studies
- Risk Assessment
- Risk Factors
- Ventricular Dysfunction, Right / epidemiology

Full text links

ELSEVIER FULL-TEXT ARTICLE Elsevier Science Free PMC article
Proceed to details
Cite
Share
1,006
Observational Study
J Infect Dis
. 2020 Jun 16;222(1):38-43.
doi: 10.1093/infdis/jiaa228.

Effect of Convalescent Plasma Therapy on Viral Shedding and Survival in Patients With Coronavirus Disease 2019

Qing-Lei Zeng [1], Zu-Jiang Yu [1], Jian-Jun Gou [1], Guang-Ming Li [2], Shu-Huan Ma [2], Guo-Fan Zhang [3], Jiang-Hai Xu [4], Wan-Bao Lin [5], Guang-Lin Cui [6], Min-Min Zhang [2], Cheng Li [2], Ze-Shuai Wang [7], Zhi-Hao Zhang [1], Zhang-Suo Liu [1]
Affiliations Expand

Affiliations

- [1] Department of Infectious Diseases, The First Affiliated Hospital of Zhengzhou University, Zhengzhou City, China.

- [2] Department of Infectious Diseases, The Sixth People's Hospital of Zhengzhou City, Zhengzhou City, China.
- [3] The Department of Infectious Diseases, The First Affiliated Hospital of Nanyang Medical College, Nanyang City, China.
- [4] Department of Infectious Diseases, The Fifth People's Hospital of Anyang City, Anyang City, China.
- [5] Department of Infectious Diseases, Xinyang Central Hospital, Xinyang City, China.
- [6] Department of Clinical Laboratory, The First Affiliated Hospital of Zhengzhou University, Zhengzhou City, China.
- [7] Department of Pulmonary and Critical Care Medicine, The First Affiliated Hospital of Zhengzhou University, Zhengzhou City, China.

- PMID: **32348485**
- PMCID: PMC7197534
- DOI: 10.1093/infdis/jiaa228

Free PMC article
Observational Study

Effect of Convalescent Plasma Therapy on Viral Shedding and Survival in Patients With Coronavirus Disease 2019

Qing-Lei Zeng et al. J Infect Dis. 2020.
Free PMC article
Show details
J Infect Dis
. 2020 Jun 16;222(1):38-43.
doi: 10.1093/infdis/jiaa228.

Authors

Qing-Lei Zeng [1], Zu-Jiang Yu [1], Jian-Jun Gou [1], Guang-Ming Li [2], Shu-Huan Ma [2], Guo-Fan Zhang [3], Jiang-Hai Xu [4], Wan-Bao Lin [5], Guang-Lin Cui [6], Min-Min Zhang [2], Cheng Li [2], Ze-Shuai Wang [7], Zhi-Hao Zhang [1], Zhang-Suo Liu [1]

Affiliations

- [1] Department of Infectious Diseases, The First Affiliated Hospital of Zhengzhou University, Zhengzhou City, China.
- [2] Department of Infectious Diseases, The Sixth People's Hospital of Zhengzhou City, Zhengzhou City, China.
- [3] The Department of Infectious Diseases, The First Affiliated Hospital of Nanyang Medical College, Nanyang City, China.
- [4] Department of Infectious Diseases, The Fifth People's Hospital of Anyang City, Anyang City, China.
- [5] Department of Infectious Diseases, Xinyang Central Hospital, Xinyang City, China.

- [6] Department of Clinical Laboratory, The First Affiliated Hospital of Zhengzhou University, Zhengzhou City, China.
- [7] Department of Pulmonary and Critical Care Medicine, The First Affiliated Hospital of Zhengzhou University, Zhengzhou City, China.

- PMID: **32348485**
- PMCID: PMC7197534
- DOI: 10.1093/infdis/jiaa228

Abstract

Currently, coronavirus disease 2019 (COVID-19), caused by severe acute respiratory syndrome coronavirus 2 (SARS-CoV-2), has been reported in almost all countries globally. No effective therapy has been documented for COVID-19, and the role of convalescent plasma therapy is unknown. In the current study, 6 patients with COVID-19 and respiratory failure received convalescent plasma a median of 21.5 days after viral shedding was first detected, all tested negative for SARS-CoV-2 RNA within 3 days after infusion, and 5 eventually died. In conclusion, convalescent plasma treatment can end SARS-CoV-2 shedding but cannot reduce the mortality rate in critically ill patients with end-stage COVID-19, and treatment should be initiated earlier.

Keywords: Convalescent plasma therapy; Coronavirus disease 2019 (COVID-19); Fatality; Severe acute respiratory syndrome coronavirus 2 (SARS-CoV-2); Survival rate; Viral shedding.

© The Author(s) 2020. Published by Oxford University Press for the Infectious Diseases Society of America. All rights reserved. For permissions, e-mail: journals.permissions@oup.com.

Comment in

- Therapeutic Plasma Exchange-Neutralizing Antibody Combination Therapy for Severe Coronavirus Disease 2019.
  Malkovsky M. Malkovsky M. J Infect Dis. 2020 Jul 6;222(3):509-510. doi: 10.1093/infdis/jiaa312. J Infect Dis. 2020. PMID: 32473010 Free PMC article. No abstract available.
- Reply to Rabelo-da-Ponte et al.
  Zeng QL, Yu ZJ, Ji F. Zeng QL, et al. J Infect Dis. 2020 Oct 1;222(9):1578. doi: 10.1093/infdis/jiaa510. J Infect Dis. 2020. PMID: 32777045 Free PMC article. No abstract available.

Supplementary info

Publication types, MeSH terms, Substances, Supplementary concepts Expand

Publication types

- Observational Study

MeSH terms

- Adult

- Aged
- Antibodies, Viral / therapeutic use*
- Betacoronavirus / genetics*
- Blood Donors
- COVID-19
- China
- Coronavirus Infections / mortality*
- Coronavirus Infections / therapy*
- Coronavirus Infections / virology
- Critical Illness
- Female
- Humans
- Immunization, Passive / adverse effects
- Male
- Middle Aged
- Pandemics
- Pneumonia, Viral / mortality*
- Pneumonia, Viral / therapy*
- Pneumonia, Viral / virology
- RNA, Viral / genetics
- Real-Time Polymerase Chain Reaction
- Retrospective Studies
- SARS-CoV-2
- Survival Rate
- Treatment Outcome
- Virus Shedding / immunology*

Substances

- Antibodies, Viral
- RNA, Viral

Supplementary concepts

- COVID-19 serotherapy

Full text links

OXFORD ACADEMIC Silverchair Information Systems Free PMC article
Proceed to details
Cite
Share

☐ 1,007
Observational Study
Intern Emerg Med
. 2021 Jun;16(4):843-852.
doi: 10.1007/s11739-020-02600-z. Epub 2021 Jan 5.

Anakinra after treatment with corticosteroids alone or with tocilizumab in patients with severe COVID-19 pneumonia and moderate hyperinflammation. A retrospective cohort study

Ismael Francisco Aomar-Millán [1], Juan Salvatierra [# 2], Úrsula Torres-Parejo [# 3], Naya Faro-Miguez [4], José Luis Callejas-Rubio [5], Ángel Ceballos-Torres [5], María Teresa Cruces-Moreno [6], Francisco Javier Gómez-Jiménez [5], José Hernández-Quero [4], Francisco Anguita-Santos [# 4]
Affiliations Expand

Affiliations

- [1] Department of Internal Medicine, San Cecilio University Hospital, Hospital Universitario San Cecilio, Avda. del Conocimiento s/n, 18016, Granada, Spain. iaomarmillan@hotmail.com.
- [2] Department of Rheumatology, San Cecilio University Hospital, Granada, Spain.
- [3] Department of Statistics and Operational Research, University of Granada, Granada, Spain.
- [4] Department of Infectious Diseases, San Cecilio University Hospital, Granada, Spain.
- [5] Department of Internal Medicine, San Cecilio University Hospital, Hospital Universitario San Cecilio, Avda. del Conocimiento s/n, 18016, Granada, Spain.
- [6] Department of Intensive Care Unit, San Cecilio University Hospital, Granada, Spain.

[#] Contributed equally.

- PMID: **33400157**
- PMCID: PMC7782569
- DOI: 10.1007/s11739-020-02600-z

Free PMC article
Observational Study

Anakinra after treatment with corticosteroids alone or with tocilizumab in patients with severe COVID-19 pneumonia and moderate

hyperinflammation. A retrospective cohort study

Ismael Francisco Aomar-Millán et al. Intern Emerg Med. 2021 Jun.
Free PMC article
Show details
Intern Emerg Med
. 2021 Jun;16(4):843-852.
doi: 10.1007/s11739-020-02600-z. Epub 2021 Jan 5.

Authors

Ismael Francisco Aomar-Millán [1], Juan Salvatierra [# 2], Úrsula Torres-Parejo [# 3], Naya Faro-Miguez [4], José Luis Callejas-Rubio [5], Ángel Ceballos-Torres [5], María Teresa Cruces-Moreno [6], Francisco Javier Gómez-Jiménez [5], José Hernández-Quero [4], Francisco Anguita-Santos [# 4]

Affiliations

- [1] Department of Internal Medicine, San Cecilio University Hospital, Hospital Universitario San Cecilio, Avda. del Conocimiento s/n, 18016, Granada, Spain. iaomarmillan@hotmail.com.
- [2] Department of Rheumatology, San Cecilio University Hospital, Granada, Spain.
- [3] Department of Statistics and Operational Research, University of Granada, Granada, Spain.
- [4] Department of Infectious Diseases, San Cecilio University Hospital, Granada, Spain.
- [5] Department of Internal Medicine, San Cecilio University Hospital, Hospital Universitario San Cecilio, Avda. del Conocimiento s/n, 18016, Granada, Spain.
- [6] Department of Intensive Care Unit, San Cecilio University Hospital, Granada, Spain.

[#] Contributed equally.

- PMID: **33400157**
- PMCID: PMC7782569
- DOI: 10.1007/s11739-020-02600-z

Abstract

Introduction: Little evidence appears to exist for the use of anakinra, a recombinant interleukin-1 receptor antagonist, after non-response to treatment with corticosteroids alone or combined with tocilizumab in patients with severe COVID-19 pneumonia and moderate hyperinflammatory state.

Patients and methods: A retrospective observational cohort study was carried out involving 143 patients with severe COVID-19 pneumonia and moderate hyperinflammation. They received standard therapy along with pulses of methylprednisolone (group 1) or methylprednisolone plus tocilizumab (group 2), with the possibility of receiving anakinra (group 3) according to protocol. The aim of this study was to assess the role of anakinra in the clinical course (death, admission to the intensive care ward) during the first 60 days after the first corticosteroid pulse. Clinical, laboratory, and imaging characteristics as well as infectious complications were also analyzed.

Results: 74 patients (51.7%) in group 1, 59 (41.3%) patients in group 2, and 10 patients (7%) in group 3 were included. 8 patients (10.8%) in group 1 died, 6 (10.2%) in group 2, and 0 (0%) in group 3. After adjustment for age and clinical severity indices, treatment with anakinra was associated with a reduced risk of mortality (adjusted hazard ratio 0.518, 95% CI 0.265-0.910; p = 0.0437). Patients in group 3 had a lower mean CD4 count after 3 days of treatment. No patients in this group presented infectious complications.

Conclusions: In patients with moderate hyperinflammatory state associated with severe COVID-19 pneumonia, treatment with anakinra after non-response to corticosteroids or corticosteroids plus tocilizumab therapy may be an option for the management of these patients and may improve their prognosis.

Keywords: Anakinra; COVID-19; Cytokine storm syndrome; Hyperinflammation; Methylprednisolone; Tocilizumab.

Conflict of interest statement

The authors declare that they have no conflict of interest.

Comment in

- Anakinra after treatment with corticosteroids alone or with tocilizumab in patients with severe COVID-19 pneumonia and moderate hyperinflammation. A retrospective cohort study: comment.
  Yurttutan S, Gullu UU, Ipek S. Yurttutan S, et al. Intern Emerg Med. 2021 Jun;16(4):1101-1103. doi: 10.1007/s11739-021-02663-6. Epub 2021 Feb 18. Intern Emerg Med. 2021. PMID: 33598871 Free PMC article. No abstract available.

- 40 references
- 2 figures

Supplementary info

Publication types, MeSH terms, Substances, Supplementary concepts Expand

Publication types

- Observational Study

MeSH terms

- Aged
- Aged, 80 and over
- Antibodies, Monoclonal, Humanized / administration & dosage*
- Antirheumatic Agents / administration & dosage
- COVID-19 / complications*
- COVID-19 / drug therapy*
- COVID-19 / mortality

- Drug Therapy, Combination
- Female
- Glucocorticoids / administration & dosage*
- Humans
- Interleukin 1 Receptor Antagonist Protein / administration & dosage*
- Male
- Methylprednisolone / administration & dosage
- Middle Aged
- Retrospective Studies
- Spain
- Survival Rate
- Treatment Outcome

Substances

- Antibodies, Monoclonal, Humanized
- Antirheumatic Agents
- Glucocorticoids
- Interleukin 1 Receptor Antagonist Protein
- tocilizumab
- Methylprednisolone

Supplementary concepts

- COVID-19 drug treatment

Full text links

SpringerLink FULL-TEXT ARTICLE Springer Free PMC article
Proceed to details
Cite
Share
☐ 1,008
Observational Study
Crit Care
. 2021 May 17;25(1):171.
doi: 10.1186/s13054-021-03570-0.

Dead space estimates may not be independently associated with 28-day mortality in COVID-19 ARDS

Luis Morales-Quinteros [1 2 3], Ary Serpa Neto [4 5 6 7], Antonio Artigas [8 9 10 11], Lluis Blanch [8 9 10 11], Michela Botta [4], David A Kaufman [12], Marcus J Schultz [4 13 14], Anissa M Tsonas [4], Frederique Paulus [4], Lieuwe D Bos [4], PRoVENT-COVID Study Group

Collaborators, Affiliations Expand

Collaborators

- **PRoVENT-COVID Study Group**: Luis Morales-Quinteros, Ary Serpa Neto, Antonio Artigas, Lluis Blanch, Michela Botta, David A Kaufman, Marcus J Schultz, Anissa M Tsonas, Frederique Paulus, Lieuwe D Bos, A G Algera, L S Boers, L D J Bos, J Pillay, D A Dongelmans, M W Hollmann, J Horn, A P Vlaar, J P van Akkeren, A G Algera, C K Algoe, R B van Amstel, O L Baur, P van de Berg, A E van den Berg, D C J J Bergmans, D I van den Bersselaar, F A Bertens, A J G H Bindels, M M de Boer, S den Boer, L S Boers, M Bogerd, J S Breel, H de Bruin, S de Bruin, C L Bruna, L A Buiteman-Kruizinga, O Cremer, R M Determann, W Dieperink, D A Dongelmans, H S Franke, M S Galek-Aldridge, M J de Graaff, L A Hagens, J J Haringman, S T van der Heide, P L J van der Heiden, N F L Heijnen, S J P Hiel, L L Hoeijmakers, L Hol, M W Hollmann, M E Hoogendoorn, J Horn, R van der Horst, E L K Ie, D Ivanov, N P Juffermans, E Kho, E S de Klerk, A W M M Koopman-van Gemert, M Koopmans, S Kucukcelebi, M A Kuiper, D W de Lange, N van Mourik, S G Nijbroek, M Onrust, E A N Oostdijk, C J Pennartz, J Pillay, L Pisani, I M Purmer, T C D Rettig, J P Roozeman, M T U Schuijt, M E Sleeswijk, M R Smit, P E Spronk, W Stilma, A C Strang, P R Tuinman, C M A Valk, F L Veen-Schra, L I Veldhuis, P van Velzen, W H van der Ven, A P J Vlaar, P van Vliet, P H J van der Voort, L van Welie, H J F T Wesselink, H H van der Wier-Lubbers, B van Wijk, T Winters, W Y Wong, A R H van Zanten

Affiliations

- [1] Intensive Care Unit, Hospital Universitari General de Catalunya, Grupo Quironsalud, Carrer Pedro i Pons, 1, 08195, Sant Cugat del Vallès, Barcelona, Spain. luchomq2077@gmail.com.
- [2] Universidad Autonoma de Barcelona, Barcelona, Spain. luchomq2077@gmail.com.
- [3] Institut D'Investigació, Innovació Parc Taulí I3PT, Sabadell, Spain. luchomq2077@gmail.com.
- [4] Department of Intensive Care & Laboratory of Experimental Intensive Care and Anaesthesiology (L·E·I·C·A), Amsterdam UMC Location AMC, Amsterdam, The Netherlands.
- [5] Department of Critical Care Medicine, Hospital Israelita Albert Einstein, São Paulo, Brazil.
- [6] Australian and New Zealand Intensive Care Research Centre (ANZIC-RC), Monash University, Melbourne, Australia.
- [7] Data Analytics Research and Evaluation (DARE) Centre, Austin Hospital and University of Melbourne, Melbourne, Australia.

- [8] Universidad Autonoma de Barcelona, Barcelona, Spain.
- [9] Institut D'Investigació, Innovació Parc Taulí I3PT, Sabadell, Spain.
- [10] Critical Care Center, Corporacion Sanitaria Universitaria Parc Taulí, Sabadell, Spain.
- [11] CIBER Enfermedades Respiratorias (ISCiii), Madrid, Spain.
- [12] Division of Pulmonary, Critical Care, and Sleep Medicine, NYU School of Medicine, New York, NY, USA.
- [13] Nuffield Department of Medicine, Oxford University, Oxford, UK.
- [14] Mahidol-Oxford Tropical Medicine Research Unit (MORU), Mahidol University, Bangkok, Thailand.

- PMID: **34001222**
- PMCID: PMC8127435
- DOI: 10.1186/s13054-021-03570-0

Free PMC article
Observational Study

Dead space estimates may not be independently associated with 28-day mortality in COVID-19 ARDS

Luis Morales-Quinteros et al. Crit Care. 2021.
Free PMC article

Show details

Crit Care

. 2021 May 17;25(1):171.
doi: 10.1186/s13054-021-03570-0.

Authors

Luis Morales-Quinteros [1, 2, 3], Ary Serpa Neto [4, 5, 6, 7], Antonio Artigas [8, 9, 10, 11], Lluis Blanch [8, 9, 10, 11], Michela Botta [4], David A Kaufman [12], Marcus J Schultz [4, 13, 14], Anissa M Tsonas [4], Frederique Paulus [4], Lieuwe D Bos [4], PRoVENT-COVID Study Group

Collaborators

- **PRoVENT-COVID Study Group**: Luis Morales-Quinteros, Ary Serpa Neto, Antonio Artigas, Lluis Blanch, Michela Botta, David A Kaufman, Marcus J Schultz, Anissa M Tsonas, Frederique Paulus, Lieuwe D Bos, A G Algera, L S Boers, L D J Bos, J Pillay, D A Dongelmans, M W Hollmann, J Horn, A P Vlaar, J P van Akkeren, A G Algera, C K Algoe, R B van Amstel, O L Baur, P van de Berg, A E van den Berg, D C J J Bergmans, D I van den Bersselaar, F A Bertens, A J G H Bindels, M M de Boer, S den Boer, L S Boers, M Bogerd, J S Breel, H de Bruin, S de Bruin, C L Bruna, L A Buiteman-Kruizinga, O Cremer, R M Determann, W Dieperink, D A Dongelmans, H S Franke, M S Galek-Aldridge, M J de Graaff, L A Hagens, J J Haringman, S T van der Heide, P L J van der Heiden, N F L Heijnen, S J P Hiel, L L Hoeijmakers, L Hol, M W Hollmann, M E Hoogendoorn, J Horn, R van der Horst, E L K

Ie, D Ivanov, N P Juffermans, E Kho, E S de Klerk, A W M M Koopman-van Gemert, M Koopmans, S Kucukcelebi, M A Kuiper, D W de Lange, N van Mourik, S G Nijbroek, M Onrust, E A N Oostdijk, C J Pennartz, J Pillay, L Pisani, I M Purmer, T C D Rettig, J P Roozeman, M T U Schuijt, M E Sleeswijk, M R Smit, P E Spronk, W Stilma, A C Strang, P R Tuinman, C M A Valk, F L Veen-Schra, L I Veldhuis, P van Velzen, W H van der Ven, A P J Vlaar, P van Vliet, P H J van der Voort, L van Welie, H J F T Wesselink, H H van der Wier-Lubbers, B van Wijk, T Winters, W Y Wong, A R H van Zanten

Affiliations

- [1] Intensive Care Unit, Hospital Universitari General de Catalunya, Grupo Quironsalud, Carrer Pedro i Pons, 1, 08195, Sant Cugat del Vallès, Barcelona, Spain. luchomq2077@gmail.com.
- [2] Universidad Autonoma de Barcelona, Barcelona, Spain. luchomq2077@gmail.com.
- [3] Institut D'Investigació, Innovació Parc Taulí I3PT, Sabadell, Spain. luchomq2077@gmail.com.
- [4] Department of Intensive Care & Laboratory of Experimental Intensive Care and Anaesthesiology (L·E·I·C·A), Amsterdam UMC Location AMC, Amsterdam, The Netherlands.
- [5] Department of Critical Care Medicine, Hospital Israelita Albert Einstein, São Paulo, Brazil.
- [6] Australian and New Zealand Intensive Care Research Centre (ANZIC-RC), Monash University, Melbourne, Australia.
- [7] Data Analytics Research and Evaluation (DARE) Centre, Austin Hospital and University of Melbourne, Melbourne, Australia.
- [8] Universidad Autonoma de Barcelona, Barcelona, Spain.
- [9] Institut D'Investigació, Innovació Parc Taulí I3PT, Sabadell, Spain.
- [10] Critical Care Center, Corporacion Sanitaria Universitaria Parc Taulí, Sabadell, Spain.
- [11] CIBER Enfermedades Respiratorias (ISCiii), Madrid, Spain.
- [12] Division of Pulmonary, Critical Care, and Sleep Medicine, NYU School of Medicine, New York, NY, USA.
- [13] Nuffield Department of Medicine, Oxford University, Oxford, UK.
- [14] Mahidol-Oxford Tropical Medicine Research Unit (MORU), Mahidol University, Bangkok, Thailand.

- PMID: **34001222**
- PMCID: PMC8127435
- DOI: 10.1186/s13054-021-03570-0

Abstract

Background: Estimates for dead space ventilation have been shown to be independently associated with an increased risk of mortality in the acute respiratory distress syndrome and small case series of COVID-19-related ARDS.

Methods: Secondary analysis from the PRoVENT-COVID study. The PRoVENT-COVID is a national, multicenter, retrospective observational study done at 22 intensive care units in the Netherlands. Consecutive patients aged at least 18 years were eligible for participation if they had received invasive ventilation for COVID-19 at a participating ICU during the first month of the national outbreak in the Netherlands. The aim was to quantify the dynamics and determine the

prognostic value of surrogate markers of wasted ventilation in patients with COVID-19-related ARDS.

Results: A total of 927 consecutive patients admitted with COVID-19-related ARDS were included in this study. Estimations of wasted ventilation such as the estimated dead space fraction (by Harris-Benedict and direct method) and ventilatory ratio were significantly higher in non-survivors than survivors at baseline and during the following days of mechanical ventilation ($p < 0.001$). The end-tidal-to-arterial PCO_2 ratio was lower in non-survivors than in survivors ($p < 0.001$). As ARDS severity increased, mortality increased with successive tertiles of dead space fraction by Harris-Benedict and by direct estimation, and with an increase in the VR. The same trend was observed with decreased levels in the tertiles for the end-tidal-to-arterial PCO_2 ratio. After adjustment for a base risk model that included chronic comorbidities and ventilation- and oxygenation-parameters, none of the dead space estimates measured at the start of ventilation or the following days were significantly associated with 28-day mortality.

Conclusions: There is significant impairment of ventilation in the early course of COVID-19-related ARDS but quantification of this impairment does not add prognostic information when added to a baseline risk model.

Trial registration: ISRCTN04346342. Registered 15 April 2020. Retrospectively registered.

Keywords: ARDS; Acute respiratory distress syndrome; COVID-19; Dead space; Mortality; Prognostication; Respiratory dead space; Ventilatory ratio.

Conflict of interest statement

Dr Bos receives funding from the Dutch lung foundation (longfonds), from the Innovative Medicine Initiative and from Amsterdam UMC via the AUMC fellowship. The remaining authors have disclosed that they do not have any potential conflicts of interest.

- 35 references
- 4 figures

Supplementary info

Publication types, MeSH terms, Substances Expand

Publication types

- Multicenter Study
- Observational Study

MeSH terms

- Adult
- Biomarkers
- COVID-19 / complications
- COVID-19 / mortality*
- COVID-19 / physiopathology

- Female
- Humans
- Intensive Care Units
- Male
- Patient Acuity*
- Prognosis
- ROC Curve
- Respiration, Artificial*
- Respiratory Dead Space*
- Respiratory Distress Syndrome / etiology
- Respiratory Distress Syndrome / therapy*
- Respiratory Function Tests
- Respiratory Mechanics
- Retrospective Studies

Substances

- Biomarkers

Full text links

Read free full text at BMC BioMed Central Free PMC article
Proceed to details
Cite
Share
1,009
Observational Study
Acta Biomed
. 2020 Nov 30;91(12-S):e2020012.
doi: 10.23750/abm.v91i12-S.10814.

Healthcare personnel exposure to COVID - 19: an observational study on quarantined positive workers

Ivan Rubbi [1], Gianandrea Pasquinelli [2], Aura Brighenti [3], Marcella Fanelli [4], Patrizia Gualandi [5], Eleonora Nanni [6], Viviana D'Antoni [7], Cristina Fabbri [8]
Affiliations Expand

Affiliations

- [1] Corso di Laurea in Infermieristica Università di Bologna - Faenza. ivan.rubbi@auslromagna.it.
- [2] Department of Experimental, Diagnostic and Specialty Medicine, University of Bologna, S. Orsola-Malpighi Hospital, Bologna,Italy. gianandr.pasquinelli@unibo.it.
- [3] Medical Direction AUSL Romagna - Ravenna, Italy. aura.brighenti@auslromagna.it.
- [4] Quality and Clinical Governance AUSL Romagna - Ravenna, Italy. marcella.fanelli@auslromagna.it.
- [5] NSO Care Safety and Risk Management AUSL Romagna - Ravenna, Italy. patrizia.gualandi@auslromagna.it.
- [6] Surgery Department AUSL Romagna - Ravenna, Italy. eleonora.nanni@auslromagna.it.
- [7] Occupational Medicine AUSL Romagna - Ravenna, Italy. viviana.dantoni@auslromagna.it.
- [8] Nursing and Technical Direction AUSL Romagna - Ravenna, Italy. cristina.fabbri@auslromagna.it.

- PMID: **33263344**
- PMCID: PMC8023106
- DOI: 10.23750/abm.v91i12-S.10814

Free PMC article
Observational Study

Healthcare personnel exposure to COVID - 19: an observational study on quarantined positive workers

Ivan Rubbi et al. Acta Biomed. 2020.
Free PMC article
Show details
Acta Biomed
. 2020 Nov 30;91(12-S):e2020012.
doi: 10.23750/abm.v91i12-S.10814.

Authors

Ivan Rubbi [1], Gianandrea Pasquinelli [2], Aura Brighenti [3], Marcella Fanelli [4], Patrizia Gualandi [5], Eleonora Nanni [6], Viviana D'Antoni [7], Cristina Fabbri [8]

Affiliations

- [1] Corso di Laurea in Infermieristica Università di Bologna - Faenza. ivan.rubbi@auslromagna.it.
- [2] Department of Experimental, Diagnostic and Specialty Medicine, University of Bologna, S. Orsola-Malpighi Hospital, Bologna,Italy. gianandr.pasquinelli@unibo.it.
- [3] Medical Direction AUSL Romagna - Ravenna, Italy. aura.brighenti@auslromagna.it.
- [4] Quality and Clinical Governance AUSL Romagna - Ravenna, Italy. marcella.fanelli@auslromagna.it.

- [5] NSO Care Safety and Risk Management AUSL Romagna - Ravenna, Italy. patrizia.gualandi@auslromagna.it.
- [6] Surgery Department AUSL Romagna - Ravenna, Italy. eleonora.nanni@auslromagna.it.
- [7] Occupational Medicine AUSL Romagna - Ravenna, Italy. viviana.dantoni@auslromagna.it.
- [8] Nursing and Technical Direction AUSL Romagna - Ravenna, Italy. cristina.fabbri@auslromagna.it.

- PMID: **33263344**
- PMCID: PMC8023106
- DOI: 10.23750/abm.v91i12-S.10814

Abstract

Background and aim of the study: COVID-19 is characterized by super spread events occurring in communities, e.g., hospitals. To limit virus diffusion among healthcare workers the use of personal protective equipment and screening tests are highly advised; also, isolation of virus positive professionals while monitoring their health condition is recommended. This study aims to assess, in a cohort of COVID-19 positive quarantined healthcare workers, the perceived source of infection and exposure risk as well as the clinical evolution of the disease through a surveillance interview.

Methods: A retrospective observational study accounting 896 observations on 93 healthcare professionals tested positive for COVID-19. Data were collected from the Nursing and Technical Directorate of Romagna, Ravenna, Local Health Company, Italy.

Results: 99.5% of the positive workers accepted phone interviews with management staff. 2.6% of workers were positive with increasing records in the specialist medical area. Nurses and social health professionals were mostly affected. Patient exposure at a distance <1 m and a contact time > 2 hours was the first cause of positivity. In COVID-19 and territorial emergency departments, the first cause was the contact with colleagues. At the time of the infection, most of the staff wore a surgical mask. Cough, asthenia, fever, anosmia, dysgeusia, and rhinitis were common symptoms. Asymptomatic percentage was about 10%. The self-perceived physical condition was high (>7) and improved during the observation period.

Conclusions: The diffusion rate of COVID-19 among healthcare workers is relatively low, probably due to the use of personal protective equipment. The distancing, also among colleagues, is a fundamental measure to reduce the possibility of infection. Symptoms are mild and can be controlled by surveillance measures. Constant contact with the organization is an essential strategy for promoting recovering of workers and reducing the spread of the virus within the healthcare organization.

Conflict of interest statement

Each author declares that he or she has no commercial associations (e.g. consultancies, stock ownership, equity interest, patent/licensing arrangement etc.) that might pose a conflict of interest in connection with the submitted article

- 57 references
- 3 figures

Supplementary info

Publication types, MeSH terms Expand

Publication types

- Observational Study

MeSH terms

- COVID-19 / prevention & control*
- COVID-19 / transmission*
- Female
- Health Personnel / statistics & numerical data*
- Humans
- Italy / epidemiology
- Male
- Middle Aged
- Occupational Exposure / adverse effects*
- Pandemics
- Personal Protective Equipment / statistics & numerical data
- Physical Distancing
- Quarantine*
- Retrospective Studies

Full text links

Free PMC article
Proceed to details
Cite
Share
1,010
Clinical Trial
J Allergy Clin Immunol
. 2021 Jan;147(1):72-80.e8.
doi: 10.1016/j.jaci.2020.09.018. Epub 2020 Sep 30.

IL-6 serum levels predict severity and response to tocilizumab in COVID-19: An observational study

José María Galván-Román [1], Sebastián C Rodríguez-García [2], Emilia Roy-Vallejo [1], Ana Marcos-Jiménez [3], Santiago Sánchez-Alonso [3], Carlos Fernández-Díaz [2], Ana Alcaraz-Serna [3], Tamara Mateu-Albero [3], Pablo Rodríguez-Cortes [1], Ildefonso Sánchez-Cerrillo [3], Laura Esparcia [3], Pedro Martínez-Fleta [3], Celia López-Sanz [3], Ligia Gabrie [3], Luciana Del Campo Guerola [3], Carmen Suárez-Fernández [1], Julio Ancochea [4], Alfonso Canabal [5], Patricia Albert [5], Diego A Rodríguez-Serrano [5], Juan Mariano Aguilar [6], Carmen Del Arco [6], Ignacio de Los Santos [1], Lucio García-Fraile [1], Rafael de la Cámara [7], José María Serra [8], Esther Ramírez [8], Tamara Alonso [4], Pedro Landete [4], Joan B Soriano [4], Enrique Martín-Gayo [3], Arturo Fraile Torres [9], Nelly Daniela Zurita Cruz [9], Rosario García-Vicuña [2], Laura Cardeñoso [9], Francisco Sánchez-Madrid [10], Arantzazu Alfranca [3], Cecilia Muñoz-Calleja [3], Isidoro González-Álvaro [11], REINMUN-COVID Group

Collaborators, Affiliations Expand

Collaborators

- **REINMUN-COVID Group**:
  Teresa Alvarado, Pablo Martínez, Francisco Javier de la Cuerda Llorente, Carmen Del Arco, Juan Mariano Aguilar, Natalia Villalba, Mónica Negro, Elvira Contreras, Ana Del Rey, Cristina Santiago, Manuel Junquera, Raquel Caminero, Francisco Javier Val, Sonia González, Marta Caño, Isabel López, Andrés von Wernitz, Bárbara Retana, Iñigo Guerra, Jorge Sorando, Lydia Chao, María José Cárdenas, Verónica Espiga, Pablo Chicharro, Pedro Rodríguez, Iñigo Hernando Alday, Miguel Sampedro, Jorge Prada, Eukene Rojo Aldama, Yolanda Real, María Caldas, Sergio Casabona, Aitor Lanas-Gimeno, Rafael de la Camara, Angela Figuera Alvárez, Beatriz Aguadol, Alberto Morell, Esther Ramírez, Amparo Ibáñez Zurriaga, María Pérez Abanades, Silvia Ruiz García, Tomás Gallego Aranda, María Ruiz, Concepción Martínez Nieto, José María Serra, Francisco Sánchez-Madrid, Cecilia Muñoz-Calleja, Arantzazu Alfranca, Javier Aspa, Ana Marcos-Jiménez, Santiago Sánchez-Alonso, Ana Alcaraz-Serna, Tamara Mateu-Albero, Ildefonso Sánchez-Cerrillo, Laura Esparcia, Pedro Martínez-Fleta, Celia López-Sanz, Ligia Gabrie, Luciana Del Campo Guerola, Elena Fernández, Ma José Calzada, Reyes Tejedor, Alfonso Canabal, Patricia Albert, Diego A Rodríguez-Serrano, Judit Iglesias, Fernando Suarez, Juan Antonio Sánchez, Beatriz Abad, Carmen Suarez, Ignacio de Los Santos, José María Galván-Román, Emilia Roy, Pablo Rodríguez-Cortes, Lucio García-Fraile, Jesus Sanz, Eduardo Sanchez, Fernando Moldenhauer, Pedro Casado, Jose Curbelo, Angela Gutierrez, Azucena Bautista, Nuria Ruiz Giménez, Angelica Fernandez, Pedro Parra, Berta Moyano, Ana Barrios, Diego Real de Asua, Beatriz Sanchez, Carmen Saez, Marianela Ciudad, Desiré Navas, Laura Cardeñoso Domingo, María Del Carmen Cuevas Torresano, Diego Domingo García, Teresa Alarcón Cavero, Alicia García Blanco, Alexandra Martín Ramírez, María Auxiliadora Semiglia Chong, Ainhoa Gutiérrez Cobos, Nelly Daniela Zurita Cruz, Arturo Manuel Fraile Torres, Carmen Sanchez-Gonzalez, Antonio Fernádez Perpén, Carolina Díaz Pérez, Julio Ancochea, Tamara Alonso, Pedro Landete, Joan Soriano, Carolina Cisneros, Elena García Castillo, Francisco Javier García Pérez, Rosa María Girón, Celeste Marcos, Enrique Zamora, Patricia García García, Santos Castañeda, Rosario García-Vicuña, Isidoro González-Álvaro, Sebastián Rodríguez-García, Carlos Fernández-Díaz, Irene Llorente Cubas, Eva G Tomero, Noelia García Castañeda, Ana Ma Ortiz, Cristina Valero, Miren Uriarte, Nuria Montes

Affiliations

- [1] Internal Medicine Service, Hospital Universitario de la Princesa, Universidad Autónoma Madrid, Instituto de Investigación Sanitaria Princesa (IIS-IP), Madrid, Spain.
- [2] Rheumatology Service, Hospital Universitario de la Princesa, Universidad Autónoma Madrid, Instituto de Investigación Sanitaria Princesa (IIS-IP), Madrid, Spain.
- [3] Immunology Service, Hospital Universitario de la Princesa, Universidad Autónoma Madrid, Instituto de Investigación Sanitaria Princesa (IIS-IP), Madrid, Spain.
- [4] Pneumology Service, Hospital Universitario de la Princesa, Universidad Autónoma Madrid, Instituto de Investigación Sanitaria Princesa (IIS-IP), Madrid, Spain.
- [5] Intensive Care Unit, Hospital Universitario de la Princesa, Universidad Autónoma Madrid, Instituto de Investigación Sanitaria Princesa (IIS-IP), Madrid, Spain.
- [6] Emergency Service, Hospital Universitario de la Princesa, Universidad Autónoma Madrid, Instituto de Investigación Sanitaria Princesa (IIS-IP), Madrid, Spain.
- [7] Hematology Service, Hospital Universitario de la Princesa, Universidad Autónoma Madrid, Instituto de Investigación Sanitaria Princesa (IIS-IP), Madrid, Spain.
- [8] Hospital Pharmacy Service, Hospital Universitario de la Princesa, Universidad Autónoma Madrid, Instituto de Investigación Sanitaria Princesa (IIS-IP), Madrid, Spain.
- [9] Microbiology Service, Hospital Universitario de la Princesa, Universidad Autónoma Madrid, Instituto de Investigación Sanitaria Princesa (IIS-IP), Madrid, Spain.
- [10] Immunology Service, Hospital Universitario de la Princesa, Universidad Autónoma Madrid, Instituto de Investigación Sanitaria Princesa (IIS-IP), Madrid, Spain; Centro de Investigación Biomédica en Red - Enfermedades Cardiovasculares (CIBER CV), Instituto de Salud Carlos III (ISCIII), Madrid, Spain.
- [11] Rheumatology Service, Hospital Universitario de la Princesa, Universidad Autónoma Madrid, Instituto de Investigación Sanitaria Princesa (IIS-IP), Madrid, Spain. Electronic address: isidoro.ga@ser.es.

- PMID: **33010257**
- PMCID: PMC7525244
- DOI: 10.1016/j.jaci.2020.09.018

Free PMC article
Clinical Trial

IL-6 serum levels predict severity and response to tocilizumab in COVID-19: An observational study

José María Galván-Román et al. J Allergy Clin Immunol. 2021 Jan.
Free PMC article

Show details

J Allergy Clin Immunol

. 2021 Jan;147(1):72-80.e8.
doi: 10.1016/j.jaci.2020.09.018. Epub 2020 Sep 30.

Authors

José María Galván-Román [1], Sebastián C Rodríguez-García [2], Emilia Roy-Vallejo [1], Ana Marcos-Jiménez [3], Santiago Sánchez-Alonso [3], Carlos Fernández-Díaz [2], Ana Alcaraz-Serna [3], Tamara Mateu-Albero [3], Pablo Rodríguez-Cortes [1], Ildefonso Sánchez-Cerrillo [3], Laura Esparcia [3], Pedro Martínez-Fleta [3], Celia López-Sanz [3], Ligia Gabrie [3], Luciana Del Campo Guerola [3], Carmen Suárez-Fernández [1], Julio Ancochea [4], Alfonso Canabal [5], Patricia Albert [5], Diego A Rodríguez-Serrano [5], Juan Mariano Aguilar [6], Carmen Del Arco [6], Ignacio de Los Santos [1], Lucio García-Fraile [1], Rafael de la Cámara [7], José María Serra [8], Esther Ramírez [8], Tamara Alonso [4], Pedro Landete [4], Joan B Soriano [4], Enrique Martín-Gayo [3], Arturo Fraile Torres [9], Nelly Daniela Zurita Cruz [9], Rosario García-Vicuña [2], Laura Cardeñoso [9], Francisco Sánchez-Madrid [10], Arantzazu Alfranca [3], Cecilia Muñoz-Calleja [3], Isidoro González-Álvaro [11], REINMUN-COVID Group

Collaborators

- **REINMUN-COVID Group**:
Teresa Alvarado, Pablo Martínez, Francisco Javier de la Cuerda Llorente, Carmen Del Arco, Juan Mariano Aguilar, Natalia Villalba, Mónica Negro, Elvira Contreras, Ana Del Rey, Cristina Santiago, Manuel Junquera, Raquel Caminero, Francisco Javier Val, Sonia González, Marta Caño, Isabel López, Andrés von Wernitz, Bárbara Retana, Iñigo Guerra, Jorge Sorando, Lydia Chao, María José Cárdenas, Verónica Espiga, Pablo Chicharro, Pedro Rodríguez, Iñigo Hernando Alday, Miguel Sampedro, Jorge Prada, Eukene Rojo Aldama, Yolanda Real, María Caldas, Sergio Casabona, Aitor Lanas-Gimeno, Rafael de la Camara, Angela Figuera Alvárez, Beatriz Aguadol, Alberto Morell, Esther Ramírez, Amparo Ibáñez Zurriaga, María Pérez Abanades, Silvia Ruiz García, Tomás Gallego Aranda, María Ruiz, Concepción Martínez Nieto, José María Serra, Francisco Sánchez-Madrid, Cecilia Muñoz-Calleja, Arantzazu Alfranca, Javier Aspa, Ana Marcos-Jiménez, Santiago Sánchez-Alonso, Ana Alcaraz-Serna, Tamara Mateu-Albero, Ildefonso Sánchez-Cerrillo, Laura Esparcia, Pedro Martínez-Fleta, Celia López-Sanz, Ligia Gabrie, Luciana Del Campo Guerola, Elena Fernández, Ma José Calzada, Reyes Tejedor, Alfonso Canabal, Patricia Albert, Diego A Rodríguez-Serrano, Judit Iglesias, Fernando Suarez, Juan Antonio Sánchez, Beatriz Abad, Carmen Suarez, Ignacio de Los Santos, José María Galván-Román, Emilia Roy, Pablo Rodríguez-Cortes, Lucio García-Fraile, Jesus Sanz, Eduardo Sanchez, Fernando Moldenhauer, Pedro Casado, Jose Curbelo, Angela Gutierrez, Azucena Bautista, Nuria Ruiz Giménez, Angelica Fernandez, Pedro Parra, Berta Moyano, Ana Barrios, Diego Real de Asua, Beatriz Sanchez, Carmen Saez, Marianela Ciudad, Desiré Navas, Laura Cardeñoso Domingo, María Del Carmen Cuevas Torresano, Diego Domingo García, Teresa Alarcón Cavero, Alicia García Blanco, Alexandra Martín Ramírez, María Auxiliadora Semiglia Chong, Ainhoa Gutiérrez Cobos, Nelly Daniela Zurita Cruz, Arturo Manuel Fraile Torres, Carmen Sanchez-Gonzalez, Antonio Fernádez Perpén, Carolina Díaz Pérez, Julio Ancochea, Tamara Alonso, Pedro Landete, Joan Soriano, Carolina Cisneros, Elena García Castillo, Francisco Javier García Pérez, Rosa María Girón, Celeste Marcos, Enrique Zamora, Patricia García García, Santos Castañeda, Rosario García-Vicuña, Isidoro González-Álvaro, Sebastián Rodríguez-García, Carlos Fernández-Díaz, Irene Llorente Cubas, Eva G Tomero, Noelia García Castañeda, Ana Ma Ortiz, Cristina Valero, Miren Uriarte, Nuria Montes

Affiliations

- [1] Internal Medicine Service, Hospital Universitario de la Princesa, Universidad Autónoma Madrid, Instituto de Investigación Sanitaria Princesa (IIS-IP), Madrid, Spain.

- [2] Rheumatology Service, Hospital Universitario de la Princesa, Universidad Autónoma Madrid, Instituto de Investigación Sanitaria Princesa (IIS-IP), Madrid, Spain.
- [3] Immunology Service, Hospital Universitario de la Princesa, Universidad Autónoma Madrid, Instituto de Investigación Sanitaria Princesa (IIS-IP), Madrid, Spain.
- [4] Pneumology Service, Hospital Universitario de la Princesa, Universidad Autónoma Madrid, Instituto de Investigación Sanitaria Princesa (IIS-IP), Madrid, Spain.
- [5] Intensive Care Unit, Hospital Universitario de la Princesa, Universidad Autónoma Madrid, Instituto de Investigación Sanitaria Princesa (IIS-IP), Madrid, Spain.
- [6] Emergency Service, Hospital Universitario de la Princesa, Universidad Autónoma Madrid, Instituto de Investigación Sanitaria Princesa (IIS-IP), Madrid, Spain.
- [7] Hematology Service, Hospital Universitario de la Princesa, Universidad Autónoma Madrid, Instituto de Investigación Sanitaria Princesa (IIS-IP), Madrid, Spain.
- [8] Hospital Pharmacy Service, Hospital Universitario de la Princesa, Universidad Autónoma Madrid, Instituto de Investigación Sanitaria Princesa (IIS-IP), Madrid, Spain.
- [9] Microbiology Service, Hospital Universitario de la Princesa, Universidad Autónoma Madrid, Instituto de Investigación Sanitaria Princesa (IIS-IP), Madrid, Spain.
- [10] Immunology Service, Hospital Universitario de la Princesa, Universidad Autónoma Madrid, Instituto de Investigación Sanitaria Princesa (IIS-IP), Madrid, Spain; Centro de Investigación Biomédica en Red - Enfermedades Cardiovasculares (CIBER CV), Instituto de Salud Carlos III (ISCIII), Madrid, Spain.
- [11] Rheumatology Service, Hospital Universitario de la Princesa, Universidad Autónoma Madrid, Instituto de Investigación Sanitaria Princesa (IIS-IP), Madrid, Spain. Electronic address: isidoro.ga@ser.es.

- PMID: **33010257**
- PMCID: PMC7525244
- DOI: 10.1016/j.jaci.2020.09.018

Erratum in

- Corrigendum.
  [No authors listed] [No authors listed] J Allergy Clin Immunol. 2021 Jul;148(1):281. doi: 10.1016/j.jaci.2021.03.002. J Allergy Clin Immunol. 2021. PMID: 34238504 Free PMC article. No abstract available.

Abstract

Background: Patients with coronavirus disaese 2019 (COVID-19) can develop a cytokine release syndrome that eventually leads to acute respiratory distress syndrome requiring invasive mechanical ventilation (IMV). Because IL-6 is a relevant cytokine in acute respiratory distress syndrome, the blockade of its receptor with tocilizumab (TCZ) could reduce mortality and/or morbidity in severe COVID-19.

Objective: We sought to determine whether baseline IL-6 serum levels can predict the need for IMV and the response to TCZ.

Methods: A retrospective observational study was performed in hospitalized patients diagnosed with COVID-19. Clinical information and laboratory findings, including IL-6 levels, were collected approximately 3 and 9 days after admission to be matched with preadministration and postadministration of TCZ. Multivariable logistic and linear regressions and survival analysis

were performed depending on outcomes: need for IMV, evolution of arterial oxygen tension/fraction of inspired oxygen ratio, or mortality.

Results: One hundred forty-six patients were studied, predominantly males (66%); median age was 63 years. Forty-four patients (30%) required IMV, and 58 patients (40%) received treatment with TCZ. IL-6 levels greater than 30 pg/mL was the best predictor for IMV (odds ratio, 7.1; P < .001). Early administration of TCZ was associated with improvement in oxygenation (arterial oxygen tension/fraction of inspired oxygen ratio) in patients with high IL-6 (P = .048). Patients with high IL-6 not treated with TCZ showed high mortality (hazard ratio, 4.6; P = .003), as well as those with low IL-6 treated with TCZ (hazard ratio, 3.6; P = .016). No relevant serious adverse events were observed in TCZ-treated patients.

Conclusions: Baseline IL-6 greater than 30 pg/mL predicts IMV requirement in patients with COVID-19 and contributes to establish an adequate indication for TCZ administration.

Keywords: COVID-19; IL-6; invasive mechanical ventilation; tocilizumab.

Copyright © 2020 American Academy of Allergy, Asthma & Immunology. Published by Elsevier Inc. All rights reserved.

- 33 references
- 7 figures

Supplementary info

Publication types, MeSH terms, Substances Expand

Publication types

- Clinical Trial
- Observational Study
- Research Support, Non-U.S. Gov't

MeSH terms

- Adult
- Aged
- Aged, 80 and over
- Antibodies, Monoclonal, Humanized / administration & dosage*
- COVID-19* / blood
- COVID-19* / drug therapy
- COVID-19* / mortality
- Cytokine Release Syndrome* / blood
- Cytokine Release Syndrome* / drug therapy
- Cytokine Release Syndrome* / mortality
- Disease-Free Survival
- Female

- Humans
- Interleukin-6 / blood*
- Male
- Middle Aged
- Retrospective Studies
- SARS-CoV-2*
- Survival Rate

Substances

- Antibodies, Monoclonal, Humanized
- IL6 protein, human
- Interleukin-6
- tocilizumab

Full text links

ELSEVIER FULL-TEXT ARTICLE Elsevier Science Free PMC article
Proceed to details
Cite
Share
☐ 1,011
Observational Study
Eur J Ophthalmol
. 2021 Nov;31(6):3490-3493.
doi: 10.1177/11206721211001315. Epub 2021 Mar 15.

Impact of COVID-19 on keratoconus patients waiting for corneal cross linking

Haider Shah [1,2], Luca Pagano [1,3], Anuj Vakharia [1], Giulia Coco [1,4], Kunal A Gadhvi [1], Stephen B Kaye [1,5], Vito Romano [1,5]
Affiliations Expand

Affiliations

- [1] Department of Ophthalmology, Royal Liverpool University Hospital, Liverpool, UK.
- [2] Department of Corneal and External Eye Diseases, St Paul's Eye Unit, Royal Liverpool University Hospital, Liverpool, UK.
- [3] Department of Biomedical Sciences, Humanitas University, Pieve Emanuele, Milan, Italy.
- [4] Department of Clinical Sciences and Translational Medicine, University of Tor Vergata, Rome, Italy.
- [5] Department of Eye and Vision Science, University of Liverpool, Liverpool, UK.

- PMID: **33719638**
- DOI: 10.1177/11206721211001315

Observational Study

Impact of COVID-19 on keratoconus patients waiting for corneal cross linking

Haider Shah et al. Eur J Ophthalmol. 2021 Nov.

Show details

Eur J Ophthalmol

. 2021 Nov;31(6):3490-3493.
doi: 10.1177/11206721211001315. Epub 2021 Mar 15.

Authors

Haider Shah [1, 2], Luca Pagano [1, 3], Anuj Vakharia [1], Giulia Coco [1, 4], Kunal A Gadhvi [1], Stephen B Kaye [1, 5], Vito Romano [1, 5]

Affiliations

- [1] Department of Ophthalmology, Royal Liverpool University Hospital, Liverpool, UK.
- [2] Department of Corneal and External Eye Diseases, St Paul's Eye Unit, Royal Liverpool University Hospital, Liverpool, UK.
- [3] Department of Biomedical Sciences, Humanitas University, Pieve Emanuele, Milan, Italy.
- [4] Department of Clinical Sciences and Translational Medicine, University of Tor Vergata, Rome, Italy.
- [5] Department of Eye and Vision Science, University of Liverpool, Liverpool, UK.

- PMID: **33719638**
- DOI: 10.1177/11206721211001315

Abstract

Purpose: Royal College of Ophthalmologist recent guidance recommended delaying cross-linking services during the COVID-19 pandemic. This study investigates the effects of such delays in the delivery of cross-linking services in patients with keratoconus progression.

Methods: Retrospective observational study of 46 patients with keratoconus progression, whose cross-linking was delayed due to the COVID-19 pandemic. Demographic and clinical details were obtained from assessments on the day of listing, and subsequent review on the day of the procedure. Topographic indices included keratometry of the posterior and anterior corneal surface, maximum keratometry (K_{max}), thinnest corneal thickness, ABCD progression and progression based on standard criteria recommendations (1.5 D K_{max} & 20 microns thinning).

Results: A total of 46 eyes were analysed with an average time between being listed for CXL and having the procedure done was 182 ± 65 days. The delay due to COVID-19 was of 3 months. In this time period they had a significant worsening of all keratometric indices and lost almost one

line of visual acuity (0.19 ± 0.19 to 0.26 ± 0.18 LogMAR, *p*: 0.03). Thirty two eyes (70%) demonstrated progression in accordance with the ABCD progression criteria, while 18 eyes (39%) showed either an increase in K_{max} of more than 1.5D or a thinning in corneal thickness of at least 20 µm.

Conclusions: The treatment delay for the keratoconus patients caused further progression and vision worsening. We recommend that corneal collagen crosslinking needs to be considered as a high priority intervention.

Keywords: Corneal dystrophies; cornea/external disease; corneal procedures for astigmatism; corneal topography; corneal topography/imaging systems; epidemiology/biostatistics; examination techniques: corneal topography/keratometry; lens/cataract; refractive surgery.

Supplementary info

Publication types, MeSH terms, Substances Expand

Publication types

- Observational Study

MeSH terms

- COVID-19*
- Collagen / therapeutic use
- Corneal Topography
- Cross-Linking Reagents / therapeutic use
- Humans
- Keratoconus* / drug therapy
- Pandemics
- Photochemotherapy*
- Photosensitizing Agents / therapeutic use
- Riboflavin / therapeutic use
- SARS-CoV-2
- Ultraviolet Rays

Substances

- Cross-Linking Reagents
- Photosensitizing Agents
- Collagen
- Riboflavin

Full text links

SAGE journals Atypon
Proceed to details
Cite
Share
☐ 1,012
Observational Study
J Am Coll Cardiol
. 2020 Nov 3;76(18):2060-2072.
doi: 10.1016/j.jacc.2020.08.070.

Registry of Arterial and Venous Thromboembolic Complications in Patients With COVID-19

Gregory Piazza [1], Umberto Campia [2], Shelley Hurwitz [3], Julia E Snyder [2], Samantha M Rizzo [2], Mariana B Pfeferman [2], Ruth B Morrison [2], Orly Leiva [4], John Fanikos [5], Victor Nauffal [2], Zaid Almarzooq [2], Samuel Z Goldhaber [2]
Affiliations Expand

Affiliations

- [1] Division of Cardiovascular Medicine, Department of Medicine, Brigham and Women's Hospital, Harvard Medical School, Boston, Massachusetts. Electronic address: gpiazza@partners.org.
- [2] Division of Cardiovascular Medicine, Department of Medicine, Brigham and Women's Hospital, Harvard Medical School, Boston, Massachusetts.
- [3] Division of Infectious Diseases, Department of Medicine, Brigham and Women's Hospital, Harvard Medical School, Boston, Massachusetts.
- [4] Department of Medicine, Brigham and Women's Hospital, Harvard Medical School, Boston, Massachusetts.
- [5] Department of Pharmacy, Brigham and Women's Hospital, Harvard Medical School, Boston, Massachusetts.

- PMID: **33121712**
- PMCID: PMC7588178
- DOI: 10.1016/j.jacc.2020.08.070

Free PMC article
Observational Study

Registry of Arterial and Venous Thromboembolic Complications in Patients With COVID-19

Gregory Piazza et al. J Am Coll Cardiol. 2020.
Free PMC article
Show details
J Am Coll Cardiol
. 2020 Nov 3;76(18):2060-2072.
doi: 10.1016/j.jacc.2020.08.070.

Authors

Gregory Piazza [1], Umberto Campia [2], Shelley Hurwitz [3], Julia E Snyder [2], Samantha M Rizzo [2], Mariana B Pfeferman [2], Ruth B Morrison [2], Orly Leiva [4], John Fanikos [5], Victor Nauffal [2], Zaid Almarzooq [2], Samuel Z Goldhaber [2]

Affiliations

- [1] Division of Cardiovascular Medicine, Department of Medicine, Brigham and Women's Hospital, Harvard Medical School, Boston, Massachusetts. Electronic address: gpiazza@partners.org.
- [2] Division of Cardiovascular Medicine, Department of Medicine, Brigham and Women's Hospital, Harvard Medical School, Boston, Massachusetts.
- [3] Division of Infectious Diseases, Department of Medicine, Brigham and Women's Hospital, Harvard Medical School, Boston, Massachusetts.
- [4] Department of Medicine, Brigham and Women's Hospital, Harvard Medical School, Boston, Massachusetts.
- [5] Department of Pharmacy, Brigham and Women's Hospital, Harvard Medical School, Boston, Massachusetts.

- PMID: **33121712**
- PMCID: PMC7588178
- DOI: 10.1016/j.jacc.2020.08.070

Abstract

Background: Cardiovascular complications, including myocardial infarction, ischemic stroke, and pulmonary embolism, represent an important source of adverse outcomes in coronavirus disease-2019 (COVID-19).

Objectives: To assess the frequency of arterial and venous thromboembolic disease, risk factors, prevention and management patterns, and outcomes in patients with COVID-19, the authors designed a multicenter, observational cohort study.

Methods: We analyzed a retrospective cohort of 1,114 patients with COVID-19 diagnosed through our Mass General Brigham integrated health network. The total cohort was analyzed by

site of care: intensive care (n = 170); hospitalized nonintensive care (n = 229); and outpatient (n = 715). The primary study outcome was a composite of adjudicated major arterial or venous thromboembolism.

Results: Patients with COVID-19 were 22.3% Hispanic/Latinx and 44.2% non-White. Cardiovascular risk factors of hypertension (35.8%), hyperlipidemia (28.6%), and diabetes (18.0%) were common. Prophylactic anticoagulation was prescribed in 89.4% of patients with COVID-19 in the intensive care cohort and 84.7% of those in the hospitalized nonintensive care setting. Frequencies of major arterial or venous thromboembolism, major cardiovascular adverse events, and symptomatic venous thromboembolism were highest in the intensive care cohort (35.3%, 45.9%, and 27.0 %, respectively) followed by the hospitalized nonintensive care cohort (2.6%, 6.1%, and 2.2%, respectively) and the outpatient cohort (0% for all).

Conclusions: Major arterial or venous thromboembolism, major adverse cardiovascular events, and symptomatic venous thromboembolism occurred with high frequency in patients with COVID-19, especially in the intensive care setting, despite a high utilization rate of thromboprophylaxis.

Keywords: COVID-19; anticoagulation; cardiovascular disease; coronavirus; deep venous thrombosis; myocardial infarction; pulmonary embolism; stroke; thromboembolism.

Copyright © 2020 The Authors. Published by Elsevier Inc. All rights reserved.

Comment in

- Thromboembolism and the Pandemic.
  McBane RD 2nd. McBane RD 2nd. J Am Coll Cardiol. 2020 Nov 3;76(18):2073-2075. doi: 10.1016/j.jacc.2020.09.543. J Am Coll Cardiol. 2020. PMID: 33121713 Free PMC article.

- 33 references
- 3 figures

Supplementary info

Publication types, MeSH terms, Substances Expand

Publication types

- Observational Study

MeSH terms

- Adult
- Aged
- Anticoagulants / therapeutic use*
- Betacoronavirus
- COVID-19
- Coronavirus Infections / complications*
- Coronavirus Infections / mortality

- Female
- Humans
- Intensive Care Units / statistics & numerical data
- Male
- Massachusetts / epidemiology
- Middle Aged
- Pandemics
- Pneumonia, Viral / complications*
- Pneumonia, Viral / mortality
- Registries*
- Retrospective Studies
- Risk Factors
- SARS-CoV-2
- Thromboembolism / epidemiology
- Thromboembolism / prevention & control
- Thromboembolism / virology*

Substances

- Anticoagulants

Full text links

ELSEVIER OPEN ACCESS Elsevier Science Free PMC article

Proceed to details

Cite

Share

1,013

Observational Study

J Clin Apher
. 2021 Jun;36(3):313-321.
doi: 10.1002/jca.21861. Epub 2020 Dec 15.

Direct hemoperfusion using a polymyxin B-immobilized polystyrene column for COVID-19

Daisuke Katagiri [1], Masahiro Ishikane [2], Yusuke Asai [3], Shinyu Izumi [4], Jin Takasaki [4], Hiyori Katsuoka [5], Isao Kondo [1], Satoshi Ide [2], Keiji Nakamura [2], Takato Nakamoto [2], Hidetoshi Nomoto [2], Yutaro Akiyama [2], Yusuke Miyazato [2], Tetsuya Suzuki [2], Noriko Kinoshita [2], Tatsunori Ogawa [5], Tomiteru Togano [6], Manabu Suzuki [4], Masao Hashimoto [4], Keita

Sakamoto [4], Yusaku Kusaba [4], Takashi Katsuno [4], Takashi Fukaya [5], Masayuki Hojo [4], Masaya Sugiyama [7], Masashi Mizokami [7], Tatsuya Okamoto [8], Akio Kimura [9], Eisei Noiri [10], Norio Ohmagari [2], Fumihiko Hinoshita [1], Haruhito Sugiyama [10]
Affiliations Expand

Affiliations

- [1] Department of Nephrology, National Center for Global Health and Medicine, Tokyo, Japan.
- [2] Disease Control and Prevention Center, National Center for Global Health and Medicine, Tokyo, Japan.
- [3] Antimicrobial Resistance Clinical Reference Center, Disease Control and Prevention Center, National Center for Global Health and Medicine, Tokyo, Japan.
- [4] Department of Respiratory Medicine, National Center for Global Health and Medicine, Tokyo, Japan.
- [5] Medical Equipment Management Office, National Center for Global Health and Medicine, Tokyo, Japan.
- [6] Department of Hematology, National Center for Global Health and Medicine, Tokyo, Japan.
- [7] Genome Medical Sciences Project, Research Institute, National Center for Global Health and Medicine, Ichikawa, Japan.
- [8] Department of Intensive Care Medicine, National Center for Global Health and Medicine, Tokyo, Japan.
- [9] Department of Emergency Medicine and Critical Care, National Center for Global Health and Medicine, Tokyo, Japan.
- [10] National Center Biobank Network, National Center for Global Health and Medicine, Tokyo, Japan.

- PMID: **33325084**
- PMCID: PMC8246724
- DOI: 10.1002/jca.21861

Free PMC article
Observational Study

Direct hemoperfusion using a polymyxin B-immobilized polystyrene column for COVID-19

Daisuke Katagiri et al. J Clin Apher. 2021 Jun.
Free PMC article
Show details
J Clin Apher
. 2021 Jun;36(3):313-321.
doi: 10.1002/jca.21861. Epub 2020 Dec 15.

Authors

Daisuke Katagiri [1], Masahiro Ishikane [2], Yusuke Asai [3], Shinyu Izumi [4], Jin Takasaki [4], Hiyori Katsuoka [5], Isao Kondo [1], Satoshi Ide [2], Keiji Nakamura [2], Takato Nakamoto [2], Hidetoshi Nomoto [2], Yutaro Akiyama [2], Yusuke Miyazato [2], Tetsuya Suzuki [2], Noriko Kinoshita [2], Tatsunori Ogawa [5], Tomiteru Togano [6], Manabu Suzuki [4], Masao Hashimoto [4], Keita Sakamoto [4], Yusaku Kusaba [4], Takashi Katsuno [4], Takashi Fukaya [5], Masayuki Hojo [4], Masaya Sugiyama [7], Masashi Mizokami [7], Tatsuya Okamoto [8], Akio Kimura [9], Eisei Noiri [10], Norio Ohmagari [2], Fumihiko Hinoshita [1], Haruhito Sugiyama [10]

Affiliations

- [1] Department of Nephrology, National Center for Global Health and Medicine, Tokyo, Japan.
- [2] Disease Control and Prevention Center, National Center for Global Health and Medicine, Tokyo, Japan.
- [3] Antimicrobial Resistance Clinical Reference Center, Disease Control and Prevention Center, National Center for Global Health and Medicine, Tokyo, Japan.
- [4] Department of Respiratory Medicine, National Center for Global Health and Medicine, Tokyo, Japan.
- [5] Medical Equipment Management Office, National Center for Global Health and Medicine, Tokyo, Japan.
- [6] Department of Hematology, National Center for Global Health and Medicine, Tokyo, Japan.
- [7] Genome Medical Sciences Project, Research Institute, National Center for Global Health and Medicine, Ichikawa, Japan.
- [8] Department of Intensive Care Medicine, National Center for Global Health and Medicine, Tokyo, Japan.
- [9] Department of Emergency Medicine and Critical Care, National Center for Global Health and Medicine, Tokyo, Japan.
- [10] National Center Biobank Network, National Center for Global Health and Medicine, Tokyo, Japan.

- PMID: **33325084**
- PMCID: PMC8246724
- DOI: 10.1002/jca.21861

Abstract

Objective: To evaluate the efficacy and safety of direct hemoperfusion using a polymyxin B-immobilized polystyrene column (PMX-DHP) in severe acute respiratory syndrome coronavirus 2 (SARS-CoV-2)-positive pneumonia patients.

Methods: This study was a case series conducted at a designated infectious diseases hospital. Twelve SARS-CoV-2-positive patients with partial pressure of arterial oxygen/percentage of inspired oxygen (P/F) ratio < 300 were treated with PMX-DHP on two consecutive days each during hospitalization. We defined day 1 as the first day when PMX-DHP was performed. PMX-DHP efficacy was assessed on days 7 and 14 after the first treatment based on eight categories.

Subsequently, improvement in P/F ratio and urinary biomarkers on days 4 and 8, malfunctions, and ventilator and extracorporeal membrane oxygenation avoidance rates were also evaluated.

Results: On day 14 after the first treatment, disease severity decreased in 58.3% of the patients. P/F ratio increased while urine β2-microglobulin decreased on days 4 and 8. Cytokine measurement pre- and post-PMX-DHP revealed decreased levels of interleukin-6 and the factors involved in vascular endothelial injury, including vascular endothelial growth factor. Twenty-two PMX-DHPs were performed, of which seven and five PMX-DHPs led to increased inlet pressure and membrane coagulation, respectively. When the membranes coagulated, the circuitry needed to be reconfigured. Circuit problems were usually observed when D-dimer and fibrin degradation product levels were high before PMX-DHP.

Conclusions: Future studies are expected to determine the therapeutic effect of PMX-DHP on COVID-19. Because of the relatively high risk of circuit coagulation, coagulation capacity should be assessed beforehand.

Keywords: cytokine; pneumonia; steroids.

© 2020 The Authors. Journal of Clinical Apheresis published by Wiley Periodicals LLC.

- 42 references
- 3 figures

Supplementary info

Publication types, MeSH terms, Substances, Grant support Expand

Publication types

- Observational Study

MeSH terms

- Adult
- Aged
- Aged, 80 and over
- Arteries / metabolism
- Biomarkers / urine
- Blood Gas Analysis
- COVID-19 / therapy*
- Cytokines / blood
- Endothelium, Vascular / metabolism
- Female
- Hemoperfusion / instrumentation*
- Hemoperfusion / methods*
- Hospitalization
- Humans

- Male
- Middle Aged
- Oxygen / metabolism
- Polymyxin B / chemistry*
- Polystyrenes / chemistry*
- Respiration, Artificial
- Retrospective Studies
- Risk
- beta 2-Microglobulin / urine

Substances

- Biomarkers
- Cytokines
- Polystyrenes
- beta 2-Microglobulin
- Polymyxin B
- Oxygen

Grant support

- 20A-3002/Grants-in-Aid for Research from the National Center for Global Health and Medicine
- 20he0822003j0001/Japan Agency for Medical Research and Development

Full text links

WILEY Full Text Article Wiley Free PMC article

Proceed to details

Cite

Share

☐ 1,014

Observational Study

Diabetes Res Clin Pract

. 2020 Sep;167:108354.

doi: 10.1016/j.diabres.2020.108354. Epub 2020 Jul 30.

The impact of strict COVID-19 lockdown in Spain on glycemic profiles in patients with type 1 Diabetes prone to hypoglycemia using standalone continuous glucose monitoring

Alex Mesa [1], Clara Viñals [1], Irene Pueyo 1st [1], Daria Roca [1], Mercè Vidal [1], Marga Giménez [2], Ignacio Conget [3]
Affiliations Expand

Affiliations

- [1] Diabetes Unit, Endocrinology and Nutrition Department, Hospital Clínic i Universitari, Barcelona, Spain.
- [2] Diabetes Unit, Endocrinology and Nutrition Department, Hospital Clínic i Universitari, Barcelona, Spain; CIBERDEM, Centro de Investigación Biomédica en Red de Diabetes y Enfermedades Metabólicas, Madrid, Spain; IDIBAPS, Institut d'Investigacions Biomèdiques August Pi i Sunyer, Barcelona, Spain.
- [3] Diabetes Unit, Endocrinology and Nutrition Department, Hospital Clínic i Universitari, Barcelona, Spain; CIBERDEM, Centro de Investigación Biomédica en Red de Diabetes y Enfermedades Metabólicas, Madrid, Spain; IDIBAPS, Institut d'Investigacions Biomèdiques August Pi i Sunyer, Barcelona, Spain. Electronic address: iconget@clinic.cat.

- PMID: **32739380**
- PMCID: PMC7392049
- DOI: 10.1016/j.diabres.2020.108354

Free PMC article
Observational Study

The impact of strict COVID-19 lockdown in Spain on glycemic profiles in patients with type 1 Diabetes prone to hypoglycemia using standalone continuous glucose monitoring

Alex Mesa et al. Diabetes Res Clin Pract. 2020 Sep.
Free PMC article
Show details
Diabetes Res Clin Pract

. 2020 Sep;167:108354.
doi: 10.1016/j.diabres.2020.108354. Epub 2020 Jul 30.

Authors

Alex Mesa [1], Clara Viñals [1], Irene Pueyo 1st [1], Daria Roca [1], Mercè Vidal [1], Marga Giménez [2], Ignacio Conget [3]

Affiliations

- [1] Diabetes Unit, Endocrinology and Nutrition Department, Hospital Clínic i Universitari, Barcelona, Spain.
- [2] Diabetes Unit, Endocrinology and Nutrition Department, Hospital Clínic i Universitari, Barcelona, Spain; CIBERDEM, Centro de Investigación Biomédica en Red de Diabetes y Enfermedades Metabólicas, Madrid, Spain; IDIBAPS, Institut d'Investigacions Biomèdiques August Pi i Sunyer, Barcelona, Spain.
- [3] Diabetes Unit, Endocrinology and Nutrition Department, Hospital Clínic i Universitari, Barcelona, Spain; CIBERDEM, Centro de Investigación Biomédica en Red de Diabetes y Enfermedades Metabólicas, Madrid, Spain; IDIBAPS, Institut d'Investigacions Biomèdiques August Pi i Sunyer, Barcelona, Spain. Electronic address: iconget@clinic.cat.

- PMID: **32739380**
- PMCID: PMC7392049
- DOI: 10.1016/j.diabres.2020.108354

Abstract

Aims: Spain has been one of the worst affected countries by the COVID-19 pandemic. A very strict lockdown at home was imposed with a tough restriction of mobility. We aimed to evaluate the impact of this exceptional scenario on glucose profile of patients with T1D prone to hypoglycemia using standalone continuous glucose monitoring.

Methods: Patients with T1D prone to hypoglycemia using multiple daily injections and either a Dexcom G5® or a Free Style Libre® CGM systems for at least 6 months under the funding of National Health Service were included in an observational, retrospective study. Data were collected in two periods: pre-lockdown (PL), February 23rd-March 7th and within lockdown (WL), April 1st-14th 2020. The primary outcome was the difference in the proportion of time in target glucose range of 70-180 mg/dL (TIR). Additional glucometric data were also analysed.

Results: 92 patients were included: 40 women, age 42.8 ± 3.9 years, disease duration of 23.1 ± 12.6 years. Seventeen patients used Dexcom G5® and 75 Free Style Libre®. TIR 70-180 mg/dL (59.3 ± 16.2 vs 62.6 ± 15.2%), time > 180 (34.4 ± 18.0 vs 30.7 ± 16.9%), >250 (11.1 ± 10.6 vs 9.2 ± 9.7%) and Glucose Management Indicator (7.2 ± 0.8 vs 7.0 ± 0.8%) significantly improved (PL vs WL, respectively, $p < 0.05$). Time in hypoglycemia remained unchanged.

Conclusions: Lockdown conditions imposed by the COVID-19 pandemic may be managed successfully in terms of glycemic control by population with T1D prone to hypoglycemia using CGM. The strict daily routine at home could probably explain the improvement in the time in glycemic target without increasing the time in hypoglycemia.

Keywords: COVID-19 lockdown; Hypoglycemia; Standalone continuous glucose monitoring; Type 1 diabetes mellitus.

Copyright © 2020 Elsevier B.V. All rights reserved.

Conflict of interest statement

Declaration of Competing Interest Dr Viñals has received lecturing fees from NovoNordisk A/S, Medtronic Inc., Sanofi-Aventis and MSD. Dr Giménez has received lecturing and consulting fees from Medtronic Inc., Eli Lilly & Co., NovoNordisk A/S, Sanofi-Aventis, Astra Zeneca and MSD. Dr Conget reported receiving lecturing and consulting fees from Medtronic Inc., Bayer AG, GlaxoSmithKline, Eli Lilly & Co., NovoNordisk A/S, Sanofi-Aventis, Novartis, Astra Zeneca and MSD.

- 15 references

Supplementary info

Publication types, MeSH terms, Substances Expand

Publication types

- Observational Study

MeSH terms

- Adult
- Betacoronavirus
- Blood Glucose
- Blood Glucose Self-Monitoring*
- COVID-19
- Communicable Disease Control*
- Coronavirus Infections / epidemiology*
- Diabetes Mellitus, Type 1 / drug therapy*
- Diabetes Mellitus, Type 1 / metabolism
- Female
- Humans
- Hypoglycemia / chemically induced*
- Hypoglycemic Agents / therapeutic use*
- Injections
- Insulin / therapeutic use*
- Insulin, Long-Acting / therapeutic use
- Insulin, Short-Acting / therapeutic use
- Longitudinal Studies
- Male
- Middle Aged
- Monitoring, Ambulatory*
- Pandemics
- Pneumonia, Viral / epidemiology*

- Retrospective Studies
- SARS-CoV-2
- Spain / epidemiology
- State Medicine

Substances

- Blood Glucose
- Hypoglycemic Agents
- Insulin
- Insulin, Long-Acting
- Insulin, Short-Acting

Full text links

ELSEVIER FULL-TEXT ARTICLE Elsevier Science Free PMC article
Proceed to details
Cite
Share
1,015
Curr Med Sci
. 2021 Feb;41(1):39-45.
doi: 10.1007/s11596-021-2315-4. Epub 2021 Feb 13.

Infection-associated Hemophagocytic Syndrome in Critically Ill Patients with COVID-19

Kun Yang [# 1], Ming-You Xing [# 2], Ling-Yu Jiang [3], Yan-Ping Cai [4], Li-Li Yang [5], Na-Na Xie [2], Jia Chen [2], Wen-Xia Wang [2], Li Wang [2], Ji-Ling Zhu [6], Ding-Yu Zhang [4], Qiu-Rong Ruan [7, 8], Jian-Xin Song [9]
Affiliations Expand

Affiliations

- [1] Department of Dermatology and Venereology, Tongji Hospital, Tongji Medical College, Huazhong University of Science and Technology, Wuhan, 430030, China.
- [2] Department of Infectious Diseases, Tongji Hospital, Tongji Medical College, Huazhong University of Science and Technology, Wuhan, 430030, China.
- [3] Department of Clinical Immunology, Tongji Hospital, Tongji Medical College, Huazhong University of Science and Technology, Wuhan, 430030, China.
- [4] Wuhan Jinyintan Hospital, Wuhan, 430011, China.

- [5] Institute of Pathology, Tongji Hospital, Tongji Medical College, Huazhong University of Science and Technology, Wuhan, 430030, China.
- [6] Department of Infectious Diseases, Renmin Hospital of Wuhan University, Wuhan, 430060, China.
- [7] Institute of Pathology, Tongji Hospital, Tongji Medical College, Huazhong University of Science and Technology, Wuhan, 430030, China. ruanqiurong@sina.com.
- [8] Department of Pathology, School of Basic Medicine, Tongji Medical College, Huazhong University of Science and Technology, Wuhan, 430030, China. ruanqiurong@sina.com.
- [9] Department of Infectious Diseases, Tongji Hospital, Tongji Medical College, Huazhong University of Science and Technology, Wuhan, 430030, China. jxsong@tjh.tjmu.edu.cn.

[#] Contributed equally.

- PMID: **33582903**
- PMCID: PMC7881909
- DOI: 10.1007/s11596-021-2315-4

Free PMC article

Infection-associated Hemophagocytic Syndrome in Critically Ill Patients with COVID-19

Kun Yang et al. Curr Med Sci. 2021 Feb.
Free PMC article
Show details
Curr Med Sci

. 2021 Feb;41(1):39-45.
doi: 10.1007/s11596-021-2315-4. Epub 2021 Feb 13.

Authors

Kun Yang [#1], Ming-You Xing [#2], Ling-Yu Jiang [3], Yan-Ping Cai [4], Li-Li Yang [5], Na-Na Xie [2], Jia Chen [2], Wen-Xia Wang [2], Li Wang [2], Ji-Ling Zhu [6], Ding-Yu Zhang [4], Qiu-Rong Ruan [7] [8], Jian-Xin Song [9]

Affiliations

- [1] Department of Dermatology and Venereology, Tongji Hospital, Tongji Medical College, Huazhong University of Science and Technology, Wuhan, 430030, China.
- [2] Department of Infectious Diseases, Tongji Hospital, Tongji Medical College, Huazhong University of Science and Technology, Wuhan, 430030, China.
- [3] Department of Clinical Immunology, Tongji Hospital, Tongji Medical College, Huazhong University of Science and Technology, Wuhan, 430030, China.
- [4] Wuhan Jinyintan Hospital, Wuhan, 430011, China.
- [5] Institute of Pathology, Tongji Hospital, Tongji Medical College, Huazhong University of Science and Technology, Wuhan, 430030, China.

- [6] Department of Infectious Diseases, Renmin Hospital of Wuhan University, Wuhan, 430060, China.
- [7] Institute of Pathology, Tongji Hospital, Tongji Medical College, Huazhong University of Science and Technology, Wuhan, 430030, China. ruanqiurong@sina.com.
- [8] Department of Pathology, School of Basic Medicine, Tongji Medical College, Huazhong University of Science and Technology, Wuhan, 430030, China. ruanqiurong@sina.com.
- [9] Department of Infectious Diseases, Tongji Hospital, Tongji Medical College, Huazhong University of Science and Technology, Wuhan, 430030, China. jxsong@tjh.tjmu.edu.cn.

[#] Contributed equally.

- PMID: **33582903**
- PMCID: PMC7881909
- DOI: 10.1007/s11596-021-2315-4

Abstract

Infection-associated hemophagocytic syndrome (IAHS), a severe complication of various infections, is potentially fatal. This study aims to determine whether IAHS occurs in critically ill patients with coronavirus disease 2019 (COVID-19). We conducted a retrospective observational study on 268 critically ill patients with COVID-19 between February 1st, 2020 and February 26th, 2020. Demographics, clinical characteristics, laboratory results, information on concurrent treatments and outcomes were collected. A diagnosis of secondary hemophagocytic lymphohistiocytosis (sHLH) was made when the patients had an HScore greater than 169. Histopathological examinations were performed to confirm the presence of hemophagocytosis. Of 268 critically ill patients with confirmed SARS-CoV-2 infection, 17 (6.3%) patients had an HScore greater than 169. All the 17 patients with sHLH died. The interval from the onset of symptom of COVID-19 to the time of a diagnosis of sHLH made was 19 days and the interval from the diagnosis of sHLH to death was 4 days. Ten (59%) patients were infected with only SARS-CoV-2. Hemophagocytosis in the spleen and the liver, as well as lymphocyte infiltration in the liver on histopathological examinations, was found in 3 sHLH autopsy patients. Mortality in sHLH patients with COVID-19 is high. And SARS-CoV-2 is a potential trigger for sHLH. Prompt recognition of IAHS in critically ill patients with COVID-19 could be beneficial for improving clinical outcomes.

Keywords: coronavirus disease 2019; infection-associated hemophagocytic syndrome; severe acute respiratory coronavirus 2.

- 27 references

Supplementary info

MeSH terms Expand

MeSH terms

- Adult
- Aged
- COVID-19 / complications*
- COVID-19 / mortality

- Critical Illness
- Female
- Humans
- Lymphohistiocytosis, Hemophagocytic / etiology
- Lymphohistiocytosis, Hemophagocytic / mortality*
- Male
- Middle Aged
- Mortality
- Prognosis
- Retrospective Studies

Full text links

Springer Free PMC article

Proceed to details

Cite

Share

1,016

Observational Study

Am J Gastroenterol
. 2021 Apr;116(4):838-840.
doi: 10.14309/ajg.0000000000000978.

Gastrointestinal Hemorrhages in Patients With COVID-19 Managed With Transarterial Embolization

Anna Maria Ierardi [1], Costantino Del Giudice, Andrea Coppola, Aldo Carnevale, Melchiore Giganti, Matteo Renzulli, Vania Tacher, Jose Urbano, Hicham Kobeiter, Romaric Loffroy, Marc Sapoval, Gianpaolo Carrafiello

Affiliations Expand

Affiliation

- [1] 1UOC Radiologia, Fondazione IRCCS Ca' Granda Ospedale Maggiore Policlinico, Milan, Itlay; 2Vascular and Oncological Interventional Radiology, Hopital Georges Pompidou, Paris, France; 3UO Radiologia, ASST Settelaghi, Ospedale di Circolo e Fondazione Macchi, Varese, Italy; 4Radiology Department, University Radiology Unit, Sant'Anna University Hospital, Ferrara, Italy; 5Department of Experimental, Diagnostic and Speciality Medicine, UO Radiologia, Sant'Orsola Hospital, University of Bologna, Bologna, Italy; 6Hôpitaux de Paris (AP-HP), Service d'Imagerie Médicale, CHU Henri Mondor, Créteil, France; 7Instituto de Investigaciones Sanitarias RyC, Research coordinator at Vascular and Interventional Service, Ramón y Cajal University Hospital, Madrid, Spain; 8CHU Djon,

Dijon, France; 9Department of Health Sciences, Università degli Studi di Milano, Milan, Italy.

- PMID: **33982964**
- PMCID: PMC7553031
- DOI: 10.14309/ajg.0000000000000978

Free PMC article
Observational Study

Gastrointestinal Hemorrhages in Patients With COVID-19 Managed With Transarterial Embolization

Anna Maria Ierardi et al. Am J Gastroenterol. 2021 Apr.
Free PMC article
Show details
Am J Gastroenterol
. 2021 Apr;116(4):838-840.
doi: 10.14309/ajg.0000000000000978.

Authors

Anna Maria Ierardi [1], Costantino Del Giudice, Andrea Coppola, Aldo Carnevale, Melchiore Giganti, Matteo Renzulli, Vania Tacher, Jose Urbano, Hicham Kobeiter, Romaric Loffroy, Marc Sapoval, Gianpaolo Carrafiello

Affiliation

- [1] 1UOC Radiologia, Fondazione IRCCS Ca' Granda Ospedale Maggiore Policlinico, Milan, Itlay; 2Vascular and Oncological Interventional Radiology, Hopital Georges Pompidou, Paris, France; 3UO Radiologia, ASST Settelaghi, Ospedale di Circolo e Fondazione Macchi, Varese, Italy; 4Radiology Department, University Radiology Unit, Sant'Anna University Hospital, Ferrara, Italy; 5Department of Experimental, Diagnostic and Speciality Medicine, UO Radiologia, Sant'Orsola Hospital, University of Bologna, Bologna, Italy; 6Hôpitaux de Paris (AP-HP), Service d'Imagerie Médicale, CHU Henri Mondor, Créteil, France; 7Instituto de Investigaciones Sanitarias RyC, Research coordinator at Vascular and Interventional Service, Ramón y Cajal University Hospital, Madrid, Spain; 8CHU Djon, Dijon, France; 9Department of Health Sciences, Università degli Studi di Milano, Milan, Italy.

- PMID: **33982964**
- PMCID: PMC7553031
- DOI: 10.14309/ajg.0000000000000978

No abstract available

Conflict of interest statement

Guarantor of the article: Anna Maria Ierardi, MD.

Specific author contributions: A.M.I. and G.C.: study concept and design. A.M.I., C.D.G., A. Coppola, H.K., V.T., J.U., and R.L.: acquisition of data. A.M.I. and A.Coppola: statistical analysis. A.M.I., M.G., and M.R.: interpretation of data. A.M.I., A. Coppola, and A. Carnevale: drafting of the manuscript. All coauthors: critical revision of the manuscript for important intellectual content. A.M.I. and G.C.: study supervision. All authors approved the final draft submitted.

Financial support: None to report.

Potential competing interests: None to report.

- 5 references

Supplementary info

Publication types, MeSH terms Expand

Publication types

- Letter
- Multicenter Study
- Observational Study

MeSH terms

- Aged
- Arteries
- COVID-19 / complications*
- Embolization, Therapeutic* / methods
- Female
- Gastrointestinal Hemorrhage / etiology*
- Gastrointestinal Hemorrhage / therapy*
- Humans
- Male
- Middle Aged
- Retrospective Studies

Full text links

Wolters Kluwer Wolters Kluwer Free PMC article
Proceed to details
Cite

Share

1,017

Observational Study

Clin Chim Acta

. 2020 Oct;509:135-138.

doi: 10.1016/j.cca.2020.06.012. Epub 2020 Jun 9.

Lactate dehydrogenase and C-reactive protein as predictors of respiratory failure in CoVID-19 patients

Erika Poggiali [1], Domenica Zaino [2], Paolo Immovilli [2], Luca Rovero [1], Giulia Losi [1], Alessandro Dacrema [1], Marzia Nuccetelli [3], Giovanni Battista Vadacca [4], Donata Guidetti [2], Andrea Vercelli [1], Andrea Magnacavallo [1], Sergio Bernardini [3], Chiara Terracciano [5]

Affiliations Expand

Affiliations

- [1] Emergency Department, "Guglielmo da Saliceto" Hospital, Piacenza, Italy.
- [2] Neurology Unit, "Guglielmo da Saliceto" Hospital, Piacenza, Italy.
- [3] Laboratory Medicine, Department of Experimental Medicine and Surgery, Tor Vergata University Hospital, Rome, Italy.
- [4] Biochemestry Unit, Clinical Pathology Department, "Guglielmo da Saliceto" Hospital, Piacenza, Italy.
- [5] Neurology Unit, "Guglielmo da Saliceto" Hospital, Piacenza, Italy. Electronic address: c.terracciano@ausl.pc.it.

- PMID: **32531257**
- PMCID: PMC7282743
- DOI: 10.1016/j.cca.2020.06.012

Free PMC article

Observational Study

Lactate dehydrogenase and C-reactive protein as predictors of respiratory failure in CoVID-19 patients

Erika Poggiali et al. Clin Chim Acta. 2020 Oct.

Free PMC article

Show details

Clin Chim Acta

. 2020 Oct;509:135-138.

doi: 10.1016/j.cca.2020.06.012. Epub 2020 Jun 9.

Authors

Erika Poggiali [1], Domenica Zaino [2], Paolo Immovilli [2], Luca Rovero [1], Giulia Losi [1], Alessandro Dacrema [1], Marzia Nuccetelli [3], Giovanni Battista Vadacca [4], Donata Guidetti [2], Andrea Vercelli [1], Andrea Magnacavallo [1], Sergio Bernardini [3], Chiara Terracciano [5]

Affiliations

- [1] Emergency Department, "Guglielmo da Saliceto" Hospital, Piacenza, Italy.
- [2] Neurology Unit, "Guglielmo da Saliceto" Hospital, Piacenza, Italy.
- [3] Laboratory Medicine, Department of Experimental Medicine and Surgery, Tor Vergata University Hospital, Rome, Italy.
- [4] Biochemestry Unit, Clinical Pathology Department, "Guglielmo da Saliceto" Hospital, Piacenza, Italy.
- [5] Neurology Unit, "Guglielmo da Saliceto" Hospital, Piacenza, Italy. Electronic address: c.terracciano@ausl.pc.it.

- PMID: **32531257**
- PMCID: PMC7282743
- DOI: 10.1016/j.cca.2020.06.012

Abstract

Objective: The dramatic worldwide CoVID-19 infection requires the identification of a reliable and inexpensive tool to quickly discriminate patients with a more unfavorable outcome.

Methods: We performed routine laboratory tests suitable to identify tissue damage and inflammatory status in 123 consecutive CoVID-19 patients admitted to the Emergency Department of the hospital of Piacenza (Emilia-Romagna, Northern Italy). The results were correlated with patients' respiratory function evaluated by the partial pressure of arterial oxygen to fraction of inspired oxygen ratio (PaO2/FiO2).

Results: The most common laboratory abnormalities were lymphocytopenia and elevated values of C-reactive protein (CRP) and lactate dehydrogenase (LDH). Aspartate aminotransferase (AST), alanine aminotransferase (ALT) and creatine kinase (CK) were also increased. The respiratory performance (PaO2/FiO2) showed a strong inverse correlation with LDH ($r = 0.62$, r^2 0.38, p value < 0.0001) and CRP ($r = 0.55$, r^2 0.31, p value < 0.0001). PaO2/FiO2 values also showed a significant inverse correlation with age ($r = -0.37$, $p < 0.0001$), AST ($r = -0.31$, $p < 0.01$), WBC ($r = -0.49$, $p < 0.0001$), neutrophils count ($r = -0.5$, $p < 0.001$). ROC curves showed a sensitivity of 75% and specificity of 70% for the LDH cut-off value of 450 U/L and a sensitivity of 72% and specificity of 71% for the CRP cut-off value of 11 mg/dl in identifying CoVID-19 with moderate-severe ARDS.

Conclusions: LDH and CRP may be related to respiratory function (PaO2/FiO2) and be a predictor of respiratory failure in CoVID-19 patients. LDH and CRP should be considered a useful test for the early identification of patients who require closer respiratory monitoring and more aggressive supportive therapies to avoid poor prognosis.

Keywords: Acute respiratory failure; CRP; CoVID-19; CoVID-19 pneumonia; Italian epidemic; LDH.

Copyright © 2020 Elsevier B.V. All rights reserved.

Conflict of interest statement

Declaration of Competing Interest The authors declare that they have no conflict of interest.

- 21 references
- 2 figures

Supplementary info

Publication types, MeSH terms, Substances Expand

Publication types

- Observational Study

MeSH terms

- Adult
- Aged
- Aged, 80 and over
- Betacoronavirus*
- Biomarkers / blood
- C-Reactive Protein / metabolism*
- COVID-19
- Clinical Laboratory Techniques / methods
- Clinical Laboratory Techniques / trends
- Coronavirus Infections / blood*
- Coronavirus Infections / diagnosis
- Coronavirus Infections / epidemiology
- Female
- Humans
- Italy / epidemiology
- L-Lactate Dehydrogenase / blood*
- Male
- Middle Aged
- Pandemics
- Patient Admission / trends
- Pneumonia, Viral / blood*
- Pneumonia, Viral / diagnosis
- Pneumonia, Viral / epidemiology

- Predictive Value of Tests
- Respiratory Insufficiency / blood*
- Respiratory Insufficiency / diagnosis
- Respiratory Insufficiency / epidemiology
- Retrospective Studies
- SARS-CoV-2
- Young Adult

Substances

- Biomarkers
- C-Reactive Protein
- L-Lactate Dehydrogenase

Full text links

ELSEVIER FULL-TEXT ARTICLE Elsevier Science Free PMC article
Proceed to details
Cite
Share
1,018
Observational Study
BMC Emerg Med
. 2021 May 1;21(1):56.
doi: 10.1186/s12873-021-00449-9.

Delayed emergency healthcare seeking behaviour by Dutch emergency department visitors during the first COVID-19 wave: a mixed methods retrospective observational study

Maaike Nab [1], Robyn van Vehmendahl [1], Inne Somers [1], Yvonne Schoon [2], Gijs Hesselink [3] [4]
Affiliations Expand

Affiliations

- [1] Department of Emergency Medicine, Radboud Institute for Health Sciences, Radboud University Medical Center, Nijmegen, The Netherlands.
- [2] Department of Geriatrics, Radboud Institute for Health Sciences, Radboud University Medical Center, Nijmegen, The Netherlands.

- [3] Department of Emergency Medicine, Radboud Institute for Health Sciences, Radboud University Medical Center, Nijmegen, The Netherlands. gijs.hesselink@radboudumc.nl.
- [4] Scientific Center for Quality of Healthcare (IQ healthcare), Radboud Institute for Health Sciences, Radboud University Medical Center, Nijmegen, The Netherlands. gijs.hesselink@radboudumc.nl.

- PMID: **33932988**
- PMCID: PMC8087882
- DOI: 10.1186/s12873-021-00449-9

Free PMC article
Observational Study

Delayed emergency healthcare seeking behaviour by Dutch emergency department visitors during the first COVID-19 wave: a mixed methods retrospective observational study

Maaike Nab et al. BMC Emerg Med. 2021.
Free PMC article
Show details
BMC Emerg Med
. 2021 May 1;21(1):56.
doi: 10.1186/s12873-021-00449-9.

Authors

Maaike Nab [1], Robyn van Vehmendahl [1], Inne Somers [1], Yvonne Schoon [2], Gijs Hesselink [3] [4]

Affiliations

- [1] Department of Emergency Medicine, Radboud Institute for Health Sciences, Radboud University Medical Center, Nijmegen, The Netherlands.
- [2] Department of Geriatrics, Radboud Institute for Health Sciences, Radboud University Medical Center, Nijmegen, The Netherlands.
- [3] Department of Emergency Medicine, Radboud Institute for Health Sciences, Radboud University Medical Center, Nijmegen, The Netherlands. gijs.hesselink@radboudumc.nl.
- [4] Scientific Center for Quality of Healthcare (IQ healthcare), Radboud Institute for Health Sciences, Radboud University Medical Center, Nijmegen, The Netherlands. gijs.hesselink@radboudumc.nl.

- PMID: **33932988**
- PMCID: PMC8087882
- DOI: 10.1186/s12873-021-00449-9

Abstract

Background: Emergency department (ED) visits due to non-coronavirus disease 2019 (COVID-19) conditions have drastically decreased since the outbreak of the COVID-19 pandemic. This study aimed to identify the magnitude, characteristics and underlying motivations of ED visitors with delayed healthcare seeking behaviour during the first wave of the pandemic.

Methods: Between March 9 and July 92,020, adults visiting the ED of an academic hospital in the East of the Netherlands received an online questionnaire to collect self-reported data on delay in seeking emergency care and subsequent motivations for this delay. Telephone interviews were held with a subsample of respondents to better understand the motivations for delay as described in the questionnaire. Quantitative data were analysed using descriptive statistics. Qualitative data were thematically analysed.

Results: One thousand three hundred thirty-eight questionnaires were returned (34.0% response). One in five respondents reported a delay in seeking emergency care. Almost half of these respondents (n = 126; 45.4%) reported that the pandemic influenced the delay. Respondents reporting delay were mainly older adults (mean 61.6; ±13.1 years), referred to the ED by the general practitioner (GP; 35.1%) or a medical specialist (34.7%), visiting the ED with cardiac problems (39.7%). The estimated median time of delay in receiving ED care was 3 days (inter quartile range 8 days). Respectively 46 (16.5%) and 26 (9.4%) respondents reported that their complaints would be either less severe or preventable if they had sought for emergency care earlier. Delayed care seeking behaviour was frequently motivated by: fear of contamination, not wanting to burden professionals, perceiving own complaints less urgent relative to COVID-19 patients, limited access to services, and by stay home instructions from referring professionals.

Conclusions: A relatively large proportion of ED visitors reported delay in seeking emergency care during the first wave. Delay was often driven by misperceptions of the accessibility of services and the legitimacy for seeking emergency care. Public messaging and close collaboration between the ED and referring professionals could help reduce delayed care for acute needs during future COVID-19 infection waves.

Keywords: COVID-19 pandemic; Delayed care; Emergency department.

Conflict of interest statement

The authors declare that they have no competing interests.

- 24 references
- 1 figure

Supplementary info

Publication types, MeSH terms Expand

Publication types

- Observational Study

MeSH terms

- Age Factors
- Aged
- Attitude to Health*
- COVID-19 / psychology
- COVID-19 / therapy*
- Emergency Medical Services
- Emergency Service, Hospital / statistics & numerical data*
- Health Services Needs and Demand
- Help-Seeking Behavior*
- Humans
- Male
- Middle Aged
- Netherlands
- Patient Acceptance of Health Care / psychology
- Patient Acceptance of Health Care / statistics & numerical data*
- Retrospective Studies

Full text links

Read free full text at BMC BioMed Central Free PMC article
Proceed to details
Cite
Share
☐ 1,019
Observational Study
Aten Primaria
. 2021 Feb;53(2):101957.
doi: 10.1016/j.aprim.2020.10.005. Epub 2020 Dec 13.

[Characteristics and evolution of COVID-19 in an urban Health Center at the pandemic beginning']

[Article in Spanish]
Karen Ruth Barroso López [1], Paloma Peñasco García [2], Clara Isabel Soria López [2], María Carmen Pérez Fernández [2], José Generoso Gómez Cruz [2], Yolanda González Silva [3]
Affiliations Expand

Affiliations

- [1] Centro de Salud Sisinio de Castro, Gerencia de Atención Primaria de Salamanca, España. Electronic address: krbarroso@saludcastillayleon.es.
- [2] Centro de Salud Sisinio de Castro, Gerencia de Atención Primaria de Salamanca, España.
- [3] Servicio de Urgencias de Atención Primaria de San Juan, Gerencia de Atención Primaria de Salamanca, España.

- PMID: **33423880**
- PMCID: PMC7733679
- DOI: 10.1016/j.aprim.2020.10.005

Free PMC article
Observational Study

[Characteristics and evolution of COVID-19 in an urban Health Center at the pandemic beginning']

[Article in Spanish]
Karen Ruth Barroso López et al. Aten Primaria. 2021 Feb.
Free PMC article
Show details
Aten Primaria
. 2021 Feb;53(2):101957.
doi: 10.1016/j.aprim.2020.10.005. Epub 2020 Dec 13.

Authors

Karen Ruth Barroso López [1], Paloma Peñasco García [2], Clara Isabel Soria López [2], María Carmen Pérez Fernández [2], José Generoso Gómez Cruz [2], Yolanda González Silva [3]

Affiliations

- [1] Centro de Salud Sisinio de Castro, Gerencia de Atención Primaria de Salamanca, España. Electronic address: krbarroso@saludcastillayleon.es.
- [2] Centro de Salud Sisinio de Castro, Gerencia de Atención Primaria de Salamanca, España.
- [3] Servicio de Urgencias de Atención Primaria de San Juan, Gerencia de Atención Primaria de Salamanca, España.

- PMID: **33423880**
- PMCID: PMC7733679
- DOI: 10.1016/j.aprim.2020.10.005

Abstract
in English, Spanish

Objetive: To evaluate SAR-COV-2 pacients' features. To analyse de diferences between those who required hospital care and those who didn't.

Design: Observational, descriptive and retrospective study.

Setting: Twomedical practices of an urban health center in Salamanca (Spain).

Participants: ≥18 years diagnosed with SAR-CoV-2 between March 11th and April 20th.

Main measurements: clinical-epidemiological chatacteristics, diagnosis, treatment and outcome at the end of study RESULTS: 122 patients (63.9% female), 19.7% social and health care workers y 4.9% from nursing homes. Predominant age group: 46-60 years. 67.2% without comorbility. Predomint symptoms: low-grade fever (73.5%), cough (65.2%) y fever (43%). Average age of the patients requiring hospital care was higher: 59.85 (DE16.22) versus 50.78 (DE17.88) P=.013. 63.6% of all the patients monitored by Primary Health Care and 14.1% of patients that required assistance did not present dyspnea P=.001. Only 2.5% of the hospital-assisted patients, compared to 61.5% of Primary Health Care, were not tested P=.0001. 26 patients were attendedn at an emergency room: 11(9%) stayed and 2 (1.6%) passed away. No antibiotic or inhaler treatment for 52.5% and 70.5% respectively. The most used antipyretic treatment was paracetamol (78.7%).

Conclusions: Prevalence in females, comorbility-free patients and in age range: 46-60 years. Complementary and confirmatory test were performed mainly in hospital care. Predominance of mild symptoms and favourable evolution. Highliting the role played by Primary Health Care in detection, early intervention and monitoring of severe cases.

Objetivo: Evaluar las características de los pacientes con SARS-CoV-2 y analizar diferencias entre los que requirieron asistencia hospitalaria y los seguidos ambulatoriamente.

Diseño: Estudio observacional, descriptivo, retrospectivo.

Emplazamiento: 2 unidades básicas asistenciales de un centro de salud urbano en Salamanca (España).

Participantes: Pacientes ≥ 18 años diagnosticados de SARS-CoV-2 entre el 11 de marzo y el 20 de abril.

Mediciones principales: Características clínico-epidemiológicas, diagnóstico, tratamiento y desenlace a fecha fin de estudio.

Resultados: Ciento veintidós pacientes (63,9% mujeres), 19,7% trabajadores sociosanitarios y 4,9% institucionalizados. Franja etaria predominante: 46-60 años, edad media: 52,1(DE 17,85). El 67,2% sin comorbilidad. Síntomas más prevalentes: febrícula (73,5%), tos (65,2%) y fiebre (43%). La edad media de los que requieren atención hospitalaria es mayor a los seguidos ambulatoriamente: 59,85 años (DE 16,22) vs. 50,78 (DE 17,88); p = 0,013. El 63,6% del total seguidos por Atención Primaria no presentó disnea, frente a 17 (14,1%) de los que acudieron a Urgencias; p = 0,001. No se realizaron pruebas confirmatorias al 2,5% de los que visitaron el hospital, frente al 61,5% de los seguidos ambulatoriamente; p = 0,0001; 26 acuden a Urgencias: 11 (9%) ingresaron y 2 (1,6%) fallecieron. El 52,5% no necesitó antibioterapia y el 70.5% no requirió inhaladores. El antipirético más empleado fue paracetamol (78.7%).

Conclusiones: Prevalencia en mujeres, personas sin comorbilidad y en la franja de edad de 46-60 años. Las pruebas complementarias y confirmatorias se realizaron mayoritariamente en asistencia hospitalaria. Predominancia de sintomatología leve y evolución favorable. Destacamos el papel de Atención Primaria en la detección, la intervención temprana y el seguimiento en casos graves.

Keywords: Atención Primaria de Salud; Atención ambulatoria; Coronavirus infections; Epidemiology; Epidemiología; Infecciones por coronavirus; Outpatient Care; Primary Health Care.

Copyright © 2020 The Authors. Publicado por Elsevier España, S.L.U. All rights reserved.

- 20 references
- 1 figure

Supplementary info

Publication types, MeSH terms Expand

Publication types

- Observational Study

MeSH terms

- Adolescent
- Adult
- Aged
- Aged, 80 and over
- COVID-19* / diagnosis
- COVID-19* / epidemiology
- COVID-19* / therapy
- Female
- Humans
- Male
- Middle Aged
- Pandemics
- Retrospective Studies
- Spain / epidemiology
- Treatment Outcome
- Urban Health / statistics & numerical data*
- Urban Health Services / statistics & numerical data*
- Young Adult

Full text links

ELSEVIER OPEN ACCESS Elsevier Science Free PMC article

Proceed to details

Cite

Share

☐ 1,020
Observational Study
Ther Adv Respir Dis
. Jan-Dec 2020;14:1753466620963019.
doi: 10.1177/1753466620963019.

Definition and retrospective application of a clinical scoring system for COVID-19 triage at presentation

Jun Duan [1], Mei Liang [2], Yongpu Li [3], Dan Wu [3], Ying Chen [3], Shui Gao [3], Ping Jia [3], Mei Yang [3], Wei Xia [3], Xiaolan Wu [4], Quan Li [5], Fulin Zuo [6], Yahong Zhang [6], Yongfang He [6], Jianghua Nie [6], Wenxiu Zhou [7], Xueqin Fu [7], Xiaobin Peng [8], Zhoujun Ma [8], Xiaofeng Fu [2], Lingwei Zeng [9], Wenyi You [9], Yuan Fang [10], Lingmei Zhu [10], Ping Liu [11]
Affiliations Expand

Affiliations

- [1] Department of Respiratory and Critical Care Medicine, the First Affiliated Hospital of Chongqing Medical University, Youyi Road 1, Yuzhong District, Chongqing, 400016, China.
- [2] Department of Respiratory and Critical Care Medicine, the People's Hospital of Yubei District, Chongqing, China.
- [3] Department of Respiratory and Critical Care Medicine, the People's Hospital of Changshou District, Chongqing, China.
- [4] Department of Infectious Disease, the People's Hospital of Changshou District, Chongqing, China.
- [5] Department of Laboratory Medicine, the People's Hospital of Changshou District, Chongqing, China.
- [6] Department of Radiology, the People's Hospital of Changshou District, Chongqing, China.
- [7] Department of Outpatient Service, the People's Hospital of Changshou District, Chongqing, China.
- [8] Centers for Disease Control of Changshou District, Chongqing, China.
- [9] Department of Infectious Disease, the People's Hospital of Yubei District, Chongqing, China.
- [10] Department of Radiology, the People's Hospital of Yubei District, Chongqing, China.
- [11] Department of Respiratory and Critical Care Medicine, the People's Hospital of Changshou District, Beiguan Road 16, Fengcheng Street, Changshou District, Chongqing, 401220, China.

- PMID: **33054697**
- PMCID: PMC7570777
- DOI: 10.1177/1753466620963019

Free PMC article
Observational Study

Definition and retrospective application of a clinical scoring system for COVID-19 triage at presentation

Jun Duan et al. Ther Adv Respir Dis. Jan-Dec 2020.
Free PMC article
Show details
Ther Adv Respir Dis
. Jan-Dec 2020;14:1753466620963019.
doi: 10.1177/1753466620963019.

Authors

Jun Duan [1], Mei Liang [2], Yongpu Li [3], Dan Wu [3], Ying Chen [3], Shui Gao [3], Ping Jia [3], Mei Yang [3], Wei Xia [3], Xiaolan Wu [4], Quan Li [5], Fulin Zuo [6], Yahong Zhang [6], Yongfang He [6], Jianghua Nie [6], Wenxiu Zhou [7], Xueqin Fu [7], Xiaobin Peng [8], Zhoujun Ma [8], Xiaofeng Fu [2], Lingwei Zeng [9], Wenyi You [9], Yuan Fang [10], Lingmei Zhu [10], Ping Liu [11]

Affiliations

- [1] Department of Respiratory and Critical Care Medicine, the First Affiliated Hospital of Chongqing Medical University, Youyi Road 1, Yuzhong District, Chongqing, 400016, China.
- [2] Department of Respiratory and Critical Care Medicine, the People's Hospital of Yubei District, Chongqing, China.
- [3] Department of Respiratory and Critical Care Medicine, the People's Hospital of Changshou District, Chongqing, China.
- [4] Department of Infectious Disease, the People's Hospital of Changshou District, Chongqing, China.
- [5] Department of Laboratory Medicine, the People's Hospital of Changshou District, Chongqing, China.
- [6] Department of Radiology, the People's Hospital of Changshou District, Chongqing, China.
- [7] Department of Outpatient Service, the People's Hospital of Changshou District, Chongqing, China.
- [8] Centers for Disease Control of Changshou District, Chongqing, China.
- [9] Department of Infectious Disease, the People's Hospital of Yubei District, Chongqing, China.
- [10] Department of Radiology, the People's Hospital of Yubei District, Chongqing, China.
- [11] Department of Respiratory and Critical Care Medicine, the People's Hospital of Changshou District, Beiguan Road 16, Fengcheng Street, Changshou District, Chongqing, 401220, China.

- PMID: **33054697**
- PMCID: PMC7570777
- DOI: 10.1177/1753466620963019

Abstract

Background: A simple scoring system for triage of suspected patients with COVID-19 is lacking.

Methods: A multi-disciplinary team developed a screening score taking into account epidemiology history, clinical feature, radiographic feature, and routine blood test. At fever clinics, the screening score was used to identify the patients with moderate to high probability of COVID-19 among all the suspected patients. The patients with moderate to high probability of COVID-19 were allocated to a single room in an isolation ward with level-3 protection. And those with low probability were allocated to a single room in a general ward with level-2 protection. At the isolation ward, the screening score was used to identify the confirmed and probable cases after two consecutive real-time reverse transcription polymerase chain reaction (RT-PCR) tests. The data in the People's Hospital of Changshou District were used for internal validation and those in the People's Hospital of Yubei District for external validation.

Results: We enrolled 76 and 40 patients for internal and external validation, respectively. In the internal validation cohort, the area under the curve of receiver operating characteristics (AUC) was 0.96 [95% confidence interval (CI): 0.89-0.99] for the diagnosis of moderate to high probability of cases among all the suspected patients. Using 60 as cut-off value, the sensitivity and specificity were 88% and 93%, respectively. In the isolation ward, the AUC was 0.94 (95% CI: 0.83-0.99) for the diagnosis of confirmed and probable cases. Using 90 as cut-off value, the sensitivity and specificity were 78% and 100%, respectively. These results were confirmed in the validation cohort.

Conclusion: The scoring system provides a reference on COVID-19 triage in fever clinics to reduce misdiagnosis and consumption of protective supplies.*The reviews of this paper are available via the supplemental material section.*

Keywords: coronavirus; diagnosis; sensitivity; specificity.

Conflict of interest statement

Conflict of interest statement: The authors declare that there is no conflict of interest.

- 23 references
- 3 figures

Supplementary info

Publication types, MeSH terms Expand

Publication types

- Observational Study

MeSH terms

- Adult
- Aged
- Betacoronavirus*

- COVID-19
- Coronavirus Infections / complications
- Coronavirus Infections / diagnosis*
- Coronavirus Infections / therapy*
- Female
- Humans
- Male
- Middle Aged
- Pandemics
- Pneumonia, Viral / complications
- Pneumonia, Viral / diagnosis*
- Pneumonia, Viral / therapy*
- Retrospective Studies
- SARS-CoV-2
- Sensitivity and Specificity
- Severity of Illness Index
- Triage*

Full text links

SAGE journals Open access full text Atypon Free PMC article
Proceed to details
Cite
Share
1,021
Observational Study
Endocrinol Metab (Seoul)
. 2021 Aug;36(4):800-809.
doi: 10.3803/EnM.2021.1040. Epub 2021 Aug 20.

High Fibrosis-4 Index Is Related with Worse Clinical Outcome in Patients with Coronavirus Disease 2019 and Diabetes Mellitus: A Multicenter Observational Study

Sung-Woo Kim [1], Jae-Han Jeon [2], Jun Sung Moon [3], Mi Kyung Kim [4]
Affiliations Expand

Affiliations

- [1] Department of Internal Medicine, Daegu Catholic University Hospital, Daegu Catholic University School of Medicine, Daegu, Korea.
- [2] Department of Internal Medicine, Kyungpook National University Chilgok Hospital, School of Medicine, Kyungpook National University, Daegu, Korea.
- [3] Department of Internal Medicine, Yeungnam University Hospital, Yeungnam University College of Medicine, Daegu, Korea.
- [4] Department of Internal Medicine, Keimyung University Dongsan Hospital, Keimyung University School of Medicine, Daegu, Korea.

- PMID: **34418914**
- PMCID: PMC8419603
- DOI: 10.3803/EnM.2021.1040

Free PMC article
Observational Study

High Fibrosis-4 Index Is Related with Worse Clinical Outcome in Patients with Coronavirus Disease 2019 and Diabetes Mellitus: A Multicenter Observational Study

Sung-Woo Kim et al. Endocrinol Metab (Seoul). 2021 Aug.
Free PMC article
Show details
Endocrinol Metab (Seoul)
. 2021 Aug;36(4):800-809.
doi: 10.3803/EnM.2021.1040. Epub 2021 Aug 20.

Authors

Sung-Woo Kim [1], Jae-Han Jeon [2], Jun Sung Moon [3], Mi Kyung Kim [4]

Affiliations

- [1] Department of Internal Medicine, Daegu Catholic University Hospital, Daegu Catholic University School of Medicine, Daegu, Korea.
- [2] Department of Internal Medicine, Kyungpook National University Chilgok Hospital, School of Medicine, Kyungpook National University, Daegu, Korea.
- [3] Department of Internal Medicine, Yeungnam University Hospital, Yeungnam University College of Medicine, Daegu, Korea.
- [4] Department of Internal Medicine, Keimyung University Dongsan Hospital, Keimyung University School of Medicine, Daegu, Korea.

- PMID: **34418914**
- PMCID: PMC8419603
- DOI: 10.3803/EnM.2021.1040

Abstract

Background: Based on recent evidence on the importance of the presence of diabetes mellitus (DM) and fibrosis-4 (FIB-4) index in coronavirus disease 2019 (COVID-19) mortality, we analyzed whether these factors could additively predict such mortality.

Methods: This multicenter observational study included 1,019 adult inpatients admitted to university hospitals in Daegu. The demographic and laboratory findings, mortality, prevalence of severe disease, and duration of quarantine were compared between patients with and without DM and/or a high FIB-4 index. The mortality risk and corresponding hazard ratio (HR) were analyzed using the Kaplan-Meier method and Cox proportional hazard models.

Results: The patients with DM (n=217) exhibited significantly higher FIB-4 index and mortality compared to those without DM. Although DM (HR, 2.66; 95% confidence interval [CI], 1.63 to 4.33) and a high FIB-4 index (HR, 4.20; 95% CI, 2.21 to 7.99) were separately identified as risk factors for COVID-19 mortality, the patients with both DM and high FIB-4 index had a significantly higher mortality (HR, 9.54; 95% CI, 4.11 to 22.15). Higher FIB-4 indices were associated with higher mortality regardless of DM. A high FIB-4 index with DM was more significantly associated with a severe clinical course with mortality (odds ratio, 11.24; 95% CI, 5.90 to 21.41) than a low FIB-4 index without DM, followed by a high FIB-4 index alone and DM alone. The duration of quarantine and hospital stay also tended to be longer in those with both DM and high FIB-4 index.

Conclusion: Both DM and high FIB-4 index are independent and additive risk factors for COVID-19 mortality.

Keywords: COVID-19; Diabetes mellitus; Mortality; Non-alcoholic fatty liver disease.

Conflict of interest statement

CONFLICTS OF INTEREST

No potential conflict of interest relevant to this article was reported.

- 33 references
- 5 figures

Supplementary info

Publication types, MeSH terms Expand

Publication types

- Multicenter Study
- Observational Study

MeSH terms

- Adult
- Aged

- COVID-19 / diagnosis*
- COVID-19 / mortality*
- COVID-19 / therapy
- Diabetes Mellitus / diagnosis*
- Diabetes Mellitus / mortality*
- Diabetes Mellitus / therapy
- Female
- Humans
- Liver Cirrhosis / diagnosis*
- Liver Cirrhosis / mortality*
- Liver Cirrhosis / therapy
- Male
- Middle Aged
- Retrospective Studies
- Risk Factors
- Treatment Outcome

Full text links

M2PI Free PMC article
Proceed to details
Cite
Share
1,022
Observational Study
Open Heart
. 2021 Feb;8(1):e001497.
doi: 10.1136/openhrt-2020-001497.

COVID-19 pandemic is associated with mechanical complications in patients with ST-elevation myocardial infarction

Satoshi Kitahara[1 2], Masashi Fujino[3], Satoshi Honda[1], Yasuhide Asaumi[1], Yu Kataoka[1], Fumiyuki Otsuka[1], Michio Nakanishi[1], Yoshio Tahara[1], Soshiro Ogata[4], Daisuke Onozuka[4], Kunihiro Nishimura[4], Tomoyuki Fujita[5], Kenichi Tsujita[6], Hisao Ogawa[1], Teruo Noguchi[1 2]

Affiliations Expand

Affiliations

- [1] Department of Cardiovascular Medicine, National Cerebral and Cardiovascular Center, Suita, Osaka, Japan.
- [2] Department of Advanced Cardiovascular Medicine, Graduate School of Medical Sciences, Kumamoto University, Kumamoto, Japan.
- [3] Department of Cardiovascular Medicine, National Cerebral and Cardiovascular Center, Suita, Osaka, Japan fujinom@ncvc.go.jp.
- [4] Department of Preventive Medicine and Epidemiology, National Cerebral and Cardiovascular Center, Suita, Osaka, Japan.
- [5] Department of Cardiovascular Surgery, National Cerebral and Cardiovascular Center, Suita, Osaka, Japan.
- [6] Department of Cardiovascular Medicine, Graduate School of Medical Sciences, Kumamoto University, Kumamoto, Japan.

- PMID: **33547221**
- PMCID: PMC7871043
- DOI: 10.1136/openhrt-2020-001497

Free PMC article
Observational Study

COVID-19 pandemic is associated with mechanical complications in patients with ST-elevation myocardial infarction

Satoshi Kitahara et al. Open Heart. 2021 Feb.
Free PMC article
Show details
Open Heart
. 2021 Feb;8(1):e001497.
doi: 10.1136/openhrt-2020-001497.

Authors

Satoshi Kitahara [1, 2], Masashi Fujino [3], Satoshi Honda [1], Yasuhide Asaumi [1], Yu Kataoka [1], Fumiyuki Otsuka [1], Michio Nakanishi [1], Yoshio Tahara [1], Soshiro Ogata [4], Daisuke Onozuka [4], Kunihiro Nishimura [4], Tomoyuki Fujita [5], Kenichi Tsujita [6], Hisao Ogawa [1], Teruo Noguchi [1, 2]

Affiliations

- [1] Department of Cardiovascular Medicine, National Cerebral and Cardiovascular Center, Suita, Osaka, Japan.
- [2] Department of Advanced Cardiovascular Medicine, Graduate School of Medical Sciences, Kumamoto University, Kumamoto, Japan.
- [3] Department of Cardiovascular Medicine, National Cerebral and Cardiovascular Center, Suita, Osaka, Japan fujinom@ncvc.go.jp.

- [4] Department of Preventive Medicine and Epidemiology, National Cerebral and Cardiovascular Center, Suita, Osaka, Japan.
- [5] Department of Cardiovascular Surgery, National Cerebral and Cardiovascular Center, Suita, Osaka, Japan.
- [6] Department of Cardiovascular Medicine, Graduate School of Medical Sciences, Kumamoto University, Kumamoto, Japan.

- PMID: **33547221**
- PMCID: PMC7871043
- DOI: 10.1136/openhrt-2020-001497

Abstract

Objective: Although there are regional reports that the COVID-19 pandemic is associated with a reduction in acute myocardial infarction presentations and primary percutaneous coronary intervention (PCI) procedures, little is known about the impact of the COVID-19 pandemic on mechanical complications resulting from ST-segment elevation myocardial infarction (STEMI) and mortality.

Methods: This single-centre retrospective cohort study analysed presentations, incidence of mechanical complications, and mortality in patients with STEMI before and after a state of emergency was declared due to the COVID-19 pandemic by the Japanese government on 7 April 2020.

Results: We analysed 359 patients with STEMI hospitalised before the declaration and 63 patients hospitalised after the declaration. The proportion of patients with late presentation was significantly higher after the declaration than before (25.4% vs 14.2%, p=0.03). The incidence of late presentation was significantly higher during the COVID-19 pandemic than before (incidence rate ratio (IRR), 2.41; 95% CI, 1.37 to 4.05; p=0.001, even after adjusting for month (IRR, 2.61; 95% CI, 1.33 to 5.13; p<0.01). Primary PCI was performed significantly less often after the declaration than before (68.3% vs 82.5%, p=0.009). The mechanical complication resulting from STEMI occurred in 13 of 359 (3.6%) patients before the declaration and 9 of 63 (14.3%) patients after the declaration (p<0.001). However, the incidence of in-hospital death (before, 6.2% vs after, 6.4%, p=0.95) was comparable.

Conclusions: Following the COVID-19 pandemic, an increased incidence of mechanical complications resulting from STEMI was observed. Instructing people to stay at home, without effectively educating them to immediately seek medical attention when suffering symptoms of a heart attack, may worsen outcomes in patients with STEMI.

Keywords: acute coronary syndrome; epidemiology; myocardial infarction.

© Author(s) (or their employer(s)) 2021. Re-use permitted under CC BY-NC. No commercial re-use. See rights and permissions. Published by BMJ.

Conflict of interest statement

Competing interests: None declared.

- 21 references
- 1 figure

Supplementary info

Publication types, MeSH terms Expand

Publication types

- Comparative Study
- Observational Study
- Research Support, Non-U.S. Gov't

MeSH terms

- Aged
- Aged, 80 and over
- COVID-19*
- Female
- Hospital Mortality
- Hospitalization
- Humans
- Japan
- Male
- Middle Aged
- Patient Acceptance of Health Care*
- Percutaneous Coronary Intervention* / adverse effects
- Percutaneous Coronary Intervention* / mortality
- Registries
- Retrospective Studies
- Risk Assessment
- Risk Factors
- ST Elevation Myocardial Infarction / diagnosis
- ST Elevation Myocardial Infarction / mortality
- ST Elevation Myocardial Infarction / physiopathology
- ST Elevation Myocardial Infarction / therapy*
- Time Factors
- Time-to-Treatment*
- Treatment Outcome

Full text links

BMJ Open Access Full Text HighWire Free PMC article
Proceed to details
Cite

Share

1,023

Observational Study

Chest

. 2021 Jun;159(6):2417-2427.

doi: 10.1016/j.chest.2021.01.017. Epub 2021 Jan 16.

Impact of High-Dose Prophylactic Anticoagulation in Critically Ill Patients With COVID-19 Pneumonia

Charles Tacquard [1], Alexandre Mansour [2], Alexandre Godon [3], Julien Godet [4], Julien Poissy [5], Delphine Garrigue [6], Eric Kipnis [7], Sophie Rym Hamada [8], Paul Michel Mertes [1], Annick Steib [1], Mathilde Ulliel-Roche [3], Bélaïd Bouhemad [9], Maxime Nguyen [9], Florian Reizine [10], Isabelle Gouin-Thibault [11], Marie Charlotte Besse [12], Nived Collercandy [12], Stefan Mankikian [12], Jerrold H Levy [13], Yves Gruel [14], Pierre Albaladejo [3], Sophie Susen [15], Anne Godier [8], French Working Group on Perioperative Hemostasis

Collaborators, Affiliations Expand

Collaborators

- **French Working Group on Perioperative Hemostasis**: P Albaladejo, N Blais, F Bonhomme, A Borel-Derlon, A Cohen, J-P Collet, E de Maistre, P Fontana, D Garrigue Huet, A Godier, Y Gruel, A Godon, B Ickx, S Laporte, D Lasne, J Llau, G Le Gal, T Lecompte, S Lessire, J H Levy, D Longrois, S Madi-Jebara, A Mansour, M Mazighi, P Mismetti, P E Morange, S Motte, F Mullier, N Nathan, P Nguyen, G Pernod, N Rosencher, S Roullet, P M Roy, S Schlumberger, P Sié, A Steib, S Susen, C A Tacquard, S Testa, A Vincentelli, P Zufferey

Affiliations

- [1] Department of Anesthesiology and Intensive Care, Hôpital Civil, Hôpitaux Universitaires de Strasbourg, Strasbourg, France.
- [2] Department of Anesthesiology Critical Care Medicine and Perioperative Medicine, CHU de Rennes, Rennes, France.
- [3] Department of Anesthesiology and Critical Care, Grenoble Alpes University Hospital, Grenoble, France.
- [4] Groupe Méthodes en Recherche Clinique, Hôpital Civil, Hôpitaux Universitaires de Strasbourg, Strasbourg, France.
- [5] University of Lille, Inserm U1285, CHU Lille, Pôle de Réanimation, CNRS, UMR 8576 - UGSF - Unité de Glycobiologie Structurale et Fonctionnelle, Lille, France.
- [6] Department of Anesthesiology and Critical Care, Surgical Critical Care, Centre Hospitalier Universitaire Lille, Lille, France.
- [7] University of Lille, CNRS, Inserm, CHU Lille, Surgical Critical Care, Department of Anesthesiology and Critical Care, Institut Pasteur de Lille, U1019-UMR 9017-CIIL-Center for Infection and Immunity of Lille, Lille, France.

- [8] Department of Anesthesiology and Critical Care, European Georges Pompidou Hospital, Assistance Publique-Hôpitaux de Paris, Paris University, Paris, France.
- [9] Department of Anesthesiology and Intensive Care, Dijon University Hospital and University of Burgundy, Lipness Team, INSERM Research Center LNC-UMR1231 and LabExLipSTIC, Dijon, France.
- [10] Service des Maladies Infectieuses et Réanimation Médicale, Rennes University Hospital, Rennes, France.
- [11] Department of Hematology-Hemostasis, Rennes University Hospital, Rennes, France.
- [12] Service de Médecine Intensive-Réanimation, CHU de Tours, Tours, France.
- [13] Departments of Anesthesiology, Critical Care, and Surgery, Duke University School of Medicine, Durham, NC.
- [14] Department of Hematology-Hemostasis, Tours University Hospital, Tours, France.
- [15] Hemostasis Department, Heart and Lung Institute, CHU Lille, Lille, France. Electronic address: sophiesusen@aol.com.

- PMID: **33465342**
- PMCID: PMC7832130
- DOI: 10.1016/j.chest.2021.01.017

Free PMC article
Observational Study

Impact of High-Dose Prophylactic Anticoagulation in Critically Ill Patients With COVID-19 Pneumonia

Charles Tacquard et al. Chest. 2021 Jun.
Free PMC article
Show details
Chest

. 2021 Jun;159(6):2417-2427.
doi: 10.1016/j.chest.2021.01.017. Epub 2021 Jan 16.

Authors

Charles Tacquard [1], Alexandre Mansour [2], Alexandre Godon [3], Julien Godet [4], Julien Poissy [5], Delphine Garrigue [6], Eric Kipnis [7], Sophie Rym Hamada [8], Paul Michel Mertes [1], Annick Steib [1], Mathilde Ulliel-Roche [3], Bélaïd Bouhemad [9], Maxime Nguyen [9], Florian Reizine [10], Isabelle Gouin-Thibault [11], Marie Charlotte Besse [12], Nived Collercandy [12], Stefan Mankikian [12], Jerrold H Levy [13], Yves Gruel [14], Pierre Albaladejo [3], Sophie Susen [15], Anne Godier [8], French Working Group on Perioperative Hemostasis

Collaborators

- **French Working Group on Perioperative Hemostasis**: P Albaladejo, N Blais, F Bonhomme, A Borel-Derlon, A Cohen, J-P Collet, E de Maistre, P Fontana, D Garrigue Huet, A Godier, Y Gruel, A Godon, B Ickx, S Laporte, D Lasne, J

Llau, G Le Gal, T Lecompte, S Lessire, J H Levy, D Longrois, S Madi-Jebara, A Mansour, M Mazighi, P Mismetti, P E Morange, S Motte, F Mullier, N Nathan, P Nguyen, G Pernod, N Rosencher, S Roullet, P M Roy, S Schlumberger, P Sié, A Steib, S Susen, C A Tacquard, S Testa, A Vincentelli, P Zufferey

Affiliations

- [1] Department of Anesthesiology and Intensive Care, Hôpital Civil, Hôpitaux Universitaires de Strasbourg, Strasbourg, France.
- [2] Department of Anesthesiology Critical Care Medicine and Perioperative Medicine, CHU de Rennes, Rennes, France.
- [3] Department of Anesthesiology and Critical Care, Grenoble Alpes University Hospital, Grenoble, France.
- [4] Groupe Méthodes en Recherche Clinique, Hôpital Civil, Hôpitaux Universitaires de Strasbourg, Strasbourg, France.
- [5] University of Lille, Inserm U1285, CHU Lille, Pôle de Réanimation, CNRS, UMR 8576 - UGSF - Unité de Glycobiologie Structurale et Fonctionnelle, Lille, France.
- [6] Department of Anesthesiology and Critical Care, Surgical Critical Care, Centre Hospitalier Universitaire Lille, Lille, France.
- [7] University of Lille, CNRS, Inserm, CHU Lille, Surgical Critical Care, Department of Anesthesiology and Critical Care, Institut Pasteur de Lille, U1019-UMR 9017-CIIL-Center for Infection and Immunity of Lille, Lille, France.
- [8] Department of Anesthesiology and Critical Care, European Georges Pompidou Hospital, Assistance Publique-Hôpitaux de Paris, Paris University, Paris, France.
- [9] Department of Anesthesiology and Intensive Care, Dijon University Hospital and University of Burgundy, Lipness Team, INSERM Research Center LNC-UMR1231 and LabExLipSTIC, Dijon, France.
- [10] Service des Maladies Infectieuses et Réanimation Médicale, Rennes University Hospital, Rennes, France.
- [11] Department of Hematology-Hemostasis, Rennes University Hospital, Rennes, France.
- [12] Service de Médecine Intensive-Réanimation, CHU de Tours, Tours, France.
- [13] Departments of Anesthesiology, Critical Care, and Surgery, Duke University School of Medicine, Durham, NC.
- [14] Department of Hematology-Hemostasis, Tours University Hospital, Tours, France.
- [15] Hemostasis Department, Heart and Lung Institute, CHU Lille, Lille, France. Electronic address: sophiesusen@aol.com.

- PMID: **33465342**
- PMCID: PMC7832130
- DOI: 10.1016/j.chest.2021.01.017

Abstract

Background: Because of the high risk of thrombotic complications (TCs) during SARS-CoV-2 infection, several scientific societies have proposed to increase the dose of preventive anticoagulation, although arguments in favor of this strategy are inconsistent.

Research question: What is the incidence of TC in critically ill patients with COVID-19 and what is the relationship between the dose of anticoagulant therapy and the incidence of TC?

Study design and methods: All consecutive patients referred to eight French ICUs for COVID-19 were included in this observational study. Clinical and laboratory data were collected from ICU admission to day 14, including anticoagulation status and thrombotic and hemorrhagic events. The effect of high-dose prophylactic anticoagulation (either at intermediate or equivalent to therapeutic dose), defined using a standardized protocol of classification, was assessed using a time-varying exposure model using inverse probability of treatment weight.

Results: Of 538 patients included, 104 patients experienced a total of 122 TCs with an incidence of 22.7% (95% CI, 19.2%-26.3%). Pulmonary embolism accounted for 52% of the recorded TCs. High-dose prophylactic anticoagulation was associated with a significant reduced risk of TC (hazard ratio, 0.81; 95% CI, 0.66-0.99) without increasing the risk of bleeding (HR, 1.11; 95% CI, 0.70-1.75).

Interpretation: High-dose prophylactic anticoagulation is associated with a reduction in thrombotic complications in critically ill patients with COVID-19 without an increased risk of hemorrhage. Randomized controlled trials comparing prophylaxis with higher doses of anticoagulants are needed to confirm these results.

Trial registry: ClinicalTrials.gov; No.: NCT04405869; URL: www.clinicaltrials.gov.

Keywords: COVID-19; anticoagulation; bleeding; thrombosis.

Copyright © 2021 American College of Chest Physicians. Published by Elsevier Inc. All rights reserved.

Comment in

- Clotting and COVID-19.
  Bull TM. Bull TM. Chest. 2021 Jun;159(6):2151-2152. doi: 10.1016/j.chest.2021.02.067. Chest. 2021. PMID: 34099126 Free PMC article. No abstract available.
- High-Dose Prophylactic Anticoagulation in Severe COVID-19 Pneumonia.
  Nadeem R, Kamat S. Nadeem R, et al. Chest. 2021 Jul;160(1):e94-e95. doi: 10.1016/j.chest.2021.02.036. Chest. 2021. PMID: 34246406 Free PMC article. No abstract available.
- Response.
  Tacquard C, Godon A, Mansour A, Gruel Y, Susen S, Godier A. Tacquard C, et al. Chest. 2021 Jul;160(1):e95-e96. doi: 10.1016/j.chest.2021.02.035. Chest. 2021. PMID: 34246407 Free PMC article. No abstract available.
- COVID-19 in the Critically Ill: Too Risky for High-Dose Anticoagulation?
  Paez Vargas JJ, Vidal González A, Pérez-Calvo C, Flandes J. Paez Vargas JJ, et al. Chest. 2021 Aug;160(2):e249. doi: 10.1016/j.chest.2021.03.028. Chest. 2021. PMID: 34366058 Free PMC article. No abstract available.
- Response.
  Tacquard C, Godon A, Mansour A, Gruel Y, Susen S, Godier A. Tacquard C, et al. Chest. 2021 Aug;160(2):e250. doi: 10.1016/j.chest.2021.03.029. Chest. 2021. PMID: 34366060 Free PMC article. No abstract available.

- 21 references
- 1 figure

Supplementary info

Publication types, MeSH terms, Substances, Associated data Expand

Publication types

- Letter
- Observational Study
- Research Support, Non-U.S. Gov't

MeSH terms

- Aged
- Anticoagulants / administration & dosage*
- COVID-19 / complications*
- COVID-19 / therapy*
- Critical Care*
- Female
- France
- Humans
- Incidence
- Male
- Middle Aged
- Pulmonary Embolism / epidemiology
- Retrospective Studies
- Thrombosis / epidemiology*
- Thrombosis / prevention & control*
- Venous Thromboembolism / epidemiology

Substances

- Anticoagulants

Associated data

- ClinicalTrials.gov/NCT04405869

Full text links

ELSEVIER FULL-TEXT ARTICLE Elsevier Science Free PMC article
Proceed to details
Cite
Share
☐ 1,024
Observational Study
Anaesthesia
. 2020 Dec;75(12):1596-1604.

doi: 10.1111/anae.15281. Epub 2020 Oct 22.

Peri-operative COVID-19 infection in urgent elective surgery during a pandemic surge period: a retrospective observational cohort study

A D Kane [1], J Paterson [1], S Pokhrel [1], S K Berry [1], D Monkhouse [2], J W Brand [3], M Ingram [3], G R Danjoux [3, 4]
Affiliations Expand

Affiliations

- [1] Department of Anaesthesia, James Cook University Hospital, Middlesbrough, UK.
- [2] Department of Intensive Care Medicine, James Cook University Hospital, Middlesbrough, UK.
- [3] Department of Cardiothoracic Intensive Care Medicine and Anaesthesia, James Cook University Hospital, Middlesbrough, UK.
- [4] Hull York Medical School and School of Health and Social Science, Teesside University, Middlesbrough, UK.

- PMID: **33090469**
- DOI: 10.1111/anae.15281

Free article
Observational Study

Peri-operative COVID-19 infection in urgent elective surgery during a pandemic surge period: a retrospective observational cohort study

A D Kane et al. Anaesthesia. 2020 Dec.
Free article
Show details
Anaesthesia
. 2020 Dec;75(12):1596-1604.
doi: 10.1111/anae.15281. Epub 2020 Oct 22.

Authors

A D Kane [1], J Paterson [1], S Pokhrel [1], S K Berry [1], D Monkhouse [2], J W Brand [3], M Ingram [3], G R Danjoux [3] [4]

Affiliations

- [1] Department of Anaesthesia, James Cook University Hospital, Middlesbrough, UK.
- [2] Department of Intensive Care Medicine, James Cook University Hospital, Middlesbrough, UK.
- [3] Department of Cardiothoracic Intensive Care Medicine and Anaesthesia, James Cook University Hospital, Middlesbrough, UK.
- [4] Hull York Medical School and School of Health and Social Science, Teesside University, Middlesbrough, UK.

- PMID: **33090469**
- DOI: 10.1111/anae.15281

Abstract

Maintaining safe elective surgical activity during the global coronavirus disease 2019 (COVID-19) pandemic is challenging and it is not clear how COVID-19 may impact peri-operative morbidity and mortality in this population. Therefore, adaptations to normal care pathways are required. Here, we establish if implementation of a bespoke peri-operative care bundle for urgent elective surgery during a pandemic surge period can deliver a low COVID-19-associated complication profile. We present a single-centre retrospective cohort study from a tertiary care hospital of patients planned for urgent elective surgery during the initial COVID-19 surge in the UK between 29 March and 12 June 2020. Patients asymptomatic for COVID-19 were screened by oronasal swab and chest imaging (chest X-ray or computed tomography if aged ≥ 18 years), proceeding to surgery if negative. COVID-19 positive patients at screening were delayed. Postoperatively, patients transitioning to COVID-19 positive status by reverse transcriptase polymerase chain reaction testing were identified by an in-house tracking system and monitored for complications and death within 30 days of surgery. Out of 557 patients referred for surgery (230 (41.3%) women; median (IQR [range]) age 61 (48-72 [1-89])), 535 patients (96%) had COVID-19 screening, of which 13 were positive (2.4%, 95%CI 1.4-4.1%). Out of 512 patients subsequently undergoing surgery, 7 (1.4%) developed COVID-19 positive status (1.4%, 95%CI 0.7-2.8%) with one COVID-19-related death (0.2%, 95%CI 0.0-1.1%) within 30 days. Out of these seven patients, four developed pneumonia, of which two required invasive ventilation including one patient with acute respiratory distress syndrome. Low rates of COVID-19 infection and mortality in the elective surgical population can be achieved within a targeted care bundle. This should provide reassurance that elective surgery can continue, where possible, despite high community rates of COVID-19.

Keywords: COVID-19; SARS-CoV-2; coronavirus disease 2; elective surgery; pandemic.

© 2020 Association of Anaesthetists.

Comment in

- Should we be re-starting elective surgery?
  Simoes J, Bhangu A; CovidSurg Collaborative. Simoes J, et al. Anaesthesia. 2020 Dec;75(12):1563-1565. doi: 10.1111/anae.15296. Epub 2020 Nov 6. Anaesthesia. 2020. PMID: 33156535 No abstract available.

- 30 references

Supplementary info

Publication types, MeSH terms Expand

Publication types

- Observational Study

MeSH terms

- Adolescent
- Adult
- Aged
- Aged, 80 and over
- COVID-19
- Child
- Child, Preschool
- Cohort Studies
- Coronavirus Infections / diagnosis
- Coronavirus Infections / epidemiology*
- Coronavirus Infections / transmission
- Elective Surgical Procedures*
- Female
- Humans
- Infant
- Male
- Mass Screening
- Middle Aged
- Pandemics
- Perioperative Period*
- Pneumonia / epidemiology
- Pneumonia / etiology
- Pneumonia, Viral / diagnosis
- Pneumonia, Viral / epidemiology*
- Pneumonia, Viral / transmission
- Postoperative Complications / epidemiology
- Postoperative Complications / mortality
- Respiration, Artificial
- Retrospective Studies
- Tertiary Care Centers

- Young Adult

Full text links

WILEY Free Full Text Wiley
Proceed to details
Cite
Share
1,025
Observational Study
Aging (Albany NY)
. 2020 Aug 15;12(15):15771-15783.
doi: 10.18632/aging.103839. Epub 2020 Aug 15.

The effect of emergency surgery on acute abdomen patients with COVID-19 pneumonia: a retrospective observational study

Ning Zhao[1], Liang Wu[1], Yifeng Cheng[2], Hai Zheng[3], Ping Hu[3], Chaojie Hu[3], Ding Chen[3], Peng Xu[3], Qingyong Chen[3], Ping Cheng[3], Jinhuang Chen[3], Gang Zhao[3]
Affiliations Expand

Affiliations

- [1] Department of Gastrointestinal Surgery, Union Hospital, Tongji Medical College, Huazhong University of Science and Technology, Wuhan 430022, China.
- [2] Department of Gastrointestinal Surgery, Union Hospital West Campus, Tongji Medical College, Huazhong University of Science and Technology, Wuhan 430056, China.
- [3] Department of Emergency Surgery, Union Hospital, Tongji Medical College, Huazhong University of Science and Technology, Wuhan 430022, China.

- PMID: **32805726**
- PMCID: PMC7467361
- DOI: 10.18632/aging.103839

Free PMC article
Observational Study

The effect of emergency surgery on acute abdomen patients with COVID-19

pneumonia: a retrospective observational study

Ning Zhao et al. Aging (Albany NY). 2020.
Free PMC article
Show details
Aging (Albany NY)
. 2020 Aug 15;12(15):15771-15783.
doi: 10.18632/aging.103839. Epub 2020 Aug 15.

Authors

Ning Zhao [1], Liang Wu [1], Yifeng Cheng [2], Hai Zheng [3], Ping Hu [3], Chaojie Hu [3], Ding Chen [3], Peng Xu [3], Qingyong Chen [3], Ping Cheng [3], Jinhuang Chen [3], Gang Zhao [3]

Affiliations

- [1] Department of Gastrointestinal Surgery, Union Hospital, Tongji Medical College, Huazhong University of Science and Technology, Wuhan 430022, China.
- [2] Department of Gastrointestinal Surgery, Union Hospital West Campus, Tongji Medical College, Huazhong University of Science and Technology, Wuhan 430056, China.
- [3] Department of Emergency Surgery, Union Hospital, Tongji Medical College, Huazhong University of Science and Technology, Wuhan 430022, China.

- PMID: **32805726**
- PMCID: PMC7467361
- DOI: 10.18632/aging.103839

Abstract

During the COVID-19 outbreak, some patients with COVID-19 pneumonia also suffered from acute abdomen requiring surgical treatment; however, there is no consensus for the treatment of such patients. In this study, we retrospectively reviewed 34 patients with acute abdomen who underwent emergency surgery during the COVID-19 outbreak. Among the 34 patients with acute abdomen, a total of six cases were found with COVID-19 pneumonia (clinical classification for COVID-19 pneumonia: all were the common type). On the premise of similar demographics between both groups, patients with COVID-19 pneumonia had worse indicators of liver and coagulation function. Compared with acute abdomen patients without COVID-19, patients with COVID-19 pneumonia had a longer hospital stay, but there were no significant differences in postsurgical complications ($P = 0.58$) or clinical outcomes ($P = 0.56$). In addition, an obvious resolution of lung inflammation after surgery was observed in five COVID-19 patients (83.3%). No new COVID-19 cases occurred during the patients' hospital stays. Therefore, for the common type of COVID-19 pneumonia, emergency surgery could not only improve the outcomes of COVID-19 pneumonia patients with acute abdomen, but also benefit the resolution of pulmonary inflammation.

Keywords: COVID-19; acute abdomen; emergency surgery.

Conflict of interest statement

CONFLICTS OF INTEREST: The authors have declared that no conflicts of interest exist.

- 26 references
- 6 figures

Supplementary info

Publication types, MeSH terms Expand

Publication types

- Observational Study
- Research Support, Non-U.S. Gov't

MeSH terms

- Abdomen, Acute* / diagnosis
- Abdomen, Acute* / epidemiology
- Abdomen, Acute* / etiology
- Abdomen, Acute* / surgery
- Aged
- Betacoronavirus / isolation & purification
- Blood Coagulation Tests / methods
- COVID-19
- China / epidemiology
- Comorbidity
- Coronavirus Infections* / diagnosis
- Coronavirus Infections* / epidemiology
- Coronavirus Infections* / physiopathology
- Emergency Treatment* / methods
- Emergency Treatment* / statistics & numerical data
- Female
- Gastrointestinal Diseases* / epidemiology
- Gastrointestinal Diseases* / physiopathology
- Humans
- Length of Stay / statistics & numerical data
- Liver Function Tests / methods
- Male
- Middle Aged
- Outcome and Process Assessment, Health Care
- Pandemics*

- Pneumonia, Viral* / diagnosis
- Pneumonia, Viral* / epidemiology
- Pneumonia, Viral* / etiology
- Pneumonia, Viral* / physiopathology
- Pneumonia, Viral* / therapy
- SARS-CoV-2
- Surgical Procedures, Operative* / methods
- Surgical Procedures, Operative* / trends

Full text links

AGING FREE FULL TEXT Impact Journals, LLC Free PMC article
Proceed to details
Cite
Share
1,026
Observational Study
J Stroke Cerebrovasc Dis
. 2021 Oct;30(10):106028.
doi: 10.1016/j.jstrokecerebrovasdis.2021.106028. Epub 2021 Jul 31.

Impact of the SARS-COV-2 Pandemic on the Endovascular Treatment of Acute Stroke - an Italian Single-Center Experience

Guglielmo Pero [1], Hugo Mota Dória [2], Matteo Giavarini [3], Luca Quilici [4], Amedeo Cervo [5], Antonio Macera [6], Mariangela Piano [7]
Affiliations Expand

Affiliations

- [1] Department of Neuroradiology, ASST Grande Ospedale Metropolitano Niguarda (Niguarda Ca' Granda), Milan, Italy. Electronic address: guglielmo.pero@ospedaleniguarda.it.
- [2] Department of Neuroradiology, ASST Grande Ospedale Metropolitano Niguarda (Niguarda Ca' Granda), Milan, Italy; Department of Neuroradiology, Centro Hospitalar Universitário do Porto, Porto, Portugal; Department of Neuroradiology, Hospital Central do Funchal, Rua Nova do Comboio, N. 13, Funchal, Madeira 9050-054, Portugal. Electronic address: motadoria@gmail.com.
- [3] Facoltà di Medicina e Chirurgia dell'Università Statale di Milano, Milan, Italy.
- [4] Department of Neuroradiology, ASST Ospedale Papa Giovanni XXIII, Bergamo, Italy. Electronic address: lquilici@asst-pg23.it.

- [5] Department of Neuroradiology, ASST Grande Ospedale Metropolitano Niguarda (Niguarda Ca' Granda), Milan, Italy. Electronic address: amedeo.cervo@ospedaleniguarda.it.
- [6] Department of Neuroradiology, ASST Grande Ospedale Metropolitano Niguarda (Niguarda Ca' Granda), Milan, Italy.
- [7] Department of Neuroradiology, ASST Grande Ospedale Metropolitano Niguarda (Niguarda Ca' Granda), Milan, Italy. Electronic address: mariangela.piano@ospedaleniguarda.it.

- PMID: **34392026**
- PMCID: PMC8324420
- DOI: 10.1016/j.jstrokecerebrovasdis.2021.106028

Free PMC article
Observational Study

Impact of the SARS-COV-2 Pandemic on the Endovascular Treatment of Acute Stroke - an Italian Single-Center Experience

Guglielmo Pero et al. J Stroke Cerebrovasc Dis. 2021 Oct.
Free PMC article
Show details
J Stroke Cerebrovasc Dis
. 2021 Oct;30(10):106028.
doi: 10.1016/j.jstrokecerebrovasdis.2021.106028. Epub 2021 Jul 31.

Authors

Guglielmo Pero [1], Hugo Mota Dória [2], Matteo Giavarini [3], Luca Quilici [4], Amedeo Cervo [5], Antonio Macera [6], Mariangela Piano [7]

Affiliations

- [1] Department of Neuroradiology, ASST Grande Ospedale Metropolitano Niguarda (Niguarda Ca' Granda), Milan, Italy. Electronic address: guglielmo.pero@ospedaleniguarda.it.
- [2] Department of Neuroradiology, ASST Grande Ospedale Metropolitano Niguarda (Niguarda Ca' Granda), Milan, Italy; Department of Neuroradiology, Centro Hospitalar Universitário do Porto, Porto, Portugal; Department of Neuroradiology, Hospital Central do Funchal, Rua Nova do Comboio, N. 13, Funchal, Madeira 9050-054, Portugal. Electronic address: motadoria@gmail.com.
- [3] Facoltà di Medicina e Chirurgia dell'Università Statale di Milano, Milan, Italy.
- [4] Department of Neuroradiology, ASST Ospedale Papa Giovanni XXIII, Bergamo, Italy. Electronic address: lquilici@asst-pg23.it.

- [5] Department of Neuroradiology, ASST Grande Ospedale Metropolitano Niguarda (Niguarda Ca' Granda), Milan, Italy. Electronic address: amedeo.cervo@ospedaleniguarda.it.
- [6] Department of Neuroradiology, ASST Grande Ospedale Metropolitano Niguarda (Niguarda Ca' Granda), Milan, Italy.
- [7] Department of Neuroradiology, ASST Grande Ospedale Metropolitano Niguarda (Niguarda Ca' Granda), Milan, Italy. Electronic address: mariangela.piano@ospedaleniguarda.it.

- PMID: **34392026**
- PMCID: PMC8324420
- DOI: 10.1016/j.jstrokecerebrovasdis.2021.106028

Abstract

Objectives: The SARS-CoV-2 pandemic greatly influenced the overall quality of healthcare. The purpose of this study was to compare the time variables for acute stroke treatment and evaluate differences in the pre-hospital and in-hospital care before and during the SARS-CoV-2 pandemic, as well as between the first and second waves.

Materials and methods: Observational and retrospective study from an Italian hospital, including patients who underwent thrombectomy between January 1st 2019 and December 31st 2020.

Results: Out of a total of 594 patients, 301 were treated in 2019 and 293 in 2020. The majority observed in 2019 came from spoke centers (67,1%), while in 2020 more than half (52%, $p < 0.01$) were evaluated at the hospital's emergency room directly (ER-NCGH). When compared to 2019, time metrics were globally increased in 2020, particularly in the ER-NCGH groups during the period of the first wave (N = 24 and N = 56, respectively): "Onset-to-door":50,5 vs 88,5, $p < 0,01$; "Arrival in Neuroradiology - groin":13 vs 25, $p < 0,01$; "Door-to-groin":118 vs 143,5, $p = 0,02$; "Onset-to-groin":180 vs 244,5, $p < 0,01$; "Groin-to-recanalization": 41 vs 49,5, $p = 0,03$. When comparing ER-NCGH groups between the first (N = 56) and second (N = 49) waves, there was an overall improvement in times, namely in the "Door-to-CT" (47,5 vs 37, $p < 0,01$), "Arrival in Neuroradiology - groin" (25 vs 20, $p = 0,03$) and "Onset-to-groin" (244,5 vs 227,5, $p = 0,02$).

Conclusions: During the SARS-CoV-2 pandemic, treatment for stroke patients was delayed, particularly during the first wave. Reallocation of resources and the shutting down of spoke centers may have played a determinant role.

Keywords: Endovascular; Italy; Pandemic; SARS-CoV-2; Stroke; Thrombectomy.

Copyright © 2021 Elsevier Inc. All rights reserved.

Conflict of interest statement

Declaration of Competing Interest None.

- 19 references
- 2 figures

Supplementary info

Publication types, MeSH terms Expand

Publication types

- Comparative Study
- Observational Study

MeSH terms

- Aged
- Aged, 80 and over
- COVID-19*
- Databases, Factual
- Delivery of Health Care, Integrated / trends*
- Emergency Medical Services / trends
- Endovascular Procedures / trends*
- Female
- Health Care Rationing / trends
- Health Services Needs and Demand / trends
- Humans
- Italy
- Male
- Middle Aged
- Patient Admission / trends
- Retrospective Studies
- Stroke / diagnosis
- Stroke / therapy*
- Thrombectomy / trends*
- Time Factors
- Time-to-Treatment / trends*
- Treatment Outcome

Full text links

ELSEVIER FULL-TEXT ARTICLE Elsevier Science Free PMC article
Proceed to details
Cite
Share
1,027
Observational Study
Rheumatol Int
. 2020 Dec;40(12):2015-2021.
doi: 10.1007/s00296-020-04699-x. Epub 2020 Sep 18.

High rates of severe disease and death due to SARS-CoV-2 infection in rheumatic disease patients treated with rituximab: a descriptive study

Jesús Loarce-Martos [1], Antía García-Fernández [2], Fernando López-Gutiérrez [2], Verónica García-García [2], Laura Calvo-Sanz [2], Iván Del Bosque-Granero [2], M Andreína Terán-Tinedo [2], Alina Boteanu [2], Javier Bachiller-Corral [2], Mónica Vázquez-Díaz [2]
Affiliations Expand

Affiliations

- [1] Rheumatology Department, Hospital Universitario Ramón y Cajal, Carretera de Colmenar Viejo, 9, 1 km, 28043, Madrid, Spain. jesus.loarce@gmail.com.
- [2] Rheumatology Department, Hospital Universitario Ramón y Cajal, Carretera de Colmenar Viejo, 9, 1 km, 28043, Madrid, Spain.

- PMID: **32945944**
- PMCID: PMC7499013
- DOI: 10.1007/s00296-020-04699-x

Free PMC article
Observational Study

High rates of severe disease and death due to SARS-CoV-2 infection in rheumatic disease patients treated with rituximab: a descriptive study

Jesús Loarce-Martos et al. Rheumatol Int. 2020 Dec.
Free PMC article
Show details
Rheumatol Int
. 2020 Dec;40(12):2015-2021.
doi: 10.1007/s00296-020-04699-x. Epub 2020 Sep 18.

Authors

Jesús Loarce-Martos [1], Antía García-Fernández [2], Fernando López-Gutiérrez [2], Verónica García-García [2], Laura Calvo-Sanz [2], Iván Del Bosque-Granero [2], M Andreína Terán-Tinedo [2], Alina Boteanu [2], Javier Bachiller-Corral [2], Mónica Vázquez-Díaz [2]

Affiliations

- [1] Rheumatology Department, Hospital Universitario Ramón y Cajal, Carretera de Colmenar Viejo, 9, 1 km, 28043, Madrid, Spain. jesus.loarce@gmail.com.
- [2] Rheumatology Department, Hospital Universitario Ramón y Cajal, Carretera de Colmenar Viejo, 9, 1 km, 28043, Madrid, Spain.

- PMID: **32945944**
- PMCID: PMC7499013
- DOI: 10.1007/s00296-020-04699-x

Abstract

The objective of this study is to describe the characteristics and outcomes of rheumatic and musculoskeletal disease (RMD) patients who were treated with rituximab and had suspected or confirmed severe acute respiratory syndrome coronavirus 2 (SARS-CoV-2) infection. In this descriptive study, RMD patients who were treated with rituximab in the last 12 months at the Rheumatology Department of our hospital were screened for SARS-CoV-2 infection via telephone interview and a comprehensive review of clinical health records (01/02/2020-26/05/2020). Those with probable or confirmed SARS-CoV-2 infection were included. In total, 76 patients were screened. Of these, 13 (17.1%) had suspected or confirmed SARS-CoV-2 infection. With regard to these 13 patients, the median age at coronavirus disease (COVID-19) diagnosis was 68 years (range 28-76 years) and 8 (61.5%) were female. Five patients had rheumatoid arthritis, three had systemic vasculitis, two had Sjögren syndrome, and two had systemic lupus erythematosus. Additionally, seven patients (53.8%) had pulmonary involvement secondary to RMD. Eight patients (61.5%) developed severe disease leading to hospitalization, and seven developed bilateral pneumonia and respiratory insufficiency. Of the eight hospitalized patients, five (62.5%) fulfilled the acute respiratory distress syndrome criteria and three developed a critical disease and died. Our cohort had a high rate of severe disease requiring hospitalization (61.5%), with bilateral pneumonia and hyperinflammation leading to a high mortality rate (23.1%). Treatment with rituximab should be considered a possible risk factor for unfavorable outcomes in COVID-19 patients with RMD. However, further study is required to confirm this association.

Keywords: COVID-19; Rheumatic diseases; Rituximab; SARS-CoV-2.

Conflict of interest statement

Dr. Loarce-Martos reports personal fees from Celgene S.L.U., outside the submitted work; Dr. García-Fernández has nothing to disclose; Dr. López-Gutiérrez has nothing to disclose; Dr. García-García has nothing to disclose; Dr. Calvo-Sanz has nothing to disclose; Dr. del Bosque-Granero has nothing to disclose; Dr. Terán-Tinedo has nothing to disclose; Dr. Boteanu has nothing to disclose; Dr. Bachiller-Corral has nothing to disclose; Dr. Vázquez-Díaz reports personal fees and non-financial support from Sandoz, personal fees and non-financial support from Pfizer, personal fees and non-financial support from Merck Sharp and Dohme, outside the submitted work.

Comment in

- Use of rituximab and the risk of adverse clinical outcomes in COVID-19 patients with systemic rheumatic disease.

Kow CS, Hasan SS. Kow CS, et al. Rheumatol Int. 2020 Dec;40(12):2117-2118. doi: 10.1007/s00296-020-04715-0. Epub 2020 Oct 12. Rheumatol Int. 2020. PMID: 33044704
Free PMC article. No abstract available.

- 24 references

Supplementary info

Publication types, MeSH terms, Substances Expand

Publication types

- Observational Study

MeSH terms

- Adult
- Aged
- Arthritis, Rheumatoid / complications
- Arthritis, Rheumatoid / drug therapy*
- Betacoronavirus
- COVID-19
- Contraindications, Drug
- Coronavirus Infections / diagnosis
- Coronavirus Infections / mortality*
- Female
- Humans
- Immunologic Factors / administration & dosage
- Immunologic Factors / adverse effects*
- Lupus Erythematosus, Systemic / complications
- Lupus Erythematosus, Systemic / drug therapy*
- Male
- Middle Aged
- Pandemics
- Pneumonia, Viral / diagnosis
- Pneumonia, Viral / mortality*
- Retrospective Studies
- Rituximab / administration & dosage
- Rituximab / adverse effects*
- SARS-CoV-2
- Severity of Illness Index

Substances

- Immunologic Factors
- Rituximab

Full text links

SpringerLink FULL-TEXT ARTICLE Springer Free PMC article
Proceed to details
Cite
Share
1,028
Observational Study
Hypertens Res
. 2020 Nov;43(11):1257-1266.
doi: 10.1038/s41440-020-00535-8. Epub 2020 Aug 21.

Renin-angiotensin system inhibitors and the severity of coronavirus disease 2019 in Kanagawa, Japan: a retrospective cohort study

Yasushi Matsuzawa [1], Hisao Ogawa [2], Kazuo Kimura [3], Masaaki Konishi [4], Jin Kirigaya [5], Kazuki Fukui [6], Kengo Tsukahara [7], Hiroyuki Shimizu [8], Keisuke Iwabuchi [9], Yu Yamada [10], Kenichiro Saka [11], Ichiro Takeuchi [5], Toshio Hirano [12], Kouichi Tamura [4]
Affiliations Expand

Affiliations

- [1] Division of Cardiology, Yokohama City University Medical Center, Yokohama, Japan. matsu@yokohama-cu.ac.jp.
- [2] National Cerebral and Cardiovascular Center, Suita, Japan.
- [3] Division of Cardiology, Yokohama City University Medical Center, Yokohama, Japan.
- [4] Department of Medical Science and Cardiorenal Medicine, Yokohama City University Graduate School of Medicine, Yokohama, Japan.
- [5] Department of Emergency Medicine, Yokohama City University Graduate School of Medicine, Yokohama, Japan.
- [6] Department of Cardiology, Kanagawa Cardiovascular and Respiratory Center, Yokohama, Japan.
- [7] Division of Cardiology, Fujisawa City Hospital, Fujisawa, Japan.
- [8] Department of Clinical Laboratory Medicine, Fujisawa City Hospital, Fujisawa, Japan.
- [9] Department of General Medicine, Kanagawa Prefectural Ashigarakami Hospital, Ashigara, Japan.

- [10] Division of Cardiology, Kanagawa Prefectural Ashigarakami Hospital, Ashigara, Japan.
- [11] Division of Cardiology, Yokosuka City Hospital, Yokosuka, Japan.
- [12] Headquarters, National Institutes for Quantum and Radiological Science and Technology, Chiba, Japan.

- PMID: **32820236**
- DOI: 10.1038/s41440-020-00535-8

Observational Study

Renin-angiotensin system inhibitors and the severity of coronavirus disease 2019 in Kanagawa, Japan: a retrospective cohort study

Yasushi Matsuzawa et al. Hypertens Res. 2020 Nov.

Show details

Hypertens Res

. 2020 Nov;43(11):1257-1266.
doi: 10.1038/s41440-020-00535-8. Epub 2020 Aug 21.

Authors

Yasushi Matsuzawa [1], Hisao Ogawa [2], Kazuo Kimura [3], Masaaki Konishi [4], Jin Kirigaya [5], Kazuki Fukui [6], Kengo Tsukahara [7], Hiroyuki Shimizu [8], Keisuke Iwabuchi [9], Yu Yamada [10], Kenichiro Saka [11], Ichiro Takeuchi [5], Toshio Hirano [12], Kouichi Tamura [4]

Affiliations

- [1] Division of Cardiology, Yokohama City University Medical Center, Yokohama, Japan. matsu@yokohama-cu.ac.jp.
- [2] National Cerebral and Cardiovascular Center, Suita, Japan.
- [3] Division of Cardiology, Yokohama City University Medical Center, Yokohama, Japan.
- [4] Department of Medical Science and Cardiorenal Medicine, Yokohama City University Graduate School of Medicine, Yokohama, Japan.
- [5] Department of Emergency Medicine, Yokohama City University Graduate School of Medicine, Yokohama, Japan.
- [6] Department of Cardiology, Kanagawa Cardiovascular and Respiratory Center, Yokohama, Japan.
- [7] Division of Cardiology, Fujisawa City Hospital, Fujisawa, Japan.
- [8] Department of Clinical Laboratory Medicine, Fujisawa City Hospital, Fujisawa, Japan.
- [9] Department of General Medicine, Kanagawa Prefectural Ashigarakami Hospital, Ashigara, Japan.
- [10] Division of Cardiology, Kanagawa Prefectural Ashigarakami Hospital, Ashigara, Japan.
- [11] Division of Cardiology, Yokosuka City Hospital, Yokosuka, Japan.

- [12] Headquarters, National Institutes for Quantum and Radiological Science and Technology, Chiba, Japan.

- PMID: **32820236**
- DOI: 10.1038/s41440-020-00535-8

Abstract

Since the beginning of the coronavirus disease 2019 (COVID-19) outbreak initiated on the Diamond Princess Cruise Ship at Yokohama harbor in February 2020, we have been doing our best to treat COVID-19 patients. In animal experiments, angiotensin converting enzyme inhibitors (ACEIs) and angiotensin II type-1 receptor blockers (ARBs) are reported to suppress the downregulation of angiotensin converting enzyme 2 (ACE2), and they may inhibit the worsening of pathological conditions. We aimed to examine whether preceding use of ACEIs and ARBs affected the clinical manifestations and prognosis of COVID-19 patients. One hundred fifty-one consecutive patients (mean age 60 ± 19 years) with polymerase-chain-reaction proven severe acute respiratory syndrome coronavirus 2 (SARS-CoV-2) infection who were admitted to six hospitals in Kanagawa Prefecture, Japan, were analyzed in this multicenter retrospective observational study. Among all COVID-19 patients, in the multiple regression analysis, older age (age ≥ 65 years) was significantly associated with the primary composite outcome (odds ratio (OR) 6.63, 95% confidence interval (CI) 2.28-22.78, $P < 0.001$), which consisted of (i) in-hospital death, (ii) extracorporeal membrane oxygenation, (iii) mechanical ventilation, including invasive and noninvasive methods, and (iv) admission to the intensive care unit. In COVID-19 patients with hypertension, preceding ACEI/ARB use was significantly associated with a lower occurrence of new-onset or worsening mental confusion (OR 0.06, 95% CI 0.002-0.69, $P = 0.02$), which was defined by the confusion criterion, which included mild disorientation or hallucination with an estimation of medical history of mental status, after adjustment for age, sex, and diabetes. In conclusion, older age was a significant contributor to a worse prognosis in COVID-19 patients, and ACEIs/ARBs could be beneficial for the prevention of confusion in COVID-19 patients with hypertension.

Keywords: Angiotensin II type-1 receptor blockers; Angiotensin converting enzyme inhibitors; COVID-19.

- 22 references

Supplementary info

Publication types, MeSH terms, Substances Expand

Publication types

- Multicenter Study
- Observational Study
- Research Support, Non-U.S. Gov't

MeSH terms

- Adult

- Age Factors
- Aged
- Aged, 80 and over
- Angiotensin II Type 1 Receptor Blockers / therapeutic use*
- Angiotensin-Converting Enzyme Inhibitors / therapeutic use*
- Betacoronavirus*
- COVID-19
- Confusion / prevention & control
- Coronavirus Infections / mortality*
- Coronavirus Infections / psychology
- Coronavirus Infections / therapy
- Female
- Humans
- Hypertension / complications
- Hypertension / drug therapy*
- Male
- Middle Aged
- Pandemics
- Pneumonia, Viral / mortality*
- Pneumonia, Viral / psychology
- Pneumonia, Viral / therapy
- Retrospective Studies
- SARS-CoV-2
- Severity of Illness Index

Substances

- Angiotensin II Type 1 Receptor Blockers
- Angiotensin-Converting Enzyme Inhibitors

Proceed to details
Cite
Share
☐ 1,029
Observational Study
Ann Palliat Med
. 2020 Jul;9(4):2118-2130.
doi: 10.21037/apm-20-1273. Epub 2020 Jul 20.

Clinical characteristics and treatment of critically ill patients with COVID-19 in Hebei

Yuhong Chen [1], Kun Zhang [2], Guijun Zhu [2], Lixia Liu [2], Xixin Yan [3], Zhigang Cai [3], Zhongheng Zhang [4], Haijun Zhi [5], Zhenjie Hu [6]
Affiliations Expand

Affiliations

- [1] Department of Intensive Care Unit, Hebei Medical University Fourth Affiliated Hospital and Hebei Provincial Tumor Hospital, Shijiazhuang, China. yuhong_apple@126.com.
- [2] Department of Intensive Care Unit, Hebei Medical University Fourth Affiliated Hospital and Hebei Provincial Tumor Hospital, Shijiazhuang, China.
- [3] Department of Respiration, Hebei Medical University Second Affiliated Hospital, Shijiazhuang, China.
- [4] Department of Emergency Medicine, Sir Run Run Shaw Hospital, Zhejiang University School of Medicine, Hangzhou, China.
- [5] Emergency Department, Cangzhou Central Hospital, Cangzhou, China.
- [6] Department of Intensive Care Unit, Hebei Medical University Fourth Affiliated Hospital and Hebei Provincial Tumor Hospital, Shijiazhuang, China. syicu@vip.sina.com.

- PMID: **32692230**
- DOI: 10.21037/apm-20-1273

Free article
Observational Study

Clinical characteristics and treatment of critically ill patients with COVID-19 in Hebei

Yuhong Chen et al. Ann Palliat Med. 2020 Jul.
Free article
Show details
Ann Palliat Med
. 2020 Jul;9(4):2118-2130.
doi: 10.21037/apm-20-1273. Epub 2020 Jul 20.

Authors

Yuhong Chen [1], Kun Zhang [2], Guijun Zhu [2], Lixia Liu [2], Xixin Yan [3], Zhigang Cai [3], Zhongheng Zhang [4], Haijun Zhi [5], Zhenjie Hu [6]

Affiliations

- [1] Department of Intensive Care Unit, Hebei Medical University Fourth Affiliated Hospital and Hebei Provincial Tumor Hospital, Shijiazhuang, China. yuhong_apple@126.com.
- [2] Department of Intensive Care Unit, Hebei Medical University Fourth Affiliated Hospital and Hebei Provincial Tumor Hospital, Shijiazhuang, China.
- [3] Department of Respiration, Hebei Medical University Second Affiliated Hospital, Shijiazhuang, China.

- [4] Department of Emergency Medicine, Sir Run Run Shaw Hospital, Zhejiang University School of Medicine, Hangzhou, China.
- [5] Emergency Department, Cangzhou Central Hospital, Cangzhou, China.
- [6] Department of Intensive Care Unit, Hebei Medical University Fourth Affiliated Hospital and Hebei Provincial Tumor Hospital, Shijiazhuang, China. syicu@vip.sina.com.

- PMID: **32692230**
- DOI: 10.21037/apm-20-1273

Abstract

Background: In December, 2019, a novel coronavirus disease 2019 (COVID-19) emerged in Wuhan, China. We aimed to clarify the epidemiology, laboratory examinations, imaging findings, and treatment of critically ill patients with COVID-19 in Hebei province, China.

Methods: In this retrospective study, the demographic, laboratory and imaging, and treatment data of patients with severe COVID-19 treated in 13 designated hospitals in Hebei were collected and analyzed.

Results: A total of 319 severe COVID-19 patients were treated at the 13 designated hospitals between 22 January, 2020 and 25 March, 2020. Eventually, 51 critically ill (31 severe cases and 20 critically severe cases) patients were included in the analysis. The patients had an average age of 58.9±13.7 years, and 27 (52.9%) were men. Twenty-one (41.2%) were familial cluster, and 33 (64.7%) had chronic illnesses. The patients in critically severe group had longer duration from symptom to confirmation, more severe infections, more severe lung injury, and a lower percentage of lymphocytes. All 51 patients received antiviral drugs, 47 (92.2%) received antibacterial agents, 49 (96.1%) received traditional Chinese drugs, and 46 (90.2%) received methylprednisolone. The critically severe patients received more fluid and more diuretic treatment; 14 (70.0%) required invasive mechanical ventilation, and 13 (65.0%) developed extrapulmonary complications.

Conclusions: COVID-19 patients who had underlying diseases and longer confirmation times were more likely to progress to critically severe COVID-19. These patients also presented with a higher risk of respiratory depression, circulatory collapse, extrapulmonary complications, and infection.

Keywords: 2019 novel coronavirus; 2019-nCoV; SARS-CoV-2; critically ill; novel coronavirus disease 2019 (COVID-19).

Supplementary info

Publication types, MeSH terms Expand

Publication types

- Multicenter Study
- Observational Study

MeSH terms

- Aged

- COVID-19
- China / epidemiology
- Coronavirus Infections / diagnosis*
- Coronavirus Infections / epidemiology
- Coronavirus Infections / therapy*
- Critical Care
- Critical Illness
- Female
- Humans
- Intensive Care Units
- Male
- Middle Aged
- Pandemics
- Pneumonia, Viral / diagnosis*
- Pneumonia, Viral / epidemiology
- Pneumonia, Viral / therapy*
- Retrospective Studies

Full text links

APM FULL TEXT AME Publishing Company
Proceed to details
Cite
Share
☐ 1,030
Observational Study
J Clin Pharmacol
. 2021 Mar;61(3):406-411.
doi: 10.1002/jcph.1787. Epub 2020 Nov 29.

Treatment With Tocilizumab for Patients With COVID-19 Infections: A Case-Series Study

Yoonsun Mo [1] [2], Obed Adarkwah [2], John Zeibeq [2], Evgeny Pinelis [2], Jose Orsini [2], James Gasperino [2]
Affiliations Expand

Affiliations

- [1] Arnold & Marie Schwartz College of Pharmacy and Health Sciences, Long Island University, Brooklyn, New York, USA.

- [2] Center for Critical Care Services, Department of Medicine, The Brooklyn Hospital Center, Brooklyn, New York, USA.

- PMID: **33180360**
- DOI: 10.1002/jcph.1787

Observational Study

Treatment With Tocilizumab for Patients With COVID-19 Infections: A Case-Series Study

Yoonsun Mo et al. J Clin Pharmacol. 2021 Mar.
Show details
J Clin Pharmacol
. 2021 Mar;61(3):406-411.
doi: 10.1002/jcph.1787. Epub 2020 Nov 29.

Authors

Yoonsun Mo [1, 2], Obed Adarkwah [2], John Zeibeq [2], Evgeny Pinelis [2], Jose Orsini [2], James Gasperino [2]

Affiliations

- [1] Arnold & Marie Schwartz College of Pharmacy and Health Sciences, Long Island University, Brooklyn, New York, USA.
- [2] Center for Critical Care Services, Department of Medicine, The Brooklyn Hospital Center, Brooklyn, New York, USA.

- PMID: **33180360**
- DOI: 10.1002/jcph.1787

Abstract

Tocilizumab (TCZ), a humanized monoclonal antibody targeting the interleukin-6 receptor, holds the potential for treating coronavirus disease 2019 (COVID-19) patients, particularly those at high risk of cytokine storm syndrome. However, data regarding the clinical impact of treatment with TCZ in patients with COVID-19 are limited. This study was conducted to evaluate the safety and effectiveness of TCZ as an adjunct therapy for the treatment of severe COVID-19 infection. This was a retrospective observational chart review of confirmed COVID-19 patients who received TCZ, along with other COVID-19 therapies. The outcomes of interest included changes in vital signs such as temperature and laboratory biomarkers, duration of mechanical ventilation, adverse events possibly associated with TCZ, and intensive care unit and hospital lengths of stay. This study included 38 patients with an average age of 63 years (IQR, 48-70 years). The average dose of TCZ given was 519 ± 61 mg. Median C-reactive protein significantly decreased following TCZ administration (189.9 vs 54.8 mg/L, P = .003). Nineteen of all febrile patients before the initiation

of TCZ (73%) became fever free on the fourth day of TCZ treatment. Following TCZ treatment, 11 patients developed infections because of multidrug-resistant bacteria, and elevated liver transaminases were observed in 6 patients. The preliminary findings of this study suggested TCZ appeared to ameliorate COVID-19-related cytokine storm syndrome. However, large randomized, controlled trials are needed to investigate whether treatment with TCZ is associated with better outcomes in COVID-19.

Keywords: COVID-19; adverse drug events; effectiveness; safety; tocilizumab.

© 2020, The American College of Clinical Pharmacology.

- 22 references

Supplementary info

Publication types, MeSH terms, Substances Expand

Publication types

- Observational Study

MeSH terms

- Aged
- Aged, 80 and over
- Antibodies, Monoclonal, Humanized / pharmacology
- Antibodies, Monoclonal, Humanized / therapeutic use*
- COVID-19 / blood*
- COVID-19 / diagnosis
- COVID-19 / drug therapy*
- Cytokines / antagonists & inhibitors*
- Cytokines / blood*
- Female
- Humans
- Male
- Middle Aged
- Receptors, Interleukin-6 / antagonists & inhibitors
- Receptors, Interleukin-6 / blood
- Retrospective Studies

Substances

- Antibodies, Monoclonal, Humanized
- Cytokines
- IL6R protein, human

- Receptors, Interleukin-6
- tocilizumab

Full text links

WILEY Full Text Article Wiley

Proceed to details

Cite

Share

1,031

Observational Study

JAMA
. 2021 Mar 2;325(9):855-864.
doi: 10.1001/jama.2021.0694.

Association of Intravenous Immunoglobulins Plus Methylprednisolone vs Immunoglobulins Alone With Course of Fever in Multisystem Inflammatory Syndrome in Children

Naïm Ouldali [1] [2] [3], Julie Toubiana [4] [5], Denise Antona [6], Etienne Javouhey [7] [8], Fouad Madhi [9], Mathie Lorrot [10], Pierre-Louis Léger [11], Caroline Galeotti [12], Caroline Claude [13], Arnaud Wiedemann [14] [15], Noémie Lachaume [16], Caroline Ovaert [17] [18], Morgane Dumortier [19], Jean-Emmanuel Kahn [20], Alexis Mandelcwajg [21], Lucas Percheron [22], Blandine Biot [23], Jeanne Bordet [24], Marie-Laure Girardin [25], David Dawei Yang [26], Marion Grimaud [27], Mehdi Oualha [27], Slimane Allali [4], Fanny Bajolle [28], Constance Beyler [29], Ulrich Meinzer [1] [30], Michael Levy [31], Ana-Maria Paulet [32], Corinne Levy [2] [33], Robert Cohen [2] [33], Alexandre Belot [34], François Angoulvant [26] [35], French Covid-19 Paediatric Inflammation Consortium

Collaborators, Affiliations Expand

Collaborators

- **French Covid-19 Paediatric Inflammation Consortium**: Cinthia Rames, Aurelie Donzeau, Sophie Lety, Cristian Fedorczuk, Marion Lajus, Philippe Bensaid, Yacine Laoudi, Charlotte Pons, Camille Beaucourt, Loïc De Pontual, Camille Aupiais, Alain Lefevre-Utile, Muriel Richard, Etienne Goisque, Xavier Iriart, Olivier Brissaud, Marion Bailhache, Pierre Segretin, Julie Molimard, Marie-Clothilde Orcel, Gregoire Benoist, Elsa Amouyal, Margaux Guerder, Robin Pouyau, Jean-Marie De Guillebon De Resnes, Ellia Mezgueldi, Fleur Cour-Andlauer, Come Horvat, Pierre Poinsot, Cecile Frachette, Antoine Ouziel, Yves Gillet, Catherine Barrey, Jacques Brouard, Caroline Faucon, Henri Ginies, Vathanaksambath Ro, Narcisse Elanga, Vincent Gajdos, Romain Basmaci, Nevena Danekova, Hadile Mutar, Sébastien Rouget, Xavier Torterüe, Elodie Nattes, Isabelle Hau, Sandra Biscardi, Houmam El Jurdi, Camille Jung, Ralph Epaud, Céline Delestrain, Adèle Carlier-Gonod, Camille Chavy, Benoît

Colomb, Stéphanie Litzler-Renault, Denis Semama, Frederic Huet, Mayssa Sarakbi, Guillaume Mortamet, Cécile Bost-Bru, Charlotte Kevorkian-Verguet, Matthias Lachaud, Caroline Vinit, Véronique Hentgen, Pascal Leroux, Valérie Bertrand, Caroline Parrod, Irina Craiu, Isabelle Kone-Paut, Philippe Durand, Pierre Tissiere, Luc Morin, Jordi Miatello, Guillaume Morelle, Tamazoust Guiddir, Charlotte Borocco, Camille Guillot, Stéphane Leteurtre, François Dubos, Mylene Jouancastay, Morgan Recher, Alain Martinot, Valentine Voeusler, Jeanne Languepin, Aurélie Morand, Emmanuelle Bosdure, Violaine Bresson, Noémie Vanel, Fabrice Ughetto, Fabrice Michel, Caujolle Marie, Renaud Blonde, Jacqueline Nguyen, Nathalie Garrec, Arnaud Chalvon-Demersay, Caroline Masserot-Lureau, Anne-Sophie Colas, Claire Ferrua, Anis Larakeb, Sakina Benkaddouss, Laurence Mathivon, Marie Monfort, Sanaa Naji, Aurelia Carbasse, Christophe Milesi, Cyril Schweitzer, Nathan Giroux, Noël Boussard, Benedicte Romefort, Elise Launay, Christèle Gras-Le Guen, Ahmed Ali, Nathalie Blot, Antoine Tran, Anne Rancurel, Hervé Haas, Mickael Afanetti, Julie Bernardor, Deborah Talmud, Imen Jhaouat, Françoise Monceaux, Anaïs Chosidow, Anne-Sophie Romain, Emmanuel Grimprel, Jérôme Rambaud, Sandrine Jean, Julie Starck, Yaël Levy, Romain Guedj, Ricardo Carbajal, Pauline Parisot, Géraldine Poncelet, Richard Wolff, Boris Lacarra, Arielle Maroni, Jérôme Naudin, Guillaume Geslin, Laure Maurice, Anna Deho, Fleur Lebourgeois, Marilyne Chomton, Stephane Dauger, Mathieu Genuini, Chérine Benzouid, Johanna Lokmer, Ronan Bonnefoy, Isabelle Melki, Glory Dingulu, Jean Gaschignard, Camille Ducrocq, Marie Pouletty, Olivier Corseri, Albert Faye, Alexis Rybak, Luigi Titomanlio, Marie-Françoise Hurtaux, Guislaine Garcelain, Stéphane Bonacorsi, Philippe Bidet, André Birgy, Sylvain Renolleau, Fabrice Lesage, Florence Moulin, Laurent Dupic, Laure de Saint Blanquat, Claire Heilbronner, Meryl Vedrenne-Cloquet, Elodie Salvador, Matthieu Bendavid, Charles De Marcellus, Judith Chareyre, Yael Pinhas, Joséphine Brisse, Melissa Taylor, Agathe Debray, Pauline Adnot, Martin Chalumeau, Véronique Abadie, Pierre Frange, Jeremie F Cohen, William Curtis, Hélène Chappuy, Zahra Belhadjer, Johanne Auriau, Mathilde Méot, Lucile Houyel, Damien Bonnet, Christophe Delacourt, David Drummond, Brigitte Bader-Meunier, Pierre Quartier, Frédérique Delion, Philippe Blanc, Elisabeth Caron, Natacha Maledon, Blandine Robert, Letitia Pantalone, Hanane Kouider, Camille Loeile, Gauthier Loron, Cécile Vittot, Thierry Blanc, Didier Pinquier, François Buisson, Hugues Flodrops, Jamal-Bey Karim, Raphaëlle Sarton, Fadhila Mokraoui, Simon Escoda, Nina Deschamps, Laurent Bonnemains, Sarah-Louisa Mahi, Clara Mertes, Joelle Terzic, Charlotte Idier, Ariane Benezech, Thomas Simon, Stephane Decramer, Clement Karsenty, Camille Brehin, Soraya Chenichene, Nicoleta-Magdolena Ursulescu, Céline Manteau, Marie Delattre, Bérengère Dalichoux

Affiliations

- [1] Assistance Publique-Hôpitaux de Paris, Department of General Paediatrics, Paediatric Infectious Disease and Internal Medicine, Robert Debré University Hospital, Université de Paris, Paris, France.
- [2] ACTIV, Association Clinique et Thérapeutique Infantile du Val-de-Marne, Créteil, France.
- [3] Université de Paris, INSERM UMR 1123, ECEVE, Paris, France.
- [4] Assistance Publique-Hôpitaux de Paris, Department of General Paediatrics and Paediatric Infectious Diseases, Necker-Enfants-Malades University Hospital, Université de Paris, Paris, France.
- [5] Institut Pasteur, Biodiversity and Epidemiology of Bacterial Pathogens, Paris, France.
- [6] Santé Publique France, Agence Nationale de Santé Publique, Saint-Maurice, France.

- [7] Hospices Civils de Lyon, Paediatric Intensive Care Unit, Hopital Femme, Mère Enfant, University of Lyon, Bron, France.
- [8] EA 7426 Pathophysiology of Injury-Induced Immunosuppression, University Claude Bernard Lyon 1, Hospices Civils of Lyon, Lyon, France.
- [9] Centre Hospitalier Intercommunal, Paediatric Department, Université Paris Est, IMRB-GRC GEMINI, Créteil, France.
- [10] Assistance Publique-Hôpitaux de Paris, Department of General Paediatric, Armand Trousseau University Hospital, Sorbonne Université, Paris, France.
- [11] Assistance Publique-Hôpitaux de Paris, Paediatric Intensive Care Unit, Armand Trousseau University Hospital, Sorbonne Université, Paris, France.
- [12] Assistance Publique-Hôpitaux de Paris, Department of Paediatric Rheumatology, Reference Centre for Autoinflammatory Diseases and Amyloidosis (CEREMAIA), Bicêtre University Hospital, Université de Paris Saclay, Le Kremlin-Bicêtre, France.
- [13] Assistance Publique-Hôpitaux de Paris, Paediatric Intensive Care Unit, Bicêtre University Hospital, Université de Paris Saclay, Le Kremlin-Bicêtre, France.
- [14] Children's Hospital, University Hospital of Nancy, Paediatric Department, Université de Lorraine, Vandoeuvre les Nancy, France.
- [15] INSERM UMRS 1256 NGERE, Nutrition, Genetics, and Environmental Risk Exposure, National Center of Inborn Errors of Metabolism, Université de Lorraine, Vandoeuvre les Nancy, France.
- [16] Assistance Publique-Hôpitaux de Paris, Paediatric Emergency Departement, Louis Mourier University Hospital, Colombes, France.
- [17] Assistance Publique-Hôpitaux de Marseille, Paediatric and Congenital Cardiology, Timone Hospital Marseille, University Hospital, Marseille, France.
- [18] INSERM, Marseille Medical Genetics, UMR 1251, Aix Marseille Université, Marseille, France.
- [19] Hôpital Femme Enfant Adolescent, Department of Paediatrics and Paediatric Emergency, University Hospital, Nantes, France.
- [20] Assistance Publique-Hôpitaux de Paris, Internal Medicine Department, Ambroise Paré University Hospital, Université Versailles-Saint Quentin-en-Yvelines, Boulogne-Billancourt, France.
- [21] Paediatric Department, Hôpital Delafontaine, Saint Denis, France.
- [22] Hôpital des Enfants, Paediatric Nephrology Department, Purpan University Hospital, Toulouse, France.
- [23] Paediatric Department, Hôpital de Valence, Valence, France.
- [24] Strasbourg University Hospital, Paediatric Cardiology Department, Hautepierre University Hospital, Strasbourg, France.
- [25] Paediatric Intensive Care Unit, Strasbourg University Hospital, Hautepierre University Hospital, Strasbourg, France.
- [26] Assistance Publique-Hôpitaux de Paris, Paediatric Emergency Department, Necker-Enfants Malades University Hospital, Université de Paris, Paris, France.
- [27] Assistance Publique-Hôpitaux de Paris, Paediatric Intensive Care Unit, Necker-Enfants Malades University Hospital, EA7323, Université de Paris, Paris, France.
- [28] Assistance Publique-Hôpitaux de Paris, M3C Department, Necker-Enfants Malades University Hospital, Université de Paris, Paris, France.
- [29] Assistance Publique-Hôpitaux de Paris, Cardiopaediatric Unit, Robert Debré University Hospital, Université de Paris, Paris, France.
- [30] Centre for Research on Inflammation, UMR1149, INSERM, Paris, France.

- [31] Assistance Publique-Hôpitaux de Paris, Paediatric Intensive Care Unit, Robert Debré University Hospital, Université de Paris, Paris, France.
- [32] Hôpital Nord Franche-Comté, Paediatric Department, Trévenans, France.
- [33] Centre Hospitalier Intercommunal, Research Centre, Université Paris Est, IMRB-GRC GEMINI, Créteil, France.
- [34] Hospices Civils de Lyon, Paediatric Nephrology, Rheumatology, Dermatology, Hopital Femme, Mère Enfant, Centre International de Recherche en Infectiologie/INSERM U1111, Bron, France.
- [35] INSERM, Centre de Recherche des Cordeliers, UMRS 1138, Sorbonne Université, Université de Paris, Paris, France.

- PMID: **33523115**
- PMCID: PMC7851757
- DOI: 10.1001/jama.2021.0694

Free PMC article
Observational Study

Association of Intravenous Immunoglobulins Plus Methylprednisolone vs Immunoglobulins Alone With Course of Fever in Multisystem Inflammatory Syndrome in Children

Naïm Ouldali et al. JAMA. 2021.
Free PMC article

Show details

JAMA
. 2021 Mar 2;325(9):855-864.
doi: 10.1001/jama.2021.0694.

Authors

Naïm Ouldali [1, 2, 3], Julie Toubiana [4, 5], Denise Antona [6], Etienne Javouhey [7, 8], Fouad Madhi [9], Mathie Lorrot [10], Pierre-Louis Léger [11], Caroline Galeotti [12], Caroline Claude [13], Arnaud Wiedemann [14, 15], Noémie Lachaume [16], Caroline Ovaert [17, 18], Morgane Dumortier [19], Jean-Emmanuel Kahn [20], Alexis Mandelcwajg [21], Lucas Percheron [22], Blandine Biot [23], Jeanne Bordet [24], Marie-Laure Girardin [25], David Dawei Yang [26], Marion Grimaud [27], Mehdi Oualha [27], Slimane Allali [4], Fanny Bajolle [28], Constance Beyler [29], Ulrich Meinzer [1, 30], Michael Levy [31], Ana-Maria Paulet [32], Corinne Levy [2, 33], Robert Cohen [2, 33], Alexandre Belot [34], François Angoulvant [26, 35], French Covid-19 Paediatric Inflammation Consortium

Collaborators

- **French Covid-19 Paediatric Inflammation Consortium**:
Cinthia Rames, Aurelie Donzeau, Sophie Lety, Cristian Fedorczuk, Marion Lajus, Philippe Bensaid, Yacine Laoudi, Charlotte Pons, Camille Beaucourt, Loïc De Pontual, Camille

Aupiais, Alain Lefevre-Utile, Muriel Richard, Etienne Goisque, Xavier Iriart, Olivier Brissaud, Marion Bailhache, Pierre Segretin, Julie Molimard, Marie-Clothilde Orcel, Gregoire Benoist, Elsa Amouyal, Margaux Guerder, Robin Pouyau, Jean-Marie De Guillebon De Resnes, Ellia Mezgueldi, Fleur Cour-Andlauer, Come Horvat, Pierre Poinsot, Cecile Frachette, Antoine Ouziel, Yves Gillet, Catherine Barrey, Jacques Brouard, Caroline Faucon, Henri Ginies, Vathanaksambath Ro, Narcisse Elanga, Vincent Gajdos, Romain Basmaci, Nevena Danekova, Hadile Mutar, Sébastien Rouget, Xavier Torterüe, Elodie Nattes, Isabelle Hau, Sandra Biscardi, Houmam El Jurdi, Camille Jung, Ralph Epaud, Céline Delestrain, Adèle Carlier-Gonod, Camille Chavy, Benoît Colomb, Stéphanie Litzler-Renault, Denis Semama, Frederic Huet, Mayssa Sarakbi, Guillaume Mortamet, Cécile Bost-Bru, Charlotte Kevorkian-Verguet, Matthias Lachaud, Caroline Vinit, Véronique Hentgen, Pascal Leroux, Valérie Bertrand, Caroline Parrod, Irina Craiu, Isabelle Kone-Paut, Philippe Durand, Pierre Tissiere, Luc Morin, Jordi Miatello, Guillaume Morelle, Tamazoust Guiddir, Charlotte Borocco, Camille Guillot, Stéphane Leteurtre, François Dubos, Mylene Jouancastay, Morgan Recher, Alain Martinot, Valentine Voeusler, Jeanne Languepin, Aurélie Morand, Emmanuelle Bosdure, Violaine Bresson, Noémie Vanel, Fabrice Ughetto, Fabrice Michel, Caujolle Marie, Renaud Blonde, Jacqueline Nguyen, Nathalie Garrec, Arnaud Chalvon-Demersay, Caroline Masserot-Lureau, Anne-Sophie Colas, Claire Ferrua, Anis Larakeb, Sakina Benkaddouss, Laurence Mathivon, Marie Monfort, Sanaa Naji, Aurelia Carbasse, Christophe Milesi, Cyril Schweitzer, Nathan Giroux, Noël Boussard, Benedicte Romefort, Elise Launay, Christèle Gras-Le Guen, Ahmed Ali, Nathalie Blot, Antoine Tran, Anne Rancurel, Hervé Haas, Mickael Afanetti, Julie Bernardor, Deborah Talmud, Imen Jhaouat, Françoise Monceaux, Anaïs Chosidow, Anne-Sophie Romain, Emmanuel Grimprel, Jérôme Rambaud, Sandrine Jean, Julie Starck, Yaël Levy, Romain Guedj, Ricardo Carbajal, Pauline Parisot, Géraldine Poncelet, Richard Wolff, Boris Lacarra, Arielle Maroni, Jérôme Naudin, Guillaume Geslin, Laure Maurice, Anna Deho, Fleur Lebourgeois, Marilyne Chomton, Stephane Dauger, Mathieu Genuini, Chérine Benzouid, Johanna Lokmer, Ronan Bonnefoy, Isabelle Melki, Glory Dingulu, Jean Gaschignard, Camille Ducrocq, Marie Pouletty, Olivier Corseri, Albert Faye, Alexis Rybak, Luigi Titomanlio, Marie-Françoise Hurtaux, Guislaine Garcelain, Stéphane Bonacorsi, Philippe Bidet, André Birgy, Sylvain Renolleau, Fabrice Lesage, Florence Moulin, Laurent Dupic, Laure de Saint Blanquat, Claire Heilbronner, Meryl Vedrenne-Cloquet, Elodie Salvador, Matthieu Bendavid, Charles De Marcellus, Judith Chareyre, Yael Pinhas, Joséphine Brisse, Melissa Taylor, Agathe Debray, Pauline Adnot, Martin Chalumeau, Véronique Abadie, Pierre Frange, Jeremie F Cohen, William Curtis, Hélène Chappuy, Zahra Belhadjer, Johanne Auriau, Mathilde Méot, Lucile Houyel, Damien Bonnet, Christophe Delacourt, David Drummond, Brigitte Bader-Meunier, Pierre Quartier, Frédérique Delion, Philippe Blanc, Elisabeth Caron, Natacha Maledon, Blandine Robert, Letitia Pantalone, Hanane Kouider, Camille Loeile, Gauthier Loron, Cécile Vittot, Thierry Blanc, Didier Pinquier, François Buisson, Hugues Flodrops, Jamal-Bey Karim, Raphaëlle Sarton, Fadhila Mokraoui, Simon Escoda, Nina Deschamps, Laurent Bonnemains, Sarah-Louisa Mahi, Clara Mertes, Joelle Terzic, Charlotte Idier, Ariane Benezech, Thomas Simon, Stephane Decramer, Clement Karsenty, Camille Brehin, Soraya Chenichene, Nicoleta-Magdolena Ursulescu, Céline Manteau, Marie Delattre, Bérengère Dalichoux

Affiliations

- [1] Assistance Publique-Hôpitaux de Paris, Department of General Paediatrics, Paediatric Infectious Disease and Internal Medicine, Robert Debré University Hospital, Université de Paris, Paris, France.

- [2] ACTIV, Association Clinique et Thérapeutique Infantile du Val-de-Marne, Créteil, France.
- [3] Université de Paris, INSERM UMR 1123, ECEVE, Paris, France.
- [4] Assistance Publique-Hôpitaux de Paris, Department of General Paediatrics and Paediatric Infectious Diseases, Necker-Enfants-Malades University Hospital, Université de Paris, Paris, France.
- [5] Institut Pasteur, Biodiversity and Epidemiology of Bacterial Pathogens, Paris, France.
- [6] Santé Publique France, Agence Nationale de Santé Publique, Saint-Maurice, France.
- [7] Hospices Civils de Lyon, Paediatric Intensive Care Unit, Hopital Femme, Mère Enfant, University of Lyon, Bron, France.
- [8] EA 7426 Pathophysiology of Injury-Induced Immunosuppression, University Claude Bernard Lyon 1, Hospices Civils of Lyon, Lyon, France.
- [9] Centre Hospitalier Intercommunal, Paediatric Department, Université Paris Est, IMRB-GRC GEMINI, Créteil, France.
- [10] Assistance Publique-Hôpitaux de Paris, Department of General Paediatric, Armand Trousseau University Hospital, Sorbonne Université, Paris, France.
- [11] Assistance Publique-Hôpitaux de Paris, Paediatric Intensive Care Unit, Armand Trousseau University Hospital, Sorbonne Université, Paris, France.
- [12] Assistance Publique-Hôpitaux de Paris, Department of Paediatric Rheumatology, Reference Centre for Autoinflammatory Diseases and Amyloidosis (CEREMAIA), Bicêtre University Hospital, Université de Paris Saclay, Le Kremlin-Bicêtre, France.
- [13] Assistance Publique-Hôpitaux de Paris, Paediatric Intensive Care Unit, Bicêtre University Hospital, Université de Paris Saclay, Le Kremlin-Bicêtre, France.
- [14] Children's Hospital, University Hospital of Nancy, Paediatric Department, Université de Lorraine, Vandoeuvre les Nancy, France.
- [15] INSERM UMRS 1256 NGERE, Nutrition, Genetics, and Environmental Risk Exposure, National Center of Inborn Errors of Metabolism, Université de Lorraine, Vandoeuvre les Nancy, France.
- [16] Assistance Publique-Hôpitaux de Paris, Paediatric Emergency Departement, Louis Mourier University Hospital, Colombes, France.
- [17] Assistance Publique-Hôpitaux de Marseille, Paediatric and Congenital Cardiology, Timone Hospital Marseille, University Hospital, Marseille, France.
- [18] INSERM, Marseille Medical Genetics, UMR 1251, Aix Marseille Université, Marseille, France.
- [19] Hôpital Femme Enfant Adolescent, Department of Paediatrics and Paediatric Emergency, University Hospital, Nantes, France.
- [20] Assistance Publique-Hôpitaux de Paris, Internal Medicine Department, Ambroise Paré University Hospital, Université Versailles-Saint Quentin-en-Yvelines, Boulogne-Billancourt, France.
- [21] Paediatric Department, Hôpital Delafontaine, Saint Denis, France.
- [22] Hôpital des Enfants, Paediatric Nephrology Department, Purpan University Hospital, Toulouse, France.
- [23] Paediatric Department, Hôpital de Valence, Valence, France.
- [24] Strasbourg University Hospital, Paediatric Cardiology Department, Hautepierre University Hospital, Strasbourg, France.
- [25] Paediatric Intensive Care Unit, Strasbourg University Hospital, Hautepierre University Hospital, Strasbourg, France.
- [26] Assistance Publique-Hôpitaux de Paris, Paediatric Emergency Department, Necker-Enfants Malades University Hospital, Université de Paris, Paris, France.

- [27] Assistance Publique-Hôpitaux de Paris, Paediatric Intensive Care Unit, Necker-Enfants Malades University Hospital, EA7323, Université de Paris, Paris, France.
- [28] Assistance Publique-Hôpitaux de Paris, M3C Department, Necker-Enfants Malades University Hospital, Université de Paris, Paris, France.
- [29] Assistance Publique-Hôpitaux de Paris, Cardiopaediatric Unit, Robert Debré University Hospital, Université de Paris, Paris, France.
- [30] Centre for Research on Inflammation, UMR1149, INSERM, Paris, France.
- [31] Assistance Publique-Hôpitaux de Paris, Paediatric Intensive Care Unit, Robert Debré University Hospital, Université de Paris, Paris, France.
- [32] Hôpital Nord Franche-Comté, Paediatric Department, Trévenans, France.
- [33] Centre Hospitalier Intercommunal, Research Centre, Université Paris Est, IMRB-GRC GEMINI, Créteil, France.
- [34] Hospices Civils de Lyon, Paediatric Nephrology, Rheumatology, Dermatology, Hopital Femme, Mère Enfant, Centre International de Recherche en Infectiologie/INSERM U1111, Bron, France.
- [35] INSERM, Centre de Recherche des Cordeliers, UMRS 1138, Sorbonne Université, Université de Paris, Paris, France.

- PMID: **33523115**
- PMCID: PMC7851757
- DOI: 10.1001/jama.2021.0694

Erratum in

- Addition of Nonauthor Collaborator Names of the French Covid-19 Paediatric Inflammation Consortium.
  [No authors listed] [No authors listed] JAMA. 2021 Jul 6;326(1):90. doi: 10.1001/jama.2021.7427. JAMA. 2021. PMID: 34228084 Free PMC article. No abstract available.

Abstract

Importance: Multisystem inflammatory syndrome in children (MIS-C) is the most severe pediatric disease associated with severe acute respiratory syndrome coronavirus 2 infection, potentially life-threatening, but the optimal therapeutic strategy remains unknown.

Objective: To compare intravenous immunoglobulins (IVIG) plus methylprednisolone vs IVIG alone as initial therapy in MIS-C.

Design, setting, and participants: Retrospective cohort study drawn from a national surveillance system with propensity score-matched analysis. All cases with suspected MIS-C were reported to the French National Public Health Agency. Confirmed MIS-C cases fulfilling the World Health Organization definition were included. The study started on April 1, 2020, and follow-up ended on January 6, 2021.

Exposures: IVIG and methylprednisolone vs IVIG alone.

Main outcomes and measures: The primary outcome was persistence of fever 2 days after the introduction of initial therapy or recrudescence of fever within 7 days, which defined treatment failure. Secondary outcomes included a second-line therapy, hemodynamic support, acute left

ventricular dysfunction after first-line therapy, and length of stay in the pediatric intensive care unit. The primary analysis involved propensity score matching with a minimum caliper of 0.1.

Results: Among 181 children with suspected MIS-C, 111 fulfilled the World Health Organization definition (58 females [52%]; median age, 8.6 years [interquartile range, 4.7 to 12.1]). Five children did not receive either treatment. Overall, 3 of 34 children (9%) in the IVIG and methylprednisolone group and 37 of 72 (51%) in the IVIG alone group did not respond to treatment. Treatment with IVIG and methylprednisolone vs IVIG alone was associated with lower risk of treatment failure (absolute risk difference, -0.28 [95% CI, -0.48 to -0.08]; odds ratio [OR], 0.25 [95% CI, 0.09 to 0.70]; P = .008). IVIG and methylprednisolone therapy vs IVIG alone was also significantly associated with lower risk of use of second-line therapy (absolute risk difference, -0.22 [95% CI, -0.40 to -0.04]; OR, 0.19 [95% CI, 0.06 to 0.61]; P = .004), hemodynamic support (absolute risk difference, -0.17 [95% CI, -0.34 to -0.004]; OR, 0.21 [95% CI, 0.06 to 0.76]), acute left ventricular dysfunction occurring after initial therapy (absolute risk difference, -0.18 [95% CI, -0.35 to -0.01]; OR, 0.20 [95% CI, 0.06 to 0.66]), and duration of stay in the pediatric intensive care unit (median, 4 vs 6 days; difference in days, -2.4 [95% CI, -4.0 to -0.7]).

Conclusions and relevance: Among children with MIS-C, treatment with IVIG and methylprednisolone vs IVIG alone was associated with a more favorable fever course. Study interpretation is limited by the observational design.

Conflict of interest statement

Conflict of Interest Disclosures: Dr Javouhey reported receiving grants from CSL Behring. Dr C. Levy reported receiving grants from GlaxoSmithKline, Merck Sharp & Dohme, and Sanofi and personal fees from Pfizer and Merck. Dr Cohen reported receiving personal fees from GlaxoSmithKline, Pfizer, Sanofi, and Merck Sharp & Dohme. No other disclosures were reported.

- 2 figures

Supplementary info

Publication types, MeSH terms, Substances, Supplementary concepts Expand

Publication types

- Comparative Study
- Observational Study
- Research Support, Non-U.S. Gov't

MeSH terms

- Adolescent
- COVID-19 / complications
- COVID-19 / drug therapy
- COVID-19 / therapy*
- Child
- Child, Preschool

- Combined Modality Therapy
- Female
- Fever / etiology
- France
- Glucocorticoids / adverse effects
- Glucocorticoids / therapeutic use*
- Humans
- Immunoglobulins, Intravenous / therapeutic use*
- Intensive Care Units, Pediatric
- Length of Stay
- Male
- Methylprednisolone / adverse effects
- Methylprednisolone / therapeutic use*
- Propensity Score
- Recurrence
- Retrospective Studies
- Systemic Inflammatory Response Syndrome / complications
- Systemic Inflammatory Response Syndrome / drug therapy
- Systemic Inflammatory Response Syndrome / therapy*
- Treatment Outcome

Substances

- Glucocorticoids
- Immunoglobulins, Intravenous
- Methylprednisolone

Supplementary concepts

- pediatric multisystem inflammatory disease, COVID-19 related

Full text links

JAMA FULL TEXT Silverchair Information Systems Free PMC article

Proceed to details

Cite

Share

☐ 1,032

Observational Study

Adv Respir Med

. 2021;89(4):378-385.
doi: 10.5603/ARM.a2021.0087.

Epidemiological characteristics and outcomes from 187 patients with COVID-19 admitted to 6 reference centers in Greece: an observational study during the first wave of the COVID-19 pandemic

Argyris Tzouvelekis [1] [2], Karolina Akinosoglou [2], Theodoros Karampitsakos [3] [4], Vassiliki Panou [5], Ioannis Tomos [6], Georgios Tsoukalas [7], Magdalini Stratiki [7], Katerina Dimakou [8], Serafeim Chrysikos [8], Ourania Papaioannou [1] [8], Georgios Hillas [8], Petros Bakakos [5], Grigoris Stratakos [5], Aris Anagnostopoulos [5], Athanasios Koromilias [5], Afroditi Boutou [9], Ioannis Kioumis [10], Diamantis Chloros [11], Theodoros Kontakiotis [11], Despoina Papakosta [11], Spyridon Papiris [6], Effrosyni Manali [6], Elvira-Markela Antonogiannaki [6], Nikolaos Koulouris [5], Demosthenes Bouros [5], Stylianos Loukides [6], Charalampos Gogos [2]

Affiliations Expand

Affiliations

- [1] Department of Respiratory Medicine, University Hospital of Patras, University of Patras, Patras, Greece, Greece.
- [2] Department of Internal Medicine, University Hospital of Patras, University of Patras, Patras, Greece.
- [3] Department of Respiratory Medicine, University Hospital of Patras, University of Patras, Patras, Greece, Greece. THODORISKARAMPITSAKOS@GMAIL.COM.
- [4] Department of Internal Medicine, University Hospital of Patras, University of Patras, Patras, Greece. THODORISKARAMPITSAKOS@GMAIL.COM.
- [5] 1st Academic Department of Respiratory Medicine, SOTIRIA General Hospital for Thoracic Diseases, National and Kapodistrian University of Athens, Athens, Greece.
- [6] 2nd Academic Department of Respiratory Medicine, ATTIKON General Hospital, National and Kapodistrian University of Athens, Athens, Greece.
- [7] 4th Department of Respiratory Medicine, SOTIRIA General Hospital, Athens, Greece.
- [8] 5th Department of Respiratory Medicine, SOTIRIA General Hospital, Athens, Greece.
- [9] Department of Respiratory Medicine, Papanikolaou General Hospital, Thessaloniki, Greece.
- [10] Department of Respiratory Failure, Aristotle University of Thessaloniki, Thessaloniki, Greece.
- [11] Department of Respiratory Medicine, Aristotle University of Thessaloniki, Thessaloniki, Greece.

- PMID: **34494241**
- DOI: 10.5603/ARM.a2021.0087

Free article
Observational Study

Epidemiological characteristics and outcomes from 187 patients with COVID-19 admitted to 6 reference centers in Greece: an observational study during the first wave of the COVID-19 pandemic

Argyris Tzouvelekis et al. Adv Respir Med. 2021.
Free article
Show details
Adv Respir Med
. 2021;89(4):378-385.
doi: 10.5603/ARM.a2021.0087.

Authors

Argyris Tzouvelekis [1, 2], Karolina Akinosoglou [2], Theodoros Karampitsakos [3, 4], Vassiliki Panou [5], Ioannis Tomos [6], Georgios Tsoukalas [7], Magdalini Stratiki [7], Katerina Dimakou [8], Serafeim Chrysikos [8], Ourania Papaioannou [1, 8], Georgios Hillas [8], Petros Bakakos [5], Grigoris Stratakos [5], Aris Anagnostopoulos [5], Athanasios Koromilias [5], Afroditi Boutou [9], Ioannis Kioumis [10], Diamantis Chloros [11], Theodoros Kontakiotis [11], Despoina Papakosta [11], Spyridon Papiris [6], Effrosyni Manali [6], Elvira-Markela Antonogiannaki [6], Nikolaos Koulouris [5], Demosthenes Bouros [5], Stylianos Loukides [6], Charalampos Gogos [2]

Affiliations

- [1] Department of Respiratory Medicine, University Hospital of Patras, University of Patras, Patras, Greece, Greece.
- [2] Department of Internal Medicine, University Hospital of Patras, University of Patras, Patras, Greece.
- [3] Department of Respiratory Medicine, University Hospital of Patras, University of Patras, Patras, Greece, Greece. THODORISKARAMPITSAKOS@GMAIL.COM.
- [4] Department of Internal Medicine, University Hospital of Patras, University of Patras, Patras, Greece. THODORISKARAMPITSAKOS@GMAIL.COM.
- [5] 1st Academic Department of Respiratory Medicine, SOTIRIA General Hospital for Thoracic Diseases, National and Kapodistrian University of Athens, Athens, Greece.
- [6] 2nd Academic Department of Respiratory Medicine, ATTIKON General Hospital, National and Kapodistrian University of Athens, Athens, Greece.
- [7] 4th Department of Respiratory Medicine, SOTIRIA General Hospital, Athens, Greece.
- [8] 5th Department of Respiratory Medicine, SOTIRIA General Hospital, Athens, Greece.
- [9] Department of Respiratory Medicine, Papanikolaou General Hospital, Thessaloniki, Greece.
- [10] Department of Respiratory Failure, Aristotle University of Thessaloniki, Thessaloniki, Greece.

- [11] Department of Respiratory Medicine, Aristotle University of Thessaloniki, Thessaloniki, Greece.

- PMID: **34494241**
- DOI: 10.5603/ARM.a2021.0087

Abstract

Introduction: Epidemiological data from patients with COVID-19 has been recently published in several countries. Nationwide data of hospitalized patients with COVID-19 in Greece remain scarce.

Material and methods: This was an observational, retrospective study from 6 reference centers between February 26 and May 15, 2020.

Results: The patients were mostly males (65.7%) and never smokers (57.2%) of median age 60 (95% CI: 57.6-64) years. The majority of the subjects (98%) were treated with the standard-of-care therapeutic regimen at that time, including hydroxychlo-roquine and azithromycin. Median time of hospitalization was 10 days (95% CI: 10-12). Twenty-five (13.3%) individuals were intubated and 8 died (4.2%). The patients with high neutrophil-to-lymphocyte ratio (NLR) (> 3.58) exhibited more severe disease as indicated by significantly increased World Health Organization (WHO) R&D ordinal scale (4; 95% CI: 4-4 vs 3; 95% CI: 3-4, p = 0.0001) and MaxFiO2% (50; 95% CI: 38.2-50 vs 29.5; 95% CI: 21-31, p < 0.0001). The patients with increased lactate dehydrogenase (LDH) levels (> 270 IU/ml) also exhibited more advanced disease compared to the low LDH group (< 270 IU/ml) as indicated by both WHO R&D ordinal scale (4; 95% CI: 4-4 vs 4; 95% CI: 3-4, p = 0.0001) and MaxFiO2% (50; 95% CI: 35-60 vs 28; 95% CI: 21-31, p < 0.0001).

Conclusion: We present the first epidemiological report from a low-incidence and mortality COVID-19 country. NLR and LDH may represent reliable disease prognosticators leading to timely treatment decisions.

Keywords: COVID-19; LDH; neutrophil-to-lymphocyte ratio; prognosticators; severity.

Supplementary info

Publication types, MeSH terms Expand

Publication types

- Observational Study

MeSH terms

- Adult
- COVID-19 / diagnosis*
- COVID-19 / therapy*
- Critical Care / methods*
- Female

- Greece
- Humans
- Male
- Middle Aged
- Respiration, Artificial / statistics & numerical data
- Severity of Illness Index*

Full text links

Full-text Via Medica Medical Publishers
Proceed to details
Cite
Share
1,033

Eur J Hosp Pharm
. 2021 Sep;28(5):242-247.
doi: 10.1136/ejhpharm-2020-002449. Epub 2020 Nov 25.

Prescribing practices of lopinavir/ritonavir, hydroxychloroquine and azithromycin during the COVID-19 epidemic crisis and pharmaceutical interventions in a French teaching hospital

Benedicte Gourieux [1], Fanny Reisz [2], Anne Sophie Belmas [2], François Danion [3], Marion Fourtage [2], Thierry Nai [2], Aurélie Reiter-Schatz [2], Yvon Ruch [3], Julia Walther [2], Yasmine Nivoix [2], Bruno Michel [4]
Affiliations Expand

Affiliations

- [1] Pharmacy, Hopitaux universitaires de Strasbourg, Strasbourg, Alsace, France.
- [2] Service de Pharmacie, Hopitaux universitaires de Strasbourg, Strasbourg, Alsace, France.
- [3] Service des Maladies infectieuses et tropicales, Hopitaux universitaires de Strasbourg, Strasbourg, Alsace, France.
- [4] Service de Pharmacie, Hopitaux universitaires de Strasbourg, Strasbourg, Alsace, France bruno.michel@chru-strasbourg.fr.

- PMID: **33239282**
- PMCID: PMC7689541
- DOI: 10.1136/ejhpharm-2020-002449

Free PMC article

Prescribing practices of lopinavir/ritonavir, hydroxychloroquine and azithromycin during the COVID-19 epidemic crisis and pharmaceutical interventions in a French teaching hospital

Benedicte Gourieux et al. Eur J Hosp Pharm. 2021 Sep.
Free PMC article
Show details
Eur J Hosp Pharm
. 2021 Sep;28(5):242-247.
doi: 10.1136/ejhpharm-2020-002449. Epub 2020 Nov 25.

Authors

Benedicte Gourieux [1], Fanny Reisz [2], Anne Sophie Belmas [2], François Danion [3], Marion Fourtage [2], Thierry Nai [2], Aurélie Reiter-Schatz [2], Yvon Ruch [3], Julia Walther [2], Yasmine Nivoix [2], Bruno Michel [4]

Affiliations

- [1] Pharmacy, Hopitaux universitaires de Strasbourg, Strasbourg, Alsace, France.
- [2] Service de Pharmacie, Hopitaux universitaires de Strasbourg, Strasbourg, Alsace, France.
- [3] Service des Maladies infectieuses et tropicales, Hopitaux universitaires de Strasbourg, Strasbourg, Alsace, France.
- [4] Service de Pharmacie, Hopitaux universitaires de Strasbourg, Strasbourg, Alsace, France bruno.michel@chru-strasbourg.fr.

- PMID: **33239282**
- PMCID: PMC7689541
- DOI: 10.1136/ejhpharm-2020-002449

Abstract

Objective: The aims of this study were to describe prescribing practices of lopinavir/ritonavir, hydroxychloroquine and azithromycin during the COVID-19 epidemic crisis (primary endpoint), then to characterise pharmaceutical interventions (PIs) targeted to these medications and evaluate the impact of these PIs on prescribers' practices (secondary end-points).

Methods: This retrospective observational study was carried out at the University Hospital of Strasbourg (France) from March to April 2020. The analysed population excluded patients from intensive care units but included all other adult patients with COVID-19 who received at least one dose of lopinavir/ritonavir combination, hydroxychloroquine or azithromycin, while inpatients. Analyses were performed by using data extracted from electronic medical records.

Result: During the study period, 278 patients were included. A rapid decrease in lopinavir/ritonavir prescriptions was observed. This was accompanied by an increase in hydroxychloroquine and azithromycin prescriptions until the end of March, followed by a decrease leading to the disappearance of these two medications in April. The pharmaceutical analysis of the prescriptions resulted in 59 PIs of which 21 were associated with lopinavir/ritonavir, 32 with hydroxychloroquine and 6 with azithromycin. Regarding the medication-related problems, the most frequent ones were incorrect treatment durations (n=32 (54.2%)), drug interactions with potential torsadogenic reactions (n=14 (23.7%)) and incorrect dosing (n=6 (10.2%)). From the 59 PIs, 48 (81.4%) were accepted and physicians adjusted the medication regimens in a timely manner.

Conclusion: This study demonstrated the value-even more meaningful in a crisis situation-of a strong synergy between physicians and pharmacists for patient-safety focused practices.

Keywords: drug misuse; drug-related side effects and adverse reactions; evidence-based medicine; hospital; pharmacy service; quality of health care.

© European Association of Hospital Pharmacists 2021. No commercial re-use. See rights and permissions. Published by BMJ.

Conflict of interest statement

Competing interests: None declared.

- 23 references
- 1 figure

Supplementary info

Publication types, MeSH terms, Substances Expand

Publication types

- Research Support, Non-U.S. Gov't

MeSH terms

- Adult
- Aged
- Antiviral Agents / adverse effects
- Antiviral Agents / therapeutic use*
- Azithromycin / adverse effects
- Azithromycin / therapeutic use*
- COVID-19 / drug therapy*
- Drug Combinations
- Drug Prescriptions / statistics & numerical data*
- Female
- France

- Hospitals, Teaching / statistics & numerical data*
- Humans
- Hydroxychloroquine / adverse effects
- Hydroxychloroquine / therapeutic use*
- Lopinavir / adverse effects
- Lopinavir / therapeutic use*
- Male
- Middle Aged
- Pandemics*
- Patient Safety
- Pharmacists
- Physicians
- Retrospective Studies
- Ritonavir / adverse effects
- Ritonavir / therapeutic use*

Substances

- Antiviral Agents
- Drug Combinations
- lopinavir-ritonavir drug combination
- Lopinavir
- Hydroxychloroquine
- Azithromycin
- Ritonavir

Full text links

BMJ Full Text HighWire Free PMC article
Proceed to details
Cite
Share
☐ 1,034
Observational Study
J Am Heart Assoc
. 2020 Dec 15;9(24):e018475.
doi: 10.1161/JAHA.120.018475. Epub 2020 Oct 23.

Statin Use and In-Hospital Mortality in Patients With Diabetes Mellitus and COVID-19

Omar Saeed [1], Francesco Castagna [1], Ilir Agalliu [2], Xiaonan Xue [2], Snehal R Patel [1], Yogita Rochlani [1], Rachna Kataria [1], Sasa Vukelic [1], Daniel B Sims [1], Chikezie Alvarez [1], Mercedes Rivas-Lasarte [1], Mario J Garcia [1], Ulrich P Jorde [1]
Affiliations Expand

Affiliations

- [1] Division of Cardiology Department of Medicine Montefiore Medical CenterAlbert Einstein College of Medicine New York NY.
- [2] Department of Epidemiology and Population Health Albert Einstein College of Medicine New York NY.

- PMID: **33092446**
- PMCID: PMC7955378
- DOI: 10.1161/JAHA.120.018475

Free PMC article
Observational Study

Statin Use and In-Hospital Mortality in Patients With Diabetes Mellitus and COVID-19

Omar Saeed et al. J Am Heart Assoc. 2020.
Free PMC article
Show details
J Am Heart Assoc
. 2020 Dec 15;9(24):e018475.
doi: 10.1161/JAHA.120.018475. Epub 2020 Oct 23.

Authors

Omar Saeed [1], Francesco Castagna [1], Ilir Agalliu [2], Xiaonan Xue [2], Snehal R Patel [1], Yogita Rochlani [1], Rachna Kataria [1], Sasa Vukelic [1], Daniel B Sims [1], Chikezie Alvarez [1], Mercedes Rivas-Lasarte [1], Mario J Garcia [1], Ulrich P Jorde [1]

Affiliations

- [1] Division of Cardiology Department of Medicine Montefiore Medical CenterAlbert Einstein College of Medicine New York NY.

- [2] Department of Epidemiology and Population Health Albert Einstein College of Medicine New York NY.

- PMID: **33092446**
- PMCID: PMC7955378
- DOI: 10.1161/JAHA.120.018475

Abstract

Background Severe coronavirus disease 2019 (COVID-19) is characterized by a proinflammatory state with high mortality. Statins have anti-inflammatory effects and may attenuate the severity of COVID-19. Methods and Results An observational study of all consecutive adult patients with COVID-19 admitted to a single center located in Bronx, New York, was conducted from March 1, 2020, to May 2, 2020. Patients were grouped as those who did and those who did not receive a statin, and in-hospital mortality was compared by competing events regression. In addition, propensity score matching and inverse probability treatment weighting were used in survival models to examine the association between statin use and death during hospitalization. A total of 4252 patients were admitted with COVID-19. Diabetes mellitus modified the association between statin use and in-hospital mortality. Patients with diabetes mellitus on a statin (n=983) were older (69±11 versus 67±14 years; *P*<0.01), had lower inflammatory markers (C-reactive protein, 10.2; interquartile range, 4.5-18.4 versus 12.9; interquartile range, 5.9-21.4 mg/dL; *P*<0.01) and reduced cumulative in-hospital mortality (24% versus 39%; *P*<0.01) than those not on a statin (n=1283). No difference in hospital mortality was noted in patients without diabetes mellitus on or off statin (20% versus 21%; *P*=0.82). Propensity score matching (hazard ratio, 0.88; 95% CI, 0.83-0.94; *P*<0.01) and inverse probability treatment weighting (HR, 0.88; 95% CI, 0.84-0.92; *P*<0.01) showed a 12% lower risk of death during hospitalization for statin users than for nonusers. Conclusions Statin use was associated with reduced in-hospital mortality from COVID-19 in patients with diabetes mellitus. These findings, if validated, may further reemphasize administration of statins to patients with diabetes mellitus during the COVID-19 era.

Keywords: COVID-19; diabetes mellitus; hospitalization; statin.

Conflict of interest statement

None.

- 27 references
- 1 figure

Supplementary info

Publication types, MeSH terms, Substances, Grant support Expand

Publication types

- Observational Study
- Research Support, N.I.H., Extramural

MeSH terms

- Aged
- Aged, 80 and over
- COVID-19 / diagnosis
- COVID-19 / mortality*
- COVID-19 / therapy
- Diabetes Mellitus / diagnosis
- Diabetes Mellitus / mortality*
- Dyslipidemias / diagnosis
- Dyslipidemias / drug therapy*
- Dyslipidemias / mortality
- Female
- Hospital Mortality*
- Humans
- Hydroxymethylglutaryl-CoA Reductase Inhibitors / therapeutic use*
- Male
- Middle Aged
- New York / epidemiology
- Prognosis
- Protective Factors
- Retrospective Studies
- Risk Assessment
- Risk Factors
- Time Factors

Substances

- Hydroxymethylglutaryl-CoA Reductase Inhibitors

Grant support

- K23 HL145140/HL/NHLBI NIH HHS/United States
- UL1 TR002556/TR/NCATS NIH HHS/United States
- UL1 TR001073/TR/NCATS NIH HHS/United States

Full text links

OPEN ACCESS Atypon Free PMC article
Proceed to details
Cite
Share
☐ 1,035
Observational Study
Balkan Med J

. 2021 Sep;38(5):296-303.
doi: 10.5152/balkanmedj.2021.21188.

Clinical Outcomes and Independent Risk Factors for 90-Day Mortality in Critically Ill Patients with Respiratory Failure Infected with SARS-CoV-2: A Multicenter Study in Turkish Intensive Care Units

Kürşat Gündoğan [1], İsmail Hakkı Akbudak [2], Pervin Hancı [3], Burçin Halaçlı [4], Şahin Temel [1], Zuhal Güllü [5], Kamil İnci [5], Yeliz Bilir [6], Firdevs Tuğba Bozkurt [7], Fatma Yıldırım [8], Meltem Şimşek [8], Recep Civan Yüksel [9], Esma Eren [9], Neriman Defne Altıntaş [10], Leyla Talan [10], Gülseren Elay [11], Göksel Güven [12], İskender Kara [13], Emre Aydın [14], Seda Yılmaz [15], Tuğçe Mengi [16], Sema Sarı [16], Türkay Akbaş [17], Burcu Acar Cinleti [18], Nazire Ateş Ayhan [19], Deniz Aral Özbek [4], Taha Koray Şahin [4], Aslı Açıkgöz [13], Ali Ümit Esbah [17], Ahmet Fırat [19], Ferhan Aydemir [20], Mehmet Çağatay Gürkök [20], Avşar Zerman [21], Ayça Gümüş [21], Melda Türkoğlu [22], Müge Aydoğdu [22], Ramazan Ulu [23], Jale Bengi Çelik [13], Canan Balcı [15], Cenk Kıraklı [18], Emre Karakoç [19], Ezgi Özyılmaz [24], Ebru Ortaç Ersoy [4], Serpil Öcal [4], İrem Akın Şen [3], İbrahim Hakkı Tor [3], Bilgin Cömert [20], Begüm Ergan [25], Kemal Tolga Saraçoğlu [6], Jülide Ergil [8], Ümmü Gülsüm Yüksel [12], Nuri Tutar [26], Murat Sungur [1], Arzu Topeli [4]
Affiliations Expand

Affiliations

- [1] Division of Intensive Care Medicine, Department of Internal Medicine, Erciyes University School of Medicine, Kayseri, Turkey.
- [2] Division of Intensive Care Medicine, Department of Internal Medicine, Pamukkale University School of Medicine, Denizli, Turkey.
- [3] Division of Intensive Care Medicine, Department of Chest Diseases, Ministry of Health, Intensive Care Unit, Erzurum Training and Research Hospital, Erzurum, Turkey and Trakya University School of Medicine, Edirne, Turkey.
- [4] Division of Intensive Care Medicine, Department of Internal Medicine, Hacettepe University School of Medicine, Ankara, Turkey.
- [5] Intensive Care Unit, Ministry of Health, Ankara Yenimahalle Training and Research Hospital, Ankara, Turkey.
- [6] Intensive Unit, Ministry of Health, İstanbul Lütfi Kırdar Training and Research Hospital, İstanbul, Turkey.
- [7] Intensive Care Unit, Ministry of Health, Mehmet Akif İnan Training and Research Hospital, Şanlıurfa, Turkey.
- [8] Intensive Care Unit, Ministry of Health, Ankara Dışkapı Training and Research Hospital, Ankara, Turkey.
- [9] Intensive Care Unit, Ministry of Health, Kayseri City Hospital, Kayseri, Turkey.
- [10] Division of Intensive Care Medicine, Department of Internal Medicine, Ankara University School of Medicine, Ankara, Turkey.

- [11] Division of Intensive Care Medicine, Department of Internal Medicine, Gaziantep University School of Medicine, Gaziantep, Turkey.
- [12] Division of Intensive Care Medicine, Department of Internal Medicine, Ministry of Health, Intensive Care Unit, Tokat State Hospital, Tokat and Hacettepe University School of Medicine, Ankara, Turkey.
- [13] Department of Anesthesiology, Intensive Care Unit, Selçuk University School of Medicine, Konya, Turkey.
- [14] Division of Intensive Care Medicine, Department of Internal Medicine, Dicle University School of Medicine, Diyarbakır, Turkey.
- [15] Anesthesiology Intensive Care Unit, Kütahya Health Science University, Kütahya, Turkey.
- [16] Intensive Care Unit, Ministry of Health, Nigde Omer Halisdemir University, Nigde, Turkey.
- [17] Division of Intensive Care Medicine, Department of Internal Medicine, Düzce University School of Medicine, Düzce, Turkey.
- [18] Intensive Care Unit, Ministry of Health, İzmir Suat Seren Training and Research Hospital, İzmir, Turkey.
- [19] Division of Intensive Care Medicine, Department of Internal Medicine, Çukurova University School of Medicine, Adana, Turkey.
- [20] Division of Intensive Care Medicine, Department of Internal Medicine, Dokuz Eylül University School of Medicine, İzmir, Turkey.
- [21] Intensive Care Unit, Kırşehir Ahi Evran University, Kırşehir, Turkey.
- [22] Division of Intensive Care Medicine, Department of Internal Medicine, Gazi University School of Medicine, Ankara, Turkey.
- [23] Division of Intensive Care Medicine, Department of Internal Medicine, Fırat University School of Medicine, Elazığ, Turkey.
- [24] Division of Intensive Care Medicine, Department of Chest Diseases, Çukurova University School of Medicine, Adana, Turkey.
- [25] Division of Intensive Care Medicine, Department of Chest Diseases, Dokuz Eylül University School of Medicine, İzmir, Turkey.
- [26] Division of Intensive Care Medicine, Department of Chest Diseases, Erciyes University School of Medicine, Kayseri, Turkey

- PMID: **34558415**
- PMCID: PMC8880837
- DOI: 10.5152/balkanmedj.2021.21188

Free PMC article
Observational Study

Clinical Outcomes and Independent Risk Factors for 90-Day Mortality in Critically Ill Patients with Respiratory Failure Infected with SARS-CoV-2: A Multicenter Study in Turkish Intensive Care Units

Kürşat Gündoğan et al. Balkan Med J. 2021 Sep.
Free PMC article
Show details
Balkan Med J
. 2021 Sep;38(5):296-303.
doi: 10.5152/balkanmedj.2021.21188.

Authors

Kürşat Gündoğan [1], İsmail Hakkı Akbudak [2], Pervin Hancı [3], Burçin Halaçlı [4], Şahin Temel [1], Zuhal Güllü [5], Kamil İnci [5], Yeliz Bilir [6], Firdevs Tuğba Bozkurt [7], Fatma Yıldırım [8], Meltem Şimşek [8], Recep Civan Yüksel [9], Esma Eren [9], Neriman Defne Altıntaş [10], Leyla Talan [10], Gülseren Elay [11], Göksel Güven [12], İskender Kara [13], Emre Aydın [14], Seda Yılmaz [15], Tuğçe Mengi [16], Sema Sarı [16], Türkay Akbaş [17], Burcu Acar Cinleti [18], Nazire Ateş Ayhan [19], Deniz Aral Özbek [4], Taha Koray Şahin [4], Aslı Açıkgöz [13], Ali Ümit Esbah [17], Ahmet Fırat [19], Ferhan Aydemir [20], Mehmet Çağatay Gürkök [20], Avşar Zerman [21], Ayça Gümüş [21], Melda Türkoğlu [22], Müge Aydoğdu [22], Ramazan Ulu [23], Jale Bengi Çelik [13], Canan Balcı [15], Cenk Kıraklı [18], Emre Karakoç [19], Ezgi Özyılmaz [24], Ebru Ortaç Ersoy [4], Serpil Öcal [4], İrem Akın Şen [3], İbrahim Hakkı Tor [3], Bilgin Cömert [20], Begüm Ergan [25], Kemal Tolga Saraçoğlu [6], Jülide Ergil [8], Ümmü Gülsüm Yüksel [12], Nuri Tutar [26], Murat Sungur [1], Arzu Topeli [4]

Affiliations

- [1] Division of Intensive Care Medicine, Department of Internal Medicine, Erciyes University School of Medicine, Kayseri, Turkey.
- [2] Division of Intensive Care Medicine, Department of Internal Medicine, Pamukkale University School of Medicine, Denizli, Turkey.
- [3] Division of Intensive Care Medicine, Department of Chest Diseases, Ministry of Health, Intensive Care Unit, Erzurum Training and Research Hospital, Erzurum, Turkey and Trakya University School of Medicine, Edirne, Turkey.
- [4] Division of Intensive Care Medicine, Department of Internal Medicine, Hacettepe University School of Medicine, Ankara, Turkey.
- [5] Intensive Care Unit, Ministry of Health, Ankara Yenimahalle Training and Research Hospital, Ankara, Turkey.
- [6] Intensive Unit, Ministry of Health, İstanbul Lütfi Kırdar Training and Research Hospital, İstanbul, Turkey.
- [7] Intensive Care Unit, Ministry of Health, Mehmet Akif İnan Training and Research Hospital, Şanlıurfa, Turkey.

- [8] Intensive Care Unit, Ministry of Health, Ankara Dışkapı Training and Research Hospital, Ankara, Turkey.
- [9] Intensive Care Unit, Ministry of Health, Kayseri City Hospital, Kayseri, Turkey.
- [10] Division of Intensive Care Medicine, Department of Internal Medicine, Ankara University School of Medicine, Ankara, Turkey.
- [11] Division of Intensive Care Medicine, Department of Internal Medicine, Gaziantep University School of Medicine, Gaziantep, Turkey.
- [12] Division of Intensive Care Medicine, Department of Internal Medicine, Ministry of Health, Intensive Care Unit, Tokat State Hospital, Tokat and Hacettepe University School of Medicine, Ankara, Turkey.
- [13] Department of Anesthesiology, Intensive Care Unit, Selçuk University School of Medicine, Konya, Turkey.
- [14] Division of Intensive Care Medicine, Department of Internal Medicine, Dicle University School of Medicine, Diyarbakır, Turkey.
- [15] Anesthesiology Intensive Care Unit, Kütahya Health Science University, Kütahya, Turkey.
- [16] Intensive Care Unit, Ministry of Health, Nigde Omer Halisdemir University, Nigde, Turkey.
- [17] Division of Intensive Care Medicine, Department of Internal Medicine, Düzce University School of Medicine, Düzce, Turkey.
- [18] Intensive Care Unit, Ministry of Health, İzmir Suat Seren Training and Research Hospital, İzmir, Turkey.
- [19] Division of Intensive Care Medicine, Department of Internal Medicine, Çukurova University School of Medicine, Adana, Turkey.
- [20] Division of Intensive Care Medicine, Department of Internal Medicine, Dokuz Eylül University School of Medicine, İzmir, Turkey.
- [21] Intensive Care Unit, Kırşehir Ahi Evran University, Kırşehir, Turkey.
- [22] Division of Intensive Care Medicine, Department of Internal Medicine, Gazi University School of Medicine, Ankara, Turkey.
- [23] Division of Intensive Care Medicine, Department of Internal Medicine, Fırat University School of Medicine, Elazığ, Turkey.
- [24] Division of Intensive Care Medicine, Department of Chest Diseases, Çukurova University School of Medicine, Adana, Turkey.
- [25] Division of Intensive Care Medicine, Department of Chest Diseases, Dokuz Eylül University School of Medicine, İzmir, Turkey.
- [26] Division of Intensive Care Medicine, Department of Chest Diseases, Erciyes University School of Medicine, Kayseri, Turkey

- PMID: **34558415**
- PMCID: PMC8880837
- DOI: 10.5152/balkanmedj.2021.21188

Erratum in

- Erratum.
  [No authors listed] [No authors listed] Balkan Med J. 2021 Nov;38(6):397. doi: 10.5152/balkanmedj.2021.20888. Balkan Med J. 2021. PMID: 34860169 Free PMC article. No abstract available.

Abstract

Background: There are limited data on the long-term outcomes of COVID-19 from different parts of the world.

Aims: To determine risk factors of 90-day mortality in critically ill patients in Turkish intensive care units (ICUs), with respiratory failure.

Study design: Retrospective, observational cohort.

Methods: Patients with laboratory-confirmed COVID-19 and who had been followed up in the ICUs with respiratory failure for more than 24 hours were included in the study. Their demographics, clinical characteristics, laboratory variables, treatment protocols, and survival data were recorded.

Results: A total of 421 patients were included. The median age was 67 (IQR: 57-76) years, and 251 patients (59.6%) were men. The 90-day mortality rate was 55.1%. The factors independently associated with 90-day mortality were invasive mechanical ventilation (IMV) (HR 4.09 [95% CI: [2.20-7.63], $P < .001$), lactate level >2 mmol/L (2.78 [1.93-4.01], $P < .001$), age ≥60 years (2.45 [1.48-4.06)], $P < .001$), cardiac arrhythmia during ICU stay (2.01 [1.27-3.20], $P = .003$), vasopressor treatment (1.94 [1.32-2.84], $P = .001$), positive fluid balance of ≥600 mL/day (1.68 [1.21-2.34], $P = .002$), PaO2/FiO2 ratio of ≤150 mmHg (1.66 [1.18-2.32], $P = .003$), and ECOG score ≥1 (1.42 [1.00-2.02], $P = .050$).

Conclusion: Long-term mortality was high in critically ill patients with COVID-19 hospitalized in intensive care units in Turkey. Invasive mechanical ventilation, lactate level, age, cardiac arrhythmia, vasopressor therapy, positive fluid balance, severe hypoxemia and ECOG score were the independent risk factors for 90-day mortality.

Conflict of interest statement

Conflict of Interest: The authors have no conflicts of interest to declare.

- 43 references
- 5 figures

Supplementary info

Publication types, MeSH terms Expand

Publication types

- Multicenter Study
- Observational Study

MeSH terms

- Adult
- Aged
- Aged, 80 and over

- COVID-19 / complications*
- COVID-19 / diagnosis
- COVID-19 / mortality*
- COVID-19 / therapy
- Critical Care
- Critical Illness
- Female
- Follow-Up Studies
- Humans
- Intensive Care Units
- Male
- Middle Aged
- Prognosis
- Respiratory Insufficiency / diagnosis
- Respiratory Insufficiency / mortality*
- Respiratory Insufficiency / therapy
- Respiratory Insufficiency / virology*
- Retrospective Studies
- Risk Factors
- Survival Analysis
- Turkey / epidemiology

Full text links

Galenos Yayinevi Free PMC article

Proceed to details

Cite

Share

1,036

Observational Study

Emergencias

. 2020 Ago;32(4):253-257.

Clinical findings, risk factors, and final outcome in patients diagnosed with pulmonary thromboembolism and COVID-19 in hospital emergency departments

[Article in English, Spanish]

Sònia Jiménez Hernández [1], Laura Lozano Polo [2], Guillem Suñen Cuquerella [3], Bárbara Peña Pardo [4], Begoña Espinosa [4], Carlos Cardozo [1], Alfons Aguirre Tejedo [5], Pere Llorens Soriano [4], Òscar Miró [1]
Affiliations Expand

Affiliations

- [1] Area de Urgencias, Hospital Clínic. Grupo UPyP, Área 1, IDIBAPS, Barcelona, España.
- [2] Servicio de Urgencias, Hospital de la Santa Creu i Sant Pau, Barcelona, España.
- [3] Servicio de Medicina Interna, Hospital del Mar, Barcelona, España.
- [4] Servicio de Urgencias, Hospital General de Alicante, Universidad Miguel Hernández, Elche, Alicante, España.
- [5] Servicio de Urgencias, Hospital del Mar, Barcelona, España.

- PMID: **32692002**

Free article
Observational Study

Clinical findings, risk factors, and final outcome in patients diagnosed with pulmonary thromboembolism and COVID-19 in hospital emergency departments

[Article in English, Spanish]
Sònia Jiménez Hernández et al. Emergencias. 2020 Ago.
Free article
Show details
Emergencias
. 2020 Ago;32(4):253-257.

Authors

Sònia Jiménez Hernández [1], Laura Lozano Polo [2], Guillem Suñen Cuquerella [3], Bárbara Peña Pardo [4], Begoña Espinosa [4], Carlos Cardozo [1], Alfons Aguirre Tejedo [5], Pere Llorens Soriano [4], Òscar Miró [1]

Affiliations

- [1] Area de Urgencias, Hospital Clínic. Grupo UPyP, Área 1, IDIBAPS, Barcelona, España.
- [2] Servicio de Urgencias, Hospital de la Santa Creu i Sant Pau, Barcelona, España.
- [3] Servicio de Medicina Interna, Hospital del Mar, Barcelona, España.
- [4] Servicio de Urgencias, Hospital General de Alicante, Universidad Miguel Hernández, Elche, Alicante, España.
- [5] Servicio de Urgencias, Hospital del Mar, Barcelona, España.

- PMID: **32692002**

Abstract
in English, Spanish

Objectives: To analyze clinical, laboratory, and radiologic findings and final health outcomes in patients with pulmonary embolism and coronavirus disease 2019 (COVID-19). To compare them to findings and outcomes in patients with pulmonary embolism without COVID-19.

Material and methods: Multicenter, observational, retrospective study in 4 Spanish hospital emergency departments (EDs) from January 15 to April 15, 2020. Cases were located by reviewing all ED requests for pulmonary computed tomography angiography (CTA) procedures. Clinical, laboratory, and radiologic findings; medical histories and comorbidity; risk factors; and outcomes were compared between the 2 groups of patients (with or without COVID-19).

Results: A total of 399 CTAs were ordered; 88 pulmonary embolisms were diagnosed, 28 of them (32%) in patients with COVID-19. This group had more men, and a history of thromboembolic disease was more common. We found no between-group differences in clinical presentation, laboratory, or radiologic findings; nor were there differences in final outcomes. In-hospital mortality was 7% (2 cases) in patients with COVID-19 and 17% (10 cases) in patients without the virus (odds ratio for death in patients with pulmonary embolism and COVID-19, 0.38; 95% CI, 0.08-1.89).

Conclusion: We found no clinically important differences in the clinical, laboratory, or radiologic findings between patients with or without COVID-19 who were treated for pulmonary embolism in our hospital EDs. Final outcomes also did not differ.

Objetivo: Analizar las características clínicas, analíticas, radiológicas y los resultados finales de salud en una cohorte de pacientes con embolia pulmonar (EP) y COVID-19, y compararlas con un grupo de pacientes con EP sin COVID-19.

Metodo: Estudio multicéntrico, observacional y retrospectivo realizado en cuatro servicios de urgencias hospitalarios (SUH) españoles del 15 de enero al 15 de abril de 2020. La detección de EP se hizo mediante la revisión de todas las angiografías por tomografía computarizada (angioTC) pulmonares solicitadas desde los SUH. Se han analizado dos grupos de pacientes con EP, con o sin enfermedad por COVID-19, y se comparan las características clínicas, analíticas, radiológicas, antecedentes y comorbilidad, factores de riesgo y resultados finales.

Resultados: Se realizaron 399 angioTC y se diagnosticaron 88 EP, 28 (32%) en pacientes con COVID-19. Estos pacientes fueron con más frecuencia hombres y tenían más antecedentes de enfermedad tromboembólica previa. No hubo diferencias en la presentación clínica, características analíticas o radiológicas ni en los resultados finales entre ambos grupos. La mortalidad intrahospitalaria fue del 7% (2 casos) en pacientes COVID y del 17% (10 casos) en pacientes sin COVID (OR de muerte en pacientes COVID con EP: 0,38, IC 95%: 0,08-1,89).

Conclusiones: No se han observado diferencias clínicamente relevantes en las características clínicas, analíticas, radiológicas y los resultados finales de salud en una cohorte de pacientes con EP y COVID-19 respecto a los pacientes con EP sin esta enfermedad atendidos en los SUH.

Keywords: COVID-19; Embolia de pulmón; Hospital emergency department; Pulmonary embolism; Servicio de urgencias.

Supplementary info

Publication types, MeSH terms Expand

Publication types

- Multicenter Study
- Observational Study

MeSH terms

- Aged
- Betacoronavirus*
- COVID-19
- Comorbidity
- Computed Tomography Angiography / statistics & numerical data
- Coronavirus Infections / blood
- Coronavirus Infections / complications*
- Coronavirus Infections / diagnosis
- Coronavirus Infections / mortality
- Emergency Service, Hospital / statistics & numerical data
- Female
- Hospital Mortality
- Humans
- Intensive Care Units
- Intubation, Intratracheal
- Length of Stay
- Male
- Odds Ratio
- Pandemics
- Pneumonia, Viral / blood
- Pneumonia, Viral / complications*
- Pneumonia, Viral / diagnosis
- Pneumonia, Viral / mortality
- Pulmonary Embolism / blood
- Pulmonary Embolism / diagnostic imaging*
- Pulmonary Embolism / etiology
- Pulmonary Embolism / mortality
- Retrospective Studies
- Risk Factors
- SARS-CoV-2
- Spain / epidemiology

Full text links

EMERGENCIAS Grupo Saned

Proceed to details

Cite

Share

1,037

Observational Study

Ups J Med Sci

. 2020 Nov;125(4):293-296.

doi: 10.1080/03009734.2020.1822960. Epub 2020 Sep 29.

Correlation analysis of coagulation dysfunction and liver damage in patients with novel coronavirus pneumonia: a single-center, retrospective, observational study

Sai Chen [1], Hanting Liu [1], Tie Li [2], Rong Huang [1], Rong Gui [1], Junhua Zhang [1]

Affiliations Expand

Affiliations

- [1] Department of Blood Transfusion, the Third Xiangya Hospital of Central South University, Changsha, China.
- [2] Department of Clinical Laboratory, the First People's Hospital of Yueyang, Yueyang, China.

- PMID: **32990149**
- PMCID: PMC7594753
- DOI: 10.1080/03009734.2020.1822960

Free PMC article

Observational Study

Correlation analysis of coagulation dysfunction and liver damage in patients with novel coronavirus pneumonia: a single-center, retrospective, observational study

Sai Chen et al. Ups J Med Sci. 2020 Nov.

Free PMC article

Show details
Ups J Med Sci
. 2020 Nov;125(4):293-296.
doi: 10.1080/03009734.2020.1822960. Epub 2020 Sep 29.

Authors

Sai Chen [1], Hanting Liu [1], Tie Li [2], Rong Huang [1], Rong Gui [1], Junhua Zhang [1]

Affiliations

- [1] Department of Blood Transfusion, the Third Xiangya Hospital of Central South University, Changsha, China.
- [2] Department of Clinical Laboratory, the First People's Hospital of Yueyang, Yueyang, China.

- PMID: **32990149**
- PMCID: PMC7594753
- DOI: 10.1080/03009734.2020.1822960

Abstract

Background: The novel coronavirus disease 2019 (COVID-19) is currently breaking out worldwide. COVID-19 patients may have different degrees of coagulopathy, but the mechanism is not yet clear. We aimed to analyse the relationship between coagulation dysfunction and liver damage in patients with COVID-19.

Methods: A retrospective analysis of 74 patients with COVID-19 admitted to the First People's Hospital of Yueyang from 1 January to 30 March 2020 was carried out. According to the coagulation function, 27 cases entered the coagulopathy group and 47 cases entered the control group. A case control study was conducted to analyse the correlation between the occurrence of coagulation dysfunction and liver damage in COVID-19 patients.

Results: Alanine aminotransferase (ALT) and aspartate aminotransferase (AST), markers of liver damage, were positively correlated with coagulopathy ($p = 0.039$, OR 2.960, 95% CI 1.055-8.304; and $p = 0.028$, OR 3.352, 95% CI 1.137-9.187). Alkaline phosphatase (ALP), γ-glutamyl transpeptidase (γ-GT), and total bilirubin (TBIL) were not statistically correlated with coagulopathy. According to the diagnosis and treatment plan, the included cases were classified into mild, moderate, severe, and critical. The results showed that the occurrence of coagulation dysfunction had no statistical correlation with the severity of COVID-19.

Conclusion: Coagulation dysfunction in patients with COVID-19 is closely related to liver damage. A longer course of the disease may cause a vicious circle of coagulopathy and liver damage. Clinicians need to closely monitor coagulation and liver function tests and to give prophylactic or supportive therapy when needed.

Keywords: Blood coagulation dysfunction; COVID-19; SARS-CoV-2; liver damage; pneumonia.

Conflict of interest statement

The authors declare that they have no conflicts of interest.

- 19 references

Supplementary info

Publication types, MeSH terms, Substances Expand

Publication types

- Observational Study

MeSH terms

- Adult
- Alanine Transaminase / blood
- Aspartate Aminotransferases / blood
- Betacoronavirus*
- Biomarkers / blood
- Blood Coagulation Disorders / etiology*
- Blood Coagulation Disorders / physiopathology
- COVID-19
- Case-Control Studies
- China
- Coronavirus Infections / complications*
- Coronavirus Infections / epidemiology
- Coronavirus Infections / physiopathology
- Female
- Humans
- Liver Diseases / etiology*
- Liver Diseases / physiopathology
- Male
- Middle Aged
- Pandemics
- Pneumonia, Viral / complications*
- Pneumonia, Viral / epidemiology
- Pneumonia, Viral / physiopathology
- Retrospective Studies
- Risk Factors
- SARS-CoV-2

Substances

- Biomarkers

- Aspartate Aminotransferases
- Alanine Transaminase

Full text links

View full text Taylor & Francis Free PMC article
Proceed to details
Cite
Share
1,038
Observational Study
Eur J Cancer
. 2021 Sep;154:246-252.
doi: 10.1016/j.ejca.2021.06.029. Epub 2021 Jun 30.

Coronavirus disease 2019 in patients with neuroendocrine neoplasms: Preliminary results of the INTENSIVE study

Nicola Fazio [1], Lorenzo Gervaso [2], Thorvardur R Halfdanarson [3], Anna La Salvia [4], Johannes Hofland [5], Jorge Hernando [6], Mohamad B Sonbol [7], Rocio Garcia-Carbonero [4], Jaume Capdevila [6], Wouter W de Herder [5], Anna Koumarianou [8], Gregory Kaltsas [9], Maura Rossi [10], Simona Grozinsky-Glasberg [11], Kira Oleinikov [11], Sabrina Boselli [12], Darina Tamayo [12], Vincenzo Bagnardi [13], Alice Laffi [14], Manila Rubino [14], Francesca Spada [14]
Affiliations Expand

Affiliations

- [1] Division of Gastrointestinal Medical Oncology and Neuroendocrine Tumors, European Institute of Oncology (IEO) IRCCS, Milan, Italy. Electronic address: nicola.fazio@ieo.it.
- [2] Division of Gastrointestinal Medical Oncology and Neuroendocrine Tumors, European Institute of Oncology (IEO) IRCCS, Milan, Italy; Molecular Medicine Program, University of Pavia, Pavia, Italy.
- [3] Division of Medical Oncology Mayo Clinic, Rochester, MN, USA.
- [4] Medical Oncology Department, Hospital Universitario Doce de Octubre, Imas12, UCM, Madrid, Spain.
- [5] Department of Internal Medicine, Sector Endocrinology, Rotterdam, the Netherlands.
- [6] Vall Hebron University Hospital and Vall Hebron Institute of Oncology (VHIO), Barcelona, Spain.
- [7] Department of Hematology and Oncology Mayo Clinic, Phoenix, AZ, USA.
- [8] Hematology-Oncology Unit, Fourth Department of Internal Medicine, Attikon Hospital, Medical School, National and Kapodistrian University of Athens, Athens, Greece.
- [9] 1st Department of Propaedeutic Internal Medicine, Endocrine Unit, National and Kapodistrian, University of Athens, 11527 Athens, Greece.

- [10] Oncology Unit and Centro Documentazione Osteonecrosi, Azienda Ospedaliera SS Antonio e Biagio e Cesare Arrigo, Alessandria, Italy.
- [11] Neuroendocrine Tumors Unit, ENETS Center of Excellence, Department of Endocrinology and Metabolism Service, Hadassah-Hebrew University Medical Center, Jerusalem, Israel.
- [12] Data Management-Clinical Trial Office. Scientific Direction. European Institute of Oncology (IEO) IRCCS, Milan, Italy.
- [13] Department of Statistics and Quantitative Methods, University of Milan-Bicocca, Milan, Italy.
- [14] Division of Gastrointestinal Medical Oncology and Neuroendocrine Tumors, European Institute of Oncology (IEO) IRCCS, Milan, Italy.

- PMID: **34298375**
- PMCID: PMC8241688
- DOI: 10.1016/j.ejca.2021.06.029

Free PMC article
Observational Study

Coronavirus disease 2019 in patients with neuroendocrine neoplasms: Preliminary results of the INTENSIVE study

Nicola Fazio et al. Eur J Cancer. 2021 Sep.
Free PMC article
Show details
Eur J Cancer
. 2021 Sep;154:246-252.
doi: 10.1016/j.ejca.2021.06.029. Epub 2021 Jun 30.

Authors

Nicola Fazio [1], Lorenzo Gervaso [2], Thorvardur R Halfdanarson [3], Anna La Salvia [4], Johannes Hofland [5], Jorge Hernando [6], Mohamad B Sonbol [7], Rocio Garcia-Carbonero [4], Jaume Capdevila [6], Wouter W de Herder [5], Anna Koumarianou [8], Gregory Kaltsas [9], Maura Rossi [10], Simona Grozinsky-Glasberg [11], Kira Oleinikov [11], Sabrina Boselli [12], Darina Tamayo [12], Vincenzo Bagnardi [13], Alice Laffi [14], Manila Rubino [14], Francesca Spada [14]

Affiliations

- [1] Division of Gastrointestinal Medical Oncology and Neuroendocrine Tumors, European Institute of Oncology (IEO) IRCCS, Milan, Italy. Electronic address: nicola.fazio@ieo.it.
- [2] Division of Gastrointestinal Medical Oncology and Neuroendocrine Tumors, European Institute of Oncology (IEO) IRCCS, Milan, Italy; Molecular Medicine Program, University of Pavia, Pavia, Italy.
- [3] Division of Medical Oncology Mayo Clinic, Rochester, MN, USA.

- [4] Medical Oncology Department, Hospital Universitario Doce de Octubre, Imas12, UCM, Madrid, Spain.
- [5] Department of Internal Medicine, Sector Endocrinology, Rotterdam, the Netherlands.
- [6] Vall Hebron University Hospital and Vall Hebron Institute of Oncology (VHIO), Barcelona, Spain.
- [7] Department of Hematology and Oncology Mayo Clinic, Phoenix, AZ, USA.
- [8] Hematology-Oncology Unit, Fourth Department of Internal Medicine, Attikon Hospital, Medical School, National and Kapodistrian University of Athens, Athens, Greece.
- [9] 1st Department of Propaedeutic Internal Medicine, Endocrine Unit, National and Kapodistrian, University of Athens, 11527 Athens, Greece.
- [10] Oncology Unit and Centro Documentazione Osteonecrosi, Azienda Ospedaliera SS Antonio e Biagio e Cesare Arrigo, Alessandria, Italy.
- [11] Neuroendocrine Tumors Unit, ENETS Center of Excellence, Department of Endocrinology and Metabolism Service, Hadassah-Hebrew University Medical Center, Jerusalem, Israel.
- [12] Data Management-Clinical Trial Office. Scientific Direction. European Institute of Oncology (IEO) IRCCS, Milan, Italy.
- [13] Department of Statistics and Quantitative Methods, University of Milan-Bicocca, Milan, Italy.
- [14] Division of Gastrointestinal Medical Oncology and Neuroendocrine Tumors, European Institute of Oncology (IEO) IRCCS, Milan, Italy.

- PMID: **34298375**
- PMCID: PMC8241688
- DOI: 10.1016/j.ejca.2021.06.029

Erratum in

- Corrigendum to 'Coronavirus disease 2019 in patients with neuroendocrine neoplasms: Preliminary results of the INTENSIVE study' [European Journal of Cancer 154 (2021) 246-252].
  Fazio N, Gervaso L, Halfdanarson TR, La Salvia A, Hofland J, Hernando J, Sonbol MB, Garcia-Carbonero R, Capdevila J, de Herder WW, Koumarianou A, Kaltsas G, Rossi M, Grozinsky-Glasberg S, Oleinikov K, Boselli S, Tamayo D, Bagnardi V, Laffi A, Rubino M, Spada F. Fazio N, et al. Eur J Cancer. 2022 Jan;160:289-290. doi: 10.1016/j.ejca.2021.10.001. Epub 2021 Oct 21. Eur J Cancer. 2022. PMID: 34690038 Free PMC article. No abstract available.

Abstract

Background: Specific data regarding coronavirus disease 2019 (COVID-19) in patients with neuroendocrine neoplasms (NENs) are lacking. The aim of this study is to describe the characteristics of patients with NENs who tested severe acute respiratory syndrome coronavirus 2 (SARS-CoV-2) positive.

Material and methods: This is a worldwide study collecting cases of patients with NENs along with a positive nasopharyngeal swab reverse transcriptase-polymerase chain reaction (RT-PCR) test for SARS-CoV-2 between June 1, 2020, and March 31, 2021. Centres treating patients with NENs were directly contacted by the principal investigator. Patients with NENs of any primary

site, grade and stage were included, excluding small-cell lung carcinoma and mixed adenoneuroendocrine carcinoma.

Results: Among 81 centres directly contacted, 88.8% responded and 48.6% of them declined due to lack of cases or interest. On March 31st, 2021, eight recruiting centres enrolled 89 patients. The median age was 64 years at the time of COVID-19 diagnosis. Most patients had metastatic, non-functioning, low-/intermediate-grade gastroenteropancreatic NENs on treatment with somatostatin analogues and radioligand therapy. Most of them had comorbidities. Only 8% of patients had high-grade NENs and 12% were receiving chemotherapy. Most patients had symptoms or signs of COVID-19, mainly fever and cough. Only 3 patients underwent sub-intensive treatment, whereas most of them received medical therapies, mostly antibiotics. In two third of cases, no changes occurred for the anti-NEN therapy. More than 80% of patients completely recovered without sequelae, whereas 7.8% patients died due to COVID-19.

Conclusions: Patients included in this study reflect the typical NEN population regardless of SARS-CoV-2. In most cases, they overcome COVID-19 without need of intensive care, short-term sequelae and discontinuation of systemic oncological therapy.

Keywords: COVID-19; Coronavirus; Neuroendocrine neoplasms; Neuroendocrine tumours; SARS-CoV-2.

Copyright © 2021 Elsevier Ltd. All rights reserved.

Conflict of interest statement

Conflict of interest statement The authors declare that they have no known competing financial interests or personal relationships that could have appeared to influence the work reported in this article.

- 41 references
- 1 figure

Supplementary info

Publication types, MeSH terms Expand

Publication types

- Multicenter Study
- Observational Study

MeSH terms

- Adult
- Aged
- COVID-19 / diagnosis
- COVID-19 / immunology
- COVID-19 / therapy*
- COVID-19 / virology

- Carcinoma, Neuroendocrine / diagnosis
- Carcinoma, Neuroendocrine / immunology
- Carcinoma, Neuroendocrine / therapy*
- Comorbidity
- Female
- Global Health*
- Humans
- Male
- Middle Aged
- Preliminary Data
- Prospective Studies
- Retrospective Studies
- Risk Factors
- Time Factors
- Treatment Outcome
- Young Adult

Full text links

ELSEVIER FULL-TEXT ARTICLE Elsevier Science Free PMC article
Proceed to details
Cite
Share
☐ 1,039
Observational Study
Emerg Med Australas
. 2021 Feb;33(1):149-151.
doi: 10.1111/1742-6723.13668. Epub 2020 Oct 26.

Impact of COVID-19 'circuit-breaker' measures on emergency medical services utilisation and out-of-hospital cardiac arrest outcomes in Singapore

Qin Xiang Ng [1], Ebenezer Zh Lee [1], Joey Am Tay [1], Shalini Arulanandam [1]
Affiliations Expand

Affiliation

- [1] Emergency Medical Services Department, Singapore Civil Defence Force Headquarters, Singapore.

- PMID: **33074594**
- DOI: 10.1111/1742-6723.13668

Observational Study

Impact of COVID-19 'circuit-breaker' measures on emergency medical services utilisation and out-of-hospital cardiac arrest outcomes in Singapore

Qin Xiang Ng et al. Emerg Med Australas. 2021 Feb.
Show details
Emerg Med Australas
. 2021 Feb;33(1):149-151.
doi: 10.1111/1742-6723.13668. Epub 2020 Oct 26.

Authors

Qin Xiang Ng [1], Ebenezer Zh Lee [1], Joey Am Tay [1], Shalini Arulanandam [1]

Affiliation

- [1] Emergency Medical Services Department, Singapore Civil Defence Force Headquarters, Singapore.

- PMID: **33074594**
- DOI: 10.1111/1742-6723.13668

Abstract

Objective: To understand the impact of COVID-19 restrictions on emergency medical services (EMSs) utilisation and out-of-hospital cardiac arrest outcomes in Singapore.

Methods: This was a retrospective observational study comparing data collected by the Singapore Civil Defence Force on EMS utilisation in Singapore from 1 April to 31 May 2020 to previous figures.

Results: Overall, EMS call volume and total out-of-hospital cardiac arrests remained comparable to past years. There was an appreciable decline in prehospital return of spontaneous circulation rates, albeit not statistically lower than pre-COVID periods ($P = 0.078$).

Conclusion: The findings contribute to a growing body of literature internationally on the potential impact of the COVID-19 pandemic on EMS utilisation and outcomes.

Keywords: COVID-19; emergency medical services; out-of-hospital cardiac arrest; pandemic; resuscitation; return of spontaneous circulation.

© 2020 Australasian College for Emergency Medicine.

- 5 references

Supplementary info

Publication types, MeSH terms Expand

Publication types

- Observational Study

MeSH terms

- Adolescent
- Aged
- Aged, 80 and over
- COVID-19 / epidemiology*
- Emergency Medical Services / statistics & numerical data*
- Humans
- Middle Aged
- Out-of-Hospital Cardiac Arrest / epidemiology*
- Out-of-Hospital Cardiac Arrest / therapy
- Patient Acceptance of Health Care / statistics & numerical data*
- Retrospective Studies
- Singapore / epidemiology
- Treatment Outcome

Full text links

WILEY Full Text Article Wiley
Proceed to details
Cite
Share
1,040
Observational Study
J Nephrol
. 2021 Apr;34(2):305-314.
doi: 10.1007/s40620-021-00997-0. Epub 2021 Mar 3.

Role of blood pressure dysregulation on kidney and mortality outcomes in COVID-19.

Kidney, blood pressure and mortality in SARS-CoV-2 infection

Chiara Lanzani [#] [1] [2], Marco Simonini [#] [1] [2], Teresa Arcidiacono [1] [2], Elisabetta Messaggio [1], Romina Bucci [1] [2], Paolo Betti [1] [2], Monica Avino [1] [2], Giulia Magni [1] [2], Chiara Maggioni [1] [2], Caterina Conte [2] [3], Patrizia Rovere Querini [2] [4], Fabio Ciceri [2] [5], Antonella Castagna [2] [6], Giuseppe Vezzoli [7] [8], Paolo Manunta [1] [2], Bio Angels for COVID-BioB Study Group
Collaborators, Affiliations Expand

Collaborators

- **Bio Angels for COVID-BioB Study Group**: Nicola Farina, Luigi De Filippo, Marco Battista, Domenico Grosso, Francesca Gorgoni, Carlo Di Biase, Alessio Grazioli Moretti, Lucio Granata, Filippo Bonaldi, Giulia Bettinelli, Elena Delmastro, Damiano Salvato, Chiara Maggioni, Giulia Magni, Monica Avino, Paolo Betti, Romina Bucci, Iulia Dumoa, Simona Bossolasco, Federica Morselli

Affiliations

- [1] Nephrology and Dialysis Unit, Genomics of Renal Diseases and Hypertension Unit, IRCCS San Raffaele Scientific Institute, Via Olgettina 60, 20132, Milan, Italy.
- [2] Vita-Salute San Raffaele University, Milan, Italy.
- [3] Division of Transplantation, Immunology and Transplantation Diseases, IRCCS San Raffaele Scientific Institute, Milan, Italy.
- [4] Internal Medicine, Diabetes and Endocrinology Unit, IRCCS San Raffaele Scientific Institute, Milan, Italy.
- [5] Hematology and Bone Marrow Transplant Unit, IRCCS San Raffaele Scientific Institute, Milan, Italy.
- [6] Department of Infectious Diseases, IRCCS San Raffaele Scientific Institute, Milan, Italy.
- [7] Nephrology and Dialysis Unit, Genomics of Renal Diseases and Hypertension Unit, IRCCS San Raffaele Scientific Institute, Via Olgettina 60, 20132, Milan, Italy. vezzoli.giuseppe@hsr.it.
- [8] Vita-Salute San Raffaele University, Milan, Italy. vezzoli.giuseppe@hsr.it.

[#] Contributed equally.

- PMID: **33656707**
- PMCID: PMC7926195
- DOI: 10.1007/s40620-021-00997-0

Free PMC article
Observational Study

Role of blood pressure dysregulation on kidney and mortality outcomes in COVID-19.

Kidney, blood pressure and mortality in SARS-CoV-2 infection

Chiara Lanzani et al. J Nephrol. 2021 Apr.
Free PMC article
Show details
J Nephrol
. 2021 Apr;34(2):305-314.
doi: 10.1007/s40620-021-00997-0. Epub 2021 Mar 3.

Authors

Chiara Lanzani [#] [1] [2], Marco Simonini [#] [1] [2], Teresa Arcidiacono [1] [2], Elisabetta Messaggio [1], Romina Bucci [1] [2], Paolo Betti [1] [2], Monica Avino [1] [2], Giulia Magni [1] [2], Chiara Maggioni [1] [2], Caterina Conte [2] [3], Patrizia Rovere Querini [2] [4], Fabio Ciceri [2] [5], Antonella Castagna [2] [6], Giuseppe Vezzoli [7] [8], Paolo Manunta [1] [2], Bio Angels for COVID-BioB Study Group

Collaborators

- **Bio Angels for COVID-BioB Study Group**: Nicola Farina, Luigi De Filippo, Marco Battista, Domenico Grosso, Francesca Gorgoni, Carlo Di Biase, Alessio Grazioli Moretti, Lucio Granata, Filippo Bonaldi, Giulia Bettinelli, Elena Delmastro, Damiano Salvato, Chiara Maggioni, Giulia Magni, Monica Avino, Paolo Betti, Romina Bucci, Iulia Dumoa, Simona Bossolasco, Federica Morselli

Affiliations

- [1] Nephrology and Dialysis Unit, Genomics of Renal Diseases and Hypertension Unit, IRCCS San Raffaele Scientific Institute, Via Olgettina 60, 20132, Milan, Italy.
- [2] Vita-Salute San Raffaele University, Milan, Italy.
- [3] Division of Transplantation, Immunology and Transplantation Diseases, IRCCS San Raffaele Scientific Institute, Milan, Italy.
- [4] Internal Medicine, Diabetes and Endocrinology Unit, IRCCS San Raffaele Scientific Institute, Milan, Italy.
- [5] Hematology and Bone Marrow Transplant Unit, IRCCS San Raffaele Scientific Institute, Milan, Italy.
- [6] Department of Infectious Diseases, IRCCS San Raffaele Scientific Institute, Milan, Italy.
- [7] Nephrology and Dialysis Unit, Genomics of Renal Diseases and Hypertension Unit, IRCCS San Raffaele Scientific Institute, Via Olgettina 60, 20132, Milan, Italy. vezzoli.giuseppe@hsr.it.
- [8] Vita-Salute San Raffaele University, Milan, Italy. vezzoli.giuseppe@hsr.it.

[#] Contributed equally.

- PMID: **33656707**
- PMCID: PMC7926195
- DOI: 10.1007/s40620-021-00997-0

Abstract

Background: In February 2020 the corona virus disease 2019 (COVID-19) infection started spreading throughout Italy, hitting the Lombardy region very hard. Despite the high diffusion, only a subset of patients developed severe COVID-19: around 25% of them developed acute kidney injury (AKI) and one-third of them died. Elderly patients and patients with high comorbidities were identified as being at higher risk of severe COVID-19.

Methods: Our prospective observational cohort study includes 392 consecutive patients hospitalized for COVID-19 in Milan (median age 67 years, 75% male). We evaluated the relationship between blood pressure at presentation, presence of AKI at Emergency Department admission and during hospitalization, and total in-hospital mortality (24%).

Results: Although 58% of our study patients reported a history of hypertension (HYP) (86% on treatment), 30% presented with low blood pressure levels. Only 5.5% were diagnosed with AKI on admission; 75% of hypertensive patients discontinued therapy during hospitalization (only 20% were on treatment at discharge). Gender and hypertension were strongly associated with AKI at admission (odds ratio 11). Blood pressure was inversely correlated with increased risk of AKI upon admission, regardless of the severity of respiratory distress. Age over 65, history of hypertension, and severity of respiratory distress were the main predictors of AKI, which developed in 34.7% of cases during hospitalization. AKI was associated with increased in-hospital mortality. Hypertension and low blood pressure at presentation were the main predictors of in-hospital mortality, together with age over 65, baseline pulmonary involvement, and severity of illness.

Conclusions: In patients hospitalized for COVID-19, hypertension and low blood pressure at presentation are important risk factors for AKI and mortality. Early reduction of antihypertensive therapy may improve outcomes in patients with SARS-CoV-2 infection.

Keywords: AKI; Blood pressure; COVID-19; Hypertension; Mortality; SARS-CoV-2 infection.

Conflict of interest statement

The authors declare that they have no conflict of interest.

- 25 references
- 3 figures

Supplementary info

Publication types, MeSH terms Expand

Publication types

- Observational Study

MeSH terms

- Acute Kidney Injury / epidemiology
- Acute Kidney Injury / etiology

- Acute Kidney Injury / physiopathology*
- Aged
- Blood Pressure / physiology*
- COVID-19 / epidemiology*
- Female
- Follow-Up Studies
- Hospital Mortality / trends
- Humans
- Intensive Care Units
- Italy / epidemiology
- Male
- Prospective Studies
- Retrospective Studies
- Risk Factors
- Survival Rate / trends

Full text links

SpringerLink FULL-TEXT ARTICLE Springer Free PMC article
Proceed to details
Cite
Share
1,041
Observational Study
Pediatrics
. 2022 Feb 1;149(2):e2021053079.
doi: 10.1542/peds.2021-053079.

COVID-19 and Antibiotic Prescribing in Pediatric Primary Care

Lauren Dutcher [1, 2], Yun Li [2, 3, 4], Giyoung Lee [4], Robert Grundmeier [3, 5], Keith W Hamilton [1], Jeffrey S Gerber [2, 3, 4, 6]
Affiliations Expand

Affiliations

- [1] Division of Infectious Diseases, Department of Medicine.
- [2] Departments of Biostatistics, Epidemiology, and Informatics.
- [3] Pediatrics, University of Pennsylvania Perelman School of Medicine, Philadelphia, Pennsylvania.
- [4] Center for Pediatric Clinical Effectiveness.
- [5] Department of Biomedical and Health Informatics.

- [6] Division of Infectious Diseases, Children's Hospital of Philadelphia, Philadelphia, Pennsylvania.

- PMID: **35102416**
- DOI: 10.1542/peds.2021-053079

Observational Study

COVID-19 and Antibiotic Prescribing in Pediatric Primary Care

Lauren Dutcher et al. Pediatrics. 2022.
Show details
Pediatrics
. 2022 Feb 1;149(2):e2021053079.
doi: 10.1542/peds.2021-053079.

Authors

Lauren Dutcher [1, 2], Yun Li [2, 3, 4], Giyoung Lee [4], Robert Grundmeier [3, 5], Keith W Hamilton [1], Jeffrey S Gerber [2, 3, 4, 6]

Affiliations

- [1] Division of Infectious Diseases, Department of Medicine.
- [2] Departments of Biostatistics, Epidemiology, and Informatics.
- [3] Pediatrics, University of Pennsylvania Perelman School of Medicine, Philadelphia, Pennsylvania.
- [4] Center for Pediatric Clinical Effectiveness.
- [5] Department of Biomedical and Health Informatics.
- [6] Division of Infectious Diseases, Children's Hospital of Philadelphia, Philadelphia, Pennsylvania.

- PMID: **35102416**
- DOI: 10.1542/peds.2021-053079

Abstract

Background and objectives: With the onset of the coronavirus disease 2019 (COVID-19) pandemic, pediatric ambulatory encounter volume and antibiotic prescribing both decreased; however, the durability of these reductions in pediatric primary care in the United States has not been assessed.

Methods: We conducted a retrospective observational study to assess the impact of the COVID-19 pandemic and associated public health measures on antibiotic prescribing in 27 pediatric primary care practices. Encounters from January 1, 2018, through June 30, 2021, were included. The primary outcome was monthly antibiotic prescriptions per 1000 patients. Interrupted time series analysis was performed.

Results: There were 69 327 total antibiotic prescriptions from April through December in 2019 and 18 935 antibiotic prescriptions during the same months in 2020, a 72.7% reduction. The reduction in prescriptions at visits for respiratory tract infection (RTI) accounted for 87.3% of this decrease. Using interrupted time series analysis, overall antibiotic prescriptions decreased from 31.6 to 6.4 prescriptions per 1000 patients in April 2020 (difference of -25.2 prescriptions per 1000 patients; 95% CI: -32.9 to -17.5). This was followed by a nonsignificant monthly increase in antibiotic prescriptions, with prescribing beginning to rebound from April to June 2021. Encounter volume also immediately decreased, and while overall encounter volume quickly started to recover, RTI encounter volume returned more slowly.

Conclusions: Reductions in antibiotic prescribing in pediatric primary care during the COVID-19 pandemic were sustained, only beginning to rise in 2021, primarily driven by reductions in RTI encounters. Reductions in viral RTI transmission likely played a substantial role in reduced RTI visits and antibiotic prescriptions.

Copyright © 2022 by the American Academy of Pediatrics.

Conflict of interest statement

FINANCIAL DISCLOSURE: The authors have indicated they have no financial relationships relevant to this article to disclose.

Supplementary info

Publication types, MeSH terms, Substances Expand

Publication types

- Observational Study
- Research Support, U.S. Gov't, P.H.S.

MeSH terms

- Anti-Bacterial Agents / therapeutic use*
- COVID-19 / epidemiology*
- Child
- Drug Prescriptions / statistics & numerical data*
- Female
- Humans
- Interrupted Time Series Analysis
- Male
- Pandemics
- Pediatrics
- Philadelphia / epidemiology
- Practice Patterns, Physicians' / statistics & numerical data*
- Primary Health Care*
- Retrospective Studies

Substances

- Anti-Bacterial Agents

Full text links

AAP Publications Silverchair Information Systems
Proceed to details
Cite
Share
1,042
Observational Study
JMIR Public Health Surveill
. 2021 Feb 8;7(2):e25452.
doi: 10.2196/25452.

Clinical Characterization of Patients With COVID-19 in Primary Care in Catalonia: Retrospective Observational Study

Miguel Angel Mayer [1], Josep Vidal-Alaball [2, 3], Anna Puigdellívol-Sánchez [4, 5, 6], Francesc X Marín Gomez [2, 3], Angela Leis [1], Jacobo Mendioroz Peña [2, 7]
Affiliations Expand

Affiliations

- [1] Research Programme on Biomedical Informatics, Hospital del Mar Medical Research Institute, Faculty of Health and Life Sciences, Universitat Pompeu Fabra, Barcelona, Spain.
- [2] Health Promotion in Rural Areas Research Group, Gerència Territorial de la Catalunya Central, Institut Català de la Salut, Sant Fruitós de Bages, Spain.
- [3] Unitat de Suport a la Recerca de la Catalunya Central, Fundació Institut Universitari per a la recerca a l'Atenció Primària de Salut Jordi Gol i Gurina, Sant Fruitós de Bages, Spain.
- [4] Facultat de Medicina, Universitat de Vic-Universitat Central de Catalunya, Vic, Spain.
- [5] CAP Anton de Borja, Consorci Sanitari de Terrassa, Terrassa, Spain.
- [6] Unitat d'Anatomia Humana Facultat de Medicina, Universitat de Barcelona, Barcelona, Spain.
- [7] COVID-19 Response Unit, Department of Health, Generalitat de Catalunya, Barcelona, Spain.

- PMID: **33496668**
- PMCID: PMC7871981
- DOI: 10.2196/25452

Free PMC article
Observational Study

Clinical Characterization of Patients With COVID-19 in Primary Care in Catalonia: Retrospective Observational Study

Miguel Angel Mayer et al. JMIR Public Health Surveill. 2021.
Free PMC article
Show details
JMIR Public Health Surveill
. 2021 Feb 8;7(2):e25452.
doi: 10.2196/25452.

Authors

Miguel Angel Mayer [1], Josep Vidal-Alaball [2, 3], Anna Puigdellívol-Sánchez [4, 5, 6], Francesc X Marín Gomez [2, 3], Angela Leis [1], Jacobo Mendioroz Peña [2, 7]

Affiliations

- [1] Research Programme on Biomedical Informatics, Hospital del Mar Medical Research Institute, Faculty of Health and Life Sciences, Universitat Pompeu Fabra, Barcelona, Spain.
- [2] Health Promotion in Rural Areas Research Group, Gerència Territorial de la Catalunya Central, Institut Català de la Salut, Sant Fruitós de Bages, Spain.
- [3] Unitat de Suport a la Recerca de la Catalunya Central, Fundació Institut Universitari per a la recerca a l'Atenció Primària de Salut Jordi Gol i Gurina, Sant Fruitós de Bages, Spain.
- [4] Facultat de Medicina, Universitat de Vic-Universitat Central de Catalunya, Vic, Spain.
- [5] CAP Anton de Borja, Consorci Sanitari de Terrassa, Terrassa, Spain.
- [6] Unitat d'Anatomia Humana Facultat de Medicina, Universitat de Barcelona, Barcelona, Spain.
- [7] COVID-19 Response Unit, Department of Health, Generalitat de Catalunya, Barcelona, Spain.

- PMID: **33496668**
- PMCID: PMC7871981
- DOI: 10.2196/25452

Abstract

Background: The country of Spain has one of the highest incidences of COVID-19, with more than 1,000,000 cases as of the end of October 2020. Patients with a history of chronic conditions, obesity, and cancer are at greater risk from COVID-19; moreover, concerns surrounding the use of angiotensin-converting enzyme inhibitors (ACEIs) and angiotensin type II receptor blockers (ARBs) and its relationship to COVID-19 susceptibility have increased since the beginning of the pandemic.

Objective: The objectives of this study were to compare the characteristics of patients diagnosed with COVID-19 to those of patients without COVID-19 in primary care; to determine the risk

factors associated with the outcome of mortality; and to determine the potential influence of certain medications, such as ACEIs and ARBs, on the mortality of patients with COVID-19.

Methods: An observational retrospective study of patients diagnosed with COVID-19 in the Catalan Central Region of Spain between March 1 and August 17, 2020, was conducted. The data were obtained from the Primary Care Services Information Technologies System of the Catalan Institute of Health in Barcelona, Spain.

Results: The study population included 348,596 patients (aged >15 years) registered in the Primary Care Services Information Technologies System of the Catalan Central Region. The mean age of the patients was 49.53 years (SD 19.42), and 31.17% of the patients were aged ≥60 years. 175,484/348,596 patients (50.34%) were women. A total of 23,844/348,596 patients (6.84%) in the population studied were diagnosed with COVID-19 during the study period, and the most common clinical conditions of these patients were hypertension (5267 patients, 22.1%) and obesity (5181 patients, 21.7%). Overall, 2680/348,596 patients in the study population (0.77%) died during the study period. The number of deaths among patients without COVID-19 was 1825/324,752 (0.56%; mean age 80.6 years, SD 13.3), while among patients diagnosed with COVID-19, the number of deaths was 855/23,844 (3.58%; mean age 83.0 years, SD 10.80) with an OR of 6.58 (95% CI 6.06-7.15).

Conclusions: We observed that women were more likely to contract COVID-19 than men. In addition, our study did not show that hypertension, obesity, or being treated with ACEIs or ARBs was linked to an increase in mortality in patients with COVID-19. Age is the main factor associated with mortality in patients infected with SARS-CoV-2.

Keywords: COVID-19; Spain; angiotensin II type 2 receptor blockers; angiotensin-converting enzyme inhibitors; characteristic; mortality; observational; primary health care; retrospective; risk; risk factors.

©Miguel Angel Mayer, Josep Vidal-Alaball, Anna Puigdellívol-Sánchez, Francesc X Marín Gomez, Angela Leis, Jacobo Mendioroz Peña. Originally published in JMIR Public Health and Surveillance (http://publichealth.jmir.org), 08.02.2021.

Conflict of interest statement

Conflicts of Interest: None declared.

- 39 references
- 1 figure

Supplementary info

Publication types, MeSH terms, Substances Expand

Publication types

- Observational Study

MeSH terms

- Adolescent

- Adult
- Aged
- Aged, 80 and over
- Angiotensin II Type 2 Receptor Blockers / adverse effects
- Angiotensin II Type 2 Receptor Blockers / therapeutic use
- Angiotensin-Converting Enzyme Inhibitors / adverse effects
- Angiotensin-Converting Enzyme Inhibitors / therapeutic use
- COVID-19 / epidemiology
- COVID-19 / mortality
- COVID-19 / therapy*
- Female
- Humans
- Male
- Middle Aged
- Primary Health Care*
- Retrospective Studies
- Risk Factors
- Spain / epidemiology
- Treatment Outcome
- Young Adult

Substances

- Angiotensin II Type 2 Receptor Blockers
- Angiotensin-Converting Enzyme Inhibitors

Full text links

JMIR Publications Free PMC article

Proceed to details

Cite

Share

☐ 1,043

Observational Study

Am J Phys Med Rehabil

. 2022 Jan 1;101(1):48-52.

doi: 10.1097/PHM.0000000000001910.

Post-acute COVID-19 Syndrome Negatively Impacts Physical Function, Cognitive

Function, Health-Related Quality of Life, and Participation

Laura Tabacof[1], Jenna Tosto-Mancuso, Jamie Wood, Mar Cortes, Amy Kontorovich, Dayna McCarthy, Dahlia Rizk, Gabriela Rozanski, Erica Breyman, Leila Nasr, Christopher Kellner, Joseph E Herrera, David Putrino
Affiliations Expand

Affiliation

- [1] From the Abilities Research Center, Department of Rehabilitation and Human Performance, Icahn School of Medicine at Mount Sinai, New York, New York (LT, JT-M, JW, MC, DM, GR, EB, LN, JEH, DP); Zena and Michael A. Weiner Cardiovascular Institute, Icahn School of Medicine at Mount Sinai, New York, New York (AK); The Mindich Child Health and Development Institute, Icahn School of Medicine at Mount Sinai, New York, New York (AK); Division of Hospital Medicine, Mount Sinai Beth Israel, New York, New York (DR); and Department of Neurosurgery, Icahn School of Medicine at Mount Sinai, New York, New York (CK).

- PMID: **34686631**
- PMCID: PMC8667685
- DOI: 10.1097/PHM.0000000000001910

Free PMC article
Observational Study

Post-acute COVID-19 Syndrome Negatively Impacts Physical Function, Cognitive Function, Health-Related Quality of Life, and Participation

Laura Tabacof et al. Am J Phys Med Rehabil. 2022.
Free PMC article
Show details
Am J Phys Med Rehabil
. 2022 Jan 1;101(1):48-52.
doi: 10.1097/PHM.0000000000001910.

Authors

Laura Tabacof[1], Jenna Tosto-Mancuso, Jamie Wood, Mar Cortes, Amy Kontorovich, Dayna McCarthy, Dahlia Rizk, Gabriela Rozanski, Erica Breyman, Leila Nasr, Christopher Kellner, Joseph E Herrera, David Putrino

Affiliation

- [1] From the Abilities Research Center, Department of Rehabilitation and Human Performance, Icahn School of Medicine at Mount Sinai, New York, New York (LT, JT-M, JW, MC, DM, GR, EB, LN, JEH, DP); Zena and Michael A. Weiner Cardiovascular Institute, Icahn School of Medicine at Mount Sinai, New York, New York (AK); The Mindich Child Health and Development Institute, Icahn School of Medicine at Mount Sinai, New York, New York (AK); Division of Hospital Medicine, Mount Sinai Beth Israel, New York, New York (DR); and Department of Neurosurgery, Icahn School of Medicine at Mount Sinai, New York, New York (CK).

- PMID: **34686631**
- PMCID: PMC8667685
- DOI: 10.1097/PHM.0000000000001910

Abstract

Objective: This report describes persistent symptoms associated with post-acute COVID-19 syndrome (PACS) and the impact of these symptoms on physical function, cognitive function, health-related quality of life, and participation.

Design: This study used a cross-sectional observational study design. Patients attending Mount Sinai's post-acute COVID-19 syndrome clinic completed surveys containing patient-reported outcomes.

Results: A total of 156 patients completed the survey, at a median (range) time of 351 days (82-457 days) after COVID-19 infection. All patients were prevaccination. The most common persistent symptoms reported were fatigue (n = 128, 82%), brain fog (n = 105, 67%), and headache (n = 94, 60%). The most common triggers of symptom exacerbation were physical exertion (n = 134, 86%), stress (n = 107, 69%), and dehydration (n = 77, 49%). Increased levels of fatigue (Fatigue Severity Scale) and dyspnea (Medical Research Council) were reported, alongside reductions in levels of regularly completed physical activity. Ninety-eight patients (63%) scored for at least mild cognitive impairment (Neuro-Qol), and the domain of the EuroQol: 5 dimension, 5 level most impacted was Self-care, Anxiety/Depression and Usual Activities.

Conclusions: Persistent symptoms associated with post-acute COVID-19 syndrome seem to impact physical and cognitive function, health-related quality of life, and participation in society. More research is needed to further clarify the relationship between COVID-19 infection and post-acute COVID-19 syndrome symptoms, the underlying mechanisms, and treatment options.

Copyright © 2021 The Author(s). Published by Wolters Kluwer Health, Inc.

Conflict of interest statement

Financial disclosure statements have been obtained, and no conflicts of interest have been reported by the authors or by any individuals in control of the content of this article.

- 32 references
- 4 figures

Supplementary info

Publication types, MeSH terms, Supplementary concepts Expand

Publication types

- Observational Study

MeSH terms

- COVID-19 / complications*
- COVID-19 / physiopathology
- Cognition Disorders / virology*
- Cross-Sectional Studies
- Humans
- Physical Functional Performance*
- Quality of Life*
- Retrospective Studies
- Social Participation*
- Surveys and Questionnaires

Supplementary concepts

- post-acute COVID-19 syndrome

Full text links

Wolters Kluwer Wolters Kluwer Free PMC article

Proceed to details

Cite

Share

1,044

Observational Study

Radiology

. 2021 Aug;300(2):E328-E336.

doi: 10.1148/radiol.2021204141. Epub 2021 Mar 16.

CT-derived Chest Muscle Metrics for Outcome Prediction in Patients with COVID-19

Simone Schiaffino [1], Domenico Albano [1], Andrea Cozzi [1], Carmelo Messina [1], Roberto Arioli [1], Claudio Bnà [1], Antonio Bruno [1], Luca A Carbonaro [1], Alessandro Carriero [1], Serena Carriero

[1], Pietro S C Danna [1], Elisa D'Ascoli [1], Claudia De Berardinis [1], Gianmarco Della Pepa [1], Zeno Falaschi [1], Salvatore Gitto [1], Alexis E Malavazos [1], Giovanni Mauri [1], Lorenzo Monfardini [1], Alessio Paschè [1], Roberto Rizzati [1], Francesco Secchi [1], Angelo Vanzulli [1], Valeria Tombini [1], Ilaria Vicentin [1], Domenico Zagaria [1], Francesco Sardanelli [1], Luca M Sconfienza [1]
Affiliations Expand

Affiliation

- [1] From the Unit of Radiology (S.S., L.A.C., F. Secchi, F. Sardanelli) and High Specialty Center for Dietetics, Nutritional Education and Cardiometabolic Prevention (A.E.M.), Istituto di Ricovero e Cura a Carattere Scientifico Policlinico San Donato, Via Rodolfo Morandi 30, 20097 San Donato Milanese, Milan, Italy; Department of Biomedicine, Neurosciences and Advanced Diagnostics, Section of Radiological Sciences, Università degli Studi di Palermo, Palermo, Italy (D.A.); Unit of Radiology, Istituto di Ricovero e Cura a Carattere Scientifico Istituto Ortopedico Galeazzi, Milan, Italy (D.A., C.M., L.M.S.); Department of Biomedical Sciences for Health (A. Cozzi, S.G., F. Secchi, F. Sardanelli, L.M.S.), Postgraduate School in Radiodiagnostics (S.C., E.D., C.D.B., G.D.P.), and Department of Oncology and Hematology-Oncology (G.M., A.V.), Università degli Studi di Milano, Milan, Italy; Division of Radiodiagnostics, Department of Diagnosis and Treatment Services, Azienda Ospedaliero Universitaria Maggiore della Carità, Novara, Italy (R.A., A. Carriero, P.S.C.D., Z.F., A.P., D.Z.); Department of Radiology, Fondazione Poliambulanza Istituto Ospedaliero, Brescia, Italy (C.B., L.M.); Department of Radiology, Ospedale Santissima Annunziata, Cento, Italy (A.B., R.R.); Department of Translational Medicine, Università degli Studi del Piemonte Orientale, Novara, Italy (A. Carriero); Division of Interventional Radiology, Istituto di Ricovero e Cura a Carattere Scientifico Istituto Europeo di Oncologia, Milan, Italy (G.M.); and Azienda Socio-Sanitaria Territoriale (ASST) Grande Ospedale Metropolitano Niguarda, Milan, Italy (A.V., V.T., I.V.).

- PMID: **33724065**
- PMCID: PMC7971428
- DOI: 10.1148/radiol.2021204141

Free PMC article
Observational Study

CT-derived Chest Muscle Metrics for Outcome Prediction in Patients with COVID-19

Simone Schiaffino et al. Radiology. 2021 Aug.
Free PMC article
Show details
Radiology
. 2021 Aug;300(2):E328-E336.
doi: 10.1148/radiol.2021204141. Epub 2021 Mar 16.

Authors

Simone Schiaffino [1], Domenico Albano [1], Andrea Cozzi [1], Carmelo Messina [1], Roberto Arioli [1], Claudio Bnà [1], Antonio Bruno [1], Luca A Carbonaro [1], Alessandro Carriero [1], Serena Carriero [1], Pietro S C Danna [1], Elisa D'Ascoli [1], Claudia De Berardinis [1], Gianmarco Della Pepa [1], Zeno Falaschi [1], Salvatore Gitto [1], Alexis E Malavazos [1], Giovanni Mauri [1], Lorenzo Monfardini [1], Alessio Paschè [1], Roberto Rizzati [1], Francesco Secchi [1], Angelo Vanzulli [1], Valeria Tombini [1], Ilaria Vicentin [1], Domenico Zagaria [1], Francesco Sardanelli [1], Luca M Sconfienza [1]

Affiliation

- [1] From the Unit of Radiology (S.S., L.A.C., F. Secchi, F. Sardanelli) and High Specialty Center for Dietetics, Nutritional Education and Cardiometabolic Prevention (A.E.M.), Istituto di Ricovero e Cura a Carattere Scientifico Policlinico San Donato, Via Rodolfo Morandi 30, 20097 San Donato Milanese, Milan, Italy; Department of Biomedicine, Neurosciences and Advanced Diagnostics, Section of Radiological Sciences, Università degli Studi di Palermo, Palermo, Italy (D.A.); Unit of Radiology, Istituto di Ricovero e Cura a Carattere Scientifico Istituto Ortopedico Galeazzi, Milan, Italy (D.A., C.M., L.M.S.); Department of Biomedical Sciences for Health (A. Cozzi, S.G., F. Secchi, F. Sardanelli, L.M.S.), Postgraduate School in Radiodiagnostics (S.C., E.D., C.D.B., G.D.P.), and Department of Oncology and Hematology-Oncology (G.M., A.V.), Università degli Studi di Milano, Milan, Italy; Division of Radiodiagnostics, Department of Diagnosis and Treatment Services, Azienda Ospedaliero Universitaria Maggiore della Carità, Novara, Italy (R.A., A. Carriero, P.S.C.D., Z.F., A.P., D.Z.); Department of Radiology, Fondazione Poliambulanza Istituto Ospedaliero, Brescia, Italy (C.B., L.M.); Department of Radiology, Ospedale Santissima Annunziata, Cento, Italy (A.B., R.R.); Department of Translational Medicine, Università degli Studi del Piemonte Orientale, Novara, Italy (A. Carriero); Division of Interventional Radiology, Istituto di Ricovero e Cura a Carattere Scientifico Istituto Europeo di Oncologia, Milan, Italy (G.M.); and Azienda Socio-Sanitaria Territoriale (ASST) Grande Ospedale Metropolitano Niguarda, Milan, Italy (A.V., V.T., I.V.).

- PMID: **33724065**
- PMCID: PMC7971428
- DOI: 10.1148/radiol.2021204141

Abstract

Background Lower muscle mass is a known predictor of unfavorable outcomes, but its prognostic impact on patients with COVID-19 is unknown. Purpose To investigate the contribution of CT-derived muscle status in predicting clinical outcomes in patients with COVID-19. Materials and Methods Clinical or laboratory data and outcomes (intensive care unit [ICU] admission and death) were retrospectively retrieved for patients with reverse transcriptase polymerase chain reaction-confirmed SARS-CoV-2 infection, who underwent chest CT on admission in four hospitals in Northern Italy from February 21 to April 30, 2020. The extent and type of pulmonary involvement, mediastinal lymphadenopathy, and pleural effusion were assessed. Cross-sectional areas and attenuation by paravertebral muscles were measured on axial CT images at the T5 and T12 vertebral level. Multivariable linear and binary logistic regression, including calculation of odds ratios (ORs) with 95% CIs, were used to build four models to predict ICU admission and death, which were tested and compared by using receiver operating characteristic curve analysis. Results A total of 552 patients (364 men and 188 women; median age, 65 years [interquartile range, 54-75 years]) were included. In a CT-based model, lower-than-median T5 paravertebral muscle areas showed the highest ORs for ICU admission (OR, 4.8; 95% CI: 2.7, 8.5; $P < .001$) and death (OR, 2.3; 95% CI: 1.0, 2.9; $P = .03$). When clinical variables were included in the

model, lower-than-median T5 paravertebral muscle areas still showed the highest ORs for both ICU admission (OR, 4.3; 95%: CI: 2.5, 7.7; $P < .001$) and death (OR, 2.3; 95% CI: 1.3, 3.7; $P = .001$). At receiver operating characteristic analysis, the CT-based model and the model including clinical variables showed the same area under the receiver operating characteristic curve (AUC) for ICU admission prediction (AUC, 0.83; $P = .38$) and were not different in terms of predicting death (AUC, 0.86 vs AUC, 0.87, respectively; $P = .28$). Conclusion In hospitalized patients with COVID-19, lower muscle mass on CT images was independently associated with intensive care unit admission and in-hospital mortality. © RSNA, 2021 *Online supplemental material is available for this article.*

- 35 references
- 5 figures

Supplementary info

Publication types, MeSH terms Expand

Publication types

- Multicenter Study
- Observational Study

MeSH terms

- Aged
- COVID-19 / complications*
- Female
- Humans
- Italy
- Male
- Middle Aged
- Muscle, Skeletal / diagnostic imaging
- Predictive Value of Tests
- Radiography, Thoracic / methods*
- Retrospective Studies
- SARS-CoV-2
- Sarcopenia / complications*
- Sarcopenia / diagnostic imaging*
- Tomography, X-Ray Computed / methods*

Full text links

Radiology FULL TEXT Atypon Free PMC article
Proceed to details
Cite

Share

☐ 1,045

Observational Study

Stroke

. 2021 Jan;52(1):48-56.

doi: 10.1161/STROKEAHA.120.031668. Epub 2020 Dec 7.

Acute Cerebrovascular Events With COVID-19 Infection

Mandip S Dhamoon [1], Alison Thaler [1], Kapil Gururangan [1], Amit Kohli [1], Daniella Sisniega [1], Danielle Wheelwright [1], Connor Mensching [1], Johanna T Fifi [1], Michael G Fara [1], Nathalie Jette [1], Ella Cohen [1], Priya Dave [1], Aislyn C DiRisio [1], Jonathan Goldstein [1], Emma M Loebel [1], Naomi A Mayman [1], Akarsh Sharma [1], Daniel S Thomas [1], Ruben D Vega Perez [1], Mark R Weingarten [1], Huei Hsun Wen [1], Stanley Tuhrim [1], Laura K Stein [1], Mount Sinai Stroke Investigators*

Collaborators, Affiliations Expand

Collaborators

- **Mount Sinai Stroke Investigators***:
  Jesse Weinberger, Deborah Horowitz, Kara Sheinart, Benjamin Kummer, Qing Hao, Tara Roche, Mahalet Gizaw, Kamil Stefanowski, Vaibhav Goswami, J Mocco, Thomas Oxley, Shahram Majidi, Inder Paul Singh, Hazem Shoirah, Reade DeLeacy, Christopher Kellner, Tomoyoshi Shimegatsu, Benjamin Yim, Travis Ladner, Kurt Yaeger, Maryna Skliut, Irene Boniece, Carolyn Brockington, Punam Dass, Eli Nasrallah, Steven Rudolph, Holly Morhaim, John Liang, Alexandra Reynolds, Neha Dengayach, Cappi Lay, Kate Reilly, Helen Cheung, Daniel Chiu, Veronica Peschansky, Sarah Levy, John Erdman, Rebecca Baron, Daniel Charytonowicz, Caroline Gentile, Marcia Lange, Jacob Lurie, Rio O'Mary, Akila Pai, Dahniel Sastow, Himanshu Sharma, Charlotte Solmssen, Ruben Vega Perez

Affiliation

- [1] Department of Neurology, Icahn School of Medicine at Mount Sinai, New York.

- PMID: **33280551**
- DOI: 10.1161/STROKEAHA.120.031668

Free article

Observational Study

Acute Cerebrovascular Events With COVID-19 Infection

Mandip S Dhamoon et al. Stroke. 2021 Jan.

Free article

Show details

Stroke

. 2021 Jan;52(1):48-56.
doi: 10.1161/STROKEAHA.120.031668. Epub 2020 Dec 7.

Authors

Mandip S Dhamoon [1], Alison Thaler [1], Kapil Gururangan [1], Amit Kohli [1], Daniella Sisniega [1], Danielle Wheelwright [1], Connor Mensching [1], Johanna T Fifi [1], Michael G Fara [1], Nathalie Jette [1], Ella Cohen [1], Priya Dave [1], Aislyn C DiRisio [1], Jonathan Goldstein [1], Emma M Loebel [1], Naomi A Mayman [1], Akarsh Sharma [1], Daniel S Thomas [1], Ruben D Vega Perez [1], Mark R Weingarten [1], Huei Hsun Wen [1], Stanley Tuhrim [1], Laura K Stein [1], Mount Sinai Stroke Investigators*

Collaborators

- **Mount Sinai Stroke Investigators***:
  Jesse Weinberger, Deborah Horowitz, Kara Sheinart, Benjamin Kummer, Qing Hao, Tara Roche, Mahalet Gizaw, Kamil Stefanowski, Vaibhav Goswami, J Mocco, Thomas Oxley, Shahram Majidi, Inder Paul Singh, Hazem Shoirah, Reade DeLeacy, Christopher Kellner, Tomoyoshi Shimegatsu, Benjamin Yim, Travis Ladner, Kurt Yaeger, Maryna Skliut, Irene Boniece, Carolyn Brockington, Punam Dass, Eli Nasrallah, Steven Rudolph, Holly Morhaim, John Liang, Alexandra Reynolds, Neha Dengayach, Cappi Lay, Kate Reilly, Helen Cheung, Daniel Chiu, Veronica Peschansky, Sarah Levy, John Erdman, Rebecca Baron, Daniel Charytonowicz, Caroline Gentile, Marcia Lange, Jacob Lurie, Rio O'Mary, Akila Pai, Dahniel Sastow, Himanshu Sharma, Charlotte Solmssen, Ruben Vega Perez

Affiliation

- [1] Department of Neurology, Icahn School of Medicine at Mount Sinai, New York.

- PMID: **33280551**
- DOI: 10.1161/STROKEAHA.120.031668

Abstract

Background and purpose: Coronavirus disease 2019 (COVID-19) has been associated with an increased incidence of thrombotic events, including stroke. However, characteristics and outcomes of COVID-19 patients with stroke are not well known.

Methods: We conducted a retrospective observational study of risk factors, stroke characteristics, and short-term outcomes in a large health system in New York City. We included consecutively admitted patients with acute cerebrovascular events from March 1, 2020 through April 30, 2020. Data were stratified by COVID-19 status, and demographic variables, medical comorbidities, stroke characteristics, imaging results, and in-hospital outcomes were examined. Among COVID-19-positive patients, we also summarized laboratory test results.

Results: Of 277 patients with stroke, 105 (38.0%) were COVID-19-positive. Compared with COVID-19-negative patients, COVID-19-positive patients were more likely to have a cryptogenic (51.8% versus 22.3%, *P*<0.0001) stroke cause and were more likely to suffer ischemic stroke in the temporal (*P*=0.02), parietal (*P*=0.002), occipital (*P*=0.002), and cerebellar (*P*=0.028) regions. In COVID-19-positive patients, mean coagulation markers were slightly elevated (prothrombin time 15.4±3.6 seconds, partial thromboplastin time 38.6±24.5 seconds, and international normalized ratio 1.4±1.3). Outcomes were worse among COVID-19-positive patients, including longer length of stay (*P*<0.0001), greater percentage requiring intensive care unit care (*P*=0.017), and greater rate of neurological worsening during admission (*P*<0.0001); additionally, more COVID-19-positive patients suffered in-hospital death (33% versus 12.9%, *P*<0.0001).

Conclusions: Baseline characteristics in patients with stroke were similar comparing those with and without COVID-19. However, COVID-19-positive patients were more likely to experience stroke in a lobar location, more commonly had a cryptogenic cause, and had worse outcomes.

Keywords: epidemiology; incidence; intracerebral hemorrhage; ischemic stroke; length of stay; risk factors; subarachnoid hemorrhage.

Supplementary info

Publication types, MeSH terms Expand

Publication types

- Observational Study

MeSH terms

- Adult
- Aged
- Aged, 80 and over
- COVID-19 / complications*
- Female
- Humans
- Incidence
- Male
- Middle Aged
- New York City / epidemiology
- Risk Factors
- SARS-CoV-2
- Stroke / epidemiology*
- Treatment Outcome

Full text links

FREE FULL TEXT Atypon

Proceed to details

Cite

Share

☐ 1,046

Observational Study

Clin Neurol Neurosurg

. 2021 Feb;201:106436.

doi: 10.1016/j.clineuro.2020.106436. Epub 2020 Dec 15.

Decline in mild stroke presentations and intravenous thrombolysis during the COVID-19 pandemic: The Society of Vascular and Interventional Neurology Multicenter Collaboration

Santiago Ortega-Gutierrez [1], Mudassir Farooqui [2], Alicia Zha [3], Alexandra Czap [3], Jacob Sebaugh [3], Shashvat Desai [4], Ashutosh Jadhav [4], Nirav Vora [5], Vivek Rai [5], Tudor G Jovin [6], Jesse M Thon [6], Mark Heslin [6], Lauren Thau [2], Cynthia Zevallos [2], Darko Quispe-Orozco [2], Dinesh V Jillella [7], Fadi Nahab [8], Mahmoud H Mohammaden [9], Raul G Nogueira [7], Diogo C Haussen [7], Thanh N Nguyen [10], Jose Rafael Romero [10], Hugo J Aparicio [10], Mohamed Osman [11], Israr Ul Haq [11], David Liebeskind [12], Ameer E Hassan [13], Osama Zaidat [11], James E Siegler [6], SVIN COVID-19 Multinational Registry and Task Force

Affiliations Expand

Affiliations

- [1] Department of Neurology, University of Iowa Hospitals and Clinics, Iowa City, IA, 52242, USA. Electronic address: santy-ortega@uiowa.edu.
- [2] Department of Neurology, University of Iowa Hospitals and Clinics, Iowa City, IA, 52242, USA.
- [3] Institute of Stroke and Cerebrovascular Disease, Department of Neurology, University of Texas McGovern Medical School, Houston TX, 77030, USA.
- [4] University of Pittsburgh Medical Center Mercy Hospital, Pittsburgh, PA, 15219, USA; University of Pittsburgh Medical Center Presbyterian Medical Center, Pittsburgh, PA, 15213, USA.
- [5] Ohio Health Neuroscience Center, Riverside Methodist Hospital, Columbus, OH, 43214, USA.
- [6] Cooper Neurological Institute, Cooper University Hospital, Camden, NJ, 08103, USA.
- [7] Department of Neurology, Emory University School of Medicine, Atlanta, GA, 30322, USA; Marcus Stroke & Neuroscience Center, Grady Memorial Hospital, Atlanta, GA, 30303, USA.
- [8] Department of Neurology & Pediatrics, Emory University, Atlanta, GA, 30319, USA.
- [9] Marcus Stroke & Neuroscience Center, Grady Memorial Hospital, Atlanta, GA, 30303, USA.

- [10] Department of Neurology, Boston Medical Center, Boston University School of Medicine, MA, 02118, USA.
- [11] Department of Neurology, Mercy Health St. Vincent Hospital, Toledo, OH, 43608, USA.
- [12] Department of Neurology, Ronald Reagan University of California at Los Angeles, Los Angeles, CA, 90095, USA.
- [13] Department of Neurology, University of Texas Rio Grande Valley, Valley Baptist Medical Center, Harlingen, TX, 78550, USA.

- PMID: **33383463**
- PMCID: PMC7836428
- DOI: 10.1016/j.clineuro.2020.106436

Free PMC article
Observational Study

Decline in mild stroke presentations and intravenous thrombolysis during the COVID-19 pandemic: The Society of Vascular and Interventional Neurology Multicenter Collaboration

Santiago Ortega-Gutierrez et al. Clin Neurol Neurosurg. 2021 Feb.
Free PMC article
Show details
Clin Neurol Neurosurg
. 2021 Feb;201:106436.
doi: 10.1016/j.clineuro.2020.106436. Epub 2020 Dec 15.

Authors

Santiago Ortega-Gutierrez [1], Mudassir Farooqui [2], Alicia Zha [3], Alexandra Czap [3], Jacob Sebaugh [3], Shashvat Desai [4], Ashutosh Jadhav [4], Nirav Vora [5], Vivek Rai [5], Tudor G Jovin [6], Jesse M Thon [6], Mark Heslin [6], Lauren Thau [2], Cynthia Zevallos [2], Darko Quispe-Orozco [2], Dinesh V Jillella [7], Fadi Nahab [8], Mahmoud H Mohammaden [9], Raul G Nogueira [7], Diogo C Haussen [7], Thanh N Nguyen [10], Jose Rafael Romero [10], Hugo J Aparicio [10], Mohamed Osman [11], Israr Ul Haq [11], David Liebeskind [12], Ameer E Hassan [13], Osama Zaidat [11], James E Siegler [6], SVIN COVID-19 Multinational Registry and Task Force

Affiliations

- [1] Department of Neurology, University of Iowa Hospitals and Clinics, Iowa City, IA, 52242, USA. Electronic address: santy-ortega@uiowa.edu.
- [2] Department of Neurology, University of Iowa Hospitals and Clinics, Iowa City, IA, 52242, USA.

- [3] Institute of Stroke and Cerebrovascular Disease, Department of Neurology, University of Texas McGovern Medical School, Houston TX, 77030, USA.
- [4] University of Pittsburgh Medical Center Mercy Hospital, Pittsburgh, PA, 15219, USA; University of Pittsburgh Medical Center Presbyterian Medical Center, Pittsburgh, PA, 15213, USA.
- [5] Ohio Health Neuroscience Center, Riverside Methodist Hospital, Columbus, OH, 43214, USA.
- [6] Cooper Neurological Institute, Cooper University Hospital, Camden, NJ, 08103, USA.
- [7] Department of Neurology, Emory University School of Medicine, Atlanta, GA, 30322, USA; Marcus Stroke & Neuroscience Center, Grady Memorial Hospital, Atlanta, GA, 30303, USA.
- [8] Department of Neurology & Pediatrics, Emory University, Atlanta, GA, 30319, USA.
- [9] Marcus Stroke & Neuroscience Center, Grady Memorial Hospital, Atlanta, GA, 30303, USA.
- [10] Department of Neurology, Boston Medical Center, Boston University School of Medicine, MA, 02118, USA.
- [11] Department of Neurology, Mercy Health St. Vincent Hospital, Toledo, OH, 43608, USA.
- [12] Department of Neurology, Ronald Reagan University of California at Los Angeles, Los Angeles, CA, 90095, USA.
- [13] Department of Neurology, University of Texas Rio Grande Valley, Valley Baptist Medical Center, Harlingen, TX, 78550, USA.

- PMID: **33383463**
- PMCID: PMC7836428
- DOI: 10.1016/j.clineuro.2020.106436

Abstract

Background: To evaluate overall ischemic stroke volumes and rates, specific subtypes, and clinical presentation during the COVID-19 pandemic in a multicenter observational study from eight states across US.

Methods: We compared all ischemic strokes admitted between January 2019 and May 2020, grouped as; March-May 2020 (COVID-19 period) and March-May 2019 (seasonal pre-COVID-19 period). Primary outcome was stroke severity at admission measured by NIHSS stratified as mild (0-7), moderate [8-14], and severe (>14). Secondary outcomes were volume of large vessel occlusions (LVOs), stroke etiology, IV-tPA rates, and discharge disposition.

Results: Of the 7969 patients diagnosed with acute ischemic stroke during the study period, 933 (12 %) presented in the COVID-19 period while 1319 (17 %) presented in the seasonal pre-COVID-19 period. Significant decline was observed in the mean weekly volumes of newly diagnosed ischemic strokes (98 ± 3 vs 50 ± 20,p = 0.003), LVOs (16.5 ± 3.8 vs 8.3 ± 5.9,p = 0.008), and IV-tPA (10.9 ± 3.4 vs 5.3 ± 2.9,p = 0.0047), whereas the mean weekly proportion of LVOs (18 % ±5 vs 16 % ±7,p = 0.24) and IV-tPA (10.4 % ±4.5 vs. 9.9 % ±2.4,p = 0.66) remained the same, when compared to the seasonal pre-COVID-19 period. Additionally, an increased proportion of patients presented with a severe disease (NIHSS > 14) during the COVID-19 period (29.7 % vs 24.5 %,p < 0.025). The odds of being discharged to home were 26 % greater in the COVID-19 period when compared to seasonal pre-COVID-19 period (OR:1.26, 95 % CI:1.07-1.49,p = 0.016).

Conclusions: During COVID-19 period there was a decrease in volume of newly diagnosed ischemic stroke cases and IV-tPA administration. Patients admitted to the hospital had severe neurological clinical presentation and were more likely to discharge home.

Keywords: COVID-19; Coronavirus; Ischemic stroke; Large vessel occlusion; NIHSS.

Copyright © 2020 Elsevier B.V. All rights reserved.

- 37 references
- 3 figures

Supplementary info

Publication types, MeSH terms, Substances Expand

Publication types

- Multicenter Study
- Observational Study

MeSH terms

- Administration, Intravenous
- Adult
- Aged
- Aged, 80 and over
- COVID-19 / diagnosis
- COVID-19 / epidemiology*
- Cohort Studies
- Female
- Humans
- Male
- Middle Aged
- Neurology / trends*
- Pandemics
- Retrospective Studies
- Societies, Medical / trends*
- Stroke / diagnosis
- Stroke / drug therapy*
- Stroke / epidemiology*
- Thrombolytic Therapy / trends*
- Tissue Plasminogen Activator / administration & dosage
- United States / epidemiology
- Vascular Diseases / drug therapy
- Vascular Diseases / epidemiology

Substances

- Tissue Plasminogen Activator

Full text links

ELSEVIER FULL-TEXT ARTICLE Elsevier Science Free PMC article
Proceed to details
Cite
Share
1,047

Observational Study

Am J Otolaryngol
. Mar-Apr 2021;42(2):102865.
doi: 10.1016/j.amjoto.2020.102865. Epub 2021 Jan 9.

ENT emergencies during the first wave of COVID-19 pandemic in Spain: Our experience

Jesús Herranz-Larrañeta [1], Alejandro Klein-Rodríguez [2], María Menéndez-Riera [2], Lara Mejuto-Torreiro [2], Aldán López-Eiroa [2], Juan Carlos Vázquez-Barro [3], Jesús Herranz González-Botas [4], Miguel Mayo-Yáñez [5]
Affiliations Expand

Affiliations

- [1] Otorhinolaryngology - Head and Neck Surgery Department, Complexo Hospitalario Universitario A Coruña (CHUAC), 15006 A Coruña, Galicia, Spain; Clinical Research in Medicine, International Center for Doctorate and Advanced Studies (CIEDUS), Universidade de Santiago de Compostela (USC), 15782, Santiago de Compostela, Galicia, Spain. Electronic address: jesus.herranz.larraneta@sergas.es.
- [2] Otorhinolaryngology - Head and Neck Surgery Department, Complexo Hospitalario Universitario A Coruña (CHUAC), 15006 A Coruña, Galicia, Spain.
- [3] Otorhinolaryngology - Head and Neck Surgery Department, Complexo Hospitalario Universitario A Coruña (CHUAC), 15006 A Coruña, Galicia, Spain; School of Educational Sciences and Speech Therapy, Universidade da Coruña (UDC), A Coruña, Galicia, Spain.
- [4] Otorhinolaryngology - Head and Neck Surgery Department, Complexo Hospitalario Universitario A Coruña (CHUAC), 15006 A Coruña, Galicia, Spain; School of Medicine and Odontology, Universidade de Santiago de Compostela (USC), Santiago de Compostela, Galicia, Spain.
- [5] Otorhinolaryngology - Head and Neck Surgery Department, Complexo Hospitalario Universitario A Coruña (CHUAC), 15006 A Coruña, Galicia, Spain; Clinical Research in Medicine, International Center for Doctorate and Advanced Studies (CIEDUS),

Universidade de Santiago de Compostela (USC), 15782, Santiago de Compostela, Galicia, Spain.

- PMID: **33450479**
- PMCID: PMC7794058
- DOI: 10.1016/j.amjoto.2020.102865

Free PMC article
Observational Study

ENT emergencies during the first wave of COVID-19 pandemic in Spain: Our experience

Jesús Herranz-Larrañeta et al. Am J Otolaryngol. Mar-Apr 2021.
Free PMC article
Show details
Am J Otolaryngol
. Mar-Apr 2021;42(2):102865.
doi: 10.1016/j.amjoto.2020.102865. Epub 2021 Jan 9.

Authors

Jesús Herranz-Larrañeta [1], Alejandro Klein-Rodríguez [2], María Menéndez-Riera [2], Lara Mejuto-Torreiro [2], Aldán López-Eiroa [2], Juan Carlos Vázquez-Barro [3], Jesús Herranz González-Botas [4], Miguel Mayo-Yáñez [5]

Affiliations

- [1] Otorhinolaryngology - Head and Neck Surgery Department, Complexo Hospitalario Universitario A Coruña (CHUAC), 15006 A Coruña, Galicia, Spain; Clinical Research in Medicine, International Center for Doctorate and Advanced Studies (CIEDUS), Universidade de Santiago de Compostela (USC), 15782, Santiago de Compostela, Galicia, Spain. Electronic address: jesus.herranz.larraneta@sergas.es.
- [2] Otorhinolaryngology - Head and Neck Surgery Department, Complexo Hospitalario Universitario A Coruña (CHUAC), 15006 A Coruña, Galicia, Spain.
- [3] Otorhinolaryngology - Head and Neck Surgery Department, Complexo Hospitalario Universitario A Coruña (CHUAC), 15006 A Coruña, Galicia, Spain; School of Educational Sciences and Speech Therapy, Universidade da Coruña (UDC), A Coruña, Galicia, Spain.
- [4] Otorhinolaryngology - Head and Neck Surgery Department, Complexo Hospitalario Universitario A Coruña (CHUAC), 15006 A Coruña, Galicia, Spain; School of Medicine and Odontology, Universidade de Santiago de Compostela (USC), Santiago de Compostela, Galicia, Spain.
- [5] Otorhinolaryngology - Head and Neck Surgery Department, Complexo Hospitalario Universitario A Coruña (CHUAC), 15006 A Coruña, Galicia, Spain; Clinical Research in Medicine, International Center for Doctorate and Advanced Studies (CIEDUS),

Universidade de Santiago de Compostela (USC), 15782, Santiago de Compostela, Galicia, Spain.

- PMID: **33450479**
- PMCID: PMC7794058
- DOI: 10.1016/j.amjoto.2020.102865

Abstract

Objectives: To analyze the characteristics of the visits attended to in an ENT Emergency Department (ENT-ED) during the first wave of COVID-19, comparing them with the emergencies attended to during the same period of time in 2019.

Methods: Descriptive and analytical observational retrospective study of all emergency consultations between March 1, 2020, and May 21, 2020, carried out by the Otorhinolaryngology-Head and Neck Surgery Department of a tertiary university hospital. The adequacy of consultations was assessed with the Hospital Emergency Suitability Protocol (HESP). The correlation between the emergencies and the SARS-CoV-2 confirmed cases was assessed with a generalized linear model.

Results: Although there was a decrease of almost 50% in ENT-ED visits during the first wave of COVID-19, the pattern of most cases remained similar to the pre-COVID-19 era: non-urgent consultations, not previously assessed by Primary Care (PC), being considered inadequate by the HESP. The three main reasons for consultation were otalgia, odynophagia, and epistaxis. The number of ENT-ED visits and the total number of confirmed cases of SARS-CoV-2 in the health area were correlated.

Conclusions: SARS-CoV-2 pandemic was a challenge for the Spanish health system. The critical epidemiological situation experienced during March, April, and May explains the reduction in the number of visits to the ENT-ED. However, this condition did not affect the predominant pattern of visits with respect to the pre-COVID-19 era, which were mostly inadequate. A strengthening of PC and an improvement in the population's health education is essential.

Keywords: Ambulatory care; COVID-19; Emergency medicine; Hospital administration; Otolaryngology; Primary health care.

Copyright © 2021 Elsevier Inc. All rights reserved.

Conflict of interest statement

The author(s) declare(s) that there is no conflict of interest.

- 17 references
- 1 figure

Supplementary info

Publication types, MeSH terms Expand

Publication types

- Observational Study

MeSH terms

- Adolescent
- Adult
- Aged
- Aged, 80 and over
- COVID-19 / epidemiology*
- Child
- Child, Preschool
- Emergency Service, Hospital*
- Female
- Humans
- Infant
- Male
- Middle Aged
- Otorhinolaryngologic Diseases / epidemiology*
- Pandemics
- Referral and Consultation
- Retrospective Studies
- Spain / epidemiology
- Young Adult

Full text links

ELSEVIER FULL-TEXT ARTICLE Elsevier Science Free PMC article

Proceed to details

Cite

Share

☐ 1,048

Observational Study

Eur J Intern Med

. 2020 Dec;82:38-47.

doi: 10.1016/j.ejim.2020.08.019. Epub 2020 Aug 25.

Use of hydroxychloroquine in hospitalised COVID-19 patients is associated with reduced mortality: Findings from the observational multicentre Italian CORIST study

COVID-19 RISK and Treatments (CORIST) Collaboration

Collaborators, Affiliations Expand

Collaborators

- **COVID-19 RISK and Treatments (CORIST) Collaboration**: Augusto Di Castelnuovo [1], Simona Costanzo [2], Andrea Antinori [3], Nausicaa Berselli [4], Lorenzo Blandi [5], Raffaele Bruno [6], Roberto Cauda [7], Giovanni Guaraldi [8], Lorenzo Menicanti [5], Ilaria My [9], Giustino Parruti [10], Giuseppe Patti [11], Stefano Perlini [12], Francesca Santilli [13], Carlo Signorelli [14], Enrico Spinoni [11], Giulio G Stefanini [9], Alessandra Vergori [15], Walter Ageno [16], Antonella Agodi [17], Luca Aiello [18], Piergiuseppe Agostoni [19], Samir Al Moghazi [20], Marinella Astuto [17], Filippo Aucella [21], Greta Barbieri [22], Alessandro Bartoloni [23], Marialaura Bonaccio [2], Paolo Bonfanti [24], Francesco Cacciatore [25], Lucia Caiano [16], Francesco Cannata [9], Laura Carrozzi [26], Antonio Cascio [27], Arturo Ciccullo [28], Antonella Cingolani [7], Francesco Cipollone [13], Claudia Colomba [27], Francesca Crosta [10], Chiara Dal Pra [29], Gian Battista Danzi [30], Damiano D'Ardes [13], Katleen de Gaetano Donati [28], Paola Del Giacomo [28], Francesco Di Gennaro [31], Giuseppe Di Tano [30], Giampiero D'Offizi [32], Tommaso Filippini [4], Francesco Maria Fusco [33], Ivan Gentile [34], Alessandro Gialluisi [2], Giancarlo Gini [16], Elvira Grandone [21], Leonardo Grisafi [11], Gabriella Guarnieri [35], Silvia Lamonica [28], Francesco Landi [18], Armando Leone [36], Gloria Maccagni [30], Sandro Maccarella [37], Andrea Madaro [38], Massimo Mapelli [19], Riccardo Maragna [19], Lorenzo Marra [36], Giulio Maresca [39], Claudia Marotta [31], Franco Mastroianni [38], Maria Mazzitelli [40], Alessandro Mengozzi [22], Francesco Menichetti [22], Marianna Meschiari [8], Filippo Minutolo [41], Arturo Montineri [42], Roberta Mussinelli [14], Cristina Mussini [8], Maria Musso [43], Anna Odone [14], Marco Olivieri [44], Emanuela Pasi [45], Francesco Petri [46], Biagio Pinchera [34], Carlo A Pivato [9], Venerino Poletti [47], Claudia Ravaglia [47], Massimo Rinaldi [38], Andrea Rognoni [11], Marco Rossato [29], Ilaria Rossi [13], Marianna Rossi [46], Anna Sabena [48], Francesco Salinaro [48], Vincenzo Sangiovanni [33], Carlo Sanrocco [10], Laura Scorzolini [49], Raffaella Sgariglia [39], Paola Giustina Simeone [10], Michele Spinicci [23], Enrico Maria Trecarichi [40], Amedeo Venezia [38], Giovanni Veronesi [16], Roberto Vettor [29], Andrea Vianello [35], Marco Vinceti [50], Laura Vocciante [39], Raffaele De Caterina [31], Licia Iacoviello [51]

Affiliations

- [1] Mediterranea Cardiocentro, Napoli, Italy.
- [2] Department of Epidemiology and Prevention, IRCCS Neuromed, Pozzilli (IS), Italy.
- [3] UOC Immunodeficienze Virali, National Institute for Infectious Diseases "L. Spallanzani", IRCCS. Roma, Italy.
- [4] Section of Public Health, Department of Biomedical, Metabolic and Neural Sciences, University of Modena and Reggio Emilia, Modena, Italy.
- [5] IRCCS Policlinico San Donato, San Donato Milanese, Italy.
- [6] Division of Infectious Diseases I, Fondazione IRCCS Policlinico San Matteo, Pavia, Italy; Department of Clinical, Surgical, Diagnostic, and Paediatric Sciences, University of Pavia, Pavia, Italy.

- [7] Fondazione Policlinico Universitario A. Gemelli IRCCS, Roma, Italy; Università Cattolica del Sacro Cuore- Dipartimento di Sicurezza e Bioetica Sede di Roma, Roma, Italy.
- [8] Infectious Disease Unit, Department of Surgical, Medical, Dental and Morphological Sciences, University of Modena and Reggio Emilia, Modena, Italy.
- [9] Humanitas Clinical and Research Hospital IRCCS, Rozzano-Milano, Italy.
- [10] Department of Infectious Disease, Azienda Sanitaria Locale (AUSL) di Pescara, Pescara, Italy.
- [11] University of Eastern Piedmont, Maggiore della Carità Hospital, Novara, Italy.
- [12] Emergency Department, IRCCS Policlinico San Matteo Foundation, Pavia, Italy; Department of Internal Medicine, University of Pavia, Pavia, Italy.
- [13] Department of Medicine and Aging, Clinica Medica, "SS. Annunziata" Hospital and University of Chieti, Chieti, Italy.
- [14] School of Medicine, Vita-Salute San Raffaele University, Milano, Italy.
- [15] HIV/AIDS Department, National Institute for Infectious Diseases "Lazzaro Spallanzani"-IRCCS, Roma, Italy.
- [16] Department of Medicine and Surgery, University of Insubria, Varese, Italy.
- [17] Department of Medical and Surgical Sciences and Advanced Technologies "G.F. Ingrassia", University of Catania; AOU Policlinico-Vittorio Emanuele, Catania, Italy.
- [18] UOC. Anestesia e Rianimazione. Dipartimento di Chirurgia Generale Ospedale Morgagni-Pierantoni, Forlì, Italy.
- [19] Centro Cardiologico Monzino IRCCS, Milano, Italy; Department of Clinical Sciences and Community Health, Cardiovascular Section, University of Milano, Milano, Italy.
- [20] Infezioni Sistemiche dell'Immunodepresso, National Institute for Infectious Diseases L. Spallanzani, IRCCS, Roma, Italy.
- [21] Fondazione I.R.C.C.S "Casa Sollievo della Sofferenza", San Giovanni Rotondo, Foggia, Italy.
- [22] Department of Clinical and Experimental Medicine, Azienda Ospedaliero-Universitaria Pisana, and University of Pisa, Pisa, Italy.
- [23] Department of Experimental and Clinical Medicine. University of Florence, Firenze, Italy.
- [24] UOC Malattie Infettive, Ospedale San Gerardo, ASST Monza, Monza, Italy; School of Medicine and Surgery, University of Milano-Bicocca, Milano, Italy.
- [25] Department of Translational Medical Sciences. University of Naples, Federico II, Napoli, Italy.
- [26] Cardiovascular and Thoracic Department, Azienda Ospedaliero-Universitaria Pisana, and University of Pisa, Pisa, Italy.
- [27] Infectious and Tropical Diseases Unit- Department of Health Promotion, Mother and Child Care, Internal Medicine and Medical Specialties (PROMISE) - University of Palermo, Palermo, Italy.
- [28] Fondazione Policlinico Universitario A. Gemelli IRCCS, Roma, Italy.
- [29] Clinica Medica 3, Department of Medicine - DIMED, University hospital of Padova, Padova, Italy.
- [30] Department of Cardiology, Ospedale di Cremona, Cremona, Italy.
- [31] Medical Direction, IRCCS Neuromed, Pozzilli (IS), Italy.
- [32] UOC Malattie Infettive-Epatologia, National Institute for Infectious Diseases L. Spallanzani, IRCCS, Roma, Italy.
- [33] UOC Infezioni Sistemiche e dell'Immunodepresso, Azienda Ospedaliera dei Colli, Ospedale Cotugno. Napoli, Italy.

- [34] Department of Clinical Medicine and Surgery. University of Naples "Federico II", Napoli, Italy.
- [35] Respiratory Pathophysiology Division, Department of Cardiologic, Thoracic and Vascular Sciences, University of Padova, Padova, Italy.
- [36] UOC di Pneumologia, P.O. San Giuseppe Moscati, Taranto, Italy.
- [37] ASST Milano Nord - Ospedale Edoardo Bassini, Cinisello Balsamo, Italy.
- [38] COVID-19 Unit. EE Ospedale Regionale F. Miulli, Acquaviva delle Fonti (BA), Italy.
- [39] UOC Medicina - PO S. Maria di Loreto Nuovo -ASL Napoli 1 Centro. Napoli.
- [40] Infectious and Tropical Diseases Unit. Deparment of Medical and Surgical Sciences "Magna Graecia" University, Catanzaro, Italy.
- [41] Dipartimento di Farmacia, Università di Pisa, Pisa, Italy.
- [42] U.O. C. Malattie Infettive e Tropicali, P.O. "San Marco", AOU Policlinico-Vittorio Emanuele, Catania, Italy.
- [43] UOC Malattie Infettive-Apparato Respiratorio, National Institute for Infectious Diseases "L. Spallanzani", IRCCS, Roma, Italy.
- [44] Computer Service, University of Molise, Campobasso. Italy.
- [45] Medicina Interna. Ospedale di Ravenna. AUSL della Romagna, Ravenna, Italy.
- [46] UOC Malattie Infettive, Ospedale San Gerardo, ASST Monza, Monza, Italy.
- [47] UOC Pneumologia. Dipartimento di Malattie Apparato Respiratorio e Torace. Ospedale Morgagni-Pierantoni Forlì, Forlì, Italy.
- [48] Emergency Department, IRCCS Policlinico San Matteo Foundation, Pavia, Italy.
- [49] UOC Malattie Infettive ad Alta Intensità di Cura, National Institute for Infectious Diseases "L. Spallanzani", IRCCS, Roma, Italy.
- [50] Section of Public Health, Department of Biomedical, Metabolic and Neural Sciences, University of Modena and Reggio Emilia, Modena, Italy; Department of Epidemiology, Boston University School of Public Health, Boston. USA.
- [51] Department of Epidemiology and Prevention, IRCCS Neuromed, Pozzilli (IS), Italy; Department of Medicine and Surgery, University of Insubria, Varese, Italy. Electronic address: licia.iacoviello@moli-sani.org.

- PMID: **32859477**
- PMCID: PMC7446618
- DOI: 10.1016/j.ejim.2020.08.019

Free PMC article
Observational Study

Use of hydroxychloroquine in hospitalised COVID-19 patients is associated with reduced mortality: Findings from the observational multicentre Italian CORIST study

COVID-19 RISK and Treatments (CORIST) Collaboration. Eur J Intern Med. 2020 Dec.
Free PMC article
Show details

Eur J Intern Med
. 2020 Dec;82:38-47.
doi: 10.1016/j.ejim.2020.08.019. Epub 2020 Aug 25.

Author

COVID-19 RISK and Treatments (CORIST) Collaboration

Collaborators

- **COVID-19 RISK and Treatments (CORIST) Collaboration**: Augusto Di Castelnuovo [1], Simona Costanzo [2], Andrea Antinori [3], Nausicaa Berselli [4], Lorenzo Blandi [5], Raffaele Bruno [6], Roberto Cauda [7], Giovanni Guaraldi [8], Lorenzo Menicanti [5], Ilaria My [9], Giustino Parruti [10], Giuseppe Patti [11], Stefano Perlini [12], Francesca Santilli [13], Carlo Signorelli [14], Enrico Spinoni [11], Giulio G Stefanini [9], Alessandra Vergori [15], Walter Ageno [16], Antonella Agodi [17], Luca Aiello [18], Piergiuseppe Agostoni [19], Samir Al Moghazi [20], Marinella Astuto [17], Filippo Aucella [21], Greta Barbieri [22], Alessandro Bartoloni [23], Marialaura Bonaccio [2], Paolo Bonfanti [24], Francesco Cacciatore [25], Lucia Caiano [16], Francesco Cannata [9], Laura Carrozzi [26], Antonio Cascio [27], Arturo Ciccullo [28], Antonella Cingolani [7], Francesco Cipollone [13], Claudia Colomba [27], Francesca Crosta [10], Chiara Dal Pra [29], Gian Battista Danzi [30], Damiano D'Ardes [13], Katleen de Gaetano Donati [28], Paola Del Giacomo [28], Francesco Di Gennaro [31], Giuseppe Di Tano [30], Giampiero D'Offizi [32], Tommaso Filippini [4], Francesco Maria Fusco [33], Ivan Gentile [34], Alessandro Gialluisi [2], Giancarlo Gini [16], Elvira Grandone [21], Leonardo Grisafi [11], Gabriella Guarnieri [35], Silvia Lamonica [28], Francesco Landi [18], Armando Leone [36], Gloria Maccagni [30], Sandro Maccarella [37], Andrea Madaro [38], Massimo Mapelli [19], Riccardo Maragna [19], Lorenzo Marra [36], Giulio Maresca [39], Claudia Marotta [31], Franco Mastroianni [38], Maria Mazzitelli [40], Alessandro Mengozzi [22], Francesco Menichetti [22], Marianna Meschiari [8], Filippo Minutolo [41], Arturo Montineri [42], Roberta Mussinelli [14], Cristina Mussini [8], Maria Musso [43], Anna Odone [14], Marco Olivieri [44], Emanuela Pasi [45], Francesco Petri [46], Biagio Pinchera [34], Carlo A Pivato [9], Venerino Poletti [47], Claudia Ravaglia [47], Massimo Rinaldi [38], Andrea Rognoni [11], Marco Rossato [29], Ilaria Rossi [13], Marianna Rossi [46], Anna Sabena [48], Francesco Salinaro [48], Vincenzo Sangiovanni [33], Carlo Sanrocco [10], Laura Scorzolini [49], Raffaella Sgariglia [39], Paola Giustina Simeone [10], Michele Spinicci [23], Enrico Maria Trecarichi [40], Amedeo Venezia [38], Giovanni Veronesi [16], Roberto Vettor [29], Andrea Vianello [35], Marco Vinceti [50], Laura Vocciante [39], Raffaele De Caterina [31], Licia Iacoviello [51]

Affiliations

- [1] Mediterranea Cardiocentro, Napoli, Italy.
- [2] Department of Epidemiology and Prevention, IRCCS Neuromed, Pozzilli (IS), Italy.
- [3] UOC Immunodeficienze Virali, National Institute for Infectious Diseases "L. Spallanzani", IRCCS. Roma, Italy.
- [4] Section of Public Health, Department of Biomedical, Metabolic and Neural Sciences, University of Modena and Reggio Emilia, Modena, Italy.

- [5] IRCCS Policlinico San Donato, San Donato Milanese, Italy.
- [6] Division of Infectious Diseases I, Fondazione IRCCS Policlinico San Matteo, Pavia, Italy; Department of Clinical, Surgical, Diagnostic, and Paediatric Sciences, University of Pavia, Pavia, Italy.
- [7] Fondazione Policlinico Universitario A. Gemelli IRCCS, Roma, Italy; Università Cattolica del Sacro Cuore- Dipartimento di Sicurezza e Bioetica Sede di Roma, Roma, Italy.
- [8] Infectious Disease Unit, Department of Surgical, Medical, Dental and Morphological Sciences, University of Modena and Reggio Emilia, Modena, Italy.
- [9] Humanitas Clinical and Research Hospital IRCCS, Rozzano-Milano, Italy.
- [10] Department of Infectious Disease, Azienda Sanitaria Locale (AUSL) di Pescara, Pescara, Italy.
- [11] University of Eastern Piedmont, Maggiore della Carità Hospital, Novara, Italy.
- [12] Emergency Department, IRCCS Policlinico San Matteo Foundation, Pavia, Italy; Department of Internal Medicine, University of Pavia, Pavia, Italy.
- [13] Department of Medicine and Aging, Clinica Medica, "SS. Annunziata" Hospital and University of Chieti, Chieti, Italy.
- [14] School of Medicine, Vita-Salute San Raffaele University, Milano, Italy.
- [15] HIV/AIDS Department, National Institute for Infectious Diseases "Lazzaro Spallanzani"-IRCCS, Roma, Italy.
- [16] Department of Medicine and Surgery, University of Insubria, Varese, Italy.
- [17] Department of Medical and Surgical Sciences and Advanced Technologies "G.F. Ingrassia", University of Catania; AOU Policlinico-Vittorio Emanuele, Catania, Italy.
- [18] UOC. Anestesia e Rianimazione. Dipartimento di Chirurgia Generale Ospedale Morgagni-Pierantoni, Forlì, Italy.
- [19] Centro Cardiologico Monzino IRCCS, Milano, Italy; Department of Clinical Sciences and Community Health, Cardiovascular Section, University of Milano, Milano, Italy.
- [20] Infezioni Sistemiche dell'Immunodepresso, National Institute for Infectious Diseases L. Spallanzani, IRCCS, Roma, Italy.
- [21] Fondazione I.R.C.C.S "Casa Sollievo della Sofferenza", San Giovanni Rotondo, Foggia, Italy.
- [22] Department of Clinical and Experimental Medicine, Azienda Ospedaliero-Universitaria Pisana, and University of Pisa, Pisa, Italy.
- [23] Department of Experimental and Clinical Medicine. University of Florence, Firenze, Italy.
- [24] UOC Malattie Infettive, Ospedale San Gerardo, ASST Monza, Monza, Italy; School of Medicine and Surgery, University of Milano-Bicocca, Milano, Italy.
- [25] Department of Translational Medical Sciences. University of Naples, Federico II, Napoli, Italy.
- [26] Cardiovascular and Thoracic Department, Azienda Ospedaliero-Universitaria Pisana, and University of Pisa, Pisa, Italy.
- [27] Infectious and Tropical Diseases Unit- Department of Health Promotion, Mother and Child Care, Internal Medicine and Medical Specialties (PROMISE) - University of Palermo, Palermo, Italy.
- [28] Fondazione Policlinico Universitario A. Gemelli IRCCS, Roma, Italy.
- [29] Clinica Medica 3, Department of Medicine - DIMED, University hospital of Padova, Padova, Italy.
- [30] Department of Cardiology, Ospedale di Cremona, Cremona, Italy.
- [31] Medical Direction, IRCCS Neuromed, Pozzilli (IS), Italy.

- [32] UOC Malattie Infettive-Epatologia, National Institute for Infectious Diseases L. Spallanzani, IRCCS, Roma, Italy.
- [33] UOC Infezioni Sistemiche e dell'Immunodepresso, Azienda Ospedaliera dei Colli, Ospedale Cotugno. Napoli, Italy.
- [34] Department of Clinical Medicine and Surgery. University of Naples "Federico II", Napoli, Italy.
- [35] Respiratory Pathophysiology Division, Department of Cardiologic, Thoracic and Vascular Sciences, University of Padova, Padova, Italy.
- [36] UOC di Pneumologia, P.O. San Giuseppe Moscati, Taranto, Italy.
- [37] ASST Milano Nord - Ospedale Edoardo Bassini, Cinisello Balsamo, Italy.
- [38] COVID-19 Unit. EE Ospedale Regionale F. Miulli, Acquaviva delle Fonti (BA), Italy.
- [39] UOC Medicina - PO S. Maria di Loreto Nuovo -ASL Napoli 1 Centro. Napoli.
- [40] Infectious and Tropical Diseases Unit. Deparment of Medical and Surgical Sciences "Magna Graecia" University, Catanzaro, Italy.
- [41] Dipartimento di Farmacia, Università di Pisa, Pisa, Italy.
- [42] U.O. C. Malattie Infettive e Tropicali, P.O. "San Marco", AOU Policlinico-Vittorio Emanuele, Catania, Italy.
- [43] UOC Malattie Infettive-Apparato Respiratorio, National Institute for Infectious Diseases "L. Spallanzani", IRCCS, Roma, Italy.
- [44] Computer Service, University of Molise, Campobasso. Italy.
- [45] Medicina Interna. Ospedale di Ravenna. AUSL della Romagna, Ravenna, Italy.
- [46] UOC Malattie Infettive, Ospedale San Gerardo, ASST Monza, Monza, Italy.
- [47] UOC Pneumologia. Dipartimento di Malattie Apparato Respiratorio e Torace. Ospedale Morgagni-Pierantoni Forlì, Forlì, Italy.
- [48] Emergency Department, IRCCS Policlinico San Matteo Foundation, Pavia, Italy.
- [49] UOC Malattie Infettive ad Alta Intensità di Cura, National Institute for Infectious Diseases "L. Spallanzani", IRCCS, Roma, Italy.
- [50] Section of Public Health, Department of Biomedical, Metabolic and Neural Sciences, University of Modena and Reggio Emilia, Modena, Italy; Department of Epidemiology, Boston University School of Public Health, Boston. USA.
- [51] Department of Epidemiology and Prevention, IRCCS Neuromed, Pozzilli (IS), Italy; Department of Medicine and Surgery, University of Insubria, Varese, Italy. Electronic address: licia.iacoviello@moli-sani.org.

- PMID: **32859477**
- PMCID: PMC7446618
- DOI: 10.1016/j.ejim.2020.08.019

Abstract

Background: Hydroxychloroquine (HCQ) was proposed as potential treatment for COVID-19.

Objective: We set-up a multicenter Italian collaboration to investigate the relationship between HCQ therapy and COVID-19 in-hospital mortality.

Methods: In a retrospective observational study, 3,451 unselected patients hospitalized in 33 clinical centers in Italy, from February 19, 2020 to May 23, 2020, with laboratory-confirmed SARS-CoV-2 infection, were analyzed. The primary end-point in a time-to event analysis was in-hospital death, comparing patients who received HCQ with patients who did not. We used

multivariable Cox proportional-hazards regression models with inverse probability for treatment weighting by propensity scores, with the addition of subgroup analyses.

Results: Out of 3,451 COVID-19 patients, 76.3% received HCQ. Death rates (per 1,000 person-days) for patients receiving or not HCQ were 8.9 and 15.7, respectively. After adjustment for propensity scores, we found 30% lower risk of death in patients receiving HCQ (HR=0.70; 95% CI: 0.59 to 0.84; E-value=1.67). Secondary analyses yielded similar results. The inverse association of HCQ with inpatient mortality was particularly evident in patients having elevated C-reactive protein at entry.

Conclusions: HCQ use was associated with a 30% lower risk of death in COVID-19 hospitalized patients. Within the limits of an observational study and awaiting results from randomized controlled trials, these data do not discourage the use of HCQ in inpatients with COVID-19.

Keywords: COVID-19; Disease severity; Hydroxychloroquine; Inflammation; Mortality.

Copyright © 2020 European Federation of Internal Medicine. Published by Elsevier B.V. All rights reserved.

Conflict of interest statement

None.

Comment in

- Does hydroxychloroquine reduce mortality for COVID-19?
  Gallus S, Clavenna A, Lugo A. Gallus S, et al. Eur J Intern Med. 2020 Dec;82:21-22. doi: 10.1016/j.ejim.2020.10.015. Epub 2020 Oct 16. Eur J Intern Med. 2020. PMID: 33127218 Free PMC article. No abstract available.
- Large-scale use of hydroxychloroquine for COVID-19 confirms safety, if not effectiveness.
  Cavalli G, Dagna L. Cavalli G, et al. Eur J Intern Med. 2020 Dec;82:23-24. doi: 10.1016/j.ejim.2020.10.023. Epub 2020 Oct 28. Eur J Intern Med. 2020. PMID: 33132004 Free PMC article. No abstract available.
- Hydroxychloroquine for COVID-19: Balancing contrasting claims.
  Horby PW, Emberson JR. Horby PW, et al. Eur J Intern Med. 2020 Dec;82:25-26. doi: 10.1016/j.ejim.2020.11.018. Epub 2020 Nov 23. Eur J Intern Med. 2020. PMID: 33243609 Free PMC article. No abstract available.

- 42 references
- 2 figures

Supplementary info

Publication types, MeSH terms, Substances Expand

Publication types

- Multicenter Study
- Observational Study

MeSH terms

- Aged
- Aged, 80 and over
- COVID-19 / drug therapy*
- COVID-19 / mortality*
- Female
- Hospital Mortality
- Humans
- Hydroxychloroquine / therapeutic use*
- Italy
- Male
- Middle Aged
- Retrospective Studies
- Treatment Outcome

Substances

- Hydroxychloroquine

Full text links

ELSEVIER FULL-TEXT ARTICLE Elsevier Science Free PMC article
Proceed to details
Cite
Share
1,049
Observational Study
Minerva Med
. 2021 Dec;112(6):779-785.
doi: 10.23736/S0026-4806.21.07585-6. Epub 2021 Jun 18.

Pneumomediastinum associated with severe pneumonia related to COVID-19: diagnosis and management

Corinna Gandolfo [1], Monica Bonfiglio [1], Giulia Spinetto [1], Gianluca Ferraioli [2], Cornelius Barlascini [3], Antonello Nicolini [4], Paolo Solidoro [5, 6]
Affiliations Expand

Affiliations

- [1] COVID-19 Intensive Care Unit, General Hospital of Sestri Levante, Sestri Levante, Genoa, Italy.
- [2] COVID-19 Unit, General Hospital of Sestri Levante, Sestri Levante, Genoa, Italy.
- [3] Unit of Hygiene and Health Medicine, General Hospital of Sestri Levante, Sestri Levante, Genoa, Italy.
- [4] Unit of Respiratory Diseases, General Hospital of Sestri Levante, Sestri Levante, Genoa, Italy - antonellonicolini@gmail.com.
- [5] Department of Medical Sciences, University of Turin, Turin, Italy.
- [6] Division of Respiratory Diseases, Cardiovascular and Thoracic Department, Città della Salute e della Scienza, Turin, Italy.

- PMID: **34142771**
- DOI: 10.23736/S0026-4806.21.07585-6

Observational Study

Pneumomediastinum associated with severe pneumonia related to COVID-19: diagnosis and management

Corinna Gandolfo et al. Minerva Med. 2021 Dec.

Show details

Minerva Med

. 2021 Dec;112(6):779-785.
doi: 10.23736/S0026-4806.21.07585-6. Epub 2021 Jun 18.

Authors

Corinna Gandolfo [1], Monica Bonfiglio [1], Giulia Spinetto [1], Gianluca Ferraioli [2], Cornelius Barlascini [3], Antonello Nicolini [4], Paolo Solidoro [5] [6]

Affiliations

- [1] COVID-19 Intensive Care Unit, General Hospital of Sestri Levante, Sestri Levante, Genoa, Italy.
- [2] COVID-19 Unit, General Hospital of Sestri Levante, Sestri Levante, Genoa, Italy.
- [3] Unit of Hygiene and Health Medicine, General Hospital of Sestri Levante, Sestri Levante, Genoa, Italy.
- [4] Unit of Respiratory Diseases, General Hospital of Sestri Levante, Sestri Levante, Genoa, Italy - antonellonicolini@gmail.com.
- [5] Department of Medical Sciences, University of Turin, Turin, Italy.
- [6] Division of Respiratory Diseases, Cardiovascular and Thoracic Department, Città della Salute e della Scienza, Turin, Italy.

- PMID: **34142771**
- DOI: 10.23736/S0026-4806.21.07585-6

Abstract

Background: Pneumomediastinum (PNM) can develop as a severe complication of severe COVID-19 and may be correlated with greater morbidity and mortality. PNM is a rarely reported complication in COVID-19 patients and usually associated with endotracheal intubation.

Methods: Our aim was to describe the characteristics of patients with PNM in twenty-one patients with COVID-19 related pneumonia and acute respiratory failure in a retrospective case series.

Results: Twenty-one patients were diagnosed, four were treated with high-flow nasal cannula, thirteen with non invasive ventilation and four with invasive mechanical ventilation. In five cases PNM was massive and associated to subcutaneous emphysema; more rarely PNM was associated with pneumothorax. Conservative management was the most used therapeutic strategy.

Conclusions: PNM is a serious and not extremely rare complication of severe forms of pulmonary involvement of COVID-19. The clinician should consider this rare complication; moreover, we suggest being careful when clinicians start mechanical ventilation.

Supplementary info

Publication types, MeSH terms Expand

Publication types

- Observational Study

MeSH terms

- Adult
- COVID-19 / complications*
- Female
- Humans
- Male
- Mediastinal Emphysema / diagnosis
- Mediastinal Emphysema / etiology*
- Mediastinal Emphysema / therapy
- Middle Aged
- Pneumonia / complications
- Pneumonia / etiology*
- Retrospective Studies
- Severity of Illness Index
- Young Adult

Full text links

FULL TEXT article at minervamedica.it Minerva Medica

Proceed to details

Cite

Share

☐ 1,050

Case Reports

Med Clin (Barc)

. 2020 Aug 28;155(4):159-161.

doi: 10.1016/j.medcli.2020.04.018. Epub 2020 May 27.

Effectiveness of corticoid pulses in patients with cytokine storm syndrome induced by SARS-CoV-2 infection

[Article in English, Spanish]

José Luis Callejas Rubio [1], Juan de Dios Luna Del Castillo [2], Javier de la Hera Fernández [3], Emilio Guirao Arrabal [4], Manuel Colmenero Ruiz [5], Norberto Ortego Centeno [6]

Affiliations Expand

Affiliations

- [1] Unidad de Enfermedades Sistémicas, Servicio de Medicina Interna, Hospital Universitario Clínico San Cecilio, Granada, España. Electronic address: jlcalleja@telefonica.net.
- [2] Departamento de Bioestadística, Facultad de Medicina, Universidad de Granada, Granada, España.
- [3] Servicio de Medicina Interna, Hospital Universitario Clínico San Cecilio, Granada, España.
- [4] Unidad de Enfermedades Infecciosas, Hospital Universitario Clínico San Cecilio, Granada, España.
- [5] Servicio de Cuidados Intensivos, Hospital Universitario Clínico San Cecilio, Granada, España.
- [6] Unidad de Enfermedades Sistémicas, Servicio de Medicina Interna, Hospital Universitario Clínico San Cecilio, Granada, España.

- PMID: **32532461**
- PMCID: PMC7250763
- DOI: 10.1016/j.medcli.2020.04.018

Free PMC article

Case Reports

Effectiveness of corticoid pulses in patients with cytokine storm syndrome induced by SARS-CoV-2 infection

[Article in English, Spanish]
José Luis Callejas Rubio et al. Med Clin (Barc). 2020.
Free PMC article
Show details
Med Clin (Barc)
. 2020 Aug 28;155(4):159-161.
doi: 10.1016/j.medcli.2020.04.018. Epub 2020 May 27.

Authors

José Luis Callejas Rubio [1], Juan de Dios Luna Del Castillo [2], Javier de la Hera Fernández [3], Emilio Guirao Arrabal [4], Manuel Colmenero Ruiz [5], Norberto Ortego Centeno [6]

Affiliations

- [1] Unidad de Enfermedades Sistémicas, Servicio de Medicina Interna, Hospital Universitario Clínico San Cecilio, Granada, España. Electronic address: jlcalleja@telefonica.net.
- [2] Departamento de Bioestadística, Facultad de Medicina, Universidad de Granada, Granada, España.
- [3] Servicio de Medicina Interna, Hospital Universitario Clínico San Cecilio, Granada, España.
- [4] Unidad de Enfermedades Infecciosas, Hospital Universitario Clínico San Cecilio, Granada, España.
- [5] Servicio de Cuidados Intensivos, Hospital Universitario Clínico San Cecilio, Granada, España.
- [6] Unidad de Enfermedades Sistémicas, Servicio de Medicina Interna, Hospital Universitario Clínico San Cecilio, Granada, España.

- PMID: **32532461**
- PMCID: PMC7250763
- DOI: 10.1016/j.medcli.2020.04.018

Abstract

Introduction: Cytokine storm syndrome (CSS) is a serious complication of COVID-19 patients. Treatment is tocilizumab. The use of glucocorticoids (GC) is controversial. In other very similar CSS, such as macrophage activation syndrome (MAS) and hemophagocytic syndrome (HFS), the main treatment are corticosteroids. Our objective is to evaluate the efficacy of GC in the CSS by COVID-19.

Patients: We included 92 patients with CSS associated to COVID-19 who received GC, GC, and tocilizumab and only tocilizumab. We determine CSS markers. We evaluated mortality, intubation, and a combined variable.

Results: In all cases the percentages of events were lower in the group of patients with GC was administered. The hazard ratio of the final variables with GC versus the group in which only tocilizumab was administered was lower as CGs were considered, with statistical significance for survival.

Discussion: The early use of GC pulses could control SLC, with a lower requirement to use tocilizumab and a decrease in events such as intubation and death.

Keywords: Coronavirus COVID-19; Cytokine release synddrome; Hemofagocítico; Hemophagocytic; Síndrome de liberación de citocinas.

Copyright © 2020 Elsevier España, S.L.U. All rights reserved.

- 10 references

Supplementary info

Publication types, MeSH terms, Substances Expand

Publication types

- Case Reports
- Observational Study

MeSH terms

- Adrenal Cortex Hormones / administration & dosage*
- Adult
- Aged
- Aged, 80 and over
- Antibodies, Monoclonal, Humanized / administration & dosage*
- Betacoronavirus*
- COVID-19
- Coronavirus Infections / complications*
- Coronavirus Infections / immunology
- Coronavirus Infections / mortality
- Cytokine Release Syndrome / drug therapy*
- Cytokine Release Syndrome / etiology
- Cytokine Release Syndrome / mortality
- Drug Administration Schedule
- Female
- Humans
- Intubation, Intratracheal / statistics & numerical data
- Kaplan-Meier Estimate
- Male
- Middle Aged
- Pandemics
- Pneumonia, Viral / complications*
- Pneumonia, Viral / immunology

- Pneumonia, Viral / mortality
- Retrospective Studies
- SARS-CoV-2

Substances

- Adrenal Cortex Hormones
- Antibodies, Monoclonal, Humanized
- tocilizumab

Full text links

Full text at MEDICINA CLINICA Ediciones Doyma, S.L. Free PMC article
Proceed to details
Cite
Share

☐ 1,051
Observational Study
Arch Pediatr
. 2020 Jul;27(5):235-238.
doi: 10.1016/j.arcped.2020.05.010. Epub 2020 Jun 4.

Severe and fatal forms of COVID-19 in children

M Oualha[1], M Bendavid[2], L Berteloot[3], A Corsia[2], F Lesage[2], M Vedrenne[2], E Salvador[2], M Grimaud[2], J Chareyre[2], C de Marcellus[2], L Dupic[2], L de Saint Blanquat[2], C Heilbronner[2], D Drummond[4], M Castelle[5], R Berthaud[6], F Angoulvant[7], J Toubiana[8], Y Pinhas[8], P Frange[9], G Chéron[10], J Fourgeaud[11], F Moulin[2], S Renolleau[2]
Affiliations Expand

Affiliations

- [1] Pediatric Intensive Care Unit, Necker-Enfants-Malades University Hospital, APHP, Centre-Université de Paris, Paris, France. Electronic address: mehdi.oualha@aphp.fr.
- [2] Pediatric Intensive Care Unit, Necker-Enfants-Malades University Hospital, APHP, Centre-Université de Paris, Paris, France.
- [3] Pediatric Radiology Department, Necker-Enfants-Malades University Hospital, APHP, Centre-Université de Paris, Paris, France; INSERM U1163, Université Paris Descartes-Sorbonne Paris Cité, Institut Imagine, France.
- [4] Department of pediatric pneumology and allergology, Necker-Enfants-Malades University Hospital, APHP, Centre-Université de Paris, Paris, France; INSERM UMR 1138, Université de Paris, Paris, France.

- [5] Department of pediatric Immuno-hematology and rhumatology, Necker-Enfants Malades University Hospital, APHP, Centre-Université de Paris, Paris, France.
- [6] Department of Pediatric Nephrology, Necker-Enfants Malades University Hospital, APHP, Centre-Université de Paris, Paris, France.
- [7] Pediatric Emergency Department, Necker-Enfants-Malades University Hospital, APHP, Centre-Université de Paris, Paris, France; INSERM, Centre de Recherche des Cordeliers, UMRS 1138, Université de Paris, Paris, France.
- [8] Department of General Paediatrics and Paediatric Infectious Diseases, Necker-Enfants-Malades University Hospital, APHP, Centre-Université de Paris, Paris, France.
- [9] Clinical microbiology laboratory, Necker-Enfants-Malades University Hospital, APHP, Centre-Université de Paris, Paris, France; EHU 7328 PACT, Imagine institute, Université de Paris, Paris, France.
- [10] Pediatric Emergency Department, Necker-Enfants-Malades University Hospital, APHP, Centre-Université de Paris, Paris, France.
- [11] Virology laboratory, Necker-Enfants-Malades University Hospital, APHP, Centre-Université de Paris, EHU 7328 PACT, Imagine Institute, University of Paris, Paris, France.

- PMID: **32518045**
- PMCID: PMC7269941
- DOI: 10.1016/j.arcped.2020.05.010

Free PMC article
Observational Study

Severe and fatal forms of COVID-19 in children

M Oualha et al. Arch Pediatr. 2020 Jul.
Free PMC article
Show details
Arch Pediatr
. 2020 Jul;27(5):235-238.
doi: 10.1016/j.arcped.2020.05.010. Epub 2020 Jun 4.

Authors

M Oualha [1], M Bendavid [2], L Berteloot [3], A Corsia [2], F Lesage [2], M Vedrenne [2], E Salvador [2], M Grimaud [2], J Chareyre [2], C de Marcellus [2], L Dupic [2], L de Saint Blanquat [2], C Heilbronner [2], D Drummond [4], M Castelle [5], R Berthaud [6], F Angoulvant [7], J Toubiana [8], Y Pinhas [8], P Frange [9], G Chéron [10], J Fourgeaud [11], F Moulin [2], S Renolleau [2]

Affiliations

- [1] Pediatric Intensive Care Unit, Necker-Enfants-Malades University Hospital, APHP, Centre-Université de Paris, Paris, France. Electronic address: mehdi.oualha@aphp.fr.
- [2] Pediatric Intensive Care Unit, Necker-Enfants-Malades University Hospital, APHP, Centre-Université de Paris, Paris, France.

- [3] Pediatric Radiology Department, Necker-Enfants-Malades University Hospital, APHP, Centre-Université de Paris, Paris, France; INSERM U1163, Université Paris Descartes-Sorbonne Paris Cité, Institut Imagine, France.
- [4] Department of pediatric pneumology and allergology, Necker-Enfants-Malades University Hospital, APHP, Centre-Université de Paris, Paris, France; INSERM UMR 1138, Université de Paris, Paris, France.
- [5] Department of pediatric Immuno-hematology and rhumatology, Necker-Enfants Malades University Hospital, APHP, Centre-Université de Paris, Paris, France.
- [6] Department of Pediatric Nephrology, Necker-Enfants Malades University Hospital, APHP, Centre-Université de Paris, Paris, France.
- [7] Pediatric Emergency Department, Necker-Enfants-Malades University Hospital, APHP, Centre-Université de Paris, Paris, France; INSERM, Centre de Recherche des Cordeliers, UMRS 1138, Université de Paris, Paris, France.
- [8] Department of General Paediatrics and Paediatric Infectious Diseases, Necker-Enfants-Malades University Hospital, APHP, Centre-Université de Paris, Paris, France.
- [9] Clinical microbiology laboratory, Necker-Enfants-Malades University Hospital, APHP, Centre-Université de Paris, Paris, France; EHU 7328 PACT, Imagine institute, Université de Paris, Paris, France.
- [10] Pediatric Emergency Department, Necker-Enfants-Malades University Hospital, APHP, Centre-Université de Paris, Paris, France.
- [11] Virology laboratory, Necker-Enfants-Malades University Hospital, APHP, Centre-Université de Paris, EHU 7328 PACT, Imagine Institute, University of Paris, Paris, France.

- PMID: **32518045**
- PMCID: PMC7269941
- DOI: 10.1016/j.arcped.2020.05.010

Abstract

Objectives: The aim of this study was to describe severe forms of novel coronavirus disease 2019 in children, including patient characteristics, clinical, laboratory, and imaging findings, as well as the disease management and outcomes.

Methods: This was a retrospective, single-center, observational study conducted in a pediatric intensive and high-dependency care unit (PICU, HDU) in an urban hospital in Paris. All patients, aged from 1 month to 18 years, admitted for confirmed or highly suspected SARS-CoV-2 were included.

Results: We analyzed the data of 27 children. Comorbidities (n=19, 70%) were mainly neurological (n=7), respiratory, (n=4), or sickle cell disease (n=4). SARS-CoV-2 PCR results were positive in 24 children (nasopharyngeal swabs). The three remaining children had a chest CT scan consistent with COVID-19. Respiratory involvement was observed in 24 patients (89%). Supportive treatments were invasive mechanical ventilation (n=9), catecholamine (n=4), erythropheresis (n=4), renal replacement therapy (n=1), and extracorporeal membrane oxygenation (n=1). Five children died, of whom three were without past medical history.

Conclusion: This study highlighted the large spectrum of clinical presentation and time course of disease progression as well as the non-negligible occurrence of pediatric life-threatening and fatal cases of COVID-19 mostly in patients with comorbidities. Additional laboratory investigations are needed to further analyze the mechanism underlying the variability of SARS-Cov-2 pathogenicity in children.

Keywords: COVID-19; Coronavirus; SARS-Cov-2.

Copyright © 2020 French Society of Pediatrics. Published by Elsevier Masson SAS. All rights reserved.

Comment in

- Demographic and Clinical Profile of Mortality Cases of COVID-19 in Children in New Delhi.
  Singh A, Saini I, Meena SK, Gera R. Singh A, et al. Indian J Pediatr. 2021 Jun;88(6):610. doi: 10.1007/s12098-021-03687-8. Epub 2021 Mar 10. Indian J Pediatr. 2021. PMID: 33689110 Free PMC article. No abstract available.

- 17 references
- 1 figure

Supplementary info

Publication types, MeSH terms Expand

Publication types

- Observational Study

MeSH terms

- Adolescent
- Betacoronavirus* / isolation & purification
- COVID-19
- COVID-19 Testing
- Child
- Child, Preschool
- Clinical Laboratory Techniques
- Comorbidity
- Coronavirus Infections / diagnosis*
- Coronavirus Infections / epidemiology
- Coronavirus Infections / mortality*
- Coronavirus Infections / therapy
- Disease Progression
- Female
- Humans
- Infant
- Male
- Pandemics
- Paris / epidemiology

- Pneumonia, Viral / diagnosis*
- Pneumonia, Viral / epidemiology
- Pneumonia, Viral / mortality*
- Pneumonia, Viral / therapy
- Prognosis
- Retrospective Studies
- Risk Factors
- SARS-CoV-2
- Severity of Illness Index

Full text links

ELSEVIER FULL-TEXT ARTICLE Elsevier Science Free PMC article
Proceed to details
Cite
Share
1,052
Observational Study
Medicine (Baltimore)
. 2020 Jun 26;99(26):e21012.
doi: 10.1097/MD.0000000000021012.

Critically ill patients with COVID-19 with ECMO and artificial liver plasma exchange: A retrospective study

Jian Liu [1], Yong-Quan Dong [2], Jie Yin [3], Guojun He [1], Xiaoxin Wu [4], Jianping Li [5], Yunqing Qiu [4], Xuelin He [6]
Affiliations Expand

Affiliations

- [1] Department of Intensive Care Unit, the First Affiliated Hospital, College of Medicine, Zhejiang University, Hangzhou.
- [2] Department of Respiratory Disease, Yinzhou No.2 Hospital, Ningbo.
- [3] Department of Radiation Oncology.
- [4] Department of Infectious Diseases.
- [5] Department of Traditional Chinese Medicine.
- [6] Department of Kidney Disease Center, the First Affiliated Hospital, College of Medicine, Zhejiang University, Hangzhou, Zhejiang Province, China.

- PMID: 32590819
- PMCID: PMC7328989
- DOI: 10.1097/MD.0000000000021012

Free PMC article
Observational Study

Critically ill patients with COVID-19 with ECMO and artificial liver plasma exchange: A retrospective study

Jian Liu et al. Medicine (Baltimore). 2020.
Free PMC article
Show details
Medicine (Baltimore)
. 2020 Jun 26;99(26):e21012.
doi: 10.1097/MD.0000000000021012.

Authors

Jian Liu [1], Yong-Quan Dong [2], Jie Yin [3], Guojun He [1], Xiaoxin Wu [4], Jianping Li [5], Yunqing Qiu [4], Xuelin He [6]

Affiliations

- [1] Department of Intensive Care Unit, the First Affiliated Hospital, College of Medicine, Zhejiang University, Hangzhou.
- [2] Department of Respiratory Disease, Yinzhou No.2 Hospital, Ningbo.
- [3] Department of Radiation Oncology.
- [4] Department of Infectious Diseases.
- [5] Department of Traditional Chinese Medicine.
- [6] Department of Kidney Disease Center, the First Affiliated Hospital, College of Medicine, Zhejiang University, Hangzhou, Zhejiang Province, China.

- PMID: **32590819**
- PMCID: PMC7328989
- DOI: 10.1097/MD.0000000000021012

Abstract

COVID-19 is an emerging infectious disease capable of causing severe pneumonia. We aimed to characterize a group of critically ill patients in a single-center study.This was a retrospective case series of 23 patients with confirmed COVID-19-related critical illness in the intensive care unit (ICU) of a hospital in Hangzhou Zhejiang Province between January 22 and March 20, 2020.Of the 23 critically ill patients, the median age was 66 years (interquartile range [IQR] 59-80 years). The median time from disease onset to ICU admission was 10 days (IQR 6-11 days), to mechanical ventilation (MV) was 11 days (IQR 7.75-13 days), to artificial liver plasma exchange was 12 days (IQR 9.75-14.75 days), and to extracorporeal membrane oxygenation (ECMO) was 22 days (IQR 17.5-30 days). Nine patients required high flow oxygen. Fourteen patients received MV. Six required ECMO. Nine received artificial liver plasma exchange. Mortality was 0 at day 28.Mortality was 0 at day 28 in our single-center study. Extracorporeal membrane oxygenation

reduced the requirements for ventilator support. Artificial liver plasma exchange significantly reduced inflammatory cytokine levels. These supportive therapies helped to extend the patients' survival times and increase the chance of follow-up treatments.

Conflict of interest statement

JL, Y-QD, and JY contributed equally to this study. The authors report no conflicts of interest.

- 28 references
- 2 figures

Supplementary info

Publication types, MeSH terms Expand

Publication types

- Observational Study

MeSH terms

- Aged
- Aged, 80 and over
- COVID-19
- Coronavirus Infections / complications
- Coronavirus Infections / therapy*
- Critical Illness
- Extracorporeal Membrane Oxygenation*
- Female
- Humans
- Liver, Artificial*
- Male
- Middle Aged
- Pandemics
- Pneumonia, Viral / complications
- Pneumonia, Viral / therapy*
- Retrospective Studies

Full text links

Wolters Kluwer Wolters Kluwer Free PMC article
Proceed to details
Cite
Share
☐ 1,053

Observational Study

Front Endocrinol (Lausanne)
. 2020 Jul 14;11:478.
doi: 10.3389/fendo.2020.00478. eCollection 2020.

Association Between Diabetes and COVID-19: A Retrospective Observational Study With a Large Sample of 1,880 Cases in Leishenshan Hospital, Wuhan

Zeming Liu [1], Jinpeng Li [2], Jianglong Huang [1], Liang Guo [1], Rongfen Gao [3], Kuan Luo [4], Guang Zeng [5], Tingbao Zhang [6], Meilin Yi [7], Yihui Huang [1], Jincao Chen [6], Yibin Yang [8], Xiaohui Wu [6]

Affiliations Expand

Affiliations

- [1] Department of Plastic Surgery, Zhongnan Hospital of Wuhan University, Wuhan, China.
- [2] Department of Thyroid and Breast Surgery, Zhongnan Hospital of Wuhan University, Wuhan, China.
- [3] Department of Rheumatology and Immunology, Tongji Medical College, Tongji Hospital, Huazhong University of Science and Technology, Wuhan, China.
- [4] Department of Neurosurgery, Wuhan Puren Hospital, Wuhan, China.
- [5] Department of Urology, Zhongnan Hospital of Wuhan University, Wuhan, China.
- [6] Department of Neurosurgery, Zhongnan Hospital of Wuhan University, Wuhan, China.
- [7] Department of Burn and Plastic Surgery, College of Traditional Chinese Medicine, Three Gorges University, Yichang Hospital of Traditional Chinese Medicine, Yichang, China.
- [8] Department of Respiratory and Critical Care Medicine, Zhongnan Hospital of Wuhan University, Wuhan, China.

- PMID: **32760350**
- PMCID: PMC7371935
- DOI: 10.3389/fendo.2020.00478

Free PMC article
Observational Study

Association Between Diabetes and COVID-19: A Retrospective Observational Study With a Large Sample of 1,880 Cases in Leishenshan Hospital, Wuhan

Zeming Liu et al. Front Endocrinol (Lausanne). 2020.
Free PMC article
Show details
Front Endocrinol (Lausanne)
. 2020 Jul 14;11:478.
doi: 10.3389/fendo.2020.00478. eCollection 2020.

Authors

Zeming Liu [1], Jinpeng Li [2], Jianglong Huang [1], Liang Guo [1], Rongfen Gao [3], Kuan Luo [4], Guang Zeng [5], Tingbao Zhang [6], Meilin Yi [7], Yihui Huang [1], Jincao Chen [6], Yibin Yang [8], Xiaohui Wu [6]

Affiliations

- [1] Department of Plastic Surgery, Zhongnan Hospital of Wuhan University, Wuhan, China.
- [2] Department of Thyroid and Breast Surgery, Zhongnan Hospital of Wuhan University, Wuhan, China.
- [3] Department of Rheumatology and Immunology, Tongji Medical College, Tongji Hospital, Huazhong University of Science and Technology, Wuhan, China.
- [4] Department of Neurosurgery, Wuhan Puren Hospital, Wuhan, China.
- [5] Department of Urology, Zhongnan Hospital of Wuhan University, Wuhan, China.
- [6] Department of Neurosurgery, Zhongnan Hospital of Wuhan University, Wuhan, China.
- [7] Department of Burn and Plastic Surgery, College of Traditional Chinese Medicine, Three Gorges University, Yichang Hospital of Traditional Chinese Medicine, Yichang, China.
- [8] Department of Respiratory and Critical Care Medicine, Zhongnan Hospital of Wuhan University, Wuhan, China.

- PMID: **32760350**
- PMCID: PMC7371935
- DOI: 10.3389/fendo.2020.00478

Abstract

Aims: This study aimed to investigate the clinical courses and outcomes of diabetes mellitus patients with coronavirus disease 2019 (COVID-19) in Wuhan. **Methods:** This study enrolled 1,880 consecutive patients with confirmed COVID-19 in Leishenshan Hospital. We collected and analyzed their data, including demographic data, history of comorbidity, clinical symptoms, laboratory tests, chest computed tomography (CT) images, treatment options, and survival. **Results:** The percentages of patients with diabetes among the severe and critical COVID-19 cases were higher than those among the mild or general cases (89.2%, 10.8 vs. 0%, $p = 0.001$). However, patients with and without diabetes showed no difference in the follow-up period ($p = 0.993$). The mortality rate in patients with or without diabetes was 2.9% ($n = 4$) and 1.1% ($n = 9$), respectively ($p = 0.114$). Univariate and multivariate Cox regression analyses and the Kaplan-Meier curves did not show any statistically significant differences between patients with and without diabetes (all $p > 0.05$). **Conclusions:** Our study results suggested that diabetes had no effect on the prognosis of COVID-19 patients but had a negative association with their clinical courses. These results may be useful for clinicians in the management of diabetic patients with COVID-19.

Keywords: Coronavirus disease 2019 (COVID-19); clinical courses; comorbidity; diabetes mellitus; prognosis.

Copyright © 2020 Liu, Li, Huang, Guo, Gao, Luo, Zeng, Zhang, Yi, Huang, Chen, Yang and Wu.

- 27 references
- 2 figures

Supplementary info

Publication types, MeSH terms Expand

Publication types

- Observational Study

MeSH terms

- Adult
- Betacoronavirus / isolation & purification*
- COVID-19
- China / epidemiology
- Comorbidity
- Coronavirus Infections / complications
- Coronavirus Infections / epidemiology*
- Coronavirus Infections / virology*
- Diabetes Complications
- Diabetes Mellitus / physiopathology*
- Female
- Follow-Up Studies
- Humans
- Male
- Middle Aged
- Pandemics
- Pneumonia, Viral / complications
- Pneumonia, Viral / epidemiology*
- Pneumonia, Viral / virology*
- Prognosis
- Retrospective Studies
- Risk Factors
- SARS-CoV-2

Full text links

Frontiers Media SA Free PMC article
Proceed to details
Cite
Share
1,054
Observational Study
Tidsskr Nor Laegeforen
. 2020 Apr 10;140(7).
doi: 10.4045/tidsskr.20.0301. Print 2020 May 5.

COVID-19: Symptoms, course of illness and use of clinical scoring systems for the first 42 patients admitted to a Norwegian local hospital

[Article in English, Norwegian]
Håkon Ihle-Hansen, Trygve Berge, Anders Tveita, Else Johanne Rønning, Per Erik Ernø, Elizabeth Lyster Andersen, Christian Hjorth Wang, Arnljot Tveit, Marius Myrstad

- PMID: **32378844**
- DOI: 10.4045/tidsskr.20.0301

Free article
Observational Study

COVID-19: Symptoms, course of illness and use of clinical scoring systems for the first 42 patients admitted to a Norwegian local hospital

[Article in English, Norwegian]
Håkon Ihle-Hansen et al. Tidsskr Nor Laegeforen. 2020.
Free article
Show details
Tidsskr Nor Laegeforen
. 2020 Apr 10;140(7).
doi: 10.4045/tidsskr.20.0301. Print 2020 May 5.

Authors

Håkon Ihle-Hansen, Trygve Berge, Anders Tveita, Else Johanne Rønning, Per Erik Ernø, Elizabeth Lyster Andersen, Christian Hjorth Wang, Arnljot Tveit, Marius Myrstad

- PMID: **32378844**
- DOI: 10.4045/tidsskr.20.0301

Abstract

Background: The COVID-19 outbreak is presenting the health system with new challenges, and there is a great need for knowledge about symptoms, clinical findings and course of illness in patients admitted to Norwegian hospitals with COVID-19.

Material and method: In this observational qualitative study, all patients admitted to a Norwegian local hospital (Bærum Hospital) with proven COVID-19 infection were included consecutively from the start of the outbreak. We present here patient characteristics, symptoms, clinical findings, experience of using clinical scoring systems and course of illness based on data in medical records.

Results: In the period 9-31 March 2020, 42 patients, of whom 28 (67 %) were men, were admitted to hospital with COVID-19 infection. The median age was 72.5 years (range 30-95). Fever (79 %), reduced general condition (79 %), dyspnoea (69 %) and cough (67 %) were the most common symptoms. A total of nine patients (21 %) had a critical course of illness with treatment in the Intensive Care Department and/or death during their stay in hospital. Patients with a critical course had a higher average score on National Early Warning Score 2 (NEWS2) on admission (7.6 vs 3.3). Only one of the most severely ill patients scored ≥ 2 on the quick Sepsis-related Organ Failure Assessment (qSOFA) on admission.

Interpretation: Most patients admitted to our hospital with COVID-19 had a fever and respiratory tract symptoms. A high percentage of patients had a critical course of illness. A NEWS2 score of ≥ 5 on admission may be a useful aid in identifying patients at risk of a critical course of illness, while CRB-65 and qSOFA score ≥ 2 proved to be of little usefulness for this purpose in our material.

Supplementary info

Publication types, MeSH terms Expand

Publication types

- Observational Study

MeSH terms

- Adult
- Aged
- Aged, 80 and over
- Betacoronavirus
- COVID-19
- COVID-19 Testing
- Clinical Laboratory Techniques
- Coronavirus Infections* / complications

- Coronavirus Infections* / diagnosis
- Coronavirus Infections* / epidemiology
- Cough / etiology
- Critical Illness*
- Dyspnea / etiology
- Emergency Service, Hospital
- Fever / etiology
- Humans
- Male
- Middle Aged
- Norway / epidemiology
- Organ Dysfunction Scores
- Pandemics*
- Pneumonia, Viral* / complications
- Pneumonia, Viral* / diagnosis
- Pneumonia, Viral* / epidemiology
- Retrospective Studies
- SARS-CoV-2
- Severity of Illness Index*

Full text links

Free Tidsskriftet Norwegian Medical Association
Proceed to details
Cite
Share
1,055
Observational Study
Intern Emerg Med
. 2020 Nov;15(8):1399-1407.
doi: 10.1007/s11739-020-02425-w. Epub 2020 Jul 10.

Impact of COVID-19 on liver function: results from an internal medicine unit in Northern Italy

Marco Vincenzo Lenti [#] [1], Federica Borrelli de Andreis [#] [1], Ivan Pellegrino [1], Catherine Klersy [2], Stefania Merli [1], Emanuela Miceli [1], Nicola Aronico [1], Caterina Mengoli [1], Michele Di Stefano [1], Sara Cococcia [1], Giovanni Santacroce [1], Simone Soriano [1], Federica Melazzini [1], Mariangela Delliponti [1], Fausto Baldanti [3], Antonio Triarico [4], Gino Roberto Corazza [1], Massimo Pinzani [5], Antonio Di Sabatino [6] [7], Internal Medicine Covid-19 Team

Collaborators, Affiliations Expand

Collaborators

- **Internal Medicine Covid-19 Team**: Gaetano Bergamaschi, Giampiera Bertolino, Silvia Codega, Filippo Costanzo, Roberto Cresci, Giuseppe Derosa, Francesco Falaschi, Carmine Iadarola, Elisabetta Lovati, Pietro Carlo Lucotti, Alessandra Martignoni, Amedeo Mugellini, Chiara Muggia, Patrizia Noris, Elisabetta Pagani, Ilaria Palumbo, Alessandro Pecci, Tiziano Perrone, Carla Pieresca, Paola Stefania Preti, Mariaconcetta Russo, Carmelo Sgarlata, Luisa Siciliani, Andrea Staniscia, Francesca Torello Vjera, Giovanna Achilli, Andrea Agostinelli, Valentina Antoci, Alessia Ballesio, Francesco Banfi, Chiara Barteselli, Irene Benedetti, Michele Brattoli, Francesca Calabretta, Ginevra Cambiè, Roberta Canta, Federico Conca, Luigi Coppola, Elisa Maria Cremonte, Gabriele Croce, Virginia Del Rio, Francesco Di Terlizzi, Maria Giovanna Ferrari, Sara Ferrari, Anna Fiengo, Tommaso Forni, Giulia Freddi, Chiara Frigerio, Federica Fumoso, Alessandra Fusco, Margherita Gabba, Matteo Garolfi, Antonella Gentile, Giulia Gori, Giacomo Grandi, Paolo Grimaldi, Alice Lampugnani, Francesco Lapia, Federica Lepore, Gianluca Lettieri, Jacopo Mambella, Chiara Mercanti, Francesco Mordà, Alba Nardone, Luca Pace, Lucia Padovini, Alessandro Parodi, Lavinia Pitotti, Margherita Reduzzi, Giovanni Rigano, Giorgio Rotola, Umberto Sabatini, Lucia Salvi, Giovanni Santacroce, Jessica Savioli, Simone Soriano, Carmine Spataro, Debora Stefani

Affiliations

- [1] Department of Internal Medicine, San Matteo Hospital Foundation, University of Pavia, Pavia, Italy.
- [2] Biometry and Clinical Epidemiology Service, San Matteo Hospital Foundation, Pavia, Italy.
- [3] Molecular Virology Unit, Microbiology and Virology Department, San Matteo Hospital Foundation, Pavia, Italy.
- [4] Chief Medical Direction, San Matteo Hospital Foundation, Pavia, Italy.
- [5] UCL Institute for Liver and Digestive Health and Sheila Sherlock Liver Unit, Royal Free Hospital and UCL, London, UK.
- [6] Department of Internal Medicine, San Matteo Hospital Foundation, University of Pavia, Pavia, Italy. a.disabatino@smatteo.pv.it.
- [7] Clinica Medica, Fondazione IRCCS Policlinico San Matteo, Università di Pavia, Viale Golgi 19, 27100, Pavia, Italy. a.disabatino@smatteo.pv.it.

[#] Contributed equally.

- PMID: **32651938**
- PMCID: PMC7348571
- DOI: 10.1007/s11739-020-02425-w

Free PMC article
Observational Study

Impact of COVID-19 on liver function: results from an internal medicine unit in Northern Italy

Marco Vincenzo Lenti et al. Intern Emerg Med. 2020 Nov.
Free PMC article
Show details
Intern Emerg Med
. 2020 Nov;15(8):1399-1407.
doi: 10.1007/s11739-020-02425-w. Epub 2020 Jul 10.

Authors

Marco Vincenzo Lenti [#] [1], Federica Borrelli de Andreis [#] [1], Ivan Pellegrino [1], Catherine Klersy [2], Stefania Merli [1], Emanuela Miceli [1], Nicola Aronico [1], Caterina Mengoli [1], Michele Di Stefano [1], Sara Cococcia [1], Giovanni Santacroce [1], Simone Soriano [1], Federica Melazzini [1], Mariangela Delliponti [1], Fausto Baldanti [3], Antonio Triarico [4], Gino Roberto Corazza [1], Massimo Pinzani [5], Antonio Di Sabatino [6] [7], Internal Medicine Covid-19 Team

Collaborators

- **Internal Medicine Covid-19 Team**: Gaetano Bergamaschi, Giampiera Bertolino, Silvia Codega, Filippo Costanzo, Roberto Cresci, Giuseppe Derosa, Francesco Falaschi, Carmine Iadarola, Elisabetta Lovati, Pietro Carlo Lucotti, Alessandra Martignoni, Amedeo Mugellini, Chiara Muggia, Patrizia Noris, Elisabetta Pagani, Ilaria Palumbo, Alessandro Pecci, Tiziano Perrone, Carla Pieresca, Paola Stefania Preti, Mariaconcetta Russo, Carmelo Sgarlata, Luisa Siciliani, Andrea Staniscia, Francesca Torello Vjera, Giovanna Achilli, Andrea Agostinelli, Valentina Antoci, Alessia Ballesio, Francesco Banfi, Chiara Barteselli, Irene Benedetti, Michele Brattoli, Francesca Calabretta, Ginevra Cambiè, Roberta Canta, Federico Conca, Luigi Coppola, Elisa Maria Cremonte, Gabriele Croce, Virginia Del Rio, Francesco Di Terlizzi, Maria Giovanna Ferrari, Sara Ferrari, Anna Fiengo, Tommaso Forni, Giulia Freddi, Chiara Frigerio, Federica Fumoso, Alessandra Fusco, Margherita Gabba, Matteo Garolfi, Antonella Gentile, Giulia Gori, Giacomo Grandi, Paolo Grimaldi, Alice Lampugnani, Francesco Lapia, Federica Lepore, Gianluca Lettieri, Jacopo Mambella, Chiara Mercanti, Francesco Mordà, Alba Nardone, Luca Pace, Lucia Padovini, Alessandro Parodi, Lavinia Pitotti, Margherita Reduzzi, Giovanni Rigano, Giorgio Rotola, Umberto Sabatini, Lucia Salvi, Giovanni Santacroce, Jessica Savioli, Simone Soriano, Carmine Spataro, Debora Stefani

Affiliations

- [1] Department of Internal Medicine, San Matteo Hospital Foundation, University of Pavia, Pavia, Italy.
- [2] Biometry and Clinical Epidemiology Service, San Matteo Hospital Foundation, Pavia, Italy.

- [3] Molecular Virology Unit, Microbiology and Virology Department, San Matteo Hospital Foundation, Pavia, Italy.
- [4] Chief Medical Direction, San Matteo Hospital Foundation, Pavia, Italy.
- [5] UCL Institute for Liver and Digestive Health and Sheila Sherlock Liver Unit, Royal Free Hospital and UCL, London, UK.
- [6] Department of Internal Medicine, San Matteo Hospital Foundation, University of Pavia, Pavia, Italy. a.disabatino@smatteo.pv.it.
- [7] Clinica Medica, Fondazione IRCCS Policlinico San Matteo, Università di Pavia, Viale Golgi 19, 27100, Pavia, Italy. a.disabatino@smatteo.pv.it.

[#] Contributed equally.

- PMID: **32651938**
- PMCID: PMC7348571
- DOI: 10.1007/s11739-020-02425-w

Abstract

Little is known regarding coronavirus disease 2019 (COVID-19) clinical spectrum in non-Asian populations. We herein describe the impact of COVID-19 on liver function in 100 COVID-19 consecutive patients (median age 70 years, range 25-97; 79 males) who were admitted to our internal medicine unit in March 2020. We retrospectively assessed liver function tests, taking into account demographic characteristics and clinical outcome. A patient was considered as having liver injury when alanine aminotransferase (ALT) was > 50 mU/ml, gamma-glutamyl transpeptidase (GGT) > 50 mU/ml, or total bilirubin > 1.1 mg/dl. Spearman correlation coefficient for laboratory data and bivariable analysis for mortality and/or need for intensive care were assessed. A minority of patients (18.6%) were obese, and most patients were non- or moderate-drinkers (88.5%). Liver function tests were altered in 62.4% of patients, and improved during follow-up. None of the seven patients with known chronic liver disease had liver decompensation. Only one patient developed acute liver failure. In patients with altered liver function tests, $PaO_2/FiO_2 < 200$ was associated with greater mortality and need for intensive care (HR 2.34, 95% CI 1.07-5.11, p = 0.033). To conclude, a high prevalence of altered liver function tests was noticed in Italian patients with COVID-19, and this was associated with worse outcomes when developing severe acute respiratory distress syndrome.

Keywords: Acute respiratory failure; Coronavirus; Hepatitis; Severe acute respiratory syndrome.

Conflict of interest statement

The authors declare that they have no conflict of interest.

- 37 references
- 1 figure

Supplementary info

Publication types, MeSH terms Expand

Publication types

- Observational Study

MeSH terms

- Adult
- Aged
- Aged, 80 and over
- COVID-19
- Coronavirus Infections / complications*
- Coronavirus Infections / epidemiology
- Coronavirus Infections / physiopathology
- Female
- Hospitalization / statistics & numerical data
- Humans
- Internal Medicine / methods
- Internal Medicine / trends
- Italy / epidemiology
- Liver / physiopathology
- Liver Failure / complications*
- Liver Failure / epidemiology
- Liver Failure / physiopathology
- Male
- Middle Aged
- Pandemics
- Patients' Rooms / organization & administration
- Pneumonia, Viral / complications*
- Pneumonia, Viral / epidemiology
- Pneumonia, Viral / physiopathology
- Retrospective Studies

Full text links

SpringerLink FULL-TEXT ARTICLE Springer Free PMC article
Proceed to details
Cite
Share
☐ 1,056
Observational Study
PLoS One
. 2020 Jun 24;15(6):e0235248.
doi: 10.1371/journal.pone.0235248. eCollection 2020.

Predictors of severe or lethal COVID-19, including Angiotensin Converting Enzyme inhibitors and Angiotensin II Receptor Blockers, in a sample of infected Italian citizens

Francesca Bravi [1], Maria Elena Flacco [2], Tiziano Carradori [1], Carlo Alberto Volta [1] [3], Giuseppe Cosenza [4], Aldo De Togni [4], Cecilia Acuti Martellucci [5], Giustino Parruti [6], Lorenzo Mantovani [7] [8], Lamberto Manzoli [1] [2]

Affiliations Expand

Affiliations

- [1] "Sant'Anna" University Hospital of Ferrara, Ferrara, Italy.
- [2] Department of Medical Sciences, University of Ferrara, Ferrara, Italy.
- [3] Department of Morphology, Surgery and Experimental Medicine, University of Ferrara, Ferrara, Italy.
- [4] Local Health Authority of Ferrara, Ferrara, Italy.
- [5] Department of Biomedical Sciences and Public Health, University of the Marche Region, Ancona, Italy.
- [6] Local Health Authority of Pescara, Cerveteri, Italy.
- [7] Center for Public Health Research, University of Milan-Bicocca, Milan, Italy.
- [8] IRCCS Multimedica, Sesto San Giovanni, Italy.

- PMID: **32579597**
- PMCID: PMC7314008
- DOI: 10.1371/journal.pone.0235248

Free PMC article
Observational Study

Predictors of severe or lethal COVID-19, including Angiotensin Converting Enzyme inhibitors and Angiotensin II Receptor Blockers, in a sample of infected Italian citizens

Francesca Bravi et al. PLoS One. 2020.
Free PMC article
Show details

PLoS One
. 2020 Jun 24;15(6):e0235248.
doi: 10.1371/journal.pone.0235248. eCollection 2020.

Authors

Francesca Bravi [1], Maria Elena Flacco [2], Tiziano Carradori [1], Carlo Alberto Volta [1, 3], Giuseppe Cosenza [4], Aldo De Togni [4], Cecilia Acuti Martellucci [5], Giustino Parruti [6], Lorenzo Mantovani [7, 8], Lamberto Manzoli [1, 2]

Affiliations

- [1] "Sant'Anna" University Hospital of Ferrara, Ferrara, Italy.
- [2] Department of Medical Sciences, University of Ferrara, Ferrara, Italy.
- [3] Department of Morphology, Surgery and Experimental Medicine, University of Ferrara, Ferrara, Italy.
- [4] Local Health Authority of Ferrara, Ferrara, Italy.
- [5] Department of Biomedical Sciences and Public Health, University of the Marche Region, Ancona, Italy.
- [6] Local Health Authority of Pescara, Cerveteri, Italy.
- [7] Center for Public Health Research, University of Milan-Bicocca, Milan, Italy.
- [8] IRCCS Multimedica, Sesto San Giovanni, Italy.

- PMID: **32579597**
- PMCID: PMC7314008
- DOI: 10.1371/journal.pone.0235248

Abstract

Aims: This retrospective case-control study was aimed at identifying potential independent predictors of severe/lethal COVID-19, including the treatment with Angiotensin-Converting Enzyme inhibitors (ACEi) and/or Angiotensin II Receptor Blockers (ARBs).

Methods and results: All adults with SARS-CoV-2 infection in two Italian provinces were followed for a median of 24 days. ARBs and/or ACEi treatments, and hypertension, diabetes, cancer, COPD, renal and major cardiovascular diseases (CVD) were extracted from clinical charts and electronic health records, up to two years before infection. The sample consisted of 1603 subjects (mean age 58.0y; 47.3% males): 454 (28.3%) had severe symptoms, 192 (12.0%) very severe or lethal disease (154 deaths; mean age 79.3 years; 70.8% hypertensive, 42.2% with CVD). The youngest deceased person aged 44 years. Among hypertensive subjects (n = 543), the proportion of those treated with ARBs or ACEi were 88.4%, 78.7% and 80.6% among patients with mild, severe and very severe/lethal disease, respectively. At multivariate analysis, no association was observed between therapy and disease severity (Adjusted OR for very severe/lethal COVID-19: 0.87; 95% CI: 0.50-1.49). Significant predictors of severe disease were older age (with AORs largely increasing after 70 years of age), male gender (AOR: 1.76; 1.40-2.23), diabetes (AOR: 1.52; 1.05-2.18), CVD (AOR: 1.88; 1.32-2.70) and COPD (AOR: 1.88; 1.11-3.20). Only gender, age and diabetes also predicted very severe/lethal disease.

Conclusion: No association was found between COVID-19 severity and treatment with ARBs and/or ACEi, supporting the recommendation to continue medication for all patients unless otherwise advised by their physicians.

Conflict of interest statement

The authors have declared that no competing interests exist.

- 44 references

Supplementary info

Publication types, MeSH terms, Substances, Grant support Expand

Publication types

- Observational Study

MeSH terms

- Angiotensin Receptor Antagonists / adverse effects
- Angiotensin Receptor Antagonists / therapeutic use*
- Angiotensin-Converting Enzyme Inhibitors / therapeutic use*
- Antihypertensive Agents / therapeutic use*
- Betacoronavirus / physiology
- COVID-19
- Case-Control Studies
- Coronavirus Infections / complications*
- Coronavirus Infections / drug therapy
- Coronavirus Infections / epidemiology
- Female
- Guidelines as Topic
- Humans
- Hypertension / drug therapy
- Italy / epidemiology
- Male
- Middle Aged
- Pandemics
- Pneumonia, Viral / complications*
- Pneumonia, Viral / drug therapy
- Pneumonia, Viral / epidemiology
- Retrospective Studies
- SARS-CoV-2
- Severity of Illness Index

Substances

- Angiotensin Receptor Antagonists
- Angiotensin-Converting Enzyme Inhibitors
- Antihypertensive Agents

Grant support

The authors received no specific funding for this work

Full text links

OPEN ACCESS TO FULL TEXT PLOS ONE Public Library of Science Free PMC article
Proceed to details
Cite
Share
☐ 1,057
Observational Study
Am J Respir Crit Care Med
. 2020 Jun 1;201(11):1372-1379.
doi: 10.1164/rccm.202003-0543OC.

Clinical Features of 85 Fatal Cases of COVID-19 from Wuhan. A Retrospective Observational Study

Yingzhen Du [1], Lei Tu [2], Pingjun Zhu [1], Mi Mu [1], Runsheng Wang [1], Pengcheng Yang [3] [4], Xi Wang [5], Chao Hu [6], Rongyu Ping [6], Peng Hu [6], Tianzhi Li [6], Feng Cao [6], Christopher Chang [7] [8], Qinyong Hu [3] [4], Yang Jin [9], Guogang Xu [6]
Affiliations Expand

Affiliations

- [1] Department of Respiratory Medicine, the Second Medical Center & National Clinical Research Center for Geriatric Diseases, Medical School of Chinese People's Liberation Army (PLA).
- [2] Division of Gastroenterology, Wuhan Union Hospital, Tongji Medical College and.
- [3] Cancer Center, Renmin Hospital of Wuhan University, Wuhan, China.
- [4] Wuhan Hannan Hospital, Wuhan, China.
- [5] Department of Cardiology, the Second Medical Center & National Clinical Research Center for Geriatric Diseases, and.
- [6] The Second Medical Center & National Clinical Research Center for Geriatric Diseases, Chinese PLA General Hospital, Beijing, China.

- [7] Division of Rheumatology, Allergy, and Clinical Immunology, University of California Davis, Davis, California; and.
- [8] Division of Pediatric Immunology and Allergy, Joe DiMaggio Children's Hospital, Hollywood, Florida.
- [9] Department of Respiratory and Critical Care Medicine, National Health Commission Key Laboratory of Pulmonary Diseases, Wuhan Union Hospital, Tongji Medical College, Huazhong University of Science & Technology, Wuhan, China.

- PMID: **32242738**
- PMCID: PMC7258652
- DOI: 10.1164/rccm.202003-0543OC

Free PMC article
Observational Study

Clinical Features of 85 Fatal Cases of COVID-19 from Wuhan. A Retrospective Observational Study

Yingzhen Du et al. Am J Respir Crit Care Med. 2020.
Free PMC article
Show details
Am J Respir Crit Care Med
. 2020 Jun 1;201(11):1372-1379.
doi: 10.1164/rccm.202003-0543OC.

Authors

Yingzhen Du [1], Lei Tu [2], Pingjun Zhu [1], Mi Mu [1], Runsheng Wang [1], Pengcheng Yang [3] [4], Xi Wang [5], Chao Hu [6], Rongyu Ping [6], Peng Hu [6], Tianzhi Li [6], Feng Cao [6], Christopher Chang [7] [8], Qinyong Hu [3] [4], Yang Jin [9], Guogang Xu [6]

Affiliations

- [1] Department of Respiratory Medicine, the Second Medical Center & National Clinical Research Center for Geriatric Diseases, Medical School of Chinese People's Liberation Army (PLA).
- [2] Division of Gastroenterology, Wuhan Union Hospital, Tongji Medical College and.
- [3] Cancer Center, Renmin Hospital of Wuhan University, Wuhan, China.
- [4] Wuhan Hannan Hospital, Wuhan, China.
- [5] Department of Cardiology, the Second Medical Center & National Clinical Research Center for Geriatric Diseases, and.
- [6] The Second Medical Center & National Clinical Research Center for Geriatric Diseases, Chinese PLA General Hospital, Beijing, China.
- [7] Division of Rheumatology, Allergy, and Clinical Immunology, University of California Davis, Davis, California; and.

- [8] Division of Pediatric Immunology and Allergy, Joe DiMaggio Children's Hospital, Hollywood, Florida.
- [9] Department of Respiratory and Critical Care Medicine, National Health Commission Key Laboratory of Pulmonary Diseases, Wuhan Union Hospital, Tongji Medical College, Huazhong University of Science & Technology, Wuhan, China.

- PMID: **32242738**
- PMCID: PMC7258652
- DOI: 10.1164/rccm.202003-0543OC

Abstract

Rationale: The global death toll from coronavirus disease (COVID-19) virus as of May 12, 2020, exceeds 286,000. The risk factors for death were attributed to advanced age and comorbidities but have not been accurately defined.**Objectives:** To report the clinical features of 85 fatal cases of COVID-19 in two hospitals in Wuhan.**Methods:** Medical records were collected of 85 fatal cases of COVID-19 between January 9, 2020, and February 15, 2020. Information recorded included medical history, exposure history, comorbidities, symptoms, signs, laboratory findings, computed tomographic scans, and clinical management.**Measurements and Main Results:** The median age of the patients was 65.8 years, and 72.9% were male. Common symptoms were fever (78 [91.8%]), shortness of breath (50 [58.8%]), fatigue (50 [58.8%]), and dyspnea (60 [70.6%]). Hypertension, diabetes, and coronary heart disease were the most common comorbidities. Notably, 81.2% of patients had very low eosinophil counts on admission. Complications included respiratory failure (80 [94.1%]), shock (69 [81.2%]), acute respiratory distress syndrome (63 [74.1%]), and arrhythmia (51 [60%]), among others. Most patients received antibiotic (77 [90.6%]), antiviral (78 [91.8%]), and glucocorticoid (65 [76.5%]) treatments. A total of 38 (44.7%) and 33 (38.8%) patients received intravenous immunoglobulin and IFN-α2b, respectively.**Conclusions:** In this depictive study of 85 fatal cases of COVID-19, most cases were males aged over 50 years with noncommunicable chronic diseases. The majority of the patients died of multiple organ failure. Early onset of shortness of breath may be used as an observational symptom for COVID-19 exacerbations. Eosinophilopenia may indicate a poor prognosis. A combination of antimicrobial drugs did not offer considerable benefit to the outcome of this group of patients.

Keywords: copathogen; coronavirus disease 2019; eosinophilopenia; fatal cases; severe acute respiratory syndrome coronavirus 2.

Comment in

- COVID-19: First Do No Harm.
  Waterer GW, Rello J, Wunderink RG. Waterer GW, et al. Am J Respir Crit Care Med. 2020 Jun 1;201(11):1324-1325. doi: 10.1164/rccm.202004-1153ED. Am J Respir Crit Care Med. 2020. PMID: 32311297 Free PMC article. No abstract available.
- Are Patients with COVID-19 Dying of or with Cardiac Injury?
  Tsolaki V, Zakynthinos GE. Tsolaki V, et al. Am J Respir Crit Care Med. 2020 Jul 15;202 (2):300-301. doi: 10.1164/rccm.202004-1083LE. Am J Respir Crit Care Med. 2020. PMID: 32432894 Free PMC article. No abstract available.

- 27 references
- 2 figures

Supplementary info

Publication types, MeSH terms Expand

Publication types

- Multicenter Study
- Observational Study
- Research Support, Non-U.S. Gov't

MeSH terms

- Adolescent
- Adult
- Aged
- Aged, 80 and over
- Betacoronavirus
- COVID-19
- China / epidemiology
- Comorbidity
- Coronary Disease / epidemiology
- Coronavirus Infections / mortality*
- Diabetes Mellitus / epidemiology
- Female
- Humans
- Hypertension / epidemiology
- Male
- Middle Aged
- Multiple Organ Failure / virology
- Pandemics
- Pneumonia, Viral / mortality*
- Retrospective Studies
- SARS-CoV-2
- Tomography, X-Ray Computed
- Young Adult

Full text links

AJRCCM Final Version Atypon Free PMC article
Proceed to details
Cite
Share

☐ 1,058
Observational Study
Rev Esp Cardiol (Engl Ed)
. 2020 Dec;73(12):994-1002.
doi: 10.1016/j.rec.2020.08.002. Epub 2020 Sep 8.

Impact of COVID-19 on ST-segment elevation myocardial infarction care. The Spanish experience

[Article in English, Spanish]

Oriol Rodríguez-Leor [1], Belén Cid-Álvarez [2], Armando Pérez de Prado [3], Xavier Rossello [4], Soledad Ojeda [5], Ana Serrador [6], Ramón López-Palop [7], Javier Martín-Moreiras [8], José Ramón Rumoroso [9], Ángel Cequier [10], Borja Ibáñez [11], Ignacio Cruz-González [8], Rafael Romaguera [10], Raúl Moreno [12], Working Group on the Infarct Code of the Interventional Cardiology Association of the Spanish Society of Cardiology Investigators; Manuel Villa [13], Rafael Ruíz-Salmerón [14], Francisco Molano [15], Carlos Sánchez [16], Erika Muñoz-García [17], Luís Íñigo [18], Juan Herrador [19], Antonio Gómez-Menchero [20], Antonio Gómez-Menchero [21], Juan Caballero [22], Soledad Ojeda [23], Mérida Cárdenas [24], Livia Gheorghe [25], Jesús Oneto [26], Francisco Morales [27], Félix Valencia [28], José Ramón Ruíz [29], José Antonio Diarte [30], Pablo Avanzas [31], Juan Rondán [32], Vicente Peral [33], Lucía Vera Pernasetti [34], Julio Hernández [35], Francisco Bosa [36], Pedro Luís Martín Lorenzo [37], Francisco Jiménez [38], José M de la Torre Hernández [39], Jesús Jiménez-Mazuecos [40], Fernando Lozano [41], José Moreu [42], Enrique Novo [43], Javier Robles [44], Javier Martín Moreiras [45], Felipe Fernández-Vázquez [46], Ignacio J Amat-Santos [47], Joan Antoni Gómez-Hospital [48], Joan García-Picart [49], Bruno García Del Blanco [50], Ander Regueiro [51], Xavier Carrillo-Suárez [52], Helena Tizón [53], Mohsen Mohandes [54], Juan Casanova [55], Víctor Agudelo-Montañez [56], Juan Francisco Muñoz [57], Juan Franco [58], Roberto Del Castillo [59], Pablo Salinas [60], Jaime Elizaga [61], Fernando Sarnago [62], Santiago Jiménez-Valero [63], Fernando Rivero [64], Juan Francisco Oteo [65], Eduardo Alegría-Barrero [66], Ángel Sánchez-Recalde [67], Valeriano Ruíz [68], Eduardo Pinar [69], Eduardo Pinar [70], Ana Planas [71], Bernabé López Ledesma [72], Alberto Berenguer [73], Agustín Fernández-Cisnal [74], Pablo Aguar [75], Francisco Pomar [76], Miguel Jerez [77], Francisco Torres [78], Ricardo García [79], Araceli Frutos [80], Juan Miguel Ruíz Nodar [81], Koldobika García [82], Roberto Sáez [83], Alfonso Torres [84], Miren Tellería [85], Mario Sadaba [86], José Ramón López Mínguez [87], Juan Carlos Rama Merchán [88], Javier Portales [89], Ramiro Trillo [90], Guillermo Aldama [91], Saleta Fernández [92], Melisa Santás [93], María Pilar Portero Pérez [94]
Affiliations Expand

Affiliations

- [1] Institut del Cor, Hospital Universitari Germans Trias i Pujol, Badalona, Barcelona, Spain; Centro de Investigación Biomédica en Red Enfermedades Cardiovaculares (CIBERCV), Instituto de Salud Carlos III, Madrid, Spain; Institut de Recerca en Ciències de la Salut Germans Trias i Pujol, Badalona, Barcelona, Spain. Electronic address: oriolrodriguez@gmail.com.

- [2] Servicio de Cardiología, Hospital Clínico de Santiago de Compostela, Santiago de Compostela, A Coruña, Spain.
- [3] Servicio de Cardiología, Hospital de León, León, España.
- [4] Centro Nacional de Investigaciones Cardiovasculares Carlos III (CNIC), Madrid, Spain; Servicio de Cardiología, Institut d'Investigació Sanitària de les Illes Balears (IdISBa), Hospital Universitari Son Espases, Palma de Mallorca, Islas Baleares, Spain; Centro de Investigación Biomédica en Red Enfermedades Cardiovaculares (CIBERCV), Instituto de Salud Carlos III, Madrid, Spain.
- [5] Servicio de Cardiología, Hospital Universitario Reina Sofía, Instituto Maimónides de Investigación Biomédica de Córdoba (IMIBIC), Universidad de Córdoba, Córdoba, Spain.
- [6] Servicio de Cardiología, Hospital Clínico de Valladolid, Valladolid, Spain; Centro de Investigación Biomédica en Red Enfermedades Cardiovaculares (CIBERCV), Instituto de Salud Carlos III, Madrid, Spain.
- [7] Servicio de Cardiología, Hospital Virgen de la Arrixaca, El Palmar, Murcia, Spain.
- [8] Servicio de Cardiología, Hospital Universitario de Salamanca, Instituto de Investigación Biomédica de Salamanca (IBSAL), Salamanca, Spain; Centro de Investigación Biomédica en Red Enfermedades Cardiovaculares (CIBERCV), Instituto de Salud Carlos III, Madrid, Spain.
- [9] Servicio de Cardiología, Hospital de Galdakao-Usansolo, Galdakao, Vizcaya, Spain.
- [10] Servicio de Cardiología, Hospital de Bellvitge-Instituto de Investigación Biomédica de Bellvitge (IDIBELL), Universitat de Barcelona, L'Hospitalet de Llobregat, Barcelona, Spain.
- [11] Centro de Investigación Biomédica en Red Enfermedades Cardiovaculares (CIBERCV), Instituto de Salud Carlos III, Madrid, Spain; Centro Nacional de Investigaciones Cardiovasculares Carlos III (CNIC), Madrid, Spain; Servicio de Cardiología, Hospital Universitario IIS-Fundación Jiménez Díaz, Madrid, Spain.
- [12] Servicio de Cardiología, Hospital Universitario La Paz, Madrid, Spain; Centro de Investigación Biomédica en Red Enfermedades Cardiovaculares (CIBERCV), Instituto de Salud Carlos III, Madrid, Spain.
- [13] Hospital Universitario Virgen del Rocío.
- [14] Hospital Universitario Virgen Macarena.
- [15] Hospital Universitario Virgen de Valme.
- [16] Hospital Universitario General de Málaga.
- [17] Hospital Universitario Virgen de la Victoria.
- [18] Hospital Costa del Sol.
- [19] Hospital Universitario de Jaén.
- [20] Hospital Universitario Juan Ramón Jiménez.
- [21] Eduardo Molina, Hospital Universitario Virgen de las Nieves.
- [22] Hospital Universitario San Cecilio.
- [23] Hospital Universitario Reina Sofía.
- [24] Hospital Punta de Europa.
- [25] Hospital Universitario Puerta del Mar.
- [26] Hospital Universitario de Jerez de la Frontera.
- [27] Hospital Universitario de Puerto Real.
- [28] Hospital Universitario Torrecárdenas.
- [29] Hospital Clínico Universitario Lozano Blesa.
- [30] Hospital Universitario Miguel Servet.
- [31] Hospital Universitario Central de Asturias.

- [32] Hospital Universitario de Cabueñes.
- [33] Hospital Universitari Son Espases.
- [34] Policlínica Nuestra Señora del Rosario.
- [35] Hospital Universitario Nuestra Señora de Candelaria.
- [36] Hospital Universitario de Canarias.
- [37] Hospital Universitario de Gran Canaria Doctor Negrín.
- [38] Hospital Insular de Gran Canaria.
- [39] Hospital Universitario Marqués de Valdecilla de Santander.
- [40] Hospital General Universitario de Albacete.
- [41] Hospital General Universitario de Ciudad Real.
- [42] Complejo Hospitalario de Toledo.
- [43] Hospital Universitario de Guadalajara.
- [44] Hospital Universitario de Burgos.
- [45] Hospital de Universitario de Salamanca.
- [46] Hospital de León.
- [47] Hospital Clínico Universitario de Valladolid, CIBERCV.
- [48] Hospital Universitari de Bellvitge.
- [49] Hospital de la Santa Creu i Sant Pau.
- [50] Hospital Universitari Vall d'Hebron.
- [51] Hospital Clínic de Barcelona.
- [52] Hospital Universitari Germans Trias i Pujol.
- [53] Hospital del Mar.
- [54] Hospital Universitari Joan XXIII.
- [55] Hospital Universitari Arnau de Vilanova.
- [56] Hospital Universitari de Girona Josep Trueta.
- [57] Hospital Universitari Mútua de Tarrassa.
- [58] Hospital Universitario Fundación Jiménez Díaz.
- [59] Hospital Universitario Fundació Alcorcón.
- [60] Hospital Clínico San Carlos y Hospital Príncipe de Asturias.
- [61] Hospital General Universitario Gregorio Marañón.
- [62] Hospital Universitario 12 de Octubre.
- [63] Hospital Universitario La Paz.
- [64] Hospital Universitario de La Princesa.
- [65] Hospital Universitario Puerta de Hierro Majadahonda.
- [66] Hospital Univesitario de Torrejó-Universidad Francisco de Vitoria.
- [67] Hospital Ramón y Cajal.
- [68] Complejo Hospitalario de Navarra.
- [69] Hospital Virgen de la Arrixaca.
- [70] Luciano Consuegra-Sánchez, Hospital Universitario Santa Lucía de Cartagena.
- [71] Hospital General Universitario de Castellón.
- [72] Hospital Universitario y Politécnico La Fe.
- [73] Hospital General Universitario de Valencia.
- [74] Hospital Clínico Universitario de Valencia.
- [75] Hospital Universitario Dr. Peset.
- [76] Hospital Universitario de la Ribera.
- [77] Hospital de Manises.

- [78] Hospitales de Torrevieja-Elche-Vinalopó.
- [79] Hospital General Universitario de Elche.
- [80] Hospital General Universitario de San Juan de Alicante.
- [81] Hospital General Universitario de Alicante.
- [82] Hospital Universitario de Cruces.
- [83] Hospital de Basurto.
- [84] Hospital Universitario Araba.
- [85] Hospital Universitario Donostia.
- [86] Hospital de Galdakao-Usansolo.
- [87] Complejo Hospitalario Universitario de Badajoz.
- [88] Hospital de Mérida.
- [89] Complejo Hospitalario Universitario de Cáceres.
- [90] Hospital Clínico Universitario Santiago de Compostela.
- [91] Complejo Hospitalario Universitario de A Coruña.
- [92] Complejo Hospitalario Universitario de Vigo.
- [93] Hospital Universitario Lucus Augusti.
- [94] Hospital San Pedro de Logroño.

- PMID: **32917566**
- PMCID: PMC7834732
- DOI: 10.1016/j.rec.2020.08.002

Free PMC article
Observational Study

Impact of COVID-19 on ST-segment elevation myocardial infarction care. The Spanish experience

[Article in English, Spanish]
Oriol Rodríguez-Leor et al. Rev Esp Cardiol (Engl Ed). 2020 Dec.
Free PMC article
Show details
Rev Esp Cardiol (Engl Ed)
. 2020 Dec;73(12):994-1002.
doi: 10.1016/j.rec.2020.08.002. Epub 2020 Sep 8.

Authors

Oriol Rodríguez-Leor [1], Belén Cid-Álvarez [2], Armando Pérez de Prado [3], Xavier Rossello [4], Soledad Ojeda [5], Ana Serrador [6], Ramón López-Palop [7], Javier Martín-Moreiras [8], José Ramón Rumoroso [9], Ángel Cequier [10], Borja Ibáñez [11], Ignacio Cruz-González [8], Rafael Romaguera [10], Raúl Moreno [12], Working Group on the Infarct Code of the Interventional Cardiology Association of the Spanish Society of Cardiology Investigators; Manuel Villa [13], Rafael Ruíz-Salmerón [14], Francisco Molano [15], Carlos Sánchez [16], Erika Muñoz-García

[17], Luís Íñigo [18], Juan Herrador [19], Antonio Gómez-Menchero [20], Antonio Gómez-Menchero [21], Juan Caballero [22], Soledad Ojeda [23], Mérida Cárdenas [24], Livia Gheorghe [25], Jesús Oneto [26], Francisco Morales [27], Félix Valencia [28], José Ramón Ruíz [29], José Antonio Diarte [30], Pablo Avanzas [31], Juan Rondán [32], Vicente Peral [33], Lucía Vera Pernasetti [34], Julio Hernández [35], Francisco Bosa [36], Pedro Luís Martín Lorenzo [37], Francisco Jiménez [38], José M de la Torre Hernández [39], Jesús Jiménez-Mazuecos [40], Fernando Lozano [41], José Moreu [42], Enrique Novo [43], Javier Robles [44], Javier Martín Moreiras [45], Felipe Fernández-Vázquez [46], Ignacio J Amat-Santos [47], Joan Antoni Gómez-Hospital [48], Joan García-Picart [49], Bruno García Del Blanco [50], Ander Regueiro [51], Xavier Carrillo-Suárez [52], Helena Tizón [53], Mohsen Mohandes [54], Juan Casanova [55], Víctor Agudelo-Montañez [56], Juan Francisco Muñoz [57], Juan Franco [58], Roberto Del Castillo [59], Pablo Salinas [60], Jaime Elizaga [61], Fernando Sarnago [62], Santiago Jiménez-Valero [63], Fernando Rivero [64], Juan Francisco Oteo [65], Eduardo Alegría-Barrero [66], Ángel Sánchez-Recalde [67], Valeriano Ruíz [68], Eduardo Pinar [69], Eduardo Pinar [70], Ana Planas [71], Bernabé López Ledesma [72], Alberto Berenguer [73], Agustín Fernández-Cisnal [74], Pablo Aguar [75], Francisco Pomar [76], Miguel Jerez [77], Francisco Torres [78], Ricardo García [79], Araceli Frutos [80], Juan Miguel Ruíz Nodar [81], Koldobika García [82], Roberto Sáez [83], Alfonso Torres [84], Miren Tellería [85], Mario Sadaba [86], José Ramón López Mínguez [87], Juan Carlos Rama Merchán [88], Javier Portales [89], Ramiro Trillo [90], Guillermo Aldama [91], Saleta Fernández [92], Melisa Santás [93], María Pilar Portero Pérez [94]

Affiliations

- [1] Institut del Cor, Hospital Universitari Germans Trias i Pujol, Badalona, Barcelona, Spain; Centro de Investigación Biomédica en Red Enfermedades Cardiovaculares (CIBERCV), Instituto de Salud Carlos III, Madrid, Spain; Institut de Recerca en Ciències de la Salut Germans Trias i Pujol, Badalona, Barcelona, Spain. Electronic address: oriolrodriguez@gmail.com.
- [2] Servicio de Cardiología, Hospital Clínico de Santiago de Compostela, Santiago de Compostela, A Coruña, Spain.
- [3] Servicio de Cardiología, Hospital de León, León, España.
- [4] Centro Nacional de Investigaciones Cardiovasculares Carlos III (CNIC), Madrid, Spain; Servicio de Cardiología, Institut d'Investigació Sanitària de les Illes Balears (IdISBa), Hospital Universitari Son Espases, Palma de Mallorca, Islas Baleares, Spain; Centro de Investigación Biomédica en Red Enfermedades Cardiovaculares (CIBERCV), Instituto de Salud Carlos III, Madrid, Spain.
- [5] Servicio de Cardiología, Hospital Universitario Reina Sofía, Instituto Maimónides de Investigación Biomédica de Córdoba (IMIBIC), Universidad de Córdoba, Córdoba, Spain.
- [6] Servicio de Cardiología, Hospital Clínico de Valladolid, Valladolid, Spain; Centro de Investigación Biomédica en Red Enfermedades Cardiovaculares (CIBERCV), Instituto de Salud Carlos III, Madrid, Spain.
- [7] Servicio de Cardiología, Hospital Virgen de la Arrixaca, El Palmar, Murcia, Spain.
- [8] Servicio de Cardiología, Hospital Universitario de Salamanca, Instituto de Investigación Biomédica de Salamanca (IBSAL), Salamanca, Spain; Centro de Investigación Biomédica en Red Enfermedades Cardiovaculares (CIBERCV), Instituto de Salud Carlos III, Madrid, Spain.
- [9] Servicio de Cardiología, Hospital de Galdakao-Usansolo, Galdakao, Vizcaya, Spain.

- [10] Servicio de Cardiología, Hospital de Bellvitge-Instituto de Investigación Biomédica de Bellvitge (IDIBELL), Universitat de Barcelona, L'Hospitalet de Llobregat, Barcelona, Spain.
- [11] Centro de Investigación Biomédica en Red Enfermedades Cardiovaculares (CIBERCV), Instituto de Salud Carlos III, Madrid, Spain; Centro Nacional de Investigaciones Cardiovasculares Carlos III (CNIC), Madrid, Spain; Servicio de Cardiología, Hospital Universitario IIS-Fundación Jiménez Díaz, Madrid, Spain.
- [12] Servicio de Cardiología, Hospital Universitario La Paz, Madrid, Spain; Centro de Investigación Biomédica en Red Enfermedades Cardiovaculares (CIBERCV), Instituto de Salud Carlos III, Madrid, Spain.
- [13] Hospital Universitario Virgen del Rocío.
- [14] Hospital Universitario Virgen Macarena.
- [15] Hospital Universitario Virgen de Valme.
- [16] Hospital Universitario General de Málaga.
- [17] Hospital Universitario Virgen de la Victoria.
- [18] Hospital Costa del Sol.
- [19] Hospital Universitario de Jaén.
- [20] Hospital Universitario Juan Ramón Jiménez.
- [21] Eduardo Molina, Hospital Universitario Virgen de las Nieves.
- [22] Hospital Universitario San Cecilio.
- [23] Hospital Universitario Reina Sofía.
- [24] Hospital Punta de Europa.
- [25] Hospital Universitario Puerta del Mar.
- [26] Hospital Universitario de Jerez de la Frontera.
- [27] Hospital Universitario de Puerto Real.
- [28] Hospital Universitario Torrecárdenas.
- [29] Hospital Clínico Universitario Lozano Blesa.
- [30] Hospital Universitario Miguel Servet.
- [31] Hospital Universitario Central de Asturias.
- [32] Hospital Universitario de Cabueñes.
- [33] Hospital Universitari Son Espases.
- [34] Policlínica Nuestra Señora del Rosario.
- [35] Hospital Universitario Nuestra Señora de Candelaria.
- [36] Hospital Universitario de Canarias.
- [37] Hospital Universitario de Gran Canaria Doctor Negrín.
- [38] Hospital Insular de Gran Canaria.
- [39] Hospital Universitario Marqués de Valdecilla de Santander.
- [40] Hospital General Universitario de Albacete.
- [41] Hospital General Universitario de Ciudad Real.
- [42] Complejo Hospitalario de Toledo.
- [43] Hospital Universitario de Guadalajara.
- [44] Hospital Universitario de Burgos.
- [45] Hospital de Universitario de Salamanca.
- [46] Hospital de León.
- [47] Hospital Clínico Universitario de Valladolid, CIBERCV.
- [48] Hospital Universitari de Bellvitge.
- [49] Hospital de la Santa Creu i Sant Pau.

- [50] Hospital Universitari Vall d'Hebron.
- [51] Hospital Clínic de Barcelona.
- [52] Hospital Universitari Germans Trias i Pujol.
- [53] Hospital del Mar.
- [54] Hospital Universitari Joan XXIII.
- [55] Hospital Universitari Arnau de Vilanova.
- [56] Hospital Universitari de Girona Josep Trueta.
- [57] Hospital Universitari Mútua de Tarrassa.
- [58] Hospital Universitario Fundación Jiménez Díaz.
- [59] Hospital Universitario Fundació Alcorcón.
- [60] Hospital Clínico San Carlos y Hospital Príncipe de Asturias.
- [61] Hospital General Universitario Gregorio Marañón.
- [62] Hospital Universitario 12 de Octubre.
- [63] Hospital Universitario La Paz.
- [64] Hospital Universitario de La Princesa.
- [65] Hospital Universitario Puerta de Hierro Majadahonda.
- [66] Hospital Univesitario de Torrejó-Universidad Francisco de Vitoria.
- [67] Hospital Ramón y Cajal.
- [68] Complejo Hospitalario de Navarra.
- [69] Hospital Virgen de la Arrixaca.
- [70] Luciano Consuegra-Sánchez, Hospital Universitario Santa Lucía de Cartagena.
- [71] Hospital General Universitario de Castellón.
- [72] Hospital Universitario y Politécnico La Fe.
- [73] Hospital General Universitario de Valencia.
- [74] Hospital Clínico Universitario de Valencia.
- [75] Hospital Universitario Dr. Peset.
- [76] Hospital Universitario de la Ribera.
- [77] Hospital de Manises.
- [78] Hospitales de Torrevieja-Elche-Vinalopó.
- [79] Hospital General Universitario de Elche.
- [80] Hospital General Universitario de San Juan de Alicante.
- [81] Hospital General Universitario de Alicante.
- [82] Hospital Universitario de Cruces.
- [83] Hospital de Basurto.
- [84] Hospital Universitario Araba.
- [85] Hospital Universitario Donostia.
- [86] Hospital de Galdakao-Usansolo.
- [87] Complejo Hospitalario Universitario de Badajoz.
- [88] Hospital de Mérida.
- [89] Complejo Hospitalario Universitario de Cáceres.
- [90] Hospital Clínico Universitario Santiago de Compostela.
- [91] Complejo Hospitalario Universitario de A Coruña.
- [92] Complejo Hospitalario Universitario de Vigo.
- [93] Hospital Universitario Lucus Augusti.
- [94] Hospital San Pedro de Logroño.

- PMID: **32917566**
- PMCID: PMC7834732
- DOI: 10.1016/j.rec.2020.08.002

Abstract
in English, Spanish

Introduction and objectives: The COVID-19 outbreak has had an unclear impact on the treatment and outcomes of patients with ST-segment elevation myocardial infarction (STEMI). The aim of this study was to assess changes in STEMI management during the COVID-19 outbreak.

Methods: Using a multicenter, nationwide, retrospective, observational registry of consecutive patients who were managed in 75 specific STEMI care centers in Spain, we compared patient and procedural characteristics and in-hospital outcomes in 2 different cohorts with 30-day follow-up according to whether the patients had been treated before or after COVID-19.

Results: Suspected STEMI patients treated in STEMI networks decreased by 27.6% and patients with confirmed STEMI fell from 1305 to 1009 (22.7%). There were no differences in reperfusion strategy (> 94% treated with primary percutaneous coronary intervention in both cohorts). Patients treated with primary percutaneous coronary intervention during the COVID-19 outbreak had a longer ischemic time (233 [150-375] vs 200 [140-332] minutes, P<.001) but showed no differences in the time from first medical contact to reperfusion. In-hospital mortality was higher during COVID-19 (7.5% vs 5.1%; unadjusted OR, 1.50; 95%CI, 1.07-2.11; P <.001); this association remained after adjustment for confounders (risk-adjusted OR, 1.88; 95%CI, 1.12-3.14; P=.017). In the 2020 cohort, there was a 6.3% incidence of confirmed SARS-CoV-2 infection during hospitalization.

Conclusions: The number of STEMI patients treated during the current COVID-19 outbreak fell vs the previous year and there was an increase in the median time from symptom onset to reperfusion and a significant 2-fold increase in the rate of in-hospital mortality. No changes in reperfusion strategy were detected, with primary percutaneous coronary intervention performed for the vast majority of patients. The co-existence of STEMI and SARS-CoV-2 infection was relatively infrequent.

Introducción y objetivos: El impacto del brote de COVID-19 en el tratamiento del infarto agudo de miocardio con elevación del segmento ST (IAMCEST) no está claro. El objetivo de este estudio es evaluar los cambios en el tratamiento del IAMCEST durante el brote de COVID-19.

Métodos: Se utilizó un registro multicéntrico, nacional, retrospectivo y observacional de pacientes consecutivos atendidos en 75 centros, se compararon las características de los pacientes y de los procedimientos y los resultados hospitalarios en 2 cohortes según se los hubiera tratado antes o durante la COVID-19.

Resultados: Los casos con sospecha de IAMCEST disminuyeron el 27,6% y los pacientes con IAMCEST confirmado se redujeron de 1.305 a 1.009 (22,7%). No hubo diferencias en la estrategia de reperfusión (más del 94% tratados con angioplastia primaria). El tiempo de isquemia fue más largo durante la COVID-19 (233 [150-375] frente a 200 [140-332] min; p < 0,001), sin diferencias en el tiempo primer contacto médico-reperfusión. La mortalidad hospitalaria fue mayor durante la COVID-19 (el 7,5 frente al 5,1%; OR bruta = 1,50; IC95%, 1,07-2,11; p < 0,001); esta asociación se mantuvo tras ajustar por factores de confusión (OR ajustada = 1,88;

IC95%, 1,12-3,14; p = 0,017). La incidencia de infección confirmada por SARS-CoV-2 fue del 6,3%.

Conclusiones: El brote de COVID-19 ha implicado una disminución en el número de pacientes con IAMCEST, un aumento del tiempo entre el inicio de los síntomas y la reperfusión y un aumento en la mortalidad hospitalaria. No se han detectado cambios en la estrategia de reperfusión. La combinación de infección por SARS-CoV-2 e IAMCEST fue relativamente infrecuente.

Keywords: Angioplastia primaria; COVID-19; IAMCEST; Primary angioplasty; Red de atención al infarto; STEMI; STEMI network.

Copyright © 2020 Sociedad Española de Cardiología. Published by Elsevier España, S.L.U. All rights reserved.

Comment in

- Myocardial infarction in times of COVID-19.
  Ibáñez B. Ibáñez B. Rev Esp Cardiol (Engl Ed). 2020 Dec;73(12):975-977. doi: 10.1016/j.rec.2020.09.023. Epub 2020 Oct 31. Rev Esp Cardiol (Engl Ed). 2020. PMID: 33139226 Free PMC article. No abstract available.

- 26 references
- 4 figures

Supplementary info

Publication types, MeSH terms Expand

Publication types

- Multicenter Study
- Observational Study

MeSH terms

- COVID-19 / epidemiology*
- Comorbidity
- Disease Management*
- Female
- Follow-Up Studies
- Hospital Mortality / trends
- Humans
- Male
- Middle Aged
- Pandemics*
- Percutaneous Coronary Intervention / methods*

- Registries*
- Retrospective Studies
- SARS-CoV-2*
- ST Elevation Myocardial Infarction / epidemiology
- ST Elevation Myocardial Infarction / surgery*
- Spain / epidemiology

Full text links

FULL TEXT AT REVISTA ESPAÑOLA DE CARDIOLOGIA Ediciones Doyma, S.L. Free PMC article
Proceed to details
Cite
Share
1,059
Observational Study
JAMA Neurol
. 2020 Sep 1;77(9):1079-1088.
doi: 10.1001/jamaneurol.2020.2581.

Clinical Characteristics and Outcomes in Patients With Coronavirus Disease 2019 and Multiple Sclerosis

Céline Louapre [1], Nicolas Collongues [2], Bruno Stankoff [1] [3], Claire Giannesini [3], Caroline Papeix [1], Caroline Bensa [4], Romain Deschamps [4], Alain Créange [5], Abir Wahab [5], Jean Pelletier [6], Olivier Heinzlef [7], Pierre Labauge [8], Laurent Guilloton [9], Guido Ahle [10], Mathilde Goudot [11], Kevin Bigaut [2], David-Axel Laplaud [12], Sandra Vukusic [13], Catherine Lubetzki [1], Jérôme De Sèze [2], Covisep investigators; Fayçal Derouiche, Ayman Tourbah, Guillaume Mathey, Marie Théaudin, François Sellal, Marie-Hélène Dugay, Helene Zéphir, Patrick Vermersch, Françoise Durand-Dubief, Romain Françoise, Géraldine Androdias-Condemine, Julie Pique, Pékès Codjia, Caroline Tilikete, Véronique Marcaud, Christine Lebrun-Frenay, Mikael Cohen, Aurelian Ungureanu, Elisabeth Maillart, Ysoline Beigneux, Thomas Roux, Jean-Christophe Corvol, Amandine Bordet, Yanica Mathieu, Frédérique Le Breton, Dalia Dimitri Boulos, Olivier Gout, Antoine Guéguen, Antoine Moulignier, Marine Boudot, Audrey Chardain, Sarah Coulette, Eric Manchon, Samar S. Ayache, Thibault Moreau, Pierre-Yves Garcia, Deiva Kumaran, Giovanni Castelnovo, Eric Thouvenot, Frederic Taithe, Julien Poupart, Arnaud Kwiatkowski, Gilles Defer, Nathalie Derache, Pierre Branger, Damien Biotti, Jonathan Ciron, Christine Clerc, Mathieu Vaillant, Laurent Magy, Alexis Montcuquet, Philippe Kerschen, Marc Coustans, Anne-Marie Guennoc, Bruno Brochet, Jean-Christophe Ouallet, Aurélie Ruet, Cécile Dulau, Sandrine Wiertlewski, Eric Berger, Dan Buch, Bertrand Bourre, Maud Pallix-Guiot, Aude Maurousset, Bertrand Audoin, Audrey Rico, Adil Maarouf, Gilles Edan, Jérémie Papassin, Dorothée Videt
Affiliations Expand

Affiliations

- [1] Institut du Cerveau et de la Moelle Épinière, Assistance Publique des Hôpitaux de Paris, Hôpital de la Pitié Salpêtrière, Sorbonne Université, Institut National de la Santé et de la Recherche Médicale, Centre National de la Recherche Scientifique, Clinical Investigation Center Neuroscience, Paris, France.
- [2] Service de Neurologie, Clinical Investigation Center Institut National de la Santé et de la Recherche Médicale 1434, Centre Hospitalier Universitaire de Strasbourg, Strasbourg, France.
- [3] Hôpital St Antoine, Assistance Publique des Hôpitaux de Paris, Paris, France.
- [4] Département de Neurologie, Hôpital Fondation Adolphe de Rothschild, Paris, France.
- [5] Service de Neurologie, Centre de Ressources et de Compétences-Sclérose en Plaques, Assistance Publique des Hôpitaux de Paris, Groupe Hospitalier Henri Mondor, Université Paris-Est Créteil, Créteil, France.
- [6] Service de Neurologie, Pôle de Neurosciences Cliniques, Assistance Publique-Hôpitaux de Marseille, Hôpital de la Timone, Aix Marseille Université, Marseille, France.
- [7] Département de Neurologie, Centre Hospitalier de Poissy, St Germain, France.
- [8] Département de Neurologie, Centre Hospitalier Universitaire de Montpellier, Montpellier, France.
- [9] Association des Neurologues Libéraux de Langue Française, Bergerac, France.
- [10] Département de Neurologie, Hôpitaux Civils de Colmar, Colmar, France.
- [11] Service de Neurologie, Groupe Hospitalier de la Region de Mulhouse, Mulhouse, France.
- [12] Centre de Recherche en Transplantation et Immunologie-Institut National de la Santé et de la Recherche Médicale U1064, Service Neurologie, Clinical Investigation Center 1413, Centre Hospitalier Universitaire Nantes, Nantes, France.
- [13] Hôpital Neurologique Pierre Wertheimer, Hospices Civils de Lyon, Lyon/Bron, France.

- PMID: **32589189**
- PMCID: PMC7320356
- DOI: 10.1001/jamaneurol.2020.2581

Free PMC article
Observational Study

Clinical Characteristics and Outcomes in Patients With Coronavirus Disease 2019 and Multiple Sclerosis

Céline Louapre et al. JAMA Neurol. 2020.
Free PMC article
Show details
JAMA Neurol

. 2020 Sep 1;77(9):1079-1088.
doi: 10.1001/jamaneurol.2020.2581.

Authors

Céline Louapre [1], Nicolas Collongues [2], Bruno Stankoff [1] [3], Claire Giannesini [3], Caroline Papeix [1], Caroline Bensa [4], Romain Deschamps [4], Alain Créange [5], Abir Wahab [5], Jean Pelletier [6], Olivier Heinzlef [7], Pierre Labauge [8], Laurent Guilloton [9], Guido Ahle [10], Mathilde Goudot [11], Kevin Bigaut [2], David-Axel Laplaud [12], Sandra Vukusic [13], Catherine Lubetzki [1], Jérôme De Sèze [2], Covisep investigators; Fayçal Derouiche, Ayman Tourbah, Guillaume Mathey, Marie Théaudin, François Sellal, Marie-Hélène Dugay, Helene Zéphir, Patrick Vermersch, Françoise Durand-Dubief, Romain Françoise, Géraldine Androdias-Condemine, Julie Pique, Pékès Codjia, Caroline Tilikete, Véronique Marcaud, Christine Lebrun-Frenay, Mikael Cohen, Aurelian Ungureanu, Elisabeth Maillart, Ysoline Beigneux, Thomas Roux, Jean-Christophe Corvol, Amandine Bordet, Yanica Mathieu, Frédérique Le Breton, Dalia Dimitri Boulos, Olivier Gout, Antoine Guéguen, Antoine Moulignier, Marine Boudot, Audrey Chardain, Sarah Coulette, Eric Manchon, Samar S. Ayache, Thibault Moreau, Pierre-Yves Garcia, Deiva Kumaran, Giovanni Castelnovo, Eric Thouvenot, Frederic Taithe, Julien Poupart, Arnaud Kwiatkowski, Gilles Defer, Nathalie Derache, Pierre Branger, Damien Biotti, Jonathan Ciron, Christine Clerc, Mathieu Vaillant, Laurent Magy, Alexis Montcuquet, Philippe Kerschen, Marc Coustans, Anne-Marie Guennoc, Bruno Brochet, Jean-Christophe Ouallet, Aurélie Ruet, Cécile Dulau, Sandrine Wiertlewski, Eric Berger, Dan Buch, Bertrand Bourre, Maud Pallix-Guiot, Aude Maurousset, Bertrand Audoin, Audrey Rico, Adil Maarouf, Gilles Edan, Jérémie Papassin, Dorothée Videt

Affiliations

- [1] Institut du Cerveau et de la Moelle Épinière, Assistance Publique des Hôpitaux de Paris, Hôpital de la Pitié Salpêtrière, Sorbonne Université, Institut National de la Santé et de la Recherche Médicale, Centre National de la Recherche Scientifique, Clinical Investigation Center Neuroscience, Paris, France.
- [2] Service de Neurologie, Clinical Investigation Center Institut National de la Santé et de la Recherche Médicale 1434, Centre Hospitalier Universitaire de Strasbourg, Strasbourg, France.
- [3] Hôpital St Antoine, Assistance Publique des Hôpitaux de Paris, Paris, France.
- [4] Département de Neurologie, Hôpital Fondation Adolphe de Rothschild, Paris, France.
- [5] Service de Neurologie, Centre de Ressources et de Compétences-Sclérose en Plaques, Assistance Publique des Hôpitaux de Paris, Groupe Hospitalier Henri Mondor, Université Paris-Est Créteil, Créteil, France.
- [6] Service de Neurologie, Pôle de Neurosciences Cliniques, Assistance Publique-Hôpitaux de Marseille, Hôpital de la Timone, Aix Marseille Université, Marseille, France.
- [7] Département de Neurologie, Centre Hospitalier de Poissy, St Germain, France.
- [8] Département de Neurologie, Centre Hospitalier Universitaire de Montpellier, Montpellier, France.
- [9] Association des Neurologues Libéraux de Langue Française, Bergerac, France.
- [10] Département de Neurologie, Hôpitaux Civils de Colmar, Colmar, France.
- [11] Service de Neurologie, Groupe Hospitalier de la Region de Mulhouse, Mulhouse, France.
- [12] Centre de Recherche en Transplantation et Immunologie-Institut National de la Santé et de la Recherche Médicale U1064, Service Neurologie, Clinical Investigation Center 1413, Centre Hospitalier Universitaire Nantes, Nantes, France.
- [13] Hôpital Neurologique Pierre Wertheimer, Hospices Civils de Lyon, Lyon/Bron, France.

- PMID: **32589189**
- PMCID: PMC7320356
- DOI: 10.1001/jamaneurol.2020.2581

Abstract

Importance: Risk factors associated with the severity of coronavirus disease 2019 (COVID-19) in patients with multiple sclerosis (MS) are unknown. Disease-modifying therapies (DMTs) may modify the risk of developing a severe COVID-19 infection, beside identified risk factors such as age and comorbidities.

Objective: To describe the clinical characteristics and outcomes in patients with MS and COVID-19 and identify factors associated with COVID-19 severity.

Design, setting, and participants: The Covisep registry is a multicenter, retrospective, observational cohort study conducted in MS expert centers and general hospitals and with neurologists collaborating with MS expert centers and members of the Société Francophone de la Sclérose en Plaques. The study included patients with MS presenting with a confirmed or highly suspected diagnosis of COVID-19 between March 1, 2020, and May 21, 2020.

Exposures: COVID-19 diagnosed with a polymerase chain reaction test on a nasopharyngeal swab, thoracic computed tomography, or typical symptoms.

Main outcomes and measures: The main outcome was COVID-19 severity assessed on a 7-point ordinal scale (ranging from 1 [not hospitalized with no limitations on activities] to 7 [death]) with a cutoff at 3 (hospitalized and not requiring supplemental oxygen). We collected demographics, neurological history, Expanded Disability Severity Scale score (EDSS; ranging from 0 to 10, with cutoffs at 3 and 6), comorbidities, COVID-19 characteristics, and outcomes. Univariate and multivariate logistic regression models were used to estimate the association of collected variables with COVID-19 outcomes.

Results: A total of 347 patients (mean [SD] age, 44.6 [12.8] years, 249 women; mean [SD] disease duration, 13.5 [10.0] years) were analyzed. Seventy-three patients (21.0%) had a COVID-19 severity score of 3 or more, and 12 patients (3.5%) died of COVID-19. The median EDSS was 2.0 (range, 0-9.5), and 284 patients (81.8%) were receiving DMT. There was a higher proportion of patients with a COVID-19 severity score of 3 or more among patients with no DMT relative to patients receiving DMTs (46.0% vs 15.5%; $P < .001$). Multivariate logistic regression models determined that age (odds ratio per 10 years: 1.9 [95% CI, 1.4-2.5]), EDSS (OR for EDSS ≥6, 6.3 [95% CI. 2.8-14.4]), and obesity (OR, 3.0 [95% CI, 1.0-8.7]) were independent risk factors for a COVID-19 severity score of 3 or more (indicating hospitalization or higher severity). The EDSS was associated with the highest variability of COVID-19 severe outcome (R2, 0.2), followed by age (R2, 0.06) and obesity (R2, 0.01).

Conclusions and relevance: In this registry-based cohort study of patients with MS, age, EDSS, and obesity were independent risk factors for severe COVID-19; there was no association found between DMTs exposure and COVID-19 severity. The identification of these risk factors should provide the rationale for an individual strategy regarding clinical management of patients with MS during the COVID-19 pandemic.

Conflict of interest statement

Conflict of Interest Disclosures: Dr Louapre has received consulting or travel fees from Biogen, Novartis, Roche, Sanofi, Teva and Merck Serono, outside the submitted work. Dr Collongues serves on scientific advisory boards for and has received honoraria from Biogen Idec, Merck Serono, Sanofi-Genzyme, Bayer Schering Pharma and Alexion Pharmaceutical, and personal fees and nonfinancial support from Roche, Biogen, Novartis, Sanofi, and MedDay outside the submitted work outside the submitted work. Dr Stankoff has received fees for advisory boards and

lectures from Genzyme, Novartis, Teva, and Biogen and research support from Roche, Sanofi-Genzyme, and Merck-Serono outside the submitted work. Dr Papeix has received consulting and lecturing fees and travel grants from Biogen, Genzyme, Novartis, Merck, Roche, Sanofi, and Teva Pharma outside the submitted work. Dr Bensa has received consulting fees for advisory boards from Biogen, Novartis, Roche, and Sanofi Genzyme outside the submitted work. Dr Deschamps has received travel grants from Biogen outside the submitted work. Dr Créange has received departmental research grants from Biogen, GeNeuro, MedDay, Novartis, Octapharma, and Roche; completed expert testimony with Biogen, Novartis, GeNeuro, and Roche; and received grants and personal fees from MedDay outside the submitted work. Dr Wahab has received expert testimony fees from Sanofi-Genzyme and Roche and travel grants from Biogen and Roche outside the submitted work. Dr Pelletier has received consulting or travel fees from Biogen, Novartis, Roche, Sanofi, Teva, MedDay, and Merck Serono outside the submitted work. Dr Heinzlef has received consulting or travel fees from Biogen, Novartis, Roche, Sanofi, Teva, Merck Serono, and MedDay outside the submitted work. Dr Guilloton has received consulting or travel fees from Biogen, Merck Serono, Novartis, Sanofi, and Teva outside the submitted work. Dr Ahle has received consulting or travel fees from AbbVie, Biogen, Novartis, Roche, and Sanofi outside the submitted work and reported grants from Roche, Novartis, Biogen, and Sanofi during the conduct of the study. Dr Bigaut has received travel grants from Biogen Idec and Sanofi-Genzyme outside the submitted work. Dr Laplaud has received grants from the ARSEP Foundation and MedDay; personal fees from Biogen, Sanofi-Genzyme, Merck, Celgene, Roche, MedDay, and Teva; grants and personal fees from Novartis; and consulting and lecturing fees, travel grants, and unconditional research support from Biogen, Genzyme, Novartis, Merck Serono, Roche, Sanofi-Aventis, and Teva Pharma outside the submitted work. Dr Vukusic has received grants, personal fees and nonfinancial support from Biogen, Merck, Novartis, Sanofi-Genzyme, Roche, Teva, Celgene, and MedDay and grants from Novartis, Roche, Sanofi, and Teva outside the submitted work. Dr Lubetzki reports grants and personal fees from Biogen and personal fees from Merck-Serono, Roche, Rewind, and Ipsen outside the submitted work. Dr De Sèze has received consulting fees from Biogen, Roche, Novartis, Teva, Cellgen, Jansen, and Sanofi-Genzyme and contracted research from Novartis and Sanofi-Genzyme outside the submitted work.

- 4 figures

Supplementary info

Publication types, MeSH terms Expand

Publication types

- Multicenter Study
- Observational Study
- Research Support, Non-U.S. Gov't

MeSH terms

- Adult
- Betacoronavirus*
- COVID-19
- Cohort Studies
- Coronavirus Infections / epidemiology*

- Coronavirus Infections / therapy*
- Female
- France / epidemiology
- Humans
- Male
- Middle Aged
- Multiple Sclerosis / epidemiology*
- Multiple Sclerosis / therapy*
- Pandemics
- Pneumonia, Viral / epidemiology*
- Pneumonia, Viral / therapy*
- Registries
- Retrospective Studies
- SARS-CoV-2
- Treatment Outcome

Full text links

Silverchair Information Systems Free PMC article
Proceed to details
Cite
Share
1,060
Observational Study
Crit Care Med
. 2020 Dec;48(12):e1211-e1217.
doi: 10.1097/CCM.0000000000004605.

Prevalence and Impact of Hyponatremia in Patients With Coronavirus Disease 2019 in New York City

Jennifer A Frontera [1], Eduard Valdes [1], Joshua Huang [2], Ariane Lewis [1], Aaron S Lord [1], Ting Zhou [1], D Ethan Kahn [1], Kara Melmed [1], Barry M Czeisler [1], Shadi Yaghi [1], Erica Scher [1], Thomas Wisniewski [1 3 4], Laura Balcer [1], Elizabeth Hammer [5]
Affiliations Expand

Affiliations

- [1] Department of Neurology, NYU Grossman School of Medicine, New York, NY.
- [2] Department of Medical Center Information Technology, NYU Grossman School of Medicine, New York, NY.

- [3] Department of Pathology, NYU Grossman School of Medicine, New York, NY.
- [4] Department of Psychiatry, NYU Grossman School of Medicine, New York, NY.
- [5] Department of Medicine, NYU Grossman School of Medicine, New York, NY.

- PMID: **32826430**
- PMCID: PMC7467047
- DOI: 10.1097/CCM.0000000000004605

Free PMC article
Observational Study

Prevalence and Impact of Hyponatremia in Patients With Coronavirus Disease 2019 in New York City

Jennifer A Frontera et al. Crit Care Med. 2020 Dec.
Free PMC article
Show details
Crit Care Med
. 2020 Dec;48(12):e1211-e1217.
doi: 10.1097/CCM.0000000000004605.

Authors

Jennifer A Frontera [1], Eduard Valdes [1], Joshua Huang [2], Ariane Lewis [1], Aaron S Lord [1], Ting Zhou [1], D Ethan Kahn [1], Kara Melmed [1], Barry M Czeisler [1], Shadi Yaghi [1], Erica Scher [1], Thomas Wisniewski [1 3 4], Laura Balcer [1], Elizabeth Hammer [5]

Affiliations

- [1] Department of Neurology, NYU Grossman School of Medicine, New York, NY.
- [2] Department of Medical Center Information Technology, NYU Grossman School of Medicine, New York, NY.
- [3] Department of Pathology, NYU Grossman School of Medicine, New York, NY.
- [4] Department of Psychiatry, NYU Grossman School of Medicine, New York, NY.
- [5] Department of Medicine, NYU Grossman School of Medicine, New York, NY.

- PMID: **32826430**
- PMCID: PMC7467047
- DOI: 10.1097/CCM.0000000000004605

Abstract

Objectives: Hyponatremia occurs in up to 30% of patients with pneumonia and is associated with increased morbidity and mortality. The prevalence of hyponatremia associated with coronavirus disease 2019 and the impact on outcome is unknown. We aimed to identify the prevalence,

predictors, and impact on outcome of mild, moderate, and severe admission hyponatremia compared with normonatremia among coronavirus disease 2019 patients.

Design: Retrospective, multicenter, observational cohort study.

Setting: Four New York City hospitals that are part of the same health network.

Patients: Hospitalized, laboratory-confirmed adult coronavirus disease 2019 patients admitted between March 1, 2020, and May 13, 2020.

Interventions: None.

Measurements and main results: Hyponatremia was categorized as mild (sodium: 130-134 mmol/L), moderate (sodium: 121-129 mmol/L), or severe (sodium: ≤ 120 mmol/L) versus normonatremia (135-145 mmol/L). The primary outcome was the association of increasing severity of hyponatremia and in-hospital mortality assessed using multivariable logistic regression analysis. Secondary outcomes included encephalopathy, acute renal failure, mechanical ventilation, and discharge home compared across sodium levels using Kruskal-Wallis and chi-square tests. In exploratory analysis, the association of sodium levels and interleukin-6 levels (which has been linked to nonosmotic release of vasopressin) was assessed. Among 4,645 patient encounters, hyponatremia (sodium < 135 mmol/L) occurred in 1,373 (30%) and 374 of 1,373 (27%) required invasive mechanical ventilation. Mild, moderate, and severe hyponatremia occurred in 1,032 (22%), 305 (7%), and 36 (1%) patients, respectively. Each level of worsening hyponatremia conferred 43% increased odds of in-hospital death after adjusting for age, gender, race, body mass index, past medical history, admission laboratory abnormalities, admission Sequential Organ Failure Assessment score, renal failure, encephalopathy, and mechanical ventilation (adjusted odds ratio, 1.43; 95% CI, 1.08-1.88; p = 0.012). Increasing severity of hyponatremia was associated with encephalopathy, mechanical ventilation, and decreased probability of discharge home (all p < 0.001). Higher interleukin-6 levels correlated with lower sodium levels (p = 0.017).

Conclusions: Hyponatremia occurred in nearly a third of coronavirus disease 2019 patients, was an independent predictor of in-hospital mortality, and was associated with increased risk of encephalopathy and mechanical ventilation.

Conflict of interest statement

Dr. Frontera's institution received funding from National Institutes of Health (NIH)/National Institute on Aging, and she received support for article research from the NIH. Dr. Yaghi's institution received funding from Medtronic, and he received funding from Mediasphere and Massachusetts General Hospital. The remaining authors have disclosed that they do not have any potential conflicts of interest.

Comment in

- Understanding the Underlying Mechanisms of Hyponatremia in Coronavirus Disease 2019 Is Critical Since Treatment Varies Based on Etiology: Let Us Not Forget Critical Illness-Related Corticosteroid Insufficiency As the Treatment Is Very Different and Often Lifesaving!
  Honore PM, Redant S, Preseau T, Kaefer K, Barreto Gutierrez L, Attou R, Gallerani A, De Bels D. Honore PM, et al. Crit Care Med. 2021 Jul 1;49(7):e724-e725. doi: 10.1097/CCM.0000000000005006. Crit Care Med. 2021. PMID: 33870921 No abstract available.

- The author replies.
  Frontera JA. Frontera JA. Crit Care Med. 2021 Jul 1;49(7):e725-e726. doi: 10.1097/CCM.0000000000005084. Crit Care Med. 2021. PMID: 33883457 No abstract available.

- 28 references
- 2 figures

Supplementary info

Publication types, MeSH terms, Substances, Grant support Expand

Publication types

- Multicenter Study
- Observational Study

MeSH terms

- Adolescent
- Adult
- Age Factors
- Aged
- Aged, 80 and over
- Body Mass Index
- COVID-19 / epidemiology*
- COVID-19 / mortality
- Female
- Hospital Mortality / trends
- Humans
- Hyponatremia / epidemiology*
- Interleukin-6 / blood
- Kaplan-Meier Estimate
- Logistic Models
- Male
- Middle Aged
- New York City / epidemiology
- Pandemics
- Patient Discharge / statistics & numerical data
- Prevalence
- Respiration, Artificial / statistics & numerical data
- Retrospective Studies
- Severity of Illness Index
- Sex Factors

- Young Adult

Substances

- Interleukin-6

Grant support

- P30 AG066512/AG/NIA NIH HHS/United States
- U24 NS113844/NS/NINDS NIH HHS/United States

Full text links

Wolters Kluwer Wolters Kluwer Free PMC article
Proceed to details
Cite
Share
1,061
Observational Study
Crit Care Med
. 2021 Nov 1;49(11):e1151-e1156.
doi: 10.1097/CCM.0000000000005093.

Endothelial Dysfunction as a Component of Severe Acute Respiratory Syndrome Coronavirus 2-Related Multisystem Inflammatory Syndrome in Children With Shock

Delphine Borgel [1 2], Richard Chocron [3 4], Marion Grimaud [5], Aurélien Philippe [6 7], Judith Chareyre [5 8], Charlyne Brakta [1], Dominique Lasne [1 2], Damien Bonnet [9], Julie Toubiana [10], François Angoulvant [11 12], Maximilien Desvages [1 2], Sylvain Renolleau [5 8], David M Smadja [6 7], Mehdi Oualha [5 8]
Affiliations Expand

Affiliations

- [1] Department of Biological Hematology, Necker Hospital, AP-HP Centre-Université de Paris, Paris, France.
- [2] HITh, UMR_S 1176, INSERM, Univ. Paris-Saclay, Le Kremlin-Bicêtre, France.
- [3] Emergency Department, Georges Pompidou European Hospital, AP-HP Centre Université de Paris, Paris, France.

- [4] Université de Paris, PARCC, INSERM, Paris, France.
- [5] Pediatric Intensive Care Unit, Necker Hospital, AP-HP Centre Université de Paris, Paris, France.
- [6] Hematology Department and Biosurgical Research Lab (Carpentier Foundation), Georges Pompidou European Hospital, AP-HP Centre Université de Paris, Paris, France.
- [7] Université de Paris, Innovative Therapies in Hemostasis, INSERM, Paris, France.
- [8] Université de Paris, EA7323, Paris, France.
- [9] M3C-Necker, Congenital and Pediatric Cardiology, Necker Hospital, AP-HP Centre-Université de Paris, Paris, France.
- [10] Department of General Pediatrics and Pediatric Infectious Diseases, Necker Hospital, AP-HP Centre-Université de Paris, Paris, France.
- [11] Pediatric Emergency Department, Necker Hospital, AP-HP Centre Université de Paris, Paris, France.
- [12] INSERM, Centre de Recherche des Cordeliers, UMRS 1138, Sorbonne Université, Université de Paris, Paris, France.

- PMID: **34049308**
- PMCID: PMC8507588
- DOI: 10.1097/CCM.0000000000005093

Free PMC article
Observational Study

Endothelial Dysfunction as a Component of Severe Acute Respiratory Syndrome Coronavirus 2-Related Multisystem Inflammatory Syndrome in Children With Shock

Delphine Borgel et al. Crit Care Med. 2021.
Free PMC article

Show details

Crit Care Med
. 2021 Nov 1;49(11):e1151-e1156.
doi: 10.1097/CCM.0000000000005093.

Authors

Delphine Borgel [1 2], Richard Chocron [3 4], Marion Grimaud [5], Aurélien Philippe [6 7], Judith Chareyre [5 8], Charlyne Brakta [1], Dominique Lasne [1 2], Damien Bonnet [9], Julie Toubiana [10], François Angoulvant [11 12], Maximilien Desvages [1 2], Sylvain Renolleau [5 8], David M Smadja [6 7], Mehdi Oualha [5 8]

Affiliations

- [1] Department of Biological Hematology, Necker Hospital, AP-HP Centre-Université de Paris, Paris, France.
- [2] HITh, UMR_S 1176, INSERM, Univ. Paris-Saclay, Le Kremlin-Bicêtre, France.
- [3] Emergency Department, Georges Pompidou European Hospital, AP-HP Centre Université de Paris, Paris, France.
- [4] Université de Paris, PARCC, INSERM, Paris, France.
- [5] Pediatric Intensive Care Unit, Necker Hospital, AP-HP Centre Université de Paris, Paris, France.
- [6] Hematology Department and Biosurgical Research Lab (Carpentier Foundation), Georges Pompidou European Hospital, AP-HP Centre Université de Paris, Paris, France.
- [7] Université de Paris, Innovative Therapies in Hemostasis, INSERM, Paris, France.
- [8] Université de Paris, EA7323, Paris, France.
- [9] M3C-Necker, Congenital and Pediatric Cardiology, Necker Hospital, AP-HP Centre-Université de Paris, Paris, France.
- [10] Department of General Pediatrics and Pediatric Infectious Diseases, Necker Hospital, AP-HP Centre-Université de Paris, Paris, France.
- [11] Pediatric Emergency Department, Necker Hospital, AP-HP Centre Université de Paris, Paris, France.
- [12] INSERM, Centre de Recherche des Cordeliers, UMRS 1138, Sorbonne Université, Université de Paris, Paris, France.

- PMID: **34049308**
- PMCID: PMC8507588
- DOI: 10.1097/CCM.0000000000005093

Abstract

Trial registration: NCT04420468.

Objectives: Severe acute respiratory syndrome coronavirus 2-related multisystem inflammatory syndrome in children is frequently associated with shock; endothelial involvement may be one of the underlying mechanisms. We sought to describe endothelial dysfunction during multisystem inflammatory syndrome in children with shock and then assess the relationship between the degree of endothelial involvement and the severity of shock.

Design: Observational study.

Setting: A PICU in a tertiary hospital.

Patients: Patients aged under 18 (n = 28) with severe acute respiratory syndrome coronavirus 2-related multisystem inflammatory syndrome in children and shock, according to the Centers for Disease Control and Prevention criteria.

Interventions: None.

Measurements and main results: Correlations between endothelial marker levels and shock severity were assessed using Spearman coefficient. The median (interquartile range) age was 9 years (7.5-11.2 yr). Sixteen children presented with cardiogenic and distributive shock, 10 presented with cardiogenic shock only, and two presented with distributive shock only. The median left ventricular ejection fraction, troponin level, and lactate level were, respectively, 40% (35-45%), 261 ng/mL (131-390 ng/mL), and 3.2 mmol/L (2-4.2 mmol/L). Twenty-five children

received inotropes and/or vasopressors; the median Vasoactive and Inotropic Score was 8 (5-28). Plasma levels of angiopoietin-2 (6,426 pg/mL [2,814-11,836 pg/mL]), sE-selectin (130,405 pg/mL [92,987-192,499 pg/mL]), von Willebrand factor antigen (344% [288-378%]), and the angiopoietin-2/angiopoietin-1 ratio (1.111 [0.472-1.524]) were elevated and significantly correlated with the Vasoactive and Inotropic Score (r = 0.45, p = 0.016; r = 0.53, p = 0.04; r = 0.46, p = 0.013; and r = 0.46, p = 0.012, respectively).

Conclusions: Endothelial dysfunction is associated with severe acute respiratory syndrome coronavirus 2-related multisystem inflammatory syndrome in children with shock and may constitute one of the underlying mechanisms.

Copyright © 2021 by the Society of Critical Care Medicine and Wolters Kluwer Health, Inc. All Rights Reserved.

Conflict of interest statement

Dr. Borgel's institution received funding from Leo PHARMA and ROCHE Financial. The remaining authors have disclosed that they do not have any potential conflicts of interest.

- 20 references
- 1 figure

Supplementary info

Publication types, MeSH terms, Substances, Supplementary concepts, Associated data Expand

Publication types

- Observational Study

MeSH terms

- Adrenal Cortex Hormones / therapeutic use
- Angiopoietin-2 / blood
- Biomarkers
- C-Reactive Protein / analysis
- COVID-19 / complications*
- COVID-19 / drug therapy
- COVID-19 / pathology
- Cardiotonic Agents / therapeutic use
- Child
- Female
- Humans
- Immunoglobulins / therapeutic use
- Intensive Care Units, Pediatric
- Interleukin-6 / blood
- Lactic Acid / blood

- Male
- Respiration, Artificial
- Retrospective Studies
- SARS-CoV-2
- Severity of Illness Index
- Shock / pathology*
- Shock, Cardiogenic / pathology
- Systemic Inflammatory Response Syndrome / drug therapy
- Systemic Inflammatory Response Syndrome / pathology*
- Troponin / blood
- Vasoconstrictor Agents / therapeutic use
- Ventricular Function, Left

Substances

- Adrenal Cortex Hormones
- Angiopoietin-2
- Biomarkers
- Cardiotonic Agents
- Immunoglobulins
- Interleukin-6
- Troponin
- Vasoconstrictor Agents
- Lactic Acid
- C-Reactive Protein

Supplementary concepts

- pediatric multisystem inflammatory disease, COVID-19 related

Associated data

- ClinicalTrials.gov/NCT04420468

Full text links

Wolters Kluwer Wolters Kluwer Free PMC article

Proceed to details

Cite

Share

☐ 1,062

Observational Study

Thromb Haemost

. 2021 Dec;121(12):1610-1621.
doi: 10.1055/a-1477-3829. Epub 2021 Apr 8.

Platelet Count Rose While D-Dimer Levels Dropped as Deaths and Thrombosis Declined-An Observational Study on Anticoagulation Shift in COVID-19

Anna Sjöström [1, 2], Johanna Dehlsen Wersäll [2], Anna Warnqvist [3], Maria Farm [1, 2], Maria Magnusson [1, 4, 5], Anders Oldner [6, 7], Anna Ågren [1, 5, 8], Jovan Antovic [1, 2], Maria Bruzelius [1, 5, 9]

Affiliations Expand

Affiliations

- [1] Department of Molecular Medicine and Surgery, Karolinska Institutet, Stockholm, Sweden.
- [2] Clinical Chemistry, Karolinska University Laboratory, Stockholm, Sweden.
- [3] Division of Biostatistics, Institute of Environmental Medicine, Karolinska Institutet, Stockholm, Sweden.
- [4] Department of Clinical Science, Intervention and Technology, Karolinska Institutet, Stockholm, Sweden.
- [5] Coagulation Unit, Department of Hematology, Karolinska University Hospital, Stockholm, Sweden.
- [6] Department of Physiology and Pharmacology, Karolinska Institutet, Stockholm, Sweden.
- [7] Department of Perioperative Medicine and Intensive Care, Karolinska University Hospital, Stockholm, Sweden.
- [8] Department of Clinical Sciences, Danderyd Hospital, Karolinska Institutet, Stockholm, Sweden.
- [9] Department of Medicine, Solna, Karolinska Institutet, Stockholm, Sweden.

- PMID: **33831964**
- DOI: 10.1055/a-1477-3829

Observational Study

Platelet Count Rose While D-Dimer Levels Dropped as Deaths and Thrombosis Declined-An Observational Study on Anticoagulation Shift in COVID-19

Anna Sjöström et al. Thromb Haemost. 2021 Dec.

Show details

Thromb Haemost

. 2021 Dec;121(12):1610-1621.
doi: 10.1055/a-1477-3829. Epub 2021 Apr 8.

Authors

Anna Sjöström [1, 2], Johanna Dehlsen Wersäll [2], Anna Warnqvist [3], Maria Farm [1, 2], Maria Magnusson [1, 4, 5], Anders Oldner [6, 7], Anna Ågren [1, 5, 8], Jovan Antovic [1, 2], Maria Bruzelius [1, 5, 9]

Affiliations

- [1] Department of Molecular Medicine and Surgery, Karolinska Institutet, Stockholm, Sweden.
- [2] Clinical Chemistry, Karolinska University Laboratory, Stockholm, Sweden.
- [3] Division of Biostatistics, Institute of Environmental Medicine, Karolinska Institutet, Stockholm, Sweden.
- [4] Department of Clinical Science, Intervention and Technology, Karolinska Institutet, Stockholm, Sweden.
- [5] Coagulation Unit, Department of Hematology, Karolinska University Hospital, Stockholm, Sweden.
- [6] Department of Physiology and Pharmacology, Karolinska Institutet, Stockholm, Sweden.
- [7] Department of Perioperative Medicine and Intensive Care, Karolinska University Hospital, Stockholm, Sweden.
- [8] Department of Clinical Sciences, Danderyd Hospital, Karolinska Institutet, Stockholm, Sweden.
- [9] Department of Medicine, Solna, Karolinska Institutet, Stockholm, Sweden.

- PMID: **33831964**
- DOI: 10.1055/a-1477-3829

Abstract

Background: High levels of D-dimer and low platelet counts are associated with poor outcome in coronavirus disease 2019 (COVID-19). As anticoagulation appeared to improve survival, hospital-wide recommendations regarding higher doses of anticoagulation were implemented on April 9, 2020.

Objectives: To investigate if trends in D-dimer levels and platelet counts were associated with death, thrombosis, and the shift in anticoagulation.

Methods: Retrospective cohort study of 429 patients with COVID-19 at Karolinska University Hospital. Information on D-dimer levels and platelet counts was obtained from laboratory databases and clinical data from medical records.

Results: Thirty-day mortality and thrombosis rates were 19% and 18%, respectively. Pulmonary embolism was common, 65/83 (78%). Increased D-dimer levels in the first week in hospital were significantly associated with death and thrombosis (odds ratio [OR]: 6.06; 95% confidence interval [CL]: 2.10-17.5 and 3.11; 95% CI: 1.20-8.10, respectively). If platelet count increased

more than 35 × 10^9/L per day, the mortality and thrombotic risk decreased (OR: 0.16; 95% CI: 0.06-0.41, and OR: 0.36; 95% CI: 0.17-0.80). After implementation of updated hospital-wide recommendations, the daily mean significantly decreased regarding D-dimer levels while platelet counts rose; -1.93; 95% CI: -1.00-2.87 mg/L FEU (fibrinogen-equivalent unit) and 65; 95% CI: 54-76 ×10^9/L, and significant risk reductions for death and thrombosis were observed; OR: 0.48; 95% CI: 0.25-0.92 and 0.35; 95% CI: 0.17-0.72.

Conclusion: In contrast to D-dimer levels, increase of platelet count over the first week in hospital was associated with improved survival and reduced thrombotic risk. The daily mean levels of D-dimer dropped while the platelet counts rose, coinciding with increased anticoagulation and a decline in thrombotic burden and mortality.

Thieme. All rights reserved.

Conflict of interest statement

J.A. has received research grants from Shire, honoraria from Stago, Siemens, Sysmex, Roche, Baxter, and Sobi, and acts on advisory boards for Sobi and Novo Nordisk. M.B. acts on the advisory board for CSL Behringer and Sobi and has had consultant assignments for Novo Nordisk and received lecturer honoraria from Sobi.A.O. and M.B. were both supported by funds from Stockholm County Council. A.O. was supported by Swedish Carnegie Hero Funds. A.S., J.D.W., A.W., A.Å., and M.M. declare no competing financial interests.

Supplementary info

Publication types, MeSH terms, Substances, Supplementary concepts Expand

Publication types

- Multicenter Study
- Observational Study

MeSH terms

- Administration, Oral
- Aged
- Anticoagulants / administration & dosage*
- Anticoagulants / adverse effects
- Biomarkers / blood
- Blood Platelets / drug effects*
- Blood Platelets / metabolism
- COVID-19 / blood
- COVID-19 / diagnosis
- COVID-19 / drug therapy*
- COVID-19 / mortality
- Female
- Fibrin Fibrinogen Degradation Products / metabolism*

- Hospital Mortality
- Humans
- Male
- Middle Aged
- Retrospective Studies
- Sweden / epidemiology
- Thrombosis / blood
- Thrombosis / diagnosis
- Thrombosis / drug therapy*
- Thrombosis / mortality
- Time Factors
- Treatment Outcome

Substances

- Anticoagulants
- Biomarkers
- Fibrin Fibrinogen Degradation Products
- fibrin fragment D

Supplementary concepts

- COVID-19 drug treatment

Full text links

Full text THIEME CONNECT Georg Thieme Verlag Stuttgart, New York
Proceed to details
Cite
Share
☐ 1,063
PLoS One
. 2021 Jul 27;16(7):e0253778.
doi: 10.1371/journal.pone.0253778. eCollection 2021.

High sedation needs of critically ill COVID-19 ARDS patients-A monocentric observational study

Armin Niklas Flinspach [1], Hendrik Booke [1], Kai Zacharowski [1], Ümniye Balaban [2], Eva Herrmann [2], Elisabeth Hannah Adam [1]

Affiliations Expand

Affiliations

- [1] Department of Anaesthesiology, Intensive Care Medicine and Pain Therapy, University Hospital Frankfurt, Goethe-University Frankfurt, Hessen, Germany.
- [2] Department of Biostatistics and Mathematical Modelling, Goethe-University Frankfurt, Hessen, Germany.

- PMID: **34314422**
- PMCID: PMC8315516
- DOI: 10.1371/journal.pone.0253778

Free PMC article

High sedation needs of critically ill COVID-19 ARDS patients-A monocentric observational study

Armin Niklas Flinspach et al. PLoS One. 2021.
Free PMC article
Show details
PLoS One

. 2021 Jul 27;16(7):e0253778.
doi: 10.1371/journal.pone.0253778. eCollection 2021.

Authors

Armin Niklas Flinspach [1], Hendrik Booke [1], Kai Zacharowski [1], Ümniye Balaban [2], Eva Herrmann [2], Elisabeth Hannah Adam [1]

Affiliations

- [1] Department of Anaesthesiology, Intensive Care Medicine and Pain Therapy, University Hospital Frankfurt, Goethe-University Frankfurt, Hessen, Germany.
- [2] Department of Biostatistics and Mathematical Modelling, Goethe-University Frankfurt, Hessen, Germany.

- PMID: **34314422**
- PMCID: PMC8315516
- DOI: 10.1371/journal.pone.0253778

Abstract

Background: Therapy of severely affected coronavirus patient, requiring intubation and sedation is still challenging. Recently, difficulties in sedating these patients have been discussed. This study

aims to describe sedation practices in patients with 2019 coronavirus disease (COVID-19) -induced acute respiratory distress syndrome (ARDS).

Methods: We performed a retrospective monocentric analysis of sedation regimens in critically ill intubated patients with respiratory failure who required sedation in our mixed 32-bed university intensive care unit. All mechanically ventilated adults with COVID-19-induced ARDS requiring continuously infused sedative therapy admitted between April 4, 2020, and June 30, 2020 were included. We recorded demographic data, sedative dosages, prone positioning, sedation levels and duration. Descriptive data analysis was performed; for additional analysis, a logistic regression with mixed effect was used.

Results: In total, 56 patients (mean age 67 (±14) years) were included. The mean observed sedation period was 224 (±139) hours. To achieve the prescribed sedation level, we observed the need for two or three sedatives in 48.7% and 12.8% of the cases, respectively. In cases with a triple sedation regimen, the combination of clonidine, esketamine and midazolam was observed in most cases (75.7%). Analgesia was achieved using sufentanil in 98.6% of the cases. The analysis showed that the majority of COVID-19 patients required an unusually high sedation dose compared to those available in the literature.

Conclusion: The global pandemic continues to affect patients severely requiring ventilation and sedation, but optimal sedation strategies are still lacking. The findings of our observation suggest unusual high dosages of sedatives in mechanically ventilated patients with COVID-19. Prescribed sedation levels appear to be achievable only with several combinations of sedatives in most critically ill patients suffering from COVID-19-induced ARDS and a potential association to the often required sophisticated critical care including prone positioning and ECMO treatment seems conceivable.

Conflict of interest statement

KZ has received honoraria for participation in advisory board meetings for Haemonetics and Vifor and received speaker fees from CSL Behring and GE Healthcare. KZ is the Principal Investigator of the EU-Horizon 2020 project ENVISION (Intelligent plug-and-play digital tool for real-time surveillance of COVID-19 patients and smart decision-making in Intensive Care Units). None of the Competing Interests mentioned is related to the present work. The author confirms that the disclosed conflicts of interest of KZ does not alter the adherence to PLOS ONE policies on sharing data and materials. There are no patents, products in development or marketed products associated with this research to declare.

- 45 references
- 4 figures

Supplementary info

MeSH terms, Substances, Grant support Expand

MeSH terms

- Adult
- Aged
- COVID-19 / complications*
- Critical Illness*

- Dose-Response Relationship, Drug
- Female
- Humans
- Hypnotics and Sedatives / pharmacology*
- Hypnotics and Sedatives / therapeutic use
- Male
- Middle Aged
- Respiration, Artificial
- Respiratory Distress Syndrome / complications*
- Respiratory Distress Syndrome / drug therapy*
- Retrospective Studies
- Time Factors

Substances

- Hypnotics and Sedatives

Grant support

The author(s) received no specific funding for this work.

Full text links

OPEN ACCESS TO FULL TEXT PLOS ONE Public Library of Science Free PMC article
Proceed to details
Cite
Share
☐ 1,064
Observational Study
Rev Esp Enferm Dig
. 2021 Feb;113(2):98-102.
doi: 10.17235/reed.2020.7543/2020.

Consequences and management of COVID-19 on the care activity of an Inflammatory Bowel Disease Unit

Ismael El Hajra [1], Marta Calvo [2], Elena Santos Pérez [2], Santiago Blanco Rey [2], Irene González Partida [2], Virginia Matallana [2], Yago González-Lama [1], María Isabel Vera [2]
Affiliations Expand

Affiliations

- [1] Aparato Digestivo, Hospital Universitario Puerta de Hierro, España.
- [2] Aparato Digestivo, Hospital Universitario Puerta de Hierro.

- PMID: **33342217**
- DOI: 10.17235/reed.2020.7543/2020

Free article
Observational Study

Consequences and management of COVID-19 on the care activity of an Inflammatory Bowel Disease Unit

Ismael El Hajra et al. Rev Esp Enferm Dig. 2021 Feb.
Free article
Show details
Rev Esp Enferm Dig
. 2021 Feb;113(2):98-102.
doi: 10.17235/reed.2020.7543/2020.

Authors

Ismael El Hajra [1], Marta Calvo [2], Elena Santos Pérez [2], Santiago Blanco Rey [2], Irene González Partida [2], Virginia Matallana [2], Yago González-Lama [1], María Isabel Vera [2]

Affiliations

- [1] Aparato Digestivo, Hospital Universitario Puerta de Hierro, España.
- [2] Aparato Digestivo, Hospital Universitario Puerta de Hierro.

- PMID: **33342217**
- DOI: 10.17235/reed.2020.7543/2020

Abstract

Introduction: COVID-19 has altered the usual practice of medicine and the state of emergency declared in Spain on March 14th has considerably changed the activity of inflammatory bowel disease (IBD) units. The aim of this study was to evaluate the consequences of COVID-19 on the IBD Unit's activity and provide information on restructuring with available resources.

Methods: an observational study was performed in a referral hospital in Madrid (Spain). Type of appointment, loss of follow-up, hospital admission, treatment changes, endoscopic activity, surgeries and blood tests were evaluated between March 15th and May 15th, 2020. This data was compared with the usual activity a year before.

Results: among the 510 patients included, 476 (93.33 %) received had a remote consultation, representing an increase of 92.38 % compared with the previous year (0.95 %). There was a loss of follow-up in 26 patients (5.1 %) vs 15 (3.58 %) the previous year. A total of 60 (35.09 %) blood tests, 64 (76.19 %) endoscopies and all scheduled surgeries were suspended. Besides, 484 (94.9 %) patients remained adherent vs 417/419 (99.5 %) in the pre-pandemic period and 48 (9.41 %) reported symptoms of an IBD flare. Thirty-nine (7.6 %) patients developed symptoms suggestive of COVID-19.

Conclusion: a large number of tests and on-site outpatient visit consultations were suspended. However, a rapid adaptation to telemedicine allowed these patients to be closely followed up. Although it was possible to maintain therapeutic compliance, with a loss to follow-up slightly higher than the previous year, suspensions and delays of tests could have significant negative consequences in the long term.

Comment in

- The COVID-19 pandemic shows us in which way the care of patients with inflammatory bowel disease should move.
  Hernández Ramírez V, López Serrano P. Hernández Ramírez V, et al. Rev Esp Enferm Dig. 2021 Feb;113(2):153-154. doi: 10.17235/reed.2021.7765/2020. Rev Esp Enferm Dig. 2021. PMID: 33467860

Supplementary info

Publication types, MeSH terms Expand

Publication types

- Observational Study

MeSH terms

- Adult
- COVID-19* / epidemiology
- Delivery of Health Care
- Female
- Hospital Units
- Humans
- Inflammatory Bowel Diseases / therapy*
- Male
- Middle Aged
- Retrospective Studies
- Spain / epidemiology

Full text links

REED.es Free full text Aran ediciones, S.L.

Proceed to details
Cite
Share
☐ 1,065
Observational Study
Wound Manag Prev
. 2021 Jan;67(1):12-17.

Telemedicine in Patients With an Ostomy During the COVID-19 Pandemic: A Retrospective Observational Study

Vincenza Paola Dinuzzi [1], Giuseppe Palomba [1], Maria Minischetti [1], Alfonso Amendola [1], Pierluigi Aprea [1], Gaetano Luglio [1], Giovanni Domenico De Palma [1], Giovanni Aprea [1]
Affiliations Expand

Affiliation

- [1] University of Naples, Federico II, Naples, Italy.

- PMID: **33448938**

Free article
Observational Study

Telemedicine in Patients With an Ostomy During the COVID-19 Pandemic: A Retrospective Observational Study

Vincenza Paola Dinuzzi et al. Wound Manag Prev. 2021 Jan.
Free article
Show details
Wound Manag Prev
. 2021 Jan;67(1):12-17.

Authors

Vincenza Paola Dinuzzi [1], Giuseppe Palomba [1], Maria Minischetti [1], Alfonso Amendola [1], Pierluigi Aprea [1], Gaetano Luglio [1], Giovanni Domenico De Palma [1], Giovanni Aprea [1]

Affiliation

- [1] University of Naples, Federico II, Naples, Italy.

- PMID: **33448938**

Abstract

Background: Italy instituted a lockdown from March 10 to May 3, 2020, due to the coronavirus disease-19 pandemic. All nonessential businesses were closed during this time, and health care services were reorganized. On March 11, the Stoma Care Center started providing telehealth services for patients with a stoma.

Purpose: This retrospective observational study describes the experience of the Stoma Care Center of the University Hospital Federico II, Naples, Italy, before and during the lockdown.

Methods: Consultation records from January 1 through April 29 were retrieved, patient demographics and reasons for consultation abstracted, and pre-lockdown (January 1 to February 29) and lockdown (March 1 to April 29) information was compared. Patients who used telehealth services were also asked to rate their satisfaction with these services on a scale of 0 (extremely dissatisfied) to 4 (extremely satisfied). The authors analyzed all consultations provided from January to April 2020 and evaluated the use of telemedicine services for patients with an ostomy. Consultations were divided into 2 groups. Group A included consultations provided from January 1 to February 29. Group B included consultations provided from March 1 to April 29, which included the lockdown period. Group B included both in-person and telemedicine consultations.

Results: During the pre-lockdown period, 240 in-person consultations were provided. During the lockdown period, 181 in-person and 99 telemedicine consultations were provided. The number of in-person consultations for mechanical bowel preparation and transanal irrigation system training was lower (12.5% vs 6.6% [P = .046] and 3.3% vs 0% [P = .03]), whereas the number of consults for stoma care follow-up and stoma complications was higher (202 [84.1%] vs 266 [95%]). Of the 65 patients who completed the questionnaire, 82% indicated being extremely satisfied.

Conclusions: The reorganization of stoma care services, including the availability of telemedicine, did not result in a decrease in the number of consultations provided. The results suggest that stoma care services using telemedicine may provide valid support for patients with an ostomy in the future.

Supplementary info

Publication types, MeSH terms Expand

Publication types

- Observational Study

MeSH terms

- Adult
- COVID-19 / complications*
- COVID-19 / transmission
- Female
- Humans

- Italy
- Male
- Middle Aged
- Ostomy / nursing*
- Pandemics / prevention & control
- Patient Satisfaction
- Quarantine / trends*
- Retrospective Studies
- Telemedicine / methods

Full text links

WOUND MANAGEMENT & PREVENTION HMP Communications, LLC
Proceed to details
Cite
Share
1,066

Observational Study
J Korean Med Sci
. 2020 Jun 29;35(25):e234.
doi: 10.3346/jkms.2020.35.e234.

Prognostic Accuracy of the SIRS, qSOFA, and NEWS for Early Detection of Clinical Deterioration in SARS-CoV-2 Infected Patients

Jong Geol Jang[# 1], Jian Hur[# 2], Kyung Soo Hong[1], Wonhwa Lee[3], June Hong Ahn[4]
Affiliations Expand

Affiliations

- [1] Division of Pulmonology and Allergy, Department of Internal Medicine, College of Medicine, Yeungnam University and Regional Center for Respiratory Diseases, Yeungnam University Medical Center, Daegu, Korea.
- [2] Division of Infection, Department of Internal Medicine, College of Medicine, Yeungnam University, Yeungnam University Medical Center, Daegu, Korea.
- [3] Aging Research Center, Korea Research Institute of Bioscience and Biotechnology, Daejeon, Korea. wonhwalee@kribb.re.kr.
- [4] Division of Pulmonology and Allergy, Department of Internal Medicine, College of Medicine, Yeungnam University and Regional Center for Respiratory Diseases, Yeungnam University Medical Center, Daegu, Korea. fireajh@gmail.com.

# Contributed equally.

- PMID: **32597046**
- PMCID: PMC7324266
- DOI: 10.3346/jkms.2020.35.e234

Free PMC article
Observational Study

Prognostic Accuracy of the SIRS, qSOFA, and NEWS for Early Detection of Clinical Deterioration in SARS-CoV-2 Infected Patients

Jong Geol Jang et al. J Korean Med Sci. 2020.
Free PMC article
Show details
J Korean Med Sci
. 2020 Jun 29;35(25):e234.
doi: 10.3346/jkms.2020.35.e234.

Authors

Jong Geol Jang [#] [1], Jian Hur [#] [2], Kyung Soo Hong [1], Wonhwa Lee [3], June Hong Ahn [4]

Affiliations

- [1] Division of Pulmonology and Allergy, Department of Internal Medicine, College of Medicine, Yeungnam University and Regional Center for Respiratory Diseases, Yeungnam University Medical Center, Daegu, Korea.
- [2] Division of Infection, Department of Internal Medicine, College of Medicine, Yeungnam University, Yeungnam University Medical Center, Daegu, Korea.
- [3] Aging Research Center, Korea Research Institute of Bioscience and Biotechnology, Daejeon, Korea. wonhwalee@kribb.re.kr.
- [4] Division of Pulmonology and Allergy, Department of Internal Medicine, College of Medicine, Yeungnam University and Regional Center for Respiratory Diseases, Yeungnam University Medical Center, Daegu, Korea. fireajh@gmail.com.

[#] Contributed equally.

- PMID: **32597046**
- PMCID: PMC7324266
- DOI: 10.3346/jkms.2020.35.e234

Abstract

Background: The case fatality rate of coronavirus disease 2019 (COVID-19) is estimated to be between 4.3% and 11.0%. Currently there is no effective antiviral treatment for COVID-19. Thus, early recognition of patients at high risk is important.

Methods: We performed a retrospective observational study of 110 patients with severe acute respiratory syndrome coronavirus 2 infection. We compared the effectiveness of three scoring systems: the Systemic Inflammatory Response Syndrome (SIRS), quick Sequential Organ Failure Assessment (qSOFA), and National Early Warning Score (NEWS) systems, for predicting the prognosis of COVID-19. The area under the receiver operating characteristic curve (AUROC) was used for these assessments, and Kaplan-Meier survival curves were used to identify the cumulative risk for 28-day mortality according to the NEWS stratification.

Results: For predicting 28-day mortality, NEWS was superior to qSOFA (AUROC, 0.867 vs. 0.779, $P < 0.001$), while there was no significant difference between NEWS and SIRS (AUROC, 0.867 vs. 0.639, $P = 0.100$). For predicting critical outcomes, NEWS was superior to both SIRS (AUROC, 0.918 vs. 0.744, $P = 0.032$) and qSOFA (AUROC, 0.918 vs. 0.760, $P = 0.012$). Survival time was significantly shorter for patients with NEWS ≥ 7 than for patients with NEWS < 7.

Conclusion: Calculation of the NEWS at the time of hospital admission can predict critical outcomes in patients with COVID-19. Early intervention for high-risk patients can thereby improve clinical outcomes in COVID-19 patients.

Keywords: COVID-19; NEWS; Outcome; Prediction.

© 2020 The Korean Academy of Medical Sciences.

Conflict of interest statement

The authors have no potential conflicts of interest to disclose.

Comment in

- Letter to the Editor: Discussion of the Article "Prognostic Accuracy of the SIRS, qSOFA, and NEWS for Early Detection of Clinical Deterioration in SARS-CoV-2 Infected Patients". Kim EJ, Hong HL. Kim EJ, et al. J Korean Med Sci. 2020 Aug 3;35(30):e274. doi: 10.3346/jkms.2020.35.e274. J Korean Med Sci. 2020. PMID: 32743993 Free PMC article. No abstract available.

- 21 references
- 2 figures

Supplementary info

Publication types, MeSH terms Expand

Publication types

- Comparative Study
- Observational Study

MeSH terms

- Aged
- Betacoronavirus
- COVID-19
- Clinical Deterioration*
- Coronavirus Infections / diagnosis*
- Coronavirus Infections / mortality*
- Coronavirus Infections / pathology
- Early Diagnosis
- Female
- Humans
- Kaplan-Meier Estimate
- Male
- Middle Aged
- Multiple Organ Failure / diagnosis*
- Organ Dysfunction Scores*
- Pandemics
- Pneumonia, Viral / diagnosis*
- Pneumonia, Viral / mortality*
- Pneumonia, Viral / pathology
- Prognosis
- Retrospective Studies
- SARS-CoV-2

Full text links

JKMS FULL TEXT FREE Journal of korean Medical Science Korean Academy of Medical Sciences Free PMC article

Proceed to details

Cite

Share

☐ 1,067

Observational Study

Int J Colorectal Dis

. 2021 Mar;36(3):611-615.

doi: 10.1007/s00384-021-03847-4. Epub 2021 Jan 26.

Are colorectal cancer patients at risk for COVID-19 infection during the postoperative period? The Covid-GRECCAR study

Jean-Jacques Tuech [1], Gilles Manceau [2], Mehdi Ouaissi [3], Christine Denet [4], Amélie Chau [5], Alex Kartheuser [6], Véronique Desfourneaux [7], Emilie Duchalais [8], Martin Bertrand [9], Bogdan Badic [10], Arnaud Alves [11], Cecilia Ceribelli [12], Aurelien Venara [13], Diane Mege [14], François Mauvais [15], Fréderic Dumont [16], Jean-Yves Mabrut [17], Zaher Lakkis [18], Eddy Cotte [19], Helene Meillat [20], French Research Group of Rectal Cancer Surgery (GRECCAR)

Affiliations Expand

Affiliations

- [1] Department of Digestive Surgery, Rouen University Hospital, 1 rue de Germont, F-76031, Rouen cedex, France. jean-jacques.tuech@chu-rouen.fr.
- [2] Department of Digestive and Hepato-Pancreato-Biliary Surgery, Sorbonne University, Assistance Publique Hôpitaux de Paris, Pitié-Salpêtrière Hospital, Paris, France.
- [3] Department of Digestive, Oncological, Endocrine, and Hepatic Surgery, and Hepatic Transplantation, Trousseau Hospital, CHRU Trouseau, Tours, France.
- [4] Service de Chirurgie Digestive, Institut Mutualiste Montsouris, Paris, France.
- [5] Polyclinique d'Hénin-Beaumont, Route de Courrières, 62110, Hénin-Beaumont, France.
- [6] Colorectal Surgery Unit, Department of Abdominal Surgery and Transplantation Cliniques Universitaires Saint-Luc, Université Catholique de Louvain (UCL), Brussels, Belgium.
- [7] Department of Hepatobiliary and Digestive Surgery, CHU Rennes, 2 rue Henri Le Guilloux, Rennes, France.
- [8] Chirurgie Cancérologique, Digestive et Endocrinienne (CCDE), Institut des Maladies de l'Appareil Digestif (IMAD), Centre Hospitalo-universitaire de Nantes (CHU) Hôtel-Dieu, Place Alexis Ricordeau, 44093, Nantes, France.
- [9] Digestive surgery & digestive cancerology, CHU Carémeau, université de Montpellier, place du Professeur-Robert-Debré, 30029, Nîmes cedex 9, France.
- [10] CHRU de Brest, Brest, France.
- [11] Service de chirurgie digestive CHU Caen, registre des tumeurs digestive du calvados, Inserm U1086 ANTICIPE, 14000, Caen, France.
- [12] Department of Surgical Oncology, Institut de Cancérologie de Lorraine, Université de Lorraine, 54519, Vandoeuvre-lès-Nancy, France.
- [13] Department of Endocrinal and Visceral Surgery, Angers University Hospital, 49933, Angers, France.
- [14] Department of Digestive Surgery, Assistance Publique Hôpitaux de Marseille, Timone University Hospital, Marseille, France.
- [15] Digestive Surgery Department, Beauvais Hospital, Beauvais, France.
- [16] Department of Surgical Oncology, Comprehensive Cancer Center, Institut de Cancérologie de l'Ouest, Saint-Herblain, France.
- [17] Department of Digestive Surgery and Transplantation, University Hospital Croix Rousse, Hospices Civils de Lyon, University of Lyon I, Lyon, France.
- [18] Department of Surgical Oncology, University Hospital Jean Minjoz, Besançon, France.
- [19] Department of Gastrointestinal Surgery, Hospices Civils de Lyon, Université de Lyon, Centre Hospitalier Lyon-Sud, 165 chemin du grand Revoyet, 69495, Pierre Bénite, France.
- [20] Department of Digestive Surgical Oncology, Department of Mini Invasive Interventions (DIMI), Paoli Calmettes Institute, Marseille, France.

- PMID: **33495872**
- PMCID: PMC7835106
- DOI: 10.1007/s00384-021-03847-4

Free PMC article
Observational Study

Are colorectal cancer patients at risk for COVID-19 infection during the postoperative period? The Covid-GRECCAR study

Jean-Jacques Tuech et al. Int J Colorectal Dis. 2021 Mar.
Free PMC article
Show details
Int J Colorectal Dis
. 2021 Mar;36(3):611-615.
doi: 10.1007/s00384-021-03847-4. Epub 2021 Jan 26.

Authors

Jean-Jacques Tuech [1], Gilles Manceau [2], Mehdi Ouaissi [3], Christine Denet [4], Amélie Chau [5], Alex Kartheuser [6], Véronique Desfourneaux [7], Emilie Duchalais [8], Martin Bertrand [9], Bogdan Badic [10], Arnaud Alves [11], Cecilia Ceribelli [12], Aurelien Venara [13], Diane Mege [14], François Mauvais [15], Fréderic Dumont [16], Jean-Yves Mabrut [17], Zaher Lakkis [18], Eddy Cotte [19], Helene Meillat [20], French Research Group of Rectal Cancer Surgery (GRECCAR)

Affiliations

- [1] Department of Digestive Surgery, Rouen University Hospital, 1 rue de Germont, F-76031, Rouen cedex, France. jean-jacques.tuech@chu-rouen.fr.
- [2] Department of Digestive and Hepato-Pancreato-Biliary Surgery, Sorbonne University, Assistance Publique Hôpitaux de Paris, Pitié-Salpêtrière Hospital, Paris, France.
- [3] Department of Digestive, Oncological, Endocrine, and Hepatic Surgery, and Hepatic Transplantation, Trousseau Hospital, CHRU Trouseau, Tours, France.
- [4] Service de Chirurgie Digestive, Institut Mutualiste Montsouris, Paris, France.
- [5] Polyclinique d'Hénin-Beaumont, Route de Courrières, 62110, Hénin-Beaumont, France.
- [6] Colorectal Surgery Unit, Department of Abdominal Surgery and Transplantation Cliniques Universitaires Saint-Luc, Université Catholique de Louvain (UCL), Brussels, Belgium.
- [7] Department of Hepatobiliary and Digestive Surgery, CHU Rennes, 2 rue Henri Le Guilloux, Rennes, France.
- [8] Chirurgie Cancérologique, Digestive et Endocrinienne (CCDE), Institut des Maladies de l'Appareil Digestif (IMAD), Centre Hospitalo-universitaire de Nantes (CHU) Hôtel-Dieu, Place Alexis Ricordeau, 44093, Nantes, France.
- [9] Digestive surgery & digestive cancerology, CHU Carémeau, université de Montpellier, place du Professeur-Robert-Debré, 30029, Nîmes cedex 9, France.
- [10] CHRU de Brest, Brest, France.
- [11] Service de chirurgie digestive CHU Caen, registre des tumeurs digestive du calvados, Inserm U1086 ANTICIPE, 14000, Caen, France.
- [12] Department of Surgical Oncology, Institut de Cancérologie de Lorraine, Université de Lorraine, 54519, Vandoeuvre-lès-Nancy, France.

- [13] Department of Endocrinal and Visceral Surgery, Angers University Hospital, 49933, Angers, France.
- [14] Department of Digestive Surgery, Assistance Publique Hôpitaux de Marseille, Timone University Hospital, Marseille, France.
- [15] Digestive Surgery Department, Beauvais Hospital, Beauvais, France.
- [16] Department of Surgical Oncology, Comprehensive Cancer Center, Institut de Cancérologie de l'Ouest, Saint-Herblain, France.
- [17] Department of Digestive Surgery and Transplantation, University Hospital Croix Rousse, Hospices Civils de Lyon, University of Lyon I, Lyon, France.
- [18] Department of Surgical Oncology, University Hospital Jean Minjoz, Besançon, France.
- [19] Department of Gastrointestinal Surgery, Hospices Civils de Lyon, Université de Lyon, Centre Hospitalier Lyon-Sud, 165 chemin du grand Revoyet, 69495, Pierre Bénite, France.
- [20] Department of Digestive Surgical Oncology, Department of Mini Invasive Interventions (DIMI), Paoli Calmettes Institute, Marseille, France.

- PMID: **33495872**
- PMCID: PMC7835106
- DOI: 10.1007/s00384-021-03847-4

Abstract

Introduction: During the COVID-19 pandemic, cancer patients have been regarded as having a high risk of severe events if they are infected with SARS-CoV-2, particularly those under medical or surgical treatment. The aim of this study was to assess the posttreatment risk of infection by SARS-CoV-2 in a population of patients operated on for colorectal cancer 3 months before the COVID-19 outbreak and who after hospitalization returned to an environment where the virus was circulating.

Materials and methods: This French, multicenter cohort study included consecutive patients undergoing elective surgery for colorectal cancer between January 1 and March 31, 2020, at 19 GRECCAR hospitals. The outcome was the rate of COVID-19 infection in this group of patients who were followed until June 15, 2020.

Results: This study included 448 patients, 262 male (58.5%) and 186 female (41.5%), who underwent surgery for colon cancer (n = 290, 64.7%), rectal cancer (n = 155, 34.6%), or anal cancer (n = 3, 0.7%). The median age was 68 years (19-95). Comorbidities were present in nearly half of the patients, 52% were at least overweight, and the median BMI was 25 (12-42). At the end of the study, 448 were alive. Six patients (1.3%) developed COVID-19 infection; among them, 3 were hospitalized in the conventional ward, and none of them died.

Conclusion: The results are reassuring, with only a 1.3% infection rate and no deaths related to COVID-19. We believe that we can operate on colorectal cancer patients without additional mortality from COVID-19, applying all measures aimed at reducing the risk of infection.

Keywords: COVID-19 outbreak; Cancer; Colorectal surgery; Oncology; Pandemic.

- 13 references

Supplementary info

Publication types, MeSH terms Expand

Publication types

- Multicenter Study
- Observational Study

MeSH terms

- Adult
- Aged
- Aged, 80 and over
- COVID-19 / epidemiology*
- Colorectal Neoplasms / surgery*
- Comorbidity
- Elective Surgical Procedures
- Female
- France / epidemiology
- Humans
- Male
- Middle Aged
- Postoperative Period
- Retrospective Studies
- Risk Assessment
- Young Adult

Full text links

SpringerLink FULL-TEXT ARTICLE Springer Free PMC article
Proceed to details
Cite
Share
1,068
Observational Study
Circ Arrhythm Electrophysiol
. 2020 Sep;13(9):e008774.
doi: 10.1161/CIRCEP.120.008774. Epub 2020 Jul 23.

Impact of the COVID-19 Pandemic on a Tertiary-Level Electrophysiology Laboratory in Italy

Paolo Compagnucci [1] [2], Giovanni Volpato [1] [2], Riccardo Pascucci [1] [2], Umberto Falanga [1] [2], Agostino Misiani [1], Silvano Molini [1], Giulio Spinucci [1], Laura Cipolletta [1], Manuel Conti [1] [3], Gino Grifoni [1], Laura Carboni [4], Gian Piero Perna [5], Christopher Munch [4], Marco Di Eusanio [6], Alessandro Capucci [1] [2], Michela Casella [1], Federico Guerra [1] [2], Antonio Dello Russo [1] [2]
Affiliations Expand

Affiliations

- [1] Cardiology and Arrhythmology Clinic (P.C., G.V., R.P., U.F., A.M., S.M., G.S., L. Cipolletta, M.C., G.G., A.C., M.C., F.G., A.D.R.), University Hospital "Ospedali Riuniti", Ancona, Italy.
- [2] Department of Biomedical Sciences and Public Health (P.C., G.V., R.P., U.F., A.C., F.G., A.D.R.), Marche Polytechnic University, Ancona, Italy.
- [3] Department of Clinical, Special and Dental Sciences (M.C.), Marche Polytechnic University, Ancona, Italy.
- [4] Cardiac Surgery Anesthesia and Critical Care Unit (L. Carboni, C.M.), University Hospital "Ospedali Riuniti", Ancona, Italy.
- [5] Cardiology and Coronary Care Unit (G.P.P.), University Hospital "Ospedali Riuniti", Ancona, Italy.
- [6] Cardiac Surgery Unit, University Hospital "Ospedali Riuniti", Marche Polytechnic University, Ancona, Italy (M.D.E.).

- PMID: **32703011**
- PMCID: PMC7482615
- DOI: 10.1161/CIRCEP.120.008774

Free PMC article
Observational Study

Impact of the COVID-19 Pandemic on a Tertiary-Level Electrophysiology Laboratory in Italy

Paolo Compagnucci et al. Circ Arrhythm Electrophysiol. 2020 Sep.
Free PMC article
Show details
Circ Arrhythm Electrophysiol
. 2020 Sep;13(9):e008774.
doi: 10.1161/CIRCEP.120.008774. Epub 2020 Jul 23.

Authors

Paolo Compagnucci [1] [2], Giovanni Volpato [1] [2], Riccardo Pascucci [1] [2], Umberto Falanga [1] [2], Agostino Misiani [1], Silvano Molini [1], Giulio Spinucci [1], Laura Cipolletta [1], Manuel Conti [1] [3], Gino Grifoni [1], Laura Carboni [4], Gian Piero Perna [5], Christopher Munch [4], Marco Di

Eusanio [6], Alessandro Capucci [1] [2], Michela Casella [1], Federico Guerra [1] [2], Antonio Dello Russo [1] [2]

Affiliations

- [1] Cardiology and Arrhythmology Clinic (P.C., G.V., R.P., U.F., A.M., S.M., G.S., L. Cipolletta, M.C., G.G., A.C., M.C., F.G., A.D.R.), University Hospital "Ospedali Riuniti", Ancona, Italy.
- [2] Department of Biomedical Sciences and Public Health (P.C., G.V., R.P., U.F., A.C., F.G., A.D.R.), Marche Polytechnic University, Ancona, Italy.
- [3] Department of Clinical, Special and Dental Sciences (M.C.), Marche Polytechnic University, Ancona, Italy.
- [4] Cardiac Surgery Anesthesia and Critical Care Unit (L. Carboni, C.M.), University Hospital "Ospedali Riuniti", Ancona, Italy.
- [5] Cardiology and Coronary Care Unit (G.P.P.), University Hospital "Ospedali Riuniti", Ancona, Italy.
- [6] Cardiac Surgery Unit, University Hospital "Ospedali Riuniti", Marche Polytechnic University, Ancona, Italy (M.D.E.).

- PMID: **32703011**
- PMCID: PMC7482615
- DOI: 10.1161/CIRCEP.120.008774

No abstract available

Keywords: arrhythmias; catheter ablation; coronavirus; epidemiology; viruses.

Conflict of interest statement

None.

- 5 references

Supplementary info

Publication types, MeSH terms Expand

Publication types

- Letter
- Observational Study

MeSH terms

- COVID-19
- Cohort Studies
- Coronavirus Infections / epidemiology
- Coronavirus Infections / prevention & control*

- Cross Infection / prevention & control*
- Electrophysiologic Techniques, Cardiac / methods
- Electrophysiologic Techniques, Cardiac / statistics & numerical data*
- Female
- Hospitals, University
- Humans
- Infection Control / methods*
- Italy
- Laboratories, Hospital / organization & administration*
- Male
- Pandemics / prevention & control*
- Pandemics / statistics & numerical data
- Pneumonia, Viral / epidemiology
- Pneumonia, Viral / prevention & control*
- Retrospective Studies
- Tertiary Care Centers

Full text links

FULL TEXT Atypon Free PMC article
Proceed to details
Cite
Share
1,069
Observational Study
J Clin Anesth
. 2020 Dec;67:110005.
doi: 10.1016/j.jclinane.2020.110005. Epub 2020 Jul 20.

Clinical comorbidities, characteristics, and outcomes of mechanically ventilated patients in the State of Michigan with SARS-CoV-2 pneumonia

Sandeep Krishnan [1], Kinjal Patel [2], Ronak Desai [3], Anupam Sule [4], Peter Paik [5], Ashley Miller [6], Alicia Barclay [7], Adam Cassella [8], Jon Lucaj [9], Yvonne Royster [10], Joffer Hakim [11], Zulfiqar Ahmed [12], Farhad Ghoddoussi [13]
Affiliations Expand

Affiliations

- [1] Department of Anesthesiology, Wayne State University School of Medicine, Detroit, MI 48201, USA; Department of Anesthesiology, St. Joseph Mercy Oakland Hospital, Pontiac, MI 48341, USA; Department of Anesthesiology, Wayne State University School of Medicine, St. Joseph Mercy Oakland Medical Office Building, 44555 Woodward Avenue, Suite 308, Pontiac, MI 48341, USA. Electronic address: sakrishna@med.wayne.edu.
- [2] Department of Anesthesiology, Cooper Medical School of Rowan University, Cooper University Healthcare, One Cooper Plaza, Camden, NJ 08103, USA; Department of Anesthesiology, Cooper Medical School of Rowan University, Cooper University Health Care, 1 Cooper Plaza, Camden, NJ 08103, USA. Electronic address: patel-kinjal@cooperhealth.edu.
- [3] Department of Anesthesiology, Cooper Medical School of Rowan University, Cooper University Healthcare, One Cooper Plaza, Camden, NJ 08103, USA; Department of Anesthesiology, Cooper Medical School of Rowan University, Cooper University Health Care, 1 Cooper Plaza, Camden, NJ 08103, USA. Electronic address: desai-ronak@cooperhealth.edu.
- [4] Department of Internal Medicine, St. Joseph Mercy Oakland Hospital, Pontiac, MI 48341, USA; Chief Medical Informatics Officer and Medical Director of Outcomes, Admin Ste, St. Joseph Mercy Oakland, Pontiac, MI 48341, USA. Electronic address: Anupam.a.sule@stjoeshealth.org.
- [5] Department of Anesthesiology, Wayne State University School of Medicine, Detroit, MI 48201, USA; Department of Anesthesiology, St. Joseph Mercy Oakland Hospital, Pontiac, MI 48341, USA; Department of Anesthesiology, St. Joseph Mercy Oakland Hospital, St. Joseph Mercy Oakland Medical Office Building, 44555 Woodward Avenue, Suite 308, Pontiac, MI 48341, USA. Electronic address: Peterpaik@wayne.edu.
- [6] Department of Anesthesiology, Wayne State University School of Medicine, Detroit, MI 48201, USA; Department of Anesthesiology, St. Joseph Mercy Oakland Hospital, Pontiac, MI 48341, USA; Department of Anesthesiology, St. Joseph Mercy Oakland Hospital, St. Joseph Mercy Oakland Medical Office Building, 44555 Woodward Avenue, Suite 308, Pontiac, MI 48341, USA. Electronic address: gs2642@wayne.edu.
- [7] Department of Anesthesiology, Wayne State University School of Medicine, Detroit, MI 48201, USA; Department of Anesthesiology, St. Joseph Mercy Oakland Hospital, Pontiac, MI 48341, USA; Department of Anesthesiology, Wayne State University School of Medicine, St. Joseph Mercy Oakland Medical Office Building, 44555 Woodward Avenue, Suite 308, Pontiac, MI 48341, USA. Electronic address: abarclay@wayne.edu.
- [8] Department of Anesthesiology, Wayne State University School of Medicine, Detroit, MI 48201, USA; Department of Anesthesiology, St. Joseph Mercy Oakland Hospital, Pontiac, MI 48341, USA; Department of Anesthesiology, Wayne State University School of Medicine, St. Joseph Mercy Oakland Medical Office Building, 44555 Woodward Avenue, Suite 308, Pontiac, MI 48341, USA. Electronic address: a.cassella@wayne.edu.
- [9] Department of Anesthesiology, Wayne State University School of Medicine, Detroit, MI 48201, USA; Department of Anesthesiology, St. Joseph Mercy Oakland Hospital, Pontiac, MI 48341, USA; Department of Anesthesiology, Wayne State University School of Medicine, St. Joseph Mercy Oakland Medical Office Building, 44555 Woodward Avenue, Suite 308, Pontiac, MI 48341, USA. Electronic address: jon_lucaj@wayne.edu.
- [10] Department of Cardiovascular Surgery, St. Joseph Mercy Oakland Hospital, Pontiac, MI 48341, USA; Department of Cardiothoracic Surgery, St. Joseph Mercy Oakland, Medical Office Building, 44555 Woodward Ave, Suite #506, Pontiac, MI 48341, USA. Electronic address: Yvonne.royster@stjoeshealth.org.
- [11] Department of Anesthesiology, Wayne State University School of Medicine, Detroit, MI 48201, USA; Department of Anesthesiology, St. Joseph Mercy Oakland Hospital, Pontiac, MI 48341, USA; Department of Anesthesiology, Wayne State University School of

Medicine, St. Joseph Mercy Oakland Medical Office Building, 44555 Woodward Avenue, Suite 308, Pontiac, MI 48341, USA. Electronic address: jhhakim@med.wayne.edu.

- [12] Department of Anesthesiology, Wayne State University School of Medicine, Detroit, MI 48201, USA; Department of Anesthesiology, St. Joseph Mercy Oakland Hospital, Pontiac, MI 48341, USA; Department of Anesthesiology, Wayne State University School of Medicine, St. Joseph Mercy Oakland Medical Office Building, 44555 Woodward Avenue, Suite 308, Pontiac, MI 48341, USA. Electronic address: zahmed@a4anes.net.
- [13] Department of Anesthesiology, Wayne State University School of Medicine, Detroit, MI 48201, USA; Department of Anesthesiology, Wayne State University School of Medicine, University Health Center (UHC) Bldg., Section 4J, Room #28, Detroit, MI 48201, USA. Electronic address: fghoddoussi@med.wayne.edu.

- PMID: **32707517**
- PMCID: PMC7369577
- DOI: 10.1016/j.jclinane.2020.110005

Free PMC article
Observational Study

Clinical comorbidities, characteristics, and outcomes of mechanically ventilated patients in the State of Michigan with SARS-CoV-2 pneumonia

Sandeep Krishnan et al. J Clin Anesth. 2020 Dec.
Free PMC article

Show details

J Clin Anesth

. 2020 Dec;67:110005.
doi: 10.1016/j.jclinane.2020.110005. Epub 2020 Jul 20.

Authors

Sandeep Krishnan [1], Kinjal Patel [2], Ronak Desai [3], Anupam Sule [4], Peter Paik [5], Ashley Miller [6], Alicia Barclay [7], Adam Cassella [8], Jon Lucaj [9], Yvonne Royster [10], Joffer Hakim [11], Zulfiqar Ahmed [12], Farhad Ghoddoussi [13]

Affiliations

- [1] Department of Anesthesiology, Wayne State University School of Medicine, Detroit, MI 48201, USA; Department of Anesthesiology, St. Joseph Mercy Oakland Hospital, Pontiac, MI 48341, USA; Department of Anesthesiology, Wayne State University School of Medicine, St. Joseph Mercy Oakland Medical Office Building, 44555 Woodward Avenue, Suite 308, Pontiac, MI 48341, USA. Electronic address: sakrishna@med.wayne.edu.
- [2] Department of Anesthesiology, Cooper Medical School of Rowan University, Cooper University Healthcare, One Cooper Plaza, Camden, NJ 08103, USA; Department of Anesthesiology, Cooper Medical School of Rowan University, Cooper University Health

Care, 1 Cooper Plaza, Camden, NJ 08103, USA. Electronic address: patel-kinjal@cooperhealth.edu.

- [3] Department of Anesthesiology, Cooper Medical School of Rowan University, Cooper University Healthcare, One Cooper Plaza, Camden, NJ 08103, USA; Department of Anesthesiology, Cooper Medical School of Rowan University, Cooper University Health Care, 1 Cooper Plaza, Camden, NJ 08103, USA. Electronic address: desai-ronak@cooperhealth.edu.
- [4] Department of Internal Medicine, St. Joseph Mercy Oakland Hospital, Pontiac, MI 48341, USA; Chief Medical Informatics Officer and Medical Director of Outcomes, Admin Ste, St. Joseph Mercy Oakland, Pontiac, MI 48341, USA. Electronic address: Anupam.a.sule@stjoeshealth.org.
- [5] Department of Anesthesiology, Wayne State University School of Medicine, Detroit, MI 48201, USA; Department of Anesthesiology, St. Joseph Mercy Oakland Hospital, Pontiac, MI 48341, USA; Department of Anesthesiology, St. Joseph Mercy Oakland Hospital, St. Joseph Mercy Oakland Medical Office Building, 44555 Woodward Avenue, Suite 308, Pontiac, MI 48341, USA. Electronic address: Peterpaik@wayne.edu.
- [6] Department of Anesthesiology, Wayne State University School of Medicine, Detroit, MI 48201, USA; Department of Anesthesiology, St. Joseph Mercy Oakland Hospital, Pontiac, MI 48341, USA; Department of Anesthesiology, St. Joseph Mercy Oakland Hospital, St. Joseph Mercy Oakland Medical Office Building, 44555 Woodward Avenue, Suite 308, Pontiac, MI 48341, USA. Electronic address: gs2642@wayne.edu.
- [7] Department of Anesthesiology, Wayne State University School of Medicine, Detroit, MI 48201, USA; Department of Anesthesiology, St. Joseph Mercy Oakland Hospital, Pontiac, MI 48341, USA; Department of Anesthesiology, Wayne State University School of Medicine, St. Joseph Mercy Oakland Medical Office Building, 44555 Woodward Avenue, Suite 308, Pontiac, MI 48341, USA. Electronic address: abarclay@wayne.edu.
- [8] Department of Anesthesiology, Wayne State University School of Medicine, Detroit, MI 48201, USA; Department of Anesthesiology, St. Joseph Mercy Oakland Hospital, Pontiac, MI 48341, USA; Department of Anesthesiology, Wayne State University School of Medicine, St. Joseph Mercy Oakland Medical Office Building, 44555 Woodward Avenue, Suite 308, Pontiac, MI 48341, USA. Electronic address: a.cassella@wayne.edu.
- [9] Department of Anesthesiology, Wayne State University School of Medicine, Detroit, MI 48201, USA; Department of Anesthesiology, St. Joseph Mercy Oakland Hospital, Pontiac, MI 48341, USA; Department of Anesthesiology, Wayne State University School of Medicine, St. Joseph Mercy Oakland Medical Office Building, 44555 Woodward Avenue, Suite 308, Pontiac, MI 48341, USA. Electronic address: jon_lucaj@wayne.edu.
- [10] Department of Cardiovascular Surgery, St. Joseph Mercy Oakland Hospital, Pontiac, MI 48341, USA; Department of Cardiothoracic Surgery, St. Joseph Mercy Oakland, Medical Office Building, 44555 Woodward Ave, Suite #506, Pontiac, MI 48341, USA. Electronic address: Yvonne.royster@stjoeshealth.org.
- [11] Department of Anesthesiology, Wayne State University School of Medicine, Detroit, MI 48201, USA; Department of Anesthesiology, St. Joseph Mercy Oakland Hospital, Pontiac, MI 48341, USA; Department of Anesthesiology, Wayne State University School of Medicine, St. Joseph Mercy Oakland Medical Office Building, 44555 Woodward Avenue, Suite 308, Pontiac, MI 48341, USA. Electronic address: jhhakim@med.wayne.edu.
- [12] Department of Anesthesiology, Wayne State University School of Medicine, Detroit, MI 48201, USA; Department of Anesthesiology, St. Joseph Mercy Oakland Hospital, Pontiac, MI 48341, USA; Department of Anesthesiology, Wayne State University School of Medicine, St. Joseph Mercy Oakland Medical Office Building, 44555 Woodward Avenue, Suite 308, Pontiac, MI 48341, USA. Electronic address: zahmed@a4anes.net.

- [13] Department of Anesthesiology, Wayne State University School of Medicine, Detroit, MI 48201, USA; Department of Anesthesiology, Wayne State University School of Medicine, University Health Center (UHC) Bldg., Section 4J, Room #28, Detroit, MI 48201, USA. Electronic address: fghoddoussi@med.wayne.edu.

- PMID: **32707517**
- PMCID: PMC7369577
- DOI: 10.1016/j.jclinane.2020.110005

Abstract

- Retrospective multi-center study of patients with confirmed coronavirus (SARS-CoV-2).

- Consecutive patients requiring mechanical ventilation from March 10 to April 15, 2020 enrolled.

- Increased age, hypertension, statin use, increased fluid administration were associated with increased mortality.

- Need for continuous renal replacement therapy (CRRT), and vasopressor use were associated with increased mortality.

- Decreased risk of mortality in patients treated with steroids & vitamin C, & in patients with greater urine output.

- 5 references

Supplementary info

Publication types, MeSH terms Expand

Publication types

- Letter
- Multicenter Study
- Observational Study

MeSH terms

- Aged

- COVID-19 / complications*
- COVID-19 / mortality
- COVID-19 / therapy*
- Comorbidity
- Demography
- Female
- Humans
- Male
- Michigan / epidemiology
- Middle Aged
- Respiration, Artificial*
- Retrospective Studies
- Treatment Outcome

Full text links

ELSEVIER FULL-TEXT ARTICLE Elsevier Science Free PMC article
Proceed to details
Cite
Share
1,070
Observational Study
CPT Pharmacometrics Syst Pharmacol
. 2021 Oct;10(10):1161-1170.
doi: 10.1002/psp4.12685. Epub 2021 Aug 17.

Population pharmacokinetics of favipiravir in patients with COVID-19

Kei Irie[1 2], Atsushi Nakagawa[3], Hirotoshi Fujita[1], Ryo Tamura[1], Masaaki Eto[4], Hiroaki Ikesue[1], Nobuyuki Muroi[1], Shoji Fukushima[2], Keisuke Tomii[3], Tohru Hashida[1]
Affiliations Expand

Affiliations

- [1] Department of Pharmacy, Kobe City Hospital Organization, Kobe City Medical Center General Hospital, Kobe, Japan.
- [2] Faculty of Pharmaceutical Science, Kobe Gakuin University, Kobe, Japan.
- [3] Department of Respiratory Medicine, Kobe City Hospital Organization, Kobe City Medical Center General Hospital, Kobe, Japan.
- [4] Department of Clinical Laboratory, Kobe City Hospital Organization, Kobe City Medical Center General Hospital, Kobe, Japan.

- PMID: **34292670**

- PMCID: PMC8420316
- DOI: 10.1002/psp4.12685

Free PMC article
Observational Study

Population pharmacokinetics of favipiravir in patients with COVID-19

Kei Irie et al. CPT Pharmacometrics Syst Pharmacol. 2021 Oct.
Free PMC article

Show details

CPT Pharmacometrics Syst Pharmacol
. 2021 Oct;10(10):1161-1170.
doi: 10.1002/psp4.12685. Epub 2021 Aug 17.

Authors

Kei Irie [1, 2], Atsushi Nakagawa [3], Hirotoshi Fujita [1], Ryo Tamura [1], Masaaki Eto [4], Hiroaki Ikesue [1], Nobuyuki Muroi [1], Shoji Fukushima [2], Keisuke Tomii [3], Tohru Hashida [1]

Affiliations

- [1] Department of Pharmacy, Kobe City Hospital Organization, Kobe City Medical Center General Hospital, Kobe, Japan.
- [2] Faculty of Pharmaceutical Science, Kobe Gakuin University, Kobe, Japan.
- [3] Department of Respiratory Medicine, Kobe City Hospital Organization, Kobe City Medical Center General Hospital, Kobe, Japan.
- [4] Department of Clinical Laboratory, Kobe City Hospital Organization, Kobe City Medical Center General Hospital, Kobe, Japan.

- PMID: **34292670**
- PMCID: PMC8420316
- DOI: 10.1002/psp4.12685

Abstract

The antiretroviral drug favipiravir (FPV) inhibits RNA-dependent RNA polymerase. It has been developed for the treatment of the novel coronavirus (severe acute respiratory syndrome coronavirus 2) infection disease, coronavirus disease 2019 (COVID-19). However, its pharmacokinetics in patients with COVID-19 is poorly understood. In this study, we measured FPV serum concentration by liquid chromatography-tandem mass spectrometry and conducted population pharmacokinetic analysis. A total of 39 patients were enrolled in the study: 33 were administered FPV 1600 mg twice daily (b.i.d.) on the first day followed by 600 mg b.i.d., and 6 were administered FPV 1800 mg b.i.d. on the first day followed by 800 mg or 600 mg b.i.d. The median age was 68 years (range, 27-89 years), 31 (79.5%) patients were men, median body surface area (BSA) was 1.72 m^2 (range, 1.11-2.2 m^2), and 10 (25.6%) patients required invasive mechanical ventilation (IMV) at the start of FPV. A total of 204 serum concentrations were

available for pharmacokinetic analysis. A one-compartment model with first-order elimination was used to describe the pharmacokinetics. The estimated mean clearance/bioavailability (CL/F) and distribution volume/bioavailability (V/F) were 5.11 L/h and 41.6 L, respectively. Covariate analysis revealed that CL/F was significantly related to dosage, IMV use, and BSA. A simulation study showed that the 1600 mg/600 mg b.i.d. regimen was insufficient for the treatment of COVID-19 targeting the 50% effective concentration (9.7 µg/mL), especially in patients with larger BSA and/or IMV. A higher FPV dosage is required for COVID-19, but dose-dependent nonlinear pharmacokinetics may cause an unexpected significant pharmacokinetic change and drug toxicity. Further studies are warranted to explore the optimal FPV regimen.

© 2021 The Authors. CPT: Pharmacometrics & Systems Pharmacology published by Wiley Periodicals LLC on behalf of American Society for Clinical Pharmacology and Therapeutics.

Conflict of interest statement

The authors declared no competing interests for this work.

- 19 references
- 3 figures

Supplementary info

Publication types, MeSH terms, Substances Expand

Publication types

- Observational Study

MeSH terms

- Adult
- Aged
- Aged, 80 and over
- Amides / administration & dosage*
- Amides / pharmacokinetics
- Antiviral Agents / administration & dosage*
- Antiviral Agents / pharmacokinetics
- COVID-19 / blood
- COVID-19 / drug therapy*
- Chromatography, Liquid
- Dose-Response Relationship, Drug
- Drug Administration Schedule
- Female
- Humans
- Male
- Middle Aged

- Models, Theoretical
- Pyrazines / administration & dosage*
- Pyrazines / pharmacokinetics
- Retrospective Studies
- Tandem Mass Spectrometry
- Treatment Outcome

Substances

- Amides
- Antiviral Agents
- Pyrazines
- favipiravir

Full text links

WILEY Free Full Text Wiley Free PMC article
Proceed to details
Cite
Share
1,071
Observational Study
Crit Care
. 2021 Aug 19;25(1):299.
doi: 10.1186/s13054-021-03729-9.

Continuous renal replacement therapy in COVID-19-associated AKI: adding heparin to citrate to extend filter life-a retrospective cohort study

Eduardo de Oliveira Valle [1], Carla Paulina Sandoval Cabrera [1], Claudia Coimbra César de Albuquerque [1], Giovanio Vieira da Silva [1], Márcia Fernanda Arantes de Oliveira [1], Gabriel Teixeira Montezuma Sales [1], Igor Smolentzov [1], Bernardo Vergara Reichert [1], Lucia Andrade [1], Victor Faria Seabra [1], Paulo Ricardo Gessolo Lins [1], Camila Eleuterio Rodrigues [2]
Affiliations Expand

Affiliations

- [1] Hospital das Clínicas, University of São Paulo School of Medicine, Av. Dr. Arnaldo, 455, 3º andar, sala 3310, São Paulo, SP, CEP 01246-903, Brazil.

- [2] Hospital das Clínicas, University of São Paulo School of Medicine, Av. Dr. Arnaldo, 455, 3° andar, sala 3310, São Paulo, SP, CEP 01246-903, Brazil. camila.eleuterio@hc.fm.usp.br.

- PMID: **34412667**
- PMCID: PMC8375288
- DOI: 10.1186/s13054-021-03729-9

Free PMC article
Observational Study

Continuous renal replacement therapy in COVID-19-associated AKI: adding heparin to citrate to extend filter life-a retrospective cohort study

Eduardo de Oliveira Valle et al. Crit Care. 2021.
Free PMC article

Show details

Crit Care
. 2021 Aug 19;25(1):299.
doi: 10.1186/s13054-021-03729-9.

Authors

Eduardo de Oliveira Valle [1], Carla Paulina Sandoval Cabrera [1], Claudia Coimbra César de Albuquerque [1], Giovanio Vieira da Silva [1], Márcia Fernanda Arantes de Oliveira [1], Gabriel Teixeira Montezuma Sales [1], Igor Smolentzov [1], Bernardo Vergara Reichert [1], Lucia Andrade [1], Victor Faria Seabra [1], Paulo Ricardo Gessolo Lins [1], Camila Eleuterio Rodrigues [2]

Affiliations

- [1] Hospital das Clínicas, University of São Paulo School of Medicine, Av. Dr. Arnaldo, 455, 3° andar, sala 3310, São Paulo, SP, CEP 01246-903, Brazil.
- [2] Hospital das Clínicas, University of São Paulo School of Medicine, Av. Dr. Arnaldo, 455, 3° andar, sala 3310, São Paulo, SP, CEP 01246-903, Brazil. camila.eleuterio@hc.fm.usp.br.

- PMID: **34412667**
- PMCID: PMC8375288
- DOI: 10.1186/s13054-021-03729-9

Abstract

Background: Coronavirus disease 2019 (COVID-19) may predispose patients to thrombotic events. The best anticoagulation strategy for continuous renal replacement therapy (CRRT) in such patients is still under debate. The purpose of this study was to evaluate the impact that different anticoagulation protocols have on filter clotting risk.

Methods: This was a retrospective observational study comparing two different anticoagulation strategies (citrate only and citrate plus intravenous infusion of unfractionated heparin) in patients with acute kidney injury (AKI), associated or not with COVID-19 (COV + AKI and COV - AKI, respectively), who were submitted to CRRT. Filter clotting risks were compared among groups.

Results: Between January 2019 and July 2020, 238 patients were evaluated: 188 in the COV + AKI group and 50 in the COV - AKI group. Filter clotting during the first filter use occurred in 111 patients (46.6%). Heparin use conferred protection against filter clotting (HR = 0.37, 95% CI 0.25-0.55), resulting in longer filter survival. Bleeding events and the need for blood transfusion were similar between the citrate only and citrate plus unfractionated heparin strategies. In-hospital mortality was higher among the COV + AKI patients than among the COV - AKI patients, although it was similar between the COV + AKI patients who received heparin and those who did not. Filter clotting was more common in patients with D-dimer levels above the median (5990 ng/ml). In the multivariate analysis, heparin was associated with a lower risk of filter clotting (HR = 0.28, 95% CI 0.18-0.43), whereas an elevated D-dimer level and high hemoglobin were found to be risk factors for circuit clotting. A diagnosis of COVID-19 was marginally associated with an increased risk of circuit clotting (HR = 2.15, 95% CI 0.99-4.68).

Conclusions: In COV + AKI patients, adding systemic heparin to standard regional citrate anticoagulation may prolong CRRT filter patency by reducing clotting risk with a low risk of complications.

Keywords: Acute kidney injury; COVID-19; Citrate; Continuous renal replacement therapy; D-Dimer; Filter lifespan; Heparin.

© 2021. The Author(s).

Conflict of interest statement

C.E.R. received fees from Medtronic for providing instruction in catheter insertion. All remaining authors have nothing to disclose.

- 25 references
- 5 figures

Supplementary info

Publication types, MeSH terms, Substances, Grant support Expand

Publication types

- Observational Study
- Research Support, Non-U.S. Gov't

MeSH terms

- Acute Kidney Injury / drug therapy*
- Acute Kidney Injury / epidemiology
- Acute Kidney Injury / etiology
- Adult

- COVID-19 / complications
- COVID-19 / epidemiology
- Citric Acid / adverse effects
- Citric Acid / pharmacology*
- Citric Acid / therapeutic use
- Cohort Studies
- Continuous Renal Replacement Therapy / instrumentation*
- Continuous Renal Replacement Therapy / methods
- Continuous Renal Replacement Therapy / statistics & numerical data
- Female
- Heparin / adverse effects
- Heparin / pharmacology*
- Heparin / therapeutic use
- Humans
- Kaplan-Meier Estimate
- Male
- Micropore Filters / standards*
- Micropore Filters / statistics & numerical data
- Middle Aged
- Proportional Hazards Models
- Retrospective Studies

Substances

- Citric Acid
- Heparin

Grant support

- CG 19.108/Faculdade de Medicina da Universidade de São Paulo

Full text links

Read free full text at BMC BioMed Central Free PMC article

Proceed to details

Cite

Share

1,072

Observational Study

J Stroke Cerebrovasc Dis
. 2021 Jun;30(6):105733.
doi: 10.1016/j.jstrokecerebrovasdis.2021.105733. Epub 2021 Mar 4.

Cerebral Venous Sinus Thrombosis in COVID-19 Patients: A Multicenter Study and Review of Literature

Mohamad Abdalkader [1], Shamsh P Shaikh [2], James E Siegler [3], Anna M Cervantes-Arslanian [4], Cristina Tiu [5], Razvan Alexandru Radu [5], Vlad Eugen Tiu [5], Dinesh V Jillella [6], Ossama Yassin Mansour [7], Víctor Vera [8], Ángel Chamorro [8], Jordi Blasco [8], Antonio López [8], Mudassir Farooqui [9], Lauren Thau [3], Ainsley Smith [3], Santiago Ortega Gutierrez [10], Thanh N Nguyen [11], Tudor G Jovin [3]
Affiliations Expand

Affiliations

- [1] Department of Radiology, Boston Medical Center, Boston, Massachusetts, USA. Electronic address: mohamad.abdalkader@bmc.org.
- [2] Department of Neurology, Boston Medical Center, Boston, Massachusetts, USA.
- [3] Department of Neurosurgery, Boston Medical Center, Boston, Massachusetts, USA.
- [4] Department of Neurology, Boston Medical Center, Boston, Massachusetts, USA; Department of Neurology, Cooper University Hospital, Camden, New Jersey, USA.
- [5] Department of Neurology, Elias University Emergency Hospital, Bucharest, Romania.
- [6] Department of Neurology, Emory University Hospital, Atlanta, Georgia, USA.
- [7] Department of Neurology, Alexandria University School of Medicine, Alexandria, Egypt.
- [8] Department of Radiology, Hospital Clínic de Barcelona, Barcelona, Spain.
- [9] Department of Neurology, University of Iowa, Iowa City, Iowa, USA.
- [10] Department of Neurosurgery, University of Iowa, Iowa City, Iowa, USA.
- [11] Department of Radiology, Boston Medical Center, Boston, Massachusetts, USA; Department of Neurology, Boston Medical Center, Boston, Massachusetts, USA; Department of Neurology, Cooper University Hospital, Camden, New Jersey, USA.

- PMID: **33743411**
- PMCID: PMC7931726
- DOI: 10.1016/j.jstrokecerebrovasdis.2021.105733

Free PMC article
Observational Study

Cerebral Venous Sinus Thrombosis in COVID-19 Patients: A Multicenter Study and Review of Literature

Mohamad Abdalkader et al. J Stroke Cerebrovasc Dis. 2021 Jun.
Free PMC article
Show details

J Stroke Cerebrovasc Dis
. 2021 Jun;30(6):105733.
doi: 10.1016/j.jstrokecerebrovasdis.2021.105733. Epub 2021 Mar 4.

Authors

Mohamad Abdalkader [1], Shamsh P Shaikh [2], James E Siegler [3], Anna M Cervantes-Arslanian [4], Cristina Tiu [5], Razvan Alexandru Radu [5], Vlad Eugen Tiu [5], Dinesh V Jillella [6], Ossama Yassin Mansour [7], Víctor Vera [8], Ángel Chamorro [8], Jordi Blasco [8], Antonio López [8], Mudassir Farooqui [9], Lauren Thau [3], Ainsley Smith [3], Santiago Ortega Gutierrez [10], Thanh N Nguyen [11], Tudor G Jovin [3]

Affiliations

- [1] Department of Radiology, Boston Medical Center, Boston, Massachusetts, USA. Electronic address: mohamad.abdalkader@bmc.org.
- [2] Department of Neurology, Boston Medical Center, Boston, Massachusetts, USA.
- [3] Department of Neurosurgery, Boston Medical Center, Boston, Massachusetts, USA.
- [4] Department of Neurology, Boston Medical Center, Boston, Massachusetts, USA; Department of Neurology, Cooper University Hospital, Camden, New Jersey, USA.
- [5] Department of Neurology, Elias University Emergency Hospital, Bucharest, Romania.
- [6] Department of Neurology, Emory University Hospital, Atlanta, Georgia, USA.
- [7] Department of Neurology, Alexandria University School of Medicine, Alexandria, Egypt.
- [8] Department of Radiology, Hospital Clínic de Barcelona, Barcelona, Spain.
- [9] Department of Neurology, University of Iowa, Iowa City, Iowa, USA.
- [10] Department of Neurosurgery, University of Iowa, Iowa City, Iowa, USA.
- [11] Department of Radiology, Boston Medical Center, Boston, Massachusetts, USA; Department of Neurology, Boston Medical Center, Boston, Massachusetts, USA; Department of Neurology, Cooper University Hospital, Camden, New Jersey, USA.

- PMID: **33743411**
- PMCID: PMC7931726
- DOI: 10.1016/j.jstrokecerebrovasdis.2021.105733

Abstract

Background: COVID-19 infection has been known to predispose patients to both arterial and venous thromboembolic events such as deep venous thrombosis, pulmonary embolism, myocardial infarction, and stroke. A few reports from the literature suggest that Cerebral Venous Sinus Thrombosis (CVSTs) may be a direct complication of COVID-19.

Objective: To review the clinical and radiological presentation of COVID-19 positive patients diagnosed with CVST.

Methods: This was a multicenter, cross-sectional, retrospective study of patients diagnosed with CVST and COVID-19 reviewed from March 1, 2020 to November 8, 2020. We evaluated their clinical presentations, risk factors, clinical management, and outcome. We reviewed all published cases of CVST in patients with COVID-19 infection from January 1, 2020 to November 13, 2020.

Results: There were 8 patients diagnosed with CVST and COVID-19 during the study period at 7 out of 31 participating centers. Patients in our case series were mostly female (7/8, 87.5%). Most patients presented with non-specific symptoms such as headache (50%), fever (50%), and gastrointestinal symptoms (75%). Several patients presented with focal neurologic deficits (2/8, 25%) or decreased consciousness (2/8, 25%). D-dimer and inflammatory biomarkers were significantly elevated relative to reference ranges in patients with available laboratory data. The superior sagittal and transverse sinuses were the most common sites for acute CVST formation (6/8, 75%). Median time to onset of focal neurologic deficit from initial COVID-19 diagnosis was 3 days (interquartile range 0.75-3 days). Median time from onset of COVID-19 symptoms to CVST radiologic diagnosis was 11 days (interquartile range 6-16.75 days). Mortality was low in this cohort (1/8 or 12.5%).

Conclusions: Clinicians should consider the risk of acute CVST in patients positive for COVID-19, especially if neurological symptoms develop.

Keywords: COVID-19; CVST; Cerebral venous sinus thrombosis; SARS-CoV-2; Stroke.

Copyright © 2021 Elsevier Inc. All rights reserved.

Comment in

- Elevated Lipoprotein(a) and Cerebral Venous Sinus Thrombosis in COVID-19.
  Vuorio A, Kaste M, Kovanen PT. Vuorio A, et al. J Stroke Cerebrovasc Dis. 2021 Oct;30 (10):105865. doi: 10.1016/j.jstrokecerebrovasdis.2021.105865. Epub 2021 May 10. J Stroke Cerebrovasc Dis. 2021. PMID: 34039523 Free PMC article. No abstract available.
- Cerebral Venous Thrombosis following COVID-19 Vaccination.
  Kow CS, Hasan SS. Kow CS, et al. J Stroke Cerebrovasc Dis. 2021 Oct;30(10):105866. doi: 10.1016/j.jstrokecerebrovasdis.2021.105866. Epub 2021 May 10. J Stroke Cerebrovasc Dis. 2021. PMID: 34045111 Free PMC article. No abstract available.

- 57 references
- 1 figure

Supplementary info

Publication types, MeSH terms, Substances Expand

Publication types

- Multicenter Study
- Observational Study

MeSH terms

- Adult
- Aged
- Aged, 80 and over
- Biomarkers / analysis
- COVID-19 / complications*

- COVID-19 / epidemiology*
- COVID-19 / mortality
- Cranial Sinuses / pathology
- Cross-Sectional Studies
- Female
- Humans
- Male
- Middle Aged
- Nervous System Diseases / etiology
- Registries
- Retrospective Studies
- Risk Factors
- Sinus Thrombosis, Intracranial / epidemiology*
- Sinus Thrombosis, Intracranial / etiology*
- Sinus Thrombosis, Intracranial / mortality
- Tomography, X-Ray Computed
- Treatment Outcome

Substances

- Biomarkers

Full text links

ELSEVIER FULL-TEXT ARTICLE Elsevier Science Free PMC article

Proceed to details

Cite

Share

☐ 1,073

Observational Study

Med Clin (Barc)
. 2021 Feb 26;156(4):166-171.
doi: 10.1016/j.medcli.2020.10.006. Epub 2020 Nov 6.

Incidence of COVID-19 in patients under chronic treatment with hydroxychloroquine

[Article in English, Spanish]

Francisco Tejada Cifuentes [1], Ángeles Lloret Callejo [2], María José Tirado Peláez [2], Olga Rubio Pulido [3], Marta Ruiz-Morote Aragón [4], Rocío Fernández Urrusuno [5], María Isabel Muñoz Carreras [6], María Isabel Méndez Esteban [7], Victoria Maestre Sánchez [8], Antonio García Bonilla [9], José Manuel Paredero Dominguez [10], Virginia Arroyo Pineda [11], Esther Marco Tejón

[12], Gregorio Romero Candel [13], Ana Isabel Fernández Marchante [14], José Marco Del Rio [15], Teresa Ortiz Martín [16], Piedad López Sánchez [17]
Affiliations Expand

Affiliations

- [1] Gerencia de Atención Integrada de Albacete, Albacete, España. Electronic address: ftejada@sescam.jccm.es.
- [2] Gerencia de Atención Integrada de Albacete, Albacete, España.
- [3] Gerencia de Atención Primaria de Toledo, Toledo, España.
- [4] Gerencia de Atención Integrada. de Puertollano, Puertollano, España.
- [5] Distrito Sanitario Aljarafe, Sevilla, España.
- [6] Gerencia de Atención Integrada, Alcázar de San Juan, Alcázar de San Juan, España.
- [7] Área de Gestión Sanitaria Campo de Gibraltar, Cádiz, España.
- [8] Distrito Sanitario Condado Campiña, Huelva, España.
- [9] Área de Gestión Sanitaria Jerez, Costa Noroeste y Sierra de Cádiz, Jerez, España.
- [10] Gerencia de Atención Integrada, de Guadalajara, Guadalajara, España.
- [11] Gerencia de Atención Integrada Talavera de la Reina, Talavera de la Reina, España.
- [12] Gerencia de Atención Integrada de Cuenca, Cuenca, España.
- [13] Gerencia de Atención Integrada de Hellín, Hellín, España.
- [14] Gerencia de Atención Integrada de Villarrobledo, Villarrobledo, España.
- [15] Gerencia de Atención Integrada de Almansa, Almansa, España.
- [16] Gerencia de Atención Integrada de Valdepeñas, Valdepeñas, España.
- [17] Gerencia de Atención Integrada de Tomelloso, Tomelloso, España.

- PMID: **33308853**
- PMCID: PMC7833866
- DOI: 10.1016/j.medcli.2020.10.006

Free PMC article
Observational Study

Incidence of COVID-19 in patients under chronic treatment with hydroxychloroquine

[Article in English, Spanish]
Francisco Tejada Cifuentes et al. Med Clin (Barc). 2021.
Free PMC article
Show details
Med Clin (Barc)
. 2021 Feb 26;156(4):166-171.
doi: 10.1016/j.medcli.2020.10.006. Epub 2020 Nov 6.

Authors

Francisco Tejada Cifuentes [1], Ángeles Lloret Callejo [2], María José Tirado Peláez [2], Olga Rubio Pulido [3], Marta Ruiz-Morote Aragón [4], Rocío Fernández Urrusuno [5], María Isabel Muñoz Carreras [6], María Isabel Méndez Esteban [7], Victoria Maestre Sánchez [8], Antonio García Bonilla [9], José Manuel Paredero Dominguez [10], Virginia Arroyo Pineda [11], Esther Marco Tejón [12], Gregorio Romero Candel [13], Ana Isabel Fernández Marchante [14], José Marco Del Rio [15], Teresa Ortiz Martín [16], Piedad López Sánchez [17]

Affiliations

- [1] Gerencia de Atención Integrada de Albacete, Albacete, España. Electronic address: ftejada@sescam.jccm.es.
- [2] Gerencia de Atención Integrada de Albacete, Albacete, España.
- [3] Gerencia de Atención Primaria de Toledo, Toledo, España.
- [4] Gerencia de Atención Integrada. de Puertollano, Puertollano, España.
- [5] Distrito Sanitario Aljarafe, Sevilla, España.
- [6] Gerencia de Atención Integrada, Alcázar de San Juan, Alcázar de San Juan, España.
- [7] Área de Gestión Sanitaria Campo de Gibraltar, Cádiz, España.
- [8] Distrito Sanitario Condado Campiña, Huelva, España.
- [9] Área de Gestión Sanitaria Jerez, Costa Noroeste y Sierra de Cádiz, Jerez, España.
- [10] Gerencia de Atención Integrada, de Guadalajara, Guadalajara, España.
- [11] Gerencia de Atención Integrada Talavera de la Reina, Talavera de la Reina, España.
- [12] Gerencia de Atención Integrada de Cuenca, Cuenca, España.
- [13] Gerencia de Atención Integrada de Hellín, Hellín, España.
- [14] Gerencia de Atención Integrada de Villarrobledo, Villarrobledo, España.
- [15] Gerencia de Atención Integrada de Almansa, Almansa, España.
- [16] Gerencia de Atención Integrada de Valdepeñas, Valdepeñas, España.
- [17] Gerencia de Atención Integrada de Tomelloso, Tomelloso, España.

- PMID: **33308853**
- PMCID: PMC7833866
- DOI: 10.1016/j.medcli.2020.10.006

Abstract

Objective: To analyze the incidence of Covid-19 in patients who are chronic users of hydroxychloroquine.

Patients and methods: Cross-sectional retrospective observational multicenter study in health areas and districts from Castilla La-Mancha and Andalucia. Of the 4451 participants included in the first recruitment, 3817 with valid data were selected. The main variable of the study is the presence or absence of Covid-19 infection by clinical, serological or polymerase chain reaction diagnosis. Sociodemographic and clinical variables and treatment and concomitant comorbidities were recorded.

Results: 169 (4,45%) patients had Covid-19 infection, of which 12 (7.1%) died and 32 (18.9%) required hospital admission. Previous respiratory pathology was related to Covid-19 infection (P<.05). Maculopathy appears in 5.3% of patients and is significantly related to the dose of hydroxychloroquine consumed (P<.05).

Conclusion: There is no relationship between chronic use of hydroxychloroquine and the incidence of Covid-19.

Keywords: COVID-19; Coronavirus; Hidroxicloroquina; Hydroxychloroquine; Incidence; Incidencia; Tratamiento; Treatment.

Copyright © 2020 Elsevier España, S.L.U. All rights reserved.

- 22 references

Supplementary info

Publication types, MeSH terms, Substances Expand

Publication types

- Multicenter Study
- Observational Study

MeSH terms

- Adult
- Aged
- Aged, 80 and over
- Antirheumatic Agents / therapeutic use*
- Arthritis, Rheumatoid / complications
- Arthritis, Rheumatoid / drug therapy*
- COVID-19 / complications
- COVID-19 / diagnosis
- COVID-19 / epidemiology*
- COVID-19 Testing
- Chronic Disease
- Cross-Sectional Studies
- Female
- Humans
- Hydroxychloroquine / therapeutic use*
- Incidence
- Lupus Erythematosus, Systemic / complications
- Lupus Erythematosus, Systemic / drug therapy*
- Male
- Middle Aged
- Prognosis
- Protective Factors
- Retrospective Studies

- Risk Factors
- Spain / epidemiology

Substances

- Antirheumatic Agents
- Hydroxychloroquine

Full text links

Full text at MEDICINA CLINICA Ediciones Doyma, S.L. Free PMC article
Proceed to details
Cite
Share
1,074
Observational Study
Muscle Nerve
. 2021 Sep;64(3):357-361.
doi: 10.1002/mus.27348. Epub 2021 Jun 22.

Changes in motor function in Duchenne muscular dystrophy patients after travel restrictions due to COVID-19

Hitomi Nishizawa [1], Akinori Nakamura [2] [3]
Affiliations Expand

Affiliations

- [1] Faculty of Health Sciences, Department of Medicine, Shinshu University, Matsumoto, Japan.
- [2] Department of Neurology, National Hospital Organization Matsumoto Medical Center, Matsumoto, Japan.
- [3] Third Department of Internal Medicine, School of Medicine, Shinshu University, Matsumoto, Japan.

- PMID: **34105182**
- PMCID: PMC8242363
- DOI: 10.1002/mus.27348

Free PMC article
Observational Study

Changes in motor function in Duchenne muscular dystrophy patients after travel restrictions due to COVID-19

Hitomi Nishizawa et al. Muscle Nerve. 2021 Sep.
Free PMC article
Show details
Muscle Nerve
. 2021 Sep;64(3):357-361.
doi: 10.1002/mus.27348. Epub 2021 Jun 22.

Authors

Hitomi Nishizawa [1], Akinori Nakamura [2, 3]

Affiliations

- [1] Faculty of Health Sciences, Department of Medicine, Shinshu University, Matsumoto, Japan.
- [2] Department of Neurology, National Hospital Organization Matsumoto Medical Center, Matsumoto, Japan.
- [3] Third Department of Internal Medicine, School of Medicine, Shinshu University, Matsumoto, Japan.

- PMID: **34105182**
- PMCID: PMC8242363
- DOI: 10.1002/mus.27348

Abstract

Introduction/aim: This retrospective study aimed to quantify the changes in motor function in patients with Duchenne muscular dystrophy (DMD) due to the government-imposed travel restrictions associated with the coronavirus disease 2019 (COVID-19) pandemic.

Methods: Twelve DMD patients were enrolled in this investigation (mean ± SD age: 9.8 ± 3.6 y). Their physical characteristics and motor function were evaluated approximately 3 mo before, immediately before, and approximately 3 mo after the travel restrictions were decreed. Statistical comparisons were performed of the changes in motor function before and after the travel restrictions.

Results: The change in range of motion (ROM) of ankle dorsiflexion was significantly decreased after the travel restrictions. Changes in body mass index and other motor function parameters were not significant.

Discussion: An apparent decrease in the amount of physical activity due to travel restrictions in response to COVID-19 negatively affected ankle dorsiflexion ROM but not other motor functions. A more sedentary lifestyle and lack of regular physical therapy services most likely contributed to

this reduction. The use of remote rehabilitation tools with the involvement of physiotherapists may help mitigate such changes and prevent more severe physical decline.

Keywords: COVID-19; Duchenne muscular dystrophy; contracture; motor function; travel restrictions.

© 2021 Wiley Periodicals LLC.

Conflict of interest statement

None of the authors have any conflict of interest to disclose.

- 12 references
- 1 figure

Supplementary info

Publication types, MeSH terms Expand

Publication types

- Observational Study
- Research Support, Non-U.S. Gov't

MeSH terms

- Adolescent
- COVID-19 / prevention & control*
- Child
- Child, Preschool
- Communicable Disease Control / methods*
- Communicable Disease Control / trends
- Female
- Humans
- Male
- Motor Skills / physiology*
- Muscular Dystrophy, Duchenne / diagnosis
- Muscular Dystrophy, Duchenne / physiopathology
- Muscular Dystrophy, Duchenne / therapy*
- Physical Therapy Modalities* / trends
- Range of Motion, Articular / physiology
- Retrospective Studies
- Travel* / trends

Full text links

WILEY Full Text Article Wiley Free PMC article
Proceed to details
Cite
Share
1,075

Observational Study
J Am Geriatr Soc
. 2021 Oct;69(10):2752-2758.
doi: 10.1111/jgs.17357. Epub 2021 Jul 9.

Baricitinib reduces 30-day mortality in older adults with moderate-to-severe COVID-19 pneumonia

Pedro Abizanda [1, 2, 3], Juan María Calbo Mayo [4], Marta Mas Romero [1], Elisa Belén Cortés Zamora [1, 2], María Teresa Tabernero Sahuquillo [1], Luis Romero Rizos [1, 2, 3], Pedro Manuel Sánchez-Jurado [1, 2, 3], Ginés Sánchez-Nievas [5], Carlos Campayo Escolano [4], Alba Ochoa Serrano [4], Victoria Sánchez-Flor Alfaro [1], Rita López Bru [1], Cristina Gómez Ballesteros [1], David Caldevilla Bernardo [6], Francisco Javier Callejas González [7], Fernando Andrés-Pretel [8], Volker Martin Lauschke [9], Justin Stebbing [10]
Affiliations Expand

Affiliations

- [1] Department of Geriatrics, Complejo Hospitalario Universitario de Albacete, Albacete, Spain.
- [2] CIBERFES, Ministerio de Economía y Competitividad, Madrid, Spain.
- [3] Facultad de Medicina, Universidad de Castilla-La Mancha, Albacete, Spain.
- [4] Department of Internal Medicine, Complejo Hospitalario Universitario de Albacete, Albacete, Spain.
- [5] Department of Rheumatology, Complejo Hospitalario Universitario de Albacete, Albacete, Spain.
- [6] Department of Radiology, Complejo Hospitalario Universitario of Albacete, Albacete, Spain.
- [7] Department of Neumology, Complejo Hospitalario Universitario of Albacete, Albacete, Spain.
- [8] Department of Statistics, Foundation of the National Paraplegics Hospital of Toledo, Toledo, Spain.
- [9] Department of Physiology and Pharmacology, Karolinska Institutet, Stockholm, Sweden.
- [10] Department of Surgery and Cancer, Imperial College, Hammersmith Hospital, ICTEM Building, London, UK.

- PMID: **34235720**
- PMCID: PMC8447356
- DOI: 10.1111/jgs.17357

Free PMC article
Observational Study

Baricitinib reduces 30-day mortality in older adults with moderate-to-severe COVID-19 pneumonia

Pedro Abizanda et al. J Am Geriatr Soc. 2021 Oct.
Free PMC article
Show details
J Am Geriatr Soc
. 2021 Oct;69(10):2752-2758.
doi: 10.1111/jgs.17357. Epub 2021 Jul 9.

Authors

Pedro Abizanda [1, 2, 3], Juan María Calbo Mayo [4], Marta Mas Romero [1], Elisa Belén Cortés Zamora [1, 2], María Teresa Tabernero Sahuquillo [1], Luis Romero Rizos [1, 2, 3], Pedro Manuel Sánchez-Jurado [1, 2, 3], Ginés Sánchez-Nievas [5], Carlos Campayo Escolano [4], Alba Ochoa Serrano [4], Victoria Sánchez-Flor Alfaro [1], Rita López Bru [1], Cristina Gómez Ballesteros [1], David Caldevilla Bernardo [6], Francisco Javier Callejas González [7], Fernando Andrés-Pretel [8], Volker Martin Lauschke [9], Justin Stebbing [10]

Affiliations

- [1] Department of Geriatrics, Complejo Hospitalario Universitario de Albacete, Albacete, Spain.
- [2] CIBERFES, Ministerio de Economía y Competitividad, Madrid, Spain.
- [3] Facultad de Medicina, Universidad de Castilla-La Mancha, Albacete, Spain.
- [4] Department of Internal Medicine, Complejo Hospitalario Universitario de Albacete, Albacete, Spain.
- [5] Department of Rheumatology, Complejo Hospitalario Universitario de Albacete, Albacete, Spain.
- [6] Department of Radiology, Complejo Hospitalario Universitario of Albacete, Albacete, Spain.
- [7] Department of Neumology, Complejo Hospitalario Universitario of Albacete, Albacete, Spain.
- [8] Department of Statistics, Foundation of the National Paraplegics Hospital of Toledo, Toledo, Spain.
- [9] Department of Physiology and Pharmacology, Karolinska Institutet, Stockholm, Sweden.
- [10] Department of Surgery and Cancer, Imperial College, Hammersmith Hospital, ICTEM Building, London, UK.

- PMID: **34235720**
- PMCID: PMC8447356
- DOI: 10.1111/jgs.17357

Abstract

Background: Older adults are at the highest risk of severe disease and death due to COVID-19. Randomized data have shown that baricitinib improves outcomes in these patients, but focused stratified analyses of geriatric cohorts are lacking. Our objective was to analyze the efficacy of baricitinib in older adults with COVID-19 moderate-to-severe pneumonia.

Methods: This is a propensity score [PS]-matched retrospective cohort study. Patients from the COVID-AGE and Alba-Score cohorts, hospitalized for moderate-to-severe COVID-19 pneumonia, were categorized in two age brackets of age <70 years old (86 with baricitinib and 86 PS-matched controls) or ≥70 years old (78 on baricitinib and 78 PS-matched controls). Thirty-day mortality rates were analyzed with Kaplan-Meier and Cox proportional hazard models.

Results: Mean age was 79.1 for those ≥70 years and 58.9 for those <70. Exactly 29.6% were female. Treatment with baricitinib resulted in a significant reduction in death from any cause by 48% in patients aged 70 or older, an 18.5% reduction in 30-day absolute mortality risk (n/N: 16/78 [20.5%] baricitinib, 30/78 [38.5%] in PS-matched controls, $p < 0.001$) and a lower 30-day adjusted fatality rate (HR 0.21; 95% CI 0.09-0.47; $p < 0.001$). Beneficial effects on mortality were also observed in the age group <70 (8.1% reduction in 30-day absolute mortality risk; HR 0.14; 95% CI 0.03-0.64; $p = 0.011$).

Conclusions: Baricitinib is associated with an absolute mortality risk reduction of 18.5% in adults older than 70 years hospitalized with COVID-19 pneumonia.

Keywords: COVID-19; baricitinib; mortality; older adults.

© 2021 The American Geriatrics Society.

Conflict of interest statement

All authors declare that there are no conflicts of interest, except. V.M.L. declares no conflict of interest according to the ICMJE Uniform Requirements but discloses the following financial relationship: CEO and shareholder of HepaPredict AB; co-founder and chairman of the board PersoMedix AB; consultancy work for Enginzyme AB. JS declares his conflict at: https://www.nature.com/onc/editors and none are relevant here.

- 24 references
- 1 figure

Supplementary info

Publication types, MeSH terms, Substances, Grant support Expand

Publication types

- Observational Study
- Research Support, Non-U.S. Gov't

MeSH terms

- Age Factors
- Aged
- Aged, 80 and over
- Antiviral Agents / administration & dosage
- Antiviral Agents / adverse effects
- Azetidines* / administration & dosage
- Azetidines* / adverse effects
- COVID-19* / drug therapy
- COVID-19* / mortality
- COVID-19* / physiopathology
- Female
- Hospital Mortality
- Humans
- Janus Kinase Inhibitors / administration & dosage
- Janus Kinase Inhibitors / adverse effects
- Male
- Mortality
- Outcome and Process Assessment, Health Care
- Pneumonia, Viral* / diagnosis
- Pneumonia, Viral* / drug therapy
- Purines* / administration & dosage
- Purines* / adverse effects
- Pyrazoles* / administration & dosage
- Pyrazoles* / adverse effects
- SARS-CoV-2 / isolation & purification
- Severity of Illness Index
- Spain / epidemiology
- Sulfonamides* / administration & dosage
- Sulfonamides* / adverse effects

Substances

- Antiviral Agents
- Azetidines
- Janus Kinase Inhibitors
- Purines
- Pyrazoles
- Sulfonamides
- baricitinib

Grant support

- CB16/10/00408/Centro de Investigación Biomédica en Red Fragilidad y Envejecimiento Saludable
- COV20/00004/Instituto de Salud Carlos III

Full text links

WILEY Full Text Article Wiley Free PMC article
Proceed to details
Cite
Share
1,076
Observational Study
Emerg Nurse
. 2022 Jan 4;30(1):25-31.
doi: 10.7748/en.2021.e2091. Epub 2021 Aug 26.

Using vital signs measurements to identify patients with COVID-19 who require early continuous positive airway pressure

Chiara Colombo [1], Andrea Albani [1], Francesco Banfi [1], Maria Antonietta Maltana [1], Luca Meroni [2], Paolo Villa [2], Anna Maria Brambilla [1]
Affiliations Expand

Affiliations

- [1] emergency department, ASST Fatebenefratelli Sacco, Milan, Italy.
- [2] ASST Fatebenefratelli Sacco, Milan, Italy.

- PMID: **34435478**
- DOI: 10.7748/en.2021.e2091

Observational Study

Using vital signs measurements to identify patients with COVID-19 who require early continuous positive airway pressure

Chiara Colombo et al. Emerg Nurse. 2022.
Show details
Emerg Nurse
. 2022 Jan 4;30(1):25-31.
doi: 10.7748/en.2021.e2091. Epub 2021 Aug 26.

Authors

Chiara Colombo [1], Andrea Albani [1], Francesco Banfi [1], Maria Antonietta Maltana [1], Luca Meroni [2], Paolo Villa [2], Anna Maria Brambilla [1]

Affiliations

- [1] emergency department, ASST Fatebenefratelli Sacco, Milan, Italy.
- [2] ASST Fatebenefratelli Sacco, Milan, Italy.

- PMID: **34435478**
- DOI: 10.7748/en.2021.e2091

Abstract

Background: The coronavirus disease 2019 (COVID-19) can result in severe pneumonia, leading to acute respiratory distress syndrome, which are treated using continuous positive airway pressure (CPAP). Patients must be evaluated quickly to commence early CPAP if required.

Aim: To identify patients with COVID-19 in the emergency department (ED) who require early CPAP, using vital signs measurements during triage.

Method: This was a retrospective, observational, single-centre cohort study of patients with COVID-19 admitted to the ED of a university hospital in Lombardy, Italy, between 21 February 2020 and 30 April 2020. These patients were divided into two groups: those who required CPAP and those did not require CPAP. Recordings of their vital signs were retrieved from triage medical records. The vital signs values recorded in the two groups on their arrival at the ED were compared.

Results: Of 601 patients, 120 (20%) required CPAP. It was identified that the typical characteristics of patients requiring early CPAP were: male (P=.013) with a median age of 68 years (P=.000), oxygen saturation of 92% (P=.000), temperature ≥38°C (P=.008), respiratory rate of 26 breaths per minute (P=.000) and had received pre-hospital oxygen therapy before arriving at the ED (P=.000). The CPAP group was divided into two subgroups: patients who had received pre-hospital oxygen therapy and those who had not. The median respiratory rate values between the two subgroups presented a statistically significant difference (P=.004).

Conclusion: This study identified the characteristics of a typical patient with COVID-19 who requires early CPAP. Based on the results, the authors have devised a triage flow chart that uses selected vital signs measurements (oxygen saturation, respiratory rate and receipt of pre-hospital oxygen therapy) to identify patients requiring early CPAP. This flow chart should be trialled in a prospective study before it is used to inform clinical decision-making.

Keywords: COVID-19; accident and emergency; cardiorespiratory; clinical; coronavirus; coronavirus symptoms; emergency care; non-invasive ventilation; respiratory; triage; ventilation.

© 2021 RCN Publishing Company Ltd. All rights reserved. Not to be copied, transmitted or recorded in any way, in whole or part, without prior permission of the publishers.

Conflict of interest statement

None declared

Supplementary info

Publication types, MeSH terms Expand

Publication types

- Observational Study

MeSH terms

- Adult
- Aged
- COVID-19* / diagnosis
- COVID-19* / therapy
- Cohort Studies
- Continuous Positive Airway Pressure*
- Female
- Humans
- Male
- Middle Aged
- Oxygen Saturation
- Prospective Studies
- Vital Signs*

Proceed to details

Cite

Share

☐ 1,077

J Thromb Thrombolysis

. 2022 Jan;53(1):58-66.

doi: 10.1007/s11239-021-02514-3. Epub 2021 Jun 26.

Pragmatic study of a thromboprophylaxis algorithm in critically ill patients with SARS-COV-2 infection

Maurizio Fattorutto [1], Yves Bouckaert [2], Jonathan Brauner [3], Stéphane Franck [2], Fabrice Bouton [2], Danielle Heuse [2], Charlotte Bouckaert [4], Arnaud Bruyneel [2] [5]

Affiliations Expand

Affiliations

- [1] Department of Anesthesiology, Centre Hospitalier Universitaire Tivoli, Avenue Max Buset 34, 7100, La Louvière, Belgium. maurizio.fattorutto@chu-tivoli.be.
- [2] Intensive Care Unit, Centre Hospitalier Universitaire Tivoli, La Louvière, Belgium.
- [3] Department of Clinical Biology/Blood Bank, Centre Hospitalier Universitaire Tivoli, La Louvière, Belgium.
- [4] Werfen N.V./S.A., Excelsiorlaan 48-50 boite 8, Zaventem, Belgium.
- [5] School of Public Health, Université Libre Bruxelles, Brussels, Belgium.

- PMID: **34173169**
- PMCID: PMC8233177
- DOI: 10.1007/s11239-021-02514-3

Free PMC article

Pragmatic study of a thromboprophylaxis algorithm in critically ill patients with SARS-COV-2 infection

Maurizio Fattorutto et al. J Thromb Thrombolysis. 2022 Jan.
Free PMC article
Show details
J Thromb Thrombolysis
. 2022 Jan;53(1):58-66.
doi: 10.1007/s11239-021-02514-3. Epub 2021 Jun 26.

Authors

Maurizio Fattorutto [1], Yves Bouckaert [2], Jonathan Brauner [3], Stéphane Franck [2], Fabrice Bouton [2], Danielle Heuse [2], Charlotte Bouckaert [4], Arnaud Bruyneel [2] [5]

Affiliations

- [1] Department of Anesthesiology, Centre Hospitalier Universitaire Tivoli, Avenue Max Buset 34, 7100, La Louvière, Belgium. maurizio.fattorutto@chu-tivoli.be.
- [2] Intensive Care Unit, Centre Hospitalier Universitaire Tivoli, La Louvière, Belgium.
- [3] Department of Clinical Biology/Blood Bank, Centre Hospitalier Universitaire Tivoli, La Louvière, Belgium.
- [4] Werfen N.V./S.A., Excelsiorlaan 48-50 boite 8, Zaventem, Belgium.
- [5] School of Public Health, Université Libre Bruxelles, Brussels, Belgium.

- PMID: **34173169**
- PMCID: PMC8233177
- DOI: 10.1007/s11239-021-02514-3

Abstract

The optimal thromboprophylactic strategy for patients affected by Coronavirus disease 2019 (COVID-19) has been debated among experts. This study evaluated the safety and efficacy of a thromboprophylaxis algorithm. This was a retrospective, single-center study in critically ill patients admitted to the intensive care unit (University affiliated Hospital) for acute respiratory failure due to Severe Acute Respiratory Syndrome-Coronavirus 2 (SARS-CoV-2). From March 16 to April 9, 2020, thromboprophylaxis was adjusted according to weight (control group, n = 19) and after this date, thromboprophylaxis depended on an algorithm based on thrombotic and hemorrhagic risk factors (protocol group, n = 13). With regard to safety (number of major bleeding events and blood transfusions), the groups were not significantly different. With regard to efficacy, the number of thrombotic events decreased from 37 to 0%, p = 0.025 after implementation of the algorithm. Also, peak fibrinogen dropped from 8.6 (7.2-9.3) to 6.5 (4.6-8.4) g/L, p = 0.041 and D-dimers from 2194 (1464-3763) to 1486 (900-2582) ng/mL, p = 0.0001. In addition, length of stay declined from 19 (10-31) to 5 (3-19) days, p = 0.009. In conclusion, a tailored thromboprophylaxis algorithm (risk stratification based on clinical parameters and biological markers) reduce thrombotic phenomena in critically ill COVID-19 patients without increasing major bleeding.

Keywords: Anticoagulation; COVID-19; Hypercoagulability; SARS-CoV-2; Thrombosis.

© 2021. The Author(s), under exclusive licence to Springer Science+Business Media, LLC, part of Springer Nature.

Conflict of interest statement

The authors declare no conflicts of interest/competing interests. Authors declare to adhere to the minimum reporting guidelines hosted by the EQUATOR Network when preparing their observational study (STROBE statement).

- 62 references
- 1 figure

Supplementary info

MeSH terms, Substances Expand

MeSH terms

- Algorithms*
- Anticoagulants / therapeutic use*
- COVID-19* / complications
- Critical Illness
- Hemorrhage / chemically induced
- Humans
- Retrospective Studies
- Thrombosis* / etiology
- Thrombosis* / prevention & control

Substances

- Anticoagulants

Full text links

Springer Free PMC article
Proceed to details
Cite
Share
1,078
Observational Study
J Cancer Res Ther
. Apr-Jun 2021;17(2):547-550.
doi: 10.4103/jcrt.JCRT_1689_20.

Impact of the COVID-19 pandemic on health care activities at a Uruguayan mastology unit

Cecilia Castillo [1], Natalia Camejo [1], Dahiana Amarillo [1], Florencia Rodriguez [1], Florencia Vitureira [1], Gabriel Krygier [1], Lucía Delgado [1]
Affiliations Expand

Affiliation

- [1] Department of Clinical Oncology, Hospital de Clínicas "Dr. Manuel Quintela", School of Medicine, University of Uruguay, Montevideo, Uruguay.

- PMID: **34121706**
- DOI: 10.4103/jcrt.JCRT_1689_20

Free article
Observational Study

Impact of the COVID-19 pandemic on health care activities at a Uruguayan mastology unit

Cecilia Castillo et al. J Cancer Res Ther. Apr-Jun 2021.
Free article
Show details
J Cancer Res Ther
. Apr-Jun 2021;17(2):547-550.
doi: 10.4103/jcrt.JCRT_1689_20.

Authors

Cecilia Castillo [1], Natalia Camejo [1], Dahiana Amarillo [1], Florencia Rodriguez [1], Florencia Vitureira [1], Gabriel Krygier [1], Lucía Delgado [1]

Affiliation

- [1] Department of Clinical Oncology, Hospital de Clínicas "Dr. Manuel Quintela", School of Medicine, University of Uruguay, Montevideo, Uruguay.

- PMID: **34121706**
- DOI: 10.4103/jcrt.JCRT_1689_20

Abstract

Purpose: Health emergency due to COVID-19 started in Uruguay on March 13, 2020; our mastology unit tried to ensure adequate oncological care, and protect patients from the virus infection and complications.

Objective: To assess the health care activities in the "peak" of the pandemic during 3 months.

Materials and methods: we collected data from the electronic health record.

Results: There were a total of 293 medical appointments from 131 patients (221 face-to-face), that decreased by 16.7% compared to the same period in 2019 (352 appointments). The medical appointments were scheduled to evaluate the continuity of systemic treatment or modifications (95 patients; 72.5%), follow-up (17; 12.9%), first-time consultation (12; 9.1%), and assess paraclinical studies (7; 5.3%). The patients were on hormone therapy (81 patients; 74%), chemotherapy (CT) (21; 19%), and anti-HER2 therapies (9; 8%). New twenty treatments were initiated. Of the 14 patients that were on adjuvant/neoadjuvant CT, 9 (64.3%) continued with the same regimen with the addition of prophylactic granulocyte-colony-stimulating factors (G-CSF), and 5 (35.7%), who were receiving weekly paclitaxel, continued the treatment with no changes. Of the seven patients that were on palliative CT, 2 (28.5%) continued the treatment with the addition of G-CSF, 3 (42.8%) continued with weekly capecitabine or paclitaxel with no treatment changes, and 2 (28.5%) changed their treatment regimen (a less myelosuppressive regimen was selected for one and due to progression of the disease in the other patient). The ninety patients who were receiving adjuvant, neoadjuvant, or palliative criteria hormone therapy and/or anti-HER2 therapies, continued the treatment with no changes.

Conclusions: The evidence suggests that, although medical appointments decreased by approximately 17%, we could maintain healthcare activities, continued most of the treatments while the most modified was CT with G-CSF to avoid myelosuppression.

Keywords: Breast cancer; COVID 19; pandemic; systemic treatment.

Conflict of interest statement

None

Supplementary info

Publication types, MeSH terms, Substances Expand

Publication types

- Observational Study

MeSH terms

- Adult
- Aged
- Aged, 80 and over
- Antineoplastic Combined Chemotherapy Protocols / adverse effects
- Bone Marrow / drug effects
- Breast Neoplasms / complications
- Breast Neoplasms / diagnosis
- Breast Neoplasms / drug therapy*
- Breast Neoplasms / immunology
- COVID-19 / epidemiology*
- COVID-19 / immunology
- COVID-19 / prevention & control
- COVID-19 / transmission
- Communicable Disease Control / standards
- Continuity of Patient Care / organization & administration
- Continuity of Patient Care / statistics & numerical data*
- Delivery of Health Care / organization & administration
- Delivery of Health Care / standards
- Delivery of Health Care / statistics & numerical data*
- Electronic Health Records / statistics & numerical data
- Female
- Granulocyte Colony-Stimulating Factor / administration & dosage
- Hematopoiesis / drug effects
- Hematopoiesis / immunology
- Humans
- Medical Oncology / organization & administration
- Medical Oncology / standards
- Medical Oncology / statistics & numerical data*
- Middle Aged
- Pandemics / prevention & control
- Referral and Consultation / standards
- Referral and Consultation / statistics & numerical data
- Retrospective Studies
- Telemedicine / organization & administration
- Telemedicine / standards
- Telemedicine / statistics & numerical data

- Triage / organization & administration
- Triage / standards
- Uruguay / epidemiology

Substances

- Granulocyte Colony-Stimulating Factor

Full text links

Get Free Full Text Wolters Kluwer | Medknow Medknow Publications and Media Pvt Ltd
Proceed to details
Cite
Share
1,079
Observational Study
Blood Adv
. 2020 Oct 27;4(20):4981-4989.
doi: 10.1182/bloodadvances.2020002623.

The association of ABO blood group with indices of disease severity and multiorgan dysfunction in COVID-19

Ryan L Hoiland [1] [2], Nicholas A Fergusson [3] [4], Anish R Mitra [5], Donald E G Griesdale [1] [4] [5] [6], Dana V Devine [7] [8] [9], Sophie Stukas [7], Jennifer Cooper [7], Sonny Thiara [5], Denise Foster [5], Luke Y C Chen [10], Agnes Y Y Lee [10], Edward M Conway [9] [10], Cheryl L Wellington [7] [11] [12] [13], Mypinder S Sekhon [5]
Affiliations Expand

Affiliations

- [1] Department of Anesthesiology, Pharmacology, and Therapeutics, University of British Columbia, Vancouver, BC, Canada.
- [2] Centre for Heart, Lung, and Vascular Health, School of Health and Exercise Sciences, University of British Columbia-Okanagan, Kelowna, BC, Canada.
- [3] MD Undergraduate Program, University of British Columbia, Vancouver, BC, Canada.
- [4] Medicine, Quality, and Safety, Vancouver Coastal Health, Vancouver, BC, Canada.
- [5] Division of Critical Care Medicine, Department of Medicine, University of British Columbia, Vancouver, BC, Canada.
- [6] Center for Clinical Epidemiology and Evaluation, Vancouver Coastal Health Research Institute, Vancouver, BC, Canada.
- [7] Department of Pathology and Laboratory Medicine, Faculty of Medicine, University of British Columbia, Vancouver, BC, Canada.

- [8] Canadian Blood Services, Ottawa, ON, Canada; and.
- [9] Centre for Blood Research, Life Sciences Institute.
- [10] Division of Hematology, Department of Medicine.
- [11] Djavad Mowafaghian Centre for Brain Health.
- [12] School of Biomedical Engineering, and.
- [13] International Collaboration on Repair Discoveries (ICORD), University of British Columbia, Vancouver, BC, Canada.

- PMID: **33057633**
- PMCID: PMC7594392
- DOI: 10.1182/bloodadvances.2020002623

Free PMC article
Observational Study

The association of ABO blood group with indices of disease severity and multiorgan dysfunction in COVID-19

Ryan L Hoiland et al. Blood Adv. 2020.
Free PMC article

Show details

Blood Adv

. 2020 Oct 27;4(20):4981-4989.
doi: 10.1182/bloodadvances.2020002623.

Authors

Ryan L Hoiland [1] [2], Nicholas A Fergusson [3] [4], Anish R Mitra [5], Donald E G Griesdale [1] [4] [5] [6], Dana V Devine [7] [8] [9], Sophie Stukas [7], Jennifer Cooper [7], Sonny Thiara [5], Denise Foster [5], Luke Y C Chen [10], Agnes Y Y Lee [10], Edward M Conway [9] [10], Cheryl L Wellington [7] [11] [12] [13], Mypinder S Sekhon [5]

Affiliations

- [1] Department of Anesthesiology, Pharmacology, and Therapeutics, University of British Columbia, Vancouver, BC, Canada.
- [2] Centre for Heart, Lung, and Vascular Health, School of Health and Exercise Sciences, University of British Columbia-Okanagan, Kelowna, BC, Canada.
- [3] MD Undergraduate Program, University of British Columbia, Vancouver, BC, Canada.
- [4] Medicine, Quality, and Safety, Vancouver Coastal Health, Vancouver, BC, Canada.
- [5] Division of Critical Care Medicine, Department of Medicine, University of British Columbia, Vancouver, BC, Canada.
- [6] Center for Clinical Epidemiology and Evaluation, Vancouver Coastal Health Research Institute, Vancouver, BC, Canada.

- [7] Department of Pathology and Laboratory Medicine, Faculty of Medicine, University of British Columbia, Vancouver, BC, Canada.
- [8] Canadian Blood Services, Ottawa, ON, Canada; and.
- [9] Centre for Blood Research, Life Sciences Institute.
- [10] Division of Hematology, Department of Medicine.
- [11] Djavad Mowafaghian Centre for Brain Health.
- [12] School of Biomedical Engineering, and.
- [13] International Collaboration on Repair Discoveries (ICORD), University of British Columbia, Vancouver, BC, Canada.

- PMID: **33057633**
- PMCID: PMC7594392
- DOI: 10.1182/bloodadvances.2020002623

Abstract

Studies on severe acute respiratory syndrome coronavirus 1 (SARS-CoV-1) suggest a protective effect of anti-A antibodies against viral cell entry that may hold relevance for SARS-CoV-2 infection. Therefore, we aimed to determine whether ABO blood groups are associated with different severities of COVID-19. We conducted a multicenter retrospective analysis and nested prospective observational substudy of critically ill patients with COVID-19. We collected data pertaining to age, sex, comorbidities, dates of symptom onset, hospital admission, intensive care unit (ICU) admission, mechanical ventilation, continuous renal replacement therapy (CRRT), standard laboratory parameters, and serum inflammatory cytokines. National (N = 398 671; P = .38) and provincial (n = 62 246; P = .60) ABO blood group distributions did not differ from our cohort (n = 95). A higher proportion of COVID-19 patients with blood group A or AB required mechanical ventilation (P = .02) and CRRT (P = .004) and had a longer ICU stay (P = .03) compared with patients with blood group O or B. Blood group A or AB also had an increased probability of requiring mechanical ventilation and CRRT after adjusting for age, sex, and presence of ≥1 comorbidity. Inflammatory cytokines did not differ between patients with blood group A or AB (n = 11) vs O or B (n = 14; P > .10 for all cytokines). Collectively, our data indicate that critically ill COVID-19 patients with blood group A or AB are at increased risk for requiring mechanical ventilation, CRRT, and prolonged ICU admission compared with patients with blood group O or B. Further work is needed to understand the underlying mechanisms.

© 2020 by The American Society of Hematology.

Conflict of interest statement

Conflict-of-interest disclosure: The authors declare no competing financial interests.

Comment in

- Association of ABO blood group with indices of disease severity and multiorgan dysfunction in COVID-19.
  Tonon D, Simeone P, Lagier D, Bourenne J, Velly L. Tonon D, et al. Anaesth Crit Care Pain Med. 2021 Apr;40(2):100812. doi: 10.1016/j.accpm.2021.100812. Epub 2021 Feb 6. Anaesth Crit Care Pain Med. 2021. PMID: 33556587 Free PMC article. No abstract available.

- 38 references

- 3 figures

Supplementary info

Publication types, MeSH terms, Substances Expand

Publication types

- Multicenter Study
- Observational Study
- Research Support, Non-U.S. Gov't

MeSH terms

- ABO Blood-Group System / blood*
- Aged
- Betacoronavirus / isolation & purification*
- COVID-19
- Coronavirus Infections / blood*
- Coronavirus Infections / epidemiology
- Coronavirus Infections / therapy
- Critical Illness / epidemiology
- Critical Illness / therapy
- Cytokines / blood
- Female
- Humans
- Male
- Middle Aged
- Pandemics
- Pneumonia, Viral / blood*
- Pneumonia, Viral / epidemiology
- Pneumonia, Viral / therapy
- Prospective Studies
- Respiration, Artificial
- Retrospective Studies
- Risk Factors
- SARS-CoV-2
- Severity of Illness Index

Substances

- ABO Blood-Group System

- Cytokines

Full text links

FREE Full Text blood advances Silverchair Information Systems Free PMC article
Proceed to details
Cite
Share
1,080
Observational Study
Arch Cardiovasc Dis
. 2021 May;114(5):394-406.
doi: 10.1016/j.acvd.2021.04.002. Epub 2021 May 21.

Characteristics and impact of cardiovascular comorbidities on coronavirus disease 2019 in women: A multicentre cohort study

Orianne Weizman [1], Delphine Mika [2], Joffrey Cellier [3], Laura Geneste [4], Antonin Trimaille [5], Thibaut Pommier [6], Vassili Panagides [7], Wassima Marsou [8], Antoine Deney [9], Sabir Attou [10], Thomas Delmotte [11], Sophie Ribeyrolles [12], Pascale Chemaly [13], Clément Karsenty [9], Gauthier Giordano [14], Alexandre Gautier [13], Corentin Chaumont [15], Pierre Guilleminot [6], Audrey Sagnard [6], Julie Pastier [6], Baptiste Duceau [16], Willy Sutter [16], Charles Fauvel [15], Théo Pezel [17], Guillaume Bonnet [18], Ariel Cohen [19], Victor Waldmann [20], Critical COVID-19 France Investigators
Affiliations Expand

Affiliations

- [1] Centre Hospitalier Régional Universitaire de Nancy, 54511 Vandœuvre-Les-Nancy, France; Université de Paris, PARCC, INSERM, 75015 Paris, France.
- [2] Université Paris-Saclay, INSERM, UMR-S 1180, 92296 Châtenay-Malabry, France.
- [3] Hôpital Européen Georges-Pompidou, AP-HP, Université de Paris, 75015 Paris, France.
- [4] Centre Hospitalier Universitaire d'Amiens-Picardie, 80000 Amiens, France.
- [5] Nouvel Hôpital Civil, Centre Hospitalier Régional Universitaire de Strasbourg, 67000 Strasbourg, France.
- [6] Centre Hospitalier Universitaire de Dijon, 21000 Dijon, France.
- [7] Centre Hospitalier Universitaire de Marseille, 13005 Marseille, France.
- [8] GCS-Groupement des Hôpitaux de l'Institut Catholique de Lille, Faculté de Médecine et de Maïeutique, Université Catholique de Lille, Lille, France.
- [9] Centre Hospitalier Universitaire de Toulouse, 31400 Toulouse, France.
- [10] Centre Hospitalier Universitaire de Caen-Normandie, 14000 Caen, France.
- [11] Centre Hospitalier Universitaire de Reims, 51100 Reims, France.
- [12] Institut Mutualiste Montsouris, 75014 Paris, France.

- [13] Institut Cardiovasculaire Paris Sud, 91300 Massy, France.
- [14] Centre Hospitalier Régional Universitaire de Nancy, 54511 Vandœuvre-Les-Nancy, France.
- [15] Rouen University Hospital, FHU REMOD-VHF, 76000 Rouen, France.
- [16] Université de Paris, PARCC, INSERM, 75015 Paris, France.
- [17] Hôpital Lariboisiere, AP-HP, University of Paris, 75010 Paris, France.
- [18] Université de Paris, PARCC, INSERM, 75015 Paris, France; University of Bordeaux, Hôpital Cardiologique Haut-Lévêque, Centre Hospitalier Universitaire de Bordeaux, Pessac, France.
- [19] Hôpital Saint-Antoine, AP-HP, 75012 Paris, France. Electronic address: ariel.cohen@aphp.fr.
- [20] Université de Paris, PARCC, INSERM, 75015 Paris, France; Hôpital Européen Georges-Pompidou, AP-HP, Université de Paris, 75015 Paris, France.

- PMID: **34154954**
- PMCID: PMC8139232
- DOI: 10.1016/j.acvd.2021.04.002

Free PMC article
Observational Study

Characteristics and impact of cardiovascular comorbidities on coronavirus disease 2019 in women: A multicentre cohort study

Orianne Weizman et al. Arch Cardiovasc Dis. 2021 May.
Free PMC article
Show details
Arch Cardiovasc Dis
. 2021 May;114(5):394-406.
doi: 10.1016/j.acvd.2021.04.002. Epub 2021 May 21.

Authors

Orianne Weizman [1], Delphine Mika [2], Joffrey Cellier [3], Laura Geneste [4], Antonin Trimaille [5], Thibaut Pommier [6], Vassili Panagides [7], Wassima Marsou [8], Antoine Deney [9], Sabir Attou [10], Thomas Delmotte [11], Sophie Ribeyrolles [12], Pascale Chemaly [13], Clément Karsenty [9], Gauthier Giordano [14], Alexandre Gautier [13], Corentin Chaumont [15], Pierre Guilleminot [6], Audrey Sagnard [6], Julie Pastier [6], Baptiste Duceau [16], Willy Sutter [16], Charles Fauvel [15], Théo Pezel [17], Guillaume Bonnet [18], Ariel Cohen [19], Victor Waldmann [20], Critical COVID-19 France Investigators

Affiliations

- [1] Centre Hospitalier Régional Universitaire de Nancy, 54511 Vandœuvre-Les-Nancy, France; Université de Paris, PARCC, INSERM, 75015 Paris, France.

- [2] Université Paris-Saclay, INSERM, UMR-S 1180, 92296 Châtenay-Malabry, France.
- [3] Hôpital Européen Georges-Pompidou, AP-HP, Université de Paris, 75015 Paris, France.
- [4] Centre Hospitalier Universitaire d'Amiens-Picardie, 80000 Amiens, France.
- [5] Nouvel Hôpital Civil, Centre Hospitalier Régional Universitaire de Strasbourg, 67000 Strasbourg, France.
- [6] Centre Hospitalier Universitaire de Dijon, 21000 Dijon, France.
- [7] Centre Hospitalier Universitaire de Marseille, 13005 Marseille, France.
- [8] GCS-Groupement des Hôpitaux de l'Institut Catholique de Lille, Faculté de Médecine et de Maïeutique, Université Catholique de Lille, Lille, France.
- [9] Centre Hospitalier Universitaire de Toulouse, 31400 Toulouse, France.
- [10] Centre Hospitalier Universitaire de Caen-Normandie, 14000 Caen, France.
- [11] Centre Hospitalier Universitaire de Reims, 51100 Reims, France.
- [12] Institut Mutualiste Montsouris, 75014 Paris, France.
- [13] Institut Cardiovasculaire Paris Sud, 91300 Massy, France.
- [14] Centre Hospitalier Régional Universitaire de Nancy, 54511 Vandœuvre-Les-Nancy, France.
- [15] Rouen University Hospital, FHU REMOD-VHF, 76000 Rouen, France.
- [16] Université de Paris, PARCC, INSERM, 75015 Paris, France.
- [17] Hôpital Lariboisiere, AP-HP, University of Paris, 75010 Paris, France.
- [18] Université de Paris, PARCC, INSERM, 75015 Paris, France; University of Bordeaux, Hôpital Cardiologique Haut-Lévêque, Centre Hospitalier Universitaire de Bordeaux, Pessac, France.
- [19] Hôpital Saint-Antoine, AP-HP, 75012 Paris, France. Electronic address: ariel.cohen@aphp.fr.
- [20] Université de Paris, PARCC, INSERM, 75015 Paris, France; Hôpital Européen Georges-Pompidou, AP-HP, Université de Paris, 75015 Paris, France.

- PMID: **34154954**
- PMCID: PMC8139232
- DOI: 10.1016/j.acvd.2021.04.002

Abstract
in English, French

Background: Although women account for up to half of patients hospitalized for coronavirus disease 2019 (COVID-19), no specific data have been reported in this population.

Aims: To assess the burden and impact of cardiovascular comorbidities in women with COVID-19.

Methods: All consecutive patients hospitalized for COVID-19 across 24 hospitals from 26 February to 20 April 2020 were included. The primary composite outcome was transfer to an intensive care unit or in-hospital death.

Results: Among 2878 patients, 1212 (42.1%) were women. Women were older (68.3±18.0 vs. 65.4±16.0 years; P<0.001), but had less prevalent cardiovascular comorbidities than men. Among women, 276 (22.8%) experienced the primary outcome, including 161 (13.3%) transfers to an intensive care unit and 115 (9.5%) deaths without transfer to intensive care unit. The rate of in-hospital death or transfer to an intensive care unit was lower in women versus men (crude hazard

ratio [HR]: 0.62, 95% confidence interval [CI]: 0.53-0.72). Age (adjusted HR: 1.05 per 5-year increase, 95% CI: 1.01-1.10), body mass index (adjusted HR: 1.06 per 2-unit increase, 95% CI: 1.02-1.10), chronic kidney disease (adjusted HR: 1.57, 95% CI: 1.11-2.22) and heart failure (adjusted HR: 1.52, 95% CI: 1.04-2.22) were independently associated with the primary outcome in women. Elevated B-type natriuretic peptide/N-terminal prohormone of B-type natriuretic peptide (adjusted HR: 2.41, 95% CI: 1.70-3.44) and troponin (adjusted HR: 2.00, 95% CI: 1.39-2.88) concentrations at admission were also associated with the primary outcome, even in women free of previous coronary artery disease or heart failure.

Conclusions: Although female sex was associated with a lower risk of transfer to an intensive care unit or in-hospital death, COVID-19 remained associated with considerable morbimortality in women, especially in those with cardiovascular diseases.

Justification: Bien que les femmes constituent près de la moitié des patients hospitalisés pour une infection COVID-19, il n'a pas été reporté de données spécifiques dans cette population.

Objectifs: Évaluer la fréquence et l'impact des comorbidités cardiovasculaires chez les femmes atteintes de la COVID-19.

Méthode: Tous les patients hospitalisés de façon consécutive pour une infection COVID-19 dans 24 hôpitaux ont été inclus consécutivement entre le 29 février et le 20 avril 2020. Le critère de jugement principal est le transfert en unité de soins intensifs ou al survenue d'un décès intra-hospitalier.

Résultats: Parmi les 2878 patients, 1212 (42,1 %) étaient des femmes. Les femmes étaient plus âgées (68,3 ± 18,0 vs 65,4 ± 16,0 ans, $p < 0,001$) mais présentaient moins de comorbidités cardiovasculaires par rapport aux hommes. Parmi les femmes, 176 (22,8 %) ont présenté un évènement incluant 161 patients (13,3 %) ont dû être transférées en unité de soins intensifs et 115 (9,5%) sont décédées, sans transfert dans une unité de soins intensifs. Le taux de décès intra-hospitaliers ou de transferts dans une unité de soins intensifs était moindre chez les femmes comparativement aux hommes (HR brut : 0,62, IC95 % : 0,53–0,72). L'âge (HR ajusté : 1,05 par tranche de 5 ans, IC95 %: 1,02–1,1), l'insuffisance rénale chronique (HR ajusté : 1,57, IC95 % : 1,11–2,22) et l'insuffisance cardiaque (HR ajusté: 1,52, IC95 % : 1,02–2,22) étaient indépendamment associés avec la survenue du critère de jugement principal chez la femme. Une élévation du peptide natriurétique (BNP) ou du NT-pro-BNP (HR ajusté : 2,41, IC95 % : 1,73 –3,44) et une élévation de la troponine (HR ajusté : 2,00, IC95 % : 1,39–2,88) à l'admission étaient également associés avec la survenue du critère de jugement principal, y compris chez les femmes n'ayant pas de maladie coronaire ou d'insuffisance cardiaque avérée.

Conclusion: Bien que le sexe féminin soit associé avec un risque moindre de transfert en unité de soins intensifs ou de survenue de décès hospitalier, la COVID-19 associée à une augmentation de la morbi-mortalité chez la femme en particulier chez celle ayant une maladie cardiovasculaire sous-jacente.

Keywords: COVID-19; Facteurs de risque; Femmes; Outcomes; Risk factors; Résultats; SARS-CoV-2; Women.

Copyright © 2021. Published by Elsevier Masson SAS.

- 40 references
- 4 figures

Supplementary info

Publication types, MeSH terms, Substances Expand

Publication types

- Multicenter Study
- Observational Study

MeSH terms

- Aged
- Asthma / epidemiology
- Biomarkers
- COVID-19 / epidemiology*
- Cardiovascular Diseases / blood
- Cardiovascular Diseases / epidemiology*
- Comorbidity
- Diabetes Mellitus / epidemiology
- Female
- France / epidemiology
- Hospital Mortality
- Humans
- Intensive Care Units / statistics & numerical data
- Length of Stay / statistics & numerical data
- Male
- Middle Aged
- Natriuretic Peptide, Brain / blood
- Peptide Fragments / blood
- Proportional Hazards Models
- Retrospective Studies
- Risk Assessment
- Risk Factors
- Sex Distribution
- Smoking / epidemiology
- Troponin / blood

Substances

- Biomarkers
- Peptide Fragments
- Troponin
- Natriuretic Peptide, Brain

Full text links

Elsevier Science Free PMC article
Proceed to details
Cite
Share
1,081
Observational Study
Am J Respir Crit Care Med
. 2021 Dec 1;204(11):1274-1285.
doi: 10.1164/rccm.202105-1302OC.

Latent Class Analysis Reveals COVID-19-related Acute Respiratory Distress Syndrome Subgroups with Differential Responses to Corticosteroids

Pratik Sinha [1], David Furfaro [2], Matthew J Cummings [2], Darryl Abrams [2], Kevin Delucchi [3], Manoj V Maddali [4], June He [1], Alison Thompson [2], Michael Murn [2], John Fountain [5], Amanda Rosen [5], Shelief Y Robbins-Juarez [6], Matthew A Adan [6], Tejus Satish [6], Mahesh Madhavan [7], Aakriti Gupta [7], Alexander K Lyashchenko [8], Cara Agerstrand [2], Natalie H Yip [2], Kristin M Burkart [2], Jeremy R Beitler [2], Matthew R Baldwin [2], Carolyn S Calfee [9] [10] [11], Daniel Brodie [2], Max R O'Donnell [2] [12]
Affiliations Expand

Affiliations

- [1] Department of Anesthesiology, Washington University Medical School, Saint Louis, Missouri.
- [2] Division of Pulmonary, Allergy, and Critical Care Medicine.
- [3] Department of Psychiatry & Behavioral Sciences.
- [4] Department of Medicine.
- [5] Department of Internal Medicine, and.
- [6] Vagelos College of Physicians and Surgeons, Columbia University, New York, New York.
- [7] Division of Cardiology, Department of Medicine.
- [8] Department of Pathology and Cell Biology, Columbia University Irving Medical Center and NewYork-Presbyterian Hospital, New York, New York.
- [9] Department of Medicine, Division of Pulmonary, Critical Care, Allergy and Sleep Medicine.
- [10] Cardiovascular Research Institute, and.
- [11] Department of Anesthesia, University of California, San Francisco, San Francisco, California; and.
- [12] Department of Epidemiology, Mailman School of Public Health, and.

- PMID: **34543591**
- PMCID: PMC8786071
- DOI: 10.1164/rccm.202105-1302OC

Free PMC article
Observational Study

Latent Class Analysis Reveals COVID-19-related Acute Respiratory Distress Syndrome Subgroups with Differential Responses to Corticosteroids

Pratik Sinha et al. Am J Respir Crit Care Med. 2021.
Free PMC article
Show details
Am J Respir Crit Care Med
. 2021 Dec 1;204(11):1274-1285.
doi: 10.1164/rccm.202105-1302OC.

Authors

Pratik Sinha [1], David Furfaro [2], Matthew J Cummings [2], Darryl Abrams [2], Kevin Delucchi [3], Manoj V Maddali [4], June He [1], Alison Thompson [2], Michael Murn [2], John Fountain [5], Amanda Rosen [5], Shelief Y Robbins-Juarez [6], Matthew A Adan [6], Tejus Satish [6], Mahesh Madhavan [7], Aakriti Gupta [7], Alexander K Lyashchenko [8], Cara Agerstrand [2], Natalie H Yip [2], Kristin M Burkart [2], Jeremy R Beitler [2], Matthew R Baldwin [2], Carolyn S Calfee [9] [10] [11], Daniel Brodie [2], Max R O'Donnell [2] [12]

Affiliations

- [1] Department of Anesthesiology, Washington University Medical School, Saint Louis, Missouri.
- [2] Division of Pulmonary, Allergy, and Critical Care Medicine.
- [3] Department of Psychiatry & Behavioral Sciences.
- [4] Department of Medicine.
- [5] Department of Internal Medicine, and.
- [6] Vagelos College of Physicians and Surgeons, Columbia University, New York, New York.
- [7] Division of Cardiology, Department of Medicine.
- [8] Department of Pathology and Cell Biology, Columbia University Irving Medical Center and NewYork-Presbyterian Hospital, New York, New York.
- [9] Department of Medicine, Division of Pulmonary, Critical Care, Allergy and Sleep Medicine.
- [10] Cardiovascular Research Institute, and.
- [11] Department of Anesthesia, University of California, San Francisco, San Francisco, California; and.

- [12] Department of Epidemiology, Mailman School of Public Health, and.

- PMID: **34543591**
- PMCID: PMC8786071
- DOI: 10.1164/rccm.202105-1302OC

Abstract

Rationale: Two distinct subphenotypes have been identified in acute respiratory distress syndrome (ARDS), but the presence of subgroups in ARDS associated with coronavirus disease (COVID-19) is unknown. **Objectives:** To identify clinically relevant, novel subgroups in COVID-19-related ARDS and compare them with previously described ARDS subphenotypes. **Methods:** Eligible participants were adults with COVID-19 and ARDS at Columbia University Irving Medical Center. Latent class analysis was used to identify subgroups with baseline clinical, respiratory, and laboratory data serving as partitioning variables. A previously developed machine learning model was used to classify patients as the hypoinflammatory and hyperinflammatory subphenotypes. Baseline characteristics and clinical outcomes were compared between subgroups. Heterogeneity of treatment effect for corticosteroid use in subgroups was tested. **Measurements and Main Results:** From March 2, 2020, to April 30, 2020, 483 patients with COVID-19-related ARDS met study criteria. A two-class latent class analysis model best fit the population (P = 0.0075). Class 2 (23%) had higher proinflammatory markers, troponin, creatinine, and lactate, lower bicarbonate, and lower blood pressure than class 1 (77%). Ninety-day mortality was higher in class 2 versus class 1 (75% vs. 48%; $P < 0.0001$). Considerable overlap was observed between these subgroups and ARDS subphenotypes. Severe acute respiratory syndrome coronavirus 2 (SARS-CoV-2) RT-PCR cycle threshold was associated with mortality in the hypoinflammatory but not the hyperinflammatory phenotype. Heterogeneity of treatment effect to corticosteroids was observed (P = 0.0295), with improved mortality in the hyperinflammatory phenotype and worse mortality in the hypoinflammatory phenotype, with the caveat that corticosteroid treatment was not randomized. **Conclusions:** We identified two COVID-19-related ARDS subgroups with differential outcomes, similar to previously described ARDS subphenotypes. SARS-CoV-2 PCR cycle threshold had differential value for predicting mortality in the subphenotypes. The subphenotypes had differential treatment responses to corticosteroids.

Keywords: ARDS; COVID-19; latent class analysis; phenotyping.

Comment in

- COVID-19-related Acute Respiratory Distress Syndrome Subphenotypes and Differential Response to Corticosteroids: Time for More Precision?
  Reddy K, Hardin CC, McAuley DF. Reddy K, et al. Am J Respir Crit Care Med. 2021 Dec 1;204(11):1241-1243. doi: 10.1164/rccm.202109-2213ED. Am J Respir Crit Care Med. 2021. PMID: 34705609 Free PMC article. No abstract available.
- Time to Tailor the One-Size-Fits-All Approach?
  Kuindersma M, Spronk PE. Kuindersma M, et al. Am J Respir Crit Care Med. 2022 Feb 15;205(4):479-480. doi: 10.1164/rccm.202110-2317LE. Am J Respir Crit Care Med. 2022. PMID: 34818118 Free PMC article. No abstract available.

- 47 references
- 5 figures

Supplementary info

Publication types, MeSH terms, Substances, Grant support Expand

Publication types

- Observational Study
- Research Support, N.I.H., Extramural
- Research Support, Non-U.S. Gov't
- Research Support, U.S. Gov't, Non-P.H.S.

MeSH terms

- Adrenal Cortex Hormones / therapeutic use*
- Aged
- COVID-19 / complications
- COVID-19 / drug therapy*
- Cohort Studies
- Female
- Humans
- Latent Class Analysis*
- Male
- Middle Aged
- Respiratory Distress Syndrome / classification
- Respiratory Distress Syndrome / drug therapy*
- Respiratory Distress Syndrome / etiology
- Retrospective Studies

Substances

- Adrenal Cortex Hormones

Grant support

- R21 HL145506/HL/NHLBI NIH HHS/United States
- F32 AI147528/AI/NIAID NIH HHS/United States
- R35 HL140026/HL/NHLBI NIH HHS/United States
- R35 GM142992/GM/NIGMS NIH HHS/United States
- K23 HL133489/HL/NHLBI NIH HHS/United States

- UL1 TR001873/TR/NCATS NIH HHS/United States

Show all 6 grants

Full text links

AJRCCM Final Version Atypon Free PMC article
Proceed to details
Cite
Share
1,082
Observational Study
Stroke
. 2020 Aug;51(8):2593-2596.
doi: 10.1161/STROKEAHA.120.030794. Epub 2020 Jul 1.

Acute Stroke Management During the COVID-19 Pandemic: Does Confinement Impact Eligibility for Endovascular Therapy?

Steven D Hajdu [1], Valerie Pittet [2], Francesco Puccinelli [1], Wagih Ben Hassen [3], Malek Ben Maacha [4], Raphaël Blanc [4], Sandra Bracco [5], Gabriel Broocks [6], Bruno Bartolini [1], Tommaso Casseri [5], Frederic Clarençon [7], Olivier Naggara [3], François Eugène [8], Jean-Christophe Ferré [8], Alexis Guédon [9], Emmanuel Houdart [9], Timo Krings [10], Pierre Lehmann [11], Nicola Limbucci [12], Paolo Machi [13], Juan Macho [14], Nicolo Mandruzzato [13], Sergio Nappini [12], Marie Teresa Nawka, Patrick Nicholson [6] [10], João Pedro Marto [15], Vitor Pereira [10], Manuel A Correia [16], Teresa Pinho-E-Melo [16], João Nuno Ramos [15], Eytan Raz [17], Patrícia Ferreira [18], João Reis [18], Maksim Shapiro [17], Eimad Shotar [7], Noel van Horn [6], Michel Piotin [4], Guillaume Saliou [1]
Affiliations Expand

Affiliations

- [1] Department of Interventional and Diagnostic Radiology, Lausanne University Hospital, Switzerland (S.D.H., F.P., B.B., G.S.).
- [2] Center for Primary Care and Public Health, University of Lausanne, Switzerland (V.P.).
- [3] Department of Neuroradiology, Sainte Anne Hospital, Paris, France (W.B.H., O.N.).
- [4] Interventional Neuroradiology, Fondation Rothschild Hospital, Paris, France (M.B.M., R.B., M.P.).
- [5] Unit of Neuroimaging and Neurointervention, University Hospital of Siena, Italy (S.B., T.C.).
- [6] Department of Diagnostic and Interventional Neuroradiology, University Medical Center Hamburg Eppendorf, Hamburg, Germany (G.B., M.T.N., N.v.H.).
- [7] Department of Neuroradiology, Pitié-Salpêtrière Hospital, Paris, France (F.C., E.S.).
- [8] Department of Neuroradiology, Centre Hospitalier Universitaire de Rennes, Rennes, France (F.E., J.-C.F.).
- [9] Department of Neuroradiology, Lariboisière Hospital, Paris, France (A.G., E.H.).
- [10] Division of Neuroradiology, Joint Department of Medical Imaging, Toronto Western Hospital, Canada (T.K., P.N., V.P.).
- [11] Department of Neuroradiology, Marseille Hospital, France (P.L.).

- [12] Department of Interventional Neuroradiology, Careggi University Hospital, Florence, Italy (N.L., S.N.).
- [13] Service of diagnostic and interventional neuroradiology, Geneva University Hospital, Switzerland (P.M., N.M.).
- [14] Radiology Department, Hospital Clinic, Barcelona, Spain (J.M.).
- [15] Departments of Neurology and Neuroradiology, Hospital de Egas Moniz, Centro Hospitalar Lisboa Ocidental, Lisbon, Portugal (J.P.M., J.N.R.).
- [16] Departments of Neurology and Neuroradiology, Hospital Santa Maria, Centro Hospitalar Lisboa Norte, Lisbon, Portugal (M.A.C., T.P.-e.-M.).
- [17] NYU Langone Health, New York (E.R., M.S.).
- [18] Stroke Unit and Department of Neuroradiology, Hospital São José, Centro Hospitalar Lisboa Central, Lisbon, Portugal (P.F., J.R.).

- PMID: **32716828**
- PMCID: PMC7340133
- DOI: 10.1161/STROKEAHA.120.030794

Free PMC article
Observational Study

Acute Stroke Management During the COVID-19 Pandemic: Does Confinement Impact Eligibility for Endovascular Therapy?

Steven D Hajdu et al. Stroke. 2020 Aug.
Free PMC article
Show details
Stroke
. 2020 Aug;51(8):2593-2596.
doi: 10.1161/STROKEAHA.120.030794. Epub 2020 Jul 1.

Authors

Steven D Hajdu [1], Valerie Pittet [2], Francesco Puccinelli [1], Wagih Ben Hassen [3], Malek Ben Maacha [4], Raphaël Blanc [4], Sandra Bracco [5], Gabriel Broocks [6], Bruno Bartolini [1], Tommaso Casseri [5], Frederic Clarençon [7], Olivier Naggara [3], François Eugène [8], Jean-Christophe Ferré [8], Alexis Guédon [9], Emmanuel Houdart [9], Timo Krings [10], Pierre Lehmann [11], Nicola Limbucci [12], Paolo Machi [13], Juan Macho [14], Nicolo Mandruzzato [13], Sergio Nappini [12], Marie Teresa Nawka, Patrick Nicholson [6] [10], João Pedro Marto [15], Vitor Pereira [10], Manuel A Correia [16], Teresa Pinho-E-Melo [16], João Nuno Ramos [15], Eytan Raz [17], Patrícia Ferreira [18], João Reis [18], Maksim Shapiro [17], Eimad Shotar [7], Noel van Horn [6], Michel Piotin [4], Guillaume Saliou [1]

Affiliations

- [1] Department of Interventional and Diagnostic Radiology, Lausanne University Hospital, Switzerland (S.D.H., F.P., B.B., G.S.).

- [2] Center for Primary Care and Public Health, University of Lausanne, Switzerland (V.P.).
- [3] Department of Neuroradiology, Sainte Anne Hospital, Paris, France (W.B.H., O.N.).
- [4] Interventional Neuroradiology, Fondation Rothschild Hospital, Paris, France (M.B.M., R.B., M.P.).
- [5] Unit of Neuroimaging and Neurointervention, University Hospital of Siena, Italy (S.B., T.C.).
- [6] Department of Diagnostic and Interventional Neuroradiology, University Medical Center Hamburg Eppendorf, Hamburg, Germany (G.B., M.T.N., N.v.H.).
- [7] Department of Neuroradiology, Pitié-Salpêtrière Hospital, Paris, France (F.C., E.S.).
- [8] Department of Neuroradiology, Centre Hospitalier Universitaire de Rennes, Rennes, France (F.E., J.-C.F.).
- [9] Department of Neuroradiology, Lariboisière Hospital, Paris, France (A.G., E.H.).
- [10] Division of Neuroradiology, Joint Department of Medical Imaging, Toronto Western Hospital, Canada (T.K., P.N., V.P.).
- [11] Department of Neuroradiology, Marseille Hospital, France (P.L.).
- [12] Department of Interventional Neuroradiology, Careggi University Hospital, Florence, Italy (N.L., S.N.).
- [13] Service of diagnostic and interventional neuroradiology, Geneva University Hospital, Switzerland (P.M., N.M.).
- [14] Radiology Department, Hospital Clinic, Barcelona, Spain (J.M.).
- [15] Departments of Neurology and Neuroradiology, Hospital de Egas Moniz, Centro Hospitalar Lisboa Ocidental, Lisbon, Portugal (J.P.M., J.N.R.).
- [16] Departments of Neurology and Neuroradiology, Hospital Santa Maria, Centro Hospitalar Lisboa Norte, Lisbon, Portugal (M.A.C., T.P.-e.-M.).
- [17] NYU Langone Health, New York (E.R., M.S.).
- [18] Stroke Unit and Department of Neuroradiology, Hospital São José, Centro Hospitalar Lisboa Central, Lisbon, Portugal (P.F., J.R.).

- PMID: **32716828**
- PMCID: PMC7340133
- DOI: 10.1161/STROKEAHA.120.030794

Abstract

During the coronavirus disease 2019 (COVID-19) pandemic, the World Health Organization recommended measures to mitigate the outbreak such as social distancing and confinement. Since these measures have been put in place, anecdotal reports describe a decrease in the number of endovascular therapy (EVT) treatments for acute ischemic stroke due to large vessel occlusion. The purpose of our study was to determine the effect on EVT for patients with acute ischemic stroke during the COVID-19 confinement. In this retrospective, observational study, data were collected from November 1, 2019, to April 15, 2020, at 17 stroke centers in countries where confinement measures have been in place since March 2020 for the COVID-19 pandemic (Switzerland, Italy, France, Spain, Portugal, Germany, Canada, and United States). This study included 1600 patients treated by EVT for acute ischemic stroke. Date of EVT and symptom onset-to-groin puncture time were collected. Mean number of EVTs performed per hospital per 2-week interval and mean stroke onset-to-groin puncture time were calculated before confinement measures and after confinement measures. Distributions (non-normal) between the 2 groups (before COVID-19 confinement versus after COVID-19 confinement) were compared using 2-sample Wilcoxon rank-sum test. The results show a significant decrease in mean number of EVTs performed per hospital per 2-week interval between before COVID-19 confinement (9.0

[95% CI, 7.8-10.1]) and after COVID-19 confinement (6.1 [95% CI, 4.5-7.7]), (*P*<0.001). In addition, there is a significant increase in mean stroke onset-to-groin puncture time (*P*<0.001), between before COVID-19 confinement (300.3 minutes [95% CI, 285.3-315.4]) and after COVID-19 confinement (354.5 minutes [95% CI, 316.2-392.7]). Our preliminary analysis indicates a 32% reduction in EVT procedures and an estimated 54-minute increase in symptom onset-to-groin puncture time after confinement measures for COVID-19 pandemic were put into place.

Keywords: COVID-19; goal; groin; pandemic; standard of care.

Comment in

- Letter by Kwan et al Regarding Article, "Acute Stroke Management During the COVID-19 Pandemic: Does Confinement Impact Eligibility for Endovascular Therapy?".
  Kwan J, Lobotesis K, Banerjee S. Kwan J, et al. Stroke. 2020 Nov;51(11):e338-e339. doi: 10.1161/STROKEAHA.120.031638. Epub 2020 Oct 26. Stroke. 2020. PMID: 33104467 No abstract available.
- Response by Hajdu et al to Letter Regarding Article, "Acute Stroke Management During the COVID-19 Pandemic: Does Confinement Impact Eligibility for Endovascular Therapy?".
  Hajdu SD, Marto JP, Saliou G. Hajdu SD, et al. Stroke. 2020 Nov;51(11):e340-e341. doi: 10.1161/STROKEAHA.120.032096. Epub 2020 Oct 26. Stroke. 2020. PMID: 33104477 No abstract available.

- 13 references
- 1 figure

Supplementary info

Publication types, MeSH terms Expand

Publication types

- Multicenter Study
- Observational Study

MeSH terms

- Brain Ischemia / therapy
- COVID-19
- Coronavirus Infections*
- Disease Management*
- Eligibility Determination
- Endovascular Procedures / statistics & numerical data*
- Female
- Humans
- Male
- Middle Aged

- Pandemics*
- Pneumonia, Viral*
- Quarantine*
- Retrospective Studies
- Spain
- Stroke / therapy*
- Time-to-Treatment
- Treatment Outcome

Full text links

FREE FULL TEXT Atypon Free PMC article
Proceed to details
Cite
Share
1,083
Observational Study
BJOG
. 2021 Apr;128(5):917-920.
doi: 10.1111/1471-0528.16482. Epub 2020 Oct 7.

Impact of changes to national UK Guidance on testing for gestational diabetes screening during a pandemic: a single-centre observational study

Y van-de-l'Isle [1], P J Steer [2], I Watt Coote [1], M Cauldwell [1]
Affiliations Expand

Affiliations

- [1] Department of Obstetrics, Maternal Medicine Service, St George's Hospital, London, UK.
- [2] Academic Department of Obstetrics and Gynaecology, Chelsea and Westminster Hospital, London, UK.

- PMID: **32888369**
- DOI: 10.1111/1471-0528.16482

Observational Study

Impact of changes to national UK Guidance on testing for gestational diabetes screening during a pandemic: a single-centre observational study

Y van-de-l'Isle et al. BJOG. 2021 Apr.
Show details
BJOG
. 2021 Apr;128(5):917-920.
doi: 10.1111/1471-0528.16482. Epub 2020 Oct 7.

Authors

Y van-de-l'Isle [1], P J Steer [2], I Watt Coote [1], M Cauldwell [1]

Affiliations

- [1] Department of Obstetrics, Maternal Medicine Service, St George's Hospital, London, UK.
- [2] Academic Department of Obstetrics and Gynaecology, Chelsea and Westminster Hospital, London, UK.

- PMID: **32888369**
- DOI: 10.1111/1471-0528.16482

Abstract

Objective: To examine the differences in detection rate for gestational diabetes (GDM) comparing the methodology recommended by the National Institute for Health and Clinical Excellence (NICE) compared with testing described as appropriate during the Covid-19 pandemic by the Royal College of Obstetricians and Gynaecologists (RCOG).

Design: Cohort study of women delivering between 1 January 2016 and 1 July 2020.

Setting: London Teaching Hospital.

Population: All women delivering between 1 January 2016 and 13 May 2020 and follow up of women screening negative between 1 April 2020 and 13 May 2020.

Methods: Retrospective study of prospectively collected data.

Main outcome measures: Detection rate of gestational diabetes.

Results: Using the RCOG guidance, the overall rate of women identified as having gestational diabetes fell from 7.7% (1853/24168) to 4.2% (35/831)(P = 0.0003). Of 230 women who tested negative according to the RCOG criteria from 1 April to 13 May but who subsequently had an oral glucose tolerance test, 47 (20.4%) were diagnosed as having gestational diabetes according to the NICE criteria.

Conclusions: In our setting, the RCOG Covid-19 gestational diabetes screening regime failed to detect 47 of 82 (57%) women subsequently identified as gestational diabetics, and therefore cannot be recommended for general use.

Tweetable abstract: Screening for GDM using RCOG Covid criteria reduced detection rates.

Keywords: Covid-19; gestational diabetes; screen.

© 2020 John Wiley & Sons Ltd.

- 11 references

Supplementary info

Publication types, MeSH terms, Substances Expand

Publication types

- Observational Study

MeSH terms

- Adult
- Blood Glucose / analysis
- COVID-19* / epidemiology
- COVID-19* / prevention & control
- Cohort Studies
- Diabetes, Gestational* / diagnosis
- Diabetes, Gestational* / epidemiology
- Diagnostic Screening Programs* / organization & administration
- Diagnostic Screening Programs* / standards
- Female
- Humans
- Mass Screening* / methods
- Mass Screening* / trends
- Organizational Innovation
- Practice Guidelines as Topic / standards*
- Pregnancy
- Program Evaluation
- Reproducibility of Results
- SARS-CoV-2
- State Medicine / standards
- United Kingdom / epidemiology

Substances

- Blood Glucose

Full text links

WILEY Full Text Article Wiley
Proceed to details
Cite
Share
1,084
Observational Study
J Gastroenterol Hepatol
. 2021 Mar;36(3):700-709.
doi: 10.1111/jgh.15205. Epub 2020 Aug 16.

Impact of COVID-19 outbreak on the care of patients with inflammatory bowel disease: A comparison before and after the outbreak in South China

Ying-Fan Zhang [1], Yun Qiu [1], Jin-Shen He [1], Jin-Yu Tan [1], Xiao-Zhi Li [1], Liang-Ru Zhu [2], Yan Chen [3], Zhan-Ju Liu [4], Marietta Iacucci [5], Bai-Li Chen [1], Yao He [1], Shomron Ben-Horin [1] [6], Bo Shen [7], Zhi-Rong Zeng [1], Subrata Ghosh [5], Min-Hu Chen [1], Ren Mao [1]
Affiliations Expand

Affiliations

- [1] Department of Gastroenterology, The First Affiliated Hospital of Sun Yat-sen University, Guangzhou, China.
- [2] Division of Gastroenterology, Union Hospital, Tongji Medical College, Huazhong University of Science and Technology, Wuhan, China.
- [3] Center for Inflammatory Bowel Diseases, Department of Gastroenterology, the Second Affiliated Hospital, Zhejiang University School of Medicine, Hangzhou, China.
- [4] Department of Gastroenterology, Shanghai Tenth People's Hospital of Tongji University, Shanghai, China.
- [5] NIHR Biomedical Research Institute, Institute of Translational Medicine, University of Birmingham, University, Hospitals Birmingham NHS Foundation Trust, Birmingham, UK.
- [6] Department of Gastroenterology, Sheba Medical Center, Affiliated with the Sackler Faculty of Medicine, Tel Aviv University, Tel Aviv, Israel.
- [7] Center for Inflammatory Bowel Disease, Columbia University Irving Medical Center/NewYork-Presbyterian Hospital, New York, New York, USA.

- PMID: **32738060**

- PMCID: PMC7436411
- DOI: 10.1111/jgh.15205

Free PMC article
Observational Study

Impact of COVID-19 outbreak on the care of patients with inflammatory bowel disease: A comparison before and after the outbreak in South China

Ying-Fan Zhang et al. J Gastroenterol Hepatol. 2021 Mar.
Free PMC article
Show details
J Gastroenterol Hepatol
. 2021 Mar;36(3):700-709.
doi: 10.1111/jgh.15205. Epub 2020 Aug 16.

Authors

Ying-Fan Zhang [1], Yun Qiu [1], Jin-Shen He [1], Jin-Yu Tan [1], Xiao-Zhi Li [1], Liang-Ru Zhu [2], Yan Chen [3], Zhan-Ju Liu [4], Marietta Iacucci [5], Bai-Li Chen [1], Yao He [1], Shomron Ben-Horin [1] [6], Bo Shen [7], Zhi-Rong Zeng [1], Subrata Ghosh [5], Min-Hu Chen [1], Ren Mao [1]

Affiliations

- [1] Department of Gastroenterology, The First Affiliated Hospital of Sun Yat-sen University, Guangzhou, China.
- [2] Division of Gastroenterology, Union Hospital, Tongji Medical College, Huazhong University of Science and Technology, Wuhan, China.
- [3] Center for Inflammatory Bowel Diseases, Department of Gastroenterology, the Second Affiliated Hospital, Zhejiang University School of Medicine, Hangzhou, China.
- [4] Department of Gastroenterology, Shanghai Tenth People's Hospital of Tongji University, Shanghai, China.
- [5] NIHR Biomedical Research Institute, Institute of Translational Medicine, University of Birmingham, University, Hospitals Birmingham NHS Foundation Trust, Birmingham, UK.
- [6] Department of Gastroenterology, Sheba Medical Center, Affiliated with the Sackler Faculty of Medicine, Tel Aviv University, Tel Aviv, Israel.
- [7] Center for Inflammatory Bowel Disease, Columbia University Irving Medical Center/NewYork-Presbyterian Hospital, New York, New York, USA.

- PMID: **32738060**
- PMCID: PMC7436411
- DOI: 10.1111/jgh.15205

Abstract

Background and aims: Epidemics pose a great challenge to health care of patients. However, the impact of unprecedented situation of COVID-19 outbreak on health care of inflammatory bowel disease (IBD) patients in real-world setting has seldom been investigated.

Methods: We performed an observational study in a tertiary referral IBD center in China. The mode of health care and medication use was compared before and after COVID-19 outbreak. Electronic questionnaire surveys were performed among gastroenterologists and IBD patients to investigate the impact of COVID-19 outbreak on their attitudes towards telemedicine.

Results: COVID-19 outbreak resulted in substantial decrease of patients participating in standard face-to-face visit during 1 month post-outbreak (n = 51) than pre-outbreak (n = 249), whereas the participation in telemedicine was significantly higher than comparable period in 2019 (414 vs 93). During the 1 month after COVID-19 outbreak, 39 (39/56, 69.6%) patients had their infliximab infusion postponed with the mean delay of 3 weeks. The immunomodulator use was similar between pre-outbreak and post-outbreak. Six elective surgeries were postponed for a median of 43 days. In post-outbreak period, 193 (193/297, 64.98%) of the surveyed physicians have used telemedicine with an increase of 18.9% compared with 46.13% (137/292) in the pre-outbreak period ($P < 0.001$); 331 (331/505, 65.54%) of the surveyed IBD patients supported that the use of telemedicine should be increased in future health care.

Conclusion: COVID-19 outbreak resulted in a great change in health-care access among IBD patients including decrease in standard face-to-face visit and delay of biologics use. There was an increased use and need of telemedicine after COVID-19 outbreak.

Keywords: COVID-19; health care; inflammatory bowel disease.

© 2020 Journal of Gastroenterology and Hepatology Foundation and John Wiley & Sons Australia, Ltd.

- 12 references
- 5 figures

Supplementary info

Publication types, MeSH terms Expand

Publication types

- Comparative Study
- Observational Study

MeSH terms

- Attitude of Health Personnel*
- Attitude to Health*
- COVID-19* / epidemiology
- COVID-19* / prevention & control

- China / epidemiology
- Disease Outbreaks
- Health Care Rationing / trends
- Health Services Accessibility / trends*
- Humans
- Inflammatory Bowel Diseases / therapy*
- Practice Patterns, Physicians' / trends*
- Retrospective Studies
- Telemedicine / trends*

Full text links

WILEY Full Text Article Wiley Free PMC article

Proceed to details

Cite

Share

☐ 1,085

Observational Study

Travel Med Infect Dis
. May-Jun 2020;35:101653.
doi: 10.1016/j.tmaid.2020.101653. Epub 2020 Apr 2.

Clinical features of the first cases and a cluster of Coronavirus Disease 2019 (COVID-19) in Bolivia imported from Italy and Spain

Juan Pablo Escalera-Antezana [1], Nicolas Freddy Lizon-Ferrufino [2], Americo Maldonado-Alanoca [3], Gricel Alarcón-De-la-Vega [4], Lucia Elena Alvarado-Arnez [5], María Alejandra Balderrama-Saavedra [5], D Katterine Bonilla-Aldana [6], Alfonso J Rodríguez-Morales [7], LANCOVID [8]

Affiliations Expand

Affiliations

- [1] National Responsible for Telehealth Program, Ministry of Health, La Paz, Bolivia; Universidad Privada Franz Tamayo/UNIFRANZ, Cochabamba, Bolivia.
- [2] Interim Direction Epidemiology Unit, Ministry of Health, La Paz, Bolivia.
- [3] National Coordination of Laboratories, Ministry of Health, La Paz, Bolivia.
- [4] Rodent-Borne Diseases Program, Epidemiology Unit, Ministry of Health, La Paz, Bolivia.
- [5] Universidad Privada Franz Tamayo/UNIFRANZ, Cochabamba, Bolivia.
- [6] Incubator in Zoonosis (SIZOO), Biodiversity and Ecosystem Conservation Research Group (BIOECOS), Fundación Universitaria Autónoma de las Américas, Sede Pereira,

Pereira, Risaralda, Colombia; Public Health and infection Research Group, Faculty of Health Sciences, Universidad Tecnológica de Pereira, Pereira, Risaralda, Colombia.

- [7] Universidad Privada Franz Tamayo/UNIFRANZ, Cochabamba, Bolivia; Public Health and infection Research Group, Faculty of Health Sciences, Universidad Tecnológica de Pereira, Pereira, Risaralda, Colombia; Grupo de Investigación Biomedicina, Faculty of Medicine, Fundación Universitaria Autónoma de las Américas, Pereira, Risaralda, Colombia. Electronic address: arodriguezm@utp.edu.co.
- [8] Latin American Network of Coronavirus Disease 2019-COVID-19 Research (LANCOVID-19)(1), Colombia.

- PMID: **32247926**
- PMCID: PMC7129170
- DOI: 10.1016/j.tmaid.2020.101653

Free PMC article
Observational Study

Clinical features of the first cases and a cluster of Coronavirus Disease 2019 (COVID-19) in Bolivia imported from Italy and Spain

Juan Pablo Escalera-Antezana et al. Travel Med Infect Dis. May-Jun 2020.
Free PMC article
Show details
Travel Med Infect Dis
. May-Jun 2020;35:101653.
doi: 10.1016/j.tmaid.2020.101653. Epub 2020 Apr 2.

Authors

Juan Pablo Escalera-Antezana [1], Nicolas Freddy Lizon-Ferrufino [2], Americo Maldonado-Alanoca [3], Gricel Alarcón-De-la-Vega [4], Lucia Elena Alvarado-Arnez [5], María Alejandra Balderrama-Saavedra [5], D Katterine Bonilla-Aldana [6], Alfonso J Rodríguez-Morales [7], LANCOVID [8]

Affiliations

- [1] National Responsible for Telehealth Program, Ministry of Health, La Paz, Bolivia; Universidad Privada Franz Tamayo/UNIFRANZ, Cochabamba, Bolivia.
- [2] Interim Direction Epidemiology Unit, Ministry of Health, La Paz, Bolivia.
- [3] National Coordination of Laboratories, Ministry of Health, La Paz, Bolivia.
- [4] Rodent-Borne Diseases Program, Epidemiology Unit, Ministry of Health, La Paz, Bolivia.
- [5] Universidad Privada Franz Tamayo/UNIFRANZ, Cochabamba, Bolivia.
- [6] Incubator in Zoonosis (SIZOO), Biodiversity and Ecosystem Conservation Research Group (BIOECOS), Fundación Universitaria Autónoma de las Américas, Sede Pereira, Pereira, Risaralda, Colombia; Public Health and infection Research Group, Faculty of Health Sciences, Universidad Tecnológica de Pereira, Pereira, Risaralda, Colombia.

- [7] Universidad Privada Franz Tamayo/UNIFRANZ, Cochabamba, Bolivia; Public Health and infection Research Group, Faculty of Health Sciences, Universidad Tecnológica de Pereira, Pereira, Risaralda, Colombia; Grupo de Investigación Biomedicina, Faculty of Medicine, Fundación Universitaria Autónoma de las Américas, Pereira, Risaralda, Colombia. Electronic address: arodriguezm@utp.edu.co.
- [8] Latin American Network of Coronavirus Disease 2019-COVID-19 Research (LANCOVID-19)(1), Colombia.

- PMID: **32247926**
- PMCID: PMC7129170
- DOI: 10.1016/j.tmaid.2020.101653

Abstract

Introduction: In March 2020, Coronavirus Disease 2019 (COVID-19) arrived in Bolivia. Here, we report the main clinical findings, and epidemiological features of the first series of cases, and a cluster, confirmed in Bolivia.

Methods: For this observational, retrospective and cross-sectional study, information was obtained from the Hospitals and the Ministry of Health for the cases that were laboratory-diagnosed and related, during March 2020. rRT-PCR was used for the detection of the RNA of SARS-CoV-2 following the protocol Charité, Berlin, Germany, from nasopharyngeal swabs.

Results: Among 152 suspected cases investigated, 12 (7.9%) were confirmed with SARS-CoV-2 infected by rRT-PCR. The median age was 39 years (IQR 25-43), six of them male. Two cases proceed from Italy and three from Spain. Nine patients presented fever, and cough, five sore throat, and myalgia, among other symptoms. Only a 60 y-old woman with hypertension was hospitalized. None of the patients required ICU nor fatalities occurred in this group.

Conclusions: This is the first report of surveillance of COVID-19 in Bolivia, with patients managed mainly with home isolation. Preparedness for a significant epidemic, as is going on in other countries, and the deployment of response plans for it, in the country is now taking place to mitigate the impact of the COVID-19 pandemic in the population.

Keywords: Bolivia; Coronavirus disease 2019 (COVID-19); Epidemiology; Latin America; Severe acute respiratory syndrome coronavirus 2 (SARS-CoV-2).

Copyright © 2020 Elsevier Ltd. All rights reserved.

Conflict of interest statement

Declaration of competing interest All authors report no potential conflicts.

- 82 references
- 1 figure

Supplementary info

Publication types, MeSH terms, Substances Expand

Publication types

- Observational Study

MeSH terms

- Adolescent
- Adult
- Betacoronavirus / genetics*
- Bolivia / epidemiology
- COVID-19
- Cluster Analysis
- Communicable Diseases, Imported / epidemiology*
- Communicable Diseases, Imported / virology
- Coronavirus Infections / epidemiology*
- Coronavirus Infections / physiopathology*
- Coronavirus Infections / virology
- Cough
- Cross-Sectional Studies
- Epidemiological Monitoring*
- Female
- Fever
- Humans
- Italy
- Male
- Middle Aged
- Pandemics
- Patient Isolation / methods
- Pneumonia, Viral / epidemiology*
- Pneumonia, Viral / physiopathology*
- Pneumonia, Viral / virology
- RNA, Viral / genetics
- Retrospective Studies
- Reverse Transcriptase Polymerase Chain Reaction
- SARS-CoV-2
- Spain
- Young Adult

Substances

- RNA, Viral

Full text links

ELSEVIER FULL-TEXT ARTICLE Elsevier Science Free PMC article
Proceed to details
Cite
Share
1,086
Observational Study
Cerebrovasc Dis Extra
. 2021;11(2):55-60.
doi: 10.1159/000516641. Epub 2021 May 11.

Cerebral Venous Thrombosis Associated with COVID-19 Infection: An Observational, Multicenter Study

Sajid Hameed [1], Mohammad Wasay [1], Bashir A Soomro [2], Ossama Mansour [3], Foad Abd-Allah [4], Tianming Tu [5], Raja Farhat [6], Naila Shahbaz [7], Husnain Hashim [8], Wasim Alamgir [9], Athar Iqbal [10], Maria Khan [11]
Affiliations Expand

Affiliations

- [1] Department of Medicine, Aga Khan University, Karachi, Pakistan.
- [2] Department of Neurology, Ziauddin University Hospital, Karachi, Pakistan.
- [3] Department of Medicine, Alexandria University, Alexandria, Egypt.
- [4] Department of Neurology, Kasar Alainy School of Medicine, Cairo University Hospital, Cairo, Egypt.
- [5] Department of Neurology, National Neuroscience Institute, Singapore, Singapore.
- [6] Shifa International Hospital, Islamabad, Pakistan.
- [7] Dow University of Health Sciences, Karachi, Pakistan.
- [8] Fouji Foundation Hospital, Rawalpindi, Pakistan.
- [9] Military Hospital, Rawalpindi, Pakistan.
- [10] Sheikh Zayed Federal Postgraduate Medical Institute/Hospital, Lahore, Pakistan.
- [11] Rashid Hospital, Dubai, United Arab Emirates.

- PMID: **33975306**
- PMCID: PMC8215987
- DOI: 10.1159/000516641

Free PMC article
Observational Study

Cerebral Venous Thrombosis Associated with COVID-19 Infection: An Observational, Multicenter Study

Sajid Hameed et al. Cerebrovasc Dis Extra. 2021.
Free PMC article
Show details
Cerebrovasc Dis Extra
. 2021;11(2):55-60.
doi: 10.1159/000516641. Epub 2021 May 11.

Authors

Sajid Hameed [1], Mohammad Wasay [1], Bashir A Soomro [2], Ossama Mansour [3], Foad Abd-Allah [4], Tianming Tu [5], Raja Farhat [6], Naila Shahbaz [7], Husnain Hashim [8], Wasim Alamgir [9], Athar Iqbal [10], Maria Khan [11]

Affiliations

- [1] Department of Medicine, Aga Khan University, Karachi, Pakistan.
- [2] Department of Neurology, Ziauddin University Hospital, Karachi, Pakistan.
- [3] Department of Medicine, Alexandria University, Alexandria, Egypt.
- [4] Department of Neurology, Kasar Alainy School of Medicine, Cairo University Hospital, Cairo, Egypt.
- [5] Department of Neurology, National Neuroscience Institute, Singapore, Singapore.
- [6] Shifa International Hospital, Islamabad, Pakistan.
- [7] Dow University of Health Sciences, Karachi, Pakistan.
- [8] Fouji Foundation Hospital, Rawalpindi, Pakistan.
- [9] Military Hospital, Rawalpindi, Pakistan.
- [10] Sheikh Zayed Federal Postgraduate Medical Institute/Hospital, Lahore, Pakistan.
- [11] Rashid Hospital, Dubai, United Arab Emirates.

- PMID: **33975306**
- PMCID: PMC8215987
- DOI: 10.1159/000516641

Abstract

Background and purpose: Coronavirus disease 2019 (CO-VID-19) has an increased propensity for systemic hypercoagulability and thromboembolism. An association with cerebrovascular diseases, especially cerebral venous thrombosis (CVT), has been reported among these patients. The objective of the present study was to identify risk factors for CVT as well as its presentation and outcome in COVID-19 patients.

Methods: This is a multicenter and multinational observational study. Ten centers in 4 countries (Pakistan, Egypt, Singapore, and the United Arab Emirates) participated in this study. The study included patients (aged >18 years) with symptomatic CVT and recent COVID-19 infection.

Results: Twenty patients (70% men) were included. Their mean age was 42.4 years, with a male-to-female ratio of 2.3:1. Headache (85%) and seizures (65%) were the common presenting symptoms, with a mean admission Glasgow Coma Scale (GCS) score of 13. CVT was the presenting feature in 13 cases (65%), while 7 patients (35%) developed CVT while being treated for COVID-19 infection. Respiratory symptoms were absent in 45% of the patients. The most common imaging finding was infarction (65%), followed by hemorrhage (20%). The superior sagittal sinus (65%) was the most common site of thrombosis. Acute inflammatory markers were raised, including elevated serum D-dimer (87.5%), erythrocyte sedimentation rate (69%), and C-reactive protein (47%) levels. Homocysteine was elevated in half of the tested cases. The mortality rate was 20% (4 patients). A good functional outcome was seen in the surviving patients, with a mean modified Rankin Scale score at discharge of 1.3. Nine patients (45%) had a modified Rankin Scale score of 0-1 at discharge.

Conclusion: COVID-19-related CVT is more common among males at older ages when compared to previously reported non-COVID-19-related CVT cases. CVT should be suspected in COVID-19 patients presenting with headache or seizures. Mortality is high, but functional neurological outcome is good among survivors.

Keywords: COVID-19; Cerebral venous thrombosis; Coagulopathy; Hemorrhage; Stroke.

© 2021 The Author(s) Published by S. Karger AG, Basel.

Conflict of interest statement

The authors have no conflict of interest to declare.

- 17 references

Supplementary info

Publication types, MeSH terms Expand

Publication types

- Multicenter Study
- Observational Study

MeSH terms

- Adult
- COVID-19 / complications*
- COVID-19 / diagnosis*
- COVID-19 / therapy
- Egypt
- Female

- Humans
- Intracranial Thrombosis / diagnosis
- Intracranial Thrombosis / epidemiology*
- Intracranial Thrombosis / virology*
- Male
- Middle Aged
- Pakistan
- Retrospective Studies
- Risk Factors
- Singapore
- United Arab Emirates
- Venous Thrombosis / diagnosis
- Venous Thrombosis / epidemiology*
- Venous Thrombosis / virology*

Full text links

Karger Free Final Version S. Karger AG, Basel, Switzerland Free PMC article

Proceed to details

Cite

Share

1,087

Observational Study

Stroke Vasc Neurol

. 2021 Dec;6(4):542-552.

doi: 10.1136/svn-2020-000695. Epub 2021 Mar 26.

Decline in subarachnoid haemorrhage volumes associated with the first wave of the COVID-19 pandemic

Thanh N Nguyen [#] [1], Diogo C Haussen [2], Muhammad M Qureshi [3], Hiroshi Yamagami [4], Toshiyuki Fujinaka [5], Ossama Y Mansour [6], Mohamad Abdalkader [7], Michael Frankel [2], Zhongming Qiu [8], Allan Taylor [9], Pedro Lylyk [10], Omer F Eker [11], Laura Mechtouff [12], Michel Piotin [13], Fabricio Oliveira Lima [14], Francisco Mont'Alverne [15], Wazim Izzath [16], Nobuyuki Sakai [17], Mahmoud Mohammaden [2], Alhamza R Al-Bayati [2], Leonardo Renieri [18], Salvatore Mangiafico [18], David Ozretic [19], Vanessa Chalumeau [20], Saima Ahmad [21], Umair Rashid [21], Syed Irteza Hussain [22], Seby John [22], Emma Griffin [23], John Thornton [23], Jose Antonio Fiorot [24], Rodrigo Rivera [25], Nadia Hammami [26], Anna M Cervantes-Arslanian [27], Hormuzdiyar H Dasenbrock [28], Huynh Le Vu [29], Viet Quy Nguyen [29], Steven Hetts [30] [31], Romain Bourcier [32], Romain Guile [32], Melanie Walker [33], Malveeka Sharma [34], Don Frei [35], Pascal Jabbour [36], Nabeel Herial [36], Fawaz Al-Mufti [37], Atilla Ozcan Ozdemir [38], Ozlem

Aykac [38], Dheeraj Gandhi [39], Chandril Chugh [40], Charles Matouk [41], Pascale Lavoie [42], Randall Edgell [43], Andre Beer-Furlan [44], Michael Chen [44], Monika Killer-Oberpfalzer [45], Vitor Mendes Pereira [46], Patrick Nicholson [46], Vikram Huded [47], Nobuyuki Ohara [48], Daisuke Watanabe [49], Dong Hun Shin [50], Pedro Sc Magalhaes [51], Raghid Kikano [52], Santiago Ortega-Gutierrez [53], Mudassir Farooqui [53], Amal Abou-Hamden [54], Tatsuo Amano [55], Ryoo Yamamoto [56], Adrienne Weeks [57], Elena A Cora [58], Rotem Sivan-Hoffmann [59], Roberto Crosa [60], Markus Möhlenbruch [61], Simon Nagel [62], Hosam Al-Jehani [63], Sunil A Sheth [64], Victor S Lopez Rivera [64], James E Siegler [65], Achmad Fidaus Sani [66], Ajit S Puri [67], Anna Luisa Kuhn [67], Gianmarco Bernava [68], Paolo Machi [68], Daniel G Abud [69], Octavio M Pontes-Neto [70], Ajay K Wakhloo [71], Barbara Voetsch [72], Eytan Raz [73], Shadi Yaghi [74], Brijesh P Mehta [75], Naoto Kimura [76], Mamoru Murakami [77], Jin Soo Lee [78], Ji Man Hong [78], Robert Fahed [79], Gregory Walker [79], Eiji Hagashi [80], Steve M Cordina [81], Hong Gee Roh [82], Ken Wong [83], Juan F Arenillas [84], Mario Martinez-Galdamez [85], Jordi Blasco [86], Alejandro Rodriguez Vasquez [87], Luisa Fonseca [88], M Luis Silva [89], Teddy Y Wu [90], Simon John [91], Alex Brehm [92], Marios Psychogios [92], William J Mack [93], Matthew Tenser [93], Tatemi Todaka [94], Miki Fujimura [95], Roberta Novakovic [96], Jun Deguchi [97], Yuri Sugiura [98], Hiroshi Tokimura [99], Rakesh Khatri [100], Michael Kelly [101], Lissa Peeling [101], Yuichi Murayama [102], Hugh Stephen Winters [103], Johnny Wong [104], Mohamed Teleb [105], Jeremy Payne [105], Hiroki Fukuda [106], Kosuke Miyake [107], Junsuke Shimbo [108], Yusuke Sugimura [109], Masaaki Uno [110], Yohei Takenobu [111], Yuji Matsumaru [112], Satoshi Yamada [113], Ryuhei Kono [114], Takuya Kanamaru [115], Masafumi Morimoto [116], Junichi Iida [117], Vasu Saini [118], Dileep Yavagal [118], Saif Bushnaq [119], Wenguo Huang [120], Italo Linfante [121], Jawad Kirmani [122], David S Liebeskind [123], Viktor Szeder [124], Ruchir Shah [125], Thomas G Devlin [125], Lee Birnbaum [126], Jun Luo [127], Anchalee Churojana [128], Hesham E Masoud [129], Carlos Ynigo Lopez [129], Brendan Steinfort [130], Alice Ma [130], Ameer E Hassan [131], Amal Al Hashmi [132], Mollie McDermott [133], Maxim Mokin [134], Alex Chebl [135], Odysseas Kargiotis [136], Georgios Tsivgoulis [137], Jane G Morris [138], Clifford J Eskey [139], Jesse Thon [65], Leticia Rebello [140], Dorothea Altschul [141], Oriana Cornett [142], Varsha Singh [142], Jeyaraj Pandian [143], Anirudh Kulkarni [143], Pablo M Lavados [144], Veronica V Olavarria [144], Kenichi Todo [145], Yuki Yamamoto [146], Gisele Sampaio Silva [147], Serdar Geyik [148], Jasmine Johann [35], Sumeet Multani [149], Artem Kaliaev [7], Kazutaka Sonoda [150], Hiroyuki Hashimoto [151], Adel Alhazzani [152], David Y Chung [27], Stephan A Mayer [37], Johanna T Fifi [153], Michael D Hill [154], Hao Zhang [155], Zhengzhou Yuan [156], Xianjin Shang [157], Alicia C Castonguay [158], Rishi Gupta [159], Tudor G Jovin [65], Jean Raymond [160], Osama O Zaidat [119], Raul G Nogueira [#] [161], SVIN COVID-19 Registry, the Middle East North Africa Stroke and Interventional Neurotherapies Organization (MENA-SINO); Japanese Society of Vascular and Interventional Neurology Society (JVIN)

Affiliations Expand

Affiliations

- [1] Neurology, Radiology, Boston Medical Center, Boston, Massachusetts, USA.
- [2] Neurology, Marcus Stroke & Neuroscience Center, Grady Memorial Hospital, Emory University School of Medicine, Atlanta, GA, USA.
- [3] Radiology, Radiation Oncology, Boston University School of Medicine, Boston, Massachusetts, USA.
- [4] Neurology, National Hospital Organization Osaka National Hospital, Osaka, Japan.

- [5] Neurosurgery, National Hospital Organization Osaka National Hospital, Osaka, Japan.
- [6] Neurology, Alexandria University, Alexandria, Egypt.
- [7] Radiology, Boston Medical Center, Boston, Massachusetts, USA.
- [8] Department of Neurology, Xinqiao Hospital, Chongqing, China.
- [9] Neurosurgery, University of Cape Town, Rondebosch, Western Cape, South Africa.
- [10] Neurosurgery, Interventional Neuroradiology, Clinica La Sagrada Familia, Buenos Aires, Argentina.
- [11] Neuroradiologie, Neurologie Vasculaire, Hospices Civils de Lyon, Lyon, Auvergne-Rhône-Alpes, France.
- [12] Neurologie Vasculaire, Hospices Civils de Lyon, Lyon, Auvergne-Rhône-Alpes, France.
- [13] Interventional Neuroradiology, Fondation Ophtalmologique Adolphe de Rothschild, Paris, Île-de-France, France.
- [14] Neurology, Hospital Geral de Fortaleza, Fortaleza, Brazil.
- [15] Interventional Neuroradiology, Hospital Geral de Fortaleza, Fortaleza, Brazil.
- [16] Neuroradiology, Nottingham University Hospitals NHS Trust, Nottingham, UK.
- [17] Department of Neurosurgery, Kobe City Medical Center General Hospital, Kobe, Hyogo, Japan.
- [18] Interventional Neurovascular Unit, University Hospital Careggi, Firenze, Toscana, Italy.
- [19] Neuroradiology, University Hospital Centre Zagreb, Zagreb, Croatia.
- [20] Interventional Neuroradiology, Hopital Bicetre, Le Kremlin-Bicetre, France.
- [21] Stroke and Interventional Neuroradiology, Lahore General Hospital, Lahore, Pakistan.
- [22] Neurological Institute, Cleveland Clinic Abu Dhabi, Abu Dhabi, UAE.
- [23] Department of Radiology, Beaumont Hospital, Dublin, Ireland.
- [24] Neurology, Stroke Unit, Hospital-Estadual Central, Vitoria, Brazil.
- [25] Neuroradiology, Instituto de Neurocirugia Dr Asengo, Santiago, Chile.
- [26] Interventional Neuroradiology, Institut National de Neurologie, Tunis, Tunisia.
- [27] Neurology, Boston Medical Center, Boston, Massachusetts, USA.
- [28] Neurosurgery, Boston Medical Center, Boston, MA, USA.
- [29] Stroke Center, Hue Central Hospital, Hue, Thua Thien Hue, Vietnam.
- [30] Radiology, University of California San Francisco, San Francisco, California, USA.
- [31] Interventional Neuroradiology, University of California San Francisco, San Francisco, California, USA.
- [32] Neuroradiologie Diagnostique et Interventionnelle, Hôpital Guillaume & René Laennec, CHU Nantes, Nantes, France.
- [33] Neurological Surgery, University of Washington School of Medicine, Seattle, Washington, USA.
- [34] Neurology, University of Washington School of Medicine, Seattle, Washington, USA.
- [35] Radiology, Swedish Medical Center, Englewood, Colorado, USA.
- [36] Neurosurgery, Thomas Jefferson University Hospital, Philadelphia, Pennsylvania, USA.
- [37] Neurology, Neurosurgery, Westchester Medical Center Health Network, Valhalla, New York, USA.
- [38] Stroke and Neurointervention Unit, Eskisehir OsmangaziUniversity, Eskisehir, Turkey.
- [39] Radiology, Neurology, Neurosurgery, University of Maryland School of Medicine, Baltimore, Maryland, USA.
- [40] Interventional Neurology, MAX Superspecialty Hospital, Saket, New Delhi, India.
- [41] Neurosurgery, Yale School of Medicine, New Haven, Connecticut, USA.

- [42] Neurosurgery, Centre Hospitalier Universitaire de Québec-Université Laval, Quebec, Quebec, Canada.
- [43] Neurology, St Louis University School of Medicine, St Louis, Missouri, USA.
- [44] Neurological Surgery, Rush University Medical Center, Chicago, IL, USA.
- [45] Neurology, Research Institute of Neurointervention, University Hospital Salzburg /Paracelsus Medical University, Salzburg, Austria.
- [46] Neurosurgery, Medical Imaging, Surgery, University of Toronto, Toronto, Ontario, Canada.
- [47] Neurology, NH Mazumdar Shah Medical Center, Bangalore, India.
- [48] Neurology, Kobe City Medical Center General Hospital, Kobe, Hyogo, Japan.
- [49] Stroke and Neurovascular Surgery, IMS Tokyo-Katsushika General Hospital, Tokyo, Japan.
- [50] Gachon University, Seongnam, Korea (the Republic of).
- [51] Stroke Unit, Hospital Municipal Sao Jose, Joinville, Santa Catarina, Brazil.
- [52] Interventional Neuroradiology, Lau Medical Center, Beirut, Lebanon.
- [53] Neurology, University of Iowa Hospitals and Clinics, Iowa City, Iowa, USA.
- [54] Neurosurgery, Royal Adelaide Hospital, Adelaide, South Australia, Australia.
- [55] Stroke and Cerebrovascular Medicine, Kyorin University, Mitaka, Tokyo, Japan.
- [56] Neurology, Yokohama Brain and Spine Center, Yokohama, Japan.
- [57] Neurosurgery, Dalhousie University, Halifax, Nova Scotia, Canada.
- [58] Radiology, QEII Health Sciences Centre, Dalhousie University, Dalhousie, Nova Scotia, Canada.
- [59] Interventional Neuroradiology, Rambam Health Care Campus, Haifa, Haifa, Israel.
- [60] Centro Endovascular Neurologico Medica Uruguaya, Montevideo, Uruguay.
- [61] Neuroradiology, Heidelberg University Hospital, Heidelberg, Baden-Württemberg, Germany.
- [62] Neurology, Heidelberg University Hospital, Heidelberg, Baden-Württemberg, Germany.
- [63] Neurosurgery, Interventional Radiology and Critical Care Medicine, King Fahad Hospital of the University, Imam Abdulrahman bin Faisal University, Alkhobar, Saudi Arabia.
- [64] Neurology, University of Texas McGovern Medical School, Houston, Texas, USA.
- [65] Neurology, Cooper University Hospital, Cooper Medical School of Rowan University, Camden, New Jersey, USA.
- [66] Airlangga University, Surabaya, Jawa Timur, Indonesia.
- [67] Neurointerventional Radiology, University of Massachusetts Medical School, Worcester, Massachusetts, USA.
- [68] Interventional Neuroradiology, University Hospitals Geneva, Geneva, Switzerland.
- [69] Interventional Neuroradiology, Ribeirão Preto Medical School, University of São Paulo, São Paulo, Brazil.
- [70] Neuroscience and Behavioral Sciences, Ribeirão Preto Medical School, University of São Paulo, São Paulo, Brazil.
- [71] Interventional Neuroradiology, Beth Israel Lahey Health, Burlington, Massachusetts, USA.
- [72] Neurology, Beth Israel Lahey Health, Burlington, Massachusetts, USA.
- [73] Radiology, NYU Langone Health, NYU Grossman School of Medicine, New York, New York, USA.
- [74] Neurology, NYU Langone Health, NYU Grossman School of Medicine, New York, New York, USA.
- [75] Memorial Neuroscience Institute, Pembroke Pines, Florida, USA.

- [76] Neurosurgery, Iwate Prefectural Central Hospital, Morioka, Iwate, Japan.
- [77] Neurosurgery, Kyoto Second Red Cross Hospital, Kyoto, Japan.
- [78] Ajou University Hospital, Suwon, Gyeonggi-do, South Korea.
- [79] Neurology, University of Ottawa, Ottawa, Ontario, Canada.
- [80] Cerebrovascular Medicine, Saga-ken Medical Centre Koseikan, Saga, Japan.
- [81] Neurology, Neurosurgery, Radiology, University of South Alabama, Mobile, Alabama, USA.
- [82] Konkuk University, Gwangjin-gu, Seoul, South Korea.
- [83] Interventional Neuroradiology, Royal London Hospital, Barts Health NHS Trust, London, UK.
- [84] Neurology, Hospital Clinico Universitario de Valladolid, Valladolid, Castilla y León, Spain.
- [85] Interventional Neuroradiology, Hospital Clínico Universitario, Universidad de Valladolid, Valladolid, Spain.
- [86] INR, Hospital Clinic de Barcelona, Barcelona, Catalunya, Spain.
- [87] Neurology, Hospital Clinic de Barcelona, Barcelona, Catalunya, Spain.
- [88] Stroke Unit, Department of Medicine, Centro Hospitalar Universitário de São João, Porto, Portugal.
- [89] Neuroradiology, Centro Hospitalar Universitário de São João, Porto, Portugal.
- [90] Neurology, Christchurch Hospital, Christchurch, New Zealand.
- [91] Neurosurgery, Christchurch Hospital, Christchurch, New Zealand.
- [92] Interventional and Diagnostic Neuroradiology, University Hospital Basel, Basel, Switzerland.
- [93] Neurosurgery, University of Southern California, Los Angeles, California, USA.
- [94] Neurosurgery, Japanese Red Cross Kumamoto Hospital, Kumamoto, Kumamoto, Japan.
- [95] Neurosurgery, Kohnan Hospital, Sendai, Miyagi, Japan.
- [96] Radiology, Neurology, UT Southwestern, Dallas, Texas, USA.
- [97] Endovascular Neurosurgery, Nara City Hospital, Nara, Nara, Japan.
- [98] Neurology, Toyonaka Municipal Hospital, Toyonaka, Osaka, Japan.
- [99] Neurosurgery and Stroke Center, Kagoshima City Hospital, Kagoshima, Kagoshima, Japan.
- [100] Texas Tech University System, Lubbock, Texas, USA.
- [101] Neurosurgery, University of Saskatchewan, Saskatoon, Saskatchewan, Canada.
- [102] Neurosurgery, Jikei University School of Medicine, Minato-ku, Tokyo, Japan.
- [103] Neurology, Royal Prince Alfred Hospital, Camperdown, New South Wales, Australia.
- [104] Neurosurgery, Royal Prince Alfred Hospital, Camperdown, New South Wales, Australia.
- [105] Neurosciences, Banner Desert Medical Center, Mesa, Arizona, USA.
- [106] Neurology, Japanese Red Cross Matsue Hospital, Shimane, Japan.
- [107] Neurology, Shiroyama Hospital, Habikino, Osaka, Japan.
- [108] Cerebrovascular Medicine, Niigata City General Hospital, Niigata, Niigata, Japan.
- [109] Neurology, Sugimura Hospital, Kumamoto, Japan.
- [110] Department of Neurosurgery, Kawasaki Medical School, Kurashiki, Japan.
- [111] Neurology, Osaka Red Cross Hospital, Osaka, Japan.
- [112] Neurosurgery, University of Tsukuba, Tsukuba, Ibaraki, Japan.
- [113] Neurology, Stroke Center and Neuroendovascular Therapy, Saiseikai Central Hospital, Minato-ku, Tokyo, Japan.
- [114] Neurology, Kinikyo Chuo Hospital, Sapporo, Hokkaido, Japan.

- [115] Cerebrovascular Medicine, NTT Medical Center Tokyo, Tokyo, Japan.
- [116] Neurosurgery, Yokohama Shintoshi Neurosurgical Hospital, Yokohama, Japan.
- [117] Neurosurgery, Osaka General Medical Center, Osaka, Japan.
- [118] Neurology, Neurosurgery, University of Miami School of Medicine, Miami, Florida, USA.
- [119] Neurology, Bon Secours Mercy Health System, Toledo, Ohio, USA.
- [120] Neurology, Maoming City Hospital, Guandong, China.
- [121] Interventional Neuroradiology, Endovascular Neurosurgery, Miami Cardiac & Vascular Institute, Miami, Florida, USA.
- [122] Neurology, Hackensack Meridian Health, Edison, New Jersey, USA.
- [123] Neurology, University of California Los Angeles, Los Angeles, California, USA.
- [124] Interventional Neuroradiology, University of California Los Angeles, Los Angeles, California, USA.
- [125] Neurology, Erlanger Medical Center, University of Tennessee, Chattanooga, Tennessee, USA.
- [126] Neurology, Neurosurgery, Radiology, University of Texas Health San Antonio, San Antonio, Texas, USA.
- [127] Neurology, Mianyang 404 Hospital, Mianyang, Sichuan, China.
- [128] Radiology, Siriraj Hosital, Mahidol University, Bangkok, Thailand.
- [129] Neurology, Neurosurgery, Radiology, SUNY Upstate Medical University, Syracuse, New York, USA.
- [130] Neurosurgery, Royal North Shore Hospital, Sydney, New South Wales, Australia.
- [131] Neurosciences, The University of Texas Rio Grande Valley, Harlingen, Texas, USA.
- [132] Central Stroke Unit, Directorate of Neuroscience, Khoula Hospital, Ministry of Health, Muscat, Oman.
- [133] Neurology, University of Michigan, Ann Arbor, Michigan, USA.
- [134] Neurosurgery, University of South Florida, Tampa, Florida, USA.
- [135] Neurology, Henry Ford Health System, Detroit, Michigan, USA.
- [136] Stroke Unit, Metropolitan Hospital, Piraeus, Greece.
- [137] Faculty of Medicine, National and Kapodistrian University of Athens, Athens, Greece.
- [138] Neurology, Maine Medical Center, Portland, Maine, USA.
- [139] Neuroradiology, Dartmouth Hitchcock Medical Center, Lebanon, New Hampshire, USA.
- [140] Neurology, Hospital Universitario de Brasilia, Brasilia, Distrito Federal, Brazil.
- [141] Neurointerventional Neurosurgery, The Valley Hospital, Ridgewood, New Jersey, USA.
- [142] Neurosciences, Stroke Program, St Joseph's University Medical Center, Paterson, New Jersey, USA.
- [143] Neurology, Christian Medical College and Hospital Ludhiana, Ludhiana, Punjab, India.
- [144] Vascular Neurology Unit, Clínica Alemana, Universidad del Desarrollo, Santiago, Chile.
- [145] Neurology, Osaka University Graduate School of Medicine, Osaka, Japan.
- [146] Neurology, Tokushima University Hospital, Tokushima, Japan.
- [147] Neurology, Universidade Federal de Sao Paulo, Sao Paulo, Sao Paulo, Brazil.
- [148] Istanbul Aydin University, Istanbul, İstanbul, Turkey.
- [149] Neurology, Bayhealth Medical Center, Dover, Delaware, USA.
- [150] Neurology, Saiseikai Fukuoka General Hospital, Fukuoka, Japan.
- [151] Division of Stroke, Department of Internal Medicine, Osaka Rosai Hospital, Sakai, Osaka, Japan.

- [152] Neurology Division, Department of Medicine, King Saud University, Riyadh, Riyadh Province, Saudi Arabia.
- [153] Neurology, Mount Sinai Health System, New York, New York, USA.
- [154] Neurology, Clinical Neurosciences and Hotchkiss Brain Institute, University of Calgary, Calgary, Alberta, Canada.
- [155] Neurology, Affiliated Hangzhou First People's Hospital, Zhejiang University School of Medicine, Hangzhou, Zhejiang, China.
- [156] Neurology, The Affiliated Hospital of Southwest Medical University, Luzhou, Sichuan, China.
- [157] Neurology, Yijishan Hospital of Wannan Medical College, Wuhu, Anhui, China.
- [158] The University of Toledo, Toledo, Ohio, USA.
- [159] Neuroscience, WellStar Health System, Marietta, Georgia, USA.
- [160] Neuroradiologie Interventionelle, Centre Hospitalier de l'Université de Montréal, Montreal, Quebec, Canada.
- [161] Neurology, Marcus Stroke & Neuroscience Center, Grady Memorial Hospital, Emory University School of Medicine, Atlanta, GA, USA raul.g.nogueira@emory.edu.

[#] Contributed equally.

- PMID: **33771936**
- PMCID: PMC8006491
- DOI: 10.1136/svn-2020-000695

Free PMC article
Observational Study

Decline in subarachnoid haemorrhage volumes associated with the first wave of the COVID-19 pandemic

Thanh N Nguyen et al. Stroke Vasc Neurol. 2021 Dec.
Free PMC article

Show details

Stroke Vasc Neurol
. 2021 Dec;6(4):542-552.
doi: 10.1136/svn-2020-000695. Epub 2021 Mar 26.

Authors

Thanh N Nguyen [#] [1], Diogo C Haussen [2], Muhammad M Qureshi [3], Hiroshi Yamagami [4], Toshiyuki Fujinaka [5], Ossama Y Mansour [6], Mohamad Abdalkader [7], Michael Frankel [2], Zhongming Qiu [8], Allan Taylor [9], Pedro Lylyk [10], Omer F Eker [11], Laura Mechtouff [12], Michel Piotin [13], Fabricio Oliveira Lima [14], Francisco Mont'Alverne [15], Wazim Izzath [16], Nobuyuki Sakai [17], Mahmoud Mohammaden [2], Alhamza R Al-Bayati [2], Leonardo Renieri [18], Salvatore Mangiafico [18], David Ozretic [19], Vanessa Chalumeau [20], Saima Ahmad [21], Umair Rashid [21], Syed Irteza Hussain [22], Seby John [22], Emma Griffin [23], John Thornton [23], Jose

Antonio Fiorot [24], Rodrigo Rivera [25], Nadia Hammami [26], Anna M Cervantes-Arslanian [27], Hormuzdiyar H Dasenbrock [28], Huynh Le Vu [29], Viet Quy Nguyen [29], Steven Hetts [30] [31], Romain Bourcier [32], Romain Guile [32], Melanie Walker [33], Malveeka Sharma [34], Don Frei [35], Pascal Jabbour [36], Nabeel Herial [36], Fawaz Al-Mufti [37], Atilla Ozcan Ozdemir [38], Ozlem Aykac [38], Dheeraj Gandhi [39], Chandril Chugh [40], Charles Matouk [41], Pascale Lavoie [42], Randall Edgell [43], Andre Beer-Furlan [44], Michael Chen [44], Monika Killer-Oberpfalzer [45], Vitor Mendes Pereira [46], Patrick Nicholson [46], Vikram Huded [47], Nobuyuki Ohara [48], Daisuke Watanabe [49], Dong Hun Shin [50], Pedro Sc Magalhaes [51], Raghid Kikano [52], Santiago Ortega-Gutierrez [53], Mudassir Farooqui [53], Amal Abou-Hamden [54], Tatsuo Amano [55], Ryoo Yamamoto [56], Adrienne Weeks [57], Elena A Cora [58], Rotem Sivan-Hoffmann [59], Roberto Crosa [60], Markus Möhlenbruch [61], Simon Nagel [62], Hosam Al-Jehani [63], Sunil A Sheth [64], Victor S Lopez Rivera [64], James E Siegler [65], Achmad Fidaus Sani [66], Ajit S Puri [67], Anna Luisa Kuhn [67], Gianmarco Bernava [68], Paolo Machi [68], Daniel G Abud [69], Octavio M Pontes-Neto [70], Ajay K Wakhloo [71], Barbara Voetsch [72], Eytan Raz [73], Shadi Yaghi [74], Brijesh P Mehta [75], Naoto Kimura [76], Mamoru Murakami [77], Jin Soo Lee [78], Ji Man Hong [78], Robert Fahed [79], Gregory Walker [79], Eiji Hagashi [80], Steve M Cordina [81], Hong Gee Roh [82], Ken Wong [83], Juan F Arenillas [84], Mario Martinez-Galdamez [85], Jordi Blasco [86], Alejandro Rodriguez Vasquez [87], Luisa Fonseca [88], M Luis Silva [89], Teddy Y Wu [90], Simon John [91], Alex Brehm [92], Marios Psychogios [92], William J Mack [93], Matthew Tenser [93], Tatemi Todaka [94], Miki Fujimura [95], Roberta Novakovic [96], Jun Deguchi [97], Yuri Sugiura [98], Hiroshi Tokimura [99], Rakesh Khatri [100], Michael Kelly [101], Lissa Peeling [101], Yuichi Murayama [102], Hugh Stephen Winters [103], Johnny Wong [104], Mohamed Teleb [105], Jeremy Payne [105], Hiroki Fukuda [106], Kosuke Miyake [107], Junsuke Shimbo [108], Yusuke Sugimura [109], Masaaki Uno [110], Yohei Takenobu [111], Yuji Matsumaru [112], Satoshi Yamada [113], Ryuhei Kono [114], Takuya Kanamaru [115], Masafumi Morimoto [116], Junichi Iida [117], Vasu Saini [118], Dileep Yavagal [118], Saif Bushnaq [119], Wenguo Huang [120], Italo Linfante [121], Jawad Kirmani [122], David S Liebeskind [123], Viktor Szeder [124], Ruchir Shah [125], Thomas G Devlin [125], Lee Birnbaum [126], Jun Luo [127], Anchalee Churojana [128], Hesham E Masoud [129], Carlos Ynigo Lopez [129], Brendan Steinfort [130], Alice Ma [130], Ameer E Hassan [131], Amal Al Hashmi [132], Mollie McDermott [133], Maxim Mokin [134], Alex Chebl [135], Odysseas Kargiotis [136], Georgios Tsivgoulis [137], Jane G Morris [138], Clifford J Eskey [139], Jesse Thon [65], Leticia Rebello [140], Dorothea Altschul [141], Oriana Cornett [142], Varsha Singh [142], Jeyaraj Pandian [143], Anirudh Kulkarni [143], Pablo M Lavados [144], Veronica V Olavarria [144], Kenichi Todo [145], Yuki Yamamoto [146], Gisele Sampaio Silva [147], Serdar Geyik [148], Jasmine Johann [35], Sumeet Multani [149], Artem Kaliaev [7], Kazutaka Sonoda [150], Hiroyuki Hashimoto [151], Adel Alhazzani [152], David Y Chung [27], Stephan A Mayer [37], Johanna T Fifi [153], Michael D Hill [154], Hao Zhang [155], Zhengzhou Yuan [156], Xianjin Shang [157], Alicia C Castonguay [158], Rishi Gupta [159], Tudor G Jovin [65], Jean Raymond [160], Osama O Zaidat [119], Raul G Nogueira [#] [161], SVIN COVID-19 Registry, the Middle East North Africa Stroke and Interventional Neurotherapies Organization (MENA-SINO); Japanese Society of Vascular and Interventional Neurology Society (JVIN)

Affiliations

- [1] Neurology, Radiology, Boston Medical Center, Boston, Massachusetts, USA.
- [2] Neurology, Marcus Stroke & Neuroscience Center, Grady Memorial Hospital, Emory University School of Medicine, Atlanta, GA, USA.

- [3] Radiology, Radiation Oncology, Boston University School of Medicine, Boston, Massachusetts, USA.
- [4] Neurology, National Hospital Organization Osaka National Hospital, Osaka, Japan.
- [5] Neurosurgery, National Hospital Organization Osaka National Hospital, Osaka, Japan.
- [6] Neurology, Alexandria University, Alexandria, Egypt.
- [7] Radiology, Boston Medical Center, Boston, Massachusetts, USA.
- [8] Department of Neurology, Xinqiao Hospital, Chongqing, China.
- [9] Neurosurgery, University of Cape Town, Rondebosch, Western Cape, South Africa.
- [10] Neurosurgery, Interventional Neuroradiology, Clinica La Sagrada Familia, Buenos Aires, Argentina.
- [11] Neuroradiologie, Neurologie Vasculaire, Hospices Civils de Lyon, Lyon, Auvergne-Rhône-Alpes, France.
- [12] Neurologie Vasculaire, Hospices Civils de Lyon, Lyon, Auvergne-Rhône-Alpes, France.
- [13] Interventional Neuroradiology, Fondation Ophtalmologique Adolphe de Rothschild, Paris, Île-de-France, France.
- [14] Neurology, Hospital Geral de Fortaleza, Fortaleza, Brazil.
- [15] Interventional Neuroradiology, Hospital Geral de Fortaleza, Fortaleza, Brazil.
- [16] Neuroradiology, Nottingham University Hospitals NHS Trust, Nottingham, UK.
- [17] Department of Neurosurgery, Kobe City Medical Center General Hospital, Kobe, Hyogo, Japan.
- [18] Interventional Neurovascular Unit, University Hospital Careggi, Firenze, Toscana, Italy.
- [19] Neuroradiology, University Hospital Centre Zagreb, Zagreb, Croatia.
- [20] Interventional Neuroradiology, Hopital Bicetre, Le Kremlin-Bicetre, France.
- [21] Stroke and Interventional Neuroradiology, Lahore General Hospital, Lahore, Pakistan.
- [22] Neurological Institute, Cleveland Clinic Abu Dhabi, Abu Dhabi, UAE.
- [23] Department of Radiology, Beaumont Hospital, Dublin, Ireland.
- [24] Neurology, Stroke Unit, Hospital-Estadual Central, Vitoria, Brazil.
- [25] Neuroradiology, Instituto de Neurocirugia Dr Asengo, Santiago, Chile.
- [26] Interventional Neuroradiology, Institut National de Neurologie, Tunis, Tunisia.
- [27] Neurology, Boston Medical Center, Boston, Massachusetts, USA.
- [28] Neurosurgery, Boston Medical Center, Boston, MA, USA.
- [29] Stroke Center, Hue Central Hospital, Hue, Thua Thien Hue, Vietnam.
- [30] Radiology, University of California San Francisco, San Francisco, California, USA.
- [31] Interventional Neuroradiology, University of California San Francisco, San Francisco, California, USA.
- [32] Neuroradiologie Diagnostique et Interventionnelle, Hôpital Guillaume & René Laennec, CHU Nantes, Nantes, France.
- [33] Neurological Surgery, University of Washington School of Medicine, Seattle, Washington, USA.
- [34] Neurology, University of Washington School of Medicine, Seattle, Washington, USA.
- [35] Radiology, Swedish Medical Center, Englewood, Colorado, USA.
- [36] Neurosurgery, Thomas Jefferson University Hospital, Philadelphia, Pennsylvania, USA.
- [37] Neurology, Neurosurgery, Westchester Medical Center Health Network, Valhalla, New York, USA.
- [38] Stroke and Neurointervention Unit, Eskisehir OsmangaziUniversity, Eskisehir, Turkey.
- [39] Radiology, Neurology, Neurosurgery, University of Maryland School of Medicine, Baltimore, Maryland, USA.

- [40] Interventional Neurology, MAX Superspecialty Hospital, Saket, New Delhi, India.
- [41] Neurosurgery, Yale School of Medicine, New Haven, Connecticut, USA.
- [42] Neurosurgery, Centre Hospitalier Universitaire de Québec-Université Laval, Quebec, Quebec, Canada.
- [43] Neurology, St Louis University School of Medicine, St Louis, Missouri, USA.
- [44] Neurological Surgery, Rush University Medical Center, Chicago, IL, USA.
- [45] Neurology, Research Institute of Neurointervention, University Hospital Salzburg /Paracelsus Medical University, Salzburg, Austria.
- [46] Neurosurgery, Medical Imaging, Surgery, University of Toronto, Toronto, Ontario, Canada.
- [47] Neurology, NH Mazumdar Shah Medical Center, Bangalore, India.
- [48] Neurology, Kobe City Medical Center General Hospital, Kobe, Hyogo, Japan.
- [49] Stroke and Neurovascular Surgery, IMS Tokyo-Katsushika General Hospital, Tokyo, Japan.
- [50] Gachon University, Seongnam, Korea (the Republic of).
- [51] Stroke Unit, Hospital Municipal Sao Jose, Joinville, Santa Catarina, Brazil.
- [52] Interventional Neuroradiology, Lau Medical Center, Beirut, Lebanon.
- [53] Neurology, University of Iowa Hospitals and Clinics, Iowa City, Iowa, USA.
- [54] Neurosurgery, Royal Adelaide Hospital, Adelaide, South Australia, Australia.
- [55] Stroke and Cerebrovascular Medicine, Kyorin University, Mitaka, Tokyo, Japan.
- [56] Neurology, Yokohama Brain and Spine Center, Yokohama, Japan.
- [57] Neurosurgery, Dalhousie University, Halifax, Nova Scotia, Canada.
- [58] Radiology, QEII Health Sciences Centre, Dalhousie University, Dalhousie, Nova Scotia, Canada.
- [59] Interventional Neuroradiology, Rambam Health Care Campus, Haifa, Haifa, Israel.
- [60] Centro Endovascular Neurologico Medica Uruguaya, Montevideo, Uruguay.
- [61] Neuroradiology, Heidelberg University Hospital, Heidelberg, Baden-Württemberg, Germany.
- [62] Neurology, Heidelberg University Hospital, Heidelberg, Baden-Württemberg, Germany.
- [63] Neurosurgery, Interventional Radiology and Critical Care Medicine, King Fahad Hospital of the University, Imam Abdulrahman bin Faisal University, Alkhobar, Saudi Arabia.
- [64] Neurology, University of Texas McGovern Medical School, Houston, Texas, USA.
- [65] Neurology, Cooper University Hospital, Cooper Medical School of Rowan University, Camden, New Jersey, USA.
- [66] Airlangga University, Surabaya, Jawa Timur, Indonesia.
- [67] Neurointerventional Radiology, University of Massachusetts Medical School, Worcester, Massachusetts, USA.
- [68] Interventional Neuroradiology, University Hospitals Geneva, Geneva, Switzerland.
- [69] Interventional Neuroradiology, Ribeirão Preto Medical School, University of São Paulo, São Paulo, Brazil.
- [70] Neuroscience and Behavioral Sciences, Ribeirão Preto Medical School, University of São Paulo, São Paulo, Brazil.
- [71] Interventional Neuroradiology, Beth Israel Lahey Health, Burlington, Massachusetts, USA.
- [72] Neurology, Beth Israel Lahey Health, Burlington, Massachusetts, USA.
- [73] Radiology, NYU Langone Health, NYU Grossman School of Medicine, New York, New York, USA.

- [74] Neurology, NYU Langone Health, NYU Grossman School of Medicine, New York, New York, USA.
- [75] Memorial Neuroscience Institute, Pembroke Pines, Florida, USA.
- [76] Neurosurgery, Iwate Prefectural Central Hospital, Morioka, Iwate, Japan.
- [77] Neurosurgery, Kyoto Second Red Cross Hospital, Kyoto, Japan.
- [78] Ajou University Hospital, Suwon, Gyeonggi-do, South Korea.
- [79] Neurology, University of Ottawa, Ottawa, Ontario, Canada.
- [80] Cerebrovascular Medicine, Saga-ken Medical Centre Koseikan, Saga, Japan.
- [81] Neurology, Neurosurgery, Radiology, University of South Alabama, Mobile, Alabama, USA.
- [82] Konkuk University, Gwangjin-gu, Seoul, South Korea.
- [83] Interventional Neuroradiology, Royal London Hospital, Barts Health NHS Trust, London, UK.
- [84] Neurology, Hospital Clinico Universitario de Valladolid, Valladolid, Castilla y León, Spain.
- [85] Interventional Neuroradiology, Hospital Clínico Universitario, Universidad de Valladolid, Valladolid, Spain.
- [86] INR, Hospital Clinic de Barcelona, Barcelona, Catalunya, Spain.
- [87] Neurology, Hospital Clinic de Barcelona, Barcelona, Catalunya, Spain.
- [88] Stroke Unit, Department of Medicine, Centro Hospitalar Universitário de São João, Porto, Portugal.
- [89] Neuroradiology, Centro Hospitalar Universitário de São João, Porto, Portugal.
- [90] Neurology, Christchurch Hospital, Christchurch, New Zealand.
- [91] Neurosurgery, Christchurch Hospital, Christchurch, New Zealand.
- [92] Interventional and Diagnostic Neuroradiology, University Hospital Basel, Basel, Switzerland.
- [93] Neurosurgery, University of Southern California, Los Angeles, California, USA.
- [94] Neurosurgery, Japanese Red Cross Kumamoto Hospital, Kumamoto, Kumamoto, Japan.
- [95] Neurosurgery, Kohnan Hospital, Sendai, Miyagi, Japan.
- [96] Radiology, Neurology, UT Southwestern, Dallas, Texas, USA.
- [97] Endovascular Neurosurgery, Nara City Hospital, Nara, Nara, Japan.
- [98] Neurology, Toyonaka Municipal Hospital, Toyonaka, Osaka, Japan.
- [99] Neurosurgery and Stroke Center, Kagoshima City Hospital, Kagoshima, Kagoshima, Japan.
- [100] Texas Tech University System, Lubbock, Texas, USA.
- [101] Neurosurgery, University of Saskatchewan, Saskatoon, Saskatchewan, Canada.
- [102] Neurosurgery, Jikei University School of Medicine, Minato-ku, Tokyo, Japan.
- [103] Neurology, Royal Prince Alfred Hospital, Camperdown, New South Wales, Australia.
- [104] Neurosurgery, Royal Prince Alfred Hospital, Camperdown, New South Wales, Australia.
- [105] Neurosciences, Banner Desert Medical Center, Mesa, Arizona, USA.
- [106] Neurology, Japanese Red Cross Matsue Hospital, Shimane, Japan.
- [107] Neurology, Shiroyama Hospital, Habikino, Osaka, Japan.
- [108] Cerebrovascular Medicine, Niigata City General Hospital, Niigata, Niigata, Japan.
- [109] Neurology, Sugimura Hospital, Kumamoto, Japan.
- [110] Department of Neurosurgery, Kawasaki Medical School, Kurashiki, Japan.
- [111] Neurology, Osaka Red Cross Hospital, Osaka, Japan.
- [112] Neurosurgery, University of Tsukuba, Tsukuba, Ibaraki, Japan.

- [113] Neurology, Stroke Center and Neuroendovascular Therapy, Saiseikai Central Hospital, Minato-ku, Tokyo, Japan.
- [114] Neurology, Kinikyo Chuo Hospital, Sapporo, Hokkaido, Japan.
- [115] Cerebrovascular Medicine, NTT Medical Center Tokyo, Tokyo, Japan.
- [116] Neurosurgery, Yokohama Shintoshi Neurosurgical Hospital, Yokohama, Japan.
- [117] Neurosurgery, Osaka General Medical Center, Osaka, Japan.
- [118] Neurology, Neurosurgery, University of Miami School of Medicine, Miami, Florida, USA.
- [119] Neurology, Bon Secours Mercy Health System, Toledo, Ohio, USA.
- [120] Neurology, Maoming City Hospital, Guandong, China.
- [121] Interventional Neuroradiology, Endovascular Neurosurgery, Miami Cardiac & Vascular Institute, Miami, Florida, USA.
- [122] Neurology, Hackensack Meridian Health, Edison, New Jersey, USA.
- [123] Neurology, University of California Los Angeles, Los Angeles, California, USA.
- [124] Interventional Neuroradiology, University of California Los Angeles, Los Angeles, California, USA.
- [125] Neurology, Erlanger Medical Center, University of Tennessee, Chattanooga, Tennessee, USA.
- [126] Neurology, Neurosurgery, Radiology, University of Texas Health San Antonio, San Antonio, Texas, USA.
- [127] Neurology, Mianyang 404 Hospital, Mianyang, Sichuan, China.
- [128] Radiology, Siriraj Hosital, Mahidol University, Bangkok, Thailand.
- [129] Neurology, Neurosurgery, Radiology, SUNY Upstate Medical University, Syracuse, New York, USA.
- [130] Neurosurgery, Royal North Shore Hospital, Sydney, New South Wales, Australia.
- [131] Neurosciences, The University of Texas Rio Grande Valley, Harlingen, Texas, USA.
- [132] Central Stroke Unit, Directorate of Neuroscience, Khoula Hospital, Ministry of Health, Muscat, Oman.
- [133] Neurology, University of Michigan, Ann Arbor, Michigan, USA.
- [134] Neurosurgery, University of South Florida, Tampa, Florida, USA.
- [135] Neurology, Henry Ford Health System, Detroit, Michigan, USA.
- [136] Stroke Unit, Metropolitan Hospital, Piraeus, Greece.
- [137] Faculty of Medicine, National and Kapodistrian University of Athens, Athens, Greece.
- [138] Neurology, Maine Medical Center, Portland, Maine, USA.
- [139] Neuroradiology, Dartmouth Hitchcock Medical Center, Lebanon, New Hampshire, USA.
- [140] Neurology, Hospital Universitario de Brasilia, Brasilia, Distrito Federal, Brazil.
- [141] Neurointerventional Neurosurgery, The Valley Hospital, Ridgewood, New Jersey, USA.
- [142] Neurosciences, Stroke Program, St Joseph's University Medical Center, Paterson, New Jersey, USA.
- [143] Neurology, Christian Medical College and Hospital Ludhiana, Ludhiana, Punjab, India.
- [144] Vascular Neurology Unit, Clínica Alemana, Universidad del Desarrollo, Santiago, Chile.
- [145] Neurology, Osaka University Graduate School of Medicine, Osaka, Japan.
- [146] Neurology, Tokushima University Hospital, Tokushima, Japan.
- [147] Neurology, Universidade Federal de Sao Paulo, Sao Paulo, Sao Paulo, Brazil.
- [148] Istanbul Aydin University, Istanbul, İstanbul, Turkey.
- [149] Neurology, Bayhealth Medical Center, Dover, Delaware, USA.
- [150] Neurology, Saiseikai Fukuoka General Hospital, Fukuoka, Japan.

- [151] Division of Stroke, Department of Internal Medicine, Osaka Rosai Hospital, Sakai, Osaka, Japan.
- [152] Neurology Division, Department of Medicine, King Saud University, Riyadh, Riyadh Province, Saudi Arabia.
- [153] Neurology, Mount Sinai Health System, New York, New York, USA.
- [154] Neurology, Clinical Neurosciences and Hotchkiss Brain Institute, University of Calgary, Calgary, Alberta, Canada.
- [155] Neurology, Affiliated Hangzhou First People's Hospital, Zhejiang University School of Medicine, Hangzhou, Zhejiang, China.
- [156] Neurology, The Affiliated Hospital of Southwest Medical University, Luzhou, Sichuan, China.
- [157] Neurology, Yijishan Hospital of Wannan Medical College, Wuhu, Anhui, China.
- [158] The University of Toledo, Toledo, Ohio, USA.
- [159] Neuroscience, WellStar Health System, Marietta, Georgia, USA.
- [160] Neuroradiologie Interventionelle, Centre Hospitalier de l'Université de Montréal, Montreal, Quebec, Canada.
- [161] Neurology, Marcus Stroke & Neuroscience Center, Grady Memorial Hospital, Emory University School of Medicine, Atlanta, GA, USA raul.g.nogueira@emory.edu.

[#] Contributed equally.

- PMID: **33771936**
- PMCID: PMC8006491
- DOI: 10.1136/svn-2020-000695

Abstract

Background: During the COVID-19 pandemic, decreased volumes of stroke admissions and mechanical thrombectomy were reported. The study's objective was to examine whether subarachnoid haemorrhage (SAH) hospitalisations and ruptured aneurysm coiling interventions demonstrated similar declines.

Methods: We conducted a cross-sectional, retrospective, observational study across 6 continents, 37 countries and 140 comprehensive stroke centres. Patients with the diagnosis of SAH, aneurysmal SAH, ruptured aneurysm coiling interventions and COVID-19 were identified by prospective aneurysm databases or by International Classification of Diseases, 10th Revision, codes. The 3-month cumulative volume, monthly volumes for SAH hospitalisations and ruptured aneurysm coiling procedures were compared for the period before (1 year and immediately before) and during the pandemic, defined as 1 March-31 May 2020. The prior 1-year control period (1 March-31 May 2019) was obtained to account for seasonal variation.

Findings: There was a significant decline in SAH hospitalisations, with 2044 admissions in the 3 months immediately before and 1585 admissions during the pandemic, representing a relative decline of 22.5% (95% CI -24.3% to -20.7%, p<0.0001). Embolisation of ruptured aneurysms declined with 1170-1035 procedures, respectively, representing an 11.5% (95%CI -13.5% to -9.8%, p=0.002) relative drop. Subgroup analysis was noted for aneurysmal SAH hospitalisation decline from 834 to 626 hospitalisations, a 24.9% relative decline (95% CI -28.0% to -22.1%, p<0.0001). A relative increase in ruptured aneurysm coiling was noted in low coiling volume hospitals of 41.1% (95% CI 32.3% to 50.6%, p=0.008) despite a decrease in SAH admissions in this tertile.

Interpretation: There was a relative decrease in the volume of SAH hospitalisations, aneurysmal SAH hospitalisations and ruptured aneurysm embolisations during the COVID-19 pandemic. These findings in SAH are consistent with a decrease in other emergencies, such as stroke and myocardial infarction.

Keywords: aneurysm; coil; haemorrhage; infection; subarachnoid.

© Author(s) (or their employer(s)) 2021. Re-use permitted under CC BY-NC. No commercial re-use. See rights and permissions. Published by BMJ.

Conflict of interest statement

Competing interests: TNN: PI CLEAR study (Medtronic). DCH: Stryker, Vesalio, Cerenovus consultant. AEH: consultant and speaker for Medtronic, Stryker, Microvention, Penumbra, Balt, Scientia, Genentech and GE Healthcare. PJ: Medtronic, Microvention, Balt, Cerenovus consultant. SO-G: Medtronic, Stryker consultant. DSL: Cerenovus, Genentech, Stryker, Medtronic consultant. TGJ: advisor/investor for Anaconda, Route92, FreeOx, and Blockade Medical; Medtronic grants, DAWN, AURORA PI (Stryker). WJM: consultant: Rebound Therapeutics, Viseon Imperative Care, Q'Apel, Stryker, Stream Biomedical, Spartan Micro; Investor: Cerebrotech, Endostream, Q'Apel, Viseon, Rebound, and Spartan Micro. RGN: Stryker; Cerenovus/Neuravi; Anaconda, Cerebrotech, Ceretrieve, Vesalio (Advisory Board); Imperative Care.

- 24 references
- 1 figure

Supplementary info

Publication types, MeSH terms, Grant support Expand

Publication types

- Observational Study

MeSH terms

- COVID-19*
- Cross-Sectional Studies
- Humans
- Intracranial Aneurysm* / diagnostic imaging
- Intracranial Aneurysm* / epidemiology
- Intracranial Aneurysm* / therapy
- Pandemics
- Prospective Studies
- Retrospective Studies
- SARS-CoV-2
- Subarachnoid Hemorrhage* / diagnostic imaging
- Subarachnoid Hemorrhage* / epidemiology

- Treatment Outcome

Grant support

- K08 NS112601/NS/NINDS NIH HHS/United States
- UL1 TR001863/TR/NCATS NIH HHS/United States

Full text links

BMJ Open Access Full Text HighWire Free PMC article
Proceed to details
Cite
Share
1,088
Observational Study
Physiol Rep
. 2021 Feb;9(3):e14715.
doi: 10.14814/phy2.14715.

Timing of VV-ECMO therapy implementation influences prognosis of COVID-19 patients

Raphaël Giraud[1 2 3], David Legouis[1 2 4 5], Benjamin Assouline[1 2 3], Amandine De Charriere[1 2 3], Dumeng Decosterd[6], Marie-Eve Brunner[6], Mallory Moret-Bochatay[7], Thierry Fumeaux[2 7], Karim Bendjelid[1 2 3]
Affiliations Expand

Affiliations

- [1] Intensive Care Unit, Geneva University Hospitals, Geneva, Switzerland.
- [2] Faculty of Medicine, University of Geneva, Geneva, Switzerland.
- [3] Geneva Hemodynamic Research Group, Geneva, Switzerland.
- [4] Laboratory of Nephrology, Department of Medicine, University Hospitals of Geneva, Geneva, Switzerland.
- [5] Department of Cell Physiology, Faculty of Medicine, University of Geneva, Geneva, Switzerland.
- [6] Intensive Care Unit, Réseau Hospitalier Neuchâtelois, Site de Pourtalès, Neuchatel, Switzerland.
- [7] Intensive Care Unit, Groupement Hospitalier de l'Ouest Lémanique, Hôpital de zone de Nyon, Nyon, Switzerland.

- PMID: **33527751**
- PMCID: PMC7851435
- DOI: 10.14814/phy2.14715

Free PMC article
Observational Study

Timing of VV-ECMO therapy implementation influences prognosis of COVID-19 patients

Raphaël Giraud et al. Physiol Rep. 2021 Feb.
Free PMC article
Show details
Physiol Rep
. 2021 Feb;9(3):e14715.
doi: 10.14814/phy2.14715.

Authors

Raphaël Giraud [1 2 3], David Legouis [1 2 4 5], Benjamin Assouline [1 2 3], Amandine De Charriere [1 2 3], Dumeng Decosterd [6], Marie-Eve Brunner [6], Mallory Moret-Bochatay [7], Thierry Fumeaux [2 7], Karim Bendjelid [1 2 3]

Affiliations

- [1] Intensive Care Unit, Geneva University Hospitals, Geneva, Switzerland.
- [2] Faculty of Medicine, University of Geneva, Geneva, Switzerland.
- [3] Geneva Hemodynamic Research Group, Geneva, Switzerland.
- [4] Laboratory of Nephrology, Department of Medicine, University Hospitals of Geneva, Geneva, Switzerland.
- [5] Department of Cell Physiology, Faculty of Medicine, University of Geneva, Geneva, Switzerland.
- [6] Intensive Care Unit, Réseau Hospitalier Neuchâtelois, Site de Pourtalès, Neuchatel, Switzerland.
- [7] Intensive Care Unit, Groupement Hospitalier de l'Ouest Lémanique, Hôpital de zone de Nyon, Nyon, Switzerland.

- PMID: **33527751**
- PMCID: PMC7851435
- DOI: 10.14814/phy2.14715

Abstract

Introduction: Current knowledge on the use of extracorporeal membrane oxygenation (ECMO) in COVID-19 remains limited to small series and registry data. In the present retrospective monocentric study, we report on our experience, our basic principles, and our results in establishing and managing ECMO in critically ill COVID-19 patients.

Methods: A cohort study was conducted in patients with severe acute respiratory distress syndrome (ARDS) related to COVID-19 pneumonia admitted to the ICU of the Geneva University

Hospitals and supported by VV-ECMO from March 14 to May 31. The VV-ECMO implementation criteria were defined according to an institutional algorithm validated by the local crisis unit and the Swiss Society of Intensive Care Medicine.

Results: Out of 137 ARDS patients admitted to our ICU, 10 patients (age 57 ± 4 years, BMI 31.5 ± 5 kg/m^2 , and SAPS II score 56 ± 3) were put on VV-ECMO. The mean duration of mechanical ventilation before ECMO and mean time under ECMO were 7 ± 3 days and 19 ± 11 days, respectively. The ICU and hospital length of stay were 26 ± 11 and 35 ± 10 days, respectively. The survival rate for patients on ECMO was 40%. The comparative analysis between survivors and non-survivors highlighted that survivors had a significantly shorter mechanical ventilation duration before ECMO (4 ± 2 days vs. 9 ± 2 days, p = 0.01). All the patients who had more than 150 h of mechanical ventilation before the application of ECMO ultimately died.

Conclusion: The present results suggest that VV-ECMO can be safely utilized in appropriately selected COVID-19 patients with refractory hypoxemia. The main information for clinicians is that late VV-ECMO therapy (i.e., beyond the seventh day of mechanical ventilation) seems futile.

Keywords: ARDS; COVID-19 pandemic; VV-ECMO.

© 2021 The Authors. Physiological Reports published by Wiley Periodicals LLC on behalf of The Physiological Society and the American Physiological Society.

Conflict of interest statement

The authors declare that they have no competing interests.

- 25 references
- 6 figures

Supplementary info

Publication types, MeSH terms Expand

Publication types

- Observational Study

MeSH terms

- COVID-19 / pathology
- COVID-19 / therapy*
- Extracorporeal Membrane Oxygenation / adverse effects
- Extracorporeal Membrane Oxygenation / methods*
- Female
- Humans
- Male
- Middle Aged
- Respiration, Artificial / methods

- Survival Analysis
- Time Factors

Full text links

WILEY Free Full Text Wiley Free PMC article

Proceed to details

Cite

Share

1,089

Observational Study

Drugs

. 2021 Apr;81(6):685-695.

doi: 10.1007/s40265-021-01498-x. Epub 2021 Mar 29.

Prior Treatment with Statins is Associated with Improved Outcomes of Patients with COVID-19: Data from the SEMI-COVID-19 Registry

José David Torres-Peña [#, 1, 2], Luis M Pérez-Belmonte [#, 3], Francisco Fuentes-Jiménez [4, 5], Mª Dolores López Carmona [3], Pablo Pérez-Martinez [1, 2], José López-Miranda [1, 2], Francisco Javier Carrasco Sánchez [6], Juan Antonio Vargas Núñez [7], Esther Del Corral Beamonte [8], Jeffrey Oskar Magallanes Gamboa [9], Andrés González García [10], Julio González Moraleja [11], Andrés Cortés Troncoso [12], María Luisa Taboada Martínez [13], María Del Pilar Del Fidalgo Montero [14], José Miguel Seguí Ripol [15], Ricardo Gil Sánchez [16], Diana Alegre González [17], Ramon Boixeda [18], Begoña Cortés Rodríguez [19], Javier Ena [20], Gema María García García [21], Ana Ventura Esteve [22], José Manuel Ramos Rincón [23], Ricardo Gómez-Huelgas [3], SEMI-COVID-19 Network

Collaborators, Affiliations Expand

- PMID: **33782908**
- PMCID: PMC8006631
- DOI: 10.1007/s40265-021-01498-x

Free PMC article

Observational Study

Prior Treatment with Statins is Associated with Improved Outcomes of Patients with

COVID-19: Data from the SEMI-COVID-19 Registry

José David Torres-Peña et al. Drugs. 2021 Apr.
Free PMC article
Show details
Drugs
. 2021 Apr;81(6):685-695.
doi: 10.1007/s40265-021-01498-x. Epub 2021 Mar 29.

- PMID: **33782908**
- PMCID: PMC8006631
- DOI: 10.1007/s40265-021-01498-x

Abstract

Background: The impact of statins on COVID-19 outcomes is important given the high prevalence of their use among individuals at risk for severe COVID-19. Our aim is to assess whether patients receiving chronic statin treatment who are hospitalized with COVID-19 have reduced in-hospital mortality if statin therapy is maintained during hospitalization.

Methods: This work is a cross-sectional, observational, retrospective multicenter study that analyzed 2921 patients who required hospital admission at 150 Spanish centers included in the nationwide SEMI-COVID-19 Network. We compared the clinical characteristics and COVID-19 disease outcomes between patients receiving chronic statin therapy who maintained this therapy during hospitalization versus those who did not. Propensity score matching was used to match each statin user whose therapy was maintained during hospitalization to a statin user whose therapy was withdrawn during hospitalization.

Results: After propensity score matching, continuation of statin therapy was associated with lower all-cause mortality (OR 0.67, 0.54-0.83, $p < 0.001$); lower incidence of acute kidney injury (AKI) (OR 0.76,0.6-0.97, $p = 0.025$), acute respiratory distress syndrome (ARDS) (OR 0.78, 0.69-0.89, $p < 0.001$), and sepsis (4.82% vs 9.85%, $p = 0.008$); and less need for invasive mechanical ventilation (IMV) (5.35% vs 8.57, $p < 0.001$) compared to patients whose statin therapy was withdrawn during hospitalization.

Conclusions: Patients previously treated with statins who are hospitalized for COVID-19 and maintain statin therapy during hospitalization have a lower mortality rate than those in whom therapy is withdrawn. In addition, statin therapy was associated with a decreased probability that patients with COVID-19 will develop AKI, ARDS, or sepsis and decreases the need for IMV.

Conflict of interest statement

The authors declare that there are no conflicts of interest.

Comment in

- Comment on: "Prior Treatment with Statins is Associated with Improved Outcomes of Patients with COVID-19: Data from the SEMI-COVID-19 Registry".

Vuorio A, Kovanen PT. Vuorio A, et al. Drugs. 2021 Jun;81(9):1125-1127. doi: 10.1007/s40265-021-01537-7. Epub 2021 May 28. Drugs. 2021. PMID: 34047956 Free PMC article. No abstract available.

- 29 references
- 1 figure

Supplementary info

Publication types, MeSH terms, Substances Expand

Publication types

- Multicenter Study
- Observational Study

MeSH terms

- Aged
- Aged, 80 and over
- COVID-19 / complications*
- COVID-19 / epidemiology*
- COVID-19 / mortality
- Cross-Sectional Studies
- Female
- Hospital Mortality / trends*
- Humans
- Hydroxymethylglutaryl-CoA Reductase Inhibitors / administration & dosage*
- Male
- Middle Aged
- Propensity Score
- Retrospective Studies
- Risk Assessment
- SARS-CoV-2
- Spain / epidemiology

Substances

- Hydroxymethylglutaryl-CoA Reductase Inhibitors

Full text links

SpringerLink FULL-TEXT ARTICLE Springer Free PMC article
Proceed to details

Cite
Share
☐ 1,090
Observational Study

Circulation
. 2021 Jun 22;143(25):2508-2510.
doi: 10.1161/CIRCULATIONAHA.121.054785. Epub 2021 Jun 7.

Reduction in Kawasaki Disease After Nonpharmaceutical Interventions in the COVID-19 Era: A Nationwide Observational Study in Korea

Ji-Man Kang[#, 1, 2], Young-Eun Kim[#, 3], Kyungmin Huh[4], Jinwook Hong[5], Dong Wook Kim[3], Min Young Kim[1, 2], Se Yong Jung[6], Jong-Hun Kim[7], Jaehun Jung[5, 8], Jong Gyun Ahn[1, 2]
Affiliations Expand

Affiliations

- [1] Department of Pediatrics, Severance Children's Hospital (J.-M.K., M.Y.K., J.G.A.), Yonsei University College of Medicine, Seoul, South Korea.
- [2] Institute for Immunology and Immunological Diseases (J.-M.K., M.Y.K., J.G.A.), Yonsei University College of Medicine, Seoul, South Korea.
- [3] Department of Big Data Strategy, National Health Insurance Service, Wonju, South Korea (Y.-E.K., D.W.K.).
- [4] Division of Infectious Diseases, Department of Medicine, Samsung Medical Center, Sungkyunkwan University School of Medicine, Seoul, South Korea (K.H.).
- [5] Artificial Intelligence and Big-Data Convergence Center, Gil Medical Center (J.H., J.J.), Gachon University College of Medicine, Incheon, South Korea.
- [6] Division of Pediatric Cardiology, Department of Pediatrics (S.Y.J.), Yonsei University College of Medicine, Seoul, South Korea.
- [7] Department of Social and Preventive Medicine, Sungkyunkwan University School of Medicine, Suwon, Gyeonggi-do, South Korea (J.-H.K.).
- [8] Department of Preventive Medicine (J.J.), Gachon University College of Medicine, Incheon, South Korea.

[#] Contributed equally.

- PMID: **34092115**
- PMCID: PMC8212882
- DOI: 10.1161/CIRCULATIONAHA.121.054785

Free PMC article
Observational Study

Reduction in Kawasaki Disease After Nonpharmaceutical Interventions in the COVID-19 Era: A Nationwide Observational Study in Korea

Ji-Man Kang et al. Circulation. 2021.
Free PMC article
Show details
Circulation
. 2021 Jun 22;143(25):2508-2510.
doi: 10.1161/CIRCULATIONAHA.121.054785. Epub 2021 Jun 7.

Authors

Ji-Man Kang [#] [1] [2], Young-Eun Kim [#] [3], Kyungmin Huh [4], Jinwook Hong [5], Dong Wook Kim [3], Min Young Kim [1] [2], Se Yong Jung [6], Jong-Hun Kim [7], Jaehun Jung [5] [8], Jong Gyun Ahn [1] [2]

Affiliations

- [1] Department of Pediatrics, Severance Children's Hospital (J.-M.K., M.Y.K., J.G.A.), Yonsei University College of Medicine, Seoul, South Korea.
- [2] Institute for Immunology and Immunological Diseases (J.-M.K., M.Y.K., J.G.A.), Yonsei University College of Medicine, Seoul, South Korea.
- [3] Department of Big Data Strategy, National Health Insurance Service, Wonju, South Korea (Y.-E.K., D.W.K.).
- [4] Division of Infectious Diseases, Department of Medicine, Samsung Medical Center, Sungkyunkwan University School of Medicine, Seoul, South Korea (K.H.).
- [5] Artificial Intelligence and Big-Data Convergence Center, Gil Medical Center (J.H., J.J.), Gachon University College of Medicine, Incheon, South Korea.
- [6] Division of Pediatric Cardiology, Department of Pediatrics (S.Y.J.), Yonsei University College of Medicine, Seoul, South Korea.
- [7] Department of Social and Preventive Medicine, Sungkyunkwan University School of Medicine, Suwon, Gyeonggi-do, South Korea (J.-H.K.).
- [8] Department of Preventive Medicine (J.J.), Gachon University College of Medicine, Incheon, South Korea.

[#] Contributed equally.

- PMID: **34092115**
- PMCID: PMC8212882
- DOI: 10.1161/CIRCULATIONAHA.121.054785

No abstract available

Keywords: COVID-19; Kawasaki disease; Republic of Korea; incidence; mucocutaneous lymph node syndrome; physical distancing.

- 5 references
- 1 figure

Supplementary info

Publication types, MeSH terms Expand

Publication types

- Letter
- Observational Study
- Research Support, Non-U.S. Gov't

MeSH terms

- Adolescent
- COVID-19 / epidemiology*
- COVID-19 / prevention & control
- Child
- Child, Preschool
- Female
- Humans
- Infant
- Infant, Newborn
- Male
- Mucocutaneous Lymph Node Syndrome / epidemiology*
- Mucocutaneous Lymph Node Syndrome / therapy*
- Republic of Korea / epidemiology
- Retrospective Studies
- Young Adult

Full text links

FREE FULL TEXT Atypon Free PMC article
Proceed to details
Cite
Share
1,091
Observational Study
BMC Pediatr
. 2020 Sep 7;20(1):427.
doi: 10.1186/s12887-020-02303-6.

COVID-19 related reduction in pediatric emergency healthcare utilization - a concerning trend

Christian Dopfer [1], Martin Wetzke [1] [2] [3], Anna Zychlinsky Scharff [4], Frank Mueller [5], Frank Dressler [1], Ulrich Baumann [1], Michael Sasse [6] [7], Gesine Hansen [1] [2] [8], Alexandra Jablonka [3] [9], Christine Happle [10] [11]
Affiliations Expand

Affiliations

- [1] Department of Pediatric Pneumology, Allergology, and Neonatology, Hannover Medical School, Carl-Neuberg-Straße 1, D - 30625, Hannover, Germany.
- [2] German Center for Lung Research, Biomedical Research in End Stage and Obstructive Lung Disease, BREATH Hannover, Hanover, Germany.
- [3] German Center for Infection Research (DZIF), partner site Hannover-Braunschweig, Braunschweig, Germany.
- [4] Department of Pediatrics, Hannover Medical School, Hannover, Germany.
- [5] Department of General Practice, University Medical Centre Goettingen, Goettingen, Germany.
- [6] Department of Pediatric Cardiology and Intensive Care, Hannover Medical School, Hannover, Germany.
- [7] Pediatric Intensive Care Network Northern Germany/ PIN, Hannover Medical School, Hannover, Germany.
- [8] Cluster of Excellence RESIST (EXC 2155), Hannover Medical School, Carl-Neuberg-Straße 1, 30625, Hannover, Germany.
- [9] Department of Rheumatology and Immunology, Hannover Medical School, Hannover, Germany.
- [10] Department of Pediatric Pneumology, Allergology, and Neonatology, Hannover Medical School, Carl-Neuberg-Straße 1, D - 30625, Hannover, Germany. happle.christine@mh-hannover.de.
- [11] German Center for Lung Research, Biomedical Research in End Stage and Obstructive Lung Disease, BREATH Hannover, Hanover, Germany. happle.christine@mh-hannover.de.

- PMID: **32894080**
- PMCID: PMC7475725
- DOI: 10.1186/s12887-020-02303-6

Free PMC article
Observational Study

COVID-19 related reduction in pediatric emergency healthcare utilization - a concerning trend

Christian Dopfer et al. BMC Pediatr. 2020.
Free PMC article
Show details
BMC Pediatr
. 2020 Sep 7;20(1):427.
doi: 10.1186/s12887-020-02303-6.

Authors

Christian Dopfer [1], Martin Wetzke [1] [2] [3], Anna Zychlinsky Scharff [4], Frank Mueller [5], Frank Dressler [1], Ulrich Baumann [1], Michael Sasse [6] [7], Gesine Hansen [1] [2] [8], Alexandra Jablonka [3] [9], Christine Happle [10] [11]

Affiliations

- [1] Department of Pediatric Pneumology, Allergology, and Neonatology, Hannover Medical School, Carl-Neuberg-Straße 1, D - 30625, Hannover, Germany.
- [2] German Center for Lung Research, Biomedical Research in End Stage and Obstructive Lung Disease, BREATH Hannover, Hanover, Germany.
- [3] German Center for Infection Research (DZIF), partner site Hannover-Braunschweig, Braunschweig, Germany.
- [4] Department of Pediatrics, Hannover Medical School, Hannover, Germany.
- [5] Department of General Practice, University Medical Centre Goettingen, Goettingen, Germany.
- [6] Department of Pediatric Cardiology and Intensive Care, Hannover Medical School, Hannover, Germany.
- [7] Pediatric Intensive Care Network Northern Germany/ PIN, Hannover Medical School, Hannover, Germany.
- [8] Cluster of Excellence RESIST (EXC 2155), Hannover Medical School, Carl-Neuberg-Straße 1, 30625, Hannover, Germany.
- [9] Department of Rheumatology and Immunology, Hannover Medical School, Hannover, Germany.
- [10] Department of Pediatric Pneumology, Allergology, and Neonatology, Hannover Medical School, Carl-Neuberg-Straße 1, D - 30625, Hannover, Germany. happle.christine@mh-hannover.de.
- [11] German Center for Lung Research, Biomedical Research in End Stage and Obstructive Lung Disease, BREATH Hannover, Hanover, Germany. happle.christine@mh-hannover.de.

- PMID: **32894080**
- PMCID: PMC7475725
- DOI: 10.1186/s12887-020-02303-6

Abstract

Background: The COVID-19 pandemic has disrupted healthcare systems worldwide. In addition to the direct impact of the virus on patient morbidity and mortality, the effect of lockdown strategies on health and healthcare utilization have become apparent. Little is known on the effect of the pandemic on pediatric and adolescent medicine. We examined the impact of the pandemic on pediatric emergency healthcare utilization.

Methods: We conducted a monocentric, retrospective analysis of n = 5,424 pediatric emergency department visits between January 1st and April 19th of 2019 and 2020, and compared healthcare utilization during the pandemic in 2020 to the same period in 2019.

Results: In the four weeks after lockdown in Germany began, we observed a massive drop of 63.8% in pediatric emergency healthcare utilization (mean daily visits 26.8 ± SEM 1.5 in 2019 vs. 9.7 ± SEM 1 in 2020, $p < 0.005$). This drop in cases occurred for both communicable and non-communicable diseases. A larger proportion of patients under one year old (daily mean of 16.6% ±SEM 1.4 in 2019 vs. 23.1% ±SEM 1.7 in 2020, $p < 0.01$) and of cases requiring hospitalisation (mean of 13.9% ±SEM 1.6 in 2019 vs. 26.6% ±SEM 3.3 in 2020, $p < 0.001$) occurred during the pandemic. During the analysed time periods, few intensive care admissions and no fatalities occurred.

Conclusions: Our data illustrate a significant decrease in pediatric emergency department visits during the COVID-19 pandemic. Public outreach is needed to encourage parents and guardians to seek medical attention for pediatric emergencies in spite of the pandemic.

Keywords: COVID-19; SARS-CoV-2; children; emergency care; emergency department; healthcare utilization; pandemic; pediatric.

Conflict of interest statement

The authors have no competing interests to report.

- 23 references
- 5 figures

Supplementary info

Publication types, MeSH terms Expand

Publication types

- Observational Study
- Research Support, Non-U.S. Gov't

MeSH terms

- Adolescent
- Betacoronavirus*
- COVID-19

- Child
- Child, Preschool
- Coronavirus Infections* / prevention & control
- Coronavirus Infections* / psychology
- Emergency Service, Hospital / trends*
- Facilities and Services Utilization / trends*
- Female
- Germany
- Health Services Accessibility / trends*
- Humans
- Infant
- Infant, Newborn
- Male
- Pandemics* / prevention & control
- Patient Acceptance of Health Care / psychology
- Patient Acceptance of Health Care / statistics & numerical data*
- Pneumonia, Viral* / prevention & control
- Pneumonia, Viral* / psychology
- Retrospective Studies
- SARS-CoV-2

Full text links

Read free full text at BMC BioMed Central Free PMC article

Proceed to details

Cite

Share

☐ 1,092

Observational Study

Microbiol Spectr

. 2021 Oct 31;9(2):e0113821.

doi: 10.1128/Spectrum.01138-21. Epub 2021 Oct 20.

COVID-19-Associated Pulmonary Aspergillosis, Fungemia, and Pneumocystosis in the Intensive Care Unit: a Retrospective Multicenter Observational Cohort during the First French Pandemic Wave

Stéphane Bretagne [1 2 3], Karine Sitbon [1], Françoise Botterel [4], Sarah Dellière [1 2 3], Valérie Letscher-Bru [5], Taieb Chouaki [6], Anne-Pauline Bellanger [7], Christine Bonnal [8], Arnault Fekkar [9], Florence Persat [10], Damien Costa [11], Nathalie Bourgeois [12], Frédéric Dalle [13], Florian Lussac-Sorton [14], André Paugam [3 15], Sophie Cassaing [16], Lilia Hasseine [17], Antoine Huguenin [18], Nadia Guennouni [19], Edith Mazars [20], Solène Le Gal [21], Milène Sasso [22], Sophie Brun [23], Lucile Cadot [24], Carole Cassagne [25], Estelle Cateau [26], Jean-Pierre Gangneux [27], Maxime Moniot [28], Anne-Laure Roux [29], Céline Tournus [30], Nicole Desbois-Nogard [31], Alain Le Coustumier [32], Olivier Moquet [33], Alexandre Alanio [1 2 3], Françoise Dromer [1], French Mycoses Study Group

Affiliations Expand

Affiliations

- [1] Institut Pasteur, Université de Paris, CNRS UMR2000, unité de Mycologie Moléculaire, Centre national de Référence Mycoses Invasives et Antifongiques, Paris, France.
- [2] Laboratoire de Parasitologie-Mycologie, Hôpital Saint Louis, Assistance Publique-Hôpitaux De Paris (AP-HP), Paris, France.
- [3] Université de Paris, Paris, France.
- [4] Assistance Publique-Hôpitaux De Paris (AP-HP), Hôpital Henri Mondor, Université Paris-Est Créteil Val-de-Marne, Créteil, France.
- [5] Service de Parasitologie et de Mycologie Médicale, CHU de Strasbourg, Strasbourg, France.
- [6] Laboratoire de Parasitologie-Mycologie, CHU Amiens-Picardie, Amiens, France.
- [7] Laboratoire de Parasitologie-Mycologie, CHU Besançon, Besançon, France.
- [8] Assistance Publique-Hôpitaux De Paris (AP-HP), Laboratoire de Parasitologie-Mycologie, Hôpital Universitaire Bichat, Paris, France.
- [9] Assistance Publique-Hôpitaux De Paris (AP-HP), Groupe Hospitalier La Pitié-Salpêtrière, Service de Parasitologie Mycologie, Sorbonne Université, Inserm, CNRS, Centre d'Immunologie et des Maladies Infectieuses (CIMI), Paris, France.
- [10] Hospices Civils de Lyongrid.413852.9, Service de Parasitologie et Mycologie Médicale, Hôpital de la Croix-Rousse, Lyon-Université Claude Bernard Lyon 1, Lyon, France.
- [11] Laboratoire de Parasitologie-Mycologie, CHU Charles-Nicolle, Rouen, France.
- [12] Laboratoire de Parasitologie-Mycologie, CHU de Montpellier, Montpellier, France.
- [13] Laboratoire de Parasitologie Mycologie, Centre Hospitalier Universitaire de Dijon-Hôpital François Mitterrand, Dijon, France.
- [14] Department of Parasitology, Bordeaux University Hospital, Bordeaux, France.
- [15] Assistance Publique-Hôpitaux De Paris (AP-HP), Hôpital Cochin, Paris, France.
- [16] Service de Parasitologie-Mycologie, Hôpital Purpan Toulouse, CHU Toulouse, Toulouse, France.
- [17] Laboratoire de Parasitologie Mycologie CHU de Nice, Nice, France.
- [18] Parasitologie Mycologie-Laboratoire de Parasitologie-Mycologie, Pôle de Biopathologie, CHU de Reims, Université de Reims Champagne Ardenne, Reims, France.
- [19] Assistance Publique-Hôpitaux De Paris (AP-HP), Service de Bactériologie, Virologie, Parasitologie et Hygiène, Hôpital Necker-Enfants Malades, IHU Imagine, Paris, France.
- [20] CH de Valenciennes, Laboratoire de Microbiologie, Valenciennes, France.
- [21] Laboratoire de Parasitologie et Mycologie, Hôpital de La Cavale Blanche, CHU de Brest, Brest, France.
- [22] Laboratoire de Parasitologie Mycologie CHU Nîmes, Nîmes, France.

- [23] Assistance Publique-Hôpitaux De Paris (AP-HP), Laboratoire de Parasitologie Mycologie Hôpital Avicenne, Bobigny, France.
- [24] Département d'Hygiène Hospitalière, CHU Montpellier, Montpellier, France.
- [25] IHU Marseille-Institut Hospitalier Universitaire Méditerranée Infection, Marseille, France.
- [26] Laboratoire de Parasitologie-Mycologie, CHU de Poitiers, Poitiers, France.
- [27] CHU de Rennes, Université de Rennes, Institut de Recherche en Santé, Environnement et Travail (IRSET), Rennes, France.
- [28] Laboratoire de Parasitologie-Mycologie, CHU Clermont-Ferrand, Clermont-Ferrand, France.
- [29] Assistance Publique-Hôpitaux De Paris (AP-HP), Hôpital Raymond Poincaré Garches, Hôpital Ambroise Paré, Boulogne Billancourt, France.
- [30] Laboratoire de Microbiologie, Centre Hospitalier de Saint-Denis, Saint-Denis, France.
- [31] Laboratoire de Parasitologie-Mycologie, Centre Hospitalier Universitaire de Martiniquegrid.412874.c, Fort-de-France, La Martinique, France.
- [32] Centre Hospitalier Intercommunal de Bigorre, Tarbes, France.
- [33] Laboratoire de Parasitologie-Mycologie, Centre Hospitalier de Beauvais, Beauvais, France.

- PMID: **34668768**
- PMCID: PMC8528108
- DOI: 10.1128/Spectrum.01138-21

Free PMC article
Observational Study

COVID-19-Associated Pulmonary Aspergillosis, Fungemia, and Pneumocystosis in the Intensive Care Unit: a Retrospective Multicenter Observational Cohort during the First French Pandemic Wave

Stéphane Bretagne et al. Microbiol Spectr. 2021.
Free PMC article
Show details
Microbiol Spectr
. 2021 Oct 31;9(2):e0113821.
doi: 10.1128/Spectrum.01138-21. Epub 2021 Oct 20.

Authors

Stéphane Bretagne [1 2 3], Karine Sitbon [1], Françoise Botterel [4], Sarah Dellière [1 2 3], Valérie Letscher-Bru [5], Taieb Chouaki [6], Anne-Pauline Bellanger [7], Christine Bonnal [8], Arnault Fekkar [9], Florence Persat [10], Damien Costa [11], Nathalie Bourgeois [12], Frédéric Dalle [13], Florian Lussac-Sorton [14], André Paugam [3 15], Sophie Cassaing [16], Lilia Hasseine [17], Antoine Huguenin

[18], Nadia Guennouni [19], Edith Mazars [20], Solène Le Gal [21], Milène Sasso [22], Sophie Brun [23], Lucile Cadot [24], Carole Cassagne [25], Estelle Cateau [26], Jean-Pierre Gangneux [27], Maxime Moniot [28], Anne-Laure Roux [29], Céline Tournus [30], Nicole Desbois-Nogard [31], Alain Le Coustumier [32], Olivier Moquet [33], Alexandre Alanio [1] [2] [3], Françoise Dromer [1], French Mycoses Study Group

Affiliations

- [1] Institut Pasteur, Université de Paris, CNRS UMR2000, unité de Mycologie Moléculaire, Centre national de Référence Mycoses Invasives et Antifongiques, Paris, France.
- [2] Laboratoire de Parasitologie-Mycologie, Hôpital Saint Louis, Assistance Publique-Hôpitaux De Paris (AP-HP), Paris, France.
- [3] Université de Paris, Paris, France.
- [4] Assistance Publique-Hôpitaux De Paris (AP-HP), Hôpital Henri Mondor, Université Paris-Est Créteil Val-de-Marne, Créteil, France.
- [5] Service de Parasitologie et de Mycologie Médicale, CHU de Strasbourg, Strasbourg, France.
- [6] Laboratoire de Parasitologie-Mycologie, CHU Amiens-Picardie, Amiens, France.
- [7] Laboratoire de Parasitologie-Mycologie, CHU Besançon, Besançon, France.
- [8] Assistance Publique-Hôpitaux De Paris (AP-HP), Laboratoire de Parasitologie-Mycologie, Hôpital Universitaire Bichat, Paris, France.
- [9] Assistance Publique-Hôpitaux De Paris (AP-HP), Groupe Hospitalier La Pitié-Salpêtrière, Service de Parasitologie Mycologie, Sorbonne Université, Inserm, CNRS, Centre d'Immunologie et des Maladies Infectieuses (CIMI), Paris, France.
- [10] Hospices Civils de Lyongrid.413852.9, Service de Parasitologie et Mycologie Médicale, Hôpital de la Croix-Rousse, Lyon-Université Claude Bernard Lyon 1, Lyon, France.
- [11] Laboratoire de Parasitologie-Mycologie, CHU Charles-Nicolle, Rouen, France.
- [12] Laboratoire de Parasitologie-Mycologie, CHU de Montpellier, Montpellier, France.
- [13] Laboratoire de Parasitologie Mycologie, Centre Hospitalier Universitaire de Dijon-Hôpital François Mitterrand, Dijon, France.
- [14] Department of Parasitology, Bordeaux University Hospital, Bordeaux, France.
- [15] Assistance Publique-Hôpitaux De Paris (AP-HP), Hôpital Cochin, Paris, France.
- [16] Service de Parasitologie-Mycologie, Hôpital Purpan Toulouse, CHU Toulouse, Toulouse, France.
- [17] Laboratoire de Parasitologie Mycologie CHU de Nice, Nice, France.
- [18] Parasitologie Mycologie-Laboratoire de Parasitologie-Mycologie, Pôle de Biopathologie, CHU de Reims, Université de Reims Champagne Ardenne, Reims, France.
- [19] Assistance Publique-Hôpitaux De Paris (AP-HP), Service de Bactériologie, Virologie, Parasitologie et Hygiène, Hôpital Necker-Enfants Malades, IHU Imagine, Paris, France.
- [20] CH de Valenciennes, Laboratoire de Microbiologie, Valenciennes, France.
- [21] Laboratoire de Parasitologie et Mycologie, Hôpital de La Cavale Blanche, CHU de Brest, Brest, France.
- [22] Laboratoire de Parasitologie Mycologie CHU Nîmes, Nîmes, France.
- [23] Assistance Publique-Hôpitaux De Paris (AP-HP), Laboratoire de Parasitologie Mycologie Hôpital Avicenne, Bobigny, France.
- [24] Département d'Hygiène Hospitalière, CHU Montpellier, Montpellier, France.
- [25] IHU Marseille-Institut Hospitalier Universitaire Méditerranée Infection, Marseille, France.

- [26] Laboratoire de Parasitologie-Mycologie, CHU de Poitiers, Poitiers, France.
- [27] CHU de Rennes, Université de Rennes, Institut de Recherche en Santé, Environnement et Travail (IRSET), Rennes, France.
- [28] Laboratoire de Parasitologie-Mycologie, CHU Clermont-Ferrand, Clermont-Ferrand, France.
- [29] Assistance Publique-Hôpitaux De Paris (AP-HP), Hôpital Raymond Poincaré Garches, Hôpital Ambroise Paré, Boulogne Billancourt, France.
- [30] Laboratoire de Microbiologie, Centre Hospitalier de Saint-Denis, Saint-Denis, France.
- [31] Laboratoire de Parasitologie-Mycologie, Centre Hospitalier Universitaire de Martiniquegrid.412874.c, Fort-de-France, La Martinique, France.
- [32] Centre Hospitalier Intercommunal de Bigorre, Tarbes, France.
- [33] Laboratoire de Parasitologie-Mycologie, Centre Hospitalier de Beauvais, Beauvais, France.

- PMID: **34668768**
- PMCID: PMC8528108
- DOI: 10.1128/Spectrum.01138-21

Abstract

The aim of this study was to evaluate diagnostic means, host factors, delay of occurrence, and outcome of patients with COVID-19 pneumonia and fungal coinfections in the intensive care unit (ICU). From 1 February to 31 May 2020, we anonymously recorded COVID-19-associated pulmonary aspergillosis (CAPA), fungemia (CA-fungemia), and pneumocystosis (CA-PCP) from 36 centers, including results on fungal biomarkers in respiratory specimens and serum. We collected data from 154 episodes of CAPA, 81 of CA-fungemia, 17 of CA-PCP, and 5 of other mold infections from 244 patients (male/female [M/F] ratio = 3.5; mean age, 64.7 ± 10.8 years). CA-PCP occurred first after ICU admission (median, 1 day; interquartile range [IQR], 0 to 3 days), followed by CAPA (9 days; IQR, 5 to 13 days), and then CA-fungemia (16 days; IQR, 12 to 23 days) ($P < 10^{-4}$). For CAPA, the presence of several mycological criteria was associated with death ($P < 10^{-4}$). Serum galactomannan was rarely positive (<20%). The mortality rates were 76.7% (23/30) in patients with host factors for invasive fungal disease, 45.2% (14/31) in those with a preexisting pulmonary condition, and 36.6% (34/93) in the remaining patients ($P = 0.001$). Antimold treatment did not alter prognosis ($P = 0.370$). Candida albicans was responsible for 59.3% of CA-fungemias, with a global mortality of 45.7%. For CA-PCP, 58.8% of the episodes occurred in patients with known host factors of PCP, and the mortality rate was 29.5%. CAPA may be in part hospital acquired and could benefit from antifungal prescription at the first positive biomarker result. CA-fungemia appeared linked to ICU stay without COVID-19 specificity, while CA-PCP may not really be a concern in the ICU. Improved diagnostic strategy for fungal markers in ICU patients with COVID-19 should support these hypotheses. **IMPORTANCE** To diagnose fungal coinfections in patients with COVID-19 in the intensive care unit, it is necessary to implement the correct treatment and to prevent them if possible. For COVID-19-associated pulmonary aspergillosis (CAPA), respiratory specimens remain the best approach since serum biomarkers are rarely positive. Timing of occurrence suggests that CAPA could be hospital acquired. The associated mortality varies from 36.6% to 76.7% when no host factors or host factors of invasive fungal diseases are present, respectively. Fungemias occurred after 2 weeks in ICUs and are associated with a mortality rate of 45.7%. Candida albicans is the first yeast species recovered, with no specificity linked to COVID-19. Pneumocystosis was mainly found in patients with known immunodepression. The diagnosis occurred at the entry in ICUs and not afterwards, suggesting that if Pneumocystis jirovecii plays a role, it is upstream of the hospitalization in the ICU.

Keywords: Aspergillus; COVID-19; France; aspergillosis; critical care; fungemia; pneumocystosis.

- 46 references
- 3 figures

Supplementary info

Publication types, MeSH terms, Substances, Grant support Expand

Publication types

- Multicenter Study
- Observational Study
- Research Support, Non-U.S. Gov't

MeSH terms

- Aged
- Antifungal Agents / therapeutic use
- COVID-19 / epidemiology*
- COVID-19 / mortality
- COVID-19 / pathology
- Coinfection / epidemiology
- Coinfection / mortality*
- Critical Care
- Female
- France / epidemiology
- Fungemia / drug therapy
- Fungemia / epidemiology*
- Fungemia / mortality
- Galactose / analogs & derivatives
- Galactose / blood
- Humans
- Intensive Care Units / statistics & numerical data
- Male
- Mannans / blood
- Middle Aged
- Pneumonia, Pneumocystis / drug therapy
- Pneumonia, Pneumocystis / epidemiology*
- Pneumonia, Pneumocystis / mortality
- Pulmonary Aspergillosis / drug therapy
- Pulmonary Aspergillosis / epidemiology*

- Pulmonary Aspergillosis / mortality
- Retrospective Studies
- SARS-CoV-2
- Treatment Outcome

Substances

- Antifungal Agents
- Mannans
- galactomannan
- Galactose

Grant support

- 20CE35000701/Agence Nationale de la Recherche (ANR)

Full text links

ASM Journals OPEN ACCESS Atypon Free PMC article
Proceed to details
Cite
Share
1,093
Observational Study
Diabetes Res Clin Pract
. 2020 Aug;166:108331.
doi: 10.1016/j.diabres.2020.108331. Epub 2020 Jul 17.

Demographic and clinical features of critically ill patients with COVID-19 in Greece: The burden of diabetes and obesity

P Halvatsiotis [1], A Kotanidou [2], K Tzannis [3], E Jahaj [2], E Magira [2], M Theodorakopoulou [4], G Konstandopoulou [4], E Gkeka [5], C Pourzitaki [5], N Kapravelos [6], S Papoti [6], M Sileli [6], C Gogos [7], D Velissaris [8], N Markou [9], E Stefanatou [9], G Vlachogianni [10], E Aimoniotou [11], A Komnos [12], T Zafeiridis [12], P Koulouvaris [13], A Armaganidis [14], A Bamias [15], G Dimopoulos [16]
Affiliations Expand

Affiliations

- [1] 2nd Propaedeutic Department of Internal Medicine, Medical School, National and Kapodistrian University of Athens, "ATTIKON" University Hospital, Rimini 1, Chaidari 124 62, Greece. Electronic address: pahalv@gmail.com.

- [2] 1st Department of Critical Care, Medical School of National and Kapodistrian University of Athens, "EVANGELISMOS" General Hospital, 45-47 Ipsilantou str, 10675 Athens, Greece.
- [3] 2nd Propaedeutic Department of Internal Medicine, Medical School, National and Kapodistrian University of Athens, "ATTIKON" University Hospital, Rimini 1, Chaidari 124 62, Greece.
- [4] 2nd Department of Critical Care, Medical School of National and Kapodistrian University of Athens, "ATTIKON" University Hospital, Rimini 1, Chaidari 124 62, Greece.
- [5] Department of Anesthesiology and Intensive Care, Faculty of Medicine, School of Health Sciences of Aristotle University of Thessaloniki, AHEPA University Hospital, Kyriakidi 1, Thessaloniki 54621, Greece.
- [6] 2nd Critical Care Department, General Hospital of Thessaloniki "G. PAPANIKOLAOU" Leof. Papanikolaou, Pilaia Chortiatis 57011, Greece.
- [7] Emergency Department and Department of Internal Medicine of Patras University, Medical School, University Hospital of Patras, Rio, Patras 26504, Greece. Electronic address: cgogos@med.upatras.gr.
- [8] Emergency Department and Department of Internal Medicine of Patras University, Medical School, University Hospital of Patras, Rio, Patras 26504, Greece.
- [9] LATSION Burn Center - Intensive Care Unit, General Hospital of Eleusis "THRIASSIO", Leof G Gennimata, Elefsina 19600, Greece.
- [10] Department of Critical Care, AGIOS DIMITRIOS General Hospital of Thessaloniki, Elenis Zografou 2, Thessaloniki 54634, Greece. Electronic address: glykav@otenet.gr.
- [11] Department of Critical Care, AGIOS DIMITRIOS General Hospital of Thessaloniki, Elenis Zografou 2, Thessaloniki 54634, Greece.
- [12] Department of Critical Care, General Hospital of Larisa "KOUTLIMPANIO", Tsakalof 1, Larisa 41221, Greece.
- [13] 1st Department of Orthopaedics, Medical School of National and Kapodistrian University of Athens, "ATTIKON" University Hospital, Rimini 1, Chaidari 124 62, Greece. Electronic address: info@drkoulouvaris.gr.
- [14] 2nd Department of Critical Care, Medical School of National and Kapodistrian University of Athens, "ATTIKON" University Hospital, Rimini 1, Chaidari 124 62, Greece. Electronic address: aarmag@med.uoa.gr.
- [15] 2nd Propaedeutic Department of Internal Medicine, Medical School, National and Kapodistrian University of Athens, "ATTIKON" University Hospital, Rimini 1, Chaidari 124 62, Greece. Electronic address: abamias@med.uoa.gr.
- [16] 2nd Department of Critical Care, Medical School of National and Kapodistrian University of Athens, "ATTIKON" University Hospital, Rimini 1, Chaidari 124 62, Greece. Electronic address: gdimop@med.uoa.gr.

- PMID: **32682810**
- PMCID: PMC7366091
- DOI: 10.1016/j.diabres.2020.108331

Free PMC article
Observational Study

Demographic and clinical features of critically ill patients with COVID-19 in Greece: The burden of diabetes and obesity

P Halvatsiotis et al. Diabetes Res Clin Pract. 2020 Aug.
Free PMC article
Show details
Diabetes Res Clin Pract
. 2020 Aug;166:108331.
doi: 10.1016/j.diabres.2020.108331. Epub 2020 Jul 17.

Authors

P Halvatsiotis [1], A Kotanidou [2], K Tzannis [3], E Jahaj [2], E Magira [2], M Theodorakopoulou [4], G Konstandopoulou [4], E Gkeka [5], C Pourzitaki [5], N Kapravelos [6], S Papoti [6], M Sileli [6], C Gogos [7], D Velissaris [8], N Markou [9], E Stefanatou [9], G Vlachogianni [10], E Aimoniotou [11], A Komnos [12], T Zafeiridis [12], P Koulouvaris [13], A Armaganidis [14], A Bamias [15], G Dimopoulos [16]

Affiliations

- [1] 2nd Propaedeutic Department of Internal Medicine, Medical School, National and Kapodistrian University of Athens, "ATTIKON" University Hospital, Rimini 1, Chaidari 124 62, Greece. Electronic address: pahalv@gmail.com.
- [2] 1st Department of Critical Care, Medical School of National and Kapodistrian University of Athens, "EVANGELISMOS" General Hospital, 45-47 Ipsilantou str, 10675 Athens, Greece.
- [3] 2nd Propaedeutic Department of Internal Medicine, Medical School, National and Kapodistrian University of Athens, "ATTIKON" University Hospital, Rimini 1, Chaidari 124 62, Greece.
- [4] 2nd Department of Critical Care, Medical School of National and Kapodistrian University of Athens, "ATTIKON" University Hospital, Rimini 1, Chaidari 124 62, Greece.
- [5] Department of Anesthesiology and Intensive Care, Faculty of Medicine, School of Health Sciences of Aristotle University of Thessaloniki, AHEPA University Hospital, Kyriakidi 1, Thessaloniki 54621, Greece.
- [6] 2nd Critical Care Department, General Hospital of Thessaloniki "G. PAPANIKOLAOU" Leof. Papanikolaou, Pilaia Chortiatis 57011, Greece.
- [7] Emergency Department and Department of Internal Medicine of Patras University, Medical School, University Hospital of Patras, Rio, Patras 26504, Greece. Electronic address: cgogos@med.upatras.gr.
- [8] Emergency Department and Department of Internal Medicine of Patras University, Medical School, University Hospital of Patras, Rio, Patras 26504, Greece.
- [9] LATSION Burn Center - Intensive Care Unit, General Hospital of Eleusis "THRIASSIO", Leof G Gennimata, Elefsina 19600, Greece.
- [10] Department of Critical Care, AGIOS DIMITRIOS General Hospital of Thessaloniki, Elenis Zografou 2, Thessaloniki 54634, Greece. Electronic address: glykav@otenet.gr.

- [11] Department of Critical Care, AGIOS DIMITRIOS General Hospital of Thessaloniki, Elenis Zografou 2, Thessaloniki 54634, Greece.
- [12] Department of Critical Care, General Hospital of Larisa "KOUTLIMPANIO", Tsakalof 1, Larisa 41221, Greece.
- [13] 1st Department of Orthopaedics, Medical School of National and Kapodistrian University of Athens, "ATTIKON" University Hospital, Rimini 1, Chaidari 124 62, Greece. Electronic address: info@drkoulouvaris.gr.
- [14] 2nd Department of Critical Care, Medical School of National and Kapodistrian University of Athens, "ATTIKON" University Hospital, Rimini 1, Chaidari 124 62, Greece. Electronic address: aarmag@med.uoa.gr.
- [15] 2nd Propaedeutic Department of Internal Medicine, Medical School, National and Kapodistrian University of Athens, "ATTIKON" University Hospital, Rimini 1, Chaidari 124 62, Greece. Electronic address: abamias@med.uoa.gr.
- [16] 2nd Department of Critical Care, Medical School of National and Kapodistrian University of Athens, "ATTIKON" University Hospital, Rimini 1, Chaidari 124 62, Greece. Electronic address: gdimop@med.uoa.gr.

- PMID: **32682810**
- PMCID: PMC7366091
- DOI: 10.1016/j.diabres.2020.108331

Abstract

Aims: The aim of the study was to investigate the association between type-2 diabetes mellitus, other underlying diseases and obesity with the outcomes of critically ill Covid-19 patients in Greece.

Methods: In this retrospective observational multi-centre study, data and outcomes of 90 RNA 2109-nCoV confirmed critically ill patients from 8 hospitals throughout Greece, were analysed. All reported information stand through April 13th 2020.

Results: The median age of the patients was 65.5 (IQR 56-73), majority were male (80%) and obesity was present in 34.4% of patients most prevalent to younger than 55 years. Hypertension was the prevailing comorbidity (50%), followed by cardiovascular diseases (21.1%) and type-2 diabetes (18.9%). At admission, common symptoms duration had a median of 8 (IQR 5-11) days. A 13.3% of the patients were discharged, 53.4% were still in the ICUs and 28.9% deceased who were hospitalised for fewer days than the survivors [6 (IQR 3-9) vs. 9 (IQR 7-14.5) respectively]. Aging was not a risk factor but diabetes deteriorates the outcomes. Obesity poses a suggestive burden as it was more notable in deceased versus survivors.

Conclusions: Type 2 diabetes and obesity may have contributed to disease severity and mortality in COVID-19 critically ill patients in Greece.

Keywords: Covid-19; Critically ill; Greece; Intensive Care Unit; Obesity; Type-2 diabetes.

Copyright © 2020 Elsevier B.V. All rights reserved.

- 24 references
- 2 figures

Supplementary info

Publication types, MeSH terms Expand

Publication types

- Multicenter Study
- Observational Study

MeSH terms

- Aged
- Betacoronavirus / isolation & purification*
- COVID-19
- Comorbidity
- Coronavirus Infections / complications
- Coronavirus Infections / epidemiology
- Coronavirus Infections / mortality*
- Coronavirus Infections / virology
- Critical Illness / mortality*
- Diabetes Mellitus / mortality*
- Diabetes Mellitus / physiopathology
- Diabetes Mellitus / virology
- Female
- Greece / epidemiology
- Hospitalization
- Humans
- Male
- Middle Aged
- Obesity / mortality*
- Obesity / physiopathology
- Obesity / virology
- Pandemics
- Pneumonia, Viral / complications
- Pneumonia, Viral / epidemiology
- Pneumonia, Viral / mortality*
- Pneumonia, Viral / virology
- Prognosis
- Retrospective Studies
- Risk Factors
- SARS-CoV-2
- Survival Rate

Full text links

ELSEVIER FULL-TEXT ARTICLE Elsevier Science Free PMC article

Proceed to details

Cite

Share

☐ 1,094

Observational Study

Rheumatol Int

. 2020 Oct;40(10):1593-1598.

doi: 10.1007/s00296-020-04676-4. Epub 2020 Aug 13.

Coronavirus disease 2019 (COVID-19) in autoimmune and inflammatory conditions: clinical characteristics of poor outcomes

Fernando Montero [1], Julia Martínez-Barrio [2], Belén Serrano-Benavente [2], Teresa González [2], Javier Rivera [2], Juan Molina Collada [2], Isabel Castrejón [2], Jose Álvaro-Gracia [2]

Affiliations Expand

Affiliations

- [1] Department of Rheumatology. Hospital General, Universitario Gregorio Marañón, 28007, Madrid, Spain. fernandojose.montero@salud.madrid.org.
- [2] Department of Rheumatology. Hospital General, Universitario Gregorio Marañón, 28007, Madrid, Spain.

- PMID: **32794113**
- PMCID: PMC7425254
- DOI: 10.1007/s00296-020-04676-4

Free PMC article

Observational Study

Coronavirus disease 2019 (COVID-19) in autoimmune and inflammatory conditions: clinical characteristics of poor outcomes

Fernando Montero et al. Rheumatol Int. 2020 Oct.

Free PMC article

Show details

Rheumatol Int

. 2020 Oct;40(10):1593-1598.

doi: 10.1007/s00296-020-04676-4. Epub 2020 Aug 13.

Authors

Fernando Montero [1], Julia Martínez-Barrio [2], Belén Serrano-Benavente [2], Teresa González [2], Javier Rivera [2], Juan Molina Collada [2], Isabel Castrejón [2], Jose Álvaro-Gracia [2]

Affiliations

- [1] Department of Rheumatology. Hospital General, Universitario Gregorio Marañón, 28007, Madrid, Spain. fernandojose.montero@salud.madrid.org.
- [2] Department of Rheumatology. Hospital General, Universitario Gregorio Marañón, 28007, Madrid, Spain.

- PMID: **32794113**
- PMCID: PMC7425254
- DOI: 10.1007/s00296-020-04676-4

Abstract

Objective: To describe clinical characteristics of patients with rheumatic and musculoskeletal diseases (RMDs) and immunosuppressive therapies with Coronavirus disease 2019 (COVID-19) at an academic rheumatology center in Madrid and to identify baseline variables associated with a severe infection requiring hospitalization.

Methods: We identified SARS-CoV-2 positive cases by polymerase chain reaction performed at our center within an updated RMDs database in our clinic. Additional RMDs patients were identified when they contacted the clinic because of a positive infection. Data extraction included diagnosis, demographics, immunosuppressive treatment, comorbidities, and laboratory tests. Comparisons between patients with or without hospitalization were performed. Multivariate logistic regression was used to analyze associations between baseline variables and need for hospitalization.

Results: A total of 62 patients with COVID-19 and underlying RMDs were identified by April 24, 2020. Median age was 60.9 years, and 42% men. Forty-two patients required hospitalization; these were more frequently men, older and with comorbidities. There were no statistically significant between-group differences for rheumatologic diagnosis and for baseline use of immunosuppressive therapy except for glucocorticoids that were more frequent in hospitalized patients. Total deaths were 10 (16%) patients. In multivariate analysis, male sex (odds ratio [OR], 8.63; $p = 0.018$), previous lung disease (OR, 27.47; $p = 0.042$), and glucocorticoids use (> 5 mg/day) (OR, 9.95; $p = 0.019$) were significantly associated to hospitalization.

Conclusion: Neither specific RMD diagnoses or exposures to DMARDs were associated with increased odds of hospitalization. Being male, previous lung disease and exposure to glucocorticoids were associated with higher odds of hospitalization in RMDs patients.

Keywords: Autoimmune diseases; COVID-19; Poor outcomes; Rheumatology.

Conflict of interest statement

The authors declare that they have no competing interest. Patient and public involvement Patients and/or the public were not involved in the design, or conduct, or reporting, or dissemination plans of this research. Patient consent for publication: waived by the local IRB.

- 19 references

Supplementary info

Publication types, MeSH terms, Substances, Supplementary concepts Expand

Publication types

- Observational Study

MeSH terms

- Aged
- Anti-Bacterial Agents / therapeutic use
- Antibodies, Monoclonal, Humanized / therapeutic use
- Antirheumatic Agents / therapeutic use
- Antiviral Agents / therapeutic use
- Arthritis, Psoriatic / complications
- Arthritis, Psoriatic / drug therapy*
- Arthritis, Psoriatic / epidemiology
- Arthritis, Rheumatoid / complications
- Arthritis, Rheumatoid / drug therapy*
- Arthritis, Rheumatoid / epidemiology
- Autoimmune Diseases / complications
- Autoimmune Diseases / drug therapy
- Autoimmune Diseases / epidemiology
- Azithromycin / therapeutic use
- Betacoronavirus
- COVID-19
- Comorbidity
- Coronavirus Infections / complications
- Coronavirus Infections / drug therapy
- Coronavirus Infections / mortality
- Coronavirus Infections / physiopathology*
- Coronavirus Infections / therapy
- Drug Combinations
- Female
- Glucocorticoids / therapeutic use*
- Hospitalization / statistics & numerical data*

- Humans
- Hydroxychloroquine / therapeutic use
- Immunosuppressive Agents / therapeutic use*
- Logistic Models
- Lopinavir / therapeutic use
- Lung Diseases / epidemiology
- Lupus Erythematosus, Systemic / complications
- Lupus Erythematosus, Systemic / drug therapy*
- Lupus Erythematosus, Systemic / epidemiology
- Male
- Middle Aged
- Multivariate Analysis
- Pandemics
- Pneumonia, Viral / complications
- Pneumonia, Viral / mortality
- Pneumonia, Viral / physiopathology*
- Pneumonia, Viral / therapy
- Retrospective Studies
- Ritonavir / therapeutic use
- SARS-CoV-2
- Severity of Illness Index
- Sex Factors
- Spain / epidemiology

Substances

- Anti-Bacterial Agents
- Antibodies, Monoclonal, Humanized
- Antirheumatic Agents
- Antiviral Agents
- Drug Combinations
- Glucocorticoids
- Immunosuppressive Agents
- lopinavir-ritonavir drug combination
- Lopinavir
- Hydroxychloroquine
- Azithromycin
- tocilizumab
- Ritonavir

Supplementary concepts

- COVID-19 drug treatment

Full text links

Springer Free PMC article

Proceed to details

Cite

Share

1,095

Observational Study

Eur Rev Med Pharmacol Sci

. 2022 Feb;26(3):1056-1064.

doi: 10.26355/eurrev_202202_28017.

The neutrophil/lymphocyte ratio as a prognostic factor in COVID-19 patients: a case-control study

G La Torre [1], M Marte, A P Massetti, S M Carli, F Romano, C M Mastroianni, M Minorenti, F Alessandri, C Ajassa, M Fusconi, M De Vincentiis, D de Meo, C Villani, M Cardi, F Pugliese, COVID-Collaborative group

Collaborators, Affiliations Expand

Collaborators

- **COVID-Collaborative group**: G La Torre, M Marte, A P Massetti, S M Carli, F Romano, C M Mastroianni, M Minorenti, F Alessandri, C Ajassa, M Fusconi, M De Vincentiis, D de Meo, C Villani, M Cardi, F Pugliese

Affiliation

- [1] Department of Public Health and Infectious Diseases, Sapienza University of Rome, Rome, Italy. giuseppe.latorre@uniroma1.it.

- PMID: **35179773**
- DOI: 10.26355/eurrev_202202_28017

Free article

Observational Study

The neutrophil/lymphocyte ratio as a prognostic factor in COVID-19 patients: a case-control study

G La Torre et al. Eur Rev Med Pharmacol Sci. 2022 Feb.
Free article
Show details
Eur Rev Med Pharmacol Sci
. 2022 Feb;26(3):1056-1064.
doi: 10.26355/eurrev_202202_28017.

Authors

G La Torre [1], M Marte, A P Massetti, S M Carli, F Romano, C M Mastroianni, M Minorenti, F Alessandri, C Ajassa, M Fusconi, M De Vincentiis, D de Meo, C Villani, M Cardi, F Pugliese, COVID-Collaborative group

Collaborators

- **COVID-Collaborative group**:
  G La Torre, M Marte, A P Massetti, S M Carli, F Romano, C M Mastroianni, M Minorenti, F Alessandri, C Ajassa, M Fusconi, M De Vincentiis, D de Meo, C Villani, M Cardi, F Pugliese

Affiliation

- [1] Department of Public Health and Infectious Diseases, Sapienza University of Rome, Rome, Italy. giuseppe.latorre@uniroma1.it.

- PMID: **35179773**
- DOI: 10.26355/eurrev_202202_28017

Abstract

Objective: SARS-CoV-2 (Severe Acute Respiratory Syndrome Coronavirus 2) has been identified in China as responsible for viral pneumonia, now called COVID-19 (Coronavirus Disease 2019). Patients infected can develop common symptoms like cough and sore throat, and, in severe cases, acute respiratory syndrome and even death. To optimize the available resources, it is necessary to identify in advance the subjects that will develop a more serious illness, therefore requiring intensive care.The neutrophil / lymphocyte ratio (NLR) parameter, resulting from the blood count, could be a significant marker for the diagnosis and management of risk stratification.

Patients and methods: A retrospective, single-center case-control observational study was conducted. The differential cell count of leukocytes, the NLR and the clinical course of patients hospitalized in intensive care with COVID-19 were analyzed, comparing them with other patients (COVID-19 and non-COVID-19) and healthy individuals selected among workers of the Teaching Hospital Policlinico Umberto I in Rome.

Results: 370 patients (145 cases and 225 controls) were included in the case-control study, 211 males (57%) and 159 females (43%). The average age of the population was 63 years (SD 16.35). In the group of cases, out of 145 patients, 57 deaths and 88 survivors were recorded, with a lethality rate of 39.3%. The group of cases has an NLR of 7.83 (SD = 8.07), a much higher value than the control group where an NLR of 2.58 was recorded (SD = 1.93) (p <0.001). The Neutrophils / Lymphocytes ratio may prove to be a diagnostic factor for COVID-19, an NLR> 3.68 revealed an OR 10.84 (95% CI = 6.47 - 18.13) (p <0.005).

Conclusions: The value of NLR considered together with the age variable allows a risk stratification and allows the development of diagnostic and treatment protocols for patients affected by COVID-19. A high neutrophil to lymphocyte ratio suggests worse survival. Risk stratification and management help alleviate the shortage of medical resources and reduce the mortality of critically ill patients.

Supplementary info

Publication types, MeSH terms, Substances Expand

Publication types

- Comparative Study
- Observational Study

MeSH terms

- Aged
- Biomarkers / blood
- COVID-19 / blood*
- COVID-19 / diagnosis*
- Case-Control Studies
- Critical Illness
- Female
- Humans
- Intensive Care Units
- Italy
- Leukocyte Count
- Logistic Models
- Lymphocytes / metabolism*
- Lymphocytes / virology*
- Male
- Middle Aged
- Neutrophils / metabolism*
- Neutrophils / virology*
- Prognosis
- ROC Curve

- Retrospective Studies
- Risk Factors
- Severity of Illness Index

Substances

- Biomarkers

Full text links

European Review for Medical and Pharmacological Sciences
Proceed to details
Cite
Share
1,096
Clinical Trial
Crit Care
. 2021 Jun 9;25(1):199.
doi: 10.1186/s13054-021-03518-4.

An appraisal of respiratory system compliance in mechanically ventilated covid-19 patients

Gianluigi Li Bassi[#,1,2,3,4,5,6], Jacky Y Suen[#,7,8], Heidi J Dalton[9], Nicole White[10], Sally Shrapnel[8], Jonathon P Fanning[7,8,11], Benoit Liquet[8,12,13], Samuel Hinton[8], Aapeli Vuorinen[8], Gareth Booth[8], Jonathan E Millar[14,15], Simon Forsyth[8], Mauro Panigada[16], John Laffey[17], Daniel Brodie[18], Eddy Fan[19,20,21,22], Antoni Torres[23,24], Davide Chiumello[24,25], Amanda Corley[7,8], Alyaa Elhazmi[26], Carol Hodgson[27,28], Shingo Ichiba[29], Carlos Luna[30], Srinivas Murthy[31], Alistair Nichol[28,32], Pauline Yeung Ng[33], Mark Ogino[34], Antonio Pesenti[16,35], Huynh Trung Trieu[36], John F Fraser[7,8,23,37,38,11], COVID-19 Critical Care Consortium
Collaborators, Affiliations Expand

- PMID: **34108029**
- PMCID: PMC8188162
- DOI: 10.1186/s13054-021-03518-4

Free PMC article
Clinical Trial

An appraisal of respiratory system compliance in mechanically ventilated covid-19 patients

Gianluigi Li Bassi et al. Crit Care. 2021.
Free PMC article
Show details
Crit Care
. 2021 Jun 9;25(1):199.
doi: 10.1186/s13054-021-03518-4.

- PMID: **34108029**
- PMCID: PMC8188162
- DOI: 10.1186/s13054-021-03518-4

Abstract

Background: Heterogeneous respiratory system static compliance (C_{RS}) values and levels of hypoxemia in patients with novel coronavirus disease (COVID-19) requiring mechanical ventilation have been reported in previous small-case series or studies conducted at a national level.

Methods: We designed a retrospective observational cohort study with rapid data gathering from the international COVID-19 Critical Care Consortium study to comprehensively describe C_{RS}-calculated as: tidal volume/[airway plateau pressure-positive end-expiratory pressure (PEEP)] -and its association with ventilatory management and outcomes of COVID-19 patients on mechanical ventilation (MV), admitted to intensive care units (ICU) worldwide.

Results: We studied 745 patients from 22 countries, who required admission to the ICU and MV from January 14 to December 31, 2020, and presented at least one value of C_{RS} within the first seven days of MV. Median (IQR) age was 62 (52-71), patients were predominantly males (68%) and from Europe/North and South America (88%). C_{RS}, within 48 h from endotracheal intubation, was available in 649 patients and was neither associated with the duration from onset of symptoms to commencement of MV ($p = 0.417$) nor with PaO_2/FiO_2 ($p = 0.100$). Females presented lower C_{RS} than males (95% CI of C_{RS} difference between females-males: - 11.8 to - 7.4 mL/cmH_2O $p < 0.001$), and although females presented higher body mass index (BMI), association of BMI with C_{RS} was marginal ($p = 0.139$). Ventilatory management varied across C_{RS} range, resulting in a significant association between C_{RS} and driving pressure (estimated decrease - 0.31 cmH_2O/L per mL/cmH_20 of C_{RS}, 95% CI - 0.48 to - 0.14, $p < 0.001$). Overall, 28-day ICU mortality, accounting for the competing risk of being discharged within the period, was 35.6% (SE 1.7). Cox proportional hazard analysis demonstrated that C_{RS} (+ 10 mL/cm H_2O) was only associated with being discharge from the ICU within 28 days (HR 1.14, 95% CI 1.02-1.28, $p = 0.018$).

Conclusions: This multicentre report provides a comprehensive account of C_{RS} in COVID-19 patients on MV. C_{RS} measured within 48 h from commencement of MV has marginal predictive value for 28-day mortality, but was associated with being discharged from ICU within the same period. Trial documentation: Available at https://www.covid-critical.com/study .

Trial registration: ACTRN12620000421932.

Keywords: ARDS; COVID-19; Compliance; Mechanical ventilation; SARS-CoV-2.

Conflict of interest statement

GLB and JF received research funds, through their affiliated institution from Fisher & Paykel. All remaining authors do not have any conflict of interest related to this report.

- 35 references
- 9 figures

Supplementary info

Publication types, MeSH terms, Associated data, Grant support Expand

Publication types

- Clinical Trial
- Multicenter Study
- Observational Study
- Research Support, Non-U.S. Gov't

MeSH terms

- Adult
- COVID-19 / complications*
- COVID-19 / therapy*
- Cohort Studies
- Critical Care / methods
- Europe
- Female
- Humans
- Intensive Care Units
- Lung Compliance / physiology*
- Male
- Middle Aged
- Respiration, Artificial / methods*
- Respiratory Distress Syndrome / etiology*
- Respiratory Distress Syndrome / therapy*
- Retrospective Studies
- Severity of Illness Index

Associated data

- ANZCTR/ACTRN12620000421932

Grant support

- T32 GM112596/GM/NIGMS NIH HHS/United States

Full text links

Read free full text at BMC BioMed Central Free PMC article
Proceed to details
Cite
Share
1,097

Observational Study

Int J Stroke
. 2021 Jun;16(4):429-436.
doi: 10.1177/1747493020968236. Epub 2020 Dec 6.

COVID-19-related strokes are associated with increased mortality and morbidity: A multicenter comparative study from Bengaluru, South India

Thomas Mathew [1], Saji K John [1], Grk Sarma [1], Raghunandan Nadig [1], Shiva Kumar R [2], Uday Murgod [3], Manjunath Mahadevappa [3], Mahendra Javali [4], Purushottam Thammaya Acharya [4], Guruprasad Hosurkar [5], Pramod Krishnan [3], Vikram Kamath [6], Sagar Badachi [1], Delon D Souza [1], Rajesh B Iyer [7], Rajesh Karalumangala Nagarajaiah [8], Bawani Anand [2], Sujit Kumar [9], Suresh Kodapala [10], Sonia Shivde [1], Amrutha Avati [1], Rohit Baddala [1], Prasanth Babu Potharlanka [1], Sravanthi Pavuluri [1], Abhinaya Varidireddy [1], Poonam Awatare [1], Nandavar Shobha [11], Umashankar Renukaradhya [11], S Praveen Kumar [12], Jayachandran Ramachandran [3], Ramesh Arumugam [1], Saikanth Deepalam [1], Sharath Kumar [1], Vikram Huded [13]
Affiliations Expand

Affiliations

- [1] Department of Neurology, St. John's Medical College Hospital, Bengaluru, Karnataka, India.
- [2] Department of Neurology, 477861Sakra World Hospital, Bengaluru, Karnataka, India.
- [3] Department of Neurology, Manipal Hospitals, Bengaluru, Karnataka, India.
- [4] Department of Neurology, Ramaiah Memorial Hospital, Bengaluru, Karnataka, India.
- [5] Department of Neurology, Columbia Asia Hospital 6/4, Bengaluru, Karnataka, India.
- [6] Department of Neurology, Apollo Hospital, Bannerghatta, Bengaluru, Karnataka, India.
- [7] Department of Neurology, Vikram Hospital, Anne's College, Bengaluru, Karnataka, India.

- [8] Department of Neurology, People Tree Hospitals, Bengaluru, Karnataka, India.
- [9] Department of Neurology, Apollo Hospitals, Sheshadripuram, Bengaluru, Karnataka, India.
- [10] Department of Neurology, Vydehi Institute of Medical Science, Bangalore, India.
- [11] Department of Neurology, Bangalore Neuro Centre, Bengaluru, Karnataka, India.
- [12] Department of Neurology, 29099Bangalore Medical College and Research Institute, Bengaluru, Karnataka, India.
- [13] Department of Neurology, NH Institute of Neurosciences, Mazumdar Shaw Medical Center, Bengaluru, Karnataka, India.

- PMID: **33034546**
- PMCID: PMC7723737
- DOI: 10.1177/1747493020968236

Free PMC article
Observational Study

COVID-19-related strokes are associated with increased mortality and morbidity: A multicenter comparative study from Bengaluru, South India

Thomas Mathew et al. Int J Stroke. 2021 Jun.
Free PMC article
Show details
Int J Stroke
. 2021 Jun;16(4):429-436.
doi: 10.1177/1747493020968236. Epub 2020 Dec 6.

Authors

Thomas Mathew [1], Saji K John [1], Grk Sarma [1], Raghunandan Nadig [1], Shiva Kumar R [2], Uday Murgod [3], Manjunath Mahadevappa [3], Mahendra Javali [4], Purushottam Thammaya Acharya [4], Guruprasad Hosurkar [5], Pramod Krishnan [3], Vikram Kamath [6], Sagar Badachi [1], Delon D Souza [1], Rajesh B Iyer [7], Rajesh Karalumangala Nagarajaiah [8], Bawani Anand [2], Sujit Kumar [9], Suresh Kodapala [10], Sonia Shivde [1], Amrutha Avati [1], Rohit Baddala [1], Prasanth Babu Potharlanka [1], Sravanthi Pavuluri [1], Abhinaya Varidireddy [1], Poonam Awatare [1], Nandavar Shobha [11], Umashankar Renukaradhya [11], S Praveen Kumar [12], Jayachandran Ramachandran [3], Ramesh Arumugam [1], Saikanth Deepalam [1], Sharath Kumar [1], Vikram Huded [13]

Affiliations

- [1] Department of Neurology, St. John's Medical College Hospital, Bengaluru, Karnataka, India.
- [2] Department of Neurology, 477861Sakra World Hospital, Bengaluru, Karnataka, India.

- [3] Department of Neurology, Manipal Hospitals, Bengaluru, Karnataka, India.
- [4] Department of Neurology, Ramaiah Memorial Hospital, Bengaluru, Karnataka, India.
- [5] Department of Neurology, Columbia Asia Hospital 6/4, Bengaluru, Karnataka, India.
- [6] Department of Neurology, Apollo Hospital, Bannerghatta, Bengaluru, Karnataka, India.
- [7] Department of Neurology, Vikram Hospital, Anne's College, Bengaluru, Karnataka, India.
- [8] Department of Neurology, People Tree Hospitals, Bengaluru, Karnataka, India.
- [9] Department of Neurology, Apollo Hospitals, Sheshadripuram, Bengaluru, Karnataka, India.
- [10] Department of Neurology, Vydehi Institute of Medical Science, Bangalore, India.
- [11] Department of Neurology, Bangalore Neuro Centre, Bengaluru, Karnataka, India.
- [12] Department of Neurology, 29099Bangalore Medical College and Research Institute, Bengaluru, Karnataka, India.
- [13] Department of Neurology, NH Institute of Neurosciences, Mazumdar Shaw Medical Center, Bengaluru, Karnataka, India.

- PMID: **33034546**
- PMCID: PMC7723737
- DOI: 10.1177/1747493020968236

Abstract

Background: COVID-19-related strokes are increasingly being diagnosed across the world. Knowledge about the clinical profile, imaging findings, and outcomes is still evolving. Here we describe the characteristics of a cohort of 62 COVID-19-related stroke patients from 13 hospitals, from Bangalore city, south India.

Objective: To describe the clinical profile, neuroimaging findings, interventions, and outcomes in COVID-19-related stroke patients.

Methods: This is a multicenter retrospective study of all COVID-19-related stroke patients from 13 hospitals from south India; 1st June 2020-31st August 2020. The demographic, clinical, laboratory, and neuroimaging data were collected along with treatment administered and outcomes. SARS-CoV-2 infection was confirmed in all cases by RT-PCR testing. The data obtained from the case records were entered in SPSS 25 for statistical analysis.

Results: During the three-month period, we had 62 COVID-19-related stroke patients, across 13 centers; 60 (97%) had ischemic strokes, while 2 (3%) had hemorrhagic strokes. The mean age of patients was 55.66 ± 13.20 years, with 34 (77.4%) males. Twenty-six percent (16/62) of patients did not have any conventional risk factors for stroke. Diabetes mellitus was seen in 54.8%, hypertension was present in 61.3%, coronary artery disease in 8%, and atrial fibrillation in 4.8%. Baseline National Institutes of Health Stroke Scale score was 12.7 ± 6.44. Stroke severity was moderate (National Institutes of Health Stroke Scale 5-15) in 27 (61.3%) patients, moderate to severe (National Institutes of Health Stroke Scale 16-20) in 13 (20.9%) patients and severe (National Institutes of Health Stroke Scale 21-42) in 11 (17.7%) patients. According to TOAST classification, 48.3% was stroke of undetermined etiology, 36.6% had large artery atherosclerosis, 10% had small vessel occlusion, and 5% had cardioembolic strokes. Three (5%) received intravenous thrombolysis with tenecteplase 0.2 mg/kg and 3 (5%) underwent mechanical thrombectomy, two endovascular and one surgical. Duration of hospital stay was 16.16 ± 6.39 days; 21% (13/62) died in hospital, while 37 (59.7%) had a modified Rankin score of 3-5 at discharge. Hypertension, atrial fibrillation, and higher baseline National Institutes of Health Stroke Scale scores were associated with increased mortality. A comparison to 111 historical controls

during the non-COVID period showed a higher proportion of strokes of undetermined etiology, higher mortality, and higher morbidity in COVID-19-related stroke patients.

Conclusion: COVID-19-related strokes are increasingly being recognized in developing countries, like India. Stroke of undetermined etiology appears to be the most common TOAST subtype of COVID-19-related strokes. COVID-19-related strokes were more severe in nature and resulted in higher mortality and morbidity. Hypertension, atrial fibrillation, and higher baseline National Institutes of Health Stroke Scale scores were associated with increased mortality.

Keywords: COVID-19; India; SARS–CoV2; ischemic stroke; morbidity; mortality; thrombolysis.

Conflict of interest statement

Declaration of conflicting interests: The author(s) declared no potential conflicts of interest with respect to the research, authorship, and/or publication of this article.

- 12 references

Supplementary info

Publication types, MeSH terms Expand

Publication types

- Comparative Study
- Multicenter Study
- Observational Study

MeSH terms

- Adult
- Aged
- Aged, 80 and over
- COVID-19 / complications*
- COVID-19 / diagnostic imaging
- COVID-19 / mortality*
- COVID-19 Testing
- Diabetes Complications / mortality
- Female
- Humans
- Hypertension / complications
- India / epidemiology
- Intracranial Hemorrhages / diagnostic imaging
- Intracranial Hemorrhages / epidemiology
- Intracranial Hemorrhages / mortality
- Ischemic Stroke / diagnostic imaging

- Ischemic Stroke / epidemiology
- Ischemic Stroke / mortality
- Male
- Middle Aged
- Neuroimaging
- Real-Time Polymerase Chain Reaction
- Retrospective Studies
- Sex Factors
- Stroke / diagnostic imaging
- Stroke / etiology*
- Stroke / mortality*
- Thrombolytic Therapy
- Treatment Outcome
- Young Adult

Full text links

SAGE journals Open access full text Atypon Free PMC article
Proceed to details
Cite
Share
1,098
Observational Study
An Pediatr (Engl Ed)
. 2021 Apr;94(4):245-251.
doi: 10.1016/j.anpedi.2020.12.003. Epub 2020 Dec 19.

[Impact of COVID-19 on the presentation and course of acute appendicitis in paediatrics]

[Article in Spanish]
Leire Bonilla [1], Carmen Gálvez [2], Lara Medrano [2], Javier Benito [3]
Affiliations Expand

Affiliations

- [1] Servicio de Urgencias de Pediatría, Hospital Universitario Cruces, Barakaldo, España.
- [2] Servicio de Cirugía Infantil, Hospital Universitario Cruces, Barakaldo, España.
- [3] Servicio de Urgencias de Pediatría, Hospital Universitario Cruces, Barakaldo, España. Electronic address: javier.benitof@osakidetza.eus.

- PMID: **33431331**
- PMCID: PMC7749640
- DOI: 10.1016/j.anpedi.2020.12.003

Free PMC article
Observational Study

[Impact of COVID-19 on the presentation and course of acute appendicitis in paediatrics]

[Article in Spanish]
Leire Bonilla et al. An Pediatr (Engl Ed). 2021 Apr.
Free PMC article
Show details
An Pediatr (Engl Ed)
. 2021 Apr;94(4):245-251.
doi: 10.1016/j.anpedi.2020.12.003. Epub 2020 Dec 19.

Authors

Leire Bonilla [1], Carmen Gálvez [2], Lara Medrano [2], Javier Benito [3]

Affiliations

- [1] Servicio de Urgencias de Pediatría, Hospital Universitario Cruces, Barakaldo, España.
- [2] Servicio de Cirugía Infantil, Hospital Universitario Cruces, Barakaldo, España.
- [3] Servicio de Urgencias de Pediatría, Hospital Universitario Cruces, Barakaldo, España. Electronic address: javier.benitof@osakidetza.eus.

- PMID: **33431331**
- PMCID: PMC7749640
- DOI: 10.1016/j.anpedi.2020.12.003

Abstract
in English, Spanish

Introduction: Acute appendicitis (AA) is the most frequent urgent surgical pathology in pediatrics. The COVID-19 pandemic has led to a decrease in emergency department (ED) visits, which can lead to a delay in health care and an increase in the severity of the pathologies. The objective is to analyze the rate of complicated AA during the pandemic, compared to the same period of the previous year.

Materials and methods: Retrospective unicenter observational cohort study that included patients under 14 years of age seen in the ED with a diagnosis of AA during the months of March to May 2019 (non-pandemic) and 2020 (pandemic).

Results: 90 patients were included (41 in non-pandemic and 49 in pandemic). No difference was found between the two periods in the time from the clinic onset until the visit to the ED (37h vs 38h, p=0.881), but there was a difference in the time from arrival at the ED until the surgery (7:00h vs 10:30h, p=0.004). The difference was accentuated when comparing the month of March with April-May 2020 (6h vs 12h; p=0.001). No significant differences were observed in the rate of complicated AA in intraoperative diagnosis (35% vs 33%; p=0.870) or anatomopathology (35%

vs 48%; p=0.222), nor in the number of postoperative complications, length of hospitalization and readmissions. An increase in the anatomopathological diagnosis of AA with periapendicitis was observed (47% vs 81%; p=0.001) CONCLUSION: During the pandemic, a delay from arrival at the ED until the surgery was observed in children diagnosed with AA. This delay resulted in an increase in the diagnosis of histologically evolved AA, but without an increase in the clinical complications of the disease.

Introducción: La apendicitis aguda (AA) es la patología quirúrgica urgente más frecuente en pediatría. La pandemia por la COVID-19 ha generado un descenso en las consultas a urgencias, pudiendo conllevar un retraso en la atención sanitaria y un aumento en la gravedad de las patologías. El objetivo es analizar la tasa de AA complicadas durante la pandemia, en comparación con el mismo periodo del año anterior.

Material y métodos: Estudio retrospectivo observacional de cohortes unicéntrico que incluyó a pacientes menores de 14 años atendidos en urgencias con diagnóstico de AA durante los meses de marzo a mayo de 2019 (no pandemia) y 2020 (pandemia).

Resultados: Se incluyeron 90 pacientes (41 en no pandemia y 49 en pandemia). No se encontraron diferencias en el tiempo desde el inicio de la clínica hasta la consulta en urgencias entre los dos periodos (37 h vs. 38 h, p = 0,881), pero sí en el tiempo desde la llegada a urgencias hasta la intervención quirúrgica (7:00 h vs. 10:30 h; p = 0,004). La diferencia se acentuó al comparar el mes de marzo con abril-mayo de 2020 (6 h vs. 12 h; p = 0,001). No se observaron diferencias significativas en la tasa de AA complicadas en el diagnóstico intraoperatorio (35% vs. 33%; p = 0,870) ni anatomopatológico (35% vs. 48%; p = 0,222), ni tampoco en el número de complicaciones postoperatorias, duración de ingreso y reingresos. Se objetivó un aumento del diagnóstico anatomopatológico de AA con periapendicitis (47% vs. 81%; p = 0,001).

Conclusión: Durante la pandemia se observó una demora desde la llegada a urgencias hasta la intervención quirúrgica en niños diagnosticados de AA. Esta demora se tradujo en un incremento del diagnóstico de AA evolucionadas histológicamente, pero sin objetivarse un aumento de las complicaciones de la enfermedad.

Keywords: Acute appendicitis; Apendicitis aguda; Apendicitis complicada; COVID-19; Complicaciones postoperatorias; Complicated appendicitis; Pandemia; Pandemic; Perforación; Perforation; Postoperative complications.

Copyright © 2020 Asociación Española de Pediatría. Publicado por Elsevier España, S.L.U. All rights reserved.

- 28 references
- 3 figures

Supplementary info

Publication types, MeSH terms Expand

Publication types

- Observational Study

MeSH terms

- Adolescent
- Appendicitis / complications*
- Appendicitis / diagnosis
- Appendicitis / epidemiology*
- Appendicitis / surgery
- COVID-19*
- Child
- Child, Preschool
- Cohort Studies
- Emergency Service, Hospital
- Female
- Humans
- Male
- Retrospective Studies
- Time-to-Treatment / statistics & numerical data*

Full text links

ELSEVIER OPEN ACCESS Elsevier Science Free PMC article

Proceed to details

Cite

Share

☐ 1,099

Observational Study

Am J Perinatol

. 2022 Jan;39(2):165-171.

doi: 10.1055/s-0041-1739292. Epub 2021 Nov 14.

Management of Critically Ill Pregnant Patients with COVID-19 Infection in a Rural State

Megan E Pagan [1], Abigail M Ramseyer [1], Dayna D Whitcombe [1], Tucker E Doiron [2], Everett F Magann [1], Adam T Sandlin [1], Dawn S Hughes [1]

Affiliations Expand

Affiliations

- [1] Division of Maternal Fetal Medicine, Department of Obstetrics and Gynecology, University of Arkansas for the Medical Sciences, Little Rock, Arkansas.
- [2] Department of Obstetrics and Gynecology, University of Arkansas for the Medical Sciences, Little Rock, Arkansas.

- PMID: **34775583**
- DOI: 10.1055/s-0041-1739292

Observational Study

Management of Critically Ill Pregnant Patients with COVID-19 Infection in a Rural State

Megan E Pagan et al. Am J Perinatol. 2022 Jan.
Show details
Am J Perinatol
. 2022 Jan;39(2):165-171.
doi: 10.1055/s-0041-1739292. Epub 2021 Nov 14.

Authors

Megan E Pagan [1], Abigail M Ramseyer [1], Dayna D Whitcombe [1], Tucker E Doiron [2], Everett F Magann [1], Adam T Sandlin [1], Dawn S Hughes [1]

Affiliations

- [1] Division of Maternal Fetal Medicine, Department of Obstetrics and Gynecology, University of Arkansas for the Medical Sciences, Little Rock, Arkansas.
- [2] Department of Obstetrics and Gynecology, University of Arkansas for the Medical Sciences, Little Rock, Arkansas.

- PMID: **34775583**
- DOI: 10.1055/s-0041-1739292

Abstract

Objective: There is limited data on the treatment of coronavirus disease 2019 (COVID-19) in pregnancy. Arkansas saw an increase in COVID-19 cases in June 2020. The first critically ill pregnant patient was admitted to our institution on May 21st, 2020. The objective of this study was to evaluate outcomes in critically ill pregnant women with COVID-19 at a single tertiary care center who received remdesivir and convalescent plasma (CCP).

Study design: This is a retrospective observational review of critically ill pregnant women with COVID-19 who received remdesivir and CCP. This study was approved by the institutional review board (#261354).

Results: Seven pregnant patients with COVID-19 were admitted to the intensive care unit (ICU). All received remdesivir and CCP. Six received dexamethasone. The median ICU length of stay (LOS) was 8 days (range 3-17). Patient 1 had multi-organ failure requiring vasopressors, renal dialysis, and had an intrauterine fetal demise. Patients 4 and 6 required mechanical ventilation, were delivered for respiratory distress and were extubated at 2 and 1 days postpartum,

respectively. The only common risk factor was obesity. There were no adverse events noted with remdesivir or CCP.

Conclusion: There is little data regarding the use of remdesivir or CCP for the treatment of COVID-19 in pregnant women. In our cohort, these were well tolerated with no adverse events. Previously reported median ICU LOS in critically ill pregnant women with COVID-19 was 8 days (range 4-15).1 Our study found a similar ICU LOS (8 days; range 3-17). Patient 1 did not receive remdesivir or CCP until transport to our facility on hospital day 3. Excluding patient 1, median ICU LOS was 6.5 days (range 3-9). Our institution's treatment of pregnant women with critical illness with remdesivir, CCP and dexamethasone combined with delivery in select cases has thus far had good outcomes.

Key points: · Combined therapy: remdesivir, CCP, dexamethasone.. · Remdesivir, CCP and dexamethasone was effective in treating critically ill pregnant women with COVID-19.. · No adverse events were associated with combined therapy.. · Delivery improved respiratory status..

Thieme. All rights reserved.

Conflict of interest statement

None declared.

Supplementary info

Publication types, MeSH terms, Supplementary concepts Expand

Publication types

- Observational Study

MeSH terms

- Adult
- COVID-19 / drug therapy*
- COVID-19 / therapy*
- Cohort Studies
- Critical Illness / therapy*
- Female
- Humans
- Immunization, Passive
- Intensive Care Units
- Pregnancy
- Pregnancy Complications, Infectious / drug therapy*

Supplementary concepts

- COVID-19 drug treatment

- COVID-19 serotherapy

Full text links

Full text THIEME CONNECT Georg Thieme Verlag Stuttgart, New York

Proceed to details

Cite

Share

1,100

Observational Study

Medicine (Baltimore)

. 2021 Jan 15;100(2):e23923.

doi: 10.1097/MD.0000000000023923.

Medical treatment of 55 patients with COVID-19 from seven cities in northeast China who fully recovered: A single-center, retrospective, observational study

Lichao Fan [1], Huan Liu [2], Na Li [3], Chang Liu [4], Ye Gu [5], Yongyu Liu [4], Yu Chen [1]

Affiliations Expand

Affiliations

- [1] Department of Tuberculosis.
- [2] Department of Respiratory.
- [3] Department of Central Laboratory.
- [4] Department of Thoracic Surgery, Shenyang Tenth People's Hospital, Shenyang Chest Hospital.
- [5] Department of Respiratory, Shenyang Sixth People's Hospital, Shenyang 110044, Liaoning, China.

- PMID: **33466134**
- PMCID: PMC7808500
- DOI: 10.1097/MD.0000000000023923

Free PMC article

Observational Study

Medical treatment of 55 patients with COVID-19 from seven cities in northeast

China who fully recovered: A single-center, retrospective, observational study

Lichao Fan et al. Medicine (Baltimore). 2021.
Free PMC article
Show details
Medicine (Baltimore)
. 2021 Jan 15;100(2):e23923.
doi: 10.1097/MD.0000000000023923.

Authors

Lichao Fan [1], Huan Liu [2], Na Li [3], Chang Liu [4], Ye Gu [5], Yongyu Liu [4], Yu Chen [1]

Affiliations

- [1] Department of Tuberculosis.
- [2] Department of Respiratory.
- [3] Department of Central Laboratory.
- [4] Department of Thoracic Surgery, Shenyang Tenth People's Hospital, Shenyang Chest Hospital.
- [5] Department of Respiratory, Shenyang Sixth People's Hospital, Shenyang 110044, Liaoning, China.

- PMID: **33466134**
- PMCID: PMC7808500
- DOI: 10.1097/MD.0000000000023923

Abstract

Coronavirus disease 2019 (COVID-19) is an emerging disease caused by severe acute respiratory syndrome coronavirus 2; no specific effective medication to treat the disease has been identified to date. We aimed to investigate the administered medications and intervention times for patients who completely recovered from COVID-19.This single-center, retrospective, observational study included 55 patients with COVID-19 who were transferred to Shenyang Sixth People's Hospital between January 20 and March 15, 2020. Data on demographics, symptoms, laboratory indicators, treatment processes, and clinical outcomes were collected. Administered drugs and intervention times were compared in 47 and 8 patients with mild and severe symptoms, respectively.All 55 patients recovered. Fifty-three patients (96.36%) received antiviral therapy, including 45 in the mild group (median treatment: 14 days; 17 received umifenovir) and all 8 severe-group patients (median treatment: 17.5 days; 4 received lopinavir/ritonavir). Twenty-nine patients (52.72%) were administered antibiotics, including 21 in the mild group (median treatment: 13.5 days; 15 received moxifloxacin) and all 8 in the severe group (median treatment: 9 days; 2 received linezolid). Moreover, 7 patients (12.72%) were treated with glucocorticoids and 9 (16.36%) with immunomodulators.Given the 100% recovery rate, early administration of antiviral drugs can be considered. Umifenovir may benefit patients with mild symptoms, while lopinavir/ritonavir may benefit those with severe symptoms. Prophylactic administration of common antibiotics may

reduce the risk of co-infection. The use of glucocorticoids is usually not necessary. Randomized, double-blind, and controlled trials remain necessary for more accurate conclusions.

Copyright © 2021 the Author(s). Published by Wolters Kluwer Health, Inc.

Conflict of interest statement

The authors have no conflicts of interest to disclose.

- 22 references
- 3 figures

Supplementary info

Publication types, MeSH terms, Substances, Grant support Expand

Publication types

- Observational Study

MeSH terms

- Anti-Bacterial Agents / therapeutic use
- Antiviral Agents / therapeutic use
- COVID-19 / drug therapy*
- China
- Female
- Glucocorticoids / therapeutic use
- Humans
- Immunologic Factors / therapeutic use
- Indoles / therapeutic use
- Linezolid / therapeutic use
- Lopinavir / therapeutic use
- Male
- Middle Aged
- Moxifloxacin / therapeutic use
- Retrospective Studies
- Ritonavir / therapeutic use

Substances

- Anti-Bacterial Agents
- Antiviral Agents
- Glucocorticoids
- Immunologic Factors

- Indoles
- Lopinavir
- umifenovir
- Linezolid
- Ritonavir
- Moxifloxacin

Grant support

- JY2020-9-018 to Y.Chen/Shenyang Major Science and Technology Innovation R&D Program

Full text links

Wolters Kluwer Wolters Kluwer Free PMC article

Proceed to details

Cite

Share

☐ 1,101

Observational Study

Updates Surg

. 2020 Dec;72(4):1263-1271.

doi: 10.1007/s13304-020-00884-6. Epub 2020 Sep 14.

Impact of lockdown for SARS-CoV-2 (COVID-19) on surgical site infection rates: a monocentric observational cohort study

Pasquale Losurdo [1], Lucia Paiano [2], Natasa Samardzic [2], Paola Germani [2], Laura Bernardi [2], Massimo Borelli [2], Barbara Pozzetto [2], Nicolò de Manzini [2], Marina Bortul [2]

Affiliations Expand

Affiliations

- [1] Division of General Surgery, Department of Medical and Surgical Sciences, Hospital of Cattinara, University of Trieste, Strada di Fiume 447, 34149, Trieste, Italy. palosurdo@gmail.com.
- [2] Division of General Surgery, Department of Medical and Surgical Sciences, Hospital of Cattinara, University of Trieste, Strada di Fiume 447, 34149, Trieste, Italy.

- PMID: **32926340**
- PMCID: PMC7488636
- DOI: 10.1007/s13304-020-00884-6

Free PMC article

Observational Study

Impact of lockdown for SARS-CoV-2 (COVID-19) on surgical site infection rates: a monocentric observational cohort study

Pasquale Losurdo et al. Updates Surg. 2020 Dec.
Free PMC article
Show details
Updates Surg
. 2020 Dec;72(4):1263-1271.
doi: 10.1007/s13304-020-00884-6. Epub 2020 Sep 14.

Authors

Pasquale Losurdo [1], Lucia Paiano [2], Natasa Samardzic [2], Paola Germani [2], Laura Bernardi [2], Massimo Borelli [2], Barbara Pozzetto [2], Nicolò de Manzini [2], Marina Bortul [2]

Affiliations

- [1] Division of General Surgery, Department of Medical and Surgical Sciences, Hospital of Cattinara, University of Trieste, Strada di Fiume 447, 34149, Trieste, Italy. palosurdo@gmail.com.
- [2] Division of General Surgery, Department of Medical and Surgical Sciences, Hospital of Cattinara, University of Trieste, Strada di Fiume 447, 34149, Trieste, Italy.

- PMID: **32926340**
- PMCID: PMC7488636
- DOI: 10.1007/s13304-020-00884-6

Abstract

Surgical site infections are the most common in-hospital acquired infections. The aim of this study and the primary endpoint is to evaluate how the measures to reduce the SARS-CoV-2 spreading affected the superficial and deep SSI rate. A total of 541 patients were included. Of those, 198 from March to April 2018, 220 from March till April 2019 and 123 in the COVID-19 era from March to April 2020. The primary endpoint occurred in 39 over 541 patients. In COVID-19 era, we reported a lower rate of global SSIs (3.3% vs. 8.4%; p 0.035), few patients developed a superficial SSIs (0.8% vs. 3.4%; p 0.018) and none experienced deep SSIs (0% vs. 3.4%; p 0.025). Comparing the previous two "COVID-19-free" years, no significative differences were reported. At multivariate analysis, the measures to reduce the SARS-CoV-2 spread (OR 0.368; p 0.05) were independently associated with the reduction for total, superficial and deep SSIs. Moreover, the presence of drains (OR 4.99; p 0.009) and a Type III-IV of SWC (OR 1.8; p 0.001) demonstrated a worse effect regarding the primary endpoint. Furthermore, the presence of the drain was not associated with an increased risk of superficial and deep SSIs. In this study, we provided important insights into the superficial and deep SSIs risk assessment for patients who underwent

surgery. Simple and easily viable precautions such as wearing surgical masks and the restriction of visitors emerged as promising tools for the reduction of SSIs risk.

Keywords: COVID-19; Italy lockdown; SARS-CoV-2; Surgical site infections.

Conflict of interest statement

There is no conflict of interest for all authors regarding the publication of this manuscript and there are no financial issues to disclose.

Comment in

- Reduction in nosocomial infections during the COVID-19 era: a lesson to be learned. Cerulli Irelli E, Morano A, Di Bonaventura C. Cerulli Irelli E, et al. Updates Surg. 2021 Apr;73(2):785-786. doi: 10.1007/s13304-020-00925-0. Epub 2020 Nov 19. Updates Surg. 2021. PMID: 33215337 Free PMC article. No abstract available.

- 40 references
- 1 figure

Supplementary info

Publication types, MeSH terms Expand

Publication types

- Observational Study

MeSH terms

- Adult
- Aged
- COVID-19 / prevention & control*
- Female
- Humans
- Infection Control*
- Italy / epidemiology
- Male
- Middle Aged
- Pandemics
- Prevalence
- Prognosis
- Retrospective Studies
- Risk Factors
- Surgery Department, Hospital / organization & administration*
- Surgical Wound Infection / epidemiology

- Surgical Wound Infection / prevention & control*

Full text links

SpringerLink Springer Free PMC article
Proceed to details
Cite
Share
☐ 1,102
Observational Study
PLoS Med
. 2020 Jun 16;17(6):e1003130.
doi: 10.1371/journal.pmed.1003130. eCollection 2020 Jun.

Clinical and epidemiological characteristics of pediatric SARS-CoV-2 infections in China: A multicenter case series

Che Zhang [1, 2], Jiaowei Gu [2], Quanjing Chen [3], Na Deng [4], Jingfeng Li [2], Li Huang [2], Xihui Zhou [1]
Affiliations Expand

Affiliations

- [1] Department of Neonatology, The First Affiliated Hospital of Xi'an Jiaotong University, Xi'an, Shaanxi, People's Republic of China.
- [2] Department of Pediatrics, Affiliated Taihe Hospital of Hubei University of Medicine, Shiyan, Hubei, People's Republic of China.
- [3] Department of Pediatrics, Dongfeng Hospital of Hubei University of Medicine, Shiyan, Hubei, People's Republic of China.
- [4] Pediatric Intensive Care Unit, Shiyan People Hospital, Shiyan, Hubei, People's Republic of China.

- PMID: **32544155**
- PMCID: PMC7297312
- DOI: 10.1371/journal.pmed.1003130

Free PMC article
Observational Study

Clinical and epidemiological characteristics of pediatric SARS-CoV-2 infections in China: A multicenter case series

Che Zhang et al. PLoS Med. 2020.
Free PMC article
Show details
PLoS Med
. 2020 Jun 16;17(6):e1003130.
doi: 10.1371/journal.pmed.1003130. eCollection 2020 Jun.

Authors

Che Zhang [1, 2], Jiaowei Gu [2], Quanjing Chen [3], Na Deng [4], Jingfeng Li [2], Li Huang [2], Xihui Zhou [1]

Affiliations

- [1] Department of Neonatology, The First Affiliated Hospital of Xi'an Jiaotong University, Xi'an, Shaanxi, People's Republic of China.
- [2] Department of Pediatrics, Affiliated Taihe Hospital of Hubei University of Medicine, Shiyan, Hubei, People's Republic of China.
- [3] Department of Pediatrics, Dongfeng Hospital of Hubei University of Medicine, Shiyan, Hubei, People's Republic of China.
- [4] Pediatric Intensive Care Unit, Shiyan People Hospital, Shiyan, Hubei, People's Republic of China.

- PMID: **32544155**
- PMCID: PMC7297312
- DOI: 10.1371/journal.pmed.1003130

Abstract

Background: As of April 18, 2020, over 2,000,000 patients had been diagnosed with coronavirus disease-2019 (COVID-19) globally, and more than 140,000 deaths had been reported. The clinical and epidemiological characteristics of adult patients have been documented recently. However, information on pediatric patients is limited. We describe the clinical and epidemiological characteristics of pediatric patients to provide valuable insight into the early diagnosis and assessment of COVID-19 in children.

Methods and findings: This retrospective, observational study involves a case series performed at 4 hospitals in West China. Thirty-four pediatric patients with COVID-19 were included from January 27 to February 23, 2020. The final follow-up visit was completed by March 16, 2020. Clinical and epidemiological characteristics were analyzed on the basis of demographic data, medical history, laboratory tests, radiological findings, and treatment information. Data analysis was performed for 34 pediatrics patients with COVID-19 aged from 1 to 144 months (median 33.00, interquartile range 10.00-94.25), among whom 14 males (41%) were included. All the

patients in the current study presented mild (18%) or moderate (82%) forms of COVID-19. A total of 48% of patients were noted to be without a history of exposure to an identified source. Mixed infections of other respiratory pathogens were reported in 16 patients (47%). Comorbidities were reported in 6 patients (18%). The most common initial symptoms were fever (76%) and cough (62%). Expectoration (21%), vomiting (12%), and diarrhea (12%) were also reported in a considerable portion of cases. A substantial increase was detected in serum amyloid A for 17 patients (among 20 patients with available data; 85%) and in high-sensitivity C-reactive protein for 17 patients (among 29 patients with available data; 59%), whereas a decrease in prealbumin was noticed in 25 patients (among 32 patients with available data; 78%). In addition, significant increases in the levels of lactate dehydrogenase and α-hydroxybutyrate dehydrogenase were detected in 28 patients (among 34 patients with available data; 82%) and 25 patients (among 34 patients with available data; 74%), respectively. Patchy lesions in lobules were detected by chest computed tomographic scans in 28 patients (82%). Ground-glass opacities, which were a typical feature in adults, were rare in pediatric patients (3%). Rapid radiologic progression and a late-onset pattern of lesions in the lobules were also noticed. Lesions in lobules still existed in 24 (among 32 patients with lesions; 75%) patients that were discharged, although the main symptoms disappeared a few days after treatment. All patients were discharged, and the median duration of hospitalization was 10.00 (8.00-14.25) days. The current study was limited by the small sample size and a lack of dynamic detection of inflammatory markers.

Conclusions: Our data systemically presented the clinical and epidemiological features, as well as the outcomes, of pediatric patients with COVID-19. Stratified analysis was performed between mild and moderate cases. The findings offer new insight into early identification and intervention in pediatric patients with COVID-19.

Conflict of interest statement

The authors have declared that no competing interests exist.

Comment in

- Pulmonary sequelae of pediatric patients after discharge for COVID-19: An observational study.
  Zhang C, Huang L, Tang X, Zhang Y, Zhou X. Zhang C, et al. Pediatr Pulmonol. 2021 May;56(5):1266-1269. doi: 10.1002/ppul.25239. Epub 2021 Feb 9. Pediatr Pulmonol. 2021. PMID: 33559979 Free PMC article. No abstract available.

- 25 references
- 4 figures

Supplementary info

Publication types, MeSH terms, Substances, Grant support Expand

Publication types

- Multicenter Study
- Observational Study

MeSH terms

- Betacoronavirus
- C-Reactive Protein / metabolism
- COVID-19
- Child
- Child, Preschool
- China / epidemiology
- Coinfection / epidemiology
- Coronavirus Infections / diagnostic imaging
- Coronavirus Infections / epidemiology*
- Coronavirus Infections / metabolism
- Coronavirus Infections / physiopathology*
- Cough / epidemiology
- Cough / physiopathology
- Diarrhea / epidemiology
- Diarrhea / physiopathology
- Female
- Fever / epidemiology
- Fever / physiopathology
- Humans
- Hydroxybutyrate Dehydrogenase / metabolism
- Infant
- L-Lactate Dehydrogenase / metabolism
- Length of Stay / statistics & numerical data
- Lung / diagnostic imaging*
- Male
- Pandemics
- Pneumonia, Viral / diagnostic imaging
- Pneumonia, Viral / epidemiology*
- Pneumonia, Viral / metabolism
- Pneumonia, Viral / physiopathology*
- Prealbumin / metabolism
- Retrospective Studies
- SARS-CoV-2
- Serum Amyloid A Protein / metabolism
- Severity of Illness Index
- Tomography, X-Ray Computed
- Vomiting / epidemiology
- Vomiting / physiopathology

Substances

- Prealbumin
- Serum Amyloid A Protein
- C-Reactive Protein
- 2-hydroxybutyrate dehydrogenase
- L-Lactate Dehydrogenase
- Hydroxybutyrate Dehydrogenase

Grant support

The authors received no specific funding for this work.

Full text links

OPEN ACCESS TO FULL TEXT PLOS MEDICINE Public Library of Science Free PMC article
Proceed to details
Cite
Share
☐ 1,103
Observational Study
Emergencias
. 2020 Nov;32(6):442-444.

Care of neurology patients in a hospital emergency department during the lockdown period for COVID-19: a comparative analysis

[Article in Spanish, English]

Sara Ballesta-Martínez [1], María Pilar Navarro-Pérez [2], Judith Espinosa-Rueda [1], Marta Marín-Gracia [1], José Alberto García-Noain [3], Elena Muñoz-Farjas [1]
Affiliations Expand

Affiliations

- [1] Servicio de Neurología, Hospital Clínico Universitario Lozano Blesa, Zaragoza, España.
- [2] Servicio de Neurología, Hospital Clínico Universitario Lozano Blesa, Zaragoza, España. Instituto de Investigación Sanitaria Aragón (IIS Aragón), Zaragoza, España.
- [3] Servicio de Urgencias, Hospital Clínico Universitario Lozano Blesa, Zaragoza, España.

- PMID: **33275371**

Free article
Observational Study

Care of neurology patients in a hospital emergency department during the lockdown period for COVID-19: a comparative analysis

[Article in Spanish, English]
Sara Ballesta-Martínez et al. Emergencias. 2020 Nov.
Free article
Show details
Emergencias
. 2020 Nov;32(6):442-444.

Authors

Sara Ballesta-Martínez [1], María Pilar Navarro-Pérez [2], Judith Espinosa-Rueda [1], Marta Marín-Gracia [1], José Alberto García-Noain [3], Elena Muñoz-Farjas [1]

Affiliations

- [1] Servicio de Neurología, Hospital Clínico Universitario Lozano Blesa, Zaragoza, España.
- [2] Servicio de Neurología, Hospital Clínico Universitario Lozano Blesa, Zaragoza, España. Instituto de Investigación Sanitaria Aragón (IIS Aragón), Zaragoza, España.
- [3] Servicio de Urgencias, Hospital Clínico Universitario Lozano Blesa, Zaragoza, España.

- PMID: **33275371**

No abstract available

Supplementary info

Publication types, MeSH terms Expand

Publication types

- Comparative Study
- Observational Study

MeSH terms

- Adult
- Aged
- COVID-19 / epidemiology
- COVID-19 / prevention & control*
- Emergency Service, Hospital / trends*
- Facilities and Services Utilization / trends*

- Female
- Health Services Accessibility / trends*
- Humans
- Male
- Middle Aged
- Nervous System Diseases / diagnosis
- Nervous System Diseases / epidemiology
- Nervous System Diseases / therapy*
- Pandemics
- Patient Acceptance of Health Care / statistics & numerical data*
- Physical Distancing*
- Retrospective Studies
- Spain / epidemiology

Full text links

EMERGENCIAS Grupo Saned
Proceed to details
Cite
Share

1,104

Observational Study

J Stroke Cerebrovasc Dis
. 2021 Apr;30(4):105603.
doi: 10.1016/j.jstrokecerebrovasdis.2021.105603. Epub 2021 Jan 8.

Intracranial Hemorrhage in COVID-19 Patients

Sanskriti Mishra [1], Murray Choueka [2], Qiang Wang [3], Chloe Hu [4], Stephanie Visone [5], Michael Silver [6], Evan G Stein [7], Steven R Levine [8], Qingliang T Wang [9]
Affiliations Expand

Affiliations

- [1] Department of Neurology, Comprehensive Stroke Center, Maimonides Medical Center, 948 48th St, 2nd Fl, Brooklyn, NY 11219, United States; Department of Neurology & Stroke Center, SUNY Downstate Health Sciences University, Brooklyn, NY, United States. Electronic address: SAmishra@maimonidesmed.org.
- [2] Department of Neurology & Stroke Center, SUNY Downstate Health Sciences University, Brooklyn, NY, United States. Electronic address: Murray.Choueka@downstate.edu.
- [3] Department of Neurology & Stroke Center, SUNY Downstate Health Sciences University, Brooklyn, NY, United States. Electronic address: Qiang.Wang@downstate.edu.

- [4] Department of Neurology, Comprehensive Stroke Center, Maimonides Medical Center, 948 48th St, 2nd Fl, Brooklyn, NY 11219, United States. Electronic address: CHu@maimonidesmed.org.
- [5] Department of Neurology, Comprehensive Stroke Center, Maimonides Medical Center, 948 48th St, 2nd Fl, Brooklyn, NY 11219, United States. Electronic address: SVisone@maimonidesmed.org.
- [6] Department of Research Administration, Maimonides Medical Center, Brooklyn, NY, United States. Electronic address: MSilver@maimonidesmed.org.
- [7] Department of Radiology, Maimonides Medical Center, Brooklyn, NY, United States. Electronic address: EGstein@maimonidesmed.org.
- [8] Department of Neurology, Comprehensive Stroke Center, Maimonides Medical Center, 948 48th St, 2nd Fl, Brooklyn, NY 11219, United States; Department of Neurology & Stroke Center, SUNY Downstate Health Sciences University, Brooklyn, NY, United States; Department of Emergency Medicine, SUNY Downstate Health Sciences University, Brooklyn, NY, United States; Department of Neurology, Kings County Hospital Center, Brooklyn, NY, United States. Electronic address: Steven.Levine@downstate.edu.
- [9] Department of Neurology, Comprehensive Stroke Center, Maimonides Medical Center, 948 48th St, 2nd Fl, Brooklyn, NY 11219, United States; Department of Surgery/Division of Neurosurgery, Maimonides Medical Center, Brooklyn, NY, United States; Department of Neurology & Stroke Center, SUNY Downstate Health Sciences University, Brooklyn, NY, United States. Electronic address: QWang@maimonidesmed.org.

- PMID: **33484980**
- PMCID: PMC7831866
- DOI: 10.1016/j.jstrokecerebrovasdis.2021.105603

Free PMC article
Observational Study

Intracranial Hemorrhage in COVID-19 Patients

Sanskriti Mishra et al. J Stroke Cerebrovasc Dis. 2021 Apr.
Free PMC article
Show details
J Stroke Cerebrovasc Dis
. 2021 Apr;30(4):105603.
doi: 10.1016/j.jstrokecerebrovasdis.2021.105603. Epub 2021 Jan 8.

Authors

Sanskriti Mishra [1], Murray Choueka [2], Qiang Wang [3], Chloe Hu [4], Stephanie Visone [5], Michael Silver [6], Evan G Stein [7], Steven R Levine [8], Qingliang T Wang [9]

Affiliations

- [1] Department of Neurology, Comprehensive Stroke Center, Maimonides Medical Center, 948 48th St, 2nd Fl, Brooklyn, NY 11219, United States; Department of Neurology &

Stroke Center, SUNY Downstate Health Sciences University, Brooklyn, NY, United States. Electronic address: SAmishra@maimonidesmed.org.

- [2] Department of Neurology & Stroke Center, SUNY Downstate Health Sciences University, Brooklyn, NY, United States. Electronic address: Murray.Choueka@downstate.edu.
- [3] Department of Neurology & Stroke Center, SUNY Downstate Health Sciences University, Brooklyn, NY, United States. Electronic address: Qiang.Wang@downstate.edu.
- [4] Department of Neurology, Comprehensive Stroke Center, Maimonides Medical Center, 948 48th St, 2nd Fl, Brooklyn, NY 11219, United States. Electronic address: CHu@maimonidesmed.org.
- [5] Department of Neurology, Comprehensive Stroke Center, Maimonides Medical Center, 948 48th St, 2nd Fl, Brooklyn, NY 11219, United States. Electronic address: SVisone@maimonidesmed.org.
- [6] Department of Research Administration, Maimonides Medical Center, Brooklyn, NY, United States. Electronic address: MSilver@maimonidesmed.org.
- [7] Department of Radiology, Maimonides Medical Center, Brooklyn, NY, United States. Electronic address: EGstein@maimonidesmed.org.
- [8] Department of Neurology, Comprehensive Stroke Center, Maimonides Medical Center, 948 48th St, 2nd Fl, Brooklyn, NY 11219, United States; Department of Neurology & Stroke Center, SUNY Downstate Health Sciences University, Brooklyn, NY, United States; Department of Emergency Medicine, SUNY Downstate Health Sciences University, Brooklyn, NY, United States; Department of Neurology, Kings County Hospital Center, Brooklyn, NY, United States. Electronic address: Steven.Levine@downstate.edu.
- [9] Department of Neurology, Comprehensive Stroke Center, Maimonides Medical Center, 948 48th St, 2nd Fl, Brooklyn, NY 11219, United States; Department of Surgery/Division of Neurosurgery, Maimonides Medical Center, Brooklyn, NY, United States; Department of Neurology & Stroke Center, SUNY Downstate Health Sciences University, Brooklyn, NY, United States. Electronic address: QWang@maimonidesmed.org.

- PMID: **33484980**
- PMCID: PMC7831866
- DOI: 10.1016/j.jstrokecerebrovasdis.2021.105603

Abstract

Objective: To describe the clinical, laboratory, temporal, radiographic, and outcome features of acute Intracranial Hemorrhage (ICH) in COVID-19 patients.

Methods: Retrospective, observational, consecutive case series of patients admitted with ICH to Maimonides Medical Center from March 1 through July 31, 2020, who had confirmed or highly suspected COVID-19. Demographic, clinical, laboratory, imaging, and outcome data were analyzed. ICH rates among all strokes were compared to the same time period in 2019 in two-week time intervals. Correlation of systolic blood pressure variability (SBPV) and neutrophil-to-lymphocyte ratio (NLR) to clinical outcomes were performed.

Results: Of 324 patients who presented with stroke, 65 (20%) were diagnosed with non-traumatic ICH: 8 had confirmed and 3 had highly suspected COVID-19. Nine (82%) had at least one associated risk factor for ICH. Three ICHs occurred during inpatient anticoagulation. More than half (6) suffered either deep or cerebellar hemorrhages; only 2 were lobar hemorrhages. Two of 8 patients with severe pneumonia survived. During the NYC COVID-19 peak period in April, ICH comprised the highest percentage of all strokes (40%), and then steadily decreased week-after-

week (p = 0.02). SBPV and NLR were moderately and weakly positively correlated to discharge modified Rankin Scale, respectively.

Conclusions: COVID-19 associated ICH is often associated with at least one known ICH risk factor and severe pneumonia. There was a suggestive relative surge in ICH among all stroke types during the first peak of the NYC pandemic. It is important to be vigilant of ICH as a possible and important manifestation of COVID-19.

Keywords: COVID-19; Hemorrhagic Stroke; Intracerebral Hemorrhage; Intracranial Hemorrhage; Subarachnoid hemorrhage.

Copyright © 2021 Elsevier Inc. All rights reserved.

- 51 references
- 2 figures

Supplementary info

Publication types, MeSH terms Expand

Publication types

- Observational Study

MeSH terms

- Adult
- Aged
- Aged, 80 and over
- COVID-19 / diagnosis
- COVID-19 / epidemiology*
- COVID-19 / therapy
- Female
- Humans
- Intracranial Hemorrhages / diagnostic imaging
- Intracranial Hemorrhages / epidemiology*
- Intracranial Hemorrhages / therapy
- Male
- New York / epidemiology
- Prevalence
- Prognosis
- Retrospective Studies
- Risk Assessment
- Risk Factors
- Severity of Illness Index
- Time Factors

Full text links

ELSEVIER FULL-TEXT ARTICLE Elsevier Science Free PMC article

Proceed to details

Cite

Share

1,105

Observational Study

Eur Heart J Cardiovasc Pharmacother
. 2021 May 23;7(3):e48-e51.
doi: 10.1093/ehjcvp/pvaa098.

The association of hypertension and diabetes pharmacotherapy with COVID-19 severity and immune signatures: an observational study

Rinkoo Dalan[1], Li Wei Ang[2], Wilnard Y T Tan[2], Siew-Wai Fong[3 4], Woo Chiao Tay[2], Yi-Hao Chan[3], Laurent Renia[3], Lisa F P Ng[3], David Chien Lye[5], Daniel E K Chew[1], Barnaby E Young[5]

Affiliations Expand

Affiliations

- [1] Department of Endocrinology, Tan Tock Seng Hospital, Lee Kong Chian School of Medicine, Singapore.
- [2] National Centre for Infectious Diseases, Singapore.
- [3] Singapore Immunology Network, Agency for Science, Technology and Research, Singapore.
- [4] Department of Biological Sciences, National University of Singapore, Singapore.
- [5] National Centre for Infectious Diseases, Tan Tock Seng Hospital, Lee Kong Chian School of Medicine, Yong Loo Lin School of Medicine, Singapore.

- PMID: **32766831**
- PMCID: PMC7454507
- DOI: 10.1093/ehjcvp/pvaa098

Free PMC article
Observational Study

The association of hypertension and diabetes pharmacotherapy with COVID-19 severity

and immune signatures: an observational study

Rinkoo Dalan et al. Eur Heart J Cardiovasc Pharmacother. 2021.
Free PMC article
Show details
Eur Heart J Cardiovasc Pharmacother
. 2021 May 23;7(3):e48-e51.
doi: 10.1093/ehjcvp/pvaa098.

Authors

Rinkoo Dalan [1], Li Wei Ang [2], Wilnard Y T Tan [2], Siew-Wai Fong [3] [4], Woo Chiao Tay [2], Yi-Hao Chan [3], Laurent Renia [3], Lisa F P Ng [3], David Chien Lye [5], Daniel E K Chew [1], Barnaby E Young [5]

Affiliations

- [1] Department of Endocrinology, Tan Tock Seng Hospital, Lee Kong Chian School of Medicine, Singapore.
- [2] National Centre for Infectious Diseases, Singapore.
- [3] Singapore Immunology Network, Agency for Science, Technology and Research, Singapore.
- [4] Department of Biological Sciences, National University of Singapore, Singapore.
- [5] National Centre for Infectious Diseases, Tan Tock Seng Hospital, Lee Kong Chian School of Medicine, Yong Loo Lin School of Medicine, Singapore.

- PMID: **32766831**
- PMCID: PMC7454507
- DOI: 10.1093/ehjcvp/pvaa098

No abstract available

Keywords: Angiotensin receptor blocker (ARB); Angiotensin-converting enzyme inhibitor (ACE-I); Diabetes; Dipeptidyl peptidase 4 inhibitor (DPP4i); Hypertension.

Supplementary info

Publication types, MeSH terms Expand

Publication types

- Observational Study
- Research Support, Non-U.S. Gov't

MeSH terms

- COVID-19 / complications*
- Cohort Studies
- Diabetes Complications / complications*
- Diabetes Mellitus / drug therapy*
- Humans
- Hypertension / complications*
- Hypertension / drug therapy*
- Retrospective Studies
- Severity of Illness Index

Full text links

OXFORD ACADEMIC Silverchair Information Systems Free PMC article
Proceed to details
Cite
Share
☐ 1,106
Observational Study
Mayo Clin Proc
. 2020 Dec;95(12):2674-2683.
doi: 10.1016/j.mayocp.2020.09.022. Epub 2020 Sep 22.

Cardiovascular Disease in Hospitalized Patients With a Diagnosis of Coronavirus From the Pre-COVID-19 Era in United States: National Analysis From 2016-2017

Manyoo A Agarwal [1], Boback Ziaeian [2], Carl J Lavie [3], Gregg C Fonarow [4]
Affiliations Expand

Affiliations

- [1] Division of Cardiovascular Medicine, University of California, Los Angeles, CA. Electronic address: manyooagarwal@mednet.ucla.edu.
- [2] Division of Cardiovascular Medicine, University of California, Los Angeles, CA; Division of Cardiology, VA Greater Los Angeles, Los Angeles, CA.
- [3] John Ochsner Heart and Vascular Institute, Ochsner Clinical School-the University of Queensland School of Medicine, New Orleans, LA.

- [4] Division of Cardiovascular Medicine, University of California, Los Angeles, CA; Ahmanson-UCLA Cardiomyopathy Center, University of California, Los Angeles, CA. Electronic address: gfonarow@mednet.ucla.edu.

- PMID: **33276839**
- PMCID: PMC7508501
- DOI: 10.1016/j.mayocp.2020.09.022

Free PMC article
Observational Study

Cardiovascular Disease in Hospitalized Patients With a Diagnosis of Coronavirus From the Pre-COVID-19 Era in United States: National Analysis From 2016-2017

Manyoo A Agarwal et al. Mayo Clin Proc. 2020 Dec.
Free PMC article
Show details
Mayo Clin Proc
. 2020 Dec;95(12):2674-2683.
doi: 10.1016/j.mayocp.2020.09.022. Epub 2020 Sep 22.

Authors

Manyoo A Agarwal [1], Boback Ziaeian [2], Carl J Lavie [3], Gregg C Fonarow [4]

Affiliations

- [1] Division of Cardiovascular Medicine, University of California, Los Angeles, CA. Electronic address: manyooagarwal@mednet.ucla.edu.
- [2] Division of Cardiovascular Medicine, University of California, Los Angeles, CA; Division of Cardiology, VA Greater Los Angeles, Los Angeles, CA.
- [3] John Ochsner Heart and Vascular Institute, Ochsner Clinical School-the University of Queensland School of Medicine, New Orleans, LA.
- [4] Division of Cardiovascular Medicine, University of California, Los Angeles, CA; Ahmanson-UCLA Cardiomyopathy Center, University of California, Los Angeles, CA. Electronic address: gfonarow@mednet.ucla.edu.

- PMID: **33276839**
- PMCID: PMC7508501
- DOI: 10.1016/j.mayocp.2020.09.022

Abstract

Objective: To analyze the cardiovascular disease (CVD) burden in hospitalized patients with a diagnosis of coronavirus from the pre-coronavirus disease 2019 era in the United States.

Patients and methods: We identified hospitalized adults with a diagnosis of coronavirus in a large US administrative database, the National (Nationwide) Inpatient Sample, from January 1, 2016, to December 3, 2017, to study patient demographic characteristics, clinical comorbidities, and outcomes (in-hospital mortality and health care resource utilization) based on the presence or absence of CVD.

Results: A total of 21,300 hospitalized adults with a diagnosis of coronavirus in 2016 and 2017 from all across the United States were included in the final analysis; the mean age was 63.6 years, 11,033 (51.8%) were female, and 15,911 (74.7%) had public insurers. Among these hospitalized patients, 11,930 (56.0%) had a diagnosis of CVD. Compared with those without CVD, the patients with CVD were older (70.1 vs 55.4 years) and had higher Charlson comorbidity index scores (2.5 vs 1.6) and Elixhauser comorbidity index scores (4.3 vs 2.4) (all P<.001). After multivariable risk adjustment, patients with CVD had higher mortality than those without CVD (5.3% [632 of 11,930] vs 1.5% [140 of 9370]; adjusted odds ratio, 2.0 [95% CI, 1.2 to 3.4]; P=.008). The mean length of hospital stay (6.9 vs 6.1 days; P=.003), hospital charges ($78,377 vs $66,538; P=.002), and discharge to nursing home (24.6% [2945 of 11,930] vs 12.9% [1208 of 9370]; P<.001) were higher in those with CVD compared with the patients without CVD.

Conclusion: Cardiovascular disease was present in a notable proportion of hospitalized patients with coronavirus in the pre-coronavirus disease 2019 era in United States and was associated with higher risk of in-hospital mortality and health care resource utilization.

Copyright © 2020 Mayo Foundation for Medical Education and Research. All rights reserved.

- 35 references
- 2 figures

Supplementary info

Publication types, MeSH terms, Grant support Expand

Publication types

- Observational Study

MeSH terms

- Adult
- Aged
- Cardiovascular Diseases / diagnosis
- Cardiovascular Diseases / epidemiology*
- Case-Control Studies
- Comorbidity
- Coronavirus Infections / diagnosis
- Coronavirus Infections / epidemiology*
- Cost of Illness*

- Databases, Factual
- Female
- Hospital Mortality
- Hospitalization / statistics & numerical data*
- Humans
- Male
- Middle Aged
- Retrospective Studies
- United States / epidemiology
- Young Adult

Grant support

- KL2 TR001882/TR/NCATS NIH HHS/United States

Full text links

ELSEVIER FULL-TEXT ARTICLE Elsevier Science Free PMC article

Proceed to details

Cite

Share

☐ 1,107

Observational Study

Nephron

. 2021;145(4):363-370.

doi: 10.1159/000515128. Epub 2021 Apr 26.

COVID-19 Infection: Viral Clearance and Antibody Response in Dialysis Patients and Renal Transplant Recipients

Paolo Ferdinando Bruno [1], Maria Cappuccilli [2], Alessandra Spazzoli [1], Matteo De Liberali [3], Brunilda Sejdiu [4], Marianna Napoli [2], Vera Minerva [2], Simona Semprini [5], Giorgio Dirani [5], Vittorio Sambri [5], Andrea Buscaroli [4], Angelo Rigotti [3], Elena Mancini [6], Paolo Masperi [7], Gaetano La Manna [2], Giovanni Mosconi [1]

Affiliations Expand

Affiliations

- [1] Nephrology and Dialysis Unit, AUSL Romagna Morgagni-Pierantoni Hospital, Forlì, Italy.

- [2] Department of Experimental Diagnostic and Specialty Medicine (DIMES), Nephrology, Dialysis and Renal Transplant Unit, S. Orsola-Malpighi Hospital, University of Bologna, Bologna, Italy.
- [3] Nephrology and Dialysis Unit, AUSL Romagna Infermi Hospital, Rimini, Italy.
- [4] Nephrology and Dialysis Unit, AUSL Romagna S. Maria Delle Croci Hospital, Ravenna, Italy.
- [5] Unit of Microbiology, AUSL Romagna Laboratory Pievesestina Cesena (FC), Cesena, Italy.
- [6] Nephrology, Dialysis and Hypertension Unit, S. Orsola-Malpighi Hospital, Bologna, Italy.
- [7] Medical Direction Unit, AUSL Romagna Morgagni-Pierantoni Hospital, Forlì, Italy.

- PMID: **33902031**
- PMCID: PMC8247803
- DOI: 10.1159/000515128

Free PMC article
Observational Study

COVID-19 Infection: Viral Clearance and Antibody Response in Dialysis Patients and Renal Transplant Recipients

Paolo Ferdinando Bruno et al. Nephron. 2021.
Free PMC article

Show details

Nephron
. 2021;145(4):363-370.
doi: 10.1159/000515128. Epub 2021 Apr 26.

Authors

Paolo Ferdinando Bruno [1], Maria Cappuccilli [2], Alessandra Spazzoli [1], Matteo De Liberali [3], Brunilda Sejdiu [4], Marianna Napoli [2], Vera Minerva [2], Simona Semprini [5], Giorgio Dirani [5], Vittorio Sambri [5], Andrea Buscaroli [4], Angelo Rigotti [3], Elena Mancini [6], Paolo Masperi [7], Gaetano La Manna [2], Giovanni Mosconi [1]

Affiliations

- [1] Nephrology and Dialysis Unit, AUSL Romagna Morgagni-Pierantoni Hospital, Forlì, Italy.
- [2] Department of Experimental Diagnostic and Specialty Medicine (DIMES), Nephrology, Dialysis and Renal Transplant Unit, S. Orsola-Malpighi Hospital, University of Bologna, Bologna, Italy.
- [3] Nephrology and Dialysis Unit, AUSL Romagna Infermi Hospital, Rimini, Italy.
- [4] Nephrology and Dialysis Unit, AUSL Romagna S. Maria Delle Croci Hospital, Ravenna, Italy.

- [5] Unit of Microbiology, AUSL Romagna Laboratory Pievesestina Cesena (FC), Cesena, Italy.
- [6] Nephrology, Dialysis and Hypertension Unit, S. Orsola-Malpighi Hospital, Bologna, Italy.
- [7] Medical Direction Unit, AUSL Romagna Morgagni-Pierantoni Hospital, Forlì, Italy.

- PMID: **33902031**
- PMCID: PMC8247803
- DOI: 10.1159/000515128

Abstract

Background/aims: The coronavirus disease 2019 (CO-VID-19) pandemic is the major current health emergency worldwide, adding a significant burden also to the community of nephrologists for the management of their patients. Here, we analyzed the impact of COVID-19 infection in renal patients to assess the time to viral clearance, together with the production and persistence of IgG and IgM antibody response, in consideration of the altered immune capacity of this fragile population.

Methods: Viral clearance and antibody kinetics were investigated in 49 renal patients recovered from COVID-19 infection: 7 of them with chronic decompensated renal failure, 31 under dialysis treatment, and 11 kidney transplant recipients.

Results: The time span between the diagnosis of infection and recovery based on laboratory testing (2 negative nasopharyngeal swabs in consecutive days) was 31.7 ± 13.3 days. Three new positive cases were detected from 8 to 13 days following recovery. At the first serological determination after swab negativization, all the patients developed IgG and IgM antibodies. The semiquantitative analysis showed a progressive increase in IgG and a slow reduction in IgM.

Discussion/conclusion: In subjects with decompensated chronic kidney disease, under dialysis and in transplant recipients, viral clearance is lengthened compared to the general population. However, in spite of their common status of immunodepression, all of them were able to produce specific antibodies. These data might provide useful insights for monitoring and planning health-care activities in the weak category of patients with compromised renal function recovered from COVID-19.

Keywords: Chronic renal failure; Coronavirus disease 2019; Dialysis; Kidney transplantation; Viral clearance.

© 2021 S. Karger AG, Basel.

Conflict of interest statement

The authors have no conflicts of interest to declare.

- 35 references

Supplementary info

Publication types, MeSH terms, Substances Expand

Publication types

- Multicenter Study
- Observational Study

MeSH terms

- Adult
- Aged
- Aged, 80 and over
- Antibodies, Viral / analysis
- COVID-19 / epidemiology
- COVID-19 / immunology*
- COVID-19 / virology*
- Female
- Glomerular Filtration Rate
- Humans
- Immunoglobulin G / analysis
- Immunoglobulin M / analysis
- Kidney Failure, Chronic / complications
- Kidney Failure, Chronic / immunology
- Kidney Failure, Chronic / therapy
- Kidney Transplantation*
- Kinetics
- Male
- Middle Aged
- Nasopharynx / immunology
- Nasopharynx / virology
- Renal Dialysis*
- Retrospective Studies
- Transplant Recipients
- Treatment Outcome

Substances

- Antibodies, Viral
- Immunoglobulin G
- Immunoglobulin M

Full text links

Karger Free Final Version S. Karger AG, Basel, Switzerland Free PMC article

Proceed to details

Cite

Share

☐ 1,108

Observational Study

Virol J

. 2021 Mar 31;18(1):67.

doi: 10.1186/s12985-021-01538-8.

Scores based on neutrophil percentage and lactate dehydrogenase with or without oxygen saturation predict hospital mortality risk in severe COVID-19 patients

Xiude Fan [#] [1], Bin Zhu [#] [2], Masoud Nouri-Vaskeh [#] [3], Chunguo Jiang [#] [4], Xiaokai Feng [#] [4], Kyle Poulsen [5], Behzad Baradaran [3], Jiansong Fang [6] [7], Erfan Ahmadi Ade [3], Akbar Sharifi [8], Zhigang Zhao [2], Qunying Han [1], Yong Zhang [9], Liming Zhang [10], Zhengwen Liu [11]

Affiliations Expand

Affiliations

- [1] Department of Infectious Diseases, First Affiliated Hospital of Xi'an Jiaotong University, No. 277 Yanta West Road, Xi'an, 710061, Shaanxi Province, People's Republic of China.
- [2] Department of Pharmacy, Beijing Tiantan Hospital, Capital Medical University, Beijing, China.
- [3] Immunology Research Center, Tabriz University of Medical Sciences, Tabriz, Iran.
- [4] Department of Respiratory and Critical Care Medicine, Beijing Institute of Respiratory Medicine, Beijing Chaoyang Hospital, Capital Medical University, Beijing, People's Republic of China.
- [5] Department of Inflammation and Immunity, Cleveland Clinic, Cleveland, OH, USA.
- [6] Science and Technology Innovation Center, Guangzhou University of Chinese Medicine, Guangzhou, Guangdong Province, China.
- [7] DME Center, Institute of Clinical Pharmacology, Guangzhou University of Chinese Medicine, Guangzhou, Guangdong Province, People's Republic of China.
- [8] Tuberculosis and Lung Disease Research Center, Tabriz University of Medical Sciences, Tabriz, Iran.
- [9] Department of Hepatobiliary Surgery, Union Hospital, Tongji Medical College, Huazhong University of Science and Technology, Wuhan, People's Republic of China. mailzhangyong@126.com.
- [10] Department of Respiratory and Critical Care Medicine, Beijing Institute of Respiratory Medicine, Beijing Chaoyang Hospital, Capital Medical University, Beijing, People's Republic of China. zhangliming@bjcyh.com.
- [11] Department of Infectious Diseases, First Affiliated Hospital of Xi'an Jiaotong University, No. 277 Yanta West Road, Xi'an, 710061, Shaanxi Province, People's Republic of China. liuzhengwen113@xjtu.edu.cn.

# Contributed equally.

- PMID: **33789703**
- PMCID: PMC8011050
- DOI: 10.1186/s12985-021-01538-8

Free PMC article
Observational Study

Scores based on neutrophil percentage and lactate dehydrogenase with or without oxygen saturation predict hospital mortality risk in severe COVID-19 patients

Xiude Fan et al. Virol J. 2021.
Free PMC article
Show details
Virol J
. 2021 Mar 31;18(1):67.
doi: 10.1186/s12985-021-01538-8.

Authors

Xiude Fan [# 1], Bin Zhu [# 2], Masoud Nouri-Vaskeh [# 3], Chunguo Jiang [# 4], Xiaokai Feng [# 4], Kyle Poulsen [5], Behzad Baradaran [3], Jiansong Fang [6 7], Erfan Ahmadi Ade [3], Akbar Sharifi [8], Zhigang Zhao [2], Qunying Han [1], Yong Zhang [9], Liming Zhang [10], Zhengwen Liu [11]

Affiliations

- [1] Department of Infectious Diseases, First Affiliated Hospital of Xi'an Jiaotong University, No. 277 Yanta West Road, Xi'an, 710061, Shaanxi Province, People's Republic of China.
- [2] Department of Pharmacy, Beijing Tiantan Hospital, Capital Medical University, Beijing, China.
- [3] Immunology Research Center, Tabriz University of Medical Sciences, Tabriz, Iran.
- [4] Department of Respiratory and Critical Care Medicine, Beijing Institute of Respiratory Medicine, Beijing Chaoyang Hospital, Capital Medical University, Beijing, People's Republic of China.
- [5] Department of Inflammation and Immunity, Cleveland Clinic, Cleveland, OH, USA.
- [6] Science and Technology Innovation Center, Guangzhou University of Chinese Medicine, Guangzhou, Guangdong Province, China.
- [7] DME Center, Institute of Clinical Pharmacology, Guangzhou University of Chinese Medicine, Guangzhou, Guangdong Province, People's Republic of China.
- [8] Tuberculosis and Lung Disease Research Center, Tabriz University of Medical Sciences, Tabriz, Iran.

- [9] Department of Hepatobiliary Surgery, Union Hospital, Tongji Medical College, Huazhong University of Science and Technology, Wuhan, People's Republic of China. mailzhangyong@126.com.
- [10] Department of Respiratory and Critical Care Medicine, Beijing Institute of Respiratory Medicine, Beijing Chaoyang Hospital, Capital Medical University, Beijing, People's Republic of China. zhangliming@bjcyh.com.
- [11] Department of Infectious Diseases, First Affiliated Hospital of Xi'an Jiaotong University, No. 277 Yanta West Road, Xi'an, 710061, Shaanxi Province, People's Republic of China. liuzhengwen113@xjtu.edu.cn.

[#] Contributed equally.

- PMID: **33789703**
- PMCID: PMC8011050
- DOI: 10.1186/s12985-021-01538-8

Abstract

Background: Risk scores are needed to predict the risk of death in severe coronavirus disease 2019 (COVID-19) patients in the context of rapid disease progression.

Methods: Using data from China (training dataset, n = 96), prediction models were developed by logistic regression and then risk scores were established. Leave-one-out cross validation was used for internal validation and data from Iran (test dataset, n = 43) was used for external validation.

Results: A NSL model (area under the curve (AUC) 0.932) and a NL model (AUC 0.903) were developed based on neutrophil percentage and lactate dehydrogenase with and without oxygen saturation (SaO_2) using the training dataset. AUCs of the NSL and NL models in the test dataset were 0.910 and 0.871, respectively. The risk scoring systems corresponding to these two models were established. The AUCs of the NSL and NL scores in the training dataset were 0.928 and 0.901, respectively. At the optimal cut-off value of NSL score, the sensitivity and specificity were 94% and 82%, respectively. The sensitivity and specificity of NL score were 94% and 75%, respectively.

Conclusions: These scores may be used to predict the risk of death in severe COVID-19 patients and the NL score could be used in regions where patients' SaO_2 cannot be tested.

Keywords: Hospital mortality; Prediction; SARS-CoV-2; Severe COVID-19.

Conflict of interest statement

The authors declare that they have no competing interests.

- 15 references
- 3 figures

Supplementary info

Publication types, MeSH terms, Substances Expand

Publication types

- Multicenter Study
- Observational Study

MeSH terms

- Aged
- COVID-19 / mortality*
- COVID-19 / therapy
- China
- Disease Progression
- Female
- Hospital Mortality*
- Humans
- Iran
- L-Lactate Dehydrogenase / blood*
- Male
- Middle Aged
- Models, Theoretical*
- Neutrophils / cytology*
- Oxygen / blood*
- Prognosis
- Retrospective Studies
- Risk Assessment

Substances

- L-Lactate Dehydrogenase
- Oxygen

Full text links

Read free full text at BMC BioMed Central Free PMC article

Proceed to details

Cite

Share

☐ 1,109

Observational Study

Circ Arrhythm Electrophysiol

. 2020 Oct;13(10):e009023.
doi: 10.1161/CIRCEP.120.009023. Epub 2020 Sep 15.

Electrocardiographic Changes and Arrhythmias in Hospitalized Patients With COVID-19

Melissa Y Y Moey [1], Prasanna M Sengodan [1], Neeraj Shah [1], Justin D McCallen [2], Oghenesuvwe Eboh [3], Rajasekhar Nekkanti [1], Blase A Carabello [1], Aditi R Naniwadekar [1]
Affiliations Expand

Affiliations

- [1] Department of Cardiovascular Disease (M.Y.Y.M., P.M.S., N.S., R.N., B.A.C., A.R.N.), Vidant Medical Center/East Carolina University.
- [2] Brody School of Medicine at East Carolina University, Greenville, NC (J.D.M.).
- [3] Department of Internal Medicine (O.E.), Vidant Medical Center/East Carolina University.

- PMID: **32931707**
- PMCID: PMC7566299
- DOI: 10.1161/CIRCEP.120.009023

Free PMC article
Observational Study

Electrocardiographic Changes and Arrhythmias in Hospitalized Patients With COVID-19

Melissa Y Y Moey et al. Circ Arrhythm Electrophysiol. 2020 Oct.
Free PMC article
Show details
Circ Arrhythm Electrophysiol
. 2020 Oct;13(10):e009023.
doi: 10.1161/CIRCEP.120.009023. Epub 2020 Sep 15.

Authors

Melissa Y Y Moey [1], Prasanna M Sengodan [1], Neeraj Shah [1], Justin D McCallen [2], Oghenesuvwe Eboh [3], Rajasekhar Nekkanti [1], Blase A Carabello [1], Aditi R Naniwadekar [1]

Affiliations

- [1] Department of Cardiovascular Disease (M.Y.Y.M., P.M.S., N.S., R.N., B.A.C., A.R.N.), Vidant Medical Center/East Carolina University.
- [2] Brody School of Medicine at East Carolina University, Greenville, NC (J.D.M.).
- [3] Department of Internal Medicine (O.E.), Vidant Medical Center/East Carolina University.

- PMID: **32931707**
- PMCID: PMC7566299
- DOI: 10.1161/CIRCEP.120.009023

No abstract available

Keywords: COVID-19; arrhythmia; hydroxychloroquine; incidence; tachycardia.

- 5 references

Supplementary info

Publication types, MeSH terms Expand

Publication types

- Letter
- Observational Study

MeSH terms

- Action Potentials
- Aged
- Arrhythmias, Cardiac / diagnosis*
- Arrhythmias, Cardiac / epidemiology
- Arrhythmias, Cardiac / physiopathology
- Arrhythmias, Cardiac / therapy
- COVID-19
- Coronavirus Infections / diagnosis*
- Coronavirus Infections / epidemiology
- Coronavirus Infections / physiopathology
- Coronavirus Infections / therapy
- Electrocardiography*
- Female
- Heart Conduction System / physiopathology*
- Heart Rate*
- Humans
- Incidence
- Male
- Middle Aged
- North Carolina / epidemiology
- Pandemics
- Patient Admission*
- Pneumonia, Viral / diagnosis*

- Pneumonia, Viral / epidemiology
- Pneumonia, Viral / physiopathology
- Pneumonia, Viral / therapy
- Predictive Value of Tests
- Retrospective Studies
- Risk Factors

Full text links

Atypon Free PMC article
Proceed to details
Cite
Share
1,110
Observational Study
Respir Med
. Nov-Dec 2021;189:106667.
doi: 10.1016/j.rmed.2021.106667. Epub 2021 Oct 28.

Impact of ketamine as an adjunct sedative in acute respiratory distress syndrome due to COVID-19 Pneumonia

Orlando Garner [1], Jonathan Patterson [2], Julieta Muñoz Mejia [3], Vijay Anand [3], Juan Deleija [4], Christopher Nemeh [4], Meghna Vallabh [2], Kristen A Staggers [5], Christopher M Howard [3], Sergio Enrique Treviño [3], Muhammad Asim Siddique [3], Christopher K Morgan [3]
Affiliations Expand

Affiliations

- [1] Baylor College of Medicine, Department of Medicine, Division of Pulmonary, Critical Care and Sleep Medicine, Baylor-St. Luke's Medical Center, 7200 Cambridge St. Suite 8A, Houston, TX, 77024, USA; Baylor St. Luke's Medical Center, Baylor-St. Luke's Medical Center, 7200 Cambridge St. Suite 8A, Houston, TX, 77024, USA. Electronic address: ogarnermd@gmail.com.
- [2] Baylor St. Luke's Medical Center, Baylor-St. Luke's Medical Center, 7200 Cambridge St. Suite 8A, Houston, TX, 77024, USA.
- [3] Baylor College of Medicine, Department of Medicine, Division of Pulmonary, Critical Care and Sleep Medicine, Baylor-St. Luke's Medical Center, 7200 Cambridge St. Suite 8A, Houston, TX, 77024, USA; Baylor St. Luke's Medical Center, Baylor-St. Luke's Medical Center, 7200 Cambridge St. Suite 8A, Houston, TX, 77024, USA.
- [4] Baylor College of Medicine, Department of Medicine, Division of Internal Medicine, Baylor-St. Luke's Medical Center, 7200 Cambridge St. Suite 8A, Houston, TX, 77024, USA.

- [5] Baylor College of Medicine, Institute for Clinical & Translational Research, Baylor-St. Luke's Medical Center, 7200 Cambridge St. Suite 8A, Houston, TX, 77024, USA.

- PMID: **34757277**
- PMCID: PMC8552750
- DOI: 10.1016/j.rmed.2021.106667

Free PMC article
Observational Study

Impact of ketamine as an adjunct sedative in acute respiratory distress syndrome due to COVID-19 Pneumonia

Orlando Garner et al. Respir Med. Nov-Dec 2021.
Free PMC article

Show details

Respir Med
. Nov-Dec 2021;189:106667.
doi: 10.1016/j.rmed.2021.106667. Epub 2021 Oct 28.

Authors

Orlando Garner [1], Jonathan Patterson [2], Julieta Muñoz Mejia [3], Vijay Anand [3], Juan Deleija [4], Christopher Nemeh [4], Meghna Vallabh [2], Kristen A Staggers [5], Christopher M Howard [3], Sergio Enrique Treviño [3], Muhammad Asim Siddique [3], Christopher K Morgan [3]

Affiliations

- [1] Baylor College of Medicine, Department of Medicine, Division of Pulmonary, Critical Care and Sleep Medicine, Baylor-St. Luke's Medical Center, 7200 Cambridge St. Suite 8A, Houston, TX, 77024, USA; Baylor St. Luke's Medical Center, Baylor-St. Luke's Medical Center, 7200 Cambridge St. Suite 8A, Houston, TX, 77024, USA. Electronic address: ogarnermd@gmail.com.
- [2] Baylor St. Luke's Medical Center, Baylor-St. Luke's Medical Center, 7200 Cambridge St. Suite 8A, Houston, TX, 77024, USA.
- [3] Baylor College of Medicine, Department of Medicine, Division of Pulmonary, Critical Care and Sleep Medicine, Baylor-St. Luke's Medical Center, 7200 Cambridge St. Suite 8A, Houston, TX, 77024, USA; Baylor St. Luke's Medical Center, Baylor-St. Luke's Medical Center, 7200 Cambridge St. Suite 8A, Houston, TX, 77024, USA.
- [4] Baylor College of Medicine, Department of Medicine, Division of Internal Medicine, Baylor-St. Luke's Medical Center, 7200 Cambridge St. Suite 8A, Houston, TX, 77024, USA.
- [5] Baylor College of Medicine, Institute for Clinical & Translational Research, Baylor-St. Luke's Medical Center, 7200 Cambridge St. Suite 8A, Houston, TX, 77024, USA.

- PMID: **34757277**

- PMCID: PMC8552750
- DOI: 10.1016/j.rmed.2021.106667

Abstract

Purpose: Deep sedation is sometimes needed in acute respiratory distress syndrome. Ketamine is a sedative that has been shown to have analgesic and sedating properties without having a detrimental impact on hemodynamics. This pharmacological profile makes ketamine an attractive sedative, potentially reducing the necessity for other sedatives and vasopressors, but there are no studies evaluating its effect on these medications in patients requiring deep sedation for acute respiratory distress syndrome.

Materials and methods: This is a retrospective, observational study in a single center, quaternary care hospital in southeast Texas. We looked at adults with COVID-19 requiring mechanical ventilation from March 2020 to September 2020.

Results: We found that patients had less propofol requirements at 72 h after ketamine initiation when compared to 24 h (median 34.2 vs 54.7 mg/kg, p = 0.003). Norepinephrine equivalents were also significantly lower at 48 h than 24 h after ketamine initiation (median 38 vs 62.8 mcg/kg, p = 0.028). There was an increase in hydromorphone infusion rates at all three time points after ketamine was introduced.

Conclusions: In this cohort of patients with COVID-19 ARDS who required mechanical ventilation receiving ketamine we found propofol sparing effects and vasopressor requirements were reduced, while opioid infusions were not.

Keywords: ARDS; COVID-19; Critical care; Ketamine; Mechanical ventilation; Sedation; Vasopressor.

Copyright © 2021 Elsevier Ltd. All rights reserved.

Conflict of interest statement

The authors have no conflict of interests to declare with regards to this submission.

- 19 references
- 2 figures

Supplementary info

Publication types, MeSH terms, Substances Expand

Publication types

- Observational Study

MeSH terms

- Adult
- Aged

- Aged, 80 and over
- Analgesics, Opioid / therapeutic use
- COVID-19 / epidemiology*
- COVID-19 / therapy
- Deep Sedation*
- Drug Utilization / statistics & numerical data
- Female
- Humans
- Hydromorphone / therapeutic use
- Hypnotics and Sedatives / administration & dosage*
- Ketamine / administration & dosage*
- Male
- Middle Aged
- Norepinephrine / therapeutic use
- Propofol / therapeutic use
- Respiration, Artificial*
- Respiratory Distress Syndrome / epidemiology*
- Respiratory Distress Syndrome / therapy
- Retrospective Studies
- Texas / epidemiology

Substances

- Analgesics, Opioid
- Hypnotics and Sedatives
- Ketamine
- Hydromorphone
- Norepinephrine
- Propofol

Full text links

ELSEVIER FULL-TEXT ARTICLE Elsevier Science Free PMC article
Proceed to details
Cite
Share
☐ 1,111
Observational Study
World Neurosurg
. 2020 Dec;144:e380-e388.
doi: 10.1016/j.wneu.2020.08.168. Epub 2020 Sep 3.

COVID-19 Impact on Neurosurgical Practice: Lockdown Attitude and Experience of a European Academic Center

Alphonse Lubansu [1], Mouhssine Assamadi [2], Sami Barrit [3], Victoria Dembour [4], Gedeon Yao [5], Salim El Hadwe [3], Olivier De Witte [3]

Affiliations Expand

Affiliations

- [1] Department of Neurosurgery, Erasme Hospital, Université Libre de Bruxelles, Belgium. Electronic address: alphonse.lubansu@erasme.ulb.ac.be.
- [2] Department of Neurosurgery, Erasme Hospital, Université Libre de Bruxelles, Belgium; Department of Neurosurgery, Ibn Tofail Hospital, Université Cadi Ayyad, CHU Mohammed Sixth, Marrakech, Morocco.
- [3] Department of Neurosurgery, Erasme Hospital, Université Libre de Bruxelles, Belgium.
- [4] Department of Neurosurgery, Delta Hospital, CHIREC, Brussels, Belgium.
- [5] Department of Neurosurgery, Erasme Hospital, Université Libre de Bruxelles, Belgium; Department of Neurosurgery, Hospital University of Yopougon, Abidjan, Ivory Coast.

- PMID: **32891850**
- PMCID: PMC7470722
- DOI: 10.1016/j.wneu.2020.08.168

Free PMC article
Observational Study

COVID-19 Impact on Neurosurgical Practice: Lockdown Attitude and Experience of a European Academic Center

Alphonse Lubansu et al. World Neurosurg. 2020 Dec.
Free PMC article
Show details
World Neurosurg
. 2020 Dec;144:e380-e388.
doi: 10.1016/j.wneu.2020.08.168. Epub 2020 Sep 3.

Authors

Alphonse Lubansu [1], Mouhssine Assamadi [2], Sami Barrit [3], Victoria Dembour [4], Gedeon Yao [5], Salim El Hadwe [3], Olivier De Witte [3]

Affiliations

- [1] Department of Neurosurgery, Erasme Hospital, Université Libre de Bruxelles, Belgium. Electronic address: alphonse.lubansu@erasme.ulb.ac.be.
- [2] Department of Neurosurgery, Erasme Hospital, Université Libre de Bruxelles, Belgium; Department of Neurosurgery, Ibn Tofail Hospital, Université Cadi Ayyad, CHU Mohammed Sixth, Marrakech, Morocco.
- [3] Department of Neurosurgery, Erasme Hospital, Université Libre de Bruxelles, Belgium.
- [4] Department of Neurosurgery, Delta Hospital, CHIREC, Brussels, Belgium.
- [5] Department of Neurosurgery, Erasme Hospital, Université Libre de Bruxelles, Belgium; Department of Neurosurgery, Hospital University of Yopougon, Abidjan, Ivory Coast.

- PMID: **32891850**
- PMCID: PMC7470722
- DOI: 10.1016/j.wneu.2020.08.168

Abstract

Background: The coronavirus disease 2019 (COVID-19) pandemic is an unprecedented challenge. Different models of reorganization have been described aiming to preserve resources and ensure optimal medical care. Limited clinical neurosurgical experience with patients with COVID-19 has been reported. We share organizational experience, attitudes, and preliminary data of patients treated at our institution.

Methods: Institutional guidelines and patient workflow are described and visualized. A cohort of all neurosurgical patients managed during the lockdown period is presented and analyzed, assessing suspected nosocomial infection risk factors. A comparative surgical subcohort from the previous year was used to investigate the impact on surgical activity.

Results: A total of 176 patients were admitted in 66 days, 20 of whom tested positive for COVID-19. Patients initially admitted to the neurosurgical ward were less likely to be suspected for a COVID-19 infection compared with patients admitted for critical emergencies, particularly with neurovascular and stroke-related diseases. The mortality of patients with COVID-19 was remarkably high (45%), and even higher in patients who underwent surgical intervention (77%). In addition to the expected decrease in surgical activity (-53%), a decrease in traumatic emergencies was noted.

Conclusions: By applying infection prevention and resource-sparing logistics measures shared by the international medical community, we were able to maintain essential neurosurgical care in a pandemic with controlled nosocomial infection risk. Special consideration should be given to medical management and surgical indications in patients infected with severe acute respiratory syndrome coronavirus 2, because they seem to show a problematic hemostatic profile that might result in an unfavorable clinical and surgical outcome.

Keywords: COVID-19; Lockdown; Neurosurgery; SARS-CoV-2.

Copyright © 2020 Elsevier Inc. All rights reserved.

Comment in

- Letter to the Editor Regarding "COVID-19 Impact on Neurosurgical Practice: Lockdown Attitude and Experience of a European Academic Center".

Goyal N, Gupta K. Goyal N, et al. World Neurosurg. 2021 Apr;148:221-223. doi: 10.1016/j.wneu.2020.09.117. Epub 2020 Sep 28. World Neurosurg. 2021. PMID: 32992061 Free PMC article. No abstract available.

- In Reply to the Letter to the Editor Regarding "COVID-19 Impact on Neurosurgical Practice: Lockdown Attitude and Experience of a European Academic Center".
  Lubansu A, El Hadwe S. Lubansu A, et al. World Neurosurg. 2021 Apr;148:224. doi: 10.1016/j.wneu.2020.10.143. Epub 2020 Nov 2. World Neurosurg. 2021. PMID: 33144209 Free PMC article. No abstract available.

- 23 references
- 3 figures

Supplementary info

Publication types, MeSH terms Expand

Publication types

- Observational Study

MeSH terms

- Academic Medical Centers*
- Acute Disease
- Adolescent
- Adult
- Aged
- Aged, 80 and over
- Belgium / epidemiology
- COVID-19 / complications
- COVID-19 / epidemiology
- COVID-19 / prevention & control*
- COVID-19 Testing
- Central Nervous System Diseases / surgery*
- Cerebrovascular Disorders / complications
- Cerebrovascular Disorders / surgery
- Child
- Child, Preschool
- Craniocerebral Trauma / surgery
- Cross Infection / prevention & control*
- Decompressive Craniectomy
- Elective Surgical Procedures
- Emergencies
- Female
- Humans

- Hydrocephalus / surgery
- Infant
- Infant, Newborn
- Infection Control
- Intracranial Hemorrhages / surgery
- Male
- Mass Screening
- Middle Aged
- Neuroendoscopy
- Neurosurgery*
- Neurosurgical Procedures / methods
- Neurosurgical Procedures / statistics & numerical data
- Organizational Policy*
- Personal Protective Equipment
- Personnel Staffing and Scheduling
- Retrospective Studies
- Spinal Injuries / surgery
- Telemedicine
- Thrombectomy
- Vascular Surgical Procedures
- Workflow*
- Young Adult

Full text links

ELSEVIER FULL-TEXT ARTICLE Elsevier Science Free PMC article
Proceed to details
Cite
Share
☐ 1,112
Observational Study
Lancet Respir Med
. 2020 May;8(5):475-481.
doi: 10.1016/S2213-2600(20)30079-5. Epub 2020 Feb 24.

Clinical course and outcomes of critically ill patients with SARS-CoV-2 pneumonia in Wuhan, China: a single-centered, retrospective, observational study

Xiaobo Yang[1], Yuan Yu[2], Jiqian Xu[2], Huaqing Shu[2], Jia'an Xia[3], Hong Liu[1], Yongran Wu[2], Lu Zhang[4], Zhui Yu[5], Minghao Fang[6], Ting Yu[3], Yaxin Wang[2], Shangwen Pan[2], Xiaojing Zou[2], Shiying Yuan[2], You Shang[7]
Affiliations Expand

Affiliations

- [1] Department of Critical Care Medicine, Tongji Medical College, Huazhong University of Science and Technology, Wuhan, China; Institute of Anesthesiology and Critical Care Medicine, Tongji Medical College, Huazhong University of Science and Technology, Wuhan, China; Jin Yin-tan Hospital, Wuhan, China.
- [2] Department of Critical Care Medicine, Tongji Medical College, Huazhong University of Science and Technology, Wuhan, China; Institute of Anesthesiology and Critical Care Medicine, Tongji Medical College, Huazhong University of Science and Technology, Wuhan, China.
- [3] Jin Yin-tan Hospital, Wuhan, China.
- [4] Department of Critical Care Medicine, Xiangyang Central Hospital, Affiliated Hospital of Hubei University of Arts and Science, Hubei, China.
- [5] Department of Critical Care Medicine, Renmin Hospital of Wuhan University, Wuhan, China.
- [6] Union Hospital, and Department of Critical Care Medicine, Tongji Hospital, Tongji Medical College, Huazhong University of Science and Technology, Wuhan, China.
- [7] Department of Critical Care Medicine, Tongji Medical College, Huazhong University of Science and Technology, Wuhan, China; Institute of Anesthesiology and Critical Care Medicine, Tongji Medical College, Huazhong University of Science and Technology, Wuhan, China; Jin Yin-tan Hospital, Wuhan, China. Electronic address: you_shang@yahoo.com.

- PMID: **32105632**
- PMCID: PMC7102538
- DOI: 10.1016/S2213-2600(20)30079-5

Free PMC article
Observational Study

Clinical course and outcomes of critically ill patients with SARS-CoV-2 pneumonia in Wuhan, China: a single-centered, retrospective, observational study

Xiaobo Yang et al. Lancet Respir Med. 2020 May.
Free PMC article
Show details
Lancet Respir Med
. 2020 May;8(5):475-481.
doi: 10.1016/S2213-2600(20)30079-5. Epub 2020 Feb 24.

Authors

Xiaobo Yang [1], Yuan Yu [2], Jiqian Xu [2], Huaqing Shu [2], Jia'an Xia [3], Hong Liu [1], Yongran Wu [2], Lu Zhang [4], Zhui Yu [5], Minghao Fang [6], Ting Yu [3], Yaxin Wang [2], Shangwen Pan [2], Xiaojing Zou [2], Shiying Yuan [2], You Shang [7]

Affiliations

- [1] Department of Critical Care Medicine, Tongji Medical College, Huazhong University of Science and Technology, Wuhan, China; Institute of Anesthesiology and Critical Care Medicine, Tongji Medical College, Huazhong University of Science and Technology, Wuhan, China; Jin Yin-tan Hospital, Wuhan, China.
- [2] Department of Critical Care Medicine, Tongji Medical College, Huazhong University of Science and Technology, Wuhan, China; Institute of Anesthesiology and Critical Care Medicine, Tongji Medical College, Huazhong University of Science and Technology, Wuhan, China.
- [3] Jin Yin-tan Hospital, Wuhan, China.
- [4] Department of Critical Care Medicine, Xiangyang Central Hospital, Affiliated Hospital of Hubei University of Arts and Science, Hubei, China.
- [5] Department of Critical Care Medicine, Renmin Hospital of Wuhan University, Wuhan, China.
- [6] Union Hospital, and Department of Critical Care Medicine, Tongji Hospital, Tongji Medical College, Huazhong University of Science and Technology, Wuhan, China.
- [7] Department of Critical Care Medicine, Tongji Medical College, Huazhong University of Science and Technology, Wuhan, China; Institute of Anesthesiology and Critical Care Medicine, Tongji Medical College, Huazhong University of Science and Technology, Wuhan, China; Jin Yin-tan Hospital, Wuhan, China. Electronic address: you_shang@yahoo.com.

- PMID: **32105632**
- PMCID: PMC7102538
- DOI: 10.1016/S2213-2600(20)30079-5

Erratum in

- Correction to Lancet Respir Med 2020; published online Feb 21. https://doi.org/10.1016/S2213-2600(20)30079-5.
  [No authors listed] [No authors listed] Lancet Respir Med. 2020 Apr;8(4):e26. doi: 10.1016/S2213-2600(20)30103-X. Epub 2020 Feb 28. Lancet Respir Med. 2020. PMID: 32119827 Free PMC article. No abstract available.

Abstract

Background: An ongoing outbreak of pneumonia associated with the severe acute respiratory coronavirus 2 (SARS-CoV-2) started in December, 2019, in Wuhan, China. Information about critically ill patients with SARS-CoV-2 infection is scarce. We aimed to describe the clinical course and outcomes of critically ill patients with SARS-CoV-2 pneumonia.

Methods: In this single-centered, retrospective, observational study, we enrolled 52 critically ill adult patients with SARS-CoV-2 pneumonia who were admitted to the intensive care unit (ICU)

of Wuhan Jin Yin-tan hospital (Wuhan, China) between late December, 2019, and Jan 26, 2020. Demographic data, symptoms, laboratory values, comorbidities, treatments, and clinical outcomes were all collected. Data were compared between survivors and non-survivors. The primary outcome was 28-day mortality, as of Feb 9, 2020. Secondary outcomes included incidence of SARS-CoV-2-related acute respiratory distress syndrome (ARDS) and the proportion of patients requiring mechanical ventilation.

Findings: Of 710 patients with SARS-CoV-2 pneumonia, 52 critically ill adult patients were included. The mean age of the 52 patients was 59·7 (SD 13·3) years, 35 (67%) were men, 21 (40%) had chronic illness, 51 (98%) had fever. 32 (61·5%) patients had died at 28 days, and the median duration from admission to the intensive care unit (ICU) to death was 7 (IQR 3-11) days for non-survivors. Compared with survivors, non-survivors were older (64·6 years [11·2] vs 51·9 years [12·9]), more likely to develop ARDS (26 [81%] patients vs 9 [45%] patients), and more likely to receive mechanical ventilation (30 [94%] patients vs 7 [35%] patients), either invasively or non-invasively. Most patients had organ function damage, including 35 (67%) with ARDS, 15 (29%) with acute kidney injury, 12 (23%) with cardiac injury, 15 (29%) with liver dysfunction, and one (2%) with pneumothorax. 37 (71%) patients required mechanical ventilation. Hospital-acquired infection occurred in seven (13·5%) patients.

Interpretation: The mortality of critically ill patients with SARS-CoV-2 pneumonia is considerable. The survival time of the non-survivors is likely to be within 1-2 weeks after ICU admission. Older patients (>65 years) with comorbidities and ARDS are at increased risk of death. The severity of SARS-CoV-2 pneumonia poses great strain on critical care resources in hospitals, especially if they are not adequately staffed or resourced.

Funding: None.

Copyright © 2020 Elsevier Ltd. All rights reserved.

Comment in

- Coronavirus Disease 2019 (COVID-19): A critical care perspective beyond China.
  Rello J, Tejada S, Userovici C, Arvaniti K, Pugin J, Waterer G. Rello J, et al. Anaesth Crit Care Pain Med. 2020 Apr;39(2):167-169. doi: 10.1016/j.accpm.2020.03.001. Epub 2020 Mar 3. Anaesth Crit Care Pain Med. 2020. PMID: 32142972 Free PMC article. No abstract available.
- Respiratory support for patients with COVID-19 infection.
  Ñamendys-Silva SA. Ñamendys-Silva SA. Lancet Respir Med. 2020 Apr;8(4):e18. doi: 10.1016/S2213-2600(20)30110-7. Epub 2020 Mar 5. Lancet Respir Med. 2020. PMID: 32145829 Free PMC article. No abstract available.
- Are patients with hypertension and diabetes mellitus at increased risk for COVID-19 infection?
  Fang L, Karakiulakis G, Roth M. Fang L, et al. Lancet Respir Med. 2020 Apr;8(4):e21. doi: 10.1016/S2213-2600(20)30116-8. Epub 2020 Mar 11. Lancet Respir Med. 2020. PMID: 32171062 Free PMC article. No abstract available.
- COVID-19, ECMO, and lymphopenia: a word of caution.
  Henry BM. Henry BM. Lancet Respir Med. 2020 Apr;8(4):e24. doi: 10.1016/S2213-2600(20)30119-3. Epub 2020 Mar 13. Lancet Respir Med. 2020. PMID: 32178774 Free PMC article. No abstract available.
- End-Stage Heart Failure With COVID-19: Strong Evidence of Myocardial Injury by 2019-nCoV.

Dong N, Cai J, Zhou Y, Liu J, Li F. Dong N, et al. JACC Heart Fail. 2020 Jun;8(6):515-517. doi: 10.1016/j.jchf.2020.04.001. Epub 2020 Apr 7. JACC Heart Fail. 2020. PMID: 32265149 Free PMC article. No abstract available.
- Coronavirus disease 2019 outbreak: is liver disease a prognostic tool?
Testino G, Fagoonee S. Testino G, et al. Panminerva Med. 2021 Dec;63(4):553-554. doi: 10.23736/S0031-0808.20.03947-6. Epub 2020 May 14. Panminerva Med. 2021. PMID: 32414233 No abstract available.

- 25 references
- 2 figures

Supplementary info

Publication types, MeSH terms Expand

Publication types

- Observational Study

MeSH terms

- Adult
- Aged
- Aged, 80 and over
- Betacoronavirus / isolation & purification
- COVID-19
- China / epidemiology
- Coronavirus Infections / epidemiology
- Coronavirus Infections / mortality*
- Coronavirus Infections / therapy*
- Critical Illness
- Female
- Humans
- Intensive Care Units / statistics & numerical data
- Male
- Middle Aged
- Pandemics
- Pneumonia, Viral / epidemiology
- Pneumonia, Viral / mortality*
- Pneumonia, Viral / therapy*
- Pneumonia, Viral / virology
- Respiration, Artificial / statistics & numerical data
- Respiratory Distress Syndrome / mortality
- Respiratory Distress Syndrome / therapy

- Respiratory Distress Syndrome / virology
- Retrospective Studies
- SARS-CoV-2
- Treatment Outcome

Full text links

ELSEVIER FULL-TEXT ARTICLE Elsevier Science Free PMC article
Proceed to details
Cite
Share
1,113
Observational Study
Cir Pediatr
. 2021 Jan 1;34(1):3-8.

Acute abdomen in COVID-19 disease: the pediatric surgeon's standpoint

[Article in English, Spanish]
C Rico Espiñeira [1], H Souto Romero [1], R Espinosa Góngora [1], M L Espinoza Vega [1], J L Alonso Calderón [1]
Affiliations Expand

Affiliation

- [1] Pediatric Surgery Department. Niño Jesús Pediatric University Hospital. Madrid (Spain).

- PMID: **33507637**

Free article
Observational Study

Acute abdomen in COVID-19 disease: the pediatric surgeon's standpoint

[Article in English, Spanish]
C Rico Espiñeira et al. Cir Pediatr. 2021.
Free article
Show details
Cir Pediatr
. 2021 Jan 1;34(1):3-8.

Authors

C Rico Espiñeira [1], H Souto Romero [1], R Espinosa Góngora [1], M L Espinoza Vega [1], J L Alonso Calderón [1]

Affiliation

- [1] Pediatric Surgery Department. Niño Jesús Pediatric University Hospital. Madrid (Spain).

- PMID: **33507637**

Abstract
in English, Spanish

Objective: To describe our experience in the diagnostic and therapeutic management of patients with acute abdomen as the main manifestation of SARS-CoV-2 infection.

Material and methods: A descriptive study of patients with clinical signs of acute abdomen diagnosed with COVID-19 and admitted at out healthcare facility from April 1 to May 10, 2020 was carried out. Clinical records were reviewed for data collection purposes.

Results: A series of 14 patients (9 male and 5 female) with a median age of 9.5 years was analyzed. All patients had abdominal pain. There were 11 patients with fever, 9 patients with vomit or diarrhea, and 9 patients with clinically suspected surgical pathology (acute appendicitis or peritonitis). Increased acute phase reactants and coagulation disorders were a common characteristic at blood tests. An abdominal ultrasonography was carried out in all patients, and a CT-scan was performed in 4 patients, which demonstrated inflammatory signs in the terminal ileum, the ileocecal valve and the ascending colon, as well as gallbladder edema. Conservative management was decided upon in all patients except one, and eight patients required intensive care admission for support treatment.

Conclusions: Gastrointestinal symptoms can be the primary manifestation of the new coronavirus infection, which simulates an acute abdomen with a potentially unfavorable evolution. For an accurate diagnosis to be achieved, a good clinical record and a comprehensive physical exploration, as well as complementary tests in search of characteristic findings of COVID-19, should be carried out.

Objetivos: Describir nuestra experiencia en el manejo diagnóstico y terapéutico de los pacientes que han presentado abdomen agudo como principal manifestación de la infección por SARS-Cov-2.

Material y metodos: Estudio descriptivo de los pacientes ingresados con clínica inicial de abdomen agudo que fueron diagnosticados de COVID-19 entre el 1 de abril y el 10 de mayo de 2020. Se ha realizado la revisión de historias clínicas para la recogida de datos.

Resultados: Describimos una serie de 14 pacientes (9 varones y 5 mujeres) con una mediana de edad de 9,5 años. Todos ellos consultaron por dolor abdominal acompañado de fiebre en 11 y vómitos o diarrea en 9, y la sospecha clínica inicial fue de patología quirúrgica (apendicitis aguda o peritonitis) en 9. En la analítica sanguínea se encontró como característica común elevación de reactantes de fase aguda y alteraciones de coagulación. Se realizó ecografía abdominal a todos los pacientes y tomografía computarizada en cuatro observándose signos inflamatorios en íleon terminal, válvula ileocecal, colon ascendente y edema de vesícula biliar. Se optó por un manejo

conservador en todos los pacientes menos uno y ocho pacientes precisaron ingreso en cuidados intensivos para tratamiento de soporte.

Conclusiones: La infección por el nuevo coronavirus puede producir síntomas gastrointestinales como principal manifestación, simulando un abdomen agudo que en algunos casos puede evolucionar de forma desfavorable. Para el diagnóstico es preciso realizar una buena historia clínica y exploración física, así como pruebas complementarias en busca de hallazgos característicos de COVID-19.

Keywords: Abdominal pain; Acute abdomen; COVID-19; Childhood; Pediatric surgery.

Supplementary info

Publication types, MeSH terms Expand

Publication types

- Observational Study

MeSH terms

- Abdomen, Acute / diagnosis*
- Abdomen, Acute / surgery
- Abdomen, Acute / virology
- Abdominal Pain / etiology*
- Abdominal Pain / virology
- Adolescent
- Appendicitis / diagnosis
- COVID-19 / complications
- COVID-19 / diagnosis*
- COVID-19 Testing*
- Child
- Child, Preschool
- Diarrhea / epidemiology
- Diarrhea / etiology
- Female
- Fever / epidemiology
- Fever / etiology
- Humans
- Intensive Care Units / statistics & numerical data
- Male
- Peritonitis / diagnosis
- Retrospective Studies
- Vomiting / epidemiology

- Vomiting / etiology

Full text links

Ergon
Proceed to details
Cite
Share
1,114
Observational Study
Emergencias
. 2020 Nov;32(6):435-436.

Experience of an emergency surgery department during the pandemic caused by SARS-CoV-2 infection

[Article in Spanish, English]
Xavier Morales [1], Víctor Turrado [1], Borja de Lacy [1], Amelia Hessheimer [1], Constantino Fondevila [1], Antonio M de Lacy [1]
Affiliations Expand

Affiliation

- [1] Servicio de Cirugía Gastrointestinal, Servicio de Cirugía Hepatobiliopancreática, Hospital Clínic, Barcelona, España.

- PMID: **33275368**

Free article
Observational Study

Experience of an emergency surgery department during the pandemic caused by SARS-CoV-2 infection

[Article in Spanish, English]
Xavier Morales et al. Emergencias. 2020 Nov.
Free article
Show details
Emergencias
. 2020 Nov;32(6):435-436.

Authors

Xavier Morales [1], Víctor Turrado [1], Borja de Lacy [1], Amelia Hessheimer [1], Constantino Fondevila [1], Antonio M de Lacy [1]

Affiliation

- [1] Servicio de Cirugía Gastrointestinal, Servicio de Cirugía Hepatobiliopancreática, Hospital Clínic, Barcelona, España.

- PMID: **33275368**

No abstract available

Supplementary info

Publication types, MeSH terms Expand

Publication types

- Comparative Study
- Observational Study

MeSH terms

- Adult
- Aged
- COVID-19* / epidemiology
- COVID-19* / prevention & control
- Emergencies
- Emergency Service, Hospital / trends*
- Facilities and Services Utilization / trends*
- Female
- Health Services Accessibility / trends*
- Humans
- Male
- Middle Aged
- Pandemics
- Patient Acceptance of Health Care / statistics & numerical data*
- Retrospective Studies
- Spain / epidemiology
- Surgery Department, Hospital / trends*
- Surgical Procedures, Operative / trends*

Full text links

[EMERGENCIAS] Grupo Saned

Proceed to details

Cite

Share

☐ 1,115

Observational Study

Stroke

. 2020 Sep;51(9):2656-2663.

doi: 10.1161/STROKEAHA.120.030397. Epub 2020 Jul 31.

Emergent Large Vessel Occlusion Stroke During New York City's COVID-19 Outbreak: Clinical Characteristics and Paraclinical Findings

Shahram Majidi [1], Johanna T Fifi [1], Travis R Ladner [1], Jacques Lara-Reyna [1], Kurt A Yaeger [1], Benjamin Yim [1], Neha Dangayach [1], Thomas J Oxley [1], Tomoyoshi Shigematsu [1], Benjamin R Kummer [2], Laura K Stein [2], Jesse Weinberger [2], Michael G Fara [2], Reade De Leacy [1], Mandip S Dhamoon [2], Stanley Tuhrim [2], J Mocco [1]

Affiliations Expand

Affiliations

- [1] Department of Neurosurgery (S.M., J.T.F., T.R.L., J.L.-R., K.A.Y., B.Y., N.D., T.J.O., T.S., R.D.L., J.M.), Icahn School of Medicine at Mount Sinai, New York, NY.
- [2] Department of Neurology (B.R.K., L.K.S., J.W., M.G.F., M.S.D., S.T.), Icahn School of Medicine at Mount Sinai, New York, NY.

- PMID: **32755349**
- PMCID: PMC7434004
- DOI: 10.1161/STROKEAHA.120.030397

Free PMC article

Observational Study

Emergent Large Vessel Occlusion Stroke During New York City's COVID-19 Outbreak: Clinical Characteristics and Paraclinical Findings

Shahram Majidi et al. Stroke. 2020 Sep.
Free PMC article
Show details
Stroke
. 2020 Sep;51(9):2656-2663.
doi: 10.1161/STROKEAHA.120.030397. Epub 2020 Jul 31.

Authors

Shahram Majidi [1], Johanna T Fifi [1], Travis R Ladner [1], Jacques Lara-Reyna [1], Kurt A Yaeger [1], Benjamin Yim [1], Neha Dangayach [1], Thomas J Oxley [1], Tomoyoshi Shigematsu [1], Benjamin R Kummer [2], Laura K Stein [2], Jesse Weinberger [2], Michael G Fara [2], Reade De Leacy [1], Mandip S Dhamoon [2], Stanley Tuhrim [2], J Mocco [1]

Affiliations

- [1] Department of Neurosurgery (S.M., J.T.F., T.R.L., J.L.-R., K.A.Y., B.Y., N.D., T.J.O., T.S., R.D.L., J.M.), Icahn School of Medicine at Mount Sinai, New York, NY.
- [2] Department of Neurology (B.R.K., L.K.S., J.W., M.G.F., M.S.D., S.T.), Icahn School of Medicine at Mount Sinai, New York, NY.

- PMID: **32755349**
- PMCID: PMC7434004
- DOI: 10.1161/STROKEAHA.120.030397

Abstract

Background and purpose: The 2019 novel coronavirus outbreak and its associated disease (coronavirus disease 2019 [COVID-19]) have created a worldwide pandemic. Early data suggest higher rate of ischemic stroke in severe COVID-19 infection. We evaluated whether a relationship exists between emergent large vessel occlusion (ELVO) and the ongoing COVID-19 outbreak.

Methods: This is a retrospective, observational case series. Data were collected from all patients who presented with ELVO to the Mount Sinai Health System Hospitals across New York City during the peak 3 weeks of hospitalization and death from COVID-19. Patients' demographic, comorbid conditions, cardiovascular risk factors, COVID-19 disease status, and clinical presentation were extracted from the electronic medical record. Comparison was made between COVID-19 positive and negative cohorts. The incidence of ELVO stroke was compared with the pre-COVID period.

Results: Forty-five consecutive ELVO patients presented during the observation period. Fifty-three percent of patients tested positive for COVID-19. Total patients' mean (±SD) age was 66 (±17). Patients with COVID-19 were significantly younger than patients without COVID-19, 59±13 versus 74±17 (odds ratio [95% CI], 0.94 [0.81-0.98]; *P*=0.004). Seventy-five percent of patients with COVID-19 were male compared with 43% of patients without COVID-19 (odds ratio [95% CI], 3.99 [1.12-14.17]; *P*=0.032). Patients with COVID-19 were less likely to be White (8% versus 38% [odds ratio (95% CI), 0.15 (0.04-0.81); *P*=0.027]). In comparison to a similar time duration before the COVID-19 outbreak, a 2-fold increase in the total number of ELVO was observed (estimate: 0.78 [95% CI, 0.47-1.08], *P*≤0.0001).

Conclusions: More than half of the ELVO stroke patients during the peak time of the New York City's COVID-19 outbreak were COVID-19 positive, and those patients with COVID-19 were younger, more likely to be male, and less likely to be White. Our findings also suggest an increase in the incidence of ELVO stroke during the peak of the COVID-19 outbreak.

Keywords: acute stroke; coronavirus disease; hospitalization; incidence; pandemics.

Conflict of interest statement

Dr Mocco has received research grants from Stryker and Penumbara and Microvention. He is a consultant to Endostream, Viseon, Imperative Care, RIST, Synchron, Viz.ai, Perflow, CVAid, and Cerebrotech. He is investor/Stockholder at Rebound, BlinkTBI, Endostream, Viseon, Imperative Care, Serenity, Cardinal Consulting, NTI, RIST, Synchron, Viz.ai, and Cerebrotech. Dr Oxley has received personal fees from Imperative Care outside the submitted work. The other authors report no conflicts.

- 19 references
- 3 figures

Supplementary info

Publication types, MeSH terms Expand

Publication types

- Observational Study

MeSH terms

- Age Factors
- Aged
- Aged, 80 and over
- Arterial Occlusive Diseases / complications
- Arterial Occlusive Diseases / epidemiology*
- Blacks / statistics & numerical data
- Brain Ischemia / complications
- Brain Ischemia / epidemiology*
- COVID-19

- Coronavirus Infections / complications
- Coronavirus Infections / epidemiology*
- Electronic Health Records
- Female
- Hospitalization
- Humans
- Incidence
- Male
- Middle Aged
- New York City
- Pandemics
- Pneumonia, Viral / complications
- Pneumonia, Viral / epidemiology*
- Retrospective Studies
- Risk Factors
- Sex Factors
- Stroke / complications
- Stroke / epidemiology*
- Whites / statistics & numerical data

Full text links

FREE FULL TEXT Atypon Free PMC article
Proceed to details
Cite
Share
1,116

Observational Study

Br J Surg
. 2020 Nov;107(12):e605-e606.
doi: 10.1002/bjs.12058. Epub 2020 Sep 14.

Appendicitis and the COVID pandemic; new challenges in the management of a familiar foe

C Toale [1], D Westby [1], M O'Callaghan [1], D Nally [1], P Burke [1], C Peirce [1], J C Coffey [1], R M Cunningham [1]
Affiliations Expand

Affiliation

- [1] Department of Surgery, University of Limerick Hospital Group, Ireland.

- PMID: **32924149**
- DOI: 10.1002/bjs.12058

Observational Study

Appendicitis and the COVID pandemic; new challenges in the management of a familiar foe

C Toale et al. Br J Surg. 2020 Nov.
Show details
Br J Surg
. 2020 Nov;107(12):e605-e606.
doi: 10.1002/bjs.12058. Epub 2020 Sep 14.

Authors

C Toale [1], D Westby [1], M O'Callaghan [1], D Nally [1], P Burke [1], C Peirce [1], J C Coffey [1], R M Cunningham [1]

Affiliation

- [1] Department of Surgery, University of Limerick Hospital Group, Ireland.

- PMID: **32924149**
- DOI: 10.1002/bjs.12058

No abstract available

- 5 references

Supplementary info

Publication types, MeSH terms Expand

Publication types

- Letter
- Observational Study

MeSH terms

- Adolescent
- Adult

- Aged
- Appendicitis / epidemiology
- Appendicitis / surgery
- Appendicitis / therapy*
- COVID-19 / epidemiology*
- Child
- Child, Preschool
- Female
- Humans
- Male
- Middle Aged
- Practice Guidelines as Topic
- Quarantine
- Retrospective Studies
- United Kingdom
- Young Adult

Full text links

OXFORD ACADEMIC Silverchair Information Systems
Proceed to details
Cite
Share
1,117
Observational Study
Am J Kidney Dis
. 2020 Oct;76(4):490-499.e1.
doi: 10.1053/j.ajkd.2020.06.008. Epub 2020 Jul 3.

Serologic Detection of SARS-CoV-2 Infections in Hemodialysis Centers: A Multicenter Retrospective Study in Wuhan, China

Hui Tang [1], Jian-Bo Tian [2], Jun-Wu Dong [3], Xiao-Tie Tang [4], Zhen-Yuan Yan [5], Yuan-Yuan Zhao [6], Fei Xiong [7], Xin Sun [1], Cai-Xia Song [5], Chang-Gang Xiang [5], Can Tu [7], Chun-Tao Lei [1], Jing Liu [1], Hua Su [1], Jing Huang [1], Yang Qiu [1], Xiao-Ping Miao [2], Chun Zhang [8]
Affiliations Expand

Affiliations

- [1] Department of Nephrology, Union Hospital, Tongji Medical College, Huazhong University of Science and Technology, Wuhan, China.

- [2] Department of Epidemiology and Biostatistics, Ministry of Education Key Lab of Environment and Health, School of Public Health, Tongji Medical College, Huazhong University of Science and Technology, Wuhan, China.
- [3] Department of Nephrology, Wuhan Fourth Hospital, Tongji Medical College, Huazhong University of Science and Technology, Wuhan, China.
- [4] Department of Nephrology, Wuhan Pu-Ren Hospital, Wuhan, China.
- [5] Department of Nephrology, Jiang-Xia District Renmin Hospital, Wuhan, China.
- [6] Department of Nephrology, Wuhan No.3 Hospital, Wuhan, China.
- [7] Department of Nephrology, Wuhan No.1 Hospital, Tongji Medical College, Huazhong University of Science and Technology, Wuhan, China.
- [8] Department of Nephrology, Union Hospital, Tongji Medical College, Huazhong University of Science and Technology, Wuhan, China. Electronic address: drzhangchun@hus//t.edu.cn.

- PMID: **32628990**
- PMCID: PMC7837208
- DOI: 10.1053/j.ajkd.2020.06.008

Free PMC article
Observational Study

Serologic Detection of SARS-CoV-2 Infections in Hemodialysis Centers: A Multicenter Retrospective Study in Wuhan, China

Hui Tang et al. Am J Kidney Dis. 2020 Oct.
Free PMC article
Show details
Am J Kidney Dis
. 2020 Oct;76(4):490-499.e1.
doi: 10.1053/j.ajkd.2020.06.008. Epub 2020 Jul 3.

Authors

Hui Tang [1], Jian-Bo Tian [2], Jun-Wu Dong [3], Xiao-Tie Tang [4], Zhen-Yuan Yan [5], Yuan-Yuan Zhao [6], Fei Xiong [7], Xin Sun [1], Cai-Xia Song [5], Chang-Gang Xiang [5], Can Tu [7], Chun-Tao Lei [1], Jing Liu [1], Hua Su [1], Jing Huang [1], Yang Qiu [1], Xiao-Ping Miao [2], Chun Zhang [8]

Affiliations

- [1] Department of Nephrology, Union Hospital, Tongji Medical College, Huazhong University of Science and Technology, Wuhan, China.
- [2] Department of Epidemiology and Biostatistics, Ministry of Education Key Lab of Environment and Health, School of Public Health, Tongji Medical College, Huazhong University of Science and Technology, Wuhan, China.
- [3] Department of Nephrology, Wuhan Fourth Hospital, Tongji Medical College, Huazhong University of Science and Technology, Wuhan, China.

- [4] Department of Nephrology, Wuhan Pu-Ren Hospital, Wuhan, China.
- [5] Department of Nephrology, Jiang-Xia District Renmin Hospital, Wuhan, China.
- [6] Department of Nephrology, Wuhan No.3 Hospital, Wuhan, China.
- [7] Department of Nephrology, Wuhan No.1 Hospital, Tongji Medical College, Huazhong University of Science and Technology, Wuhan, China.
- [8] Department of Nephrology, Union Hospital, Tongji Medical College, Huazhong University of Science and Technology, Wuhan, China. Electronic address: drzhangchun@hus//t.edu.cn.

- PMID: **32628990**
- PMCID: PMC7837208
- DOI: 10.1053/j.ajkd.2020.06.008

Abstract

Rationale & objective: Patients receiving maintenance hemodialysis (MHD) are highly vulnerable to infection with severe acute respiratory syndrome coronavirus 2 (SARS-CoV-2). The current study was designed to evaluate the prevalence of SARS-CoV-2 infection based on both nucleic acid testing (NAT) and antibody testing in Chinese patients receiving MHD.

Study design: Cross-sectional study.

Setting & participants: From December 1, 2019, to March 31, 2020, a total of 1,027 MHD patients in 5 large hemodialysis centers in Wuhan, China, were enrolled. Patients were screened for SARS-CoV-2 infection by symptoms and initial computed tomography (CT) of the chest. If patients developed symptoms after the initial screening was negative, repeat CT was performed. Patients suspected of being infected with SARS-CoV-2 were tested with 2 consecutive throat swabs for viral RNA. In mid-March 2020, antibody testing for SARS-CoV-2 was obtained for all MHD patients.

Exposure: NAT and antibody testing results for SARS-CoV-2.

Outcomes: Morbidity, clinical features, and laboratory and radiologic findings.

Analytical approach: Differences between groups were examined using t test or Mann-Whitney U test, comparing those not infected with those infected and comparing those with infection detected using NAT with those with infection detected by positive serology test results.

Results: Among 1,027 patients receiving MHD, 99 were identified as having SARS-CoV-2 infection, for a prevalence of 9.6%. Among the 99 cases, 52 (53%) were initially diagnosed with SARS-CoV-2 infection by positive NAT; 47 (47%) were identified later by positive immunoglobulin G (IgG) or IgM antibodies against SARS-CoV-2. There was a spectrum of antibody profiles in these 47 patients: IgM antibodies in 5 (11%), IgG antibodies in 35 (74%), and both IgM and IgG antibodies in 7 (15%). Of the 99 cases, 51% were asymptomatic during the epidemic; 61% had ground-glass or patchy opacities on CT of the chest compared with 11.6% among uninfected patients ($P<0.001$). Patients with hypertensive kidney disease were more often found to have SARS-CoV-2 infection and were more likely to be symptomatic than patients with another primary cause of kidney failure.

Limitations: Possible false-positive and false-negative results for both NAT and antibody testing; possible lack of generalizability to other dialysis populations.

Conclusions: Half the SARS-CoV-2 infections in patients receiving MHD were subclinical and were not identified by universal CT of the chest and selective NAT. Serologic testing may help evaluate the overall prevalence and understand the diversity of clinical courses among patients receiving MHD who are infected with SARS-CoV-2.

Keywords: SARS-CoV-2 infection; Severe acute respiratory syndrome coronavirus 2 (SARS-CoV-2); antibody testing; asymptomatic infection; chest computed tomography; chronic kidney disease (CKD); coronavirus disease 2019 (COVID-19); dialysis unit; disease surveillance; end-stage kidney disease (ESKD); hemodialysis; infection detection; nucleic acid testing (NAT); pandemic; serology; subclinical infection.

Copyright © 2020 National Kidney Foundation, Inc. Published by Elsevier Inc. All rights reserved.

- 28 references
- 3 figures

Supplementary info

Publication types, MeSH terms, Substances Expand

Publication types

- Multicenter Study
- Observational Study
- Research Support, Non-U.S. Gov't

MeSH terms

- Antibodies, Viral / analysis*
- Betacoronavirus / immunology*
- COVID-19
- China / epidemiology
- Comorbidity
- Coronavirus Infections / diagnosis*
- Coronavirus Infections / epidemiology
- Cross-Sectional Studies
- Female
- Humans
- Kidney Failure, Chronic / epidemiology
- Kidney Failure, Chronic / therapy*
- Male
- Middle Aged
- Pandemics
- Pneumonia, Viral / diagnosis*
- Pneumonia, Viral / epidemiology

- Prevalence
- Renal Dialysis*
- Retrospective Studies
- SARS-CoV-2
- Serologic Tests / methods
- Tomography, X-Ray Computed

Substances

- Antibodies, Viral

Full text links

ELSEVIER FULL-TEXT ARTICLE Elsevier Science Free PMC article
Proceed to details
Cite
Share
1,118
Observational Study
Medicine (Baltimore)
. 2020 Oct 23;99(43):e22766.
doi: 10.1097/MD.0000000000022766.

The importance of overweight in COVID-19: A retrospective analysis in a single center of Wuhan, China

Xinrui Rao [1], Chuangyan Wu [2], Sihua Wang [2], Song Tong [2], Geng Wang [3], Gang Wu [1], Rui Zhou [1]
Affiliations Expand

Affiliations

- [1] Cancer center.
- [2] Department of Thoracic Surgery.
- [3] Department of Gastrointestinal Surgery, Union Hospital, Tongji Medical College, Huazhong University of Science and Technology, Wuhan, China.

- PMID: **33120785**
- PMCID: PMC7581045
- DOI: 10.1097/MD.0000000000022766

Free PMC article
Observational Study

The importance of overweight in COVID-19: A retrospective analysis in a single center of Wuhan, China

Xinrui Rao et al. Medicine (Baltimore). 2020.
Free PMC article
Show details
Medicine (Baltimore)
. 2020 Oct 23;99(43):e22766.
doi: 10.1097/MD.0000000000022766.

Authors

Xinrui Rao [1], Chuangyan Wu [2], Sihua Wang [2], Song Tong [2], Geng Wang [3], Gang Wu [1], Rui Zhou [1]

Affiliations

- [1] Cancer center.
- [2] Department of Thoracic Surgery.
- [3] Department of Gastrointestinal Surgery, Union Hospital, Tongji Medical College, Huazhong University of Science and Technology, Wuhan, China.

- PMID: **33120785**
- PMCID: PMC7581045
- DOI: 10.1097/MD.0000000000022766

Abstract

The aim of this study was to evaluate the association between overweight and severity, drug response, and clinical outcomes of novel coronavirus disease 2019 (COVID-19).In this retrospective cohort study, we reviewed medical records of 240 COVID-19 patients admitted to Union Hospital in Wuhan, China, between December 24, 2019, and March 25, 2020. Physical, clinical, laboratory, radiological characteristics, treatment, and outcome data were abstracted. Patients who were obese [body mass index (BMI) ≥28 kg/m], underweight (BMI < 18.5 kg/m), under 18 years old, pregnant, or still in hospital were excluded. Disease severity was classified as moderate or severe pneumonia based on the World Health Organization interim guidance. Overweight was defined as BMI ≥24 kg/m and <28 kg/m. Patients were followed for discharge or death through April 10, 2020. We used logistic regression models to identify risk factors for severe disease, Cox proportional hazard models to explore associations between medications and patient outcomes (discharge or in-hospital death), and Kaplan-Meier survival curves and Cox regression models to evaluate risk factors for in-hospital death.One-half of patients (120, 50.0%) had severe pneumonia, while nearly one-half (114, 47.5%) were overweight. Among patients over 45 years old, overweight patients had significantly lower rates of fatigue, higher rates of headache, and higher median C-reactive protein levels. Patients under 45 years old had higher rates of cough and myalgia and higher proportions of increased alanine aminotransferase and lactic dehydrogenase, as well as more pulmonary lobes involved in the pneumonia revealed by chest

computed tomography scans. Overweight patients were at higher risk of developing severe pneumonia. Although weight was not a risk factor for in-hospital death, overweight patients showed different responses to medications compared with normal weight patients. Intravenous interferon-α, intravenous glucocorticoids, and antifungal drugs were associated with reduced mortality in overweight patients. Intravenous immunoglobulin, oseltamivir, and ribavirin were associated with reduced mortality in normal weight patients.Overweight is a worldwide health problem. We found overweight to be related to the COVID-19 severity but not to in-hospital death. Clinicians should be aware that overweight COVID-19 patients require increased attention for different clinical features and treatment response.

Conflict of interest statement

The authors report no conflicts of interest.

- 34 references
- 5 figures

Supplementary info

Publication types, MeSH terms, Substances, Supplementary concepts Expand

Publication types

- Observational Study

MeSH terms

- Adult
- Aged
- Aged, 80 and over
- Antiviral Agents / therapeutic use
- Betacoronavirus*
- COVID-19
- China / epidemiology
- Coronavirus Infections / complications
- Coronavirus Infections / diagnosis*
- Coronavirus Infections / drug therapy
- Coronavirus Infections / mortality
- Female
- Follow-Up Studies
- Hospital Mortality
- Humans
- Male
- Middle Aged
- Overweight / complications*

- Pandemics
- Pneumonia, Viral / complications
- Pneumonia, Viral / diagnosis*
- Pneumonia, Viral / drug therapy
- Pneumonia, Viral / mortality
- Prognosis
- Retrospective Studies
- Risk Factors
- SARS-CoV-2
- Severity of Illness Index
- Survival Analysis

Substances

- Antiviral Agents

Supplementary concepts

- COVID-19 drug treatment

Full text links

Wolters Kluwer Wolters Kluwer Free PMC article

Proceed to details

Cite

Share

☐ 1,119

Observational Study

Stroke Vasc Neurol

. 2020 Sep;5(3):279-284.

doi: 10.1136/svn-2020-000431. Epub 2020 Jul 2.

Acute cerebrovascular disease following COVID-19: a single center, retrospective, observational study

Yanan Li [# 1], Man Li [# 1], Mengdie Wang [# 1], Yifan Zhou [# 1], Jiang Chang [# 2], Ying Xian [3], David Wang [4], Ling Mao [1], Huijuan Jin [5], Bo Hu [5]

Affiliations Expand

Affiliations

- [1] Department of Neurology Union Hospital, Tongji Medical College, Huazhong University of Science and Technology, Wuhan, China.
- [2] Department of Epidemiology and Biostatistics, Key Laboratory for Environment and Health, School of Public Health, Tongji Medical College, Huazhong University of Science and Technology, Wuhan, China.
- [3] Duke Clinical Research Institute and Department of Neurology, Duke University Medical Center, Durham, North Carolina, USA.
- [4] Neurovascular Division, Department of Neurology, Barrow Neurological Institute/Saint Joseph Hospital Medical Center, Phoenix, Arizona, USA.
- [5] Department of Neurology Union Hospital, Tongji Medical College, Huazhong University of Science and Technology, Wuhan, China hubo@mail.hust.edu.cn jinhuijuan1983@163.com.

[#] Contributed equally.

- PMID: **32616524**
- PMCID: PMC7371480
- DOI: 10.1136/svn-2020-000431

Free PMC article
Observational Study

Acute cerebrovascular disease following COVID-19: a single center, retrospective, observational study

Yanan Li et al. Stroke Vasc Neurol. 2020 Sep.
Free PMC article
Show details
Stroke Vasc Neurol
. 2020 Sep;5(3):279-284.
doi: 10.1136/svn-2020-000431. Epub 2020 Jul 2.

Authors

Yanan Li [# 1], Man Li [# 1], Mengdie Wang [# 1], Yifan Zhou [# 1], Jiang Chang [# 2], Ying Xian [3], David Wang [4], Ling Mao [1], Huijuan Jin [5], Bo Hu [5]

Affiliations

- [1] Department of Neurology Union Hospital, Tongji Medical College, Huazhong University of Science and Technology, Wuhan, China.
- [2] Department of Epidemiology and Biostatistics, Key Laboratory for Environment and Health, School of Public Health, Tongji Medical College, Huazhong University of Science and Technology, Wuhan, China.
- [3] Duke Clinical Research Institute and Department of Neurology, Duke University Medical Center, Durham, North Carolina, USA.

- [4] Neurovascular Division, Department of Neurology, Barrow Neurological Institute/Saint Joseph Hospital Medical Center, Phoenix, Arizona, USA.
- [5] Department of Neurology Union Hospital, Tongji Medical College, Huazhong University of Science and Technology, Wuhan, China hubo@mail.hust.edu.cn jinhuijuan1983@163.com.

[#] Contributed equally.

- PMID: **32616524**
- PMCID: PMC7371480
- DOI: 10.1136/svn-2020-000431

Abstract

Background and purpose: COVID-19 is an infectious disease caused by Severe Acute Respiratory Syndrome Coronavirus 2 (SARS-CoV-2). Apart from respiratory complications, acute cerebrovascular disease (CVD) has been observed in some patients with COVID-19. Therefore, we described the clinical characteristics, laboratory features, treatment and outcomes of CVD complicating SARS-CoV-2 infection.

Materials and methods: Demographic and clinical characteristics, laboratory findings, treatments and clinical outcomes were collected and analysed. Clinical characteristics and laboratory findings of patients with COVID-19 with or without new-onset CVD were compared.

Results: Of 219 patients with COVID-19, 10 (4.6%) developed acute ischaemic stroke and 1 (0.5%) had intracerebral haemorrhage. COVID-19 with new onset of CVD were significantly older (75.7±10.8 years vs 52.1±15.3 years, p<0.001), more likely to present with severe COVID-19 (81.8% vs 39.9%, p<0.01) and were more likely to have cardiovascular risk factors, including hypertension, diabetes and medical history of CVD (all p<0.05). In addition, they were more likely to have increased inflammatory response and hypercoagulable state as reflected in C reactive protein (51.1 (1.3-127.9) vs 12.1 (0.1-212.0) mg/L, p<0.05) and D-dimer (6.9 (0.3-20.0) vs 0.5 (0.1-20.0) mg/L, p<0.001). Of 10 patients with ischemic stroke; 6 received antiplatelet treatment with aspirin or clopidogrel; and 3 of them died. The other four patients received anticoagulant treatment with enoxaparin and 2 of them died. As of 24 March 2020, six patients with CVD died (54.5%).

Conclusion: Acute CVD is not uncommon in COVID-19. Our findings suggest that older patients with risk factors are more likely to develop CVD. The development of CVD is an important negative prognostic factor which requires further study to identify optimal management strategy to combat the COVID-19 outbreak.

Keywords: Anticoagulation therapy; COVID-19; Cerebrovascular disease; Thromboembolic events; stroke.

© Author(s) (or their employer(s)) 2020. Re-use permitted under CC BY-NC. No commercial re-use. See rights and permissions. Published by BMJ.

Conflict of interest statement

Competing interests: None declared.

- 26 references

- 1 figure

Supplementary info

Publication types, MeSH terms, Substances Expand

Publication types

- Observational Study
- Research Support, Non-U.S. Gov't

MeSH terms

- Acute Disease
- Aged
- Aged, 80 and over
- Anticoagulants / therapeutic use
- Betacoronavirus / pathogenicity*
- COVID-19
- Cerebrovascular Disorders / diagnosis
- Cerebrovascular Disorders / drug therapy
- Cerebrovascular Disorders / mortality
- Cerebrovascular Disorders / virology*
- China
- Coronavirus Infections / diagnosis
- Coronavirus Infections / mortality
- Coronavirus Infections / therapy
- Coronavirus Infections / virology*
- Female
- Host-Pathogen Interactions
- Humans
- Male
- Middle Aged
- Pandemics
- Platelet Aggregation Inhibitors / therapeutic use
- Pneumonia, Viral / diagnosis
- Pneumonia, Viral / mortality
- Pneumonia, Viral / therapy
- Pneumonia, Viral / virology*
- Retrospective Studies
- Risk Assessment
- Risk Factors

- SARS-CoV-2
- Treatment Outcome

Substances

- Anticoagulants
- Platelet Aggregation Inhibitors

Full text links

BMJ Open Access Full Text HighWire Free PMC article
Proceed to details
Cite
Share
1,120

Observational Study
Diabetes Metab Syndr
. Nov-Dec 2021;15(6):102322.
doi: 10.1016/j.dsx.2021.102322. Epub 2021 Oct 27.

COVID-19 associated mucormycosis: A Descriptive Multisite Study from India

Ritesh Gupta [1], Jothydev Kesavadev [2], Gopika Krishnan [2], Sanjay Agarwal [3], Banshi Saboo [4], Meet Shah [5], Atul Mittal [6], Suhail Durani [6], Atul Luthra [6], Anuj Singhal [7], Muhammed Rasheed [8], G V S Rao [9], Vidit Tripathi [10], Alka Jha [10], Amerta Ghosh [11], V Mohan [12], Awadhesh K Singh [13], Sanjeev Phatak [14], John Panicker [15], Sanjay Kumar Bhadada [16], Shashank Joshi [17], Rimesh Pal [16], Ambrish Mithal [18], Naval Vikram [19], Anoop Misra [11]
Affiliations Expand

Affiliations

- [1] Fortis CDOC Hospital, New Delhi, India. Electronic address: riteshgupta72@gmail.com.
- [2] Jothydev's Diabetes Research Centre, Trivandrum, Kerala, India.
- [3] Jehangir Hospital, Pune, Maharashtra, India.
- [4] Diacare Clinic, Ahmedabad, Gujarat, India.
- [5] GMERS, Himmatnagar, Gujarat, India.
- [6] Fortis Memorial Research Institute, Gurugram, Haryana, India.
- [7] Fortis Escorts Heart Institute, New Delhi, India.
- [8] KIMS Hospital, Trivandrum, India.
- [9] Dr Rao's ENT Centre, Hyderabad, Telangana, India.
- [10] Fortis Rajan Dhall Hospital, New Delhi, India.
- [11] Fortis CDOC Hospital, New Delhi, India.

- [12] Dr Mohan's Diabetes Specialities Centre and Madras Diabetes Research Foundation, Chennai, Tamilnadu, India.
- [13] GD Hospital and Diabetes Institute, Kolkata, India.
- [14] Vijayratna Diabetes Centre, Ahmedabad, Gujarat, India.
- [15] Santhwana Hospital, Trivandrum, India.
- [16] Postgraduate Institute of Medical Education and Research, Chandigarh, India.
- [17] Lilavati Hospital and Research Centre, Mumbai, India.
- [18] Max Healthcare, New Delhi, India.
- [19] All India Institute of Medical Sciences, New Delhi, India.

- PMID: **34717135**
- PMCID: PMC8635309
- DOI: 10.1016/j.dsx.2021.102322

Free PMC article
Observational Study

COVID-19 associated mucormycosis: A Descriptive Multisite Study from India

Ritesh Gupta et al. Diabetes Metab Syndr. Nov-Dec 2021.
Free PMC article
Show details
Diabetes Metab Syndr
. Nov-Dec 2021;15(6):102322.
doi: 10.1016/j.dsx.2021.102322. Epub 2021 Oct 27.

Authors

Ritesh Gupta [1], Jothydev Kesavadev [2], Gopika Krishnan [2], Sanjay Agarwal [3], Banshi Saboo [4], Meet Shah [5], Atul Mittal [6], Suhail Durani [6], Atul Luthra [6], Anuj Singhal [7], Muhammed Rasheed [8], G V S Rao [9], Vidit Tripathi [10], Alka Jha [10], Amerta Ghosh [11], V Mohan [12], Awadhesh K Singh [13], Sanjeev Phatak [14], John Panicker [15], Sanjay Kumar Bhadada [16], Shashank Joshi [17], Rimesh Pal [16], Ambrish Mithal [18], Naval Vikram [19], Anoop Misra [11]

Affiliations

- [1] Fortis CDOC Hospital, New Delhi, India. Electronic address: riteshgupta72@gmail.com.
- [2] Jothydev's Diabetes Research Centre, Trivandrum, Kerala, India.
- [3] Jehangir Hospital, Pune, Maharashtra, India.
- [4] Diacare Clinic, Ahmedabad, Gujarat, India.
- [5] GMERS, Himmatnagar, Gujarat, India.
- [6] Fortis Memorial Research Institute, Gurugram, Haryana, India.
- [7] Fortis Escorts Heart Institute, New Delhi, India.
- [8] KIMS Hospital, Trivandrum, India.
- [9] Dr Rao's ENT Centre, Hyderabad, Telangana, India.

- [10] Fortis Rajan Dhall Hospital, New Delhi, India.
- [11] Fortis CDOC Hospital, New Delhi, India.
- [12] Dr Mohan's Diabetes Specialities Centre and Madras Diabetes Research Foundation, Chennai, Tamilnadu, India.
- [13] GD Hospital and Diabetes Institute, Kolkata, India.
- [14] Vijayratna Diabetes Centre, Ahmedabad, Gujarat, India.
- [15] Santhwana Hospital, Trivandrum, India.
- [16] Postgraduate Institute of Medical Education and Research, Chandigarh, India.
- [17] Lilavati Hospital and Research Centre, Mumbai, India.
- [18] Max Healthcare, New Delhi, India.
- [19] All India Institute of Medical Sciences, New Delhi, India.

- PMID: **34717135**
- PMCID: PMC8635309
- DOI: 10.1016/j.dsx.2021.102322

Abstract

Background and aims: Mucormycosis is an invasive fungal infection and carries a significant morbidity and mortality. A number of cases of mucormycosis have been reported in association with COVID-19. In this study, a consortium of clinicians from various parts of India studied clinical profile of COVID-19 associated mucormycosis (CAM) and this analysis is presented here.

Methods: Investigators from multiple sites in India were involved in this study. Clinical details included the treatment and severity of COVID-19, associated morbidities, as well as the diagnosis, treatment and prognosis of mucormycosis. These data were collected using google spreadsheet at one centre. Descriptive analysis was done.

Results: There were 115 patients with CAM. Importantly, all patients had received corticosteroids. Diabetes was present in 85.2% of patients and 13.9% of patients had newly detected diabetes. The most common site of involvement was rhino-orbital. Mortality occurred in 25 (21.7%) patients. On logistic regression analysis, CT scan-based score for severity of lung involvement was associated with mortality.

Conclusion: Universal administration of corticosteroids in our patients is notable. A large majority of patients had diabetes, while mortality was seen in ~1/5th of patients, lower as compared to recently published data.

Copyright © 2021 Diabetes India. Published by Elsevier Ltd. All rights reserved.

Conflict of interest statement

Declaration of competing interest There is no conflict of interest of any author regarding the manuscript “COVID-19 Associated Mucormycosis: A Descriptive Multisite Study from India”.

- 22 references
- 1 figure

Supplementary info

Publication types, MeSH terms, Substances Expand

Publication types

- Multicenter Study
- Observational Study

MeSH terms

- Adrenal Cortex Hormones / adverse effects*
- Adult
- Aged
- COVID-19 / complications*
- COVID-19 / drug therapy
- Comorbidity
- Diabetes Complications / mortality
- Diabetes Complications / virology*
- Female
- Humans
- India / epidemiology
- Male
- Middle Aged
- Mucormycosis / chemically induced
- Mucormycosis / mortality
- Mucormycosis / virology*
- Retrospective Studies
- Risk Factors

Substances

- Adrenal Cortex Hormones

Full text links

ELSEVIER FULL-TEXT ARTICLE Elsevier Science Free PMC article
Proceed to details
Cite
Share
☐ 1,121
Observational Study
Stroke
. 2020 Jul;51(7):2002-2011.
doi: 10.1161/STROKEAHA.120.030335. Epub 2020 May 20.

SARS-CoV-2 and Stroke in a New York Healthcare System

Shadi Yaghi [1], Koto Ishida [1], Jose Torres [1], Brian Mac Grory [2], Eytan Raz [3], Kelley Humbert [1], Nils Henninger [4], Tushar Trivedi [1], Kaitlyn Lillemoe [1], Shazia Alam [1], Matthew Sanger [1], Sun Kim [1], Erica Scher [1], Seena Dehkharghani [3], Michael Wachs [5], Omar Tanweer [6], Frank Volpicelli [7], Brian Bosworth [7], Aaron Lord [1], Jennifer Frontera [1]

Affilliations Expand

Affiliations

- [1] Department of Neurology (S.Y., K.I., J.T., K.H., T.T., K.L., S.A., M.S., S.K., E.S., A.L., J.F.), NYU Langone Health, New York, NY.
- [2] Department of Neurology, Brown University, Providence, RI (B.M.G.).
- [3] Department of Radiology (E.R., S.D.), NYU Langone Health, New York, NY.
- [4] Department of Neurology, University of Massachusetts, Worcester (N.H.).
- [5] Department of Operational Projects and Analytics (M.W.), NYU Langone Health, New York, NY.
- [6] Department of Neurosurgery (O.T.), NYU Langone Health, New York, NY.
- [7] Department of Internal Medicine (F.V., B.B.), NYU Langone Health, New York, NY.

- PMID: **32432996**
- PMCID: PMC7258764
- DOI: 10.1161/STROKEAHA.120.030335

Free PMC article
Observational Study

SARS-CoV-2 and Stroke in a New York Healthcare System

Shadi Yaghi et al. Stroke. 2020 Jul.
Free PMC article
Show details
Stroke
. 2020 Jul;51(7):2002-2011.
doi: 10.1161/STROKEAHA.120.030335. Epub 2020 May 20.

Authors

Shadi Yaghi [1], Koto Ishida [1], Jose Torres [1], Brian Mac Grory [2], Eytan Raz [3], Kelley Humbert [1], Nils Henninger [4], Tushar Trivedi [1], Kaitlyn Lillemoe [1], Shazia Alam [1], Matthew Sanger [1], Sun Kim [1], Erica Scher [1], Seena Dehkharghani [3], Michael Wachs [5], Omar Tanweer [6], Frank Volpicelli [7], Brian Bosworth [7], Aaron Lord [1], Jennifer Frontera [1]

Affiliations

- [1] Department of Neurology (S.Y., K.I., J.T., K.H., T.T., K.L., S.A., M.S., S.K., E.S., A.L., J.F.), NYU Langone Health, New York, NY.
- [2] Department of Neurology, Brown University, Providence, RI (B.M.G.).
- [3] Department of Radiology (E.R., S.D.), NYU Langone Health, New York, NY.
- [4] Department of Neurology, University of Massachusetts, Worcester (N.H.).
- [5] Department of Operational Projects and Analytics (M.W.), NYU Langone Health, New York, NY.
- [6] Department of Neurosurgery (O.T.), NYU Langone Health, New York, NY.
- [7] Department of Internal Medicine (F.V., B.B.), NYU Langone Health, New York, NY.

- PMID: **32432996**
- PMCID: PMC7258764
- DOI: 10.1161/STROKEAHA.120.030335

Erratum in

- Correction to: SARS2-CoV-2 and Stroke in a New York Healthcare System.
  [No authors listed] [No authors listed] Stroke. 2020 Aug;51(8):e179. doi: 10.1161/STR.0000000000000342. Epub 2020 Jul 27. Stroke. 2020. PMID: 32716821 Free PMC article. No abstract available.

Abstract

Background and purpose: With the spread of coronavirus disease 2019 (COVID-19) during the current worldwide pandemic, there is mounting evidence that patients affected by the illness may develop clinically significant coagulopathy with thromboembolic complications including ischemic stroke. However, there is limited data on the clinical characteristics, stroke mechanism, and outcomes of patients who have a stroke and COVID-19.

Methods: We conducted a retrospective cohort study of consecutive patients with ischemic stroke who were hospitalized between March 15, 2020, and April 19, 2020, within a major health system in New York, the current global epicenter of the pandemic. We compared the clinical characteristics of stroke patients with a concurrent diagnosis of COVID-19 to stroke patients without COVID-19 (contemporary controls). In addition, we compared patients to a historical cohort of patients with ischemic stroke discharged from our hospital system between March 15, 2019, and April 15, 2019 (historical controls).

Results: During the study period in 2020, out of 3556 hospitalized patients with diagnosis of COVID-19 infection, 32 patients (0.9%) had imaging proven ischemic stroke. Cryptogenic stroke was more common in patients with COVID-19 (65.6%) as compared to contemporary controls (30.4%, P=0.003) and historical controls (25.0%, P<0.001). When compared with contemporary controls, COVID-19 positive patients had higher admission National Institutes of Health Stroke Scale score and higher peak D-dimer levels. When compared with historical controls, COVID-19 positive patients were more likely to be younger men with elevated troponin, higher admission National Institutes of Health Stroke Scale score, and higher erythrocyte sedimentation rate. Patients with COVID-19 and stroke had significantly higher mortality than historical and contemporary controls.

Conclusions: We observed a low rate of imaging-confirmed ischemic stroke in hospitalized patients with COVID-19. Most strokes were cryptogenic, possibly related to an acquired hypercoagulability, and mortality was increased. Studies are needed to determine the utility of therapeutic anticoagulation for stroke and other thrombotic event prevention in patients with COVID-19.

Keywords: COVID-19; coronavirus; diagnosis; pandemic; troponin.

Comment in

- Ischemic Stroke Epidemiology During the COVID-19 Pandemic: Navigating Uncharted Waters With Changing Tides.
  Tsivgoulis G, Katsanos AH, Ornello R, Sacco S. Tsivgoulis G, et al. Stroke. 2020 Jul;51(7):1924-1926. doi: 10.1161/STROKEAHA.120.030791. Epub 2020 Jun 4. Stroke. 2020. PMID: 32496937 Free PMC article. No abstract available.
- Response by Yaghi et al to Letter Regarding Article, "SARS-CoV-2 and Stroke in a New York Healthcare System".
  Yaghi S, Lord A, Frontera J. Yaghi S, et al. Stroke. 2020 Nov;51(11):e312-e313. doi: 10.1161/STROKEAHA.120.031676. Epub 2020 Aug 5. Stroke. 2020. PMID: 32755453 Free PMC article. No abstract available.
- Letter by Albiero and Seresini Regarding Article, "SARS-CoV-2 and Stroke in a New York Healthcare System".
  Albiero R, Seresini G. Albiero R, et al. Stroke. 2020 Nov;51(11):e310-e311. doi: 10.1161/STROKEAHA.120.031093. Epub 2020 Aug 5. Stroke. 2020. PMID: 32755455 Free PMC article. No abstract available.
- COVID-19 erhöht Schlaganfallrisiko : Coronavirus-Pandemie.
  Diener HC. Diener HC. MMW Fortschr Med. 2020 Aug;162(14):29. doi: 10.1007/s15006-020-0739-6. MMW Fortschr Med. 2020. PMID: 32780387 Free PMC article. Review. German.
- Letter by Sajeev et al Regarding Article, "SARS-CoV-2 and Stroke in a New York Healthcare System".
  Sajeev JK, Burrell LM, Teh AW. Sajeev JK, et al. Stroke. 2020 Nov;51(11):e314-e315. doi: 10.1161/STROKEAHA.120.031113. Epub 2020 Oct 26. Stroke. 2020. PMID: 33104463 No abstract available.
- Response by Ishida et al Regarding Article, "SARS-CoV-2 and Stroke in a New York Healthcare System".
  Ishida K, Torres J, Yaghi S. Ishida K, et al. Stroke. 2020 Nov;51(11):e316-e317. doi: 10.1161/STROKEAHA.120.031606. Epub 2020 Oct 26. Stroke. 2020. PMID: 33104484 No abstract available.
- Response by Mac Grory and Yaghi to Letter Regarding Article, "SARS-CoV-2 and Stroke in a New York Healthcare System".
  Mac Grory B, Yaghi S. Mac Grory B, et al. Stroke. 2020 Dec;51(12):e373-e374. doi: 10.1161/STROKEAHA.120.032519. Epub 2020 Nov 23. Stroke. 2020. PMID: 33226920 No abstract available.
- Letter by Bayona et al Regarding Article, "SARS-CoV-2 and Stroke in a New York Healthcare System".
  Bayona H, Valencia-Enciso N, Zafra-Sierra M. Bayona H, et al. Stroke. 2020 Dec;51(12):e371-e372. doi: 10.1161/STROKEAHA.120.031838. Epub 2020 Nov 23. Stroke. 2020. PMID: 33226930 No abstract available.

- 16 references
- 2 figures

Supplementary info

Publication types, MeSH terms, Substances, Grant support Expand

Publication types

- Comparative Study
- Multicenter Study
- Observational Study
- Research Support, N.I.H., Extramural

MeSH terms

- Adult
- Aged
- Betacoronavirus*
- Biomarkers
- Blood Sedimentation
- Brain Ischemia / blood
- Brain Ischemia / epidemiology*
- Brain Ischemia / etiology
- Brain Ischemia / therapy
- COVID-19
- Causality
- Cerebral Small Vessel Diseases / complications
- Cerebral Small Vessel Diseases / diagnostic imaging
- Cerebral Small Vessel Diseases / epidemiology
- Comorbidity
- Coronavirus Infections / blood
- Coronavirus Infections / complications
- Coronavirus Infections / epidemiology*
- Female
- Fibrin Fibrinogen Degradation Products / analysis
- Humans
- Incidence
- Male
- Middle Aged
- Neuroimaging
- New York City / epidemiology
- Pandemics*
- Patient Admission / statistics & numerical data
- Pneumonia, Viral / blood

- Pneumonia, Viral / complications
- Pneumonia, Viral / epidemiology*
- Retrospective Studies
- SARS-CoV-2
- Severity of Illness Index
- Stroke / blood
- Stroke / epidemiology*
- Stroke / etiology
- Stroke / therapy
- Thrombophilia / etiology
- Troponin / blood

Substances

- Biomarkers
- Fibrin Fibrinogen Degradation Products
- Troponin
- fibrin fragment D

Grant support

- K08 NS091499/NS/NINDS NIH HHS/United States

Full text links

FREE FULL TEXT Atypon Free PMC article
Proceed to details
Cite
Share
☐ 1,122
Observational Study
J Orthop Surg Res
. 2021 Feb 24;16(1):155.
doi: 10.1186/s13018-021-02301-z.

The impact of COVID-19 on the management and outcomes of patients with proximal femoral fractures: a multi-centre study of 580 patients

Alice Wignall [1], Vasileios Giannoudis [2], Chiranjit De [3], Andrea Jimenez [2], Simon Sturdee [2], Sohail Nisar [4], Hemant Pandit [4], Aashish Gulati [3], Jeya Palan [4]
Affiliations Expand

Affiliations

- [1] Leeds Teaching Hospitals NHS Trust, Leeds, UK. alice.wignall@nhs.net.
- [2] Huddersfield Royal Infirmary, Huddersfield, UK.
- [3] Sandwell & West Birmingham Hospitals NHS Trust, Lyndon, UK.
- [4] Leeds Teaching Hospitals NHS Trust, Leeds, UK.

- PMID: **33627153**
- PMCID: PMC7903025
- DOI: 10.1186/s13018-021-02301-z

Free PMC article
Observational Study

The impact of COVID-19 on the management and outcomes of patients with proximal femoral fractures: a multi-centre study of 580 patients

Alice Wignall et al. J Orthop Surg Res. 2021.
Free PMC article
Show details
J Orthop Surg Res
. 2021 Feb 24;16(1):155.
doi: 10.1186/s13018-021-02301-z.

Authors

Alice Wignall [1], Vasileios Giannoudis [2], Chiranjit De [3], Andrea Jimenez [2], Simon Sturdee [2], Sohail Nisar [4], Hemant Pandit [4], Aashish Gulati [3], Jeya Palan [4]

Affiliations

- [1] Leeds Teaching Hospitals NHS Trust, Leeds, UK. alice.wignall@nhs.net.
- [2] Huddersfield Royal Infirmary, Huddersfield, UK.
- [3] Sandwell & West Birmingham Hospitals NHS Trust, Lyndon, UK.
- [4] Leeds Teaching Hospitals NHS Trust, Leeds, UK.

- PMID: **33627153**
- PMCID: PMC7903025
- DOI: 10.1186/s13018-021-02301-z

Abstract

Background: On the 11th March 2020, the World Health Organization declared the COVID-19 outbreak a pandemic. Multiple new guidelines were proposed and existing models of social, domestic and hospital care altered. Most healthcare systems were largely unprepared for this, and the pandemic has tested their adaptability. This study aimed to assess the impact of COVID-19 on the demographics, presentation, clinical management and outcomes of patients with proximal femoral (hip) fractures comparing them to a similar cohort of patients admitted a year earlier.

Methods: This retrospective multi-centre cohort study compared all patients admitted with hip fractures between 1st March and 30th May 2019 (group PC: pre-COVID-19) with hip fracture patients admitted over the same time period during the pandemic in 2020 (group C: COVID-19). The data was obtained from the hospitals' local and National Hip Fracture Databases. Mortality data was checked with the Office for National Statistics (ONS). Primary outcomes were time to theatre, in-patient length of stay and 30-day mortality.

Results: A total of 580 patients were included (304 group PC, 276 group C). Patient demographics including Charlson Comorbidity Index and Nottingham Hip Fracture Scores were broadly similar across the two cohorts. There was a significant reduction in the percentage of total hip replacements (11 to 5%, $p = 0.006$) in group C. There was an increase in conservative management (1 to 5%, $p = 0.002$) in group C. Time to theatre was significantly delayed in group C (43.7 h) vs group PC (34.6 h) ($p \leq 0.001$). The overall length of hospital stay was significantly longer in group PC (16.6 days) vs group C (15 days) ($p = 0.025$). The 30-day mortality rate in group C was 9.8% compared to 8.2% in group PC ($p = 0.746$), but for COVID-19 (+) patients, it was significantly higher at 38.2% vs 5.8% in COVID-19 (-) patients ($p < 0.001$).

Conclusion: This is one of the largest multi-centre comparative cohort study in the literature to date examining the impact of the COVID-19 pandemic on the management of hip fracture patients. Whilst mortality rates were similar in both groups, COVID-19-positive patients were almost seven times more likely to die, reflecting the seriousness of the COVID-19 infection and its sequelae in such elderly, vulnerable patients.

Keywords: 30-day mortality; COVID-19; Extracapsular hip fracture; Intracapsular hip fracture; Length of stay; Proximal femoral (hip) fracture; Time to theatre.

Conflict of interest statement

The authors declare that they have no competing interests.

- 37 references

Supplementary info

Publication types, MeSH terms Expand

Publication types

- Comparative Study
- Multicenter Study
- Observational Study

MeSH terms

- Aged
- Aged, 80 and over
- Arthroplasty, Replacement, Hip / statistics & numerical data*
- COVID-19*
- Female
- Hemiarthroplasty / statistics & numerical data*
- Hip Fractures / surgery*
- Humans
- Male
- Pandemics*
- Retrospective Studies

Full text links

Read free full text at BMC BioMed Central Free PMC article

Proceed to details

Cite

Share

☐ 1,123

Observational Study

Vascul Pharmacol

. 2020 Dec;135:106805.

doi: 10.1016/j.vph.2020.106805. Epub 2020 Sep 28.

RAAS inhibitors are not associated with mortality in COVID-19 patients: Findings from an observational multicenter study in Italy and a meta-analysis of 19 studies

COVID-19 RISk and Treatments (CORIST) Collaboration

Collaborators, Affiliations Expand

Collaborators

- **COVID-19 RISk and Treatments (CORIST) Collaboration**: Augusto Di Castelnuovo [1], Simona Costanzo [2], Andrea Antinori [3], Nausicaa Berselli [4], Lorenzo Blandi [5], Marialaura Bonaccio [2], Roberto Cauda [6], Alessandro Gialluisi [2], Giovanni Guaraldi [7], Lorenzo Menicanti [5], Marco Mennuni [8], Roberta Mussinelli [9], Ilaria My [10], Giustino Parruti [11], Giuseppe Patti [8], Stefano Perlini [12], Francesca Santilli [13], Carlo Signorelli [14], Giulio G Stefanini [10], Alessandra Vergori [15], Pasquale Abete

[16], Walter Ageno [17], Piergiuseppe Agostoni [18], Luca Aiello [19], Samir Al Moghazi [20], Rosa Arboretti [21], Filippo Aucella [22], Greta Barbieri [23], Martina Barchitta [24], Alessandro Bartoloni [25], Paolo Bonfanti [26], Francesco Cacciatore [16], Lucia Caiano [17], Laura Carrozzi [27], Antonio Cascio [28], Giacomo Castiglione [29], Stefania Cianfrone [13], Arturo Ciccullo [30], Antonella Cingolani [6], Francesco Cipollone [13], Claudia Colomba [28], Crizia Colombo [8], Ottavia Cozzi [10], Annalisa Crisetti [22], Francesca Crosta [11], Gian Battista Danzi [31], Damiano D'Ardes [13], Katleen de Gaetano Donati [30], Francesco Di Gennaro [32], Giuseppe Di Tano [31], Gianpiero D'Offizi [33], Francesco Maria Fusco [34], Ivan Gentile [35], Emauele Graziani [36], Gabriella Guarnieri [37], Giovanni Larizza [38], Armando Leone [39], Veronica Lio [8], Mothanje Barbara Lucia [6], Gloria Maccagni [31], Ferruccio Madaro [38], Stefano Maitan [19], Sandro Mancarella [40], Rosa Manuele [41], Massimo Mapelli [18], Riccardo Maragna [18], Rossella Marcucci [25], Giulio Maresca [42], Silvia Marongiu [43], Claudia Marotta [32], Lorenzo Marra [39], Franco Mastroianni [38], Maria Mazzitelli [44], Alessandro Mengozzi [23], Francesco Menichetti [23], Marianna Meschiari [7], Jovana Milic [7], Filippo Minutolo [45], Beatrice Molena [37], Cristina Mussini [7], Maria Musso [46], Anna Odone [14], Marco Olivieri [47], Antonella Palimodde [43], Emanuela Pasi [36], Raffaele Pesavento [48], Francesco Petri [49], Biagio Pinchera [35], Carlo A Pivato [10], Venerino Poletti [50], Claudia Ravaglia [50], Marco Rossato [48], Marianna Rossi [49], Anna Sabena [51], Francesco Salinaro [51], Vincenzo Sangiovanni [34], Carlo Sanrocco [11], Giancarlo Scoppettuolo [30], Laura Scorzolini [52], Raffaella Sgariglia [40], Paola Giustina Simeone [11], Enrico Maria Trecarichi [44], Roberto Vettor [48], Andrea Vianello [37], Marco Vinceti [53], Alexandra Virano [17], Laura Vocciante [42], Raffaele De Caterina [54], Licia Iacoviello [55]

Affiliations

- [1] Mediterranea Cardiocentro, Napoli, Italy.
- [2] Department of Epidemiology and Prevention, IRCCS Neuromed, Pozzilli (IS), Italy.
- [3] UOC Immunodeficienze Virali, National Institute for Infectious Diseases "L. Spallanzani", IRCCS, Roma, Italy.
- [4] Section of Public Health, Department of Biomedical, Metabolic and Neural Sciences, University of Modena and Reggio Emilia, Modena, Italy.
- [5] IRCCS Policlinico San Donato, San Donato Milanese (MI), Italy.
- [6] Fondazione Policlinico Universitario A, Gemelli IRCCS, Roma, Italy; Università Cattolica del Sacro Cuore- Dipartimento di Sicurezza e Bioetica Sede di Roma, Roma, Italy.
- [7] Infectious Disease Unit, Department of Surgical, Medical, Dental and Morphological Sciences, University of Modena and Reggio Emilia, Modena, Italy.
- [8] University of Eastern Piedmont, Maggiore della Carità Hospital, Novara, Italy.
- [9] Department of Internal Medicine, University of Pavia, Pavia, Italy.
- [10] Humanitas Clinical and Research Center - IRCCS, Rozzano (Mi), Italy; Humanitas University, Department of Biomedical Sciences, Milano, Italy.
- [11] Department of Infectious Disease, Azienda Sanitaria Locale (AUSL) di Pescara, Pescara, Italy.
- [12] Emergency Department, IRCCS Policlinico San Matteo Foundation, Pavia, Italy; Department of Internal Medicine, University of Pavia, Pavia, Italy.
- [13] Department of Medicine and Aging, Clinica Medica, "SS, Annunziata" Hospital and University of Chieti, Chieti, Italy.
- [14] School of Medicine, Vita-Salute San Raffaele University, Milano, Italy.

- [15] HIV/AIDS Department, National Institute for Infectious Diseases "Lazzaro Spallanzani"-IRCCS, Roma, Italy.
- [16] Dipartimento di Scienze Mediche Traslazionali, Università degli studi di Napoli "Federico II", Napoli, Italy.
- [17] Department of Medicine and Surgery, University of Insubria, Varese, Italy.
- [18] Centro Cardiologico Monzino IRCCS, Milano, Italy; Department of Clinical Sciences and Community Health, Cardiovascular Section, University of Milano, Milano, Italy.
- [19] UOC, Anestesia e Rianimazione, Dipartimento di Chirurgia Generale Ospedale Morgagni-Pierantoni, Forlì, Italy.
- [20] UOC Infezioni Sistemiche dell'Immunodepresso, National Institute for Infectious Diseases L. Spallanzani, IRCCS, Rome, Italy.
- [21] Department of Civil Environmental and Architectural Engineering, University of Padova, Padova, Italy.
- [22] Fondazione I.R.C.C.S "Casa Sollievo della Sofferenza", San Giovanni Rotondo, Foggia, Italy.
- [23] Emergency Medicine Unit, University Hospital of Pisa, Department of Surgical, Medical and Molecular Medicine and Critical Care, University of Pisa, Pisa, Italy.
- [24] Department of Medical and Surgical Sciences and Advanced Technologies "G.F, Ingrassia", University of Catania, Italy.
- [25] Department of Experimental and Clinical Medicine, University of Florence and Azienda Ospedaliero-Universitaria Careggi, Firenze, Italy.
- [26] UOC Malattie Infettive, Ospedale San Gerardo, ASST, Monza, Monza, Italy; School of Medicine and Surgery, University of Milano-Bicocca, Milano, Italy.
- [27] Cardiovascular and Thoracic Department, Azienda Ospedaliero-Universitaria Pisana and University of Pisa, Pisa, Italy.
- [28] Infectious and Tropical Diseases Unit- Department of Health Promotion, Mother and Child Care, Internal Medicine and Medical Specialties (PROMISE) - University of Palermo, Palermo, Italy.
- [29] Servizio di Anestesia e Rianimazione II UO Rianimazione Ospedale San Marco, AOU Policlinico-Vittorio Emanuele, Catania, Italy.
- [30] Fondazione Policlinico Universitario A, Gemelli IRCCS, Roma, Italy.
- [31] Department of Cardiology, Ospedale di Cremona, Cremona, Italy.
- [32] Medical Direction, IRCCS Neuromed, Pozzilli (IS), Italy.
- [33] UOC Malattie Infettive-Epatologia, National Institute for Infectious Diseases L. Spallanzani, IRCCS, Roma, Italy.
- [34] UOC Infezioni Sistemiche e dell'Immunodepresso, Azienda Ospedaliera dei Colli, Ospedale Cotugno, Napoli, Italy.
- [35] Department of Clinical Medicine and Surgery, University of Naples "Federico II", Napoli, Italy.
- [36] Medicina Interna, Ospedale di Ravenna, AUSL della Romagna, Ravenna, Italy.
- [37] Respiratory Pathophysiology Division, Department of Cardiologic, Thoracic and Vascular Sciences, University of Padova, Padova, Italy.
- [38] COVID-19 Unit, EE Ospedale Regionale F, Miulli, Acquaviva delle Fonti (BA), Italy.
- [39] UOC di Pneumologia, P.O, San Giuseppe Moscati, Taranto, Italy.
- [40] ASST Milano Nord - Ospedale Edoardo Bassini Cinisello Balsamo (MI), Italy.
- [41] U.O, C, Malattie Infettive e Tropicali, P.O, "San Marco", AOU Policlinico-Vittorio Emanuele, Catania, Italy.
- [42] UOC di Medicina - Presidio Ospedaliero S, Maria di Loreto Nuovo, Napoli, Italy.

- [43] Ospedale Santissima Trinità, Cagliari, Italy.
- [44] Infectious and Tropical Diseases Unit, Deparment of Medical and Surgical Sciences "Magna Graecia" University, Catanzaro, Italy.
- [45] Dipartimento di Farmacia, Università di Pisa, Pisa, Italy.
- [46] UOC Malattie Infettive-Apparato Respiratorio, National Institute for Infectious Diseases "L. Spallanzani", IRCCS, Roma, Italy.
- [47] Computer Service, University of Molise, Campobasso, Italy.
- [48] Clinica Medica 3, Department of Medicine - DIMED, University Hospital of Padova, Padova, Italy.
- [49] UOC Malattie Infettive, Ospedale San Gerardo, ASST, Monza, Monza, Italy.
- [50] UOC Pneumologia, Dipartimento di Malattie Apparato Respiratorio e Torace, Ospedale Morgagni-Pierantoni, Forlì, Italy.
- [51] Emergency Department, IRCCS Policlinico San Matteo Foundation, Pavia, Italy.
- [52] UOC Malattie Infettive ad Alta Intensità di Cura, National Institute for Infectious Diseases "L. Spallanzani". IRCCS, Rome, Italy.
- [53] Section of Public Health, Department of Biomedical, Metabolic and Neural Sciences, University of Modena and Reggio Emilia, Modena, Italy; Department of Epidemiology, Boston University School of Public Health, Boston, USA.
- [54] Cardiovascular and Thoracic Department, Azienda Ospedaliero-Universitaria Pisana and University of Pisa, Pisa, Italy. Electronic address: raffaele.decaterina@unipi.it.
- [55] Department of Epidemiology and Prevention, IRCCS Neuromed, Pozzilli (IS), Italy; Department of Medicine and Surgery, University of Insubria, Varese, Italy.

- PMID: **32992048**
- PMCID: PMC7521934
- DOI: 10.1016/j.vph.2020.106805

Free PMC article
Observational Study

RAAS inhibitors are not associated with mortality in COVID-19 patients: Findings from an observational multicenter study in Italy and a meta-analysis of 19 studies

COVID-19 RISk and Treatments (CORIST) Collaboration. Vascul Pharmacol. 2020 Dec.
Free PMC article
Show details
Vascul Pharmacol
. 2020 Dec;135:106805.
doi: 10.1016/j.vph.2020.106805. Epub 2020 Sep 28.

Author

COVID-19 RISk and Treatments (CORIST) Collaboration

Collaborators

- **COVID-19 RISk and Treatments (CORIST) Collaboration**: Augusto Di Castelnuovo [1], Simona Costanzo [2], Andrea Antinori [3], Nausicaa Berselli [4], Lorenzo Blandi [5], Marialaura Bonaccio [2], Roberto Cauda [6], Alessandro Gialluisi [2], Giovanni Guaraldi [7], Lorenzo Menicanti [5], Marco Mennuni [8], Roberta Mussinelli [9], Ilaria My [10], Giustino Parruti [11], Giuseppe Patti [8], Stefano Perlini [12], Francesca Santilli [13], Carlo Signorelli [14], Giulio G Stefanini [10], Alessandra Vergori [15], Pasquale Abete [16], Walter Ageno [17], Piergiuseppe Agostoni [18], Luca Aiello [19], Samir Al Moghazi [20], Rosa Arboretti [21], Filippo Aucella [22], Greta Barbieri [23], Martina Barchitta [24], Alessandro Bartoloni [25], Paolo Bonfanti [26], Francesco Cacciatore [16], Lucia Caiano [17], Laura Carrozzi [27], Antonio Cascio [28], Giacomo Castiglione [29], Stefania Cianfrone [13], Arturo Ciccullo [30], Antonella Cingolani [6], Francesco Cipollone [13], Claudia Colomba [28], Crizia Colombo [8], Ottavia Cozzi [10], Annalisa Crisetti [22], Francesca Crosta [11], Gian Battista Danzi [31], Damiano D'Ardes [13], Katleen de Gaetano Donati [30], Francesco Di Gennaro [32], Giuseppe Di Tano [31], Gianpiero D'Offizi [33], Francesco Maria Fusco [34], Ivan Gentile [35], Emauele Graziani [36], Gabriella Guarnieri [37], Giovanni Larizza [38], Armando Leone [39], Veronica Lio [8], Mothanje Barbara Lucia [6], Gloria Maccagni [31], Ferruccio Madaro [38], Stefano Maitan [19], Sandro Mancarella [40], Rosa Manuele [41], Massimo Mapelli [18], Riccardo Maragna [18], Rossella Marcucci [25], Giulio Maresca [42], Silvia Marongiu [43], Claudia Marotta [32], Lorenzo Marra [39], Franco Mastroianni [38], Maria Mazzitelli [44], Alessandro Mengozzi [23], Francesco Menichetti [23], Marianna Meschiari [7], Jovana Milic [7], Filippo Minutolo [45], Beatrice Molena [37], Cristina Mussini [7], Maria Musso [46], Anna Odone [14], Marco Olivieri [47], Antonella Palimodde [43], Emanuela Pasi [36], Raffaele Pesavento [48], Francesco Petri [49], Biagio Pinchera [35], Carlo A Pivato [10], Venerino Poletti [50], Claudia Ravaglia [50], Marco Rossato [48], Marianna Rossi [49], Anna Sabena [51], Francesco Salinaro [51], Vincenzo Sangiovanni [34], Carlo Sanrocco [11], Giancarlo Scoppettuolo [30], Laura Scorzolini [52], Raffaella Sgariglia [40], Paola Giustina Simeone [11], Enrico Maria Trecarichi [44], Roberto Vettor [48], Andrea Vianello [37], Marco Vinceti [53], Alexandra Virano [17], Laura Vocciante [42], Raffaele De Caterina [54], Licia Iacoviello [55]

Affiliations

- [1] Mediterranea Cardiocentro, Napoli, Italy.
- [2] Department of Epidemiology and Prevention, IRCCS Neuromed, Pozzilli (IS), Italy.
- [3] UOC Immunodeficienze Virali, National Institute for Infectious Diseases "L. Spallanzani", IRCCS, Roma, Italy.
- [4] Section of Public Health, Department of Biomedical, Metabolic and Neural Sciences, University of Modena and Reggio Emilia, Modena, Italy.
- [5] IRCCS Policlinico San Donato, San Donato Milanese (MI), Italy.
- [6] Fondazione Policlinico Universitario A, Gemelli IRCCS, Roma, Italy; Università Cattolica del Sacro Cuore- Dipartimento di Sicurezza e Bioetica Sede di Roma, Roma, Italy.
- [7] Infectious Disease Unit, Department of Surgical, Medical, Dental and Morphological Sciences, University of Modena and Reggio Emilia, Modena, Italy.
- [8] University of Eastern Piedmont, Maggiore della Carità Hospital, Novara, Italy.
- [9] Department of Internal Medicine, University of Pavia, Pavia, Italy.

- [10] Humanitas Clinical and Research Center - IRCCS, Rozzano (Mi), Italy; Humanitas University, Department of Biomedical Sciences, Milano, Italy.
- [11] Department of Infectious Disease, Azienda Sanitaria Locale (AUSL) di Pescara, Pescara, Italy.
- [12] Emergency Department, IRCCS Policlinico San Matteo Foundation, Pavia, Italy; Department of Internal Medicine, University of Pavia, Pavia, Italy.
- [13] Department of Medicine and Aging, Clinica Medica, "SS, Annunziata" Hospital and University of Chieti, Chieti, Italy.
- [14] School of Medicine, Vita-Salute San Raffaele University, Milano, Italy.
- [15] HIV/AIDS Department, National Institute for Infectious Diseases "Lazzaro Spallanzani"-IRCCS, Roma, Italy.
- [16] Dipartimento di Scienze Mediche Traslazionali, Università degli studi di Napoli "Federico II", Napoli, Italy.
- [17] Department of Medicine and Surgery, University of Insubria, Varese, Italy.
- [18] Centro Cardiologico Monzino IRCCS, Milano, Italy; Department of Clinical Sciences and Community Health, Cardiovascular Section, University of Milano, Milano, Italy.
- [19] UOC, Anestesia e Rianimazione, Dipartimento di Chirurgia Generale Ospedale Morgagni-Pierantoni, Forlì, Italy.
- [20] UOC Infezioni Sistemiche dell'Immunodepresso, National Institute for Infectious Diseases L. Spallanzani, IRCCS, Rome, Italy.
- [21] Department of Civil Environmental and Architectural Engineering, University of Padova, Padova, Italy.
- [22] Fondazione I.R.C.C.S "Casa Sollievo della Sofferenza", San Giovanni Rotondo, Foggia, Italy.
- [23] Emergency Medicine Unit, University Hospital of Pisa, Department of Surgical, Medical and Molecular Medicine and Critical Care, University of Pisa, Pisa, Italy.
- [24] Department of Medical and Surgical Sciences and Advanced Technologies "G.F, Ingrassia", University of Catania, Italy.
- [25] Department of Experimental and Clinical Medicine, University of Florence and Azienda Ospedaliero-Universitaria Careggi, Firenze, Italy.
- [26] UOC Malattie Infettive, Ospedale San Gerardo, ASST, Monza, Monza, Italy; School of Medicine and Surgery, University of Milano-Bicocca, Milano, Italy.
- [27] Cardiovascular and Thoracic Department, Azienda Ospedaliero-Universitaria Pisana and University of Pisa, Pisa, Italy.
- [28] Infectious and Tropical Diseases Unit- Department of Health Promotion, Mother and Child Care, Internal Medicine and Medical Specialties (PROMISE) - University of Palermo, Palermo, Italy.
- [29] Servizio di Anestesia e Rianimazione II UO Rianimazione Ospedale San Marco, AOU Policlinico-Vittorio Emanuele, Catania, Italy.
- [30] Fondazione Policlinico Universitario A, Gemelli IRCCS, Roma, Italy.
- [31] Department of Cardiology, Ospedale di Cremona, Cremona, Italy.
- [32] Medical Direction, IRCCS Neuromed, Pozzilli (IS), Italy.
- [33] UOC Malattie Infettive-Epatologia, National Institute for Infectious Diseases L. Spallanzani, IRCCS, Roma, Italy.
- [34] UOC Infezioni Sistemiche e dell'Immunodepresso, Azienda Ospedaliera dei Colli, Ospedale Cotugno, Napoli, Italy.
- [35] Department of Clinical Medicine and Surgery, University of Naples "Federico II", Napoli, Italy.
- [36] Medicina Interna, Ospedale di Ravenna, AUSL della Romagna, Ravenna, Italy.

- [37] Respiratory Pathophysiology Division, Department of Cardiologic, Thoracic and Vascular Sciences, University of Padova, Padova, Italy.
- [38] COVID-19 Unit, EE Ospedale Regionale F, Miulli, Acquaviva delle Fonti (BA), Italy.
- [39] UOC di Pneumologia, P.O, San Giuseppe Moscati, Taranto, Italy.
- [40] ASST Milano Nord - Ospedale Edoardo Bassini Cinisello Balsamo (MI), Italy.
- [41] U.O, C, Malattie Infettive e Tropicali, P.O, "San Marco", AOU Policlinico-Vittorio Emanuele, Catania, Italy.
- [42] UOC di Medicina - Presidio Ospedaliero S, Maria di Loreto Nuovo, Napoli, Italy.
- [43] Ospedale Santissima Trinità, Cagliari, Italy.
- [44] Infectious and Tropical Diseases Unit, Deparment of Medical and Surgical Sciences "Magna Graecia" University, Catanzaro, Italy.
- [45] Dipartimento di Farmacia, Università di Pisa, Pisa, Italy.
- [46] UOC Malattie Infettive-Apparato Respiratorio, National Institute for Infectious Diseases "L. Spallanzani", IRCCS, Roma, Italy.
- [47] Computer Service, University of Molise, Campobasso, Italy.
- [48] Clinica Medica 3, Department of Medicine - DIMED, University Hospital of Padova, Padova, Italy.
- [49] UOC Malattie Infettive, Ospedale San Gerardo, ASST, Monza, Monza, Italy.
- [50] UOC Pneumologia, Dipartimento di Malattie Apparato Respiratorio e Torace, Ospedale Morgagni-Pierantoni, Forlì, Italy.
- [51] Emergency Department, IRCCS Policlinico San Matteo Foundation, Pavia, Italy.
- [52] UOC Malattie Infettive ad Alta Intensità di Cura, National Institute for Infectious Diseases "L. Spallanzani". IRCCS, Rome, Italy.
- [53] Section of Public Health, Department of Biomedical, Metabolic and Neural Sciences, University of Modena and Reggio Emilia, Modena, Italy; Department of Epidemiology, Boston University School of Public Health, Boston, USA.
- [54] Cardiovascular and Thoracic Department, Azienda Ospedaliero-Universitaria Pisana and University of Pisa, Pisa, Italy. Electronic address: raffaele.decaterina@unipi.it.
- [55] Department of Epidemiology and Prevention, IRCCS Neuromed, Pozzilli (IS), Italy; Department of Medicine and Surgery, University of Insubria, Varese, Italy.

- PMID: **32992048**
- PMCID: PMC7521934
- DOI: 10.1016/j.vph.2020.106805

Abstract

Objective: The hypothesis that been set forward that use of Renin Angiotensin Aldosterone System (RAAS) inhibitors is associated with COVID-19 severity. We set-up a multicenter Italian collaboration (CORIST Project, ClinicalTrials.gov ID: NCT04318418) to retrospectively investigate the relationship between RAAS inhibitors and COVID-19 in-hospital mortality. We also carried out an updated meta-analysis on the relevant studies.

Methods: We analyzed 4069 unselected patients with laboratory-confirmed SARS-CoV-2 infection and hospitalized in 34 clinical centers in Italy from February 19, 2020 to May 23, 2020. The primary end-point in a time-to event analysis was in-hospital death, comparing patients who received angiotensin-converting-enzyme inhibitors (ACEI) or angiotensin-receptor blockers (ARB) with patients who did not. Articles for the meta-analysis were retrieved until July 13th,

2020 by searching in web-based libraries, and data were combined using the general variance-based method.

Results: Out of 4069 COVID-19 patients, 13.5% and 13.3% received ACE-I or ARB, respectively. Use of neither ACE-I nor ARB was associated with mortality (multivariable hazard ratio (HR) adjusted also for COVID-19 treatments: 0.96, 95% confidence interval 0.77-1.20 and HR = 0.89, 0.67-1.19 for ACE-I and ARB, respectively). Findings were similar restricting the analysis to hypertensive (N = 2057) patients (HR = 1.00, 0.78-1.26 and HR = 0.88, 0.65-1.20) or when ACE-I or ARB were considered as a single group. Results from the meta-analysis (19 studies, 29,057 COVID-19 adult patients, 9700 with hypertension) confirmed the absence of association.

Conclusions: In this observational study and meta-analysis of the literature, ACE-I or ARB use was not associated with severity or in-hospital mortality in COVID-19 patients.

Keywords: ACE-I; ARB; Angiotensin converting enzyme inhibitors; Angiotensin receptor blockers; COVID−19; Mortality; Sartans.

Copyright © 2020 Elsevier Inc. All rights reserved.

- 38 references
- 1 figure

Supplementary info

Publication types, MeSH terms, Substances, Associated data Expand

Publication types

- Multicenter Study
- Observational Study

MeSH terms

- Aged
- Aged, 80 and over
- Angiotensin Receptor Antagonists / adverse effects
- Angiotensin Receptor Antagonists / therapeutic use*
- Angiotensin-Converting Enzyme Inhibitors / adverse effects
- Angiotensin-Converting Enzyme Inhibitors / therapeutic use*
- Antihypertensive Agents / adverse effects
- Antihypertensive Agents / therapeutic use*
- COVID-19 / diagnosis
- COVID-19 / mortality*
- COVID-19 / therapy
- Female
- Hospital Mortality*

- Hospitalization
- Humans
- Hypertension / drug therapy*
- Hypertension / epidemiology
- Hypertension / physiopathology
- Incidence
- Italy / epidemiology
- Male
- Meta-Analysis as Topic
- Middle Aged
- Renin-Angiotensin System / drug effects*
- Risk Assessment
- Risk Factors
- Severity of Illness Index

Substances

- Angiotensin Receptor Antagonists
- Angiotensin-Converting Enzyme Inhibitors
- Antihypertensive Agents

Associated data

- ClinicalTrials.gov/NCT04318418

Full text links

ELSEVIER FULL-TEXT ARTICLE Elsevier Science Free PMC article
Proceed to details
Cite
Share
1,124
Observational Study
Magnes Res
. 2021 Aug 1;34(3):93-102.
doi: 10.1684/mrh.2021.0485.

Relationship of magnesemia with myocardial damage and mortality in patients with COVID-19

Seyda Gunay [1], Serhat Caliskan [2], Deniz Sigirli [3]

Affiliations Expand

Affiliations

- [1] Bursa Uludag University Faculty of Medicine, Department of Cardiology, Turkey.
- [2] Istanbul Bahcelievler State Hospital Department of Cardiology, Turkey.
- [3] Bursa Uludag University Faculty of Medicine, Department of Biostatistics, Turkey.

- PMID: **34704948**
- DOI: 10.1684/mrh.2021.0485

Observational Study

Relationship of magnesemia with myocardial damage and mortality in patients with COVID-19

Seyda Gunay et al. Magnes Res. 2021.

Show details

Magnes Res

. 2021 Aug 1;34(3):93-102.

doi: 10.1684/mrh.2021.0485.

Authors

Seyda Gunay [1], Serhat Caliskan [2], Deniz Sigirli [3]

Affiliations

- [1] Bursa Uludag University Faculty of Medicine, Department of Cardiology, Turkey.
- [2] Istanbul Bahcelievler State Hospital Department of Cardiology, Turkey.
- [3] Bursa Uludag University Faculty of Medicine, Department of Biostatistics, Turkey.

- PMID: **34704948**
- DOI: 10.1684/mrh.2021.0485

Abstract

Magnesium (Mg) is the second most abundant intracellular cation and plays a significant role in immune system and cardiac protection. Mg deficiency contributes to chronic low-grade inflammation leading to cardiovascular diseases, and low Mg level exacerbates virus-induced inflammation. The aim of the study was to investigate whether serum magnesium level is associated with myocardial damage and prognosis of COVID-19. This was a single-center, observational retrospective study of patients with COVID-19. The study population was divided into two groups according to in-hospital mortality: a survivor group (SG) and a non-survivor group (NSG). Myocardial damage was defined as blood levels of cardiac troponin I (cTnI) above

the 99th percentile upper reference limit. Magnesium, variables regarding inflammation, and myocardial damage were compared between the groups. A total of 629 patients with COVID-19 were included. Mortality rate was 11.85% (n = 82). There were 61 (74.4%) and 294 male patients (53.7%) in NSG and SG, respectively (p = 0.001). The median age of NSG was 64.5 years (min-max: 37-93) and the median age of SG was 56.0 years (min-max: 22-92) (p < 0.001). Median serum magnesium levels of NSG and SG were 1.94 mg/dL (min-max: 1.04-2.87) and 2.03 mg/dL (min-max: 1.18-2.88), respectively (p = 0.027). Median cTnI levels of NSG and SG were 25.20 pg/mL (min-max: 2.10-2240.80) and 4.50 pg/mL (min-max: 0.50-984.3), respectively (p < 0.001). The cTnI levels were lower in those patients whose serum Mg levels were higher than 1.94. Although serum magnesium level was not a predictor for in-hospital mortality, there was a significant negative correlation between magnesemia and myocardial damage.

Keywords: COVID-19; damage; magnesium; mortality; myocardium; troponin.

Supplementary info

Publication types, MeSH terms, Substances Expand

Publication types

- Observational Study

MeSH terms

- Adult
- Aged
- Aged, 80 and over
- COVID-19 / blood*
- COVID-19 / complications*
- COVID-19 / mortality
- Cardiomyopathies / blood*
- Cardiomyopathies / complications*
- Female
- Humans
- Inflammation
- Magnesium Deficiency / blood*
- Magnesium Deficiency / complications*
- Male
- Middle Aged
- Myocardium / pathology
- Prognosis
- Proportional Hazards Models
- Reference Values
- Retrospective Studies
- Treatment Outcome

- Troponin I / blood

Substances

- Troponin I

Full text links

Magnesium RESEARCH John Libbey Eurotext
Proceed to details
Cite
Share
1,125
Observational Study
Ann Vasc Surg
. 2021 Jul;74:73-79.
doi: 10.1016/j.avsg.2021.01.072. Epub 2021 Feb 5.

COVID-19 Impact on Vascular Surgery Practice: Experience From an Italian University Regional Hub Center for Vascular Pathology

Gian Antonio Boschetti [1], Sara Di Gregorio [2], Jorge Miguel Mena Vera [3], Bianca Pane [2], Giovanni Spinella [2], Domenico Palombo [2], Giovanni Pratesi [2]
Affiliations Expand

Affiliations

- [1] Vascular and Endovascular Surgery Unit, Ospedale Policlinico San Martino - IRCCS, Genoa, Italy; Department of Surgical Sciences and Integrated Diagnostic (DISC), University of Genoa, Genoa, Italy. Electronic address: gianantonioboschetti@gmail.com.
- [2] Vascular and Endovascular Surgery Unit, Ospedale Policlinico San Martino - IRCCS, Genoa, Italy; Department of Surgical Sciences and Integrated Diagnostic (DISC), University of Genoa, Genoa, Italy.
- [3] Vascular and Endovascular Surgery Unit, Ospedale Policlinico San Martino - IRCCS, Genoa, Italy.

- PMID: **33549797**
- PMCID: PMC7862030
- DOI: 10.1016/j.avsg.2021.01.072

Free PMC article
Observational Study

COVID-19 Impact on Vascular Surgery Practice: Experience From an Italian University Regional Hub Center for Vascular Pathology

Gian Antonio Boschetti et al. Ann Vasc Surg. 2021 Jul.
Free PMC article
Show details
Ann Vasc Surg
. 2021 Jul;74:73-79.
doi: 10.1016/j.avsg.2021.01.072. Epub 2021 Feb 5.

Authors

Gian Antonio Boschetti [1], Sara Di Gregorio [2], Jorge Miguel Mena Vera [3], Bianca Pane [2], Giovanni Spinella [2], Domenico Palombo [2], Giovanni Pratesi [2]

Affiliations

- [1] Vascular and Endovascular Surgery Unit, Ospedale Policlinico San Martino - IRCCS, Genoa, Italy; Department of Surgical Sciences and Integrated Diagnostic (DISC), University of Genoa, Genoa, Italy. Electronic address: gianantonioboschetti@gmail.com.
- [2] Vascular and Endovascular Surgery Unit, Ospedale Policlinico San Martino - IRCCS, Genoa, Italy; Department of Surgical Sciences and Integrated Diagnostic (DISC), University of Genoa, Genoa, Italy.
- [3] Vascular and Endovascular Surgery Unit, Ospedale Policlinico San Martino - IRCCS, Genoa, Italy.

- PMID: **33549797**
- PMCID: PMC7862030
- DOI: 10.1016/j.avsg.2021.01.072

Abstract

Background: The aim of the study is to evaluate the impact of COVID-19 pandemic on vascular surgery practice in a regional hub center for complex vascular disease.

Methods: This is an observational single-center study in which we collected clinical and surgical data during (P1) and after (P2) the COVID-19 outbreak and the lockdown measures implemented in Northern Italy. We compared those data with the two-month period before the pandemic (P0).

Results: Compared to P0, ambulatory activities were severely reduced during P1 and limited to hospitalized patients and outpatients with urgent criteria. We performed 61 operations (18 urgent and 43 elective), with a decrease in both aortic (-17.8%), cerebrovascular (-53.3%), and peripheral artery (-42.6%) disease treatments. We also observed a greater drop in open procedures (-53.2%) than in endovascular ones (-22%). All the elective patients were treated for notdeferrable conditions and they were COVID-19 negative at the ward admission screening; despite this one of

them developed COVID19 during the hospital stay. Four COVID-19 positive patients were treated in urgent setting for acute limb ischemia. Throughout P2 we gradually rescheduled elective ambulatory (+155.5%) and surgical (+18%) activities, while remaining substantially lower than during P0 (respectively -45.6% and -25.7%).

Conclusions: Despite COVID-19 pandemic, our experience shows that with careful patient's selection, dedicated prehospitalization protocol and proper use of personal protective equipment it is possible to guarantee continuity of care.

Copyright © 2021. Published by Elsevier Inc.

- 22 references
- 2 figures

Supplementary info

Publication types, MeSH terms Expand

Publication types

- Comparative Study
- Observational Study

MeSH terms

- Academic Medical Centers / trends*
- Aged
- Aged, 80 and over
- COVID-19*
- Clinical Decision-Making
- Continuity of Patient Care / trends
- Female
- Humans
- Italy
- Male
- Middle Aged
- Patient Selection
- Practice Patterns, Physicians' / trends*
- Regional Health Planning / trends
- Retrospective Studies
- Surgeons / trends*
- Time Factors
- Vascular Surgical Procedures / trends*

Full text links

ELSEVIER FULL-TEXT ARTICLE Elsevier Science Free PMC article
Proceed to details
Cite
Share
1,126

Observational Study

Anaesth Crit Care Pain Med
. 2021 Feb;40(1):100780.
doi: 10.1016/j.accpm.2020.10.014. Epub 2020 Nov 13.

A novel, automated, quantification of abnormal lung parenchyma in patients with COVID-19 infection: Initial description of feasibility and association with clinical outcome

Eric Noll[1], Luc Soler[2], Mickael Ohana[3], Pierre-Olivier Ludes[4], Julien Pottecher[4], Elliott Bennett-Guerrero[5], Francis Veillon[6], Bernard Goichot[7], Francis Schneider[8], Nicolas Meyer[9], Pierre Diemunsch[10]
Affiliations Expand

Affiliations

- [1] Department of Anesthesiology and Intensive Care, Hautepierre Hospital, Strasbourg University Hospital, France. Electronic address: eric.noll@chru-strasbourg.fr.
- [2] Digestive and Endocrine Surgery Department, Nouvel Hôpital Civil, Strasbourg, France; Visible Patient, Strasbourg, France.
- [3] Department of Radiology, Nouvel Hôpital Civil, Strasbourg University Hospital, France.
- [4] Department of Anesthesiology and Intensive Care, Hautepierre Hospital, Strasbourg University Hospital, France; Equipe d'Accueil 3072, Medical School, Strasbourg University, Strasbourg, France.
- [5] Department of Anesthesiology, Stony Brook Medicine, New-York, USA.
- [6] Department of Radiology, Hautepierre Hospital, Strasbourg University Hospital, France.
- [7] Department of Internal Medicine, Hautepierre Hospital, Strasbourg University Hospital, France.
- [8] Médecine Intensive-Réanimation, Hautepierre Hospital, Strasbourg University Hospital, Strasbourg, France.
- [9] Department of Biostatistics, Strasbourg University Hospital, France.
- [10] Department of Anesthesiology and Intensive Care, Hautepierre Hospital, Strasbourg University Hospital, France; Institut Hospitalo-Universitaire "Image-Guided Surgery", Strasbourg University Hospital, Strasbourg, France; Equipe d'Accueil 3072, Medical School, Strasbourg University, Strasbourg, France.

- PMID: **33197638**

- PMCID: PMC7664353
- DOI: 10.1016/j.accpm.2020.10.014

Free PMC article
Observational Study

A novel, automated, quantification of abnormal lung parenchyma in patients with COVID-19 infection: Initial description of feasibility and association with clinical outcome

Eric Noll et al. Anaesth Crit Care Pain Med. 2021 Feb.
Free PMC article
Show details
Anaesth Crit Care Pain Med
. 2021 Feb;40(1):100780.
doi: 10.1016/j.accpm.2020.10.014. Epub 2020 Nov 13.

Authors

Eric Noll [1], Luc Soler [2], Mickael Ohana [3], Pierre-Olivier Ludes [4], Julien Pottecher [4], Elliott Bennett-Guerrero [5], Francis Veillon [6], Bernard Goichot [7], Francis Schneider [8], Nicolas Meyer [9], Pierre Diemunsch [10]

Affiliations

- [1] Department of Anesthesiology and Intensive Care, Hautepierre Hospital, Strasbourg University Hospital, France. Electronic address: eric.noll@chru-strasbourg.fr.
- [2] Digestive and Endocrine Surgery Department, Nouvel Hôpital Civil, Strasbourg, France; Visible Patient, Strasbourg, France.
- [3] Department of Radiology, Nouvel Hôpital Civil, Strasbourg University Hospital, France.
- [4] Department of Anesthesiology and Intensive Care, Hautepierre Hospital, Strasbourg University Hospital, France; Equipe d'Accueil 3072, Medical School, Strasbourg University, Strasbourg, France.
- [5] Department of Anesthesiology, Stony Brook Medicine, New-York, USA.
- [6] Department of Radiology, Hautepierre Hospital, Strasbourg University Hospital, France.
- [7] Department of Internal Medicine, Hautepierre Hospital, Strasbourg University Hospital, France.
- [8] Médecine Intensive-Réanimation, Hautepierre Hospital, Strasbourg University Hospital, Strasbourg, France.
- [9] Department of Biostatistics, Strasbourg University Hospital, France.
- [10] Department of Anesthesiology and Intensive Care, Hautepierre Hospital, Strasbourg University Hospital, France; Institut Hospitalo-Universitaire "Image-Guided Surgery",

Strasbourg University Hospital, Strasbourg, France; Equipe d'Accueil 3072, Medical School, Strasbourg University, Strasbourg, France.

- PMID: **33197638**
- PMCID: PMC7664353
- DOI: 10.1016/j.accpm.2020.10.014

Abstract

Objective: Ground-glass opacities are the most frequent radiologic features of COVID-19 patients. We aimed to determine the feasibility of automated lung volume measurements, including ground-glass volumes, on the CT of suspected COVID-19 patients. Our goal was to create an automated and quantitative measure of ground-glass opacities from lung CT images that could be used clinically for diagnosis, triage and research.

Design: Single centre, retrospective, observational study.

Measurements: Demographic data, respiratory support treatment (synthetised in the maximal respiratory severity score) and CT-images were collected. Volume of abnormal lung parenchyma was measured with conventional semi-automatic software and with a novel automated algorithm based on voxels X-Ray attenuation. We looked for the relationship between the automated and semi-automated evaluations. The association between the ground-glass opacities volume and the maximal respiratory severity score was assessed.

Main results: Thirty-seven patients were included in the main outcome analysis. The mean duration of automated and semi-automated volume measurement process were 15 (2) and 93 (41) min, respectively ($p=8.05*10^{-8}$). The intraclass correlation coefficient between the semi-automated and automated measurement of ground-glass opacities and restricted normally aerated lung were both superior to 0.99. The association between the automated measured lung volume and the maximal clinical severity score was statistically significant for the restricted normally aerated (p=0.0097, effect-size: -385mL) volumes and for the ratio of ground-glass opacities/restricted normally aerated volumes (p=0.027, effect-size: 3.3).

Conclusion: The feasibility and preliminary validity of automated impaired lung volume measurements in a high-density COVID-19 cluster was confirmed by our results.

Keywords: ARDS; COVID-19; CT-scan; Infectious disease; Severity assessment; Triage.

Copyright © 2020 Société française d'anesthésie et de réanimation (Sfar). Published by Elsevier Masson SAS. All rights reserved.

- 30 references
- 3 figures

Supplementary info

Publication types, MeSH terms Expand

Publication types

- Observational Study

MeSH terms

- Algorithms
- Automation
- COVID-19 / diagnostic imaging*
- Feasibility Studies
- Female
- Humans
- Lung / diagnostic imaging*
- Lung Volume Measurements / methods*
- Male
- Middle Aged
- Reproducibility of Results
- Retrospective Studies
- Severity of Illness Index
- Software
- Supine Position
- Time Factors
- Tomography, X-Ray Computed / methods*
- Treatment Outcome
- Triage

Full text links

ELSEVIER FULL-TEXT ARTICLE Elsevier Science Free PMC article

Proceed to details

Cite

Share

☐ 1,127

Observational Study

Clin Dermatol

. Jul-Aug 2021;39(4):710-713.

doi: 10.1016/j.clindermatol.2020.11.006. Epub 2020 Nov 25.

Syphilis and the COVID-19 pandemic: Did the lockdown stop risky sexual behavior?

Filippo Bonato [1], Caterina Ferreli [2], Roberta Satta [3], Franco Rongioletti [4], Laura Atzori [5]

Affiliations Expand

Affiliations

- [1] Dermatology Clinic, Department of Medical Sciences and Public Health, University of Cagliari, Cagliari, Italy. Electronic address: filippomaria.bonato@gmail.com.
- [2] Dermatology Clinic, Department of Medical Sciences and Public Health, University of Cagliari, Cagliari, Italy. Electronic address: ferreli@unica.it.
- [3] Dermatology Clinic, Department of Medical Sciences and Public Health, University of Cagliari, Cagliari, Italy. Electronic address: roalesat@yahoo.it.
- [4] Dermatology Clinic, Department of Medical Sciences and Public Health, University of Cagliari, Cagliari, Italy. Electronic address: rongioletti@unica.it.
- [5] Dermatology Clinic, Department of Medical Sciences and Public Health, University of Cagliari, Cagliari, Italy. Electronic address: atzoril@unica.it.

- PMID: **34809778**
- PMCID: PMC7685935
- DOI: 10.1016/j.clindermatol.2020.11.006

Free PMC article
Observational Study

Syphilis and the COVID-19 pandemic: Did the lockdown stop risky sexual behavior?

Filippo Bonato et al. Clin Dermatol. Jul-Aug 2021.
Free PMC article
Show details
Clin Dermatol
. Jul-Aug 2021;39(4):710-713.
doi: 10.1016/j.clindermatol.2020.11.006. Epub 2020 Nov 25.

Authors

Filippo Bonato [1], Caterina Ferreli [2], Roberta Satta [3], Franco Rongioletti [4], Laura Atzori [5]

Affiliations

- [1] Dermatology Clinic, Department of Medical Sciences and Public Health, University of Cagliari, Cagliari, Italy. Electronic address: filippomaria.bonato@gmail.com.
- [2] Dermatology Clinic, Department of Medical Sciences and Public Health, University of Cagliari, Cagliari, Italy. Electronic address: ferreli@unica.it.
- [3] Dermatology Clinic, Department of Medical Sciences and Public Health, University of Cagliari, Cagliari, Italy. Electronic address: roalesat@yahoo.it.
- [4] Dermatology Clinic, Department of Medical Sciences and Public Health, University of Cagliari, Cagliari, Italy. Electronic address: rongioletti@unica.it.
- [5] Dermatology Clinic, Department of Medical Sciences and Public Health, University of Cagliari, Cagliari, Italy. Electronic address: atzoril@unica.it.

- PMID: **34809778**
- PMCID: PMC7685935
- DOI: 10.1016/j.clindermatol.2020.11.006

Abstract

Restrictive measures to contain the coronavirus disease 2019 (COVID-19) pandemic might produce different effects on other infective diseases, especially those affecting the most intimate sphere of sexuality. The epidemiology of syphilis could reflect the consequences of whether people are avoiding or not risky behaviors. To understand the course of syphilis during the COVID-19 outbreak, we performed a retrospective observational study of all new diagnoses observed at the STDs Service of the Dermatology Clinic at Cagliari, part of the Italian sentinel surveillance system. All incident cases diagnosed during the first 6 months of each year, from 2016 to 2020, thus including the recent lockdown period, were retrieved from the database. Of the 87 cases studied, 18 occurred during the first 6 months of 2020, almost all patients (88%) presenting with early phases of the disease and reporting unprotected sexual intercourses in spite of community containment and social distancing. Comparison with the previous 4 years found no significant statistical differences that hospital access and management limitations had not impaired the management of patients with syphilis. We alert the medical community of the possible increase of sexually transmitted diseases, as society returns to normal.

Copyright © 2020 Elsevier Inc. All rights reserved.

Conflict of interest statement

Conflict of interest The authors declare that they have no known competing financial interests or personal relationships that could have appeared to influence the work reported in this paper.

Comment in

- STIs during the first and second wave of COVID-19 in Denmark.
  Heerfordt IM. Heerfordt IM. Sex Transm Infect. 2022 Mar;98(2):150-151. doi: 10.1136/sextrans-2021-055021. Epub 2021 Mar 18. Sex Transm Infect. 2022. PMID: 33737449 Free PMC article. No abstract available.

- 12 references
- 2 figures

Supplementary info

Publication types, MeSH terms Expand

Publication types

- Observational Study

MeSH terms

- COVID-19*
- Communicable Disease Control
- Humans
- Pandemics
- SARS-CoV-2

- Sexual Behavior
- Syphilis* / diagnosis
- Syphilis* / epidemiology
- Syphilis* / prevention & control

Full text links

ELSEVIER FULL-TEXT ARTICLE Elsevier Science Free PMC article
Proceed to details
Cite
Share
1,128
Observational Study
Actas Dermosifiliogr (Engl Ed)
. 2020 Oct;111(8):629-638.
doi: 10.1016/j.ad.2020.05.001. Epub 2020 May 11.

[Estimated Effect of COVID-19 Lockdown on Skin Tumor Size and Survival: An Exponential Growth Model]

[Article in Spanish]
A Tejera-Vaquerizo [1], J Cañueto [2], A Toll [3], J Santos-Juanes [4], A Jaka [5], C Ferrandiz-Pulido [6], O Sanmartín [7], S Ribero [8], D Moreno-Ramírez [9], F Almazán [10], M J Fuente [5], S Podlipnik [3], E Nagore [7]
Affiliations Expand

Affiliations

- [1] Servicio de Dermatología, Instituto Dermatológico GlobalDerm, Palma del Río, Córdoba, España. Electronic address: antoniotejera@aedv.es.
- [2] Servicio de Dermatología, Hospital Universitario de Salamanca, Salamanca, España.
- [3] Servicio de Dermatología, Hospital Clìnic de Barcelona, Barcelona, España.
- [4] Servicio de Dermatología, Hospital Universitario Central de Asturias, Oviedo, Asturias, España.
- [5] Servicio de Dermatología, Hospital Germans Trial i Pujol, Badalona, Barcelona, España.
- [6] Servicio de Dermatología, Hospital Universitario Vall d'Hebron, Barcelona, España.
- [7] Servicio de Dermatología, Instituto Valenciano de Oncología, Valencia, España.
- [8] Departamento de Dermatología, Hospital Universitario de Turín, Turín, Italia.
- [9] Unidad de Melanoma, Servicio de Dermatología Médico-Quirúrgica, Hospital Universitario Virgen Macarena, Sevilla, España.
- [10] Unidad de Gestión Clínica de Dermatología, Hospital Universitario San Cecilio, Granada, España.

- PMID: **32513393**
- PMCID: PMC7211725
- DOI: 10.1016/j.ad.2020.05.001

Free PMC article
Observational Study

[Estimated Effect of COVID-19 Lockdown on Skin Tumor Size and Survival: An Exponential Growth Model]

[Article in Spanish]
A Tejera-Vaquerizo et al. Actas Dermosifiliogr (Engl Ed). 2020 Oct.
Free PMC article
Show details
Actas Dermosifiliogr (Engl Ed)
. 2020 Oct;111(8):629-638.
doi: 10.1016/j.ad.2020.05.001. Epub 2020 May 11.

Authors

A Tejera-Vaquerizo [1], J Cañueto [2], A Toll [3], J Santos-Juanes [4], A Jaka [5], C Ferrandiz-Pulido [6], O Sanmartín [7], S Ribero [8], D Moreno-Ramírez [9], F Almazán [10], M J Fuente [5], S Podlipnik [3], E Nagore [7]

Affiliations

- [1] Servicio de Dermatología, Instituto Dermatológico GlobalDerm, Palma del Río, Córdoba, España. Electronic address: antoniotejera@aedv.es.
- [2] Servicio de Dermatología, Hospital Universitario de Salamanca, Salamanca, España.
- [3] Servicio de Dermatología, Hospital Clìnic de Barcelona, Barcelona, España.
- [4] Servicio de Dermatología, Hospital Universitario Central de Asturias, Oviedo, Asturias, España.
- [5] Servicio de Dermatología, Hospital Germans Trial i Pujol, Badalona, Barcelona, España.
- [6] Servicio de Dermatología, Hospital Universitario Vall d'Hebron, Barcelona, España.
- [7] Servicio de Dermatología, Instituto Valenciano de Oncología, Valencia, España.
- [8] Departamento de Dermatología, Hospital Universitario de Turín, Turín, Italia.
- [9] Unidad de Melanoma, Servicio de Dermatología Médico-Quirúrgica, Hospital Universitario Virgen Macarena, Sevilla, España.
- [10] Unidad de Gestión Clínica de Dermatología, Hospital Universitario San Cecilio, Granada, España.

- PMID: **32513393**
- PMCID: PMC7211725
- DOI: 10.1016/j.ad.2020.05.001

Abstract
in English, Spanish

Background and objectives: Spain is in a situation of indefinite lockdown due to the ongoing coronavirus disease 2019 (COVID-19) pandemic. One of the consequences of this lockdown is delays in medical and surgical procedures for common diseases. The aim of this study was to model the impact on survival of tumor growth caused by such delays in patients with squamous cell carcinoma (SCC) and melanoma.

Material and methods: Multicenter, retrospective, observational cohort study. We constructed an exponential growth model for both SCC and melanoma to estimate tumor growth between patient-reported onset and surgical excision at different time points.

Results: Data from 200 patients with SCC of the head and neck and 1000 patients with cutaneous melanoma were included. An exponential growth curve was calculated for each tumor type and we estimated tumor size after 1, 2, and 3 months of potential surgical delay. The proportion of patients with T3 SCC (diameter >4cm or thickness >6 mm) increased from 41.5% (83 patients) in the initial study group to an estimated 58.5%, 70.5%, and 72% after 1, 2, and 3 months of delay. Disease-specific survival at 2, 5, and 10 years in patients whose surgery was delayed by 3 months decreased by 6.2%, 8.2%, and 5.2%, respectively. The proportion of patients with ultrathick melanoma (>6 mm) increased from 6.9% in the initial study group to 21.9%, 30.2%, and 30.2% at 1, 2, and 3 months. Five- and 10-year disease-specific survival both decreased by 14.4% in patients treated after a potential delay of 3 months.

Conclusions: In the absence of adequate diagnosis and treatment of SCC and melanoma in the current lockdown situation in Spain, we can expect to see to a considerable increase in large and thick SCCs and melanomas. Efforts must be taken to encourage self-examination and facilitate access to dermatologists in order to prevent further delays.

Antecedentes y objetivos: La pandemia del coronavirus SARS-CoV-2 ha provocado un confinamiento indefinido. Una posible consecuencia de esta situación es un retraso en los procedimientos asistenciales de las enfermedades oncológicas. El objetivo de este estudio es estimar el hipotético impacto en la supervivencia que tendría el aumento del tamaño tanto para los carcinomas de células escamosas (CCE) como de los melanomas.

Material y método: Estudio observacional retrospectivo de cohorte multicéntrico. Se desarrolló un modelo de crecimiento exponencial para cada tumor basado en el tiempo de evolución que refiere el paciente.

Resultados: Se incluyeron un total de 200 pacientes con CCE localizados en la cabeza y el cuello y 1.000 pacientes con melanoma cutáneo. Se calculó una curva de crecimiento exponencial para cada tumor y se estimó el tamaño del tumor tras 1, 2 y 3 meses tras el diagnóstico. En la muestra, los CCE mayores de 4 cm o > 6 mm de grosor (definidos como T3) pasaron de 83 (41,5%) en el grupo de estudio real a una estimación del 58,5, 70,5 y 72% tras 1, 2 y 3 meses de retraso quirúrgico estimado, respectivamente. Se estimó una disminución de la supervivencia específica de enfermedad (SEE) de un 6,2, 8,2 y 5,2% a los 2, 5 y 10 años, respectivamente, tras 3 meses de retraso. Para los melanomas ultragruesos (> 6 mm de Breslow) pasaron del 6,9% en el grupo de estudio al 21,9, 30,2 y 30,2% tras 1, 2 y 3 meses de demora. La SEE a los 5 y 10 años del grupo de estudio descendió un 14,4% en ambos tiempos.

Conclusiones: En ausencia de un adecuado diagnóstico y tratamiento de los pacientes con CCE y melanoma en la actual situación de confinamiento en España, podemos llegar a asistir a un considerable aumento de los casos de CCE y melanomas gruesos y de gran tamaño. Se deben

fomentar los esfuerzos para promocionar la autoexploración y facilitar el acceso a los dermatólogos para no aumentar la demora de estos pacientes.

Keywords: COVID-19 virus disease; Cutaneous squamous cell carcinoma; Early diagnosis; Lockdown; Melanoma; Prognosis.

© 2020 Published by Elsevier España, S.L.U. on behalf of AEDV.

- 19 references
- 4 figures

Supplementary info

Publication types, MeSH terms Expand

Publication types

- Multicenter Study
- Observational Study

MeSH terms

- Age Factors
- Algorithms
- Betacoronavirus*
- COVID-19
- Carcinoma, Squamous Cell / mortality
- Carcinoma, Squamous Cell / pathology*
- Coronavirus Infections / epidemiology*
- Delayed Diagnosis / adverse effects
- Delayed Diagnosis / statistics & numerical data
- Female
- Head and Neck Neoplasms / mortality
- Head and Neck Neoplasms / pathology*
- Health Services Accessibility
- Humans
- Male
- Melanoma / mortality
- Melanoma / pathology*
- Pandemics
- Pneumonia, Viral / epidemiology*
- Public Health Surveillance / methods
- Quarantine
- Retrospective Studies
- SARS-CoV-2

- Sex Factors
- Skin Neoplasms / mortality
- Skin Neoplasms / pathology*
- Spain / epidemiology
- Time Factors
- Time-to-Treatment
- Tumor Burden*

Full text links

ELSEVIER OPEN ACCESS Elsevier Science Free PMC article
Proceed to details
Cite
Share
1,129
Observational Study
PLoS One
. 2020 Oct 15;15(10):e0239389.
doi: 10.1371/journal.pone.0239389. eCollection 2020.

The first wave of COVID-19 in Malta; a national cross-sectional study

Sarah Micallef [1], Tonio V Piscopo [2], Ramon Casha [2], Denise Borg [2], Chantal Vella [2], Maria-Alessandra Zammit [2], Janice Borg [1], Daniela Mallia [3], James Farrugia [2], Sarah Marie Vella [2], Thelma Xerri [2], Anette Portelli [2], Manuel Fenech [2], Claudia Fsadni [2], Charles Mallia Azzopardi [2]
Affiliations Expand

Affiliations

- [1] Department of Medicine, Mater Dei Hospital, Msida, Malta.
- [2] Department of Infectious Diseases, Mater Dei Hospital, Msida, Malta.
- [3] Department of Pharmacy, Mater Dei Hospital, Msida, Malta.

- PMID: **33057434**
- PMCID: PMC7561161
- DOI: 10.1371/journal.pone.0239389

Free PMC article
Observational Study

The first wave of COVID-19 in Malta; a national cross-sectional study

Sarah Micallef et al. PLoS One. 2020.
Free PMC article
Show details
PLoS One
. 2020 Oct 15;15(10):e0239389.
doi: 10.1371/journal.pone.0239389. eCollection 2020.

Authors

Sarah Micallef [1], Tonio V Piscopo [2], Ramon Casha [2], Denise Borg [2], Chantal Vella [2], Maria-Alessandra Zammit [2], Janice Borg [1], Daniela Mallia [3], James Farrugia [2], Sarah Marie Vella [2], Thelma Xerri [2], Anette Portelli [2], Manuel Fenech [2], Claudia Fsadni [2], Charles Mallia Azzopardi [2]

Affiliations

- [1] Department of Medicine, Mater Dei Hospital, Msida, Malta.
- [2] Department of Infectious Diseases, Mater Dei Hospital, Msida, Malta.
- [3] Department of Pharmacy, Mater Dei Hospital, Msida, Malta.

- PMID: **33057434**
- PMCID: PMC7561161
- DOI: 10.1371/journal.pone.0239389

Erratum in

- Correction: The first wave of COVID-19 in Malta; a national cross-sectional study.
  Micallef S, Piscopo TV, Casha R, Borg D, Vella C, Zammit MA, Borg J, Mallia D, Farrugia J, Vella SM, Xerri T, Portelli A, Fenech M, Fsadni C, Azzopardi CM. Micallef S, et al. PLoS One. 2021 Aug 3;16(8):e0255881. doi: 10.1371/journal.pone.0255881. eCollection 2021. PLoS One. 2021. PMID: 34343217 Free PMC article.

Abstract

Introduction: The COVID-19 pandemic has posed major challenges to all aspects of healthcare. Malta's population density, large proportion of elderly and high prevalence of diabetes and obesity put the country at risk of uncontrolled viral transmission and high mortality. Despite this, Malta achieved low mortality rates compared to figures overseas. The aim of this paper is to identify key factors that contributed to these favorable outcomes.

Methods: This is a retrospective, observational, nationwide study which evaluates outcomes of patients during the first wave of the pandemic in Malta, from the 7th of March to the 24th of April 2020. Data was collected on demographics and mode of transmission. Hospitalization rates to Malta's main general hospital, Mater Dei Hospital, length of in-hospital stay, intensive care unit admissions and 30-day mortality were also analyzed.

Results: There were 447 confirmed cases in total; 19.5% imported, 74.2% related to community transmission and 6.3% nosocomially transmitted. Ninety-three patients (20.8%) were hospitalized, of which 4 were children. Patients with moderate-severe disease received hydroxychloroquine and azithromycin, in line with evidence available at the time. A total of 4 deaths were recorded, resulting in an all-cause mortality of 0.89%. Importantly, all admitted patients with moderate-severe disease survived to 30-day follow up.

Conclusion: Effective public health interventions, widespread testing, remote surveillance of patients in the community and a low threshold for admission are likely to have contributed to these favorable outcomes. Hospital infection control measures were key in preventing significant nosocomial spread. These concepts can potentially be applied to stem future outbreaks of viral diseases. Patients with moderate-severe disease had excellent outcomes with no deaths reported at 30-day follow up.

Conflict of interest statement

The authors have declared that no competing interests exist

- 27 references
- 4 figures

Supplementary info

Publication types, MeSH terms, Substances, Grant support Expand

Publication types

- Observational Study

MeSH terms

- Adult
- Aged
- Antiviral Agents / administration & dosage
- Antiviral Agents / therapeutic use
- Azithromycin / administration & dosage
- Azithromycin / therapeutic use
- COVID-19
- Coronavirus Infections / epidemiology*
- Coronavirus Infections / mortality
- Coronavirus Infections / therapy
- Drug Utilization / statistics & numerical data
- Female
- Hospitalization / statistics & numerical data
- Humans
- Hydroxychloroquine / administration & dosage

- Hydroxychloroquine / therapeutic use
- Intensive Care Units / statistics & numerical data
- Length of Stay / statistics & numerical data
- Male
- Malta
- Middle Aged
- Pandemics
- Pneumonia, Viral / epidemiology*
- Pneumonia, Viral / mortality
- Pneumonia, Viral / therapy
- Survival Analysis

Substances

- Antiviral Agents
- Hydroxychloroquine
- Azithromycin

Grant support

The authors received no specific funding for this work.

Full text links

OPEN ACCESS TO FULL TEXT PLOS ONE Public Library of Science Free PMC article

Proceed to details

Cite

Share

☐ 1,130

Observational Study

Respir Med

. 2020 Aug;169:106023.

doi: 10.1016/j.rmed.2020.106023. Epub 2020 May 13.

D-dimer in patients infected with COVID-19 and suspected pulmonary embolism

Ignasi Garcia-Olivé [1], Helena Sintes [2], Joaquim Radua [3], Jorge Abad Capa [4], Antoni Rosell [5]

Affiliations Expand

Affiliations

- [1] Servei de Pneumologia. Hospital Universitari Germans Trias i Pujol, Badalona, Barcelona, Spain; CibeRes, Ciber de Enfermedades Respiratorias, Bunyola, Spain; Germans Trias i Pujol Research Institute (IGTP), Badalona, Barcelona, Spain.
- [2] Servei de Pneumologia. Hospital Universitari Germans Trias i Pujol, Badalona, Barcelona, Spain; Departament de Medicina. Universitat Autònoma de Barcelona, Barcelona, Spain.
- [3] Imaging of Mood- and Anxiety-Related Disorders (IMARD) group, Institut d'Investigacions Biomèdiques August Pi i Sunyer (IDIBAPS), Barcelona, Spain; CIBERSAM, Madrid, Spain; Early Psychosis: Interventions and Clinical-detection (EPIC) lab, Institute of Psychiatry, Psychology and Neuroscience, King's College London, London, UK; Department of Clinical Neuroscience, Stockholm Health Care Services, Stockholm County Council, Karolinska Institutet, Stockholm, Sweden.
- [4] Servei de Pneumologia. Hospital Universitari Germans Trias i Pujol, Badalona, Barcelona, Spain; CibeRes, Ciber de Enfermedades Respiratorias, Bunyola, Spain; Germans Trias i Pujol Research Institute (IGTP), Badalona, Barcelona, Spain; Departament de Medicina. Universitat Autònoma de Barcelona, Barcelona, Spain. Electronic address: jabadc.germanstrias@gencat.cat.
- [5] Servei de Pneumologia. Hospital Universitari Germans Trias i Pujol, Badalona, Barcelona, Spain; CibeRes, Ciber de Enfermedades Respiratorias, Bunyola, Spain; Germans Trias i Pujol Research Institute (IGTP), Badalona, Barcelona, Spain; Departament de Medicina. Universitat Autònoma de Barcelona, Barcelona, Spain.

- PMID: **32454268**
- PMCID: PMC7219417
- DOI: 10.1016/j.rmed.2020.106023

Free PMC article
Observational Study

D-dimer in patients infected with COVID-19 and suspected pulmonary embolism

Ignasi Garcia-Olivé et al. Respir Med. 2020 Aug.
Free PMC article

Show details

Respir Med
. 2020 Aug;169:106023.
doi: 10.1016/j.rmed.2020.106023. Epub 2020 May 13.

Authors

Ignasi Garcia-Olivé [1], Helena Sintes [2], Joaquim Radua [3], Jorge Abad Capa [4], Antoni Rosell [5]

Affiliations

- [1] Servei de Pneumologia. Hospital Universitari Germans Trias i Pujol, Badalona, Barcelona, Spain; CibeRes, Ciber de Enfermedades Respiratorias, Bunyola, Spain; Germans Trias i Pujol Research Institute (IGTP), Badalona, Barcelona, Spain.

- [2] Servei de Pneumologia. Hospital Universitari Germans Trias i Pujol, Badalona, Barcelona, Spain; Departament de Medicina. Universitat Autònoma de Barcelona, Barcelona, Spain.
- [3] Imaging of Mood- and Anxiety-Related Disorders (IMARD) group, Institut d'Investigacions Biomèdiques August Pi i Sunyer (IDIBAPS), Barcelona, Spain; CIBERSAM, Madrid, Spain; Early Psychosis: Interventions and Clinical-detection (EPIC) lab, Institute of Psychiatry, Psychology and Neuroscience, King's College London, London, UK; Department of Clinical Neuroscience, Stockholm Health Care Services, Stockholm County Council, Karolinska Institutet, Stockholm, Sweden.
- [4] Servei de Pneumologia. Hospital Universitari Germans Trias i Pujol, Badalona, Barcelona, Spain; CibeRes, Ciber de Enfermedades Respiratorias, Bunyola, Spain; Germans Trias i Pujol Research Institute (IGTP), Badalona, Barcelona, Spain; Departament de Medicina. Universitat Autònoma de Barcelona, Barcelona, Spain. Electronic address: jabadc.germanstrias@gencat.cat.
- [5] Servei de Pneumologia. Hospital Universitari Germans Trias i Pujol, Badalona, Barcelona, Spain; CibeRes, Ciber de Enfermedades Respiratorias, Bunyola, Spain; Germans Trias i Pujol Research Institute (IGTP), Badalona, Barcelona, Spain; Departament de Medicina. Universitat Autònoma de Barcelona, Barcelona, Spain.

- PMID: **32454268**
- PMCID: PMC7219417
- DOI: 10.1016/j.rmed.2020.106023

Abstract

Objective: To analyze the risk factors for pulmonary embolism (PE) in patients infected with COVID-19.

Methods: We conducted an observational, retrospective study. Patients with severe infection with COVID-19 and suspected PE were included.

Results: Patients with higher levels of D-dimer and those requiring intubation were at a higher risk of developing PE. Higher D-dimer levels were associated with a greater probability of PE 3, 6, 9 and 12 days after determining D-dimer levels with an OR of 1.7, 2.0, 2.4 and 2.4, respectively.

Conclusion: In conclusion, patients infected with COVID-19 requiring OTI with higher levels of D-dimer have an increased risk of developing PE.

Keywords: COVID-19; D-dimer; Pulmonary embolism.

Copyright © 2020 Elsevier Ltd. All rights reserved.

Conflict of interest statement

The authors do not have any financial or personal relationships with people or organizations that may have inappropriately influenced their work in the present article.

The authors declare that they have no known competing financial interests or personal relationships that could have appeared to influence the work reported in this paper.

- 10 references
- 1 figure

Supplementary info

Publication types, MeSH terms, Substances Expand

Publication types

- Observational Study

MeSH terms

- Aged
- Betacoronavirus
- COVID-19
- Coronavirus Infections / blood
- Coronavirus Infections / epidemiology*
- Coronavirus Infections / metabolism
- Coronavirus Infections / therapy
- Female
- Fibrin Fibrinogen Degradation Products / metabolism*
- Humans
- Intubation, Intratracheal / statistics & numerical data*
- Male
- Middle Aged
- Pandemics
- Pneumonia, Viral / blood
- Pneumonia, Viral / epidemiology*
- Pneumonia, Viral / metabolism
- Pneumonia, Viral / therapy
- Pulmonary Embolism / blood
- Pulmonary Embolism / epidemiology*
- Pulmonary Embolism / metabolism
- Respiration, Artificial
- Retrospective Studies
- Risk Factors
- SARS-CoV-2
- Severity of Illness Index
- Spain / epidemiology

Substances

- Fibrin Fibrinogen Degradation Products
- fibrin fragment D

Full text links

ELSEVIER FULL-TEXT ARTICLE Elsevier Science Free PMC article

Proceed to details

Cite

Share

1,131

Observational Study

Crit Care

. 2021 Sep 13;25(1):331.

doi: 10.1186/s13054-021-03727-x.

The evolution of the ventilatory ratio is a prognostic factor in mechanically ventilated COVID-19 ARDS patients

Antoni Torres[#, 1, 2, 3], Anna Motos[#, 4, 5], Jordi Riera[6], Laia Fernández-Barat[#, 4, 5], Adrián Ceccato[#, 4], Raquel Pérez-Arnal[7], Dario García-Gasulla[7], Oscar Peñuelas[4, 8], José Angel Lorente[4, 8], Alejandro Rodriguez[9], David de Gonzalo-Calvo[4, 10], Raquel Almansa[11, 12], Albert Gabarrús[5], Rosario Menéndez[13], Jesús F Bermejo-Martin[11, 12], Ricard Ferrer[6], Rosario Amaya Villar[14], José M Añón[4, 15], Carme Barberà[16], José Barberán[17], Aaron Blandino Ortiz[18], Elena Bustamante-Munguira[19], Jesús Caballero[20], Cristina Carbajales[21], Nieves Carbonell[22], Mercedes Catalán-González[23], Cristóbal Galbán[24], Víctor D Gumucio-Sanguino[25, 26], Maria Del Carmen de la Torre[27], Emili Díaz[28, 29], Ángel Estella[30], Elena Gallego[31], José Luis García Garmendia[32], José Garnacho-Montero[33], José M Gómez[34], Arturo Huerta[35], Ruth Noemí Jorge García[36], Ana Loza-Vázquez[37], Judith Marin-Corral[38], Amalia Martínez de la Gándara[39], Ignacio Martínez Varela[40], Juan López Messa[41], Guillermo M Albaiceta[4, 42, 43], Mariana Andrea Novo[44], Yhivian Peñasco[45], Juan Carlos Pozo-Laderas[46], Pilar Ricart[47], Inmaculada Salvador-Adell[48], Angel Sánchez-Miralles[49], Susana Sancho Chinesta[50], Lorenzo Socias[51], Jordi Solé-Violan[52], Fernando Suares Sipmann[53], Luis Tamayo Lomas[54], José Trenado[55], Ferran Barbé[4, 10], CIBERESUCICOVID Project (COV20/00110, ISCIII)

Collaborators, Affiliations Expand

Collaborators

- **CIBERESUCICOVID Project (COV20/00110, ISCIII)**: Berta Adell-Serrano, Alexander Agrifoglio, María Aguilar Cabello, Luciano Aguilera, Victoria Alcaraz-Serrano, Cesar Aldecoa, Cynthia Alegre, Sergio Álvarez, Antonio Álvarez Ruiz, Rut Andrea, José Ángel, Marta Arrieta, J Ignacio Ayestarán, Joan Ramon Badia, Mariona Badía, Orville Báez Pravia, Ana Balan Mariño, Begoña Balsera, Laura Barbena, Enric Barbeta, Tommaso Bardi, Patricia Barral Segade, Marta Barroso, José Ángel Berezo García, Judit Bigas, Rafael Blancas, María Luisa Blasco Cortés, María Boado, María Bodi Saera, Neus Bofill, María Teresa Bouza Vieiro, Leticia Bueno, Juan Bustamante-Munguira, Lucia Cachafeiro, David Campi

Hermoso, Sandra Campos Fernández, Iosune Cano, Maria Luisa Cantón-Bulnes, Pablo Cardina Fernández, Laura Carrión García, Sula Carvalho, Núria Casacuberta-Barberà, Manuel Castellà, Andrea Castellví, Pedro Castro, Ramon Cicuendez Ávila, Catia Cillóniz, Luisa Clar, Cristina Climent, Jordi Codina, Pamela Conde, Sofía Contreras, María Cruz Martin, Raul de Pablo Sánchez, Diego De Mendoza, Cecilia Del Busto Martínez, Yolanda Díaz, María Digna Rivas Vilas, Cristina Dólera Moreno, Irene Dot, Pedro Enríquez Giraudo, Inés Esmorís Arijón, Teresa Farre Monjo, Javier Fernández, Carlos Ferrando, Albert Figueras, Eva Forcadell-Ferreres, Lorena Forcelledo Espina, Nieves Franco, Àngels Furro, Felipe García, Beatriz García, Emilio García Prieto, Carlos García Redruello, Amaia García Sagastume, Maria Luisa Gascón Castillo, Gemma Gomà, Vanesa Gómez Casal, Silvia Gómez, Carmen Gómez Gonzalez, Jessica González, Federico Gordo, Maria Pilar Gracia, Alba Herraiz, Rubén Herrán-Monge, Mercedes Ibarz, Silvia Iglesias, Maria Teresa Janer, Gabriel Jiménez, Mar Juan Díaz, Karsa Kiarostami, Juan I Lazo Álvarez, Miguel León, Alexandre López-Gavín, Ana López Lago, Desire Macias Guerrero, Nuria Mamolar Herrera, Rafael Mañez Mendiluce, Cecilia L Mantellini, Gregorio Marco Naya, Pilar Marcos, Enrique Marmol Peis, Paula Martín Vicente, María Martínez, Carmen Eulalia Martínez Fernández, Maria Dolores Martínez Juan, Juan Fernando Masa Jimenez, Joan Ramon Masclans, Emilio Maseda, Eva María Menor Fernández, Mar Miralbés, Josman Monclou, Juan Carlos Montejo-González, Neus Montserrat, María Mora Aznar, Pedro Moral-Parras, Dulce Morales, Sara Guadalupe Moreno Cano, David Mosquera Rodríguez, Rosana Muñoz-Bermúdez, José María Nicolás, Ramon Nogue Bou, Rafaela Nogueras Salinas, Marta Ocón, Ana Ortega, Sergio Ossa, Pablo Pagliarani, Anna Parera Pous, Francisco Parrilla, Leire Pérez Bastida, Purificación Pérez, Gloria Pérez Planelles, Eva Pérez Rubio, David Pestaña Laguna, Àngels Piñol-Tena, Javier Prados, Andrés Pujol, Núria Ramon Coll, Gloria Renedo Sanchez-Giron, Ferran Roche-Campo, Laura Rodriguez, Felipe Rodríguez de Castro, Silvia Rodríguez, Covadonga Rodríguez Ruiz, Jorge Rubio, Alberto Rubio López, Miriam Ruiz Miralles, Pablo Ryan Murúa, Eva Saborido Paz, Ana Salazar Degracia, Miguel Sanchez, Ana Sánchez, Bitor Santacoloma, Maria Teresa Sariñena, Marta Segura Pensado, Lidia Serra, Mireia Serra-Fortuny, Ainhoa Serrano Lázaro, Lluís Servià, Laura Soliva, Carla Speziale, Daniel Tognetti, Adrián Tormos, Mateu Torres, Sandra Trefler, Javier Trujillano, Alejandro Úbeda, Luis Urrelo-Cerrón, Estela Val, Luis Valdivia Ruiz, Montserrat Vallverdú, Maria Van der Hofstadt Martin-Montalvo, Sabela Vara Adrio, Nil Vázquez, Javier Vengoechea, Pablo Vidal Cortes, Clara Vilà-Vilardel, Judit Vilanova, Tatiana Villada Warrington, Hua Yang, Minlan Yang, Ana Zapatero

Affiliations

- [1] Centro de Investigación Biomedica En Red - Enfermedades Respiratorias (CIBERES), Barcelona, Spain. atorres@clinic.cat.
- [2] Institut d'Investigacions August Pi i Sunyer (IDIBAPS), Universitat de Barcelona, Barcelona, Spain. atorres@clinic.cat.
- [3] Servei de Pneumologia i Al·lèrgia Respiratòria, Hospital Clínic, Villarroel 170, Esc 6/8 Planta 2, 08036, Barcelona, Spain. atorres@clinic.cat.
- [4] Centro de Investigación Biomedica En Red - Enfermedades Respiratorias (CIBERES), Barcelona, Spain.
- [5] Institut d'Investigacions August Pi i Sunyer (IDIBAPS), Universitat de Barcelona, Barcelona, Spain.
- [6] Intensive Care Department, Hospital Universitari Vall d'Hebron, Vall d'Hebron Institut de Recerca, Barcelona, Spain.

- [7] Barcelona Supercomputing Center (BSC), Barcelona, Spain.
- [8] Hospital Universitario de Getafe, Universidad Europea, Madrid, Spain.
- [9] Critical Care Department, Hospital Joan XXIII, Tarragona, Spain.
- [10] Translational Research in Respiratory Medicine, Respiratory Department, Hospital Universitari Aranu de Vilanova and Santa Maria, IRBLleida, Lleida, Spain.
- [11] Hospital Universitario Río Hortega de Valladolid, Valladolid, Spain.
- [12] Instituto de Investigación Biomédica de Salamanca (IBSAL), Gerencia Regional de Salud de Castilla y León, Salamanca, Spain.
- [13] Pulmonary Department, University and Polytechnic Hospital La Fe, Valencia, Spain.
- [14] Intensive Care Clinical Unit, Hospital Universitario Virgen de Rocío, Sevilla, Spain.
- [15] Servicio de Medicina Intensiva, Hospital Universitario La Paz, IdiPAZ, Madrid, Spain.
- [16] Hospital Santa Maria, IRBLleida, Lleida, Spain.
- [17] Hospital Universitario HM Montepríncipe, Universidad San Pablo-CEU, Madrid, Spain.
- [18] Servicio de Medicina Intensiva, Hospital Universitario Ramón y Cajal, Madrid, Spain.
- [19] Department of Intensive Care Medicine, Hospital Clínico Universitario Valladolid, Valladolid, Spain.
- [20] Critical Care Department, Hospital Universitari Arnau de Vilanova, IRBLleida, Lleida, Spain.
- [21] Hospital Álvaro Cunqueiro, Vigo, Spain.
- [22] Intensive Care Unit, Hospital Clínico y Universitario de Valencia, Valencia, Spain.
- [23] Department of Intensive Care Medicine, Hospital Universitario, 12 de Octubre, Madrid, Spain.
- [24] Department of Medicine, CHUS, Complejo Hospitalario Universitario de Santiago, Santiago de Compostela, Spain.
- [25] Department of Intensive Care, Hospital Universitari de Bellvitge, L'Hospitalet de Llobregat, Barcelona, Spain.
- [26] Bellvitge Biomedical Research Institute (IDIBELL), L'Hospitalet de Llobregat, Barcelona, Spain.
- [27] Hospital de Mataró de Barcelona, Barcelona, Spain.
- [28] Department of Medicine, Universitat Autònoma de Barcelona (UAB), Barcelona, Spain.
- [29] Critical Care Department, Corporació Sanitària Parc Taulí, Sabadell, Barcelona, Spain.
- [30] Departamento Medicina Facultad Medicina, Universidad de Cádiz, Hospital Universitario de Jerez, Jerez de la Frontera, Spain.
- [31] Unidad de Cuidados Intensivos, Hospital San Pedro de Alcántara, Cáceres, Spain.
- [32] Intensive Care Unit, Hospital San Juan de Dios del Aljarafe, Sevilla, Spain.
- [33] Intensive Care Clinical Unit, Hospital Universitario Virgen Macarena, Seville, Spain.
- [34] Hospital General Universitario Gregorio Marañón, Madrid, Spain.
- [35] Pulmonary and Critical Care Division, Emergency Department, Clínica Sagrada Família, Barcelona, Spain.
- [36] Intensive Care Department, Hospital Nuestra Señora de Gracia, Zaragoza, Spain.
- [37] Unidad de Medicina Intensiva, Hospital Universitario Virgen de Valme, Sevilla, Spain.
- [38] Critical Care Department, Hospital del Mar-IMIM, Barcelona, Spain.
- [39] Department of Intensive Medicine, Hospital Universitario Infanta Leonor, Madrid, Spain.
- [40] Critical Care Department, Hospital Universitario Lucus Augusti, Lugo, Spain.
- [41] Complejo Asistencial Universitario de Palencia, Palencia, Spain.
- [42] Departamento de Biología Funcional, Instituto Universitario de Oncología del Principado de Asturias, Universidad de Oviedo, Oviedo, Spain.

- [43] Instituto de Investigación Sanitaria del Principado de Asturias, Hospital Central de Asturias, Oviedo, Spain.
- [44] Servei de Medicina Intensiva, Hospital Universitari Son Espases, Palma de Mallorca, Illes Balears, Spain.
- [45] Servicio de Medicina Intensiva, Hospital Universitario Marqués de Valdecilla, Santander, Spain.
- [46] UGC-Medicina Intensiva, Hospital Universitario Reina Sofia, Instituto Maimonides IMIBIC, Córdoba, Spain.
- [47] Servei de medicina intensiva, Hospital Universitari Germans Trias, Badalona, Spain.
- [48] Hospital Verge de La Cinta, Tortosa, Tarragona, Spain.
- [49] Hospital de Sant Joan d'Alacant, Alacant, Spain.
- [50] Servicio de medicina intensiva, Hospital Universitario y Politécnico La Fe, Valencia, Spain.
- [51] Intensive Care Unit, Hospital Son Llàtzer, Palma de Mallorca, Illes Balears, Spain.
- [52] Critical Care Department, Hospital Dr. Negrín Gran Canaria, Las Palmas, Gran Canaria, Spain.
- [53] Intensive Care Unit, Hospital Universitario La Princesa, Madrid, Spain.
- [54] Critical Care Department, Hospital Universitario Río Hortega de Valladolid, Valladolid, Spain.
- [55] Servicio de Medicina Intensiva, Hospital Universitario Mútua de Terrassa, Terrassa, Barcelona, Spain.

[#] Contributed equally.

- PMID: **34517881**
- PMCID: PMC8436582
- DOI: 10.1186/s13054-021-03727-x

Free PMC article
Observational Study

The evolution of the ventilatory ratio is a prognostic factor in mechanically ventilated COVID-19 ARDS patients

Antoni Torres et al. Crit Care. 2021.
Free PMC article
Show details
Crit Care
. 2021 Sep 13;25(1):331.
doi: 10.1186/s13054-021-03727-x.

Authors

Antoni Torres [#,1,2,3], Anna Motos [#,4,5], Jordi Riera [6], Laia Fernández-Barat [#,4,5], Adrián Ceccato [#,4], Raquel Pérez-Arnal [7], Dario García-Gasulla [7], Oscar Peñuelas [4,8], José Angel

Lorente [4, 8], Alejandro Rodriguez [9], David de Gonzalo-Calvo [4, 10], Raquel Almansa [11, 12], Albert Gabarrús [5], Rosario Menéndez [13], Jesús F Bermejo-Martin [11, 12], Ricard Ferrer [6], Rosario Amaya Villar [14], José M Añón [4, 15], Carme Barberà [16], José Barberán [17], Aaron Blandino Ortiz [18], Elena Bustamante-Munguira [19], Jesús Caballero [20], Cristina Carbajales [21], Nieves Carbonell [22], Mercedes Catalán-González [23], Cristóbal Galbán [24], Víctor D Gumucio-Sanguino [25, 26], Maria Del Carmen de la Torre [27], Emili Díaz [28, 29], Ángel Estella [30], Elena Gallego [31], José Luis García Garmendia [32], José Garnacho-Montero [33], José M Gómez [34], Arturo Huerta [35], Ruth Noemí Jorge García [36], Ana Loza-Vázquez [37], Judith Marin-Corral [38], Amalia Martínez de la Gándara [39], Ignacio Martínez Varela [40], Juan López Messa [41], Guillermo M Albaiceta [4, 42, 43], Mariana Andrea Novo [44], Yhivian Peñasco [45], Juan Carlos Pozo-Laderas [46], Pilar Ricart [47], Inmaculada Salvador-Adell [48], Angel Sánchez-Miralles [49], Susana Sancho Chinesta [50], Lorenzo Socias [51], Jordi Solé-Violan [52], Fernando Suares Sipmann [53], Luis Tamayo Lomas [54], José Trenado [55], Ferran Barbé [4, 10], CIBERESUCICOVID Project (COV20/00110, ISCIII)

Collaborators

- **CIBERESUCICOVID Project (COV20/00110, ISCIII)**: Berta Adell-Serrano, Alexander Agrifoglio, María Aguilar Cabello, Luciano Aguilera, Victoria Alcaraz-Serrano, Cesar Aldecoa, Cynthia Alegre, Sergio Álvarez, Antonjo Álvarez Ruiz, Rut Andrea, José Ángel, Marta Arrieta, J Ignacio Ayestarán, Joan Ramon Badia, Mariona Badía, Orville Báez Pravia, Ana Balan Mariño, Begoña Balsera, Laura Barbena, Enric Barbeta, Tommaso Bardi, Patricia Barral Segade, Marta Barroso, José Ángel Berezo García, Judit Bigas, Rafael Blancas, María Luisa Blasco Cortés, María Boado, María Bodi Saera, Neus Bofill, María Teresa Bouza Vieiro, Leticia Bueno, Juan Bustamante-Munguira, Lucia Cachafeiro, David Campi Hermoso, Sandra Campos Fernández, Iosune Cano, Maria Luisa Cantón-Bulnes, Pablo Cardina Fernández, Laura Carrión García, Sula Carvalho, Núria Casacuberta-Barberà, Manuel Castellà, Andrea Castellví, Pedro Castro, Ramon Cicuendez Ávila, Catia Cillóniz, Luisa Clar, Cristina Climent, Jordi Codina, Pamela Conde, Sofía Contreras, María Cruz Martin, Raul de Pablo Sánchez, Diego De Mendoza, Cecilia Del Busto Martínez, Yolanda Díaz, María Digna Rivas Vilas, Cristina Dólera Moreno, Irene Dot, Pedro Enríquez Giraudo, Inés Esmorís Arijón, Teresa Farre Monjo, Javier Fernández, Carlos Ferrando, Albert Figueras, Eva Forcadell-Ferreres, Lorena Forcelledo Espina, Nieves Franco, Àngels Furro, Felipe García, Beatriz García, Emilio García Prieto, Carlos García Redruello, Amaia García Sagastume, Maria Luisa Gascón Castillo, Gemma Gomà, Vanesa Gómez Casal, Silvia Gómez, Carmen Gómez Gonzalez, Jessica González, Federico Gordo, Maria Pilar Gracia, Alba Herraiz, Rubén Herrán-Monge, Mercedes Ibarz, Silvia Iglesias, Maria Teresa Janer, Gabriel Jiménez, Mar Juan Díaz, Karsa Kiarostami, Juan I Lazo Álvarez, Miguel León, Alexandre López-Gavín, Ana López Lago, Desire Macias Guerrero, Nuria Mamolar Herrera, Rafael Mañez Mendiluce, Cecilia L Mantellini, Gregorio Marco Naya, Pilar Marcos, Enrique Marmol Peis, Paula Martín Vicente, María Martínez, Carmen Eulalia Martínez Fernández, Maria Dolores Martínez Juan, Juan Fernando Masa Jimenez, Joan Ramon Masclans, Emilio Maseda, Eva María Menor Fernández, Mar Miralbés, Josman Monclou, Juan Carlos Montejo-González, Neus Montserrat, María Mora Aznar, Pedro Moral-Parras, Dulce Morales, Sara Guadalupe Moreno Cano, David Mosquera Rodríguez, Rosana Muñoz-Bermúdez, José María Nicolás, Ramon Nogue Bou, Rafaela Nogueras Salinas, Marta Ocón, Ana Ortega, Sergio Ossa, Pablo Pagliarani, Anna Parera Pous, Francisco Parrilla, Leire Pérez Bastida, Purificación Pérez, Gloria Pérez Planelles, Eva Pérez Rubio, David Pestaña Laguna, Àngels Piñol-Tena, Javier Prados, Andrés Pujol, Núria

Ramon Coll, Gloria Renedo Sanchez-Giron, Ferran Roche-Campo, Laura Rodriguez, Felipe Rodríguez de Castro, Silvia Rodríguez, Covadonga Rodríguez Ruiz, Jorge Rubio, Alberto Rubio López, Miriam Ruiz Miralles, Pablo Ryan Murúa, Eva Saborido Paz, Ana Salazar Degracia, Miguel Sanchez, Ana Sánchez, Bitor Santacoloma, Maria Teresa Sariñena, Marta Segura Pensado, Lidia Serra, Mireia Serra-Fortuny, Ainhoa Serrano Lázaro, Lluís Servià, Laura Soliva, Carla Speziale, Daniel Tognetti, Adrián Tormos, Mateu Torres, Sandra Trefler, Javier Trujillano, Alejandro Úbeda, Luis Urrelo-Cerrón, Estela Val, Luis Valdivia Ruiz, Montserrat Vallverdú, Maria Van der Hofstadt Martin-Montalvo, Sabela Vara Adrio, Nil Vázquez, Javier Vengoechea, Pablo Vidal Cortes, Clara Vilà-Vilardel, Judit Vilanova, Tatiana Villada Warrington, Hua Yang, Minlan Yang, Ana Zapatero

Affiliations

- [1] Centro de Investigación Biomedica En Red - Enfermedades Respiratorias (CIBERES), Barcelona, Spain. atorres@clinic.cat.
- [2] Institut d'Investigacions August Pi i Sunyer (IDIBAPS), Universitat de Barcelona, Barcelona, Spain. atorres@clinic.cat.
- [3] Servei de Pneumologia i Al·lèrgia Respiratòria, Hospital Clínic, Villarroel 170, Esc 6/8 Planta 2, 08036, Barcelona, Spain. atorres@clinic.cat.
- [4] Centro de Investigación Biomedica En Red - Enfermedades Respiratorias (CIBERES), Barcelona, Spain.
- [5] Institut d'Investigacions August Pi i Sunyer (IDIBAPS), Universitat de Barcelona, Barcelona, Spain.
- [6] Intensive Care Department, Hospital Universitari Vall d'Hebron, Vall d'Hebron Institut de Recerca, Barcelona, Spain.
- [7] Barcelona Supercomputing Center (BSC), Barcelona, Spain.
- [8] Hospital Universitario de Getafe, Universidad Europea, Madrid, Spain.
- [9] Critical Care Department, Hospital Joan XXIII, Tarragona, Spain.
- [10] Translational Research in Respiratory Medicine, Respiratory Department, Hospital Universitari Aranu de Vilanova and Santa Maria, IRBLleida, Lleida, Spain.
- [11] Hospital Universitario Río Hortega de Valladolid, Valladolid, Spain.
- [12] Instituto de Investigación Biomédica de Salamanca (IBSAL), Gerencia Regional de Salud de Castilla y León, Salamanca, Spain.
- [13] Pulmonary Department, University and Polytechnic Hospital La Fe, Valencia, Spain.
- [14] Intensive Care Clinical Unit, Hospital Universitario Virgen de Rocío, Sevilla, Spain.
- [15] Servicio de Medicina Intensiva, Hospital Universitario La Paz, IdiPAZ, Madrid, Spain.
- [16] Hospital Santa Maria, IRBLleida, Lleida, Spain.
- [17] Hospital Universitario HM Montepríncipe, Universidad San Pablo-CEU, Madrid, Spain.
- [18] Servicio de Medicina Intensiva, Hospital Universitario Ramón y Cajal, Madrid, Spain.
- [19] Department of Intensive Care Medicine, Hospital Clínico Universitario Valladolid, Valladolid, Spain.
- [20] Critical Care Department, Hospital Universitari Arnau de Vilanova, IRBLleida, Lleida, Spain.
- [21] Hospital Álvaro Cunqueiro, Vigo, Spain.
- [22] Intensive Care Unit, Hospital Clínico y Universitario de Valencia, Valencia, Spain.
- [23] Department of Intensive Care Medicine, Hospital Universitario, 12 de Octubre, Madrid, Spain.

- [24] Department of Medicine, CHUS, Complejo Hospitalario Universitario de Santiago, Santiago de Compostela, Spain.
- [25] Department of Intensive Care, Hospital Universitari de Bellvitge, L'Hospitalet de Llobregat, Barcelona, Spain.
- [26] Bellvitge Biomedical Research Institute (IDIBELL), L'Hospitalet de Llobregat, Barcelona, Spain.
- [27] Hospital de Mataró de Barcelona, Barcelona, Spain.
- [28] Department of Medicine, Universitat Autònoma de Barcelona (UAB), Barcelona, Spain.
- [29] Critical Care Department, Corporació Sanitària Parc Taulí, Sabadell, Barcelona, Spain.
- [30] Departamento Medicina Facultad Medicina, Universidad de Cádiz, Hospital Universitario de Jerez, Jerez de la Frontera, Spain.
- [31] Unidad de Cuidados Intensivos, Hospital San Pedro de Alcántara, Cáceres, Spain.
- [32] Intensive Care Unit, Hospital San Juan de Dios del Aljarafe, Sevilla, Spain.
- [33] Intensive Care Clinical Unit, Hospital Universitario Virgen Macarena, Seville, Spain.
- [34] Hospital General Universitario Gregorio Marañón, Madrid, Spain.
- [35] Pulmonary and Critical Care Division, Emergency Department, Clínica Sagrada Família, Barcelona, Spain.
- [36] Intensive Care Department, Hospital Nuestra Señora de Gracia, Zaragoza, Spain.
- [37] Unidad de Medicina Intensiva, Hospital Universitario Virgen de Valme, Sevilla, Spain.
- [38] Critical Care Department, Hospital del Mar-IMIM, Barcelona, Spain.
- [39] Department of Intensive Medicine, Hospital Universitario Infanta Leonor, Madrid, Spain.
- [40] Critical Care Department, Hospital Universitario Lucus Augusti, Lugo, Spain.
- [41] Complejo Asistencial Universitario de Palencia, Palencia, Spain.
- [42] Departamento de Biología Funcional, Instituto Universitario de Oncología del Principado de Asturias, Universidad de Oviedo, Oviedo, Spain.
- [43] Instituto de Investigación Sanitaria del Principado de Asturias, Hospital Central de Asturias, Oviedo, Spain.
- [44] Servei de Medicina Intensiva, Hospital Universitari Son Espases, Palma de Mallorca, Illes Balears, Spain.
- [45] Servicio de Medicina Intensiva, Hospital Universitario Marqués de Valdecilla, Santander, Spain.
- [46] UGC-Medicina Intensiva, Hospital Universitario Reina Sofia, Instituto Maimonides IMIBIC, Córdoba, Spain.
- [47] Servei de medicina intensiva, Hospital Universitari Germans Trias, Badalona, Spain.
- [48] Hospital Verge de La Cinta, Tortosa, Tarragona, Spain.
- [49] Hospital de Sant Joan d'Alacant, Alacant, Spain.
- [50] Servicio de medicina intensiva, Hospital Universitario y Politécnico La Fe, Valencia, Spain.
- [51] Intensive Care Unit, Hospital Son Llàtzer, Palma de Mallorca, Illes Balears, Spain.
- [52] Critical Care Department, Hospital Dr. Negrín Gran Canaria, Las Palmas, Gran Canaria, Spain.
- [53] Intensive Care Unit, Hospital Universitario La Princesa, Madrid, Spain.
- [54] Critical Care Department, Hospital Universitario Río Hortega de Valladolid, Valladolid, Spain.
- [55] Servicio de Medicina Intensiva, Hospital Universitario Mútua de Terrassa, Terrassa, Barcelona, Spain.

[#] Contributed equally.

- PMID: **34517881**
- PMCID: PMC8436582
- DOI: 10.1186/s13054-021-03727-x

Erratum in

- Correction to: The evolution of the ventilatory ratio is a prognostic factor in mechanically ventilated COVID-19 ARDS patients.
Torres A, Motos A, Riera J, Fernández-Barat L, Ceccato A, Pérez-Arnal R, García-Gasulla D, Peñuelas O, Lorente JA, Rodriguez A, de Gonzalo-Calvo D, Almansa R, Gabarrús A, Menéndez R, Bermejo-Martin JF, Ferrer R, Amaya Villar R, Añón JM, Barberà C, Barberán J, Blandino Ortiz A, Bustamante-Munguira E, Caballero J, Carbajales C, Carbonell N, Catalán-González M, Galbán C, Gumucio-Sanguino VD, de la Torre MDC, Díaz E, Estella Á, Gallego E, García Garmendia JL, Garnacho-Montero J, Gómez JM, Huerta A, Jorge García RN, Loza-Vázquez A, Marin-Corral J, Martínez de la Gándara A, Martínez Varela I, López Messa J, M Albaiceta G, Novo MA, Peñasco Y, Pozo-Laderas JC, Ricart P, Salvador-Adell I, Sánchez-Miralles A, Sancho Chinesta S, Socias L, Solé-Violan J, Suares Sipmann F, Tamayo Lomas L, Trenado J, Barbé F; CIBERESUCICOVID Project (COV20/00110, ISCIII). Torres A, et al. Crit Care. 2021 Dec 17;25(1):435. doi: 10.1186/s13054-021-03849-2. Crit Care. 2021. PMID: 34920738 Free PMC article. No abstract available.

Abstract

Background: Mortality due to COVID-19 is high, especially in patients requiring mechanical ventilation. The purpose of the study is to investigate associations between mortality and variables measured during the first three days of mechanical ventilation in patients with COVID-19 intubated at ICU admission.

Methods: Multicenter, observational, cohort study includes consecutive patients with COVID-19 admitted to 44 Spanish ICUs between February 25 and July 31, 2020, who required intubation at ICU admission and mechanical ventilation for more than three days. We collected demographic and clinical data prior to admission; information about clinical evolution at days 1 and 3 of mechanical ventilation; and outcomes.

Results: Of the 2,095 patients with COVID-19 admitted to the ICU, 1,118 (53.3%) were intubated at day 1 and remained under mechanical ventilation at day three. From days 1 to 3, PaO_2/FiO_2 increased from 115.6 [80.0-171.2] to 180.0 [135.4-227.9] mmHg and the ventilatory ratio from 1.73 [1.33-2.25] to 1.96 [1.61-2.40]. In-hospital mortality was 38.7%. A higher increase between ICU admission and day 3 in the ventilatory ratio (OR 1.04 [CI 1.01-1.07], $p = 0.030$) and creatinine levels (OR 1.05 [CI 1.01-1.09], $p = 0.005$) and a lower increase in platelet counts (OR 0.96 [CI 0.93-1.00], $p = 0.037$) were independently associated with a higher risk of death. No association between mortality and the PaO_2/FiO_2 variation was observed (OR 0.99 [CI 0.95 to 1.02], $p = 0.47$).

Conclusions: Higher ventilatory ratio and its increase at day 3 is associated with mortality in patients with COVID-19 receiving mechanical ventilation at ICU admission. No association was found in the PaO_2/FiO_2 variation.

Keywords: COVID-19; Coronavirus; Mechanical ventilation; SARS-CoV-2; Ventilatory ratio.

© 2021. The Author(s).

Conflict of interest statement

The authors have disclosed that they do not have any conflicts of interest.

- 33 references
- 2 figures

Supplementary info

Publication types, MeSH terms, Grant support Expand

Publication types

- Multicenter Study
- Observational Study
- Research Support, Non-U.S. Gov't

MeSH terms

- Aged
- Aged, 80 and over
- COVID-19 / epidemiology
- COVID-19 / physiopathology
- COVID-19 / therapy*
- Cohort Studies
- Critical Care / methods
- Critical Care / trends
- Female
- Hospital Mortality / trends
- Humans
- Intensive Care Units / trends
- Male
- Middle Aged
- Prognosis
- Prospective Studies
- Pulmonary Ventilation / physiology
- Respiration, Artificial / methods*
- Respiration, Artificial / trends
- Respiratory Distress Syndrome / epidemiology
- Respiratory Distress Syndrome / physiopathology
- Respiratory Distress Syndrome / therapy*
- Retrospective Studies
- Spain / epidemiology

- Ventilation-Perfusion Ratio / physiology*

Grant support

- COV20/00110/instituto de salud carlos iii
- Miguel Servet 2020:CP20/00041/instituto de salud carlos iii

Full text links

Read free full text at BMC BioMed Central Free PMC article
Proceed to details
Cite
Share
1,132
Observational Study
J Am Soc Echocardiogr
. 2020 Jul;33(7):895-899.
doi: 10.1016/j.echo.2020.05.005. Epub 2020 May 11.

Tablet-Based Limited Echocardiography to Reduce Sonographer Scan and Decontamination Time during the COVID-19 Pandemic

Sean R McMahon [1], Garrett De Francis [2], Sara Schwartz [2], William L Duvall [2], Bhaskar Arora [2], David I Silverman [2]
Affiliations Expand

Affiliations

- [1] Hartford Hospital Division of Cardiology and Heart and Vascular Institute, Hartford, Connecticut. Electronic address: sean.mcmahon@hhchealth.org.
- [2] Hartford Hospital Division of Cardiology and Heart and Vascular Institute, Hartford, Connecticut.

- PMID: **32624089**
- PMCID: PMC7211571
- DOI: 10.1016/j.echo.2020.05.005

Free PMC article
Observational Study

Tablet-Based Limited Echocardiography to Reduce Sonographer Scan and Decontamination Time during the COVID-19 Pandemic

Sean R McMahon et al. J Am Soc Echocardiogr. 2020 Jul.
Free PMC article
Show details
J Am Soc Echocardiogr
. 2020 Jul;33(7):895-899.
doi: 10.1016/j.echo.2020.05.005. Epub 2020 May 11.

Authors

Sean R McMahon [1], Garrett De Francis [2], Sara Schwartz [2], William L Duvall [2], Bhaskar Arora [2], David I Silverman [2]

Affiliations

- [1] Hartford Hospital Division of Cardiology and Heart and Vascular Institute, Hartford, Connecticut. Electronic address: sean.mcmahon@hhchealth.org.
- [2] Hartford Hospital Division of Cardiology and Heart and Vascular Institute, Hartford, Connecticut.

- PMID: **32624089**
- PMCID: PMC7211571
- DOI: 10.1016/j.echo.2020.05.005

Abstract

Background: Limited assessments with handheld ultrasound have found meaningful clinical use in the care of acutely ill patients. However, there are limited data on incorporating handheld-based limited echocardiography into the echocardiography laboratory. The purpose of this study was to assess the efficacy of limited handheld tablet echocardiography as an alternative to traditional echocardiography during the coronavirus disease 2019 (COVID-19) pandemic as a means to limit exposure while providing essential clinical information.

Methods: Ninety consecutive inpatients with known or suspected COVID-19 were scanned according to laboratory COVID-19 guidelines using a limited 11- to 20-clip protocol on a tablet sonograph. The primary assessment was length of study time. Comparison data were drawn from comprehensive echocardiographic examinations ordered on intensive care patients not under COVID-19 precautions.

Results: Over a 36-day time period, a total of 91 requests were deemed to be appropriate for echocardiography on patients with suspected or confirmed COVID-19 (average age, 67 years; 64% men; mean body mass index, 32 kg/m^2). Of these, 90 (99%) examinations were performed using a handheld device, and all were deemed diagnostic and provided sufficient information for

the clinical care team. Sonographer scan time decreased from an average of 24 ± 6.8 min on a traditional platform to 5.4 ± 1.9 min on a tablet.

Conclusions: Limited handheld echocardiography can be successfully implemented in the echocardiography laboratory for screening of COVID-19-related cardiac conditions. The protocol performed with handheld tablet ultrasound provides adequate diagnostic information of major cardiac complications of COVID-19 while decreasing sonographer contact and simplifying decontamination.

Keywords: COVID-19; Handheld echocardiography; Limited echocardiography.

Copyright © 2020 American Society of Echocardiography. Published by Elsevier Inc. All rights reserved.

- 13 references
- 1 figure

Supplementary info

Publication types, MeSH terms Expand

Publication types

- Observational Study

MeSH terms

- Aged
- Betacoronavirus*
- COVID-19
- Computers, Handheld*
- Connecticut / epidemiology
- Coronavirus Infections / complications
- Coronavirus Infections / epidemiology*
- Coronavirus Infections / transmission
- Decontamination / methods*
- Disease Transmission, Infectious / prevention & control*
- Echocardiography / instrumentation*
- Equipment Design
- Female
- Follow-Up Studies
- Heart Diseases / complications
- Heart Diseases / diagnosis*
- Humans
- Incidence
- Male

- Middle Aged
- Pandemics
- Pneumonia, Viral / complications
- Pneumonia, Viral / epidemiology*
- Pneumonia, Viral / transmission
- Reproducibility of Results
- Retrospective Studies
- SARS-CoV-2
- Time Factors

Full text links

ELSEVIER FULL-TEXT ARTICLE Elsevier Science Free PMC article
Proceed to details
Cite
Share
1,133
Observational Study
Alcohol Clin Exp Res
. 2021 Apr;45(4):802-807.
doi: 10.1111/acer.14555. Epub 2021 Mar 5.

Abstinence Among Alcohol Use Disorder Patients During the COVID-19 Pandemic: Insights From Spain

Pablo Barrio [1], Nuria Baldaquí [1], Magalí Andreu [1], Carolin Kilian [2], Jürgen Rehm [2 3 4 5 6 7 8 9], Antoni Gual [1], Jakob Manthey [2 9 10]
Affiliations Expand

Affiliations

- [1] Grup Recerca Addiccions Clínic (GRAC-GRE), Institut d'Investigacions Biomèdiques August Pi I Sunyer (IDIBAPS), Hospital Clínic Barcelona, Barcelona, Spain.
- [2] Institute of Clinical Psychology and Psychotherapy, Technische Universität Dresden, Dresden, Germany.
- [3] Centre for Addiction and Mental Health, Institute for Mental Health Policy Research, Toronto, Ontario, Canada.
- [4] Dalla Lana School of Public Health, University of Toronto, Toronto, Ontario, Canada.
- [5] Faculty of Medicine, Institute of Medical Science, University of Toronto, Toronto, Ontario, Canada.
- [6] Campbell Family Mental Health Research Institute, Centre for Addiction and Mental Health, Toronto, Ontario, Canada.

- [7] Department of Psychiatry, University of Toronto, Toronto, Ontario, Canada.
- [8] I.M. Sechenov First Moscow State Medical University (Sechenov University), Moscow, Russian Federation.
- [9] Center for Interdisciplinary Addiction Research (ZIS), Department of Psychiatry and Psychotherapy, University Medical Center Hamburg-Eppendorf (UKE), Hamburg, Germany.
- [10] Department of Psychiatry, Medical Faculty, University of Leipzig, Leipzig, Germany.

- PMID: **33667019**
- DOI: 10.1111/acer.14555

Observational Study

Abstinence Among Alcohol Use Disorder Patients During the COVID-19 Pandemic: Insights From Spain

Pablo Barrio et al. Alcohol Clin Exp Res. 2021 Apr.

Show details

Alcohol Clin Exp Res

. 2021 Apr;45(4):802-807.

doi: 10.1111/acer.14555. Epub 2021 Mar 5.

Authors

Pablo Barrio [1], Nuria Baldaquí [1], Magalí Andreu [1], Carolin Kilian [2], Jürgen Rehm [2] [3] [4] [5] [6] [7] [8] [9], Antoni Gual [1], Jakob Manthey [2] [9] [10]

Affiliations

- [1] Grup Recerca Addiccions Clínic (GRAC-GRE), Institut d'Investigacions Biomèdiques August Pi I Sunyer (IDIBAPS), Hospital Clínic Barcelona, Barcelona, Spain.
- [2] Institute of Clinical Psychology and Psychotherapy, Technische Universität Dresden, Dresden, Germany.
- [3] Centre for Addiction and Mental Health, Institute for Mental Health Policy Research, Toronto, Ontario, Canada.
- [4] Dalla Lana School of Public Health, University of Toronto, Toronto, Ontario, Canada.
- [5] Faculty of Medicine, Institute of Medical Science, University of Toronto, Toronto, Ontario, Canada.
- [6] Campbell Family Mental Health Research Institute, Centre for Addiction and Mental Health, Toronto, Ontario, Canada.
- [7] Department of Psychiatry, University of Toronto, Toronto, Ontario, Canada.
- [8] I.M. Sechenov First Moscow State Medical University (Sechenov University), Moscow, Russian Federation.

- [9] Center for Interdisciplinary Addiction Research (ZIS), Department of Psychiatry and Psychotherapy, University Medical Center Hamburg-Eppendorf (UKE), Hamburg, Germany.
- [10] Department of Psychiatry, Medical Faculty, University of Leipzig, Leipzig, Germany.

- PMID: **33667019**
- DOI: 10.1111/acer.14555

Abstract

Background: Patients with alcohol use disorder (AUD) are likely to suffer disproportionate harms related to the COVID-19 pandemic and related policy measures. While many surveys have been conducted, most are focused on drinking changes in the general population and validation with biological markers is lacking.

Method: We performed a retrospective cohort study among patients with AUD attending a urine drug screening program. With mixed-effects logistic regression models, we assessed the probability of screening positive for ethyl glucuronide according to patients' main clinical characteristics and time of analysis (either prior to or after a lockdown was implemented in Spain).

Results: A total of 362 patients provided 2,040 urine samples (1,295 prior to lockdown, 745 during lockdown). The mean age of participants was 52.0 years (SD 12.6), and 69.2% were men. Of the 43% of patients tested for other drugs 22% screened positive. After adjusting for all covariates, the odds of screening positive for ethyl glucuronide during lockdown almost doubled (OR = 1.99, 95% CI 1.20 to 3.33, p = 0.008). Other significant covariates included testing positive for other drugs (OR = 10.79, 95% CI 4.60 to 26.97) and length of treatment (OR = 0.59, 95% CI 0.47 to 0.74).

Conclusions: Our data support an association between the lockdown due to COVID-19 and increased alcohol use in patients with AUD. Thus, addiction healthcare systems could face significant challenges ahead. In light of these findings, it is essential to evaluate prospectively how patients with AUD are affected by the pandemic and how health systems respond to their needs.

Keywords: Abstinence; Alcohol; Alcohol Use Disorder; COVID-19; Relapse.

© 2021 The Authors. Alcoholism: Clinical & Experimental Research published by Wiley Periodicals LLC on behalf of Research Society on Alcoholism.

- 45 references

Supplementary info

Publication types, MeSH terms Expand

Publication types

- Observational Study

MeSH terms

- Adolescent
- Adult
- Aged
- Alcohol Abstinence / psychology
- Alcohol Abstinence / trends*
- Alcoholism / epidemiology*
- Alcoholism / psychology
- COVID-19 / epidemiology*
- COVID-19 / psychology
- Cohort Studies
- Female
- Humans
- Male
- Middle Aged
- Pandemics
- Quarantine / psychology
- Quarantine / trends*
- Retrospective Studies
- Spain / epidemiology
- Young Adult

Full text links

WILEY Full Text Article Wiley

Proceed to details

Cite

Share

1,134

Observational Study

Int J Stroke

. 2021 Jun;16(4):437-447.

doi: 10.1177/1747493020959216. Epub 2020 Sep 30.

Cerebrovascular events and outcomes in hospitalized patients with COVID-19: The SVIN COVID-19 Multinational Registry

James E Siegler [1, 2], Pere Cardona [3], Juan F Arenillas [4, 5], Blanca Talavera [3], Ana N Guillen [3], Alba Chavarría-Miranda [3], Mercedes de Lera [3], Priyank Khandelwal [5], Ivo Bach [6], Pratit Patel [5], Amit Singla [7], Manuel Requena [8, 9], Marc Ribo [8, 9], Dinesh V Jillella [10], Srikant Rangaraju [10], Raul G Nogueira [10, 11], Diogo C Haussen [10, 11], Alejandro R Vazquez [12], Xabier

Urra[12 13], Ángel Chamorro[12 13], Luis S Román[14], Jesse M Thon[1 2], Ryna Then[1 2], Emma Sanborn[1 2], Natalia P de la Ossa[15], Mònica Millàn[15], Isaac N Ruiz[15], Ossama Y Mansour[16], Mohammed Megahed[17], Cristina Tiu[18 19], Elena O Terecoasa[18 19], Răzvan A Radu[18], Thanh N Nguyen[20 21 22], Gioacchino Curiale[20], Artem Kaliaev[21], Alexandra L Czap[23], Jacob Sebaugh[23], Alicia M Zha[23], David S Liebeskind[24], Santiago Ortega-Gutierrez[25], Mudassir Farooqui[25], Ameer E Hassan[26 27], Laurie Preston[26], Mary S Patterson[28], Saif Bushnaq[28], Osama Zaidat[28], Tudor G Jovin[1 2]

Affiliations Expand

Affiliations

- [1] Cooper Neurologic Institute, Cooper University Hospital, Camden, NJ, USA.
- [2] Cooper Medical School of Rowan University, Camden NJ, USA.
- [3] Department of Neurology, Hospital Universitari, Bellvitge, Barcelona, Spain.
- [4] Department of Neurology, Hospital Clínico Universitario, Valladolid, Spain.
- [5] Neurovascular Research Laboratory, Instituto de Biología y Genética Molecular, Universidad de Valladolid, Consejo Superior de Investigaciones Científicas, Madrid, Spain.
- [6] Department of Neurology, Robert Wood Johnson University Hospital, New Brunswick, NJ, USA.
- [7] New Jersey Medical School, Newark, NJ, USA.
- [8] Department of Neurosurgery, Robert Wood Johnson University Hospital, New Brunswick, NJ, USA.
- [9] Stroke Unit, Department of Neurology, Vall d'Hebron Research Institute, Barcelona, Spain.
- [10] Departament de Medicina, Universitat Autónoma de Barcelona, Barcelona, Spain.
- [11] Department of Neurology, Emory University School of Medicine, Atlanta, GA, USA.
- [12] Department of Neurology, Grady Memorial Hospital, Atlanta, GA, USA.
- [13] Department of Neurology, Hospital Clínic, Barcelona, Spain.
- [14] Area of Neuroscience, Institut d'Investigacions Biomèdiques August Pi I Sunyer (IDIBAPS), Barcelona, Spain.
- [15] Department of Radiology, Hospital Clínic, Barcelona, Spain.
- [16] Stroke Unit, Neuroscience Department, Hospital Universitari Germans Trias i Pujol, Carretera Canyet s/n, Badalona, Barcelona, Spain.
- [17] Department of Neurology, Stroke and Neurointervention division, Alexandria University, Alexandria, Egypt.
- [18] Department of Critical Care Medicine, Alexandria University, Alexandria, Egypt.
- [19] Department of Neurology, University Emergency Hospital Bucharest, Bucharest, Romania.
- [20] "Carol Davila" University of Medicine and Pharmacy, Bucharest, Romania.
- [21] Department of Neurology, Boston Medical Center, Boston University School of Medicine, MA, USA.
- [22] Department of Radiology, Boston Medical Center, Boston University School of Medicine, MA, USA.
- [23] Department of Neurosurgery, Boston Medical Center, Boston University School of Medicine, MA, USA.
- [24] Department of Neurology, McGovern Medical School, University of Texas Health Science Center, Houston, TX, USA.

- [25] Department of Neurology, Ronald Reagan UCLA Medical Center, Los Angeles, CA, USA.
- [26] Department of Neurology, Neurosurgery and Radiology, University of Iowa Hospitals and Clinics, Iowa City, IA, USA.
- [27] Department of Clinical Neuroscience Research, Valley Baptist Medical Center, Harlingen, TX, USA.
- [28] Department of Neurology, University of Texas Rio Grande Valley, Harlingen, TX, USA.

- PMID: **32852257**
- PMCID: PMC7533468
- DOI: 10.1177/1747493020959216

Free PMC article
Observational Study

Cerebrovascular events and outcomes in hospitalized patients with COVID-19: The SVIN COVID-19 Multinational Registry

James E Siegler et al. Int J Stroke. 2021 Jun.
Free PMC article
Show details
Int J Stroke
. 2021 Jun;16(4):437-447.
doi: 10.1177/1747493020959216. Epub 2020 Sep 30.

Authors

James E Siegler [1, 2], Pere Cardona [3], Juan F Arenillas [4, 5], Blanca Talavera [3], Ana N Guillen [3], Alba Chavarría-Miranda [3], Mercedes de Lera [3], Priyank Khandelwal [5], Ivo Bach [6], Pratit Patel [5], Amit Singla [7], Manuel Requena [8, 9], Marc Ribo [8, 9], Dinesh V Jillella [10], Srikant Rangaraju [10], Raul G Nogueira [10, 11], Diogo C Haussen [10, 11], Alejandro R Vazquez [12], Xabier Urra [12, 13], Ángel Chamorro [12, 13], Luis S Román [14], Jesse M Thon [1, 2], Ryna Then [1, 2], Emma Sanborn [1, 2], Natalia P de la Ossa [15], Mònica Millàn [15], Isaac N Ruiz [15], Ossama Y Mansour [16], Mohammed Megahed [17], Cristina Tiu [18, 19], Elena O Terecoasa [18, 19], Răzvan A Radu [18], Thanh N Nguyen [20, 21, 22], Gioacchino Curiale [20], Artem Kaliaev [21], Alexandra L Czap [23], Jacob Sebaugh [23], Alicia M Zha [23], David S Liebeskind [24], Santiago Ortega-Gutierrez [25], Mudassir Farooqui [25], Ameer E Hassan [26, 27], Laurie Preston [26], Mary S Patterson [28], Saif Bushnaq [28], Osama Zaidat [28], Tudor G Jovin [1, 2]

Affiliations

- [1] Cooper Neurologic Institute, Cooper University Hospital, Camden, NJ, USA.
- [2] Cooper Medical School of Rowan University, Camden NJ, USA.
- [3] Department of Neurology, Hospital Universitari, Bellvitge, Barcelona, Spain.
- [4] Department of Neurology, Hospital Clínico Universitario, Valladolid, Spain.

- [5] Neurovascular Research Laboratory, Instituto de Biología y Genética Molecular, Universidad de Valladolid, Consejo Superior de Investigaciones Científicas, Madrid, Spain.
- [6] Department of Neurology, Robert Wood Johnson University Hospital, New Brunswick, NJ, USA.
- [7] New Jersey Medical School, Newark, NJ, USA.
- [8] Department of Neurosurgery, Robert Wood Johnson University Hospital, New Brunswick, NJ, USA.
- [9] Stroke Unit, Department of Neurology, Vall d'Hebron Research Institute, Barcelona, Spain.
- [10] Departament de Medicina, Universitat Autónoma de Barcelona, Barcelona, Spain.
- [11] Department of Neurology, Emory University School of Medicine, Atlanta, GA, USA.
- [12] Department of Neurology, Grady Memorial Hospital, Atlanta, GA, USA.
- [13] Department of Neurology, Hospital Clínic, Barcelona, Spain.
- [14] Area of Neuroscience, Institut d'Investigacions Biomèdiques August Pi I Sunyer (IDIBAPS), Barcelona, Spain.
- [15] Department of Radiology, Hospital Clínic, Barcelona, Spain.
- [16] Stroke Unit, Neuroscience Department, Hospital Universitari Germans Trias i Pujol, Carretera Canyet s/n, Badalona, Barcelona, Spain.
- [17] Department of Neurology, Stroke and Neurointervention division, Alexandria University, Alexandria, Egypt.
- [18] Department of Critical Care Medicine, Alexandria University, Alexandria, Egypt.
- [19] Department of Neurology, University Emergency Hospital Bucharest, Bucharest, Romania.
- [20] "Carol Davila" University of Medicine and Pharmacy, Bucharest, Romania.
- [21] Department of Neurology, Boston Medical Center, Boston University School of Medicine, MA, USA.
- [22] Department of Radiology, Boston Medical Center, Boston University School of Medicine, MA, USA.
- [23] Department of Neurosurgery, Boston Medical Center, Boston University School of Medicine, MA, USA.
- [24] Department of Neurology, McGovern Medical School, University of Texas Health Science Center, Houston, TX, USA.
- [25] Department of Neurology, Ronald Reagan UCLA Medical Center, Los Angeles, CA, USA.
- [26] Department of Neurology, Neurosurgery and Radiology, University of Iowa Hospitals and Clinics, Iowa City, IA, USA.
- [27] Department of Clinical Neuroscience Research, Valley Baptist Medical Center, Harlingen, TX, USA.
- [28] Department of Neurology, University of Texas Rio Grande Valley, Harlingen, TX, USA.

- PMID: **32852257**
- PMCID: PMC7533468
- DOI: 10.1177/1747493020959216

Abstract

Background: Severe acute respiratory syndrome-coronavirus-2 (SARS-CoV-2) has been associated with a significant risk of thrombotic events in critically ill patients.

Aim: To summarize the findings of a multinational observational cohort of patients with SARS-CoV-2 and cerebrovascular disease.

Methods: Retrospective observational cohort of consecutive adults evaluated in the emergency department and/or admitted with coronavirus disease 2019 (COVID-19) across 31 hospitals in four countries (1 February 2020-16 June 2020). The primary outcome was the incidence rate of cerebrovascular events, inclusive of acute ischemic stroke, intracranial hemorrhages (ICH), and cortical vein and/or sinus thrombosis (CVST).

Results: Of the 14,483 patients with laboratory-confirmed SARS-CoV-2, 172 were diagnosed with an acute cerebrovascular event (1.13% of cohort; 1130/100,000 patients, 95%CI 970-1320/100,000), 68/171 (40.5%) were female and 96/172 (55.8%) were between the ages 60 and 79 years. Of these, 156 had acute ischemic stroke (1.08%; 1080/100,000 95%CI 920-1260/100,000), 28 ICH (0.19%; 190/100,000 95%CI 130-280/100,000), and 3 with CVST (0.02%; 20/100,000, 95%CI 4-60/100,000). The in-hospital mortality rate for SARS-CoV-2-associated stroke was 38.1% and for ICH 58.3%. After adjusting for clustering by site and age, baseline stroke severity, and all predictors of in-hospital mortality found in univariate regression ($p < 0.1$: male sex, tobacco use, arrival by emergency medical services, lower platelet and lymphocyte counts, and intracranial occlusion), cryptogenic stroke mechanism (aOR 5.01, 95%CI 1.63-15.44, $p < 0.01$), older age (aOR 1.78, 95%CI 1.07-2.94, $p = 0.03$), and lower lymphocyte count on admission (aOR 0.58, 95%CI 0.34-0.98, $p = 0.04$) were the only independent predictors of mortality among patients with stroke and COVID-19.

Conclusions: COVID-19 is associated with a small but significant risk of clinically relevant cerebrovascular events, particularly ischemic stroke. The mortality rate is high for COVID-19-associated cerebrovascular complications; therefore, aggressive monitoring and early intervention should be pursued to mitigate poor outcomes.

Keywords: All cerebrovascular diseases/stroke; COVID-19; cerebral venous thrombosis; intracranial hemorrhage.

Conflict of interest statement

Declaration of conflicting interests: The author(s) declared the following potential conflicts of interest with respect to the research, authorship, and/or publication of this article: RGN reports consulting fees for advisory roles with Anaconda, Biogen, Cerenovus, Genentech, Imperative Care, Medtronic, Phenox, Prolong Pharmaceuticals, Stryker Neurovascular and stock options for advisory roles with Astrocyte, Brainomix, Cerebrotech, Ceretrieve, Corindus Vascular Robotics, Vesalio, Viz-AI, and Perfuze. No other authors report any competing financial interests.

- 22 references
- 2 figures

Supplementary info

Publication types, MeSH terms Expand

Publication types

- Multicenter Study
- Observational Study

MeSH terms

- Adult
- Age Factors
- Aged
- Aged, 80 and over
- COVID-19 / complications
- COVID-19 / epidemiology*
- COVID-19 / therapy
- Cerebrovascular Disorders / epidemiology*
- Cerebrovascular Disorders / etiology
- Cerebrovascular Disorders / therapy
- Cohort Studies
- Female
- Hospital Mortality
- Humans
- Intracranial Hemorrhages / epidemiology
- Ischemic Stroke / epidemiology
- Ischemic Stroke / etiology
- Ischemic Stroke / therapy
- Lymphocyte Count
- Male
- Middle Aged
- Prevalence
- Registries
- Retrospective Studies
- Risk Factors
- Sex Factors
- Thrombosis / etiology
- Tobacco Use
- Young Adult

Full text links

SAGE journals Open access full text Atypon Free PMC article
Proceed to details
Cite
Share
☐ 1,135
Observational Study
Respir Res
. 2021 Mar 21;22(1):88.

doi: 10.1186/s12931-021-01685-0.

The impact of the involvement of a healthcare professional on the usage of an eHealth platform: a retrospective observational COPD study

Cathelijne M van Zelst [1, 2], Marise J Kasteleyn [3, 4, 5], Esther M J van Noort [3], Maureen P M H Rutten-van Molken [6], Gert-Jan Braunstahl [7, 8], Niels H Chavannes [3, 4], Johannes C C M In 't Veen [7]
Affiliations Expand

Affiliations

- [1] Department of Pulmonology, Franciscus Gasthuis en Vlietland, Kleiweg 500, Rotterdam, 3045 PM, The Netherlands. c.zelst@franciscus.nl.
- [2] Department of Pulmonology, Erasmus Medical Center, Rotterdam, The Netherlands. c.zelst@franciscus.nl.
- [3] Department of Public Health and Primary Care, Leiden University Medical Center, Leiden, The Netherlands.
- [4] National eHealth Living Lab, Leiden University Medical Center, Leiden, The Netherlands.
- [5] Department of Pulmonology, Leiden University Medical Center, Leiden, The Netherlands.
- [6] Erasmus School of Health Policy & Management, Erasmus University, Rotterdam, The Netherlands.
- [7] Department of Pulmonology, Franciscus Gasthuis en Vlietland, Kleiweg 500, Rotterdam, 3045 PM, The Netherlands.
- [8] Department of Pulmonology, Erasmus Medical Center, Rotterdam, The Netherlands.

- PMID: **33743686**
- PMCID: PMC7981385
- DOI: 10.1186/s12931-021-01685-0

Free PMC article
Observational Study

The impact of the involvement of a healthcare professional on the usage of an eHealth platform: a retrospective observational COPD study

Cathelijne M van Zelst et al. Respir Res. 2021.
Free PMC article

Show details

Respir Res
. 2021 Mar 21;22(1):88.
doi: 10.1186/s12931-021-01685-0.

Authors

Cathelijne M van Zelst [1, 2], Marise J Kasteleyn [3, 4, 5], Esther M J van Noort [3], Maureen P M H Rutten-van Molken [6], Gert-Jan Braunstahl [7, 8], Niels H Chavannes [3, 4], Johannes C C M In 't Veen [7]

Affiliations

- [1] Department of Pulmonology, Franciscus Gasthuis en Vlietland, Kleiweg 500, Rotterdam, 3045 PM, The Netherlands. c.zelst@franciscus.nl.
- [2] Department of Pulmonology, Erasmus Medical Center, Rotterdam, The Netherlands. c.zelst@franciscus.nl.
- [3] Department of Public Health and Primary Care, Leiden University Medical Center, Leiden, The Netherlands.
- [4] National eHealth Living Lab, Leiden University Medical Center, Leiden, The Netherlands.
- [5] Department of Pulmonology, Leiden University Medical Center, Leiden, The Netherlands.
- [6] Erasmus School of Health Policy & Management, Erasmus University, Rotterdam, The Netherlands.
- [7] Department of Pulmonology, Franciscus Gasthuis en Vlietland, Kleiweg 500, Rotterdam, 3045 PM, The Netherlands.
- [8] Department of Pulmonology, Erasmus Medical Center, Rotterdam, The Netherlands.

- PMID: **33743686**
- PMCID: PMC7981385
- DOI: 10.1186/s12931-021-01685-0

Abstract

Background: Ehealth platforms, since the outbreak of COVID-19 more important than ever, can support self-management in patients with Chronic Obstructive Pulmonary Disease (COPD). The aim of this observational study is to explore the impact of healthcare professional involvement on the adherence of patients to an eHealth platform. We evaluated the usage of an eHealth platform by patients who used the platform individually compared with patients in a blended setting, where healthcare professionals were involved.

Methods: In this observational cohort study, log data from September 2011 until January 2018 were extracted from the eHealth platform Curavista. Patients with COPD who completed at least one Clinical COPD Questionnaire (CCQ) were included for analyses (n = 299). In 57% (n = 171) of the patients, the eHealth platform was used in a blended setting, either in hospital (n = 128) or primary care (n = 29). To compare usage of the platform between patients who used the platform independently or with a healthcare professional, we applied propensity score matching and performed adjusted Poisson regression analysis on CCQ-submission rate.

Results: Using the eHealth platform in a blended setting was associated with a 3.25 higher CCQ-submission rate compared to patients using the eHealth platform independently. Within the

blended setting, the CCQ-submission rate was 1.83 higher in the hospital care group than in the primary care group.

Conclusion: It is shown that COPD patients used the platform more frequently in a blended care setting compared to patients who used the eHealth platform independently, adjusted for age, sex and disease burden. Blended care seems essential for adherence to eHealth programs in COPD, which in turn may improve self-management.

Keywords: Adherence; CCQ; COPD; EHealth.

Conflict of interest statement

CZ: No competing interests related to the topic. MK: No competing interests related to the topic. EN: Co-owner of the eHealth platform Curavista. MR: No competing interests related to the topic. GB: No competing interests related to the topic. NC: No competing interests related to the topic. JV: No competing interests related to the topic.

- 28 references
- 4 figures

Supplementary info

Publication types, MeSH terms Expand

Publication types

- Observational Study

MeSH terms

- Aged
- COVID-19 / epidemiology
- COVID-19 / psychology*
- COVID-19 / therapy
- Cohort Studies
- Female
- Health Personnel / psychology*
- Humans
- Male
- Middle Aged
- Patient Acceptance of Health Care / psychology*
- Professional Role / psychology*
- Pulmonary Disease, Chronic Obstructive / epidemiology
- Pulmonary Disease, Chronic Obstructive / psychology*
- Pulmonary Disease, Chronic Obstructive / therapy
- Retrospective Studies

- Telemedicine / methods*

Full text links

Read free full text at BMC BioMed Central Free PMC article
Proceed to details
Cite
Share
1,136
Observational Study
Lancet Haematol
. 2020 Oct;7(10):e737-e745.
doi: 10.1016/S2352-3026(20)30251-9. Epub 2020 Aug 13.

Clinical characteristics and risk factors associated with COVID-19 severity in patients with haematological malignancies in Italy: a retrospective, multicentre, cohort study

Francesco Passamonti [1], Chiara Cattaneo [2], Luca Arcaini [3], Riccardo Bruna [4], Michele Cavo [5], Francesco Merli [6], Emanuele Angelucci [7], Mauro Krampera [8], Roberto Cairoli [9], Matteo Giovanni Della Porta [10], Nicola Fracchiolla [11], Marco Ladetto [12], Carlo Gambacorti Passerini [13], Marco Salvini [14], Monia Marchetti [12], Roberto Lemoli [15], Alfredo Molteni [16], Alessandro Busca [17], Antonio Cuneo [18], Alessandra Romano [19], Nicola Giuliani [20], Sara Galimberti [21], Alessandro Corso [22], Alessandro Morotti [23], Brunangelo Falini [24], Atto Billio [25], Filippo Gherlinzoni [26], Giuseppe Visani [27], Maria Chiara Tisi [28], Agostino Tafuri [29], Patrizia Tosi [30], Francesco Lanza [31], Massimo Massaia [32], Mauro Turrini [33], Felicetto Ferrara [34], Carmela Gurrieri [35], Daniele Vallisa [36], Maurizio Martelli [37], Enrico Derenzini [38], Attilio Guarini [39], Annarita Conconi [40], Annarosa Cuccaro [41], Laura Cudillo [42], Domenico Russo [43], Fabrizio Ciambelli [44], Anna Maria Scattolin [45], Mario Luppi [46], Carmine Selleri [47], Elettra Ortu La Barbera [48], Celestino Ferrandina [49], Nicola Di Renzo [50], Attilio Olivieri [51], Monica Bocchia [52], Massimo Gentile [53], Francesco Marchesi [54], Pellegrino Musto [55], Augusto Bramante Federici [56], Anna Candoni [57], Adriano Venditti [58], Carmen Fava [23], Antonio Pinto [59], Piero Galieni [60], Luigi Rigacci [61], Daniele Armiento [62], Fabrizio Pane [63], Margherita Oberti [2], Patrizia Zappasodi [64], Carlo Visco [8], Matteo Franchi [65], Paolo Antonio Grossi [14], Lorenza Bertù [14], Giovanni Corrao [65], Livio Pagano [66], Paolo Corradini [67], ITA-HEMA-COV Investigators
Affiliations Expand

Affiliations

- [1] Department of Medicine and Surgery, University of Insubria and ASST Sette Laghi, Ospedale di Circolo of Varese, Varese, Italy. Electronic address: francesco.passamonti@uninsubria.it.

- [2] Haematology, ASST-Spedali Civili, Brescia, Italy.
- [3] Department of Molecular Medicine, University of Pavia, Pavia, Italy; Division of Hematology, Fondazione IRCCS Policlinico San Matteo, Pavia, Italy.
- [4] Division of Hematology, Department of Translational Medicine, University of Eastern Piedmont and Ospedale Maggiore della Carità, Novara, Italy.
- [5] Seràgnoli Institute of Hematology, Department of Experimental, Diagnostic and Specialty Medicine, Bologna University School of Medicine, Bologna, Italy.
- [6] Hematology, Azienda USL-IRCCS Reggio Emilia, Reggio Emilia, Italy.
- [7] Hematology, Ospedale Policlinico San Martino, Genoa, Italy.
- [8] Department of Medicine, Section of Hematology, University of Verona, Verona, Italy.
- [9] Hematology, ASST Grande Ospedale Metropolitano Niguarda, Milan, Italy.
- [10] Humanitas Clinical and Research Hospital-IRCCS and Department of Biomedical Sciences, Humanitas University, Milan, Italy.
- [11] Fondazione IRCCS Ca' Granda-Ospedale Maggiore Policlinico, Milan, Italy.
- [12] Hematology, Azienda Ospedaliera SS Antonio e Biagio e Cesare Arrigo, Alessandria, Italy.
- [13] Department of Hematology, Università degli Studi di Milano-Bicocca, Milan, Italy.
- [14] Department of Medicine and Surgery, University of Insubria and ASST Sette Laghi, Ospedale di Circolo of Varese, Varese, Italy.
- [15] Dipartimento di Medicina interna e Specialità mediche, University of Genoa, Genoa, Italy.
- [16] Hematology, ASST Cremona, Cremona, Italy.
- [17] Stem Cell Transplant Center, AOU Citta' della Salute e della Scienza, Turin, Italy.
- [18] Hematology, Azienda Ospedaliero Universitaria Sant'Anna, Ferrara, Italy.
- [19] Hematology, Dipartimento di Chirurgia e Specialità Medico Chirurgiche, Università degli Studi di Catania, Catania, Italy.
- [20] Dipartimento di Medicina e Chirurgia, University of Parma, Parma, Italy.
- [21] Department of Clinical and Experimental Medicine, University of Pisa, Pisa, Italy.
- [22] Hematology, ASST Ovest Milanese, Milan, Italy.
- [23] Department of Clinical and Biological Sciences, Università di Torino, Turin, Italy.
- [24] Department of Medicine, University of Perugia, Perugia, Italy.
- [25] Ospedale di Bolzano, Bolzano, Italy.
- [26] Hematology, Ospedale Ca' Foncello, Treviso, Italy.
- [27] Dipartimento di Onco- Ematologia, Azienda Ospedaliera Ospedali Riuniti Marche Nord, Pesaro, Italy.
- [28] Hematology, Ospedale San Bortolo, Vicenza, Italy.
- [29] Hematology, University Hospital Sant'Andrea, Sapienza, Rome, Italy; Department of Clinical and Molecular Medicine, Sapienza, University of Rome, Rome, Italy.
- [30] Hematology, Ospedale degli Infermi di Rimini, Rimini, Italy.
- [31] Hematology, Santa Maria delle Croci, Ravenna, Italy.
- [32] Hematology, Santa Croce Hospital, Cuneo, Italy.
- [33] Hematology, Ospedale Valduce, Como, Italy.
- [34] Hematology, Ospedale Antonio Cardarelli, Naples, Italy.
- [35] Dipartimento Strutturale Aziendale Medicina, University of Padova, Padova, Italy.
- [36] Hematology, Ospedale di Piacenza, Piacenza, Italy.
- [37] Hematology, Department of Translational and Precision Medicine, Sapienza, University of Rome, Rome, Italy.

- [38] Hematology, Istituto Europeo di Oncologia, Milan, Italy.
- [39] Hematology, Istituto Tumori Giovanni Paolo II, Bari, Italy.
- [40] Hematology, Ospedale degli Infermi, Biella, Italy.
- [41] Ematologia, USL 6 Livorno, Livorno, Italy.
- [42] Hematology, San Giovanni Addolorata Hospital, Rome, Italy.
- [43] Dipartimento di Scienze Cliniche e Sperimentali, University of Brescia, Brescia, Italy.
- [44] Dipartimento Oncologico, ASST Valle Olona, Busto Arsizio, Italy.
- [45] Hematology, Ospedale dell'Angelo di Mestre, Venice, Italy.
- [46] Dipartimento di Scienze Mediche e Chirurgiche Materno-Infantili e dell'Adulto, University of Modena and Reggio Emilia, Azienda Ospedaliera Universitaria, Modena, Italy.
- [47] Hematology, Ospedale San Giovanni di Dio e Ruggi D'Aragona, Salerno, Italy.
- [48] UOC Ematologia con Trapianto, Ospedale Santa Maria Goretti, Latina, Italy; Hematology, Ospedale Santa Maria Goretti, Latina, Italy.
- [49] Hematology, Ospedali Riuniti Azienda Ospedaliera Universitaria di Foggia, Foggia, Italy.
- [50] Hematology and Transplant Unit, Ospedale Vito Fazzi, Lecce, Italy.
- [51] Hematology, Ospedali Riuniti di Ancona, Ancona, Italy.
- [52] Hematology Unit, University of Siena, Azienda Ospedaliero Universitaria Senese, Siena, Italy.
- [53] Hematology, Ospedale SS Annunziata, Taranto, Italy.
- [54] Hematology and Stem Cell Transplant Unit, IRCCS Regina Elena National Cancer Institute, Rome, Italy.
- [55] Department of Emergency and Organ Transplantation, "Aldo Moro" University School of Medicine and Unit of Hematology and Stem Cell Transplantation, AOU Consorziale Policlinico, Bari, Italy.
- [56] Hematology and Transfusion Medicine, L Sacco University Hospital, Milan, Italy.
- [57] Dipartimento di Medicina Specialistica, University of Udine, Udine, Italy.
- [58] Hematology, Fondazione Policlinico Tor Vergata, Rome, Italy.
- [59] Hematology, Istituto Nazionale Tumori IRCCS "Fondazione G Pascale", Naples, Italy.
- [60] Hematology, Mazzoni Hospital, Ascoli Piceno, Italy.
- [61] Hematology, Camillo-Forlanini Hospital, Rome, Italy.
- [62] Unit of Hematology, Stem Cell Transplantation, University Campus Bio-Medico, Rome, Italy.
- [63] Department of Clinical Medicine and Surgery, Federico II Hospital, Naples, Italy.
- [64] Division of Hematology, Fondazione IRCCS Policlinico San Matteo, Pavia, Italy.
- [65] Laboratory of Healthcare Research & Pharmacoepidemiology, Department of Statistics and Quantitative Methods, Università degli Studi di Milano-Bicocca, Milan, Italy; National Centre for Healthcare Research and Pharmacoepidemiology, Milan, Italy.
- [66] Dipartimento di Scienze Radiologiche ed Ematologiche, Fondazione Policlinico Universitario A Gemelli-IRCCS-Università Cattolica del Sacro Cuore, Rome, Italy.
- [67] Hematology, Fondazione IRCCS Istituto Nazionale dei Tumori, University of Milano.

- PMID: **32798473**
- PMCID: PMC7426107
- DOI: 10.1016/S2352-3026(20)30251-9

Free PMC article
Observational Study

Clinical characteristics and risk factors associated with COVID-19 severity in patients with haematological malignancies in Italy: a retrospective, multicentre, cohort study

Francesco Passamonti et al. Lancet Haematol. 2020 Oct.
Free PMC article
Show details
Lancet Haematol
. 2020 Oct;7(10):e737-e745.
doi: 10.1016/S2352-3026(20)30251-9. Epub 2020 Aug 13.

Authors

Francesco Passamonti [1], Chiara Cattaneo [2], Luca Arcaini [3], Riccardo Bruna [4], Michele Cavo [5], Francesco Merli [6], Emanuele Angelucci [7], Mauro Krampera [8], Roberto Cairoli [9], Matteo Giovanni Della Porta [10], Nicola Fracchiolla [11], Marco Ladetto [12], Carlo Gambacorti Passerini [13], Marco Salvini [14], Monia Marchetti [12], Roberto Lemoli [15], Alfredo Molteni [16], Alessandro Busca [17], Antonio Cuneo [18], Alessandra Romano [19], Nicola Giuliani [20], Sara Galimberti [21], Alessandro Corso [22], Alessandro Morotti [23], Brunangelo Falini [24], Atto Billio [25], Filippo Gherlinzoni [26], Giuseppe Visani [27], Maria Chiara Tisi [28], Agostino Tafuri [29], Patrizia Tosi [30], Francesco Lanza [31], Massimo Massaia [32], Mauro Turrini [33], Felicetto Ferrara [34], Carmela Gurrieri [35], Daniele Vallisa [36], Maurizio Martelli [37], Enrico Derenzini [38], Attilio Guarini [39], Annarita Conconi [40], Annarosa Cuccaro [41], Laura Cudillo [42], Domenico Russo [43], Fabrizio Ciambelli [44], Anna Maria Scattolin [45], Mario Luppi [46], Carmine Selleri [47], Elettra Ortu La Barbera [48], Celestino Ferrandina [49], Nicola Di Renzo [50], Attilio Olivieri [51], Monica Bocchia [52], Massimo Gentile [53], Francesco Marchesi [54], Pellegrino Musto [55], Augusto Bramante Federici [56], Anna Candoni [57], Adriano Venditti [58], Carmen Fava [23], Antonio Pinto [59], Piero Galieni [60], Luigi Rigacci [61], Daniele Armiento [62], Fabrizio Pane [63], Margherita Oberti [2], Patrizia Zappasodi [64], Carlo Visco [8], Matteo Franchi [65], Paolo Antonio Grossi [14], Lorenza Bertù [14], Giovanni Corrao [65], Livio Pagano [66], Paolo Corradini [67], ITA-HEMA-COV Investigators

Affiliations

- [1] Department of Medicine and Surgery, University of Insubria and ASST Sette Laghi, Ospedale di Circolo of Varese, Varese, Italy. Electronic address: francesco.passamonti@uninsubria.it.
- [2] Haematology, ASST-Spedali Civili, Brescia, Italy.
- [3] Department of Molecular Medicine, University of Pavia, Pavia, Italy; Division of Hematology, Fondazione IRCCS Policlinico San Matteo, Pavia, Italy.
- [4] Division of Hematology, Department of Translational Medicine, University of Eastern Piedmont and Ospedale Maggiore della Carità, Novara, Italy.
- [5] Seràgnoli Institute of Hematology, Department of Experimental, Diagnostic and Specialty Medicine, Bologna University School of Medicine, Bologna, Italy.

- [6] Hematology, Azienda USL-IRCCS Reggio Emilia, Reggio Emilia, Italy.
- [7] Hematology, Ospedale Policlinico San Martino, Genoa, Italy.
- [8] Department of Medicine, Section of Hematology, University of Verona, Verona, Italy.
- [9] Hematology, ASST Grande Ospedale Metropolitano Niguarda, Milan, Italy.
- [10] Humanitas Clinical and Research Hospital-IRCCS and Department of Biomedical Sciences, Humanitas University, Milan, Italy.
- [11] Fondazione IRCCS Ca' Granda-Ospedale Maggiore Policlinico, Milan, Italy.
- [12] Hematology, Azienda Ospedaliera SS Antonio e Biagio e Cesare Arrigo, Alessandria, Italy.
- [13] Department of Hematology, Università degli Studi di Milano-Bicocca, Milan, Italy.
- [14] Department of Medicine and Surgery, University of Insubria and ASST Sette Laghi, Ospedale di Circolo of Varese, Varese, Italy.
- [15] Dipartimento di Medicina interna e Specialità mediche, University of Genoa, Genoa, Italy.
- [16] Hematology, ASST Cremona, Cremona, Italy.
- [17] Stem Cell Transplant Center, AOU Citta' della Salute e della Scienza, Turin, Italy.
- [18] Hematology, Azienda Ospedaliero Universitaria Sant'Anna, Ferrara, Italy.
- [19] Hematology, Dipartimento di Chirurgia e Specialità Medico Chirurgiche, Università degli Studi di Catania, Catania, Italy.
- [20] Dipartimento di Medicina e Chirurgia, University of Parma, Parma, Italy.
- [21] Department of Clinical and Experimental Medicine, University of Pisa, Pisa, Italy.
- [22] Hematology, ASST Ovest Milanese, Milan, Italy.
- [23] Department of Clinical and Biological Sciences, Università di Torino, Turin, Italy.
- [24] Department of Medicine, University of Perugia, Perugia, Italy.
- [25] Ospedale di Bolzano, Bolzano, Italy.
- [26] Hematology, Ospedale Ca' Foncello, Treviso, Italy.
- [27] Dipartimento di Onco- Ematologia, Azienda Ospedaliera Ospedali Riuniti Marche Nord, Pesaro, Italy.
- [28] Hematology, Ospedale San Bortolo, Vicenza, Italy.
- [29] Hematology, University Hospital Sant'Andrea, Sapienza, Rome, Italy; Department of Clinical and Molecular Medicine, Sapienza, University of Rome, Rome, Italy.
- [30] Hematology, Ospedale degli Infermi di Rimini, Rimini, Italy.
- [31] Hematology, Santa Maria delle Croci, Ravenna, Italy.
- [32] Hematology, Santa Croce Hospital, Cuneo, Italy.
- [33] Hematology, Ospedale Valduce, Como, Italy.
- [34] Hematology, Ospedale Antonio Cardarelli, Naples, Italy.
- [35] Dipartimento Strutturale Aziendale Medicina, University of Padova, Padova, Italy.
- [36] Hematology, Ospedale di Piacenza, Piacenza, Italy.
- [37] Hematology, Department of Translational and Precision Medicine, Sapienza, University of Rome, Rome, Italy.
- [38] Hematology, Istituto Europeo di Oncologia, Milan, Italy.
- [39] Hematology, Istituto Tumori Giovanni Paolo II, Bari, Italy.
- [40] Hematology, Ospedale degli Infermi, Biella, Italy.
- [41] Ematologia, USL 6 Livorno, Livorno, Italy.
- [42] Hematology, San Giovanni Addolorata Hospital, Rome, Italy.
- [43] Dipartimento di Scienze Cliniche e Sperimentali, University of Brescia, Brescia, Italy.
- [44] Dipartimento Oncologico, ASST Valle Olona, Busto Arsizio, Italy.

- [45] Hematology, Ospedale dell'Angelo di Mestre, Venice, Italy.
- [46] Dipartimento di Scienze Mediche e Chirurgiche Materno-Infantili e dell'Adulto, University of Modena and Reggio Emilia, Azienda Ospedaliera Universitaria, Modena, Italy.
- [47] Hematology, Ospedale San Giovanni di Dio e Ruggi D'Aragona, Salerno, Italy.
- [48] UOC Ematologia con Trapianto, Ospedale Santa Maria Goretti, Latina, Italy; Hematology, Ospedale Santa Maria Goretti, Latina, Italy.
- [49] Hematology, Ospedali Riuniti Azienda Ospedaliera Universitaria di Foggia, Foggia, Italy.
- [50] Hematology and Transplant Unit, Ospedale Vito Fazzi, Lecce, Italy.
- [51] Hematology, Ospedali Riuniti di Ancona, Ancona, Italy.
- [52] Hematology Unit, University of Siena, Azienda Ospedaliero Universitaria Senese, Siena, Italy.
- [53] Hematology, Ospedale SS Annunziata, Taranto, Italy.
- [54] Hematology and Stem Cell Transplant Unit, IRCCS Regina Elena National Cancer Institute, Rome, Italy.
- [55] Department of Emergency and Organ Transplantation, "Aldo Moro" University School of Medicine and Unit of Hematology and Stem Cell Transplantation, AOU Consorziale Policlinico, Bari, Italy.
- [56] Hematology and Transfusion Medicine, L Sacco University Hospital, Milan, Italy.
- [57] Dipartimento di Medicina Specialistica, University of Udine, Udine, Italy.
- [58] Hematology, Fondazione Policlinico Tor Vergata, Rome, Italy.
- [59] Hematology, Istituto Nazionale Tumori IRCCS "Fondazione G Pascale", Naples, Italy.
- [60] Hematology, Mazzoni Hospital, Ascoli Piceno, Italy.
- [61] Hematology, Camillo-Forlanini Hospital, Rome, Italy.
- [62] Unit of Hematology, Stem Cell Transplantation, University Campus Bio-Medico, Rome, Italy.
- [63] Department of Clinical Medicine and Surgery, Federico II Hospital, Naples, Italy.
- [64] Division of Hematology, Fondazione IRCCS Policlinico San Matteo, Pavia, Italy.
- [65] Laboratory of Healthcare Research & Pharmacoepidemiology, Department of Statistics and Quantitative Methods, Università degli Studi di Milano-Bicocca, Milan, Italy; National Centre for Healthcare Research and Pharmacoepidemiology, Milan, Italy.
- [66] Dipartimento di Scienze Radiologiche ed Ematologiche, Fondazione Policlinico Universitario A Gemelli-IRCCS-Università Cattolica del Sacro Cuore, Rome, Italy.
- [67] Hematology, Fondazione IRCCS Istituto Nazionale dei Tumori, University of Milano.

- PMID: **32798473**
- PMCID: PMC7426107
- DOI: 10.1016/S2352-3026(20)30251-9

Abstract

Background: Several small studies on patients with COVID-19 and haematological malignancies are available showing a high mortality in this population. The Italian Hematology Alliance on COVID-19 aimed to collect data from adult patients with haematological malignancies who required hospitalisation for COVID-19.

Methods: This multicentre, retrospective, cohort study included adult patients (aged ≥18 years) with diagnosis of a WHO-defined haematological malignancy admitted to 66 Italian hospitals between Feb 25 and May 18, 2020, with laboratory-confirmed and symptomatic COVID-19. Data

cutoff for this analysis was June 22, 2020. The primary outcome was mortality and evaluation of potential predictive parameters of mortality. We calculated standardised mortality ratios between observed death in the study cohort and expected death by applying stratum-specific mortality rates of the Italian population with COVID-19 and an Italian cohort of 31 993 patients with haematological malignancies without COVID-19 (data up to March 1, 2019). Multivariable Cox proportional hazards model was used to identify factors associated with overall survival. This study is registered with ClinicalTrials.gov, NCT04352556, and the prospective part of the study is ongoing.

Findings: We enrolled 536 patients with a median follow-up of 20 days (IQR 10-34) at data cutoff, 85 (16%) of whom were managed as outpatients. 440 (98%) of 451 hospitalised patients completed their hospital course (were either discharged alive or died). 198 (37%) of 536 patients died. When compared with the general Italian population with COVID-19, the standardised mortality ratio was 2·04 (95% CI 1·77-2·34) in our whole study cohort and 3·72 (2·86-4·64) in individuals younger than 70 years. When compared with the non-COVID-19 cohort with haematological malignancies, the standardised mortality ratio was 41·3 (38·1-44·9). Older age (hazard ratio 1·03, 95% CI 1·01-1·05); progressive disease status (2·10, 1·41-3·12); diagnosis of acute myeloid leukaemia (3·49, 1·56-7·81), indolent non-Hodgin lymphoma (2·19, 1·07-4·48), aggressive non-Hodgkin lymphoma (2·56, 1·34-4·89), or plasma cell neoplasms (2·48, 1·31-4·69), and severe or critical COVID-19 (4·08, 2·73-6·09) were associated with worse overall survival.

Interpretation: This study adds to the evidence that patients with haematological malignancies have worse outcomes than both the general population with COVID-19 and patients with haematological malignancies without COVID-19. The high mortality among patients with haematological malignancies hospitalised with COVID-19 highlights the need for aggressive infection prevention strategies, at least until effective vaccination or treatment strategies are available.

Funding: Associazione italiana contro le leucemie, linfomi e mieloma-Varese Onlus.

Copyright © 2020 Elsevier Ltd. All rights reserved.

Comment in

- COVID-19 and haematological malignancy: navigating a narrow strait.
  Rubinstein SM, Warner JL. Rubinstein SM, et al. Lancet Haematol. 2020 Oct;7(10):e701-e703. doi: 10.1016/S2352-3026(20)30252-0. Epub 2020 Aug 13. Lancet Haematol. 2020. PMID: 32798474 Free PMC article. No abstract available.

- 31 references
- 3 figures

Supplementary info

Publication types, MeSH terms, Supplementary concepts, Associated data Expand

Publication types

- Multicenter Study
- Observational Study

MeSH terms

- Adult
- Aged
- Aged, 80 and over
- Betacoronavirus*
- COVID-19
- Comorbidity
- Coronavirus Infections / drug therapy
- Coronavirus Infections / epidemiology*
- Female
- Follow-Up Studies
- Hematologic Neoplasms / epidemiology*
- Hematologic Neoplasms / therapy
- Humans
- Inpatients
- Italy / epidemiology
- Leukemia / epidemiology
- Leukemia / therapy
- Lymphoma, Non-Hodgkin / epidemiology
- Lymphoma, Non-Hodgkin / therapy
- Male
- Middle Aged
- Myeloproliferative Disorders / epidemiology
- Myeloproliferative Disorders / therapy
- Neoplasms, Plasma Cell / epidemiology
- Neoplasms, Plasma Cell / therapy
- Pandemics*
- Pneumonia, Viral / epidemiology*
- Retrospective Studies
- Risk Factors
- SARS-CoV-2
- Young Adult

Supplementary concepts

- COVID-19 drug treatment

Associated data

- ClinicalTrials.gov/NCT04352556

Full text links

ELSEVIER FULL-TEXT ARTICLE Elsevier Science Free PMC article

Proceed to details

Cite

Share

1,137

Observational Study

Respir Res

. 2020 Sep 21;21(1):241.

doi: 10.1186/s12931-020-01510-0.

The effect of vascular risk factor burden on the severity of COVID-19 illness, a retrospective cohort study

Houwei Du [1, 2], Xiaobin Pan [3], Nan Liu [4, 5], Junnian Chen [6], Xiaoling Chen [7], David J Werring [8], Gareth Ambler [9], Xiaoqing Li [10], Ronghua Chen [4, 11], Yixian Zhang [5], Huayao Huang [5], Feifei Lin [4], Pincang Xia [10], Chao Chen [12], Zhenyang Zheng [4, 11], Sangru Wu [4], Hanhan Lei [4], Lei Gao [13], Mingxu Huang [14], Kexu Lin [14], Xiaoping Xu [15], Yukun Luo [16], Ziwen Zhao [16], Chen Li [17], Hailong Lin [18], Yu Lin [19], Zhenghui Huang [20], Rongxiang Cao [20], Limin Chen [20], Fujian Medical Team Support Wuhan for COVID-19

Affiliations Expand

Affiliations

- [1] Stroke Research Center, Department of Neurology, Fujian Medical University Union Hospital, 29 Xinquan Road, Gulou District, Fuzhou, 350001, China. duhouwei@outlook.com.
- [2] Institute of Clinical Neurology, Fujian Medical University, Fuzhou, China. duhouwei@outlook.com.
- [3] Department of Critical Care Medicine, Fujian Provincial Hospital South Branch, Fuzhou, China.
- [4] Stroke Research Center, Department of Neurology, Fujian Medical University Union Hospital, 29 Xinquan Road, Gulou District, Fuzhou, 350001, China.
- [5] Department of Rehabilitation, Fujian Medical University Union Hospital, Fuzhou, China.
- [6] Department of Critical Care Medicine, Fujian Medical University Union Hospital, Fuzhou, China.
- [7] Department of Infectious Disease, Fujian Medical University Union Hospital, Fuzhou, China.
- [8] UCL Queen Square Institute of Neurology, London, UK.
- [9] Statistical Science, University College London, London, UK.
- [10] Fujian Center for Disease Control and Prevention, Fuzhou, China.
- [11] Institute of Clinical Neurology, Fujian Medical University, Fuzhou, China.

- [12] Department of Neurology, Fuzhou Second Hospital Affiliated to Xiamen University, Fuzhou, China.
- [13] Department of Thoracic Surgery, Fujian Medical University Union Hospital, Fuzhou, China.
- [14] Department of Emergency Medicine, Fujian Medical University Union Hospital, Fuzhou, China.
- [15] Department of Anesthesiology, Fujian Medical University Union Hospital, Fuzhou, China.
- [16] Department of Cardiology, Fujian Medical University Union Hospital, Fuzhou, China.
- [17] Department of Otolaryngology, Fujian Medical University Union Hospital, Fuzhou, China.
- [18] Department of Radiology, Fujian Medical University Union Hospital, Fuzhou, China.
- [19] Department of Colorectal Surgery, Fujian Medical University Union Hospital, Fuzhou, China.
- [20] Department of Respiratory Medicine, Fujian Medical University Union Hospital, Fuzhou, China.

- PMID: **32957997**
- PMCID: PMC7503438
- DOI: 10.1186/s12931-020-01510-0

Free PMC article
Observational Study

The effect of vascular risk factor burden on the severity of COVID-19 illness, a retrospective cohort study

Houwei Du et al. Respir Res. 2020.
Free PMC article

Show details

Respir Res
. 2020 Sep 21;21(1):241.
doi: 10.1186/s12931-020-01510-0.

Authors

Houwei Du [1, 2], Xiaobin Pan [3], Nan Liu [4, 5], Junnian Chen [6], Xiaoling Chen [7], David J Werring [8], Gareth Ambler [9], Xiaoqing Li [10], Ronghua Chen [4, 11], Yixian Zhang [5], Huayao Huang [5], Feifei Lin [4], Pincang Xia [10], Chao Chen [12], Zhenyang Zheng [4, 11], Sangru Wu [4], Hanhan Lei [4], Lei Gao [13], Mingxu Huang [14], Kexu Lin [14], Xiaoping Xu [15], Yukun Luo [16], Ziwen Zhao [16], Chen Li [17], Hailong Lin [18], Yu Lin [19], Zhenghui Huang [20], Rongxiang Cao [20], Limin Chen [20], Fujian Medical Team Support Wuhan for COVID-19

Affiliations

- [1] Stroke Research Center, Department of Neurology, Fujian Medical University Union Hospital, 29 Xinquan Road, Gulou District, Fuzhou, 350001, China. duhouwei@outlook.com.
- [2] Institute of Clinical Neurology, Fujian Medical University, Fuzhou, China. duhouwei@outlook.com.
- [3] Department of Critical Care Medicine, Fujian Provincial Hospital South Branch, Fuzhou, China.
- [4] Stroke Research Center, Department of Neurology, Fujian Medical University Union Hospital, 29 Xinquan Road, Gulou District, Fuzhou, 350001, China.
- [5] Department of Rehabilitation, Fujian Medical University Union Hospital, Fuzhou, China.
- [6] Department of Critical Care Medicine, Fujian Medical University Union Hospital, Fuzhou, China.
- [7] Department of Infectious Disease, Fujian Medical University Union Hospital, Fuzhou, China.
- [8] UCL Queen Square Institute of Neurology, London, UK.
- [9] Statistical Science, University College London, London, UK.
- [10] Fujian Center for Disease Control and Prevention, Fuzhou, China.
- [11] Institute of Clinical Neurology, Fujian Medical University, Fuzhou, China.
- [12] Department of Neurology, Fuzhou Second Hospital Affiliated to Xiamen University, Fuzhou, China.
- [13] Department of Thoracic Surgery, Fujian Medical University Union Hospital, Fuzhou, China.
- [14] Department of Emergency Medicine, Fujian Medical University Union Hospital, Fuzhou, China.
- [15] Department of Anesthesiology, Fujian Medical University Union Hospital, Fuzhou, China.
- [16] Department of Cardiology, Fujian Medical University Union Hospital, Fuzhou, China.
- [17] Department of Otolaryngology, Fujian Medical University Union Hospital, Fuzhou, China.
- [18] Department of Radiology, Fujian Medical University Union Hospital, Fuzhou, China.
- [19] Department of Colorectal Surgery, Fujian Medical University Union Hospital, Fuzhou, China.
- [20] Department of Respiratory Medicine, Fujian Medical University Union Hospital, Fuzhou, China.

- PMID: **32957997**
- PMCID: PMC7503438
- DOI: 10.1186/s12931-020-01510-0

Abstract

Background: Patients with cardiovascular comorbidities are at high risk of poor outcome from COVID-19. However, how the burden (number) of vascular risk factors influences the risk of severe COVID-19 disease remains unresolved. Our aim was to investigate the association of severe COVID-19 illness with vascular risk factor burden.

Methods: We included 164 (61.8 ± 13.6 years) patients with COVID-19 in this retrospective study. We compared the difference in clinical characteristics, laboratory findings and chest computed tomography (CT) findings between patients with severe and non-severe COVID-19

illness. We evaluated the association between the number of vascular risk factors and the development of severe COVID-19 disease, using a Cox regression model.

Results: Sixteen (9.8%) patients had no vascular risk factors; 38 (23.2%) had 1; 58 (35.4%) had 2; 34 (20.7%) had 3; and 18 (10.9%) had ≥4 risk factors. Twenty-nine patients (17.7%) experienced severe COVID-19 disease with a median (14 [7-27] days) duration between onset to developing severe COVID-19 disease, an event rate of 4.47 per 1000-patient days (95%CI 3.10-6.43). Kaplan-Meier curves showed a gradual increase in the risk of severe COVID-19 illness (log-rank $P < 0.001$) stratified by the number of vascular risk factors. After adjustment for age, sex, and comorbidities as potential confounders, vascular risk factor burden remained associated with an increasing risk of severe COVID-19 illness.

Conclusions: Patients with increasing vascular risk factor burden have an increasing risk of severe COVID-19 disease, and this population might benefit from specific COVID-19 prevention (e.g., self-isolation) and early hospital treatment measures.

Keywords: Coronavirus disease 2019; Prognosis; Vascular risk factor.

Conflict of interest statement

None.

- 33 references
- 1 figure

Supplementary info

Publication types, MeSH terms, Grant support Expand

Publication types

- Comparative Study
- Observational Study

MeSH terms

- Aged
- Betacoronavirus / pathogenicity
- COVID-19
- China / epidemiology
- Comorbidity
- Coronavirus Infections / diagnosis
- Coronavirus Infections / epidemiology*
- Coronavirus Infections / virology
- Female
- Host-Pathogen Interactions
- Humans

- Male
- Middle Aged
- Pandemics
- Pneumonia, Viral / diagnosis
- Pneumonia, Viral / epidemiology*
- Pneumonia, Viral / virology
- Prognosis
- Retrospective Studies
- Risk Assessment
- Risk Factors
- SARS-CoV-2
- Severity of Illness Index
- Time Factors
- Vascular Diseases / diagnosis
- Vascular Diseases / epidemiology*

Grant support

- 2016B014/Fujian Provincial Special Foundation for Natural Science Innovation Project
- 2019Y9099/the Joint Funds for the Innovation of Science and Technology, Fujian Province

Full text links

Read free full text at BMC BioMed Central Free PMC article
Proceed to details
Cite
Share
1,138
Observational Study
Eur Rev Med Pharmacol Sci
. 2021 Dec;25(23):7218-7222.
doi: 10.26355/eurrev_202112_27414.

Sick leave request following anti-COVID-19 vaccine administration is low among healthcare workers: results from a retrospective cross-sectional monocentric study

A Schianchi [1], N Ughi, G Cassano, F Del Gaudio, A Dicuonzo, F Scaglione, P M Alberti, C Rossetti, G Micheloni, L Zoppini, G Bellavia, S Giroldi, M Moreno, A Russo, M Bosio, O M Epis

Affiliations Expand

Affiliation

- [1] Division of Chemical-Clinical and Microbiological Analyses, Department of Laboratory Medicine, ASST Grande Ospedale Metropolitano Niguarda, Milan, Italy. nicola.ughi@ospedaleniguarda.it.

- PMID: **34919220**
- DOI: 10.26355/eurrev_202112_27414

Free article
Observational Study

Sick leave request following anti-COVID-19 vaccine administration is low among healthcare workers: results from a retrospective cross-sectional monocentric study

A Schianchi et al. Eur Rev Med Pharmacol Sci. 2021 Dec.
Free article
Show details
Eur Rev Med Pharmacol Sci
. 2021 Dec;25(23):7218-7222.
doi: 10.26355/eurrev_202112_27414.

Authors

A Schianchi [1], N Ughi, G Cassano, F Del Gaudio, A Dicuonzo, F Scaglione, P M Alberti, C Rossetti, G Micheloni, L Zoppini, G Bellavia, S Giroldi, M Moreno, A Russo, M Bosio, O M Epis

Affiliation

- [1] Division of Chemical-Clinical and Microbiological Analyses, Department of Laboratory Medicine, ASST Grande Ospedale Metropolitano Niguarda, Milan, Italy. nicola.ughi@ospedaleniguarda.it.

- PMID: **34919220**
- DOI: 10.26355/eurrev_202112_27414

Abstract

Objective: Anti-COVID-19 vaccines were mainly associated with non-serious adverse events (AEs), whose prevalence was reported to be up to 70% in healthcare workers (HCWs). This may

lead to sick leave requests, but this impact has never been quantified. This study aimed to investigate the absence from work among HCWs following anti-COVID-19 vaccination. Its association with age and previous COVID-19 infection was also assessed.

Patients and methods: This is a retrospective observational cross-sectional study on administrative data about sick leave requests after anti-COVID-19 vaccination. All the HCWs employed at the Niguarda Hospital (Milan, Italy) who received the vaccine from December 27, 2020 to February 28, 2021 were included.

Results: In total, 4,088 HCWs received the first dose of the vaccine and 4,043 completed the vaccination cycle. After the first injection, 1.6% of HCWs requested sick leave, while after the second injection, the number of requests significantly increased (+6.1%, $p<0.001$). A significant increase in sick leave was detected for those who have had SARS-CoV-2 infection after the first injection (+2.3%, $p<0.001$). After the second dose, a significant increase in sick leave was observed in the 20-30-year-old group compared to >30 years (+3.6%, $p=0.017$), if HCWs without a history of SARS-CoV-2 infection were considered.

Conclusions: The requests for sick leave among HCWs following the anti-COVID-19 vaccine were limited and higher after the second injection. This may help the management of the human resources when the large-scale administration of the anti-COVID-19 vaccines will involve other categories of workers.

Supplementary info

Publication types, MeSH terms, Substances Expand

Publication types

- Observational Study

MeSH terms

- Adult
- Age Factors
- BNT162 Vaccine / administration & dosage*
- BNT162 Vaccine / adverse effects
- COVID-19 / prevention & control*
- Cross-Sectional Studies
- Female
- Health Personnel / statistics & numerical data*
- Humans
- Italy / epidemiology
- Male
- Middle Aged
- Prevalence
- Retrospective Studies
- Sick Leave / statistics & numerical data*

- Young Adult

Substances

- BNT162 Vaccine

Full text links

FREE FULL TEXT European Review for Medical and Pharmacological Sciences
Proceed to details
Cite
Share
1,139
Observational Study
Nephron
. 2021;145(3):256-264.
doi: 10.1159/000514064. Epub 2021 Mar 29.

Risk Factors for Development of Acute Kidney Injury in COVID-19 Patients: A Retrospective Observational Cohort Study

Yong Pey See [1], Barnaby Edward Young [2 3 4], Li Wei Ang [3], Xi Yan Ooi [1], Chi Peng Chan [1], Wan Limm Looi [1], See Cheng Yeo [1 4], David Chien Lye [2 3 4 5]
Affiliations Expand

Affiliations

- [1] Department of Renal Medicine, Tan Tock Seng Hospital, Singapore, Singapore.
- [2] Department of Infectious Disease, Tan Tock Seng Hospital, Singapore, Singapore.
- [3] National Centre for Infectious Disease, Singapore, Singapore.
- [4] Lee Kong Chian School of Medicine, Singapore, Singapore.
- [5] Yong Loo Lin School of Medicine, Singapore, Singapore.

- PMID: **33780937**
- PMCID: PMC8089436
- DOI: 10.1159/000514064

Free PMC article
Observational Study

Risk Factors for Development of Acute Kidney Injury in COVID-19 Patients: A Retrospective Observational Cohort Study

Yong Pey See et al. Nephron. 2021.
Free PMC article
Show details
Nephron
. 2021;145(3):256-264.
doi: 10.1159/000514064. Epub 2021 Mar 29.

Authors

Yong Pey See [1], Barnaby Edward Young [2 3 4], Li Wei Ang [3], Xi Yan Ooi [1], Chi Peng Chan [1], Wan Limm Looi [1], See Cheng Yeo [1 4], David Chien Lye [2 3 4 5]

Affiliations

- [1] Department of Renal Medicine, Tan Tock Seng Hospital, Singapore, Singapore.
- [2] Department of Infectious Disease, Tan Tock Seng Hospital, Singapore, Singapore.
- [3] National Centre for Infectious Disease, Singapore, Singapore.
- [4] Lee Kong Chian School of Medicine, Singapore, Singapore.
- [5] Yong Loo Lin School of Medicine, Singapore, Singapore.

- PMID: **33780937**
- PMCID: PMC8089436
- DOI: 10.1159/000514064

Abstract

Introduction: Acute kidney injury (AKI) in coronavirus infection disease (COVID-19) is associated with disease severity. We aimed to evaluate risk factors associated with AKI beyond COVID-19 severity.

Methods: A retrospective observational study of COVID-19 patients admitted to a tertiary hospital in Singapore. Logistic regression was used to evaluate associations between risk factors and AKI (based on Kidney Disease Improving Global Outcomes criteria). Dominance analysis was performed to evaluate the relative importance of individual factors.

Results: Seven hundred seven patients were included. Median age was 46 years (interquartile range [IQR]: 29-57) and 57% were male with few comorbidities (93%, Charlson Comorbidity Index [CCI] <1). AKI occurred in 57 patients (8.1%); 39 were in AKI stage 1 (68%), 9 in stage 2 (16%), and 9 in stage 3 (16%). Older age (adjusted odds ratio [aOR] 1.04; 95% confidence interval [CI]: 1.01-1.07), baseline use of angiotensin-converting enzyme inhibitor (ACE-I) or angiotensin receptor blocker (ARB) (aOR 2.86; 95% CI: 1.20-6.83), exposure to vancomycin (aOR 5.84; 95% CI: 2.10-16.19), use of nonsteroidal anti-inflammatory drugs (NSAIDs) (aOR 3.04; 95% CI: 1.15-8.05), and severe COVID-19 with hypoxia (aOR 13.94; 95% CI: 6.07-31.98)

were associated with AKI in the multivariable logistic regression model. The 3 highest ranked predictors were severe COVID-19 with hypoxia, vancomycin exposure, and age, accounting for 79.6% of the predicted variance (41.6, 23.1, and 14.9%, respectively) on dominance analysis.

Conclusion: Severe COVID-19 is independently associated with increased risk of AKI beyond premorbid conditions and age. Appropriate avoidance of vancomycin and NSAIDs are potentially modifiable means to prevent AKI in patients with COVID-19.

Keywords: Acute renal failure; Chronic kidney disease; Creatinine.

© 2021 S. Karger AG, Basel.

Conflict of interest statement

Yong Pey See, Xi Yan Ooi, Wan Limm Looi, Chi Peng Chan, Li Wei Ang, See Cheng Yeo, and David Chien Lye have nothing to disclose. Barnaby Edwards Young reports personal fees from Sanofi and personal fees from Roche outside of submitted work.

- 32 references
- 1 figure

Supplementary info

Publication types, MeSH terms, Substances Expand

Publication types

- Observational Study

MeSH terms

- Acute Kidney Injury / epidemiology*
- Acute Kidney Injury / etiology*
- Adult
- Age Factors
- Aged
- Anti-Bacterial Agents / adverse effects
- Anti-Inflammatory Agents, Non-Steroidal / adverse effects
- COVID-19 / complications*
- COVID-19 / epidemiology*
- Cohort Studies
- Comorbidity
- Drug-Related Side Effects and Adverse Reactions / epidemiology
- Female
- Humans
- Hypoxia / epidemiology
- Hypoxia / etiology

- Male
- Middle Aged
- Retrospective Studies
- Risk Factors
- Treatment Outcome
- Vancomycin / adverse effects

Substances

- Anti-Bacterial Agents
- Anti-Inflammatory Agents, Non-Steroidal
- Vancomycin

Full text links

Karger Free Final Version S. Karger AG, Basel, Switzerland Free PMC article
Proceed to details
Cite
Share
☐ 1,140
Observational Study
JAMA Cardiol
. 2020 Jul 1;5(7):811-818.
doi: 10.1001/jamacardio.2020.1017.

Cardiovascular Implications of Fatal Outcomes of Patients With Coronavirus Disease 2019 (COVID-19)

Tao Guo [1], Yongzhen Fan [1], Ming Chen [1], Xiaoyan Wu [1], Lin Zhang [1], Tao He [1], Hairong Wang [1], Jing Wan [1], Xinghuan Wang [2], Zhibing Lu [1]
Affiliations Expand

Affiliations

- [1] Department of Cardiology, Zhongnan Hospital of Wuhan University, Wuhan, China.
- [2] Department of Urology, Zhongnan Hospital of Wuhan University, Wuhan, China.

- PMID: **32219356**
- PMCID: PMC7101506
- DOI: 10.1001/jamacardio.2020.1017

Free PMC article
Observational Study

Cardiovascular Implications of Fatal Outcomes of Patients With Coronavirus Disease 2019 (COVID-19)

Tao Guo et al. JAMA Cardiol. 2020.
Free PMC article
Show details
JAMA Cardiol
. 2020 Jul 1;5(7):811-818.
doi: 10.1001/jamacardio.2020.1017.

Authors

Tao Guo [1], Yongzhen Fan [1], Ming Chen [1], Xiaoyan Wu [1], Lin Zhang [1], Tao He [1], Hairong Wang [1], Jing Wan [1], Xinghuan Wang [2], Zhibing Lu [1]

Affiliations

- [1] Department of Cardiology, Zhongnan Hospital of Wuhan University, Wuhan, China.
- [2] Department of Urology, Zhongnan Hospital of Wuhan University, Wuhan, China.

- PMID: **32219356**
- PMCID: PMC7101506
- DOI: 10.1001/jamacardio.2020.1017

Erratum in

- Error in Text.
  [No authors listed] [No authors listed] JAMA Cardiol. 2020 Jul 1;5(7):848. doi: 10.1001/jamacardio.2020.1722. JAMA Cardiol. 2020. PMID: 32432683 Free PMC article. No abstract available.

Abstract

Importance: Increasing numbers of confirmed cases and mortality rates of coronavirus disease 2019 (COVID-19) are occurring in several countries and continents. Information regarding the impact of cardiovascular complication on fatal outcome is scarce.

Objective: To evaluate the association of underlying cardiovascular disease (CVD) and myocardial injury with fatal outcomes in patients with COVID-19.

Design, setting, and participants: This retrospective single-center case series analyzed patients with COVID-19 at the Seventh Hospital of Wuhan City, China, from January 23, 2020, to February 23, 2020. Analysis began February 25, 2020.

Main outcomes and measures: Demographic data, laboratory findings, comorbidities, and treatments were collected and analyzed in patients with and without elevation of troponin T (TnT) levels.

Results: Among 187 patients with confirmed COVID-19, 144 patients (77%) were discharged and 43 patients (23%) died. The mean (SD) age was 58.50 (14.66) years. Overall, 66 (35.3%) had underlying CVD including hypertension, coronary heart disease, and cardiomyopathy, and 52 (27.8%) exhibited myocardial injury as indicated by elevated TnT levels. The mortality during hospitalization was 7.62% (8 of 105) for patients without underlying CVD and normal TnT levels, 13.33% (4 of 30) for those with underlying CVD and normal TnT levels, 37.50% (6 of 16) for those without underlying CVD but elevated TnT levels, and 69.44% (25 of 36) for those with underlying CVD and elevated TnTs. Patients with underlying CVD were more likely to exhibit elevation of TnT levels compared with the patients without CVD (36 [54.5%] vs 16 [13.2%]). Plasma TnT levels demonstrated a high and significantly positive linear correlation with plasma high-sensitivity C-reactive protein levels ($\beta = 0.530$, $P < .001$) and N-terminal pro-brain natriuretic peptide (NT-proBNP) levels ($\beta = 0.613$, $P < .001$). Plasma TnT and NT-proBNP levels during hospitalization (median [interquartile range (IQR)], 0.307 [0.094-0.600]; 1902.00 [728.35-8100.00]) and impending death (median [IQR], 0.141 [0.058-0.860]; 5375 [1179.50-25695.25]) increased significantly compared with admission values (median [IQR], 0.0355 [0.015-0.102]; 796.90 [401.93-1742.25]) in patients who died ($P = .001$; $P < .001$), while no significant dynamic changes of TnT (median [IQR], 0.010 [0.007-0.019]; 0.013 [0.007-0.022]; 0.011 [0.007-0.016]) and NT-proBNP (median [IQR], 352.20 [174.70-636.70]; 433.80 [155.80-1272.60]; 145.40 [63.4-526.50]) was observed in survivors ($P = .96$; $P = .16$). During hospitalization, patients with elevated TnT levels had more frequent malignant arrhythmias, and the use of glucocorticoid therapy (37 [71.2%] vs 69 [51.1%]) and mechanical ventilation (31 [59.6%] vs 14 [10.4%]) were higher compared with patients with normal TnT levels. The mortality rates of patients with and without use of angiotensin-converting enzyme inhibitors/angiotensin receptor blockers was 36.8% (7 of 19) and 21.4% (36 of 168) ($P = .13$).

Conclusions and relevance: Myocardial injury is significantly associated with fatal outcome of COVID-19, while the prognosis of patients with underlying CVD but without myocardial injury is relatively favorable. Myocardial injury is associated with cardiac dysfunction and arrhythmias. Inflammation may be a potential mechanism for myocardial injury. Aggressive treatment may be considered for patients at high risk of myocardial injury.

Conflict of interest statement

Conflict of Interest Disclosures: None reported.

Comment in

- Letter by Golomb and Hall Regarding Article, "Temporary Emergency Guidance to US Stroke Centers During the COVID-19 Pandemic".
  Golomb MR, Hall GC. Golomb MR, et al. Stroke. 2020 Jul;51(7):e138. doi: 10.1161/STROKEAHA.120.030251. Epub 2020 May 18. Stroke. 2020. PMID: 32421393 Free PMC article. No abstract available.
- Subclinical coronary artery disease in COVID-19 patients.
  Nai Fovino L, Cademartiri F, Tarantini G. Nai Fovino L, et al. Eur Heart J Cardiovasc Imaging. 2020 Sep 1;21(9):1055-1056. doi: 10.1093/ehjci/jeaa202. Eur Heart J Cardiovasc Imaging. 2020. PMID: 32671381 Free PMC article. No abstract available.
- Acute Fulminant Myocarditis in a Pediatric Patient With COVID-19 Infection.
  Lara D, Young T, Del Toro K, Chan V, Ianiro C, Hunt K, Kleinmahon J. Lara D, et al. Pediatrics. 2020 Aug;146(2):e20201509. doi: 10.1542/peds.2020-1509. Pediatrics. 2020. PMID: 32747591
- Electrocardiography Holter monitoring in critically ill patients with coronavirus disease 2019 (COVID-19).

Laleh Far V, Mehrakizadeh A, Eslami M, Shirazi S, Mohammadi M, Mollazadeh R. Laleh Far V, et al. Pol Arch Intern Med. 2020 Nov 30;130(11):1010-1012. doi: 10.20452/pamw.15601. Epub 2020 Sep 15. Pol Arch Intern Med. 2020. PMID: 32930544 No abstract available.

- Myocardial Injury in COVID-19-Can We Successfully Target Inflammation?
  Giannopoulos G, Vrachatis DA, Deftereos SG. Giannopoulos G, et al. JAMA Cardiol. 2020 Sep 1;5(9):1069-1070. doi: 10.1001/jamacardio.2020.2569. JAMA Cardiol. 2020. PMID: 32936267 No abstract available.
- McConnell's sign assessed by point-of-care cardiac ultrasound associated with in-hospital mortality of COVID-19 patients with respiratory failure.
  Doi S, Izumo M, Shiokawa N, Teramoto K, Ishibashi Y, Higuma T, Fujitani S, Akashi YJ. Doi S, et al. J Echocardiogr. 2021 Mar;19(1):67-69. doi: 10.1007/s12574-020-00507-4. Epub 2021 Jan 16. J Echocardiogr. 2021. PMID: 33452995 Free PMC article. No abstract available.

- 15 references
- 3 figures

Supplementary info

Publication types, MeSH terms, Substances Expand

Publication types

- Observational Study
- Research Support, Non-U.S. Gov't

MeSH terms

- Adult
- Aged
- Betacoronavirus*
- COVID-19
- Cardiovascular Diseases / blood
- Cardiovascular Diseases / epidemiology*
- China
- Coronavirus Infections / complications*
- Coronavirus Infections / mortality*
- Coronavirus Infections / therapy
- Female
- Humans
- Male
- Middle Aged
- Natriuretic Peptide, Brain / blood
- Pandemics
- Peptide Fragments / blood

- Pneumonia, Viral / complications*
- Pneumonia, Viral / mortality*
- Pneumonia, Viral / therapy
- Retrospective Studies
- Risk Factors
- SARS-CoV-2
- Survival Rate
- Troponin T / blood

Substances

- Peptide Fragments
- Troponin T
- pro-brain natriuretic peptide (1-76)
- Natriuretic Peptide, Brain

Full text links

FULL TEXT JAMA Cardiology Silverchair Information Systems Free PMC article
Proceed to details
Cite
Share
1,141
Observational Study
J Laryngol Otol
. 2021 Mar;135(3):246-249.
doi: 10.1017/S002221512100061X. Epub 2021 Feb 24.

Risk of disease transmission from flexible nasoendoscopy during the coronavirus disease 2019 pandemic

F G Kavanagh [1 2], C Connolly [2], E Farrell [3], D Callanan [2], D Brinkman [2], A Affendi [2], E Lang [3], P Sheahan [2 4 5]
Affiliations Expand

Affiliations

- [1] Institute of Research, Royal College of Surgeons in Ireland, Dublin, Republic of Ireland.
- [2] Department of Otolaryngology, Head and Neck Surgery, South Infirmary Victoria University Hospital, Cork, Republic of Ireland.

- [3] Department of Otolaryngology, Head and Neck Surgery, University Hospital Waterford, Waterford, Republic of Ireland.
- [4] Ear, Nose, Throat and Oral ('ENTO') Research Unit, College of Medicine and Health, University College Cork, Cork, Republic of Ireland.
- [5] Department of Surgery, University College Cork, Republic of Ireland.

- PMID: **33622427**
- PMCID: PMC7948103
- DOI: 10.1017/S002221512100061X

Free PMC article
Observational Study

Risk of disease transmission from flexible nasoendoscopy during the coronavirus disease 2019 pandemic

F G Kavanagh et al. J Laryngol Otol. 2021 Mar.
Free PMC article
Show details
J Laryngol Otol

. 2021 Mar;135(3):246-249.
doi: 10.1017/S002221512100061X. Epub 2021 Feb 24.

Authors

F G Kavanagh [1, 2], C Connolly [2], E Farrell [3], D Callanan [2], D Brinkman [2], A Affendi [2], E Lang [3], P Sheahan [2, 4, 5]

Affiliations

- [1] Institute of Research, Royal College of Surgeons in Ireland, Dublin, Republic of Ireland.
- [2] Department of Otolaryngology, Head and Neck Surgery, South Infirmary Victoria University Hospital, Cork, Republic of Ireland.
- [3] Department of Otolaryngology, Head and Neck Surgery, University Hospital Waterford, Waterford, Republic of Ireland.
- [4] Ear, Nose, Throat and Oral ('ENTO') Research Unit, College of Medicine and Health, University College Cork, Cork, Republic of Ireland.
- [5] Department of Surgery, University College Cork, Republic of Ireland.

- PMID: **33622427**
- PMCID: PMC7948103
- DOI: 10.1017/S002221512100061X

Abstract

Background: Concerns have emerged regarding infection transmission during flexible nasoendoscopy.

Methods: Information was gathered prospectively on flexible nasoendoscopy procedures performed between March and June 2020. Patients and healthcare workers were followed up to assess for coronavirus disease 2019 development. One-sided 97.5 per cent Poisson confidence intervals were calculated for upper limits of risk where zero events were observed.

Results: A total of 286 patients were recruited. The most common indication for flexible nasoendoscopy was investigation of 'red flag' symptoms (67 per cent). Forty-seven patients (16 per cent, 95 per cent confidence interval = 13-21 per cent) had suspicious findings on flexible nasoendoscopy requiring further investigation. Twenty patients (7.1 per cent, 95 per cent confidence interval = 4.4-11 per cent) had new cancer diagnoses. Zero coronavirus disease 2019 infections were recorded in the 273 patients. No. 27 endoscopists (the doctors and nurses who carried out the procedures) were followed up.The risk of developing coronavirus disease 2019 after flexible nasoendoscopy was determined to be 0-1.3 per cent.

Conclusion: The risk of coronavirus disease 2019 transmission associated with performing flexible nasoendoscopy in asymptomatic patients, while using appropriate personal protective equipment, is very low. Additional data are required to confirm these findings in the setting of further disease surges.

Keywords: COVID-19; Flexible Nasoendoscopy; Laryngology; Patient Safety.

- 14 references

Supplementary info

Publication types, MeSH terms Expand

Publication types

- Observational Study

MeSH terms

- Adult
- COVID-19 / epidemiology*
- COVID-19 / prevention & control
- COVID-19 / transmission*
- Endoscopy / adverse effects*
- Endoscopy / instrumentation
- Female
- Humans
- Infection Control / organization & administration*
- Infectious Disease Transmission, Patient-to-Professional / prevention & control*
- Infectious Disease Transmission, Patient-to-Professional / statistics & numerical data*
- Ireland

- Male
- Patient Selection
- Personal Protective Equipment
- Prospective Studies
- Retrospective Studies
- Risk Assessment

Full text links

Cambridge University Press Free PMC article
Proceed to details
Cite
Share
1,142
Observational Study
Nutr Metab Cardiovasc Dis
. 2020 Oct 30;30(11):1899-1913.
doi: 10.1016/j.numecd.2020.07.031. Epub 2020 Jul 31.

Common cardiovascular risk factors and in-hospital mortality in 3,894 patients with COVID-19: survival analysis and machine learning-based findings from the multicentre Italian CORIST Study

Augusto Di Castelnuovo [1], Marialaura Bonaccio [2], Simona Costanzo [2], Alessandro Gialluisi [2], Andrea Antinori [3], Nausicaa Berselli [4], Lorenzo Blandi [5], Raffaele Bruno [6], Roberto Cauda [7], Giovanni Guaraldi [8], Ilaria My [9], Lorenzo Menicanti [5], Giustino Parruti [10], Giuseppe Patti [11], Stefano Perlini [12], Francesca Santilli [13], Carlo Signorelli [14], Giulio G Stefanini [9], Alessandra Vergori [15], Amina Abdeddaim [16], Walter Ageno [17], Antonella Agodi [18], Piergiuseppe Agostoni [19], Luca Aiello [20], Samir Al Moghazi [21], Filippo Aucella [22], Greta Barbieri [23], Alessandro Bartoloni [24], Carolina Bologna [25], Paolo Bonfanti [26], Serena Brancati [27], Francesco Cacciatore [28], Lucia Caiano [17], Francesco Cannata [9], Laura Carrozzi [29], Antonio Cascio [30], Antonella Cingolani [7], Francesco Cipollone [13], Claudia Colomba [30], Annalisa Crisetti [22], Francesca Crosta [10], Gian B Danzi [31], Damiano D'Ardes [13], Katleen de Gaetano Donati [32], Francesco Di Gennaro [33], Gisella Di Palma [34], Giuseppe Di Tano [31], Massimo Fantoni [7], Tommaso Filippini [4], Paola Fioretto [35], Francesco M Fusco [36], Ivan Gentile [37], Leonardo Grisafi [11], Gabriella Guarnieri [38], Francesco Landi [20], Giovanni Larizza [39], Armando Leone [40], Gloria Maccagni [31], Sandro Maccarella [41], Massimo Mapelli [19], Riccardo Maragna [42], Rossella Marcucci [24], Giulio Maresca [43], Claudia Marotta [33], Lorenzo Marra [40], Franco Mastroianni [39], Alessandro Mengozzi [23], Francesco Menichetti [23], Jovana Milic [8], Rita Murri [7], Arturo Montineri [44], Roberta

Mussinelli [45], Cristina Mussini [8], Maria Musso [46], Anna Odone [14], Marco Olivieri [47], Emanuela Pasi [48], Francesco Petri [49], Biagio Pinchera [37], Carlo A Pivato [9], Roberto Pizzi [17], Venerino Poletti [50], Francesca Raffaelli [32], Claudia Ravaglia [50], Giulia Righetti [39], Andrea Rognoni [51], Marco Rossato [35], Marianna Rossi [49], Anna Sabena [52], Francesco Salinaro [52], Vincenzo Sangiovanni [36], Carlo Sanrocco [10], Antonio Scarafino [39], Laura Scorzolini [53], Raffaella Sgariglia [41], Paola G Simeone [10], Enrico Spinoni [11], Carlo Torti [54], Enrico M Trecarichi [54], Francesca Vezzani [13], Giovanni Veronesi [17], Roberto Vettor [35], Andrea Vianello [38], Marco Vinceti [55], Raffaele De Caterina [29], Licia Iacoviello [56], COvid-19 RISk and Treatments (CORIST) collaboration

Affiliations Expand

Affiliations

- [1] Mediterranea Cardiocentro, Napoli, Italy.
- [2] Department of Epidemiology and Prevention, IRCCS Neuromed, Pozzilli, IS, Italy.
- [3] UOC Immunodeficienze Virali, National Institute for Infectious Diseases "L. Spallanzani", IRCCS, Rome, Italy.
- [4] Section of Public Health, Department of Biomedical, Metabolic and Neural Sciences, University of Modena and Reggio Emilia, Modena, Italy.
- [5] IRCCS Policlinico San Donato, San Donato Milanese, Italy.
- [6] Division of Infectious Diseases I, Fondazione IRCCS Policlinico San Matteo, Pavia, Italy; Department of Clinical, Surgical, Diagnostic, and Paediatric Sciences, University of Pavia, Pavia, Italy.
- [7] Fondazione Policlinico Universitario A. Gemelli IRCCS, Roma, Italy; Università Cattolica del Sacro Cuore- Dipartimento di Sicurezza e Bioetica Sede di Roma, Italy.
- [8] Infectious Disease Unit, Department of Surgical, Medical, Dental and Morphological Sciences, University of Modena and Reggio Emilia, Modena, Italy.
- [9] Humanitas Clinical and Research Hospital IRCCS, Rozzano-Milano, Italy.
- [10] Department of Infectious Disease, Azienda Sanitaria Locale (AUSL) di Pescara, Pescara, Italy.
- [11] University of Eastern Piedmont, Maggiore della Carità Hospital, Novara, Italy.
- [12] Emergency Department, IRCCS Policlinico San Matteo Foundation, Pavia, Italy; Department of Internal Medicine, University of Pavia, Pavia, Italy.
- [13] Department of Medicine and Aging, Clinica Medica, "SS. Annunziata" Hospital and University of Chieti, Chieti, Italy.
- [14] School of Medicine, Vita-Salute San Raffaele University, Milano, Italy.
- [15] HIV/AIDS Department, National Institute for Infectious Diseases "Lazzaro Spallanzani"-IRCCS, Roma, Italy.
- [16] UOC Malattie Infettive-Epatologia, National Institute for Infectious Diseases L. Spallanzani, IRCCS, Rome, Italy.
- [17] Department of Medicine and Surgery, University of Insubria, Varese, Italy.
- [18] Department of Medical and Surgical Sciences and Advanced Technologies "G.F. Ingrassia", University of Catania, AOU Policlinico "G. Rodolico - San Marco", Catania, Italy.
- [19] Centro Cardiologico Monzino IRCCS, Milano, Italy; Department of Clinical Sciences and Community Health, Cardiovascular Section, University of Milano, Milano, Italy.
- [20] UOC Anestesia e Rianimazione. Dipartimento di Chirurgia Generale Ospedale Morgagni-Pierantoni Forlì, Italy.

- [21] UOC Infezioni Sistemiche dell'Immunodepresso, National Institute for Infectious Diseases L. Spallanzani, IRCCS, Rome, Italy.
- [22] Fondazione I.R.C.C.S "Casa Sollievo della Sofferenza", San Giovanni Rotondo, Foggia, Italy.
- [23] Department of Clinical and Experimental Medicine, Azienda Ospedaliero-Universitaria Pisana, and University of Pisa, Pisa, Italy.
- [24] Department of Experimental and Clinical Medicine, University of Florence, Firenze, Italy.
- [25] Ospedale del Mare, ASL NA1, Naples, Italy.
- [26] UOC Malattie Infettive, Ospedale San Gerardo, ASST Monza, Monza, Italy; School of Medicine and Surgery, University of Milano-Bicocca, Milano, Italy.
- [27] Department of General Surgery and Medical-Surgical Specialties, University of Catania, Catania, Italy.
- [28] Department of Translational Medical Sciences. University of Naples, Federico II, Naples, Italy.
- [29] Cardiovascular and Thoracic Department, Azienda Ospedaliero-Universitaria Pisana, and University of Pisa, Pisa, Italy.
- [30] Infectious and Tropical Diseases Unit- Department of Health Promotion, Mother and Child Care, Internal Medicine and Medical Specialties (PROMISE) - University of Palermo, Palermo, Italy.
- [31] Department of Cardiology, Ospedale di Cremona, Cremona, Italy.
- [32] Fondazione Policlinico Universitario A. Gemelli IRCCS, Roma, Italy.
- [33] Medical Direction, IRCCS Neuromed, Pozzilli, IS, Italy.
- [34] UOC Medicina - PO S. Maria di Loreto Nuovo -ASL Napoli 1 Centro, Napoli, Italy.
- [35] Clinica Medica 3, Department of Medicine - DIMED, University hospital of Padova, Padova, Italy.
- [36] UOC Infezioni Sistemiche e dell'Immunodepresso, Azienda Ospedaliera dei Colli, Ospedale Cotugno, Napoli, Italy.
- [37] Department of Clinical Medicine and Surgery, University of Naples "Federico II". Napoli, Italy.
- [38] Respiratory Pathophysiology Division, Department of Cardiologic, Thoracic and Vascular Sciences, University of Padova, Padova, Italy.
- [39] COVID-19 Unit. EE Ospedale Regionale F. Miulli, Acquaviva delle Fonti, BA, Italy.
- [40] UOC di Pneumologia, P.O. San Giuseppe Moscati, Taranto, Italy.
- [41] ASST Milano Nord - Ospedale Edoardo Bassini, Cinisello Balsamo, Italy.
- [42] Department of Clinical Sciences and Community Health, Cardiovascular Section, University of Milano, Milano, Italy.
- [43] Centro Cardiologico Monzino IRCCS, Milano, Italy; UOC Medicina - PO S. Maria di Loreto Nuovo -ASL Napoli 1 Centro, Napoli, Italy.
- [44] U.O. C. Malattie Infettive e Tropicali, P.O. "San Marco", AOU Policlinico "G. Rodolico - San Marco", Catania, Italy.
- [45] Department of Internal Medicine, University of Pavia, Pavia, Italy.
- [46] UOC Malattie Infettive-Apparato Respiratorio, National Institute for Infectious Diseases "L. Spallanzani", IRCCS, Rome, Italy.
- [47] Computer Service, University of Molise, Campobasso, Italy.
- [48] Medicina Interna. Ospedale di Ravenna. AUSL della Romagna, Ravenna, Italy.
- [49] UOC Malattie Infettive, Ospedale San Gerardo, ASST Monza, Monza, Italy.

- [50] UOC Pneumologia, Dipartimento di Malattie Apparato Respiratorio e Torace, Ospedale Morgagni-Pierantoni Forlì, Forlì, Italy.
- [51] Coronary Care Unit and Catheterization Laboratory, A.O.U. Maggiore della Carità, Novara, Italy.
- [52] Emergency Department, IRCCS Policlinico San Matteo Foundation, Pavia, Italy.
- [53] UOC Malattie Infettive ad Alta Intensità di Cura, National Institute for Infectious Diseases "L. Spallanzani", IRCCS, Rome, Italy.
- [54] Infectious and Tropical Diseases Unit, Department of Medical and Surgical Sciences "Magna Graecia" University, Catanzaro, Italy.
- [55] Section of Public Health, Department of Biomedical, Metabolic and Neural Sciences, University of Modena and Reggio Emilia, Modena, Italy; Department of Epidemiology, Boston University School of Public Health, Boston, USA.
- [56] Department of Epidemiology and Prevention, IRCCS Neuromed, Pozzilli, IS, Italy; Department of Medicine and Surgery, University of Insubria, Varese, Italy. Electronic address: licia.iacoviello@moli-sani.org.

- PMID: **32912793**
- PMCID: PMC7833278
- DOI: 10.1016/j.numecd.2020.07.031

Free PMC article
Observational Study

Common cardiovascular risk factors and in-hospital mortality in 3,894 patients with COVID-19: survival analysis and machine learning-based findings from the multicentre Italian CORIST Study

Augusto Di Castelnuovo et al. Nutr Metab Cardiovasc Dis. 2020.
Free PMC article
Show details
Nutr Metab Cardiovasc Dis
. 2020 Oct 30;30(11):1899-1913.
doi: 10.1016/j.numecd.2020.07.031. Epub 2020 Jul 31.

Authors

Augusto Di Castelnuovo [1], Marialaura Bonaccio [2], Simona Costanzo [2], Alessandro Gialluisi [2], Andrea Antinori [3], Nausicaa Berselli [4], Lorenzo Blandi [5], Raffaele Bruno [6], Roberto Cauda [7], Giovanni Guaraldi [8], Ilaria My [9], Lorenzo Menicanti [5], Giustino Parruti [10], Giuseppe Patti [11], Stefano Perlini [12], Francesca Santilli [13], Carlo Signorelli [14], Giulio G Stefanini [9], Alessandra Vergori [15], Amina Abdeddaim [16], Walter Ageno [17], Antonella Agodi [18], Piergiuseppe Agostoni [19], Luca Aiello [20], Samir Al Moghazi [21], Filippo Aucella [22], Greta Barbieri [23], Alessandro Bartoloni [24], Carolina Bologna [25], Paolo Bonfanti [26], Serena Brancati [27], Francesco Cacciatore

[28], Lucia Caiano [17], Francesco Cannata [9], Laura Carrozzi [29], Antonio Cascio [30], Antonella Cingolani [7], Francesco Cipollone [13], Claudia Colomba [30], Annalisa Crisetti [22], Francesca Crosta [10], Gian B Danzi [31], Damiano D'Ardes [13], Katleen de Gaetano Donati [32], Francesco Di Gennaro [33], Gisella Di Palma [34], Giuseppe Di Tano [31], Massimo Fantoni [7], Tommaso Filippini [4], Paola Fioretto [35], Francesco M Fusco [36], Ivan Gentile [37], Leonardo Grisafi [11], Gabriella Guarnieri [38], Francesco Landi [20], Giovanni Larizza [39], Armando Leone [40], Gloria Maccagni [31], Sandro Maccarella [41], Massimo Mapelli [19], Riccardo Maragna [42], Rossella Marcucci [24], Giulio Maresca [43], Claudia Marotta [33], Lorenzo Marra [40], Franco Mastroianni [39], Alessandro Mengozzi [23], Francesco Menichetti [23], Jovana Milic [8], Rita Murri [7], Arturo Montineri [44], Roberta Mussinelli [45], Cristina Mussini [8], Maria Musso [46], Anna Odone [14], Marco Olivieri [47], Emanuela Pasi [48], Francesco Petri [49], Biagio Pinchera [37], Carlo A Pivato [9], Roberto Pizzi [17], Venerino Poletti [50], Francesca Raffaelli [32], Claudia Ravaglia [50], Giulia Righetti [39], Andrea Rognoni [51], Marco Rossato [35], Marianna Rossi [49], Anna Sabena [52], Francesco Salinaro [52], Vincenzo Sangiovanni [36], Carlo Sanrocco [10], Antonio Scarafino [39], Laura Scorzolini [53], Raffaella Sgariglia [41], Paola G Simeone [10], Enrico Spinoni [11], Carlo Torti [54], Enrico M Trecarichi [54], Francesca Vezzani [13], Giovanni Veronesi [17], Roberto Vettor [35], Andrea Vianello [38], Marco Vinceti [55], Raffaele De Caterina [29], Licia Iacoviello [56], COvid-19 RISk and Treatments (CORIST) collaboration

Affiliations

- [1] Mediterranea Cardiocentro, Napoli, Italy.
- [2] Department of Epidemiology and Prevention, IRCCS Neuromed, Pozzilli, IS, Italy.
- [3] UOC Immunodeficienze Virali, National Institute for Infectious Diseases "L. Spallanzani", IRCCS, Rome, Italy.
- [4] Section of Public Health, Department of Biomedical, Metabolic and Neural Sciences, University of Modena and Reggio Emilia, Modena, Italy.
- [5] IRCCS Policlinico San Donato, San Donato Milanese, Italy.
- [6] Division of Infectious Diseases I, Fondazione IRCCS Policlinico San Matteo, Pavia, Italy; Department of Clinical, Surgical, Diagnostic, and Paediatric Sciences, University of Pavia, Pavia, Italy.
- [7] Fondazione Policlinico Universitario A. Gemelli IRCCS, Roma, Italy; Università Cattolica del Sacro Cuore- Dipartimento di Sicurezza e Bioetica Sede di Roma, Italy.
- [8] Infectious Disease Unit, Department of Surgical, Medical, Dental and Morphological Sciences, University of Modena and Reggio Emilia, Modena, Italy.
- [9] Humanitas Clinical and Research Hospital IRCCS, Rozzano-Milano, Italy.
- [10] Department of Infectious Disease, Azienda Sanitaria Locale (AUSL) di Pescara, Pescara, Italy.
- [11] University of Eastern Piedmont, Maggiore della Carità Hospital, Novara, Italy.
- [12] Emergency Department, IRCCS Policlinico San Matteo Foundation, Pavia, Italy; Department of Internal Medicine, University of Pavia, Pavia, Italy.
- [13] Department of Medicine and Aging, Clinica Medica, "SS. Annunziata" Hospital and University of Chieti, Chieti, Italy.
- [14] School of Medicine, Vita-Salute San Raffaele University, Milano, Italy.
- [15] HIV/AIDS Department, National Institute for Infectious Diseases "Lazzaro Spallanzani"-IRCCS, Roma, Italy.
- [16] UOC Malattie Infettive-Epatologia, National Institute for Infectious Diseases L. Spallanzani, IRCCS, Rome, Italy.

- [17] Department of Medicine and Surgery, University of Insubria, Varese, Italy.
- [18] Department of Medical and Surgical Sciences and Advanced Technologies "G.F. Ingrassia", University of Catania, AOU Policlinico "G. Rodolico - San Marco", Catania, Italy.
- [19] Centro Cardiologico Monzino IRCCS, Milano, Italy; Department of Clinical Sciences and Community Health, Cardiovascular Section, University of Milano, Milano, Italy.
- [20] UOC Anestesia e Rianimazione. Dipartimento di Chirurgia Generale Ospedale Morgagni-Pierantoni Forlì, Italy.
- [21] UOC Infezioni Sistemiche dell'Immunodepresso, National Institute for Infectious Diseases L. Spallanzani, IRCCS, Rome, Italy.
- [22] Fondazione I.R.C.C.S "Casa Sollievo della Sofferenza", San Giovanni Rotondo, Foggia, Italy.
- [23] Department of Clinical and Experimental Medicine, Azienda Ospedaliero-Universitaria Pisana, and University of Pisa, Pisa, Italy.
- [24] Department of Experimental and Clinical Medicine, University of Florence, Firenze, Italy.
- [25] Ospedale del Mare, ASL NA1, Naples, Italy.
- [26] UOC Malattie Infettive, Ospedale San Gerardo, ASST Monza, Monza, Italy; School of Medicine and Surgery, University of Milano-Bicocca, Milano, Italy.
- [27] Department of General Surgery and Medical-Surgical Specialties, University of Catania, Catania, Italy.
- [28] Department of Translational Medical Sciences. University of Naples, Federico II, Naples, Italy.
- [29] Cardiovascular and Thoracic Department, Azienda Ospedaliero-Universitaria Pisana, and University of Pisa, Pisa, Italy.
- [30] Infectious and Tropical Diseases Unit- Department of Health Promotion, Mother and Child Care, Internal Medicine and Medical Specialties (PROMISE) - University of Palermo, Palermo, Italy.
- [31] Department of Cardiology, Ospedale di Cremona, Cremona, Italy.
- [32] Fondazione Policlinico Universitario A. Gemelli IRCCS, Roma, Italy.
- [33] Medical Direction, IRCCS Neuromed, Pozzilli, IS, Italy.
- [34] UOC Medicina - PO S. Maria di Loreto Nuovo -ASL Napoli 1 Centro, Napoli, Italy.
- [35] Clinica Medica 3, Department of Medicine - DIMED, University hospital of Padova, Padova, Italy.
- [36] UOC Infezioni Sistemiche e dell'Immunodepresso, Azienda Ospedaliera dei Colli, Ospedale Cotugno, Napoli, Italy.
- [37] Department of Clinical Medicine and Surgery, University of Naples "Federico II". Napoli, Italy.
- [38] Respiratory Pathophysiology Division, Department of Cardiologic, Thoracic and Vascular Sciences, University of Padova, Padova, Italy.
- [39] COVID-19 Unit. EE Ospedale Regionale F. Miulli, Acquaviva delle Fonti, BA, Italy.
- [40] UOC di Pneumologia, P.O. San Giuseppe Moscati, Taranto, Italy.
- [41] ASST Milano Nord - Ospedale Edoardo Bassini, Cinisello Balsamo, Italy.
- [42] Department of Clinical Sciences and Community Health, Cardiovascular Section, University of Milano, Milano, Italy.
- [43] Centro Cardiologico Monzino IRCCS, Milano, Italy; UOC Medicina - PO S. Maria di Loreto Nuovo -ASL Napoli 1 Centro, Napoli, Italy.
- [44] U.O. C. Malattie Infettive e Tropicali, P.O. "San Marco", AOU Policlinico "G. Rodolico - San Marco", Catania, Italy.

- [45] Department of Internal Medicine, University of Pavia, Pavia, Italy.
- [46] UOC Malattie Infettive-Apparato Respiratorio, National Institute for Infectious Diseases "L. Spallanzani", IRCCS, Rome, Italy.
- [47] Computer Service, University of Molise, Campobasso, Italy.
- [48] Medicina Interna. Ospedale di Ravenna. AUSL della Romagna, Ravenna, Italy.
- [49] UOC Malattie Infettive, Ospedale San Gerardo, ASST Monza, Monza, Italy.
- [50] UOC Pneumologia, Dipartimento di Malattie Apparato Respiratorio e Torace, Ospedale Morgagni-Pierantoni Forlì, Forlì, Italy.
- [51] Coronary Care Unit and Catheterization Laboratory, A.O.U. Maggiore della Carità, Novara, Italy.
- [52] Emergency Department, IRCCS Policlinico San Matteo Foundation, Pavia, Italy.
- [53] UOC Malattie Infettive ad Alta Intensità di Cura, National Institute for Infectious Diseases "L. Spallanzani", IRCCS, Rome, Italy.
- [54] Infectious and Tropical Diseases Unit, Department of Medical and Surgical Sciences "Magna Graecia" University, Catanzaro, Italy.
- [55] Section of Public Health, Department of Biomedical, Metabolic and Neural Sciences, University of Modena and Reggio Emilia, Modena, Italy; Department of Epidemiology, Boston University School of Public Health, Boston, USA.
- [56] Department of Epidemiology and Prevention, IRCCS Neuromed, Pozzilli, IS, Italy; Department of Medicine and Surgery, University of Insubria, Varese, Italy. Electronic address: licia.iacoviello@moli-sani.org.

- PMID: **32912793**
- PMCID: PMC7833278
- DOI: 10.1016/j.numecd.2020.07.031

Abstract

Background and aims: There is poor knowledge on characteristics, comorbidities and laboratory measures associated with risk for adverse outcomes and in-hospital mortality in European Countries. We aimed at identifying baseline characteristics predisposing COVID-19 patients to in-hospital death.

Methods and results: Retrospective observational study on 3894 patients with SARS-CoV-2 infection hospitalized from February 19th to May 23rd, 2020 and recruited in 30 clinical centres distributed throughout Italy. Machine learning (random forest)-based and Cox survival analysis. 61.7% of participants were men (median age 67 years), followed up for a median of 13 days. In-hospital mortality exhibited a geographical gradient, Northern Italian regions featuring more than twofold higher death rates as compared to Central/Southern areas (15.6% vs 6.4%, respectively). Machine learning analysis revealed that the most important features in death classification were impaired renal function, elevated C reactive protein and advanced age. These findings were confirmed by multivariable Cox survival analysis (hazard ratio (HR): 8.2; 95% confidence interval (CI) 4.6-14.7 for age ≥85 vs 18-44 y); HR = 4.7; 2.9-7.7 for estimated glomerular filtration rate levels <15 vs ≥ 90 mL/min/1.73 m^2; HR = 2.3; 1.5-3.6 for C-reactive protein levels ≥10 vs ≤ 3 mg/L). No relation was found with obesity, tobacco use, cardiovascular disease and related-comorbidities. The associations between these variables and mortality were substantially homogenous across all sub-groups analyses.

Conclusions: Impaired renal function, elevated C-reactive protein and advanced age were major predictors of in-hospital death in a large cohort of unselected patients with COVID-19, admitted to 30 different clinical centres all over Italy.

Keywords: COVID-19; Epidemiology; In-hospital mortality; Risk factors.

Copyright © 2020 The Italian Diabetes Society, the Italian Society for the Study of Atherosclerosis, the Italian Society of Human Nutrition and the Department of Clinical Medicine and Surgery, Federico II University. Published by Elsevier B.V. All rights reserved.

Conflict of interest statement

Declaration of Competing Interest All Authors declare no competing interests.

- 47 references
- 2 figures

Supplementary info

Publication types, MeSH terms, Substances Expand

Publication types

- Multicenter Study
- Observational Study

MeSH terms

- Adolescent
- Adult
- Age Factors
- Aged
- Aged, 80 and over
- Betacoronavirus*
- C-Reactive Protein / analysis
- COVID-19
- Cardiovascular Diseases / etiology*
- Coronavirus Infections / mortality*
- Female
- Glomerular Filtration Rate
- Hospital Mortality*
- Humans
- Machine Learning*
- Male
- Middle Aged
- Pandemics

- Pneumonia, Viral / mortality*
- Retrospective Studies
- Risk Factors
- SARS-CoV-2
- Survival Analysis
- Young Adult

Substances

- C-Reactive Protein

Full text links

ELSEVIER FULL-TEXT ARTICLE Elsevier Science Free PMC article
Proceed to details
Cite
Share
☐ 1,143
Observational Study
J Hosp Med
. 2020 Dec;15(12):709-715.
doi: 10.12788/jhm.3475.

Comparison of Resident, Advanced Practice Clinician, and Hospitalist Teams in an Academic Medical Center: Association With Clinical Outcomes and Resource Utilization

Stacy A Johnson[1], Claire E Ciarkowski[1], Katie L Lappe[1 2], David R Kendrick[1], Adrienne Smith[1], Santosh P Reddy[1]
Affiliations Expand

Affiliations

- [1] Division of General Internal Medicine, Department of Internal Medicine, School of Medicine, University of Utah, Salt Lake City, Utah.
- [2] Division of General Internal Medicine, Department of Internal Medicine, George E. Wahlen VA Hospital, Salt Lake City, Utah.

- PMID: **33231541**
- DOI: 10.12788/jhm.3475

Observational Study

Comparison of Resident, Advanced Practice Clinician, and Hospitalist Teams in an Academic Medical Center: Association With Clinical Outcomes and Resource Utilization

Stacy A Johnson et al. J Hosp Med. 2020 Dec.
Show details
J Hosp Med
. 2020 Dec;15(12):709-715.
doi: 10.12788/jhm.3475.

Authors

Stacy A Johnson [1], Claire E Ciarkowski [1], Katie L Lappe [1] [2], David R Kendrick [1], Adrienne Smith [1], Santosh P Reddy [1]

Affiliations

- [1] Division of General Internal Medicine, Department of Internal Medicine, School of Medicine, University of Utah, Salt Lake City, Utah.
- [2] Division of General Internal Medicine, Department of Internal Medicine, George E. Wahlen VA Hospital, Salt Lake City, Utah.

- PMID: **33231541**
- DOI: 10.12788/jhm.3475

Abstract

Background: Academic medical centers have expanded their inpatient medicine services with advanced practice clinicians (APCs) or nonteaching hospitalists in response to patient volumes, residency work hour restrictions, and recently, COVID-19. Reports of clinical outcomes, cost, and resource utilization differ among inpatient team structures.

Objective: Directly compare outcomes among resident, APC, and solo hospitalist inpatient general medicine teams.

Design: Retrospective cohort study using multivariable analysis adjusted for time of admission, interhospital transfer, and comorbidities that compares clinical outcomes, cost, and resource utilization.

Subjects: Patients 18 years or older discharged from an inpatient medicine service between July 2015 and July 2018 (N = 12,716).

Main measures: Length of stay (LOS), 30-day readmission, inpatient mortality, normalized total direct cost, discharge time, and consultation utilization.

Key results: Resident teams admitted fewer patients at night (32.0%; P < .001) than did APC (49.5%) and hospitalist (48.6%) teams. APCs received nearly 4% more outside transfer patients (P = .015). Hospitalists discharged patients 26 minutes earlier than did residents (mean hours after midnight [95% CI], 14.58 [14.44-14.72] vs 15.02 [14.97-15.08]). Adjusted consult utilization was 15% higher for APCs (adjusted mean consults per admission [95% CI], 1.00 [0.96-1.03]) and 8% higher for residents (0.93 [0.90-0.95]) than it was for hospitalists (0.85 [0.80-0.90]). No differences in LOS, readmission, mortality, or cost were observed between the teams.

Conclusion: We observed similar costs, LOS, 30-day readmission, and mortality among hospitalist, APC, and resident teams. Our results suggest clinical outcomes are not significantly affected by team structure. The addition of APC or hospitalist teams represent safe and effective alternatives to traditional inpatient resident teams.

Supplementary info

Publication types, MeSH terms Expand

Publication types

- Comparative Study
- Observational Study

MeSH terms

- Academic Medical Centers*
- Female
- Health Resources / economics*
- Hospitalists / economics*
- Humans
- Internal Medicine* / economics
- Internal Medicine* / education
- Internship and Residency*
- Length of Stay / statistics & numerical data
- Male
- Middle Aged
- Patient Outcome Assessment*
- Patient Readmission
- Quality of Health Care / statistics & numerical data
- Retrospective Studies

Full text links

Journal of Hospital Medicine Frontline Medical Communications Inc

Proceed to details

Cite

Share
1,144
Observational Study
Nutrients
. 2020 Jul 7;12(7):2016.
doi: 10.3390/nu12072016.

Changes in Weight and Nutritional Habits in Adults with Obesity during the "Lockdown" Period Caused by the COVID-19 Virus Emergency

Marianna Pellegrini [1], Valentina Ponzo [1], Rosalba Rosato [2], Elena Scumaci [1], Ilaria Goitre [1], Andrea Benso [1], Sara Belcastro [1], Chiara Crespi [1], Franco De Michieli [1 3], Ezio Ghigo [1 3], Fabio Broglio [1 3], Simona Bo [1 3]
Affiliations Expand

Affiliations

- [1] Department of Medical Sciences, University of Torino, c.so AM Dogliotti 14, 10126 Torino, Italy.
- [2] Department of Psychology, University of Torino, c.so AM Dogliotti 14, 10126 Torino, Italy.
- [3] Diabetes and Metabolic Diseases Clinic, "Città della Salute e della Scienza" Hospital of Torino, 10126 Torino, Italy.

- PMID: **32645970**
- PMCID: PMC7400808
- DOI: 10.3390/nu12072016

Free PMC article
Observational Study

Changes in Weight and Nutritional Habits in Adults with Obesity during the "Lockdown" Period Caused by the COVID-19 Virus Emergency

Marianna Pellegrini et al. Nutrients. 2020.
Free PMC article
Show details
Nutrients

. 2020 Jul 7;12(7):2016.
doi: 10.3390/nu12072016.

Authors

Marianna Pellegrini [1], Valentina Ponzo [1], Rosalba Rosato [2], Elena Scumaci [1], Ilaria Goitre [1], Andrea Benso [1], Sara Belcastro [1], Chiara Crespi [1], Franco De Michieli [1] [3], Ezio Ghigo [1] [3], Fabio Broglio [1] [3], Simona Bo [1] [3]

Affiliations

- [1] Department of Medical Sciences, University of Torino, c.so AM Dogliotti 14, 10126 Torino, Italy.
- [2] Department of Psychology, University of Torino, c.so AM Dogliotti 14, 10126 Torino, Italy.
- [3] Diabetes and Metabolic Diseases Clinic, "Città della Salute e della Scienza" Hospital of Torino, 10126 Torino, Italy.

- PMID: **32645970**
- PMCID: PMC7400808
- DOI: 10.3390/nu12072016

Abstract

Our aim is evaluating the changes in weight and dietary habits in a sample of outpatients with obesity after 1 month of enforced lockdown during the COVID-19 pandemic in Northern Italy. In this observational retrospective study, the patients of our Obesity Unit were invited to answer to a 12-question multiple-choice questionnaire relative to weight changes, working activity, exercise, dietary habits, and conditions potentially impacting on nutritional choices. A multivariate regression analysis was performed to evaluate the associations among weight/BMI changes and the analyzed variables. A total of 150 subjects (91.5%) completed the questionnaire. Mean self-reported weight gain was ≈1.5 kg ($p < 0.001$). Lower exercise, self-reported boredom/solitude, anxiety/depression, enhanced eating, consumption of snacks, unhealthy foods, cereals, and sweets were correlated with a significantly higher weight gain. Multiple regression analyses showed that increased education (inversely, β = -1.15; 95%CI -2.13, -0.17, $p = 0.022$), self-reported anxiety/depression (β = 1.61; 0.53, 2.69, $p = 0.004$), and not consuming healthy foods (β = 1.48; 0.19, 2.77, $p = 0.026$) were significantly associated with increased weight gain. The estimated direct effect of self-reported anxiety/depression on weight was 2.07 kg (1.07, 3.07, $p < 0.001$). Individuals with obesity significantly gained weight 1 month after the beginning of the quarantine. The adverse mental burden linked to the COVID-19 pandemic was greatly associated with increased weight gain.

Keywords: COVID-19 infection; dietary habits; lockdown; obesity.

Conflict of interest statement

The authors declare no conflict of interest.

- 28 references

Supplementary info

Publication types, MeSH terms Expand

Publication types

- Observational Study

MeSH terms

- Adult
- Betacoronavirus
- Body Mass Index
- COVID-19
- Coronavirus Infections / epidemiology
- Coronavirus Infections / prevention & control*
- Feeding Behavior / psychology*
- Female
- Humans
- Italy / epidemiology
- Male
- Middle Aged
- Obesity / physiopathology
- Obesity / psychology*
- Obesity / virology
- Pandemics / prevention & control*
- Pneumonia, Viral / epidemiology
- Pneumonia, Viral / prevention & control*
- Quarantine / psychology*
- Regression Analysis
- Retrospective Studies
- SARS-CoV-2
- Weight Gain*

Full text links

FULL TEXT OPEN ACCESS MDPI Multidisciplinary Digital Publishing Institute (MDPI) Free PMC article

Proceed to details

Cite

Share

☐ 1,145

Observational Study

Korean J Intern Med

. 2021 Mar;36(Suppl 1):S123-S131.
doi: 10.3904/kjim.2020.390. Epub 2021 Feb 10.

Effects of renin-angiotensin system blockers on the risk and outcomes of severe acute respiratory syndrome coronavirus 2 infection in patients with hypertension

Jinwoo Lee [1], Seong Jin Jo [2], Youngjin Cho [3], Ji Hyun Lee [3], Il-Young Oh [3], Jin Joo Park [3], Young-Seok Cho [3], Dong-Ju Choi [3]
Affiliations Expand

Affiliations

- [1] Division of Pulmonary and Critical Care Medicine, Department of Internal Medicine, Seoul National University College of Medicine, Seoul, Korea.
- [2] Department of Dermatology, Seoul National University College of Medicine, Seoul, Korea.
- [3] Division of Cardiology, Department of Internal Medicine, Seoul National University Bundang Hospital, Seongnam, Korea.

- PMID: **32872731**
- PMCID: PMC8009159
- DOI: 10.3904/kjim.2020.390

Free PMC article
Observational Study

Effects of renin-angiotensin system blockers on the risk and outcomes of severe acute respiratory syndrome coronavirus 2 infection in patients with hypertension

Jinwoo Lee et al. Korean J Intern Med. 2021 Mar.
Free PMC article
Show details
Korean J Intern Med
. 2021 Mar;36(Suppl 1):S123-S131.
doi: 10.3904/kjim.2020.390. Epub 2021 Feb 10.

Authors

Jinwoo Lee [1], Seong Jin Jo [2], Youngjin Cho [3], Ji Hyun Lee [3], Il-Young Oh [3], Jin Joo Park [3], Young-Seok Cho [3], Dong-Ju Choi [3]

Affiliations

- [1] Division of Pulmonary and Critical Care Medicine, Department of Internal Medicine, Seoul National University College of Medicine, Seoul, Korea.
- [2] Department of Dermatology, Seoul National University College of Medicine, Seoul, Korea.
- [3] Division of Cardiology, Department of Internal Medicine, Seoul National University Bundang Hospital, Seongnam, Korea.

- PMID: **32872731**
- PMCID: PMC8009159
- DOI: 10.3904/kjim.2020.390

Abstract

Background/aims: There are concerns that the use of renin-angiotensin system (RAS) blockers may increase the risk of being infected with severe acute respiratory syndrome coronavirus 2 (SARS-CoV-2) or progressing to a severe clinical course after infection. This this study aimed to investigate the influence of RAS blockers on the risk and severity of SARS-CoV-2 infection.

Methods: We conducted a retrospective cohort study analyzing nationwide claims data of 215,184 adults who underwent SARS-CoV-2 tests in South Korea. The SARS-CoV-2 positive rates and clinical outcomes were evaluated according to the use of RAS blockers in patients with hypertension (n = 64,243).

Results: In total, 38,919 patients with hypertension were on RAS blockers. The SARS-CoV-2 positive rates were significantly higher in the RAS blocker group than in the control group after adjustments (adjusted odds ratio [OR], 1.22; 95% confidence interval [CI], 1.10 to 1.36; $p < 0.001$), and matching by propensity score (adjusted OR, 1.16; 95% CI, 1.03 to 1.32; $p = 0.017$). Among the 1,609 SARS-CoV-2-positive patients with hypertension, the use of RAS blockers was not associated with poor outcomes, such as mortality (adjusted OR, 0.81; 95% CI, 0.56 to 1.17; $p = 0.265$), and a composite of admission to the intensive care unit and mortality (adjusted OR, 0.95; 95% CI, 0.73 to 1.22; $p = 0.669$). Analysis in the propensity scorematched population showed consistent results.

Conclusion: In this Korean nationwide claims dataset, the use of RAS blockers was associated with a higher risk to SARS-CoV-2 infection but not with higher mortality or other severe clinical courses.

Keywords: Angiotensin receptor antagonists; Angiotensin-converting enzyme inhibitor; Coronavirus infections; Hypertension.

Conflict of interest statement

Conflict of interest

No potential conflict of interest relevant to this article was reported.

- 25 references

- 2 figures

Supplementary info

Publication types, MeSH terms, Substances Expand

Publication types

- Observational Study

MeSH terms

- Administrative Claims, Healthcare
- Aged
- Aged, 80 and over
- Angiotensin Receptor Antagonists / adverse effects
- Angiotensin Receptor Antagonists / therapeutic use*
- Angiotensin-Converting Enzyme Inhibitors / adverse effects
- Angiotensin-Converting Enzyme Inhibitors / therapeutic use*
- Antihypertensive Agents / adverse effects
- Antihypertensive Agents / therapeutic use*
- COVID-19 / diagnosis
- COVID-19 / mortality
- COVID-19 / therapy*
- Databases, Factual
- Female
- Humans
- Hypertension / diagnosis
- Hypertension / drug therapy*
- Hypertension / mortality
- Hypertension / physiopathology
- Male
- Middle Aged
- Renin-Angiotensin System / drug effects*
- Retrospective Studies
- Risk Assessment
- Risk Factors
- Severity of Illness Index
- Treatment Outcome

Substances

- Angiotensin Receptor Antagonists
- Angiotensin-Converting Enzyme Inhibitors
- Antihypertensive Agents

Full text links

KJIM FREE FULL TEXT M2PI Free PMC article
Proceed to details
Cite
Share
1,146
Observational Study
J Med Virol
. 2020 Nov;92(11):2813-2820.
doi: 10.1002/jmv.26191. Epub 2020 Jun 29.

Tocilizumab in patients with severe COVID-19: A single-center observational analysis

John P Knorr [1], Veronika Colomy [1], Christine M Mauriello [1], Seung Ha [1]
Affiliations Expand

Affiliation

- [1] Department of Pharmacy, Einstein Healthcare Network, Philadelphia, Pennsylvania.

- PMID: **32628003**
- PMCID: PMC7323415
- DOI: 10.1002/jmv.26191

Free PMC article
Observational Study

Tocilizumab in patients with severe COVID-19: A single-center observational analysis

John P Knorr et al. J Med Virol. 2020 Nov.
Free PMC article
Show details
J Med Virol
. 2020 Nov;92(11):2813-2820.
doi: 10.1002/jmv.26191. Epub 2020 Jun 29.

Authors

John P Knorr [1], Veronika Colomy [1], Christine M Mauriello [1], Seung Ha [1]

Affiliation

- [1] Department of Pharmacy, Einstein Healthcare Network, Philadelphia, Pennsylvania.

- PMID: **32628003**
- PMCID: PMC7323415
- DOI: 10.1002/jmv.26191

Abstract

Patients with coronavirus disease 2019 (COVID-19) may develop severe respiratory distress, thought to be mediated by cytokine release. Elevated proinflammatory markers have been associated with disease severity. Tocilizumab, an interleukin-6 receptor antagonist, may be beneficial for severe COVID-19, when cytokine storm is suspected. This is a retrospective single-center analysis of the records of patients diagnosed with COVID-19 who received tocilizumab. Outcomes, including clinical improvement, mortality and changes in oxygen-support at 24, 48, and 72 hours, and 7, 14, and 28 days post-tocilizumab, are reported. Patients were evaluated by baseline pre-tocilizumab oxygenation status and changes in proinflammatory markers within 7 days post-tocilizumab are reported. Sixty-six patients received tocilizumab at a mean dose of 724 mg (7.4 mg/kg), 3.7 days from admission. At baseline, 53% of patients were on ventilation support and all had elevated proinflammatory markers, including c-reactive protein (CRP). Common comorbidities were diabetes mellitus (43%) and hypertension (74%). Most patients received concomitant glucocorticoids and hydroxychloroquine. Seven days after tocilizumab, ten patients (15.2%) had clinical improvement in their oxygenation status, and there was a 95% decrease in CRP. Within 14 days of treatment, 29% of patients had clinical improvement, 20% had minimal or no improvement, 17% worsened, 27% died, and 7% were transferred to an outside hospital. Ultimately, 42% of all patients that received tocilizumab expired and 49% were discharged. This study found limited clinical improvement in patients that received tocilizumab in the setting of severe COVID-19. Clinical trials are ongoing to further evaluate tocilizumab's benefit in this patient population.

Keywords: COVID-19; IL-6 antagonist; cytokine release syndrome; cytokine storm; tocilizumab.

© 2020 Wiley Periodicals LLC.

- 21 references

Supplementary info

Publication types, MeSH terms, Substances Expand

Publication types

- Observational Study

MeSH terms

- Antibodies, Monoclonal, Humanized / therapeutic use*
- Arthritis, Rheumatoid / drug therapy
- C-Reactive Protein / analysis
- COVID-19 / drug therapy*
- Cohort Studies
- Cytokine Release Syndrome / drug therapy
- Ferritins / blood
- Fibrin Fibrinogen Degradation Products / analysis
- Humans
- L-Lactate Dehydrogenase / blood
- Oxygen / administration & dosage
- Receptors, Interleukin-6 / antagonists & inhibitors
- Respiration, Artificial
- Retrospective Studies

Substances

- Antibodies, Monoclonal, Humanized
- Fibrin Fibrinogen Degradation Products
- Receptors, Interleukin-6
- fibrin fragment D
- C-Reactive Protein
- Ferritins
- L-Lactate Dehydrogenase
- tocilizumab
- Oxygen

Full text links

WILEY Full Text Article Wiley Free PMC article

Proceed to details

Cite

Share

☐ 1,147

Observational Study

Theranostics

. 2020 Jul 29;10(21):9663-9673.
doi: 10.7150/thno.47980. eCollection 2020.

Myocardial injury and COVID-19: Serum hs-cTnI level in risk stratification and the prediction of 30-day fatality in COVID-19 patients with no prior cardiovascular disease

Jiatian Cao [1], Yan Zheng [1 2 3], Zhe Luo [4], Zhendong Mei [2], Yumeng Yao [5], Zilong Liu [6], Chao Liang [7], Hongbo Yang [1], Yanan Song [1], Kaihuan Yu [8], Yan Gao [9], Chouwen Zhu [10], Zheyong Huang [1], Juying Qian [1], Junbo Ge [1]

Affiliations Expand

Affiliations

- [1] Department of Cardiology, Zhongshan Hospital, Fudan University. Shanghai Institute of Cardiovascular Diseases. 180 Feng Lin Road, Shanghai 200032, China.
- [2] State Key Laboratory of Genetic Engineering, School of Life Sciences, Fudan University, Shanghai 200438, China.
- [3] Ministry of Education Key Laboratory of Public Health Safety, School of Public Health, Fudan University, Shanghai, 200438 China.
- [4] Department of Critical Medicine, Zhongshan Hospital, Fudan University, 180 Feng Lin Road, Shanghai 200032, China.
- [5] Department of Infectious Diseases, Zhongshan Hospital, Fudan University, 180 Feng Lin Road, Shanghai 200032, China.
- [6] Department of Pulmonary and Critical Care Medicine, Zhongshan Hospital, Fudan University, 180 Feng Lin Road, Shanghai 200032, China.
- [7] Department of Anesthesiology, Zhongshan Hospital, Fudan University, 180 Feng Lin Road, Shanghai 200032, China.
- [8] Department of Hepatobiliary Surgery, Renmin Hospital of Wuhan University. Gaoxin 6th Road, Donghu high tech Development Zone, Wuhan 430200, China.
- [9] Department of Neurology, Remin Hospital of Wuhan University, 99 Ziyang Road,Wuchang District, Wuhan 430200, China.
- [10] Department of Gastroenterology, Zhongshan Hospital, Fudan University, 180 Feng Lin Road, Shanghai 200032, China.

- PMID: **32863952**
- PMCID: PMC7449913
- DOI: 10.7150/thno.47980

Free PMC article
Observational Study

Myocardial injury and COVID-19: Serum hs-cTnI level in risk stratification and the

prediction of 30-day fatality in COVID-19 patients with no prior cardiovascular disease

Jiatian Cao et al. Theranostics. 2020.
Free PMC article
Show details
Theranostics
. 2020 Jul 29;10(21):9663-9673.
doi: 10.7150/thno.47980. eCollection 2020.

Authors

Jiatian Cao [1], Yan Zheng [1] [2] [3], Zhe Luo [4], Zhendong Mei [2], Yumeng Yao [5], Zilong Liu [6], Chao Liang [7], Hongbo Yang [1], Yanan Song [1], Kaihuan Yu [8], Yan Gao [9], Chouwen Zhu [10], Zheyong Huang [1], Juying Qian [1], Junbo Ge [1]

Affiliations

- [1] Department of Cardiology, Zhongshan Hospital, Fudan University. Shanghai Institute of Cardiovascular Diseases. 180 Feng Lin Road, Shanghai 200032, China.
- [2] State Key Laboratory of Genetic Engineering, School of Life Sciences, Fudan University, Shanghai 200438, China.
- [3] Ministry of Education Key Laboratory of Public Health Safety, School of Public Health, Fudan University, Shanghai, 200438 China.
- [4] Department of Critical Medicine, Zhongshan Hospital, Fudan University, 180 Feng Lin Road, Shanghai 200032, China.
- [5] Department of Infectious Diseases, Zhongshan Hospital, Fudan University, 180 Feng Lin Road, Shanghai 200032, China.
- [6] Department of Pulmonary and Critical Care Medicine, Zhongshan Hospital, Fudan University, 180 Feng Lin Road, Shanghai 200032, China.
- [7] Department of Anesthesiology, Zhongshan Hospital, Fudan University, 180 Feng Lin Road, Shanghai 200032, China.
- [8] Department of Hepatobiliary Surgery, Renmin Hospital of Wuhan University. Gaoxin 6th Road, Donghu high tech Development Zone, Wuhan 430200, China.
- [9] Department of Neurology, Remin Hospital of Wuhan University, 99 Ziyang Road,Wuchang District, Wuhan 430200, China.
- [10] Department of Gastroenterology, Zhongshan Hospital, Fudan University, 180 Feng Lin Road, Shanghai 200032, China.

- PMID: **32863952**
- PMCID: PMC7449913
- DOI: 10.7150/thno.47980

Abstract

Introduction: To explore the involvement of the cardiovascular system in coronavirus disease 2019 (COVID-19), we investigated whether myocardial injury occurred in COVID-19 patients

and assessed the performance of serum high-sensitivity cardiac Troponin I (hs-cTnI) levels in predicting disease severity and 30-day in-hospital fatality. **Methods:** We included 244 COVID-19 patients, who were admitted to Renmin Hospital of Wuhan University with no preexisting cardiovascular disease or renal dysfunction. We analyzed the data including patients' clinical characteristics, cardiac biomarkers, severity of medical conditions, and 30-day in-hospital fatality. We performed multivariable Cox regressions and the receiver operating characteristic analysis to assess the association of cardiac biomarkers on admission with disease severity and prognosis. **Results:** In this retrospective observational study, 11% of COVID-19 patients had increased hs-cTnI levels (>40 ng/L) on admission. Of note, serum hs-cTnI levels were positively associated with the severity of medical conditions (median [interquartile range (IQR)]: 6.00 [6.00-6.00] ng/L in 91 patients with moderate conditions, 6.00 [6.00-18.00] ng/L in 107 patients with severe conditions, and 11.00 [6.00-56.75] ng/L in 46 patients with critical conditions, *P* for trend=0.001). Moreover, compared with those with normal cTnI levels, patients with increased hs-cTnI levels had higher in-hospital fatality (adjusted hazard ratio [95% CI]: 4.79 [1.46-15.69]). The receiver-operating characteristic curve analysis suggested that the inclusion of hs-cTnI levels into a panel of empirical prognostic factors substantially improved the prediction performance for severe or critical conditions (area under the curve (AUC): 0.71 (95% CI: 0.65-0.78) vs. 0.65 (0.58-0.72), *P*=0.01), as well as for 30-day fatality (AUC: 0.91 (0.85-0.96) vs. 0.77 (0.62-0.91), *P*=0.04). A cutoff value of 20 ng/L of hs-cTnI level led to the best prediction to 30-day fatality. **Conclusions:** In COVID-19 patients with no preexisting cardiovascular disease, 11% had increased hs-cTnI levels. Besides empirical prognostic factors, serum hs-cTnI levels upon admission provided independent prediction to both the severity of the medical condition and 30-day in-hospital fatality. These findings may shed important light on the clinical management of COVID-19.

Keywords: COVID-19; Troponin I; in-hospital fatality; myocardial injury.

© The author(s).

Conflict of interest statement

Competing Interests: The authors have declared that no competing interest exists.

- 47 references
- 4 figures

Supplementary info

Publication types, MeSH terms, Substances Expand

Publication types

- Observational Study
- Research Support, Non-U.S. Gov't

MeSH terms

- Aged
- COVID-19
- Cardiomyopathies / blood

- Cardiomyopathies / etiology*
- China
- Cohort Studies
- Coronavirus Infections / blood
- Coronavirus Infections / complications*
- Coronavirus Infections / mortality
- Female
- Hospitalization
- Humans
- Male
- Middle Aged
- Pandemics
- Pneumonia, Viral / blood
- Pneumonia, Viral / complications*
- Pneumonia, Viral / mortality
- Predictive Value of Tests
- Prognosis
- Retrospective Studies
- Troponin I / blood*

Substances

- TNNI1 protein, human
- Troponin I

Full text links

Ivyspring International Publisher Free PMC article
Proceed to details
Cite
Share
☐ 1,148
Observational Study
Theranostics
. 2020 May 15;10(14):6372-6383.
doi: 10.7150/thno.46833. eCollection 2020.

Risk factors for adverse clinical outcomes with COVID-19 in China: a multicenter, retrospective, observational study

Peng Peng Xu [1], Rong Hua Tian [2], Song Luo [1], Zi Yue Zu [1], Bin Fan [3], Xi Ming Wang [4], Kai Xu [5] [6], Jiang Tao Wang [7], Juan Zhu [8], Ji Chan Shi [9], Feng Chen [10], Bing Wan [11], Zhi Han Yan [12], Rong Pin Wang [13], Wen Chen [14], Wen Hui Fan [15], Can Zhang [16], Meng Jie Lu [1], Zhi Yuan Sun [1], Chang Sheng Zhou [1], Li Na Zhang [17], Fei Xia [1], Li Qi [1], Wei Zhang [1], Jing Zhong [1], Xiao Xue Liu [1], Qi Rui Zhang [1], Guang Ming Lu [1], Long Jiang Zhang [1]
Affiliations Expand

Affiliations

- [1] Department of Medical Imaging, Jinling Hospital, Medical School of Nanjing University, Nanjing, Jiangsu, 210002, China.
- [2] Department of Medical Imaging, Xiaogan Central Hospital of Wuhan University of Science and Technology, Xiaogan, Hubei, China.
- [3] Huanggang Central Hospital, No. 11, Kaopeng Road, Huangzhou District, Huanggang, Hubei, China.
- [4] Department of Medical Imaging, Shandong Provincial Hospital Affiliated to Shandong First Medical University, Jinan, Shandong, China.
- [5] Department of Radiology, the Affiliated Hospital of Xuzhou Medical University, Xuzhou, Jiangsu, China.
- [6] Institute of Medical Imaging and Digital Medicine, Xuzhou Medical University, Xuzhou, Jiangsu, China.
- [7] Department of Radiology, Xiangyang Central Hospital, Affiliated Hospital of Hubei University of Arts and Science, Xiangyang, Hubei, China.
- [8] Department of Medical Imaging, the Affiliated Anqing Hospital of Anhui Medical University, Anqing, Anhui, China.
- [9] Departments of Infectious Disease & Department of Medical Imaging, Wenzhou Central Hospital, Wenzhou, Zhejiang, China.
- [10] Department of Radiology, Hainan General Hospital, Haikou, Hainan, China.
- [11] Department of Medical Imaging, Jingzhou Central Hospital, The Second Clinical Medical College, Yangtze University, Jingzhou, Hubei, China.
- [12] Department of Medical Imaging, The Second Affiliated Hospital and Yuying Children's Hospital of Wenzhou Medical University, Wenzhou, Zhejiang, PR China.
- [13] Department of Medical Imaging, Guizhou Provincial People's Hospital, Guiyang, China.
- [14] Department of Medical Imaging, Taihe Hospital, Hubei University of Medicine, Shiyan, Hubei, China.
- [15] Department of Radiology, General Hospital of the Yangtze River Shipping, Wuhan, Hubei, China.
- [16] Department of Radiology, Yichang Central People's Hospital, Yichang, Hubei, China.
- [17] Department of Radiology, The First Hospital of China Medical University, Shenyang, Liaoning, China.

- PMID: **32483458**
- PMCID: PMC7255028
- DOI: 10.7150/thno.46833

Free PMC article
Observational Study

Risk factors for adverse clinical outcomes with COVID-19 in China: a multicenter, retrospective, observational study

Peng Peng Xu et al. Theranostics. 2020.
Free PMC article
Show details
Theranostics
. 2020 May 15;10(14):6372-6383.
doi: 10.7150/thno.46833. eCollection 2020.

Authors

Peng Peng Xu [1], Rong Hua Tian [2], Song Luo [1], Zi Yue Zu [1], Bin Fan [3], Xi Ming Wang [4], Kai Xu [5] [6], Jiang Tao Wang [7], Juan Zhu [8], Ji Chan Shi [9], Feng Chen [10], Bing Wan [11], Zhi Han Yan [12], Rong Pin Wang [13], Wen Chen [14], Wen Hui Fan [15], Can Zhang [16], Meng Jie Lu [1], Zhi Yuan Sun [1], Chang Sheng Zhou [1], Li Na Zhang [17], Fei Xia [1], Li Qi [1], Wei Zhang [1], Jing Zhong [1], Xiao Xue Liu [1], Qi Rui Zhang [1], Guang Ming Lu [1], Long Jiang Zhang [1]

Affiliations

- [1] Department of Medical Imaging, Jinling Hospital, Medical School of Nanjing University, Nanjing, Jiangsu, 210002, China.
- [2] Department of Medical Imaging, Xiaogan Central Hospital of Wuhan University of Science and Technology, Xiaogan, Hubei, China.
- [3] Huanggang Central Hospital, No. 11, Kaopeng Road, Huangzhou District, Huanggang, Hubei, China.
- [4] Department of Medical Imaging, Shandong Provincial Hospital Affiliated to Shandong First Medical University, Jinan, Shandong, China.
- [5] Department of Radiology, the Affiliated Hospital of Xuzhou Medical University, Xuzhou, Jiangsu, China.
- [6] Institute of Medical Imaging and Digital Medicine, Xuzhou Medical University, Xuzhou, Jiangsu, China.
- [7] Department of Radiology, Xiangyang Central Hospital, Affiliated Hospital of Hubei University of Arts and Science, Xiangyang, Hubei, China.
- [8] Department of Medical Imaging, the Affiliated Anqing Hospital of Anhui Medical University, Anqing, Anhui, China.
- [9] Departments of Infectious Disease & Department of Medical Imaging, Wenzhou Central Hospital, Wenzhou, Zhejiang, China.
- [10] Department of Radiology, Hainan General Hospital, Haikou, Hainan, China.
- [11] Department of Medical Imaging, Jingzhou Central Hospital, The Second Clinical Medical College, Yangtze University, Jingzhou, Hubei, China.
- [12] Department of Medical Imaging, The Second Affiliated Hospital and Yuying Children's Hospital of Wenzhou Medical University, Wenzhou, Zhejiang, PR China.
- [13] Department of Medical Imaging, Guizhou Provincial People's Hospital, Guiyang, China.

- [14] Department of Medical Imaging, Taihe Hospital, Hubei University of Medicine, Shiyan, Hubei, China.
- [15] Department of Radiology, General Hospital of the Yangtze River Shipping, Wuhan, Hubei, China.
- [16] Department of Radiology, Yichang Central People's Hospital, Yichang, Hubei, China.
- [17] Department of Radiology, The First Hospital of China Medical University, Shenyang, Liaoning, China.

- PMID: **32483458**
- PMCID: PMC7255028
- DOI: 10.7150/thno.46833

Abstract

Background: The risk factors for adverse events of Coronavirus Disease-19 (COVID-19) have not been well described. We aimed to explore the predictive value of clinical, laboratory and CT imaging characteristics on admission for short-term outcomes of COVID-19 patients. **Methods:** This multicenter, retrospective, observation study enrolled 703 laboratory-confirmed COVID-19 patients admitted to 16 tertiary hospitals from 8 provinces in China between January 10, 2020 and March 13, 2020. Demographic, clinical, laboratory data, CT imaging findings on admission and clinical outcomes were collected and compared. The primary endpoint was in-hospital death, the secondary endpoints were composite clinical adverse outcomes including in-hospital death, admission to intensive care unit (ICU) and requiring invasive mechanical ventilation support (IMV). Multivariable Cox regression, Kaplan-Meier plots and log-rank test were used to explore risk factors related to in-hospital death and in-hospital adverse outcomes. **Results:** Of 703 patients, 55 (8%) developed adverse outcomes (including 33 deceased), 648 (92%) discharged without any adverse outcome. Multivariable regression analysis showed risk factors associated with in-hospital death included ≥ 2 comorbidities (hazard ratio [HR], 6.734; 95% CI; 3.239-14.003, $p < 0.001$), leukocytosis (HR, 9.639; 95% CI, 4.572-20.321, $p < 0.001$), lymphopenia (HR, 4.579; 95% CI, 1.334-15.715, $p = 0.016$) and CT severity score > 14 (HR, 2.915; 95% CI, 1.376-6.177, $p = 0.005$) on admission, while older age (HR, 2.231; 95% CI, 1.124-4.427, $p = 0.022$), ≥ 2 comorbidities (HR, 4.778; 95% CI; 2.451-9.315, $p < 0.001$), leukocytosis (HR, 6.349; 95% CI; 3.330-12.108, $p < 0.001$), lymphopenia (HR, 3.014; 95% CI; 1.356-6.697, $p = 0.007$) and CT severity score > 14 (HR, 1.946; 95% CI; 1.095-3.459, $p = 0.023$) were associated with increased odds of composite adverse outcomes. **Conclusion:** The risk factors of older age, multiple comorbidities, leukocytosis, lymphopenia and higher CT severity score could help clinicians identify patients with potential adverse events.

Keywords: COVID-19; Coronavirus; Mortality; Pneumonia; Risk factor.

© The author(s).

Conflict of interest statement

Competing Interests: The authors have declared that no competing interest exists.

- 35 references
- 5 figures

Supplementary info

Publication types, MeSH terms Expand

Publication types

- Multicenter Study
- Observational Study
- Research Support, Non-U.S. Gov't

MeSH terms

- Adolescent
- Adult
- Age Factors
- Aged
- Aged, 80 and over
- Betacoronavirus*
- COVID-19
- Child
- Child, Preschool
- China / epidemiology
- Comorbidity
- Coronavirus Infections / diagnosis*
- Coronavirus Infections / epidemiology
- Coronavirus Infections / mortality
- Female
- Hospital Mortality
- Humans
- Infant
- Kaplan-Meier Estimate
- Male
- Middle Aged
- Pandemics
- Pneumonia, Viral / diagnosis*
- Pneumonia, Viral / epidemiology
- Pneumonia, Viral / mortality
- Prognosis
- Proportional Hazards Models
- Retrospective Studies
- Risk Factors
- SARS-CoV-2
- Theranostic Nanomedicine
- Thorax / diagnostic imaging

- Tomography, X-Ray Computed
- Young Adult

Full text links

Theranostics Ivyspring International Publisher Free PMC article
Proceed to details
Cite
Share
☐ 1,149
Observational Study
Open Heart
. 2021 Aug;8(2):e001716.
doi: 10.1136/openhrt-2021-001716.

Review of Irish patients meeting ST elevation criteria during the COVID-19 pandemic

Luke Byrne [1], Roisin Gardiner [2], Patrick Devitt [2], Caleb Powell [2], Richard Armstrong [2], Sinead Teehan [2], Stephen O'Connor [2]
Affiliations Expand

Affiliations

- [1] Department of Cardiology, Saint James's Hospital, Dublin, Ireland lubyrne@tcd.ie.
- [2] Department of Cardiology, Saint James's Hospital, Dublin, Ireland.

- PMID: **34344723**
- PMCID: PMC8338315
- DOI: 10.1136/openhrt-2021-001716

Free PMC article
Observational Study

Review of Irish patients meeting ST elevation criteria during the COVID-19 pandemic

Luke Byrne et al. Open Heart. 2021 Aug.
Free PMC article
Show details
Open Heart
. 2021 Aug;8(2):e001716.
doi: 10.1136/openhrt-2021-001716.

Authors

Luke Byrne [1], Roisin Gardiner [2], Patrick Devitt [2], Caleb Powell [2], Richard Armstrong [2], Sinead Teehan [2], Stephen O'Connor [2]

Affiliations

- [1] Department of Cardiology, Saint James's Hospital, Dublin, Ireland lubyrne@tcd.ie.
- [2] Department of Cardiology, Saint James's Hospital, Dublin, Ireland.

- PMID: **34344723**
- PMCID: PMC8338315
- DOI: 10.1136/openhrt-2021-001716

Abstract

Introduction: The COVID-19 pandemic has seen the introduction of important public health measures to minimise the spread of the virus. We aim to identify the impact government restrictions and hospital-based infection control procedures on ST elevation myocardial infarction (STEMI) care during the COVID-19 pandemic.

Methods: Patients meeting ST elevation criteria and undergoing primary percutaneous coronary intervention from 27 March 2020, the day initial national lockdown measures were announced in Ireland, were included in the study. Patients presenting after the lockdown period, from 18 May to 31 June 2020, were also examined. Time from symptom onset to first medical contact (FMC), transfer time and time of wire cross was noted. Additionally, patient characteristics, left ventricular ejection fraction, mortality and biochemical parameters were documented. Outcomes and characteristics were compared against a control group of patients meeting ST elevation criteria during the month of January.

Results: A total of 42 patients presented with STEMI during the lockdown period. A significant increase in total ischaemic time (TIT) was noted versus controls (8.81 hours (±16.4) vs 2.99 hours (±1.39), p=0.03), with increases driven largely by delays in seeking FMC (7.13 hours (±16.4) vs 1.98 hours (±1.46), p=0.049). TIT remained significantly elevated during the postlockdown period (6.1 hours (±5.3), p=0.05), however, an improvement in patient delays was seen versus the control group (3.99 hours (±4.5), p=0.06). There was no difference seen in transfer times and door to wire cross time during lockdown, however, a significant increase in transfer times was seen postlockdown versus controls (1.81 hours (±1.0) vs 1.1 hours (±0.87), p=0.004).

Conclusion: A significant increase in TIT was seen during the lockdown period driven mainly by patient factors highlighting the significance of public health messages on public perception. Additionally, a significant delay in transfer times to our centre was seen postlockdown.

Keywords: COVID-19; cardiac catheterization; delivery of health care; global burden of disease; myocardial infarction.

© Author(s) (or their employer(s)) 2021. Re-use permitted under CC BY-NC. No commercial re-use. See rights and permissions. Published by BMJ.

Conflict of interest statement

Competing interests: None declared.

- 14 references
- 2 figures

Supplementary info

Publication types, MeSH terms Expand

Publication types

- Comparative Study
- Observational Study

MeSH terms

- Aged
- COVID-19*
- Databases, Factual
- Female
- Humans
- Infection Control / trends
- Ireland
- Male
- Middle Aged
- Outcome and Process Assessment, Health Care / trends*
- Patient Acceptance of Health Care
- Patient Transfer / trends
- Percutaneous Coronary Intervention / adverse effects
- Percutaneous Coronary Intervention / mortality
- Percutaneous Coronary Intervention / trends*
- Retrospective Studies
- ST Elevation Myocardial Infarction / diagnostic imaging
- ST Elevation Myocardial Infarction / mortality
- ST Elevation Myocardial Infarction / therapy*
- Time Factors
- Time-to-Treatment / trends
- Treatment Outcome

Full text links

BMJ Open Access Full Text HighWire Free PMC article

Proceed to details

Cite

Share

☐ 1,150
Observational Study
J Stroke Cerebrovasc Dis
. 2021 Oct;30(10):106051.
doi: 10.1016/j.jstrokecerebrovasdis.2021.106051. Epub 2021 Aug 13.

Impact of Coronavirus Disease 2019 on Time Delay and Functional Outcome of Mechanical Thrombectomy in Tokyo, Japan

Masahiro Katsumata [1], Takahiro Ota [2], Junya Kaneko [3], Hiroyuki Jimbo [4], Rie Aoki [5], Shigeta Fujitani [6], Masahiko Ichijo [7], Masato Inoue [8], Keigo Shigeta [9], Yoshifumi Miyauchi [10], Yu Sakai [11], Hideki Arakawa [12], Yoshinobu Otsuka [13], Kenichi Ariyada [14], Yoshiaki Kuroshima [15], Takahisa Fuse [16], Yoshiaki Shiokawa [17], Teruyuki Hirano [18]
Affiliations Expand

Affiliations

- [1] Department of Neurology, Keio University School of Medicine, 35 Shinanomachi, Shinjuku-ku, Tokyo 160-8582, Japan. Electronic address: ktmasahiro@gmail.com.
- [2] Department of Neurosurgery, Tokyo Metropolitan Tama Medical Center, Tokyo, Japan.
- [3] Department of Emergency and Critical Care Medicine, Nippon Medical School Tama Nagayama Hospital, Tokyo, Japan.
- [4] Department of Neurosurgery, Tokyo Medical University Hachioji Medical Center, Tokyo, Japan.
- [5] Department of Neurosurgery, Tokai University Hachioji Hospital, Tokyo, Japan.
- [6] Department of Endovascular Neurosurgery, Toranomon Hospital, Tokyo, Japan.
- [7] Department of Neurology, Musashino Red Cross Hospital, Tokyo, Japan.
- [8] Department of Neurosurgery, Center Hospital of the National Center for Global Health and Medicine, Tokyo, Japan.
- [9] Department of Neurosurgery, National Hospital Organization Disaster Medical Center, Tokyo, Japan.
- [10] Department of Neurology, Showa University Koto Toyosu Hospital, Tokyo, Japan.
- [11] Department of Neurosurgery, Showa General Hospital, Tokyo, Japan.
- [12] Department of Neurosurgery, Omori Red Cross Hospital, Tokyo, Japan.
- [13] Department of Neurology, Machida Municipal Hospital, Tokyo, Japan.
- [14] Department of Neurosurgery, Tokyo Metropolitan Bokuto Hospital, Tokyo, Japan.
- [15] Department of Neurosurgery, Hino Municipal Hospital, Tokyo, Japan.
- [16] Department of Neurosurgery, Public Fussa Hospital, Tokyo, Japan.
- [17] Department of Neurosurgery, Kyorin University, Tokyo, Japan.
- [18] Department of Stroke and Cerebrovascular Medicine, Kyorin University, Tokyo, Japan.

- PMID: **34419835**
- PMCID: PMC8361142
- DOI: 10.1016/j.jstrokecerebrovasdis.2021.106051

Free PMC article
Observational Study

Impact of Coronavirus Disease 2019 on Time Delay and Functional Outcome of Mechanical Thrombectomy in Tokyo, Japan

Masahiro Katsumata et al. J Stroke Cerebrovasc Dis. 2021 Oct.
Free PMC article
Show details
J Stroke Cerebrovasc Dis
. 2021 Oct;30(10):106051.
doi: 10.1016/j.jstrokecerebrovasdis.2021.106051. Epub 2021 Aug 13.

Authors

Masahiro Katsumata [1], Takahiro Ota [2], Junya Kaneko [3], Hiroyuki Jimbo [4], Rie Aoki [5], Shigeta Fujitani [6], Masahiko Ichijo [7], Masato Inoue [8], Keigo Shigeta [9], Yoshifumi Miyauchi [10], Yu Sakai [11], Hideki Arakawa [12], Yoshinobu Otsuka [13], Kenichi Ariyada [14], Yoshiaki Kuroshima [15], Takahisa Fuse [16], Yoshiaki Shiokawa [17], Teruyuki Hirano [18]

Affiliations

- [1] Department of Neurology, Keio University School of Medicine, 35 Shinanomachi, Shinjuku-ku, Tokyo 160-8582, Japan. Electronic address: ktmasahiro@gmail.com.
- [2] Department of Neurosurgery, Tokyo Metropolitan Tama Medical Center, Tokyo, Japan.
- [3] Department of Emergency and Critical Care Medicine, Nippon Medical School Tama Nagayama Hospital, Tokyo, Japan.
- [4] Department of Neurosurgery, Tokyo Medical University Hachioji Medical Center, Tokyo, Japan.
- [5] Department of Neurosurgery, Tokai University Hachioji Hospital, Tokyo, Japan.
- [6] Department of Endovascular Neurosurgery, Toranomon Hospital, Tokyo, Japan.
- [7] Department of Neurology, Musashino Red Cross Hospital, Tokyo, Japan.
- [8] Department of Neurosurgery, Center Hospital of the National Center for Global Health and Medicine, Tokyo, Japan.
- [9] Department of Neurosurgery, National Hospital Organization Disaster Medical Center, Tokyo, Japan.
- [10] Department of Neurology, Showa University Koto Toyosu Hospital, Tokyo, Japan.
- [11] Department of Neurosurgery, Showa General Hospital, Tokyo, Japan.
- [12] Department of Neurosurgery, Omori Red Cross Hospital, Tokyo, Japan.
- [13] Department of Neurology, Machida Municipal Hospital, Tokyo, Japan.
- [14] Department of Neurosurgery, Tokyo Metropolitan Bokuto Hospital, Tokyo, Japan.
- [15] Department of Neurosurgery, Hino Municipal Hospital, Tokyo, Japan.
- [16] Department of Neurosurgery, Public Fussa Hospital, Tokyo, Japan.
- [17] Department of Neurosurgery, Kyorin University, Tokyo, Japan.

- [18] Department of Stroke and Cerebrovascular Medicine, Kyorin University, Tokyo, Japan.

- PMID: **34419835**
- PMCID: PMC8361142
- DOI: 10.1016/j.jstrokecerebrovasdis.2021.106051

Abstract

Objectives: An association has been reported between delays in the onset-to-door (O2D) time for mechanical thrombectomy (MT) and outbreaks of coronavirus disease 2019 (COVID-19). However, the association between other MT time courses or functional outcomes and COVID-19 outbreaks remains unclear. We compared the time courses of stroke pathways or functional outcomes in 2020 (the COVID-19 era) with those in 2019 (the pre-COVID-19 era) in Tokyo, Japan.

Materials and methods: This retrospective observational study used data from the Tokyo-tama-REgistry of Acute endovascular Thrombectomy (TREAT), a multicenter registry of MT for acute large vessel occlusion in the Tokyo Metropolitan Area. Patients who had undergone acute MT from January 2019 to December 2020 were included. Patients were classified by the year they had undergone MT (2019 or 2020).

Results: In total, 477 patients were analyzed. O2D time was significantly longer in 2020 (146.0 min) than in 2019 (105.0 min; $p = 0.034$). No significant difference in door-to-puncture time (D2P) time or modified Rankin Scale (mRS) score 0-2 at 90 days was seen between 2019 and 2020. In the subgroup analysis, O2D time was significantly longer in the first half of 2020 compared with 2019. Multivariable logistic regression analysis revealed that the year 2020 was a independent predictor of longer O2D time, but not for mRS score 0-2 at 90 days.

Conclusions: Although O2D time was significantly longer in the COVID-19 compared with the pre-COVID-19 era, D2P may not be significantly delayed and functional outcomes may not be different, despite the COVID-19 pandemic.

Keywords: Acute ischemic stroke; COVID-19; Epidemiology; Thrombectomy.

Copyright © 2021 Elsevier Inc. All rights reserved.

Conflict of interest statement

Declaration of Competing Interest Yoshiaki Shiokawa has received research grants from AbbVie GK and ONO Pharmaceitocal CO., LTD. Teruyuki Hirano has received honoraria from Bayer, Boehringer-Ingelheim, Bristol-Myers Squibb, Daiichi-Sankyo, Otsuka Pharma, Pfizer, and Sanofi. All the other authors have nothing to disclosure.

- 18 references
- 2 figures

Supplementary info

Publication types, MeSH terms Expand

Publication types

- Multicenter Study
- Observational Study

MeSH terms

- COVID-19*
- Health Care Rationing / trends
- Health Services Needs and Demand / trends
- Humans
- Practice Patterns, Physicians' / trends*
- Registries
- Retrospective Studies
- Stroke / diagnosis
- Stroke / therapy*
- Thrombectomy / trends*
- Time Factors
- Time-to-Treatment / trends*
- Tokyo
- Treatment Outcome

Full text links

ELSEVIER FULL-TEXT ARTICLE Elsevier Science Free PMC article
Proceed to details
Cite
Share
1,151
Observational Study
S Afr Med J
. 2020 Nov 5;110(12):1201-1205.
doi: 10.7196/SAMJ.2020.v110i12.15294.

The impact of COVID-19 on routine patient care from a laboratory perspective

E C Kruger [1], R Banderker, R T Erasmus, A E Zemlin
Affiliations Expand

Affiliation

- [1] Division of Chemical Pathology, Department of Pathology, National Health Laboratory Service, Tygerberg Hospital and Faculty of Medicine and Health Sciences, Stellenbosch University, Cape Town, South Africa. elsie.kruger@nhls.ac.za.

- PMID: **33403966**
- DOI: 10.7196/SAMJ.2020.v110i12.15294

Observational Study

The impact of COVID-19 on routine patient care from a laboratory perspective

E C Kruger et al. S Afr Med J. 2020.

Show details

S Afr Med J

. 2020 Nov 5;110(12):1201-1205.
doi: 10.7196/SAMJ.2020.v110i12.15294.

Authors

E C Kruger [1], R Banderker, R T Erasmus, A E Zemlin

Affiliation

- [1] Division of Chemical Pathology, Department of Pathology, National Health Laboratory Service, Tygerberg Hospital and Faculty of Medicine and Health Sciences, Stellenbosch University, Cape Town, South Africa. elsie.kruger@nhls.ac.za.

- PMID: **33403966**
- DOI: 10.7196/SAMJ.2020.v110i12.15294

Abstract

Background: Globally, few studies have examined the effect of the COVID-19 pandemic on routine patient care and follow-up.

Objectives: To evaluate the effect of the COVID-19 response on biochemical test requests received from outpatient departments (OPDs) and peripheral clinics serviced by the National Health Laboratory Service Chemical Pathology Laboratory at Tygerberg Hospital, Cape Town, South Africa (SA). Request volumes were used as a measure of the routine care of patients, as clinical information was not readily available.

Methods: A retrospective audit was conducted. The numbers of requests received from OPDs and peripheral clinics for creatinine, glycated haemoglobin (HbA1c), lipid profiles, thyroid-stimulating hormone (TSH), free thyroxine, free tri-iodothyronine (fT3), serum and urine protein electrophoresis, serum free light chains and neonatal total serum bilirubin were obtained from 1 March to 30 June for 2017, 2018, 2019 and 2020.

Results: The biggest impact was seen on lipids, creatinine, HbA1c, TSH and fT3. The percentage reduction between 1 March and 30 June 2019 and between 1 March and 30 June 2020 was 59% for lipids, 64% for creatinine and HbA1c, 80% for TSH and 81% for fT3. There was a noteworthy decrease in overall analyte testing from March to April 2020, coinciding with initiation of level 5 lockdown. Although an increase in testing was observed during June 2020, the number of requests was still lower than in June 2019.

Conclusions: This study, focusing on the short-term consequences of the SA response to the COVID-19 pandemic, found that routine follow-up of patients with communicable and non-communicable diseases was affected. Future studies are necessary to evaluate the long-term consequences of the pandemic for these patient groups.

Supplementary info

Publication types, MeSH terms, Substances Expand

Publication types

- Observational Study

MeSH terms

- Ambulatory Care
- Bilirubin / blood
- Blood Chemical Analysis / trends
- Blood Protein Electrophoresis
- COVID-19*
- Clinical Laboratory Services / trends*
- Clinical Laboratory Techniques / trends*
- Creatinine / blood
- Delivery of Health Care*
- Electrophoresis / trends
- Glycated Hemoglobin A / metabolism
- Humans
- Lipids / blood
- Retrospective Studies
- SARS-CoV-2
- Thyroid Function Tests / statistics & numerical data
- Thyrotropin / blood
- Thyroxine / blood
- Triiodothyronine / blood
- Urinalysis / trends

Substances

- Glycated Hemoglobin A
- Lipids
- Triiodothyronine
- Thyrotropin
- Creatinine
- Thyroxine
- Bilirubin

Proceed to details
Cite
Share
☐ 1,152
Observational Study
Clin J Am Soc Nephrol
. 2020 Oct 7;15(10):1394-1402.
doi: 10.2215/CJN.04650420. Epub 2020 Sep 22.

The Incidence, Risk Factors, and Prognosis of Acute Kidney Injury in Adult Patients with Coronavirus Disease 2019

Yichun Cheng[1], Ran Luo[1], Xu Wang[2], Kun Wang[1], Nanhui Zhang[1], Meng Zhang[1], Zhixiang Wang[1], Lei Dong[1], Junhua Li[1], Rui Zeng[1], Ying Yao[1], Shuwang Ge[3], Gang Xu[3]
Affiliations Expand

Affiliations

- [1] Department of Nephrology, Tongji Hospital Affiliated to Tongji Medical College, Huazhong University of Science and Technology, Wuhan, China.
- [2] Department of Laboratory Medicine, Tongji Hospital Affiliated to Tongji Medical College, Huazhong University of Science and Technology, Wuhan, China.
- [3] Department of Nephrology, Tongji Hospital Affiliated to Tongji Medical College, Huazhong University of Science and Technology, Wuhan, China geshuwang@tjh.tjmu.edu.cn xugang@tjh.tjmu.edu.cn.

- PMID: **32963018**
- PMCID: PMC7536762
- DOI: 10.2215/CJN.04650420

Free PMC article
Observational Study

The Incidence, Risk Factors, and Prognosis of Acute Kidney Injury in Adult Patients with Coronavirus Disease 2019

Yichun Cheng et al. Clin J Am Soc Nephrol. 2020.
Free PMC article
Show details
Clin J Am Soc Nephrol
. 2020 Oct 7;15(10):1394-1402.
doi: 10.2215/CJN.04650420. Epub 2020 Sep 22.

Authors

Yichun Cheng [1], Ran Luo [1], Xu Wang [2], Kun Wang [1], Nanhui Zhang [1], Meng Zhang [1], Zhixiang Wang [1], Lei Dong [1], Junhua Li [1], Rui Zeng [1], Ying Yao [1], Shuwang Ge [3], Gang Xu [3]

Affiliations

- [1] Department of Nephrology, Tongji Hospital Affiliated to Tongji Medical College, Huazhong University of Science and Technology, Wuhan, China.
- [2] Department of Laboratory Medicine, Tongji Hospital Affiliated to Tongji Medical College, Huazhong University of Science and Technology, Wuhan, China.
- [3] Department of Nephrology, Tongji Hospital Affiliated to Tongji Medical College, Huazhong University of Science and Technology, Wuhan, China geshuwang@tjh.tjmu.edu.cn xugang@tjh.tjmu.edu.cn.

- PMID: **32963018**
- PMCID: PMC7536762
- DOI: 10.2215/CJN.04650420

Abstract

Background and objectives: Since December 2019, coronavirus disease 2019 (COVID-19) outbreak occurred and has rapidly spread worldwide. However, little information is available about the AKI in COVID-19. We aimed to evaluate the incidence, risk factors, and prognosis of AKI in adult patients with COVID-19.

Design, setting, participants, & measurements: This was a retrospective cohort study of 1392 patients with COVID-19 admitted to a tertiary teaching hospital. Clinical characteristics and laboratory data were extracted from electronic hospitalization and laboratory databases. AKI was defined and staged according to the 2012 Kidney Disease: Improving Global Outcomes criteria. Risk factors for AKI and the association of AKI with in-hospital mortality were assessed.

Results: A total of 7% (99 of 1392) of patients developed AKI during hospitalization, 40% (40 of 99) of which occurred within 1 week of admission. Factors associated with a higher risk of AKI include severe disease (odds ratio [OR], 2.25; 95% confidence interval [CI], 1.37 to 3.67), higher

baseline serum creatinine (OR, 2.19; 95% CI, 1.17 to 4.11), lymphopenia (OR, 1.99; 95% CI, 1.12 to 3.53), and elevated D-dimer level (OR, 2.68; 95% CI, 1.07 to 6.70). The in-hospital mortality in patients with AKI stage 1, stage 2, and stage 3 was 62%, 77%, and 80%, respectively. AKI was associated with in-hospital mortality even after adjustment for confounders (OR, 5.12; 95% CI, 2.70 to 9.72).

Conclusions: AKI is uncommon but carries high in-hospital mortality in patients with COVID-19.

Keywords: COVID-19; acute kidney injury; mortality; prognosis; risk factors.

Copyright © 2020 by the American Society of Nephrology.

Comment in

- COVID-19-Associated Acute Kidney Injury: An Evolving Picture.
  Siew ED, Birkelo BC. Siew ED, et al. Clin J Am Soc Nephrol. 2020 Oct 7;15(10):1383-1385. doi: 10.2215/CJN.13600820. Epub 2020 Sep 22. Clin J Am Soc Nephrol. 2020. PMID: 32969342 Free PMC article. No abstract available.

- 5 figures

Supplementary info

Publication types, MeSH terms Expand

Publication types

- Observational Study
- Research Support, Non-U.S. Gov't

MeSH terms

- Acute Kidney Injury / diagnosis
- Acute Kidney Injury / mortality*
- Acute Kidney Injury / therapy
- Acute Kidney Injury / virology
- Aged
- Betacoronavirus / pathogenicity*
- COVID-19
- China
- Coronavirus Infections / diagnosis
- Coronavirus Infections / mortality*
- Coronavirus Infections / therapy
- Coronavirus Infections / virology
- Female
- Hospital Mortality*
- Host-Pathogen Interactions

- Humans
- Incidence
- Male
- Middle Aged
- Pandemics
- Pneumonia, Viral / diagnosis
- Pneumonia, Viral / mortality*
- Pneumonia, Viral / therapy
- Pneumonia, Viral / virology
- Prognosis
- Retrospective Studies
- Risk Factors
- SARS-CoV-2
- Time Factors

Full text links

Final Version FREE Clin J Am Soc Nephrol HighWire Free PMC article
Proceed to details
Cite
Share
1,153
Observational Study
Clin Microbiol Infect
. 2020 Nov;26(11):1560.e5-1560.e8.
doi: 10.1016/j.cmi.2020.08.018. Epub 2020 Aug 21.

More than loss of taste and smell: burning watering eyes in coronavirus disease 2019

Alexander C Rokohl [1], Niklas Loreck [2], Philomena A Wawer Matos [2], Sarah Zwingelberg [2], Max Augustin [3], Felix Dewald [4], Rafael S Grajewski [2], Florian Klein [4], Clara Lehmann [3], Ludwig M Heindl [2]
Affiliations Expand

Affiliations

- [1] Department of Ophthalmology, University of Cologne, Faculty of Medicine and University Hospital of Cologne, Cologne, Germany. Electronic address: alexander.rokohl@uk.koeln.de.
- [2] Department of Ophthalmology, University of Cologne, Faculty of Medicine and University Hospital of Cologne, Cologne, Germany.

- [3] University of Cologne, Department I of Internal Medicine, Division of Infectious Diseases, Cologne, Germany; German Centre for Infection Research (DZIF), Partner Site Bonn-Cologne, Cologne, Germany; University of Cologne, Centre for Molecular Medicine Cologne, Cologne, Germany.
- [4] Laboratory of Experimental Immunology, Institute of Virology, University of Cologne, Faculty of Medicine and University Hospital Cologne, Cologne, Germany.

- PMID: **32835793**
- PMCID: PMC7442009
- DOI: 10.1016/j.cmi.2020.08.018

Free PMC article
Observational Study

More than loss of taste and smell: burning watering eyes in coronavirus disease 2019

Alexander C Rokohl et al. Clin Microbiol Infect. 2020 Nov.
Free PMC article
Show details
Clin Microbiol Infect
. 2020 Nov;26(11):1560.e5-1560.e8.
doi: 10.1016/j.cmi.2020.08.018. Epub 2020 Aug 21.

Authors

Alexander C Rokohl [1], Niklas Loreck [2], Philomena A Wawer Matos [2], Sarah Zwingelberg [2], Max Augustin [3], Felix Dewald [4], Rafael S Grajewski [2], Florian Klein [4], Clara Lehmann [3], Ludwig M Heindl [2]

Affiliations

- [1] Department of Ophthalmology, University of Cologne, Faculty of Medicine and University Hospital of Cologne, Cologne, Germany. Electronic address: alexander.rokohl@uk.koeln.de.
- [2] Department of Ophthalmology, University of Cologne, Faculty of Medicine and University Hospital of Cologne, Cologne, Germany.
- [3] University of Cologne, Department I of Internal Medicine, Division of Infectious Diseases, Cologne, Germany; German Centre for Infection Research (DZIF), Partner Site Bonn-Cologne, Cologne, Germany; University of Cologne, Centre for Molecular Medicine Cologne, Cologne, Germany.
- [4] Laboratory of Experimental Immunology, Institute of Virology, University of Cologne, Faculty of Medicine and University Hospital Cologne, Cologne, Germany.

- PMID: **32835793**
- PMCID: PMC7442009
- DOI: 10.1016/j.cmi.2020.08.018

Abstract

Objectives: To evaluate ocular symptoms in European non-hospitalized patients with severe acute respiratory syndrome-related coronavirus 2 (SARS-CoV-2) and to investigate associations with the demographic data as well as nasal and general physical symptoms.

Methods: In this prospective, observational study, 108 non-hospitalized patients with PCR-confirmed SARS-CoV-2 infection not requiring intensive care were asked about disease-associated ocular symptoms, demographic data, as well as general physical and nasal symptoms using a standardized questionnaire. Total ocular symptom score (TOSS) was evaluated during and, retrospectively, before development of coronavirus disease 2019 (COVID-19). Associations between TOSS and demographic data as well as general and nasal symptoms were evaluated.

Results: Seventy-five of the 108 COVID-19 patients (69.4%) had at least one ocular symptom during COVID-19. The most common symptoms included burning sensations in 39 (36.1%), epiphora in 37 (34.3%) and redness in 28 (25.9%), compatible with conjunctivitis. These symptoms occurred 1.96 ± 3.17 days after the beginning of COVID-19 and were mild. TOSS was significantly higher during COVID-19 (1.27 ± 1.85) than before the infection (0.33 ± 1.04; $p < 0.001$). There were no significant associations between TOSS and gender (β coefficient -0.108; p 0.302), age (-0.024; p 0.816), rhinorrhoea (-0.127; p 0.353), nasal itching (-0.026; p 0.803), sneezing (0.099; p 0.470), nasal congestion (-0.012; p 0.930), cough (-0.079; p 0.450), headache (0.102; p 0.325), sore throat (0.208; p 0.052), or fever (0.094; p 0.361).

Conclusions: Ocular involvement in European non-hospitalized individuals with COVID-19 seems to be highly underestimated. Overall, these ocular symptoms, including burning sensations, epiphora and redness, seem to be mild and to not need treatment.

Keywords: Burning sensations; Conjunctivitis; Coronavirus; Coronavirus disease 2019; Severe acute respiratory syndrome coronavirus 2; Watering.

Copyright © 2020 European Society of Clinical Microbiology and Infectious Diseases. Published by Elsevier Ltd. All rights reserved.

- 10 references

Supplementary info

Publication types, MeSH terms Expand

Publication types

- Observational Study

MeSH terms

- Adolescent
- Adult
- Aged
- Aged, 80 and over

- Betacoronavirus
- COVID-19
- Coronavirus Infections / complications*
- Coronavirus Infections / epidemiology
- Coronavirus Infections / pathology
- Europe / epidemiology
- Eye Diseases / epidemiology
- Eye Diseases / etiology*
- Eye Diseases / pathology*
- Female
- Humans
- Male
- Middle Aged
- Pandemics
- Pneumonia, Viral / complications*
- Pneumonia, Viral / epidemiology
- Pneumonia, Viral / pathology
- Prevalence
- Prospective Studies
- SARS-CoV-2
- Young Adult

Full text links

ELSEVIER OPEN ACCESS Elsevier Science Free PMC article

Proceed to details

Cite

Share

1,154

Diabet Med

. 2021 Jan;38(1):e14380.

doi: 10.1111/dme.14380. Epub 2020 Sep 21.

Approaches to screening for hyperglycaemia in pregnant women during and after the COVID-19 pandemic

C L Meek [1, 2, 3], R S Lindsay [4], E M Scott [5], C E Aiken [1, 2], J Myers [6], R M Reynolds [7], D Simmons [8], J M Yamamoto [9], D R McCance [10], H R Murphy [2, 11, 12]

Affiliations Expand

Affiliations

- [1] Wellcome Trust-MRC Institute of Metabolic Science, Metabolic Research Laboratories, University of Cambridge, Cambridge, UK.
- [2] Diabetes in Pregnancy Team, Cambridge University Hospitals, Cambridge, UK.
- [3] Department of Clinical Biochemistry, Cambridge University Hospitals, Addenbrookes's Hospital, Cambridge, UK.
- [4] Institute of Cardiovascular and Medical Sciences, British Heart Foundation Glasgow Cardiovascular Research Centre, University of Glasgow, Glasgow, UK.
- [5] Department of Population and Clinical Sciences, Leeds Institute of Cardiovascular and Metabolic Medicine, University of Leeds, Leeds, UK.
- [6] Maternal and Fetal Health Research Centre, University of Manchester, St Mary's Hospital, Manchester, UK.
- [7] Centre for Cardiovascular Science, Queen's Medical Research Institute, Edinburgh, UK.
- [8] School of Medicine, Western Sydney University, Campbelltown, NSW, Australia.
- [9] Departments of Medicine and Obstetrics and Gynaecology, University of Calgary, Calgary, Canada.
- [10] Regional Centre for Endocrinology and Diabetes, Belfast, UK.
- [11] Norwich Medical School, Bob Champion Research and Education Building, University of East Anglia, Norwich, UK.
- [12] Division of Women's Health, Kings College London, London, UK.

- PMID: **32750184**
- PMCID: PMC7436759
- DOI: 10.1111/dme.14380

Free PMC article

Approaches to screening for hyperglycaemia in pregnant women during and after the COVID-19 pandemic

C L Meek et al. Diabet Med. 2021 Jan.
Free PMC article

Show details

Diabet Med
. 2021 Jan;38(1):e14380.
doi: 10.1111/dme.14380. Epub 2020 Sep 21.

Authors

C L Meek [1] [2] [3], R S Lindsay [4], E M Scott [5], C E Aiken [1] [2], J Myers [6], R M Reynolds [7], D Simmons [8], J M Yamamoto [9], D R McCance [10], H R Murphy [2] [11] [12]

Affiliations

- [1] Wellcome Trust-MRC Institute of Metabolic Science, Metabolic Research Laboratories, University of Cambridge, Cambridge, UK.
- [2] Diabetes in Pregnancy Team, Cambridge University Hospitals, Cambridge, UK.
- [3] Department of Clinical Biochemistry, Cambridge University Hospitals, Addenbrookes's Hospital, Cambridge, UK.
- [4] Institute of Cardiovascular and Medical Sciences, British Heart Foundation Glasgow Cardiovascular Research Centre, University of Glasgow, Glasgow, UK.
- [5] Department of Population and Clinical Sciences, Leeds Institute of Cardiovascular and Metabolic Medicine, University of Leeds, Leeds, UK.
- [6] Maternal and Fetal Health Research Centre, University of Manchester, St Mary's Hospital, Manchester, UK.
- [7] Centre for Cardiovascular Science, Queen's Medical Research Institute, Edinburgh, UK.
- [8] School of Medicine, Western Sydney University, Campbelltown, NSW, Australia.
- [9] Departments of Medicine and Obstetrics and Gynaecology, University of Calgary, Calgary, Canada.
- [10] Regional Centre for Endocrinology and Diabetes, Belfast, UK.
- [11] Norwich Medical School, Bob Champion Research and Education Building, University of East Anglia, Norwich, UK.
- [12] Division of Women's Health, Kings College London, London, UK.

- PMID: **32750184**
- PMCID: PMC7436759
- DOI: 10.1111/dme.14380

Abstract

Aim: To evaluate the diagnostic and prognostic performance of alternative diagnostic strategies to oral glucose tolerance tests, including random plasma glucose, fasting plasma glucose and HbA_{1c} , during the COVID-19 pandemic.

Methods: Retrospective service data (Cambridge, UK; 17 736 consecutive singleton pregnancies, 2004-2008; 826 consecutive gestational diabetes pregnancies, 2014-2019) and 361 women with ≥1 gestational diabetes risk factor (OPHELIA prospective observational study, UK) were included. Pregnancy outcomes included gestational diabetes (National Institute of Health and Clinical Excellence or International Association of Diabetes and Pregnancy Study Groups criteria), diabetes in pregnancy (WHO criteria), Caesarean section, large-for-gestational age infant, neonatal hypoglycaemia and neonatal intensive care unit admission. Receiver-operating characteristic curves and unadjusted logistic regression were used to compare random plasma glucose, fasting plasma glucose and HbA_{1c} performance.

Results: Gestational diabetes diagnosis was significantly associated with random plasma glucose at 12 weeks [area under the receiver-operating characteristic curve for both criteria 0.81 (95% CI 0.79-0.83)], fasting plasma glucose [National Institute of Health and Clinical Excellence: area under the receiver-operating characteristic curve 0.75 (95% CI 0.65-0.85); International Association of Diabetes and Pregnancy Study Groups: area under the receiver-operating characteristic curve 0.92 (95% CI 0.85-0.98)] and HbA_{1c} at 28 weeks' gestation [National Institute of Health and Clinical Excellence: 0.83 (95% CI 0.75-0.90); International Association of Diabetes and Pregnancy Study Groups: 0.84 (95% CI 0.77-0.91)]. Each measure predicts some, but not all, pregnancy outcomes studied. At 12 weeks, ~5% of women would be identified using random plasma glucose ≥8.5 mmol/l (sensitivity 42%; specificity 96%) and at 28 weeks using HbA_{1c} ≥39

mmol/mol (sensitivity 26%; specificity 96%) or fasting plasma glucose ≥5.2-5.4 mmol/l (sensitivity 18-41%; specificity 97-98%).

Conclusions: Random plasma glucose at 12 weeks, and fasting plasma glucose or HbA_{1c} at 28 weeks identify women with hyperglycaemia at risk of suboptimal pregnancy outcomes. These opportunistic laboratory tests perform adequately for risk stratification when oral glucose tolerance testing is not available.

© 2020 The Authors. Diabetic Medicine published by John Wiley & Sons Ltd on behalf of Diabetes UK.

Comment in

- HbA_{1c} and fasting plasma glucose cannot replace oral glucose tolerance test in order to screen for hyperglycaemia in pregnancy.
  Nachtergaele C, Vicaut E, Bihan H, Pinto S, Sal M, Berkane N, Carbillon L, Cosson E.
  Nachtergaele C, et al. Diabet Med. 2021 Sep;38(9):e14604. doi: 10.1111/dme.14604. Epub 2021 Jun 8. Diabet Med. 2021. PMID: 34021631 No abstract available.

- 31 references
- 1 figure

Supplementary info

Publication types, MeSH terms, Substances, Grant support Expand

Publication types

- Research Support, Non-U.S. Gov't

MeSH terms

- Adult
- Blood Glucose / analysis
- COVID-19 / epidemiology
- COVID-19 / prevention & control*
- Comorbidity
- Diabetes, Gestational / diagnosis*
- Diabetes, Gestational / epidemiology
- Fasting / blood
- Female
- Gestational Age
- Glucose Tolerance Test
- Glycated Hemoglobin A / analysis
- Humans
- Hyperglycemia / diagnosis*
- Mass Screening / methods*

- Pandemics
- Pregnancy
- Pregnancy Outcome / epidemiology
- Retrospective Studies
- Risk Factors
- SARS-CoV-2*
- Sensitivity and Specificity
- United Kingdom / epidemiology

Substances

- Blood Glucose
- Glycated Hemoglobin A

Grant support

- European Foundation for the Study of Diabetes-Sanofi/International
- CDF-2013-06-035/NIHR/International
- NNF19SA058974/EFSD-Novo Nordisk Foundation/International
- DUK-HKF 17/0005712/DUK_/Diabetes UK/United Kingdom
- RE/18/5/34216/BHF_/British Heart Foundation/United Kingdom

Full text links

WILEY Full Text Article Wiley Free PMC article
Proceed to details
Cite
Share
☐ 1,155
Observational Study
J Cardiovasc Pharmacol
. 2020 Nov;76(5):540-548.
doi: 10.1097/FJC.0000000000000909.

In-Hospital Management and Outcomes of Acute Myocardial Infarction Before and During the Coronavirus Disease 2019 Pandemic

Bing Huang [1 2 3 4], Changwu Xu [1 3 4], Huafen Liu [1 3 4], Wei Deng [1 3 4], Zheng Yang [1 3 4], Jun Wan [1 3 4], Hui Yan [2], Guiqiu Cao [2], Jing Chen [1 3 4], Hong Jiang [1 3 4]
Affiliations Expand

Affiliations

- [1] Department of Cardiology, Renmin Hospital of Wuhan University, Wuhan, Hubei, China.
- [2] Department of Cardiology, Fifth Affiliated Hospital of Xinjiang Medical University, Urumqi, Xinjiang, China.
- [3] Cardiovascular Research Institute, Wuhan University, Wuhan, Hubei, China; and.
- [4] Hubei Key Laboratory of Cardiology, Wuhan, Hubei, China.

- PMID: **33170591**
- DOI: [10.1097/FJC.0000000000000909](10.1097/FJC.0000000000000909)

Observational Study

In-Hospital Management and Outcomes of Acute Myocardial Infarction Before and During the Coronavirus Disease 2019 Pandemic

Bing Huang et al. J Cardiovasc Pharmacol. 2020 Nov.

Show details

J Cardiovasc Pharmacol

. 2020 Nov;76(5):540-548.
doi: 10.1097/FJC.0000000000000909.

Authors

Bing Huang [1, 2, 3, 4], Changwu Xu [1, 3, 4], Huafen Liu [1, 3, 4], Wei Deng [1, 3, 4], Zheng Yang [1, 3, 4], Jun Wan [1, 3, 4], Hui Yan [2], Guiqiu Cao [2], Jing Chen [1, 3, 4], Hong Jiang [1, 3, 4]

Affiliations

- [1] Department of Cardiology, Renmin Hospital of Wuhan University, Wuhan, Hubei, China.
- [2] Department of Cardiology, Fifth Affiliated Hospital of Xinjiang Medical University, Urumqi, Xinjiang, China.
- [3] Cardiovascular Research Institute, Wuhan University, Wuhan, Hubei, China; and.
- [4] Hubei Key Laboratory of Cardiology, Wuhan, Hubei, China.

- PMID: **33170591**
- DOI: [10.1097/FJC.0000000000000909](10.1097/FJC.0000000000000909)

Abstract

The outbreak of coronavirus disease 2019 (COVID-19) has rapidly spread worldwide. This study sought to share our experiences with in-hospital management and outcomes of acute myocardial infarction (AMI) during the COVID-19 pandemic. We retrospectively analyzed consecutive AMI

patients, including those with ST-elevation myocardial infarction (STEMI) and non-STEMI (NSTEMI), from February 1, 2020, to April 15, 2020 (during the COVID-19 pandemic), and from January 1, 2019, to December 31, 2019 (before the COVID-19 pandemic), respectively. Fifty-three AMI patients (31 STEMI, 22 NSTEMI) during the COVID-19 pandemic were matched to 53 AMI patients before the pandemic. Baseline characteristics were comparable between the matched patients. STEMI patients during the COVID-19 pandemic had a longer delay time, less primary or remedial PCI and more emergency thrombolysis than those before the pandemic. Less coronary angiography and stenting were performed in AMI patients during the COVID-19 pandemic than before the pandemic. There were no statistically significant differences in the clinical outcomes between the matched patients. However, STEMI patients during the COVID-19 pandemic had a 4-fold (12.9% vs. 3.2%) increase in all-cause mortality rate compared with those before the pandemic. AMI combined with COVID-19 infection was associated with higher rates of mortality than AMI alone. This study demonstrates that the COVID-19 pandemic results in significant reperfusion delays in STEMI patients and has a marked impact on the treatment options selection in AMI patients. The mortality rate of STEMI patients exhibits an increasing trend during the pandemic of COVID-19.

- 23 references

Supplementary info

Publication types, MeSH terms Expand

Publication types

- Comparative Study
- Observational Study
- Research Support, Non-U.S. Gov't

MeSH terms

- Aged
- COVID-19
- Cardiology Service, Hospital / trends*
- China
- Coronary Angiography / trends
- Coronavirus Infections* / diagnosis
- Coronavirus Infections* / mortality
- Coronavirus Infections* / transmission
- Female
- Humans
- Male
- Middle Aged
- Non-ST Elevated Myocardial Infarction / diagnosis
- Non-ST Elevated Myocardial Infarction / mortality
- Non-ST Elevated Myocardial Infarction / therapy*

- Outcome and Process Assessment, Health Care / trends*
- Pandemics*
- Patient Admission
- Percutaneous Coronary Intervention / adverse effects
- Percutaneous Coronary Intervention / instrumentation
- Percutaneous Coronary Intervention / trends*
- Pneumonia, Viral* / diagnosis
- Pneumonia, Viral* / mortality
- Pneumonia, Viral* / transmission
- Retrospective Studies
- Risk Factors
- ST Elevation Myocardial Infarction / diagnosis
- ST Elevation Myocardial Infarction / mortality
- ST Elevation Myocardial Infarction / therapy*
- Thrombolytic Therapy / adverse effects
- Thrombolytic Therapy / mortality
- Thrombolytic Therapy / trends*
- Time Factors
- Time-to-Treatment / trends*
- Treatment Outcome

Full text links

Wolters Kluwer Wolters Kluwer
Proceed to details
Cite
Share
☐ 1,156
Observational Study
Br J Surg
. 2020 Jun;107(7):e183-e185.
doi: 10.1002/bjs.11643. Epub 2020 Apr 27.

Performing abdominal surgery during the COVID-19 epidemic in Wuhan, China: a single-centred, retrospective, observational study

M Cai [1], G Wang [1], L Zhang [2], J Gao [1], Z Xia [1], P Zhang [1], Z Wang [1], K Cai [1], G Wang [1], K Tao [1]

Affiliations Expand

Affiliations

- [1] Departments of Gastrointestinal Surgery, Wuhan, China.
- [2] Radiology, Union Hospital, Tongji Medical College, Huazhong University of Science and Technology, Wuhan, China.

- PMID: **32339259**
- PMCID: PMC7267650
- DOI: 10.1002/bjs.11643

Free PMC article
Observational Study

Performing abdominal surgery during the COVID-19 epidemic in Wuhan, China: a single-centred, retrospective, observational study

M Cai et al. Br J Surg. 2020 Jun.
Free PMC article
Show details
Br J Surg

. 2020 Jun;107(7):e183-e185.
doi: 10.1002/bjs.11643. Epub 2020 Apr 27.

Authors

M Cai [1], G Wang [1], L Zhang [2], J Gao [1], Z Xia [1], P Zhang [1], Z Wang [1], K Cai [1], G Wang [1], K Tao [1]

Affiliations

- [1] Departments of Gastrointestinal Surgery, Wuhan, China.
- [2] Radiology, Union Hospital, Tongji Medical College, Huazhong University of Science and Technology, Wuhan, China.

- PMID: **32339259**
- PMCID: PMC7267650
- DOI: 10.1002/bjs.11643

No abstract available

- 2 references
- 1 figure

Supplementary info

Publication types, MeSH terms, Grant support Expand

Publication types

- Letter
- Observational Study

MeSH terms

- Abdomen / surgery*
- Adult
- Aged
- Betacoronavirus* / isolation & purification
- COVID-19
- COVID-19 Testing
- Case-Control Studies
- China / epidemiology
- Clinical Laboratory Techniques
- Coronavirus Infections / complications*
- Coronavirus Infections / diagnosis
- Coronavirus Infections / mortality
- Digestive System Diseases / mortality
- Digestive System Diseases / surgery*
- Digestive System Diseases / virology
- Digestive System Surgical Procedures* / mortality
- Female
- Humans
- Male
- Middle Aged
- Pandemics
- Pneumonia, Viral / complications*
- Pneumonia, Viral / diagnosis
- Pneumonia, Viral / mortality
- Postoperative Cognitive Complications / diagnosis
- Postoperative Cognitive Complications / epidemiology
- Postoperative Cognitive Complications / virology*
- Retrospective Studies
- Risk Factors
- SARS-CoV-2

Grant support

- 81700488/National Natural Science Foundation of China
- 81902703/National Natural Science Foundation of China
- 81972881/National Natural Science Foundation of China
- 2019CFB514/Natural Science Foundation of Hubei Province of China

Full text links

OXFORD ACADEMIC Silverchair Information Systems Free PMC article
Proceed to details
Cite
Share
1,157
Observational Study
Sci Rep
. 2021 Mar 11;11(1):5745.
doi: 10.1038/s41598-021-84810-9.

The effect of ABO blood group and antibody class on the risk of COVID-19 infection and severity of clinical outcomes

Marwa Ali Almadhi [1], Abdulkarim Abdulrahman [1] [2], Abdulla Alawadhi [1] [3], Ali A Rabaan [4], Stephen Atkin [5], Manaf AlQahtani [6] [7] [8]
Affiliations Expand

Affiliations

- [1] National Taskforce for Combating the Coronavirus (COVID-19), Riffa, Bahrain.
- [2] Mohammed Bin Khalifa Cardiac Centre, Awali, Bahrain.
- [3] Bahrain Defence Force Hospital, Riffa, Bahrain.
- [4] Molecular Diagnostic Laboratory, John Hopkins Aramco Healthcare, Dhahran, Saudi Arabia.
- [5] Royal College of Surgeons in Ireland, Busaiteen, Bahrain.
- [6] National Taskforce for Combating the Coronavirus (COVID-19), Riffa, Bahrain. drmanaf@gmail.com.
- [7] Bahrain Defence Force Hospital, Riffa, Bahrain. drmanaf@gmail.com.
- [8] Royal College of Surgeons in Ireland, Busaiteen, Bahrain. drmanaf@gmail.com.

- PMID: **33707451**
- PMCID: PMC7952683
- DOI: 10.1038/s41598-021-84810-9

Free PMC article
Observational Study

The effect of ABO blood group and antibody class on the risk of COVID-19 infection and severity of clinical outcomes

Marwa Ali Almadhi et al. Sci Rep. 2021.
Free PMC article
Show details
Sci Rep
. 2021 Mar 11;11(1):5745.
doi: 10.1038/s41598-021-84810-9.

Authors

Marwa Ali Almadhi [1], Abdulkarim Abdulrahman [1] [2], Abdulla Alawadhi [1] [3], Ali A Rabaan [4], Stephen Atkin [5], Manaf AlQahtani [6] [7] [8]

Affiliations

- [1] National Taskforce for Combating the Coronavirus (COVID-19), Riffa, Bahrain.
- [2] Mohammed Bin Khalifa Cardiac Centre, Awali, Bahrain.
- [3] Bahrain Defence Force Hospital, Riffa, Bahrain.
- [4] Molecular Diagnostic Laboratory, John Hopkins Aramco Healthcare, Dhahran, Saudi Arabia.
- [5] Royal College of Surgeons in Ireland, Busaiteen, Bahrain.
- [6] National Taskforce for Combating the Coronavirus (COVID-19), Riffa, Bahrain. drmanaf@gmail.com.
- [7] Bahrain Defence Force Hospital, Riffa, Bahrain. drmanaf@gmail.com.
- [8] Royal College of Surgeons in Ireland, Busaiteen, Bahrain. drmanaf@gmail.com.

- PMID: **33707451**
- PMCID: PMC7952683
- DOI: 10.1038/s41598-021-84810-9

Abstract

The COVID-19 pandemic has affected more than 100 million cases and caused immense burdens on governments and healthcare systems worldwide. Since its emergence in December 2019, research has been focused on treating the infected, identifying those at risk and preventing spread. There is currently no known biological biomarker that predicts the risk of infection. Several studies emerged suggesting an association between ABO blood group and the risk of COVID-19 infection. In this study, we used retrospective observational data in Bahrain to investigate the association between ABO blood group and risk of infection, as well as susceptibility to severe ICU-requiring infection. We found a higher risk associated with blood group B, and a lower risk with blood group AB. No association was observed between blood group and the risk of a severe ICU-requiring infection. We extended the analysis to study the association by antibodies; anti-a (blood groups B and O) and anti-b (blood groups A and O). No association between antibodies and both risk of infection or susceptibility to severe infection was found. The current study, along

with the variation in blood group association results, indicates that blood group may not be an ideal biomarker to predict risk of COVID-19 infection.

Conflict of interest statement

The authors declare no competing interests.

- 15 references
- 2 figures

Supplementary info

Publication types, MeSH terms, Substances Expand

Publication types

- Observational Study

MeSH terms

- ABO Blood-Group System*
- COVID-19 / immunology*
- Critical Care / statistics & numerical data
- Cross-Sectional Studies
- Humans

Substances

- ABO Blood-Group System

Full text links

Nature Publishing Group Free PMC article
Proceed to details
Cite
Share
☐ 1,158
Observational Study
Dig Dis Sci
. 2021 Jun;66(6):1845-1851.
doi: 10.1007/s10620-021-06940-4. Epub 2021 Mar 23.

Endoscopic Retrograde Cholangiopancreatography During the

COVID-19 Pandemic: Effects of Enhanced Personal Protective Equipment

Tolga Düzenli [1], Hüseyin Köseoğlu [2]
Affiliations Expand

Affiliations

- [1] Department of Gastroenterology, Hitit University Erol Olcok Training and Research Hospital, Corum, Turkey. tolgaduzenli@yahoo.com.
- [2] Department of Gastroenterology, Hitit University Erol Olcok Training and Research Hospital, Corum, Turkey.

- PMID: **33755824**
- PMCID: PMC7985571
- DOI: 10.1007/s10620-021-06940-4

Free PMC article
Observational Study

Endoscopic Retrograde Cholangiopancreatography During the COVID-19 Pandemic: Effects of Enhanced Personal Protective Equipment

Tolga Düzenli et al. Dig Dis Sci. 2021 Jun.
Free PMC article
Show details
Dig Dis Sci
. 2021 Jun;66(6):1845-1851.
doi: 10.1007/s10620-021-06940-4. Epub 2021 Mar 23.

Authors

Tolga Düzenli [1], Hüseyin Köseoğlu [2]

Affiliations

- [1] Department of Gastroenterology, Hitit University Erol Olcok Training and Research Hospital, Corum, Turkey. tolgaduzenli@yahoo.com.
- [2] Department of Gastroenterology, Hitit University Erol Olcok Training and Research Hospital, Corum, Turkey.

- PMID: **33755824**

- PMCID: PMC7985571
- DOI: 10.1007/s10620-021-06940-4

Abstract

Background and aims: Personal protective equipment (PPE) decreases the risk of disease contagion, and because of the COVID-19 pandemic, enhanced PPE (EPPE) is widely used during endoscopic procedures including endoscopic retrograde cholangiopancreatography (ERCP). The aim of this study was to investigate the effects of EPPE on ERCP success parameters compared to standard PPE (SPPE).

Methods: ERCP procedures were evaluated retrospectively and ERCP outcomes were compared for similar time periods as before and after the COVID-19 pandemic. Primary outcomes were cannulation time, number of cannulation attempts, cannulation success rate, difficult cannulation rate, undesired pancreatic duct cannulation rate, ERCP-related adverse events, and length of hospital stay.

Results: Three hundred and eighty ERCP procedures were examined. One hundred and fifty-nine procedures were excluded due to missing data, previous sphincterotomy or altered anatomy. Of the final eligible sample size of 221 ERCPs, 93 were performed using SPPE and 128 were performed under EPPE. Indications of ERCP and demographic parameters were similar between groups. The majority of the ERCP cases included were for benign biliary obstruction of common bile duct stones (88.7%). No significant differences were detected in overall technical success (91.4% vs 92.2%, p = 0.832), cannulation success rates (94.6% vs 96.8%, p = 0.403), cannulation times (median times of both groups were 3 min, p = 0.824), difficult cannulation rates (37.6% vs 33.6%, p = 0.523), undesired pancreatic duct cannulation rates (29% vs 22.7%, p = 0.593), number of cannulation attempts (2.80 vs 2.71, p = 0.731), ERCP-related adverse events (9.7% vs 10.9%, p = 0.762), and length of hospital stay (6.63 vs 6.92 days, p = 0.768) between SPPE and EPPE groups, respectively.

Conclusion: Biliary obstructions of common bile duct stones were the major indication of ERCP in the current study. The use of EPPE had no negative effects on ERCP performance in this patient group. ERCP can be effectively performed under EPPE.

Keywords: COVID-19; ERCP; Endoscopic retrograde cholangiopancreatography; Pandemic; Personal protective equipment.

Conflict of interest statement

The authors declare that there is no conflict of interest with regard to the authorship and/or publication of this article. The authors received no financial support for the research and/or authorship of this article.

- 19 references
- 2 figures

Supplementary info

Publication types, MeSH terms Expand

Publication types

- Comparative Study
- Observational Study

MeSH terms

- Aged
- Aged, 80 and over
- COVID-19 / prevention & control*
- COVID-19 / transmission
- Cholangiopancreatography, Endoscopic Retrograde* / adverse effects
- Female
- Humans
- Infection Control / instrumentation*
- Infectious Disease Transmission, Patient-to-Professional / prevention & control*
- Male
- Middle Aged
- Occupational Exposure / adverse effects
- Occupational Exposure / prevention & control*
- Occupational Health
- Personal Protective Equipment*
- Protective Factors
- Retrospective Studies
- Risk Assessment
- Risk Factors

Full text links

SpringerLink FULL-TEXT ARTICLE Springer Free PMC article
Proceed to details
Cite
Share
☐ 1,159
Observational Study
Gastroenterol Hepatol
. Aug-Sep 2021;44(7):481-488.
doi: 10.1016/j.gastrohep.2020.11.018. Epub 2021 Jan 27.

Impact of the COVID-19 pandemic on the activity of advanced-practice nurses on a reference unit for inflammatory bowel disease

[Article in English, Spanish]

Ester Navarro-Correal [1], Natalia Borruel [2], Virginia Robles [2], Claudia Herrera-de Guise [2], Luis Fernando Mayorga Ayala [2], Zahira Pérez Martínez [2], Arantxa Ibarz Casas [2], Sandra Agustino Rodríguez [3], Irene Joana Batuecas Duelt [4], Jorge García Alcaide [4], Sara López Branchadell [4], Esperanza Zuriguel-Perez [4], Francesc Casellas [2]
Affiliations Expand

Affiliations

- [1] Nurse and Health, Nurse and Physiotherapy, Department at Universitat de Lleida, Doctoral School, Lleida, Spain; Crohn-Colitis Care Unit, Vall d'Hebron Hospital Universitari, Barcelona, Spain. Electronic address: enavarro@vhebron.net.
- [2] Crohn-Colitis Care Unit, Vall d'Hebron Hospital Universitari, Barcelona, Spain.
- [3] Department of Gastroenterology, Vall d'Hebron Hospital Universitari, Barcelona, Spain.
- [4] Management, Knowledge and Evaluation Unit, Vall d'Hebron Hospital Universitari, Barcelona, Spain.

- PMID: **33515625**
- PMCID: PMC7839383
- DOI: 10.1016/j.gastrohep.2020.11.018

Free PMC article
Observational Study

Impact of the COVID-19 pandemic on the activity of advanced-practice nurses on a reference unit for inflammatory bowel disease

[Article in English, Spanish]

Ester Navarro-Correal et al. Gastroenterol Hepatol. Aug-Sep 2021.
Free PMC article
Show details
Gastroenterol Hepatol
. Aug-Sep 2021;44(7):481-488.
doi: 10.1016/j.gastrohep.2020.11.018. Epub 2021 Jan 27.

Authors

Ester Navarro-Correal [1], Natalia Borruel [2], Virginia Robles [2], Claudia Herrera-de Guise [2], Luis Fernando Mayorga Ayala [2], Zahira Pérez Martínez [2], Arantxa Ibarz Casas [2], Sandra Agustino

Rodríguez [3], Irene Joana Batuecas Duelt [4], Jorge García Alcaide [4], Sara López Branchadell [4], Esperanza Zuriguel-Perez [4], Francesc Casellas [2]

Affiliations

- [1] Nurse and Health, Nurse and Physiotherapy, Department at Universitat de Lleida, Doctoral School, Lleida, Spain; Crohn-Colitis Care Unit, Vall d'Hebron Hospital Universitari, Barcelona, Spain. Electronic address: enavarro@vhebron.net.
- [2] Crohn-Colitis Care Unit, Vall d'Hebron Hospital Universitari, Barcelona, Spain.
- [3] Department of Gastroenterology, Vall d'Hebron Hospital Universitari, Barcelona, Spain.
- [4] Management, Knowledge and Evaluation Unit, Vall d'Hebron Hospital Universitari, Barcelona, Spain.

- PMID: **33515625**
- PMCID: PMC7839383
- DOI: 10.1016/j.gastrohep.2020.11.018

Abstract
in English, Spanish

Objective: To report the impact of the COVID-19 pandemic on the activity of nurses working on an inflammatory bowel disease (IBD) unit and to identify reasons for telehealth care and its relationship to certain characteristics.

Background: The COVID-19 pandemic had led to an increase in demand for remote care in patients with inflammatory bowel disease who require monitoring and frequent access to health services.

Design - methods: A retrospective study of all activity (in person and by phone call or email) done on the unit during the acute phase of the pandemic at a reference hospital in Spain. Numbers of activities done by nurses, reasons for telehealth care and sociodemographic and clinical data were collected. Statistical analysis was performed using frequency, chi-squared and analysis of variance tests.

Results: A total of 1095 activities for 561 patients who received care were reported. Among them, 1042 (95.2%) were telemedicine activities, amounting to a 47.3% increase over the prior year. COVID-19-related activities numbered 588 (59.5%). Consultations due to disease flare-up numbered 134 (13.7%), representing a 145% increase compared to 2019. Significant differences were found between reasons for using telemedicine and diagnosis, occupational status, contact week and treatment.

Conclusion: The acute phase of the pandemic has changed the activity managed by the nursing staff on the unit. Identifying and analysing these changes has yielded valuable information to achieve more efficient management and better care quality for patients in special situations.

Objetivos: Describir el impacto de la pandemia por COVID-19 en la actividad de la enfermera, en enfermedad inflamatoria intestinal (EII) de la unidad, e identificar las razones de la asistencia telemática y la relación con las variables recogidas.

Antecedentes: La pandemia de COVID-19 ha dado lugar a un aumento en la demanda de atención remota en pacientes con EII que requieren un seguimiento y un acceso frecuente a los servicios de salud.

Diseño y métodos: Estudio retrospectivo de toda la actividad (presencia, llamada telefónica o correo electrónico), realizado en la unidad durante la fase aguda de la pandemia, en un hospital de referencia en España. Se recogió el número de actividades llevadas a cabo por la enfermera, motivo de asistencia telemática y datos sociodemográficos y clínicos. El análisis estadístico se realizó utilizando la prueba de frecuencia, χ^2 y el análisis de varianza.

Resultados: Fueron registradas 1.095 actividades por 561 pacientes atendidos, siendo 1.042 (95,2%) actividades de telemedicina, lo que supuso un incremento del 47,3% con respecto al año anterior. Las demandas relacionadas con COVID-19 fueron 588 (59,5%). Por otro lado, 134 (13,7%) fueron consultas por brote de su enfermedad, un 145% más que en 2019. Se han encontrado diferencias significativas entre los motivos del uso de la telemedicina y el diagnóstico, la situación laboral, la semana en que se realizó el contacto y el tratamiento.

Conclusión: La fase aguda de la pandemia ha cambiado la actividad gestionada por enfermería en la unidad. Identificar y analizar estos cambios nos ha proporcionado información para conseguir una gestión más eficiente y de calidad al cuidado de los pacientes en situaciones excepcionales.

Keywords: Advanced-practice nurses; COVID-19; Colitis ulcerosa; Crohn's disease; Enfermedad de Crohn; Enfermedad inflamatoria intestinal; Enfermeras de práctica avanzada; Inflammatory bowel disease; Telehealth; Telemedicina; Telemedicine; Telesalud; Ulcerative colitis.

Copyright © 2021 The Author(s). Publicado por Elsevier España, S.L.U. All rights reserved.

- 30 references
- 3 figures

Supplementary info

Publication types, MeSH terms Expand

Publication types

- Observational Study

MeSH terms

- Adult
- Analysis of Variance
- COVID-19 / epidemiology*
- Chi-Square Distribution
- Colitis, Ulcerative / nursing*
- Crohn Disease / nursing*
- Electronic Mail / statistics & numerical data*
- Female
- Humans

- Male
- Middle Aged
- Pandemics*
- Retrospective Studies
- Spain / epidemiology
- Symptom Flare Up
- Telemedicine / methods
- Telemedicine / statistics & numerical data*
- Telephone / statistics & numerical data*

Full text links

Full text at GASTROENTEROLOGIA Y HEPATOLOGIA Ediciones Doyma, S.L. Free PMC article

Proceed to details

Cite

Share

1,160

Observational Study

Ther Adv Cardiovasc Dis

. Jan-Dec 2020;14:1753944720977732.

doi: 10.1177/1753944720977732.

The missing acute coronary syndromes in the COVID-19 era

Neil Ruparelia [1], Vasileios Panoulas [2, 3]

Affiliations Expand

Affiliations

- [1] Department of Cardiology, Royal Berkshire Hospital, London Road, Reading, Berkshire, RG1 5AN, UK.
- [2] Harefield Hospital, London, UK.
- [3] Imperial College London, UK.

- PMID: **33274695**
- PMCID: PMC7720298
- DOI: 10.1177/1753944720977732

Free PMC article

Observational Study

The missing acute coronary syndromes in the COVID-19 era

Neil Ruparelia et al. Ther Adv Cardiovasc Dis. Jan-Dec 2020.
Free PMC article
Show details
Ther Adv Cardiovasc Dis
. Jan-Dec 2020;14:1753944720977732.
doi: 10.1177/1753944720977732.

Authors

Neil Ruparelia [1], Vasileios Panoulas [2] [3]

Affiliations

- [1] Department of Cardiology, Royal Berkshire Hospital, London Road, Reading, Berkshire, RG1 5AN, UK.
- [2] Harefield Hospital, London, UK.
- [3] Imperial College London, UK.

- PMID: **33274695**
- PMCID: PMC7720298
- DOI: 10.1177/1753944720977732

Abstract

Aims: To determine whether the number of patients presenting with acute coronary syndromes has reduced during the COVID-19 pandemic.

Methods: Numbers of primary percutaneous coronary intervention (PPCI) activations, ST elevation myocardial infarctions (STEMIs) and non-ST elevation myocardial infarctions (NSTEMIs) in a large tertiary Greater London centre and a large district general hospital, both of which have on-site heart attack centres, were collected. We compared the number of PPCI activations, STEMI, NSTEMIs and all MIs prior to the COVID-19 era (January to third week of February 2020), after the start of some COVID-19 restrictions taking place (fourth week of February 2020) and after formal instruction by the United Kingdom Government that all citizens were to observe strict social distancing measures (20 March 2020). We further obtained data for the corresponding weekly figures from 2019.

Results: The average weekly figure of all myocardial infarction in 2020, prior to the COVID-19 social distancing restrictions/awareness in the UK (beginning of January to third week of February), did not differ when compared with corresponding weeks in 2019 (23.3 ± 5.4 in 2019 *versus* 21.13 ± 3.5, $p = 0.411$).With increased media reporting and associated public awareness of the threat of COVID-19 (last week of February), there was a significant reduction in all myocardial infarction (27.1 ± 4.7 in 2019 *versus* 15.9 ± 3.6 in 2020, $p < 0.001$). Following official governmental instruction that mandated strict social distancing and the 'stay at home' campaign, the weekly figures of STEMI (15 ± 3.5 in 2019 *versus* 10 ± 4.4 in 2020, $p = 0.013$), NSTEMI (13

± 2.6 in 2019 *versus* 4.7 ± 2.3 in 2020, $p = 0.038$) and all myocardial infarction (28 ± 6.1 in 2019 *versus* 14.7 ± 5.7 in 2020, $p = 0.008$) have remained significantly reduced.

Conclusion: We have observed an unexpected major decline in presentations (and treatment) of the entire spectrum of acute coronary syndromes following the beginning of the COVID-19 pandemic and nationwide public-health measures that have promoted the importance of strict social distancing and self-quarantine.

Keywords: COVID-19; NSTEMI; STEMI; acute coronary syndrome; myocardial infarction.

Conflict of interest statement

Conflict of interest statement: The authors declare that there is no conflict of interest.

- 12 references
- 2 figures

Supplementary info

Publication types, MeSH terms Expand

Publication types

- Comparative Study
- Multicenter Study
- Observational Study

MeSH terms

- Acute Coronary Syndrome / diagnosis
- Acute Coronary Syndrome / epidemiology*
- Acute Coronary Syndrome / therapy
- Aged
- Aged, 80 and over
- COVID-19 / diagnosis
- COVID-19 / epidemiology*
- Female
- Heart Disease Risk Factors
- Humans
- London / epidemiology
- Male
- Non-ST Elevated Myocardial Infarction / diagnosis
- Non-ST Elevated Myocardial Infarction / epidemiology*
- Non-ST Elevated Myocardial Infarction / therapy
- Percutaneous Coronary Intervention / trends
- Retrospective Studies

- ST Elevation Myocardial Infarction / diagnosis
- ST Elevation Myocardial Infarction / epidemiology*
- ST Elevation Myocardial Infarction / therapy
- Time Factors

Full text links

SAGE journals Open access full text Atypon Free PMC article
Proceed to details
Cite
Share
1,161

Observational Study
Int J Antimicrob Agents
. 2021 Feb;57(2):106247.
doi: 10.1016/j.ijantimicag.2020.106247. Epub 2020 Nov 28.

Hydroxychloroquine lung pharmacokinetics in critically ill patients with COVID-19

S Ruiz [1], D Concordet [2], T Lanot [3], B Georges [4], P Goudy [4], S Baklouti [3], C Mané [3], E Losha [3], H Vinour [5], D Rousset [6], M Lavit [3], V Minville [4], J-M Conil [4], P Gandia [7]
Affiliations Expand

Affiliations

- [1] CHU de Toulouse, Réanimation Polyvalente Hôpital Rangueil, Pôle d'Anesthésie-Réanimation, 1 avenue du Professeur Jean Poulhès, 31059, Toulouse cedex 9, France. Electronic address: ruiz.stephanie@chu-toulouse.fr.
- [2] INTHERES, Université de Toulouse, INRA, ENVT, 23 Chemin des Capelles, BP 87614, 31076, Toulouse cedex 3, France.
- [3] CHU de Toulouse, Laboratoire de Pharmacocinétique et Toxicologie Clinique, Institut Fédératif de Biologie, 330 avenue de Grande-Bretagne, 31059, Toulouse cedex 9, France.
- [4] CHU de Toulouse, Réanimation Polyvalente Hôpital Rangueil, Pôle d'Anesthésie-Réanimation, 1 avenue du Professeur Jean Poulhès, 31059, Toulouse cedex 9, France.
- [5] CHU de Toulouse, Réanimation Polyvalente URM, Pôle d'Anesthésie-Réanimation, 330 avenue de Grande-Bretagne, 31059, Toulouse cedex 9, France.
- [6] CHU de Toulouse, Réanimation Neurochirurgicale, Pôle d'Anesthésie-Réanimation, 330 avenue de Grande-Bretagne, 31059, Toulouse cedex 9, France.
- [7] INTHERES, Université de Toulouse, INRA, ENVT, 23 Chemin des Capelles, BP 87614, 31076, Toulouse cedex 3, France; CHU de Toulouse, Laboratoire de Pharmacocinétique et Toxicologie Clinique, Institut Fédératif de Biologie, 330 avenue de Grande-Bretagne, 31059, Toulouse cedex 9, France.

- PMID: **33259916**
- PMCID: PMC7698654

- DOI: 10.1016/j.ijantimicag.2020.106247

Free PMC article
Observational Study

Hydroxychloroquine lung pharmacokinetics in critically ill patients with COVID-19

S Ruiz et al. Int J Antimicrob Agents. 2021 Feb.
Free PMC article
Show details
Int J Antimicrob Agents
. 2021 Feb;57(2):106247.
doi: 10.1016/j.ijantimicag.2020.106247. Epub 2020 Nov 28.

Authors

S Ruiz [1], D Concordet [2], T Lanot [3], B Georges [4], P Goudy [4], S Baklouti [3], C Mané [3], E Losha [3], H Vinour [5], D Rousset [6], M Lavit [3], V Minville [4], J-M Conil [4], P Gandia [7]

Affiliations

- [1] CHU de Toulouse, Réanimation Polyvalente Hôpital Rangueil, Pôle d'Anesthésie-Réanimation, 1 avenue du Professeur Jean Poulhès, 31059, Toulouse cedex 9, France. Electronic address: ruiz.stephanie@chu-toulouse.fr.
- [2] INTHERES, Université de Toulouse, INRA, ENVT, 23 Chemin des Capelles, BP 87614, 31076, Toulouse cedex 3, France.
- [3] CHU de Toulouse, Laboratoire de Pharmacocinétique et Toxicologie Clinique, Institut Fédératif de Biologie, 330 avenue de Grande-Bretagne, 31059, Toulouse cedex 9, France.
- [4] CHU de Toulouse, Réanimation Polyvalente Hôpital Rangueil, Pôle d'Anesthésie-Réanimation, 1 avenue du Professeur Jean Poulhès, 31059, Toulouse cedex 9, France.
- [5] CHU de Toulouse, Réanimation Polyvalente URM, Pôle d'Anesthésie-Réanimation, 330 avenue de Grande-Bretagne, 31059, Toulouse cedex 9, France.
- [6] CHU de Toulouse, Réanimation Neurochirurgicale, Pôle d'Anesthésie-Réanimation, 330 avenue de Grande-Bretagne, 31059, Toulouse cedex 9, France.
- [7] INTHERES, Université de Toulouse, INRA, ENVT, 23 Chemin des Capelles, BP 87614, 31076, Toulouse cedex 3, France; CHU de Toulouse, Laboratoire de Pharmacocinétique et Toxicologie Clinique, Institut Fédératif de Biologie, 330 avenue de Grande-Bretagne, 31059, Toulouse cedex 9, France.

- PMID: **33259916**
- PMCID: PMC7698654
- DOI: 10.1016/j.ijantimicag.2020.106247

Abstract

Different dosage regimens of hydroxychloroquine (HCQ) have been used to manage COVID-19 (coronavirus disease 2019) patients, with no information on lung exposure in this population. The

aim of our study was to evaluate HCQ concentrations in the lung epithelial lining fluid (ELF) in patients infected with SARS-CoV-2 (severe acute respiratory syndrome coronavirus 2), the virus that causes COVID-19. This was a retrospective, observational, multicentre, pharmacokinetic study of HCQ in critically ill COVID-19 patients. No additional interventions or additional samples compared with standard care of these patients were conducted in our teaching hospital. We included all intubated COVID-19 patients treated with crushed HCQ tablets, regardless of the dosage administered by nasogastric tube. Blood and bronchoalveolar lavage samples (n = 28) were collected from 22 COVID-19 patients and total HCQ concentrations in ELF were estimated. Median (interquartile range) HCQ plasma concentrations were 0.09 (0.06-0.14) mg/L and 0.07 (0.05-0.08) mg/L for 400 mg × 1/day and 200 mg × 3/day, respectively. Median HCQ ELF concentrations were 3.74 (1.10-7.26) mg/L and 1.81 (1.20-7.25) for 400 mg × 1/day and 200 mg × 3/day, respectively. The median ratio of ELF/plasma concentrations was 40.0 (7.3-162.7) and 21.2 (18.4-109.5) for 400 mg × 1/day and 200 mg × 3/day, respectively. ELF exposure is likely to be underestimated from HCQ concentrations in plasma. In clinical practice, low plasma concentrations should not induce an increase in drug dosage because lung exposure may already be high.

Keywords: BAL; Bronchoalveolar lavage; COVID-19; Hydroxychloroquine; Plasma drug monitoring.

Copyright © 2020 Elsevier Ltd and International Society of Antimicrobial Chemotherapy. All rights reserved.

- 29 references
- 1 figure

Supplementary info

Publication types, MeSH terms, Substances Expand

Publication types

- Multicenter Study
- Observational Study

MeSH terms

- Adult
- Aged
- Aged, 80 and over
- Antiviral Agents / administration & dosage
- Antiviral Agents / blood
- Antiviral Agents / pharmacokinetics*
- Bronchoalveolar Lavage Fluid / chemistry
- COVID-19 / drug therapy*
- Critical Illness
- Female
- Humans

- Hydroxychloroquine / administration & dosage
- Hydroxychloroquine / blood
- Hydroxychloroquine / pharmacokinetics*
- Intubation, Gastrointestinal
- Lung / drug effects
- Lung / virology
- Male
- Middle Aged
- Retrospective Studies
- Tablets / administration & dosage
- Tablets / pharmacokinetics

Substances

- Antiviral Agents
- Tablets
- Hydroxychloroquine

Full text links

ELSEVIER FULL-TEXT ARTICLE Elsevier Science Free PMC article

Proceed to details

Cite

Share

☐ 1,162

Observational Study

Br J Anaesth

. 2020 Dec;125(6):e480-e483.

doi: 10.1016/j.bja.2020.08.047. Epub 2020 Sep 3.

Outcomes in mechanically ventilated patients with hypoxaemic respiratory failure caused by COVID-19

Luigi Camporota [1], Barnaby Sanderson [2], Alison Dixon [2], Francesco Vasques [2], Andrew Jones [2], Manu Shankar-Hari [3]

Affiliations Expand

Affiliations

- [1] Guy's and St Thomas' NHS Foundation Trust, St Thomas' Hospital, London, UK; Centre of Human Applied Physiological Sciences, King's College London, London, UK. Electronic address: luigi.camporota@gstt.nhs.uk.
- [2] Guy's and St Thomas' NHS Foundation Trust, St Thomas' Hospital, London, UK.
- [3] Guy's and St Thomas' NHS Foundation Trust, St Thomas' Hospital, London, UK; School of Immunology & Microbial Sciences, King's College London, London, UK.

- PMID: **32962855**
- PMCID: PMC7470709
- DOI: 10.1016/j.bja.2020.08.047

Free PMC article
Observational Study

Outcomes in mechanically ventilated patients with hypoxaemic respiratory failure caused by COVID-19

Luigi Camporota et al. Br J Anaesth. 2020 Dec.
Free PMC article
Show details
Br J Anaesth
. 2020 Dec;125(6):e480-e483.
doi: 10.1016/j.bja.2020.08.047. Epub 2020 Sep 3.

Authors

Luigi Camporota [1], Barnaby Sanderson [2], Alison Dixon [2], Francesco Vasques [2], Andrew Jones [2], Manu Shankar-Hari [3]

Affiliations

- [1] Guy's and St Thomas' NHS Foundation Trust, St Thomas' Hospital, London, UK; Centre of Human Applied Physiological Sciences, King's College London, London, UK. Electronic address: luigi.camporota@gstt.nhs.uk.
- [2] Guy's and St Thomas' NHS Foundation Trust, St Thomas' Hospital, London, UK.
- [3] Guy's and St Thomas' NHS Foundation Trust, St Thomas' Hospital, London, UK; School of Immunology & Microbial Sciences, King's College London, London, UK.

- PMID: **32962855**
- PMCID: PMC7470709
- DOI: 10.1016/j.bja.2020.08.047

No abstract available

Keywords: ARDS; COVID-10; SARS-CoV-2; critical care; mechanical ventilation; outcomes; respiratory failure.

- 8 references
- 1 figure

Supplementary info

Publication types, MeSH terms Expand

Publication types

- Letter
- Observational Study

MeSH terms

- COVID-19
- Coronavirus Infections / complications*
- Coronavirus Infections / mortality
- Coronavirus Infections / therapy*
- Female
- Hospital Mortality
- Humans
- Hypoxia / etiology*
- Hypoxia / mortality
- Hypoxia / therapy*
- Intensive Care Units
- Male
- Middle Aged
- Pandemics
- Pneumonia, Viral / complications*
- Pneumonia, Viral / mortality
- Pneumonia, Viral / therapy*
- Respiration, Artificial / methods*
- Respiratory Distress Syndrome / complications
- Respiratory Distress Syndrome / therapy
- Respiratory Function Tests
- Respiratory Insufficiency / etiology*
- Respiratory Insufficiency / mortality
- Respiratory Insufficiency / therapy*
- Retrospective Studies
- Treatment Outcome

Full text links

ELSEVIER OPEN ACCESS Elsevier Science Free PMC article
Proceed to details
Cite
Share
1,163

Observational Study
Eur J Vasc Endovasc Surg
. 2021 Apr;61(4):688-697.
doi: 10.1016/j.ejvs.2021.01.037. Epub 2021 Mar 13.

Regional Survey in Lombardy, Northern Italy, on Vascular Surgery Intervention Outcomes During The COVID-19 Pandemic

Raffaello Bellosta [1], Gabriele Piffaretti [2], Stefano Bonardelli [3], Patrizio Castelli [4], Roberto Chiesa [5], Dalmazio Frigerio [6], Gaetano Lanza [7], Stefano Pirrelli [8], Giovanni Rossi [9], Santi Trimarchi [10], Lombardy Covid-19 Vascular Study Group
Collaborators, Affiliations Expand

Collaborators

- **Lombardy Covid-19 Vascular Study Group**:
Franco Briolini [8], Pietro Cefali [8], Roberto Caronno [11], Aldo Arzini [12], Domenico Diaco [12], Vittorio Baratta [13], Stefano Aiello [13], Alessandro C L Molinari [14], Francesca Giovannini [15], Anna Maria Socrate [16], Matteo Ferraris [16], Antonino Silvestro [17], Gianluca Canu [18], Emidio Costantini [19], Davide Logaldo [19], Federico Romani [20], Alfredo Lista [20], Cristina Busoni [21], Marco Setti [22], Roberto Mezzetti [23], Piergiorgio Sala [24], Luca Bassi [24], Luca Luzzani [25], Matteo A Pegorer [25], Luca Attisani [25], Claudio Carugati [26], Monica Vescovi [26], Piero Trabattoni [27], Stefano Zoli [27], Andrea Rignano [28], Clara Magri [28], Pierluigi Vandone [29], Sergio Losa [30], Efrem Civilini [31], Giovanni Nano [32], Daniela Mazzaccaro [32], Valerio Tolva [33], Jessica Lanza [34], Ruggiero Curci [35], Giovanna Simonetti [35], Chiara Lomazzi [36], Viviana Grassi [36], Daniele Bissacco [36], Andrea Kahlberg [37], Daniele Mascia [37], Raffaello Dallatana [38], Michele Carmo [38], Franco Ragni [39], Enrico M Marone [39], Antonio Bozzani [39], Matteo Tozzi [40], Marco Franchin [40], Gianluca Lussardi [41], Vittorio Segramora [42], Gaetano Deleo [42], Matteo Crippa [43], Tiziano Porretta [44], Marco Viani [44], Silvia Stegher [44], Davide Foresti [44], Giovanni Bonalumi [45]

Affiliations

- [1] Vascular Surgery, Cardiovascular Department, Poliambulanza Foundation, Brescia, Italy.
- [2] Vascular Surgery - Department of Medicine and Surgery, University of Insubria School of Medicine, ASST Settelaghi Circolo University Teaching Hospital, via F. Guicciardini 9, 21100, Varese, Italy. Electronic address: gabriele.piffaretti@uninsubria.it.

- [3] Vascular Surgery, Department of Surgery, Spedali Civili University Teaching Hospital, University of Brescia School of Medicine, Brescia, Italy.
- [4] Vascular Surgery - Department of Medicine and Surgery, University of Insubria School of Medicine, ASST Settelaghi Circolo University Teaching Hospital, via F. Guicciardini 9, 21100, Varese, Italy.
- [5] Vascular Surgery, University "Vita Salute" San Raffaele Institute, Milan, Italy.
- [6] Vascular Surgery, ASST Vimercate, Vimercate, Italy.
- [7] Vascular Surgery, IRCCS Multimedica, Castellanza, Italy.
- [8] Vascular Surgery - Cardiovascular Department, ASST Papa Giovanni XXIII, Bergamo, Italy.
- [9] Vascular Surgery, Cardiovascular Department, "Alessandro Manzoni" Hospital, Lecco, Italy.
- [10] Vascular Surgery, Fondazione IRCCS Cà Granda, Ospedale Maggiore Policlinico, Milan, Italy; Department of Clinical and Community Sciences, University of Milano, Milan, Italy.
- [11] Vascular Surgery - ASST Lariana Como "Sant'Anna, Como, Italy.
- [12] Vascular Surgery - ASST Crema "Ospedale Maggiore, Crema, Italy.
- [13] Vascular Surgery - ASST di Cremona, Cremona, Italy.
- [14] Vascular Surgery - Cardiovascular Department, "Alessandro Manzoni" Hospital, Lecco, Italy.
- [15] Vascular Surgery - ASST Mantova "Carlo Poma, Mantova, Italy.
- [16] Vascular Surgery - ASST Ovest Milano Legnano "Civile", Legnano, Italy.
- [17] Vascular Surgery - ASST Rhodense, Rho Milano, Italy.
- [18] Vascular Surgery - ASST Valtellina Sondalo, Sondalo, Italy.
- [19] Vascular Surgery - ASST Valle Olona, Busto Arsizio, Italy.
- [20] Vascular Surgery - ASST Grande Ospedale Metropolitano Niguarda, Milano, Italy.
- [21] Vascular Surgery - ASST Vimercate, Vimercate, Italy.
- [22] Vascular Surgery - IRCCS Humanitas Gavazzeni, Bergamo, Italy.
- [23] Vascular Surgery - IRCCS Policlinico "San Marco", Zingonia, Italy.
- [24] Vascular Surgery - Clinica "Sant' Anna", Brescia, Italy.
- [25] Vascular Surgery - Cardiovascular Department, Poliambulanza Foundation, Brescia, Italy.
- [26] Vascular Surgery - Clinica "San Carlo", Paderno Dugnano, Italy.
- [27] Vascular Surgery - IRCCS Centro Cardiologico Monzino, Milano, Italy.
- [28] Vascular Surgery - IRCCS Istituto Clinico "Sant'Ambrogio", Milano, Italy.
- [29] Vascular Surgery - IRCCS Istituto Ortopedico Galeazzi, Milano, Italy.
- [30] Vascular Surgery - IRCCS Multimedica, Sesto San Giovanni, Italy.
- [31] Vascular Surgery - IRCCS Humanitas, Rozzano, Italy.
- [32] Vascular Surgery - IRCCS Policlinico San Donato, San Donato Milanese, Italy.
- [33] Vascular Surgery - Ospedale Policlinico, Monza, Italy.
- [34] Vascular Surgery - IRCCS Multimedica, Castellanza, Italy.
- [35] Vascular Surgery - ASST "Ospedale Maggiore", Lodi, Italy.
- [36] Vascular Surgery - Fondazione IRCCS Cà Granda, Ospedale Maggiore Policlinico, Milan, Italy.
- [37] Vascular Surgery - University "Vita Salute" San Raffaele Institute, Milan, Italy.
- [38] Vascular Surgery - Ospedale "San Carlo Borromeo", Milan, Italy.
- [39] Vascular Surgery - Fondazione IRCCS Policlinico San Matteo, Pavia, Italy.

- [40] Vascular Surgery - Department of Medicine and Surgery, University of Insubria School of Medicine, Varese, Italy.
- [41] Vascular Surgery - Department of Surgery, Spedali Civili University Teaching Hospital, University of Brescia School of Medicine, Brescia, Italy.
- [42] Vascular Surgery - ASST Monza Ospedale "San Gerardo", Monza Brianza, Italy.
- [43] Vascular Surgery - ASST "Santi Paolo e Carlo", Milan, Italy.
- [44] Vascular Surgery - ASST Fatebenefratelli Sacco, Milan, Italy.
- [45] Vascular Surgery - Istituto di Cura Città di Pavia, Pavia, Italy.

- PMID: **33722483**
- DOI: 10.1016/j.ejvs.2021.01.037

Observational Study

Regional Survey in Lombardy, Northern Italy, on Vascular Surgery Intervention Outcomes During The COVID-19 Pandemic

Raffaello Bellosta et al. Eur J Vasc Endovasc Surg. 2021 Apr.

Show details

Eur J Vasc Endovasc Surg

. 2021 Apr;61(4):688-697.
doi: 10.1016/j.ejvs.2021.01.037. Epub 2021 Mar 13.

Authors

Raffaello Bellosta [1], Gabriele Piffaretti [2], Stefano Bonardelli [3], Patrizio Castelli [4], Roberto Chiesa [5], Dalmazio Frigerio [6], Gaetano Lanza [7], Stefano Pirrelli [8], Giovanni Rossi [9], Santi Trimarchi [10], Lombardy Covid-19 Vascular Study Group

Collaborators

- **Lombardy Covid-19 Vascular Study Group**: Franco Briolini [8], Pietro Cefali [8], Roberto Caronno [11], Aldo Arzini [12], Domenico Diaco [12], Vittorio Baratta [13], Stefano Aiello [13], Alessandro C L Molinari [14], Francesca Giovannini [15], Anna Maria Socrate [16], Matteo Ferraris [16], Antonino Silvestro [17], Gianluca Canu [18], Emidio Costantini [19], Davide Logaldo [19], Federico Romani [20], Alfredo Lista [20], Cristina Busoni [21], Marco Setti [22], Roberto Mezzetti [23], Piergiorgio Sala [24], Luca Bassi [24], Luca Luzzani [25], Matteo A Pegorer [25], Luca Attisani [25], Claudio Carugati [26], Monica Vescovi [26], Piero Trabattoni [27], Stefano Zoli [27], Andrea Rignano [28], Clara Magri [28], Pierluigi Vandone [29], Sergio Losa [30], Efrem Civilini [31], Giovanni Nano [32], Daniela Mazzaccaro [32], Valerio Tolva [33], Jessica Lanza [34], Ruggiero Curci [35], Giovanna Simonetti [35], Chiara Lomazzi [36], Viviana Grassi [36], Daniele Bissacco [36], Andrea Kahlberg [37], Daniele Mascia [37], Raffaello Dallatana [38], Michele Carmo [38], Franco Ragni [39], Enrico M Marone [39], Antonio Bozzani [39], Matteo Tozzi [40], Marco

Franchin[40], Gianluca Lussardi[41], Vittorio Segramora[42], Gaetano Deleo[42], Matteo Crippa[43], Tiziano Porretta[44], Marco Viani[44], Silvia Stegher[44], Davide Foresti [44], Giovanni Bonalumi[45]

Affiliations

- [1] Vascular Surgery, Cardiovascular Department, Poliambulanza Foundation, Brescia, Italy.
- [2] Vascular Surgery - Department of Medicine and Surgery, University of Insubria School of Medicine, ASST Settelaghi Circolo University Teaching Hospital, via F. Guicciardini 9, 21100, Varese, Italy. Electronic address: gabriele.piffaretti@uninsubria.it.
- [3] Vascular Surgery, Department of Surgery, Spedali Civili University Teaching Hospital, University of Brescia School of Medicine, Brescia, Italy.
- [4] Vascular Surgery - Department of Medicine and Surgery, University of Insubria School of Medicine, ASST Settelaghi Circolo University Teaching Hospital, via F. Guicciardini 9, 21100, Varese, Italy.
- [5] Vascular Surgery, University "Vita Salute" San Raffaele Institute, Milan, Italy.
- [6] Vascular Surgery, ASST Vimercate, Vimercate, Italy.
- [7] Vascular Surgery, IRCCS Multimedica, Castellanza, Italy.
- [8] Vascular Surgery - Cardiovascular Department, ASST Papa Giovanni XXIII, Bergamo, Italy.
- [9] Vascular Surgery, Cardiovascular Department, "Alessandro Manzoni" Hospital, Lecco, Italy.
- [10] Vascular Surgery, Fondazione IRCCS Cà Granda, Ospedale Maggiore Policlinico, Milan, Italy; Department of Clinical and Community Sciences, University of Milano, Milan, Italy.
- [11] Vascular Surgery - ASST Lariana Como "Sant'Anna, Como, Italy.
- [12] Vascular Surgery - ASST Crema "Ospedale Maggiore, Crema, Italy.
- [13] Vascular Surgery - ASST di Cremona, Cremona, Italy.
- [14] Vascular Surgery - Cardiovascular Department, "Alessandro Manzoni" Hospital, Lecco, Italy.
- [15] Vascular Surgery - ASST Mantova "Carlo Poma, Mantova, Italy.
- [16] Vascular Surgery - ASST Ovest Milano Legnano "Civile", Legnano, Italy.
- [17] Vascular Surgery - ASST Rhodense, Rho Milano, Italy.
- [18] Vascular Surgery - ASST Valtellina Sondalo, Sondalo, Italy.
- [19] Vascular Surgery - ASST Valle Olona, Busto Arsizio, Italy.
- [20] Vascular Surgery - ASST Grande Ospedale Metropolitano Niguarda, Milano, Italy.
- [21] Vascular Surgery - ASST Vimercate, Vimercate, Italy.
- [22] Vascular Surgery - IRCCS Humanitas Gavazzeni, Bergamo, Italy.
- [23] Vascular Surgery - IRCCS Policlinico "San Marco", Zingonia, Italy.
- [24] Vascular Surgery - Clinica "Sant' Anna", Brescia, Italy.
- [25] Vascular Surgery - Cardiovascular Department, Poliambulanza Foundation, Brescia, Italy.
- [26] Vascular Surgery - Clinica "San Carlo", Paderno Dugnano, Italy.
- [27] Vascular Surgery - IRCCS Centro Cardiologico Monzino, Milano, Italy.
- [28] Vascular Surgery - IRCCS Istituto Clinico "Sant'Ambrogio", Milano, Italy.
- [29] Vascular Surgery - IRCCS Istituto Ortopedico Galeazzi, Milano, Italy.
- [30] Vascular Surgery - IRCCS Multimedica, Sesto San Giovanni, Italy.
- [31] Vascular Surgery - IRCCS Humanitas, Rozzano, Italy.

- [32] Vascular Surgery - IRCCS Policlinico San Donato, San Donato Milanese, Italy.
- [33] Vascular Surgery - Ospedale Policlinico, Monza, Italy.
- [34] Vascular Surgery - IRCCS Multimedica, Castellanza, Italy.
- [35] Vascular Surgery - ASST "Ospedale Maggiore", Lodi, Italy.
- [36] Vascular Surgery - Fondazione IRCCS Cà Granda, Ospedale Maggiore Policlinico, Milan, Italy.
- [37] Vascular Surgery - University "Vita Salute" San Raffaele Institute, Milan, Italy.
- [38] Vascular Surgery - Ospedale "San Carlo Borromeo", Milan, Italy.
- [39] Vascular Surgery - Fondazione IRCCS Policlinico San Matteo, Pavia, Italy.
- [40] Vascular Surgery - Department of Medicine and Surgery, University of Insubria School of Medicine, Varese, Italy.
- [41] Vascular Surgery - Department of Surgery, Spedali Civili University Teaching Hospital, University of Brescia School of Medicine, Brescia, Italy.
- [42] Vascular Surgery - ASST Monza Ospedale "San Gerardo", Monza Brianza, Italy.
- [43] Vascular Surgery - ASST "Santi Paolo e Carlo", Milan, Italy.
- [44] Vascular Surgery - ASST Fatebenefratelli Sacco, Milan, Italy.
- [45] Vascular Surgery - Istituto di Cura Città di Pavia, Pavia, Italy.

- PMID: **33722483**
- DOI: 10.1016/j.ejvs.2021.01.037

Abstract

Objective: The characteristics and outcomes of patients undergoing vascular surgery hospitalised and managed in Lombardy are described with a comparison of patients tested positive for COVID-19 (CV19-pos) vs. those tested negative (CV19-neg).

Methods: This was a multicentre, retrospective, observational cohort study which involved all vascular surgery services in Lombardy, Northern Italy. Data were retrospectively merged into a combined dataset covering the nine weeks of the Italian COVID-19 pandemic phase 1 (8 March 2020 to 3 May 2020). The primary outcome was freedom from in hospital death, secondary outcomes were re-thrombosis rate after peripheral revascularisation, and freedom from post-operative complication.

Results: Among 674 patients managed during the outbreak, 659 (97.8%) were included in the final analysis: 121 (18.4%) were CV19-pos. CV19-pos status was associated with a higher rate of complications (OR 4.5; $p < .001$, 95% CI 2.64 - 7.84), and a higher rate of re-thrombosis after peripheral arterial revascularisation (OR 2.2; $p = .004$, 95% CI 1.29 - 3.88). In hospital mortality was higher in CV19-pos patients (24.8% vs. 5.6%; OR 5.4, $p < .001$;95% CI 2.86 - 8.92). Binary logistic regression analysis identified CV19-pos status (OR 7.6; $p < .001$, 95% CI 3.75 - 15.28) and age > 80 years (OR 3.2; $p = .001$, 95% CI 1.61 - 6.57) to be predictors of in hospital death.

Conclusion: In this experience of the vascular surgery group of Lombardy, COVID-19 infection was a marker of poor outcomes in terms of mortality and post-operative complications for patients undergoing vascular surgery treatments.

Keywords: COVID-19; acute limb ischaemia; vascular surgery activities.

Copyright © 2021 European Society for Vascular Surgery. Published by Elsevier B.V. All rights reserved.

Comment in

- Riding the First Covid-19 Wave: Vascular Surgery Lessons from Lombardy, Italy.
  Prendes CF, Bellmunt-Montoya S. Prendes CF, et al. Eur J Vasc Endovasc Surg. 2021 Apr;61(4):698. doi: 10.1016/j.ejvs.2021.01.001. Epub 2021 Jan 9. Eur J Vasc Endovasc Surg. 2021. PMID: 33483218 Free PMC article. No abstract available.

Supplementary info

Publication types, MeSH terms Expand

Publication types

- Multicenter Study
- Observational Study

MeSH terms

- Aged
- Aged, 80 and over
- COVID-19*
- Cohort Studies
- Female
- Health Care Surveys
- Humans
- Italy
- Male
- Middle Aged
- Postoperative Complications / epidemiology*
- Retrospective Studies
- Treatment Outcome
- Vascular Surgical Procedures*

Full text links

ELSEVIER FULL-TEXT ARTICLE Elsevier Science
Proceed to details
Cite
Share
☐ 1,164
Observational Study
Cancer
. 2020 Sep 1;126(17):4023-4031.
doi: 10.1002/cncr.33042. Epub 2020 Jun 23.

Outcomes of novel coronavirus disease 2019 (COVID-19) infection in 107 patients with cancer from Wuhan, China

Hongyan Zhang [1, 2], Linwei Wang [1, 2], Yuanyuan Chen [1, 2], Qiuji Wu [1, 2], Gaili Chen [1, 2], Xiaokun Shen [3], Qun Wang [4], Youqin Yan [5], Yi Yu [6], Yahua Zhong [1, 2], Xinghuan Wang [7, 8], Melvin L K Chua [1, 2, 9, 10, 11], Conghua Xie [1, 2]
Affiliations Expand

Affiliations

- [1] Department of Radiation and Medical Oncology, Zhongnan Hospital of Wuhan University, Wuhan, China.
- [2] Hubei Key Laboratory of Tumor Biological Behaviors, Hubei Cancer Clinical Study Center, Wuhan, China.
- [3] Convalife (Shanghai) Company Ltd, Shanghai, China.
- [4] Department of Oncology, Fifth Hospital of Wuhan, Wuhan, China.
- [5] Department of Infection, Seventh Hospital of Wuhan, Wuhan, China.
- [6] Department of Oncology, Han Kou Hospital of Wuhan, Wuhan, China.
- [7] Center for Evidence-based and Translational Medicine, Zhongnan Hospital of Wuhan University, Wuhan, China.
- [8] Department of Urology, Zhongnan Hospital of Wuhan University, Wuhan, China.
- [9] Division of Radiation Oncology, National Cancer Center Singapore, Singapore, Singapore.
- [10] Division of Medical Sciences, National Cancer Center Singapore, Singapore, Singapore.
- [11] Oncology Academic Programme, Duke-NUS Medical School, Singapore, Singapore.

- PMID: **32573776**
- PMCID: PMC7361610
- DOI: 10.1002/cncr.33042

Free PMC article
Observational Study

Outcomes of novel coronavirus disease 2019 (COVID-19) infection in 107 patients with cancer from Wuhan, China

Hongyan Zhang et al. Cancer. 2020.
Free PMC article
Show details
Cancer
. 2020 Sep 1;126(17):4023-4031.
doi: 10.1002/cncr.33042. Epub 2020 Jun 23.

Authors

Hongyan Zhang [1, 2], Linwei Wang [1, 2], Yuanyuan Chen [1, 2], Qiuji Wu [1, 2], Gaili Chen [1, 2], Xiaokun Shen [3], Qun Wang [4], Youqin Yan [5], Yi Yu [6], Yahua Zhong [1, 2], Xinghuan Wang [7, 8], Melvin L K Chua [1, 2, 9, 10, 11], Conghua Xie [1, 2]

Affiliations

- [1] Department of Radiation and Medical Oncology, Zhongnan Hospital of Wuhan University, Wuhan, China.
- [2] Hubei Key Laboratory of Tumor Biological Behaviors, Hubei Cancer Clinical Study Center, Wuhan, China.
- [3] Convalife (Shanghai) Company Ltd, Shanghai, China.
- [4] Department of Oncology, Fifth Hospital of Wuhan, Wuhan, China.
- [5] Department of Infection, Seventh Hospital of Wuhan, Wuhan, China.
- [6] Department of Oncology, Han Kou Hospital of Wuhan, Wuhan, China.
- [7] Center for Evidence-based and Translational Medicine, Zhongnan Hospital of Wuhan University, Wuhan, China.
- [8] Department of Urology, Zhongnan Hospital of Wuhan University, Wuhan, China.
- [9] Division of Radiation Oncology, National Cancer Center Singapore, Singapore, Singapore.
- [10] Division of Medical Sciences, National Cancer Center Singapore, Singapore, Singapore.
- [11] Oncology Academic Programme, Duke-NUS Medical School, Singapore, Singapore.

- PMID: **32573776**
- PMCID: PMC7361610
- DOI: 10.1002/cncr.33042

Abstract

Background: Patients with cancer have a higher risk of coronavirus disease 2019 (COVID-19) than noncancer patients. The authors conducted a multicenter retrospective study to investigate the clinical manifestations and outcomes of patients with cancer who are diagnosed with COVID-19.

Methods: The authors reviewed the medical records of hospitalized patients who were treated at 5 hospitals in Wuhan City, China, between January 5 and March 18, 2020. Clinical parameters relating to cancer history (type and treatment) and COVID-19 were collected. The primary outcome was overall survival (OS). Secondary analyses were the association between clinical factors and severe COVID-19 and OS.

Results: A total of 107 patients with cancer were diagnosed with COVID-19, with a median age of 66 years (range, 37-98 years). Lung (21 patients; 19.6%), gastrointestinal (20 patients; 18.7%), and genitourinary (20 patients; 18.7%) cancers were the most common cancer diagnoses. A total of 37 patients (34.6%) were receiving active anticancer treatment when diagnosed with COVID-19, whereas 70 patients (65.4%) were on follow-up. Overall, 52.3% of patients (56 patients) developed severe COVID-19; this rate was found to be higher among patients receiving anticancer treatment than those on follow-up (64.9% vs 45.7%), which corresponded to an inferior OS in the former subgroup of patients (hazard ratio, 3.365; 95% CI, 1.455-7.782 [P = .005]). The detrimental effect of anticancer treatment on OS was found to be independent of exposure to systemic therapy (case fatality rate of 33.3% [systemic therapy] vs 43.8% [nonsystemic therapy]).

Conclusions: The results of the current study demonstrated that >50.0% of infected patients with cancer are susceptible to severe COVID-19. This risk is aggravated by simultaneous anticancer treatment and portends for a worse survival, despite treatment for COVID-19.

Keywords: anticancer treatment; cancer; case fatality rate; coronavirus disease 2019 (COVID-19); systemic therapy.

© 2020 The Authors. Cancer published by Wiley Periodicals LLC on behalf of American Cancer Society.

Conflict of interest statement

Conghua Xie is supported by the Health Commission of Hubei Province Scientific Research Project (WJ2019H002); the Health Commission of Hubei Province Medical Leading Talent Project; Fundamental Research Funds for the Central Universities (2042018kf1037 and 2042019kf0329); the Medical Science Advancement Program (Basic Medical Sciences) of Wuhan University (TFJC2018005); and the Zhongnan Hospital of Wuhan University Science, Technology and Innovation Seed Fund (znpy2017049 and znpy2018070). Melvin L. K. Chua has received grants and personal fees from Ferring Singapore; has acted as a paid member of the advisory board for Janssen, Astellas, Merck, and Illumina; has acted as a paid member of the advisory board and received hardware for research from Varian; has received nonfinancial support (provision of an artificial intelligence contouring work station for research) from PAVmed Inc; and has received nonfinancial support from MedLever Inc and Decipher Biosciences for work performed outside of the current study. Dr. Chua also is supported by the National Medical Research Council Clinician-scientist award (NMRC/CSA/0027/2018). Xiaokun Shen is the founder and Chief Executive Officer of Convalife (Shanghai) Company Ltd and has an equity interest. Xiaokun Shen was supported in part by the National Science and Technology Major Projects for Major New Drugs Innovation and Development (2019ZX09301010) and Pudong New Area Science and Technology Development Foundation (PKX2019-S08). The other authors made no disclosures.

Comment in

- Colorectal cancer and COVID-19: Do we need to raise awareness and vigilance?
  Niu P, Lei F, Gu J. Niu P, et al. Cancer. 2021 Mar 15;127(6):979-980. doi: 10.1002/cncr.33217. Epub 2021 Jan 26. Cancer. 2021. PMID: 33498093 Free PMC article. No abstract available.

- 21 references
- 2 figures

Supplementary info

Publication types, MeSH terms, Substances, Grant support Expand

Publication types

- Multicenter Study
- Observational Study
- Research Support, Non-U.S. Gov't

MeSH terms

- Adult
- Aged
- Aged, 80 and over
- Antineoplastic Agents / therapeutic use
- Antiviral Agents / therapeutic use
- Betacoronavirus / genetics*
- COVID-19
- China / epidemiology
- Coronavirus Infections / drug therapy
- Coronavirus Infections / epidemiology*
- Coronavirus Infections / mortality*
- Coronavirus Infections / virology
- Female
- Humans
- Immunoglobulins, Intravenous / therapeutic use
- Incidence
- Male
- Middle Aged
- Neoplasms / drug therapy
- Neoplasms / epidemiology*
- Neoplasms / mortality*
- Pandemics
- Pneumonia, Viral / drug therapy
- Pneumonia, Viral / epidemiology*
- Pneumonia, Viral / mortality*
- Pneumonia, Viral / virology
- Retrospective Studies
- Risk
- SARS-CoV-2
- Severity of Illness Index
- Steroids / therapeutic use
- Survival Rate
- Treatment Outcome

Substances

- Antineoplastic Agents
- Antiviral Agents
- Immunoglobulins, Intravenous
- Steroids

Grant support

- 2042018kf1037/Health Commission of Hubei Province Medical Leading Talent Project/International
- 2042019kf0329/Health Commission of Hubei Province Medical Leading Talent Project/International
- WJ2019H002/Health Commission of Hubei Province Scientific Research Project/International

Full text links

WILEY Free Full Text Wiley Free PMC article
Proceed to details
Cite
Share
☐ 1,165
Observational Study
Aust Health Rev
. 2020 Sep;44(5):741-747.
doi: 10.1071/AH20180.

Description of the effect of patient flow, junior doctor supervision and pandemic preparation on the ability of emergency physicians to provide direct patient care

Andy Lim [1], Namankit Gupta [2], Alvin Lim [3], Wei Hong [4], Katie Walker [5]
Affiliations Expand

Affiliations

- [1] Department of Emergency Medicine, Monash Medical Centre, 246 Clayton Road, Vic. 3168, Australia; and School of Clinical Sciences at Monash Health, Monash University, Wellington Road, Clayton, Vic. 3800, Australia. Email: ngup27@student.monash.edu; and Corresponding author. Email: andy.lim2@monash.edu.
- [2] School of Clinical Sciences at Monash Health, Monash University, Wellington Road, Clayton, Vic. 3800, Australia. Email: ngup27@student.monash.edu.
- [3] Department of Medicine, University of Queensland, St Lucia, Qld 4072, Australia. Email: a.lim.syuen@gmail.com.
- [4] Department of Oncology, St Vincent's Hospital, 41 Victoria Parade, Fitzroy, Vic. 3065, Australia. Email: wei.hong@svha.org.au.
- [5] School of Clinical Sciences at Monash Health, Monash University, Wellington Road, Clayton, Vic. 3800, Australia. Email: ngup27@student.monash.edu; and Department of Emergency Medicine, Casey Hospital, 62-70 Kangan Drive, Berwick, Vic. 3806, Australia. Email: katie_walker01@yahoo.com.au.

- PMID: **32862832**
- DOI: 10.1071/AH20180

Observational Study

Description of the effect of patient flow, junior doctor supervision and pandemic preparation on the ability of emergency physicians to provide direct patient care

Andy Lim et al. Aust Health Rev. 2020 Sep.

Show details

Aust Health Rev

. 2020 Sep;44(5):741-747.
doi: 10.1071/AH20180.

Authors

Andy Lim [1], Namankit Gupta [2], Alvin Lim [3], Wei Hong [4], Katie Walker [5]

Affiliations

- [1] Department of Emergency Medicine, Monash Medical Centre, 246 Clayton Road, Vic. 3168, Australia; and School of Clinical Sciences at Monash Health, Monash University, Wellington Road, Clayton, Vic. 3800, Australia. Email: ngup27@student.monash.edu; and Corresponding author. Email: andy.lim2@monash.edu.
- [2] School of Clinical Sciences at Monash Health, Monash University, Wellington Road, Clayton, Vic. 3800, Australia. Email: ngup27@student.monash.edu.
- [3] Department of Medicine, University of Queensland, St Lucia, Qld 4072, Australia. Email: a.lim.syuen@gmail.com.
- [4] Department of Oncology, St Vincent's Hospital, 41 Victoria Parade, Fitzroy, Vic. 3065, Australia. Email: wei.hong@svha.org.au.
- [5] School of Clinical Sciences at Monash Health, Monash University, Wellington Road, Clayton, Vic. 3800, Australia. Email: ngup27@student.monash.edu; and Department of Emergency Medicine, Casey Hospital, 62-70 Kangan Drive, Berwick, Vic. 3806, Australia. Email: katie_walker01@yahoo.com.au.

- PMID: **32862832**
- DOI: 10.1071/AH20180

Abstract

Objective A pilot study to: (1) describe the ability of emergency physicians to provide primary consults at an Australian, major metropolitan, adult emergency department (ED) during the COVID-19 pandemic when compared with historical performance; and (2) to identify the effect of system and process factors on productivity. Methods A retrospective cross-sectional description of

shifts worked between 1 and 29 February 2020, while physicians were carrying out their usual supervision, flow and problem-solving duties, as well as undertaking additional COVID-19 preparation, was documented. Effect of supervisory load, years of Australian registration and departmental flow factors were evaluated. Descriptive statistical methods were used and regression analyses were performed. Results A total of 188 shifts were analysed. Productivity was 4.07 patients per 9.5-h shift (95% CI 3.56-4.58) or 0.43 patients per h, representing a 48.5% reduction from previously published data (P<0.0001). Working in a shift outside of the resuscitation area or working a day shift was associated with a reduction in individual patient load. There was a 2.2% (95% CI: 1.1-3.4, P<0.001) decrease in productivity with each year after obtaining Australian medical registration. There was a 10.6% (95% CI: 5.4-15.6, P<0.001) decrease in productivity for each junior physician supervised. Bed access had no statistically significant effect on productivity. Conclusions Emergency physicians undertake multiple duties. Their ability to manage their own patients varies depending on multiple ED operational factors, particularly their supervisory load. COVID-19 preparations reduced their ability to see their own patients by half. What is known about the topic? An understanding of emergency physician productivity is essential in planning clinical operations. Medical productivity, however, is challenging to define, and is controversial to measure. Although baseline data exist, few studies examine the effect of patient flow and supervision requirements on the emergency physician's ability to perform primary consults. No studies describe these metrics during COVID-19. What does this paper add? This pilot study provides a novel cross-sectional description of the effect of COVID-19 preparations on the ability of emergency physicians to provide direct patient care. It also examines the effect of selected system and process factors in a physician's ability to complete primary consults. What are the implications for practitioners? When managing an emergency medical workforce, the contribution of emergency physicians to the number of patients requiring consults should take into account the high volume of alternative duties required. Increasing alternative duties can decrease primary provider tasks that can be completed. COVID-19 pandemic preparation has significantly reduced the ability of emergency physicians to manage their own patients.

Supplementary info

Publication types, MeSH terms Expand

Publication types

- Comparative Study
- Observational Study

MeSH terms

- Adult
- Aged
- Aged, 80 and over
- Appointments and Schedules
- Australia
- Betacoronavirus / pathogenicity
- COVID-19
- Coronavirus Infections / diagnosis*

- Coronavirus Infections / therapy*
- Cross-Sectional Studies
- Efficiency, Organizational / statistics & numerical data*
- Emergency Service, Hospital / organization & administration*
- Emergency Service, Hospital / statistics & numerical data
- Emergency Service, Hospital / trends*
- Female
- Forecasting
- Humans
- Male
- Medical Staff, Hospital / organization & administration*
- Medical Staff, Hospital / statistics & numerical data
- Medical Staff, Hospital / trends
- Middle Aged
- Pandemics / statistics & numerical data
- Patient-Centered Care / organization & administration*
- Patient-Centered Care / statistics & numerical data
- Pilot Projects
- Pneumonia, Viral / diagnosis*
- Pneumonia, Viral / therapy*
- Retrospective Studies
- SARS-CoV-2

Proceed to details

Cite

Share

1,166

Clinical Trial

Thromb Haemost
. 2021 Aug;121(8):1054-1065.
doi: 10.1055/a-1347-6070. Epub 2021 Jan 7.

Heparin in COVID-19 Patients Is Associated with Reduced In-Hospital Mortality: The Multicenter Italian CORIST Study

Augusto Di Castelnuovo [#] [1], Simona Costanzo [#] [2], Andrea Antinori [3], Nausicaa Berselli [4], Lorenzo Blandi [5], Marialaura Bonaccio [2], Roberto Cauda [6] [7], Giovanni Guaraldi [8], Lorenzo Menicanti [9], Marco Mennuni [10], Giustino Parruti [11], Giuseppe Patti [10], Francesca Santilli [12], Carlo Signorelli [13], Alessandra Vergori [14], Pasquale Abete [15], Walter Ageno [16], Antonella Agodi [17], Piergiuseppe Agostoni [18] [19], Luca Aiello [20], Samir Al Moghazi [21], Rosa Arboretti [22], Marinella Astuto [23], Filippo Aucella [24], Greta Barbieri [25], Alessandro Bartoloni [26], Paolo

Bonfanti [27, 28], Francesco Cacciatore [15], Lucia Caiano [16], Laura Carrozzi [29], Antonio Cascio [30], Arturo Cicullo [6], Antonella Cingolani [6, 7], Francesco Cipollone [12], Claudia Colomba [30], Crizia Colombo [10], Francesca Crosta [11], Gian Battista Danzi [31], Damiano D'Ardes [12], Katleen de Gaetano Donati [6], Francesco Di Gennaro [32], Giuseppe Di Tano [31], Gianpiero D'Offizi [33], Massimo Fantoni [6, 7], Francesco Maria Fusco [34], Ivan Gentile [35], Francesco Gianfagna [1, 16], Elvira Grandone [24], Emauele Graziani [36], Leonardo Grisafi [10], Gabriella Guarnieri [37], Giovanni Larizza [38], Armando Leone [39], Gloria Maccagni [31], Ferruccio Madaro [38], Stefano Maitan [20], Sandro Mancarella [40], Massimo Mapelli [18, 19], Riccardo Maragna [18, 19], Rossella Marcucci [26], Giulio Maresca [41], Silvia Marongiu [42], Claudia Marotta [32], Lorenzo Marra [39], Franco Mastroianni [38], Maria Mazzitelli [43], Alessandro Mengozzi [44], Francesco Menichetti [44], Marianna Meschiari [8], Jovana Milic [8], Filippo Minutolo [45], Beatrice Molena [37], Arturo Montineri [46], Cristina Mussini [8], Maria Musso [47], Daniela Niola [12], Anna Odone [5], Marco Olivieri [48], Antonella Palimodde [42], Roberta Parisi [2], Emanuela Pasi [36], Raffaele Pesavento [49], Francesco Petri [27], Biagio Pinchera [35], Venerino Poletti [50, 51], Claudia Ravaglia [50], Andrea Rognoni [10], Marco Rossato [49], Marianna Rossi [27], Vincenzo Sangiovanni [34], Carlo Sanrocco [11], Laura Scorzolini [52], Raffaella Sgariglia [40], Paola Giustina Simeone [11], Eleonora Taddei [6], Carlo Torti [43], Roberto Vettor [49], Andrea Vianello [37], Marco Vinceti [4, 53], Alexandra Virano [16], Laura Vocciante [41], Raffaele De Caterina [29], Licia Iacoviello [2, 16]

Affiliations Expand

Affiliations

- [1] Mediterranea Cardiocentro, Napoli, Italy.
- [2] Department of Epidemiology and Prevention, IRCCS Neuromed, Pozzilli, Isernia, Italy.
- [3] UOC Immunodeficienze Virali, National Institute for Infectious Diseases "L. Spallanzani," IRCCS, Rome, Italy.
- [4] Section of Public Health, Department of Biomedical, Metabolic and Neural Sciences, University of Modena and Reggio Emilia, Modena, Italy.
- [5] Università di Pavia, Pavia, Italy.
- [6] Fondazione Policlinico Universitario A, Gemelli IRCCS, Rome, Italy.
- [7] Dipartimento di Sicurezza e Bioetica Sede di Roma, Università Cattolica del Sacro Cuore, Rome, Italy.
- [8] Infectious Disease Unit, Department of Surgical, Medical, Dental and Morphological Sciences, University of Modena and Reggio Emilia, Modena, Italy.
- [9] IRCCS Policlinico San Donato, San Donato, Milanese (MI), Italy.
- [10] University of Eastern Piedmont, Maggiore della Carità Hospital, Novara, Italy.
- [11] Department of Infectious Disease, Azienda Sanitaria Locale (AUSL) di Pescara, Pescara, Italy.
- [12] Department of Medicine and Aging, Clinica Medica, "SS. Annunziata" Hospital and University of Chieti, Chieti, Italy.
- [13] School of Medicine, Vita-Salute San Raffaele University, Milan, Italy.
- [14] Department of HIV/AIDS, National Institute for Infectious Diseases "Lazzaro Spallanzani"-IRCCS, Rome, Italy.
- [15] Dipartimento di Scienze Mediche Traslazionali, Università degli studi di Napoli "Federico II," Napoli, Italy.
- [16] Department of Medicine and Surgery, University of Insubria, Varese, Italy.

- [17] Department of Medical and Surgical Sciences and Advanced Technologies "G.F. Ingrassia," University of Catania; AOU Policlinico "G. Rodolico - San Marco," Catania, Italy.
- [18] Centro Cardiologico Monzino IRCCS, Milan, Italy.
- [19] Section of Cardiovascular, Department of Clinical Sciences and Community Health, University of Milano, Milan, Italy.
- [20] UOC, Anestesia e Rianimazione, Dipartimento di Chirurgia Generale Ospedale Morgagni-Pierantoni, Forlì, Italy.
- [21] UOC Infezioni Sistemiche dell'Immunodepresso, National Institute for Infectious Diseases L. Spallanzani, IRCCS, Rome, Italy.
- [22] Department of Civil Environmental and Architectural Engineering, University of Padova, Padova, Italy.
- [23] Department of General Surgery and Medical-Surgical Specialties, University of Catania, U.O. Anestesia, Rianimazione 1. P.O. "G. Rodolico," AOU Policlinico "G. Rodolico - San Marco," Catania, Italy.
- [24] Fondazione I.R.C.C.S "Casa Sollievo della Sofferenza," San Giovanni Rotondo, Foggia, Italy.
- [25] Department of Surgical, Medical and Molecular Medicine and Critical Care, Azienda Ospedaliera Universitaria Pisana, University of Pisa, Pisa, Italy.
- [26] Department of Experimental and Clinical Medicine, University of Florence and Azienda Ospedaliero-Universitaria Careggi, Firenze, Italy.
- [27] UOC Malattie Infettive, Ospedale San Gerardo, ASST Monza, Monza, Italy.
- [28] School of Medicine and Surgery, University of Milano-Bicocca, Milan, Italy.
- [29] Cardiovascular and Thoracic Department, Azienda Ospedaliero-Universitaria Pisana and University of Pisa, Pisa, Italy.
- [30] Infectious and Tropical Diseases Unit, Department of Health Promotion, Mother and Child Care, Internal Medicine and Medical Specialties (PROMISE), University of Palermo, Palermo, Italy.
- [31] Department of Cardiology, Ospedale di Cremona, Cremona, Italy.
- [32] Medical Direction, IRCCS Neuromed, Pozzilli, Isernia, Italy.
- [33] UOC Malattie Infettive-Epatologia, National Institute for Infectious Diseases L, Spallanzani, IRCCS, Roma, Italy.
- [34] UOC Infezioni Sistemiche e dell'Immunodepresso, Azienda Ospedaliera dei Colli, Ospedale Cotugno, Napoli, Italy.
- [35] Department of Clinical Medicine and Surgery, University of Naples Federico II, Napoli, Italy.
- [36] Medicina Interna, Ospedale di Ravenna, AUSL della Romagna, Ravenna, Italy.
- [37] Division of Respiratory Pathophysiology, Department of Cardiologic, Thoracic and Vascular Sciences, University of Padova, Padova, Italy.
- [38] COVID-19 Unit, EE Ospedale Regionale F. Miulli, Acquaviva delle Fonti, Bari, Italy.
- [39] UOC di Pneumologia, P.O. San Giuseppe Moscati, Taranto, Italy.
- [40] ASST Milano Nord, Ospedale Edoardo Bassini Cinisello Balsamo, Milan, Italy.
- [41] UOC di Medicina - Presidio Ospedaliero S. Maria di Loreto Nuovo. ASL Napoli 1 Centro. Napoli. Italy.
- [42] P.O. Santissima Trinità di Cagliari, Cagliari, Italy.
- [43] Infectious and Tropical Diseases Unit, Department of Medical and Surgical Sciences "Magna Graecia" University, Catanzaro, Italy.
- [44] Department of Clinical and Experimental Medicine, Azienda Ospedaliera Universitaria Pisana, University of Pisa, Pisa, Italy.

- [45] Dipartimento di Farmacia, Università di Pisa, Pisa, Italy.
- [46] U.O. C. Malattie Infettive e Tropicali, P.O. "San Marco." AOU Policlinico "G. Rodolico - San Marco," Catania, Italy.
- [47] UOC Malattie Infettive-Apparato Respiratorio, National Institute for Infectious Diseases "L. Spallanzani," IRCCS, Rome, Italy.
- [48] Computer Service, University of Molise, Campobasso, Italy.
- [49] Clinica Medica 3, Department of Medicine - DIMED, University hospital of Padova, Padova, Italy.
- [50] UOC Pneumologia, Dipartimento di Malattie Apparato Respiratorio e Torace, Ospedale Morgagni-Pierantoni, Forlì, Italy.
- [51] Department of Respiratory Diseases and Allergy, Aarhus University Hospital, Aarhus, Denmark.
- [52] UOC Malattie Infettive ad Alta Intensità di Cura, National Institute for Infectious Diseases "L. Spallanzani," IRCCS, Rome, Italy.
- [53] Department of Epidemiology, Boston University School of Public Health, Boston, Massachusetts, United States.

[#] Contributed equally.

- PMID: **33412596**
- DOI: 10.1055/a-1347-6070

Clinical Trial

Heparin in COVID-19 Patients Is Associated with Reduced In-Hospital Mortality: The Multicenter Italian CORIST Study

Augusto Di Castelnuovo et al. Thromb Haemost. 2021 Aug.

Show details

Thromb Haemost

. 2021 Aug;121(8):1054-1065.
doi: 10.1055/a-1347-6070. Epub 2021 Jan 7.

Authors

Augusto Di Castelnuovo [# 1], Simona Costanzo [# 2], Andrea Antinori [3], Nausicaa Berselli [4], Lorenzo Blandi [5], Marialaura Bonaccio [2], Roberto Cauda [6 7], Giovanni Guaraldi [8], Lorenzo Menicanti [9], Marco Mennuni [10], Giustino Parruti [11], Giuseppe Patti [10], Francesca Santilli [12], Carlo Signorelli [13], Alessandra Vergori [14], Pasquale Abete [15], Walter Ageno [16], Antonella Agodi [17], Piergiuseppe Agostoni [18 19], Luca Aiello [20], Samir Al Moghazi [21], Rosa Arboretti [22], Marinella Astuto [23], Filippo Aucella [24], Greta Barbieri [25], Alessandro Bartoloni [26], Paolo Bonfanti [27 28], Francesco Cacciatore [15], Lucia Caiano [16], Laura Carrozzi [29], Antonio Cascio [30], Arturo Ciccullo [6], Antonella Cingolani [6 7], Francesco Cipollone [12], Claudia Colomba [30], Crizia Colombo [10], Francesca Crosta [11], Gian Battista Danzi [31], Damiano D'Ardes [12], Katleen de Gaetano Donati [6], Francesco Di Gennaro [32], Giuseppe Di Tano [31], Gianpiero

D'Offizi [33], Massimo Fantoni [6, 7], Francesco Maria Fusco [34], Ivan Gentile [35], Francesco Gianfagna [1, 16], Elvira Grandone [24], Emauele Graziani [36], Leonardo Grisafi [10], Gabriella Guarnieri [37], Giovanni Larizza [38], Armando Leone [39], Gloria Maccagni [31], Ferruccio Madaro [38], Stefano Maitan [20], Sandro Mancarella [40], Massimo Mapelli [18, 19], Riccardo Maragna [18, 19], Rossella Marcucci [26], Giulio Maresca [41], Silvia Marongiu [42], Claudia Marotta [32], Lorenzo Marra [39], Franco Mastroianni [38], Maria Mazzitelli [43], Alessandro Mengozzi [44], Francesco Menichetti [44], Marianna Meschiari [8], Jovana Milic [8], Filippo Minutolo [45], Beatrice Molena [37], Arturo Montineri [46], Cristina Mussini [8], Maria Musso [47], Daniela Niola [12], Anna Odone [5], Marco Olivieri [48], Antonella Palimodde [42], Roberta Parisi [2], Emanuela Pasi [36], Raffaele Pesavento [49], Francesco Petri [27], Biagio Pinchera [35], Venerino Poletti [50, 51], Claudia Ravaglia [50], Andrea Rognoni [10], Marco Rossato [49], Marianna Rossi [27], Vincenzo Sangiovanni [34], Carlo Sanrocco [11], Laura Scorzolini [52], Raffaella Sgariglia [40], Paola Giustina Simeone [11], Eleonora Taddei [6], Carlo Torti [43], Roberto Vettor [49], Andrea Vianello [37], Marco Vinceti [4, 53], Alexandra Virano [16], Laura Vocciante [41], Raffaele De Caterina [29], Licia Iacoviello [2, 16]

Affiliations

- [1] Mediterranea Cardiocentro, Napoli, Italy.
- [2] Department of Epidemiology and Prevention, IRCCS Neuromed, Pozzilli, Isernia, Italy.
- [3] UOC Immunodeficienze Virali, National Institute for Infectious Diseases "L. Spallanzani," IRCCS, Rome, Italy.
- [4] Section of Public Health, Department of Biomedical, Metabolic and Neural Sciences, University of Modena and Reggio Emilia, Modena, Italy.
- [5] Università di Pavia, Pavia, Italy.
- [6] Fondazione Policlinico Universitario A, Gemelli IRCCS, Rome, Italy.
- [7] Dipartimento di Sicurezza e Bioetica Sede di Roma, Università Cattolica del Sacro Cuore, Rome, Italy.
- [8] Infectious Disease Unit, Department of Surgical, Medical, Dental and Morphological Sciences, University of Modena and Reggio Emilia, Modena, Italy.
- [9] IRCCS Policlinico San Donato, San Donato, Milanese (MI), Italy.
- [10] University of Eastern Piedmont, Maggiore della Carità Hospital, Novara, Italy.
- [11] Department of Infectious Disease, Azienda Sanitaria Locale (AUSL) di Pescara, Pescara, Italy.
- [12] Department of Medicine and Aging, Clinica Medica, "SS. Annunziata" Hospital and University of Chieti, Chieti, Italy.
- [13] School of Medicine, Vita-Salute San Raffaele University, Milan, Italy.
- [14] Department of HIV/AIDS, National Institute for Infectious Diseases "Lazzaro Spallanzani"-IRCCS, Rome, Italy.
- [15] Dipartimento di Scienze Mediche Traslazionali, Università degli studi di Napoli "Federico II," Napoli, Italy.
- [16] Department of Medicine and Surgery, University of Insubria, Varese, Italy.
- [17] Department of Medical and Surgical Sciences and Advanced Technologies "G.F. Ingrassia," University of Catania; AOU Policlinico "G. Rodolico - San Marco," Catania, Italy.
- [18] Centro Cardiologico Monzino IRCCS, Milan, Italy.
- [19] Section of Cardiovascular, Department of Clinical Sciences and Community Health, University of Milano, Milan, Italy.

- [20] UOC, Anestesia e Rianimazione, Dipartimento di Chirurgia Generale Ospedale Morgagni-Pierantoni, Forlì, Italy.
- [21] UOC Infezioni Sistemiche dell'Immunodepresso, National Institute for Infectious Diseases L. Spallanzani, IRCCS, Rome, Italy.
- [22] Department of Civil Environmental and Architectural Engineering, University of Padova, Padova, Italy.
- [23] Department of General Surgery and Medical-Surgical Specialties, University of Catania, U.O. Anestesia, Rianimazione 1. P.O. "G. Rodolico," AOU Policlinico "G. Rodolico - San Marco," Catania, Italy.
- [24] Fondazione I.R.C.C.S "Casa Sollievo della Sofferenza," San Giovanni Rotondo, Foggia, Italy.
- [25] Department of Surgical, Medical and Molecular Medicine and Critical Care, Azienda Ospedaliera Universitaria Pisana, University of Pisa, Pisa, Italy.
- [26] Department of Experimental and Clinical Medicine, University of Florence and Azienda Ospedaliero-Universitaria Careggi, Firenze, Italy.
- [27] UOC Malattie Infettive, Ospedale San Gerardo, ASST Monza, Monza, Italy.
- [28] School of Medicine and Surgery, University of Milano-Bicocca, Milan, Italy.
- [29] Cardiovascular and Thoracic Department, Azienda Ospedaliero-Universitaria Pisana and University of Pisa, Pisa, Italy.
- [30] Infectious and Tropical Diseases Unit, Department of Health Promotion, Mother and Child Care, Internal Medicine and Medical Specialties (PROMISE), University of Palermo, Palermo, Italy.
- [31] Department of Cardiology, Ospedale di Cremona, Cremona, Italy.
- [32] Medical Direction, IRCCS Neuromed, Pozzilli, Isernia, Italy.
- [33] UOC Malattie Infettive-Epatologia, National Institute for Infectious Diseases L, Spallanzani, IRCCS, Roma, Italy.
- [34] UOC Infezioni Sistemiche e dell'Immunodepresso, Azienda Ospedaliera dei Colli, Ospedale Cotugno, Napoli, Italy.
- [35] Department of Clinical Medicine and Surgery, University of Naples Federico II, Napoli, Italy.
- [36] Medicina Interna, Ospedale di Ravenna, AUSL della Romagna, Ravenna, Italy.
- [37] Division of Respiratory Pathophysiology, Department of Cardiologic, Thoracic and Vascular Sciences, University of Padova, Padova, Italy.
- [38] COVID-19 Unit, EE Ospedale Regionale F. Miulli, Acquaviva delle Fonti, Bari, Italy.
- [39] UOC di Pneumologia, P.O. San Giuseppe Moscati, Taranto, Italy.
- [40] ASST Milano Nord, Ospedale Edoardo Bassini Cinisello Balsamo, Milan, Italy.
- [41] UOC di Medicina - Presidio Ospedaliero S. Maria di Loreto Nuovo. ASL Napoli 1 Centro. Napoli. Italy.
- [42] P.O. Santissima Trinità di Cagliari, Cagliari, Italy.
- [43] Infectious and Tropical Diseases Unit, Department of Medical and Surgical Sciences "Magna Graecia" University, Catanzaro, Italy.
- [44] Department of Clinical and Experimental Medicine, Azienda Ospedaliera Universitaria Pisana, University of Pisa, Pisa, Italy.
- [45] Dipartimento di Farmacia, Università di Pisa, Pisa, Italy.
- [46] U.O. C. Malattie Infettive e Tropicali, P.O. "San Marco." AOU Policlinico "G. Rodolico - San Marco," Catania, Italy.
- [47] UOC Malattie Infettive-Apparato Respiratorio, National Institute for Infectious Diseases "L. Spallanzani," IRCCS, Rome, Italy.

- [48] Computer Service, University of Molise, Campobasso, Italy.
- [49] Clinica Medica 3, Department of Medicine - DIMED, University hospital of Padova, Padova, Italy.
- [50] UOC Pneumologia, Dipartimento di Malattie Apparato Respiratorio e Torace, Ospedale Morgagni-Pierantoni, Forlì, Italy.
- [51] Department of Respiratory Diseases and Allergy, Aarhus University Hospital, Aarhus, Denmark.
- [52] UOC Malattie Infettive ad Alta Intensità di Cura, National Institute for Infectious Diseases "L. Spallanzani," IRCCS, Rome, Italy.
- [53] Department of Epidemiology, Boston University School of Public Health, Boston, Massachusetts, United States.

[#] Contributed equally.

- PMID: **33412596**
- DOI: 10.1055/a-1347-6070

Abstract

Introduction: A hypercoagulable condition was described in patients with coronavirus disease 2019 (COVID-19) and proposed as a possible pathogenic mechanism contributing to disease progression and lethality.

Aim: We evaluated if in-hospital administration of heparin improved survival in a large cohort of Italian COVID-19 patients.

Methods: In a retrospective observational study, 2,574 unselected patients hospitalized in 30 clinical centers in Italy from February 19, 2020 to June 5, 2020 with laboratory-confirmed severe acute respiratory syndrome coronavirus-2 infection were analyzed. The primary endpoint in a time-to event analysis was in-hospital death, comparing patients who received heparin (low-molecular-weight heparin [LMWH] or unfractionated heparin [UFH]) with patients who did not. We used multivariable Cox proportional-hazards regression models with inverse probability for treatment weighting by propensity scores.

Results: Out of 2,574 COVID-19 patients, 70.1% received heparin. LMWH was largely the most used formulation (99.5%). Death rates for patients receiving heparin or not were 7.4 and 14.0 per 1,000 person-days, respectively. After adjustment for propensity scores, we found a 40% lower risk of death in patients receiving heparin (hazard ratio = 0.60; 95% confidence interval: 0.49-0.74; E-value = 2.04). This association was particularly evident in patients with a higher severity of disease or strong coagulation activation.

Conclusion: In-hospital heparin treatment was associated with a lower mortality, particularly in severely ill COVID-19 patients and in those with strong coagulation activation. The results from randomized clinical trials are eagerly awaited to provide clear-cut recommendations.

Thieme. All rights reserved.

Conflict of interest statement

None declared.

Supplementary info

Publication types, MeSH terms, Substances Expand

Publication types

- Clinical Trial
- Multicenter Study
- Observational Study

MeSH terms

- Aged
- Anticoagulants / therapeutic use*
- Blood Coagulation / drug effects
- COVID-19 / blood
- COVID-19 / complications*
- COVID-19 / drug therapy
- Female
- Heparin / therapeutic use*
- Heparin, Low-Molecular-Weight / therapeutic use*
- Hospital Mortality
- Humans
- Italy / epidemiology
- Male
- Middle Aged
- Retrospective Studies
- Survival Analysis
- Thrombophilia / blood
- Thrombophilia / etiology*
- Thrombophilia / prevention & control*

Substances

- Anticoagulants
- Heparin, Low-Molecular-Weight
- Heparin

Full text links

Full text THIEME CONNECT Georg Thieme Verlag Stuttgart, New York
Proceed to details

Cite
Share
1,167

PLoS One
. 2021 Mar 31;16(3):e0243291.
doi: 10.1371/journal.pone.0243291. eCollection 2021.

Clinical characteristics and outcomes for 7,995 patients with SARS-CoV-2 infection

Jacob McPadden [1, 2], Frederick Warner [2, 3], H Patrick Young [2, 4], Nathan C Hurley [5], Rebecca A Pulk [6], Avinainder Singh [4], Thomas J S Durant [2, 7], Guannan Gong [2, 8], Nihar Desai [2], Adrian Haimovich [9], Richard Andrew Taylor [10], Murat Gunel [11, 12, 13, 14], Charles S Dela Cruz [15], Shelli F Farhadian [16], Jonathan Siner [15], Merceditas Villanueva [4, 17], Keith Churchwell [18], Allen Hsiao [1, 19], Charles J Torre Jr [7, 19], Eric J Velazquez [3], Roy S Herbst [20], Akiko Iwasaki [21, 22], Albert I Ko [23], Bobak J Mortazavi [2, 3, 5, 24], Harlan M Krumholz [2, 3, 25], Wade L Schulz [2, 7]
Affiliations Expand

Affiliations

- [1] Department of Pediatrics, Yale School of Medicine, New Haven, Connecticut, United States of America.
- [2] Center for Outcomes Research and Evaluation, Yale-New Haven Hospital, New Haven, Connecticut, United States of America.
- [3] Department of Internal Medicine, Section of Cardiovascular Medicine, Yale School of Medicine, New Haven, Connecticut, United States of America.
- [4] Department of Internal Medicine, Yale University School of Medicine, New Haven, Connecticut, United States of America.
- [5] Department of Computer Science and Engineering, Texas A&M University, College Station, Texas, United States of America.
- [6] Corporate Pharmacy Services, Yale New Haven Health, New Haven, Connecticut, United States of America.
- [7] Department of Laboratory Medicine, Yale University School of Medicine, New Haven, Connecticut, United States of America.
- [8] Interdepartmental Program in Computational Biology and Bioinformatics, Yale University School of Medicine, New Haven, Connecticut, United States of America.
- [9] Yale School of Medicine, New Haven, Connecticut, United States of America.
- [10] Department of Emergency Medicine, Yale School of Medicine, New Haven, Connecticut, United States of America.
- [11] Department of Genetics, Yale University School of Medicine, New Haven, Connecticut, United States of America.
- [12] Medical Scientist Training Program, Yale University School of Medicine, New Haven, Connecticut, United States of America.
- [13] Yale Center for Genome Analysis, Yale University School of Medicine, New Haven, Connecticut, United States of America.

- [14] Department of Neurosurgery, Yale University School of Medicine, New Haven, Connecticut, United States of America.
- [15] Department of Internal Medicine, Pulmonary, Critical Care and Sleep Medicine, Yale School of Medicine, New Haven, Connecticut, United States of America.
- [16] Department of Internal Medicine, Section of Infectious Diseases, Yale School of Medicine, New Haven, Connecticut, United States of America.
- [17] Center for Interdisciplinary Research on AIDS, Yale School of Public Health, New Haven, Connecticut, United States of America.
- [18] Yale New Haven Hospital, New Haven, Connecticut, United States of America.
- [19] Information Technology Services, Yale New Haven Health, New Haven, Connecticut, United States of America.
- [20] Yale Comprehensive Cancer Center, Yale School of Medicine, New Haven, Connecticut, United States of America.
- [21] Department of Immunobiology, Yale University School of Medicine, New Haven, Connecticut, United States of America.
- [22] Howard Hughes Medical Institute, Chevy Chase, Maryland, United States of America.
- [23] Department of Epidemiology of Microbial Diseases, Yale School of Public Health, New Haven, Connecticut, United States of America.
- [24] Center for Remote Health Technologies and Systems, Texas A&M University, College Station, Texas, United States of America.
- [25] Department of Health Policy and Management, Yale School of Public Health, New Haven, Connecticut, United States of America.

- PMID: **33788846**
- PMCID: PMC8011821
- DOI: 10.1371/journal.pone.0243291

Free PMC article

Clinical characteristics and outcomes for 7,995 patients with SARS-CoV-2 infection

Jacob McPadden et al. PLoS One. 2021.
Free PMC article

Show details

PLoS One

. 2021 Mar 31;16(3):e0243291.
doi: 10.1371/journal.pone.0243291. eCollection 2021.

Authors

Jacob McPadden [1, 2], Frederick Warner [2, 3], H Patrick Young [2, 4], Nathan C Hurley [5], Rebecca A Pulk [6], Avinainder Singh [4], Thomas J S Durant [2, 7], Guannan Gong [2, 8], Nihar Desai [2], Adrian Haimovich [9], Richard Andrew Taylor [10], Murat Gunel [11, 12, 13, 14], Charles S Dela Cruz [15], Shelli F Farhadian [16], Jonathan Siner [15], Merceditas Villanueva [4, 17], Keith Churchwell [18], Allen Hsiao [1, 19], Charles J Torre Jr [7, 19], Eric J Velazquez [3], Roy S Herbst [20], Akiko

Iwasaki [21, 22], Albert I Ko [23], Bobak J Mortazavi [2, 3, 5, 24], Harlan M Krumholz [2, 3, 25], Wade L Schulz [2, 7]

Affiliations

- [1] Department of Pediatrics, Yale School of Medicine, New Haven, Connecticut, United States of America.
- [2] Center for Outcomes Research and Evaluation, Yale-New Haven Hospital, New Haven, Connecticut, United States of America.
- [3] Department of Internal Medicine, Section of Cardiovascular Medicine, Yale School of Medicine, New Haven, Connecticut, United States of America.
- [4] Department of Internal Medicine, Yale University School of Medicine, New Haven, Connecticut, United States of America.
- [5] Department of Computer Science and Engineering, Texas A&M University, College Station, Texas, United States of America.
- [6] Corporate Pharmacy Services, Yale New Haven Health, New Haven, Connecticut, United States of America.
- [7] Department of Laboratory Medicine, Yale University School of Medicine, New Haven, Connecticut, United States of America.
- [8] Interdepartmental Program in Computational Biology and Bioinformatics, Yale University School of Medicine, New Haven, Connecticut, United States of America.
- [9] Yale School of Medicine, New Haven, Connecticut, United States of America.
- [10] Department of Emergency Medicine, Yale School of Medicine, New Haven, Connecticut, United States of America.
- [11] Department of Genetics, Yale University School of Medicine, New Haven, Connecticut, United States of America.
- [12] Medical Scientist Training Program, Yale University School of Medicine, New Haven, Connecticut, United States of America.
- [13] Yale Center for Genome Analysis, Yale University School of Medicine, New Haven, Connecticut, United States of America.
- [14] Department of Neurosurgery, Yale University School of Medicine, New Haven, Connecticut, United States of America.
- [15] Department of Internal Medicine, Pulmonary, Critical Care and Sleep Medicine, Yale School of Medicine, New Haven, Connecticut, United States of America.
- [16] Department of Internal Medicine, Section of Infectious Diseases, Yale School of Medicine, New Haven, Connecticut, United States of America.
- [17] Center for Interdisciplinary Research on AIDS, Yale School of Public Health, New Haven, Connecticut, United States of America.
- [18] Yale New Haven Hospital, New Haven, Connecticut, United States of America.
- [19] Information Technology Services, Yale New Haven Health, New Haven, Connecticut, United States of America.
- [20] Yale Comprehensive Cancer Center, Yale School of Medicine, New Haven, Connecticut, United States of America.
- [21] Department of Immunobiology, Yale University School of Medicine, New Haven, Connecticut, United States of America.
- [22] Howard Hughes Medical Institute, Chevy Chase, Maryland, United States of America.
- [23] Department of Epidemiology of Microbial Diseases, Yale School of Public Health, New Haven, Connecticut, United States of America.

- [24] Center for Remote Health Technologies and Systems, Texas A&M University, College Station, Texas, United States of America.
- [25] Department of Health Policy and Management, Yale School of Public Health, New Haven, Connecticut, United States of America.

- PMID: **33788846**
- PMCID: PMC8011821
- DOI: 10.1371/journal.pone.0243291

Abstract

Objective: Severe acute respiratory syndrome virus (SARS-CoV-2) has infected millions of people worldwide. Our goal was to identify risk factors associated with admission and disease severity in patients with SARS-CoV-2.

Design: This was an observational, retrospective study based on real-world data for 7,995 patients with SARS-CoV-2 from a clinical data repository.

Setting: Yale New Haven Health (YNHH) is a five-hospital academic health system serving a diverse patient population with community and teaching facilities in both urban and suburban areas.

Populations: The study included adult patients who had SARS-CoV-2 testing at YNHH between March 1 and April 30, 2020.

Main outcome and performance measures: Primary outcomes were admission and in-hospital mortality for patients with SARS-CoV-2 infection as determined by RT-PCR testing. We also assessed features associated with the need for respiratory support.

Results: Of the 28605 patients tested for SARS-CoV-2, 7995 patients (27.9%) had an infection (median age 52.3 years) and 2154 (26.9%) of these had an associated admission (median age 66.2 years). Of admitted patients, 2152 (99.9%) had a discharge disposition at the end of the study period. Of these, 329 (15.3%) required invasive mechanical ventilation and 305 (14.2%) expired. Increased age and male sex were positively associated with admission and in-hospital mortality (median age 80.7 years), while comorbidities had a much weaker association with the risk of admission or mortality. Black race (OR 1.43, 95%CI 1.14-1.78) and Hispanic ethnicity (OR 1.81, 95%CI 1.50-2.18) were identified as risk factors for admission, but, among discharged patients, age-adjusted in-hospital mortality was not significantly different among racial and ethnic groups.

Conclusions: This observational study identified, among people testing positive for SARS-CoV-2 infection, older age and male sex as the most strongly associated risks for admission and in-hospital mortality in patients with SARS-CoV-2 infection. While minority racial and ethnic groups had increased burden of disease and risk of admission, age-adjusted in-hospital mortality for discharged patients was not significantly different among racial and ethnic groups. Ongoing studies will be needed to continue to evaluate these risks, particularly in the setting of evolving treatment guidelines.

Conflict of interest statement

H.M.K. works under contract with the Centers for Medicare & Medicaid Services to support quality measurement programs; was a recipient of a research grant, through Yale, from Medtronic and the U.S. Food and Drug Administration to develop methods for post-market surveillance of

medical devices; was a recipient of a research grant from Johnson & Johnson, through Yale University, to support clinical trial data sharing; was a recipient of a research agreement, through Yale University, from the Shenzhen Center for Health Information for work to advance intelligent disease prevention and health promotion; collaborates with the National Center for Cardiovascular Diseases in Beijing; receives payment from the Arnold & Porter Law Firm for work related to the Sanofi clopidogrel litigation, from the Martin Baughman Law Firm for work related to the Cook Celect IVC filter litigation, and from the Siegfried and Jensen Law Firm for work related to Vioxx litigation; chairs a Cardiac Scientific Advisory Board for UnitedHealth; was a member of the IBM Watson Health Life Sciences Board; is a member of the Advisory Board for Element Science, the Advisory Board for Facebook, and the Physician Advisory Board for Aetna; and is the co-founder of HugoHealth, a personal health information platform, and cofounder of Refactor Health, a healthcare AI-augmented data management company. W.L.S. was an investigator for a research agreement, through Yale University, from the Shenzhen Center for Health Information for work to advance intelligent disease prevention and health promotion; collaborates with the National Center for Cardiovascular Diseases in Beijing; is a technical consultant to HugoHealth, a personal health information platform, and cofounder of Refactor Health, an AI-augmented data management platform for healthcare; is a consultant for Interpace Diagnostics Group, a molecular diagnostics company. This does not alter our adherence to PLOS ONE policies on sharing data and materials. There are no patents, products in development or marketed products associated with this research to declare.

Update of

- Clinical Characteristics and Outcomes for 7,995 Patients with SARS-CoV-2 Infection. McPadden J, Warner F, Young HP, Hurley NC, Pulk RA, Singh A, Durant TJ, Gong G, Desai N, Haimovich A, Taylor RA, Gunel M, Cruz CSD, Farhadian SF, Siner J, Villanueva M, Churchwell K, Hsiao A, Torre CJ Jr, Velazquez EJ, Herbst RS, Iwasaki A, Ko AI, Mortazavi BJ, Krumholz HM, Schulz WL. McPadden J, et al. medRxiv. 2020 Nov 8:2020.07.19.20157305. doi: 10.1101/2020.07.19.20157305. Preprint. medRxiv. 2020. PMID: 32743602 Free PMC article. Updated.

- 34 references
- 4 figures

Supplementary info

Publication types, MeSH terms, Grant support Expand

Publication types

- Research Support, N.I.H., Extramural
- Research Support, Non-U.S. Gov't

MeSH terms

- Adolescent
- Adult
- Aged
- Aged, 80 and over

- COVID-19 / diagnosis
- COVID-19 / epidemiology*
- COVID-19 / mortality
- COVID-19 / therapy
- COVID-19 Testing
- Cohort Studies
- Female
- Hospital Mortality
- Humans
- Male
- Middle Aged
- Prognosis
- Retrospective Studies
- Treatment Outcome
- Young Adult

Grant support

- T15 LM007056/LM/NLM NIH HHS/United States
- UL1 TR001863/TR/NCATS NIH HHS/United States

Full text links

OPEN ACCESS TO FULL TEXT PLOS ONE Public Library of Science Free PMC article

Proceed to details

Cite

Share

☐ 1,168

Observational Study

J Korean Med Sci

. 2021 Jul 12;36(27):e196.

doi: 10.3346/jkms.2021.36.e196.

Emergency Department Utilization by In-hospital Healthcare Workers after COVID-19 Vaccination

Min Ji Park [1], Yoo Jin Choi [2], Sangchun Choi [1]

Affiliations Expand

Affiliations

- [1] Department of Emergency Medicine, Ajou University School of Medicine, Suwon, Korea.
- [2] Department of Emergency Medicine, Ajou University School of Medicine, Suwon, Korea. choiyj0729@naver.com.

- PMID: **34254475**
- PMCID: PMC8275460
- DOI: 10.3346/jkms.2021.36.e196

Free PMC article
Observational Study

Emergency Department Utilization by In-hospital Healthcare Workers after COVID-19 Vaccination

Min Ji Park et al. J Korean Med Sci. 2021.
Free PMC article
Show details
J Korean Med Sci
. 2021 Jul 12;36(27):e196.
doi: 10.3346/jkms.2021.36.e196.

Authors

Min Ji Park [1], Yoo Jin Choi [2], Sangchun Choi [1]

Affiliations

- [1] Department of Emergency Medicine, Ajou University School of Medicine, Suwon, Korea.
- [2] Department of Emergency Medicine, Ajou University School of Medicine, Suwon, Korea. choiyj0729@naver.com.

- PMID: **34254475**
- PMCID: PMC8275460
- DOI: 10.3346/jkms.2021.36.e196

Abstract

Background: This is an observational study to analyze an emergency department (ED) utilization pattern of coronavirus disease 2019 (COVID-19) vaccinated in-hospital healthcare workers (HCWs).

Methods: We included 4,703 HCWs who were administered the first dose of the COVID-19 vaccine between March 4 and April 2, 2021, in a tertiary hospital in Korea where fast-track and post-vaccination cohort zone (PVCZ) were introduced in ED. We analyzed data of participants' age, sex, occupation, date and type of vaccination, and their clinical information using SPSS v25.0.

Results: The sample comprised HCWs, who received either the ChAdOx1 (n = 4,458) or the BNT162B2 (n = 245) vaccines; most participants were female (73.5%), and 81.1% were under 50 years old. Further, 153 (3.3%) visited the ED and reported experiencing fever (66.9%) and myalgia (56.1%). Additionally, 91 (59.5%) of them were in their 20s, and 106 (67.5%) were assigned to the PVCZ. Lastly, 107 (68.2%) of the patients received parenteral management. No patient required hospitalization.

Conclusion: In conclusion, vaccinated HCWs who visited the ED with adverse events had a high incidence of fever and a low likelihood of developing serious illnesses. As the COVID-19 vaccination program for Korean citizens continues to expand, strategies to minimize unnecessary ED overcrowding should be put into effect.

Keywords: COVID-19, Korea; Emergency Department; Vaccination.

© 2021 The Korean Academy of Medical Sciences.

Conflict of interest statement

The authors have no potential conflicts of interest to disclose.

- 11 references
- 1 figure

Supplementary info

Publication types, MeSH terms, Substances Expand

Publication types

- Observational Study

MeSH terms

- Adult
- Antiemetics / therapeutic use
- Antipyretics / therapeutic use
- BNT162 Vaccine
- COVID-19 Testing / statistics & numerical data
- COVID-19 Vaccines / adverse effects*
- ChAdOx1 nCoV-19
- Chills / chemically induced
- Chills / epidemiology
- Clinical Protocols
- Emergency Service, Hospital / organization & administration
- Emergency Service, Hospital / statistics & numerical data*
- Female
- Fever / chemically induced

- Fever / drug therapy
- Fever / epidemiology
- Headache / chemically induced
- Headache / epidemiology
- Humans
- Male
- Middle Aged
- Myalgia / chemically induced
- Myalgia / epidemiology
- Nausea / chemically induced
- Nausea / drug therapy
- Nausea / epidemiology
- Patient Acceptance of Health Care / statistics & numerical data*
- Patient Readmission / statistics & numerical data
- Personnel, Hospital / statistics & numerical data*
- Republic of Korea
- Retrospective Studies
- Software Design
- Tertiary Care Centers / statistics & numerical data
- Triage
- Vaccination / adverse effects*
- Young Adult

Substances

- Antiemetics
- Antipyretics
- COVID-19 Vaccines
- ChAdOx1 nCoV-19
- BNT162 Vaccine

Full text links

JKMS FULL TEXT FREE Journal of korean Medical Science Korean Academy of Medical Sciences Free PMC article

Proceed to details

Cite

Share

1,169

Observational Study

Crit Care

. 2021 Apr 22;25(1):155.
doi: 10.1186/s13054-021-03543-3.

Safety profile of enhanced thromboprophylaxis strategies for critically ill COVID-19 patients during the first wave of the pandemic: observational report from 28 European intensive care units

Andrea Lavinio [1], Ari Ercole [# 1], Denise Battaglini [# 2], Sandra Magnoni [3], Rafael Badenes [4], Fabio Silvio Taccone [5], Raimund Helbok [6], William Thomas [7], Paolo Pelosi [2 8], Chiara Robba [9 10], collaborators
Collaborators, Affiliations Expand

Collaborators

- **collaborators**:
Nicole Innerhofer, Sara Miori, Alberto Librizzi, Rita Bertuetti, Nicolas Figueiredo Faria, Lorenzo Peluso, Giorgia Montrucchio, Gabriele Sales, Luca Brazzi, Daniela Alampi, Maria Beatrice Manca, Lilia Sepe, Giuseppe Natalini, Antonio Bellino, Maria Grazia Bocci, Chiara Mattana, Francesco Corradi, Francesco Forfori, Francesco Cundari, Emilio Bonvecchio, Zara Busani, Andrea Bianchin, Carla Federico, Anna Santoro, Federico Bilotta, Giorgio Rajani, Berta Moleon Lopez, Raffaele Aspide, Merola Raffaele, Luca Cabrini, Alessandro Motta, Lara Frattini, Alexandre Godon, Pierre Bouzat, Elena Grappa, Alberto Bonvecchio, Nicole Innerhofer, Dietmar Fries, Christian Preuss Hernandez, Claudius Thomé, Sebastian Klein, Michael Joannidis, Paolo Pelosi, Lorenzo Ball, Nicolo' Patroniti, Iole Brunetti, Matteo Bassetti, Daniele Roberto Giacobbe, Antonio Vena, Alberto Valbusa, Italo Porto, Roberta Della Bona

Affiliations

- [1] Neurosciences and Trauma Critical Care Unit, Addenbrookes Hospital Cambridge, Cambridge, UK.
- [2] San Martino Policlinico Hospital, IRCCS for Oncology and Neurosciences, Genoa, Italy.
- [3] Anestesia e Rianimazione Ospedale Santa Chiara, APSS, Trento, Italy.
- [4] Department of Anesthesia and Intensive Care, Hospital Clinic Universitari, University of Valencia, INCLIVA Research Health Institute, Valencia, Spain. rafaelbadenes@gmail.com.
- [5] Department of Intensive Care, Hopital Erasme, Université Libre de Bruxelles, Brussels, Belgium.
- [6] Department of Neurology, Neurocritical Care Unit, Medical University of Innsbruck, Innsbruck, Austria.
- [7] Hematology Department, Addenbrookes Hospital, Cambridge, UK.
- [8] Department of Surgical Sciences and Integrated Diagnostics (DISC), University of Genova, Genoa, Italy.
- [9] San Martino Policlinico Hospital, IRCCS for Oncology and Neurosciences, Genoa, Italy. kiarobba@gmail.com.
- [10] Department of Surgical Sciences and Integrated Diagnostics (DISC), University of Genova, Genoa, Italy. kiarobba@gmail.com.

# Contributed equally.

- PMID: **33888132**
- PMCID: PMC8061459
- DOI: 10.1186/s13054-021-03543-3

Free PMC article
Observational Study

Safety profile of enhanced thromboprophylaxis strategies for critically ill COVID-19 patients during the first wave of the pandemic: observational report from 28 European intensive care units

Andrea Lavinio et al. Crit Care. 2021.
Free PMC article
Show details
Crit Care
. 2021 Apr 22;25(1):155.
doi: 10.1186/s13054-021-03543-3.

Authors

Andrea Lavinio [1], Ari Ercole [# 1], Denise Battaglini [# 2], Sandra Magnoni [3], Rafael Badenes [4], Fabio Silvio Taccone [5], Raimund Helbok [6], William Thomas [7], Paolo Pelosi [2 8], Chiara Robba [9 10], collaborators

Collaborators

- **collaborators**: Nicole Innerhofer, Sara Miori, Alberto Librizzi, Rita Bertuetti, Nicolas Figueiredo Faria, Lorenzo Peluso, Giorgia Montrucchio, Gabriele Sales, Luca Brazzi, Daniela Alampi, Maria Beatrice Manca, Lilia Sepe, Giuseppe Natalini, Antonio Bellino, Maria Grazia Bocci, Chiara Mattana, Francesco Corradi, Francesco Forfori, Francesco Cundari, Emilio Bonvecchio, Zara Busani, Andrea Bianchin, Carla Federico, Anna Santoro, Federico Bilotta, Giorgio Rajani, Berta Moleon Lopez, Raffaele Aspide, Merola Raffaele, Luca Cabrini, Alessandro Motta, Lara Frattini, Alexandre Godon, Pierre Bouzat, Elena Grappa, Alberto Bonvecchio, Nicole Innerhofer, Dietmar Fries, Christian Preuss Hernandez, Claudius Thomé, Sebastian Klein, Michael Joannidis, Paolo Pelosi, Lorenzo Ball, Nicolo' Patroniti, Iole Brunetti, Matteo Bassetti, Daniele Roberto Giacobbe, Antonio Vena, Alberto Valbusa, Italo Porto, Roberta Della Bona

Affiliations

- [1] Neurosciences and Trauma Critical Care Unit, Addenbrookes Hospital Cambridge, Cambridge, UK.
- [2] San Martino Policlinico Hospital, IRCCS for Oncology and Neurosciences, Genoa, Italy.
- [3] Anestesia e Rianimazione Ospedale Santa Chiara, APSS, Trento, Italy.
- [4] Department of Anesthesia and Intensive Care, Hospital Clinic Universitari, University of Valencia, INCLIVA Research Health Institute, Valencia, Spain. rafaelbadenes@gmail.com.
- [5] Department of Intensive Care, Hopital Erasme, Université Libre de Bruxelles, Brussels, Belgium.
- [6] Department of Neurology, Neurocritical Care Unit, Medical University of Innsbruck, Innsbruck, Austria.
- [7] Hematology Department, Addenbrookes Hospital, Cambridge, UK.
- [8] Department of Surgical Sciences and Integrated Diagnostics (DISC), University of Genova, Genoa, Italy.
- [9] San Martino Policlinico Hospital, IRCCS for Oncology and Neurosciences, Genoa, Italy. kiarobba@gmail.com.
- [10] Department of Surgical Sciences and Integrated Diagnostics (DISC), University of Genova, Genoa, Italy. kiarobba@gmail.com.

[#] Contributed equally.

- PMID: **33888132**
- PMCID: PMC8061459
- DOI: 10.1186/s13054-021-03543-3

Abstract

Introduction: Critical illness from SARS-CoV-2 infection (COVID-19) is associated with a high burden of pulmonary embolism (PE) and thromboembolic events despite standard thromboprophylaxis. Available guidance is discordant, ranging from standard care to the use of therapeutic anticoagulation for enhanced thromboprophylaxis (ET). Local ET protocols have been empirically determined and are generally intermediate between standard prophylaxis and full anticoagulation. Concerns have been raised in regard to the potential risk of haemorrhage associated with therapeutic anticoagulation. This report describes the prevalence and safety of ET strategies in European Intensive Care Unit (ICUs) and their association with outcomes during the first wave of the COVID pandemic, with particular focus on haemorrhagic complications and ICU mortality.

Methods: Retrospective, observational, multi-centre study including adult critically ill COVID-19 patients. Anonymised data included demographics, clinical characteristics, thromboprophylaxis and/or anticoagulation treatment. Critical haemorrhage was defined as intracranial haemorrhage or bleeding requiring red blood cells transfusion. Survival was collected at ICU discharge. A multivariable mixed effects generalised linear model analysis matched for the propensity for receiving ET was constructed for both ICU mortality and critical haemorrhage.

Results: A total of 852 (79% male, age 66 [37-85] years) patients were included from 28 ICUs. Median body mass index and ICU length of stay were 27.7 (25.1-30.7) Kg/m^2 and 13 (7-22) days, respectively. Thromboembolic events were reported in 146 patients (17.1%), of those 78 (9.2%) were PE. ICU mortality occurred in 335/852 (39.3%) patients. ET was used in 274 (32.1%) patients, and it was independently associated with significant reduction in ICU mortality (log odds = 0.64 [95% CIs 0.18-1.1; p = 0.0069]) but not an increased risk of critical haemorrhage (log odds = 0.187 [95%CI - 0.591 to - 0.964; p = 0.64]).

Conclusions: In a cohort of critically ill patients with a high prevalence of thromboembolic events, ET was associated with reduced ICU mortality without an increased burden of haemorrhagic complications. This study suggests ET strategies are safe and associated with favourable outcomes. Whilst full anticoagulation has been questioned for prophylaxis in these patients, our results suggest that there may nevertheless be a role for enhanced / intermediate levels of prophylaxis. Clinical trials investigating causal relationship between intermediate thromboprophylaxis and clinical outcomes are urgently needed.

Keywords: Anticoagulation; COVID-19; Heparin; Intensive care medicine; Prophylaxis; Thrombosis.

Conflict of interest statement

None.

- 26 references
- 1 figure

Supplementary info

Publication types, MeSH terms, Substances Expand

Publication types

- Observational Study

MeSH terms

- Adult
- Aged
- Aged, 80 and over
- Anticoagulants / adverse effects*
- COVID-19 / drug therapy*
- COVID-19 / epidemiology
- Critical Care / methods*
- Critical Illness
- Europe / epidemiology
- Female
- Humans
- Intensive Care Units
- Male
- Middle Aged
- Pandemics*
- Treatment Outcome
- Venous Thromboembolism / prevention & control*

Substances

- Anticoagulants

Full text links

Read free full text at BMC BioMed Central Free PMC article
Proceed to details
Cite
Share
1,170
Observational Study
J Orthop Trauma
. 2020 Sep;34(9):e325-e329.
doi: 10.1097/BOT.0000000000001889.

Thirty-Day Mortality Rate of Patients With Hip Fractures During the COVID-19 Pandemic: A Single Centre Prospective Study in the United Kingdom

Amit Thakrar [1], Karen Chui, Akhil Kapoor, John Hambidge
Affiliations Expand

Affiliation

- [1] Trauma and Orthopedic Department, Queen's Hospital, Barking, Havering and Redbridge University Hospitals NHS Trust, Romford, United Kingdom.

- PMID: **32815846**
- PMCID: PMC7446986
- DOI: 10.1097/BOT.0000000000001889

Free PMC article
Observational Study

Thirty-Day Mortality Rate of Patients With Hip Fractures During the COVID-19 Pandemic: A Single Centre Prospective Study in the United Kingdom

Amit Thakrar et al. J Orthop Trauma. 2020 Sep.
Free PMC article
Show details
J Orthop Trauma
. 2020 Sep;34(9):e325-e329.
doi: 10.1097/BOT.0000000000001889.

Authors

Amit Thakrar [1], Karen Chui, Akhil Kapoor, John Hambidge

Affiliation

- [1] Trauma and Orthopedic Department, Queen's Hospital, Barking, Havering and Redbridge University Hospitals NHS Trust, Romford, United Kingdom.

- PMID: **32815846**
- PMCID: PMC7446986
- DOI: 10.1097/BOT.0000000000001889

Abstract

Objectives: During the COVID-19 pandemic, the care of hip fracture patients remains a clinical priority. Our study aims to investigate the 30-day mortality rate of hip fracture patients during the first 30 days of the pandemic in the United Kingdom.

Methods: A single-center, observational, prospective study of patients presenting with hip fractures. Data collection started from "day 0" of the COVID-19 pandemic in the United Kingdom and continued for 30 days. We collected data on time to surgery, Clinical Frailty Scale score, Nottingham Hip Fracture Score, COVID-19 infection status, 30-day mortality, and cause of death. For comparison, we collected retrospective data during the same 30-day period in 2018, 2019, and the previous 6 months (Control groups A, B, and C, respectively).

Results: Forty-three patients were included in the study. There was no difference in age or gender between the Study and Control groups. The 30-day mortality rate of the Study group was 16.3%, which was higher than Control groups A ($P = 0.022$), B ($P = 0.003$) and C ($P = 0.001$). The prevalence of COVID-19 infection in our Study group was 26%. Of the 7 mortalities recorded, 4 patients tested positive for COVID-19 infection. In our Study group, COVID-19 infection correlated significantly with 30-day mortality ($P = 0.002$, odds ratio 2.4).

Conclusions: Our study demonstrated a significant increase in 30-day mortality among hip fracture patients during the first 30 days of the COVID-19 pandemic in the United Kingdom. A positive COVID-19 test result in patients with hip fractures is associated with a 2.4-fold increase in risk of 30-day mortality.

Level of evidence: Prognostic Level II. See Instructions for Authors for a complete description of levels of evidence.

Conflict of interest statement

The authors report no conflict of interest.

- 14 references

Supplementary info

Publication types, MeSH terms Expand

Publication types

- Observational Study

MeSH terms

- Aged
- Aged, 80 and over
- Arthroplasty, Replacement, Hip
- Betacoronavirus*
- COVID-19
- Coronavirus Infections / diagnosis
- Coronavirus Infections / epidemiology*
- Female
- Fracture Fixation, Internal
- Hip Fractures / complications
- Hip Fractures / mortality*
- Hip Fractures / surgery
- Hospital Mortality
- Humans
- Male
- Middle Aged
- Pandemics
- Pneumonia, Viral / diagnosis
- Pneumonia, Viral / epidemiology*
- Prospective Studies
- Risk Factors
- SARS-CoV-2
- Survival Rate
- Time Factors
- United Kingdom

Full text links

Wolters Kluwer Wolters Kluwer Free PMC article
Proceed to details
Cite

Share

☐ 1,171

Observational Study

J Infect Dis

. 2021 Jun 4;223(11):1879-1886.

doi: 10.1093/infdis/jiaa626.

Comparing Clinical Characteristics of Influenza and Common Coronavirus Infections Using Electronic Health Records

Dadong Li [1], Donna M Wolk [2], Michael N Cantor [1]

Affiliations Expand

Affiliations

- [1] Regeneron Genetics Center, Tarrytown, New York, USA.
- [2] Geisinger Health System, Danville, Pennsylvania, USA.

- PMID: **33011809**
- PMCID: PMC7797744
- DOI: 10.1093/infdis/jiaa626

Free PMC article

Observational Study

Comparing Clinical Characteristics of Influenza and Common Coronavirus Infections Using Electronic Health Records

Dadong Li et al. J Infect Dis. 2021.

Free PMC article

Show details

J Infect Dis

. 2021 Jun 4;223(11):1879-1886.

doi: 10.1093/infdis/jiaa626.

Authors

Dadong Li [1], Donna M Wolk [2], Michael N Cantor [1]

Affiliations

- [1] Regeneron Genetics Center, Tarrytown, New York, USA.
- [2] Geisinger Health System, Danville, Pennsylvania, USA.

- PMID: **33011809**
- PMCID: PMC7797744
- DOI: 10.1093/infdis/jiaa626

Abstract

Background: We compared outcomes in inpatients and outpatients, pre-COVID-19, who were infected with either coronavirus or influenza.

Methods: Using deidentified electronic health records data from the Geisinger-Regeneron partnership, we compared patients with RT-PCR-positive tests for the 4 common coronaviruses (229E, HKU1, NL63, OC43) or influenza (A and B) from June 2016 to February 2019.

Results: Overall, 52 833 patients were tested for coronaviruses and influenza. For patients ≥21 years old, 1555 and 3991 patient encounters had confirmed positive coronavirus and influenza tests, respectively. Both groups had similar intensive care unit (ICU) admission rates (7.2% vs 6.1%, P = .12), although patients with coronavirus had significantly more pneumonia (15% vs 7.4%, P < .001) and higher death rate within 30 days (4.9% vs 3.0%, P < .001). After controlling for other covariates, coronavirus infection still had a higher risk of death and pneumonia than influenza (odds ratio, 1.64 and 2.05, P < .001), with no significant difference in ICU admission rates.

Conclusions: Common coronaviruses cause significant morbidity, with potentially worse outcomes than influenza. Identifying a subset of patients who are more susceptible to poor outcomes from common coronavirus infections may help plan clinical interventions in patients with suspected infections.

Keywords: common coronavirus; electronic health records; epidemiology; influenza.

© The Author(s) 2020. Published by Oxford University Press for the Infectious Diseases Society of America. All rights reserved. For permissions, e-mail: journals.permissions@oup.com.

Comment in

- An Opportunity to Better Understand the Impact of Coronaviruses on Immunocompromised Patients.
  Parisi C. Parisi C. J Infect Dis. 2021 Jul 15;224(2):372-373. doi: 10.1093/infdis/jiaa720. J Infect Dis. 2021. PMID: 33220053 Free PMC article. No abstract available.

- 17 references
- 2 figures

Supplementary info

Publication types, MeSH terms Expand

Publication types

- Comparative Study
- Observational Study

MeSH terms

- Adult
- Age Factors
- Aged
- Coronavirus Infections / mortality
- Coronavirus Infections / pathology*
- Electronic Health Records* / statistics & numerical data
- Hospitalization / statistics & numerical data
- Humans
- Influenza, Human / mortality
- Influenza, Human / pathology*
- Intensive Care Units / statistics & numerical data
- Middle Aged
- Retrospective Studies
- Risk Factors

Full text links

OXFORD ACADEMIC Silverchair Information Systems Free PMC article

Proceed to details

Cite

Share

1,172

Observational Study

Radiology

. 2020 Dec;297(3):E313-E323.

doi: 10.1148/radiol.2020202422. Epub 2020 Jul 17.

Retrospective Observational Study of Brain MRI Findings in Patients with Acute SARS-CoV-2 Infection and Neurologic Manifestations

Lydia Chougar [1], Natalia Shor [1], Nicolas Weiss [1], Damien Galanaud [1], Delphine Leclercq [1], Bertrand Mathon [1], Samia Belkacem [1], Sebastian Ströer [1], Sonia Burrel [1], David Boutolleau [1], Alexandre Demoule [1], Charlotte Rosso [1], Cécile Delorme [1], Danielle Seilhean [1], Didier Dormont [1], Elise Morawiec [1], Mathieu Raux [1], Sophie Demeret [1], Sophie Gerber [1], Stéphanie

Trunet [1], Thomas Similowski [1], Vincent Degos [1], Pierre Rufat [1], Jean-Christophe Corvol [1], Stéphane Lehéricy [1], Nadya Pyatigorskaya [1], CoCo Neurosciences Study Group [1]
Affiliations Expand

Affiliation

- [1] From the Sorbonne Université, Inserm, CNRS, Institut du Cerveau-Paris Brain Institute (ICM), F-75013 Paris, France (L.C., D.G., B.M., C.R., D.D., J.C.C., S.L., N.P.); Sorbonne Université, INSERM 75013 Paris, France (L.C., N.S., N.W., D.G., B.M., S. Burrel, D.B., A.D., C.R., D.S., D.D., E.M., M.R., T.S., V.D., J.C.C., S.L., N.P.); Paris Brain Institute - ICM, Movement Investigations and Therapeutics Team (MOV'IT), Paris, France (L.C., S.L., N.P.); ICM, Centre de NeuroImagerie de Recherche-CENIR, Paris, France (L.C., D.G., S.L., N.P.); Assistance Publique Hôpitaux de Paris, Hôpital Pitié-Salpêtrière, Service de Neuroradiologie (L.C., N.S., D.G., D.L., S. Belkacem, S.S., D.D., S.G., S.T., S.L., N.P.), Médecine Intensive Réanimation Neurologique (N.W.), Service de Neurochirurgie (B.M.), Service de Virologie, Centre d'Investigation Clinique Neurosciences (S. Burrel, D.B.), Service de Pneumologie, Médecine Intensive et Réanimation (A.D., E.M., T.S.), Urgences Cérébro-Vasculaires (C.R.), Département de Neurologie, Centre d'Investigation Clinique Neurosciences (C.D., J.C.C.), Département de Neuropathologie (D.S.), Department of Anesthesia, Critical Care and Peri-Operative Medicine (M.R., V.D.), Paris, France; Brain Liver Pitié-Salpêtrière Study Group, INSERM UMR S 938, Centre de Recherche Saint-Antoine, Maladies Métaboliques, biliaires et fibro-inflammatoire du foie, Institute of Cardiometabolism and Nutrition (N.W., S.D.); CNR Herpèsvirus (laboratoire associé HSV), SU-INSERM UMR_S 1136 Team 3 THERAVIR IPLESP (S. Burrel, D.B.); ICM, Stroke Network, STAR Team, Paris, France (C.R.); Assistance Publique Hôpitaux de Paris, Hôpital Pitié-Salpêtrière,; ICM, INRIA, ARAMIS project-team, Paris, France (D.D., M.R.); Clinical Research Group ARPE, Sorbonne University, Paris, France (V.D.); INSERM UMR 1141, Paris France (V.D.); and Assistance Publique Hôpitaux de Paris, DMU ESPRIT, Paris, France (P.R.).

- PMID: **32677875**
- PMCID: PMC7370354
- DOI: 10.1148/radiol.2020202422

Free PMC article
Observational Study

Retrospective Observational Study of Brain MRI Findings in Patients with Acute SARS-CoV-2 Infection and Neurologic Manifestations

Lydia Chougar et al. Radiology. 2020 Dec.
Free PMC article
Show details
Radiology
. 2020 Dec;297(3):E313-E323.

doi: 10.1148/radiol.2020202422. Epub 2020 Jul 17.

Authors

Lydia Chougar [1], Natalia Shor [1], Nicolas Weiss [1], Damien Galanaud [1], Delphine Leclercq [1], Bertrand Mathon [1], Samia Belkacem [1], Sebastian Ströer [1], Sonia Burrel [1], David Boutolleau [1], Alexandre Demoule [1], Charlotte Rosso [1], Cécile Delorme [1], Danielle Seilhean [1], Didier Dormont [1], Elise Morawiec [1], Mathieu Raux [1], Sophie Demeret [1], Sophie Gerber [1], Stéphanie Trunet [1], Thomas Similowski [1], Vincent Degos [1], Pierre Rufat [1], Jean-Christophe Corvol [1], Stéphane Lehéricy [1], Nadya Pyatigorskaya [1], CoCo Neurosciences Study Group [1]

Affiliation

- [1] From the Sorbonne Université, Inserm, CNRS, Institut du Cerveau-Paris Brain Institute (ICM), F-75013 Paris, France (L.C., D.G., B.M., C.R., D.D., J.C.C., S.L., N.P.); Sorbonne Université, INSERM 75013 Paris, France (L.C., N.S., N.W., D.G., B.M., S. Burrel, D.B., A.D., C.R., D.S., D.D., E.M., M.R., T.S., V.D., J.C.C., S.L., N.P.); Paris Brain Institute - ICM, Movement Investigations and Therapeutics Team (MOV'IT), Paris, France (L.C., S.L., N.P.); ICM, Centre de NeuroImagerie de Recherche-CENIR, Paris, France (L.C., D.G., S.L., N.P.); Assistance Publique Hôpitaux de Paris, Hôpital Pitié-Salpêtrière, Service de Neuroradiologie (L.C., N.S., D.G., D.L., S. Belkacem, S.S., D.D., S.G., S.T., S.L., N.P.), Médecine Intensive Réanimation Neurologique (N.W.), Service de Neurochirurgie (B.M.), Service de Virologie, Centre d'Investigation Clinique Neurosciences (S. Burrel, D.B.), Service de Pneumologie, Médecine Intensive et Réanimation (A.D., E.M., T.S.), Urgences Cérébro-Vasculaires (C.R.), Département de Neurologie, Centre d'Investigation Clinique Neurosciences (C.D., J.C.C.), Département de Neuropathologie (D.S.), Department of Anesthesia, Critical Care and Peri-Operative Medicine (M.R., V.D.), Paris, France; Brain Liver Pitié-Salpêtrière Study Group, INSERM UMR S 938, Centre de Recherche Saint-Antoine, Maladies Métaboliques, biliaires et fibro-inflammatoire du foie, Institute of Cardiometabolism and Nutrition (N.W., S.D.); CNR Herpèsvirus (laboratoire associé HSV), SU-INSERM UMR_S 1136 Team 3 THERAVIR IPLESP (S. Burrel, D.B.); ICM, Stroke Network, STAR Team, Paris, France (C.R.); Assistance Publique Hôpitaux de Paris, Hôpital Pitié-Salpêtrière,; ICM, INRIA, ARAMIS project-team, Paris, France (D.D., M.R.); Clinical Research Group ARPE, Sorbonne University, Paris, France (V.D.); INSERM UMR 1141, Paris France (V.D.); and Assistance Publique Hôpitaux de Paris, DMU ESPRIT, Paris, France (P.R.).

- PMID: **32677875**
- PMCID: PMC7370354
- DOI: 10.1148/radiol.2020202422

Abstract

Background This study provides a detailed imaging assessment in a large series of patients infected with coronavirus disease 2019 (COVID-19) and presenting with neurologic manifestations. Purpose To review the MRI findings associated with acute neurologic manifestations in patients with COVID-19. Materials and Methods This was a cross-sectional study conducted between March 23 and May 7, 2020, at the Pitié-Salpêtrière Hospital, a reference center for COVID-19 in the Paris area. Adult patients were included if they had a diagnosis of severe acute respiratory syndrome coronavirus 2 (SARS-CoV-2) infection with acute neurologic manifestations and referral for brain MRI. Patients with a prior history of neurologic disease were

excluded. The characteristics and frequency of different MRI features were investigated. The findings were analyzed separately in patients in intensive care units (ICUs) and other departments (non-ICU). Results During the inclusion period, 1176 patients suspected of having COVID-19 were hospitalized. Of 308 patients with acute neurologic symptoms, 73 met the inclusion criteria and were included (23.7%): thirty-five patients were in the ICU (47.9%) and 38 were not (52.1%). The mean age was 58.5 years ± 15.6 [standard deviation], with a male predominance (65.8% vs 34.2%). Forty-three patients had abnormal MRI findings 2-4 weeks after symptom onset (58.9%), including 17 with acute ischemic infarct (23.3%), one with a deep venous thrombosis (1.4%), eight with multiple microhemorrhages (11.3%), 22 with perfusion abnormalities (47.7%), and three with restricted diffusion foci within the corpus callosum consistent with cytotoxic lesions of the corpus callosum (4.1%). Multifocal white matter-enhancing lesions were seen in four patients in the ICU (5%). Basal ganglia abnormalities were seen in four other patients (5%). Cerebrospinal fluid analyses were negative for SARS-CoV-2 in all patients tested (n = 39). Conclusion In addition to cerebrovascular lesions, perfusion abnormalities, cytotoxic lesions of the corpus callosum, and intensive care unit-related complications, we identified two patterns including white matter-enhancing lesions and basal ganglia abnormalities that could be related to severe acute respiratory syndrome coronavirus 2 infection. © RSNA, 2020 *Online supplemental material is available for this article.*

- 38 references
- 6 figures

Supplementary info

Publication types, MeSH terms Expand

Publication types

- Observational Study
- Research Support, Non-U.S. Gov't

MeSH terms

- Acute Disease
- Adult
- Aged
- Aged, 80 and over
- Betacoronavirus
- Brain / diagnostic imaging*
- Brain / physiopathology
- COVID-19
- Cerebrovascular Disorders / complications*
- Cerebrovascular Disorders / diagnostic imaging*
- Cerebrovascular Disorders / physiopathology
- Coronavirus Infections / complications*
- Coronavirus Infections / physiopathology
- Cross-Sectional Studies

- Female
- Humans
- Magnetic Resonance Imaging / methods*
- Male
- Middle Aged
- Pandemics
- Pneumonia, Viral / complications*
- Pneumonia, Viral / physiopathology
- Retrospective Studies
- SARS-CoV-2

Full text links

Radiology FULL TEXT Atypon Free PMC article
Proceed to details
Cite
Share
1,173
Observational Study
Respir Med
. Aug-Sep 2020;170:106062.
doi: 10.1016/j.rmed.2020.106062. Epub 2020 Jun 8.

Impact of SARS-CoV-2 infection in patients with cystic fibrosis in Spain: Incidence and results of the national CF-COVID19-Spain survey

Pedro Mondejar-Lopez [1], Esther Quintana-Gallego [2], Rosa M Giron-Moreno [3], Isidoro Cortell-Aznar [4], Marta Ruiz de Valbuena-Maiz [5], Layla Diab-Caceres [6], Concepcion Prados-Sanchez [7], Antonio Alvarez-Fernandez [8], Patricia W Garcia-Marcos [9], Carlos Peñalver-Mellado [10], M Dolores Pastor-Vivero [11], Casilda Olveira [12], Alejandro Lopez-Neyra [13], Silvia Castillo-Corullon [14], Samara Palma-Milla [15], Estela Perez-Ruiz [16], Amparo Sole-Jover [17], M Isabel Barrio [5], Manuel Sanchez-Solis [18], Óscar Asensio de la Cruz [19], CF-COVID19-Spain Registry Group
Collaborators, Affiliations Expand

Collaborators

- **CF-COVID19-Spain Registry Group**: Antonio J Aguilar-Fernández [20], Anselmo Andres-Martin [21], Marina Blanco-Aparicio [22], Josep Sirvent-Gomez [22], Verisima Barajas-Sanchez [23], Felix Baranda [24], Ainhoa

Gomez-Bonilla [24], Carlos Bousoño-Garcia [25], Marta Garcia-Clemente [25], Catalina Bover-Bauza [26], Joan Figuerola-Mulet [26], M Jesús Cabero-Perez [27], David Iturbe-Fernandez [27], Laura Carrasco-Hernandez [28], Isabel Delgado-Pecellin [28], Francisco Casas-Maldonado [29], Silvia Merlos-Navarro [29], Alfredo Valenzuela-Soria [29], J Antonio Cascante-Rodrigo [30], Laura Moreno-Galarraga [30], Maria Cols-Roig [31], Ofelia Fernandez de la Cruz [31], Francisco J Gomez de Terreros [31], Carlos Garcia-Magan [32], Silvia Gartner [33], David Gomez-Pastrana [34], Estela Gonzalez-Castro [35], Esperanza Jimenez-Nogueira [35], M Ines Herrero-Labarga [36], Carlos Martín de Vicente [36], Mercedes Juste-Ruiz [37], Adelaida Lamas-Ferreiro [38], Luis Maiz-Carro [38], M Carmen Luna-Paredes [39], Orlando Mesa-Medina [40], Concepcion Monton-Soler [41], Cristina Ramos-Hernandez [42], M Jesus Rodriguez-Saez [42], J Alejandro Romero-Albillos [43], Veronica Sanz-Santiago [44], J Ramón Villa-Asensi [44], Javier Torres-Borrego [45], J Manuel Vaquero-Barrios [45], Rosa Velasco-Bernardo [46], Valle Velasco-Gonzalez [47]

Affiliations

- [1] Pediatric Pulmonology and Cystic Fibrosis Unit, Hospital Clinico Universitario Virgen de la Arrixaca, Murcia, Spain; Department of Surgery, Paediatrics, Obstetrics and Genecology, Universidad de Murcia, Spain. Electronic address: pedroe.mondejar@carm.es.
- [2] Cystic Fibrosis Unit, Medical-Surgical Unit for Respiratory Diseases, Instituto de Biomedicina de Sevilla (IBiS), Hospital Universitario Virgen Del Rocío, Sevilla, Spain; Centre for Biomedical Research Network of Respiratory Diseases (CIBERES), Instituto de Salud Carlos III, Madrid, Spain.
- [3] Cystic Fibrosis and Bronchiectasis Unit, Instituto de Investigacion, Hospital Universitario de la Princesa, Madrid, Spain.
- [4] Pediatric Pulmonology and Allergy Unit, Cystic Fibrosis Unit, Hospital Universitari i Politecnic La Fe, Valencia, Spain.
- [5] Pediatric Pulmonology and Cystic Fibrosis Unit, Hospital Universitario La Paz, Madrid, Spain.
- [6] Adult Cystic Fibrosis Unit, Pulmonology Service, Hospital Universitario 12 de Octubre, Madrid, Spain.
- [7] Pulmonology Service, Cystic Fibrosis and Bronchiectasis Unit, Hospital Universitario La Paz, Madrid, Spain.
- [8] Adult Cystic Fibrosis Unit, Pulmonology Service, Hospital Vall D'Hebron, Barcelona, Spain; Vall D'Hebron Institut de Recerca, Barcelona, Spain.
- [9] Pediatric Pulmonology and Cystic Fibrosis Unit, Hospital Clinico Universitario Virgen de la Arrixaca, Murcia, Spain.
- [10] Pulmonology Service. Hospital Clinico Universitario Virgen de La Arrixaca, Murcia, Spain.
- [11] Osakidetza. Organización Sanitaria Integrada Ezkerraldea-Enkarterri-Cruces, Hospital Universitario Cruces, Paediatrics, Cystic Fibrosis Unit, Bizkaia, Spain.
- [12] Cystic Fibrosis and Bronchiectasis Unit, Pulmonology Service, Hospital Regional Universitario, Malaga, Spain; Biomedical Research Institute of Malaga (IBIMA), Universidad de Malaga, Spain.
- [13] Cystic Fibrosis Unit, Pediatrics Service, Hospital Universitario Ramón y Cajal, Madrid, Spain.
- [14] Pediatric Pulmonology and Cystic Fibrosis Unit, Hospital Clínico Universitario, Valencia, Spain.

- [15] Endocrinology and Nutrition Service, Cystic Fibrosis Unit, Hospital Universitario La Paz, Madrid, Spain.
- [16] Cystic Fibrosis and Bronchiectasis Unit, Pulmonology Service, Hospital Regional Universitario, Malaga, Spain; Pediatric Pulmonology and Cystic Fibrosis Unit, UGC de Pediatría, Hospital Regional Universitario, Málaga, Spain.
- [17] Lung Transplantation and Cystic Fibrosis Unit, Hospital Universitario La Fe, Universidad de Valencia, Valencia, Spain.
- [18] Pediatric Pulmonology and Cystic Fibrosis Unit, Hospital Clinico Universitario Virgen de la Arrixaca, Murcia, Spain; Department of Surgery, Paediatrics, Obstetrics and Genecology, Universidad de Murcia, Spain; Biomedical Research Institute of Murcia (IMIB), Murcia, Spain.
- [19] Cystic Fibrosis Unit, Pediatric Pulmonology and Allergy Unit, Hospital Universitari Parc Tauli, Sabadell, Barcelona, Spain; Faculty of Medicine, Universidad Autonoma de Barcelona, Spain.
- [20] Hospital Materno-Infantil, Las Palmas de Gran Canaria, Spain.
- [21] Hospital Universitario Virgen Macarena, Sevilla, Spain.
- [22] Hospital Universitario A Coruña, Spain.
- [23] Complejo Asistencial Universitario de Salamanca, Spain.
- [24] Hospital Universitario Cruces, Barakaldo, Vizcaya, Spain.
- [25] Hospital Central de Asturias, Oviedo, Spain.
- [26] Hospital Universitari Son Espases, Palma de Mallorca, Spain.
- [27] Hospital Universitario Marques de Valdecilla, Santander, Spain.
- [28] Hospital Universitario Virgen Del Rocio, Sevilla, Spain.
- [29] Hospital Universitario Virgen de las Nieves, Granada, Spain.
- [30] Complejo Hospitalario de Navarra, Pamplona, Spain.
- [31] Hospital Sant Joan de Deu, Barcelona, Spain.
- [32] Hospital Universitario de Santiago de Compostela, A Coruña, Spain.
- [33] Hospital Universitario Vall D'Hebron, Barcelona, Spain.
- [34] Hospital Universitario de Jerez de la Frontera, Cadiz, Spain.
- [35] Hospital Universitario Torrecardenas, Almeria, Spain.
- [36] Hospital Universitario Miguel Servet, Zaragoza, Spain.
- [37] Hospital Clinico Universitario San Juan de Alicante, Spain.
- [38] Hospital Universitario Ramon y Cajal, Madrid, Spain.
- [39] Hospital Universitario 12 de Octubre, Madrid, Spain.
- [40] Hospital Universitario Nuestra Señora de Candelaria, Santa Cruz de Tenerife, Spain.
- [41] Hospital Universitario Parc Tauli, Sabadell, Barcelona, Spain.
- [42] Hospital Alvaro Cunqueiro, Vigo, Pontevedra, Spain.
- [43] Hospital Universitario de Badajoz, Spain.
- [44] Hospital Universitario Infantil Niño Jesus, Madrid, Spain.
- [45] Hospital Universitario Reina Sofia, Cordoba, Spain.
- [46] Hospital Virgen de la Salud, Toledo, Spain.
- [47] Hospital Universitario de Canarias, La Laguna, Santa Cruz de Tenerife, Spain.

- PMID: **32843180**
- PMCID: PMC7831988
- DOI: 10.1016/j.rmed.2020.106062

Free PMC article

Observational Study

Impact of SARS-CoV-2 infection in patients with cystic fibrosis in Spain: Incidence and results of the national CF-COVID19-Spain survey

Pedro Mondejar-Lopez et al. Respir Med. Aug-Sep 2020.
Free PMC article
Show details
Respir Med
. Aug-Sep 2020;170:106062.
doi: 10.1016/j.rmed.2020.106062. Epub 2020 Jun 8.

Authors

Pedro Mondejar-Lopez [1], Esther Quintana-Gallego [2], Rosa M Giron-Moreno [3], Isidoro Cortell-Aznar [4], Marta Ruiz de Valbuena-Maiz [5], Layla Diab-Caceres [6], Concepcion Prados-Sanchez [7], Antonio Alvarez-Fernandez [8], Patricia W Garcia-Marcos [9], Carlos Peñalver-Mellado [10], M Dolores Pastor-Vivero [11], Casilda Olveira [12], Alejandro Lopez-Neyra [13], Silvia Castillo-Corullon [14], Samara Palma-Milla [15], Estela Perez-Ruiz [16], Amparo Sole-Jover [17], M Isabel Barrio [5], Manuel Sanchez-Solis [18], Óscar Asensio de la Cruz [19], CF-COVID19-Spain Registry Group

Collaborators

- **CF-COVID19-Spain Registry Group**:
  Antonio J Aguilar-Fernández [20], Anselmo Andres-Martin [21], Marina Blanco-Aparicio [22], Josep Sirvent-Gomez [22], Verisima Barajas-Sanchez [23], Felix Baranda [24], Ainhoa Gomez-Bonilla [24], Carlos Bousoño-Garcia [25], Marta Garcia-Clemente [25], Catalina Bover-Bauza [26], Joan Figuerola-Mulet [26], M Jesús Cabero-Perez [27], David Iturbe-Fernandez [27], Laura Carrasco-Hernandez [28], Isabel Delgado-Pecellin [28], Francisco Casas-Maldonado [29], Silvia Merlos-Navarro [29], Alfredo Valenzuela-Soria [29], J Antonio Cascante-Rodrigo [30], Laura Moreno-Galarraga [30], Maria Cols-Roig [31], Ofelia Fernandez de la Cruz [31], Francisco J Gomez de Terreros [31], Carlos Garcia-Magan [32], Silvia Gartner [33], David Gomez-Pastrana [34], Estela Gonzalez-Castro [35], Esperanza Jimenez-Nogueira [35], M Ines Herrero-Labarga [36], Carlos Martín de Vicente [36], Mercedes Juste-Ruiz [37], Adelaida Lamas-Ferreiro [38], Luis Maiz-Carro [38], M Carmen Luna-Paredes [39], Orlando Mesa-Medina [40], Concepcion Monton-Soler [41], Cristina Ramos-Hernandez [42], M Jesus Rodriguez-Saez [42], J Alejandro Romero-Albillos [43], Veronica Sanz-Santiago [44], J Ramón Villa-Asensi [44], Javier Torres-Borrego [45], J Manuel Vaquero-Barrios [45], Rosa Velasco-Bernardo [46], Valle Velasco-Gonzalez [47]

Affiliations

- [1] Pediatric Pulmonology and Cystic Fibrosis Unit, Hospital Clinico Universitario Virgen de la Arrixaca, Murcia, Spain; Department of Surgery, Paediatrics, Obstetrics and Genecology, Universidad de Murcia, Spain. Electronic address: pedroe.mondejar@carm.es.
- [2] Cystic Fibrosis Unit, Medical-Surgical Unit for Respiratory Diseases, Instituto de Biomedicina de Sevilla (IBiS), Hospital Universitario Virgen Del Rocío, Sevilla, Spain; Centre for Biomedical Research Network of Respiratory Diseases (CIBERES), Instituto de Salud Carlos III, Madrid, Spain.
- [3] Cystic Fibrosis and Bronchiectasis Unit, Instituto de Investigacion, Hospital Universitario de la Princesa, Madrid, Spain.
- [4] Pediatric Pulmonology and Allergy Unit, Cystic Fibrosis Unit, Hospital Universitari i Politecnic La Fe, Valencia, Spain.
- [5] Pediatric Pulmonology and Cystic Fibrosis Unit, Hospital Universitario La Paz, Madrid, Spain.
- [6] Adult Cystic Fibrosis Unit, Pulmonology Service, Hospital Universitario 12 de Octubre, Madrid, Spain.
- [7] Pulmonology Service, Cystic Fibrosis and Bronchiectasis Unit, Hospital Universitario La Paz, Madrid, Spain.
- [8] Adult Cystic Fibrosis Unit, Pulmonology Service, Hospital Vall D'Hebron, Barcelona, Spain; Vall D'Hebron Institut de Recerca, Barcelona, Spain.
- [9] Pediatric Pulmonology and Cystic Fibrosis Unit, Hospital Clinico Universitario Virgen de la Arrixaca, Murcia, Spain.
- [10] Pulmonology Service. Hospital Clinico Universitario Virgen de La Arrixaca, Murcia, Spain.
- [11] Osakidetza. Organización Sanitaria Integrada Ezkerraldea-Enkarterri-Cruces, Hospital Universitario Cruces, Paediatrics, Cystic Fibrosis Unit, Bizkaia, Spain.
- [12] Cystic Fibrosis and Bronchiectasis Unit, Pulmonology Service, Hospital Regional Universitario, Malaga, Spain; Biomedical Research Institute of Malaga (IBIMA), Universidad de Malaga, Spain.
- [13] Cystic Fibrosis Unit, Pediatrics Service, Hospital Universitario Ramón y Cajal, Madrid, Spain.
- [14] Pediatric Pulmonology and Cystic Fibrosis Unit, Hospital Clínico Universitario, Valencia, Spain.
- [15] Endocrinology and Nutrition Service, Cystic Fibrosis Unit, Hospital Universitario La Paz, Madrid, Spain.
- [16] Cystic Fibrosis and Bronchiectasis Unit, Pulmonology Service, Hospital Regional Universitario, Malaga, Spain; Pediatric Pulmonology and Cystic Fibrosis Unit, UGC de Pediatría, Hospital Regional Universitario, Málaga, Spain.
- [17] Lung Transplantation and Cystic Fibrosis Unit, Hospital Universitario La Fe, Universidad de Valencia, Valencia, Spain.
- [18] Pediatric Pulmonology and Cystic Fibrosis Unit, Hospital Clinico Universitario Virgen de la Arrixaca, Murcia, Spain; Department of Surgery, Paediatrics, Obstetrics and Genecology, Universidad de Murcia, Spain; Biomedical Research Institute of Murcia (IMIB), Murcia, Spain.
- [19] Cystic Fibrosis Unit, Pediatric Pulmonology and Allergy Unit, Hospital Universitari Parc Tauli, Sabadell, Barcelona, Spain; Faculty of Medicine, Universidad Autonoma de Barcelona, Spain.
- [20] Hospital Materno-Infantil, Las Palmas de Gran Canaria, Spain.

- [21] Hospital Universitario Virgen Macarena, Sevilla, Spain.
- [22] Hospital Universitario A Coruña, Spain.
- [23] Complejo Asistencial Universitario de Salamanca, Spain.
- [24] Hospital Universitario Cruces, Barakaldo, Vizcaya, Spain.
- [25] Hospital Central de Asturias, Oviedo, Spain.
- [26] Hospital Universitari Son Espases, Palma de Mallorca, Spain.
- [27] Hospital Universitario Marques de Valdecilla, Santander, Spain.
- [28] Hospital Universitario Virgen Del Rocio, Sevilla, Spain.
- [29] Hospital Universitario Virgen de las Nieves, Granada, Spain.
- [30] Complejo Hospitalario de Navarra, Pamplona, Spain.
- [31] Hospital Sant Joan de Deu, Barcelona, Spain.
- [32] Hospital Universitario de Santiago de Compostela, A Coruña, Spain.
- [33] Hospital Universitario Vall D'Hebron, Barcelona, Spain.
- [34] Hospital Universitario de Jerez de la Frontera, Cadiz, Spain.
- [35] Hospital Universitario Torrecardenas, Almeria, Spain.
- [36] Hospital Universitario Miguel Servet, Zaragoza, Spain.
- [37] Hospital Clinico Universitario San Juan de Alicante, Spain.
- [38] Hospital Universitario Ramon y Cajal, Madrid, Spain.
- [39] Hospital Universitario 12 de Octubre, Madrid, Spain.
- [40] Hospital Universitario Nuestra Señora de Candelaria, Santa Cruz de Tenerife, Spain.
- [41] Hospital Universitario Parc Tauli, Sabadell, Barcelona, Spain.
- [42] Hospital Alvaro Cunqueiro, Vigo, Pontevedra, Spain.
- [43] Hospital Universitario de Badajoz, Spain.
- [44] Hospital Universitario Infantil Niño Jesus, Madrid, Spain.
- [45] Hospital Universitario Reina Sofia, Cordoba, Spain.
- [46] Hospital Virgen de la Salud, Toledo, Spain.
- [47] Hospital Universitario de Canarias, La Laguna, Santa Cruz de Tenerife, Spain.

- PMID: **32843180**
- PMCID: PMC7831988
- DOI: 10.1016/j.rmed.2020.106062

Abstract

Background: Given the high incidence of confirmed infection by SARS-CoV-2 and mortality by COVID-19 in the Spanish population, its impact was analysed among persons with Cystic Fibrosis (CF) as a group at risk of a worse evolution. The possible causes of the incidence observed in them are explained and how CF Units have faced this health challenge is detailed.

Methods: Retrospective descriptive observational study, for which a Spanish CF Patients with Confirmed COVID-19 Registry is created, requesting information on number of people affected between 8 March-16 May 2020 and their clinical-demographic characteristics from the CF Units participating in the European Cystic Fibrosis Society Patient Registry (ECFSPR). The accumulated incidence is calculated, compared with that of the general population. Additionally, a survey (CF-COVID19-Spain) is carried out on prevention of SARS-CoV-2 infection, workings of CF Units and possible reasons for the incidence observed.

Results: COVID-19 was diagnosed in eight CF patients, one of whom had received a lung transplant. The accumulated incidence was 32/10000 in CF patients and 49/10000 in the general population. General death rate was 5.85/10000 while no CF patients included in the ECFSPR died. The characteristics of those affected and the results of the survey are described.

Conclusions: Despite being considered a disease at high risk of severe COVID-19, the low incidence and mortality in CF patients in Spain contrasts with the figures for the general population. The possible factors that would explain such findings are discussed, with the help of the results of the CF-COVID19-Spain survey.

Keywords: COVID-19; Coronavirus; Cystic fibrosis; SARS-CoV-2.

Copyright © 2020 Elsevier Ltd. All rights reserved.

Conflict of interest statement

The authors have no conflicts of interest to declare relating to this work.

- 35 references

Supplementary info

Publication types, MeSH terms Expand

Publication types

- Observational Study

MeSH terms

- Adult
- Betacoronavirus / isolation & purification*
- COVID-19
- COVID-19 Testing
- Clinical Laboratory Techniques / methods
- Clinical Laboratory Techniques / statistics & numerical data
- Coronavirus Infections* / diagnosis
- Coronavirus Infections* / epidemiology
- Coronavirus Infections* / prevention & control
- Coronavirus Infections* / therapy
- Cystic Fibrosis / epidemiology*
- Female
- Humans
- Incidence
- Male
- Mortality

- Pandemics* / prevention & control
- Pneumonia, Viral* / epidemiology
- Pneumonia, Viral* / prevention & control
- Pneumonia, Viral* / therapy
- Registries / statistics & numerical data
- Retrospective Studies
- Risk Assessment
- SARS-CoV-2
- Spain / epidemiology

Full text links

ELSEVIER FULL-TEXT ARTICLE Elsevier Science Free PMC article
Proceed to details
Cite
Share
1,174
Observational Study
Heart Rhythm
. 2020 Nov;17(11):1960-1966.
doi: 10.1016/j.hrthm.2020.06.033. Epub 2020 Jul 1.

Incidence of arrhythmias and electrocardiographic abnormalities in symptomatic pediatric patients with PCR-positive SARS-CoV-2 infection, including drug-induced changes in the corrected QT interval

Sharmeen Samuel [1], Richard A Friedman [2], Chetan Sharma [2], Madhusudan Ganigara [2], Elizabeth Mitchell [2], Charles Schleien [3], Andrew D Blaufox [2]
Affiliations Expand

Affiliations

- [1] Section of Pediatric Cardiology and; Department of Pediatrics, Cohen Children's Medical Center, Northwell Health System, Donald and Barbara Zucker School of Medicine at Hofstra University, New Hyde Park, New York. Electronic address: ssamuel21@northwell.edu.

- [2] Section of Pediatric Cardiology and; Department of Pediatrics, Cohen Children's Medical Center, Northwell Health System, Donald and Barbara Zucker School of Medicine at Hofstra University, New Hyde Park, New York.
- [3] Department of Pediatrics, Cohen Children's Medical Center, Northwell Health System, Donald and Barbara Zucker School of Medicine at Hofstra University, New Hyde Park, New York.

- PMID: **32621881**
- PMCID: PMC7328618
- DOI: 10.1016/j.hrthm.2020.06.033

Free PMC article
Observational Study

Incidence of arrhythmias and electrocardiographic abnormalities in symptomatic pediatric patients with PCR-positive SARS-CoV-2 infection, including drug-induced changes in the corrected QT interval

Sharmeen Samuel et al. Heart Rhythm. 2020 Nov.
Free PMC article
Show details
Heart Rhythm
. 2020 Nov;17(11):1960-1966.
doi: 10.1016/j.hrthm.2020.06.033. Epub 2020 Jul 1.

Authors

Sharmeen Samuel [1], Richard A Friedman [2], Chetan Sharma [2], Madhusudan Ganigara [2], Elizabeth Mitchell [2], Charles Schleien [3], Andrew D Blaufox [2]

Affiliations

- [1] Section of Pediatric Cardiology and; Department of Pediatrics, Cohen Children's Medical Center, Northwell Health System, Donald and Barbara Zucker School of Medicine at Hofstra University, New Hyde Park, New York. Electronic address: ssamuel21@northwell.edu.
- [2] Section of Pediatric Cardiology and; Department of Pediatrics, Cohen Children's Medical Center, Northwell Health System, Donald and Barbara Zucker School of Medicine at Hofstra University, New Hyde Park, New York.
- [3] Department of Pediatrics, Cohen Children's Medical Center, Northwell Health System, Donald and Barbara Zucker School of Medicine at Hofstra University, New Hyde Park, New York.

- PMID: **32621881**
- PMCID: PMC7328618
- DOI: 10.1016/j.hrthm.2020.06.033

Abstract

Background: There is limited data regarding the electrophysiological abnormalities and arrhythmias in children with COVID-19, including those associated with treatment using potentially proarrhythmic hydroxychloroquine (HCQ) and azithromycin (AZN).

Objectives: To describe the electrophysiologic findings and arrhythmias associated with pediatric COVID-19 and its treatment.

Methods: A single-center retrospective chart review was undertaken and included all patients with (1) symptoms of COVID-19 and (2) PCR-positive nasopharyngeal swabs for SARS-CoV-2 who were placed on continuous telemetry for the duration of their hospitalization during March through May, 2020.

Results: Thirty-six patients were included in the study. Significant arrhythmias were found in 6 (nonsustained ventricular tachycardia in 5 and sustained atrial tachycardia in 1). All were self-resolving and half prompted prophylactic antiarrhythmic therapy. Patients with significant arrhythmias were likely to have noncardiac comorbidities (4/6), but these were not more common than in patients without arrhythmias (20/30, P = 1). The use of HCQ was associated with statistically significant QTc prolongation (413 ± 19 ms vs 425 ± 16 ms, P =.005). QTc was not statistically different in patients with and without arrhythmias (425 ± 15 ms vs 425 ± 15 ms, P = 1).

Conclusions: In pediatric patients with PCR-positive active COVID-19 infection, significant arrhythmias are infrequent, but are more common than expected in a general pediatric population. Comorbidities are not more common in patients with arrhythmias than in patients without arrhythmias. COVID-19 treatment using HCQ is associated with QTc prolongation but was not associated with arrhythmias in pediatric patients.

Keywords: Arrhythmias; COVID-19; Drug-induced prolonged QT; Hydroxychloroquine; Pediatric.

Copyright © 2020 Heart Rhythm Society. Published by Elsevier Inc. All rights reserved.

- 20 references
- 1 figure

Supplementary info

Publication types, MeSH terms, Substances Expand

Publication types

- Observational Study

MeSH terms

- Anti-Infective Agents / administration & dosage
- Anti-Infective Agents / adverse effects
- Arrhythmias, Cardiac* / diagnosis
- Arrhythmias, Cardiac* / epidemiology
- Azithromycin* / administration & dosage
- Azithromycin* / adverse effects
- Betacoronavirus / isolation & purification
- COVID-19
- COVID-19 Testing
- Child
- Clinical Laboratory Techniques / methods
- Coronavirus Infections* / diagnosis
- Coronavirus Infections* / drug therapy
- Coronavirus Infections* / epidemiology
- Coronavirus Infections* / physiopathology
- Electrocardiography* / methods
- Electrocardiography* / statistics & numerical data
- Female
- Humans
- Hydroxychloroquine* / administration & dosage
- Hydroxychloroquine* / adverse effects
- Incidence
- Long QT Syndrome* / chemically induced
- Long QT Syndrome* / diagnosis
- Male
- New York City / epidemiology
- Outcome and Process Assessment, Health Care
- Pandemics*
- Pneumonia, Viral* / diagnosis
- Pneumonia, Viral* / drug therapy
- Pneumonia, Viral* / epidemiology
- Pneumonia, Viral* / physiopathology
- Retrospective Studies
- Risk Factors
- SARS-CoV-2

Substances

- Anti-Infective Agents
- Hydroxychloroquine
- Azithromycin

Full text links

ELSEVIER FULL-TEXT ARTICLE Elsevier Science Free PMC article
Proceed to details
Cite
Share
1,175
Observational Study
PLoS One
. 2020 Dec 3;15(12):e0243346.
doi: 10.1371/journal.pone.0243346. eCollection 2020.

Retinal changes in COVID-19 hospitalized cases

Rafael Lani-Louzada [1], Carolina do Val Ferreira Ramos [2], Ricardo Mello Cordeiro [3] [4], Alfredo A Sadun [5]
Affiliations Expand

Affiliations

- [1] Hospital Nossa Senhora da Saúde, Santa Casa da Misericórdia do Rio de Janeiro (Hospital da Gamboa), Instituto de Oftalmologia do Rio de Janeiro, Rio de Janeiro, Rio de Janeiro, Brazil.
- [2] Clínica Nossa Senhora da Paz, Rio de Janeiro, Rio de Janeiro, Brazil.
- [3] Departamento de Clínica Médica, Hospital de Clínicas de Jacarepaguá, Rio de Janeiro, Rio de Janeiro, Brazil.
- [4] Departamento de Clínica Médica, Hospital de Clínicas Mário Lioni, Rio de Janeiro, Rio de Janeiro, Brazil.
- [5] Department of Ophthalmology, David Geffen School of Medicine at UCLA, Doheny Eye Institute, Los Angeles, California, United States of America.

- PMID: **33270751**
- PMCID: PMC7714146
- DOI: 10.1371/journal.pone.0243346

Free PMC article
Observational Study

Retinal changes in COVID-19 hospitalized cases

Rafael Lani-Louzada et al. PLoS One. 2020.
Free PMC article
Show details

PLoS One
. 2020 Dec 3;15(12):e0243346.
doi: 10.1371/journal.pone.0243346. eCollection 2020.

Authors

Rafael Lani-Louzada [1], Carolina do Val Ferreira Ramos [2], Ricardo Mello Cordeiro [3] [4], Alfredo A Sadun [5]

Affiliations

- [1] Hospital Nossa Senhora da Saúde, Santa Casa da Misericórdia do Rio de Janeiro (Hospital da Gamboa), Instituto de Oftalmologia do Rio de Janeiro, Rio de Janeiro, Rio de Janeiro, Brazil.
- [2] Clínica Nossa Senhora da Paz, Rio de Janeiro, Rio de Janeiro, Brazil.
- [3] Departamento de Clínica Médica, Hospital de Clínicas de Jacarepaguá, Rio de Janeiro, Rio de Janeiro, Brazil.
- [4] Departamento de Clínica Médica, Hospital de Clínicas Mário Lioni, Rio de Janeiro, Rio de Janeiro, Brazil.
- [5] Department of Ophthalmology, David Geffen School of Medicine at UCLA, Doheny Eye Institute, Los Angeles, California, United States of America.

- PMID: **33270751**
- PMCID: PMC7714146
- DOI: 10.1371/journal.pone.0243346

Abstract

The main objective of this study was to evaluate the retinas of severely or critically ill COVID-19 patients during their hospital stay, at varying time points after symptoms onset. This was a case series observed during May 2020 in two referral centers for COVID-19 treatment in Rio de Janeiro, Brazil. 47 eyes from 25 hospitalized patients with severe or critical confirmed illness were evaluated. A handheld retinal camera was used to acquire bilateral fundus images at several time points after symptoms onset. Electronic health records were retrospectively analyzed and clinical data collected. Severe and critical diseases were noticed in 52% (13/25) and 48% (12/25) of enrolled patients, respectively. Retinal changes were present in 12% (3/25) of patients: a 35 year-old male demonstrated bilateral nerve fiber layer infarcts and microhemorrhages in the papillomacular bundle, but required mechanical ventilation and developed severe anemia and systemic hypotension, acute kidney injury and neurologic symptoms during the course of the disease (critical illness); a 56 year-old male, who required full enoxaparin anticoagulation due to particularly elevated D-dimer (>5.0 mcg/mL), demonstrated unilateral and isolated flame-shaped hemorrhages; and a 49 year-old hypertensive male showed bilateral and discrete retinal dot and blot microhemorrhages. The other 22 patients evaluated did not demonstrate convincing retinal changes upon examination. There was no correlation between disease severity and admission serum levels of CRP, D-dimer and ferritin. This was the first study to show that vascular retinal changes may be present in not insignificant numbers of severe or critical COVID-19 inpatients. These retinal changes, only seen after morbid developments, were likely secondary to clinical intercurrences or comorbidities instead of a direct damage by SARS-CoV-2, and may be important and easily accessible outcome measures of therapeutic interventions and sentinels of neurologic and systemic diseases during COVID-19 pandemic.

Conflict of interest statement

The authors have declared that no competing interests exist.

- 42 references
- 3 figures

Supplementary info

Publication types, MeSH terms, Grant support Expand

Publication types

- Observational Study

MeSH terms

- Adult
- Aged
- COVID-19 / complications*
- COVID-19 / pathology
- Female
- Fundus Oculi
- Humans
- Inpatients / statistics & numerical data
- Male
- Middle Aged
- Retinal Hemorrhage / epidemiology*
- Retinal Hemorrhage / etiology
- Retinal Hemorrhage / pathology
- Retinal Vessels / pathology

Grant support

The authors received no specific funding for this work.

Full text links

OPEN ACCESS TO FULL TEXT PLOS ONE Public Library of Science Free PMC article
Proceed to details
Cite
Share
☐ 1,176
Observational Study
J Clin Psychiatry

. 2021 Mar 3;82(2):20m13685.
doi: 10.4088/JCP.20m13685.

Changes in Diagnostic and Demographic Characteristics of Patients Seeking Mental Health Care During the Early COVID-19 Pandemic in a Large, Community-Based Health Care System

Kathryn K Ridout [1] [2] [3], Mubarika Alavi [2], Samuel J Ridout [1], Maria T Koshy [1], Brooke Harris [4], Inderpreet Dhillon [1], Sameer Awsare [1], Constance M Weisner [2], Cynthia I Campbell [2], Esti Iturralde [2]

Affiliations Expand

Affiliations

- [1] The Permanente Medical Group, Kaiser Permanente Northern California, Oakland, California.
- [2] Division of Research, Kaiser Permanente Northern California, Oakland, California.
- [3] Corresponding author: Kathryn K. Ridout, MD, PhD, 401 Bicentennial Way. Santa Rosa, CA 95403 (Kathryn.Erickson-Ridout@KP.org).
- [4] Kaiser Foundation Hospitals, Kaiser Permanente Northern California, Oakland, California.

- PMID: **33979485**
- DOI: 10.4088/JCP.20m13685

Observational Study

Changes in Diagnostic and Demographic Characteristics of Patients Seeking Mental Health Care During the Early COVID-19 Pandemic in a Large, Community-Based Health Care System

Kathryn K Ridout et al. J Clin Psychiatry. 2021.

Show details

J Clin Psychiatry

. 2021 Mar 3;82(2):20m13685.
doi: 10.4088/JCP.20m13685.

Authors

Kathryn K Ridout [1] [2] [3], Mubarika Alavi [2], Samuel J Ridout [1], Maria T Koshy [1], Brooke Harris [4], Inderpreet Dhillon [1], Sameer Awsare [1], Constance M Weisner [2], Cynthia I Campbell [2], Esti Iturralde [2]

Affiliations

- [1] The Permanente Medical Group, Kaiser Permanente Northern California, Oakland, California.
- [2] Division of Research, Kaiser Permanente Northern California, Oakland, California.
- [3] Corresponding author: Kathryn K. Ridout, MD, PhD, 401 Bicentennial Way. Santa Rosa, CA 95403 (Kathryn.Erickson-Ridout@KP.org).
- [4] Kaiser Foundation Hospitals, Kaiser Permanente Northern California, Oakland, California.

- PMID: **33979485**
- DOI: 10.4088/JCP.20m13685

Abstract

Objective: The early COVID-19 pandemic resulted in great psychosocial disruption and stress, raising speculation that psychiatric disorders may worsen. This study aimed to identify patients vulnerable to worsening mental health during the COVID-19 pandemic.

Methods: This retrospective observational study used electronic health records from March 9 to May 31 in 2019 (n = 94,720) and 2020 (n = 94,589) in a large, community-based health care system. Percent change analysis compared variables standardized to the average patient population for the respective time periods.

Results: Compared to 2019, psychiatric visits increased significantly (P < .0001) in 2020, with the majority being telephone/video-based (+264%). Psychiatric care volume increased overall (7%), with the greatest increases in addiction (+42%), behavioral health in primary care (+17%), and adult psychiatry (+5%) clinics. While patients seeking care with preexisting psychiatric diagnoses were mainly stable (−2%), new patients declined (−42%). Visits for substance use (+51%), adjustment (+15%), anxiety (+12%), bipolar (+9%), and psychotic (+6%) disorder diagnoses, and for patients aged 18–25 years (+4%) and 26–39 years (+4%), increased. Child/adolescent and older adult patient visits decreased (−22.7% and −5.5%, respectively), and fewer patients identifying as White (−3.8%) or male (−5.0) or with depression (−3%) or disorders of childhood (−2%) sought care.

Conclusions: The early COVID-19 pandemic was associated with dramatic changes in psychiatric care facilitated by a rapid telehealth care transition. Patient volume, demographic, and diagnostic changes may reflect comfort with telehealth or navigating the psychiatric care system. These data can inform health system resource management and guide future work examining how care delivery changes impact psychiatric care quality and access.

© Copyright 2021 Physicians Postgraduate Press, Inc.

Supplementary info

Publication types, MeSH terms Expand

Publication types

- Observational Study
- Research Support, Non-U.S. Gov't

MeSH terms

- Adolescent
- Adult
- COVID-19*
- Child
- Community Health Services / statistics & numerical data*
- Electronic Health Records
- Female
- Humans
- Male
- Mental Disorders / diagnosis
- Mental Disorders / epidemiology*
- Mental Disorders / therapy*
- Mental Health Services / statistics & numerical data*
- Middle Aged
- Patient Acceptance of Health Care / statistics & numerical data*
- Primary Health Care / statistics & numerical data*
- Retrospective Studies
- Telemedicine / statistics & numerical data*
- Young Adult

Full text links

J CLIN PSYCHIATRY FULL TEXT ONLINE AT PSYCHIATRIST.COM Physicians Postgraduate Press, Inc.

Proceed to details

Cite

Share

☐ 1,177

Observational Study

Rev Esp Geriatr Gerontol

. Sep-Oct 2020;55(5):286-288.

doi: 10.1016/j.regg.2020.05.004. Epub 2020 Jun 3.

[Evolution and treatment of storm cytoquine syndrome associated to SARS-CoV-2 infection among octogenarians]

[Article in Spanish]

José Luis Callejas Rubio [1], Ismael Aomar Millán [2], Manuela Moreno Higueras [2], Leopoldo Muñoz Medina [3], María López López [4], Ángel Ceballos Torres [2]

Affiliations Expand

Affiliations

- [1] Unidad de Enfermedades Sistémicas, Servicio de Medicina Interna, Hospital Universitario San Cecilio, Granada, España. Electronic address: jlcalleja@telefonica.net.
- [2] Servicio de Medicina Interna, Hospital Universitario San Cecilio, Granada, España.
- [3] Unidad de Enfermedades Infecciosas, Hospital Universitario San Cecilio, Granada, España.
- [4] Servicio de Neumología, Hospital Universitario San Cecilio, Granada, España.

- PMID: **32564984**
- PMCID: PMC7266759
- DOI: 10.1016/j.regg.2020.05.004

Free PMC article
Observational Study

[Evolution and treatment of storm cytoquine syndrome associated to SARS-CoV-2 infection among octogenarians]

[Article in Spanish]

José Luis Callejas Rubio et al. Rev Esp Geriatr Gerontol. Sep-Oct 2020.

Free PMC article

Show details

Rev Esp Geriatr Gerontol
. Sep-Oct 2020;55(5):286-288.
doi: 10.1016/j.regg.2020.05.004. Epub 2020 Jun 3.

Authors

José Luis Callejas Rubio [1], Ismael Aomar Millán [2], Manuela Moreno Higueras [2], Leopoldo Muñoz Medina [3], María López López [4], Ángel Ceballos Torres [2]

Affiliations

- [1] Unidad de Enfermedades Sistémicas, Servicio de Medicina Interna, Hospital Universitario San Cecilio, Granada, España. Electronic address: jlcalleja@telefonica.net.
- [2] Servicio de Medicina Interna, Hospital Universitario San Cecilio, Granada, España.
- [3] Unidad de Enfermedades Infecciosas, Hospital Universitario San Cecilio, Granada, España.
- [4] Servicio de Neumología, Hospital Universitario San Cecilio, Granada, España.

- PMID: **32564984**
- PMCID: PMC7266759
- DOI: 10.1016/j.regg.2020.05.004

Abstract
in English, Spanish

Introduction: Cytokine storm syndrome (CTS) is a serious complication of patients with SARS-CoV-2 infection. Treatment and evolution in octogenarians are not well defined. Our objective is to describe its clinical characteristics, the treatments and its clinical evolution.

Patients and method: Retrospective observational study of consecutive patients admitted in the period between March 23 and April 12, 2020 with confirmed SARS-CoV-2 infection, with pneumonia by radiological study or chest tomography, whith STC criteria and who received treatment. We classified patients as those who received only glucocorticoid (GC) pulses, or GC and tocilizumab pulses. We determined serum levels of ferritin, CRP and D-dimers. The final variable was survival.

Results: 21 patients, (80-88 years). The mean ferritin was 1056 microg/L (317-3,553), CRP 115.8mg/dL (22-306) and D-dimers 2.9m/L (0.45-17.5). All patients received GC pulses and in 2 cases simultaneously tocilizumab. The mean follow-up time was 13.7 days (8-21). The overall mortality was 38.1% (8/21 patients). The 2 patients who received tocilizumab died. The deceased had significantly higher levels of ferritin (1,254 vs. 925microg/L; P=.045) and CRP (197.6 vs. 76mg / dL; P=.007). At the end of the follow-up, a decrease in the biochemical parameters was observed with ferritin of 727microg/L, CRP of 27mg/dl and D-dimers of 1.18mg/L. In 13/21 patients (61.9%), the CTS was controlled without the need to add other treatments.

Conclusions: STC mortality from SARS-CoV-2 is high despite treatment. A greater inflammatory response was associated with a higher mortality. Although it seems that the early use of GC pulses could control it, and the use of other treatments such as tocilizumab shouldo be, with the study design and its limitations, this conclusion cannot be stablished.

Introducción: El síndrome de tormenta de citoquinas (STC) es una complicación muy grave de los pacientes con infección por SARS-CoV-2. El tratamiento y la evolución no están bien definidos. Nuestro objetivo es describir sus características clínicas, los tratamientos empleados y su evolución clínica.

Pacientes y método: Estudio retrospectivo observacional de pacientes consecutivos ingresados en el período comprendido entre el 23 de marzo y el 12 de abril de 2020 con infección por SARS-CoV-2 confirmada, con neumonía por estudio radiológico o tomografía de tórax, que cumplían criterios de STC y que recibieron tratamiento. Clasificamos a los pacientes en los que recibieron solo pulsos de glucocorticoides (GC), o pulsos de GC y tocilizumab. Determinamos niveles séricos de ferritina, PCR y dímeros-D. La variable final fue la supervivencia.

Resultados: Veintiún pacientes con una edad de 83 años (80-88 años). La ferritina media fue de 1.056 microg/L (317-3.553), la PCR de 115,8 mg/dL (22-306) y los dímeros-D de 2,9 mg/L (0,45-17,5). Todos los pacientes recibieron pulsos de GC y en 2 casos simultáneamente tocilizumab. El tiempo medio de seguimiento fue de 13,7 días (8-21). La mortalidad global fue del 38,1% (8/21pacientes). Los 2 pacientes que recibieron tocilizumab fallecieron. Los fallecidos presentaron niveles significativamente más elevados de ferritina (1.254 vs. 925 microg/L; p = 0,045) y PCR (197,6 vs. 76 mg/dL; p = 0,007). Al final del seguimiento se observó una disminución en los parámetros bioquímicos con ferritina de 727 microg/L, PCR de 27 mg/dl y dímeros-D de 1,18 mg/L. En 13/21 pacientes (61,9%) el STC se controló sin necesidad de añadir otros tratamientos.

Conclusiones: La mortalidad del STC por SARS-CoV-2 es alta a pesar del tratamiento. Una mayor respuesta inflamatoria se asoció con una mayor mortalidad. Aunque parece que el uso precoz de pulsos de GC puede controlarlo, pudiendo disminuir la necesidad de uso de otros tratamientos, con el diseño del estudio y sus limitaciones, no se puede establecer esta conclusión.

Keywords: Coronavirus; Hemophagocytic syndrome; Storm cytokine syndrome.

Copyright © 2020 SEGG. Publicado por Elsevier España, S.L.U. All rights reserved.

- 11 references

Supplementary info

Publication types, MeSH terms, Substances Expand

Publication types

- Observational Study

MeSH terms

- Aged, 80 and over
- Antibodies, Monoclonal, Humanized / therapeutic use*
- COVID-19
- Coronavirus Infections / complications*
- Coronavirus Infections / immunology*
- Cytokines / immunology*
- Female
- Glucocorticoids / therapeutic use*
- Humans
- Immunoproliferative Disorders / drug therapy*
- Immunoproliferative Disorders / virology*
- Male
- Pandemics
- Pneumonia, Viral / complications*
- Pneumonia, Viral / immunology*
- Retrospective Studies

- Syndrome

Substances

- Antibodies, Monoclonal, Humanized
- Cytokines
- Glucocorticoids
- tocilizumab

Full text links

FULL TEXT AT: Geriatría y Gerontología Ediciones Doyma, S.L. Free PMC article
Proceed to details
Cite
Share
1,178
Observational Study
Indian J Ophthalmol
. 2021 Jul;69(7):1670-1692.
doi: 10.4103/ijo.IJO_1565_21.

Epidemiology, clinical profile, management, and outcome of COVID-19-associated rhino-orbital-cerebral mucormycosis in 2826 patients in India - Collaborative OPAI-IJO Study on Mucormycosis in COVID-19 (COSMIC), Report 1

Mrittika Sen [1], Santosh G Honavar [1], Rolika Bansal [1], Sabyasachi Sengupta [2], Raksha Rao [3], Usha Kim [4], Mukesh Sharma [5], Mahipal Sachdev [6], Ashok K Grover [7], Abhidnya Surve [8], Abhishek Budharapu [9], Abhishek K Ramadhin [10], Abhishek Kumar Tripathi [11], Adit Gupta [12], Aditya Bhargava [13], Animesh Sahu [14], Anjali Khairnar [15], Anju Kochar [16], Ankita Madhavani [17], Ankur K Shrivastava [18], Anuja K Desai [19], Anujeet Paul [20], Anuradha Ayyar [21], Aparna Bhatnagar [22], Aparna Singhal [23], Archana Sunil Nikose [24], Arun Bhargava [14], Arvind L Tenagi [25], Ashish Kamble [26], Ashiyana Nariani [27], Bhavin Patel [28], Bibbhuti Kashyap [29], Bodhraj Dhawan [30], Busaraben Vohra [31], Charuta Mandke [32], Chinmayee Thrishulamurthy [33], Chitra Sambare [34], Deepayan Sarkar [35], Devanshi Shirishbhai Mankad [17], Dhwani Maheshwari [36], Dilip Lalwani [37], Dipti Kanani [17], Diti Patel [31], Fairooz P Manjandavida [38], Frenali Godhani [39], Garima Amol Agarwal [40], Gayatri Ravulaparthi [41], Gondhi Vijay Shilpa [42], Gunjan Deshpande [43], Hansa Thakkar [40], Hardik Shah [44], Hare Ram Ojha [45], Harsha Jani

[46], Jyoti Gontia [47], Jyotika P Mishrikotkar [48], Kamalpreet Likhari [49], Kamini Prajapati [40], Kavita Porwal [50], Kirthi Koka [51], Kulveer Singh Dharawat [52], Lakshmi B Ramamurthy [53], Mainak Bhattacharyya [54], Manorama Saini [23], Marem C Christy [1], Mausumi Das [20], Maya Hada [52], Mehul Panchal [55], Modini Pandharpurkar [42], Mohammad Osman Ali [42], Mukesh Porwal [56], Nagaraju Gangashetappa [33], Neelima Mehrotra [57], Neha Bijlani [58], Nidhi Gajendragadkar [29], Nitin M Nagarkar [59], Palak Modi [40], Parveen Rewri [23], Piyushi Sao [60], Prajakta Salunkhe Patil [61], Pramod Giri [43], Priti Kapadia [62], Priti Yadav [47], Purvi Bhagat [40], Ragini Parekh [63], Rajashekhar Dyaberi [53], Rajender Singh Chauhan [64], Rajwinder Kaur [65], Ram Kishan Duvesh [66], Ramesh Murthy [67], Ravi Varma Dandu [68], Ravija Kathiara [69], Renu Beri [70], Rinal Pandit [71], Rita Hepsi Rani [72], Roshmi Gupta [3], Ruchi Pherwani [73], Rujuta Sapkal [69], Rupa Mehta [70], Sameeksha Tadepalli [74], Samra Fatima [42], Sandeep Karmarkar [75], Sandeep Suresh Patil [76], Sanjana Shah [31], Sankit Shah [77], Sapan Shah [19], Sarika Dubey [52], Saurin Gandhi [78], Savitha Kanakpur [53], Shalini Mohan [79], Sharad Bhomaj [80], Sheela Kerkar [27], Shivani Jariwala [62], Shivati Sahu [47], Shruthi Tara [81], Shruti Kochar Maru [50], Shubha Jhavar [82], Shubhda Sharma [83], Shweta Gupta [83], Shwetha Kumari [84], Sima Das [85], Smita Menon [27], Snehal Burkule [86], Sonam Poonam Nisar [51], Subashini Kaliaperumal [83], Subramanya Rao [33], Sudipto Pakrasi [83], Sujatha Rathod [33], Sunil G Biradar [60], Suresh Kumar [87], Susheen Dutt [88], Svati Bansal [83], Swati Amulbhai Ravani [40], Sweta Lohiya [89], Syed Wajahat Ali Rizvi [90], Tanmay Gokhale [91], Tatyarao P Lahane [63], Tejaswini Vukkadala [92], Triveni Grover [93], Trupti Bhesaniya [62], Urmil Chawla [64], Usha Singh [74], Vaishali L Une [15], Varsha Nandedkar [82], Venkata Subramaniam [94], Vidya Eswaran [84], Vidya Nair Chaudhry [95], Viji Rangarajan [96], Vipin Dehane [97], Vivek M Sahasrabudhe [86], Yarra Sowjanya [98], Yashaswini Tupkary [99], Yogita Phadke [69], members of the Collaborative OPAI-IJO Study on Mucormycosis in COVID-19 (COSMIC) Study Group

Affiliations Expand

Affiliations

- [1] Centre for Sight, Hyderabad, Telangana, India.
- [2] Future Vision Eye Care and Research Centre, Mumbai, Maharashtra, India.
- [3] Narayana Netralaya, Bengaluru, Karnataka, India.
- [4] Aravind Eye Hospital, Madurai, Tamil Nadu, India.
- [5] Centre for Sight, Jaipur, Rajasthan, India.
- [6] Centre for Sight, New Delhi, India.
- [7] Department of Ophthalmology, Sir Ganga Ram Hospital and Vision Eye Centres, New Delhi, India.
- [8] Department of Ophthalmology, Dr. Hedgewar Rugnalaya, Aurangabad, Maharashtra, India.
- [9] Department of Head and Neck Surgery, Apollo Cancer Hospital, Hyderabad, Telangana, India.
- [10] Department of Otorhinolaryngology, Dr Abhishek K. Ramadhin Hospital and Avyaan Research Centre, Ranchi, Jharkhand, India.
- [11] Department of Ophthalmology, Bharati Vidyapeeth Hospital, Sangli, Maharashtra, India.
- [12] Mumbai Eye Plastic Surgery, Mumbai, Maharashtra, India.
- [13] Department of Otorhinolaryngology, Post Graduate Institute of Medical Sciences, Rohtak, Haryana, India.
- [14] Retina Speciality Hospital, Indore, Madhya Pradesh, India.

- [15] Department of Ophthalmology, Shree Bhausaheb Hire Government Medical College, Dhule, Maharashtra, India.
- [16] Department of Ophthalmology, Sardar Patel Medical College, Bikaner, Rajasthan, India.
- [17] Department of Ophthalmology, Pandit Deendayal Upadhyay Medical College, Rajkot, Gujarat, India.
- [18] Department of Ophthalmology, All India Institute of Medical Sciences, Raipur, Chhattisgarh, India.
- [19] Department of Ophthalmology, Kusum Dhirajlal Hospital, Ahmedabad, Gujarat, India.
- [20] Department of Ophthalmology, Mahatma Gandhi Medical College and Research Institute, Puducherry, India.
- [21] Oases Eye Care Centre, Thane, Maharashtra, India.
- [22] Department of Ophthalmology, Apollo Specialty Hospitals, Chennai, Tamil Nadu, India.
- [23] Department of Ophthalmology, Maharaja Agrasen Medical College, Agroha, Haryana, India.
- [24] Department of Ophthalmology, NKP Salve Institute of Medical Sciences and Research Centre, Nagpur, Maharashtra, India.
- [25] Department of Ophthalmology, Jawaharlal Nehru Medical College, Karnataka Lingayat Education Academy of Higher Education and Research, Belagavi, Karnataka, India.
- [26] Department of Ophthalmology, Kingsway Hospital, Nagpur, Maharashtra, India.
- [27] Department of Ophthalmology, King Edward Memorial Hospital and Seth Gordhandas Sunderdas Medical College, Mumbai, Maharashtra, India.
- [28] Department of Otorhinolaryngology, Kiran Super Multi-Speciality Hospital, Surat, Gujarat, India.
- [29] The Kashyap Memorial Eye Hospital, Ranchi, Jharkhand, India.
- [30] Department of Ophthalmology, Alexis Hospital, Nagpur, Maharashtra, India.
- [31] Department of Ophthalmology, Medical College Baroda and Sir Sayajirao General Hospital, Vadodara, Gujarat, India.
- [32] Department of Ophthalmology, Hinduhridaysamrat Balasaheb Thackeray Medical College & Dr. R. N. Cooper Municipal Hospital, Mumbai, Maharashtra, India.
- [33] Department of Ophthalmology, Bangalore Medical College and Research Institute, Bengaluru, Karnataka, India.
- [34] Department of Ophthalmology, Jehangir Hospital, Pune, Maharashtra, India.
- [35] Department of Ophthalmology, All India Institute of Medical Sciences, Bhopal, Madhya Pradesh, India.
- [36] Department of Ophthalmology, Sir Sayajirao Gaekwad Hospital, Vadodra, Gujarat, India.
- [37] Bhaskar Eye Care, Raipur, Chhattisgarh, India.
- [38] Horus Specialty Eye Care, Bengaluru, Karnataka, India.
- [39] Department of Ophthalmology, Jagjivan Ram Railway Hospital, Mumbai, Maharashtra, India.
- [40] M and J Western Regional Institute of Ophthalmology, Byramjee Jeejeebhoy Medical College, Ahmedabad, Gujarat, India.
- [41] Department of Ophthalmology, Mamata Academy of Medical Sciences, Hyderabad, Telangana, India.
- [42] Sarojini Devi Eye Hospital, Hyderabad, Telangana, India.
- [43] Max Vision Eye Hospital, Nagpur, Maharashtra, India.
- [44] Department of Otorhinolaryngology, Kusum Dhirajlal Hospital, Ahmedabad, Gujarat, India.
- [45] Raj Eye Hospital, Gorakhpur, Uttar Pradesh, India.

- [46] Department of Ophthalmology, Pramukh Swami Medical College, Karamsad, Gujarat, India.
- [47] Department of Ophthalmology, Maharaja Yeshwantrao Hospital, Mahatma Gandhi Memorial Medical College, Indore, Madhya Pradesh, India.
- [48] ADepartment of Ophthalmology, Mahatma Gandhi Mission Medical College and Hospital, Aurangabad, Maharashtra, India.
- [49] Ratan Jyoti Netralaya, Gwalior, Madhya Pradesh, India.
- [50] Department of Ophthalmology, Convenient Hospitals Limited (CHL) - Hospitals, Indore, Madhya Pradesh, India.
- [51] Sankara Nethralaya, Chennai, Tamil Nadu, India.
- [52] Department of Ophthalmology, Sawai Man Singh Medical College, Jaipur, Rajasthan, India.
- [53] Department of Ophthalmology, Karnataka Institute of Medical Sciences, Hubli, Karnataka, India.
- [54] Eye-Q and Max Group of Hospitals, New Delhi, India.
- [55] Department of Microbiology, Kiran Super Multi-Speciality Hospital, Surat, Gujarat, India.
- [56] Porwal Eye Clinic, Rajkot, Gujarat, India.
- [57] Department of Ophthalmology, Shri Ram Murti Smarak Institute of Medical Sciences, Bareilly, Uttar Pradesh, India.
- [58] Vision Care & Research Centre, Bhopal, Madhya Pradesh, India.
- [59] Department of Otorhinolaryngology, All India Institute of Medical Sciences, Raipur, Chhattisgarh, India.
- [60] Department of Ophthalmology, Shri Mallanagouda Basanagouda Patil Medical College, BLDE University, Vijayapura, Karnataka, India.
- [61] Department of Ophthalmology, Krishna Institute of Medical Sciences, Karad, Maharashtra, India.
- [62] Department of Ophthalmology, Government Medical College, Surat, Gujarat, India.
- [63] Department of Ophthalmology, Grant Medical College and Sir Jamshedjee Jeejeebhoy Group of Hospitals, Mumbai, Maharashtra, India.
- [64] Regional Institute of Ophthalmology, Post Graduate Institute of Medical Sciences, Rohtak, Haryana, India.
- [65] Department of Ophthalmology, Adesh Institute of Medical Sciences and Research, Bhatinda, Punjab, India.
- [66] Department of Ophthalmology, Vardhaman Mahavir Medical College and Safdarjung Hospital, New Delhi, India.
- [67] Axis Eye Clinic, Pune, Maharashtra, India.
- [68] Department of Neuroradiology, Citi Neuro Centre, Hyderabad, Telangana, India.
- [69] Department of Ophthalmology, Mahatma Gandhi Mission Medical College and Hospital, Aurangabad, Maharashtra, India.
- [70] Department of Ophthalmology, Civil Hospital Ambala Cantonment, Haryana, India.
- [71] Department of Ophthalmology, Choithram Hospital & Research Centre, Indore, Madhya Pradesh, India.
- [72] Department of Ophthalmology, Tirunelveli Medical College, Tirunelveli, Tamil Nadu, India.
- [73] Department of Ophthalmology, SMBT Institute of Medical Sciences and Research Centre, Nashik, Maharashtra, India.

- [74] Advanced Eye Centre, Post Graduate Institute of Medical Education and Research, Chandigarh, India.
- [75] Department of Otorhinolaryngology, Ruby Hall Clinic, Pune, Maharashtra, India.
- [76] Department of Ophthalmology, Sakra World Hospital, Bengaluru, Karnataka, India.
- [77] Department of Ophthalmology, Kiran Super Multi-Speciality Hospital, Surat, Gujarat, India.
- [78] Eye Plasty Centre, Surat, Gujarat, India.
- [79] Department of Ophthalmology, Ganesh Shankar Vidyarthi Memorial Medical College, Kanpur, Uttar Pradesh, India.
- [80] Shanti Saroj Netralaya, Miraj, Maharashtra, India.
- [81] Sankara Eye Hospital, Coimbatore, Tamil Nadu, India.
- [82] Department of Ophthalmology, Government Medical College, Aurangabad, Maharashtra, India.
- [83] Department of Ophthalmology, Medanta - The Medicity, Gurugram, Haryana, India.
- [84] Department of Ophthalmology, Bowring and Lady Curzon Hospital, Bangalore Medical College and Research Institute, Bengaluru, Karnataka, India.
- [85] Dr. Shroff Charity Eye Hospital, New Delhi, India.
- [86] Department of Ophthalmology, Dr. Shankarrao Chavan Government Medical College, Nanded, Maharashtra, India.
- [87] Department of Ophthalmology, Government Medical College, Chandigarh, India.
- [88] Department of Otorhinolaryngology, Rangadore Memorial Hospital, Bengaluru, Karnataka, India.
- [89] Department of Otorhinolaryngology, Kingsway Hospital, Nagpur, Maharashtra, India.
- [90] Department of Ophthalmology, Jawaharlal Nehru Medical College, Aligarh Muslim University, Aligarh, Uttar Pradesh, India.
- [91] Department of Ophthalmology, Jawaharlal Institute of Postgraduate Medical Education and Research, Puducherry, India.
- [92] Department of Ophthalmology, Virinchi Hospital, Hyderabad, Telangana, India.
- [93] Department of Ophthalmology, Fortis Hospital, Shalimar Bagh, New Delhi, India.
- [94] Rangalakshmi Netralaya, Bengaluru, Karnataka, India.
- [95] Department of Ophthalmology, Aakash Healthcare Super-Specialty Hospital, New Delhi, India.
- [96] Aravind Eye Hospital, Coimbatore, Tamil Nadu, India.
- [97] Department of Oral and Maxillofacial Surgery, Kingsway Hospital, Nagpur, Maharashtra, India.
- [98] Sankara Eye Hospital, Guntur, Andhra PradeshSankara Eye Hospital, Guntur, Andhra Pradesh, India.
- [99] Department of Medicine, Dr. Hedgewar Rugnalaya, Aurangabad, Maharashtra, India.

- PMID: **34156034**
- PMCID: PMC8374756
- DOI: 10.4103/ijo.IJO_1565_21

Free PMC article
Observational Study

Epidemiology, clinical profile, management, and outcome of COVID-19-associated rhino-orbital-cerebral mucormycosis in 2826 patients in India - Collaborative OPAI-IJO Study on Mucormycosis in COVID-19 (COSMIC), Report 1

Mrittika Sen et al. Indian J Ophthalmol. 2021 Jul.
Free PMC article
Show details
Indian J Ophthalmol
. 2021 Jul;69(7):1670-1692.
doi: 10.4103/ijo.IJO_1565_21.

Authors

Mrittika Sen [1], Santosh G Honavar [1], Rolika Bansal [1], Sabyasachi Sengupta [2], Raksha Rao [3], Usha Kim [4], Mukesh Sharma [5], Mahipal Sachdev [6], Ashok K Grover [7], Abhidnya Surve [8], Abhishek Budharapu [9], Abhishek K Ramadhin [10], Abhishek Kumar Tripathi [11], Adit Gupta [12], Aditya Bhargava [13], Animesh Sahu [14], Anjali Khairnar [15], Anju Kochar [16], Ankita Madhavani [17], Ankur K Shrivastava [18], Anuja K Desai [19], Anujeet Paul [20], Anuradha Ayyar [21], Aparna Bhatnagar [22], Aparna Singhal [23], Archana Sunil Nikose [24], Arun Bhargava [14], Arvind L Tenagi [25], Ashish Kamble [26], Ashiyana Nariani [27], Bhavin Patel [28], Bibbhuti Kashyap [29], Bodhraj Dhawan [30], Busaraben Vohra [31], Charuta Mandke [32], Chinmayee Thrishulamurthy [33], Chitra Sambare [34], Deepayan Sarkar [35], Devanshi Shirishbhai Mankad [17], Dhwani Maheshwari [36], Dilip Lalwani [37], Dipti Kanani [17], Diti Patel [31], Fairooz P Manjandavida [38], Frenali Godhani [39], Garima Amol Agarwal [40], Gayatri Ravulaparthi [41], Gondhi Vijay Shilpa [42], Gunjan Deshpande [43], Hansa Thakkar [40], Hardik Shah [44], Hare Ram Ojha [45], Harsha Jani [46], Jyoti Gontia [47], Jyotika P Mishrikotkar [48], Kamalpreet Likhari [49], Kamini Prajapati [40], Kavita Porwal [50], Kirthi Koka [51], Kulveer Singh Dharawat [52], Lakshmi B Ramamurthy [53], Mainak Bhattacharyya [54], Manorama Saini [23], Marem C Christy [1], Mausumi Das [20], Maya Hada [52], Mehul Panchal [55], Modini Pandharpurkar [42], Mohammad Osman Ali [42], Mukesh Porwal [56], Nagaraju Gangashetappa [33], Neelima Mehrotra [57], Neha Bijlani [58], Nidhi Gajendragadkar [29], Nitin M Nagarkar [59], Palak Modi [40], Parveen Rewri [23], Piyushi Sao [60], Prajakta Salunkhe Patil [61], Pramod Giri [43], Priti Kapadia [62], Priti Yadav [47], Purvi Bhagat [40], Ragini Parekh [63], Rajashekhar Dyaberi [53], Rajender Singh Chauhan [64], Rajwinder Kaur [65], Ram Kishan Duvesh [66], Ramesh Murthy [67], Ravi Varma Dandu [68], Ravija Kathiara [69], Renu Beri [70], Rinal Pandit [71], Rita Hepsi Rani [72], Roshmi Gupta [3], Ruchi Pherwani [73], Rujuta Sapkal [69], Rupa Mehta [70], Sameeksha Tadepalli [74], Samra Fatima [42], Sandeep Karmarkar [75], Sandeep Suresh Patil [76], Sanjana Shah [31], Sankit Shah [77], Sapan Shah [19], Sarika Dubey [52], Saurin Gandhi [78], Savitha Kanakpur [53], Shalini Mohan [79], Sharad Bhomaj [80], Sheela Kerkar [27], Shivani Jariwala [62], Shivati Sahu [47], Shruthi Tara [81], Shruti Kochar Maru [50], Shubha Jhavar [82], Shubhda

Sharma [83], Shweta Gupta [83], Shwetha Kumari [84], Sima Das [85], Smita Menon [27], Snehal Burkule [86], Sonam Poonam Nisar [51], Subashini Kaliaperumal [83], Subramanya Rao [33], Sudipto Pakrasi [83], Sujatha Rathod [33], Sunil G Biradar [60], Suresh Kumar [87], Susheen Dutt [88], Svati Bansal [83], Swati Amulbhai Ravani [40], Sweta Lohiya [89], Syed Wajahat Ali Rizvi [90], Tanmay Gokhale [91], Tatyarao P Lahane [63], Tejaswini Vukkadala [92], Triveni Grover [93], Trupti Bhesaniya [62], Urmil Chawla [64], Usha Singh [74], Vaishali L Une [15], Varsha Nandedkar [82], Venkata Subramaniam [94], Vidya Eswaran [84], Vidya Nair Chaudhry [95], Viji Rangarajan [96], Vipin Dehane [97], Vivek M Sahasrabudhe [86], Yarra Sowjanya [98], Yashaswini Tupkary [99], Yogita Phadke [69], members of the Collaborative OPAI-IJO Study on Mucormycosis in COVID-19 (COSMIC) Study Group

Affiliations

- [1] Centre for Sight, Hyderabad, Telangana, India.
- [2] Future Vision Eye Care and Research Centre, Mumbai, Maharashtra, India.
- [3] Narayana Netralaya, Bengaluru, Karnataka, India.
- [4] Aravind Eye Hospital, Madurai, Tamil Nadu, India.
- [5] Centre for Sight, Jaipur, Rajasthan, India.
- [6] Centre for Sight, New Delhi, India.
- [7] Department of Ophthalmology, Sir Ganga Ram Hospital and Vision Eye Centres, New Delhi, India.
- [8] Department of Ophthalmology, Dr. Hedgewar Rugnalaya, Aurangabad, Maharashtra, India.
- [9] Department of Head and Neck Surgery, Apollo Cancer Hospital, Hyderabad, Telangana, India.
- [10] Department of Otorhinolaryngology, Dr Abhishek K. Ramadhin Hospital and Avyaan Research Centre, Ranchi, Jharkhand, India.
- [11] Department of Ophthalmology, Bharati Vidyapeeth Hospital, Sangli, Maharashtra, India.
- [12] Mumbai Eye Plastic Surgery, Mumbai, Maharashtra, India.
- [13] Department of Otorhinolaryngology, Post Graduate Institute of Medical Sciences, Rohtak, Haryana, India.
- [14] Retina Speciality Hospital, Indore, Madhya Pradesh, India.
- [15] Department of Ophthalmology, Shree Bhausaheb Hire Government Medical College, Dhule, Maharashtra, India.
- [16] Department of Ophthalmology, Sardar Patel Medical College, Bikaner, Rajasthan, India.
- [17] Department of Ophthalmology, Pandit Deendayal Upadhyay Medical College, Rajkot, Gujarat, India.
- [18] Department of Ophthalmology, All India Institute of Medical Sciences, Raipur, Chhattisgarh, India.
- [19] Department of Ophthalmology, Kusum Dhirajlal Hospital, Ahmedabad, Gujarat, India.
- [20] Department of Ophthalmology, Mahatma Gandhi Medical College and Research Institute, Puducherry, India.
- [21] Oases Eye Care Centre, Thane, Maharashtra, India.
- [22] Department of Ophthalmology, Apollo Specialty Hospitals, Chennai, Tamil Nadu, India.
- [23] Department of Ophthalmology, Maharaja Agrasen Medical College, Agroha, Haryana, India.
- [24] Department of Ophthalmology, NKP Salve Institute of Medical Sciences and Research Centre, Nagpur, Maharashtra, India.

- [25] Department of Ophthalmology, Jawaharlal Nehru Medical College, Karnataka Lingayat Education Academy of Higher Education and Research, Belagavi, Karnataka, India.
- [26] Department of Ophthalmology, Kingsway Hospital, Nagpur, Maharashtra, India.
- [27] Department of Ophthalmology, King Edward Memorial Hospital and Seth Gordhandas Sunderdas Medical College, Mumbai, Maharashtra, India.
- [28] Department of Otorhinolaryngology, Kiran Super Multi-Speciality Hospital, Surat, Gujarat, India.
- [29] The Kashyap Memorial Eye Hospital, Ranchi, Jharkhand, India.
- [30] Department of Ophthalmology, Alexis Hospital, Nagpur, Maharashtra, India.
- [31] Department of Ophthalmology, Medical College Baroda and Sir Sayajirao General Hospital, Vadodara, Gujarat, India.
- [32] Department of Ophthalmology, Hinduhridaysamrat Balasaheb Thackeray Medical College & Dr. R. N. Cooper Municipal Hospital, Mumbai, Maharashtra, India.
- [33] Department of Ophthalmology, Bangalore Medical College and Research Institute, Bengaluru, Karnataka, India.
- [34] Department of Ophthalmology, Jehangir Hospital, Pune, Maharashtra, India.
- [35] Department of Ophthalmology, All India Institute of Medical Sciences, Bhopal, Madhya Pradesh, India.
- [36] Department of Ophthalmology, Sir Sayajirao Gaekwad Hospital, Vadodra, Gujarat, India.
- [37] Bhaskar Eye Care, Raipur, Chhattisgarh, India.
- [38] Horus Specialty Eye Care, Bengaluru, Karnataka, India.
- [39] Department of Ophthalmology, Jagjivan Ram Railway Hospital, Mumbai, Maharashtra, India.
- [40] M and J Western Regional Institute of Ophthalmology, Byramjee Jeejeebhoy Medical College, Ahmedabad, Gujarat, India.
- [41] Department of Ophthalmology, Mamata Academy of Medical Sciences, Hyderabad, Telangana, India.
- [42] Sarojini Devi Eye Hospital, Hyderabad, Telangana, India.
- [43] Max Vision Eye Hospital, Nagpur, Maharashtra, India.
- [44] Department of Otorhinolaryngology, Kusum Dhirajlal Hospital, Ahmedabad, Gujarat, India.
- [45] Raj Eye Hospital, Gorakhpur, Uttar Pradesh, India.
- [46] Department of Ophthalmology, Pramukh Swami Medical College, Karamsad, Gujarat, India.
- [47] Department of Ophthalmology, Maharaja Yeshwantrao Hospital, Mahatma Gandhi Memorial Medical College, Indore, Madhya Pradesh, India.
- [48] ADepartment of Ophthalmology, Mahatma Gandhi Mission Medical College and Hospital, Aurangabad, Maharashtra, India.
- [49] Ratan Jyoti Netralaya, Gwalior, Madhya Pradesh, India.
- [50] Department of Ophthalmology, Convenient Hospitals Limited (CHL) - Hospitals, Indore, Madhya Pradesh, India.
- [51] Sankara Nethralaya, Chennai, Tamil Nadu, India.
- [52] Department of Ophthalmology, Sawai Man Singh Medical College, Jaipur, Rajasthan, India.
- [53] Department of Ophthalmology, Karnataka Institute of Medical Sciences, Hubli, Karnataka, India.
- [54] Eye-Q and Max Group of Hospitals, New Delhi, India.

- [55] Department of Microbiology, Kiran Super Multi-Speciality Hospital, Surat, Gujarat, India.
- [56] Porwal Eye Clinic, Rajkot, Gujarat, India.
- [57] Department of Ophthalmology, Shri Ram Murti Smarak Institute of Medical Sciences, Bareilly, Uttar Pradesh, India.
- [58] Vision Care & Research Centre, Bhopal, Madhya Pradesh, India.
- [59] Department of Otorhinolaryngology, All India Institute of Medical Sciences, Raipur, Chhattisgarh, India.
- [60] Department of Ophthalmology, Shri Mallanagouda Basanagouda Patil Medical College, BLDE University, Vijayapura, Karnataka, India.
- [61] Department of Ophthalmology, Krishna Institute of Medical Sciences, Karad, Maharashtra, India.
- [62] Department of Ophthalmology, Government Medical College, Surat, Gujarat, India.
- [63] Department of Ophthalmology, Grant Medical College and Sir Jamshedjee Jeejeebhoy Group of Hospitals, Mumbai, Maharashtra, India.
- [64] Regional Institute of Ophthalmology, Post Graduate Institute of Medical Sciences, Rohtak, Haryana, India.
- [65] Department of Ophthalmology, Adesh Institute of Medical Sciences and Research, Bhatinda, Punjab, India.
- [66] Department of Ophthalmology, Vardhaman Mahavir Medical College and Safdarjung Hospital, New Delhi, India.
- [67] Axis Eye Clinic, Pune, Maharashtra, India.
- [68] Department of Neuroradiology, Citi Neuro Centre, Hyderabad, Telangana, India.
- [69] Department of Ophthalmology, Mahatma Gandhi Mission Medical College and Hospital, Aurangabad, Maharashtra, India.
- [70] Department of Ophthalmology, Civil Hospital Ambala Cantonment, Haryana, India.
- [71] Department of Ophthalmology, Choithram Hospital & Research Centre, Indore, Madhya Pradesh, India.
- [72] Department of Ophthalmology, Tirunelveli Medical College, Tirunelveli, Tamil Nadu, India.
- [73] Department of Ophthalmology, SMBT Institute of Medical Sciences and Research Centre, Nashik, Maharashtra, India.
- [74] Advanced Eye Centre, Post Graduate Institute of Medical Education and Research, Chandigarh, India.
- [75] Department of Otorhinolaryngology, Ruby Hall Clinic, Pune, Maharashtra, India.
- [76] Department of Ophthalmology, Sakra World Hospital, Bengaluru, Karnataka, India.
- [77] Department of Ophthalmology, Kiran Super Multi-Speciality Hospital, Surat, Gujarat, India.
- [78] Eye Plasty Centre, Surat, Gujarat, India.
- [79] Department of Ophthalmology, Ganesh Shankar Vidyarthi Memorial Medical College, Kanpur, Uttar Pradesh, India.
- [80] Shanti Saroj Netralaya, Miraj, Maharashtra, India.
- [81] Sankara Eye Hospital, Coimbatore, Tamil Nadu, India.
- [82] Department of Ophthalmology, Government Medical College, Aurangabad, Maharashtra, India.
- [83] Department of Ophthalmology, Medanta - The Medicity, Gurugram, Haryana, India.
- [84] Department of Ophthalmology, Bowring and Lady Curzon Hospital, Bangalore Medical College and Research Institute, Bengaluru, Karnataka, India.

- [85] Dr. Shroff Charity Eye Hospital, New Delhi, India.
- [86] Department of Ophthalmology, Dr. Shankarrao Chavan Government Medical College, Nanded, Maharashtra, India.
- [87] Department of Ophthalmology, Government Medical College, Chandigarh, India.
- [88] Department of Otorhinolaryngology, Rangadore Memorial Hospital, Bengaluru, Karnataka, India.
- [89] Department of Otorhinolaryngology, Kingsway Hospital, Nagpur, Maharashtra, India.
- [90] Department of Ophthalmology, Jawaharlal Nehru Medical College, Aligarh Muslim University, Aligarh, Uttar Pradesh, India.
- [91] Department of Ophthalmology, Jawaharlal Institute of Postgraduate Medical Education and Research, Puducherry, India.
- [92] Department of Ophthalmology, Virinchi Hospital, Hyderabad, Telangana, India.
- [93] Department of Ophthalmology, Fortis Hospital, Shalimar Bagh, New Delhi, India.
- [94] Rangalakshmi Netralaya, Bengaluru, Karnataka, India.
- [95] Department of Ophthalmology, Aakash Healthcare Super-Specialty Hospital, New Delhi, India.
- [96] Aravind Eye Hospital, Coimbatore, Tamil Nadu, India.
- [97] Department of Oral and Maxillofacial Surgery, Kingsway Hospital, Nagpur, Maharashtra, India.
- [98] Sankara Eye Hospital, Guntur, Andhra PradeshSankara Eye Hospital, Guntur, Andhra Pradesh, India.
- [99] Department of Medicine, Dr. Hedgewar Rugnalaya, Aurangabad, Maharashtra, India.

- PMID: **34156034**
- PMCID: PMC8374756
- DOI: 10.4103/ijo.IJO_1565_21

Abstract

Purpose: COVID-19-associated rhino-orbital-cerebral mucormycosis (ROCM) has reached epidemic proportion during India's second wave of COVID-19 pandemic, with several risk factors being implicated in its pathogenesis. This study aimed to determine the patient demographics, risk factors including comorbidities, and medications used to treat COVID-19, presenting symptoms and signs, and the outcome of management.

Methods: This was a retrospective, observational study of patients with COVID-19-associated ROCM managed or co-managed by ophthalmologists in India from January 1, 2020 to May 26, 2021.

Results: Of the 2826 patients, the states of Gujarat (22%) and Maharashtra (21%) reported the highest number of ROCM. The mean age of patients was 51.9 years with a male preponderance (71%). While 57% of the patients needed oxygen support for COVID-19 infection, 87% of the patients were treated with corticosteroids, (21% for > 10 days). Diabetes mellitus (DM) was present in 78% of all patients. Most of the cases showed onset of symptoms of ROCM between day 10 and day 15 from the diagnosis of COVID-19, 56% developed within 14 days after COVID-19 diagnosis, while 44% had delayed onset beyond 14 days. Orbit was involved in 72% of patients, with stage 3c forming the bulk (27%). Overall treatment included intravenous amphotericin B in 73%, functional endoscopic sinus surgery (FESS)/paranasal sinus (PNS) debridement in 56%, orbital exenteration in 15%, and both FESS/PNS debridement and orbital exenteration in 17%. Intraorbital injection of amphotericin B was administered in 22%. At final

follow-up, mortality was 14%. Disease stage >3b had poorer prognosis. Paranasal sinus debridement and orbital exenteration reduced the mortality rate from 52% to 39% in patients with stage 4 disease with intracranial extension ($p < 0.05$).

Conclusion: : Corticosteroids and DM are the most important predisposing factors in the development of COVID-19-associated ROCM. COVID-19 patients must be followed up beyond recovery. Awareness of red flag symptoms and signs, high index of clinical suspicion, prompt diagnosis, and early initiation of treatment with amphotericin B, aggressive surgical debridement of the PNS, and orbital exenteration, where indicated, are essential for successful outcome.

Keywords: COVID-19; COVID-19-associated ROCM; Corticosteroids; diabetes mellitus; mucormycosis; orbital exenteration; paransal sinus debridement; rhino-orbital-cerebral mucormycosis; staging of rhino-orbital-cerebral mucormycosis.

Conflict of interest statement

There are no conflicts of interest.

- 54 references
- 19 figures

Supplementary info

Publication types, MeSH terms, Substances Expand

Publication types

- Observational Study

MeSH terms

- Antifungal Agents / therapeutic use
- COVID-19 Testing
- COVID-19*
- Eye Infections, Fungal* / diagnosis
- Eye Infections, Fungal* / epidemiology
- Eye Infections, Fungal* / therapy
- Humans
- India / epidemiology
- Male
- Middle Aged
- Mucormycosis* / diagnosis
- Mucormycosis* / epidemiology
- Mucormycosis* / therapy
- Orbital Diseases* / diagnosis
- Orbital Diseases* / epidemiology
- Orbital Diseases* / therapy

- Pandemics
- SARS-CoV-2

Substances

- Antifungal Agents

Full text links

Get Free Full Text Wolters Kluwer | Medknow Medknow Publications and Media Pvt Ltd Free PMC article

Proceed to details

Cite

Share

☐ 1,179

Observational Study

Acta Diabetol

. 2021 Mar;58(3):383-388.

doi: 10.1007/s00592-020-01625-2. Epub 2020 Oct 30.

Management of glucose profile throughout strict COVID-19 lockdown by patients with type 1 diabetes prone to hypoglycaemia using sensor-augmented pump

Clara Viñals[# 1], Alex Mesa[# 2], Daria Roca[2], Merce Vidal[2], Irene Pueyo[2], Ignacio Conget[2 3 4], Marga Giménez[2 3 4]

Affiliations Expand

Affiliations

- [1] Diabetes Unit. Endocrinology and Nutrition Department, Hospital Clínic i Universitari, Villarroel 170, 08036, Barcelona, Spain. vinals@clinic.cat.
- [2] Diabetes Unit. Endocrinology and Nutrition Department, Hospital Clínic i Universitari, Villarroel 170, 08036, Barcelona, Spain.
- [3] CIBERDEM, Centro de Investigación Biomédica en Red de Diabetes y Enfermedades Metabólicas, Madrid, Spain.
- [4] IDIBAPS, Institut d'Investigacions Biomèdiques August Pi i Sunyer, Barcelona, Spain.

[#] Contributed equally.

- PMID: **33125525**
- PMCID: PMC7596617
- DOI: 10.1007/s00592-020-01625-2

Free PMC article
Observational Study

Management of glucose profile throughout strict COVID-19 lockdown by patients with type 1 diabetes prone to hypoglycaemia using sensor-augmented pump

Clara Viñals et al. Acta Diabetol. 2021 Mar.
Free PMC article
Show details
Acta Diabetol
. 2021 Mar;58(3):383-388.
doi: 10.1007/s00592-020-01625-2. Epub 2020 Oct 30.

Authors

Clara Viñals [#] [1], Alex Mesa [#] [2], Daria Roca [2], Merce Vidal [2], Irene Pueyo [2], Ignacio Conget [2] [3] [4], Marga Giménez [2] [3] [4]

Affiliations

- [1] Diabetes Unit. Endocrinology and Nutrition Department, Hospital Clínic i Universitari, Villarroel 170, 08036, Barcelona, Spain. vinals@clinic.cat.
- [2] Diabetes Unit. Endocrinology and Nutrition Department, Hospital Clínic i Universitari, Villarroel 170, 08036, Barcelona, Spain.
- [3] CIBERDEM, Centro de Investigación Biomédica en Red de Diabetes y Enfermedades Metabólicas, Madrid, Spain.
- [4] IDIBAPS, Institut d'Investigacions Biomèdiques August Pi i Sunyer, Barcelona, Spain.

[#] Contributed equally.

- PMID: **33125525**
- PMCID: PMC7596617
- DOI: 10.1007/s00592-020-01625-2

Abstract

Aims: Spain has been one of the worst affected countries by the COVID-19 pandemic. A very strict lockdown at home was imposed with a tough restriction of mobility. We aimed to evaluate the impact of this exceptional scenario on glucose profile of patients with type 1 diabetes (T1D) prone to hypoglycaemia using sensor-augmented pump (SAP).

Methods: Patients with T1D prone to hypoglycaemia using SAP (640G Medtronic-Minimed®) for at least 6 months under the funding of a National Health Service were included in an observational, retrospective study. Data were collected in two periods: pre-lockdown (PL),

February 23rd-March 7th and within lockdown (WL), April 1st to 14th 2020. The primary outcome was the difference in the proportion of time in target glucose range of 70-180 mg/dL (TIR). Additional glucometric data and total daily insulin were also analysed.

Results: Fifty-nine patients were included: 33 women, age 46.17 ± 13.0 years and disease duration of 30.2 ± 12.0 years. TIR 70-180 mg/dL (67.6 ± 11.8 vs. 69.8 ± 12.0%), time > 180 (28.1 ± 13.6 vs. 25.5 ± 13.1%), time > 250 (6.9 ± 6.1 vs. 5.1 ± 4.8) and estimated HbA_{1c} (6.94 ± 0.8 vs. 6.75 ± 0.7%) significantly improved (PL vs. WL, respectively, $p < 0.05$). Time in hypoglycaemia, coefficient of variation, sensor usage and total daily insulin dose remained unchanged.

Conclusions: Lockdown conditions imposed by the COVID-19 pandemic may be managed successfully in terms of glycaemia control by population with DT1 prone to hypoglycaemia using SAP. The strict daily routine at home could probably explain the improvement in the time in glycemic target without increasing the time hypoglycaemia.

Keywords: COVID-19 lockdown, sensor-augmented pump; Hypoglycaemia; Type 1 diabetes.

Conflict of interest statement

Dr Viñals has received lecturing fees from NovoNordisk A/S, Medtronic Inc., Sanofi-Aventis and MSD. Dr Giménez has received lecturing and consulting fees from Medtronic Inc., Eli Lilly & Co., NovoNordisk A/S, Sanofi-Aventis, Astra Zeneca and MSD. Dr Conget reported receiving lecturing and consulting fees from Medtronic Inc., Bayer AG, GlaxoSmithKline, Eli Lilly & Co., NovoNordisk A/S, Sanofi-Aventis, Novartis, Astra Zeneca and MSD.

- 20 references

Supplementary info

Publication types, MeSH terms, Substances Expand

Publication types

- Observational Study

MeSH terms

- Adult
- Aged
- Blood Glucose / analysis
- COVID-19*
- Diabetes Mellitus, Type 1 / blood
- Diabetes Mellitus, Type 1 / drug therapy*
- Female
- Glycemic Control / methods*
- Humans
- Hypoglycemia / blood*
- Hypoglycemic Agents / administration & dosage

- Hypoglycemic Agents / therapeutic use
- Insulin / administration & dosage
- Insulin / therapeutic use
- Insulin Infusion Systems*
- Male
- Middle Aged
- Pandemics*
- Quarantine*
- Retrospective Studies
- Treatment Outcome

Substances

- Blood Glucose
- Hypoglycemic Agents
- Insulin

Full text links

SpringerLink FULL-TEXT ARTICLE Springer Free PMC article
Proceed to details
Cite
Share
1,180
Observational Study
Crit Care Med
. 2020 Oct;48(10):e971-e975.
doi: 10.1097/CCM.0000000000004504.

Venous Thromboembolism Events Following Venovenous Extracorporeal Membrane Oxygenation for Severe Acute Respiratory Syndrome Coronavirus 2 Based on CT Scans

Gabriel Parzy [1, 2], Florence Daviet [1, 2], Basile Puech [3], Aude Sylvestre [1, 2], Christophe Guervilly [1, 2], Alizée Porto [4], Sami Hraiech [1, 2], Kathia Chaumoitre [3], Laurent Papazian [1, 2], Jean-Marie Forel [1, 2]
Affiliations Expand

Affiliations

- [1] Médecine Intensive Réanimation Détresses Respiratoires et Infection Sévères, AP-HM, CHU Nord, Marseille, France.
- [2] CEReSS - Center for Studies and Research on Health Services and Quality of Life EA3279, Aix-Marseille University, Marseille, France.
- [3] Service d'Imagerie Médicale, AP-HM, CHU Nord, Marseille, France.
- [4] Département de Chirurgie Cardiaque, AP-HM, CHU Timone, Marseille, France.

- PMID: **32618700**
- PMCID: PMC7328443
- DOI: 10.1097/CCM.0000000000004504

Free PMC article
Observational Study

Venous Thromboembolism Events Following Venovenous Extracorporeal Membrane Oxygenation for Severe Acute Respiratory Syndrome Coronavirus 2 Based on CT Scans

Gabriel Parzy et al. Crit Care Med. 2020 Oct.
Free PMC article
Show details
Crit Care Med
. 2020 Oct;48(10):e971-e975.
doi: 10.1097/CCM.0000000000004504.

Authors

Gabriel Parzy [1, 2], Florence Daviet [1, 2], Basile Puech [3], Aude Sylvestre [1, 2], Christophe Guervilly [1, 2], Alizée Porto [4], Sami Hraiech [1, 2], Kathia Chaumoitre [3], Laurent Papazian [1, 2], Jean-Marie Forel [1, 2]

Affiliations

- [1] Médecine Intensive Réanimation Détresses Respiratoires et Infection Sévères, AP-HM, CHU Nord, Marseille, France.
- [2] CEReSS - Center for Studies and Research on Health Services and Quality of Life EA3279, Aix-Marseille University, Marseille, France.
- [3] Service d'Imagerie Médicale, AP-HM, CHU Nord, Marseille, France.
- [4] Département de Chirurgie Cardiaque, AP-HM, CHU Timone, Marseille, France.

- PMID: **32618700**
- PMCID: PMC7328443
- DOI: 10.1097/CCM.0000000000004504

Abstract

Objectives: The main objective of the study was to determine the prevalence of venous thromboembolism events in patients infected with severe acute respiratory syndrome coronavirus 2 requiring venovenous extracorporeal membrane oxygenation. The secondary objective was to compare venous thromboembolism events and coagulation variables in patients requiring venovenous extracorporeal membrane oxygenation according to the pathogen.

Design: Retrospective observational analysis at a single center.

Setting: Tertiary referral university teaching hospital.

Patients: Patients with severe acute respiratory syndrome coronavirus 2-related severe acute respiratory distress syndrome requiring venovenous extracorporeal membrane oxygenation therapy with an injected CT scan performed after extracorporeal membrane oxygenation retrieval.

Interventions: None.

Measurements and main results: We included 13 severe acute respiratory syndrome coronavirus 2 patients requiring venovenous extracorporeal membrane oxygenation. All of these patients experienced venous thromboembolism: 10 patients (76.9%) had isolated cannula-associated deep vein thrombosis, two patients (15.4%) had isolated pulmonary embolism, and one patient (7.7%) had both cannula-associated deep vein thrombosis and pulmonary embolism. Eleven patients (84.6%) had cannula-associated deep vein thrombosis. A jugular associated cannula-associated deep vein thrombosis was identified in seven patients (53.8%), a femoral associated cannula-associated deep vein thrombosis was identified in 10 patients (76.9%), and six patients (46.2%) had both femoral and jugular cannula-associated deep vein thrombosis. A pulmonary embolism was found in three patients (23.1%). No patient had central venous catheter-related deep vein thrombosis. One patient had thrombotic occlusion of the centrifugal pump, and one had oxygenator thrombosis requiring circuit replacement. Three patients (23.1%) had significant bleeding. Three patients (23.1%) had laboratory-confirmed heparin-induced thrombocytopenia, and all of them developed cannula-associated deep vein thrombosis. These three patients had femoral cannula-associated deep vein thrombosis, and two had an oxygenator or pump thrombosis. The mean activated partial thromboplastin time ratio was higher in the severe acute respiratory syndrome coronavirus 2 group than in the influenza group and the community-acquired pneumonia group (1.91 vs 1.48 vs 1.53; $p = 0.001$), which was also found in regard to the percentage of patients with an activated partial thromboplastin time ratio greater than 1.8 (47.8% vs 20% vs 20.9%; $p = 0.003$) and the mean prothrombin ratio (86.3 vs 61.6 vs 67.1; $p = 0.003$). There was no difference in baseline characteristics or venous thromboembolism events.

Conclusions: We report a 100% occurrence of venous thromboembolism in critically ill patients supported by venovenous extracorporeal membrane oxygenation for severe acute respiratory syndrome coronavirus 2-related acute respiratory distress syndrome using CT scan imaging despite a high target and close monitoring of anticoagulation.

Conflict of interest statement

Dr. Guervilly received funding from Xenios Fresenius Medical Care. Dr. Papazian’s institution received funding from Sedana (grant for a study), and he received funding from Lowenstein and Hamilton. The remaining authors have disclosed that they do not have any potential conflicts of interest.

- 11 references

Supplementary info

Publication types, MeSH terms, Substances Expand

Publication types

- Observational Study

MeSH terms

- Anticoagulants / administration & dosage*
- COVID-19
- Cohort Studies
- Coronavirus Infections / diagnosis
- Coronavirus Infections / therapy*
- Critical Care / methods
- Critical Illness / mortality
- Critical Illness / therapy
- Extracorporeal Membrane Oxygenation / adverse effects*
- Extracorporeal Membrane Oxygenation / methods
- Female
- France
- Hospital Mortality / trends
- Hospitals, University
- Humans
- Intensive Care Units
- Male
- Pandemics
- Pneumonia, Viral / diagnosis
- Pneumonia, Viral / therapy*
- Retrospective Studies
- Risk Assessment
- Severe Acute Respiratory Syndrome / diagnostic imaging*
- Severe Acute Respiratory Syndrome / mortality
- Severe Acute Respiratory Syndrome / therapy*
- Survival Rate
- Tertiary Care Centers
- Tomography, X-Ray Computed / methods
- Treatment Outcome
- Venous Thromboembolism / drug therapy*
- Venous Thromboembolism / etiology
- Venous Thromboembolism / mortality

Substances

- Anticoagulants

Full text links

Wolters Kluwer Wolters Kluwer Free PMC article
Proceed to details
Cite
Share
1,181
Observational Study
PLoS One
. 2020 Oct 14;15(10):e0240645.
doi: 10.1371/journal.pone.0240645. eCollection 2020.

Continuous Positive Airway Pressure (CPAP) face-mask ventilation is an easy and cheap option to manage a massive influx of patients presenting acute respiratory failure during the SARS-CoV-2 outbreak: A retrospective cohort study

Sophie Alviset [1], Quentin Riller [1], Jérôme Aboab [1], Kelly Dilworth [2], Pierre-Antoine Billy [3], Yannis Lombardi [1], Mathilde Azzi [1], Luis Ferreira Vargas [1], Laurent Laine [1], Mathilde Lermuzeaux [1], Nathalie Mémain [1], Daniel Silva [1], Tona Tchoubou [1], Daria Ushmorova [1], Hanane Dabbagh [4], Simon Escoda [5], Rémi Lefrançois [6], Annelyse Nardi [7], Armand Ngima [8], Vincent Ioos [1]
Affiliations Expand

Affiliations

- [1] Service de Médecine Intensive Réanimation, Hôpital Delafontaine, Saint-Denis, France.
- [2] Service d'Anesthésie, Centre Hospitalier Universitaire de Grenoble, La Tronche, France.
- [3] Laboratoire de Microbiologie, Hôpital Delafontaine, Saint-Denis, France.
- [4] Service d'Anesthésie, Hôpital Delafontaine, Saint-Denis, France.
- [5] Service de Pédiatrie, Hôpital Delafontaine, Saint-Denis, France.
- [6] Service des Maladies infectieuses, Hôpital Delafontaine, Saint-Denis, France.
- [7] Service de Pneumologie, Hôpital Delafontaine, Saint-Denis, France.
- [8] Service des Urgences, Hôpital Delafontaine, Saint-Denis, France.

- PMID: **33052968**
- PMCID: PMC7556440
- DOI: 10.1371/journal.pone.0240645

Free PMC article
Observational Study

Continuous Positive Airway Pressure (CPAP) face-mask ventilation is an easy and cheap option to manage a massive influx of patients presenting acute respiratory failure during the SARS-CoV-2 outbreak: A retrospective cohort study

Sophie Alviset et al. PLoS One. 2020.
Free PMC article
Show details
PLoS One
. 2020 Oct 14;15(10):e0240645.
doi: 10.1371/journal.pone.0240645. eCollection 2020.

Authors

Sophie Alviset [1], Quentin Riller [1], Jérôme Aboab [1], Kelly Dilworth [2], Pierre-Antoine Billy [3], Yannis Lombardi [1], Mathilde Azzi [1], Luis Ferreira Vargas [1], Laurent Laine [1], Mathilde Lermuzeaux [1], Nathalie Mémain [1], Daniel Silva [1], Tona Tchoubou [1], Daria Ushmorova [1], Hanane Dabbagh [4], Simon Escoda [5], Rémi Lefrançois [6], Annelyse Nardi [7], Armand Ngima [8], Vincent Ioos [1]

Affiliations

- [1] Service de Médecine Intensive Réanimation, Hôpital Delafontaine, Saint-Denis, France.
- [2] Service d'Anesthésie, Centre Hospitalier Universitaire de Grenoble, La Tronche, France.
- [3] Laboratoire de Microbiologie, Hôpital Delafontaine, Saint-Denis, France.
- [4] Service d'Anesthésie, Hôpital Delafontaine, Saint-Denis, France.
- [5] Service de Pédiatrie, Hôpital Delafontaine, Saint-Denis, France.
- [6] Service des Maladies infectieuses, Hôpital Delafontaine, Saint-Denis, France.
- [7] Service de Pneumologie, Hôpital Delafontaine, Saint-Denis, France.
- [8] Service des Urgences, Hôpital Delafontaine, Saint-Denis, France.

- PMID: **33052968**
- PMCID: PMC7556440
- DOI: 10.1371/journal.pone.0240645

Abstract

Introduction: Because of the COVID-19 pandemic, intensive care units (ICU) can be overwhelmed by the number of hypoxemic patients.

Material and methods: This single centre retrospective observational cohort study took place in a French hospital where the number of patients exceeded the ICU capacity despite an increase from 18 to 32 beds. Because of this, 59 (37%) of the 159 patients requiring ICU care were referred to other hospitals. From 27th March to 23rd April, consecutive patients who had respiratory failure or were unable to maintain an SpO2 > 90%, despite receiving 10-15 l/min of oxygen with a non-rebreather mask, were treated by continuous positive airway pressure (CPAP) unless the ICU physician judged that immediate intubation was indicated. We describe the characteristics, clinical course, and outcomes of these patients. The main outcome under study was CPAP discontinuation.

Results: CPAP was initiated in 49 patients and performed out of ICU in 41 (84%). Median age was 65 years (IQR = 54-71) and 36 (73%) were men. Median respiratory rate before CPAP was 36 (30-40) and median SpO2 was 92% (90-95) under 10 to 15 L/min oxygen flow. Median duration of CPAP was 3 days (IQR = 1-5). Reasons for discontinuation of CPAP were: intubation in 25 (51%), improvement in 16 (33%), poor tolerance in 6 (12%) and death in 2 (4%) patients. A decision not to intubate had been taken for 8 patients, including the 2 who died while on CPAP. Two patients underwent less than one hour CPAP for poor tolerance. In the end, 15 (38%) out of 39 evaluable patients recovered with only CPAP whereas 24 (62%) were intubated.

Conclusions: CPAP is feasible in a non-ICU environment in the context of massive influx of patients. In our cohort up to 1/3 of the patients presenting with acute respiratory failure recovered without intubation.

Conflict of interest statement

The authors have declared that no competing interests exist.

- 44 references
- 3 figures

Supplementary info

Publication types, MeSH terms, Grant support Expand

Publication types

- Observational Study

MeSH terms

- Aged
- COVID-19
- Continuous Positive Airway Pressure / economics
- Continuous Positive Airway Pressure / instrumentation
- Continuous Positive Airway Pressure / methods*

- Coronavirus Infections / economics
- Coronavirus Infections / epidemiology
- Coronavirus Infections / therapy*
- Costs and Cost Analysis
- Female
- France
- Hospital Bed Capacity / statistics & numerical data
- Humans
- Intensive Care Units / statistics & numerical data
- Male
- Middle Aged
- Pandemics / economics
- Patient Admission / statistics & numerical data
- Pneumonia, Viral / economics
- Pneumonia, Viral / epidemiology
- Pneumonia, Viral / therapy*

Grant support

The author(s) received no specific funding for this work.

Full text links

OPEN ACCESS TO FULL TEXT PLOS ONE Public Library of Science Free PMC article

Proceed to details

Cite

Share

1,182

Observational Study

Radiology
. 2020 Nov;297(2):E242-E251.
doi: 10.1148/radiol.2020202222. Epub 2020 Jun 16.

Brain MRI Findings in Severe COVID-19: A Retrospective Observational Study

Stéphane Kremer[#,1,2], François Lersy[#,1,2], Jérome de Sèze[1,2], Jean-Christophe Ferré[1,2], Adel Maamar[1,2], Béatrice Carsin-Nicol[1,2], Olivier Collange[1,2], Fabrice Bonneville[1,2], Gilles Adam[1,2], Guillaume Martin-Blondel[1,2], Marie Rafiq[1,2], Thomas Geeraerts[1,2], Louis Delamarre[1,2], Sylvie Grand[1,2], Alexandre Krainik[1,2], Sophie Caillard[1,2], Jean Marc Constans[1,2], Serge Metanbou[1,2], Adrien Heintz[1,2], Julie Helms[1,2], Maleka Schenck[1,2], Nicolas Lefèbvre[1,2], Claire Boutet[1,2], Xavier Fabre[1,2], Géraud Forestier[1,2], Isaure de Beaurepaire[1,2], Grégoire Bornet[1,2], Audrey Lacalm[1,2], Hélène Oesterlé[1,2], Federico

Bolognini [1] [2], Julien Messié [1] [2], Ghazi Hmeydia [1] [2], Joseph Benzakoun [1] [2], Catherine Oppenheim [1] [2], Blanche Bapst [1] [2], Imen Megdiche [1] [2], Marie-Cécile Henry Feugeas [1] [2], Antoine Khalil [1] [2], Augustin Gaudemer [1] [2], Lavinia Jager [1] [2], Patrick Nesser [1] [2], Yannick Talla Mba [1] [2], Céline Hemmert [1] [2], Philippe Feuerstein [1] [2], Nathan Sebag [1] [2], Sophie Carré [1] [2], Manel Alleg [1] [2], Claire Lecocq [1] [2], Emmanuelle Schmitt [1] [2], René Anxionnat [1] [2], François Zhu [1] [2], Pierre-Olivier Comby [1] [2], Frédéric Ricolfi [1] [2], Pierre Thouant [1] [2], Hubert Desal [1] [2], Grégoire Boulouis [1] [2], Jérome Berge [1] [2], Apolline Kazémi [1] [2], Nadya Pyatigorskaya [1] [2], Augustin Lecler [1] [2], Suzana Saleme [1] [2], Myriam Edjlali-Goujon [1] [2], Basile Kerleroux [1] [2], Pierre-Emmanuel Zorn [1] [2], Muriel Matthieu [1] [2], Seyyid Baloglu [1] [2], François-Daniel Ardellier [1] [2], Thibault Willaume [1] [2], Jean Christophe Brisset [1] [2], Clotilde Boulay [1] [2], Véronique Mutschler [1] [2], Yves Hansmann [1] [2], Paul-Michel Mertes [1] [2], Francis Schneider [1] [2], Samira Fafi-Kremer [1] [2], Mickael Ohana [1] [2], Ferhat Meziani [1] [2], Jean-Stéphane David [1] [2], Nicolas Meyer [1] [2], Mathieu Anheim [1] [2], François Cotton [1] [2]

Affiliations Expand

Affiliations

- [1] From the Hôpitaux Universitaires de Strasbourg, Service d'Imagerie 2, Hôpital de Hautepierre, Strasbourg, France (S.K.).
- [2] Author affiliations: Hôpitaux Universitaires de Strasbourg, Service d’Imagerie 2, Hôpital de Hautepierre, Strasbourg, France (S.K., F.L., S.B., F.D.A., T.W.); Engineering Science, Computer Science and Imaging Laboratory (ICube), Integrative Multimodal Imaging in Healthcare, UMR 7357, University of Strasbourg-CNRS, Strasbourg, France (S.K.); Service de Neurologie, Hôpitaux Universitaires de Strasbourg, Strasbourg, France (J.d.S., C. Boulay, V.M., M. Anheim); CHU Rennes, Department of Neuroradiology, Rennes, France (J.C.F., B.C.N.); Service de Maladies Infectieuses et Réanimation Médicale, CHU Rennes, France (A.M.); Hôpitaux Universitaires de Strasbourg, Service d’Anesthésie-Réanimation, Nouvel Hôpital Civil, Strasbourg, France (O.C., P.M.M.); Service de Neuroradiologie, CHU Toulouse, Toulouse, France (F. Bonneville., G.A.); Department of Infectious and Tropical Diseases, Toulouse University Hospital, Toulouse, France (G.M.B.); Department of Neurology, Toulouse University Hospital, Toulouse, France (M.R.); Department of Anesthesia and Critical Care, Toulouse University Hospital University Toulouse 3-Paul Sabatier, Toulouse, France (T.G., L.D.); Service de Neuroradiologie Diagnostique et Interventionnelle, Centre Hospitalier Universitaire des Alpes, Grenoble, France (S.G., A. Krainik); Nephrology and Transplantation Department, Hôpitaux Universitaires de Strasbourg, Inserm UMR S1109, LabEx Transplantex, Fédération de Médecine Translationnelle de Strasbourg (FMTS), Université de Strasbourg, Strasbourg, France (S. Caillard); EA CHIMERE 7516, Université de Picardie Jules Verne, Amiens, France; Service de NeuroRadiologie, Pôle Imagerie Médicale, Centre Hospitalo-Universitaire d’Amiens, Amiens, France (J.M.C., A.H.); Service de Neuro Radiologie, Pôle Imagerie Médicale, Centre Hospitalo-Universitaire d’Amiens, Amiens, France (S.M.); Hôpitaux Universitaires de Strasbourg, Service de Médecine Intensive Réanimation, Nouvel Hôpital Civil, Strasbourg, France (J.H., F.M.); Immuno-Rhumatologie Moléculaire, INSERM UMR_S1109, LabEx TRANSPLANTEX, Centre de Recherche d’Immunologie et d’Hématologie, Faculté de Médecine, Fédération Hospitalo-Universitaire (FHU) OMICARE, Fédération de Médecine Translationnelle de Strasbourg (FMTS), Université de Strasbourg (UNISTRA), Strasbourg, France (J.H.); Hôpitaux Universitaires de Strasbourg, Service de Médecine Intensive Réanimation, Hautepierre, Strasbourg, France (M.S., F.S.); Service de Maladies Infectieuses, NHC, CHU de Strasbourg, Strasbourg, France (N.L., Y.H.); Service de Radiologie, CHU de Saint-Etienne, Saint-Etienne, France (C. Boutet);

Service de Réanimation, CH de Roanne, Roanne, France (X.F.); University Hospital of Limoges, Neuroradiology Department, Limoges, France (G.F., S.S.); Radiology Department, Hôpital Privé d'Antony, Antony, France (I.d.B., G. Bornet); Service d'Imagerie Pédiatrique et Fœtale, Hôpital Femme Mère Enfant, HCL, Lyon, France (A. Lacalm); Service de Neuroradiologie, Hôpitaux Civils de Colmar, Colmar, France (H.O., F. Bolognini, J.M.); INSERM U1266, Service d'Imagerie Morphologique et Fonctionnelle, GHU Psychiatrie et Neurosciences, Site Sainte-Anne, Paris, France (G.H., J. Benzakoun, C.O., G. Boulouis, M.E.G., B.K.); Service de Neuroradiologie, CHU Henri Mondor, Créteil, France (B.B., I.M.); Neuroradiology Unit, Department of Radiology, Assistance Publique –Hôpitaux de Paris (APHP), Bichat University Hospital, Paris, France (M.C.H.F., A.G.); Department of Radiology, Assistance Publique–Hôpitaux de Paris (APHP), Denis Diderot University and Medical School, Bichat University Hospital, Paris, France (A. Khalil); CHIC Unisanté, Hôpital Marie Madeleine, Forbach, France (L.J., P.N., Y.T.M.); Service de Radiologie 1, GHR Mulhouse Sud Alsace, Hôpital Mère Enfants, Mulhouse, France (C.H., P.F., N.S.); Service de Neurologie, Centre Hospitalier de Haguenau, Haguenau, France (S. Carré, C.L.); Service de Radiologie, Centre Hospitalier de Haguenau, Haguenau, France (M. Alleg); Service de Neuroradiologie, Hôpital Central, CHU de Nancy, Nancy, France (E.S., R.A., F.Z.); Department of Neuroradiology, University Hospital of Dijon, Hôpital François Mitterrand, Dijon, France (P.O.C., F.R., P.T.); Department of Diagnostic and Interventional Neuroradiology, University Hospital, Nantes, France (H.D.); Neuroradiology Department, CHU de Bordeaux, Bordeaux, France (J. Berge); Service de Neuroradiologie, CHU de Lille, Lille, France (A. Kazémi); Assistance Publique Hôpitaux de Paris, Service de Neuroradiologie, Hôpital Pitié-Salpêtrière, Paris, France Sorbonne Université, Univ Paris 06, UMR S 1127, CNRS UMR 7225, ICM, F-75013, Paris, France (N.P.); Neuroradiology Department, Fondation A. Rothschild Hospital, Paris, France (A. Lecler); Hôpitaux Universitaires de Strasbourg, UCIEC, Pôle d'Imagerie, Strasbourg, France (P.E.Z., M.M.); Observatoire Français de la Sclérose en Plaques, Lyon, France (J.C.B.); Hôpitaux Universitaires de Strasbourg, Laboratoire de Virologie Médicale, Strasbourg, France (S.F.K.); Radiology Department, Nouvel Hôpital Civil, Strasbourg University Hospital, Strasbourg, France (M.O.); INSERM (French National Institute of Health and Medical Research), UMR 1260, Regenerative Nanomedicine (RNM), FMTS, Strasbourg, France (F.M.); Department of Anaesthesia and Intensive Care, Lyon-Sud Hospital, Hospices Civils de Lyon, F-69495 Pierre Benite; University Claude Bernard Lyon 1, Lyon, France (J.S.D.); CHU de Strasbourg, Service de Santé Publique, GMRC, F-67091 Strasbourg, France (N.M.); Institut de Génétique et de Biologie Moléculaire et Cellulaire (IGBMC), INSERM-U964/CNRS-UMR7104/Université de Strasbourg, Illkirch, France (M. Anheim); MRI Center, Centre Hospitalier Lyon Sud, Hospices Civils de Lyon, Lyon, France (F.C.); and Université Lyon 1, CREATIS-LRMN, CNRS/UMR/5220-INSERM U630, Villeurbanne, France (F.C.).

[#] Contributed equally.

- PMID: **32544034**
- PMCID: PMC7301613
- DOI: 10.1148/radiol.2020202222

Free PMC article
Observational Study

Brain MRI Findings in Severe COVID-19: A Retrospective Observational Study

Stéphane Kremer et al. Radiology. 2020 Nov.
Free PMC article
Show details
Radiology
. 2020 Nov;297(2):E242-E251.
doi: 10.1148/radiol.2020202222. Epub 2020 Jun 16.

Authors

Stéphane Kremer[#,1,2], François Lersy[#,1,2], Jérome de Sèze[1,2], Jean-Christophe Ferré[1,2], Adel Maamar[1,2], Béatrice Carsin-Nicol[1,2], Olivier Collange[1,2], Fabrice Bonneville[1,2], Gilles Adam[1,2], Guillaume Martin-Blondel[1,2], Marie Rafiq[1,2], Thomas Geeraerts[1,2], Louis Delamarre[1,2], Sylvie Grand[1,2], Alexandre Krainik[1,2], Sophie Caillard[1,2], Jean Marc Constans[1,2], Serge Metanbou[1,2], Adrien Heintz[1,2], Julie Helms[1,2], Maleka Schenck[1,2], Nicolas Lefèbvre[1,2], Claire Boutet[1,2], Xavier Fabre[1,2], Géraud Forestier[1,2], Isaure de Beaurepaire[1,2], Grégoire Bornet[1,2], Audrey Lacalm[1,2], Hélène Oesterlé[1,2], Federico Bolognini[1,2], Julien Messié[1,2], Ghazi Hmeydia[1,2], Joseph Benzakoun[1,2], Catherine Oppenheim[1,2], Blanche Bapst[1,2], Imen Megdiche[1,2], Marie-Cécile Henry Feugeas[1,2], Antoine Khalil[1,2], Augustin Gaudemer[1,2], Lavinia Jager[1,2], Patrick Nesser[1,2], Yannick Talla Mba[1,2], Céline Hemmert[1,2], Philippe Feuerstein[1,2], Nathan Sebag[1,2], Sophie Carré[1,2], Manel Alleg[1,2], Claire Lecocq[1,2], Emmanuelle Schmitt[1,2], René Anxionnat[1,2], François Zhu[1,2], Pierre-Olivier Comby[1,2], Frédéric Ricolfi[1,2], Pierre Thouant[1,2], Hubert Desal[1,2], Grégoire Boulouis[1,2], Jérome Berge[1,2], Apolline Kazémi[1,2], Nadya Pyatigorskaya[1,2], Augustin Lecler[1,2], Suzana Saleme[1,2], Myriam Edjlali-Goujon[1,2], Basile Kerleroux[1,2], Pierre-Emmanuel Zorn[1,2], Muriel Matthieu[1,2], Seyyid Baloglu[1,2], François-Daniel Ardellier[1,2], Thibault Willaume[1,2], Jean Christophe Brisset[1,2], Clotilde Boulay[1,2], Véronique Mutschler[1,2], Yves Hansmann[1,2], Paul-Michel Mertes[1,2], Francis Schneider[1,2], Samira Fafi-Kremer[1,2], Mickael Ohana[1,2], Ferhat Meziani[1,2], Jean-Stéphane David[1,2], Nicolas Meyer[1,2], Mathieu Anheim[1,2], François Cotton[1,2]

Affiliations

- [1] From the Hôpitaux Universitaires de Strasbourg, Service d'Imagerie 2, Hôpital de Hautepierre, Strasbourg, France (S.K.).
- [2] Author affiliations: Hôpitaux Universitaires de Strasbourg, Service d'Imagerie 2, Hôpital de Hautepierre, Strasbourg, France (S.K., F.L., S.B., F.D.A., T.W.); Engineering Science, Computer Science and Imaging Laboratory (ICube), Integrative Multimodal Imaging in Healthcare, UMR 7357, University of Strasbourg-CNRS, Strasbourg, France (S.K.); Service de Neurologie, Hôpitaux Universitaires de Strasbourg, Strasbourg, France (J.d.S., C. Boulay, V.M., M. Anheim); CHU Rennes, Department of Neuroradiology, Rennes, France (J.C.F., B.C.N.); Service de Maladies Infectieuses et Réanimation Médicale, CHU Rennes, France (A.M.); Hôpitaux Universitaires de Strasbourg, Service d'Anesthésie-Réanimation, Nouvel Hôpital Civil, Strasbourg, France (O.C., P.M.M.); Service de Neuroradiologie, CHU

Toulouse, Toulouse, France (F. Bonneville., G.A.); Department of Infectious and Tropical Diseases, Toulouse University Hospital, Toulouse, France (G.M.B.); Department of Neurology, Toulouse University Hospital, Toulouse, France (M.R.); Department of Anesthesia and Critical Care, Toulouse University Hospital University Toulouse 3-Paul Sabatier, Toulouse, France (T.G., L.D.); Service de Neuroradiologie Diagnostique et Interventionnelle, Centre Hospitalier Universitaire des Alpes, Grenoble, France (S.G., A. Krainik); Nephrology and Transplantation Department, Hôpitaux Universitaires de Strasbourg, Inserm UMR S1109, LabEx Transplantex, Fédération de Médecine Translationnelle de Strasbourg (FMTS), Université de Strasbourg, Strasbourg, France (S. Caillard); EA CHIMERE 7516, Université de Picardie Jules Verne, Amiens, France; Service de NeuroRadiologie, Pôle Imagerie Médicale, Centre Hospitalo-Universitaire d'Amiens, Amiens, France (J.M.C., A.H.); Service de Neuro Radiologie, Pôle Imagerie Médicale, Centre Hospitalo-Universitaire d'Amiens, Amiens, France (S.M.); Hôpitaux Universitaires de Strasbourg, Service de Médecine Intensive Réanimation, Nouvel Hôpital Civil, Strasbourg, France (J.H., F.M.); Immuno-Rhumatologie Moléculaire, INSERM UMR_S1109, LabEx TRANSPLANTEX, Centre de Recherche d'Immunologie et d'Hématologie, Faculté de Médecine, Fédération Hospitalo-Universitaire (FHU) OMICARE, Fédération de Médecine Translationnelle de Strasbourg (FMTS), Université de Strasbourg (UNISTRA), Strasbourg, France (J.H.); Hôpitaux Universitaires de Strasbourg, Service de Médecine Intensive Réanimation, Hautepierre, Strasbourg, France (M.S., F.S.); Service de Maladies Infectieuses, NHC, CHU de Strasbourg, Strasbourg, France (N.L., Y.H.); Service de Radiologie, CHU de Saint-Etienne, Saint-Etienne, France (C. Boutet); Service de Réanimation, CH de Roanne, Roanne, France (X.F.); University Hospital of Limoges, Neuroradiology Department, Limoges, France (G.F., S.S.); Radiology Department, Hôpital Privé d'Antony, Antony, France (I.d.B., G. Bornet); Service d'Imagerie Pédiatrique et Fœtale, Hôpital Femme Mère Enfant, HCL, Lyon, France (A. Lacalm); Service de Neuroradiologie, Hôpitaux Civils de Colmar, Colmar, France (H.O., F. Bolognini, J.M.); INSERM U1266, Service d'Imagerie Morphologique et Fonctionnelle, GHU Psychiatrie et Neurosciences, Site Sainte-Anne, Paris, France (G.H., J. Benzakoun, C.O., G. Boulouis, M.E.G., B.K.); Service de Neuroradiologie, CHU Henri Mondor, Créteil, France (B.B., I.M.); Neuroradiology Unit, Department of Radiology, Assistance Publique –Hôpitaux de Paris (APHP), Bichat University Hospital, Paris, France (M.C.H.F., A.G.); Department of Radiology, Assistance Publique–Hôpitaux de Paris (APHP), Denis Diderot University and Medical School, Bichat University Hospital, Paris, France (A. Khalil); CHIC Unisanté, Hôpital Marie Madeleine, Forbach, France (L.J., P.N., Y.T.M.); Service de Radiologie 1, GHR Mulhouse Sud Alsace, Hôpital Mère Enfants, Mulhouse, France (C.H., P.F., N.S.); Service de Neurologie, Centre Hospitalier de Haguenau, Haguenau, France (S. Carré, C.L.); Service de Radiologie, Centre Hospitalier de Haguenau, Haguenau, France (M. Alleg); Service de Neuroradiologie, Hôpital Central, CHU de Nancy, Nancy, France (E.S., R.A., F.Z.); Department of Neuroradiology, University Hospital of Dijon, Hôpital François Mitterrand, Dijon, France (P.O.C., F.R., P.T.); Department of Diagnostic and Interventional Neuroradiology, University Hospital, Nantes, France (H.D.); Neuroradiology Department, CHU de Bordeaux, Bordeaux, France (J. Berge); Service de Neuroradiologie, CHU de Lille, Lille, France (A. Kazémi); Assistance Publique Hôpitaux de Paris, Service de Neuroradiologie, Hôpital Pitié-Salpêtrière, Paris, France Sorbonne Université, Univ Paris 06, UMR S 1127, CNRS UMR 7225, ICM, F-75013, Paris, France (N.P.); Neuroradiology Department, Fondation A. Rothschild Hospital, Paris, France (A. Lecler); Hôpitaux Universitaires de Strasbourg, UCIEC, Pôle d'Imagerie, Strasbourg, France (P.E.Z., M.M.); Observatoire Français de la Sclérose en Plaques, Lyon, France (J.C.B.); Hôpitaux Universitaires de Strasbourg, Laboratoire de Virologie Médicale, Strasbourg, France (S.F.K.); Radiology Department, Nouvel Hôpital Civil, Strasbourg University Hospital, Strasbourg, France (M.O.); INSERM (French National Institute of Health and Medical Research), UMR 1260, Regenerative Nanomedicine (RNM), FMTS, Strasbourg, France

(F.M.); Department of Anaesthesia and Intensive Care, Lyon-Sud Hospital, Hospices Civils de Lyon, F-69495 Pierre Benite; University Claude Bernard Lyon 1, Lyon, France (J.S.D.); CHU de Strasbourg, Service de Santé Publique, GMRC, F-67091 Strasbourg, France (N.M.); Institut de Génétique et de Biologie Moléculaire et Cellulaire (IGBMC), INSERM-U964/CNRS-UMR7104/Université de Strasbourg, Illkirch, France (M. Anheim); MRI Center, Centre Hospitalier Lyon Sud, Hospices Civils de Lyon, Lyon, France (F.C.); and Université Lyon 1, CREATIS-LRMN, CNRS/UMR/5220-INSERM U630, Villeurbanne, France (F.C.).

[#] Contributed equally.

- PMID: **32544034**
- PMCID: PMC7301613
- DOI: 10.1148/radiol.2020202222

Abstract

Background Brain MRI parenchymal signal abnormalities have been associated with severe acute respiratory syndrome coronavirus 2 (SARS-CoV-2). Purpose To describe the neuroimaging findings (excluding ischemic infarcts) in patients with severe coronavirus disease 2019 (COVID-19) infection. Materials and Methods This was a retrospective study of patients evaluated from March 23, 2020, to April 27, 2020, at 16 hospitals. Inclusion criteria were (*a*) positive nasopharyngeal or lower respiratory tract reverse transcriptase polymerase chain reaction assays, (*b*) severe COVID-19 infection defined as a requirement for hospitalization and oxygen therapy, (*c*) neurologic manifestations, and (*d*) abnormal brain MRI findings. Exclusion criteria were patients with missing or noncontributory data regarding brain MRI or brain MRI showing ischemic infarcts, cerebral venous thrombosis, or chronic lesions unrelated to the current event. Categorical data were compared using the Fisher exact test. Quantitative data were compared using the Student t test or Wilcoxon test. $P < .05$ represented a significant difference. Results Thirty men (81%) and seven women (19%) met the inclusion criteria, with a mean age of 61 years ± 12 (standard deviation) (age range, 8-78 years). The most common neurologic manifestations were alteration of consciousness (27 of 37, 73%), abnormal wakefulness when sedation was stopped (15 of 37, 41%), confusion (12 of 37, 32%), and agitation (seven of 37, 19%). The most frequent MRI findings were signal abnormalities located in the medial temporal lobe in 16 of 37 patients (43%; 95% confidence interval [CI]: 27%, 59%), nonconfluent multifocal white matter hyperintense lesions seen with fluid-attenuated inversion recovery and diffusion-weighted sequences with variable enhancement, with associated hemorrhagic lesions in 11 of 37 patients (30%; 95% CI: 15%, 45%), and extensive and isolated white matter microhemorrhages in nine of 37 patients (24%; 95% CI: 10%, 38%). A majority of patients (20 of 37, 54%) had intracerebral hemorrhagic lesions with a more severe clinical presentation and a higher admission rate in intensive care units (20 of 20 patients [100%] vs 12 of 17 patients without hemorrhage [71%], $P = .01$) and development of the acute respiratory distress syndrome (20 of 20 patients [100%] vs 11 of 17 patients [65%], $P = .005$). Only one patient had SARS-CoV-2 RNA in the cerebrospinal fluid. Conclusion Patients with severe coronavirus disease 2019 and without ischemic infarcts had a wide range of neurologic manifestations that were associated with abnormal brain MRI scans. Eight distinctive neuroradiologic patterns were described. © RSNA, 2020.

- 28 references
- 6 figures

Supplementary info

Publication types, MeSH terms Expand

Publication types

- Multicenter Study
- Observational Study

MeSH terms

- Adolescent
- Adult
- Aged
- Betacoronavirus*
- Brain / diagnostic imaging*
- Brain / pathology*
- COVID-19
- Child
- Cohort Studies
- Coronavirus Infections / diagnostic imaging*
- Coronavirus Infections / pathology*
- Female
- Humans
- Magnetic Resonance Imaging / methods*
- Male
- Middle Aged
- Pandemics
- Pneumonia, Viral / diagnostic imaging*
- Pneumonia, Viral / pathology*
- Retrospective Studies
- SARS-CoV-2
- Young Adult

Full text links

Radiology FULL TEXT Atypon Free PMC article
Proceed to details
Cite
Share
☐ 1,183
Observational Study
J Pediatr
. 2020 Sep;224:24-29.
doi: 10.1016/j.jpeds.2020.06.045. Epub 2020 Jun 14.

Multisystem Inflammatory Syndrome in Children Associated with Severe Acute Respiratory Syndrome Coronavirus 2 Infection (MIS-C): A Multi-institutional Study from New York City

Shubhi Kaushik [1], Scott I Aydin [2], Kim R Derespina [3], Prerna B Bansal [4], Shanna Kowalsky [5], Rebecca Trachtman [6], Jennifer K Gillen [1], Michelle M Perez [3], Sara H Soshnick [3], Edward E Conway Jr [7], Asher Bercow [7], Howard S Seiden [4], Robert H Pass [4], Henry M Ushay [3], George Ofori-Amanfo [2], Shivanand S Medar [8]
Affilations Expand

Affiliations

- [1] Department of Pediatrics, Division of Pediatric Critical Care Medicine, Mount Sinai Kravis Children's Hospital, Icahn School of Medicine at Mount Sinai, New York, NY.
- [2] Department of Pediatrics, Division of Pediatric Critical Care Medicine, Mount Sinai Kravis Children's Hospital, Icahn School of Medicine at Mount Sinai, New York, NY; Department of Pediatrics, Division of Pediatric Cardiology, Mount Sinai Kravis Children's Hospital, Icahn School of Medicine at Mount Sinai, New York, NY.
- [3] Department of Pediatrics, Division of Pediatric Critical Care Medicine, Albert Einstein College of Medicine, Children's Hospital at Montefiore, New York, NY.
- [4] Department of Pediatrics, Division of Pediatric Cardiology, Mount Sinai Kravis Children's Hospital, Icahn School of Medicine at Mount Sinai, New York, NY.
- [5] Department of Pediatrics, Division of Infectious Diseases, Mount Sinai Kravis Children's Hospital, Icahn School of Medicine at Mount Sinai, New York, NY.
- [6] Department of Pediatrics, Division of Clinical Immunology and Pediatric Rheumatology, Mount Sinai Kravis Children's Hospital, Icahn School of Medicine at Mount Sinai, New York, NY.
- [7] Department of Pediatrics, Jacobi Medical Center, Albert Einstein College of Medicine, New York, NY.
- [8] Department of Pediatrics, Division of Pediatric Critical Care Medicine, Albert Einstein College of Medicine, Children's Hospital at Montefiore, New York, NY; Department of Pediatrics, Division of Pediatric Cardiology, Albert Einstein College of Medicine, Children's Hospital at Montefiore, New York, NY. Electronic address: smedar@montefiore.org.

- PMID: **32553861**
- PMCID: PMC7293760
- DOI: 10.1016/j.jpeds.2020.06.045

Free PMC article
Observational Study

Multisystem Inflammatory Syndrome in Children Associated with Severe Acute Respiratory Syndrome Coronavirus 2 Infection (MIS-C): A Multi-institutional Study from New York City

Shubhi Kaushik et al. J Pediatr. 2020 Sep.
Free PMC article
Show details
J Pediatr
. 2020 Sep;224:24-29.
doi: 10.1016/j.jpeds.2020.06.045. Epub 2020 Jun 14.

Authors

Shubhi Kaushik [1], Scott I Aydin [2], Kim R Derespina [3], Prerna B Bansal [4], Shanna Kowalsky [5], Rebecca Trachtman [6], Jennifer K Gillen [1], Michelle M Perez [3], Sara H Soshnick [3], Edward E Conway Jr [7], Asher Bercow [7], Howard S Seiden [4], Robert H Pass [4], Henry M Ushay [3], George Ofori-Amanfo [2], Shivanand S Medar [8]

Affiliations

- [1] Department of Pediatrics, Division of Pediatric Critical Care Medicine, Mount Sinai Kravis Children's Hospital, Icahn School of Medicine at Mount Sinai, New York, NY.
- [2] Department of Pediatrics, Division of Pediatric Critical Care Medicine, Mount Sinai Kravis Children's Hospital, Icahn School of Medicine at Mount Sinai, New York, NY; Department of Pediatrics, Division of Pediatric Cardiology, Mount Sinai Kravis Children's Hospital, Icahn School of Medicine at Mount Sinai, New York, NY.
- [3] Department of Pediatrics, Division of Pediatric Critical Care Medicine, Albert Einstein College of Medicine, Children's Hospital at Montefiore, New York, NY.
- [4] Department of Pediatrics, Division of Pediatric Cardiology, Mount Sinai Kravis Children's Hospital, Icahn School of Medicine at Mount Sinai, New York, NY.
- [5] Department of Pediatrics, Division of Infectious Diseases, Mount Sinai Kravis Children's Hospital, Icahn School of Medicine at Mount Sinai, New York, NY.
- [6] Department of Pediatrics, Division of Clinical Immunology and Pediatric Rheumatology, Mount Sinai Kravis Children's Hospital, Icahn School of Medicine at Mount Sinai, New York, NY.
- [7] Department of Pediatrics, Jacobi Medical Center, Albert Einstein College of Medicine, New York, NY.
- [8] Department of Pediatrics, Division of Pediatric Critical Care Medicine, Albert Einstein College of Medicine, Children's Hospital at Montefiore, New York, NY; Department of Pediatrics, Division of Pediatric Cardiology, Albert Einstein College of Medicine, Children's Hospital at Montefiore, New York, NY. Electronic address: smedar@montefiore.org.

- PMID: **32553861**
- PMCID: PMC7293760
- DOI: 10.1016/j.jpeds.2020.06.045

Abstract

Objective: To assess clinical characteristics and outcomes of severe acute respiratory syndrome coronavirus 2-associated multisystem inflammatory syndrome in children (MIS-C).

Study design: Children with MIS-C admitted to pediatric intensive care units in New York City between April 23 and May 23, 2020, were included. Demographic and clinical data were collected.

Results: Of 33 children with MIS-C, the median age was 10 years; 61% were male; 45% were Hispanic/Latino; and 39% were black. Comorbidities were present in 45%. Fever (93%) and vomiting (69%) were the most common presenting symptoms. Depressed left ventricular ejection fraction was found in 63% of patients with median ejection fraction of 46.6% (IQR, 39.5-52.8). C-reactive protein, procalcitonin, d-dimer, and pro-B-type natriuretic peptide levels were elevated in all patients. For treatment, intravenous immunoglobulin was used in 18 (54%), corticosteroids in 17 (51%), tocilizumab in 12 (36%), remdesivir in 7 (21%), vasopressors in 17 (51%), mechanical ventilation in 5 (15%), extracorporeal membrane oxygenation in 1 (3%), and intra-aortic balloon pump in 1 (3%). The left ventricular ejection fraction normalized in 95% of those with a depressed ejection fraction. All patients were discharged home with median duration of pediatric intensive care unit stay of 4.7 days (IQR, 4-8 days) and a hospital stay of 7.8 days (IQR, 6.0-10.1 days). One patient (3%) died after withdrawal of care secondary to stroke while on extracorporeal membrane oxygenation.

Conclusions: Critically ill children with coronavirus disease-2019-associated MIS-C have a spectrum of severity broader than described previously but still require careful supportive intensive care. Rapid, complete clinical and myocardial recovery was almost universal.

Copyright © 2020 Elsevier Inc. All rights reserved.

Comment in

- Reply.
  Kaushik S, Derespina KR, Medar SS. Kaushik S, et al. J Pediatr. 2020 Nov;226:315. doi: 10.1016/j.jpeds.2020.07.064. Epub 2020 Jul 24. J Pediatr. 2020. PMID: 32712286 Free PMC article. No abstract available.
- Epidemiologic trends in Kawasaki disease during coronavirus disease-19 in Singapore.
  Yung CF, Nadua KD, Oh BK, Thoon KC. Yung CF, et al. J Pediatr. 2020 Nov;226:314-315. doi: 10.1016/j.jpeds.2020.07.063. Epub 2020 Jul 24. J Pediatr. 2020. PMID: 32717229 Free PMC article. No abstract available.
- Reply.
  Kaushik S, Jhaveri S, Derespina KR, Medar SS, Stern K, Aydin SI. Kaushik S, et al. J Pediatr. 2021 Jan;228:315-316. doi: 10.1016/j.jpeds.2020.09.055. Epub 2020 Nov 2. J Pediatr. 2021. PMID: 32979385 No abstract available.
- Use of tocilizumab in multisystem inflammatory syndrome in children associated with severe acute respiratory syndrome coronavirus 2.
  Banday AZ, Vignesh P. Banday AZ, et al. J Pediatr. 2021 Jan;228:315. doi: 10.1016/j.jpeds.2020.09.054. Epub 2020 Oct 26. J Pediatr. 2021. PMID: 32979386 Free PMC article. No abstract available.

- Coronavirus disease 2019, multisystem inflammatory syndrome in children, apolipoprotein E4, and race.
  Goldstein MR, Poland GA, Graeber CW. Goldstein MR, et al. J Pediatr. 2021 Feb;229:313-314. doi: 10.1016/j.jpeds.2020.10.072. Epub 2020 Oct 30. J Pediatr. 2021. PMID: 33137315 Free PMC article. No abstract available.

- 15 references

Supplementary info

Publication types, MeSH terms, Substances, Supplementary concepts Expand

Publication types

- Multicenter Study
- Observational Study

MeSH terms

- Adolescent
- Betacoronavirus
- C-Reactive Protein / analysis
- COVID-19
- Child
- Child, Preschool
- Coronavirus Infections / complications*
- Coronavirus Infections / drug therapy
- Female
- Fibrin Fibrinogen Degradation Products / analysis
- Humans
- Infant
- Intensive Care Units, Pediatric
- Male
- Natriuretic Peptide, Brain / blood
- New York City
- Pandemics
- Pneumonia, Viral / complications*
- Procalcitonin / analysis
- Retrospective Studies
- SARS-CoV-2
- Systemic Inflammatory Response Syndrome / diagnosis*
- Systemic Inflammatory Response Syndrome / therapy
- Treatment Outcome
- Ventricular Function, Left

- Young Adult

Substances

- Fibrin Fibrinogen Degradation Products
- Procalcitonin
- fibrin fragment D
- Natriuretic Peptide, Brain
- C-Reactive Protein

Supplementary concepts

- COVID-19 drug treatment
- pediatric multisystem inflammatory disease, COVID-19 related

Full text links

ELSEVIER FULL-TEXT ARTICLE Elsevier Science Free PMC article
Proceed to details
Cite
Share
1,184
Observational Study
Am J Transplant
. 2020 Jul;20(7):1849-1858.
doi: 10.1111/ajt.15929. Epub 2020 May 10.

COVID-19 in solid organ transplant recipients: A single-center case series from Spain

Mario Fernández-Ruiz [1], Amado Andrés [2], Carmelo Loinaz [3], Juan F Delgado [4] [5], Francisco López-Medrano [1], Rafael San Juan [1], Esther González [2], Natalia Polanco [2], María D Folgueira [6], Antonio Lalueza [7], Carlos Lumbreras [7], José M Aguado [1]
Affiliations Expand

Affiliations

- [1] Unit of Infectious Diseases, Hospital Universitario "12 de Octubre", Instituto de Investigación Sanitaria Hospital "12 de Octubre" (imas12), Madrid, Spain.
- [2] Department of Nephrology, Hospital Universitario "12 de Octubre", Instituto de Investigación Sanitaria Hospital "12 de Octubre" (imas12), Madrid, Spain.

- [3] Department of General Surgery, Digestive Tract and Abdominal Organ Transplantation, Hospital Universitario "12 de Octubre", Instituto de Investigación Sanitaria Hospital "12 de Octubre" (imas12), Madrid, Spain.
- [4] Department of Cardiology, Hospital Universitario "12 de Octubre", Instituto de Investigación Sanitaria Hospital "12 de Octubre" (imas12), Madrid, Spain.
- [5] CIBER de Enfermedades Cardiovasculares (CIBERCV), Madrid, Spain.
- [6] Department of Microbiology, Hospital Universitario "12 de Octubre", Instituto de Investigación Sanitaria Hospital "12 de Octubre" (imas12), Madrid, Spain.
- [7] Department of Internal Medicine, Hospital Universitario "12 de Octubre", Instituto de Investigación Sanitaria Hospital "12 de Octubre" (imas12), Madrid, Spain.

- PMID: **32301155**
- DOI: 10.1111/ajt.15929

Free article
Observational Study

COVID-19 in solid organ transplant recipients: A single-center case series from Spain

Mario Fernández-Ruiz et al. Am J Transplant. 2020 Jul.
Free article
Show details
Am J Transplant
. 2020 Jul;20(7):1849-1858.
doi: 10.1111/ajt.15929. Epub 2020 May 10.

Authors

Mario Fernández-Ruiz [1], Amado Andrés [2], Carmelo Loinaz [3], Juan F Delgado [4] [5], Francisco López-Medrano [1], Rafael San Juan [1], Esther González [2], Natalia Polanco [2], María D Folgueira [6], Antonio Lalueza [7], Carlos Lumbreras [7], José M Aguado [1]

Affiliations

- [1] Unit of Infectious Diseases, Hospital Universitario "12 de Octubre", Instituto de Investigación Sanitaria Hospital "12 de Octubre" (imas12), Madrid, Spain.
- [2] Department of Nephrology, Hospital Universitario "12 de Octubre", Instituto de Investigación Sanitaria Hospital "12 de Octubre" (imas12), Madrid, Spain.
- [3] Department of General Surgery, Digestive Tract and Abdominal Organ Transplantation, Hospital Universitario "12 de Octubre", Instituto de Investigación Sanitaria Hospital "12 de Octubre" (imas12), Madrid, Spain.
- [4] Department of Cardiology, Hospital Universitario "12 de Octubre", Instituto de Investigación Sanitaria Hospital "12 de Octubre" (imas12), Madrid, Spain.
- [5] CIBER de Enfermedades Cardiovasculares (CIBERCV), Madrid, Spain.

- [6] Department of Microbiology, Hospital Universitario "12 de Octubre", Instituto de Investigación Sanitaria Hospital "12 de Octubre" (imas12), Madrid, Spain.
- [7] Department of Internal Medicine, Hospital Universitario "12 de Octubre", Instituto de Investigación Sanitaria Hospital "12 de Octubre" (imas12), Madrid, Spain.

- PMID: **32301155**
- DOI: 10.1111/ajt.15929

Abstract

The clinical characteristics, management, and outcome of coronavirus disease 2019 (COVID-19) caused by severe acute respiratory syndrome coronavirus 2 (SARS-CoV-2) after solid organ transplant (SOT) remain unknown. We report our preliminary experience with 18 SOT (kidney [44.4%], liver [33.3%], and heart [22.2%]) recipients diagnosed with COVID-19 by March 23, 2020 at a tertiary-care center at Madrid. Median age at diagnosis was 71.0 ± 12.8 years, and the median interval since transplantation was 9.3 years. Fever (83.3%) and radiographic abnormalities in form of unilateral or bilateral/multifocal consolidations (72.2%) were the most common presentations. Lopinavir/ritonavir (usually associated with hydroxychloroquine) was used in 50.0% of patients and had to be prematurely discontinued in 2 of them. Other antiviral regimens included hydroxychloroquine monotherapy (27.8%) and interferon-β (16.7%). As of April 4, the case-fatality rate was 27.8% (5/18). After a median follow-up of 18 days from symptom onset, 30.8% (4/13) of survivors developed progressive respiratory failure, 7.7% (1/13) showed stable clinical condition or improvement, and 61.5% (8/13) had been discharged home. C-reactive protein levels at various points were significantly higher among recipients who experienced unfavorable outcome. In conclusion, this frontline report suggests that SARS-CoV-2 infection has a severe course in SOT recipients.

Keywords: COVID-19; SARS-CoV-2; coronavirus; outcome; solid organ transplantation; treatment.

© 2020 The American Society of Transplantation and the American Society of Transplant Surgeons.

Comment in

- Coronavirus disease 2019 and transplantation: The combination of lopinavir/ritonavir and hydroxychloroquine is responsible for excessive tacrolimus trough level and unfavorable outcome.
  Xia T, Wang Y. Xia T, et al. Am J Transplant. 2020 Sep;20(9):2630-2631. doi: 10.1111/ajt.15992. Epub 2020 Jun 12. Am J Transplant. 2020. PMID: 32400965 Free PMC article.
- Clinical outcome in solid organ transplant recipients with COVID-19: A single-center experience.
  Travi G, Rossotti R, Merli M, Sacco A, Perricone G, Lauterio A, Colombo VG, De Carlis L, Frigerio M, Minetti E, Belli LS, Puoti M. Travi G, et al. Am J Transplant. 2020 Sep;20(9):2628-2629. doi: 10.1111/ajt.16069. Epub 2020 Jun 8. Am J Transplant. 2020. PMID: 32436646 Free PMC article. No abstract available.

- 29 references

Supplementary info

Publication types, MeSH terms, Substances, Grant support Expand

Publication types

- Observational Study
- Research Support, Non-U.S. Gov't

MeSH terms

- Aged
- Antiviral Agents / administration & dosage
- Betacoronavirus
- COVID-19
- Coronavirus Infections / complications*
- Coronavirus Infections / mortality*
- Coronavirus Infections / therapy*
- Drug Combinations
- Female
- Fever
- Humans
- Hydroxychloroquine / administration & dosage
- Immunosuppressive Agents / administration & dosage
- Immunosuppressive Agents / adverse effects
- Interferon-beta / administration & dosage
- Lopinavir / administration & dosage
- Male
- Middle Aged
- Organ Transplantation*
- Pandemics
- Pneumonia, Viral / complications*
- Pneumonia, Viral / mortality*
- Pneumonia, Viral / therapy*
- Radiography, Thoracic
- Retrospective Studies
- Ritonavir / administration & dosage
- SARS-CoV-2
- Spain / epidemiology
- Transplant Recipients*

Substances

- Antiviral Agents

- Drug Combinations
- Immunosuppressive Agents
- lopinavir-ritonavir drug combination
- Lopinavir
- Hydroxychloroquine
- Interferon-beta
- Ritonavir

Grant support

- Spanish Ministry of Science and Innovation/International
- 13/00045/Instituto de Salud Carlos III/International
- CP 18/00073/Instituto de Salud Carlos III/International
- COV 20/00181/Instituto de Salud Carlos III/International

Full text links

Wiley

Proceed to details

Cite

Share

1,185

Observational Study

J Am Geriatr Soc

. 2021 Jul;69(7):1713-1721.

doi: 10.1111/jgs.17227. Epub 2021 May 14.

Emergency department visits for emergent conditions among older adults during the COVID-19 pandemic

Alexander T Janke [1], Snigdha Jain [2], Ula Hwang [1, 3], Mark Rosenberg [4, 5], Kevin Biese [6], Sandra Schneider [5], Pawan Goyal [5], Arjun K Venkatesh [1, 7]

Affiliations Expand

Affiliations

- [1] Department of Emergency Medicine, Yale University School of Medicine, New Haven, Connecticut, USA.
- [2] Section of Geriatrics and Section of Pulmonary, Critical Care and Sleep Medicine, Yale University School of Medicine, New Haven, Connecticut, USA.
- [3] Geriatrics Research, Education and Clinical Center, James J. Peters VAMC, Bronx, New York, USA.
- [4] St. Joseph's Health, Paterson, New Jersey, USA.

- [5] American College of Emergency Physicians, Irving, Texas, USA.
- [6] Department of Emergency Medicine, University of North Carolina School of Medicine, Chapel Hill, North Carolina, USA.
- [7] Center for Outcomes Research and Evaluation, Yale University School of Medicine, New Haven, Connecticut, USA.

- PMID: **33955546**
- PMCID: PMC8242842
- DOI: 10.1111/jgs.17227

Free PMC article
Observational Study

Emergency department visits for emergent conditions among older adults during the COVID-19 pandemic

Alexander T Janke et al. J Am Geriatr Soc. 2021 Jul.
Free PMC article
Show details
J Am Geriatr Soc
. 2021 Jul;69(7):1713-1721.
doi: 10.1111/jgs.17227. Epub 2021 May 14.

Authors

Alexander T Janke [1], Snigdha Jain [2], Ula Hwang [1] [3], Mark Rosenberg [4] [5], Kevin Biese [6], Sandra Schneider [5], Pawan Goyal [5], Arjun K Venkatesh [1] [7]

Affiliations

- [1] Department of Emergency Medicine, Yale University School of Medicine, New Haven, Connecticut, USA.
- [2] Section of Geriatrics and Section of Pulmonary, Critical Care and Sleep Medicine, Yale University School of Medicine, New Haven, Connecticut, USA.
- [3] Geriatrics Research, Education and Clinical Center, James J. Peters VAMC, Bronx, New York, USA.
- [4] St. Joseph's Health, Paterson, New Jersey, USA.
- [5] American College of Emergency Physicians, Irving, Texas, USA.
- [6] Department of Emergency Medicine, University of North Carolina School of Medicine, Chapel Hill, North Carolina, USA.
- [7] Center for Outcomes Research and Evaluation, Yale University School of Medicine, New Haven, Connecticut, USA.

- PMID: **33955546**
- PMCID: PMC8242842
- DOI: 10.1111/jgs.17227

Abstract

Background/objective: Emergency department (ED) visits have declined while excess mortality, not attributable to COVID-19, has grown. It is not known whether older adults are accessing emergency care differently from their younger counterparts. Our objective was to determine patterns of ED visit counts for emergent conditions during the COVID-19 pandemic for older adults.

Design: Retrospective, observational study.

Setting: Observational analysis of ED sites enrolled in a national clinical quality registry.

Participants: One hundred and sixty-four ED sites in 33 states from January 1, 2019 to November 15, 2020.

Main outcome and measures: We measured daily ED visit counts for acute myocardial infarction (AMI), stroke, sepsis, fall, and hip fracture, as well as deaths in the ED, by age categories. We estimated Poisson regression models comparing early and post-early pandemic periods (defined by the Centers for Disease Control and Prevention) to the pre-pandemic period. We report incident rate ratios to summarize changes in visit incidence.

Results: For AMI, stroke, and sepsis, the older (75-84) and oldest old (85+ years) had the greatest decline in visit counts initially and the smallest recovery in the post-early pandemic periods. For falls, visits declined early and partially recovered uniformly across age categories. In contrast, hip fractures exhibited less change in visit rates across time periods. Deaths in the ED increased during the early pandemic period, but then fell and were persistently lower than baseline, especially for the older (75-84) and oldest old (85+ years).

Conclusions: The decline in ED visits for emergent conditions among older adults has been more pronounced and persistent than for younger patients, with fewer deaths in the ED. This is concerning given the greater prevalence and risk of poor outcomes for emergent conditions in this age group that are amenable to time-sensitive ED diagnosis and treatment, and may in part explain excess mortality during the COVID-19 era among older adults.

Keywords: ED visits; care-seeking; emergency care; older adults.

© 2021 The American Geriatrics Society.

Conflict of interest statement

The authors have no conflicts of interest to report.

- 30 references
- 3 figures

Supplementary info

Publication types, MeSH terms, Grant support Expand

Publication types

- Observational Study

- Research Support, N.I.H., Extramural
- Research Support, Non-U.S. Gov't

MeSH terms

- Accidental Falls / statistics & numerical data*
- Aged
- Aged, 80 and over
- Aging* / physiology
- Aging* / psychology
- COVID-19 / epidemiology*
- COVID-19 / prevention & control
- Emergencies / epidemiology
- Emergency Medical Services / methods
- Emergency Medical Services / statistics & numerical data
- Emergency Service, Hospital / statistics & numerical data*
- Humans
- Mortality
- Myocardial Infarction* / diagnosis
- Myocardial Infarction* / mortality
- Patient Acceptance of Health Care / statistics & numerical data
- SARS-CoV-2
- Sepsis* / diagnosis
- Sepsis* / mortality
- Stroke* / diagnosis
- Stroke* / mortality
- United States / epidemiology

Grant support

- T32 AG019134/AG/NIA NIH HHS/United States
- John A. Hartford Foundation
- National Academy of Medicine
- R61AG069822/NIH Clinical Center
- R61 AG069822/AG/NIA NIH HHS/United States

- KL2 TR001862/TR/NCATS NIH HHS/United States
- HHSM-500-2013-13018I-T0001 Modification 000002/Centers for Medicare and Medicaid Services
- T32AG1934/AG/NIA NIH HHS/United States
- KL2TR001862/TR/NCATS NIH HHS/United States
- UL1 TR001863/TR/NCATS NIH HHS/United States
- Gary and Mary West Health Institute
- R33AG058926/NIH Clinical Center
- R33 AG058926/AG/NIA NIH HHS/United States

Show all 13 grants

Full text links

Wiley Full Text Article Wiley Europe PMC FREE FULL TEXT Europe PubMed Central
Proceed to details
Cite
Share
1,186
Observational Study
Open Heart
. 2020 Oct;7(2):e001432.
doi: 10.1136/openhrt-2020-001432.

COVID-19 pandemic and STEMI: pathway activation and outcomes from the pan-London heart attack group

Callum D Little [1] [2], Tushar Kotecha [3], Luciano Candilio [3], Richard J Jabbour [4], George B Collins [5], Asrar Ahmed [6], Michelle Connolly [7], Ritesh Kanyal [8], Ozan M Demir [9], Lucy O Lawson [3], Brian Wang [4], Sam Firoozi [7], James C Spratt [7], Divaka Perera [9], Philip MacCarthy [8], Miles Dalby [6], Ajay Jain [5], Simon J Wilson [7], Iqbal Malik [4], Roby Rakhit [3] [2]
Affiliations Expand

Affiliations

- [1] Department of Cardiology, Royal Free London NHS Foundation Trust, London, United Kingdom callumlittle@nhs.net.
- [2] Institute of Cardiovascular Science, University College London, London, United Kingdom.
- [3] Department of Cardiology, Royal Free London NHS Foundation Trust, London, United Kingdom.
- [4] Department of Cardiology, Imperial College Healthcare NHS Trust, London, United Kingdom.
- [5] Department of Cardiology, Barts Health NHS Trust, London, United Kingdom.
- [6] Department of Cardiology, Royal Brompton & Harefield NHS Foundation Trust, London, United Kingdom.
- [7] Department of Cardiology, St George's University Hospitals NHS Foundation Trust, London, United Kingdom.
- [8] Department of Cardiology, King's College Hospital NHS Foundation Trust, London, United Kingdom.
- [9] Department of Cardiology, Guy's and St Thomas' NHS Foundation Trust, London, United Kingdom.

- PMID: **33106441**

- PMCID: PMC7592245
- DOI: 10.1136/openhrt-2020-001432

Free PMC article
Observational Study

COVID-19 pandemic and STEMI: pathway activation and outcomes from the pan-London heart attack group

Callum D Little et al. Open Heart. 2020 Oct.
Free PMC article
Show details
Open Heart
. 2020 Oct;7(2):e001432.
doi: 10.1136/openhrt-2020-001432.

Authors

Callum D Little [1] [2], Tushar Kotecha [3], Luciano Candilio [3], Richard J Jabbour [4], George B Collins [5], Asrar Ahmed [6], Michelle Connolly [7], Ritesh Kanyal [8], Ozan M Demir [9], Lucy O Lawson [3], Brian Wang [4], Sam Firoozi [7], James C Spratt [7], Divaka Perera [9], Philip MacCarthy [8], Miles Dalby [6], Ajay Jain [5], Simon J Wilson [7], Iqbal Malik [4], Roby Rakhit [3] [2]

Affiliations

- [1] Department of Cardiology, Royal Free London NHS Foundation Trust, London, United Kingdom callumlittle@nhs.net.
- [2] Institute of Cardiovascular Science, University College London, London, United Kingdom.
- [3] Department of Cardiology, Royal Free London NHS Foundation Trust, London, United Kingdom.
- [4] Department of Cardiology, Imperial College Healthcare NHS Trust, London, United Kingdom.
- [5] Department of Cardiology, Barts Health NHS Trust, London, United Kingdom.
- [6] Department of Cardiology, Royal Brompton & Harefield NHS Foundation Trust, London, United Kingdom.
- [7] Department of Cardiology, St George's University Hospitals NHS Foundation Trust, London, United Kingdom.
- [8] Department of Cardiology, King's College Hospital NHS Foundation Trust, London, United Kingdom.
- [9] Department of Cardiology, Guy's and St Thomas' NHS Foundation Trust, London, United Kingdom.

- PMID: **33106441**
- PMCID: PMC7592245
- DOI: 10.1136/openhrt-2020-001432

Abstract

Objectives: To understand the impact of COVID-19 on delivery and outcomes of primary percutaneous coronary intervention (PPCI). Furthermore, to compare clinical presentation and outcomes of patients with ST-segment elevation myocardial infarction (STEMI) with active COVID-19 against those without COVID-19.

Methods: We systematically analysed 348 STEMI cases presenting to the PPCI programme in London during the peak of the pandemic (1 March to 30 April 2020) and compared with 440 cases from the same period in 2019. Outcomes of interest included ambulance response times, timeliness of revascularisation, angiographic and procedural characteristics, and in-hospital clinical outcomes RESULTS: There was a 21% reduction in STEMI admissions and longer ambulance response times (87 (62-118) min in 2020 vs 75 (57-95) min in 2019, p<0.001), but that this was not associated with a delays in achieving revascularisation once in hospital (48 (34-65) min in 2020 vs 48 (35-70) min in 2019, p=0.35) or increased mortality (10.9% (38) in 2020 vs 8.6% (38) in 2019, p=0.28). 46 patients with active COVID-19 were more thrombotic and more likely to have intensive care unit admissions (32.6% (15) vs 9.3% (28), OR 5.74 (95%CI 2.24 to 9.89), p<0.001). They also had increased length of stay (4 (3-9) days vs 3 (2-4) days, p<0.001) and a higher mortality (21.7% (10) vs 9.3% (28), OR 2.72 (95% CI 1.25 to 5.82), p=0.012) compared with patients having PPCI without COVID-19.

Conclusion: These findings suggest that PPCI pathways can be maintained during unprecedented healthcare emergencies but confirms the high mortality of STEMI in the context of concomitant COVID-19 infection characterised by a heightened state of thrombogenicity.

Keywords: acute coronary syndrome; chest pain; myocardial infarction; percutaneous coronary intervention.

© Author(s) (or their employer(s)) 2020. Re-use permitted under CC BY. Published by BMJ.

Conflict of interest statement

Competing interests: None declared.

Comment in

- Herzinfarkte in Zeiten von COVID-19 : Versorgung und Prognose.
  Facharztmagazine R. Facharztmagazine R. MMW Fortschr Med. 2020 Nov;162(Suppl 3):12. doi: 10.1007/s15006-020-4569-3. MMW Fortschr Med. 2020. PMID: 33164170 Free PMC article. German. No abstract available.

- 30 references
- 1 figure

Supplementary info

Publication types, MeSH terms, Grant support Expand

Publication types

- Multicenter Study

- Observational Study
- Research Support, Non-U.S. Gov't

MeSH terms

- Aged
- Ambulances / organization & administration
- COVID-19
- Coronavirus Infections* / diagnosis
- Coronavirus Infections* / mortality
- Coronavirus Infections* / therapy
- Coronavirus Infections* / transmission
- Critical Pathways / organization & administration*
- Databases, Factual
- Delivery of Health Care, Integrated / organization & administration*
- Female
- Hospital Mortality
- Humans
- Length of Stay
- London / epidemiology
- Male
- Middle Aged
- Outcome and Process Assessment, Health Care / organization & administration*
- Pandemics*
- Patient Admission
- Patient Safety
- Percutaneous Coronary Intervention* / adverse effects
- Percutaneous Coronary Intervention* / mortality
- Pneumonia, Viral* / diagnosis
- Pneumonia, Viral* / mortality
- Pneumonia, Viral* / therapy
- Pneumonia, Viral* / transmission
- Retrospective Studies
- Risk Assessment
- Risk Factors
- ST Elevation Myocardial Infarction / diagnosis
- ST Elevation Myocardial Infarction / mortality
- ST Elevation Myocardial Infarction / therapy*
- Thrombosis / mortality
- Thrombosis / therapy
- Time Factors
- Time-to-Treatment / organization & administration

- Treatment Outcome

Grant support

- WT_/Wellcome Trust/United Kingdom
- 203145Z/16/Z/WT_/Wellcome Trust/United Kingdom

Full text links

BMJ Open Access Full Text HighWire Free PMC article
Proceed to details
Cite
Share
1,187
Observational Study
J Autoimmun
. 2020 Nov;114:102512.
doi: 10.1016/j.jaut.2020.102512. Epub 2020 Jul 3.

Clinical characteristics and predictors of survival in adults with coronavirus disease 2019 receiving tocilizumab

Austin R Morrison [1], Joseph M Johnson [1], Kristin M Griebe [1], Mathew C Jones [1], John J Stine [1], Laura N Hencken [1], Long To [1], Monica L Bianchini [2], Amit T Vahia [3], Jennifer Swiderek [4], Mayur S Ramesh [3], Michael A Peters [1], Zachary R Smith [5]
Affiliations Expand

Affiliations

- [1] Department of Pharmacy, Henry Ford Hospital, 2799 West Grand Blvd, Detroit, MI, 48202, USA.
- [2] Department of Clinical Pharmacy, University of Colorado Anschutz Medical Campus, 13001 E 17th Pl, Aurora, CO, 80045, USA.
- [3] Division of Infectious Diseases, Henry Ford Hospital, 2799 West Grand Blvd, Detroit, MI, 48202, USA.
- [4] Division of Pulmonary & Critical Care Medicine, Henry Ford Hospital, 2799 West Grand Blvd, Detroit, MI, 48202, USA.
- [5] Department of Pharmacy, Henry Ford Hospital, 2799 West Grand Blvd, Detroit, MI, 48202, USA. Electronic address: zsmith1@hfhs.org.

- PMID: **32646770**
- PMCID: PMC7332925
- DOI: 10.1016/j.jaut.2020.102512

Free PMC article

Observational Study

Clinical characteristics and predictors of survival in adults with coronavirus disease 2019 receiving tocilizumab

Austin R Morrison et al. J Autoimmun. 2020 Nov.
Free PMC article
Show details
J Autoimmun
. 2020 Nov;114:102512.
doi: 10.1016/j.jaut.2020.102512. Epub 2020 Jul 3.

Authors

Austin R Morrison [1], Joseph M Johnson [1], Kristin M Griebe [1], Mathew C Jones [1], John J Stine [1], Laura N Hencken [1], Long To [1], Monica L Bianchini [2], Amit T Vahia [3], Jennifer Swiderek [4], Mayur S Ramesh [3], Michael A Peters [1], Zachary R Smith [5]

Affiliations

- [1] Department of Pharmacy, Henry Ford Hospital, 2799 West Grand Blvd, Detroit, MI, 48202, USA.
- [2] Department of Clinical Pharmacy, University of Colorado Anschutz Medical Campus, 13001 E 17th Pl, Aurora, CO, 80045, USA.
- [3] Division of Infectious Diseases, Henry Ford Hospital, 2799 West Grand Blvd, Detroit, MI, 48202, USA.
- [4] Division of Pulmonary & Critical Care Medicine, Henry Ford Hospital, 2799 West Grand Blvd, Detroit, MI, 48202, USA.
- [5] Department of Pharmacy, Henry Ford Hospital, 2799 West Grand Blvd, Detroit, MI, 48202, USA. Electronic address: zsmith1@hfhs.org.

- PMID: **32646770**
- PMCID: PMC7332925
- DOI: 10.1016/j.jaut.2020.102512

Abstract

Coronavirus disease 2019 (COVID-19) can progress to cytokine storm that is associated with organ dysfunction and death. The purpose of the present study is to determine clinical characteristics associated with 28 day in-hospital survival in patients with coronavirus disease 2019 (COVID-19) that received tocilizumab. This was a retrospective observational cohort study conducted at a five hospital health system in Michigan, United States. Adult patients with confirmed COVID-19 that were admitted to the hospital and received tocilizumab for cytokine storm from March 1, 2020 through April 3, 2020 were included. Patients were grouped into survivors and non-survivors based on 28 day in-hospital mortality. Study day 0 was defined as the day tocilizumab was administered. Factors independently associated with in-hospital survival at

28 days after tocilizumab administration were assessed. Epidemiologic, demographic, laboratory, prognostic scores, treatment, and outcome data were collected and analyzed. Clinical response was collected and defined as a decline of two levels on a six-point ordinal scale of clinical status or discharged alive from the hospital. Of the 81 patients included, the median age was 64 (58-71) years and 56 (69.1%) were male. The 28 day in-hospital mortality was 43.2%. There were 46 (56.8%) patients in the survivors and 35 (43.2%) in the non-survivors group. On study day 0 no differences were noted in demographics, clinical characteristics, severity of illness scores, or treatments received between survivors and non-survivors. C-reactive protein was significantly higher in the non-survivors compared to survivors. Compared to non-survivors, recipients of tocilizumab within 12 days of symptom onset was independently associated with survival (adjusted OR: 0.296, 95% CI: 0.098-0.889). SOFA score ≥8 on day 0 was independently associated with mortality (adjusted OR: 2.842, 95% CI: 1.042-7.753). Clinical response occurred more commonly in survivors than non-survivors (80.4% vs. 5.7%; $p < 0.001$). Improvements in the six-point ordinal scale and SOFA score were observed in survivors after tocilizumab. Early receipt of tocilizumab in patients with severe COVID-19 was an independent predictor for in-hospital survival at 28 days.

Keywords: COVID-19; Critical illness; Cytokine release syndrome; Interleukin-6; Severe acute respiratory syndrome coronavirus 2; Tocilizumab.

Copyright © 2020 Elsevier Ltd. All rights reserved.

- 39 references
- 2 figures

Supplementary info

Publication types, MeSH terms, Substances, Supplementary concepts Expand

Publication types

- Multicenter Study
- Observational Study

MeSH terms

- Adult
- Aged
- Antibodies, Monoclonal, Humanized / administration & dosage*
- Betacoronavirus / immunology
- C-Reactive Protein / analysis*
- COVID-19
- Coronavirus Infections / blood
- Coronavirus Infections / drug therapy*
- Coronavirus Infections / immunology
- Coronavirus Infections / mortality
- Cytokine Release Syndrome / blood
- Cytokine Release Syndrome / drug therapy*

- Cytokine Release Syndrome / immunology
- Cytokine Release Syndrome / mortality
- Female
- Hospital Mortality
- Humans
- Infusions, Intravenous
- Interleukin-6 / immunology
- Interleukin-6 / metabolism
- Male
- Michigan / epidemiology
- Middle Aged
- Organ Dysfunction Scores
- Pandemics
- Pneumonia, Viral / blood
- Pneumonia, Viral / drug therapy*
- Pneumonia, Viral / immunology
- Pneumonia, Viral / mortality
- Prognosis
- Receptors, Interleukin-6 / antagonists & inhibitors
- Receptors, Interleukin-6 / metabolism
- Retrospective Studies
- SARS-CoV-2
- Survival Analysis
- Time Factors
- Time-to-Treatment
- Treatment Outcome

Substances

- Antibodies, Monoclonal, Humanized
- IL6 protein, human
- IL6R protein, human
- Interleukin-6
- Receptors, Interleukin-6
- C-Reactive Protein
- tocilizumab

Supplementary concepts

- COVID-19 drug treatment

Full text links

ELSEVIER FULL-TEXT ARTICLE Elsevier Science Free PMC article

Proceed to details

Cite

Share

☐ 1,188

Observational Study

BMJ Open Respir Res

. 2020 Jul;7(1):e000639.

doi: 10.1136/bmjresp-2020-000639.

Is continuous positive airway pressure (CPAP) a new standard of care for type 1 respiratory failure in COVID-19 patients? A retrospective observational study of a dedicated COVID-19 CPAP service

Rebecca Nightingale [1, 2], Nneka Nwosu [2], Farheen Kutubudin [2], Tom Fletcher [3, 2], Joe Lewis [3, 2, 4], Frederick Frost [2, 4], Kathryn Haigh [2], Ryan Robinson [3, 2], Ayesha Kumar [2], Gareth Jones [2], Deborah Brown [2], Michael Abouyannis [3, 2, 4], Mike Beadsworth [3, 2], Peter Hampshire [2], Stephen Aston [2, 4], Manish Gautam [2], Hassan Burhan [2]

Affiliations Expand

Affiliations

- [1] Department of Clinical Sciences, Liverpool School of Tropical Medicine, Liverpool, UK rebecca.nightingale@lstmed.ac.uk.
- [2] Department of Respiratory Medicine, Tropical and Infectious Disease Unit, Intensive Care Unit, Liverpool University Hospitals NHS Foundation Trust, Liverpool, UK.
- [3] Department of Clinical Sciences, Liverpool School of Tropical Medicine, Liverpool, UK.
- [4] Institute of Infection and Global Health, University of Liverpool, Liverpool, Merseyside, UK.

- PMID: **32624495**
- PMCID: PMC7337881
- DOI: 10.1136/bmjresp-2020-000639

Free PMC article

Observational Study

Is continuous positive airway pressure (CPAP) a new standard of care for type 1 respiratory failure in COVID-19 patients? A retrospective observational study of a dedicated COVID-19 CPAP service

Rebecca Nightingale et al. BMJ Open Respir Res. 2020 Jul.
Free PMC article
Show details
BMJ Open Respir Res
. 2020 Jul;7(1):e000639.
doi: 10.1136/bmjresp-2020-000639.

Authors

Rebecca Nightingale [1, 2], Nneka Nwosu [2], Farheen Kutubudin [2], Tom Fletcher [3, 2], Joe Lewis [3, 2, 4], Frederick Frost [2, 4], Kathryn Haigh [2], Ryan Robinson [3, 2], Ayesha Kumar [2], Gareth Jones [2], Deborah Brown [2], Michael Abouyannis [3, 2, 4], Mike Beadsworth [3, 2], Peter Hampshire [2], Stephen Aston [2, 4], Manish Gautam [2], Hassan Burhan [2]

Affiliations

- [1] Department of Clinical Sciences, Liverpool School of Tropical Medicine, Liverpool, UK rebecca.nightingale@lstmed.ac.uk.
- [2] Department of Respiratory Medicine, Tropical and Infectious Disease Unit, Intensive Care Unit, Liverpool University Hospitals NHS Foundation Trust, Liverpool, UK.
- [3] Department of Clinical Sciences, Liverpool School of Tropical Medicine, Liverpool, UK.
- [4] Institute of Infection and Global Health, University of Liverpool, Liverpool, Merseyside, UK.

- PMID: **32624495**
- PMCID: PMC7337881
- DOI: 10.1136/bmjresp-2020-000639

Abstract

The aim of this case series is to describe and evaluate our experience of continuous positive airway pressure (CPAP) to treat type 1 respiratory failure in patients with COVID-19. CPAP was delivered in negative pressure rooms in the newly repurposed infectious disease unit. We report a cohort of 24 patients with type 1 respiratory failure and COVID-19 admitted to the Royal Liverpool Hospital between 1 April and 30 April 2020. Overall, our results were positive; we were able to safely administer CPAP outside the walls of a critical care or high dependency unit environment and over half of patients (58%) avoided mechanical ventilation and a total of 19 out of 24 (79%) have survived and been discharged from our care.

Keywords: non invasive ventilation; respiratory Infection.

© Author(s) (or their employer(s)) 2020. Re-use permitted under CC BY-NC. No commercial re-use. See rights and permissions. Published by BMJ.

Conflict of interest statement

Competing interests: None declared.

- 9 references

Supplementary info

Publication types, MeSH terms Expand

Publication types

- Observational Study

MeSH terms

- Betacoronavirus / isolation & purification
- COVID-19
- Continuous Positive Airway Pressure / methods*
- Coronavirus Infections* / epidemiology
- Coronavirus Infections* / physiopathology
- Coronavirus Infections* / therapy
- Critical Pathways / trends
- Female
- Humans
- Male
- Medical Records / statistics & numerical data
- Middle Aged
- Outcome Assessment, Health Care
- Oxygen Consumption
- Pandemics*
- Pneumonia, Viral* / epidemiology
- Pneumonia, Viral* / physiopathology
- Pneumonia, Viral* / therapy
- Procedures and Techniques Utilization / statistics & numerical data*
- Respiratory Care Units* / methods
- Respiratory Care Units* / organization & administration
- Respiratory Insufficiency* / etiology
- Respiratory Insufficiency* / mortality

- Respiratory Insufficiency* / physiopathology
- Respiratory Insufficiency* / therapy
- SARS-CoV-2
- Survival Analysis
- United Kingdom / epidemiology

Full text links

BMJ Free Full Text HighWire Free PMC article
Proceed to details
Cite
Share
1,189
Observational Study
BMJ Open
. 2021 Apr 1;11(4):e049066.
doi: 10.1136/bmjopen-2021-049066.

Variables associated with COVID-19 severity: an observational study of non-paediatric confirmed cases from the general population of the Basque Country, Spain

Kalliopi Vrotsou [1] [2], Rafael Rotaeche [3], Maider Mateo-Abad [2], Mónica Machón [4] [2], Itziar Vergara [4] [2]
Affiliations Expand

Affiliations

- [1] Primary Care Group, Biodonostia Institute for Health Research, Donostia-San Sebastián, Spain kalliopi.vrotsoukanari@osakidetza.eus.
- [2] Research Network in Health Services in Chronic Diseases (REDISSEC), Kronikgune Health Services Research Institute, Baracaldo, Spain.
- [3] Alza Health Center, Osakidetza-Basque Health Service, Donostia-San Sebastian, Spain.
- [4] Primary Care Group, Biodonostia Institute for Health Research, Donostia-San Sebastián, Spain.

- PMID: **33795313**
- PMCID: PMC8024058
- DOI: 10.1136/bmjopen-2021-049066

Free PMC article
Observational Study

Variables associated with COVID-19 severity: an observational study of non-paediatric confirmed cases from the general population of the Basque Country, Spain

Kalliopi Vrotsou et al. BMJ Open. 2021.
Free PMC article
Show details
BMJ Open
. 2021 Apr 1;11(4):e049066.
doi: 10.1136/bmjopen-2021-049066.

Authors

Kalliopi Vrotsou [1] [2], Rafael Rotaeche [3], Maider Mateo-Abad [2], Mónica Machón [4] [2], Itziar Vergara [4] [2]

Affiliations

- [1] Primary Care Group, Biodonostia Institute for Health Research, Donostia-San Sebastián, Spain kalliopi.vrotsoukanari@osakidetza.eus.
- [2] Research Network in Health Services in Chronic Diseases (REDISSEC), Kronikgune Health Services Research Institute, Baracaldo, Spain.
- [3] Alza Health Center, Osakidetza-Basque Health Service, Donostia-San Sebastian, Spain.
- [4] Primary Care Group, Biodonostia Institute for Health Research, Donostia-San Sebastián, Spain.

- PMID: **33795313**
- PMCID: PMC8024058
- DOI: 10.1136/bmjopen-2021-049066

Abstract

Objectives: To investigate which were the most relevant sociodemographic and clinical variables associated with COVID-19 severity, and uncover how their inter-relations may have affected such severity.

Design: A retrospective observational study based on electronic health record data.

Participants: Individuals ≥14 years old with a positive PCR or serology test, between 28 February and 31 May 2020, belonging to the Basque Country (Spain) public health system. Institutionalised and individuals admitted to a hospital at home unit were excluded from the study.

Main outcome measure: Three severity categories were established: primary care, hospital/intensive care unit admission and death.

Results: A total of n=14 197 cases fulfilled the inclusion criteria. Most variables presented statistically significant associations with the outcome ($p<0.0001$). The Classification and Regression Trees recursive partitioning methodology (based on n=13 792) suggested that among all associations, those with, age, sex, stratification of patient healthcare complexity, chronic consumption of blood and blood-forming organ, and nervous system drugs, as well as the total number of chronic Anatomical Therapeutic Chemical types were the most relevant. Psychosis also emerged as a potential factor.

Conclusions: Older cases are more likely to experience more severe outcomes. However, the sex, underlying health status and chronic drug consumption may interfere and alter the ageing effect. Understanding the factors related to the outcome severity is of key importance when designing and promoting public health intervention plans for the COVID-19 pandemic.

Keywords: COVID-19; public health; statistics & research methods.

© Author(s) (or their employer(s)) 2021. Re-use permitted under CC BY-NC. No commercial re-use. See rights and permissions. Published by BMJ.

Conflict of interest statement

Competing interests: None declared.

- 43 references
- 2 figures

Supplementary info

Publication types, MeSH terms Expand

Publication types

- Observational Study

MeSH terms

- Adult
- Aged
- Aged, 80 and over
- COVID-19 / epidemiology*
- COVID-19 / pathology
- Humans
- Middle Aged
- Pandemics*
- Spain / epidemiology
- Treatment Outcome

Full text links

BMJ Free Full Text HighWire Free PMC article
Proceed to details
Cite
Share
1,190
Observational Study
Medicine (Baltimore)
. 2020 Oct 30;99(44):e23064.
doi: 10.1097/MD.0000000000023064.

Comparison of clinical, laboratory, and radiological characteristics between SARS-CoV-2 infection and community-acquired pneumonia caused by influenza virus: A cross-sectional retrospective study

Yi-Hua Lin [1, 2], Wen Luo [1], Ding-Hui Wu [3], Fang Lu [1], Su-Xian Hu [1], Xiang-Yang Yao [3], Zhan-Xiang Wang [2, 4], Yong-Hong Shi [1, 2]
Affiliations Expand

Affiliations

- [1] Department of Respiratory and Critical Care Medicine, the First Affiliated Hospital of Xiamen University, Xiamen.
- [2] Department of Clinical Medicine, Fujian Medical University.
- [3] Department of pulmonary diseases, the First Affiliated Hospital of Xiamen University.
- [4] the First Affiliated Hospital of Xiamen University, Xiamen, Fujian, China.

- PMID: **33126398**
- PMCID: PMC7598784
- DOI: 10.1097/MD.0000000000023064

Free PMC article
Observational Study

Comparison of clinical, laboratory, and radiological characteristics between SARS-CoV-2 infection and community-acquired

pneumonia caused by influenza virus: A cross-sectional retrospective study

Yi-Hua Lin et al. Medicine (Baltimore). 2020.
Free PMC article
Show details
Medicine (Baltimore)
. 2020 Oct 30;99(44):e23064.
doi: 10.1097/MD.0000000000023064.

Authors

Yi-Hua Lin [1] [2], Wen Luo [1], Ding-Hui Wu [3], Fang Lu [1], Su-Xian Hu [1], Xiang-Yang Yao [3], Zhan-Xiang Wang [2] [4], Yong-Hong Shi [1] [2]

Affiliations

- [1] Department of Respiratory and Critical Care Medicine, the First Affiliated Hospital of Xiamen University, Xiamen.
- [2] Department of Clinical Medicine, Fujian Medical University.
- [3] Department of pulmonary diseases, the First Affiliated Hospital of Xiamen University.
- [4] the First Affiliated Hospital of Xiamen University, Xiamen, Fujian, China.

- PMID: **33126398**
- PMCID: PMC7598784
- DOI: 10.1097/MD.0000000000023064

Abstract

Coronavirus disease 2019 (COVID-19) is the most important global public health issue that we currently face. We aimed to explore the clinical features of patients with COVID-19 and compared them with those of hospitalized community-acquired pneumonia (CAP) patients caused by influenza virus during the same period.From Jan 1, to Mar 4, 2020, patients with COVID-19 or CAP caused by influenza virus who were admitted to the First Affiliated Hospital of Xiamen University were consecutively screened for enrollment.A total of 35 COVID-19 patients and 22 CAP patients caused by influenza virus were included in this study. Most of COVID-19 patients had characteristics of familial clustering (63%), however, in the other group, there was no similar finding. The percentages of patients with a high fever (the highest recorded temperature was ≥39.0°C; 11% vs 45% [COVID-19 vs CAP groups, respectively]), dyspnea (9% vs 59%), leukocytosis (3% vs 32%), elevated C-reactive protein concentrations (>10 mg/L, 48% vs 86%), elevated procalcitonin levels (>0.1 ng/ml, 15% vs 73%), PaO2/FiO2 <200 mm Hg (4% vs 22%), and infiltration on imaging (29% vs 68%) in the COVID-19 group were less than those same indices in the hospitalized CAP patients caused by influenza virus. Ground-glass opacity with reticular pattern (63%) and interlobular septal thickening (71%) in chest CT were commonly observed in the COVID-19 group.COVID-19 and CAP caused by influenza virus appear to share some similarities in clinical manifestaions but they definitely have major distinctions. Influenza infection remains a health problem even during COVID-19 pandemic.

Conflict of interest statement

The authors have no conflicts of interests to disclose.

- 13 references

Supplementary info

Publication types, MeSH terms, Supplementary concepts Expand

Publication types

- Comparative Study
- Observational Study

MeSH terms

- Adult
- Aged
- Aged, 80 and over
- COVID-19
- China / epidemiology
- Community-Acquired Infections
- Coronavirus Infections / blood
- Coronavirus Infections / diagnostic imaging
- Coronavirus Infections / drug therapy
- Coronavirus Infections / epidemiology*
- Coronavirus Infections / therapy
- Cross-Sectional Studies
- Female
- Humans
- Influenza, Human / blood
- Influenza, Human / diagnostic imaging
- Influenza, Human / epidemiology*
- Influenza, Human / therapy
- Male
- Middle Aged
- Pandemics
- Pneumonia, Viral / blood
- Pneumonia, Viral / diagnostic imaging
- Pneumonia, Viral / epidemiology*
- Pneumonia, Viral / therapy
- Radiography, Thoracic

- Retrospective Studies

Supplementary concepts

- COVID-19 drug treatment

Full text links

Wolters Kluwer Free PMC article
Proceed to details
Cite
Share
1,191
Observational Study
Epidemiol Infect
. 2020 Jun 30;148:e129.
doi: 10.1017/S0950268820001442.

Laboratory findings and a combined multifactorial approach to predict death in critically ill patients with COVID-19: a retrospective study

Q Liu [1], N C Song [1], Z K Zheng [1], J S Li [1], S K Li [1]
Affiliations Expand

Affiliation

- [1] Department of Thoracic Surgery, Union Hospital, Tongji Medical College, Huazhong University of Science and Technology, Wuhan, 430022, China.

- PMID: **32600484**
- PMCID: PMC7343973
- DOI: 10.1017/S0950268820001442

Free PMC article
Observational Study

Laboratory findings and a combined multifactorial approach to predict death in

critically ill patients with COVID-19: a retrospective study

Q Liu et al. Epidemiol Infect. 2020.
Free PMC article
Show details
Epidemiol Infect
. 2020 Jun 30;148:e129.
doi: 10.1017/S0950268820001442.

Authors

Q Liu [1], N C Song [1], Z K Zheng [1], J S Li [1], S K Li [1]

Affiliation

- [1] Department of Thoracic Surgery, Union Hospital, Tongji Medical College, Huazhong University of Science and Technology, Wuhan, 430022, China.

- PMID: **32600484**
- PMCID: PMC7343973
- DOI: 10.1017/S0950268820001442

Abstract

To describe the laboratory findings of cases of death with coronavirus disease 2019 (COVID-19) and to establish a scoring system for predicting death, we conducted this single-centre, retrospective, observational study including 336 adult patients (≥18 years old) with severe or critically ill COVID-19 admitted in two wards of Union Hospital, Tongji Medical College, Huazhong University of Science and Technology in Wuhan, who had definite outcomes (death or discharge) between 1 February 2020 and 13 March 2020. Single variable and multivariable logistic regression analyses were performed to identify mortality-related factors. We combined multiple factors to predict mortality, which was validated by receiver operating characteristic curves. As a result, in a total of 336 patients, 34 (10.1%) patients died during hospitalisation. Through multivariable logistic regression, we found that decreased lymphocyte ratio (Lymr, %) (odds ratio, OR 0.574, $P < 0.001$), elevated blood urea nitrogen (BUN) (OR 1.513, $P = 0.009$), and raised D-dimer (DD) (OR 1.334, $P = 0.002$) at admission were closely related to death. The combined prediction model was developed by these factors with a sensitivity of 100.0% and specificity of 97.2%. In conclusion, decreased Lymr, elevated BUN, and raised DD were found to be in association with death outcomes in critically ill patients with COVID-19. A scoring system was developed to predict the clinical outcome of these patients.

Keywords: Blood urea nitrogen (BUN); D-dimer (DD); coronavirus disease 2019 (COVID-19); death; lymphocyte ratio.

Conflict of interest statement

None.

- 22 references
- 3 figures

Supplementary info

Publication types, MeSH terms, Substances Expand

Publication types

- Observational Study

MeSH terms

- Age Factors
- Aged
- Area Under Curve
- Blood Chemical Analysis
- Blood Urea Nitrogen
- C-Reactive Protein / analysis
- COVID-19
- Causality
- Coronavirus Infections / blood*
- Coronavirus Infections / complications
- Coronavirus Infections / epidemiology
- Coronavirus Infections / mortality*
- Critical Illness
- Female
- Fibrin Fibrinogen Degradation Products / analysis
- Humans
- Logistic Models
- Lymphocyte Count
- Male
- Middle Aged
- Pandemics
- Pneumonia, Viral / blood*
- Pneumonia, Viral / complications
- Pneumonia, Viral / epidemiology
- Pneumonia, Viral / mortality*
- ROC Curve
- Respiration, Artificial
- Retrospective Studies

Substances

- Fibrin Fibrinogen Degradation Products
- fibrin fragment D
- C-Reactive Protein

Full text links

Cambridge University Press Free PMC article

Proceed to details

Cite

Share

1,192

Observational Study

Diabetes Metab J

. 2020 Aug;44(4):602-613.

doi: 10.4093/dmj.2020.0146. Epub 2020 Aug 12.

The Clinical Characteristics and Outcomes of Patients with Moderate-to-Severe Coronavirus Disease 2019 Infection and Diabetes in Daegu, South Korea

Mi Kyung Kim[#,1], Jae Han Jeon[#,2], Sung Woo Kim[3], Jun Sung Moon[4], Nan Hee Cho[1], Eugene Han[1], Ji Hong You[1], Ji Yeon Lee[1], Miri Hyun[1], Jae Seok Park[1], Yong Shik Kwon[1], Yeon Kyung Choi[2], Ki Tae Kwon[2], Shin Yup Lee[2], Eon Ju Jeon[3], Jin Woo Kim[3], Hyo Lim Hong[3], Hyun Hee Kwon[3], Chi Young Jung[3], Yin Young Lee[4], Eunyeoung Ha[4], Seung Min Chung[4], Jian Hur[4], June Hong Ahn[4], Na Young Kim[5], Shin Woo Kim[5], Hyun Ha Chang[5], Yong Hoon Lee[5], Jaehee Lee[5], Keun Gyu Park[5], Hyun Ah Kim[6], Ji Hyun Lee[7]

Affiliations Expand

Affiliations

- [1] Department of Internal Medicine, Keimyung University Dongsan Hospital, Keimyung University School of Medicine, Daegu, Korea.
- [2] Department of Internal Medicine, Kyungpook National University Chilgok Hospital, School of Medicine, Kyungpook National University, Daegu, Korea.
- [3] Department of Internal Medicine, Daegu Catholic University Hospital, Daegu Catholic University School of Medicine, Daegu, Korea.
- [4] Department of Internal Medicine, Yeungnam University Hospital, Yeungnam University College of Medicine, Daegu, Korea.
- [5] Department of Internal Medicine, Kyungpook National University Hospital, School of Medicine, Kyungpook National University, Daegu, Korea.

- [6] Department of Internal Medicine, Keimyung University Dongsan Hospital, Keimyung University School of Medicine, Daegu, Korea. hyunah1118@dsmc.or.kr.
- [7] Department of Internal Medicine, Daegu Catholic University Hospital, Daegu Catholic University School of Medicine, Daegu, Korea. jhlee9@cu.ac.kr.

[#] Contributed equally.

- PMID: **32794386**
- PMCID: PMC7453989
- DOI: 10.4093/dmj.2020.0146

Free PMC article
Observational Study

The Clinical Characteristics and Outcomes of Patients with Moderate-to-Severe Coronavirus Disease 2019 Infection and Diabetes in Daegu, South Korea

Mi Kyung Kim et al. Diabetes Metab J. 2020 Aug.
Free PMC article
Show details
Diabetes Metab J
. 2020 Aug;44(4):602-613.
doi: 10.4093/dmj.2020.0146. Epub 2020 Aug 12.

Authors

Mi Kyung Kim [# 1], Jae Han Jeon [# 2], Sung Woo Kim [3], Jun Sung Moon [4], Nan Hee Cho [1], Eugene Han [1], Ji Hong You [1], Ji Yeon Lee [1], Miri Hyun [1], Jae Seok Park [1], Yong Shik Kwon [1], Yeon Kyung Choi [2], Ki Tae Kwon [2], Shin Yup Lee [2], Eon Ju Jeon [3], Jin Woo Kim [3], Hyo Lim Hong [3], Hyun Hee Kwon [3], Chi Young Jung [3], Yin Young Lee [4], Eunyeoung Ha [4], Seung Min Chung [4], Jian Hur [4], June Hong Ahn [4], Na Young Kim [5], Shin Woo Kim [5], Hyun Ha Chang [5], Yong Hoon Lee [5], Jaehee Lee [5], Keun Gyu Park [5], Hyun Ah Kim [6], Ji Hyun Lee [7]

Affiliations

- [1] Department of Internal Medicine, Keimyung University Dongsan Hospital, Keimyung University School of Medicine, Daegu, Korea.
- [2] Department of Internal Medicine, Kyungpook National University Chilgok Hospital, School of Medicine, Kyungpook National University, Daegu, Korea.
- [3] Department of Internal Medicine, Daegu Catholic University Hospital, Daegu Catholic University School of Medicine, Daegu, Korea.
- [4] Department of Internal Medicine, Yeungnam University Hospital, Yeungnam University College of Medicine, Daegu, Korea.

- [5] Department of Internal Medicine, Kyungpook National University Hospital, School of Medicine, Kyungpook National University, Daegu, Korea.
- [6] Department of Internal Medicine, Keimyung University Dongsan Hospital, Keimyung University School of Medicine, Daegu, Korea. hyunah1118@dsmc.or.kr.
- [7] Department of Internal Medicine, Daegu Catholic University Hospital, Daegu Catholic University School of Medicine, Daegu, Korea. jhlee9@cu.ac.kr.

[#] Contributed equally.

- PMID: **32794386**
- PMCID: PMC7453989
- DOI: 10.4093/dmj.2020.0146

Abstract

Background: Coronavirus disease 2019 (COVID-19) is a global pandemic that had affected more than eight million people worldwide by June 2020. Given the importance of the presence of diabetes mellitus (DM) for host immunity, we retrospectively evaluated the clinical characteristics and outcomes of moderate-to-severe COVID-19 in patients with diabetes.

Methods: We conducted a multi-center observational study of 1,082 adult inpatients (aged ≥18 years) who were admitted to one of five university hospitals in Daegu because of the severity of their COVID-19-related disease. The demographic, laboratory, and radiologic findings, and the mortality, prevalence of severe disease, and duration of quarantine were compared between patients with and without DM. In addition, 1:1 propensity score (PS)-matching was conducted with the DM group.

Results: Compared with the non-DM group (n=847), patients with DM (n=235) were older, exhibited higher mortality, and required more intensive care. Even after PS-matching, patients with DM exhibited more severe disease, and DM remained a prognostic factor for higher mortality (hazard ratio, 2.40; 95% confidence interval, 1.38 to 4.15). Subgroup analysis revealed that the presence of DM was associated with higher mortality, especially in older people (≥70 years old). Prior use of a dipeptidyl peptidase-4 inhibitor or a renin-angiotensin system inhibitor did not affect mortality or the clinical severity of the disease.

Conclusion: DM is a significant risk factor for COVID-19 severity and mortality. Our findings imply that COVID-19 patients with DM, especially if elderly, require special attention and prompt intensive care.

Keywords: COVID-19; Diabetes mellitus; Mortality; Prognosis.

Copyright © 2020 Korean Diabetes Association.

Conflict of interest statement

No potential conflict of interest relevant to this article was reported.

- 29 references
- 3 figures

Supplementary info

Publication types, MeSH terms, Substances, Grant support Expand

Publication types

- Observational Study
- Research Support, Non-U.S. Gov't

MeSH terms

- Adult
- Aged
- Aged, 80 and over
- Alanine Transaminase / metabolism
- Angiotensin-Converting Enzyme Inhibitors / therapeutic use
- Aspartate Aminotransferases / metabolism
- Betacoronavirus
- C-Reactive Protein / metabolism
- COVID-19
- Case-Control Studies
- Comorbidity
- Coronavirus Infections / metabolism
- Coronavirus Infections / mortality*
- Coronavirus Infections / physiopathology
- Diabetes Mellitus / drug therapy
- Diabetes Mellitus / epidemiology*
- Diabetes Mellitus / metabolism
- Dipeptidyl-Peptidase IV Inhibitors / therapeutic use
- Female
- Humans
- Length of Stay / statistics & numerical data
- Logistic Models
- Lymphocytosis
- Male
- Middle Aged
- Multivariate Analysis
- Pandemics
- Pneumonia, Viral / metabolism
- Pneumonia, Viral / mortality*
- Pneumonia, Viral / physiopathology
- Prognosis
- Propensity Score
- Proportional Hazards Models

- Quarantine / statistics & numerical data
- Republic of Korea / epidemiology
- Risk Factors
- SARS-CoV-2
- Severity of Illness Index
- Thrombocytopenia

Substances

- Angiotensin-Converting Enzyme Inhibitors
- Dipeptidyl-Peptidase IV Inhibitors
- C-Reactive Protein
- Aspartate Aminotransferases
- Alanine Transaminase

Grant support

- Daegu Medical Association COVID-19 Scientific Committee/International

Full text links

Korean Diabetes Association Free PMC article
Proceed to details
Cite
Share
1,193
Observational Study
Rev Cardiovasc Med
. 2021 Jun 30;22(2):271-276.
doi: 10.31083/j.rcm2202034.

Impact of the COVID-19 pandemic on the management of chronic heart failure

Melanie McGinlay [1], Sam Straw [2], Jacob Jagger [2], Bako Nouri [2], John Gierula [2], Klaus K Witte [2]
Affiliations Expand

Affiliations

- [1] Department of Cardiology, Leeds Teaching Hospitals NHS Trust, LS9 7TF Leeds, UK.
- [2] Leeds Institute of Cardiovascular and Metabolic Medicine, University of Leeds, LS2 9JT Leeds, UK.

- PMID: **34258895**
- DOI: 10.31083/j.rcm2202034

Free article
Observational Study

Impact of the COVID-19 pandemic on the management of chronic heart failure

Melanie McGinlay et al. Rev Cardiovasc Med. 2021.
Free article
Show details
Rev Cardiovasc Med
. 2021 Jun 30;22(2):271-276.
doi: 10.31083/j.rcm2202034.

Authors

Melanie McGinlay [1], Sam Straw [2], Jacob Jagger [2], Bako Nouri [2], John Gierula [2], Klaus K Witte [2]

Affiliations

- [1] Department of Cardiology, Leeds Teaching Hospitals NHS Trust, LS9 7TF Leeds, UK.
- [2] Leeds Institute of Cardiovascular and Metabolic Medicine, University of Leeds, LS2 9JT Leeds, UK.

- PMID: **34258895**
- DOI: 10.31083/j.rcm2202034

Abstract

The coronavirus disease 2019 (COVID-19) pandemic is an unprecedented challenge. Meeting this has resulted in changes to working practices and the impact on the management of patients with heart failure with reduced ejection fraction (HFrEF) is largely unknown. We performed a retrospective, observational study contrasting patients diagnosed with HFrEF attending specialist heart failure clinics at a UK hospital, whose subsequent period of optimisation of medical therapy was during the COVID-19 pandemic, with patients diagnosed the previous year. The primary outcome was the change in equivalent dosing of ramipril and bisoprolol at 6-months. Secondary outcomes were the number and type of follow-up consultations, hospitalisation for heart failure and all-cause mortality. In total, 60 patients were diagnosed with HFrEF between 1 December 2019 and 30 April 2020, compared to 54 during the same period of the previous year. The absolute number of consultations was higher (390 vs 270; $p = 0.69$), driven by increases in telephone consultations, with a reduction in appointments with hospital nurse specialists. After 6-months, we observed lower equivalent dosing of ramipril (3.1 ± 3.0 mg vs 4.4 ± 0.5 mg; $p = 0.035$) and similar dosing of bisoprolol (4.1 ± 0.5 mg vs 4.9 ± 0.5 mg; $p = 0.27$), which persisted for ramipril (mean difference 1.0 mg, 95% CI 0.018-2.09; $p = 0.046$) and bisoprolol (mean difference 0.52 mg, 95% CI -0.23-1.28; $p = 0.17$) after adjustment for baseline dosing. We

observed no differences in the proportion of patients who died (5.0% vs 7.4%; $p = 0.59$) or were hospitalised with heart failure (13.3% vs 9.3%; $p = 0.49$). Our study suggests the transition to telephone appointments and re-deployment of heart failure nurse specialists was associated with less successful optimisation of medical therapy, especially renin-angiotensin inhibitors, compared with usual care.

Keywords: ACE-inhibitors; Heart failure; Specialist nursing; β-blockers.

© 2021 The Author(s). Published by IMR Press.

Conflict of interest statement

KKW has received speakers' fees and honoraria from Medtronic, Cardiac Dimensions, Novartis, Abbott, BMS, Pfizer, Bayer and has received an unconditional research grant from Medtronic. JG has received honoraria from Abbott, Medtronic and Microport and has received an unrestricted research grant from Medtronic. SS is funded by a British Heart Foundation Clinical Research Training Fellowship. None of the other authors have any disclosures.

Supplementary info

Publication types, MeSH terms, Substances, Grant support Expand

Publication types

- Observational Study

MeSH terms

- Adrenergic beta-1 Receptor Antagonists / administration & dosage*
- Adrenergic beta-1 Receptor Antagonists / adverse effects
- Aged
- Angiotensin-Converting Enzyme Inhibitors / administration & dosage*
- Angiotensin-Converting Enzyme Inhibitors / adverse effects
- Bisoprolol / administration & dosage*
- Bisoprolol / adverse effects
- COVID-19*
- Chronic Disease
- Female
- Heart Failure / diagnosis
- Heart Failure / drug therapy*
- Heart Failure / mortality
- Heart Failure / physiopathology
- Humans
- Male
- Ramipril / administration & dosage*
- Ramipril / adverse effects

- Retrospective Studies
- Time Factors
- Treatment Outcome

Substances

- Adrenergic beta-1 Receptor Antagonists
- Angiotensin-Converting Enzyme Inhibitors
- Ramipril
- Bisoprolol

Grant support

- FS/CRTF/20/24071/British Heart Foundation

Full text links

IMR Press Free Full Text Online IMR Press
Proceed to details
Cite
Share
1,194
Observational Study
Lancet
. 2020 Aug 29;396(10251):603-611.
doi: 10.1016/S0140-6736(20)31757-8. Epub 2020 Aug 18.

Effects of a major deletion in the SARS-CoV-2 genome on the severity of infection and the inflammatory response: an observational cohort study

Barnaby E Young [1], Siew-Wai Fong [2], Yi-Hao Chan [3], Tze-Minn Mak [4], Li Wei Ang [5], Danielle E Anderson [6], Cheryl Yi-Pin Lee [3], Siti Naqiah Amrun [3], Bernett Lee [7], Yun Shan Goh [3], Yvonne C F Su [6], Wycliffe E Wei [5], Shirin Kalimuddin [8], Louis Yi Ann Chai [9], Surinder Pada [10], Seow Yen Tan [11], Louisa Sun [12], Purnima Parthasarathy [13], Yuan Yi Constance Chen [14], Timothy Barkham [15], Raymond Tzer Pin Lin [4], Sebastian Maurer-Stroh [16], Yee-Sin Leo [17], Lin-Fa Wang [6], Laurent Renia [3], Vernon J Lee [18], Gavin J D Smith [19], David Chien Lye [20], Lisa F P Ng [21]
Affiliations Expand

Affiliations

- [1] National Centre for Infectious Diseases, Singapore; Department of Infectious Diseases, Tan Tock Seng Hospital, Singapore; Lee Kong Chian School of Medicine, Nanyang Technological University, Singapore.
- [2] Infectious Diseases Horizontal Technology Centre, Agency for Science, Technology, and Research, Singapore; Singapore Immunology Network, Agency for Science, Technology, and Research, Singapore; Department of Biological Sciences, National University of Singapore, Singapore.
- [3] Infectious Diseases Horizontal Technology Centre, Agency for Science, Technology, and Research, Singapore; Singapore Immunology Network, Agency for Science, Technology, and Research, Singapore.
- [4] National Public Health Laboratory, Singapore.
- [5] National Public Health and Epidemiology Unit, National Centre for Infectious Diseases, Singapore.
- [6] Duke-NUS Medical School, National University of Singapore, Singapore.
- [7] Singapore Immunology Network, Agency for Science, Technology, and Research, Singapore.
- [8] Duke-NUS Medical School, National University of Singapore, Singapore; Department of Infectious Diseases, Singapore General Hospital, Singapore.
- [9] Yong Loo Lin School of Medicine, National University of Singapore, Singapore; Department of Medicine, National University Health System, Singapore.
- [10] Department of Medicine, Infectious Diseases Service, Ng Teng Fong General Hospital, Singapore.
- [11] Department of Infectious Diseases, Changi General Hospital, Singapore.
- [12] Alexandra Hospital, Singapore.
- [13] Department of General Medicine, Khoo Teck Puat Hospital, Singapore.
- [14] Department of Laboratory Medicine, Tan Tock Seng Hospital, Singapore.
- [15] Department of Laboratory Medicine, Tan Tock Seng Hospital, Singapore; Yong Loo Lin School of Medicine, National University of Singapore, Singapore.
- [16] National Centre for Infectious Diseases, Singapore; Bioinformatics Institute, Agency for Science, Technology, and Research, Singapore; Department of Biological Sciences, National University of Singapore, Singapore; Global Initiative on Sharing All Influenza Data, Munich, Germany.
- [17] National Centre for Infectious Diseases, Singapore; Department of Infectious Diseases, Tan Tock Seng Hospital, Singapore; Lee Kong Chian School of Medicine, Nanyang Technological University, Singapore; Yong Loo Lin School of Medicine, National University of Singapore, Singapore; Saw Swee Hock School of Public Health, National University of Singapore, Singapore.
- [18] Saw Swee Hock School of Public Health, National University of Singapore, Singapore; Ministry of Health, Singapore.
- [19] Duke-NUS Medical School, National University of Singapore, Singapore. Electronic address: gavin.smith@duke-nus.edu.sg.
- [20] National Centre for Infectious Diseases, Singapore; Department of Infectious Diseases, Tan Tock Seng Hospital, Singapore; Lee Kong Chian School of Medicine, Nanyang Technological University, Singapore; Yong Loo Lin School of Medicine, National University of Singapore, Singapore.

- [21] Infectious Diseases Horizontal Technology Centre, Agency for Science, Technology, and Research, Singapore; Singapore Immunology Network, Agency for Science, Technology, and Research, Singapore. Electronic address: lisa_ng@immunol.a-star.edu.sg.

- PMID: **32822564**
- PMCID: PMC7434477
- DOI: 10.1016/S0140-6736(20)31757-8

Free PMC article
Observational Study

Effects of a major deletion in the SARS-CoV-2 genome on the severity of infection and the inflammatory response: an observational cohort study

Barnaby E Young et al. Lancet. 2020.
Free PMC article

Show details

Lancet
. 2020 Aug 29;396(10251):603-611.
doi: 10.1016/S0140-6736(20)31757-8. Epub 2020 Aug 18.

Authors

Barnaby E Young [1], Siew-Wai Fong [2], Yi-Hao Chan [3], Tze-Minn Mak [4], Li Wei Ang [5], Danielle E Anderson [6], Cheryl Yi-Pin Lee [3], Siti Naqiah Amrun [3], Bernett Lee [7], Yun Shan Goh [3], Yvonne C F Su [6], Wycliffe E Wei [5], Shirin Kalimuddin [8], Louis Yi Ann Chai [9], Surinder Pada [10], Seow Yen Tan [11], Louisa Sun [12], Purnima Parthasarathy [13], Yuan Yi Constance Chen [14], Timothy Barkham [15], Raymond Tzer Pin Lin [4], Sebastian Maurer-Stroh [16], Yee-Sin Leo [17], Lin-Fa Wang [6], Laurent Renia [3], Vernon J Lee [18], Gavin J D Smith [19], David Chien Lye [20], Lisa F P Ng [21]

Affiliations

- [1] National Centre for Infectious Diseases, Singapore; Department of Infectious Diseases, Tan Tock Seng Hospital, Singapore; Lee Kong Chian School of Medicine, Nanyang Technological University, Singapore.
- [2] Infectious Diseases Horizontal Technology Centre, Agency for Science, Technology, and Research, Singapore; Singapore Immunology Network, Agency for Science, Technology, and Research, Singapore; Department of Biological Sciences, National University of Singapore, Singapore.
- [3] Infectious Diseases Horizontal Technology Centre, Agency for Science, Technology, and Research, Singapore; Singapore Immunology Network, Agency for Science, Technology, and Research, Singapore.
- [4] National Public Health Laboratory, Singapore.

- [5] National Public Health and Epidemiology Unit, National Centre for Infectious Diseases, Singapore.
- [6] Duke-NUS Medical School, National University of Singapore, Singapore.
- [7] Singapore Immunology Network, Agency for Science, Technology, and Research, Singapore.
- [8] Duke-NUS Medical School, National University of Singapore, Singapore; Department of Infectious Diseases, Singapore General Hospital, Singapore.
- [9] Yong Loo Lin School of Medicine, National University of Singapore, Singapore; Department of Medicine, National University Health System, Singapore.
- [10] Department of Medicine, Infectious Diseases Service, Ng Teng Fong General Hospital, Singapore.
- [11] Department of Infectious Diseases, Changi General Hospital, Singapore.
- [12] Alexandra Hospital, Singapore.
- [13] Department of General Medicine, Khoo Teck Puat Hospital, Singapore.
- [14] Department of Laboratory Medicine, Tan Tock Seng Hospital, Singapore.
- [15] Department of Laboratory Medicine, Tan Tock Seng Hospital, Singapore; Yong Loo Lin School of Medicine, National University of Singapore, Singapore.
- [16] National Centre for Infectious Diseases, Singapore; Bioinformatics Institute, Agency for Science, Technology, and Research, Singapore; Department of Biological Sciences, National University of Singapore, Singapore; Global Initiative on Sharing All Influenza Data, Munich, Germany.
- [17] National Centre for Infectious Diseases, Singapore; Department of Infectious Diseases, Tan Tock Seng Hospital, Singapore; Lee Kong Chian School of Medicine, Nanyang Technological University, Singapore; Yong Loo Lin School of Medicine, National University of Singapore, Singapore; Saw Swee Hock School of Public Health, National University of Singapore, Singapore.
- [18] Saw Swee Hock School of Public Health, National University of Singapore, Singapore; Ministry of Health, Singapore.
- [19] Duke-NUS Medical School, National University of Singapore, Singapore. Electronic address: gavin.smith@duke-nus.edu.sg.
- [20] National Centre for Infectious Diseases, Singapore; Department of Infectious Diseases, Tan Tock Seng Hospital, Singapore; Lee Kong Chian School of Medicine, Nanyang Technological University, Singapore; Yong Loo Lin School of Medicine, National University of Singapore, Singapore.
- [21] Infectious Diseases Horizontal Technology Centre, Agency for Science, Technology, and Research, Singapore; Singapore Immunology Network, Agency for Science, Technology, and Research, Singapore. Electronic address: lisa_ng@immunol.a-star.edu.sg.

- PMID: **32822564**
- PMCID: PMC7434477
- DOI: 10.1016/S0140-6736(20)31757-8

Abstract

Background: Severe acute respiratory syndrome coronavirus 2 (SARS-CoV-2) variants with a 382-nucleotide deletion (Δ382) in the open reading frame 8 (ORF8) region of the genome have been detected in Singapore and other countries. We investigated the effect of this deletion on the clinical features of infection.

Methods: We retrospectively identified patients who had been screened for the Δ382 variant and recruited to the PROTECT study-a prospective observational cohort study conducted at seven public hospitals in Singapore. We collected clinical, laboratory, and radiological data from patients' electronic medical records and serial blood and respiratory samples taken during hospitalisation and after discharge. Individuals infected with the Δ382 variant were compared with those infected with wild-type SARS-CoV-2. Exact logistic regression was used to examine the association between the infection groups and the development of hypoxia requiring supplemental oxygen (an indicator of severe COVID-19, the primary endpoint). Follow-up for the study's primary endpoint is completed.

Findings: Between Jan 22 and March 21, 2020, 278 patients with PCR-confirmed SARS-CoV-2 infection were screened for the Δ382 deletion and 131 were enrolled onto the study, of whom 92 (70%) were infected with the wild-type virus, ten (8%) had a mix of wild-type and Δ382-variant viruses, and 29 (22%) had only the Δ382 variant. Development of hypoxia requiring supplemental oxygen was less frequent in the Δ382 variant group (0 [0%] of 29 patients) than in the wild-type only group (26 [28%] of 92; absolute difference 28% [95% CI 14-28]). After adjusting for age and presence of comorbidities, infection with the Δ382 variant only was associated with lower odds of developing hypoxia requiring supplemental oxygen (adjusted odds ratio 0·07 [95% CI 0·00-0·48]) compared with infection with wild-type virus only.

Interpretation: The Δ382 variant of SARS-CoV-2 seems to be associated with a milder infection. The observed clinical effects of deletions in ORF8 could have implications for the development of treatments and vaccines.

Funding: National Medical Research Council Singapore.

Copyright © 2020 Elsevier Ltd. All rights reserved.

- 25 references
- 3 figures

Supplementary info

Publication types, MeSH terms, Grant support Expand

Publication types

- Observational Study
- Research Support, Non-U.S. Gov't

MeSH terms

- Adult
- Aged
- Betacoronavirus
- COVID-19
- Coronavirus Infections / complications
- Coronavirus Infections / epidemiology
- Coronavirus Infections / virology*

- Gene Deletion*
- Genome, Viral / genetics*
- Humans
- Hypoxia / etiology
- Hypoxia / therapy
- Middle Aged
- Open Reading Frames
- Pandemics
- Pneumonia, Viral / complications
- Pneumonia, Viral / epidemiology
- Pneumonia, Viral / virology*
- Prospective Studies
- Respiratory Therapy
- SARS-CoV-2
- Severity of Illness Index
- Singapore / epidemiology
- Virus Replication

Grant support

- HHSN272201400006C/AI/NIAID NIH HHS/United States

Full text links

THE LANCET FULL-TEXT ARTICLE Elsevier Science Free PMC article

Proceed to details

Cite

Share

☐ 1,195

Observational Study

Swiss Med Wkly

. 2020 Oct 16;150:w20361.

doi: 10.4414/smw.2020.20361. eCollection 2020 Oct 5.

Flattening the curve in 52 days: characterisation of the COVID-19 pandemic in the Principality of Liechtenstein - an observational study

Sarah Lucia Thiel [1], Myriam Carol Weber [1], Lorenz Risch [2], Nadia Wohlwend [3], Thomas Lung [4], Dorothea Hillmann [4], Michael Ritzler [4], Martin Risch [5], Philipp Kohler [6], Pietro Vernazza

[6], Christian R Kahlert [7], Felix Fleisch [8], Alexia Cusini [8], Tomas V Karajan [1], Sandra Copeland [1], Matthias Paprotny [1]
Affiliations Expand

Affiliations

- [1] Department of General Internal Medicine, Landesspital Liechtenstein, Vaduz, Liechtenstein.
- [2] Labormedizinisches Zentrum Dr Risch, Vaduz, Liechtenstein / Centre of Laboratory Medicine, University Institute of Clinical Chemistry, University of Bern, Switzerland / Private University of the Principality of Liechtenstein, Triesen, Liechtenstein.
- [3] Private University of the Principality of Liechtenstein, Triesen, Liechtenstein.
- [4] Labormedizinisches Zentrum Dr Risch, Vaduz, Liechtenstein.
- [5] Central Laboratory, Kantonsspital Graubünden, Chur, Switzerland.
- [6] Division of Infectious Diseases and Hospital Epidemiology, Kantonsspital St Gallen, Switzerland.
- [7] Division of Infectious Diseases and Hospital Epidemiology, Kantonsspital St Gallen, Switzerland / Department of Infectious Diseases and Hospital Epidemiology, Children's Hospital of Eastern Switzerland, St Gallen, Switzerland.
- [8] Division of Infectious Diseases and Hospital Epidemiology, Kantonsspital Graubünden, Chur, Switzerland.

- PMID: **33105020**
- DOI: 10.4414/smw.2020.20361

Free article
Observational Study

Flattening the curve in 52 days: characterisation of the COVID-19 pandemic in the Principality of Liechtenstein - an observational study

Sarah Lucia Thiel et al. Swiss Med Wkly. 2020.
Free article
Show details
Swiss Med Wkly
. 2020 Oct 16;150:w20361.
doi: 10.4414/smw.2020.20361. eCollection 2020 Oct 5.

Authors

Sarah Lucia Thiel [1], Myriam Carol Weber [1], Lorenz Risch [2], Nadia Wohlwend [3], Thomas Lung [4], Dorothea Hillmann [4], Michael Ritzler [4], Martin Risch [5], Philipp Kohler [6], Pietro Vernazza

[6], Christian R Kahlert [7], Felix Fleisch [8], Alexia Cusini [8], Tomas V Karajan [1], Sandra Copeland [1], Matthias Paprotny [1]

Affiliations

- [1] Department of General Internal Medicine, Landesspital Liechtenstein, Vaduz, Liechtenstein.
- [2] Labormedizinisches Zentrum Dr Risch, Vaduz, Liechtenstein / Centre of Laboratory Medicine, University Institute of Clinical Chemistry, University of Bern, Switzerland / Private University of the Principality of Liechtenstein, Triesen, Liechtenstein.
- [3] Private University of the Principality of Liechtenstein, Triesen, Liechtenstein.
- [4] Labormedizinisches Zentrum Dr Risch, Vaduz, Liechtenstein.
- [5] Central Laboratory, Kantonsspital Graubünden, Chur, Switzerland.
- [6] Division of Infectious Diseases and Hospital Epidemiology, Kantonsspital St Gallen, Switzerland.
- [7] Division of Infectious Diseases and Hospital Epidemiology, Kantonsspital St Gallen, Switzerland / Department of Infectious Diseases and Hospital Epidemiology, Children's Hospital of Eastern Switzerland, St Gallen, Switzerland.
- [8] Division of Infectious Diseases and Hospital Epidemiology, Kantonsspital Graubünden, Chur, Switzerland.

- PMID: **33105020**
- DOI: 10.4414/smw.2020.20361

Abstract

Background: The principality of Liechtenstein had its first COVID-19 case at the beginning of March 2020. After exponential growth, the pandemic’s first wave was contained, with the last case being diagnosed 52 days after the initial occurrence.

Aim: To characterise the COVID-19 pandemic in Liechtenstein.

Methods: All patients diagnosed in Liechtenstein were followed up until recovery and again 6–8 weeks after symptom onset. They were contacted every 2 days to record their clinical status until the resolution of their symptoms. The diagnosis of COVID-19 was based on clinical symptoms and molecular testing. Household and close workplace contacts were included in the follow-up, which also comprised antibody testing. In addition, public health measures installed during the pandemic in Liechtenstein are summarised.

Results: During the first wave, 5% of the population obtained a reverse transcriptase polymerase chain reaction test. A total of 95 patients (median age 39 years) were diagnosed with COVID-19 (82 who resided in Liechtenstein), resulting in an incidence in Liechtenstein of 0.211%. One patient, aged 94, died (mortality rate 1%). Only 62% of patients could retrospectively identify a potential source of infection. Testing the patients’ household and close workplace contacts (n = 170) with antibody tests revealed that 25% of those tested were additional COVID-19 cases, a quarter of whom were asymptomatic. Those households which adhered to strict isolation measures had a significantly lower rate of affected household members than those who didn’t follow such measures. The national public health measures never restricted free movement of residents. Masks were only mandatory in healthcare settings. The use of home working for the general workforce was promoted. Gatherings were prohibited. Schools, universities, certain public spaces (like sports facilities and playgrounds), childcare facilities, nonessential shops, restaurants and

bars were closed. Social distancing, hygienic measures, solidarity and supporting individuals who were at risk were the main pillars of the public health campaigns.

Conclusion: The close collaboration of all relevant stakeholders allowed for the complete workup of all COVID-19 patients nationwide. A multitude of factors (e.g., young age of the patients, low-threshold access to testing, close monitoring of cases, high alertness and adherence to public health measures by the population) led to the early containment of the first wave of the pandemic, with a very low rate of serious outcomes. Antibody testing for SARS-CoV-2 revealed a substantial proportion of undiagnosed COVID-19 cases among close contacts of the patients.

Supplementary info

Publication types, MeSH terms Expand

Publication types

- Observational Study

MeSH terms

- Adult
- Asymptomatic Diseases / epidemiology
- Betacoronavirus / isolation & purification
- COVID-19
- COVID-19 Testing
- Clinical Laboratory Techniques / methods
- Clinical Laboratory Techniques / statistics & numerical data
- Communicable Disease Control* / methods
- Communicable Disease Control* / organization & administration
- Contact Tracing
- Coronavirus Infections* / diagnosis
- Coronavirus Infections* / epidemiology
- Coronavirus Infections* / prevention & control
- Coronavirus Infections* / therapy
- Female
- Humans
- Incidence
- Liechtenstein / epidemiology
- Male
- Monitoring, Physiologic / methods*
- Pandemics* / prevention & control
- Pneumonia, Viral* / epidemiology
- Pneumonia, Viral* / prevention & control
- Pneumonia, Viral* / therapy

- SARS-CoV-2

Full text links

Open access to full text on Swiss Medical Weekly EMH Swiss Medical Publishers Ltd.
Proceed to details
Cite
Share
☐ 1,196
Observational Study
JACC Cardiovasc Imaging
. 2020 Nov;13(11):2330-2339.
doi: 10.1016/j.jcmg.2020.05.004. Epub 2020 May 12.

Cardiac Involvement in Patients Recovered From COVID-2019 Identified Using Magnetic Resonance Imaging

Lu Huang [1], Peijun Zhao [1], Dazhong Tang [1], Tong Zhu [1], Rui Han [2], Chenao Zhan [1], Weiyong Liu [3], Hesong Zeng [4], Qian Tao [5], Liming Xia [6]
Affiliations Expand

Affiliations

- [1] Department of Radiology, Tongji Hospital, Tongji Medical College, Huazhong University of Science and Technology, Wuhan, China.
- [2] Department of Radiology, Wuhan No.1 Hospital, Wuhan, China.
- [3] Department of Laboratory Medicine, Tongji Hospital, Tongji Medical College, Huazhong University of Science and Technology, Wuhan, China.
- [4] Department of Cardiology, Tongji Hospital, Tongji Medical College, Huazhong University of Science and Technology, Wuhan, China. Electronic address: zenghs@tjh.tjmu.edu.cn.
- [5] Division of Imaging Processing, Department of Radiology, Leiden University Medical Center, Leiden, the Netherlands. Electronic address: q.tao@lumc.nl.
- [6] Department of Radiology, Tongji Hospital, Tongji Medical College, Huazhong University of Science and Technology, Wuhan, China. Electronic address: lmxia@tjh.tjmu.edu.cn.

- PMID: **32763118**
- PMCID: PMC7214335
- DOI: 10.1016/j.jcmg.2020.05.004

Free PMC article
Observational Study

Cardiac Involvement in Patients Recovered From COVID-2019 Identified Using Magnetic Resonance Imaging

Lu Huang et al. JACC Cardiovasc Imaging. 2020 Nov.
Free PMC article
Show details
JACC Cardiovasc Imaging
. 2020 Nov;13(11):2330-2339.
doi: 10.1016/j.jcmg.2020.05.004. Epub 2020 May 12.

Authors

Lu Huang [1], Peijun Zhao [1], Dazhong Tang [1], Tong Zhu [1], Rui Han [2], Chenao Zhan [1], Weiyong Liu [3], Hesong Zeng [4], Qian Tao [5], Liming Xia [6]

Affiliations

- [1] Department of Radiology, Tongji Hospital, Tongji Medical College, Huazhong University of Science and Technology, Wuhan, China.
- [2] Department of Radiology, Wuhan No.1 Hospital, Wuhan, China.
- [3] Department of Laboratory Medicine, Tongji Hospital, Tongji Medical College, Huazhong University of Science and Technology, Wuhan, China.
- [4] Department of Cardiology, Tongji Hospital, Tongji Medical College, Huazhong University of Science and Technology, Wuhan, China. Electronic address: zenghs@tjh.tjmu.edu.cn.
- [5] Division of Imaging Processing, Department of Radiology, Leiden University Medical Center, Leiden, the Netherlands. Electronic address: q.tao@lumc.nl.
- [6] Department of Radiology, Tongji Hospital, Tongji Medical College, Huazhong University of Science and Technology, Wuhan, China. Electronic address: lmxia@tjh.tjmu.edu.cn.

- PMID: **32763118**
- PMCID: PMC7214335
- DOI: 10.1016/j.jcmg.2020.05.004

Abstract

Objectives: This study evaluated cardiac involvement in patients recovered from coronavirus disease-2019 (COVID-19) using cardiac magnetic resonance (CMR).

Background: Myocardial injury caused by COVID-19 was previously reported in hospitalized patients. It is unknown if there is sustained cardiac involvement after patients' recovery from COVID-19.

Methods: Twenty-six patients recovered from COVID-19 who reported cardiac symptoms and underwent CMR examinations were retrospectively included. CMR protocols consisted of conventional sequences (cine, T2-weighted imaging, and late gadolinium enhancement [LGE])

and quantitative mapping sequences (T1, T2, and extracellular volume [ECV] mapping). Edema ratio and LGE were assessed in post-COVID-19 patients. Cardiac function, native T1/T2, and ECV were quantitatively evaluated and compared with controls.

Results: Fifteen patients (58%) had abnormal CMR findings on conventional CMR sequences: myocardial edema was found in 14 (54%) patients and LGE was found in 8 (31%) patients. Decreased right ventricle functional parameters including ejection fraction, cardiac index, and stroke volume/body surface area were found in patients with positive conventional CMR findings. Using quantitative mapping, global native T1, T2, and ECV were all found to be significantly elevated in patients with positive conventional CMR findings, compared with patients without positive findings and controls (median [interquartile range]: native T1 1,271 ms [1,243 to 1,298 ms] vs. 1,237 ms [1,216 to 1,262 ms] vs. 1,224 ms [1,217 to 1,245 ms]; mean ± SD: T2 42.7 ± 3.1 ms vs. 38.1 ms ± 2.4 vs. 39.1 ms ± 3.1; median [interquartile range]: 28.2% [24.8% to 36.2%] vs. 24.8% [23.1% to 25.4%] vs. 23.7% [22.2% to 25.2%]; $p = 0.002$; $p < 0.001$, and $p = 0.002$, respectively).

Conclusions: Cardiac involvement was found in a proportion of patients recovered from COVID-19. CMR manifestation included myocardial edema, fibrosis, and impaired right ventricle function. Attention should be paid to the possible myocardial involvement in patients recovered from COVID-19 with cardiac symptoms.

Keywords: ACE2, angiotensin-converting enzyme 2; AHA, American Heart Association; BSA, body surface area; CI, cardiac index; CMR, cardiac magnetic resonance; CO, cardiac output; COVID-19, coronavirus disease-2019; ECV, extracellular volume; EDV, end-diastolic volume; EF, ejection fraction; ER, edema ratio; ESV, end-systolic volume; FA, flip angle; FOV, field of view; IQR, interquartile range; LGE, late gadolinium enhancement; LV, left ventricle; LVEF, left ventricular ejection fraction; PSIR, phase-sensitive inversion-recovery; RT-PCR, reverse transcription and polymerase chain reaction; RV, right ventricle; RVEF, right ventricular ejection fraction; SARS-CoV-2, severe acute respiratory syndrome-coronavirus-2; SI, signal intensity; SSFP, steady state free precession; STIR, short tau inversion recovery; SV, stroke volume; T2WI, T2-weighted imaging; TE, echo time; TR, repetition time; cardiac involvement; cardiac magnetic resonance imaging; coronavirus disease-2019; hs-cTnI, high-sensitive cardiac troponin I.

© 2020 by the American College of Cardiology Foundation. Published by Elsevier.

Conflict of interest statement

This work was supported in part by the National Natural Science Foundation of China (81471637 and 81873889), the National Mega Project on Major Infectious Disease Prevention (2017ZX10103005-007), and the National Key Research and Development Program of China (2018YFE0204500). All authors have reported that they have no relationships relevant to the contents of this paper to disclose.

Comment in

- CMR in the Era of COVID-19: Evaluation of Myocarditis in the Subacute Phase.
  Salerno M, Kwong RY. Salerno M, et al. JACC Cardiovasc Imaging. 2020 Nov;13 (11):2340-2342. doi: 10.1016/j.jcmg.2020.06.013. Epub 2020 Jul 3. JACC Cardiovasc Imaging. 2020. PMID: 32771570 Free PMC article.
- Cardiac Involvement in the COVID-19 Pandemic: Hazy Lessons From Cardiac Imaging?
  Sengupta PP, Chandrashekhar YS. Sengupta PP, et al. JACC Cardiovasc Imaging. 2020 Nov;13(11):2480-2483. doi: 10.1016/j.jcmg.2020.10.001. Epub 2020 Oct 10. JACC Cardiovasc Imaging. 2020. PMID: 33153538 Free PMC article. No abstract available.

- 29 references
- 4 figures

Supplementary info

Publication types, MeSH terms Expand

Publication types

- Observational Study
- Research Support, Non-U.S. Gov't

MeSH terms

- Adult
- COVID-19
- China
- Coronavirus Infections / complications
- Coronavirus Infections / diagnosis
- Coronavirus Infections / therapy*
- Edema, Cardiac / diagnostic imaging*
- Edema, Cardiac / etiology
- Edema, Cardiac / pathology
- Female
- Fibrosis
- Humans
- Magnetic Resonance Imaging, Cine*
- Male
- Middle Aged
- Myocardium / pathology
- Pandemics
- Pneumonia, Viral / complications
- Pneumonia, Viral / diagnosis
- Pneumonia, Viral / therapy*
- Predictive Value of Tests
- Remission Induction
- Retrospective Studies
- Ventricular Dysfunction, Right / diagnostic imaging*
- Ventricular Dysfunction, Right / etiology
- Ventricular Dysfunction, Right / physiopathology
- Ventricular Function, Right

Full text links

ELSEVIER OPEN ACCESS Elsevier Science Free PMC article

Proceed to details

Cite

Share

☐ 1,197

Observational Study

Bone Joint J
. 2021 Apr;103-B(4):672-680.
doi: 10.1302/0301-620X.103B.BJJ-2021-0104.R1. Epub 2021 Mar 23.

The number of patients "worse than death" while waiting for a hip or knee arthroplasty has nearly doubled during the COVID-19 pandemic

Nick D Clement [1], Chloe E H Scott [1 2], James R D Murray [3], Colin R Howie [1 2], David J Deehan [4], IMPACT-Restart Collaboration

Affiliations Expand

Affiliations

- [1] Edinburgh Orthopaedics, Royal Infirmary of Edinburgh, Edinburgh, UK.
- [2] Department of Orthopaedics, University of Edinburgh, Edinburgh, UK.
- [3] Avon Orthopaedic Centre, Southmead Hospital & University of Bristol, Bristol, UK.
- [4] Department of Orthopaedics, Freeman Hospital, Newcastle, UK.

- PMID: **33752468**
- DOI: 10.1302/0301-620X.103B.BJJ-2021-0104.R1

Observational Study

The number of patients "worse than death" while waiting for a hip or knee arthroplasty has nearly doubled during the COVID-19 pandemic

Nick D Clement et al. Bone Joint J. 2021 Apr.

Show details

Bone Joint J
. 2021 Apr;103-B(4):672-680.
doi: 10.1302/0301-620X.103B.BJJ-2021-0104.R1. Epub 2021 Mar 23.

Authors

Nick D Clement [1], Chloe E H Scott [1] [2], James R D Murray [3], Colin R Howie [1] [2], David J Deehan [4], IMPACT-Restart Collaboration

Affiliations

- [1] Edinburgh Orthopaedics, Royal Infirmary of Edinburgh, Edinburgh, UK.
- [2] Department of Orthopaedics, University of Edinburgh, Edinburgh, UK.
- [3] Avon Orthopaedic Centre, Southmead Hospital & University of Bristol, Bristol, UK.
- [4] Department of Orthopaedics, Freeman Hospital, Newcastle, UK.

- PMID: **33752468**
- DOI: 10.1302/0301-620X.103B.BJJ-2021-0104.R1

Abstract

Aims: The aim of this study was to assess the quality of life of patients on the waiting list for a total hip (THA) or knee arthroplasty (KA) during the COVID-19 pandemic. Secondary aims were to assess whether length of time on the waiting list influenced quality of life and rate of deferral of surgery.

Methods: During the study period (August and September 2020) 843 patients (THA n = 394, KA n = 449) from ten centres in the UK reported their EuroQol five dimension (EQ-5D) scores and completed a waiting list questionnaire (2020 group). Patient demographic details, procedure, and date when listed were recorded. Patients scoring less than zero for their EQ-5D score were defined to be in a health state "worse than death" (WTD). Data from a retrospective cohort (January 2014 to September 2017) were used as the control group.

Results: The 2020 group had a significantly worse EQ-5D score compared to the control group for both THA ($p < 0.001$) and KA ($p < 0.001$). Over one-third (35.0%, n = 138/394) of patients waiting for a THA and nearly a quarter (22.3%, n = 100/449) for KA were in a health state WTD, which was significantly greater than the control group (odds ratio 2.30 (95% confidence interval (CI) 1.83 to 2.93) and 2.08 (95% CI 1.61 to 2.70), respectively; $p < 0.001$). Over 80% (n = 680/843) of the 2020 group felt that their quality of life had deteriorated while waiting. Each additional month spent on the waiting list was independently associated with a decrease in quality of life (EQ-5D: -0.0135, p = 0.004). There were 117 (13.9%) patients who wished to defer their surgery and the main reason for this was health concerns for themselves and or their family (99.1%, n = 116/117).

Conclusion: Over one-third of patients waiting for THA and nearly one-quarter waiting for a KA were in a state WTD, which was approaching double that observed prior to the pandemic. Increasing length of time on the waiting list was associated with decreasing quality of life. Level of evidence: Level III retrospective case control study Cite this article: *Bone Joint J* 2021;103-B (4):672-680.

Keywords: Arthroplasty; COVID-19; Quality of life; Waiting list; Worse than death.

Comment in

- Orthopaedic surgical prioritisation: can it be made fairer to minimise clinical harm?
  Iyengar KP, Monga P, Elbana H, Singh B. Iyengar KP, et al. Postgrad Med J. 2022 Feb;98 (e1):e61-e62. doi: 10.1136/postgradmedj-2021-140272. Postgrad Med J. 2022. PMID: 35105778 No abstract available.

Supplementary info

Publication types, MeSH terms Expand

Publication types

- Multicenter Study
- Observational Study

MeSH terms

- Adult
- Aged
- Aged, 80 and over
- Arthroplasty, Replacement, Hip*
- Arthroplasty, Replacement, Knee*
- COVID-19* / epidemiology
- COVID-19* / prevention & control
- COVID-19* / psychology
- Cross-Sectional Studies
- Female
- Health Services Accessibility*
- Health Status Indicators*
- Humans
- Linear Models
- Male
- Medical Audit
- Middle Aged
- Multivariate Analysis
- Pandemics
- Patient Acceptance of Health Care
- Quality Improvement
- Quality of Life / psychology*
- Time Factors
- United Kingdom / epidemiology
- Waiting Lists*

Full text links

The Bone & Joint Journal Full Text Available Atypon
Proceed to details
Cite
Share
1,198
Observational Study
Front Biosci (Elite Ed)
. 2021 Dec 20;13(2):259-271.
doi: 10.52586/E883.

The influence of COVID-19 lockdowns on presentation with spontaneous posterior vitreous detachment to the emergency department in Italy

Marco Zeppieri [1], Maria Letizia Salvetat [2], Carlo Salati [1]
Affiliations Expand

Affiliations

- [1] Department of Ophthalmology, University Hospital of Udine, 33100 Udine, Italy.
- [2] Department of Ophthalmology, Azienda Sanitaria "Friuli Occidentale", 33170 Pordenone, Italy.

- PMID: **34937313**
- DOI: 10.52586/E883

Free article
Observational Study

The influence of COVID-19 lockdowns on presentation with spontaneous posterior vitreous detachment to the emergency department in Italy

Marco Zeppieri et al. Front Biosci (Elite Ed). 2021.
Free article
Show details
Front Biosci (Elite Ed)

. 2021 Dec 20;13(2):259-271.
doi: 10.52586/E883.

Authors

Marco Zeppieri [1], Maria Letizia Salvetat [2], Carlo Salati [1]

Affiliations

- [1] Department of Ophthalmology, University Hospital of Udine, 33100 Udine, Italy.
- [2] Department of Ophthalmology, Azienda Sanitaria "Friuli Occidentale", 33170 Pordenone, Italy.

- PMID: **34937313**
- DOI: 10.52586/E883

Abstract

The purpose of the study was to analyze the frequency of the spontaneous posterior vitreous detachment (PVD) in patients admitted to an Emergency Eye Department in Italy (EED) during the COVID-19 pandemic national lockdown in 2020 compared with the similar time period in 2019. In this retrospective observational study, patient records for ophthalmology EED patients in the month of April 2020 during the COVID-19 Italian national lockdown, were compared with those for an equivalent one-month period in 2019. Diagnoses, gender, and age were assessed. Unpaired Student *t*-tests were used for continuous variables. Poisson regression was used for count analysis to compare categorical variables. Chi-square test was applied to asses proportion differences. In comparison with the 2019 equivalent period, there was a significant decrease in the overall number of EED visits and in the number of patients presenting with a spontaneous PVD during the 2020 lockdown (-41.6% and -49%, respectively). During the 2020 lockdown, all diagnostic categories showed less patient admittance, however, the proportions remained stable when considering the entire cohort. The proportion of urgent visits was 90% in 2020 and 86% in 2019 ($p = 0.66$). The proportion of EED patients affected by spontaneous PVD was comparable between the two study periods (8.4% in 2020 vs. 9.6% in 2019, $p = 0.34$). Patients presenting with spontaneous PVD in both periods were significantly older when compared to patients with other pathologies (mean age of 63years in 2020 and 64years in 2019, $p < 0.001$). There was a significant bias in female gender (61.2% in 2019 and 60% in 2020, $p < 0.05$). There was a significant decrease of accesses to the EED during COVID-19 2020 lockdown. Patients affected by spontaneous PVD were about 50% less compared with the same period of 2019. Risk factors for the development of spontaneous PVD were older age and female gender. PVD represents a potentially visual function threatening condition because it can cause retinal ruptures and retinal detachment. Patients need to be educated to get urgent ophthalmic assessments in the presence of important acute signs and symptoms, like floaters and flashes, even in the presence of a lockdown.

Keywords: COVID-19; Emergency eye department; Myodesopsia; Pandemic; Photopsia; Retinal detachment; Spontaneous posterior vitreous detachment.

© 2021 The Author(s). Published by BRI.

Supplementary info

Publication types, MeSH terms Expand

Publication types

- Observational Study

MeSH terms

- COVID-19 / epidemiology*
- COVID-19 / prevention & control*
- Emergency Service, Hospital / statistics & numerical data*
- Female
- Humans
- Italy / epidemiology
- Male
- Middle Aged
- Pandemics
- Quarantine / statistics & numerical data*
- Vitreous Detachment / diagnosis*
- Vitreous Detachment / epidemiology*

Full text links

IMR Press Free Full Text Online IMR Press
Proceed to details
Cite
Share
☐ 1,199
Observational Study
Front Endocrinol (Lausanne)
. 2021 Jan 8;11:593179.
doi: 10.3389/fendo.2020.593179. eCollection 2020.

The Adrenal Cortex, an Underestimated Site of SARS-CoV-2 Infection

Yanfei Mao [1], Bo Xu [1], Wenbin Guan [2], Dunfeng Xu [1], Feng Li [3], Rongrong Ren [1], Xiaoyan Zhu [4], Yuan Gao [5], Lai Jiang [1]
Affiliations Expand

Affiliations

- [1] Department of Anesthesiology and Surgical Intensive Care Unit, Xinhua Hospital, Shanghai Jiaotong University School of Medicine, Shanghai, China.

- [2] Department of Pathology, Xinhua Hospital, Shanghai Jiaotong University School of Medicine, Shanghai, China.
- [3] Department of Respiratory and Critical Care Medicine, Shanghai Public Health Clinical Center Affiliated to Fudan University, Shanghai, China.
- [4] Department of Physiology, Navy Medical University, Shanghai, China.
- [5] Department of Critical Care Medicine, Renji Hospital, Shanghai Jiaotong University School of Medicine, Shanghai, China.

- PMID: **33488517**
- PMCID: PMC7820749
- DOI: 10.3389/fendo.2020.593179

Free PMC article
Observational Study

The Adrenal Cortex, an Underestimated Site of SARS-CoV-2 Infection

Yanfei Mao et al. Front Endocrinol (Lausanne). 2021.
Free PMC article
Show details
Front Endocrinol (Lausanne)
. 2021 Jan 8;11:593179.
doi: 10.3389/fendo.2020.593179. eCollection 2020.

Authors

Yanfei Mao [1], Bo Xu [1], Wenbin Guan [2], Dunfeng Xu [1], Feng Li [3], Rongrong Ren [1], Xiaoyan Zhu [4], Yuan Gao [5], Lai Jiang [1]

Affiliations

- [1] Department of Anesthesiology and Surgical Intensive Care Unit, Xinhua Hospital, Shanghai Jiaotong University School of Medicine, Shanghai, China.
- [2] Department of Pathology, Xinhua Hospital, Shanghai Jiaotong University School of Medicine, Shanghai, China.
- [3] Department of Respiratory and Critical Care Medicine, Shanghai Public Health Clinical Center Affiliated to Fudan University, Shanghai, China.
- [4] Department of Physiology, Navy Medical University, Shanghai, China.
- [5] Department of Critical Care Medicine, Renji Hospital, Shanghai Jiaotong University School of Medicine, Shanghai, China.

- PMID: **33488517**
- PMCID: PMC7820749
- DOI: 10.3389/fendo.2020.593179

Abstract

Background: The majority of the critically ill patients may have critical illness-related corticosteroid insufficiency (CIRCI). The therapeutic effect of dexamethasone may be related to its ability to improve cortical function. Recent study showed that dexamethasone can reduce COVID-19 deaths by up to one third in critically ill patients. The aim of this article is to investigate whether SARS-CoV-2 can attack the adrenal cortex to aggravate the relative adrenal insufficiency.

Methods: We summarized the clinical features of COVID-19 reported in currently available observational studies. ACE2 and TMPRSS2 expression was examined in human adrenal glands by immunohistochemical staining. We retrospectively analyzed serum cortisol levels in critically ill patients with or without COVID-19.

Results: High percentage of critically ill patients with SARS-COV-2 infection in the study were treated with vasopressors. ACE2 receptor and TMPRSS2 serine protease were colocalized in adrenocortical cells in zona fasciculata and zona reticularis. We collected plasma cortisol concentrations in nine critically ill patients with COVID-19. The cortisol levels of critically ill patients with COVID-19 were lower than those in non-COVID-19 critically ill group. Six of the nine COVID-19 critically ill patients had random plasma cortisol concentrations below 10 μg/dl, which met the criteria for the diagnosis of CIRCI.

Conclusion: We demonstrate that ACE2 and TMPRSS2 are colocalized in adrenocortical cells, and that the cortisol levels are lower in critically ill patients with COVID-19 as compared to those of non-COVID-19 critically ill patients. Based on our findings, we recommend measuring plasma cortisol level to guide hormonal therapy.

Keywords: adrenal cortex; adrenal insufficiency; coronavirus disease 2019; critically ill patients; severe acute respiratory syndrome coronavirus 2.

Copyright © 2021 Mao, Xu, Guan, Xu, Li, Ren, Zhu, Gao and Jiang.

Conflict of interest statement

The authors declare that the research was conducted in the absence of any commercial or financial relationships that could be construed as a potential conflict of interest.

- 49 references
- 6 figures

Supplementary info

Publication types, MeSH terms, Substances Expand

Publication types

- Observational Study

MeSH terms

- Adrenal Cortex / enzymology
- Adrenal Cortex / virology*
- Adrenal Cortex Diseases / drug therapy*
- Adrenal Cortex Diseases / virology*
- Adrenal Insufficiency / etiology
- Adrenal Insufficiency / therapy
- Adult
- Aged
- Aged, 80 and over
- Angiotensin-Converting Enzyme 2 / metabolism
- COVID-19 / drug therapy
- COVID-19 / virology*
- Critical Illness
- Dexamethasone / therapeutic use
- Female
- Humans
- Hydrocortisone / blood
- Male
- Middle Aged
- Serine Endopeptidases / metabolism
- Vasoconstrictor Agents / therapeutic use
- Zona Fasciculata / metabolism
- Zona Reticularis / metabolism

Substances

- Vasoconstrictor Agents
- Dexamethasone
- ACE2 protein, human
- Angiotensin-Converting Enzyme 2
- Serine Endopeptidases
- TMPRSS2 protein, human
- Hydrocortisone

Full text links

Frontiers Media SA Free PMC article

Proceed to details

Cite

Share

☐ 1,200

Observational Study

Emerg Med Australas
. 2020 Dec;32(6):1027-1033.
doi: 10.1111/1742-6723.13606. Epub 2020 Aug 29.

Impact of COVID-19 State of Emergency restrictions on presentations to two Victorian emergency departments

Rob D Mitchell [1, 2], Gerard M O'Reilly [1, 2, 3], Biswadev Mitra [1, 2, 3], De Villiers Smit [1, 2, 3], Jean-Philippe Miller [1], Peter A Cameron [1, 2, 3]
Affiliations Expand

Affiliations

- [1] Emergency and Trauma Centre, The Alfred Hospital, Melbourne, Victoria, Australia.
- [2] School of Public Health and Preventive Medicine, Monash University, Melbourne, Victoria, Australia.
- [3] National Trauma Research Institute, The Alfred Hospital, Melbourne, Victoria, Australia.

- PMID: **32748481**
- PMCID: PMC7436380
- DOI: 10.1111/1742-6723.13606

Free PMC article
Observational Study

Impact of COVID-19 State of Emergency restrictions on presentations to two Victorian emergency departments

Rob D Mitchell et al. Emerg Med Australas. 2020 Dec.
Free PMC article
Show details
Emerg Med Australas
. 2020 Dec;32(6):1027-1033.
doi: 10.1111/1742-6723.13606. Epub 2020 Aug 29.

Authors

Rob D Mitchell [1, 2], Gerard M O'Reilly [1, 2, 3], Biswadev Mitra [1, 2, 3], De Villiers Smit [1, 2, 3], Jean-Philippe Miller [1], Peter A Cameron [1, 2, 3]

Affiliations

- [1] Emergency and Trauma Centre, The Alfred Hospital, Melbourne, Victoria, Australia.
- [2] School of Public Health and Preventive Medicine, Monash University, Melbourne, Victoria, Australia.
- [3] National Trauma Research Institute, The Alfred Hospital, Melbourne, Victoria, Australia.

- PMID: **32748481**
- PMCID: PMC7436380
- DOI: 10.1111/1742-6723.13606

Abstract

Objective: To determine if COVID-19 State of Emergency (SOE) restrictions were associated with a reduction in presentations to two urban EDs in Melbourne, Victoria.

Methods: This retrospective observational study included adult patients presenting to The Alfred and Sandringham Hospital EDs during the first month of stage 2 and 3 SOE restrictions (26 March-25 April 2020). Patients transferred from other hospitals or diagnosed with COVID-19 were excluded. The primary outcome was the average number of presentations per day. Secondary outcomes included the average daily number of presentations for pre-specified subgroups defined by triage category and diagnosis. The independent impact of SOE restrictions, adjusted for underlying trends in attendance, was determined using negative binomial regression and reported as an incident rate ratio (IRR) with a 95% confidence interval (CI).

Results: Average daily attendance during the exposure period was 174.7. In the absence of SOE restrictions, 278.8 presentations per day were predicted, a reduction of 37.3% (IRR 0.63, 95% CI 0.59-0.67). Attendance was lower than anticipated for all triage categories (especially category 5 [IRR 0.51, 95% CI 0.44-0.59]) and diagnostic groups (including circulatory problems [IRR 0.62, 95% CI 0.50-0.76] and injury [IRR 0.58, 95% CI 0.53-0.63]). There were fewer than predicted presentations for several sentinel diagnoses, including gastroenteritis (IRR 0.27, 95% CI 0.17-0.42) and renal colic (IRR 0.55, 95% CI 0.33-0.92).

Conclusions: SOE restrictions were associated with a significant reduction in ED presentations across a range of triage categories and diagnoses. Public health messaging should emphasise the importance of timely ED attendance for acute illness and injury.

Keywords: COVID-19; emergency care; pandemic; public health.

© 2020 Australasian College for Emergency Medicine.

- 23 references

Supplementary info

Publication types, MeSH terms Expand

Publication types

- Observational Study

MeSH terms

- Aged
- COVID-19
- Coronavirus Infections / epidemiology*
- Coronavirus Infections / prevention & control
- Coronavirus Infections / therapy
- Emergency Service, Hospital / statistics & numerical data*
- Female
- Humans
- Infection Control* / statistics & numerical data
- Male
- Pandemics / prevention & control
- Patient Acceptance of Health Care / statistics & numerical data
- Pneumonia, Viral / epidemiology*
- Pneumonia, Viral / prevention & control
- Pneumonia, Viral / therapy
- Retrospective Studies
- Victoria / epidemiology

Full text links

WILEY Full Text Article Wiley Free PMC article

Proceed to details

Cite

Share

1,388 results

Show more results

[x]

Cite

Copy

Download .nbib

Format: NLM

[x]

Share

-
-

Permalink

Copy

first first First

previous previous Prev

Page

6

of 7

Next next next

Last last last

Send To

- Clipboard
- Email
- Save
- My Bibliography
- Collections
- Citation Manager

[x]

- Article type
- Species
- Language
- Sex
- Journal
- Age

- ☐ Address
- ☐ Autobiography
- ☐ Bibliography
- ☐ Biography
- ☐ Case Reports
- ☐ Classical Article
- ☐ Clinical Conference
- ☐ Clinical Study
- ☐ Clinical Trial Protocol
- ☐ Clinical Trial, Phase I
- ☐ Clinical Trial, Phase II
- ☐ Clinical Trial, Phase III
- ☐ Clinical Trial, Phase IV
- ☐ Clinical Trial, Veterinary
- ☐ Comment
- ☐ Comparative Study
- ☐ Congress
- ☐ Consensus Development Conference
- ☐ Consensus Development Conference, NIH
- ☐ Controlled Clinical Trial
- ☐ Corrected and Republished Article
- ☐ Dataset
- ☐ Dictionary
- ☐ Directory
- ☐ Duplicate Publication
- ☐ Editorial
- ☐ Electronic Supplementary Materials
- ☐ English Abstract

- ☐ Evaluation Study
- ☐ Festschrift
- ☐ Government Publication
- ☐ Guideline
- ☐ Historical Article
- ☐ Interactive Tutorial
- ☐ Interview
- ☐ Introductory Journal Article
- ☐ Lecture
- ☐ Legal Case
- ☐ Legislation
- ☐ Letter
- ☐ Multicenter Study
- ☐ News
- ☐ Newspaper Article
- ☐ Observational Study
- ☐ Observational Study, Veterinary
- ☐ Overall
- ☐ Patient Education Handout
- ☐ Periodical Index
- ☐ Personal Narrative
- ☐ Portrait
- ☐ Practice Guideline
- ☐ Pragmatic Clinical Trial
- ☐ Preprint
- ☐ Published Erratum
- ☐ Research Support, American Recovery and Reinvestment Act
- ☐ Research Support, N.I.H., Extramural
- ☐ Research Support, N.I.H., Intramural
- ☐ Research Support, Non-U.S. Gov't
- ☐ Research Support, U.S. Gov't, Non-P.H.S.
- ☐ Research Support, U.S. Gov't, P.H.S.
- ☐ Research Support, U.S. Gov't
- ☐ Retracted Publication
- ☐ Retraction of Publication
- ☐ Scientific Integrity Review
- ☐ Technical Report
- ☐ Twin Study
- ☐ Validation Study
- ☐ Video-Audio Media
- ☐ Webcast

- ☐ Humans
- ☐ Other Animals

- ☐ Afrikaans
- ☐ Albanian
- ☐ Arabic
- ☐ Armenian

- ☐ Azerbaijani
- ☐ Bosnian
- ☐ Bulgarian
- ☐ Catalan
- ☐ Chinese
- ☐ Croatian
- ☐ Czech
- ☐ Danish
- ☐ Dutch
- ☐ English
- ☐ Esperanto
- ☐ Estonian
- ☐ Finnish
- ☐ French
- ☐ Georgian
- ☐ German
- ☐ Greek, Modern
- ☐ Hebrew
- ☐ Hindi
- ☐ Hungarian
- ☐ Icelandic
- ☐ Indonesian
- ☐ Italian
- ☐ Japanese
- ☐ Kinyarwanda
- ☐ Korean
- ☐ Latin
- ☐ Latvian
- ☐ Lithuanian
- ☐ Macedonian
- ☐ Malay
- ☐ Malayalam
- ☐ Maori
- ☐ Multiple Languages
- ☐ Norwegian
- ☐ Persian
- ☐ Polish
- ☐ Portuguese
- ☐ Pushto
- ☐ Romanian
- ☐ Russian
- ☐ Sanskrit
- ☐ Scottish gaelic
- ☐ Serbian
- ☐ Slovak
- ☐ Slovenian
- ☐ Spanish
- ☐ Swedish

- ☐ Thai
- ☐ Turkish
- ☐ Ukrainian
- ☐ Undetermined
- ☐ Vietnamese
- ☐ Welsh

- ☐ Female
- ☐ Male

- ☐ MEDLINE

- ☐ Child: birth-18 years
- ☐ Newborn: birth-1 month
- ☐ Infant: birth-23 months
- ☐ Infant: 1-23 months
- ☐ Preschool Child: 2-5 years
- ☐ Child: 6-12 years
- ☐ Adolescent: 13-18 years
- ☐ Adult: 19+ years
- ☐ Young Adult: 19-24 years
- ☐ Adult: 19-44 years
- ☐ Middle Aged + Aged: 45+ years
- ☐ Middle Aged: 45-64 years
- ☐ Aged: 65+ years
- ☐ 80 and over: 80+ years

Cancel Show
Close dialog
Back to Top
Jump to page Close dialog
7 of 7
Jump

NCBI Literature Resources

MeSH PMC Bookshelf Disclaimer

Follow NCBI

[Connect with NLM]

•

-
-

National Library of Medicine
8600 Rockville Pike
Bethesda, MD 20894

Web Policies
FOIA
HHS Vulnerability Disclosure

Help
Accessibility
Careers

- NLM
- NIH
- HHS
- USA.gov

ERREUR p
du site :
Domaine
