# Supplementary material for: COVID-19 treatment of hospital patients worldwide at the onset of the pandemic in 2020: a systematic review
Source: BMC Infect Dis. 2025 Dec 17;26:107. doi: 10.1186/s12879-025-12368-2 (PMC12822144; doi:10.1186/s12879-025-12368-2)
Supplement: Supplementary file 4 — Supplementary Material 4 [file 12879_2025_12368_MOESM4_ESM.zip › 12879_2025_12368_MOESM4_ESM/Search Pubmed 2022 03 28 retrospective observational study hospital treatment covid 1201-1388.pdf]

Skip to main page content

COVID-19 Information

Public health information (CDC)

Research information (NIH)

SARS-CoV-2 data (NCBI)

Prevention and treatment information (HHS)

Español

Close

Account

Logged in as:
username

- Dashboard
- Publications
- Account settings
- Log out

Access keys NCBI Homepage MyNCBI Homepage Main Content Main Navigation

Search Page

pubmed

Search: retrospective observation Search
Advanced Create alert Create RSS Clipboard
User Guide
Filters 0 Timeline
Sorted by: Best match
Sorted by: Best match Display options
Save Email
Send to

Save citations to file

Selection: All results on this page

Format: Summary (text)
Create file Cancel

# Email citations

Subject: retrospective observational study hospital treatm - PubMed
To: antoine.bosquet@lmr.apl
Selection: All results on this page
Format: Summary
MeSH and other data
Send email Cancel

Send citations to clipboard

Selection: All results on this page
Send Cancel

Add to Collections

Selection: All results on this page

- Create a new collection
- Add to an existing collection

Name your collection:
Name must be less than 100 characters
Choose a collection:
Unable to load your collection due to an error
Please try again
Add Cancel

Add to My Bibliography

Selection: All results on this page

- My Bibliography

Unable to load your delegates due to an error
Please try again
Add Cancel

Create a file for external citation management software

Selection: All results on this page
Create file Cancel

Your saved search

Name of saved search: retrospective observation

Search terms: retrospective observational study

Test search terms

Would you like email updates of new search results?

Saved Search Alert Radio Buttons

- Yes
- No

Email: antoine.bosquet@lmr.aphp.fr (change)

Frequency: Monthly

Which day? The first Sunday

Which day? Sunday

Report format: Summary

Send at most: 5 items

Send even when there aren't any new results

Optional text in email:

Save Cancel

Your RSS Feed

Name of RSS Feed: retrospective observation

Number of items displayed: 15

Create RSS Cancel

RSS Link Your RSS Feed Link Copy

My NCBI Filters

- All (1,388)
- Assistance Publique Hopitaux de Paris (0)
- clinical trial (17)
- Review (1)

Show Fewer

Results by year Expand/collapse timeline

Reset

Table representation of search results timeline featuring number of search results per year.

Year	Number of Results
2020	548
2021	893
2022	147

Text availability

- Abstract
- Free full text
- Full text

Article attribute

- Associated data

Article type

- Books and Documents
- Clinical Trial
- Meta-Analysis
- Randomized Controlled Trial
- Review
- Systematic Review

Publication date

- 1 year
- 5 years
- 10 years
- Custom Range

Additional filters Reset all filters

Search Results

clear all

1,388 results

first first previous previous

Page

7

of 7

next next last last

Use COVID-19 filters from PubMed Clinical Queries to refine your search

- Treatment
- Mechanism
- Transmission
- More filters

See more SARS-CoV-2 literature, sequence, and clinical content from NCBI

Results by year Expand/collapse timeline

Reset

Filters applied: . *Clear all* Select search result to email or save

Page 7

☐ 1,201

Observational Study

Diabetes Res Clin Pract

. 2020 Aug;166:108302.

doi: 10.1016/j.diabres.2020.108302. Epub 2020 Jul 3.

School and pre-school children with type 1 diabetes during Covid-19 quarantine: The synergic effect of parental care and technology

Riccardo Schiaffini [1], Fabrizio Barbetti [2], Novella Rapini [3], Elena Inzaghi [3], Annalisa Deodati [3], Ippolita P Patera [3], Maria C Matteoli [3], Paolo Ciampalini [3], Chiara Carducci [3], Antonella Lorubbio [3], Gabriele Schiaffini [4], Stefano Cianfarani [5]

Affiliations Expand

Affiliations

- [1] Dipartimento Pediatrico Universitario Ospedaliero "Bambino Gesù" Children's Hospital - Tor Vergata University, Rome, Italy. Electronic address: riccardo.schiaffini@opbg.net.
- [2] Department of Experimental Medicine, Tor Vergata University, Rome, Italy.
- [3] Dipartimento Pediatrico Universitario Ospedaliero "Bambino Gesù" Children's Hospital - Tor Vergata University, Rome, Italy.
- [4] Department of Translational and Precision Medicine, Sapienza University of Rome, Italy.
- [5] Dipartimento Pediatrico Universitario Ospedaliero "Bambino Gesù" Children's Hospital - Tor Vergata University, Rome, Italy; Department of Women's and Children's Health, Karolinska Institutet and University Hospital, Stockholm, Sweden.

- PMID: **32623034**
- PMCID: PMC7332425
- DOI: 10.1016/j.diabres.2020.108302

Free PMC article
Observational Study

School and pre-school children with type 1 diabetes during Covid-19 quarantine: The synergic effect of parental care and technology

Riccardo Schiaffini et al. Diabetes Res Clin Pract. 2020 Aug.
Free PMC article
Show details
Diabetes Res Clin Pract
. 2020 Aug;166:108302.
doi: 10.1016/j.diabres.2020.108302. Epub 2020 Jul 3.

Authors

Riccardo Schiaffini [1], Fabrizio Barbetti [2], Novella Rapini [3], Elena Inzaghi [3], Annalisa Deodati [3], Ippolita P Patera [3], Maria C Matteoli [3], Paolo Ciampalini [3], Chiara Carducci [3], Antonella Lorubbio [3], Gabriele Schiaffini [4], Stefano Cianfarani [5]

Affiliations

- [1] Dipartimento Pediatrico Universitario Ospedaliero "Bambino Gesù" Children's Hospital - Tor Vergata University, Rome, Italy. Electronic address: riccardo.schiaffini@opbg.net.
- [2] Department of Experimental Medicine, Tor Vergata University, Rome, Italy.
- [3] Dipartimento Pediatrico Universitario Ospedaliero "Bambino Gesù" Children's Hospital - Tor Vergata University, Rome, Italy.
- [4] Department of Translational and Precision Medicine, Sapienza University of Rome, Italy.
- [5] Dipartimento Pediatrico Universitario Ospedaliero "Bambino Gesù" Children's Hospital - Tor Vergata University, Rome, Italy; Department of Women's and Children's Health, Karolinska Institutet and University Hospital, Stockholm, Sweden.

- PMID: **32623034**
- PMCID: PMC7332425
- DOI: 10.1016/j.diabres.2020.108302

Abstract

Introduction: Management of Type 1 Diabetes (T1D) poses numerous challenges, especially for young children and their families. Parental care positively influencesthe outcomesofchildren with T1D, while there are often criticisms in school environment. The COVID-19 pandemic has forced children and parents to spend many hours at home and diabetes care has returned mainly in the hands of parents.

Aim of the study: To evaluate the effectiveness of exclusive return to parental care in pre-school and school children with T1D treated with Tandem Basal IQ system during the COVID-19 pandemic.

Patients and methods: 22 children (M:F = 14:8) with T1D have been evaluated. We compared insulin and CGM data (TIR, TBR and TAR) of two periods: PRE-COV and IN-COV, in which children have transitioned from normal school attendance to the exclusive care of their parents.

Results: During the IN-COV period a significantly ($p < 0.001$) higher median value of TIR (66,41%) was observed as compared to PRE-COV period (61,45%). Patients also showed a statistically significant difference ($p < 0.002$) between the IN-COV period and the PRE-COV period as concerning the TAR metric: respectively 29,86 ± 10,6% vs 34,73 ± 12,8%. The difference between the bolus insulin doses was statistically significant (PRE-COV 5,3 IU/day, IN-COV 7,9 IU/day - $p < 0.05$).

Conclusion: Our observational real-life study confirms the positive effect of parental care in T1D very young children and demonstrates that during the COVID-19 pandemic it was possible to obtain a good glycometabolic compensation despite the significant change in lifestyle.

Copyright © 2020 Elsevier B.V. All rights reserved.

Conflict of interest statement

Declaration of Competing Interest The authors declare that they have no known competing financial interests or personal relationships that could have appeared to influence the work reported in this paper.

- 20 references
- 1 figure

Supplementary info

Publication types, MeSH terms, Substances Expand

Publication types

- Observational Study

MeSH terms

- Adolescent
- Betacoronavirus / isolation & purification*
- COVID-19
- Child
- Child, Preschool
- Coronavirus Infections / complications
- Coronavirus Infections / epidemiology
- Coronavirus Infections / prevention & control*
- Coronavirus Infections / virology

- Diabetes Mellitus, Type 1 / drug therapy*
- Diabetes Mellitus, Type 1 / physiopathology
- Diabetes Mellitus, Type 1 / virology
- Female
- Humans
- Hypoglycemic Agents / therapeutic use*
- Infant
- Infant, Newborn
- Insulin / therapeutic use*
- Italy / epidemiology
- Male
- Pandemics / prevention & control*
- Parents / psychology*
- Pneumonia, Viral / complications
- Pneumonia, Viral / epidemiology
- Pneumonia, Viral / prevention & control*
- Pneumonia, Viral / virology
- Prognosis
- Quarantine / methods*
- Retrospective Studies
- SARS-CoV-2

Substances

- Hypoglycemic Agents
- Insulin

Full text links

ELSEVIER FULL-TEXT ARTICLE Elsevier Science Free PMC article
Proceed to details
Cite
Share
☐ 1,202
Observational Study
AIDS
. 2020 Sep 1;34(11):1696-1697.
doi: 10.1097/QAD.0000000000002608.

Coronavirus disease 2019 in patients with HIV in the province of Araba, Basque Country, Spain

Estibaliz Molina-Iturritza [1 2], Irene San-José-Muñiz [1 2], Maite Ganchegui-Aguirre [1 2], Leire Balerdi-Sarasola [1 2], Zuriñe Ortiz-de-Zárate-Ibarra [1 2], Juan C Gainzarain-Arana [1 2], Joseba Portu-Zapirain [1 2]
Affiliations Expand

Affiliations

- [1] Bioaraba, Infectious Diseases Research Group.
- [2] Internal Medicine Department, Osakidetza Basque Health Service, Araba University Hospital, Vitoria-Gasteiz, Spain.

- PMID: **32769770**
- DOI: 10.1097/QAD.0000000000002608

Observational Study

Coronavirus disease 2019 in patients with HIV in the province of Araba, Basque Country, Spain

Estibaliz Molina-Iturritza et al. AIDS. 2020.
Show details
AIDS
. 2020 Sep 1;34(11):1696-1697.
doi: 10.1097/QAD.0000000000002608.

Authors

Estibaliz Molina-Iturritza [1 2], Irene San-José-Muñiz [1 2], Maite Ganchegui-Aguirre [1 2], Leire Balerdi-Sarasola [1 2], Zuriñe Ortiz-de-Zárate-Ibarra [1 2], Juan C Gainzarain-Arana [1 2], Joseba Portu-Zapirain [1 2]

Affiliations

- [1] Bioaraba, Infectious Diseases Research Group.
- [2] Internal Medicine Department, Osakidetza Basque Health Service, Araba University Hospital, Vitoria-Gasteiz, Spain.

- PMID: **32769770**
- DOI: 10.1097/QAD.0000000000002608

No abstract available

- 1 reference

Supplementary info

Publication types, MeSH terms, Substances, Supplementary concepts Expand

Publication types

- Letter
- Observational Study

MeSH terms

- Adult
- Aged
- Anti-Retroviral Agents / therapeutic use
- Antiviral Agents / therapeutic use
- Betacoronavirus
- CD4 Lymphocyte Count
- COVID-19
- Coinfection
- Coronavirus Infections / drug therapy
- Coronavirus Infections / epidemiology*
- Coronavirus Infections / immunology
- Coronavirus Infections / physiopathology
- Female
- HIV Infections / drug therapy
- HIV Infections / epidemiology*
- HIV Infections / immunology
- Hepatitis C, Chronic / drug therapy
- Hepatitis C, Chronic / epidemiology
- Humans
- Hydroxychloroquine / therapeutic use
- Interferon beta-1b / therapeutic use
- Lopinavir / therapeutic use
- Male
- Middle Aged
- Pandemics
- Pneumonia, Viral / epidemiology*
- Pneumonia, Viral / immunology
- Pneumonia, Viral / physiopathology

- Protective Factors
- Retrospective Studies
- Risk Factors
- SARS-CoV-2
- Severity of Illness Index
- Spain / epidemiology
- Sustained Virologic Response
- Viral Load

Substances

- Anti-Retroviral Agents
- Antiviral Agents
- Interferon beta-1b
- Lopinavir
- Hydroxychloroquine

Supplementary concepts

- COVID-19 drug treatment

Full text links

Wolters Kluwer Wolters Kluwer
Proceed to details
Cite
Share
1,203
Observational Study
Biomedica
. 2020 Oct 30;40(Supl. 2):116-130.
doi: 10.7705/biomedica.5764.

Prognostic factors in hospitalized patients diagnosed with SARS-CoV-2 infection, Bogotá, Colombia

[Article in English, Spanish]
Juan Camilo Motta [1], Danny Julian Novoa [2], Carmen Cecilia Gómez [3], Julian Mauricio Moreno [4], Lina Vargas [5], Jairo Pérez [6], Henry Millán [7], Álvaro Ignacio Arango [8]
Affiliations Expand

Affiliations

- [1] Escuela de Medicina y Ciencias de la Salud, Universidad del Rosario, Bogotá, D.C., Colombia. juankamo19@gmail.com.
- [2] Servicio de Medicina Interna, Fundación Cardioinfantil, Bogotá, D.C., Colombia. dnovoa@cardioinfantil.org.
- [3] Servicio de Medicina Interna, Fundación Cardioinfantil, Bogotá, D.C., Colombia. ccgom@cardioinfantil.org.
- [4] Servicio de Medicina Interna, Fundación Cardioinfantil, Bogotá, D.C., Colombia. julianmoreno88@yahoo.es.
- [5] Servicio de Medicina Interna, Fundación Cardioinfantil, Bogotá, D.C., Colombia. linapccvargas@hotmail.com.
- [6] Servicio de Infectología, Fundación Cardioinfantil, Bogotá, D.C., Colombia. jperezf@cardioinfantil.org.
- [7] Servicio de Infectología, Fundación Cardioinfantil, Bogotá, D.C., Colombia. drhenrymillan@hotmail.com.
- [8] Servicio de Infectología, Fundación Cardioinfantil, Bogotá, D.C., Colombia. aarango@cardioinfantil.org.

- PMID: **33152195**
- PMCID: PMC7676839
- DOI: 10.7705/biomedica.5764

Free PMC article
Observational Study

Prognostic factors in hospitalized patients diagnosed with SARS-CoV-2 infection, Bogotá, Colombia

[Article in English, Spanish]
Juan Camilo Motta et al. Biomedica. 2020.
Free PMC article
Show details
Biomedica

. 2020 Oct 30;40(Supl. 2):116-130.
doi: 10.7705/biomedica.5764.

Authors

Juan Camilo Motta [1], Danny Julian Novoa [2], Carmen Cecilia Gómez [3], Julian Mauricio Moreno [4], Lina Vargas [5], Jairo Pérez [6], Henry Millán [7], Álvaro Ignacio Arango [8]

Affiliations

- [1] Escuela de Medicina y Ciencias de la Salud, Universidad del Rosario, Bogotá, D.C., Colombia. juankamo19@gmail.com.

- [2] Servicio de Medicina Interna, Fundación Cardioinfantil, Bogotá, D.C., Colombia. dnovoa@cardioinfantil.org.
- [3] Servicio de Medicina Interna, Fundación Cardioinfantil, Bogotá, D.C., Colombia. ccgom@cardioinfantil.org.
- [4] Servicio de Medicina Interna, Fundación Cardioinfantil, Bogotá, D.C., Colombia. julianmoreno88@yahoo.es.
- [5] Servicio de Medicina Interna, Fundación Cardioinfantil, Bogotá, D.C., Colombia. linapccvargas@hotmail.com.
- [6] Servicio de Infectología, Fundación Cardioinfantil, Bogotá, D.C., Colombia. jperezf@cardioinfantil.org.
- [7] Servicio de Infectología, Fundación Cardioinfantil, Bogotá, D.C., Colombia. drhenrymillan@hotmail.com.
- [8] Servicio de Infectología, Fundación Cardioinfantil, Bogotá, D.C., Colombia. aarango@cardioinfantil.org.

- PMID: **33152195**
- PMCID: PMC7676839
- DOI: 10.7705/biomedica.5764

Abstract
in English, Spanish

Introduction: Infection with the new SARS-Cov-2 coronavirus is a worldwide public health emergency; its diagnosis is based on molecular tests, while its prognosis depends on the patient's history and on some paraclinical tests. In Colombia, forecasts are not yet counted. Objective: To assess the factors associated with the development of severe disease in hospitalized patients diagnosed with SARS-CoV-2 infection, as well as the prognostic factors for the outcome of mortality. Materials and methods: We conducted an ambispective cohort study in hospitalized patients at the Fundación Cadioinfantil from March to June, 2020. Results: Of the 104 patients analyzed, 31.7% (n=33) had a severe presentation and 9.6% (n=10) had a mortality outcome. For mortality, the most important prognostic factor was the development of severe disease followed by age over 60 years and malnutrition. For the development of the severe disease, prognostic factors were a history of hemodialysis (HR=135), diabetes (HR=4.4), and an increased level of lactate dehydrogenase (LDH) (HR=1,004), while the lymphocyte count over 1,064 was a protective factor (HR=0.9). In the classification of patients, the National Early Warning Score (NEWS2) score in the high and low-risk categories corresponded to the best performance. There was no difference between the treatments administered. Conclusions: The most important prognostic factors for mortality were being over 60 years of age, hypertension, diabetes, and cirrhosis, while for the development of severe disease they were chronic kidney disease with hemodialysis, NEWS2 with high risk at admission, increased levels of LDH and C reactive protein (CRP), and leukocytosis.

Introducción. La infección por el nuevo coronavirus SARS-Cov-2 es una emergencia de salud pública en todo el mundo; su diagnóstico se basa en pruebas moleculares, en tanto que su pronóstico depende de los antecedentes del paciente y de algunos exámenes paraclínicos. En Colombia aún no se cuenta con datos de pronóstico en una población local. Objetivo. Evaluar los factores asociados con el desarrollo de la enfermedad grave en pacientes hospitalizados con diagnóstico de infección por SARS-CoV-2, así como los factores pronósticos de la mortalidad. Materiales y métodos. Se hizo un estudio de cohorte ambispectivo en pacientes hospitalizados en la Fundación Cardioinfantil entre marzo y junio de 2020. Resultados. De los 104 pacientes analizados, en el 31,7 % (n=33) la infección fue grave y en el 9,6 % (n=10) se produjo la muerte.

El factor pronóstico más importante de la mortalidad fue el desarrollo de la enfermedad grave, seguido de una edad de más de 60 años y la desnutrición. Para el desarrollo de la enfermedad grave los factores pronósticos fueron los antecedentes de hemodiálisis (hazard ratio, HR=135), diabetes (HR=4,4) y el aumento en el nivel de la lactato deshidrogenasa (LDH) (HR=1,004), en tanto que un conteo de linfocitos superior a 1.064 fue un factor protector (HR=0,9). El puntaje del National Early Warning Score (NEWS2) correspondiente a las categorías de alto y bajo riesgo fue el que mejor rendimiento tuvo. No hubo diferencia entre los tratamientos administrados. Conclusiones. Los factores pronósticos más importantes para la mortalidad fueron tener más de 60 años, hipertensión, diabetes y cirrosis, en tanto que para el desarrollo de la enfermedad grave fueron la enfermedad renal crónica con hemodiálisis, un puntaje de NEWS2 de alto riesgo al ingreso, y aumento en los niveles de LDH y proteína C reactiva, y leucocitosis.

Keywords: Coronavirus infections; severe acute respiratory syndrome; mortality; prognosis; inpatients.

Comment in

- Letter to the editor "Prognostic factors in hospitalized patients diagnosed with SARS-CoV-2 infection, Bogotá, Colombia".
  Ramos-Vera CA, Motta JC. Ramos-Vera CA, et al. Biomedica. 2021 Jun 29;41(2):374-377. Biomedica. 2021. PMID: 34214276 Free PMC article. English, Spanish. No abstract available.

- 31 references
- 4 figures

Supplementary info

Publication types, MeSH terms, Substances Expand

Publication types

- Observational Study

MeSH terms

- Adult
- Aged
- Betacoronavirus*
- Blood Group Antigens
- Body Mass Index
- COVID-19
- Cardiovascular Diseases / epidemiology
- Colombia / epidemiology
- Comorbidity
- Coronavirus Infections / blood
- Coronavirus Infections / diagnostic imaging

- Coronavirus Infections / mortality*
- Coronavirus Infections / therapy
- Diabetes Mellitus / epidemiology
- Female
- Hospital Mortality
- Humans
- Inpatients / statistics & numerical data
- Kaplan-Meier Estimate
- Male
- Middle Aged
- Pandemics*
- Pneumonia, Viral / blood
- Pneumonia, Viral / diagnostic imaging
- Pneumonia, Viral / mortality*
- Pneumonia, Viral / therapy
- Prognosis
- Proportional Hazards Models
- Prospective Studies
- Renal Insufficiency, Chronic / epidemiology
- Respiratory Distress Syndrome / etiology
- Respiratory Distress Syndrome / mortality
- Retrospective Studies
- Risk Factors
- SARS-CoV-2
- Smoking / epidemiology

Substances

- Blood Group Antigens

Full text links

Full text article at revistabiomedica.org Colombian National Health Institute Free PMC article

Proceed to details

Cite

Share

☐ 1,204

Observational Study

J Fr Ophtalmol

. 2021 Mar;44(3):307-312.
doi: 10.1016/j.jfo.2020.12.002. Epub 2021 Feb 10.

An algorithm in ophthalmic emergencies to evaluate the necessity of physical consultation during COVID-19 lockdown in Paris: Experience of the first 100 patients

H Bourdon [1], A Herbaut [2], L Trinh [2], E Tuil [3], J F Girmens [3], C Baudouin [2]
Affiliations Expand

Affiliations

- [1] Department of Ophthalmology III, CHNO des Quinze-Vingts, IHU FOReSIGHT, 28, rue de Charenton, 75012 Paris, France. Electronic address: bourdonhugo@gmail.com.
- [2] Department of Ophthalmology III, CHNO des Quinze-Vingts, IHU FOReSIGHT, 28, rue de Charenton, 75012 Paris, France.
- [3] Department of Ophthalmology IV, CHNO des Quinze-Vingts, IHU FOReSIGHT, 28, rue de Charenton, 75012 Paris, France.

- PMID: **33612327**
- PMCID: PMC7874947
- DOI: 10.1016/j.jfo.2020.12.002

Free PMC article
Observational Study

An algorithm in ophthalmic emergencies to evaluate the necessity of physical consultation during COVID-19 lockdown in Paris: Experience of the first 100 patients

H Bourdon et al. J Fr Ophtalmol. 2021 Mar.
Free PMC article
Show details
J Fr Ophtalmol
. 2021 Mar;44(3):307-312.
doi: 10.1016/j.jfo.2020.12.002. Epub 2021 Feb 10.

Authors

H Bourdon [1], A Herbaut [2], L Trinh [2], E Tuil [3], J F Girmens [3], C Baudouin [2]

Affiliations

- [1] Department of Ophthalmology III, CHNO des Quinze-Vingts, IHU FOReSIGHT, 28, rue de Charenton, 75012 Paris, France. Electronic address: bourdonhugo@gmail.com.
- [2] Department of Ophthalmology III, CHNO des Quinze-Vingts, IHU FOReSIGHT, 28, rue de Charenton, 75012 Paris, France.
- [3] Department of Ophthalmology IV, CHNO des Quinze-Vingts, IHU FOReSIGHT, 28, rue de Charenton, 75012 Paris, France.

- PMID: **33612327**
- PMCID: PMC7874947
- DOI: 10.1016/j.jfo.2020.12.002

Abstract
in English, French

Purpose: This study aimed to evaluate the ability of a freely accessible internet algorithm to correctly identify the need for emergency ophthalmologic consultation for correct diagnosis and management.

Method: This retrospective observational cohort study was based on the first 100 patients who requested recommendations on the necessity of breaking the lockdown for emergency ophthalmology consultation during the period from March to May 2020.

Results: Ninety-one patients completed questionnaires. Forty-nine were directed to emergency consultation and 42 to differed scheduled visits or telemedicine visits. One patient sent for emergency consultation had an overestimated severity and could have been seen later, while two patients initially recommended for a scheduled visit were considered appropriate for emergency consultation. However, these patients' management did not suffer as a consequence of the delay. The sensitivity of the algorithm, defined as the number of emergency consultations suggested by the algorithm divided by the total number of emergency consultations deemed appropriate by the practitioner's final evaluation, was 96.0%. The specificity of the algorithm, defined as the number of patients recommended for delayed consultation by the algorithm divided by the number of patients deemed clinically appropriate for this approach, was 97.5%. The positive predictive value, defined as the number of appropriate emergency consultations divided by the total number of emergency consultations suggested by the algorithm, was 97.9%. Finally, the negative predictive value, defined as the number of appropriately deferred patients divided by the number of deferred patients recommended by the algorithm, was 95.2%.

Conclusion: This study demonstrates the reliability of an algorithm based on patients' past medical history and symptoms to classify patients and direct them to either emergency consultation or to a more appropriate deferred, scheduled appointment. This algorithm might allow reduction of walk-in visits by half and thus help control patient flow into ophthalmologic emergency departments.

Objectif: L'objectif de cette étude était d'évaluer la capacité d'un algorithme en libre accès sur internet à indiquer correctement la nécessité d'une consultation ophtalmologique en urgence pour une prise en charge et un traitement approprié.

Méthode: Il s'agit d'une étude observationnelle rétrospective reprenant les 100 premiers questionnaires patients évaluant la nécessité d'une consultation en service d'urgence ophtalmologiques durant le confinement de mars à mai 2020.

Résultats: Au total, 91 patients ont rempli les questionnaire complètements. Quarante-neuf ont été orientés vers une consultation immédiate et 42 vers une consultation programmée ou une téléconsultation. Un patient, orienté aux urgences, avait une gravité surestimée et aurait pu être orienté en consultation différée et deux patients, orientés en consultation différée, relevaient d'une consultation d'urgence. Cependant, aucune perte de chance n'a été identifiée durant la prise en charge. La sensibilité de l'algorithme, définie comme le nombre de consultations en urgence recommandées par l'algorithme parmi les consultations en urgence appropriées dans l'évaluation finale était de 96,0 %. La spécificité, définie comme le nombre de patients orientés par l'algorithme en consultation différée, parmi les patients requérant en effet de cette prise en charge, était de 97,5 %. La valeur prédictive positive, définie comme le nombre de consultation en urgences appropriées parmi le nombre de consultations en urgence recommandées par l'algorithme était de 97,9 %. Finalement, la valeur prédictive négative, définie comme le nombre de consultations différées appropriées parmi le nombre de consultation différées recommandées par l'algorithme était de 95,2 %.

Conclusion: Cette étude montre la fiabilité d'un algorithme basé sur les antécédents médicaux du patient, son histoire clinique et ses symptômes afin de classer et orienter les patients vers une consultation aux urgences ou programmée. Cet algorithme permet de réduire de moitié les consultations non programmées et ainsi réguler la fréquentation des services d'urgences ophtalmologiques.

Keywords: COVID-19; Confinement; Distanciation sociale; Emergency ophthalmology; Lockdown; Ophtalmologie d'urgence; Social distancing; Teleophthalmology; Téléophtalmologie.

Copyright © 2021 Elsevier Masson SAS. All rights reserved.

- 14 references
- 1 figure

Supplementary info

Publication types, MeSH terms Expand

Publication types

- Observational Study

MeSH terms

- Adult
- Aged
- Aged, 80 and over
- Algorithms*
- Appointments and Schedules*
- COVID-19 / epidemiology*
- Cohort Studies
- Communicable Disease Control / standards
- Emergencies* / epidemiology

- Emergency Medical Services / organization & administration
- Emergency Service, Hospital / organization & administration
- Emergency Service, Hospital / standards
- Eye Diseases / epidemiology
- Eye Diseases / therapy*
- Female
- Humans
- Male
- Middle Aged
- Ophthalmology / organization & administration*
- Paris / epidemiology
- Quarantine*
- Referral and Consultation / organization & administration
- Referral and Consultation / standards
- Reproducibility of Results
- Retrospective Studies
- Surveys and Questionnaires
- Telemedicine / organization & administration
- Telemedicine / standards
- Young Adult

Full text links

ELSEVIER FULL-TEXT ARTICLE Elsevier Science Free PMC article

Proceed to details

Cite

Share

☐ 1,205

Observational Study

Kidney Int

. 2020 Jul;98(1):27-34.

doi: 10.1016/j.kint.2020.04.031. Epub 2020 May 11.

COVID-19: clinical course and outcomes of 36 hemodialysis patients in Spain

Marian Goicoechea [1], Luis Alberto Sánchez Cámara [2], Nicolás Macías [3], Alejandra Muñoz de Morales [3], Ángela González Rojas [3], Arturo Bascuñana [3], David Arroyo [3], Almudena Vega [3], Soraya Abad [3], Eduardo Verde [3], Ana María García Prieto [3], Úrsula Verdalles [3], Diego Barbieri [3], Andrés Felipe Delgado [3], Javier Carbayo [3], Antonia Mijaylova [3], Adriana Acosta [3], Rosa Melero [3], Alberto Tejedor [3], Patrocinio Rodriguez Benitez [3], Ana Pérez de José [3], María

Luisa Rodriguez Ferrero [3], Fernando Anaya [3], Manuel Rengel [3], Daniel Barraca [3], José Luño [3], Inés Aragoncillo [4]
Affiliations Expand

Affiliations

- [1] Department of Nephrology, Hospital General Universitario Gregorio Marañón, Madrid, Spain; Red de Investigación Renal Instituto de Salud Carlos III (ISCIII) Red temática de investigación cooperativa en salud (RETIC) Red de Investigación Renal (REDINREN) RD016/009 (FEDER funds), Madrid, Spain. Electronic address: marian.goicoechea@gmail.com.
- [2] Department of Nephrology, Instituto de Investigación Sanitaria Gregorio Marañón, Madrid, Spain.
- [3] Department of Nephrology, Hospital General Universitario Gregorio Marañón, Madrid, Spain.
- [4] Red de Investigación Renal Instituto de Salud Carlos III (ISCIII) Red temática de investigación cooperativa en salud (RETIC) Red de Investigación Renal (REDINREN) RD016/009 (FEDER funds), Madrid, Spain; Department of Nephrology, Instituto de Investigación Sanitaria Gregorio Marañón, Madrid, Spain.

- PMID: **32437770**
- PMCID: PMC7211728
- DOI: 10.1016/j.kint.2020.04.031

Free PMC article
Observational Study

COVID-19: clinical course and outcomes of 36 hemodialysis patients in Spain

Marian Goicoechea et al. Kidney Int. 2020 Jul.
Free PMC article
Show details
Kidney Int
. 2020 Jul;98(1):27-34.
doi: 10.1016/j.kint.2020.04.031. Epub 2020 May 11.

Authors

Marian Goicoechea [1], Luis Alberto Sánchez Cámara [2], Nicolás Macías [3], Alejandra Muñoz de Morales [3], Ángela González Rojas [3], Arturo Bascuñana [3], David Arroyo [3], Almudena Vega [3], Soraya Abad [3], Eduardo Verde [3], Ana María García Prieto [3], Úrsula Verdalles [3], Diego Barbieri [3], Andrés Felipe Delgado [3], Javier Carbayo [3], Antonia Mijaylova [3], Adriana Acosta [3], Rosa Melero [3], Alberto Tejedor [3], Patrocinio Rodriguez Benitez [3], Ana Pérez de José [3], María Luisa Rodriguez Ferrero [3], Fernando Anaya [3], Manuel Rengel [3], Daniel Barraca [3], José Luño [3], Inés Aragoncillo [4]

Affiliations

- [1] Department of Nephrology, Hospital General Universitario Gregorio Marañón, Madrid, Spain; Red de Investigación Renal Instituto de Salud Carlos III (ISCIII) Red temática de investigación cooperativa en salud (RETIC) Red de Investigación Renal (REDINREN) RD016/009 (FEDER funds), Madrid, Spain. Electronic address: marian.goicoechea@gmail.com.
- [2] Department of Nephrology, Instituto de Investigación Sanitaria Gregorio Marañón, Madrid, Spain.
- [3] Department of Nephrology, Hospital General Universitario Gregorio Marañón, Madrid, Spain.
- [4] Red de Investigación Renal Instituto de Salud Carlos III (ISCIII) Red temática de investigación cooperativa en salud (RETIC) Red de Investigación Renal (REDINREN) RD016/009 (FEDER funds), Madrid, Spain; Department of Nephrology, Instituto de Investigación Sanitaria Gregorio Marañón, Madrid, Spain.

- PMID: **32437770**
- PMCID: PMC7211728
- DOI: 10.1016/j.kint.2020.04.031

Abstract

Severe acute respiratory syndrome coronavirus 2 (SARS-CoV-2) pneumonia emerged in Wuhan, China in December 2019. Unfortunately, there is a lack of evidence about the optimal management of novel coronavirus disease 2019 (COVID-19), and even less is available in patients on maintenance hemodialysis therapy than in the general population. In this retrospective, observational, single-center study, we analyzed the clinical course and outcomes of all maintenance hemodialysis patients hospitalized with COVID-19 from March 12th to April 10th, 2020 as confirmed by real-time polymerase chain reaction. Baseline features, clinical course, laboratory data, and different therapies were compared between survivors and nonsurvivors to identify risk factors associated with mortality. Among the 36 patients, 11 (30.5%) died, and 7 were able to be discharged within the observation period. Clinical and radiological evolution during the first week of admission were predictive of mortality. Among the 36 patients, 18 had worsening of their clinical status, as defined by severe hypoxia with oxygen therapy requirements greater than 4 L/min and radiological worsening. Significantly, 11 of those 18 patients (61.1%) died. None of the classical cardiovascular risk factors in the general population were associated with higher mortality. Compared to survivors, nonsurvivors had significantly longer dialysis vintage, increased lactate dehydrogenase (490 U/l ± 120 U/l vs. 281 U/l ± 151 U/l, P = 0.008) and C-reactive protein levels (18.3 mg/dl ± 13.7 mg/dl vs. 8.1 mg/dl ± 8.1 mg/dl, P = 0.021), and a lower lymphocyte count ($0.38 \times 10^3/\mu l \pm 0.14 \times 10^3/\mu l$ vs. $0.76 \times 10^3/\mu l \pm 0.48 \times 10^3/\mu l$, P = 0.04) 1 week after clinical onset. Thus, the mortality among hospitalized hemodialysis patients diagnosed with COVID-19 is high. Certain laboratory tests can be used to predict a worsening clinical course.

Keywords: COVID-19; SARS-CoV-2; coronavirus; hemodialysis; mortality.

Copyright © 2020 International Society of Nephrology. Published by Elsevier Inc. All rights reserved.

- 24 references
- 2 figures

Supplementary info

Publication types, MeSH terms, Substances Expand

Publication types

- Observational Study

MeSH terms

- Adult
- Aged
- Aged, 80 and over
- Anti-Bacterial Agents / therapeutic use
- Antimalarials / therapeutic use
- Azithromycin / therapeutic use
- COVID-19
- Coronavirus Infections / complications
- Coronavirus Infections / diagnosis
- Coronavirus Infections / drug therapy
- Coronavirus Infections / mortality*
- Drug Combinations
- Female
- Hospital Mortality
- Humans
- Hydroxychloroquine / therapeutic use
- Kidney Failure, Chronic / complications*
- Kidney Failure, Chronic / therapy
- Lopinavir / therapeutic use
- Male
- Middle Aged
- Pandemics
- Pneumonia, Viral / complications
- Pneumonia, Viral / diagnosis
- Pneumonia, Viral / drug therapy
- Pneumonia, Viral / mortality*
- Prognosis
- Renal Dialysis
- Retrospective Studies
- Ritonavir / therapeutic use
- Spain / epidemiology

Substances

- Anti-Bacterial Agents
- Antimalarials
- Drug Combinations
- lopinavir-ritonavir drug combination
- Lopinavir
- Hydroxychloroquine
- Azithromycin
- Ritonavir

Full text links

ELSEVIER FULL-TEXT ARTICLE Elsevier Science Free PMC article
Proceed to details
Cite
Share
☐ 1,206
Observational Study
J Am Coll Radiol
. 2020 Aug;17(8):1011-1013.
doi: 10.1016/j.jacr.2020.06.002. Epub 2020 Jun 28.

Acute Appendicitis During Coronavirus Disease 2019 (COVID-19): Changes in Clinical Presentation and CT Findings

Javier Romero [1], Sergio Valencia [2], Andres Guerrero [1]
Affiliations Expand

Affiliations

- [1] Fundación Santa Fe de Bogotá, Bogotá, Colombia.
- [2] Fundación Santa Fe de Bogotá, Bogotá, Colombia. Electronic address: sevava92@gmail.com.

- PMID: **32610104**
- PMCID: PMC7321660
- DOI: 10.1016/j.jacr.2020.06.002

Free PMC article
Observational Study

Acute Appendicitis During Coronavirus Disease 2019 (COVID-19): Changes in Clinical Presentation and CT Findings

Javier Romero et al. J Am Coll Radiol. 2020 Aug.
Free PMC article
Show details
J Am Coll Radiol
. 2020 Aug;17(8):1011-1013.
doi: 10.1016/j.jacr.2020.06.002. Epub 2020 Jun 28.

Authors

Javier Romero [1], Sergio Valencia [2], Andres Guerrero [1]

Affiliations

- [1] Fundación Santa Fe de Bogotá, Bogotá, Colombia.
- [2] Fundación Santa Fe de Bogotá, Bogotá, Colombia. Electronic address: sevava92@gmail.com.

- PMID: **32610104**
- PMCID: PMC7321660
- DOI: 10.1016/j.jacr.2020.06.002

Erratum in

- Erratum.
  [No authors listed] [No authors listed] J Am Coll Radiol. 2021 Nov;18(11):1473. doi: 10.1016/j.jacr.2021.09.007. Epub 2021 Sep 22. J Am Coll Radiol. 2021. PMID: 34562413 Free PMC article. No abstract available.

Abstract

Background: Quarantine and stay-at-home orders are strategies that many countries used during the acute pandemic period of coronavirus disease 2019 (COVID-19) to prevent disease dissemination, health system overload, and mortality. However, there are concerns that patients did not seek necessary health care because of these mandates.

Purpose: To evaluate the differences in the clinical presentation of acute appendicitis and CT findings related to these cases between the COVID-19 acute pandemic period and nonpandemic period.

Materials and methods: A retrospective observational study was performed to compare the acute pandemic period (March 23, 2020, to May 4, 2020) versus the same period the year before (March 23, 2019, to May 4, 2019). The proportion of appendicitis diagnosed by CT and level of severity of the disease were reviewed in each case. Univariate and bivariate analyses were performed to identify significant differences between the two groups.

Results: A total of 196 abdominal CT scans performed due to suspected acute appendicitis were evaluated: 55 from the acute pandemic period and 141 from the nonpandemic period. The proportion of acute appendicitis diagnosed by abdominal CT was higher in the acute pandemic period versus the nonpandemic period: 45.5% versus 29.8% (P = .038). The severity of the diagnosed appendicitis was higher during the acute pandemic period: 92% versus 57.1% (P = .003).

Conclusion: During the acute COVID-19 pandemic period, fewer patients presented with acute appendicitis to the emergency room, and those who did presented at a more severe stage of the disease.

Keywords: Abdominal CT; COVID-19; SARS-CoV-2; acute appendicitis; nonquarantine; quarantine.

Copyright © 2020 American College of Radiology. Published by Elsevier Inc. All rights reserved.

Comment in

- Handle With Care: Use of Proportions to Assess Changes in Acute Appendicitis During the 2020 COVID-19 "Surge".
  Neufeld MY, Sanchez SE, Drake FT. Neufeld MY, et al. J Am Coll Radiol. 2021 Jul;18 (7):893-894. doi: 10.1016/j.jacr.2021.02.027. Epub 2021 Mar 23. J Am Coll Radiol. 2021. PMID: 33775654 Free PMC article. No abstract available.

- 7 references
- 2 figures

Supplementary info

Publication types, MeSH terms Expand

Publication types

- Comparative Study
- Observational Study

MeSH terms

- Analysis of Variance
- Appendicitis / diagnostic imaging*
- Appendicitis / epidemiology*
- COVID-19
- Cohort Studies
- Coronavirus Infections / epidemiology
- Coronavirus Infections / prevention & control*
- Emergency Service, Hospital / statistics & numerical data
- Female
- Humans

- Incidence
- Infection Control / organization & administration*
- Male
- Multivariate Analysis
- Pandemics / prevention & control*
- Pandemics / statistics & numerical data
- Pneumonia, Viral / epidemiology
- Pneumonia, Viral / prevention & control*
- Quarantine / statistics & numerical data
- Retrospective Studies
- Risk Assessment
- Tomography, X-Ray Computed / methods
- Tomography, X-Ray Computed / statistics & numerical data*
- United States

Full text links

ELSEVIER FULL-TEXT ARTICLE Elsevier Science Free PMC article

Proceed to details

Cite

Share

1,207

Observational Study

Infect Dis Poverty

. 2020 Aug 3;9(1):108.

doi: 10.1186/s40249-020-00723-1.

Abnormal immunity of non-survivors with COVID-19: predictors for mortality

Yang Zhao [1], Han-Xiang Nie [2], Ke Hu [2], Xiao-Jun Wu [2], Yun-Ting Zhang [2], Meng-Mei Wang [2], Tao Wang [2], Zhi-Shui Zheng [2], Xiao-Chen Li [2], Shao-Lin Zeng [2]

Affiliations Expand

Affiliations

- [1] Department of Respiratory Medicine, Renmin Hospital of Wuhan University, 238 Jiefang Road, Wuchang District, Wuhan, 430060, China. zhaoyangrm@whu.edu.cn.
- [2] Department of Respiratory Medicine, Renmin Hospital of Wuhan University, 238 Jiefang Road, Wuchang District, Wuhan, 430060, China.

- PMID: **32746940**
- PMCID: PMC7396941
- DOI: 10.1186/s40249-020-00723-1

Free PMC article
Observational Study

Abnormal immunity of non-survivors with COVID-19: predictors for mortality

Yang Zhao et al. Infect Dis Poverty. 2020.
Free PMC article
Show details
Infect Dis Poverty
. 2020 Aug 3;9(1):108.
doi: 10.1186/s40249-020-00723-1.

Authors

Yang Zhao [1], Han-Xiang Nie [2], Ke Hu [2], Xiao-Jun Wu [2], Yun-Ting Zhang [2], Meng-Mei Wang [2], Tao Wang [2], Zhi-Shui Zheng [2], Xiao-Chen Li [2], Shao-Lin Zeng [2]

Affiliations

- [1] Department of Respiratory Medicine, Renmin Hospital of Wuhan University, 238 Jiefang Road, Wuchang District, Wuhan, 430060, China. zhaoyangrm@whu.edu.cn.
- [2] Department of Respiratory Medicine, Renmin Hospital of Wuhan University, 238 Jiefang Road, Wuchang District, Wuhan, 430060, China.

- PMID: **32746940**
- PMCID: PMC7396941
- DOI: 10.1186/s40249-020-00723-1

Abstract

Background: The number of coronavirus disease 2019 (COVID-19) cases has rapidly increased all over the world. Specific information about immunity in non-survivors with COVID-19 is scarce. This study aimed to analyse the clinical characteristics and abnormal immunity of the confirmed COVID-19 non-survivors.

Methods: In this single-centered, retrospective, observational study, we enrolled 125 patients with COVID-19 who were died between January 13 and March 4, 2020 in Renmin Hospital of Wuhan University. A total of 414 randomly recruited patients with confirmed COVID-19 who were discharged from the same hospital during the same period served as control. The demographic, clinical characteristics and laboratory findings at admission, and treatment used in these patients were collected. The immunity-related risk factors associated with in-hospital death were tested by logistic regression models and Receiver Operating Characteristic (ROC) curve.

Results: Non-survivors (70 years, IQR: 61.5-80) were significantly older than survivors (54 years, IQR: 37-65) ($P < 0.001$). 56.8% of non-survivors was male. Nearly half of the patients (44.9%) had chronic medical illness. In non-survivors, hypertension (49.6%) was the most common comorbidity, followed by diabetes (20.0%) and coronary heart disease (16.0%). The common signs and symptoms at admission of non-survivors were fever (88%), followed by cough (64.8%),

dyspnea (62.4%), fatigue (62.4%) and chest tightness (58.4%). Compared with survivors, non-survivors had higher white blood cell (WBC) count (7.85 vs 5.07 × 10^9/L), more elevated neutrophil count (6.41 vs 3.08 × 10^9/L), smaller lymphocyte count (0.69 vs 1.20 × 10^9/L) and lower platelet count (172 vs 211 × 10^9/L), raised concentrations of procalcitonin (0.21 vs 0.06 ng/mL) and CRP (70.5 vs 7.2 mg/L) ($P < 0.001$). This was accompanied with significantly decreased levels of $CD3^+$ T cells (277 vs 814 cells/µl), $CD4^+$ T cells (172 vs 473 cells/µl), $CD8^+$ T cells (84 vs 262.5 cells/µl, $P < 0.001$), $CD19^+$ T cells (88 vs 141 cells/µl) and $CD16^+$ 56^+ T cells (79 vs 128.5 cells/µl) ($P < 0.001$). The concentrations of immunoglobulins (Ig) G (13.30 vs 11.95 g/L), IgA (2.54 vs 2.21 g/L), and IgE (71.30 vs 42.25 IU/ml) were increased, whereas the levels of complement proteins (C)3 (0.89 vs 0.99 g/L) and C4 (0.22 vs 0.24 g/L) were decreased in non-survivors when compared with survivors (all $P < 0.05$). The non-survivors presented lower levels of oximetry saturation (90 vs 97%) at rest and lactate (2.40 vs 1.90 mmol/L) ($P < 0.001$). Old age, comorbidity of malignant tumor, neutrophilia, lymphocytopenia, low $CD4^+$ T cells, decreased C3, and low oximetry saturation were the risk factors of death in patients with confirmed COVID-19. The frequency of $CD4^+$ T cells positively correlated with the numbers of lymphocytes ($r = 0.787$) and the level of oximetry saturation ($r = 0.295$), Whereas $CD4^+$ T cells were negatively correlated with age ($r =-0.323$) and the numbers of neutrophils ($r = - 0.244$) (all $P < 0.001$).

Conclusions: Abnormal cellular immunity and humoral immunity were key features of non-survivors with COVID-19. Neutrophilia, lymphocytopenia, low $CD4^+$ T cells, and decreased C3 were immunity-related risk factors predicting mortality of patients with COVID-19.

Keywords: COVID-19; Cellular immunity; Humoral immunity; Mortality.

Conflict of interest statement

No conflicts of interest are declared by the authors.

- 43 references
- 2 figures

Supplementary info

Publication types, MeSH terms, Grant support Expand

Publication types

- Observational Study

MeSH terms

- Adolescent
- Adult
- Aged
- Aged, 80 and over
- Betacoronavirus / isolation & purification
- CD4-Positive T-Lymphocytes / immunology
- CD8-Positive T-Lymphocytes / immunology

- COVID-19
- China / epidemiology
- Coronavirus Infections / blood
- Coronavirus Infections / epidemiology
- Coronavirus Infections / immunology*
- Coronavirus Infections / mortality*
- Female
- Humans
- Leukocyte Count
- Logistic Models
- Male
- Middle Aged
- Neutrophils / immunology
- Pandemics
- Pneumonia, Viral / blood
- Pneumonia, Viral / epidemiology
- Pneumonia, Viral / immunology*
- Pneumonia, Viral / mortality*
- ROC Curve
- Retrospective Studies
- Risk Factors
- SARS-CoV-2
- Young Adult

Grant support

- 81500022/National Natural Science Foundation of China
- 2020FCA002/Natural Science Foundation of Hubei Province

Full text links

Read free full text at BMC BioMed Central Free PMC article

Proceed to details

Cite

Share

☐ 1,208

Observational Study

Kidney Int

. 2020 Jul;98(1):209-218.

doi: 10.1016/j.kint.2020.05.006. Epub 2020 May 16.

Acute kidney injury in patients hospitalized with COVID-19

Jamie S Hirsch [1], Jia H Ng [2], Daniel W Ross [2], Purva Sharma [2], Hitesh H Shah [2], Richard L Barnett [2], Azzour D Hazzan [2], Steven Fishbane [2], Kenar D Jhaveri [3], Northwell COVID-19 Research Consortium; Northwell Nephrology COVID-19 Research Consortium

Collaborators, Affiliations Expand

Collaborators

- Mersema Abate, Hugo Paz Andrade, Richard L Barnett, Alessandro Bellucci, Madhu C Bhaskaran, Antonio G Corona, Bessy Flores Chang, Mark Finger, Steven Fishbane, Michael Gitman, Candice Halinski, Shamir Hasan, Azzour D Hazzan, Jamie S Hirsch, Susana Hong, Kenar D Jhaveri, Yuriy Khanin, Aireen Kuan, Varun Madireddy, Deepa Malieckal, Abdulrahman Muzib, Gayatri Nair, Vinay V Nair, Jia H Ng, Rushang Parikh, Daniel W Ross, Vipulbhai Sakhiya, Mala Sachdeva, Richard Schwarz, Hitesh H Shah, Purva Sharma, Pravin C Singhal, Nupur N Uppal, Rimda Wanchoo, Bessy Suyin Flores Chang, Jia Hwei Ng

Affiliations

- [1] Division of Kidney Diseases and Hypertension, Department of Medicine, Donald and Barbara Zucker School of Medicine at Hofstra/Northwell, Great Neck, New York, USA; Institute of Health Innovations and Outcomes Research, Feinstein Institutes for Medical Research, Manhasset, New York, USA; Department of Information Services, Northwell Health, New Hyde Park, New York, USA.
- [2] Division of Kidney Diseases and Hypertension, Department of Medicine, Donald and Barbara Zucker School of Medicine at Hofstra/Northwell, Great Neck, New York, USA.
- [3] Division of Kidney Diseases and Hypertension, Department of Medicine, Donald and Barbara Zucker School of Medicine at Hofstra/Northwell, Great Neck, New York, USA. Electronic address: kjhaveri@northwell.edu.

- PMID: **32416116**
- PMCID: PMC7229463
- DOI: 10.1016/j.kint.2020.05.006

Free PMC article
Observational Study

Acute kidney injury in patients hospitalized with COVID-19

Jamie S Hirsch et al. Kidney Int. 2020 Jul.
Free PMC article

Show details

Kidney Int

. 2020 Jul;98(1):209-218.
doi: 10.1016/j.kint.2020.05.006. Epub 2020 May 16.

Authors

Jamie S Hirsch [1], Jia H Ng [2], Daniel W Ross [2], Purva Sharma [2], Hitesh H Shah [2], Richard L Barnett [2], Azzour D Hazzan [2], Steven Fishbane [2], Kenar D Jhaveri [3], Northwell COVID-19 Research Consortium; Northwell Nephrology COVID-19 Research Consortium

Collaborators

- Mersema Abate, Hugo Paz Andrade, Richard L Barnett, Alessandro Bellucci, Madhu C Bhaskaran, Antonio G Corona, Bessy Flores Chang, Mark Finger, Steven Fishbane, Michael Gitman, Candice Halinski, Shamir Hasan, Azzour D Hazzan, Jamie S Hirsch, Susana Hong, Kenar D Jhaveri, Yuriy Khanin, Aireen Kuan, Varun Madireddy, Deepa Malieckal, Abdulrahman Muzib, Gayatri Nair, Vinay V Nair, Jia H Ng, Rushang Parikh, Daniel W Ross, Vipulbhai Sakhiya, Mala Sachdeva, Richard Schwarz, Hitesh H Shah, Purva Sharma, Pravin C Singhal, Nupur N Uppal, Rimda Wanchoo, Bessy Suyin Flores Chang, Jia Hwei Ng

Affiliations

- [1] Division of Kidney Diseases and Hypertension, Department of Medicine, Donald and Barbara Zucker School of Medicine at Hofstra/Northwell, Great Neck, New York, USA; Institute of Health Innovations and Outcomes Research, Feinstein Institutes for Medical Research, Manhasset, New York, USA; Department of Information Services, Northwell Health, New Hyde Park, New York, USA.
- [2] Division of Kidney Diseases and Hypertension, Department of Medicine, Donald and Barbara Zucker School of Medicine at Hofstra/Northwell, Great Neck, New York, USA.
- [3] Division of Kidney Diseases and Hypertension, Department of Medicine, Donald and Barbara Zucker School of Medicine at Hofstra/Northwell, Great Neck, New York, USA. Electronic address: kjhaveri@northwell.edu.

- PMID: **32416116**
- PMCID: PMC7229463
- DOI: 10.1016/j.kint.2020.05.006

Abstract

The rate of acute kidney injury (AKI) associated with patients hospitalized with Covid-19, and associated outcomes are not well understood. This study describes the presentation, risk factors and outcomes of AKI in patients hospitalized with Covid-19. We reviewed the health records for all patients hospitalized with Covid-19 between March 1, and April 5, 2020, at 13 academic and community hospitals in metropolitan New York. Patients younger than 18 years of age, with end stage kidney disease or with a kidney transplant were excluded. AKI was defined according to KDIGO criteria. Of 5,449 patients admitted with Covid-19, AKI developed in 1,993 (36.6%). The peak stages of AKI were stage 1 in 46.5%, stage 2 in 22.4% and stage 3 in 31.1%. Of these, 14.3% required renal replacement therapy (RRT). AKI was primarily seen in Covid-19 patients with respiratory failure, with 89.7% of patients on mechanical ventilation developing AKI compared to 21.7% of non-ventilated patients. 276/285 (96.8%) of patients requiring RRT were on ventilators. Of patients who required ventilation and developed AKI, 52.2% had the onset of AKI within 24

hours of intubation. Risk factors for AKI included older age, diabetes mellitus, cardiovascular disease, black race, hypertension and need for ventilation and vasopressor medications. Among patients with AKI, 694 died (35%), 519 (26%) were discharged and 780 (39%) were still hospitalized. AKI occurs frequently among patients with Covid-19 disease. It occurs early and in temporal association with respiratory failure and is associated with a poor prognosis.

Keywords: AKI; COVID-19; continuous RRT; dialysis; renal failure.

Copyright © 2020 International Society of Nephrology. Published by Elsevier Inc. All rights reserved.

Comment in

- Diabetes begünstigt Nierenversagen bei COVID-19-Patienten : Corona-Pandemie -- Autor: E. Fritschka.
  Fritschka E. Fritschka E. MMW Fortschr Med. 2020 Oct;162(18):31. doi: 10.1007/s15006-020-4481-x. MMW Fortschr Med. 2020. PMID: 33074500 Free PMC article. Review. German. No abstract available.
- The authors reply.
  Ng JH, Hirsch JS, Jhaveri KD, Fishbane S. Ng JH, et al. Kidney Int. 2020 Nov;98(5):1348-1349. doi: 10.1016/j.kint.2020.07.048. Kidney Int. 2020. PMID: 33126980 No abstract available.
- Time-dependent effect, immortal bias, and competing risk: 3 components that should be handled to assess the impact of covariates on occurrence of acute kidney injury.
  Jamme M, Geri G. Jamme M, et al. Kidney Int. 2020 Nov;98(5):1348. doi: 10.1016/j.kint.2020.07.049. Kidney Int. 2020. PMID: 33126981 No abstract available.
- Wayne State University Nephrology service experience in the constraint of COVID-19.
  Ghandour M, Bhat ZY, Rossi NF, Ciccotelli G, Osman-Malik Y. Ghandour M, et al. Ther Apher Dial. 2021 Dec;25(6):1012-1013. doi: 10.1111/1744-9987.13632. Epub 2021 Mar 13. Ther Apher Dial. 2021. PMID: 33528080 Free PMC article. No abstract available.

Dataset use reported in

- Role of pediatric nephrologists in managing adults with AKI due to COVID-19.
  Lipton M, Kavanagh CR, Mahajan R, Jain NG, Uy NS, Dogra S, Lin F. Lipton M, et al. Pediatr Nephrol. 2020 Nov;35(11):2019-2022. doi: 10.1007/s00467-020-04680-7. Epub 2020 Jun 25. Pediatr Nephrol. 2020. PMID: 32588224 Free PMC article. No abstract available.

- 35 references
- 6 figures

Supplementary info

Publication types, MeSH terms, Grant support Expand

Publication types

- Multicenter Study
- Observational Study

MeSH terms

- Acute Kidney Injury / epidemiology
- Acute Kidney Injury / virology*
- Aged
- COVID-19
- Coronavirus Infections / complications*
- Coronavirus Infections / epidemiology
- Female
- Humans
- Inpatients / statistics & numerical data
- Male
- Middle Aged
- New York City / epidemiology
- Pandemics
- Pneumonia, Viral / complications*
- Pneumonia, Viral / epidemiology
- Respiratory Insufficiency / complications*
- Respiratory Insufficiency / virology
- Retrospective Studies

Grant support

- R01 DK118017/DK/NIDDK NIH HHS/United States

Full text links

ELSEVIER FULL-TEXT ARTICLE Elsevier Science Free PMC article
Proceed to details
Cite
Share
☐ 1,209
Clinical Trial
PLoS One
. 2021 Jul 29;16(7):e0255263.
doi: 10.1371/journal.pone.0255263. eCollection 2021.

Long-term effects of coronavirus disease 2019 on the cardiovascular system, CV COVID registry: A structured summary of a study protocol

Victor Arévalos [1], Luis Ortega-Paz [1], Diego Fernandez-Rodríguez [2], Víctor Alfonso Jiménez-Díaz [3], Jordi Bañeras Rius [4], Gianluca Campo [5], Miguel Rodríguez-Santamarta [6], Armando Pérez de Prado [6], Antonio Gómez-Menchero [7], José Francisco Díaz Fernández [7], Claudia Scardino [8], Nieves Gonzalo [9], Alberto Pernigotti [10], Fernando Alfonso [11], Ignacio Jesús Amat-Santos [12], Antonio Silvestro [13], Alfonso Ielasi [14], José María de la Torre [15], Gabriela Bastidas [16], Josep Gómez-Lara [17], Manel Sabaté [1], Salvatore Brugaletta [1], CV COVID-19 Registry Investigators
Affiliations Expand

Affiliations

- [1] Department of Cardiology, Clinic Cardiovascular Institute, Hospital Universitari Clinic, Barcelona, Spain.
- [2] Department of Cardiology, Hospital Universitari Arnau de Vilanova, Lérida, Spain.
- [3] Department of Cardiology, Hospital Universitario de Vigo, Vigo, Spain.
- [4] Department of Cardiology, Hospital Universitari Vall d'Hebron, Barcelona, Spain.
- [5] Department of Cardiology, Azienda Ospedaliero-Universitaria di Ferrara, Ferrara, Italy.
- [6] Department of Cardiology, Hospital Universitario de León, León, Spain.
- [7] Department of Cardiology, Hospital Universitario Juan Ramón Jiménez, Huelva, Spain.
- [8] Department of Cardiology, Hospital Universitari Joan XXIII, Tarragona, Spain.
- [9] Department of Cardiology, Hospital Universitario Clínico San Carlos Madrid, Madrid, Spain.
- [10] Department of Cardiology, Hospital de Tortosa Verge de la Cinta, Tarragona, Spain.
- [11] Department of Cardiology, Hospital Universitario La Princesa, Madrid, Spain.
- [12] Department of Cardiology, Hospital Clínico Universitario de Valladolid, Valladolid, Spain.
- [13] Department of Cardiology, Ospedale Bolognini di Seriate, Bérgamo, Italy.
- [14] Department of Cardiology, Istituto Clinico Sant'Ambrogio, Milano, Italy.
- [15] Department of Cardiology, Hospital Marqués de Valdecilla, Santander, Spain.
- [16] Department of Cardiology, Hospital Universitari Sagrat Cor, Barcelona, Spain.
- [17] Department of Cardiology, Hospital Universitari de Bellvitge, Barcelona, Spain.

- PMID: **34324524**
- PMCID: PMC8320971
- DOI: 10.1371/journal.pone.0255263

Free PMC article
Clinical Trial

Long-term effects of coronavirus disease 2019 on the cardiovascular system, CV COVID registry: A structured summary of a study protocol

Victor Arévalos et al. PLoS One. 2021.
Free PMC article
Show details
PLoS One
. 2021 Jul 29;16(7):e0255263.
doi: 10.1371/journal.pone.0255263. eCollection 2021.

Authors

Victor Arévalos [1], Luis Ortega-Paz [1], Diego Fernandez-Rodríguez [2], Víctor Alfonso Jiménez-Díaz [3], Jordi Bañeras Rius [4], Gianluca Campo [5], Miguel Rodríguez-Santamarta [6], Armando Pérez de Prado [6], Antonio Gómez-Menchero [7], José Francisco Díaz Fernández [7], Claudia Scardino [8], Nieves Gonzalo [9], Alberto Pernigotti [10], Fernando Alfonso [11], Ignacio Jesús Amat-Santos [12], Antonio Silvestro [13], Alfonso Ielasi [14], José María de la Torre [15], Gabriela Bastidas [16], Josep Gómez-Lara [17], Manel Sabaté [1], Salvatore Brugaletta [1], CV COVID-19 Registry Investigators

Affiliations

- [1] Department of Cardiology, Clinic Cardiovascular Institute, Hospital Universitari Clinic, Barcelona, Spain.
- [2] Department of Cardiology, Hospital Universitari Arnau de Vilanova, Lérida, Spain.
- [3] Department of Cardiology, Hospital Universitario de Vigo, Vigo, Spain.
- [4] Department of Cardiology, Hospital Universitari Vall d'Hebron, Barcelona, Spain.
- [5] Department of Cardiology, Azienda Ospedaliero-Universitaria di Ferrara, Ferrara, Italy.
- [6] Department of Cardiology, Hospital Universitario de León, León, Spain.
- [7] Department of Cardiology, Hospital Universitario Juan Ramón Jiménez, Huelva, Spain.
- [8] Department of Cardiology, Hospital Universitari Joan XXIII, Tarragona, Spain.
- [9] Department of Cardiology, Hospital Universitario Clínico San Carlos Madrid, Madrid, Spain.
- [10] Department of Cardiology, Hospital de Tortosa Verge de la Cinta, Tarragona, Spain.
- [11] Department of Cardiology, Hospital Universitario La Princesa, Madrid, Spain.
- [12] Department of Cardiology, Hospital Clínico Universitario de Valladolid, Valladolid, Spain.
- [13] Department of Cardiology, Ospedale Bolognini di Seriate, Bérgamo, Italy.
- [14] Department of Cardiology, Istituto Clinico Sant'Ambrogio, Milano, Italy.
- [15] Department of Cardiology, Hospital Marqués de Valdecilla, Santander, Spain.
- [16] Department of Cardiology, Hospital Universitari Sagrat Cor, Barcelona, Spain.
- [17] Department of Cardiology, Hospital Universitari de Bellvitge, Barcelona, Spain.

- PMID: **34324524**
- PMCID: PMC8320971
- DOI: 10.1371/journal.pone.0255263

Abstract

Background: Patients presenting with the coronavirus-2019 disease (COVID-19) may have a high risk of cardiovascular adverse events, including death from cardiovascular causes. The long-term cardiovascular outcomes of these patients are entirely unknown. We aim to perform a registry of patients who have undergone a diagnostic nasopharyngeal swab for SARS-CoV-2 and to determine their long-term cardiovascular outcomes.

Study and design: This is a multicenter, observational, retrospective registry to be conducted at 17 centers in Spain and Italy (ClinicalTrials.gov number: NCT04359927). Consecutive patients older than 18 years, who underwent a real-time reverse transcriptase-polymerase chain reaction (RT-PCR) for SARS-CoV2 in the participating institutions, will be included since March 2020, to August 2020. Patients will be classified into two groups, according to the results of the RT-PCR: COVID-19 positive or negative. The primary outcome will be cardiovascular mortality at 1 year. The secondary outcomes will be acute myocardial infarction, stroke, heart failure hospitalization, pulmonary embolism, and serious cardiac arrhythmias, at 1 year. Outcomes will be compared between the two groups. Events will be adjudicated by an independent clinical event committee.

Conclusion: The results of this registry will contribute to a better understanding of the long-term cardiovascular implications of the COVID19.

Conflict of interest statement

The authors have declared that no competing interests exist.

- 18 references
- 1 figure

Supplementary info

Publication types, MeSH terms, Associated data, Grant support Expand

Publication types

- Clinical Trial
- Multicenter Study
- Observational Study
- Research Support, Non-U.S. Gov't

MeSH terms

- Arrhythmias, Cardiac / etiology*
- Arrhythmias, Cardiac / virology
- COVID-19 / complications*

- Cardiovascular System / virology*
- Female
- Heart Failure / etiology*
- Heart Failure / virology
- Humans
- Italy
- Male
- Myocardial Infarction / etiology*
- Myocardial Infarction / virology
- Pulmonary Embolism / etiology
- Pulmonary Embolism / virology
- Registries
- Retrospective Studies
- Spain
- Stroke / etiology*
- Stroke / virology
- Time Factors
- Treatment Outcome

Associated data

- ClinicalTrials.gov/NCT04359927

Grant support

SB. Research grant (COV20/00040) from the Carlos III Institute, Madrid, Spain. https://www.isciii.es/Paginas/Inicio.aspx The funders had and will not have a role in study design, data collection and analysis, decision to publish, or preparation of the manuscript.

Full text links

OPEN ACCESS TO FULL TEXT PLOS ONE Public Library of Science Free PMC article

Proceed to details

Cite

Share

☐ 1,210

Observational Study

Am J Trop Med Hyg

. 2020 Jun;102(6):1198-1202.

doi: 10.4269/ajtmh.20-0280.

Point-of-Care Lung Ultrasound Findings in Patients with COVID-19 Pneumonia

Kosuke Yasukawa [1], Taro Minami [2] [3]
Affiliations Expand

Affiliations

- [1] Division of Hospital Medicine, Department of Medicine, MedStar Washington Hospital Center, Washington, District of Columbia.
- [2] Division of Pulmonary, Critical Care, and Sleep Medicine, Department of Medicine, The Warren Alpert Medical School of Brown University, Providence, Rhode Island.
- [3] Division of Pulmonary and Sleep Medicine, Care New England Medical Group, Pawtucket, Rhode Island.

- PMID: **32333544**
- PMCID: PMC7253090
- DOI: 10.4269/ajtmh.20-0280

Free PMC article
Observational Study

Point-of-Care Lung Ultrasound Findings in Patients with COVID-19 Pneumonia

Kosuke Yasukawa et al. Am J Trop Med Hyg. 2020 Jun.
Free PMC article
Show details
Am J Trop Med Hyg
. 2020 Jun;102(6):1198-1202.
doi: 10.4269/ajtmh.20-0280.

Authors

Kosuke Yasukawa [1], Taro Minami [2] [3]

Affiliations

- [1] Division of Hospital Medicine, Department of Medicine, MedStar Washington Hospital Center, Washington, District of Columbia.
- [2] Division of Pulmonary, Critical Care, and Sleep Medicine, Department of Medicine, The Warren Alpert Medical School of Brown University, Providence, Rhode Island.
- [3] Division of Pulmonary and Sleep Medicine, Care New England Medical Group, Pawtucket, Rhode Island.

- PMID: **32333544**

- PMCID: PMC7253090
- DOI: 10.4269/ajtmh.20-0280

Abstract

Patients with novel coronavirus disease (COVID-19) typically present with bilateral multilobar ground-glass opacification with a peripheral distribution. The utility of point-of-care ultrasound has been suggested, but detailed descriptions of lung ultrasound findings are not available. We evaluated lung ultrasound findings in 10 patients admitted to the internal medicine ward with COVID-19. All of the patients had characteristic glass rockets with or without the Birolleau variant (white lung). Thick irregular pleural lines and confluent B lines were also present in all of the patients. Five of the 10 patients had small subpleural consolidations. Point-of-care lung ultrasound has multiple advantages, including lack of radiation exposure and repeatability. Also, lung ultrasound has been shown to be more sensitive than a chest radiograph in detecting alveolar-interstitial syndrome. The utilization of lung ultrasound may also reduce exposure of healthcare workers to severe acute respiratory syndrome-coronavirus-2 and may mitigate the shortage of personal protective equipment. Further studies are needed to evaluate the utility of lung ultrasound in the diagnosis and management of COVID-19.

Conflict of interest statement

Disclosure: T. M. reports personal fees and nonfinancial support from Consultant of FUJIFILM, Japan International Cooperation Agency (JICA) outside the submitted work. T. M. is a consultant of FUJIFILM Corp, Japan, in association with the project funded by Japan International Cooperation Agency (JICA) concerning the "SDGs Business Verification Survey with the Private Sector for Point of Care Ultrasound through Professional Capacity Development in Kenya."

Comment in

- Lung ultrasound cannot be used to screen for Covid-19 in children.
  Scheier E, Guri A, Balla U. Scheier E, et al. Eur Rev Med Pharmacol Sci. 2020 May;24 (9):4623-4624. doi: 10.26355/eurrev_202005_21145. Eur Rev Med Pharmacol Sci. 2020. PMID: 32432724 No abstract available.

- 18 references
- 6 figures

Supplementary info

Publication types, MeSH terms, Substances Expand

Publication types

- Observational Study

MeSH terms

- Adult
- Aged

- Asthma / diagnostic imaging*
- Asthma / pathology
- Asthma / therapy
- Asthma / virology
- Betacoronavirus / genetics
- Betacoronavirus / pathogenicity*
- COVID-19
- Coronavirus Infections / diagnostic imaging*
- Coronavirus Infections / pathology
- Coronavirus Infections / therapy
- Coronavirus Infections / virology
- Female
- Humans
- Hypertension / diagnostic imaging*
- Hypertension / pathology
- Hypertension / therapy
- Hypertension / virology
- Lung / diagnostic imaging*
- Lung / drug effects
- Lung / pathology
- Lung / virology
- Male
- Middle Aged
- Obesity / diagnostic imaging*
- Obesity / pathology
- Obesity / therapy
- Obesity / virology
- Oxygen / therapeutic use
- Pandemics
- Pneumonia, Viral / diagnostic imaging*
- Pneumonia, Viral / pathology
- Pneumonia, Viral / therapy
- Pneumonia, Viral / virology
- Point-of-Care Systems
- Retrospective Studies
- Reverse Transcriptase Polymerase Chain Reaction
- SARS-CoV-2
- Ultrasonography

Substances

- Oxygen

Full text links

Full Text Sheridan PubFactory Free PMC article
Proceed to details
Cite
Share
1,211
Observational Study
Int J Med Sci
. 2020 Sep 21;17(17):2653-2662.
doi: 10.7150/ijms.51159. eCollection 2020.

Temporal changes of CT findings between non-severe and severe cases of COVID-19 pneumonia: a multi-center, retrospective, longitudinal Study

Meng Dai [1, 2], Xiaoming Liu [1, 2], Xiqi Zhu [3], Tiejun Liu [4], Cihao Xu [1, 2], Fang Ye [5], Lian Yang [1, 2], Yu Zhang [1, 2]
Affiliations Expand

Affiliations

- [1] Department of Radiology, Union Hospital, Tongji Medical College, Huazhong University of Science and Technology, Wuhan, Hubei, China.
- [2] Hubei Province Key Laboratory of Molecular Imaging, Wuhan, Hubei, China.
- [3] Department of Radiology, Nanxishan Hospital, Guangxi Zhuang Autonomous Region, No. 46 Chongxin Road, Guilin 541002 Guangxi, People's Republic of China.
- [4] Department of Radiology, Liuzhou People's Hospital, No. 8, Wenchang Road, Liuzhou 545006, Guangxi, People's Republic of China.
- [5] Department of Occupational and Environmental Health and Ministry of Education Key Lab for Environment and Health, School of Public Health, Tongji Medical College, Huazhong University of Science and Technology, Wuhan, Hubei, China.

- PMID: **33162793**
- PMCID: PMC7645333
- DOI: 10.7150/ijms.51159

Free PMC article
Observational Study

Temporal changes of CT findings between non-severe and severe cases of COVID-19

pneumonia: a multi-center, retrospective, longitudinal Study

Meng Dai et al. Int J Med Sci. 2020.
Free PMC article
Show details
Int J Med Sci
. 2020 Sep 21;17(17):2653-2662.
doi: 10.7150/ijms.51159. eCollection 2020.

Authors

Meng Dai [1, 2], Xiaoming Liu [1, 2], Xiqi Zhu [3], Tiejun Liu [4], Cihao Xu [1, 2], Fang Ye [5], Lian Yang [1, 2], Yu Zhang [1, 2]

Affiliations

- [1] Department of Radiology, Union Hospital, Tongji Medical College, Huazhong University of Science and Technology, Wuhan, Hubei, China.
- [2] Hubei Province Key Laboratory of Molecular Imaging, Wuhan, Hubei, China.
- [3] Department of Radiology, Nanxishan Hospital, Guangxi Zhuang Autonomous Region, No. 46 Chongxin Road, Guilin 541002 Guangxi, People's Republic of China.
- [4] Department of Radiology, Liuzhou People's Hospital, No. 8, Wenchang Road, Liuzhou 545006, Guangxi, People's Republic of China.
- [5] Department of Occupational and Environmental Health and Ministry of Education Key Lab for Environment and Health, School of Public Health, Tongji Medical College, Huazhong University of Science and Technology, Wuhan, Hubei, China.

- PMID: **33162793**
- PMCID: PMC7645333
- DOI: 10.7150/ijms.51159

Abstract

Background and aim: To perform a longitudinal analysis of serial CT findings over time in patients with COVID-19 pneumonia. **Methods:** From February 5 to March 8, 2020, 73 patients (male to female, ratio of 43:30; mean age, 51 years) with COVID-19 pneumonia were retrospectively enrolled and followed up until discharge from three institutions in China. The patients were divided into the severe and non-severe groups according to treatment option. The patterns and distribution of lung abnormalities, total CT scores, single ground-glass opacity (GGO) CT scores, single consolidation CT scores, single reticular CT scores and the amounts of zones involved were reviewed by 2 radiologists. These features were analyzed for temporal changes. **Results:** In non-severe group, total CT scores (median, 9.5) and the amounts of zones involved were slowly increased and peaked in disease week 2. In the severe group, the increase was faster, with scores also peaking at 2 weeks (median, 20). In both groups, the later parameters began to decrease in week 4 (median values of 9 and 19 in the non-severe and severe groups, respectively). In the severe group, the dominant residual lung lesions were reticular (median single reticular CT score, 10) and consolidation (median single consolidation CT score, 7). In the non-

severe group, the dominant residual lung lesions were GGO (median single GGO CT score, 7) and reticular (median single reticular CT score, 4). In both non-severe and severe groups, the GGO pattern was dominant in week 1, with a higher proportion in the severe group compared with the non-severe group (72% vs. 65%). The consolidation pattern peaked in week 2, with 9 (32%) and 19 (73%) in the non-severe and severe groups, respectively; the reticular pattern became dominant from week 4 (both group >40%). **Conclusion:** The extent of CT abnormalities in the severe and non-severe groups peaked in disease week 2. The temporal changes of CT manifestations followed a specific pattern, which might indicate disease progression and recovery.

Keywords: COVID-19; CT abnormalities; CT score; Ground-glass opacity; non-severe; severe.

© The author(s).

Conflict of interest statement

Competing Interests: The authors have declared that no competing interest exists.

- 32 references
- 4 figures

Supplementary info

Publication types, MeSH terms Expand

Publication types

- Multicenter Study
- Observational Study

MeSH terms

- Adult
- Aged
- Aged, 80 and over
- Betacoronavirus / pathogenicity
- COVID-19
- China
- Coronavirus Infections / diagnostic imaging*
- Coronavirus Infections / physiopathology
- Coronavirus Infections / virology
- Disease Progression
- Female
- Humans
- Longitudinal Studies
- Lung / diagnostic imaging*
- Lung / physiopathology

- Lung / virology
- Male
- Middle Aged
- Pandemics*
- Pneumonia / diagnostic imaging*
- Pneumonia / physiopathology
- Pneumonia / virology
- Pneumonia, Viral / diagnostic imaging*
- Pneumonia, Viral / physiopathology
- Pneumonia, Viral / virology
- SARS-CoV-2
- Tomography, X-Ray Computed

Full text links

Ivyspring International Publisher Free PMC article

Proceed to details

Cite

Share

☐ 1,212

Observational Study

Front Immunol

. 2020 Oct 6;11:581338.

doi: 10.3389/fimmu.2020.581338. eCollection 2020.

Pro- and Anti-Inflammatory Responses in Severe COVID-19-Induced Acute Respiratory Distress Syndrome-An Observational Pilot Study

Quirin Notz [1], Marc Schmalzing [2], Florian Wedekink [3], Tobias Schlesinger [1], Michael Gernert [2], Johannes Herrmann [1], Lena Sorger [3], Dirk Weismann [4], Benedikt Schmid [1], Magdalena Sitter [1], Nicolas Schlegel [5], Peter Kranke [1], Jörg Wischhusen [3], Patrick Meybohm [1], Christopher Lotz [1]

Affiliations Expand

Affiliations

- [1] Department of Anesthesiology and Intensive Care Medicine, University Hospital Würzburg, Würzburg, Germany.
- [2] Department of Medicine II, Rheumatology and Clinical Immunology, University Hospital Würzburg, Würzburg, Germany.

- [3] Department of Gynecology, Section for Experimental Tumor Immunology, University Hospital Würzburg, Würzburg, Germany.
- [4] Department of Internal Medicine I, University Hospital Würzburg, Würzburg, Germany.
- [5] Department of General, Visceral, Vascular and Pediatric Surgery (Surgery I), University Hospital Würzburg, Würzburg, Germany.

- PMID: **33123167**
- PMCID: PMC7573122
- DOI: 10.3389/fimmu.2020.581338

Free PMC article
Observational Study

Pro- and Anti-Inflammatory Responses in Severe COVID-19-Induced Acute Respiratory Distress Syndrome-An Observational Pilot Study

Quirin Notz et al. Front Immunol. 2020.
Free PMC article
Show details
Front Immunol
. 2020 Oct 6;11:581338.
doi: 10.3389/fimmu.2020.581338. eCollection 2020.

Authors

Quirin Notz [1], Marc Schmalzing [2], Florian Wedekink [3], Tobias Schlesinger [1], Michael Gernert [2], Johannes Herrmann [1], Lena Sorger [3], Dirk Weismann [4], Benedikt Schmid [1], Magdalena Sitter [1], Nicolas Schlegel [5], Peter Kranke [1], Jörg Wischhusen [3], Patrick Meybohm [1], Christopher Lotz [1]

Affiliations

- [1] Department of Anesthesiology and Intensive Care Medicine, University Hospital Würzburg, Würzburg, Germany.
- [2] Department of Medicine II, Rheumatology and Clinical Immunology, University Hospital Würzburg, Würzburg, Germany.
- [3] Department of Gynecology, Section for Experimental Tumor Immunology, University Hospital Würzburg, Würzburg, Germany.
- [4] Department of Internal Medicine I, University Hospital Würzburg, Würzburg, Germany.
- [5] Department of General, Visceral, Vascular and Pediatric Surgery (Surgery I), University Hospital Würzburg, Würzburg, Germany.

- PMID: **33123167**
- PMCID: PMC7573122

- DOI: 10.3389/fimmu.2020.581338

Abstract

Objectives: The severity of Coronavirus Disease 2019 (COVID-19) is largely determined by the immune response. First studies indicate altered lymphocyte counts and function. However, interactions of pro- and anti-inflammatory mechanisms remain elusive. In the current study we characterized the immune responses in patients suffering from severe COVID-19-induced acute respiratory distress syndrome (ARDS).

Methods: This was a single-center retrospective study in patients admitted to the intensive care unit (ICU) with confirmed COVID-19 between March 14th and May 28th 2020 (n = 39). Longitudinal data were collected within routine clinical care, including flow-cytometry of lymphocyte subsets, cytokine analysis and growth differentiation factor 15 (GDF-15). Antibody responses against the receptor binding domain (RBD) of Severe Acute Respiratory Syndrome Coronavirus 2 (SARS-CoV-2) Spike protein were analyzed.

Results: All patients suffered from severe ARDS, 30.8% died. Interleukin (IL)-6 was massively elevated at every time-point. The anti-inflammatory cytokine IL-10 was concomitantly upregulated with IL-6. The cellular response was characterized by lymphocytopenia with low counts of CD8+ T cells, natural killer (NK) and naïve T helper cells. CD8+ T and NK cells recovered after 8 to 14 days. The B cell system was largely unimpeded. This coincided with a slight increase in anti-SARS-CoV-2-Spike-RBD immunoglobulin (Ig) G and a decrease in anti-SARS-CoV-2-Spike-RBD IgM. GDF-15 levels were elevated throughout ICU treatment.

Conclusions: Massively elevated levels of IL-6 and a delayed cytotoxic immune defense characterized severe COVID-19-induced ARDS. The B cell response and antibody production were largely unimpeded. No obvious imbalance of pro- and anti-inflammatory mechanisms was observed, with elevated GDF-15 levels suggesting increased tissue resilience.

Keywords: Coronavirus Disease 2019; Severe Acute Respiratory Syndrome Coronavirus 2; acute respiratory distress syndrome; cytokines; growth differentiation factor 15; immune response; inflammation.

Copyright © 2020 Notz, Schmalzing, Wedekink, Schlesinger, Gernert, Herrmann, Sorger, Weismann, Schmid, Sitter, Schlegel, Kranke, Wischhusen, Meybohm and Lotz.

- 54 references
- 8 figures

Supplementary info

Publication types, MeSH terms, Substances Expand

Publication types

- Observational Study

MeSH terms

- Aged
- Antibodies, Viral / blood
- Antibodies, Viral / immunology
- Betacoronavirus / immunology*
- COVID-19
- Coronavirus Infections / immunology
- Coronavirus Infections / pathology*
- Cytokine Release Syndrome / immunology
- Cytokine Release Syndrome / pathology*
- Female
- Growth Differentiation Factor 15 / blood
- Humans
- Immunoglobulin G / blood
- Immunoglobulin G / immunology
- Intensive Care Units
- Interleukin-10 / blood
- Interleukin-6 / blood
- Longitudinal Studies
- Lymphopenia
- Male
- Middle Aged
- Pandemics
- Pilot Projects
- Pneumonia, Viral / immunology
- Pneumonia, Viral / pathology*
- Retrospective Studies
- SARS-CoV-2
- Severe Acute Respiratory Syndrome / immunology
- Severe Acute Respiratory Syndrome / pathology*
- Spike Glycoprotein, Coronavirus / immunology

Substances

- Antibodies, Viral
- GDF15 protein, human
- Growth Differentiation Factor 15
- IL10 protein, human
- IL6 protein, human
- Immunoglobulin G
- Interleukin-6
- Spike Glycoprotein, Coronavirus
- spike protein, SARS-CoV-2

- Interleukin-10

Full text links

Frontiers Media SA Free PMC article
Proceed to details
Cite
Share
1,213
Observational Study
Nutrients
. 2020 Sep 11;12(9):2775.
doi: 10.3390/nu12092775.

Impact of Vitamin D Deficiency on COVID-19-A Prospective Analysis from the CovILD Registry

Alex Pizzini[1], Magdalena Aichner[1], Sabina Sahanic[1], Anna Böhm[1], Alexander Egger[2], Gregor Hoermann[2 3 4], Katharina Kurz[1], Gerlig Widmann[5], Rosa Bellmann-Weiler[1], Günter Weiss[1 6], Ivan Tancevski[1], Thomas Sonnweber[1], Judith Löffler-Ragg[1]
Affiliations Expand

Affiliations

- [1] Department of Internal Medicine II, Infectious Diseases, Pneumology, Rheumatology, Medical University of Innsbruck, 6020 Innsbruck, Austria.
- [2] Central Institute of Medical and Chemical Laboratory Diagnostics, Medical University of Innsbruck, 6020 Innsbruck, Austria.
- [3] Department of Laboratory Medicine, Medical University of Vienna, 1090 Vienna, Austria.
- [4] MLL Munich Leukemia Laboratory, Klinikum Großhadern, 81377 Munich, Germany.
- [5] Department of Radiology, Medical University of Innsbruck, 6020 Innsbruck, Austria.
- [6] Christian Doppler Laboratory for Iron Metabolism and Anemia Research, Medical University of Innsbruck, 6020 Innsbruck, Austria.

- PMID: **32932831**
- PMCID: PMC7551662
- DOI: 10.3390/nu12092775

Free PMC article
Observational Study

Impact of Vitamin D Deficiency on COVID-19-A Prospective Analysis from the CovILD Registry

Alex Pizzini et al. Nutrients. 2020.
Free PMC article
Show details
Nutrients
. 2020 Sep 11;12(9):2775.
doi: 10.3390/nu12092775.

Authors

Alex Pizzini [1], Magdalena Aichner [1], Sabina Sahanic [1], Anna Böhm [1], Alexander Egger [2], Gregor Hoermann [2 3 4], Katharina Kurz [1], Gerlig Widmann [5], Rosa Bellmann-Weiler [1], Günter Weiss [1 6], Ivan Tancevski [1], Thomas Sonnweber [1], Judith Löffler-Ragg [1]

Affiliations

- [1] Department of Internal Medicine II, Infectious Diseases, Pneumology, Rheumatology, Medical University of Innsbruck, 6020 Innsbruck, Austria.
- [2] Central Institute of Medical and Chemical Laboratory Diagnostics, Medical University of Innsbruck, 6020 Innsbruck, Austria.
- [3] Department of Laboratory Medicine, Medical University of Vienna, 1090 Vienna, Austria.
- [4] MLL Munich Leukemia Laboratory, Klinikum Großhadern, 81377 Munich, Germany.
- [5] Department of Radiology, Medical University of Innsbruck, 6020 Innsbruck, Austria.
- [6] Christian Doppler Laboratory for Iron Metabolism and Anemia Research, Medical University of Innsbruck, 6020 Innsbruck, Austria.

- PMID: **32932831**
- PMCID: PMC7551662
- DOI: 10.3390/nu12092775

Abstract

The novel Coronavirus disease 2019 (COVID-19) caused by severe acute respiratory syndrome coronavirus type 2 (SARS-CoV-2) is a global health concern. Vitamin D (VITD) deficiency has been suggested to alter SARS-CoV-2 susceptibility and the course of disease. Thus, we aimed to investigate associations of VITD status to disease presentation within the CovILD registry. This prospective, multicenter, observational study on long-term sequelae includes patients with COVID-19 after hospitalization or outpatients with persistent symptoms. Eight weeks after PCR confirmed diagnosis, a detailed questionnaire, a clinical examination, and laboratory testing, including VITD status, were evaluated. Furthermore, available laboratory specimens close to hospital admission were used to retrospectively analyze 25-hydroxyvitamin D levels at disease onset. A total of 109 patients were included in the analysis (60% males, 40% females), aged 58 ± 14 years. Eight weeks after the onset of COVID-19, a high proportion of patients presented with impaired VITD metabolism and elevated parathyroid hormone (PTH) levels. PTH concentrations

were increased in patients who needed intensive care unit (ICU) treatment, while VITD levels were not significantly different between disease severity groups. Low VITD levels at disease onset or at eight-week follow-up were not related to persistent symptom burden, lung function impairment, ongoing inflammation, or more severe CT abnormalities. VITD deficiency is frequent among COVID-19 patients but not associated with disease outcomes. However, individuals with severe disease display a disturbed parathyroid-vitamin-D axis within their recovery phase. The proposed significance of VITD supplementation in the clinical management of COVID-19 remains elusive.

Keywords: COVID-19; PTH; SARS-CoV-2; VITD; parathyroid hormone; vitamin D.

Conflict of interest statement

The authors declare no conflict of interest.

- 35 references
- 2 figures

Supplementary info

Publication types, MeSH terms, Substances Expand

Publication types

- Multicenter Study
- Observational Study

MeSH terms

- Aged
- Betacoronavirus*
- COVID-19
- Coronavirus Infections / blood*
- Coronavirus Infections / complications
- Coronavirus Infections / virology
- Female
- Humans
- Male
- Middle Aged
- Nutritional Status
- Pandemics
- Parathyroid Hormone / blood
- Pneumonia, Viral / blood*
- Pneumonia, Viral / complications
- Pneumonia, Viral / virology
- Prospective Studies

- Registries
- Risk Factors
- SARS-CoV-2
- Severity of Illness Index
- Vitamin D / analogs & derivatives*
- Vitamin D / blood
- Vitamin D Deficiency / blood
- Vitamin D Deficiency / epidemiology
- Vitamin D Deficiency / virology*

Substances

- Parathyroid Hormone
- Vitamin D
- 25-hydroxyvitamin D

Full text links

FULL TEXT OPEN ACCESS MDPI Multidisciplinary Digital Publishing Institute (MDPI) Free PMC article

Proceed to details

Cite

Share

☐ 1,214

Observational Study

Neurol Neuroimmunol Neuroinflamm

. 2021 Jun 24;8(5):e1024.

doi: 10.1212/NXI.0000000000001024. Print 2021 Jul.

SARS-CoV-2 Infection in Multiple Sclerosis: Results of the Spanish Neurology Society Registry

Georgina Arrambide [1], Miguel Ángel Llaneza-González [1], Lucienne Costa-Frossard França [1], Virginia Meca-Lallana [2], Eva Fernández- Díaz [1], Irene Moreno-Torres [1], Jose Manuel García-Domínguez [1], Gloria Ortega-Suero [1], Lucía Ayuso-Peralta [1], Mayra Gómez-Moreno [1], Javier J Sotoca-Fernández [1], Ana Belén Caminero-Rodríguez [1], Luis A Rodríguez de Antonio [1], Marcial Corujo-Suárez [1], María A Otano-Martínez [1], Francisco Carlos Pérez-Miralles [1], Virginia Reyes-Garrido [1], Teresa Ayuso-Blanco [1], José Jesús Balseiro-Gómez [1], Mercedes Muñoz-Pasadas [1], Inmaculada Pérez-Molina [1], Carmen Arnal-García [1], Ángela Domingo-Santos [1], Cristina Guijarro-Castro [1], Cristina Íñiguez-Martínez [1], Nieves Téllez Lara [1], Fernando Castellanos-Pinedo [1], Tamara Castillo-Triviño [1], Debora María Cerdán-Santacruz [1], Ángel Pérez-Sempere [1], Berta Sebastián Torres [1], Amaya Álvarez de Arcaya [1], Eva Costa-Arpín [1], Eduardo Durán-

Ferreras [1], Marta Fragoso-Martínez [1], Montserrat González-Platas [1], Lamberto Landete Pascual [1], Jorge Millán-Pascual [1], Celia Oreja-Guevara [1], José E Meca-Lallana [2]
Affiliations Expand

Affiliations

- [1] From the Centro de Esclerosis Múltiple de Cataluña (G.A.), (Cemcat), Instituto de Investigación Vall d'Hebron, Hospital Universitario Vall d'Hebron, Universidad Autónoma de Barcelona; Complejo Hospitalario Universitario de Ferrol (M.Á.L.-G.); Hospital Universitario Ramón y Cajal (L.C.-F.F.), Madrid; Hospital Universitario de la Princesa (V.M.L.), Madrid; Complejo Hospitalario Universitario de Albacete, (E.F.D.); Hospital Universitario Fundación Jiménez Díaz (I.M.T.), Madrid; Hospital General Universitario Gregorio Marañón (J.M.G.-D.), Madrid; Hospital La Mancha Centro (G.O.-S., Á.D.S.), Alcázar de San Juan; Hospital Universitario Príncipe de Asturias (L.A.P.), Alcalá de Henares; Hospital Universitario Infanta Leonor (M.G.M.), Madrid; Hospital Universitario Mútua Terrasa (J.J.S.-F.); Complejo Asistencial de Ávila (A.B.C.-R.), Ávila; Hospital Universitario de Fuenlabrada (L.A.R.A.), Madrid; Hospital Universitario Son Espases, Palma de Mallorca (M.C.S.); Complejo Hospitalario de Navarra (M.A.O.-M., T.A.B.), Pamplona; Hospital Universitario y Politécnico La Fe (F.C.P.-M.), Valencia; Hospital Regional Universitario de Málaga (V.R.G.); Hospital Universitario de Getafe (J.J.B.-G.), Madrid; Hospital Santa Barbara (M.M.P.), Puertollano; Hospital Virgen de la Salud (I.P.M.), Toledo; Hospital Universitario Virgen de las Nieves (C.A.G.), Granada; Hospital Universitario Puerta del Sur (CINAC), Madrid (C.G.C.); Hospital Clínico Universitario Zaragoza (C.Í.M.), Zaragoza; Hospital Clínico Universitario Valladolid (N.T.L.,), Valladolid; Hospital Virgen del Puerto (F.C.P.), Plasencia; Hospital Universitario Donostia (T.C.T.), San Sebastian; Hospital General de Segovia (D.M.C.-S.), Segovia; Hospital General Universitario de Alicante (Á.P.S.), Alicante; Hospital Universitario Miguel Servet (IIS Aragón) (B.S.T.), Zaragoza; Hospital Universitario Araba (A.Á.A.), Vitoria; Hospital Clínico Universitario de Santiago de Compostela (E.C.A.), Santiago de Compostela; Hospital Universitario Juan Ramón Jiménez (E.D.-F.), Huelva; Hospital de Terrasa (M.F.M.), Terrasa; Hospital Universitario de Canarias (M.G.P.), San Cristobal de La Laguna; Hospital Universitario Dr Peset de Valencia (L.L.P.), Valencia; Complejo Hospitalario Universitario de Cartagena (J.M.P.), Murcia; Hospital Clínico San Carlos (C.O.-G.),Facultad de Medicina, Universidad Complutense de Madrid, IdISSC, Madrid; and CSUR Unidad de Esclerosis Múltiple y Neuroinmunología Clínica (J.E.M.-L.), Hospital Clínico Universitario Virgen de la Arrixaca, IMIB-Arrixaca, Cátedra de Esclerosis Múltiple y Neuroinmunología Clínica, UCAM, Universidad Católica San Antonio, Murcia, Spain.
- [2] From the Centro de Esclerosis Múltiple de Cataluña (G.A.), (Cemcat), Instituto de Investigación Vall d'Hebron, Hospital Universitario Vall d'Hebron, Universidad Autónoma de Barcelona; Complejo Hospitalario Universitario de Ferrol (M.Á.L.-G.); Hospital Universitario Ramón y Cajal (L.C.-F.F.), Madrid; Hospital Universitario de la Princesa (V.M.L.), Madrid; Complejo Hospitalario Universitario de Albacete, (E.F.D.); Hospital Universitario Fundación Jiménez Díaz (I.M.T.), Madrid; Hospital General Universitario Gregorio Marañón (J.M.G.-D.), Madrid; Hospital La Mancha Centro (G.O.-S., Á.D.S.), Alcázar de San Juan; Hospital Universitario Príncipe de Asturias (L.A.P.), Alcalá de Henares; Hospital Universitario Infanta Leonor (M.G.M.), Madrid; Hospital Universitario Mútua Terrasa (J.J.S.-F.); Complejo Asistencial de Ávila (A.B.C.-R.), Ávila; Hospital Universitario de Fuenlabrada (L.A.R.A.), Madrid; Hospital Universitario Son Espases, Palma de Mallorca (M.C.S.); Complejo Hospitalario de Navarra (M.A.O.-M., T.A.B.), Pamplona; Hospital Universitario y Politécnico La Fe (F.C.P.-M.), Valencia; Hospital Regional Universitario de Málaga (V.R.G.); Hospital Universitario de Getafe (J.J.B.-G.), Madrid; Hospital Santa Barbara (M.M.P.), Puertollano; Hospital Virgen de la Salud

(I.P.M.), Toledo; Hospital Universitario Virgen de las Nieves (C.A.G.), Granada; Hospital Universitario Puerta del Sur (CINAC), Madrid (C.G.C.); Hospital Clínico Universitario Zaragoza (C.Í.M.), Zaragoza; Hospital Clínico Universitario Valladolid (N.T.L.,), Valladolid; Hospital Virgen del Puerto (F.C.P.), Plasencia; Hospital Universitario Donostia (T.C.T.), San Sebastian; Hospital General de Segovia (D.M.C.-S.), Segovia; Hospital General Universitario de Alicante (Á.P.S.), Alicante; Hospital Universitario Miguel Servet (IIS Aragón) (B.S.T.), Zaragoza; Hospital Universitario Araba (A.Á.A.), Vitoria; Hospital Clínico Universitario de Santiago de Compostela (E.C.A.), Santiago de Compostela; Hospital Universitario Juan Ramón Jiménez (E.D.-F.), Huelva; Hospital de Terrasa (M.F.M.), Terrasa; Hospital Universitario de Canarias (M.G.P.), San Cristobal de La Laguna; Hospital Universitario Dr Peset de Valencia (L.L.P.), Valencia; Complejo Hospitalario Universitario de Cartagena (J.M.P.), Murcia; Hospital Clínico San Carlos (C.O.-G.),Facultad de Medicina, Universidad Complutense de Madrid, IdISSC, Madrid; and CSUR Unidad de Esclerosis Múltiple y Neuroinmunología Clínica (J.E.M.-L.), Hospital Clínico Universitario Virgen de la Arrixaca, IMIB-Arrixaca, Cátedra de Esclerosis Múltiple y Neuroinmunología Clínica, UCAM, Universidad Católica San Antonio, Murcia, Spain. pmecal@gmail.com.

- PMID: **34168057**
- PMCID: PMC8225011
- DOI: 10.1212/NXI.0000000000001024

Free PMC article
Observational Study

SARS-CoV-2 Infection in Multiple Sclerosis: Results of the Spanish Neurology Society Registry

Georgina Arrambide et al. Neurol Neuroimmunol Neuroinflamm. 2021.
Free PMC article

Show details

Neurol Neuroimmunol Neuroinflamm
. 2021 Jun 24;8(5):e1024.
doi: 10.1212/NXI.0000000000001024. Print 2021 Jul.

Authors

Georgina Arrambide [1], Miguel Ángel Llaneza-González [1], Lucienne Costa-Frossard França [1], Virginia Meca-Lallana [2], Eva Fernández- Díaz [1], Irene Moreno-Torres [1], Jose Manuel García-Domínguez [1], Gloria Ortega-Suero [1], Lucía Ayuso-Peralta [1], Mayra Gómez-Moreno [1], Javier J Sotoca-Fernández [1], Ana Belén Caminero-Rodríguez [1], Luis A Rodríguez de Antonio [1], Marcial Corujo-Suárez [1], María A Otano-Martínez [1], Francisco Carlos Pérez-Miralles [1], Virginia Reyes-Garrido [1], Teresa Ayuso-Blanco [1], José Jesús Balseiro-Gómez [1], Mercedes Muñoz-Pasadas [1], Inmaculada Pérez-Molina [1], Carmen Arnal-García [1], Ángela Domingo-Santos [1], Cristina Guijarro-Castro [1], Cristina Íñiguez-Martínez [1], Nieves Téllez Lara [1], Fernando Castellanos-Pinedo [1], Tamara Castillo-Triviño [1], Debora María Cerdán-Santacruz [1], Ángel Pérez-Sempere

[1], Berta Sebastián Torres [1], Amaya Álvarez de Arcaya [1], Eva Costa-Arpín [1], Eduardo Durán-Ferreras [1], Marta Fragoso-Martínez [1], Montserrat González-Platas [1], Lamberto Landete Pascual [1], Jorge Millán-Pascual [1], Celia Oreja-Guevara [1], José E Meca-Lallana [2]

Affiliations

- [1] From the Centro de Esclerosis Múltiple de Cataluña (G.A.), (Cemcat), Instituto de Investigación Vall d'Hebron, Hospital Universitario Vall d'Hebron, Universidad Autónoma de Barcelona; Complejo Hospitalario Universitario de Ferrol (M.Á.L.-G.); Hospital Universitario Ramón y Cajal (L.C.-F.F.), Madrid; Hospital Universitario de la Princesa (V.M.L.), Madrid; Complejo Hospitalario Universitario de Albacete, (E.F.D.); Hospital Universitario Fundación Jiménez Díaz (I.M.T.), Madrid; Hospital General Universitario Gregorio Marañón (J.M.G.-D.), Madrid; Hospital La Mancha Centro (G.O.-S., Á.D.S.), Alcázar de San Juan; Hospital Universitario Príncipe de Asturias (L.A.P.), Alcalá de Henares; Hospital Universitario Infanta Leonor (M.G.M.), Madrid; Hospital Universitario Mútua Terrasa (J.J.S.-F.); Complejo Asistencial de Ávila (A.B.C.-R.), Ávila; Hospital Universitario de Fuenlabrada (L.A.R.A.), Madrid; Hospital Universitario Son Espases, Palma de Mallorca (M.C.S.); Complejo Hospitalario de Navarra (M.A.O.-M., T.A.B.), Pamplona; Hospital Universitario y Politécnico La Fe (F.C.P.-M.), Valencia; Hospital Regional Universitario de Málaga (V.R.G.); Hospital Universitario de Getafe (J.J.B.-G.), Madrid; Hospital Santa Barbara (M.M.P.), Puertollano; Hospital Virgen de la Salud (I.P.M.), Toledo; Hospital Universitario Virgen de las Nieves (C.A.G.), Granada; Hospital Universitario Puerta del Sur (CINAC), Madrid (C.G.C.); Hospital Clínico Universitario Zaragoza (C.Í.M.), Zaragoza; Hospital Clínico Universitario Valladolid (N.T.L.,), Valladolid; Hospital Virgen del Puerto (F.C.P.), Plasencia; Hospital Universitario Donostia (T.C.T.), San Sebastian; Hospital General de Segovia (D.M.C.-S.), Segovia; Hospital General Universitario de Alicante (Á.P.S.), Alicante; Hospital Universitario Miguel Servet (IIS Aragón) (B.S.T.), Zaragoza; Hospital Universitario Araba (A.Á.A.), Vitoria; Hospital Clínico Universitario de Santiago de Compostela (E.C.A.), Santiago de Compostela; Hospital Universitario Juan Ramón Jiménez (E.D.-F.), Huelva; Hospital de Terrasa (M.F.M.), Terrasa; Hospital Universitario de Canarias (M.G.P.), San Cristobal de La Laguna; Hospital Universitario Dr Peset de Valencia (L.L.P.), Valencia; Complejo Hospitalario Universitario de Cartagena (J.M.P.), Murcia; Hospital Clínico San Carlos (C.O.-G.),Facultad de Medicina, Universidad Complutense de Madrid, IdISSC, Madrid; and CSUR Unidad de Esclerosis Múltiple y Neuroinmunología Clínica (J.E.M.-L.), Hospital Clínico Universitario Virgen de la Arrixaca, IMIB-Arrixaca, Cátedra de Esclerosis Múltiple y Neuroinmunología Clínica, UCAM, Universidad Católica San Antonio, Murcia, Spain.
- [2] From the Centro de Esclerosis Múltiple de Cataluña (G.A.), (Cemcat), Instituto de Investigación Vall d'Hebron, Hospital Universitario Vall d'Hebron, Universidad Autónoma de Barcelona; Complejo Hospitalario Universitario de Ferrol (M.Á.L.-G.); Hospital Universitario Ramón y Cajal (L.C.-F.F.), Madrid; Hospital Universitario de la Princesa (V.M.L.), Madrid; Complejo Hospitalario Universitario de Albacete, (E.F.D.); Hospital Universitario Fundación Jiménez Díaz (I.M.T.), Madrid; Hospital General Universitario Gregorio Marañón (J.M.G.-D.), Madrid; Hospital La Mancha Centro (G.O.-S., Á.D.S.), Alcázar de San Juan; Hospital Universitario Príncipe de Asturias (L.A.P.), Alcalá de Henares; Hospital Universitario Infanta Leonor (M.G.M.), Madrid; Hospital Universitario Mútua Terrasa (J.J.S.-F.); Complejo Asistencial de Ávila (A.B.C.-R.), Ávila; Hospital Universitario de Fuenlabrada (L.A.R.A.), Madrid; Hospital Universitario Son Espases, Palma de Mallorca (M.C.S.); Complejo Hospitalario de Navarra (M.A.O.-M., T.A.B.), Pamplona; Hospital Universitario y Politécnico La Fe (F.C.P.-M.), Valencia; Hospital Regional Universitario de Málaga (V.R.G.); Hospital Universitario de Getafe (J.J.B.-G.), Madrid; Hospital Santa Barbara (M.M.P.), Puertollano; Hospital Virgen de la Salud

(I.P.M.), Toledo; Hospital Universitario Virgen de las Nieves (C.A.G.), Granada; Hospital Universitario Puerta del Sur (CINAC), Madrid (C.G.C.); Hospital Clínico Universitario Zaragoza (C.Í.M.), Zaragoza; Hospital Clínico Universitario Valladolid (N.T.L.,), Valladolid; Hospital Virgen del Puerto (F.C.P.), Plasencia; Hospital Universitario Donostia (T.C.T.), San Sebastian; Hospital General de Segovia (D.M.C.-S.), Segovia; Hospital General Universitario de Alicante (Á.P.S.), Alicante; Hospital Universitario Miguel Servet (IIS Aragón) (B.S.T.), Zaragoza; Hospital Universitario Araba (A.Á.A.), Vitoria; Hospital Clínico Universitario de Santiago de Compostela (E.C.A.), Santiago de Compostela; Hospital Universitario Juan Ramón Jiménez (E.D.-F.), Huelva; Hospital de Terrasa (M.F.M.), Terrasa; Hospital Universitario de Canarias (M.G.P.), San Cristobal de La Laguna; Hospital Universitario Dr Peset de Valencia (L.L.P.), Valencia; Complejo Hospitalario Universitario de Cartagena (J.M.P.), Murcia; Hospital Clínico San Carlos (C.O.-G.),Facultad de Medicina, Universidad Complutense de Madrid, IdISSC, Madrid; and CSUR Unidad de Esclerosis Múltiple y Neuroinmunología Clínica (J.E.M.-L.), Hospital Clínico Universitario Virgen de la Arrixaca, IMIB-Arrixaca, Cátedra de Esclerosis Múltiple y Neuroinmunología Clínica, UCAM, Universidad Católica San Antonio, Murcia, Spain. pmecal@gmail.com.

- PMID: **34168057**
- PMCID: PMC8225011
- DOI: 10.1212/NXI.0000000000001024

Abstract

Objective: To understand COVID-19 characteristics in people with multiple sclerosis (MS) and identify high-risk individuals due to their immunocompromised state resulting from the use of disease-modifying treatments.

Methods: Retrospective and multicenter registry in patients with MS with suspected or confirmed COVID-19 diagnosis and available disease course (mild = ambulatory; severe = hospitalization; and critical = intensive care unit/death). Cases were analyzed for associations between MS characteristics and COVID-19 course and for identifying risk factors for a fatal outcome.

Results: Of the 326 patients analyzed, 120 were cases confirmed by real-time PCR, 34 by a serologic test, and 205 were suspected. Sixty-nine patients (21.3%) developed severe infection, 10 (3%) critical, and 7 (2.1%) died. Ambulatory patients were higher in relapsing MS forms, treated with injectables and oral first-line agents, whereas more severe cases were observed in patients on pulsed immunosuppressors and critical cases among patients with no therapy. Severe and critical infections were more likely to affect older males with comorbidities, with progressive MS forms, a longer disease course, and higher disability. Fifteen of 33 patients treated with rituximab were hospitalized. Four deceased patients have progressive MS, 5 were not receiving MS therapy, and 2 were treated (natalizumab and rituximab). Multivariate analysis showed age (OR 1.09, 95% CI, 1.04-1.17) as the only independent risk factor for a fatal outcome.

Conclusions: This study has not demonstrated the presumed critical role of MS therapy in the course of COVID-19 but evidenced that people with MS with advanced age and disease, in progressive course, and those who are more disabled have a higher probability of severe and even fatal disease.

Copyright © 2021 The Author(s). Published by Wolters Kluwer Health, Inc. on behalf of the American Academy of Neurology.

- 18 references

- 2 figures

Supplementary info

Publication types, MeSH terms, Substances Expand

Publication types

- Multicenter Study
- Observational Study
- Research Support, Non-U.S. Gov't

MeSH terms

- Adult
- Age Factors
- COVID-19 / epidemiology
- COVID-19 / physiopathology*
- Comorbidity
- Female
- Humans
- Immunocompromised Host*
- Immunosuppressive Agents / administration & dosage*
- Immunosuppressive Agents / adverse effects
- Male
- Middle Aged
- Multiple Sclerosis, Chronic Progressive / drug therapy*
- Multiple Sclerosis, Chronic Progressive / epidemiology
- Multiple Sclerosis, Relapsing-Remitting / drug therapy*
- Multiple Sclerosis, Relapsing-Remitting / epidemiology
- Neurology
- Registries*
- Retrospective Studies
- Risk Factors
- Severity of Illness Index*
- Sex Factors
- Societies, Medical
- Spain

Substances

- Immunosuppressive Agents

Full text links

Neurology Neuroimmunology & Neuroinflammation HighWire Free PMC article
Proceed to details
Cite
Share
1,215
Observational Study
Neurol Neuroimmunol Neuroinflamm
. 2021 Jul 14;8(5):e1035.
doi: 10.1212/NXI.0000000000001035. Print 2021 Sep.

Ocrelizumab Extended Interval Dosing in Multiple Sclerosis in Times of COVID-19

Leoni Rolfes [1], Marc Pawlitzki [1], Steffen Pfeuffer [1], Christopher Nelke [1], Anke Lux [1], Refik Pul [1], Christoph Kleinschnitz [1], Konstanze Kleinschnitz [1], Rebeca Rogall [1], Katrin Pape [1], Stefan Bittner [1], Frauke Zipp [1], Clemens Warnke [1], Yasemin Goereci [1], Michael Schroeter [1], Jens Ingwersen [1], Orhan Aktas [1], Luisa Klotz [1], Tobias Ruck [1], Heinz Wiendl [1], Sven G Meuth [2]
Affiliations Expand

Affiliations

- [1] From the Department of Neurology with Institute of Translational Neurology (L.R., M.P., S.P., C.N., L.K., H.W.), University Hospital Muenster, Germany; Institute for Biometrics and Bioinformatic (A.L.), Otto-von-Guericke University, Magdeburg, Germany; Department for Neurology (R.P., C.K., K.K., R.R.), University Hospital Essen, Germany; Focus Program Translational Neurosciences (FTN) and Immunology (FZI) (K.P., S.B., F.Z.), Rhine Main Neuroscience Network (rmn2), Department of Neurology, University Medical Center of the Johannes Gutenberg University Mainz, Germany; Department of Neurology (C.W., Y.G., M.S.), University Hospital Cologne, Germany; and Department of Neurology (J.I., O.A., T.R., S.G.M.), Heinrich-Heine University, Duesseldorf, Germany.
- [2] From the Department of Neurology with Institute of Translational Neurology (L.R., M.P., S.P., C.N., L.K., H.W.), University Hospital Muenster, Germany; Institute for Biometrics and Bioinformatic (A.L.), Otto-von-Guericke University, Magdeburg, Germany; Department for Neurology (R.P., C.K., K.K., R.R.), University Hospital Essen, Germany; Focus Program Translational Neurosciences (FTN) and Immunology (FZI) (K.P., S.B., F.Z.), Rhine Main Neuroscience Network (rmn2), Department of Neurology, University Medical Center of the Johannes Gutenberg University Mainz, Germany; Department of Neurology (C.W., Y.G., M.S.), University Hospital Cologne, Germany; and Department of Neurology (J.I., O.A., T.R., S.G.M.), Heinrich-Heine University, Duesseldorf, Germany. sven.meuth@uni-duesseldorf.de.

- PMID: **34261812**
- PMCID: PMC8362352
- DOI: 10.1212/NXI.0000000000001035

Free PMC article
Observational Study

Ocrelizumab Extended Interval Dosing in Multiple Sclerosis in Times of COVID-19

Leoni Rolfes et al. Neurol Neuroimmunol Neuroinflamm. 2021.
Free PMC article
Show details
Neurol Neuroimmunol Neuroinflamm
. 2021 Jul 14;8(5):e1035.
doi: 10.1212/NXI.0000000000001035. Print 2021 Sep.

Authors

Leoni Rolfes[1], Marc Pawlitzki[1], Steffen Pfeuffer[1], Christopher Nelke[1], Anke Lux[1], Refik Pul[1], Christoph Kleinschnitz[1], Konstanze Kleinschnitz[1], Rebeca Rogall[1], Katrin Pape[1], Stefan Bittner[1], Frauke Zipp[1], Clemens Warnke[1], Yasemin Goereci[1], Michael Schroeter[1], Jens Ingwersen[1], Orhan Aktas[1], Luisa Klotz[1], Tobias Ruck[1], Heinz Wiendl[1], Sven G Meuth[2]

Affiliations

- [1] From the Department of Neurology with Institute of Translational Neurology (L.R., M.P., S.P., C.N., L.K., H.W.), University Hospital Muenster, Germany; Institute for Biometrics and Bioinformatic (A.L.), Otto-von-Guericke University, Magdeburg, Germany; Department for Neurology (R.P., C.K., K.K., R.R.), University Hospital Essen, Germany; Focus Program Translational Neurosciences (FTN) and Immunology (FZI) (K.P., S.B., F.Z.), Rhine Main Neuroscience Network (rmn2), Department of Neurology, University Medical Center of the Johannes Gutenberg University Mainz, Germany; Department of Neurology (C.W., Y.G., M.S.), University Hospital Cologne, Germany; and Department of Neurology (J.I., O.A., T.R., S.G.M.), Heinrich-Heine University, Duesseldorf, Germany.
- [2] From the Department of Neurology with Institute of Translational Neurology (L.R., M.P., S.P., C.N., L.K., H.W.), University Hospital Muenster, Germany; Institute for Biometrics and Bioinformatic (A.L.), Otto-von-Guericke University, Magdeburg, Germany; Department for Neurology (R.P., C.K., K.K., R.R.), University Hospital Essen, Germany; Focus Program Translational Neurosciences (FTN) and Immunology (FZI) (K.P., S.B., F.Z.), Rhine Main Neuroscience Network (rmn2), Department of Neurology, University Medical Center of the Johannes Gutenberg University Mainz, Germany; Department of Neurology (C.W., Y.G., M.S.), University Hospital Cologne, Germany; and Department of Neurology (J.I., O.A., T.R., S.G.M.), Heinrich-Heine University, Duesseldorf, Germany. sven.meuth@uni-duesseldorf.de.

- PMID: **34261812**
- PMCID: PMC8362352
- DOI: 10.1212/NXI.0000000000001035

Abstract

Objective: To evaluate the clinical consequences of extended interval dosing (EID) of ocrelizumab in relapsing-remitting multiple sclerosis (RRMS) during the coronavirus disease 2019 (COVID-19) pandemic.

Methods: In our retrospective, multicenter cohort study, we compared patients with RRMS on EID (defined as ≥4-week delay of dose interval) with a control group on standard interval dosing (SID) at the same period (January to December 2020).

Results: Three hundred eighteen patients with RRMS were longitudinally evaluated in 5 German centers. One hundred sixteen patients received ocrelizumab on EID (median delay [interquartile range 8.68 [5.09-13.07] weeks). Three months after the last ocrelizumab infusion, 182 (90.1%) patients following SID and 105 (90.5%) EID patients remained relapse free ($p = 0.903$). Three-month confirmed progression of disability was observed in 18 SID patients (8.9%) and 11 EID patients (9.5%, $p = 0.433$). MRI progression was documented in 9 SID patients (4.5%) and 8 EID patients (6.9%) at 3-month follow-up ($p = 0.232$). Multivariate logistic regression showed no association between treatment regimen and no evidence of disease activity status at follow-up (OR: 1.266 [95% CI: 0.695-2.305]; $p = 0.441$). Clinical stability was accompanied by persistent peripheral $CD19^+$ B-cell depletion in both groups (SID vs EID: 82.6% vs 83.3%, $p = 0.463$). Disease activity in our cohort was not associated with $CD19^+$ B-cell repopulation.

Conclusion: Our data support EID of ocrelizumab as potential risk mitigation strategy in times of the COVID-19 pandemic.

Classification of evidence: This study provides Class IV evidence that for patients with RRMS, an EID of at least 4 weeks does not diminish effectiveness of ocrelizumab.

Copyright © 2021 The Author(s). Published by Wolters Kluwer Health, Inc. on behalf of the American Academy of Neurology.

- 34 references
- 3 figures

Supplementary info

Publication types, MeSH terms, Substances Expand

Publication types

- Multicenter Study
- Observational Study

MeSH terms

- Adult
- Antibodies, Monoclonal, Humanized / administration & dosage*
- Antibodies, Monoclonal, Humanized / therapeutic use*
- Antigens, CD19

- B-Lymphocytes / immunology
- COVID-19 / complications*
- Disability Evaluation
- Female
- Humans
- Lymphocyte Count
- Magnetic Resonance Imaging
- Male
- Middle Aged
- Multiple Sclerosis, Relapsing-Remitting / complications*
- Multiple Sclerosis, Relapsing-Remitting / drug therapy*
- Pandemics
- Retrospective Studies
- Treatment Outcome

Substances

- Antibodies, Monoclonal, Humanized
- Antigens, CD19
- ocrelizumab

Full text links

Neurology Neuroimmunology & Neuroinflammation HighWire Free PMC article

Proceed to details

Cite

Share

☐ 1,216

Observational Study

Dis Markers

. 2021 May 13;2021:8863053.

doi: 10.1155/2021/8863053. eCollection 2021.

Simple Parameters from Complete Blood Count Predict In-Hospital Mortality in COVID-19

Mattia Bellan [1 2], Danila Azzolina [1], Eyal Hayden [1 2], Gianluca Gaidano [1 2], Mario Pirisi [1 2], Antonio Acquaviva [1 2], Gianluca Aimaretti [1 2], Paolo Aluffi Valletti [1 2], Roberto Angilletta [3], Roberto Arioli [1 2], Gian Carlo Avanzi [1 2], Gianluca Avino [1 2], Piero Emilio Balbo [2], Giulia Baldon [1 2], Francesca Baorda [1 4], Emanuela Barbero [1 2], Alessio Baricich [1 2], Michela Barini

[2], Francesco Barone-Adesi [1], Sofia Battistini [1, 2], Michela Beltrame [1, 2], Matteo Bertoli [1, 2], Stephanie Bertolin [1, 2], Marinella Bertolotti [3], Marta Betti [3], Flavio Bobbio [2], Paolo Boffano [1, 2], Lucio Boglione [1, 4], Silvio Borrè [4], Matteo Brucoli [1, 2], Elisa Calzaducca [1, 2], Edoardo Cammarata [1, 2], Vincenzo Cantaluppi [1, 2], Roberto Cantello [1, 2], Andrea Capponi [2], Alessandro Carriero [1, 2], Giuseppe Francesco Casciaro [1, 2], Luigi Mario Castello [1, 2], Federico Ceruti [1, 2], Guido Chichino [3], Emilio Chirico [1, 2], Carlo Cisari [1, 2], Micol Giulia Cittone [1, 2], Crizia Colombo [1, 2], Cristoforo Comi [1, 4], Eleonora Croce [1, 4], Tommaso Daffara [1, 2], Pietro Danna [1, 2], Francesco Della Corte [1, 2], Simona De Vecchi [1, 2], Umberto Dianzani [1, 2], Davide Di Benedetto [1, 2], Elia Esposto [1, 2], Fabrizio Faggiano [1], Zeno Falaschi [1, 2], Daniela Ferrante [1], Alice Ferrero [1, 2], Ileana Gagliardi [1, 2], Alessandra Galbiati [1, 2], Silvia Gallo [1, 4], Pietro Luigi Garavelli [2], Clara Ada Gardino [1, 2], Massimiliano Garzaro [1, 2], Maria Luisa Gastaldello [1, 2], Francesco Gavelli [1, 2], Alessandra Gennari [1, 2], Greta Maria Giacomini [1, 2], Irene Giacone [1, 4], Valentina Giai Via [1, 2], Francesca Giolitti [1, 2], Laura Cristina Gironi [1, 2], Carla Gramaglia [1, 2], Leonardo Grisafi [1, 2], Ilaria Inserra [1, 2], Marco Invernizzi [1, 2], Marco Krengli [1, 2], Emanuela Labella [1, 2], Irene Cecilia Landi [1, 2], Raffaella Landi [1, 2], Ilaria Leone [1, 2], Veronica Lio [1, 2], Luca Lorenzini [1, 2], Antonio Maconi [3], Mario Malerba [1, 4], Giulia Francesca Manfredi [1, 2], Maria Martelli [1, 2], Letizia Marzari [1, 2], Paolo Marzullo [1, 2], Marco Mennuni [1, 2], Claudia Montabone [1, 4], Umberto Morosini [1, 2], Marco Mussa [3], Ilaria Nerici [1, 2], Alessandro Nuzzo [1, 2], Carlo Olivieri [1, 4], Samuel Alberto Padelli [1, 4], Massimiliano Panella [1], Andrea Parisini [3], Alessio Paschè [1, 2], Filippo Patrucco [1, 2], Giuseppe Patti [1, 2], Alberto Pau [1, 2], Anita Rebecca Pedrinelli [1, 2], Ilaria Percivale [1, 2], Luca Ragazzoni [1], Roberta Re [1, 4], Cristina Rigamonti [1, 2], Eleonora Rizzi [1, 2], Andrea Rognoni [1, 2], Annalisa Roveta [3], Luigia Salamina [2], Matteo Santagostino [1, 2], Massimo Saraceno [1, 2], Paola Savoia [1, 2], Marco Sciarra [3], Andrea Schimmenti [3], Lorenza Scotti [1], Enrico Spinoni [1, 2], Carlo Smirne [1, 2], Vanessa Tarantino [1, 2], Paolo Amedeo Tillio [1, 4], Stelvio Tonello [1], Rosanna Vaschetto [1, 2], Veronica Vassia [1, 2], Domenico Zagaria [1, 2], Elisa Zavattaro [1, 2], Patrizia Zeppegno [1, 2], Francesca Zottarelli [1, 2], Pier Paolo Sainaghi [1, 2]

Affiliations Expand

Affiliations

- [1] Università del Piemonte Orientale UPO, Novara, Italy.
- [2] "AOU Maggiore della Carità", Novara, Italy.
- [3] Azienda Ospedaliera SS. Antonio e Biagio e Cesare Arrigo, Alessandria, Italy.
- [4] "Sant'Andrea" Hospital, Vercelli, Italy.

- PMID: **34055104**
- PMCID: PMC8123088
- DOI: 10.1155/2021/8863053

Free PMC article
Observational Study

Simple Parameters from Complete Blood Count Predict In-Hospital Mortality in COVID-19

Mattia Bellan et al. Dis Markers. 2021.
Free PMC article
Show details
Dis Markers
. 2021 May 13;2021:8863053.
doi: 10.1155/2021/8863053. eCollection 2021.

Authors

Mattia Bellan [1,2], Danila Azzolina [1], Eyal Hayden [1,2], Gianluca Gaidano [1,2], Mario Pirisi [1,2], Antonio Acquaviva [1,2], Gianluca Aimaretti [1,2], Paolo Aluffi Valletti [1,2], Roberto Angilletta [3], Roberto Arioli [1,2], Gian Carlo Avanzi [1,2], Gianluca Avino [1,2], Piero Emilio Balbo [2], Giulia Baldon [1,2], Francesca Baorda [1,4], Emanuela Barbero [1,2], Alessio Baricich [1,2], Michela Barini [2], Francesco Barone-Adesi [1], Sofia Battistini [1,2], Michela Beltrame [1,2], Matteo Bertoli [1,2], Stephanie Bertolin [1,2], Marinella Bertolotti [3], Marta Betti [3], Flavio Bobbio [2], Paolo Boffano [1,2], Lucio Boglione [1,4], Silvio Borrè [4], Matteo Brucoli [1,2], Elisa Calzaducca [1,2], Edoardo Cammarata [1,2], Vincenzo Cantaluppi [1,2], Roberto Cantello [1,2], Andrea Capponi [2], Alessandro Carriero [1,2], Giuseppe Francesco Casciaro [1,2], Luigi Mario Castello [1,2], Federico Ceruti [1,2], Guido Chichino [3], Emilio Chirico [1,2], Carlo Cisari [1,2], Micol Giulia Cittone [1,2], Crizia Colombo [1,2], Cristoforo Comi [1,4], Eleonora Croce [1,4], Tommaso Daffara [1,2], Pietro Danna [1,2], Francesco Della Corte [1,2], Simona De Vecchi [1,2], Umberto Dianzani [1,2], Davide Di Benedetto [1,2], Elia Esposto [1,2], Fabrizio Faggiano [1], Zeno Falaschi [1,2], Daniela Ferrante [1], Alice Ferrero [1,2], Ileana Gagliardi [1,2], Alessandra Galbiati [1,2], Silvia Gallo [1,4], Pietro Luigi Garavelli [2], Clara Ada Gardino [1,2], Massimiliano Garzaro [1,2], Maria Luisa Gastaldello [1,2], Francesco Gavelli [1,2], Alessandra Gennari [1,2], Greta Maria Giacomini [1,2], Irene Giacone [1,4], Valentina Giai Via [1,2], Francesca Giolitti [1,2], Laura Cristina Gironi [1,2], Carla Gramaglia [1,2], Leonardo Grisafi [1,2], Ilaria Inserra [1,2], Marco Invernizzi [1,2], Marco Krengli [1,2], Emanuela Labella [1,2], Irene Cecilia Landi [1,2], Raffaella Landi [1,2], Ilaria Leone [1,2], Veronica Lio [1,2], Luca Lorenzini [1,2], Antonio Maconi [3], Mario Malerba [1,4], Giulia Francesca Manfredi [1,2], Maria Martelli [1,2], Letizia Marzari [1,2], Paolo Marzullo [1,2], Marco Mennuni [1,2], Claudia Montabone [1,4], Umberto Morosini [1,2], Marco Mussa [3], Ilaria Nerici [1,2], Alessandro Nuzzo [1,2], Carlo Olivieri [1,4], Samuel Alberto Padelli [1,4], Massimiliano Panella [1], Andrea Parisini [3], Alessio Paschè [1,2], Filippo Patrucco [1,2], Giuseppe Patti [1,2], Alberto Pau [1,2], Anita Rebecca Pedrinelli [1,2], Ilaria Percivale [1,2], Luca Ragazzoni [1], Roberta Re [1,4], Cristina Rigamonti [1,2], Eleonora Rizzi [1,2], Andrea Rognoni [1,2], Annalisa Roveta [3], Luigia Salamina [2], Matteo Santagostino [1,2], Massimo Saraceno [1,2], Paola Savoia [1,2], Marco Sciarra [3], Andrea Schimmenti [3], Lorenza Scotti [1], Enrico Spinoni [1,2], Carlo Smirne [1,2], Vanessa Tarantino [1,2], Paolo Amedeo Tillio [1,4], Stelvio Tonello [1], Rosanna Vaschetto [1,2], Veronica Vassia [1,2], Domenico Zagaria [1,2], Elisa Zavattaro [1,2], Patrizia Zeppegno [1,2], Francesca Zottarelli [1,2], Pier Paolo Sainaghi [1,2]

Affiliations

- [1] Università del Piemonte Orientale UPO, Novara, Italy.
- [2] "AOU Maggiore della Carità", Novara, Italy.
- [3] Azienda Ospedaliera SS. Antonio e Biagio e Cesare Arrigo, Alessandria, Italy.
- [4] "Sant'Andrea" Hospital, Vercelli, Italy.

- PMID: **34055104**
- PMCID: PMC8123088
- DOI: 10.1155/2021/8863053

Abstract

Introduction: The clinical course of Coronavirus Disease 2019 (COVID-19) is highly heterogenous, ranging from asymptomatic to fatal forms. The identification of clinical and laboratory predictors of poor prognosis may assist clinicians in monitoring strategies and therapeutic decisions.

Materials and methods: In this study, we retrospectively assessed the prognostic value of a simple tool, the complete blood count, on a cohort of 664 patients (*F* 260; 39%, median age 70 (56-81) years) hospitalized for COVID-19 in Northern Italy. We collected demographic data along with complete blood cell count; moreover, the outcome of the hospital in-stay was recorded.

Results: At data cut-off, 221/664 patients (33.3%) had died and 453/664 (66.7%) had been discharged. Red cell distribution width (RDW) (χ^2 10.4; $p < 0.001$), neutrophil-to-lymphocyte (NL) ratio (χ^2 7.6; $p = 0.006$), and platelet count (χ^2 5.39; $p = 0.02$), along with age (χ^2 87.6; $p < 0.001$) and gender (χ^2 17.3; $p < 0.001$), accurately predicted in-hospital mortality. Hemoglobin levels were not associated with mortality. We also identified the best cut-off for mortality prediction: a NL ratio > 4.68 was characterized by an odds ratio for in-hospital mortality (OR) = 3.40 (2.40-4.82), while the OR for a RDW > 13.7% was 4.09 (2.87-5.83); a platelet count > 166,000/μL was, conversely, protective (OR: 0.45 (0.32-0.63)).

Conclusion: Our findings arise the opportunity of stratifying COVID-19 severity according to simple lab parameters, which may drive clinical decisions about monitoring and treatment.

Copyright © 2021 Mattia Bellan et al.

Conflict of interest statement

The authors have no conflict of interest to declare.

- 42 references

Supplementary info

Publication types, MeSH terms Expand

Publication types

- Observational Study

MeSH terms

- Adult
- Aged
- Aged, 80 and over
- Blood Cell Count*
- COVID-19 / blood*
- COVID-19 / diagnosis
- COVID-19 / mortality*
- Clinical Decision Rules*
- Female
- Hospital Mortality*
- Humans
- Italy / epidemiology
- Male
- Middle Aged
- Multivariate Analysis
- Prognosis
- Retrospective Studies
- Severity of Illness Index*

Full text links

Hindawi Limited Free PMC article
Proceed to details
Cite
Share
1,217
Observational Study
PLoS One
. 2020 Sep 22;15(9):e0239401.
doi: 10.1371/journal.pone.0239401. eCollection 2020.

Second week methyl-prednisolone pulses improve prognosis in patients with severe coronavirus disease 2019 pneumonia: An observational comparative study using routine care data

Guillermo Ruiz-Irastorza[1 2 3], Jose-Ignacio Pijoan[4 2 5], Elena Bereciartua[6 2 3], Susanna Dunder[7 2], Jokin Dominguez[7 2], Paula Garcia-Escudero[8 2], Alejandro Rodrigo[7 2], Carlota Gomez-Carballo[7 2], Jimena Varona[7 2], Laura Guio[6 2 3], Marta Ibarrola[6 2], Amaia Ugarte[1 2], Agustin Martinez-Berriotxoa[7 2 3], Cruces COVID Study Group
Affiliations Expand

Affiliations

- [1] Autoimmune Diseases Research Unit, Service of Internal Medicine, Hospital Universitario Cruces, Barakaldo, Bizkaia, Spain.
- [2] Infectious Diseases Unit, Hospital Universitario Cruces, Barakaldo, Bizkaia, Spain.
- [3] Service of Internal Medicine, Hospital Universitario Cruces, Barakaldo, Bizkaia, Spain.
- [4] Biocruces Bizkaia Health Research Institute, Barakaldo, Bizkaia, Spain.
- [5] Service of Rheumatology, Hospital Universitario Cruces, Barakaldo, Bizkaia, Spain.
- [6] University of the Basque Country (UPV/EHU), Leioa, BI, Spain.
- [7] Clinical Epidemiology Unit, Hospital Universitario Cruces, Barakaldo, Bizkaia, Spain.
- [8] CIBER of Epidemiology and Public Health (CIBERESP), Madrid, Spain.

- PMID: **32960899**
- PMCID: PMC7508405
- DOI: 10.1371/journal.pone.0239401

Free PMC article
Observational Study

Second week methyl-prednisolone pulses improve prognosis in patients with severe coronavirus disease 2019 pneumonia: An observational comparative study using routine care data

Guillermo Ruiz-Irastorza et al. PLoS One. 2020.
Free PMC article
Show details
PLoS One
. 2020 Sep 22;15(9):e0239401.
doi: 10.1371/journal.pone.0239401. eCollection 2020.

Authors

Guillermo Ruiz-Irastorza[1 2 3], Jose-Ignacio Pijoan[4 2 5], Elena Bereciartua[6 2 3], Susanna Dunder[7 2], Jokin Dominguez[7 2], Paula Garcia-Escudero[8 2], Alejandro Rodrigo[7 2], Carlota Gomez-Carballo[7 2], Jimena Varona[7 2], Laura Guio[6 2 3], Marta Ibarrola[6 2], Amaia Ugarte[1 2], Agustin Martinez-Berriotxoa[7 2 3], Cruces COVID Study Group

Affiliations

- [1] Autoimmune Diseases Research Unit, Service of Internal Medicine, Hospital Universitario Cruces, Barakaldo, Bizkaia, Spain.
- [2] Infectious Diseases Unit, Hospital Universitario Cruces, Barakaldo, Bizkaia, Spain.
- [3] Service of Internal Medicine, Hospital Universitario Cruces, Barakaldo, Bizkaia, Spain.
- [4] Biocruces Bizkaia Health Research Institute, Barakaldo, Bizkaia, Spain.
- [5] Service of Rheumatology, Hospital Universitario Cruces, Barakaldo, Bizkaia, Spain.
- [6] University of the Basque Country (UPV/EHU), Leioa, BI, Spain.
- [7] Clinical Epidemiology Unit, Hospital Universitario Cruces, Barakaldo, Bizkaia, Spain.
- [8] CIBER of Epidemiology and Public Health (CIBERESP), Madrid, Spain.

- PMID: **32960899**
- PMCID: PMC7508405
- DOI: 10.1371/journal.pone.0239401

Abstract

Objective: To analyze the effects of a short course of methyl-prednisolone pulses (MP) during the second week of disease (week-2) in patients with severe coronavirus disease 2019 (COVID-19) pneumonia.

Methods: Comparative observational study using data collected from routine care at Hospital Universitario Cruces, Barakaldo, Bizkaia, Spain in patients with COVID-19 pneumonia. We compared patients who received week-2-MP (125-250 mg/d x3) with those who did not, with the end-points time to death and time to death or endotracheal intubation.

Results: We included 242 patients with COVID-19 pneumonia and elevated inflammatory markers at admission. Sixty-one patients (25%) received week-2-MP. Twenty-two patients (9%) died and 31 (12.8%) suffered death or intubation. The adjusted HRs for death and death or intubation for patients in the week-2-MP group were 0.35 (95%CI 0.11 to 1.06, p = 0.064) and 0.33 (95%CI 0.13 to 0.84, p = 0.020), respectively. These differences were specifically seen in the subcohort of patients with a SpO2/FiO2 at day 7 lower than 353 (adjusted HR 0.31, 95% CI 0.08 to 1.12, p = 0.073 and HR 0.34, 95%CI 0.12 to 0.94, p = 0.038, respectively) but not in patients with higher SpO2/FiO2. Patients receiving out-of-week-2-MP, non-pulse glucocorticoids or no glucocorticoids had an increased adjusted risk for both outcomes compared with week-2-MP group: HR 5.04 (95% CI 0.91-27.86), HR 10.09 (95% CI 2.14-47.50), HR 4.14 (95% CI 0.81-21.23), respectively, for death; HR 7.38 (95% CI 1.86-29.29), HR 13.71 (95% CI 3.76-50.07), HR 3.58 (95% CI 0.89-14.32), respectively, for death or intubation. These differences were significant only in the subgroup with low SpO2/FiO2.

Conclusions: Week-2-MP are effective in improving the prognosis of patients with COVID-19 pneumonia with features of inflammatory activity and respiratory deterioration entering the second week of disease. The recognition of this high-risk population should prompt early use of MP at this point.

Conflict of interest statement

The authors have declared that no competing interests exist.

- 20 references

- 2 figures

Supplementary info

Publication types, MeSH terms, Substances, Grant support Expand

Publication types

- Comparative Study
- Observational Study

MeSH terms

- Aged
- COVID-19
- Coronavirus Infections / diagnosis*
- Coronavirus Infections / pathology
- Female
- Glucocorticoids / therapeutic use
- Humans
- Inflammation
- Intubation, Intratracheal
- Male
- Methylprednisolone / administration & dosage*
- Methylprednisolone / pharmacology
- Methylprednisolone / therapeutic use
- Middle Aged
- Oxygen / blood
- Pandemics
- Pneumonia, Viral / diagnosis*
- Pneumonia, Viral / pathology
- Prognosis
- Retrospective Studies
- Risk
- Risk Factors
- Spain
- Time Factors

Substances

- Glucocorticoids
- Oxygen

- Methylprednisolone

Grant support

The authors received no specific funding for this work.

Full text links

OPEN ACCESS TO FULL TEXT PLOS ONE Public Library of Science Free PMC article
Proceed to details
Cite
Share
1,218
Observational Study
J Int Med Res
. 2020 Sep;48(9):300060520955037.
doi: 10.1177/0300060520955037.

Thrombo-inflammatory features predicting mortality in patients with COVID-19: The FAD-85 score

Junhong Wang [1], Hua Zhang [2], Rui Qiao [3], Qinggang Ge [4], Shuisheng Zhang [5], Zongxuan Zhao [6], Ci Tian [1], Qingbian Ma [1], Ning Shen [7]
Affiliations Expand

Affiliations

- [1] Emergency Department, Peking University Third Hospital, Beijing, China.
- [2] Clinical Epidemiology Research Center, Peking University Third Hospital, Beijing, China.
- [3] Department of Clinical Laboratory Medicine, Peking University Third Hospital, Beijing, China.
- [4] Department of Critical Care Medicine, Peking University Third Hospital, Beijing, China.
- [5] Department of General Surgery, Peking University Third Hospital, Beijing, China.
- [6] The Third School of Clinical Medicine, Peking University, Beijing, China.
- [7] Department of Respiratory and Critical Medicine, Peking University Third Hospital, Beijing, China.

- PMID: **32960106**
- PMCID: PMC7511832
- DOI: 10.1177/0300060520955037

Free PMC article
Observational Study

Thrombo-inflammatory features predicting mortality in patients with COVID-19: The FAD-85 score

Junhong Wang et al. J Int Med Res. 2020 Sep.
Free PMC article
Show details
J Int Med Res
. 2020 Sep;48(9):300060520955037.
doi: 10.1177/0300060520955037.

Authors

Junhong Wang [1], Hua Zhang [2], Rui Qiao [3], Qinggang Ge [4], Shuisheng Zhang [5], Zongxuan Zhao [6], Ci Tian [1], Qingbian Ma [1], Ning Shen [7]

Affiliations

- [1] Emergency Department, Peking University Third Hospital, Beijing, China.
- [2] Clinical Epidemiology Research Center, Peking University Third Hospital, Beijing, China.
- [3] Department of Clinical Laboratory Medicine, Peking University Third Hospital, Beijing, China.
- [4] Department of Critical Care Medicine, Peking University Third Hospital, Beijing, China.
- [5] Department of General Surgery, Peking University Third Hospital, Beijing, China.
- [6] The Third School of Clinical Medicine, Peking University, Beijing, China.
- [7] Department of Respiratory and Critical Medicine, Peking University Third Hospital, Beijing, China.

- PMID: **32960106**
- PMCID: PMC7511832
- DOI: 10.1177/0300060520955037

Abstract

Background: The roles of inflammation and hypercoagulation in predicting outcomes of coronavirus disease 2019 (COVID-19) are unclear.

Methods: Adult patients diagnosed with COVID-19 from 28 January 2020 to 4 March 2020 in Tongji Hospital, Wuhan were recruited. Data on related parameters were collected. Univariate analysis and multivariable binary logistic regression were used to explore predictors of critical illness and mortality.

Results: In total, 199 and 44 patients were enrolled in the training and testing sets, respectively. Elevated ferritin, tumor necrosis factor-α and D-dimer and decreased albumin concentration were associated with disease severity. Older age, elevated ferritin and elevated interleukin-6 were associated with 28-day mortality. The FAD-85 score, defined as age + 0.01 * ferritin +D-dimer, was used to predict risk of mortality. The sensitivity, specificity and accuracy of FAD-85 were

86.4%, 81.8% and 86.4%, respectively. A nomogram was established using age, ferritin and D-dimer to predict the risk of 28-day mortality.

Conclusions: Thrombo-inflammatory parameters provide key information on the severity and prognosis of COVID-19 and can be used as references for clinical treatment to correct inflammatory and coagulation abnormalities.

Keywords: COVID-19; coagulation; inflammation; model; mortality; predictor.

- 31 references
- 3 figures

Supplementary info

Publication types, MeSH terms, Substances Expand

Publication types

- Observational Study

MeSH terms

- Adult
- Aged
- Betacoronavirus / pathogenicity*
- Biomarkers / blood
- COVID-19
- Coronavirus Infections / complications
- Coronavirus Infections / diagnosis
- Coronavirus Infections / mortality*
- Coronavirus Infections / virology
- Disseminated Intravascular Coagulation / complications
- Disseminated Intravascular Coagulation / diagnosis
- Disseminated Intravascular Coagulation / mortality*
- Disseminated Intravascular Coagulation / virology
- Female
- Ferritins / blood
- Fibrin Fibrinogen Degradation Products / metabolism
- Humans
- Interleukin-6 / blood
- Logistic Models
- Male
- Middle Aged
- Pandemics
- Pneumonia, Viral / complications

- Pneumonia, Viral / diagnosis
- Pneumonia, Viral / mortality*
- Pneumonia, Viral / virology
- Prognosis
- Research Design
- Retrospective Studies
- SARS-CoV-2
- Serum Albumin / metabolism
- Severity of Illness Index
- Survival Analysis
- Thrombosis / complications
- Thrombosis / diagnosis
- Thrombosis / mortality*
- Thrombosis / virology
- Tumor Necrosis Factor-alpha / blood

Substances

- Biomarkers
- Fibrin Fibrinogen Degradation Products
- IL6 protein, human
- Interleukin-6
- Serum Albumin
- Tumor Necrosis Factor-alpha
- fibrin fragment D
- Ferritins

Full text links

SAGE journals Open access full text Atypon Free PMC article
Proceed to details
Cite
Share
☐ 1,219
Observational Study
J Am Soc Nephrol
. 2020 Jul;31(7):1387-1397.
doi: 10.1681/ASN.2020030354. Epub 2020 May 8.

Clinical Characteristics of and Medical Interventions for COVID-19 in Hemodialysis Patients in Wuhan, China

Fei Xiong [1], Hui Tang [2], Li Liu [3], Can Tu [1], Jian-Bo Tian [3], Chun-Tao Lei [2], Jing Liu [2], Jun-Wu Dong [4], Wen-Li Chen [5], Xiao-Hui Wang [6], Dan Luo [7], Ming Shi [8], Xiao-Ping Miao [3], Chun Zhang [9]
Affiliations Expand

Affiliations

- [1] Department of Nephrology, Wuhan No. 1 Hospital, Wuhan, China.
- [2] Department of Nephrology, Union Hospital, Tongji Medical College, Huazhong University of Science and Technology, Wuhan, China.
- [3] Department of Epidemiology and Biostatistics, Ministry of Education Key Laboratory of Environment and Health, School of Public Health, Tongji Medical College, Huazhong University of Science and Technology, Wuhan, China.
- [4] Department of Nephrology, Wuhan Fourth Hospital, Tongji Medical College, Huazhong University of Science and Technology, Wuhan, China.
- [5] Department of Nephrology, The Central Hospital of Wuhan, Tongji Medical College, Huazhong University of Science and Technology, Wuhan, China.
- [6] Department of Nephrology, Wuhan No. 5 Hospital, Wuhan, China.
- [7] Department of Nephrology, Wuhan Third Hospital, Wuhan, China.
- [8] Department of Nephrology, Renmin Hospital of Wuhan University, Wuhan, China.
- [9] Department of Nephrology, Union Hospital, Tongji Medical College, Huazhong University of Science and Technology, Wuhan, China drzhangchun@hust.edu.cn.

- PMID: **32385130**
- PMCID: PMC7350995
- DOI: 10.1681/ASN.2020030354

Free PMC article
Observational Study

Clinical Characteristics of and Medical Interventions for COVID-19 in Hemodialysis Patients in Wuhan, China

Fei Xiong et al. J Am Soc Nephrol. 2020 Jul.
Free PMC article
Show details
J Am Soc Nephrol
. 2020 Jul;31(7):1387-1397.
doi: 10.1681/ASN.2020030354. Epub 2020 May 8.

Authors

Fei Xiong [1], Hui Tang [2], Li Liu [3], Can Tu [1], Jian-Bo Tian [3], Chun-Tao Lei [2], Jing Liu [2], Jun-Wu Dong [4], Wen-Li Chen [5], Xiao-Hui Wang [6], Dan Luo [7], Ming Shi [8], Xiao-Ping Miao [3], Chun Zhang [9]

Affiliations

- [1] Department of Nephrology, Wuhan No. 1 Hospital, Wuhan, China.
- [2] Department of Nephrology, Union Hospital, Tongji Medical College, Huazhong University of Science and Technology, Wuhan, China.
- [3] Department of Epidemiology and Biostatistics, Ministry of Education Key Laboratory of Environment and Health, School of Public Health, Tongji Medical College, Huazhong University of Science and Technology, Wuhan, China.
- [4] Department of Nephrology, Wuhan Fourth Hospital, Tongji Medical College, Huazhong University of Science and Technology, Wuhan, China.
- [5] Department of Nephrology, The Central Hospital of Wuhan, Tongji Medical College, Huazhong University of Science and Technology, Wuhan, China.
- [6] Department of Nephrology, Wuhan No. 5 Hospital, Wuhan, China.
- [7] Department of Nephrology, Wuhan Third Hospital, Wuhan, China.
- [8] Department of Nephrology, Renmin Hospital of Wuhan University, Wuhan, China.
- [9] Department of Nephrology, Union Hospital, Tongji Medical College, Huazhong University of Science and Technology, Wuhan, China drzhangchun@hust.edu.cn.

- PMID: **32385130**
- PMCID: PMC7350995
- DOI: 10.1681/ASN.2020030354

Abstract

Background: Reports indicate that those most vulnerable to developing severe coronavirus disease 2019 (COVID-19) are older adults and those with underlying illnesses, such as diabetes mellitus, hypertension, or cardiovascular disease, which are common comorbidities among patients undergoing maintenance hemodialysis. However, there is limited information about the clinical characteristics of hemodialysis patients with COVID-19 or about interventions to control COVID-19 in hemodialysis centers.

Methods: We collected data retrospectively through an online registration system that includes all patients receiving maintenance hemodialysis at 65 centers in Wuhan, China. We reviewed epidemiologic and clinical data of patients with laboratory-confirmed COVID-19 between January 1, 2020 and March 10, 2020.

Results: Of 7154 patients undergoing hemodialysis, 154 had laboratory-confirmed COVID-19. The mean age of the 131 patients in our analysis was 63.2 years; 57.3% were men. Many had underlying comorbidities, with cardiovascular disease (including hypertension) being the most common (68.7%). Only 51.9% of patients manifested fever; 21.4% of infected patients were asymptomatic. The most common finding on chest computed tomography (CT) was ground-grass or patchy opacity (82.1%). After initiating comprehensive interventions-including entrance screening of body temperature and symptoms, universal chest CT and blood tests, and other

measures-new patients presenting with COVID-19 peaked at 10 per day on January 30, decreasing to 4 per day on February 11. No new cases occurred between February 26 and March 10, 2020.

Conclusions: We found that patients receiving maintenance hemodialysis were susceptible to COVID-19 and that hemodialysis centers were high-risk settings during the epidemic. Increasing prevention efforts, instituting universal screening, and isolating patients with COVID-19 and directing them to designated hemodialysis centers were effective in preventing the spread of COVID-19 in hemodialysis centers.

Keywords: COVID-19; Clinical characteristics; Interventions; hemodialysis.

Copyright © 2020 by the American Society of Nephrology.

- 3 figures

Supplementary info

Publication types, MeSH terms Expand

Publication types

- Multicenter Study
- Observational Study

MeSH terms

- Age Factors
- Aged
- COVID-19
- Chi-Square Distribution
- China / epidemiology
- Comorbidity
- Coronavirus Infections / diagnosis
- Coronavirus Infections / epidemiology*
- Coronavirus Infections / therapy
- Disease Susceptibility / epidemiology*
- Female
- Humans
- Kidney Failure, Chronic / diagnosis
- Kidney Failure, Chronic / epidemiology*
- Kidney Failure, Chronic / therapy
- Male
- Middle Aged
- Pandemics
- Pneumonia, Viral / diagnosis
- Pneumonia, Viral / epidemiology*

- Pneumonia, Viral / therapy
- Prevalence
- Radiography, Thoracic / methods
- Registries*
- Renal Dialysis / methods*
- Renal Dialysis / statistics & numerical data
- Retrospective Studies
- Risk Assessment
- Sex Factors
- Statistics, Nonparametric
- Survival Analysis
- Tomography, X-Ray Computed / methods

Full text links

Final Version FREE J Am Soc Nephrol HighWire Free PMC article

Proceed to details

Cite

Share

☐ 1,220

Observational Study

Clin Pharmacokinet

. 2020 Oct;59(10):1251-1260.

doi: 10.1007/s40262-020-00933-8.

Comparative Population Pharmacokinetics of Darunavir in SARS-CoV-2 Patients vs. HIV Patients: The Role of Interleukin-6

Pier Giorgio Cojutti [1] [2], Angela Londero [3], Paola Della Siega [3], Filippo Givone [3], Martina Fabris [4], Jessica Biasizzo [4], Carlo Tascini [3], Federico Pea [5] [6]

Affiliations Expand

Affiliations

- [1] Department of Medicine, University of Udine, Udine, Italy.
- [2] Institute of Clinical Pharmacology, Santa Maria della Misericordia University Hospital of Udine, ASUFC, P.le S. Maria della Misericordia 3, 33100, Udine, Italy.
- [3] Clinic of Infectious Diseases, Santa Maria della Misericordia University Hospital of Udine, ASUFC, Udine, Italy.
- [4] Institute of Clinical Pathology, Santa Maria della Misericordia University Hospital of Udine, ASUFC, Udine, Italy.
- [5] Department of Medicine, University of Udine, Udine, Italy. federico.pea@uniud.it.

- [6] Institute of Clinical Pharmacology, Santa Maria della Misericordia University Hospital of Udine, ASUFC, P.le S. Maria della Misericordia 3, 33100, Udine, Italy. federico.pea@uniud.it.

- PMID: **32856282**
- PMCID: PMC7453069
- DOI: 10.1007/s40262-020-00933-8

Free PMC article
Observational Study

Comparative Population Pharmacokinetics of Darunavir in SARS-CoV-2 Patients vs. HIV Patients: The Role of Interleukin-6

Pier Giorgio Cojutti et al. Clin Pharmacokinet. 2020 Oct.
Free PMC article
Show details
Clin Pharmacokinet
. 2020 Oct;59(10):1251-1260.
doi: 10.1007/s40262-020-00933-8.

Authors

Pier Giorgio Cojutti [1 2], Angela Londero [3], Paola Della Siega [3], Filippo Givone [3], Martina Fabris [4], Jessica Biasizzo [4], Carlo Tascini [3], Federico Pea [5 6]

Affiliations

- [1] Department of Medicine, University of Udine, Udine, Italy.
- [2] Institute of Clinical Pharmacology, Santa Maria della Misericordia University Hospital of Udine, ASUFC, P.le S. Maria della Misericordia 3, 33100, Udine, Italy.
- [3] Clinic of Infectious Diseases, Santa Maria della Misericordia University Hospital of Udine, ASUFC, Udine, Italy.
- [4] Institute of Clinical Pathology, Santa Maria della Misericordia University Hospital of Udine, ASUFC, Udine, Italy.
- [5] Department of Medicine, University of Udine, Udine, Italy. federico.pea@uniud.it.
- [6] Institute of Clinical Pharmacology, Santa Maria della Misericordia University Hospital of Udine, ASUFC, P.le S. Maria della Misericordia 3, 33100, Udine, Italy. federico.pea@uniud.it.

- PMID: **32856282**
- PMCID: PMC7453069
- DOI: 10.1007/s40262-020-00933-8

Abstract

Background: Darunavir is an anti-HIV protease inhibitor repurposed for SARS-CoV-2 treatment.

Objective: The aim of this study was to assess the population pharmacokinetics of darunavir in SARS-CoV-2 patients compared with HIV patients.

Methods: Two separate models were created by means of a nonlinear mixed-effect approach. The influence of clinical covariates on each basic model was tested and the association of significant covariates with darunavir parameters was assessed at multivariate regression and classification and regression tree (CART) analyses. Monte Carlo simulation assessed the influence of covariates on the darunavir concentration versus time profile.

Results: A one-compartment model well-described darunavir concentrations in both groups. In SARS-CoV-2 patients (n = 30), interleukin (IL)-6 and body surface area were covariates associated with darunavir oral clearance (CL/F) and volume of distribution (V_d), respectively; no covariates were identified in HIV patients (n = 25). Darunavir CL/F was significantly lower in SARS-CoV-2 patients compared with HIV patients (4.1 vs. 10.3 L/h; $p < 0.001$). CART analysis found that an IL-6 level of 18 pg/mL may split the SARS-CoV-2 population in patients with low versus high darunavir CL/F (mean ± standard deviation 3.47 ± 1.90 vs. 8.03 ± 3.24 L/h; proportion of reduction in error = 0.46). Median (interquartile range) darunavir CL/F was significantly lower in SARS-CoV-2 patients with IL-6 levels ≥ 18 pg/mL than in SARS-CoV-2 patients with IL-6 levels < 18 pg/mL or HIV patients (2.78 [2.16-4.47] vs. 7.24 [5.88-10.38] vs. 9.75 [8.45-13.79] L/h, respectively; $p < 0.0001$). Increasing IL-6 levels affected darunavir concentration versus time simulated profiles. We hypothesized that increases in IL-6 levels associated with severe SARS-CoV-2 disease may downregulate the cytochrome P450 (CYP) 3A4-mediated metabolism of darunavir.

Conclusions: This is a proof-of-concept of SARS-CoV-2 disease-drug interactions, and may support the need for optimal dose selection of sensitive CYP3A4 substrates in severe SARS-CoV-2 patients.

Conflict of interest statement

F.P. participated in speaker bureau for Angelini, Basilea Pharmaceutica, Gilead, Hikma, Merck Sharp & Dohme, Nordic Pharma, Pfizer and Sanofi Aventis, and in advisory board for Angelini, Basilea Pharmaceutica, Correvio, Gilead, Hikma, Merck Sharp & Dohme, Nordic Pharma, Novartis, Pfizer, Shionogi and Thermo-Fisher. A.L. participated in speaker bureau for Viiv, Gilead, Merck Sharp & Dohme and Janssen. C.T. participated in speaker bureau and received unconditional grants from Angelini, Biomerieux, Biotest, Gilead, Correvio, Hikma, Merck Sharp & Dohme, Pfizer, Shionogi, Thermo-Fisher, and Nordic Pharma. All other authors have no conflict of interest.

Comment in

- Comment on "Comparative Population Pharmacokinetics of Darunavir in SARS-CoV-2 Patients vs. HIV Patients: The Role of Interleukin-6".
  Cattaneo D, Corbellino M, Cozzi V, Fusi M, Gervasoni C. Cattaneo D, et al. Clin Pharmacokinet. 2021 Jun;60(6):829-831. doi: 10.1007/s40262-021-00992-5. Epub 2021 Apr 17. Clin Pharmacokinet. 2021. PMID: 33864625 Free PMC article. No abstract available.

- 29 references
- 4 figures

Supplementary info

Publication types, MeSH terms, Substances Expand

Publication types

- Observational Study

MeSH terms

- Adult
- Age Factors
- Aged
- Betacoronavirus
- Body Weights and Measures
- COVID-19
- Comorbidity
- Coronavirus Infections / drug therapy*
- Cytochrome P-450 CYP3A
- Darunavir / pharmacokinetics*
- Darunavir / therapeutic use
- Dose-Response Relationship, Drug
- Female
- HIV Infections / drug therapy*
- HIV Protease Inhibitors / pharmacokinetics*
- HIV Protease Inhibitors / therapeutic use
- Humans
- Interleukin-6 / blood*
- Male
- Metabolic Clearance Rate
- Middle Aged
- Models, Biological
- Monte Carlo Method
- Pandemics
- Pneumonia, Viral / drug therapy*
- Retrospective Studies
- SARS-CoV-2
- Sex Factors

Substances

- HIV Protease Inhibitors

- Interleukin-6
- Cytochrome P-450 CYP3A
- Darunavir

Full text links

Springer Free PMC article
Proceed to details
Cite
Share
1,221
Observational Study
Dermatol Surg
. 2022 Feb 1;48(2):252-253.
doi: 10.1097/DSS.0000000000003291.

Reasons for Mohs Micrographic Surgery Cancellation: A Retrospective 1-Year Analysis in a Tertiary Care Clinic Before the COVID-19 Pandemic

Ardeshir Edward Nadimi [1], Samantha R Spierling Bagsic [2], Megan Prosser [3], Hubert T Greenway [1]
Affiliations Expand

Affiliations

- [1] Scripps Green Hospital, Bighorn Mohs Surgery and Dermatology Center, San Diego, California.
- [2] Scripps Whittier Diabetes Institute, Scripps Health, San Diego, California.
- [3] Bighorn Mohs Surgery and Dermatology Center, San Diego, California.

- PMID: **34889216**
- DOI: 10.1097/DSS.0000000000003291

Observational Study

Reasons for Mohs Micrographic Surgery Cancellation: A Retrospective 1-Year Analysis in a Tertiary Care Clinic Before the COVID-19 Pandemic

Ardeshir Edward Nadimi et al. Dermatol Surg. 2022.
Show details
Dermatol Surg
. 2022 Feb 1;48(2):252-253.
doi: 10.1097/DSS.0000000000003291.

Authors

Ardeshir Edward Nadimi [1], Samantha R Spierling Bagsic [2], Megan Prosser [3], Hubert T Greenway [1]

Affiliations

- [1] Scripps Green Hospital, Bighorn Mohs Surgery and Dermatology Center, San Diego, California.
- [2] Scripps Whittier Diabetes Institute, Scripps Health, San Diego, California.
- [3] Bighorn Mohs Surgery and Dermatology Center, San Diego, California.

- PMID: **34889216**
- DOI: 10.1097/DSS.0000000000003291

No abstract available

- 4 references

Supplementary info

Publication types, MeSH terms, Grant support Expand

Publication types

- Observational Study
- Research Support, N.I.H., Extramural

MeSH terms

- Aged
- Aged, 80 and over
- Appointments and Schedules*

- Biopsy
- Carcinoma, Basal Cell / pathology
- Carcinoma, Basal Cell / radiotherapy
- Carcinoma, Basal Cell / surgery*
- Carcinoma, Squamous Cell / pathology
- Carcinoma, Squamous Cell / radiotherapy
- Carcinoma, Squamous Cell / surgery*
- Female
- Health Status
- Humans
- Male
- Middle Aged
- Mohs Surgery / statistics & numerical data*
- Outpatient Clinics, Hospital / statistics & numerical data
- Referral and Consultation
- Retrospective Studies
- Skin / pathology
- Skin Neoplasms / pathology
- Skin Neoplasms / radiotherapy
- Skin Neoplasms / surgery*
- Tertiary Care Centers
- Time-to-Treatment

Grant support

- UL1 TR002550/TR/NCATS NIH HHS/United States

Full text links

Wolters Kluwer Wolters Kluwer
Proceed to details
Cite
Share
☐ 1,222
In Vivo
. Nov-Dec 2020;34(6):3735-3746.
doi: 10.21873/invivo.12223.

Chest Computed Tomography Scoring in Patients With Novel Coronavirus-infected

Pneumonia: Correlation With Clinical and Laboratory Features and Disease Outcome

Luca Pugliese [1], Francesco Paolo Sbordone [1], Francesco Grimaldi [1], Francesca Ricci [1], Federica DI Tosto [1], Luigi Spiritigliozzi [1], Carlo DI Donna [1], Matteo Presicce [1], Vincenzo DE Stasio [1], Leonardo Benelli [1], Francesca D'Errico [1], Monia Pasqualetto [1], Jacopo Maria Legramante [2], Marco Materazzo [3], Marco Pellicciaro [4], Oreste Claudio Buonomo [4], Gianluca Vanni [4], Stefano Rizza [5], Alfonso Bellia [5], Roberto Floris [1], Francesco Garaci [1], Marcello Chiocchi [1]
Affiliations Expand

Affiliations

- [1] Department of Biomedicine and Prevention, Division of Diagnostic Imaging, Tor Vergata University, and Unit of Diagnostic Imaging, Policlinico Tor Vergata, Rome, Italy.
- [2] Department of System Medicine, Tor Vergata University, and Emergency Department, Rome, Italy.
- [3] Breast Unit, Department of Surgical Science, Policlinico Tor Vergata University, Rome, Italy mrcmaterazzo@gmail.com.
- [4] Breast Unit, Department of Surgical Science, Policlinico Tor Vergata University, Rome, Italy.
- [5] Department of System Medicine, Tor Vergata University, and Department of Medical Sciences, Policlinico Tor Vergata, Rome, Italy.

- PMID: **33144492**
- PMCID: PMC7811662
- DOI: 10.21873/invivo.12223

Free PMC article

Chest Computed Tomography Scoring in Patients With Novel Coronavirus-infected Pneumonia: Correlation With Clinical and Laboratory Features and Disease Outcome

Luca Pugliese et al. In Vivo. Nov-Dec 2020.
Free PMC article
Show details
In Vivo
. Nov-Dec 2020;34(6):3735-3746.
doi: 10.21873/invivo.12223.

Authors

Luca Pugliese [1], Francesco Paolo Sbordone [1], Francesco Grimaldi [1], Francesca Ricci [1], Federica DI Tosto [1], Luigi Spiritigliozzi [1], Carlo DI Donna [1], Matteo Presicce [1], Vincenzo DE Stasio [1], Leonardo Benelli [1], Francesca D'Errico [1], Monia Pasqualetto [1], Jacopo Maria Legramante [2], Marco Materazzo [3], Marco Pellicciaro [4], Oreste Claudio Buonomo [4], Gianluca Vanni [4], Stefano Rizza [5], Alfonso Bellia [5], Roberto Floris [1], Francesco Garaci [1], Marcello Chiocchi [1]

Affiliations

- [1] Department of Biomedicine and Prevention, Division of Diagnostic Imaging, Tor Vergata University, and Unit of Diagnostic Imaging, Policlinico Tor Vergata, Rome, Italy.
- [2] Department of System Medicine, Tor Vergata University, and Emergency Department, Rome, Italy.
- [3] Breast Unit, Department of Surgical Science, Policlinico Tor Vergata University, Rome, Italy mrcmaterazzo@gmail.com.
- [4] Breast Unit, Department of Surgical Science, Policlinico Tor Vergata University, Rome, Italy.
- [5] Department of System Medicine, Tor Vergata University, and Department of Medical Sciences, Policlinico Tor Vergata, Rome, Italy.

- PMID: **33144492**
- PMCID: PMC7811662
- DOI: 10.21873/invivo.12223

Abstract

Background/aim: This study investigated the correlation of chest computed tomography (CT), findings, graded using two different scoring methods, with clinical and laboratory features and disease outcome, including a novel clinical predictive score, in patients with novel coronavirus-infected pneumonia (NCIP).

Patients and methods: In this retrospective, observational study, CT scan of 92 NCIP patients admitted to Policlinico Tor Vergata, were analyzed using a quantitative, computed-based and a semiquantitative, radiologist-assessed scoring system. Correlations of the two radiological scores with clinical and laboratory features, the CALL score, and their association with a composite adverse outcome were assessed.

Results: The two scores correlated significantly with each other ($\rho=0.637$, $p<0.0001$) and were independently associated with age, LDH, estimated glomerular filtration rate, diabetes, and with the composite outcome, which occurred in 24 patients.

Conclusion: In NCIP patients, two different radiological scores correlated with each other and with several clinical, laboratory features, and the CALL score. The quantitative score was a better independent predictor of the composite adverse outcome than the semiquantitative score.

Keywords: Novel coronavirus-infected pneumonia; computer tomography; death; intensive care unit; radiological scores.

Copyright© 2020, International Institute of Anticancer Research (Dr. George J. Delinasios), All rights reserved.

Conflict of interest statement

The Authors declare no conflicts of interest regarding this study.

- 4 figures

Supplementary info

MeSH terms Expand

MeSH terms

- Aged
- Aged, 80 and over
- Betacoronavirus / pathogenicity
- COVID-19
- Coronavirus Infections / diagnostic imaging*
- Coronavirus Infections / mortality
- Coronavirus Infections / physiopathology
- Coronavirus Infections / therapy
- Coronavirus Infections / virology
- Female
- Hospitalization
- Humans
- Intensive Care Units
- Male
- Middle Aged
- Pandemics
- Pneumonia / diagnostic imaging*
- Pneumonia / mortality
- Pneumonia / physiopathology
- Pneumonia / virology
- Pneumonia, Viral / diagnostic imaging*
- Pneumonia, Viral / physiopathology
- Pneumonia, Viral / therapy
- Pneumonia, Viral / virology
- SARS-CoV-2
- Thorax / diagnostic imaging*
- Thorax / physiopathology
- Thorax / virology
- Tomography, X-Ray Computed

Full text links

Full Text In Vivo HighWire Free PMC article
Proceed to details
Cite
Share
1,223
Retracted article
See the retraction notice
Observational Study
PLoS One
. 2020 Sep 3;15(9):e0238679.
doi: 10.1371/journal.pone.0238679. eCollection 2020.

Lung ultrasound score in establishing the timing of intubation in COVID-19 interstitial pneumonia: A preliminary retrospective observational study

Xiao Lu [1, 2], Mao Zhang [1], Anyu Qian [1, 2], Luping Tang [1, 2], Shanxiang Xu [1, 2]
Affiliations Expand

Affiliations

- [1] Department of Emergency Medicine, Second Affiliated Hospital, Zhejiang University School of Medicine, Hangzhou, China.
- [2] The Wuhan Critical Care Medical Team from Second Affiliated Hospital, Zhejiang University School of Medicine, Hangzhou, China.

- PMID: **32881950**
- PMCID: PMC7470341
- DOI: 10.1371/journal.pone.0238679

Free PMC article
Observational Study

Lung ultrasound score in establishing the timing of intubation in COVID-19 interstitial pneumonia: A preliminary retrospective observational study

Xiao Lu et al. PLoS One. 2020.
Free PMC article

Show details

PLoS One
. 2020 Sep 3;15(9):e0238679.
doi: 10.1371/journal.pone.0238679. eCollection 2020.

Authors

Xiao Lu [1 2], Mao Zhang [1], Anyu Qian [1 2], Luping Tang [1 2], Shanxiang Xu [1 2]

Affiliations

- [1] Department of Emergency Medicine, Second Affiliated Hospital, Zhejiang University School of Medicine, Hangzhou, China.
- [2] The Wuhan Critical Care Medical Team from Second Affiliated Hospital, Zhejiang University School of Medicine, Hangzhou, China.

- PMID: **32881950**
- PMCID: PMC7470341
- DOI: 10.1371/journal.pone.0238679

Retraction in

- Retraction: Lung ultrasound score in establishing the timing of intubation in COVID-19 interstitial pneumonia: A preliminary retrospective observational study.
  PLOS ONE Editors. PLOS ONE Editors. PLoS One. 2020 Dec 31;15(12):e0245032. doi: 10.1371/journal.pone.0245032. eCollection 2020. PLoS One. 2020. PMID: 33382851 Free PMC article. No abstract available.

Expression of concern in

- Expression of Concern: Lung ultrasound score in establishing the timing of intubation in COVID-19 interstitial pneumonia: A preliminary retrospective observational study.
  PLOS ONE Editors. PLOS ONE Editors. PLoS One. 2020 Nov 30;15(11):e0243267. doi: 10.1371/journal.pone.0243267. eCollection 2020. PLoS One. 2020. PMID: 33253279 Free PMC article. No abstract available.

Abstract

Purpose: To investigate the role of lung ultrasound score (LUS) in assessing intubation timing for patients with severe acute respiratory syndrome coronavirus 2 (SARS-CoV-2) pneumonia.

Materials and methods: Seventy-two patients with critical coronavirus disease 2019 (COVID-19) were admitted to a makeshift intensive care unit (ICU). All patients underwent bedside lung ultrasonography one to two times per day. The patients were either intubated, treated with noninvasive ventilation (NIV), or given high-flow nasal cannula (HFNC) after a discussion with the multidisciplinary group after their conditions worsened. Bedside lung ultrasound was performed daily after intubation, and patients received mechanical ventilation. Lung ultrasound was performed on days 1, 2, 3, 5, and 7 after patients were admitted to the ICU; if the patient was intubated, LUS determination was performed before intubation within 24 h (T1) and on days 1, 2, 5, and 7 after intubation (T2, T3, T4, and T5, respectively).The goal of this study was to evaluate

the severity of lung aeration loss in intubated and non-intubated patients with SARS-CoV-2 pneumonia by ultrasound at different time points within one week.

Results: A total of 16 patients were included in this study, including nine who were intubated and mechanically ventilated and seven patients without intubation. The number of elderly individuals in the intubated group was higher than in the non-intubated group ($P < 0.05$). In addition, there were more male than female patients in both groups. Patient characteristics (BMI, SOFA, and PaO2/FiO2 value) were similar between the two groups ($P > 0.05$). The 28-day mortality rate of intubated patients was higher than that of non-intubated patients; six patients in the intubated group and two patients in the non-intubated group died. Nine intubated patients showed changes in LUS within seven days (n = 9). The mean LUS within 24 h before intubation was 12.8 ± 1.3. LUS was significantly higher on T1 than on T5 ($P < 0.05$), and did not significantly differ from T1 to T4. Comparing LUS between intubated and non-intubated patients on T1 showed that the LUS of intubated patients was significantly higher than that of non-intubated patients ($P < 0.05$). Between the two patient groups, oxygenation index was 140.1 ± 7.7 vs. 137.8 ± 5.9 on T1, and the respiratory rate of the two groups was 26 ± 5 vs. 28 ± 4 breaths/min. Neither oxygenation index nor RR significantly differed between the two groups.

Conclusion: LUS may be an effective tool for assessing intubation timing in critically ill patients with Covid-19 interstitial pneumonia.

Conflict of interest statement

The author(s) received no specific funding for this work.

- 23 references
- 4 figures

Supplementary info

Publication types, MeSH terms, Grant support Expand

Publication types

- Observational Study
- Retracted Publication

MeSH terms

- Aged
- COVID-19
- Coronavirus Infections / diagnostic imaging
- Coronavirus Infections / therapy*
- Female
- Humans
- Intubation, Intratracheal / adverse effects
- Intubation, Intratracheal / methods*
- Lung / diagnostic imaging*

- Male
- Middle Aged
- Pandemics
- Pneumonia, Viral / diagnostic imaging
- Pneumonia, Viral / therapy*
- Respiration, Artificial / adverse effects
- Respiration, Artificial / methods*
- Time Factors
- Ultrasonography / methods*

Grant support

The author(s) received no specific funding for this work.

Full text links

OPEN ACCESS TO FULL TEXT PLOS ONE Public Library of Science Free PMC article
Proceed to details
Cite
Share
1,224
Observational Study
BMJ Open
. 2020 Sep 29;10(9):e042867.
doi: 10.1136/bmjopen-2020-042867.

Effect of COVID-19 lockdown on child protection medical assessments: a retrospective observational study in Birmingham, UK

Joanna Garstang [1] [2], Geoff Debelle [2] [3], Indu Anand [1], Jane Armstrong [1], Emily Botcher [1], Helen Chaplin [1], Nutmeg Hallett [2], Clare Morgans [1], Malcolm Price [2], Ern Ern Henna Tan [1], Emily Tudor [1], Julie Taylor [4] [3]
Affiliations Expand

Affiliations

- [1] Birmingham Community Healthcare NHS Trust, Birmingham, UK.
- [2] College of Medical and Dental Sciences, University of Birmingham, Birmingham, UK.
- [3] Birmingham Women's and Children's Hospital NHS Foundation Trust, Birmingham, UK.

- [4] College of Medical and Dental Sciences, University of Birmingham, Birmingham, UK j.taylor.1@bham.ac.uk.

- PMID: **32994262**
- PMCID: PMC7526028
- DOI: 10.1136/bmjopen-2020-042867

Free PMC article
Observational Study

Effect of COVID-19 lockdown on child protection medical assessments: a retrospective observational study in Birmingham, UK

Joanna Garstang et al. BMJ Open. 2020.
Free PMC article
Show details
BMJ Open
. 2020 Sep 29;10(9):e042867.
doi: 10.1136/bmjopen-2020-042867.

Authors

Joanna Garstang [1] [2], Geoff Debelle [2] [3], Indu Anand [1], Jane Armstrong [1], Emily Botcher [1], Helen Chaplin [1], Nutmeg Hallett [2], Clare Morgans [1], Malcolm Price [2], Ern Ern Henna Tan [1], Emily Tudor [1], Julie Taylor [4] [3]

Affiliations

- [1] Birmingham Community Healthcare NHS Trust, Birmingham, UK.
- [2] College of Medical and Dental Sciences, University of Birmingham, Birmingham, UK.
- [3] Birmingham Women's and Children's Hospital NHS Foundation Trust, Birmingham, UK.
- [4] College of Medical and Dental Sciences, University of Birmingham, Birmingham, UK j.taylor.1@bham.ac.uk.

- PMID: **32994262**
- PMCID: PMC7526028
- DOI: 10.1136/bmjopen-2020-042867

Abstract

Objectives: To determine any change in referral patterns and outcomes in children (0-18) referred for child protection medical examination (CPME) during the COVID-19 pandemic compared with previous years.

Design: Retrospective observational study, analysing routinely collected clinical data from CPME reports in a rapid response to the pandemic lockdown.

Setting: Birmingham Community Healthcare NHS Trust, which provides all routine CPME for Birmingham, England, population 1.1 million including 288 000 children.

Participants: Children aged under 18 years attending CPME during an 18-week period from late February to late June during the years 2018-2020.

Main outcome measures: Numbers of referrals, source of disclosure and outcomes from CPME.

Results: There were 78 CPME referrals in 2018, 75 in 2019 and 47 in 2020, this was a 39.7% (95% CI 12.4% to 59.0%) reduction in referrals from 2018 to 2020, and a 37.3% (95% CI 8.6% to 57.4%) reduction from 2019 to 2020. There were fewer CPME referrals initiated by school staff in 2020, 12 (26%) compared with 36 (47%) and 38 (52%) in 2018 and 2019, respectively. In all years 75.9% of children were known to social care prior to CPME, and 94% of CPME concluded that there were significant safeguarding concerns.

Conclusions: School closure due to COVID-19 may have harmed children as child abuse has remained hidden. There needs to be either mandatory attendance at schools in future or viable alternatives found. There may be a significant increase in safeguarding referrals when schools fully reopen as children disclose the abuse they have experienced at home.

Keywords: child protection; community child health; non-accidental injury.

© Author(s) (or their employer(s)) 2020. Re-use permitted under CC BY-NC. No commercial re-use. See rights and permissions. Published by BMJ.

Conflict of interest statement

Competing interests: None declared.

- 19 references
- 2 figures

Supplementary info

Publication types, MeSH terms Expand

Publication types

- Observational Study
- Research Support, Non-U.S. Gov't

MeSH terms

- Betacoronavirus
- COVID-19
- Child
- Child Abuse* / prevention & control

- Child Abuse* / psychology
- Child Abuse* / statistics & numerical data
- Child Protective Services* / methods
- Child Protective Services* / statistics & numerical data
- Child Welfare* / statistics & numerical data
- Child Welfare* / trends
- Communicable Disease Control* / methods
- Communicable Disease Control* / statistics & numerical data
- Coronavirus Infections* / epidemiology
- Coronavirus Infections* / prevention & control
- Coronavirus Infections* / psychology
- Female
- Humans
- Male
- Pandemics* / prevention & control
- Pneumonia, Viral* / epidemiology
- Pneumonia, Viral* / prevention & control
- Pneumonia, Viral* / psychology
- Population
- SARS-CoV-2
- School Health Services / statistics & numerical data*
- Social Isolation
- Social Work / methods
- Social Work / statistics & numerical data
- United Kingdom / epidemiology

Full text links

BMJ Free Full Text HighWire Free PMC article

Proceed to details

Cite

Share

☐ 1,225

Observational Study

PLoS One

. 2020 Oct 14;15(10):e0239570.
doi: 10.1371/journal.pone.0239570. eCollection 2020.

Residual clinical damage after COVID-19: A retrospective and prospective observational cohort study

Rebecca De Lorenzo [1], Caterina Conte [1 2], Chiara Lanzani [3], Francesco Benedetti [1 4], Luisa Roveri [5], Mario G Mazza [1 4], Elena Brioni [3], Giacomo Giacalone [5], Valentina Canti [2], Valentina Sofia [1], Marta D'Amico [1], Davide Di Napoli [6], Alberto Ambrosio [6], Paolo Scarpellini [2], Antonella Castagna [1 2], Giovanni Landoni [1 7], Alberto Zangrillo [1 7], Emanuele Bosi [1 2], Moreno Tresoldi [8], Fabio Ciceri [1 2], Patrizia Rovere-Querini [1 2]
Affiliations Expand

Affiliations

- [1] School of Medicine, Vita-Salute San Raffaele University, Milan, Italy.
- [2] Division of Immunology, Transplantation and Infectious Diseases, IRCCS San Raffaele Scientific Institute, Milan, Italy.
- [3] Unit of Nephrology, IRCCS San Raffaele Scientific Institute, Milan, Italy.
- [4] Unit of Psychiatry and Clinical Psychobiology, Division of Neuroscience, IRCCS San Raffaele Scientific Institute, Milan, Italy.
- [5] Department of Neuroscience, INSPE, IRCCS San Raffaele Scientific Institute, Milan, Italy.
- [6] Clinical Governance Division, IRCCS San Raffaele Scientific Institute, Milan, Italy.
- [7] Department of Anaesthesia and Intensive Care, IRCCS San Raffaele Scientific Institute, Milan, Italy.
- [8] Unit of General Medicine and Advanced Care, IRCCS San Raffaele Scientific Institute, Milan, Italy.

- PMID: **33052920**
- PMCID: PMC7556454
- DOI: 10.1371/journal.pone.0239570

Free PMC article
Observational Study

Residual clinical damage after COVID-19: A retrospective and prospective observational cohort study

Rebecca De Lorenzo et al. PLoS One. 2020.
Free PMC article
Show details
PLoS One
. 2020 Oct 14;15(10):e0239570.
doi: 10.1371/journal.pone.0239570. eCollection 2020.

Authors

Rebecca De Lorenzo [1], Caterina Conte [1 2], Chiara Lanzani [3], Francesco Benedetti [1 4], Luisa Roveri [5], Mario G Mazza [1 4], Elena Brioni [3], Giacomo Giacalone [5], Valentina Canti [2], Valentina Sofia [1], Marta D'Amico [1], Davide Di Napoli [6], Alberto Ambrosio [6], Paolo Scarpellini [2], Antonella Castagna [1 2], Giovanni Landoni [1 7], Alberto Zangrillo [1 7], Emanuele Bosi [1 2], Moreno Tresoldi [8], Fabio Ciceri [1 2], Patrizia Rovere-Querini [1 2]

Affiliations

- [1] School of Medicine, Vita-Salute San Raffaele University, Milan, Italy.
- [2] Division of Immunology, Transplantation and Infectious Diseases, IRCCS San Raffaele Scientific Institute, Milan, Italy.
- [3] Unit of Nephrology, IRCCS San Raffaele Scientific Institute, Milan, Italy.
- [4] Unit of Psychiatry and Clinical Psychobiology, Division of Neuroscience, IRCCS San Raffaele Scientific Institute, Milan, Italy.
- [5] Department of Neuroscience, INSPE, IRCCS San Raffaele Scientific Institute, Milan, Italy.
- [6] Clinical Governance Division, IRCCS San Raffaele Scientific Institute, Milan, Italy.
- [7] Department of Anaesthesia and Intensive Care, IRCCS San Raffaele Scientific Institute, Milan, Italy.
- [8] Unit of General Medicine and Advanced Care, IRCCS San Raffaele Scientific Institute, Milan, Italy.

- PMID: **33052920**
- PMCID: PMC7556454
- DOI: 10.1371/journal.pone.0239570

Abstract

Data on residual clinical damage after Coronavirus disease-2019 (COVID-19) are lacking. The aims of this study were to investigate whether COVID-19 leaves behind residual dysfunction, and identify patients who might benefit from post-discharge monitoring. All patients aged ≥18 years admitted to the Emergency Department (ED) for COVID-19, and evaluated at post-discharge follow-up between 7 April and 7 May, 2020, were enrolled. Primary outcome was need of follow-up, defined as the presence at follow-up of at least one among: respiratory rate (RR) >20 breaths/min, uncontrolled blood pressure (BP) requiring therapeutic change, moderate to very severe dyspnoea, malnutrition, or new-onset cognitive impairment, according to validated scores. Post-traumatic stress disorder (PTSD) served as secondary outcome. 185 patients were included. Median [interquartile range] time from hospital discharge to follow-up was 23 [20-29] days. 109 (58.9%) patients needed follow-up. At follow-up evaluation, 58 (31.3%) patients were dyspnoeic, 41 (22.2%) tachypnoeic, 10 (5.4%) malnourished, 106 (57.3%) at risk for malnutrition. Forty (21.6%) patients had uncontrolled BP requiring therapeutic change, and 47 (25.4%) new-onset cognitive impairment. PTSD was observed in 41 (22.2%) patients. At regression tree analysis, the ratio of arterial oxygen partial pressure to fractional inspired oxygen (PaO2/FiO2) and body mass index (BMI) at ED presentation, and age emerged as independent predictors of the need of follow-up. Patients with PaO2/FiO2 <324 and BMI ≥33 Kg/m2 had the highest odds to require follow-up. Among hospitalised patients, age ≥63 years, or age <63 plus non-invasive ventilation or diabetes identified those with the highest probability to need follow-up. PTSD was independently predicted by female gender and hospitalisation, the latter being protective (odds ratio, OR, 4.03, 95%

confidence interval, CI, 1.76 to 9.47, p 0.0011; OR 0.37, 95% CI 0.14 to 0.92, p 0.033, respectively). COVID-19 leaves behind physical and psychological dysfunctions. Follow-up programmes should be implemented for selected patients.

Conflict of interest statement

The authors have declared that no competing interests exist.

- 47 references
- 4 figures

Supplementary info

Publication types, MeSH terms, Grant support Expand

Publication types

- Observational Study

MeSH terms

- Aftercare / statistics & numerical data
- Aged
- COVID-19
- Cognitive Dysfunction / epidemiology*
- Coronavirus Infections / complications
- Coronavirus Infections / epidemiology*
- Coronavirus Infections / rehabilitation
- Dyspnea / epidemiology*
- Female
- Follow-Up Studies
- Humans
- Male
- Malnutrition / epidemiology*
- Middle Aged
- Pandemics
- Pneumonia, Viral / complications
- Pneumonia, Viral / epidemiology*
- Pneumonia, Viral / rehabilitation
- Stress Disorders, Post-Traumatic / epidemiology*

Grant support

The authors received no specific funding for this work.

Full text links

PLOS ONE Public Library of Science Free PMC article
Proceed to details
Cite
Share
1,226
Observational Study
J Am Coll Radiol
. 2020 Nov;17(11):1443-1449.
doi: 10.1016/j.jacr.2020.08.010. Epub 2020 Aug 20.

Collateral Damage: The Impact of the COVID-19 Pandemic on Acute Abdominal Emergency Presentations

Ciara M O'Brien [1], Katherine Jung [2], Wilfred Dang [3], Hyun-Jung Jang [4], Ania Z Kielar [4]
Affiliations Expand

Affiliations

- [1] Department of Medical Imaging, University Health Network, University of Toronto, Toronto, Ontario, Canada. Electronic address: ciara.obrien@uhn.ca.
- [2] Department of Physiology and Psychology, University of Toronto, Toronto, Ontario, Canada.
- [3] Faculty of Medicine, University of Ottawa, Ottawa, Ontario, Canada.
- [4] Department of Medical Imaging, University Health Network, University of Toronto, Toronto, Ontario, Canada.

- PMID: **32905786**
- PMCID: PMC7439814
- DOI: 10.1016/j.jacr.2020.08.010

Free PMC article
Observational Study

Collateral Damage: The Impact of the COVID-19 Pandemic on Acute Abdominal Emergency Presentations

Ciara M O'Brien et al. J Am Coll Radiol. 2020 Nov.
Free PMC article
Show details

J Am Coll Radiol
. 2020 Nov;17(11):1443-1449.
doi: 10.1016/j.jacr.2020.08.010. Epub 2020 Aug 20.

Authors

Ciara M O'Brien [1], Katherine Jung [2], Wilfred Dang [3], Hyun-Jung Jang [4], Ania Z Kielar [4]

Affiliations

- [1] Department of Medical Imaging, University Health Network, University of Toronto, Toronto, Ontario, Canada. Electronic address: ciara.obrien@uhn.ca.
- [2] Department of Physiology and Psychology, University of Toronto, Toronto, Ontario, Canada.
- [3] Faculty of Medicine, University of Ottawa, Ottawa, Ontario, Canada.
- [4] Department of Medical Imaging, University Health Network, University of Toronto, Toronto, Ontario, Canada.

- PMID: **32905786**
- PMCID: PMC7439814
- DOI: 10.1016/j.jacr.2020.08.010

Abstract

Introduction: In March 2020, the World Health Organization declared a pandemic caused by a novel coronavirus. Public information created awareness as well as concern in the general population. There has been a reported decrease in the number of patients attending emergency departments (ED) during the pandemic. This is the first study to determine differences in the types of presenting illnesses, severity, and rate of resultant surgical intervention during the pandemic.

Methods and materials: We carried out a retrospective, observational cohort study comparing two groups of patients attending the ED at our tertiary-care academic hospital. A historical comparison cohort was obtained by reviewing the number of patients referred by the ED for abdominal CT between March 15 and April 15, 2020, compared with March 15 and April 15, 2019. CT reports were reviewed; primary pathologies, complications, and subsequent surgical intervention were documented and compared between the two groups.

Results: In all, 733 patients were included in the 2019 cohort, and 422 patients were included in the 2020 cohort. In 2019, 32.7% had positive CT findings, increasing to 50.5% in 2020. The number of complications increased from 7.9% to 19.7%. The rate requiring surgical intervention increased from 26.3% to 47.6% in 2020.

Conclusion: To date, there is little published data regarding the presentation and severity of illnesses during the coronavirus disease 2019 pandemic. This information has important public health implications, highlighting the need to educate patients to continue to present to hospital services during such crises, including if a purported second wave of COVID-19 arises.

Keywords: Abdominal; COVID-19; CT; emergency department; pandemic.

Crown Copyright © 2020. Published by Elsevier Inc. All rights reserved.

- 11 references

- 4 figures

Supplementary info

Publication types, MeSH terms Expand

Publication types

- Comparative Study
- Observational Study

MeSH terms

- Abdomen, Acute / complications
- Abdomen, Acute / diagnostic imaging*
- Abdomen, Acute / surgery
- Adult
- COVID-19 / epidemiology*
- Emergency Service, Hospital*
- Female
- Humans
- Male
- Middle Aged
- Pandemics
- Severity of Illness Index
- Tomography, X-Ray Computed*

Full text links

ELSEVIER FULL-TEXT ARTICLE Elsevier Science Free PMC article
Proceed to details
Cite
Share
1,227
Observational Study
BMJ Health Care Inform
. 2021 May;28(1):e100310.
doi: 10.1136/bmjhci-2020-100310.

Smartphone-based remote monitoring of vision in macular disease enables early

detection of worsening pathology and need for intravitreal therapy

Meriam Islam[1], Stafford Sansome[1], Radha Das[1], Marko Lukic[1], Kelvin Yi Chong Teo[2,3], Gavin Tan[2], Konstantinos Balaskas[1], Peter B M Thomas[1], Lucas M Bachmann[4], Andrew M Schimel[5], Dawn A Sim[6]
Affiliations Expand

Affiliations

- [1] NIHR Biomedical Research Centre for Ophthalmology, Moorfields Eye Hospital NHS Foundation Trust and UCL Institute of Ophthalmology, Moorfields Eye Hospital NHS Foundation Trust, London, UK.
- [2] Department of Ophthalmology, Singapore National Eye Centre, Singapore.
- [3] Department of Ophthalmology, NUS Medical School, Singapore.
- [4] Department of Clinical Epidemiology, University of Zurich, Zurich, Switzerland.
- [5] Department of Ophthalmology, Centre for Excellence in Eye Care, Miami, Florida, USA.
- [6] NIHR Biomedical Research Centre for Ophthalmology, Moorfields Eye Hospital NHS Foundation Trust and UCL Institute of Ophthalmology, Moorfields Eye Hospital NHS Foundation Trust, London, UK dawnsim@nhs.net.

- PMID: **34035050**
- PMCID: PMC8154994
- DOI: 10.1136/bmjhci-2020-100310

Free PMC article
Observational Study

Smartphone-based remote monitoring of vision in macular disease enables early detection of worsening pathology and need for intravitreal therapy

Meriam Islam et al. BMJ Health Care Inform. 2021 May.
Free PMC article
Show details
BMJ Health Care Inform
. 2021 May;28(1):e100310.
doi: 10.1136/bmjhci-2020-100310.

Authors

Meriam Islam [1], Stafford Sansome [1], Radha Das [1], Marko Lukic [1], Kelvin Yi Chong Teo [2] [3], Gavin Tan [2], Konstantinos Balaskas [1], Peter B M Thomas [1], Lucas M Bachmann [4], Andrew M Schimel [5], Dawn A Sim [6]

Affiliations

- [1] NIHR Biomedical Research Centre for Ophthalmology, Moorfields Eye Hospital NHS Foundation Trust and UCL Institute of Ophthalmology, Moorfields Eye Hospital NHS Foundation Trust, London, UK.
- [2] Department of Ophthalmology, Singapore National Eye Centre, Singapore.
- [3] Department of Ophthalmology, NUS Medical School, Singapore.
- [4] Department of Clinical Epidemiology, University of Zurich, Zurich, Switzerland.
- [5] Department of Ophthalmology, Centre for Excellence in Eye Care, Miami, Florida, USA.
- [6] NIHR Biomedical Research Centre for Ophthalmology, Moorfields Eye Hospital NHS Foundation Trust and UCL Institute of Ophthalmology, Moorfields Eye Hospital NHS Foundation Trust, London, UK dawnsim@nhs.net.

- PMID: **34035050**
- PMCID: PMC8154994
- DOI: 10.1136/bmjhci-2020-100310

Abstract

Background/aims: To assess the outcomes of home monitoring of distortion caused by macular diseases using a smartphone-based application (app), and to examine them with hospital-based assessments of visual acuity (VA), optical coherence tomography-derived central macular thickness (CMT) and the requirement of intravitreal injection therapy.

Design: Observational study with retrospective analysis of data.

Methods: Participants were trained in the correct use of the app (Alleye, Oculocare, Zurich, Switzerland) in person or by using video and telephone consultations. Automated threshold-based alerts were communicated based on a traffic light system. A 'threshold alarm' was defined as three consecutive 'red' scores, and turned into a 'persistent alarm' if present for greater than a 7-day period. Changes of VA and CMT, and the requirement for intravitreal therapy after an alarm were examined.

Results: 245 patients performing a total of 11 592 tests (mean 46.9 tests per user) were included and 85 eyes (164 alarms) examined. Mean drop in VA from baseline was -4.23 letters (95% CI: -6.24 to -2.22; p<0.001) and mean increase in CMT was 29.5 µm (95% CI: -0.08 to 59.13; p=0.051). Sixty-six eyes (78.5%) producing alarms either had a drop in VA, increase in CMT or both and 60.0% received an injection. Eyes with persistent alarms had a greater loss of VA, -4.79 letters (95% CI: -6.73 to -2.85; p<0.001) or greater increase in CMT, +87.8 µm (95% CI: 5.2 to 170.4; p=0.038).

Conclusion: Smartphone-based self-tests for macular disease may serve as reliable indicators for the worsening of pathology and the need for treatment.

Keywords: COVID-19; health care sector; information management; patient care.

© Author(s) (or their employer(s)) 2021. Re-use permitted under CC BY-NC. No commercial re-use. See rights and permissions. Published by BMJ.

Conflict of interest statement

Competing interests: LMB is a founding member of Oculocare Medical, which develops innovative products in eye care, such as the self-monitoring test described in this paper.

- 23 references
- 3 figures

Supplementary info

Publication types, MeSH terms Expand

Publication types

- Observational Study

MeSH terms

- Aged
- Female
- Humans
- Intravitreal Injections / statistics & numerical data*
- Macular Degeneration* / diagnosis
- Macular Degeneration* / pathology
- Male
- Mobile Applications
- Remote Consultation / statistics & numerical data*
- Retrospective Studies
- Smartphone*
- Tomography, Optical Coherence
- Visual Acuity / physiology*

Full text links

BMJ Free Full Text HighWire Free PMC article

Proceed to details

Cite

Share

☐ 1,228

BMC Endocr Disord

. 2021 Nov 15;21(1):228.

doi: 10.1186/s12902-021-00896-2.

Suppression of the hypothalamic-pituitary-thyroid axis is associated with the severity of prognosis in hospitalized patients with COVID-19

Juan Zheng [#] [1] [2], Zhenhai Cui [#] [1] [2], Ningjie Shi [#] [1] [2], Shenghua Tian [1] [2], Ting Chen [1] [2], Xueyu Zhong [1] [2], Kangli Qiu [1] [2], Jiaoyue Zhang [1] [2], Tianshu Zeng [3] [4], Lulu Chen [5] [6], Huiqing Li [7] [8]
Affiliations Expand

Affiliations

- [1] Department of Endocrinology, Union Hospital, Tongji Medical College, Huazhong University of Science and Technology, Wuhan, 430022, China.
- [2] Hubei provincial Clinical Research Center for Diabetes and Metabolic Disorders, Wuhan, China.
- [3] Department of Endocrinology, Union Hospital, Tongji Medical College, Huazhong University of Science and Technology, Wuhan, 430022, China. tszeng@126.com.
- [4] Hubei provincial Clinical Research Center for Diabetes and Metabolic Disorders, Wuhan, China. tszeng@126.com.
- [5] Department of Endocrinology, Union Hospital, Tongji Medical College, Huazhong University of Science and Technology, Wuhan, 430022, China. cheria_chen@126.com.
- [6] Hubei provincial Clinical Research Center for Diabetes and Metabolic Disorders, Wuhan, China. cheria_chen@126.com.
- [7] Department of Endocrinology, Union Hospital, Tongji Medical College, Huazhong University of Science and Technology, Wuhan, 430022, China. lhqing5@126.com.
- [8] Hubei provincial Clinical Research Center for Diabetes and Metabolic Disorders, Wuhan, China. lhqing5@126.com.

[#] Contributed equally.

- PMID: **34781943**
- PMCID: PMC8591433
- DOI: 10.1186/s12902-021-00896-2

Free PMC article

Suppression of the hypothalamic-pituitary-thyroid axis is associated with the severity of prognosis in hospitalized patients with COVID-19

Juan Zheng et al. BMC Endocr Disord. 2021.
Free PMC article
Show details
BMC Endocr Disord
. 2021 Nov 15;21(1):228.
doi: 10.1186/s12902-021-00896-2.

Authors

Juan Zheng [#,1,2], Zhenhai Cui [#,1,2], Ningjie Shi [#,1,2], Shenghua Tian [1,2], Ting Chen [1,2], Xueyu Zhong [1,2], Kangli Qiu [1,2], Jiaoyue Zhang [1,2], Tianshu Zeng [3,4], Lulu Chen [5,6], Huiqing Li [7,8]

Affiliations

- [1] Department of Endocrinology, Union Hospital, Tongji Medical College, Huazhong University of Science and Technology, Wuhan, 430022, China.
- [2] Hubei provincial Clinical Research Center for Diabetes and Metabolic Disorders, Wuhan, China.
- [3] Department of Endocrinology, Union Hospital, Tongji Medical College, Huazhong University of Science and Technology, Wuhan, 430022, China. tszeng@126.com.
- [4] Hubei provincial Clinical Research Center for Diabetes and Metabolic Disorders, Wuhan, China. tszeng@126.com.
- [5] Department of Endocrinology, Union Hospital, Tongji Medical College, Huazhong University of Science and Technology, Wuhan, 430022, China. cheria_chen@126.com.
- [6] Hubei provincial Clinical Research Center for Diabetes and Metabolic Disorders, Wuhan, China. cheria_chen@126.com.
- [7] Department of Endocrinology, Union Hospital, Tongji Medical College, Huazhong University of Science and Technology, Wuhan, 430022, China. lhqing5@126.com.
- [8] Hubei provincial Clinical Research Center for Diabetes and Metabolic Disorders, Wuhan, China. lhqing5@126.com.

[#] Contributed equally.

- PMID: **34781943**
- PMCID: PMC8591433
- DOI: 10.1186/s12902-021-00896-2

Abstract

Background: The outbreak of severe acute respiratory syndrome novel coronavirus 2 (SARS-CoV-2) has spread rapidly worldwide. SARS-CoV-2 has been found to cause multiple organ damage; however, little attention has been paid to the damage to the endocrine system caused by this virus, and the subsequent impact on prognosis. This may be the first research on the hypothalamic-pituitary-thyroid (HPT) axis and prognosis in coronavirus disease 2019 (COVID-19).

Methods: In this retrospective observational study, 235 patients were admitted to the hospital with laboratory-confirmed SARS-CoV-2 infection from 22 January to 17 March 2020. Clinical characteristics, laboratory findings, and treatments were obtained from electronic medical records

with standard data collection forms and compared among patients with different thyroid function status.

Results: Among 235 patients, 17 (7.23%) had subclinical hypothyroidism, 11 (4.68%) severe non-thyroidal illness syndrome (NTIS), and 23 (9.79%) mild to moderate NTIS. Composite endpoint events of each group, including mortality, admission to the ICU, and using IMV were observed. Compared with normal thyroid function, the hazard ratios (HRs) of composite endpoint events for mild to moderate NTIS, severe NTIS, subclinical hypothyroidism were 27.3 (95% confidence interval [CI] 7.07-105.7), 23.1 (95% CI 5.75-92.8), and 4.04 (95% CI 0.69-23.8) respectively. The multivariate-adjusted HRs for acute cardiac injury among patients with NTF, subclinical hypothyroidism, severe NTIS, and mild to moderate NTIS were 1.00, 1.68 (95% CI 0.56-5.05), 4.68 (95% CI 1.76-12.4), and 2.63 (95% CI 1.09-6.36) respectively.

Conclusions: Our study shows that the suppression of the HPT axis could be a common complication in COVID-19 patients and an indicator of the severity of prognosis. Among the three different types of thyroid dysfunction with COVID-19, mild to moderate NTIS and severe NTIS have a higher risk of severe outcomes compared with subclinical hypothyroidism.

Keywords: Coronavirus disease 2019; Hypothalamic-pituitary-thyroid axis; Non-thyroidal illness syndrome; Subclinical hypothyroidism.

© 2021. The Author(s).

Conflict of interest statement

The authors declare that they have no conflict of interest.

- 32 references

Supplementary info

MeSH terms, Substances Expand

MeSH terms

- Adult
- Age Factors
- Aged
- Aged, 80 and over
- COVID-19 Vaccines / adverse effects*
- Euthyroid Sick Syndromes / etiology*
- Female
- Humans
- Hypertension / etiology*
- Male
- Middle Aged
- Odds Ratio
- Retrospective Studies

- Sex Factors

Substances

- COVID-19 Vaccines

Full text links

Read free full text at BMC BioMed Central Free PMC article

Proceed to details

Cite

Share

1,229

Observational Study

J Stroke Cerebrovasc Dis

. 2020 Nov;29(11):105314.

doi: 10.1016/j.jstrokecerebrovasdis.2020.105314. Epub 2020 Sep 11.

Characteristics of a Diverse Cohort of Stroke Patients with SARS-CoV-2 and Outcome by Sex

G Trifan [1], F D Goldenberg [2], F Z Caprio [3], J Biller [4], M Schneck [5], A Khaja [6], T Terna [7], J Brorson [8], C Lazaridis [9], Z Bulwa [10], R Alvarado Dyer [11], F G Saleh Velez [12], S Prabhakaran [13], E M Liotta [14], A Batra [15], N J Reish [16], S Ruland [17], M Teitcher [18], W Taylor [19], P De la Pena [20], J J Conners [21], P K Grewal [22], P Pinna [23], R M Dafer [24], N D Osteraas [25], I DaSilva [26], J P Hall [27], S John [28], N Shafi [29], K Miller [30], B Moustafa [31], A Vargas [32], P B Gorelick [33], F D Testai [34]

Affiliations Expand

Affiliations

- [1] Department of Neurology and Rehabilitation, University of Illinois at Chicago, Chicago, IL 60612, U.S.A.. Electronic address: gtrifan@uic.edu.
- [2] Department of Neurology, University of Chicago Hospital, Chicago, IL 60612, U.S.A.. Electronic address: fgoldenb@neurology.bsd.uchicago.edu.
- [3] Department of Neurology, Northwestern University, 633 Clark St, Evanston, IL 60208, U.S.A.. Electronic address: Caprio@nm.org.
- [4] Department of Neurology, Loyola University Health System, 2160 S 1st Ave, Maywood, IL 60153, U.S.A.. Electronic address: jbiller@lumc.edu.
- [5] Department of Neurology, Loyola University Health System, 2160 S 1st Ave, Maywood, IL 60153, U.S.A.. Electronic address: mschneck@lumc.edu.
- [6] AMITA Health - Alexian Brothers Hospital, 800 Biesterfield Rd, IL 60007, U.S.A.. Electronic address: akhaja@northwestneuro.com.

- [7] AMITA Health - Alexian Brothers Hospital, 800 Biesterfield Rd, IL 60007, U.S.A.. Electronic address: Theresa.terna@amitahealth.org.
- [8] Department of Neurology, University of Chicago Hospital, Chicago, IL 60612, U.S.A.
- [9] Department of Neurology, University of Chicago Hospital, Chicago, IL 60612, U.S.A.. Electronic address: lazaridis@uchicago.edu.
- [10] Department of Neurology, University of Chicago Hospital, Chicago, IL 60612, U.S.A.. Electronic address: Zachary.Bulwa@uchospitals.edu.
- [11] Department of Neurology, University of Chicago Hospital, Chicago, IL 60612, U.S.A.. Electronic address: Ronald.AlvaradoDyer@uchospitals.edu.
- [12] Department of Neurology, University of Chicago Hospital, Chicago, IL 60612, U.S.A.. Electronic address: Faddi.SalehVelez@uchospitals.edu.
- [13] Department of Neurology, University of Chicago Hospital, Chicago, IL 60612, U.S.A.. Electronic address: shyam1@neurology.bsd.uchicago.edu.
- [14] Department of Neurology, Northwestern University, 633 Clark St, Evanston, IL 60208, U.S.A.. Electronic address: eric.liotta@northwestern.edu.
- [15] Department of Neurology, Northwestern University, 633 Clark St, Evanston, IL 60208, U.S.A.. Electronic address: ayush.batra@nm.org.
- [16] Department of Neurology, Northwestern University, 633 Clark St, Evanston, IL 60208, U.S.A.. Electronic address: nicholas.reish@northwestern.edu.
- [17] Department of Neurology, Loyola University Health System, 2160 S 1st Ave, Maywood, IL 60153, U.S.A.. Electronic address: sruland@lumc.edu.
- [18] Department of Neurology, Loyola University Health System, 2160 S 1st Ave, Maywood, IL 60153, U.S.A.. Electronic address: michael.teitcher@lumc.edu.
- [19] Department of Neurology, Loyola University Health System, 2160 S 1st Ave, Maywood, IL 60153, U.S.A.. Electronic address: william.taylor@lumc.ed.
- [20] Department of Neurology, Loyola University Health System, 2160 S 1st Ave, Maywood, IL 60153, U.S.A.. Electronic address: paula.delapena@lumc.edu.
- [21] Department of Neurological Sciences, Rush University Medical Center, 1620 W Harrison St, Chicago, IL 60612, U.S.A.. Electronic address: james_conners@rush.edu.
- [22] Department of Neurological Sciences, Rush University Medical Center, 1620 W Harrison St, Chicago, IL 60612, U.S.A.. Electronic address: parneet_k_grewal@rush.edu.
- [23] Department of Neurological Sciences, Rush University Medical Center, 1620 W Harrison St, Chicago, IL 60612, U.S.A.. Electronic address: pranusha_pinna@rush.edu.
- [24] Department of Neurological Sciences, Rush University Medical Center, 1620 W Harrison St, Chicago, IL 60612, U.S.A.. Electronic address: rima_dafer@rush.edu.
- [25] Department of Neurological Sciences, Rush University Medical Center, 1620 W Harrison St, Chicago, IL 60612, U.S.A.. Electronic address: nicholas_d_osteraas@rush.edu.
- [26] Department of Neurological Sciences, Rush University Medical Center, 1620 W Harrison St, Chicago, IL 60612, U.S.A.. Electronic address: ivan_dasilva@rush.edu.
- [27] Department of Neurological Sciences, Rush University Medical Center, 1620 W Harrison St, Chicago, IL 60612, U.S.A.. Electronic address: julianne_p_hall@rush.edu.
- [28] Department of Neurological Sciences, Rush University Medical Center, 1620 W Harrison St, Chicago, IL 60612, U.S.A.. Electronic address: sayona_john@rush.edu.
- [29] Department of Neurology and Rehabilitation, University of Illinois at Chicago, Chicago, IL 60612, U.S.A.. Electronic address: nshafi@uic.edu.
- [30] Department of Neurology and Rehabilitation, University of Illinois at Chicago, Chicago, IL 60612, U.S.A.. Electronic address: kmille25@uic.edu.
- [31] Department of Neurology and Rehabilitation, University of Illinois at Chicago, Chicago, IL 60612, U.S.A.. Electronic address: bmoust2@uic.edu.

- [32] Department of Neurological Sciences, Rush University Medical Center, 1620 W Harrison St, Chicago, IL 60612, U.S.A.. Electronic address: alejandro_vargas@rush.edu.
- [33] Department of Neurology, Northwestern University, 633 Clark St, Evanston, IL 60208, U.S.A.. Electronic address: pgorelick@thorek.org.
- [34] Department of Neurology and Rehabilitation, University of Illinois at Chicago, Chicago, IL 60612, U.S.A.. Electronic address: testai@uic.edu.

- PMID: **32951959**
- PMCID: PMC7486061
- DOI: 10.1016/j.jstrokecerebrovasdis.2020.105314

Free PMC article
Observational Study

Characteristics of a Diverse Cohort of Stroke Patients with SARS-CoV-2 and Outcome by Sex

G Trifan et al. J Stroke Cerebrovasc Dis. 2020 Nov.
Free PMC article
Show details
J Stroke Cerebrovasc Dis
. 2020 Nov;29(11):105314.
doi: 10.1016/j.jstrokecerebrovasdis.2020.105314. Epub 2020 Sep 11.

Authors

G Trifan [1], F D Goldenberg [2], F Z Caprio [3], J Biller [4], M Schneck [5], A Khaja [6], T Terna [7], J Brorson [8], C Lazaridis [9], Z Bulwa [10], R Alvarado Dyer [11], F G Saleh Velez [12], S Prabhakaran [13], E M Liotta [14], A Batra [15], N J Reish [16], S Ruland [17], M Teitcher [18], W Taylor [19], P De la Pena [20], J J Conners [21], P K Grewal [22], P Pinna [23], R M Dafer [24], N D Osteraas [25], I DaSilva [26], J P Hall [27], S John [28], N Shafi [29], K Miller [30], B Moustafa [31], A Vargas [32], P B Gorelick [33], F D Testai [34]

Affiliations

- [1] Department of Neurology and Rehabilitation, University of Illinois at Chicago, Chicago, IL 60612, U.S.A.. Electronic address: gtrifan@uic.edu.
- [2] Department of Neurology, University of Chicago Hospital, Chicago, IL 60612, U.S.A.. Electronic address: fgoldenb@neurology.bsd.uchicago.edu.
- [3] Department of Neurology, Northwestern University, 633 Clark St, Evanston, IL 60208, U.S.A.. Electronic address: Caprio@nm.org.
- [4] Department of Neurology, Loyola University Health System, 2160 S 1st Ave, Maywood, IL 60153, U.S.A.. Electronic address: jbiller@lumc.edu.
- [5] Department of Neurology, Loyola University Health System, 2160 S 1st Ave, Maywood, IL 60153, U.S.A.. Electronic address: mschneck@lumc.edu.

- [6] AMITA Health - Alexian Brothers Hospital, 800 Biesterfield Rd, IL 60007, U.S.A.. Electronic address: akhaja@northwestneuro.com.
- [7] AMITA Health - Alexian Brothers Hospital, 800 Biesterfield Rd, IL 60007, U.S.A.. Electronic address: Theresa.terna@amitahealth.org.
- [8] Department of Neurology, University of Chicago Hospital, Chicago, IL 60612, U.S.A.
- [9] Department of Neurology, University of Chicago Hospital, Chicago, IL 60612, U.S.A.. Electronic address: lazaridis@uchicago.edu.
- [10] Department of Neurology, University of Chicago Hospital, Chicago, IL 60612, U.S.A.. Electronic address: Zachary.Bulwa@uchospitals.edu.
- [11] Department of Neurology, University of Chicago Hospital, Chicago, IL 60612, U.S.A.. Electronic address: Ronald.AlvaradoDyer@uchospitals.edu.
- [12] Department of Neurology, University of Chicago Hospital, Chicago, IL 60612, U.S.A.. Electronic address: Faddi.SalehVelez@uchospitals.edu.
- [13] Department of Neurology, University of Chicago Hospital, Chicago, IL 60612, U.S.A.. Electronic address: shyam1@neurology.bsd.uchicago.edu.
- [14] Department of Neurology, Northwestern University, 633 Clark St, Evanston, IL 60208, U.S.A.. Electronic address: eric.liotta@northwestern.edu.
- [15] Department of Neurology, Northwestern University, 633 Clark St, Evanston, IL 60208, U.S.A.. Electronic address: ayush.batra@nm.org.
- [16] Department of Neurology, Northwestern University, 633 Clark St, Evanston, IL 60208, U.S.A.. Electronic address: nicholas.reish@northwestern.edu.
- [17] Department of Neurology, Loyola University Health System, 2160 S 1st Ave, Maywood, IL 60153, U.S.A.. Electronic address: sruland@lumc.edu.
- [18] Department of Neurology, Loyola University Health System, 2160 S 1st Ave, Maywood, IL 60153, U.S.A.. Electronic address: michael.teitcher@lumc.edu.
- [19] Department of Neurology, Loyola University Health System, 2160 S 1st Ave, Maywood, IL 60153, U.S.A.. Electronic address: william.taylor@lumc.ed.
- [20] Department of Neurology, Loyola University Health System, 2160 S 1st Ave, Maywood, IL 60153, U.S.A.. Electronic address: paula.delapena@lumc.edu.
- [21] Department of Neurological Sciences, Rush University Medical Center, 1620 W Harrison St, Chicago, IL 60612, U.S.A.. Electronic address: james_conners@rush.edu.
- [22] Department of Neurological Sciences, Rush University Medical Center, 1620 W Harrison St, Chicago, IL 60612, U.S.A.. Electronic address: parneet_k_grewal@rush.edu.
- [23] Department of Neurological Sciences, Rush University Medical Center, 1620 W Harrison St, Chicago, IL 60612, U.S.A.. Electronic address: pranusha_pinna@rush.edu.
- [24] Department of Neurological Sciences, Rush University Medical Center, 1620 W Harrison St, Chicago, IL 60612, U.S.A.. Electronic address: rima_dafer@rush.edu.
- [25] Department of Neurological Sciences, Rush University Medical Center, 1620 W Harrison St, Chicago, IL 60612, U.S.A.. Electronic address: nicholas_d_osteraas@rush.edu.
- [26] Department of Neurological Sciences, Rush University Medical Center, 1620 W Harrison St, Chicago, IL 60612, U.S.A.. Electronic address: ivan_dasilva@rush.edu.
- [27] Department of Neurological Sciences, Rush University Medical Center, 1620 W Harrison St, Chicago, IL 60612, U.S.A.. Electronic address: julianne_p_hall@rush.edu.
- [28] Department of Neurological Sciences, Rush University Medical Center, 1620 W Harrison St, Chicago, IL 60612, U.S.A.. Electronic address: sayona_john@rush.edu.
- [29] Department of Neurology and Rehabilitation, University of Illinois at Chicago, Chicago, IL 60612, U.S.A.. Electronic address: nshafi@uic.edu.
- [30] Department of Neurology and Rehabilitation, University of Illinois at Chicago, Chicago, IL 60612, U.S.A.. Electronic address: kmille25@uic.edu.

- [31] Department of Neurology and Rehabilitation, University of Illinois at Chicago, Chicago, IL 60612, U.S.A.. Electronic address: bmoust2@uic.edu.
- [32] Department of Neurological Sciences, Rush University Medical Center, 1620 W Harrison St, Chicago, IL 60612, U.S.A.. Electronic address: alejandro_vargas@rush.edu.
- [33] Department of Neurology, Northwestern University, 633 Clark St, Evanston, IL 60208, U.S.A.. Electronic address: pgorelick@thorek.org.
- [34] Department of Neurology and Rehabilitation, University of Illinois at Chicago, Chicago, IL 60612, U.S.A.. Electronic address: testai@uic.edu.

- PMID: **32951959**
- PMCID: PMC7486061
- DOI: 10.1016/j.jstrokecerebrovasdis.2020.105314

Abstract

Background and purpose: Severe Acute Respiratory Syndrome Coronavirus 2 (SARS-CoV-2) infection is associated with stroke. The role of sex on stroke outcome has not been investigated. To objective of this paper is to describe the characteristics of a diverse cohort of acute stroke patients with COVID-19 disease and determine the role of sex on outcome.

Methods: This is a retrospective study of patients with acute stroke and SARS-CoV-2 infection admitted between March 15 to May 15, 2020 to one of the six participating comprehensive stroke centers. Baseline characteristics, stroke subtype, workup, treatment and outcome are presented as total number and percentage or median and interquartile range. Outcome at discharge was determined by the modified Rankin Scale Score (mRS). Variables and outcomes were compared for males and females using univariate and multivariate analysis.

Results: The study included 83 patients, 47% of which were Black, 28% Hispanics/Latinos, and 16% whites. Median age was 64 years. Approximately 89% had at least one preexisting vascular risk factor (VRF). The most common complications were respiratory failure (59%) and septic shock (34%). Compared with females, a higher proportion of males experienced severe SARS-CoV-2 symptoms requiring ICU hospitalization (73% vs. 49%; $p = 0.04$). When divided by stroke subtype, there were 77% ischemic, 19% intracerebral hemorrhage and 3% subarachnoid hemorrhage. The most common ischemic stroke etiologies were cryptogenic (39%) and cardioembolic (27%). Compared with females, males had higher mortality (38% vs. 13%; $p = 0.02$) and were less likely to be discharged home (12% vs. 33%; $p = 0.04$). After adjustment for age, race/ethnicity, and number of VRFs, mRS was higher in males than in females (OR = 1.47, 95% CI = 1.03-2.09).

Conclusion: In this cohort of SARS-CoV-2 stroke patients, most had clinical evidence of coronavirus infection on admission and preexisting VRFs. Severe in-hospital complications and worse outcomes after ischemic strokes were higher in males, than females.

Keywords: COVID-19; Outcome; Race; Sex; Stroke.

Copyright © 2020 Elsevier Inc. All rights reserved.

- 19 references

Supplementary info

Publication types, MeSH terms Expand

Publication types

- Comparative Study
- Multicenter Study
- Observational Study

MeSH terms

- Aged
- Aged, 80 and over
- Brain Ischemia / diagnosis
- Brain Ischemia / epidemiology*
- Brain Ischemia / therapy
- COVID-19
- Chicago / epidemiology
- Coronavirus Infections / diagnosis
- Coronavirus Infections / epidemiology*
- Coronavirus Infections / therapy
- Female
- Health Status Disparities*
- Humans
- Intracranial Hemorrhages / diagnosis
- Intracranial Hemorrhages / epidemiology*
- Intracranial Hemorrhages / therapy
- Male
- Middle Aged
- Pandemics
- Pneumonia, Viral / diagnosis
- Pneumonia, Viral / epidemiology*
- Pneumonia, Viral / therapy
- Prognosis
- Retrospective Studies
- Risk Assessment
- Risk Factors
- Sex Factors
- Stroke / diagnosis
- Stroke / epidemiology*
- Stroke / therapy
- Time Factors

Full text links

ELSEVIER FULL-TEXT ARTICLE Elsevier Science Free PMC article
Proceed to details
Cite
Share
1,230

Observational Study

Eur Respir J
. 2020 Oct 15;56(4):2002767.
doi: 10.1183/13993003.02767-2020. Print 2020 Oct.

Utility and safety of bronchoscopy during the SARS-CoV-2 outbreak in Italy: a retrospective, multicentre study

Michele Mondoni [1], Giuseppe Francesco Sferrazza Papa [2] [3], Rocco Rinaldo [1], Paola Faverio [4], Almerico Marruchella [4], Francesca D'Arcangelo [4], Alberto Pesci [4], Simone Pasini [5], Sonia Henchi [6], Giuseppe Cipolla [6], Francesco Tarantini [7], Lisa Giuliani [8], Fabiano Di Marco [8], Laura Saracino [9], Stefano Tomaselli [9], Angelo Corsico [10], Stefano Gasparini [11], Martina Bonifazi [11], Lina Zuccatosta [11], Laura Saderi [12], Giulia Pellegrino [2] [3], Matteo Davì [1], Paolo Carlucci [1], Stefano Centanni [1], Giovanni Sotgiu [12]
Affiliations Expand

Affiliations

- [1] Respiratory Unit, ASST Santi Paolo e Carlo, San Paolo Hospital, Dept of Health Sciences, Università degli Studi di Milano, Milan, Italy.
- [2] Dept of Health Sciences, Università degli Studi di Milano, Milan, Italy.
- [3] Dipartimento di Scienze Neuroriabilitative, Casa di Cura del Policlinico, Milan, Italy.
- [4] School of Medicine and Surgery, University of Milano Bicocca, Respiratory Unit, San Gerardo Hospital, ASST Monza, Monza, Italy.
- [5] ASST Lodi, UOC Medicina Interna, Lodi, Italy.
- [6] ASST Lodi, UOC Pneumologia, Lodi, Italy.
- [7] Respiratory Unit, Papa Giovanni XXIII Hospital, Bergamo, Italy.
- [8] Respiratory Unit, Papa Giovanni XXIII Hospital, Dept of Health Sciences, Università degli Studi di Milano, Bergamo, Italy.
- [9] Respiratory Diseases Unit, Fondazione IRCCS Policlinico San Matteo, Pavia, Italy.
- [10] Respiratory Diseases Unit, Fondazione IRCCS Policlinico San Matteo, Dept of Internal Medicine and Therapeutics, University of Pavia, Pavia, Italy.
- [11] Pulmonary Disease Unit, Dept of Internal Medicine, Azienda Ospedali Riuniti, Dept of Biomedical Sciences and Public Health, Università Politecnica delle Marche, Ancona, Italy.
- [12] Clinical Epidemiology and Medical Statistics Unit, Dept of Medical, Surgical and Experimental Medicine, University of Sassari, Sassari, Italy.

- PMID: **32859682**
- PMCID: PMC7453732

- DOI: 10.1183/13993003.02767-2020

Free PMC article
Observational Study

Utility and safety of bronchoscopy during the SARS-CoV-2 outbreak in Italy: a retrospective, multicentre study

Michele Mondoni et al. Eur Respir J. 2020.
Free PMC article
Show details
Eur Respir J
. 2020 Oct 15;56(4):2002767.
doi: 10.1183/13993003.02767-2020. Print 2020 Oct.

Authors

Michele Mondoni [1], Giuseppe Francesco Sferrazza Papa [2] [3], Rocco Rinaldo [1], Paola Faverio [4], Almerico Marruchella [4], Francesca D'Arcangelo [4], Alberto Pesci [4], Simone Pasini [5], Sonia Henchi [6], Giuseppe Cipolla [6], Francesco Tarantini [7], Lisa Giuliani [8], Fabiano Di Marco [8], Laura Saracino [9], Stefano Tomaselli [9], Angelo Corsico [10], Stefano Gasparini [11], Martina Bonifazi [11], Lina Zuccatosta [11], Laura Saderi [12], Giulia Pellegrino [2] [3], Matteo Davì [1], Paolo Carlucci [1], Stefano Centanni [1], Giovanni Sotgiu [12]

Affiliations

- [1] Respiratory Unit, ASST Santi Paolo e Carlo, San Paolo Hospital, Dept of Health Sciences, Università degli Studi di Milano, Milan, Italy.
- [2] Dept of Health Sciences, Università degli Studi di Milano, Milan, Italy.
- [3] Dipartimento di Scienze Neuroriabilitative, Casa di Cura del Policlinico, Milan, Italy.
- [4] School of Medicine and Surgery, University of Milano Bicocca, Respiratory Unit, San Gerardo Hospital, ASST Monza, Monza, Italy.
- [5] ASST Lodi, UOC Medicina Interna, Lodi, Italy.
- [6] ASST Lodi, UOC Pneumologia, Lodi, Italy.
- [7] Respiratory Unit, Papa Giovanni XXIII Hospital, Bergamo, Italy.
- [8] Respiratory Unit, Papa Giovanni XXIII Hospital, Dept of Health Sciences, Università degli Studi di Milano, Bergamo, Italy.
- [9] Respiratory Diseases Unit, Fondazione IRCCS Policlinico San Matteo, Pavia, Italy.
- [10] Respiratory Diseases Unit, Fondazione IRCCS Policlinico San Matteo, Dept of Internal Medicine and Therapeutics, University of Pavia, Pavia, Italy.
- [11] Pulmonary Disease Unit, Dept of Internal Medicine, Azienda Ospedali Riuniti, Dept of Biomedical Sciences and Public Health, Università Politecnica delle Marche, Ancona, Italy.
- [12] Clinical Epidemiology and Medical Statistics Unit, Dept of Medical, Surgical and Experimental Medicine, University of Sassari, Sassari, Italy.

- PMID: **32859682**
- PMCID: PMC7453732
- DOI: 10.1183/13993003.02767-2020

Abstract

Utility and safety of bronchoscopy during the SARS-CoV-2 outbreak https://bit.ly/3ish52k

Conflict of interest statement

Conflict of interest: M. Mondoni has nothing to disclose. Conflict of interest: G.F. Sferrazza Papa has nothing to disclose. Conflict of interest: R. Rinaldo has nothing to disclose. Conflict of interest: P. Faverio has nothing to disclose. Conflict of interest: A. Marruchella has nothing to disclose. Conflict of interest: F. D'Arcangelo has nothing to disclose. Conflict of interest: A. Pesci has nothing to disclose. Conflict of interest: S. Pasini has nothing to disclose. Conflict of interest: S. Henchi has nothing to disclose. Conflict of interest: G. Cipolla has nothing to disclose. Conflict of interest: F. Tarantini has nothing to disclose. Conflict of interest: L. Giuliani has nothing to disclose. Conflict of interest: F. Di Marco has nothing to disclose. Conflict of interest: L. Saracino has nothing to disclose. Conflict of interest: S. Tomaselli has nothing to disclose. Conflict of interest: A. Corsico has nothing to disclose. Conflict of interest: S. Gasparini has nothing to disclose. Conflict of interest: M. Bonifazi has nothing to disclose. Conflict of interest: L. Zuccatosta has nothing to disclose. Conflict of interest: L. Saderi has nothing to disclose. Conflict of interest: G. Pellegrino has nothing to disclose. Conflict of interest: M. Davì has nothing to disclose. Conflict of interest: P. Carlucci has nothing to disclose. Conflict of interest: S. Centanni has nothing to disclose. Conflict of interest: G. Sotgiu has nothing to disclose.

- 16 references
- 1 figure

Supplementary info

Publication types, MeSH terms Expand

Publication types

- Letter
- Multicenter Study
- Observational Study

MeSH terms

- Betacoronavirus / isolation & purification*
- Bronchoalveolar Lavage Fluid / virology*
- Bronchoscopy* / adverse effects
- Bronchoscopy* / methods
- Bronchoscopy* / statistics & numerical data
- COVID-19

- COVID-19 Testing
- Clinical Laboratory Techniques / methods*
- Coinfection / diagnosis
- Coronavirus Infections* / diagnosis
- Coronavirus Infections* / epidemiology
- Coronavirus Infections* / physiopathology
- Coronavirus Infections* / virology
- Disease Transmission, Infectious / prevention & control
- Early Diagnosis
- Female
- Humans
- Infection Control / methods
- Italy / epidemiology
- Male
- Middle Aged
- Outcome Assessment, Health Care
- Pandemics*
- Pneumonia, Viral* / diagnosis
- Pneumonia, Viral* / epidemiology
- Pneumonia, Viral* / physiopathology
- Pneumonia, Viral* / virology
- Procedures and Techniques Utilization*
- SARS-CoV-2
- Symptom Assessment / methods
- Symptom Assessment / statistics & numerical data

Full text links

Free full text at ersjournals.com HighWire Free PMC article

Proceed to details

Cite

Share

☐ 1,231

EClinicalMedicine

. 2021 Jul;37:100941.

doi: 10.1016/j.eclinm.2021.100941. Epub 2021 Jun 9.

A simple, home-therapy algorithm to prevent hospitalisation for COVID-19 patients: A

retrospective observational matched-cohort study

Fredy Suter [1], Elena Consolaro [2], Stefania Pedroni [2], Chiara Moroni [2], Elena Pastò [2], Maria Vittoria Paganini [2], Grazia Pravettoni [3], Umberto Cantarelli [4], Nadia Rubis [5], Norberto Perico [5], Annalisa Perna [5], Tobia Peracchi [5], Piero Ruggenenti [1] [5], Giuseppe Remuzzi [5]
Affiliations Expand

Affiliations

- [1] Azienda Socio-Sanitaria Territoriale (ASST) Papa Giovanni XXIII, Bergamo, Italy.
- [2] ATS Insubria, Varese, Italy.
- [3] Ospedale Circolo di Busto Arsizio, Varese, Italy.
- [4] ASL Teramo, Teramo, Italy.
- [5] Istituto di Ricerche Farmacologiche Mario Negri IRCCS, Bergamo, Italy.

- PMID: **34127959**
- PMCID: PMC8189543
- DOI: 10.1016/j.eclinm.2021.100941

Free PMC article

A simple, home-therapy algorithm to prevent hospitalisation for COVID-19 patients: A retrospective observational matched-cohort study

Fredy Suter et al. EClinicalMedicine. 2021 Jul.
Free PMC article
Show details
EClinicalMedicine
. 2021 Jul;37:100941.
doi: 10.1016/j.eclinm.2021.100941. Epub 2021 Jun 9.

Authors

Fredy Suter [1], Elena Consolaro [2], Stefania Pedroni [2], Chiara Moroni [2], Elena Pastò [2], Maria Vittoria Paganini [2], Grazia Pravettoni [3], Umberto Cantarelli [4], Nadia Rubis [5], Norberto Perico [5], Annalisa Perna [5], Tobia Peracchi [5], Piero Ruggenenti [1] [5], Giuseppe Remuzzi [5]

Affiliations

- [1] Azienda Socio-Sanitaria Territoriale (ASST) Papa Giovanni XXIII, Bergamo, Italy.

- [2] ATS Insubria, Varese, Italy.
- [3] Ospedale Circolo di Busto Arsizio, Varese, Italy.
- [4] ASL Teramo, Teramo, Italy.
- [5] Istituto di Ricerche Farmacologiche Mario Negri IRCCS, Bergamo, Italy.

- PMID: **34127959**
- PMCID: PMC8189543
- DOI: 10.1016/j.eclinm.2021.100941

Abstract

Background: Effective home treatment algorithms implemented based on a pathophysiologic and pharmacologic rationale to accelerate recovery and prevent hospitalisation of patients with early coronavirus disease 2019 (COVID-19) would have major implications for patients and health system.

Methods: This academic, matched-cohort study compared outcomes of 90 consecutive consenting patients with mild COVID-19 treated at home by their family physicians between October 2020 and January 2021 in Northern and Central Italy, according to the proposed recommendation algorithm, with outcomes for 90 age-, sex-, and comorbidities-matched patients who received other therapeutic regimens. Primary outcome was time to resolution of major symptoms. Secondary outcomes included prevention of hospitalisation. Analyses were by intention-to-treat.

Findings: All patients achieved complete remission. The median [IQR] time to resolution of major symptoms was 18 [14-23] days in the 'recommended schedule' cohort and 14 [7-30] days in the matched 'control' cohort ($p = 0{\cdot}033$). Other symptoms persisted in a lower percentage of patients in the 'recommended' than in the 'control' cohort (23·3% versus 73·3%, respectively, $p<0{\cdot}0001$) and for a shorter period ($p = 0{\cdot}0107$). Two patients in the 'recommended' cohort were hospitalised compared to 13 (14·4%) controls ($p = 0{\cdot}0103$). The prevention algorithm reduced the days and cumulative costs of hospitalisation by >90%.

Interpretation: Implementation of an early home treatment algorithm failed to accelerate recovery from major symptoms of COVID-19, but reduced the risk of hospitalisation and related treatment costs. Given the study design, additional research would be required to consolidate the proposed treatment recommendations.

Funding: Fondazione Cav.Lav. Carlo Pesenti.

Keywords: COVID-19; Early symptoms at home; Family physicians; Matched-cohort observational study; SARS-CoV-2; Simple home-therapy algorithm.

© 2021 The Authors.

Conflict of interest statement

We declare that we have no conflicts of interest.

- 45 references
- 3 figures

Full text links

Elsevier Science Free PMC article

Proceed to details

Cite

Share

1,232

Case Reports

Med Clin (Barc)

. 2021 Dec 28;S0025-7753(21)00723-5.

doi: 10.1016/j.medcli.2021.11.016. Online ahead of print.

Clinical characteristics of children hospitalized for COVID-19

[Article in English, Spanish]

Rosa María Luz Romero [1], Marta Illán Ramos [2], Arantxa Berzosa Sánchez [2], Belén Joyanes Abancens [2], Elvira Baos Muñoz [3], José Tomás Ramos Amador [2]

Affiliations Expand

Affiliations

- [1] Servicio de Pediatría, Hospital Clínico San Carlos, Madrid, España. Electronic address: rosamaria.luz@salud.madrid.org.
- [2] Servicio de Pediatría, Hospital Clínico San Carlos, Madrid, España.
- [3] Servicio de Microbiología Clínica, Hospital Clínico San Carlos, Madrid, España.

- PMID: **35039168**
- PMCID: PMC8712264
- DOI: 10.1016/j.medcli.2021.11.016

Free PMC article

Case Reports

Clinical characteristics of children hospitalized for COVID-19

[Article in English, Spanish]

Rosa María Luz Romero et al. Med Clin (Barc). 2021.

Free PMC article

Show details

Med Clin (Barc)

. 2021 Dec 28;S0025-7753(21)00723-5.

doi: 10.1016/j.medcli.2021.11.016. Online ahead of print.

Authors

Rosa María Luz Romero [1], Marta Illán Ramos [2], Arantxa Berzosa Sánchez [2], Belén Joyanes Abancens [2], Elvira Baos Muñoz [3], José Tomás Ramos Amador [2]

Affiliations

- [1] Servicio de Pediatría, Hospital Clínico San Carlos, Madrid, España. Electronic address: rosamaria.luz@salud.madrid.org.
- [2] Servicio de Pediatría, Hospital Clínico San Carlos, Madrid, España.
- [3] Servicio de Microbiología Clínica, Hospital Clínico San Carlos, Madrid, España.

- PMID: **35039168**
- PMCID: PMC8712264
- DOI: 10.1016/j.medcli.2021.11.016

Abstract

Introduction: Most SARS-CoV-2 infections in the pediatric population are asymptomatic or with mild symptoms, with a minimal proportion of severe cases described as SARS-CoV-2-associated multi-system inflammatory syndrome (MIS-C). The objective was to describe the clinical and epidemiological characteristics of pediatric patients admitted with confirmed diagnosis of SARS-CoV-2 infection from the beginning of the pandemic until May 2021.

Methods: Retrospective observational study of pediatric patients hospitalized with confirmed COVID-19, in a tertiary hospital. Epidemiological and clinical data, additional tests, treatments administered and evolution were collected.

Results: 30 patients were included, classified into 3 groups according to diagnosis: respiratory infection, MIS-C and compatible symptoms. The patients with pneumonia were associated with age older, comorbidities and lymphopenia. MIS-C were more serious patients, with marked laboratory involvement and greater admission to PICU. Most of these were secondary cases of contact in the family environment.

Discussion: The most frequent clinical manifestations of COVID-19 in children are mild-moderate respiratory with good evolution. MIS-C is another form of expression of SARS-CoV-2 infection of greater severity, but usually with good prognosis after early diagnosis and frequent PICU admission.

Keywords: COVID-19; Hospitalización; Hospitalization; Pediatric; Pediatría.

Copyright © 2021 Elsevier España, S.L.U. All rights reserved.

- 10 references

Supplementary info

Publication types Expand

Publication types

- Case Reports

Full text links

MEDICINA CLINICA Ediciones Doyma, S.L. Free PMC article
Proceed to details
Cite
Share
1,233
Observational Study
Ann Med
. 2021 Dec;53(1):1863-1874.
doi: 10.1080/07853890.2021.1992495.

An observational cohort study of the performance of the REDS score compared to the SIRS criteria, NEWS2, CURB65, SOFA, MEDS and PIRO scores to risk-stratify emergency department suspected sepsis

Narani Sivayoham [1], Adil N Hussain [1], Luke Shabbo [1], Dylon Christie [1]
Affiliations Expand

Affiliation

- [1] Department of Emergency Medicine, St George's University Hospitals NHS Foundation Trust, London, UK.

- PMID: **34686088**
- PMCID: PMC8547872
- DOI: 10.1080/07853890.2021.1992495

Free PMC article
Observational Study

An observational cohort study of the performance of the REDS score compared to the SIRS criteria, NEWS2, CURB65, SOFA, MEDS and PIRO scores to risk-stratify emergency department suspected sepsis

Narani Sivayoham et al. Ann Med. 2021 Dec.

Free PMC article

Show details

Ann Med

. 2021 Dec;53(1):1863-1874.

doi: 10.1080/07853890.2021.1992495.

Authors

Narani Sivayoham [1], Adil N Hussain [1], Luke Shabbo [1], Dylon Christie [1]

Affiliation

- [1] Department of Emergency Medicine, St George's University Hospitals NHS Foundation Trust, London, UK.

- PMID: **34686088**
- PMCID: PMC8547872
- DOI: 10.1080/07853890.2021.1992495

Abstract

Objective: To compare the performance of the Risk-stratification of Emergency Department suspected Sepsis (REDS) score to the SIRS criteria, NEWS2, CURB65, SOFA, MEDS and PIRO scores, to risk-stratify Emergency Department (ED) suspected sepsis patients for mortality.

Method: A retrospective observational cohort study of prospectively collected data. Adult patients admitted from the ED after receiving intravenous antibiotics for suspected sepsis in the year 2020, were studied. Patients with COVID-19 were excluded. The scores stated above were calculated for each patient. Receiver operator characteristics (ROC) curves were constructed for each score for the primary outcome measure, all-cause in-hospital mortality. The area under the ROC (AUROC) curves and cut-off points were identified by the statistical software. Scores above the cut-off point were deemed high-risk. The test characteristics of the high-risk groups were calculated. Comparisons were based on the AUROC curve and sensitivity for mortality of the high-risk groups. Previously published cut-off points were also studied. Calibration was also studied.

Results: Of the 2594 patients studied, 332 (12.8%) died. The AUROC curve for the REDS score 0.73 (95% confidence interval [CI] 0.72-0.75) was significantly greater than the AUROC curve for the SIRS criteria 0.51 (95% CI 0.49-0.53), $p < .0001$ and the NEWS2 score 0.69 (95% CI 0.67-0.70), $p = .005$, and similar to all other scores studied. Sensitivity for mortality at the respective cut-off points identified (REDS ≥3, NEWS2 ≥ 8, CURB65 ≥ 3, SOFA ≥3, MEDS ≥10 and PIRO ≥10) was greatest for the REDS score at 80.1% (95% CI 75.4-84.3) and significantly greater than the other scores. The sensitivity for mortality for an increase of two points from baseline in the SOFA score was 63% (95% CI 57.5-68.2).

Conclusions: In this single centre study, the REDS score had either a greater AUROC curve or sensitivity for mortality compared to the comparator scores, at the respective cut-off points identified.KEY MESSAGESThe REDS score is a simple and objective scoring system to risk-stratify for mortality in emergency department (MED) patients with suspected sepsis.The REDS score is better or equivalent to existing scoring systems in its discrimination for mortality.

Keywords: Clinical prediction rule; discrimination; emergency department; prognosis; sepsis; septic shock.

Conflict of interest statement

The authors declare that they have no competing interests.

- 40 references
- 3 figures

Supplementary info

Publication types, MeSH terms, Substances Expand

Publication types

- Comparative Study
- Observational Study

MeSH terms

- Administration, Intravenous
- Aged
- Aged, 80 and over
- Anti-Bacterial Agents / administration & dosage*
- Emergency Service, Hospital / statistics & numerical data*
- Female
- Hospital Mortality
- Humans
- Intensive Care Units / statistics & numerical data*
- Male
- Middle Aged
- Prognosis
- Prospective Studies
- ROC Curve
- Retrospective Studies
- Risk Assessment / methods
- Sepsis / diagnosis
- Sepsis / drug therapy
- Sepsis / mortality*
- Severity of Illness Index*

Substances

- Anti-Bacterial Agents

Full text links

View full text Taylor & Francis Free PMC article

Proceed to details

Cite

Share

1,234

Observational Study

Arch Endocrinol Metab
. 2021 Nov 1;65(2):164-171.
doi: 10.20945/2359-3997000000332. Epub 2021 Feb 24.

Hypoglycemia frequency and treatment satisfaction in patients receiving insulin analogues for treatment of type 1 diabetes mellitus

Gabriela Berlanda [1, 2], Gabriela H Telo [3], Bárbara Côrrea Krug [4], Rafael Selbach Scheffel [1, 5], Bruna Pasinato [6], Fernando Iorra [6], João Gabbardo Dos Reis [7], Paulo Dornelles Picon [5, 4], Beatriz D Schaan [1, 5]

Affiliations Expand

Affiliations

- [1] Programa de Pós-Graduação em Endocrinologia, Universidade Federal do Rio Grande do Sul (UFRGS), Porto Alegre, RS, Brasil.
- [2] Hospital de Clínicas de Porto Alegre, Porto Alegre, RS, Brasil, gabrielaberlanda@yahoo.com.br.
- [3] Departamento de Medicina Interna, Escola de Medicina da Pontifícia Universidade Católica do Rio Grande do Sul, Porto Alegre, RS, Brasil.
- [4] Secretaria Estadual da Saúde do Rio Grande do Sul, Porto Alegre, RS, Brasil.
- [5] Hospital de Clínicas de Porto Alegre, Porto Alegre, RS, Brasil.
- [6] Universidade Federal do Rio Grande do Sul (UFRGS), Porto Alegre, RS, Brasil.
- [7] Coordenação Executiva do Comitê de Contingência de Combate ao COVID-19 do Governo do Estado de São Paulo, SP, Brasil.

- PMID: **33905628**
- DOI: 10.20945/2359-3997000000332

Observational Study

Hypoglycemia frequency and treatment satisfaction in patients receiving insulin analogues for treatment of type 1 diabetes mellitus

Gabriela Berlanda et al. Arch Endocrinol Metab. 2021.
Show details
Arch Endocrinol Metab
. 2021 Nov 1;65(2):164-171.
doi: 10.20945/2359-3997000000332. Epub 2021 Feb 24.

Authors

Gabriela Berlanda [1] [2], Gabriela H Telo [3], Bárbara Côrrea Krug [4], Rafael Selbach Scheffel [1] [5], Bruna Pasinato [6], Fernando Iorra [6], João Gabbardo Dos Reis [7], Paulo Dornelles Picon [5] [4], Beatriz D Schaan [1] [5]

Affiliations

- [1] Programa de Pós-Graduação em Endocrinologia, Universidade Federal do Rio Grande do Sul (UFRGS), Porto Alegre, RS, Brasil.
- [2] Hospital de Clínicas de Porto Alegre, Porto Alegre, RS, Brasil, gabrielaberlanda@yahoo.com.br.
- [3] Departamento de Medicina Interna, Escola de Medicina da Pontifícia Universidade Católica do Rio Grande do Sul, Porto Alegre, RS, Brasil.
- [4] Secretaria Estadual da Saúde do Rio Grande do Sul, Porto Alegre, RS, Brasil.
- [5] Hospital de Clínicas de Porto Alegre, Porto Alegre, RS, Brasil.
- [6] Universidade Federal do Rio Grande do Sul (UFRGS), Porto Alegre, RS, Brasil.
- [7] Coordenação Executiva do Comitê de Contingência de Combate ao COVID-19 do Governo do Estado de São Paulo, SP, Brasil.

- PMID: **33905628**
- DOI: 10.20945/2359-3997000000332

Abstract

Objective: The aim of this study was to evaluate the frequency of hypoglycemia and the treatment satisfaction in patients with type 1 diabetes (T1D) using insulin analogues.

Methods: This observational retrospective study included 516 adult patients with T1D from 38 cities in Southern Brazil. Demographics and clinical data were collected using a self-report questionnaire. Hypoglycemia was defined as an event based on either symptoms or self-monitored blood glucose < 70 mg/dL. Treatment satisfaction was evaluated using the Diabetes Treatment Satisfaction Questionnaire status version (DTSQs) and with a specific question with scores ranging from 0-10. Common mental disorders were assessed using the General Health Questionnaire (GHQ-12).

Results: Overall, the mean age was 38 ± 14 years and 52% of the participants were women. The median diabetes duration was 18 years. The scores for insulin analogue treatment satisfaction were higher than those for previous treatments. DTSQ scores had a median value of 32 (interquartile range 29-35) and remained unchanged over time. The percentage of patients with hypoglycemia (including severe and nocturnal) was comparable across groups divided according to duration of use of insulin analogues. Most patients (n=395, 77%) screened positive for common mental disorders.

Conclusion: Patient satisfaction with insulin analogue treatment was high and remained unchanged with time. Episodes of hypoglycemia also remained unchanged over time among patients using insulin analogues.

Keywords: Type 1 diabetes; hypoglycemia; insulin analogues; treatment satisfaction.

Supplementary info

Publication types, MeSH terms, Substances Expand

Publication types

- Observational Study

MeSH terms

- Adult
- Blood Glucose
- Diabetes Mellitus, Type 1* / drug therapy
- Female
- Glycated Hemoglobin A / analysis
- Humans
- Hypoglycemia* / chemically induced
- Hypoglycemic Agents* / therapeutic use
- Insulins* / therapeutic use
- Male
- Middle Aged
- Patient Satisfaction
- Retrospective Studies
- Young Adult

Substances

- Blood Glucose
- Glycated Hemoglobin A
- Hypoglycemic Agents
- Insulins

Proceed to details

Cite

Share

☐ 1,235

Observational Study

Int Immunopharmacol

. 2020 Dec;89(Pt B):107093.

doi: 10.1016/j.intimp.2020.107093. Epub 2020 Oct 19.

Acute generalized exanthematous pustulosis with a focus on hydroxychloroquine: A 10-year experience in a skin hospital

Ali Nili [1], Ehsan Zarei [2], Azin Ghamari [3], Ali Salehi Farid [1], Soheil Tavakolpour [4], Maryam Daneshpazhooh [1], Hamidreza Mahmoudi [5]

Affiliations Expand

Affiliations

- [1] Autoimmune Bullous Diseases Research Center, Tehran University of Medical Sciences, Tehran 63911-11996, Iran.
- [2] Tehran University of Medical Sciences, School of Medicine, Tehran 14176-13151, Iran.
- [3] Autoimmune Bullous Diseases Research Center, Tehran University of Medical Sciences, Tehran 63911-11996, Iran; Growth and Development Research Center, Children's Medical Center, Tehran University of Medical Sciences, Tehran 14197-33151, Iran.
- [4] Autoimmune Bullous Diseases Research Center, Tehran University of Medical Sciences, Tehran 63911-11996, Iran; Dana-Farber Cancer Institute, Harvard Medical School, Boston, MA 02215, USA.
- [5] Autoimmune Bullous Diseases Research Center, Tehran University of Medical Sciences, Tehran 63911-11996, Iran. Electronic address: hr_mahmoody@yahoo.com.

- PMID: **33091817**
- PMCID: PMC7572086
- DOI: 10.1016/j.intimp.2020.107093

Free PMC article

Observational Study

Acute generalized exanthematous pustulosis with a focus on hydroxychloroquine: A 10-year experience in a skin hospital

Ali Nili et al. Int Immunopharmacol. 2020 Dec.

Free PMC article

Show details

Int Immunopharmacol
. 2020 Dec;89(Pt B):107093.
doi: 10.1016/j.intimp.2020.107093. Epub 2020 Oct 19.

Authors

Ali Nili [1], Ehsan Zarei [2], Azin Ghamari [3], Ali Salehi Farid [1], Soheil Tavakolpour [4], Maryam Daneshpazhooh [1], Hamidreza Mahmoudi [5]

Affiliations

- [1] Autoimmune Bullous Diseases Research Center, Tehran University of Medical Sciences, Tehran 63911-11996, Iran.
- [2] Tehran University of Medical Sciences, School of Medicine, Tehran 14176-13151, Iran.
- [3] Autoimmune Bullous Diseases Research Center, Tehran University of Medical Sciences, Tehran 63911-11996, Iran; Growth and Development Research Center, Children's Medical Center, Tehran University of Medical Sciences, Tehran 14197-33151, Iran.
- [4] Autoimmune Bullous Diseases Research Center, Tehran University of Medical Sciences, Tehran 63911-11996, Iran; Dana-Farber Cancer Institute, Harvard Medical School, Boston, MA 02215, USA.
- [5] Autoimmune Bullous Diseases Research Center, Tehran University of Medical Sciences, Tehran 63911-11996, Iran. Electronic address: hr_mahmoody@yahoo.com.

- PMID: **33091817**
- PMCID: PMC7572086
- DOI: 10.1016/j.intimp.2020.107093

Abstract

Objective: Acute generalized exanthematous pustulosis (AGEP) is a severe skin pustular drug reaction that can lead to life-threatening consequences. In this study, we have investigated the characteristics and outcomes of patients with AGEP in a tertiary skin hospital.

Methods: From March 2007 to December 2019, medical records of all patients diagnosed with AGEP, were assessed. Demographic data, culprit drug, past medical history, laboratory tests, recurrence, and systemic organ involvement were all documented as well.

Results: Seventy-four patients, including 54 women (73%) and 20 men (27%), with a mean age of 44.3 ± 16.5 years were evaluated. The most common comorbidities among the patients were rheumatoid arthritis and diabetes. In addition, hydroxychloroquine, cephalosporin, and amoxicillin were found as the three most common medications associated with AGEP induction. Among the study group, seventeen (23%) patients had systemic organ involvement (nine (12.2%), six (8.1%), and five (6.8%) had hepatic, renal and pulmonary involvement, respectively). All patients responded to oral prednisolone within a median of five days (IQR = 4; ranged 2-14). The median duration of treatment was significantly longer in hydroxychloroquine group compared to other drugs (8 versus 5 days; HR 0.57,95%CI 0·35-0.91). Likewise, the median duration of treatment was significantly longer in febrile patients compared to the afebrile ones (7 versus 4 days; HR 0.46, 95%CI 0.25-0.85). Recurrence occurred in six patients after resuming treatment with the same medication. The mean Naranjo score was 7.6 ± 0.9 denoting a probable causal relationship.

Conclusion: In this study, we found that using hydroxychloroquine and presence of fever are the risk factors potentially leading to a prolonged treatment duration of AGEP.

Keywords: AGEP; Acute generalized exanthematous pustulosis; COVID-19; Drug reaction; Hydroxychloroquine; Rituximab.

Copyright © 2020 Elsevier B.V. All rights reserved.

Conflict of interest statement

The authors declare that they have no known competing financial interests or personal relationships that could have appeared to influence the work reported in this paper.

- 20 references
- 1 figure

Supplementary info

Publication types, MeSH terms, Substances Expand

Publication types

- Observational Study

MeSH terms

- Acute Generalized Exanthematous Pustulosis / etiology*
- Adult
- Aged
- Female
- Humans
- Hydroxychloroquine / adverse effects*
- Male
- Middle Aged
- Retrospective Studies

Substances

- Hydroxychloroquine

Full text links

ELSEVIER FULL-TEXT ARTICLE Elsevier Science Free PMC article
Proceed to details
Cite
Share

☐ 1,236

Infect Dis Clin Pract (Baltim Md)
. 2021 Sep;29(5):e282-e286.
doi: 10.1097/IPC.0000000000001023. Epub 2021 Apr 15.

Early Use of Remdesivir in Patients Hospitalized With COVID-19 Improves Clinical Outcomes: A Retrospective Observational Study

Neha Paranjape [1], Mir Husain [2], Jennifer Priestley [3], Yashila Koonjah [3], Christopher Watts [4], Joseph Havlik [2]
Affiliations Expand

Affiliations

- [1] Department of Infectious Disease, Wellstar Kennestone Hospital.
- [2] Department of Infectious Disease, Wellstar Cobb Hospital, Marietta.
- [3] Analytics and Data Science Institute, Kennesaw State University, Kennesaw.
- [4] Department of Infectious Disease, Wellstar Douglas Hospital, Marietta, GA.

- PMID: **34539162**
- PMCID: PMC8436815
- DOI: 10.1097/IPC.0000000000001023

Free PMC article

Early Use of Remdesivir in Patients Hospitalized With COVID-19 Improves Clinical Outcomes: A Retrospective Observational Study

Neha Paranjape et al. Infect Dis Clin Pract (Baltim Md). 2021 Sep.
Free PMC article
Show details

Infect Dis Clin Pract (Baltim Md)
. 2021 Sep;29(5):e282-e286.
doi: 10.1097/IPC.0000000000001023. Epub 2021 Apr 15.

Authors

Neha Paranjape [1], Mir Husain [2], Jennifer Priestley [3], Yashila Koonjah [3], Christopher Watts [4], Joseph Havlik [2]

Affiliations

- [1] Department of Infectious Disease, Wellstar Kennestone Hospital.
- [2] Department of Infectious Disease, Wellstar Cobb Hospital, Marietta.
- [3] Analytics and Data Science Institute, Kennesaw State University, Kennesaw.
- [4] Department of Infectious Disease, Wellstar Douglas Hospital, Marietta, GA.

- PMID: **34539162**
- PMCID: PMC8436815
- DOI: 10.1097/IPC.0000000000001023

Abstract

Background: Remdesivir treatment, like most antiviral drugs, is likely to be most effective when used early in the course of coronavirus disease 2019 (COVID-19). Optimal timing of remdesivir for the treatment of COVID-19 remains unclear.

Objectives: The aim of this study was to determine whether early treatment with remdesivir improves clinical outcomes: length of stay, need for mechanical ventilation, and death.

Methods: We conducted a retrospective observational study of patients hospitalized with COVID-19 who received remdesivir therapy within 10 days of symptom onset at a large health system in Georgia, United States.

Results: We identified a total of 475 patients. Initiation of therapy 3 days or less from first positive SARS-CoV-2 improved length of stay (15.7 days) compared with those started on therapy more than 3 days after a positive test (19.3 days) ($P = 0.03$). In the ≤3 day group, further reduction in length of stay was seen in those with lower oxygen requirement at baseline ($P < 0.0001$). Length of stay was lower in the ≤3 day group both with and without the use of corticosteroids ($P = 0.0003$). The odds of requiring mechanical ventilation were higher for the >3 day group compared with the ≤3 day group (odds ratio, 1.5; 95% confidence interval, 0.8-2.7), and the odds of death were higher for the >3 day group versus the ≤3 day group (odds ratio, 1.74; 95% confidence interval, 0.9-3.2).

Conclusions: Our data show that early treatment with remdesivir in patients hospitalized with COVID-19 shortened length of stay.

Keywords: COVID-19; SARS-CoV-2; clinical outcomes; length of stay; remdesivir.

Copyright © 2021 The Author(s). Published by Wolters Kluwer Health, Inc.

Conflict of interest statement

The authors have no funding or conflicts of interest to disclose.

- 13 references

Full text links

Free PMC article
Proceed to details
Cite
Share
1,237
Observational Study
Sci Rep
. 2021 Sep 9;11(1):18023.
doi: 10.1038/s41598-021-97595-8.

Upward trends in new, rifampicin-resistant and concurrent extrapulmonary tuberculosis cases in northern Guizhou Province of China

Ling Chen [1], Xuefeng Fu [1], Peng Tian [1], Qing Li [1], Dan Lei [1], Zhangli Peng [1], Quanxian Liu [1], Nana Li [1], Jianyong Zhang [2], Peng Xu [3], Hong Zhang [4 5]
Affiliations Expand

Affiliations

- [1] Tuberculosis Division of Respiratory and Critical Care Medicine, Affiliated Hospital of Zunyi Medical University, Zunyi, 563003, China.
- [2] Tuberculosis Division of Respiratory and Critical Care Medicine, Affiliated Hospital of Zunyi Medical University, Zunyi, 563003, China. zjy9453@163.com.
- [3] Institute of Life Sciences, Zunyi Medical University, Zunyi, 563003, China. derek_zmc@163.com.
- [4] Tuberculosis Division of Respiratory and Critical Care Medicine, Affiliated Hospital of Zunyi Medical University, Zunyi, 563003, China. hzhang@zbiomed.com.
- [5] Z-BioMed, Inc., Rockville, MD, 20855, USA. hzhang@zbiomed.com.

- PMID: **34504296**
- PMCID: PMC8429731
- DOI: 10.1038/s41598-021-97595-8

Free PMC article
Observational Study

Upward trends in new, rifampicin-resistant and concurrent extrapulmonary tuberculosis cases in northern Guizhou Province of China

Ling Chen et al. Sci Rep. 2021.
Free PMC article
Show details

Sci Rep
. 2021 Sep 9;11(1):18023.
doi: 10.1038/s41598-021-97595-8.

Authors

Ling Chen [1], Xuefeng Fu [1], Peng Tian [1], Qing Li [1], Dan Lei [1], Zhangli Peng [1], Quanxian Liu [1], Nana Li [1], Jianyong Zhang [2], Peng Xu [3], Hong Zhang [4] [5]

Affiliations

- [1] Tuberculosis Division of Respiratory and Critical Care Medicine, Affiliated Hospital of Zunyi Medical University, Zunyi, 563003, China.
- [2] Tuberculosis Division of Respiratory and Critical Care Medicine, Affiliated Hospital of Zunyi Medical University, Zunyi, 563003, China. zjy9453@163.com.
- [3] Institute of Life Sciences, Zunyi Medical University, Zunyi, 563003, China. derek_zmc@163.com.
- [4] Tuberculosis Division of Respiratory and Critical Care Medicine, Affiliated Hospital of Zunyi Medical University, Zunyi, 563003, China. hzhang@zbiomed.com.
- [5] Z-BioMed, Inc., Rockville, MD, 20855, USA. hzhang@zbiomed.com.

- PMID: **34504296**
- PMCID: PMC8429731
- DOI: 10.1038/s41598-021-97595-8

Abstract

Similar to global trends, the incidence rate of tuberculosis (TB) in China declined from 2000 to 2018. In this study, we aimed to evaluate TB trends in northern Guizhou Province and identify risk factors associated with rifampicin-resistant (RR) and concurrent extrapulmonary TB (EPTB). We analyzed data of TB patients hospitalized in Affiliated Hospital of Zunyi Medical University from 2011 to 2018, and assessed correlations between demographic characteristics of patients and RR-TB as well as concurrent EPTB. Our results showed that numbers of new, retreated, RR-TB and concurrent EPTB cases increased gradually from 2011 to 2018. Retreated patients had the highest odds of RR-TB but a lower likelihood of concurrent EPTB compared to new patients. Patients between 21 and 40 years of age had a higher likelihood of RR-TB compared to those 20 years and younger. Female patients and patients from Bijie city as well as the Miao ethnic minority had higher odds of concurrent EPTB. In summary, our data demonstrate upward trends in new, rifampicin-resistant and concurrent extrapulmonary TB cases in northern Guizhou Province of China, which should not be overlooked especially during and post the COVID-19 pandemic because TB is a greater long-term global health threat than COVID-19.

© 2021. The Author(s).

Conflict of interest statement

All authors except HZ report no potential conflicts. HZ is employed by and has shares in Z-BioMed, Inc.

- 35 references

- 2 figures

Supplementary info

Publication types, MeSH terms, Substances Expand

Publication types

- Observational Study
- Research Support, Non-U.S. Gov't

MeSH terms

- Adolescent
- Adult
- Aged
- Aged, 80 and over
- Antibiotics, Antitubercular / therapeutic use*
- Child
- China / epidemiology
- Drug Resistance, Multiple, Bacterial / physiology
- Expert Systems
- Female
- Humans
- Incidence
- Male
- Middle Aged
- Mycobacterium tuberculosis / drug effects*
- Mycobacterium tuberculosis / genetics
- Mycobacterium tuberculosis / isolation & purification
- Retrospective Studies
- Rifampin / therapeutic use*
- Risk Factors
- Tuberculosis, Pulmonary / drug therapy*
- Tuberculosis, Pulmonary / epidemiology*
- Young Adult

Substances

- Antibiotics, Antitubercular
- Rifampin

Full text links

Nature Publishing Group Free PMC article
Proceed to details
Cite
Share
1,238
J Clin Med

. 2021 Dec 24;11(1):70.
doi: 10.3390/jcm11010070.

Ongoing Use of SSRIs Does Not Alter Outcome in Hospitalized COVID-19 Patients: A Retrospective Analysis

Steven H Rauchman[1], Sherri G Mendelson[2], Courtney Rauchman[1], Lora J Kasselman[3], Aaron Pinkhasov[3], Allison B Reiss[3]
Affiliations Expand

Affiliations

- [1] The Fresno Institute of Neuroscience, Fresno, CA 93730, USA.
- [2] Providence Southern California Region, Irvine, CA 92612, USA.
- [3] Department of Medicine and Biomedical Research Institute, NYU Long Island School of Medicine, Mineola, NY 11501, USA.

- PMID: **35011811**
- PMCID: PMC8745642
- DOI: 10.3390/jcm11010070

Free PMC article

Ongoing Use of SSRIs Does Not Alter Outcome in Hospitalized COVID-19 Patients: A Retrospective Analysis

Steven H Rauchman et al. J Clin Med. 2021.
Free PMC article
Show details
J Clin Med

. 2021 Dec 24;11(1):70.
doi: 10.3390/jcm11010070.

Authors

Steven H Rauchman [1], Sherri G Mendelson [2], Courtney Rauchman [1], Lora J Kasselman [3], Aaron Pinkhasov [3], Allison B Reiss [3]

Affiliations

- [1] The Fresno Institute of Neuroscience, Fresno, CA 93730, USA.
- [2] Providence Southern California Region, Irvine, CA 92612, USA.
- [3] Department of Medicine and Biomedical Research Institute, NYU Long Island School of Medicine, Mineola, NY 11501, USA.

- PMID: **35011811**
- PMCID: PMC8745642
- DOI: 10.3390/jcm11010070

Abstract

SARS-CoV-2 continues to have devastating consequences worldwide. Though vaccinations have helped reduce spread, new strains still pose a threat. Therefore, it is imperative to identify treatments that prevent severe COVID-19 infection. Recently, acute use of SSRI antidepressants in COVID+ patients was shown to reduce symptom severity. The aim of this retrospective observational study was to determine whether COVID+ patients already on SSRIs upon hospital admission had reduced mortality compared to COVID+ patients not on chronic SSRI treatment. Electronic medical records of 9044 patients with laboratory-confirmed COVID-19 from six hospitals were queried for demographic and clinical information. Using R, a logistic regression model was run with mortality as the outcome and SSRI status as the exposure. In this sample, no patients admitted on SSRIs had them discontinued. There was no significant difference in the odds of dying between COVID+ patients on chronic SSRIs vs. those not taking SSRIs, after controlling for age category, gender, and race. This study shows the utility of large clinical databases in determining what commonly prescribed drugs might be useful in treating COVID-19. During pandemics due to novel infectious agents, it is critical to evaluate safety and efficacy of drugs that might be repurposed for treatment.

Keywords: COVID-19; SSRI; antidepressant; clinical presentation; disease severity.

Conflict of interest statement

The authors declare no conflict of interest.

- 51 references
- 1 figure

Full text links

FULL TEXT OPEN ACCESS MDPI Multidisciplinary Digital Publishing Institute (MDPI) Free PMC article
Proceed to details

Cite

Share

☐ 1,239

Med Clin (Engl Ed)
. 2021 Mar 12;156(5):221-228.
doi: 10.1016/j.medcle.2020.11.006. Epub 2021 Feb 6.

Effectiveness of glucocorticoids in patients hospitalized for severe SARS-CoV-2 pneumonia

José Francisco Pascual Pareja [1] [2], Rebeca García-Caballero [1], Llanos Soler Rangel [1] [2], Miguel Angel Vázquez-Ronda [1] [2], Silvia Roa Franco [1], Gema Navarro Jiménez [1], Miguel Angel Moreno Palanco [1] [2], Patricia González-Ruano [1] [2], Ramiro López-Menchaca [1], Pilar Ruíz-Seco [1] [2], Bárbara Pagán Muñoz [1], Alejandro Gómez Gómez [1], Beatriz Pérez-Monte [1], Rebeca Fuerte Martínez [1], Jose Luis Valle López [1], Arturo Muñoz Blanco [1], Isabel Rábago Lorite [1], Patricia Martínez Martín [1], Gonzalo Serralta San Martín [1] [2], Jorge Francisco Gómez-Cerezo [1] [2], HUIS-COVID-19 working group
Affiliations Expand

Affiliations

- [1] Hospital Universitario Infanta Sofía, San Sebastián de los Reyes, Madrid, Spain.
- [2] Universidad Europea de Madrid Villaviciosa de Odón, Madrid, Spain.

- PMID: **33585689**
- PMCID: PMC7867393
- DOI: 10.1016/j.medcle.2020.11.006

Free PMC article

Effectiveness of glucocorticoids in patients hospitalized for severe SARS-CoV-2 pneumonia

José Francisco Pascual Pareja et al. Med Clin (Engl Ed). 2021.
Free PMC article
Show details

Med Clin (Engl Ed)
. 2021 Mar 12;156(5):221-228.
doi: 10.1016/j.medcle.2020.11.006. Epub 2021 Feb 6.

Authors

José Francisco Pascual Pareja [1, 2], Rebeca García-Caballero [1], Llanos Soler Rangel [1, 2], Miguel Angel Vázquez-Ronda [1, 2], Silvia Roa Franco [1], Gema Navarro Jiménez [1], Miguel Angel Moreno Palanco [1, 2], Patricia González-Ruano [1, 2], Ramiro López-Menchaca [1], Pilar Ruíz-Seco [1, 2], Bárbara Pagán Muñoz [1], Alejandro Gómez Gómez [1], Beatriz Pérez-Monte [1], Rebeca Fuerte Martínez [1], Jose Luis Valle López [1], Arturo Muñoz Blanco [1], Isabel Rábago Lorite [1], Patricia Martínez Martín [1], Gonzalo Serralta San Martín [1, 2], Jorge Francisco Gómez-Cerezo [1, 2], HUIS-COVID-19 working group

Affiliations

- [1] Hospital Universitario Infanta Sofía, San Sebastián de los Reyes, Madrid, Spain.
- [2] Universidad Europea de Madrid Villaviciosa de Odón, Madrid, Spain.

- PMID: **33585689**
- PMCID: PMC7867393
- DOI: 10.1016/j.medcle.2020.11.006

Abstract
in English, Spanish

Background: Several studies have reported the beneficial effect of glucocorticoids in the treatment of cytokine storm that occurs in patients with severe COVID-19. Various glucocorticoids regimens have been proposed.

Methods: Retrospective observational study that includes patients with severe SARS-CoV-2 pneumonia and compares admission to an Intensive Care Unit (ICU) or death during hospitalization in three groups of patients: no glucocorticoids treatment, use of glucocorticoids doses equivalent to less than 250 mg of prednisone daily and use of equivalent doses greater than or equal to 250 mg of prednisone daily. Multivariate analysis was performed using logistic regression, using the propensity index as a covariant.

Results: Of the 259 patients enrolled in the study, 67 (25.9%) had an unfavorable evolution, dying or requiring ICU admission. Comparative analyzes between different glucocorticoids treatments and the association with ICU admission or death were: glucocorticoids treatment (any dose) versus no glucocorticoids treatment (OR: 0.71 [0.30-1.66]), treatment with glucocorticoids (≥250 mg prednisone daily) versus no glucocorticoids treatment (OR: 0.35 [0.11-1.08]) and glucocorticoids treatment (≥250 mg prednisone daily) versus patients with glucocorticoids doses <250 mg prednisone daily or without glucocorticoids treatment (OR: 0.30 [0.10-0.88]).

Conclusion: The results of this study show that patients with severe SARS-CoV-2 pneumonia treated with glucocorticoids pulses with equivalent doses of prednisone greater than or equal to 250 mg have a more favorable evolution (less mortality and less admission to ICU).

Introducción: Se han comunicado varios trabajos donde se ha demostrado un efecto beneficioso de los glucocorticoides como tratamiento de la tormenta de citocinas que se asocia a los cuadros graves por SARS-CoV-2, plateándose diferentes pautas de glucocorticoides.

Métodos: Estudio observacional retrospectivo que incluye pacientes con neumonía grave por SARS-CoV-2 y compara el ingreso en una unidad de cuidados intensivos (UCI) o fallecimiento durante la hospitalización en 3 grupos de pacientes: sin tratamiento con glucocorticoides, uso de dosis diarias de glucocorticoides equivalentes menores a 250 mg de prednisona y dosis diarias

equivalentes mayores o iguales a 250 mg de prednisona. Se realizó un análisis multivariante mediante regresión logística, utilizando el índice de propensión como covariante.

Resultados: De los 259 pacientes incorporados al estudio 67 (25,9%) tuvieron una evolución desfavorable, falleciendo o precisando ingreso en UCI. Los análisis comparativos entre diferentes tratamientos con glucocorticoides, y la asociación con ingreso en UCI o fallecimiento fueron: tratamiento con glucocorticoides (cualquier dosis) versus sin tratamiento con glucocorticoides (OR: 0,71 [0,30–1,66]), tratamiento con glucocorticoides (≥250 mg de prednisona al día) versus sin tratamiento con glucocorticoides (OR: 0,35 [0,11–1,08]) y tratamiento con glucocorticoides (≥250 mg de prednisona al día) versus pacientes con dosis de glucocorticoides < 250 mg de prednisona o sin tratamiento con glucocorticoides (OR: 0,30 [0,10–0,88]).

Conclusión: Los resultados de este estudio muestran que los paciente con neumonía grave por SARS-CoV-2 tratados con pulsos con glucocorticoides con dosis equivalentes de prednisona mayor o igual de 250 mg tienen una evolución más favorable (menos mortalidad e ingreso en UCI).

Keywords: Coronavirus infection disease 2019 (COVID-19); Corticosteroids; Propensity index; Severe acute respiratory syndrome coronavirus 2 (SARS-CoV-2).

© 2020 Elsevier España, S.L.U. All rights reserved.

- 23 references
- 1 figure

Full text links

Free PMC article
Proceed to details
Cite
Share
☐ 1,240
Open Forum Infect Dis
. 2020 Sep 13;7(10):ofaa432.
doi: 10.1093/ofid/ofaa432. eCollection 2020 Oct.

Epidemiological and Clinical Findings of Short-Term Recurrence of Severe Acute Respiratory Syndrome Coronavirus 2 Ribonucleic Acid Polymerase Chain Reaction Positivity in 1282 Discharged Coronavirus Disease 2019 Cases: A Multicenter, Retrospective, Observational Study

Sheng-Long Chen [1], Hui Xu [1] [2], Hui-Ying Feng [3], Jiu-Feng Sun [4], Xin Li [1], Lin Zhou [3], Wen-Liang Song [1] [5], Shan-Shan Huang [3], Jun-Lei He [3], Yi-Yu Deng [1], Rui-Jie Wang [1] [6], Ming Fang [1] [5] [6]
Affiliations Expand

Affiliations

- [1] Guangdong Provincial People's Hospital, Guangdong Academy of Medical Sciences, Guangzhou, People's Republic of China.
- [2] Shantou University Medical College, Shantou, People's Republic of China.
- [3] Center for Tuberculosis Control of Guangdong Province, Guangzhou, People's Republic of China.
- [4] Guangdong Provincial Institute of Public Health, Guangdong Provincial Center for Disease Control and Prevention, Guangzhou, People's Republic of China.
- [5] The Second School of Clinical Medicine, Southern Medical University, Guangzhou, People's Republic of China.
- [6] School of Medicine, South China University of Technology, Guangzhou, People's Republic of China.

- PMID: **33123610**
- PMCID: PMC7543527
- DOI: 10.1093/ofid/ofaa432

Free PMC article

Epidemiological and Clinical Findings of Short-Term Recurrence of Severe Acute Respiratory Syndrome Coronavirus 2 Ribonucleic Acid Polymerase Chain Reaction Positivity in 1282 Discharged Coronavirus Disease 2019 Cases: A Multicenter, Retrospective, Observational Study

Sheng-Long Chen et al. Open Forum Infect Dis. 2020.
Free PMC article
Show details
Open Forum Infect Dis
. 2020 Sep 13;7(10):ofaa432.
doi: 10.1093/ofid/ofaa432. eCollection 2020 Oct.

Authors

Sheng-Long Chen [1], Hui Xu [1, 2], Hui-Ying Feng [3], Jiu-Feng Sun [4], Xin Li [1], Lin Zhou [3], Wen-Liang Song [1, 5], Shan-Shan Huang [3], Jun-Lei He [3], Yi-Yu Deng [1], Rui-Jie Wang [1, 6], Ming Fang [1, 5, 6]

Affiliations

- [1] Guangdong Provincial People's Hospital, Guangdong Academy of Medical Sciences, Guangzhou, People's Republic of China.
- [2] Shantou University Medical College, Shantou, People's Republic of China.
- [3] Center for Tuberculosis Control of Guangdong Province, Guangzhou, People's Republic of China.
- [4] Guangdong Provincial Institute of Public Health, Guangdong Provincial Center for Disease Control and Prevention, Guangzhou, People's Republic of China.
- [5] The Second School of Clinical Medicine, Southern Medical University, Guangzhou, People's Republic of China.
- [6] School of Medicine, South China University of Technology, Guangzhou, People's Republic of China.

- PMID: **33123610**
- PMCID: PMC7543527
- DOI: 10.1093/ofid/ofaa432

Abstract

Background: Short-term recurrence of positive severe acute respiratory syndrome coronavirus 2 (SARS-CoV-2) ribonucleic acid (RNA) polymerase chain reaction (PCR) in discharged coronavirus disease 2019 (COVID-19) patients attracts the public's concern. This study aimed to determine the clinical and epidemiological results of such patients.

Methods: This retrospective study was conducted on 32 designated hospitals for COVID-19 patients discharged from January 14 to March 10, 2020. After 28-day followed-up, patients who tested positive again for SARS-CoV-2 RNA and confirmed by reverse-transcriptase polymerase chain reaction were re-admitted to hospital for further treatments. All of the close contacts of patients who tested positive again were asked to self-segregate for 14 days. Data of epidemiology, symptoms, laboratory tests, and treatments were analyzed in those patients, and their close contacts were investigated.

Results: Of 1282 discharged patients, 189 (14.74%) tested positive again for SARS-CoV-2 RNA during 28-day follow-up. The median time from discharge to the next positive test was 8 days (interquartile range [IQR], 5-13). Patients in the group that tested positive again were younger (34 vs 45 years, $P < .001$) with a higher proportion of moderate symptoms (95.77% vs 84.35%, $P < .001$) in the first hospitalization than in the negative group. During the second hospitalization, all patients who tested positive again showed normal peripheral white blood cells and lymphocytes and no new symptoms of COVID-19; 78.31% further improved on chest computed tomography scan compared with the first discharge, yet 25.93% accepted antiviral therapy. The median time of re-positive to negative test was 8 days (IQR, 4-15). None of the close contacts developed COVID-19.

Conclusions: Our data suggest that the short-term recurrence of positive SARS-CoV-2 RNA in discharged patients is not a relapse of COVID-19, and the risk of onward transmission is very low. This provides important information for managing COVID-19 patients.

Keywords: COVID-19; communicable; recurrence; relapse; retrospective.

© The Author(s) 2020. Published by Oxford University Press on behalf of Infectious Diseases Society of America.

- 28 references
- 3 figures

Full text links

OXFORD ACADEMIC Silverchair Information Systems Free PMC article
Proceed to details
Cite
Share
1,241
Case Reports
Med Clin (Engl Ed)
. 2022 Feb 21.
doi: 10.1016/j.medcle.2021.11.004. Online ahead of print.

Clinical characteristics of children hospitalized for COVID-19

Rosa María Luz Romero [1], Marta Illán Ramos [1], Arantxa Berzosa Sánchez [1], Belén Joyanes Abancens [1], Elvira Baos Muñoz [2], José Tomás Ramos Amador [1]
Affiliations Expand

Affiliations

- [1] Servicio de Pediatría, Hospital Clínico San Carlos, Madrid, Spain.
- [2] Servicio de Microbiología Clínica, Hospital Clínico San Carlos, Madrid, Spain.

- PMID: **35224201**
- PMCID: PMC8858688
- DOI: 10.1016/j.medcle.2021.11.004

Free PMC article
Case Reports

Clinical characteristics of children hospitalized for COVID-19

Rosa María Luz Romero et al. Med Clin (Engl Ed). 2022.
Free PMC article
Show details

Med Clin (Engl Ed)
. 2022 Feb 21.
doi: 10.1016/j.medcle.2021.11.004. Online ahead of print.

Authors

Rosa María Luz Romero [1], Marta Illán Ramos [1], Arantxa Berzosa Sánchez [1], Belén Joyanes Abancens [1], Elvira Baos Muñoz [2], José Tomás Ramos Amador [1]

Affiliations

- [1] Servicio de Pediatría, Hospital Clínico San Carlos, Madrid, Spain.
- [2] Servicio de Microbiología Clínica, Hospital Clínico San Carlos, Madrid, Spain.

- PMID: **35224201**
- PMCID: PMC8858688
- DOI: 10.1016/j.medcle.2021.11.004

Abstract
in English, Spanish

Introduction: Most SARS-CoV2 infections in the pediatric population are asymptomatic or with mild symptoms, with a minimal proportion of severe cases described as SARS-CoV2-associated multi-system inflammatory syndrome (MIS-C).The objective was to describe the clinical and epidemiological characteristics of pediatric patients admitted with confirmed diagnosis of SARS-CoV2 infection from the beginning of the pandemic until May 2021.

Methods: Retrospective observational study of pediatric patients hospitalized with confirmed COVID-19, in a tertiary hospital. Epidemiological and clinical data, additional tests, treatments administered and evolution were collected.

Results: 30 patients were included, classified into 3 groups according to diagnosis: respiratory infection, MIS-C and compatible symptoms. The patients with pneumonia were associated with age older, comorbidities and lymphopenia. MIS-C were more serious patients, with marked laboratory involvement and greater admission to PICU. Most of these were secondary cases of contact in the family environment.

Discussion: The most frequent clinical manifestations of COVID-19 in children are mild-moderate respiratory with good evolution. MIS-C is another form of expression of SARS-COV2 infection of greater severity, but usually with good prognosis after early diagnosis and frequent PICU admission.

Introducción: La mayoría de infecciones por SARS-CoV2 en población pediátrica cursan asintomáticas o con síntomas leves, con porcentaje mínimo de casos graves descritos como síndrome inflamatorio multisistémico asociado al SARS-CoV2 (SIM-PEDs).El objetivo fue describir las características clínico epidemiológicas de aquellos pacientes pediátricos ingresados, con diagnóstico confirmado de SARS-CoV2 desde el inicio de la pandemia hasta mayo 2021.

Métodos: Estudio retrospectivo observacional de pacientes pediátricos ingresados con diagnóstico de COVID-19, de un hospital terciario. Se recogieron datos demográficos, clínicos, pruebas complementarias, tratamiento administrado y evolución.

Resultados: Se incluyeron 30 pacientes, clasificándose en 3 grupos según diagnóstico: Infección respiratoria, SIM-PEDs y síntomas compatibles. Los pacientes con neumonía asociaban mayor edad, comorbilidades y linfopenia. SIM-PEDs fueron pacientes más graves, con afectación analítica marcada y mayor ingreso en UCIP. La mayoría eran casos secundarios de contacto en el entorno familiar.

Discusión: Los cuadros clínicos de COVID-19 más frecuentes en niños son respiratorios leves-moderados con buena evolución. SIM-PEDs es otra forma de expresión de infección por SARS-COV2 de mayor gravedad, pero habitualmente con buen pronóstico tras diagnóstico precoz y requiriendo frecuentemente ingreso en UCIP.

Keywords: COVID-19; Hospitalization; Pediatric.

© 2021 Elsevier España, S.L.U. All rights reserved.

- 10 references

Supplementary info

Publication types Expand

Publication types

- Case Reports

Full text links

Free PMC article
Proceed to details
Cite
Share
☐ 1,242
Mediterr J Hematol Infect Dis
. 2021 Nov 1;13(1):e2021061.
doi: 10.4084/MJHID.2021.061. eCollection 2021.

Early Treatment with Bamlanivimab Alone does not Prevent COVID-19 Hospitalization and Its Post-Acute Sequelae. A Real Experience in Umbria, Italy

Elisabetta Schiaroli [1], Giuseppe Vittorio De Socio [1], Laura Martinelli [2], Lisa Malincarne [1], Martina Savoia [3], Anna Laura Spinelli [4], Daniela Francisci [1]
Affiliations Expand

Affiliations

- [1] Unit of Infectious Diseases, Department of Medicine and Surgery, University of Perugia, Perugia, Italy.
- [2] Internal Medicine of Citta di Castello Hospital, USL Umbria 1, Italy.
- [3] Hospital Pharmacy of Foligno Hospital, USL Umbria 2, Italy.
- [4] Internal Medicine of Spoleto Hospital, USL Umbria 2, Italy.

- PMID: **34804435**
- PMCID: PMC8577560
- DOI: 10.4084/MJHID.2021.061

Free PMC article

Early Treatment with Bamlanivimab Alone does not Prevent COVID-19 Hospitalization and Its Post-Acute Sequelae. A Real Experience in Umbria, Italy

Elisabetta Schiaroli et al. Mediterr J Hematol Infect Dis. 2021.
Free PMC article
Show details
Mediterr J Hematol Infect Dis
. 2021 Nov 1;13(1):e2021061.
doi: 10.4084/MJHID.2021.061. eCollection 2021.

Authors

Elisabetta Schiaroli [1], Giuseppe Vittorio De Socio [1], Laura Martinelli [2], Lisa Malincarne [1], Martina Savoia [3], Anna Laura Spinelli [4], Daniela Francisci [1]

Affiliations

- [1] Unit of Infectious Diseases, Department of Medicine and Surgery, University of Perugia, Perugia, Italy.
- [2] Internal Medicine of Citta di Castello Hospital, USL Umbria 1, Italy.
- [3] Hospital Pharmacy of Foligno Hospital, USL Umbria 2, Italy.
- [4] Internal Medicine of Spoleto Hospital, USL Umbria 2, Italy.

- PMID: **34804435**
- PMCID: PMC8577560
- DOI: 10.4084/MJHID.2021.061

Abstract

Background and objective: The use of monoclonal antibodies to the SARS-Cov-2 spike protein for early treatment of COVID-19 disease is being evaluated, with only phase 2 studies available to date. The emergency authorization of bamlanivimab monotherapy was obtained in November 2020 by the FDA and in March 2021 by Italian agency AIFA. Its use was then revoked in April 2021 by both. This study reports the results of bamlanivimab utilization in monotherapy in Umbria (Italian region) to verify whether, in a population with multiple risk factors, comparable results to the phase 2 BLAZE1 trial had been obtained.

Methods: Between March and April 2021, a retrospective observational study was performed on patients treated with bamlanivimab. Demographic and clinical characteristics before and after infusion were evaluated. Moreover, a telephone interview was conducted about 30 days after the infusion to evaluate the overall course.

Results: All patients had an early infection (mean 4±1.73 days), almost all by alpha variant (97%). No adverse events to treatment were observed. Altogether within 30 days, the hospitalization rate was 20%, 15% for COVID-19 related pathologies, versus 4% at 11 days in the BLAZE1 phase 2 study. In addition, worsening of some symptoms observed at baseline such as asthenia (77 vs. 51.3%), shortness of breath (38 vs. 23%) was registered, as well as the onset of non-restorative sleep (41%).

Conclusion: The clinical outcome after bamlanivimab monotherapy was far below the expectation despite the patients had been infected by a theoretically sensitive viral variant.

Keywords: Bamlanivimab; COVID-19 hospitalization; Umbria; real experience.

Conflict of interest statement

Competing interests: The authors declare no conflict of Interest.

- 9 references
- 1 figure

Full text links

Catholic University in Rome, Institute of Hematology Free PMC article

Proceed to details

Cite

Share

☐ 1,243

J Travel Med

. 2022 Feb 5;taab195.

doi: 10.1093/jtm/taab195. Online ahead of print.

Melatonin does not reduce mortality in adult hospitalized patients with COVID-19: a multicenter retrospective observational study

Marina Sánchez-Rico [1] [2], Pedro de la Muela [1] [2], Juan J Herrera-Morueco [1] [2], Pierre A Geoffroy [3] [4] [5], Frédéric Limosin [1] [6] [7], Nicolas Hoertel [1] [6] [7], AP-HP/Université de Paris/INSERM COVID-19 Research Collaboration/AP-HP COVID CDR Initiative/Entrepôt de Données de Santé AP-HP Consortium

Affiliations Expand

Affiliations

- [1] AP-HP Centre, DMU Psychiatrie et Addictologie, Corentin Celton Hospital, Issy-les-Moulineaux, France.
- [2] Campus de Somosaguas, Universidad Complutense de Madrid, Pozuelo de Alarcón, Spain.
- [3] Département de psychiatrie et d'addictologie, AP-HP, GHU Paris Nord, DMU Neurosciences, Hopital Bichat - Claude Bernard, F-75018 Paris, France.
- [4] GHU Paris - Psychiatry & Neurosciences, 1 rue Cabanis, 75014 Paris, France.
- [5] NeuroDiderot, Inserm, FHU I2-D2, Université de Paris, F-75019 Paris, France.
- [6] INSERM U1266, Paris, France.
- [7] Université de Paris, Paris, France.

- PMID: **35137210**
- PMCID: PMC8903398
- DOI: 10.1093/jtm/taab195

Free PMC article

Melatonin does not reduce mortality in adult hospitalized patients with COVID-19: a multicenter retrospective observational study

Marina Sánchez-Rico et al. J Travel Med. 2022.
Free PMC article
Show details
J Travel Med
. 2022 Feb 5;taab195.
doi: 10.1093/jtm/taab195. Online ahead of print.

Authors

Marina Sánchez-Rico [1] [2], Pedro de la Muela [1] [2], Juan J Herrera-Morueco [1] [2], Pierre A Geoffroy [3] [4] [5], Frédéric Limosin [1] [6] [7], Nicolas Hoertel [1] [6] [7], AP-HP/Université de Paris/INSERM COVID-19 Research Collaboration/AP-HP COVID CDR Initiative/Entrepôt de Données de Santé AP-HP Consortium

Affiliations

- [1] AP-HP Centre, DMU Psychiatrie et Addictologie, Corentin Celton Hospital, Issy-les-Moulineaux, France.

- [2] Campus de Somosaguas, Universidad Complutense de Madrid, Pozuelo de Alarcón, Spain.
- [3] Département de psychiatrie et d'addictologie, AP-HP, GHU Paris Nord, DMU Neurosciences, Hopital Bichat - Claude Bernard, F-75018 Paris, France.
- [4] GHU Paris - Psychiatry & Neurosciences, 1 rue Cabanis, 75014 Paris, France.
- [5] NeuroDiderot, Inserm, FHU I2-D2, Université de Paris, F-75019 Paris, France.
- [6] INSERM U1266, Paris, France.
- [7] Université de Paris, Paris, France.

- PMID: **35137210**
- PMCID: PMC8903398
- DOI: 10.1093/jtm/taab195

Abstract

In this multicenter retrospective observational study involving 58 562 adult patients hospitalized for COVID-19, melatonin use ($N = 272$) at a mean daily dose of 2.6 mg was not associated with reduced mortality.

Keywords: COVID-19; SARS-CoV-2; death; efficacy; melatonin; mortality; treatment.

- 10 references

Full text links

OXFORD ACADEMIC Silverchair Information Systems Free PMC article

Proceed to details

Cite

Share

☐ 1,244

Med Clin (Barc)

. 2021 Jul 24;S0025-7753(21)00431-0.

doi: 10.1016/j.medcli.2021.07.003. Online ahead of print.

Influence of statin treatment in a cohort of patients admitted for COVID-19

[Article in English, Spanish]

Juan R Rey [1], José Luis Merino Llorens [2], Ángel Manuel Iniesta Manjavacas [2], Sandra Ofelia Rosillo Rodríguez [2], Sergio Castrejón-Castrejón [2], Emilio Arbas-Redondo [2], Isabel Dolores Poveda-Pinedo [2], Daniel Tebar-Márquez [2], Andrea Severo-Sánchez [2], Borja Rivero-Santana [2], Víctor Juárez-Olmos [2], Marcel Martínez-Cossiani [2], Antonio Buño-Soto [3], Luis Gonzalez-Valle [4], Alicia Herrero-Ambrosio [4], Esteban López-de-Sá [2], Juan Caro-Codón [2]

Affiliations Expand

Affiliations

- [1] Servicio de Cardiología, Hospital Universitario La Paz, Madrid, España. Electronic address: juanr.rey@salud.madrid.org.
- [2] Servicio de Cardiología, Hospital Universitario La Paz, Madrid, España.
- [3] Servicio de Análisis Clínicos, Hospital Universitario La Paz, Madrid, España.
- [4] Servicio de Farmacia Hospitalaria, Hospital Universitario La Paz, Madrid, España.

- PMID: **34511251**
- PMCID: PMC8302844
- DOI: 10.1016/j.medcli.2021.07.003

Free PMC article

Influence of statin treatment in a cohort of patients admitted for COVID-19

[Article in English, Spanish]
Juan R Rey et al. Med Clin (Barc). 2021.
Free PMC article
Show details
Med Clin (Barc)
. 2021 Jul 24;S0025-7753(21)00431-0.
doi: 10.1016/j.medcli.2021.07.003. Online ahead of print.

Authors

Juan R Rey [1], José Luis Merino Llorens [2], Ángel Manuel Iniesta Manjavacas [2], Sandra Ofelia Rosillo Rodríguez [2], Sergio Castrejón-Castrejón [2], Emilio Arbas-Redondo [2], Isabel Dolores Poveda-Pinedo [2], Daniel Tebar-Márquez [2], Andrea Severo-Sánchez [2], Borja Rivero-Santana [2], Víctor Juárez-Olmos [2], Marcel Martínez-Cossiani [2], Antonio Buño-Soto [3], Luis Gonzalez-Valle [4], Alicia Herrero-Ambrosio [4], Esteban López-de-Sá [2], Juan Caro-Codón [2]

Affiliations

- [1] Servicio de Cardiología, Hospital Universitario La Paz, Madrid, España. Electronic address: juanr.rey@salud.madrid.org.
- [2] Servicio de Cardiología, Hospital Universitario La Paz, Madrid, España.
- [3] Servicio de Análisis Clínicos, Hospital Universitario La Paz, Madrid, España.
- [4] Servicio de Farmacia Hospitalaria, Hospital Universitario La Paz, Madrid, España.

- PMID: **34511251**
- PMCID: PMC8302844
- DOI: 10.1016/j.medcli.2021.07.003

Abstract

Aims and objectives: Statins have been proposed as potentially useful agents for modulating the host response in COVID-19. However, solid evidence-based recommendations are still lacking.

Our aim was to study the association between statin use and clinical outcomes in a large cohort of hospitalized patients with SARS-CoV-2 infection, as well as the specific consequences of chronic treatment withdrawal during hospital admission.

Material and methods: Retrospective observational study including 2191 hospitalized patients with confirmed SARS-CoV-2 infection.

Results: Mean age was 68.0±17.8 years and 597 (27.3%) patients died during follow-up. A total of 827 patients (37.7% of the whole sample), received chronic treatment with statins. Even though they underwent more frequent admissions in critical care units, chronic treatment with statins was not independently associated with all-cause mortality [HR 0.95 (0.72-1.25)]. During the whole hospital admission, 371 patients (16.9%) received at least one dose of statin. Although these patients had a significantly worse clinical profile, both treatment with statins during admission [HR 1.03 (0.78-1.35)] and withdrawal of chronic statin treatment [HR 1.01 (0.78-1.30)] showed a neutral effect in mortality. However, patients treated with statins presented more frequently hepatic cytolysis, rhabdomyolysis and thrombotic/hemorrhagic events.

Conclusions: In this large cohort of hospitalized COVID-19 patients, statins were not independently associated with all-cause mortality during follow-up. Clinically relevant statin-associated adverse effects should be carefully monitored during hospital admission.

Keywords: Adverse effects; COVID-19; Efectos adversos; Estatinas; Morbidity; Morbilidad; Mortalidad; Mortality; Retirada medicación; Statins; Treatment withdrawal.

Copyright © 2021 Elsevier España, S.L.U. All rights reserved.

- 27 references
- 2 figures

Full text links

Full text at MEDICINA CLINICA Ediciones Doyma, S.L. Free PMC article
Proceed to details
Cite
Share
☐ 1,245
New Microbes New Infect
. 2021 Sep;43:100915.
doi: 10.1016/j.nmni.2021.100915. Epub 2021 Jul 7.

Early COVID-19 therapy with azithromycin plus nitazoxanide, ivermectin or hydroxychloroquine in outpatient settings significantly improved COVID-19 outcomes

# compared to known outcomes in untreated patients

F A Cadegiani [1, 2], A Goren [2], C G Wambier [3], J McCoy [2]
Affiliations Expand

Affiliations

- [1] Corpometria Institute, Brasília, DF, Brazil.
- [2] Applied Biology, Inc., Irvine, CA, USA.
- [3] Department of Dermatology, The Alpert Medical School of Brown University, RI, USA.

- PMID: **34249367**
- PMCID: PMC8262389
- DOI: 10.1016/j.nmni.2021.100915

Free PMC article

Early COVID-19 therapy with azithromycin plus nitazoxanide, ivermectin or hydroxychloroquine in outpatient settings significantly improved COVID-19 outcomes compared to known outcomes in untreated patients

F A Cadegiani et al. New Microbes New Infect. 2021 Sep.
Free PMC article
Show details
New Microbes New Infect
. 2021 Sep;43:100915.
doi: 10.1016/j.nmni.2021.100915. Epub 2021 Jul 7.

Authors

F A Cadegiani [1, 2], A Goren [2], C G Wambier [3], J McCoy [2]

Affiliations

- [1] Corpometria Institute, Brasília, DF, Brazil.
- [2] Applied Biology, Inc., Irvine, CA, USA.
- [3] Department of Dermatology, The Alpert Medical School of Brown University, RI, USA.

- PMID: **34249367**
- PMCID: PMC8262389
- DOI: 10.1016/j.nmni.2021.100915

Abstract

In a prospective observational study (pre-AndroCoV Trial), the use of nitazoxanide, ivermectin and hydroxychloroquine demonstrated unexpected improvements in COVID-19 outcomes when compared to untreated patients. The apparent yet likely positive results raised ethical concerns on the employment of further full placebo controlled studies in early-stage COVID-19. The present analysis aimed to elucidate, through a comparative analysis with two control groups, whether full placebo-control randomized clinical trials (RCTs) on early-stage COVID-19 are still ethically acceptable. The Active group (AG) consisted of patients enrolled in the Pre-AndroCoV-Trial (n = 585). Control Group 1 (CG1) consisted of a retrospectively obtained group of untreated patients of the same population (n = 137), and Control Group 2 (CG2) resulted from a precise prediction of clinical outcomes based on a thorough and structured review of indexed articles and official statements. Patients were matched for sex, age, comorbidities and disease severity at baseline. Compared to CG1 and CG2, AG showed reduction of 31.5-36.5% in viral shedding ($p < 0.0001$), 70-85% in disease duration ($p < 0.0001$), and 100% in respiratory complications, hospitalization, mechanical ventilation, deaths and post-COVID manifestations ($p < 0.0001$ for all). For every 1000 confirmed cases for COVID-19, at least 70 hospitalizations, 50 mechanical ventilations and five deaths were prevented. Benefits from the combination of early COVID-19 detection and early pharmacological approaches were consistent and overwhelming when compared to untreated groups, which, together with the well-established safety profile of the drug combinations tested in the Pre-AndroCoV Trial, precluded our study from continuing employing full placebo in early COVID-19.

Keywords: Antiandrogen; COVID-19; SARS-CoV-2; clinical equipoise; dutasteride; hydroxychloroquine; ivermectin; nitazoxanide; proxalutamide; spironolactone.

© 2021 The Author(s).

- 70 references
- 2 figures

Full text links

Free PMC article
Proceed to details
Cite
Share
☐ 1,246
Cureus
. 2021 Jan 7;13(1):e12552.
doi: 10.7759/cureus.12552.

Rhabdomyolysis in COVID-19 Patients: A Retrospective Observational Study

Magued W Haroun [1], Vladyslav Dieiev [1], John Kang [1], Mali Barbi [1], Seyed Farzad Marashi Nia [1], Mohamed Gabr [1], Gerardo Eman [1], Grace Kajita [1], Kristin Swedish [1]
Affiliations Expand

Affiliation

- [1] Internal Medicine, Montefiore Medical Center, Wakefield Campus, Bronx, USA.

- PMID: **33575135**
- PMCID: PMC7869906
- DOI: 10.7759/cureus.12552

Free PMC article

Rhabdomyolysis in COVID-19 Patients: A Retrospective Observational Study

Magued W Haroun et al. Cureus. 2021.
Free PMC article
Show details
Cureus
. 2021 Jan 7;13(1):e12552.
doi: 10.7759/cureus.12552.

Authors

Magued W Haroun [1], Vladyslav Dieiev [1], John Kang [1], Mali Barbi [1], Seyed Farzad Marashi Nia [1], Mohamed Gabr [1], Gerardo Eman [1], Grace Kajita [1], Kristin Swedish [1]

Affiliation

- [1] Internal Medicine, Montefiore Medical Center, Wakefield Campus, Bronx, USA.

- PMID: **33575135**
- PMCID: PMC7869906
- DOI: 10.7759/cureus.12552

Abstract

Background The coronavirus disease 2019 (COVID-19) pandemic has caused significant morbidity and mortality worldwide. Knowledge about the pathophysiology of the disease and its effect on multiple systems is growing. Kidney injury has been a topic of focus, and rhabdomyolysis is suspected to be one of the contributing mechanisms. However, information on rhabdomyolysis in patients affected by COVID-19 is limited. We aim to describe the incidence, clinical characteristics, and outcomes of patients hospitalized with COVID-19 who developed rhabdomyolysis. Materials and methods A retrospective observational cohort consisted of patients who were admitted and had an outcome between March 16 to May 27, 2020, inclusive of those dates at a single center in the Bronx, New York City. All consecutive inpatients with lab-

confirmed COVID-19 were identified. Patients with peak total creatine kinase (CK) over 1,000 U/L were reviewed; 140 patients were included in the study. The main outcomes during hospitalization were new-onset renal replacement therapy and in-hospital mortality. Results The median age was 68 years (range: 21-93); 64% were males. The most common comorbidities were hypertension (73%), diabetes mellitus (47%), and chronic kidney disease (24%). Median CK on admission was 1,323 U/L (interquartile range [IQR]: 775 - 2,848). Median CK on discharge among survivors was 852 (IQR: 170 - 1,788). Median creatinine on admission was 1.78 mg/dL (IQR: 1.23 - 3.06). During hospitalization, 49 patients (35%) received invasive mechanical ventilation, 24 patients (17.1%) were treated with renal replacement therapy (RRT), and 66 (47.1%) died. Conclusions Rhabdomyolysis was a common finding among hospitalized patients with COVID-19 in our hospital in the Bronx. The incidence of new-onset renal replacement therapy and in-hospital mortality is higher in patients who develop rhabdomyolysis. McMahon score, rather than isolated creatine kinase levels, was a statistically significant predictor of new-onset RRT. Clinicians should maintain a high level of suspicion for rhabdomyolysis in COVID-19 patients throughout their admission and use validated scores like McMahon score to devise their treatment plan accordingly.

Keywords: acute kidney injury; covid-19; hemodialysis; muscle breakdown; rhabdomyolysis.

Copyright © 2021, Haroun et al.

Conflict of interest statement

The authors have declared that no competing interests exist.

- 20 references

Full text links

Free PMC article
Proceed to details
Cite
Share
☐ 1,247
Case Reports
Med Clin (Engl Ed)
. 2021 Apr 23;156(8):386-389.
doi: 10.1016/j.medcle.2020.11.016. Epub 2021 Apr 17.

Early prone positioning therapy for patients with mild COVID-19 disease

Xiaoyi Liu [1], Hui Liu [2], Qing Lan [1], Xiangde Zheng [1], Jun Duan [3], Fanwei Zeng [4]
Affiliations Expand

Affiliations

- [1] Department of Critical Care Medicine, The Central Hospital of Dazhou, Dazhou, Sichuan, PR China.

- [2] Ophthalmology, The Central Hospital of Dazhou, Dazhou, Sichuan, PR China.
- [3] Department of Respiratory and Critical Care Medicine, The First Affiliated Hospital of Chongqing Medical University, Chongqing, PR China.
- [4] Orthopedics, The Central Hospital of Dazhou, Dazhou, Sichuan, PR China.

- PMID: **33898754**
- PMCID: PMC8052860
- DOI: 10.1016/j.medcle.2020.11.016

Free PMC article
Case Reports

Early prone positioning therapy for patients with mild COVID-19 disease

Xiaoyi Liu et al. Med Clin (Engl Ed). 2021.
Free PMC article

Show details

Med Clin (Engl Ed)
. 2021 Apr 23;156(8):386-389.
doi: 10.1016/j.medcle.2020.11.016. Epub 2021 Apr 17.

Authors

Xiaoyi Liu [1], Hui Liu [2], Qing Lan [1], Xiangde Zheng [1], Jun Duan [3], Fanwei Zeng [4]

Affiliations

- [1] Department of Critical Care Medicine, The Central Hospital of Dazhou, Dazhou, Sichuan, PR China.
- [2] Ophthalmology, The Central Hospital of Dazhou, Dazhou, Sichuan, PR China.
- [3] Department of Respiratory and Critical Care Medicine, The First Affiliated Hospital of Chongqing Medical University, Chongqing, PR China.
- [4] Orthopedics, The Central Hospital of Dazhou, Dazhou, Sichuan, PR China.

- PMID: **33898754**
- PMCID: PMC8052860
- DOI: 10.1016/j.medcle.2020.11.016

Abstract
in English, Spanish

Objective: In December 2019, Wuhan, China, experienced an outbreak of coronavirus disease 2019 (COVID-19). Some patients admitted to our hospital were treated with early prone positioning (PP). Here, we analyzed its clinical significance.

Methods: This was a retrospective observational study. We defined the early PP group as mild COVID-19 patients who were placed into a prone position within 24 h of admission; others served

as the control group. We recorded basic data and outcomes of early PP and compared the results to those of controls.

Results: After 1 day of treatment, oxygenation was greater in the early PP group than in the control group (P/F: 421.6 ± 39.74 vs. 382.1 ± 38.84 mmHg [1 mmHg = 0.133 kPa], $p < 0.01$). And early PP group spent less total time in prone position (11.1 ± 4.17 vs. 16.9 ± 5.20 days, $p < 0.01$), and required shorter hospitalization duration (12.2 ± 4.49 vs. 23.2 ± 4.83 days, $p < 0.001$).

Conclusions: Early PP treatment can improve hypoxia and shorten the prone position time and hospitalization duration in mild COVID-19 patients. It is a potential clinically applicable intervention.

Objetivo: En diciembre de 2019, Wuhan, China, experimentó un brote de enfermedad por coronavirus 2019 (COVID-19). Algunos pacientes ingresados en nuestro hospital fueron tratados con posicionamiento temprano en decúbito prono (PP). En este estudio analizamos su significación clínica.

Métodos: Estudio retrospectivo observacional en el que definimos el PP temprano como aquellos pacientes con COVID-19 que fueron posicionados en decúbito prono dentro de las 24 horas siguientes a su ingreso, sirviendo el resto de los pacientes como grupo control. Registramos los datos básicos y los resultados de PP temprano, comparando dichos resultados con los de los controles.

Resultados: Tras un día de tratamiento, la oxigenación fue más alta en el grupo PP temprano que en el grupo control (P/F: 421,6 ± 39,74 vs. 382,1 ± 38,84 mmHg [1 mmHg = 0,133 kPa], p < 0,01). El grupo PP temprano pasó menor tiempo total en posición de decúbito prono (11,1 ± 4,17 vs. 16,9 ± 5,20 días, p < 0,01), y requirió menor tiempo de hospitalización (12,2 ± 4,49 vs. 23,2 ± 4,83 días, p < 0,001).

Conclusiones: El tratamiento de PP temprano puede mejorar la hipoxia y reducir el tiempo de posición en decúbito prono en pacientes con COVID-19 leve. Se trata de una intervención potencialmente aplicable desde el punto de vista clínico.

Keywords: ARDS; COVID-19; Hypoxemia; Prone position; SARS-CoV-2.

© 2020 Elsevier España, S.L.U. All rights reserved.

- 10 references
- 2 figures

Supplementary info

Publication types Expand

Publication types

- Case Reports

Full text links

Free PMC article
Proceed to details

Cite
Share
1,248
Ann Med Surg (Lond)
. 2021 Mar;63:102151.
doi: 10.1016/j.amsu.2021.01.099. Epub 2021 Feb 4.

COVID-19 pandemic and breast cancer management: A retrospective observational clinical study from Pakistan

Lubna M Vohra [1], Dua Jabeen [2], Narmeen Asif [3], Abdul Ahad [3]
Affiliations Expand

Affiliations

- [1] Department of Breast Surgery, Aga Khan University Hospital, Karachi, Pakistan.
- [2] Jinnah Sindh Medical University, Rafiqi H.J, Iqbal Shaheed Rd, Karachi Cantonment, Karachi, 75510, Pakistan.
- [3] Aga Khan University Hospital, National Stadium Rd, Aga Khan University Hospital, Karachi, 74800, Pakistan.

- PMID: **33564461**
- PMCID: PMC7860941
- DOI: 10.1016/j.amsu.2021.01.099

Free PMC article

COVID-19 pandemic and breast cancer management: A retrospective observational clinical study from Pakistan

Lubna M Vohra et al. Ann Med Surg (Lond). 2021 Mar.
Free PMC article
Show details
Ann Med Surg (Lond)
. 2021 Mar;63:102151.
doi: 10.1016/j.amsu.2021.01.099. Epub 2021 Feb 4.

Authors

Lubna M Vohra [1], Dua Jabeen [2], Narmeen Asif [3], Abdul Ahad [3]

Affiliations

- [1] Department of Breast Surgery, Aga Khan University Hospital, Karachi, Pakistan.
- [2] Jinnah Sindh Medical University, Rafiqi H.J, Iqbal Shaheed Rd, Karachi Cantonment, Karachi, 75510, Pakistan.
- [3] Aga Khan University Hospital, National Stadium Rd, Aga Khan University Hospital, Karachi, 74800, Pakistan.

- PMID: **33564461**
- PMCID: PMC7860941
- DOI: 10.1016/j.amsu.2021.01.099

Abstract

Background: COVID-19 outbreak has adversely affected care of breast cancer patients world-wide. There is paucity of available data on cancer management in lower-middle income countries during this pandemic, we sought to determine the institutional approach towards management of breast cancer patients and the outcomes during COVID-19 pandemic at our institution.

Materials and methods: Clinicopathological and treatment record of cancer patients who presented to the Breast Clinic from 15th March to 31st December 2020 was retrieved from the institutional database for this retrospective clinical observational study.

Results: A total of 292 patients were qualified for the analysis in which 206 patients (70.5%) underwent breast cancer surgeries. Only 10 of them (4.9%) were identified to be COVID-19 virus positive on routine pre-operative RT-PCR test before elective surgeries. All were asymptomatic, received home-based care via telecommunication and were operated at a later date when test turned out negative. None of them developed any complications post-operatively. Another group of 86 patients (29.5%) were referred for Neoadjuvant/Systemic chemotherapy in which COVID-19 infection was detected in only 4 patients (4.6%) while receiving cycles. Two patients shown symptoms of cough and fever hence hospitalised but were not candidates for Intensive Care Unit admission while other two patient were asymptomatic and isolated at home. All patients recovered well and chemotherapy was commenced again after negative RT-PCR test. No mortality was observed.

Conclusion: Despite of being a global crisis particularly for cancer patients we observed infectivity, complications and fatality much lower among breast cancer cases. Further research is needed in this regard including public and private sector hospitals for better understanding behavior of COVID-19 disease and achieve common goal to combat COVID-19 and cancer together.

Keywords: Breast cancer; COVID-19; Neoadjuvant therapy; Pandemic.

© 2021 The Authors.

Conflict of interest statement

The authors have no conflicts of interest to declare.

- 16 references
- 2 figures

Full text links

ELSEVIER OPEN ACCESS Elsevier Science Free PMC article

Proceed to details

Cite

Share

1,249

Observational Study

Ann Clin Transl Neurol

. 2021 Feb;8(2):385-394.

doi: 10.1002/acn3.51282. Epub 2020 Dec 25.

Real-world experience of ocrelizumab in multiple sclerosis in a Spanish population

Eva Fernandez-Diaz [1], Jose A Perez-Vicente [2], Ramon Villaverde-Gonzalez [3], Leticia Berenguer-Ruiz [4], Antonio Candeliere Merlicco [5], Maria Luisa Martinez-Navarro [6], Julia Gracia Gil [1], Carlos M Romero-Sanchez [1], Arantxa Alfaro-Saez [7] [8], Inmaculada Diaz [2], Juana Gimenez-Martinez [9], Maria Angeles Mendez-Miralles [10] [11], Jorge Millan-Pascual [2], Javier Jimenez-Pancho [7], Santiago Mola [7], Angel P Sempere [9] [12] [13]

Affiliations Expand

Affiliations

- [1] Neurology Department, Complejo Hospitalario Universitario de Albacete, Albacete, Spain.
- [2] Neurology Department, Hospital Universitario Santa Lucía, Cartagena, Spain.
- [3] Section of Neurology, Hospital Morales y Meseguer, Murcia, Spain.
- [4] Section of Neurology, Hospital Marina Baixa, La Vila-Joiosa, Spain.
- [5] Section of Neurology, Hospital Rafael Méndez, Lorca, Spain.
- [6] Section of Neurology, Hospital Reina Sofia, Murcia, Spain.
- [7] Section of Neurology, Hospital Vega Baja, Orihuela, Spain.
- [8] Center for Biomedical Research in the Network in Bioengineering, Biomaterials and Nanomedicine (CIBER-BBN), Elche, Spain.
- [9] Hospital General Universitario de Alicante, Alicante, Spain.
- [10] Section of Neurology, Hospital Universitario Los Arcos del Mar Menor, Murcia, Spain.
- [11] Universidad Católica de Murcia (UCAM), Murcia, Spain.
- [12] Department of Clinical Medicine, Miguel Hernández University, San Juan de Alicante, Spain.
- [13] ISABIAL, Alicante, Spain.

- PMID: **33369288**
- PMCID: PMC7886031
- DOI: 10.1002/acn3.51282

Free PMC article

Observational Study

Real-world experience of ocrelizumab in multiple sclerosis in a Spanish population

Eva Fernandez-Diaz et al. Ann Clin Transl Neurol. 2021 Feb.
Free PMC article
Show details
Ann Clin Transl Neurol
. 2021 Feb;8(2):385-394.
doi: 10.1002/acn3.51282. Epub 2020 Dec 25.

Authors

Eva Fernandez-Diaz [1], Jose A Perez-Vicente [2], Ramon Villaverde-Gonzalez [3], Leticia Berenguer-Ruiz [4], Antonio Candeliere Merlicco [5], Maria Luisa Martinez-Navarro [6], Julia Gracia Gil [1], Carlos M Romero-Sanchez [1], Arantxa Alfaro-Saez [7] [8], Inmaculada Diaz [2], Juana Gimenez-Martinez [9], Maria Angeles Mendez-Miralles [10] [11], Jorge Millan-Pascual [2], Javier Jimenez-Pancho [7], Santiago Mola [7], Angel P Sempere [9] [12] [13]

Affiliations

- [1] Neurology Department, Complejo Hospitalario Universitario de Albacete, Albacete, Spain.
- [2] Neurology Department, Hospital Universitario Santa Lucía, Cartagena, Spain.
- [3] Section of Neurology, Hospital Morales y Meseguer, Murcia, Spain.
- [4] Section of Neurology, Hospital Marina Baixa, La Vila-Joiosa, Spain.
- [5] Section of Neurology, Hospital Rafael Méndez, Lorca, Spain.
- [6] Section of Neurology, Hospital Reina Sofia, Murcia, Spain.
- [7] Section of Neurology, Hospital Vega Baja, Orihuela, Spain.
- [8] Center for Biomedical Research in the Network in Bioengineering, Biomaterials and Nanomedicine (CIBER-BBN), Elche, Spain.
- [9] Hospital General Universitario de Alicante, Alicante, Spain.
- [10] Section of Neurology, Hospital Universitario Los Arcos del Mar Menor, Murcia, Spain.
- [11] Universidad Católica de Murcia (UCAM), Murcia, Spain.
- [12] Department of Clinical Medicine, Miguel Hernández University, San Juan de Alicante, Spain.
- [13] ISABIAL, Alicante, Spain.

- PMID: **33369288**
- PMCID: PMC7886031
- DOI: 10.1002/acn3.51282

Abstract

Objective: Pivotal trial have shown that patients with multiple sclerosis (MS) receiving ocrelizumab had better outcomes. However, data on ocrelizumab in clinical practice are limited. The aim of this study was to evaluate the preliminary safety profile and effectiveness of ocrelizumab treatment for multiple sclerosis (MS) in a real-world clinical setting.

Methods: We conducted a retrospective study including consecutive patients from nine public hospitals in south-eastern Spain who received ocrelizumab after it was approved.

Results: A total of 228 MS patients were included (144 with relapsing-remitting MS [RRMS], 25 secondary progressive MS [SPMS], and 59 primary progressive MS [PPMS]). Median follow-up period was 12 months (range, 1-32). No evidence of disease activity (NEDA) status at year 1 was achieved in 91.2% of the relapsing MS (RMS) population, while disability progression was detected in 37.5% of the PPMS patients (median follow-up period, 19 months). The most common adverse events reported were infusion-related reactions and infections, with the most common infections being urinary tract infections followed by upper respiratory infections and COVID-19.

Interpretation: The preliminary results in our real-world setting show that ocrelizumab presented excellent results in suppressing disease activity with a favorable and consistent safety profile.

© 2020 The Authors. Annals of Clinical and Translational Neurology published by Wiley Periodicals LLC on behalf of American Neurological Association.

Conflict of interest statement

E. Fernández-Díaz EFD has received research support, compensation for participating on advisory boards, speaking fees, and/or funding for travel from Almirall, Bayer, Biogen, Genzyme-Sanofi, Merck, Novartis, and Roche. R. Villaverde-Gonzalez: consulting or lectura fees or grant and research support from Roche, Biogen Idec, and Merck-Serono. L. Berenguer-Ruiz has received personal compensation for consulting, serving on a scientific advisory board, or speaking with Almirall, Biogen Idec, Merck Serono, Novartis, Sanofi-Aventis, and Teva. Julia Gracia-Gil has received research support, compensation for participating on advisory boards, lecture fees, and/or travel support from Almirall, Bayer, Biogen, Genzyme-Sanofi, Novartis, Roche, and Teva. CM Romero-Sanchez reports personal fees from Merck, Sanofi, Roche, and Bayer. J Giménez-Martínez has received personal compensation from Almirall, Biogen Idec, Merck Serono, and Sanofi-Aventis for serving on a scientific advisory board or speaking. AP Sempere has received personal compensation for consulting, serving on a scientific advisory board or speaking with Almirall, Biogen Idec, Bayer Schering Pharma, Merck Serono, Novartis, Roche, Sanofi-Aventis, and Teva. JA Pérez-Vicente, A. Candeliere-Merlicco, ML Martínez-Navarro, A. Alfaro-Saez, I. Diaz, MA Méndez-Miralles, J. Millán-Pascual, J. Jiménez-Pancho, and S. Mola report no disclosures.

- 31 references
- 2 figures

Supplementary info

Publication types, MeSH terms, Substances Expand

Publication types

- Observational Study

MeSH terms

- Adult
- Antibodies, Monoclonal, Humanized / therapeutic use*
- Brain / diagnostic imaging
- Disease Progression
- Female
- Humans
- Immunologic Factors / therapeutic use*
- Injection Site Reaction
- Magnetic Resonance Imaging
- Male
- Middle Aged
- Multiple Sclerosis, Chronic Progressive / diagnostic imaging
- Multiple Sclerosis, Chronic Progressive / drug therapy*
- Multiple Sclerosis, Chronic Progressive / physiopathology
- Multiple Sclerosis, Relapsing-Remitting / diagnostic imaging
- Multiple Sclerosis, Relapsing-Remitting / drug therapy*
- Multiple Sclerosis, Relapsing-Remitting / physiopathology
- Retrospective Studies
- Spain
- Spinal Cord / diagnostic imaging
- Treatment Outcome

Substances

- Antibodies, Monoclonal, Humanized
- Immunologic Factors
- ocrelizumab

Full text links

Free PMC article
Proceed to details
Cite
Share
☐ 1,250
Dig Liver Dis
. 2021 Dec 27;S1590-8658(21)00923-3.
doi: 10.1016/j.dld.2021.12.014. Online ahead of print.

Hepatocellular liver injury in hospitalized patients affected by COVID-19: Presence of different risk factors at different time points

M Leo [1], A Galante [2], A Pagnamenta [3], L Ruinelli [4], F R Ponziani [5], A Gasbarrini [5], A De Gottardi [6]
Affiliations Expand

Affiliations

- [1] Gastroenterology and Hepatology, Ente Ospedaliero Cantonale, Lugano, Switzerland; Internal Medicine and Gastroenterology - Hepatology Unit, Fondazione Policlinico Universitario Agostino Gemelli IRCCS, Rome, Italy. Electronic address: massimo.leo@aphp.fr.
- [2] Gastroenterology and Hepatology, Ente Ospedaliero Cantonale, Lugano, Switzerland.
- [3] Clinical Trial Unit, Ente Ospedaliero Cantonale, Bellinzona, Switzerland; Department of Intensive Care, Ente Ospedaliero Cantonale, Bellinzona, Switzerland; Division of Pneumology, University of Geneva, Geneva, Switzerland.
- [4] ICT (Information and Communication Technologies), Ente Ospedaliero Cantonale, Bellinzona, Switzerland.
- [5] Internal Medicine and Gastroenterology - Hepatology Unit, Fondazione Policlinico Universitario Agostino Gemelli IRCCS, Rome, Italy; Catholic University of the Sacred Heart, Rome, Italy.
- [6] Gastroenterology and Hepatology, Ente Ospedaliero Cantonale, Lugano, Switzerland; Faculty of Biomedical Sciences, Università della Svizzera Italiana, Lugano, Switzerland.

- PMID: **35093272**
- PMCID: PMC8710398
- DOI: 10.1016/j.dld.2021.12.014

Free PMC article

Hepatocellular liver injury in hospitalized patients affected by COVID-19: Presence of different risk factors at different time points

M Leo et al. Dig Liver Dis. 2021.
Free PMC article
Show details
Dig Liver Dis
. 2021 Dec 27;S1590-8658(21)00923-3.
doi: 10.1016/j.dld.2021.12.014. Online ahead of print.

Authors

M Leo [1], A Galante [2], A Pagnamenta [3], L Ruinelli [4], F R Ponziani [5], A Gasbarrini [5], A De Gottardi [6]

Affiliations

- [1] Gastroenterology and Hepatology, Ente Ospedaliero Cantonale, Lugano, Switzerland; Internal Medicine and Gastroenterology - Hepatology Unit, Fondazione Policlinico Universitario Agostino Gemelli IRCCS, Rome, Italy. Electronic address: massimo.leo@aphp.fr.
- [2] Gastroenterology and Hepatology, Ente Ospedaliero Cantonale, Lugano, Switzerland.
- [3] Clinical Trial Unit, Ente Ospedaliero Cantonale, Bellinzona, Switzerland; Department of Intensive Care, Ente Ospedaliero Cantonale, Bellinzona, Switzerland; Division of Pneumology, University of Geneva, Geneva, Switzerland.
- [4] ICT (Information and Communication Technologies), Ente Ospedaliero Cantonale, Bellinzona, Switzerland.
- [5] Internal Medicine and Gastroenterology - Hepatology Unit, Fondazione Policlinico Universitario Agostino Gemelli IRCCS, Rome, Italy; Catholic University of the Sacred Heart, Rome, Italy.
- [6] Gastroenterology and Hepatology, Ente Ospedaliero Cantonale, Lugano, Switzerland; Faculty of Biomedical Sciences, Università della Svizzera Italiana, Lugano, Switzerland.

- PMID: **35093272**
- PMCID: PMC8710398
- DOI: 10.1016/j.dld.2021.12.014

Abstract

Background: Prevalence and clinical impact of increased liver function tests in patients affected by Coronavirus disease 2019 (COVID-19) is controversial.

Aims: This observational study evaluates the prevalence of transaminases elevation in hospitalized patients affected by COVID-19 and investigates the presence of factors associated with hepatocellular injury and with mortality.

Methods: Data of 292 adult patients with confirmed COVID-19 admitted to the Ente Ospedaliero Cantonale (Switzerland) were retrospectively analyzed.

Results: Transaminases were increased in about one-third of patients on hospital admission and two-thirds of patients during the hospital stay. On hospital admission, transaminases were more commonly elevated in younger patients, who also reported elevated C reactive protein and a higher degree of respiratory failure. Independent factors associated with abnormal transaminases during hospitalization were drugs, in particular paracetamol (OR=2.67; 95% CI=1.38-5.18; p = 0.004) and remdesivir (OR=5.16; 95% CI=1.10-24.26; p = 0.04). Mortality was independently associated to age (OR = 1.09; 95% CI=1.05-1.13; p<0.001), admission to intensive care unit (OR=5.22; 95% CI=2.28-11.90; p<0.001) and alkaline phosphatase peak (OR=1.01; 95% CI=1.00- 1.01; p = 0.01).

Conclusions: On hospital admission, factors associated with liver damage were linked to demographic and clinical characteristics (age, inflammation and hypoxia) while, during

hospitalization, drug treatment was related to development and progression of hepatocellular damage. Mortality was associated with alkaline phosphate peak value.

Keywords: Liver function tests; Liver injury; Mortality; SARS-CoV-2.

Copyright © 2021. Published by Elsevier Ltd.

Conflict of interest statement

Conflict of Interest Massimo Leo, Antonio Galante, Alberto Pagnamenta, Lorenzo Ruinelli, Francesca Romana Ponziani, Antonio Gasbarrini and Andrea De Gottardi declare that there are no conflicts of interest.

- 32 references
- 1 figure

Full text links

ELSEVIER FULL-TEXT ARTICLE Elsevier Science Free PMC article

Proceed to details

Cite

Share

☐ 1,251

Front Med (Lausanne)
. 2020 Nov 27;7:569567.
doi: 10.3389/fmed.2020.569567. eCollection 2020.

Thromboprofilaxys With Fondaparinux vs. Enoxaparin in Hospitalized COVID-19 Patients: A Multicenter Italian Observational Study

Vincenzo Russo [1], Giuseppe Cardillo [2], Giuseppe Vito Viggiano [3], Sara Mangiacapra [4], Antonella Cavalli [5], Andrea Fontanella [6], Federica Agrusta [3], Annamaria Bellizzi [5], Maria Amitrano [4], Mariateresa Iannuzzo [6], Clara Sacco [7], Corrado Lodigiani [7], Giampiero Castaldo [6], Pierpaolo Di Micco [6]

Affiliations Expand

Affiliations

- [1] Department of Translational Medical Sciences, Monaldi Hospital, University of Campania "Luigi Vanvitelli", Naples, Italy.
- [2] Medylab, Advanced Biochemistry Unit, Naples, Italy.
- [3] Emergency Medicine Unit, Marazzini Hospital, Modena, Italy.
- [4] Internal Medicine Unit, Moscati Hospital, Avellino, Italy.

- [5] Internal Medicine Unit, Frangipane Hospital, Ariano Irpino, Italy.
- [6] Internal Medicine Unit, Fatebenefratelli Hospital, Naples, Italy.
- [7] Thrombosis and Hemorragic Center, Humanitas Research Hospital and University, Rozzano, Italy.

- PMID: **33330530**
- PMCID: PMC7729125
- DOI: 10.3389/fmed.2020.569567

Free PMC article

Thromboprofilaxys With Fondaparinux vs. Enoxaparin in Hospitalized COVID-19 Patients: A Multicenter Italian Observational Study

Vincenzo Russo et al. Front Med (Lausanne). 2020.
Free PMC article
Show details
Front Med (Lausanne)
. 2020 Nov 27;7:569567.
doi: 10.3389/fmed.2020.569567. eCollection 2020.

Authors

Vincenzo Russo [1], Giuseppe Cardillo [2], Giuseppe Vito Viggiano [3], Sara Mangiacapra [4], Antonella Cavalli [5], Andrea Fontanella [6], Federica Agrusta [3], Annamaria Bellizzi [5], Maria Amitrano [4], Mariateresa Iannuzzo [6], Clara Sacco [7], Corrado Lodigiani [7], Giampiero Castaldo [6], Pierpaolo Di Micco [6]

Affiliations

- [1] Department of Translational Medical Sciences, Monaldi Hospital, University of Campania "Luigi Vanvitelli", Naples, Italy.
- [2] Medylab, Advanced Biochemistry Unit, Naples, Italy.
- [3] Emergency Medicine Unit, Marazzini Hospital, Modena, Italy.
- [4] Internal Medicine Unit, Moscati Hospital, Avellino, Italy.
- [5] Internal Medicine Unit, Frangipane Hospital, Ariano Irpino, Italy.
- [6] Internal Medicine Unit, Fatebenefratelli Hospital, Naples, Italy.
- [7] Thrombosis and Hemorragic Center, Humanitas Research Hospital and University, Rozzano, Italy.

- PMID: **33330530**
- PMCID: PMC7729125
- DOI: 10.3389/fmed.2020.569567

Abstract

Importance: The use of anticoagulant therapy with heparins decreased mortality in hospitalized patients with severe coronavirus disease 2019 (COVID-19). Even if enoxaparin and fondaparinux have the same clinical indication for venous thromboembolism (VTE) prevention; to date, there are no data about the use of fondaparinux in terms of safety, effectiveness, and impact on clinical prognosis among COVID-19 patients. **Objective:** To evaluate the safety, effectiveness, and clinical impact of VTE prophylaxis with fondaparinux and enoxaparin among COVID-19 patients hospitalized in internal medicine units. **Design, Setting, and Participants:** This was a retrospective multicenter observation study, including consecutive symptomatic patients with laboratory-proven COVID-19 admitted to internal medicine units of five Italian hospitals from 15th February to 15th March 2020. **Main Outcomes and Measures:** The primary safety outcome was the composite of major bleeding and clinically relevant non-major bleeding; the primary effectiveness outcome was the composite of all events classified as pulmonary embolism and deep venous thrombosis. The secondary effectiveness outcome included acute respiratory distress syndrome and all-cause death. **Results:** Among 120 COVID-19 patients enrolled in the study, 74 were taking enoxaparin (4,000 or 6,000 units/day) and 46 fondaparinux (2.5 units/day). No statistically significant difference in demographic and laboratory and clinical characteristics between the two groups has been shown. During a median follow-up of 32 (interquartile range: 14-51) days, the cumulative incidence rates of VTE and bleeding events on pharmacological thromboprophylaxis with heparins were 19% and 8%, respectively. The incidence of both VTE (6.5 vs. 13.5%; $P = 0.36$) and bleeding events (6.5 vs. 4.1%; $P = 0.68$) did not show a significant difference between COVID-19 patients on fondaparinux compared with those on enoxaparin therapy. The regression model for the risk of outcome events according to different VTE prophylaxis drugs did not show significant differences. **Conclusions and Relevance:** Although these results need confirmation by prospective studies including a larger population, our study provides preliminary evidence of a safe and efficacy use of fondaparinux for VTE prophylaxis in hospitalized COVID-19 patients.

Keywords: COVID-19; acute respiratory distress syndrome; deep venous thrombosis; enoxaparin; fondaparinux; major bleedings; pulmonary embolism; thromboprofylaxis.

Copyright © 2020 Russo, Cardillo, Viggiano, Mangiacapra, Cavalli, Fontanella, Agrusta, Bellizzi, Amitrano, Iannuzzo, Sacco, Lodigiani, Castaldo and Di Micco.

Conflict of interest statement

The authors declare that the research was conducted in the absence of any commercial or financial relationships that could be construed as a potential conflict of interest.

- 20 references
- 1 figure

Full text links

Frontiers Media SA Free PMC article

Proceed to details

Cite

Share

☐ 1,252

Cancers (Basel)
. 2020 Aug 20;12(9):2352.
doi: 10.3390/cancers12092352.

Low Incidence of SARS-CoV-2 in Patients with Solid Tumours on Active Treatment: An Observational Study at a Tertiary Cancer Centre in Lombardy, Italy

Alexia Francesca Bertuzzi [1], Andrea Marrari [1 2], Nicolò Gennaro [2 3], Umberto Cariboni [4], Michele Ciccarelli [5], Laura Giordano [6], Vittorio Lorenzo Quagliuolo [7], Armando Santoro [1 2]
Affiliations Expand

Affiliations

- [1] Medical Oncology and Hematology Unit, Humanitas Clinical and Research Center, IRCCS, 20089 Milan, Italy.
- [2] Department of Biomedical Sciences, Humanitas University, 20072 Milan, Italy.
- [3] Radiology Unit, Humanitas Clinical and Research Center, IRCCS, 20089 Milan, Italy.
- [4] Thoracic Surgery Unit, Humanitas Clinical and Research Center, IRCCS, 20089 Milan, Italy.
- [5] Pneumology Unit, Humanitas Clinical and Research Center, IRCCS, 20089 Milan, Italy.
- [6] Biostatistics Unit, Humanitas Clinical and Research Center, IRCCS, 20089 Milan, Italy.
- [7] Sarcoma, Melanoma and Rare Tumors Surgery Unit, Humanitas Clinical and Research Center, IRCCS, 20089 Milan, Italy.

- PMID: **32825295**
- PMCID: PMC7564537
- DOI: 10.3390/cancers12092352

Free PMC article

Low Incidence of SARS-CoV-2 in Patients with Solid Tumours on Active Treatment: An Observational Study at a Tertiary Cancer Centre in Lombardy, Italy

Alexia Francesca Bertuzzi et al. Cancers (Basel). 2020.
Free PMC article
Show details
Cancers (Basel)
. 2020 Aug 20;12(9):2352.

doi: 10.3390/cancers12092352.

Authors

Alexia Francesca Bertuzzi [1], Andrea Marrari [1 2], Nicolò Gennaro [2 3], Umberto Cariboni [4], Michele Ciccarelli [5], Laura Giordano [6], Vittorio Lorenzo Quagliuolo [7], Armando Santoro [1 2]

Affiliations

- [1] Medical Oncology and Hematology Unit, Humanitas Clinical and Research Center, IRCCS, 20089 Milan, Italy.
- [2] Department of Biomedical Sciences, Humanitas University, 20072 Milan, Italy.
- [3] Radiology Unit, Humanitas Clinical and Research Center, IRCCS, 20089 Milan, Italy.
- [4] Thoracic Surgery Unit, Humanitas Clinical and Research Center, IRCCS, 20089 Milan, Italy.
- [5] Pneumology Unit, Humanitas Clinical and Research Center, IRCCS, 20089 Milan, Italy.
- [6] Biostatistics Unit, Humanitas Clinical and Research Center, IRCCS, 20089 Milan, Italy.
- [7] Sarcoma, Melanoma and Rare Tumors Surgery Unit, Humanitas Clinical and Research Center, IRCCS, 20089 Milan, Italy.

- PMID: **32825295**
- PMCID: PMC7564537
- DOI: 10.3390/cancers12092352

Abstract

Background: The incidence and prognosis of SARS-CoV-2-positive cancer patients on active oncologic treatment remain unknown. Retrospective data from China reported higher incidence and poorer outcomes with respect to the general population. We aimed to describe the real-word incidence of SARS-CoV-2 in cancer patients and the impact of oncologic therapies on the infection. **Materials & Methods:** In this study, we analysed all consecutive cancer patients with solid tumours undergoing active intravenous treatment (chemotherapy, immunotherapy, targeted therapy, alone or in combination) between 21 February and 30 April 2020, in a high-volume cancer centre in Lombardy, Italy. We focused on SARS-CoV-2-positive patients, reporting on the clinical characteristics of the cancer and the infection. **Results:** We registered 17 SARS-CoV-2-positive patients among 1267 cancer patients on active treatment, resulting in an incidence of 1.3%. The median age was 69.5 years (range 43-79). Fourteen patients (82%) required hospitalisation for COVID-19 with a median in-hospital stay of 11.5 days (range 3-58). Fourteen of the seventeen (82%) were treated for locally advanced or metastatic disease. We could not demonstrate any correlation between SARS-CoV-2 infection and tumour or treatment type. The COVID-19-related fatality rate was 29% (5/17), which was higher than that of the general population cared for in our centre (20%). **Conclusions:** Active oncologic treatments do not represent a risk factor for SARS-CoV-2 infection in cancer patients. However, the prognosis of infected cancer patients appears to be worse compared with that of the non-oncologic population. Given the low number of SARS-CoV-2-positive cases and the uncertainties in risk factors that may have an impact on the prognosis, we advocate for the continuum of cancer care even during the current pandemic.

Keywords: COVID-19; SARS-CoV-2; cancer care; chemotherapy; immunotherapy; treatment safety.

Conflict of interest statement

The authors declare no potential conflicts of interest. A.S. reports personal fees and others from BMS, Servier, Gilead, Pfizer, Eisai, Bayer, MSD, and ArQule; others from Takeda, Roche, AbbVie, Amgen, Celgene, AstraZeneca, Lilly, Sandoz, and Novartis, outside the submitted work.

- 16 references

Full text links

FULL TEXT OPEN ACCESS MDPI Multidisciplinary Digital Publishing Institute (MDPI) Free PMC article
Proceed to details
Cite
Share
☐ 1,253
Neth Heart J
. 2020 Jul;28(7-8):418-423.
doi: 10.1007/s12471-020-01462-6.

The risk of QTc-interval prolongation in COVID-19 patients treated with chloroquine

F S Sinkeler[1], F A Berger[2], H J Muntinga[3], M M P M Jansen[4]
Affiliations Expand

Affiliations

- [1] Department of Clinical Pharmacy, Elisabeth-TweeSteden Hospital, Tilburg, The Netherlands. f.sinkeler@etz.nl.
- [2] Department of Clinical Pharmacy, Meander Medical Centre, Amersfoort, The Netherlands.
- [3] Department of Cardiology, Elisabeth-TweeSteden Hospital, Tilburg, The Netherlands.
- [4] Department of Clinical Pharmacy, Elisabeth-TweeSteden Hospital, Tilburg, The Netherlands.

- PMID: **32648153**
- PMCID: PMC7346846
- DOI: 10.1007/s12471-020-01462-6

Free PMC article

The risk of QTc-interval prolongation in COVID-19 patients treated with chloroquine

F S Sinkeler et al. Neth Heart J. 2020 Jul.
Free PMC article

Show details

Neth Heart J
. 2020 Jul;28(7-8):418-423.
doi: 10.1007/s12471-020-01462-6.

Authors

F S Sinkeler[1], F A Berger[2], H J Muntinga[3], M M P M Jansen[4]

Affiliations

- [1] Department of Clinical Pharmacy, Elisabeth-TweeSteden Hospital, Tilburg, The Netherlands. f.sinkeler@etz.nl.
- [2] Department of Clinical Pharmacy, Meander Medical Centre, Amersfoort, The Netherlands.
- [3] Department of Cardiology, Elisabeth-TweeSteden Hospital, Tilburg, The Netherlands.
- [4] Department of Clinical Pharmacy, Elisabeth-TweeSteden Hospital, Tilburg, The Netherlands.

- PMID: **32648153**
- PMCID: PMC7346846
- DOI: 10.1007/s12471-020-01462-6

Abstract

Background: Chloroquine, a quinolone antimalarial drug, is known to potentially inhibit pH-dependent viral replication of the SARS-CoV-2 infection. Therefore, chloroquine is considered as a treatment option for coronavirus disease 2019 (COVID-19). Chloroquine is known for prolonging the QT interval, but limited data are available on the extent of this QT-prolonging effect.

Objective: To assess the QTc-prolonging potential of chloroquine in COVID-19 patients and to evaluate whether this prolongation increases with the cumulative dose of chloroquine and is associated with the peak plasma concentration of chloroquine. Furthermore, the number of patients who prematurely discontinued treatment or had an adjustment in dose due to QTc-interval prolongation was established.

Methods: A retrospective, observational study was performed in patients aged over 18 years, hospitalised for a suspected or proven infection with COVID-19, and therefore treated with chloroquine, with a baseline electrocardiogram (ECG) performed prior to the start of treatment and at least one ECG after starting the treatment.

Results: In total, 397 patients were included. The mean increase in QTc interval throughout the treatment with chloroquine was 33 ms. Nineteen out of 344 patients unnecessarily had their treatment prematurely discontinued or adjusted due to a prolonged QTc interval based on the computerised interpretation of the ECG.

Conclusion: Chloroquine treatment in COVID-19 patients gradually increased the QTc interval. Due to a significant number of overestimated QTc intervals by computer analysis, it is advisable to measure the QTc interval manually before adjusting the dose or withdrawing this potentially beneficial medication.

Keywords: COVID-19; Chloroquine; Electrocardiography; QTc interval.

Conflict of interest statement

F.S. Sinkeler, F.A. Berger, H.J. Muntinga and M.M.P.M. Jansen declare that they have no competing interests.

- 14 references
- 3 figures

Full text links

SpringerLink FULL-TEXT ARTICLE Springer Free PMC article
Proceed to details
Cite
Share
1,254
Clin Nutr
. 2021 Jun 4;S0261-5614(21)00274-0.
doi: 10.1016/j.clnu.2021.05.024. Online ahead of print.

Impact of nutritional therapy during the first wave of the COVID-19 pandemic in intensive care patients: A retrospective observational study

Aude de Watteville [1], Florencia Montalbano [2], Hannah Wozniak [2], Tinh-Hai Collet [3], Cyril Jaksic [4], Christophe Le Terrier [2], Jérôme Pugin [2], Laurence Genton [3], Claudia Paula Heidegger [5]

Affiliations Expand

Affiliations

- [1] Clinical Nutrition, Division of Endocrinology, Diabetes, Nutrition and Therapeutic Education, Department of Medicine and the University of Geneva Faculty of Medicine Geneva, Switzerland; Division of Intensive Care, Department of Acute Medicine, Geneva University Hospitals and the University of Geneva Faculty of Medicine Geneva, Switzerland.
- [2] Division of Intensive Care, Department of Acute Medicine, Geneva University Hospitals and the University of Geneva Faculty of Medicine Geneva, Switzerland.
- [3] Clinical Nutrition, Division of Endocrinology, Diabetes, Nutrition and Therapeutic Education, Department of Medicine and the University of Geneva Faculty of Medicine Geneva, Switzerland.
- [4] CRC & Division of Clinical-Epidemiology, Department of Health and Community Medicine, University of Geneva & University Hospitals of Geneva, Switzerland.

- [5] Division of Intensive Care, Department of Acute Medicine, Geneva University Hospitals and the University of Geneva Faculty of Medicine Geneva, Switzerland. Electronic address: claudia-paula.heidegger@hcuge.ch.

- PMID: **34134917**
- PMCID: PMC8176891
- DOI: 10.1016/j.clnu.2021.05.024

Free PMC article

Impact of nutritional therapy during the first wave of the COVID-19 pandemic in intensive care patients: A retrospective observational study

Aude de Watteville et al. Clin Nutr. 2021.
Free PMC article

Show details

Clin Nutr
. 2021 Jun 4;S0261-5614(21)00274-0.
doi: 10.1016/j.clnu.2021.05.024. Online ahead of print.

Authors

Aude de Watteville [1], Florencia Montalbano [2], Hannah Wozniak [2], Tinh-Hai Collet [3], Cyril Jaksic [4], Christophe Le Terrier [2], Jérôme Pugin [2], Laurence Genton [3], Claudia Paula Heidegger [5]

Affiliations

- [1] Clinical Nutrition, Division of Endocrinology, Diabetes, Nutrition and Therapeutic Education, Department of Medicine and the University of Geneva Faculty of Medicine Geneva, Switzerland; Division of Intensive Care, Department of Acute Medicine, Geneva University Hospitals and the University of Geneva Faculty of Medicine Geneva, Switzerland.
- [2] Division of Intensive Care, Department of Acute Medicine, Geneva University Hospitals and the University of Geneva Faculty of Medicine Geneva, Switzerland.
- [3] Clinical Nutrition, Division of Endocrinology, Diabetes, Nutrition and Therapeutic Education, Department of Medicine and the University of Geneva Faculty of Medicine Geneva, Switzerland.
- [4] CRC & Division of Clinical-Epidemiology, Department of Health and Community Medicine, University of Geneva & University Hospitals of Geneva, Switzerland.
- [5] Division of Intensive Care, Department of Acute Medicine, Geneva University Hospitals and the University of Geneva Faculty of Medicine Geneva, Switzerland. Electronic address: claudia-paula.heidegger@hcuge.ch.

- PMID: **34134917**
- PMCID: PMC8176891
- DOI: 10.1016/j.clnu.2021.05.024

Abstract

Background & aims: The COVID-19 pandemic has caused major organizational challenges to healthcare systems concerning staff, material and bed availability. Nutrition was not a priority in the intensive care unit (ICU) at the beginning of the pandemic with the need for simplified protocols. We aimed to assess the impact of a simplified nutritional protocol for critically ill COVID-19 patients during the pandemic first wave.

Methods: We included all patients with SARS-CoV-2 infections, admitted to the ICU of the Geneva University Hospitals for at least 4 days from March 9 to May 19, 2020. Data on the route and solution of nutritional therapy, prescribed and received volume, calorie and protein intake, amount of insulin, propofol and glucose administered were collected daily during the entire ICU stay. We compared nutritional outcomes between patients admitted to the ICU before and after implementing the simplified nutritional protocol using unpaired t-test.

Results: Out of 119 patients, 48 were hospitalized in the ICU before, 47 across and 24 after the implementation of the nutritional protocol. The mean age was 63.2 (±12.7) years and 76% were men without significant difference between before and after group. The nutritional protocol implementation led to an increase in caloric intake (1070 vs. 1357 kcal/day, $p = 0.018$) and in the percentage of days within 80-100% of the energy target (11 vs. 20%, $p = 0.021$). The protein debt decreased significantly from 48 g/day to 37 g/day ($p = 0.015$). No significant difference in the percentage of days within the protein target (80-100%) was observed.

Conclusions: Calorie and protein coverage improved after the implementation of the simplified nutritional protocol in critically ill COVID-19 patients. Further studies are needed to assess the impact of such an approach on patients' clinical outcomes.

Keywords: COVID-19; ICU; Nutrition protocol; Nutrition therapy; Pandemic; SARS-CoV-2.

Copyright © 2021 The Authors. Published by Elsevier Ltd.. All rights reserved.

Conflict of interest statement

Conflicts of Interest The authors have no potential conflict of interest to declare.

- 31 references
- 2 figures

Full text links

ELSEVIER OPEN ACCESS Elsevier Science Free PMC article

Proceed to details

Cite

Share

☐ 1,255

Clin Exp Med

. 2021 Sep 20;1-12.

doi: 10.1007/s10238-021-00755-3. Online ahead of print.

Neither inflammatory bowel disease nor immunosuppressants are associated with an increased risk of severe COVID-19: an observational Dutch cohort study

Lennard P L Gilissen [1], Stefan G H Heinen [2], Lotte Rijpma-Jacobs [3], Erik Schoon, Ramon-Michel Schreuder [3], Anne-Marie Wensing [3], Mirjam C M van der Ende-van Loon [3], Johanne G Bloemen [4], Janneke M Stapelbroek [5], Arnold Stronkhorst [3]
Affiliations Expand

Affiliations

- [1] Department of Gastroenterology and Hepatology, Catharina Hospital Eindhoven, Michelangelolaan 2, 5623 EJ, Eindhoven, The Netherlands. lennard.gilissen@catharinaziekenhuis.nl.
- [2] Department of Quality and Safety, Catharina Hospital Eindhoven, Michelangelolaan 2, 5623 EJ, Eindhoven, The Netherlands.
- [3] Department of Gastroenterology and Hepatology, Catharina Hospital Eindhoven, Michelangelolaan 2, 5623 EJ, Eindhoven, The Netherlands.
- [4] Department of Surgery, Catharina Hospital Eindhoven, Michelangelolaan 2, 5623 EJ, Eindhoven, The Netherlands.
- [5] Department of Pediatrics, Catharina Hospital Eindhoven, Michelangelolaan 2, 5623 EJ, Eindhoven, The Netherlands.

- PMID: **34542781**
- PMCID: PMC8450711
- DOI: 10.1007/s10238-021-00755-3

Free PMC article

Neither inflammatory bowel disease nor immunosuppressants are associated with an increased risk of severe COVID-19: an observational Dutch cohort study

Lennard P L Gilissen et al. Clin Exp Med. 2021.
Free PMC article

Show details

Clin Exp Med

. 2021 Sep 20;1-12.

doi: 10.1007/s10238-021-00755-3. Online ahead of print.

Authors

Lennard P L Gilissen [1], Stefan G H Heinen [2], Lotte Rijpma-Jacobs [3], Erik Schoon, Ramon-Michel Schreuder [3], Anne-Marie Wensing [3], Mirjam C M van der Ende-van Loon [3], Johanne G Bloemen [4], Janneke M Stapelbroek [5], Arnold Stronkhorst [3]

Affiliations

- [1] Department of Gastroenterology and Hepatology, Catharina Hospital Eindhoven, Michelangelolaan 2, 5623 EJ, Eindhoven, The Netherlands. lennard.gilissen@catharinaziekenhuis.nl.
- [2] Department of Quality and Safety, Catharina Hospital Eindhoven, Michelangelolaan 2, 5623 EJ, Eindhoven, The Netherlands.
- [3] Department of Gastroenterology and Hepatology, Catharina Hospital Eindhoven, Michelangelolaan 2, 5623 EJ, Eindhoven, The Netherlands.
- [4] Department of Surgery, Catharina Hospital Eindhoven, Michelangelolaan 2, 5623 EJ, Eindhoven, The Netherlands.
- [5] Department of Pediatrics, Catharina Hospital Eindhoven, Michelangelolaan 2, 5623 EJ, Eindhoven, The Netherlands.

- PMID: **34542781**
- PMCID: PMC8450711
- DOI: 10.1007/s10238-021-00755-3

Abstract

Conflicting data about inflammatory bowel disease [IBD] and immunosuppressants are risk factors for severe COVID-19 confuse patients and healthcare providers. Clinical reports with longer follow-up are lacking. A retrospective search was performed for severe COVID-19 (hospital admission and/or mortality) one year after the SARS-CoV-2 outbreak in an IBD cohort from one of the most affected Dutch regions. Cohort characteristics were explored by value-based healthcare data, including immunotherapy. COVID-19 cases were detected by ICD-10 codes and further examined for IBD determinants (including medication) and COVID-19 characteristics (intensive care admission, respiratory support, treatment, mortality). The national mortality register was consulted, ensuring detection of patients that died without admission. Results were compared with regional and national general population registries. The IBD cohort consisted of 1453 patients (51% Crohn's disease, 54% women, 39.9% using immunotherapy), including children. Biologics use increased during the study. Eight cases (0.55%) had severe COVID-19: seven were hospitalized (0.48%, 95% confidence interval [CI] 0.21-1.04), and two died (0.14%, CI 0.002-0.55). Six patients had comorbidity, one used immunotherapy, and four had no medication. Both deceased patients were older than 80 years, had severe comorbidity, but used no immunotherapy. Hospitalization occurred significantly more in the IBD cohort than regionally (0.18%, CI 0.17-0.19, p = 0.015), but not significantly more than nationally (0.28%, CI 0.279-0.284). Mortality was equal in IBD patients, regionally (0.11%, CI 0.10-0.12) and nationally (0.13%, CI 0.125-0.128). Neither IBD nor immunosuppressants are associated with increased risks of severe COVID-19 in an observational study with one-year follow-up.

Keywords: Biological; COVID-19; IBD; Inflammatory bowel disease; SARS-CoV-2; Thiopurine.

© 2021. The Author(s), under exclusive licence to Springer Nature Switzerland AG.

Conflict of interest statement

The authors have no conflicts of interest to declare that are relevant to the content of this article.

- 40 references

Full text links

Springer Free PMC article
Proceed to details
Cite
Share
1,256
An Pediatr (Engl Ed)
. 2020 Aug;93(2):118-122.
doi: 10.1016/j.anpede.2020.04.010. Epub 2020 Jul 1.

Influence of the coronavirus 2 (SARS-Cov-2) pandemic on acute appendicitis

M Velayos [1], A J Muñoz-Serrano [1], K Estefanía-Fernández [1], M C Sarmiento Caldas [1], L Moratilla Lapeña [1], M López-Santamaría [1], J C López-Gutiérrez [1]
Affiliations Expand

Affiliation

- [1] Servicio de Cirugía Pediátrica, Hospital Universitario La Paz, Madrid, Spain.

- PMID: **32837965**
- PMCID: PMC7328590
- DOI: 10.1016/j.anpede.2020.04.010

Free PMC article

Influence of the coronavirus 2 (SARS-Cov-2) pandemic on acute appendicitis

M Velayos et al. An Pediatr (Engl Ed). 2020 Aug.
Free PMC article
Show details
An Pediatr (Engl Ed)
. 2020 Aug;93(2):118-122.
doi: 10.1016/j.anpede.2020.04.010. Epub 2020 Jul 1.

Authors

M Velayos [1], A J Muñoz-Serrano [1], K Estefanía-Fernández [1], M C Sarmiento Caldas [1], L Moratilla Lapeña [1], M López-Santamaría [1], J C López-Gutiérrez [1]

Affiliation

- [1] Servicio de Cirugía Pediátrica, Hospital Universitario La Paz, Madrid, Spain.

- PMID: **32837965**
- PMCID: PMC7328590
- DOI: 10.1016/j.anpede.2020.04.010

Abstract
in English, Spanish

Introduction: Acute appendicitis (AA) is the most common abdominal surgical emergency. No specific studies have been found that evaluate the impact of the coronavirus 2 (SARS-Cov-2) pandemic on AA and its surgical management. An analysis was made on the influence of this new pathology on the clinical course of AA.

Material and methods: Retrospective observational study was conducted on patients operated on for AA from January to April 2020. They were classified according to the time of the appendectomy, before the declaration of the state of alarm (Pre-COVID-19), and after its declaration (Post-COVID-19) in Spain, one the most affected countries in the world. An evaluation was made of demographic variables, duration of symptoms, type of appendicitis, surgical time, hospital stay, and postoperative complications.

Results: The study included 66 patients (41 Pre-COVID-19; 25 Post-COVID-19) with mean age of 10.7 ± 3 and 9.3 ± 3.1; $P = .073$, respectively. Fever was found in a higher number of post-COVID-19 patients (52 vs 19.5%; $P = .013$), as well as a higher CRP (72.7 ± 96.2 vs 31.3 ± 36.2 mg/dL; $P = .042$). This group presented with a higher proportion of complicated appendicitis when compared to Pre-COVID-19 (32 vs 7.3%; $P = .015$). The mean hospital stay was longer in the Post-COVID-19 group (5.6 ± 5.9 vs 3.2 ± 4.3 days; $P = .041$). No differences were found in the time of onset of symptoms or surgical time.

Conclusions: The SARS-Cov-2 pandemic influenced the time of diagnosis of appendicitis, as well as its course, and mean hospital stay. Peritonitis was more frequently seen. As a result of the significant circumstances, delaying diagnosis and treatment of AA during SARS-Cov-2 pandemic, inappropriate management of this common surgical disorder has been noticed.

Introducción: La apendicitis aguda (AA) es la urgencia quirúrgica abdominal más frecuente. No encontramos estudios específicos que evalúen el impacto de la pandemia causada por el coronavirus 2 (SARS-Cov-2) sobre la AA y su tratamiento quirúrgico. Analizamos la influencia de esta nueva patología sobre la AA.

Material y métodos: Estudio observacional retrospectivo en pacientes intervenidos por AA desde enero hasta abril de 2020. Fueron clasificados según el momento de la apendicectomía, antes de la declaración del estado de alarma (Pre-COVID19) y después de la declaración del estado de alarma (Post-COVID19) en España. Se evaluaron variables demográficas, duración de la sintomatología, tipo de apendicitis, tiempo quirúrgico, estancia hospitalaria y complicaciones postoperatorias.

Resultados: Se incluyeron 66 pacientes (41 Pre-COVID19; 25 Post-COVID19 con edad media de 10.7 ± 3 y 9.3 ± 3.1; *P* = .073; respectivamente). La fiebre se encontró en un mayor número de pacientes post-COVID19 (52 vs 19.5%; *P* = .013), así como una PCR más elevada (72.7 ± 96.2 vs 31.3 ± 36.2 mg/dl; *P* = .042). Este grupo presentó una mayor proporción de apendicitis complicada al compararle con el Pre-COVID19 (32 vs 7.3%; *P* = .015). La estancia media hospitalaria fue mayor en el grupo Post-COVID19 (5.6 ± 5.9 vs 3.3 ± 4.3 días; *P* = .041). No se encontraron diferencias en el tiempo de evolución de los síntomas ni en el tiempo quirúrgico.

Conclusiones: La pandemia por SARS-Cov-2 influye en el momento de diagnóstico de la apendicitis, así como en su grado de evolución y estancia hospitalaria. La peritonitis fue lo más frecuentemente observado. Una sospecha y orientación clínica más temprana, es necesaria para evitar un manejo inadecuado de este trastorno quirúrgico común.

Keywords: Acute appendicitis; Appendectomy; Paediatric patient; Post-surgical complications; SARS-Cov-2.

© 2020 Asociación Española de Pediatría. Published by Elsevier España, S.L.U.

- 14 references
- 1 figure

Full text links

ELSEVIER OPEN ACCESS Elsevier Science Free PMC article

Proceed to details

Cite

Share

☐ 1,257

Front Cardiovasc Med
. 2021 Dec 24;8:792804.
doi: 10.3389/fcvm.2021.792804. eCollection 2021.

Impact of RAAS Inhibitors on Clinical Outcome and Mortality in Patients With STEMI During the COVID-19 Era: A Multicenter Observational Study

Lucia Barbieri [1], Daniela Trabattoni [2], Giulio G Stefanini [3] [4], Enrico Vizzardi [5], Gabriele Tumminello [1], Emilio Assanelli [2], Marianna Adamo [5], Carlo A Pivato [3], Giovanni Provenzale [1], Domitilla Gentile [1], Marco Metra [5], Stefano Carugo [1]
Affiliations Expand

Affiliations

- [1] Cardiology Unit, Fondazione IRCCS Ca' Granda Ospedale Maggiore Policlinico, Milan, Italy.

- [2] Centro Cardiologico Monzino, IRCCS, Milan, Italy.
- [3] Department of Biomedical Sciences, Humanitas University, Milan, Italy.
- [4] IRCCS Humanitas Research Hospital, Milan, Italy.
- [5] Cardiology, ASST Spedali Civili, Department of Medical and Surgical Specialties, Radiological Sciences, and Public Health, University of Brescia, Brescia, Italy.

- PMID: **35004902**
- PMCID: PMC8739948
- DOI: 10.3389/fcvm.2021.792804

Free PMC article

Impact of RAAS Inhibitors on Clinical Outcome and Mortality in Patients With STEMI During the COVID-19 Era: A Multicenter Observational Study

Lucia Barbieri et al. Front Cardiovasc Med. 2021.
Free PMC article

Show details

Front Cardiovasc Med

. 2021 Dec 24;8:792804.
doi: 10.3389/fcvm.2021.792804. eCollection 2021.

Authors

Lucia Barbieri [1], Daniela Trabattoni [2], Giulio G Stefanini [3] [4], Enrico Vizzardi [5], Gabriele Tumminello [1], Emilio Assanelli [2], Marianna Adamo [5], Carlo A Pivato [3], Giovanni Provenzale [1], Domitilla Gentile [1], Marco Metra [5], Stefano Carugo [1]

Affiliations

- [1] Cardiology Unit, Fondazione IRCCS Ca' Granda Ospedale Maggiore Policlinico, Milan, Italy.
- [2] Centro Cardiologico Monzino, IRCCS, Milan, Italy.
- [3] Department of Biomedical Sciences, Humanitas University, Milan, Italy.
- [4] IRCCS Humanitas Research Hospital, Milan, Italy.
- [5] Cardiology, ASST Spedali Civili, Department of Medical and Surgical Specialties, Radiological Sciences, and Public Health, University of Brescia, Brescia, Italy.

- PMID: **35004902**
- PMCID: PMC8739948
- DOI: 10.3389/fcvm.2021.792804

Abstract

Conflicting results are available regarding the influence of ACEi/ARBs on the risk of COVID-19 infection, while less is known about their impact on the clinical outcome of patients with STEMI diagnosed with COVID-19. Our aim was to evaluate the impact of ACEi/ARBs therapy on in-hospital mortality and clinical outcomes of patients with STEMI during the COVID-19 pandemic. We retrospectively analyzed consecutive patients with STEMI hospitalized from February 20 to May 10, 2020 in four Hospitals in Lombardy. SARS-COV-2 diagnosis was performed by nasopharyngeal swab test. Procedural outcome, respiratory complications, and in-hospital mortality were reported. Univariate and multivariate analyses were performed by logistic regressions. Our population was represented by 182 patients with STEMI, 76.9% of which were males, and mean age was 67 ± 12.5. Hypertension was reported in 53.3%, and 29.1% was treated with ACEi/ARBs. COVID-19 diagnosis was confirmed in 17.1% of the patients. In-hospital mortality (13.2%) was significantly higher in patients with COVID-19 (31 vs. 10%, $p = 0.003$), even if ejection fraction [OR 0.93 (95% CI) 0.87-0.99; $p = 0.03$] and respiratory complications [OR 9.39 (95% CI) 1.91-45.9; $p = 0.006$] were the only two independent predictors. The incidence of COVID-19 infection was not influenced by ACEi/ARBs (16.5 in naïve vs. 18.8%) whose presence on admission did not correlate with respiratory complications or mortality both in the case of discontinuation and maintenance. In conclusion, in a high-risk population, such as that of patients with STEMI, the potential benefit of ACEi/ARB discontinuation in patients with COVID-19 is overcome by its detrimental effect. Intensive care, additional preventive respiratory investigations, regardless of swab test result, should be suggested for all patients admitted for STEMI during the pandemic.

Keywords: COVID-19; RAAS inhibitors; STEMI patients; mortality; outcome.

Copyright © 2021 Barbieri, Trabattoni, Stefanini, Vizzardi, Tumminello, Assanelli, Adamo, Pivato, Provenzale, Gentile, Metra and Carugo.

Conflict of interest statement

The authors declare that the research was conducted in the absence of any commercial or financial relationships that could be construed as a potential conflict of interest.

- 27 references

Full text links

Frontiers Media SA Free PMC article

Proceed to details

Cite

Share

☐ 1,258

Lung India

. Sep-Oct 2021;38(5):448-453.

doi: 10.4103/lungindia.lungindia_935_20.

Utility of various inflammatory markers in predicting outcomes of hospitalized patients with COVID-19 pneumonia: A single-center experience

Aishwarya K Marimuthu [1], Monisha Anandhan [2], Lakshmikanthan Sundararajan [2], Jagadeesh Chandrasekaran [1], Balasubramaniam Ramakrishnan [3]
Affiliations Expand

Affiliations

- [1] Department of Internal Medicine, Apollo Hospitals, Greams Road, Chennai, Tamil Nadu, India.
- [2] Department of Respiratory Medicine, Apollo Hospitals, Greams Road, Chennai, Tamil Nadu, India.
- [3] Department of Biostatistics, Apollo Hospitals, Greams Road, Chennai, Tamil Nadu, India.

- PMID: **34472523**
- PMCID: PMC8509176
- DOI: 10.4103/lungindia.lungindia_935_20

Free PMC article

Utility of various inflammatory markers in predicting outcomes of hospitalized patients with COVID-19 pneumonia: A single-center experience

Aishwarya K Marimuthu et al. Lung India. Sep-Oct 2021.
Free PMC article
Show details
Lung India
. Sep-Oct 2021;38(5):448-453.
doi: 10.4103/lungindia.lungindia_935_20.

Authors

Aishwarya K Marimuthu [1], Monisha Anandhan [2], Lakshmikanthan Sundararajan [2], Jagadeesh Chandrasekaran [1], Balasubramaniam Ramakrishnan [3]

Affiliations

- [1] Department of Internal Medicine, Apollo Hospitals, Greams Road, Chennai, Tamil Nadu, India.
- [2] Department of Respiratory Medicine, Apollo Hospitals, Greams Road, Chennai, Tamil Nadu, India.
- [3] Department of Biostatistics, Apollo Hospitals, Greams Road, Chennai, Tamil Nadu, India.

- PMID: **34472523**
- PMCID: PMC8509176
- DOI: 10.4103/lungindia.lungindia_935_20

Abstract

Aim: The aim of the study is to study the utility of various inflammatory markers in predicting outcomes of hospitalized patients with coronavirus disease 2019 (COVID-19) pneumonia.

Primary objective: The primary objective of the study is to analyze the correlation between various inflammatory markers and in-hospital mortality.

Secondary objectives: The secondary objective of the study is to assess the correlation between the inflammatory markers and clinical category of patients, and other outcomes such as length of hospital stay and need for invasive ventilation.

Methods: A retrospective cross-sectional observational study was done in 221 hospitalized patients who were diagnosed with COVID-19 pneumonia in a tertiary care hospital in South India from May 2020 to July 2020. Clinical and laboratory data of patients diagnosed with COVID-19 pneumonia were collected. This included epidemiological data, clinical data, laboratory parameter (neutrophil: lymphocyte [N: L] ratio, C-reactive protein [CRP], ferritin, interleukin-6 [IL-6], lactate dehydrogenase, D-dimer, and procalcitonin), treatment details, and outcomes.

Results: IL-6 levels >60.5 pg/mL and D-dimer levels >0.5 mcg/mL predicted in-hospital mortality with sensitivities of 80% and 76.7%, respectively. N: L ratio and CRP levels had good correlation with the need for oxygen supplementation and/or invasive ventilation.

Conclusions: Judicious use of COVID-19 biomarkers could help in disease prognostication and thereby provide guidance to devise appropriate management strategies.

Keywords: C-reactive protein; Coronavirus Disease 2019 pneumonia; Coronavirus disease 2019 biomarkers; D-dimer; ferritin; interleukin-6; lactate dehydrogenase; neutrophil: lymphocyte ratio; procalcitonin.

Conflict of interest statement

None

- 26 references
- 1 figure

Full text links

Medknow Publications and Media Pvt Ltd Free PMC article

Proceed to details

Cite

Share

☐ 1,259

J Clin Med

. 2021 Feb 10;10(4):686.

doi: 10.3390/jcm10040686.

The Use of Antiviral Agents against SARS-CoV-2: Ineffective or Time and Age Dependent Result? A Retrospective, Observational Study among COVID-19 Older Adults

Antonio Desai [1, 2], Giuseppe Caltagirone [1, 2], Sharon Sari [3], Daria Pocaterra [4], Maria Kogan [1], Elena Azzolini [2, 5], Victor Savevski [6], Filippo Martinelli-Boneschi [7, 8], Antonio Voza [1], On Behalf Of The Humanitas Covid-Task Force [1]

Affiliations Expand

Affiliations

- [1] Emergency Department, Humanitas Clinical and Research Center, IRCCS, 20089 Milan, Italy.
- [2] Department of Biomedical Sciences, Humanitas University, 20090 Pieve Emanuele, Italy.
- [3] Internal Medicine Department, Geriatrics, Santa Margherita Rehabilitation and Cure Institute, ASP, 27100 Pavia, Italy.
- [4] Department of Infectious Diseases, Humanitas Clinical and Research Center, IRCCS, 20089 Milan, Italy.
- [5] Health Directorate, Humanitas Clinical and Research Center, IRCCS, 20089 Milan, Italy.
- [6] Artificial Intelligence Center, Humanitas Clinical and Research Center, IRCCS, 20089 Milan, Italy.
- [7] Dino Ferrari Centre, Neuroscience Section, Department of Pathophysiology and Transplantation (DEPT), University of Milan, 20122 Milan, Italy.
- [8] Neurology Unit and MS Centre, Fondazione IRCCS Ca' Granda Ospedale Maggiore, Policlinico, 20122 Milan, Italy.

- PMID: **33578922**
- PMCID: PMC7916694
- DOI: 10.3390/jcm10040686

Free PMC article

The Use of Antiviral Agents against SARS-CoV-2: Ineffective or Time and Age Dependent Result? A Retrospective, Observational Study among COVID-19 Older Adults

Antonio Desai et al. J Clin Med. 2021.
Free PMC article
Show details
J Clin Med
. 2021 Feb 10;10(4):686.
doi: 10.3390/jcm10040686.

Authors

Antonio Desai [1, 2], Giuseppe Caltagirone [1, 2], Sharon Sari [3], Daria Pocaterra [4], Maria Kogan [1], Elena Azzolini [2, 5], Victor Savevski [6], Filippo Martinelli-Boneschi [7, 8], Antonio Voza [1], On Behalf Of The Humanitas Covid-Task Force [1]

Affiliations

- [1] Emergency Department, Humanitas Clinical and Research Center, IRCCS, 20089 Milan, Italy.
- [2] Department of Biomedical Sciences, Humanitas University, 20090 Pieve Emanuele, Italy.
- [3] Internal Medicine Department, Geriatrics, Santa Margherita Rehabilitation and Cure Institute, ASP, 27100 Pavia, Italy.
- [4] Department of Infectious Diseases, Humanitas Clinical and Research Center, IRCCS, 20089 Milan, Italy.
- [5] Health Directorate, Humanitas Clinical and Research Center, IRCCS, 20089 Milan, Italy.
- [6] Artificial Intelligence Center, Humanitas Clinical and Research Center, IRCCS, 20089 Milan, Italy.
- [7] Dino Ferrari Centre, Neuroscience Section, Department of Pathophysiology and Transplantation (DEPT), University of Milan, 20122 Milan, Italy.
- [8] Neurology Unit and MS Centre, Fondazione IRCCS Ca' Granda Ospedale Maggiore, Policlinico, 20122 Milan, Italy.

- PMID: **33578922**
- PMCID: PMC7916694
- DOI: 10.3390/jcm10040686

Abstract

Background: Our aim was to investigate the impact of therapeutics with antiviral activity against severe acute respiratory syndrome coronavirus 2 (SARS-CoV-2) on mortality of older adults

affected by coronavirus disease 2019 (COVID-19), taking into consideration the time interval from symptoms onset to drugs administration.

Methods: Data from 143 COVID-19 patients over 65 years of age admitted to the Humanitas Clinical and Research Center Emergency Department (Milan, Italy) and treated with Lopinavir/ritonavir (LPV/r) or Darunavir/cobicistat (DVR/c) associated to Hydroxychloroquine (HCQ) were retrospectively analyzed. Statistical analysis was performed by using a logistic regression model and survival analysis to assess the role of different predictors of in-hospital mortality, including an early (<6 days from symptoms onset) vs. late treatment onset, signs and symptoms at COVID-19 presentation, type of antiviral treatment (LPV/r or DVR/c) and patients' age (65-80 vs. >80 years old).

Results: Multivariate analysis showed that an older age (OR: 2.54) and dyspnea as presenting symptom (OR: 2.01) were associated with higher mortality rate, whereas cough as presenting symptom (OR: 0.53) and a timely drug administration (OR: 0.44) were associated with lower mortality. Survival analysis demonstrated that the timing of drug administration had an impact on mortality in 65-80 years-old patients ($p = 0.02$), whereas no difference was seen in those >80 years-old. This impact was more evident in patients with dyspnea as primary symptom of COVID-19, in whom mortality decreased from 57.1% to 38.3% due to timely drug administration (OR: 0.5; $p = 0.04$).

Conclusions: There was a significant association between the use of a combined antiviral regimen and HCQ and lower mortality, when timely-administered, in COVID-19 patients aged 65-80 years. Our findings support timely treatment onset as a key component in the treatment of COVID-19.

Keywords: COVID-19; SARS-CoV-2; age; antivirals; darunavir/cobicistat; geriatric; gerontology; hydroxychloroquine; lopinavir/ritonavir; timing.

Conflict of interest statement

The authors declare no conflict of interest.

- 47 references
- 3 figures

Full text links

FULL TEXT OPEN ACCESS MDPI Multidisciplinary Digital Publishing Institute (MDPI) Free PMC article

Proceed to details

Cite

Share

☐ 1,260

Front Med (Lausanne)

. 2020 Oct 19;7:576457.

doi: 10.3389/fmed.2020.576457. eCollection 2020.

Clinical Findings of COVID-19 Patients Admitted to Intensive Care Units in

Guangdong Province, China: A Multicenter, Retrospective, Observational Study

Yonghao Xu [1], Zhiheng Xu [1], Xuesong Liu [1], Lihua Cai [2], Haichong Zheng [1], Yongbo Huang [1], Lixin Zhou [3], Linxi Huang [4], Yun Ling [5], Liehua Deng [6], Jianwei Li [7], Sibei Chen [1], Dongdong Liu [1], Zhimin Lin [1], Liang Zhou [1], Weiqun He [1], Nanshan Zhong [1], Xiaoqing Liu [1], Yimin Li [1]

Affiliations Expand

Affiliations

- [1] State Key Laboratory of Respiratory Diseases, Department of Critical Care Medicine, Guangzhou Institute of Respiratory Health, First Affiliated Hospital of Guangzhou Medical University, Guangzhou, China.
- [2] Department of Critical Care Medicine, Dongguan People's Hospital, Dongguan, China.
- [3] Department of Critical Care Medicine, Foshan First People's Hospital, Foshan, China.
- [4] Department of Critical Care Medicine, The First Affiliated Hospital of Shantou University Medical College, Shantou, China.
- [5] Department of Critical Care Medicine, Huizhou Municipal Central Hospital, Huizhou, China.
- [6] Department of Critical Care Medicine, Affiliated Hospital of Guangdong Medical University, Zhanjiang, China.
- [7] Department of Critical Care Medicine, Zhongshan City People's Hospital, Zhongshan, China.

- PMID: **33195325**
- PMCID: PMC7604321
- DOI: 10.3389/fmed.2020.576457

Free PMC article

Clinical Findings of COVID-19 Patients Admitted to Intensive Care Units in Guangdong Province, China: A Multicenter, Retrospective, Observational Study

Yonghao Xu et al. Front Med (Lausanne). 2020.
Free PMC article
Show details
Front Med (Lausanne)
. 2020 Oct 19;7:576457.
doi: 10.3389/fmed.2020.576457. eCollection 2020.

Authors

Yonghao Xu [1], Zhiheng Xu [1], Xuesong Liu [1], Lihua Cai [2], Haichong Zheng [1], Yongbo Huang [1], Lixin Zhou [3], Linxi Huang [4], Yun Ling [5], Liehua Deng [6], Jianwei Li [7], Sibei Chen [1], Dongdong Liu [1], Zhimin Lin [1], Liang Zhou [1], Weiqun He [1], Nanshan Zhong [1], Xiaoqing Liu [1], Yimin Li [1]

Affiliations

- [1] State Key Laboratory of Respiratory Diseases, Department of Critical Care Medicine, Guangzhou Institute of Respiratory Health, First Affiliated Hospital of Guangzhou Medical University, Guangzhou, China.
- [2] Department of Critical Care Medicine, Dongguan People's Hospital, Dongguan, China.
- [3] Department of Critical Care Medicine, Foshan First People's Hospital, Foshan, China.
- [4] Department of Critical Care Medicine, The First Affiliated Hospital of Shantou University Medical College, Shantou, China.
- [5] Department of Critical Care Medicine, Huizhou Municipal Central Hospital, Huizhou, China.
- [6] Department of Critical Care Medicine, Affiliated Hospital of Guangdong Medical University, Zhanjiang, China.
- [7] Department of Critical Care Medicine, Zhongshan City People's Hospital, Zhongshan, China.

- PMID: **33195325**
- PMCID: PMC7604321
- DOI: 10.3389/fmed.2020.576457

Abstract

Background: Information about critically ill patients with coronavirus disease 2019 (COVID-19) in China but outside of Wuhan is scarce. We aimed to describe the clinical features, treatment, and outcomes of patients with COVID-19 admitted to the intensive care unit (ICU) in Guangdong Province. **Methods:** In this multicenter, retrospective, observational study, we enrolled consecutive patients with COVID-19 who were admitted to seven ICUs in Guangdong Province. Demographic data, symptoms, laboratory findings, comorbidities, treatment, and outcomes were collected. Data were compared between patients with and without intubation. **Results:** A total of 45 COVID-19 patients required ICU admission in the study hospitals [mean age 56.7 ± 15.4 years, 29 males (64.4%)]. The most common symptoms at onset were fever and cough. Most patients presented with lymphopenia and elevated lactate dehydrogenase. Treatment with antiviral drugs was initiated in all patients. Thirty-six patients (80%) developed acute respiratory distress syndrome at ICU admission, and 15 (33.3%) septic shock. Twenty patients (44.4%) were intubated, and 10 (22.2%) received extracorporeal membrane oxygenation. The 60-day mortality was 4.4% (2 of 45). **Conclusion:** COVID-19 patients admitted to ICU were characterized by fever, lymphopenia, acute respiratory failure, and multiple organ dysfunction. The mortality of ICU patients in Guangdong Province was relatively low with a small sample size.

Keywords: COVID-19; SARS-CoV-2; critically ill; intensive care unit; mortality.

Copyright © 2020 Xu, Xu, Liu, Cai, Zheng, Huang, Zhou, Huang, Ling, Deng, Li, Chen, Liu, Lin, Zhou, He, Zhong, Liu and Li.

- 35 references
- 1 figure

Full text links

Frontiers Media SA Free PMC article
Proceed to details
Cite
Share
1,261
Infect Control Hosp Epidemiol
. 2021 Apr 19;1-6.
doi: 10.1017/ice.2021.175. Online ahead of print.

The role of procalcitonin results in antibiotic decision-making in coronavirus disease 2019 (COVID-19)

Valeria Fabre [1, 2], Sara Karaba [1], Joe Amoah [3], Matthew Robinson [1], George Jones [1], Kathryn Dzintars [2], Morgan Katz [1], B Mark Landrum [4], Sarojini Qasba [5], Pooja Gupta [6], Eili Klein [7, 8], Sara E Cosgrove [1, 2]
Affiliations Expand

Affiliations

- [1] Division of Infectious Diseases, Department of Medicine, Johns Hopkins University School of Medicine, Baltimore, Maryland.
- [2] Department of Antimicrobial Stewardship, The Johns Hopkins Hospital, Baltimore, Maryland.
- [3] Division of Infectious Diseases, Department of Pediatrics, Johns Hopkins University School of Medicine, Baltimore, Maryland.
- [4] Howard County General Hospital, Columbia, Maryland.
- [5] Suburban Hospital, Bethesda, Maryland.
- [6] Sibley Memorial Hospital, Washington, DC.
- [7] Department of Emergency Medicine, Johns Hopkins University School of Medicine, Baltimore, Maryland.
- [8] Center for Disease Dynamics, Economics & Policy, Washington, DC.

- PMID: **33866995**
- PMCID: PMC8485015
- DOI: 10.1017/ice.2021.175

Free PMC article

The role of procalcitonin results in antibiotic decision-making in coronavirus disease 2019 (COVID-19)

Valeria Fabre et al. Infect Control Hosp Epidemiol. 2021.
Free PMC article
Show details
Infect Control Hosp Epidemiol
. 2021 Apr 19;1-6.
doi: 10.1017/ice.2021.175. Online ahead of print.

Authors

Valeria Fabre[1 2], Sara Karaba[1], Joe Amoah[3], Matthew Robinson[1], George Jones[1], Kathryn Dzintars[2], Morgan Katz[1], B Mark Landrum[4], Sarojini Qasba[5], Pooja Gupta[6], Eili Klein[7 8], Sara E Cosgrove[1 2]

Affiliations

- [1] Division of Infectious Diseases, Department of Medicine, Johns Hopkins University School of Medicine, Baltimore, Maryland.
- [2] Department of Antimicrobial Stewardship, The Johns Hopkins Hospital, Baltimore, Maryland.
- [3] Division of Infectious Diseases, Department of Pediatrics, Johns Hopkins University School of Medicine, Baltimore, Maryland.
- [4] Howard County General Hospital, Columbia, Maryland.
- [5] Suburban Hospital, Bethesda, Maryland.
- [6] Sibley Memorial Hospital, Washington, DC.
- [7] Department of Emergency Medicine, Johns Hopkins University School of Medicine, Baltimore, Maryland.
- [8] Center for Disease Dynamics, Economics & Policy, Washington, DC.

- PMID: **33866995**
- PMCID: PMC8485015
- DOI: 10.1017/ice.2021.175

Abstract

Objective: To evaluate the role of procalcitonin (PCT) results in antibiotic decisions for COVID-19 patients at hospital presentation.

Design, setting, and participants: Multicenter retrospective observational study of patients ≥18 years hospitalized due to COVID-19 at the Johns Hopkins Health system. Patients who were transferred from another facility with >24 hours stay and patients who died within 48 hours of hospitalization were excluded.

Methods: Elevated PCT values were determined based on each hospital's definition. Antibiotic therapy and PCT results were evaluated for patients with no evidence of bacterial community-acquired pneumonia (bCAP) and patients with confirmed, probable, or possible bCAP. The added value of PCT testing to clinical criteria in detecting bCAP was evaluated using receiving operating curve characteristics (ROC).

Results: Of 962 patients, 611 (64%) received a PCT test. ROC curves for clinical criteria and clinical criteria plus PCT test were similar (at 0.5 ng/mL and 0.25 ng/mL). By bCAP group, median initial PCT values were 0.58 ng/mL (interquartile range [IQR], 0.24-1.14), 0.23 ng/mL (IQR, 0.1-0.63), and 0.15 ng/mL (IQR, 0.09-0.35) for proven/probable, possible, and no bCAP groups, respectively. Among patients without bCAP, an elevated PCT level was associated with 1.8 additional days of CAP therapy (95% CI, 1.01-2.75; $P < .01$) compared to patients with a negative PCT result after adjusting for potential confounders. Duration of CAP therapy was similar between patients without a PCT test ordered and a low PCT level for no bCAP and possible bCAP groups.

Conclusions: PCT results may be abnormal in COVID-19 patients without bCAP and may result in receipt of unnecessary antibiotics.

- 16 references
- 1 figure

Full text links

Cambridge University Press Free PMC article

Proceed to details

Cite

Share

☐ 1,262

J Nippon Med Sch

. 2021 Sep 14.
doi: 10.1272/jnms.JNMS.2022_89-210. Online ahead of print.

Machine Learning Prediction for Supplemental Oxygen Requirement in Patients with COVID-19

Yutaka Igarashi[1], Kan Nishimura[2], Kei Ogawa[2], Nodoka Miyake[1], Taiki Mizobuchi[1], Kenta Shigeta[1], Hirofumi Obinata[1,3], Yasuhiro Takayama[1,4], Takashi Tagami[1,5], Masahiro Seike[6], Hayato Ohwada[2], Shoji Yokobori[1]

Affiliations Expand

Affiliations

- [1] Department of Emergency and Critical Care Medicine, Nippon Medical School.
- [2] Department of Industrial Administration, Tokyo University of Science.
- [3] Department of Anesthesiology, Self-Defense Forces Central Hospital.

- [4] Emergency Department, Flowers and Forest Tokyo Hospital.
- [5] Department of Emergency and Critical Care Medicine, Nippon Medical School Musashi Kosugi Hospital.
- [6] Department of Pulmonary Medicine and Oncology, Nippon Medical School.

- PMID: **34526457**
- DOI: 10.1272/jnms.JNMS.2022_89-210

Free article

Machine Learning Prediction for Supplemental Oxygen Requirement in Patients with COVID-19

Yutaka Igarashi et al. J Nippon Med Sch. 2021.
Free article

Show details

J Nippon Med Sch
. 2021 Sep 14.
doi: 10.1272/jnms.JNMS.2022_89-210. Online ahead of print.

Authors

Yutaka Igarashi [1], Kan Nishimura [2], Kei Ogawa [2], Nodoka Miyake [1], Taiki Mizobuchi [1], Kenta Shigeta [1], Hirofumi Obinata [1 3], Yasuhiro Takayama [1 4], Takashi Tagami [1 5], Masahiro Seike [6], Hayato Ohwada [2], Shoji Yokobori [1]

Affiliations

- [1] Department of Emergency and Critical Care Medicine, Nippon Medical School.
- [2] Department of Industrial Administration, Tokyo University of Science.
- [3] Department of Anesthesiology, Self-Defense Forces Central Hospital.
- [4] Emergency Department, Flowers and Forest Tokyo Hospital.
- [5] Department of Emergency and Critical Care Medicine, Nippon Medical School Musashi Kosugi Hospital.
- [6] Department of Pulmonary Medicine and Oncology, Nippon Medical School.

- PMID: **34526457**
- DOI: 10.1272/jnms.JNMS.2022_89-210

Abstract

Background: The coronavirus disease (COVID-19) poses an urgent threat to global public health and is characterized by rapid disease progression even in mild cases. In this study, we investigated whether machine learning can be used to predict which patients will have a deteriorated condition and require oxygenation in asymptomatic or mild cases of COVID-19.

Methods: This single-center, retrospective, observational study included COVID-19 patients admitted to the hospital from February 1, 2020, to May 31, 2020, and who were either asymptomatic or presented with mild symptoms and did not require oxygen support on admission. Data on patient characteristics and vital signs were collected upon admission. We used seven machine learning algorithms, assessed their capability to predict exacerbation, and analyzed important influencing features using the best algorithm.

Results: In total, 210 patients were included in the study. Among them, 43 (19%) required oxygen therapy. Of all the models, the logistic regression model had the highest accuracy and precision. Logistic regression analysis showed that the model had an accuracy of 0.900, precision of 0.893, and recall of 0.605. The most important parameter for predictive capability was SpO_2, followed by age, respiratory rate, and systolic blood pressure.

Conclusion: In this study, we developed a machine learning model that can be used as a triage tool by clinicians to detect high-risk patients and disease progression earlier. Prospective validation studies are needed to verify the application of the tool in clinical practice.

Keywords: COVID-19; Oxygen Inhalation Therapy; SARS-CoV-2; machine learning; pneumonia.

Full text links

J-STAGE, Japan Science and Technology Information Aggregator, Electronic
Proceed to details
Cite
Share
1,263

EClinicalMedicine
. 2021 Jul;37:100936.
doi: 10.1016/j.eclinm.2021.100936. Epub 2021 Jun 4.

The influence of selection bias on identifying an association between allergy medication use and SARS-CoV-2 infection

Lindsay A Thompson [1, 2], Matthew J Gurka [1, 2], Stephanie L Filipp [2], Desmond A Schatz [1], Rebeccah E Mercado [1], David A Ostrov [3], Mark A Atkinson [1, 3], Sonja A Rasmussen [1, 4, 5]
Affiliations Expand

Affiliations

- [1] Department of Pediatrics, University of Florida College of Medicine, Gainesville, FL USA.
- [2] Department of Health Outcomes and Biomedical Informatics, University of Florida College of Medicine, Gainesville, Florida USA.
- [3] Department of Pathology, University of Florida College of Medicine, Gainesville, Florida USA.

- [4] Department of Epidemiology, University of Florida College of Public Health and Health Professions and College of Medicine, Gainesville, Florida USA.
- [5] Department of Obstetrics and Gynecology, University of Florida College of Medicine, Gainesville, Florida USA.

- PMID: **34104879**
- PMCID: PMC8175126
- DOI: 10.1016/j.eclinm.2021.100936

Free PMC article

The influence of selection bias on identifying an association between allergy medication use and SARS-CoV-2 infection

Lindsay A Thompson et al. EClinicalMedicine. 2021 Jul.
Free PMC article
Show details
EClinicalMedicine
. 2021 Jul;37:100936.
doi: 10.1016/j.eclinm.2021.100936. Epub 2021 Jun 4.

Authors

Lindsay A Thompson [1] [2], Matthew J Gurka [1] [2], Stephanie L Filipp [2], Desmond A Schatz [1], Rebeccah E Mercado [1], David A Ostrov [3], Mark A Atkinson [1] [3], Sonja A Rasmussen [1] [4] [5]

Affiliations

- [1] Department of Pediatrics, University of Florida College of Medicine, Gainesville, FL USA.
- [2] Department of Health Outcomes and Biomedical Informatics, University of Florida College of Medicine, Gainesville, Florida USA.
- [3] Department of Pathology, University of Florida College of Medicine, Gainesville, Florida USA.
- [4] Department of Epidemiology, University of Florida College of Public Health and Health Professions and College of Medicine, Gainesville, Florida USA.
- [5] Department of Obstetrics and Gynecology, University of Florida College of Medicine, Gainesville, Florida USA.

- PMID: **34104879**
- PMCID: PMC8175126
- DOI: 10.1016/j.eclinm.2021.100936

Abstract

Background: Medications to prevent and treat SARS-CoV-2 infection are needed to complement emerging vaccinations. Recent in vitro and electronic health record (EHR) studies suggested that certain allergy medications could prevent SARS-CoV-2 infection. We sought to carefully examine the potential selection bias associated with utilizing EHRs in these settings.

Methods: We analyzed associations of three allergy medications (cetirizine, diphenhydramine or hydroxyzine) with testing negative for SARS-CoV-2, measuring the potential effect of selection bias on these associations. We used a retrospective cohort of EHR data from 230,376 patients (18 years+) who visited outpatient clinicians in a single, large academic center at least once but were never hospitalized (10/1/2019-6/1/2020). Main exposures included EHR documentation of three allergy medications and allergy, with an intermediate outcome of receipt of a SARS-CoV-2 test, and the primary outcome as testing negative.

Findings: SARS-CoV-2 testing rates varied by sex, age, race/ethnicity and insurance. Increasing age and public insurance were associated with a higher adjusted odds of test negativity, while being Black or Hispanic was significantly associated with test positivity. Allergy diagnosis and use of any of three allergy medications were each associated with a higher likelihood of receiving a test (e.g. diphenhydramine - Odds Ratio (OR) 2.99, 95% Confidence Interval (CI) 2.73, 3.28; cetirizine 1.75 (95% CI 1.60, 1.92)). Among those tested, only use of diphenhydramine was associated with a negative SARS-CoV-2 test (adjusted OR = 2.23, 95% CI 1.10, 4.55). However, analyses revealed that selection bias may be responsible for the apparent protective effect of diphenhydramine.

Interpretation: Diphenhydramine use was associated with more SARS-CoV-2 testing and subsequent higher odds for negative tests. While EHR-based observational studies can inform a need for interventional trials, this study revealed limitations of EHR data. The finding that diphenhydramine documentation conferred a higher odds of testing negative for SARS-CoV-2 must be interpreted with caution due to probable selection bias.*Abbreviations*: SARS-CoV-2, ACE2, COVID-19, EHR.

Keywords: COVID-19; Observational study; SARS-CoV-2; Selection bias.

© 2021 The Author(s).

Conflict of interest statement

The following authors have interests not directly related to this study but worth noting: Dr. Thompson is an editor for the patient pages of JAMA Pediatrics and as such receives an annual stipend. Dr. Ostrov has two patents: T18131 for methods to prevent and treat COVID-19 and T18371 for Diphenhydramine and Lactoferrin for prevention and treatment of COVID-19. Dr. Rasmussen has served on advisory committees for the Teva Pregnancy Registry and Solriamfetol Pregnancy Registry and has consulted for F. Hoffmann-La Roche AG as a litigation expert. All other authors have nothing to declare.

- 32 references
- 1 figure

Full text links

ELSEVIER OPEN ACCESS Elsevier Science Free PMC article

Proceed to details

Cite

Share

☐ 1,264

Med Clin (Barc)
. 2021 May 6;S0025-7753(21)00210-4.
doi: 10.1016/j.medcli.2021.03.005. Online ahead of print.

Effect of tocilizumab versus standard of care in adults hospitalized with moderate-severe COVID-19 pneumonia

[Article in English, Spanish]

Ignacio Cardona-Pascual [1], David Berlana [2], Ferran Martinez-Valle [3], David Campany-Herrero [1], José Bruno Montoro-Ronsano [1]

Affiliations Expand

Affiliations

- [1] Pharmacy Department, Vall Hebron Barcelona Campus Hospital, Barcelona, Spain.
- [2] Pharmacy Department, Vall Hebron Barcelona Campus Hospital, Barcelona, Spain. Electronic address: dberlana@vhebron.net.
- [3] Internal Medicine Department, Vall Hebron Barcelona Campus Hospital, Barcelona, Spain.

- PMID: **34147248**
- PMCID: PMC8101781
- DOI: 10.1016/j.medcli.2021.03.005

Free PMC article

Effect of tocilizumab versus standard of care in adults hospitalized with moderate-severe COVID-19 pneumonia

[Article in English, Spanish]
Ignacio Cardona-Pascual et al. Med Clin (Barc). 2021.
Free PMC article

Show details

Med Clin (Barc)
. 2021 May 6;S0025-7753(21)00210-4.
doi: 10.1016/j.medcli.2021.03.005. Online ahead of print.

Authors

Ignacio Cardona-Pascual [1], David Berlana [2], Ferran Martinez-Valle [3], David Campany-Herrero [1], José Bruno Montoro-Ronsano [1]

Affiliations

- [1] Pharmacy Department, Vall Hebron Barcelona Campus Hospital, Barcelona, Spain.
- [2] Pharmacy Department, Vall Hebron Barcelona Campus Hospital, Barcelona, Spain. Electronic address: dberlana@vhebron.net.
- [3] Internal Medicine Department, Vall Hebron Barcelona Campus Hospital, Barcelona, Spain.

- PMID: **34147248**
- PMCID: PMC8101781
- DOI: 10.1016/j.medcli.2021.03.005

Abstract
in English, Spanish

Introduction and objectives: Tocilizumab is an interleukin-6 receptor-blocking agent proposed for the treatment of severe COVID-19; however, limited data are available on their efficacy. The aim of this study was to assess the effect of tocilizumab on the outcomes of patients with COVID-19 pneumonia by using propensity-score-matching (PSM) analysis.

Methods: A retrospective observational analysis of hospitalized COVID-19 adult patients admitted to the Vall d'Hebron Hospital was performed between March and April 2020. We used the logistic regression to analyze the effect of tocilizumab on mortality, as main outcome, and PSM analysis to further validate their effect. Secondary outcomes were length-of-stay (LOS) and intensive-care-unit (ICU) stay. Same outcomes were also assessed for early tocilizumab administration, within 72h after admission. Patients were selected by matching their individual propensity for receiving therapy with tocilizumab, conditional on their demographic and clinical variables.

Results: A total of 544 COVID-19 patients were included, 197 (36.2%) were treated with tocilizumab of whom 147 were treated within the first 72h after admission; and 347 were included in the control group. After PSM analyses, the results showed no association between tocilizumab use and overall mortality (OR=1.03, 95%CI: 0.63-1.68). However, shorter ICU-stay in the tocilizumab group was found compared to the control group (Coefficient -4.27 95%CI: -6.63 to -1.92). Similar results were found in the early tocilizumab cohort.

Conclusions: The administration of tocilizumab in patients with moderate to severe COVID-19 did not reduce the risk of mortality in our cohort of patients, regardless of the time of administration.

Introducción y objetivos: El tocilizumab es un agente bloqueador del receptor de la interleucina 6 propuesto para el tratamiento de la COVID-19 grave; sin embargo, se dispone de datos limitados sobre su eficacia. El objetivo de este estudio fue evaluar el efecto de tocilizumab en los resultados de los pacientes con neumonía por COVID-19 mediante un análisis de emparejamiento por *propensity-score-matching* (PSM, «puntuación de propensión»).

Métodos: Se realizó un análisis observacional retrospectivo de los pacientes adultos con COVID-19 ingresados en el Hospital Vall d'Hebron entre marzo y abril de 2020. Se utilizó la regresión

logística para analizar el efecto de tocilizumab en la mortalidad, como resultado principal, y el análisis PSM para validar aún más su efecto. Los resultados secundarios fueron la duración de la estancia y la estancia en la unidad de cuidados intensivos (UCI). También se evaluaron los mismos resultados para la administración temprana de tocilizumab, dentro de las 72 h posteriores al ingreso. Los pacientes se seleccionaron mediante el emparejamiento de su propensión individual a recibir tratamiento con tocilizumab, condicionado a sus variables demográficas y clínicas.

Resultados: Se incluyeron 544 pacientes de COVID-19, 197 (36,2%) fueron tratados con tocilizumab, de los cuales 147 fueron tratados dentro de las primeras 72 h tras el ingreso; y 347 fueron incluidos en el grupo control. Tras los análisis PSM, los resultados no mostraron ninguna asociación entre el uso de tocilizumab y la mortalidad global (OR = 1,03; IC del 95%: 0,63-1,68). Sin embargo, se encontró una menor estancia en la UCI en el grupo de tocilizumab en comparación con el grupo de control (coeficiente −4,27; IC del 95%: −6,63 − −1,92). Se encontraron resultados similares en la cohorte de tocilizumab temprano.

Conclusiones: La administración de tocilizumab en pacientes con COVID-19 moderada a grave no redujo el riesgo de mortalidad en nuestra cohorte de pacientes, independientemente del momento de la administración.

Keywords: COVID-19; Estudio observacional; Mortalidad; Mortality; Neumonía; Observational study; Pneumonia; Propensity-score-matched; Tocilizumab.

Copyright © 2021 Elsevier España, S.L.U. All rights reserved.

- 20 references
- 1 figure

Full text links

Ediciones Doyma, S.L. Free PMC article
Proceed to details

Cite

Share

☐ 1,265

Observational Study

BMC Infect Dis
. 2021 Jan 19;21(1):84.
doi: 10.1186/s12879-021-05771-y.

Critically ill patients with diabetes and Middle East respiratory syndrome: a multi-center observational study

Jesna Jose[1 2], Hasan M Al-Dorzi[3], Awad Al-Omari[4], Yasser Mandourah[5], Fahad Al-Hameed[6], Musharaf Sadat[3], Eman Al Qasim[3], Basem Alraddadi[7 8], Abdulrahman Al Harthy[9], Ghaleb A Al Mekhlafi[10], Abdullah Almotairi[11], Kasim Al Khatib[12], Ahmed Abdulmomen

[13], Ismael Qushmaq [14], Anees A Sindi [15], Ahmed Mady [9] [16], Othman Solaiman [17], Rajaa Al-Raddadi [18], Khalid Maghrabi [17], Ahmed Ragab [19], Ayman Kharaba [20], Sarah Shalhoub [21] [22], Abdulsalam M Al-Aithan [23], Gajendra K Vishwakarma [2], Atanu Bhattacharjee [24], Yaseen M Arabi [25], Saudi Critical Care Trials Group

Affiliations Expand

Affiliations

- [1] Department of Biostatistics and Bioinformatics, King Abdullah International Medical Research Center, Riyadh, Saudi Arabia.
- [2] Department of Mathematics & Computing, Indian Institute of Technology (ISM), Dhanbad, Jharkhand, 826004, India.
- [3] Intensive Care Department, Ministry of National Guard Health Affairs, King Abdullah International Medical Research Center and King Saud Bin Abdulaziz University for Health Sciences, Riyadh, Saudi Arabia.
- [4] Department of Intensive Care, College of Medicine, Alfaisal University, Dr Sulaiman Al-Habib Group Hospitals, Riyadh, Saudi Arabia.
- [5] Military Medical Services, Ministry of Defense, Prince Sultan Military Medical City, Riyadh, Saudi Arabia.
- [6] Department of Intensive Care, College of Medicine, King Saud bin Abdulaziz University for Health Sciences, King Abdullah International Medical Research Center, King Abdulaziz Medical City, Jeddah, Saudi Arabia.
- [7] Department of Medicine, King Faisal Specialist Hospital and Research Center, Jeddah, Saudi Arabia.
- [8] Department of Medicine, University of Jeddah, Jeddah, Saudi Arabia.
- [9] Intensive Care Department, King Saud Medical City, Riyadh, Saudi Arabia.
- [10] Department of Intensive Care Services, Prince Sultan Military Medical City, Riyadh, Saudi Arabia.
- [11] Department of Critical Care Medicine, King Fahad Medical City, Riyadh, Saudi Arabia.
- [12] Intensive Care Department, Al-Noor Specialist Hospital, Makkah, Saudi Arabia.
- [13] Department of Critical Care Medicine, King Saud University, Riyadh, Saudi Arabia.
- [14] Section of Critical Care Medicine, Department of Medicine, King Faisal Specialist Hospital and Research Center, Jeddah, Saudi Arabia.
- [15] Department of Anesthesia and Critical Care, Faculty of Medicine, King Abdulaziz University, Jeddah, Saudi Arabia.
- [16] Tanta University Hospitals, Tanta, Egypt.
- [17] Intensive Care Department, King Faisal Specialist Hospital and Research Center, Riyadh, Saudi Arabia.
- [18] Department of Community Medicine, Faculty of Medicine, King Abdulaziz University, Jeddah, Saudi Arabia.
- [19] Intensive Care Department, King Fahd Hospital, Jeddah, Saudi Arabia.
- [20] Department of Critical Care, King Fahad Hospital, Ohoud Hospital, Al-Madinah, Saudi Arabia.
- [21] Department of Medicine, Division of Infectious Diseases, University of Western Ontario, London, Canada.
- [22] King Fahad Armed Forces Hospital, Jeddah, Saudi Arabia.
- [23] Department of Medicine, Critical Care Division, King Abdulaziz Hospital, Al Ahsa, Saudi Arabia.

- [24] Homi Bhaba National Institute, Section of Biostatistics, Centre for Cancer Epidemiology, Tata Memorial Centre, Navi Mumbai, India.
- [25] Intensive Care Department, Ministry of National Guard Health Affairs, King Abdullah International Medical Research Center and King Saud Bin Abdulaziz University for Health Sciences, Riyadh, Saudi Arabia. arabi@ngha.med.sa.

- PMID: **33468070**
- PMCID: PMC7814976
- DOI: 10.1186/s12879-021-05771-y

Free PMC article
Observational Study

Critically ill patients with diabetes and Middle East respiratory syndrome: a multi-center observational study

Jesna Jose et al. BMC Infect Dis. 2021.
Free PMC article
Show details
BMC Infect Dis
. 2021 Jan 19;21(1):84.
doi: 10.1186/s12879-021-05771-y.

Authors

Jesna Jose [1, 2], Hasan M Al-Dorzi [3], Awad Al-Omari [4], Yasser Mandourah [5], Fahad Al-Hameed [6], Musharaf Sadat [3], Eman Al Qasim [3], Basem Alraddadi [7, 8], Abdulrahman Al Harthy [9], Ghaleb A Al Mekhlafi [10], Abdullah Almotairi [11], Kasim Al Khatib [12], Ahmed Abdulmomen [13], Ismael Qushmaq [14], Anees A Sindi [15], Ahmed Mady [9, 16], Othman Solaiman [17], Rajaa Al-Raddadi [18], Khalid Maghrabi [17], Ahmed Ragab [19], Ayman Kharaba [20], Sarah Shalhoub [21, 22], Abdulsalam M Al-Aithan [23], Gajendra K Vishwakarma [2], Atanu Bhattacharjee [24], Yaseen M Arabi [25], Saudi Critical Care Trials Group

Affiliations

- [1] Department of Biostatistics and Bioinformatics, King Abdullah International Medical Research Center, Riyadh, Saudi Arabia.
- [2] Department of Mathematics & Computing, Indian Institute of Technology (ISM), Dhanbad, Jharkhand, 826004, India.
- [3] Intensive Care Department, Ministry of National Guard Health Affairs, King Abdullah International Medical Research Center and King Saud Bin Abdulaziz University for Health Sciences, Riyadh, Saudi Arabia.
- [4] Department of Intensive Care, College of Medicine, Alfaisal University, Dr Sulaiman Al-Habib Group Hospitals, Riyadh, Saudi Arabia.
- [5] Military Medical Services, Ministry of Defense, Prince Sultan Military Medical City, Riyadh, Saudi Arabia.

- [6] Department of Intensive Care, College of Medicine, King Saud bin Abdulaziz University for Health Sciences, King Abdullah International Medical Research Center, King Abdulaziz Medical City, Jeddah, Saudi Arabia.
- [7] Department of Medicine, King Faisal Specialist Hospital and Research Center, Jeddah, Saudi Arabia.
- [8] Department of Medicine, University of Jeddah, Jeddah, Saudi Arabia.
- [9] Intensive Care Department, King Saud Medical City, Riyadh, Saudi Arabia.
- [10] Department of Intensive Care Services, Prince Sultan Military Medical City, Riyadh, Saudi Arabia.
- [11] Department of Critical Care Medicine, King Fahad Medical City, Riyadh, Saudi Arabia.
- [12] Intensive Care Department, Al-Noor Specialist Hospital, Makkah, Saudi Arabia.
- [13] Department of Critical Care Medicine, King Saud University, Riyadh, Saudi Arabia.
- [14] Section of Critical Care Medicine, Department of Medicine, King Faisal Specialist Hospital and Research Center, Jeddah, Saudi Arabia.
- [15] Department of Anesthesia and Critical Care, Faculty of Medicine, King Abdulaziz University, Jeddah, Saudi Arabia.
- [16] Tanta University Hospitals, Tanta, Egypt.
- [17] Intensive Care Department, King Faisal Specialist Hospital and Research Center, Riyadh, Saudi Arabia.
- [18] Department of Community Medicine, Faculty of Medicine, King Abdulaziz University, Jeddah, Saudi Arabia.
- [19] Intensive Care Department, King Fahd Hospital, Jeddah, Saudi Arabia.
- [20] Department of Critical Care, King Fahad Hospital, Ohoud Hospital, Al-Madinah, Saudi Arabia.
- [21] Department of Medicine, Division of Infectious Diseases, University of Western Ontario, London, Canada.
- [22] King Fahad Armed Forces Hospital, Jeddah, Saudi Arabia.
- [23] Department of Medicine, Critical Care Division, King Abdulaziz Hospital, Al Ahsa, Saudi Arabia.
- [24] Homi Bhaba National Institute, Section of Biostatistics, Centre for Cancer Epidemiology, Tata Memorial Centre, Navi Mumbai, India.
- [25] Intensive Care Department, Ministry of National Guard Health Affairs, King Abdullah International Medical Research Center and King Saud Bin Abdulaziz University for Health Sciences, Riyadh, Saudi Arabia. arabi@ngha.med.sa.

- PMID: **33468070**
- PMCID: PMC7814976
- DOI: 10.1186/s12879-021-05771-y

Abstract

Background: Diabetes is a risk factor for infection with coronaviruses. This study describes the demographic, clinical data, and outcomes of critically ill patients with diabetes and Middle East Respiratory Syndrome (MERS).

Methods: This retrospective cohort study was conducted at 14 hospitals in Saudi Arabia (September 2012-January 2018). We compared the demographic characteristics, underlying medical conditions, presenting symptoms and signs, management and clinical course, and outcomes of critically ill patients with MERS who had diabetes compared to those with no

diabetes. Multivariable logistic regression analysis was performed to determine if diabetes was an independent predictor of 90-day mortality.

Results: Of the 350 critically ill patients with MERS, 171 (48.9%) had diabetes. Patients with diabetes were more likely to be older, and have comorbid conditions, compared to patients with no diabetes. They were more likely to present with respiratory failure requiring intubation, vasopressors, and corticosteroids. The median time to clearance of MERS-CoV RNA was similar (23 days (Q1, Q3: 17, 36) in patients with diabetes and 21.0 days (Q1, Q3: 10, 33) in patients with no diabetes). Mortality at 90 days was higher in patients with diabetes (78.9% versus 54.7%, $p < 0.0001$). Multivariable regression analysis showed that diabetes was an independent risk factor for 90-day mortality (odds ratio, 2.09; 95% confidence interval, 1.18-3.72).

Conclusions: Half of the critically ill patients with MERS have diabetes; which is associated with more severe disease. Diabetes is an independent predictor of mortality among critically patients with MERS.

Keywords: Acute respiratory distress syndrome; Coronavirus; Diabetes; Middle East respiratory syndrome.

Conflict of interest statement

Yaseen Arabi provided nonpaid consultations on therapeutics for MERS for Gilead Sciences and SAB Biotherapeutics and he is a Board Member of the International Severe Acute Respiratory and Emerging Infection Consortium (ISARIC). He is the Lead-Co Chair of the Think-20 Saudi Arabia (T20) Taskforce for COVID-19. Other authors declared that they have no competing interests.

- 38 references
- 1 figure

Supplementary info

Publication types, MeSH terms, Substances Expand

Publication types

- Multicenter Study
- Observational Study

MeSH terms

- Adrenal Cortex Hormones
- Adult
- Age Factors
- Aged
- Bronchoalveolar Lavage Fluid / virology
- Cohort Studies
- Comorbidity
- Coronavirus Infections / complications*

- Coronavirus Infections / epidemiology
- Coronavirus Infections / mortality
- Critical Illness
- Diabetes Complications / epidemiology*
- Diabetes Mellitus / epidemiology*
- Female
- Humans
- Male
- Middle Aged
- Middle East Respiratory Syndrome Coronavirus / genetics
- Middle East Respiratory Syndrome Coronavirus / isolation & purification
- Nasopharynx / virology
- Respiratory Insufficiency / etiology
- Respiratory Insufficiency / mortality
- Retrospective Studies
- Risk Factors
- Saudi Arabia / epidemiology
- Sputum / virology
- Trachea / virology

Substances

- Adrenal Cortex Hormones

Full text links

Read free full text at BMC BioMed Central Free PMC article
Proceed to details
Cite
Share
☐ 1,266
Front Med (Lausanne)
. 2022 Feb 23;9:846525.
doi: 10.3389/fmed.2022.846525. eCollection 2022.

Machine-Learning Approaches for Predicting the Need of Oxygen Therapy in Early-Stage COVID-19 in Japan: Multicenter Retrospective Observational Study

Syunsuke Yamanaka [1], Koji Morikawa [2], Hiroyuki Azuma [3], Maki Yamanaka [4], Yoshimitsu Shimada [5], Toru Wada [6], Hideyuki Matano [7], Naoki Yamada [1], Osamu Yamamura [8], Hiroyuki Hayashi [1]
Affiliations Expand

Affiliations

- [1] Department of Emergency Medicine and General Internal Medicine, University of Fukui Hospital, Fukui, Japan.
- [2] Connect Inc., Tokyo, Japan.
- [3] Department of Emergency Medicine, Fukui Prefectural Hospital, Fukui, Japan.
- [4] Department of Emergency Medicine, Tannan Regional Medical Center, Sabae, Japan.
- [5] Department of Emergency Medicine, Japanese Red Cross Fukui Hospital, Fukui, Japan.
- [6] Department of Emergency Medicine, Sugita Genpaku Memorial Obama Municipal Hospital, Obama, Japan.
- [7] Department of Emergency Medicine, Fukui-ken Saiseikai Hospital, Fukui, Japan.
- [8] Department of Community Medicine, Faculty of Medicine, University of Fukui Hospital, Fukui, Japan.

- PMID: **35280897**
- PMCID: PMC8904892
- DOI: 10.3389/fmed.2022.846525

Free PMC article

Machine-Learning Approaches for Predicting the Need of Oxygen Therapy in Early-Stage COVID-19 in Japan: Multicenter Retrospective Observational Study

Syunsuke Yamanaka et al. Front Med (Lausanne). 2022.
Free PMC article
Show details
Front Med (Lausanne)
. 2022 Feb 23;9:846525.
doi: 10.3389/fmed.2022.846525. eCollection 2022.

Authors

Syunsuke Yamanaka [1], Koji Morikawa [2], Hiroyuki Azuma [3], Maki Yamanaka [4], Yoshimitsu Shimada [5], Toru Wada [6], Hideyuki Matano [7], Naoki Yamada [1], Osamu Yamamura [8], Hiroyuki Hayashi [1]

Affiliations

- [1] Department of Emergency Medicine and General Internal Medicine, University of Fukui Hospital, Fukui, Japan.
- [2] Connect Inc., Tokyo, Japan.
- [3] Department of Emergency Medicine, Fukui Prefectural Hospital, Fukui, Japan.
- [4] Department of Emergency Medicine, Tannan Regional Medical Center, Sabae, Japan.
- [5] Department of Emergency Medicine, Japanese Red Cross Fukui Hospital, Fukui, Japan.
- [6] Department of Emergency Medicine, Sugita Genpaku Memorial Obama Municipal Hospital, Obama, Japan.
- [7] Department of Emergency Medicine, Fukui-ken Saiseikai Hospital, Fukui, Japan.
- [8] Department of Community Medicine, Faculty of Medicine, University of Fukui Hospital, Fukui, Japan.

- PMID: **35280897**
- PMCID: PMC8904892
- DOI: 10.3389/fmed.2022.846525

Abstract

Background: Early prediction of oxygen therapy in patients with coronavirus disease 2019 (COVID-19) is vital for triage. Several machine-learning prognostic models for COVID-19 are currently available. However, external validation of these models has rarely been performed. Therefore, most reported predictive performance is optimistic and has a high risk of bias. This study aimed to develop and validate a model that predicts oxygen therapy needs in the early stages of COVID-19 using a sizable multicenter dataset.

Methods: This multicenter retrospective study included consecutive COVID-19 hospitalized patients confirmed by a reverse transcription chain reaction in 11 medical institutions in Fukui, Japan. We developed and validated seven machine-learning models (e.g., penalized logistic regression model) using routinely collected data (e.g., demographics, simple blood test). The primary outcome was the need for oxygen therapy (≥1 L/min or $SpO_2 \leq 94\%$) during hospitalization. C-statistics, calibration slope, and association measures (e.g., sensitivity) evaluated the performance of the model using the test set (randomly selected 20% of data for internal validation). Among these seven models, the machine-learning model that showed the best performance was re-evaluated using an external dataset. We compared the model performances using the A-DROP criteria (modified version of CURB-65) as a conventional method.

Results: Of the 396 patients with COVID-19 for the model development, 102 patients (26%) required oxygen therapy during hospitalization. For internal validation, machine-learning models, except for the *k*-point nearest neighbor, had a higher discrimination ability than the A-DORP criteria ($P < 0.01$). The XGboost had the highest c-statistic in the internal validation (0.92 vs. 0.69 in A-DROP criteria; $P < 0.001$). For the external validation with 728 temporal independent datasets (106 patients [15%] required oxygen therapy), the XG boost model had a higher c-statistic (0.88 vs. 0.69 in A-DROP criteria; $P < 0.001$).

Conclusions: Machine-learning models demonstrated a more significant performance in predicting the need for oxygen therapy in the early stages of COVID-19.

Keywords: COVID-19; PROBAST; TRIPOD; machine learning; medical triage; multicenter; prognostic model.

Copyright © 2022 Yamanaka, Morikawa, Azuma, Yamanaka, Shimada, Wada, Matano, Yamada, Yamamura and Hayashi.

Conflict of interest statement

KM was employed by Connect Inc. The remaining authors declare that the research was conducted in the absence of any commercial or financial relationships that could be construed as a potential conflict of interest.

- 33 references
- 2 figures

Full text links

Frontiers Media SA Free PMC article
Proceed to details
Cite
Share
1,267
Future Sci OA
. 2021 Jun 15;7(8):FSO739.
doi: 10.2144/fsoa-2021-0064. eCollection 2021 Sep.

An observational study of a cohort of citizens receiving the AZD1222 vaccine against SARS-CoV-2

Pellegrino Cerino [1], Annachiara Coppola [1 2], Biancamaria Pierri [1 3], Palmiero Volzone [1], Dario Bruzzese [4], Andrea Pierri [1], Amedeo Ferro [1], Daniela Schiavi [1], Antonio Coppola [5], Stefano Miniero [5], Luigi Atripaldi [6], Caterina Pirozzi [6], Pasquale Rusciano [6], Alessandra Macri [6], Rita Boenzi [6], Silvia Sale [6], Gianfranco Brambilla [7], Carlo Buonerba [1]
Affiliations Expand

Affiliations

- [1] Centro di Referenza Nazionale per l'Analisi e Studio di Correlazione tra Ambiente, Animale e Uomo, Istituto Zooprofilattico Sperimentale del Mezzogiorno, Portici, 80055, Italy.
- [2] Dipartimento di Medicina Sperimentale, Università degli studi della Campania 'L Vanvitelli', Naples, 80138, Italy.
- [3] Department of Medicine, Surgery & Dentistry (Scuola Medica Salernitana), University of Salerno, Baronissi, 84081, Italy.
- [4] Department of Public Health, University of Naples 'Federico II', Naples, 80131, Italy.
- [5] ASL Napoli 3 SUD, Torre del Greco, 80059, Italy.
- [6] Cotugno Hospital, AORN Ospedali dei Colli, Naples, 80131, Italy.
- [7] Food Safety, Nutrition & Veterinary Public Health Department, Istituto Superiore di Sanità, Rome, 00161, Italy.

- PMID: **34290884**
- PMCID: PMC8216649
- DOI: 10.2144/fsoa-2021-0064

Free PMC article

An observational study of a cohort of citizens receiving the AZD1222 vaccine against SARS-CoV-2

Pellegrino Cerino et al. Future Sci OA. 2021.
Free PMC article
Show details
Future Sci OA
. 2021 Jun 15;7(8):FSO739.
doi: 10.2144/fsoa-2021-0064. eCollection 2021 Sep.

Authors

Pellegrino Cerino [1], Annachiara Coppola [1] [2], Biancamaria Pierri [1] [3], Palmiero Volzone [1], Dario Bruzzese [4], Andrea Pierri [1], Amedeo Ferro [1], Daniela Schiavi [1], Antonio Coppola [5], Stefano Miniero [5], Luigi Atripaldi [6], Caterina Pirozzi [6], Pasquale Rusciano [6], Alessandra Macri [6], Rita Boenzi [6], Silvia Sale [6], Gianfranco Brambilla [7], Carlo Buonerba [1]

Affiliations

- [1] Centro di Referenza Nazionale per l'Analisi e Studio di Correlazione tra Ambiente, Animale e Uomo, Istituto Zooprofilattico Sperimentale del Mezzogiorno, Portici, 80055, Italy.
- [2] Dipartimento di Medicina Sperimentale, Università degli studi della Campania 'L Vanvitelli', Naples, 80138, Italy.
- [3] Department of Medicine, Surgery & Dentistry (Scuola Medica Salernitana), University of Salerno, Baronissi, 84081, Italy.
- [4] Department of Public Health, University of Naples 'Federico II', Naples, 80131, Italy.
- [5] ASL Napoli 3 SUD, Torre del Greco, 80059, Italy.
- [6] Cotugno Hospital, AORN Ospedali dei Colli, Naples, 80131, Italy.
- [7] Food Safety, Nutrition & Veterinary Public Health Department, Istituto Superiore di Sanità, Rome, 00161, Italy.

- PMID: **34290884**
- PMCID: PMC8216649
- DOI: 10.2144/fsoa-2021-0064

Abstract

In this retrospective study, a cohort of 67 subjects vaccinated with AZD1222 was retrospectively observed. Consistently with published findings, no serious adverse event was reported, and all adverse events reported (fever, muscle ache and/or pain in the site of injection) had resolved by day 8. Of note, some citizens were prescribed low-dose aspirin and even heparin for thrombosis prevention. We also found variations in laboratory test results (full blood count and chemistry) on day 1 compared with day 8. Physicians should be aware that no prevention therapy for thrombosis is currently recommended, given the very low incidence of this side effect. Additional studies are warranted to interpret our findings.

Keywords: AZD1222; COVID-19; SARS-CoV-2; adverse events; coagulation.

© 2021 The Authors.

Conflict of interest statement

Financial & competing interests disclosure The authors have no relevant affiliationsor financial involvement with any organization or entity with a financial interest in or financial conflict with the subject matter or materials discussed in the manuscript. This includes employment, consultancies, honoraria, stock ownership or options, expert testimony, grants or patents received or pending, or royalties. No writing assistance was utilized in the production of this manuscript.

- 12 references

Full text links

Future Science Free PMC article

Proceed to details

Cite

Share

☐ 1,268

J Clin Med

. 2021 Dec 15;10(24):5891.
doi: 10.3390/jcm10245891.

Hydroxyzine Use and Mortality in Patients Hospitalized for COVID-19: A Multicenter Observational Study

Marina Sánchez-Rico [1, 2], Frédéric Limosin [1, 3, 4], Raphaël Vernet [5], Nathanaël Beeker [6], Antoine Neuraz [7, 8], Carlos Blanco [9], Mark Olfson [10], Cédric Lemogne [1, 3, 4], Pierre Meneton [11], Christel Daniel [12, 13], Nicolas Paris [12, 14], Alexandre Gramfort [15], Guillaume Lemaitre [15], Pedro De La Muela [1, 2], Elisa Salamanca [16], Mélodie Bernaux [17], Ali Bellamine [18], Anita Burgun [7], Nicolas Hoertel [1, 3, 4], On Behalf Of Ap-Hp/Université de Paris/Inserm Covid-Research Collaboration/Ap-Hp Covid Cdr Initiative/Entrepôt de Données de Santé Ap-Hp Consortium

Affiliations Expand

Affiliations

- [1] Département de Psychiatrie, Hôpital Corentin-Celton, AP-HP.Centre-Université de Paris, 92130 Issy-les-Moulineaux, France.
- [2] Department of Psychobiology & Behavioural Sciences Methods, Faculty of Psychology, Campus de Somosaguas Universidad Complutense de Madrid, 28223 Pozuelo de Alarcon, Spain.
- [3] Institut de Psychiatrie et Neurosciences de Paris, Université de Paris, UMR_S1266, INSERM, 75014 Paris, France.
- [4] UFR de Médecine, Faculté de Santé, Université de Paris, 75006 Paris, France.
- [5] Hôpital Européen Georges Pompidou, Medical Informatics, Biostatistics and Public Health Department, AP-HP.Centre-Université de Paris, 75015 Paris, France.
- [6] Unité de Recherche Clinique, Hopital Cochin, Assistance Publique-Hopitaux de Paris, 75004 Paris, France.
- [7] Cordeliers Research Center, Université de Paris, UMRS 1138, INSERM, 75006 Paris, France.
- [8] Department of Medical Informatics, Necker-Enfants Malades Hospital, AP-HP, Centre-Université de Paris, 75015 Paris, France.
- [9] Division of Epidemiology, Services and Prevention Research, National Institute on Drug Abuse, 6001 Executive Boulevard, Bethesda, MD 20852, USA.
- [10] Department of Psychiatry, New York State Psychiatric Institute, Columbia University, 1051 Riverside Drive, Unit 69, New York, NY 10032, USA.
- [11] Laboratoire d'Informatique Médicale et d'Ingénierie des Connaissances en e-Santé, UMR 1142, INSERM, Sorbonne Université, Université Paris 13, 93017 Paris, France.
- [12] AP-HP, DSI-WIND (Web Innovation Données), 75184 Paris, France.
- [13] Laboratoire d'Informatique Médicale et d'Ingénierie des Connaissances en e-Santé, Sorbonne University, University Paris 13, Sorbonne Paris Cité, INSERM UMRS 1142, 75012 Paris, France.
- [14] LIMSI, CNRS, Université Paris-Sud, Université Paris-Saclay, 91405 Orsay, France.
- [15] Institut National de Recherche en Sciences et Technologies du Numérique (INRIA), Université Paris-Saclay, INRIA, CEA, 75012 Palaiseau, France.
- [16] Banque Nationale de Données Maladies Rares (BNDMR), Campus Picpus, Département WIND (Web Innovation Données), AP-HP, 75012 Paris, France.
- [17] Direction de la Stratégie et de la Transformation, AP-HP, 75004 Paris, France.
- [18] Unité de Recherche Clinique, Hôpital Cochin, AP-HP, Centre-Université de Paris, 75014 Paris, France.

- PMID: **34945186**
- PMCID: PMC8707307
- DOI: 10.3390/jcm10245891

Free PMC article

Hydroxyzine Use and Mortality in Patients Hospitalized for COVID-19: A Multicenter Observational Study

Marina Sánchez-Rico et al. J Clin Med. 2021.
Free PMC article
Show details
J Clin Med
. 2021 Dec 15;10(24):5891.
doi: 10.3390/jcm10245891.

Authors

Marina Sánchez-Rico[1, 2], Frédéric Limosin[1, 3, 4], Raphaël Vernet[5], Nathanaël Beeker[6], Antoine Neuraz[7, 8], Carlos Blanco[9], Mark Olfson[10], Cédric Lemogne[1, 3, 4], Pierre Meneton[11], Christel Daniel[12, 13], Nicolas Paris[12, 14], Alexandre Gramfort[15], Guillaume Lemaitre[15], Pedro De La Muela[1, 2], Elisa Salamanca[16], Mélodie Bernaux[17], Ali Bellamine[18], Anita Burgun[7], Nicolas Hoertel[1, 3, 4], On Behalf Of Ap-Hp/Université de Paris/Inserm Covid-Research Collaboration/Ap-Hp Covid Cdr Initiative/Entrepôt de Données de Santé Ap-Hp Consortium

Affiliations

- [1] Département de Psychiatrie, Hôpital Corentin-Celton, AP-HP.Centre-Université de Paris, 92130 Issy-les-Moulineaux, France.
- [2] Department of Psychobiology & Behavioural Sciences Methods, Faculty of Psychology, Campus de Somosaguas Universidad Complutense de Madrid, 28223 Pozuelo de Alarcon, Spain.
- [3] Institut de Psychiatrie et Neurosciences de Paris, Université de Paris, UMR_S1266, INSERM, 75014 Paris, France.
- [4] UFR de Médecine, Faculté de Santé, Université de Paris, 75006 Paris, France.
- [5] Hôpital Européen Georges Pompidou, Medical Informatics, Biostatistics and Public Health Department, AP-HP.Centre-Université de Paris, 75015 Paris, France.
- [6] Unité de Recherche Clinique, Hopital Cochin, Assistance Publique-Hopitaux de Paris, 75004 Paris, France.
- [7] Cordeliers Research Center, Université de Paris, UMRS 1138, INSERM, 75006 Paris, France.
- [8] Department of Medical Informatics, Necker-Enfants Malades Hospital, AP-HP, Centre-Université de Paris, 75015 Paris, France.
- [9] Division of Epidemiology, Services and Prevention Research, National Institute on Drug Abuse, 6001 Executive Boulevard, Bethesda, MD 20852, USA.
- [10] Department of Psychiatry, New York State Psychiatric Institute, Columbia University, 1051 Riverside Drive, Unit 69, New York, NY 10032, USA.
- [11] Laboratoire d'Informatique Médicale et d'Ingénierie des Connaissances en e-Santé, UMR 1142, INSERM, Sorbonne Université, Université Paris 13, 93017 Paris, France.
- [12] AP-HP, DSI-WIND (Web Innovation Données), 75184 Paris, France.
- [13] Laboratoire d'Informatique Médicale et d'Ingénierie des Connaissances en e-Santé, Sorbonne University, University Paris 13, Sorbonne Paris Cité, INSERM UMRS 1142, 75012 Paris, France.
- [14] LIMSI, CNRS, Université Paris-Sud, Université Paris-Saclay, 91405 Orsay, France.
- [15] Institut National de Recherche en Sciences et Technologies du Numérique (INRIA), Université Paris-Saclay, INRIA, CEA, 75012 Palaiseau, France.

- [16] Banque Nationale de Données Maladies Rares (BNDMR), Campus Picpus, Département WIND (Web Innovation Données), AP-HP, 75012 Paris, France.
- [17] Direction de la Stratégie et de la Transformation, AP-HP, 75004 Paris, France.
- [18] Unité de Recherche Clinique, Hôpital Cochin, AP-HP, Centre-Université de Paris, 75014 Paris, France.

- PMID: **34945186**
- PMCID: PMC8707307
- DOI: 10.3390/jcm10245891

Abstract

(1) Background: Based on its antiviral activity, anti-inflammatory properties, and functional inhibition effects on the acid sphingomyelinase/ceramide system (FIASMA), we sought to examine the potential usefulness of the H1 antihistamine hydroxyzine in patients hospitalized for COVID-19. (2) Methods: In a multicenter observational study, we included 15,103 adults hospitalized for COVID-19, of which 164 (1.1%) received hydroxyzine within the first 48 h of hospitalization, administered orally at a median daily dose of 25.0 mg (SD = 29.5). We compared mortality rates between patients who received hydroxyzine at hospital admission and those who did not, using a multivariable logistic regression model adjusting for patients' characteristics, medical conditions, and use of other medications. (3) Results: This analysis showed a significant association between hydroxyzine use and reduced mortality (AOR, 0.51; 95%CI, 0.29-0.88, p = 0.016). This association was similar in multiple sensitivity analyses. (4) Conclusions: In this retrospective observational multicenter study, the use of the FIASMA hydroxyzine was associated with reduced mortality in patients hospitalized for COVID-19. Double-blind placebo-controlled randomized clinical trials of hydroxyzine for COVID-19 are needed to confirm these results, as are studies to examine the potential usefulness of this medication for outpatients and as post-exposure prophylaxis for individuals at high risk for severe COVID-19.

Keywords: COVID-19; FIASMA; SARS-CoV-2; death; hydroxyzine; inpatients; mortality; treatment.

Conflict of interest statement

All authors have completed the Unified Competing Interest form (available on request from the corresponding author) and declare: no support from any organization for the submitted work; N. H. has received personal fees and non-financial support from Lundbeck, outside the submitted work. F. L. has received speaker and consulting fees from Janssen-Cilag, Euthérapie-Servier, and Lundbeck, outside the submitted work. C. L. reports personal fees and non-financial support from Janssen-Cilag, Lundbeck, Otsuka Pharmaceutical, and Boehringer Ingelheim, outside the submitted work. Other authors declare no financial relationships with any organisation that might have an interest in the submitted work in the previous three years; no other relationships or activities that could appear to have influenced the submitted work.

- 51 references
- 2 figures

Full text links

FULL TEXT OPEN ACCESS MDPI Multidisciplinary Digital Publishing Institute (MDPI) Free PMC article
Proceed to details

Cite

Share

☐ 1,269

Lancet Reg Health West Pac

. 2020 Dec;5:100061.

doi: 10.1016/j.lanwpc.2020.100061. Epub 2020 Nov 27.

Presenting characteristics and clinical outcome of patients with COVID-19 in South Korea: A nationwide retrospective observational study

Hyun-Young Park [1], Jung Hyun Lee [1], Nam-Kyoo Lim [1], Do Sang Lim [1], Sung Ok Hong [1], Mi-Jin Park [1], Seon Young Lee [1], Geehyuk Kim [1], Jae Kyung Park [1], Dae Sub Song [1], Hee Youl Chai [1], Sung Soo Kim [1], Yeon-Kyeng Lee [1], Hye Kyung Park [1], Jun-Wook Kwon [1], Eun Kyeong Jeong [1]

Affiliations Expand

Affiliation

- [1] COVID-19 National Emergency Response Center, Korea Centers for Disease Control and Prevention, Cheongju city 28159, South Korea.

- PMID: **34173605**
- PMCID: PMC7691821
- DOI: 10.1016/j.lanwpc.2020.100061

Free PMC article

Presenting characteristics and clinical outcome of patients with COVID-19 in South Korea: A nationwide retrospective observational study

Hyun-Young Park et al. Lancet Reg Health West Pac. 2020 Dec.

Free PMC article

Show details

Lancet Reg Health West Pac

. 2020 Dec;5:100061.

doi: 10.1016/j.lanwpc.2020.100061. Epub 2020 Nov 27.

Authors

Hyun-Young Park[1], Jung Hyun Lee[1], Nam-Kyoo Lim[1], Do Sang Lim[1], Sung Ok Hong[1], Mi-Jin Park[1], Seon Young Lee[1], Geehyuk Kim[1], Jae Kyung Park[1], Dae Sub Song[1], Hee Youl Chai[1], Sung Soo Kim[1], Yeon-Kyeng Lee[1], Hye Kyung Park[1], Jun-Wook Kwon[1], Eun Kyeong Jeong[1]

Affiliation

- [1] COVID-19 National Emergency Response Center, Korea Centers for Disease Control and Prevention, Cheongju city 28159, South Korea.

- PMID: **34173605**
- PMCID: PMC7691821
- DOI: 10.1016/j.lanwpc.2020.100061

Abstract

Background: More than 13,000 cases were reported to be infected with COVID-19 by RT-PCR in South Korea. Most studies report clinical characteristics of hospitalized patients with COVID-19; the full spectrum of disease severity has thus not yet been well described.

Methods: Using retrospective observational methods, this study analyzed factors affecting early clinical symptoms, clinical progress, and severity of disease for COVID-19 positive patients released from quarantine to provide information on establishing optimized care for new patients. The medical data of 7803 laboratory-confirmed patients who had been discharged or died by April 30, 2020 were analyzed using multivariate logistic regression analysis.

Findings: On admission, 7383 (94•5%) patients were asymptomatic or showed mild illness, and 372 (4•8%) patients were severe illness. Also, 48 (0 0•6%) were hospitalized with critically ill when diagnosed. Most patients with asymptomatic or mild illness on admission remained mild until discharge, 253 (3•4%) progressed to severe illness, and 83 (1•1%) died in hospital. However, the case fatality were 29•8% and 62•5% in severe and critically ill patients, respectively. At admission, 73•0% of hospitalized patients had symptoms; most common were cough (42•5%), sputum (28•8%), and fever (20•1%). Only 35•2% of laboratory confirmed patients admitted to the temporary care facility complained of symptoms. Increasing odds of being critically ill was associated with older age (OR 28•93, 95% CI 13•34-62•75 for age >70y, vs. age <50 y; *p*<0•0001), being male (OR 2•15, 95% CI1•59-2•89; *p*<0•0001), fever (OR 2•52, 95% CI 1.84-3•45; *p*<0•0001), and shortness of breath (OR 7•40, 95% CI 5•37-10•19; *p*<0•0001). Comorbid illness significantly increased risk of critical illness or death.

Interpretation: Most cases were discharged as asymptomatic or recovered from mild illness, and only 9•7% developed severe disease requiring oxygen therapy or more. Case fatality rate was 2•9%, and markedly increased in those over age 50. Risk factors such as age, sex, fever, shortness of breath, and underlying disease can be useful in predicting future clinical severity. Additionally, the number of confirmed asymptomatic COVID-19 patients significantly contribute to continued spread.

Funding: none.

© 2020 The Authors. Published by Elsevier Ltd.

Conflict of interest statement

None.

- 21 references
- 2 figures

Full text links

ELSEVIER OPEN ACCESS Elsevier Science Free PMC article
Proceed to details
Cite
Share
☐ 1,270
BMJ Evid Based Med
. 2020 Dec 11;bmjebm-2020-111549.
doi: 10.1136/bmjebm-2020-111549. Online ahead of print.

Inactivated trivalent influenza vaccination is associated with lower mortality among patients with COVID-19 in Brazil

Günther Fink [1] [2], Nina Orlova-Fink [3] [2], Tobias Schindler [3] [2], Sandra Grisi [4], Ana Paula S Ferrer [4], Claudia Daubenberger [3] [2], Alexandra Brentani [4]
Affiliations Expand

Affiliations

- [1] University of Basel, Basel, Switzerland guenther.fink@swisstph.ch.
- [2] Swiss Tropical and Public Health Institute, Basel, Basel-Stadt, Switzerland.
- [3] University of Basel, Basel, Switzerland.
- [4] Department of Pediatrics, University of São Paulo Medical School, São Paulo, Brazil.

- PMID: **33310766**
- PMCID: PMC7735072
- DOI: 10.1136/bmjebm-2020-111549

Free PMC article

Inactivated trivalent influenza vaccination is associated with lower mortality among patients with COVID-19 in Brazil

Günther Fink et al. BMJ Evid Based Med. 2020.
Free PMC article
Show details
BMJ Evid Based Med
. 2020 Dec 11;bmjebm-2020-111549.
doi: 10.1136/bmjebm-2020-111549. Online ahead of print.

Authors

Günther Fink [1, 2], Nina Orlova-Fink [3, 2], Tobias Schindler [3, 2], Sandra Grisi [4], Ana Paula S Ferrer [4], Claudia Daubenberger [3, 2], Alexandra Brentani [4]

Affiliations

- [1] University of Basel, Basel, Switzerland guenther.fink@swisstph.ch.
- [2] Swiss Tropical and Public Health Institute, Basel, Basel-Stadt, Switzerland.
- [3] University of Basel, Basel, Switzerland.
- [4] Department of Pediatrics, University of São Paulo Medical School, São Paulo, Brazil.

- PMID: **33310766**
- PMCID: PMC7735072
- DOI: 10.1136/bmjebm-2020-111549

Abstract

Objective: To estimate associations between trivalent influenza vaccination and COVID-19 mortality as well as severe clinical outcomes among hospitalised patients.

Design: Retrospective observational study.

Setting: This study was conducted among hospitalised patients with COVID-19 in Brazil.

Participants: We analysed all hospitalised patients with COVID-19 with available vaccination information captured in Brazil's national electronic respiratory infection data system between 1 January 2020 and 23 June 2020.

Main outcome measures: The primary outcomes were age-specific mortality rates of hospitalised patients with COVID-19 with and without recent inactivated trivalent influenza vaccination.

Results: A total of 53 752 clinically confirmed COVID-19 cases were analysed. Controlling for health facility of treatment, comorbidities as well as an extensive range of sociodemographic factors, patients who received a recent influenza vaccine experienced on average 7% lower odds of needing intensive care treatment (95% CI 0.87 to 0.98), 17% lower odds of requiring invasive respiratory support (95% CI 0.77 to 0.88) and 16% lower odds of death (95% CI 0.78 to 0.90). Protective effects were larger when the vaccine was administered after onset of symptoms as well as among younger patients.

Conclusion: Patients with COVID-19 with recent inactivated influenza vaccination experience significantly better health outcomes than non-vaccinated patients in Brazil. Beneficial off-target effects of influenza vaccination through trained innate immune responses seem plausible and need

to be further explored. Large-scale promotion of influenza vaccines seems advisable, especially in populations at high risk for severe COVID-19 disease progression.

Keywords: immunisation; infectious disease medicine.

© Author(s) (or their employer(s)) 2020. No commercial re-use. See rights and permissions. Published by BMJ.

Conflict of interest statement

Competing interests: None declared.

- 41 references
- 2 figures

Full text links

BMJ Online First HighWire Free PMC article

Proceed to details

Cite

Share

☐ 1,271

SICOT J

. 2021;7:5.

doi: 10.1051/sicotj/2021001. Epub 2021 Feb 17.

Older patients with proximal femur fractures and SARS-CoV-2 infection - An observational study

Abdus S Burahee [1], Veronica E Barry [1], Robert P Sutcliffe [2], Sabreena Mahroof [1]

Affiliations Expand

Affiliations

- [1] Department of Orthopaedic Surgery, Royal Wolverhampton NHS Trust, WV8 1DN Wolverhampton, UK.
- [2] Liver Unit, Queen Elizabeth Hospital, Birmingham B15 2WB, UK.

- PMID: **33595434**
- PMCID: PMC7888253
- DOI: 10.1051/sicotj/2021001

Free PMC article

Older patients with proximal femur fractures and SARS-CoV-2 infection - An observational study

Abdus S Burahee et al. SICOT J. 2021.
Free PMC article
Show details
SICOT J
. 2021;7:5.
doi: 10.1051/sicotj/2021001. Epub 2021 Feb 17.

Authors

Abdus S Burahee [1], Veronica E Barry [1], Robert P Sutcliffe [2], Sabreena Mahroof [1]

Affiliations

- [1] Department of Orthopaedic Surgery, Royal Wolverhampton NHS Trust, WV8 1DN Wolverhampton, UK.
- [2] Liver Unit, Queen Elizabeth Hospital, Birmingham B15 2WB, UK.

- PMID: **33595434**
- PMCID: PMC7888253
- DOI: 10.1051/sicotj/2021001

Abstract

Background: Older patients are at increased risk of severe COVID-19 infection and associated mortality. There are limited data evaluating the outcome of older patients with hip fractures treated during the COVID-19 pandemic, and it has been suggested that these patients should be treated non-operatively due to high mortality risk. The aim of this study was to report the outcomes of COVID-19 infected hip fracture patients treated at a single centre.

Methods: This was a retrospective cohort study. Data were collected from February 2020 (after the first confirmed COVID-19 infected patient was reported in the Midlands region of the UK). All patients admitted to the hospital with femoral neck fractures were included. Patient demographics, comorbidity, COVID-19 status, and short-term clinical outcomes were obtained by review of electronic medical records. The outcomes of COVID-19 infected patients were compared with non-COVID-19 patients treated during the study period.

Results: Twenty-nine patients were included (mean age of 80 years), of whom 14 (48%) were tested positive for COVID-19 infection in the postoperative period. Overall, 26 patients (90%) underwent surgical treatment. COVID-19 infected patients had significantly higher Charlson comorbidity scores compared to the control group (5 vs. 4; $p = 0.047$). Only 5 COVID-19 infected patients (36%) required supplemental oxygen therapy in the postoperative period, and no patients required respiratory or other organ support. The 30-day mortality rate in COVID-19 patients was 14% compared to 0% in the negative controls ($p = 0.22$).

Interpretation: COVID-19 infection did not increase the mortality rate of older patients undergoing surgery for hip fractures during the pandemic. The authors recommend careful assessment of patient fitness and prompt surgical treatment. In addition, it was noted that nearly all admissions were either given large boluses of Vitamin D or were on maintenance supplementation, which may have affected the severity of the response to COVID-19 infections.

Keywords: COVID-19; Elderly Care; Proximal femur fracture; SARS-CoV-2; Vitamin D.

© The Authors, published by EDP Sciences, 2021.

- 18 references

Full text links

EDP Sciences Free PMC article

Proceed to details

Cite

Share

☐ 1,272

J Acute Care Phys Ther

. 2022 Jan;13(1):2-7.

doi: 10.1097/JAT.0000000000000163. Epub 2021 Apr 20.

Patient Characteristics and Acute PT and OT Utilization During the Initial Surge of COVID-19: A Retrospective Observational Study

Adele Myszenski [1, 2, 2, 2], Romina Bello [1, 2, 2, 2], Cynthia Melican [1, 2, 2, 2], Nanette Pfitzenmaier [1, 2, 2, 2]

Affiliations Expand

Affiliations

- [1] Rehabilitation Services, Henry Ford Hospital, A-Basement, 2799 W Grand Blvd, Detroit, MI 48202 (USA). amyszen1@hfhs.org.
- [2] Rehabilitation Services, Henry Ford Hospital, Detroit, Michigan.

- PMID: **34925956**
- PMCID: PMC8670083
- DOI: 10.1097/JAT.0000000000000163

Free PMC article

Patient Characteristics and Acute PT and OT Utilization During the Initial Surge of COVID-19: A Retrospective Observational Study

Adele Myszenski et al. J Acute Care Phys Ther. 2022 Jan.
Free PMC article
Show details
J Acute Care Phys Ther
. 2022 Jan;13(1):2-7.
doi: 10.1097/JAT.0000000000000163. Epub 2021 Apr 20.

Authors

Adele Myszenski [1 2 2 2], Romina Bello [1 2 2 2], Cynthia Melican [1 2 2 2], Nanette Pfitzenmaier [1 2 2 2]

Affiliations

- [1] Rehabilitation Services, Henry Ford Hospital, A-Basement, 2799 W Grand Blvd, Detroit, MI 48202 (USA). amyszen1@hfhs.org.
- [2] Rehabilitation Services, Henry Ford Hospital, Detroit, Michigan.

- PMID: **34925956**
- PMCID: PMC8670083
- DOI: 10.1097/JAT.0000000000000163

Abstract

Objective: To describe the characteristics of patients and investigate the utilization of physical (PT) and occupational therapy (OT) intervention for those with a positive coronavirus disease-2019 (COVID-19) diagnosis compared with other patient populations during the first 6 weeks of the novel coronavirus pandemic.

Methods: A retrospective, observational study of adult inpatients with a length of stay of 1 or more days at an urban hospital in Detroit, Michigan. Individuals with a COVID-19 diagnosis were compared with a cohort within similar diagnostic categories (respiratory, fever, and sepsis) but without COVID-19. Outcome measures included PT or OT intervention on 1 or more days, the timing of initial PT or OT visit, the average number of visits and units per patient, length of stay, discharge to home, and readmission within 30 days.

Results: Individuals with COVID-19 had lower rates of discharge to home (P = .001), higher rates of readmission within 30 days of hospital discharge (P = .01), increased hospital length of stay (P = .001), and waited an average of 3.1 days longer for therapy evaluations than subjects in the comparison group (P = .001). The percentage of subjects who had one or more PT or OT visits during their hospital stays was comparable between groups. Once therapy was initiated, the

average number of visits per patient and dosing of units in 15-minute increments were similar between the 2 groups.

Conclusions: Patients acutely ill with COVID-19 hospitalized with the virus during the first 6 weeks of the pandemic remained in the intensive care unit and hospital longer than their counterparts without COVID-19 and had a delay in initiation of PT and OT intervention. PT and OT are important members of the care team for patients with the novel coronavirus. Understanding the descriptive characteristics of patients and therapy services during the initial surge could help improve utilization and patient outcomes.

© 2021 Academy of Acute Care Physical Therapy, APTA.

Conflict of interest statement

The authors have no conflicts of interest and no source of funding to declare.

- 29 references
- 1 figure

Full text links

Free PMC article
Proceed to details
Cite
Share
☐ 1,273
EXCLI J
. 2020 Nov 16;19:1533-1543.
doi: 10.17179/excli2020-2988. eCollection 2020.

Clinical characteristics and outcomes of diabetics hospitalized for COVID-19 infection: a single-centered, retrospective, observational study

Asieh Mansour [1], Sayed Mahmoud Sajjadi-Jazi [1] [2], Amir Kasaeian [3] [4], Bardia Khosravi [4], Majid Sorouri [4], Fatemeh Azizi [5], Zeinab Rajabi [5], Fatemeh Motamedi [5], Azin Sirusbakht [4], Masoud Eslahi [5], Heila Mojtabbavi [4], Ali Reza Sima [4], Amir Reza Radmard [6], Mohhamad Reza Mohajeri-Tehrani [1], Mohammad Abdollahi [4]
Affiliations Expand

Affiliations

- [1] Endocrinology and Metabolism Research Center, Endocrinology and Metabolism Clinical Sciences Institute, Tehran University of Medical Sciences, Tehran, Iran.

- [2] Cell Therapy and Regenerative Medicine Research Center, Endocrinology and Metabolism Molecular-Cellular Sciences Institute, Tehran University of Medical Sciences, Tehran, Iran.
- [3] Hematology, Oncology and Stem Cell Transplantation Research Center, Tehran University of Medical Sciences, Tehran, Iran.
- [4] Digestive Disease Research Center, Digestive Disease Research Institute, Tehran University of Medical Sciences, Tehran, Iran.
- [5] Department of Internal Medicine, Shariati Hospital, Tehran University of Medical Sciences, Tehran, Iran.
- [6] Radiology Department, Shariati Hospital, Tehran University of Medical Sciences, Tehran, Iran.

- PMID: **33343270**
- PMCID: PMC7744965
- DOI: 10.17179/excli2020-2988

Free PMC article

Clinical characteristics and outcomes of diabetics hospitalized for COVID-19 infection: a single-centered, retrospective, observational study

Asieh Mansour et al. EXCLI J. 2020.
Free PMC article

Show details

EXCLI J
. 2020 Nov 16;19:1533-1543.
doi: 10.17179/excli2020-2988. eCollection 2020.

Authors

Asieh Mansour [1], Sayed Mahmoud Sajjadi-Jazi [1, 2], Amir Kasaeian [3, 4], Bardia Khosravi [4], Majid Sorouri [4], Fatemeh Azizi [5], Zeinab Rajabi [5], Fatemeh Motamedi [5], Azin Sirusbakht [4], Masoud Eslahi [5], Heila Mojtabbavi [4], Ali Reza Sima [4], Amir Reza Radmard [6], Mohhamad Reza Mohajeri-Tehrani [1], Mohammad Abdollahi [4]

Affiliations

- [1] Endocrinology and Metabolism Research Center, Endocrinology and Metabolism Clinical Sciences Institute, Tehran University of Medical Sciences, Tehran, Iran.
- [2] Cell Therapy and Regenerative Medicine Research Center, Endocrinology and Metabolism Molecular-Cellular Sciences Institute, Tehran University of Medical Sciences, Tehran, Iran.
- [3] Hematology, Oncology and Stem Cell Transplantation Research Center, Tehran University of Medical Sciences, Tehran, Iran.

- [4] Digestive Disease Research Center, Digestive Disease Research Institute, Tehran University of Medical Sciences, Tehran, Iran.
- [5] Department of Internal Medicine, Shariati Hospital, Tehran University of Medical Sciences, Tehran, Iran.
- [6] Radiology Department, Shariati Hospital, Tehran University of Medical Sciences, Tehran, Iran.

- PMID: **33343270**
- PMCID: PMC7744965
- DOI: 10.17179/excli2020-2988

Abstract

Some debates exist regarding the association of diabetes mellitus (DM) with COVID-19 infection severity and mortality. In this study, we aimed to describe and compare the clinical characteristics and outcomes of hospitalized COVID-19 patients with and without DM. In this single-centered, retrospective, observational study, we enrolled adult patients with COVID-19 who were admitted to the Shariati hospital, Tehran, Iran, from February 25, 2020, to April 21, 2020. The clinical and paraclinical information as well as the clinical outcomes of patients were collected from inpatient medical records. A total of 353 cases were included (mean age, 61.67 years; 57.51 % male), of whom 111 patients were diabetics (mean age, 63.66 years; 55.86 % male). In comparison to those without DM, diabetic patients with COVID-19 were more likely to have other comorbidities, elevated systolic blood pressure (SBP), elevated blood sugar (BS), lower estimated glomerular filtration rate (eGFR) and elevated blood urea nitrogen (BUN). The association of DM with severe outcomes of COVID-19 infection (i.e. mechanical ventilation, median length of hospital stay and mortality) remained non-significant before and after adjustments for several factors including age, sex, body mass index (BMI), smoking status, and comorbidities. Based on our results DM has not been associated with worse outcomes in hospitalized patients for COVID-19 infection.

Keywords: COVID-19; DM; diabetes mellitus.

Copyright © 2020 Mansour et al.

- 18 references
- 5 figures

Full text links

Free PMC article
Proceed to details
Cite
Share
☐ 1,274
Mayo Clin Proc Innov Qual Outcomes
. 2021 Jun;5(3):605-613.
doi: 10.1016/j.mayocpiqo.2021.03.007. Epub 2021 Mar 27.

Anti-Inflammatory Treatment of COVID-19 Pneumonia With Tofacitinib Alone or in

Combination With Dexamethasone is Safe and Possibly Superior to Dexamethasone as a Single Agent in a Predominantly African American Cohort

Maroun E Hayek [1], Michael Mansour [1], Harrison Ndetan [2], Quentin Burkes [1], Robert Corkern [1], Ammar Dulli [1], Reya Hayek [3], Karim Parvez [1], Satwinder Singh [1]
Affiliations Expand

Affiliations

- [1] Delta Regional Medical Center, Greenville, MS.
- [2] Department of Epidemiology and Biostatistics, The University of Texas Health Science Center at Tyler, Tyler, TX.
- [3] University of Mississippi Medical School, Jackson, MS.

- PMID: **33817559**
- PMCID: PMC7998063
- DOI: 10.1016/j.mayocpiqo.2021.03.007

Free PMC article

Anti-Inflammatory Treatment of COVID-19 Pneumonia With Tofacitinib Alone or in Combination With Dexamethasone is Safe and Possibly Superior to Dexamethasone as a Single Agent in a Predominantly African American Cohort

Maroun E Hayek et al. Mayo Clin Proc Innov Qual Outcomes. 2021 Jun.
Free PMC article
Show details
Mayo Clin Proc Innov Qual Outcomes
. 2021 Jun;5(3):605-613.
doi: 10.1016/j.mayocpiqo.2021.03.007. Epub 2021 Mar 27.

Authors

Maroun E Hayek [1], Michael Mansour [1], Harrison Ndetan [2], Quentin Burkes [1], Robert Corkern [1], Ammar Dulli [1], Reya Hayek [3], Karim Parvez [1], Satwinder Singh [1]

Affiliations

- [1] Delta Regional Medical Center, Greenville, MS.
- [2] Department of Epidemiology and Biostatistics, The University of Texas Health Science Center at Tyler, Tyler, TX.
- [3] University of Mississippi Medical School, Jackson, MS.

- PMID: **33817559**
- PMCID: PMC7998063
- DOI: 10.1016/j.mayocpiqo.2021.03.007

Abstract

Objective: To explore the survival benefit of tofacitinib in addition to dexamethasone in hospitalized patients treated for coronavirus disease 2019 (COVID-19)-related pneumonia.

Patients and methods: This is a single-center retrospective observational study. All patients who were hospitalized at Delta Regional Medical Center (a regional hospital in the Mississippi Delta) with a COVID-19 diagnosis and discharged between March 1 and September 30, 2020, are included. The primary outcome was in-hospital mortality in relation to receipt of tofacitinib alone or in addition to dexamethasone (designated as the tofacitinib group), versus dexamethasone alone (designated as the dexamethasone group).

Results: Of 269 eligible patients, 138 (51.3%) received tofacitinib uniformly and 131 (48.7%) patients received dexamethasone without tofacitinib. A total of 44 patients expired: 14 (31.8%) in the tofacitinib group and 30 (68.2%) in the dexamethasone group. The proportions of death among the tofacitinib and dexamethasone groups were, respectively, 10.1% and 22.9%. This represents a 70% reduction in odds of dying among the tofacitinib group compared to the dexamethasone group after adjusting for age and clinical parameters captured at hospitalization (adjusted odds ratio: 0.30; 95% CI: 0.12 to 0.76; *P*=.01).

Conclusion: The in-patient treatment of COVID-19 pneumonia has rapidly evolved. The addition of dexamethasone has made a relevant improvement on survival. Other immunomodulators have yet to show an impact. Here we present the potential survival benefit of the Janus kinase-signal transducer and activator of transcription inhibitor tofacitinib on COVID-19 pneumonia. We found that adding tofacitinib-based anti-inflammatory therapy to a treatment regimen including dexamethasone in COVID-19 pneumonia seems to have potential benefit of improving survival when compared to dexamethasone alone.

Keywords: AOR, adjusted odds ratio; COVID-19, coronavirus disease 2019; CRP, C-reactive protein; CT, computed tomography; DRMC, Delta Regional Medical Center; IL, interleukin; JAK, Janus kinase; OR, odds ratio; PCR, polymerase chain reaction; STAT, Signal transducer and activator of transcription.

© 2021 The Authors.

- 42 references
- 2 figures

Full text links

ELSEVIER OPEN ACCESS Elsevier Science Free PMC article
Proceed to details
Cite
Share
1,275

Observational Study

Life Sci
. 2022 Mar 15;293:120324.
doi: 10.1016/j.lfs.2022.120324. Epub 2022 Jan 12.

Renin-angiotensin system blockade on angiotensin-converting enzyme 2 and TMPRSS2 in human type II pneumocytes

Mauro G Silva [1], Nora L Falcoff [2], Gerardo R Corradi [1], José Alfie [3], Rolando F Seguel [4], Gabriela C Tabaj [4], Laura I Iglesias [2], Myriam Nuñez [5], Gabriela R Guman [2], Mariela M Gironacci [6]
Affiliations Expand

Affiliations

- [1] Universidad de Buenos Aires, Facultad de Farmacia y Bioquímica, Dpto. Química Biológica, IQUIFIB (UBA-CONICET), Buenos Aires, Argentina.
- [2] Servicio Unificado de Patología Hospital Prov de Tórax "Dr. A. Cetrángolo" y Municipal de Vicente López "Prof. B. Houssay", Buenos Aires, Argentina.
- [3] Servicio de Hipertensión Arterial, Hospital Italiano, Buenos Aires, Argentina.
- [4] Servicio de Neumonología Hospital Prov de Tórax "Dr. A. Cetrángolo", Buenos Aires, Argentina.
- [5] Universidad de Buenos Aires, Facultad de Farmacia y Bioquímica, Cátedra de Matemáticas, Buenos Aires, Argentina.
- [6] Universidad de Buenos Aires, Facultad de Farmacia y Bioquímica, Dpto. Química Biológica, IQUIFIB (UBA-CONICET), Buenos Aires, Argentina. Electronic address: mariela@qb.ffyb.uba.ar.

- PMID: **35032553**
- PMCID: PMC8754457
- DOI: 10.1016/j.lfs.2022.120324

Free PMC article
Observational Study

Renin-angiotensin system blockade on angiotensin-converting enzyme 2 and TMPRSS2 in human type II pneumocytes

Mauro G Silva et al. Life Sci. 2022.
Free PMC article
Show details
Life Sci
. 2022 Mar 15;293:120324.
doi: 10.1016/j.lfs.2022.120324. Epub 2022 Jan 12.

Authors

Mauro G Silva [1], Nora L Falcoff [2], Gerardo R Corradi [1], José Alfie [3], Rolando F Seguel [4], Gabriela C Tabaj [4], Laura I Iglesias [2], Myriam Nuñez [5], Gabriela R Guman [2], Mariela M Gironacci [6]

Affiliations

- [1] Universidad de Buenos Aires, Facultad de Farmacia y Bioquímica, Dpto. Química Biológica, IQUIFIB (UBA-CONICET), Buenos Aires, Argentina.
- [2] Servicio Unificado de Patología Hospital Prov de Tórax "Dr. A. Cetrángolo" y Municipal de Vicente López "Prof. B. Houssay", Buenos Aires, Argentina.
- [3] Servicio de Hipertensión Arterial, Hospital Italiano, Buenos Aires, Argentina.
- [4] Servicio de Neumonología Hospital Prov de Tórax "Dr. A. Cetrángolo", Buenos Aires, Argentina.
- [5] Universidad de Buenos Aires, Facultad de Farmacia y Bioquímica, Cátedra de Matemáticas, Buenos Aires, Argentina.
- [6] Universidad de Buenos Aires, Facultad de Farmacia y Bioquímica, Dpto. Química Biológica, IQUIFIB (UBA-CONICET), Buenos Aires, Argentina. Electronic address: mariela@qb.ffyb.uba.ar.

- PMID: **35032553**
- PMCID: PMC8754457
- DOI: 10.1016/j.lfs.2022.120324

Abstract

Aims: Angiotensin-converting enzyme (ACE) 2 is the receptor for severe acute respiratory syndrome coronavirus 2 which causes coronavirus disease 2019 (COVID-19). Viral cellular entry requires ACE2 and transmembrane protease serine 2 (TMPRSS2). ACE inhibitors (ACEIs) or angiotensin (Ang) receptor blockers (ARBs) influence ACE2 in animals, though evidence in human lungs is lacking. We investigated ACE2 and TMPRSS2 in type II pneumocytes, the key cells that maintain lung homeostasis, in lung parenchymal of ACEI/ARB-treated subjects compared to untreated control subjects.

Main methods: Ang II and Ang-(1-7) levels and ACE2 and TMPRSS2 protein expression were measured by radioimmunoassay and immunohistochemistry, respectively.

Key findings: We found that the ratio Ang-(1-7)/Ang II, a surrogate marker of ACE2 activity, as well as the amount of ACE2-expressing type II pneumocytes were not different between ACEI/ARB-treated and untreated subjects. ACE2 protein content correlated positively with smoking habit and age. The percentage of TMPRSS2-expressing type II pneumocytes was higher in males than females and in subjects under 60 years of age but it was not different between ACEI/ARB-treated and untreated subjects. However, there was a positive association of TMPRSS2 protein content with age and smoking in ACEI/ARB-treated subjects, with high TMPRSS2 protein levels most evident in ACEI/ARB-treated older adults and smokers.

Significance: ACEI/ARB treatment influences human lung TMPRSS2 but not ACE2 protein content and this effect is dependent on age and smoking habit. This finding may help explain the increased susceptibility to COVID-19 seen in smokers and older patients with treated cardiovascular-related pathologies.

Keywords: Angiotensin; Angiotensin-converting enzyme 2; Hypertension; Lung; TMPRSS2; Type II pneumocytes.

Copyright © 2022 Elsevier Inc. All rights reserved.

Conflict of interest statement

None.

- 61 references
- 8 figures

Supplementary info

Publication types, MeSH terms, Substances Expand

Publication types

- Observational Study

MeSH terms

- Adult
- Age Factors
- Aged
- Alveolar Epithelial Cells / chemistry
- Alveolar Epithelial Cells / drug effects
- Alveolar Epithelial Cells / metabolism*
- Angiotensin I / metabolism
- Angiotensin II / metabolism
- Angiotensin Receptor Antagonists / pharmacology*

- Angiotensin-Converting Enzyme 2 / analysis
- Angiotensin-Converting Enzyme 2 / antagonists & inhibitors
- Angiotensin-Converting Enzyme 2 / metabolism*
- Angiotensin-Converting Enzyme Inhibitors / pharmacology*
- Female
- Humans
- Lung / chemistry
- Lung / drug effects
- Lung / metabolism
- Male
- Middle Aged
- Peptide Fragments / metabolism
- Renin-Angiotensin System / drug effects
- Renin-Angiotensin System / physiology*
- Retrospective Studies
- Serine Endopeptidases / analysis
- Serine Endopeptidases / metabolism*
- Smoking / metabolism
- Smoking / pathology

Substances

- Angiotensin Receptor Antagonists
- Angiotensin-Converting Enzyme Inhibitors
- Peptide Fragments
- Angiotensin II
- Angiotensin I
- ACE2 protein, human
- Angiotensin-Converting Enzyme 2
- Serine Endopeptidases
- TMPRSS2 protein, human
- angiotensin I (1-7)

Full text links

ELSEVIER FULL-TEXT ARTICLE Elsevier Science Free PMC article
Proceed to details
Cite
Share
☐ 1,276
Indian J Anaesth
. 2021 Sep;65(9):669-675.
doi: 10.4103/ija.ija_474_21. Epub 2021 Oct 8.

NUTRIC score as a predictor of outcome in COVID-19 ARDS patients: A retrospective observational study

Neeraj Kumar [1], Abhyuday Kumar [2], Ajeet Kumar [2], Arunima Pattanayak [2], Kunal Singh [2], Prabhat K Singh [3]
Affiliations Expand

Affiliations

- [1] Department of Trauma and Emergency, All India Institute of Medical Sciences, Patna, Bihar, India.
- [2] Department of Anaesthesiology, All India Institute of Medical Sciences, Patna, Bihar, India.
- [3] Director, All India Institute of Medical Sciences, Patna, Bihar, India.

- PMID: **34764502**
- PMCID: PMC8577713
- DOI: 10.4103/ija.ija_474_21

Free PMC article

NUTRIC score as a predictor of outcome in COVID-19 ARDS patients: A retrospective observational study

Neeraj Kumar et al. Indian J Anaesth. 2021 Sep.
Free PMC article
Show details
Indian J Anaesth
. 2021 Sep;65(9):669-675.
doi: 10.4103/ija.ija_474_21. Epub 2021 Oct 8.

Authors

Neeraj Kumar [1], Abhyuday Kumar [2], Ajeet Kumar [2], Arunima Pattanayak [2], Kunal Singh [2], Prabhat K Singh [3]

Affiliations

- [1] Department of Trauma and Emergency, All India Institute of Medical Sciences, Patna, Bihar, India.
- [2] Department of Anaesthesiology, All India Institute of Medical Sciences, Patna, Bihar, India.

- [3] Director, All India Institute of Medical Sciences, Patna, Bihar, India.

- PMID: **34764502**
- PMCID: PMC8577713
- DOI: 10.4103/ija.ija_474_21

Abstract

Background and aims: The Nutrition Risk in Critically ill (NUTRIC) score is an appropriate nutritional assessment tool in mechanically ventilated patients. We retrospectively observed the applicability of the NUTRIC score for predicting outcomes in coronavirus disease (COVID)-19 acute respiratory distress syndrome (ARDS) patients.

Methods: All adult COVID-19 ARDS patients admitted to the intensive care unit and requiring various forms of oxygen therapy were included in the study. The demographic characteristics and clinical information about the patients were obtained from the hospital's medical records department. The nutritional risk for each patient was assessed using the NUTRIC score at 72 hours of ICU admission. The discriminating power and ability of NUTRIC score, Sequential Organ Failure Assessment (SOFA) score, age and Acute Physiology and Chronic Health Evaluation (APACHE) II to predict the 28-day mortality and need for mechanical ventilation (MV) was calculated using receiver operating characteristic curves and area under this curve.

Results: A total of 80 COVID-19 ARDS patients fitted into the inclusion criteria. Among non-survivors, the median Glasgow Coma Score, APACHE II score, NUTRIC score and SOFA score were 10, 16, 6 and 4, respectively. The cut-off values for NUTRIC score, SOFA, and APACHE II to predict 28-day mortality and need for MV was obtained as 3.5, 3.5 and 11.5, respectively. These cut-off values of NUTRIC score, SOFA score, and APACHE II have a sensitivity of 62%, 72.5% and 75.5%, respectively, and specificity of 95%, 72% and 83% for predicting mortality.

Conclusions: Most COVID-19 ARDS patients requiring MV in the ICU are at nutritional risk, and a high NUTRIC score is associated with higher mortality.

Keywords: COVID-19; intensive care unit; nutritional assessment; respiratory distress syndrome.

Copyright: © 2021 Indian Journal of Anaesthesia.

Conflict of interest statement

There are no conflicts of interest.

- 23 references
- 2 figures

Full text links

Free PMC article
Proceed to details
Cite
Share
☐ 1,277
Cureus

. 2021 Dec 13;13(12):e20394.
doi: 10.7759/cureus.20394. eCollection 2021 Dec.

Determinants of Outcome Among Critically Ill Police Personnel With COVID-19: A Retrospective Observational Study From Andhra Pradesh, India

Limalemla Jamir [1], Mukesh Tripathi [2], Sumita Shankar [3], Rakesh Kakkar [1], Ravishankar Ayyanar [4], Rajeev Aravindakshan [1]
Affiliations Expand

Affiliations

- [1] Department of Community and Family Medicine, All India Institute of Medical Sciences (AIIMS), Mangalagiri, IND.
- [2] Department of Anesthesiology, All India Institute of Medical Sciences (AIIMS), Mangalagiri, IND.
- [3] Department of Plastic Surgery, Rangaraya Medical College, Kakinada, IND.
- [4] Law & Order, Andhra Pradesh Police Department, Mangalagiri, IND.

- PMID: **35036224**
- PMCID: PMC8754352
- DOI: 10.7759/cureus.20394

Free PMC article

Determinants of Outcome Among Critically Ill Police Personnel With COVID-19: A Retrospective Observational Study From Andhra Pradesh, India

Limalemla Jamir et al. Cureus. 2021.
Free PMC article
Show details
Cureus
. 2021 Dec 13;13(12):e20394.
doi: 10.7759/cureus.20394. eCollection 2021 Dec.

Authors

Limalemla Jamir [1], Mukesh Tripathi [2], Sumita Shankar [3], Rakesh Kakkar [1], Ravishankar Ayyanar [4], Rajeev Aravindakshan [1]

Affiliations

- [1] Department of Community and Family Medicine, All India Institute of Medical Sciences (AIIMS), Mangalagiri, IND.
- [2] Department of Anesthesiology, All India Institute of Medical Sciences (AIIMS), Mangalagiri, IND.
- [3] Department of Plastic Surgery, Rangaraya Medical College, Kakinada, IND.
- [4] Law & Order, Andhra Pradesh Police Department, Mangalagiri, IND.

- PMID: **35036224**
- PMCID: PMC8754352
- DOI: 10.7759/cureus.20394

Abstract

Background and aims: Police personnel have been key frontline workers throughout the coronavirus disease 2019 (COVID-19) pandemic. This study was conducted to assess the correlates and outcomes of critically ill police personnel.

Methods: This retrospective observational study analyzed key parameters of hospitalized police personnel who were critically ill with COVID-19 in Andhra Pradesh, India, between June and October 2020. Survival was analyzed for correlation with body mass index, ABO/Rh blood group, co-morbidities, treatment (oxygen therapy, prone positioning, mechanical ventilation, remdesivir, Ivermectin, oral and nasal topical povidone-iodine). We also performed Cox proportional hazard analysis with relevant function plots.

Results: The majority of the 266 patients were male (n = 259; 97.4%) and obese (75.2%). The overall mortality of patients was 38% (n = 101). COVID-19 mortality increased significantly with age (p = 0.019) and BMI (p = 0.030) in the bivariate analysis. There was no significant difference between blood group (p = 0.297), co-morbidity (p = 0.582) and COVID-19 outcome. Multivariable-adjusted hazard ratios (HRs) and 95% confidence intervals (CIs) of the risk factors for COVID-19 mortality were males (HR 4.89, 95% CI: 1.020-23.430) and ventilator therapy (HR 7.5, 95% CI: 4.527-12.296). The protective factors were symptom onset to reverse transcription polymerase chain reaction (RT-PCR) report interval (HR 0.36, 95% CI: 0.158-0.814), prone positioning (HR 0.43, 95% CI: 0.197-0.915), and use of povidone iodine (HR 0.43; 95% CI: 0.273-0.692).

Conclusion: COVID-19 mortality among critically ill hospitalized police personnel was reduced by time to diagnostic test result, prone positioning, and povidone-iodine use and increased with male gender and mechanical ventilation.

Keywords: covid-19; covid-19 mortality; mechanical ventilation; police personnel; povidone iodine; prone positioning; remdesivir.

Copyright © 2021, Jamir et al.

Conflict of interest statement

The authors have declared that no competing interests exist.

- 30 references
- 1 figure

Full text links

Free PMC article
Proceed to details
Cite
Share
☐ 1,278
Neurologia (Engl Ed)
. 2021 Sep 8.
doi: 10.1016/j.nrl.2021.06.008. Online ahead of print.

[Mild sensory symptoms during SARS-CoV-2 infection among healthcare professionals]

[Article in Spanish]
Diego Gayoso Cantero [1], Estefanía Cantador Pavón [2], Elia Pérez Fernández [3], María Elena Novillo López [2]
Affiliations Expand

Affiliations

- [1] Servicio de Medicina Interna, Hospital Universitario Fundación Alcorcón, Spain.
- [2] Servicio de Neurología, Hospital Universitario Fundación Alcorcón, Spain.
- [3] Apoyo Metodológico y Análisis de Datos de la Unidad de Investigación, Hospital Universitario Fundación Alcorcón, Spain.

- PMID: **34511684**
- PMCID: PMC8423987
- DOI: 10.1016/j.nrl.2021.06.008

Free PMC article

[Mild sensory symptoms during SARS-CoV-2 infection among healthcare professionals]

[Article in Spanish]
Diego Gayoso Cantero et al. Neurologia (Engl Ed). 2021.
Free PMC article
Show details
Neurologia (Engl Ed)
. 2021 Sep 8.

doi: 10.1016/j.nrl.2021.06.008. Online ahead of print.

Authors

Diego Gayoso Cantero [1], Estefanía Cantador Pavón [2], Elia Pérez Fernández [3], María Elena Novillo López [2]

Affiliations

- [1] Servicio de Medicina Interna, Hospital Universitario Fundación Alcorcón, Spain.
- [2] Servicio de Neurología, Hospital Universitario Fundación Alcorcón, Spain.
- [3] Apoyo Metodológico y Análisis de Datos de la Unidad de Investigación, Hospital Universitario Fundación Alcorcón, Spain.

- PMID: **34511684**
- PMCID: PMC8423987
- DOI: 10.1016/j.nrl.2021.06.008

Abstract
in English, Spanish

Introduction: It is not yet possible to estimate the proportion of patients with COVID-19 who present distinguishable classical neurological symptoms and syndromes.The objective of this study is to estimate the incidence of sensory symptoms (hypoaesthesia, paraesthesia, and hyperalgesia) in physicians who have presented the disease at Hospital Universitario Fundación Alcorcón (HUFA) in Madrid; to establish the relationship between sensory symptoms and the presence of other signs of infection; and to study their association with the severity of COVID-19.

Methods: We conducted a descriptive, cross-sectional, retrospective, observational study. HUFA physicians who presented SARS-CoV-2 infection between 1 March and 25 July 2020 were included in the study. A voluntary, anonymous survey was distributed via corporate email. Sociodemographic and clinical characteristics were collected from professionals with PCR- or serology-confirmed COVID-19.

Results: The survey was sent to 801 physicians and we received 89 responses. The mean age of respondents was 38.28 years. A total of 17.98% presented sensory symptoms. A significant relationship was found between the presence of paraesthesia and cough, fever, myalgia, asthaenia, and dyspnoea. A significant relationship was also found between paraesthesia and the need for treatment and admission due to COVID-19. Sensory symptoms were present from the fifth day of illness in 87.4% of cases.

Conclusions: SARS-CoV-2 infection can be associated with sensory symptoms, mostly in severe cases. Sensory symptoms often appear after a time interval, and may be caused by a parainfectious syndrome with an autoimmunity background.

Introducción: Aún no es posible estimar la proporción de pacientes con la COVID-19 que presentan síntomas y síndromes neurológicos clásicos diferenciables.

Nuestro objetivo es estimar la incidencia de síntomas sensitivos (hipoestesia, parestesias e hiperalgesia), en médicos que han sufrido la COVID-19 en el Hospital Universitario Fundación

Alcorcón de Madrid; relacionar la clínica sensitiva con la presencia de otros síntomas o signos de la infección y estudiar la asociación con la gravedad de la COVID-19.

Métodos: Estudio descriptivo, transversal, retrospectivo y de carácter observacional. La población del estudio incluyó a médicos del Hospital Universitario Fundación Alcorcón que presentaron infección por SARS-CoV-2 entre el 1 de marzo y el 25 de julio del 2020. Se hizo llegar a través del correo electrónico corporativo una encuesta voluntaria y anónima en la que se recogieron características sociodemográficas y clínicas de aquellos trabajadores con confirmación de la COVID-19 por PCR o serología.

Resultados: La encuesta se envió a 801 médicos. Contestaron 89. La edad media fue de 38,28 años; el 17,98% presentó clínica sensitiva. Se encontró una relación significativa entre la presencia de parestesias y tos, fiebre, mialgias, astenia y disnea. Se observó una relación significativa entre las parestesias y la necesidad de recibir tratamientos e ingresar por la COVID-19. El 87,4% presentó la clínica sensitiva a partir del 5.° día de la enfermedad.

Conclusiones: La infección por SARS-CoV-2 se puede acompañar de clínica sensitiva que refleje cuadros más graves; aparece diferida en el tiempo, lo que podría corresponder con un probable origen parainfeccioso en el que quizá influya un trasfondo de autoinmunidad.

Keywords: COVID-19; SARS-CoV-2; hyperalgesia; hypoaesthesia; paraesthesia; sensory symptoms.

© 2021 Published by Elsevier España, S.L.U. on behalf of Sociedad Española de Neurología.

- 18 references
- 1 figure

Supplementary info

Publication types Expand

Publication types

- English Abstract

Full text links

ELSEVIER OPEN ACCESS Elsevier Science Free PMC article

Proceed to details

Cite

Share

☐ 1,279

Rev Esp Quimioter

. 2022 Mar 10;gomez10mar2022.

doi: 10.37201/req/122.2021. Online ahead of print.

[Retrospective observational study of the persistence of SARS-CoV-2 infection in patients previously treated with rituximab]

[Article in Spanish]

M T Gómez Lluch [1], B Proy Vega, M Cabero Becerra, A Rodríguez, A Escalera Zalvide, S A Sánchez
Affiliations Expand

Affiliation

- [1] María Teresa Gómez Lluch, Servicio Farmacia Hospital General Mancha Centro, Alcázar de San Juan (Ciudad Real), Spain, tgomezlluch@gmail.com.

- PMID: **35259778**
- DOI: 10.37201/req/122.2021

Free article

[Retrospective observational study of the persistence of SARS-CoV-2 infection in patients previously treated with rituximab]

[Article in Spanish]

M T Gómez Lluch et al. Rev Esp Quimioter. 2022.
Free article
Show details
Rev Esp Quimioter
. 2022 Mar 10;gomez10mar2022.
doi: 10.37201/req/122.2021. Online ahead of print.

Authors

M T Gómez Lluch [1], B Proy Vega, M Cabero Becerra, A Rodríguez, A Escalera Zalvide, S A Sánchez

Affiliation

- [1] María Teresa Gómez Lluch, Servicio Farmacia Hospital General Mancha Centro, Alcázar de San Juan (Ciudad Real), Spain, tgomezlluch@gmail.com.

- PMID: **35259778**
- DOI: 10.37201/req/122.2021

Abstract

Objective: Rituximab-induced immunosuppression could be a risk factor for mortality from COVID-19. The aim of the study was to describe the prevalence of SARS-CoV-2 infection in patients who have received rituximab and its association with a persistent viral infection.

Methods: Retrospective observational study of patients who received rituximab in the 6 months before to the onset of the pandemic. We analyzed the presence of infection and associated them with demographic variables, pathological history related to an increased risk of developing severe COVID-19, the doses of rituximab received, the type of ventilatory support, thromboembolic events, and the treatment received. A descriptive analysis of all the variables was carried out and infected and uninfected patients were compared.

Results: We screened a total of 68 patients who had received rituximab (median cumulative dose: 4,161mg (2,611-8,187.5)). 54.4% men, mean age 60.8 years (15.7; 25-87)). C + was confirmed for 22 patients. Of these, 45.5% had high blood pressure, 36.4% Diabetes Mellitus, 31.8% smokers/ex-smoker, 22.7% lung disease, 13.6% heart disease and 4.5% obesity. There were no statistically significant differences between C+ and C-. Only 2 patients developed immunity. For 10 patients (45.5%) did not have a negative CRP until the end of the follow-up. There was no association with cumulative dose of rituximab. The mortality rate was 22.7% in the C+.

Conclusions: We observe that the persistence of the infection leads to a worse evolution of COVID-19. The use of alternatives should be considered during the pandemic, because of patients with decreased B-cell function may have high risk of fatal progression from COVID-19.

Keywords: Persistence; Rituximab; SARS-CoV-2.

©The Author 2022. Published by Sociedad Española de Quimioterapia. This article is distributed under the terms of the Creative Commons Attribution-NonCommercial 4.0 International (CC BY-NC 4.0)(https://creativecommons.org/licenses/by-nc/4.0/).

Supplementary info

Publication types Expand

Publication types

- English Abstract

Full text links

FREE Full Text www.seq.es Sociedad Espanola de Quimioterapia

Proceed to details

Cite

Share

☐ 1,280

Eur Neurol

. 2021 Sep 23;1-4.

doi: 10.1159/000519226. Online ahead of print.

Lower Cranial Nerve Palsies in the COVID-19 Pandemic: A 10-Case Series of Intensive Care Unit Patients

Pierre Decavel [1, 2], Olympe Nahmias [3], Carine Petit [3], Laurent Tatu [2, 4]
Affiliations Expand

Affiliations

- [1] Department of Rehabilitation, Hôpital Fribourgeois, Villars-sur-Glâne, Switzerland.
- [2] EA 481 Integrative and Clinical Neuroscience, University of Franche-Comté, Besançon, France.
- [3] Department of Rehabilitation, CHRU de Besançon, Besançon, France.
- [4] Department of Neuromuscular Diseases and Department of Anatomy, CHRU Besançon, University of Franche-Comté, Besançon, France.

- PMID: **34555828**
- PMCID: PMC8678256
- DOI: 10.1159/000519226

Free PMC article

Lower Cranial Nerve Palsies in the COVID-19 Pandemic: A 10-Case Series of Intensive Care Unit Patients

Pierre Decavel et al. Eur Neurol. 2021.
Free PMC article
Show details
Eur Neurol
. 2021 Sep 23;1-4.
doi: 10.1159/000519226. Online ahead of print.

Authors

Pierre Decavel [1, 2], Olympe Nahmias [3], Carine Petit [3], Laurent Tatu [2, 4]

Affiliations

- [1] Department of Rehabilitation, Hôpital Fribourgeois, Villars-sur-Glâne, Switzerland.
- [2] EA 481 Integrative and Clinical Neuroscience, University of Franche-Comté, Besançon, France.
- [3] Department of Rehabilitation, CHRU de Besançon, Besançon, France.

- [4] Department of Neuromuscular Diseases and Department of Anatomy, CHRU Besançon, University of Franche-Comté, Besançon, France.

- PMID: **34555828**
- PMCID: PMC8678256
- DOI: 10.1159/000519226

Abstract

Introduction: A number of neurological complications of COVID-19 have been identified, including cranial nerve paralyses. We present a series of 10 patients with lower cranial nerve involvement after severe COVID-19 infection requiring hospitalization in an intensive care unit.

Methods: We conducted a retrospective, observational study of patients admitted to the post-intensive care unit (p-ICU) of Besançon University Hospital (France) between March 16 and May 22, 2020. We included patients with confirmed COVID-19 and cranial neuropathy at admission to the p-ICU. All these patients were treated by orotracheal intubation, and all but one underwent prone-position ventilation therapy.

Results: Of the 88 patients admitted to the p-ICU, 10 patients (11%) presented at least 1 cranial nerve palsy. Of these 10 patients, 9 had a hypoglossal nerve palsy and 8 of these also had a deficit in another cranial nerve. The most frequent association was between hypoglossal and vagal palsies (5 patients). None of the patients developed neurological signs related to a global neuropathy. We found no correlation between the intensity of the motor limb weakness and the occurrence of lower cranial nerve palsies. All but 2 of the patients recovered within less than a month.

Conclusion: The mechanical compressive hypothesis, linked to the prone-position ventilation therapy, appears to be the major factor. The direct toxicity of SARS-CoV-2 and the context of immune dysfunction induced by the virus may be involved in a multifactorial etiology.

Keywords: COVID-19; Intensive care unit; Lower cranial nerve palsy.

© 2021 S. Karger AG, Basel.

Conflict of interest statement

The authors have no conflicts of interest to declare.

- 12 references

Supplementary info

Publication types Expand

Publication types

- News

Full text links

S. Karger AG, Basel, Switzerland Free PMC article
Proceed to details
Cite
Share
1,281
Front Neurol
. 2021 Sep 8;12:673703.
doi: 10.3389/fneur.2021.673703. eCollection 2021.

Impact of COVID-19 on Acute Stroke Presentation in a Designated COVID-19 Hospital

Qing Tan [1, 2], Qing-Jun Liu [1, 2], Wen-Hui Fan [3], Xiao-Yan Du [1, 2], Lin Wu [1, 2], Hong-Min Gong [1, 2], Jing Wei [1, 2], Rui Zhao [1, 2], Ming Lei [1, 2], Li-Bo Zhao [1, 2]
Affiliations Expand

Affiliations

- [1] Department of Neurology, Yongchuan Hospital, Chongqing Medical University, Chongqing, China.
- [2] Chongqing Key Laboratory of Cerebrovascular Disease Research, Chongqing, China.
- [3] Department of Neurology, Chongqing Ninth People's Hospital, Chongqing, China.

- PMID: **34566833**
- PMCID: PMC8456084
- DOI: 10.3389/fneur.2021.673703

Free PMC article

Impact of COVID-19 on Acute Stroke Presentation in a Designated COVID-19 Hospital

Qing Tan et al. Front Neurol. 2021.
Free PMC article
Show details
Front Neurol
. 2021 Sep 8;12:673703.
doi: 10.3389/fneur.2021.673703. eCollection 2021.

Authors

Qing Tan [1 2], Qing-Jun Liu [1 2], Wen-Hui Fan [3], Xiao-Yan Du [1 2], Lin Wu [1 2], Hong-Min Gong [1 2], Jing Wei [1 2], Rui Zhao [1 2], Ming Lei [1 2], Li-Bo Zhao [1 2]

Affiliations

- [1] Department of Neurology, Yongchuan Hospital, Chongqing Medical University, Chongqing, China.
- [2] Chongqing Key Laboratory of Cerebrovascular Disease Research, Chongqing, China.
- [3] Department of Neurology, Chongqing Ninth People's Hospital, Chongqing, China.

- PMID: **34566833**
- PMCID: PMC8456084
- DOI: 10.3389/fneur.2021.673703

Abstract

Objectives: Thousands of designated COVID-19 hospitals have been set up in China to fight the ongoing COVID-19 pandemic. Anecdotal reports indicate a falling rate of acute stroke diagnoses in these hospitals during the COVID-19 period. We conducted an exploratory single-center analysis to estimate the change in acute stroke presentation at the designated COVID-19 hospitals. **Methods:** This retrospective observational study included all patients admitted to Yongchuan Hospital Affiliated to Chongqing Medical University with acute stroke between January 24 and March 10, 2020. Patient demographics, characteristics of the stroke, treatment details, and clinical outcomes were compared with those of patients admitted in the corresponding period in the year before (2019, "the pre-COVID-19 period"). Subgroup analysis was performed in the ischemic and hemorrhagic stroke groups. **Results:** A total of 110 patients presented with acute stroke symptoms during the COVID-19 pandemic, compared with 173 patients in the pre-COVID-19 period. A higher proportion of stroke patients presented to the hospital *via* emergency medical services during the pandemic (48.2 vs. 31.8%, $p = 0.006$). There was a lower proportion of ischemic stroke patients (50.9 vs. 65.3%, $p = 0.016$) than in the preceding year. There were significantly fewer patients with 90-day modified Rankin Scale score ≥3 in the COVID-19 period compared with the pre-COVID-19 period (17.3 vs. 30.6%, $p = 0.012$). Among patients with ischemic stroke, the mean time from patient arrival to vessel puncture for emergency endovascular therapy in the COVID-19 period was shorter than that in the pre-COVID-19 period (109.18 ± 71.39 vs. 270.50 ± 161.51 min, $p = 0.002$). Among patients with hemorrhagic stroke, the rate of emergency surgical operation in the COVID-19 period was higher than that in the pre-COVID-19 period (48.1 vs. 30.0%, $p = 0.047$). The mean time from patient arrival to emergency surgical operation (15.31 ± 22.89 vs. 51.72 ± 40.47 min, $p = 0.002$) was shorter in the COVID-19 period than in the pre-COVID-19 period. **Conclusions:** Although fewer acute stroke patients sought medical care in this designated COVID-19 hospital during the COVID-19 pandemic, this type of hospital was more efficient for timely treatment of acute stroke. Recognizing how acute strokes presented in designated COVID-19 hospitals will contribute to appropriate adjustments in strategy for dealing with acute stroke during COVID-19 and future pandemics.

Keywords: COVID-19; acute stroke; coronavirus; designated hospital; stroke center.

Copyright © 2021 Tan, Liu, Fan, Du, Wu, Gong, Wei, Zhao, Lei and Zhao.

Conflict of interest statement

The authors declare that the research was conducted in the absence of any commercial or financial relationships that could be construed as a potential conflict of interest.

- 28 references

Full text links

Frontiers Media SA Free PMC article
Proceed to details
Cite
Share
☐ 1,282

Int J Infect Dis
. 2022 Feb 12;117:334-344.
doi: 10.1016/j.ijid.2022.02.019. Online ahead of print.

Reduced ADAMTS13 Activity in Correlation with Pathophysiology, Severity, and Outcome of COVID-19: A Retrospective Observational Study

Wael Hafez [1], Mohamad Azzam Ziade [2], Arun Arya [2], Husam Saleh [2], Sara Ali [2], Srinivasa Raghu Rao [2], Osman Fdl Alla [2], Mohamed Ali [2], Mouhamad Al Zouhbi [2], Ahmed Abdelrahman [3]
Affiliations Expand

Affiliations

- [1] NMC Royal Hospital, 16th Street, Khalifa City, Abu Dhabi, United Arab Emirates; Medical Research Division, Department of Internal Medicine, The National Research Centre, 33 El Buhouth St, Ad Doqi, Dokki, Cairo Governorate 12622, Egypt. Electronic address: waeelhafez@yahoo.com.
- [2] NMC Royal Hospital, 16th Street, Khalifa City, Abu Dhabi, United Arab Emirates.
- [3] NMC Royal Hospital, 16th Street, Khalifa City, Abu Dhabi, United Arab Emirates; Internal Medicine Department, Zagazig School of medicine, Zagazig, Egypt.

- PMID: **35167969**
- PMCID: PMC8839807
- DOI: 10.1016/j.ijid.2022.02.019

Free PMC article

Reduced ADAMTS13 Activity in Correlation with Pathophysiology, Severity, and Outcome of COVID-19: A Retrospective Observational Study

Wael Hafez et al. Int J Infect Dis. 2022.
Free PMC article
Show details
Int J Infect Dis
. 2022 Feb 12;117:334-344.
doi: 10.1016/j.ijid.2022.02.019. Online ahead of print.

Authors

Wael Hafez [1], Mohamad Azzam Ziade [2], Arun Arya [2], Husam Saleh [2], Sara Ali [2], Srinivasa Raghu Rao [2], Osman Fdl Alla [2], Mohamed Ali [2], Mouhamad Al Zouhbi [2], Ahmed Abdelrahman [3]

Affiliations

- [1] NMC Royal Hospital, 16th Street, Khalifa City, Abu Dhabi, United Arab Emirates; Medical Research Division, Department of Internal Medicine, The National Research Centre, 33 El Buhouth St, Ad Doqi, Dokki, Cairo Governorate 12622, Egypt. Electronic address: waeelhafez@yahoo.com.
- [2] NMC Royal Hospital, 16th Street, Khalifa City, Abu Dhabi, United Arab Emirates.
- [3] NMC Royal Hospital, 16th Street, Khalifa City, Abu Dhabi, United Arab Emirates; Internal Medicine Department, Zagazig School of medicine, Zagazig, Egypt.

- PMID: **35167969**
- PMCID: PMC8839807
- DOI: 10.1016/j.ijid.2022.02.019

Abstract

Background: Low ADAMTS13 activity has been suggested to be an interplaying factor in the pathogenesis of COVID-19, considering that it is a thromboinflammatory disease with high risk of microthrombosis.

Objectives: The study aimed to explore the correlation between ADAMTS13 activity and the pathophysiological pathway of COVID-19.

Methods: We carried out a retrospective observational study of 87 patients with COVID-19 in NMC Royal Hospital, Abu Dhabi, UAE. ADAMTS13 activity was measured and compared with patients' characteristics and clinical outcomes.

Results: Low ADAMTS13 activity was associated with pneumonia ($p = 0.007$), severity of COVID-19 ($p < 0.001$), and mechanical ventilation rates ($p = 0.018$). Death was more frequently

observed among patients (5 patients) with low ADAMTS13 activity compared with normal activity (1 patient), as well as inflammatory markers. Decreased ADAMTS13 activity increased with the risk of pneumonia, severity of COVID-19, need for mechanical ventilation, and use of anticoagulants ([OR = 4.75, 95% CI 1.54-18.02, p = 0.011], [OR = 6.50, 95% CI 2.57-17.74; p <0.001], [OR = 4.10, 95% CI 1.29-15.82; p = 0.024], [OR = 8.00, 95% CI 3.13-22.16; p <0.001], respectively). The low ADAMTS13 activity group had a slightly longer time to viral clearance than the normal ADAMTS13 activity group, but it was not statistically significant (20 days, 95% CI 16-27 days vs 17 days, 95% CI 13-22 days; p = 0.08; Log rank = 3.1).

Conclusions: Low ADAMTS13 activity has been linked to pneumonia, COVID-19 severity, use of anticoagulants, and need for mechanical ventilation but not to mortality. We propose rADAMTS13 as a novel treatment for severe COVID-19.

Keywords: ADAMTS13; Anticoagulation; COVID-19; Cytokine storm; Pneumonia; SARS-CoV-2; Thrombosis.

Copyright © 2022 The Author(s). Published by Elsevier Ltd.. All rights reserved.

- 75 references
- 5 figures

Full text links

ELSEVIER OPEN ACCESS Elsevier Science Free PMC article

Proceed to details

Cite

Share

☐ 1,283

Lancet Reg Health West Pac

. 2021 Feb;7:100088.

doi: 10.1016/j.lanwpc.2020.100088. Epub 2021 Jan 22.

Power of universal health coverage in the era of COVID-19: A nationwide observational study

Hyejin Lee [1], Jae-Ryun Lee [1], Hyemin Jung [2] [3], Jin Yong Lee [2] [3] [4]

Affiliations Expand

Affiliations

- [1] Department of Family Medicine, Seoul National University Bundang Hospital, 82 Gumi-ro, 173 Beon-gil, Bundang-gu, Seongnam-si, Gyeomggi-do 13620, Republic of Korea.
- [2] Public Healthcare Center, Seoul National University Hospital, 101 Daehak-ro, Jongno-gu, Seoul 03080, Republic of Korea.
- [3] Department of Health Policy and Management, Seoul National University College of Medicine, 101 Daehak-ro, Jongno-gu, Seoul 03080, Republic of Korea.

- [4] HIRA Research Institute, Health Insurance Review and Assessment Service, Wonju, Republic of Korea.

- PMID: **33521744**
- PMCID: PMC7826087
- DOI: 10.1016/j.lanwpc.2020.100088

Free PMC article

Power of universal health coverage in the era of COVID-19: A nationwide observational study

Hyejin Lee et al. Lancet Reg Health West Pac. 2021 Feb.
Free PMC article
Show details
Lancet Reg Health West Pac
. 2021 Feb;7:100088.
doi: 10.1016/j.lanwpc.2020.100088. Epub 2021 Jan 22.

Authors

Hyejin Lee [1], Jae-Ryun Lee [1], Hyemin Jung [2 3], Jin Yong Lee [2 3 4]

Affiliations

- [1] Department of Family Medicine, Seoul National University Bundang Hospital, 82 Gumi-ro, 173 Beon-gil, Bundang-gu, Seongnam-si, Gyeomggi-do 13620, Republic of Korea.
- [2] Public Healthcare Center, Seoul National University Hospital, 101 Daehak-ro, Jongno-gu, Seoul 03080, Republic of Korea.
- [3] Department of Health Policy and Management, Seoul National University College of Medicine, 101 Daehak-ro, Jongno-gu, Seoul 03080, Republic of Korea.
- [4] HIRA Research Institute, Health Insurance Review and Assessment Service, Wonju, Republic of Korea.

- PMID: **33521744**
- PMCID: PMC7826087
- DOI: 10.1016/j.lanwpc.2020.100088

Abstract

Background: During the COVID-19 pandemic, South Korea has achieved perfect universal health coverage (UHC)-all Koreans receive UHC regardless of their socioeconomic status. The current study investigated whether socioeconomic disparities remained in COVID-19 health outcomes under UHC.

Methods: This retrospective, observational study included all 7,590 confirmed COVID-19 patients in South Korea up to 15 May 2020. We used the official medical claim database, and socioeconomic status was estimated by insurance type (National Health Insurance Service [NHIS] beneficiaries and Medical Aid [MA] recipients). Type of insurance is a well-known indicator of socioeconomic status. Prevalence (per one million), mortality rate (per one million), and case fatality rate were calculated. To determine the factors associated with case fatality rate, multivariable logistic regressions were performed.

Findings: The nationwide prevalence, mortality rate, and case fatality rate of COVID-19 was 144•4, 4•3 and 3•0%, respectively. MA recipients had higher prevalence (424•3 vs 136.3), mortality rate (28•3 vs 3•6), and case fatality rate (6•7 vs 2•7) than NHIS beneficiaries. However, the adjusted analysis showed that the type of insurance was not associated with higher odds of case fatality.

Interpretation: We found socioeconomic disparities in COVID-19 prevalence and fatality despite UHC. However, disparities in fatality were not due to socioeconomic status, but due to the poor underlying health conditions of the people. This result can be explained by a combination of UHC, rapid early testing and treatment, transmission-reducing behaviours, and regional preparedness.

Funding: This research did not receive any funding.

Keywords: COVID-19; Case Fatality; Health disparity; Mortality; Prevalence; SARS-CoV-2.

© 2020 The Authors. Published by Elsevier Ltd.

Conflict of interest statement

The authors declare that there are no conflicts of interests.

- 30 references
- 1 figure

Full text links

ELSEVIER OPEN ACCESS Elsevier Science Free PMC article

Proceed to details

Cite

Share

☐ 1,284

Ann Med Surg (Lond)
. 2021 Mar;63:102160.
doi: 10.1016/j.amsu.2021.02.006. Epub 2021 Feb 12.

A local experience of non-operative management for an appendicitis cohort during COVID-19

Dinh Van Chi Mai [1], Alex Sagar [1], Nainika Suresh Menon [1], Oliver Claydon [1], Ji Young Park [1], Billy Down [1], Barrie David Keeler [1]
Affiliations Expand

Affiliation

- [1] General Surgery, Milton Keynes University Hospital, Standing Way, Milton Keynes, MK6 5LD, UK.

- PMID: **33614023**
- PMCID: PMC7879779
- DOI: 10.1016/j.amsu.2021.02.006

Free PMC article

A local experience of non-operative management for an appendicitis cohort during COVID-19

Dinh Van Chi Mai et al. Ann Med Surg (Lond). 2021 Mar.
Free PMC article
Show details
Ann Med Surg (Lond)
. 2021 Mar;63:102160.
doi: 10.1016/j.amsu.2021.02.006. Epub 2021 Feb 12.

Authors

Dinh Van Chi Mai [1], Alex Sagar [1], Nainika Suresh Menon [1], Oliver Claydon [1], Ji Young Park [1], Billy Down [1], Barrie David Keeler [1]

Affiliation

- [1] General Surgery, Milton Keynes University Hospital, Standing Way, Milton Keynes, MK6 5LD, UK.

- PMID: **33614023**
- PMCID: PMC7879779
- DOI: 10.1016/j.amsu.2021.02.006

Abstract

Background: During the first United Kingdom COVID-19 wave, the Royal Colleges of Surgeons initially recommended conservative management with antibiotics instead of surgery for appendicitis. This study compared local outcomes of appendicitis during this period with a pre-COVID-19 cohort.

Methods: An observational study was conducted in a district general hospital. All episodes of appendicitis were prospectively studied from 25th March 2020 until 26th May 2020 and compared with a retrospective pre-COVID cohort from 27th November 2019 until 29th January 2020. Primary outcome was 30-day treatment failure of simple appendicitis for conservatively managed cases during COVID-19 compared to surgically managed cases pre-pandemic. Treatment failure was defined as any unplanned radiological or surgical intervention.

Results: Over nine weeks, there were 39 cases of appendicitis during COVID-19 and 50 cases pre-COVID-19. Twenty-six and 50 cases underwent appendicectomy during and pre-COVID-19 respectively. There was no difference in 30-day postoperative complication rates and nor were there any peri-operative COVID-19 infections.Twelve cases of simple appendicitis underwent conservative management during COVID-19 and were compared with 23 operatively managed simple cases pre-pandemic. There was a higher failure rate in the conservative versus operative group (33.3 vs 0% OR = 24.88, 95% CI 1.21 to 512.9, *p=0.0095*). Length of stay was similar (1.5 vs 2.0 *p=0.576*).

Discussion: Locally, conservative management was more likely to fail than initial appendicectomy. We suggest that surgery should remain first line for appendicitis, with conservative management reserved for those with suspected or proven COVID-19 infection.

Keywords: Appendicectomy; Appendicitis; COVID-19; SARS-CoV-2.

Crown Copyright © 2021 Published by Elsevier Ltd on behalf of IJS Publishing Group Ltd.

Conflict of interest statement

The authors have no conflicts of interest to declare and nor were there any sources of funding for this project.

- 17 references

Full text links

ELSEVIER OPEN ACCESS Elsevier Science Free PMC article

Proceed to details

Cite

Share

☐ 1,285

J Clin Med
. 2021 Dec 14;10(24):5857.
doi: 10.3390/jcm10245857.

Efficacy of Prolonged-Release Melatonin 2 mg (PRM 2 mg) Prescribed for Insomnia in Hospitalized Patients for COVID-19: A Retrospective Observational Study

Carolina Bologna [1], Pasquale Madonna [1], Eduardo Pone [1]
Affiliations Expand

Affiliation

- [1] UO Pneumologia Subintensiva COVID Ospedale del Mare ASL Na1, 80147 Naples, Italy.

- PMID: **34945156**
- PMCID: PMC8705392
- DOI: 10.3390/jcm10245857

Free PMC article

Efficacy of Prolonged-Release Melatonin 2 mg (PRM 2 mg) Prescribed for Insomnia in Hospitalized Patients for COVID-19: A Retrospective Observational Study

Carolina Bologna et al. J Clin Med. 2021.
Free PMC article
Show details
J Clin Med
. 2021 Dec 14;10(24):5857.
doi: 10.3390/jcm10245857.

Authors

Carolina Bologna [1], Pasquale Madonna [1], Eduardo Pone [1]

Affiliation

- [1] UO Pneumologia Subintensiva COVID Ospedale del Mare ASL Na1, 80147 Naples, Italy.

- PMID: **34945156**
- PMCID: PMC8705392
- DOI: 10.3390/jcm10245857

Abstract

Background: we have observed the effect of insomnia treatment in clinical and prognostic differences of patients admitted for COVID-19 pneumonia in respiratory sub-intensive units that were administered a prolonged-release melatonin 2 mg (PRM 2 mg) therapy versus a group of patients out of therapy.

Materials and methods: We evaluated 40 patients on prolonged-release melatonin 2 mg (PRM 2 mg) therapy versus a control group of 40 patients out of therapy.

Results: patients in the PRM 2 mg group had a shorter duration of therapy with non-invasive ventilation (5.2 ± 3.0 vs. 12.5 ± 4.2; $p < 0.001$), with a shorter stay in sub-intensive care (12.3 ± 3.2 vs. 20.1 ± 6.1; $p < 0.001$), and, therefore, a shorter overall duration of hospitalization (31.3 ± 6.8 vs. 34.3 ± 6.9 $p = 0.03$). In addition, a lower incidence of delirium was found (2.2 ± 1.1 vs. 3.3 ± 1.3; $p < 0.001$).

Conclusions: A significant increase in sleep hours and a reduction in delirium episodes occurs in hospitalized insomniac patients treated with PRM 2 mg, compared to untreated patients. Based on these preliminary results, we can assume that there are benefits of prolonged-release melatonin 2 mg in COVID-19 therapy.

Keywords: COVID; SARS-CoV; delirium; insomnia; prolonged-release melatonin 2 mg.

Conflict of interest statement

The authors declare no conflict of interest.

- 25 references
- 4 figures

Full text links

FULL TEXT OPEN ACCESS MDPI Multidisciplinary Digital Publishing Institute (MDPI) Free PMC article

Proceed to details

Cite

Share

☐ 1,286

Saudi Pharm J

. 2022 Feb 3.

doi: 10.1016/j.jsps.2022.01.022. Online ahead of print.

Comparison Between Standard Vs. Escalated Dose Venous Thromboembolism (VTE) Prophylaxis in Critically Ill Patients with COVID-19: A Two centers, Observational Study

Ohoud Aljuhani [1], Khalid Alsulaiman [2] [3] [4], Awattif Hafiz [1], Khalid Eljaaly [1] [5], Aisha Alharbi [6], Rahma Algarni [6], Sarah Al Homaid [3], Khawla Al Qahtani [3], Tareq Alsulaiman [7], Ramesh Vishwakarma [4], Ghassan Al Ghamdi [4] [8] [9], Mai Alalawi [10], Ghazwa B Korayem [11]

Affiliations Expand

Affiliations

- [1] Department of Pharmacy Practice, Faculty of Pharmacy, King Abdulaziz University, Jeddah, Saudi Arabia.
- [2] Pharmaceutical Care Department, King Abdulaziz Medical City, Riyadh, Saudi Arabia.
- [3] College of Pharmacy, King Saud bin Abdulaziz University for Health Sciences, Riyadh, Saudi Arabia.
- [4] King Abdullah International Medical Research Center, Biostatistics and Bioinformatics Department, Riyadh, Saudi Arabia.
- [5] College of Pharmacy, University of Arizona, Tucson, AZ, United States.
- [6] Pharmaceutical Care Department, King Abdulaziz University Hospital, Jeddah, Saudi Arabia.
- [7] Department of Orthopedic Surgery, Imam Abdulrahman Al Faisal Hospital, Riyadh, Saudi Arabia.
- [8] College of Medicine, King Saud Bin Abdulaziz University for Health Sciences, King Abdullah International Medical Research Center.
- [9] Intensive Care Department, King Abdulaziz Medical City, Riyadh, Saudi Arabia.
- [10] Children Cancer Hospital Foundation, Cairo, Egypt.
- [11] Department of Pharmacy Practice, College of Pharmacy, Princess Nourah bint Abdulrahman University, Riyadh, Saudi Arabia.

- PMID: **35136364**
- PMCID: PMC8812085
- DOI: 10.1016/j.jsps.2022.01.022

Free PMC article

Comparison Between Standard Vs. Escalated Dose Venous Thromboembolism (VTE) Prophylaxis in Critically Ill Patients with COVID-19: A Two centers, Observational Study

Ohoud Aljuhani et al. Saudi Pharm J. 2022.
Free PMC article
Show details
Saudi Pharm J
. 2022 Feb 3.
doi: 10.1016/j.jsps.2022.01.022. Online ahead of print.

Authors

Ohoud Aljuhani [1], Khalid Alsulaiman [2, 3, 4], Awattif Hafiz [1], Khalid Eljaaly [1, 5], Aisha Alharbi [6], Rahma Algarni [6], Sarah Al Homaid [3], Khawla Al Qahtani [3], Tareq Alsulaiman [7], Ramesh Vishwakarma [4], Ghassan Al Ghamdi [4, 8, 9], Mai Alalawi [10], Ghazwa B Korayem [11]

Affiliations

- [1] Department of Pharmacy Practice, Faculty of Pharmacy, King Abdulaziz University, Jeddah, Saudi Arabia.
- [2] Pharmaceutical Care Department, King Abdulaziz Medical City, Riyadh, Saudi Arabia.
- [3] College of Pharmacy, King Saud bin Abdulaziz University for Health Sciences, Riyadh, Saudi Arabia.
- [4] King Abdullah International Medical Research Center, Biostatistics and Bioinformatics Department, Riyadh, Saudi Arabia.
- [5] College of Pharmacy, University of Arizona, Tucson, AZ, United States.
- [6] Pharmaceutical Care Department, King Abdulaziz University Hospital, Jeddah, Saudi Arabia.
- [7] Department of Orthopedic Surgery, Imam Abdulrahman Al Faisal Hospital, Riyadh, Saudi Arabia.
- [8] College of Medicine, King Saud Bin Abdulaziz University for Health Sciences, King Abdullah International Medical Research Center.
- [9] Intensive Care Department, King Abdulaziz Medical City, Riyadh, Saudi Arabia.
- [10] Children Cancer Hospital Foundation, Cairo, Egypt.
- [11] Department of Pharmacy Practice, College of Pharmacy, Princess Nourah bint Abdulrahman University, Riyadh, Saudi Arabia.

- PMID: **35136364**
- PMCID: PMC8812085
- DOI: 10.1016/j.jsps.2022.01.022

Abstract

Introduction: The risk of mortality in patients with COVID-19 was found to be significantly higher in patients who experienced thromboembolic events. Thus, several guidelines recommend using prophylactic anticoagulants in all COVID-19 hospitalized patients. However, there is uncertainty about the appropriate dosing regimen and safety of anticoagulation in critically ill COVID-19 patients. Thus, the study aims to compare the effectiveness and safety of standard and escalated dose pharmacological VTE prophylaxis in critically ill patients with COVID-19.

Methods: A two centers retrospective cohort study including critically ill patients aged ≥ 18-years with confirmed COVID-19 admitted to the ICUs at two tertiary hospitals in Saudi Arabia from March 1st, 2020, until January 31st, 2021. Patients who received either Enoxaparin 40 mg daily or UFH 5000 Unit three times daily were grouped under the "standard dose VTE prophylaxis or higher than standard dose but not as treatment dose "escalated VTE prophylaxis dose" The primary outcome was thrombosis, and the secondary outcomes included ICU-related complication (s).

Results: A total of 758 patients were screened; 565 patients were included in the study. We matched 352 patients using propensity score matching (1:1). The differences in venous thromboembolism (OR 0.75; 95% CI 0.16-3.38; *P* =0.70) and any case of thrombosis during ICU were more likely to occur (OR 1.22;95%CI 0.52-2.86; *P*=0.64). In contrast, minor bleeding was associated with the use of escalated VTE prophylaxis dose (OR 3.39; 95% CI 1.08-10.61; *P*=0.04). There was no difference in the 30-day mortality nor in-hospital mortality between the two groups (HR 1.17;95%CI0.79-1.73; *P*=0.43 and (HR 1.08;95%CI 0.76-1.53; *P*=0.83, respectively.

Conclusion: Escalated-dose pharmacological VTE prophylaxis in critically ill patients with COVID-19 was not associated with VTE, thrombosis, or mortality benefits but led to an increased risk of minor bleeding. This study supports previous study findings regarding optimal dosing VTE pharmacological prophylaxis regimen for critically ill patients with COVID-19.

Keywords: COVID-19; COVID-19, Coronavirus disease; Critically ill; DVT prophylaxis; Enoxaparin; Heparin; ICUs, Intensive care units; Intensive Care Units (ICUs); LOS, Length of Stay; MV, Mechanical ventilation; SARS-Cov-2; VTE prophylaxis; VTE, Venous thromboembolism.

© 2022 The Author(s).

Conflict of interest statement

The authors declare that they have no known competing financial interests or personal relationships that could have appeared to influence the work reported in this paper.

- 37 references
- 3 figures

Full text links

Elsevier Science Free PMC article

Proceed to details

Cite

Share

☐ 1,287

EClinicalMedicine

. 2020 Nov;28:100591.

doi: 10.1016/j.eclinm.2020.100591. Epub 2020 Oct 15.

Clinical characteristics and outcomes among hospitalized adults with severe COVID-19 admitted to a tertiary medical center and receiving antiviral, antimalarials, glucocorticoids, or immunomodulation with tocilizumab or cyclosporine: A retrospective observational study (COQUIMA cohort)

Pablo Guisado-Vasco [1], Sofia Valderas-Ortega [2], Maria Maravillas Carralón-González [1], Ana Roda-Santacruz [3], Lucia González-Cortijo [4], Gabriel Sotres-Fernández [1], Eva María Martí-Ballesteros [5], José Manuel Luque-Pinilla [1], Elena Almagro-Casado [4], Félix J La Coma-Lanuza [6], Ruth Barrena-Puertas [1], Esteban Javier Malo-Benages [7], María José Monforte-Gómez

[8], Rocío Diez-Munar [9], Esther Merino-Lanza [10], Lorena Comeche-Casanova [11], Margarita Remirez-de-Esparza-Otero [12], María Correyero-Plaza [13], Manuel Recio-Rodríguez [14], Margarita Rodríguez-López [15], María Dolores Sánchez-Manzano [1], Cristina Andreu-Vázquez [16], Israel John Thuissard-Vasallo [16], José María Echave-Sustaeta María-Tomé [11], Daniel Carnevali-Ruiz [1]
Affiliations Expand

Affiliations

- [1] Department of Internal Medicine. Hospital universitario quironsalud Madrid, Universidad Europea (Madrid), Pozuelo de Alarcon, Madrid, Spain.
- [2] Nurse. Hospital Infectious Diseases Control Unit, Hospital universitario quironsalud Madrid, Pozuelo de Alarcon, Madrid, Spain.
- [3] Research coordinator. Research and clinical trials unit, Hospital universitario quironsalud Madrid, Pozuelo de Alarcon, Madrid, Spain.
- [4] Department of Oncology. Hospital universitario quironsalud Madrid, Universidad Europea (Madrid), Pozuelo de Alarcon, Madrid, Spain.
- [5] Department of Hematology, Hospital universitario quironsalud Madrid, Pozuelo de Alarcon, Madrid, Spain.
- [6] Intensive Care Unit, Hospital universitario quironsalud Madrid, Pozuelo de Alarcon, Madrid, Spain.
- [7] Department of Angiology and Vascular Surgery, Hospital universitario quironsalud Madrid, Pozuelo de Alarcon, Madrid, Spain.
- [8] Department of Internal Medicine, Hospital quironsalud San Jose, Madrid, Spain.
- [9] Department of Anesthesiology, Hospital universitario quironsalud Madrid, Pozuelo de Alarcon, Madrid, Spain.
- [10] Department of Cardiology, Hospital universitario quironsalud Madrid, Pozuelo de Alarcon, Madrid, Spain.
- [11] Department of Pneumology, Hospital universitario quironsalud Madrid, Universidad Europea (Madrid), Pozuelo de Alarcon, Madrid, Spain.
- [12] Pharmacy Unit, Hospital universitario quironsalud Madrid, Pozuelo de Alarcon, Madrid, Spain.
- [13] Department of Rheumatology, Hospital universitario quironsalud Madrid, Pozuelo de Alarcon, Madrid, Spain.
- [14] Department of Radiology department, Hospital universitario quironsalud Madrid, Universidad Europea (Madrid), Pozuelo de Alarcon, Madrid, Spain.
- [15] Department of Endocrinology, Hospital universitario quironsalud Madrid, Pozuelo de Alarcon, Madrid, Spain.
- [16] Statistical section, Faculty of Biomedical Science and Health, Universidad Europea (Madrid), Villaciosa de Odón, Madrid, Spain.

- PMID: **33078138**
- PMCID: PMC7557296
- DOI: 10.1016/j.eclinm.2020.100591

Free PMC article

Clinical characteristics and outcomes among hospitalized adults with severe COVID-19 admitted to a tertiary medical center and receiving antiviral, antimalarials, glucocorticoids, or immunomodulation with tocilizumab or cyclosporine: A retrospective observational study (COQUIMA cohort)

Pablo Guisado-Vasco et al. EClinicalMedicine. 2020 Nov.
Free PMC article
Show details
EClinicalMedicine
. 2020 Nov;28:100591.
doi: 10.1016/j.eclinm.2020.100591. Epub 2020 Oct 15.

Authors

Pablo Guisado-Vasco [1], Sofia Valderas-Ortega [2], Maria Maravillas Carralón-González [1], Ana Roda-Santacruz [3], Lucia González-Cortijo [4], Gabriel Sotres-Fernández [1], Eva María Martí-Ballesteros [5], José Manuel Luque-Pinilla [1], Elena Almagro-Casado [4], Félix J La Coma-Lanuza [6], Ruth Barrena-Puertas [1], Esteban Javier Malo-Benages [7], María José Monforte-Gómez [8], Rocío Diez-Munar [9], Esther Merino-Lanza [10], Lorena Comeche-Casanova [11], Margarita Remirez-de-Esparza-Otero [12], María Correyero-Plaza [13], Manuel Recio-Rodríguez [14], Margarita Rodríguez-López [15], María Dolores Sánchez-Manzano [1], Cristina Andreu-Vázquez [16], Israel John Thuissard-Vasallo [16], José María Echave-Sustaeta María-Tomé [11], Daniel Carnevali-Ruiz [1]

Affiliations

- [1] Department of Internal Medicine. Hospital universitario quironsalud Madrid, Universidad Europea (Madrid), Pozuelo de Alarcon, Madrid, Spain.
- [2] Nurse. Hospital Infectious Diseases Control Unit, Hospital universitario quironsalud Madrid, Pozuelo de Alarcon, Madrid, Spain.
- [3] Research coordinator. Research and clinical trials unit, Hospital universitario quironsalud Madrid, Pozuelo de Alarcon, Madrid, Spain.
- [4] Department of Oncology. Hospital universitario quironsalud Madrid, Universidad Europea (Madrid), Pozuelo de Alarcon, Madrid, Spain.
- [5] Department of Hematology, Hospital universitario quironsalud Madrid, Pozuelo de Alarcon, Madrid, Spain.
- [6] Intensive Care Unit, Hospital universitario quironsalud Madrid, Pozuelo de Alarcon, Madrid, Spain.
- [7] Department of Angiology and Vascular Surgery, Hospital universitario quironsalud Madrid, Pozuelo de Alarcon, Madrid, Spain.

- [8] Department of Internal Medicine, Hospital quironsalud San Jose, Madrid, Spain.
- [9] Department of Anesthesiology, Hospital universitario quironsalud Madrid, Pozuelo de Alarcon, Madrid, Spain.
- [10] Department of Cardiology, Hospital universitario quironsalud Madrid, Pozuelo de Alarcon, Madrid, Spain.
- [11] Department of Pneumology, Hospital universitario quironsalud Madrid, Universidad Europea (Madrid), Pozuelo de Alarcon, Madrid, Spain.
- [12] Pharmacy Unit, Hospital universitario quironsalud Madrid, Pozuelo de Alarcon, Madrid, Spain.
- [13] Department of Rheumatology, Hospital universitario quironsalud Madrid, Pozuelo de Alarcon, Madrid, Spain.
- [14] Department of Radiology department, Hospital universitario quironsalud Madrid, Universidad Europea (Madrid), Pozuelo de Alarcon, Madrid, Spain.
- [15] Department of Endocrinology, Hospital universitario quironsalud Madrid, Pozuelo de Alarcon, Madrid, Spain.
- [16] Statistical section, Faculty of Biomedical Science and Health, Universidad Europea (Madrid), Villaciosa de Odón, Madrid, Spain.

- PMID: **33078138**
- PMCID: PMC7557296
- DOI: 10.1016/j.eclinm.2020.100591

Abstract

Background: The COVID-19 outbreak challenges the Spanish health system since March 2020. Some available therapies (antimalarials, antivirals, biological agents) were grounded on clinical case observations or basic science data. The aim of this study is to describe the characteristics and impact of different therapies on clinical outcomes in a cohort of severe COVID-19 patients.

Methods: In this retrospective, single-center, observational study, we collected sequential data on adult patients admitted to Hospital Universitario Quironsalud Madrid. Eligible patients should have a microbiological (positive test on RT-PCR assay from a nasal swab) or an epidemiological diagnosis of severe COVID-19. Demographic, baseline comorbidities, laboratory data, clinical outcomes, and treatments were compared between survivors and non-survivors. We carried out univariate and multivariate logistic regression models to assess potential risk factors for in-hospital mortality.

Findings: From March 10th to April 15th, 2020, 607 patients were included. Median age was 69 years [interquartile range, {IQR} 22; 65% male). The most common comorbidities were hypertension (276 [46·94%]), diabetes (95 [16·16%]), chronic cardiac (133 [22·62%]) and respiratory (114 [19·39%]) diseases. 141 patients (23·2%) died. In the multivariate model the risk of death increased with older age (odds ratio, for every year of age, 1·15, [95% CI 1·11 - 1·2]), tocilizumab therapy (2·4, [1·13 - 5·11]), C-reactive protein at admission (1·07, per 10 mg/L, [1·04 - 1·10]), d-dimer > 2·5 µg/mL (1·99, [1·03 - 3·86]), diabetes mellitus (2·61, [1·19 - 5·73]), and the PaO_2/FiO_2 at admission (0·99, per every 1 mmHg, [0·98 - 0·99]). Among the prescribed therapies (tocilizumab, glucocorticoids, lopinavir/ritonavir, hydroxychloroquine, cyclosporine), only cyclosporine was associated with a significant decrease in mortality (0·24, [0·12 - 0·46]; *p*<0·001).

Interpretation: In a real-clinical setting, inhibition of the calcineurin inflammatory pathway, NF-κB, could reduce the hyperinflammatory phase in COVID-19. Our findings might entail relevant

implications for the therapy of this disease and could boost the design of new clinical trials among subjects affected by severe COVID-19.

Funding: Hospital Universitario Quironsalud Madrid. Own fundings for COVID-19 research.

Keywords: Acute respiratory insufficiency; Cyclosporine a; Hyperinflammation state; Immunosuppressants; SARS-CoV-2 infection; Severe COVID-19.

© 2020 The Author(s).

Conflict of interest statement

Dr. Correyero Plaza reports grants from Procare, Pfizer, Novartis, Lilly, Abbvie, Roche and personal fees from Onclinical during the conduct of the study; grants from Procare, Abbvie, Roche and personal fees from Onclinical outside the submitted work. The rest of the authors declares no competing interest for the current work.

- 51 references
- 4 figures

Full text links

ELSEVIER OPEN ACCESS Elsevier Science Free PMC article

Proceed to details

Cite

Share

☐ 1,288

Turk J Emerg Med

. 2022 Jan 20;22(1):36-43.

doi: 10.4103/2452-2473.336106. eCollection Jan-Mar 2022.

Extracorporeal membrane oxygenation experiences during COVID-19 pandemic, third wave with younger patients: A retrospective observational study

Ahmet Oğuzhan Küçük [1], Mehtap Pehlivanlar Küçük [1], Olcay Ayçiçek [2], Gökalp Altun [3], Ahmet Coşkun Özdemir [3]

Affiliations Expand

Affiliations

- [1] Department of Chest Diseases, Division of Intensive Care Medicine, Faculty of Medicine, Karadeniz Technical University, Trabzon, Turkey.
- [2] Department of Chest Diseases, Faculty of Medicine, Karadeniz Technical University, Trabzon, Turkey.

- [3] Department of Cardiovascular Surgery, Faculty of Medicine, Karadeniz Technical University, Trabzon, Turkey.

- PMID: **35284694**
- PMCID: PMC8862797
- DOI: 10.4103/2452-2473.336106

Free PMC article

Extracorporeal membrane oxygenation experiences during COVID-19 pandemic, third wave with younger patients: A retrospective observational study

Ahmet Oğuzhan Küçük et al. Turk J Emerg Med. 2022.
Free PMC article
Show details
Turk J Emerg Med
. 2022 Jan 20;22(1):36-43.
doi: 10.4103/2452-2473.336106. eCollection Jan-Mar 2022.

Authors

Ahmet Oğuzhan Küçük [1], Mehtap Pehlivanlar Küçük [1], Olcay Ayçiçek [2], Gökalp Altun [3], Ahmet Coşkun Özdemir [3]

Affiliations

- [1] Department of Chest Diseases, Division of Intensive Care Medicine, Faculty of Medicine, Karadeniz Technical University, Trabzon, Turkey.
- [2] Department of Chest Diseases, Faculty of Medicine, Karadeniz Technical University, Trabzon, Turkey.
- [3] Department of Cardiovascular Surgery, Faculty of Medicine, Karadeniz Technical University, Trabzon, Turkey.

- PMID: **35284694**
- PMCID: PMC8862797
- DOI: 10.4103/2452-2473.336106

Abstract

Objectives: In this article, the results of severe coronavirus disease 2019 (COVID-19) cases followed with extracorporeal membrane oxygenation (ECMO) support in a 3-month period in the third wave when there were an increased number of cases of young patients in our intensive care unit (ICU) were presented.

Methods: The study was carried out with all COVID-19 patients who were given ECMO support in our tertiary referral hospital ICU after obtaining the consent of the Ministry of Health Scientific Research Platform and after the approval of the local ethics committee. Patient data were obtained retrospectively from intensive care bedside follow-up charts and computer records. The demographic and clinical characteristics of the patients were presented in average, median, and percentages. The data of the patients were evaluated and compared with the current literature.

Results: ECMO treatment was applied in seven patients who were followed up with severe COVID-19 pneumonia in the last 3 months. Venovenous extracorporeal membrane oxygenation (VV-ECMO) was applied to all patients. Five (71.5%) of seven patients were weaned from ECMO. Four (57.2%) of seven patients were discharged from the ICU and hospital in good health. While two of the patients had a cesarean section (C/S) before ECMO, one patient underwent C/S under ECMO. All three newborns were delivered via C/S and all were premature (C/S dates were 35 weeks, 32 weeks, and 27 weeks), and all were discharged from the hospital in good health.

Conclusion: Our experience shows that ECMO in COVID-19 patients is a lifesaving treatment option that can be successfully applied in severe acute respiratory distress syndrome cases who do not respond to conventional treatments.

Keywords: Coronavirus disease 2019; SARS-CoV-2; extracorporeal membrane oxygenation; mortality; pregnancy.

Copyright: © 2022 Turkish Journal of Emergency Medicine.

Conflict of interest statement

Conflicts of interest None Declared.

- 17 references
- 1 figure

Full text links

Free PMC article
Proceed to details
Cite
Share
☐ 1,289
JBMR Plus
. 2021 Nov 17;5(12):e10576.
doi: 10.1002/jbm4.10576. eCollection 2021 Dec.

Vitamin D Endocrine System and COVID-19

Roger Bouillon [1], José Manuel Quesada-Gomez [2] [3]
Affiliations Expand

Affiliations

- [1] Laboratory of Clinical and Experimental Endocrinology, Department of Chronic Diseases, Metabolism and Ageing KU Leuven Leuven Belgium.
- [2] Instituto Maimónides de Investigación Biomédica de Córdoba (IMIBIC), Hospital Universitario Reina Sofía Universidad de Córdoba, Fundación Progreso y Salud Córdoba Spain.
- [3] CIBER de Fragilidad y Envejecimiento Saludable (CIBERFES) Madrid Spain.

- PMID: **34950831**
- PMCID: PMC8674769
- DOI: 10.1002/jbm4.10576

Free PMC article

Vitamin D Endocrine System and COVID-19

Roger Bouillon et al. JBMR Plus. 2021.
Free PMC article
Show details
JBMR Plus
. 2021 Nov 17;5(12):e10576.
doi: 10.1002/jbm4.10576. eCollection 2021 Dec.

Authors

Roger Bouillon [1], José Manuel Quesada-Gomez [2, 3]

Affiliations

- [1] Laboratory of Clinical and Experimental Endocrinology, Department of Chronic Diseases, Metabolism and Ageing KU Leuven Leuven Belgium.
- [2] Instituto Maimónides de Investigación Biomédica de Córdoba (IMIBIC), Hospital Universitario Reina Sofía Universidad de Córdoba, Fundación Progreso y Salud Córdoba Spain.
- [3] CIBER de Fragilidad y Envejecimiento Saludable (CIBERFES) Madrid Spain.

- PMID: **34950831**
- PMCID: PMC8674769
- DOI: 10.1002/jbm4.10576

Abstract

Preclinical data strongly suggest that the vitamin D endocrine system (VDES) may have extraskeletal effects. Cells of the immune and cardiovascular systems and lungs can express the vitamin D receptor, and overall these cells respond in a coherent fashion when exposed to 1,25-dihydroxyvitamin D, the main metabolite of the VDES. Supplementation of vitamin D-deficient subjects may decrease the risk of upper respiratory infections. The VDES also has broad anti-inflammatory and anti-thrombotic effects, and other mechanisms argue for a potential beneficial effect of a good vitamin D status on acute respiratory distress syndrome, a major complication of this SARS-2/COVID-19 infection. Activation of the VDES may thus have beneficial effects on

the severity of COVID-19. Meta-analysis of observational data show that a better vitamin D status decreased the requirement of intensive care treatment or decreased mortality. A pilot study in Cordoba indicated that admission to intensive care was drastically reduced by administration of a high dose of calcifediol early after hospital admission for COVID-19. A large observational study in Barcelona confirmed that such therapy significantly decreased the odds ratio (OR) of mortality (OR = 0.52). This was also the conclusion of a retrospective study in five hospitals of Southern Spain. A retrospective study on all Andalusian patients hospitalized because of COVID-19, based on real-world data from the health care system, concluded that prescription of calcifediol (hazard ratio [HR] = 0.67) or vitamin D (HR = 0.75), 15 days before hospital admission decreased mortality within the first month. In conclusion, a good vitamin D status may have beneficial effects on the course of COVID-19. This needs to be confirmed by large, randomized trials, but in the meantime, we recommend (rapid) correction of 25 hydroxyvitamin D (25OHD) deficiency in subjects exposed to this coronavirus. © 2021 The Authors. *JBMR Plus* published by Wiley Periodicals LLC on behalf of American Society for Bone and Mineral Research.

Keywords: ACUTE RESPIRATORY DISTRESS SYNDROME; CALCIFEDIOL; COVID-19; INTENSIVE CARE TREATMENT; MORTALITY; VITAMIN D.

© 2021 The Authors. JBMR Plus published by Wiley Periodicals LLC on behalf of American Society for Bone and Mineral Research.

Conflict of interest statement

RB received small lecture fees from Abiogen (Italy), FAES-Farma (Spain), and Fresenius (Germany). JMQG received small lecture fees from Amgen (Spain) and FAES-Farma (Spain).

- 131 references
- 4 figures

Full text links

WILEY Free Full Text Wiley Free PMC article

Proceed to details

Cite

Share

☐ 1,290

Clin Exp Nephrol

. 2022 Mar 1;1-8.

doi: 10.1007/s10157-022-02180-6. Online ahead of print.

Disease severity and renal outcomes of patients with chronic kidney disease infected with COVID-19

Efrat Gur [1], David Levy [1], Guy Topaz [1, 2], Rawand Naser [1], Ori Wand [2, 3], Yona Kitay-Cohen [1, 2], Sydney Benchetrit [2, 4], Erez Sarel [5], Keren Cohen-Hagai [6, 7]

Affiliations Expand

Affiliations

- [1] Department of Internal Medicine C, Meir Medical Center, Kefar Sava, Israel.
- [2] Sackler Faculty of Medicine, Tel Aviv University, Tel Aviv, Israel.
- [3] Department of Pulmonology, Meir Medical Center, Kefar Sava, Israel.
- [4] Department of Nephrology and Hypertension, Meir Medical Center, 59 Tchernichovsky St., 4428164, Kefar Sava, Israel.
- [5] Department of Anesthesiology, Meir Medical Center, Kefar Sava, Israel.
- [6] Sackler Faculty of Medicine, Tel Aviv University, Tel Aviv, Israel. keren.cohen@clalit.org.il.
- [7] Department of Nephrology and Hypertension, Meir Medical Center, 59 Tchernichovsky St., 4428164, Kefar Sava, Israel. keren.cohen@clalit.org.il.

- PMID: **35230569**
- PMCID: PMC8886555
- DOI: 10.1007/s10157-022-02180-6

Free PMC article

Disease severity and renal outcomes of patients with chronic kidney disease infected with COVID-19

Efrat Gur et al. Clin Exp Nephrol. 2022.
Free PMC article
Show details
Clin Exp Nephrol
. 2022 Mar 1;1-8.
doi: 10.1007/s10157-022-02180-6. Online ahead of print.

Authors

Efrat Gur [1], David Levy [1], Guy Topaz [1, 2], Rawand Naser [1], Ori Wand [2, 3], Yona Kitay-Cohen [1, 2], Sydney Benchetrit [2, 4], Erez Sarel [5], Keren Cohen-Hagai [6, 7]

Affiliations

- [1] Department of Internal Medicine C, Meir Medical Center, Kefar Sava, Israel.
- [2] Sackler Faculty of Medicine, Tel Aviv University, Tel Aviv, Israel.
- [3] Department of Pulmonology, Meir Medical Center, Kefar Sava, Israel.
- [4] Department of Nephrology and Hypertension, Meir Medical Center, 59 Tchernichovsky St., 4428164, Kefar Sava, Israel.
- [5] Department of Anesthesiology, Meir Medical Center, Kefar Sava, Israel.
- [6] Sackler Faculty of Medicine, Tel Aviv University, Tel Aviv, Israel. keren.cohen@clalit.org.il.

- [7] Department of Nephrology and Hypertension, Meir Medical Center, 59 Tchernichovsky St., 4428164, Kefar Sava, Israel. keren.cohen@clalit.org.il.

- PMID: **35230569**
- PMCID: PMC8886555
- DOI: 10.1007/s10157-022-02180-6

Abstract

Introduction: While there is evidence of the presence of the coronavirus in the kidneys and resultant acute kidney injury (AKI), information on the effect of chronic kidney disease (CKD) on COVID-19 outcomes and its pathogenesis is currently lacking.

Methods: This retrospective, observational study evaluated the outcomes of all consecutive patients hospitalized during COVID-19 outbreaks in Meir Medical Center. Serum creatinine level was assessed before hospitalization ("baseline serum creatinine") and at admission, as well as minimum and maximum serum creatinine levels during hospitalization.

Results: Among 658 patients, 152 had eGFR < 60 ml/min (termed the CKD group), 506 patients served as controls. Patients in the CKD group were older, with higher prevalence of hypertension, diabetes mellitus and atherosclerosis. Disease severity and clinical presentation of CKD group were comparable to that of control group. Odds ratio for AKI was 5.8 (95%CI 3.8-8.7; p < 0.001) in CKD group vs. control group and 3.4 (95%CI 1.1-10.8) for renal replacement therapy (p < 0.026). Among the CKD group, 32.2% died after COVID-19 infection versus 14.8% of the controls (p < 0.001). Mortality increased as CKD stage increased (14.8% in controls, 29.6% in CKD stage 3, and 39.3% in CKD stages 4 and 5, p < 0.001).

Conclusion: Despite comparable disease severity at presentation, patients with CKD had significantly more AKI events and required more renal replacement therapy during hospitalization than control patients did. Mortality increased as CKD stage increased.

Keywords: Acute kidney injury; COVID-19 outcomes; Chronic kidney disease.

© 2022. The Author(s), under exclusive licence to The Japanese Society of Nephrology.

Conflict of interest statement

The authors declare that they have no conflicts of interest.

- 38 references
- 3 figures

Full text links

SpringerLink FULL-TEXT ARTICLE Springer Free PMC article
Proceed to details

Cite

Share

☐ 1,291

Infect Drug Resist

. 2021 Dec 10;14:5287-5291.

doi: 10.2147/IDR.S330743. eCollection 2021.

Ribavirin Treatment for Critically Ill COVID-19 Patients: An Observational Study

Yonghao Xu [#] [1], Manshu Li [#] [1], Liang Zhou [#] [1], Dongdong Liu [1], Weiqun He [1], Weibo Liang [1], Qingwen Sun [1], Huadong Sun [1], Yimin Li [1], Xiaoqing Liu [1]
Affiliations Expand

Affiliation

- [1] Department of Critical Care Medicine, State Key Laboratory of Respiratory Diseases, Guangzhou Institute of Respiratory Health, First Affiliated Hospital of Guangzhou Medical University, Guangzhou, Guangdong, 510120, People's Republic of China.

[#] Contributed equally.

- PMID: **34916812**
- PMCID: PMC8672023
- DOI: 10.2147/IDR.S330743

Free PMC article

Ribavirin Treatment for Critically Ill COVID-19 Patients: An Observational Study

Yonghao Xu et al. Infect Drug Resist. 2021.
Free PMC article
Show details
Infect Drug Resist
. 2021 Dec 10;14:5287-5291.
doi: 10.2147/IDR.S330743. eCollection 2021.

Authors

Yonghao Xu [#] [1], Manshu Li [#] [1], Liang Zhou [#] [1], Dongdong Liu [1], Weiqun He [1], Weibo Liang [1], Qingwen Sun [1], Huadong Sun [1], Yimin Li [1], Xiaoqing Liu [1]

Affiliation

- [1] Department of Critical Care Medicine, State Key Laboratory of Respiratory Diseases, Guangzhou Institute of Respiratory Health, First Affiliated Hospital of Guangzhou Medical University, Guangzhou, Guangdong, 510120, People's Republic of China.

[#] Contributed equally.

- PMID: **34916812**
- PMCID: [PMC8672023]
- DOI: [10.2147/IDR.S330743]

Abstract

Background: The coronavirus disease 2019 (COVID-19) pandemic has spread all over the world resulting in high mortality, yet no specific antiviral treatment has been recommended.

Methods: A retrospective descriptive study was conducted involving 19 consecutive critically ill patients during January 27, 2020 to April 18, 2020. Ribavirin was given at 0.15g q8h orally upon ICU admission for 7 to 21 days. Here, 28-day mortality, lower respiratory tract specimens (ETA), and ribavirin side effect on the day of ICU admission (Day 1), Day 7, Day 14 and Day 21 were analyzed.

Results: All the nineteen critically ill COVID-19 patients (14 males and 5 females, median age 56yr) survived through to the 28th day of observations with 6 patients (31.58%) being discharged from the ICU. The SARS-CoV-2 viral positivity in sputum/ETA was 100% (19/19) on Day 1, 73.68% (14/19) on Day 7, 57.89% (11/19) on Day 14 and 36.84% (7/19) on Day 21. Ribavirin side effect was not observed in these patients.

Conclusion: Ribavirin is well tolerated in critically ill patients with COVID-19 and may benefit COVID-19 patients through increasing the virus clearance.

Keywords: COVID-19; SARS-CoV-2; antiviral; ribavirin.

© 2021 Xu et al.

Conflict of interest statement

The authors report no conflicts of interest in this work.

- 18 references
- 1 figure

Supplementary info

Grant support Expand

Grant support

The study was funded by the National Natural Science Foundation of China (82070084), National Science and Technology Major Project (No. 2017ZX10204401), and the Special Project of Guangdong Science and Technology Department (2020B111105001).

Full text links

FREE full-text article Dovepress Dove Medical Press Free PMC article

Proceed to details

Cite

Share

☐ 1,292

J Clin Med Res
. 2021 May;13(5):258-267.
doi: 10.14740/jocmr4507. Epub 2021 May 25.

Low Serum Albumin Predicts Severe Outcomes in COVID-19 Infection: A Single-Center Retrospective Case-Control Study

Roshan Acharya [1], Dilli Poudel [2], Riley Bowers [3], Aakash Patel [1], Evan Schultz [1], Michael Bourgeois [1], Rishi Paswan [1], Scott Stockholm [1], Macelyn Batten [1], Smita Kafle [4], Kriti Lonial [5], Irlene Locklear [5]
Affiliations Expand

Affiliations

- [1] Department of Internal Medicine, Cape Fear Valley Medical Center, Fayetteville, NC 28304, USA.
- [2] Department of Rheumatology, Indiana Regional Medical Center, Indiana, PA 15701, USA.
- [3] Department of Pharmacy, Cape Fear Valley Medical Center, Fayetteville, NC 28304, USA.
- [4] RN-BSN Program, Fayetteville State University, Fayetteville, NC 28301, USA.
- [5] Department of Pulmonology and Critical Care, Cape Fear Valley Medical Center, Fayetteville, NC 28304, USA.

- PMID: **34104277**
- PMCID: PMC8166291
- DOI: 10.14740/jocmr4507

Free PMC article

Low Serum Albumin Predicts Severe Outcomes in COVID-19 Infection: A Single-Center Retrospective Case-Control Study

Roshan Acharya et al. J Clin Med Res. 2021 May.
Free PMC article

Show details

J Clin Med Res
. 2021 May;13(5):258-267.
doi: 10.14740/jocmr4507. Epub 2021 May 25.

Authors

Roshan Acharya [1], Dilli Poudel [2], Riley Bowers [3], Aakash Patel [1], Evan Schultz [1], Michael Bourgeois [1], Rishi Paswan [1], Scott Stockholm [1], Macelyn Batten [1], Smita Kafle [4], Kriti Lonial [5], Irlene Locklear [5]

Affiliations

- [1] Department of Internal Medicine, Cape Fear Valley Medical Center, Fayetteville, NC 28304, USA.
- [2] Department of Rheumatology, Indiana Regional Medical Center, Indiana, PA 15701, USA.
- [3] Department of Pharmacy, Cape Fear Valley Medical Center, Fayetteville, NC 28304, USA.
- [4] RN-BSN Program, Fayetteville State University, Fayetteville, NC 28301, USA.
- [5] Department of Pulmonology and Critical Care, Cape Fear Valley Medical Center, Fayetteville, NC 28304, USA.

- PMID: **34104277**
- PMCID: PMC8166291
- DOI: 10.14740/jocmr4507

Abstract

Background: Coronavirus disease 2019 (COVID-19) can cause serious complications such as multiorgan failure and death which are difficult to predict. We conducted this retrospective case-control observational study with the hypothesis that low serum albumin at presentation can predict serious outcomes in COVID-19 infection.

Methods: We included severe acute respiratory syndrome coronavirus 2 (SARS-CoV-2) reverse transcriptase-polymerase chain reaction (RT-PCR) confirmed, hospitalized patients from March to July 2020 in a tertiary care hospital in the USA. Patients were followed for 21 days for the development of the primary endpoint defined as the composite outcome which included acute encephalopathy, acute kidney injury, the requirement of new renal replacement therapy, acute hypercoagulability, acute circulatory failure, new-onset heart failure, acute cardiac injury, acute arrhythmia, acute respiratory distress syndrome (ARDS), high flow oxygen support, intensive care unit (ICU) stay, mechanical ventilation or death; and the secondary endpoint of death only. Univariate and multivariate logistic regression analyses were performed to study the effect of albumin level and outcomes.

Results: The mean age was 56.76 years vs. 55.67 years (P = 0.68) in the normal albumin vs. the low albumin group. We noticed an inverse relationship between serum albumin at presentation and serious outcomes. The low albumin group had a higher composite outcome (93.88% vs. 6.12%, P < 0.05) and higher mortality (13.87% vs. 2.38%, P < 0.05) in comparison to the normal albumin group. The multivariate logistic regression analysis revealed higher odds of having composite outcomes with lower albumin group (odds ratio (OR) 10.88, 95% confidence interval (CI) 4.74 - 24.97, P < 0.05). In the subgroup analysis, the multivariate logistic regression analysis revealed higher odds of having composite outcomes with the very low albumin group (OR 7.94, 95% CI 1.70 - 37.14, P < 0.05).

Conclusions: Low serum albumin on presentation in COVID-19 infection is associated with serious outcomes not limited to mortality. The therapeutic option of albumin infusion should be investigated.

Keywords: ARDS; Albumin; COVID-19; Hypercoagulopathy; Hypoalbuminemia; Mortality; SARS-CoV-2; Serious outcomes.

Copyright 2021, Acharya et al.

Conflict of interest statement

None to declare.

- 47 references
- 1 figure

Full text links

Free PMC article
Proceed to details
Cite
Share
☐ 1,293

Front Med (Lausanne)
. 2020 Nov 30;7:593133.
doi: 10.3389/fmed.2020.593133. eCollection 2020.

No Evidence of Re-infection or Person-to-Person Transmission in Cured COVID-19 Patients in Guangzhou, a Retrospective Observational Study

Gang Xu [1], Feng Liu [1], Min Ye [1], Jun Zhao [1], Qing Li [1], Congrui Feng [1], Yudong Hu [1], Yueping Li [2], Haiyan Shi [2], Fuchun Zhang [2], Yuwei Tong [2], Wei Ma [1]
Affiliations Expand

Affiliations

- [1] Department of Geriatric Medicine, Guangzhou First People's Hospital, School of Medicine, South China University of Technology, Guangzhou, China.
- [2] Department of Respiratory Medicine, Guangzhou Eighth People's Hospital, Guangzhou, China.

- PMID: **33330554**
- PMCID: PMC7734204
- DOI: 10.3389/fmed.2020.593133

Free PMC article

No Evidence of Re-infection or Person-to-Person Transmission in Cured COVID-19 Patients in Guangzhou, a Retrospective Observational Study

Gang Xu et al. Front Med (Lausanne). 2020.
Free PMC article
Show details
Front Med (Lausanne)
. 2020 Nov 30;7:593133.
doi: 10.3389/fmed.2020.593133. eCollection 2020.

Authors

Gang Xu [1], Feng Liu [1], Min Ye [1], Jun Zhao [1], Qing Li [1], Congrui Feng [1], Yudong Hu [1], Yueping Li [2], Haiyan Shi [2], Fuchun Zhang [2], Yuwei Tong [2], Wei Ma [1]

Affiliations

- [1] Department of Geriatric Medicine, Guangzhou First People's Hospital, School of Medicine, South China University of Technology, Guangzhou, China.
- [2] Department of Respiratory Medicine, Guangzhou Eighth People's Hospital, Guangzhou, China.

- PMID: **33330554**
- PMCID: PMC7734204
- DOI: 10.3389/fmed.2020.593133

Abstract

Objectives: To clarify the clinical characteristics of cured patients with coronavirus disease (COVID-19), and to clarify the re-infection and person-to-person transmission in the cured. **Methods:** A total of 187 cured COVID-19 patients with antibody test were followed up every 2 weeks in this retrospective observational study. Assessment for general condition, symptoms, epidemiological contact history, polymerase chain reaction (PCR) assay, and antibody tests were performed and recorded. Information from Guangzhou CDC was also screened. **Results:** There were 33 (17.6%) patients with negative results for IgG and 35 (18.7%) patients with positive results for IgM. The average days of antibody detection from disease onset were 53.0. PCR assay was positive in 10 (5.3%) patients during the follow-up. Neither IgG nor IgM results showed a relationship with PCR test results (all $P > 0.05$). Neither re-infection nor person-to-person transmission was found in the cured patients. Factors associated with appearance of antibody comprised hospitalization days (OR: 1.06, 95%CI: 1.02-1.11, $P = 0.006$) and antibiotics treatment (OR: 3.50, 95%CI: 1.40-8.77, $P = 0.007$). **Conclusions:** In our study, no evidence of person-to-person transmission was found in cured COVID-19 patients. There seemed to be no re-infection in

the cured COVID-19 patients in Guangzhou. These finding suggest that the cured do not cause the spread of disease. Additionally, neither IgG nor IgM can be used to replace the PCR test in cured patients.

Keywords: COVID-19; antibody; cured patients; person-to-person transmission; re-infection.

Copyright © 2020 Xu, Liu, Ye, Zhao, Li, Feng, Hu, Li, Shi, Zhang, Tong and Ma.

Conflict of interest statement

The authors declare that the research was conducted in the absence of any commercial or financial relationships that could be construed as a potential conflict of interest.

- 17 references
- 2 figures

Full text links

Frontiers Media SA Free PMC article
Proceed to details
Cite
Share
☐ 1,294
J Pharm Policy Pract
. 2021 Oct 25;14(1):84.
doi: 10.1186/s40545-021-00370-3.

Incidence of adverse reaction of drugs used in COVID-19 management: a retrospective, observational study

Jia Yin Lee [1], Abby Shoon Yeun Ang [2], Nurdalila Mohd Ali [2], Li Min Ang [2], Azura Omar [2]
Affiliations Expand

Affiliations

- [1] Department of Pharmacy, Sungai Buloh Hospital, Ministry of Health Malaysia, Sungai Buloh, Selangor, Malaysia. joannelee415@gmail.com.
- [2] Department of Pharmacy, Sungai Buloh Hospital, Ministry of Health Malaysia, Sungai Buloh, Selangor, Malaysia.

- PMID: **34696800**
- PMCID: PMC8543425
- DOI: 10.1186/s40545-021-00370-3

Free PMC article

Incidence of adverse reaction of drugs used in COVID-19 management: a retrospective, observational study

Jia Yin Lee et al. J Pharm Policy Pract. 2021.
Free PMC article
Show details
J Pharm Policy Pract
. 2021 Oct 25;14(1):84.
doi: 10.1186/s40545-021-00370-3.

Authors

Jia Yin Lee [1], Abby Shoon Yeun Ang [2], Nurdalila Mohd Ali [2], Li Min Ang [2], Azura Omar [2]

Affiliations

- [1] Department of Pharmacy, Sungai Buloh Hospital, Ministry of Health Malaysia, Sungai Buloh, Selangor, Malaysia. joannelee415@gmail.com.
- [2] Department of Pharmacy, Sungai Buloh Hospital, Ministry of Health Malaysia, Sungai Buloh, Selangor, Malaysia.

- PMID: **34696800**
- PMCID: PMC8543425
- DOI: 10.1186/s40545-021-00370-3

Abstract

Background: An urgent need for coronavirus infectious disease (COVID-19) treatment has resulted in off-label drug use. Although previous studies had investigated the adverse drug reaction (ADR) of the medications for COVID-19 in their respective local settings, the safety profile in a Malaysian setting remains unknown. Our study aims to establish the incidence of ADR for drugs used in COVID-19 management in a Malaysian tertiary hospital.

Methods: This retrospective observational study enrolled patients started on drugs for COVID-19 in Sungai Buloh Hospital from 1 March 2020 to 31 May 2020. The clinical staging of COVID-19 patients was decided by the treating physician in accordance with the Clinical Management of Confirmed COVID-19 Case in Adults (Annex 2E). Suspected ADRs were evaluated with a trigger tool of pre-defined laboratory values or the adverse events listed in the registered product insert. Causality assessment was conducted when an ADR was suspected using the World Health Organization-Uppsala Monitoring Centre (WHO-UMC) system, and only cases classified as certain, probable and possible ADR were considered. Data analysis was completed with descriptive, univariate and multivariate analysis.

Results: The study (N = 1,080) identified 217 patients (20.1%) who experienced ADR, with 246 adverse events detected. Most events were related to the gastrointestinal (43.5%), hepatobiliary (36.2%) and cardiac (16.3%) systems. The most commonly suspected drugs were atazanavir (52.7%), chloroquine (36.8%) and lopinavir/ritonavir (34.6%). The independent risk factors of

ADR were female (adjusted odds ratio (OR): 1.53; 95% CI 1.06-2.20; P = 0.024), diagnosis of COVID-19 stage 3 (adjusted OR: 2.58; 95% CI 1.20-5.55; P = 0.015) and stage 4 (adjusted OR: 4.17; 95% CI 1.79-9.73; P = 0.001), and the number of COVID-19 drugs (adjusted OR: 3.34; 95% CI 2.51-4.44; P < 0.001). Only 49 adverse events (19.9%) were manually reported by healthcare professionals, with hyperbilirubinaemia (65.3%) and QT prolongation (28.6%) most frequently reported.

Conclusion: Medications used in COVID-19 management had resulted in one in five patients experiencing ADR. Our study has provided an overview on incidence of ADR for off-label use of medications used in COVID-19 management, which suggests a similar safety profile when used for FDA-approved indications.

Keywords: Adverse drug reactions; Clinical pharmacy; Drug monitoring; Drug safety; Pharmaceutical care.

© 2021. The Author(s).

Conflict of interest statement

The authors declare that they have no competing interests.

- 37 references

Full text links

Read free full text at BMC BioMed Central Free PMC article

Proceed to details

Cite

Share

☐ 1,295

Front Neurol

. 2022 Jan 31;12:822342.

doi: 10.3389/fneur.2021.822342. eCollection 2021.

Telestroke for the Treatment of Ischemic Stroke in Western China During the COVID-19 Pandemic: A Multicenter Observational Study

Ning Chen [1], Xintong Wu [1], Muke Zhou [1], Rongdong Yang [2], Daofeng Chen [3], Ming Liao [4], Yongyi Deng [5], Zhen Hong [1], Dong Zhou [1], Li He [1]

Affiliations Expand

Affiliations

- [1] Department of Neurology, West China Hospital, Sichuan University, Chengdu, China.

- [2] Department of Neurology, Guangyuan Central Hospital, Guangyuan, China.
- [3] Department of Neurology, The Second People's Hospital of Yibin, Yibin, China.
- [4] Department of Neurology, The First People's Hospital of Jintang, Chengdu, China.
- [5] Department of Neurology, West China Ganzi Hospital of Sichuan University, Kangding, China.

- PMID: **35173670**
- PMCID: PMC8841424
- DOI: 10.3389/fneur.2021.822342

Free PMC article

Telestroke for the Treatment of Ischemic Stroke in Western China During the COVID-19 Pandemic: A Multicenter Observational Study

Ning Chen et al. Front Neurol. 2022.
Free PMC article
Show details
Front Neurol
. 2022 Jan 31;12:822342.
doi: 10.3389/fneur.2021.822342. eCollection 2021.

Authors

Ning Chen [1], Xintong Wu [1], Muke Zhou [1], Rongdong Yang [2], Daofeng Chen [3], Ming Liao [4], Yongyi Deng [5], Zhen Hong [1], Dong Zhou [1], Li He [1]

Affiliations

- [1] Department of Neurology, West China Hospital, Sichuan University, Chengdu, China.
- [2] Department of Neurology, Guangyuan Central Hospital, Guangyuan, China.
- [3] Department of Neurology, The Second People's Hospital of Yibin, Yibin, China.
- [4] Department of Neurology, The First People's Hospital of Jintang, Chengdu, China.
- [5] Department of Neurology, West China Ganzi Hospital of Sichuan University, Kangding, China.

- PMID: **35173670**
- PMCID: PMC8841424
- DOI: 10.3389/fneur.2021.822342

Abstract

Background: Intravenous thrombolysis is still underutilized in patients with acute ischemic stroke (AIS) in China. A promising strategy for addressing this issue, especially in situations, such as the global pandemic of coronavirus disease 2019 (COVID-19), is the telestroke mode, which remains to be widely implemented in China. The present study aimed to assess the effects of telemedicine for patients with stroke in Western China, as well as the impact of the pandemic on telestroke services in 1 year after the COVID-19 outbreak.

Methods: In this 2-year multicenter observational study, we retrospectively collected data from 10 hospitals within the Sichuan Telestroke and Telethrombolysis Network. Demographic and clinical characteristics of patients with IS and those relevant to thrombolysis were compared between the pre-telestroke and post-telestroke phases, and between the periods before and after declaration of the COVID-19 pandemic.

Results: A total of 11,449 admissions with a primary diagnosis of IS were recorded during the study period. Prior to telestroke implementation, 6.7% of patients ($n = 367$) received intravenous thrombolysis, and the proportion increased to 7.4% ($n = 443$; $p = 0.084$) in the post-telestroke phase. The thrombolysis rate was 7.4% during the COVID-19 pandemic and in the latter half of the year when the viral spread was better controlled in China. The mean door-to-needle time (DNT) was significantly shorter after implementation of the telestroke network (63.76 ± 13.50 vs. 52.66 ± 25.49 min; $p < 0.001$).

Conclusions: Telemedicine is effective in improving the thrombolysis administration among patients with IS in Western China. Implementation of the telestroke network should be promoted, especially when access to care is affected by public health emergencies, such as the COVID-19 pandemic.

Keywords: Covid-19; coronavirus; stroke; telemedicine; telestroke; thrombolysis.

Copyright © 2022 Chen, Wu, Zhou, Yang, Chen, Liao, Deng, Hong, Zhou and He.

Conflict of interest statement

The authors declare that the research was conducted in the absence of any commercial or financial relationships that could be construed as a potential conflict of interest.

- 25 references
- 2 figures

Full text links

Frontiers Media SA Free PMC article

Proceed to details

Cite

Share

☐ 1,296

Turk Thorac J

. 2021 Jan;22(1):62-66.

doi: 10.5152/TurkThoracJ.2021.20180. Epub 2021 Jan 1.

Use of Hydroxychloroquine in Patients with COVID-19: A Retrospective Observational Study

Samah Mohamad Lotfy [1], Ahmad Abbas [1], Waheed Shouman [1]
Affiliations Expand

Affiliation

- [1] Department of Chest Diseases, Zagazig University Hospitals, Zagazig, Egypt.

- PMID: **33646106**
- PMCID: PMC7919430
- DOI: 10.5152/TurkThoracJ.2021.20180

Free PMC article

Use of Hydroxychloroquine in Patients with COVID-19: A Retrospective Observational Study

Samah Mohamad Lotfy et al. Turk Thorac J. 2021 Jan.
Free PMC article
Show details
Turk Thorac J
. 2021 Jan;22(1):62-66.
doi: 10.5152/TurkThoracJ.2021.20180. Epub 2021 Jan 1.

Authors

Samah Mohamad Lotfy [1], Ahmad Abbas [1], Waheed Shouman [1]

Affiliation

- [1] Department of Chest Diseases, Zagazig University Hospitals, Zagazig, Egypt.

- PMID: **33646106**
- PMCID: PMC7919430
- DOI: 10.5152/TurkThoracJ.2021.20180

Abstract

Objective: There is no consensus on a certain drug therapy for COVID-19 infection. Growing reports argue about the potential benefits of hydroxychloroquine (HCQ) in reducing morbidity and

mortality in patients hospitalized with COVID-19, but with inconsistent results.This study aimed to assess the potential benefits of HCQ on viral conversion, reducing the need for ICU or mechanical ventilation, and its impact on mortality.

Material and methods: This retrospective observational study was conducted enrolling confirmed SARS-CoV2 patients. They were subjected to plain CXR (HRCT of chest if needed), routine laboratory tests for COVID-19 (including CBC, CRP, LDH, D-Dimer, ferritin, and blood sugar), ECG, and blood gases. They were allocated to either HCQ or non-HCQ groups. Both groups were followed-up for symptoms resolution, need for ICU admission, non-invasive or invasive ventilation, duration till conversion, and mortality.

Results: A total of 202 patients with moderate COVID-19 were enrolled with a mean age of 55.05±10.15, out of whom 80% were male patients. The most common presenting symptom was fever (87.38% in the control group versus 92% in the HCQ group), followed by cough (82.52% versus 89.9%). In total, 24.27% of patients in the control group versus 28.3% in the HCQ group deteriorated and necessitated ICU admission (p=0.52), 13.6% of the control group versus 19.2% in the HCQ group required mechanical ventilation (p=0.28), and 69.9% of the control group versus 68.9% in the HCQ group converted negative on day 7 (p=0.85). No significant mortality difference between both groups was observed (4.9% versus 6.1%, p=0.47).

Conclusion: This work did not support any benefits of using HCQ in patients with COVID-19, neither in reducing the need for ICU, mechanical ventilation, nor mortality.

Conflict of interest statement

Conflict of Interest: The authors have no conflicts of interest to declare.

Full text links

Free PMC article
Proceed to details
Cite
Share
☐ 1,297
Ann Intensive Care
. 2020 Dec 10;10(1):167.
doi: 10.1186/s13613-020-00783-4.

Protracted viral shedding and viral load are associated with ICU mortality in Covid-19 patients with acute respiratory failure

L Bitker [1 2], F Dhelft [1 2], L Chauvelot [1], E Frobert [3 4 5], L Folliet [1], M Mezidi [1 5], S Trouillet-Assant [3 6], A Belot [7 8], B Lina [3 4 5], F Wallet [9], J C Richard [10 11]
Affiliations Expand

Affiliations

- [1] Service de Médecine Intensive Réanimation, Hôpital De La Croix Rousse, Hospices Civils de Lyon, 103 Grande Rue de la Croix Rousse, 69004, Lyon, France.
- [2] Université de Lyon, Université Claude Bernard, Lyon 1, INSA-Lyon, UJM-Saint Etienne, CNRS, Inserm, CREATIS, UMR 5220, U1206, 69621, Lyon, France.
- [3] CIRI, Centre International de Recherche en Infectiologie, Univ Lyon, Inserm, U1111, Université Claude Bernard Lyon 1, CNRS, UMR5308, ENS de Lyon, Team Virpath, 69007, Lyon, France.
- [4] Laboratoire de Virologie, Institut des Agents Infectieux, Hospices Civils de Lyon, National Reference Center for Respiratory Viruses, Department of Virology, Infective Agents Institute, North Hospital Network, Lyon, France; Virpath Laboratory, International Center of Research in Infectiology, INSERM U1111, CNRS-UMR 5308, École Normale Supérieure de Lyon, Université Claude Bernard Lyon, Université Claude Bernard, Lyon, France.
- [5] Université de Lyon, Université Claude Bernard, Lyon 1, Lyon, France.
- [6] Joint Research Unit Hospices Civils de Lyon-bioMérieux, Hospices Civils de Lyon, Lyon Sud Hospital, Pierre-Bénite, France.
- [7] National Referee Centre for Rheumatic AutoImmune and Systemic Diseases in childrEn (RAISE), Lyon, France.
- [8] Pediatric Nephrology, Rheumatology, Dermatology Unit, Hospices Civils de Lyon, Pierre-Bénite, France.
- [9] Service de Réanimation Polyvalente, Centre Hospitalier Lyon Sud, Hospices Civils de Lyon, Pierre-Bénite, France.
- [10] Service de Médecine Intensive Réanimation, Hôpital De La Croix Rousse, Hospices Civils de Lyon, 103 Grande Rue de la Croix Rousse, 69004, Lyon, France. j-christophe.richard@chu-lyon.fr.
- [11] Université de Lyon, Université Claude Bernard, Lyon 1, INSA-Lyon, UJM-Saint Etienne, CNRS, Inserm, CREATIS, UMR 5220, U1206, 69621, Lyon, France. j-christophe.richard@chu-lyon.fr.

- PMID: **33301059**
- PMCID: PMC7725883
- DOI: 10.1186/s13613-020-00783-4

Free PMC article

Protracted viral shedding and viral load are associated with ICU mortality in Covid-19 patients with acute respiratory failure

L Bitker et al. Ann Intensive Care. 2020.
Free PMC article

Show details

Ann Intensive Care
. 2020 Dec 10;10(1):167.
doi: 10.1186/s13613-020-00783-4.

Authors

L Bitker[1,2], F Dhelft[1,2], L Chauvelot[1], E Frobert[3,4,5], L Folliet[1], M Mezidi[1,5], S Trouillet-Assant[3,6], A Belot[7,8], B Lina[3,4,5], F Wallet[9], J C Richard[10,11]

Affiliations

- [1] Service de Médecine Intensive Réanimation, Hôpital De La Croix Rousse, Hospices Civils de Lyon, 103 Grande Rue de la Croix Rousse, 69004, Lyon, France.
- [2] Université de Lyon, Université Claude Bernard, Lyon 1, INSA-Lyon, UJM-Saint Etienne, CNRS, Inserm, CREATIS, UMR 5220, U1206, 69621, Lyon, France.
- [3] CIRI, Centre International de Recherche en Infectiologie, Univ Lyon, Inserm, U1111, Université Claude Bernard Lyon 1, CNRS, UMR5308, ENS de Lyon, Team Virpath, 69007, Lyon, France.
- [4] Laboratoire de Virologie, Institut des Agents Infectieux, Hospices Civils de Lyon, National Reference Center for Respiratory Viruses, Department of Virology, Infective Agents Institute, North Hospital Network, Lyon, France; Virpath Laboratory, International Center of Research in Infectiology, INSERM U1111, CNRS-UMR 5308, École Normale Supérieure de Lyon, Université Claude Bernard Lyon, Université Claude Bernard, Lyon, France.
- [5] Université de Lyon, Université Claude Bernard, Lyon 1, Lyon, France.
- [6] Joint Research Unit Hospices Civils de Lyon-bioMérieux, Hospices Civils de Lyon, Lyon Sud Hospital, Pierre-Bénite, France.
- [7] National Referee Centre for Rheumatic AutoImmune and Systemic Diseases in childrEn (RAISE), Lyon, France.
- [8] Pediatric Nephrology, Rheumatology, Dermatology Unit, Hospices Civils de Lyon, Pierre-Bénite, France.
- [9] Service de Réanimation Polyvalente, Centre Hospitalier Lyon Sud, Hospices Civils de Lyon, Pierre-Bénite, France.
- [10] Service de Médecine Intensive Réanimation, Hôpital De La Croix Rousse, Hospices Civils de Lyon, 103 Grande Rue de la Croix Rousse, 69004, Lyon, France. j-christophe.richard@chu-lyon.fr.
- [11] Université de Lyon, Université Claude Bernard, Lyon 1, INSA-Lyon, UJM-Saint Etienne, CNRS, Inserm, CREATIS, UMR 5220, U1206, 69621, Lyon, France. j-christophe.richard@chu-lyon.fr.

- PMID: **33301059**
- PMCID: PMC7725883
- DOI: 10.1186/s13613-020-00783-4

Abstract

Background: Protracted viral shedding is common in hospitalized patients with COVID-19 pneumonia, and up to 40% display signs of pulmonary fibrosis on computed tomography (CT) after hospital discharge. We hypothesized that COVID-19 patients with acute respiratory failure (ARF) who die in intensive care units (ICU) have a lower viral clearance in the respiratory tract than ICU patients discharged alive, and that protracted viral shedding in respiratory samples is associated with patterns of fibroproliferation on lung CT. We, therefore, conducted a retrospective observational study, in 2 ICU of Lyon university hospital.

Results: 129 patients were included in the study, of whom 44 (34%) died in ICU. 432 RT-PCR for SARS-CoV-2 were performed and 137 CT scans were analyzed. Viral load was significantly higher in patients deceased as compared to patients alive at ICU discharge ($p < 0.001$), after adjustment for the site of viral sampling and RT-PCR technique. The median time to SARS-CoV-2 negativation on RT-PCR was 19 days [$CI_{95\,\%}$:15-21] in patients alive at ICU discharge and 26 days [$CI_{95\,\%}$:17-infinity] in non-survivors at ICU discharge. Competitive risk regression identified patients who died in ICU and age as independent risk factors for longer time to SARS-CoV-2 negativation on RT-PCR, while antiviral treatment was independently associated with shorter time. None of the CT scores exploring fibroproliferation (i.e., bronchiectasis and reticulation scores) were significantly associated with time to SARS-CoV-2 negativation.

Conclusions: Viral load in respiratory samples is significantly lower and viral shedding significantly shorter in ICU survivors of COVID-19 associated acute respiratory failure. Protracted viral shedding is unrelated to occurrence of fibrosis on lung CT.

Keywords: Acute respiratory distress syndrome; Acute respiratory failure; COVID-19; Polymerase chain reaction; SARS-COV-2; Viral load; Viral shedding.

Conflict of interest statement

The authors declare that they have no competing interests.

- 40 references
- 4 figures

Full text links

SpringerLink FULL-TEXT ARTICLE Springer Free PMC article
Proceed to details
Cite
Share
☐ 1,298
Indian J Nephrol
. Nov-Dec 2021;31(6):524-530.
doi: 10.4103/ijn.IJN_460_20. Epub 2021 Nov 9.

COVID-19 in CKD Patients: Report from India

Nikita Pawar [1], Vaibhav Tiwari [1], Anurag Gupta [1], Vinant Bhargava [1], Manish Malik [1], Ashwani Gupta [1], Anil Kumar Bhalla [1], D S Rana [1]
Affiliations Expand

Affiliation

- [1] Department of Nephrology, Sir Ganga Ram Hospital, New Delhi, India.

- PMID: **35068758**

- PMCID: PMC8722557
- DOI: 10.4103/ijn.IJN_460_20

Free PMC article

COVID-19 in CKD Patients: Report from India

Nikita Pawar et al. Indian J Nephrol. Nov-Dec 2021.
Free PMC article
Show details
Indian J Nephrol
. Nov-Dec 2021;31(6):524-530.
doi: 10.4103/ijn.IJN_460_20. Epub 2021 Nov 9.

Authors

Nikita Pawar [1], Vaibhav Tiwari [1], Anurag Gupta [1], Vinant Bhargava [1], Manish Malik [1], Ashwani Gupta [1], Anil Kumar Bhalla [1], D S Rana [1]

Affiliation

- [1] Department of Nephrology, Sir Ganga Ram Hospital, New Delhi, India.

- PMID: **35068758**
- PMCID: PMC8722557
- DOI: 10.4103/ijn.IJN_460_20

Abstract

Background: COVID-19 is a novel acute infection that is mainly manifested as acute respiratory disease. Information on coronavirus disease-2019 (COVID-19) in CKD patients who are not on dialysis is very limited. We are reporting a single-center observational study on the effect of COVID-19 in CKD patients.

Methods: A single-center retrospective study with consecutive patients who had eGFR <60 mL/min/1.73 m^2 (CKD-EPI) admitted with COVID-19, from April to July 2020 were included.

Result: A total of 30 patients were included in the study. Patients of CKD stage 5, 4 and 3 were 50%, 13.3%, and 36.6%, respectively. The mortality rate was 53.3%. Category wise, 9 were in mild; 3 in moderate, and 18 were in the severe COVID category. Twenty-five patients (83.3%) developed acute on CKD. Twenty patients (67%) required renal replacement therapy (RRT). The prognosis of patients who required RRT was poor. High LDH and IL-6 were significantly associated with mortality. Lymphopenia, present in 50% of cases was associated with fatal outcome. There was a 100% survival rate in mild to moderate cases and 11% in severe cases.

Conclusion: Mortality among hospitalized CKD patients is high.

Keywords: AKI; CKD; Coronavirus; Covid19; SARSCoV2.

Copyright: © 2021 Indian Journal of Nephrology.

Conflict of interest statement

There are no conflicts of interest.

- 34 references

Full text links

Free PMC article
Proceed to details
Cite
Share
☐ 1,299
Craniomaxillofac Trauma Reconstr
. 2022 Mar;15(1):46-50.
doi: 10.1177/19433875211007008. Epub 2021 Mar 30.

Cycling-Related Injuries During COVID-19 Lockdown: A North London Experience

Shadaab Mumtaz [1], James Cymerman [1], Deepak Komath [1]
Affiliations Expand

Affiliation

- [1] Department of Oral and Maxillofacial Surgery, Royal Free London Foundation Trust, London, UK.

- PMID: **35265277**
- PMCID: **PMC8899346** (available on 2023-03-01)
- DOI: 10.1177/19433875211007008

Cycling-Related Injuries During COVID-19 Lockdown: A North London Experience

Shadaab Mumtaz et al. Craniomaxillofac Trauma Reconstr. 2022 Mar.
Show details
Craniomaxillofac Trauma Reconstr
. 2022 Mar;15(1):46-50.
doi: 10.1177/19433875211007008. Epub 2021 Mar 30.

Authors

Shadaab Mumtaz [1], James Cymerman [1], Deepak Komath [1]

Affiliation

- [1] Department of Oral and Maxillofacial Surgery, Royal Free London Foundation Trust, London, UK.

- PMID: **35265277**
- PMCID: **PMC8899346** (available on 2023-03-01)
- DOI: 10.1177/19433875211007008

Abstract

Objectives: There has been a notable surge in cycling injuries during the COVID-19(SARS-CoV-2 virus) pandemic. Cycling in general increased during lockdown as a leisure & fitness activity along with reduction in the use of public transport for commuting. We investigated the bicycle-related maxillofacial injuries & associations presenting through our emergency department (ED) which covers more than 1.6 million of London population.

Study design/methods: A retrospective observational study was undertaken in the Barnet General Hospital ("hub") which receives all maxillofacial referrals from 6 "spoke" hospitals & other urgent primary/community care practices in North London area between 16 March 2020 & 16 July 2020. All data corresponding to cycling injuries during the lockdown period was analyzed with the aid of trauma database/trust-wide electronic patient records.

Results: Twenty-two patients (6.7%) with cycling-related injuries out of a total of 322 patients who attended during the 4 months study period with maxillofacial emergencies were identified. Average age of patient cohort was 35.4 years, mainly consisting of adult males (77%). Seven patients had minor head injury and 1 patient suffered traumatic brain injury. About 59% patients did not wear a protective helmet & 3 patients had heavy alcohol/recreational drug intoxication during the accidents. Four patients needed inpatient admission and treatment under general anesthesia.

Conclusions: Based on our humble study, we advocate the need for robust road & personal safety measures with mandatory government legislations, policing of drug intoxication & encouragement of physical & mental health improvement measures during these unprecedented times & beyond.

Keywords: COVID-19; cycling; facial injuries; head injury; helmet; lockdown; trauma.

© The Author(s) 2021.

Conflict of interest statement

Declaration of Conflicting Interests: The author(s) declared no potential conflicts of interest with respect to the research, authorship, and/or publication of this article.

Proceed to details

Cite

Share

☐ 1,300

Neth Heart J

. 2021 Apr;29(4):230-236.
doi: 10.1007/s12471-021-01554-x. Epub 2021 Mar 11.

Impact of COVID-19 outbreak on hospital admissions and outcome of acute coronary syndromes in a single high-volume centre in southeastern Europe

M Petrović [1, 2], A Milovančev [3, 4], M Kovačević [1, 2], T Miljković [1, 2], A Ilić [1, 2], A Stojšić-Milosavljević [1, 2], M Golubović [1, 2]
Affiliations Expand

Affiliations

- [1] Faculty of Medicine, University of Novi Sad, Novi Sad, Serbia.
- [2] Institute of Cardiovascular Diseases of Vojvodina, Sremska Kamenica, Serbia.
- [3] Faculty of Medicine, University of Novi Sad, Novi Sad, Serbia. aleksandra.milovancev@mf.uns.ac.rs.
- [4] Institute of Cardiovascular Diseases of Vojvodina, Sremska Kamenica, Serbia. aleksandra.milovancev@mf.uns.ac.rs.

- PMID: **33704668**
- PMCID: PMC7950419
- DOI: 10.1007/s12471-021-01554-x

Free PMC article

Impact of COVID-19 outbreak on hospital admissions and outcome of acute coronary syndromes in a single high-volume centre in southeastern Europe

M Petrović et al. Neth Heart J. 2021 Apr.
Free PMC article
Show details
Neth Heart J
. 2021 Apr;29(4):230-236.
doi: 10.1007/s12471-021-01554-x. Epub 2021 Mar 11.

Authors

M Petrović [1, 2], A Milovančev [3, 4], M Kovačević [1, 2], T Miljković [1, 2], A Ilić [1, 2], A Stojšić-Milosavljević [1, 2], M Golubović [1, 2]

Affiliations

- [1] Faculty of Medicine, University of Novi Sad, Novi Sad, Serbia.
- [2] Institute of Cardiovascular Diseases of Vojvodina, Sremska Kamenica, Serbia.
- [3] Faculty of Medicine, University of Novi Sad, Novi Sad, Serbia. aleksandra.milovancev@mf.uns.ac.rs.
- [4] Institute of Cardiovascular Diseases of Vojvodina, Sremska Kamenica, Serbia. aleksandra.milovancev@mf.uns.ac.rs.

- PMID: **33704668**
- PMCID: PMC7950419
- DOI: 10.1007/s12471-021-01554-x

Abstract

Background: As coronavirus disease 2019 (COVID-19) has reached pandemic status, authors from the most severely affected countries have reported reduced rates of hospital admissions for patients with acute coronary syndrome (ACS).

Aim: The aim of the present study was to investigate the influence of the COVID-19 outbreak on hospital admissions and outcomes in ACS patients in a single high-volume centre in southeastern Europe.

Methods: This retrospective observational study aimed to investigate the number of hospital admissions for ACS, clinical findings at admission, length of hospitalisation, major complications and in-hospital mortality during the COVID-19 outbreak and to compare the data with the same parameters during an equivalent time frame in 2019. For the ST-elevated myocardial infarction (STEMI) subgroup of patients, changes in ischaemic times were analysed as well.

Results: There was a significant reduction of 44.3% in the number of patients admitted for ACS during the COVID-19 outbreak when compared with the same period in 2019 (151 vs 271; 95% confidence interval 38.4-50.2, $p < 0.01$) with a higher mortality rate (13.2% vs 7.2%, $p = 0.03$). In 2020, patients with non-ST-elevated myocardial infarction presented more often with acute heart failure (3.3% vs 0.7%, $p = 0.04$). During the COVID-19 outbreak, we observed increases in the total ischaemic time (303 ± 163.4 vs 200.8 ± 156.8 min, $p < 0.05$) and door-to-balloon time (69.2 ± 58.4 vs 50.5 ± 31.3 min, $p < 0.01$) in STEMI patients.

Conclusions: These findings should increase the awareness of morbidity and mortality related to missed or delayed treatment of ACS among the public and the healthcare services.

Keywords: Acute coronary syndrome; COVID-19; Mortality.

Conflict of interest statement

M. Petrović, A. Milovančev,M. Kovačević, T. Miljković, A. Ilić, A. Stojšić-Milosavljević and M. Golubović declare that they have no competing interests.

- 12 references
- 1 figure

Full text links

Springer Free PMC article
Proceed to details
Cite
Share
1,301
Cureus

. 2021 Nov 18;13(11):e19723.
doi: 10.7759/cureus.19723. eCollection 2021 Nov.

Assessment of the Impact of COVID-19 on Drug Store Management in a Tertiary Care Teaching Hospital of Central India

Srikanta Padhan [1], Pugazhenthan T [2], Ramesh Chandrakar [3], Abhiruchi Galhotra [1], Nitinkumar B Borkar [4]
Affiliations Expand

Affiliations

- [1] Epidemiology and Public Health, All India Institute of Medical Sciences, Raipur, IND.
- [2] Pharmacology and Therapeutics, All India Institute of Medical Sciences, Raipur, IND.
- [3] Tranfusion Medicine and Blood Banking, All India Institute of Medical Sciences, Raipur, IND.
- [4] Paediatrics Surgery, All India Institute of Medical Sciences, Raipur, IND.

- PMID: **34934586**
- PMCID: PMC8684361
- DOI: 10.7759/cureus.19723

Free PMC article

Assessment of the Impact of COVID-19 on Drug Store Management in a Tertiary Care Teaching Hospital of Central India

Srikanta Padhan et al. Cureus. 2021.
Free PMC article
Show details
Cureus

. 2021 Nov 18;13(11):e19723.
doi: 10.7759/cureus.19723. eCollection 2021 Nov.

Authors

Srikanta Padhan [1], Pugazhenthan T [2], Ramesh Chandrakar [3], Abhiruchi Galhotra [1], Nitinkumar B Borkar [4]

Affiliations

- [1] Epidemiology and Public Health, All India Institute of Medical Sciences, Raipur, IND.
- [2] Pharmacology and Therapeutics, All India Institute of Medical Sciences, Raipur, IND.
- [3] Tranfusion Medicine and Blood Banking, All India Institute of Medical Sciences, Raipur, IND.
- [4] Paediatrics Surgery, All India Institute of Medical Sciences, Raipur, IND.

- PMID: **34934586**
- PMCID: PMC8684361
- DOI: 10.7759/cureus.19723

Abstract

Introduction: One-third of the annual hospital budget is spent on the purchase of medicines, materials, and supplies. Drug store management is a complex but critical process within the healthcare delivery system. Health supply chains, the import of active pharmaceutical ingredients, transportation, procurement, finished products have been disrupted by COVID-19.

Materials & methods: A retrospective, observational study was carried out at the Department of Hospital Administration, All India Institute of Medical Sciences (AIIMS), Raipur. Quantitative data about the pattern of consumption of 20 most commonly used drugs (10 antibiotics, three analgesics, three antipyretics, two anticoagulants, and two steroids), and 20 most frequently used consumables were sourced from existing records of the Central Pharmacy for 24 months between 1st January 2019 to 31st December 2020.

Results: A significant rise in the consumption pattern was seen in 25 drugs and consumables out of 40 total selected drugs and consumables. The maximum increase was observed in antibiotics followed by antipyretics, and the least increase was observed in analgesics followed by anticoagulants. Tablet Azithromycin 500 mg was the most frequently used antibiotic during the COVID-19 Period as compared to the Pre-COVID-19 period followed by injection Piperacillin + Tazobactam. The only antibiotic having a decline in consumption and also with the lowest consumption was tablet Metronidazole 400 mg. The highest increase in consumables occurs by 10088% in N95 Masks, followed by 573% in shoe covers, and 153% in face masks (three-layers), respectively.

Conclusion: This study will enhance education to the pharmaceutical industries, policymakers to the Government, and other hospitals on how to better manage drug stores in future pandemic-like situations. Proper drug store management played a crucial role in medication usage that improved patient outcomes and prevented the misuse of medications. The pattern of changes in the consumption of drugs and consumables in the present study can be utilized by other hospitals in the third wave of the pandemic.

Keywords: antibiotics; drug store; impact; pandemic; shortage; utilization.

Copyright © 2021, Padhan et al.

Conflict of interest statement

The authors have declared that no competing interests exist.

- 22 references
- 1 figure

Full text links

Free PMC article
Proceed to details
Cite
Share
1,302
Clin Exp Immunol
. 2021 Nov 27;uxab024.
doi: 10.1093/cei/uxab024. Online ahead of print.

Patients diagnosed with COVID-19 and treated with anakinra: a real-world study in the USA

Carly Rich [1], Daniel Eriksson [1], Fabrizio Dolfi [1], Katarzyna Jablonska [2], Firas Dabbous [3], Jameel Nazir [1]
Affiliations Expand

Affiliations

- [1] Swedish Orphan Biovitrum AB, Stockholm, Sweden.
- [2] Creativ-Ceutical, Kraków, Poland.
- [3] Creativ-Ceutical, Chicago, Illinois, USA.

- PMID: **35020840**
- PMCID: PMC8767882
- DOI: 10.1093/cei/uxab024

Free PMC article

Patients diagnosed with COVID-19 and treated with anakinra: a real-world study in the USA

Carly Rich et al. Clin Exp Immunol. 2021.
Free PMC article

Show details

Clin Exp Immunol

. 2021 Nov 27;uxab024.
doi: 10.1093/cei/uxab024. Online ahead of print.

Authors

Carly Rich [1], Daniel Eriksson [1], Fabrizio Dolfi [1], Katarzyna Jablonska [2], Firas Dabbous [3], Jameel Nazir [1]

Affiliations

- [1] Swedish Orphan Biovitrum AB, Stockholm, Sweden.
- [2] Creativ-Ceutical, Kraków, Poland.
- [3] Creativ-Ceutical, Chicago, Illinois, USA.

- PMID: **35020840**
- PMCID: PMC8767882
- DOI: 10.1093/cei/uxab024

Abstract

Anakinra, a recombinant, non-glycosylated human interleukin (IL)-1 receptor antagonist, has been used in real-world clinical practice to manage hyperinflammation in COVID-19. This retrospective, observational study analyses USA hospital inpatient data of patients diagnosed with moderate/severe COVID-19 and treated with anakinra between 1 April and 31 August 2020. Of the 119 patients included in the analysis, 63.9% were male, 48.6% were of black ethnicity and the mean (standard deviation [SD]) age was 64.7 (12.5) years. Mean (SD) time from hospital admission to anakinra initiation was 7.3 (6.1) days. Following anakinra initiation, 73.1% of patients received antibiotics, 55.5% received antithrombotics, and 91.0% received corticosteroids. Overall, 64.7% of patients required intensive care unit (ICU) admittance, and 28.6% received mechanical ventilation following admission. Patients who did not require ICU admittance or who were discharged alive experienced a significantly shorter time between hospital admission and receiving anakinra treatment compared with those admitted to the ICU (5 vs 8 days; $p = 0.002$) or those who died in hospital (6 vs 9 days; $p = 0.01$). Patients with myocardial infarction or renal conditions were six times ($p < 0.01$) and three times ($p = 0.01$), respectively, more likely to die in hospital than be discharged alive. A longer time from hospital admission until anakinra treatment was associated with significantly higher mortality ($p = 0.01$). Findings from this real-world study suggest that a shorter time from hospital admission to anakinra treatment is associated with significantly lower ICU admissions and mortality among patients with moderate/severe COVID-19.

Keywords: Anakinra; COVID-19; database studies; interleukin 1 receptor antagonist protein; real-world evidence.

© Crown copyright 2021.

Full text links

OXFORD ACADEMIC Silverchair Information Systems Free PMC article

Proceed to details
Cite
Share
☐ 1,303
Nefrologia (Engl Ed)
. 2021 Jul 12.
doi: 10.1016/j.nefro.2021.05.005. Online ahead of print.

[Remdesivir in kidney transplant patients with SARS-CoV-2 pneumonia]

[Article in Spanish]
Judit Cacho [1], Elena Burgos [1], María Molina [1], Andrés Villegas [1], Mónica Pérez [1], Laura Cañas [1], Omar Taco [1], Javier Juega [1], Ricardo Lauzurica [1]
Affiliations Expand

Affiliation

- [1] Departamento de Nefrología, Hospital Universitario Germans Trias i Pujol, Badalona, Barcelona, España.

- PMID: **34305226**
- PMCID: PMC8272977
- DOI: 10.1016/j.nefro.2021.05.005

Free PMC article

[Remdesivir in kidney transplant patients with SARS-CoV-2 pneumonia]

[Article in Spanish]
Judit Cacho et al. Nefrologia (Engl Ed). 2021.
Free PMC article
Show details
Nefrologia (Engl Ed)
. 2021 Jul 12.
doi: 10.1016/j.nefro.2021.05.005. Online ahead of print.

Authors

Judit Cacho [1], Elena Burgos [1], María Molina [1], Andrés Villegas [1], Mónica Pérez [1], Laura Cañas [1], Omar Taco [1], Javier Juega [1], Ricardo Lauzurica [1]

Affiliation

- [1] Departamento de Nefrología, Hospital Universitario Germans Trias i Pujol, Badalona, Barcelona, España.

- PMID: **34305226**
- PMCID: PMC8272977
- DOI: 10.1016/j.nefro.2021.05.005

Abstract
in English, Spanish

Background: Remdesivir is the only antiviral treatment that has been shown to be useful against SARS-CoV-2 infection. It shorts hospitalization time compared to placebo. Its effects in Kidney transplant (KT) patients are limited to some published cases.

Methods: We performed a retrospective observational study that included all KT patients admitted between August 01, 2020 and December 31, 2020 with SARS-CoV-2 pneumonia who received remdesivir.The objective of this study was to describe the experience of a cohort of KT patients treated with remdesivir.

Discussion: A total of 37 KT patients developed SARS-CoV-2 infection, 7 of them received treatment with remdesivir. The rest of the patients did not receive the drug due to either CKD-EPI less than 30mL/min or they did not present clinical criteria. In addition to remdesivir, all pacients received dexamethasone and anticoagulation therapy. 4 were men, the median age was 59 (53-71) years. Median time from transplantation was 43 (16-82) months. Chest X-rays of all patients showed pulmonary infiltrates and required low oxygen flow therapy upon admission, requiring high flow nasal therapy in 3 cases. Only 2 cases presented deterioration of the graft function, not requiring hemodialysis in any case, and all recovered renal function at hospital discharge. 2 patients rise up 1.5 times the liver function test. No patient died or required admission to the critical care unit. Median days of admission was 12 (9-27) days.

Conclusions: Our study suggests that the use of remdesivir could be useful in KT patients with SARS-CoV-2 pneumonia without side effects. Additional studies are necessary with a larger number of patients to improve the knowledge of this drug in SARS-CoV-2 infection.

Introducción: El remdesivir es el único tratamiento antiviral que ha demostrado ser útil frente al SARS-CoV-2 acortando el tiempo de hospitalización frente a placebo. Su efecto en pacientes trasplantados renales (TR) se limita a algunos casos publicados.

Material y métodos: Estudio retrospectivo observacional de los pacientes TR que ingresaron entre el 1 de agosto de 2020 hasta el 31 de diciembre de 2020 con neumonía por SARS-CoV-2 y recibieron remdesivir.

El objetivo es describir la experiencia de una cohorte de pacientes TR con neumonía por SARS-CoV-2 tratados con remdesivir.

Resultados: 37 pacientes TR ingresaron por infección secundaria a SARS-CoV-2, 7 de ellos recibieron tratamiento con remdesivir. El resto de pacientes fueron excluidos por CKD-EPI menor a 30 mL/min o por no presentar criterios clínicos. Además de remdesivir, todos recibieron dexametasona y anticoagulación. Cuatro eran hombres, siendo la mediana de edad de 59 (53-71) años. La mediana de tiempo post-trasplante fue de 43 (16-82) meses. Todos los pacientes presentaban neumonía y requirieron oxigenoterapia de bajo flujo al ingreso, precisando en tres de ellos oxigenoterapia de alto flujo durante el ingreso. Dos presentaron deterioro de la función del

injerto al diagnóstico, no precisando en ningún caso hemodiálisis, y recuperándose al alta. Dos pacientes elevaron 1,5 veces el valor normal de las transaminasas. Ningún paciente falleció ni precisó ingreso en unidad de críticos. La mediana de días de ingreso fue de 12 (9-27) días.

Conclusiones: Nuestro estudio sugiere que el uso de remdesivir podría ser útil en los pacientes TR con neumonía por SARS-CoV-2 sin presentar efectos secundarios. Son necesarios más estudios con un mayor número de pacientes para ampliar el conocimiento de este fármaco en la infección por SARS-CoV-2.

Keywords: Kidney transplantation; Remdesivir; SARS-CoV-2; acute kidney failure; coronavirus; survival; treatment.

© 2021 Published by Elsevier España, S.L.U. on behalf of Sociedad Española de Nefrología.

- 18 references
- 1 figure

Supplementary info

Publication types Expand

Publication types

- English Abstract

Full text links

FREE Fulltext at nefrología Ediciones Doyma, S.L. Free PMC article

Proceed to details

Cite

Share

1,304

Indian J Crit Care Med
. 2021 Mar;25(3):322-326.
doi: 10.5005/jp-journals-10071-23765.

Prognostic Value of "Cycle Threshold" in Confirmed COVID-19 Patients

B Rajyalakshmi [1], Srinivas Samavedam [1], P Ramakrishna Reddy [1], Narmada Aluru [1]
Affiliations Expand

Affiliation

- [1] Department of Critical Care, Virinchi Hospital, Hyderabad, Telangana, India.

- PMID: **33790515**

- PMCID: PMC7991767
- DOI: 10.5005/jp-journals-10071-23765

Free PMC article

Prognostic Value of "Cycle Threshold" in Confirmed COVID-19 Patients

B Rajyalakshmi et al. Indian J Crit Care Med. 2021 Mar.
Free PMC article
Show details
Indian J Crit Care Med
. 2021 Mar;25(3):322-326.
doi: 10.5005/jp-journals-10071-23765.

Authors

B Rajyalakshmi [1], Srinivas Samavedam [1], P Ramakrishna Reddy [1], Narmada Aluru [1]

Affiliation

- [1] Department of Critical Care, Virinchi Hospital, Hyderabad, Telangana, India.

- PMID: **33790515**
- PMCID: PMC7991767
- DOI: 10.5005/jp-journals-10071-23765

Abstract

Objective: To study the correlation between the cycle threshold (CT) of reverse transcription-polymerase chain reaction (RT-PCR) test in confirmed COVID-19 patients and the severity of disease. **Background:** RT-PCR test is a standard method for the diagnosis of severe acute respiratory syndrome coronavirus-2 (SARS-CoV-2) infections. This test is based upon the amplification of the fluorescent signal. The number of cycles that the fluorescent signal undergoes to reach the threshold is called "cycle threshold." It is inversely related to the nucleic acid content of the sample. **Patients and methods:** This is a single-centered, retrospective observational study. We have included a total of 192 patients. SARS-CoV-2 infection was confirmed by the RT-PCR test. Entire data have been collected from the electronic medical records. The primary outcome was 28-day mortality, whereas the secondary outcomes were intensive care unit (ICU) admission, invasive ventilation, acute kidney injury, renal replacement therapy (RRT), shock, and COVID-19 reporting and data system (CO-RADS) score on high-resolution computed tomography of the chest, total length of stay in the hospital, and the number of ICU days and ventilator days. **Results:** We have calculated the mean CT value for all groups and calculated the *p*-value for statistical significance. For the total length of stay in the hospital and the number of ICU days and ventilator days, we applied the Pearson correlation coefficient. The *p*-value was statistically significant for mortality, ICU admission, and shock groups. The CT values and the length of ICU stay were inversely correlated with the statistically significant *p*-value. **Conclusion:** Low CT value is associated with increased ICU admission, high mortality, shock, and increased length of ICU stay. **How to cite this article:** Rajyalakshmi B, Samavedam S, Reddy PR, Aluru N.

Prognostic Value of "Cycle Threshold" in Confirmed COVID-19 Patients. Indian J Crit Care Med 2021;25(3):322-326.

Keywords: COVID-19; Cycle threshold; RT-PCR; Viral load.

Copyright © 2021; Jaypee Brothers Medical Publishers (P) Ltd.

Conflict of interest statement

Source of support: Nil Conflict of interest: None

- 15 references
- 9 figures

Full text links

Free PMC article
Proceed to details
Cite
Share
☐ 1,305
J Clin Med
. 2022 Mar 4;11(5):1412.
doi: 10.3390/jcm11051412.

Real-World Efficacy of Regdanvimab on Clinical Outcomes in Patients with Mild to Moderate COVID-19

Taeyun Kim [1], Dong-Hyun Joo [1], Seung Woo Lee [1], Jaejun Lee [1], Sang Jin Lee [2], Jihun Kang [3]
Affiliations Expand

Affiliations

- [1] Department of Internal Medicine, The Armed Forces Goyang Hospital, Goyang 10271, Korea.
- [2] Department of Statistics, Pusan National University, Busan 46241, Korea.
- [3] Department of Family Medicine, Kosin University Gospel Hospital, Busan 46241, Korea.

- PMID: **35268503**
- PMCID: PMC8911404
- DOI: 10.3390/jcm11051412

Free PMC article

Real-World Efficacy of Regdanvimab on Clinical Outcomes in Patients with Mild to Moderate COVID-19

Taeyun Kim et al. J Clin Med. 2022.
Free PMC article
Show details
J Clin Med
. 2022 Mar 4;11(5):1412.
doi: 10.3390/jcm11051412.

Authors

Taeyun Kim [1], Dong-Hyun Joo [1], Seung Woo Lee [1], Jaejun Lee [1], Sang Jin Lee [2], Jihun Kang [3]

Affiliations

- [1] Department of Internal Medicine, The Armed Forces Goyang Hospital, Goyang 10271, Korea.
- [2] Department of Statistics, Pusan National University, Busan 46241, Korea.
- [3] Department of Family Medicine, Kosin University Gospel Hospital, Busan 46241, Korea.

- PMID: **35268503**
- PMCID: PMC8911404
- DOI: 10.3390/jcm11051412

Abstract

Background: This study aims to evaluate the real-world effectiveness of regdanvimab on clinical outcomes in patients with mild to moderate coronavirus disease 2019 (COVID-19).

Methods: This retrospective observational study included 152 patients (89 received regdanvimab and 63 did not) diagnosed with mild to moderate COVID-19 between August 2021 and October 2021 and admitted to Armed Forces Goyang Hospital. We collected information on the use of regdanvimab, remdesivir, dexamethasone, and supplemental oxygen; symptom severity score (SSS); and laboratory test results. A linear mixed-effects model was used to test the effectiveness of regdanvimab usage on SSS and the results of laboratory tests. A multivariate logistic regression model was used to calculate the odds ratio (OR) for additional therapeutic options, such as remdesivir, dexamethasone, and supplemental oxygen.

Results: The patients who received regdanvimab were older, showed a higher rate of vaccination, and had a higher Charlson comorbidity index, initial body temperature, and percentages of pneumonia at admission. The use of regdanvimab showed no interactive effects on the SSS and laboratory findings. Older age, male sex, obesity, high initial body temperature, and the presence of pneumonia at admission were associated with increased ORs for the use of these additional treatments. The use of regdanvimab reduced the probability of requiring additional therapies such as remdesivir, dexamethasone, and oxygen supplementation by 90.3% (95% confidence interval (CI), 60.3-97.6), 85.8% (95% CI, 34.2-96.9), and 89.8% (95% CI, 48.3-98), respectively.

Conclusions: Regdanvimab usage was well tolerated and was associated with a decreased probability of requiring remdesivir, dexamethasone, and oxygen therapy. However, changes in SSS were not significantly different by the drug usage.

Keywords: COVID-19; SARS-CoV-2; dexamethasone; oxygen; regdanvimab; remdesivir.

Conflict of interest statement

The authors declare no conflict of interest related to this article.

- 22 references
- 1 figure

Supplementary info

Grant support Expand

Grant support

- 2019R1G1A1099627/National Research Foundation of Korea

Full text links

FULL TEXT OPEN ACCESS MDPI Multidisciplinary Digital Publishing Institute (MDPI) Free PMC article
Proceed to details
Cite
Share
☐ 1,306
Intern Emerg Med
. 2022 Jan 1;1-9.
doi: 10.1007/s11739-021-02891-w. Online ahead of print.

Low in-hospital mortality rate in patients with COVID-19 receiving thromboprophylaxis: data from the multicentre observational START-COVID Register

Daniela Poli [1], Emilia Antonucci [2], Walter Ageno [3], Paolo Prandoni [2], Gualtiero Palareti [2], Rossella Marcucci [2, 3, 4, 5], START-COVID Investigators
Collaborators, Affiliations Expand

Collaborators

- **START-COVID Investigators**:

Rossella Marcucci, Daniela Poli, Walter Ageno, Giovanna Colombo, Chiara Ambaglio, Guido Arpaia, Giovanni Barillari, Giuseppina Bitti, Eugenio Bucherini, Antonio Chistolini, Alessandra Serrao, Egidio De Gaudenzi, Valeria De Micheli, Anna Falanga, Teresa Lerede, Luca Barcella, Laura Russo, Silvia Galliazzo, Alberto Gandolfo, Gianni Biolo, Valentina Trapletti, Giorgio Ghigliotti, Elisa Grifoni, Luca Masotti, Egidio Imbalzano, Gianfranco Lessiani, Niccolò Marchionni, Giuliana Martini, Sara Merelli, Franco Mastroianni, Giovanni Larizza, Carlo Nozzoli, Serena Panarello, Chiara Fioravanti, Simona Pedrini, Federica Bertola, Raffaele Pesavento, Filippo Pieralli, Pasquale Pignatelli, Daniele Pastori, Paola Preti, Elias Romano, Alessandro Morettini, Girolamo Sala, Fabrizio Foieni, Michela Provisone, Luca Sarti, Antonella Caronna, Federico Simonetti, Ilaria Bertaggia, Piera Sivera, Carmen Fava, Viviana Scancassani, Michele Spinicci, Alessio Bartoloni, Adriana Visonà, Beniamino Zalunardo, Sabina Villalta

Affiliations

- [1] Centro Trombosi, Azienda Ospedaliero Universitaria Careggi, Viale Morgagni, 85-50134, Firenze, Italy. polida@aou-careggi.toscana.it.
- [2] Fondazione Arianna Anticoagulazione, Bologna, Italy.
- [3] Dipartimento di Medicina e Chirurgia, Università dell'Insubria, Varese, Italy.
- [4] Department of Experimental and Clinical Medicine, University of Florence, Firenze, Italy.
- [5] Centro Trombosi, Azienda Ospedaliero Universitaria Careggi, Viale Morgagni, 85-50134, Firenze, Italy.

- PMID: **34973126**
- PMCID: PMC8720160
- DOI: 10.1007/s11739-021-02891-w

Free PMC article

Low in-hospital mortality rate in patients with COVID-19 receiving thromboprophylaxis: data from the multicentre observational START-COVID Register

Daniela Poli et al. Intern Emerg Med. 2022.
Free PMC article
Show details
Intern Emerg Med
. 2022 Jan 1;1-9.
doi: 10.1007/s11739-021-02891-w. Online ahead of print.

Authors

Daniela Poli [1], Emilia Antonucci [2], Walter Ageno [3], Paolo Prandoni [2], Gualtiero Palareti [2], Rossella Marcucci [2 3 4 5], START-COVID Investigators

Collaborators

- **START-COVID Investigators**:
  Rossella Marcucci, Daniela Poli, Walter Ageno, Giovanna Colombo, Chiara Ambaglio, Guido Arpaia, Giovanni Barillari, Giuseppina Bitti, Eugenio Bucherini, Antonio Chistolini, Alessandra Serrao, Egidio De Gaudenzi, Valeria De Micheli, Anna Falanga, Teresa Lerede, Luca Barcella, Laura Russo, Silvia Galliazzo, Alberto Gandolfo, Gianni Biolo, Valentina Trapletti, Giorgio Ghigliotti, Elisa Grifoni, Luca Masotti, Egidio Imbalzano, Gianfranco Lessiani, Niccolò Marchionni, Giuliana Martini, Sara Merelli, Franco Mastroianni, Giovanni Larizza, Carlo Nozzoli, Serena Panarello, Chiara Fioravanti, Simona Pedrini, Federica Bertola, Raffaele Pesavento, Filippo Pieralli, Pasquale Pignatelli, Daniele Pastori, Paola Preti, Elias Romano, Alessandro Morettini, Girolamo Sala, Fabrizio Foieni, Michela Provisone, Luca Sarti, Antonella Caronna, Federico Simonetti, Ilaria Bertaggia, Piera Sivera, Carmen Fava, Viviana Scancassani, Michele Spinicci, Alessio Bartoloni, Adriana Visonà, Beniamino Zalunardo, Sabina Villalta

Affiliations

- [1] Centro Trombosi, Azienda Ospedaliero Universitaria Careggi, Viale Morgagni, 85-50134, Firenze, Italy. polida@aou-careggi.toscana.it.
- [2] Fondazione Arianna Anticoagulazione, Bologna, Italy.
- [3] Dipartimento di Medicina e Chirurgia, Università dell'Insubria, Varese, Italy.
- [4] Department of Experimental and Clinical Medicine, University of Florence, Firenze, Italy.
- [5] Centro Trombosi, Azienda Ospedaliero Universitaria Careggi, Viale Morgagni, 85-50134, Firenze, Italy.

- PMID: **34973126**
- PMCID: PMC8720160
- DOI: 10.1007/s11739-021-02891-w

Abstract

COVID-19 infection causes respiratory pathology with severe interstitial pneumonia and extra-pulmonary complications; in particular, it may predispose to thromboembolic disease. The current guidelines recommend the use of thromboprophylaxis in patients with COVID-19, however, the optimal heparin dosage treatment is not well-established. We conducted a multicentre, Italian, retrospective, observational study on COVID-19 patients admitted to ordinary wards, to describe clinical characteristic of patients at admission, bleeding and thrombotic events occurring during hospital stay. The strategies used for thromboprophylaxis and its role on patient outcome were, also, described. 1091 patients hospitalized were included in the START-COVID-19 Register. During hospital stay, 769 (70.7%) patients were treated with antithrombotic drugs: low molecular weight heparin (the great majority enoxaparin), fondaparinux, or unfractioned heparin. These patients were more frequently affected by comorbidities, such as hypertension, atrial fibrillation, previous thromboembolism, neurological disease, and cancer with respect to patients who did not receive thromboprophylaxis. During hospital stay, 1.2% patients had a major bleeding event. All patients were treated with antithrombotic drugs; 5.4%, had venous thromboembolism [30.5% deep vein thrombosis (DVT), 66.1% pulmonary embolism (PE), and 3.4% patients had DVT + PE]. In our cohort the mortality rate was 18.3%. Heparin use was independently associated with survival in patients aged ≥ 59 years at multivariable analysis. We confirmed the high mortality rate of COVID-19 in hospitalized patients in ordinary wards. Treatment with antithrombotic drugs is

significantly associated with a reduction of mortality rates especially in patients older than 59 years.

Keywords: Antithrombotic; COVID-19 disease; Heparin; Mortality.

© 2021. The Author(s).

Conflict of interest statement

None declared.

- 32 references
- 1 figure

Full text links

SpringerLink FULL-TEXT ARTICLE Springer Free PMC article
Proceed to details
Cite
Share
1,307

Multidiscip Respir Med
. 2020 Sep 16;15(1):693.
doi: 10.4081/mrm.2020.693. eCollection 2020 Jan 28.

High-flow nasal cannula for Acute Respiratory Distress Syndrome (ARDS) due to COVID-19

Carolina Panadero [1], Araceli Abad-Fernández [1], Mª Teresa Rio-Ramirez [1], Carmen Maria Acosta Gutierrez [1], Mariara Calderon-Alcala [1], Cristina Lopez-Riolobos [1], Cristina Matesanz-Lopez [1], Fernando Garcia-Prieto [1], Jose Maria Diaz-Garcia [1], Beatriz Raboso-Moreno [1], Zully Vasquez-Gambasica [1], Pilar Andres-Ruzafa [1], Jose Luis Garcia-Satue [1], Sara Calero-Pardo [1], Belen Sagastizabal [2], Diego Bautista [2], Alfonso Campos [2], Marina González [2], Luis Grande [2], Marta Jimenez Fernandez [3], Jose L Santiago-Ruiz [4], Pedro Caravaca Perez [5], Andres Jose Alcaraz [2]
Affiliations Expand

Affiliations

- [1] Pulmonology Department, Hospital Universitario de Getafe, Madrid.
- [2] Department of Pediatrics, Hospital Universitario de Getafe, Madrid.
- [3] Thoracic Surgery Department, Hospital Universitario de Getafe, Madrid.
- [4] Cardiology Department, Hospital Universitario de Getafe, Madrid.

- [5] Cardiology Department, Hospital Universitario 12 de Octubre, Instituto de Investigación Sanitaria Hospital 12 de Octubre (imas12), CIBERCV, Madrid, Spain.

- PMID: **32983456**
- PMCID: PMC7512942
- DOI: 10.4081/mrm.2020.693

Free PMC article

High-flow nasal cannula for Acute Respiratory Distress Syndrome (ARDS) due to COVID-19

Carolina Panadero et al. Multidiscip Respir Med. 2020.
Free PMC article

Show details

Multidiscip Respir Med
. 2020 Sep 16;15(1):693.
doi: 10.4081/mrm.2020.693. eCollection 2020 Jan 28.

Authors

Carolina Panadero [1], Araceli Abad-Fernández [1], Mª Teresa Rio-Ramirez [1], Carmen Maria Acosta Gutierrez [1], Mariara Calderon-Alcala [1], Cristina Lopez-Riolobos [1], Cristina Matesanz-Lopez [1], Fernando Garcia-Prieto [1], Jose Maria Diaz-Garcia [1], Beatriz Raboso-Moreno [1], Zully Vasquez-Gambasica [1], Pilar Andres-Ruzafa [1], Jose Luis Garcia-Satue [1], Sara Calero-Pardo [1], Belen Sagastizabal [2], Diego Bautista [2], Alfonso Campos [2], Marina González [2], Luis Grande [2], Marta Jimenez Fernandez [3], Jose L Santiago-Ruiz [4], Pedro Caravaca Perez [5], Andres Jose Alcaraz [2]

Affiliations

- [1] Pulmonology Department, Hospital Universitario de Getafe, Madrid.
- [2] Department of Pediatrics, Hospital Universitario de Getafe, Madrid.
- [3] Thoracic Surgery Department, Hospital Universitario de Getafe, Madrid.
- [4] Cardiology Department, Hospital Universitario de Getafe, Madrid.
- [5] Cardiology Department, Hospital Universitario 12 de Octubre, Instituto de Investigación Sanitaria Hospital 12 de Octubre (imas12), CIBERCV, Madrid, Spain.

- PMID: **32983456**
- PMCID: PMC7512942
- DOI: 10.4081/mrm.2020.693

Abstract

Introduction: High-flow nasal cannula oxygen therapy (HFNC) has been shown to be a useful therapy in the treatment of patients with Acute Respiratory Distress Syndrome (ARDS), but its efficacy is still unknown in patients with COVID-19. Our objective is to describe its utility as therapy for the treatment of ARDS caused by SARS-CoV-2.

Methods: A retrospective, observational study was performed at a single centre, evaluating patients with ARDS secondary to COVID-19 treated with HFNC. The main outcome was the intubation rate at day 30, which defined failure of therapy. We also analysed the role of the ROX index to predict the need for intubation.

Results: In the study period, 196 patients with bilateral pneumonia were admitted to our pulmonology unit, 40 of whom were treated with HFNC due to the presence of ARDS. The intubation rate at day 30 was 52.5%, and overall mortality was 22.5%. After initiating HFNC, the SpO^2/FiO^2 ratio was significantly better in the group that did not require intubation (113.4±6.6 *vs* 93.7±6.7, p=0.020), as was the ROX index (5.0±1.6 *vs* 4.0±1.0, p=0.018). A ROX index less than 4.94 measured 2 to 6 h after the start of therapy was associated with increased risk of intubation (HR 4.03 [95% CI 1.18 - 13.7]; p=0.026).

Conclusion: High-flow therapy is a useful treatment in ARDS in order to avoid intubation or as a bridge therapy, and no increased mortality was observed secondary to the delay in intubation. After initiating HFNC, a ROX index below 4.94 predicts the need for intubation.

Keywords: COVID-19; acute respiratory distress syndrome; coronavirus; high-flow nasal cannula.

©Copyright: the Author(s).

- 20 references
- 2 figures

Full text links

Multidisciplinary Respiratory Medicine Pagepress Publications Free PMC article

Proceed to details

Cite

Share

☐ 1,308

J Clin Med

. 2021 Nov 15;10(22):5314.

doi: 10.3390/jcm10225314.

Effects of the COVID-19 Pandemic on Treatment Efficiency for Traumatic Brain Injury in the Emergency Department: A Multicenter Study in Taiwan

Carlos Lam [1] [2], Ju-Chuan Yen [3] [4], Chia-Chieh Wu [1] [2], Heng-Yu Lin [5], Min-Huei Hsu [6]

Affiliations Expand

Affiliations

- [1] Emergency Department, Wan Fang Hospital, Taipei Medical University, Taipei 11696, Taiwan.
- [2] Department of Emergency, School of Medicine, College of Medicine, Taipei Medical University, Taipei 11030, Taiwan.
- [3] Department of Ophthalmology, Taipei City Hospital, Renai Branch, Taipei 10629, Taiwan.
- [4] Graduate Institute of Biomedical Informatics, College of Medical Technology, Taipei Medical University, Taipei 11030, Taiwan.
- [5] School of Medicine, College of Medicine, Taipei Medical University, Taipei 11030, Taiwan.
- [6] Graduate Institute of Data Science, College of Management, Taipei Medical University, Taipei 11030, Taiwan.

- PMID: **34830592**
- PMCID: PMC8621260
- DOI: 10.3390/jcm10225314

Free PMC article

Effects of the COVID-19 Pandemic on Treatment Efficiency for Traumatic Brain Injury in the Emergency Department: A Multicenter Study in Taiwan

Carlos Lam et al. J Clin Med. 2021.
Free PMC article
Show details
J Clin Med
. 2021 Nov 15;10(22):5314.
doi: 10.3390/jcm10225314.

Authors

Carlos Lam [1, 2], Ju-Chuan Yen [3, 4], Chia-Chieh Wu [1, 2], Heng-Yu Lin [5], Min-Huei Hsu [6]

Affiliations

- [1] Emergency Department, Wan Fang Hospital, Taipei Medical University, Taipei 11696, Taiwan.
- [2] Department of Emergency, School of Medicine, College of Medicine, Taipei Medical University, Taipei 11030, Taiwan.

- [3] Department of Ophthalmology, Taipei City Hospital, Renai Branch, Taipei 10629, Taiwan.
- [4] Graduate Institute of Biomedical Informatics, College of Medical Technology, Taipei Medical University, Taipei 11030, Taiwan.
- [5] School of Medicine, College of Medicine, Taipei Medical University, Taipei 11030, Taiwan.
- [6] Graduate Institute of Data Science, College of Management, Taipei Medical University, Taipei 11030, Taiwan.

- PMID: **34830592**
- PMCID: PMC8621260
- DOI: 10.3390/jcm10225314

Abstract

The coronavirus disease 2019 (COVID-19) pandemic has impacted emergency department (ED) practice, including the treatment of traumatic brain injury (TBI), which is commonly encountered in the ED. Our study aimed to evaluate TBI treatment efficiency in the ED during the COVID-19 pandemic. A retrospective observational study was conducted using the electronic medical records from three hospitals in metropolitan Taipei, Taiwan. The time from ED arrival to brain computed tomography (CT) and the time from ED arrival to surgical management were used as measures of treatment efficiency. TBI treatment efficiencies in the ED coinciding with a small-scale local COVID-19 outbreak in 2020 (P1) and large-scale community spread in 2021 (P2) were compared against the pre-pandemic efficiency recorded in 2019. The interval between ED arrival and brain CT was significantly shortened during P1 and P2 compared with the pre-pandemic interval, and no significant delay between ED arrival and surgical management was found, indicating increased treatment efficiency for TBI in the ED during the COVID-19 pandemic. Minimizing viral spread in the community and the hospital is vital to maintaining ED treatment efficiency and capacity. The ED should retain sufficient capacity to treat older patients with serious TBI during the COVID-19 pandemic.

Keywords: COVID-19 pandemic; emergency department; traumatic brain injury; treatment efficiency.

Conflict of interest statement

The authors declare no conflict of interest.

- 35 references
- 8 figures

Supplementary info

Grant support Expand

Grant support

- 99TMU-WFH-15/Taipei Medical University-Wan Fang Hospital
- TMU108-AE1-B49/Taipei Medical University

Full text links

FULL TEXT OPEN ACCESS MDPI Multidisciplinary Digital Publishing Institute (MDPI) Free PMC article

Proceed to details

Cite

Share

1,309

Front Med (Lausanne)

. 2020 Jun 23;7:347.
doi: 10.3389/fmed.2020.00347. eCollection 2020.

Liver Injury in Critically Ill and Non-critically Ill COVID-19 Patients: A Multicenter, Retrospective, Observational Study

Saiping Jiang [1], Rongrong Wang [1], Lu Li [1], Dongsheng Hong [1], Renping Ru [2], Yuefeng Rao [1], Jing Miao [1], Na Chen [1], Xiuhua Wu [1], Ziqi Ye [1], Yunzhen Hu [1], Minghua Xie [3], Minjuan Zuo [4], Xiaoyang Lu [1], Yunqing Qiu [5], Tingbo Liang [6]

Affiliations Expand

Affiliations

- [1] Department of Pharmacy, The First Affiliated Hospital, College of Medicine, Zhejiang University, Hangzhou, China.
- [2] Department of Pharmacy, Xixi Hospital of Hangzhou, Hangzhou, China.
- [3] Department of Pharmacy, First People's Hospital of Yuhang District, Hangzhou, China.
- [4] Public Service Platform for the Evaluation of Innovative Drug Property, Hangzhou, China.
- [5] State Key Laboratory for Diagnosis and Treatment of Infectious Disease, Collaborative Innovation Center for Diagnosis and Treatment of Infectious Diseases, Zhejiang Provincial Key Laboratory for Drug Clinical Research and Evaluation, The First Affiliated Hospital, College of Medicine, Zhejiang University, Hangzhou, China.
- [6] Department of Hepatobiliary and Pancreatic Surgery, The First Affiliated Hospital, College of Medicine, Zhejiang University, Hangzhou, China.

- PMID: **32656222**
- PMCID: PMC7324794
- DOI: 10.3389/fmed.2020.00347

Free PMC article

Liver Injury in Critically Ill and Non-critically Ill COVID-19 Patients: A

Multicenter, Retrospective, Observational Study

Saiping Jiang et al. Front Med (Lausanne). 2020.
Free PMC article
Show details
Front Med (Lausanne)
. 2020 Jun 23;7:347.
doi: 10.3389/fmed.2020.00347. eCollection 2020.

Authors

Saiping Jiang [1], Rongrong Wang [1], Lu Li [1], Dongsheng Hong [1], Renping Ru [2], Yuefeng Rao [1], Jing Miao [1], Na Chen [1], Xiuhua Wu [1], Ziqi Ye [1], Yunzhen Hu [1], Minghua Xie [3], Minjuan Zuo [4], Xiaoyang Lu [1], Yunqing Qiu [5], Tingbo Liang [6]

Affiliations

- [1] Department of Pharmacy, The First Affiliated Hospital, College of Medicine, Zhejiang University, Hangzhou, China.
- [2] Department of Pharmacy, Xixi Hospital of Hangzhou, Hangzhou, China.
- [3] Department of Pharmacy, First People's Hospital of Yuhang District, Hangzhou, China.
- [4] Public Service Platform for the Evaluation of Innovative Drug Property, Hangzhou, China.
- [5] State Key Laboratory for Diagnosis and Treatment of Infectious Disease, Collaborative Innovation Center for Diagnosis and Treatment of Infectious Diseases, Zhejiang Provincial Key Laboratory for Drug Clinical Research and Evaluation, The First Affiliated Hospital, College of Medicine, Zhejiang University, Hangzhou, China.
- [6] Department of Hepatobiliary and Pancreatic Surgery, The First Affiliated Hospital, College of Medicine, Zhejiang University, Hangzhou, China.

- PMID: **32656222**
- PMCID: PMC7324794
- DOI: 10.3389/fmed.2020.00347

Abstract

Background: Liver injury commonly occurs in patients with COVID-19. There is limited data describing the course of liver injury occurrence in patients with different disease severity, and the causes and risk factors are unknown. We aim to investigate the incidence, characteristics, risk factors, and clinical outcomes of liver injury in patients with COVID-19. **Methods:** This retrospective observational study was conducted in three hospitals (Zhejiang, China). From January 19, 2020 to February 20, 2020, patients confirmed with COVID-19 (≥18 years) and without liver injury were enrolled and divided into non-critically ill and critically ill groups. The incidence and characteristics of liver injury were compared between the two groups. Demographics, clinical characteristics, treatments, and treatment outcomes between patients with or without liver injury were compared within each group. The multivariable logistic regression model was used to explore the risk factors for liver injury. **Results:** The mean age of 131 enrolled patients was 51.2 years (standard deviation *[SD]*: 16.1 years), and 70 (53.4%) patients were male.

A total of 76 patients developed liver injury (mild, 40.5%; moderate, 15.3%; severe, 2.3%) with a median occurrence time of 10.0 days. Critically ill patients had higher and earlier occurrence (81.5 vs. 51.9%, 12.0 vs. 5.0 days; $p < 0.001$), greater injury severity ($p < 0.001$), and slower recovery (50.0 vs. 61.1%) of liver function than non-critically ill patients. Multivariable regression showed that the number of concomitant medications (odds ratio [OR]: 1.12, 95% confidence interval [CI]: 1.05-1.21) and the combination treatment of lopinavir/ritonavir and arbidol (OR: 3.58, 95% CI: 1.44-9.52) were risk factors for liver injury in non-critically ill patients. The metabolism of arbidol can be significantly inhibited by lopinavir/ritonavir *in vitro* ($p < 0.005$), which may be the underlying cause of drug-related liver injury. Liver injury was related to increased length of hospital stay (mean difference [MD]: 3.2, 95% CI: 1.3-5.2) and viral shedding duration (MD: 3.0, 95% CI: 1.0-4.9). **Conclusions:** Critically ill patients with COVID-19 suffered earlier occurrence, greater injury severity, and slower recovery from liver injury than non-critically ill patients. Drug factors were related to liver injury in non-critically ill patients. Liver injury was related to prolonged hospital stay and viral shedding duration in patients with COVID-19. **Clinical Trial Registration:** World Health Organization International Clinical Trials Registry Platform, ChiCTR2000030593. Registered March 8, 2020.

Keywords: COVID-19; Incidence; disease severity; liver injury; risk factors.

Copyright © 2020 Jiang, Wang, Li, Hong, Ru, Rao, Miao, Chen, Wu, Ye, Hu, Xie, Zuo, Lu, Qiu and Liang.

- 28 references
- 3 figures

Full text links

Frontiers Media SA Free PMC article

Proceed to details

Cite

Share

1,310

J Clin Med

. 2021 Feb 25;10(5):899.

doi: 10.3390/jcm10050899.

Gender-Based Differences by Age Range in Patients Hospitalized with COVID-19: A Spanish Observational Cohort Study

Claudia Josa-Laorden [1, 2], Anxela Crestelo-Vieitez [1], María Del Mar García Andreu [1], Manuel Rubio-Rivas [3], Marcos Sánchez [4], Neera Toledo Samaniego [5], Francisco Arnalich Fernández [6], Rosario Iguaran Bermudez [7], Eva Ma Fonseca Aizpuru [8], Juan Antonio Vargas Núñez [9], Paula Maria Pesqueira Fontan [10], Jorge Serrano Ballesteros [11], Santiago Jesús Freire Castro [12], Melani Pestaña Fernández [13], Alba Viana García [14], Victoria Nuñez Rodriguez [15], Vicente Giner-Galvañ [16, 17], Francisco Javier Carrasco Sánchez [18], Almudena Hernández Milián [19], Marta Cobos-Siles [20], Jose Javier Napal Lecumberri [21], Virginia Herrero García [22], Maria de

Los Reyes Pascual Pérez [23], Jesús Millán Núñez-Cortés [5], José Manuel Casas Rojo [24], On Behalf Of The Semi-Covid-Network
Affiliations Expand

Affiliations

- [1] Internal Medicine Department, Royo Villanova Hospital, Avenida San Gregorio 30, 50015 Zaragoza, Spain.
- [2] Aragon Institute of Health Research, IIS-Aragon, 50009 Zaragoza, Spain.
- [3] Internal Medicine Department, Bellvitge University Hospital-IDIBELL, L'Hospitalet de Llobregat, 08901 Barcelona, Spain.
- [4] Internal Medicine Department, 12 de Octubre University Hospital, 28041 Madrid, Spain.
- [5] Internal Medicine Department, Gregorio Marañon University Hospital, 28007 Madrid, Spain.
- [6] Internal Medicine Department, La Paz University Hospital, 28046 Madrid, Spain.
- [7] Internal Medicine Department, San Carlos Clinical Hospital, 28040 Madrid, Spain.
- [8] Internal Medicine Department, Cabueñes Hospital, 33394 Gijón, Asturias, Spain.
- [9] Internal Medicine Department, Puerta de Hierro University Hospital, 28222 Majadahonda, Spain.
- [10] Internal Medicine Department, Santiago Clinical Hospital, 15706 Santiago de Compostela, Spain.
- [11] Internal Medicine Department, La Princesa University Hospital, 28006 Madrid, Spain.
- [12] Internal Medicine Department, A Coruña University Hospital, 15006 A Coruna, Spain.
- [13] Internal Medicine Department, Moisès Broggi Hospital, 08970 Sant Joan Despí, Spain.
- [14] Internal Medicine Department, Dr. Peset University Hospital, 45017 Valencia, Spain.
- [15] Internal Medicine Department, Costa del Sol Hospital, 29603 Málaga, Spain.
- [16] General Internal Medicine Department, San Juan de Alicante University Hospital, 03550 Alicante, Spain.
- [17] Department of Clinical Medicine, Faculty of Medicine, Miguel Hernández University, 03202 Elche, Alicante, Spain.
- [18] Internal Medicine Department, Juan Ramón Jiménez Hospital, 21005 Huelva, Spain.
- [19] Internal Medicine Department, Son Llàtzer University Hospital, 07120 Palma de Mallorca, Spain.
- [20] Internal Medicine Department, Río Hortega University Hospital, Regional Health Management of Castilla y Leon (SACYL), 47012 Valladolid, Spain.
- [21] Internal Medicine Department, Marqués de Valdecilla University Hospital, 39008 Santander, Spain.
- [22] Internal Medicine Department, Doctor José Molina Orosa Hospital, 25005 Arrecife, Spain.
- [23] Internal Medicine Department, Elda University General Hospital, 03600 Elda, Alicante, Spain.
- [24] Internal Medicine Department, Infanta Cristina University Hospital, 28981 Madrid, Spain.

- PMID: **33668766**
- PMCID: PMC7956359
- DOI: 10.3390/jcm10050899

Free PMC article

Gender-Based Differences by Age Range in Patients Hospitalized with COVID-19: A Spanish Observational Cohort Study

Claudia Josa-Laorden et al. J Clin Med. 2021.
Free PMC article
Show details
J Clin Med
. 2021 Feb 25;10(5):899.
doi: 10.3390/jcm10050899.

Authors

Claudia Josa-Laorden [1, 2], Anxela Crestelo-Vieitez [1], María Del Mar García Andreu [1], Manuel Rubio-Rivas [3], Marcos Sánchez [4], Neera Toledo Samaniego [5], Francisco Arnalich Fernández [6], Rosario Iguaran Bermudez [7], Eva Ma Fonseca Aizpuru [8], Juan Antonio Vargas Núñez [9], Paula Maria Pesqueira Fontan [10], Jorge Serrano Ballesteros [11], Santiago Jesús Freire Castro [12], Melani Pestaña Fernández [13], Alba Viana García [14], Victoria Nuñez Rodriguez [15], Vicente Giner-Galvañ [16, 17], Francisco Javier Carrasco Sánchez [18], Almudena Hernández Milián [19], Marta Cobos-Siles [20], Jose Javier Napal Lecumberri [21], Virginia Herrero García [22], Maria de Los Reyes Pascual Pérez [23], Jesús Millán Núñez-Cortés [5], José Manuel Casas Rojo [24], On Behalf Of The Semi-Covid-Network

Affiliations

- [1] Internal Medicine Department, Royo Villanova Hospital, Avenida San Gregorio 30, 50015 Zaragoza, Spain.
- [2] Aragon Institute of Health Research, IIS-Aragon, 50009 Zaragoza, Spain.
- [3] Internal Medicine Department, Bellvitge University Hospital-IDIBELL, L'Hospitalet de Llobregat, 08901 Barcelona, Spain.
- [4] Internal Medicine Department, 12 de Octubre University Hospital, 28041 Madrid, Spain.
- [5] Internal Medicine Department, Gregorio Marañon University Hospital, 28007 Madrid, Spain.
- [6] Internal Medicine Department, La Paz University Hospital, 28046 Madrid, Spain.
- [7] Internal Medicine Department, San Carlos Clinical Hospital, 28040 Madrid, Spain.
- [8] Internal Medicine Department, Cabueñes Hospital, 33394 Gijón, Asturias, Spain.
- [9] Internal Medicine Department, Puerta de Hierro University Hospital, 28222 Majadahonda, Spain.
- [10] Internal Medicine Department, Santiago Clinical Hospital, 15706 Santiago de Compostela, Spain.
- [11] Internal Medicine Department, La Princesa University Hospital, 28006 Madrid, Spain.
- [12] Internal Medicine Department, A Coruña University Hospital, 15006 A Coruna, Spain.
- [13] Internal Medicine Department, Moisès Broggi Hospital, 08970 Sant Joan Despí, Spain.
- [14] Internal Medicine Department, Dr. Peset University Hospital, 45017 Valencia, Spain.
- [15] Internal Medicine Department, Costa del Sol Hospital, 29603 Málaga, Spain.

- [16] General Internal Medicine Department, San Juan de Alicante University Hospital, 03550 Alicante, Spain.
- [17] Department of Clinical Medicine, Faculty of Medicine, Miguel Hernández University, 03202 Elche, Alicante, Spain.
- [18] Internal Medicine Department, Juan Ramón Jiménez Hospital, 21005 Huelva, Spain.
- [19] Internal Medicine Department, Son Llàtzer University Hospital, 07120 Palma de Mallorca, Spain.
- [20] Internal Medicine Department, Río Hortega University Hospital, Regional Health Management of Castilla y Leon (SACYL), 47012 Valladolid, Spain.
- [21] Internal Medicine Department, Marqués de Valdecilla University Hospital, 39008 Santander, Spain.
- [22] Internal Medicine Department, Doctor José Molina Orosa Hospital, 25005 Arrecife, Spain.
- [23] Internal Medicine Department, Elda University General Hospital, 03600 Elda, Alicante, Spain.
- [24] Internal Medicine Department, Infanta Cristina University Hospital, 28981 Madrid, Spain.

- PMID: **33668766**
- PMCID: PMC7956359
- DOI: 10.3390/jcm10050899

Abstract

There is some evidence that male gender could have a negative impact on the prognosis and severity of severe acute respiratory syndrome coronavirus 2 (SARS-CoV-2) infection. The aim of the present study was to compare the characteristics of coronavirus disease 2019 (COVID-19) between hospitalized men and women with confirmed SARS-CoV-2 infection. This multicenter, retrospective, observational study is based on the SEMI-COVID-19 Registry. We analyzed the differences between men and women for a wide variety of demographic, clinical, and treatment variables, and the sex distribution of the reported COVID-19 deaths, as well as intensive care unit (ICU) admission by age subgroups. This work analyzed 12,063 patients (56.8% men). The women in our study were older than the men, on average (67.9 vs. 65.7 years; $p < 001$). Bilateral condensation was more frequent among men than women (31.8% vs. 29.9%; $p = 0.007$). The men needed non-invasive and invasive mechanical ventilation more frequently (5.6% vs. 3.6%, $p < 0.001$, and 7.9% vs. 4.8%, $p < 0.001$, respectively). The most prevalent complication was acute respiratory distress syndrome, with severe cases in 19.9% of men ($p < 0.001$). In men, intensive care unit admission was more frequent (10% vs. 6.1%; $p < 0.001$) and the mortality rate was higher (23.1% vs. 18.9%; $p < 0.001$). Regarding mortality, the differences by gender were statistically significant in the age groups from 55 years to 89 years of age. A multivariate analysis showed that female sex was significantly and independently associated with a lower risk of mortality in our study. Male sex appears to be related to worse progress in COVID-19 patients and is an independent prognostic factor for mortality. In order to fully understand its prognostic impact, other factors associated with sex must be considered.

Keywords: COVID-19; SARS-CoV-2; Spain; coronavirus; gender differences.

Conflict of interest statement

The authors declare no conflict of interest.

- [28 references]
- [3 figures]

Full text links

FULL TEXT OPEN ACCESS MDPI Multidisciplinary Digital Publishing Institute (MDPI) Free PMC article
Proceed to details
Cite
Share
☐ 1,311
Cureus
. 2021 Dec 1;13(12):e20072.
doi: 10.7759/cureus.20072. eCollection 2021 Dec.

Epidemiology and Risk Factors of COVID-19-Related Mortality

Debarchan Barman Roy [1], Vandana Gupta [2], Shalini Tomar [1], Gaurav Gupta [1], Ashutosh Biswas [1], Piyush Ranjan [1], Upendra Baitha [1], Shivam Pandey [3], Bindoo Prakash [1], Naveet Wig [1]

Affiliations Expand

Affiliations

- [1] Medicine, All India Institute of Medical Sciences, New Delhi, New Delhi, IND.
- [2] Periodontics, All India Institute of Medical Sciences, New Delhi, New Delhi, IND.
- [3] Biostatistics, All India Institute of Medical Sciences, New Delhi, New Delhi, IND.

- PMID: **34987936**
- PMCID: PMC8719433
- DOI: 10.7759/cureus.20072

Free PMC article

Epidemiology and Risk Factors of COVID-19-Related Mortality

Debarchan Barman Roy et al. Cureus. 2021.
Free PMC article
Show details
Cureus
. 2021 Dec 1;13(12):e20072.
doi: 10.7759/cureus.20072. eCollection 2021 Dec.

Authors

Debarchan Barman Roy [1], Vandana Gupta [2], Shalini Tomar [1], Gaurav Gupta [1], Ashutosh Biswas [1], Piyush Ranjan [1], Upendra Baitha [1], Shivam Pandey [3], Bindoo Prakash [1], Naveet Wig [1]

Affiliations

- [1] Medicine, All India Institute of Medical Sciences, New Delhi, New Delhi, IND.
- [2] Periodontics, All India Institute of Medical Sciences, New Delhi, New Delhi, IND.
- [3] Biostatistics, All India Institute of Medical Sciences, New Delhi, New Delhi, IND.

- PMID: **34987936**
- PMCID: PMC8719433
- DOI: 10.7759/cureus.20072

Abstract

Introduction During the coronavirus disease 2019 (COVID-19) pandemic in India, several characteristics of hospitalized COVID-19 patients, based on demographics, mortality predictors, and presence of comorbidities, were found to be associated with poor outcomes. The objective of this study was to identify such epidemiological and clinical characteristics among the patients admitted at a tertiary-care center in India that may have predisposed them to COVID-19-related mortality. Methods This retrospective observational study conducted at the Department of Medicine, All India Institute of Medical Sciences, New Delhi, in May 2021 included 141 COVID-19 confirmed patients. The medical history, demographic characteristics, comorbidities, clinical findings, and laboratory data of each patient were obtained. The data were analyzed to identify significant clinical and laboratory parameters that led to the adverse final outcomes. Results Hypertension was the most common comorbidity and the presence of diabetes with hypertension led to poorer final outcomes. Lower oxygen saturation and requirement of oxygen supplementation at admission along with worse prognostic scores during admission led to poorer outcomes. Twenty-seven patients needed non-invasive ventilation (NIV) during the hospital course, and all ultimately landed up among the 56 patients who were managed on invasive mechanical ventilation (IMV). Multivariate logistic regression analysis performed identified COVID-19 severity at admission, co-existence of hypertension and diabetes mellitus, systolic blood pressure less than 90 mm Hg, and serum creatinine greater than 1.2 mg/dL to be associated with higher COVID-19 mortality. Conclusion COVID-19 patients having the co-existence of diabetes and hypertension constitute a high-risk group and may be targeted by prompt vaccination strategies. The presence of severe disease along with a need for oxygen therapy and other intensive care interventions ultimately led to unfavorable outcomes.

Keywords: covid-19 india; covid-19 mortality; diabetes and hypertension; epidemiology and public health; risk-factors.

Copyright © 2021, Barman Roy et al.

Conflict of interest statement

The authors have declared that no competing interests exist.

- 20 references

Full text links

Free PMC article
Proceed to details
Cite
Share
☐ 1,312
Ann Transl Med
. 2021 Apr;9(8):701.
doi: 10.21037/atm-21-1561.

Early risk factors for extrapulmonary organ injury in adult COVID-19 patients

Fang Huang [1], Wenxia Ma [2], Hui Zheng [3], Yan Ye [4], Hui Chen [1], Nan Su [5], Xiaoping Li [1], Xinyue Li [1], Yuyu Wang [1], Jun Jin [1], Zhengyuan Yu [6], Yongsheng Li [4], Jun Wang [1]
Affiliations Expand

Affiliations

- [1] Department of Intensive Care Medicine, The First Affiliated Hospital of Soochow University, Suzhou, China.
- [2] Department of Quality Management, The First Affiliated Hospital of Soochow University, Suzhou, China.
- [3] Institutes of Biology and Medical Sciences, Soochow University, Suzhou, China.
- [4] Department of Intensive Care Medicine, Tongji Hospital, Tongji Medical College, Huazhong University of Science and Technology, Wuhan, China.
- [5] Department of Respiratory Medicine, The First Affiliated Hospital of Soochow University, Suzhou, China.
- [6] Department of Oncology, The First Affiliated Hospital of Soochow University, Suzhou, China.

- PMID: **33987399**
- PMCID: PMC8106092
- DOI: 10.21037/atm-21-1561

Free PMC article

Early risk factors for extrapulmonary organ injury in adult COVID-19 patients

Fang Huang et al. Ann Transl Med. 2021 Apr.
Free PMC article
Show details
Ann Transl Med

. 2021 Apr;9(8):701.
doi: 10.21037/atm-21-1561.

Authors

Fang Huang[1], Wenxia Ma[2], Hui Zheng[3], Yan Ye[4], Hui Chen[1], Nan Su[5], Xiaoping Li[1], Xinyue Li[1], Yuyu Wang[1], Jun Jin[1], Zhengyuan Yu[6], Yongsheng Li[4], Jun Wang[1]

Affiliations

- [1] Department of Intensive Care Medicine, The First Affiliated Hospital of Soochow University, Suzhou, China.
- [2] Department of Quality Management, The First Affiliated Hospital of Soochow University, Suzhou, China.
- [3] Institutes of Biology and Medical Sciences, Soochow University, Suzhou, China.
- [4] Department of Intensive Care Medicine, Tongji Hospital, Tongji Medical College, Huazhong University of Science and Technology, Wuhan, China.
- [5] Department of Respiratory Medicine, The First Affiliated Hospital of Soochow University, Suzhou, China.
- [6] Department of Oncology, The First Affiliated Hospital of Soochow University, Suzhou, China.

- PMID: **33987399**
- PMCID: PMC8106092
- DOI: 10.21037/atm-21-1561

Abstract

Background: The novel 2019 coronavirus (COVID-19) has caused a global pandemic, and often leads to extrapulmonary organ injury. However, the risk factors for extrapulmonary organ injury are still unclear. We aim to explore the risk factors for extrapulmonary organ injury and the association between extrapulmonary organ injury and the prognosis in COVID-19 patients.

Methods: We implemented a single-center, retrospective, observational study, in which a total of 349 confirmed COVID-19 patients admitted to Tongji Hospital from January 25, 2020, to February 25, 2020, were enrolled. We collected demographic, clinical, laboratory, and treatment data from electronic medical records. Potential risk factors for extrapulmonary organ injury of COVID-19 patients were analyzed by a multivariable binary logistic model, and multivariable Cox proportional hazards regression model was used for survival analysis in the patients with extrapulmonary organ injury.

Results: The average age of the included patients was 61.73±14.64 years. In the final logistic model, variables including aged 60 or older [odds ratio (OR) 1.826, 95% confidence interval (CI): 1.060-3.142], acute respiratory distress syndrome (ARDS) (OR 2.748, 95% CI: 1.051-7.185), lymphocytes count lower than 1.1×10^9/L (OR 0.478, 95% CI: 0.240-0.949), level of interleukin-6 (IL-6) greater than 7 pg/mL (OR 1.664, 95% CI: 1.005-2.751) and D-Dimer greater than 0.5 μg/mL (OR 2.190, 95% CI: 1.176-4.084) were significantly associated with the extrapulmonary organ injury. Kaplan-Meier curve and log-rank test showed that the probabilities of survival for patients with extrapulmonary organ injury were significantly lower than those without extrapulmonary organ injury. Multivariate Cox proportional hazards model showed that only myocardial injury (P=0.000, HR: 5.068, 95% CI: 2.728-9.417) and circulatory system injury

(P=0.000, HR: 4.076, 95% CI: 2.216-7.498) were the independent factors associated with COVID-19 patients' poor prognosis.

Conclusions: Older age, lymphocytopenia, high level of D-Dimer and IL-6, and the severity of lung injury were the high-risk factors of extrapulmonary organ injury in COVID-19 patients. Myocardial and circulatory system injury were the most important risk factors related to poor outcomes of COVID-19 patients. It may help clinicians to identify extrapulmonary organ injury early and initiate appropriate treatment.

Keywords: COVID-19; SARS-CoV-2; extrapulmonary organ injury; risk factors.

2021 Annals of Translational Medicine. All rights reserved.

Conflict of interest statement

Conflicts of Interest: All authors have completed the ICMJE uniform disclosure form (available at http://dx.doi.org/10.21037/atm-21-1561). The authors have no conflicts of interest to declare.

- 5 figures

Full text links

ATM FULL TEXT AME Publishing Company Free PMC article

Proceed to details

Cite

Share

☐ 1,313

J Clin Med
. 2021 Apr 17;10(8):1752.
doi: 10.3390/jcm10081752.

Risk Factors for Mortality in Adult COVID-19 Patients Who Develop Bloodstream Infections Mostly Caused by Antimicrobial-Resistant Organisms: Analysis at a Large Teaching Hospital in Italy

Brunella Posteraro [1, 2], Giulia De Angelis [1, 3], Giulia Menchinelli [1, 3], Tiziana D'Inzeo [1, 3], Barbara Fiori [3], Flavio De Maio [3], Venere Cortazzo [1], Maurizio Sanguinetti [1, 3], Teresa Spanu [1, 3]

Affiliations Expand

Affiliations

- [1] Dipartimento di Scienze Biotecnologiche di Base, Cliniche Intensivologiche e Perioperatorie, Università Cattolica del Sacro Cuore, 00168 Roma, Italy.
- [2] Dipartimento di Scienze Mediche e Chirurgiche, Fondazione Policlinico Universitario A. Gemelli IRCCS, 00168 Roma, Italy.
- [3] Dipartimento di Scienze di Laboratorio e Infettivologiche, Fondazione Policlinico Universitario A. Gemelli IRCCS, 00168 Roma, Italy.

- PMID: **33920701**
- PMCID: PMC8073579
- DOI: 10.3390/jcm10081752

Free PMC article

Risk Factors for Mortality in Adult COVID-19 Patients Who Develop Bloodstream Infections Mostly Caused by Antimicrobial-Resistant Organisms: Analysis at a Large Teaching Hospital in Italy

Brunella Posteraro et al. J Clin Med. 2021.
Free PMC article
Show details
J Clin Med
. 2021 Apr 17;10(8):1752.
doi: 10.3390/jcm10081752.

Authors

Brunella Posteraro [1, 2], Giulia De Angelis [1, 3], Giulia Menchinelli [1, 3], Tiziana D'Inzeo [1, 3], Barbara Fiori [3], Flavio De Maio [3], Venere Cortazzo [1], Maurizio Sanguinetti [1, 3], Teresa Spanu [1, 3]

Affiliations

- [1] Dipartimento di Scienze Biotecnologiche di Base, Cliniche Intensivologiche e Perioperatorie, Università Cattolica del Sacro Cuore, 00168 Roma, Italy.
- [2] Dipartimento di Scienze Mediche e Chirurgiche, Fondazione Policlinico Universitario A. Gemelli IRCCS, 00168 Roma, Italy.
- [3] Dipartimento di Scienze di Laboratorio e Infettivologiche, Fondazione Policlinico Universitario A. Gemelli IRCCS, 00168 Roma, Italy.

- PMID: **33920701**
- PMCID: PMC8073579
- DOI: 10.3390/jcm10081752

Abstract

The aim of this study was to characterize COVID-19 (SARS-CoV-2-infected) patients who develop bloodstream infection (BSI) and to assess risk factors associated with in-hospital mortality. We conducted a retrospective observational study of adult patients admitted for ≥48 h to a large Central Italy hospital for COVID-19 (1 March to 31 May 2020) who had or had not survived at discharge. We included only patients having blood cultures drawn or other inclusion criteria satisfied. Kaplan-Meier survival or Cox regression analyses were performed of 293 COVID-19 patients studied, 46 patients (15.7%) had a hospital-acquired clinically relevant BSI secondary to SARS-CoV-2 infection, accounting for 58 episodes (49 monomicrobial and 9 polymicrobial) in total. Twelve episodes (20.7%) occurred at day 3 of hospital admission. Sixty-nine species were isolated, including *Staphylococcus aureus* (32.8%), Enterobacterales (20.7%), *Enterococcus faecalis* (17.2%), *Candida* (13.8%) and *Pseudomonas aeruginosa* (10.3%). Of 69 isolates, 27 (39.1%) were multidrug-resistant organisms. Twelve (54.5%) of 22 patients for whom empirical antimicrobial therapy was inappropriate were infected by a multidrug-resistant organism. Of 46 patients, 26 (56.5%) survived and 20 (43.5%) died. Exploring variables for association with in-hospital mortality identified > 75-year age (HR 2.97, 95% CI 1.15-7.68, p = 0.02), septic shock (HR 6.55, 95% CI 2.36-18.23, $p < 0.001$) and BSI onset ≤ 3 days (HR 4.68, 95% CI 1.40-15.63, p = 0.01) as risk factors independently associated with death. In our hospital, mortality among COVID-19 patients with BSI was high. While continued vigilance against these infections is essential, identification of risk factors for mortality may help to reduce fatal outcomes in patients with COVID-19.

Keywords: COVID-19; antimicrobial resistance; bloodstream infection; mortality; risk factors; septic shock.

Conflict of interest statement

The authors declare no conflict of interest.

- 40 references
- 3 figures

Full text links

FULL TEXT OPEN ACCESS MDPI Multidisciplinary Digital Publishing Institute (MDPI) Free PMC article

Proceed to details

Cite

Share

☐ 1,314

Bone Jt Open

. 2021 Apr;2(4):236-242.

doi: 10.1302/2633-1462.24.BJO-2021-0005.R1.

[Did COVID-19 related delays in surgical management lead to patient morbidity in the orthopaedic oncological population?]

Michael J Fitzgerald [1], Howard J Goodman [1], Samuel Kenan [1], Shachar Kenan [1]
Affiliations Expand

Affiliation

- [1] Department of Orthopaedics, North Shore-Long Island Jewish Hospital, Northwell Health Medical Center, New Hyde Park, New York, USA.

- PMID: **33870729**
- PMCID: PMC8085619
- DOI: 10.1302/2633-1462.24.BJO-2021-0005.R1

Free PMC article

Did COVID-19 related delays in surgical management lead to patient morbidity in the orthopaedic oncological population?

Michael J Fitzgerald et al. Bone Jt Open. 2021 Apr.
Free PMC article
Show details
Bone Jt Open
. 2021 Apr;2(4):236-242.
doi: 10.1302/2633-1462.24.BJO-2021-0005.R1.

Authors

Michael J Fitzgerald [1], Howard J Goodman [1], Samuel Kenan [1], Shachar Kenan [1]

Affiliation

- [1] Department of Orthopaedics, North Shore-Long Island Jewish Hospital, Northwell Health Medical Center, New Hyde Park, New York, USA.

- PMID: **33870729**
- PMCID: PMC8085619
- DOI: 10.1302/2633-1462.24.BJO-2021-0005.R1

Abstract

Aims: The aim of this study was to assess orthopaedic oncologic patient morbidity resulting from COVID-19 related institutional delays and surgical shutdowns during the first wave of the pandemic in New York, USA.

Methods: A single-centre retrospective observational study was conducted of all orthopaedic oncologic patients undergoing surgical evaluation from March to June 2020. Patients were prioritized as level 0-IV, 0 being elective and IV being emergent. Only priority levels 0 to III were included. Delay duration was measured in days and resulting morbidities were categorized into seven groups: prolonged pain/disability; unplanned preoperative radiation and/or chemotherapy; local tumour progression; increased systemic disease; missed opportunity for surgery due to progression of disease/lost to follow up; delay in diagnosis; and no morbidity.

Results: Overall, 25 patients met inclusion criteria. There were eight benign tumours, seven metastatic, seven primary sarcomas, one multiple myeloma, and two patients without a biopsy proven diagnosis. There was no priority level 0, two priority level I, six priority level II, and 17 priority level III cases. The mean duration of delay for priority level I was 114 days (84 to 143), priority level II was 88 days (63 to 133), and priority level III was 77 days (35 to 269). Prolonged pain/disability and delay in diagnosis, affecting 52% and 40%,respectively, represented the two most frequent morbidities. Local tumour progression and increased systemic disease affected 32% and 24% respectively. No patients tested positive for COVID-19.

Conclusion: COVID-19 related delays in surgical management led to major morbidity in this studied orthopaedic oncologic patient population. By understanding these morbidities through clearer hindsight, a thoughtful approach can be developed to balance the risk of COVID-19 exposure versus delay in treatment, ensuring optimal care for orthopedic oncologic patients as the pandemic continues with intermittent calls for halting surgery. Cite this article: *Bone Jt Open* 2021;2(4):236-242.

Keywords: COVID-19; Cancer; Coronavirus; Orthopaedic oncology; SARS-CoV-2.

- 15 references
- 4 figures

Full text links

Bone & Joint Open Full Text Available Atypon Free PMC article

Proceed to details

Cite

Share

☐ 1,315

Cureus

. 2021 Dec 21;13(12):e20571.
doi: 10.7759/cureus.20571. eCollection 2021 Dec.

The Impact of COVID-19 Pandemic on Orthopedic Trauma Practice: An Experience at a Tertiary Care Center in Northern India

Bhavkaran Singh [1], Rajesh Kapila [1], Kamalpreet Singh [2]
Affiliations Expand

Affiliations

- [1] Orthopedics and Traumatology, Government Medical College, Amritsar, IND.
- [2] Orthopaedics, Government Medical College, Amritsar, IND.

- PMID: **35103150**
- PMCID: PMC8773358
- DOI: 10.7759/cureus.20571

Free PMC article

The Impact of COVID-19 Pandemic on Orthopedic Trauma Practice: An Experience at a Tertiary Care Center in Northern India

Bhavkaran Singh et al. Cureus. 2021.
Free PMC article
Show details
Cureus
. 2021 Dec 21;13(12):e20571.
doi: 10.7759/cureus.20571. eCollection 2021 Dec.

Authors

Bhavkaran Singh [1], Rajesh Kapila [1], Kamalpreet Singh [2]

Affiliations

- [1] Orthopedics and Traumatology, Government Medical College, Amritsar, IND.
- [2] Orthopaedics, Government Medical College, Amritsar, IND.

- PMID: **35103150**
- PMCID: PMC8773358
- DOI: 10.7759/cureus.20571

Abstract

The Impact of Covid -19 Pandemic on Orthopedic trauma practice: An experience at a tertiary care center in Northern India Introduction: COVID-19 pandemic and associated lockdown have had drastic effects on the health care system. To dedicate all the staff, residents, interns to covid care and accommodate the escalated inflow of infected persons, most hospitals had to restructure their departments. The orthopedic department was no exception. The present study aimed to evaluate trends in orthopedic trauma cases during two waves of the Covid -19 pandemic.

Material and methods: In this retrospective observational study, the period of lockdown during the first wave of Covid (March 24, 2020, to May 31, 2020 (Period 2)) and the second wave in 2021 was compared with a similar nine weeks interval in 2019 before COVID -19 (Period 1). Demographic details and epidemiological parameters of trauma were collected and compared.

Observations: The number of admissions declined from 8.2 admissions/day to 2.3/day and 2.71/day in periods 2 and 3, respectively. Roadside accidents in 73.37% of patients in period 1 reduced to 30.43% and 59 36.2% in period 2 and 3, respectively. After soft tissue injuries, fractures around the hip joint dominated the pattern of injury during the lockdown, while polytrauma significantly decreased compared to the pre-covid era. More than 80% of patients during lockdown were treated conservatively.

Conclusion: Evaluation of differences in injury patterns and method of treatment during distinctive situations arising due to the COVID-19 pandemic will help to judicially plan and formulate protocols for more effective management of patients if similar events arise again.

Keywords: Covid-19; Lockdown; Pandemic; covid-19 pandemic; lockdown; orthopaedic; orthopedic trauma trends.; pattern; trauma.

Copyright © 2021, Singh et al.

Conflict of interest statement

The authors have declared that no competing interests exist.

- 13 references

Full text links

Free PMC article
Proceed to details
Cite
Share
☐ 1,316

J Clin Med
. 2020 Nov 20;9(11):3726.
doi: 10.3390/jcm9113726.

Phenotypic Characteristics and Development of a Hospitalization Prediction Risk Score for Outpatients with Diabetes and COVID-19: The DIABCOVID Study

Adèle Lasbleiz [1 2], Bertrand Cariou [3], Patrice Darmon [1 2], Astrid Soghomonian [1], Patricia Ancel [2], Sandrine Boullu [1 2], Marie Houssays [4], Fanny Romain [5], Jean Christophe Lagier [6], Mohamed Boucekine [7], Noémie Resseguier [7 8], Pierre Gourdy [9], Matthieu Pichelin [3], Matthieu Wargny [10], Anne Dutour [1 2], Bénédicte Gaborit [1 2]

Affiliations Expand

Affiliations

- [1] Department of Endocrinology, Metabolic Diseases and Nutrition, Pôle ENDO, APHM, 13005 Marseille, France.
- [2] Aix Marseille University, INSERM, INRAE, C2VN, 13005 Marseille, France.
- [3] L'institut du Thorax, Inserm, CNRS, UNIV Nantes, CHU Nantes, Département d'Endocrinologie, Diabétologie et Nutrition, Hôpital Guillaume et René Laennec, 44093 Nantes, France.
- [4] Assistance-Publique Hôpitaux de Marseille, Medical Evaluation Department, CIC-CPCET, 13005 Marseille, France.
- [5] Public Health and Medical Information Department, APHM, 13005 Marseille, France.
- [6] Aix Marseille University, IRD, AP-HM, MEPHI, IHU Méditerranée Infection, 13005 Marseille, France.
- [7] Aix-Marseille University, EA 3279 CEReSS-Health Service Research and Quality of Life Center, 13005 Marseille, France.
- [8] Support Unit for Clinical Research and Economic Evaluation, Assistance Publique-Hôpitaux de Marseille, 13005 Marseille, France.
- [9] Département d'Endocrinologie, Diabétologie et Nutrition, CHU Toulouse, Institut des Maladies Métaboliques et Cardiovasculaires, UMR1048 Inserm/UPS, Université de Toulouse, 31432 Toulouse, France.
- [10] CIC-EC 1413, Clinique des Données, CHU de Nantes, 44000 Nantes, France.

- PMID: **33233575**
- PMCID: PMC7699790
- DOI: 10.3390/jcm9113726

Free PMC article

Phenotypic Characteristics and Development of a Hospitalization Prediction Risk Score for Outpatients with Diabetes and COVID-19: The DIABCOVID Study

Adèle Lasbleiz et al. J Clin Med. 2020.
Free PMC article
Show details
J Clin Med

. 2020 Nov 20;9(11):3726.
doi: 10.3390/jcm9113726.

Authors

Adèle Lasbleiz [1, 2], Bertrand Cariou [3], Patrice Darmon [1, 2], Astrid Soghomonian [1], Patricia Ancel [2], Sandrine Boullu [1, 2], Marie Houssays [4], Fanny Romain [5], Jean Christophe Lagier

[6], Mohamed Boucekine [7], Noémie Resseguier [7 8], Pierre Gourdy [9], Matthieu Pichelin [3], Matthieu Wargny [10], Anne Dutour [1 2], Bénédicte Gaborit [1 2]

Affiliations

- [1] Department of Endocrinology, Metabolic Diseases and Nutrition, Pôle ENDO, APHM, 13005 Marseille, France.
- [2] Aix Marseille University, INSERM, INRAE, C2VN, 13005 Marseille, France.
- [3] L'institut du Thorax, Inserm, CNRS, UNIV Nantes, CHU Nantes, Département d'Endocrinologie, Diabétologie et Nutrition, Hôpital Guillaume et René Laennec, 44093 Nantes, France.
- [4] Assistance-Publique Hôpitaux de Marseille, Medical Evaluation Department, CIC-CPCET, 13005 Marseille, France.
- [5] Public Health and Medical Information Department, APHM, 13005 Marseille, France.
- [6] Aix Marseille University, IRD, AP-HM, MEPHI, IHU Méditerranée Infection, 13005 Marseille, France.
- [7] Aix-Marseille University, EA 3279 CEReSS-Health Service Research and Quality of Life Center, 13005 Marseille, France.
- [8] Support Unit for Clinical Research and Economic Evaluation, Assistance Publique-Hôpitaux de Marseille, 13005 Marseille, France.
- [9] Département d'Endocrinologie, Diabétologie et Nutrition, CHU Toulouse, Institut des Maladies Métaboliques et Cardiovasculaires, UMR1048 Inserm/UPS, Université de Toulouse, 31432 Toulouse, France.
- [10] CIC-EC 1413, Clinique des Données, CHU de Nantes, 44000 Nantes, France.

- PMID: **33233575**
- PMCID: PMC7699790
- DOI: 10.3390/jcm9113726

Abstract

Diabetes mellitus (DM) has been identified as a risk factor for severe COVID-19. DM is highly prevalent in the general population. Defining strategies to reduce the health care system burden and the late arrival of some patients thus seems crucial. The study aim was to compare phenotypic characteristics between in and outpatients with diabetes and infected by COVID-19, and to build an easy-to-use hospitalization prediction risk score. This was a retrospective observational study. Patients with DM and laboratory- or CT-confirmed COVID-19, who did (n = 185) and did not (n = 159) require hospitalization between 10 March and 10 April 2020, were compared. Data on diabetes duration, treatments, glycemic control, complications, anthropometrics and peripheral oxygen saturation (SpO_2) were collected from medical records. Stepwise multivariate logistic regressions and ROC analyses were performed to build the DIAB score, a score using no more than five easy-to-collect clinical parameters predicting the risk of hospitalization. The DIAB score was then validated in two external cohorts (n = 132 and n = 2036). Hospitalized patients were older (68.0 ± 12.6 vs. 55.2 ± 12.6 years, $p < 0.001$), with more class III obesity (BMI ≥ 40 kg/m^2, 9.7 vs. 3.5%, $p = 0.03$), hypertension (81.6 vs. 44.3%, $p < 0.0001$), insulin therapy (37% vs. 23.7%, $p = 0.009$), and lower SpO_2 (91.6 vs. 97.3%, $p < 0.0001$) than outpatients. Type 2 DM (T2D) was found in 94% of all patients, with 10 times more type 1 DM in the outpatient group (11.3 vs. 1.1%, $p < 0.0001$). A DIAB score > 27 points predicted hospitalization (sensitivity 77.7%, specificity 89.2%, AUC = 0.895), and death within 28 days. Its performance was validated in the two external cohorts. Outpatients with diabetes were found to be younger, with fewer

diabetic complications and less severe obesity than inpatients. DIAB score is an easy-to-use score integrating five variables to help clinicians better manage patients with DM and avert the saturation of emergency care units.

Keywords: COVID-19; DIABSCORE; diabetes; hospitalization risk score; outpatients.

Conflict of interest statement

The authors declare no conflict of interest.

- 32 references
- 3 figures

Supplementary info

Grant support Expand

Grant support

- //Fondation francophone de recherche sur le diabète
- //Fédération française des diabétiques
- //Société francophone du diabète
- //Air Liquide Health Care international

Full text links

Multidisciplinary Digital Publishing Institute (MDPI) Free PMC article

Proceed to details

Cite

Share

1,317

Int J Environ Res Public Health

. 2022 Mar 19;19(6):3665.

doi: 10.3390/ijerph19063665.

Help Seeking of Highly Specialized Mental Health Treatment before and during the COVID-19 Pandemic among Health Professionals

María Dolores Braquehais [1, 2], Esperanza L Gómez-Duran [1, 3], Gemma Nieva [1, 2, 4], Sergi Valero [1, 5], Josep Antoni Ramos-Quiroga [2, 4, 6], Eugeni Bruguera [1, 2, 4]

Affiliations Expand

Affiliations

- [1] Integral Care Program for Sick Health Professionals, Galatea Clinic, Galatea Foundation, 08017 Barcelona, Spain.
- [2] Psychiatry, Mental Health and Addiction Research Group, Networking Research on Mental Health (CIBERSAM), Vall d'Hebron Institut de Recerca (VHIR), Vall d'Hebron Hospital Universitari, Vall d'Hebron Barcelona Hospital Campus, 08035 Barcelona, Spain.
- [3] School of Medicine, Universitat Internacional de Catalunya, 08017 Barcelona, Spain.
- [4] Department of Psychiatry, Vall d'Hebron Hospital Universitari, Vall d'Hebron Barcelona Hospital Campus, 08035 Barcelona, Spain.
- [5] ACE Alzheimer Center Barcelona, Universitat Internacional de Catalunya (UIC), 08017 Barcelona, Spain.
- [6] Department of Psychiatry, School of Medicine, Universitat Autònoma de Barcelona, 08193 Barcelona, Spain.

- PMID: **35329351**
- PMCID: PMC8951467
- DOI: 10.3390/ijerph19063665

Free PMC article

Help Seeking of Highly Specialized Mental Health Treatment before and during the COVID-19 Pandemic among Health Professionals

María Dolores Braquehais et al. Int J Environ Res Public Health. 2022.
Free PMC article
Show details
Int J Environ Res Public Health

. 2022 Mar 19;19(6):3665.
doi: 10.3390/ijerph19063665.

Authors

María Dolores Braquehais [1, 2], Esperanza L Gómez-Duran [1, 3], Gemma Nieva [1, 2, 4], Sergi Valero [1, 5], Josep Antoni Ramos-Quiroga [2, 4, 6], Eugeni Bruguera [1, 2, 4]

Affiliations

- [1] Integral Care Program for Sick Health Professionals, Galatea Clinic, Galatea Foundation, 08017 Barcelona, Spain.
- [2] Psychiatry, Mental Health and Addiction Research Group, Networking Research on Mental Health (CIBERSAM), Vall d'Hebron Institut de Recerca (VHIR), Vall d'Hebron Hospital Universitari, Vall d'Hebron Barcelona Hospital Campus, 08035 Barcelona, Spain.
- [3] School of Medicine, Universitat Internacional de Catalunya, 08017 Barcelona, Spain.

- [4] Department of Psychiatry, Vall d'Hebron Hospital Universitari, Vall d'Hebron Barcelona Hospital Campus, 08035 Barcelona, Spain.
- [5] ACE Alzheimer Center Barcelona, Universitat Internacional de Catalunya (UIC), 08017 Barcelona, Spain.
- [6] Department of Psychiatry, School of Medicine, Universitat Autònoma de Barcelona, 08193 Barcelona, Spain.

- PMID: **35329351**
- PMCID: PMC8951467
- DOI: 10.3390/ijerph19063665

Abstract

(1) Background: Ongoing specialized programs for health professionals (HPs) adapted their treatment services during the COVID-19 pandemic. (2) Methods: We conducted a retrospective observational study of medical e-records of HPs with mental disorders working in Catalonia that were consecutively admitted to the Galatea Care Program Clinical Unit. The sample (N = 1461) was divided into two periods: 21.5 months before (*n* = 637) and after (*n* = 824) 14 March 2020. (3) Results: There was a significant increase (29.4%) in the number of referrals to the specialized Clinical Unit during the pandemic, especially with respect to physicians compared to nurses. The percentage of HP women at admission and the clinical severity of the first treatment episode remained without changes before and after the COVID-19 pandemic. The most prevalent main diagnoses also remained similar: adjustment disorders (41.5%), mood disorders (24.9%), anxiety disorders (14.4%), and substance use disorders (11.8%). (4) Conclusions: HPs, particularly physicians, more frequently sought voluntary help from specialized mental health programs during the COVID-19 pandemic. Future studies are needed to analyze the reasons behind this finding and the evolution of referrals to these types of programs after the COVID-19 outbreak.

Keywords: COVID-19; health professionals; mental disorders; mental health; mental health programs; substance use disorders; treatment services.

Conflict of interest statement

The authors declare no conflict of interest.

- 33 references

Full text links

FULL TEXT OPEN ACCESS MDPI Multidisciplinary Digital Publishing Institute (MDPI)

Proceed to details

Cite

Share

☐ 1,318

J Mycol Med

. 2022 Feb 1;32(2):101252.

doi: 10.1016/j.mycmed.2022.101252. Online ahead of print.

Pathogenetic factors fanning the flames of COVID-19 to cause rhino-orbito-cerebral mucormycosis: An observational study

Y Muralidhar Reddy [1], Sreekanth Yeduguri [2], Vishnu Swaroop Reddy N [3], Subhendu Parida [2], Shanti Naidu Kamatham [4], Lalitha Pidaparthi [2], Shyam K Jaiswal [2], Bhavana Sadhvani [5], Vijaya Tourani [5], Sudhir Kumar [6], Sundaram Challa [7], Jagarlapudi Mk Murthy [2]
Affiliations Expand

Affiliations

- [1] Department of Neurology, CARE Hospital, Banjara Hills, Hyderabad, India. Electronic address: muralidharnims@gmail.com.
- [2] Department of Neurology, CARE Hospital, Banjara Hills, Hyderabad, India.
- [3] Department of E.N.T, CARE Hospital, Banjara Hills, Hyderabad, India.
- [4] Department of Biochemistry, CARE Hospital, Banjara Hills, Hyderabad, India.
- [5] Department of Pathology, CARE Hospital, Banjara Hills, Hyderabad, India.
- [6] Department of Biostatistics, CARE Hospital, Banjara Hills, Hyderabad, India.
- [7] Department of Pathology, Basavatarakam Indo American Cancer Hospital & Research Institute, Hyderabad, India.

- PMID: **35134736**
- PMCID: PMC8806396
- DOI: 10.1016/j.mycmed.2022.101252

Free PMC article

Pathogenetic factors fanning the flames of COVID-19 to cause rhino-orbito-cerebral mucormycosis: An observational study

Y Muralidhar Reddy et al. J Mycol Med. 2022.
Free PMC article
Show details
J Mycol Med
. 2022 Feb 1;32(2):101252.
doi: 10.1016/j.mycmed.2022.101252. Online ahead of print.

Authors

Y Muralidhar Reddy [1], Sreekanth Yeduguri [2], Vishnu Swaroop Reddy N [3], Subhendu Parida [2], Shanti Naidu Kamatham [4], Lalitha Pidaparthi [2], Shyam K Jaiswal [2], Bhavana Sadhvani [5], Vijaya Tourani [5], Sudhir Kumar [6], Sundaram Challa [7], Jagarlapudi Mk Murthy [2]

Affiliations

- [1] Department of Neurology, CARE Hospital, Banjara Hills, Hyderabad, India. Electronic address: muralidharnims@gmail.com.
- [2] Department of Neurology, CARE Hospital, Banjara Hills, Hyderabad, India.
- [3] Department of E.N.T, CARE Hospital, Banjara Hills, Hyderabad, India.
- [4] Department of Biochemistry, CARE Hospital, Banjara Hills, Hyderabad, India.
- [5] Department of Pathology, CARE Hospital, Banjara Hills, Hyderabad, India.
- [6] Department of Biostatistics, CARE Hospital, Banjara Hills, Hyderabad, India.
- [7] Department of Pathology, Basavatarakam Indo American Cancer Hospital & Research Institute, Hyderabad, India.

- PMID: **35134736**
- PMCID: PMC8806396
- DOI: 10.1016/j.mycmed.2022.101252

Abstract

Background and aims: Published studies on coronavirus disease 19 (COVID-19) associated rhino-orbito-cerebral mucormycosis (CAROCM) were primarily descriptive. Therefore, we aimed to identify features of COVID-19 that could predispose to CAROCM and explore the pathogenic pathways.

Patients and methods: This retrospective hospital-based study was done during the first (March 2020 - January 2021) and the second (February 2021 - June 2021) waves of the COVID-19 pandemic. Subjects were grouped into four categories: first-wave CAROCM (n-4); second-wave CAROCM (n-27); first-wave non-mucor COVID (n-75), and second-wave non-mucor COVID (n-50). Data elements included age, gender, comorbidities, COVID-19 severity, steroid therapy, peak values of interleukin-6 (IL-6), serum ferritin and D-dimer, nadir values of absolute lymphocyte count (ALC), absolute neutrophil count (ANC) and platelet count (Pl. C).

Results: Thirty-one patients of CAROCM were included. The mean (SD) age was 51.26 (11.48) years. 27 (87.1%) were aged ≥ 40 years and males. Severe COVID-19 was seen more often in the second wave than the first wave (P-0.001). CAROCM group was significantly younger (P-0.008) and showed a higher incidence of uncontrolled diabetes (P-0.001) and renal dysfunction (P-0.004) than non-mucor COVID. While IL-6, ferritin and D-dimer were significantly elevated in CAROCM than non-mucor COVID, clinical severity, ANC, ALC and Pl. C showed no significant difference.

Conclusion: CAROCM is seen often in middle-aged diabetic males with uncontrolled hyperglycaemia, diabetic ketoacidosis, renal dysfunction and those infected by more transmissible delta variants and treated with steroids. IL-6, D-dimer, serum ferritin are more often elevated in CAROCM and might play a pathogenic role.

Keywords: Coronavirus disease-19; Cytokine storm; Delta variants; Diabetes; Hyperferritinemia; Rhino-orbito-cerebral mucormycosis.

Copyright © 2022 SFMM. Published by Elsevier Masson SAS. All rights reserved.

- 25 references
- 4 figures

Full text links

Elsevier Science Free PMC article
Proceed to details
Cite
Share
1,319
Ann Intensive Care
. 2021 Aug 6;11(1):123.
doi: 10.1186/s13613-021-00914-5.

Acute kidney injury prevalence, progression and long-term outcomes in critically ill patients with COVID-19: a cohort study

Nuttha Lumlertgul [1] [2] [3], Leah Pirondini [4], Enya Cooney [1], Waisun Kok [1], John Gregson [4], Luigi Camporota [1], Katie Lane [1], Richard Leach [1], Marlies Ostermann [5]
Affiliations Expand

Affiliations

- [1] Department of Critical Care, Guy's & St Thomas' Hospital NHS Foundation Hospital, 249 Westminster Bridge Road, London, SE1 7EH, UK.
- [2] Division of Nephrology and Excellence Centre for Critical Care Nephrology, King Chulalongkorn Memorial Hospital, Bangkok, Thailand.
- [3] Critical Care Nephrology Research Unit, Chulalongkorn University, Bangkok, Thailand.
- [4] Department of Medical Statistics, London School of Hygiene and Tropical Medicine, London, UK.
- [5] Department of Critical Care, Guy's & St Thomas' Hospital NHS Foundation Hospital, 249 Westminster Bridge Road, London, SE1 7EH, UK. marlies.ostermann@gstt.nhs.uk.

- PMID: **34357478**
- PMCID: PMC8343342
- DOI: 10.1186/s13613-021-00914-5

Free PMC article

Acute kidney injury prevalence, progression and long-term outcomes in critically ill patients with COVID-19: a cohort study

Nuttha Lumlertgul et al. Ann Intensive Care. 2021.
Free PMC article

Show details

Ann Intensive Care
. 2021 Aug 6;11(1):123.
doi: 10.1186/s13613-021-00914-5.

Authors

Nuttha Lumlertgul [1] [2] [3], Leah Pirondini [4], Enya Cooney [1], Waisun Kok [1], John Gregson [4], Luigi Camporota [1], Katie Lane [1], Richard Leach [1], Marlies Ostermann [5]

Affiliations

- [1] Department of Critical Care, Guy's & St Thomas' Hospital NHS Foundation Hospital, 249 Westminster Bridge Road, London, SE1 7EH, UK.
- [2] Division of Nephrology and Excellence Centre for Critical Care Nephrology, King Chulalongkorn Memorial Hospital, Bangkok, Thailand.
- [3] Critical Care Nephrology Research Unit, Chulalongkorn University, Bangkok, Thailand.
- [4] Department of Medical Statistics, London School of Hygiene and Tropical Medicine, London, UK.
- [5] Department of Critical Care, Guy's & St Thomas' Hospital NHS Foundation Hospital, 249 Westminster Bridge Road, London, SE1 7EH, UK. marlies.ostermann@gstt.nhs.uk.

- PMID: **34357478**
- PMCID: PMC8343342
- DOI: 10.1186/s13613-021-00914-5

Abstract

Background: There are limited data on acute kidney injury (AKI) progression and long-term outcomes in critically ill patients with coronavirus disease-19 (COVID-19). We aimed to describe the prevalence and risk factors for development of AKI, its subsequent clinical course and AKI progression, as well as renal recovery or dialysis dependence and survival in this group of patients.

Methods: This was a retrospective observational study in an expanded tertiary care intensive care unit in London, United Kingdom. Critically ill patients admitted to ICU between 1st March 2020 and 31st July 2020 with confirmed SARS-COV2 infection were included. Analysis of baseline characteristics, organ support, COVID-19 associated therapies and their association with mortality and outcomes at 90 days was performed.

Results: Of 313 patients (70% male, mean age 54.5 ± 13.9 years), 240 (76.7%) developed AKI within 14 days after ICU admission: 63 (20.1%) stage 1, 41 (13.1%) stage 2, 136 (43.5%) stage 3. 113 (36.1%) patients presented with AKI on ICU admission. Progression to AKI stage 2/3 occurred in 36%. Risk factors for AKI progression were mechanical ventilation [HR (hazard ratio) 4.11; 95% confidence interval (CI) 1.61-10.49] and positive fluid balance [HR 1.21 (95% CI 1.11-1.31)], while steroid therapy was associated with a reduction in AKI progression (HR 0.73 [95% CI 0.55-0.97]). Kidney replacement therapy (KRT) was initiated in 31.9%. AKI patients had a higher 90-day mortality than non-AKI patients (34% vs. 14%; $p < 0.001$). Dialysis dependence was 5% at hospital discharge and 4% at 90 days. Renal recovery was identified in 81.6% of

survivors at discharge and in 90.9% at 90 days. At 3 months, 16% of all AKI survivors had chronic kidney disease (CKD); among those without renal recovery, the CKD incidence was 44%.

Conclusions: During the first COVID-19 wave, AKI was highly prevalent among severely ill COVID-19 patients with a third progressing to severe AKI requiring KRT. The risk of developing CKD was high. This study identifies factors modifying AKI progression, including a potentially protective effect of steroid therapy. Recognition of risk factors and monitoring of renal function post-discharge might help guide future practice and follow-up management strategies. Trial registration NCT04445259.

Keywords: AKI; Acute kidney injury; COVID-19; Dialysis; Kidney replacement therapy; Recovery; SARS-CoV-2.

© 2021. The Author(s).

Conflict of interest statement

All other authors declare no conflicts of interests.

- 52 references
- 2 figures

Supplementary info

Associated data Expand

Associated data

- ClinicalTrials.gov/NCT04445259

Full text links

SpringerLink FULL-TEXT ARTICLE Springer Free PMC article
Proceed to details
Cite
Share
☐ 1,320
Arch Soc Esp Oftalmol
. 2021 Dec 3.
doi: 10.1016/j.oftal.2021.02.012. Online ahead of print.

[Results Of The Treatment Of Patients With Exudative Amd During The Covid -19 Pandemic]

[Article in Spanish]
C Arruabarrena [1, 2], R Montejano-Milner [1, 2], F De Aragón [1], G Allendes [1], M A Teus [1, 2, 3]

Affiliations Expand

Affiliations

- [1] Servicio de Oftalmología Hospital Universitario Príncipe de Asturias, Spain.
- [2] Clínica Novovisión Madrid, Spain.
- [3] Facultad de Medicina de la Universidad de Alcalá de Henares, Spain.

- PMID: **34876774**
- PMCID: PMC8639395
- DOI: 10.1016/j.oftal.2021.02.012

Free PMC article

[Results Of The Treatment Of Patients With Exudative Amd During The Covid -19 Pandemic]

[Article in Spanish]
C Arruabarrena et al. Arch Soc Esp Oftalmol. 2021.
Free PMC article
Show details
Arch Soc Esp Oftalmol
. 2021 Dec 3.
doi: 10.1016/j.oftal.2021.02.012. Online ahead of print.

Authors

C Arruabarrena [1] [2], R Montejano-Milner [1] [2], F De Aragón [1], G Allendes [1], M A Teus [1] [2] [3]

Affiliations

- [1] Servicio de Oftalmología Hospital Universitario Príncipe de Asturias, Spain.
- [2] Clínica Novovisión Madrid, Spain.
- [3] Facultad de Medicina de la Universidad de Alcalá de Henares, Spain.

- PMID: **34876774**
- PMCID: PMC8639395
- DOI: 10.1016/j.oftal.2021.02.012

Abstract
in English, Spanish

Background and objective: The SARS-CoV-2 pandemic has caused chaos in all health systems on the planet. It has been difficult to cope with COVID 19, but also to maintain the activity in other specialties. In ophthalmology, the scientific societies recommended providing urgent care,

including the intravitreal treatment of patients with active neovascular AMD, since a delay in treatment implies a potential loss of VA.The main objective of this study was to measure the impact of the coronavirus lockdown on the activity and visual results in patients with neovascular AMD in Area 3 of Madrid.

Material and method: A retrospective observational study was conducted of all patients with neovascular AMD who attended a consultation and/or received intravitreal treatment in the three months before the lockdown.

Results: In the three months before the lockdown, 144 patients with neovascular AMD were treated, of whom only 51 attended a consultation during the lockdown and, at six months after it, only 117 patients had resumed their follow-up. Mean VA before the lockdown was 58.0 ± 23.7 letters and was statistically significantly reduced to 53.0 ± 27.1 letters at six months after the lockdown. We also observed a significant decrease in the number of visits during the lockdown, despite the security measures implemented.

Conclusions: Our study shows that patients with neovascular AMD have had a statistically significant decrease in VA due to the lockdown. A VA of almost 58 letters was reduced to 53 at six months after the lockdown. The percentage of patients who lost 15 or more letters doubled. We observed a 63.3% loss of temporary follow-up during the lockdown and a 14.58% loss of permanent follow-up at six months after the lockdown.

Antecedentes y objetivo: La pandemia SARS-CoV-2 ha supuesto un caos organizativo para todos los sistemas sanitarios del planeta. No solo ha sido complicado hacer frente a la COVID 19, sino también ajustar la actividad asistencial en otras especialidades. En oftalmología las recomendaciones de las sociedades científicas eran dar asistencia urgente y dentro de esta se contemplaba el tratamiento intravítreo de los pacientes con degeneración macular asociada a la edad neovascular (DMAEn) activa, puesto que el retraso en el tratamiento supone una pérdida potencialmente irrecuperable de agudeza visual (AV).

El objetivo primario del presente estudio es medir el impacto en la actividad y los resultados visuales del confinamiento por coronavirus en los pacientes con DMAEn en el área 3 de la Comunidad de Madrid.

Material y método: Se plantea un estudio observacional retrospectivo de todos los pacientes con DMAEn que habían acudido a consulta y/o recibido tratamiento intravítreo los 3 meses previos al inicio del confinamiento.

Resultados: Los 3 meses previos al confinamiento se atendieron a 144 pacientes con DMAEn de los cuales solo 51 acudieron durante el confinamiento y a los 6 meses tras el confinamiento solo 117 pacientes han retomado su seguimiento. La AV media antes del confinamiento era de 58 ± 23,7 letras y se redujo de forma estadísticamente significativa a 53 ± 27,1 letras a los 6 meses tras el confinamiento. También observamos una disminución significativa del número de visitas durante el confinamiento a pesar de las medidas de seguridad implementadas.

Conclusiones: Nuestro estudio demuestra que los pacientes con DMAEn presentan una disminución estadísticamente significativa de la AV durante el confinamiento. De una AV de casi 58 letras, se redujo a 53 a los 6 meses del confinamiento. El porcentaje de pacientes que perdió 15 o más letras se duplicó. Observamos un 63,3% de pérdida de seguimiento temporal durante el confinamiento y un 14,6% de pérdida de seguimiento permanente a los 6 meses tras el confinamiento.

Keywords: AMD; Covid; anti-VEGF; lockdown.

© 2021 Sociedad Española de Oftalmología. Published by Elsevier España, S.L.U. All rights reserved.

- 19 references
- 2 figures

Supplementary info

Publication types Expand

Publication types

- English Abstract

Full text links

FREE FULL ENGLISH TEXT ARCHIVOS DE LA SOCIEDAD ESPAÑOLA DE OFTALMOLOGÍA Ediciones Doyma, S.L. Free PMC article

Proceed to details

Cite

Share

☐ 1,321

Cureus
. 2020 Aug 11;12(8):e9658.
doi: 10.7759/cureus.9658.

Clinical Outcomes of Early Treatment With Doxycycline for 89 High-Risk COVID-19 Patients in Long-Term Care Facilities in New York

Mohammud M Alam [1], Saborny Mahmud [2], Mohammad M Rahman [3], JoAnn Simpson [4], Sandeep Aggarwal [5], Ziauddin Ahmed [6]

Affiliations Expand

Affiliations

- [1] Department of Medicine and Infectious Diseases, Northwell University Hospital, Plainview, USA.
- [2] Department of Medicine, Johns Hopkins Bloomberg School of Public Health, Baltimore, USA.
- [3] Department of Medicine, New York University (NYU) School of Medicine, New York, USA.
- [4] Department of Nursing, Stony Brook University, Stony Brook, USA.
- [5] Department of Nephrology, University of Pennsylvania, Philadelphia, USA.

- [6] Department of Medicine and Nephrology, Temple University, Philadelphia, USA.

- PMID: **32802622**
- PMCID: PMC7419149
- DOI: 10.7759/cureus.9658

Free PMC article

Clinical Outcomes of Early Treatment With Doxycycline for 89 High-Risk COVID-19 Patients in Long-Term Care Facilities in New York

Mohammud M Alam et al. Cureus. 2020.
Free PMC article
Show details
Cureus
. 2020 Aug 11;12(8):e9658.
doi: 10.7759/cureus.9658.

Authors

Mohammud M Alam [1], Saborny Mahmud [2], Mohammad M Rahman [3], JoAnn Simpson [4], Sandeep Aggarwal [5], Ziauddin Ahmed [6]

Affiliations

- [1] Department of Medicine and Infectious Diseases, Northwell University Hospital, Plainview, USA.
- [2] Department of Medicine, Johns Hopkins Bloomberg School of Public Health, Baltimore, USA.
- [3] Department of Medicine, New York University (NYU) School of Medicine, New York, USA.
- [4] Department of Nursing, Stony Brook University, Stony Brook, USA.
- [5] Department of Nephrology, University of Pennsylvania, Philadelphia, USA.
- [6] Department of Medicine and Nephrology, Temple University, Philadelphia, USA.

- PMID: **32802622**
- PMCID: PMC7419149
- DOI: 10.7759/cureus.9658

Abstract

Rationale Due to the cluster and associated comorbidities in residents of long-term care facilities (LTCFs), COVID-19-associated morbidity and mortality are significantly increased. Multiple therapeutic options, including hydroxychloroquine (HCQ) and azithromycin (AZI), were tried

initially to treat moderate to severe COVID-19 and high-risk patients in LTCFs, but they were abandoned due to unfavorable reports. As a less toxic option, we initiated treatment with doxycycline (DOXY) very early in the course of illness. DOXY has antiviral, cardioprotective, immunomodulatory, and anti-inflammatory properties, but the efficacy of early intervention with DOXY in high-risk COVID-19 patients in LTCFs is unknown. Objective The goal of this retrospective study is to describe the clinical outcomes of high-risk COVID-19 patients with moderate to severe symptoms in LTCFs after early intervention with DOXY. Design Case-series analysis Setting LTCFs in New York Participants This observational study examines 89 patients who were diagnosed with COVID-19 from March 18 to May 13, 2020. Exposure All patients who were diagnosed with COVID-19 received DOXY and regular standard of care within 12 hours of the onset of symptoms. Additionally, four patients received meropenem, three patients received Zosyn, two patients received linezolid, and two patients received Bactrim DS. Four patients were on chronic ventilator support. No patients received any steroids or any other antiviral or immunomodulatory agents. The majority of the patients received zinc and calcium supplements as well. Main outcomes and measures Assessed measures were patients' characteristics, fever, shortness of breath (SOB), cough, oxygen saturation/pulse oximetry (POX), radiologic improvements, laboratory tests, DOXY side effects, hospital transfers, and death. Results Eighty-nine (89) high-risk patients, who developed a sudden onset of fever, cough, SOB, and hypoxia and were diagnosed with COVID-19, were treated with DOXY (100 mg PO or intravenous (IV) for seven days) and regular standard of care. Eighty-five percent (85%) of patients (n=76) demonstrated clinical recovery that is defined as resolution of fever (average 3.7 days, Coeff = -0.96, p = 0.0001), resolution of SOB (average 4.2 days), and improvement of POX: average 84% before treatment and average 95% after treatment (84.7 ± 7% vs. 95 ± 2.6%, p = 0.0001). Higher pre- and post-treatment POX is associated with lower mortality (oxygen saturation (Spo2) vs. Death, Coeff = -0.01, p = 0.023; post-Spo2 vs. Death, Coeff = -0.05, p = 0.0002). Within 10 days of symptom onset, 3% of patients (n=3) were transferred to hospital due to clinical deterioration and 11% of patients (n=10) died. The result was followed for 30 days from the onset of symptoms in each patient. Conclusion Early treatment with DOXY for high-risk patients with moderate to severe COVID-19 infections in non-hospital settings, such as LTCFs, is associated with early clinical recovery, decreased hospitalization, and decreased mortality.

Keywords: covid-19; doxycycline; long-term care facility; treatment.

Copyright © 2020, Alam et al.

Conflict of interest statement

The authors have declared that no competing interests exist.

- 30 references

Full text links

Free PMC article
Proceed to details
Cite
Share
☐ 1,322
EClinicalMedicine
. 2020 Jul 1;24:100410.
doi: 10.1016/j.eclinm.2020.100410. eCollection 2020 Jul.

Subcutaneous tocilizumab treatment in patients with severe COVID-19-related cytokine release syndrome: An observational cohort study

Antonio Mastroianni [1], Sonia Greco [1], Giovanni Apuzzo [1], Salvatore De Santis [1], Carmela Oriolo [2], Alfredo Zanolini [3], Luciana Chidichimo [1], Valeria Vangeli [1]
Affiliations Expand

Affiliations

- [1] Infectious Diseases Unit, Annunziata Hospital, Viale della Repubblica s.n.c., 87100 Cosenza, Italy.
- [2] Hospital Pharmacy, Annunziata Hospital, Cosenza, Italy.
- [3] Radiology Unit, Annunziata Hospital, Cosenza, Italy.

- PMID: **32766535**
- PMCID: PMC7329292
- DOI: 10.1016/j.eclinm.2020.100410

Free PMC article

Subcutaneous tocilizumab treatment in patients with severe COVID-19-related cytokine release syndrome: An observational cohort study

Antonio Mastroianni et al. EClinicalMedicine. 2020.
Free PMC article
Show details
EClinicalMedicine
. 2020 Jul 1;24:100410.
doi: 10.1016/j.eclinm.2020.100410. eCollection 2020 Jul.

Authors

Antonio Mastroianni [1], Sonia Greco [1], Giovanni Apuzzo [1], Salvatore De Santis [1], Carmela Oriolo [2], Alfredo Zanolini [3], Luciana Chidichimo [1], Valeria Vangeli [1]

Affiliations

- [1] Infectious Diseases Unit, Annunziata Hospital, Viale della Repubblica s.n.c., 87100 Cosenza, Italy.
- [2] Hospital Pharmacy, Annunziata Hospital, Cosenza, Italy.
- [3] Radiology Unit, Annunziata Hospital, Cosenza, Italy.

- PMID: **32766535**
- PMCID: PMC7329292
- DOI: 10.1016/j.eclinm.2020.100410

Abstract

Background: Patients with severe coronavirus disease 2019 (COVID-19) have elevated levels of acute phase reactants and inflammatory cytokines, including interleukin-6, indicative of cytokine release syndrome (CRS). The interleukin-6 receptor inhibitor tocilizumab is used for the treatment of chimeric antigen receptor T-cell therapy-induced CRS.

Methods: Patients aged 18 years or older with laboratory-confirmed COVID-19 admitted to the Annunziata Hospital in Cosenza, Italy, through March 7, 2020, who received at least one dose of tocilizumab 162 mg subcutaneously for the treatment of COVID-19-related CRS in addition to standard care were included in this retrospective observational study. The primary observation was the incidence of grade 4 CRS after tocilizumab treatment. Chest computed tomography (CT) scans were evaluated to investigate lung manifestations.

Findings: Twelve patients were included; all had fever, cough, and fatigue at presentation, and all had at least one comorbidity (hypertension, six patients; diabetes, five patients; chronic obstructive lung disease, four patients). Seven patients received high-flow nasal cannula oxygen therapy and five received non-invasive mechanical ventilation for lung complications of COVID-19. No incidence of grade 4 CRS was observed within 1 week of tocilizumab administration in all 12 patients (100%) and within 2 days of tocilizumab administration in 5 patients (42%). The predominant pattern on chest CT scans at presentation was ground-glass opacity, air bronchograms, smooth or irregular interlobular or septal thickening, and thickening of the adjacent pleura. Follow-up CT scans 7 to 10 days after tocilizumab treatment showed improvement of lung manifestations in all patients. No adverse events or new safety concerns attributable to tocilizumab were reported.

Interpretation: Tocilizumab administered subcutaneously to patients with COVID-19 and CRS is a promising treatment for reduction in disease activity and improvement in lung function. The effect of tocilizumab should be confirmed in a randomised controlled trial.

Keywords: Coronavirus disease 2019; Cytokine release syndrome; Tocilizumab.

© 2020 The Author(s).

Conflict of interest statement

The authors have no conflicts of interest to disclose.

- 39 references
- 1 figure

Full text links

ELSEVIER OPEN ACCESS Elsevier Science Free PMC article
Proceed to details
Cite
Share
1,323

Front Med (Lausanne)
. 2020 Dec 17;7:615845.
doi: 10.3389/fmed.2020.615845. eCollection 2020.

Tracheostomy in 80 COVID-19 Patients: A Multicenter, Retrospective, Observational Study

Yun Tang [1], Yongran Wu [1], Fangfang Zhu [2], Xiaobo Yang [1], Chaolin Huang [3], Guo Hou [4], Wenhao Xu [5], Ming Hu [6], Lu Zhang [7], Aiguo Cheng [8], Zhengqin Xu [9], Boyi Liu [10], Song Hu [11], Guochao Zhu [12], Xuepeng Fan [13], Xijing Zhang [14], Yadong Yang [15], Huibin Feng [16], Lixia Yu [17], Bing Wang [18], Zhiqiang Li [19], Yong Peng [20], Zubo Shen [21], Shouzhi Fu [22], Yaqi Ouyang [1], Jiqian Xu [1], Xiaojing Zou [1], Minghao Fang [23], Zhui Yu [3], Bo Hu [2], You Shang [1]

Affiliations Expand

Affiliations

- [1] Department of Critical Care Medicine, Union Hospital, Tongji Medical College, Huazhong University of Science and Technology, Wuhan, China.
- [2] Department of Critical Care Medicine, Zhongnan Hospital of Wuhan University, Wuhan, China.
- [3] Research Center for Translational Medicine, Wuhan Jinyin-Tan Hospital, Wuhan, China.
- [4] Department of Critical Care Medicine, Renmin Hospital, Wuhan University, Wuhan, China.
- [5] Department of Critical Care Medicine, Xiaogan Central Hospital, Xiaogan, China.
- [6] Department of Critical Care Medicine, Wuhan Pulmonary Hospital, Wuhan, China.
- [7] Department of Critical Care Medicine, Xiangyang Central Hospital, Affiliated Hospital of Hubei University of Arts and Science, Xiangyang, China.
- [8] Department of Critical Care, The Third People's Hospital of Yichang, Yichang, China.
- [9] Department of Critical Care Medicine, Xiangyang No.1 People's Hospital, Affiliated Hospital of Hubei University of Medicine, Xiangyang, China.
- [10] Department of Critical Care Medicine, Taihe Hospital Affiliated to Hubei University Medicine, Shiyan, China.
- [11] Department of Critical Care Medicine, Fifth Hospital of Wuhan, Wuhan, China.
- [12] Department of Critical Care Medicine, The Affiliated Hospital of Jianghan University, Wuhan, China.
- [13] Department of Critical Care, Wuhan No.1 Hospital, Wuhan, China.

- [14] Intensive Care Unit (ICU) Center of Xijing Hospital, Airforce Medical University, Xi'an, China.
- [15] Department of Critical Care Medicine, Huanggang Central Hospital, Huanggang, China.
- [16] Department of Intensive Care Unit (ICU), Huangshi Central Hospital, Affiliated Hospital of Hubei Polytechnic University, Edong Healthcare Group, Huangshi, China.
- [17] Department of Critical Care Medicine, Jingzhou Central Hospital, The Second Clinical Medical College, Yangtze University, Jingzhou, China.
- [18] Department of Critical Care Medicine, No.2 Hospital of Huangshi, Huangshi, China.
- [19] Department of Critical Care Medicine, The First People's Hospital of Jingmen, Jingmen, China.
- [20] Intensive Care Unit, Xiehe Wuhan Red Cross Hospital, Wuhan, China.
- [21] Department of Critical Care Medicine, Ezhou Central Hospital, Ezhou, China.
- [22] Department of Intensive Care Unit (ICU)/Emergency, Wuhan Third Hospital, Wuhan, China.
- [23] Department of Critical Care Medicine, Tongji Hospital, Tongji Medical College, Huazhong University of Science and Technology, Wuhan, China.

- PMID: **33425960**
- PMCID: PMC7793766
- DOI: 10.3389/fmed.2020.615845

Free PMC article

Tracheostomy in 80 COVID-19 Patients: A Multicenter, Retrospective, Observational Study

Yun Tang et al. Front Med (Lausanne). 2020.
Free PMC article
Show details
Front Med (Lausanne)
. 2020 Dec 17;7:615845.
doi: 10.3389/fmed.2020.615845. eCollection 2020.

Authors

Yun Tang [1], Yongran Wu [1], Fangfang Zhu [2], Xiaobo Yang [1], Chaolin Huang [3], Guo Hou [4], Wenhao Xu [5], Ming Hu [6], Lu Zhang [7], Aiguo Cheng [8], Zhengqin Xu [9], Boyi Liu [10], Song Hu [11], Guochao Zhu [12], Xuepeng Fan [13], Xijing Zhang [14], Yadong Yang [15], Huibin Feng [16], Lixia Yu [17], Bing Wang [18], Zhiqiang Li [19], Yong Peng [20], Zubo Shen [21], Shouzhi Fu [22], Yaqi Ouyang [1], Jiqian Xu [1], Xiaojing Zou [1], Minghao Fang [23], Zhui Yu [3], Bo Hu [2], You Shang [1]

Affiliations

- [1] Department of Critical Care Medicine, Union Hospital, Tongji Medical College, Huazhong University of Science and Technology, Wuhan, China.
- [2] Department of Critical Care Medicine, Zhongnan Hospital of Wuhan University, Wuhan, China.
- [3] Research Center for Translational Medicine, Wuhan Jinyin-Tan Hospital, Wuhan, China.
- [4] Department of Critical Care Medicine, Renmin Hospital, Wuhan University, Wuhan, China.
- [5] Department of Critical Care Medicine, Xiaogan Central Hospital, Xiaogan, China.
- [6] Department of Critical Care Medicine, Wuhan Pulmonary Hospital, Wuhan, China.
- [7] Department of Critical Care Medicine, Xiangyang Central Hospital, Affiliated Hospital of Hubei University of Arts and Science, Xiangyang, China.
- [8] Department of Critical Care, The Third People's Hospital of Yichang, Yichang, China.
- [9] Department of Critical Care Medicine, Xiangyang No.1 People's Hospital, Affiliated Hospital of Hubei University of Medicine, Xiangyang, China.
- [10] Department of Critical Care Medicine, Taihe Hospital Affiliated to Hubei University Medicine, Shiyan, China.
- [11] Department of Critical Care Medicine, Fifth Hospital of Wuhan, Wuhan, China.
- [12] Department of Critical Care Medicine, The Affiliated Hospital of Jianghan University, Wuhan, China.
- [13] Department of Critical Care, Wuhan No.1 Hospital, Wuhan, China.
- [14] Intensive Care Unit (ICU) Center of Xijing Hospital, Airforce Medical University, Xi'an, China.
- [15] Department of Critical Care Medicine, Huanggang Central Hospital, Huanggang, China.
- [16] Department of Intensive Care Unit (ICU), Huangshi Central Hospital, Affiliated Hospital of Hubei Polytechnic University, Edong Healthcare Group, Huangshi, China.
- [17] Department of Critical Care Medicine, Jingzhou Central Hospital, The Second Clinical Medical College, Yangtze University, Jingzhou, China.
- [18] Department of Critical Care Medicine, No.2 Hospital of Huangshi, Huangshi, China.
- [19] Department of Critical Care Medicine, The First People's Hospital of Jingmen, Jingmen, China.
- [20] Intensive Care Unit, Xiehe Wuhan Red Cross Hospital, Wuhan, China.
- [21] Department of Critical Care Medicine, Ezhou Central Hospital, Ezhou, China.
- [22] Department of Intensive Care Unit (ICU)/Emergency, Wuhan Third Hospital, Wuhan, China.
- [23] Department of Critical Care Medicine, Tongji Hospital, Tongji Medical College, Huazhong University of Science and Technology, Wuhan, China.

- PMID: **33425960**
- PMCID: PMC7793766
- DOI: 10.3389/fmed.2020.615845

Abstract

Background: The outbreak of coronavirus disease 2019 (COVID-19) has led to a large and increasing number of patients requiring prolonged mechanical ventilation and tracheostomy. The indication and optimal timing of tracheostomy in COVID-19 patients are still unclear, and the outcomes about tracheostomy have not been extensively reported. We aimed to describe the clinical characteristics and outcomes of patients with confirmed severe acute respiratory syndrome coronavirus 2 (SARS-CoV-2) pneumonia who underwent elective tracheostomies. **Methods:** The

multi-center, retrospective, observational study investigated all the COVID-19 patients who underwent elective tracheostomies in intensive care units (ICUs) of 23 hospitals in Hubei province, China, from January 8, 2020 to March 25, 2020. Demographic information, clinical characteristics, treatment, details of the tracheostomy procedure, successful weaning after tracheostomy, and living status were collected and analyzed. Data were compared between early tracheostomy patients (tracheostomy performed within 14 days of intubation) and late tracheostomy patients (tracheostomy performed after 14 days). **Results:** A total of 80 patients were included. The median duration from endotracheal intubation to tracheostomy was 17.5 [IQR 11.3-27.0] days. Most tracheotomies were performed by ICU physician [62 (77.5%)], and using percutaneous techniques [63 (78.8%)] at the ICU bedside [76 (95.0%)]. The most common complication was tracheostoma bleeding [14 (17.5%)], and major bleeding occurred in 4 (5.0%) patients. At 60 days after intubation, 31 (38.8%) patients experienced successful weaning from ventilator, 17 (21.2%) patients discharged from ICU, and 43 (53.8%) patients had died. Higher 60 day mortality [22 (73.3%) vs. 21 (42.0%)] were identified in patients who underwent early tracheostomy. **Conclusions:** In patients with SARS-CoV-2 pneumonia, tracheostomies were feasible to conduct by ICU physician at bedside with few major complications. Compared with tracheostomies conducted after 14 days of intubation, tracheostomies within 14 days were associated with an increased mortality rate.

Keywords: COVID-19; critically ill patients; intensive care unit; mechanical ventilation; tracheostomy.

Copyright © 2020 Tang, Wu, Zhu, Yang, Huang, Hou, Xu, Hu, Zhang, Cheng, Xu, Liu, Hu, Zhu, Fan, Zhang, Yang, Feng, Yu, Wang, Li, Peng, Shen, Fu, Ouyang, Xu, Zou, Fang, Yu, Hu and Shang.

Conflict of interest statement

The authors declare that the research was conducted in the absence of any commercial or financial relationships that could be construed as a potential conflict of interest.

- 34 references
- 1 figure

Full text links

OPEN ACCESS frontiers Frontiers Media SA Free PMC article

Proceed to details

Cite

Share

☐ 1,324

J Family Med Prim Care

. 2020 Dec 31;9(12):6267-6272.

doi: 10.4103/jfmpc.jfmpc_1198_20. eCollection 2020 Dec.

Characteristics and outcomes of 231 COVID-19 cases admitted at a tertiary facility in India: An observational cohort study

Rohit Kumar [1], Bisakh Bhattacharya [1], Ved Prakash Meena [1], Anivita Aggarwal [1], Manasi Tripathi [2], Manish Soneja [1], Ankit Mittal [1], Komal Singh [1], Nishkarsh Gupta [3], Rakesh Kumar Garg [3], Brajesh Kumar Ratre [3], Balbir Kumar [3], Shweta Arun Bhopale [3], Pavan Tiwari [4], Ankit Verma [5], Sushma Bhatnagar [3], Anant Mohan [4], Naveet Wig [1], Randeep Guleria [4]
Affiliations Expand

Affiliations

- [1] Department of Medicine, All India Institute of Medical Sciences, New Delhi, India.
- [2] Department of Ophthalmology, All India Institute of Medical Sciences, New Delhi, India.
- [3] Department of Onco-anesthesia and Palliative Medicine, All India Institute of Medical Sciences, New Delhi, India.
- [4] Department of Pulmonary Medicine and Sleep Disorders, All India Institute of Medical Sciences, New Delhi, India.
- [5] Department of Pediatrics, All India Institute of Medical Sciences, New Delhi, India.

- PMID: **33681075**
- PMCID: PMC7928080
- DOI: 10.4103/jfmpc.jfmpc_1198_20

Free PMC article

Characteristics and outcomes of 231 COVID-19 cases admitted at a tertiary facility in India: An observational cohort study

Rohit Kumar et al. J Family Med Prim Care. 2020.
Free PMC article
Show details
J Family Med Prim Care
. 2020 Dec 31;9(12):6267-6272.
doi: 10.4103/jfmpc.jfmpc_1198_20. eCollection 2020 Dec.

Authors

Rohit Kumar [1], Bisakh Bhattacharya [1], Ved Prakash Meena [1], Anivita Aggarwal [1], Manasi Tripathi [2], Manish Soneja [1], Ankit Mittal [1], Komal Singh [1], Nishkarsh Gupta [3], Rakesh Kumar Garg [3], Brajesh Kumar Ratre [3], Balbir Kumar [3], Shweta Arun Bhopale [3], Pavan Tiwari [4], Ankit Verma [5], Sushma Bhatnagar [3], Anant Mohan [4], Naveet Wig [1], Randeep Guleria [4]

Affiliations

- [1] Department of Medicine, All India Institute of Medical Sciences, New Delhi, India.
- [2] Department of Ophthalmology, All India Institute of Medical Sciences, New Delhi, India.
- [3] Department of Onco-anesthesia and Palliative Medicine, All India Institute of Medical Sciences, New Delhi, India.
- [4] Department of Pulmonary Medicine and Sleep Disorders, All India Institute of Medical Sciences, New Delhi, India.
- [5] Department of Pediatrics, All India Institute of Medical Sciences, New Delhi, India.

- PMID: **33681075**
- PMCID: PMC7928080
- DOI: 10.4103/jfmpc.jfmpc_1198_20

Abstract

Background: Ongoing pandemic because of COVID-19 has spread across countries, with varied clinical features and severity. Awareness of clinical course among asymptomatic and symptomatology in symptomatic cases is essential for patients' management as well as optimal utilization of health services (in resource limited settings) based on clinical status and risk factors. This study aimed to describe the clinical characteristics and outcomes of patients admitted with COVID-19 illness in the initial phase of the pandemic in India.

Methods: It was an observational study. Patients aged 18 years or more, with confirmed SARS-CoV-2 infection, asymptomatic or mildly ill, were included. Patients with moderate-severe disease at admission or incomplete clinical symptomatology records were excluded. Data regarding demography, comorbidities, clinical features and course, treatment, results of SARS-CoV-2 RT-PCR, chest radiographs, and laboratory parameters were obtained retrospectively from hospital records. The outcome was noted in terms of course, patients discharged, still admitted (at the time of the study), or death.

Results: Out of 231 cases, most were males (78.3%) with a mean age of 39.8 years. Comorbidities were present in 21.2% of patients, diabetes mellitus and hypertension being the most common. The most common symptoms were dry cough (81, 35%), fever (64, 27.7%), sore throat (36, 15.6%); asymptomatic infection noted in 108 (46.8%) patients. The presence of comorbidities was an independent predictor of symptomatic disease (OR-2.66; 95%CI 1.08-6.53, $P = 0{\cdot}03$). None of the patients progressed to moderate-severe COVID-19, and there were no deaths.

Conclusions: A large proportion of patients remained asymptomatic whereas those with comorbidities were more likely to be symptomatic. Most with mild disease had a stable disease course, barring few complication in those with comorbidities. The pandemic continues to grow as large number of asymptomatic cases may go undiagnosed.

Keywords: Asymptomatic; COVID-19; characteristics; outcomes.

Copyright: © 2020 Journal of Family Medicine and Primary Care.

Conflict of interest statement

There are no conflicts of interest.

- 29 references

- 3 figures

Full text links

Free PMC article
Proceed to details
Cite
Share
1,325
Trop Med Infect Dis
. 2021 Jul 19;6(3):137.
doi: 10.3390/tropicalmed6030137.

Clinical Mortality Review of COVID-19 Patients at Sukraraj Tropical and Infectious Disease Hospital, Nepal; A Retrospective Study

Anup Bastola [1], Sanjay Shrestha [2], Richa Nepal [2], Kijan Maharjan [2], Bikesh Shrestha [2], Bimal Sharma Chalise [2], Pratistha Thapa [3], Pujan Balla [3], Alisha Sapkota [4], Priyanka Shah [4]
Affiliations Expand

Affiliations

- [1] Department of Tropical Medicine, Sukraraj Tropical and Infectious Disease Hospital, Kathmandu 44600, Nepal.
- [2] Department of Internal Medicine, Sukraraj Tropical and Infectious Disease Hospital, Kathmandu 44600, Nepal.
- [3] Department of Anesthesiology, Sukraraj Tropical and Infectious Disease Hospital, Kathmandu 44600, Nepal.
- [4] Department of Internal Medicine, Nepal Medical College and Teaching Hospital, Kathmandu 44600, Nepal.

- PMID: **34287389**
- PMCID: PMC8293465
- DOI: 10.3390/tropicalmed6030137

Free PMC article

Clinical Mortality Review of COVID-19 Patients at Sukraraj Tropical and Infectious

[Disease Hospital, Nepal; A Retrospective Study]

Anup Bastola et al. Trop Med Infect Dis. 2021.
Free PMC article
Show details
Trop Med Infect Dis
. 2021 Jul 19;6(3):137.
doi: 10.3390/tropicalmed6030137.

Authors

Anup Bastola [1], Sanjay Shrestha [2], Richa Nepal [2], Kijan Maharjan [2], Bikesh Shrestha [2], Bimal Sharma Chalise [2], Pratistha Thapa [3], Pujan Balla [3], Alisha Sapkota [4], Priyanka Shah [4]

Affiliations

- [1] Department of Tropical Medicine, Sukraraj Tropical and Infectious Disease Hospital, Kathmandu 44600, Nepal.
- [2] Department of Internal Medicine, Sukraraj Tropical and Infectious Disease Hospital, Kathmandu 44600, Nepal.
- [3] Department of Anesthesiology, Sukraraj Tropical and Infectious Disease Hospital, Kathmandu 44600, Nepal.
- [4] Department of Internal Medicine, Nepal Medical College and Teaching Hospital, Kathmandu 44600, Nepal.

- PMID: **34287389**
- PMCID: PMC8293465
- DOI: 10.3390/tropicalmed6030137

Abstract

Coronavirus Disease 2019 (COVID-19) has challenged the health system worldwide, including the low and middle income countries like Nepal. In view of the rising number of infections and prediction of multiple waves of this disease, mortalities due to COVID-19 need to be critically analyzed so that every possible effort could be made to prevent COVID-19 related mortalities in future. Main aim of this research was to study about the mortalities due to COVID-19 at a tertiary level hospital, in Nepal. This was a retrospective, observational study that included all inpatients from Sukraraj Tropical and Infectious Disease Hospital, who were reverse transcriptase polymerase chain reaction positive for SARS-COV-2 and died during hospital stay from January 2020 till January 2021. Medical records of the patients were evaluated. Out of 860 total admissions in a year, there were 50 mortalities in the study center. Out of 50 mortalities, majority were males (76%) with male to female ratio of 3.17:1. Most were above 65 years of age (72%) and had two or more comorbidities (64%). The most common comorbidities among the patients who had died during hospital stay were hypertension (58%) followed by diabetes mellitus (50%) and chronic obstructive airway disease (24%). The median duration from the symptom onset to death was 18 days, ranged from the minimum of 2 days till maximum of 39 days. D-dimer was found to be >1 mg/L in 58% cases and ferritin was >500 ng/ml in 42% patients at presentation. A

total of 42% patients had thrombocytopenia, 80% patients had lymphocytopenia and 60% had Neutrophil to Lymphocyte ratio >11.75 with the mean NLR of 18.38. Of total mortalities, 16% patients also showed microbiological evidence of secondary infection; Male gender, age more than 65 years, multiple comorbidities with lymphocytopenia, elevated Neutrophil lymphocyte ratio and elevated inflammatory markers were risk factors found in majority of mortalities in our study. These findings could be utilized for early triage and risk assessment in COVID-19 patients so that aggressive treatment strategies could be employed at the earliest to reduce mortalities due to COVID-19 in future.

Keywords: COVID-19; Nepal; mortality.

Conflict of interest statement

The authors declare no conflict of interest.

- 23 references
- 8 figures

Full text links

FULL TEXT OPEN ACCESS MDPI Multidisciplinary Digital Publishing Institute (MDPI) Free PMC article

Proceed to details

Cite

Share

☐ 1,326

Semin Arthritis Rheum

. 2022 Feb 25;55:151987.

doi: 10.1016/j.semarthrit.2022.151987. Online ahead of print.

Severity and mortality of COVID-19 in patients with systemic sclerosis: a Brazilian multicenter study

Sandra Maximiano de Oliveira [1], Lucas Victória de Oliveira Martins [1], Ana Paula Lupino-Assad [2], Ana Cristina Medeiros-Ribeiro [2], Daniela Aparecida de Moraes [3], Ana Paula Toledo Del-Rio [4], Maria Carolina Oliveira [3], Percival Degrava Sampaio-Barros [2], Cristiane Kayser [5]

Affiliations Expand

Affiliations

- [1] Rheumatology Division, Escola Paulista de Medicina, Universidade Federal de São Paulo - UNIFESP, São Paulo, Brazil.
- [2] Division of Rheumatology, Hospital das Clinicas HCFMUSP, Faculdade de Medicina, Universidade de São Paulo, São Paulo, Brazil.
- [3] Internal Medicine Department, Ribeirão Preto Medical School, Universidade de São Paulo - USP, Ribeirão Preto, Brazil.

- [4] Rheumatology Division, Universidade Estadual de Campinas - UNICAMP, Campinas, Brazil.
- [5] Rheumatology Division, Escola Paulista de Medicina, Universidade Federal de São Paulo - UNIFESP, São Paulo, Brazil. Electronic address: cristiane.kayser@unifesp.br.

- PMID: **35286906**
- PMCID: PMC8875950
- DOI: 10.1016/j.semarthrit.2022.151987

Free PMC article

Severity and mortality of COVID-19 in patients with systemic sclerosis: a Brazilian multicenter study

Sandra Maximiano de Oliveira et al. Semin Arthritis Rheum. 2022.
Free PMC article
Show details
Semin Arthritis Rheum
. 2022 Feb 25;55:151987.
doi: 10.1016/j.semarthrit.2022.151987. Online ahead of print.

Authors

Sandra Maximiano de Oliveira [1], Lucas Victória de Oliveira Martins [1], Ana Paula Lupino-Assad [2], Ana Cristina Medeiros-Ribeiro [2], Daniela Aparecida de Moraes [3], Ana Paula Toledo Del-Rio [4], Maria Carolina Oliveira [3], Percival Degrava Sampaio-Barros [2], Cristiane Kayser [5]

Affiliations

- [1] Rheumatology Division, Escola Paulista de Medicina, Universidade Federal de São Paulo - UNIFESP, São Paulo, Brazil.
- [2] Division of Rheumatology, Hospital das Clinicas HCFMUSP, Faculdade de Medicina, Universidade de São Paulo, São Paulo, Brazil.
- [3] Internal Medicine Department, Ribeirão Preto Medical School, Universidade de São Paulo - USP, Ribeirão Preto, Brazil.
- [4] Rheumatology Division, Universidade Estadual de Campinas - UNICAMP, Campinas, Brazil.
- [5] Rheumatology Division, Escola Paulista de Medicina, Universidade Federal de São Paulo - UNIFESP, São Paulo, Brazil. Electronic address: cristiane.kayser@unifesp.br.

- PMID: **35286906**
- PMCID: PMC8875950
- DOI: 10.1016/j.semarthrit.2022.151987

Abstract

Introduction: COVID-19 may be associated with greater severity and mortality in patients with systemic sclerosis (SSc). The present study aimed to evaluate the prevalence, severity and mortality of COVID-19 in a Brazilian cohort of SSc patients.

Methods: This multicenter, retrospective, observational study included 1,042 SSc patients followed in four centers of São Paulo between March 2020 and June 2021. Diagnosis of COVID-19 was established by proper positive RT-PCR testing or by highly suspicious infection. Patients were grouped into mild (outpatient setting treatment and no need for oxygen support) and moderate-to-severe (hospitalization and/or need for oxygen support) COVID-19.

Results: Of the 1,042 SSc patients, 118 patients were diagnosed with COVID-19. Interstitial lung disease (SSc-ILD) was present in 65.6% of the total cohort and in 46.3% of SSc patients with COVID-19. There were 78 (66.1%) cases of mild COVID-19, and 40 (33.9%) cases of moderate-to-severe disease, with 6 (5.1%) deaths. By univariate analysis, pulmonary arterial hypertension (OR 9.50, p=0.006), SSc-ILD (OR 3.90, p=0.007), FVC <80% (OR 2.90, p=0.01), cardiac involvement (OR 5.53, p=0.003), and use of rituximab (OR 3.92, p=0.039), but not age, gender, comorbidities or use of corticosteroids, were predictors of worse outcome for COVID-19. Using multivariate analysis, only SSc-ILD was significantly associated to a higher risk of moderate-to-severe COVID-19 (OR 2.73, 95% CI 1.12-6.69, p=0.02). Forty percent of the patients remained with symptoms after presenting COVID-19, predominantly dyspnea and/or cough (17%).

Conclusion: In this cohort of patients with SSc, those with SSc-ILD were highly impacted by COVID-19, with a higher risk of moderate-to-severe COVID-19 infection and death.

Keywords: COVID-19; Interstitial lung disease; Mortality; Severity; Systemic sclerosis.

Copyright © 2022 Elsevier Inc. All rights reserved.

Conflict of interest statement

Declaration of Competing Interest The authors declare that they have no known competing financial interests or personal relationships that could have appeared to influence the work reported in this paper.

- 47 references
- 1 figure

Full text links

ELSEVIER FULL-TEXT ARTICLE Elsevier Science Free PMC article

Proceed to details

Cite

Share

☐ 1,327

J Clin Transl Res

. 2021 Oct 30;7(6):717-722.
eCollection 2021 Dec 28.

Risk of post-intubation cardiac arrest with the use of high-dose rocuronium in COVID-19 patients with acute respiratory distress syndrome: A retrospective cohort study

Natalie Kandinata [1], Roshan Acharya [1], Aakash Patel [1], Aalok Parekh [1], Jessica Santana [2], Aaron Darden [3], Yub Raj Sedhai [4], Smita Kafle [5], Usman Younus [6]
Affiliations Expand

Affiliations

- [1] Department of Internal Medicine, Cape Fear Valley Medical Center, Fayetteville, NC, 28306, United States of America.
- [2] Department of Internal Medicine, Campbell University School of Osteopathic Medicine, Buies Creek, NC, 27506, United States of America.
- [3] Department of Respiratory Therapy, Cape Fear Valley Medical Center, Fayetteville, NC, 28306, United States of America.
- [4] Department of Internal Medicine, Virginia Commonwealth University School of Medicine, Richmond, VA, 23970, United States of America.
- [5] Bachelor of Science in Nursing, Fayetteville State University School of Nursing, Fayetteville, NC, 28301, United States of America.
- [6] Department of Critical Care Medicine, Cape Fear Valley Medical Center, Fayetteville, NC, 28306, United States of America.

- PMID: **34901517**
- PMCID: PMC8654367

Free PMC article

Risk of post-intubation cardiac arrest with the use of high-dose rocuronium in COVID-19 patients with acute respiratory distress syndrome: A retrospective cohort study

Natalie Kandinata et al. J Clin Transl Res. 2021.
Free PMC article
Show details
J Clin Transl Res
. 2021 Oct 30;7(6):717-722.
eCollection 2021 Dec 28.

Authors

Natalie Kandinata [1], Roshan Acharya [1], Aakash Patel [1], Aalok Parekh [1], Jessica Santana [2], Aaron Darden [3], Yub Raj Sedhai [4], Smita Kafle [5], Usman Younus [6]

Affiliations

- [1] Department of Internal Medicine, Cape Fear Valley Medical Center, Fayetteville, NC, 28306, United States of America.
- [2] Department of Internal Medicine, Campbell University School of Osteopathic Medicine, Buies Creek, NC, 27506, United States of America.
- [3] Department of Respiratory Therapy, Cape Fear Valley Medical Center, Fayetteville, NC, 28306, United States of America.
- [4] Department of Internal Medicine, Virginia Commonwealth University School of Medicine, Richmond, VA, 23970, United States of America.
- [5] Bachelor of Science in Nursing, Fayetteville State University School of Nursing, Fayetteville, NC, 28301, United States of America.
- [6] Department of Critical Care Medicine, Cape Fear Valley Medical Center, Fayetteville, NC, 28306, United States of America.

- PMID: **34901517**
- PMCID: PMC8654367

Abstract

Background: Post-intubation cardiac arrest (PICA) is an uncommon complication of intubation, but numbers have risen to over 1.5 times the usual number since the coronavirus disease 2019 (COVID-19) pandemic. Due to expert recommendations, high-dose rocuronium (HDR) has become a commonly used pre-intubation neuromuscular blocking agent.

Aim: We conducted this retrospective case-control observational study with the hypothesis that high-dose rocuronium was not associated with higher incidence of PICA.

Methods: We included 93 patients who were intubated using the rapid sequence intubation (RSI) technique with rocuronium for acute respiratory distress syndrome (ARDS) due to confirmed COVID-19 pneumonia, admitted from March 2020 to February 2021 to a tertiary care hospital in North Carolina, USA. The patients were grouped based on high (1.5 mg/kg of ideal body weight and above) versus low (<1.5 mg/kg of ideal body weight) dose rocuronium used for RSI. The differences of the various outcomes between the groups were analyzed.

Results: The baseline demographics were similar in both groups except for higher body mass index in high-dose group 39 versus 32 (kg/m^2), p = 0.009. There was a total of six PICA events (6.45%). The HDR group had 8.0% of PICA versus 4.7% in the low-dose group. In-hospital mortality was 60.0% in the HDR group versus 72.1% in the low-dose group.

Conclusion: The incidence of PICA in COVID-19 patients with ARDS who were intubated using the RSI technique was higher than in the pre-COVID-19 era.

Relevance for patients: The use of high-dose paralytics during invasive ventilation with RSI and their consequences should be explored with the help of large-scale studies. The rate of PICA is still very low, and perhaps, the use of HDR is safe, as suggested by the expert panel.

Keywords: COVID-19; acute respiratory distress syndrome; etomidate; peri-intubation cardiac arrest; post-intubation cardiac arrest; rapid sequence intubation; rocuronium.

Copyright: © Whioce Publishing Pte. Ltd.

Conflict of interest statement

The authors declare that there are no conflicts of interest.

- 23 references

Full text links

Free PMC article
Proceed to details
Cite
Share
☐ 1,328
J Community Hosp Intern Med Perspect
. 2021 Jan 26;11(1):9-16.
doi: 10.1080/20009666.2020.1835214.

Correlation of refractory hypoxemia with biochemical markers and clinical outcomes of COVID-19 patients in a developing country: A retrospective observational study: Running head: Predictors of hypoxemia in COVID-19

Muhammad Sohaib Asghar [1], Iftekhar Ahmed [1], Haris Alvi [1], Sadia Iqbal [1], Ismail Khan [1], Rabia Seher Alvi [1], Zara Saeed [1], Saboohi Irfan [1], Maria Akhtar [1], Ibraj Fatima [1]
Affiliations Expand

Affiliation

- [1] Resident Physician, Internal Medicine, Dow University Hospital, Dow University of Health Sciences, Karachi, Pakistan.

- PMID: **33552406**
- PMCID: PMC7850320
- DOI: 10.1080/20009666.2020.1835214

Free PMC article

[Correlation of refractory hypoxemia with biochemical markers and clinical outcomes of COVID-19 patients in a developing country: A retrospective observational study: Running head: Predictors of hypoxemia in COVID-19]

Muhammad Sohaib Asghar et al. J Community Hosp Intern Med Perspect. 2021.
Free PMC article
Show details
J Community Hosp Intern Med Perspect
. 2021 Jan 26;11(1):9-16.
doi: 10.1080/20009666.2020.1835214.

Authors

Muhammad Sohaib Asghar[1], Iftekhar Ahmed[1], Haris Alvi[1], Sadia Iqbal[1], Ismail Khan[1], Rabia Seher Alvi[1], Zara Saeed[1], Saboohi Irfan[1], Maria Akhtar[1], Ibraj Fatima[1]

Affiliation

- [1] Resident Physician, Internal Medicine, Dow University Hospital, Dow University of Health Sciences, Karachi, Pakistan.

- PMID: **33552406**
- PMCID: PMC7850320
- DOI: 10.1080/20009666.2020.1835214

Abstract

Introduction: COVID-19 is mainly a respiratory illness, causing hypoxemia in the majority of those been infected. In our study, we aimed to correlate the biochemical markers with hypoxemia and predicting the prognosis of COVID-19 patients.

Materials and methods: A retrospective, observational study was conducted to include all the admitted COVID-19 patients (n = 183) diagnosed by a real-time Polymerase chain reaction and evaluated those for hypoxemia and disease outcomes by utilizing the biochemical markers.

Results: Out of the 183 patients, 117 were in the ward, 66 were in ICU, 148 of them recovered, while 35 deaths were reported, 89 patients were having persisting hypoxemia (despite oxygen therapy) during the hospital stay, and the remaining 94 were non-hypoxemic with or without supplemental oxygen therapy. There were significant differences in mean hemoglobin (p = 0.028), total leukocyte count (p = 0.005), Neutrophil-to-Lymphocyte ratio (p = 0.001), serum urea and creatinine (p = 0.002), serum potassium (p = 0.009), C-reactive protein (p = 0.001), Lactate dehydrogenase (p = 0.005), and Ferritin (p = 0.042) of the hypoxemic patients versus non-hypoxemic group. Amongst the deceased patients, there was significant leukocytosis (p = 0.008), increased Neutrophil-to-Lymphocyte ratio (p = 0.001), elevated C-reactive protein (p = 0.001),

and Lactate dehydrogenase (p = 0.009). Receiver operating characteristic curves showed Neutrophil-to-Lymphocyte ratio (p < 0.001), C-reactive protein (p < 0.001), and Lactate dehydrogenase (p < 0.001) most significantly associated with hypoxemia and death.

Conclusion: The inflammatory markers are a good guide for predicting the hypoxemia and disease outcome. The results concluded Neutrophil-to-Lymphocyte ratio, C-reactive protein, and Lactate dehydrogenase were effective biomarkers in predicting a severe course of COVID-19, but could not establish significant associations of serum Ferritin, Procalcitonin, and D-Dimer.

Keywords: COVID-19; biochemical markers; coronavirus; hypoxemia; infectious diseases; mortality; pandemic.

© 2020 The Author(s). Published by Informa UK Limited, trading as Taylor & Francis Group on behalf of Greater Baltimore Medical Center.

Conflict of interest statement

The authors declare no conflicts of interest with this article's content.

- 40 references
- 2 figures

Supplementary info

Grant support Expand

Grant support

This work is not supported by any sponsors. No funding required in this study.

Full text links

Free PMC article
Proceed to details
Cite
Share
☐ 1,329

Heart Views
. Jul-Sep 2020;21(3):166-170.
doi: 10.4103/HEARTVIEWS.HEARTVIEWS_128_20. Epub 2020 Oct 13.

The Impact of COVID-19 Pandemic on the Presentation and Hospital Management of STEMI Patients in a Tertiary Care Center in Saudi Arabia

Mohammed Ali Balghith [1]

Affiliations Expand

Affiliation

- [1] King Abdulaziz Cardiac Center, College of Medicine, King Saud Bin Abdulaziz University for Health Sciences, Riyadh, Kingdom of Saudi Arabia.

- PMID: **33688408**
- PMCID: PMC7898991
- DOI: 10.4103/HEARTVIEWS.HEARTVIEWS_128_20

Free PMC article

The Impact of COVID-19 Pandemic on the Presentation and Hospital Management of STEMI Patients in a Tertiary Care Center in Saudi Arabia

Mohammed Ali Balghith. Heart Views. Jul-Sep 2020.
Free PMC article
Show details
Heart Views
. Jul-Sep 2020;21(3):166-170.
doi: 10.4103/HEARTVIEWS.HEARTVIEWS_128_20. Epub 2020 Oct 13.

Author

Mohammed Ali Balghith [1]

Affiliation

- [1] King Abdulaziz Cardiac Center, College of Medicine, King Saud Bin Abdulaziz University for Health Sciences, Riyadh, Kingdom of Saudi Arabia.

- PMID: **33688408**
- PMCID: PMC7898991
- DOI: 10.4103/HEARTVIEWS.HEARTVIEWS_128_20

Abstract

Background: The COVID-19 Pandemic has put enormous pressure on the healthcare system globally, causing many healthcare organizations to cancel elective admission for coronary angiograms. The purpose of this study is to assess changes in ST segment elevation myocardial infarction (STEMI) practice, including the number of patients, door to balloon time and time from the onset of symptoms until reperfusion therapy in a tertiary center in Saudi Arabia.

Methods: This is a single center retrospective observational study, comparing all STEMI patients in the last five months of 2019 (Pre-COVID-19 period) with the first 5 months of 2020 (COVID -19 period) in regards to the volume of STEMI patients, symptoms onset to ER arrival time, door to balloon timing and the reperfusion therapy strategy.

Results: A total number of 173 STEMI patients were analyzed; 81 STEMI patients in the Pre-COVID-19 period and 92 STEMI patients in the COVID-19 period. When compared with pre-COVID period, there was a statistically non-significant increase in STEMI patients (12%), slight delay in the door to balloon timing; 94 vs 87 minutes. As well, there was more delay from onset of symptoms to presentation to the ER (>12 hours from symptoms onset to ER arrival (16% vs, 4% in group 1). Primary percutaneous coronary intervention (PPCI) was the main modality between the 2 groups without significant differences (100% Pre-COVID vs. 97% COVID-19 period).

Conclusion: There was some delay of STEMI patient's presentation to the hospital during Covid-19 timing, without significant changes in the medical practice of care.

Keywords: Covid-19; Door to Balloon; PPCI; STEMI.

Copyright: © 2020 Heart Views.

Conflict of interest statement

There are no conflicts of interest.

- 18 references
- 3 figures

Full text links

Free PMC article
Proceed to details
Cite
Share
☐ 1,330
Front Med (Lausanne)
. 2021 Jun 9;8:639970.
doi: 10.3389/fmed.2021.639970. eCollection 2021.

Lopinavir/Ritonavir and Darunavir/Cobicistat in Hospitalized COVID-19 Patients: Findings From the Multicenter Italian CORIST Study

Augusto Di Castelnuovo [1], Simona Costanzo [2], Andrea Antinori [3], Nausicaa Berselli [4], Lorenzo Blandi [5], Marialaura Bonaccio [2], Raffaele Bruno [6] [7], Roberto Cauda [8] [9], Alessandro Gialluisi [2], Giovanni Guaraldi [10], Lorenzo Menicanti [5], Marco Mennuni [11], Ilaria My [12], Agostino Parruti [13], Giuseppe Patti [11], Stefano Perlini [14] [15], Francesca Santilli [16], Carlo Signorelli

[17], Giulio G Stefanini [12], Alessandra Vergori [18], Walter Ageno [19], Luca Aiello [20], Piergiuseppe Agostoni [21 22], Samir Al Moghazi [23], Rosa Arboretti [24], Filippo Aucella [25], Greta Barbieri [26], Martina Barchitta [27], Alessandro Bartoloni [28], Carolina Bologna [29], Paolo Bonfanti [30 31], Lucia Caiano [19], Laura Carrozzi [32], Antonio Cascio [33], Giacomo Castiglione [34], Mauro Chiarito [12], Arturo Ciccullo [8], Antonella Cingolani [8 9], Francesco Cipollone [16], Claudia Colomba [33], Crizia Colombo [11], Francesco Crosta [13], Giovanni Dalena [35], Chiara Dal Pra [36], Gian Battista Danzi [37], Damiano D'Ardes [16], Katleen de Gaetano Donati [8], Francesco Di Gennaro [38], Giuseppe Di Tano [37], Gianpiero D'Offizi [39], Tommaso Filippini [4], Francesco Maria Fusco [40], Carlo Gaudiosi [41], Ivan Gentile [42], Giancarlo Gini [19], Elvira Grandone [25], Gabriella Guarnieri [43], Gennaro L F Lamanna [35], Giovanni Larizza [35], Armando Leone [44], Veronica Lio [11], Angela Raffaella Losito [8], Gloria Maccagni [37], Stefano Maitan [20], Sandro Mancarella [45], Rosa Manuele [46], Massimo Mapelli [21 22], Riccardo Maragna [21 22], Lorenzo Marra [44], Giulio Maresca [47], Claudia Marotta [38], Franco Mastroianni [35], Maria Mazzitelli [48], Alessandro Mengozzi [26], Francesco Menichetti [26], Jovana Milic [10], Filippo Minutolo [49], Beatrice Molena [43], R Mussinelli [15], Cristina Mussini [10], Maria Musso [50], Anna Odone [17], Marco Olivieri [51], Emanuela Pasi [52], Annalisa Perroni [16], Francesco Petri [30], Biagio Pinchera [42], Carlo A Pivato [12], Venerino Poletti [53], Claudia Ravaglia [53], Marco Rossato [36], Marianna Rossi [30], Anna Sabena [14], Francesco Salinaro [14], Vincenzo Sangiovanni [40], Carlo Sanrocco [13], Laura Scorzolini [54], Raffaella Sgariglia [45], Paola Giustina Simeone [13], Michele Spinicci [28], Enrico Maria Trecarichi [48], Giovanni Veronesi [19], Roberto Vettor [36], Andrea Vianello [43], Marco Vinceti [4 55], Elena Visconti [8], Laura Vocciante [47], Raffaele De Caterina [32], Licia Iacoviello [2 19], COVID-19 RISK and Treatments (CORIST) Collaboration

Affiliations Expand

Affiliations

- [1] Mediterranea Cardiocentro, Napoli, Italy.
- [2] Department of Epidemiology and Prevention, IRCCS Neuromed, Pozzilli, Italy.
- [3] UOC Immunodeficienze Virali, National Institute for Infectious Diseases L. Spallanzani, IRCCS, Roma, Italy.
- [4] Section of Public Health, Department of Biomedical, Metabolic and Neural Sciences, University of Modena, Modena, Italy.
- [5] IRCCS Policlinico San Donato, San Donato Milanese, Italy.
- [6] Division of Infectious Diseases I, Fondazione IRCCS Policlinico San Matteo, Pavia, Italy.
- [7] Department of Clinical, Surgical, Diagnostic, and Paediatric Sciences, University of Pavia, Pavia, Italy.
- [8] Fondazione Policlinico Universitario A. Gemelli IRCCS, Roma, Italy.
- [9] Università Cattolica del Sacro Cuore- Dipartimento di Sicurezza e Bioetica Sede di Roma, Roma, Italy.
- [10] Infectious Disease Unit, Department of Surgical, Medical, Dental and Morphological Sciences, University of Modena and Reggio Emilia, Modena, Italy.
- [11] University of Eastern Piedmont, Maggiore della Carità Hospital, Novara, Italy.
- [12] Humanitas Clinical and Research Hospital IRCCS, Rozzano, Italy.
- [13] Department of Infectious Disease, Azienda Sanitaria Locale (AUSL) di Pescara, Pescara, Italy.
- [14] Emergency Department, IRCCS Policlinico San Matteo Foundation, Pavia, Italy.
- [15] Department of Internal Medicine, University of Pavia, Pavia, Italy.

- [16] Department of Medicine and Aging, Clinica Medica, SS. Annunziata Hospital and University of Chieti, Chieti, Italy.
- [17] School of Medicine, Vita-Salute San Raffaele University, Milano, Italy.
- [18] HIV/AIDS Department, National Institute for Infectious Diseases Lazzaro Spallanzani-IRCCS, Roma, Italy.
- [19] Department of Medicine and Surgery, University of Insubria, Varese, Italy.
- [20] UOC, Anestesia e Rianimazione, Dipartimento di Chirurgia Generale Ospedale Morgagni-Pierantoni, Forlì, Italy.
- [21] Centro Cardiologico Monzino IRCCS, Milano, Italy.
- [22] Cardiovascular Section, Department of Clinical Sciences and Community Health, University of Milano, Milano, Italy.
- [23] UOC Infezioni Sistemiche dell'Immunodepresso, National Institute for Infectious Diseases L. Spallanzani, IRCCS, Rome, Italy.
- [24] Department of Civil Environmental and Architectural Engineering, University of Padova, Padova, Italy.
- [25] Fondazione IRCCS Casa Sollievo della Sofferenza, San Giovanni Rotondo, Foggia, Italy.
- [26] Department of Clinical and Experimental Medicine, Azienda Ospedaliero-Universitaria Pisana, University of Pisa, Pisa, Italy.
- [27] Department of Medical and Surgical Sciences and Advanced Technologies G.F. Ingrassia, University of Catania, Catania, Italy.
- [28] Department of Experimental and Clinical Medicine, University of Florence and Azienda Ospedaliero-Universitaria Careggi, Firenze, Italy.
- [29] Ospedale del Mare, ASL Napoli 1, Napoli, Italy.
- [30] UOC Malattie Infettive, Ospedale San Gerardo, ASST Monza, Monza, Italy.
- [31] School of Medicine and Surgery, University of Milano-Bicocca, Milano, Italy.
- [32] Cardiovascular and Thoracic Department, Azienda Ospedaliero-Universitaria Pisana, University of Pisa, Pisa, Italy.
- [33] Infectious and Tropical Diseases Unit- Department of Health Promotion, Mother and Child Care, Internal Medicine and Medical Specialties (PROMISE) - University of Palermo, Palermo, Italy.
- [34] Servizio di Anestesia e Rianimazione II UO Rianimazione Ospedale San Marco, AOU Policlinico-Vittorio Emanuele, Catania, Italy.
- [35] COVID-19 Unit, EE Ospedale Regionale F. Miulli, Acquaviva delle Fonti, Italy.
- [36] Clinica Medica 3, Department of Medicine - DIMED, University Hospital of Padova, Padova, Italy.
- [37] Department of Cardiology, Ospedale di Cremona, Cremona, Italy.
- [38] Medical Direction, IRCCS Neuromed, Pozzilli, Italy.
- [39] UOC Malattie Infettive-Epatologia, National Institute for Infectious Diseases L. Spallanzani, IRCCS, Roma, Italy.
- [40] UOC Infezioni Sistemiche e dell'Immunodepresso, Azienda Ospedaliera dei Colli, Ospedale Cotugno, Napoli, Italy.
- [41] Ospedale di Boscotrecase - ASL Napoli 3, Napoli, Italy.
- [42] Department of Clinical Medicine and Surgery, University of Naples Federico II, Napoli, Italy.
- [43] Respiratory Pathophysiology Division, Department of Cardiologic, Thoracic and Vascular Sciences, University of Padova, Padova, Italy.
- [44] UOC di Pneumologia, P.O. San Giuseppe Moscati, Taranto, Italy.
- [45] ASST Milano Nord - Ospedale Edoardo Bassini Cinisello Balsamo, Milan, Italy.

- [46] UOC Malattie Infettive e Tropicali, P.O. San Marco, AOU Policlinico-Vittorio Emanuele, Catania, Italy.
- [47] UOC di Medicina - Presidio Ospedaliero S.Maria di Loreto Nuovo, Napoli, Italy.
- [48] Infectious and Tropical Diseases Unit, Department of Medical and Surgical Sciences, Magna Graecia University, Catanzaro, Italy.
- [49] Dipartimento di Farmacia, Università di Pisa, Pisa, Italy.
- [50] UOC Malattie Infettive-Apparato Respiratorio, National Institute for Infectious Diseases L. Spallanzani, IRCCS, Roma, Italy.
- [51] Computer Service, University of Molise, Campobasso, Italy.
- [52] Medicina Interna. Ospedale di Ravenna, AUSL della Romagna, Ravenna, Italy.
- [53] UOC Pneumologia, Dipartimento di Malattie Apparato Respiratorio e Torace, Ospedale Morgagni-Pierantoni, Forlì, Italy.
- [54] UOC Malattie Infettive ad Alta Intensità di Cura, National Institute for Infectious Diseases L. Spallanzani, IRCCS, Rome, Italy.
- [55] Department of Epidemiology, Boston University School of Public Health, Boston, MA, United States.

- PMID: **34179035**
- PMCID: PMC8221239
- DOI: 10.3389/fmed.2021.639970

Free PMC article

Lopinavir/Ritonavir and Darunavir/Cobicistat in Hospitalized COVID-19 Patients: Findings From the Multicenter Italian CORIST Study

Augusto Di Castelnuovo et al. Front Med (Lausanne). 2021.
Free PMC article

Show details

Front Med (Lausanne)

. 2021 Jun 9;8:639970.
doi: 10.3389/fmed.2021.639970. eCollection 2021.

Authors

Augusto Di Castelnuovo [1], Simona Costanzo [2], Andrea Antinori [3], Nausicaa Berselli [4], Lorenzo Blandi [5], Marialaura Bonaccio [2], Raffaele Bruno [6] [7], Roberto Cauda [8] [9], Alessandro Gialluisi [2], Giovanni Guaraldi [10], Lorenzo Menicanti [5], Marco Mennuni [11], Ilaria My [12], Agostino Parruti [13], Giuseppe Patti [11], Stefano Perlini [14] [15], Francesca Santilli [16], Carlo Signorelli [17], Giulio G Stefanini [12], Alessandra Vergori [18], Walter Ageno [19], Luca Aiello [20], Piergiuseppe Agostoni [21] [22], Samir Al Moghazi [23], Rosa Arboretti [24], Filippo Aucella [25], Greta Barbieri [26], Martina Barchitta [27], Alessandro Bartoloni [28], Carolina Bologna [29], Paolo Bonfanti [30] [31], Lucia Caiano [19], Laura Carrozzi [32], Antonio Cascio [33], Giacomo Castiglione [34], Mauro

Chiarito [12], Arturo Cicculo [8], Antonella Cingolani [8, 9], Francesco Cipollone [16], Claudia Colomba [33], Crizia Colombo [11], Francesco Crosta [13], Giovanni Dalena [35], Chiara Dal Pra [36], Gian Battista Danzi [37], Damiano D'Ardes [16], Katleen de Gaetano Donati [8], Francesco Di Gennaro [38], Giuseppe Di Tano [37], Gianpiero D'Offizi [39], Tommaso Filippini [4], Francesco Maria Fusco [40], Carlo Gaudiosi [41], Ivan Gentile [42], Giancarlo Gini [19], Elvira Grandone [25], Gabriella Guarnieri [43], Gennaro L F Lamanna [35], Giovanni Larizza [35], Armando Leone [44], Veronica Lio [11], Angela Raffaella Losito [8], Gloria Maccagni [37], Stefano Maitan [20], Sandro Mancarella [45], Rosa Manuele [46], Massimo Mapelli [21, 22], Riccardo Maragna [21, 22], Lorenzo Marra [44], Giulio Maresca [47], Claudia Marotta [38], Franco Mastroianni [35], Maria Mazzitelli [48], Alessandro Mengozzi [26], Francesco Menichetti [26], Jovana Milic [10], Filippo Minutolo [49], Beatrice Molena [43], R Mussinelli [15], Cristina Mussini [10], Maria Musso [50], Anna Odone [17], Marco Olivieri [51], Emanuela Pasi [52], Annalisa Perroni [16], Francesco Petri [30], Biagio Pinchera [42], Carlo A Pivato [12], Venerino Poletti [53], Claudia Ravaglia [53], Marco Rossato [36], Marianna Rossi [30], Anna Sabena [14], Francesco Salinaro [14], Vincenzo Sangiovanni [40], Carlo Sanrocco [13], Laura Scorzolini [54], Raffaella Sgariglia [45], Paola Giustina Simeone [13], Michele Spinicci [28], Enrico Maria Trecarichi [48], Giovanni Veronesi [19], Roberto Vettor [36], Andrea Vianello [43], Marco Vinceti [4, 55], Elena Visconti [8], Laura Vocciante [47], Raffaele De Caterina [32], Licia Iacoviello [2, 19], COVID-19 RISK and Treatments (CORIST) Collaboration

Affiliations

- [1] Mediterranea Cardiocentro, Napoli, Italy.
- [2] Department of Epidemiology and Prevention, IRCCS Neuromed, Pozzilli, Italy.
- [3] UOC Immunodeficienze Virali, National Institute for Infectious Diseases L. Spallanzani, IRCCS, Roma, Italy.
- [4] Section of Public Health, Department of Biomedical, Metabolic and Neural Sciences, University of Modena, Modena, Italy.
- [5] IRCCS Policlinico San Donato, San Donato Milanese, Italy.
- [6] Division of Infectious Diseases I, Fondazione IRCCS Policlinico San Matteo, Pavia, Italy.
- [7] Department of Clinical, Surgical, Diagnostic, and Paediatric Sciences, University of Pavia, Pavia, Italy.
- [8] Fondazione Policlinico Universitario A. Gemelli IRCCS, Roma, Italy.
- [9] Università Cattolica del Sacro Cuore- Dipartimento di Sicurezza e Bioetica Sede di Roma, Roma, Italy.
- [10] Infectious Disease Unit, Department of Surgical, Medical, Dental and Morphological Sciences, University of Modena and Reggio Emilia, Modena, Italy.
- [11] University of Eastern Piedmont, Maggiore della Carità Hospital, Novara, Italy.
- [12] Humanitas Clinical and Research Hospital IRCCS, Rozzano, Italy.
- [13] Department of Infectious Disease, Azienda Sanitaria Locale (AUSL) di Pescara, Pescara, Italy.
- [14] Emergency Department, IRCCS Policlinico San Matteo Foundation, Pavia, Italy.
- [15] Department of Internal Medicine, University of Pavia, Pavia, Italy.
- [16] Department of Medicine and Aging, Clinica Medica, SS. Annunziata Hospital and University of Chieti, Chieti, Italy.
- [17] School of Medicine, Vita-Salute San Raffaele University, Milano, Italy.
- [18] HIV/AIDS Department, National Institute for Infectious Diseases Lazzaro Spallanzani-IRCCS, Roma, Italy.

- [19] Department of Medicine and Surgery, University of Insubria, Varese, Italy.
- [20] UOC, Anestesia e Rianimazione, Dipartimento di Chirurgia Generale Ospedale Morgagni-Pierantoni, Forlì, Italy.
- [21] Centro Cardiologico Monzino IRCCS, Milano, Italy.
- [22] Cardiovascular Section, Department of Clinical Sciences and Community Health, University of Milano, Milano, Italy.
- [23] UOC Infezioni Sistemiche dell'Immunodepresso, National Institute for Infectious Diseases L. Spallanzani, IRCCS, Rome, Italy.
- [24] Department of Civil Environmental and Architectural Engineering, University of Padova, Padova, Italy.
- [25] Fondazione IRCCS Casa Sollievo della Sofferenza, San Giovanni Rotondo, Foggia, Italy.
- [26] Department of Clinical and Experimental Medicine, Azienda Ospedaliero-Universitaria Pisana, University of Pisa, Pisa, Italy.
- [27] Department of Medical and Surgical Sciences and Advanced Technologies G.F. Ingrassia, University of Catania, Catania, Italy.
- [28] Department of Experimental and Clinical Medicine, University of Florence and Azienda Ospedaliero-Universitaria Careggi, Firenze, Italy.
- [29] Ospedale del Mare, ASL Napoli 1, Napoli, Italy.
- [30] UOC Malattie Infettive, Ospedale San Gerardo, ASST Monza, Monza, Italy.
- [31] School of Medicine and Surgery, University of Milano-Bicocca, Milano, Italy.
- [32] Cardiovascular and Thoracic Department, Azienda Ospedaliero-Universitaria Pisana, University of Pisa, Pisa, Italy.
- [33] Infectious and Tropical Diseases Unit- Department of Health Promotion, Mother and Child Care, Internal Medicine and Medical Specialties (PROMISE) - University of Palermo, Palermo, Italy.
- [34] Servizio di Anestesia e Rianimazione II UO Rianimazione Ospedale San Marco, AOU Policlinico-Vittorio Emanuele, Catania, Italy.
- [35] COVID-19 Unit, EE Ospedale Regionale F. Miulli, Acquaviva delle Fonti, Italy.
- [36] Clinica Medica 3, Department of Medicine - DIMED, University Hospital of Padova, Padova, Italy.
- [37] Department of Cardiology, Ospedale di Cremona, Cremona, Italy.
- [38] Medical Direction, IRCCS Neuromed, Pozzilli, Italy.
- [39] UOC Malattie Infettive-Epatologia, National Institute for Infectious Diseases L. Spallanzani, IRCCS, Roma, Italy.
- [40] UOC Infezioni Sistemiche e dell'Immunodepresso, Azienda Ospedaliera dei Colli, Ospedale Cotugno, Napoli, Italy.
- [41] Ospedale di Boscotrecase - ASL Napoli 3, Napoli, Italy.
- [42] Department of Clinical Medicine and Surgery, University of Naples Federico II, Napoli, Italy.
- [43] Respiratory Pathophysiology Division, Department of Cardiologic, Thoracic and Vascular Sciences, University of Padova, Padova, Italy.
- [44] UOC di Pneumologia, P.O. San Giuseppe Moscati, Taranto, Italy.
- [45] ASST Milano Nord - Ospedale Edoardo Bassini Cinisello Balsamo, Milan, Italy.
- [46] UOC Malattie Infettive e Tropicali, P.O. San Marco, AOU Policlinico-Vittorio Emanuele, Catania, Italy.
- [47] UOC di Medicina - Presidio Ospedaliero S.Maria di Loreto Nuovo, Napoli, Italy.
- [48] Infectious and Tropical Diseases Unit, Department of Medical and Surgical Sciences, Magna Graecia University, Catanzaro, Italy.

- [49] Dipartimento di Farmacia, Università di Pisa, Pisa, Italy.
- [50] UOC Malattie Infettive-Apparato Respiratorio, National Institute for Infectious Diseases L. Spallanzani, IRCCS, Roma, Italy.
- [51] Computer Service, University of Molise, Campobasso, Italy.
- [52] Medicina Interna. Ospedale di Ravenna, AUSL della Romagna, Ravenna, Italy.
- [53] UOC Pneumologia, Dipartimento di Malattie Apparato Respiratorio e Torace, Ospedale Morgagni-Pierantoni, Forlì, Italy.
- [54] UOC Malattie Infettive ad Alta Intensità di Cura, National Institute for Infectious Diseases L. Spallanzani, IRCCS, Rome, Italy.
- [55] Department of Epidemiology, Boston University School of Public Health, Boston, MA, United States.

- PMID: **34179035**
- PMCID: PMC8221239
- DOI: 10.3389/fmed.2021.639970

Abstract

Background: Protease inhibitors have been considered as possible therapeutic agents for COVID-19 patients. **Objectives:** To describe the association between lopinavir/ritonavir (LPV/r) or darunavir/cobicistat (DRV/c) use and in-hospital mortality in COVID-19 patients. **Study Design:** Multicenter observational study of COVID-19 patients admitted in 33 Italian hospitals. Medications, preexisting conditions, clinical measures, and outcomes were extracted from medical records. Patients were retrospectively divided in three groups, according to use of LPV/r, DRV/c or none of them. Primary outcome in a time-to event analysis was death. We used Cox proportional-hazards models with inverse probability of treatment weighting by multinomial propensity scores. **Results:** Out of 3,451 patients, 33.3% LPV/r and 13.9% received DRV/c. Patients receiving LPV/r or DRV/c were more likely younger, men, had higher C-reactive protein levels while less likely had hypertension, cardiovascular, pulmonary or kidney disease. After adjustment for propensity scores, LPV/r use was not associated with mortality (HR = 0.94, 95% CI 0.78 to 1.13), whereas treatment with DRV/c was associated with a higher death risk (HR = 1.89, 1.53 to 2.34, E-value = 2.43). This increased risk was more marked in women, in elderly, in patients with higher severity of COVID-19 and in patients receiving other COVID-19 drugs. **Conclusions:** In a large cohort of Italian patients hospitalized for COVID-19 in a real-life setting, the use of LPV/r treatment did not change death rate, while DRV/c was associated with increased mortality. Within the limits of an observational study, these data do not support the use of LPV/r or DRV/c in COVID-19 patients.

Keywords: COVID-19; SARS-CoV-2; darunavir; in-hospital mortality; lopinavir.

Copyright © 2021 Di Castelnuovo, Costanzo, Antinori, Berselli, Blandi, Bonaccio, Bruno, Cauda, Gialluisi, Guaraldi, Menicanti, Mennuni, My, Parruti, Patti, Perlini, Santilli, Signorelli, Stefanini, Vergori, Ageno, Aiello, Agostoni, Al Moghazi, Arboretti, Aucella, Barbieri, Barchitta, Bartoloni, Bologna, Bonfanti, Caiano, Carrozzi, Cascio, Castiglione, Chiarito, Ciccullo, Cingolani, Cipollone, Colomba, Colombo, Crosta, Dalena, Dal Pra, Danzi, D'Ardes, de Gaetano Donati, Di Gennaro, Di Tano, D'Offizi, Filippini, Maria Fusco, Gaudiosi, Gentile, Gini, Grandone, Guarnieri, Lamanna, Larizza, Leone, Lio, Losito, Maccagni, Maitan, Mancarella, Manuele, Mapelli, Maragna, Marra, Maresca, Marotta, Mastroianni, Mazzitelli, Mengozzi, Menichetti, Milic, Minutolo, Molena, Mussinelli, Mussini, Musso, Odone, Olivieri, Pasi, Perroni, Petri, Pinchera, Pivato, Poletti, Ravaglia, Rossato, Rossi, Sabena, Salinaro, Sangiovanni, Sanrocco, Scorzolini,

Sgariglia, Simeone, Spinicci, Trecarichi, Veronesi, Vettor, Vianello, Vinceti, Visconti, Vocciante, De Caterina, Iacoviello and The COVID-19 RISK and Treatments (CORIST) Collaboration.

Conflict of interest statement

The authors declare that the research was conducted in the absence of any commercial or financial relationships that could be construed as a potential conflict of interest.

- 40 references
- 1 figure

Full text links

Frontiers Media SA Free PMC article
Proceed to details
Cite
Share
1,331
J Diabetes Investig
. 2022 Mar 4.
doi: 10.1111/jdi.13784. Online ahead of print.

Impact of diabetes and Krebs von den Lungen-6 on coronavirus disease 2019 severity: A single-center study from Japan

Yosuke Yakushiji [1], Koka Motoyama [1], Mayu Fukuda [1], Hisako Takahashi [1], Makiko Kimura [1], Satoshi Tazoe [1], Hiromi Iida [1], Anna Tamai [1], Takeshi Sakura [1], Yoshihiro Isaka [1], Mariko Fukumoto [1], Keiko Yamagami [1], Hidenori Nakagawa [1], Michinori Shirano [1], Masayuki Hosoi [1]
Affiliations Expand

Affiliation

- [1] Department of Infectious Disease, Osaka City General Hospital, Osaka, Japan.

- PMID: **35243802**
- DOI: 10.1111/jdi.13784

Free article

Impact of diabetes and Krebs von den Lungen-6 on coronavirus disease 2019 severity: A single-center study from Japan

Yosuke Yakushiji et al. J Diabetes Investig. 2022.
Free article
Show details
J Diabetes Investig
. 2022 Mar 4.
doi: 10.1111/jdi.13784. Online ahead of print.

Authors

Yosuke Yakushiji [1], Koka Motoyama [1], Mayu Fukuda [1], Hisako Takahashi [1], Makiko Kimura [1], Satoshi Tazoe [1], Hiromi Iida [1], Anna Tamai [1], Takeshi Sakura [1], Yoshihiro Isaka [1], Mariko Fukumoto [1], Keiko Yamagami [1], Hidenori Nakagawa [1], Michinori Shirano [1], Masayuki Hosoi [1]

Affiliation

- [1] Department of Infectious Disease, Osaka City General Hospital, Osaka, Japan.

- PMID: **35243802**
- DOI: 10.1111/jdi.13784

Abstract

Aims/introduction: Diabetes mellitus is reported as a risk factor for increased coronavirus disease 2019 (COVID-19) severity and mortality, but there have been few reports from Japan. Associations between diabetes mellitus and COVID-19 severity and mortality were investigated in a single Japanese hospital.

Materials and methods: Patients aged ≥20 years admitted to Osaka City General Hospital for COVID-19 treatment between April 2020 and March 2021 were included in this retrospective, observational study. Multivariable logistic regression analysis was carried out to examine whether diabetes mellitus contributes to COVID-19-related death and severity.

Results: Of the 262 patients included, 108 (41.2%) required invasive ventilation, and 34 (13.0%) died in hospital. The diabetes group (n = 92) was significantly older, more obese, had longer hospital stays, more severe illness and higher mortality than the non-diabetes group (n = 170). On multivariable logistic regression analysis, age (odds ratio [OR] 1.054, 95% confidence interval [CI] 1.023-1.086), body mass index (OR 1.111, 95% CI 1.028-1.201), history of diabetes mellitus (OR 2.429, 95% CI 1.152-5.123), neutrophil count (OR 1.222, 95% CI 1.077-1.385), C-reactive protein (OR 1.096, 95% CI 1.030-1.166) and Krebs von den Lungen-6 (OR 1.002, 95% CI 1.000-1.003) were predictors for COVID-19 severity ($R^2 = 0.468$). Meanwhile, age (OR 1.104, 95% CI 1.037-1.175) and Krebs von den Lungen-6 (OR 1.003, 95% CI 1.001-1.005) were predictors for COVID-19-related death ($R^2 = 0.475$).

Conclusions: Diabetes mellitus was a definite risk factor for COVID-19 severity in a single Japanese hospital treating moderately-to-severely ill patients.

Keywords: COVID-19; Diabetes mellitus; Retrospective study.

© 2022 The Authors. Journal of Diabetes Investigation published by Asian Association for the Study of Diabetes (AASD) and John Wiley & Sons Australia, Ltd.

- 32 references

Full text links

WILEY Free Full Text Wiley

Proceed to details

Cite

Share

☐ 1,332

Kidney Dis (Basel)
. 2021 Mar;7(2):111-119.
doi: 10.1159/000512270. Epub 2020 Oct 26.

Risk Factors and Outcomes of Acute Kidney Injury in Critically Ill Patients with Coronavirus Disease 2019

Yichun Cheng [1], Nanhui Zhang [1], Ran Luo [1], Meng Zhang [1], Zhixiang Wang [1], Lei Dong [1], Junhua Li [1], Rui Zeng [1], Ying Yao [1], Shuwang Ge [1], Gang Xu [1]
Affiliations Expand

Affiliation

- [1] Department of Nephrology, Tongji Hospital Affiliated to Tongji Medical College, Huazhong University of Science and Technology, Wuhan, China.

- PMID: **33821208**
- PMCID: PMC7649690
- DOI: 10.1159/000512270

Free PMC article

Risk Factors and Outcomes of Acute Kidney Injury in Critically Ill Patients with Coronavirus Disease 2019

Yichun Cheng et al. Kidney Dis (Basel). 2021 Mar.
Free PMC article
Show details
Kidney Dis (Basel)
. 2021 Mar;7(2):111-119.
doi: 10.1159/000512270. Epub 2020 Oct 26.

Authors

Yichun Cheng [1], Nanhui Zhang [1], Ran Luo [1], Meng Zhang [1], Zhixiang Wang [1], Lei Dong [1], Junhua Li [1], Rui Zeng [1], Ying Yao [1], Shuwang Ge [1], Gang Xu [1]

Affiliation

- [1] Department of Nephrology, Tongji Hospital Affiliated to Tongji Medical College, Huazhong University of Science and Technology, Wuhan, China.

- PMID: **33821208**
- PMCID: PMC7649690
- DOI: 10.1159/000512270

Abstract

Background: Coronavirus disease 2019 (COVID-19) has emerged as a major global health threat with a great number of deaths worldwide. Acute kidney injury (AKI) is a common complication in patients admitted to the intensive care unit. We aimed to assess the incidence, risk factors and in-hospital outcomes of AKI in COVID-19 patients admitted to the intensive care unit.

Methods: We conducted a retrospective observational study in the intensive care unit of Tongji Hospital, which was assigned responsibility for the treatments of severe COVID-19 patients by the Wuhan government. AKI was defined and staged based on Kidney Disease: Improving Global Outcomes (KDIGO) criteria. Mild AKI was defined as stage 1, and severe AKI was defined as stage 2 or stage 3. Logistic regression analysis was used to evaluate AKI risk factors, and Cox proportional hazards model was used to assess the association between AKI and in-hospital mortality.

Results: A total of 119 patients with COVID-19 were included in our study. The median patient age was 70 years (interquartile range, 59-77) and 61.3% were male. Fifty-one (42.8%) patients developed AKI during hospitalization, corresponding to 14.3% in stage 1, 28.6% in stage 2 and 18.5% in stage 3, respectively. Compared to patients without AKI, patients with AKI had a higher proportion of mechanical ventilation mortality and higher in-hospital mortality. A total of 97.1% of patients with severe AKI received mechanical ventilation and in-hospital mortality was up to 79.4%. Severe AKI was independently associated with high in-hospital mortality (OR: 1.82; 95% CI: 1.06-3.13). Logistic regression analysis demonstrated that high serum interleukin-8 (OR: 4.21; 95% CI: 1.23-14.38), interleukin-10 (OR: 3.32; 95% CI: 1.04-10.59) and interleukin-2 receptor (OR: 4.50; 95% CI: 0.73-6.78) were risk factors for severe AKI development.

Conclusions: Severe AKI was associated with high in-hospital mortality, and inflammatory response may play a role in AKI development in critically ill patients with COVID-19.

Keywords: Acute kidney injury; Coronavirus disease 2019; Mortality; Risk factor.

Copyright © 2020 by S. Karger AG, Basel.

Conflict of interest statement

The authors have no conflicts of interest to disclose.

- 35 references
- 2 figures

Full text links

Karger Free Final Version S. Karger AG, Basel, Switzerland Free PMC article

Proceed to details

Cite

Share

☐ 1,333

Ir J Med Sci
. 2021 Jun 28;1-8.
doi: 10.1007/s11845-021-02687-z. Online ahead of print.

Impact of COVID-19 pandemic on hip fractures: the central London experience COVID-related urgent geriatric hip trauma (COUGH) study COVERT (COVid Emergency-Related Trauma and orthopaedics) collaborative

Chang Park [1], Kapil Sugand [2], Arash Aframian [3], Catrin Morgan [3], Nadia Pakroo [3], Charles Gibbons [3], Michael Fertleman [1], Dinesh Nathwani [1], Rajarshi Bhattacharya [1], Khaled M Sarraf [1]

Affiliations Expand

Affiliations

- [1] Imperial College Healthcare NHS Trust, London, UK.
- [2] Imperial College Healthcare NHS Trust, London, UK. ks704@ic.ac.uk.
- [3] Chelsea & Westminster NHS Foundation Trust, London, UK.

- PMID: **34184207**
- PMCID: PMC8238478
- DOI: 10.1007/s11845-021-02687-z

Free PMC article

Impact of COVID-19 pandemic on hip fractures: the central London experience COVID-related urgent geriatric hip trauma (COUGH) study COVERT (COVid Emergency-Related Trauma and orthopaedics) collaborative

Chang Park et al. Ir J Med Sci. 2021.
Free PMC article
Show details
Ir J Med Sci
. 2021 Jun 28;1-8.
doi: 10.1007/s11845-021-02687-z. Online ahead of print.

Authors

Chang Park [1], Kapil Sugand [2], Arash Aframian [3], Catrin Morgan [3], Nadia Pakroo [3], Charles Gibbons [3], Michael Fertleman [1], Dinesh Nathwani [1], Rajarshi Bhattacharya [1], Khaled M Sarraf [1]

Affiliations

- [1] Imperial College Healthcare NHS Trust, London, UK.
- [2] Imperial College Healthcare NHS Trust, London, UK. ks704@ic.ac.uk.
- [3] Chelsea & Westminster NHS Foundation Trust, London, UK.

- PMID: **34184207**
- PMCID: PMC8238478
- DOI: 10.1007/s11845-021-02687-z

Abstract

Introduction: COVID-19 has been recognized as the unprecedented global health crisis in modern times. The purpose of this study was to assess the impact of COVID-19 on treatment of neck of femur fractures (NOFF) against the current guidelines and meeting best practice key performance indicators (KPIs) according to the National Hip Fracture Database (NHFD) in two large central London hospitals.

Materials and methods: A multi-center, longitudinal, retrospective, observational study of NOFF patients was performed for the first 'golden' month following the lockdown measures introduced in mid-March 2020. This was compared to the same time period in 2019.

Results: A total of 78 cases were observed. NOFFs accounted for 11% more of all acute referrals during the COVID era. There were fewer overall breaches in KPIs in time to theatre in 2020 and

also for those awaiting an orthogeriatric review. Time to discharge from the trust during the pandemic was improved by 54% ($p < 0.00001$) but patients were 51% less likely to return to their usual residence ($p = 0.007$). The odds ratio was significantly higher for consultant surgeon-led operations and consultant orthogeriatric-led review in the post-COVID era. There was no significant difference in using aerosol-generating anaesthetic procedures or immortality rates between both years.

Conclusion: The impact of COVID-19 pandemic has not adversely affected the KPIs for the treatment of NOFF patients with significant improvement in numerous care domains. These findings may represent the efforts to ensure that these vulnerable patients are treated promptly to minimize their risks from the coronavirus.

Keywords: Best practice tariff; COVID-19; Epidemiology; Hip fracture; Mortality; National hip fracture database; Neck of femur fracture; Surgery.

- 20 references
- 1 figure

Full text links

SpringerLink FULL-TEXT ARTICLE Springer Free PMC article
Proceed to details
Cite
Share
☐ 1,334
Bone Jt Open
. 2021 Mar;2(3):203-210.
doi: 10.1302/2633-1462.23.BJO-2020-0193.R1.

National operating volume for primary hip and knee arthroplasty in the COVID-19 era: a study utilizing the Scottish arthroplasty project dataset

Liam Z Yapp [1, 2], Jon V Clarke [2, 3], Matthew Moran [1, 2, 4], A Hamish R W Simpson [1], Chloe E H Scott [1, 4]
Affiliations Expand

Affiliations

- [1] Department of Orthopaedics, University of Edinburgh, Edinburgh, UK.
- [2] Scottish Arthroplasty Project, NHS Public Health Scotland, Edinburgh, UK.
- [3] Department of Orthopaedics, Golden Jubilee National Hospital, Clydebank, UK.
- [4] Department of Trauma & Orthopaedics, Royal Infirmary of Edinburgh, Edinburgh, UK.

- PMID: **33739125**

- PMCID: PMC8009902
- DOI: 10.1302/2633-1462.23.BJO-2020-0193.R1

Free PMC article

National operating volume for primary hip and knee arthroplasty in the COVID-19 era: a study utilizing the Scottish arthroplasty project dataset

Liam Z Yapp et al. Bone Jt Open. 2021 Mar.
Free PMC article
Show details
Bone Jt Open
. 2021 Mar;2(3):203-210.
doi: 10.1302/2633-1462.23.BJO-2020-0193.R1.

Authors

Liam Z Yapp [1,2], Jon V Clarke [2,3], Matthew Moran [1,2,4], A Hamish R W Simpson [1], Chloe E H Scott [1,4]

Affiliations

- [1] Department of Orthopaedics, University of Edinburgh, Edinburgh, UK.
- [2] Scottish Arthroplasty Project, NHS Public Health Scotland, Edinburgh, UK.
- [3] Department of Orthopaedics, Golden Jubilee National Hospital, Clydebank, UK.
- [4] Department of Trauma & Orthopaedics, Royal Infirmary of Edinburgh, Edinburgh, UK.

- PMID: **33739125**
- PMCID: PMC8009902
- DOI: 10.1302/2633-1462.23.BJO-2020-0193.R1

Abstract

Aims: The COVID-19 pandemic led to a national suspension of "non-urgent" elective hip and knee arthroplasty. The study aims to measure the effect of the COVID-19 pandemic on total hip arthroplasty (THA) and total knee arthroplasty (TKA) volume in Scotland. Secondary objectives are to measure the success of restarting elective services and model the time required to bridge the gap left by the first period of suspension.

Methods: A retrospective observational study using the Scottish Arthroplasty Project dataset. All patients undergoing elective THAs and TKAs during the period 1 January 2008 to 31 December 2020 were included. A negative binomial regression model using historical case-volume and mid-year population estimates was built to project the future case-volume of THA and TKA in Scotland. The median monthly case volume was calculated for the period 2008 to 2019 (baseline)

and compared to the actual monthly case volume for 2020. The time taken to eliminate the deficit was calculated based upon the projected monthly workload and with a potential workload between 100% to 120% of baseline.

Results: Compared to the period 2008 to 2019, primary TKA and THA volume fell by 61.1% and 53.6%, respectively. Since restarting elective services, Scottish hospitals have achieved approximately 40% to 50% of baseline monthly activity. With no changes in current workload, by 2021 there would be a reduction of 9,180 and 10,170 for THA and TKA, respectively. Conversely, working at 120% baseline monthly output, it would take over four years to eliminate the deficit for both TKA and THA.

Conclusion: This national study demonstrates the significant impact that COVID-19 pandemic has had on overall THA and TKA volume. In the six months after resuming elective services, Scottish hospitals averaged less than 50% normal monthly output. Loss of operating capacity will increase treatment delays and likely worsen overall morbidity. Cite this article: *Bone Joint Open* 2021;2(3):203-210.

Keywords: Arthroplasty; COVID-19; Elective Operating.

Conflict of interest statement

ICMJE COI statement: The authors declare the following, all of which is unrelated to this article: J. V. Clarke reports consultancy from Zimmer-Biomet; M. Moran reports payment for lectures (including service on speakers bureaus) from Stryker; C. E. H. Scott reports board membership to the Bone & Joint Journal and Bone & Joint Research, and consultancy from Stryker; and A. H. R. W. Simpson reports employment to Bone & Joint Research, and multiple grants from RCUK, charities, and Stryker.

- 29 references
- 8 figures

Full text links

Bone & Joint Open Full Text Available Atypon Free PMC article

Proceed to details

Cite

Share

☐ 1,335

Ann Transl Med

. 2020 Jun;8(11):679.

doi: 10.21037/atm-20-2119a.

Coronavirus disease 2019 (COVID-19): chest CT characteristics benefit to early disease recognition and patient classification-a single center experience

Hua Zhou [1], Kaijin Xu [2], Yihong Shen [3], Qiang Fang [4], Feng Chen [1], Jifang Sheng [2], Feng Zhao [5], Haiyan Lou [1]
Affiliations Expand

Affiliations

- [1] Department of Radiology, The First Affiliated Hospital, Zhejiang University School of Medicine, Hangzhou, China.
- [2] The State Key Laboratory for Diagnosis and Treatment of Infectious Diseases, Collaborative Innovation Center for Diagnosis and Treatment of Infectious Diseases, The First Affiliated Hospital, Zhejiang University School of Medicine, Hangzhou, China.
- [3] Department of Respiration, The First Affiliated Hospital, Zhejiang University School of Medicine, Hangzhou, China.
- [4] Intensive Care Unit, The First Affiliated Hospital, Zhejiang University School of Medicine, Hangzhou, China.
- [5] Department of Radiation Oncology, The First Affiliated Hospital, Zhejiang University School of Medicine, Hangzhou, China.

- PMID: **32617299**
- PMCID: PMC7327362
- DOI: 10.21037/atm-20-2119a

Free PMC article

Coronavirus disease 2019 (COVID-19): chest CT characteristics benefit to early disease recognition and patient classification-a single center experience

Hua Zhou et al. Ann Transl Med. 2020 Jun.
Free PMC article
Show details
Ann Transl Med
. 2020 Jun;8(11):679.
doi: 10.21037/atm-20-2119a.

Authors

Hua Zhou [1], Kaijin Xu [2], Yihong Shen [3], Qiang Fang [4], Feng Chen [1], Jifang Sheng [2], Feng Zhao [5], Haiyan Lou [1]

Affiliations

- [1] Department of Radiology, The First Affiliated Hospital, Zhejiang University School of Medicine, Hangzhou, China.

- [2] The State Key Laboratory for Diagnosis and Treatment of Infectious Diseases, Collaborative Innovation Center for Diagnosis and Treatment of Infectious Diseases, The First Affiliated Hospital, Zhejiang University School of Medicine, Hangzhou, China.
- [3] Department of Respiration, The First Affiliated Hospital, Zhejiang University School of Medicine, Hangzhou, China.
- [4] Intensive Care Unit, The First Affiliated Hospital, Zhejiang University School of Medicine, Hangzhou, China.
- [5] Department of Radiation Oncology, The First Affiliated Hospital, Zhejiang University School of Medicine, Hangzhou, China.

- PMID: **32617299**
- PMCID: PMC7327362
- DOI: 10.21037/atm-20-2119a

Abstract

Background: The current outbreak of coronavirus disease 2019 (COVID-19), epi-centered in Wuhan, Hubei Province of the China, has become a global health emergency. Several studies from China have recently provided the evidence of epidemiological, clinical, laboratory, and outcomes of COVID-19 patients. Investigation on the role of chest CT in patient screening and management course in a large cohort remains paucity.

Methods: This was a retrospective observational study based on the data collected between January 19 and 2020 to February 15, 2020. A clinic workflow using chest CT and RT-PCR assay to screen suspected patient was reviewed. Clinical data were evaluated and patients were classified to mild, common, severe and critical group. Chest CT characteristics of each patient were evaluated and a CT scoring system was applied to grade the lung involvement.

Results: Of 98 enrolled patients, 1, 29, 51 and 17 were clinically classified into mild, common, severe and critical group, respectively. Eighty-three patients (84.7%) demonstrated ground-glass opacity (GGO), 76 patients (77.5%) demonstrated consolidation and 18 patients (18.4%) demonstrated crazy-paving pattern on chest CT. Based on the CT scoring, 2, 35, 55 and 6 patients were categorized to grade 0, grade 1, grade 2 and grade 3, respectively, which significantly consistent with clinical classification (kappa =0.638, $P < 0.05$). Twenty-nine patients admitted from fever clinic, with an average interval of 1.2 days (range, 0-4 days) between CT examination and onset of symptom. Three of these patients had negative initial RT-PCR result while abnormalities displayed on the initial chest CT.

Conclusions: Peripheral lung distributed GGO and consolidation, without subpleural sparing, are the most common manifestations on chest CT of COVID-19. Abnormalities on chest CT can occur in an early stage of COVID-19, even when RT-PCR assay negative, which may help to early recognition and rapid diagnosis of this disease.

Keywords: Coronavirus disease 2019 (COVID-19); computed tomography; coronavirus; ground-glass opacity (GGO); pneumonia.

2020 Annals of Translational Medicine. All rights reserved.

Conflict of interest statement

Conflicts of Interest: All authors have completed the ICMJE uniform disclosure form (available at http://dx.doi.org/10.21037/atm-20-2119a). FZ serves as an unpaid section editor of Annals of

Translational Medicine from Jan 2020 to Dec 2020. The other authors have no conflicts of interest to declare.

- 5 figures

Full text links

ATM FULL TEXT AME Publishing Company Free PMC article

Proceed to details

Cite

Share

☐ 1,336

J Diabetes Investig

. 2022 Jan 27.

doi: 10.1111/jdi.13758. Online ahead of print.

Living and working environments are important determinants of glycemic control in patients with diabetes during the COVID-19 pandemic: A retrospective observational study

Aiko Terakawa [1], Ryotaro Bouchi [1] [2], Noriko Kodani [1], Tomoko Hisatake [1] [3], Takehiro Sugiyama [2] [4], Michihiro Matsumoto [5], Noriko Ihana-Sugiyama [1] [2], Mitsuru Ohsugi [1] [2], Kohjiro Ueki [1] [6], Hiroshi Kajio [1]

Affiliations Expand

Affiliations

- [1] Department of Diabetes, Endocrinology and Metabolism, Center Hospital, National Center for Global Health and Medicine, Tokyo, Japan.
- [2] Diabetes and Metabolism Information Center, Research Institute, National Center for Global Health and Medicine, Tokyo, Japan.
- [3] Medical Corporation Rikkuikai Tokyo, Tokyo, Japan.
- [4] Department of Health Services Research, Faculty of Medicine, University of Tsukuba, Ibaraki, Japan.
- [5] Department of Molecular Metabolic Regulation, Diabetes Research Center, Research Institute National Center for Global Health and Medicine, Tokyo, Japan.
- [6] Diabetes Research Center, National Center for Global Health and Medicine, Tokyo, Japan.

- PMID: **35088564**
- DOI: 10.1111/jdi.13758

Free article

Living and working environments are important determinants of glycemic control in patients with diabetes during the COVID-19 pandemic: A retrospective observational study

Aiko Terakawa et al. J Diabetes Investig. 2022.
Free article
Show details
J Diabetes Investig
. 2022 Jan 27.
doi: 10.1111/jdi.13758. Online ahead of print.

Authors

Aiko Terakawa [1], Ryotaro Bouchi [1, 2], Noriko Kodani [1], Tomoko Hisatake [1, 3], Takehiro Sugiyama [2, 4], Michihiro Matsumoto [5], Noriko Ihana-Sugiyama [1, 2], Mitsuru Ohsugi [1, 2], Kohjiro Ueki [1, 6], Hiroshi Kajio [1]

Affiliations

- [1] Department of Diabetes, Endocrinology and Metabolism, Center Hospital, National Center for Global Health and Medicine, Tokyo, Japan.
- [2] Diabetes and Metabolism Information Center, Research Institute, National Center for Global Health and Medicine, Tokyo, Japan.
- [3] Medical Corporation Rikkuikai Tokyo, Tokyo, Japan.
- [4] Department of Health Services Research, Faculty of Medicine, University of Tsukuba, Ibaraki, Japan.
- [5] Department of Molecular Metabolic Regulation, Diabetes Research Center, Research Institute National Center for Global Health and Medicine, Tokyo, Japan.
- [6] Diabetes Research Center, National Center for Global Health and Medicine, Tokyo, Japan.

- PMID: **35088564**
- DOI: 10.1111/jdi.13758

Abstract

Aim: To investigate (1) the association of lifestyle changes and living and working conditions with glycemic control and (2) whether treatment was intensified appropriately in patients with diabetes under the first COVID-19 state of emergency in Japan.

Materials and methods: A total of 321 participants were included. Participants completed a questionnaire regarding lifestyle changes, including diet, physical activity, and living and working conditions during the COVID-19 pandemic. The change in hemoglobin A1c (HbA1c) levels was estimated before (June 1, 2019 to August 31, 2019) and during (June 1, 2020 to August 31, 2020)

the pandemic. Factors associated with changes in HbA1c levels were examined by multiple linear regression analysis. The proportion of patients who received treatment intensification for diabetes was compared between before and during the pandemic.

Results: There was no significant change in HbA1c levels before the pandemic and during the pandemic (7.13 ± 0.98% vs 7.18 ± 1.01%, P = 0.186). Teleworking (estimate 0.206, P = 0.004) and living with a dog (estimate -0.149, P = 0.038) were significantly associated with changes in HbA1c levels after adjusting for covariates. There was no significant difference in the proportion of patients who received treatment intensification for diabetes during the pandemic and before the pandemic in either the elderly or non-elderly patients.

Conclusions: Overall glycemic control did not worsen during the pandemic. Nonetheless, environmental factors, including telework, were found to influence glycemic control in patients with diabetes. Further studies are needed to clarify whether the COVID-19 pandemic could affect treatment intensification for diabetes.

Keywords: COVID-19; Glycemic control; Lifestyle changes.

© 2022 The Authors. Journal of Diabetes Investigation published by Asian Association for the Study of Diabetes (AASD) and John Wiley & Sons Australia, Ltd.

- 51 references

Supplementary info

Grant support Expand

Grant support

- 21CA2021/Health and Labour Sciences Research Grants

Full text links

WILEY Free Full Text Wiley

Proceed to details

Cite

Share

1,337

Front Med (Lausanne)
. 2020 Dec 18;7:599533.
doi: 10.3389/fmed.2020.599533. eCollection 2020.

COVID-19 Induced Acute Respiratory Distress Syndrome-A Multicenter Observational Study

Johannes Herrmann [1], Elisabeth Hannah Adam [2], Quirin Notz [1], Philipp Helmer [2], Michael Sonntagbauer [2], Peter Ungemach-Papenberg [3], Andreas Sanns [3], York Zausig [3], Thorsten Steinfeldt [4], Iuliu Torje [5], Benedikt Schmid [1], Tobias Schlesinger [1], Caroline Rolfes [5], Christian Reyher [5], Markus Kredel [1], Jan Stumpner [1], Alexander Brack [1], Thomas Wurmb [1], Daniel Gill-Schuster [6], Peter Kranke [1], Dirk Weismann [7], Hartwig Klinker [8], Peter Heuschmann [9] [10], Viktoria Rücker [9], Stefan Frantz [7], Georg Ertl [7], Ralf Michael Muellenbach [5], Haitham Mutlak [6], Patrick Meybohm [1], Kai Zacharowski [2], Christopher Lotz [1]
Affiliations Expand

Affiliations

- [1] Department of Anesthesiology and Critical Care, University Hospital Würzburg, Julius-Maximilians-University Würzburg, Würzburg, Germany.
- [2] Department of Anesthesiology, Intensive Care Medicine and Pain Therapy, University Hospital Frankfurt, Goethe-University, Frankfurt, Germany.
- [3] Department of Anesthesiology and Critical Care, Klinikum Aschaffenburg-Alzenau, Aschaffenburg, Germany.
- [4] Department of Anesthesiology and Critical Care, Diakoneo Diak Klinikum Schwabisch Hall, Schwabisch-Hall, Germany.
- [5] Department of Critical Care, Emergency Medicine and Anesthesiology, ARDS/ECMO-Centre, Campus Kassel of the University of Southampton, Southampton, Germany.
- [6] Department of Anesthesiology and Critical Care, Sana-Klinikum Offenbach GmbH, Offenbach, Germany.
- [7] Department of Internal Medicine I, University Hospital Würzburg, Würzburg, Germany.
- [8] Department of Internal Medicine II, University Hospital Würzburg, Würzburg, Germany.
- [9] Institute for Clinical Epidemiology and Biometry, Julius-Maximilians-University, Würzburg, Germany.
- [10] Clinical Trial Center, University Hospital Würzburg, Julius-Maximilians-University, Würzburg, Germany.

- PMID: **33392222**
- PMCID: PMC7775385
- DOI: 10.3389/fmed.2020.599533

Free PMC article

COVID-19 Induced Acute Respiratory Distress Syndrome-A Multicenter Observational Study

Johannes Herrmann et al. Front Med (Lausanne). 2020.
Free PMC article
Show details
Front Med (Lausanne)
. 2020 Dec 18;7:599533.
doi: 10.3389/fmed.2020.599533. eCollection 2020.

Authors

Johannes Herrmann [1], Elisabeth Hannah Adam [2], Quirin Notz [1], Philipp Helmer [2], Michael Sonntagbauer [2], Peter Ungemach-Papenberg [3], Andreas Sanns [3], York Zausig [3], Thorsten Steinfeldt [4], Iuliu Torje [5], Benedikt Schmid [1], Tobias Schlesinger [1], Caroline Rolfes [5], Christian Reyher [5], Markus Kredel [1], Jan Stumpner [1], Alexander Brack [1], Thomas Wurmb [1], Daniel Gill-Schuster [6], Peter Kranke [1], Dirk Weismann [7], Hartwig Klinker [8], Peter Heuschmann [9] [10], Viktoria Rücker [9], Stefan Frantz [7], Georg Ertl [7], Ralf Michael Muellenbach [5], Haitham Mutlak [6], Patrick Meybohm [1], Kai Zacharowski [2], Christopher Lotz [1]

Affiliations

- [1] Department of Anesthesiology and Critical Care, University Hospital Würzburg, Julius-Maximilians-University Würzburg, Würzburg, Germany.
- [2] Department of Anesthesiology, Intensive Care Medicine and Pain Therapy, University Hospital Frankfurt, Goethe-University, Frankfurt, Germany.
- [3] Department of Anesthesiology and Critical Care, Klinikum Aschaffenburg-Alzenau, Aschaffenburg, Germany.
- [4] Department of Anesthesiology and Critical Care, Diakoneo Diak Klinikum Schwabisch Hall, Schwabisch-Hall, Germany.
- [5] Department of Critical Care, Emergency Medicine and Anesthesiology, ARDS/ECMO-Centre, Campus Kassel of the University of Southampton, Southampton, Germany.
- [6] Department of Anesthesiology and Critical Care, Sana-Klinikum Offenbach GmbH, Offenbach, Germany.
- [7] Department of Internal Medicine I, University Hospital Würzburg, Würzburg, Germany.
- [8] Department of Internal Medicine II, University Hospital Würzburg, Würzburg, Germany.
- [9] Institute for Clinical Epidemiology and Biometry, Julius-Maximilians-University, Würzburg, Germany.
- [10] Clinical Trial Center, University Hospital Würzburg, Julius-Maximilians-University, Würzburg, Germany.

- PMID: **33392222**
- PMCID: PMC7775385
- DOI: 10.3389/fmed.2020.599533

Abstract

Background: Proportions of patients dying from the coronavirus disease-19 (COVID-19) vary between different countries. We report the characteristics; clinical course and outcome of patients requiring intensive care due to COVID-19 induced acute respiratory distress syndrome (ARDS). **Methods:** This is a retrospective, observational multicentre study in five German secondary or tertiary care hospitals. All patients consecutively admitted to the intensive care unit (ICU) in any of the participating hospitals between March 12 and May 4, 2020 with a COVID-19 induced ARDS were included. **Results:** A total of 106 ICU patients were treated for COVID-19 induced ARDS, whereas severe ARDS was present in the majority of cases. Survival of ICU treatment was 65.0%. Median duration of ICU treatment was 11 days; median duration of mechanical ventilation was 9 days. The majority of ICU treated patients (75.5%) did not receive any antiviral or anti-inflammatory therapies. Venovenous (vv) ECMO was utilized in 16.3%. ICU triage with population-level decision making was not necessary at any time. Univariate analysis associated

older age, diabetes mellitus or a higher SOFA score on admission with non-survival during ICU stay. **Conclusions:** A high level of care adhering to standard ARDS treatments lead to a good outcome in critically ill COVID-19 patients.

Keywords: ARDS (acute respiratory distress syndrome); COVID-19; Germany; intensive care medicine; pandemia.

Copyright © 2020 Herrmann, Adam, Notz, Helmer, Sonntagbauer, Ungemach-Papenberg, Sanns, Zausig, Steinfeldt, Torje, Schmid, Schlesinger, Rolfes, Reyher, Kredel, Stumpner, Brack, Wurmb, Gill-Schuster, Kranke, Weismann, Klinker, Heuschmann, Rücker, Frantz, Ertl, Muellenbach, Mutlak, Meybohm, Zacharowski and Lotz.

Conflict of interest statement

PHeu reports grants from German Ministry of Research and Education, German Research Foundation, European Union, Charité—Universitätsmedizin Berlin, Berlin Chamber of Physicians, German Parkinson Society, University Hospital Würzburg, Robert Koch Institute, German Heart Foundation, Federal Joint Committee (G-BA) within the Innovationfond, University Hospital Heidelberg (within RASUNOA-prime; supported by an unrestricted research grant to the University Hospital Heidelberg from Bayer, BMS, Boehringer-Ingelheim, Daiichi Sankyo), Charité—Universitätsmedizin Berlin (within Mondafis; supported by an unrestricted research grant to the Charité from Bayer), University Göttingen (within FIND-AF randomized; supported by an unrestricted research grant to the University Göttingen from Boehringer-Ingelheim), outside the submitted work. SF reports grants from DFG, BMBF, grants and personal fees from Abiomed, Amgen, Akzea, AstraZeneca, Bayer, Berlin-Chemie, Braun, Bristol-Myers Squibb, Boehringer, Daiichi Sankyo, MSD, Novartis, Pfizer, Sanofi-Aventis, Servier, Siemens, Zoll, outside the submitted work. GE reports grants and personal fees from Bayer, grants and personal fees from Novartis, grants and personal fees from Vifor Pharma Deutschland GmbH, outside the submitted work; PK reports other from FreseniusKabi, personal fees from BBraun, grants, personal fees and other from TEVARatiopharm, other from CSL Behring, other from Pajunk, other from APEPTICO Forschung und Entwicklung GmbH, outside the submitted work; KZ reports personal fees from Aesculap Akademie GmbH, personal fees from Affinites Sante, grants from Ashai Kasai Pharma, grants and personal fees from B. Braun AG, grants and personal fees from B. Braun Avitum AG, personal fees from Bayer AG, grants from Biotest AG, personal fees from Christian Doppler Stiftung, grants and personal fees from CSL Behring GmbH, personal fees from Cyto Sorbents GmbH, personal fees from Edward Lifescience Corporation, personal fees from Executive Insight AG, personal fees from Fresenius Kabi GmbH, personal fees from Fresenius Medical Care, personal fees from Haemonetics Corporation, personal fees from Hartmannbund Landesverband, personal fees from Health Adcances GmbH, personal fees from Heinen + Löwenstein GmbH, personal fees from Hexal AG, grants from INC Research, personal fees from Johnson and Johnson, personal fees from Josef Gassner, personal fees from Maquet GmbH, personal fees from Markus Lücke Kongress Organization, personal fees from Masimo International, personal fees from med Update GmbH, personal fees from Medizin and Markt Gesundheitswerk, personal fees from MSD Sharp and Dohme GmbH, personal fees from Nordic Group, personal fees from Nordic Pharma, grants from Novo Nordisc Pharma GmbH, grants from Pfizer Pharma GmbH, personal fees from Pharmacosmos, personal fees from Ratiopharm GmbH, personal fees from Salvia Medical GmbH, personal fees from Schering Stiftung, personal fees from Schöchl Medical Österreich, personal fees from Serumwerke, personal fees from Verlag für Printmedien und PR, Forum Sanitas, grants and personal fees from Vifor Pharma GmbH, personal fees from Wellington, personal fees from Werfen, outside the submitted work; HK served as a speaker and/or an Advisory Board Member for AbbVie, BMS, Gilead, Hexal, Janssen, MSD, Pfizer, ViiV and has received research funding from AbbVie, Arrowhaed, BMS, Gilead, Janssen, MSD, Novartis, German Liver Foundation, Hector Foundation, Virtual University of Bavaria,

Federal Ministry of Education and Research, outside the submitted work. The remaining authors declare that the research was conducted in the absence of any commercial or financial relationships that could be construed as a potential conflict of interest.

- 26 references
- 1 figure

Full text links

Frontiers Media SA Free PMC article
Proceed to details
Cite
Share
1,338
BMJ Open Ophthalmol
. 2020 Sep 18;5(1):e000560.
doi: 10.1136/bmjophth-2020-000560. eCollection 2020.

Remodelling intravitreal therapy pathways for macular disease during the COVID-19 pandemic and an Austrian national lockdown

Josef Huemer[1, 2], Julius Hienert[1], Cornelia Hirn[1], Christoph Hackl[1], Stephan M Radda[1], Oliver Findl[1]
Affiliations Expand

Affiliations

- [1] Department of Ophthalmology, Hanusch Hospital, Vienna, Austria.
- [2] NIHR Biomedical Research Center at Moorfields Eye Hospital NHS Foundation Trust and UCL Institute of Ophthalmology, London, UK.

- PMID: **34192151**
- PMCID: PMC7503197
- DOI: 10.1136/bmjophth-2020-000560

Free PMC article

Remodelling intravitreal therapy pathways for macular disease during the COVID-19 pandemic and an Austrian national lockdown

Josef Huemer et al. BMJ Open Ophthalmol. 2020.

Free PMC article

Show details

BMJ Open Ophthalmol

. 2020 Sep 18;5(1):e000560.

doi: 10.1136/bmjophth-2020-000560. eCollection 2020.

Authors

Josef Huemer [1] [2], Julius Hienert [1], Cornelia Hirn [1], Christoph Hackl [1], Stephan M Radda [1], Oliver Findl [1]

Affiliations

- [1] Department of Ophthalmology, Hanusch Hospital, Vienna, Austria.
- [2] NIHR Biomedical Research Center at Moorfields Eye Hospital NHS Foundation Trust and UCL Institute of Ophthalmology, London, UK.

- PMID: **34192151**
- PMCID: PMC7503197
- DOI: 10.1136/bmjophth-2020-000560

Abstract

Objective: To analyse the remodelling and recovery of a relocated intravitreal injection (IVI) service with an adapted treatment regimen in a tertiary referral centre during a nationwide lockdown with initial cancellation of all non-emergency treatments caused by the COVID-19 pandemic.

Methods and analysis: For this retrospective observational study at Hanusch Hospital, Vienna, between 16 March 2020 and 5 May 2020, we conducted an analysis of an appointment booking system based on prioritisation incorporating disease class, severity and fellow eye status by evidence-based impact on irreversible structural impairment and survey data from telephone interviews. Recapture time was defined as the time-to-discard the backlog of patients in need for treatment. Non-attendance was stratified as treatment refusal for personal reasons and non-attendance due to lockdown-related restrictions.

Results: Of the 1109 patients, 241 (21.7%) were considered as highly urgent, 269 (24.3%) as urgent, 402 (36.2%) as semiurgent and 197 (17.8%) as non-urgent. Recapture time was 15 days for highly urgent patients, 22 days for urgent patients, 43 days for semiurgent patients and 46 days for non-urgent patients. The proportion of patients who refused treatment due to personal reasons was 5.2%, with a mean age of 82.4 years; 29 patients (2.6%) could not attend due to lockdown-related restrictions.

Conclusion: By streamlining treatment based on urgency as well as increasing the number of bilateral IVI, recapture time was fast. We could provide a safe treatment environment for healthcare professionals and patients after resetting the injection service outside of the hospital with increased levels of protection.

Keywords: degeneration; macula; neovascularisation; retina.

© Author(s) (or their employer(s)) 2020. Re-use permitted under CC BY-NC. No commercial re-use. See rights and permissions. Published by BMJ.

Conflict of interest statement

Competing interests: None declared.

- 27 references
- 1 figure

Full text links

BMJ Free Full Text BMJ Publishing Group Free PMC article

Proceed to details

Cite

Share

☐ 1,339

Cureus

. 2021 Nov 21;13(11):e19791.

doi: 10.7759/cureus.19791. eCollection 2021 Nov.

Clinical and Laboratory Factors in Predicting Mortality Among COVID-19 RT-PCR Positive Patients: A Retrospective Observational Study From a Tertiary Care Center

Raja Sundaramurthy [1], Suryakumar Balasubramanian [2], Vithiya Ganesan [3], Pearl Aggarwal [4], Tarun Parvataneni [5], Devi Parvathy Jyothi Ramachandran Nair [6], Raja Prahadeesh Saravanan [7]

Affiliations Expand

Affiliations

- [1] Microbiology, All India Institute of Medical Sciences, Bibinagar, IND.
- [2] Critical Care Medicine, Velammal Medical College Hospital & Research Institute, Madurai, IND.
- [3] Microbiology, Velammal Medical College Hospital & Research Institute, Madurai, IND.
- [4] Internal Medicine, Golden Hospital, Zirakpur, IND.
- [5] Internal Medicine, Siddavanahalli Nijalingappa Medical College and HSK Hospital and Research Centre, Bagalkot, IND.
- [6] Internal Medicine, Tirunelveli Medical College, Tirunelveli, IND.
- [7] Internal Medicine, R.R Hospital, Tuticorin, IND.

- PMID: **34956783**

- PMCID: PMC8693567
- DOI: 10.7759/cureus.19791

Free PMC article

Clinical and Laboratory Factors in Predicting Mortality Among COVID-19 RT-PCR Positive Patients: A Retrospective Observational Study From a Tertiary Care Center

Raja Sundaramurthy et al. Cureus. 2021.
Free PMC article
Show details
Cureus
. 2021 Nov 21;13(11):e19791.
doi: 10.7759/cureus.19791. eCollection 2021 Nov.

Authors

Raja Sundaramurthy [1], Suryakumar Balasubramanian [2], Vithiya Ganesan [3], Pearl Aggarwal [4], Tarun Parvataneni [5], Devi Parvathy Jyothi Ramachandran Nair [6], Raja Prahadeesh Saravanan [7]

Affiliations

- [1] Microbiology, All India Institute of Medical Sciences, Bibinagar, IND.
- [2] Critical Care Medicine, Velammal Medical College Hospital & Research Institute, Madurai, IND.
- [3] Microbiology, Velammal Medical College Hospital & Research Institute, Madurai, IND.
- [4] Internal Medicine, Golden Hospital, Zirakpur, IND.
- [5] Internal Medicine, Siddavanahalli Nijalingappa Medical College and HSK Hospital and Research Centre, Bagalkot, IND.
- [6] Internal Medicine, Tirunelveli Medical College, Tirunelveli, IND.
- [7] Internal Medicine, R.R Hospital, Tuticorin, IND.

- PMID: **34956783**

- PMCID: PMC8693567
- DOI: 10.7759/cureus.19791

Abstract

Background: In coronavirus disease 2019 (COVID-19) patients, risk stratification based on clinical presentation, co-morbid illness, and combined laboratory parameters is essential to provide an adequate, timely intervention based on an individual's conditions to prevent mortality among cases.

Methods: A retrospective observational study was carried out from June to October 2020, including all reverse transcription-polymerase chain reaction (RT-PCR) positive COVID-19 non-survivors and control group survivors randomly selected after age and sex matching. Clinical and demographic information was collected from the medical records. Categorical variables were expressed by frequency and percentage. To explore the risk factors associated with mortality, univariable and multivariable logistic regression models were used.

Results and discussions: All non-survivors (n = 100) and 100 survivors (out of 1,018) were analyzed. Male gender (67.4%) was the independent risk factor for COVID-19 infection. Advanced age group, diabetes, cardiovascular, neurological, and hypertensive co-morbidities were statistically associated with mortality. Cardiac arrest and acute kidney injury (AKI) were the most common complications. Mortality is significantly associated with lymphopenia and raised lactate dehydrogenase (LDH), as shown by higher odds. In addition, raised neutrophils, monocytes, aspartate aminotransferase (AST), serum creatinine, interleukin 6 (IL-6), and C-reactive protein (CRP) are also significantly associated with mortality. The most common causes of death were respiratory failure (84%) and acute respiratory distress syndrome (77%). Of the non-survivors, 92% received corticosteroids, 63% were on high-flow nasal cannula oxygen therapy, 29% were mechanically ventilated, and 29% received tocilizumab.

Conclusion: Serial monitoring of neutrophils, lymphocytes, D-dimer, procalcitonin, AST, LDH, CRP, IL-6, serum creatinine, and albumin might provide a reliable and convenient method for classifying and predicting the severity and outcomes of patients with COVID-19.

Keywords: acute respiratory distress syndrome [ards]; clinical and laboratory characteristics; co-morbidities; coronavirus disease (covid-19); mortality predictors.

Copyright © 2021, Sundaramurthy et al.

Conflict of interest statement

The authors have declared that no competing interests exist.

- 17 references
- 2 figures

Full text links

Free PMC article
Proceed to details
Cite
Share
☐ 1,340
Indian J Crit Care Med
. 2021 Mar;25(3):260-266.
doi: 10.5005/jp-journals-10071-23747.

Tocilizumab: An Effective Therapy for Severely and Critically Ill COVID-19 Patients

Sudhir Bhandari [1], Govind Rankawat [1], Ajeet Singh [1]

Affiliations Expand

Affiliation

- [1] Department of General Medicine, SMS Medical College and Hospital, Jaipur, Rajasthan, India.

- PMID: **33790504**
- PMCID: PMC7991771
- DOI: 10.5005/jp-journals-10071-23747

Free PMC article

Tocilizumab: An Effective Therapy for Severely and Critically Ill COVID-19 Patients

Sudhir Bhandari et al. Indian J Crit Care Med. 2021 Mar.
Free PMC article
Show details
Indian J Crit Care Med
. 2021 Mar;25(3):260-266.
doi: 10.5005/jp-journals-10071-23747.

Authors

Sudhir Bhandari [1], Govind Rankawat [1], Ajeet Singh [1]

Affiliation

- [1] Department of General Medicine, SMS Medical College and Hospital, Jaipur, Rajasthan, India.

- PMID: **33790504**
- PMCID: PMC7991771
- DOI: 10.5005/jp-journals-10071-23747

Abstract

Background: Tocilizumab (TCZ), a monoclonal antibody against the most prevalent cytokine interleukin-6 (IL-6), is an emerging therapeutic option for COVID-19 infections. The present study was undertaken to assess the therapeutic response of TCZ therapy in severely or critically ill COVID-19 patients and its role as an effective modality of management. **Methods:** The present retrospective observational study included 30 admitted severely or critically ill COVID-19 patients, treated with TCZ therapy on behalf of raised IL-6 levels. The patients' data concerning medical history, clinical manifestation, arterial blood gas analysis, mode of oxygenation, radiological imaging, and outcome were extracted from their medical records and compared pre- and post-TCZ infusion. **Results:** All patients of the study group had symptomatic presentations with a mean PaO^2/FiO_2 (P/F) ratio of 205.41 before TCZ infusion. All patients had a raised IL-6

level (mean value 206.56 pg/mL) that was extremely elevated in 90% of patients. Infusion of TCZ dramatically reduced mean body temperature (100.78-99.32°F) and the requirement for supplemental oxygen (68-48%) and improved mean SpO_2 (86-89%) and mean P/F ratio (208-240) within 24 hours. Three patients on noninvasive ventilation were weaned off after TCZ infusion. Serum levels of IL-6 were raised initially but declined within 3-5 days of post-TCZ infusion. **Conclusion:** TCZ appears to be an effective therapeutic option in severely or critically ill COVID-19 patients with raised IL-6 levels. TCZ immediately improves the clinical status of patients by a probable mechanism of inhibition of cytokine storm and reduces COVID-19-related mortalities.
How to cite this article: Bhandari S, Rankawat G, Singh A. Tocilizumab: An Effective Therapy for Severely and Critically Ill COVID-19 Patients. Indian J Crit Care Med 2021;25(3):260-266.

Keywords: COVID-19; Cytokine storm; Interleukin-6; Tocilizumab.

Copyright © 2021; Jaypee Brothers Medical Publishers (P) Ltd.

Conflict of interest statement

Source of support: Nil Conflict of interest: None

- 15 references
- 2 figures

Full text links

Free PMC article
Proceed to details
Cite
Share
☐ 1,341
Pharmaceutics
. 2022 Mar 11;14(3):624.
doi: 10.3390/pharmaceutics14030624.

Safety of Tocilizumab in COVID-19 Patients and Benefit of Single-Dose: The Largest Retrospective Observational Study

Ayman M Al-Qaaneh [1, 2], Fuad H Al-Ghamdi [3], Sayed AbdulAzeez [2], J Francis Borgio [2, 4]
Affiliations Expand

Affiliations

- [1] Clinical Pharmacy Services Division, Pharmacy Services Department, Johns Hopkins Aramco Healthcare (JHAH), Dhahran 31311, Saudi Arabia.
- [2] Department of Genetic Research, Institute for Research and Medical Consultations (IRMC), Imam Abdulrahman Bin Faisal University, Dammam 31441, Saudi Arabia.

- [3] Pharmacy Services Department, Johns Hopkins Aramco Healthcare (JHAH), Dhahran 31311, Saudi Arabia.
- [4] Department of Epidemic Diseases Research, Institute for Research and Medical Consultations (IRMC), Imam Abdulrahman Bin Faisal University, Dammam 31441, Saudi Arabia.

- PMID: **35335998**
- DOI: 10.3390/pharmaceutics14030624

Free article

Safety of Tocilizumab in COVID-19 Patients and Benefit of Single-Dose: The Largest Retrospective Observational Study

Ayman M Al-Qaaneh et al. Pharmaceutics. 2022.
Free article
Show details
Pharmaceutics
. 2022 Mar 11;14(3):624.
doi: 10.3390/pharmaceutics14030624.

Authors

Ayman M Al-Qaaneh [1, 2], Fuad H Al-Ghamdi [3], Sayed AbdulAzeez [2], J Francis Borgio [2, 4]

Affiliations

- [1] Clinical Pharmacy Services Division, Pharmacy Services Department, Johns Hopkins Aramco Healthcare (JHAH), Dhahran 31311, Saudi Arabia.
- [2] Department of Genetic Research, Institute for Research and Medical Consultations (IRMC), Imam Abdulrahman Bin Faisal University, Dammam 31441, Saudi Arabia.
- [3] Pharmacy Services Department, Johns Hopkins Aramco Healthcare (JHAH), Dhahran 31311, Saudi Arabia.
- [4] Department of Epidemic Diseases Research, Institute for Research and Medical Consultations (IRMC), Imam Abdulrahman Bin Faisal University, Dammam 31441, Saudi Arabia.

- PMID: **35335998**
- DOI: 10.3390/pharmaceutics14030624

Abstract

Severe acute respiratory coronavirus-2 (SARS-CoV-2) still presents a public threat and puts extra strain on healthcare facilities. Without an effective antiviral drug, all available treatment options are considered supportive. Tocilizumab as a treatment option has to date shown variable results. In this retrospective study, we aimed to assess predictors of mortality of COVID-19 patients (*n* =

300) on tocilizumab and the clinical effectiveness of this drug. The results showed that ICU admission *OR* = 64.6 (95% CI: 8.2, 507.4); age of the patient *OR* = 1.1 (95% CI: 1.0, 1.1); and number of tocilizumab doses administered by the patient $OR_{(two\ doses)}$ = 4.0 (95% CI: 1.5, 10.9), $OR_{(three\ doses)}$ = 1.5 (95% CI: 0.5, 5.1), and $OR_{(four\ doses\ or\ more)}$ = 7.2 (95% CI: 2.0, 25.5) presented strong correlation factors that may be linked to COVID-19 mortality. Furthermore, our study showed the beneficial effects of early administration of tocilizumab *OR* = 1.2 (95% CI: 1.1, 1.4) and longer hospital length of stay *OR* = 0.974 (95% CI: 0.9, 1.0) in reducing COVID-19 mortalities. High blood D-dimer concentration *OR* = 1.1 (95% CI: 1.0, 1.2) and reciprocal blood phosphate concentration *OR* = 0.008 (95% CI: 0.0, 1.2) were correlated to high mortality under SARS-CoV-2 infection. The short-term effect of a single dose of tocilizumab was a significant increase in blood BUN and liver enzymes (ALT, AST, and LDH) above their normal ranges. Furthermore, it significantly reduced CRP blood concentration, but not to normal levels (13.90 to 1.40 mg/dL, *p* < 0.001). Assessing the effect of different doses of tocilizumab (in terms of the number of doses, total mg, and total mg/kg administered by the patients) indicated that administering more than one dose may lead to increases in ICU length of stay and hospital length of stay of up to 14 and 22 days after the last dose of tocilizumab (6 to 14, *p* = 0.06, and 10 to 22, *p* < 0.001), with no improvement in 28- and 90-day mortality, as confirmed by Kaplan-Meier analysis. There were also clear correlations and trends between the number of doses of tocilizumab and increased blood CO_2, MCV, RDW, and D-dimer concentrations and between number of doses of tocilizumab and decreased CRP, AST, and hemoglobin concentrations. Microbiology analysis showed a significant increase in the incidence of infection after tocilizumab administration (28 to 119, *p* < 0.001) with a median time of incidence within 6 days of the first dose of tocilizumab. A significant correlation was also found between the number of tocilizumab doses and the number of incidences of infections after tocilizumab administration r (298) = 0.396, $p = 1.028 \times 10^{-12}$. Based on these results and depending on the pharmacokinetic parameters of the drug, we recommend single-dose administration of tocilizumab as the optimal dosage for COVID-19 patients who do not have active bacterial infection or liver diseases, to be administered as soon as the patient is admitted to the hospital.

Keywords: COVID-19 patient; ICU admission; infection; monoclonal antibody; mortality; tocilizumab; treatment.

Full text links

FULL TEXT OPEN ACCESS MDPI [Multidisciplinary Digital Publishing Institute (MDPI)]

Proceed to details

Cite

Share

☐ 1,342

J Clin Med

. 2022 Feb 9;11(4):906.

doi: 10.3390/jcm11040906.

Effect of Designating Emergency Medical Centers for Critical Care on Emergency Medical Service Systems during the COVID-

19 Pandemic: A Retrospective Observational Study

Hang A Park [1], Sola Kim [1], Sang Ook Ha [2], Sangsoo Han [3], ChoungAh Lee [1]
Affiliations Expand

Affiliations

- [1] Department of Emergency Medicine, Dongtan Sacred Heart Hospital, Hwaseong-si 18450, Korea.
- [2] Department of Emergency Medicine, Hallym University Sacred Heart Hospital, Anyang-si 14068, Korea.
- [3] Department of Emergency Medicine, Soonchunhyang University Bucheon Hospital, Bucheon 14584, Korea.

- PMID: **35207182**
- PMCID: PMC8875071
- DOI: 10.3390/jcm11040906

Free PMC article

Effect of Designating Emergency Medical Centers for Critical Care on Emergency Medical Service Systems during the COVID-19 Pandemic: A Retrospective Observational Study

Hang A Park et al. J Clin Med. 2022.
Free PMC article
Show details
J Clin Med
. 2022 Feb 9;11(4):906.
doi: 10.3390/jcm11040906.

Authors

Hang A Park [1], Sola Kim [1], Sang Ook Ha [2], Sangsoo Han [3], ChoungAh Lee [1]

Affiliations

- [1] Department of Emergency Medicine, Dongtan Sacred Heart Hospital, Hwaseong-si 18450, Korea.

- [2] Department of Emergency Medicine, Hallym University Sacred Heart Hospital, Anyang-si 14068, Korea.
- [3] Department of Emergency Medicine, Soonchunhyang University Bucheon Hospital, Bucheon 14584, Korea.

- PMID: **35207182**
- PMCID: PMC8875071
- DOI: 10.3390/jcm11040906

Abstract

During the coronavirus disease 2019 (COVID-19) pandemic, prehospital times were delayed for patients who needed to arrive at the hospital in a timely manner to receive treatment. To address this, in March 2020, the Korean government designated emergency medical centers for critical care (EMC-CC). This study retrospectively analyzed whether this intervention effectively reduced ambulance diversion (AD) and shortened prehospital times using emergency medical service records from 219,763 patients from the Gyeonggi Province, collected between 1 January and 31 December 2020. We included non-traumatic patients aged 18 years or older. We used interrupted time series analysis to investigate the intervention effects on the daily AD rate and compared prehospital times before and after the intervention. Following the intervention, the proportion of patients transported 30-35 km and 50 km or more was 13.8% and 5.7%, respectively, indicating an increased distance compared to before the intervention. Although the change in the AD rate was insignificant, the daily AD rate significantly decreased after the intervention. Prehospital times significantly increased after the intervention in all patients ($p < 0.001$) and by disease group; all prehospital times except for the scene time of cardiac arrest patients increased. In order to achieve optimal treatment times for critically ill patients in a situation that pushes the limits of the medical system, such as the COVID-19 pandemic, even regional distribution of EMC-CC may be necessary, and priority should be given to the allocation of care for patients with mild symptoms.

Keywords: COVID-19; ambulance diversion; emergency medical services; prehospital; time factors.

Conflict of interest statement

The authors declare no conflict of interest. The funders had no role in the design of the study, in the collection, analyses, or interpretation of data, in the writing of the manuscript, or in the decision to publish the results.

- 37 references
- 3 figures

Full text links

FULL TEXT OPEN ACCESS MDPI Multidisciplinary Digital Publishing Institute (MDPI) Free PMC article

Proceed to details

Cite

Share

☐ 1,343

J Thorac Dis

. 2020 May;12(5):1811-1823.

doi: 10.21037/jtd-20-1914.

Clinical characteristics of COVID-19 infection in chronic obstructive pulmonary disease: a multicenter, retrospective, observational study

Fan Wu [1], Yumin Zhou [1], Zhongfang Wang [1], Min Xie [2], Zhe Shi [3], Zhiqiang Tang [4], Xiaohe Li [5], Xiaochen Li [6], Chunliang Lei [7], Yimin Li [1], Zhengyi Ni [8], Yu Hu [9], Xiaoqing Liu [1], Wenguang Yin [1], Linling Cheng [1], Feng Ye [1], Jieqi Peng [1], Lingmei Huang [10], Jia Tian [11], Lingjuan Zhang [3], Xiaoneng Mo [7], Ying Zhang [5], Ke Hu [6], Yongliang Jiang [12], Weijie Guan [1], Jie Xiang [8], Yingxia Liu [5], Yixiang Peng [13], Li Wei [14], Yahua Hu [15], Peng Peng [16], Jianming Wang [17], Jiyang Liu [18], Wei Huang [19], Ruchong Chen [1], Jianping Zhao [2], Shiyue Li [1], Nuofu Zhang [1], Jincun Zhao [1], Nanshan Zhong [1], Pixin Ran [1], Medical Treatment Expert Group for COPD and COVID-19

Affiliations Expand

Affiliations

- [1] State Key Laboratory of Respiratory Disease & National Clinical Research Center for Respiratory Disease, Guangzhou Institute of Respiratory Health, the First Affiliated Hospital of Guangzhou Medical University, Guangzhou Medical University, Guangzhou, China.
- [2] Department of Pulmonary and Critical Care Medicine, Tongji Hospital, Tongji Medical College, Huazhong University of Science and Technology, Wuhan, China.
- [3] Huizhou First Hospital, Huizhou, China.
- [4] The Second People's Hospital of Changde City, Changde, China.
- [5] Shenzhen Third People's Hospital, Shenzhen, China.
- [6] Renmin Hospital of Wuhan University, Wuhan, China.
- [7] Guangzhou Eighth People's Hospital, Guangzhou Medical University, Guangzhou, China.
- [8] Wuhan Jinyintan Hospital, Wuhan, China.
- [9] Institute of Hematology, Union Hospital, Tongji Medical College, Huazhong University of Science and Technology, Wuhan, China.
- [10] The First people's Hospital of Yueyang, Yueyang, China.
- [11] The Second People's Hospital of Hunan Province, Changsha, China.
- [12] Department of Respiratory and Critical Care Medicine, Hunan Provincial People's Hospital, Changsha, China.
- [13] The Central Hospital of Wuhan, Wuhan, China.
- [14] Wuhan No. 1 Hospital, Wuhan Hospital of Traditional Chinese and Western Medicine, Wuhan, China.
- [15] Huangshi Central Hospital of Edong Healthcare Group, Affiliated Hospital of Hubei Polytechnic University, Huangshi, China.
- [16] Wuhan Pulmonary Hospital, Wuhan, China.

- [17] Tianyou Hospital Affiliated to Wuhan University of Science and Technology, Wuhan, China.
- [18] The First Hospital of Changsha, Changsha, China.
- [19] Jiangling County People's Hospital, Jingzhou, China.

- PMID: **32642086**
- PMCID: PMC7330323
- DOI: 10.21037/jtd-20-1914

Free PMC article

Clinical characteristics of COVID-19 infection in chronic obstructive pulmonary disease: a multicenter, retrospective, observational study

Fan Wu et al. J Thorac Dis. 2020 May.
Free PMC article

Show details

J Thorac Dis

. 2020 May;12(5):1811-1823.
doi: 10.21037/jtd-20-1914.

Authors

Fan Wu [1], Yumin Zhou [1], Zhongfang Wang [1], Min Xie [2], Zhe Shi [3], Zhiqiang Tang [4], Xiaohe Li [5], Xiaochen Li [6], Chunliang Lei [7], Yimin Li [1], Zhengyi Ni [8], Yu Hu [9], Xiaoqing Liu [1], Wenguang Yin [1], Linling Cheng [1], Feng Ye [1], Jieqi Peng [1], Lingmei Huang [10], Jia Tian [11], Lingjuan Zhang [3], Xiaoneng Mo [7], Ying Zhang [5], Ke Hu [6], Yongliang Jiang [12], Weijie Guan [1], Jie Xiang [8], Yingxia Liu [5], Yixiang Peng [13], Li Wei [14], Yahua Hu [15], Peng Peng [16], Jianming Wang [17], Jiyang Liu [18], Wei Huang [19], Ruchong Chen [1], Jianping Zhao [2], Shiyue Li [1], Nuofu Zhang [1], Jincun Zhao [1], Nanshan Zhong [1], Pixin Ran [1], Medical Treatment Expert Group for COPD and COVID-19

Affiliations

- [1] State Key Laboratory of Respiratory Disease & National Clinical Research Center for Respiratory Disease, Guangzhou Institute of Respiratory Health, the First Affiliated Hospital of Guangzhou Medical University, Guangzhou Medical University, Guangzhou, China.
- [2] Department of Pulmonary and Critical Care Medicine, Tongji Hospital, Tongji Medical College, Huazhong University of Science and Technology, Wuhan, China.
- [3] Huizhou First Hospital, Huizhou, China.
- [4] The Second People's Hospital of Changde City, Changde, China.
- [5] Shenzhen Third People's Hospital, Shenzhen, China.

- [6] Renmin Hospital of Wuhan University, Wuhan, China.
- [7] Guangzhou Eighth People's Hospital, Guangzhou Medical University, Guangzhou, China.
- [8] Wuhan Jinyintan Hospital, Wuhan, China.
- [9] Institute of Hematology, Union Hospital, Tongji Medical College, Huazhong University of Science and Technology, Wuhan, China.
- [10] The First people's Hospital of Yueyang, Yueyang, China.
- [11] The Second People's Hospital of Hunan Province, Changsha, China.
- [12] Department of Respiratory and Critical Care Medicine, Hunan Provincial People's Hospital, Changsha, China.
- [13] The Central Hospital of Wuhan, Wuhan, China.
- [14] Wuhan No. 1 Hospital, Wuhan Hospital of Traditional Chinese and Western Medicine, Wuhan, China.
- [15] Huangshi Central Hospital of Edong Healthcare Group, Affiliated Hospital of Hubei Polytechnic University, Huangshi, China.
- [16] Wuhan Pulmonary Hospital, Wuhan, China.
- [17] Tianyou Hospital Affiliated to Wuhan University of Science and Technology, Wuhan, China.
- [18] The First Hospital of Changsha, Changsha, China.
- [19] Jiangling County People's Hospital, Jingzhou, China.

- PMID: **32642086**
- PMCID: PMC7330323
- DOI: 10.21037/jtd-20-1914

Abstract

Background: Coronavirus disease 2019 (COVID-19) has been a global pandemic disease, with more than 4 million cases and nearly 300,000 deaths. Little is known about COVID-19 in patients with chronic obstructive pulmonary disease (COPD). We aimed to evaluate the influence of preexisting COPD on the progress and outcomes of COVID-19.

Methods: This was a multicenter, retrospective, observational study. We enrolled 1,048 patients aged 40 years and above, including 50 patients with COPD and 998 patients without COPD, and with COVID-19 confirmed via high-throughput sequencing or real-time reverse transcription-polymerase chain reaction, between December 11, 2019 and February 20, 2020. We collected data of demographics, pathologic test results, radiologic imaging, and treatments. The primary outcomes were composite endpoints determined by admission to an intensive care unit, the use of mechanical ventilation, or death.

Results: Compared with patients who had COVID-19 but not COPD, those with COPD had higher rates of fatigue (56.0% *vs.* 40.2%), dyspnea (66.0% *vs.* 26.3%), diarrhea (16.0% *vs.* 3.6%), and unconsciousness (8.0% *vs.* 1.7%) and a significantly higher proportion of increased activated partial thromboplastin time (23.5% *vs.* 5.2%) and D-dimer (65.9% *vs.* 29.3%), as well as ground-glass opacities (77.6% *vs.* 60.3%), local patchy shadowing (61.2% *vs.* 41.4%), and interstitial abnormalities (51.0% *vs.* 19.8%) on chest computed tomography. Patients with COPD were more likely to develop bacterial or fungal coinfection (20.0% *vs.* 5.9%), acute respiratory distress syndrome (ARDS) (20.0% *vs.* 7.3%), septic shock (14.0% *vs.* 2.3%), or acute renal failure (12.0% *vs.* 1.3%). Patients with COPD and COVID-19 had a higher risk of reaching the composite endpoints [hazard ratio (HR): 2.17, 95% confidence interval (CI): 1.40-3.38; P=0.001] or death (HR: 2.28, 95% CI: 1.15-4.51; P=0.019), after adjustment.

Conclusions: In this study, patients with COPD who developed COVID-19 showed a higher risk of admission to the intensive care unit, mechanical ventilation, or death.

Keywords: Clinical characteristics; chronic obstructive pulmonary disease (COPD); coronavirus disease 2019 (COVID-19).

2020 Journal of Thoracic Disease. All rights reserved.

Conflict of interest statement

Conflicts of Interest: All authors have completed the ICMJE uniform disclosure form (available at http://dx.doi.org/10.21037/jtd-20-1914). NZ serves as the unpaid Editor-in Chief of Journal of Thoracic Disease. The other authors have no conflicts of interest to declare.

Comment in

- Coronavirus 2019 Disease (COVID-19), Systemic Inflammation, and Cardiovascular Disease.
  Inciardi RM, Solomon SD, Ridker PM, Metra M. Inciardi RM, et al. J Am Heart Assoc. 2020 Aug 18;9(16):e017756. doi: 10.1161/JAHA.120.017756. Epub 2020 Jul 17. J Am Heart Assoc. 2020. PMID: 32677478 Free PMC article. No abstract available.

- 3 figures

Full text links

JTD FREE FULL TEXT AME Publishing Company Free PMC article

Proceed to details

Cite

Share

☐ 1,344

Cureus
. 2021 Jun 22;13(6):e15833.
doi: 10.7759/cureus.15833. eCollection 2021 Jun.

Impact of the COVID-19 Pandemic on Pelvic and Acetabular Trauma: Experiences From a National Tertiary Referral Centre

Kunal Mohan [1], Patrick McCabe [1], Wafi Mohammed [1], Justin M Hintze [1], Hasnain Raza [1], Brendan O'Daly [1], Michael Leonard [1]
Affiliations Expand

Affiliation

- [1] Department of Trauma & Orthopaedics, National Centre for Pelvic and Acetabular Surgery, Tallaght University Hospital, Dublin, IRL.

- PMID: **34322330**
- PMCID: PMC8297654
- DOI: 10.7759/cureus.15833

Free PMC article

Impact of the COVID-19 Pandemic on Pelvic and Acetabular Trauma: Experiences From a National Tertiary Referral Centre

Kunal Mohan et al. Cureus. 2021.
Free PMC article
Show details
Cureus
. 2021 Jun 22;13(6):e15833.
doi: 10.7759/cureus.15833. eCollection 2021 Jun.

Authors

Kunal Mohan [1], Patrick McCabe [1], Wafi Mohammed [1], Justin M Hintze [1], Hasnain Raza [1], Brendan O'Daly [1], Michael Leonard [1]

Affiliation

- [1] Department of Trauma & Orthopaedics, National Centre for Pelvic and Acetabular Surgery, Tallaght University Hospital, Dublin, IRL.

- PMID: **34322330**
- PMCID: PMC8297654
- DOI: 10.7759/cureus.15833

Abstract

Introduction The coronavirus disease 2019 (COVID-19) pandemic has had a significant impact on daily life. Restrictions imposed to help minimise virus transmission have limited both population movement and employment, as well as altering the potential mechanisms of high-energy trauma. The objective of this study was to assess the impact of the COVID-19 pandemic on pelvic and acetabular trauma. Materials and methods A retrospective observational study of the incidence, causality, patient profile, fracture morphology, and treatment strategy of pelvic and acetabular trauma managed in a national tertiary referral specialist pelvic and acetabular centre between the 1st of March and 1st of August 2020 was undertaken and compared to corresponding time periods in the two preceding years. Results A total of 78 patients were referred for management following pelvic and acetabular trauma during the study period with a mean age of 52 years (SD +/- 24.2). Overall, 45% and 42% of patients were referred following isolated pelvic or acetabular fractures

respectively. The most frequent mechanism of injury was a fall from height (>1m) (42%), with 53% of patients suffering from concomitant injuries and 32% requiring surgical management. While there was a statistically significant difference in mechanism of injury (P=0.026), there was no significant difference in overall incidence, fracture types, incidence of concomitant injuries, or overall proportion requiring surgical intervention during the study period when compared to previous years. Conclusion While some variation in the mechanisms of injury have been observed, the overall incidence, patient, fracture, and injury profiles associated with pelvic and acetabular trauma appear to have remained consistent during the COVID-19 pandemic. Additionally, the number and proportion of those requiring surgical treatment of these fractures have remained stable. Understanding the continued burden of these potentially severe injuries may help guide injury prevention, treatment, and resource allocation as the pandemic continues.

Keywords: acetabulum; covid-19; fracture; pandemic; pelvis; trauma.

Copyright © 2021, Mohan et al.

Conflict of interest statement

The authors have declared that no competing interests exist.

- 27 references
- 5 figures

Full text links

Free PMC article
Proceed to details
Cite
Share
☐ 1,345
Front Oncol
. 2021 Nov 19;11:754838.
doi: 10.3389/fonc.2021.754838. eCollection 2021.

Management and Outcomes of Patients With Radiotherapy Interruption During the COVID-19 Pandemic

Xiaofang Ying [1], Jianping Bi [1], Yi Ding [1], Xueyan Wei [1], Wei Wei [1], Fang Xin [1], Chuangying Xiao [1], Desheng Hu [1], Vivek Verma [2], Guang Han [1]
Affiliations Expand

Affiliations

- [1] Department of Radiation Oncology, Hubei Cancer Hospital, Tongji Medical College, Huazhong University of Science and Technology, Wuhan, China.

- [2] Department of Radiation Oncology, The University of Texas MD Anderson Cancer Center, Houston, TX, United States.

- PMID: **34868962**
- PMCID: PMC8639682
- DOI: 10.3389/fonc.2021.754838

Free PMC article

Management and Outcomes of Patients With Radiotherapy Interruption During the COVID-19 Pandemic

Xiaofang Ying et al. Front Oncol. 2021.
Free PMC article
Show details
Front Oncol
. 2021 Nov 19;11:754838.
doi: 10.3389/fonc.2021.754838. eCollection 2021.

Authors

Xiaofang Ying [1], Jianping Bi [1], Yi Ding [1], Xueyan Wei [1], Wei Wei [1], Fang Xin [1], Chuangying Xiao [1], Desheng Hu [1], Vivek Verma [2], Guang Han [1]

Affiliations

- [1] Department of Radiation Oncology, Hubei Cancer Hospital, Tongji Medical College, Huazhong University of Science and Technology, Wuhan, China.
- [2] Department of Radiation Oncology, The University of Texas MD Anderson Cancer Center, Houston, TX, United States.

- PMID: **34868962**
- PMCID: PMC8639682
- DOI: 10.3389/fonc.2021.754838

Abstract

Purpose: This retrospective observational study examined patients who experienced radiotherapy (RT) interruption during the Wuhan lockdown for the novel coronavirus disease 2019 (COVID-19) pandemic.

Materials and methods: The data of all patients whose RT was interrupted during the Wuhan lockdown from January 23 to April 8, 2020 were collected. Patient-, cancer-, and treatment-related characteristics were analyzed, along with interruption time, disease progression type, and survival status. The methods employed in order to compensate for RT interruption were also described.

Results: There were altogether 129 cancer patients whose RT was interrupted. Nineteen (14.7%) patients experienced a total interruption time of at most 7 days; the interruption time was 8-14 days for 27 (20.9%) patients, and 15 or more days for 47 (36.4%) patients. The remaining 36 (27.9%) patients did not come back to our hospital for further RT. We first describe our experience with re-immobilization and/or re-planning (n = 17) as well as dose compensation/adjustment. Of the 40 definitive radiotherapy patients, 37 had squamous cell carcinoma of nasopharyngeal, lung, or cervical origin. Most patients (85/93, 91.4%) were followed up for more than one year. Among the 40 patients who received definitive radiotherapy, nine patients experienced disease progression and five patients died. Three of the seven (42.9%) patients who did not finish radiotherapy after interruption died, as compared to only two of the 33 (6.1%) patients who completed radiotherapy. EQD2 (equivalent dose in 2 Gy fractions) at the time point of RT interruption was calculated. Five of the six patients (83.3%) who received EQD2 ≤10 Gy suffered from disease progression, compared with four of the 34 (11.8%) patients who received EQD2 >10 Gy. For the seven definitive radiotherapy cases who did not finish radiotherapy, three received systemic anti-cancer treatments and three died (all of whom did not receive further systemic therapies).

Conclusions: This study provides the longest follow-up for the outcomes of RT interruption during COVID-19 pandemic to date. It cannot imply causation but implies that completing RT is important, along with the utility of having patients remain on systemic therapies if RT is to be interrupted.

Keywords: COVID-19 pandemic; distant metastasis; interruption; outcomes; radiotherapy.

Copyright © 2021 Ying, Bi, Ding, Wei, Wei, Xin, Xiao, Hu, Verma and Han.

Conflict of interest statement

The authors declare that the research was conducted in the absence of any commercial or financial relationships that could be construed as a potential conflict of interest.

- 18 references
- 3 figures

Full text links

frontiers Full text Frontiers Media SA Free PMC article

Proceed to details

Cite

Share

☐ 1,346

Lancet Rheumatol

. 2020 Oct;2(10):e603-e612.
doi: 10.1016/S2665-9913(20)30277-0. Epub 2020 Aug 14.

Tocilizumab among patients with COVID-19 in the intensive care unit: a multicentre observational study

Noa Biran [1], Andrew Ip [2], Jaeil Ahn [3], Ronaldo C Go [4], Shuqi Wang [3], Shivam Mathura [5], Brittany A Sinclaire [1], Urszula Bednarz [1], Michael Marafelias [1], Eric Hansen [5], David S Siegel [1], Andre H Goy [1], Andrew L Pecora [1], Ihor S Sawczuk [6], Lauren S Koniaris [4], Micky Simwenyi [4], Daniel W Varga [4], Lisa K Tank [4], Aaron A Stein [7], Valerie Allusson [8], George S Lin [9], William F Oser [10], Roman A Tuma [11], Joseph Reichman [12], Louis Brusco Jr [13], Kim L Carpenter [14], Eric J Costanzo [14], Vincent Vivona [15], Stuart L Goldberg [2]

Affiliations Expand

Affiliations

- [1] John Theurer Cancer Center, Hackensack, NJ, USA.
- [2] Division of Outcomes and Value Research, Hackensack, NJ, USA.
- [3] Department of Biostatistics, Bioinformatics, and Biomathematics, Georgetown University, Washington, DC, USA.
- [4] Hackensack University Medical Center, Hackensack, NJ, USA.
- [5] COTA, Boston, MA, USA.
- [6] Hackensack University Medical Center - Hackensack Meridian Health School of Medicine at Seton Hall University, Nutley, NJ, USA.
- [7] Palisades Medical Center, North Bergen, NJ, USA.
- [8] Mountainside Medical Center, Glen Ridge, NJ, USA.
- [9] Pascack Valley Medical Center, Westwood, NJ, USA.
- [10] JFK Medical Center, Edison, NJ, USA.
- [11] Bayshore Medical Center, Holmdel, NJ, USA.
- [12] Riverview Medical Center, Red Bank, NJ, USA.
- [13] Raritan Bay Medical Center, Old Bridge, and Raritan Bay Medical Center, Perth Amboy, NJ, USA.
- [14] Jersey Shore University Medical Center, Neptune, NJ, USA.
- [15] Ocean Medical Center, Brick Township, and Southern Ocean Medical Center, Stafford Township, NJ, USA.

- PMID: **32838323**
- PMCID: PMC7428303
- DOI: 10.1016/S2665-9913(20)30277-0

Free PMC article

Tocilizumab among patients with COVID-19 in the intensive care unit: a multicentre observational study

Noa Biran et al. Lancet Rheumatol. 2020 Oct.
Free PMC article
Show details
Lancet Rheumatol
. 2020 Oct;2(10):e603-e612.
doi: 10.1016/S2665-9913(20)30277-0. Epub 2020 Aug 14.

Authors

Noa Biran [1], Andrew Ip [2], Jaeil Ahn [3], Ronaldo C Go [4], Shuqi Wang [3], Shivam Mathura [5], Brittany A Sinclaire [1], Urszula Bednarz [1], Michael Marafelias [1], Eric Hansen [5], David S Siegel [1], Andre H Goy [1], Andrew L Pecora [1], Ihor S Sawczuk [6], Lauren S Koniaris [4], Micky Simwenyi [4], Daniel W Varga [4], Lisa K Tank [4], Aaron A Stein [7], Valerie Allusson [8], George S Lin [9], William F Oser [10], Roman A Tuma [11], Joseph Reichman [12], Louis Brusco Jr [13], Kim L Carpenter [14], Eric J Costanzo [14], Vincent Vivona [15], Stuart L Goldberg [2]

Affiliations

- [1] John Theurer Cancer Center, Hackensack, NJ, USA.
- [2] Division of Outcomes and Value Research, Hackensack, NJ, USA.
- [3] Department of Biostatistics, Bioinformatics, and Biomathematics, Georgetown University, Washington, DC, USA.
- [4] Hackensack University Medical Center, Hackensack, NJ, USA.
- [5] COTA, Boston, MA, USA.
- [6] Hackensack University Medical Center - Hackensack Meridian Health School of Medicine at Seton Hall University, Nutley, NJ, USA.
- [7] Palisades Medical Center, North Bergen, NJ, USA.
- [8] Mountainside Medical Center, Glen Ridge, NJ, USA.
- [9] Pascack Valley Medical Center, Westwood, NJ, USA.
- [10] JFK Medical Center, Edison, NJ, USA.
- [11] Bayshore Medical Center, Holmdel, NJ, USA.
- [12] Riverview Medical Center, Red Bank, NJ, USA.
- [13] Raritan Bay Medical Center, Old Bridge, and Raritan Bay Medical Center, Perth Amboy, NJ, USA.
- [14] Jersey Shore University Medical Center, Neptune, NJ, USA.
- [15] Ocean Medical Center, Brick Township, and Southern Ocean Medical Center, Stafford Township, NJ, USA.

- PMID: **32838323**
- PMCID: PMC7428303
- DOI: 10.1016/S2665-9913(20)30277-0

Abstract

Background: Tocilizumab, a monoclonal antibody directed against the interleukin-6 receptor, has been proposed to mitigate the cytokine storm syndrome associated with severe COVID-19. We aimed to investigate the association between tocilizumab exposure and hospital-related mortality among patients requiring intensive care unit (ICU) support for COVID-19.

Methods: We did a retrospective observational cohort study at 13 hospitals within the Hackensack Meridian Health network (NJ, USA). We included patients (aged ≥18 years) with laboratory-confirmed COVID-19 who needed support in the ICU. We obtained data from a prospective observational database and compared outcomes in patients who received tocilizumab with those who did not. We applied a multivariable Cox model with propensity score matching to reduce confounding effects. The primary endpoint was hospital-related mortality. The prospective observational database is registered on ClinicalTrials.gov, NCT04347993.

Findings: Between March 1 and April 22, 2020, 764 patients with COVID-19 required support in the ICU, of whom 210 (27%) received tocilizumab. Factors associated with receiving tocilizumab were patients' age, gender, renal function, and treatment location. 630 patients were included in the propensity score-matched population, of whom 210 received tocilizumab and 420 did not receive tocilizumab. 358 (57%) of 630 patients died, 102 (49%) who received tocilizumab and 256 (61%) who did not receive tocilizumab. Overall median survival from time of admission was not reached (95% CI 23 days-not reached) among patients receiving tocilizumab and was 19 days (16-26) for those who did not receive tocilizumab (hazard ratio [HR] 0·71, 95% CI 0·56-0·89; p=0·0027). In the primary multivariable Cox regression analysis with propensity matching, an association was noted between receiving tocilizumab and decreased hospital-related mortality (HR 0·64, 95% CI 0·47-0·87; p=0·0040). Similar associations with tocilizumab were noted among subgroups requiring mechanical ventilatory support and with baseline C-reactive protein of 15 mg/dL or higher.

Interpretation: In this observational study, patients with COVID-19 requiring ICU support who received tocilizumab had reduced mortality. Results of ongoing randomised controlled trials are awaited.

Funding: None.

© 2020 Elsevier Ltd. All rights reserved.

Comment in

- Tocilizumab in Treatment for Patients With COVID-19.
  Yang C, Liu M. Yang C, et al. JAMA Intern Med. 2021 Jul 1;181(7):1017-1018. doi: 10.1001/jamainternmed.2021.0392. JAMA Intern Med. 2021. PMID: 33818614 No abstract available.

- 29 references
- 2 figures

Supplementary info

Associated data Expand

Associated data

- ClinicalTrials.gov/NCT04347993

Full text links

Free PMC article
Proceed to details
Cite
Share
☐ 1,347
J Family Med Prim Care
. 2022 Jan;11(1):123-132.
doi: 10.4103/jfmpc.jfmpc_817_21. Epub 2022 Jan 31.

Clinical experience of tocilizumab treatment among a cohort of patients with COVID-19 infection from Western India

Prince D Surana [1], Rupesh Nayak [1], Arif Sheikh [1], Pradnya Haldankar [2], Jyoti Kale [2]
Affiliations Expand

Affiliations

- [1] Department of Internal Medicine and Critical Care, Surana Sethia Hospital, Suman Nagar Sion Trombay Road Chembur, Mumbai, India.
- [2] Department of Anesthesia and Critical Care, MPCT Hospital, Sector - 4, Sanpada, Navi Mumbai, Maharashtra, India.

- PMID: **35309657**
- PMCID: PMC8930122
- DOI: 10.4103/jfmpc.jfmpc_817_21

Free PMC article

Clinical experience of tocilizumab treatment among a cohort of patients with COVID-19 infection from Western India

Prince D Surana et al. J Family Med Prim Care. 2022 Jan.
Free PMC article
Show details
J Family Med Prim Care

. 2022 Jan;11(1):123-132.
doi: 10.4103/jfmpc.jfmpc_817_21. Epub 2022 Jan 31.

Authors

Prince D Surana [1], Rupesh Nayak [1], Arif Sheikh [1], Pradnya Haldankar [2], Jyoti Kale [2]

Affiliations

- [1] Department of Internal Medicine and Critical Care, Surana Sethia Hospital, Suman Nagar Sion Trombay Road Chembur, Mumbai, India.
- [2] Department of Anesthesia and Critical Care, MPCT Hospital, Sector - 4, Sanpada, Navi Mumbai, Maharashtra, India.

- PMID: **35309657**
- PMCID: PMC8930122
- DOI: 10.4103/jfmpc.jfmpc_817_21

Abstract

Background: Initiation of tocilizumab (TCZ) treatment in patients with coronavirus disease 2019 (COVID-19) during the early phases of cytokine storm is crucial. This study evaluated the clinical experience of TCZ use in the treatment of patients with COVID-19.

Methods: This retrospective observational study included patients (>18 years) with confirmed COVID19 treated with TCZ alone/in combination with other drugs. Data related to demographics, clinical characteristics, radiological parameters, oxygen/ventilator/vasopressor support, treatment parameters, laboratory investigations pre- and post-TCZ treatment, and clinical outcomes were retrieved from medical records.

Results: Out of 95 patients (mean age, 55 years), 68.4% and 31.6% of patients had moderate and severe COVID-19 disease, respectively. The mean time to TCZ administration from symptom onset was 8.7 days. At the time of admission, the mean oxygen saturation (SpO_2) was 90.4% and mean concentration of fraction of inspired oxygen (FiO_2) was 80.6%. The most commonly received dose of TCZ was 400 mg (84.2%) intravenously. The mean concentration of FiO_2 and SpO_2 improved significantly during the treatment ($P < 0.001$) compared to before TCZ initiation. The change in median levels of C-reactive protein (CRP) from baseline to post-treatment (63.0 vs. 4.5 mg/dL; $P < 0.001$) was significant. Post TCZ treatment, 73.6% of patients improved; whereas 26.4% of patients died. Acute respiratory distress syndrome (23.2%) and elevated transaminases (12.6%) were the most commonly reported adverse events.

Conclusion: Tocilizumab administration during earlier phase of cytokine storm syndrome leads to reversal of abnormal SpO_2 and FiO_2 concentrations to normal levels and rapid decline of elevated CRP levels in patients with COVID-19.

Keywords: Cytokine storm syndrome; TCZ treatment; inflammatory markers; moderate and severe; oxygen saturation.

Copyright: © 2022 Journal of Family Medicine and Primary Care.

Conflict of interest statement

There are no conflicts of interest.

- 16 references
- 3 figures

Full text links

Free PMC article
Proceed to details
Cite
Share
☐ 1,348
JAC Antimicrob Resist
. 2021 Nov 17;3(4):dlab174.
doi: 10.1093/jacamr/dlab174. eCollection 2021 Dec.

Cefiderocol treatment for carbapenem-resistant *Acinetobacter baumannii* infection in the ICU during the COVID-19 pandemic: a multicentre cohort study

Renato Pascale [1], Zeno Pasquini [1], Michele Bartoletti [1], Luca Caiazzo [2], Giacomo Fornaro [1], Linda Bussini [1], Francesca Volpato [1], Elisa Marchionni [1], Matteo Rinaldi [1], Filippo Trapani [1], Chiara Temperoni [2], Paolo Gaibani [3], Simone Ambretti [3], Francesco Barchiesi [2] [4], Pierluigi Viale [1], Maddalena Giannella [1]
Affiliations Expand

Affiliations

- [1] Infectious Diseases Unit, Department of Medical and Surgical Sciences, Policlinico Sant'Orsola, Bologna, Italy.
- [2] Infectious Disease Unit, Azienda Ospedaliera Ospedali Riuniti Marche Nord, Pesaro, Italy.
- [3] Operative Unit of Microbiology, University of Bologna, Policlinico Sant'Orsola, Bologna, Italy.
- [4] Department of Scienze Biomediche e Sanità Pubblica, Università Politecnica delle Marche, Ancona, Italy.

- PMID: **34806011**
- PMCID: PMC8599913
- DOI: 10.1093/jacamr/dlab174

Free PMC article

Cefiderocol treatment for carbapenem-resistant *Acinetobacter baumannii* infection in the ICU during the COVID-19 pandemic: a multicentre cohort study

Renato Pascale et al. JAC Antimicrob Resist. 2021.
Free PMC article
Show details
JAC Antimicrob Resist
. 2021 Nov 17;3(4):dlab174.
doi: 10.1093/jacamr/dlab174. eCollection 2021 Dec.

Authors

Renato Pascale [1], Zeno Pasquini [1], Michele Bartoletti [1], Luca Caiazzo [2], Giacomo Fornaro [1], Linda Bussini [1], Francesca Volpato [1], Elisa Marchionni [1], Matteo Rinaldi [1], Filippo Trapani [1], Chiara Temperoni [2], Paolo Gaibani [3], Simone Ambretti [3], Francesco Barchiesi [2] [4], Pierluigi Viale [1], Maddalena Giannella [1]

Affiliations

- [1] Infectious Diseases Unit, Department of Medical and Surgical Sciences, Policlinico Sant'Orsola, Bologna, Italy.
- [2] Infectious Disease Unit, Azienda Ospedaliera Ospedali Riuniti Marche Nord, Pesaro, Italy.
- [3] Operative Unit of Microbiology, University of Bologna, Policlinico Sant'Orsola, Bologna, Italy.
- [4] Department of Scienze Biomediche e Sanità Pubblica, Università Politecnica delle Marche, Ancona, Italy.

- PMID: **34806011**
- PMCID: PMC8599913
- DOI: 10.1093/jacamr/dlab174

Abstract

Objectives: To analyse the impact of cefiderocol use on outcome in patients admitted to the ICU for severe COVID-19 and further diagnosed with carbapenem-resistant *Acinetobacter baumannii* (CR-Ab) infection.

Methods: Retrospective multicentre observational study was performed at four Italian hospitals, from January 2020 to April 2021. Adult patients admitted to ICU for severe COVID-19 and further diagnosed with CR-Ab infections were enrolled. Patients treated with cefiderocol, as compassionate use, for at least 72 h were compared with those receiving alternative regimens. Primary endpoint was all-cause 28 day mortality. The impact of cefiderocol on mortality was evaluated by multivariable Cox regression model.

Results: In total, 107 patients were enrolled (76% male, median age 65 years). The median time from ICU admission to CR-Ab infection diagnosis was 14 (IQR 8-20) days, and the main types of CR-Ab infections were bloodstream infection (58%) and lower respiratory tract infection (41%). Cefiderocol was administered to 42 patients within a median of 2 (IQR 1-4) days after CR-Ab infection diagnosis and as monotherapy in all cases. The remaining patients received colistin, mostly (82%) administered as combination therapy. All-cause 28 day mortality rate was 57%, without differences between groups (cefiderocol 55% versus colistin 58% $P = 0.70$). In multivariable analysis, the independent risk factor for mortality was SOFA score (HR 1.24, 95% CI 1.15-1.38, $P < 0.001$). Cefiderocol was associated with a non-significant lower mortality risk (HR 0.64, 95% CI 0.38-1.08, $P = 0.10$).

Conclusions: Our study confirms the potential role of cefiderocol in the treatment of CR-Ab infection, but larger clinical studies are needed.

© The Author(s) 2021. Published by Oxford University Press on behalf of the British Society for Antimicrobial Chemotherapy.

- 24 references
- 2 figures

Full text links

Free PMC article
Proceed to details
Cite
Share
☐ 1,349
Bone Jt Open
. 2021 Feb;2(2):86-92.
doi: 10.1302/2633-1462.22.BJO-2020-0152.R1.

Trampolines injuries are bouncing back

Yahya Ibrahim [1], Sumon Huq [1], Kanatheepan Shanmuganathan [1], Helen Gille [1], Pranai Buddhdev [1]
Affiliations Expand

Affiliation

- [1] Orthopaedic Department, Broomfield Hospital, Mid Essex Hospital NHS Trust, Chelmsford, UK.

- PMID: **33573399**
- PMCID: PMC7925211
- DOI: 10.1302/2633-1462.22.BJO-2020-0152.R1

Free PMC article

Trampolines injuries are bouncing back

Yahya Ibrahim et al. Bone Jt Open. 2021 Feb.
Free PMC article
Show details
Bone Jt Open
. 2021 Feb;2(2):86-92.
doi: 10.1302/2633-1462.22.BJO-2020-0152.R1.

Authors

Yahya Ibrahim [1], Sumon Huq [1], Kanatheepan Shanmuganathan [1], Helen Gille [1], Pranai Buddhdev [1]

Affiliation

- [1] Orthopaedic Department, Broomfield Hospital, Mid Essex Hospital NHS Trust, Chelmsford, UK.

- PMID: **33573399**
- PMCID: PMC7925211
- DOI: 10.1302/2633-1462.22.BJO-2020-0152.R1

Abstract

Aims: This observational study examines the effect of the COVID-19 pandemic upon the paediatric trauma burden of a district general hospital. We aim to compare the nature and volume of the paediatric trauma during the first 2020 UK lockdown period with the same period in 2019.

Methods: Prospective data was collected from 23 March 2020 to 14 June 2020 and compared with retrospective data collected from 23 March 2019 to 14 June 2019. Patient demographics, mechanism of injury, nature of the injury, and details of any surgery were tabulated and statistically analyzed using the independent-samples *t*-test for normally distributed data and the Mann-Whitney-U test for non-parametric data. Additionally, patients were contacted by telephone to further explore the mechanism of injury where required, to gain some qualitative insight into the risk factors for injury.

Results: The 2020 lockdown resulted in 30% fewer paediatric trauma presentations (441 vs 306), but no significant change in the number of patients requiring surgery (47 vs 51; $p = 0.686$). Trampolining injuries increased in absolute numbers by 168% ($p < 0.001$), almost four times more common when considered as percentage of all injuries observed in 2020 vs 2019. There was a decrease in high energy trauma from road traffic accidents and falls from height (21.5% decrease, $p < 0.001$). Despite a shift towards more conservative treatment options, trampolining injuries continued to require surgery in similar proportions (19.4 vs 20%; $p = 0.708$). Qualitative investigation revealed that the most common risk factor for trampolining injury was concurrent usage, especially with an older child.

Conclusion: COVID-19 lockdown has resulted in a decrease in paediatric orthopaedic presentations and high energy trauma. However, due to a marked increase in home trampolining injuries, and their unchanged requirement for surgery, there has been no change in the requirement

for surgery during the lockdown period. As home exercise becomes more prevalent, a duty of public health falls upon clinicians to advise parents against trampoline usage. Cite this article: *Bone Jt Open* 2021;2(2):86-92.

Keywords: COVID; COVID-19; Coronavirus; Effect; Lockdown; Paediatric; Pandemic; Trampoline; Trauma.

- 42 references
- 2 figures

Full text links

Bone & Joint Open Full Text Available Atypon Free PMC article

Proceed to details

Cite

Share

☐ 1,350

Saudi Pharm J

. 2022 Jan 29.

doi: 10.1016/j.jsps.2022.01.021. Online ahead of print.

Incidence and risk factors of adverse drug reactions in patients with coronavirus disease 2019: A pharmacovigilance experience utilizing an ADR trigger tool

Bashayer Alshehail [1], Zainab Al Jamea [1], Royes Chacko [2], Fawaz Alotaibi [2], Nadia Ismail [1], Dhafer Alshayban [2]

Affiliations Expand

Affiliations

- [1] Pharmaceutical Care Department, King Fahd Hospital of The University, Khobar, Saudi Arabia.
- [2] Pharmacy Practice Department, College of Clinical Pharmacy, Imam Abdulrahman bin Faisal University, Dammam, Saudi Arabia.

- PMID: **35125905**
- PMCID: PMC8800535
- DOI: 10.1016/j.jsps.2022.01.021

Free PMC article

Incidence and risk factors of adverse drug reactions in patients with coronavirus disease 2019: A pharmacovigilance experience utilizing an ADR trigger tool

Bashayer Alshehail et al. Saudi Pharm J. 2022.
Free PMC article
Show details
Saudi Pharm J
. 2022 Jan 29.
doi: 10.1016/j.jsps.2022.01.021. Online ahead of print.

Authors

Bashayer Alshehail [1], Zainab Al Jamea [1], Royes Chacko [2], Fawaz Alotaibi [2], Nadia Ismail [1], Dhafer Alshayban [2]

Affiliations

- [1] Pharmaceutical Care Department, King Fahd Hospital of The University, Khobar, Saudi Arabia.
- [2] Pharmacy Practice Department, College of Clinical Pharmacy, Imam Abdulrahman bin Faisal University, Dammam, Saudi Arabia.

- PMID: **35125905**
- PMCID: PMC8800535
- DOI: 10.1016/j.jsps.2022.01.021

Abstract

Background: Since the World Health Organization declared coronavirus disease (COVID-19) as a pandemic, most countries started treating their patients with various therapies. However, the data regarding their safety and effectiveness is still lacking.

Objectives: We aimed to evaluate the adverse drug reactions (ADRs) incidence and their predisposing factors among COVID-19 patients.

Methods: A retrospective observational study that was conducted at a tertiary academic hospital from March - June 2020. Patients were included if they were ≥ 18 years old, inpatient, had a reverse transcriptase-polymerase chain reaction (PCR) positive for COVID-19, and were treated with; (lopinavir-ritonavir, hydroxychloroquine, chloroquine, favipiravir, ribavirin, or interferon-ß) either as monotherapy or combination therapy for three days or longer. The data of eligible patients were retrieved from the electronic medical records. A standardized data collection form was designed to collect patient demographics, COVID-19 severity based on the Saudi Ministry of Health management protocols, antiviral therapies, duration of therapy, and length of stay (LOS). The ADRs were identified via conducting a comprehensive review using predefined triggers and were evaluated using Naranjo Score.

Results: A total of 155 patients were included of which 123 (79.4%) were males. In our sample, the incidence proportion of ADRs per patient was 72.3%. A total of 287 ADRs were identified most of them were hepatic (n = **101**, 35.2%), gastrointestinal (n = **59**, 20.6%), hematological (n = **47**, 16%), and endocrine (n = **45**, 15%). Hydroxychloroquine was the most common drug associated with ADRs (n = **155**). The length of stay (10 - 20 days) was the only statistically significant with the ADR incidence (p-value = 0.008; 95 %CI 1.216:3.568).

Conclusions: The ADRs are prevalent among COVID-19 patients, which assure the importance of implementing active hospital-based pharmacovigilance systems.

Keywords: Adverse drug events; Adverse drug reactions; Coronavirus disease 2019; Infectious diseases; Pharmacoepidemiology; Pharmacovigilance.

© 2022 The Author(s).

Conflict of interest statement

The authors declare that they have no known competing financial interests or personal relationships that could have appeared to influence the work reported in this paper.

- 34 references
- 1 figure

Full text links

ELSEVIER OPEN ACCESS Elsevier Science Free PMC article

Proceed to details

Cite

Share

☐ 1,351

Front Med (Lausanne)

. 2020 Oct 23;7:572989.

doi: 10.3389/fmed.2020.572989. eCollection 2020.

Coagulopathy as a Prodrome of Cytokine Storm in COVID-19-Infected Patients

Hui Guo [1], Ying Sheng [2], Wei Li [3], Fei Li [4], Zongyu Xie [5], Jing Li [1], Yuhe Zhu [6], Jian Geng [6], Gang Liu [7], LeJian Wang [8], Jing Li [9], Fengchao Wang [1]

Affiliations Expand

Affiliations

- [1] Department of Laboratory Medicine, The First Affiliated Hospital of Bengbu Medical College, Bengbu, China.
- [2] School of Nursing, Indiana University, Indianapolis, IN, United States.

- [3] Department of Respiratory Disease, The First Affiliated Hospital of Bengbu Medical College, Bengbu, China.
- [4] Department of Laboratory Medicine, Taizhou Central Hospital (Taizhou University Hospital), Taizhou, China.
- [5] Department of Radiology, The First Affiliated Hospital of Bengbu Medical College, Bengbu, China.
- [6] Department of Laboratory Medicine, Bengbu Medical College, Bengbu, China.
- [7] Department of Thoracic Surgery, The Second Affiliated Hospital of Bengbu Medical College, Bengbu, China.
- [8] Department of Laboratory Medicine, Zhejiang University of Traditional Chinese Medicine Affiliated XinHua Hospital, Hangzhou, China.
- [9] Department of Surgery, University of Michigan School of Medicine, Ann Arbor, MI, United States.

- PMID: **33195321**
- PMCID: PMC7645068
- DOI: 10.3389/fmed.2020.572989

Free PMC article

Coagulopathy as a Prodrome of Cytokine Storm in COVID-19-Infected Patients

Hui Guo et al. Front Med (Lausanne). 2020.
Free PMC article
Show details
Front Med (Lausanne)
. 2020 Oct 23;7:572989.
doi: 10.3389/fmed.2020.572989. eCollection 2020.

Authors

Hui Guo [1], Ying Sheng [2], Wei Li [3], Fei Li [4], Zongyu Xie [5], Jing Li [1], Yuhe Zhu [6], Jian Geng [6], Gang Liu [7], LeJian Wang [8], Jing Li [9], Fengchao Wang [1]

Affiliations

- [1] Department of Laboratory Medicine, The First Affiliated Hospital of Bengbu Medical College, Bengbu, China.
- [2] School of Nursing, Indiana University, Indianapolis, IN, United States.
- [3] Department of Respiratory Disease, The First Affiliated Hospital of Bengbu Medical College, Bengbu, China.
- [4] Department of Laboratory Medicine, Taizhou Central Hospital (Taizhou University Hospital), Taizhou, China.
- [5] Department of Radiology, The First Affiliated Hospital of Bengbu Medical College, Bengbu, China.
- [6] Department of Laboratory Medicine, Bengbu Medical College, Bengbu, China.

- [7] Department of Thoracic Surgery, The Second Affiliated Hospital of Bengbu Medical College, Bengbu, China.
- [8] Department of Laboratory Medicine, Zhejiang University of Traditional Chinese Medicine Affiliated XinHua Hospital, Hangzhou, China.
- [9] Department of Surgery, University of Michigan School of Medicine, Ann Arbor, MI, United States.

- PMID: **33195321**
- PMCID: PMC7645068
- DOI: 10.3389/fmed.2020.572989

Abstract

Background: The rapid coronavirus disease 2019 (COVID-19) pandemic has hit hard on the world and causes panic since the virus causes serious infectious respiratory illness and easily leads to severe conditions such as immune system overactivation or cytokine storm. Due to the limited knowledge on the course of infection of this coronavirus and the lack of an effective treatment for this fatal disease, mortality remains high. The emergence of a cytokine storm in patients with a severe condition has been reported as the top reason of the death of patients with COVID-19 infection. However, the causative mechanism of cytokine storm remains elusive. Thus, we aim to observe the association of coagulopathy (D-dimer) with cytokine (i.e., IL-6) and CT imaging in COVID-19-infected patients. **Methods:** In this retrospective observational study, we systematically analyzed the comprehensive clinical laboratory data of COVID-19-positive patients in different illness groups of mild, moderate, and severe conditions according to the Chinese Clinical Guidance for COVID-19 Pneumonia Diagnosis and Treatment (7th edition). *T* tests and chi-square tests were used for two-group comparisons. One-way ANOVA was used for three-group comparisons. Pearson and Spearman correlation coefficients of the D-dimer level with IL-6 and CT imaging were computed at baseline. With regular liquid biopsy approach, D-dimer, IL-6, and neutrophil-to-lymphocyte ratio were recorded repeatedly with a time curve to investigate disease progression, along with CT imaging, and other indicators. **Results:** All the 64 patients were clinically evaluated and classified into three groups of mild (32 cases), moderate (23 cases), and severe (nine cases) conditions. The D-dimer level positively correlated with IL-6 ($R = 0.5$) at baseline when the COVID-19-infected patients were admitted. In addition, we observed that D-dimer rises earlier than the cytokine storm represented by IL-6 surge, which suggests that coagulopathy might act as a trigger to potentiate a cytokine storm. **Conclusion:** Integrated analysis revealed a positive correlation of coagulopathy with cytokine storm in COVID-19-infected patients; the D-dimer rises early, which indicates that coagulopathy acts as a prodrome of cytokine storm. Coagulopathy can be used to monitor early cytokine storm in COVID-19-infected patients.

Keywords: COVID-19; IL-6; coagulopathy; cytokine storm; d-dimer; prodrome.

Copyright © 2020 Guo, Sheng, Li, Li, Xie, Li, Zhu, Geng, Liu, Wang, Li and Wang.

- 23 references
- 3 figures

Supplementary info

Grant support Expand

Grant support

- T32 CA117865/CA/NCI NIH HHS/United States

Full text links

Frontiers Media SA Free PMC article

Proceed to details

Cite

Share

1,352

Anaesthesia
. 2022 Mar 3.
doi: 10.1111/anae.15700. Online ahead of print.

Cardiovascular subphenotypes in patients with COVID-19 pneumonitis whose lungs are mechanically ventilated: a single-centre retrospective observational study

M Chotalia [1], M Ali [2], J E Alderman [2], J M Patel [2], D Parekh [2], M N Bangash [2]
Affiliations Expand

Affiliations

- [1] Department of Anaesthesia and Critical Care Medicine, Queen Elizabeth Hospital, Birmingham, UK.
- [2] Department of Anaesthesia and Critical Care Medicine, Queen Elizabeth Hospital, UK.

- PMID: **35243617**
- DOI: 10.1111/anae.15700

Cardiovascular subphenotypes in patients with COVID-19 pneumonitis whose lungs are mechanically ventilated: a single-centre retrospective observational study

M Chotalia et al. Anaesthesia. 2022.

Show details

Anaesthesia
. 2022 Mar 3.

doi: 10.1111/anae.15700. Online ahead of print.

Authors

M Chotalia [1], M Ali [2], J E Alderman [2], J M Patel [2], D Parekh [2], M N Bangash [2]

Affiliations

- [1] Department of Anaesthesia and Critical Care Medicine, Queen Elizabeth Hospital, Birmingham, UK.
- [2] Department of Anaesthesia and Critical Care Medicine, Queen Elizabeth Hospital, UK.

- PMID: **35243617**
- DOI: 10.1111/anae.15700

Abstract

Unsupervised clustering methods of transthoracic echocardiography variables have not been used to characterise circulatory failure mechanisms in patients with COVID-19 pneumonitis. We conducted a retrospective, single-centre cohort study in ICU patients with COVID-19 pneumonitis whose lungs were mechanically ventilated and who underwent transthoracic echocardiography between March 2020 and May 2021. We performed latent class analysis of echocardiographic and haemodynamic variables. We characterised the identified subphenotypes by comparing their clinical parameters, treatment responses and 90-day mortality rates. We included 305 patients with a median (IQR [range]) age 59 (49-66 [16-83]) y. Of these, 219 (72%) were male, 199 (65%) had moderate acute respiratory distress syndrome and 113 (37%) did not survive more than 90 days. Latent class analysis identified three cardiovascular subphenotypes: class 1 (52%; normal right ventricular function); class 2 (31%; right ventricular dilation with mostly preserved systolic function); and class 3 (17%; right ventricular dilation with systolic impairment). The three subphenotypes differed in their clinical characteristics and response to prone ventilation and outcomes, with 90-day mortality rates of 22%, 42% and 73%, respectively ($p < 0.001$). We conclude that the identified subphenotypes aligned with right ventricular pathophysiology rather than the accepted definitions of right ventricular dysfunction, and these identified classifications were associated with clinical outcomes.

Keywords: acute respiratory distress syndrome; right ventricular dysfunction; right ventricular failure; transthoracic echocardiography.

© 2022 The Authors. Anaesthesia published by John Wiley & Sons Ltd on behalf of Association of Anaesthetists.

- 36 references

Full text links

WILEY Full Text Article Wiley
Proceed to details
Cite
Share
☐ 1,353

Clin Ophthalmol
. 2020 Aug 24;14:2473-2480.
doi: 10.2147/OPTH.S269179. eCollection 2020.

Clinical Experience in the Administration of Intravitreal Injection Therapy at a Tertiary University Hospital in Jordan During the COVID-19 Lockdown

Omar A Saleh [1], Hisham Jammal [1], Noor Alqudah [1], Asem Alqudah [1], Nakhleh Abu-Yaghi [2]
Affiliations Expand

Affiliations

- [1] Department of Ophthalmology, Jordan University of Science and Technology, Irbid, Jordan.
- [2] Department of Special Surgery/Ophthalmology Division, School of Medicine, The University of Jordan, Amman, Jordan.

- PMID: **32943831**
- PMCID: PMC7468368
- DOI: 10.2147/OPTH.S269179

Free PMC article

Clinical Experience in the Administration of Intravitreal Injection Therapy at a Tertiary University Hospital in Jordan During the COVID-19 Lockdown

Omar A Saleh et al. Clin Ophthalmol. 2020.
Free PMC article
Show details

Clin Ophthalmol
. 2020 Aug 24;14:2473-2480.
doi: 10.2147/OPTH.S269179. eCollection 2020.

Authors

Omar A Saleh [1], Hisham Jammal [1], Noor Alqudah [1], Asem Alqudah [1], Nakhleh Abu-Yaghi [2]

Affiliations

- [1] Department of Ophthalmology, Jordan University of Science and Technology, Irbid, Jordan.
- [2] Department of Special Surgery/Ophthalmology Division, School of Medicine, The University of Jordan, Amman, Jordan.

- PMID: **32943831**
- PMCID: PMC7468368
- DOI: 10.2147/OPTH.S269179

Abstract

Purpose: To describe the clinical experience with the delivery of intravitreal injection therapy to patients with various indications at a tertiary university hospital during the COVID-19 lockdown in Jordan.

Methods: This is a retrospective observational study of patients who received intravitreal injections between April 12th and May 9th, 2020, a period during the national COVID-19 lockdown (March 16th to June 6th, 2020). Special medical and logistic arrangements, priority and visual risk assessment and strict infection control precautions were implemented. Demographics, diagnosis, intravitreal injection history, medical history, ophthalmic examinations and optical coherence tomography data were collected and analyzed.

Results: Intravitreal injections were successfully administered to 132 patients with diabetic retinopathy, age-related macular degeneration and retinal vein occlusion. All logistic and transmission control measures were followed by the medical staff and patients with no incidents. No new exposures or COVID-19 positive cases were traced to our location or time of therapy. No complications related to the injections were recorded. The mean period of delay due to the lockdown from the original scheduled appointment was six weeks. Mean visual acuity significantly decreased from 20/55 before the lockdown to 20/70 after the lockdown, and mean central macular thickness significantly increased from 329 to 370 μ.

Conclusion: The administration of intravitreal injection therapy during the COVID-19 lockdown under special safety precautions was feasible and successful. Resumption of the essential therapies and medical services during periods of pandemic restrictions while adhering to strict transmission control measures is encouraged.

Keywords: COVID-19; age-related macular degeneration; diabetic macular edema; intravitreal injections; lockdown.

© 2020 Saleh et al.

Conflict of interest statement

The authors report no conflicts of interest for this work.

- 29 references
- 1 figure

Full text links

FREE full-text article Dovepress Dove Medical Press Free PMC article
Proceed to details
Cite
Share
1,354

Infect Drug Resist
. 2021 Oct 14;14:4217-4226.
doi: 10.2147/IDR.S335868. eCollection 2021.

Clinical Features and Factors Associated with Occult Gastrointestinal Bleeding in COVID-19 Patients

Xi Zhao [#1], Meihui Tao [#1], Chaoyue Chen [1], Ying Zhang [1], Yu Fu [1]
Affiliations Expand

Affiliation

- [1] Department of Gastroenterology, Union Hospital, Tongji Medical College, Huazhong University of Science and Technology, Wuhan, People's Republic of China.

[#] Contributed equally.

- PMID: **34703248**
- PMCID: PMC8523806
- DOI: 10.2147/IDR.S335868

Free PMC article

Clinical Features and Factors Associated with Occult Gastrointestinal Bleeding in COVID-19 Patients

Xi Zhao et al. Infect Drug Resist. 2021.
Free PMC article
Show details

Infect Drug Resist
. 2021 Oct 14;14:4217-4226.
doi: 10.2147/IDR.S335868. eCollection 2021.

Authors

Xi Zhao [#1], Meihui Tao [#1], Chaoyue Chen [1], Ying Zhang [1], Yu Fu [1]

Affiliation

- [1] Department of Gastroenterology, Union Hospital, Tongji Medical College, Huazhong University of Science and Technology, Wuhan, People's Republic of China.

[#] Contributed equally.

- PMID: **34703248**
- PMCID: PMC8523806
- DOI: 10.2147/IDR.S335868

Abstract

Background: There has been an increasing number of COVID-19 patients around the world. Since some patients developed with gastrointestinal bleeding, our study focused on the clinical features and gastroscopic findings of these patients, and factors associated with occult gastrointestinal bleeding.

Patients and methods: In this retrospective, observational study, we collected 368 COVID-19 patients who performed fecal or gastric occult blood from Wuhan Tongji Hospital, Jin Yin-tan Hospital, and Wuhan Union Hospital between February 1, 2020 and March 6, 2020. Clinical features were compared between patients with or without occult gastrointestinal bleeding, and gastroscopic findings of seven patients were described. Logistic regression analyses were performed to explore the factors associated with occult gastrointestinal bleeding.

Results: In total, 43 (11.7%) patients presented occult gastrointestinal bleeding, whereas 35 (81.4%) of severe cases. CRP level, prothrombin time and D-dimer were higher, while lymphocyte count and albumin levels were decreased in patients with occult gastrointestinal bleeding. Gastroscopy in seven COVID-19 patients showed mucosal congestion, erosion or scattered bleeding at different sites. Albumin levels (OR, 0.856 [95% CI 0.793-0.924]; $p < 0.001$), prothrombin time (OR, 1.267 [1.089-1.475]; $p = 0.002$) on admission and severe disease (OR, 4.157 [1.765-9.791]; $p = 0.001$) were independent factors associated with GIB in COVID-19 patients, while antiviral drugs and glucocorticoid therapy were not associated with it.

Conclusion: COVID-19 patients with occult gastrointestinal bleeding suffered from worse prognosis. Patients with decreased serum albumin levels or prolonged prothrombin time, and severe cases were at higher risk of occult gastrointestinal bleeding.

Keywords: COVID-19; clinical characteristics; occult gastrointestinal bleeding; related factors.

© 2021 Zhao et al.

Conflict of interest statement

None of the authors have declared any potential conflicts of interest.

- 27 references
- 1 figure

Full text links

FREE full-text article Dovepress Dove Medical Press Free PMC article
Proceed to details
Cite
Share
1,355

Lancet Reg Health Eur
. 2021 Jun;5:100081.
doi: 10.1016/j.lanepe.2021.100081. Epub 2021 Mar 18.

Pediatric admissions to emergency departments of North-Western Italy during COVID-19 pandemic: A retrospective observational study

Irene Raffaldi [1], Emanuele Castagno [1], Ilaria Fumi [2], Claudia Bondone [1], Fulvio Ricceri [3][4], Luigi Besenzon [5], Adalberto Brach Del Prever [6], Pina Capalbo [7], Gianluca Cosi [8], Enrico Felici [9], Patrizia Fusco [10], Maria Rita Gallina [11], Franco Garofalo [12], Paola Gianino [13], Andrea Guala [14], Oscar Haitink [15], Paolo Manzoni [16], Antonio Marra [17], Ivana Rabbone [18], Luca Roasio [19], Savino Santovito [20], Alberto Serra [21], Eleonora Tappi [22], Gian Maria Terragni [23], Fabio S Timeus [24], Flaminia Torielli [25], Alessandro Vigo [26], Antonio F Urbino [1], ICOPER Study Group
Affiliations Expand

Affiliations

- [1] Department of Pediatric Emergency, Regina Margherita Children's Hospital - A.O.U. Città della Salute e della Scienza di Torino, Piazza Polonia 94, 10126 Turin, Italy.
- [2] Department of Public Health and Pediatrics, University of Turin, Piazza Polonia 94, Turin, Italy.
- [3] Department of Clinical and Biological Sciences, University of Turin, Regione Gonzole 10, Orbassano, TO, Italy.
- [4] Unit of Epidemiology, Regional Health Service, ASL TO3, Via Sabaudia 164, Grugliasco, TO, Italy.
- [5] Division of Pediatrics and Neonatology, P.O. Savigliano, ASL CN1, Via Ospedali 9, Savigliano, CN, Italy.
- [6] Division of Pediatrics and Neonatology, P.O. Ciriè, ASL TO4, Via Battitore 7/9, Ciriè, TO, Italy.
- [7] Division of Pediatrics and Neonatology 3, P.O Martini, ASL Città di Torino, Via Tofane 71, Turin, Italy.
- [8] Pediatric Unit, Sant'Andrea Hospital, ASL VC, Corso Mario Abbiate 21, Vercelli, Italy.
- [9] Pediatrics and Pediatric Emergency Unit, The Children's Hospital, AO SS Antonio e Biagio e C. Arrigo, Via Spalto Marengo 46, Alessandria, Italy.
- [10] Pediatric Unit, Montis Regalis Hospital, ASL CN1, Via San Rocchetto 99, Mondovì, CN, Italy.

- [11] Division of Pediatrics and Neonatology, Beauregard Hospital, Via L. Vaccari 5, Aosta, Italy.
- [12] Pediatric Unit, P.O. Rivoli, ASL TO3, Via Rivalta 29, Rivoli, TO, Italy.
- [13] Division of Pediatrics, P.O. Cardinal Massaia, ASL AT, Corso Dante Alighieri 202, Asti, Italy.
- [14] Division of Pediatrics, Ospedale Castelli, Via Fiume 18, Pallanza, Verbania, Italy.
- [15] Pediatric Department, SS Trinità Hospital, Viale Zoppis 10, Borgomanero, NO, Italy.
- [16] Division of Pediatrics and Neonatology, Department of Maternal-Infantile Medicine, Nuovo Ospedale Degli Infermi, Via dei Ponderanesi 2, Ponderano, BI, Italy.
- [17] Pediatric Unit - NICU, S. Croce Hospital, ASLT TO5, Piazza Amedeo Ferdinando 3, Moncalieri, TO, Italy.
- [18] Division of Pediatrics, Department of Health Science, University of Piemonte Orientale, Corso G. Mazzini 18, Novara, Italy.
- [19] Pediatric Department, E. Agnelli Hospital, Via Brigata Cagliari 39, Pinerolo, TO, Italy.
- [20] Division of Pediatrics 1, Maria Vittoria Hospital, Via L. Cibrario 72, Turin, Italy.
- [21] Division of Pediatrics, Ospedale Michele e Pietro Ferrero, P.O. Alba-Bra ASL CN2, Verduno, CN, Italy.
- [22] Pediatric Unit, A.S.O. S. Croce e Carle, Via M. Coppino 26, Cuneo, Italy.
- [23] Division of Pediatrics, P.O. Chieri, ASL TO5, Via de Maria 1, Chieri, TO, Italy.
- [24] Division of Pediatrics and Neonatology, P.O. Chivasso, ASL TO4, Corso G. Ferraris 3, Chivasso, TO, Italy.
- [25] Division of Pediatrics, P.O. Novi Ligure, ASL AL, Via E. Raggio 12, Novi Ligure, AL, Italy.
- [26] Division of Pediatrics and Neonatology, P.O. Ivrea, ASL TO4, Piazza Credenza 2, Ivrea, TO, Italy.

- PMID: **34104902**
- PMCID: PMC7969147
- DOI: 10.1016/j.lanepe.2021.100081

Free PMC article

Pediatric admissions to emergency departments of North-Western Italy during COVID-19 pandemic: A retrospective observational study

Irene Raffaldi et al. Lancet Reg Health Eur. 2021 Jun.

Free PMC article

Show details

Lancet Reg Health Eur

. 2021 Jun;5:100081.
doi: 10.1016/j.lanepe.2021.100081. Epub 2021 Mar 18.

Authors

Irene Raffaldi [1], Emanuele Castagno [1], Ilaria Fumi [2], Claudia Bondone [1], Fulvio Ricceri [3] [4], Luigi Besenzon [5], Adalberto Brach Del Prever [6], Pina Capalbo [7], Gianluca Cosi [8], Enrico Felici [9], Patrizia Fusco [10], Maria Rita Gallina [11], Franco Garofalo [12], Paola Gianino [13], Andrea Guala [14], Oscar Haitink [15], Paolo Manzoni [16], Antonio Marra [17], Ivana Rabbone [18], Luca Roasio [19], Savino Santovito [20], Alberto Serra [21], Eleonora Tappi [22], Gian Maria Terragni [23], Fabio S Timeus [24], Flaminia Torielli [25], Alessandro Vigo [26], Antonio F Urbino [1], ICOPER Study Group

Affiliations

- [1] Department of Pediatric Emergency, Regina Margherita Children's Hospital - A.O.U. Città della Salute e della Scienza di Torino, Piazza Polonia 94, 10126 Turin, Italy.
- [2] Department of Public Health and Pediatrics, University of Turin, Piazza Polonia 94, Turin, Italy.
- [3] Department of Clinical and Biological Sciences, University of Turin, Regione Gonzole 10, Orbassano, TO, Italy.
- [4] Unit of Epidemiology, Regional Health Service, ASL TO3, Via Sabaudia 164, Grugliasco, TO, Italy.
- [5] Division of Pediatrics and Neonatology, P.O. Savigliano, ASL CN1, Via Ospedali 9, Savigliano, CN, Italy.
- [6] Division of Pediatrics and Neonatology, P.O. Ciriè, ASL TO4, Via Battitore 7/9, Ciriè, TO, Italy.
- [7] Division of Pediatrics and Neonatology 3, P.O Martini, ASL Città di Torino, Via Tofane 71, Turin, Italy.
- [8] Pediatric Unit, Sant'Andrea Hospital, ASL VC, Corso Mario Abbiate 21, Vercelli, Italy.
- [9] Pediatrics and Pediatric Emergency Unit, The Children's Hospital, AO SS Antonio e Biagio e C. Arrigo, Via Spalto Marengo 46, Alessandria, Italy.
- [10] Pediatric Unit, Montis Regalis Hospital, ASL CN1, Via San Rocchetto 99, Mondovì, CN, Italy.
- [11] Division of Pediatrics and Neonatology, Beauregard Hospital, Via L. Vaccari 5, Aosta, Italy.
- [12] Pediatric Unit, P.O. Rivoli, ASL TO3, Via Rivalta 29, Rivoli, TO, Italy.
- [13] Division of Pediatrics, P.O. Cardinal Massaia, ASL AT, Corso Dante Alighieri 202, Asti, Italy.
- [14] Division of Pediatrics, Ospedale Castelli, Via Fiume 18, Pallanza, Verbania, Italy.
- [15] Pediatric Department, SS Trinità Hospital, Viale Zoppis 10, Borgomanero, NO, Italy.
- [16] Division of Pediatrics and Neonatology, Department of Maternal-Infantile Medicine, Nuovo Ospedale Degli Infermi, Via dei Ponderanesi 2, Ponderano, BI, Italy.
- [17] Pediatric Unit - NICU, S. Croce Hospital, ASLT TO5, Piazza Amedeo Ferdinando 3, Moncalieri, TO, Italy.
- [18] Division of Pediatrics, Department of Health Science, University of Piemonte Orientale, Corso G. Mazzini 18, Novara, Italy.
- [19] Pediatric Department, E. Agnelli Hospital, Via Brigata Cagliari 39, Pinerolo, TO, Italy.
- [20] Division of Pediatrics 1, Maria Vittoria Hospital, Via L. Cibrario 72, Turin, Italy.
- [21] Division of Pediatrics, Ospedale Michele e Pietro Ferrero, P.O. Alba-Bra ASL CN2, Verduno, CN, Italy.

- [22] Pediatric Unit, A.S.O. S. Croce e Carle, Via M. Coppino 26, Cuneo, Italy.
- [23] Division of Pediatrics, P.O. Chieri, ASL TO5, Via de Maria 1, Chieri, TO, Italy.
- [24] Division of Pediatrics and Neonatology, P.O. Chivasso, ASL TO4, Corso G. Ferraris 3, Chivasso, TO, Italy.
- [25] Division of Pediatrics, P.O. Novi Ligure, ASL AL, Via E. Raggio 12, Novi Ligure, AL, Italy.
- [26] Division of Pediatrics and Neonatology, P.O. Ivrea, ASL TO4, Piazza Credenza 2, Ivrea, TO, Italy.

- PMID: **34104902**
- PMCID: PMC7969147
- DOI: 10.1016/j.lanepe.2021.100081

Abstract

Background: COVID-19 pandemic caused huge decrease of pediatric admissions to Emergency Department (ED), arising concerns about possible delays in diagnosis and treatment of severe disorders.

Methods: Impact of COVID-19 on Pediatric Emergency Room (ICOPER) was a retrospective multicentre observational study including 23 Italian EDs.All the children <18 years admitted, between March 9th and May 3rd 2020 stratified by age, priority code, cause of admission and outcome have been included and compared to those admitted in the same period of 2019.Our objectives were to assess the characteristics of pediatric admissions to EDs since COVID-19 outbreak until the end of lockdown, and to describe the features of critical children.

Findings: 16,426 children were admitted in 2020, compared to 55,643 in 2019 (-70·48%). Higher reduction was reported in hospitals without Pediatric Intensive Care Unit (PICU) (-73·38%) than in those with PICU (-64·08%) ($P<0·0001$). Admissions with low priority decreased more than critical ones (-82·77% vs. 44·17% respectively; $P<0·0001$). Reduction of discharged patients was observed both in hospitals with (-66·50%) and without PICU (-74·65%) ($P<0·0001$). No difference in the duration of symptoms before admission was reported between 2019 and 2020, with the majority of children accessing within 24 h (55·08% vs. 57·28% respectively; $P =$ 0·2344).

Interpretation: Admissions with low priority decreased significantly more than those with high priority; we suppose that the fear of being infected in hospital maybe overcame the concerns of caregivers. Compared to 2019, no significant referral delay by caregivers was reported. Our data suggest the need of adaptation of EDs and primary care services to different needs of children during COVID-9 pandemic.

© 2021 The Author(s).

Conflict of interest statement

Nothing to disclose.

- 28 references
- 2 figures

Full text links

ELSEVIER OPEN ACCESS Elsevier Science Free PMC article
Proceed to details
Cite
Share
1,356

J Clin Med
. 2021 Nov 28;10(23):5599.
doi: 10.3390/jcm10235599.

Pulmonary Edema in COVID-19 Treated with Furosemide and Negative Fluid Balance (NEGBAL): A Different and Promising Approach

Jose L Francisco Santos [1], Patricio Zanardi [1], Veronica Alo [1], Marcelo Rodriguez [2], Federico Magdaleno [3], Virginia De Langhe [3], Vanina Dos Santos [1], Giuliana Murialdo [1], Andrea Villoldo [1], Micaela Coria [1], Diego Quiros [4], Claudio Milicchio [4], Eduardo Garcia Saiz [4]
Affiliations Expand

Affiliations

- [1] Intensive Care Unit, Clinica Colon, Mar del Plata, Buenos Aires 7600, Argentina.
- [2] Cardiology Service, Clinica Colon, Mar del Plata, Buenos Aires 7600, Argentina.
- [3] Diagnostic Imaging Service, Hospital Privado del Sur. Bahía Blanca, Buenos Aires 8000, Argentina.
- [4] Diagnostic Imaging Service, Clinica Colon, Mar del Plata, Buenos Aires 7600, Argentina.

- PMID: **34884300**
- PMCID: PMC8658626
- DOI: 10.3390/jcm10235599

Free PMC article

Pulmonary Edema in COVID-19 Treated with Furosemide and Negative Fluid Balance (NEGBAL): A Different and Promising Approach

Jose L Francisco Santos et al. J Clin Med. 2021.
Free PMC article
Show details

J Clin Med
. 2021 Nov 28;10(23):5599.
doi: 10.3390/jcm10235599.

Authors

Jose L Francisco Santos [1], Patricio Zanardi [1], Veronica Alo [1], Marcelo Rodriguez [2], Federico Magdaleno [3], Virginia De Langhe [3], Vanina Dos Santos [1], Giuliana Murialdo [1], Andrea Villoldo [1], Micaela Coria [1], Diego Quiros [4], Claudio Milicchio [4], Eduardo Garcia Saiz [4]

Affiliations

- [1] Intensive Care Unit, Clinica Colon, Mar del Plata, Buenos Aires 7600, Argentina.
- [2] Cardiology Service, Clinica Colon, Mar del Plata, Buenos Aires 7600, Argentina.
- [3] Diagnostic Imaging Service, Hospital Privado del Sur. Bahía Blanca, Buenos Aires 8000, Argentina.
- [4] Diagnostic Imaging Service, Clinica Colon, Mar del Plata, Buenos Aires 7600, Argentina.

- PMID: **34884300**
- PMCID: PMC8658626
- DOI: 10.3390/jcm10235599

Abstract

In COVID-19, pulmonary edema has been attributed to "cytokine storm". However, it is known that SARS-CoV2 promotes angiotensin-converting enzyme 2 deficit, increases angiotensin II, and this triggers volume overload. Our report is based on COVID-19 patients with tomographic evidence of pulmonary edema and volume overload to whom established a standard treatment with diuretic (furosemide) guided by objectives: Negative Fluid Balance (NEGBAL approach). Retrospective observational study. We reviewed data from medical records: demographic, clinical, laboratory, blood gas, and chest tomography (CT) before and while undergoing NEGBAL, from 20 critically ill patients. Once the NEGBAL strategy was started, no patient required mechanical ventilation. All cases reverted to respiratory failure with NEGBAL, but subsequently two patients died from sepsis and acute myocardial infarction (AMI). The regressive analysis between PaO2/FiO2BAL and NEGBAL demonstrated correlation ($p < 0.032$). The results comparing the Pao2Fio2 between admission to NEGBAL to NEGBAL day 4, were statistically significant ($p < 0.001$). We noted between admission to NEGBAL and day 4 improvement in CT score ($p < 0.001$), decrease in the superior vena cava diameter ($p < 0.001$) and the decrease of cardiac axis ($p < 0.001$). Though our study has several limitations, we believe the promising results encourage further investigation of this different pathophysiological approach.

Keywords: COVID-19; NEGBAL; diuretic; edema; furosemide; volume overload.

Conflict of interest statement

The authors declare no conflict of interest.

- 55 references
- 2 figures

Full text links

FULL TEXT OPEN ACCESS MDPI Multidisciplinary Digital Publishing Institute (MDPI) Free PMC article
Proceed to details
Cite
Share
1,357

Evid Based Complement Alternat Med
. 2021 Oct 5;2021:4303380.
doi: 10.1155/2021/4303380. eCollection 2021.

Efficacy of Qingfei Paidu Decoction on Patients with COVID-19 Pneumonia in Wuhan, China: A Propensity Score Matching Study

Zhen Liu [1], Shan Du [2], Fei Shao [3 4], Haibin Li [5], Shuang Xu [6], Xuedi Ma [7], Zhouming Xu [7], Hao Cui [4], Changxiao Yu [4], Yang Wu [4], Feng Wang [8 9], Liyan Li [1], Rui Chen [10], Hui Qiu [11], Ziren Tang [3 4], Peng Sun [6]
Affiliations Expand

Affiliations

- [1] Department of Emergency Medicine, Beijing First Hospital of Integrated Chinese and Western Medicine, Beijing, China.
- [2] Department of Neurology, Beijing First Hospital of Integrated Chinese and Western Medicine, Beijing, China.
- [3] Department of Emergency Medicine, Beijing Chaoyang Hospital, Capital Medical University, Beijing, China.
- [4] Beijing Key Laboratory of Cardiopulmonary Cerebral Resuscitation, Beijing, China.
- [5] Department of Epidemiology and Health Statistics, School of Public Health, Capital Medical University, Beijing, China.
- [6] Department of Emergency Medicine, Union Hospital, Tongji Medical College, Huazhong University of Science and Technology, Wuhan, China.
- [7] AI Research Division, A.I. Phoenix Technology Co., Ltd., Hong Kong, China.
- [8] Department of Respiratory and Critical Care Medicine, Beijing Chaoyang Hospital, Capital Medical University, Beijing, China.
- [9] Beijing Key Laboratory of Respiratory and Pulmonary Circulation Disorders, Beijing, China.
- [10] Department of Integrated Traditional Chinese and Western Medicine, Union Hospital, Tongji Medical College, Huazhong University of Science and Technology, China.
- [11] Department of Emergency Surgery, the West Campus of Union Hospital, Tongji Medical College, Huazhong University of Science and Technology, Wuhan, China.

- PMID: **34650611**
- PMCID: PMC8510827
- DOI: 10.1155/2021/4303380

Free PMC article

Efficacy of Qingfei Paidu Decoction on Patients with COVID-19 Pneumonia in Wuhan, China: A Propensity Score Matching Study

Zhen Liu et al. Evid Based Complement Alternat Med. 2021.
Free PMC article
Show details
Evid Based Complement Alternat Med
. 2021 Oct 5;2021:4303380.
doi: 10.1155/2021/4303380. eCollection 2021.

Authors

Zhen Liu [1], Shan Du [2], Fei Shao [3, 4], Haibin Li [5], Shuang Xu [6], Xuedi Ma [7], Zhouming Xu [7], Hao Cui [4], Changxiao Yu [4], Yang Wu [4], Feng Wang [8, 9], Liyan Li [1], Rui Chen [10], Hui Qiu [11], Ziren Tang [3, 4], Peng Sun [6]

Affiliations

- [1] Department of Emergency Medicine, Beijing First Hospital of Integrated Chinese and Western Medicine, Beijing, China.
- [2] Department of Neurology, Beijing First Hospital of Integrated Chinese and Western Medicine, Beijing, China.
- [3] Department of Emergency Medicine, Beijing Chaoyang Hospital, Capital Medical University, Beijing, China.
- [4] Beijing Key Laboratory of Cardiopulmonary Cerebral Resuscitation, Beijing, China.
- [5] Department of Epidemiology and Health Statistics, School of Public Health, Capital Medical University, Beijing, China.
- [6] Department of Emergency Medicine, Union Hospital, Tongji Medical College, Huazhong University of Science and Technology, Wuhan, China.
- [7] AI Research Division, A.I. Phoenix Technology Co., Ltd., Hong Kong, China.
- [8] Department of Respiratory and Critical Care Medicine, Beijing Chaoyang Hospital, Capital Medical University, Beijing, China.
- [9] Beijing Key Laboratory of Respiratory and Pulmonary Circulation Disorders, Beijing, China.
- [10] Department of Integrated Traditional Chinese and Western Medicine, Union Hospital, Tongji Medical College, Huazhong University of Science and Technology, China.

- [11] Department of Emergency Surgery, the West Campus of Union Hospital, Tongji Medical College, Huazhong University of Science and Technology, Wuhan, China.

- PMID: **34650611**
- PMCID: PMC8510827
- DOI: 10.1155/2021/4303380

Abstract

Background: In view of the global efforts to develop effective treatments for the current worldwide coronavirus 2019 (COVID-19) pandemic, Qingfei Paidu decoction (QPD), a novel traditional Chinese medicine (TCM) prescription, was formulated as an optimized combination of constituents of classic prescriptions used to treat numerous febrile and respiratory-related diseases. This prescription has been used to treat patients with COVID-19 pneumonia in Wuhan, China. *Hypothesis/Purpose*. We hypothesized that QPD would have beneficial effects on patients with COVID-19. We aimed to prove this hypothesis by evaluating the efficacy of QPD in patients with COVID-19 pneumonia.

Methods: In this single-center, retrospective, observational study, we identified eligible participants who received a laboratory diagnosis of COVID-19 between January 15 and March 15, 2020, in the west campus of Union Hospital in Wuhan, China. QPD was supplied as an oral liquid packaged in 200-mL containers, and patients were orally administered one package twice daily 40 minutes after a meal. The primary outcome was death, which was compared between patients who did and did not receive QPD (QPD and NoQPD groups, respectively). Propensity score matching (PSM) was used to identify cohorts.

Results: In total, 239 and 522 participants were enrolled in the QPD and NoQPD groups, respectively. After PSM at a 1 : 1 ratio, 446 patients meeting the criteria were included in the analysis with 223 in each arm. In the QPD and NoQPD groups, 7 (3.2%) and 29 (13.0%) patients died, and those in the QPD group had a significantly lower risk of death (hazard ratio (HR) 0.29, 95% CI: 0.13-0.67) than those in the NoQPD group ($p = 0.004$). Furthermore, the survival time was significantly longer in the QPD group than in the NoQPD group ($p < 0.001$).

Conclusion: The use of QPD may reduce the risk of death in patients with COVID-19 pneumonia.

Copyright © 2021 Zhen Liu et al.

Conflict of interest statement

The authors declare that they have no conflicts of interest.

- 26 references
- 2 figures

Full text links

Hindawi FREE Full Text Article Hindawi Limited Free PMC article
Proceed to details
Cite
Share
☐ 1,358

J Clin Med
. 2021 Jul 26;10(15):3290.
doi: 10.3390/jcm10153290.

Differences in Inflammatory Marker Kinetics between the First and Second Wave of COVID-19 Patients Admitted to the ICU: A Retrospective, Single-Center Study

Tamas Szakmany [1, 2], William Tuckwell [2], Elsa Harte [2], Nick Wetherall [2], Saraswathi Ramachandran [2], Shannon Price [2], Henry Breen [2], Charlotte Killick [2], Yusuf Cheema [2], Charles King [2], Owen Richards [2]
Affiliations Expand

Affiliations

- [1] Critical Care Directorate, Grange University Hospital, Aneurin Bevan University Health Board, Llanyravon, Cwmbran NP44 8YN, UK.
- [2] Department of Anaesthesia, Intensive Care and Pain Medicine, Division of Population Medicine, Cardiff University, Cardiff CF14 4XN, UK.

- PMID: **34362074**
- PMCID: PMC8348515
- DOI: 10.3390/jcm10153290

Free PMC article

Differences in Inflammatory Marker Kinetics between the First and Second Wave of COVID-19 Patients Admitted to the ICU: A Retrospective, Single-Center Study

Tamas Szakmany et al. J Clin Med. 2021.
Free PMC article
Show details
J Clin Med
. 2021 Jul 26;10(15):3290.
doi: 10.3390/jcm10153290.

Authors

Tamas Szakmany [1] [2], William Tuckwell [2], Elsa Harte [2], Nick Wetherall [2], Saraswathi Ramachandran [2], Shannon Price [2], Henry Breen [2], Charlotte Killick [2], Yusuf Cheema [2], Charles King [2], Owen Richards [2]

Affiliations

- [1] Critical Care Directorate, Grange University Hospital, Aneurin Bevan University Health Board, Llanyravon, Cwmbran NP44 8YN, UK.
- [2] Department of Anaesthesia, Intensive Care and Pain Medicine, Division of Population Medicine, Cardiff University, Cardiff CF14 4XN, UK.

- PMID: **34362074**
- PMCID: PMC8348515
- DOI: 10.3390/jcm10153290

Abstract

Background: We sought to determine if there was a difference in the longitudinal inflammatory response measured by white blood cell count (WBC), C-reactive protein (CRP), procalcitonin (PCT), and ferritin levels between the first and the second COVID-19 wave of ICU patients.

Methods: In a single-center retrospective observational study, ICU patients were enrolled during the first and second waves of the COVID-19 pandemic. Data were collected on patient demographics, comorbidities, laboratory results, management strategies, and complications during the ICU stay. The inflammatory response was evaluated using WBC count, CRP, PCT, and Ferritin levels on the day of admission until Day 28, respectively. Organ dysfunction was measured by the SOFA score.

Results: 65 patients were admitted during the first and 113 patients during the second wave. WBC and ferritin levels were higher in the second wave. CRP and PCT showed markedly different longitudinal kinetics up until day 28 of ICU stay between the first and second wave, with significantly lower levels in the second wave. Steroid and immunomodulatory therapy use was significantly greater in the second wave. Mortality was similar in both waves.

Conclusions: We found that there was a significantly reduced inflammatory response in the second wave, which is likely to be attributable to the more widespread use of immunomodulatory therapies.

Keywords: C-reactive protein; COVID-19; corticosteroid; immunomodulation; procalcitonin.

Conflict of interest statement

The authors declare no conflict of interest.

- 29 references
- 4 figures

Full text links

FULL TEXT OPEN ACCESS MDPI Multidisciplinary Digital Publishing Institute (MDPI) Free PMC article
Proceed to details

Cite

Share

☐ 1,359

Cureus

. 2020 Dec 12;12(12):e12039.
doi: 10.7759/cureus.12039.

Clinical Characteristics of 47 Death Cases With COVID-19: A Retrospective Study at a Tertiary Center in Lahore

Ahmad Ussaid [1], Babar Riaz [1], Wajid Rafai [1], Sohail Anwar [2], Faisal Baig [1], Khurram Saleem [1], Farwa Pervaiz [3], Zaima Firdous [1], Shumaila A Nasir [1], Farrukh Iqbal [1]

Affiliations Expand

Affiliations

- [1] Internal Medicine, University College of Medicine and Dentistry, University of Lahore Teaching Hospital, Lahore, PAK.
- [2] Pulmonology, University College of Medicine and Dentistry, University of Lahore Teaching Hospital, Lahore, PAK.
- [3] Internal Medicine, Chaudhary Muhammad Akram Teaching and Research Hospital, Lahore, PAK.

- PMID: **33457138**
- PMCID: PMC7797435
- DOI: 10.7759/cureus.12039

Free PMC article

Clinical Characteristics of 47 Death Cases With COVID-19: A Retrospective Study at a Tertiary Center in Lahore

Ahmad Ussaid et al. Cureus. 2020.
Free PMC article

Show details

Cureus

. 2020 Dec 12;12(12):e12039.
doi: 10.7759/cureus.12039.

Authors

Ahmad Ussaid [1], Babar Riaz [1], Wajid Rafai [1], Sohail Anwar [2], Faisal Baig [1], Khurram Saleem [1], Farwa Pervaiz [3], Zaima Firdous [1], Shumaila A Nasir [1], Farrukh Iqbal [1]

Affiliations

- [1] Internal Medicine, University College of Medicine and Dentistry, University of Lahore Teaching Hospital, Lahore, PAK.
- [2] Pulmonology, University College of Medicine and Dentistry, University of Lahore Teaching Hospital, Lahore, PAK.
- [3] Internal Medicine, Chaudhary Muhammad Akram Teaching and Research Hospital, Lahore, PAK.

- PMID: **33457138**
- PMCID: PMC7797435
- DOI: 10.7759/cureus.12039

Abstract

Introduction Coronavirus disease 2019 (COVID-19) presents with a wide spectrum of symptoms, ranging from patients being asymptomatic to having life-threatening acute respiratory distress syndrome (ARDS). COVID-19 emerged as a pandemic and has led to multiple causalities worldwide. A better understanding of the clinical characteristics of the COVID-19 patients and their disease course will aid in better management of these patients and hence may positively impact their outcomes as well. Methodology This was a retrospective observational study conducted from April 15, 2020, to August 31, 2020, after gaining institutional review board approval at the University of Lahore Teaching Hospital, Lahore, Pakistan. A total of 47 patients with severe disease who had died due to COVID-19 during this period were enrolled by the consecutive method. Patients were evaluated for their epidemiological, biochemical, clinical, and radiological features. The modified Radiographic Assessment of Lung Edema (mRALE) score was used to calculate the extent of alveolar opacities and percentages of lung involvement in chest radiographs. Furthermore, patients' management plans were also evaluated. Data were analyzed using SPSS Statistics version 23 (IBM, Armonk, NY). Results The mean age of the patients was 61.53 ±13.35 years. The male-to-female ratio was 2:1, and the mean BMI was 28.05 ±3.52 kg/m^2. Diabetes was the most prevalent comorbidity among the patients (32, 68.1%), followed by hypertension (six, 12.8%), ischemic heart disease (five, 10.6%), and chronic kidney disease (four, 8.5%) respectively. The predominant symptom observed among patients was cough (95%), followed by shortness of breath (93%), fever (63%), sputum (23%), and gastrointestinal symptoms (6.4%). The mean D-dimer was 1,567.13 ±1,903.77 ng/mL, mean ferritin was 1,730.34 ±1,382.35 ng/mL, mean C-reactive protein (CRP) was 202.59 ±104.97 mg/dl, and the mean neutrophil-to-lymphocyte ratio was 10.50 ±9.58. Bilateral lung involvement was seen among 40 (85.11%) patients whereas unilateral right lung involvement was reported in three (6.38%) and unilateral left lung involvement in four (8.51%) respectively. The mean mRALE score for bilateral lung involvement was 18.78 ±4.89. The mean area radiologically involved in bilateral lung fields was 72.12 ±18.45%, followed by unilateral right lung involvement of 67.87 ±15.97%, and unilateral left lung involvement of 61.38 ±17.95% in the cohort respectively. The most common type of radiological pathology was diffuse ground-glass opacities, which was observed in 18 (38%) patients. Most patients received antibiotics (39, 63.83%), while nine (19%) received tocilizumab, four (8.5%) had antiviral therapy, and three (6.4%) were given plasma treatment. All patients received glucocorticoids and anticoagulation. The most common cause of death was ARDS, which was observed in 12 (25.5%) patients. Conclusion This study significantly demonstrated that most cases were males above 50 years of age with chronic medical comorbidities of diabetes,

hypertension, and ischemic heart disease. COVID-19 has a predilection for multisystem involvement leading to mortality. In addition, elevated D-dimer and neutrophil-to-lymphocyte ratio may be indicative of a poor prognosis. A combination of antimicrobials had no positive impact on the outcomes in this cohort. It is difficult to predict the efficacy of tocilizumab and remdesivir as only a few patients in the cohort received these drugs.

Keywords: ards; clinical features; covid-19; d-dimer; mortality; predictors.

Copyright © 2020, Ussaid et al.

Conflict of interest statement

The authors have declared that no competing interests exist.

- 23 references
- 3 figures

Full text links

Free PMC article
Proceed to details
Cite
Share
☐ 1,360
Front Med (Lausanne)
. 2021 Dec 17;8:762740.
doi: 10.3389/fmed.2021.762740. eCollection 2021.

Sedation, Analgesia, and Muscle Relaxation During VV-ECMO Therapy in Patients With Severe Acute Respiratory Syndrome Coronavirus Type 2 (SARS-CoV-2): A Single-Center, Retrospective, Observational Study

Fang Wu [1], Mingna Li [1], Zhongwei Zhang [1], Jiawei Shang [1], Yong Guo [1], Yingchuan Li [2]
Affiliations Expand

Affiliations

- [1] Department of Critical Care Medicine, Shanghai Jiao Tong University Affiliated Sixth People's Hospital, Shanghai, China.
- [2] Department of Critical Care Medicine, Tongji University Affiliated Shanghai Tenth People's Hospital, Shanghai, China.

- PMID: **34977069**
- PMCID: PMC8718548

- DOI: 10.3389/fmed.2021.762740

Free PMC article

Sedation, Analgesia, and Muscle Relaxation During VV-ECMO Therapy in Patients With Severe Acute Respiratory Syndrome Coronavirus Type 2 (SARS-CoV-2): A Single-Center, Retrospective, Observational Study

Fang Wu et al. Front Med (Lausanne). 2021.
Free PMC article
Show details
Front Med (Lausanne)
. 2021 Dec 17;8:762740.
doi: 10.3389/fmed.2021.762740. eCollection 2021.

Authors

Fang Wu [1], Mingna Li [1], Zhongwei Zhang [1], Jiawei Shang [1], Yong Guo [1], Yingchuan Li [2]

Affiliations

- [1] Department of Critical Care Medicine, Shanghai Jiao Tong University Affiliated Sixth People's Hospital, Shanghai, China.
- [2] Department of Critical Care Medicine, Tongji University Affiliated Shanghai Tenth People's Hospital, Shanghai, China.

- PMID: **34977069**
- PMCID: PMC8718548
- DOI: 10.3389/fmed.2021.762740

Abstract

Objective: The pharmacokinetics and pharmacodynamics of ECMO-supported sedative, analgesic, and muscle relaxants have changed, but there are insufficient data to determine the optimal dosing strategies for these agents. Sedation, analgesia and muscle relaxation therapy for patients with severe acute respiratory syndrome coronavirus type 2 (SARS-CoV-2) receiving ECMO support are more specific and have not been fully reported. This study observed and evaluated the use of sedative and analgesic drugs and muscle relaxants in SARS-CoV-2 patients treated with VV-ECMO. **Methods:** This study was a single-center, retrospective and observational study. Our study includes 8 SARS-CoV-2 patients treated with VV-ECMO in an intensive care unit at Shanghai Public Health Center from February to June 2020. We collected the demographic data from these patients and the dose and course of sedation, analgesia, and muscle relaxants administered during ECMO treatment. **Results:** The doses of sedative, analgesic and muscle relaxant drugs used in patients with VV-ECMO were significant. Over time, the doses of

drugs that were used were increased, and the course of muscle relaxant treatment was extended. **Conclusion:** Sedation, analgesia, and muscle relaxant use require individualized titration in patients with SARS-CoV-2 who have respiratory failure and who are receiving VV-ECMO.

Keywords: SARS-CoV-2; VV-ECMO; analgesia; muscle relaxant; sedation.

Copyright © 2021 Wu, Li, Zhang, Shang, Guo and Li.

Conflict of interest statement

The authors declare that the research was conducted in the absence of any commercial or financial relationships that could be construed as a potential conflict of interest.

- 19 references
- 1 figure

Full text links

Frontiers Media SA Free PMC article

Proceed to details

Cite

Share

1,361

Int J Nephrol Renovasc Dis
. 2021 Feb 17;14:41-51.
doi: 10.2147/IJNRD.S287455. eCollection 2021.

Clinical Characteristics and Short-Term Outcomes of Chronic Dialysis Patients Admitted for COVID-19 in Metro Manila, Philippines

Isabelle Dominique Tomacruz [1], Paolo Nikolai So [1], Renz Michael Pasilan [1], Jameel Kristine Camenforte [1], Maria Isabel Duavit [1]
Affiliations Expand

Affiliation

- [1] Division of Nephrology, Department of Medicine, Philippine General Hospital, Manila, Metro Manila, Philippines.

- PMID: **33628044**
- PMCID: PMC7898205
- DOI: 10.2147/IJNRD.S287455

Free PMC article

Clinical Characteristics and Short-Term Outcomes of Chronic Dialysis Patients Admitted for COVID-19 in Metro Manila, Philippines

Isabelle Dominique Tomacruz et al. Int J Nephrol Renovasc Dis. 2021.
Free PMC article
Show details
Int J Nephrol Renovasc Dis
. 2021 Feb 17;14:41-51.
doi: 10.2147/IJNRD.S287455. eCollection 2021.

Authors

Isabelle Dominique Tomacruz [1], Paolo Nikolai So [1], Renz Michael Pasilan [1], Jameel Kristine Camenforte [1], Maria Isabel Duavit [1]

Affiliation

- [1] Division of Nephrology, Department of Medicine, Philippine General Hospital, Manila, Metro Manila, Philippines.

- PMID: **33628044**
- PMCID: PMC7898205
- DOI: 10.2147/IJNRD.S287455

Abstract

Aim: Data published on COVID-19 in the Filipino population, particularly those with end stage kidney disease (ESKD) are still lacking.

Methods: We performed a retrospective, observational study of 68 ESKD patients admitted with COVID-19 infection at a tertiary hospital in Metro Manila, Philippines from April 1, 2020 to July 31, 2020. We compared the clinical features, baseline laboratory data, treatment strategies and short-term outcomes between those who survived and those who died. We also determined the risk factors associated with mortality from COVID-19.

Results: Mean age was 54.5 years old, 66% were male. All patients admitted were on maintenance hemodialysis (HD). The most common presenting symptoms were dyspnea (57%), fever (47%) and cough (38%). There was an equal number of patients on high flow nasal cannula (17.7%) and invasive mechanical ventilation (17.7%). ICU admission was required in 17.7% of the cohort. In-hospital death occurred in 25% of the patients. Admission PaO_2/FiO_2 (PF) ratios (162 ± 134 versus 356 ± 181; p=0.0009) were lower, and procalcitonin (6.07 ± 10.5ng/mL versus 0.73 ± 3.61 ng/mL; p=0.02), lactate dehydrogenase (396 ± 274U/L versus 282 ± 148 U/L; p=0.03), and white blood cell counts (10 ± 7.3 x 10^9/L versus 6.3 ± 4.2 x 10^9/L; p= 0.0039) were significantly higher among those who died compared to those who survived. After adjusting for

confounders, only low PF ratio (HR 1.01 for every unit decrease, 95% CI 1-1.01) and need for ventilation (HR 6.45, 95% CI 1.16-35.97) conferred a significant risk for in-hospital mortality.

Conclusion: Short-term, in-hospital mortality is high among patients on chronic hemodialysis admitted for COVID-19 infection. They present similarly with the general population. Low PF ratio on admission and need for ventilation are independent risk factors for in-hospital mortality.

Keywords: COVID-19; coronavirus; dialysis; hemodialysis; kidney failure.

© 2021 Tomacruz et al.

Conflict of interest statement

The authors report no conflicts of interest for this work.

- 26 references
- 3 figures

Full text links

FREE full-text article Dovepress Dove Medical Press Free PMC article

Proceed to details

Cite

Share

☐ 1,362

J Clin Med

. 2021 Oct 8;10(19):4610.

doi: 10.3390/jcm10194610.

Evolution of the Use of Corticosteroids for the Treatment of Hospitalised COVID-19 Patients in Spain between March and November 2020: SEMI-COVID National Registry

David Balaz [1] [2], Philip Erick Wikman-Jorgensen [1] [2], Vicente Giner Galvañ [1] [2] [3], Manuel Rubio-Rivas [4], Borja de Miguel Campo [5], Mariam Noureddine López [6], Juan Francisco López Caleya [7], Ricardo Gómez Huelgas [8], Paula María Pesqueira Fontán [9], Manuel Méndez Bailón [10], Mar Fernández-Garcés [11], Ana Fernández Cruz [12], Gema María García García [13], Nicolás Rhyman [14], Luis Corral-Gudino [15], Aquiles Lozano Rodríguez-Mancheño [16], María Navarro De La Chica [17], Andrea Torregrosa García [18], José Nicolás Alcalá [19], Pablo Díaz Jiménez [20], Leticia Esther Royo Trallero [21], Pere Comas Casanova [22], Jesús Millán Núñez-Cortés [23], José-Manuel Casas-Rojo [24], On Behalf Of The Semi-Covid-Network

Affiliations Expand

Affiliations

- [1] Department of Internal Medicine, Hospital Universitario San Juan de Alicante, 03550 Alicante, Spain.
- [2] Department of Clinical Medicine, Faculty of Medicine, Miguel Hernández University, 03202 Alicante, Spain.
- [3] Fundación para el Fomento de la Investigación Sanitaria y Biomédica de la Comunitat Valenciana (FISABIO), Conselleria de Sanitat, 46010 Valencia, Spain.
- [4] Department of Internal Medicine, Hospital Universitario de Bellvitge, 08907 Barcelona, Spain.
- [5] Department of Internal Medicine, Hospital Universitario 12 de Octubre, 28041 Madrid, Spain.
- [6] Department of Internal Medicine, Hospital Costa del Sol, 29603 Málaga, Spain.
- [7] Department of Internal Medicine, Hospital de Cabueñes, 33394 Gijón, Asturias, Spain.
- [8] Department of Internal Medicine, Hospital Regional Universitario de Málaga, 29010 Málaga, Spain.
- [9] Department of Internal Medicine, Hospital Clínico de Santiago de Compostela, 15706 A Coruña, Spain.
- [10] Department of Internal Medicine, Hospital Clínico San Carlos, 28040 Madrid, Spain.
- [11] Department of Internal Medicine, Hospital Universitario Dr. Peset, 46017 Valencia, Spain.
- [12] Department of Internal Medicine, Hospital Universitario Puerta de Hierro-Majadahonda, 28222 Madrid, Spain.
- [13] Department of Internal Medicine, Complejo Hospitalario Universitario de Badajoz, 06010 Badajoz, Spain.
- [14] Department of Internal Medicine, Hospital Moisès Broggi, Sant Joan Despí, 08970 Barcelona, Spain.
- [15] Department of Internal Medicine, Hospital Universitario Río Hortega, 47012 Valladolid, Spain.
- [16] Department of Internal Medicine, Hospital Alto Guadalquivir, 23740 Jaén, Spain.
- [17] Department of Internal Medicine, Hospital Nuestra Señora del Prado, 45600 Toledo, Spain.
- [18] Department of Internal Medicine, Hospital General Universitario de Elda, 03600 Alicante, Spain.
- [19] Department of Internal Medicine, Hospital de Pozoblanco, 14400 Córdoba, Spain.
- [20] Department of Internal Medicine, Hospital Universitario Virgen del Rocío, 41013 Sevilla, Spain.
- [21] Department of Internal Medicine, Hospital General Defensa, 50009 Zaragoza, Spain.
- [22] Department of Internal Medicine, Hospital Comarcal de Blanes, 17300 Girona, Spain.
- [23] Department of Internal Medicine, Hospital Universitario Gregorio Marañón, 28007 Madrid, Spain.
- [24] Department of Internal Medicine, Hospital Infanta Cristina University Hospital, 28981 Madrid, Spain.

- PMID: **34640628**
- PMCID: PMC8509849
- DOI: 10.3390/jcm10194610

Free PMC article

Evolution of the Use of Corticosteroids for the Treatment of Hospitalised COVID-19 Patients in Spain between March and November 2020: SEMI-COVID National Registry

David Balaz et al. J Clin Med. 2021.
Free PMC article
Show details
J Clin Med
. 2021 Oct 8;10(19):4610.
doi: 10.3390/jcm10194610.

Authors

David Balaz [1, 2], Philip Erick Wikman-Jorgensen [1, 2], Vicente Giner Galvañ [1, 2, 3], Manuel Rubio-Rivas [4], Borja de Miguel Campo [5], Mariam Noureddine López [6], Juan Francisco López Caleya [7], Ricardo Gómez Huelgas [8], Paula María Pesqueira Fontán [9], Manuel Méndez Bailón [10], Mar Fernández-Garcés [11], Ana Fernández Cruz [12], Gema María García García [13], Nicolás Rhyman [14], Luis Corral-Gudino [15], Aquiles Lozano Rodríguez-Mancheño [16], María Navarro De La Chica [17], Andrea Torregrosa García [18], José Nicolás Alcalá [19], Pablo Díaz Jiménez [20], Leticia Esther Royo Trallero [21], Pere Comas Casanova [22], Jesús Millán Núñez-Cortés [23], José-Manuel Casas-Rojo [24], On Behalf Of The Semi-Covid-Network

Affiliations

- [1] Department of Internal Medicine, Hospital Universitario San Juan de Alicante, 03550 Alicante, Spain.
- [2] Department of Clinical Medicine, Faculty of Medicine, Miguel Hernández University, 03202 Alicante, Spain.
- [3] Fundación para el Fomento de la Investigación Sanitaria y Biomédica de la Comunitat Valenciana (FISABIO), Conselleria de Sanitat, 46010 Valencia, Spain.
- [4] Department of Internal Medicine, Hospital Universitario de Bellvitge, 08907 Barcelona, Spain.
- [5] Department of Internal Medicine, Hospital Universitario 12 de Octubre, 28041 Madrid, Spain.
- [6] Department of Internal Medicine, Hospital Costa del Sol, 29603 Málaga, Spain.
- [7] Department of Internal Medicine, Hospital de Cabueñes, 33394 Gijón, Asturias, Spain.
- [8] Department of Internal Medicine, Hospital Regional Universitario de Málaga, 29010 Málaga, Spain.
- [9] Department of Internal Medicine, Hospital Clínico de Santiago de Compostela, 15706 A Coruña, Spain.
- [10] Department of Internal Medicine, Hospital Clínico San Carlos, 28040 Madrid, Spain.
- [11] Department of Internal Medicine, Hospital Universitario Dr. Peset, 46017 Valencia, Spain.

- [12] Department of Internal Medicine, Hospital Universitario Puerta de Hierro-Majadahonda, 28222 Madrid, Spain.
- [13] Department of Internal Medicine, Complejo Hospitalario Universitario de Badajoz, 06010 Badajoz, Spain.
- [14] Department of Internal Medicine, Hospital Moisès Broggi, Sant Joan Despí, 08970 Barcelona, Spain.
- [15] Department of Internal Medicine, Hospital Universitario Río Hortega, 47012 Valladolid, Spain.
- [16] Department of Internal Medicine, Hospital Alto Guadalquivir, 23740 Jaén, Spain.
- [17] Department of Internal Medicine, Hospital Nuestra Señora del Prado, 45600 Toledo, Spain.
- [18] Department of Internal Medicine, Hospital General Universitario de Elda, 03600 Alicante, Spain.
- [19] Department of Internal Medicine, Hospital de Pozoblanco, 14400 Córdoba, Spain.
- [20] Department of Internal Medicine, Hospital Universitario Virgen del Rocío, 41013 Sevilla, Spain.
- [21] Department of Internal Medicine, Hospital General Defensa, 50009 Zaragoza, Spain.
- [22] Department of Internal Medicine, Hospital Comarcal de Blanes, 17300 Girona, Spain.
- [23] Department of Internal Medicine, Hospital Universitario Gregorio Marañón, 28007 Madrid, Spain.
- [24] Department of Internal Medicine, Hospital Infanta Cristina University Hospital, 28981 Madrid, Spain.

- PMID: **34640628**
- PMCID: PMC8509849
- DOI: 10.3390/jcm10194610

Abstract

Objectives: Since the results of the RECOVERY trial, WHO recommendations about the use of corticosteroids (CTs) in COVID-19 have changed. The aim of the study is to analyse the evolutive use of CTs in Spain during the pandemic to assess the potential influence of new recommendations.

Material and methods: A retrospective, descriptive, and observational study was conducted on adults hospitalised due to COVID-19 in Spain who were included in the SEMI-COVID-19 Registry from March to November 2020.

Results: CTs were used in 6053 (36.21%) of the included patients. The patients were older (mean (SD)) (69.6 (14.6) vs. 66.0 (16.8) years; $p < 0.001$), with hypertension (57.0% vs. 47.7%; $p < 0.001$), obesity (26.4% vs. 19.3%; $p < 0.0001$), and multimorbidity prevalence (20.6% vs. 16.1%; $p < 0.001$). These patients had higher values (mean (95% CI)) of C-reactive protein (CRP) (86 (32.7-160) vs. 49.3 (16-109) mg/dL; $p < 0.001$), ferritin (791 (393-1534) vs. 470 (236-996) µg/dL; $p < 0.001$), D dimer (750 (430-1400) vs. 617 (345-1180) µg/dL; $p < 0.001$), and lower $Sp0_2/Fi0_2$ (266 (91.1) vs. 301 (101); $p < 0.001$). Since June 2020, there was an increment in the use of CTs (March vs. September; $p < 0.001$). Overall, 20% did not receive steroids, and 40% received less than 200 mg accumulated prednisone equivalent dose (APED). Severe patients are treated with higher doses. The mortality benefit was observed in patients with oxygen saturation </=90%.

Conclusions: Patients with greater comorbidity, severity, and inflammatory markers were those treated with CTs. In severe patients, there is a trend towards the use of higher doses. The mortality benefit was observed in patients with oxygen saturation </=90%.

Keywords: COVID-19; Spain; comorbidities; corticosteroids.

Conflict of interest statement

The authors declare no conflict of interest.

- 29 references
- 4 figures

Full text links

FULL TEXT OPEN ACCESS MDPI Multidisciplinary Digital Publishing Institute (MDPI) Free PMC article

Proceed to details

Cite

Share

☐ 1,363

J Clin Med

. 2021 Jun 6;10(11):2513.
doi: 10.3390/jcm10112513.

Usefulness of Respiratory Mechanics and Laboratory Parameter Trends as Markers of Early Treatment Success in Mechanically Ventilated Severe Coronavirus Disease: A Single-Center Pilot Study

Daisuke Kasugai [1], Masayuki Ozaki [1], Kazuki Nishida [2], Hiroaki Hiraiwa [1], Naruhiro Jingushi [1], Atsushi Numaguchi [1], Norihito Omote [3], Yuichiro Shindo [3], Yukari Goto [1]
Affiliations Expand

Affiliations

- [1] Department of Emergency and Critical Care Medicine, Nagoya University Graduate School of Medicine, Tsurumai-cho 65, Syowa-ku, Nagoya, Aichi 466-8550, Japan.
- [2] Department of Biostatistics Section, Center for Advanced Medicine and Clinical Research, Nagoya University Graduate School of Medicine, 65, Tsurumaicho, Showa, Nagoya, Aichi 466-8550, Japan.
- [3] Department of Respiratory Medicine, Nagoya University Graduate School of Medicine, Tsurumai-cho 65, Syowa-ku, Nagoya, Aichi 466-8550, Japan.

- PMID: **34204119**
- PMCID: PMC8201161
- DOI: 10.3390/jcm10112513

Free PMC article

Usefulness of Respiratory Mechanics and Laboratory Parameter Trends as Markers of Early Treatment Success in Mechanically Ventilated Severe Coronavirus Disease: A Single-Center Pilot Study

Daisuke Kasugai et al. J Clin Med. 2021.
Free PMC article
Show details
J Clin Med
. 2021 Jun 6;10(11):2513.
doi: 10.3390/jcm10112513.

Authors

Daisuke Kasugai [1], Masayuki Ozaki [1], Kazuki Nishida [2], Hiroaki Hiraiwa [1], Naruhiro Jingushi [1], Atsushi Numaguchi [1], Norihito Omote [3], Yuichiro Shindo [3], Yukari Goto [1]

Affiliations

- [1] Department of Emergency and Critical Care Medicine, Nagoya University Graduate School of Medicine, Tsurumai-cho 65, Syowa-ku, Nagoya, Aichi 466-8550, Japan.
- [2] Department of Biostatistics Section, Center for Advanced Medicine and Clinical Research, Nagoya University Graduate School of Medicine, 65, Tsurumaicho, Showa, Nagoya, Aichi 466-8550, Japan.
- [3] Department of Respiratory Medicine, Nagoya University Graduate School of Medicine, Tsurumai-cho 65, Syowa-ku, Nagoya, Aichi 466-8550, Japan.

- PMID: **34204119**
- PMCID: PMC8201161
- DOI: 10.3390/jcm10112513

Abstract

Whether a patient with severe coronavirus disease (COVID-19) will be successfully liberated from mechanical ventilation (MV) early is important in the COVID-19 pandemic. This study aimed to characterize the time course of parameters and outcomes of severe COVID-19 in relation to the timing of liberation from MV. This retrospective, single-center, observational study was performed using data from mechanically ventilated COVID-19 patients admitted to the ICU

between 1 March 2020 and 15 December 2020. Early liberation from ventilation (EL group) was defined as successful extubation within 10 days of MV. The trends of respiratory mechanics and laboratory data were visualized and compared between the EL and prolonged MV (PMV) groups using smoothing spline and linear mixed effect models. Of 52 admitted patients, 31 mechanically ventilated COVID-19 patients were included (EL group, 20 (69%); PMV group, 11 (31%)). The patients' median age was 71 years. While in-hospital mortality was low (6%), activities of daily living (ADL) at the time of hospital discharge were significantly impaired in the PMV group compared to the EL group (mean Barthel index (range): 30 (7.5-95) versus 2.5 (0-22.5), p = 0.048). The trends in respiratory compliance were different between patients in the EL and PMV groups. An increasing trend in the ventilatory ratio during MV until approximately 2 weeks was observed in both groups. The interaction between daily change and earlier liberation was significant in the trajectory of the thrombin-antithrombin complex, antithrombin 3, fibrinogen, C-reactive protein, lymphocyte, and positive end-expiratory pressure (PEEP) values. The indicator of physiological dead space increases during MV. The trajectory of markers of the hypercoagulation status, inflammation, and PEEP were significantly different depending on the timing of liberation from MV. These findings may provide insight into the pathophysiology of COVID-19 during treatment in the critical care setting.

Keywords: COVID-19; mechanical ventilation; respiratory failure.

Conflict of interest statement

The authors declare no conflict of interests.

- 36 references
- 4 figures

Full text links

Multidisciplinary Digital Publishing Institute (MDPI) Free PMC article
Proceed to details

Cite

Share

☐ 1,364

JMIR Form Res
. 2021 Aug 31;5(8):e21817.
doi: 10.2196/21817.

Online Search Trends Influencing Anticoagulation in Patients With COVID-19: Observational Study

Amy P Worrall [#, 1], Claire Kelly [#, 2], Aine O'Neill [1], Murray O'Doherty [1], Eoin Kelleher [3], Anne Marie Cushen [4], Cora McNally [1], Samuel McConkey [1, 5], Siobhan Glavey [2], Michelle Lavin [2, 6], Eoghan de Barra [1, 5]
Affiliations Expand

Affiliations

- [1] Department of Infectious Diseases, Beaumont Hospital, Dublin, Ireland.
- [2] Department of Haematology, Beaumont Hospital, Dublin, Ireland.
- [3] Department of Anaesthesiology, Beaumont Hospital, Dublin, Ireland.
- [4] Department of Pharmacy, Beaumont Hospital, Dublin, Ireland.
- [5] Department of International Health and Tropical Medicine, Royal College of Surgeons Ireland, Dublin, Ireland.
- [6] Irish Centre for Vascular Biology, School of Pharmacy & Biomedical Sciences, Royal College of Surgeons Ireland, Dublin, Ireland.

[#] Contributed equally.

- PMID: **34292865**
- PMCID: PMC8409499
- DOI: 10.2196/21817

Free PMC article

Online Search Trends Influencing Anticoagulation in Patients With COVID-19: Observational Study

Amy P Worrall et al. JMIR Form Res. 2021.
Free PMC article
Show details
JMIR Form Res

. 2021 Aug 31;5(8):e21817.
doi: 10.2196/21817.

Authors

Amy P Worrall [#] [1], Claire Kelly [#] [2], Aine O'Neill [1], Murray O'Doherty [1], Eoin Kelleher [3], Anne Marie Cushen [4], Cora McNally [1], Samuel McConkey [1] [5], Siobhan Glavey [2], Michelle Lavin [2] [6], Eoghan de Barra [1] [5]

Affiliations

- [1] Department of Infectious Diseases, Beaumont Hospital, Dublin, Ireland.
- [2] Department of Haematology, Beaumont Hospital, Dublin, Ireland.
- [3] Department of Anaesthesiology, Beaumont Hospital, Dublin, Ireland.
- [4] Department of Pharmacy, Beaumont Hospital, Dublin, Ireland.
- [5] Department of International Health and Tropical Medicine, Royal College of Surgeons Ireland, Dublin, Ireland.
- [6] Irish Centre for Vascular Biology, School of Pharmacy & Biomedical Sciences, Royal College of Surgeons Ireland, Dublin, Ireland.

# Contributed equally.

- PMID: **34292865**
- PMCID: PMC8409499
- DOI: 10.2196/21817

Abstract

Background: Early evidence of COVID-19-associated coagulopathy disseminated rapidly online during the first months of 2020, followed by clinical debate about how best to manage thrombotic risks in these patients. The rapid online spread of case reports was followed by online interim guidelines, discussions, and worldwide online searches for further information. The impact of global online search trends and online discussion on local approaches to coagulopathy in patients with COVID-19 has not been studied.

Objective: The goal of this study was to investigate the relationship between online search trends using Google Trends and the rate of appropriate venous thromboembolism (VTE) prophylaxis and anticoagulation therapy in a cohort of patients with COVID-19 admitted to a tertiary hospital in Ireland.

Methods: A retrospective audit of anticoagulation therapy and VTE prophylaxis among patients with COVID-19 who were admitted to a tertiary hospital was conducted between February 29 and May 31, 2020. Worldwide Google search trends of the term "COVID-19" and anticoagulation synonyms during this time period were determined and correlated against one another using a Spearman correlation. A P value of <.05 was considered significant, and analysis was completed using Prism, version 8 (GraphPad).

Results: A statistically significant Spearman correlation (P<.001, r=0.71) was found between the two data sets, showing an increase in VTE prophylaxis in patients with COVID-19 with increasing online searches worldwide. This represents a proxy for online searches and discussion, dissemination of information, and Google search trends relating to COVID-19 and clotting risk, in particular, which correlated with an increasing trend of providing thromboprophylaxis and anticoagulation therapy to patients with COVID-19 in our tertiary center.

Conclusions: We described a correlation of local change in clinical practice with worldwide online dialogue and digital search trends that influenced individual clinicians, prior to the publication of formal guidelines or a local quality-improvement intervention.

Keywords: COVID-19; anticoagulation; coronavirus; health information dissemination; online influence; online search engines; thrombosis.

©Amy P Worrall, Claire Kelly, Aine O'Neill, Murray O'Doherty, Eoin Kelleher, Anne Marie Cushen, Cora McNally, Samuel McConkey, Siobhan Glavey, Michelle Lavin, Eoghan de Barra. Originally published in JMIR Formative Research (https://formative.jmir.org), 31.08.2021.

Conflict of interest statement

Conflicts of Interest: None declared.

- 13 references
- 2 figures

Full text links

JMIR Publications Free PMC article

Proceed to details

Cite

Share

1,365

BJPsych Open

. 2020 Oct 12;6(6):e117.

doi: 10.1192/bjo.2020.104.

Patterns of use of secondary mental health services before and during COVID-19 lockdown: observational study

Samuel Tromans [1], Verity Chester [2], Hannah Harrison [3], Precina Pankhania [3], Hanna Booth [4], Nandini Chakraborty [5]

Affiliations Expand

Affiliations

- [1] Speciality Registrar in the Psychiatry of Intellectual Disability at the Agnes Unit, Leicestershire Partnership NHS Trust; and an Honorary Academic Clinical Lecturer in the Department of Health Sciences, University of Leicester, UK.
- [2] Department of Psychiatry, St John's House, Norfolk, UK; and a PhD Student at Norwich Medical School, Norwich, UK.
- [3] Clinical Studies Officer in the Department of Research and Development, Leicestershire Partnership NHS Trust, UK.
- [4] Speciality Doctor in the Psychosis Intervention and Early Recovery team, Leicestershire Partnership NHS Trust, UK.
- [5] Psychiatrist in the Psychosis Intervention and Early Recovery team, Leicestershire Partnership NHS Trust, UK.

- PMID: **33040771**
- PMCID: PMC7550872
- DOI: 10.1192/bjo.2020.104

Free PMC article

Patterns of use of secondary mental health services before and during COVID-19 lockdown: observational study

Samuel Tromans et al. BJPsych Open. 2020.
Free PMC article
Show details
BJPsych Open
. 2020 Oct 12;6(6):e117.
doi: 10.1192/bjo.2020.104.

Authors

Samuel Tromans [1], Verity Chester [2], Hannah Harrison [3], Precina Pankhania [3], Hanna Booth [4], Nandini Chakraborty [5]

Affiliations

- [1] Speciality Registrar in the Psychiatry of Intellectual Disability at the Agnes Unit, Leicestershire Partnership NHS Trust; and an Honorary Academic Clinical Lecturer in the Department of Health Sciences, University of Leicester, UK.
- [2] Department of Psychiatry, St John's House, Norfolk, UK; and a PhD Student at Norwich Medical School, Norwich, UK.
- [3] Clinical Studies Officer in the Department of Research and Development, Leicestershire Partnership NHS Trust, UK.
- [4] Speciality Doctor in the Psychosis Intervention and Early Recovery team, Leicestershire Partnership NHS Trust, UK.
- [5] Psychiatrist in the Psychosis Intervention and Early Recovery team, Leicestershire Partnership NHS Trust, UK.

- PMID: **33040771**
- PMCID: PMC7550872
- DOI: 10.1192/bjo.2020.104

Abstract

Background: The coronavirus disease 2019 (COVID-19) pandemic has had a profound impact on both the physical and mental well-being of the global population. Relatively few studies have measured the impact of lockdown on utilisation of secondary mental health services in England.

Aims: To describe secondary mental health service utilisation pre-lockdown and during lockdown within Leicestershire, UK, and the numbers of serious incidents during this time frame.

Method: Data pertaining to mental health referral and hospital admissions to adult mental health, child and adolescent mental health, intellectual disability and mental health services for older people were collated retrospectively from electronic records for both 8 weeks pre-lockdown and the first 8 weeks of lockdown in England. Serious incidents during this time frame were also analysed.

Results: Significantly ($P < 0.05$) reduced referrals to a diverse range of mental health services were observed during lockdown, including child and adolescent, adult, older people and intellectual disability services. Although admissions remained relatively stable before and during lockdown for several services, admissions to both acute adult and mental health services for older people were significantly ($P < 0.05$) reduced during lockdown. Numbers of serious incidents in the

pre-lockdown and lockdown periods were similar, with 23 incidents pre-lockdown, compared with 20 incidents in lockdown.

Conclusions: To the best of our knowledge, this is the first UK-based study reporting patterns of use of mental health services immediately prior to and during COVID-19 lockdown. Overall numbers of referrals and admissions reduced following commencement of COVID-19 lockdown. Potential reasons for these observations are discussed.

Keywords: COVID-19; Epidemiology; coronavirus; inpatient treatment; outpatient treatment.

Conflict of interest statement

None.

ICMJE forms are in the supplementary material, available online at http://doi.org/10.1192/bjo.2020.104.

- 30 references
- 2 figures

Full text links

Cambridge University Press Free PMC article

Proceed to details

Cite

Share

1,366

Crit Care Explor

. 2022 Feb 18;10(2):e0638.

doi: 10.1097/CCE.0000000000000638. eCollection 2022 Feb.

Hospital Variation in Management and Outcomes of Acute Respiratory Distress Syndrome Due to COVID-19

Shelsey W Johnson [1], Michael A Garcia [1], Emily K Q Sisson [2], Christopher R Sheldrick [2], Vishakha K Kumar [3], Karen Boman [3], Scott Bolesta [4], Vikas Bansal [5], Amos Lal [6], J P Domecq [7], Roman R Melamed [8], Amy B Christie [9], Abdurrahman Husain [10], Santiago Yus [11], Ognjen Gajic [6], Rahul Kashyap [5], Allan J Walkey [1] [12]

Affiliations Expand

Affiliations

- [1] The Pulmonary Center, Division of Pulmonary, Allergy, Sleep and Critical Care, Department of Medicine, Boston University School of Medicine, Boston, MA.

- [2] Biostatistics and Epidemiology Data Analytics Center, Boston University School of Public Health, Boston, MA.
- [3] Society of Critical Care Medicine, Mount Prospect, IL.
- [4] Department of Pharmacy Practice, Nesbitt School of Pharmacy, Wilkes University, Wilkes-Barre, PA.
- [5] Department of Anesthesia and Perioperative Medicine; Mayo Clinic, Rochester, MN.
- [6] Division of Pulmonary and Critical Care Medicine, Department of Medicine, Mayo Clinic, Rochester, MN.
- [7] Division of Nephrology and Hypertension, Department of Medicine, Mayo Clinic, Rochester, MN.
- [8] Critical Care Medicine, Abbott Northwestern Hospital, Allina Health, Minneapolis, MN.
- [9] Department of Critical Care, Atrium Health Navicent, Macon, Georgia.
- [10] Division of Pulmonary and Critical Care Medicine, UC San Diego Medical Center-Hillcrest, San Diego, CA.
- [11] Intensive Care Department, Hospital Universitario La Paz, Madrid, Spain.
- [12] Evans Center of Implementation and Improvement Sciences, Department of Medicine, Boston University School of Medicine, Boston, MA.

- PMID: **35211681**
- PMCID: PMC8860338
- DOI: 10.1097/CCE.0000000000000638

Free PMC article

Hospital Variation in Management and Outcomes of Acute Respiratory Distress Syndrome Due to COVID-19

Shelsey W Johnson et al. Crit Care Explor. 2022.
Free PMC article

Show details

Crit Care Explor
. 2022 Feb 18;10(2):e0638.
doi: 10.1097/CCE.0000000000000638. eCollection 2022 Feb.

Authors

Shelsey W Johnson [1], Michael A Garcia [1], Emily K Q Sisson [2], Christopher R Sheldrick [2], Vishakha K Kumar [3], Karen Boman [3], Scott Bolesta [4], Vikas Bansal [5], Amos Lal [6], J P Domecq [7], Roman R Melamed [8], Amy B Christie [9], Abdurrahman Husain [10], Santiago Yus [11], Ognjen Gajic [6], Rahul Kashyap [5], Allan J Walkey [1] [12]

Affiliations

- [1] The Pulmonary Center, Division of Pulmonary, Allergy, Sleep and Critical Care, Department of Medicine, Boston University School of Medicine, Boston, MA.

- [2] Biostatistics and Epidemiology Data Analytics Center, Boston University School of Public Health, Boston, MA.
- [3] Society of Critical Care Medicine, Mount Prospect, IL.
- [4] Department of Pharmacy Practice, Nesbitt School of Pharmacy, Wilkes University, Wilkes-Barre, PA.
- [5] Department of Anesthesia and Perioperative Medicine; Mayo Clinic, Rochester, MN.
- [6] Division of Pulmonary and Critical Care Medicine, Department of Medicine, Mayo Clinic, Rochester, MN.
- [7] Division of Nephrology and Hypertension, Department of Medicine, Mayo Clinic, Rochester, MN.
- [8] Critical Care Medicine, Abbott Northwestern Hospital, Allina Health, Minneapolis, MN.
- [9] Department of Critical Care, Atrium Health Navicent, Macon, Georgia.
- [10] Division of Pulmonary and Critical Care Medicine, UC San Diego Medical Center-Hillcrest, San Diego, CA.
- [11] Intensive Care Department, Hospital Universitario La Paz, Madrid, Spain.
- [12] Evans Center of Implementation and Improvement Sciences, Department of Medicine, Boston University School of Medicine, Boston, MA.

- PMID: **35211681**
- PMCID: PMC8860338
- DOI: 10.1097/CCE.0000000000000638

Abstract

Objectives: To describe hospital variation in use of "guideline-based care" for acute respiratory distress syndrome (ARDS) due to COVID-19.

Design: Retrospective, observational study.

Setting: The Society of Critical Care Medicine's Discovery Viral Infection and **RESPIRATORY ILLNESS UNIVERSAL STUDY COVID-19 REGISTRY.**

Patients: Adult patients with ARDS due to COVID-19 between February 15, 2020, and April 12, 2021.

Interventions: Hospital-level use of "guideline-based care" for ARDS including low-tidal-volume ventilation, plateau pressure less than 30 cm H_2O, and prone ventilation for a Pao_2/Fio_2 ratio less than 100.

Measurements and main results: Among 1,495 adults with COVID-19 ARDS receiving care across 42 hospitals, 50.4% ever received care consistent with ARDS clinical practice guidelines. After adjusting for patient demographics and severity of illness, hospital characteristics, and pandemic timing, hospital of admission contributed to 14% of the risk-adjusted variation in "guideline-based care." A patient treated at a randomly selected hospital with higher use of guideline-based care had a median odds ratio of 2.0 (95% CI, 1.1-3.4) for receipt of "guideline-based care" compared with a patient receiving treatment at a randomly selected hospital with low use of recommended therapies. Median-adjusted inhospital mortality was 53% (interquartile range, 47-62%), with a nonsignificantly decreased risk of mortality for patients admitted to hospitals in the highest use "guideline-based care" quartile (49%) compared with the lowest use quartile (60%) (odds ratio, 0.7; 95% CI, 0.3-1.9; $p = 0.49$).

Conclusions: During the first year of the COVID-19 pandemic, only half of patients received "guideline-based care" for ARDS management, with wide practice variation across hospitals. Strategies that improve adherence to recommended ARDS management strategies are needed.

Keywords: COVID-19; Viral Infection and Respiratory Illness Universal Study; acute respiratory distress syndrome; low-tidal-volume ventilation; prone ventilation; severe acute respiratory syndrome coronavirus-2.

Copyright © 2022 The Authors. Published by Wolters Kluwer Health, Inc. on behalf of the Society of Critical Care Medicine.

Conflict of interest statement

Dr. Gajic receives funding from the Agency of Healthcare Research and Quality R18HS 26609-2, National Institutes of Health/National Heart, Lung and Blood Institute (NHLBI): R01HL 130881 and UG3/UH3HL 141722; Department of Defense W81XWH; American Heart Association Rapid Response Grant—COVID-19; and royalties from Ambient Clinical Analytics. Dr. Kashyap receives funding from the National Institutes of Health (NIH)/National Heart, Lung and Blood Institute: R01HL 130881, UG3/UH3HL 141722; Gordon and Betty Moore Foundation, and Janssen Research & Development, LLC; and royalties from Ambient Clinical Analytics. Dr. Walkey receives funding from the National Institutes of Health/National Heart, Lung and Blood Institute grants R01HL151607, R01HL139751, and R01HL136660, Agency of Healthcare Research and Quality, R01HS026485, Boston Biomedical Innovation Center/NIH/NHLBI 5U54HL119145-07, and royalties from UptoDate. The remaining authors have disclosed that they do not have any potential conflicts of interest.

- 29 references
- 2 figures

Supplementary info

Grant support Expand

Grant support

- R01 HL130881/HL/NHLBI NIH HHS/United States
- R18 HS026609/HS/AHRQ HHS/United States
- R01 HL136660/HL/NHLBI NIH HHS/United States
- U54 HL119145/HL/NHLBI NIH HHS/United States
- R01 HL139751/HL/NHLBI NIH HHS/United States

- R01 HS026485/HS/AHRQ HHS/United States
- R01 HL151607/HL/NHLBI NIH HHS/United States
- UL1 TR002377/TR/NCATS NIH HHS/United States

Show all 8 grants

Full text links

Free PMC article
Proceed to details
Cite

Share
1,367

Leuk Lymphoma
. 2022 Feb 13;1-10.
doi: 10.1080/10428194.2022.2034157. Online ahead of print.

COVID-19 in patients with chronic lymphocytic leukemia: a Moscow observational study

Olga L Kochneva [1], Maria Kislova [2], Evgenya I Zhelnova [1], Andrei A Petrenko [3 4], Elena A Baryakh [1 3], Konstantin V Yatskov [1], Elena A Dmitrieva [2 3], Elena N Misurina [1], Konstantin E Nikitin [4], Elena J Vasilieva [5], Inna V Samsonova [1], Vadim V Ptushkin [2 3], Ancha Baranova [6 7], Eugene A Nikitin [2 3]
Affiliations Expand

Affiliations

- [1] City Clinical Hospital No. 52, Moscow, Russia.
- [2] Botkin Hospital, Moscow, Russia.
- [3] Federal State Budgetary Educational Institution of Further Professional Education "Russian Medical Academy of Continuous Professional Education" of the Ministry of Healthcare of the Russian Federation.
- [4] Department of Radio Engineering and Cybernetics, Moscow Institute of Physics and Technology, Moscow, Russia.
- [5] Davidovsky City Clinical Hospital, Moscow, Russia.
- [6] School of Systems Biology, George Mason University, Fairfax, VA, USA.
- [7] Research Center for Medical Genetics, Moscow, Russia.

- PMID: **35156528**
- DOI: 10.1080/10428194.2022.2034157

COVID-19 in patients with chronic lymphocytic leukemia: a Moscow observational study

Olga L Kochneva et al. Leuk Lymphoma. 2022.
Show details

Leuk Lymphoma
. 2022 Feb 13;1-10.
doi: 10.1080/10428194.2022.2034157. Online ahead of print.

Authors

Olga L Kochneva [1], Maria Kislova [2], Evgenya I Zhelnova [1], Andrei A Petrenko [3] [4], Elena A Baryakh [1] [3], Konstantin V Yatskov [1], Elena A Dmitrieva [2] [3], Elena N Misurina [1], Konstantin E Nikitin [4], Elena J Vasilieva [5], Inna V Samsonova [1], Vadim V Ptushkin [2] [3], Ancha Baranova [6] [7], Eugene A Nikitin [2] [3]

Affiliations

- [1] City Clinical Hospital No. 52, Moscow, Russia.
- [2] Botkin Hospital, Moscow, Russia.
- [3] Federal State Budgetary Educational Institution of Further Professional Education "Russian Medical Academy of Continuous Professional Education" of the Ministry of Healthcare of the Russian Federation.
- [4] Department of Radio Engineering and Cybernetics, Moscow Institute of Physics and Technology, Moscow, Russia.
- [5] Davidovsky City Clinical Hospital, Moscow, Russia.
- [6] School of Systems Biology, George Mason University, Fairfax, VA, USA.
- [7] Research Center for Medical Genetics, Moscow, Russia.

- PMID: **35156528**
- DOI: 10.1080/10428194.2022.2034157

Abstract

We describe a retrospective cohort, 156 patients with chronic lymphocytic leukemia (CLL) diagnosed with COVID-19, analyze factors associated with a severe disease course and the effects of various treatment regimens. Anti-SARS-CoV-2 IgG and IgM levels are significantly lower. Patients with CLL are more likely to have a severe course of COVID-19, with IL-6 levels acting as a consistent biomarker of disease severity. Ten patients had recurrent episodes, fatality rate of 20%. Overall survival did not differ between patients receiving ibrutinib monotherapy and anti-CD20 antibodies ± chemotherapy. It seems that the immunodeficiency inherent to CLL influences outcomes to a larger degree than does the treatment. Glucocorticoids are not associated with significant OS improvement whereas anti-cytokine compounds usage seemed to be beneficial in patients with mild pulmonary involvement. Our data attest to the necessity of reorganizing health care for patients with CLL. Early administration of effective antiviral compounds and tailored vaccination protocols are warranted.

Keywords: COVID-19; Chronic lymphocytic leukemia; anti-cytokine therapy; glucocorticoid therapy; specific antibody response.

Full text links

View full text Taylor & Francis

Proceed to details

Cite

Share

☐ 1,368

J Clin Med
. 2021 May 13;10(10):2096.
doi: 10.3390/jcm10102096.

Myocardial Injury in COVID-19 Patients: Association with Inflammation, Coagulopathy and In-Hospital Prognosis

Victor Arévalos [1, 2], Luis Ortega-Paz [1, 2], Juan José Rodríguez-Arias [1, 2], Margarita Calvo [1], Leticia Castrillo [1], Anthony Salazar [1], Merce Roque [1, 2], Ana Paula Dantas [2], Manel Sabaté [1, 2], Salvatore Brugaletta [1, 2]
Affiliations Expand

Affiliations

- [1] Department of Cardiology, Clinic Cardiovascular Institute, Hospital Clinic, 08036 Barcelona, Spain.
- [2] Institut d'Investigacions Biomèdiques August Pi i Sunyer (IDIBAPS), University of Barcelona, 08036 Barcelona, Spain.

- PMID: **34068127**
- PMCID: PMC8152726
- DOI: 10.3390/jcm10102096

Free PMC article

Myocardial Injury in COVID-19 Patients: Association with Inflammation, Coagulopathy and In-Hospital Prognosis

Victor Arévalos et al. J Clin Med. 2021.
Free PMC article
Show details
J Clin Med
. 2021 May 13;10(10):2096.
doi: 10.3390/jcm10102096.

Authors

Victor Arévalos [1, 2], Luis Ortega-Paz [1, 2], Juan José Rodríguez-Arias [1, 2], Margarita Calvo [1], Leticia Castrillo [1], Anthony Salazar [1], Merce Roque [1, 2], Ana Paula Dantas [2], Manel Sabaté [1, 2], Salvatore Brugaletta [1, 2]

Affiliations

- [1] Department of Cardiology, Clinic Cardiovascular Institute, Hospital Clinic, 08036 Barcelona, Spain.
- [2] Institut d'Investigacions Biomèdiques August Pi i Sunyer (IDIBAPS), University of Barcelona, 08036 Barcelona, Spain.

- PMID: **34068127**
- PMCID: PMC8152726
- DOI: 10.3390/jcm10102096

Abstract

The exact mechanisms leading to myocardial injury in the coronavirus disease 2019 (COVID-19) are still unknown. In this retrospective observational study, we include all consecutive COVID-19 patients admitted to our center. They were divided into two groups according to the presence of myocardial injury. Clinical variables, Charlson Comorbidity Index (CCI), C-reactive protein (CRP), CAC (COVID-19-associated coagulopathy), defined according to the ISTH score, treatment and in-hospital events were collected. Between March and April 2020, 331 COVID-19 patients were enrolled, 72 of them (21.8%) with myocardial injury. Patients with myocardial injury showed a higher CCI score (median (interquartile range), 5 (4-7) vs. 2 (1-4), $p = 0.001$), higher CRP values (18.3 (9.6-25.9) mg/dL vs. 12.0 (5.4-19.4) mg/dL, $p < 0.001$) and CAC score (1 (0-2) vs. 0 (0-1), $p = 0.001$), and had lower use of any anticoagulant (57 patients (82.6%) vs. 229 patients (90.9%), $p = 0.078$), than those without. In the adjusted logistic regression, CRP, myocardial injury, CCI and CAC score were positive independent predictors of mortality, whereas anticoagulants resulted as a protective factor. Myocardial injury in COVID-19 patients is associated with inflammation and coagulopathy, resulting in a worse in-hospital prognosis. Treatment with anticoagulant agents may help to improve in-hospital outcomes.

Keywords: coagulopathy; coronavirus disease 2019; mortality; myocardial injury.

Conflict of interest statement

The authors declare no conflict of interest.

- 31 references
- 2 figures

Supplementary info

Grant support Expand

Grant support

- COV20/00040/Instituto de Salud Carlos III

Full text links

FULL TEXT OPEN ACCESS MDPI Multidisciplinary Digital Publishing Institute (MDPI) Free PMC article

Proceed to details

Cite
Share
1,369

Diagnostics (Basel)
. 2022 Mar 13;12(3):703.
doi: 10.3390/diagnostics12030703.

Evaluation and Comparison of the Predictive Value of 4C Mortality Score, NEWS, and CURB-65 in Poor Outcomes in COVID-19 Patients: A Retrospective Study from a Single Center in Romania

Cosmin Citu [1], Florin Gorun [1], Andrei Motoc [2], Adrian Ratiu [1], Oana Maria Gorun [3], Bogdan Burlea [3], Octavian Neagoe [4], Ioana Mihaela Citu [5], Ovidiu Rosca [6], Felix Bratosin [6], Mirela Loredana Grigoras [2], Raul Patrascu [7], Daniel Malita [8]
Affiliations Expand

Affiliations

- [1] Department of Obstetrics and Gynecology, "Victor Babes" University of Medicine and Pharmacy Timisoara, 2 Eftimie Murgu Square, 300041 Timisoara, Romania.
- [2] Department of Anatomy and Embryology, "Victor Babes" University of Medicine and Pharmacy Timisoara, 2 Eftimie Murgu Square, 300041 Timisoara, Romania.
- [3] Department of Obstetrics and Gynecology, Municipal Emergency Clinical Hospital Timisoara, 1-3 Alexandru Odobescu Street, 300202 Timisoara, Romania.
- [4] First Department of Surgery, Second Discipline of Surgical Semiology, "Victor Babes" University of Medicine and Pharmacy, Eftimie Murgu Sq. Nr. 2, 300041 Timisoara, Romania.
- [5] Department of Internal Medicine I, "Victor Babes" University of Medicine and Pharmacy Timisoara, 2 Eftimie Murgu Square, 300041 Timisoara, Romania.
- [6] Methodological and Infectious Diseases Research Center, Department of Infectious Diseases, "Victor Babes" University of Medicine and Pharmacy, 300041 Timisoara, Romania.
- [7] Department of Functional Sciences, "Victor Babes" University of Medicine and Pharmacy Timisoara, Eftimie Murgu Square 2, 300041 Timisoara, Romania.
- [8] Department of Radiology, "Victor Babes" University of Medicine and Pharmacy Timisoara, Eftimie Murgu Square nr. 2, 300041 Timisoara, Romania.

- PMID: **35328256**
- PMCID: PMC8947715
- DOI: 10.3390/diagnostics12030703

Free PMC article

Evaluation and Comparison of the Predictive Value of 4C Mortality Score, NEWS, and CURB-65 in Poor Outcomes in COVID-19 Patients: A Retrospective Study from a Single Center in Romania

Cosmin Citu et al. Diagnostics (Basel). 2022.
Free PMC article
Show details
Diagnostics (Basel)
. 2022 Mar 13;12(3):703.
doi: 10.3390/diagnostics12030703.

Authors

Cosmin Citu [1], Florin Gorun [1], Andrei Motoc [2], Adrian Ratiu [1], Oana Maria Gorun [3], Bogdan Burlea [3], Octavian Neagoe [4], Ioana Mihaela Citu [5], Ovidiu Rosca [6], Felix Bratosin [6], Mirela Loredana Grigoras [2], Raul Patrascu [7], Daniel Malita [8]

Affiliations

- [1] Department of Obstetrics and Gynecology, "Victor Babes" University of Medicine and Pharmacy Timisoara, 2 Eftimie Murgu Square, 300041 Timisoara, Romania.
- [2] Department of Anatomy and Embryology, "Victor Babes" University of Medicine and Pharmacy Timisoara, 2 Eftimie Murgu Square, 300041 Timisoara, Romania.
- [3] Department of Obstetrics and Gynecology, Municipal Emergency Clinical Hospital Timisoara, 1-3 Alexandru Odobescu Street, 300202 Timisoara, Romania.
- [4] First Department of Surgery, Second Discipline of Surgical Semiology, "Victor Babes" University of Medicine and Pharmacy, Eftimie Murgu Sq. Nr. 2, 300041 Timisoara, Romania.
- [5] Department of Internal Medicine I, "Victor Babes" University of Medicine and Pharmacy Timisoara, 2 Eftimie Murgu Square, 300041 Timisoara, Romania.
- [6] Methodological and Infectious Diseases Research Center, Department of Infectious Diseases, "Victor Babes" University of Medicine and Pharmacy, 300041 Timisoara, Romania.
- [7] Department of Functional Sciences, "Victor Babes" University of Medicine and Pharmacy Timisoara, Eftimie Murgu Square 2, 300041 Timisoara, Romania.
- [8] Department of Radiology, "Victor Babes" University of Medicine and Pharmacy Timisoara, Eftimie Murgu Square nr. 2, 300041 Timisoara, Romania.

- PMID: **35328256**
- PMCID: PMC8947715
- DOI: 10.3390/diagnostics12030703

Abstract

To date, the COVID-19 pandemic has caused millions of deaths across the world. Prognostic scores can improve the clinical management of COVID-19 diagnosis and treatment. The objective of this study was to assess the predictive role of 4C Mortality, CURB-65, and NEWS in COVID-19 mortality among the Romanian population. A single-center, retrospective, observational study was conducted on patients with reverse transcriptase-polymerase chain reaction (RT-PCR)-proven COVID-19 admitted to the Municipal Emergency Clinical Hospital of Timisoara, Romania, between 1 October 2020 and 15 March 2021. Receiver operating characteristic (ROC) and area under the curve (AUC) analyses were performed to determine the discrimination accuracy of the three scores. The mean values of the risk scores were higher in the non-survivors group (survivors group vs. non-survivors group: 8 vs. 15 (4C Mortality Score); 3 vs. 8.5 (NEWS); 1 vs. 3 (CURB-65)). In terms of mortality risk prediction, the NEWS performed best, with an AUC of 0.86, and the CURB-65 score performed poorly, with an AUC of 0.80. CURB-65, NEWS, and 4C Mortality scores were significant mortality predictors in the analysis, with acceptable calibration. Among the scores assessed in our study, NEWS had the highest performance in predicting in-hospital mortality in COVID-19 patients. Thus, the findings from this study suggest that the use of NEWS may be beneficial to the early identification of high-risk COVID-19 patients and the provision of more aggressive care to reduce mortality associated with COVID-19.

Keywords: 4C Mortality; COVID-19; CURB-65; NEWS; mortality; prediction.

Conflict of interest statement

The authors declare no conflict of interest.

- 21 references
- 5 figures

Full text links

FULL TEXT OPEN ACCESS MDPI Multidisciplinary Digital Publishing Institute (MDPI)

Proceed to details

Cite

Share

☐ 1,370

Indian J Crit Care Med
. 2021 Dec;25(12):1395-1401.
doi: 10.5005/jp-journals-10071-24048.

Clinical Characteristics and Treatment Outcomes of 293 COVID-19 Patients Admitted to the Intensive Care Unit of a Tertiary Care Hospital of Eastern India

Deependra K Rai [1], Nishant Sahay [2], Pallavi Lohani [3]

Affiliations Expand

Affiliations

- [1] Department of Pulmonary Medicine, All India Institute of Medical Sciences, Patna, Bihar, India.
- [2] Department of Anesthesiology, All India Institute of Medical Sciences, Patna, Bihar, India.
- [3] Department of CFM, All India Institute of Medical Sciences, Patna, Bihar, India.

- PMID: **35027800**
- PMCID: PMC8693122
- DOI: 10.5005/jp-journals-10071-24048

Free PMC article

Clinical Characteristics and Treatment Outcomes of 293 COVID-19 Patients Admitted to the Intensive Care Unit of a Tertiary Care Hospital of Eastern India

Deependra K Rai et al. Indian J Crit Care Med. 2021 Dec.
Free PMC article
Show details

Indian J Crit Care Med
. 2021 Dec;25(12):1395-1401.
doi: 10.5005/jp-journals-10071-24048.

Authors

Deependra K Rai [1], Nishant Sahay [2], Pallavi Lohani [3]

Affiliations

- [1] Department of Pulmonary Medicine, All India Institute of Medical Sciences, Patna, Bihar, India.
- [2] Department of Anesthesiology, All India Institute of Medical Sciences, Patna, Bihar, India.
- [3] Department of CFM, All India Institute of Medical Sciences, Patna, Bihar, India.

- PMID: **35027800**
- PMCID: PMC8693122
- DOI: 10.5005/jp-journals-10071-24048

Abstract

Objectives of the study: The objectives of the study were to assess the mortality among critically ill coronavirus disease (COVID) patients and to look at the factors which could have a bearing on mortality of these patients presenting to our designated tertiary COVID care institute.

Method: This was a retrospective observational study involving all adult patients admitted to our intensive care unit (ICU) with coronavirus disease-2019 (COVID-19) infection between June 30, 2020, and August 31, 2020. We compared patient-related factors and laboratory test results among all survivors vs nonsurvivors in our ICU with an aim to predict the factors which could predict increased risks of mortality among sick patients admitted to our ICU.

Results and conclusion: The overall ICU mortality in our ICU during the study period was 76.69% and less than 5% of the patients requiring mechanical ventilation within 1 day of admission, survived. More than half of the deaths (54.66%) occurred within 5 days of ICU admission. The best predictors for mortality based upon the Cox proportional hazard ratio are increasing age, neutrophilia, increased D-dimer, prolonged stay in ICU for 1-2 weeks, and those requiring mechanical ventilation. Patients with one or more comorbidities were noted to have 16% of higher risk of death than those without any comorbidity.

How to cite this article: Rai DK, Sahay N, Lohani P. Clinical Characteristics and Treatment Outcomes of 293 COVID-19 Patients Admitted to the Intensive Care Unit of a Tertiary Care Hospital of Eastern India. Indian J Crit Care Med 2021;25(12):1395-1401.

Keywords: Coronavirus disease-2019; Intensive care unit; Mortality; Predictors.

Copyright © 2021; Jaypee Brothers Medical Publishers (P) Ltd.

Conflict of interest statement

Source of support: Nil Conflict of interest: None

- 19 references
- 3 figures

Full text links

Free PMC article
Proceed to details
Cite
Share
☐ 1,371
Microorganisms
. 2021 Sep 12;9(9):1941.
doi: 10.3390/microorganisms9091941.

Factors Associated with Prolonged Hospital Length of Stay in Adults with Imported Falciparum Malaria-An Observational Study

from a Tertiary Care University Hospital in Berlin, Germany

Bodo Hoffmeister [1]
Affiliations Expand

Affiliation

- [1] Department of Respiratory Medicine, Clinic-Group Ernst von Bergmann, Potsdam and Bad Belzig, 14806 Bad Belzig, Germany.

- PMID: **34576836**
- PMCID: PMC8466442
- DOI: 10.3390/microorganisms9091941

Free PMC article

Factors Associated with Prolonged Hospital Length of Stay in Adults with Imported Falciparum Malaria-An Observational Study from a Tertiary Care University Hospital in Berlin, Germany

Bodo Hoffmeister. Microorganisms. 2021.
Free PMC article
Show details
Microorganisms
. 2021 Sep 12;9(9):1941.
doi: 10.3390/microorganisms9091941.

Author

Bodo Hoffmeister [1]

Affiliation

- [1] Department of Respiratory Medicine, Clinic-Group Ernst von Bergmann, Potsdam and Bad Belzig, 14806 Bad Belzig, Germany.

- PMID: **34576836**
- PMCID: PMC8466442
- DOI: 10.3390/microorganisms9091941

Abstract

Outcome of falciparum malaria is largely influenced by the standard of care provided, which in turn depends on the available medical resources. Worldwide, the COVID-19 pandemic has had a major impact on the availability of these resources, even in resource-rich healthcare systems such as Germany's. The present study aimed to determine the under-explored factors associated with hospital length of stay (LOS) in imported falciparum malaria to identify potential targets for improving management. This retrospective observational study used multivariate Cox proportional hazard regression with time to discharge as an endpoint for adults hospitalized between 2001 and 2015 with imported falciparum malaria in the Charité University Hospital, Berlin. The median LOS of the 535 cases enrolled was 3 days (inter-quartile range, IQR, 3-4 days). The likelihood of being discharged by day 3 strongly decreased with severe malaria (hazard ratio, HR, 0.274; 95% Confidence interval, 95%CI: 0.190-0.396) and by 40% with each additional presenting complication (HR, 0.595; 95%CI: 0.510-0.694). The 55 (10.3%) severe cases required a median LOS of 7 days (IQR, 5-12 days). In multivariate analysis, occurrence of shock (adjusted HR, aHR, 0.438; 95%CI 0.220-0.873), acute pulmonary oedema or acute respiratory distress syndrome (aHR, 0.450; 95%CI: 0.223-0.874), and the need for renal replacement therapy (aHR, 0.170; 95% CI: 0.063-0.461) were independently associated with LOS. All patients survived to discharge. This study illustrates that favourable outcomes can be achieved with high-standard care in imported falciparum malaria. Early recognition of disease severity together with targeted supportive care can lead to avoidance of manifest organ failure, thereby potentially decreasing LOS and alleviating pressure on bed capacities.

Keywords: epidemiology; hospital length of stay; imported falciparum malaria; public health; severe malaria.

Conflict of interest statement

The author declares no conflict of interest.

- 43 references
- 4 figures

Full text links

FULL TEXT OPEN ACCESS MDPI Multidisciplinary Digital Publishing Institute (MDPI) Free PMC article
Proceed to details
Cite
Share
1,372
Front Pharmacol
. 2021 Dec 10;12:778386.
doi: 10.3389/fphar.2021.778386. eCollection 2021.

Increased Use of Antibiotics in the Intensive Care Unit During Coronavirus Disease (COVID-19) Pandemic in a Brazilian Hospital

Alice Ramos Oliveira Silva [1], Diamantino Ribeiro Salgado [2], Luis Phillipe Nagem Lopes [1], Débora Castanheira [3], Isabel Cristina Martins Emmerick [4], Elisangela Costa Lima [1]
Affiliations Expand

Affiliations

- [1] Pharmacy School, Federal University of Rio de Janeiro, Rio de Janeiro, Brazil.
- [2] Clementino Fraga Filho University Hospital, Federal University of Rio de Janeiro, Rio de Janeiro, Brazil.
- [3] National School of Public Health Sergio Arouca, Rio de Janeiro, Brazil.
- [4] Division of Thoracic Surgery, Department of Surgery, UMass Chan Medical School, Worcester, MA, United States.

- PMID: **34955847**
- PMCID: PMC8703131
- DOI: 10.3389/fphar.2021.778386

Free PMC article

Increased Use of Antibiotics in the Intensive Care Unit During Coronavirus Disease (COVID-19) Pandemic in a Brazilian Hospital

Alice Ramos Oliveira Silva et al. Front Pharmacol. 2021.
Free PMC article
Show details
Front Pharmacol
. 2021 Dec 10;12:778386.
doi: 10.3389/fphar.2021.778386. eCollection 2021.

Authors

Alice Ramos Oliveira Silva [1], Diamantino Ribeiro Salgado [2], Luis Phillipe Nagem Lopes [1], Débora Castanheira [3], Isabel Cristina Martins Emmerick [4], Elisangela Costa Lima [1]

Affiliations

- [1] Pharmacy School, Federal University of Rio de Janeiro, Rio de Janeiro, Brazil.
- [2] Clementino Fraga Filho University Hospital, Federal University of Rio de Janeiro, Rio de Janeiro, Brazil.

- [3] National School of Public Health Sergio Arouca, Rio de Janeiro, Brazil.
- [4] Division of Thoracic Surgery, Department of Surgery, UMass Chan Medical School, Worcester, MA, United States.

- PMID: **34955847**
- PMCID: PMC8703131
- DOI: 10.3389/fphar.2021.778386

Abstract

Background: Microbial drug resistance is one of the biggest public health problems. Antibiotic consumption is an essential factor for the emergence and spread of multiresistant bacteria. Therefore, we aimed to analyze the antibiotics consumption in the Intensive Care Unit (ICU), identifying trends in the antibiotics use profile and microbiological isolates throughout the COVID-19 pandemic. **Methods:** We performed this retrospective observational study in intensive care units of a Brazilian tertiary hospital from January 2019 to December 2020. The primary outcome was antimicrobial consumption in the ICU, measured by defined daily doses (DDDs) per 100 bed-days. As a secondary outcome, bacterial infections (microbiological isolates) were calculated in the same fashion. Outcomes trends were analyzed using Joinpoint regression models, considering constant variance (homoscedasticity) and first-order autocorrelation assumptions. A monthly percent change (MPC) was estimated for each analyzed segment. **Results:** Seven thousand and nine hundred fifty-three patients had data available on prescribed and received medications and were included in the analyses. Overall, the use of antibiotics increased over time in the ICU. The reserve group (World Health Organization Classification) had an increasing trend (MPC = 7.24) from February to April 2020. The azithromycin consumption (J01FA) increased rapidly, with a MPC of 5.21 from January to April 2020. Polymyxin B showed a relevant increase from March to June 2020 (MPC = 6.93). The peak of the antibiotic consumption of Reserve group did not overlap with the peak of the pathogenic agents they are intended to treat. **Conclusion:** Overall antimicrobial consumption in ICU has increased in the context of the COVID-19 pandemic. The peaks in the antimicrobial's use were not associated with the rise of the pathogenic agents they intended to treat, indicating an empirical use, which is especially concerning in the context of treating multidrug-resistant (MDR) infections. This fact may contribute to the depletion of the therapeutic arsenal for MDR treatment.

Keywords: COVID-19; anti-infective agents; bacterial; bacterial infection; coinfection; drug resistance; intensive care units.

Copyright © 2021 Silva, Salgado, Lopes, Castanheira, Emmerick and Lima.

Conflict of interest statement

The authors declare that the research was conducted in the absence of any commercial or financial relationships that could be construed as a potential conflict of interest.

- 63 references
- 2 figures

Full text links

Frontiers Media SA Free PMC article

Proceed to details

Cite
Share
1,373

Int J Appl Basic Med Res
. Jan-Mar 2022;12(1):30-36.
doi: 10.4103/ijabmr.ijabmr_602_21. Epub 2022 Jan 31.

Prescription-Event Monitoring Study on Safety and Efficacy of Levonadifloxacin (Oral and I.V.) in Management of Bacterial Infections: Findings of Real-World Observational Study

Yatin Mehta [1], Anand R Sutar [2], Kapil Zirpe [3], Jay Narendra Kothari [4], Chakravarthi Alapati [5], Manu Pathak [6], Vasant C Nagvekar [7], Kapil Dev Mehta [8], Khokan Debnath [8]
Affiliations Expand

Affiliations

- [1] Department of Medanta Institute of Critical Care and Anesthesiology, Medanta - The Medicity, Gurgaon, Haryana, India.
- [2] Department of Critical Care, Apollo Hospital, Bengaluru, Karnataka, India.
- [3] Department of Neuro Trauma Unit, Grant Medical Foundation, Ruby Hall Clinic, Pune, Maharashtra, India.
- [4] Department of Critical Care, Apollo Hospital International Ltd., Ahmedabad, Gujarat, India.
- [5] Department of Emergency and Critical Care Unit, Billroth Hospitals, Shenoy Nagar, Chennai, Tamil Nadu, India.
- [6] Department of Anaesthesia and Critical Care, Sips Burn and Trauma Centre, Lucknow, Uttar Pradesh, India.
- [7] Department of Physician/Internal Medicine, Infectious Diseases, Lilavati Hospital, Mumbai, Maharashtra, India.
- [8] Department of Medical Affairs, Wockhardt Ltd., BKC, Mumbai, Maharashtra, India.

- PMID: **35265478**
- PMCID: PMC8848560
- DOI: 10.4103/ijabmr.ijabmr_602_21

Free PMC article

Prescription-Event Monitoring Study on Safety and Efficacy of Levonadifloxacin (Oral

and I.V.) in Management of Bacterial Infections: Findings of Real-World Observational Study

Yatin Mehta et al. Int J Appl Basic Med Res. Jan-Mar 2022.
Free PMC article
Show details
Int J Appl Basic Med Res
. Jan-Mar 2022;12(1):30-36.
doi: 10.4103/ijabmr.ijabmr_602_21. Epub 2022 Jan 31.

Authors

Yatin Mehta [1], Anand R Sutar [2], Kapil Zirpe [3], Jay Narendra Kothari [4], Chakravarthi Alapati [5], Manu Pathak [6], Vasant C Nagvekar [7], Kapil Dev Mehta [8], Khokan Debnath [8]

Affiliations

- [1] Department of Medanta Institute of Critical Care and Anesthesiology, Medanta - The Medicity, Gurgaon, Haryana, India.
- [2] Department of Critical Care, Apollo Hospital, Bengaluru, Karnataka, India.
- [3] Department of Neuro Trauma Unit, Grant Medical Foundation, Ruby Hall Clinic, Pune, Maharashtra, India.
- [4] Department of Critical Care, Apollo Hospital International Ltd., Ahmedabad, Gujarat, India.
- [5] Department of Emergency and Critical Care Unit, Billroth Hospitals, Shenoy Nagar, Chennai, Tamil Nadu, India.
- [6] Department of Anaesthesia and Critical Care, Sips Burn and Trauma Centre, Lucknow, Uttar Pradesh, India.
- [7] Department of Physician/Internal Medicine, Infectious Diseases, Lilavati Hospital, Mumbai, Maharashtra, India.
- [8] Department of Medical Affairs, Wockhardt Ltd., BKC, Mumbai, Maharashtra, India.

- PMID: **35265478**
- PMCID: PMC8848560
- DOI: 10.4103/ijabmr.ijabmr_602_21

Abstract

Background: Levonadifloxacin is a novel broad-spectrum antibiotic belonging to the benzoquinolizine subclass of quinolones. It is available in intravenous as well as oral formulation for the treatment of infections caused by common Gram-positive bacterial pathogens including methicillin-resistant *Staphylococcus aureus* (MRSA).

Patients and methods: This study retrospectively assessed the real-world safety and efficacy of levonadifloxacin (oral and/or IV) in the treatment of 1229 patients across various clinical conditions. Study outcomes were clinical and microbiological success at the end of therapy.

Results: The mean duration of levonadifloxacin therapy was 7.2 days, with a time to clinical improvement averaging at 4 days. Three hundred and three patients received oral therapy, 875 received IV, and 51 received a combination of IV followed by oral therapy. Patients were prescribed levonadifloxacin for skin and soft-tissue infections, diabetic foot infections, septicemia, catheter-related bloodstream infections, bone and joint infections, febrile neutropenia, and respiratory infections including COVID-19 pneumonia. High clinical success rates of 98.3%, 93.7%, and 96.1% with oral, IV, and IV followed by oral levonadifloxacin, respectively, were obtained. Only 11 mild adverse events were reported in 9 patients which included constipation, diarrhea, hyperglycemia, nausea, fatigue, and vomiting. Overall, 96.3% and 97.3% of investigators rated the efficacy and safety of levonadifloxacin as "good to excellent."

Conclusions: An excellent safety and efficacy profile of levonadifloxacin was observed in this study making it a suitable treatment option for management of various bacterial infections, including those caused by resistant Gram-positive pathogens such as MRSA and quinolone-resistant *S. aureus*.

Keywords: Methicillin-resistant Staphylococcus aureus; bacterial infections; clinical success; levonadifloxacin.

Copyright: © 2022 International Journal of Applied and Basic Medical Research.

Conflict of interest statement

Nil.

- 18 references
- 1 figure

Full text links

Free PMC article
Proceed to details
Cite
Share
1,374
Ann Intensive Care
. 2021 May 31;11(1):86.
doi: 10.1186/s13613-021-00875-9.

Acute kidney injury in SARS-CoV2-related pneumonia ICU patients: a retrospective multicenter study

Guillaume Geri [#, 1, 2, 3, 4], Michael Darmon [#, 5, 6, 7], Lara Zafrani [5, 6, 8], Muriel Fartoukh [9, 10], Guillaume Voiriot [9, 10, 11], Julien Le Marec [10, 12, 13], Saafa Nemlaghi [10, 12, 13], Antoine Vieillard-Baron [14, 15, 16, 17], Elie Azoulay [5, 6, 7]
Affiliations Expand

Affiliations

- [1] Medical Intensive Care Unit, Ambroise Paré Hospital, AP-HP, 9 avenue Charles de Gaulle, 92100, Boulogne-Billancourt, France. guillaume.geri@aphp.fr.
- [2] Paris Saclay University, Gif-sur-Yvette, France. guillaume.geri@aphp.fr.
- [3] INSERM UMR 1018, CESP, Villejuif, France. guillaume.geri@aphp.fr.
- [4] FHU SEPSIS, Saclay, France. guillaume.geri@aphp.fr.
- [5] Medical Intensive Care Unit, Saint Louis Hospital, AP-HP, Paris, France.
- [6] Paris University, Paris, France.
- [7] INSERM U1153, Centre of Research in Epidemiology and Statistics, Paris, France.
- [8] INSERM U976, Immunologie Humaine, Pathophysiologie et immunothérapie, Paris, France.
- [9] Medical Intensive Care Unit, Tenon Hospital, AP-HP, Paris, France.
- [10] Paris Sorbonne University, Paris, France.
- [11] INSERM U955 (IMRB), Equipe GEIC2O, 94000, Créteil, France.
- [12] Medical Intensive Care Unit, Pitié-Salpétrière Hospital, AP-HP, Paris, France.
- [13] INSERM, UMRS1158 Neurophysiologie Respiratoire Expérimentale et Clinique, Paris, France.
- [14] Medical Intensive Care Unit, Ambroise Paré Hospital, AP-HP, 9 avenue Charles de Gaulle, 92100, Boulogne-Billancourt, France.
- [15] Paris Saclay University, Gif-sur-Yvette, France.
- [16] INSERM UMR 1018, CESP, Villejuif, France.
- [17] FHU SEPSIS, Saclay, France.

[#] Contributed equally.

- PMID: **34057648**
- PMCID: PMC8165682
- DOI: 10.1186/s13613-021-00875-9

Free PMC article

Acute kidney injury in SARS-CoV2-related pneumonia ICU patients: a retrospective multicenter study

Guillaume Geri et al. Ann Intensive Care. 2021.
Free PMC article

Show details

Ann Intensive Care
. 2021 May 31;11(1):86.
doi: 10.1186/s13613-021-00875-9.

Authors

Guillaume Geri[#] [1] [2] [3] [4], Michael Darmon[#] [5] [6] [7], Lara Zafrani [5] [6] [8], Muriel Fartoukh [9] [10], Guillaume Voiriot [9] [10] [11], Julien Le Marec [10] [12] [13], Saafa Nemlaghi [10] [12] [13], Antoine Vieillard-Baron [14] [15] [16] [17], Elie Azoulay [5] [6] [7]

Affiliations

- [1] Medical Intensive Care Unit, Ambroise Paré Hospital, AP-HP, 9 avenue Charles de Gaulle, 92100, Boulogne-Billancourt, France. guillaume.geri@aphp.fr.
- [2] Paris Saclay University, Gif-sur-Yvette, France. guillaume.geri@aphp.fr.
- [3] INSERM UMR 1018, CESP, Villejuif, France. guillaume.geri@aphp.fr.
- [4] FHU SEPSIS, Saclay, France. guillaume.geri@aphp.fr.
- [5] Medical Intensive Care Unit, Saint Louis Hospital, AP-HP, Paris, France.
- [6] Paris University, Paris, France.
- [7] INSERM U1153, Centre of Research in Epidemiology and Statistics, Paris, France.
- [8] INSERM U976, Immunologie Humaine, Pathophysiologie et immunothérapie, Paris, France.
- [9] Medical Intensive Care Unit, Tenon Hospital, AP-HP, Paris, France.
- [10] Paris Sorbonne University, Paris, France.
- [11] INSERM U955 (IMRB), Equipe GEIC2O, 94000, Créteil, France.
- [12] Medical Intensive Care Unit, Pitié-Salpétrière Hospital, AP-HP, Paris, France.
- [13] INSERM, UMRS1158 Neurophysiologie Respiratoire Expérimentale et Clinique, Paris, France.
- [14] Medical Intensive Care Unit, Ambroise Paré Hospital, AP-HP, 9 avenue Charles de Gaulle, 92100, Boulogne-Billancourt, France.
- [15] Paris Saclay University, Gif-sur-Yvette, France.
- [16] INSERM UMR 1018, CESP, Villejuif, France.
- [17] FHU SEPSIS, Saclay, France.

[#] Contributed equally.

- PMID: **34057648**
- PMCID: PMC8165682
- DOI: 10.1186/s13613-021-00875-9

Abstract

Background: While acute kidney injury (AKI) is frequent in severe SARS-CoV2-related pneumonia ICU patients, few data are still available about its risk factors.

Methods: Retrospective observational study performed in four university affiliated hospitals in Paris. AKI was defined according to the KIDGO guidelines. Factors associated with AKI were picked up using multivariable mixed-effects logistic regression. Independent risk factors of day 28 mortality were assessed using Cox model.

Results: 379 patients (median age 62 [53,69], 77% of male) were included. Half of the patients had AKI (n = 195, 52%) including 58 patients (15%) with AKI stage 1, 44 patients (12%) with AKI stage 2, and 93 patients (25% with AKI stage 3). Chronic kidney disease (OR 7.41; 95% CI 2.98-18.4), need for invasive mechanical ventilation at day 1 (OR 4.83; 95% CI 2.26-10.3), need for vasopressors at day 1 (OR 2.1; 95% CI 1.05-4.21) were associated with increased risk of AKI.

Day 28 mortality in the cohort was 26.4% and was higher in patients with AKI (37.4 vs. 14.7%, P < 0.001). Neither AKI (HR 1.35; 95% CI 0.78-2.32) nor AKI stage were associated with mortality (HR [95% CI] for stage 1, 2 and 3 when compared to no AKI of, respectively, 1.02 [0.49-2.10], 1.73 [0.81-3.68] and 1.42 [0.78-2.58]).

Conclusion: In this large cohort of SARS-CoV2-related pneumonia patients admitted to the ICU, AKI was frequent, mostly driven by preexisting chronic kidney disease and life sustaining therapies, with unclear adjusted relationship with day 28 outcome.

Keywords: Acute kidney injury; COVID-19; Renal replacement therapy.

Conflict of interest statement

The authors declare that they have no competing interests.

- 26 references
- 3 figures

Full text links

SpringerLink FULL-TEXT ARTICLE Springer Free PMC article

Proceed to details

Cite

Share

1,375

Healthcare (Basel)
. 2021 Dec 23;10(1):18.
doi: 10.3390/healthcare10010018.

Dynamics of the Third Wave of COVID-19 from the Perspective of the Emergency Department in a Large Regional Hospital-Single Center Observational Study

Tomasz Kłosiewicz [1], Weronika Szkudlarek [2], Magdalena Węglewska [2], Patryk Konieczka [3], Radosław Zalewski [1], Roland Podlewski [1], Anna Sowińska [4], Mateusz Puślecki [1] [5]

Affiliations Expand

Affiliations

- [1] Department of Medical Rescue, Faculty of Health Sciences, Poznan University of Medical Sciences, 60-608 Poznan, Poland.
- [2] Students' Scientific Circle of Emergency Medicine, Department of Medical Rescue, Faculty of Health Sciences, Poznan University of Medical Sciences, 60-608 Poznan, Poland.

- [3] Department of Emergency Medicine, Faculty of Health Sciences, Poznan University of Medical Sciences, 60-608 Poznan, Poland.
- [4] Department of Computer Science and Statistics, Poznan University of Medical Sciences, 60-608 Poznan, Poland.
- [5] Department of Cardiac Surgery and Transplantology, Medical Faculty, Poznan University of Medical Sciences, 61-848 Poznan, Poland.

- PMID: **35052182**
- PMCID: PMC8775057
- DOI: 10.3390/healthcare10010018

Free PMC article

Dynamics of the Third Wave of COVID-19 from the Perspective of the Emergency Department in a Large Regional Hospital-Single Center Observational Study

Tomasz Kłosiewicz et al. Healthcare (Basel). 2021.
Free PMC article
Show details
Healthcare (Basel)

. 2021 Dec 23;10(1):18.
doi: 10.3390/healthcare10010018.

Authors

Tomasz Kłosiewicz [1], Weronika Szkudlarek [2], Magdalena Węglewska [2], Patryk Konieczka [3], Radosław Zalewski [1], Roland Podlewski [1], Anna Sowińska [4], Mateusz Puślecki [1] [5]

Affiliations

- [1] Department of Medical Rescue, Faculty of Health Sciences, Poznan University of Medical Sciences, 60-608 Poznan, Poland.
- [2] Students' Scientific Circle of Emergency Medicine, Department of Medical Rescue, Faculty of Health Sciences, Poznan University of Medical Sciences, 60-608 Poznan, Poland.
- [3] Department of Emergency Medicine, Faculty of Health Sciences, Poznan University of Medical Sciences, 60-608 Poznan, Poland.
- [4] Department of Computer Science and Statistics, Poznan University of Medical Sciences, 60-608 Poznan, Poland.
- [5] Department of Cardiac Surgery and Transplantology, Medical Faculty, Poznan University of Medical Sciences, 61-848 Poznan, Poland.

- PMID: **35052182**
- PMCID: PMC8775057

- DOI: 10.3390/healthcare10010018

Abstract

Background: The outbreak of the Coronavirus Disease 2019 (COVID-19) pandemic has caused many significant social and economic changes. The consecutive waves of the epidemic in various countries have had dissimilar courses depending on the methods used to combat it. The aim of this study was to determine the dynamics of the third wave of COVID-19 from the perspective of emergency departments (ED).

Methods: This was a retrospective review of medical records from ED. The authors have identified the most frequent symptoms. Prognostic factors have been chosen-prognostic scales, length of stay (LOS)-and a number of resources required have been calculated.

Results: As the time passed, there were fewer patients and they presented mild symptoms. A statistically significant difference was observed in the median of blood oxygenation measurement ($p = 0.00009$), CRP level ($p = 0.0016$), and admission rate. Patients admitted to the hospital required more resources at ED. LOS was shorter in patients discharged home ($p < 0.0001$).

Conclusions: The blood oxygen saturation (SPO2) and CPR levels can be helpful in decision-making regarding medical treatment. The fast-track for patients in good clinical condition may shorten the duration of stay in ED, and reduce the number of required resources.

Keywords: COVID-19; decision making process; emergency department; fast-track; pandemic.

Conflict of interest statement

The authors declare no conflict of interest.

- 25 references
- 4 figures

Full text links

FULL TEXT OPEN ACCESS MDPI Multidisciplinary Digital Publishing Institute (MDPI) Free PMC article

Proceed to details

Cite

Share

☐ 1,376

BMC Sports Sci Med Rehabil

. 2021 Jun 30;13(1):70.

doi: 10.1186/s13102-021-00299-9.

ReCOV: recovery and rehabilitation during and after COVID-19 - a study protocol of a

longitudinal observational study on patients, next of kin and health care staff

E Rydwik[1 2], L Anmyr[3 4], M Regardt[5 6], A McAllister[7 8], R Zarenoe[3], E Åkerman[9 10], Y Orrevall[11 12], M Bragesjö[13 14], O Dahl[9 10], M K Kemani[14 15], L Nordstrand[6 14], U Ekman[14 16], L Holmström[13 14], M Nygren-Bonnier[17 5]

Affiliations Expand

Affiliations

- [1] Department of Neurobiology, Care Sciences and Society, Division of Physiotherapy, Karolinska Institutet, Huddinge, Sweden. elisabeth.rydwik@ki.se.
- [2] Women's Health and Allied Health Professionals Theme, Medical Unit Occupational Therapy and Physiotherapy, Karolinska University Hospital, Solna, Sweden. elisabeth.rydwik@ki.se.
- [3] Women's Health and Allied Health Professionals Theme, Department of Social Work in Health, Karolinska University Hospital, Solna, Sweden.
- [4] Department of CLINTEC, Karolinska Institutet, Stockholm, Sweden.
- [5] Women's Health and Allied Health Professionals Theme, Medical Unit Occupational Therapy and Physiotherapy, Karolinska University Hospital, Solna, Sweden.
- [6] Department of Neurobiology, Care Sciences and Society, Division of Occupational Therapy, Karolinska Institutet, Huddinge, Sweden.
- [7] Women's Health and Allied Health Professionals Theme, Medical Unit Speech and Language Pathology, Karolinska University Hospital, Stockholm, Sweden.
- [8] CLINTEC, Division of Speech-Language Pathology, Karolinska Institutet, Stockholm, Sweden.
- [9] Perioperative Medicine and Intensive Care Function, Department of Intensive Care, Karolinska University Hospital, Stockholm, Sweden.
- [10] Department of Neurobiology, Care Sciences and Society, Division of Nursing, Karolinska Institutet, Huddinge, Sweden.
- [11] Women's Health and Allied Health Professionals Theme, Medical Unit Clinical Nutrition, Karolinska University Hospital, Stockholm, Sweden.
- [12] Department of Biosciences and Nutrition, Karolinska Institutet, Stockholm, Sweden.
- [13] Department of Clinical Neuroscience, Division of Psychology Karolinska Institutet, Stockholm, Sweden.
- [14] Women's Health and Allied Health Professionals Theme, Medical Unit Medical Psychology Karolinska University Hospital, Solna, Sweden.
- [15] Department of Psychology, Stress Research Institute, Stockholm University, Stockholm, Sweden.
- [16] Department of Neurobiology, Care Sciences and Society, Division of Clinical Geriatrics, Karolinska Institutet, Huddinge, Sweden.
- [17] Department of Neurobiology, Care Sciences and Society, Division of Physiotherapy, Karolinska Institutet, Huddinge, Sweden.

- PMID: **34193260**
- PMCID: PMC8243048
- DOI: 10.1186/s13102-021-00299-9

Free PMC article

ReCOV: recovery and rehabilitation during and after COVID-19 - a study protocol of a longitudinal observational study on patients, next of kin and health care staff

E Rydwik et al. BMC Sports Sci Med Rehabil. 2021.
Free PMC article
Show details
BMC Sports Sci Med Rehabil
. 2021 Jun 30;13(1):70.
doi: 10.1186/s13102-021-00299-9.

Authors

E Rydwik [1] [2], L Anmyr [3] [4], M Regardt [5] [6], A McAllister [7] [8], R Zarenoe [3], E Åkerman [9] [10], Y Orrevall [11] [12], M Bragesjö [13] [14], O Dahl [9] [10], M K Kemani [14] [15], L Nordstrand [6] [14], U Ekman [14] [16], L Holmström [13] [14], M Nygren-Bonnier [17] [5]

Affiliations

- [1] Department of Neurobiology, Care Sciences and Society, Division of Physiotherapy, Karolinska Institutet, Huddinge, Sweden. elisabeth.rydwik@ki.se.
- [2] Women's Health and Allied Health Professionals Theme, Medical Unit Occupational Therapy and Physiotherapy, Karolinska University Hospital, Solna, Sweden. elisabeth.rydwik@ki.se.
- [3] Women's Health and Allied Health Professionals Theme, Department of Social Work in Health, Karolinska University Hospital, Solna, Sweden.
- [4] Department of CLINTEC, Karolinska Institutet, Stockholm, Sweden.
- [5] Women's Health and Allied Health Professionals Theme, Medical Unit Occupational Therapy and Physiotherapy, Karolinska University Hospital, Solna, Sweden.
- [6] Department of Neurobiology, Care Sciences and Society, Division of Occupational Therapy, Karolinska Institutet, Huddinge, Sweden.
- [7] Women's Health and Allied Health Professionals Theme, Medical Unit Speech and Language Pathology, Karolinska University Hospital, Stockholm, Sweden.
- [8] CLINTEC, Division of Speech-Language Pathology, Karolinska Institutet, Stockholm, Sweden.
- [9] Perioperative Medicine and Intensive Care Function, Department of Intensive Care, Karolinska University Hospital, Stockholm, Sweden.
- [10] Department of Neurobiology, Care Sciences and Society, Division of Nursing, Karolinska Institutet, Huddinge, Sweden.
- [11] Women's Health and Allied Health Professionals Theme, Medical Unit Clinical Nutrition, Karolinska University Hospital, Stockholm, Sweden.
- [12] Department of Biosciences and Nutrition, Karolinska Institutet, Stockholm, Sweden.

- [13] Department of Clinical Neuroscience, Division of Psychology Karolinska Institutet, Stockholm, Sweden.
- [14] Women's Health and Allied Health Professionals Theme, Medical Unit Medical Psychology Karolinska University Hospital, Solna, Sweden.
- [15] Department of Psychology, Stress Research Institute, Stockholm University, Stockholm, Sweden.
- [16] Department of Neurobiology, Care Sciences and Society, Division of Clinical Geriatrics, Karolinska Institutet, Huddinge, Sweden.
- [17] Department of Neurobiology, Care Sciences and Society, Division of Physiotherapy, Karolinska Institutet, Huddinge, Sweden.

- PMID: **34193260**
- PMCID: PMC8243048
- DOI: 10.1186/s13102-021-00299-9

Abstract

Background: The knowledge of the long-term consequences of covid-19 is limited. In patients, symptoms such as fatigue, decreased physical, psychological, and cognitive function, and nutritional problems have been reported. How the disease has affected next of kin, as well as staff involved in the care of patients with covid-19, is also largely unknown. The overall aim of this study is therefore three-fold: (1) to describe and evaluate predictors of patient recovery, the type of rehabilitation received and patients' experiences of specialized rehabilitation following COVID-19 infection; (2) to study how next of kin experienced the hospital care of their relative and their experiences of the psychosocial support they received as well as their psychological wellbeing; (3) to describe experiences of caring for patients with COVID-19 and evaluate psychological wellbeing, coping mechanisms and predictors for development of psychological distress over time in health care staff.

Methods: This observational longitudinal study consists of three cohorts; patients, next of kin, and health care staff. The assessments for the patients consist of physical tests (lung function, muscle strength, physical capacity) and questionnaires (communication and swallowing, nutritional status, hearing, activities of daily living, physical activity, fatigue, cognition) longitudinally at 3, 6 and 12 months. Patient records auditing (care, rehabilitation) will be done retrospectively at 12 months. Patients (3, 6 and 12 months), next of kin (6 months) and health care staff (baseline, 3, 6, 9 and 12 months) will receive questionnaires regarding, health-related quality of life, depression, anxiety, sleeping disorders, and post-traumatic stress. Staff will also answer questionnaires about burnout and coping strategies. Interviews will be conducted in all three cohorts.

Discussion: This study will be able to answer different research questions from a quantitative and qualitative perspective, by describing and evaluating long-term consequences and their associations with recovery, as well as exploring patients', next of kins' and staffs' views and experiences of the disease and its consequences. This will form a base for a deeper and better understanding of the consequences of the disease from different perspectives as well as helping the society to better prepare for a future pandemic.

Keywords: Infection; Physical function; Well-being.

Conflict of interest statement

The authors declare that they have no competing interests.

- 50 references
- 3 figures

Supplementary info

Grant support Expand

Grant support

- 2020-02789/Forte/Formas

Full text links

Read free full text at BMC BioMed Central Free PMC article
Proceed to details
Cite
Share
1,377
Ophthalmol Ther
. 2021 Dec;10(4):1015-1024.
doi: 10.1007/s40123-021-00395-6. Epub 2021 Sep 5.

Intravitreal Dexamethasone Implant in Patients Who Did Not Complete Anti-VEGF Loading Dose During the COVID-19 Pandemic: a Retrospective Observational Study

Vincenzo Scorcia [1], Giuseppe Giannaccare [1], Valentina Gatti [1], Sabrina Vaccaro [1], Gabriele Piccoli [1], Annarita Villì [1], Mario Damiano Toro [2], Angeli Christy Yu [3], Claudio Iovino [4], Francesca Simonelli [4], Adriano Carnevali [5]
Affiliations Expand

Affiliations

- [1] Department of Ophthalmology, University of Magna Graecia of Cantazaro, Viale Europa, Loc. Germaneto, 88100, Catanzaro, Calabria, Italy.
- [2] Department of Ophthalmology, University Hospital of Zurich, University of Zurich, 9081, Zurich, Switzerland.
- [3] Department of Translational Medicine, University of Ferrara, Ferrara, Italy.
- [4] Multidisciplinary Department of Medical, Surgical and Dental Sciences, Eye Clinic, University of Campania Luigi Vanvitelli School of Medicine and Surgery, Naples, Italy.

- [5] Department of Ophthalmology, University of Magna Graecia of Cantazaro, Viale Europa, Loc. Germaneto, 88100, Catanzaro, Calabria, Italy. adrianocarnevali@live.it.

- PMID: **34482532**
- PMCID: PMC8418689
- DOI: 10.1007/s40123-021-00395-6

Free PMC article

Intravitreal Dexamethasone Implant in Patients Who Did Not Complete Anti-VEGF Loading Dose During the COVID-19 Pandemic: a Retrospective Observational Study

Vincenzo Scorcia et al. Ophthalmol Ther. 2021 Dec.
Free PMC article

Show details

Ophthalmol Ther
. 2021 Dec;10(4):1015-1024.
doi: 10.1007/s40123-021-00395-6. Epub 2021 Sep 5.

Authors

Vincenzo Scorcia [1], Giuseppe Giannaccare [1], Valentina Gatti [1], Sabrina Vaccaro [1], Gabriele Piccoli [1], Annarita Villì [1], Mario Damiano Toro [2], Angeli Christy Yu [3], Claudio Iovino [4], Francesca Simonelli [4], Adriano Carnevali [5]

Affiliations

- [1] Department of Ophthalmology, University of Magna Graecia of Cantazaro, Viale Europa, Loc. Germaneto, 88100, Catanzaro, Calabria, Italy.
- [2] Department of Ophthalmology, University Hospital of Zurich, University of Zurich, 9081, Zurich, Switzerland.
- [3] Department of Translational Medicine, University of Ferrara, Ferrara, Italy.
- [4] Multidisciplinary Department of Medical, Surgical and Dental Sciences, Eye Clinic, University of Campania Luigi Vanvitelli School of Medicine and Surgery, Naples, Italy.
- [5] Department of Ophthalmology, University of Magna Graecia of Cantazaro, Viale Europa, Loc. Germaneto, 88100, Catanzaro, Calabria, Italy. adrianocarnevali@live.it.

- PMID: **34482532**
- PMCID: PMC8418689
- DOI: 10.1007/s40123-021-00395-6

Abstract

Introduction: To compare the functional and anatomic outcomes between eyes in patients with diabetic macular edema (DME) who underwent a complete anti-vascular endothelial growth factor (VEGF) loading dose with aflibercept and those who were switched to dexamethasone intravitreal (DEX) implant after an incomplete anti-VEGF treatment regimen during the coronavirus disease 2019 (COVID-19) pandemic.

Methods: This was a retrospective and comparative study conducted on patients with DME. Main outcome measures were mean change in best corrected visual acuity (BCVA) and central retinal thickness (CRT) from baseline to month 4.

Results: Forty-three eyes (23 eyes in the anti-VEGF group and 20 eyes in the DEX group) were included. Mean BCVA significantly improved from 37.7 ± 25.3 and 35.7 ± 22.0 letters at baseline to 45.4 (23.9) (mean adjusted BCVA improvement 7.6 ± 20.8 letters, p = 0.033) and 46.1 ± 26.0 (mean adjusted BCVA improvement 10.6 ± 15.9 letters, p = 0.049) at month 4 in the anti-VEGF and DEX groups, respectively, with no significant differences between study groups (mean adjusted BCVA difference 2.8 letters, 95% CI - 9.4 to 14.9 letters, p = 0.648). There were no statistically significant differences in the proportion of eyes that achieved a BCVA improvement of ≥ 5, ≥ 10, and ≥ 15 letters between groups. CRT was significantly reduced from baseline to month 4 in both DEX (mean adjusted CRT reduction 167.3 ± 148.2 µm, p = 0.012) and anti-VEGF groups (mean adjusted CRT reduction 109.9 ± 181.9 µm, p < 0.001), with no differences between them (mean adjusted CRT difference 56.1 µm, 95% CI - 46.0 to 158.2 µm, p = 0.273). Of 20 eyes in the DEX group, 16 (80.0%) and 9 (45.0%) eyes achieved a CRT reduction of ≥ 20% from baseline at 2 months and at 4 months, respectively.

Conclusions: Our results seem to suggest that DEX implant can significantly improve both functional and anatomic clinical outcomes in patients who were unable to complete anti-VEGF loading dose during the COVID-19 pandemic.

Keywords: COVID-19; Dexamethasone Intravitreal Implant; Diabetes; Diabetic Macular Edema; Diabetic Retinopathy; Vascular Endothelial Growth Factor Inhibitors.

© 2021. The Author(s).

- 30 references
- 3 figures

Full text links

SpringerLink FULL-TEXT ARTICLE Springer Free PMC article
Proceed to details
Cite
Share
☐ 1,378
JMIR Ment Health
. 2022 Mar 21;9(3):e33092.
doi: 10.2196/33092.

Treatment Interruptions and Telemedicine Utilization in Serious Mental Illness: Retrospective Longitudinal Claims Analysis

Marcy Ainslie [1], Mary F Brunette [2], Michelle Capozzoli [3]
Affiliations Expand

Affiliations

- [1] Department of Nursing, University of New Hampshire, Durham, NH, United States.
- [2] Department of Psychiatry, Geisel School of Medicine at Dartmouth, Lebanon, NH, United States.
- [3] Department of Mathematics & Statistics, University of New Hampshire, Durham, NH, United States.

- PMID: **35311673**
- DOI: 10.2196/33092

Free article

Treatment Interruptions and Telemedicine Utilization in Serious Mental Illness: Retrospective Longitudinal Claims Analysis

Marcy Ainslie et al. JMIR Ment Health. 2022.
Free article
Show details
JMIR Ment Health
. 2022 Mar 21;9(3):e33092.
doi: 10.2196/33092.

Authors

Marcy Ainslie [1], Mary F Brunette [2], Michelle Capozzoli [3]

Affiliations

- [1] Department of Nursing, University of New Hampshire, Durham, NH, United States.
- [2] Department of Psychiatry, Geisel School of Medicine at Dartmouth, Lebanon, NH, United States.
- [3] Department of Mathematics & Statistics, University of New Hampshire, Durham, NH, United States.

- PMID: **35311673**

- DOI: 10.2196/33092

Abstract

Background: Avoiding interruptions and dropout in outpatient care can prevent mental illness symptom exacerbation and costly crisis services, such as emergency room visits and inpatient psychiatric hospitalization. During the COVID-19 pandemic, to attempt to maintain care continuity, telemedicine services were increasingly utilized, despite the lack of data on efficacy in patients with serious mental illness. Patients with serious mental illness are challenging to enroll and sustain in randomized controlled trials over time due to fluctuations in disease exacerbation. However, capturing and examining utilization and efficacy data in community mental health center (CMHC) patients with serious mental illness during the pandemic is a unique opportunity to inform future clinical and policy decision-making.

Objective: We aimed to identify and describe the characteristics of CMHC patients with serious mental illness who experienced treatment interruptions and who utilized telemedicine during the pandemic.

Methods: We conducted a retrospective observational study of treatment interruptions and telemedicine use during the period from December 2019 to June 2020 (compared to the period from December 2018 to June 2019) in New Hampshire CMHC patients. The study population included all Medicaid beneficiaries with serious mental illness engaged in treatment 3 months prior to the declaration of a state of emergency in response to the COVID-19 pandemic. We used chi-square tests of independence and logistic regression to explore associations between treatment interruptions and variables (gender, age, rurality, and diagnosis). Telemedicine utilization was categorized as low (<25%), medium (25%-75%), or high (>75%) use.

Results: A total of 16,030 patients were identified. New Hampshire CMHCs demonstrated only a 4.9% increase in treatment interruptions compared with the year prior. Patients who were male (odds ratio [OR] 1.27, 95% CI 1.17-1.38; P<.001), under the age of 18 years (ages 0-12 years: OR 1.37, 95% CI 0.62-0.86, P<.001; aged 13-17 years: OR 1.49, 95% CI 0.57-0.79, P<.001), or among milder diagnostic categories, such as anxiety disorders (OR 3.77, 95% CI 3.04-4.68; P<.001) and posttraumatic stress disorder (OR 3.69, 95% CI 2.96-4.61; P<.001), were most likely to experience treatment interruptions. Patients who were female (OR 0.89, CI 0.65-0.74), 18 to 34 years old (OR 0.74, CI 0.70-0.79), or among milder diagnostic categories, such as anxiety disorder (OR 0.69, CI 0.65-0.74) or posttraumatic stress disorder (OR 0.77, CI 0.72-0.83), and with major depressive disorder (OR 0.73, CI 0.68-0.78) were less likely to be in the low telemedicine utilization group.

Conclusions: The integration of telemedicine supported care continuity for most CMHC patients; yet, retention varied by subpopulation, as did telemedicine utilization. The development of policies and clinical practice guidelines requires empirical evidence on the effectiveness and limitations of telemedicine in patients with serious mental illness.

Keywords: mental health; mental illness; retention; serious mental illness; telehealth; telemedicine.

©Marcy Ainslie, Mary F Brunette, Michelle Capozzoli. Originally published in JMIR Mental Health (https://mental.jmir.org), 21.03.2022.

Full text links

JMIR Publications

Proceed to details

Cite

Share

1,379

Observational Study

Crit Care Med

. 2022 Mar 1;50(3):e284-e293.

doi: 10.1097/CCM.0000000000005333.

Single Nucleotide Variant in FAS Associates With Organ Failure and Soluble Fas Cell Surface Death Receptor in Critical Illness

Carmen Mikacenic [1], Pavan Bhatraju [2], Cassianne Robinson-Cohen [3], Susanna Kosamo [4], Alison E Fohner [5], Victoria Dmyterko [2], S Alice Long [1], Karen Cerosaletti [1], Carolyn S Calfee [6], Michael A Matthay [6], Keith R Walley [7], James A Russell [7], Jason D Christie [8], Nuala J Meyer [8], David C Christiani [9], Mark M Wurfel [2]

Affiliations Expand

Affiliations

- [1] Translational Immunology, Benaroya Research Institute, Seattle, WA.
- [2] Division of Pulmonary, Critical Care and Sleep Medicine, University of Washington, Seattle, WA.
- [3] Division of Nephrology, Vanderbilt University Medical Center, Nashville, TN.
- [4] Disease Networks Research Unit, Faculty of Biochemistry and Molecular Medicine, University of Oulu, Oulu, Finland.
- [5] Department of Epidemiology, Institute of Public Health Genetics, University of Washington, Seattle, WA.
- [6] Division of Pulmonary, Critical Care, Allergy, and Sleep Medicine, University of California San Francisco, San Francisco, CA.
- [7] Department of Medicine, St. Paul's Hospital, University of British Columbia, Vancouver, BC, Canada.
- [8] Division of Pulmonary, Allergy, and Critical Care Medicine, Perelman School of Medicine, University of Pennsylvania, Philadelphia, PA.
- [9] Harvard University School of Public Health and Division of Pulmonary and Critical Care, Department of Epidemiology, Massachusetts General Hospital/Harvard Medical School, Boston, MA.

- PMID: **34593707**
- PMCID: **PMC8863632** (available on 2023-03-01)
- DOI: 10.1097/CCM.0000000000005333

Observational Study

Single Nucleotide Variant in FAS Associates With Organ Failure and Soluble Fas Cell Surface Death Receptor in Critical Illness

Carmen Mikacenic et al. Crit Care Med. 2022.
Show details
Crit Care Med
. 2022 Mar 1;50(3):e284-e293.
doi: 10.1097/CCM.0000000000005333.

Authors

Carmen Mikacenic [1], Pavan Bhatraju [2], Cassianne Robinson-Cohen [3], Susanna Kosamo [4], Alison E Fohner [5], Victoria Dmyterko [2], S Alice Long [1], Karen Cerosaletti [1], Carolyn S Calfee [6], Michael A Matthay [6], Keith R Walley [7], James A Russell [7], Jason D Christie [8], Nuala J Meyer [8], David C Christiani [9], Mark M Wurfel [2]

Affiliations

- [1] Translational Immunology, Benaroya Research Institute, Seattle, WA.
- [2] Division of Pulmonary, Critical Care and Sleep Medicine, University of Washington, Seattle, WA.
- [3] Division of Nephrology, Vanderbilt University Medical Center, Nashville, TN.
- [4] Disease Networks Research Unit, Faculty of Biochemistry and Molecular Medicine, University of Oulu, Oulu, Finland.
- [5] Department of Epidemiology, Institute of Public Health Genetics, University of Washington, Seattle, WA.
- [6] Division of Pulmonary, Critical Care, Allergy, and Sleep Medicine, University of California San Francisco, San Francisco, CA.
- [7] Department of Medicine, St. Paul's Hospital, University of British Columbia, Vancouver, BC, Canada.
- [8] Division of Pulmonary, Allergy, and Critical Care Medicine, Perelman School of Medicine, University of Pennsylvania, Philadelphia, PA.
- [9] Harvard University School of Public Health and Division of Pulmonary and Critical Care, Department of Epidemiology, Massachusetts General Hospital/Harvard Medical School, Boston, MA.

- PMID: **34593707**
- PMCID: **PMC8863632** (available on 2023-03-01)
- DOI: 10.1097/CCM.0000000000005333

Abstract

Objectives: Multiple organ failure in critically ill patients is associated with poor prognosis, but biomarkers contributory to pathogenesis are unknown. Previous studies support a role for Fas cell surface death receptor (Fas)-mediated apoptosis in organ dysfunction. Our objectives were to test

for associations between soluble Fas and multiple organ failure, identify protein quantitative trait loci, and determine associations between genetic variants and multiple organ failure.

Design: Retrospective observational cohort study.

Setting: Four academic ICUs at U.S. hospitals.

Patients: Genetic analyses were completed in a discovery (n = 1,589) and validation set (n = 863). Fas gene expression and flow cytometry studies were completed in outpatient research participants (n = 250).

Interventions: None.

Measurements and main results: In discovery and validation sets of critically ill patients, we tested for associations between enrollment plasma soluble Fas concentrations and Sequential Organ Failure Assessment score on day 3. We conducted a genome-wide association study of plasma soluble Fas (discovery n = 1,042) and carried forward a single nucleotide variant in the FAS gene, rs982764, for validation (n = 863). We further tested whether the single nucleotide variant in FAS (rs982764) was associated with Sequential Organ Failure Assessment score, FAS transcriptional isoforms, and Fas cell surface expression. Higher plasma soluble Fas was associated with higher day 3 Sequential Organ Failure Assessment scores in both the discovery ($\beta = 4.07$; $p < 0.001$) and validation ($\beta = 6.96$; $p < 0.001$) sets. A single nucleotide variant in FAS (rs982764G) was associated with lower plasma soluble Fas concentrations and lower day 3 Sequential Organ Failure Assessment score in meta-analysis (-0.21; $p = 0.02$). Single nucleotide variant rs982764G was also associated with a lower relative expression of the transcript for soluble as opposed to transmembrane Fas and higher cell surface expression of Fas on CD4+ T cells.

Conclusions: We found that single nucleotide variant rs982764G was associated with lower plasma soluble Fas concentrations in a discovery and validation population, and single nucleotide variant rs982764G was also associated with lower organ dysfunction on day 3. These findings support further study of the Fas pathway as a potential mediator of organ dysfunction in critically ill patients.

Copyright © 2021 by the Society of Critical Care Medicine and Wolters Kluwer Health, Inc. All Rights Reserved.

Conflict of interest statement

Dr. Mikacenic's institution received funding from the National Center for Advancing Translational Sciences (UL1 TR002319), the National Heart, Lung, and Blood Institute (NHLBI) (R01HL060710, RC2 HL101779), the National Institute on Aging (U19AG023122), the National Institute of allergy and Infectious Diseases (AI101990, AI083455), and the National Institute of Diabetes and Digestive and Kidney Issues (DK097672). Drs. Mikacenic, Bhatraju, Robinson-Cohen, Long, Cerosaletti, Calfee, Matthay, Christie, Meyer, Christiani, and Wurfel received support for article research from the National Institutes of Health (NIH). Drs. Cerosaletti's, Calfee's, and Meyer's institutions received funding from the NIH. Dr. Cerosaletti's institution received funding from the Department of Defense, the American Diabetes Association, and the Juvenile Diabetes Research Foundation. Drs. Calfee's and Matthay's institutions received funding from Gentech/Roche. Dr. Calfee's institution received funding from Bayer; she received funding from Quark, Vasomune, Gen1e Life Sciences, and Prometic. Dr. Matthay received funding from Novartis and Citius Pharmaceuticals. Dr. Russell received funding from Asahi Kesai Pharmaceuticals of America, IB Therapeutics LLC, and Ferring Pharmaceuticals; he received

funding from Grifols; he disclosed that he is the inventor of two patents owned by the University of British Colombia and Ferring, that he is a founder, director, and shareholder in Cyon Therapetutics Inc and a shareholder in Molecular You Corp, and that he is a member of the Data Safety Monitoring Board of an NIH-sponsored trial of plasma in coronavirus disease 2019 (Passive Immunity Trial for Our Nation to Treat COVID-19 in Hospitalized Adults [PassItOn]). Dr. Meyer's institution received funding from the NHLBI (HL137006, HL137915), Quantum Leap Healthcare Collaborative, Biomarck, Inc, Athersys, Inc, and The Marcus Foundation. Dr. Wurfel's institution received funding from the NHLBI. The remaining authors have disclosed that they do not have any potential conflicts of interest.

Supplementary info

Publication types, MeSH terms, Substances, Grant support Expand

Publication types

- Multicenter Study
- Observational Study

MeSH terms

- Adult
- Aged
- Apoptosis
- Biomarkers
- Critical Illness / epidemiology*
- Female
- Genome-Wide Association Study
- Genotype
- Humans
- Intensive Care Units
- Male
- Middle Aged
- Multiple Organ Failure / blood
- Multiple Organ Failure / epidemiology*
- Organ Dysfunction Scores
- Polymorphism, Single Nucleotide
- fas Receptor / blood
- fas Receptor / genetics*

Substances

- Biomarkers
- fas Receptor

Grant support

- R01 AI083455/AI/NIAID NIH HHS/United States
- R01 HL137915/HL/NHLBI NIH HHS/United States
- R56 AI083455/AI/NIAID NIH HHS/United States
- U01 AI101990/AI/NIAID NIH HHS/United States
- UL1 TR002319/TR/NCATS NIH HHS/United States

- DP3 DK097672/DK/NIDDK NIH HHS/United States
- RC2 HL101779/HL/NHLBI NIH HHS/United States
- R01 HL060710/HL/NHLBI NIH HHS/United States
- R01 HL137006/HL/NHLBI NIH HHS/United States
- U19 AG023122/AG/NIA NIH HHS/United States

Show all 10 grants

Full text links

Wolters Kluwer Wolters Kluwer

Proceed to details

Cite

Share

☐ 1,380

Clin Ophthalmol

. 2021 Feb 17;15:661-669.

doi: 10.2147/OPTH.S289068. eCollection 2021.

Delayed Intravitreal Anti-VEGF Therapy for Patients During the COVID-19 Lockdown: An Ethical Endeavor

Mutasem Elfalah [1], Saif Aldeen AlRyalat [1], Mario Damiano Toro [2] [3] [4], Robert Rejdak [4], Sandrine Zweifel [3], Rashed Nazzal [5], Mohammed Abu-Ameerh [1], Osama Ababneh [1], Almutez Gharaibeh [1], Zuhair Sharif [1], Jehad Meqbil [1], Mo'ath AlShawabkeh [1], Amal Alwreikat [6], Muawyah Al Bdour [1], Maysa Al-Hussaini [7], Yacoub A Yousef [8]

Affiliations Expand

Affiliations

- [1] Department of Special Surgery, Faculty of Medicine, The University of Jordan, Amman, Jordan.
- [2] Faculty of Medical Sciences, Collegium Medicum, Cardinal Stefan Wyszyński University, Warsaw, Poland.
- [3] Department of Ophthalmology, University Hospital of Zürich, Zürich, Switzerland.
- [4] Department of General Ophthalmology, Medical University of Lublin, Lublin, Poland.

- [5] Shami Eye Center, Amman, Jordan.
- [6] Department of Ophthalmology, Royal Medical Services, Amman, Jordan.
- [7] Department of Pathology and Laboratory Medicine, King Hussein Cancer Center, Amman, Jordan.
- [8] Department of Surgery (Ophthalmology), King Hussein Cancer Center, Amman, Jordan.

- PMID: **33628009**
- PMCID: PMC7898208
- DOI: 10.2147/OPTH.S289068

Free PMC article

Delayed Intravitreal Anti-VEGF Therapy for Patients During the COVID-19 Lockdown: An Ethical Endeavor

Mutasem Elfalah et al. Clin Ophthalmol. 2021.
Free PMC article
Show details
Clin Ophthalmol
. 2021 Feb 17;15:661-669.
doi: 10.2147/OPTH.S289068. eCollection 2021.

Authors

Mutasem Elfalah [1], Saif Aldeen AlRyalat [1], Mario Damiano Toro [2] [3] [4], Robert Rejdak [4], Sandrine Zweifel [3], Rashed Nazzal [5], Mohammed Abu-Ameerh [1], Osama Ababneh [1], Almutez Gharaibeh [1], Zuhair Sharif [1], Jehad Meqbil [1], Mo'ath AlShawabkeh [1], Amal Alwreikat [6], Muawyah Al Bdour [1], Maysa Al-Hussaini [7], Yacoub A Yousef [8]

Affiliations

- [1] Department of Special Surgery, Faculty of Medicine, The University of Jordan, Amman, Jordan.
- [2] Faculty of Medical Sciences, Collegium Medicum, Cardinal Stefan Wyszyński University, Warsaw, Poland.
- [3] Department of Ophthalmology, University Hospital of Zürich, Zürich, Switzerland.
- [4] Department of General Ophthalmology, Medical University of Lublin, Lublin, Poland.
- [5] Shami Eye Center, Amman, Jordan.
- [6] Department of Ophthalmology, Royal Medical Services, Amman, Jordan.
- [7] Department of Pathology and Laboratory Medicine, King Hussein Cancer Center, Amman, Jordan.
- [8] Department of Surgery (Ophthalmology), King Hussein Cancer Center, Amman, Jordan.

- PMID: **33628009**
- PMCID: PMC7898208

- DOI: 10.2147/OPTH.S289068

Abstract

Purpose: To assess the impact of Jordanian's Corona Virus Disease (COVID-19) lockdown on visual acuity and macular thickness in patients with macular edema receiving intravitreal injections, and to assess the ethical endeavor of lockdown among serious sight threatening conditions.

Patients and methods: This retrospective observational study included patients planned for intravitreal injections who did not complete the planned course before the lockdown (ie, before 20th of March 2020). Data included demographics, indication for the intravitreal injection, corrected distance visual acuity (CDVA), and central macular thickness on Optical Coherence Tomography (OCT) before and after the lockdown.

Results: One-hundred and sixty-six eyes of 125 patients were studied, 68 (54.4%) patients were males, and the mean (± standard deviation, SD) age was 64.79 (±9.41) years. Mean (±SD) duration of delay in the planned injection was 60.97 (±24.35) days. The change in visual acuity was statistically significant for patients with diabetic macular edema (p= 0.045 improvement), patients with central retinal vein thrombosis (CRVO) (p= 0.05 deterioration), and patients with age-related macular degeneration (AMD) (p= 0.005 deterioration). Of interest, delay of more than 2 months and the previous need for 3 or more injections were significant poor prognostic factors for visual outcome for patients with diabetic macular edema (p=0.027 and 0.045).

Conclusion: The impact of delay in the scheduled intravitreal injections resulted in variable outcomes depending on the indication. Triaging the urgency of patients should be based on the indication to support the equity principle of bioethics, where those in need are prioritized against others, depending on potential adverse outcome.

Keywords: COVID-19 lockdown; COVID-19 pandemic; ethics; intravitreal injection; macular edema.

© 2021 Elfalah et al.

Conflict of interest statement

Dr Sandrine Zweifel reports grants, personal fees from Bayer Healthcare Pharmaceuticals, grants, personal fees from Novartis Pharma AG, and personal fees from Roche Diagnostics, outside the submitted work. The authors report no other conflicts of interest in this work.

- 36 references
- 1 figure

Supplementary info

Grant support Expand

Grant support

This research received no external funding.

Full text links

Dove Medical Press Free PMC article

Proceed to details

Cite

Share

☐ 1,381

Adv Virol

. 2021 Jun 25;2021:6689669.

doi: 10.1155/2021/6689669. eCollection 2021.

Evaluation of the Risk of Clinical Deterioration among Inpatients with COVID-19

Víctor O Costa [1], Eveline M Nicolini [2], Bruna M A da Costa [3], Fabrício M Teixeira [1], Júlia P Ferreira [1], Marcos A Moura [4], Jorge Montessi [2], Rogério L Campos [5], Andrea N Guaraldo [6], Patrícia M Costa [7]

Affiliations Expand

Affiliations

- [1] Medicine, Faculdade de Ciências Médicas e da Saúde de Juiz de Fora-SUPREMA, Juiz de Fora 36033-003, Brazil.
- [2] Thoracic Surgery, Hospital Monte Sinai, Juiz de Fora 36033-318, Brazil.
- [3] Nursing, Faculdade de Ciências Médicas e da Saúde de Juiz de Fora-Suprema, Juiz de Fora 36033-003, Brazil.
- [4] Infectology, Faculdade de Ciências Médicas e da Saúde de Juiz de Fora-Suprema, Juiz de Fora 36033-003, Brazil.
- [5] Psychiatrist and Emergency, Hospital Monte Sinai, Juiz de Fora 36033-318, Brazil.
- [6] Intensive Therapy, Hospital Monte Sinai, Juiz de Fora 36033-318, Brazil.
- [7] Intensive Therapy and Dermatology Hospital Monte Sinai, Juiz de Fora 36033-318, Brazil.

- PMID: **34257657**
- PMCID: PMC8241522
- DOI: 10.1155/2021/6689669

Free PMC article

Evaluation of the Risk of Clinical Deterioration among Inpatients with COVID-19

Víctor O Costa et al. Adv Virol. 2021.
Free PMC article
Show details
Adv Virol
. 2021 Jun 25;2021:6689669.
doi: 10.1155/2021/6689669. eCollection 2021.

Authors

Víctor O Costa [1], Eveline M Nicolini [2], Bruna M A da Costa [3], Fabrício M Teixeira [1], Júlia P Ferreira [1], Marcos A Moura [4], Jorge Montessi [2], Rogério L Campos [5], Andrea N Guaraldo [6], Patrícia M Costa [7]

Affiliations

- [1] Medicine, Faculdade de Ciências Médicas e da Saúde de Juiz de Fora-SUPREMA, Juiz de Fora 36033-003, Brazil.
- [2] Thoracic Surgery, Hospital Monte Sinai, Juiz de Fora 36033-318, Brazil.
- [3] Nursing, Faculdade de Ciências Médicas e da Saúde de Juiz de Fora-Suprema, Juiz de Fora 36033-003, Brazil.
- [4] Infectology, Faculdade de Ciências Médicas e da Saúde de Juiz de Fora-Suprema, Juiz de Fora 36033-003, Brazil.
- [5] Psychiatrist and Emergency, Hospital Monte Sinai, Juiz de Fora 36033-318, Brazil.
- [6] Intensive Therapy, Hospital Monte Sinai, Juiz de Fora 36033-318, Brazil.
- [7] Intensive Therapy and Dermatology Hospital Monte Sinai, Juiz de Fora 36033-318, Brazil.

- PMID: **34257657**
- PMCID: PMC8241522
- DOI: 10.1155/2021/6689669

Abstract

This study aims to assess the risk of severe forms of COVID-19, based on clinical, laboratory, and imaging markers in patients initially admitted to the ward. This is a retrospective observational study, with data from electronic medical records of inpatients, with laboratory confirmation of COVID-19, between March and September 2020, in a hospital from Juiz de Fora-MG, Brazil. Participants (n = 74) were separated into two groups by clinical evolution: those who remained in the ward and those who progressed to the ICU. Mann-Whitney U test was taken for continuous variables and the chi-square test or Fisher's exact test for categorical variables. Comparing the proposed groups, lower values of lymphocytes (p = <0.001) and increases in serum creatinine (p = 0.009), LDH (p = 0.057), troponin (p = 0.018), IL-6 (p = 0.053), complement C4 (p = 0.040), and CRP (p = 0.053) showed significant differences or statistical tendency for clinical deterioration. The average age of the groups was 47.9 ± 16.5 and 66.5 ± 7.3 years (p = 0.001). Hypertension (p =

0.064), heart disease ($p = 0.048$), and COPD ($p = 0.039$) were more linked to ICU admission, as well as the presence of tachypnea on admission ($p = 0.051$). Ground-glass involvement >25% of the lung parenchyma or pleural effusion on chest CT showed association with evolution to ICU ($p = 0.027$), as well as bilateral opacifications ($p = 0.030$) when compared to unilateral ones. Laboratory, clinical, and imaging markers may have significant relation with worse outcomes and the need for intensive treatment, being helpful as predictive factors.

Copyright © 2021 Víctor O. Costa et al.

Conflict of interest statement

The authors declare that there are no conflicts of interest regarding the publication of this article.

- 29 references

Full text links

Hindawi Limited Free PMC article

Proceed to details

Cite

Share

☐ 1,382

Z Gesundh Wiss

. 2020 Apr 21;1-4.

doi: 10.1007/s10389-020-01291-2. Online ahead of print.

The clinical data from 19 critically ill patients with coronavirus disease 2019: a single-centered, retrospective, observational study

Jinping Zhang [#1], Peng Liu [#2], Morong Wang [1], Jie Wang [1], Jie Chen [1], Wenling Yuan [1], Mei Li [1], Zhijuan Xie [1], Wangping Dong [1], Hongye Li [1], Yan Zhao [1], Lun Wan [1], Tian Chu [1], Lu Wang [1], Hui Zhang [1], Ting Tao [1], Jing Ma [1]

Affiliations Expand

Affiliations

- [1] 1ICU, Liyuan Hospital affiliated to Tongji Medical College, Huazhong University of Science and Technology, Wuhan, 430071 China.
- [2] 2State Key Laboratory of Virology and Hubei Province Key Laboratory of Allergy and Immune-related Diseases, Department of Immunology, Wuhan University School of Basic Medical Sciences, Wuhan, 430071 China.

[#] Contributed equally.

- PMID: **32318325**

- PMCID: PMC7171052
- DOI: 10.1007/s10389-020-01291-2

Free PMC article

The clinical data from 19 critically ill patients with coronavirus disease 2019: a single-centered, retrospective, observational study

Jinping Zhang et al. Z Gesundh Wiss. 2020.
Free PMC article
Show details
Z Gesundh Wiss
. 2020 Apr 21;1-4.
doi: 10.1007/s10389-020-01291-2. Online ahead of print.

Authors

Jinping Zhang[#,1], Peng Liu[#,2], Morong Wang[1], Jie Wang[1], Jie Chen[1], Wenling Yuan[1], Mei Li[1], Zhijuan Xie[1], Wangping Dong[1], Hongye Li[1], Yan Zhao[1], Lun Wan[1], Tian Chu[1], Lu Wang[1], Hui Zhang[1], Ting Tao[1], Jing Ma[1]

Affiliations

- [1] 1ICU, Liyuan Hospital affiliated to Tongji Medical College, Huazhong University of Science and Technology, Wuhan, 430071 China.
- [2] 2State Key Laboratory of Virology and Hubei Province Key Laboratory of Allergy and Immune-related Diseases, Department of Immunology, Wuhan University School of Basic Medical Sciences, Wuhan, 430071 China.

[#] Contributed equally.

- PMID: **32318325**
- PMCID: PMC7171052
- DOI: 10.1007/s10389-020-01291-2

Abstract

Background: The objectives of this study were to analyze the clinical features of coronavirus disease 2019 (COVID-19) and evaluate the diagnosis and treatment.

Methods: A retrospective analysis of the clinical manifestation and auxiliary examination of 19 patients with COVID-19 from the Liyuan Hospital intensive care unit (ICU) between January 16, 2020 and February 20, 2020 was undertaken.

Results: There were 11 male and 8 female cases among the patients. The median (range) age was 73 (38-91) years. Of these patients, 8 (42.1%) had died and the median duration from ICU

admission to death was 2 (interquartile range (IQR): 1-10.75) days. Seven of these 8 patients had underlying diseases. The auxiliary examination showed fever (68.4%), dry cough (15.8%), dyspnea (10.5%), and diarrhea (5.3%). All 19 cases showed ground-glass changes on chest computed tomography. Serum hypersensitive C-reactive protein (hs-CRP) and serum amylase A (SAA) were clearly increased in all of the cases. Among the 19 cases, there were 16 (84.2%) cases in which the total number of lymphocytes decreased, 12 cases (63%) had reduced liver function, and 11 cases (58%) had deviant results for fibrinogen (FIB) and D-dimer, in particular, the D-dimer level was significantly higher in the non-survivors compared with the survivors.

Conclusion: There were more men than women among critically ill patients. All of the cases showed ground-glass changes on chest computed tomography and the vast majority of patients displayed fever and dry cough. The clinical laboratory indices change significantly, especially the D-dimer level among non-survivors.

Keywords: Clinical manifestations; Coronavirus disease 2019; D-dimer; Epidemiology; Retrospective.

© Springer-Verlag GmbH Germany, part of Springer Nature 2020.

Conflict of interest statement

Conflict of interestThe authors declare that they have no conflicts of interest related to this work. We declare that we do not have any commercial or associative interest that represents a conflict of interest in connection with the work submitted.

- 12 references
- 1 figure

Full text links

Free PMC article
Proceed to details
Cite
Share
☐ 1,383

J Clin Apher
. 2022 Feb 17.
doi: 10.1002/jca.21971. Online ahead of print.

Early is superior to late plasma exchange for severe multisystem inflammatory syndrome in children

Banu Katlan [1], Selman Kesici [2], Dilek Karacanoğlu [1], Pembe Derin Oygar [3], Ayse Ünal Yüksekgönül [4], Seher Şener [5], Hayrettin Hakan Aykan [4], Yasemin Özsurekçi [3], Seza Özen [5], Benan Bayrakci [2]
Affiliations Expand

Affiliations

- [1] Department of Pediatric Critical Care Medicine, Hacettepe University, Ankara, Turkey.
- [2] Life Support Practice and Research Center, Hacettepe University, Ankara, Turkey.
- [3] Department of Pediatric Infection Diseases, Hacettepe University, Ankara, Turkey.
- [4] Department of Pediatric Cardiology, Hacettepe University, Ankara, Turkey.
- [5] Department of Pediatric Rheumatology, Hacettepe University, Ankara, Turkey.

- PMID: **35174897**
- DOI: 10.1002/jca.21971

Early is superior to late plasma exchange for severe multisystem inflammatory syndrome in children

Banu Katlan et al. J Clin Apher. 2022.

Show details

J Clin Apher

. 2022 Feb 17.
doi: 10.1002/jca.21971. Online ahead of print.

Authors

Banu Katlan [1], Selman Kesici [2], Dilek Karacanoğlu [1], Pembe Derin Oygar [3], Ayse Ünal Yüksekgönül [4], Seher Şener [5], Hayrettin Hakan Aykan [4], Yasemin Özsurekçi [3], Seza Özen [5], Benan Bayrakci [2]

Affiliations

- [1] Department of Pediatric Critical Care Medicine, Hacettepe University, Ankara, Turkey.
- [2] Life Support Practice and Research Center, Hacettepe University, Ankara, Turkey.
- [3] Department of Pediatric Infection Diseases, Hacettepe University, Ankara, Turkey.
- [4] Department of Pediatric Cardiology, Hacettepe University, Ankara, Turkey.
- [5] Department of Pediatric Rheumatology, Hacettepe University, Ankara, Turkey.

- PMID: **35174897**
- DOI: 10.1002/jca.21971

Abstract

Background: Multisystem inflammatory syndrome in children (MIS-C) can be life threatening in severe cases because of uncontrolled inflammation and multi-organ failure. In this study, we report the effect of plasma exchange in the treatment of MIS-C and to emphasize the effect of its early application on outcome.

Method: In this retrospective observational study, the medical records of children with severe MIS-C admitted to pediatric intensive care unit (PICU) between April 2020 and January 2021 were reviewed. Severe MIS-C patients were treated according to protocol consisting of plasma exchange (PE), intravenous immune globulin, steroids, and anakinra which we called the "PISA" protocol referring to the initials. The patients were divided into two groups as early plasma exchange (E-PE) and late plasma exchange (L-PE) according to the elapse time between hospital admission and the administration of PE. Groups were compared in terms of outcome variables. Primary study outcome was 28-day mortality. Secondary outcome variables were acute phase response time, length of immunomodulatory treatment, frequency of patients requiring mechanical ventilation (MV) and inotropic support, length of inotropic support and MV, length of hospital and PICU stays.

Results: Eighteen pediatric patients with MIS-C were included in the study. Seventeen (95%) of the patients presented with decompensated shock and required inotropic support. One of the 17 patients needed extracorporeal membrane oxygenation support (ECMO) PISA protocol was used in all patients. There was no mortality in the E-PE group while the mortality rate was 20% in the L-PE group. Acute phase reactant response was faster in the E-PE group and immunomodulatory treatments could be reduced earlier; the frequency of patients requiring inotropic and mechanical ventilation (MV) support was lower in the E-PE group; the duration of inotropic support, duration of MV, and length of stay in hospital and PICU were significantly shorter in the E-PE group.

Conclusion: We suggest that in selected cases, timely administration of PE is a beneficial rescue therapy for MIS-C related hyperinflammation presenting with severe cardiovascular collapse.

Keywords: SARS-CoV-2; children; intensive care unit; plasma exchange; severe MIS-C.

© 2022 Wiley Periodicals LLC.

- 24 references

Full text links

WILEY Full Text Article Wiley

Proceed to details

Cite

Share

☐ 1,384

Exp Hematol Oncol
. 2020 Aug 25;9:21.
doi: 10.1186/s40164-020-00177-z. eCollection 2020.

Risk factors and outcome of COVID-19 in patients with hematological malignancies

José Luis Piñana [1] [2] [3], Rodrigo Martino [4], Irene García-García [5], Rocío Parody [6], María Dolores Morales [7], Gonzalo Benzo [8], Irene Gómez-Catalan [9], Rosa Coll [10], Ignacio De La Fuente [11], Alejandro Luna [5], Beatriz Merchán [7], Anabelle Chinea [5], Dunia de Miguel [7], Ana Serrano [9], Carmen Pérez [11], Carola Diaz [12], José Luis Lopez [13], Adolfo Jesús Saez [5], Rebeca

Bailen [14], Teresa Zudaire [15], Diana Martínez [16], Manuel Jurado [17], María Calbacho [18], Lourdes Vázquez [19], Irene Garcia-Cadenas [4], Laura Fox [20], Ana I Pimentel [21], Guiomar Bautista [22], Agustin Nieto [23], Pascual Fernandez [24], Juan Carlos Vallejo [25], Carlos Solano [26], Marta Valero [27], Ildefonso Espigado [28], Raquel Saldaña [29], Luisa Sisinni [30], Josep Maria Ribera [31], Maria Jose Jimenez [31], Maria Trabazo [32], Marta Gonzalez-Vicent [33], Noemí Fernández [34], Carme Talarn [35], Maria Carmen Montoya [9], Angel Cedillo [36], Anna Sureda [6], Infectious Complications Subcommittee of the Spanish Hematopoietic Stem Cell Transplantation and Cell Therapy Group (GETH)

Affiliations Expand

Affiliations

- [1] Hematology División, Hospital Universitario y Politécnico La Fe, Instituto de Investigación Sanitaria La Fe, Valencia, Spain.
- [2] CIBERONC, Instituto Carlos III, Madrid, Spain.
- [3] Division of Clinical Hematology, Hospital Universitario la Fe de Valencia, Avda Fernando Abril Martorell, 106 CP 46026 Valencia, Spain.
- [4] Hematology División, Hospital de la Santa Creu i Sant Pau, Barcelona, Spain.
- [5] Hematology División, Hospital Ramon y Cajal, Madrid, Spain.
- [6] Hematology División, Institut Català Oncologia-Hospital Duran i Reynals, Barcelona, Spain.
- [7] Hematology División, Hospital de Guadalajara, Guadalajara, Spain.
- [8] Hematology División, Hospital La Princesa, Madrid, Spain.
- [9] Hematology División, Hospital de Albacete, Albacete, Spain.
- [10] Hematology División, Institut Català Oncologia-Hospital Josep Trueta, Girona, Spain.
- [11] Hematology División, Hospital Clínico de Valladolid, Valladolid, Spain.
- [12] Hematology División, Hospital Carlos Haya, Malaga, Spain.
- [13] Hematology División, Hospital Fundación Jiménez Díaz, Madrid, Spain.
- [14] Hematology División, Hospital Gregorio Marañon, Madrid, Spain.
- [15] Hematology División, Hospital de Navarra, Navarra, Spain.
- [16] Hematology División, Hospital a Coruña, Coruña, Spain.
- [17] Hematology División, Hospital Virgen de la Nieves, Granada, Spain.
- [18] Hematology División, Hospital 12 de Octubre, Madrid, Spain.
- [19] Hematology División, Hospital Universitario de Salamanca, Salamanca, Spain.
- [20] Hematology División, Hospital Vall d`Hebron, Barcelona, Spain.
- [21] Hematology División, Hospital Clínico Universitario Lozano Blesa, IIS Aragon, Zaragoza, Spain.
- [22] Hematology División, Hospital Puerta de Hierro, Madrid, Spain.
- [23] Hematology División, Hospital de Vigo, Vigo, Spain.
- [24] Hematology División, Hospital General de Alicante, Alicante, Spain.
- [25] Hematology División, Hospital de Donostia, Donostia, Spain.
- [26] Hematology División, Hospital Clínico Universitario de Valencia, Valencia, Spain.
- [27] Hematology División, Hospital Arnau de Vilanova, Valencia, Spain.
- [28] Department of Hematology, University Hospital Virgen del Rocío/University of Sevilla, CSIC/Institute of Biomedicine of Sevilla, Sevilla, Spain.
- [29] Hematology División, Hospital de Jerez, Jerez, Spain.
- [30] Pediatric Hematology-Oncology División, Hospital la Paz, Madrid, Spain.

- [31] Hematology División, ICO-Hospital Germans Trias i Pujol, Josep Carreras Research Institute, Badalona, Spain.
- [32] Pediatric División, Hospital de la Santa Creu i Sant Pau, Barcelona, Spain.
- [33] Pediatric División, Hospital niño Jesús, Madrid, Spain.
- [34] Hematology División, Hospital Marqués de Valdecilla, Santander, Spain.
- [35] Hematology División, Hospital Joan XXIII, Tarragona, Spain.
- [36] Hematopoietic Stem Cell Transplantation and Cell Therapy Group (GETH), Madrid, Spain.

- PMID: **32864192**
- PMCID: PMC7445734
- DOI: 10.1186/s40164-020-00177-z

Free PMC article

Risk factors and outcome of COVID-19 in patients with hematological malignancies

José Luis Piñana et al. Exp Hematol Oncol. 2020.
Free PMC article

Show details

Exp Hematol Oncol
. 2020 Aug 25;9:21.
doi: 10.1186/s40164-020-00177-z. eCollection 2020.

Authors

José Luis Piñana [1] [2] [3], Rodrigo Martino [4], Irene García-García [5], Rocío Parody [6], María Dolores Morales [7], Gonzalo Benzo [8], Irene Gómez-Catalan [9], Rosa Coll [10], Ignacio De La Fuente [11], Alejandro Luna [5], Beatriz Merchán [7], Anabelle Chinea [5], Dunia de Miguel [7], Ana Serrano [9], Carmen Pérez [11], Carola Diaz [12], José Luis Lopez [13], Adolfo Jesús Saez [5], Rebeca Bailen [14], Teresa Zudaire [15], Diana Martínez [16], Manuel Jurado [17], María Calbacho [18], Lourdes Vázquez [19], Irene Garcia-Cadenas [4], Laura Fox [20], Ana I Pimentel [21], Guiomar Bautista [22], Agustin Nieto [23], Pascual Fernandez [24], Juan Carlos Vallejo [25], Carlos Solano [26], Marta Valero [27], Ildefonso Espigado [28], Raquel Saldaña [29], Luisa Sisinni [30], Josep Maria Ribera [31], Maria Jose Jimenez [31], Maria Trabazo [32], Marta Gonzalez-Vicent [33], Noemí Fernández [34], Carme Talarn [35], Maria Carmen Montoya [9], Angel Cedillo [36], Anna Sureda [6], Infectious Complications Subcommittee of the Spanish Hematopoietic Stem Cell Transplantation and Cell Therapy Group (GETH)

Affiliations

- [1] Hematology División, Hospital Universitario y Politécnico La Fe, Instituto de Investigación Sanitaria La Fe, Valencia, Spain.
- [2] CIBERONC, Instituto Carlos III, Madrid, Spain.
- [3] Division of Clinical Hematology, Hospital Universitario la Fe de Valencia, Avda Fernando Abril Martorell, 106 CP 46026 Valencia, Spain.

- [4] Hematology División, Hospital de la Santa Creu i Sant Pau, Barcelona, Spain.
- [5] Hematology División, Hospital Ramon y Cajal, Madrid, Spain.
- [6] Hematology División, Institut Català Oncologia-Hospital Duran i Reynals, Barcelona, Spain.
- [7] Hematology División, Hospital de Guadalajara, Guadalajara, Spain.
- [8] Hematology División, Hospital La Princesa, Madrid, Spain.
- [9] Hematology División, Hospital de Albacete, Albacete, Spain.
- [10] Hematology División, Institut Català Oncologia-Hospital Josep Trueta, Girona, Spain.
- [11] Hematology División, Hospital Clínico de Valladolid, Valladolid, Spain.
- [12] Hematology División, Hospital Carlos Haya, Malaga, Spain.
- [13] Hematology División, Hospital Fundación Jiménez Díaz, Madrid, Spain.
- [14] Hematology División, Hospital Gregorio Marañon, Madrid, Spain.
- [15] Hematology División, Hospital de Navarra, Navarra, Spain.
- [16] Hematology División, Hospital a Coruña, Coruña, Spain.
- [17] Hematology División, Hospital Virgen de la Nieves, Granada, Spain.
- [18] Hematology División, Hospital 12 de Octubre, Madrid, Spain.
- [19] Hematology División, Hospital Universitario de Salamanca, Salamanca, Spain.
- [20] Hematology División, Hospital Vall d`Hebron, Barcelona, Spain.
- [21] Hematology División, Hospital Clínico Universitario Lozano Blesa, IIS Aragon, Zaragoza, Spain.
- [22] Hematology División, Hospital Puerta de Hierro, Madrid, Spain.
- [23] Hematology División, Hospital de Vigo, Vigo, Spain.
- [24] Hematology División, Hospital General de Alicante, Alicante, Spain.
- [25] Hematology División, Hospital de Donostia, Donostia, Spain.
- [26] Hematology División, Hospital Clínico Universitario de Valencia, Valencia, Spain.
- [27] Hematology División, Hospital Arnau de Vilanova, Valencia, Spain.
- [28] Department of Hematology, University Hospital Virgen del Rocío/University of Sevilla, CSIC/Institute of Biomedicine of Sevilla, Sevilla, Spain.
- [29] Hematology División, Hospital de Jerez, Jerez, Spain.
- [30] Pediatric Hematology-Oncology División, Hospital la Paz, Madrid, Spain.
- [31] Hematology División, ICO-Hospital Germans Trias i Pujol, Josep Carreras Research Institute, Badalona, Spain.
- [32] Pediatric División, Hospital de la Santa Creu i Sant Pau, Barcelona, Spain.
- [33] Pediatric División, Hospital niño Jesús, Madrid, Spain.
- [34] Hematology División, Hospital Marqués de Valdecilla, Santander, Spain.
- [35] Hematology División, Hospital Joan XXIII, Tarragona, Spain.
- [36] Hematopoietic Stem Cell Transplantation and Cell Therapy Group (GETH), Madrid, Spain.

- PMID: **32864192**
- PMCID: PMC7445734
- DOI: 10.1186/s40164-020-00177-z

Abstract

Background: Prognostic factors of poor outcome in patients with hematological malignancies and COVID-19 are poorly defined.

Patients and methods: This was a Spanish transplant group and cell therapy (GETH) multicenter retrospective observational study, which included a large cohort of blood cancer patients with laboratory-confirmed SARS-CoV-2 infection through PCR assays from March 1st 2020 to May 15th 2020.

Results: We included 367 pediatric and adult patients with hematological malignancies, including recipients of autologous (ASCT) (n = 58) or allogeneic stem cell transplantation (allo-SCT) (n = 65) from 41 hospitals in Spain. Median age of patients was 64 years (range 1-93.8). Recipients of ASCT and allo-SCT showed lower mortality rates (17% and 18%, respectively) compared to non-SCT patients (31%) (p = 0.02). Prognostic factors identified for day 45 overall mortality (OM) by logistic regression multivariate analysis included age > 70 years [odds ratio (OR) 2.1, 95% confidence interval (CI) 1.2-3.8, p = 0.011]; uncontrolled hematological malignancy (OR 2.9, 95% CI 1.6-5.2, p < 0.0001); ECOG 3-4 (OR, 2.56, 95% CI 1.4-4.7, p = 0.003); neutropenia (< 0.5 × 10^9/L) (OR 2.8, 95% CI 1.3-6.1, p = 0.01); and a C-reactive protein (CRP) > 20 mg/dL (OR 3.3, 95% CI 1.7-6.4, p < 0.0001). In multivariate analysis of 216 patients with very severe COVID-19, treatment with azithromycin or low dose corticosteroids was associated with lower OM (OR 0.42, 95% CI 0.2-0.89 and OR 0.31, 95% CI 0.11-0.87, respectively, p = 0.02) whereas the use of hidroxycloroquine did not show significant improvement in OM (OR 0.64, 95% CI 0.37-1.1, P = 0.1).

Conclusions: In most patients with hematological malignancies COVID-19 mortality was directly driven by older age, disease status, performance status, as well as by immune (neutropenia) parameters and level of inflammation (high CRP). Use of azithromycin and low dose corticosteroids may be of value in very severe COVID-19.

© The Author(s) 2020.

Conflict of interest statement

Competing interestsThe author(s) declare that they have no conflict of interests.

- 47 references
- 3 figures

Full text links

Read free full text at BMC BioMed Central Free PMC article

Proceed to details

Cite

Share

☐ 1,385

JMIR Form Res

. 2022 Mar 15;6(3):e29967.
doi: 10.2196/29967.

Machine-Aided Self-diagnostic Prediction Models for Polycystic Ovary Syndrome: Observational Study

Angela Zigarelli [1], Ziyang Jia [1], Hyunsun Lee [1]
Affiliations Expand

Affiliation

- [1] Department of Mathematics and Statistics, University of Massachusetts Amherst, Newton, MA, United States.

- PMID: **35289757**
- DOI: 10.2196/29967

Free article

Machine-Aided Self-diagnostic Prediction Models for Polycystic Ovary Syndrome: Observational Study

Angela Zigarelli et al. JMIR Form Res. 2022.
Free article
Show details
JMIR Form Res
. 2022 Mar 15;6(3):e29967.
doi: 10.2196/29967.

Authors

Angela Zigarelli [1], Ziyang Jia [1], Hyunsun Lee [1]

Affiliation

- [1] Department of Mathematics and Statistics, University of Massachusetts Amherst, Newton, MA, United States.

- PMID: **35289757**
- DOI: 10.2196/29967

Abstract

Background: Artificial intelligence and digital health care have substantially advanced to improve and enhance medical diagnosis and treatment during the prolonged period of the COVID-

19 global pandemic. In this study, we discuss the development of prediction models for the self-diagnosis of polycystic ovary syndrome (PCOS) using machine learning techniques.

Objective: We aim to develop self-diagnostic prediction models for PCOS in potential patients and clinical providers. For potential patients, the prediction is based only on noninvasive measures such as anthropomorphic measures, symptoms, age, and other lifestyle factors so that the proposed prediction tool can be conveniently used without any laboratory or ultrasound test results. For clinical providers who can access patients' medical test results, prediction models using all predictor variables can be adopted to help health providers diagnose patients with PCOS. We compare both prediction models using various error metrics. We call the former model the patient model and the latter, the provider model throughout this paper.

Methods: In this retrospective study, a publicly available data set of 541 women's health information collected from 10 different hospitals in Kerala, India, including PCOS status, was acquired and used for analysis. We adopted the CatBoost method for classification, K-fold cross-validation for estimating the performance of models, and SHAP (Shapley Additive Explanations) values to explain the importance of each variable. In our subgroup study, we used k-means clustering and Principal Component Analysis to split the data set into 2 distinct BMI subgroups and compared the prediction results as well as the feature importance between the 2 subgroups.

Results: We achieved 81% to 82.5% prediction accuracy of PCOS status without any invasive measures in the patient models and achieved 87.5% to 90.1% prediction accuracy using both noninvasive and invasive predictor variables in the provider models. Among noninvasive measures, variables including acanthosis nigricans, acne, hirsutism, irregular menstrual cycle, length of menstrual cycle, weight gain, fast food consumption, and age were more important in the models. In medical test results, the numbers of follicles in the right and left ovaries and anti-Müllerian hormone were ranked highly in feature importance. We also reported more detailed results in a subgroup study.

Conclusions: The proposed prediction models are ultimately expected to serve as a convenient digital platform with which users can acquire pre- or self-diagnosis and counsel for the risk of PCOS, with or without obtaining medical test results. It will enable women to conveniently access the platform at home without delay before they seek further medical care. Clinical providers can also use the proposed prediction tool to help diagnose PCOS in women.

Keywords: CatBoost; Polycystic Ovary Syndrome (PCOS); SHAP values; clustering; machine learning; prediction; principal component analysis; self-diagnosis; subgroup study.

©Angela Zigarelli, Ziyang Jia, Hyunsun Lee. Originally published in JMIR Formative Research (https://formative.jmir.org), 15.03.2022.

Full text links

JMIR Publications OPEN ACCESS JMIR Publications

Proceed to details

Cite

Share

☐ 1,386

J Adv Nurs

. 2022 Feb 17.
doi: 10.1111/jan.15189. Online ahead of print.

Nurse admissions at a specialized mental health programme: A pre-Covid-19 retrospective review (2000-2019)

M Dolores Braquehais [1] [2], Xulián Mozo [1], Eva Gausachs [1], Regina Santiago [1], Enric Llavayol [1], Olga González-Irizar [1], Gemma Nieva [1] [2] [3], Sergi Valero [1] [4], José A Ramos-Quiroga [1] [2] [3] [5] [6], Eugeni Bruguera [1] [2] [3]
Affiliations Expand

Affiliations

- [1] Galatea Care Programme for Sick Health Professionals, Galatea Clinic, Galatea Foundation, Barcelona, Spain.
- [2] Department of Psychiatry, Mental Health and Addiction Research Group, Vall d'Hebron Institut de Recerca (VHIR), Vall d'Hebron Hospital Universitari, Vall d'Hebron Barcelona Hospital Campus, Barcelona, Spain.
- [3] Department of Psychiatry, Vall d'Hebron Hospital Universitari, Vall d'Hebron Barcelona Hospital Campus, Barcelona, Spain.
- [4] ACE Alzheimer Center BarcelonaResearch Center and Memory Clinic, Fundació ACE, Barcelona Alzheimer Treatment and Research Centre, Institut Català de Neurociències Aplicades, Universitat Internacional de Catalunya (UIC), Barcelona, Spain.
- [5] Biomedical Network Research Centre on Mental Health (CIBERSAM), Instituto de Salud Carlos III, Avenida de Monforte de Lemos 3-5, Madrid, Spain.
- [6] Departmen of Psychiatry, School of Medicine, Universitat Autònoma de Barcelona, Barcelona, Spain.

- PMID: **35174899**
- DOI: 10.1111/jan.15189

Nurse admissions at a specialized mental health programme: A pre-Covid-19 retrospective review (2000-2019)

M Dolores Braquehais et al. J Adv Nurs. 2022.
Show details
J Adv Nurs
. 2022 Feb 17.
doi: 10.1111/jan.15189. Online ahead of print.

Authors

M Dolores Braquehais [1] [2], Xulián Mozo [1], Eva Gausachs [1], Regina Santiago [1], Enric Llavayol [1], Olga González-Irizar [1], Gemma Nieva [1] [2] [3], Sergi Valero [1] [4], José A Ramos-Quiroga [1] [2] [3] [5] [6], Eugeni Bruguera [1] [2] [3]

Affiliations

- [1] Galatea Care Programme for Sick Health Professionals, Galatea Clinic, Galatea Foundation, Barcelona, Spain.
- [2] Department of Psychiatry, Mental Health and Addiction Research Group, Vall d'Hebron Institut de Recerca (VHIR), Vall d'Hebron Hospital Universitari, Vall d'Hebron Barcelona Hospital Campus, Barcelona, Spain.
- [3] Department of Psychiatry, Vall d'Hebron Hospital Universitari, Vall d'Hebron Barcelona Hospital Campus, Barcelona, Spain.
- [4] ACE Alzheimer Center BarcelonaResearch Center and Memory Clinic, Fundació ACE, Barcelona Alzheimer Treatment and Research Centre, Institut Català de Neurociències Aplicades, Universitat Internacional de Catalunya (UIC), Barcelona, Spain.
- [5] Biomedical Network Research Centre on Mental Health (CIBERSAM), Instituto de Salud Carlos III, Avenida de Monforte de Lemos 3-5, Madrid, Spain.
- [6] Departmen of Psychiatry, School of Medicine, Universitat Autònoma de Barcelona, Barcelona, Spain.

- PMID: **35174899**
- DOI: 10.1111/jan.15189

Abstract

Aims: Nursing is a stressful and emotionally demanding profession. To date, few mental health treatment interventions have been developed for them worldwide. This study aims to explore referral trends in nurses with mental disorders admitted to a pioneer specialized mental health programme in Europe from 2000 to 2019.

Design: A retrospective observational study of 1297 medical e-records of nurses with mental health disorders admitted to the Galatea Care Programme in Barcelona was conducted.

Methods: Three periods were analysed: 2000-2006, 2007-2012 and 2013-2019. Socio-demographic and clinical variables were compared. Diagnoses followed Diagnostic and Statistical Manual of Mental Disorders (DSM-IV-TR) criteria.

Results: Gender and age at referral did not change over time. Self-referrals grew from 85.1% in the first period to 95.3% in the last period; inpatient admissions decreased from 24.1% to 18.2%, although this was not significant; nurses were less frequently on sick leave on admission over time (59.1% vs. 45.7%); they were more likely to have a temporary contract in the second period (9.5% vs. 4.8% and 4%) and prevalence of main diagnosis changed with a considerable decrease in affective and substance use disorders after 2006 and a progressive increase in adjustment disorders during the whole period.

Conclusion: Free, voluntary, highly confidential programmes for nurses with mental disorders may enhance voluntary and earlier help seeking. These findings can be considered when implementing specialized interventions for them in other settings. WHAT PROBLEM DID THE STUDY ADDRESS?: Nursing is a stressful and emotionally demanding profession. To date, few specialized mental health services have been developed for them worldwide. This study aims to

explore referral trends in nurses with mental disorders admitted to a pioneer programme in Europe, the Galatea Care Programme in Barcelona, from 2000 to 2019. WHAT WERE THE MAIN FINDINGS?: The number of referrals to the programme grew especially after the first 7-year period. Admissions were more likely to be voluntary during the last period. Prevalence of substance use disorders at admission dropped steadily while prevalence of adjustment disorders progressively increased over the two decades. Nurses were also less likely to be on sick leave at admission. WHERE AND ON WHOM WILL THE RESEARCH HAVE IMPACT?: Free, voluntary, highly confidential programmes for nurses with mental disorders may enhance voluntary and earlier help seeking. These findings can be considered when implementing specialized interventions for them in other settings.

Keywords: addictions; help-seeking behaviour; mental disorders; mental health; mental health services; nurse; nursing; occupational health; risk to practice; self-referrals.

© 2022 John Wiley & Sons Ltd.

- 38 references

Full text links

WILEY Full Text Article Wiley
Proceed to details
Cite
Share
☐ 1,387

This article is a preprint
Preprints have not been peer reviewed.
Learn more about preprints in the NIH Preprint Pilot.

medRxiv
. 2020 Nov 8;2020.07.19.20157305.
doi: 10.1101/2020.07.19.20157305. Preprint

Clinical Characteristics and Outcomes for 7,995 Patients with SARS-CoV-2 Infection

Jacob McPadden [1], Frederick Warner [1 2], H Patrick Young [3 4], Nathan C Hurley [5], Rebecca A Pulk [6], Avinainder Singh [4], Thomas Js Durant [3 7], Guannan Gong [3 8], Nihar Desai [3], Adrian Haimovich [9], Richard Andrew Taylor [10], Murat Gunel [11 12 13 14], Charles S Dela Cruz [15], Shelli F Farhadian [16], Jonathan Siner [15], Merceditas Villanueva [4 17], Keith Churchwell [18], Allen Hsiao [1 19], Charles J Torre Jr [7 19], Eric J Velazquez [2], Roy S Herbst [20], Akiko Iwasaki [21 22], Albert I Ko [23], Bobak J Mortazavi [3 2 5 24], Harlan M Krumholz [3 2 25], Wade L Schulz [3 7]
Affiliations Expand

Affiliations

- [1] Department of Pediatrics, Yale School of Medicine, New Haven, CT.

- [2] Section of Cardiovascular Medicine, Department of Internal Medicine, Yale School of Medicine, New Haven, CT.
- [3] Center for Outcomes Research and Evaluation, Yale-New Haven Hospital, New Haven, CT.
- [4] Department of Internal Medicine, Yale University School of Medicine, New Haven, CT.
- [5] Department of Computer Science and Engineering, Texas A&M University, College Station, TX.
- [6] Corporate Pharmacy Services, Yale New Haven Health, New Haven, CT.
- [7] Department of Laboratory Medicine, Yale University School of Medicine, New Haven, CT.
- [8] Interdepartmental Program in Computational Biology and Bioinformatics, Yale University School of Medicine, New Haven, CT.
- [9] Yale School of Medicine, New Haven, CT.
- [10] Department of Emergency Medicine, Yale School of Medicine, New Haven, CT.
- [11] Department of Genetics, Yale University School of Medicine, New Haven, CT.
- [12] Medical Scientist Training Program, Yale University School of Medicine, New Haven, CT.
- [13] Yale Center for Genome Analysis, Yale University School of Medicine, New Haven, CT.
- [14] Department of Neurosurgery, Yale University School of Medicine, New Haven, CT.
- [15] Department of Internal Medicine, Pulmonary, Critical Care and Sleep Medicine, Yale School of Medicine, New Haven, CT.
- [16] Department of Internal Medicine, Section of Infectious Diseases, Yale School of Medicine, New Haven, CT.
- [17] Center for Interdisciplinary Research on AIDS, Yale School of Public Health, New Haven, CT.
- [18] Yale New Haven Hospital, New Haven, CT.
- [19] Information Technology Services, Yale New Haven Health, New Haven, CT.
- [20] Yale Comprehensive Cancer Center, Yale School of Medicine, New Haven, CT.
- [21] Department of Immunobiology, Yale University School of Medicine, New Haven, CT.
- [22] Howard Hughes Medical Institute, Chevy Chase, MD.
- [23] Department of Epidemiology of Microbial Diseases, Yale School of Public Health, New Haven, CT.
- [24] Center for Remote Health Technologies and Systems, Texas A&M University, College Station, TX.
- [25] Department of Health Policy and Management, Yale School of Public Health, New Haven, CT.

- PMID: **32743602**
- PMCID: PMC7386526
- DOI: 10.1101/2020.07.19.20157305

Free PMC article

Clinical Characteristics and Outcomes for 7,995 Patients with SARS-CoV-2 Infection

Jacob McPadden et al. medRxiv. 2020.

Free PMC article

Show details

medRxiv

. 2020 Nov 8;2020.07.19.20157305.
doi: 10.1101/2020.07.19.20157305. Preprint

Authors

Jacob McPadden [1], Frederick Warner [1] [2], H Patrick Young [3] [4], Nathan C Hurley [5], Rebecca A Pulk [6], Avinainder Singh [4], Thomas Js Durant [3] [7], Guannan Gong [3] [8], Nihar Desai [3], Adrian Haimovich [9], Richard Andrew Taylor [10], Murat Gunel [11] [12] [13] [14], Charles S Dela Cruz [15], Shelli F Farhadian [16], Jonathan Siner [15], Merceditas Villanueva [4] [17], Keith Churchwell [18], Allen Hsiao [1] [19], Charles J Torre Jr [7] [19], Eric J Velazquez [2], Roy S Herbst [20], Akiko Iwasaki [21] [22], Albert I Ko [23], Bobak J Mortazavi [3] [2] [5] [24], Harlan M Krumholz [3] [2] [25], Wade L Schulz [3] [7]

Affiliations

- [1] Department of Pediatrics, Yale School of Medicine, New Haven, CT.
- [2] Section of Cardiovascular Medicine, Department of Internal Medicine, Yale School of Medicine, New Haven, CT.
- [3] Center for Outcomes Research and Evaluation, Yale-New Haven Hospital, New Haven, CT.
- [4] Department of Internal Medicine, Yale University School of Medicine, New Haven, CT.
- [5] Department of Computer Science and Engineering, Texas A&M University, College Station, TX.
- [6] Corporate Pharmacy Services, Yale New Haven Health, New Haven, CT.
- [7] Department of Laboratory Medicine, Yale University School of Medicine, New Haven, CT.
- [8] Interdepartmental Program in Computational Biology and Bioinformatics, Yale University School of Medicine, New Haven, CT.
- [9] Yale School of Medicine, New Haven, CT.
- [10] Department of Emergency Medicine, Yale School of Medicine, New Haven, CT.
- [11] Department of Genetics, Yale University School of Medicine, New Haven, CT.
- [12] Medical Scientist Training Program, Yale University School of Medicine, New Haven, CT.
- [13] Yale Center for Genome Analysis, Yale University School of Medicine, New Haven, CT.
- [14] Department of Neurosurgery, Yale University School of Medicine, New Haven, CT.
- [15] Department of Internal Medicine, Pulmonary, Critical Care and Sleep Medicine, Yale School of Medicine, New Haven, CT.
- [16] Department of Internal Medicine, Section of Infectious Diseases, Yale School of Medicine, New Haven, CT.
- [17] Center for Interdisciplinary Research on AIDS, Yale School of Public Health, New Haven, CT.
- [18] Yale New Haven Hospital, New Haven, CT.
- [19] Information Technology Services, Yale New Haven Health, New Haven, CT.
- [20] Yale Comprehensive Cancer Center, Yale School of Medicine, New Haven, CT.
- [21] Department of Immunobiology, Yale University School of Medicine, New Haven, CT.

- [22] Howard Hughes Medical Institute, Chevy Chase, MD.
- [23] Department of Epidemiology of Microbial Diseases, Yale School of Public Health, New Haven, CT.
- [24] Center for Remote Health Technologies and Systems, Texas A&M University, College Station, TX.
- [25] Department of Health Policy and Management, Yale School of Public Health, New Haven, CT.

- PMID: **32743602**
- PMCID: PMC7386526
- DOI: 10.1101/2020.07.19.20157305

Update in

- Clinical characteristics and outcomes for 7,995 patients with SARS-CoV-2 infection. McPadden J, Warner F, Young HP, Hurley NC, Pulk RA, Singh A, Durant TJS, Gong G, Desai N, Haimovich A, Taylor RA, Gunel M, Dela Cruz CS, Farhadian SF, Siner J, Villanueva M, Churchwell K, Hsiao A, Torre CJ Jr, Velazquez EJ, Herbst RS, Iwasaki A, Ko AI, Mortazavi BJ, Krumholz HM, Schulz WL. McPadden J, et al. PLoS One. 2021 Mar 31;16(3):e0243291. doi: 10.1371/journal.pone.0243291. eCollection 2021. PLoS One. 2021. PMID: 33788846 Free PMC article.

Abstract

Objective: Severe acute respiratory syndrome virus (SARS-CoV-2) has infected millions of people worldwide. Our goal was to identify risk factors associated with admission and disease severity in patients with SARS-CoV-2.

Design: This was an observational, retrospective study based on real-world data for 7,995 patients with SARS-CoV-2 from a clinical data repository.

Setting: Yale New Haven Health (YNHH) is a five-hospital academic health system serving a diverse patient population with community and teaching facilities in both urban and suburban areas.

Populations: The study included adult patients who had SARS-CoV-2 testing at YNHH between March 1 and April 30, 2020.

Main outcome and performance measures: Primary outcomes were admission and in-hospital mortality for patients with SARS-CoV-2 infection as determined by RT-PCR testing. We also assessed features associated with the need for respiratory support.

Results: Of the 28605 patients tested for SARS-CoV-2, 7995 patients (27.9%) had an infection (median age 52.3 years) and 2154 (26.9%) of these had an associated admission (median age 66.2 years). Of admitted patients, 2152 (99.9%) had a discharge disposition at the end of the study period. Of these, 329 (15.3%) required invasive mechanical ventilation and 305 (14.2%) expired. Increased age and male sex were positively associated with admission and in-hospital mortality (median age 80.7 years), while comorbidities had a much weaker association with the risk of admission or mortality. Black race (OR 1.43, 95%CI 1.14-1.78) and Hispanic ethnicity (OR 1.81, 95%CI 1.50-2.18) were identified as risk factors for admission, but, among discharged patients, age-adjusted in-hospital mortality was not significantly different among racial and ethnic groups.

Conclusions: This observational study identified, among people testing positive for SARSCoV-2 infection, older age and male sex as the most strongly associated risks for admission and in-hospital mortality in patients with SARS-CoV-2 infection. While minority racial and ethnic groups had increased burden of disease and risk of admission, age-adjusted in-hospital mortality for discharged patients was not significantly different among racial and ethnic groups. Ongoing studies will be needed to continue to evaluate these risks, particularly in the setting of evolving treatment guidelines.

Conflict of interest statement

Competing Interests H.M.K. works under contract with the Centers for Medicare & Medicaid Services to support quality measurement programs; was a recipient of a research grant, through Yale, from Medtronic and the U.S. Food and Drug Administration to develop methods for post-market surveillance of medical devices; was a recipient of a research grant from Johnson & Johnson, through Yale University, to support clinical trial data sharing; was a recipient of a research agreement, through Yale University, from the Shenzhen Center for Health Information for work to advance intelligent disease prevention and health promotion; collaborates with the National Center for Cardiovascular Diseases in Beijing; receives payment from the Arnold & Porter Law Firm for work related to the Sanofi clopidogrel litigation, from the Martin Baughman Law Firm for work related to the Cook Celect IVC filter litigation, and from the Siegfried and Jensen Law Firm for work related to Vioxx litigation; chairs a Cardiac Scientific Advisory Board for UnitedHealth; was a member of the IBM Watson Health Life Sciences Board; is a member of the Advisory Board for Element Science, the Advisory Board for Facebook, and the Physician Advisory Board for Aetna; and is the co-founder of HugoHealth, a personal health information platform, and co-founder of Refactor Health, a healthcare AI-augmented data management company. W.L.S. was an investigator for a research agreement, through Yale University, from the Shenzhen Center for Health Information for work to advance intelligent disease prevention and health promotion; collaborates with the National Center for Cardiovascular Diseases in Beijing; is a technical consultant to HugoHealth, a personal health information platform, and cofounder of Refactor Health, an AI-augmented data management platform for healthcare; is a consultant for Interpace Diagnostics Group, a molecular diagnostics company.

- 33 references
- 4 figures

Supplementary info

Publication types, Grant support Expand

Publication types

- Preprint

Grant support

- T15 LM007056/LM/NLM NIH HHS/United States
- UL1 TR001863/TR/NCATS NIH HHS/United States

Full text links

medRxiv Cold Spring Harbor Laboratory Free PMC article
Proceed to details
Cite
Share
1,388
This article is a preprint
Preprints have not been peer reviewed.
Learn more about preprints in the NIH Preprint Pilot.
medRxiv
. 2021 Jan 23;2021.01.12.21249511.
doi: 10.1101/2021.01.12.21249511. Preprint

The National COVID Cohort Collaborative: Clinical Characterization and Early Severity Prediction

Tellen D Bennett, Richard A Moffitt, Janos G Hajagos, Benjamin Amor, Adit Anand, Mark M Bissell, Katie Rebecca Bradwell, Carolyn Bremer, James Brian Byrd, Alina Denham, Peter E DeWitt, Davera Gabriel, Brian T Garibaldi, Andrew T Girvin, Justin Guinney, Elaine L Hill, Stephanie S Hong, Hunter Jimenez, Ramakanth Kavuluru, Kristin Kostka, Harold P Lehmann, Eli Levitt, Sandeep K Mallipattu, Amin Manna, Julie A McMurry, Michele Morris, John Muschelli, Andrew J Neumann, Matvey B Palchuk, Emily R Pfaff, Zhenglong Qian, Nabeel Qureshi, Seth Russell, Heidi Spratt, Anita Walden, Andrew E Williams, Jacob T Wooldridge, Yun Jae Yoo, Xiaohan Tanner Zhang, Richard L Zhu, Christopher P Austin, Joel H Saltz, Ken R Gersing, Melissa A Haendel, Christopher G Chute

- PMID: **33469592**
- PMCID: PMC7814838
- DOI: 10.1101/2021.01.12.21249511

Free PMC article

The National COVID Cohort Collaborative: Clinical Characterization and Early Severity Prediction

Tellen D Bennett et al. medRxiv. 2021.
Free PMC article
Show details
medRxiv
. 2021 Jan 23;2021.01.12.21249511.
doi: 10.1101/2021.01.12.21249511. Preprint

Authors

Tellen D Bennett, Richard A Moffitt, Janos G Hajagos, Benjamin Amor, Adit Anand, Mark M Bissell, Katie Rebecca Bradwell, Carolyn Bremer, James Brian Byrd, Alina Denham, Peter E DeWitt, Davera Gabriel, Brian T Garibaldi, Andrew T Girvin, Justin Guinney, Elaine L Hill, Stephanie S Hong, Hunter Jimenez, Ramakanth Kavuluru, Kristin Kostka, Harold P Lehmann, Eli Levitt, Sandeep K Mallipattu, Amin Manna, Julie A McMurry, Michele Morris, John Muschelli, Andrew J Neumann, Matvey B Palchuk, Emily R Pfaff, Zhenglong Qian, Nabeel Qureshi, Seth Russell, Heidi Spratt, Anita Walden, Andrew E Williams, Jacob T Wooldridge, Yun Jae Yoo, Xiaohan Tanner Zhang, Richard L Zhu, Christopher P Austin, Joel H Saltz, Ken R Gersing, Melissa A Haendel, Christopher G Chute

- PMID: **33469592**
- PMCID: PMC7814838
- DOI: 10.1101/2021.01.12.21249511

Update in

- Clinical Characterization and Prediction of Clinical Severity of SARS-CoV-2 Infection Among US Adults Using Data From the US National COVID Cohort Collaborative.
Bennett TD, Moffitt RA, Hajagos JG, Amor B, Anand A, Bissell MM, Bradwell KR, Bremer C, Byrd JB, Denham A, DeWitt PE, Gabriel D, Garibaldi BT, Girvin AT, Guinney J, Hill EL, Hong SS, Jimenez H, Kavuluru R, Kostka K, Lehmann HP, Levitt E, Mallipattu SK, Manna A, McMurry JA, Morris M, Muschelli J, Neumann AJ, Palchuk MB, Pfaff ER, Qian Z, Qureshi N, Russell S, Spratt H, Walden A, Williams AE, Wooldridge JT, Yoo YJ, Zhang XT, Zhu RL, Austin CP, Saltz JH, Gersing KR, Haendel MA, Chute CG; National COVID Cohort Collaborative (N3C) Consortium. Bennett TD, et al. JAMA Netw Open. 2021 Jul 1;4(7):e2116901. doi: 10.1001/jamanetworkopen.2021.16901. JAMA Netw Open. 2021. PMID: 34255046 Free PMC article.

Abstract

Background: The majority of U.S. reports of COVID-19 clinical characteristics, disease course, and treatments are from single health systems or focused on one domain. Here we report the creation of the National COVID Cohort Collaborative (N3C), a centralized, harmonized, high-granularity electronic health record repository that is the largest, most representative U.S. cohort of COVID-19 cases and controls to date. This multi-center dataset supports robust evidence-based development of predictive and diagnostic tools and informs critical care and policy.

Methods and findings: In a retrospective cohort study of 1,926,526 patients from 34 medical centers nationwide, we stratified patients using a World Health Organization COVID-19 severity scale and demographics; we then evaluated differences between groups over time using multivariable logistic regression. We established vital signs and laboratory values among COVID-19 patients with different severities, providing the foundation for predictive analytics. The cohort included 174,568 adults with severe acute respiratory syndrome associated with SARS-CoV-2 (PCR >99% or antigen <1%) as well as 1,133,848 adult patients that served as lab-negative controls. Among 32,472 hospitalized patients, mortality was 11.6% overall and decreased from 16.4% in March/April 2020 to 8.6% in September/October 2020 ($p = 0.002$ monthly trend). In a multivariable logistic regression model, age, male sex, liver disease, dementia, African-American and Asian race, and obesity were independently associated with higher clinical severity. To demonstrate the utility of the N3C cohort for analytics, we used machine learning (ML) to predict clinical severity and risk factors over time. Using 64 inputs available on the first hospital day, we

predicted a severe clinical course (death, discharge to hospice, invasive ventilation, or extracorporeal membrane oxygenation) using random forest and XGBoost models (AUROC 0.86 and 0.87 respectively) that were stable over time. The most powerful predictors in these models are patient age and widely available vital sign and laboratory values. The established expected trajectories for many vital signs and laboratory values among patients with different clinical severities validates observations from smaller studies, and provides comprehensive insight into COVID-19 characterization in U.S. patients.

Conclusions: This is the first description of an ongoing longitudinal observational study of patients seen in diverse clinical settings and geographical regions and is the largest COVID-19 cohort in the United States. Such data are the foundation for ML models that can be the basis for generalizable clinical decision support tools. The N3C Data Enclave is unique in providing transparent, reproducible, easily shared, versioned, and fully auditable data and analytic provenance for national-scale patient-level EHR data. The N3C is built for intensive ML analyses by academic, industry, and citizen scientists internationally. Many observational correlations can inform trial designs and care guidelines for this new disease.

Conflict of interest statement

Declaration of interests

Benjamin Amor, Katie Rebecca Bradwell, Andrew T. Girvin, Amin Manna, and Nabeel Qureshi: employee of Palantir Technologies; Brian T. Garibaldi: Member of the FDA Pulmonary-Allergy Drugs Advisory Committee (PADAC); Matvey B. Palchuk: employee of TriNetX; Kristin Kostka: employee of IQVIA Inc.; Julie A. McMurry: and Melissa A. Haendel Cofounders of Pryzm Health; Chris P. Austin and Ken R. Gersing, employees of the National Institutes of Health.

No conflicts of interest reported for all other authors.

- 30 references
- 4 figures

Supplementary info

Publication types, Grant support Expand

Publication types

- Preprint

Grant support

- UL1 TR003096/TR/NCATS NIH HHS/United States
- UL1 TR002649/TR/NCATS NIH HHS/United States
- UL1 TR002535/TR/NCATS NIH HHS/United States
- U54 GM104942/GM/NIGMS NIH HHS/United States
- UL1 TR001420/TR/NCATS NIH HHS/United States

- UL1 TR002240/TR/NCATS NIH HHS/United States
- UL1 TR001439/TR/NCATS NIH HHS/United States
- UL1 TR001998/TR/NCATS NIH HHS/United States
- UL1 TR002537/TR/NCATS NIH HHS/United States

- UL1 TR001857/TR/NCATS NIH HHS/United States
- P30 DK092926/DK/NIDDK NIH HHS/United States
- UL1 TR002494/TR/NCATS NIH HHS/United States
- UL1 TR002736/TR/NCATS NIH HHS/United States
- UL1 TR002538/TR/NCATS NIH HHS/United States
- U54 GM115458/GM/NIGMS NIH HHS/United States
- DP5 OD021338/OD/NIH HHS/United States
- UL1 TR001453/TR/NCATS NIH HHS/United States
- UL1 TR002489/TR/NCATS NIH HHS/United States
- UL1 TR003107/TR/NCATS NIH HHS/United States
- UL1 TR003015/TR/NCATS NIH HHS/United States
- UL1 TR002733/TR/NCATS NIH HHS/United States
- U24 TR002306/TR/NCATS NIH HHS/United States
- UL1 TR001876/TR/NCATS NIH HHS/United States
- UL1 TR002003/TR/NCATS NIH HHS/United States
- UL1 TR002553/TR/NCATS NIH HHS/United States
- UL1 TR002389/TR/NCATS NIH HHS/United States
- UL1 TR002014/TR/NCATS NIH HHS/United States
- UL1 TR002373/TR/NCATS NIH HHS/United States
- UL1 TR002319/TR/NCATS NIH HHS/United States
- UL1 TR001855/TR/NCATS NIH HHS/United States
- UL1 TR002345/TR/NCATS NIH HHS/United States
- UL1 TR001450/TR/NCATS NIH HHS/United States
- UL1 TR002377/TR/NCATS NIH HHS/United States
- K23 HL128909/HL/NHLBI NIH HHS/United States
- UL1 TR002544/TR/NCATS NIH HHS/United States
- UL1 TR003098/TR/NCATS NIH HHS/United States

Show all 36 grants

Full text links

medRxiv Cold Spring Harbor Laboratory Free PMC article

Proceed to details

Cite

Share

1,388 results

[x]

Cite

Copy

Download .nbib

Format: NLM

[x]

Share

-
-

Permalink

Copy

first first First | previous previous Prev

Page

7

of 7

Next next next | Last last last

Send To

- Clipboard
- Email
- Save
- My Bibliography
- Collections
- Citation Manager

[x]

- Article type
- Species
- Language
- Sex
- Journal
- Age

- ☐ Address
- ☐ Autobiography
- ☐ Bibliography
- ☐ Biography
- ☐ Case Reports
- ☐ Classical Article
- ☐ Clinical Conference
- ☐ Clinical Study
- ☐ Clinical Trial Protocol
- ☐ Clinical Trial, Phase I
- ☐ Clinical Trial, Phase II
- ☐ Clinical Trial, Phase III
- ☐ Clinical Trial, Phase IV
- ☐ Clinical Trial, Veterinary
- ☐ Comment
- ☐ Comparative Study
- ☐ Congress
- ☐ Consensus Development Conference
- ☐ Consensus Development Conference, NIH
- ☐ Controlled Clinical Trial
- ☐ Corrected and Republished Article
- ☐ Dataset
- ☐ Dictionary
- ☐ Directory
- ☐ Duplicate Publication

- ☐ Editorial
- ☐ Electronic Supplementary Materials
- ☐ English Abstract
- ☐ Evaluation Study
- ☐ Festschrift
- ☐ Government Publication
- ☐ Guideline
- ☐ Historical Article
- ☐ Interactive Tutorial
- ☐ Interview
- ☐ Introductory Journal Article
- ☐ Lecture
- ☐ Legal Case
- ☐ Legislation
- ☐ Letter
- ☐ Multicenter Study
- ☐ News
- ☐ Newspaper Article
- ☐ Observational Study
- ☐ Observational Study, Veterinary
- ☐ Overall
- ☐ Patient Education Handout
- ☐ Periodical Index
- ☐ Personal Narrative
- ☐ Portrait
- ☐ Practice Guideline
- ☐ Pragmatic Clinical Trial
- ☐ Preprint
- ☐ Published Erratum
- ☐ Research Support, American Recovery and Reinvestment Act
- ☐ Research Support, N.I.H., Extramural
- ☐ Research Support, N.I.H., Intramural
- ☐ Research Support, Non-U.S. Gov't
- ☐ Research Support, U.S. Gov't, Non-P.H.S.
- ☐ Research Support, U.S. Gov't, P.H.S.
- ☐ Research Support, U.S. Gov't
- ☐ Retracted Publication
- ☐ Retraction of Publication
- ☐ Scientific Integrity Review
- ☐ Technical Report
- ☐ Twin Study
- ☐ Validation Study
- ☐ Video-Audio Media
- ☐ Webcast

- ☐ Humans
- ☐ Other Animals

- ☐ Afrikaans

- ☐ Albanian
- ☐ Arabic
- ☐ Armenian
- ☐ Azerbaijani
- ☐ Bosnian
- ☐ Bulgarian
- ☐ Catalan
- ☐ Chinese
- ☐ Croatian
- ☐ Czech
- ☐ Danish
- ☐ Dutch
- ☐ English
- ☐ Esperanto
- ☐ Estonian
- ☐ Finnish
- ☐ French
- ☐ Georgian
- ☐ German
- ☐ Greek, Modern
- ☐ Hebrew
- ☐ Hindi
- ☐ Hungarian
- ☐ Icelandic
- ☐ Indonesian
- ☐ Italian
- ☐ Japanese
- ☐ Kinyarwanda
- ☐ Korean
- ☐ Latin
- ☐ Latvian
- ☐ Lithuanian
- ☐ Macedonian
- ☐ Malay
- ☐ Malayalam
- ☐ Maori
- ☐ Multiple Languages
- ☐ Norwegian
- ☐ Persian
- ☐ Polish
- ☐ Portuguese
- ☐ Pushto
- ☐ Romanian
- ☐ Russian
- ☐ Sanskrit
- ☐ Scottish gaelic
- ☐ Serbian
- ☐ Slovak

- ☐ Slovenian
- ☐ Spanish
- ☐ Swedish
- ☐ Thai
- ☐ Turkish
- ☐ Ukrainian
- ☐ Undetermined
- ☐ Vietnamese
- ☐ Welsh

- ☐ Female
- ☐ Male

- ☐ MEDLINE

- ☐ Child: birth-18 years
- ☐ Newborn: birth-1 month
- ☐ Infant: birth-23 months
- ☐ Infant: 1-23 months
- ☐ Preschool Child: 2-5 years
- ☐ Child: 6-12 years
- ☐ Adolescent: 13-18 years
- ☐ Adult: 19+ years
- ☐ Young Adult: 19-24 years
- ☐ Adult: 19-44 years
- ☐ Middle Aged + Aged: 45+ years
- ☐ Middle Aged: 45-64 years
- ☐ Aged: 65+ years
- ☐ 80 and over: 80+ years

Cancel Show
Close dialog
Back to Top
Jump to page Close dialog
7 of 7
Jump

NCBI Literature Resources

MeSH PMC Bookshelf Disclaimer

Follow NCBI

Connect with NLM

-

-
-

National Library of Medicine
8600 Rockville Pike
Bethesda, MD 20894

Web Policies
FOIA
HHS Vulnerability Disclosure

Help
Accessibility
Careers

- NLM
- NIH
- HHS
- USA.gov

ERREUR p
du site :
Domaine
