# Supplementary material for: COVID-19 treatment of hospital patients worldwide at the onset of the pandemic in 2020: a systematic review
Source: BMC Infect Dis. 2025 Dec 17;26:107. doi: 10.1186/s12879-025-12368-2 (PMC12822144; doi:10.1186/s12879-025-12368-2)
Supplement: Supplementary file 4 — Supplementary Material 4 [file 12879_2025_12368_MOESM4_ESM.zip › 12879_2025_12368_MOESM4_ESM/Search Pubmed 2022 03 28 retrospective observational study hospital treatment covid 801-1000.pdf]

Skip to main page content

COVID-19 Information

Public health information (CDC)

Research information (NIH)

SARS-CoV-2 data (NCBI)

Prevention and treatment information (HHS)

Español

Close

Account

Logged in as:
username

- Dashboard
- Publications
- Account settings
- Log out

Access keys NCBI Homepage MyNCBI Homepage Main Content Main Navigation

Search Page

pubmed

Search: retrospective observation Search
Advanced Create alert Create RSS Clipboard
User Guide
Filters 0 Timeline
Sorted by: Best match
Sorted by: Best match Display options
Save Email
Send to

Save citations to file

Selection: All results on this page

Format: Summary (text)

Create file Cancel

# Email citations

Subject: retrospective observational study hospital treatm - PubMed

To: antoine.bosquet@lmr.apl

Selection: All results on this page

Format: Summary

MeSH and other data

Send email Cancel

Send citations to clipboard

Selection: All results on this page

Send Cancel

Add to Collections

Selection: All results on this page

- Create a new collection
- Add to an existing collection

Name your collection:

Name must be less than 100 characters

Choose a collection:

Unable to load your collection due to an error

Please try again

Add Cancel

Add to My Bibliography

Selection: All results on this page

- My Bibliography

Unable to load your delegates due to an error

Please try again

Add Cancel

Create a file for external citation management software

Selection: All results on this page

Create file Cancel

Your saved search

Name of saved search: retrospective observation

Search terms: retrospective observational study

Test search terms

Would you like email updates of new search results?

Saved Search Alert Radio Buttons

- Yes
- No

Email: antoine.bosquet@lmr.aphp.fr (change)

Frequency: Monthly

Which day? The first Sunday

Which day? Sunday

Report format: Summary

Send at most: 5 items

Send even when there aren't any new results

Optional text in email:

Save Cancel

Your RSS Feed

Name of RSS Feed: retrospective observation

Number of items displayed: 15

Create RSS Cancel

RSS Link Your RSS Feed Link Copy

My NCBI Filters

- All (1,388)
- Assistance Publique Hopitaux de Paris (0)
- clinical trial (17)
- Review (1)

Show Fewer

Results by year Expand/collapse timeline

Reset

Table representation of search results timeline featuring number of search results per year.

Year	Number of Results
2020	548
2021	893
2022	147

Text availability

- Abstract
- Free full text
- Full text

Article attribute

- Associated data

Article type

- Books and Documents
- Clinical Trial
- Meta-Analysis
- Randomized Controlled Trial
- Review
- Systematic Review

Publication date

- 1 year
- 5 years
- 10 years
- Custom Range

Additional filters Reset all filters

Search Results

clear all
1,388 results

first first previous previous

Page
5
of 7

next next last last

Use COVID-19 filters from PubMed Clinical Queries to refine your search

- Treatment
- Mechanism
- Transmission
- More filters

See more SARS-CoV-2 literature, sequence, and clinical content from NCBI

Results by year Expand/collapse timeline

Reset

Filters applied: . *Clear all* Select search result to email or save

Page 5

☐ 801

Observational Study

PLoS Negl Trop Dis
. 2020 Oct 16;14(10):e0008806.
doi: 10.1371/journal.pntd.0008806. eCollection 2020 Oct.

Clinical course and potential predictive factors for pneumonia of adult patients with Coronavirus Disease 2019 (COVID-19): A retrospective observational analysis of 193 confirmed cases in Thailand

Wannarat A Pongpirul [1], Surasak Wiboonchutikul [1], Lantharita Charoenpong [1], Nayot Panitantum [1], Apichart Vachiraphan [1], Sumonmal Uttayamakul [1], Krit Pongpirul [2, 3], Weerawat Manosuthi [1], Wisit Prasithsirikul [1]

Affiliations Expand

Affiliations

- [1] Bamrasnaradura Infectious Diseases Institute, Department of Disease Control, Ministry of Public Health, Nonthaburi, Thailand.
- [2] Department of Preventive and Social Medicine, Faculty of Medicine, Chulalongkorn University, Bangkok, Thailand.
- [3] Department of International Health, Johns Hopkins Bloomberg School of Public Health, Baltimore, Maryland, United States of America.

- PMID: **33064734**
- PMCID: PMC7592908
- DOI: 10.1371/journal.pntd.0008806

Free PMC article
Observational Study

Clinical course and potential predictive factors for pneumonia of adult patients with Coronavirus Disease 2019 (COVID-19): A retrospective observational analysis of 193 confirmed cases in Thailand

Wannarat A Pongpirul et al. PLoS Negl Trop Dis. 2020.
Free PMC article
Show details
PLoS Negl Trop Dis
. 2020 Oct 16;14(10):e0008806.
doi: 10.1371/journal.pntd.0008806. eCollection 2020 Oct.

Authors

Wannarat A Pongpirul [1], Surasak Wiboonchutikul [1], Lantharita Charoenpong [1], Nayot Panitantum [1], Apichart Vachiraphan [1], Sumonmal Uttayamakul [1], Krit Pongpirul [2] [3], Weerawat Manosuthi [1], Wisit Prasithsirikul [1]

Affiliations

- [1] Bamrasnaradura Infectious Diseases Institute, Department of Disease Control, Ministry of Public Health, Nonthaburi, Thailand.
- [2] Department of Preventive and Social Medicine, Faculty of Medicine, Chulalongkorn University, Bangkok, Thailand.
- [3] Department of International Health, Johns Hopkins Bloomberg School of Public Health, Baltimore, Maryland, United States of America.

- PMID: **33064734**
- PMCID: PMC7592908
- DOI: 10.1371/journal.pntd.0008806

Abstract

Clinical spectrum of Coronavirus Disease 2019 (COVID-19) remains unclear, especially with regard to the presence of pneumonia. We aimed to describe the clinical course and final outcomes of adult patients with laboratory-confirmed COVID-19 in the full spectrum of disease severity. We also aimed to identify potential predictive factors for COVID-19 pneumonia. We conducted a retrospective study among adult patients with laboratory-confirmed COVID-19 who were hospitalized at Bamrasnaradura Infectious Diseases Institute, Thailand, between January 8 and April 16, 2020. One-hundred-and-ninety-three patients were included. The median (IQR) age was 37.0 (29.0-53.0) years, and 58.5% were male. The median (IQR) incubation period was 5.5

(3.0-8.0) days. More than half (56%) of the patients were mild disease severity, 22% were moderate, 14% were severe, and 3% were critical. Asymptomatic infection was found in 5%. The final clinical outcomes in 189 (97.9%) were recovered and 4 (2.1%) were deceased. The incidence of pneumonia was 39%. The median (IQR) time from onset of illness to pneumonia detection was 7.0 (5.0-9.0) days. Bilateral pneumonia was more prevalent than unilateral pneumonia. In multivariable logistic regression, increasing age (OR 2.55 per 10-year increase from 30 years old; 95% CI, 1.67-3.90; p<0.001), obesity (OR 8.74; 95%CI, 2.06-37.18; p = 0.003), and higher temperature at presentation (OR 4.59 per 1°C increase from 37.2°C; 95% CI, 2.30-9.17; p<0.001) were potential predictive factors for COVID-19 pneumonia. Across the spectrum of disease severities, most patients with COVID-19 in our cohort had good final clinical outcomes. COVID-19 pneumonia was found in one-third of them. Older age, obesity, and higher fever at presentation were independent predictors of COVID-19 pneumonia.

Conflict of interest statement

The authors have declared that no competing interests exist.

- 38 references
- 1 figure

Supplementary info

Publication types, MeSH terms, Grant support Expand

Publication types

- Observational Study

MeSH terms

- Adult
- Age Factors
- Aged
- Betacoronavirus
- COVID-19
- Coronavirus Infections / diagnosis*
- Disease Progression*
- Female
- Fever / etiology
- Hospitalization
- Humans
- Male
- Middle Aged
- Obesity / complications
- Pandemics
- Pneumonia, Viral / diagnosis*

- Prognosis
- Real-Time Polymerase Chain Reaction
- Reverse Transcriptase Polymerase Chain Reaction
- Risk Factors
- SARS-CoV-2
- Symptom Assessment
- Thailand / epidemiology
- Young Adult

Grant support

The authors received no specific funding for this work.

Full text links

OPEN ACCESS TO FULL TEXT PLOS NEGLECTED TROPICAL DISEASES Public Library of Science Free PMC article

Proceed to details

Cite

Share

☐ 802

Observational Study

Emerg Med J

. 2021 Aug;38(8):587-593.

doi: 10.1136/emermed-2020-210783. Epub 2021 Jun 3.

Prognostic accuracy of emergency department triage tools for adults with suspected COVID-19: the PRIEST observational cohort study

Ben Thomas[1], Steve Goodacre[2], Ellen Lee[2], Laura Sutton[2], Matthew Bursnall[2], Amanda Loban[2], Simon Waterhouse[2], Richard Simmonds[2], Katie Biggs[2], Carl Marincowitz[2], José Schutter[2], Sarah Connelly[2], Elena Sheldon[2], Jamie Hall[2], Emma Young[2], Andrew Bentley[3], Kirsty Challen[4], Chris Fitzsimmons[5] [6], Tim Harris[7], Fiona Lecky[2], Andrew Lee[2], Ian Maconochie[8], Darren Walter[9]

Affiliations Expand

Affiliations

- [1] ScHARR, The University of Sheffield, Sheffield, UK b.d.thomas@sheffield.ac.uk.
- [2] ScHARR, The University of Sheffield, Sheffield, UK.

- [3] Acute intensive Care Unit, Wythenshawe Hospital, Manchester University NHS Foundation Trust, Manchester, UK.
- [4] Emergency Department, Lancashire Teaching Hospitals NHS Foundation Trust, Chorley, UK.
- [5] Sheffield Children's Hospital NHS Foundation Trust, Sheffield, UK.
- [6] Emergency Department, Sheffield Children's Hospital NHS Foundation Trust, Sheffield, UK.
- [7] Department of Emergency Medicine, Royal London Hospital, London, UK.
- [8] Paediatric ED, Imperial College Healthcare NHS Trust, London, UK.
- [9] Emergency Department, University Hospital of South Manchester NHS Foundation Trust, Manchester, UK.

- PMID: **34083427**
- PMCID: PMC8182747
- DOI: 10.1136/emermed-2020-210783

Free PMC article
Observational Study

Prognostic accuracy of emergency department triage tools for adults with suspected COVID-19: the PRIEST observational cohort study

Ben Thomas et al. Emerg Med J. 2021 Aug.
Free PMC article
Show details
Emerg Med J
. 2021 Aug;38(8):587-593.
doi: 10.1136/emermed-2020-210783. Epub 2021 Jun 3.

Authors

Ben Thomas [1], Steve Goodacre [2], Ellen Lee [2], Laura Sutton [2], Matthew Bursnall [2], Amanda Loban [2], Simon Waterhouse [2], Richard Simmonds [2], Katie Biggs [2], Carl Marincowitz [2], José Schutter [2], Sarah Connelly [2], Elena Sheldon [2], Jamie Hall [2], Emma Young [2], Andrew Bentley [3], Kirsty Challen [4], Chris Fitzsimmons [5] [6], Tim Harris [7], Fiona Lecky [2], Andrew Lee [2], Ian Maconochie [8], Darren Walter [9]

Affiliations

- [1] ScHARR, The University of Sheffield, Sheffield, UK b.d.thomas@sheffield.ac.uk.
- [2] ScHARR, The University of Sheffield, Sheffield, UK.
- [3] Acute intensive Care Unit, Wythenshawe Hospital, Manchester University NHS Foundation Trust, Manchester, UK.

- [4] Emergency Department, Lancashire Teaching Hospitals NHS Foundation Trust, Chorley, UK.
- [5] Sheffield Children's Hospital NHS Foundation Trust, Sheffield, UK.
- [6] Emergency Department, Sheffield Children's Hospital NHS Foundation Trust, Sheffield, UK.
- [7] Department of Emergency Medicine, Royal London Hospital, London, UK.
- [8] Paediatric ED, Imperial College Healthcare NHS Trust, London, UK.
- [9] Emergency Department, University Hospital of South Manchester NHS Foundation Trust, Manchester, UK.

- PMID: **34083427**
- PMCID: PMC8182747
- DOI: 10.1136/emermed-2020-210783

Abstract

Background: The WHO and National Institute for Health and Care Excellence recommend various triage tools to assist decision-making for patients with suspected COVID-19. We aimed to compare the accuracy of triage tools for predicting severe illness in adults presenting to the ED with suspected COVID-19.

Methods: We undertook a mixed prospective and retrospective observational cohort study in 70 EDs across the UK. We collected data from people attending with suspected COVID-19 and used presenting data to determine the results of assessment with the WHO algorithm, National Early Warning Score version 2 (NEWS2), CURB-65, CRB-65, Pandemic Modified Early Warning Score (PMEWS) and the swine flu adult hospital pathway (SFAHP). We used 30-day outcome data (death or receipt of respiratory, cardiovascular or renal support) to determine prognostic accuracy for adverse outcome.

Results: We analysed data from 20 891 adults, of whom 4611 (22.1%) died or received organ support (primary outcome), with 2058 (9.9%) receiving organ support and 2553 (12.2%) dying without organ support (secondary outcomes). C-statistics for the primary outcome were: CURB-65 0.75; CRB-65 0.70; PMEWS 0.77; NEWS2 (score) 0.77; NEWS2 (rule) 0.69; SFAHP (6-point rule) 0.70; SFAHP (7-point rule) 0.68; WHO algorithm 0.61. All triage tools showed worse prediction for receipt of organ support and better prediction for death without organ support. At the recommended threshold, PMEWS and the WHO criteria showed good sensitivity (0.97 and 0.95, respectively) at the expense of specificity (0.30 and 0.27, respectively). The NEWS2 score showed similar sensitivity (0.96) and specificity (0.28) when a lower threshold than recommended was used.

Conclusion: CURB-65, PMEWS and the NEWS2 score provide good but not excellent prediction for adverse outcome in suspected COVID-19, and predicted death without organ support better than receipt of organ support. PMEWS, the WHO criteria and NEWS2 (using a lower threshold than usually recommended) provide good sensitivity at the expense of specificity.

Trial registration number: ISRCTN56149622.

Keywords: clinical assessment; emergency care systems; emergency department; infectious diseases; triage; viral.

© Author(s) (or their employer(s)) 2021. Re-use permitted under CC BY-NC. No commercial re-use. See rights and permissions. Published by BMJ.

Conflict of interest statement

Competing interests: None declared.

- 23 references
- 3 figures

Supplementary info

Publication types, MeSH terms Expand

Publication types

- Comparative Study
- Multicenter Study
- Observational Study

MeSH terms

- Aged
- COVID-19 / epidemiology
- COVID-19 / therapy*
- Early Warning Score
- Emergency Service, Hospital*
- Female
- Humans
- Male
- Middle Aged
- Pandemics
- Pneumonia, Viral / epidemiology
- Pneumonia, Viral / therapy*
- Pneumonia, Viral / virology
- Predictive Value of Tests
- Prognosis
- Prospective Studies
- Retrospective Studies
- SARS-CoV-2
- Triage / methods*
- United Kingdom

Full text links

BMJ Full Text HighWire Free PMC article
Proceed to details

Cite
Share
803
Observational Study
Endocr Res
. 2021 Nov;46(4):170-177.
doi: 10.1080/07435800.2021.1924770. Epub 2021 May 20.

Association between Thyroid Function and Prognosis of COVID-19: A Retrospective Observational Study

Shan Lang [1], Ye Liu [1], Xue Qu [1], Ran Lu [1], Wei Fu [1], Wenhui Zhang [1], Haining Wang [1], Tianpei Hong [1]
Affiliations Expand

Affiliation

- [1] Department of Endocrinology and Metabolism, Peking University Third Hospital, Beijing, China.

- PMID: **34014139**
- DOI: 10.1080/07435800.2021.1924770

Observational Study

Association between Thyroid Function and Prognosis of COVID-19: A Retrospective Observational Study

Shan Lang et al. Endocr Res. 2021 Nov.
Show details
Endocr Res
. 2021 Nov;46(4):170-177.
doi: 10.1080/07435800.2021.1924770. Epub 2021 May 20.

Authors

Shan Lang [1], Ye Liu [1], Xue Qu [1], Ran Lu [1], Wei Fu [1], Wenhui Zhang [1], Haining Wang [1], Tianpei Hong [1]

Affiliation

- [1] Department of Endocrinology and Metabolism, Peking University Third Hospital, Beijing, China.

- PMID: **34014139**
- DOI: 10.1080/07435800.2021.1924770

Abstract

Background: Coronavirus disease 2019 (COVID-19) is a severe infectious illness. It has been reported that COVID-19 has an effect on thyroid function. However, the association between thyroid function and prognosis of COVID-19 is still unclear.**Methods**: This retrospective study included patients with COVID-19 admitted to Tongji Hospital in Wuhan from January 28 to April 4, 2020. Demographic, epidemiological, clinical, laboratory, treatment, and outcome data were collected from patients with laboratory-confirmed COVID-19. Patients without history of thyroid disease who had a thyroid function test at admission were enrolled in the final analysis. Risk factors of in-hospital death were explored using univariable and multivariable Cox regression analyses. Survival differences were assessed with Kaplan-Meier curves and log-rank test.**Results**: A total of 127 patients were included in this study, with 116 survivors and 11 non-survivors. The serum levels of thyroid stimulating hormone (TSH) [0.8 (0.5-1.7) *vs*. 1.9 (1.0-3.1) µIU/mL, $P = .031$] and free triiodothyronine (FT_3) [2.9 (2.8-3.1) *vs*. 4.2 (3.5-4.7) pmol/L, $P < .001$] were lower in non-survivors than in survivors, and a low FT_3 state (defined as $FT_3 < 3.1$ pmol/L) at admission accounted for a higher proportion in non-survivors than in survivors (72.7% *vs*. 11.2%, $P < .001$). Univariate Cox regression analysis showed that FT_3 level (HR 0.213, 95% CI: 0.101-0.451, $P < .001$) and the low FT_3 state (HR 14.607, 95% CI: 3.873-55.081, $P < .001$) were negatively and positively associated with the risk of in-hospital death, respectively. Furthermore, multivariate Cox regression analysis revealed that a low FT_3 state was associated with an increased risk of in-hospital death after adjusting for confounding factors (HR 13.288, 95% CI: 1.089-162.110, $P = .043$). Moreover, Kaplan-Meier curves indicated a lower survival probability in COVID-19 patients with a low FT_3 status.**Conclusion**: Serum FT_3 level is lower in non-survivors among moderate-to-critical patients with COVID-19, and the low FT_3 state is associated with an increased risk of in-hospital mortality of COVID-19.

Keywords: COVID-19; euthyroid sick syndrome; free triiodothyronine; mortality; thyroid hormone.

Supplementary info

Publication types, MeSH terms, Substances Expand

Publication types

- Observational Study

MeSH terms

- Aged
- COVID-19 / epidemiology
- COVID-19 / mortality*
- COVID-19 / physiopathology*

- China / epidemiology
- Female
- Hospital Mortality
- Humans
- Male
- Middle Aged
- Prognosis*
- Proportional Hazards Models
- Retrospective Studies
- Risk Factors
- SARS-CoV-2*
- Thyroid Gland / physiopathology*
- Thyrotropin / blood
- Thyroxine / blood
- Triiodothyronine / blood

Substances

- Triiodothyronine
- Thyrotropin
- Thyroxine

Full text links

View full text Taylor & Francis

Proceed to details

Cite

Share

804

Observational Study

PLoS One

. 2020 Aug 13;15(8):e0237693.

doi: 10.1371/journal.pone.0237693. eCollection 2020.

Hydroxychloroquine and tocilizumab therapy in COVID-19 patients-An observational study

Andrew Ip [1], Donald A Berry [2] [3], Eric Hansen [4], Andre H Goy [5], Andrew L Pecora [5], Brittany A Sinclaire [5], Urszula Bednarz [5], Michael Marafelias [5], Scott M Berry [2], Nicholas S Berry [2], Shivam Mathura [4], Ihor S Sawczuk [6], Noa Biran [5], Ronaldo C Go [6], Steven Sperber [6], Julia A Piwoz [6], Bindu Balani [6], Cristina Cicogna [6], Rani Sebti [6], Jerry Zuckerman [6], Keith M Rose [6], Lisa Tank [6], Laurie G Jacobs [6], Jason Korcak [6], Sarah L Timmapuri [6], Joseph P Underwood

[6], Gregory Sugalski [6], Carol Barsky [6], Daniel W Varga [6], Arif Asif [6], Joseph C Landolfi [6], Stuart L Goldberg [1]
Affiliations Expand

Affiliations

- [1] Division of Outcomes and Value Research, John Theurer Cancer Center at Hackensack University Medical Center, Hackensack, New Jersey, United States of America.
- [2] Berry Consultants LLC, Austin, Texas, United States of America.
- [3] M.D. Anderson Cancer Center of the University of Texas, Houston, Texas, United States of America.
- [4] COTA, Boston, Massachusetts, United States of America.
- [5] John Theurer Cancer Center at Hackensack University Medical Center, Hackensack, New Jersey, United States of America.
- [6] Hackensack Meridian Health, Hackensack New Jersey and Hackensack Meridian School of Medicine, Nutley, New Jersey, United States of America.

- PMID: **32790733**
- PMCID: PMC7425928
- DOI: 10.1371/journal.pone.0237693

Free PMC article
Observational Study

Hydroxychloroquine and tocilizumab therapy in COVID-19 patients-An observational study

Andrew Ip et al. PLoS One. 2020.
Free PMC article
Show details
PLoS One
. 2020 Aug 13;15(8):e0237693.
doi: 10.1371/journal.pone.0237693. eCollection 2020.

Authors

Andrew Ip [1], Donald A Berry [2] [3], Eric Hansen [4], Andre H Goy [5], Andrew L Pecora [5], Brittany A Sinclaire [5], Urszula Bednarz [5], Michael Marafelias [5], Scott M Berry [2], Nicholas S Berry [2], Shivam Mathura [4], Ihor S Sawczuk [6], Noa Biran [5], Ronaldo C Go [6], Steven Sperber [6], Julia A Piwoz [6], Bindu Balani [6], Cristina Cicogna [6], Rani Sebti [6], Jerry Zuckerman [6], Keith M Rose [6], Lisa Tank [6], Laurie G Jacobs [6], Jason Korcak [6], Sarah L Timmapuri [6], Joseph P Underwood [6], Gregory Sugalski [6], Carol Barsky [6], Daniel W Varga [6], Arif Asif [6], Joseph C Landolfi [6], Stuart L Goldberg [1]

Affiliations

- [1] Division of Outcomes and Value Research, John Theurer Cancer Center at Hackensack University Medical Center, Hackensack, New Jersey, United States of America.
- [2] Berry Consultants LLC, Austin, Texas, United States of America.
- [3] M.D. Anderson Cancer Center of the University of Texas, Houston, Texas, United States of America.
- [4] COTA, Boston, Massachusetts, United States of America.
- [5] John Theurer Cancer Center at Hackensack University Medical Center, Hackensack, New Jersey, United States of America.
- [6] Hackensack Meridian Health, Hackensack New Jersey and Hackensack Meridian School of Medicine, Nutley, New Jersey, United States of America.

- PMID: **32790733**
- PMCID: PMC7425928
- DOI: 10.1371/journal.pone.0237693

Abstract

Hydroxychloroquine has been touted as a potential COVID-19 treatment. Tocilizumab, an inhibitor of IL-6, has also been proposed as a treatment of critically ill patients. In this retrospective observational cohort study drawn from electronic health records we sought to describe the association between mortality and hydroxychloroquine or tocilizumab therapy among hospitalized COVID-19 patients. Patients were hospitalized at a 13-hospital network spanning New Jersey USA between March 1, 2020 and April 22, 2020 with positive polymerase chain reaction results for SARS-CoV-2. Follow up was through May 5, 2020. Among 2512 hospitalized patients with COVID-19 there have been 547 deaths (22%), 1539 (61%) discharges and 426 (17%) remain hospitalized. 1914 (76%) received at least one dose of hydroxychloroquine and 1473 (59%) received hydroxychloroquine with azithromycin. After adjusting for imbalances via propensity modeling, compared to receiving neither drug, there were no significant differences in associated mortality for patients receiving any hydroxychloroquine during the hospitalization (HR, 0.99 [95% CI, 0.80-1.22]), hydroxychloroquine alone (HR, 1.02 [95% CI, 0.83-1.27]), or hydroxychloroquine with azithromycin (HR, 0.98 [95% CI, 0.75-1.28]). The 30-day unadjusted mortality for patients receiving hydroxychloroquine alone, azithromycin alone, the combination or neither drug was 25%, 20%, 18%, and 20%, respectively. Among 547 evaluable ICU patients, including 134 receiving tocilizumab in the ICU, an exploratory analysis found a trend towards an improved survival association with tocilizumab treatment (adjusted HR, 0.76 [95% CI, 0.57-1.00]), with 30 day unadjusted mortality with and without tocilizumab of 46% versus 56%. This observational cohort study suggests hydroxychloroquine, either alone or in combination with azithromycin, was not associated with a survival benefit among hospitalized COVID-19 patients. Tocilizumab demonstrated a trend association towards reduced mortality among ICU patients. Our findings are limited to hospitalized patients and must be interpreted with caution while awaiting results of randomized trials. Trial Registration: Clinicaltrials.gov Identifier: NCT04347993.

Conflict of interest statement

I have read the journal's policy and the authors of this manuscript have the following competing interests: DAB, SMB, and NSB are employed by Berry Consultants LLC which provided statistical support for the study. EH, AHG, ALP, SM, and SLG are employed or have ownership interest in COTA Inc which provided statistical support for the study. AHG discloses consulting fees from Physicians' Education Resource, LLC for his contributions to COVID-19 and Cancer Care. This does not alter our adherence to PLOS ONE policies on sharing data and materials.

- 35 references
- 3 figures

Supplementary info

Publication types, MeSH terms, Substances, Supplementary concepts, Associated data, Grant support Expand

Publication types

- Multicenter Study
- Observational Study

MeSH terms

- Adolescent
- Adult
- Aged
- Aged, 80 and over
- Antibodies, Monoclonal, Humanized / pharmacology
- Antibodies, Monoclonal, Humanized / therapeutic use*
- Antimalarials / therapeutic use*
- Azithromycin / therapeutic use
- Betacoronavirus*
- COVID-19
- Child
- Child, Preschool
- Coronavirus Infections / drug therapy*
- Coronavirus Infections / mortality
- Coronavirus Infections / virology
- Drug Therapy, Combination
- Female
- Follow-Up Studies
- Hospitalization
- Humans
- Hydroxychloroquine / therapeutic use*
- Infant
- Infant, Newborn
- Intensive Care Units
- Interleukin-6 / antagonists & inhibitors
- Kaplan-Meier Estimate
- Male
- Middle Aged

- Pandemics
- Pneumonia, Viral / drug therapy*
- Pneumonia, Viral / mortality
- Pneumonia, Viral / virology
- Retrospective Studies
- SARS-CoV-2
- Treatment Outcome
- Young Adult

Substances

- Antibodies, Monoclonal, Humanized
- Antimalarials
- IL6 protein, human
- Interleukin-6
- Hydroxychloroquine
- Azithromycin
- tocilizumab

Supplementary concepts

- COVID-19 drug treatment

Associated data

- ClinicalTrials.gov/NCT04347993

Grant support

- P30 CA016672/CA/NCI NIH HHS/United States
- UL1 TR003167/TR/NCATS NIH HHS/United States

Full text links

OPEN ACCESS TO FULL TEXT PLOS ONE Public Library of Science Free PMC article
Proceed to details
Cite
Share
☐ 805
Observational Study
Eur J Neurol
. 2021 Oct;28(10):3461-3466.
doi: 10.1111/ene.14612. Epub 2020 Nov 27.

Outcomes of coronavirus disease 2019 in patients with neuromyelitis optica and associated disorders

Céline Louapre [1], Elisabeth Maillart [1], Caroline Papeix [1], Sinead Zeidan [1], Damien Biotti [2], Zoé Lepine [2], Abir Wahab [3], Mickael Zedet [4], Pierre Labauge [5], Caroline Tilikete [6], Julie Pique [7], Ayman Tourbah [8], Guillaume Mathey [9] [10], Dalia Dimitri Boulos [11], Pierre Branger [12], Laurent Daniel Kremer [13], Romain Marignier [7], Nicolas Collongues [13], Jérôme De Seze [13]
Affiliations Expand

Affiliations

- [1] Centre de Référence des Maladies Inflammatoires Rares du Cerveau et de la Moelle, Institut du Cerveau, CIC Neuroscience, ICM, Hôpital de la Pitié Salpêtrière, Sorbonne Université, Paris, France.
- [2] Pole des Neurosciences, B4 Neurology Unit, Centre de ressources et de compétences Sclérose en plaques, CHU Purpan, Toulouse, France.
- [3] Service de Neurologie et CRC SEP, Groupe Hospitalier Henri Mondor, APHP, UPEC Université, Créteil, France.
- [4] Unité de neurologie inflammatoire, Département de Neurologie, Hôpital Roger Salengro, Chu de Lille, Lille, France.
- [5] Département de neurologie, CHU de Montpellier, Montpellier, France.
- [6] Equipe impact, Service de Neurocognition et Neuro-ophtalmologie, Groupe Hospitalier Est, Centre de Recherche en Neurosciences de Lyon, Hospices Civils de Lyon, Université de Lyon, Lyon, France.
- [7] Centre de référence des maladies inflammatoires rares du cerveau et de la moelle (MIRCEM), Service de neurologie, sclérose en plaques, Pathologies de la myéline et neuro-inflammation, Hôpital Neurologique Pierre Wertheimer Hospices Civils de Lyon, Lyon, France.
- [8] Service de Neurologie, Hôpital Raymond Poincaré, UFR Simone Veil UVSQ, Université Paris Saclay, Garches, France.
- [9] Service de neurologie, Centre Régional Hospitalo-Universitaire de Nancy, Hôpital Central, Nancy, France.
- [10] Université de Lorraine, Vandoeuvre-lès-Nancy, France.
- [11] Service de neurologie, CHU Bicêtre, Le Kremlin Bicêtre, France.
- [12] Service de Neurologie, CHU de Caen Normandie, Caen, France.
- [13] Service de Neurologie and CIC INSERM 1434, CHU de Strasbourg, Strasbourg, France.

- PMID: **33103295**
- DOI: 10.1111/ene.14612

Observational Study

Outcomes of coronavirus disease 2019 in patients with neuromyelitis optica and associated disorders

Céline Louapre et al. Eur J Neurol. 2021 Oct.
Show details
Eur J Neurol
. 2021 Oct;28(10):3461-3466.
doi: 10.1111/ene.14612. Epub 2020 Nov 27.

Authors

Céline Louapre [1], Elisabeth Maillart [1], Caroline Papeix [1], Sinead Zeidan [1], Damien Biotti [2], Zoé Lepine [2], Abir Wahab [3], Mickael Zedet [4], Pierre Labauge [5], Caroline Tilikete [6], Julie Pique [7], Ayman Tourbah [8], Guillaume Mathey [9] [10], Dalia Dimitri Boulos [11], Pierre Branger [12], Laurent Daniel Kremer [13], Romain Marignier [7], Nicolas Collongues [13], Jérôme De Seze [13]

Affiliations

- [1] Centre de Référence des Maladies Inflammatoires Rares du Cerveau et de la Moelle, Institut du Cerveau, CIC Neuroscience, ICM, Hôpital de la Pitié Salpêtrière, Sorbonne Université, Paris, France.
- [2] Pole des Neurosciences, B4 Neurology Unit, Centre de ressources et de compétences Sclérose en plaques, CHU Purpan, Toulouse, France.
- [3] Service de Neurologie et CRC SEP, Groupe Hospitalier Henri Mondor, APHP, UPEC Université, Créteil, France.
- [4] Unité de neurologie inflammatoire, Département de Neurologie, Hôpital Roger Salengro, Chu de Lille, Lille, France.
- [5] Département de neurologie, CHU de Montpellier, Montpellier, France.
- [6] Equipe impact, Service de Neurocognition et Neuro-ophtalmologie, Groupe Hospitalier Est, Centre de Recherche en Neurosciences de Lyon, Hospices Civils de Lyon, Université de Lyon, Lyon, France.
- [7] Centre de référence des maladies inflammatoires rares du cerveau et de la moelle (MIRCEM), Service de neurologie, sclérose en plaques, Pathologies de la myéline et neuro-inflammation, Hôpital Neurologique Pierre Wertheimer Hospices Civils de Lyon, Lyon, France.
- [8] Service de Neurologie, Hôpital Raymond Poincaré, UFR Simone Veil UVSQ, Université Paris Saclay, Garches, France.
- [9] Service de neurologie, Centre Régional Hospitalo-Universitaire de Nancy, Hôpital Central, Nancy, France.
- [10] Université de Lorraine, Vandoeuvre-lès-Nancy, France.
- [11] Service de neurologie, CHU Bicêtre, Le Kremlin Bicêtre, France.
- [12] Service de Neurologie, CHU de Caen Normandie, Caen, France.
- [13] Service de Neurologie and CIC INSERM 1434, CHU de Strasbourg, Strasbourg, France.

- PMID: **33103295**

- DOI: 10.1111/ene.14612

Abstract

Background: Outcomes of coronavirus disease 2019 (COVID-19) in patients with neuromyelitis optica spectrum disorders (NMOSD) or myelin oligodendrocyte glycoprotein antibody-associated disease (MOGAD), often treated with immunosuppressive therapies, are still unknown.

Methods: We conducted a multicenter, retrospective, observational cohort study among all French expert centers for neuromyelitis optica and related disorders. Patients with NMOSD or MOGAD included in the study received a confirmed or highly suspected diagnosis of COVID-19 between 1 March 2020 and 30 June 2020. Main outcome was COVID-19 severity score assessed on a seven-point ordinal scale ranging from 1 (not hospitalized with no limitations on activities) to 7 (death).

Results: Fifteen cases (mean [SD] age: 39.3 [14.3] years, 11 female) were included. Five patients (33.3%) were hospitalized, all receiving rituximab. A 24-year-old patient with positive aquaporine-4 antibody, with obesity as comorbidity, needed mechanical ventilation. Outpatients were receiving anti-CD20 (5), mycophenolate mofetil (3) or azathioprine (3). They were younger (mean [SD] age: 37.0 [13.4] years), with a longer disease duration (mean [SD]: 8.3 [6.3] years) and had a lower expanded disability severity score (EDSS) score (median [range] EDSS: 2.5 [0-4]) relative to patients requiring hospitalization (mean [SD] age: 44.0 [16.4] years, mean [SD] disease duration: 5.8 [5.5] years, median [range] EDSS: 4 [0-6.5]).

Conclusions: COVID-19 outcome was overall favorable in this cohort. Larger international studies are needed to identify risk factors of severe COVID-19; however, we recommend personal protective measures to reduce risk of SARS-CoV-2 infection in this immunocompromised population.

Keywords: COVID-19; MOGAD; NMOSD; immunosuppressant.

© 2020 European Academy of Neurology.

- 13 references

Supplementary info

Publication types, MeSH terms, Substances Expand

Publication types

- Multicenter Study
- Observational Study

MeSH terms

- Adult
- Aquaporin 4
- COVID-19*

- Female
- Humans
- Neuromyelitis Optica* / drug therapy
- Neuromyelitis Optica* / epidemiology
- Retrospective Studies
- Rituximab
- SARS-CoV-2
- Young Adult

Substances

- Aquaporin 4
- Rituximab

Full text links

Wiley Full Text Article Wiley
Proceed to details
Cite
Share
☐ 806
Observational Study
ASAIO J
. 2021 Mar 1;67(3):254-262.
doi: 10.1097/MAT.0000000000001380.

Viscoelastic Coagulation Monitor as a Novel Device to Assess Coagulation at the Bedside. A Single-Center Experience During the COVID-19 Pandemic

Mauro Panigada [1], Andrea Meli [1], Eleonora Scotti [1], Paolo Properzi [1], Matteo Brioni [1], Shady Kamel [1] [2], Stefano Ghirardello [3], Luigia Scudeller [4], Heidi J Dalton [5], Giacomo Grasselli [1] [6]
Affiliations Expand

Affiliations

- [1] From the Department of Anaesthesia and Critical Care, Fondazione IRCCS Ca' Granda, Ospedale Maggiore Policlinico, Milan, Italy.
- [2] Betamed srl, Roma, Italy.
- [3] Neonatal Intensive Care Unit, Fondazione IRCCS Ca' Granda, Ospedale Maggiore Policlinico, Milan, Italy.

- [4] Clinical Trial Center, Fondazione IRCCS Ca' Granda Ospedale Maggiore Policlinico, Milan, Italy.
- [5] Departments of Pediatrics; Heart and Vascular Institute, INOVA Fairfax Hospital, Falls Church, Virginia.
- [6] Department of Pathophysiology and Transplantation, University of Milan, Italy.

- PMID: **33627598**
- DOI: 10.1097/MAT.0000000000001380

Observational Study

Viscoelastic Coagulation Monitor as a Novel Device to Assess Coagulation at the Bedside. A Single-Center Experience During the COVID-19 Pandemic

Mauro Panigada et al. ASAIO J. 2021.

Show details

ASAIO J

. 2021 Mar 1;67(3):254-262.
doi: 10.1097/MAT.0000000000001380.

Authors

Mauro Panigada [1], Andrea Meli [1], Eleonora Scotti [1], Paolo Properzi [1], Matteo Brioni [1], Shady Kamel [1] [2], Stefano Ghirardello [3], Luigia Scudeller [4], Heidi J Dalton [5], Giacomo Grasselli [1] [6]

Affiliations

- [1] From the Department of Anaesthesia and Critical Care, Fondazione IRCCS Ca' Granda, Ospedale Maggiore Policlinico, Milan, Italy.
- [2] Betamed srl, Roma, Italy.
- [3] Neonatal Intensive Care Unit, Fondazione IRCCS Ca' Granda, Ospedale Maggiore Policlinico, Milan, Italy.
- [4] Clinical Trial Center, Fondazione IRCCS Ca' Granda Ospedale Maggiore Policlinico, Milan, Italy.
- [5] Departments of Pediatrics; Heart and Vascular Institute, INOVA Fairfax Hospital, Falls Church, Virginia.
- [6] Department of Pathophysiology and Transplantation, University of Milan, Italy.

- PMID: **33627598**
- DOI: 10.1097/MAT.0000000000001380

Abstract

Viscoelastic coagulation monitor (VCM) is a portable device developed to evaluate the viscoelastic properties of whole blood activated by contact with glass. In this study, VCM was employed to analyze the viscoelastic profiles of 36 COVID-19 intensive care patients. Full anticoagulant dose heparin (unfractionated [UFH]; low molecular weight [LMWH]) was administrated to all patients. The association between VCM and laboratory parameters was retrospectively analyzed. The administration of UFH-influenced VCM parameters prolonging clotting time (CT) and clot formation time (CFT) and reducing angle (alpha) and amplitudes of the VCM tracings (A10, A20, and maximum clot firmness [MCF]) compared with LMWH therapy. A tendency toward hypercoagulation was observed by short CT and CFT in patients receiving LMWH. Clotting time was correlated with UFH dose (Spearman's rho = 0.48, $p \leq 0.001$), and no correlation was found between CT and LMWH. All VCM tracings failed to show lysis at 30 and 45 minutes, indicating the absence of fibrinolysis. A10, A20, and MCF exhibited very-good to good diagnostic accuracy for detecting platelet count and fibrinogen above the upper reference limit of the laboratory. In conclusion, VCM provided reliable results in COVID-19 patients and was easy to perform with minimal training at the bedside.

Copyright © ASAIO 2020.

Conflict of interest statement

Disclosure: Dr. Dalton is a consultant for Innovative ECMO Concepts. The other authors have no conflicts of interest to report.

- 39 references

Supplementary info

Publication types, MeSH terms Expand

Publication types

- Observational Study

MeSH terms

- Adult
- Blood Coagulation
- COVID-19 / blood*
- COVID-19 / complications
- Female
- Humans
- Male
- Middle Aged
- Monitoring, Physiologic / instrumentation*
- Monitoring, Physiologic / methods
- Point-of-Care Systems*
- Retrospective Studies

- SARS-CoV-2
- Thrombelastography / instrumentation*
- Thrombelastography / methods
- Thrombosis / diagnosis
- Thrombosis / virology

Full text links

Wolters Kluwer Wolters Kluwer
Proceed to details
Cite
Share
☐ 807
Observational Study
Am J Emerg Med
. 2021 Mar;41:51-54.
doi: 10.1016/j.ajem.2020.12.047. Epub 2020 Dec 30.

Impact of a shelter-in-place order during the COVID-19 pandemic on the incidence of opioid overdoses

Jennifer Rosenbaum [1], Nicole Lucas [2], Gregory Zandrow [3], Wayne A Satz [4], Derek Isenberg [5], Joseph D'Orazio [6], Nina T Gentile [7], Kraftin E Schreyer [8]
Affiliations Expand

Affiliations

- [1] Department of Emergency Medicine, Temple University Hospital, 1316 W. Ontario Street, 10(th) Floor Jones Hall, Philadelphia, PA 19140, United States. Electronic address: Jennifer.Rosenbaum@tuhs.temple.edu.
- [2] Department of Emergency Medicine, Temple University Hospital, 1316 W. Ontario Street, 10(th) Floor Jones Hall, Philadelphia, PA 19140, United States. Electronic address: Nicole.Lucas@tuhs.temple.edu.
- [3] Department of Emergency Medicine, Temple University Hospital, 1316 W. Ontario Street, 10(th) Floor Jones Hall, Philadelphia, PA 19140, United States. Electronic address: Gregory.Zandrow@tuhs.temple.edu.
- [4] Department of Emergency Medicine, Temple University Hospital, 1316 W. Ontario Street, 10(th) Floor Jones Hall, Philadelphia, PA 19140, United States. Electronic address: Wayne.Satz@tuhs.temple.edu.
- [5] Department of Emergency Medicine, Temple University Hospital, 1316 W. Ontario Street, 10(th) Floor Jones Hall, Philadelphia, PA 19140, United States. Electronic address: Derek.Isenberg@tuhs.temple.edu.

- [6] Department of Emergency Medicine, Temple University Hospital, 1316 W. Ontario Street, 10(th) Floor Jones Hall, Philadelphia, PA 19140, United States. Electronic address: Orazio@tuhs.temple.edu.
- [7] Department of Emergency Medicine, Temple University Hospital, 1316 W. Ontario Street, 10(th) Floor Jones Hall, Philadelphia, PA 19140, United States. Electronic address: Nina.Gentile@tuhs.temple.edu.
- [8] Department of Emergency Medicine, Temple University Hospital, 1316 W. Ontario Street, 10(th) Floor Jones Hall, Philadelphia, PA 19140, United States. Electronic address: Kraftin.schreyer@tuhs.temple.edu.

- PMID: **33387928**
- PMCID: PMC7836717
- DOI: 10.1016/j.ajem.2020.12.047

Free PMC article
Observational Study

Impact of a shelter-in-place order during the COVID-19 pandemic on the incidence of opioid overdoses

Jennifer Rosenbaum et al. Am J Emerg Med. 2021 Mar.
Free PMC article
Show details
Am J Emerg Med
. 2021 Mar;41:51-54.
doi: 10.1016/j.ajem.2020.12.047. Epub 2020 Dec 30.

Authors

Jennifer Rosenbaum [1], Nicole Lucas [2], Gregory Zandrow [3], Wayne A Satz [4], Derek Isenberg [5], Joseph D'Orazio [6], Nina T Gentile [7], Kraftin E Schreyer [8]

Affiliations

- [1] Department of Emergency Medicine, Temple University Hospital, 1316 W. Ontario Street, 10(th) Floor Jones Hall, Philadelphia, PA 19140, United States. Electronic address: Jennifer.Rosenbaum@tuhs.temple.edu.
- [2] Department of Emergency Medicine, Temple University Hospital, 1316 W. Ontario Street, 10(th) Floor Jones Hall, Philadelphia, PA 19140, United States. Electronic address: Nicole.Lucas@tuhs.temple.edu.
- [3] Department of Emergency Medicine, Temple University Hospital, 1316 W. Ontario Street, 10(th) Floor Jones Hall, Philadelphia, PA 19140, United States. Electronic address: Gregory.Zandrow@tuhs.temple.edu.
- [4] Department of Emergency Medicine, Temple University Hospital, 1316 W. Ontario Street, 10(th) Floor Jones Hall, Philadelphia, PA 19140, United States. Electronic address: Wayne.Satz@tuhs.temple.edu.

- [5] Department of Emergency Medicine, Temple University Hospital, 1316 W. Ontario Street, 10(th) Floor Jones Hall, Philadelphia, PA 19140, United States. Electronic address: Derek.Isenberg@tuhs.temple.edu.
- [6] Department of Emergency Medicine, Temple University Hospital, 1316 W. Ontario Street, 10(th) Floor Jones Hall, Philadelphia, PA 19140, United States. Electronic address: Orazio@tuhs.temple.edu.
- [7] Department of Emergency Medicine, Temple University Hospital, 1316 W. Ontario Street, 10(th) Floor Jones Hall, Philadelphia, PA 19140, United States. Electronic address: Nina.Gentile@tuhs.temple.edu.
- [8] Department of Emergency Medicine, Temple University Hospital, 1316 W. Ontario Street, 10(th) Floor Jones Hall, Philadelphia, PA 19140, United States. Electronic address: Kraftin.schreyer@tuhs.temple.edu.

- PMID: **33387928**
- PMCID: PMC7836717
- DOI: 10.1016/j.ajem.2020.12.047

Abstract

Introduction: Since the beginning of the novel coronavirus (COVID-19) pandemic in the United States, there have been concerns about the potential impact of the pandemic on persons with opioid use disorder. Shelter-in-place (SIP) orders, which aimed to reduce the spread and scope of the virus, likely also impacted this patient population. This study aims to assess the role of the COVID-19 pandemic on the incidence of opioid overdose before and after a SIP order.

Methods: A retrospective review of the incidence of opioid overdoses in an urban three-hospital system was conducted. Comparisons were made between the first 100 days of a city-wide SIP order during the COVID-19 pandemic and the 100 days during the COVID-19 pandemic preceding the SIP order (Pre-SIP). Differences in observed incidence and expected incidence during the SIP period were evaluated using a Fisher's Exact test.

Results: Total patient visits decreased 22% from 46,078 during the Pre-SIP period to 35,971 during the SIP period. A total of 1551 opioid overdoses were evaluated during the SIP period, compared to 1665 opioid overdoses during the Pre-SIP period, consistent with a 6.8% decline. A Fisher's Exact Test demonstrated a $p < 0.0001$, with a corresponding Odds Ratio of 1.20 with a 95% confidence interval (1.12;1.29).

Conclusion: The COVID-19 pandemic and the associated SIP order were associated with a statistically and clinically significant increase in the proportion of opioid overdoses in relation to the overall change in total ED visits.

Keywords: COVID-19; Opiate overdose; Opioid epidemic; Pandemics; Shelter in place.

Copyright © 2020 Elsevier Inc. All rights reserved.

Conflict of interest statement

Declaration of Competing Interest None.

- 36 references
- 1 figure

Supplementary info

Publication types, MeSH terms Expand

Publication types

- Observational Study

MeSH terms

- COVID-19 / epidemiology*
- Emergency Service, Hospital / statistics & numerical data
- Facilities and Services Utilization
- Humans
- Incidence
- Opiate Overdose / epidemiology*
- Opiate Overdose / mortality
- Pandemics*
- Philadelphia / epidemiology
- Physical Distancing
- Quarantine*
- Retrospective Studies
- SARS-CoV-2

Full text links

ELSEVIER FULL-TEXT ARTICLE Elsevier Science Free PMC article

Proceed to details

Cite

Share

808

Observational Study

Rev Esp Salud Publica

. 2021 Nov 22;95:e202111109.

[Controlled supply of tocilizumab during the COVID-19 pandemic and its influence on the treatment of rheumatological patients]

[Article in Spanish]

Sara Ortiz Pérez [1], José Manuel Caro Teller [1], Carmen García Muñoz [1], Paola Herraiz Robles [1], Fernando Lozano Morillo [2], José Luis Pablos Álvarez [2], José Miguel Ferrari Piquero [2]

Affiliations Expand

Affiliations

- [1] Servicio de Farmacia. Hospital Universitario 12 de octubre. Madrid. España.
- [2] Servicio de Reumatología. Hospital Universitario 12 de octubre. Madrid. España.

- PMID: **34803159**

Free article
Observational Study

[Controlled supply of tocilizumab during the COVID-19 pandemic and its influence on the treatment of rheumatological patients]

[Article in Spanish]
Sara Ortiz Pérez et al. Rev Esp Salud Publica. 2021.
Free article
Show details
Rev Esp Salud Publica
. 2021 Nov 22;95:e202111109.

Authors

Sara Ortiz Pérez [1], José Manuel Caro Teller [1], Carmen García Muñoz [1], Paola Herraiz Robles [1], Fernando Lozano Morillo [2], José Luis Pablos Álvarez [2], José Miguel Ferrari Piquero [2]

Affiliations

- [1] Servicio de Farmacia. Hospital Universitario 12 de octubre. Madrid. España.
- [2] Servicio de Reumatología. Hospital Universitario 12 de octubre. Madrid. España.

- PMID: **34803159**

Abstract
in English, Spanish

Objective: Intravenous (IV) tocilizumab has been used to stop the inflammatory phase of SARS-CoV-2 infection. To preserve the largest number of IV units for this use, the Spanish Agency for Medicines and Health Products (AEMPS) carried out a controlled supply of it and recommended the change to a subcutaneous presentation (SC) of tocilizumab or sarilumab in all those patients in IV tocilizumab treatment for rheumatologic indications. The objective of this study was to evaluate the change from IV tocilizumab to SC presentation due to its controlled supply during the COVID-19 pandemic.

Methods: Retrospective observational study of adult patients (>18 years old) under treatment with IV tocilizumab follow-up by the Rheumatology Service of the Hospital 12 de Octubre. The follow-up period was 3 months (March 2020-June 2020) and 39 patients were included in the study. Variables related to the patients and their treatment were collected. A descriptive analysis of the data was carried out.

Results: In 69.23% (n=27) of the patients, treatment was changed to SC tocilizumab (n=23) or sarilumab (n=4). 44% of patients (n=12) switched back to their original IV tocilizumab treatment. The reasons for stopping treatment with SC tocilizumab were: drug intolerance (n=4), disease worsening (n=4), and patient preference (n=1). Regarding sarilumab, the reasons were drug intolerance (n=2) and patient preference (n=1).

Conclusions: Almost half of the patients had to return to the original treatment. The main reason was intolerance to the new treatment, followed by ineffectiveness and patient preferences.

Objetivo: El tocilizumab intravenoso (IV) ha sido empleado para frenar la fase inflamatoria de la infección por SARS-CoV-2. Para reservar el mayor número de unidades IV para este uso, la Agencia Española de Medicamentos y Productos Sanitarios (AEMPS) realizó una distribución controlada del mismo y recomendó el cambio a una presentación subcutánea (SC), fuera tocilizumab o sarilumab, en todos aquellos pacientes en tratamiento con tocilizumab IV para indicaciones reumatológicas. El objetivo de este trabajo fue evaluar el cambio de tocilizumab IV a una presentación SC debido a su suministro controlado durante la pandemia de COVID-19.

Metodos: Se realizó un estudio observacional retrospectivo de pacientes adultos (mayores de 18 años) en tratamiento con tocilizumab IV en seguimiento por el Servicio de Reumatología del Hospital 12 de octubre (Madrid). El periodo de seguimiento fue de tres meses (marzo 2020-junio 2020) y se incluyeron 39 pacientes en el estudio. Se recogieron variables relacionadas con el paciente y su tratamiento. Se realizó un análisis descriptivo de los datos.

Resultados: En el 69,23% (n=27) de los pacientes se cambió el tratamiento a tocilizumab SC (n=23) o sarilumab (n=4). El 44% (n=12) de los pacientes volvieron a cambiar a su tratamiento original con tocilizumab IV. Los motivos de interrupción de tratamiento con tocilizumab SC fueron: intolerancia al fármaco (n=4), empeoramiento de la enfermedad (n=4) y preferencia del paciente (n=1). Respecto al sarilumab, los motivos fueron intolerancia al fármaco (n=2) y preferencia del paciente (n=1).

Conclusiones: Casi la mitad de los pacientes tuvieron que volver al tratamiento original. El principal motivo fue intolerancia al nuevo tratamiento, seguido de ineficacia y preferencias del paciente.

Keywords: COVID-19; Intravenous; Rheumatology; Shortage; Spain; Subcutaneous; tocilizumab.

Supplementary info

Publication types, MeSH terms, Substances, Supplementary concepts Expand

Publication types

- Observational Study

MeSH terms

- Adolescent
- Adult
- Antibodies, Monoclonal, Humanized
- COVID-19* / drug therapy
- Humans
- Pandemics
- Rheumatic Diseases*
- SARS-CoV-2
- Spain / epidemiology
- Treatment Outcome

Substances

- Antibodies, Monoclonal, Humanized
- tocilizumab

Supplementary concepts

- COVID-19 drug treatment

Full text links

Revista Española de Salud Pública FULL TEXT articles Revista Espanola de Salud Publica

Proceed to details

Cite

Share

☐ 809

Psychiatry Res
. 2020 Sep;291:113254.
doi: 10.1016/j.psychres.2020.113254. Epub 2020 Jun 24.

Psychotic symptoms in COVID-19 patients. A retrospective descriptive study

A Parra [1], A Juanes [1], C P Losada [2], S Álvarez-Sesmero [1], V D Santana [1], I Martí [1], J Urricelqui [1], D Rentero [3]
Affiliations Expand

Affiliations

- [1] Servicio de Psiquiatría, Hospital Universitario 12 de Octubre, Madrid, Spain.

- [2] Servicio de Psiquiatría, Hospital Universitario 12 de Octubre, Madrid, Spain; Centro de Investigación Biomédica en Red de Salud Mental (CIBERSAM), Vigo, Spain; MRcPsych. Royal College of Psychiatrist, London, United Kingdom.
- [3] Servicio de Psiquiatría, Hospital Universitario 12 de Octubre, Madrid, Spain; Instituto de Investigación Sanitaria Hospital Universitario 12 de Octubre (i+12), Madrid, Spain; Centro de Investigación Biomédica en Red de Salud Mental (CIBERSAM), Madrid, Spain. Electronic address: david.rentero@salud.madrid.org.

- PMID: **32603930**
- PMCID: PMC7311337
- DOI: 10.1016/j.psychres.2020.113254

Free PMC article

Psychotic symptoms in COVID-19 patients. A retrospective descriptive study

A Parra et al. Psychiatry Res. 2020 Sep.
Free PMC article
Show details
Psychiatry Res
. 2020 Sep;291:113254.
doi: 10.1016/j.psychres.2020.113254. Epub 2020 Jun 24.

Authors

A Parra [1], A Juanes [1], C P Losada [2], S Álvarez-Sesmero [1], V D Santana [1], I Martí [1], J Urricelqui [1], D Rentero [3]

Affiliations

- [1] Servicio de Psiquiatría, Hospital Universitario 12 de Octubre, Madrid, Spain.
- [2] Servicio de Psiquiatría, Hospital Universitario 12 de Octubre, Madrid, Spain; Centro de Investigación Biomédica en Red de Salud Mental (CIBERSAM), Vigo, Spain; MRcPsych. Royal College of Psychiatrist, London, United Kingdom.
- [3] Servicio de Psiquiatría, Hospital Universitario 12 de Octubre, Madrid, Spain; Instituto de Investigación Sanitaria Hospital Universitario 12 de Octubre (i+12), Madrid, Spain; Centro de Investigación Biomédica en Red de Salud Mental (CIBERSAM), Madrid, Spain. Electronic address: david.rentero@salud.madrid.org.

- PMID: **32603930**
- PMCID: PMC7311337
- DOI: 10.1016/j.psychres.2020.113254

Abstract

Psychotic symptoms have been related to other coronavirus infections. We conducted a single-centre retrospective and observational study to describe new-onset psychotic episodes in COVID-

19 patients. Ten patients infected by the novel coronavirus with psychotic symptoms and no previous history of psychosis were identified by the emergency and liaison psychiatry departments. Nine of the cases presented with psychotic symptoms at least two weeks after the first somatic manifestations attributed to COVID-19 and receiving pharmacological treatment. Structured delusions mixed with confusional features were the most frequent clinical presentations. Hence, COVID-19 patients can develop psychotic symptoms as a consequence of multiple concurrent factors.

Keywords: Coronavirus; Psychotic disorders; SARS-CoV-2.

Copyright © 2020 Elsevier B.V. All rights reserved.

Conflict of interest statement

Declaration of Competing Interest None

Comment in

- Impact of social distancing due to coronavirus disease 2019 in old age psychiatry.
  Piacenza F, Ong SK. Piacenza F, et al. Psychogeriatrics. 2021 Mar;21(2):258-259. doi: 10.1111/psyg.12657. Epub 2021 Jan 24. Psychogeriatrics. 2021. PMID: 33486862 Free PMC article. No abstract available.

- 37 references

Supplementary info

MeSH terms Expand

MeSH terms

- Adult
- Betacoronavirus*
- COVID-19
- Coronavirus Infections / complications*
- Coronavirus Infections / psychology
- Female
- Humans
- Male
- Middle Aged
- Pandemics
- Pneumonia, Viral / complications*
- Pneumonia, Viral / psychology
- Psychotic Disorders / complications*
- Psychotic Disorders / psychology
- Retrospective Studies
- SARS-CoV-2

Full text links

Elsevier Science Free PMC article
Proceed to details
Cite
Share
810
Observational Study
Clin Interv Aging
. 2021 Jun 3;16:1037-1046.
doi: 10.2147/CIA.S313028. eCollection 2021.

Remdesivir, Renal Function and Short-Term Clinical Outcomes in Elderly COVID-19 Pneumonia Patients: A Single-Centre Study

Edoardo Biancalana [#] [1], Martina Chiriacò [#] [1], Paolo Sciarrone [1], Alessandro Mengozzi [1], Sandra Mechelli [2], Stefano Taddei [1], Anna Solini [3]
Affiliations Expand

Affiliations

- [1] Department of Clinical and Experimental Medicine, University of Pisa, Pisa, Italy.
- [2] Section of General Medicine IV AUOP, University of Pisa, Pisa, Italy.
- [3] Department of Surgical, Medical, Molecular and Critical Area Pathology, University of Pisa, Pisa, Italy.

[#] Contributed equally.

- PMID: **34113086**
- PMCID: PMC8184369
- DOI: 10.2147/CIA.S313028

Free PMC article
Observational Study

Remdesivir, Renal Function and Short-Term Clinical Outcomes in Elderly COVID-19 Pneumonia Patients: A Single-Centre Study

Edoardo Biancalana et al. Clin Interv Aging. 2021.
Free PMC article
Show details

Clin Interv Aging
. 2021 Jun 3;16:1037-1046.
doi: 10.2147/CIA.S313028. eCollection 2021.

Authors

Edoardo Biancalana [#] [1], Martina Chiriacò [#] [1], Paolo Sciarrone [1], Alessandro Mengozzi [1], Sandra Mechelli [2], Stefano Taddei [1], Anna Solini [3]

Affiliations

- [1] Department of Clinical and Experimental Medicine, University of Pisa, Pisa, Italy.
- [2] Section of General Medicine IV AUOP, University of Pisa, Pisa, Italy.
- [3] Department of Surgical, Medical, Molecular and Critical Area Pathology, University of Pisa, Pisa, Italy.

[#] Contributed equally.

- PMID: **34113086**
- PMCID: PMC8184369
- DOI: 10.2147/CIA.S313028

Abstract

Background: Remdesivir, an antiviral agent able to reduce inflammatory cascade accompanying severe, life-threatening pneumonia, became the first drug approved by the Food and Drug Administration for the treatment of hospitalized patients with coronavirus 2 related severe acute respiratory syndrome (SARS CoV2). As from its previously known clinical indications, the use of remdesivir in the presence of severe renal impairment is contraindicated; however, the impact of remdesivir on renal function in aging patients has not been elucidated.

Subjects and methods: This retrospective observational study involved 109 individuals consecutively admitted in internal medicine section, Azienda Ospedaliero Universitaria Pisana hospital, in November-December 2020 due to a confirmed diagnosis of SARS CoV2 and receiving remdesivir according to international inclusion criteria. Biochemical variables at admission were evaluated, together with slopes of estimated glomerular filtration rate (eGFR) built during remdesivir treatment. Participants were followed until discharge or exitus.

Results: Patients were stratified according to age (80 formed the study cohort and 29 served as controls); CKD stage III was present in 46% of them. No patients showed any sign of deteriorated renal function during remdesivir. Fourteen patients in the elderly cohort deceased; their eGFR at baseline was significantly lower. Recovered patients were characterized by a relevant eGFR gaining during remdesivir treatment.

Conclusion: We show here for the first time as remdesivir does not influence eGFR in a cohort of elderly people hospitalized for SARS CoV2, and that eGFR gain during such treatment is coupled with a better prognosis.

Keywords: SARS CoV2; aging; chronic kidney disease; glomerular filtration rate; remdesivir.

© 2021 Biancalana et al.

Conflict of interest statement

The authors report no conflicts of interest in this work.

- 39 references
- 1 figure

Supplementary info

Publication types, MeSH terms, Substances, Supplementary concepts Expand

Publication types

- Observational Study

MeSH terms

- Adenosine Monophosphate / adverse effects
- Adenosine Monophosphate / analogs & derivatives*
- Adenosine Monophosphate / therapeutic use
- Aged
- Aged, 80 and over
- Aging / physiology*
- Alanine / adverse effects
- Alanine / analogs & derivatives*
- Alanine / therapeutic use
- Antiviral Agents / adverse effects
- Antiviral Agents / therapeutic use*
- COVID-19 / drug therapy*
- COVID-19 / mortality
- Cohort Studies
- Female
- Glomerular Filtration Rate
- Humans
- Male
- Retrospective Studies
- SARS-CoV-2
- Severity of Illness Index
- Treatment Outcome

Substances

- Antiviral Agents

- remdesivir
- Adenosine Monophosphate
- Alanine

Supplementary concepts

- COVID-19 drug treatment

Full text links

FREE full-text article Dovepress Dove Medical Press Free PMC article

Proceed to details

Cite

Share

☐ 811

Clinical Trial

Neurol Sci

. 2020 Jun;41(6):1355-1359.

doi: 10.1007/s10072-020-04450-1. Epub 2020 May 19.

An Italian multicenter retrospective-prospective observational study on neurological manifestations of COVID-19 (NEUROCOVID)

Carlo Ferrarese [1], Vincenzo Silani [2], Alberto Priori [3], Stefania Galimberti [4], Elio Agostoni [5], Salvatore Monaco [6], Alessandro Padovani [7], Gioacchino Tedeschi [8], Italian Society of Neurology (SIN)

Affiliations Expand

Affiliations

- [1] Department of Medicine and Surgery and Milan Center for Neuroscience (NeuroMI), University of Milano-Bicocca and San Gerardo Hospital, ASST-Monza, Via Pergolesi 33, Monza, Italy. carlo.ferrarese@unimib.it.
- [2] Istituto Auxologico Italiano, IRCCS, Department of Neurology and Laboratory of Neuroscience - Department of Pathophysiology and Transplantation, "Dino Ferrari" Center, Università degli Studi di Milano, Milan, Italy.
- [3] Clinica Neurologica III, "Centro Aldo Ravelli" per le Terapie Neurologiche Sperimentali, Dipartimento di Scienze della Salute, Polo Universitario San Paolo, Università degli Studi di Milano, ASST Santi Paolo e Carlo, Milano, Italy.
- [4] Bicocca Bioinformatics Biostatistics and Bioimaging Centre, Department of Medicine and Surgery, University of Milano - Bicocca, Monza, Italy.

- [5] S.C. Neurologia e Stroke Unit ASST Grande Ospedale Metropolitano Niguarda, Milan, Italy.
- [6] Department of Neuroscience, Biomedicine and Movement Sciences, University of Verona, Verona, Italy.
- [7] Department of Neurology, ASST Spedali Civili and University of Brescia, Brescia, Italy.
- [8] Department of Advanced Medical and Surgical Sciences, University of Campania "L. Vanvitelli", Naples, Italy.

- PMID: **32430621**
- PMCID: PMC7235538
- DOI: 10.1007/s10072-020-04450-1

Free PMC article
Clinical Trial

An Italian multicenter retrospective-prospective observational study on neurological manifestations of COVID-19 (NEUROCOVID)

Carlo Ferrarese et al. Neurol Sci. 2020 Jun.
Free PMC article
Show details
Neurol Sci
. 2020 Jun;41(6):1355-1359.
doi: 10.1007/s10072-020-04450-1. Epub 2020 May 19.

Authors

Carlo Ferrarese [1], Vincenzo Silani [2], Alberto Priori [3], Stefania Galimberti [4], Elio Agostoni [5], Salvatore Monaco [6], Alessandro Padovani [7], Gioacchino Tedeschi [8], Italian Society of Neurology (SIN)

Affiliations

- [1] Department of Medicine and Surgery and Milan Center for Neuroscience (NeuroMI), University of Milano-Bicocca and San Gerardo Hospital, ASST-Monza, Via Pergolesi 33, Monza, Italy. carlo.ferrarese@unimib.it.
- [2] Istituto Auxologico Italiano, IRCCS, Department of Neurology and Laboratory of Neuroscience - Department of Pathophysiology and Transplantation, "Dino Ferrari" Center, Università degli Studi di Milano, Milan, Italy.
- [3] Clinica Neurologica III, "Centro Aldo Ravelli" per le Terapie Neurologiche Sperimentali, Dipartimento di Scienze della Salute, Polo Universitario San Paolo, Università degli Studi di Milano, ASST Santi Paolo e Carlo, Milano, Italy.
- [4] Bicocca Bioinformatics Biostatistics and Bioimaging Centre, Department of Medicine and Surgery, University of Milano - Bicocca, Monza, Italy.

- [5] S.C. Neurologia e Stroke Unit ASST Grande Ospedale Metropolitano Niguarda, Milan, Italy.
- [6] Department of Neuroscience, Biomedicine and Movement Sciences, University of Verona, Verona, Italy.
- [7] Department of Neurology, ASST Spedali Civili and University of Brescia, Brescia, Italy.
- [8] Department of Advanced Medical and Surgical Sciences, University of Campania "L. Vanvitelli", Naples, Italy.

- PMID: **32430621**
- PMCID: PMC7235538
- DOI: 10.1007/s10072-020-04450-1

Abstract

Neurological manifestations of COVID-19 have been described in both single case reports and retrospective scanty case series. They may be linked to the potential neurotropism of the SARS-COV-2 virus, as previously demonstrated for other coronaviruses. We report here the description of a multicenter retrospective-prospective observational study promoted by the Italian Society of Neurology (SIN), involving the Italian Neurological Departments, who will consecutively recruit patients with neurological symptoms and/or signs, occurred at the onset or as a complication of COVID-19. Hospitalized patients will be recruited either in neurological wards or in COVID wards; in the latter cases, they will be referred from other specialists to participant neurologists. Outpatients with clinical signs of COVID and neurological manifestations will be also referred to participating neurologists from primary care physicians. A comprehensive data collection, in the form of electronic case report form (eCRF), will register all possible neurological manifestations involving central nervous systems, peripheral nerves, and muscles, together with clinical, laboratory (including cerebrospinal fluid, if available), imaging, neurological, neurophysiological, and neuropsychological data. A follow-up at hospital discharge (in hospitalized patients), and for all patients after 3 and 6 months, is also planned. We believe that this study may help to intercept the full spectrum of neurological manifestations of COVID-19 and, given the large diffusion at national level, can provide a large cohort of patients available for future more focused investigations. Similar observational studies might also be proposed at international level to better define the neurological involvement of COVID-19.

Keywords: COVID-19; NEUROCOVID; Neurological manifestations; Observational study.

Conflict of interest statement

CF is in the Editorial Board of Parkinson Disease and Neurological Sciences; Coordinator of PRIN 2017, Grant: 2017CY3J3W, received grants and compensations for consulting services from Biogen, DOCPharma, Merck, Roche.

VS is in the Editorial Board of Amyotroph Lateral Sclerosis, European Neurology, American Journal of Neurodegenerative Diseases, Frontiers in Neurology; received compensation for consulting services and/or speaking activities from AveXis, Cytokinetics, Italfarmaco, and Zambon; and receives or has received research supports from the Italian Minsitry of Health (Grant RF-201302355764), Fondazione Italiana di Ricerca per la SLA – AriSLA (Grants exomafals and Novals), Fondazione Regionale per la Ricerca Biomedica regione Lombarda (Project nr. 2015-0023), and E-RARE JTC 2018 (Project Repetomics).

APr, APa, EA, GT, SG, and SM have nothing to disclose.

- 14 references

Supplementary info

Publication types, MeSH terms Expand

Publication types

- Clinical Trial
- Multicenter Study
- Observational Study

MeSH terms

- Betacoronavirus*
- COVID-19
- Coronavirus Infections / diagnosis
- Coronavirus Infections / epidemiology*
- Coronavirus Infections / therapy*
- Humans
- Italy / epidemiology
- Nervous System Diseases / diagnosis
- Nervous System Diseases / epidemiology*
- Nervous System Diseases / therapy*
- Pandemics
- Pneumonia, Viral / diagnosis
- Pneumonia, Viral / epidemiology*
- Pneumonia, Viral / therapy*
- Prospective Studies
- Retrospective Studies
- SARS-CoV-2

Full text links

SpringerLink FULL-TEXT ARTICLE Springer Free PMC article
Proceed to details
Cite
Share
☐ 812
Observational Study
Medicine (Baltimore)
. 2021 Mar 5;100(9):e24604.
doi: 10.1097/MD.0000000000024604.

Extra-pulmonary complications of 45 critically ill patients with COVID-19 in Yichang, Hubei province, China: A single-centered, retrospective, observation study

Peng Wang [1], Xiang Tan [1], Qian Li [2], Min Qian [1], Aiguo Cheng [2], Baohua Ma [3], Peng Wan [1], Xinli Zhang [1], Changyun Guo [1], Mengting Sheng [1], Mengqiu Yi [1], Min Yu [1]
Affiliations Expand

Affiliations

- [1] Department of Critical Care Medicine, The People's Hospital of China Three Gorges University, The First People's Hospital of Yichang.
- [2] Department of Pulmonary and Critical Care Medicine, The Third People's Hospital of Yichang, China Three Gorges University Third People's Hospital.
- [3] Medical Department, The People's Hospital of China Three Gorges University, The First People's Hospital of Yichang, Yichang, Hubei, China.

- PMID: **33655925**
- PMCID: PMC7939178
- DOI: 10.1097/MD.0000000000024604

Free PMC article
Observational Study

Extra-pulmonary complications of 45 critically ill patients with COVID-19 in Yichang, Hubei province, China: A single-centered, retrospective, observation study

Peng Wang et al. Medicine (Baltimore). 2021.
Free PMC article
Show details
Medicine (Baltimore)
. 2021 Mar 5;100(9):e24604.
doi: 10.1097/MD.0000000000024604.

Authors

Peng Wang [1], Xiang Tan [1], Qian Li [2], Min Qian [1], Aiguo Cheng [2], Baohua Ma [3], Peng Wan [1], Xinli Zhang [1], Changyun Guo [1], Mengting Sheng [1], Mengqiu Yi [1], Min Yu [1]

Affiliations

- [1] Department of Critical Care Medicine, The People's Hospital of China Three Gorges University, The First People's Hospital of Yichang.
- [2] Department of Pulmonary and Critical Care Medicine, The Third People's Hospital of Yichang, China Three Gorges University Third People's Hospital.
- [3] Medical Department, The People's Hospital of China Three Gorges University, The First People's Hospital of Yichang, Yichang, Hubei, China.

- PMID: **33655925**
- PMCID: PMC7939178
- DOI: 10.1097/MD.0000000000024604

Abstract

Mortality of critically ill patients with coronavirus disease 2019 (COVID-19) was high. Aims to examine whether time from symptoms onset to intensive care unit (ICU) admission affects incidence of extra-pulmonary complications and prognosis in order to provide a new insight for reducing the mortality. A single-centered, retrospective, observational study investigated 45 critically ill patients with COVID-19 hospitalized in ICU of The Third People's Hospital of Yichang from January 17 to March 29, 2020. Patients were divided into 2 groups according to time from symptoms onset to ICU admission (>7 and ≤7 days) and into 2 groups according to prognosis (survivors and non-survivors). Epidemiological, clinical, laboratory, radiological characteristics and treatment data were studied. Compared with patients who admitted to the ICU since symptoms onset ≤7 days (55.6%), patients who admitted to the ICU since symptoms onset >7 days (44.4%) were more likely to have extra-pulmonary complications (19 [95.0%] vs 16 [64.0%], P = .034), including acute kidney injury, cardiac injury, acute heart failure, liver dysfunction, gastrointestinal hemorrhage, hyperamylasemia, and hypernatremia. The incidence rates of acute respiratory distress syndrome, pneumothorax, and hospital-acquired pneumonia had no difference between the 2 groups. Except activated partial thromboplastin and Na+ concentration, the laboratory findings were worse in group of time from symptoms onset to ICU admission >7 days. There was no difference in mortality between the 2 groups. Of the 45 cases in the ICU, 19 (42.2%) were non-survivors, and 16 (35.6%) were with hospital-acquired pneumonia. Among these non-survivors, hospital-acquired pneumonia was up to 12 (63.2%) besides higher incidence of extra-pulmonary complications. However, hospital-acquired pneumonia occurred in only 4 (15.4%) survivors. Critically ill patients with COVID-19 who admitted to ICU at once might get benefit from intensive care via lower rate of extra-pulmonary complications.

Copyright © 2021 the Author(s). Published by Wolters Kluwer Health, Inc.

Conflict of interest statement

All the authors state that there are no conflicts of interest related to this study.

- 24 references
- 2 figures

Supplementary info

Publication types, MeSH terms Expand

Publication types

- Observational Study

MeSH terms

- Acute Kidney Injury / diagnosis
- Acute Kidney Injury / etiology
- COVID-19* / complications
- COVID-19* / diagnosis
- COVID-19* / mortality
- COVID-19* / physiopathology
- China / epidemiology
- Critical Care* / methods
- Critical Care* / statistics & numerical data
- Critical Illness* / mortality
- Critical Illness* / therapy
- Digestive System Diseases / diagnosis
- Digestive System Diseases / etiology
- Female
- Healthcare-Associated Pneumonia / diagnosis
- Healthcare-Associated Pneumonia / mortality
- Heart Diseases / diagnosis
- Humans
- Hyperamylasemia / diagnosis
- Hyperamylasemia / etiology
- Hypernatremia / diagnosis
- Hypernatremia / etiology
- Male
- Middle Aged
- Outcome and Process Assessment, Health Care
- Prognosis
- SARS-CoV-2 / isolation & purification
- Survival Analysis
- Symptom Assessment* / methods
- Symptom Assessment* / statistics & numerical data
- Time-to-Treatment / statistics & numerical data*

Full text links

Wolters Kluwer Wolters Kluwer Free PMC article
Proceed to details

Cite
Share
813
Observational Study
Clin Nutr ESPEN
. 2021 Oct;45:184-191.
doi: 10.1016/j.clnesp.2021.08.030. Epub 2021 Sep 7.

mNUTRIC tool is capable to predict nutritional needs and mortality early in patients suffering from severe pneumonia

Selen Acehan [1], Muge Gulen [2], Cem Isıkber [3], Nurdan Unlu [4], Hılmı Erdem Sumbul [5], Erdinc Gulumsek [6], Salim Satar [7]
Affiliations Expand

Affiliations

- [1] Adana City Training and Research Hospital, Department of Emergency Medicine, Adana, Turkey. Electronic address: selenacehan@hotmail.com.
- [2] Adana City Training and Research Hospital, Department of Emergency Medicine, Adana, Turkey. Electronic address: muge-gulen@hotmail.com.
- [3] Adana City Training and Research Hospital, Department of Emergency Medicine, Adana, Turkey. Electronic address: cemisikber@gmail.com.
- [4] Adana City Training and Research Hospital, Department of Anesthesiology and Intensive Care, Adana, Turkey. Electronic address: kondunurdan@gmail.com.
- [5] Adana City Training and Research Hospital, Department of İnternal Medicine, Adana, Turkey. Electronic address: erdemsumbul@gmail.com.
- [6] Adana City Training and Research Hospital, Department of Gastroenterology, Adana, Turkey. Electronic address: drerd84@yahoo.com.tr.
- [7] Adana City Training and Research Hospital, Department of Emergency Medicine, Adana, Turkey. Electronic address: salim.satar@yahoo.com.

- PMID: **34620315**
- PMCID: PMC8441546
- DOI: 10.1016/j.clnesp.2021.08.030

Free PMC article
Observational Study

mNUTRIC tool is capable to predict nutritional needs and mortality early in patients suffering from severe pneumonia

Selen Acehan et al. Clin Nutr ESPEN. 2021 Oct.
Free PMC article
Show details
Clin Nutr ESPEN
. 2021 Oct;45:184-191.
doi: 10.1016/j.clnesp.2021.08.030. Epub 2021 Sep 7.

Authors

Selen Acehan [1], Muge Gulen [2], Cem Isıkber [3], Nurdan Unlu [4], Hılmı Erdem Sumbul [5], Erdinc Gulumsek [6], Salim Satar [7]

Affiliations

- [1] Adana City Training and Research Hospital, Department of Emergency Medicine, Adana, Turkey. Electronic address: selenacehan@hotmail.com.
- [2] Adana City Training and Research Hospital, Department of Emergency Medicine, Adana, Turkey. Electronic address: muge-gulen@hotmail.com.
- [3] Adana City Training and Research Hospital, Department of Emergency Medicine, Adana, Turkey. Electronic address: cemisikber@gmail.com.
- [4] Adana City Training and Research Hospital, Department of Anesthesiology and Intensive Care, Adana, Turkey. Electronic address: kondunurdan@gmail.com.
- [5] Adana City Training and Research Hospital, Department of İnternal Medicine, Adana, Turkey. Electronic address: erdemsumbul@gmail.com.
- [6] Adana City Training and Research Hospital, Department of Gastroenterology, Adana, Turkey. Electronic address: drerd84@yahoo.com.tr.
- [7] Adana City Training and Research Hospital, Department of Emergency Medicine, Adana, Turkey. Electronic address: salim.satar@yahoo.com.

- PMID: **34620315**
- PMCID: PMC8441546
- DOI: 10.1016/j.clnesp.2021.08.030

Abstract

Objective: This retrospective observational study aims to evaluate the prognostic accuracy of Modified Nutrition Risk in Critically ill (mNUTRIC) compared to Nutrition Risk Score-2002 (NRS-2002) in patients hospitalized in the intensive care unit due to severe pneumonia during the pandemic period.

Methods: RT-PCR test and Chest CT was performed in all patients in the emergency department pandemic area. The CURB-65 at the time of admission to the emergency department and Acute Physiology and Chronic Health Evaluation II (APACHE II), Sequential organ failure assessment score (SOFA), NRS-2002 and mNUTRIC scores 24 h after hospitalization in the intensive care unit were calculated. The analysis of the data was made in IBM SPSS Statistics Base 22.0 package program.

Results: One hundred and twenty-five patients found to have severe pneumonia based on the chest CT taken in the emergency department pandemic area and hospitalized in the intensive care unit were included in the study. A real-time reverse transcription PCR (RT-PCR) test was positive

in 30.4% (n: 38) of the patients. Additional nutrition treatment was initiated in 54.4% of the patients. In the analytical evaluation to predict nutritional treatment needs, mNUTRIC's AUC value (AUC: 0.681, 95% 0.582-0.780, $p < 0.001$) was higher than NRS-2002. While 64.8% (n: 81) of the patients were discharged, 35.2% (n: 44) died. In the analytical evaluation to predict mortality, the AUC value of mNUTRIC had the highest value (AUC: 0.875, 95% CI 0.814-0.935, $p < 0.001$).

Conclusion: The mNUTRIC score can predict at an early period the nutritional needs and mortality of patients with severe pneumonia during the Covid-19 pandemic.

Keywords: Covid-19; Intensive care unıt; NRS-2002; Severe pneumonia; mNUTRIC.

Copyright © 2021 European Society for Clinical Nutrition and Metabolism. Published by Elsevier Ltd. All rights reserved.

Conflict of interest statement

Declaration of competing interest The authors declare no conflicts of interest.

- 32 references
- 3 figures

Supplementary info

Publication types, MeSH terms Expand

Publication types

- Observational Study

MeSH terms

- COVID-19*
- Critical Illness
- Humans
- Pandemics
- Pneumonia* / diagnosis
- SARS-CoV-2

Full text links

ELSEVIER FULL-TEXT ARTICLE Elsevier Science Free PMC article

Proceed to details

Cite

Share

☐ 814

Observational Study

S Afr Med J

. 2021 Apr 6;111(6):550-553.

Corticosteroids in critical COVID-19: Are all corticosteroids equal?

E M Du Plessis [1], U Lalla, B W Allwood, E H Louw, A Nortje, A Parker, J J Taljaard, B T Ayele, P S Nyasulu, C F N Koegelenberg
Affiliations Expand

Affiliation

- [1] Division of Pulmonology, Department of Medicine, Faculty of Medicine and Health Sciences, Stellenbosch University and Tygerberg Academic Hospital, Cape Town, South Africa. imbalidp@gmail.com.

- PMID: **34382564**

Free article
Observational Study

Corticosteroids in critical COVID-19: Are all corticosteroids equal?

E M Du Plessis et al. S Afr Med J. 2021.
Free article
Show details
S Afr Med J
. 2021 Apr 6;111(6):550-553.

Authors

E M Du Plessis [1], U Lalla, B W Allwood, E H Louw, A Nortje, A Parker, J J Taljaard, B T Ayele, P S Nyasulu, C F N Koegelenberg

Affiliation

- [1] Division of Pulmonology, Department of Medicine, Faculty of Medicine and Health Sciences, Stellenbosch University and Tygerberg Academic Hospital, Cape Town, South Africa. imbalidp@gmail.com.

- PMID: **34382564**

Abstract

The hyperinflammation seen as part of a dysregulated immune response to SARS-CoV-2 in its most severe form leads to acute respiratory distress syndrome (ARDS), multiorgan failure and

death. Corticosteroid therapy targets this hyperinflammation, otherwise known as a cytokine storm. It is the only therapeutic agent to date with a mortality benefit, with clear guidelines from national and international health authorities guiding its use. Objectives. To compare severity-of-illness indices, survival, length of intensive care unit (ICU) stay and potential ICU complications in patients treated with different corticosteroid regimens (high-dose hydrocortisone, high-dose methylprednisolone and lower-dose dexamethasone). Methods. In this single-centre descriptive retrospective observational study of a cohort of patients with severe COVID-19 admitted to a COVID-dedicated ICU, we compared patients treated with the three different corticosteroid regimens. Results. In 242 cases we could not demonstrate any statistically or clinically significant difference in the outcome of patients with critical COVID-19 treated with high-dose intravenous hydrocortisone (n=88) or methylprednisolone (n=46) compared with a relatively lower dose of dexamethasone (n=108). The survival rates were 38.6%, 39.1% and 33.3%, respectively (p=0.68). Patients treated with methylprednisolone tended to have a shorter length of ICU stay (median (interquartile range) 6 (4 - 10), 4 (2 - 8) and 5 (2 - 8) days; p=0.015) and fewer episodes of nosocomial sepsis (47.7%, 32.6% and 48.1%; p=0.01). Conclusions. Hydrocortisone or methylprednisolone can be given as an alternative to dexamethasone in the management of critical COVID-19, and this is a feasible alternative, especially in resource-constrained settings.

Supplementary info

Publication types, MeSH terms, Substances Expand

Publication types

- Comparative Study
- Observational Study

MeSH terms

- Adult
- COVID-19 / complications
- COVID-19 / drug therapy*
- COVID-19 / mortality
- Cohort Studies
- Cytokine Release Syndrome / drug therapy
- Cytokine Release Syndrome / virology
- Dexamethasone / administration & dosage*
- Dose-Response Relationship, Drug
- Female
- Glucocorticoids / administration & dosage*
- Humans
- Hydrocortisone / administration & dosage*
- Intensive Care Units
- Length of Stay
- Male
- Methylprednisolone / administration & dosage*

- Middle Aged
- Retrospective Studies
- Severity of Illness Index
- Survival Rate

Substances

- Glucocorticoids
- Dexamethasone
- Hydrocortisone
- Methylprednisolone

Full text links

Health and Medical Publishing Group
Proceed to details
Cite
Share
815
Observational Study
Respiration
. 2020;99(12):1145-1153.
doi: 10.1159/000511376. Epub 2020 Dec 14.

Use of Intravenous Immunoglobulin (Prevagen or Octagam) for the Treatment of COVID-19: Retrospective Case Series

Felix J F Herth [1], George Sakoulas [2], Fadi Haddad [3]
Affiliations Expand

Affiliations

- [1] Department of Pneumology and Critical Care Medicine, Thoraxklinik, and Translational Lung Research Center Heidelberg, University of Heidelberg, Heidelberg, Germany.
- [2] Sharp Memorial Hospital and the University of California, San Diego, California, USA, george.sakoulas@sharp.com.
- [3] Sharp Grossmont Hospital, La Mesa, California, USA.

- PMID: **33316806**
- PMCID: PMC7801971
- DOI: 10.1159/000511376

Free PMC article

Observational Study

Use of Intravenous Immunoglobulin (Prevagen or Octagam) for the Treatment of COVID-19: Retrospective Case Series

Felix J F Herth et al. Respiration. 2020.
Free PMC article
Show details
Respiration
. 2020;99(12):1145-1153.
doi: 10.1159/000511376. Epub 2020 Dec 14.

Authors

Felix J F Herth [1], George Sakoulas [2], Fadi Haddad [3]

Affiliations

- [1] Department of Pneumology and Critical Care Medicine, Thoraxklinik, and Translational Lung Research Center Heidelberg, University of Heidelberg, Heidelberg, Germany.
- [2] Sharp Memorial Hospital and the University of California, San Diego, California, USA, george.sakoulas@sharp.com.
- [3] Sharp Grossmont Hospital, La Mesa, California, USA.

- PMID: **33316806**
- PMCID: PMC7801971
- DOI: 10.1159/000511376

Abstract

Treatment with immunomodulators, such as intravenous immunoglobulin (IVIG), may attenuate inflammatory responses observed in the severe stages of acute respiratory distress syndrome (ARDS) caused by coronavirus disease 19 (COVID-19). We retrospectively evaluated the clinical courses of 12 COVID-19 patients who received IVIG at various stages of their illness, including within the first 72 h of clinical presentation, after initiation of mechanical ventilation, and after prolonged ventilation and ICU stay. The patients included 9 men and 3 women with a median age of 50 years (range 23-74), median Charlson Comorbidity Score of 2 (range 0-7), and median Acute Physiology and Chronic Health Evaluation Score of 13 (range 5-33) at the time of IVIG. The IVIG total dose ranged from 0.5 to 2.0 g/kg (median 1.25 g/kg) distributed over 1-4 daily doses. The most common regimen received was 0.5 g/kg daily for 3 days. The median time to IVIG administration was 9 days (range 0-48 days) after admission. The median time from first IVIG dose administration to hospital discharge was 14 days (range 3-48). The 5 patients who received IVIG ≤4 days of admission demonstrated a significantly shorter length of hospital stay after treatment (median 7 days, range 3-14 days) than the 7 patients who received it >7 days after admission (median 33 days, range 8-48 days, $p = 0.03$, Mann-Whitney U test). These cases demonstrate that IVIG may improve the clinical state of patients with moderate to severe COVID-

19 infection. Despite very high illness severity scores, all patients survived hospital discharge. No thrombotic events occurred and IVIG was well tolerated, despite most cases demonstrating very elevated D-dimer suggestive of active intravascular fibrinolysis. We believe that IVIG warrants immediate clinical trial evaluation in COVID-19 to confirm its role as a mainstay treatment of moderate to severe COVID-19 infection as a means to reduce hospital stay and utilization of ICU resources, including mechanical ventilation, and potentially reduce mortality.

Keywords: Acute respiratory distress syndrome; Coronavirus disease 19; Intravenous immunoglobulin.

© 2020 The Author(s) Published by S. Karger AG, Basel.

Conflict of interest statement

G.S. has received research funding from Octapharma. F.J.F.H. and F.H. have no conflicts of interest to declare.

Comment in

- Intravenous Immunoglobulin for the Treatment of COVID-19: A Promising Tool.
  Tzilas V, Manali E, Papiris S, Bouros D. Tzilas V, et al. Respiration. 2020;99(12):1087-1089. doi: 10.1159/000512727. Epub 2020 Nov 19. Respiration. 2020. PMID: 33212437 Free PMC article. No abstract available.

- 6 figures

Supplementary info

Publication types, MeSH terms, Substances, Supplementary concepts Expand

Publication types

- Observational Study

MeSH terms

- APACHE
- Adenosine Monophosphate / analogs & derivatives
- Adenosine Monophosphate / therapeutic use
- Adrenal Cortex Hormones / therapeutic use
- Adult
- Aged
- Alanine / analogs & derivatives
- Alanine / therapeutic use
- Anti-Bacterial Agents / therapeutic use
- Antibodies, Monoclonal, Humanized / therapeutic use
- Antiviral Agents / therapeutic use

- Azithromycin / therapeutic use
- COVID-19 / drug therapy
- COVID-19 / therapy*
- Doxycycline / therapeutic use
- Enzyme Inhibitors / therapeutic use
- Extracorporeal Membrane Oxygenation*
- Female
- Humans
- Hydroxychloroquine / therapeutic use
- Immunoglobulins, Intravenous / therapeutic use*
- Immunologic Factors / therapeutic use*
- Intensive Care Units*
- Length of Stay*
- Lopinavir / therapeutic use
- Male
- Middle Aged
- Respiration, Artificial*
- Retrospective Studies
- Severity of Illness Index
- Time Factors
- Young Adult

Substances

- Adrenal Cortex Hormones
- Anti-Bacterial Agents
- Antibodies, Monoclonal, Humanized
- Antiviral Agents
- Enzyme Inhibitors
- Immunoglobulins, Intravenous
- Immunologic Factors
- Octagam
- Lopinavir
- remdesivir
- Adenosine Monophosphate
- Hydroxychloroquine
- Azithromycin
- tocilizumab
- Doxycycline
- Alanine

Supplementary concepts

- COVID-19 drug treatment

Full text links

S. Karger AG, Basel, Switzerland Free PMC article
Proceed to details
Cite
Share
816
Observational Study
Eur J Pediatr
. 2021 Jun;180(6):1895-1906.
doi: 10.1007/s00431-021-03967-7. Epub 2021 Feb 5.

A single-center observational study on clinical features and outcomes of 21 SARS-CoV-2-infected neonates from India

Ruchi Nanavati [1], Dwayne Mascarenhas [1], Medha Goyal [2], Anitha Haribalakrishna [1], Gita Nataraj [3]
Affiliations Expand

Affiliations

- [1] Department of Neonatology, Seth GS Medical College and King Edward Memorial Hospital, Parel, Mumbai, 400 012, India.
- [2] Department of Neonatology, Seth GS Medical College and King Edward Memorial Hospital, Parel, Mumbai, 400 012, India. medha_kv@yahoo.com.
- [3] Department of Microbiology, Seth GS Medical College KEM Hospital, Mumbai, India.

- PMID: **33544233**
- PMCID: PMC7862853
- DOI: 10.1007/s00431-021-03967-7

Free PMC article
Observational Study

A single-center observational study on clinical features and outcomes of 21 SARS-CoV-2-infected neonates from India

Ruchi Nanavati et al. Eur J Pediatr. 2021 Jun.
Free PMC article
Show details
Eur J Pediatr
. 2021 Jun;180(6):1895-1906.
doi: 10.1007/s00431-021-03967-7. Epub 2021 Feb 5.

Authors

Ruchi Nanavati [1], Dwayne Mascarenhas [1], Medha Goyal [2], Anitha Haribalakrishna [1], Gita Nataraj [3]

Affiliations

- [1] Department of Neonatology, Seth GS Medical College and King Edward Memorial Hospital, Parel, Mumbai, 400 012, India.
- [2] Department of Neonatology, Seth GS Medical College and King Edward Memorial Hospital, Parel, Mumbai, 400 012, India. medha_kv@yahoo.com.
- [3] Department of Microbiology, Seth GS Medical College KEM Hospital, Mumbai, India.

- PMID: **33544233**
- PMCID: PMC7862853
- DOI: 10.1007/s00431-021-03967-7

Abstract

Coronavirus disease-19 (COVID-19) caused by severe acute respiratory syndrome coronavirus-2 (SARS-CoV-2) is an ongoing pandemic with significant morbidity and mortality. Neonates represent a vulnerable population, in which we have limited knowledge of its natural history, optimal management, and outcomes. In this retrospective observational study from a low-middle-income setting, clinical characteristics and outcomes of neonatal SARS-CoV-2 infection were evaluated. We report an incidence of 10.6% of SARS-CoV-2 infection (21 neonates), among a group of 198 neonates with suspected infection. Most of the SARS-CoV-2-infected neonates were term (80.9%) and none required any resuscitation. The infection was detected by a positive nasopharyngeal swab reverse transcriptase-polymerase chain reaction (RT-PCR) for SARS-CoV-2. Neonatal COVID-19 manifestations developed in one-third (33.3%) of the infected neonates. Most of them demonstrated the involvement of respiratory (33.3%) and gastrointestinal systems (4.8%). Laboratory parameters suggested multi-systemic involvement, with elevated creatine kinase (CK) (76.2%), creatine kinase-myocardial band (CK-MB) (76.2%), and lactate dehydrogenase (LDH) (71.4%) levels. Supportive treatment was given to infected neonates with intensive care required in six neonates (28.6%). This included four preterm and two term neonates, of which two received non-invasive and one received invasive ventilation with intra-tracheal surfactant instillation. IgM antibodies against COVID-19 were detected in one neonate.

All neonates with COVID-19 improved and were successfully discharged.Conclusion: SARS-CoV-2 in neonates has a wide clinical spectrum. Further studies are needed which are adequately powered to completely understand the course of this infection in neonates, its implications not only in the neonatal period but also on long-term follow-up. What is Known: • SARS-CoV-2 infection has a predilection for all age groups but with limited literature on clinical profile, outcomes, and long-term follow-up in neonates. What is New: • SARS-CoV-2 infection in neonates has a wide clinical spectrum and displays a significant overlap with common neonatal conditions. • Most neonates with COVID-19 improved with supportive care, though a subset required intensive care, emphasizing the need for cautious monitoring and management.

Keywords: Antibodies; COVID-19; Coronavirus; Low-middle income; Newborn; RT-PCR.

Conflict of interest statement

The authors declare no conflict of interest.

- 37 references
- 1 figure

Supplementary info

Publication types, MeSH terms Expand

Publication types

- Observational Study

MeSH terms

- COVID-19*
- Female
- Humans
- India / epidemiology
- Infant, Newborn
- Infectious Disease Transmission, Vertical
- Pregnancy
- Pregnancy Complications, Infectious*
- SARS-CoV-2

Full text links

SpringerLink FULL-TEXT ARTICLE Springer Free PMC article
Proceed to details
Cite
Share
☐ 817
Observational Study

Br J Dermatol
. 2020 Aug;183(2):373-374.
doi: 10.1111/bjd.19158. Epub 2020 May 28.

The impact of the COVID-19 pandemic on patients with chronic plaque psoriasis being treated with biological therapy: the Northern Italy experience

P Gisondi [1], P Facheris [2, 3], P Dapavo [4], S Piaserico [5], A Conti [6], L Naldi [7, 8], S Cazzaniga [8, 9], P Malagoli [10], A Costanzo [2]
Affiliations Expand

Affiliations

- [1] Department of Medicine, Section of Dermatology and Venereology, University of Verona, Italy.
- [2] Dermatology Unit, Department of Biomedical Sciences, Humanitas University, Pieve Emanuele, Italy.
- [3] Humanitas Clinical and Research Center, IRCCS, Rozzano, Italy.
- [4] Department of Biomedical Science and Human Oncology, Second Dermatologic Clinic, University of Turin, Turin, Italy.
- [5] Section of Dermatology, Department of Medicine, University of Padua, Padova, Italy.
- [6] Department of Surgical, Medical, Dental and Morphological Sciences related to Transplant, Oncology and Regenerative Medicine, Dermatology Unit, University of Modena and Reggio Emilia, Modena, Italy.
- [7] Department of Dermatology, San Bortolo Hospital, Vicenza, Italy.
- [8] Centro Studi GISED, Bergamo, Italy.
- [9] Department of Dermatology, Inselspital University Hospital of Bern, Bern, Switzerland.
- [10] Dermatology Unit, Azienda Ospedaliera San Donato Milanese, Milan, Italy.

- PMID: **32343839**
- PMCID: PMC7267283
- DOI: 10.1111/bjd.19158

Free PMC article
Observational Study

The impact of the COVID-19 pandemic on patients with chronic plaque psoriasis being treated with biological therapy: the Northern Italy experience

P Gisondi et al. Br J Dermatol. 2020 Aug.
Free PMC article
Show details
Br J Dermatol
. 2020 Aug;183(2):373-374.
doi: 10.1111/bjd.19158. Epub 2020 May 28.

Authors

P Gisondi [1], P Facheris [2, 3], P Dapavo [4], S Piaserico [5], A Conti [6], L Naldi [7, 8], S Cazzaniga [8, 9], P Malagoli [10], A Costanzo [2]

Affiliations

- [1] Department of Medicine, Section of Dermatology and Venereology, University of Verona, Italy.
- [2] Dermatology Unit, Department of Biomedical Sciences, Humanitas University, Pieve Emanuele, Italy.
- [3] Humanitas Clinical and Research Center, IRCCS, Rozzano, Italy.
- [4] Department of Biomedical Science and Human Oncology, Second Dermatologic Clinic, University of Turin, Turin, Italy.
- [5] Section of Dermatology, Department of Medicine, University of Padua, Padova, Italy.
- [6] Department of Surgical, Medical, Dental and Morphological Sciences related to Transplant, Oncology and Regenerative Medicine, Dermatology Unit, University of Modena and Reggio Emilia, Modena, Italy.
- [7] Department of Dermatology, San Bortolo Hospital, Vicenza, Italy.
- [8] Centro Studi GISED, Bergamo, Italy.
- [9] Department of Dermatology, Inselspital University Hospital of Bern, Bern, Switzerland.
- [10] Dermatology Unit, Azienda Ospedaliera San Donato Milanese, Milan, Italy.

- PMID: **32343839**
- PMCID: PMC7267283
- DOI: 10.1111/bjd.19158

No abstract available

Comment in

- Psoriasis, COVID-19, and acute respiratory distress syndrome: Focusing on the risk of concomitant biological treatment.

Magnano M, Balestri R, Bardazzi F, Mazzatenta C, Girardelli CR, Rech G. Magnano M, et al. Dermatol Ther. 2020 Jul;33(4):e13706. doi: 10.1111/dth.13706. Epub 2020 Jun 19. Dermatol Ther. 2020. PMID: 32475056 Free PMC article. No abstract available.

- 6 references

Supplementary info

Publication types, MeSH terms, Substances Expand

Publication types

- Letter
- Multicenter Study
- Observational Study

MeSH terms

- Adult
- Aged
- Betacoronavirus / immunology*
- Betacoronavirus / isolation & purification
- Biological Products / adverse effects*
- COVID-19
- Comorbidity
- Coronavirus Infections / epidemiology*
- Coronavirus Infections / immunology
- Coronavirus Infections / virology
- Female
- Hospital Mortality
- Hospitalization / statistics & numerical data*
- Humans
- Italy / epidemiology
- Male
- Middle Aged
- Pandemics
- Pneumonia, Viral / epidemiology*
- Pneumonia, Viral / immunology
- Pneumonia, Viral / virology
- Psoriasis / drug therapy*
- Psoriasis / epidemiology
- Psoriasis / immunology
- Retrospective Studies

- SARS-CoV-2

Substances

- Biological Products

Full text links

WILEY Full Text Article Wiley Free PMC article
Proceed to details
Cite
Share
818
Observational Study
Int J Radiat Oncol Biol Phys
. 2021 Jul 15;110(4):947-956.
doi: 10.1016/j.ijrobp.2021.02.022. Epub 2021 Feb 17.

Systematic Screening of COVID-19 Disease Based on Chest CT and RT-PCR for Cancer Patients Undergoing Radiation Therapy in a Coronavirus French Hotspot

Roger Sun [1], Samir Achkar [2], Samy Ammari [3], Sophie Bockel [2], Emmanuelle Gallois [4], Arnaud Bayle [5], Enzo Battistella [6], Flore Salviat [7], Mansouria Merad [5], Adrien Laville [2], Kanta Ka [2], Franck Griscelli [4], Laurence Albiges [5], Fabrice Barlesi [5], Alberto Bossi [2], Sofia Rivera [2], Cyrus Chargari [1], Eric Deutsch [8]
Affiliations Expand

Affiliations

- [1] Department of Radiation Oncology, Gustave Roussy, Paris-Saclay University, Villejuif, France; Radiothérapie Moléculaire et Innovation Thérapeutique, Paris-Saclay University, Gustave Roussy, Villejuif, France.
- [2] Department of Radiation Oncology, Gustave Roussy, Paris-Saclay University, Villejuif, France.
- [3] Department of Radiology, Gustave Roussy, Paris-Saclay University, Villejuif, France; BioMaps (UMR1281), Université Paris-Saclay, CNRS, INSERM, CEA, Orsay and Gustave Roussy, Villejuif, France.
- [4] Department of Biopathology, Gustave Roussy, Paris-Saclay University, Villejuif, France.
- [5] Department of Cancer Medicine, Gustave Roussy, Paris-Saclay University, Villejuif, France.

- [6] Radiothérapie Moléculaire et Innovation Thérapeutique, Paris-Saclay University, Gustave Roussy, Villejuif, France.
- [7] Department of Biostatistics, Gustave Roussy, Paris-Saclay University, Villejuif, France.
- [8] Department of Radiation Oncology, Gustave Roussy, Paris-Saclay University, Villejuif, France; Radiothérapie Moléculaire et Innovation Thérapeutique, Paris-Saclay University, Gustave Roussy, Villejuif, France. Electronic address: eric.deutsch@gustaveroussy.fr.

- PMID: **33609591**
- PMCID: PMC7887448
- DOI: 10.1016/j.ijrobp.2021.02.022

Free PMC article
Observational Study

Systematic Screening of COVID-19 Disease Based on Chest CT and RT-PCR for Cancer Patients Undergoing Radiation Therapy in a Coronavirus French Hotspot

Roger Sun et al. Int J Radiat Oncol Biol Phys. 2021.
Free PMC article
Show details
Int J Radiat Oncol Biol Phys
. 2021 Jul 15;110(4):947-956.
doi: 10.1016/j.ijrobp.2021.02.022. Epub 2021 Feb 17.

Authors

Roger Sun [1], Samir Achkar [2], Samy Ammari [3], Sophie Bockel [2], Emmanuelle Gallois [4], Arnaud Bayle [5], Enzo Battistella [6], Flore Salviat [7], Mansouria Merad [5], Adrien Laville [2], Kanta Ka [2], Franck Griscelli [4], Laurence Albiges [5], Fabrice Barlesi [5], Alberto Bossi [2], Sofia Rivera [2], Cyrus Chargari [1], Eric Deutsch [8]

Affiliations

- [1] Department of Radiation Oncology, Gustave Roussy, Paris-Saclay University, Villejuif, France; Radiothérapie Moléculaire et Innovation Thérapeutique, Paris-Saclay University, Gustave Roussy, Villejuif, France.
- [2] Department of Radiation Oncology, Gustave Roussy, Paris-Saclay University, Villejuif, France.
- [3] Department of Radiology, Gustave Roussy, Paris-Saclay University, Villejuif, France; BioMaps (UMR1281), Université Paris-Saclay, CNRS, INSERM, CEA, Orsay and Gustave Roussy, Villejuif, France.
- [4] Department of Biopathology, Gustave Roussy, Paris-Saclay University, Villejuif, France.
- [5] Department of Cancer Medicine, Gustave Roussy, Paris-Saclay University, Villejuif, France.

- [6] Radiothérapie Moléculaire et Innovation Thérapeutique, Paris-Saclay University, Gustave Roussy, Villejuif, France.
- [7] Department of Biostatistics, Gustave Roussy, Paris-Saclay University, Villejuif, France.
- [8] Department of Radiation Oncology, Gustave Roussy, Paris-Saclay University, Villejuif, France; Radiothérapie Moléculaire et Innovation Thérapeutique, Paris-Saclay University, Gustave Roussy, Villejuif, France. Electronic address: eric.deutsch@gustaveroussy.fr.

- PMID: **33609591**
- PMCID: PMC7887448
- DOI: 10.1016/j.ijrobp.2021.02.022

Abstract

Purpose: Patients with cancer are presumed to be more vulnerable to COVID-19. We evaluated a screening strategy combining chest computed tomography (CT) and reverse-transcription polymerase chain reaction (RT-PCR) for patients treated with radiation therapy at our cancer center located in a COVID-19 French hotspot during the first wave of the pandemic.

Methods and materials: Chest CT images were proposed during radiation therapy CT simulation. Images were reviewed by an expert radiologist according to the COVID-19 Reporting and Data System classification. Nasal swabs with RT-PCR assay were initially proposed in cases of suspicious imaging or clinical context and were eventually integrated into the systematic screening. A dedicated radiation therapy workflow was proposed for COVID-19 patients to limit the risk of contamination.

Results: From March 18, 2020 to May 1, 2020, 480 patients were screened by chest CT, and 313 patients had both chest CT and RT-PCR (65%). The cumulative incidence of COVID-19 was 5.4% (95% confidence interval [CI], 3.6-7.8; 26 of 480 patients). Diagnosis of COVID-19 was made before radiation therapy for 22 patients (84.6%) and during RT for 4 patients (15.3%). Chest CT directly aided the diagnosis of 7 cases in which the initial RT-PCR was negative or not feasible, out of a total of 480 patients (1.5%) and 517 chest CT acquisitions. Four patients with COVID-19 at the time of the chest CT screening had a false negative CT. Sensitivity and specificity of chest CT screening in patients with both RT-PCR and chest CT testing were estimated at 0.82 (95% CI, 0.60-0.95) and 0.98 (95% CI, 0.96-0.99), respectively. Adaptation of the radiation therapy treatment was made for all patients, with 7 postponed treatments (median: 5 days; interquartile range, 1.5-14.8).

Conclusions: The benefit of systematic use of chest CT screening during CT simulation for patients undergoing radiation therapy during the COVID-19 pandemic seemed limited.

Copyright © 2021 Elsevier Inc. All rights reserved.

- 20 references
- 2 figures

Supplementary info

Publication types, MeSH terms Expand

Publication types

- Observational Study

MeSH terms

- Adolescent
- Adult
- Aged
- COVID-19 / complications
- COVID-19 / diagnosis*
- COVID-19 / diagnostic imaging
- COVID-19 / epidemiology
- COVID-19 Nucleic Acid Testing*
- Cancer Care Facilities
- Child
- Confidence Intervals
- Female
- France / epidemiology
- Humans
- Incidence
- Male
- Middle Aged
- Multidetector Computed Tomography*
- Neoplasms / complications
- Neoplasms / radiotherapy*
- Radiography, Thoracic / methods
- Retrospective Studies
- Sensitivity and Specificity
- Tomography, Spiral Computed
- Young Adult

Full text links

ELSEVIER FULL-TEXT ARTICLE Elsevier Science Free PMC article

Proceed to details

Cite

Share

☐ 819

Observational Study

Clin Dermatol

. Sep-Oct 2021;39(5):911-919.

doi: 10.1016/j.clindermatol.2021.05.015. Epub 2021 May 27.

The Effect of the COVID-19 Lockdown on Melanoma Diagnosis in Italy

Giulio Gualdi [1], Annamaria Porreca [2], Giuseppe Fabrizio Amoruso [3], Laura Atzori [4], Piergiacomo Calzavara-Pinton [5], Michele De Tursi [6], Andrea Di Buduo [4], Pietro Di Marino [6], Gabriella Fabroncini [7], Fabrizio Fantini [8], Maria Concetta Fargnoli [9], Gaetano Giannotta [10], Francesco Lacarruba [11], Andrea Lotesoriere [12], Martina Merli [13], Giuseppe Micali [11], Andrea Paradisi [14], Mario Puviani [15], Pietro Quaglino [13], Franco Rongioletti [4], Marco Rubatto [13], Paolo Sbano [16], Massimiliano Scalvenzi [7], Simone Soglia [5], Laura Sollima [17], Alessia Villani [7], Marta Di Nicola [2], Paolo Amerio [12]

Affiliations Expand

Affiliations

- [1] Department of Dermatology, Università G. D'Annunzio Chieti-Pescara, Chieti, Italy. Electronic address: giulio.gualdi@unich.it.
- [2] Biostatistic, Department of Medical, Oral and Biotechnological Sciences, Università G. D'Annunzio Chieti-Pescara, Italy.
- [3] Dermatology Unit, "Azienda Ospedaliera di Cosenza," Cosenza, Italy.
- [4] Dermatologic Clinic, Department of Public Health, Università di Cagliari, Cagliari, Italy.
- [5] Department of Dermatology, Università di Brescia, Brescia, Italy.
- [6] Oncologic Clinic, Department of Medical, Oral Sciences and Biotechnologies, Università G. D'Annunzio Chieti-Pescara, Italy.
- [7] Department of Clinical Medicine and Surgery, Università degli Studi di Napoli Federico II, Napoli, Italy.
- [8] Department of Dermatology, Ospedale "A. Manzoni," Lecco, Italy.
- [9] Department of Dermatology, Department of Biotechnological and Applied Clinical Sciences, Università dell'Aquila, Italy.
- [10] Pathology Unit, "Azienda Ospedaliera di Cosenza," Cosenza, Italy.
- [11] Dermatology Clinic, Università di Catania, Catania, Italy.
- [12] Department of Dermatology, Università G. D'Annunzio Chieti-Pescara, Chieti, Italy.
- [13] Department of Dermatology, Università di Torino, Torino, Italy.
- [14] Dermatology Unit, Ospedale Generale "Cristo Re," Rome, Italy.
- [15] Department of Dermatology, Ospedale di Sassuolo, Sassuolo, Italy.
- [16] Dermatology Unit, Ospedale Generale "Belcolle," Viterbo, Italy.
- [17] Pathology Unit, Ospedale San Salvatore, L'Aquila, Italy.

- PMID: **34785022**
- PMCID: PMC8156913
- DOI: 10.1016/j.clindermatol.2021.05.015

Free PMC article
Observational Study

The Effect of the COVID-19 Lockdown on Melanoma Diagnosis in Italy

Giulio Gualdi et al. Clin Dermatol. Sep-Oct 2021.
Free PMC article
Show details
Clin Dermatol
. Sep-Oct 2021;39(5):911-919.
doi: 10.1016/j.clindermatol.2021.05.015. Epub 2021 May 27.

Authors

Giulio Gualdi [1], Annamaria Porreca [2], Giuseppe Fabrizio Amoruso [3], Laura Atzori [4], Piergiacomo Calzavara-Pinton [5], Michele De Tursi [6], Andrea Di Buduo [4], Pietro Di Marino [6], Gabriella Fabroncini [7], Fabrizio Fantini [8], Maria Concetta Fargnoli [9], Gaetano Giannotta [10], Francesco Lacarruba [11], Andrea Lotesoriere [12], Martina Merli [13], Giuseppe Micali [11], Andrea Paradisi [14], Mario Puviani [15], Pietro Quaglino [13], Franco Rongioletti [4], Marco Rubatto [13], Paolo Sbano [16], Massimiliano Scalvenzi [7], Simone Soglia [5], Laura Sollima [17], Alessia Villani [7], Marta Di Nicola [2], Paolo Amerio [12]

Affiliations

- [1] Department of Dermatology, Università G. D'Annunzio Chieti-Pescara, Chieti, Italy. Electronic address: giulio.gualdi@unich.it.
- [2] Biostatistic, Department of Medical, Oral and Biotechnological Sciences, Università G. D'Annunzio Chieti-Pescara, Italy.
- [3] Dermatology Unit, "Azienda Ospedaliera di Cosenza," Cosenza, Italy.
- [4] Dermatologic Clinic, Department of Public Health, Università di Cagliari, Cagliari, Italy.
- [5] Department of Dermatology, Università di Brescia, Brescia, Italy.
- [6] Oncologic Clinic, Department of Medical, Oral Sciences and Biotechnologies, Università G. D'Annunzio Chieti-Pescara, Italy.
- [7] Department of Clinical Medicine and Surgery, Università degli Studi di Napoli Federico II, Napoli, Italy.
- [8] Department of Dermatology, Ospedale "A. Manzoni," Lecco, Italy.
- [9] Department of Dermatology, Department of Biotechnological and Applied Clinical Sciences, Università dell'Aquila, Italy.
- [10] Pathology Unit, "Azienda Ospedaliera di Cosenza," Cosenza, Italy.
- [11] Dermatology Clinic, Università di Catania, Catania, Italy.
- [12] Department of Dermatology, Università G. D'Annunzio Chieti-Pescara, Chieti, Italy.
- [13] Department of Dermatology, Università di Torino, Torino, Italy.
- [14] Dermatology Unit, Ospedale Generale "Cristo Re," Rome, Italy.
- [15] Department of Dermatology, Ospedale di Sassuolo, Sassuolo, Italy.
- [16] Dermatology Unit, Ospedale Generale "Belcolle," Viterbo, Italy.
- [17] Pathology Unit, Ospedale San Salvatore, L'Aquila, Italy.

- PMID: **34785022**

- PMCID: PMC8156913
- DOI: 10.1016/j.clindermatol.2021.05.015

Abstract

The coronavirus disease 2019 (COVID-19) pandemic has led to lockdowns for much of the world. In Italy, all health procedures not directly related to COVID-19 were reduced or suspended, thus limiting patient access to hospitals. Any delay in cancer treatment presents the additional risk of tumors progressing from being curable to incurable. Specifically, melanoma survival rate strictly depends on tumor thickness, which, in turn, is a function of time. To estimate the impact on melanoma progression caused by the reduction in dermatologic services during the COVID-19 lockdown, a retrospective observational cohort study was conducted. This study was designed to compare the clinical and histologic characteristics of the primary melanomas removed in the first 2 months after the end of the lockdown (May-July 2020) in 12 Italian centers characterized by different COVID-19 case frequencies. The control group was represented by the melanomas removed during the same period in the previous 3 years. Overall, 1,124 melanomas were considered: 237 as part of the study group and 887 from the control group (average, 295), with a 20% reduction. Breslow thickness, as well as high-risk histotypes and melanomas with vertical growth, increased for all melanomas. Ulcerated and high mitotic index melanomas increased, particularly in northern Italy. In Italy, the lockdown led to a significant worsening of melanoma severity, causing a staging jump, with a consequent worsening of outcomes.

Copyright © 2021 Elsevier Inc. All rights reserved.

Conflict of interest statement

Conflict of interest The authors declare no conflict of interest.

- 26 references
- 6 figures

Supplementary info

Publication types, MeSH terms Expand

Publication types

- Observational Study

MeSH terms

- COVID-19*
- Communicable Disease Control
- Humans
- Italy / epidemiology
- Melanoma* / diagnosis
- Melanoma* / epidemiology
- Retrospective Studies

- SARS-CoV-2
- Skin Neoplasms* / diagnosis
- Skin Neoplasms* / epidemiology

Full text links

ELSEVIER FULL-TEXT ARTICLE Elsevier Science Free PMC article
Proceed to details
Cite
Share
820
Observational Study
Eur Rev Med Pharmacol Sci
. 2020 Jul;24(13):7519-7523.
doi: 10.26355/eurrev_202007_21926.

The impact of SARS-CoV-2 pandemic on Oncologic and Degenerative Spine Surgery Department activity: the experience of Rizzoli Orthopaedic Institute under COVID-19 lockdown

R Ghermandi [1], V Pipola, S Terzi, G Tedesco, C Cavallari, S Bandiera, G Barbanti Bròdano, G Evangelisti, M Girolami, A Gasbarrini
Affiliations Expand

Affiliation

- [1] Department of Oncologic and Degenerative Spine Surgery (CVOD), IRCCS Istituto Ortopedico Rizzoli, Bologna, Italy. alessandro.gasbarrini@ior.it.

- PMID: **32706094**
- DOI: 10.26355/eurrev_202007_21926

Free article
Observational Study

The impact of SARS-CoV-2 pandemic on Oncologic and Degenerative Spine Surgery Department activity: the experience of Rizzoli

Orthopaedic Institute under COVID-19 lockdown

R Ghermandi et al. Eur Rev Med Pharmacol Sci. 2020 Jul.
Free article
Show details
Eur Rev Med Pharmacol Sci
. 2020 Jul;24(13):7519-7523.
doi: 10.26355/eurrev_202007_21926.

Authors

R Ghermandi [1], V Pipola, S Terzi, G Tedesco, C Cavallari, S Bandiera, G Barbanti Bròdano, G Evangelisti, M Girolami, A Gasbarrini

Affiliation

- [1] Department of Oncologic and Degenerative Spine Surgery (CVOD), IRCCS Istituto Ortopedico Rizzoli, Bologna, Italy. alessandro.gasbarrini@ior.it.

- PMID: **32706094**
- DOI: 10.26355/eurrev_202007_21926

Abstract

Objective: Experience of Department of Oncologic and Degenerative Spine Surgery of Rizzoli Orthopaedic Institute during SARS-CoV-2 pandemic lockdown.

Patients and methods: Retrospective observational study of surgically treated patients from 09th March 2020 to 04th May 2020.

Data collected: age, sex, type of disease, neurological status, days of hospitalization, complications and type of discharge. A comparison analysis with same period of the last year was performed in order to evaluate the impact of COVID-19 spreading on daily surgical activity.

Results: A total of 107 surgical procedures in 102 patients were performed from 09th March 2020 to 04th May 2020. Analysis showed a statistically significant difference in age, sex, ASIA class and type of treated disease compared to the same period of the last year (p=0.042, 0.006, 0.022 and 0.007, respectively). No statistically significant differences were observed in type of discharge, length of hospitalization and complications (p= 0.447, 0.261 and 0.127, respectively). 3 COVID-19 infections have been identified in hospitalized patients. 1 COVID-19 patient wad admitted from Emergency Department and was managed according to a dedicated path.

Conclusions: Surgical activity was paradoxically increased during SARS-CoV-2 pandemic lockdown through the management of urgent and non-deferrable spinal disease with a low rate (3,9%) of COVID-19 infections.

Supplementary info

Publication types, MeSH terms Expand

Publication types

- Observational Study
- Research Support, Non-U.S. Gov't

MeSH terms

- Adolescent
- Adult
- Aged
- Aged, 80 and over
- Betacoronavirus / isolation & purification*
- COVID-19
- Child
- Coronavirus Infections / surgery*
- Coronavirus Infections / virology*
- Female
- Humans
- Italy
- Male
- Middle Aged
- Neoplasms / surgery*
- Neoplasms / virology*
- Pandemics
- Pneumonia, Viral / surgery*
- Pneumonia, Viral / virology*
- Retrospective Studies
- SARS-CoV-2
- Spine / surgery*
- Spine / virology*
- Young Adult

Full text links

FREE FULL TEXT European Review for Medical and Pharmacological Sciences

Proceed to details

Cite

Share

☐ 821

Observational Study

J Thromb Thrombolysis
. 2021 May;51(4):897-901.
doi: 10.1007/s11239-021-02413-7. Epub 2021 Mar 5.

Incidence of Venous Thromboembolism and Mortality in Patients with Initial Presentation of COVID-19

Dimitrios Giannis [1], Matthew A Barish [2], Mark Goldin [1 2], Stuart L Cohen [1 3], Nina Kohn [1], Eugenia Gianos [3 4], Saurav Chatterjee [2 3], Martin Lesser [1 3], Kevin Coppa [5], Jamie S Hirsch [1 3 5], Thomas McGinn [1 3], Alex C Spyropoulos [6 7 8 9], COVID-19 Consortium Group
Affiliations Expand

Affiliations

- [1] Feinstein Institutes for Medical Research, Northwell Health, Manhasset, NY, USA.
- [2] North Shore University Hospital, Northwell Health, Manhasset, NY, USA.
- [3] Donald and Barbara Zucker School of Medicine at Hofstra/Northwell, Northwell Health, Hempstead, NY, USA.
- [4] Division of Cardiology, Lenox Hill Hospital, Northwell Health, New York, NY, USA.
- [5] Department of Information Services, Northwell Health, New Hyde Park, NY, USA.
- [6] Feinstein Institutes for Medical Research, Northwell Health, Manhasset, NY, USA. aspyropoul@northwell.edu.
- [7] Donald and Barbara Zucker School of Medicine at Hofstra/Northwell, Northwell Health, Hempstead, NY, USA. aspyropoul@northwell.edu.
- [8] Zucker School of Medicine at Hofstra/Northwell, The Feinstein Institutes for Medical Research, 130 E 77th St, New York, NY, 10075, USA. aspyropoul@northwell.edu.
- [9] Department of Medicine, Anticoagulation and Clinical Thrombosis Services, Northwell Health at Lenox Hill Hospital, 130 E 77th St, New York, NY, 10075, USA. aspyropoul@northwell.edu.

- PMID: **33665766**
- PMCID: PMC7932762
- DOI: 10.1007/s11239-021-02413-7

Free PMC article
Observational Study

Incidence of Venous Thromboembolism and Mortality in Patients with Initial Presentation of COVID-19

Dimitrios Giannis et al. J Thromb Thrombolysis. 2021 May.
Free PMC article

Show details

J Thromb Thrombolysis
. 2021 May;51(4):897-901.
doi: 10.1007/s11239-021-02413-7. Epub 2021 Mar 5.

Authors

Dimitrios Giannis [1], Matthew A Barish [2], Mark Goldin [1 2], Stuart L Cohen [1 3], Nina Kohn [1], Eugenia Gianos [3 4], Saurav Chatterjee [2 3], Martin Lesser [1 3], Kevin Coppa [5], Jamie S Hirsch [1 3 5], Thomas McGinn [1 3], Alex C Spyropoulos [6 7 8 9], COVID-19 Consortium Group

Affiliations

- [1] Feinstein Institutes for Medical Research, Northwell Health, Manhasset, NY, USA.
- [2] North Shore University Hospital, Northwell Health, Manhasset, NY, USA.
- [3] Donald and Barbara Zucker School of Medicine at Hofstra/Northwell, Northwell Health, Hempstead, NY, USA.
- [4] Division of Cardiology, Lenox Hill Hospital, Northwell Health, New York, NY, USA.
- [5] Department of Information Services, Northwell Health, New Hyde Park, NY, USA.
- [6] Feinstein Institutes for Medical Research, Northwell Health, Manhasset, NY, USA. aspyropoul@northwell.edu.
- [7] Donald and Barbara Zucker School of Medicine at Hofstra/Northwell, Northwell Health, Hempstead, NY, USA. aspyropoul@northwell.edu.
- [8] Zucker School of Medicine at Hofstra/Northwell, The Feinstein Institutes for Medical Research, 130 E 77th St, New York, NY, 10075, USA. aspyropoul@northwell.edu.
- [9] Department of Medicine, Anticoagulation and Clinical Thrombosis Services, Northwell Health at Lenox Hill Hospital, 130 E 77th St, New York, NY, 10075, USA. aspyropoul@northwell.edu.

- PMID: **33665766**
- PMCID: PMC7932762
- DOI: 10.1007/s11239-021-02413-7

Abstract

Venous thromboembolism (VTE) has emerged as an important issue in patients with COVID-19. The purpose of this study is to identify the incidence of VTE and mortality in COVID-19 patients initially presenting to a large health system. Our retrospective study included adult patients (excluding patients presenting with obstetric/gynecologic conditions) across a multihospital health system in the New York Metropolitan Region from March 1-April 27, 2020. VTE and mortality rates within 8 h of assessment were described. In 10,871 adults with COVID-19, 118 patients (1.09%) were diagnosed with symptomatic VTE (101 pulmonary embolism, 17 deep vein thrombosis events) and 28 patients (0.26%) died during initial assessment. Among these 146 patients, 64.4% were males, 56.8% were 60 years or older, 15.1% had a BMI > 35, and 11.6% were admitted to the intensive care unit. Comorbidities included hypertension (46.6%), diabetes (24.7%), hyperlipidemia (14.4%), chronic lung disease (12.3%), coronary artery disease (11.0%), and prior VTE (7.5%). Key medications included corticosteroids (22.6%), statins (21.2%), antiplatelets (20.6%), and anticoagulants (20.6%). Highest D-Dimer was greater than six times the upper limit of normal in 51.4%. Statin and antiplatelet use were associated with decreased VTE or

mortality (each p < 0.01). In COVID-19 patients who initially presented to a large multihospital health system, the overall symptomatic VTE and mortality rate was over 1.0%. Statin and antiplatelet use were associated with decreased VTE or mortality. The potential benefits of antithrombotics in high risk COVID-19 patients during the pre-hospitalization period deserves study.

Keywords: COVID-19; Outpatient; Thrombosis; Venous thromboembolism.

- 13 references

Supplementary info

Publication types, MeSH terms, Substances Expand

Publication types

- Observational Study

MeSH terms

- COVID-19 / complications*
- COVID-19 / epidemiology
- COVID-19 / physiopathology
- COVID-19 / therapy
- Female
- Fibrin Fibrinogen Degradation Products / analysis
- Humans
- Hydroxymethylglutaryl-CoA Reductase Inhibitors / therapeutic use
- Incidence
- Intensive Care Units / statistics & numerical data
- Male
- Middle Aged
- Mortality
- New York / epidemiology
- Outcome and Process Assessment, Health Care
- Platelet Aggregation Inhibitors / therapeutic use
- Protective Factors
- Pulmonary Embolism* / blood
- Pulmonary Embolism* / diagnosis
- Pulmonary Embolism* / etiology
- Pulmonary Embolism* / mortality
- Retrospective Studies
- Risk Factors
- SARS-CoV-2 / isolation & purification

- Venous Thrombosis* / blood
- Venous Thrombosis* / diagnosis
- Venous Thrombosis* / etiology
- Venous Thrombosis* / mortality

Substances

- Fibrin Fibrinogen Degradation Products
- Hydroxymethylglutaryl-CoA Reductase Inhibitors
- Platelet Aggregation Inhibitors
- fibrin fragment D

Full text links

SpringerLink FULL-TEXT ARTICLE Springer Free PMC article
Proceed to details
Cite
Share
822
Medicine (Baltimore)
. 2021 Feb 5;100(5):e24409.
doi: 10.1097/MD.0000000000024409.

Postoperative complications and mortality following emergency digestive surgery during the COVID-19 pandemic: A multicenter collaborative retrospective cohort study protocol (COVID-CIR)

Zoilo Madrazo [1], Javier Osorio [1], Aurema Otero [2], Sebastiano Biondo [1], Sebastian Videla [2, 3], COVID-CIR Collaborative Group
Affiliations Expand

Affiliations

- [1] Department of General and Digestive Surgery, Bellvitge University Hospital.
- [2] Clinical Research Support Unit, Clinical Pharmacology Department, Bellvitge University Hospital/Bellvitge Biomedical Research Institute (IDIBELL).
- [3] Department of Pathology and Experimental Therapeutics, Faculty of Medicine, Universitat de Barcelona, L'Hospitalet de Llobregat, Barcelona, Spain.

- PMID: **33592888**

- PMCID: PMC7870207
- DOI: 10.1097/MD.0000000000024409

Free PMC article

Postoperative complications and mortality following emergency digestive surgery during the COVID-19 pandemic: A multicenter collaborative retrospective cohort study protocol (COVID-CIR)

Zoilo Madrazo et al. Medicine (Baltimore). 2021.
Free PMC article
Show details
Medicine (Baltimore)
. 2021 Feb 5;100(5):e24409.
doi: 10.1097/MD.0000000000024409.

Authors

Zoilo Madrazo [1], Javier Osorio [1], Aurema Otero [2], Sebastiano Biondo [1], Sebastian Videla [2, 3], COVID-CIR Collaborative Group

Affiliations

- [1] Department of General and Digestive Surgery, Bellvitge University Hospital.
- [2] Clinical Research Support Unit, Clinical Pharmacology Department, Bellvitge University Hospital/Bellvitge Biomedical Research Institute (IDIBELL).
- [3] Department of Pathology and Experimental Therapeutics, Faculty of Medicine, Universitat de Barcelona, L'Hospitalet de Llobregat, Barcelona, Spain.

- PMID: **33592888**
- PMCID: PMC7870207
- DOI: 10.1097/MD.0000000000024409

Abstract

Infection with the SARS-CoV-2 virus seems to contribute significantly to increased postoperative complications and mortality after emergency surgical procedures. Additionally, the fear of COVID-19 contagion delays the consultation of patients, resulting in the deterioration of their acute diseases by the time of consultation. In the specific case of urgent digestive surgery patients, both factors significantly worsen the postoperative course and prognosis. Main working hypothesis: infection by COVID-19 increases postoperative 30-day-mortality for any cause in patients submitted to emergency/urgent general or gastrointestinal surgery. Likewise, hospital collapse during the first wave of the COVID-19 pandemic increased 30-day-mortality for any

cause. Hence, the main objective of this study is to estimate the cumulative incidence of mortality at 30-days-after-surgery. Secondary objectives are: to estimate the cumulative incidence of postoperative complications and to develop a specific postoperative risk propensity model for COVID-19-infected patients.A multicenter, observational retrospective cohort study (COVID-CIR-study) will be carried out in consecutive patients operated on for urgent digestive pathology. Two cohorts will be defined: the "pandemic" cohort, which will include all patients (classified as COVID-19-positive or -negative) operated on for emergency digestive pathology during the months of March to June 2020; and the "control" cohort, which will include all patients operated on for emergency digestive pathology during the months of March to June 2019. Information will be gathered on demographic characteristics, clinical and analytical parameters, scores on the usual prognostic scales for quality management in a General Surgery service (POSSUM, P-POSSUM and LUCENTUM scores), prognostic factors applicable to all patients, specific prognostic factors for patients infected with SARS-CoV-2, postoperative morbidity and mortality (at 30 and 90 postoperative days). The main objective is to estimate the cumulative incidence of mortality at 30 days after surgery. As secondary objectives, to estimate the cumulative incidence of postoperative complications and to develop a specific postoperative risk propensity model for SARS-CoV-2 infected patients.The protocol (version1.0, April 20th 2020) was approved by the local Institutional Review Board (Ethic-and-Clinical-Investigation-Committee, code PR169/20, date 05/05/20). The study findings will be submitted to peer-reviewed journals and presented at relevant national and international scientific meetings.ClinicalTrials.gov Identifier: NCT04479150 (July 21, 2020).

Copyright © 2021 the Author(s). Published by Wolters Kluwer Health, Inc.

Conflict of interest statement

The authors have no conflicts of interests to disclose.

- 72 references
- 1 figure

Supplementary info

MeSH terms, Associated data Expand

MeSH terms

- Adult
- COVID-19* / epidemiology
- COVID-19* / prevention & control
- Digestive System Diseases* / diagnosis
- Digestive System Diseases* / epidemiology
- Digestive System Diseases* / mortality
- Digestive System Diseases* / surgery
- Digestive System Surgical Procedures* / adverse effects
- Digestive System Surgical Procedures* / methods
- Digestive System Surgical Procedures* / mortality
- Emergencies / epidemiology

- Emergency Treatment* / adverse effects
- Emergency Treatment* / methods
- Emergency Treatment* / mortality
- Female
- Humans
- Incidence
- Infection Control* / methods
- Infection Control* / statistics & numerical data
- Male
- Mortality
- Multicenter Studies as Topic
- Observational Studies as Topic
- Postoperative Complications* / diagnosis
- Postoperative Complications* / epidemiology
- Postoperative Complications* / etiology
- Research Design
- Risk Assessment / methods
- Time-to-Treatment*

Associated data

- ClinicalTrials.gov/NCT04479150

Full text links

Wolters Kluwer Wolters Kluwer Free PMC article
Proceed to details
Cite
Share
823
Observational Study
Am J Infect Control
. 2022 Jan;50(1):32-38.
doi: 10.1016/j.ajic.2021.09.018. Epub 2021 Sep 23.

Impact of the COVID-19 pandemic on the incidence of multidrug-resistant bacterial infections in an acute care hospital in Brazil

Matheus Polly [1], Bianca L de Almeida [2], Robert P Lennon [3], Marina Farrel Cortês [4], Silvia F Costa [5], Thais Guimarães [2]
Affiliations Expand

Affiliations

- [1] Department of Infectious Diseases, Hospital das Clínicas, University of São Paulo, São Paulo, Brazil. Electronic address: matheus.polly@hc.fm.usp.br.
- [2] Infection Control Department, Hospital das Clínicas, University of São Paulo, São Paulo, Brazil.
- [3] Department of Family and Community Medicine, Penn State College of Medicine, Hershey, PA, USA.
- [4] Laboratory of Medical Investigation , University of São Paulo, São Paulo, Brazil.
- [5] Department of Infectious Diseases, Hospital das Clínicas, University of São Paulo, São Paulo, Brazil.

- PMID: **34562526**
- PMCID: PMC8457917
- DOI: 10.1016/j.ajic.2021.09.018

Free PMC article
Observational Study

Impact of the COVID-19 pandemic on the incidence of multidrug-resistant bacterial infections in an acute care hospital in Brazil

Matheus Polly et al. Am J Infect Control. 2022 Jan.
Free PMC article
Show details
Am J Infect Control
. 2022 Jan;50(1):32-38.
doi: 10.1016/j.ajic.2021.09.018. Epub 2021 Sep 23.

Authors

Matheus Polly [1], Bianca L de Almeida [2], Robert P Lennon [3], Marina Farrel Cortês [4], Silvia F Costa [5], Thais Guimarães [2]

Affiliations

- [1] Department of Infectious Diseases, Hospital das Clínicas, University of São Paulo, São Paulo, Brazil. Electronic address: matheus.polly@hc.fm.usp.br.
- [2] Infection Control Department, Hospital das Clínicas, University of São Paulo, São Paulo, Brazil.
- [3] Department of Family and Community Medicine, Penn State College of Medicine, Hershey, PA, USA.
- [4] Laboratory of Medical Investigation , University of São Paulo, São Paulo, Brazil.
- [5] Department of Infectious Diseases, Hospital das Clínicas, University of São Paulo, São Paulo, Brazil.

- PMID: **34562526**
- PMCID: PMC8457917
- DOI: 10.1016/j.ajic.2021.09.018

Abstract

Background: The impact of COVID-19 on healthcare- associated infections (HCAI) caused by multidrug-resistant (MDR) bacteria that contribute to higher mortality is a growing area of study METHODS: This retrospective observational study compares the incidence density (ID) of HCAI caused by MDR bacteria (CRE, CRAB, CRP, MRSA and VRE) pre-COVID (2017-2019) and during the COVID-19 pandemic (2020) in overall hospitalized patients and in intensive care (ICU) units.

Results: We identified 8,869 HCAI, of which 2,641 (29.7%) were caused by bacterial MDR, and 1,257 (14.1%) were from ICUs. The overall ID of MDR infections increased 23% (P < .005) during COVID-19. The overall per-pathogen analysis shows significant increases in infections by CRAB and MRSA (+108.1%, p<0.005; +94.7%, p<0.005, respectively), but not in CRE, CRP, or VRE. In the ICU, the overall ID of MDR infections decreased during COVID, but that decline was not significant (-6.5%, P = .26). The ICU per-pathogen analysis of ID of infection showed significant increases in CRAB and MRSA (+42.0%, P = .001; +46.2%, P = .04), significant decreases in CRE and CRP (-26.4%, P = .002; -44.2%, P = 0.003, respectively) and no change in VRE.

Conclusions: The COVID-19 pandemic correlates to an increase in ID of CRAB and MRSA both in ICU and non-ICU setting, and a decrease in ID of CRE and CRP in the ICU setting. Infection control teams should be aware of possible outbreaks of CRAB and MRSA and promote rigorous adherence to infection control measures as practices change to accommodate changes in healthcare needs during and after the pandemic.

Keywords: Carbapenem-resistant Acinetobacter baumannii infection; Epidemiology; Healthcare-associated infections; Methicillin-resistant Staphylococcus aureus infection; Multidrug resistant bacteria; Multidrug resistant infection.

Copyright © 2021 Association for Professionals in Infection Control and Epidemiology, Inc. Published by Elsevier Inc. All rights reserved.

Comment in

- Transitional impact on Acinetobacter baumannii MDR infections in 5 Brazilian ICUs in 2020.
  Silva AROD, Salgado DR, Emmerick ICM, Lima EDC. Silva AROD, et al. Am J Infect Control. 2022 Feb;50(2):239-240. doi: 10.1016/j.ajic.2021.10.013. Am J Infect Control. 2022. PMID: 35101182 No abstract available.

- 38 references
- 4 figures

Supplementary info

Publication types, MeSH terms, Substances Expand

Publication types

- Observational Study

MeSH terms

- Anti-Bacterial Agents / pharmacology
- Anti-Bacterial Agents / therapeutic use
- Bacterial Infections* / epidemiology
- Brazil / epidemiology
- COVID-19*
- Cross Infection* / drug therapy
- Cross Infection* / epidemiology
- Drug Resistance, Multiple, Bacterial
- Hospitals
- Humans
- Incidence
- Intensive Care Units
- Methicillin-Resistant Staphylococcus aureus*
- Pandemics
- SARS-CoV-2
- Staphylococcal Infections* / epidemiology

Substances

- Anti-Bacterial Agents

Full text links

ELSEVIER FULL-TEXT ARTICLE Elsevier Science Free PMC article
Proceed to details
Cite
Share
☐ 824
Observational Study
Infect Control Hosp Epidemiol
. 2021 Jul;42(7):810-816.
doi: 10.1017/ice.2020.1291. Epub 2020 Oct 26.

Changes in antimicrobial utilization during the coronavirus disease 2019 (COVID-19)

pandemic after implementation of a multispecialty clinical guidance team

Milner B Staub [#] [1] [2], Ronald M Beaulieu [#] [1], John Graves [3], George E Nelson [1]
Affiliations Expand

Affiliations

- [1] Vanderbilt University Medical Center, Division of Infectious Diseases, Nashville, Tennessee.
- [2] Veterans Health Administration, Tennessee Valley Healthcare System, Geriatric Research Education and Clinical Center, Nashville, Tennessee.
- [3] Vanderbilt University School of Medicine, Department of Medicine, Nashville, Tennessee.

[#] Contributed equally.

- PMID: **33100250**
- PMCID: PMC7683821
- DOI: 10.1017/ice.2020.1291

Free PMC article
Observational Study

Changes in antimicrobial utilization during the coronavirus disease 2019 (COVID-19) pandemic after implementation of a multispecialty clinical guidance team

Milner B Staub et al. Infect Control Hosp Epidemiol. 2021 Jul.
Free PMC article
Show details
Infect Control Hosp Epidemiol
. 2021 Jul;42(7):810-816.
doi: 10.1017/ice.2020.1291. Epub 2020 Oct 26.

Authors

Milner B Staub [#] [1] [2], Ronald M Beaulieu [#] [1], John Graves [3], George E Nelson [1]

Affiliations

- [1] Vanderbilt University Medical Center, Division of Infectious Diseases, Nashville, Tennessee.

- [2] Veterans Health Administration, Tennessee Valley Healthcare System, Geriatric Research Education and Clinical Center, Nashville, Tennessee.
- [3] Vanderbilt University School of Medicine, Department of Medicine, Nashville, Tennessee.

[#] Contributed equally.

- PMID: **33100250**
- PMCID: PMC7683821
- DOI: 10.1017/ice.2020.1291

Abstract

Objective: Evaluate changes in antimicrobial use during COVID-19 and after implementation of a multispecialty COVID-19 clinical guidance team compared to pre-COVID-19 antimicrobial use.

Design: Retrospective observational study.

Setting: Tertiary-care academic medical center.

Participants: Internal medicine and medical intensive care unit (MICU) provider teams and hospitalized COVID-19 patients.

Methods: Difference-in-differences analyses of antibiotic days of therapy per 1,000 patient days present (DOT) for internal medicine and MICU teams treating COVID-19 patients versus teams that did not were performed for 3 periods: before COVID-19, initial COVID-19 period, and after implementation of a multispecialty COVID-19 clinical guidance team which included daily, patient-specific antimicrobial stewardship recommendations. Patient characteristics associated with antibiotic DOT were evaluated using multivariable Poisson regression.

Results: In the initial COVID-19 period, compared to the pre-COVID-19 period, internal medicine and MICU teams increased weekly antimicrobial use by 145.3 DOT (95% CI, 35.1-255.5) and 204.0 DOT (95% CI, -16.9 to 424.8), respectively, compared to non-COVID-19 teams. In the intervention period, internal medicine and MICU COVID-19 teams both had significant weekly decreases of 362.3 DOT (95% CI, -443.3 to -281.2) and 226.3 DOT (95% CI, -381.2 to -71.3). Of 131 patients hospitalized with COVID-19, 86 (65.6%) received antibiotics; no specific patient factors were significantly associated with an expected change in antibiotic days.

Conclusions: Antimicrobial use initially increased for COVID-19 patient care teams compared to pre-COVID-19 levels but significantly decreased after implementation of a multispecialty clinical guidance team, which may be an effective strategy to reduce unnecessary antimicrobial use.

- 39 references
- 2 figures

Supplementary info

Publication types, MeSH terms, Substances Expand

Publication types

- Observational Study

MeSH terms

- Anti-Bacterial Agents / therapeutic use
- Anti-Infective Agents* / therapeutic use
- COVID-19*
- Humans
- Pandemics
- SARS-CoV-2

Substances

- Anti-Bacterial Agents
- Anti-Infective Agents

Full text links

Cambridge University Press Free PMC article
Proceed to details
Cite
Share
825
Observational Study
Biosci Trends
. 2021 Sep 22;15(4):219-230.
doi: 10.5582/bst.2021.01194. Epub 2021 Jul 15.

The impact of COVID-19 pandemic on the utilization of ambulatory care for patients with chronic neurological diseases in Japan: Evaluation of an administrative claims database

Kenichiro Sato [1, 2], Tatsuo Mano [2], Yoshiki Niimi [3], Atsushi Iwata [4], Tatsushi Toda [2], Takeshi Iwatsubo [1, 3]
Affiliations Expand

Affiliations

- [1] Department of Neuropathology, Graduate School of Medicine, University of Tokyo, Tokyo, Japan.
- [2] Department of Neurology, The University of Tokyo Hospital, Tokyo, Japan.

- [3] Unit for Early and Exploratory Clinical Development, The University of Tokyo Hospital, Tokyo, Japan.
- [4] Department of Neurology, Tokyo Metropolitan Geriatric Center Hospital, Tokyo, Japan.

- PMID: **34261836**
- DOI: 10.5582/bst.2021.01194

Free article
Observational Study

The impact of COVID-19 pandemic on the utilization of ambulatory care for patients with chronic neurological diseases in Japan: Evaluation of an administrative claims database

Kenichiro Sato et al. Biosci Trends. 2021.
Free article
Show details
Biosci Trends
. 2021 Sep 22;15(4):219-230.
doi: 10.5582/bst.2021.01194. Epub 2021 Jul 15.

Authors

Kenichiro Sato [1, 2], Tatsuo Mano [2], Yoshiki Niimi [3], Atsushi Iwata [4], Tatsushi Toda [2], Takeshi Iwatsubo [1, 3]

Affiliations

- [1] Department of Neuropathology, Graduate School of Medicine, University of Tokyo, Tokyo, Japan.
- [2] Department of Neurology, The University of Tokyo Hospital, Tokyo, Japan.
- [3] Unit for Early and Exploratory Clinical Development, The University of Tokyo Hospital, Tokyo, Japan.
- [4] Department of Neurology, Tokyo Metropolitan Geriatric Center Hospital, Tokyo, Japan.

- PMID: **34261836**
- DOI: 10.5582/bst.2021.01194

Abstract

The COVID-19 pandemic has affected not only the emergency medical system, but also patients' regular ambulatory care, as such decrease in the number of patients visiting outpatient clinics decreased in 2020 than in 2019, or the ban lifting of subsequent visits by telephone for outpatient

clinics since March 2020 in lieu of ambulatory care for chronic diseases. In this context, we investigate the impact of the COVID-19 pandemic on ambulatory care at Japanese outpatient clinics for patients with chronic neurological diseases during 2020. We collected data from the administrative claims database (DeSC database) covering more than 1 million individuals. Serial changes in the frequency of subsequent outpatient visits to clinics or hospitals (excluding large hospitals) for chronic ambulatory care of epilepsy, migraine, Parkinson's disease (PD), and Alzheimer's disease (AD) in 2020 were measured. As a result, since April 2020, the monthly outpatient visits for epilepsy, PD, and AD decreased slightly but significantly (approximately 0.90 in relative risk [RR]) but visits for migraine increased (RR = 1.15). Telephone visit was most frequently used in April-May, in less than 5% of monthly outpatient clinic visits for the examined neurological diseases. Outpatient visits for migraine treatment were more likely to be done by telephone than in case of other diseases (adjusted Odds ratio = 2.08). These results suggest that the impact of COVID-19 pandemic on regular ambulatory care for several chronic neurological diseases yielded different effect depending on the disease, in terms of the frequency or type of outpatient visits.

Keywords: COVID-19; administrative claims data; ambulatory care; chronic neurological disease; telemedicine.

Supplementary info

Publication types, MeSH terms Expand

Publication types

- Observational Study

MeSH terms

- Administrative Claims, Healthcare
- Aged
- Ambulatory Care*
- COVID-19 / epidemiology*
- Chronic Disease / therapy
- Communicable Disease Control*
- Female
- Humans
- Japan
- Male
- Middle Aged
- Nervous System Diseases / therapy*
- Pandemics*
- Retrospective Studies
- Telephone

Full text links

J-STAGE FREE J-STAGE, Japan Science and Technology Information Aggregator, Electronic
Proceed to details
Cite
Share
☐ 826
Clinical Trial
Am J Hematol
. 2021 Apr 1;96(4):471-479.
doi: 10.1002/ajh.26102. Epub 2021 Feb 22.

Intermediate-dose anticoagulation, aspirin, and in-hospital mortality in COVID-19: A propensity score-matched analysis

Matthew L Meizlish [1], George Goshua [2], Yiwen Liu [3], Rebecca Fine [4], Kejal Amin [5], Eric Chang [2], Nicholas DeFilippo [5 6], Craig Keating [7], Yuxin Liu [2], Michael Mankbadi [4], Dayna McManus [5], Stephen Y Wang [4], Christina Price [8], Robert D Bona [2], Cassius Iyad Ochoa Chaar [9], Hyung J Chun [10], Alexander B Pine [2], Henry M Rinder [2 11], Jonathan M Siner [12], Donna S Neuberg [3], Kent A Owusu [5 13], Alfred Ian Lee [2]
Affilliations Expand

Affiliations

- [1] Yale School of Medicine, New Haven, Connecticut, USA.
- [2] Section of Hematology, Department of Medicine, Yale School of Medicine, New Haven, Connecticut, USA.
- [3] Dana-Farber Cancer Institute, Boston, Massachusetts, USA.
- [4] Department of Medicine, Yale School of Medicine, New Haven, Connecticut, USA.
- [5] Department of Pharmacy, Yale-New Haven Hospital, New Haven, Connecticut, USA.
- [6] School of Pharmacy, University of Connecticut, Storrs, Connecticut, USA.
- [7] Joint Data Analytics Team, Yale New Haven Hospital, New Haven, Connecticut, USA.
- [8] Section of Allergy and Immunology, Department of Medicine, Yale School of Medicine, New Haven, Connecticut, USA.
- [9] Section of Vascular Surgery, Department of Surgery, Yale School of Medicine, New Haven, Connecticut, USA.
- [10] Section of Cardiology, Department of Medicine, Yale School of Medicine, New Haven, Connecticut, USA.
- [11] Department of Laboratory Medicine, Yale School of Medicine, New Haven, Connecticut, USA.
- [12] Section of Pulmonary, Critical Care, and Sleep Medicine, Department of Medicine, Yale School of Medicine, New Haven, Connecticut, USA.
- [13] Clinical Redesign, Yale New Haven Health, New Haven, Connecticut, USA.

- PMID: **33476420**
- PMCID: PMC8013588

- DOI: 10.1002/ajh.26102

Free PMC article
Clinical Trial

Intermediate-dose anticoagulation, aspirin, and in-hospital mortality in COVID-19: A propensity score-matched analysis

Matthew L Meizlish et al. Am J Hematol. 2021.
Free PMC article
Show details
Am J Hematol
. 2021 Apr 1;96(4):471-479.
doi: 10.1002/ajh.26102. Epub 2021 Feb 22.

Authors

Matthew L Meizlish [1], George Goshua [2], Yiwen Liu [3], Rebecca Fine [4], Kejal Amin [5], Eric Chang [2], Nicholas DeFilippo [5] [6], Craig Keating [7], Yuxin Liu [2], Michael Mankbadi [4], Dayna McManus [5], Stephen Y Wang [4], Christina Price [8], Robert D Bona [2], Cassius Iyad Ochoa Chaar [9], Hyung J Chun [10], Alexander B Pine [2], Henry M Rinder [2] [11], Jonathan M Siner [12], Donna S Neuberg [3], Kent A Owusu [5] [13], Alfred Ian Lee [2]

Affiliations

- [1] Yale School of Medicine, New Haven, Connecticut, USA.
- [2] Section of Hematology, Department of Medicine, Yale School of Medicine, New Haven, Connecticut, USA.
- [3] Dana-Farber Cancer Institute, Boston, Massachusetts, USA.
- [4] Department of Medicine, Yale School of Medicine, New Haven, Connecticut, USA.
- [5] Department of Pharmacy, Yale-New Haven Hospital, New Haven, Connecticut, USA.
- [6] School of Pharmacy, University of Connecticut, Storrs, Connecticut, USA.
- [7] Joint Data Analytics Team, Yale New Haven Hospital, New Haven, Connecticut, USA.
- [8] Section of Allergy and Immunology, Department of Medicine, Yale School of Medicine, New Haven, Connecticut, USA.
- [9] Section of Vascular Surgery, Department of Surgery, Yale School of Medicine, New Haven, Connecticut, USA.
- [10] Section of Cardiology, Department of Medicine, Yale School of Medicine, New Haven, Connecticut, USA.
- [11] Department of Laboratory Medicine, Yale School of Medicine, New Haven, Connecticut, USA.
- [12] Section of Pulmonary, Critical Care, and Sleep Medicine, Department of Medicine, Yale School of Medicine, New Haven, Connecticut, USA.
- [13] Clinical Redesign, Yale New Haven Health, New Haven, Connecticut, USA.

- PMID: **33476420**
- PMCID: PMC8013588
- DOI: 10.1002/ajh.26102

Abstract

Thrombotic complications occur at high rates in hospitalized patients with COVID-19, yet the impact of intensive antithrombotic therapy on mortality is uncertain. We examined in-hospital mortality with intermediate- compared to prophylactic-dose anticoagulation, and separately with in-hospital aspirin compared to no antiplatelet therapy, in a large, retrospective study of 2785 hospitalized adult COVID-19 patients. In this analysis, we established two separate, nested cohorts of patients (a) who received intermediate- or prophylactic-dose anticoagulation ("anticoagulation cohort", N = 1624), or (b) who were not on home antiplatelet therapy and received either in-hospital aspirin or no antiplatelet therapy ("aspirin cohort", N = 1956). To minimize bias and adjust for confounding factors, we incorporated propensity score matching and multivariable regression utilizing various markers of illness severity and other patient-specific covariates, yielding treatment groups with well-balanced covariates in each cohort. The primary outcome was cumulative incidence of in-hospital death. Among propensity score-matched patients in the anticoagulation cohort (N = 382), in a multivariable regression model, intermediate- compared to prophylactic-dose anticoagulation was associated with a significantly lower cumulative incidence of in-hospital death (hazard ratio 0.518 [0.308-0.872]). Among propensity-score matched patients in the aspirin cohort (N = 638), in a multivariable regression model, in-hospital aspirin compared to no antiplatelet therapy was associated with a significantly lower cumulative incidence of in-hospital death (hazard ratio 0.522 [0.336-0.812]). In this propensity score-matched, observational study of COVID-19, intermediate-dose anticoagulation and aspirin were each associated with a lower cumulative incidence of in-hospital death.

© 2021 Wiley Periodicals LLC.

Conflict of interest statement

No conflict of interest exists for any author on this manuscript. This work was supported by a gift donation from Jack Levin and a separate anonymous donation to the Benign Hematology program at Yale, the DeLuca Foundation to fund hematology research at Yale, and the National Institutes of Health (grant HL142818 to H.J.C., and GM136651 and HL139116 to M.L.M.).

Update of

- Intermediate-dose anticoagulation, aspirin, and in-hospital mortality in COVID-19: a propensity score-matched analysis.
  Meizlish ML, Goshua G, Liu Y, Fine R, Amin K, Chang E, DeFilippo N, Keating C, Liu Y, Mankbadi M, McManus D, Wang S, Price C, Bona RD, Chaar CIO, Chun HJ, Pine AB, Rinder HM, Siner J, Neuberg DS, Owusu KA, Lee AI. Meizlish ML, et al. medRxiv. 2021 Jan 15:2021.01.12.21249577. doi: 10.1101/2021.01.12.21249577. Preprint. medRxiv. 2021. PMID: 33469595 Free PMC article. Updated.

- 48 references
- 1 figure

Supplementary info

Publication types, MeSH terms, Substances, Grant support Expand

Publication types

- Clinical Trial
- Multicenter Study
- Research Support, N.I.H., Extramural
- Research Support, Non-U.S. Gov't

MeSH terms

- Adult
- Aged
- Anticoagulants / administration & dosage*
- Aspirin / administration & dosage*
- COVID-19* / drug therapy
- COVID-19* / mortality
- Female
- Hospital Mortality*
- Humans
- Incidence
- Male
- Middle Aged
- Platelet Aggregation Inhibitors / administration & dosage*
- Retrospective Studies
- SARS-CoV-2*

Substances

- Anticoagulants
- Platelet Aggregation Inhibitors
- Aspirin

Grant support

- American Society of Hematology
- F31 HL139116/HL/NHLBI NIH HHS/United States
- GM136651/NH/NIH HHS/United States
- HL139116/NH/NIH HHS/United States
- Hemostasis and Thrombosis Research Society

- UL1 TR001863/TR/NCATS NIH HHS/United States
- T32 GM136651/GM/NIGMS NIH HHS/United States
- R01 HL142818/HL/NHLBI NIH HHS/United States

- HL142818/NH/NIH HHS/United States

Show all 9 grants

Full text links

WILEY Full Text Article Wiley Free PMC article

Proceed to details

Cite

Share

☐ 827

Observational Study

Int Urol Nephrol

. 2022 Jan;54(1):193-199.

doi: 10.1007/s11255-021-02920-9. Epub 2021 Jun 16.

Evaluation of central venous catheter and other risk factors for mortality in chronic hemodialysis patients with COVID-19 in Brazil

Jocemir Ronaldo Lugon[1], Precil Diego Miranda de Menezes Neves[2 3], Andrea Pio-Abreu[2], Marcelo Mazza do Nascimento[4], Ricardo Sesso[5], COVID-19 HD-Brazil Investigators

Affiliations Expand

Affiliations

- [1] Universidade Federal Fluminense, Niterói, RJ, Brazil.
- [2] Hospital das Clínicas da Faculdade de Medicina da USP, São Paulo, SP, Brazil.
- [3] Hospital Alemão Oswaldo Cruz, São Paulo, SP, Brazil.
- [4] Universidade Federal Do Paraná, Curitiba, PR, Brazil.
- [5] Division of Nephrology, Escola Paulista de Medicina, Federal University of São Paulo, Rua Botucatu 740, São Paulo, SP, 04023-900, Brazil. rsesso@unifesp.br.

- PMID: **34132971**
- PMCID: PMC8207494
- DOI: 10.1007/s11255-021-02920-9

Free PMC article

Observational Study

Evaluation of central venous catheter and other risk factors for mortality in chronic

hemodialysis patients with COVID-19 in Brazil

Jocemir Ronaldo Lugon et al. Int Urol Nephrol. 2022 Jan.
Free PMC article
Show details
Int Urol Nephrol
. 2022 Jan;54(1):193-199.
doi: 10.1007/s11255-021-02920-9. Epub 2021 Jun 16.

Authors

Jocemir Ronaldo Lugon [1], Precil Diego Miranda de Menezes Neves [2] [3], Andrea Pio-Abreu [2], Marcelo Mazza do Nascimento [4], Ricardo Sesso [5], COVID-19 HD-Brazil Investigators

Affiliations

- [1] Universidade Federal Fluminense, Niterói, RJ, Brazil.
- [2] Hospital das Clínicas da Faculdade de Medicina da USP, São Paulo, SP, Brazil.
- [3] Hospital Alemão Oswaldo Cruz, São Paulo, SP, Brazil.
- [4] Universidade Federal Do Paraná, Curitiba, PR, Brazil.
- [5] Division of Nephrology, Escola Paulista de Medicina, Federal University of São Paulo, Rua Botucatu 740, São Paulo, SP, 04023-900, Brazil. rsesso@unifesp.br.

- PMID: **34132971**
- PMCID: PMC8207494
- DOI: 10.1007/s11255-021-02920-9

Abstract

Purpose: Hemodialysis patients with COVID-19 are at increased risk of death. We aimed to describe the characteristics of a cohort of Brazilian hemodialysis patients with COVID-19 and assess their mortality rate and risk factors for death.

Methods: Retrospective cohort study of 741 Brazilian hemodialysis patients with confirmed COVID-19 from Feb-Dec/2020, of 52 dialysis centers of the country. We analyzed comorbid conditions, sociodemographic factors, and dialysis-related parameters. To detect risk factors for mortality in hemodialysis patients, we performed multivariable Cox proportional hazard regression analysis. Survival was analyzed by Kaplan-Meier.

Results: From 9877 hemodialysis patients, 741 were diagnosed with COVID-19. Mean age was 57 ± 16 years, 61% were male, and 51% white. The most frequent symptoms were fever (54.1%), cough (50.9%), and dyspnea (37.2%); 14.2% were asymptomatic. There were 139 deaths (18.8%), with 66% within the disease's first 15 days. 333 patients (44.9%) required hospitalization, and 211 (28.5%) were admitted to an intensive care unit. The cumulative probability of survival at 90 days of diagnosis was 79% (95% CI 76-82%). In the fully adjusted multivariate model, the risk factors significantly associated with death were diabetes mellitus (HR 1.52, 95% CI 1.05-2.19, P = 0.026), use of a central venous catheter (CVC) (HR 1.79, 95% CI 1.22-2.64, P = 0.003), age (HR

1.03, 95% CI 1.01-1.04, P < 0.001), and origin from the North vs. Southeast region (HR 2.60, 95% CI 1.01-6.68, P = 0.047).

Conclusions: Hemodialysis patients using a CVC as the vascular access, aside from diabetic and elderly ones, should be closely monitored due to their high risk of death in the course of the COVID-19.

Keywords: Brazil; COVID-19; Central venous catheter; Hemodialysis; Mortality; Risk factors.

© 2021. The Author(s), under exclusive licence to Springer Nature B.V.

Conflict of interest statement

The authors of this study have no conflict of interest.

- 30 references
- 1 figure

Supplementary info

Publication types, MeSH terms Expand

Publication types

- Multicenter Study
- Observational Study

MeSH terms

- Brazil / epidemiology
- COVID-19 / epidemiology*
- Catheter-Related Infections / epidemiology
- Catheter-Related Infections / etiology*
- Central Venous Catheters / adverse effects*
- Comorbidity
- Female
- Follow-Up Studies
- Humans
- Incidence
- Kidney Failure, Chronic / epidemiology
- Kidney Failure, Chronic / therapy*
- Male
- Middle Aged
- Renal Dialysis / adverse effects*
- Retrospective Studies
- SARS-CoV-2

- Survival Rate / trends
- Time Factors

Full text links

Springer Free PMC article
Proceed to details
Cite
Share
828
Observational Study
Med Gas Res
. Apr-Jun 2022;12(2):60-66.
doi: 10.4103/2045-9912.326002.

Prediction of diagnosis and prognosis of COVID-19 disease by blood gas parameters using decision trees machine learning model: a retrospective observational study

Mehmet Tahir Huyut [1], Hilal Üstündağ [2]
Affiliations Expand

Affiliations

- [1] Department of Biostatistics and Medical Informatics, Faculty of Medicine, Erzincan Binali Yıldırım University, Erzincan, Turkey.
- [2] Department of Physiology, Faculty of Medicine, Erzincan Binali Yıldırım University, Erzincan, Turkey.

- PMID: **34677154**
- PMCID: PMC8562394
- DOI: 10.4103/2045-9912.326002

Free PMC article
Observational Study

Prediction of diagnosis and prognosis of COVID-19 disease by blood gas parameters using decision trees machine learning model: a retrospective observational study

Mehmet Tahir Huyut et al. Med Gas Res. Apr-Jun 2022.
Free PMC article
Show details
Med Gas Res
. Apr-Jun 2022;12(2):60-66.
doi: 10.4103/2045-9912.326002.

Authors

Mehmet Tahir Huyut [1], Hilal Üstündağ [2]

Affiliations

- [1] Department of Biostatistics and Medical Informatics, Faculty of Medicine, Erzincan Binali Yıldırım University, Erzincan, Turkey.
- [2] Department of Physiology, Faculty of Medicine, Erzincan Binali Yıldırım University, Erzincan, Turkey.

- PMID: **34677154**
- PMCID: PMC8562394
- DOI: 10.4103/2045-9912.326002

Abstract

The coronavirus disease 2019 (COVID-19) epidemic went down in history as a pandemic caused by corona-viruses that emerged in 2019 and spread rapidly around the world. The different symptoms of COVID-19 made it difficult to understand which variables were more influential on the diagnosis, course and mortality of the disease. Machine learning models can accurately assess hidden patterns among risk factors by analyzing large-datasets to quickly predict diagnosis, prognosis and mortality of diseases. Because of this advantage, the use of machine learning models as decision support systems in health services is increasing. The aim of this study is to determine the diagnosis and prognosis of COVID-19 disease with blood-gas data using the Chi-squared Automatic Interaction Detector (CHAID) decision-tree-model, one of the machine learning methods, which is a subfield of artificial intelligence. This study was carried out on a total of 686 patients with COVID-19 (n = 343) and non-COVID-19 (n = 343) treated at Erzincan-Mengücek-Gazi-Training and Research-Hospital between April 1, 2020 and March 1, 2021. Arterial blood gas values of all patients were obtained from the hospital registry system. While the total-accuracyratio of the decision-tree-model was 65.0% in predicting the prognosis of the disease, it was 68.2% in the diagnosis of the disease. According to the results obtained, the low ionized-calcium value (< 1.10 mM) significantly predicted the need for intensive care of COVID-19 patients. At admission, low-carboxyhemoglobin (< 1.00%), high-pH (> 7.43), low-sodium (< 135.0 mM), hematocrit (< 40.0%), and methemoglobin (< 1.30%) values are important biomarkers

in the diagnosis of COVID-19 and the results were promising. The findings in the study may aid in the early-diagnosis of the disease and the intensive-care treatment of patients who are severe. The study was approved by the Ministry of Health and Erzincan University Faculty of Medicine Clinical Research Ethics Committee.

Keywords: COVID-19; SARS-CoV-2; arterial blood gases; artificial intelligence; carboxyhemoglobin; decision trees; ionized calcium; machine learning models.

Conflict of interest statement

None

- 32 references
- 3 figures

Supplementary info

Publication types, MeSH terms Expand

Publication types

- Observational Study

MeSH terms

- Artificial Intelligence*
- COVID-19*
- Decision Trees
- Humans
- Machine Learning
- Prognosis
- SARS-CoV-2

Full text links

Medknow Publications and Media Pvt Ltd Free PMC article
Proceed to details
Cite
Share
☐ 829
Observational Study
Respir Med
. 2021 Oct;187:106556.
doi: 10.1016/j.rmed.2021.106556. Epub 2021 Aug 4.

Galectin-3 as prognostic biomarker in patients with COVID-19 acute respiratory failure

Andrea Portacci [1], Fabrizio Diaferia [2], Carla Santomasi [3], Silvano Dragonieri [4], Esterina Boniello [5], Francesca Di Serio [6], Giovanna Elisiana Carpagnano [7]
Affiliations Expand

Affiliations

- [1] Institute of Respiratory Disease, Cardio-Thoracic Department, University of Medicine "Aldo Moro", Bari, Italy. Electronic address: andrea.portacci01@ateneopv.it.
- [2] Institute of Respiratory Disease, Cardio-Thoracic Department, University of Medicine "Aldo Moro", Bari, Italy. Electronic address: fabrizio.diaferia@gmail.com.
- [3] Institute of Respiratory Disease, Cardio-Thoracic Department, University of Medicine "Aldo Moro", Bari, Italy. Electronic address: carlasantomasi@gmail.com.
- [4] Institute of Respiratory Disease, Cardio-Thoracic Department, University of Medicine "Aldo Moro", Bari, Italy. Electronic address: silvano.dragonieri@uniba.it.
- [5] Institute of Respiratory Disease, Cardio-Thoracic Department, University of Medicine "Aldo Moro", Bari, Italy. Electronic address: esterboniello@hotmail.com.
- [6] Institute of Clinical Pathology, University of Medicine "Aldo Moro", Bari, Italy. Electronic address: francesca.diserio@policlinico.ba.it.
- [7] Institute of Respiratory Disease, Cardio-Thoracic Department, University of Medicine "Aldo Moro", Bari, Italy. Electronic address: elisiana.carpagnano@uniba.it.

- PMID: **34375925**
- PMCID: PMC8332745
- DOI: 10.1016/j.rmed.2021.106556

Free PMC article
Observational Study

Galectin-3 as prognostic biomarker in patients with COVID-19 acute respiratory failure

Andrea Portacci et al. Respir Med. 2021 Oct.
Free PMC article
Show details
Respir Med
. 2021 Oct;187:106556.
doi: 10.1016/j.rmed.2021.106556. Epub 2021 Aug 4.

Authors

Andrea Portacci [1], Fabrizio Diaferia [2], Carla Santomasi [3], Silvano Dragonieri [4], Esterina Boniello [5], Francesca Di Serio [6], Giovanna Elisiana Carpagnano [7]

Affiliations

- [1] Institute of Respiratory Disease, Cardio-Thoracic Department, University of Medicine "Aldo Moro", Bari, Italy. Electronic address: andrea.portacci01@ateneopv.it.
- [2] Institute of Respiratory Disease, Cardio-Thoracic Department, University of Medicine "Aldo Moro", Bari, Italy. Electronic address: fabrizio.diaferia@gmail.com.
- [3] Institute of Respiratory Disease, Cardio-Thoracic Department, University of Medicine "Aldo Moro", Bari, Italy. Electronic address: carlasantomasi@gmail.com.
- [4] Institute of Respiratory Disease, Cardio-Thoracic Department, University of Medicine "Aldo Moro", Bari, Italy. Electronic address: silvano.dragonieri@uniba.it.
- [5] Institute of Respiratory Disease, Cardio-Thoracic Department, University of Medicine "Aldo Moro", Bari, Italy. Electronic address: esterboniello@hotmail.com.
- [6] Institute of Clinical Pathology, University of Medicine "Aldo Moro", Bari, Italy. Electronic address: francesca.diserio@policlinico.ba.it.
- [7] Institute of Respiratory Disease, Cardio-Thoracic Department, University of Medicine "Aldo Moro", Bari, Italy. Electronic address: elisiana.carpagnano@uniba.it.

- PMID: **34375925**
- PMCID: PMC8332745
- DOI: 10.1016/j.rmed.2021.106556

Abstract

Objectives: Galectin-3 is β-galactoside-binding lectin with several roles in immune-inflammatory response. To date, there is no evidence of Galectin-3 role as a prognostic biomarker in COVID-19 disease. The aim of this study is to clarify the prognostic role of Galectin-3 in patients with COVID 19 acute respiratory failure.

Methods: We enrolled 156 consecutive patients with COVID-19 disease. Routine laboratory test, arterial blood gas, chest X-ray or Computed Tomography and Galectin-3 dosage were performed. The primary outcome was to assess Galectin-3 predictive power for 30-day mortality. Secondary outcomes were 30-day Intensive Care Unit admission and Acute Respiratory Distress Syndrome stratification according to Galectin-3 dosage. We performed Mann-Whitney U and Kruskal-Wallis tests for continuous variables comparison. Fisher's exact test or Chi-square test were used for categorical variables analysis. Receiver Operating Characteristic curves estimated Galectin-3 predictive power for the endpoints. With a fixed cut-off of 35.3 ng/ml, Kaplan-Meier with Log-Rank test and Cox Regression were performed to assess mortality and Intensive Care Unit admission risk.

Results: Galectin-3 correlated with many other prognostic predictors tested in our analysis. Moreover, patients with serum levels of Galectin-3 above 35.3 ng/ml had increased risk for mortality, Intensive Care Unit admission and severe Acute Respiratory Distress Syndrome.

Conclusions: Our study demonstrates the role of Galectin-3 as a predictor of mortality, Intensive Care Unit access and ARDS stratification in patients with COVID 19 acute respiratory failure.

Keywords: ARDS; ICU; Inflammation; Lung injury; Viral pneumonia.

Copyright © 2021 Elsevier Ltd. All rights reserved.

Conflict of interest statement

None.

- 34 references
- 8 figures

Supplementary info

Publication types, MeSH terms, Substances Expand

Publication types

- Observational Study

MeSH terms

- Aged
- Aged, 80 and over
- Biomarkers / blood
- Blood Proteins
- COVID-19 / blood*
- COVID-19 / complications
- COVID-19 / mortality*
- Critical Care
- Female
- Galectins / blood*
- Hospitalization
- Humans
- Male
- Middle Aged
- Predictive Value of Tests
- Prognosis
- Respiratory Distress Syndrome / blood*
- Respiratory Distress Syndrome / mortality
- Respiratory Distress Syndrome / virology*
- Retrospective Studies
- Survival Rate

Substances

- Biomarkers
- Blood Proteins
- Galectins
- LGALS3 protein, human

Full text links

ELSEVIER FULL-TEXT ARTICLE Elsevier Science Free PMC article
Proceed to details
Cite
Share
830
Observational Study
Head Neck
. 2020 Jul;42(7):1374-1381.
doi: 10.1002/hed.26261. Epub 2020 May 19.

Safe and effective management of tracheostomy in COVID-19 patients

Xiaomeng Zhang [1], Qiling Huang [1], Xun Niu [1], Tao Zhou [1], Zhen Xie [1], Yi Zhong [1], Hongjun Xiao [1]
Affiliations Expand

Affiliation

- [1] Department of Otorhinolaryngology, Union Hospital, Tongji Medical College, Huazhong University of Science and Technology, Wuhan, China.

- PMID: **32427403**
- PMCID: PMC7276854
- DOI: 10.1002/hed.26261

Free PMC article
Observational Study

Safe and effective management of tracheostomy in COVID-19 patients

Xiaomeng Zhang et al. Head Neck. 2020 Jul.
Free PMC article
Show details

Head Neck

. 2020 Jul;42(7):1374-1381.
doi: 10.1002/hed.26261. Epub 2020 May 19.

Authors

Xiaomeng Zhang [1], Qiling Huang [1], Xun Niu [1], Tao Zhou [1], Zhen Xie [1], Yi Zhong [1], Hongjun Xiao [1]

Affiliation

- [1] Department of Otorhinolaryngology, Union Hospital, Tongji Medical College, Huazhong University of Science and Technology, Wuhan, China.

- PMID: **32427403**
- PMCID: PMC7276854
- DOI: 10.1002/hed.26261

Abstract

Background: An increasing number of COVID-19 patients worldwide will probably need tracheostomy in an emergency or at the recovering stage of COVID-19. We explored the safe and effective management of tracheostomy in COVID-19 patients, to benefit patients and protect health care workers at the same time.

Methods: We retrospectively analyzed 11 hospitalized COVID-19 patients undergoing tracheostomy. Clinical features of patients, ventilator withdrawal after tracheostomy, surgical complications, and nosocomial infection of the health care workers associated with the tracheostomy were analyzed.

Results: The tracheostomy of all the 11 cases (100%) was performed successfully, including percutaneous tracheostomy of 6 cases (54.5%) and conventional open tracheostomy of 5 cases (45.5%). No severe postoperative complications occurred, and no health care workers associated with the tracheostomy are confirmed to be infected by SARS-CoV-2.

Conclusion: Comprehensive evaluation before tracheostomy, optimized procedures during tracheostomy, and special care after tracheostomy can make the tracheostomy safe and beneficial in COVID-19 patients.

Keywords: COVID-19; nosocomial infection; open; percutaneous; tracheostomy.

© 2020 Wiley Periodicals, Inc.

Conflict of interest statement

The authors declare no conflicts of interest.

- 15 references
- 3 figures

Supplementary info

Publication types, MeSH terms Expand

Publication types

- Observational Study

MeSH terms

- Adult
- Aged
- Aged, 80 and over
- COVID-19
- China
- Cohort Studies
- Coronavirus Infections / epidemiology*
- Coronavirus Infections / prevention & control*
- Cross Infection / prevention & control*
- Female
- Humans
- Infectious Disease Transmission, Patient-to-Professional / prevention & control*
- Intubation, Intratracheal
- Male
- Middle Aged
- Minimally Invasive Surgical Procedures
- Occupational Health*
- Pandemics / prevention & control*
- Pandemics / statistics & numerical data
- Pneumonia, Viral / epidemiology*
- Pneumonia, Viral / prevention & control*
- Retrospective Studies
- Risk Assessment
- Tertiary Care Centers
- Tracheostomy / methods*

Full text links

WILEY Full Text Article Wiley Free PMC article

Proceed to details

Cite

Share

☐ 831
Observational Study
Lancet Digit Health
. 2021 Jun;3(6):e349-e359.
doi: 10.1016/S2589-7500(21)00059-5.

Associations between changes in population mobility in response to the COVID-19 pandemic and socioeconomic factors at the city level in China and country level worldwide: a retrospective, observational study

Yonghong Liu [1], Zengmiao Wang [1], Benjamin Rader [2], Bingying Li [1], Chieh-Hsi Wu [3], Jason D Whittington [4], Pai Zheng [5], Nils Chr Stenseth [4], Ottar N Bjornstad [6], John S Brownstein [7], Huaiyu Tian [8]
Affiliations Expand

Affiliations

- [1] State Key Laboratory of Remote Sensing Science, Center for Global Change and Public Health, College of Global Change and Earth System Science, Beijing Normal University, Beijing, China.
- [2] Computational Epidemiology Lab, Boston Children's Hospital, Boston MA, USA; Department of Epidemiology, Boston University School of Public Health, Boston MA, USA.
- [3] School of Mathematical Sciences, University of Southampton, Southampton, UK.
- [4] Department of Biosciences, Centre for Ecological and Evolutionary Synthesis, University of Oslo, Oslo, Norway.
- [5] Department of Occupational and Environmental Health Sciences, School of Public Health, Peking University, China.
- [6] Department of Biosciences, Centre for Ecological and Evolutionary Synthesis, University of Oslo, Oslo, Norway; Department of Biology, Center for Infectious Disease Dynamics, Pennsylvania State University, PA, USA; Department of Entomology, College of Agricultural Sciences, Pennsylvania State University, PA, USA.
- [7] Computational Epidemiology Lab, Boston Children's Hospital, Boston MA, USA; Harvard Medical School, Harvard University, Boston, MA, USA.
- [8] State Key Laboratory of Remote Sensing Science, Center for Global Change and Public Health, College of Global Change and Earth System Science, Beijing Normal University, Beijing, China. Electronic address: tianhuaiyu@gmail.com.

- PMID: **34045001**
- PMCID: PMC8143730
- DOI: 10.1016/S2589-7500(21)00059-5

Free PMC article
Observational Study

Associations between changes in population mobility in response to the COVID-19 pandemic and socioeconomic factors at the city level in China and country level worldwide: a retrospective, observational study

Yonghong Liu et al. Lancet Digit Health. 2021 Jun.
Free PMC article
Show details
Lancet Digit Health
. 2021 Jun;3(6):e349-e359.
doi: 10.1016/S2589-7500(21)00059-5.

Authors

Yonghong Liu [1], Zengmiao Wang [1], Benjamin Rader [2], Bingying Li [1], Chieh-Hsi Wu [3], Jason D Whittington [4], Pai Zheng [5], Nils Chr Stenseth [4], Ottar N Bjornstad [6], John S Brownstein [7], Huaiyu Tian [8]

Affiliations

- [1] State Key Laboratory of Remote Sensing Science, Center for Global Change and Public Health, College of Global Change and Earth System Science, Beijing Normal University, Beijing, China.
- [2] Computational Epidemiology Lab, Boston Children's Hospital, Boston MA, USA; Department of Epidemiology, Boston University School of Public Health, Boston MA, USA.
- [3] School of Mathematical Sciences, University of Southampton, Southampton, UK.
- [4] Department of Biosciences, Centre for Ecological and Evolutionary Synthesis, University of Oslo, Oslo, Norway.
- [5] Department of Occupational and Environmental Health Sciences, School of Public Health, Peking University, China.
- [6] Department of Biosciences, Centre for Ecological and Evolutionary Synthesis, University of Oslo, Oslo, Norway; Department of Biology, Center for Infectious Disease Dynamics, Pennsylvania State University, PA, USA; Department of Entomology, College of Agricultural Sciences, Pennsylvania State University, PA, USA.
- [7] Computational Epidemiology Lab, Boston Children's Hospital, Boston MA, USA; Harvard Medical School, Harvard University, Boston, MA, USA.

- [8] State Key Laboratory of Remote Sensing Science, Center for Global Change and Public Health, College of Global Change and Earth System Science, Beijing Normal University, Beijing, China. Electronic address: tianhuaiyu@gmail.com.

- PMID: **34045001**
- PMCID: PMC8143730
- DOI: 10.1016/S2589-7500(21)00059-5

Abstract

Background: Until broad vaccination coverage is reached and effective therapeutics are available, controlling population mobility (ie, changes in the spatial location of a population that affect the spread and distribution of pathogens) is one of the major interventions used to reduce transmission of SARS-CoV-2. However, population mobility differs across locations, which could reduce the effectiveness of pandemic control measures. Here we assess the extent to which socioeconomic factors are associated with reductions in population mobility during the COVID-19 pandemic, at both the city level in China and at the country level worldwide.

Methods: In this retrospective, observational study, we obtained anonymised daily mobile phone location data for 358 Chinese cities from Baidu, and for 121 countries from Google COVID-19 Community Mobility Reports. We assessed the intra-city movement intensity, inflow intensity, and outflow intensity of each Chinese city between Jan 25 (when the national emergency response was implemented) and Feb 18, 2020 (when population mobility was lowest) and compared these data to the corresponding lunar calendar period from the previous year (Feb 5 to March 1, 2019). Chinese cities were classified into four socioeconomic index (SEI) groups (high SEI, high-middle SEI, middle SEI, and low SEI) and the association between socioeconomic factors and changes in population mobility were assessed using univariate and multivariable linear regression. At the country level, we compared six types of mobility (residential, transit stations, workplaces, retail and recreation, parks, and groceries and pharmacies) 35 days after the implementation of the national emergency response in each country and compared these to data from the same day of the week in the baseline period (Jan 3 to Feb 6, 2020). We assessed associations between changes in the six types of mobility and the country's sociodemographic index using univariate and multivariable linear regression.

Findings: The reduction in intra-city movement intensity in China was stronger in cities with a higher SEI than in those with a lower SEI (r=-0·47, p<0·0001). However, reductions in inter-city movement flow (both inflow and outflow intensity) were not associated with SEI and were only associated with government control measures. In the country-level analysis, countries with higher sociodemographic and Universal Health Coverage indexes had greater reductions in population mobility (ie, in transit stations, workplaces, and retail and recreation) following national emergency declarations than those with lower sociodemographic and Universal Health Coverage indexes. A higher sociodemographic index showed a greater reduction in mobility in transit stations (r=-0·27, p=0·0028), workplaces (r=-0·34, p=0·0002), and areas retail and recreation (rxs=-0·30, p=0·0012) than those with a lower sociodemographic index.

Interpretation: Although COVID-19 outbreaks are more frequently reported in larger cities, our analysis shows that future policies should prioritise the reduction of risks in areas with a low socioeconomic level-eg, by providing financial assistance and improving public health messaging. However, our study design only allows us to assess associations, and a long-term study is needed to decipher causality.

Funding: Chinese Ministry of Science and Technology, Research Council of Norway, Beijing Municipal Science & Technology Commission, Beijing Natural Science Foundation, Beijing

Advanced Innovation Program for Land Surface Science, National Natural Science Foundation of China, China Association for Science and Technology.

Copyright © 2021 The Author(s). Published by Elsevier Ltd. This is an Open Access article under the CC BY 4.0 license. Published by Elsevier Ltd.. All rights reserved.

Conflict of interest statement

Declaration of interests We declare no competing interests.

- 29 references
- 4 figures

Supplementary info

Publication types, MeSH terms Expand

Publication types

- Observational Study
- Research Support, Non-U.S. Gov't

MeSH terms

- Adult
- COVID-19*
- Cell Phone
- China
- Cities
- Global Health
- Humans
- Physical Distancing
- Population Dynamics* / trends
- Population Surveillance / methods
- Retrospective Studies
- SARS-CoV-2
- Socioeconomic Factors*
- Travel*

Full text links

ELSEVIER OPEN ACCESS Elsevier Science Free PMC article

Proceed to details

Cite

Share

☐ 832
Observational Study
Neurol Sci
. 2021 Feb;42(2):399-406.
doi: 10.1007/s10072-020-04914-4. Epub 2020 Nov 21.

Impact of COVID-19 pandemic on acute stroke care: facing an epidemiological paradox with a paradigm shift

Matteo Paolucci [1, 2], Sara Biguzzi [3], Francesco Cordici [3], Enrico Maria Lotti [4], Simonetta Morresi [3], Michele Romoli [3, 4], Silvia Strumia [5], Rossana Terlizzi [3], Simone Vidale [4], Maurizio Menarini [6], Maria Ruggiero [7], Alessandro Valentino [8], Marco Longoni [3, 5]
Affiliations Expand

Affiliations

- [1] Neurology Unit, "M. Bufalini" hospital, AUSL Romagna, Viale Giovanni Ghirotti, 286, 47521, Cesena, FC, Italy. matteo.paolucci@auslromagna.it.
- [2] Headache and Neurosonology Unit, Campus Bio-Medico University, Rome, Italy. matteo.paolucci@auslromagna.it.
- [3] Neurology Unit, "M. Bufalini" hospital, AUSL Romagna, Viale Giovanni Ghirotti, 286, 47521, Cesena, FC, Italy.
- [4] Neurology Unit, "Infermi" hospital, AUSL Romagna, Rimini, RN, Italy.
- [5] Neurology Unit, "Morgagni-Pierantoni" hospital, AUSL Romagna, Forlì, FC, Italy.
- [6] Centrale Operativa 118 Romagna e Emergenza Territoriale della Provincia di Ravenna, AUSL Romagna, Ravenna, Italy.
- [7] Neuroradiology Unit, "M. Bufalini" hospital, AUSL Romagna, Cesena, FC, Italy.
- [8] Pronto Soccorso e Medicina d'Urgenza, "M. Bufalini" hospital, AUSL Romagna, Cesena, FC, Italy.

- PMID: **33222101**
- PMCID: PMC7680213
- DOI: 10.1007/s10072-020-04914-4

Free PMC article
Observational Study

Impact of COVID-19 pandemic on acute stroke care: facing an epidemiological paradox with a paradigm shift

Matteo Paolucci et al. Neurol Sci. 2021 Feb.
Free PMC article

Show details

Neurol Sci

. 2021 Feb;42(2):399-406.
doi: 10.1007/s10072-020-04914-4. Epub 2020 Nov 21.

Authors

Matteo Paolucci [1] [2], Sara Biguzzi [3], Francesco Cordici [3], Enrico Maria Lotti [4], Simonetta Morresi [3], Michele Romoli [3] [4], Silvia Strumia [5], Rossana Terlizzi [3], Simone Vidale [4], Maurizio Menarini [6], Maria Ruggiero [7], Alessandro Valentino [8], Marco Longoni [3] [5]

Affiliations

- [1] Neurology Unit, "M. Bufalini" hospital, AUSL Romagna, Viale Giovanni Ghirotti, 286, 47521, Cesena, FC, Italy. matteo.paolucci@auslromagna.it.
- [2] Headache and Neurosonology Unit, Campus Bio-Medico University, Rome, Italy. matteo.paolucci@auslromagna.it.
- [3] Neurology Unit, "M. Bufalini" hospital, AUSL Romagna, Viale Giovanni Ghirotti, 286, 47521, Cesena, FC, Italy.
- [4] Neurology Unit, "Infermi" hospital, AUSL Romagna, Rimini, RN, Italy.
- [5] Neurology Unit, "Morgagni-Pierantoni" hospital, AUSL Romagna, Forlì, FC, Italy.
- [6] Centrale Operativa 118 Romagna e Emergenza Territoriale della Provincia di Ravenna, AUSL Romagna, Ravenna, Italy.
- [7] Neuroradiology Unit, "M. Bufalini" hospital, AUSL Romagna, Cesena, FC, Italy.
- [8] Pronto Soccorso e Medicina d'Urgenza, "M. Bufalini" hospital, AUSL Romagna, Cesena, FC, Italy.

- PMID: **33222101**
- PMCID: PMC7680213
- DOI: 10.1007/s10072-020-04914-4

Abstract

Background: During the coronavirus disease 2019 (COVID-19) outbreak, a decrease of stroke's hospital admissions and reperfusion therapy has been reported worldwide. This retrospective observational study assessed the volume of stroke cases managed in the Emergency Department (ED) and reperfusion therapies in an Italian stroke network with a high incidence of COVID-19, particularly to evaluate if the in-hospital rerouting and the switch from a drip-and-ship to a mothership model could assure an adequate volume of acute treatments.

Methods: We compared data from March 2020 with those from previous years and formulated five PICO questions regarding (1) incidence of stroke cases in the ED; (2) relation between stroke cases and COVID-19; (3) differences in the number of reperfusion therapies, (4) in the call-to-needle and door-to-needle times for intravenous thrombolysis, and (5) in the call-to-groin and door-to-groin times for thrombectomy.

Results: We found (1) a 28% decreased of confirmed stroke cases managed in the ED, (2) a negative correlation between stroke cases in ED and COVID-19 progression (r_s = - .390, p = .030), and (3) a similar number of treatments in March 2020 and March 2019. The adoption of the mothership model (4) did not delay alteplase infusion (median call-to-needle p = .126, median

door-to-needle p = .142) but led to (5) a significant reduction in median call-to-groin (p = .018) and door-to-groin times (p = .010).

Conclusion: The "hospital avoidance" of stroke patients during the "stay-at-home" appeals needs to be considered for future public health campaigns. A prompt reorganization of the stroke network can guarantee optimal performances at times of crisis.

Keywords: COVID-19; SARS-CoV-2; Stroke incidence; Thrombectomy; Thrombolysis.

Conflict of interest statement

None.

- 15 references
- 4 figures

Supplementary info

Publication types, MeSH terms Expand

Publication types

- Multicenter Study
- Observational Study

MeSH terms

- COVID-19* / prevention & control
- Emergency Service, Hospital / statistics & numerical data*
- Humans
- Italy
- Models, Organizational
- Outcome Assessment, Health Care
- Physical Distancing*
- Reperfusion / statistics & numerical data*
- Retrospective Studies
- Stroke / therapy*
- Thrombectomy / statistics & numerical data*
- Thrombolytic Therapy / statistics & numerical data*
- Time Factors

Full text links

SpringerLink FULL-TEXT ARTICLE Springer Free PMC article
Proceed to details
Cite

Share

833

Observational Study

J Cardiovasc Electrophysiol

. 2020 Dec;31(12):3077-3085.

doi: 10.1111/jce.14770. Epub 2020 Oct 14.

Outcomes and mortality associated with atrial arrhythmias among patients hospitalized with COVID-19

Bradley Peltzer [1], Kevin K Manocha [1], Xiaohan Ying [1], Jared Kirzner [1], James E Ip [1], George Thomas [1], Christopher F Liu [1], Steven M Markowitz [1], Bruce B Lerman [1], Monika M Safford [2], Parag Goyal [1], Jim W Cheung [1]

Affiliations Expand

Affiliations

- [1] Department of Medicine, Weill Cornell Medicine-New York Presbyterian Hospital, New York, New York, USA.
- [2] Division of General Internal Medicine, Department of Medicine, Weill Cornell Medicine-New York Presbyterian Hospital, New York, New York, USA.

- PMID: **33017083**
- PMCID: PMC7675597
- DOI: 10.1111/jce.14770

Free PMC article

Observational Study

Outcomes and mortality associated with atrial arrhythmias among patients hospitalized with COVID-19

Bradley Peltzer et al. J Cardiovasc Electrophysiol. 2020 Dec.

Free PMC article

Show details

J Cardiovasc Electrophysiol

. 2020 Dec;31(12):3077-3085.

doi: 10.1111/jce.14770. Epub 2020 Oct 14.

Authors

Bradley Peltzer [1], Kevin K Manocha [1], Xiaohan Ying [1], Jared Kirzner [1], James E Ip [1], George Thomas [1], Christopher F Liu [1], Steven M Markowitz [1], Bruce B Lerman [1], Monika M Safford [2], Parag Goyal [1], Jim W Cheung [1]

Affiliations

- [1] Department of Medicine, Weill Cornell Medicine-New York Presbyterian Hospital, New York, New York, USA.
- [2] Division of General Internal Medicine, Department of Medicine, Weill Cornell Medicine-New York Presbyterian Hospital, New York, New York, USA.

- PMID: **33017083**
- PMCID: PMC7675597
- DOI: 10.1111/jce.14770

Abstract

Introduction: The impact of atrial arrhythmias on coronavirus disease 2019 (COVID-19) -associated outcomes are unclear. We sought to identify prevalence, risk factors and outcomes associated with atrial arrhythmias among patients hospitalized with COVID-19.

Methods: An observational cohort study of 1053 patients with severe acute respiratory syndrome coronavirus 2 infection admitted to a quaternary care hospital and a community hospital was conducted. Data from electrocardiographic and telemetry were collected to identify atrial fibrillation (AF) or atrial flutter/tachycardia (AFL). The association between atrial arrhythmias and 30-day mortality was assessed with multivariable analysis.

Results: Mean age of patients was 62 ± 17 years and 62% were men. Atrial arrhythmias were identified in 166 (15.8%) patients, with AF in 154 (14.6%) patients and AFL in 40 (3.8%) patients. Newly detected atrial arrhythmias occurred in 101 (9.6%) patients. Age, male sex, prior AF, renal disease, and hypoxia on presentation were independently associated with AF/AFL occurrence. Compared with patients without AF/AFL, patients with AF/AFL had significantly higher levels of troponin, B-type natriuretic peptide, C-reactive protein, ferritin and d-dimer. Mortality was significantly higher among patients with AF/AFL (39.2%) compared to patients without (13.4%; $p < .001$). After adjustment for age and co-morbidities, AF/AFL (adjusted odds ratio [OR]: 1.93; $p = .007$) and newly detected AF/AFL (adjusted OR: 2.87; $p < .001$) were independently associated with 30-day mortality.

Conclusion: Atrial arrhythmias are common among patients hospitalized with COVID-19. The presence of AF/AFL tracked with markers of inflammation and cardiac injury. Atrial arrhythmias were independently associated with increased mortality.

Keywords: COVID-19; atrial fibrillation; atrial flutter; mortality; outcomes.

© 2020 Wiley Periodicals LLC.

Comment in

- Atrial fibrillation and flutter in patients hospitalized for COVID-19: The challenging role of digoxin.

Siniorakis E, Arvanitakis S, Katsianis A, Elkouris M. Siniorakis E, et al. J Cardiovasc Electrophysiol. 2021 Mar;32(3):878-879. doi: 10.1111/jce.14894. Epub 2021 Feb 1. J Cardiovasc Electrophysiol. 2021. PMID: 33522637 Free PMC article. No abstract available.

- 15 references
- 3 figures

Supplementary info

Publication types, MeSH terms, Grant support Expand

Publication types

- Multicenter Study
- Observational Study
- Research Support, N.I.H., Extramural
- Research Support, Non-U.S. Gov't

MeSH terms

- Adult
- Aged
- Aged, 80 and over
- Atrial Fibrillation / diagnosis
- Atrial Fibrillation / mortality*
- Atrial Fibrillation / therapy
- Atrial Flutter / diagnosis
- Atrial Flutter / mortality*
- Atrial Flutter / therapy
- COVID-19 / diagnosis
- COVID-19 / mortality*
- COVID-19 / therapy
- Female
- Hospital Mortality*
- Hospitalization*
- Humans
- Incidence
- Male
- Middle Aged
- New York City / epidemiology
- Prevalence
- Prognosis
- Retrospective Studies
- Risk Assessment

- Risk Factors
- Time Factors

Grant support

- UL1 TR000457/TR/NCATS NIH HHS/United States
- UL1 TR002384/TR/NCATS NIH HHS/United States
- 18IPA34170185/American Heart Association/International
- 20CDA35310455/American Heart Association/International

Full text links

WILEY Full Text Article Wiley Free PMC article

Proceed to details

Cite

Share

☐ 834

Observational Study

Am Surg

. 2020 Nov;86(11):1508-1512.

doi: 10.1177/0003134820972098. Epub 2020 Nov 6.

Impact of COVID-19 Outbreak on the Emergency Presentation of Acute Appendicitis

Mariana Kumaira Fonseca [1], Eduardo N Trindade [1], Omero P Costa Filho [1], Miguel P Nácul [1], Artur P Seabra [1]

Affiliations Expand

Affiliation

- [1] Hospital Moinhos de Vento, Porto Alegre, Brazil.

- PMID: **33156694**
- PMCID: PMC8685472
- DOI: 10.1177/0003134820972098

Free PMC article

Observational Study

Impact of COVID-19 Outbreak on the Emergency Presentation of Acute Appendicitis

Mariana Kumaira Fonseca et al. Am Surg. 2020 Nov.
Free PMC article
Show details
Am Surg
. 2020 Nov;86(11):1508-1512.
doi: 10.1177/0003134820972098. Epub 2020 Nov 6.

Authors

Mariana Kumaira Fonseca [1], Eduardo N Trindade [1], Omero P Costa Filho [1], Miguel P Nácul [1], Artur P Seabra [1]

Affiliation

- [1] Hospital Moinhos de Vento, Porto Alegre, Brazil.

- PMID: **33156694**
- PMCID: PMC8685472
- DOI: 10.1177/0003134820972098

Abstract

Background: The global crisis resulting from the coronavirus pandemic has imposed a large burden on health systems worldwide. Nonetheless, acute abdominal surgical emergencies are major causes for nontrauma-related hospital admissions and their incidences were expected to remain unchanged. Surprisingly, a significant decrease in volume and a higher proportion of complicated cases are being observed worldwide.

Methods: The present study assesses the local impact of the coronavirus pandemic on the emergency presentation of acute appendicitis in a Brazilian hospital. A retrospective analysis was conducted on patients undergoing emergency surgery for the clinically suspected diagnosis of acute appendicitis during the 2-month period of March and April 2020 and the same time interval in the previous year. Data on demographics, timing of symptom onset and hospital presentation, intraoperative details, postoperative complications, hospital length of stay, and histological examination of the specimen were retrieved from individual registries.

Results: The number of appendectomies during the pandemic was 36, which represents a 56% reduction compared to the 82 patients operated during the same period in 2019. The average time of symptom onset to hospital arrival was significantly higher in 2020 (40.6 vs. 28.2 hours, $P = .02$). The classification of appendicitis revealed a significant higher proportion of complicated cases than the previous year (33.3% vs. 15.2%, $P = .04$). The rate of postoperative complications and the average length of stay were not statistically different between the groups.

Conclusion: Further assessment of patients' concerns and systematic monitoring of emergency presentations are expected to help us understand and adequately address this issue.

Keywords: COVID-19; appendicitis; emergency service; hospital; surgery department.

Conflict of interest statement

Declaration of Conflicting Interests: The author(s) declared no potential conflicts of interest with respect to the research, authorship, and/or publication of this article.

- 25 references

Supplementary info

Publication types, MeSH terms Expand

Publication types

- Observational Study

MeSH terms

- Acute Disease
- Adolescent
- Adult
- Aged
- Aged, 80 and over
- Appendectomy / methods*
- Appendicitis / epidemiology
- Appendicitis / surgery*
- Brazil / epidemiology
- COVID-19 / epidemiology*
- Child
- Comorbidity
- Emergencies*
- Emergency Service, Hospital / statistics & numerical data*
- Female
- Humans
- Male
- Middle Aged
- Retrospective Studies
- SARS-CoV-2*
- Time Factors
- Treatment Outcome
- Young Adult

Full text links

SAGE journals Atypon Free PMC article

Proceed to details

Cite

Share

835

Observational Study

Gynecol Oncol

. 2020 Jul;158(1):37-43.

doi: 10.1016/j.ygyno.2020.05.019. Epub 2020 May 18.

Wait-time for hysterectomy and survival of women with early-stage cervical cancer: A clinical implication during the coronavirus pandemic

Koji Matsuo [1], Hilary Novatt [2], Shinya Matsuzaki [3], Marianne S Hom [4], Antonio V Castaneda [5], Ernesto Licon [3], David J Nusbaum [2], Lynda D Roman [6]

Affiliations Expand

Affiliations

- [1] Division of Gynecologic Oncology, Department of Obstetrics and Gynecology, University of Southern California, Los Angeles, CA, USA; Norris Comprehensive Cancer Center, University of Southern California, Los Angeles, CA, USA. Electronic address: koji.matsuo@med.usc.edu.
- [2] Division of Gynecologic Oncology, Department of Obstetrics and Gynecology, University of Southern California, Los Angeles, CA, USA; Keck School of Medicine, University of Southern California, Los Angeles, CA, USA.
- [3] Division of Gynecologic Oncology, Department of Obstetrics and Gynecology, University of Southern California, Los Angeles, CA, USA.
- [4] Division of Gynecologic Oncology, Department of Obstetrics and Gynecology, University of California, San Diego, San Diego, CA, USA.
- [5] Division of Gynecologic Oncology, Department of Obstetrics and Gynecology, The Ohio State University College of Medicine, Columbus, OH, USA.
- [6] Division of Gynecologic Oncology, Department of Obstetrics and Gynecology, University of Southern California, Los Angeles, CA, USA; Norris Comprehensive Cancer Center, University of Southern California, Los Angeles, CA, USA.

- PMID: **32425268**
- PMCID: PMC7231758
- DOI: 10.1016/j.ygyno.2020.05.019

Free PMC article

Observational Study

Wait-time for hysterectomy and survival of women with early-stage cervical cancer: A clinical implication during the coronavirus pandemic

Koji Matsuo et al. Gynecol Oncol. 2020 Jul.
Free PMC article
Show details
Gynecol Oncol
. 2020 Jul;158(1):37-43.
doi: 10.1016/j.ygyno.2020.05.019. Epub 2020 May 18.

Authors

Koji Matsuo [1], Hilary Novatt [2], Shinya Matsuzaki [3], Marianne S Hom [4], Antonio V Castaneda [5], Ernesto Licon [3], David J Nusbaum [2], Lynda D Roman [6]

Affiliations

- [1] Division of Gynecologic Oncology, Department of Obstetrics and Gynecology, University of Southern California, Los Angeles, CA, USA; Norris Comprehensive Cancer Center, University of Southern California, Los Angeles, CA, USA. Electronic address: koji.matsuo@med.usc.edu.
- [2] Division of Gynecologic Oncology, Department of Obstetrics and Gynecology, University of Southern California, Los Angeles, CA, USA; Keck School of Medicine, University of Southern California, Los Angeles, CA, USA.
- [3] Division of Gynecologic Oncology, Department of Obstetrics and Gynecology, University of Southern California, Los Angeles, CA, USA.
- [4] Division of Gynecologic Oncology, Department of Obstetrics and Gynecology, University of California, San Diego, San Diego, CA, USA.
- [5] Division of Gynecologic Oncology, Department of Obstetrics and Gynecology, The Ohio State University College of Medicine, Columbus, OH, USA.
- [6] Division of Gynecologic Oncology, Department of Obstetrics and Gynecology, University of Southern California, Los Angeles, CA, USA; Norris Comprehensive Cancer Center, University of Southern California, Los Angeles, CA, USA.

- PMID: **32425268**
- PMCID: PMC7231758
- DOI: 10.1016/j.ygyno.2020.05.019

Abstract

Objective: A global pandemic caused by a novel coronavirus (Covid-19) has created unique challenges to providing timely care for cancer patients. In early-stage cervical cancer, postponing

hysterectomy for 6-8 weeks is suggested as a possible option in the Covid-19 burdened hospitals. Yet, literature examining the impact of surgery wait-time on survival in early-stage cervical cancer remains scarce. This study examined the association between surgery wait-time of 8 weeks and oncologic outcome in women with early-stage cervical cancer.

Methods: This is a single institution retrospective observational study at a tertiary referral medical center examining women who underwent primary hysterectomy or trachelectomy for clinical stage IA-IIA invasive cervical cancer between 2000 and 2017 (N = 217). Wait-time from the diagnosis of invasive cervical cancer via biopsy to definitive surgery was categorized as: short wait-time (<8 weeks; n = 110) versus long wait-time (≥8 weeks; n = 107). Propensity score inverse probability of treatment weighting was used to balance the measured demographics between the two groups, and disease-free survival (DFS) and overall survival (OS) were assessed. A systematic literature review with meta-analysis was additionally performed.

Results: In a weighted model (median follow-up, 4.6 years), women in the long wait-time group had DFS (4.5-year rates, 91.2% versus 90.7%, hazard ratio [HR] 1.11, 95% confidence interval [CI] 0.47-2.59, P = 0.818) and OS (95.0% versus 97.4%, HR 1.47, 95%CI 0.50-4.31, P = 0.487) similar to those in the short wait-time group. Three studies were examined for meta-analysis, and a pooled HR for surgery wait-time of ≥8 weeks on DFS was 0.96 (95%CI 0.59-1.55).

Conclusion: Our study suggests that wait-time of 8 weeks for hysterectomy may not be associated with short-term disease recurrence in women with early-stage cervical cancer.

Keywords: Cervical cancer; Early stage; Hysterectomy; Surgery; Survival; Wait time.

Copyright © 2020 Elsevier Inc. All rights reserved.

Conflict of interest statement

Declaration of competing interest Consultant, Quantgene (L.D.R.); honorarium, Chugai, textbook editorial expense, Springer, and investigator meeting attendance expense, VBL therapeutics (K.M.); research grant, MSD (S.M.); none for others.

- 43 references
- 4 figures

Supplementary info

Publication types, MeSH terms Expand

Publication types

- Observational Study
- Research Support, Non-U.S. Gov't

MeSH terms

- Adult
- Aged
- Betacoronavirus / isolation & purification

- COVID-19
- California / epidemiology
- Coronavirus Infections / epidemiology*
- Female
- Humans
- Hysterectomy / methods
- Hysterectomy / statistics & numerical data*
- Middle Aged
- Pandemics
- Pneumonia, Viral / epidemiology*
- Propensity Score
- Retrospective Studies
- SARS-CoV-2
- Tertiary Care Centers / statistics & numerical data
- Time-to-Treatment / statistics & numerical data*
- Uterine Cervical Neoplasms / mortality
- Uterine Cervical Neoplasms / surgery*

Full text links

ELSEVIER FULL-TEXT ARTICLE Elsevier Science Free PMC article
Proceed to details
Cite
Share
836
Observational Study
Phytomedicine
. 2021 May;85:153531.
doi: 10.1016/j.phymed.2021.153531. Epub 2021 Feb 28.

Association between use of Qingfei Paidu Tang and mortality in hospitalized patients with COVID-19: A national retrospective registry study

Lihua Zhang[1], Xin Zheng[1], Xueke Bai[1], Qing Wang[1], Bowang Chen[1], Haibo Wang[2], Jiapeng Lu[1], Shuang Hu[1], Xiaoyan Zhang[1], Haibo Zhang[1], Jiamin Liu[1], Ying Shi[3], Zhiye Zhou[3], Lanxia Gan[3], Xi Li[4], Jing Li[5]
Affiliations Expand

Affiliations

- [1] National Clinical Research Center for Cardiovascular Diseases, State Key, Laboratory of Cardiovascular Disease, Fuwai Hospital, National Center for, Cardiovascular Diseases, Chinese Academy of Medical Sciences and Peking Union, Medical College, Beijing, People's Republic of China.
- [2] Clinical Trial Unit, First Affiliated Hospital of Sun Yat-Sen University, Guangzhou Province, People's Republic of China.
- [3] China Standard Medical Information Research Center, Shenzhen, People's Republic of China.
- [4] National Clinical Research Center for Cardiovascular Diseases, State Key, Laboratory of Cardiovascular Disease, Fuwai Hospital, National Center for, Cardiovascular Diseases, Chinese Academy of Medical Sciences and Peking Union, Medical College, Beijing, People's Republic of China; Central China Subcenter of the National Center for Cardiovascular Diseases, Zhengzhou, People's Republic of China. Electronic address: xi.li@fwoxford.org.
- [5] National Clinical Research Center for Cardiovascular Diseases, State Key, Laboratory of Cardiovascular Disease, Fuwai Hospital, National Center for, Cardiovascular Diseases, Chinese Academy of Medical Sciences and Peking Union, Medical College, Beijing, People's Republic of China; Central China Subcenter of the National Center for Cardiovascular Diseases, Zhengzhou, People's Republic of China; Fuwai Hospital, Chinese Academy of Medical Sciences, Shenzhen, People's Republic of China. Electronic address: jing.li@fwoxford.org.

- PMID: **33799224**
- PMCID: PMC7914374
- DOI: 10.1016/j.phymed.2021.153531

Free PMC article
Observational Study

Association between use of Qingfei Paidu Tang and mortality in hospitalized patients with COVID-19: A national retrospective registry study

Lihua Zhang et al. Phytomedicine. 2021 May.
Free PMC article

Show details

Phytomedicine

. 2021 May;85:153531.
doi: 10.1016/j.phymed.2021.153531. Epub 2021 Feb 28.

Authors

Lihua Zhang [1], Xin Zheng [1], Xueke Bai [1], Qing Wang [1], Bowang Chen [1], Haibo Wang [2], Jiapeng Lu [1], Shuang Hu [1], Xiaoyan Zhang [1], Haibo Zhang [1], Jiamin Liu [1], Ying Shi [3], Zhiye Zhou [3], Lanxia Gan [3], Xi Li [4], Jing Li [5]

Affiliations

- [1] National Clinical Research Center for Cardiovascular Diseases, State Key, Laboratory of Cardiovascular Disease, Fuwai Hospital, National Center for, Cardiovascular Diseases, Chinese Academy of Medical Sciences and Peking Union, Medical College, Beijing, People's Republic of China.
- [2] Clinical Trial Unit, First Affiliated Hospital of Sun Yat-Sen University, Guangzhou Province, People's Republic of China.
- [3] China Standard Medical Information Research Center, Shenzhen, People's Republic of China.
- [4] National Clinical Research Center for Cardiovascular Diseases, State Key, Laboratory of Cardiovascular Disease, Fuwai Hospital, National Center for, Cardiovascular Diseases, Chinese Academy of Medical Sciences and Peking Union, Medical College, Beijing, People's Republic of China; Central China Subcenter of the National Center for Cardiovascular Diseases, Zhengzhou, People's Republic of China. Electronic address: xi.li@fwoxford.org.
- [5] National Clinical Research Center for Cardiovascular Diseases, State Key, Laboratory of Cardiovascular Disease, Fuwai Hospital, National Center for, Cardiovascular Diseases, Chinese Academy of Medical Sciences and Peking Union, Medical College, Beijing, People's Republic of China; Central China Subcenter of the National Center for Cardiovascular Diseases, Zhengzhou, People's Republic of China; Fuwai Hospital, Chinese Academy of Medical Sciences, Shenzhen, People's Republic of China. Electronic address: jing.li@fwoxford.org.

- PMID: **33799224**
- PMCID: PMC7914374
- DOI: 10.1016/j.phymed.2021.153531

Abstract

Background: Qingfei Paidu Tang (QPT), a formula of traditional Chinese medicine, which was suggested to be able to ease symptoms in patients with Coronavirus Disease 2019 (COVID-19), has been recommended by clinical guidelines and widely used to treat COVID-19 in China. However, whether it decreases mortality remains unknown.

Purpose: We aimed to explore the association between QPT use and in-hospital mortality among patients hospitalized for COVID-19.

Study design: A retrospective study based on a real-world database was conducted.

Methods: We identified patients consecutively hospitalized with COVID-19 in 15 hospitals from a national retrospective registry in China, from January through May 2020. Data on patients' characteristics, treatments, and outcomes were extracted from the electronic medical records. The association of QPT use with COVID-19 related mortality was evaluated using Cox proportional hazards models based on propensity score analysis.

Results: Of the 8939 patients included, 28.7% received QPT. The COVID-19 related mortality was 1.2% (95% confidence interval [CI] 0.8% to 1.7%) among the patients receiving QPT and 4.8% (95% CI 4.3% to 5.3%) among those not receiving QPT. After adjustment for patient characteristics and concomitant treatments, QPT use was associated with a relative reduction of 50% in-hospital COVID-19 related mortality (hazard ratio, 0.50; 95% CI, 0.37 to 0.66 $p < 0.001$). This association was consistent across subgroups by sex and age. Meanwhile, the incidences of acute liver injury (8.9% [95% CI, 7.8% to 10.1%] vs. 9.9% [95% CI, 9.2% to 10.7%]; odds ratio, 0.96 [95% CI, 0.81% to 1.14%], p = 0.658) and acute kidney injury (1.6% [95% CI, 1.2% to 2.2%] vs. 3.0% [95% CI, 2.6% to 3.5%]; odds ratio, 0.85 [95% CI, 0.62 to 1.17], p = 0.318) were comparable between patients receiving QPT and those not receiving QPT. The major study limitations included that the study was an observational study based on real-world data rather than a randomized control trial, and the quality of data could be affected by the accuracy and completeness of medical records.

Conclusions: QPT was associated with a substantially lower risk of in-hospital mortality, without extra risk of acute liver injury or acute kidney injury among patients hospitalized with COVID-19.

Keywords: COVID-19; Mortality; Qingfei Paidu Tang.

Copyright © 2021 The Author(s). Published by Elsevier GmbH.. All rights reserved.

Conflict of interest statement

Dr. Jing Li discloses that she is a recipient of research grants from the government of China, through Fuwai Hospital, for research to improve the management of hypertension and blood lipids, and to improve care quality and patient outcomes of cardiovascular disease; is a recipient of research agreements with Amgen, through National Center for Cardiovascular Diseases (NCCD) and Fuwai Hospital, for a multi-center trial to assess the efficacy and safety of Omecamtiv Mecarbil, and for dyslipidaemia patient registration; is a recipient of a research agreement with Sanofi, through Fuwai Hospital, for a multi-center trial on the effects of sotagliflozin; is a recipient of a research agreement with University of Oxford, through Fuwai Hospital, for a multi-center trial of empagliflozin; and was a recipient of a research agreement, through NCCD, from AstraZeneca for clinical research methods training. The authors declared no other relevant conflict of interest.

- 22 references
- 4 figures

Supplementary info

Publication types, MeSH terms, Substances Expand

Publication types

- Observational Study

MeSH terms

- Acute Kidney Injury
- Adult

- Aged
- COVID-19 / drug therapy*
- COVID-19 / mortality*
- Chemical and Drug Induced Liver Injury
- China
- Drugs, Chinese Herbal / therapeutic use*
- Female
- Hospital Mortality
- Humans
- Incidence
- Male
- Medicine, Chinese Traditional
- Middle Aged
- Proportional Hazards Models
- Registries
- Retrospective Studies

Substances

- Drugs, Chinese Herbal
- qing fei pai du tang

Full text links

ELSEVIER OPEN ACCESS Elsevier Science Free PMC article

Proceed to details

Cite

Share

837

Observational Study

Arch Dis Child

. 2021 Dec;106(12):1218-1225.

doi: 10.1136/archdischild-2020-320388. Epub 2021 Mar 16.

Post-COVID-19 paediatric inflammatory multisystem syndrome: association of ethnicity, key worker and socioeconomic status with risk and severity

Jonathan Broad [1] [2], Julia Forman [3], James Brighouse [4], Adebola Sobande [5], Alysha McIntosh [6], Claire Watterson [6], Elizabeth Boot [7], Felicity Montgomery [5], Iona Gilmour [5], Joy Tan

[5], Mary Johanna Fogarty [8], Xabier Gomez [7], Ronny Cheung [5], Jon Lillie [7], Vinay Shivamurthy [4], Jenny Handforth [6], Owen Miller [8], PIMS-TS study group
Collaborators, Affiliations Expand

Collaborators

- **PIMS-TS study group**:
Julia Kenny, Kevin Meesters, Nuria Martinez-Alier, Alicia Demirjian, Marc Tebruegge, Alejandro Alonso, Tish Shah, Marie White, Anna Finemore, Fran Blackburn, Emma Parish, Bianca Tiesman, Nadia Trecchi, John Jackman, Mark Butler, Rohana Ramachandran, Alice Roueche, Chloe Macaulay, Claire Lemer, Debbie Sobande, Ajanta Kamal, Nick Wilkinson, Sara Arenas, Nanna Christiansen, Mandy Wan, Asia Rashed, Sujeev Mathur, James Wong, Paraskevi Theocharis, Kirsty Stewart, Saleha Kabir, Kelly Peacock, Kuberan Pushparajah, Alex Savis, Will Regan, Emma Pascall, Aoife Cleary, Mirasol Uy, Hannah Heard, Michael Carter, Shane Tibby, Shelley Riphagen, Marilyn MacDougall, Ben Griffths Gareth Waters, Federico Minen, Andrew Nyman, Miriam Fine Goulden, Ken MacGruer, Mario Sa, Ming Lim, Susan Bryne, Jill Cadwgan, J P Lim, Rahul Singh, Shan Tang, Dan Lumsden, Sam Senior, Sarah McMurtrie, Matthew Norridge, Stephanie Emberson, Stacey Marr, Victoria Felton, Chris Reid, Shazia Adalat, Ramnath Balasubramanian, Helen Jones, Jay Alamelu, Baba Inusa

Affiliations

- [1] Department of Paediatric Infectious Disease, Evelina London Children's Healthcare, London, UK jonathanb.broad@gmail.com.
- [2] Kellogg College, University of Oxford, Oxford, UK.
- [3] Department of Women and Children's Health, King's College London, London, UK.
- [4] Department of Paediatric Rheumatology, Evelina London Children's Healthcare, London, UK.
- [5] Department of General Paediatrics, Evelina London Children's Healthcare, London, UK.
- [6] Department of Paediatric Infectious Disease, Evelina London Children's Healthcare, London, UK.
- [7] Department of Paediatric Intensive Care, Evelina London Children's Healthcare, London, UK.
- [8] Department of Paediatric Cardiology, Evelina London Children's Healthcare, London, UK.

- PMID: **33727312**
- PMCID: PMC7977079
- DOI: 10.1136/archdischild-2020-320388

Free PMC article
Observational Study

Post-COVID-19 paediatric inflammatory multisystem syndrome: association of ethnicity, key worker and socioeconomic status with risk and severity

Jonathan Broad et al. Arch Dis Child. 2021 Dec.
Free PMC article
Show details
Arch Dis Child
. 2021 Dec;106(12):1218-1225.
doi: 10.1136/archdischild-2020-320388. Epub 2021 Mar 16.

Authors

Jonathan Broad [1] [2], Julia Forman [3], James Brighouse [4], Adebola Sobande [5], Alysha McIntosh [6], Claire Watterson [6], Elizabeth Boot [7], Felicity Montgomery [5], Iona Gilmour [5], Joy Tan [5], Mary Johanna Fogarty [8], Xabier Gomez [7], Ronny Cheung [5], Jon Lillie [7], Vinay Shivamurthy [4], Jenny Handforth [6], Owen Miller [8], PIMS-TS study group

Collaborators

- **PIMS-TS study group**: Julia Kenny, Kevin Meesters, Nuria Martinez-Alier, Alicia Demirjian, Marc Tebruegge, Alejandro Alonso, Tish Shah, Marie White, Anna Finemore, Fran Blackburn, Emma Parish, Bianca Tiesman, Nadia Trecchi, John Jackman, Mark Butler, Rohana Ramachandran, Alice Roueche, Chloe Macaulay, Claire Lemer, Debbie Sobande, Ajanta Kamal, Nick Wilkinson, Sara Arenas, Nanna Christiansen, Mandy Wan, Asia Rashed, Sujeev Mathur, James Wong, Paraskevi Theocharis, Kirsty Stewart, Saleha Kabir, Kelly Peacock, Kuberan Pushparajah, Alex Savis, Will Regan, Emma Pascall, Aoife Cleary, Mirasol Uy, Hannah Heard, Michael Carter, Shane Tibby, Shelley Riphagen, Marilyn MacDougall, Ben Griffths Gareth Waters, Federico Minen, Andrew Nyman, Miriam Fine Goulden, Ken MacGruer, Mario Sa, Ming Lim, Susan Bryne, Jill Cadwgan, J P Lim, Rahul Singh, Shan Tang, Dan Lumsden, Sam Senior, Sarah McMurtrie, Matthew Norridge, Stephanie Emberson, Stacey Marr, Victoria Felton, Chris Reid, Shazia Adalat, Ramnath Balasubramanian, Helen Jones, Jay Alamelu, Baba Inusa

Affiliations

- [1] Department of Paediatric Infectious Disease, Evelina London Children's Healthcare, London, UK jonathanb.broad@gmail.com.
- [2] Kellogg College, University of Oxford, Oxford, UK.
- [3] Department of Women and Children's Health, King's College London, London, UK.
- [4] Department of Paediatric Rheumatology, Evelina London Children's Healthcare, London, UK.
- [5] Department of General Paediatrics, Evelina London Children's Healthcare, London, UK.

- [6] Department of Paediatric Infectious Disease, Evelina London Children's Healthcare, London, UK.
- [7] Department of Paediatric Intensive Care, Evelina London Children's Healthcare, London, UK.
- [8] Department of Paediatric Cardiology, Evelina London Children's Healthcare, London, UK.

- PMID: **33727312**
- PMCID: PMC7977079
- DOI: 10.1136/archdischild-2020-320388

Abstract

Objectives: Patients from ethnic minority groups and key workers are over-represented among adults hospitalised or dying from COVID-19. In this population-based retrospective cohort, we describe the association of ethnicity, socioeconomic and family key worker status with incidence and severity of Paediatric Inflammatory Multisystem Syndrome Temporally associated with SARS-CoV-2 (PIMS-TS).

Setting: Evelina London Children's Hospital (ELCH), the tertiary paediatric hospital for the South Thames Retrieval Service (STRS) region.

Participants: 70 children with PIMS-TS admitted 14 February 2020-2 June 2020.

Outcome measures: Incidence and crude ORs are presented, comparing ethnicity and socioeconomic status of our cohort and the catchment population, using census data and Index of Multiple Deprivation (IMD). Regression is used to estimate the association of ethnicity and IMD with admission duration and requirement for intensive care, inotropes and ventilation.

Results: Incidence was significantly higher in children from black (25.0 cases per 100 000 population), Asian (6.4/100 000) and other (17.8/100 000) ethnic groups, compared with 1.6/100 000 in white ethnic groups (ORs 15.7, 4.0 and 11.2, respectively). Incidence was higher in the three most deprived quintiles compared with the least deprived quintile (eg, 8.1/100 000 in quintile 1 vs 1.6/100 000 in quintile 5, OR 5.2). Proportions of families with key workers (50%) exceeded catchment proportions. Admission length of stay was 38% longer in children from black ethnic groups than white (95% CI 4% to 82%; median 8 days vs 6 days). 9/10 children requiring ventilation were from black ethnic groups.

Conclusions: Children in ethnic minority groups, living in more deprived areas and in key worker families are over-represented. Children in black ethnic groups had longer admissions; ethnicity may be associated with ventilation requirement.This project was registered with the ELCH audit and service evaluation team, ref. no 11186.

Keywords: adolescent health; cardiology; epidemiology; statistics; virology.

© Author(s) (or their employer(s)) 2021. Re-use permitted under CC BY-NC. No commercial re-use. See rights and permissions. Published by BMJ.

Conflict of interest statement

Competing interests: None declared.

- 42 references
- 4 figures

Supplementary info

Publication types, MeSH terms, Supplementary concepts Expand

Publication types

- Observational Study

MeSH terms

- COVID-19 / complications*
- COVID-19 / economics
- COVID-19 / epidemiology
- COVID-19 / ethnology
- England / epidemiology
- Ethnicity*
- Health Personnel
- Humans
- Incidence
- Length of Stay
- Poverty Areas
- Retrospective Studies
- Risk Factors
- Severity of Illness Index
- Social Class*
- Systemic Inflammatory Response Syndrome / economics*
- Systemic Inflammatory Response Syndrome / epidemiology
- Systemic Inflammatory Response Syndrome / ethnology*

Supplementary concepts

- pediatric multisystem inflammatory disease, COVID-19 related

Full text links

BMJ Open Access Full Text HighWire Free PMC article

Proceed to details

Cite

Share

☐ 838

AIDS Res Ther

. 2020 Oct 4;17(1):59.
doi: 10.1186/s12981-020-00314-y.

[Consequences of the COVID-19 pandemic on the continuum of care in a cohort of people living with HIV followed in a single center of Northern Italy]

Eugenia Quiros-Roldan [1 2], Paola Magro [3 4], Canio Carriero [1 2], Annacarla Chiesa [1 2], Issa El Hamad [2], Elena Tratta [5], Raffaella Fazio [5], Beatrice Formenti [2], Francesco Castelli [1 2]
Affiliations Expand

Affiliations

- [1] Department of Infectious Diseases, University of Brescia, Brescia, Italy.
- [2] Division of Infectious Diseases, ASST Spedali Civili, Brescia, Italy.
- [3] Department of Infectious Diseases, University of Brescia, Brescia, Italy. magropao@gmail.com.
- [4] Division of Infectious Diseases, ASST Spedali Civili, Brescia, Italy. magropao@gmail.com.
- [5] Central Pharmacy, ASST Spedali Civili, Brescia, Italy.

- PMID: **33012282**
- PMCID: PMC7533114
- DOI: 10.1186/s12981-020-00314-y

Free PMC article

Consequences of the COVID-19 pandemic on the continuum of care in a cohort of people living with HIV followed in a single center of Northern Italy

Eugenia Quiros-Roldan et al. AIDS Res Ther. 2020.
Free PMC article
Show details
AIDS Res Ther
. 2020 Oct 4;17(1):59.
doi: 10.1186/s12981-020-00314-y.

Authors

Eugenia Quiros-Roldan [1 2], Paola Magro [3 4], Canio Carriero [1 2], Annacarla Chiesa [1 2], Issa El Hamad [2], Elena Tratta [5], Raffaella Fazio [5], Beatrice Formenti [2], Francesco Castelli [1 2]

Affiliations

- [1] Department of Infectious Diseases, University of Brescia, Brescia, Italy.
- [2] Division of Infectious Diseases, ASST Spedali Civili, Brescia, Italy.
- [3] Department of Infectious Diseases, University of Brescia, Brescia, Italy. magropao@gmail.com.
- [4] Division of Infectious Diseases, ASST Spedali Civili, Brescia, Italy. magropao@gmail.com.
- [5] Central Pharmacy, ASST Spedali Civili, Brescia, Italy.

- PMID: **33012282**
- PMCID: PMC7533114
- DOI: 10.1186/s12981-020-00314-y

Abstract

Introduction: During the COVID-19 pandemic, hospitals faced increasing pressure, where people living with HIV risked to either acquire SARS-CoV-2 and to interrupt the HIV continuum of care.

Methods: This is a retrospective, observational study. We compared the numbers of medical visits performed, antiretroviral drugs dispensed and the number of new HIV diagnosis and of hospitalizations in a cohort of people living with HIV (PLWH) followed by the Spedali Civili of Brescia between the bimester of the COVID-19 pandemic peak and the bimester of October-November 2019. Data were retrieved from administrative files and from paper and electronic clinical charts. Categorical variables were described using frequencies and percentages, while continuous variables were described using mean, median, and interquartile range (IQR) values. Means for continuous variables were compared using Student's t-tests and the Mann-Whitney test. Proportions for categorical variables were compared using the χ^2 test.

Results: As of December 31st, 2019, a total of 3875 PLWH were followed in our clinic. Mean age was 51.4 ± 13 years old, where 28% were females and 18.8% non-Italian. Overall, 98.9% were on ART (n = 3834), 93% were viro-suppressed. A total of 1217 and 1162 patients had their visit scheduled at our out-patient HIV clinic during the two bimesters of 2019 and 2020, respectively. Comparing the two periods, we observed a raise of missed visits from 5 to 8% ($p < 0.01$), a reduction in the number of new HIV diagnosis from 6.4 in 2019 to 2.5 per month in 2020 ($p = 0.01$), a drop in ART dispensation and an increase of hospitalized HIV patients due to COVID-19. ART regimens including protease inhibitors (PIs) had a smaller average drop than ART not including PIs (16.6 vs 21.6%, $p < 0.05$). Whether this may be due to the perception of a possible efficacy of PIs on COVID19 is not known.

Conclusions: Our experience highlights the importance of a resilient healthcare system and the need to implement new strategies in order to guarantee the continuum of HIV care even in the context of emergency.

Keywords: Adherence; COVID-19; Follow-up; HIV continuum of care; Public health; SARS-CoV-2.

Conflict of interest statement

FC reports acting as a principle investigator of company-sponsored clinical trials in the field of HIV infection (ViiV Healthcare, GlaxoSmithKline, Gilead Sciences and Janssen – Cilag). EQR.

received travel grants from Bristol-Myers Squibb, Gilead Sciences, ViiV Healthcare, Janssen-Cilaq Merck Sharp & Dohme and consultancy fees from Janssen -Cilag, ViiV Healthcare and Merck Sharp & Dohme. The other authors have no other relevant affiliations or financial involvement with any organization or entity with a financial interest in or financial conflict with the subject matter or materials discussed in the manuscript apart from those disclosed.

- 22 references
- 3 figures

Supplementary info

MeSH terms, Substances Expand

MeSH terms

- Adult
- Anti-HIV Agents / administration & dosage
- Anti-Retroviral Agents / administration & dosage
- Betacoronavirus / isolation & purification
- COVID-19
- Cohort Studies
- Continuity of Patient Care
- Coronavirus Infections / epidemiology
- Coronavirus Infections / virology*
- Female
- HIV Infections / drug therapy*
- HIV Infections / epidemiology
- HIV Infections / virology*
- Hospitalization
- Humans
- Italy / epidemiology
- Longitudinal Studies
- Male
- Middle Aged
- Pandemics
- Pneumonia, Viral / epidemiology
- Pneumonia, Viral / virology*
- Public Health
- Retrospective Studies
- SARS-CoV-2
- Statistics, Nonparametric

Substances

- Anti-HIV Agents
- Anti-Retroviral Agents

Full text links

Read free full text at BMC BioMed Central Free PMC article

Proceed to details

Cite

Share

☐ 839

Observational Study

PLoS One

. 2020 Oct 28;15(10):e0240960.

doi: 10.1371/journal.pone.0240960. eCollection 2020.

Investigating the association between ethnicity and health outcomes in SARS-CoV-2 in a London secondary care population

Aatish Patel [1], Ahmed Abdulaal [1], David Ariyanayagam [2], Kieran Killington [1], Sarah J Denny [1], Nabeela Mughal [1 2 3], Stephen Hughes [1], Nupur Goel [1 3], Gary W Davies [1 2], Luke S P Moore [1 3 4], Esmita Charani [4]

Affiliations Expand

Affiliations

- [1] Chelsea and Westminster NHS Foundation Trust, London, United Kingdom.
- [2] Imperial College London, Kensington, London, United Kingdom.
- [3] North West London Pathology, Imperial College Healthcare NHS Trust, London, United Kingdom.
- [4] Imperial College London, NIHR Health Protection Research Unit in Healthcare Associated Infections and Antimicrobial Resistance, Hammersmith Campus, London, United Kingdom.

- PMID: **33112892**
- PMCID: PMC7592846
- DOI: 10.1371/journal.pone.0240960

Free PMC article

Observational Study

Investigating the association between ethnicity and health outcomes in SARS-CoV-2 in a London secondary care population

Aatish Patel et al. PLoS One. 2020.
Free PMC article
Show details
PLoS One
. 2020 Oct 28;15(10):e0240960.
doi: 10.1371/journal.pone.0240960. eCollection 2020.

Authors

Aatish Patel [1], Ahmed Abdulaal [1], David Ariyanayagam [2], Kieran Killington [1], Sarah J Denny [1], Nabeela Mughal [1 2 3], Stephen Hughes [1], Nupur Goel [1 3], Gary W Davies [1 2], Luke S P Moore [1 3 4], Esmita Charani [4]

Affiliations

- [1] Chelsea and Westminster NHS Foundation Trust, London, United Kingdom.
- [2] Imperial College London, Kensington, London, United Kingdom.
- [3] North West London Pathology, Imperial College Healthcare NHS Trust, London, United Kingdom.
- [4] Imperial College London, NIHR Health Protection Research Unit in Healthcare Associated Infections and Antimicrobial Resistance, Hammersmith Campus, London, United Kingdom.

- PMID: **33112892**
- PMCID: PMC7592846
- DOI: 10.1371/journal.pone.0240960

Abstract

Background: Black, Asian and minority ethnic (BAME) populations are emerging as a vulnerable group in the severe acute respiratory syndrome coronavirus disease (SARS-CoV-2) pandemic. We investigated the relationship between ethnicity and health outcomes in SARS-CoV-2.

Methods and findings: We conducted a retrospective, observational analysis of SARS-CoV-2 patients across two London teaching hospitals during March 1 -April 30, 2020. Routinely collected clinical data were extracted and analysed for 645 patients who met the study inclusion criteria. Within this hospitalised cohort, the BAME population were younger relative to the white population (61.70 years, 95% CI 59.70-63.73 versus 69.3 years, 95% CI 67.17-71.43, p<0.001). When adjusted for age, sex and comorbidity, ethnicity was not a predictor for ICU admission. The mean age at death was lower in the BAME population compared to the white population (71.44 years, 95% CI 69.90-72.90 versus, 77.40 years, 95% CI 76.1-78.70 respectively, p<0.001). When

adjusted for age, sex and comorbidities, Asian patients had higher odds of death (OR 1.99: 95% CI 1.22-3.25, p<0.006).

Conclusions: BAME patients were more likely to be admitted younger, and to die at a younger age with SARS-CoV-2. Within the BAME cohort, Asian patients were more likely to die but despite this, there was no difference in rates of admission to ICU. The reasons for these disparities are not fully understood and need to be addressed. Investigating ethnicity as a clinical risk factor remains a high public health priority. Studies that consider ethnicity as part of the wider socio-cultural determinant of health are urgently needed.

Conflict of interest statement

The authors have read the journal's policy and have the following competing interests: All authors have completed the ICMJE uniform disclosure form at (www.icmje.org/coi_disclosure.pdf). EC has received speaker fees from bioMerieux (2019). NM has received speaker fees from Beyer (2016) and Pfizer (2019) and received educational support from Eumedica (2016) and Baxter (2017). LSPM has consulted for bioMerieux (2013), DNAelectronics (2015–18), Dairy Crest (2017–2018), Umovis Lab (2020), and Pfizer (2018–2020), received speaker fees from Profile Pharma (2018–2019), received research grants from the National Institute for Health Research (2013–2020), CW+ Charity (2018–2019), and Leo Pharma (2016), and received educational support from Eumedica (2016–2018). This does not alter our adherence to PLOS ONE policies on sharing data and materials. There are no patents, products in development or marketed products associated with this research to declare. AP, AA, DA, KK, SP, SD, SH, NG and GD none to declare.

- 21 references
- 2 figures

Supplementary info

Publication types, MeSH terms, Grant support Expand

Publication types

- Multicenter Study
- Observational Study

MeSH terms

- Adolescent
- Adult
- Aged
- Aged, 80 and over
- Asians / statistics & numerical data
- Betacoronavirus*
- Blacks / statistics & numerical data
- COVID-19
- Child

- Child, Preschool
- Coronavirus Infections / epidemiology
- Coronavirus Infections / ethnology*
- Coronavirus Infections / therapy
- Ethnicity / statistics & numerical data*
- Female
- Hospital Mortality
- Hospitals, Teaching / statistics & numerical data
- Humans
- Infant
- Infant, Newborn
- Length of Stay / statistics & numerical data
- London / epidemiology
- Male
- Middle Aged
- Minority Groups / statistics & numerical data
- Pandemics*
- Pneumonia, Viral / epidemiology
- Pneumonia, Viral / ethnology*
- Pneumonia, Viral / therapy
- Retrospective Studies
- SARS-CoV-2
- Secondary Care / ethnology
- Secondary Care / statistics & numerical data
- Socioeconomic Factors
- Survival Analysis
- Treatment Outcome
- Young Adult

Grant support

The authors received no specific funding for this work. However, EC is supported by Economic and Social Science Research Council (ESRC) and the National Institute for Health Research, UK Department of Health [HPRU–2012–10047] in partnership with Public Health England. LSPM acknowledges support from the National Institute of Health Research (NIHR) Imperial Biomedical Research Centre (BRC) and the National Institute for Health Research Health Protection Research Unit (HPRU) in Healthcare Associated Infection and Antimicrobial Resistance at Imperial College London in partnership with Public Health England. The views expressed in this publication are those of the authors and not necessarily those of the NHS, the National Institute for Health Research, or the UK Department of Health.

Full text links

OPEN ACCESS TO FULL TEXT PLOS ONE Public Library of Science Free PMC article

Proceed to details

Cite

Share

☐ 840

Observational Study

Am J Transplant

. 2020 Nov;20(11):3030-3041.

doi: 10.1111/ajt.16246. Epub 2020 Sep 23.

Clinical characteristics and risk factors for severe COVID-19 in hospitalized kidney transplant recipients: A multicentric cohort study

Alexandre Favà [1] [2], David Cucchiari [3], Nuria Montero [1] [2], Nestor Toapanta [4], Francisco J Centellas [5], Anna Vila-Santandreu [6], Ana Coloma [1] [2], Maria Meneghini [1] [2], Anna Manonelles [1] [2], Joana Sellarés [4], Irina Torres [4], Rosana Gelpi [6], Inmaculada Lorenzo [4], Pedro Ventura-Aguiar [3], Frederic Cofan [3], Jose V Torregrosa [3], Manel Perelló [4], Carme Facundo [6], Daniel Seron [4], Federico Oppenheimer [3], Oriol Bestard [1] [2], Josep M Cruzado [1] [2], Francesc Moreso [4], Edoardo Melilli [1] [2]

Affiliations Expand

Affiliations

- [1] Nephrology Department, Hospital Universitari de Bellvitge, Barcelona, Spain.
- [2] Biomedical Research Institute (IDIBELL), Hospital Duran i Reynals, Barcelona, Spain.
- [3] Nephrology Department, Hospital Clínic de Barcelona, Barcelona, Spain.
- [4] Nephrology Department, Hospital de Vall d' Hebron, Barcelona, Spain.
- [5] Nephrology Department, Complejo Hospitalario Universitario de Albacete, Albacete, Spain.
- [6] Nephrology Department, Fundació Puigvert, Barcelona, Spain.

- PMID: **32777153**
- PMCID: PMC7436908
- DOI: 10.1111/ajt.16246

Free PMC article

Observational Study

Clinical characteristics and risk factors for severe COVID-19 in hospitalized kidney

transplant recipients: A multicentric cohort study

Alexandre Favà et al. Am J Transplant. 2020 Nov.
Free PMC article
Show details
Am J Transplant
. 2020 Nov;20(11):3030-3041.
doi: 10.1111/ajt.16246. Epub 2020 Sep 23.

Authors

Alexandre Favà [1] [2], David Cucchiari [3], Nuria Montero [1] [2], Nestor Toapanta [4], Francisco J Centellas [5], Anna Vila-Santandreu [6], Ana Coloma [1] [2], Maria Meneghini [1] [2], Anna Manonelles [1] [2], Joana Sellarés [4], Irina Torres [4], Rosana Gelpi [6], Inmaculada Lorenzo [4], Pedro Ventura-Aguiar [3], Frederic Cofan [3], Jose V Torregrosa [3], Manel Perelló [4], Carme Facundo [6], Daniel Seron [4], Federico Oppenheimer [3], Oriol Bestard [1] [2], Josep M Cruzado [1] [2], Francesc Moreso [4], Edoardo Melilli [1] [2]

Affiliations

- [1] Nephrology Department, Hospital Universitari de Bellvitge, Barcelona, Spain.
- [2] Biomedical Research Institute (IDIBELL), Hospital Duran i Reynals, Barcelona, Spain.
- [3] Nephrology Department, Hospital Clínic de Barcelona, Barcelona, Spain.
- [4] Nephrology Department, Hospital de Vall d' Hebron, Barcelona, Spain.
- [5] Nephrology Department, Complejo Hospitalario Universitario de Albacete, Albacete, Spain.
- [6] Nephrology Department, Fundació Puigvert, Barcelona, Spain.

- PMID: **32777153**
- PMCID: PMC7436908
- DOI: 10.1111/ajt.16246

Abstract

Kidney transplant recipients might be at higher risk for severe coronavirus disease 2019 (COVID-19). However, risk factors for relevant outcomes remain uncertain in this population. This is a multicentric kidney transplant cohort including 104 hospitalized patients between March 4 and April 17, 2020. Risk factors for death and acute respiratory distress syndrome (ARDS) were investigated, and clinical and laboratory data were analyzed. The mean age was 60 years. Forty-seven patients (54.8%) developed ARDS. Obesity was associated to ARDS development (OR 2.63; P = .04). Significant age differences were not found among patients developing and not developing ARDS (61.3 vs 57.8 years, P = .16). Seventy-six (73%) patients were discharged, and 28 (27%) died. Death was more common among the elderly (55 and 70.8 years, P < .001) and those with preexisting pulmonary disease (OR 2.89, P = .009). At admission, higher baseline lactate dehydrogenase (257 vs 358 IU/mL, P = .001) or ARDS conferred higher risk of death (HR 2.09, P = .044). In our cohort, ARDS was equally present among young and old kidney recipients.

However, the elderly might be at higher risk of death, along with those showing higher baseline LDH at admission.

Keywords: clinical research/practice; complication: infectious; epidemiology; infectious disease; kidney transplantation/nephrology; patient survival.

© 2020 The American Society of Transplantation and the American Society of Transplant Surgeons.

- 30 references

Supplementary info

Publication types, MeSH terms, Grant support Expand

Publication types

- Multicenter Study
- Observational Study
- Research Support, Non-U.S. Gov't

MeSH terms

- COVID-19 / epidemiology*
- Comorbidity
- Female
- Follow-Up Studies
- Humans
- Inpatients*
- Intensive Care Units / statistics & numerical data
- Kidney Transplantation*
- Male
- Middle Aged
- Renal Insufficiency / epidemiology
- Renal Insufficiency / surgery*
- Retrospective Studies
- Risk Assessment / methods*
- Risk Factors
- SARS-CoV-2*
- Spain / epidemiology
- Transplant Recipients*

Grant support

- CERCA Program/Generalitat de Catalunya/International

- RD16/0009/0003/ISCIII RETICS RedinRen/International

Full text links

WILEY Free Full Text Wiley Free PMC article

Proceed to details

Cite

Share

841

Observational Study

Crit Care

. 2020 Nov 10;24(1):643.

doi: 10.1186/s13054-020-03340-4.

Corticosteroid therapy for coronavirus disease 2019-related acute respiratory distress syndrome: a cohort study with propensity score analysis

Chaomin Wu [1, 2], Dongni Hou [2], Chunling Du [1], Yanping Cai [3], Junhua Zheng [4], Jie Xu [5], Xiaoyan Chen [2], Cuicui Chen [2], Xianglin Hu [2], Yuye Zhang [2], Juan Song [2], Lu Wang [2], Yen-Cheng Chao [2], Yun Feng [6], Weining Xiong [7], Dechang Chen [8], Ming Zhong [9], Jie Hu [2], Jinjun Jiang [2], Chunxue Bai [2], Xin Zhou [10], Jinfu Xu [11], Yuanlin Song [12, 13, 14, 15, 16], Fengyun Gong [17]

Affiliations Expand

Affiliations

- [1] Department of Pulmonary and Critical Care Medicine, QingPu Branch of Zhongshan Hospital Affiliated to Fudan University, Shanghai, China.
- [2] Department of Pulmonary and Critical Care Medicine, Zhongshan Hospital, Fudan University, Shanghai, China.
- [3] Infection Division, Wuhan Jin Yin-Tan Hospital, Wuhan, China.
- [4] Department of Urology, Shanghai General Hospital, Shanghai Jiao Tong University School of Medicine, Shanghai, China.
- [5] Department of Infectious Diseases, Fengxian Guhua Hospital, Shanghai, China.
- [6] Department of Gastroenterology, Shanghai First People's Hospital, Shanghai Jiao Tong University School of Medicine, Shanghai, China.
- [7] Department of Respiratory and Critical Care Medicine, Shanghai Ninth People's Hospital, Shanghai Jiao Tong University School of Medicine, Shanghai, China.
- [8] Department of Critical Care Medicine, Ruijin Hospital, Shanghai Jiao Tong University School of Medicine, Shanghai, China.
- [9] Department of Critical Care Medicine, Zhongshan Hospital, Fudan University, Shanghai, China.

- [10] Department of Pulmonary Medicine, Shanghai First People's Hospital, Shanghai Jiao Tong University School of Medicine, Shanghai, China.
- [11] Department of Respiratory and Critical Care Medicine, Shanghai Pulmonary Hospital, Shanghai, China.
- [12] Department of Pulmonary and Critical Care Medicine, QingPu Branch of Zhongshan Hospital Affiliated to Fudan University, Shanghai, China. ylsong70@163.com.
- [13] Department of Pulmonary and Critical Care Medicine, Zhongshan Hospital, Fudan University, Shanghai, China. ylsong70@163.com.
- [14] Shanghai Respiratory Research Institute, Shanghai, China. ylsong70@163.com.
- [15] National Clinical Research Center for Aging and Medicine, Huashan Hospital, Fudan University, Shanghai, China. ylsong70@163.com.
- [16] Jinshan Hospital of Fudan University, Shanghai, China. ylsong70@163.com.
- [17] Infection Division, Wuhan Jin Yin-Tan Hospital, Wuhan, China. gfy.yuyingzi@163.com.

- PMID: **33172477**
- PMCID: PMC7655069
- DOI: 10.1186/s13054-020-03340-4

Free PMC article
Observational Study

Corticosteroid therapy for coronavirus disease 2019-related acute respiratory distress syndrome: a cohort study with propensity score analysis

Chaomin Wu et al. Crit Care. 2020.
Free PMC article
Show details
Crit Care
. 2020 Nov 10;24(1):643.
doi: 10.1186/s13054-020-03340-4.

Authors

Chaomin Wu [1, 2], Dongni Hou [2], Chunling Du [1], Yanping Cai [3], Junhua Zheng [4], Jie Xu [5], Xiaoyan Chen [2], Cuicui Chen [2], Xianglin Hu [2], Yuye Zhang [2], Juan Song [2], Lu Wang [2], Yen-Cheng Chao [2], Yun Feng [6], Weining Xiong [7], Dechang Chen [8], Ming Zhong [9], Jie Hu [2], Jinjun Jiang [2], Chunxue Bai [2], Xin Zhou [10], Jinfu Xu [11], Yuanlin Song [12, 13, 14, 15, 16], Fengyun Gong [17]

Affiliations

- [1] Department of Pulmonary and Critical Care Medicine, QingPu Branch of Zhongshan Hospital Affiliated to Fudan University, Shanghai, China.

- [2] Department of Pulmonary and Critical Care Medicine, Zhongshan Hospital, Fudan University, Shanghai, China.
- [3] Infection Division, Wuhan Jin Yin-Tan Hospital, Wuhan, China.
- [4] Department of Urology, Shanghai General Hospital, Shanghai Jiao Tong University School of Medicine, Shanghai, China.
- [5] Department of Infectious Diseases, Fengxian Guhua Hospital, Shanghai, China.
- [6] Department of Gastroenterology, Shanghai First People's Hospital, Shanghai Jiao Tong University School of Medicine, Shanghai, China.
- [7] Department of Respiratory and Critical Care Medicine, Shanghai Ninth People's Hospital, Shanghai Jiao Tong University School of Medicine, Shanghai, China.
- [8] Department of Critical Care Medicine, Ruijin Hospital, Shanghai Jiao Tong University School of Medicine, Shanghai, China.
- [9] Department of Critical Care Medicine, Zhongshan Hospital, Fudan University, Shanghai, China.
- [10] Department of Pulmonary Medicine, Shanghai First People's Hospital, Shanghai Jiao Tong University School of Medicine, Shanghai, China.
- [11] Department of Respiratory and Critical Care Medicine, Shanghai Pulmonary Hospital, Shanghai, China.
- [12] Department of Pulmonary and Critical Care Medicine, QingPu Branch of Zhongshan Hospital Affiliated to Fudan University, Shanghai, China. ylsong70@163.com.
- [13] Department of Pulmonary and Critical Care Medicine, Zhongshan Hospital, Fudan University, Shanghai, China. ylsong70@163.com.
- [14] Shanghai Respiratory Research Institute, Shanghai, China. ylsong70@163.com.
- [15] National Clinical Research Center for Aging and Medicine, Huashan Hospital, Fudan University, Shanghai, China. ylsong70@163.com.
- [16] Jinshan Hospital of Fudan University, Shanghai, China. ylsong70@163.com.
- [17] Infection Division, Wuhan Jin Yin-Tan Hospital, Wuhan, China. gfy.yuyingzi@163.com.

- PMID: **33172477**
- PMCID: PMC7655069
- DOI: 10.1186/s13054-020-03340-4

Abstract

Background: The impact of corticosteroid therapy on outcomes of patients with coronavirus disease 2019 (COVID-19) is highly controversial. We aimed to compare the risk of death between COVID-19-related ARDS patients with corticosteroid treatment and those without.

Methods: In this single-center retrospective observational study, patients with ARDS caused by COVID-19 between January 20, 2020, and February 24, 2020, were enrolled. The primary outcome was 60-day in-hospital death. The exposure was prescribed systemic corticosteroids or not. Time-dependent Cox regression models were used to calculate hazard ratios (HRs) and 95% confidence intervals (CIs) for 60-day in-hospital mortality.

Results: A total of 382 patients [60.7 ± 14.1 years old (mean ± SD), 61.3% males] were analyzed. The median of sequential organ failure assessment (SOFA) score was 2.0 (IQR 2.0-3.0). Of these cases, 94 (24.6%) patients had invasive mechanical ventilation. The number of patients received systemic corticosteroids was 226 (59.2%), and 156 (40.8%) received standard treatment. The maximum dose of corticosteroids was 80.0 (IQR 40.0-80.0) mg equivalent methylprednisolone per day, and duration of corticosteroid treatment was 7.0 (4.0-12.0) days in total. In Cox regression

analysis using corticosteroid treatment as a time-varying variable, corticosteroid treatment was associated with a significant reduction in risk of in-hospital death within 60 days after adjusting for age, sex, SOFA score at hospital admission, propensity score of corticosteroid treatment, comorbidities, antiviral treatment, and respiratory supports (HR 0.42; 95% CI 0.21, 0.85; p = 0.0160). Corticosteroids were not associated with delayed viral RNA clearance in our cohort.

Conclusion: In this clinical practice setting, low-dose corticosteroid treatment was associated with reduced risk of in-hospital death within 60 days in COVID-19 patients who developed ARDS.

Keywords: Coronavirus disease 2019; Corticosteroids; Methylprednisolone; Mortality; Propensity score; Severe acute respiratory syndrome coronavirus 2.

Conflict of interest statement

The authors declare that there is no conflict of interest that could be perceived as prejudicing the impartiality of the research reported.

- 37 references
- 2 figures

Supplementary info

Publication types, MeSH terms, Substances, Grant support Expand

Publication types

- Observational Study
- Research Support, Non-U.S. Gov't

MeSH terms

- Adrenal Cortex Hormones / administration & dosage*
- Aged
- Betacoronavirus*
- COVID-19
- Cohort Studies
- Coronavirus Infections / drug therapy*
- Coronavirus Infections / mortality*
- Dexamethasone / administration & dosage
- Female
- Hospitalization / trends
- Humans
- Male
- Methylprednisolone / administration & dosage
- Middle Aged
- Pandemics

- Pneumonia, Viral / drug therapy*
- Pneumonia, Viral / mortality*
- Propensity Score*
- Respiratory Distress Syndrome / drug therapy*
- Respiratory Distress Syndrome / mortality*
- Retrospective Studies
- SARS-CoV-2
- Survival Rate / trends

Substances

- Adrenal Cortex Hormones
- Dexamethasone
- Methylprednisolone

Grant support

- 82041003/National Natural Science Foundation of China/International
- 81630001/National Natural Science Foundation of China/International
- 81770075/National Natural Science Foundation of China/International
- 81800008/National Natural Science Foundation of China/International
- 81870035/National Natural Science Foundation of China/International

- 20411950402/Science and Technology Commission of Shanghai Municipality/International
- shslczdzk02201/Shanghai Municipal Key Clinical Specialty/International
- 2017ZZ02013/Shanghai Top-Priority Clinical Key Disciplines Construction Project/International
- ZK2019B06/Shanghai key discipline of medicine/International
- 18YF1404300/Shanghai Sailing Program/International
- WD2019-36/Academic Leader of Shanghai Qingpu District Healthcare Commission/International
- YZK 2019-04/Sub-specialist project of Qingpu Branch of Zhongshan Hospital, Fudan University/International

Show all 12 grants

Full text links

Read free full text at BMC BioMed Central Free PMC article
Proceed to details
Cite
Share
☐ 842
Observational Study
Rev Invest Clin
. 2020;72(3):151-158.
doi: 10.24875/RIC.20000207.

IMPACT OF COMORBIDITIES IN MEXICAN SARS-COV-2-POSITIVE PATIENTS: A RETROSPECTIVE ANALYSIS IN A NATIONAL COHORT

Ashuin Kammar-García[1 2], José de J Vidal-Mayo[1], Juan M Vera-Zertuche[3], Martín Lazcano-Hernández[4], Obdulia Vera-López[4], Orietta Segura-Badilla[5], Patricia Aguilar-Alonso[4], Addi R Navarro-Cruz[4]
Affiliations Expand

Affiliations

- [1] Emergency Department, Instituto Nacional de Ciencias Médicas y Nutrición Salvador Zubirán (INCMNSZ), Mexico City, Mexico.
- [2] Sección de Estudios de Posgrado e Investigación, Escuela Superior de Medicina, Instituto Politécnico Nacional, Mexico City, Mexico.
- [3] Department of Endocrinology, Obesity Clinic, INCMNSZ, Mexico City, Mexico.
- [4] Deparment of Biochemistry and Foods, Faculty of Chemical Sciences, Benemérita Universidad Autónoma de Puebla, Pue., Mexico.
- [5] Department of Nutrition and Public Health, Faculty of Health and Food Sciences, "Programa Universidad Bío-Bío (UBB) Saludable," Universidad del Bío-Bío, Concepción, Chile.

- PMID: **32584330**
- DOI: 10.24875/RIC.20000207

Observational Study

IMPACT OF COMORBIDITIES IN MEXICAN SARS-COV-2-POSITIVE PATIENTS: A RETROSPECTIVE ANALYSIS IN A NATIONAL COHORT

Ashuin Kammar-García et al. Rev Invest Clin. 2020.
Show details
Rev Invest Clin
. 2020;72(3):151-158.
doi: 10.24875/RIC.20000207.

Authors

Ashuin Kammar-García [1] [2], José de J Vidal-Mayo [1], Juan M Vera-Zertuche [3], Martín Lazcano-Hernández [4], Obdulia Vera-López [4], Orietta Segura-Badilla [5], Patricia Aguilar-Alonso [4], Addi R Navarro-Cruz [4]

Affiliations

- [1] Emergency Department, Instituto Nacional de Ciencias Médicas y Nutrición Salvador Zubirán (INCMNSZ), Mexico City, Mexico.
- [2] Sección de Estudios de Posgrado e Investigación, Escuela Superior de Medicina, Instituto Politécnico Nacional, Mexico City, Mexico.
- [3] Department of Endocrinology, Obesity Clinic, INCMNSZ, Mexico City, Mexico.
- [4] Deparment of Biochemistry and Foods, Faculty of Chemical Sciences, Benemérita Universidad Autónoma de Puebla, Pue., Mexico.
- [5] Department of Nutrition and Public Health, Faculty of Health and Food Sciences, "Programa Universidad Bío-Bío (UBB) Saludable," Universidad del Bío-Bío, Concepción, Chile.

- PMID: **32584330**
- DOI: 10.24875/RIC.20000207

Abstract

Background: The coronavirus disease 2019 outbreak is a significant challenge for health-care systems around the world.

Objective: The objective of the study was to assess the impact of comorbidities on the case fatality rate (CFR) and the development of adverse events in patients positive for severe acute respiratory syndrome coronavirus 2 (SARS-CoV-2) in the Mexican population.

Materials and methods: We analyzed the data from 13,842 laboratory-confirmed SARS-CoV-2 patients in Mexico between January 1, 2020, and April 25, 2020. We investigated the risk of death and the development of adverse events (hospitalization, pneumonia, orotracheal intubation, and intensive care unit [ICU] admission), comparing the number of comorbidities of each patient.

Results: The patient mean age was 46.6 ± 15.6 years, 42.3% (n = 5853) of the cases were women, 38.8% of patients were hospitalized, 4.4% were intubated, 29.6% developed pneumonia, and 4.4% had critical illness. The CFR was 9.4%. The risk of hospitalization (odds ratio [OR] = 3.1, 95% confidence interval [CI]: 2.7-3.7), pneumonia (OR = 3.02, 95% CI: 2.6-3.5), ICU admission (OR = 2, 95% CI: 1.5-2.7), and CFR (hazard ratio = 3.5, 95% CI: 2.9-4.2) was higher in patients with three or more comorbidities than in patients with 1, 2, or with no comorbidities.

Conclusions: The number of comorbidities may be a determining factor in the clinical course and its outcomes in SARS-CoV-2-positive patients.

Keywords: Adverse events; Comorbidities; Coronavirus disease 2019; Demographic characteristic; Mortality; Severe acute respiratory syndrome coronavirus 2.

Copyright: © 2020 Permanyer.

Supplementary info

Publication types, MeSH terms Expand

Publication types

- Multicenter Study
- Observational Study

MeSH terms

- Adolescent
- Adult
- Aged
- Aged, 80 and over
- Asthma / epidemiology
- Betacoronavirus*
- COVID-19
- Cardiovascular Diseases / epidemiology
- Comorbidity
- Coronavirus Infections / epidemiology*
- Critical Care / statistics & numerical data
- Critical Illness
- Diabetes Mellitus / epidemiology
- Female
- Hospitalization / statistics & numerical data
- Humans
- Immunocompromised Host
- Male
- Mexico / epidemiology
- Middle Aged
- Obesity / epidemiology
- Pandemics*
- Pneumonia, Viral / epidemiology*
- Pregnancy
- Pregnancy Complications, Infectious / epidemiology
- Proportional Hazards Models
- Pulmonary Disease, Chronic Obstructive / epidemiology
- Renal Insufficiency, Chronic / epidemiology
- Respiration, Artificial / statistics & numerical data
- Retrospective Studies
- SARS-CoV-2
- Smoking / epidemiology
- Young Adult

Proceed to details

Cite

Share

☐ 843

Observational Study

Nutr J

. 2021 May 25;20(1):46.

doi: 10.1186/s12937-021-00702-8.

Nutritional screening based on objective indices at admission predicts in-hospital mortality in patients with COVID-19

Feier Song[#1], Huan Ma[#2], Shouhong Wang[#3], Tiehe Qin[3], Qing Xu[4], Huiqing Yuan[5], Fei Li[6], Zhonghua Wang[3], Youwan Liao[3], Xiaoping Tan[7], Xiuchan Song[8], Qing Zhang[9], Daozheng Huang[10]

Affiliations Expand

Affiliations

- [1] Department of Emergency and Critical Care Medicine, Guangdong Provincial People's Hospital, Guangdong Academy of Medical Sciences, Guangzhou, 510080, China.
- [2] Department of Cardiology, Guangdong Provincial People's Hospital, Guangdong Academy of Medical Sciences, Guangdong Provincial Cardiovascular Institute, Guangzhou, 510080, China.
- [3] Department of Critical Care Medicine, Guangdong Provincial People's Hospital, Guangdong Academy of Medical Sciences, Guangdong Provincial Geriatrics Institute, Guangzhou, 510080, China.
- [4] Department of Emergency Medicine, Shanghai Jiao Tong University Affiliated Sixth People's Hospital, Shanghai, 200233, China.
- [5] Department of Respiratory and Critical Care Medicine, the First People's Hospital of Shaoguan, Shaoguan, 512000, China.
- [6] Department of Emergency, the First Affiliated Hospital of Jingzhou, Jingzhou, 434000, China.
- [7] Department of Gastroenterology, the First Affiliated Hospital of Yangtze University, Jingzhou, 434000, China.
- [8] Department of Critical Care Medicine, Dongguan Eighth People's Hospital, Dongguan Children's Hospital, Dongguan, 523000, China. songxiuchan0769@163.com.
- [9] Department of Gastroenterology, the First Affiliated Hospital of Yangtze University, Jingzhou, 434000, China. zqcn@qq.com.
- [10] Department of Critical Care Medicine, Guangdong Provincial People's Hospital, Guangdong Academy of Medical Sciences, Guangdong Provincial Geriatrics Institute, Guangzhou, 510080, China. hdzdoctor@139.com.

[#] Contributed equally.

- PMID: **34034769**
- PMCID: PMC8145188
- DOI: 10.1186/s12937-021-00702-8

Free PMC article
Observational Study

Nutritional screening based on objective indices at admission predicts in-hospital mortality in patients with COVID-19

Feier Song et al. Nutr J. 2021.
Free PMC article
Show details
Nutr J
. 2021 May 25;20(1):46.
doi: 10.1186/s12937-021-00702-8.

Authors

Feier Song [#1], Huan Ma [#2], Shouhong Wang [#3], Tiehe Qin [3], Qing Xu [4], Huiqing Yuan [5], Fei Li [6], Zhonghua Wang [3], Youwan Liao [3], Xiaoping Tan [7], Xiuchan Song [8], Qing Zhang [9], Daozheng Huang [10]

Affiliations

- [1] Department of Emergency and Critical Care Medicine, Guangdong Provincial People's Hospital, Guangdong Academy of Medical Sciences, Guangzhou, 510080, China.
- [2] Department of Cardiology, Guangdong Provincial People's Hospital, Guangdong Academy of Medical Sciences, Guangdong Provincial Cardiovascular Institute, Guangzhou, 510080, China.
- [3] Department of Critical Care Medicine, Guangdong Provincial People's Hospital, Guangdong Academy of Medical Sciences, Guangdong Provincial Geriatrics Institute, Guangzhou, 510080, China.
- [4] Department of Emergency Medicine, Shanghai Jiao Tong University Affiliated Sixth People's Hospital, Shanghai, 200233, China.
- [5] Department of Respiratory and Critical Care Medicine, the First People's Hospital of Shaoguan, Shaoguan, 512000, China.
- [6] Department of Emergency, the First Affiliated Hospital of Jingzhou, Jingzhou, 434000, China.
- [7] Department of Gastroenterology, the First Affiliated Hospital of Yangtze University, Jingzhou, 434000, China.
- [8] Department of Critical Care Medicine, Dongguan Eighth People's Hospital, Dongguan Children's Hospital, Dongguan, 523000, China. songxiuchan0769@163.com.
- [9] Department of Gastroenterology, the First Affiliated Hospital of Yangtze University, Jingzhou, 434000, China. zqcn@qq.com.

- [10] Department of Critical Care Medicine, Guangdong Provincial People's Hospital, Guangdong Academy of Medical Sciences, Guangdong Provincial Geriatrics Institute, Guangzhou, 510080, China. hdzdoctor@139.com.

[#] Contributed equally.

- PMID: **34034769**
- PMCID: PMC8145188
- DOI: 10.1186/s12937-021-00702-8

Abstract

Background: Could nutritional status serve as prognostic factors for coronavirus disease 2019 (COVID-19)? The present study evaluated the clinical and nutritional characteristics of COVID-19 patients and explored the relationship between risk for malnutrition at admission and in-hospital mortality.

Methods: A retrospective, observational study was conducted in two hospitals in Hubei, China. Confirmed cases of COVID-19 were typed as mild/moderate, severe, or critically ill. Clinical data and in-hospital death were collected. The risk for malnutrition was assessed using the geriatric nutritional risk index (GNRI), the prognostic nutritional index (PNI), and the Controlling Nutritional Status (CONUT) via objective parameters at admission.

Results: Two hundred ninety-five patients were enrolled, including 66 severe patients and 41 critically ill patients. Twenty-five deaths were observed, making 8.47% in the whole population and 37.88% in the critically ill subgroup. Patients had significant differences in nutrition-related parameters and inflammatory biomarkers among three types of disease severity. Patients with lower GNRI and PNI, as well as higher CONUT scores, had a higher risk of in-hospital mortality. The receiver operating characteristic curves demonstrated the good prognostic implication of GNRI and CONUT score. The multivariate logistic regression showed that baseline nutritional status, assessed by GNRI, PNI, or CONUT score, was a prognostic indicator for in-hospital mortality.

Conclusions: Despite variant screening tools, poor nutritional status was associated with in-hospital death in patients infected with COVID-19. This study highlighted the importance of nutritional screening at admission and the new insight of nutritional monitoring or therapy.

Keywords: COVID-19; Coronavirus; Malnutrition; Mortality; Nutrition.

Conflict of interest statement

The author declares that there is no conflict of interests.

- 48 references
- 1 figure

Supplementary info

Publication types, MeSH terms, Grant support Expand

Publication types

- Observational Study
- Research Support, Non-U.S. Gov't

MeSH terms

- Adult
- Aged
- COVID-19 / epidemiology*
- China / epidemiology
- Comorbidity
- Critical Illness / mortality
- Female
- Hospital Mortality*
- Humans
- Male
- Malnutrition / epidemiology*
- Middle Aged
- Nutrition Assessment*
- Nutritional Status*
- Prognosis
- Retrospective Studies
- Risk Factors
- SARS-CoV-2*
- Severity of Illness Index
- Thorax / diagnostic imaging
- Tomography, X-Ray Computed

Grant support

- 2012-649/National Clinical Key Specialty Construction Project of China
- 2013-544/National Clinical Key Specialty Construction Project of China

Full text links

Read free full text at BMC BioMed Central Free PMC article

Proceed to details

Cite

Share

☐ 844

Observational Study

J Stroke Cerebrovasc Dis

. 2021 Aug;30(8):105857.
doi: 10.1016/j.jstrokecerebrovasdis.2021.105857. Epub 2021 May 4.

Decline in Rehab Transfers Among Rehab-Eligible Stroke Patients During the COVID-19 Pandemic

Lauren Thau [1], Taylor Siegal [1], Mark E Heslin [1], Ameena Rana [1], Siyuan Yu [1], Scott Kamen [1], Austin Chen [1], Nicholas Vigilante [1], Sheri Gallagher [2], Kevin Wegner [2], Jesse M Thon [3], Ryna Then [3], Pratit Patel [3], Terri Yeager [2], Tudor G Jovin [3], Rohini J Kumar [2], David E Owens [2], James E Siegler [4]
Affiliations Expand

Affiliations

- [1] Cooper Medical School of Rowan University, Camden, New Jersey, 08103.
- [2] Department of Physical Medicine and Rehabilitation, Cooper University Hospital, Camden, New Jersey, 08103.
- [3] Cooper Neurological Institute, Cooper University Hospital, Camden, New Jersey, 08103.
- [4] Cooper Neurological Institute, Cooper University Hospital, Camden, New Jersey, 08103. Electronic address: siegler-james@cooperhealth.edu.

- PMID: **34022581**
- PMCID: PMC8769561
- DOI: 10.1016/j.jstrokecerebrovasdis.2021.105857

Free PMC article
Observational Study

Decline in Rehab Transfers Among Rehab-Eligible Stroke Patients During the COVID-19 Pandemic

Lauren Thau et al. J Stroke Cerebrovasc Dis. 2021 Aug.
Free PMC article
Show details
J Stroke Cerebrovasc Dis

. 2021 Aug;30(8):105857.
doi: 10.1016/j.jstrokecerebrovasdis.2021.105857. Epub 2021 May 4.

Authors

Lauren Thau [1], Taylor Siegal [1], Mark E Heslin [1], Ameena Rana [1], Siyuan Yu [1], Scott Kamen [1], Austin Chen [1], Nicholas Vigilante [1], Sheri Gallagher [2], Kevin Wegner [2], Jesse M Thon

[3], Ryna Then [3], Pratit Patel [3], Terri Yeager [2], Tudor G Jovin [3], Rohini J Kumar [2], David E Owens [2], James E Siegler [4]

Affiliations

- [1] Cooper Medical School of Rowan University, Camden, New Jersey, 08103.
- [2] Department of Physical Medicine and Rehabilitation, Cooper University Hospital, Camden, New Jersey, 08103.
- [3] Cooper Neurological Institute, Cooper University Hospital, Camden, New Jersey, 08103.
- [4] Cooper Neurological Institute, Cooper University Hospital, Camden, New Jersey, 08103. Electronic address: siegler-james@cooperhealth.edu.

- PMID: **34022581**
- PMCID: PMC8769561
- DOI: 10.1016/j.jstrokecerebrovasdis.2021.105857

Abstract

Objective: To characterize differences in disposition arrangement among rehab-eligible stroke patients at a Comprehensive Stroke Center before and during the COVID-19 pandemic.

Materials and methods: We retrospectively analyzed a prospective registry for demographics, hospital course, and discharge dispositions of rehab-eligible acute stroke survivors admitted 6 months prior to (10/2019-03/2020) and during (04/2020-09/2020) the COVID-19 pandemic. The primary outcome was discharge to an inpatient rehabilitation facility (IRF) as opposed to other facilities using descriptive statistics, and IRF versus home using unadjusted and adjusted backward stepwise logistic regression.

Results: Of the 507 rehab-eligible stroke survivors, there was no difference in age, premorbid disability, or stroke severity between study periods (p>0.05). There was a 9% absolute decrease in discharges to an IRF during the pandemic (32.1% vs. 41.1%, p=0.04), which translated to 38% lower odds of being discharged to IRF versus home in unadjusted regression (OR 0.62, 95%CI 0.42-0.92, p=0.016). The lower odds of discharge to IRF persisted in the multivariable model (aOR 0.16, 95%CI 0.09-0.31, p<0.001) despite a significant increase in discharge disability (median discharge mRS 4 [IQR 2-4] vs. 2 [IQR 1-3], p<0.001) during the pandemic.

Conclusions: Admission for stroke during the COVID-19 pandemic was associated with a significantly lower probability of being discharged to an IRF. This effect persisted despite adjustment for predictors of IRF disposition, including functional disability at discharge. Potential reasons for this disparity are explored.

Keywords: COVID-19; Ischemic stroke; Recovery of function; Rehabilitation.

Copyright © 2021 Elsevier Inc. All rights reserved.

- 20 references
- 1 figure

Supplementary info

Publication types, MeSH terms Expand

Publication types

- Comparative Study
- Observational Study

MeSH terms

- Aged
- COVID-19*
- Disability Evaluation
- Female
- Humans
- Male
- Middle Aged
- New Jersey
- Patient Discharge / trends*
- Patient Transfer / trends*
- Practice Patterns, Physicians' / trends*
- Recovery of Function
- Registries
- Retrospective Studies
- Stroke / diagnosis
- Stroke / physiopathology
- Stroke / therapy*
- Stroke Rehabilitation / trends*
- Time Factors

Full text links

ELSEVIER FULL-TEXT ARTICLE Elsevier Science Free PMC article
Proceed to details
Cite
Share
845
Observational Study
Clin Otolaryngol
. 2022 Jan;47(1):120-130.
doi: 10.1111/coa.13869. Epub 2021 Nov 3.

Acute paediatric mastoiditis in the UK before and during the COVID-19 pandemic: A national observational study

Matthew E Smith [1], G Huw Jones [2], John C Hardman [3], Jaya Nichani [4], Sadie Khwaja [5], INTEGRATE (The UK ENT Trainee Research Network) UK Acute Paediatric Mastoiditis Audit Collaborators; Iain A Bruce [6], Peter Rea [7]

Collaborators, Affiliations Expand

Collaborators

- **INTEGRATE (The UK ENT Trainee Research Network) UK Acute Paediatric Mastoiditis Audit Collaborators**: Samuel Dewhurst, Emma J Whitehall, Gavin Donaldson, Noshin Siddiqi, Nicola Wooles, Srinish Gopala Krishnan, David Selwyn, James D Constable, Billy Wong, Ahmed Yousef, Beverley Yu, Fiona McClenaghan, Andrew Mowat, Mohamed Elmorsy, Marina Brimioulle, Georgios Sakagiannis, Bassem Mattias, Olivia Kenyon, Anna I Kaleva, Anna Harrison, Elizabeth Mathew, Oliver J Wright, Richard A Steven, Rachael Collins, Darren Yap, Priya Sethukumar, Aria Amir Ghasemi, Alison Conybeare, Guled M Jama, Alison Liu, Lucy Qian Li, Anas Gomati, William Flynn, Christopher Connon, Soo Oh, Brendan Wright, Craig James McCaffer, Hannah R Lancer, Emily Lowe, Dylan Chew, Dimitrios Ioannidis, Anastasha Herman, Arshad Zubair, Cillian T Forde, Emma Watts, Anna Loroch, Laura Burton, Alexander Yao, Hannah Emerson, Safdar Sarwar, Faiza Ali, Manohar L Bance, Madhankumar Krishnan, Cathy Smyth, Cheka Spencer, Yohanna Takwoingi, David Strachan, Philip J Robinson, Laura Harding, Robert Temple, Jayant Dugar, Robert Nash, Arshad Janjua, George Thomas, Ian Bottrill, Ananth Vijendren, Pranay Singh, Alison Hunt, Alistair Mitchell-Innes, Patrick M Spielmann, John Phillips, Mrinal Supriya, Anooj Majithia, Eleanor Sproson, Patrick Lee, Gaurav Kumar, Dilip Nair, Alok Sharma, David Wynne, Sanjiv Kumar Bhimrao, Conor Jackson, Claire McLarnon, Matthew Weller, Marcel Geyer, Sanjeev Gupta, Timothy Mitchell, Mamoona Khalid-Raja, Kay Seymour, Azhar Shaida, Emma Hoskison, Arunachalam Iyer, Andrew Hall, Mark Simmons, Ahmed Allam, Phillip Moore

Affiliations

- [1] Department of Otolaryngology, Salford Royal Hospital, Manchester, UK.
- [2] Department of Otolaryngology, Gloucestershire Hospitals NHS Foundation Trust, Gloucester, UK.
- [3] The Head and Neck Unit, The Royal Marsden Hospital, London, UK.
- [4] Paediatric Otolaryngology Department, Royal Manchester Children's Hospital, University of Manchester, UK.
- [5] Department of Otolaryngology, Manchester University Foundation Trust, Manchester, UK.
- [6] Paediatric ENT Department, Royal Manchester Children's Hospital, MAHSC, University of Manchester, UK.
- [7] Department of Otolaryngology, Leicester Royal Infirmary, Leicester, UK.

- PMID: **34606691**
- PMCID: PMC8652842
- DOI: 10.1111/coa.13869

Free PMC article
Observational Study

Acute paediatric mastoiditis in the UK before and during the COVID-19 pandemic: A national observational study

Matthew E Smith et al. Clin Otolaryngol. 2022 Jan.
Free PMC article
Show details
Clin Otolaryngol
. 2022 Jan;47(1):120-130.
doi: 10.1111/coa.13869. Epub 2021 Nov 3.

Authors

Matthew E Smith [1], G Huw Jones [2], John C Hardman [3], Jaya Nichani [4], Sadie Khwaja [5], INTEGRATE (The UK ENT Trainee Research Network) UK Acute Paediatric Mastoiditis Audit Collaborators; Iain A Bruce [6], Peter Rea [7]

Collaborators

- **INTEGRATE (The UK ENT Trainee Research Network) UK Acute Paediatric Mastoiditis Audit Collaborators**: Samuel Dewhurst, Emma J Whitehall, Gavin Donaldson, Noshin Siddiqi, Nicola Wooles, Srinish Gopala Krishnan, David Selwyn, James D Constable, Billy Wong, Ahmed Yousef, Beverley Yu, Fiona McClenaghan, Andrew Mowat, Mohamed Elmorsy, Marina Brimioulle, Georgios Sakagiannis, Bassem Mattias, Olivia Kenyon, Anna I Kaleva, Anna Harrison, Elizabeth Mathew, Oliver J Wright, Richard A Steven, Rachael Collins, Darren Yap, Priya Sethukumar, Aria Amir Ghasemi, Alison Conybeare, Guled M Jama, Alison Liu, Lucy Qian Li, Anas Gomati, William Flynn, Christopher Connon, Soo Oh, Brendan Wright, Craig James McCaffer, Hannah R Lancer, Emily Lowe, Dylan Chew, Dimitrios Ioannidis, Anastasha Herman, Arshad Zubair, Cillian T Forde, Emma Watts, Anna Loroch, Laura Burton, Alexander Yao, Hannah Emerson, Safdar Sarwar, Faiza Ali, Manohar L Bance, Madhankumar Krishnan, Cathy Smyth, Cheka Spencer, Yohanna Takwoingi, David Strachan, Philip J Robinson, Laura Harding, Robert Temple, Jayant Dugar, Robert Nash, Arshad Janjua, George Thomas, Ian Bottrill, Ananth Vijendren, Pranay Singh, Alison Hunt, Alistair Mitchell-Innes, Patrick M Spielmann, John Phillips, Mrinal Supriya, Anooj Majithia, Eleanor Sproson, Patrick Lee, Gaurav Kumar, Dilip Nair, Alok Sharma, David Wynne, Sanjiv Kumar Bhimrao, Conor Jackson, Claire McLarnon, Matthew Weller, Marcel Geyer, Sanjeev Gupta, Timothy Mitchell, Mamoona Khalid-Raja, Kay Seymour, Azhar Shaida, Emma Hoskison, Arunachalam Iyer, Andrew Hall, Mark Simmons, Ahmed Allam, Phillip Moore

Affiliations

- [1] Department of Otolaryngology, Salford Royal Hospital, Manchester, UK.
- [2] Department of Otolaryngology, Gloucestershire Hospitals NHS Foundation Trust, Gloucester, UK.
- [3] The Head and Neck Unit, The Royal Marsden Hospital, London, UK.
- [4] Paediatric Otolaryngology Department, Royal Manchester Children's Hospital, University of Manchester, UK.
- [5] Department of Otolaryngology, Manchester University Foundation Trust, Manchester, UK.
- [6] Paediatric ENT Department, Royal Manchester Children's Hospital, MAHSC, University of Manchester, UK.
- [7] Department of Otolaryngology, Leicester Royal Infirmary, Leicester, UK.

- PMID: **34606691**
- PMCID: PMC8652842
- DOI: 10.1111/coa.13869

Abstract

Objectives: To explore the impact of COVID-19 on the management and outcomes of acute paediatric mastoiditis across the UK.

Design: National retrospective and prospective audit.

Setting: 48 UK secondary care ENT departments.

Participants: Consecutive children aged 18 years or under, referred to ENT with a clinical diagnosis of mastoiditis.

Main outcome measures: Cases were divided into Period 1 (01/11/19-15/03/20), before the UK population were instructed to reduce social contact, and Period 2 (16/03/20-30/04/21), following this. Periods 1 and 2 were compared for population variables, management and outcomes. Secondary analyses compared outcomes by primary treatment (medical/needle aspiration/surgical).

Results: 286 cases met criteria (median 4 per site, range 0-24). 9.4 cases were recorded per week in period 1 versus 2.0 in period 2, with no winter increase in cases in December 2020-Febraury 2021. Patient age differed between periods 1 and 2 (3.2 vs 4.7 years respectively, $p < 0.001$). 85% of children in period 2 were tested for COVID-19 with a single positive test. In period, 2 cases associated with P. aeruginosa significantly increased. 48.6% of children were scanned in period 1 vs 41.1% in period 2. Surgical management was used more frequently in period 1 (43.0% vs 24.3%, $p = 0.001$). Treatment success was high, with failure of initial management in 6.3%, and 30-day re-admission for recurrence in 2.1%. The adverse event rate (15.7% overall) did not vary by treatment modality or between periods 1& 2.

Conclusion: The COVID-19 pandemic led to a significant change in the presentation and case mix of acute paediatric mastoiditis in the UK.

Keywords: COVID; acute mastoiditis; antibiotic; paediatric; surgery.

© 2021 John Wiley & Sons Ltd.

- 25 references
- 3 figures

Supplementary info

Publication types, MeSH terms, Grant support Expand

Publication types

- Observational Study

MeSH terms

- Acute Disease
- Adolescent
- COVID-19 / epidemiology*
- Child
- Child, Preschool
- Female
- Humans
- Incidence
- Infant
- Male
- Mastoiditis / epidemiology*
- Pandemics
- Prospective Studies
- Retrospective Studies
- SARS-CoV-2
- Seasons
- United Kingdom / epidemiology

Grant support

- NA/The British Association for Paediatric Otolaryngology
- NA/ENTUK (The professional membership body for Ear Nose and Throat surgery in the UK)
- NA/The British Society of Otology
- NA/The North West ENT Research Fund

Full text links

WILEY Full Text Article Wiley Free PMC article
Proceed to details
Cite

Share

☐ 846

Observational Study

BMJ Open
. 2020 Dec 23;10(12):e044726.
doi: 10.1136/bmjopen-2020-044726.

Changes in demand for emergency ambulances during a nationwide lockdown that resulted in elimination of COVID-19: an observational study from New Zealand

Bridget Dicker [1] [2], Andrew Swain [3] [4], Verity Frances Todd [3] [2], Bronwyn Tunnage [3] [2], Emma McConachy [3], Haydn Drake [3], Michelle Brett [2], Dan Spearing [2], Graham John Howie [3] [2]

Affiliations Expand

Affiliations

- [1] Paramedicine Department, Auckland University of Technology, Auckland, New Zealand bridget.dicker@stjohn.org.nz.
- [2] Clinical Audit and Research, St John New Zealand, Auckland, New Zealand.
- [3] Paramedicine Department, Auckland University of Technology, Auckland, New Zealand.
- [4] Wellington Free Ambulance, Wellington, New Zealand.

- PMID: **33361171**
- PMCID: PMC7759754
- DOI: 10.1136/bmjopen-2020-044726

Free PMC article

Observational Study

Changes in demand for emergency ambulances during a nationwide lockdown that resulted in elimination of COVID-19: an observational study from New Zealand

Bridget Dicker et al. BMJ Open. 2020.

Free PMC article

Show details

BMJ Open
. 2020 Dec 23;10(12):e044726.
doi: 10.1136/bmjopen-2020-044726.

Authors

Bridget Dicker [1, 2], Andrew Swain [3, 4], Verity Frances Todd [3, 2], Bronwyn Tunnage [3, 2], Emma McConachy [3], Haydn Drake [3], Michelle Brett [2], Dan Spearing [2], Graham John Howie [3, 2]

Affiliations

- [1] Paramedicine Department, Auckland University of Technology, Auckland, New Zealand bridget.dicker@stjohn.org.nz.
- [2] Clinical Audit and Research, St John New Zealand, Auckland, New Zealand.
- [3] Paramedicine Department, Auckland University of Technology, Auckland, New Zealand.
- [4] Wellington Free Ambulance, Wellington, New Zealand.

- PMID: **33361171**
- PMCID: PMC7759754
- DOI: 10.1136/bmjopen-2020-044726

Abstract

Objective: To examine the impact of a 5-week national lockdown on ambulance service demand during the COVID-19 pandemic in New Zealand.

Design: A descriptive cross-sectional, observational study.

Setting: High-quality data from ambulance electronic clinical records, New Zealand.

Participants: Ambulance records were obtained from 588 690 attendances during pre-lockdown (prior to 17 February 2020) and from 36 238 records during the lockdown period (23 March to 26 April 2020).

Main outcome measures: Ambulance service utilisation during lockdown was compared with pre-lockdown: (a) descriptive analyses of ambulance events and proportions of event types for each period, (b) absolute rates of ambulance attendance (event types/week) for each period.

Results: During lockdown, ambulance patients were more likely to be attended at home and less likely to be aged between 16 and 25 years. There was a significant increase in the proportion of lower acuity patients (Status 3 and Status 4) attended ($p<0.001$) and a corresponding increase in patients not transported from scene ($p<0.001$). Road traffic crashes ($p<0.001$) and alcohol-related incidents ($p<0.001$) significantly decreased. There was a decrease in the absolute number of weekly ambulance attendances (ratio (95% CI), 0.89 (0.87 to 0.91), $p<0.001$), attendances to respiratory conditions (0.74 (0.61 to 0.86), $p=0.01$), and trauma (0.81 (0.77 to 0.85), $p<0.001$). However, there was a significant increase in ambulance attendances for mental health conditions (1.37 (1.22 to 1.51), $p=0.005$).

Conclusions: Despite the relative absence of COVID-19 in the community during the 5-week nationwide lockdown, there were significant differences in ambulance utilisation during this period. The lockdown was associated with an increase in ambulance attendances for mental health conditions and is of concern. In considering future lockdowns, the potential implications on a population's mental well-being will need to be seriously considered against the benefits of elimination of virus transmission.

Keywords: COVID-19; accident & emergency medicine; health policy; mental health; primary care; public health.

© Author(s) (or their employer(s)) 2020. Re-use permitted under CC BY-NC. No commercial re-use. See rights and permissions. Published by BMJ.

Conflict of interest statement

Competing interests: None declared.

- 28 references
- 2 figures

Supplementary info

Publication types, MeSH terms Expand

Publication types

- Multicenter Study
- Observational Study

MeSH terms

- Adolescent
- Adult
- Aged
- Ambulances / standards*
- COVID-19 / epidemiology
- COVID-19 / therapy*
- Child
- Child, Preschool
- Communicable Disease Control / methods*
- Cross-Sectional Studies
- Emergency Service, Hospital*
- Female
- Humans
- Infant
- Infant, Newborn
- Male
- Middle Aged
- New Zealand / epidemiology
- Pandemics / prevention & control*
- Patient Acuity
- Quarantine*

- Retrospective Studies
- SARS-CoV-2*
- Young Adult

Full text links

BMJ Free Full Text HighWire Free PMC article
Proceed to details
Cite
Share
847
Observational Study
J Rheumatol
. 2021 Jul;48(7):1098-1102.
doi: 10.3899/jrheum.200755. Epub 2021 Mar 15.

Risk of Severe COVID-19 Infection in Patients With Inflammatory Rheumatic Diseases

Javier Bachiller-Corral [1], Alina Boteanu [2], Maria Jesus Garcia-Villanueva [3], Carlos de la Puente [3], Marcelino Revenga [4], M Consuelo Diaz-Miguel [3], Ana Rodriguez-Garcia [3], Jose Luis Morell-Hita [3], Marta Valero [3], Carmen Larena [3], Maria Blazquez-Cañamero [3], Carlos A Guillen-Astete [3], Sandra Garrote [3], Cristina Sobrino [3], Carmen Medina-Quiñones [3], Mónica Vazquez-Diaz [2]
Affiliations Expand

Affiliations

- [1] J. Bachiller-Corral, MD, Assistant Head, A. Boteanu, MD, Assistant Head, M. Vazquez-Diaz, MD, Department Head, Department of Rheumatology, Hospital Universitario Ramón y Cajal, and Irycis (Instituto Ramón y Cajal de investigación sanitaria), Madrid; fbachiller@salud.madrid.org.
- [2] J. Bachiller-Corral, MD, Assistant Head, A. Boteanu, MD, Assistant Head, M. Vazquez-Diaz, MD, Department Head, Department of Rheumatology, Hospital Universitario Ramón y Cajal, and Irycis (Instituto Ramón y Cajal de investigación sanitaria), Madrid.
- [3] M.J. Garcia-Villanueva, MD, Assistant Head, C. de la Puente, MD, Assistant Head, M.C. Diaz-Miguel, MD, Assistant Head, A. Rodriguez-Garcia, MD, Assistant Head, J.L. Morell-Hita, MD, Assistant Head, M. Valero, MD, Assistant Head, C. Larena, MD, Assistant Head, M. Blazquez-Cañamero, MD, Assistant Head, C.A. Guillen-Astete, MD, PhD, Assistant Head, S. Garrote, MD, Assistant Head, C. Sobrino, MD, Assistant Head, C. Medina-Quiñones, MD, Assistant Head, Department of Rheumatology, Hospital Universitario Ramón y Cajal, Madrid.
- [4] M. Revenga, MD, PhD, Department of Rheumatology, Hospital Universitario Ramón y Cajal, Madrid, and Facultad de Medicina. Universidad de Alcalá, Alcalá de Henares, Spain.

- PMID: **33722949**
- DOI: 10.3899/jrheum.200755

Free article
Observational Study

Risk of Severe COVID-19 Infection in Patients With Inflammatory Rheumatic Diseases

Javier Bachiller-Corral et al. J Rheumatol. 2021 Jul.
Free article
Show details
J Rheumatol
. 2021 Jul;48(7):1098-1102.
doi: 10.3899/jrheum.200755. Epub 2021 Mar 15.

Authors

Javier Bachiller-Corral [1], Alina Boteanu [2], Maria Jesus Garcia-Villanueva [3], Carlos de la Puente [3], Marcelino Revenga [4], M Consuelo Diaz-Miguel [3], Ana Rodriguez-Garcia [3], Jose Luis Morell-Hita [3], Marta Valero [3], Carmen Larena [3], Maria Blazquez-Cañamero [3], Carlos A Guillen-Astete [3], Sandra Garrote [3], Cristina Sobrino [3], Carmen Medina-Quiñones [3], Mónica Vazquez-Diaz [2]

Affiliations

- [1] J. Bachiller-Corral, MD, Assistant Head, A. Boteanu, MD, Assistant Head, M. Vazquez-Diaz, MD, Department Head, Department of Rheumatology, Hospital Universitario Ramón y Cajal, and Irycis (Instituto Ramón y Cajal de investigación sanitaria), Madrid; fbachiller@salud.madrid.org.
- [2] J. Bachiller-Corral, MD, Assistant Head, A. Boteanu, MD, Assistant Head, M. Vazquez-Diaz, MD, Department Head, Department of Rheumatology, Hospital Universitario Ramón y Cajal, and Irycis (Instituto Ramón y Cajal de investigación sanitaria), Madrid.
- [3] M.J. Garcia-Villanueva, MD, Assistant Head, C. de la Puente, MD, Assistant Head, M.C. Diaz-Miguel, MD, Assistant Head, A. Rodriguez-Garcia, MD, Assistant Head, J.L. Morell-Hita, MD, Assistant Head, M. Valero, MD, Assistant Head, C. Larena, MD, Assistant Head, M. Blazquez-Cañamero, MD, Assistant Head, C.A. Guillen-Astete, MD, PhD, Assistant Head, S. Garrote, MD, Assistant Head, C. Sobrino, MD, Assistant Head, C. Medina-Quiñones, MD, Assistant Head, Department of Rheumatology, Hospital Universitario Ramón y Cajal, Madrid.
- [4] M. Revenga, MD, PhD, Department of Rheumatology, Hospital Universitario Ramón y Cajal, Madrid, and Facultad de Medicina. Universidad de Alcalá, Alcalá de Henares, Spain.

- PMID: **33722949**
- DOI: 10.3899/jrheum.200755

Abstract

Objective: To describe the cohort of patients with inflammatory rheumatic diseases (IRD) hospitalized due to SARS-CoV-2 infection in the Ramón y Cajal Hospital, and to determine the increased risk of severe coronavirus disease 2019 (COVID-19) in patients with no IRD.

Methods: This is a retrospective single-center observational study of patients with IRD actively monitored in the Department of Rheumatology who were hospitalized due to COVID-19.

Results: Forty-one (1.8%) out of 2315 patients admitted due to severe SARS-CoV-2 pneumonia suffered from an IRD. The admission OR for patients with IRD was 1.91 against the general population, and it was considerably higher in patients with Sjögren syndrome, vasculitis, and systemic lupus erythematosus. Twenty-seven patients were receiving treatment for IRD with corticosteroids, 23 with conventional DMARDs, 12 with biologics (7 rituximab [RTX], 4 anti-tumor necrosis factor [anti-TNF], and 1 abatacept), and 1 with Janus kinase inhibitors. Ten deaths were registered among patients with IRD. A higher hospitalization rate and a higher number of deaths were observed in patients treated with RTX (OR 12.9) but not in patients treated with anti-TNF (OR 0.9).

Conclusion: Patients with IRD, especially autoimmune diseases and patients treated with RTX, may be at higher risk of severe pneumonia due to SARS-CoV-2 compared to the general population. More studies are needed to analyze this association further in order to help manage these patients during the pandemic.

Keywords: autoimmune diseases; biologic therapy; infection; rheumatic diseases; tumor necrosis factor inhibitor.

Copyright © 2021 by the Journal of Rheumatology.

Supplementary info

Publication types, MeSH terms, Substances Expand

Publication types

- Observational Study

MeSH terms

- COVID-19* / diagnosis
- Humans
- Retrospective Studies
- Rheumatic Diseases* / complications
- Rheumatic Diseases* / drug therapy
- Risk Factors
- Tumor Necrosis Factor Inhibitors / therapeutic use

Substances

- Tumor Necrosis Factor Inhibitors

Full text links

HighWire
Proceed to details
Cite
Share
848
Observational Study
Blood Purif
. 2021;50(4-5):531-538.
doi: 10.1159/000510557. Epub 2020 Dec 22.

SARS-CoV-2 Infection in a Spanish Cohort of CKD-5D Patients: Prevalence, Clinical Presentation, Outcomes, and De-Isolation Results

José Jesús Broseta[1], Diana Rodríguez-Espinosa[2], Elena Cuadrado[2], Elena Guillén-Olmos[2], Evelyn Hermida[2], Enrique Montagud-Marrahi[2], Lida Rodas[2], Manel Vera[2], Néstor Fontseré[2], Marta Arias[2], Aleix Cases[2], Francisco Maduell[2]
Affiliations Expand

Affiliations

- [1] Department of Nephrology and Renal Transplantation, Hospital Clínic of Barcelona, Barcelona, Spain, jjbroseta@clinic.cat.
- [2] Department of Nephrology and Renal Transplantation, Hospital Clínic of Barcelona, Barcelona, Spain.

- PMID: **33352569**
- DOI: 10.1159/000510557

Observational Study

SARS-CoV-2 Infection in a Spanish Cohort of CKD-5D Patients: Prevalence, Clinical

Presentation, Outcomes, and De-Isolation Results

José Jesús Broseta et al. Blood Purif. 2021.
Show details
Blood Purif
. 2021;50(4-5):531-538.
doi: 10.1159/000510557. Epub 2020 Dec 22.

Authors

José Jesús Broseta [1], Diana Rodríguez-Espinosa [2], Elena Cuadrado [2], Elena Guillén-Olmos [2], Evelyn Hermida [2], Enrique Montagud-Marrahi [2], Lida Rodas [2], Manel Vera [2], Néstor Fontseré [2], Marta Arias [2], Aleix Cases [2], Francisco Maduell [2]

Affiliations

- [1] Department of Nephrology and Renal Transplantation, Hospital Clínic of Barcelona, Barcelona, Spain, jjbroseta@clinic.cat.
- [2] Department of Nephrology and Renal Transplantation, Hospital Clínic of Barcelona, Barcelona, Spain.

- PMID: **33352569**
- DOI: 10.1159/000510557

Abstract

Introduction: COVID-19 is a highly contagious disease that has easily spread worldwide. Outpatient maintenance hemodialysis seems to entail an increased risk of contagion, and previous reports inform of increased mortality among this population.

Methods: We retrospectively analyzed clinical and laboratory parameters, outcomes, and management once discharged of CKD-5D patients infected with SARS-CoV-2 from our health area.

Results: Out of the 429 CKD-5D population, 36 were diagnosed with SARS-CoV-2 infection (8%): 34 on in-center hemodialysis and 2 on peritoneal dialysis. Five were asymptomatic. The most common symptom was fever (70%), followed by dyspnea and cough. History of cardiovascular disease and elevation of LDH and C-reactive protein during admission were associated with higher mortality. Thirteen patients died (36%), 8 patients were admitted to an ICU, and survival was low (38%) among the latter. The mean time to death was 12 days. Most discharged patients got negative rRT-PCR in nasopharyngeal swabs within 26 days of diagnosis. However, there is a portion of cured patients that continue to have positive results even more than 2 months after the initial presentation.

Conclusions: Patients on dialysis have an increased mortality risk if infected with SARS-CoV-2. Preventive measures have proven useful. Thus, proper ones, such as universal screening of the population and isolation when required, need to be generalized. Better de-isolation criteria are necessary to ensure an appropriate use of public health resources.

Keywords: COVID-19; De-isolation; Hemodialysis; Peritoneal dialysis; SARS-CoV-2.

© 2020 S. Karger AG, Basel.

Supplementary info

Publication types, MeSH terms Expand

Publication types

- Observational Study

MeSH terms

- Aftercare
- Aged
- Aged, 80 and over
- Asymptomatic Infections / epidemiology
- COVID-19 / diagnosis
- COVID-19 / epidemiology*
- COVID-19 / prevention & control
- COVID-19 / transmission
- COVID-19 Testing
- Cardiovascular Diseases / epidemiology
- Comorbidity
- Diabetes Mellitus / epidemiology
- Dyslipidemias / epidemiology
- Female
- Fever / etiology
- Hospital Mortality
- Humans
- Immunocompromised Host
- Male
- Middle Aged
- Patient Isolation*
- Peritoneal Dialysis
- Prevalence
- Prognosis
- Renal Dialysis
- Renal Insufficiency, Chronic / epidemiology*
- Renal Insufficiency, Chronic / therapy
- SARS-CoV-2*
- Severity of Illness Index

- Smoking / epidemiology
- Spain / epidemiology
- Survivors

Full text links

Karger Final Version S. Karger AG, Basel, Switzerland
Proceed to details
Cite
Share
849
Observational Study
Am J Emerg Med
. 2021 Oct;48:238-242.
doi: 10.1016/j.ajem.2021.05.014. Epub 2021 May 6.

Influence of post-COVID-19 deconfinement on psychiatric visits to the emergency department

Julien Flament [1], Nathan Scius [2], Nicolas Zdanowicz [3], Maxime Regnier [4], Louis De Cannière [2], Henri Thonon [2]
Affiliations Expand

Affiliations

- [1] Emergency Department, CHU UCL Namur, Yvoir, Belgium. Electronic address: julien.flament@uclouvain.be.
- [2] Emergency Department, CHU UCL Namur, Yvoir, Belgium.
- [3] Psychopathology and Psychosomatic Unit, CHU UCL Namur, Yvoir, Belgium.
- [4] Statistician, from the Scientific Support Unit of the CHU UCL Namur, Yvoir, Belgium.

- PMID: **33991973**
- PMCID: PMC8101003
- DOI: 10.1016/j.ajem.2021.05.014

Free PMC article
Observational Study

Influence of post-COVID-19 deconfinement on psychiatric visits to the emergency department

Julien Flament et al. Am J Emerg Med. 2021 Oct.
Free PMC article
Show details
Am J Emerg Med
. 2021 Oct;48:238-242.
doi: 10.1016/j.ajem.2021.05.014. Epub 2021 May 6.

Authors

Julien Flament [1], Nathan Scius [2], Nicolas Zdanowicz [3], Maxime Regnier [4], Louis De Cannière [2], Henri Thonon [2]

Affiliations

- [1] Emergency Department, CHU UCL Namur, Yvoir, Belgium. Electronic address: julien.flament@uclouvain.be.
- [2] Emergency Department, CHU UCL Namur, Yvoir, Belgium.
- [3] Psychopathology and Psychosomatic Unit, CHU UCL Namur, Yvoir, Belgium.
- [4] Statistician, from the Scientific Support Unit of the CHU UCL Namur, Yvoir, Belgium.

- PMID: **33991973**
- PMCID: PMC8101003
- DOI: 10.1016/j.ajem.2021.05.014

Abstract

Objective: During the deconfinement period after the coronavirus disease-2019 (COVID-19) pandemic, the number and characteristics of psychiatric visits changed in our emergency department (ED). We aimed to assess changes in the number of visits and characterize the profiles of these patients.

Methods: In this retrospective observational study, we examined the number of psychiatric ED visits and their proportion among the total number of ED visits. We also evaluated psychiatric visits characteristics during a one-month period after the declaration of deconfinement, and we compared those characteristics to characteristics observed during the same month over the previous 4 years.

Results: The number of psychiatric visits to our emergency department during deconfinement was similar to the number observed in the same month of previous years. However, the proportion of psychiatric visits to our emergency department among all visits to the ED rose during deconfinement to a level never before observed. The mean proportion of psychiatric admissions to all ED admissions rose from 3.5% in past years to 5.3% during deconfinement ($p = 0.013$). Moreover, during deconfinement, more visits (80%) were without an acute intoxication compared

to past years (58.5%; p = 0.031). Also, in the deconfinement period, more visits lacked a follow-up consultation organized at discharge (40%) compared to the historical period (25%, p = 0.036).

Conclusions: The deconfinement period after the first wave COVID-19 changed the number and type of psychiatric emergency medicine consultations at our hospital, suggesting a psychiatric impact of confinement during this pandemic. These findings will be of interest to practitioners and politicians in the coming months.

Keywords: COVID-19; Deconfinement; Emergency; Psychiatric.

Copyright © 2021 The Authors. Published by Elsevier Inc. All rights reserved.

Conflict of interest statement

Declaration of Competing Interest The authors have no competing interests to declare.

- 21 references
- 2 figures

Supplementary info

Publication types, MeSH terms Expand

Publication types

- Observational Study

MeSH terms

- Adult
- Aftercare
- Alcoholic Intoxication / epidemiology
- Anxiety / epidemiology*
- Belgium / epidemiology
- COVID-19*
- Communicable Disease Control*
- Depression / epidemiology*
- Emergencies
- Emergency Service, Hospital / statistics & numerical data*
- Female
- Humans
- Male
- Mental Disorders / epidemiology*
- Personality Disorders / epidemiology
- Public Policy*
- Retrospective Studies

- SARS-CoV-2
- Substance-Related Disorders / epidemiology
- Suicide, Attempted / statistics & numerical data*

Full text links

ELSEVIER OPEN ACCESS Elsevier Science Free PMC article
Proceed to details
Cite
Share
☐ 850
Observational Study
Crit Care
. 2020 Sep 18;24(1):561.
doi: 10.1186/s13054-020-03260-3.

Thrombotic and haemorrhagic complications in critically ill patients with COVID-19: a multicentre observational study

Akshay Shah [1, 2], Killian Donovan [3], Anna McHugh [4], Manish Pandey [5], Louise Aaron [3], Charlotte A Bradbury [6], Simon J Stanworth [7, 8], Raza Alikhan [9], Stephen Von Kier [10], Keith Maher [10], Nicola Curry [8, 11], Susan Shapiro [8, 11], Matthew J Rowland [3, 12], Matt Thomas [4], Richard Mason [4], Matthew Holland [4], Tom Holmes [5], Michael Ware [5], Stefan Gurney [13], Stuart R McKechnie [3]
Affiliations Expand

Affiliations

- [1] Radcliffe Department of Medicine, Level 4 Academic Block, University of Oxford, John Radcliffe Hospital, Headley Way, Oxford, OX3 9DU, UK. akshay.shah@linacre.ox.ac.uk.
- [2] Adult Intensive Care Unit, John Radcliffe Hospital, Oxford University Hospitals NHS Foundation Trust, Oxford, UK. akshay.shah@linacre.ox.ac.uk.
- [3] Adult Intensive Care Unit, John Radcliffe Hospital, Oxford University Hospitals NHS Foundation Trust, Oxford, UK.
- [4] Intensive Care Unit, North Bristol NHS Trust, Bristol, UK.
- [5] Adult Intensive Care Unit, University Hospital of Wales, Cardiff, Wales, UK.
- [6] Faculty of Health Sciences, University of Bristol, Bristol, UK.
- [7] Radcliffe Department of Medicine, Level 4 Academic Block, University of Oxford, John Radcliffe Hospital, Headley Way, Oxford, OX3 9DU, UK.
- [8] Haematology Theme, NIHR Oxford Biomedical Research Centre, Oxford, UK.
- [9] Haemostasis and Thrombosis, Department of Haematology, University Hospital of Wales, Cardiff, UK.

- [10] Blood Management and Conservation Service, Oxford University Hospitals NHS Foundation Trust, Oxford, UK.
- [11] Oxford Haemophilia & Thrombosis Centre, Department of Haematology, Churchill Hospital, Oxford University Hospitals NHS Foundation, Oxford, UK.
- [12] Kadoorie Centre for Critical Care Research, Nuffield Department of Clinical Neurosciences, University of Oxford, Oxford, UK.
- [13] Intensive Care Unit, Bristol Royal Infirmary, University Hospitals Bristol NHS Trust, Bristol, UK.

- PMID: **32948243**
- PMCID: PMC7499016
- DOI: 10.1186/s13054-020-03260-3

Free PMC article
Observational Study

Thrombotic and haemorrhagic complications in critically ill patients with COVID-19: a multicentre observational study

Akshay Shah et al. Crit Care. 2020.
Free PMC article
Show details
Crit Care
. 2020 Sep 18;24(1):561.
doi: 10.1186/s13054-020-03260-3.

Authors

Akshay Shah [1] [2], Killian Donovan [3], Anna McHugh [4], Manish Pandey [5], Louise Aaron [3], Charlotte A Bradbury [6], Simon J Stanworth [7] [8], Raza Alikhan [9], Stephen Von Kier [10], Keith Maher [10], Nicola Curry [8] [11], Susan Shapiro [8] [11], Matthew J Rowland [3] [12], Matt Thomas [4], Richard Mason [4], Matthew Holland [4], Tom Holmes [5], Michael Ware [5], Stefan Gurney [13], Stuart R McKechnie [3]

Affiliations

- [1] Radcliffe Department of Medicine, Level 4 Academic Block, University of Oxford, John Radcliffe Hospital, Headley Way, Oxford, OX3 9DU, UK. akshay.shah@linacre.ox.ac.uk.
- [2] Adult Intensive Care Unit, John Radcliffe Hospital, Oxford University Hospitals NHS Foundation Trust, Oxford, UK. akshay.shah@linacre.ox.ac.uk.
- [3] Adult Intensive Care Unit, John Radcliffe Hospital, Oxford University Hospitals NHS Foundation Trust, Oxford, UK.
- [4] Intensive Care Unit, North Bristol NHS Trust, Bristol, UK.
- [5] Adult Intensive Care Unit, University Hospital of Wales, Cardiff, Wales, UK.
- [6] Faculty of Health Sciences, University of Bristol, Bristol, UK.

- [7] Radcliffe Department of Medicine, Level 4 Academic Block, University of Oxford, John Radcliffe Hospital, Headley Way, Oxford, OX3 9DU, UK.
- [8] Haematology Theme, NIHR Oxford Biomedical Research Centre, Oxford, UK.
- [9] Haemostasis and Thrombosis, Department of Haematology, University Hospital of Wales, Cardiff, UK.
- [10] Blood Management and Conservation Service, Oxford University Hospitals NHS Foundation Trust, Oxford, UK.
- [11] Oxford Haemophilia & Thrombosis Centre, Department of Haematology, Churchill Hospital, Oxford University Hospitals NHS Foundation, Oxford, UK.
- [12] Kadoorie Centre for Critical Care Research, Nuffield Department of Clinical Neurosciences, University of Oxford, Oxford, UK.
- [13] Intensive Care Unit, Bristol Royal Infirmary, University Hospitals Bristol NHS Trust, Bristol, UK.

- PMID: **32948243**
- PMCID: PMC7499016
- DOI: 10.1186/s13054-020-03260-3

Abstract

Background: Optimal prophylactic and therapeutic management of thromboembolic disease in patients with COVID-19 remains a major challenge for clinicians. The aim of this study was to define the incidence of thrombotic and haemorrhagic complications in critically ill patients with COVID-19. In addition, we sought to characterise coagulation profiles using thromboelastography and explore possible biological differences between patients with and without thrombotic complications.

Methods: We conducted a multicentre retrospective observational study evaluating all the COVID-19 patients received in four intensive care units (ICUs) of four tertiary hospitals in the UK between March 15, 2020, and May 05, 2020. Clinical characteristics, laboratory data, thromboelastography profiles and clinical outcome data were evaluated between patients with and without thrombotic complications.

Results: A total of 187 patients were included. Their median (interquartile (IQR)) age was 57 (49-64) years and 124 (66.3%) patients were male. Eighty-one (43.3%) patients experienced one or more clinically relevant thrombotic complications, which were mainly pulmonary emboli (n = 42 (22.5%)). Arterial embolic complications were reported in 25 (13.3%) patients. ICU length of stay was longer in patients with thrombotic complications when compared with those without. Fifteen (8.0%) patients experienced haemorrhagic complications, of which nine (4.8%) were classified as major bleeding. Thromboelastography demonstrated a hypercoagulable profile in patients tested but lacked discriminatory value between those with and without thrombotic complications. Patients who experienced thrombotic complications had higher D-dimer, ferritin, troponin and white cell count levels at ICU admission compared with those that did not.

Conclusion: Critically ill patients with COVID-19 experience high rates of venous and arterial thrombotic complications. The rates of bleeding may be higher than previously reported and re-iterate the need for randomised trials to better understand the risk-benefit ratio of different anticoagulation strategies.

Keywords: COVID-19; Haemorrhage; Heparin; Thrombosis.

Conflict of interest statement

None.

- 40 references
- 1 figure

Supplementary info

Publication types, MeSH terms Expand

Publication types

- Multicenter Study
- Observational Study
- Research Support, Non-U.S. Gov't

MeSH terms

- Betacoronavirus
- COVID-19
- Coronavirus Infections / complications*
- Coronavirus Infections / therapy
- Critical Illness*
- Female
- Hemorrhage / etiology*
- Hemorrhage / therapy
- Humans
- Intensive Care Units
- Male
- Middle Aged
- Pandemics
- Pneumonia, Viral / complications*
- Pneumonia, Viral / therapy
- Retrospective Studies
- SARS-CoV-2
- Thrombelastography
- Thrombosis / etiology*
- Thrombosis / therapy
- United Kingdom

Full text links

Read free full text at BMC BioMed Central Free PMC article
Proceed to details
Cite
Share
851

Observational Study

J Cyst Fibros
. 2021 Jul;20(4):566-577.
doi: 10.1016/j.jcf.2021.03.017. Epub 2021 Apr 18.

Incidence of SARS-CoV-2 in people with cystic fibrosis in Europe between February and June 2020

Lutz Naehrlich [1], Annalisa Orenti [2], Fiona Dunlevy [3], Irena Kasmi [4], Satenik Harutyunyan [5], Andreas Pfleger [6], Svetlana Keegan [7], Géraldine Daneau [8], Guergana Petrova [9], Duška Tješić-Drinković [10], Panayiotis Yiallouros [11], Alena Bilkova [12], Hanne Vebert Olesen [13], Pierre-Régis Burgel [14], Tsitsino Parulava [15], Filia Diamantea [16], Andrea Párniczky [17], Edward F McKone [18], Meir Mei-Zahav [19], Marco Salvatore [20], Carla Colombo [21], Elina Aleksejeva [22], Kestutis Malakauskas [23], Marc Schlesser [24], Stojka Fustik [25], Oxana Turcu [26], Domenique Zomer-van Ommen [27], Anita Senstad Wathne [28], Łukasz Woźniacki [29], Luísa Pereira [30], Liviu Pop [31], Nataliya Kashirskaya [32], Milan Rodić [33], Hana Kayserova [34], Uro Krivecs [35], Pedro Mondejar-Lopez [36], Isabelle de Monestrol [37], Deniz Dogru [38], Halyna Makukh [39], Rebecca Cosgriff [40], Silke van Koningsbruggen-Rietschel [41], Andreas Jung [42], European Cystic Fibrosis COVID project group

Collaborators, Affiliations Expand

Collaborators

- **European Cystic Fibrosis COVID project group**: Vladimir Bobrovnichy [7], Ivan Bambir [10], Andrea Dugac Vukic [10], Pavel Drevinek [43], Milan Macek Jr [44], Harriet Corvol [45], Lydie Lemonnier-Videau [46], Elpis Hatziagorou [47], Godfrey Fletcher [48], Rita Padoan [49], Vincent Gulmans [27], Egil Bakkeheim [28], Elena Kondratyeva [50], Elena Amelina [28], Elena Zhekaite [51], Olga Simonova [52], Maria Dolores Pastor-Vivero [53], Anders Lindblad [54], Yasemin Gökdemir [55], Sevgi Pekcan [56], Keith Brownlee [40], Elliott McClenaghan [40], Siobhán Carr [8], Elise Lammertyn [57], Anna Zolin [2], Alice Fox [58], Marko Krasnyk [58], Jacqui Van Rens [58]

Affiliations

- [1] Universities of Giessen and Marburg Lung Center, German Center of Lung Research, Justus-Liebig-University Giessen, Giessen, Germany.
- [2] Department of Clinical Sciences and Community Health, Laboratory of Medical Statistics, Epidemiology and Biometry G. A. Maccacaro, University of Milan, Milan, Italy.

- [3] European Cystic Fibrosis Society, Karup, Denmark.
- [4] "Mother Thereza" Hospital Center, Department of Paediatrics, Tirana, Albania.
- [5] Yerevan University CF Centre, Muratsan Hospital, Yerevan, Armenia.
- [6] Department of Pediatrics and Adolescent Medicine, Division of Pediatric Pulmonology and Allergology, Medical University of Graz, Graz, Austria.
- [7] Belarusian Republic Children's Center of Pulmonology and Cystic Fibrosis, Pulmonary Department, 3rd City Children's Clinical Hospital, Minsk, Belarus.
- [8] Sciensano, Epidemiology and public health, Health services research, Brussels, Belgium.
- [9] Alexandrovska University Hospital, Pediatric Clinic, Sofia, Bulgaria.
- [10] University Hospital Centre Zagreb, Cystic Fibrosis Centre - Paediatrics and Adults, Zagreb, Croatia.
- [11] Medical School, University of Cyprus, Nicosia, Cyprus.
- [12] Cystic Fibrosis Registry of the Czech Republic, Prague, Czech Republic.
- [13] Department of Pediatrics and Adolescent Medicine, Cystic Fibrosis Center, Aarhus University Hospital, Aarhus, Denmark.
- [14] Respiratory Medicine and National Cystic Fibrosis Reference Center, Cochin Hospital, Assistance Publique-Hôpitaux de Paris, Université de Paris, Institut Cochin, INSERM U1016, Paris, France.
- [15] I. Tsitsishvili Children's Clinic, CF Centre, Tblisi, Georgia.
- [16] Sismanoglio General Hospital of Attica, Adult Cystic Fibrosis Unit, Athens, Greece.
- [17] Heim Pál National Pediatric Institute, Budapest, Hungary; Institute for Translational Medicine, University of Pécs, Medical School, Pécs, Hungary.
- [18] St. Vincent's University Hospital & University College Dublin School of Medicine, Dublin, Ireland.
- [19] Pulmonary Institute, Schneider Children's Medical Center of Israel, Petah Tikva, Israel; Sackler Faculty of Medicine, Tel Aviv University, Tel Aviv, Israel.
- [20] Undiagnosed Rare Diseases Interdepartmental Unit, National Center Rare Diseases, Istituto Superiore di Sanità, Rome, Italy.
- [21] Cystic Fibrosis Regional Reference Center, Fondazione IRCCS Ca' Granda Ospedale Maggiore Policlinico, University of Milan, Department of Pathophysiology and Transplantation, Milan, Italy.
- [22] Department of Pneumology, Children's Clinical University Hospital, Rīga Stradinš University, Riga, Latvia.
- [23] Adult Cystic Fibrosis center, Department of Pulmonology, Lithuanian University of Health Sciences, Kaunas, Lithuania.
- [24] Department of Pulmonology, Hôpital Robert Schuman, Luxembourg, Luxembourg.
- [25] Centre for Cystic Fibrosis, University Children's Hospital, Skopje, North Macedonia.
- [26] Ambulatory Cystic Fibrosis and Other Rare Diseases Center, Institute for Maternal and Child Healthcare, State University of Medicine and Pharmacy "Nicolae Testemitanu", Department of Pediatrics, Chisinau, Republic of Moldova.
- [27] Dutch Cystic Fibrosis Foundation (NCFS), Baarn, The Netherlands.
- [28] Norwegian Resource Centre for Cystic Fibrosis, Oslo University Hospital, Oslo, Norway.
- [29] Dziekanow Paediatric Hospital, Cystic Fibrosis Centre, Institute of Mother and Child, Warsaw, Poland.
- [30] Centre for Cystic Fibrosis, Hospital de Santa Maria, Lisbon, Portugal.
- [31] Victor Babes University of Medicine and Pharmacy Timisoara, National Cystic Fibrosis Centre Timisoara, Romania.

- [32] Laboratory of genetic epidemiology, "Research Centre for Medical Genetics", Moscow, Russian Federation.
- [33] National Centre for Cystic Fibrosis, Mother and Child Health Institute of Serbia "Dr Vukan Čupić", Belgrade, Serbia.
- [34] Cystic Fibrosis Centre, University Hospital of Bratislava, Bratislava, Slovakia.
- [35] Department of Paediatric Pulmonology, University Children's Hospital, Ljubljana University Medical Centre, Ljubljana, Slovenia.
- [36] Pediatric Pulmonology and Cystic Fibrosis Unit, Virgen de la Arrixaca Clinic University Hospital, Murcia, Spain.
- [37] Stockholm CF centre, Karolinska University Hospital, Karolinska Institutet, Stockholm, Sweden.
- [38] Cystic Fibrosis Registry of Turkey, Ankara, Turkey.
- [39] Institute of Hereditary Pathology Ukrainian National Academy of Medical Sciences, Lviv, Ukraine.
- [40] Cystic Fibrosis Trust, London, UK.
- [41] CF-Center, University of Cologne, Faculty of Medicine, Cologne, Germany.
- [42] Paediatric Pulmonology, University Children`s Hospital Zurich, Zurich, Switzerland.
- [43] Department of Medical Microbiology, Second Faculty of Medicine, Charles University and Motol University Hospital, Prague, Czech Republic.
- [44] Department of Biology and Medical Genetics, Second Faculty of Medicine, Charles University and Motol University Hospital, Prague, Czech Republic.
- [45] Sorbonne Université, Centre de Recherche Saint-Antoine, Inserm UMR_S938, Assistance Publique-Hôpitaux de Paris, Hôpital Trousseau, Pediatric Pulmonology Department and Cystic Fibrosis Center, Paris, France.
- [46] Vaincre la Mucoviscidose, Paris, France.
- [47] Cystic Fibrosis Unit, Hippokration General Hospital, Aristotle University of Thessaloniki, Thessaloniki, Greece.
- [48] The Cystic Fibrosis Registry of Ireland, Dublin, Ireland.
- [49] Cystic Fibrosis Regional Support Centre, Department of Paediatrics, University of Brescia, Brescia, Italy.
- [50] Clinical research department of cystic fibrosis "Research Centre for Medical Genetics", Moscow, Russian Federation.
- [51] Cystic Fibrosis Department, Pulmonology Research Institute of the Federal Medical and Biological Agency of Russia, Moscow, Russian Federation.
- [52] Center for rare disease National Medical Research Center for Children's Health, Moscow, Russia; Morozov State Pediatric Teaching Hospital, Moscow Healthcare Department; I.M. Sechenov First Moscow State Medical University (Sechenov University), Healthcare Ministry of Russia, Moscow, Russian Federation.
- [53] Pediatric Pneumology and Cystic Fibrosis Unit, Osakidetza, Hospital Universitario Cruces, Bizkaia, Spain.
- [54] Gothenburg CF Centre, Queen Silvia Children's Hospital, The Sahlgrenska Academy at the University of Gothenburg, Gothenburg, Sweden.
- [55] Division of Pulmonology, Marmara University Faculty of Medicine, Istanbul, Turkey.
- [56] Division of Pediatric Pulmonolgy, Meram Faculty of Medicine, Necmettin Erbakan University, Konya, Turkey.
- [57] Cystic Fibrosis Europe, Brussels; Association Muco A.S.B.L. - Mucovereniging V.Z.W., Brussels, Belgium.
- [58] European Cystic Fibrosis Society Patient Registry, Karup, Denmark.

- PMID: **34016559**
- PMCID: PMC8053246
- DOI: 10.1016/j.jcf.2021.03.017

Free PMC article
Observational Study

Incidence of SARS-CoV-2 in people with cystic fibrosis in Europe between February and June 2020

Lutz Naehrlich et al. J Cyst Fibros. 2021 Jul.
Free PMC article
Show details
J Cyst Fibros
. 2021 Jul;20(4):566-577.
doi: 10.1016/j.jcf.2021.03.017. Epub 2021 Apr 18.

Authors

Lutz Naehrlich [1], Annalisa Orenti [2], Fiona Dunlevy [3], Irena Kasmi [4], Satenik Harutyunyan [5], Andreas Pfleger [6], Svetlana Keegan [7], Géraldine Daneau [8], Guergana Petrova [9], Duška Tješić-Drinković [10], Panayiotis Yiallouros [11], Alena Bilkova [12], Hanne Vebert Olesen [13], Pierre-Régis Burgel [14], Tsitsino Parulava [15], Filia Diamantea [16], Andrea Párniczky [17], Edward F McKone [18], Meir Mei-Zahav [19], Marco Salvatore [20], Carla Colombo [21], Elina Aleksejeva [22], Kestutis Malakauskas [23], Marc Schlesser [24], Stojka Fustik [25], Oxana Turcu [26], Domenique Zomer-van Ommen [27], Anita Senstad Wathne [28], Łukasz Woźniacki [29], Luísa Pereira [30], Liviu Pop [31], Nataliya Kashirskaya [32], Milan Rodić [33], Hana Kayserova [34], Uro Krivecs [35], Pedro Mondejar-Lopez [36], Isabelle de Monestrol [37], Deniz Dogru [38], Halyna Makukh [39], Rebecca Cosgriff [40], Silke van Koningsbruggen-Rietschel [41], Andreas Jung [42], European Cystic Fibrosis COVID project group

Collaborators

- **European Cystic Fibrosis COVID project group**: Vladimir Bobrovnichy [7], Ivan Bambir [10], Andrea Dugac Vukic [10], Pavel Drevinek [43], Milan Macek Jr [44], Harriet Corvol [45], Lydie Lemonnier-Videau [46], Elpis Hatziagorou [47], Godfrey Fletcher [48], Rita Padoan [49], Vincent Gulmans [27], Egil Bakkeheim [28], Elena Kondratyeva [50], Elena Amelina [28], Elena Zhekaite [51], Olga Simonova [52], Maria Dolores Pastor-Vivero [53], Anders Lindblad [54], Yasemin Gökdemir [55], Sevgi Pekcan [56], Keith Brownlee [40], Elliott McClenaghan [40], Siobhán Carr [8], Elise Lammertyn [57], Anna Zolin [2], Alice Fox [58], Marko Krasnyk [58], Jacqui Van Rens [58]

Affiliations

- [1] Universities of Giessen and Marburg Lung Center, German Center of Lung Research, Justus-Liebig-University Giessen, Giessen, Germany.
- [2] Department of Clinical Sciences and Community Health, Laboratory of Medical Statistics, Epidemiology and Biometry G. A. Maccacaro, University of Milan, Milan, Italy.
- [3] European Cystic Fibrosis Society, Karup, Denmark.
- [4] "Mother Thereza" Hospital Center, Department of Paediatrics, Tirana, Albania.
- [5] Yerevan University CF Centre, Muratsan Hospital, Yerevan, Armenia.
- [6] Department of Pediatrics and Adolescent Medicine, Division of Pediatric Pulmonology and Allergology, Medical University of Graz, Graz, Austria.
- [7] Belarusian Republic Children's Center of Pulmonology and Cystic Fibrosis, Pulmonary Department, 3rd City Children's Clinical Hospital, Minsk, Belarus.
- [8] Sciensano, Epidemiology and public health, Health services research, Brussels, Belgium.
- [9] Alexandrovska University Hospital, Pediatric Clinic, Sofia, Bulgaria.
- [10] University Hospital Centre Zagreb, Cystic Fibrosis Centre - Paediatrics and Adults, Zagreb, Croatia.
- [11] Medical School, University of Cyprus, Nicosia, Cyprus.
- [12] Cystic Fibrosis Registry of the Czech Republic, Prague, Czech Republic.
- [13] Department of Pediatrics and Adolescent Medicine, Cystic Fibrosis Center, Aarhus University Hospital, Aarhus, Denmark.
- [14] Respiratory Medicine and National Cystic Fibrosis Reference Center, Cochin Hospital, Assistance Publique-Hôpitaux de Paris, Université de Paris, Institut Cochin, INSERM U1016, Paris, France.
- [15] I. Tsitsishvili Children's Clinic, CF Centre, Tblisi, Georgia.
- [16] Sismanoglio General Hospital of Attica, Adult Cystic Fibrosis Unit, Athens, Greece.
- [17] Heim Pál National Pediatric Institute, Budapest, Hungary; Institute for Translational Medicine, University of Pécs, Medical School, Pécs, Hungary.
- [18] St. Vincent's University Hospital & University College Dublin School of Medicine, Dublin, Ireland.
- [19] Pulmonary Institute, Schneider Children's Medical Center of Israel, Petah Tikva, Israel; Sackler Faculty of Medicine, Tel Aviv University, Tel Aviv, Israel.
- [20] Undiagnosed Rare Diseases Interdepartmental Unit, National Center Rare Diseases, Istituto Superiore di Sanità, Rome, Italy.
- [21] Cystic Fibrosis Regional Reference Center, Fondazione IRCCS Ca' Granda Ospedale Maggiore Policlinico, University of Milan, Department of Pathophysiology and Transplantation, Milan, Italy.
- [22] Department of Pneumology, Children's Clinical University Hospital, Rīga Stradinš University, Riga, Latvia.
- [23] Adult Cystic Fibrosis center, Department of Pulmonology, Lithuanian University of Health Sciences, Kaunas, Lithuania.
- [24] Department of Pulmonology, Hôpital Robert Schuman, Luxembourg, Luxembourg.
- [25] Centre for Cystic Fibrosis, University Children's Hospital, Skopje, North Macedonia.
- [26] Ambulatory Cystic Fibrosis and Other Rare Diseases Center, Institute for Maternal and Child Healthcare, State University of Medicine and Pharmacy "Nicolae Testemitanu", Department of Pediatrics, Chisinau, Republic of Moldova.
- [27] Dutch Cystic Fibrosis Foundation (NCFS), Baarn, The Netherlands.
- [28] Norwegian Resource Centre for Cystic Fibrosis, Oslo University Hospital, Oslo, Norway.
- [29] Dziekanow Paediatric Hospital, Cystic Fibrosis Centre, Institute of Mother and Child, Warsaw, Poland.

- [30] Centre for Cystic Fibrosis, Hospital de Santa Maria, Lisbon, Portugal.
- [31] Victor Babes University of Medicine and Pharmacy Timisoara, National Cystic Fibrosis Centre Timisoara, Romania.
- [32] Laboratory of genetic epidemiology, "Research Centre for Medical Genetics", Moscow, Russian Federation.
- [33] National Centre for Cystic Fibrosis, Mother and Child Health Institute of Serbia "Dr Vukan Čupić", Belgrade, Serbia.
- [34] Cystic Fibrosis Centre, University Hospital of Bratislava, Bratislava, Slovakia.
- [35] Department of Paediatric Pulmonology, University Children's Hospital, Ljubljana University Medical Centre, Ljubljana, Slovenia.
- [36] Pediatric Pulmonology and Cystic Fibrosis Unit, Virgen de la Arrixaca Clinic University Hospital, Murcia, Spain.
- [37] Stockholm CF centre, Karolinska University Hospital, Karolinska Institutet, Stockholm, Sweden.
- [38] Cystic Fibrosis Registry of Turkey, Ankara, Turkey.
- [39] Institute of Hereditary Pathology Ukrainian National Academy of Medical Sciences, Lviv, Ukraine.
- [40] Cystic Fibrosis Trust, London, UK.
- [41] CF-Center, University of Cologne, Faculty of Medicine, Cologne, Germany.
- [42] Paediatric Pulmonology, University Children`s Hospital Zurich, Zurich, Switzerland.
- [43] Department of Medical Microbiology, Second Faculty of Medicine, Charles University and Motol University Hospital, Prague, Czech Republic.
- [44] Department of Biology and Medical Genetics, Second Faculty of Medicine, Charles University and Motol University Hospital, Prague, Czech Republic.
- [45] Sorbonne Université, Centre de Recherche Saint-Antoine, Inserm UMR_S938, Assistance Publique-Hôpitaux de Paris, Hôpital Trousseau, Pediatric Pulmonology Department and Cystic Fibrosis Center, Paris, France.
- [46] Vaincre la Mucoviscidose, Paris, France.
- [47] Cystic Fibrosis Unit, Hippokration General Hospital, Aristotle University of Thessaloniki, Thessaloniki, Greece.
- [48] The Cystic Fibrosis Registry of Ireland, Dublin, Ireland.
- [49] Cystic Fibrosis Regional Support Centre, Department of Paediatrics, University of Brescia, Brescia, Italy.
- [50] Clinical research department of cystic fibrosis "Research Centre for Medical Genetics", Moscow, Russian Federation.
- [51] Cystic Fibrosis Department, Pulmonology Research Institute of the Federal Medical and Biological Agency of Russia, Moscow, Russian Federation.
- [52] Center for rare disease National Medical Research Center for Children's Health, Moscow, Russia; Morozov State Pediatric Teaching Hospital, Moscow Healthcare Department; I.M. Sechenov First Moscow State Medical University (Sechenov University), Healthcare Ministry of Russia, Moscow, Russian Federation.
- [53] Pediatric Pneumology and Cystic Fibrosis Unit, Osakidetza, Hospital Universitario Cruces, Bizkaia, Spain.
- [54] Gothenburg CF Centre, Queen Silvia Children's Hospital, The Sahlgrenska Academy at the University of Gothenburg, Gothenburg, Sweden.
- [55] Division of Pulmonology, Marmara University Faculty of Medicine, Istanbul, Turkey.
- [56] Division of Pediatric Pulmonolgy, Meram Faculty of Medicine, Necmettin Erbakan University, Konya, Turkey.

- [57] Cystic Fibrosis Europe, Brussels; Association Muco A.S.B.L. - Mucovereniging V.Z.W., Brussels, Belgium.
- [58] European Cystic Fibrosis Society Patient Registry, Karup, Denmark.

- PMID: **34016559**
- PMCID: PMC8053246
- DOI: 10.1016/j.jcf.2021.03.017

Abstract

Background: Viral infections can cause significant morbidity in cystic fibrosis (CF). The current Severe Acute Respiratory Syndrome Coronavirus 2 (SARS-CoV-2) pandemic could therefore have a serious impact on the health of people with CF (pwCF).

Methods: We used the 38-country European Cystic Fibrosis Society Patient Registry (ECFSPR) to collect case data about pwCF and SARS-CoV-2 infection.

Results: Up to 30 June 2020, 16 countries reported 130 SARS-CoV-2 cases in people with CF, yielding an incidence of 2.70/1000 pwCF. Incidence was higher in lung-transplanted patients (n=23) versus non-transplanted patients (n=107) (8.43 versus 2.36 cases/1000). Incidence was higher in pwCF versus the age-matched general population in the age groups <15, 15-24, and 25-49 years (p<0.001), with similar trends for pwCF with and without lung transplant. Compared to the general population, pwCF (regardless of transplantation status) had significantly higher rates of admission to hospital for all age groups with available data, and higher rates of intensive care, although not statistically significant. Most pwCF recovered (96.2%), however 5 died, of whom 3 were lung transplant recipients. The case fatality rate for pwCF (3.85%, 95% CI: 1.26-8.75) was non-significantly lower than that of the general population (7.46%; p=0.133).

Conclusions: SARS-CoV-2 infection can result in severe illness and death for pwCF, even for younger patients and especially for lung transplant recipients. PwCF should continue to shield from infection and should be prioritized for vaccination.

Keywords: Covid-19; Cystic fibrosis; Epidemiology; Europe; Incidence; SARS-CoV-2.

Copyright © 2021. Published by Elsevier B.V.

Conflict of interest statement

Declaration of Competing Interest Dr. Naehrlich reports that he has received institutional fees for site participation in clinical trials from Vertex Pharmaceuticals and Boehringer Ingelheim; Dr. Orenti has nothing to disclose; Dr. Dunlevy reports institutional grants from Chiesi, during the conduct of the study; Dr. Kasmi has nothing to disclose; Dr. Harutyunyan has nothing to disclose; Dr. Pfleger has nothing to disclose; Dr. Bobrovnichy has nothing to disclose;Dr. Keegan has nothing to disclose; Dr. Daneau has nothing to disclose; Dr. Petrova has nothing to disclose; Dr. Bambir has nothing to disclose; Dr. Vukić Dugac has nothing to disclose; Dr. Tješić-Drinković has nothing to disclose; Dr. Yiallouros has nothing to disclose; Dr. Drevinek reports personal fees from Vertex Pharmaceuticals, outside the submitted work; Prof. Milan Macek reports grants from Vertex Pharmaceuticals, outside the submitted workr r; Mrs. Bilkova has nothing to disclose; Dr. Olesen has nothing to disclose; Dr. Burgel reports personal fees from Astra-Zeneca, personal fees from Boehringer Ingelheim, personal fees from Chiesi, personal fees from GSK, personal fees from Insmed, personal fees from Novartis, personal fees from Pfizer, grants and personal fees from Vertex, personal fees from Zambon, outside the submitted work; Dr. Corvol has nothing to

disclose; Ms. Lemmonier has nothing to disclose; Dr. Parulava has nothing to disclose; Dr. Hatziagorou has nothing to disclose; Dr. Diamantea has nothing to disclose; Dr. Párniczky has nothing to disclose; G. Fletcher has nothing to disclose; Prof. McKone reports travel support from A Menarini, speaker fees from Roche Pharmaceuticals, consultancy fees from Insmed, consultancy fees from Janssen Pharmaceuticals, grants to institution and consultancy fees from Vertex, outside the submitted work; Dr. Mei-Zahav has nothing to disclose; Dr. Padoan has nothing to disclose; Dr. Salvatore has nothing to disclose; Dr. Colombo has nothing to disclose; Dr. Aleksejeva has nothing to disclose; Dr. Malakauskas has nothing to disclose; Dr. Schlesser has nothing to disclose; Dr. Fustik has nothing to disclose; Dr. Turcu has nothing to disclose; V. Gulmans has nothing to disclose; D. Zomer-van Ommen has nothing to disclose; Dr. Wathne has nothing to disclose; Dr. Bakkeheim has nothing to disclose; Dr. Wozniacki has nothing to disclose; Dr. Pereira has nothing to disclose; Dr. Pop has nothing to disclose; Dr. Kondratyeva has nothing to disclose; Dr. Amelina has nothing to disclose; Dr. Zhekaite has nothing to disclose; Dr. O. Simonova has nothing to disclose; Dr. Kashirskaya has nothing to disclose; Dr. Rodic has nothing to disclose; Dr. Kayserova has nothing to disclose; Dr. Krivec has nothing to disclose; Dr. Mondejar-Lopez has nothing to disclose; Dr. Pastor-Vivero has nothing to disclose; Dr. de Monestrol reports grants from Vertex, outside the submitted work; Dr. Lindblad has nothing to disclose; Dr. Dogru has nothing to disclose; Dr. Gokdemir has nothing to disclose; Dr. Pekcan has nothing to disclose; Dr. Makukh has nothing to disclose; Dr. Brownlee has nothing to disclose; Ms. Cosgriff has nothing to disclose; Mr. McClenaghan has nothing to disclose; Dr. Carr reports personal fees from Chiesi Pharmaceuticals, personal fees and non-financial support from Vertex, personal fees from Zambon, personal fees from Insmed, outside the submitted work; Dr. Lammertyn has nothing to disclose; Dr. Zolin has nothing to disclose; Ms.. Fox reports grants from ECFS, during the conduct of the study; Mr Krasnyk has nothing to disclose; Mrs. Van Rens has nothing to disclose; Dr. van Koningsbruggen-Rietschel reports grants and personal fees from Algipharma (HORIZON2020), personal fees from Deutsches Zentrum für Infektionsforschung, personal fees from Antabio, personal fees from Proteostasis, personal fees from Roche, personal fees from Vertex, outside the submitted work; Dr. Jung reports grants from Chiesi Pharmaceuticals, during the conduct of the study.

- 33 references
- 3 figures

Supplementary info

Publication types, MeSH terms Expand

Publication types

- Observational Study

MeSH terms

- Adolescent
- Adult
- COVID-19 / diagnosis
- COVID-19 / epidemiology*
- COVID-19 / therapy
- Child

- Child, Preschool
- Critical Care
- Cystic Fibrosis / complications*
- Cystic Fibrosis / mortality
- Cystic Fibrosis / therapy
- Europe / epidemiology
- Female
- Hospitalization
- Humans
- Incidence
- Infant
- Infant, Newborn
- Lung Transplantation
- Male
- Middle Aged
- Registries
- Retrospective Studies
- Young Adult

Full text links

ELSEVIER OPEN ACCESS Elsevier Science Free PMC article
Proceed to details
Cite
Share
☐ 852
Observational Study
J Thromb Thrombolysis
. 2021 Nov;52(4):1061-1067.
doi: 10.1007/s11239-021-02477-5. Epub 2021 May 8.

The characteristics and outcomes of critically Ill patients with COVID-19 who received systemic thrombolysis for presumed pulmonary embolism: an observational study

Matsuo So [1], David J Steiger [2], Mai Takahashi [1], Natalia N Egorova [3], Toshiki Kuno [4]
Affiliations Expand

Affiliations

- [1] Department of Medicine, Icahn School of Medicine at Mount Sinai, Mount Sinai Beth Israel, First Avenue, 16th Street, New York City, NY, 10003, USA.
- [2] Department of Pulmonary, Critical Care and Sleep Medicine, Icahn School of Medicine at Mount Sinai, Mount Sinai Beth Israel, New York City, NY, USA.
- [3] Department of Population Health Science and Policy, Icahn School of Medicine at Mount Sinai, New York City, NY, USA.
- [4] Department of Medicine, Icahn School of Medicine at Mount Sinai, Mount Sinai Beth Israel, First Avenue, 16th Street, New York City, NY, 10003, USA. toshiki.kuno@mountsinai.org.

- PMID: **33966157**
- PMCID: PMC8106515
- DOI: 10.1007/s11239-021-02477-5

Free PMC article
Observational Study

The characteristics and outcomes of critically Ill patients with COVID-19 who received systemic thrombolysis for presumed pulmonary embolism: an observational study

Matsuo So et al. J Thromb Thrombolysis. 2021 Nov.
Free PMC article
Show details
J Thromb Thrombolysis
. 2021 Nov;52(4):1061-1067.
doi: 10.1007/s11239-021-02477-5. Epub 2021 May 8.

Authors

Matsuo So [1], David J Steiger [2], Mai Takahashi [1], Natalia N Egorova [3], Toshiki Kuno [4]

Affiliations

- [1] Department of Medicine, Icahn School of Medicine at Mount Sinai, Mount Sinai Beth Israel, First Avenue, 16th Street, New York City, NY, 10003, USA.
- [2] Department of Pulmonary, Critical Care and Sleep Medicine, Icahn School of Medicine at Mount Sinai, Mount Sinai Beth Israel, New York City, NY, USA.
- [3] Department of Population Health Science and Policy, Icahn School of Medicine at Mount Sinai, New York City, NY, USA.
- [4] Department of Medicine, Icahn School of Medicine at Mount Sinai, Mount Sinai Beth Israel, First Avenue, 16th Street, New York City, NY, 10003, USA. toshiki.kuno@mountsinai.org.

- PMID: **33966157**

- PMCID: PMC8106515
- DOI: 10.1007/s11239-021-02477-5

Abstract

Coronavirus disease 2019 (COVID-19) is associated with abnormal hemostasis, autopsy evidence of systemic microthrombosis, and a high prevalence of venous thromboembolic disease. Tissue plasminogen activator (tPA) has been used in patients with critically ill COVID-19 with high clinical suspicion of pulmonary embolism (PE). A retrospective cohort study of 6095 hospitalized COVID-19 patients at 5 acute care hospitals in New York was conducted. 57 patients received tPA for presumed PE during March 10th to April 27th. The mean age was 60.8 ± 10.8 years, and 71.9% (41/57) were male. We defined strongly suspected PE among 75.4% (43/57) of patients who had acute worsening of hypoxia and acute hypotension requiring pressors. The findings suggestive of PE included right ventricular (RV) strain in 15.8% (9/57), deep venous thrombosis (DVT) in 7.0% (4/57), increased dead space ventilation (Vd) in 31.6% (18/57) of patients, respectively. RV strain and RV thrombus were present in 3.5% (2/57), RV strain and DVT in 5.3% (3/57), RV strain and increased Vd in 8.8% (5/57), and DVT and increased Vd in 3.5% (2/57) of patients. Chest CT Angiography was not performed in any of the patients. Following tPA infusion, 49.1% (28/57) of patients demonstrated improvement. Six patients (10.5%) survived to discharge, of whom 2 received extracorporeal membrane oxygenation and were transferred to other facilities for lung transplant, 2 were discharged home, and 2 were discharged to a rehabilitation facility. However, overall mortality was 89.5%. The utility of tPA for critically ill patients with COVID-19 and presumed PE warrants further studies.

Keywords: COVID-19; Pulmonary embolism; Respiratory insufficiency; Shock; Tissue plasminogen activator.

© 2021. The Author(s), under exclusive licence to Springer Science+Business Media, LLC, part of Springer Nature.

Conflict of interest statement

None of the authors have conflict of interest.

- 36 references

Supplementary info

Publication types, MeSH terms, Substances Expand

Publication types

- Multicenter Study
- Observational Study

MeSH terms

- Aged
- COVID-19* / complications

- COVID-19* / mortality
- Critical Illness
- Female
- Humans
- Male
- Middle Aged
- New York City
- Pulmonary Embolism* / drug therapy
- Retrospective Studies
- Thrombolytic Therapy*
- Thrombosis* / drug therapy
- Tissue Plasminogen Activator

Substances

- Tissue Plasminogen Activator

Full text links

Springer Free PMC article
Proceed to details
Cite
Share
853
Observational Study
PLoS One
. 2021 Aug 12;16(8):e0256116.
doi: 10.1371/journal.pone.0256116. eCollection 2021.

Efficacy of a four-tier infection response system in the emergency department during the coronavirus disease-2019 outbreak

Arom Choi [1], Ha Yan Kim [2], Ara Cho [1], Jiyoung Noh [3], Incheol Park [1], Hyun Soo Chung [1] [3]
Affiliations Expand

Affiliations

- [1] Department of Emergency Medicine, Yonsei University College of Medicine, Seoul, South Korea.
- [2] Department of Biomedical Systems Informatics, Biostatistics Collaboration Unit, Yonsei University College of Medicine, Seoul, South Korea.

- [3] Center for Disaster Relief, Training, and Research, Severance Hospital, Yonsei University College of Medicine, Seoul, South Korea.

- PMID: **34383840**
- PMCID: PMC8360518
- DOI: 10.1371/journal.pone.0256116

Free PMC article
Observational Study

Efficacy of a four-tier infection response system in the emergency department during the coronavirus disease-2019 outbreak

Arom Choi et al. PLoS One. 2021.
Free PMC article

Show details

PLoS One

. 2021 Aug 12;16(8):e0256116.
doi: 10.1371/journal.pone.0256116. eCollection 2021.

Authors

Arom Choi [1], Ha Yan Kim [2], Ara Cho [1], Jiyoung Noh [3], Incheol Park [1], Hyun Soo Chung [1] [3]

Affiliations

- [1] Department of Emergency Medicine, Yonsei University College of Medicine, Seoul, South Korea.
- [2] Department of Biomedical Systems Informatics, Biostatistics Collaboration Unit, Yonsei University College of Medicine, Seoul, South Korea.
- [3] Center for Disaster Relief, Training, and Research, Severance Hospital, Yonsei University College of Medicine, Seoul, South Korea.

- PMID: **34383840**
- PMCID: PMC8360518
- DOI: 10.1371/journal.pone.0256116

Abstract

Introduction: The coronavirus disease (COVID-19) pandemic has delayed the management of other serious medical conditions. This study presents an efficient method to prevent the degradation of the quality of diagnosis and treatment of other critical diseases during the pandemic.

Methods: We performed a retrospective observational study. The primary outcome was ED length of stay (ED LOS). The secondary outcomes were the door-to-balloon time in patients with suspected ST-segment elevation myocardial infarction and door-to-brain computed tomography

time for patients with suspected stroke. The outcome measures were compared between patients who were treated in the red and orange zones designated as the changeable isolation unit and those who were treated in the non-isolation care unit. To control confounding factors, we performed propensity score matching, following which, outcomes were analyzed for non-inferiority.

Results: The mean ED LOS for hospitalized patients in the isolation and non-isolation care units were 406.5 min (standard deviation [SD], 237.9) and 360.2 min (SD, 226.4), respectively. The mean difference between the groups indicated non-inferiority of the isolation care unit ($p = 0.037$) but not in the patients discharged from the ED ($p>0.999$). The mean difference in the ED LOS for patients admitted to the ICU between the isolation and non-isolation care units was -22.0 min ($p = 0.009$). The mean difference in the door-to-brain computed tomography time between patients with suspected stroke in the isolation and non-isolation care units was 7.4 min for those with confirmed stroke ($p = 0.013$), and -20.1 min for those who were discharged ($p = 0.012$). The mean difference in the door-to-balloon time between patients who underwent coronary angiography in the isolation and non-isolation care units was -2.1 min ($p<0.001$).

Conclusions: Appropriate and efficient handling of a properly planned ED plays a key role in improving the quality of medical care for other critical diseases during the COVID-19 outbreak.

Conflict of interest statement

The authors have declared that no competing interests exist.

- 38 references
- 4 figures

Supplementary info

Publication types, MeSH terms, Grant support Expand

Publication types

- Observational Study

MeSH terms

- Adult
- Aged
- Aged, 80 and over
- COVID-19*
- Disease Outbreaks
- Emergency Service, Hospital / organization & administration*
- Female
- Humans
- Length of Stay*
- Male
- Middle Aged
- Myocardial Infarction / diagnosis*

- Myocardial Infarction / therapy
- Retrospective Studies
- Stroke / diagnostic imaging*
- Stroke / therapy

Grant support

The author(s) received no specific funding for this work.

Full text links

OPEN ACCESS TO FULL TEXT PLOS ONE Public Library of Science Free PMC article
Proceed to details
Cite
Share
☐ 854
Observational Study
Hypertension
. 2021 Mar 3;77(3):856-867.
doi: 10.1161/HYPERTENSIONAHA.120.16563. Epub 2020 Dec 30.

Impact of Arterial Stiffness on All-Cause Mortality in Patients Hospitalized With COVID-19 in Spain

Enrique Rodilla [1] [2], Maria Dolores López-Carmona [3], Xavi Cortes [1] [2], Lidia Cobos-Palacios [3], Sergio Canales [1] [2], Maria Carmen Sáez [1] [2], Samara Campos Escudero [4], Manuel Rubio-Rivas [5], Jesus Díez Manglano [6], Santiago J Freire Castro [7], Nuria Vázquez Piqueras [8], Elisabeth Mateo Sanchis [9], Paula Maria Pesqueira Fontan [10], Jeffrey Oskar Magallanes Gamboa [11], Andrés González García [12] [13], Victor Madrid Romero [14], Lara Tamargo Chamorro [15], Julio González Moraleja [16], Javier Villanueva Martínez [17], Amara González Noya [18], Ana Suárez-Lombraña [19], Anyuli Gracia Gutiérrez [12] [13], Manuel Lorenzo López Reboiro [20], José Manuel Ramos Rincón [21], Ricardo Gómez Huelgas [3], SEMI-COVID-19 Network
Affiliations Expand

Affiliations

- [1] From the Internal Medicine Department, Hypertension and Vascular Risk Unit, Sagunto University Hospital, Sagunto (Valencia), Spain (E.R., X.C., S.C., M.C.S.).
- [2] Department of Medicine, Universidad Cardenal Herrera-CEU, CEU Universities, Valencia, Spain (E.R., X.C., S.C., M.C.S.).
- [3] Internal Medicine Department, Regional University Hospital of Málaga, Biomedical Research Institute of Málaga (IBIMA), University of Málaga (UMA), Spain (M.D.L.-C., L.C.-P., R.G.H.).

- [4] Internal Medicine Department, 12 de Octubre University Hospital, Madrid, Spain (S.C.E.).
- [5] Internal Medicine Department, Bellvitge University Hospital-IDIBELL, L'Hospitalet de Llobregat (Barcelona), Spain (M.R.-R.).
- [6] Internal Medicine Department, Royo Villanova Hospital, Zaragoza, Spain (J.D.M.).
- [7] Internal Medicine Department, A Coruña University Hospital, Spain (S.J.F.C.).
- [8] Internal Medicine Department, Consorci Sanitari Integral, Moisès Broggi Hospital Sant Joan Despí (Barcelona), Spain (N.V.P.).
- [9] Internal Medicine Department, Dr. Peset University Hospital, Valencia, Spain (E.M.S.).
- [10] Internal Medicine Department, Santiago Clinical Hospital, Santiago de Compostela (A Coruña), Spain (P.M.P.F.).
- [11] Internal Medicine Department, Nuestra Señora del Prado Hospital, Talavera de la Reina (Toledo), Spain (J.O.M.G.).
- [12] Internal Medicine Department, Ramón y Cajal University Hospital, Madrid, Spain (A.G.G.).
- [13] Internal Medicine Department, Defensa General Hospital, Zaragoza, Spain (A.G.G.).
- [14] Internal Medicine Department, Zamora Hospital Complex, Spain (V.M.R.).
- [15] Internal Medicine Department, Cabueñes Hospital, Gijón (Asturias), Spain (L.T.C.).
- [16] Internal Medicine Department, Virgen de la Salud Hospital, Toledo, Spain (J.G.M.).
- [17] Internal Medicine Department, Infanta Cristina University Hospital, Parla (Madrid), Spain (J.V.M.).
- [18] Internal Medicine Department, Ourense University Hospital Complex, Ourense, Spain (A.G.N.).
- [19] Internal Medicine Department, Platón Hospital, Barcelona, Spain (A.S.-L.).
- [20] Internal Medicine Department, Monforte de Lemos Hospital, Spain (M.L.L.R.).
- [21] Department of Clinical Medicine, Miguel Hernandez University of Elche, Alicante, Spain (J.M.R.R.).

- PMID: **33377393**
- PMCID: PMC7884247
- DOI: 10.1161/HYPERTENSIONAHA.120.16563

Free PMC article
Observational Study

Impact of Arterial Stiffness on All-Cause Mortality in Patients Hospitalized With COVID-19 in Spain

Enrique Rodilla et al. Hypertension. 2021.
Free PMC article
Show details
Hypertension

. 2021 Mar 3;77(3):856-867.
doi: 10.1161/HYPERTENSIONAHA.120.16563. Epub 2020 Dec 30.

Authors

Enrique Rodilla [1] [2], Maria Dolores López-Carmona [3], Xavi Cortes [1] [2], Lidia Cobos-Palacios [3], Sergio Canales [1] [2], Maria Carmen Sáez [1] [2], Samara Campos Escudero [4], Manuel Rubio-Rivas [5], Jesus Díez Manglano [6], Santiago J Freire Castro [7], Nuria Vázquez Piqueras [8], Elisabeth Mateo Sanchis [9], Paula Maria Pesqueira Fontan [10], Jeffrey Oskar Magallanes Gamboa [11], Andrés González García [12] [13], Victor Madrid Romero [14], Lara Tamargo Chamorro [15], Julio González Moraleja [16], Javier Villanueva Martínez [17], Amara González Noya [18], Ana Suárez-Lombraña [19], Anyuli Gracia Gutiérrez [12] [13], Manuel Lorenzo López Reboiro [20], José Manuel Ramos Rincón [21], Ricardo Gómez Huelgas [3], SEMI-COVID-19 Network

Affiliations

- [1] From the Internal Medicine Department, Hypertension and Vascular Risk Unit, Sagunto University Hospital, Sagunto (Valencia), Spain (E.R., X.C., S.C., M.C.S.).
- [2] Department of Medicine, Universidad Cardenal Herrera-CEU, CEU Universities, Valencia, Spain (E.R., X.C., S.C., M.C.S.).
- [3] Internal Medicine Department, Regional University Hospital of Málaga, Biomedical Research Institute of Málaga (IBIMA), University of Málaga (UMA), Spain (M.D.L.-C., L.C.-P., R.G.H.).
- [4] Internal Medicine Department, 12 de Octubre University Hospital, Madrid, Spain (S.C.E.).
- [5] Internal Medicine Department, Bellvitge University Hospital-IDIBELL, L'Hospitalet de Llobregat (Barcelona), Spain (M.R.-R.).
- [6] Internal Medicine Department, Royo Villanova Hospital, Zaragoza, Spain (J.D.M.).
- [7] Internal Medicine Department, A Coruña University Hospital, Spain (S.J.F.C.).
- [8] Internal Medicine Department, Consorci Sanitari Integral, Moisès Broggi Hospital Sant Joan Despí (Barcelona), Spain (N.V.P.).
- [9] Internal Medicine Department, Dr. Peset University Hospital, Valencia, Spain (E.M.S.).
- [10] Internal Medicine Department, Santiago Clinical Hospital, Santiago de Compostela (A Coruña), Spain (P.M.P.F.).
- [11] Internal Medicine Department, Nuestra Señora del Prado Hospital, Talavera de la Reina (Toledo), Spain (J.O.M.G.).
- [12] Internal Medicine Department, Ramón y Cajal University Hospital, Madrid, Spain (A.G.G.).
- [13] Internal Medicine Department, Defensa General Hospital, Zaragoza, Spain (A.G.G.).
- [14] Internal Medicine Department, Zamora Hospital Complex, Spain (V.M.R.).
- [15] Internal Medicine Department, Cabueñes Hospital, Gijón (Asturias), Spain (L.T.C.).
- [16] Internal Medicine Department, Virgen de la Salud Hospital, Toledo, Spain (J.G.M.).
- [17] Internal Medicine Department, Infanta Cristina University Hospital, Parla (Madrid), Spain (J.V.M.).
- [18] Internal Medicine Department, Ourense University Hospital Complex, Ourense, Spain (A.G.N.).
- [19] Internal Medicine Department, Platón Hospital, Barcelona, Spain (A.S.-L.).
- [20] Internal Medicine Department, Monforte de Lemos Hospital, Spain (M.L.L.R.).
- [21] Department of Clinical Medicine, Miguel Hernandez University of Elche, Alicante, Spain (J.M.R.R.).

- PMID: **33377393**

- PMCID: PMC7884247
- DOI: 10.1161/HYPERTENSIONAHA.120.16563

Abstract

Older age and cardiovascular comorbidities are well-known risk factors for all-cause mortality in patients with coronavirus disease 2019 (COVID-19). Hypertension and age are the 2 principal determinants of arterial stiffness (AS). This study aimed to estimate AS in patients with COVID-19 requiring hospitalization and analyze its association with all-cause in-hospital mortality. This observational, retrospective, multicenter cohort study analyzed 12 170 patients admitted to 150 Spanish centers included in the SEMI-COVID-19 Network. We compared AS, defined as pulse pressure ≥60 mm Hg, and clinical characteristics between survivors and nonsurvivors. Mean age was 67.5 (±16.1) years and 42.5% were women. Overall, 2606 (21.4%) subjects died. Admission systolic blood pressure (BP) <120 and ≥140 mm Hg was a predictor of higher all-cause mortality (23.5% and 22.8%, respectively, *P*<0.001), compared with systolic BP between 120 and 140 mm Hg (18.6%). The 4379 patients with AS (36.0%) were older and had higher systolic and lower diastolic BP. Multivariate analysis showed that AS and systolic BP <120 mm Hg significantly and independently predicted all-cause in-hospital mortality (adjusted odds ratio [ORadj]: 1.27, *P*=0.0001; ORadj: 1.48, *P*=0.0001, respectively) after adjusting for sex (males, ORadj: 1.6, *P*=0.0001), age tertiles (second and third tertiles, ORadj: 2.0 and 4.7, *P*=0.0001), Charlson Comorbidity Index (second and third tertiles, ORadj: 4.8 and 8.6, *P*=0.0001), heart failure, and previous and in-hospital antihypertensive treatment. Our data show that AS and admission systolic BP <120 mm Hg had independent prognostic value for all-cause mortality in patients with COVID-19 requiring hospitalization.

Keywords: COVID-19; arterial stiffness; blood pressure; heart failure; hypertension; pulse pressure.

Conflict of interest statement

None.

- 42 references
- 3 figures

Supplementary info

Publication types, MeSH terms Expand

Publication types

- Multicenter Study
- Observational Study

MeSH terms

- Age Factors
- Aged
- Aged, 80 and over

- Blood Pressure
- COVID-19 / epidemiology*
- COVID-19 / mortality
- Cardiovascular Diseases / epidemiology
- Cause of Death
- Comorbidity
- Female
- Hospital Mortality*
- Humans
- Hypertension / epidemiology*
- Kaplan-Meier Estimate
- Male
- Middle Aged
- Obesity / epidemiology
- Odds Ratio
- Pandemics*
- Prognosis
- Pulmonary Disease, Chronic Obstructive / epidemiology
- Registries
- Retrospective Studies
- SARS-CoV-2*
- Spain / epidemiology
- Vascular Stiffness*

Full text links

FREE FULL TEXT Atypon Free PMC article
Proceed to details
Cite
Share
☐ 855
Observational Study
Saudi J Kidney Dis Transpl
. Mar-Apr 2021;32(2):468-480.
doi: 10.4103/1319-2442.335459.

Severe acute respiratory syndrome coronavirus 2 and risk of inhospital mortality among end-stage renal disease patients with rheumatoid arthritis: A scientific perspective

Ahmed M El-Malky [1], Yazeed Ali S Albalawi [2], Saif Marzouq Alanazi [2], Muflih Abdullah Saed Albalawi [2], Amal Naif Althobaiti [2], Zahraa Abbas A Kassarah [2], Hussam Ali I Alzahrani [2], Amal Sulaiman A Al-Balawi [2]
Affiliations Expand

Affiliations

- [1] Morbidity and Mortality Review Unit, King Saud University Medical City; Department of Public Health, Research Chair of Evidence-Based Healthcare and Knowledge Translation, College of Medicine, King Saud University, Riyadh, Saudi Arabia.
- [2] Department of Medicine, Faculty of Medicine, Tabuk University, Tabuk, Saudi Arabia.

- PMID: **35017341**
- DOI: 10.4103/1319-2442.335459

Free article
Observational Study

Severe acute respiratory syndrome coronavirus 2 and risk of inhospital mortality among end-stage renal disease patients with rheumatoid arthritis: A scientific perspective

Ahmed M El-Malky et al. Saudi J Kidney Dis Transpl. Mar-Apr 2021.
Free article
Show details
Saudi J Kidney Dis Transpl
. Mar-Apr 2021;32(2):468-480.
doi: 10.4103/1319-2442.335459.

Authors

Ahmed M El-Malky [1], Yazeed Ali S Albalawi [2], Saif Marzouq Alanazi [2], Muflih Abdullah Saed Albalawi [2], Amal Naif Althobaiti [2], Zahraa Abbas A Kassarah [2], Hussam Ali I Alzahrani [2], Amal Sulaiman A Al-Balawi [2]

Affiliations

- [1] Morbidity and Mortality Review Unit, King Saud University Medical City; Department of Public Health, Research Chair of Evidence-Based Healthcare and Knowledge Translation, College of Medicine, King Saud University, Riyadh, Saudi Arabia.
- [2] Department of Medicine, Faculty of Medicine, Tabuk University, Tabuk, Saudi Arabia.

- PMID: **35017341**
- DOI: 10.4103/1319-2442.335459

Abstract

According to the elevated infection mortality risks, the incidence of coronavirus disease 2019 (COVID-19) could be raised in rheumatoid arthritis patients with end-stage renal disease (ESRD). Our objectives are to describe the impact of COVID-19 infection on rheumatoid arthritis patients with end-stage renal disease and to identify the risk of in-hospital mortality, comorbid conditions. and the proper way to deal with this category. It was a retrospective analysis of COVID-19 patients in Saudi Arabia from March 1, 2020 to April 27, 2020 and from May 27, 2020 to August 20, 2020. Of 10,482 patients with COVID-19, 419 had ESRD. We assessed main (in-hospital death) outcomes and secondary (mechanical breathing and residence) outcomes. Patients with ESRD were aged and more comorbid disorders. Rheumatoid arthritis patients with ESRD were aged. ESRD rheumatoid arthritis patients have a higher hospital mortality risk relative to rheumatoid arthritis patients not getting complicated with ESRD (31.7% vs. 25.4%, chances 1.38, and 95% trust range 1.12-1.70). After population and comorbid conditions had changed, the rate of rise stayed the same (changed chances: 1.37, 1.09-1.73). In both the crude and modified study (1.62, 1.26-2.07; vs. 1.57, 1.22-2.02), chances for the period of stay of seven or more days have been higher inside a group than in the non-ESRD group. Old age, respiratory support, lymphopenia, and elevated blood urea nitrogen and low serum ferritin were the independent contributing factors for the in-hospital mortality of ESRD rheumatoid arthritis patients infected with severe acute respiratory syndrome coronavirus 2.

Supplementary info

Publication types, MeSH terms Expand

Publication types

- Observational Study

MeSH terms

- Adult
- Aged
- Aged, 80 and over
- Arthritis, Rheumatoid / complications*
- Arthritis, Rheumatoid / epidemiology
- COVID-19 / complications*
- COVID-19 / mortality

- China / epidemiology
- Female
- Hospital Mortality*
- Humans
- Kidney Failure, Chronic / complications*
- Kidney Failure, Chronic / mortality
- Kidney Failure, Chronic / therapy
- Male
- Middle Aged
- Renal Dialysis
- Retrospective Studies
- Risk Factors
- SARS-CoV-2

Full text links

Get Free Full Text Wolters Kluwer | Medknow Medknow Publications and Media Pvt Ltd
Proceed to details
Cite
Share
☐ 856
Observational Study
Int Immunopharmacol
. 2021 Sep;98:107891.
doi: 10.1016/j.intimp.2021.107891. Epub 2021 Jun 14.

A single center experience of intravenous immunoglobulin treatment in Covid-19

Ahmet Omma [1], Abdulsamet Erden [1], Berkan Armağan [1], Serdar Can Güven [2], Özlem Karakaş [1], Enes Seyda Şahiner [3], Deniz Erdem [4], Seval İzdeş [5], İhsan Ateş [6], Orhan Küçükşahin [7]
Affiliations Expand

Affiliations

- [1] Ministry of Health Ankara City Hospital, Clinic of Rheumatology, Ankara 06800, Turkey.
- [2] Ministry of Health Ankara City Hospital, Clinic of Rheumatology, Ankara 06800, Turkey. Electronic address: drserdarguven@gmail.com.
- [3] Ministry of Health Ankara City Hospital, Clinic of Internal Medicine, Ankara 06800, Turkey.
- [4] University of Health Sciences, School of Medicine, Ankara City Hospital, Department of Anesthesia and Resuscitation, Ankara 06800, Turkey.

- [5] Yıldırım Beyazıt University, School of Medicine, Department of Anesthesiology and Reanimation-Critical Care, Ankara 06800, Turkey.
- [6] University of Health Sciences, School of Medicine, Ankara City Hospital, Department of Internal Medicine, Ankara 06800, Turkey.
- [7] Yıldırım Beyazıt University, School of Medicine, Department of Internal Medicine, Division of Rheumatology, Ankara 06800, Turkey.

- PMID: **34153671**
- PMCID: PMC8200303
- DOI: 10.1016/j.intimp.2021.107891

Free PMC article
Observational Study

A single center experience of intravenous immunoglobulin treatment in Covid-19

Ahmet Omma et al. Int Immunopharmacol. 2021 Sep.
Free PMC article

Show details

Int Immunopharmacol
. 2021 Sep;98:107891.
doi: 10.1016/j.intimp.2021.107891. Epub 2021 Jun 14.

Authors

Ahmet Omma [1], Abdulsamet Erden [1], Berkan Armağan [1], Serdar Can Güven [2], Özlem Karakaş [1], Enes Seyda Şahiner [3], Deniz Erdem [4], Seval İzdeş [5], İhsan Ateş [6], Orhan Küçükşahin [7]

Affiliations

- [1] Ministry of Health Ankara City Hospital, Clinic of Rheumatology, Ankara 06800, Turkey.
- [2] Ministry of Health Ankara City Hospital, Clinic of Rheumatology, Ankara 06800, Turkey. Electronic address: drserdarguven@gmail.com.
- [3] Ministry of Health Ankara City Hospital, Clinic of Internal Medicine, Ankara 06800, Turkey.
- [4] University of Health Sciences, School of Medicine, Ankara City Hospital, Department of Anesthesia and Resuscitation, Ankara 06800, Turkey.
- [5] Yıldırım Beyazıt University, School of Medicine, Department of Anesthesiology and Reanimation-Critical Care, Ankara 06800, Turkey.
- [6] University of Health Sciences, School of Medicine, Ankara City Hospital, Department of Internal Medicine, Ankara 06800, Turkey.
- [7] Yıldırım Beyazıt University, School of Medicine, Department of Internal Medicine, Division of Rheumatology, Ankara 06800, Turkey.

- PMID: **34153671**
- PMCID: PMC8200303
- DOI: 10.1016/j.intimp.2021.107891

Abstract

Background: Intravenous immunoglobulins (IVIg) have been used in management of severe Covid-19. Here in this study, we report our single-center experience regarding IVIg treatment in management of severe Covid-19.

Materials and method: Among hospitalized adult Covid-19 patients between April 1 and December 31, 2020, patients with confirmed diagnosis of Covid-19 who had Brescia-COVID respiratory severity scale score ≥ 3, hyperinflammation and received IVIg treatment in addition to standard of care were retrospectively investigated. We grouped IVIg recipients into three according to reasons for IVIg administration: Group 1 patients requiring anti-inflammatory treatment but complicated with secondary infection and/or sepsis , group 2 patients with Covid-19 related complications including progressive disease refractory to other anti-inflammatory agents, myocarditis, adult multisystem inflammatory syndrome, hemophagocytic lymphohystiocytosis like syndrome and group 3 patients with other complications non-specific to Covid-19. Mortality and clinical data was compared among groups.

Results: A total of 46 IVIg recipients were enrolled. Group 1 comprised 17 (36.9%), group 2 comprised 18 (39.1%) and group 3 comprised 11 (23.9%) patients. No significant differences in means of age, gender and comorbidities were observed among groups. Mortality was significantly lower in group 3 when compared to group 1 (64.7% vs 18.2%, p = 0.016) and close to significance when compared to group 2 (50% vs 18.2% p = 0.087).

Conclusions: IVIg seemed to be used mostly in severe, refractory and complicated cases in our population. As a rescue agent in severe cases refractory to other anti-inflammatory strategies, 33.7% survival rate was observed with IVIg.

Keywords: Covid-19; Cytokine storm; Intravenous immunoglobulin; Mortality.

Copyright © 2021 Elsevier B.V. All rights reserved.

Conflict of interest statement

The authors declare that they have no known competing financial interests or personal relationships that could have appeared to influence the work reported in this paper.

- 43 references
- 1 figure

Supplementary info

Publication types, MeSH terms, Substances, Supplementary concepts Expand

Publication types

- Observational Study

MeSH terms

- Administration, Intravenous

- Adult
- Aged
- Aged, 80 and over
- COVID-19 / diagnosis
- COVID-19 / drug therapy*
- COVID-19 / mortality
- COVID-19 / virology
- Cross-Sectional Studies
- Female
- Humans
- Immunoglobulins, Intravenous / administration & dosage*
- Immunoglobulins, Intravenous / adverse effects
- Male
- Middle Aged
- Retrospective Studies
- Severity of Illness Index
- Time Factors
- Treatment Outcome
- Turkey

Substances

- Immunoglobulins, Intravenous

Supplementary concepts

- COVID-19 drug treatment

Full text links

ELSEVIER FULL-TEXT ARTICLE Elsevier Science Free PMC article
Proceed to details
Cite
Share
☐ 857
Br J Anaesth
. 2020 Nov;125(5):730-738.
doi: 10.1016/j.bja.2020.07.049. Epub 2020 Sep 3.

Frailty and outcomes from pneumonia in critical illness: a population-based cohort study

Jai N Darvall [1], Rinaldo Bellomo [2], Michael Bailey [3], Eldho Paul [4], Paul J Young [5], Kenneth Rockwood [6], David Pilcher [7]
Affiliations Expand

Affiliations

- [1] Department of Intensive Care, Royal Melbourne Hospital, Melbourne, VIC, Australia; Centre for Integrated Critical Care, The University of Melbourne, Melbourne, VIC, Australia. Electronic address: jai.darvall@mh.org.au.
- [2] Department of Intensive Care, Royal Melbourne Hospital, Melbourne, VIC, Australia; Centre for Integrated Critical Care, The University of Melbourne, Melbourne, VIC, Australia; Australian and New Zealand Intensive Care Research Centre, Department of Epidemiology and Preventive Medicine, Monash University, Melbourne, VIC, Australia; Data Analytics Research and Evaluation Centre, The University of Melbourne and Austin Hospital, Melbourne, VIC, Australia.
- [3] Centre for Integrated Critical Care, The University of Melbourne, Melbourne, VIC, Australia; Australian and New Zealand Intensive Care Research Centre, Department of Epidemiology and Preventive Medicine, Monash University, Melbourne, VIC, Australia.
- [4] Australian and New Zealand Intensive Care Research Centre, Department of Epidemiology and Preventive Medicine, Monash University, Melbourne, VIC, Australia.
- [5] Medical Research Institute of New Zealand, Wellington, New Zealand.
- [6] Geriatric Medicine Research, Nova Scotia Health Authority, Halifax, Nova Scotia, Canada; Divisions of Geriatric Medicine & Neurology, Dalhousie University & Nova Scotia Health Authority, Halifax, Nova Scotia, Canada.
- [7] Australian and New Zealand Intensive Care Research Centre, Department of Epidemiology and Preventive Medicine, Monash University, Melbourne, VIC, Australia; Department of Intensive Care, Alfred Hospital, Melbourne, VIC, Australia; Centre for Outcome and Resource Evaluation, Australian and New Zealand Intensive Care Society, Melbourne, VIC, Australia.

- PMID: **32891413**
- PMCID: PMC7467940
- DOI: 10.1016/j.bja.2020.07.049

Free PMC article

Frailty and outcomes from pneumonia in critical illness: a population-based cohort study

Jai N Darvall et al. Br J Anaesth. 2020 Nov.

Free PMC article

Show details

Br J Anaesth

. 2020 Nov;125(5):730-738.
doi: 10.1016/j.bja.2020.07.049. Epub 2020 Sep 3.

Authors

Jai N Darvall [1], Rinaldo Bellomo [2], Michael Bailey [3], Eldho Paul [4], Paul J Young [5], Kenneth Rockwood [6], David Pilcher [7]

Affiliations

- [1] Department of Intensive Care, Royal Melbourne Hospital, Melbourne, VIC, Australia; Centre for Integrated Critical Care, The University of Melbourne, Melbourne, VIC, Australia. Electronic address: jai.darvall@mh.org.au.
- [2] Department of Intensive Care, Royal Melbourne Hospital, Melbourne, VIC, Australia; Centre for Integrated Critical Care, The University of Melbourne, Melbourne, VIC, Australia; Australian and New Zealand Intensive Care Research Centre, Department of Epidemiology and Preventive Medicine, Monash University, Melbourne, VIC, Australia; Data Analytics Research and Evaluation Centre, The University of Melbourne and Austin Hospital, Melbourne, VIC, Australia.
- [3] Centre for Integrated Critical Care, The University of Melbourne, Melbourne, VIC, Australia; Australian and New Zealand Intensive Care Research Centre, Department of Epidemiology and Preventive Medicine, Monash University, Melbourne, VIC, Australia.
- [4] Australian and New Zealand Intensive Care Research Centre, Department of Epidemiology and Preventive Medicine, Monash University, Melbourne, VIC, Australia.
- [5] Medical Research Institute of New Zealand, Wellington, New Zealand.
- [6] Geriatric Medicine Research, Nova Scotia Health Authority, Halifax, Nova Scotia, Canada; Divisions of Geriatric Medicine & Neurology, Dalhousie University & Nova Scotia Health Authority, Halifax, Nova Scotia, Canada.
- [7] Australian and New Zealand Intensive Care Research Centre, Department of Epidemiology and Preventive Medicine, Monash University, Melbourne, VIC, Australia; Department of Intensive Care, Alfred Hospital, Melbourne, VIC, Australia; Centre for Outcome and Resource Evaluation, Australian and New Zealand Intensive Care Society, Melbourne, VIC, Australia.

- PMID: **32891413**
- PMCID: PMC7467940
- DOI: 10.1016/j.bja.2020.07.049

Abstract

Background: A threshold Clinical Frailty Scale (CFS) of 5 (indicating mild frailty) has been proposed to guide ICU admission for UK patients with coronavirus disease 2019 (COVID-19) pneumonia. However, the impact of frailty on mortality with (non-COVID-19) pneumonia in critical illness is unknown. We examined the triage utility of the CFS in patients with pneumonia requiring ICU.

Methods: We conducted a retrospective cohort study of adult patients admitted with pneumonia to 170 ICUs in Australia and New Zealand from January 1, 2018 to September 31, 2019. We classified patients as: non-frail (CFS 1-4) frail (CFS 5-8), mild/moderately frail (CFS 5-6),and severe/very severely frail (CFS 7-8). We evaluated mortality (primary outcome) adjusting for site, age, sex, mechanical ventilation, pneumonia type and illness severity. We also compared the proportion of ICU bed-days occupied between frailty categories.

Results: 1852/5607 (33%) patients were classified as frail, including1291/3056 (42%) of patients aged >65 yr, who would potentially be excluded from ICU admission under UK-based COVID-19 triage guidelines. Only severe/very severe frailty scores were associated with mortality (adjusted odds ratio [aOR] for CFS=7: 3.2; 95% confidence interval [CI]: 1.3-7.8; CFS=8 [aOR: 7.2; 95% CI: 2.6-20.0]). These patients accounted for 7% of ICU bed days. Vulnerability (CFS=4) and mild frailty (CFS=5) were associated with a similar mortality risk (CFS=4 [OR: 1.6; 95% CI: 0.7-3.8]; CFS=5 [OR: 1.6; 95% CI: 0.7-3.9]).

Conclusions: Patients with severe and very severe frailty account for relatively few ICU bed days as a result of pneumonia, whilst adjusted mortality analysis indicated little difference in risk between patients in vulnerable, mild, and moderate frailty categories. These data do not support CFS ≥5 to guide ICU admission for pneumonia.

Keywords: COVID-19; frailty; intensive care unit; mortality; observational study; pneumonia; respiratory failure.

Copyright © 2020 British Journal of Anaesthesia. Published by Elsevier Ltd. All rights reserved.

Comment in

- Prognostication in older ICU patients: mission impossible?
  Flaatten H, Beil M, Guidet B. Flaatten H, et al. Br J Anaesth. 2020 Nov;125(5):655-657. doi: 10.1016/j.bja.2020.08.005. Epub 2020 Aug 14. Br J Anaesth. 2020. PMID: 32868042 Free PMC article. No abstract available.
- Association of frailty and mortality in patients with COVID-19: a meta-analysis.
  Kow CS, Hasan SS, Thiruchelvam K, Aldeyab M. Kow CS, et al. Br J Anaesth. 2021 Mar;126(3):e108-e110. doi: 10.1016/j.bja.2020.12.002. Epub 2020 Dec 5. Br J Anaesth. 2021. PMID: 33358046 Free PMC article. No abstract available.

- 28 references
- 3 figures

Supplementary info

Publication types, MeSH terms Expand

Publication types

- Research Support, Non-U.S. Gov't

MeSH terms

- Aged
- Australia / epidemiology

- Betacoronavirus*
- COVID-19
- Cohort Studies
- Coronavirus Infections / epidemiology*
- Critical Illness
- Female
- Frail Elderly / statistics & numerical data*
- Geriatric Assessment / methods
- Geriatric Assessment / statistics & numerical data*
- Humans
- Length of Stay
- Male
- Middle Aged
- New Zealand / epidemiology
- Pandemics
- Patient Outcome Assessment*
- Pneumonia, Viral / epidemiology*
- Retrospective Studies
- SARS-CoV-2

Full text links

ELSEVIER OPEN ACCESS Elsevier Science Free PMC article

Proceed to details

Cite

Share

☐ 858

Observational Study

Acta Diabetol

. 2021 Feb;58(2):231-237.

doi: 10.1007/s00592-020-01614-5. Epub 2020 Oct 16.

Impact of COVID-19 lockdown on flash and real-time glucose sensor users with type 1 diabetes in England

Joshi Prabhu Navis[#, 1], Lalantha Leelarathna[#, 1, 2], Womba Mubita[1], Andrea Urwin[1], Martin K Rutter[1, 2], Jonathan Schofield[1], Hood Thabit[3, 4]

Affiliations Expand

Affiliations

- [1] Diabetes, Endocrinology and Metabolism Centre, Manchester University NHS Foundation Trust, Manchester Academic Health Science Centre, Manchester, M13 9WL, UK.
- [2] Division of Diabetes, Endocrinology and Gastroenterology, Faculty of Biology, Medicine and Health, University of Manchester, Manchester,, M13 9WL, UK.
- [3] Diabetes, Endocrinology and Metabolism Centre, Manchester University NHS Foundation Trust, Manchester Academic Health Science Centre, Manchester, M13 9WL, UK. hood.thabit@mft.nhs.uk.
- [4] Division of Diabetes, Endocrinology and Gastroenterology, Faculty of Biology, Medicine and Health, University of Manchester, Manchester,, M13 9WL, UK. hood.thabit@mft.nhs.uk.

[#] Contributed equally.

- PMID: **33067723**
- PMCID: PMC7567414
- DOI: 10.1007/s00592-020-01614-5

Free PMC article
Observational Study

Impact of COVID-19 lockdown on flash and real-time glucose sensor users with type 1 diabetes in England

Joshi Prabhu Navis et al. Acta Diabetol. 2021 Feb.
Free PMC article
Show details
Acta Diabetol
. 2021 Feb;58(2):231-237.
doi: 10.1007/s00592-020-01614-5. Epub 2020 Oct 16.

Authors

Joshi Prabhu Navis [#] [1], Lalantha Leelarathna [#] [1] [2], Womba Mubita [1], Andrea Urwin [1], Martin K Rutter [1] [2], Jonathan Schofield [1], Hood Thabit [3] [4]

Affiliations

- [1] Diabetes, Endocrinology and Metabolism Centre, Manchester University NHS Foundation Trust, Manchester Academic Health Science Centre, Manchester, M13 9WL, UK.
- [2] Division of Diabetes, Endocrinology and Gastroenterology, Faculty of Biology, Medicine and Health, University of Manchester, Manchester,, M13 9WL, UK.
- [3] Diabetes, Endocrinology and Metabolism Centre, Manchester University NHS Foundation Trust, Manchester Academic Health Science Centre, Manchester, M13 9WL, UK. hood.thabit@mft.nhs.uk.

- [4] Division of Diabetes, Endocrinology and Gastroenterology, Faculty of Biology, Medicine and Health, University of Manchester, Manchester,, M13 9WL, UK. hood.thabit@mft.nhs.uk.

[#] Contributed equally.

- PMID: **33067723**
- PMCID: PMC7567414
- DOI: 10.1007/s00592-020-01614-5

Abstract

Aims: People with type 1 diabetes (T1D) face the daily task of implementing self-management strategies to achieve their glycaemic goals. The UK COVID-19 lockdown has had an impact on day-to-day behaviour, which may affect diabetes self-management and outcomes. We assessed whether sensor-based outcomes pre- and during lockdown periods were different in a cohort of glucose sensor users with T1D.

Methods: Data were collected from Freestyle Libre (FSL) or Dexcom G6 sensor users who remotely shared their data with the diabetes clinic web platform. Sensor metrics according to international consensus were analysed and compared between pre-lockdown period and 2 and 3 weeks into lockdown (periods 1 and 2).

Results: Two hundred and sixty-nine T1D patients (baseline HbA1c 57 ± 14 mmol/mol) were identified as FSL (n = 190) or Dexcom G6 (n = 79) users. In patients with sensor use > 70% (N = 223), compared to pre-lockdown period percentage TIR 3.9-10 mM (TIR) significantly increased during period 1 (59.6 ± 18.2 vs. 57.5 ± 17.2%, p = 0.002) and period 2 (59.3 ± 18.3 vs. 57.5 ± 17.2%, p = 0.035). The proportion of patients achieving TIR ≥ 70% increased from 23.3% pre-lockdown to 27.8% in period 1 and 30.5% in period 2. A higher proportion also achieved the recommended time below and above range, and coefficient of variation in periods 1 and 2. Dexcom G6 users had significantly lower % time below range (< 3.9 mM) compared to FSL users during both lockdown periods (period 1: Dexcom G6 vs. FSL: 1.8% vs. 4%; period 2: 1.4% vs. 4%, p < 0.005 for both periods).

Conclusion: Sensor-based glycaemic outcomes in people with T1D in the current cohort improved during COVID-19 lockdown, which may be associated with positive changes in self-management strategies. Further work is required to evaluate long-term sustainability and support.

Keywords: COVID-19 lockdown; Flash glucose monitoring; Real-time continuous glucose monitoring; Type 1 diabetes.

Conflict of interest statement

L.L. reports having received speaker honoraria from Animas, Abbott, Insulet, Medtronic, Novo Nordisk, Roche and Sanofi; having served on advisory panels for Animas, Abbott, Novo Nordisk, Dexcom, Medtronic, Sanofi and Roche; and having received research support from Novo Nordisk and Dexcom. MKR has received educational grant support from MSD and Novo Nordisk; has modest stock ownership in GSK; and has consulted for Roche. HT received research support from Dexcom. No other potential conflicts of interest relevant to this article were reported.

- 24 references

Supplementary info

Publication types, MeSH terms, Substances Expand

Publication types

- Observational Study

MeSH terms

- Adult
- Blood Glucose / analysis*
- Blood Glucose / metabolism
- Blood Glucose Self-Monitoring / instrumentation
- Blood Glucose Self-Monitoring / methods
- COVID-19 / epidemiology*
- Clinical Audit
- Communicable Disease Control / methods
- Computer Systems
- Diabetes Mellitus, Type 1 / blood*
- Diabetes Mellitus, Type 1 / drug therapy
- Diabetes Mellitus, Type 1 / epidemiology
- England / epidemiology
- Female
- Health Services Accessibility / organization & administration
- Health Services Accessibility / standards
- Hospitals, Teaching
- Humans
- Insulin / administration & dosage
- Insulin Infusion Systems
- Male
- Middle Aged
- Pandemics
- Quarantine*
- Remote Sensing Technology / instrumentation*
- Remote Sensing Technology / standards
- Retrospective Studies
- SARS-CoV-2 / physiology
- Telemedicine* / instrumentation
- Telemedicine* / organization & administration
- Telemedicine* / standards

Substances

- Blood Glucose
- Insulin

Full text links

SpringerLink FULL-TEXT ARTICLE Springer Free PMC article
Proceed to details
Cite
Share
859
Clinical Trial
PLoS One
. 2021 Oct 21;16(10):e0258916.
doi: 10.1371/journal.pone.0258916. eCollection 2021.

Mortality in association with antipsychotic medication use and clinical outcomes among geriatric psychiatry outpatients with COVID-19

Bienvenida Austria [1, 2], Rehana Haque [1, 2], Sukriti Mittal [1, 2], Jamie Scott [1, 2], Aninditha Vengassery [1, 2], Daniel Maltz [3], Wentian Li [4], Blaine Greenwald [1, 2], Yun Freudenberg-Hua [1, 2, 5]

Affiliations Expand

Affiliations

- [1] Division of Geriatric Psychiatry, Zucker Hillside Hospital, Glen Oaks, NY, United States of America.
- [2] Donald and Barbara Zucker School of Medicine at Hofstra/Northwell, Hempstead, NY, United States of America.
- [3] Information Services, Product Services and Management, Northwell Health, Lake Success, NY, United States of America.
- [4] Center for Genomics and Human Genetics, The Feinstein Institutes for Medical Research, Manhasset, NY, United States of America.
- [5] Litwin-Zucker Center for Alzheimer's Disease, The Feinstein Institutes for Medical Research, Manhasset, NY, United States of America.

- PMID: **34673821**
- PMCID: PMC8530340
- DOI: 10.1371/journal.pone.0258916

Free PMC article
Clinical Trial

Mortality in association with antipsychotic medication use and clinical outcomes among geriatric psychiatry outpatients with COVID-19

Bienvenida Austria et al. PLoS One. 2021.
Free PMC article
Show details
PLoS One
. 2021 Oct 21;16(10):e0258916.
doi: 10.1371/journal.pone.0258916. eCollection 2021.

Authors

Bienvenida Austria [1, 2], Rehana Haque [1, 2], Sukriti Mittal [1, 2], Jamie Scott [1, 2], Aninditha Vengassery [1, 2], Daniel Maltz [3], Wentian Li [4], Blaine Greenwald [1, 2], Yun Freudenberg-Hua [1, 2, 5]

Affiliations

- [1] Division of Geriatric Psychiatry, Zucker Hillside Hospital, Glen Oaks, NY, United States of America.
- [2] Donald and Barbara Zucker School of Medicine at Hofstra/Northwell, Hempstead, NY, United States of America.
- [3] Information Services, Product Services and Management, Northwell Health, Lake Success, NY, United States of America.
- [4] Center for Genomics and Human Genetics, The Feinstein Institutes for Medical Research, Manhasset, NY, United States of America.
- [5] Litwin-Zucker Center for Alzheimer's Disease, The Feinstein Institutes for Medical Research, Manhasset, NY, United States of America.

- PMID: **34673821**
- PMCID: PMC8530340
- DOI: 10.1371/journal.pone.0258916

Abstract

Objectives: Older adults are particularly vulnerable to the negative consequences of antipsychotic exposure and are disproportionally affected by higher mortality from coronavirus disease 2019 (COVID-19). Our goal was to determine whether concurrent antipsychotic medication use was associated with increased COVID-19 mortality in older patients with preexisting behavioral health problems. We also report on findings from post-COVID follow-ups.

Design: Retrospective observational study.

Participants: Outpatients at a geriatric psychiatric clinic in New York City.

Measurements: Demographic and clinical data including medication, diagnosis and Clinical Global Impression Severity (CGI-S) scales on outpatients who had COVID-19 between February 28th and October 1st 2020 were extracted from the electronic health records (EHR) from the hospital.

Results: A total of 56 patients were diagnosed with COVID-19 (mean age 76 years; median age 75 years) and 13 (23.2%) died. We found an increased mortality risk for patients who were prescribed at least one antipsychotic medication at the time of COVID-19 infection (Fisher's exact test P = 0.009, OR = 11.1, 95% confidence interval: 1.4-96.0). This result remains significant after adjusting for age, gender, housing context and dementia (Logistic regression P = 0.035, Beta = 2.4). Furthermore, we found that most patients who survived COVID-19 (88.4%) recovered to pre-COVID baseline in terms of psychiatric symptoms. Comparison of pre- and post-COVID assessments of CGI-S for 33 patients who recovered from COVID-19 were not significantly different.

Conclusion: We observed a higher COVID-19 mortality associated with concurrent antipsychotics use in older patients receiving behavioral health services. The majority of patients in our geriatric clinic who recovered from COVID-19 appeared to return to their pre-COVID psychiatric function. More precise estimates of the risk associated with antipsychotic treatment in older patients with COVID-19 and other underlying factors will come from larger datasets and meta-analyses.

Conflict of interest statement

No authors have competing interests.

- 32 references
- 2 figures

Supplementary info

Publication types, MeSH terms, Substances, Grant support Expand

Publication types

- Clinical Trial
- Observational Study
- Research Support, N.I.H., Extramural

MeSH terms

- Aged
- Aged, 80 and over
- Antipsychotic Agents / administration & dosage
- Antipsychotic Agents / adverse effects*

- COVID-19 / mortality*
- Female
- Geriatric Psychiatry
- Humans
- Male
- Mental Disorders* / drug therapy
- Mental Disorders* / epidemiology
- Mental Disorders* / mortality
- New York City / epidemiology
- Outpatients*
- Retrospective Studies
- SARS-CoV-2*

Substances

- Antipsychotic Agents

Grant support

- K08 AG054727/AG/NIA NIH HHS/United States

Full text links

OPEN ACCESS TO FULL TEXT PLOS ONE Public Library of Science Free PMC article
Proceed to details
Cite
Share
☐ 860
Observational Study
J Perinat Med
. 2020 Nov 3;49(3):263-268.
doi: 10.1515/jpm-2020-0446. Print 2021 Mar 26.

Initial review of pregnancy and neonatal outcomes of pregnant women with COVID-19 infection

Ifeoma Ogamba [1], Andrea Kliss [1], Nicole Rainville [1], Linus Chuang [2], Erin Panarelli [1], Joann Petrini [1], Dimitry Zilberman [2]
Affiliations Expand

Affiliations

- [1] Department of Obstetrics & Gynecology, Danbury Hospital, Danbury, CT, USA.
- [2] Department of Obstetrics and Gynecology, Danbury and Norwalk Hospital, Danbury/Norwalk, CT, USA.

- PMID: **33141109**
- DOI: 10.1515/jpm-2020-0446

Observational Study

Initial review of pregnancy and neonatal outcomes of pregnant women with COVID-19 infection

Ifeoma Ogamba et al. J Perinat Med. 2020.

Show details

J Perinat Med

. 2020 Nov 3;49(3):263-268.
doi: 10.1515/jpm-2020-0446. Print 2021 Mar 26.

Authors

Ifeoma Ogamba [1], Andrea Kliss [1], Nicole Rainville [1], Linus Chuang [2], Erin Panarelli [1], Joann Petrini [1], Dimitry Zilberman [2]

Affiliations

- [1] Department of Obstetrics & Gynecology, Danbury Hospital, Danbury, CT, USA.
- [2] Department of Obstetrics and Gynecology, Danbury and Norwalk Hospital, Danbury/Norwalk, CT, USA.

- PMID: **33141109**
- DOI: 10.1515/jpm-2020-0446

Abstract

Objectives: Data regarding the pathogenesis and clinical manifestations of severe acute respiratory syndrome coronavirus 2 (SARS-CoV-2) continue to emerge, however, there's limited data in regard to maternal and neonatal outcomes. Therefore, we conducted a retrospective analysis of all pregnant women who tested positive for SARS-CoV-2 within Nuvance Health system.

Methods: Data were abstracted from the medical records of each patient and descriptive analysis was performed. Variables included demographics, COVID testing results, symptoms, management, labor course, neonatal information, and complications.

Results: Total of 40 patients were identified. Average age was 29.6 years old, 35% were Hispanic, and approximately one in three patients had comorbidities. Of the patients who had repeated testing, the average number of days between first positive test and negative test was 36.8 days (± 19.9 days). Three out of four women reported symptoms. Of the 40 pregnant women who were positive for SARS-CoV-2, 25 of them delivered. About 84% of the women delivered after 37 weeks. Twelve percent of the women delivered under 33 and 6/7 weeks. Most patients had vaginal deliveries (68%) and the remaining had cesarean deliveries. Neonatal outcomes included: mean 1 and 5 min Apgar scores of 8 and 8.8, respectively and the mean birth weight was 3212 g. Twenty neonates were tested for SARS-CoV-2 and were all found to be negative.

Conclusions: Overall, with routine prenatal care and preventive measures, pregnant patients and neonates in our study had good outcomes. At this time, there appears to be no evidence of vertical transmission.

Keywords: COVID in pregnancy; COVID-19; COVID-19 in neonates; coronavirus; ethics; fetus; maternal outcomes; neonatal outcomes; synthesis.

© 2020 Walter de Gruyter GmbH, Berlin/Boston.

- 16 references

Supplementary info

Publication types, MeSH terms Expand

Publication types

- Observational Study

MeSH terms

- Adolescent
- Adult
- Apgar Score
- Birth Weight
- COVID-19 Testing*
- COVID-19* / diagnosis
- COVID-19* / epidemiology
- COVID-19* / therapy
- COVID-19* / transmission
- Female
- Humans
- Infant, Newborn
- Infectious Disease Transmission, Vertical* / prevention & control
- Infectious Disease Transmission, Vertical* / statistics & numerical data
- Male
- New York / epidemiology

- Perinatal Care / methods*
- Pregnancy
- Pregnancy Complications, Infectious* / diagnosis
- Pregnancy Complications, Infectious* / epidemiology
- Pregnancy Complications, Infectious* / therapy
- Pregnancy Outcome
- Retrospective Studies
- Treatment Outcome
- Young Adult

Full text links

De Gruyter
Proceed to details
Cite
Share
861

Observational Study
JAMA Intern Med
. 2020 Oct 1;180(10):1345-1355.
doi: 10.1001/jamainternmed.2020.3539.

Risk Factors Associated With Mortality Among Patients With COVID-19 in Intensive Care Units in Lombardy, Italy

Giacomo Grasselli [1, 2], Massimiliano Greco [3, 4], Alberto Zanella [1, 2], Giovanni Albano [5], Massimo Antonelli [6, 7], Giacomo Bellani [8, 9], Ezio Bonanomi [10], Luca Cabrini [11], Eleonora Carlesso [2], Gianpaolo Castelli [12], Sergio Cattaneo [13], Danilo Cereda [14], Sergio Colombo [15], Antonio Coluccello [16], Giuseppe Crescini [16], Andrea Forastieri Molinari [17], Giuseppe Foti [8, 9], Roberto Fumagalli [8, 18], Giorgio Antonio Iotti [19, 20], Thomas Langer [8, 18], Nicola Latronico [13, 21], Ferdinando Luca Lorini [10], Francesco Mojoli [19, 20], Giuseppe Natalini [22], Carla Maria Pessina [23], Vito Marco Ranieri [24], Roberto Rech [25], Luigia Scudeller [26], Antonio Rosano [22], Enrico Storti [27], B Taylor Thompson [28], Marcello Tirani [14, 29], Pier Giorgio Villani [27], Antonio Pesenti [1, 2], Maurizio Cecconi [3, 4], COVID-19 Lombardy ICU Network
Collaborators, Affiliations Expand

Collaborators

- **COVID-19 Lombardy ICU Network**: Emiliano Agosteo, Giovanni Albano, Andrea Albertin, Armando Alborghetti, Giorgio Aldegheri, Benvenuto Antonini, Enrico Barbara, Giulia Bardelloni, Sabrina Basilico, Nicolangela Belgiorno, Giacomo Bellani, Enrico Beretta, Angela

Berselli, Leonardo Bianciardi, Ezio Bonanomi, Stefano Bonazzi, Massimo Borelli, Nicola Bottino, Nicola Bronzini, Serena Brusatori, Luca Cabrini, Carlo Capra, Livio Carnevale, Gianpaolo Castelli, Emanuele Catena, Sergio Cattaneo, Maurizio Cecconi, Simona Celotti, Stefania Cerutti, Davide Chiumello, Silvia Cirri, Giuseppe Citerio, Sergio Colombo, Antonio Coluccello, Davide Coppini, Alberto Corona, Paolo Cortellazzi, Elena Costantini, Remo Daniel Covello, Giuseppe Crescini, Gianluca De Filippi, Marco Dei Poli, Paolo Dughi, Fulvia Fieni, Gaetano Florio, Andrea Forastieri Molinari, Giuseppe Foti, Roberto Fumagalli, Marco Galletti, Giorgio Antonio Gallioli, Hedwige Gay, Marco Gemma, Paolo Gnesin, Giacomo Grasselli, Stefano Greco, Massimiliano Greco, Paolo Grosso, Luca Guatteri, Davide Guzzon, Giorgio Antonio Iotti, Roberto Keim, Thomas Langer, Nicola Latronico, Andrea Lombardo, Ferdinando Luca Lorini, Filippo Mamprin, Giovanni Marino, Francesco Marino, Guido Merli, Antonio Micucci, Carmine Rocco Militano, Francesco Mojoli, Giacomo Monti, Stefano Muttini, Samantha Nadalin, Giuseppe Natalini, Paolo Perazzo, Giovanni Battista Perego, Luciano Perotti, Antonio Pesenti, Carla Maria Pessina, Nicola Petrucci, Angelo Pezzi, Simone Piva, Gina Portella, Alessandro Protti, Milena Racagni, Danilo Radrizzani, Maurizio Raimondi, Marco Ranucci, Roberto Rech, Mario Riccio, Antonio Rosano, Patrizia Ruggeri, Giuseppe Sala, Luca Salvi, Pietro Sebastiano, Paolo Severgnini, Donato Sigurtà, Nino Stocchetti, Enrico Storti, Matteo Subert, Mario Tavola, Serena Todaro, Francesca Torriglia, Daniela Tubiolo, Roberto Valsecchi, Pier Giorgio Villani, Uberto Viola, Giovanni Vitale, Massimo Zambon, Alberto Zanella, Elena Zoia

Affiliations

- [1] Dipartimento di Anestesia, Rianimazione e Emergenza-Urgenza, Fondazione IRCCS (Istituto di Ricovero e Cura a Carattere Scientifico) Ca' Granda Ospedale Maggiore Policlinico, Milan, Italy.
- [2] Department of Pathophysiology and Transplantation, University of Milan, Milan, Italy.
- [3] Department of Anaesthesia and Intensive Care Medicine, Humanitas Clinical and Research Center-IRCCS, Rozzano, Italy.
- [4] Department of Biomedical Sciences, Humanitas University, Milan, Italy.
- [5] Humanitas Gavazzeni, Bergamo, Italy.
- [6] Department of Anesthesiology, Intensive Care and Emergency Medicine, Fondazione Policlinico Universitario A. Gemelli IRCCS, Rome, Italy.
- [7] Dipartimento di Scienze biotecnologiche di base, cliniche intensivologiche e perioperatorie, Università Cattolica del Sacro Cuore, Rome, Italy.
- [8] Department of Medicine and Surgery, University of Milano-Bicocca, Monza, Italy.
- [9] Department of Anesthesia and Intensive Care Medicine, Azienda Socio Sanitaria Territoriale (ASST) Monza-Ospedale San Gerardo, Monza, Italy.
- [10] Department of Anaesthesia and Intensive Care, ASST Papa Giovanni XXIII, Bergamo, Italy.
- [11] Università degli Studi dell'Insubria, Azienda Ospedaliera Ospedale di Circolo e Fondazione Macchi, Varese, Italy.
- [12] Department of Anesthesiology and Intensive Care, ASST Mantova-Ospedale Carlo Poma, Mantova, Italy.
- [13] Department of Anaesthesiology, Intensive Care and Perioperative Medicine, Spedali Civili University Hospital, Brescia, Italy.
- [14] Direzione Generale (DG) Welfare, Lombardy Region, Milan, Italy.

- [15] Department of Anesthesia and Intensive Care, IRCCS San Raffaele Scientific Institute, Milan, Italy.
- [16] Department of Anesthesiology and Intensive Care, ASST Cremona-Ospedale di Cremona, Cremona, Italy.
- [17] Department of Anesthesiology and Intensive Care, ASST Lecco-Ospedale di Lecco, Lecco, Italy.
- [18] Dipartimento di Anestesia e Rianimazione, Grande Ospedale Metropolitano Niguarda, Milan, Italy.
- [19] Department of Intensive Medicine, Fondazione IRCCS Policlinico San Matteo, Pavia, Italy.
- [20] Department of Clinical-Diagnostic, Surgical and Pediatric Sciences, University of Pavia, Pavia, Italy.
- [21] Department of Medical and Surgical Specialties, Radiological Sciences, and Public Health, University of Brescia, Brescia, Italy.
- [22] Department of Anesthesia and Intensive Care, Fondazione Poliambulanza Hospital, Brescia, Italy.
- [23] Department of Anesthesia and Intensive Care, ASST Rhodense-Presidio di Rho, Milano, Italy.
- [24] Anesthesia and Intensive Care Medicine, Policlinico di Sant'Orsola, Alma Mater Studiorum University of Bologna, Bologna, Italy.
- [25] Department of Anesthesiology and Intensive Care, ASST Fatebenefratelli Sacco, Luigi Sacco Hospital, Polo Universitario, University of Milan, Milan, Italy.
- [26] Direzione Scientifica, Fondazione IRCCS Ca' Granda Ospedale Maggiore Policlinico, Milan, Italy.
- [27] Dipartimento Emergenza Urgenza, Unità Operativa Complessa (UOC) Anestesia e Rianimazione, ASST, Lodi, Italy.
- [28] Division of Pulmonary and Critical Medicine, Massachusetts General Hospital, Boston.
- [29] Health Protection Agency of Pavia, Pavia, Italy.

- PMID: **32667669**
- PMCID: PMC7364371
- DOI: 10.1001/jamainternmed.2020.3539

Free PMC article
Observational Study

Risk Factors Associated With Mortality Among Patients With COVID-19 in Intensive Care Units in Lombardy, Italy

Giacomo Grasselli et al. JAMA Intern Med. 2020.
Free PMC article
Show details
JAMA Intern Med
. 2020 Oct 1;180(10):1345-1355.
doi: 10.1001/jamainternmed.2020.3539.

Authors

Giacomo Grasselli [1, 2], Massimiliano Greco [3, 4], Alberto Zanella [1, 2], Giovanni Albano [5], Massimo Antonelli [6, 7], Giacomo Bellani [8, 9], Ezio Bonanomi [10], Luca Cabrini [11], Eleonora Carlesso [2], Gianpaolo Castelli [12], Sergio Cattaneo [13], Danilo Cereda [14], Sergio Colombo [15], Antonio Coluccello [16], Giuseppe Crescini [16], Andrea Forastieri Molinari [17], Giuseppe Foti [8, 9], Roberto Fumagalli [8, 18], Giorgio Antonio Iotti [19, 20], Thomas Langer [8, 18], Nicola Latronico [13, 21], Ferdinando Luca Lorini [10], Francesco Mojoli [19, 20], Giuseppe Natalini [22], Carla Maria Pessina [23], Vito Marco Ranieri [24], Roberto Rech [25], Luigia Scudeller [26], Antonio Rosano [22], Enrico Storti [27], B Taylor Thompson [28], Marcello Tirani [14, 29], Pier Giorgio Villani [27], Antonio Pesenti [1, 2], Maurizio Cecconi [3, 4], COVID-19 Lombardy ICU Network

Collaborators

- **COVID-19 Lombardy ICU Network**: Emiliano Agosteo, Giovanni Albano, Andrea Albertin, Armando Alborghetti, Giorgio Aldegheri, Benvenuto Antonini, Enrico Barbara, Giulia Bardelloni, Sabrina Basilico, Nicolangela Belgiorno, Giacomo Bellani, Enrico Beretta, Angela Berselli, Leonardo Bianciardi, Ezio Bonanomi, Stefano Bonazzi, Massimo Borelli, Nicola Bottino, Nicola Bronzini, Serena Brusatori, Luca Cabrini, Carlo Capra, Livio Carnevale, Gianpaolo Castelli, Emanuele Catena, Sergio Cattaneo, Maurizio Cecconi, Simona Celotti, Stefania Cerutti, Davide Chiumello, Silvia Cirri, Giuseppe Citerio, Sergio Colombo, Antonio Coluccello, Davide Coppini, Alberto Corona, Paolo Cortellazzi, Elena Costantini, Remo Daniel Covello, Giuseppe Crescini, Gianluca De Filippi, Marco Dei Poli, Paolo Dughi, Fulvia Fieni, Gaetano Florio, Andrea Forastieri Molinari, Giuseppe Foti, Roberto Fumagalli, Marco Galletti, Giorgio Antonio Gallioli, Hedwige Gay, Marco Gemma, Paolo Gnesin, Giacomo Grasselli, Stefano Greco, Massimiliano Greco, Paolo Grosso, Luca Guatteri, Davide Guzzon, Giorgio Antonio Iotti, Roberto Keim, Thomas Langer, Nicola Latronico, Andrea Lombardo, Ferdinando Luca Lorini, Filippo Mamprin, Giovanni Marino, Francesco Marino, Guido Merli, Antonio Micucci, Carmine Rocco Militano, Francesco Mojoli, Giacomo Monti, Stefano Muttini, Samantha Nadalin, Giuseppe Natalini, Paolo Perazzo, Giovanni Battista Perego, Luciano Perotti, Antonio Pesenti, Carla Maria Pessina, Nicola Petrucci, Angelo Pezzi, Simone Piva, Gina Portella, Alessandro Protti, Milena Racagni, Danilo Radrizzani, Maurizio Raimondi, Marco Ranucci, Roberto Rech, Mario Riccio, Antonio Rosano, Patrizia Ruggeri, Giuseppe Sala, Luca Salvi, Pietro Sebastiano, Paolo Severgnini, Donato Sigurtà, Nino Stocchetti, Enrico Storti, Matteo Subert, Mario Tavola, Serena Todaro, Francesca Torriglia, Daniela Tubiolo, Roberto Valsecchi, Pier Giorgio Villani, Uberto Viola, Giovanni Vitale, Massimo Zambon, Alberto Zanella, Elena Zoia

Affiliations

- [1] Dipartimento di Anestesia, Rianimazione e Emergenza-Urgenza, Fondazione IRCCS (Istituto di Ricovero e Cura a Carattere Scientifico) Ca' Granda Ospedale Maggiore Policlinico, Milan, Italy.
- [2] Department of Pathophysiology and Transplantation, University of Milan, Milan, Italy.
- [3] Department of Anaesthesia and Intensive Care Medicine, Humanitas Clinical and Research Center-IRCCS, Rozzano, Italy.
- [4] Department of Biomedical Sciences, Humanitas University, Milan, Italy.

- [5] Humanitas Gavazzeni, Bergamo, Italy.
- [6] Department of Anesthesiology, Intensive Care and Emergency Medicine, Fondazione Policlinico Universitario A. Gemelli IRCCS, Rome, Italy.
- [7] Dipartimento di Scienze biotecnologiche di base, cliniche intensivologiche e perioperatorie, Università Cattolica del Sacro Cuore, Rome, Italy.
- [8] Department of Medicine and Surgery, University of Milano-Bicocca, Monza, Italy.
- [9] Department of Anesthesia and Intensive Care Medicine, Azienda Socio Sanitaria Territoriale (ASST) Monza-Ospedale San Gerardo, Monza, Italy.
- [10] Department of Anaesthesia and Intensive Care, ASST Papa Giovanni XXIII, Bergamo, Italy.
- [11] Università degli Studi dell'Insubria, Azienda Ospedaliera Ospedale di Circolo e Fondazione Macchi, Varese, Italy.
- [12] Department of Anesthesiology and Intensive Care, ASST Mantova-Ospedale Carlo Poma, Mantova, Italy.
- [13] Department of Anaesthesiology, Intensive Care and Perioperative Medicine, Spedali Civili University Hospital, Brescia, Italy.
- [14] Direzione Generale (DG) Welfare, Lombardy Region, Milan, Italy.
- [15] Department of Anesthesia and Intensive Care, IRCCS San Raffaele Scientific Institute, Milan, Italy.
- [16] Department of Anesthesiology and Intensive Care, ASST Cremona-Ospedale di Cremona, Cremona, Italy.
- [17] Department of Anesthesiology and Intensive Care, ASST Lecco-Ospedale di Lecco, Lecco, Italy.
- [18] Dipartimento di Anestesia e Rianimazione, Grande Ospedale Metropolitano Niguarda, Milan, Italy.
- [19] Department of Intensive Medicine, Fondazione IRCCS Policlinico San Matteo, Pavia, Italy.
- [20] Department of Clinical-Diagnostic, Surgical and Pediatric Sciences, University of Pavia, Pavia, Italy.
- [21] Department of Medical and Surgical Specialties, Radiological Sciences, and Public Health, University of Brescia, Brescia, Italy.
- [22] Department of Anesthesia and Intensive Care, Fondazione Poliambulanza Hospital, Brescia, Italy.
- [23] Department of Anesthesia and Intensive Care, ASST Rhodense-Presidio di Rho, Milano, Italy.
- [24] Anesthesia and Intensive Care Medicine, Policlinico di Sant'Orsola, Alma Mater Studiorum University of Bologna, Bologna, Italy.
- [25] Department of Anesthesiology and Intensive Care, ASST Fatebenefratelli Sacco, Luigi Sacco Hospital, Polo Universitario, University of Milan, Milan, Italy.
- [26] Direzione Scientifica, Fondazione IRCCS Ca' Granda Ospedale Maggiore Policlinico, Milan, Italy.
- [27] Dipartimento Emergenza Urgenza, Unità Operativa Complessa (UOC) Anestesia e Rianimazione, ASST, Lodi, Italy.
- [28] Division of Pulmonary and Critical Medicine, Massachusetts General Hospital, Boston.
- [29] Health Protection Agency of Pavia, Pavia, Italy.

- PMID: **32667669**
- PMCID: PMC7364371
- DOI: 10.1001/jamainternmed.2020.3539

Erratum in

- Addition of Group Members Supplement.
  [No authors listed] [No authors listed] JAMA Intern Med. 2021 Jul 1;181(7):1021. doi: 10.1001/jamainternmed.2021.1229. JAMA Intern Med. 2021. PMID: 33843945 Free PMC article. No abstract available.

Abstract

Importance: Many patients with coronavirus disease 2019 (COVID-19) are critically ill and require care in the intensive care unit (ICU).

Objective: To evaluate the independent risk factors associated with mortality of patients with COVID-19 requiring treatment in ICUs in the Lombardy region of Italy.

Design, setting, and participants: This retrospective, observational cohort study included 3988 consecutive critically ill patients with laboratory-confirmed COVID-19 referred for ICU admission to the coordinating center (Fondazione IRCCS [Istituto di Ricovero e Cura a Carattere Scientifico] Ca' Granda Ospedale Maggiore Policlinico, Milan, Italy) of the COVID-19 Lombardy ICU Network from February 20 to April 22, 2020. Infection with severe acute respiratory syndrome coronavirus 2 was confirmed by real-time reverse transcriptase-polymerase chain reaction assay of nasopharyngeal swabs. Follow-up was completed on May 30, 2020.

Exposures: Baseline characteristics, comorbidities, long-term medications, and ventilatory support at ICU admission.

Main outcomes and measures: Time to death in days from ICU admission to hospital discharge. The independent risk factors associated with mortality were evaluated with a multivariable Cox proportional hazards regression.

Results: Of the 3988 patients included in this cohort study, the median age was 63 (interquartile range [IQR] 56-69) years; 3188 (79.9%; 95% CI, 78.7%-81.1%) were men, and 1998 of 3300 (60.5%; 95% CI, 58.9%-62.2%) had at least 1 comorbidity. At ICU admission, 2929 patients (87.3%; 95% CI, 86.1%-88.4%) required invasive mechanical ventilation (IMV). The median follow-up was 44 (95% CI, 40-47; IQR, 11-69; range, 0-100) days; median time from symptoms onset to ICU admission was 10 (95% CI, 9-10; IQR, 6-14) days; median length of ICU stay was 12 (95% CI, 12-13; IQR, 6-21) days; and median length of IMV was 10 (95% CI, 10-11; IQR, 6-17) days. Cumulative observation time was 164 305 patient-days. Hospital and ICU mortality rates were 12 (95% CI, 11-12) and 27 (95% CI, 26-29) per 1000 patients-days, respectively. In the subgroup of the first 1715 patients, as of May 30, 2020, 865 (50.4%) had been discharged from the ICU, 836 (48.7%) had died in the ICU, and 14 (0.8%) were still in the ICU; overall, 915 patients (53.4%) died in the hospital. Independent risk factors associated with mortality included older age (hazard ratio [HR], 1.75; 95% CI, 1.60-1.92), male sex (HR, 1.57; 95% CI, 1.31-1.88), high fraction of inspired oxygen (Fio2) (HR, 1.14; 95% CI, 1.10-1.19), high positive end-expiratory pressure (HR, 1.04; 95% CI, 1.01-1.06) or low Pao2:Fio2 ratio (HR, 0.80; 95% CI, 0.74-0.87) on ICU admission, and history of chronic obstructive pulmonary disease (HR, 1.68; 95% CI, 1.28-2.19), hypercholesterolemia (HR, 1.25; 95% CI, 1.02-1.52), and type 2 diabetes (HR, 1.18; 95% CI, 1.01-1.39). No medication was independently associated with mortality (angiotensin-converting enzyme inhibitors HR, 1.17; 95% CI, 0.97-1.42; angiotensin receptor blockers HR, 1.05; 95% CI, 0.85-1.29).

Conclusions and relevance: In this retrospective cohort study of critically ill patients admitted to ICUs in Lombardy, Italy, with laboratory-confirmed COVID-19, most patients required IMV. The mortality rate and absolute mortality were high.

Conflict of interest statement

Conflict of Interest Disclosures: Dr Grasselli reported receiving personal fees from Getinge Group, Biotest, Draeger Medical Systems, Inc, Thermo Fisher Scientific, and Fisher & Paykel outside the submitted work. Dr Zanella reported holding patents to WO2016189427 and WO2015IB55837 (licensed). Dr Bellani reported receiving grants and personal fees from Draeger Medical Systems, Inc, and Dimar SRL and personal fees from Hamilton Medical Products, Inc, Getinge Group, GE Healthcare, and Intersurgical outside the submitted work. Dr Iotti reported receiving personal fees from Hamilton Medical Products, Inc, Intersurgical, Maquet Italia, Cinisello Balsamo Eurosets, and Burke & Burke outside the submitted work. Dr Mojoli reported receiving fees for lectures from Hamilton Medical Products, Inc, GE Healthcare, and Seda SpA and a consultancy agreement between University of Pavia and Hamilton Medical Products, Inc. Dr Thompson reported receiving personal fees from Bayer AG outside the submitted work. Dr Pesenti reported receiving personal fees from Maquet Italia, Novalung/Xenios AG, Baxter International, Inc, and Boehringer Ingelheim outside the submitted work. Dr Cecconi reported receiving personal fees from Edwards Lifesciences, Directed Systems, and Cheetah Medical, Inc, outside the submitted work. No other disclosures were reported.

- 1 figure

Supplementary info

Publication types, MeSH terms Expand

Publication types

- Observational Study
- Research Support, Non-U.S. Gov't

MeSH terms

- Betacoronavirus / isolation & purification
- COVID-19
- COVID-19 Testing
- COVID-19 Vaccines
- Clinical Laboratory Techniques / methods
- Clinical Laboratory Techniques / statistics & numerical data
- Coronavirus Infections* / diagnosis
- Coronavirus Infections* / mortality
- Coronavirus Infections* / therapy
- Critical Illness* / mortality
- Critical Illness* / therapy
- Female

- Hospital Mortality
- Hospitalization / statistics & numerical data*
- Humans
- Intensive Care Units / statistics & numerical data*
- Italy / epidemiology
- Male
- Middle Aged
- Mortality
- Pandemics*
- Pneumonia, Viral* / mortality
- Pneumonia, Viral* / therapy
- Respiration, Artificial / statistics & numerical data*
- Retrospective Studies
- Risk Factors
- SARS-CoV-2

Full text links

FULL TEXT JAMA Internal Medicine Silverchair Information Systems Free PMC article

Proceed to details

Cite

Share

☐ 862

Observational Study

Medicine (Baltimore)

. 2021 Dec 3;100(48):e27881.

doi: 10.1097/MD.0000000000027881.

Emergency air evacuation of patients with acute respiratory failure due to SARS-CoV-2 from Mayotte to Reunion Island

Hamza Berguigua [1], Ludovic Iche [2], Philippe Roche [2], Cyril Aubert [1], Renaud Blondé [3], Antoine Legrand [4], Bérénice Puech [4], Chloé Combe [4], Charles Vidal [4], Margot Caron [4], Marie-Christine Jaffar-Bandjee [5], Christophe Caralp [2], Nora Oulehri [2], Hugo Kerambrun [3], Jérôme Allyn [4 6], Yvonnick Boué [3], Nicolas Allou [4 6]

Affiliations Expand

Affiliations

- [1] Department of Emergency, Center Hospitalier Universitaire Felix Guyon, Saint Denis, France.

- [2] Department of Emergency, Center Hospitalier de Mayotte, Mamoudzou, France.
- [3] Réanimation Polyvalente, Center Hospitalier de Mayotte, Mamoudzou, France.
- [4] Réanimation polyvalente, Center Hospitalier Universitaire Felix Guyon Allée des Topazes Saint Denis, France.
- [5] Microbiologie, Centre Hospitalier Universitaire Felix Guyon Allée des Topazes Saint Denis, France.
- [6] Département d'Informatique Clinique, Centre Hospitalier Universitaire Felix Guyon Allée des Topazes, Saint Denis, France.

- PMID: **35049190**
- DOI: 10.1097/MD.0000000000027881

Free article
Observational Study

Emergency air evacuation of patients with acute respiratory failure due to SARS-CoV-2 from Mayotte to Reunion Island

Hamza Berguigua et al. Medicine (Baltimore). 2021.
Free article

Show details

Medicine (Baltimore)
. 2021 Dec 3;100(48):e27881.
doi: 10.1097/MD.0000000000027881.

Authors

Hamza Berguigua [1], Ludovic Iche [2], Philippe Roche [2], Cyril Aubert [1], Renaud Blondé [3], Antoine Legrand [4], Bérénice Puech [4], Chloé Combe [4], Charles Vidal [4], Margot Caron [4], Marie-Christine Jaffar-Bandjee [5], Christophe Caralp [2], Nora Oulehri [2], Hugo Kerambrun [3], Jérôme Allyn [4] [6], Yvonnick Boué [3], Nicolas Allou [4] [6]

Affiliations

- [1] Department of Emergency, Center Hospitalier Universitaire Felix Guyon, Saint Denis, France.
- [2] Department of Emergency, Center Hospitalier de Mayotte, Mamoudzou, France.
- [3] Réanimation Polyvalente, Center Hospitalier de Mayotte, Mamoudzou, France.
- [4] Réanimation polyvalente, Center Hospitalier Universitaire Felix Guyon Allée des Topazes Saint Denis, France.
- [5] Microbiologie, Centre Hospitalier Universitaire Felix Guyon Allée des Topazes Saint Denis, France.
- [6] Département d'Informatique Clinique, Centre Hospitalier Universitaire Felix Guyon Allée des Topazes, Saint Denis, France.

- PMID: **35049190**

- DOI: 10.1097/MD.0000000000027881

Abstract

In February 2021, an explosion of cases of severe acute respiratory syndrome coronavirus 2 (SARS-CoV-2) pneumonia overwhelmed the only hospital in Mayotte. To report a case series of patients with acute respiratory failure (ARF) due to SARS-CoV-2 who were evacuated by air from Mayotte to Reunion Island.This retrospective observational study evaluated all consecutive patients with ARF due to SARS-CoV-2 who were evacuated by air from Mayotte Hospital to the intensive care unit (ICU) of Félix Guyon University Hospital in Reunion Island between February 2, and March 5, 2021.A total of 43 patients with SARS-CoV-2 pneumonia were evacuated by air, for a total flight time of 2 hours and a total travel time of 6 hours. Of these, 38 patients (88.4%) with a median age of 55 (46-65) years presented with ARF and were hospitalized in our ICU. Fifteen patients were screened for the SARS-CoV-2 501Y.V2 variant, all of whom tested positive. Thirteen patients (34.2%) developed an episode of severe hypoxemia during air transport, and the median paO2/FiO2 ratio was lower on ICU admission (140 [102-192] mmHg) than on departure (165 [150-200], P = .022). Factors associated with severe hypoxemia during air transport was lack of treatment with curare (P = .012) and lack of invasive mechanical ventilation (P = .003). Nine patients (23.7%) received veno-venous extracorporeal membrane oxygenation support in our ICU. Seven deaths (18.4%) occurred in hospital.Emergency air evacuation of patients with ARF due to SARS-CoV-2 was associated with severe hypoxemia but remained feasible. In cases of ARF due to SARS-CoV-2 requiring emergency air evacuation, sedated patients receiving invasive mechanical ventilation and curare should be prioritized over nonintubated patients. It is noteworthy that patients with SARS-CoV-2 pneumonia related to the 501Y.V2 variant were very severe despite their young age.

Copyright © 2021 the Author(s). Published by Wolters Kluwer Health, Inc.

Conflict of interest statement

The authors report no conflicts of interest.

- 26 references

Supplementary info

Publication types, MeSH terms, Substances, Supplementary concepts Expand

Publication types

- Observational Study

MeSH terms

- Aged
- Air Ambulances*
- Aircraft
- COVID-19 / complications*
- COVID-19 / diagnosis

- Comoros
- Curare
- Humans
- Hypoxia / etiology*
- Middle Aged
- Respiratory Distress Syndrome* / etiology
- Respiratory Distress Syndrome* / therapy
- Respiratory Insufficiency* / etiology
- Respiratory Insufficiency* / therapy
- Reunion / epidemiology
- SARS-CoV-2
- Transportation of Patients*

Substances

- Curare

Supplementary concepts

- SARS-CoV-2 variants

Full text links

Wolters Kluwer Wolters Kluwer

Proceed to details

Cite

Share

863

Observational Study

Eur Heart J

. 2020 Jun 7;41(22):2058-2066.

doi: 10.1093/eurheartj/ehaa433.

Association of hypertension and antihypertensive treatment with COVID-19 mortality: a retrospective observational study

Chao Gao [1, 2], Yue Cai [1, 3], Kan Zhang [1, 3], Lei Zhou [3, 4], Yao Zhang [3, 5], Xijing Zhang [3, 6], Qi Li [3, 7], Weiqin Li [3, 8], Shiming Yang [3, 9], Xiaoyan Zhao [3, 10], Yuying Zhao [3, 11], Hui Wang [3, 12], Yi Liu [1], Zhiyong Yin [1], Ruining Zhang [1], Rutao Wang [1, 2], Ming Yang [13], Chen Hui [14], William Wijns [2], J William McEvoy [2], Osama Soliman [2], Yoshinobu Onuma [2], Patrick W Serruys [2, 15], Ling Tao [1], Fei Li [1, 3]

Affiliations Expand

Affiliations

- [1] Department of Cardiology, Xijing Hospital, Changle West Road, Xi'an, 710032, China.
- [2] Department of Cardiology, National University of Ireland Galway, Galway, Ireland.
- [3] Huo shen shan Hospital, Wuhan, China.
- [4] Clinical Laboratory, Xijing Hospital, Xi'an, China.
- [5] Nursing Center, Xijing Hospital, Xi'an, China.
- [6] ICU, Xijing Hospital, Xi'an, China.
- [7] Pulmonary and Critical Care Medicine center, Xinqiao Hospital, Chongqing, China.
- [8] Department of Critical Care Medicine, Jinling hospital, Nanjing, China.
- [9] Department of Gastroenterology, Xinqiao Hospital, Chongqing, China.
- [10] Department of Cardiology, The 942 Hospital, Yinchuan, China.
- [11] Department of Cardiovascular Medicine, The 980 Hospital, Shijiazhuang, China.
- [12] Department of Pulmonary and Critical Care, The 905 Hospital of Shanghai, Shanghai, China.
- [13] The Central Hospital of Wuhan, Tongji Medical College, Huazhong University, Wuhan, China.
- [14] Department of Logistics Support, The 940 Hospital, Lanzhou, China.
- [15] NHLI, Imperial College London, London, UK.

- PMID: **32498076**
- PMCID: PMC7314067
- DOI: 10.1093/eurheartj/ehaa433

Free PMC article
Observational Study

Association of hypertension and antihypertensive treatment with COVID-19 mortality: a retrospective observational study

Chao Gao et al. Eur Heart J. 2020.
Free PMC article
Show details
Eur Heart J
. 2020 Jun 7;41(22):2058-2066.
doi: 10.1093/eurheartj/ehaa433.

Authors

Chao Gao [1, 2], Yue Cai [1, 3], Kan Zhang [1, 3], Lei Zhou [3, 4], Yao Zhang [3, 5], Xijing Zhang [3, 6], Qi Li [3, 7], Weiqin Li [3, 8], Shiming Yang [3, 9], Xiaoyan Zhao [3, 10], Yuying Zhao [3, 11], Hui Wang [3, 12], Yi Liu [1], Zhiyong Yin [1], Ruining Zhang [1], Rutao Wang [1, 2], Ming Yang [13], Chen

Hui [14], William Wijns [2], J William McEvoy [2], Osama Soliman [2], Yoshinobu Onuma [2], Patrick W Serruys [2, 15], Ling Tao [1], Fei Li [1, 3]

Affiliations

- [1] Department of Cardiology, Xijing Hospital, Changle West Road, Xi'an, 710032, China.
- [2] Department of Cardiology, National University of Ireland Galway, Galway, Ireland.
- [3] Huo shen shan Hospital, Wuhan, China.
- [4] Clinical Laboratory, Xijing Hospital, Xi'an, China.
- [5] Nursing Center, Xijing Hospital, Xi'an, China.
- [6] ICU, Xijing Hospital, Xi'an, China.
- [7] Pulmonary and Critical Care Medicine center, Xinqiao Hospital, Chongqing, China.
- [8] Department of Critical Care Medicine, Jinling hospital, Nanjing, China.
- [9] Department of Gastroenterology, Xinqiao Hospital, Chongqing, China.
- [10] Department of Cardiology, The 942 Hospital, Yinchuan, China.
- [11] Department of Cardiovascular Medicine, The 980 Hospital, Shijiazhuang, China.
- [12] Department of Pulmonary and Critical Care, The 905 Hospital of Shanghai, Shanghai, China.
- [13] The Central Hospital of Wuhan, Tongji Medical College, Huazhong University, Wuhan, China.
- [14] Department of Logistics Support, The 940 Hospital, Lanzhou, China.
- [15] NHLI, Imperial College London, London, UK.

- PMID: **32498076**
- PMCID: PMC7314067
- DOI: 10.1093/eurheartj/ehaa433

Abstract

Aims: It remains unknown whether the treatment of hypertension influences the mortality of patients diagnosed with coronavirus disease 2019 (COVID-19).

Methods and results: This is a retrospective observational study of all patients admitted with COVID-19 to Huo Shen Shan Hospital. The hospital was dedicated solely to the treatment of COVID-19 in Wuhan, China. Hypertension and the treatments were stratified according to the medical history or medications administrated prior to the infection. Among 2877 hospitalized patients, 29.5% (850/2877) had a history of hypertension. After adjustment for confounders, patients with hypertension had a two-fold increase in the relative risk of mortality as compared with patients without hypertension [4.0% vs. 1.1%, adjusted hazard ratio (HR) 2.12, 95% confidence interval (CI) 1.17-3.82, P = 0.013]. Patients with a history of hypertension but without antihypertensive treatment (n = 140) were associated with a significantly higher risk of mortality compared with those with antihypertensive treatments (n = 730) (7.9% vs. 3.2%, adjusted HR 2.17, 95% CI 1.03-4.57, P = 0.041). The mortality rates were similar between the renin-angiotensin-aldosterone system (RAAS) inhibitor (4/183) and non-RAAS inhibitor (19/527) cohorts (2.2% vs. 3.6%, adjusted HR 0.85, 95% CI 0.28-2.58, P = 0.774). However, in a study-level meta-analysis of four studies, the result showed that patients with RAAS inhibitor use tend to have a lower risk of mortality (relative risk 0.65, 95% CI 0.45-0.94, P = 0.20).

Conclusion: While hypertension and the discontinuation of antihypertensive treatment are suspected to be related to increased risk of mortality, in this retrospective observational analysis,

we did not detect any harm of RAAS inhibitors in patients infected with COVID-19. However, the results should be considered as exploratory and interpreted cautiously.

Keywords: Angiotensin receptor blockers (ARBs); Angiotensin-converting enzyme inhibitors (ACEIs); Antihypertensive regimen; COVID-19.

Published on behalf of the European Society of Cardiology. All rights reserved. © The Author(s) 2020. For permissions, please email: journals.permissions@oup.com.

Comment in

- Renin-angiotensin system inhibitors in the COVID-19 pandemic: consequences of antihypertensive drugs.
  Ruilope LM, Tamargo J, Ruiz-Hurtado G. Ruilope LM, et al. Eur Heart J. 2020 Jun 7;41(22):2067-2069. doi: 10.1093/eurheartj/ehaa487. Eur Heart J. 2020. PMID: 32498078 Free PMC article. No abstract available.
- The saga continues: is COVID-19 a cardiopulmonary disease?
  Lüscher TF. Lüscher TF. Eur Heart J. 2020 Jun 7;41(22):2041-2044. doi: 10.1093/eurheartj/ehaa502. Eur Heart J. 2020. PMID: 33216882 Free PMC article. No abstract available.

- 30 references
- 4 figures

Supplementary info

Publication types, MeSH terms, Substances Expand

Publication types

- Observational Study

MeSH terms

- Adult
- Aged
- Aged, 80 and over
- Antihypertensive Agents / therapeutic use*
- Betacoronavirus*
- COVID-19
- China / epidemiology
- Coronavirus Infections / complications
- Coronavirus Infections / mortality*
- Coronavirus Infections / therapy
- Female
- Follow-Up Studies
- Humans

- Hypertension / complications
- Hypertension / drug therapy*
- Hypertension / mortality
- Male
- Middle Aged
- Pandemics
- Pneumonia, Viral / complications
- Pneumonia, Viral / mortality*
- Pneumonia, Viral / therapy
- Retrospective Studies
- SARS-CoV-2

Substances

- Antihypertensive Agents

Full text links

OXFORD ACADEMIC Silverchair Information Systems Free PMC article

Proceed to details

Cite

Share

☐ 864

Observational Study

Am J Med Sci

. 2021 Feb;361(2):208-215.

doi: 10.1016/j.amjms.2020.11.005. Epub 2020 Nov 9.

Tocilizumab in the Management of COVID-19: A Preliminary Report

Michael Li [1], Erika J Yoo [2], Michael Baram [2], Melanie McArthur [1], Connor Skeehan [1], Bharat Awsare [2], Gautam George [2], Ross Summer [2], John Zurlo [3], Jack Jallo [4], Jesse Roman [5]

Affiliations Expand

Affiliations

- [1] Enterprise Analytics, Jefferson Health.
- [2] Division of Pulmonary, Allergy and Critical Care Medicine, Sidney Kimmel College of Medicine; Jane & Leonard Korman Respiratory Institute, Jefferson Health.
- [3] Division of Infectious Diseases, Sidney Kimmel College of Medicine.
- [4] Department of Neurosurgery; Thomas Jefferson University, Philadelphia, PA, United States.

- [5] Enterprise Analytics, Jefferson Health; Jane & Leonard Korman Respiratory Institute, Jefferson Health. Electronic address: jesse.roman@jefferson.edu.

- PMID: **33358502**
- PMCID: PMC7649658
- DOI: 10.1016/j.amjms.2020.11.005

Free PMC article
Observational Study

Tocilizumab in the Management of COVID-19: A Preliminary Report

Michael Li et al. Am J Med Sci. 2021 Feb.
Free PMC article

Show details

Am J Med Sci
. 2021 Feb;361(2):208-215.
doi: 10.1016/j.amjms.2020.11.005. Epub 2020 Nov 9.

Authors

Michael Li [1], Erika J Yoo [2], Michael Baram [2], Melanie McArthur [1], Connor Skeehan [1], Bharat Awsare [2], Gautam George [2], Ross Summer [2], John Zurlo [3], Jack Jallo [4], Jesse Roman [5]

Affiliations

- [1] Enterprise Analytics, Jefferson Health.
- [2] Division of Pulmonary, Allergy and Critical Care Medicine, Sidney Kimmel College of Medicine; Jane & Leonard Korman Respiratory Institute, Jefferson Health.
- [3] Division of Infectious Diseases, Sidney Kimmel College of Medicine.
- [4] Department of Neurosurgery; Thomas Jefferson University, Philadelphia, PA, United States.
- [5] Enterprise Analytics, Jefferson Health; Jane & Leonard Korman Respiratory Institute, Jefferson Health. Electronic address: jesse.roman@jefferson.edu.

- PMID: **33358502**
- PMCID: PMC7649658
- DOI: 10.1016/j.amjms.2020.11.005

Abstract

Importance: Pneumonia due to COVID-19 can lead to respiratory failure and death due to the development of the acute respiratory distress syndrome. Tocilizumab, a monoclonal antibody targeting the interleukin-6 receptor, is being administered off-label to some patients with COVID-19, and although early small studies suggested a benefit, there are no conclusive data proving its usefulness.

Objective: To evaluate outcomes in hospitalized patients with COVID-19 with or without treatment with Tocilizumab.

Design, setting, participants: Retrospective study of 1938 patients with confirmed COVID-19 pneumonia admitted to hospitals within the Jefferson Health system in Philadelphia, Pennsylvania, between March 25, 2020 and June 17, 2020, of which 307 received Tocilizumab.

Exposures: Confirmed COVID-19 pneumonia.

Main outcomes and measures: Outcomes data related to length of stay, admission to intensive care unit (ICU), requirement of mechanical ventilation, and mortality were collected and analyzed.

Results: The average age was 65.2, with 47% women; 36.4% were African-American. The average length of stay was 22 days with 26.3% of patients requiring admission to the ICU and 14.9% requiring mechanical ventilation. The overall mortality was 15.3%. Older age, admission to an ICU, and requirement for mechanical ventilation were associated with higher mortality. Treatment with Tocilizumab was also associated with higher mortality, which was mainly observed in subjects not requiring care in an ICU with estimated odds ratio (OR) of 2.9 ($p = 0.0004$). Tocilizumab treatment was also associated with higher likelihood of admission to an ICU ($OR = 4.8$, $p < 0.0001$), progression to requiring mechanical ventilation ($OR = 6.6$, $p < 0.0001$), and increased length of stay ($OR = 16.2$, $p < 0.0001$).

Conclusion and relevance: Our retrospective analysis revealed an association between Tocilizumab administration and increased mortality, ICU admission, mechanical ventilation, and length of stay in subjects with COVID-19. Prospective trials are needed to evaluate the true effect of Tocilizumab in this condition.

Keywords: COVID-19; IL-6; Outcomes; Treatment.

Copyright © 2020 Southern Society for Clinical Investigation. Published by Elsevier Inc. All rights reserved.

- 28 references
- 2 figures

Supplementary info

Publication types, MeSH terms, Substances, Supplementary concepts Expand

Publication types

- Observational Study

MeSH terms

- Aged
- Aged, 80 and over
- Antibodies, Monoclonal, Humanized / therapeutic use*
- COVID-19 / diagnosis
- COVID-19 / drug therapy*

- COVID-19 / mortality
- Disease Management*
- Female
- Humans
- Male
- Middle Aged
- Mortality / trends
- Retrospective Studies

Substances

- Antibodies, Monoclonal, Humanized
- tocilizumab

Supplementary concepts

- COVID-19 drug treatment

Full text links

ELSEVIER FULL-TEXT ARTICLE Elsevier Science Free PMC article
Proceed to details
Cite
Share
865
Observational Study
Int J Psychiatry Clin Pract
. 2021 Jun;25(2):135-139.
doi: 10.1080/13651501.2020.1859120. Epub 2020 Dec 21.

Effects of lockdown on emergency room admissions for psychiatric evaluation: an observational study from the AUSL Romagna, Italy

Massimiliano Beghi [1], Riccardo Brandolini [2 3], Ilaria Casolaro [4], Ettore Beghi [5], Cesare Maria Cornaggia [6], Carlo Fraticelli [4], Giovanni De Paoli [1], Claudio Ravani [7], Giulio Castelpietra [8 9], Silvia Ferrari [10 11]
Affiliations Expand

Affiliations

- [1] Department of Mental Health, AUSL Romagna, Cesena, Italy.
- [2] Department of Mental Health and Pathological Dependencies, AUSL Romagna, Ravenna, Italy.
- [3] Department of Biomedical and NeuroMotor Sciences, Psychiatry Unit, Alma Mater Studiorum, Bologna, Italy.
- [4] Department of Mental Health and Addictions, ASST Lariana, Como, Italy.
- [5] Department of Neurosciences, IRSST Mario Negri, Milan, Italy.
- [6] School of Medicine and Surgery, University of Milano-Bicocca, Monza, Italy.
- [7] Department of Mental Health, AUSL Romagna, Forlì, Italy.
- [8] Central Health Directorate, Friuli Venezia Giulia Region, Trieste, Italy.
- [9] Department of Medicine (DAME), University of Udine, Udine, Italy.
- [10] Department of Biomedical Metabolic and Neural Sciences, University of Modena & Reggio Emilia, Modena, Italy.
- [11] Department of Mental Health, AUSL Modena, Modena, Italy.

- PMID: **33346685**
- DOI: 10.1080/13651501.2020.1859120

Observational Study

Effects of lockdown on emergency room admissions for psychiatric evaluation: an observational study from the AUSL Romagna, Italy

Massimiliano Beghi et al. Int J Psychiatry Clin Pract. 2021 Jun.

Show details

Int J Psychiatry Clin Pract

. 2021 Jun;25(2):135-139.

doi: 10.1080/13651501.2020.1859120. Epub 2020 Dec 21.

Authors

Massimiliano Beghi [1], Riccardo Brandolini [2] [3], Ilaria Casolaro [4], Ettore Beghi [5], Cesare Maria Cornaggia [6], Carlo Fraticelli [4], Giovanni De Paoli [1], Claudio Ravani [7], Giulio Castelpietra [8] [9], Silvia Ferrari [10] [11]

Affiliations

- [1] Department of Mental Health, AUSL Romagna, Cesena, Italy.
- [2] Department of Mental Health and Pathological Dependencies, AUSL Romagna, Ravenna, Italy.

- [3] Department of Biomedical and NeuroMotor Sciences, Psychiatry Unit, Alma Mater Studiorum, Bologna, Italy.
- [4] Department of Mental Health and Addictions, ASST Lariana, Como, Italy.
- [5] Department of Neurosciences, IRSST Mario Negri, Milan, Italy.
- [6] School of Medicine and Surgery, University of Milano-Bicocca, Monza, Italy.
- [7] Department of Mental Health, AUSL Romagna, Forlì, Italy.
- [8] Central Health Directorate, Friuli Venezia Giulia Region, Trieste, Italy.
- [9] Department of Medicine (DAME), University of Udine, Udine, Italy.
- [10] Department of Biomedical Metabolic and Neural Sciences, University of Modena & Reggio Emilia, Modena, Italy.
- [11] Department of Mental Health, AUSL Modena, Modena, Italy.

- PMID: **33346685**
- DOI: 10.1080/13651501.2020.1859120

Abstract

Objectives: An observation of the admissions to the emergency room (ER) requiring psychiatric evaluation during the lockdown and investigation of the demographic and clinical variables.

Methods: Retrospective longitudinal observational study of ER accesses for psychiatric evaluation was performed, comparing two periods (9 March-3 May 2020 vs. 9 March-3 May 2019). Data (number of admissions, key baseline demographic and clinical variables) were extracted from the ER databases of referral centres in a well-defined geographic area of North-Eastern Italy (Cesena, Ravenna, Forlì, and Rimini).

Results: A 15% reduction of psychiatric referrals was observed, together with a 17% reduction in the total number of patients referring to the ER. This reduction was most evident in the first month of the lockdown period (almost 25% reduction of both referrals and patients). Female gender (OR: 1.52: 95%, CI: 1.12-2.06) and being a local resident (OR: 1.54: 95%CI: 1.02-2.34) were factors associated with the decrease.

Conclusions: Lockdown changed dramatically health priorities in the local population, including people with mental health. We speculate that our observations do not only refer to the confinement due to the lockdown regime but also to fear of contagion and adoption of different coping strategies, especially in women.Key-pointsDuring lockdown 15% reduction of psychiatric visits and >17% reduction in the number of psychiatric patients referring to the ER was observed.in the first four weeks of the lockdown almost 25% reduction of both visits and patients was observedFemale gender and being a local resident were factors associated with the decrease.

Keywords: COVID-19; Lockdown; consultation; mental health; outbreak; pandemic.

Supplementary info

Publication types, MeSH terms Expand

Publication types

- Observational Study

MeSH terms

- Adolescent
- Adult
- Aged
- Aged, 80 and over
- COVID-19 / prevention & control*
- Emergency Service, Hospital / statistics & numerical data*
- Female
- Humans
- Italy / epidemiology
- Longitudinal Studies
- Male
- Mental Disorders / diagnosis
- Mental Disorders / epidemiology*
- Mental Disorders / therapy
- Middle Aged
- Patient Admission / statistics & numerical data*
- Quarantine / psychology
- Quarantine / statistics & numerical data*
- Referral and Consultation / statistics & numerical data
- Retrospective Studies
- Risk Factors
- Sex Factors
- Young Adult

Full text links

View full text Taylor & Francis
Proceed to details
Cite
Share
866
Observational Study
Eur J Phys Rehabil Med
. 2021 Jun;57(3):451-457.
doi: 10.23736/S1973-9087.21.06678-8. Epub 2021 Feb 4.

The consequences of COVID-19 outbreak on outpatient rehabilitation services: a single-center experience in Slovenia

Breda Jesenšek Papež [1] [2] [3], Luka Šošić [4], Vida Bojnec [4] [5]
Affiliations Expand

Affiliations

- [1] Institute of Physical and Rehabilitation Medicine, University Medical Center Maribor, Maribor, Slovenia - breda.jesensek@gmail.com.
- [2] Faculty of Medicine, University of Maribor, Maribor, Slovenia - breda.jesensek@gmail.com.
- [3] Alma Mater Europaea ECM, Maribor, Slovenia - breda.jesensek@gmail.com.
- [4] Institute of Physical and Rehabilitation Medicine, University Medical Center Maribor, Maribor, Slovenia.
- [5] Faculty of Medicine, University of Maribor, Maribor, Slovenia.

- PMID: **33541043**
- DOI: 10.23736/S1973-9087.21.06678-8

Free article
Observational Study

The consequences of COVID-19 outbreak on outpatient rehabilitation services: a single-center experience in Slovenia

Breda Jesenšek Papež et al. Eur J Phys Rehabil Med. 2021 Jun.
Free article
Show details
Eur J Phys Rehabil Med
. 2021 Jun;57(3):451-457.
doi: 10.23736/S1973-9087.21.06678-8. Epub 2021 Feb 4.

Authors

Breda Jesenšek Papež [1] [2] [3], Luka Šošić [4], Vida Bojnec [4] [5]

Affiliations

- [1] Institute of Physical and Rehabilitation Medicine, University Medical Center Maribor, Maribor, Slovenia - breda.jesensek@gmail.com.

- [2] Faculty of Medicine, University of Maribor, Maribor, Slovenia - breda.jesensek@gmail.com.
- [3] Alma Mater Europaea ECM, Maribor, Slovenia - breda.jesensek@gmail.com.
- [4] Institute of Physical and Rehabilitation Medicine, University Medical Center Maribor, Maribor, Slovenia.
- [5] Faculty of Medicine, University of Maribor, Maribor, Slovenia.

- PMID: **33541043**
- DOI: 10.23736/S1973-9087.21.06678-8

Abstract

Background: The COVID-19 pandemic was the reason for closing down all non-urgent outpatient services in hospitals treating COVID-19 patients. The lockdown and reorganization of medical units also altered the accessibility to outpatient rehabilitation services.

Aim: The focus of interest in our report lies in the evaluation of the outpatient rehabilitation treatment accessibility at our center in the time of the COVID-19 pandemic.

Design: Cross-sectional observational study.

Setting: Outpatients Rehabilitation Unit at University Medical Centre Maribor (UMC Maribor), Slovenia.

Population: Patients with diverse pathologies referred to outpatient rehabilitation.

Methods: The data were gathered retrospectively at the Institute of Physical and Rehabilitation Medicine (IPRM) at the UMC Maribor. The search included all the patients treated at IPRM in the pre-COVID and COVID period from March 16 to August 31 in 2019 and 2020. The data for the period including the lockdown (March 16 to August 31, 2020) and the period after the lockdown (June 1 to August 31, 2020) was analyzed and compared to the same timeframes in 2019. We were interested in the magnitude of decline in the total number of patients, the number of the first and follow-up visits, the number of sessions and in the profile and pathologies of patients comparing pre-COVID and COVID period. The χ^2 and Fisher's Exact test were used in the analysis.

Results: With the lockdown period included there was a 44% decline in the total number of patients, a 71.1% decline in the number of sessions, a 42% decline of the first visits and a 60.9% decline of follow-up visits. When comparing the pre-COVID and COVID period after the lockdown, a 28.5% decline in the number of patients treated in 2020 compared to 2019 was observed. By analyzing the number of sessions in the pre-COVID and COVID period after the lockdown there was a 46.6% decline. No statistically significant difference was found in the age groups between the two periods (X^2=9.466; P=0.05). The difference for the first and follow-up visits in 2019 and 2020 proved to be statistically significant (P<0.001), as well as the difference in percentage of patients in the acute and chronic group (P=0.037).

Conclusions: Our findings showed how COVID-19 outbreak hindered the accessibility to outpatient rehabilitation service. Not only has the number of patients substantially reduced in the year 2020 compared to 2019, but also the number of sessions and number of first and follow-up visits declined. The demographic structure of the patients remained the same.

Clinical rehabilitation impact: This study adds evidence at the level of health services about lower standard of care in the physical and rehabilitation medicine field for patients experiencing disabling conditions in the time of COVID-19.

Supplementary info

Publication types, MeSH terms Expand

Publication types

- Observational Study

MeSH terms

- Adolescent
- Adult
- Aged
- Ambulatory Care*
- COVID-19 / epidemiology*
- Child
- Child, Preschool
- Cross-Sectional Studies
- Female
- Health Services Accessibility*
- Humans
- Infant
- Infant, Newborn
- Male
- Middle Aged
- Pandemics
- Physical and Rehabilitation Medicine*
- SARS-CoV-2
- Slovenia / epidemiology
- Young Adult

Full text links

FREE FULL TEXT article at minervamedica.it Minerva Medica

Proceed to details

Cite

Share

867

Observational Study

Arch Pathol Lab Med

. 2020 Dec 1;144(12):1457-1464.
doi: 10.5858/arpa.2020-0389-SA.

A Comprehensive Appraisal of Laboratory Biochemistry Tests as Major Predictors of COVID-19 Severity

Elena Aloisio [1], Mariia Chibireva [1], Ludovica Serafini [1], Sara Pasqualetti [1], Felicia S Falvella [1], Alberto Dolci [1 2], Mauro Panteghini [1 2]
Affiliations Expand

Affiliations

- [1] From the Clinical Pathology Unit, ASST Fatebenefratelli-Sacco, Milan, Italy (Aloisio, Chibireva, Serafini, Pasqualetti, Falvella, Dolci, Panteghini).
- [2] The Department of Biomedical and Clinical Sciences "Luigi Sacco," University of Milan, Milan, Italy (Dolci, Panteghini).

- PMID: **32649222**
- DOI: 10.5858/arpa.2020-0389-SA

Free article
Observational Study

A Comprehensive Appraisal of Laboratory Biochemistry Tests as Major Predictors of COVID-19 Severity

Elena Aloisio et al. Arch Pathol Lab Med. 2020.
Free article
Show details
Arch Pathol Lab Med
. 2020 Dec 1;144(12):1457-1464.
doi: 10.5858/arpa.2020-0389-SA.

Authors

Elena Aloisio [1], Mariia Chibireva [1], Ludovica Serafini [1], Sara Pasqualetti [1], Felicia S Falvella [1], Alberto Dolci [1 2], Mauro Panteghini [1 2]

Affiliations

- [1] From the Clinical Pathology Unit, ASST Fatebenefratelli-Sacco, Milan, Italy (Aloisio, Chibireva, Serafini, Pasqualetti, Falvella, Dolci, Panteghini).

- [2] The Department of Biomedical and Clinical Sciences "Luigi Sacco," University of Milan, Milan, Italy (Dolci, Panteghini).

- PMID: **32649222**
- DOI: 10.5858/arpa.2020-0389-SA

Abstract

Context.—: A relevant portion of coronavirus disease 2019 (COVID-19) patients develop severe disease with negative outcomes. Several biomarkers have been proposed to predict COVID-19 severity, but no definite interpretative criteria have been established to date for stratifying risk.

Objective.—: To evaluate 6 serum biomarkers (C-reactive protein, lactate dehydrogenase, D-dimer, albumin, ferritin, and cardiac troponin T) for predicting COVID-19 severity and to define related cutoffs able to aid clinicians in risk stratification of hospitalized patients.

Design.—: A retrospective study of 427 COVID-19 patients was performed. Patients were divided into groups based on their clinical outcome: nonsurvivors versus survivors and patients admitted to an intensive care unit versus others. Receiver operating characteristic curves and likelihood ratios were employed to define predictive cutoffs for evaluated markers.

Results.—: Marker concentrations at peak were significantly different between groups for both selected outcomes. At univariate logistic regression analysis, all parameters were significantly associated with higher odds of death and intensive care. At the multivariate analysis, high concentrations of lactate dehydrogenase and low concentrations of albumin in serum remained significantly associated with higher odds of death, whereas only low lactate dehydrogenase activities remained associated with lower odds of intensive care admission. The best cutoffs for death prediction were greater than 731 U/L for lactate dehydrogenase and 18 g/L or lower for albumin, whereas a lactate dehydrogenase activity lower than 425 U/L was associated with a negative likelihood ratio of 0.10 for intensive treatment.

Conclusions.—: Our study identifies which biochemistry tests represent major predictors of COVID-19 severity and defines the best cutoffs for their use.

© 2020 College of American Pathologists.

Supplementary info

Publication types, MeSH terms, Substances Expand

Publication types

- Observational Study

MeSH terms

- Adolescent
- Adult
- Aged
- Aged, 80 and over

- Biomarkers / blood*
- COVID-19 / blood
- COVID-19 / diagnosis*
- COVID-19 / mortality
- COVID-19 / therapy
- COVID-19 Testing / methods*
- Critical Care
- Female
- Humans
- Italy / epidemiology
- Logistic Models
- Male
- Middle Aged
- Prognosis
- ROC Curve
- Retrospective Studies
- Severity of Illness Index*
- Survival Analysis
- Young Adult

Substances

- Biomarkers

Full text links

ALLEN PRESS Open Access Silverchair Information Systems

Proceed to details

Cite

Share

☐ 868

Observational Study

J Anesth

. 2020 Aug;34(4):599-606.
doi: 10.1007/s00540-020-02778-8. Epub 2020 Apr 17.

Summary of 20 tracheal intubation by anesthesiologists for patients with severe COVID-19 pneumonia: retrospective case series

Li Zhang [1], Jiyong Li [1], Mingxing Zhou [1], Zhijun Chen [2]
Affiliations Expand

Affiliations

- [1] Department of Anesthesiology, Wuhan No.1 Hospital, Zhongshan Avenue 215#, Wuhan, 430022, Hubei, China.
- [2] Department of Anesthesiology, Wuhan No.1 Hospital, Zhongshan Avenue 215#, Wuhan, 430022, Hubei, China. doctorczj@163.com.

- PMID: **32303885**
- PMCID: PMC7164839
- DOI: 10.1007/s00540-020-02778-8

Free PMC article
Observational Study

Summary of 20 tracheal intubation by anesthesiologists for patients with severe COVID-19 pneumonia: retrospective case series

Li Zhang et al. J Anesth. 2020 Aug.
Free PMC article
Show details
J Anesth
. 2020 Aug;34(4):599-606.
doi: 10.1007/s00540-020-02778-8. Epub 2020 Apr 17.

Authors

Li Zhang [1], Jiyong Li [1], Mingxing Zhou [1], Zhijun Chen [2]

Affiliations

- [1] Department of Anesthesiology, Wuhan No.1 Hospital, Zhongshan Avenue 215#, Wuhan, 430022, Hubei, China.

- [2] Department of Anesthesiology, Wuhan No.1 Hospital, Zhongshan Avenue 215#, Wuhan, 430022, Hubei, China. doctorczj@163.com.

- PMID: **32303885**
- PMCID: PMC7164839
- DOI: 10.1007/s00540-020-02778-8

Abstract

SARS-CoV-2 pandemic is announced and it is very important to share our experience to the critical care community in the early stage. Urgent intubation team was organized by anesthesiologists and was dispatched upon request. We have retrospectively reviewed medical charts of 20 critically ill patients with Covid-19 pneumonia who required tracheal intubation from February 17 to March 19 in Wuhan No.1 hospital, China. We collected their demographics, vital signs, blood gas analysis before and after tracheal intubation, and 7-day outcome after tracheal intubation. Out of 20 patients, 90% were over 60 years old and 15 were with at least one comorbidity. All meet the indication for tracheal intubation announced by treatment expert group. We had successfully intubated all patients using personal protective equipment without circulatory collapse during tracheal intubation. During the observational period, none of 17 anesthesiologists were infected. Although intubation improved SPO_2, reduced $PaCO_2$ and blood lactate, seven of 20 patients died within 7-days after tracheal intubation. Non-survivors showed significantly lower SPO_2 and higher $PaCO_2$ and blood lactate compared to survivors. For those who are anticipated to deteriorate severe pneumonia with poor prognosis, earlier respiratory support with tracheal intubation may be advised to improve outcome.

Keywords: Blood gas analysis; COVID-19; SARS-CoV-2; Tracheal intubation.

Conflict of interest statement

The author(s) declare that they have no conflict of interest.

- 8 references

Supplementary info

Publication types, MeSH terms, Substances Expand

Publication types

- Observational Study

MeSH terms

- Aged
- Aged, 80 and over
- Anesthesiologists
- Betacoronavirus*
- Blood Gas Analysis

- COVID-19
- China / epidemiology
- Coronavirus Infections / blood
- Coronavirus Infections / complications*
- Coronavirus Infections / mortality
- Critical Care
- Critical Illness
- Female
- Hospital Mortality
- Humans
- Intubation, Intratracheal*
- Male
- Middle Aged
- Oxygen / blood
- Pandemics
- Pneumonia, Viral / blood
- Pneumonia, Viral / complications
- Pneumonia, Viral / etiology
- Pneumonia, Viral / mortality
- Pneumonia, Viral / therapy*
- Respiratory Insufficiency / etiology
- Respiratory Insufficiency / therapy*
- Retrospective Studies
- SARS-CoV-2
- Survival Rate
- Time-to-Treatment

Substances

- Oxygen

Full text links

SpringerLink FULL-TEXT ARTICLE Springer Free PMC article
Proceed to details
Cite
Share
869
Observational Study
Eur Heart J Cardiovasc Pharmacother
. 2022 Feb 16;8(2):157-164.
doi: 10.1093/ehjcvp/pvaa128.

Effect of statin therapy on SARS-CoV-2 infection-related mortality in hospitalized patients

Lluís Masana [1], Eudald Correig [2], Cèlia Rodríguez-Borjabad [1], Eva Anoro [3], Juan Antonio Arroyo [4], Carlos Jericó [5], Angels Pedragosa [6], Marcel la Miret [7], Silvia Näf [8], Anna Pardo [9], Verónica Perea [10], Rosa Pérez-Bernalte [11], Núria Plana [1], Rafael Ramírez-Montesinos [12], Meritxell Royuela [13], Cristina Soler [14], Maria Urquizu-Padilla [15], Alberto Zamora [16], Juan Pedro-Botet [17]

Affiliations Expand

Affiliations

- [1] CIBERDEM, Universitat Rovira i Virgili, LIPIDCAS, University Hospital Sant Joan IISPV, Reus, Spain.
- [2] Universitat Rovira i Virgili, Statistics Department, Institut Investigació Sanitaria Pere Virgili, Reus, Spain.
- [3] LIPIDCAS, Pius Hospital Valls, Valls, Spain.
- [4] Lipid Unit, University Hospital Santa Creu i Sant Pau, Barcelona Autonomous University, Barcelona, Spain.
- [5] Lipid Unit, Hospital Moises Broggi, Consorci Sanitari Integral, Sant Joan Despí, Spain.
- [6] Lipid Unit, Consorci Sanitari Terrassa, Spain.
- [7] LIPIDCAS, Endocrinology Department, Hospital Verge de la Cinta, Tortosa, Spain.
- [8] LIPIDCAS, Endocrinology Department, University Hospital Joan XXIII, IISPV, CIBERDEM, Universitat Rovira i Virgili, Tarragona, Spain.
- [9] Internal Medicine Department, Hospital Delfos, Barcelona, Spain.
- [10] Lipid Unit, Hospital Mutua Terrasa, Barcelona, Spain.
- [11] LIPIDCAS, Hospital del Vendrell, El Vendrell, Spain.
- [12] LIPIDCAS, Hospital Sant Pau i Santa Tecla, Tarragona, Spain.
- [13] Lipid Unit, ALTHAIA, Xarxa Assistencial Universitària de Manresa, Spain.
- [14] Lipid Unit, Hospital Santa Caterina, Girona, Spain.
- [15] Lipid Unit, University Hospital Vall d'Hebron, Barcelona Autonomous University, Barcelona, Spain.
- [16] Lipid Unit, Corporació de Salut del Maresme i la Selva, Hospital de Blanes, Spain.
- [17] Lipid Unit, University Hospital del Mar, Barcelona Autonomous University, Barcelona, Spain.

- PMID: **33135047**
- PMCID: PMC7665420
- DOI: 10.1093/ehjcvp/pvaa128

Free PMC article
Observational Study

Effect of statin therapy on SARS-CoV-2 infection-related mortality in hospitalized patients

Lluís Masana et al. Eur Heart J Cardiovasc Pharmacother. 2022.
Free PMC article
Show details
Eur Heart J Cardiovasc Pharmacother
. 2022 Feb 16;8(2):157-164.
doi: 10.1093/ehjcvp/pvaa128.

Authors

Lluís Masana [1], Eudald Correig [2], Cèlia Rodríguez-Borjabad [1], Eva Anoro [3], Juan Antonio Arroyo [4], Carlos Jericó [5], Angels Pedragosa [6], Marcel la Miret [7], Silvia Näf [8], Anna Pardo [9], Verónica Perea [10], Rosa Pérez-Bernalte [11], Núria Plana [1], Rafael Ramírez-Montesinos [12], Meritxell Royuela [13], Cristina Soler [14], Maria Urquizu-Padilla [15], Alberto Zamora [16], Juan Pedro-Botet [17]

Affiliations

- [1] CIBERDEM, Universitat Rovira i Virgili, LIPIDCAS, University Hospital Sant Joan IISPV, Reus, Spain.
- [2] Universitat Rovira i Virgili, Statistics Department, Institut Investigació Sanitaria Pere Virgili, Reus, Spain.
- [3] LIPIDCAS, Pius Hospital Valls, Valls, Spain.
- [4] Lipid Unit, University Hospital Santa Creu i Sant Pau, Barcelona Autonomous University, Barcelona, Spain.
- [5] Lipid Unit, Hospital Moises Broggi, Consorci Sanitari Integral, Sant Joan Despí, Spain.
- [6] Lipid Unit, Consorci Sanitari Terrassa, Spain.
- [7] LIPIDCAS, Endocrinology Department, Hospital Verge de la Cinta, Tortosa, Spain.
- [8] LIPIDCAS, Endocrinology Department, University Hospital Joan XXIII, IISPV, CIBERDEM, Universitat Rovira i Virgili, Tarragona, Spain.
- [9] Internal Medicine Department, Hospital Delfos, Barcelona, Spain.
- [10] Lipid Unit, Hospital Mutua Terrasa, Barcelona, Spain.
- [11] LIPIDCAS, Hospital del Vendrell, El Vendrell, Spain.
- [12] LIPIDCAS, Hospital Sant Pau i Santa Tecla, Tarragona, Spain.
- [13] Lipid Unit, ALTHAIA, Xarxa Assistencial Universitària de Manresa, Spain.
- [14] Lipid Unit, Hospital Santa Caterina, Girona, Spain.
- [15] Lipid Unit, University Hospital Vall d'Hebron, Barcelona Autonomous University, Barcelona, Spain.
- [16] Lipid Unit, Corporació de Salut del Maresme i la Selva, Hospital de Blanes, Spain.
- [17] Lipid Unit, University Hospital del Mar, Barcelona Autonomous University, Barcelona, Spain.

- PMID: **33135047**
- PMCID: PMC7665420
- DOI: 10.1093/ehjcvp/pvaa128

Abstract

Aim: Assessing the effect of statin therapy (ST) at hospital admission for COVID-19 on in-hospital mortality.

Methods and results: Retrospective observational study. Patients taking statins were 11 years older and had significantly more comorbidities than patients who were not taking statins. A genetic matching (GM) procedure was performed prior to analysis of the mortality risk. A Cox proportional hazards model was used for the cause-specific hazard (CSH) function, and a competing-risks Fine and Gray (FG) model was also used to study the direct effects of statins on risk. Data from reverse transcription-polymerase chain reaction-confirmed 2157 SARS-CoV-2-infected patients [1234 men, 923 women; age: 67 y/o (IQR 54-78)] admitted to the hospital were retrieved from the clinical records in anonymized manner. Three hundred and fifty-three deaths occurred. Five hundred and eighty-one patients were taking statins. Univariate test after GM showed a significantly lower mortality rate in patients on ST than the matched non-statin group (19.8% vs. 25.4%, $\chi 2$ with Yates continuity correction: P = 0.027). The mortality rate was even lower in patients (n = 336) who maintained their statin treatments during hospitalization compared with the GM non-statin group (17.4%; P = 0.045). The Cox model applied to the CSH function [HR = 0.58(CI: 0.39-0.89); P = 0.01] and the competing-risks FG model [HR = 0.60 (CI: 0.39-0.92); P = 0.02] suggest that statins are associated with reduced COVID-19-related mortality.

Conclusions: A lower SARS-CoV-2 infection-related mortality was observed in patients treated with ST prior to hospitalization. Statin therapy should not be discontinued due to the global concern of the pandemic or in patients hospitalized for COVID-19.

Keywords: COVID-19; Cardiovascular risk; Mortality; SARS-CoV-2; Statins.

Published on behalf of the European Society of Cardiology. All rights reserved. © The Author(s) 2020. For permissions, please email: journals.permissions@oup.com.

Supplementary info

Publication types, MeSH terms, Substances, Grant support Expand

Publication types

- Observational Study
- Research Support, Non-U.S. Gov't

MeSH terms

- Aged
- COVID-19*
- Female
- Hospital Mortality

- Humans
- Hydroxymethylglutaryl-CoA Reductase Inhibitors* / adverse effects
- Male
- Pandemics
- SARS-CoV-2

Substances

- Hydroxymethylglutaryl-CoA Reductase Inhibitors

Grant support

- Institut de Investigació Sanitària Pere Virgili
- IISPV
- Xarxa de Unitat de Lipids i Aterioesclerosis
- XULA

Full text links

OXFORD ACADEMIC Silverchair Information Systems Free PMC article
Proceed to details
Cite
Share
870
Observational Study
Resuscitation
. 2020 Nov;156:84-91.
doi: 10.1016/j.resuscitation.2020.08.124. Epub 2020 Sep 9.

Predicting intensive care unit admission and death for COVID-19 patients in the emergency department using early warning scores

Marcello Covino [1], Claudio Sandroni [2], Michele Santoro [1], Luca Sabia [1], Benedetta Simeoni [1], Maria Grazia Bocci [3], Veronica Ojetti [4], Marcello Candelli [1], Massimo Antonelli [5], Antonio Gasbarrini [6], Francesco Franceschi [7]
Affiliations Expand

Affiliations

- [1] Emergency Department, Fondazione Policlinico Universitario A. Gemelli, IRCCS, Rome, Italy.

- [2] Department of Intensive Care, Emergency Medicine and Anaesthesiology, Fondazione Policlinico Universitario A. Gemelli-IRCCS, Rome, Italy; Institute of Anaesthesiology and Intensive Care Medicine, Università Cattolica del Sacro Cuore, Rome, Italy. Electronic address: claudio.sandroni@policlinicogemelli.it.
- [3] Department of Intensive Care, Emergency Medicine and Anaesthesiology, Fondazione Policlinico Universitario A. Gemelli-IRCCS, Rome, Italy.
- [4] Emergency Department, Fondazione Policlinico Universitario A. Gemelli, IRCCS, Rome, Italy; Department of Internal Medicine and Gastroenterology, Fondazione Policlinico Universitario A. Gemelli, IRCCS, Rome, Italy.
- [5] Department of Intensive Care, Emergency Medicine and Anaesthesiology, Fondazione Policlinico Universitario A. Gemelli-IRCCS, Rome, Italy; Department of Internal Medicine and Gastroenterology, Fondazione Policlinico Universitario A. Gemelli, IRCCS, Rome, Italy.
- [6] Department of Internal Medicine and Gastroenterology, Fondazione Policlinico Universitario A. Gemelli, IRCCS, Rome, Italy; Institute of Internal Medicine and Gastroenterology, Università Cattolica del Sacro Cuore, Rome, Italy.
- [7] Emergency Department, Fondazione Policlinico Universitario A. Gemelli, IRCCS, Rome, Italy; Institute of Emergency Medicine, Università Cattolica del Sacro Cuore, Rome, Italy.

- PMID: **32918985**
- PMCID: PMC7480278
- DOI: 10.1016/j.resuscitation.2020.08.124

Free PMC article
Observational Study

Predicting intensive care unit admission and death for COVID-19 patients in the emergency department using early warning scores

Marcello Covino et al. Resuscitation. 2020 Nov.
Free PMC article
Show details
Resuscitation
. 2020 Nov;156:84-91.
doi: 10.1016/j.resuscitation.2020.08.124. Epub 2020 Sep 9.

Authors

Marcello Covino [1], Claudio Sandroni [2], Michele Santoro [1], Luca Sabia [1], Benedetta Simeoni [1], Maria Grazia Bocci [3], Veronica Ojetti [4], Marcello Candelli [1], Massimo Antonelli [5], Antonio Gasbarrini [6], Francesco Franceschi [7]

Affiliations

- [1] Emergency Department, Fondazione Policlinico Universitario A. Gemelli, IRCCS, Rome, Italy.
- [2] Department of Intensive Care, Emergency Medicine and Anaesthesiology, Fondazione Policlinico Universitario A. Gemelli-IRCCS, Rome, Italy; Institute of Anaesthesiology and Intensive Care Medicine, Università Cattolica del Sacro Cuore, Rome, Italy. Electronic address: claudio.sandroni@policlinicogemelli.it.
- [3] Department of Intensive Care, Emergency Medicine and Anaesthesiology, Fondazione Policlinico Universitario A. Gemelli-IRCCS, Rome, Italy.
- [4] Emergency Department, Fondazione Policlinico Universitario A. Gemelli, IRCCS, Rome, Italy; Department of Internal Medicine and Gastroenterology, Fondazione Policlinico Universitario A. Gemelli, IRCCS, Rome, Italy.
- [5] Department of Intensive Care, Emergency Medicine and Anaesthesiology, Fondazione Policlinico Universitario A. Gemelli-IRCCS, Rome, Italy; Department of Internal Medicine and Gastroenterology, Fondazione Policlinico Universitario A. Gemelli, IRCCS, Rome, Italy.
- [6] Department of Internal Medicine and Gastroenterology, Fondazione Policlinico Universitario A. Gemelli, IRCCS, Rome, Italy; Institute of Internal Medicine and Gastroenterology, Università Cattolica del Sacro Cuore, Rome, Italy.
- [7] Emergency Department, Fondazione Policlinico Universitario A. Gemelli, IRCCS, Rome, Italy; Institute of Emergency Medicine, Università Cattolica del Sacro Cuore, Rome, Italy.

- PMID: **32918985**
- PMCID: PMC7480278
- DOI: 10.1016/j.resuscitation.2020.08.124

Abstract

Aims: To identify the most accurate early warning score (EWS) for predicting an adverse outcome in COVID-19 patients admitted to the emergency department (ED).

Methods: In adult consecutive patients admitted (March 1-April 15, 2020) to the ED of a major referral centre for COVID-19, we retrospectively calculated NEWS, NEWS2, NEWS-C, MEWS, qSOFA, and REMS from physiological variables measured on arrival. Sensitivity, specificity, positive (PPV) and negative predictive value (NPV), and the area under the receiver operating characteristic (AUROC) curve of each EWS for predicting admission to the intensive care unit (ICU) and death at 48 h and 7 days were calculated.

Results: We included 334 patients (119 [35.6%] females, median age 66 [54-78] years). At 7 days, the rates of ICU admission and death were 56/334 (17%) and 26/334 (7.8%), respectively. NEWS was the most accurate predictor of ICU admission within 7 days (AUROC 0.783 [95% CI, 0.735-0.826]; sensitivity 71.4 [57.8-82.7]%; NPV 93.1 [89.8-95.3]%), while REMS was the most accurate predictor of death within 7 days (AUROC 0.823 [0.778-0.863]; sensitivity 96.1 [80.4-99.9]%; NPV 99.4[96.2-99.9]%). Similar results were observed for ICU admission and death at 48 h. NEWS and REMS were as accurate as the triage system used in our ED. MEWS and qSOFA had the lowest overall accuracy for both outcomes.

Conclusion: In our single-centre cohort of COVID-19 patients, NEWS and REMS measured on ED arrival were the most sensitive predictors of 7-day ICU admission or death. EWS could be useful to identify patients with low risk of clinical deterioration.

Keywords: COVID-19; Early warning scores; MEWS; NEWS; NEWS2; REMS; qSOFA.

Copyright © 2020 Elsevier B.V. All rights reserved.

Comment in

- Performance of NEWS and NEWS-C in acute medical no COVID-19 patients.
  Accordino S, Sozzi F, Canetta C. Accordino S, et al. Resuscitation. 2021 Sep;166:83-84. doi: 10.1016/j.resuscitation.2021.07.017. Epub 2021 Jul 22. Resuscitation. 2021. PMID: 34302923 Free PMC article. No abstract available.

- 36 references
- 1 figure

Supplementary info

Publication types, MeSH terms Expand

Publication types

- Multicenter Study
- Observational Study

MeSH terms

- Aged
- Betacoronavirus*
- COVID-19
- Coronavirus Infections / epidemiology
- Coronavirus Infections / therapy*
- Early Warning Score
- Female
- Hospital Mortality / trends
- Humans
- Intensive Care Units / statistics & numerical data*
- Italy / epidemiology
- Male
- Middle Aged
- Pandemics
- Patient Admission / statistics & numerical data*
- Pneumonia, Viral / epidemiology
- Pneumonia, Viral / therapy*
- ROC Curve
- Retrospective Studies
- Risk Assessment / methods*
- SARS-CoV-2

- Survival Rate / trends
- Triage

Full text links

ELSEVIER FULL-TEXT ARTICLE Elsevier Science Free PMC article

Proceed to details

Cite

Share

☐ 871

Observational Study

J Nephrol
. 2021 Feb;34(1):173-183.
doi: 10.1007/s40620-020-00875-1. Epub 2020 Oct 6.

Kidney disease and all-cause mortality in patients with COVID-19 hospitalized in Genoa, Northern Italy

Elisa Russo [1], Pasquale Esposito [1], Lucia Taramasso [2], Laura Magnasco [2], Michela Saio [1], Federica Briano [2], Chiara Russo [2], Silvia Dettori [2], Antonio Vena [2], Antonio Di Biagio [2], Giacomo Garibotto [1], Matteo Bassetti [2], Francesca Viazzi [3], GECOVID working group

Collaborators, Affiliations Expand

Collaborators

- **GECOVID working group**: Anna Alessandrini, Marco Camera, Emanuele Delfino, Andrea De Maria, Chiara Dentone, Antonio Di Biagio, Ferdinando Dodi, Antonio Ferrazin, Giovanni Mazzarello, Malgorzata Mikulska, Laura Ambra Nicolini, Federica Toscanini, Daniele Roberto Giacobbe, Antonio Vena, Lucia Taramasso, Elisa Balletto, Federica Portunato, Eva Schenone, Nirmala Rosseti, Federico Baldi, Marco Berruti, Federica Briano, Silvia Dettori, Laura Labate, Laura Magnasco, Michele Mirabella, Rachele Pincino, Chiara Russo, Giovanni Sarteschi, Chiara Sepulcri, Stefania Tutino, Roberto Pontremoli, Valentina Beccati, Salvatore Casciaro, Massimo Casu, Francesco Gavaudan, Maria Ghinatti, Elisa Gualco, Giovanna Leoncini, Paola Pitto, Kassem Salam, Angelo Gratarola, Mattia Bixio, Annalisa Amelia, Andrea Balestra, Paola Ballarino, Nicholas Bardi, Roberto Boccafogli, Francesca Fezza, Elisa Calzolari, Marta Castelli, Elisabetta Cenni, Paolo Cortese, Giuseppe Cuttone, Sara Feltrin, Stefano Giovinazzo, Patrizia Giuntini, Letizia Natale, Davide Orsi, Matteo Pastorino, Tommaso Perazzo, Fabio Pescetelli, Federico Schenone, Maria Grazia Serra, Marco Sottano, Roberto Tallone, Massimo Amelotti, Marie Jeanne Majabò, Massimo Merlini, Federica Perazzo, Nidal Ahamd, Paolo Barbera, Marta Bovio, Paola Vacca, Andrea Collidà, Ombretta Cutuli, Agnese Lomeo, Francesca Fezza Nicola Gentilucci, Nadia Hussein, Emanuele Malvezzi, Laura Massobrio, Giula Motta, Laura Pastorino, Nicoletta Pollicardo, Stefano Sartini, Paola Vacca Valentina Virga, Italo Porto, Giampaolo Bezante, Roberta Della Bona, Giovanni La Malfa, Alberto

Valbusa, Vered Gil Ad, Emanuela Barisione, Michele Bellotti, Aloe' Teresita, Alessandro Blanco, Marco Grosso, Maria Grazia Piroddi, Paolo Moscatelli, Paola Ballarino, Matteo Caiti, Elisabetta Cenni, Patrizia Giuntini, Ottavia Magnani, Samir Sukkar, Ludovica Cogorno, Raffaella Gradaschi, Erica Guiddo, Eleonora Martino, Livia Pisciotta, Bruno Cavaliere, Rossi Cristina, Farina Francesca, Giacomo Garibotto, Pasquale Esposito, Giovanni Passalacqua, Diego Bagnasco, Fulvio Braido, Annamaria Riccio, Elena Tagliabue, Claudio Gustavino, Antonella Ferraiolo, Salvatore Giuffrida, Nicola Rosso, Alessandra Morando, Riccardo Papalia, Donata Passerini, Gabriella Tiberio, Giovanni Orengo, Alberto Battaglini, Silvano Ruffoni, Sergio Caglieris, Mauro Giacomini, Sara Mora

Affiliations

- [1] Department of Internal Medicine, Clinica Nefrologica Dialisi e Trapianto, University of Genoa, Hospital Policlinico San Martino, IRCCS, Viale Benedetto XV, 16132, Genoa, Italy.
- [2] Department of Health Sciences, Infectious Diseases Clinic, University of Genoa, Hospital Policlinico San Martino-IRCCS, Genoa, Italy.
- [3] Department of Internal Medicine, Clinica Nefrologica Dialisi e Trapianto, University of Genoa, Hospital Policlinico San Martino, IRCCS, Viale Benedetto XV, 16132, Genoa, Italy. francesca.viazzi@unige.it.

- PMID: **33025516**
- PMCID: PMC7538179
- DOI: 10.1007/s40620-020-00875-1

Free PMC article
Observational Study

Kidney disease and all-cause mortality in patients with COVID-19 hospitalized in Genoa, Northern Italy

Elisa Russo et al. J Nephrol. 2021 Feb.
Free PMC article
Show details
J Nephrol
. 2021 Feb;34(1):173-183.
doi: 10.1007/s40620-020-00875-1. Epub 2020 Oct 6.

Authors

Elisa Russo [1], Pasquale Esposito [1], Lucia Taramasso [2], Laura Magnasco [2], Michela Saio [1], Federica Briano [2], Chiara Russo [2], Silvia Dettori [2], Antonio Vena [2], Antonio Di Biagio [2], Giacomo Garibotto [1], Matteo Bassetti [2], Francesca Viazzi [3], GECOVID working group

Collaborators

- **GECOVID working group**:

Anna Alessandrini, Marco Camera, Emanuele Delfino, Andrea De Maria, Chiara Dentone, Antonio Di Biagio, Ferdinando Dodi, Antonio Ferrazin, Giovanni Mazzarello, Malgorzata Mikulska, Laura Ambra Nicolini, Federica Toscanini, Daniele Roberto Giacobbe, Antonio Vena, Lucia Taramasso, Elisa Balletto, Federica Portunato, Eva Schenone, Nirmala Rosseti, Federico Baldi, Marco Berruti, Federica Briano, Silvia Dettori, Laura Labate, Laura Magnasco, Michele Mirabella, Rachele Pincino, Chiara Russo, Giovanni Sarteschi, Chiara Sepulcri, Stefania Tutino, Roberto Pontremoli, Valentina Beccati, Salvatore Casciaro, Massimo Casu, Francesco Gavaudan, Maria Ghinatti, Elisa Gualco, Giovanna Leoncini, Paola Pitto, Kassem Salam, Angelo Gratarola, Mattia Bixio, Annalisa Amelia, Andrea Balestra, Paola Ballarino, Nicholas Bardi, Roberto Boccafogli, Francesca Fezza, Elisa Calzolari, Marta Castelli, Elisabetta Cenni, Paolo Cortese, Giuseppe Cuttone, Sara Feltrin, Stefano Giovinazzo, Patrizia Giuntini, Letizia Natale, Davide Orsi, Matteo Pastorino, Tommaso Perazzo, Fabio Pescetelli, Federico Schenone, Maria Grazia Serra, Marco Sottano, Roberto Tallone, Massimo Amelotti, Marie Jeanne Majabò, Massimo Merlini, Federica Perazzo, Nidal Ahamd, Paolo Barbera, Marta Bovio, Paola Vacca, Andrea Collidà, Ombretta Cutuli, Agnese Lomeo, Francesca Fezza Nicola Gentilucci, Nadia Hussein, Emanuele Malvezzi, Laura Massobrio, Giula Motta, Laura Pastorino, Nicoletta Pollicardo, Stefano Sartini, Paola Vacca Valentina Virga, Italo Porto, Giampaolo Bezante, Roberta Della Bona, Giovanni La Malfa, Alberto Valbusa, Vered Gil Ad, Emanuela Barisione, Michele Bellotti, Aloe' Teresita, Alessandro Blanco, Marco Grosso, Maria Grazia Piroddi, Paolo Moscatelli, Paola Ballarino, Matteo Caiti, Elisabetta Cenni, Patrizia Giuntini, Ottavia Magnani, Samir Sukkar, Ludovica Cogorno, Raffaella Gradaschi, Erica Guiddo, Eleonora Martino, Livia Pisciotta, Bruno Cavaliere, Rossi Cristina, Farina Francesca, Giacomo Garibotto, Pasquale Esposito, Giovanni Passalacqua, Diego Bagnasco, Fulvio Braido, Annamaria Riccio, Elena Tagliabue, Claudio Gustavino, Antonella Ferraiolo, Salvatore Giuffrida, Nicola Rosso, Alessandra Morando, Riccardo Papalia, Donata Passerini, Gabriella Tiberio, Giovanni Orengo, Alberto Battaglini, Silvano Ruffoni, Sergio Caglieris, Mauro Giacomini, Sara Mora

Affiliations

- [1] Department of Internal Medicine, Clinica Nefrologica Dialisi e Trapianto, University of Genoa, Hospital Policlinico San Martino, IRCCS, Viale Benedetto XV, 16132, Genoa, Italy.
- [2] Department of Health Sciences, Infectious Diseases Clinic, University of Genoa, Hospital Policlinico San Martino-IRCCS, Genoa, Italy.
- [3] Department of Internal Medicine, Clinica Nefrologica Dialisi e Trapianto, University of Genoa, Hospital Policlinico San Martino, IRCCS, Viale Benedetto XV, 16132, Genoa, Italy. francesca.viazzi@unige.it.

- PMID: **33025516**
- PMCID: PMC7538179
- DOI: 10.1007/s40620-020-00875-1

Abstract

Background: The prevalence of kidney involvement during SARS-CoV-2 infection has been reported to be high. Nevertheless, data are lacking about the determinants of acute kidney injury (AKI) and the combined effect of chronic kidney disease (CKD) and AKI in COVID-19 patients.

Methods: We collected data on patient demographics, comorbidities, chronic medications, vital signs, baseline laboratory test results and in-hospital treatment in patients with COVID-19

consecutively admitted to our Institution. Chronic kidney disease was defined as eGFR < 60 mL/min per 1.73 m^2 or proteinuria at urinalysis within 180 days prior to hospital admission. AKI was defined according to KDIGO criteria. The primary and secondary outcomes were the development of AKI and death.

Results: Of 777 patients eligible for the study, acute kidney injury developed in 176 (22.6%). Of these, 79 (45%) showed an acute worsening of a preexisting CKD, and 21 (12%) required kidney replacement therapy. Independent associates of AKI were chronic kidney disease, C-reactive protein (CRP) and ventilation support. Among patients with acute kidney injury, 111 died (63%) and its occurrence increased the risk of death by 60% (HR 1.60 [95% IC 1.21-2.49] p = 0.002) independently of potential confounding factors including hypertension, preexisting kidney damage, and comorbidities. Patients with AKI showed a significantly higher rate of deaths attributed to bleeding compared to CKD and the whole population (7.5 vs 1.5 vs 3.5%, respectively).

Conclusion: Awareness of kidney function, both preexisting CKD and development of acute kidney injury, may help to identify those patients at increased risk of death.

Keywords: Acute kidney injury; COVID-19; Chronic kidney disease; Mortality; Proteinuria.

Conflict of interest statement

The authors declare they have no conflict of interest.

- 37 references
- 2 figures

Supplementary info

Publication types, MeSH terms Expand

Publication types

- Observational Study

MeSH terms

- Acute Kidney Injury / mortality*
- Acute Kidney Injury / therapy
- Acute Kidney Injury / virology
- Aged
- COVID-19 / complications*
- COVID-19 / mortality*
- COVID-19 / therapy
- Female
- Hospital Mortality
- Hospitalization
- Humans

- Italy
- Male
- Middle Aged
- Prevalence
- Renal Insufficiency, Chronic / mortality*
- Renal Insufficiency, Chronic / therapy
- Renal Insufficiency, Chronic / virology
- Renal Replacement Therapy
- Retrospective Studies
- Risk Factors
- Survival Rate

Full text links

SpringerLink FULL-TEXT ARTICLE Springer Free PMC article
Proceed to details
Cite
Share
872
Observational Study
Hypertens Res
. 2020 Aug;43(8):824-831.
doi: 10.1038/s41440-020-0485-2. Epub 2020 Jun 1.

COVID-19 patients with hypertension have more severe disease: a multicenter retrospective observational study

Songjiang Huang[# 1 2], Jianwen Wang[# 3], Fen Liu[# 4], Jiacheng Liu[1 2], Guijuan Cao[4], Chongtu Yang[1 2], Wei Liu[3], Chao Tu[5], Muxin Zhu[3], Bin Xiong[6 7]
Affiliations Expand

Affiliations

- [1] Department of Radiology, Union Hospital, Tongji Medical College, Huazhong University of Science and Technology, Wuhan, China.
- [2] Hubei Province Key Laboratory of Molecular Imaging, Wuhan, China.
- [3] Tuberculosis and Respiratory Department, Wuhan Jinyintan Hospital, Wuhan, China.
- [4] Department of Radiology, the Central Hospital of Wuhan, Tongji Medical College, Huazhong University of Science and Technology, Wuhan, Hubei, China.
- [5] Department of Critical Care Medicine, Wuhan Jinyintan Hospital, Wuhan, Hubei, China.
- [6] Department of Radiology, Union Hospital, Tongji Medical College, Huazhong University of Science and Technology, Wuhan, China. herr_xiong@126.com.

- [7] Hubei Province Key Laboratory of Molecular Imaging, Wuhan, China. herr_xiong@126.com.

[#] Contributed equally.

- PMID: **32483311**
- PMCID: PMC7261650
- DOI: 10.1038/s41440-020-0485-2

Free PMC article
Observational Study

COVID-19 patients with hypertension have more severe disease: a multicenter retrospective observational study

Songjiang Huang et al. Hypertens Res. 2020 Aug.
Free PMC article
Show details
Hypertens Res
. 2020 Aug;43(8):824-831.
doi: 10.1038/s41440-020-0485-2. Epub 2020 Jun 1.

Authors

Songjiang Huang [#] [1] [2], Jianwen Wang [#] [3], Fen Liu [#] [4], Jiacheng Liu [1] [2], Guijuan Cao [4], Chongtu Yang [1] [2], Wei Liu [3], Chao Tu [5], Muxin Zhu [3], Bin Xiong [6] [7]

Affiliations

- [1] Department of Radiology, Union Hospital, Tongji Medical College, Huazhong University of Science and Technology, Wuhan, China.
- [2] Hubei Province Key Laboratory of Molecular Imaging, Wuhan, China.
- [3] Tuberculosis and Respiratory Department, Wuhan Jinyintan Hospital, Wuhan, China.
- [4] Department of Radiology, the Central Hospital of Wuhan, Tongji Medical College, Huazhong University of Science and Technology, Wuhan, Hubei, China.
- [5] Department of Critical Care Medicine, Wuhan Jinyintan Hospital, Wuhan, Hubei, China.
- [6] Department of Radiology, Union Hospital, Tongji Medical College, Huazhong University of Science and Technology, Wuhan, China. herr_xiong@126.com.
- [7] Hubei Province Key Laboratory of Molecular Imaging, Wuhan, China. herr_xiong@126.com.

[#] Contributed equally.

- PMID: **32483311**
- PMCID: PMC7261650
- DOI: 10.1038/s41440-020-0485-2

Abstract

This study aims to explore the effect of hypertension on disease progression and prognosis in patients with coronavirus disease 2019 (COVID-19). A total of 310 patients diagnosed with COVID-19 were studied. A comparison was made between two groups of patients, those with hypertension and those without hypertension. Their demographic data, clinical manifestations, laboratory indicators, and treatment methods were collected and analyzed. A total of 310 patients, including 113 patients with hypertension and 197 patients without hypertension, were included in the analysis. Compared with patients without hypertension, patients with hypertension were older, were more likely to have diabetes and cerebrovascular disease, and were more likely to be transferred to the intensive care unit. The neutrophil count and lactate dehydrogenase, fibrinogen, and D-dimer levels in hypertensive patients were significantly higher than those in nonhypertensive patients ($P < 0.05$). However, multivariate analysis (adjusted for age and sex) failed to show that hypertension was an independent risk factor for COVID-19 mortality or severity. COVID-19 patients with hypertension were more likely than patients without hypertension to have severe pneumonia, excessive inflammatory reactions, organ and tissue damage, and deterioration of the disease. Patients with hypertension should be given additional attention to prevent worsening of their condition.

Keywords: 2019 novel coronavirus disease; Coexistent disease; Hypertension; Prognosis; SARS-COV-2.

Conflict of interest statement

The authors declare that they have no conflict of interest.

- 21 references

Supplementary info

Publication types, MeSH terms Expand

Publication types

- Multicenter Study
- Observational Study

MeSH terms

- Adult
- Aged
- Betacoronavirus
- COVID-19
- China / epidemiology
- Coronavirus Infections / complications*
- Coronavirus Infections / epidemiology
- Coronavirus Infections / therapy

- Female
- Humans
- Hypertension / complications*
- Male
- Middle Aged
- Pandemics
- Pneumonia, Viral / complications*
- Pneumonia, Viral / epidemiology
- Pneumonia, Viral / therapy
- Retrospective Studies
- SARS-CoV-2

Full text links

Free PMC article
Proceed to details
Cite
Share
873
J Healthc Eng
. 2021 Jun 25;2021:5556207.
doi: 10.1155/2021/5556207. eCollection 2021.

Disentangling the Association of Hydroxychloroquine Treatment with Mortality in Covid-19 Hospitalized Patients through Hierarchical Clustering

Augusto Di Castelnuovo [1], Alessandro Gialluisi [2], Andrea Antinori [3], Nausicaa Berselli [4], Lorenzo Blandi [5], Marialaura Bonaccio [2], Raffaele Bruno [6 7], Roberto Cauda [8 9], Simona Costanzo [2], Giovanni Guaraldi [10], Lorenzo Menicanti [11], Marco Mennuni [12], Ilaria My [13], Giustino Parruti [14], Giuseppe Patti [12], Stefano Perlini [15 16], Francesca Santilli [17], Carlo Signorelli [18], Giulio Stefanini [13], Alessandra Vergori [19], Walter Ageno [20], Antonella Agodi [21], Piergiuseppe Agostoni [22 23], Luca Aiello [24], Samir Al Moghazi [25], Rosa Arboretti [26], Filippo Aucella [27], Greta Barbieri [28], Martina Barchitta [29], Paolo Bonfanti [30 31], Francesco Cacciatore [32], Lucia Caiano [20], Francesco Cannata [13], Laura Carrozzi [33], Antonio Cascio [34], Giacomo Castiglione [35], Arturo Ciccullo [8], Antonella Cingolani [8 9], Francesco Cipollone [17], Claudia Colomba [34], Crizia Colombo [12], Annalisa Crisetti [27], Francesca Crosta [14], Gian Battista Danzi [36], Damiano D'Ardes [17], Katleen de Gaetano Donati [8 9], Francesco Di Gennaro [37], Giuseppe Di Tano [36], Gianpiero D'Offizi [38], Francesco Maria Fusco [39], Carlo Gaudiosi [40], Ivan Gentile [41], Francesco Gianfagna [20], Gabriele Giuliano [8], Emauele Graziani [42], Gabriella Guarnieri [43], Valerio Langella [44], Giovanni Larizza [45], Armando Leone [46], Gloria Maccagni [36], Federica

Magni [20], Stefano Maitan [24], Sandro Mancarella [47], Rosa Manuele [48], Massimo Mapelli [22, 23], Riccardo Maragna [22, 23], Rossella Marcucci [49], Giulio Maresca [44], Silvia Marongiu [50], Claudia Marotta [37], Lorenzo Marra [46], Franco Mastroianni [45], Alessandro Mengozzi [51], Marianna Meschiari [10], Jovana Milic [10], Filippo Minutolo [52], Roberta Mussinelli [16], Cristina Mussini [10], Maria Musso [53], Anna Odone [5], Marco Olivieri [54], Antonella Palimodde [50], Emanuela Pasi [42], Raffaele Pesavento [55], Francesco Petri [30], Carlo A Pivato [13], Venerino Poletti [56, 57], Claudia Ravaglia [56], Giulia Righetti [45], Andrea Rognoni [12], Marco Rossato [55], Ilaria Rossi [17], Marianna Rossi [30], Anna Sabena [15], Francesco Salinaro [15], Vincenzo Sangiovanni [39], Carlo Sanrocco [14], Nicola Schiano Moriello [41], Laura Scorzolini [58], Raffaella Sgariglia [47], Paola Giustina Simeone [14], Michele Spinicci [49], Enrica Tamburrini [8], Carlo Torti [59], Enrico Maria Trecarichi [59], Roberto Vettor [55], Andrea Vianello [43], Marco Vinceti [4, 60], Agostino Virdis [51], Raffaele De Caterina [33], Licia Iacoviello [2, 20]

Affiliations Expand

Affiliations

- [1] Mediterranea Cardiocentro, Napoli, Italy.
- [2] Department of Epidemiology and Prevention, IRCCS Neuromed, Pozzilli, Italy.
- [3] UOC Immunodeficienze Virali, National Institute for Infectious Diseases "L. Spallanzani" IRCCS, Rome, Italy.
- [4] Section of Public Health, Department of Biomedical, Metabolic and Neural Sciences, University of Modena and Reggio Emilia, Modena, Italy.
- [5] Università di Pavia, Pavia, Italy.
- [6] Division of Infectious Diseases I, Fondazione IRCCS Policlinico San Matteo, Pavia, Italy.
- [7] Department of Clinical, Surgical Diagnostic and Paediatric Sciences, University of Pavia, Pavia, Italy.
- [8] Fondazione Policlinico Universitario A. Gemelli IRCCS, Roma, Italy.
- [9] Università Cattolica Del Sacro Cuore- Dipartimento di Sicurezza e Bioetica Sede di Roma, Roma, Italy.
- [10] Infectious Disease Unit, Department of Surgical, Medical Dental and Morphological Sciences, University of Modena and Reggio Emilia, Modena, Italy.
- [11] IRCCS Policlinico San Donato, San Donato Milanese, Milan, Italy.
- [12] University of Eastern Piedmont, Maggiore Della Carità Hospital, Novara, Italy.
- [13] Humanitas Clinical and Research Hospital IRCCS, Rozzano, Milano, Italy.
- [14] Department of Infectious Disease, Azienda Sanitaria Locale (AUSL) di Pescara, Pescara, Italy.
- [15] Emergency Department, IRCCS Policlinico San Matteo Foundation, Pavia, Italy.
- [16] Department of Internal Medicine, University of Pavia, Pavia, Italy.
- [17] Department of Medicine and Aging, Clinica Medica, "SS. Annunziata" Hospital and University of Chieti, Chieti, Italy.
- [18] School of Medicine, Vita-Salute San Raffaele University, Milano, Italy.
- [19] HIV/AIDS Department, National Institute for Infectious Diseases Lazzaro SpallanzaniIRCCS, Roma, Italy.
- [20] Department of Medicine and Surgery, University of Insubria, Varese, Italy.
- [21] Department of Medical and Surgical Sciences and Advanced Technologies G.F. Ingrassia, University of Catania, AOU Policlinico G.Rodolico - San Marco, Catania, Italy.
- [22] Centro Cardiologico Monzino IRCCS, Milano, Italy.

- [23] Department of Clinical Sciences and Community Health, Cardiovascular Section, University of Milano, Milan, Italy.
- [24] UOC. Anestesia e Rianimazione, Dipartimento di Chirurgia Generale Ospedale Morgagni-Pierantoni, Forlì, Italy.
- [25] UOC Infezioni Sistemiche Dell'Immunodepresso, National Institute for Infectious Diseases L. Spallanzani IRCCS, Rome, Italy.
- [26] Department of Civil Environmental and Architectural Engineering, University of Padova, Padova, Italy.
- [27] Fondazione I.R.C.C.S Casa Sollievo Della Sofferenza, San Giovanni Rotondo, Foggia, Italy.
- [28] Department of Surgical Medical and Molecular Medicine and Critical Care, Azienda Ospedaliera Universitaria Pisana and University of Pisa, Pisa, Italy.
- [29] Department of Medical and Surgical Sciences and Advanced Technologies G.F. Ingrassia, University of Catania, Catania, Italy.
- [30] UOC Malattie Infettive, Ospedale San Gerardo, ASST Monza, Monza, Italy.
- [31] School of Medicine and Surgery, University of Milano-Bicocca, Milano, Italy.
- [32] Department of Translational Medical Sciences, University of Naples, Federico II, Naples, Italy.
- [33] Cardiovascular and Thoracic Department, Azienda Ospedaliero-Universitaria Pisana and University of Pisa, Pisa, Italy.
- [34] Infectious and Tropical Diseases Unit- Department of Health Promotion, Mother and Child Care, Internal Medicine and Medical Specialties (PROMISE) - University of Palermo, Palermo, Italy.
- [35] Servizio di Anestesia e Rianimazione II UO Rianimazione Ospedale San Marco, AOU Policlinico G. Rodolico, San Marco, Catania, Italy.
- [36] Department of Cardiology, Ospedale di Cremona, Cremona, Italy.
- [37] Medical Direction IRCCS Neuromed, Pozzilli, Italy.
- [38] UOC Malattie Infettive-Epatologia, National Institute for Infectious Diseases L. Spallanzani IRCCS, Roma, Italy.
- [39] UOC Infezioni Sistemiche e Dell'Immunodepresso, Azienda Ospedaliera Dei Colli Ospedale Cotugno, Napoli, Italy.
- [40] ASL Napoli3 Sud COVID HOSPITAL, Boscotrecase, Napoli, Italy.
- [41] Department of Clinical Medicine and Surgery, University of Naples Federico II, Napoli, Italy.
- [42] Medicina Interna, Ospedale di Ravenna, AUSL Della Romagna, Ravenna, Italy.
- [43] Respiratory Pathophysiology Division, Department of Cardiologic, Thoracic and Vascular Sciences, University of Padova, Padova, Italy.
- [44] UOC Medicina COVID- PO S. Maria di Loreto Nuovo, ASL Na 1 Centro, Napoli, Italy.
- [45] COVID-19 Unit. EE Ospedale Regionale F. Miulli, Acquaviva Delle Fonti, Bari, Italy.
- [46] UOC di Pneumologia P.O. San Giuseppe Moscati, Taranto, Italy.
- [47] ASST Milano Nord - Ospedale Edoardo Bassini, Cinisello Balsamo, Italy.
- [48] U.O. C. Malattie Infettive e Tropicali, P.O. "San Marco" AOU Policlinico "G. Rodolico - San Marco", Catania, Italy.
- [49] Department of Experimental and Clinical Medicine, University of Florence and Azienda Ospedaliero-Universitaria Careggi, Firenze, Italy.
- [50] Santissima Trinità di Cagliari, Cagliari, Italy.
- [51] Department of Clinical and Experimental Medicine, Azienda Ospedaliera Universitaria Pisana University of Pisa, Pisa, Italy.

- [52] Dipartimento di Farmacia, Università di Pisa, Pisa, Italy.
- [53] UOC Malattie Infettive-Apparato Respiratorio, National Institute for Infectious Diseases L. Spallanzani IRCCS, Rome, Italy.
- [54] Computer Service, University of Molise, Campobasso, Italy.
- [55] Clinica Medica 3. Department of Medicine - DIMED, University Hospital of Padova, Padova, Italy.
- [56] UOC Pneumologia. Dipartimento di Malattie Apparato Respiratorio e Torace, Ospedale Morgagni-Pierantoni Forlì, Forlì, Italy.
- [57] Department of Respiratory Diseases & Allergy Aarhus University Hospital, Aarhus, Denmark.
- [58] UOC Malattie Infettive Ad Alta Intensità di Cura, National Institute for Infectious Diseases L. Spallanzani IRCCS, Rome, Italy.
- [59] Infectious and Tropical Diseases Unit, Deparment of Medical and Surgical Sciences, Magna Graecia University, Catanzaro, Italy.
- [60] Department of Epidemiology, Boston University School of Public Health, Boston, USA.

- PMID: **34336157**
- PMCID: PMC8238578
- DOI: 10.1155/2021/5556207

Free PMC article

Disentangling the Association of Hydroxychloroquine Treatment with Mortality in Covid-19 Hospitalized Patients through Hierarchical Clustering

Augusto Di Castelnuovo et al. J Healthc Eng. 2021.
Free PMC article

Show details

J Healthc Eng
. 2021 Jun 25;2021:5556207.
doi: 10.1155/2021/5556207. eCollection 2021.

Authors

Augusto Di Castelnuovo [1], Alessandro Gialluisi [2], Andrea Antinori [3], Nausicaa Berselli [4], Lorenzo Blandi [5], Marialaura Bonaccio [2], Raffaele Bruno [6 7], Roberto Cauda [8 9], Simona Costanzo [2], Giovanni Guaraldi [10], Lorenzo Menicanti [11], Marco Mennuni [12], Ilaria My [13], Giustino Parruti [14], Giuseppe Patti [12], Stefano Perlini [15 16], Francesca Santilli [17], Carlo Signorelli [18], Giulio Stefanini [13], Alessandra Vergori [19], Walter Ageno [20], Antonella Agodi [21], Piergiuseppe Agostoni [22 23], Luca Aiello [24], Samir Al Moghazi [25], Rosa Arboretti [26], Filippo Aucella [27], Greta Barbieri [28], Martina Barchitta [29], Paolo Bonfanti [30 31], Francesco Cacciatore [32], Lucia Caiano [20], Francesco Cannata [13], Laura Carrozzi [33], Antonio Cascio [34], Giacomo Castiglione [35], Arturo Ciccullo [8], Antonella Cingolani [8 9], Francesco Cipollone [17], Claudia

Colomba [34], Crizia Colombo [12], Annalisa Crisetti [27], Francesca Crosta [14], Gian Battista Danzi [36], Damiano D'Ardes [17], Katleen de Gaetano Donati [8, 9], Francesco Di Gennaro [37], Giuseppe Di Tano [36], Gianpiero D'Offizi [38], Francesco Maria Fusco [39], Carlo Gaudiosi [40], Ivan Gentile [41], Francesco Gianfagna [20], Gabriele Giuliano [8], Emaauele Graziani [42], Gabriella Guarnieri [43], Valerio Langella [44], Giovanni Larizza [45], Armando Leone [46], Gloria Maccagni [36], Federica Magni [20], Stefano Maitan [24], Sandro Mancarella [47], Rosa Manuele [48], Massimo Mapelli [22, 23], Riccardo Maragna [22, 23], Rossella Marcucci [49], Giulio Maresca [44], Silvia Marongiu [50], Claudia Marotta [37], Lorenzo Marra [46], Franco Mastroianni [45], Alessandro Mengozzi [51], Marianna Meschiari [10], Jovana Milic [10], Filippo Minutolo [52], Roberta Mussinelli [16], Cristina Mussini [10], Maria Musso [53], Anna Odone [5], Marco Olivieri [54], Antonella Palimodde [50], Emanuela Pasi [42], Raffaele Pesavento [55], Francesco Petri [30], Carlo A Pivato [13], Venerino Poletti [56, 57], Claudia Ravaglia [56], Giulia Righetti [45], Andrea Rognoni [12], Marco Rossato [55], Ilaria Rossi [17], Marianna Rossi [30], Anna Sabena [15], Francesco Salinaro [15], Vincenzo Sangiovanni [39], Carlo Sanrocco [14], Nicola Schiano Moriello [41], Laura Scorzolini [58], Raffaella Sgariglia [47], Paola Giustina Simeone [14], Michele Spinicci [49], Enrica Tamburrini [8], Carlo Torti [59], Enrico Maria Trecarichi [59], Roberto Vettor [55], Andrea Vianello [43], Marco Vinceti [4, 60], Agostino Virdis [51], Raffaele De Caterina [33], Licia Iacoviello [2, 20]

Affiliations

- [1] Mediterranea Cardiocentro, Napoli, Italy.
- [2] Department of Epidemiology and Prevention, IRCCS Neuromed, Pozzilli, Italy.
- [3] UOC Immunodeficienze Virali, National Institute for Infectious Diseases "L. Spallanzani" IRCCS, Rome, Italy.
- [4] Section of Public Health, Department of Biomedical, Metabolic and Neural Sciences, University of Modena and Reggio Emilia, Modena, Italy.
- [5] Università di Pavia, Pavia, Italy.
- [6] Division of Infectious Diseases I, Fondazione IRCCS Policlinico San Matteo, Pavia, Italy.
- [7] Department of Clinical, Surgical Diagnostic and Paediatric Sciences, University of Pavia, Pavia, Italy.
- [8] Fondazione Policlinico Universitario A. Gemelli IRCCS, Roma, Italy.
- [9] Università Cattolica Del Sacro Cuore- Dipartimento di Sicurezza e Bioetica Sede di Roma, Roma, Italy.
- [10] Infectious Disease Unit, Department of Surgical, Medical Dental and Morphological Sciences, University of Modena and Reggio Emilia, Modena, Italy.
- [11] IRCCS Policlinico San Donato, San Donato Milanese, Milan, Italy.
- [12] University of Eastern Piedmont, Maggiore Della Carità Hospital, Novara, Italy.
- [13] Humanitas Clinical and Research Hospital IRCCS, Rozzano, Milano, Italy.
- [14] Department of Infectious Disease, Azienda Sanitaria Locale (AUSL) di Pescara, Pescara, Italy.
- [15] Emergency Department, IRCCS Policlinico San Matteo Foundation, Pavia, Italy.
- [16] Department of Internal Medicine, University of Pavia, Pavia, Italy.
- [17] Department of Medicine and Aging, Clinica Medica, "SS. Annunziata" Hospital and University of Chieti, Chieti, Italy.
- [18] School of Medicine, Vita-Salute San Raffaele University, Milano, Italy.
- [19] HIV/AIDS Department, National Institute for Infectious Diseases Lazzaro SpallanzaniIRCCS, Roma, Italy.

- [20] Department of Medicine and Surgery, University of Insubria, Varese, Italy.
- [21] Department of Medical and Surgical Sciences and Advanced Technologies G.F. Ingrassia, University of Catania, AOU Policlinico G.Rodolico - San Marco, Catania, Italy.
- [22] Centro Cardiologico Monzino IRCCS, Milano, Italy.
- [23] Department of Clinical Sciences and Community Health, Cardiovascular Section, University of Milano, Milan, Italy.
- [24] UOC. Anestesia e Rianimazione, Dipartimento di Chirurgia Generale Ospedale Morgagni-Pierantoni, Forlì, Italy.
- [25] UOC Infezioni Sistemiche Dell'Immunodepresso, National Institute for Infectious Diseases L. Spallanzani IRCCS, Rome, Italy.
- [26] Department of Civil Environmental and Architectural Engineering, University of Padova, Padova, Italy.
- [27] Fondazione I.R.C.C.S Casa Sollievo Della Sofferenza, San Giovanni Rotondo, Foggia, Italy.
- [28] Department of Surgical Medical and Molecular Medicine and Critical Care, Azienda Ospedaliera Universitaria Pisana and University of Pisa, Pisa, Italy.
- [29] Department of Medical and Surgical Sciences and Advanced Technologies G.F. Ingrassia, University of Catania, Catania, Italy.
- [30] UOC Malattie Infettive, Ospedale San Gerardo, ASST Monza, Monza, Italy.
- [31] School of Medicine and Surgery, University of Milano-Bicocca, Milano, Italy.
- [32] Department of Translational Medical Sciences, University of Naples, Federico II, Naples, Italy.
- [33] Cardiovascular and Thoracic Department, Azienda Ospedaliero-Universitaria Pisana and University of Pisa, Pisa, Italy.
- [34] Infectious and Tropical Diseases Unit- Department of Health Promotion, Mother and Child Care, Internal Medicine and Medical Specialties (PROMISE) - University of Palermo, Palermo, Italy.
- [35] Servizio di Anestesia e Rianimazione II UO Rianimazione Ospedale San Marco, AOU Policlinico G. Rodolico, San Marco, Catania, Italy.
- [36] Department of Cardiology, Ospedale di Cremona, Cremona, Italy.
- [37] Medical Direction IRCCS Neuromed, Pozzilli, Italy.
- [38] UOC Malattie Infettive-Epatologia, National Institute for Infectious Diseases L. Spallanzani IRCCS, Roma, Italy.
- [39] UOC Infezioni Sistemiche e Dell'Immunodepresso, Azienda Ospedaliera Dei Colli Ospedale Cotugno, Napoli, Italy.
- [40] ASL Napoli3 Sud COVID HOSPITAL, Boscotrecase, Napoli, Italy.
- [41] Department of Clinical Medicine and Surgery, University of Naples Federico II, Napoli, Italy.
- [42] Medicina Interna, Ospedale di Ravenna, AUSL Della Romagna, Ravenna, Italy.
- [43] Respiratory Pathophysiology Division, Department of Cardiologic, Thoracic and Vascular Sciences, University of Padova, Padova, Italy.
- [44] UOC Medicina COVID- PO S. Maria di Loreto Nuovo, ASL Na 1 Centro, Napoli, Italy.
- [45] COVID-19 Unit. EE Ospedale Regionale F. Miulli, Acquaviva Delle Fonti, Bari, Italy.
- [46] UOC di Pneumologia P.O. San Giuseppe Moscati, Taranto, Italy.
- [47] ASST Milano Nord - Ospedale Edoardo Bassini, Cinisello Balsamo, Italy.
- [48] U.O. C. Malattie Infettive e Tropicali, P.O. "San Marco" AOU Policlinico "G. Rodolico - San Marco", Catania, Italy.

- [49] Department of Experimental and Clinical Medicine, University of Florence and Azienda Ospedaliero-Universitaria Careggi, Firenze, Italy.
- [50] Santissima Trinità di Cagliari, Cagliari, Italy.
- [51] Department of Clinical and Experimental Medicine, Azienda Ospedaliera Universitaria Pisana University of Pisa, Pisa, Italy.
- [52] Dipartimento di Farmacia, Università di Pisa, Pisa, Italy.
- [53] UOC Malattie Infettive-Apparato Respiratorio, National Institute for Infectious Diseases L. Spallanzani IRCCS, Rome, Italy.
- [54] Computer Service, University of Molise, Campobasso, Italy.
- [55] Clinica Medica 3. Department of Medicine - DIMED, University Hospital of Padova, Padova, Italy.
- [56] UOC Pneumologia. Dipartimento di Malattie Apparato Respiratorio e Torace, Ospedale Morgagni-Pierantoni Forlì, Forlì, Italy.
- [57] Department of Respiratory Diseases & Allergy Aarhus University Hospital, Aarhus, Denmark.
- [58] UOC Malattie Infettive Ad Alta Intensità di Cura, National Institute for Infectious Diseases L. Spallanzani IRCCS, Rome, Italy.
- [59] Infectious and Tropical Diseases Unit, Deparment of Medical and Surgical Sciences, Magna Graecia University, Catanzaro, Italy.
- [60] Department of Epidemiology, Boston University School of Public Health, Boston, USA.

- PMID: **34336157**
- PMCID: PMC8238578
- DOI: 10.1155/2021/5556207

Abstract

The efficacy of hydroxychloroquine (HCQ) in treating SARS-CoV-2 infection is harshly debated, with observational and experimental studies reporting contrasting results. To clarify the role of HCQ in Covid-19 patients, we carried out a retrospective observational study of 4,396 unselected patients hospitalized for Covid-19 in Italy (February-May 2020). Patients' characteristics were collected at entry, including age, sex, obesity, smoking status, blood parameters, history of diabetes, cancer, cardiovascular and chronic pulmonary diseases, and medications in use. These were used to identify subtypes of patients with similar characteristics through hierarchical clustering based on Gower distance. Using multivariable Cox regressions, these clusters were then tested for association with mortality and modification of effect by treatment with HCQ. We identified two clusters, one of 3,913 younger patients with lower circulating inflammation levels and better renal function, and one of 483 generally older and more comorbid subjects, more prevalently men and smokers. The latter group was at increased death risk adjusted by HCQ (HR [CI95%] = 3.80[3.08-4.67]), while HCQ showed an independent inverse association (0.51 [0.43-0.61]), as well as a significant influence of cluster∗HCQ interaction ($p < 0.001$). This was driven by a differential association of HCQ with mortality between the high (0.89[0.65-1.22]) and the low risk cluster (0.46[0.39-0.54]). These effects survived adjustments for additional medications in use and were concordant with associations with disease severity and outcome. These findings suggest a particularly beneficial effect of HCQ within low risk Covid-19 patients and may contribute to clarifying the current controversy on HCQ efficacy in Covid-19 treatment.

Copyright © 2021 Augusto Di Castelnuovo et al.

Conflict of interest statement

The authors declare that they have no conflicts of interest.

- 40 references
- 2 figures

Supplementary info

MeSH terms, Substances, Supplementary concepts Expand

MeSH terms

- Aged
- Aged, 80 and over
- Antimalarials / adverse effects*
- Antimalarials / therapeutic use*
- COVID-19 / drug therapy*
- COVID-19 / mortality*
- COVID-19 / physiopathology
- Cluster Analysis
- Female
- Hospital Mortality*
- Humans
- Hydroxychloroquine / adverse effects*
- Hydroxychloroquine / therapeutic use*
- Italy
- Male
- Middle Aged
- Retrospective Studies
- SARS-CoV-2 / drug effects
- Severity of Illness Index
- Treatment Outcome

Substances

- Antimalarials
- Hydroxychloroquine

Supplementary concepts

- COVID-19 drug treatment

Full text links

Hindawi Limited Free PMC article
Proceed to details
Cite
Share
874
Observational Study
Clin Microbiol Infect
. 2020 Oct;26(10):1395-1399.
doi: 10.1016/j.cmi.2020.06.025. Epub 2020 Jun 27.

Bacterial and fungal coinfection among hospitalized patients with COVID-19: a retrospective cohort study in a UK secondary-care setting

S Hughes [1], O Troise [2], H Donaldson [3], N Mughal [3], L S P Moore [3]
Affiliations Expand

Affiliations

- [1] Chelsea and Westminster NHS Foundation Trust, Hammersmith Campus, London, UK. Electronic address: stephen.hughes2@chelwest.nhs.uk.
- [2] Chelsea and Westminster NHS Foundation Trust, Hammersmith Campus, London, UK.
- [3] Chelsea and Westminster NHS Foundation Trust, Hammersmith Campus, London, UK; North West London Pathology, Imperial College Healthcare NHS Trust, Hammersmith Campus, London, UK; National Institute for Health Research Health Protection Research Unit in Healthcare Associated Infections and Antimicrobial Resistance, Imperial College London, Hammersmith Campus, London, UK.

- PMID: **32603803**
- PMCID: PMC7320692
- DOI: 10.1016/j.cmi.2020.06.025

Free PMC article
Observational Study

Bacterial and fungal coinfection among hospitalized patients with COVID-19: a

[retrospective cohort study in a UK secondary-care setting]

S Hughes et al. Clin Microbiol Infect. 2020 Oct.
Free PMC article
Show details
Clin Microbiol Infect
. 2020 Oct;26(10):1395-1399.
doi: 10.1016/j.cmi.2020.06.025. Epub 2020 Jun 27.

Authors

S Hughes [1], O Troise [2], H Donaldson [3], N Mughal [3], L S P Moore [3]

Affiliations

- [1] Chelsea and Westminster NHS Foundation Trust, Hammersmith Campus, London, UK. Electronic address: stephen.hughes2@chelwest.nhs.uk.
- [2] Chelsea and Westminster NHS Foundation Trust, Hammersmith Campus, London, UK.
- [3] Chelsea and Westminster NHS Foundation Trust, Hammersmith Campus, London, UK; North West London Pathology, Imperial College Healthcare NHS Trust, Hammersmith Campus, London, UK; National Institute for Health Research Health Protection Research Unit in Healthcare Associated Infections and Antimicrobial Resistance, Imperial College London, Hammersmith Campus, London, UK.

- PMID: **32603803**
- PMCID: PMC7320692
- DOI: 10.1016/j.cmi.2020.06.025

Abstract

Objectives: To investigate the incidence of bacterial and fungal coinfection of hospitalized patients with confirmed severe acute respiratory syndrome coronavirus 2 (SARS-CoV-2) in this retrospective observational study across two London hospitals during the first UK wave of coronavirus disease 2019 (COVID-19).

Methods: A retrospective case series of hospitalized patients with confirmed SARS-CoV-2 by PCR was analysed across two acute NHS hospitals (20 February-20 April 2020; each isolate reviewed independently in parallel). This was contrasted to a control group of influenza-positive patients admitted during the 2019-2020 flu season. Patient demographics, microbiology and clinical outcomes were analysed.

Results: A total of 836 patients with confirmed SARS-CoV-2 were included; 27 (3.2%) of 836 had early confirmed bacterial isolates identified (0-5 days after admission), rising to 51 (6.1%) of 836 throughout admission. Blood cultures, respiratory samples, pneumococcal or Legionella urinary antigens and respiratory viral PCR panels were obtained from 643 (77%), 110 (13%), 249 (30%), 246 (29%) and 250 (30%) COVID-19 patients, respectively. A positive blood culture was identified in 60 patients (7.1%), of which 39 were classified as contaminants. Bacteraemia resulting from respiratory infection was confirmed in two cases (one each community-acquired

Klebsiella pneumoniae and ventilator-associated Enterobacter cloacae). Line-related bacteraemia was identified in six patients (three Candida, two Enterococcus spp. and one Pseudomonas aeruginosa). All other community-acquired bacteraemias (n = 16) were attributed to nonrespiratory infection. Zero concomitant pneumococcal, Legionella or influenza infection was detected. A low yield of positive respiratory cultures was identified; Staphylococcus aureus was the most common respiratory pathogen isolated in community-acquired coinfection (4/24; 16.7%), with pseudomonas and yeast identified in late-onset infection. Invasive fungal infections (n = 3) were attributed to line-related infections. Comparable rates of positive coinfection were identified in the control group of confirmed influenza infection; clinically relevant bacteraemias (2/141; 1.4%), respiratory cultures (10/38; 26.3%) and pneumococcal-positive antigens (1/19; 5.3%) were low.

Conclusions: We found a low frequency of bacterial coinfection in early COVID-19 hospital presentation, and no evidence of concomitant fungal infection, at least in the early phase of COVID-19.

Keywords: Antimicrobial Stewardship; Bacterial co-infection; Coronavirus; Pneumonia; SARS-CoV-2.

Copyright © 2020 European Society of Clinical Microbiology and Infectious Diseases. Published by Elsevier Ltd. All rights reserved.

- 15 references

Supplementary info

Publication types, MeSH terms Expand

Publication types

- Observational Study

MeSH terms

- Age Factors
- Aged
- Aged, 80 and over
- Bacterial Infections / diagnosis
- Bacterial Infections / epidemiology*
- Bacterial Infections / microbiology
- Bacterial Infections / virology
- Betacoronavirus / pathogenicity*
- COVID-19
- Coinfection
- Community-Acquired Infections
- Coronavirus Infections / diagnosis
- Coronavirus Infections / epidemiology*

- Coronavirus Infections / microbiology
- Coronavirus Infections / virology
- Female
- Hospitalization
- Humans
- Influenza, Human / diagnosis
- Influenza, Human / epidemiology*
- Influenza, Human / microbiology
- Influenza, Human / virology
- Male
- Middle Aged
- Mycoses / diagnosis
- Mycoses / epidemiology*
- Mycoses / microbiology
- Mycoses / virology
- Pandemics*
- Pneumonia, Viral / diagnosis
- Pneumonia, Viral / epidemiology*
- Pneumonia, Viral / microbiology
- Pneumonia, Viral / virology
- Respiratory Tract Infections / diagnosis
- Respiratory Tract Infections / epidemiology*
- Respiratory Tract Infections / microbiology
- Respiratory Tract Infections / virology
- Retrospective Studies
- SARS-CoV-2
- Severity of Illness Index
- United Kingdom / epidemiology

Full text links

ELSEVIER OPEN ACCESS Elsevier Science Free PMC article

Proceed to details

Cite

Share

☐ 875

Observational Study

Lancet Psychiatry

. 2020 Dec;7(12):1054-1063.
doi: 10.1016/S2215-0366(20)30434-X. Epub 2020 Oct 5.

Prevalence, management, and outcomes of SARS-CoV-2 infections in older people and those with dementia in mental health wards in London, UK: a retrospective observational study

Gill Livingston [1], Hossein Rostamipour [2], Paul Gallagher [3], Chris Kalafatis [4], Abhishek Shastri [5], Lauren Huzzey [6], Kathy Liu [7], Andrew Sommerlad [7], Louise Marston [8]
Affiliations Expand

Affiliations

- [1] Division of Psychiatry, University College London, London, UK; Camden and Islington NHS Foundation Trust, St Pancras Hospital, London, UK. Electronic address: g.livingston@ucl.ac.uk.
- [2] Camden and Islington NHS Foundation Trust, St Pancras Hospital, London, UK.
- [3] East London NHS Foundation Trust, London, UK.
- [4] South London and Maudsley NHS Foundation Trust, Bethlem Royal Hospital, Beckenham, UK; Department of Old Age Psychiatry, Institute of Psychiatry, Psychology & Neuroscience, King's College London, London, UK.
- [5] Central and North West London NHS Foundation Trust, London, UK.
- [6] Barnet, Enfield and Haringey MH NHS Trust, St Ann's Hospital, London, UK.
- [7] Division of Psychiatry, University College London, London, UK; Camden and Islington NHS Foundation Trust, St Pancras Hospital, London, UK.
- [8] Primary Care and Population Health, Institute of Epidemiology and Health, Faculty of Population Health Sciences, University College London, London, UK.

- PMID: **33031760**
- PMCID: PMC7535621
- DOI: 10.1016/S2215-0366(20)30434-X

Free PMC article
Observational Study

Prevalence, management, and outcomes of SARS-CoV-2 infections in older people and those with dementia in mental health wards in London, UK: a retrospective observational study

Gill Livingston et al. Lancet Psychiatry. 2020 Dec.
Free PMC article
Show details
Lancet Psychiatry
. 2020 Dec;7(12):1054-1063.
doi: 10.1016/S2215-0366(20)30434-X. Epub 2020 Oct 5.

Authors

Gill Livingston [1], Hossein Rostamipour [2], Paul Gallagher [3], Chris Kalafatis [4], Abhishek Shastri [5], Lauren Huzzey [6], Kathy Liu [7], Andrew Sommerlad [7], Louise Marston [8]

Affiliations

- [1] Division of Psychiatry, University College London, London, UK; Camden and Islington NHS Foundation Trust, St Pancras Hospital, London, UK. Electronic address: g.livingston@ucl.ac.uk.
- [2] Camden and Islington NHS Foundation Trust, St Pancras Hospital, London, UK.
- [3] East London NHS Foundation Trust, London, UK.
- [4] South London and Maudsley NHS Foundation Trust, Bethlem Royal Hospital, Beckenham, UK; Department of Old Age Psychiatry, Institute of Psychiatry, Psychology & Neuroscience, King's College London, London, UK.
- [5] Central and North West London NHS Foundation Trust, London, UK.
- [6] Barnet, Enfield and Haringey MH NHS Trust, St Ann's Hospital, London, UK.
- [7] Division of Psychiatry, University College London, London, UK; Camden and Islington NHS Foundation Trust, St Pancras Hospital, London, UK.
- [8] Primary Care and Population Health, Institute of Epidemiology and Health, Faculty of Population Health Sciences, University College London, London, UK.

- PMID: **33031760**
- PMCID: PMC7535621
- DOI: 10.1016/S2215-0366(20)30434-X

Abstract

Background: People living in group situations or with dementia are more vulnerable to infection with severe acute respiratory syndrome coronavirus 2 (SARS-CoV-2). Older people and those with multimorbidity have higher mortality if they become infected than the general population. However, no systematic study exists of COVID-19-related outcomes in older inpatients in psychiatric units, who comprise people from these high-risk groups. We aimed to describe the period prevalence, demographics, symptoms (and asymptomatic cases), management, and survival outcomes of COVID-19 in the older inpatient psychiatric population and people with young-onset dementia in five National Health Service Trusts in London, UK, from March 1 to April 30, 2020.

Methods: In this retrospective observational study, we collected demographic data, mental health diagnoses, clinical diagnosis of COVID-19, symptoms, management, and COVID-19-related outcome data of inpatients aged 65 years or older or with dementia who were already inpatients or admitted as inpatients to five London mental health Trusts between March 1 and April 30, 2020, and information about available COVID-19-related resources (ie, testing and personal protective equipment). Patients were determined to have COVID-19 if they had a positive SARS-CoV-2

PCR test, or had relevant symptoms indicative of COVID-19, as determined by their treating physician. We calculated period prevalence of COVID-19 and analysed patients' characteristics, treatments, and outcomes.

Findings: Of 344 inpatients, 131 (38%) were diagnosed with COVID-19 during the study period (period prevalence 38% [95% CI 33-43]). The mean age of patients who had COVID-19 was 75·3 years (SD 8·2); 68 (52%) were women and 47 (36%) from ethnic minority groups. 16 (12%) of 131 patients were asymptomatic and 121 (92%) had one or more disease-related comorbidity. 108 (82%) patients were compulsorily detained. 74 (56%) patients had dementia, of whom 13 (18%) had young-onset dementia. On average, sites received COVID-19 testing kits 4·5 days after the first clinical COVID-19 presentation. 19 (15%) patients diagnosed with COVID-19 died during the study period, and their deaths were determined to be COVID-19 related.

Interpretation: Patients in psychiatric inpatient settings who were admitted without known SARS-CoV-2 infection had a high risk of infection with SARS-CoV-2 compared with those in the community and had a higher proportion of deaths from COVID-19 than in the community. Implementation of the long-standing policy of parity of esteem for mental health and planning for future COVID-19 waves in psychiatric hospitals is urgent.

Funding: None.

Copyright © 2020 Elsevier Ltd. All rights reserved.

- 44 references

Supplementary info

Publication types, MeSH terms, Grant support Expand

Publication types

- Observational Study
- Research Support, Non-U.S. Gov't

MeSH terms

- Aged
- Aged, 80 and over
- COVID-19 / epidemiology*
- COVID-19 / mortality
- COVID-19 / physiopathology
- COVID-19 / therapy*
- Comorbidity
- Dementia / epidemiology*
- Dementia / physiopathology
- Dementia / therapy*
- Female
- Humans

- London / epidemiology
- Male
- Outcome Assessment, Health Care / statistics & numerical data*
- Prevalence
- Psychiatric Department, Hospital / statistics & numerical data*
- Retrospective Studies

Grant support

- WT_/Wellcome Trust/United Kingdom

Full text links

ELSEVIER FULL-TEXT ARTICLE Elsevier Science Free PMC article
Proceed to details
Cite
Share
876
Observational Study
Ann Intern Med
. 2020 Nov 17;173(10):855-858.
doi: 10.7326/M20-2730. Epub 2020 Jul 6.

Obesity and COVID-19 in New York City: A Retrospective Cohort Study

Parag Goyal [1], Joanna Bryan Ringel [1], Mangala Rajan [1], Justin J Choi [1], Laura C Pinheiro [1], Han A Li [2], Graham T Wehmeyer [2], Mark N Alshak [2], Assem Jabri [1], Edward J Schenck [1], Ruijun Chen [3], Michael J Satlin [1], Thomas R Campion Jr [1], Musarrat Nahid [1], Maria Plataki [1], Katherine L Hoffman [1], Evgeniya Reshetnyak [1], Nathaniel Hupert [1], Evelyn M Horn [1], Fernando J Martinez [1], Roy M Gulick [1], Monika M Safford [1]
Affiliations Expand

Affiliations

- [1] Weill Cornell Medicine, New York, New York (P.G., J.B.R., M.R., J.J.C., L.C.P., A.J., E.J.S., M.J.S., T.R.C., M.N., M.P., K.L.H., E.R., N.H., E.M.H., F.J.M., R.M.G., M.M.S.).
- [2] Weill Cornell Medical College, New York, New York (H.A.L., G.T.W., M.N.A.).
- [3] Weill Cornell Medicine and Columbia University, New York, New York (R.C.).

- PMID: **32628537**
- PMCID: PMC7384267
- DOI: 10.7326/M20-2730

Free PMC article

Observational Study

Obesity and COVID-19 in New York City: A Retrospective Cohort Study

Parag Goyal et al. Ann Intern Med. 2020.
Free PMC article
Show details
Ann Intern Med
. 2020 Nov 17;173(10):855-858.
doi: 10.7326/M20-2730. Epub 2020 Jul 6.

Authors

Parag Goyal [1], Joanna Bryan Ringel [1], Mangala Rajan [1], Justin J Choi [1], Laura C Pinheiro [1], Han A Li [2], Graham T Wehmeyer [2], Mark N Alshak [2], Assem Jabri [1], Edward J Schenck [1], Ruijun Chen [3], Michael J Satlin [1], Thomas R Campion Jr [1], Musarrat Nahid [1], Maria Plataki [1], Katherine L Hoffman [1], Evgeniya Reshetnyak [1], Nathaniel Hupert [1], Evelyn M Horn [1], Fernando J Martinez [1], Roy M Gulick [1], Monika M Safford [1]

Affiliations

- [1] Weill Cornell Medicine, New York, New York (P.G., J.B.R., M.R., J.J.C., L.C.P., A.J., E.J.S., M.J.S., T.R.C., M.N., M.P., K.L.H., E.R., N.H., E.M.H., F.J.M., R.M.G., M.M.S.).
- [2] Weill Cornell Medical College, New York, New York (H.A.L., G.T.W., M.N.A.).
- [3] Weill Cornell Medicine and Columbia University, New York, New York (R.C.).

- PMID: **32628537**
- PMCID: PMC7384267
- DOI: 10.7326/M20-2730

No abstract available

Conflict of interest statement

Disclosures: Disclosures can be viewed at www.acponline.org/authors/icmje/ConflictOfInterestForms.do?msNum=M20-2730.

- 5 references
- 1 figure

Supplementary info

Publication types, MeSH terms, Grant support Expand

Publication types

- Letter
- Observational Study

MeSH terms

- Adolescent
- Adult
- Aged
- Betacoronavirus
- Body Mass Index
- COVID-19
- Cohort Studies
- Coronavirus Infections / epidemiology*
- Female
- Hospital Mortality
- Hospitalization
- Humans
- Male
- Middle Aged
- New York City / epidemiology
- Obesity / epidemiology*
- Pandemics
- Pneumonia, Viral / epidemiology*
- Respiratory Insufficiency / epidemiology
- Respiratory Insufficiency / virology*
- Retrospective Studies
- Risk Factors
- SARS-CoV-2
- Young Adult

Grant support

- R03 AG056446/AG/NIA NIH HHS/United States

Full text links

Annals FREE FULL TEXT Atypon Free PMC article
Proceed to details
Cite
Share
☐ 877
Observational Study
Clin Transplant

. 2020 Dec;34(12):e14105.
doi: 10.1111/ctr.14105. Epub 2020 Oct 19.

Outpatient COVID-19 surveillance testing in orthotopic heart transplant recipients

Sandra A Carey [1], Aasim Afzal [1] [2], Aayla Jamil [3], Sarah Williams [1], Robert L Gottlieb [1] [2] [3] [4] [5]

Affiliations Expand

Affiliations

- [1] Center for Advanced Heart and Lung Disease, Baylor University Medical Center, Dallas, Texas, USA.
- [2] Baylor Scott & White The Heart Hospital - Plano, Plano, Texas, USA.
- [3] Baylor Scott & White Research Institute, Dallas, Texas, USA.
- [4] Division of Precision Medicine, Baylor Scott & White Health, Dallas, Texas, USA.
- [5] Texas A&M University College of Medicine Health Science Center, Dallas, Texas, USA.

- PMID: **32978777**
- PMCID: PMC7536909
- DOI: 10.1111/ctr.14105

Free PMC article
Observational Study

Outpatient COVID-19 surveillance testing in orthotopic heart transplant recipients

Sandra A Carey et al. Clin Transplant. 2020 Dec.
Free PMC article
Show details
Clin Transplant
. 2020 Dec;34(12):e14105.
doi: 10.1111/ctr.14105. Epub 2020 Oct 19.

Authors

Sandra A Carey [1], Aasim Afzal [1] [2], Aayla Jamil [3], Sarah Williams [1], Robert L Gottlieb [1] [2] [3] [4] [5]

Affiliations

- [1] Center for Advanced Heart and Lung Disease, Baylor University Medical Center, Dallas, Texas, USA.

- [2] Baylor Scott & White The Heart Hospital - Plano, Plano, Texas, USA.
- [3] Baylor Scott & White Research Institute, Dallas, Texas, USA.
- [4] Division of Precision Medicine, Baylor Scott & White Health, Dallas, Texas, USA.
- [5] Texas A&M University College of Medicine Health Science Center, Dallas, Texas, USA.

- PMID: **32978777**
- PMCID: PMC7536909
- DOI: 10.1111/ctr.14105

Abstract

COVID-19 case fatality rate in the United States is currently reported at 4.8% based on the confirmed cases of COVID-19. However, there are conflicting reports of estimated deaths in the post-cardiac transplantation patient population associated with COVID-19.

Methods: Observational, retrospective analysis of a large cohort of post Orthotopic Heart Transplantation (OHT) patients in a high volume heart transplantation program in Dallas, Texas underwent outpatient COVID-19 screening and testing for both SARS-CoV-2 nasopharyngeal RT-PCR and anti-SARS-CoV2 IgG serology as a result of a clinic protocol to facilitate re-opening of face-to-face outpatient clinical visits.

Results: The full outpatient cohort tested at time of their clinic visit tested negative for COVID-19 by nasopharyngeal RT-PCR. Only 2 patients tested seropositive for anti-SARS-COV2 IgG. Five positive inpatient cases were also identified and all, but one recovered.

Conclusion: A COVID-19 surveillance protocol can be easily instituted in this high-risk population and facilitate safe transplant clinic operation. As the cases and prevalence increase across the United States, further strategies will need to be developed to determine the best course of action to help manage this select population while minimizing their exposure to the ongoing pandemic.

Keywords: cardiac transplantation; clinical decision-making; complication: infectious; infection and infectious agents; risk assessment/risk stratification; viral.

© 2020 John Wiley & Sons A/S. Published by John Wiley & Sons Ltd.

- 7 references

Supplementary info

Publication types, MeSH terms Expand

Publication types

- Observational Study

MeSH terms

- Adult
- Aged

- Aged, 80 and over
- Ambulatory Care / methods*
- COVID-19 / diagnosis*
- COVID-19 / epidemiology
- COVID-19 / etiology
- COVID-19 Testing / methods*
- Clinical Protocols
- Feasibility Studies
- Female
- Heart Transplantation*
- Humans
- Male
- Middle Aged
- Postoperative Complications / diagnosis*
- Postoperative Complications / epidemiology
- Public Health Surveillance / methods*
- Retrospective Studies
- Texas / epidemiology

Full text links

WILEY Full Text Article Wiley Free PMC article

Proceed to details

Cite

Share

☐ 878

Observational Study

Intern Med J
. 2020 Dec;50(12):1457-1467.
doi: 10.1111/imj.15091.

Aeromedical retrieval diagnostic trends during a period of Coronavirus 2019 lockdown

Fergus W Gardiner [1, 2], Marianne Gillam [3], Leonid Churilov [4], Pritish Sharma [1], Mardi Steere [5], Michelle Hannan [6], Andrew Hooper [7], Frank Quinlan [1]
Affiliations Expand

Affiliations

- [1] Federation Office, The Royal Flying Doctor Service, Canberra, Australian Capital Territory, Australia.
- [2] The Rural Clinical School of Western Australia, The University of Western Australia, Perth, Western Australia, Australia.
- [3] Department of Rural Health, University of South Australia, Adelaide, South Australia, Australia.
- [4] Department of Medicine (Austin Health) and Melbourne Brain Centre at Royal Melbourne Hospital, Melbourne Medical School, The University of Melbourne, Melbourne, Victoria, Australia.
- [5] Central Operations, The Royal Flying Doctor Service, Adelaide, South Australia, Australia.
- [6] Queensland Section, The Royal Flying Doctor Service, Adelaide, South Australia, Australia.
- [7] Western Operations, The Royal Flying Doctor Service, Adelaide, South Australia, Australia.

- PMID: **33040422**
- PMCID: PMC7675287
- DOI: 10.1111/imj.15091

Free PMC article
Observational Study

Aeromedical retrieval diagnostic trends during a period of Coronavirus 2019 lockdown

Fergus W Gardiner et al. Intern Med J. 2020 Dec.
Free PMC article
Show details
Intern Med J
. 2020 Dec;50(12):1457-1467.
doi: 10.1111/imj.15091.

Authors

Fergus W Gardiner [1, 2], Marianne Gillam [3], Leonid Churilov [4], Pritish Sharma [1], Mardi Steere [5], Michelle Hannan [6], Andrew Hooper [7], Frank Quinlan [1]

Affiliations

- [1] Federation Office, The Royal Flying Doctor Service, Canberra, Australian Capital Territory, Australia.
- [2] The Rural Clinical School of Western Australia, The University of Western Australia, Perth, Western Australia, Australia.
- [3] Department of Rural Health, University of South Australia, Adelaide, South Australia, Australia.

- [4] Department of Medicine (Austin Health) and Melbourne Brain Centre at Royal Melbourne Hospital, Melbourne Medical School, The University of Melbourne, Melbourne, Victoria, Australia.
- [5] Central Operations, The Royal Flying Doctor Service, Adelaide, South Australia, Australia.
- [6] Queensland Section, The Royal Flying Doctor Service, Adelaide, South Australia, Australia.
- [7] Western Operations, The Royal Flying Doctor Service, Adelaide, South Australia, Australia.

- PMID: **33040422**
- PMCID: PMC7675287
- DOI: 10.1111/imj.15091

Abstract

Background: Little is known on the trends of aeromedical retrieval (AR) during social isolation.

Aim: To compare the pre, lockdown, and post-lockdown AR patient characteristics during a period of Coronavirus 2019 (COVID-19) social isolation.

Methods: An observational study with retrospective data collection, consisting of AR between 26 January and 23 June 2020.

Results: There were 16 981 AR consisting of 1983 (11.7%) primary evacuations and 14 998 (88.3%) inter-hospital transfers, with a population median age of 52 years (interquartile range 29.0-69.0), with 49.0% (n = 8283) of the cohort being male and 38.0% (n = 6399) being female. There were six confirmed and 230 suspected cases of COVID-19, with the majority of cases (n = 134; 58.3%) in the social isolation period. As compared to pre-restriction, the odds of retrieval for the restriction and post-restriction period differed across time between the major diagnostic groups. This included, an increase in cardiovascular retrieval for both restriction and post-restriction periods (odds ratio (OR) 1.12, 95% confidence interval (CI) 1.02-1.24 and OR 1.18 95%, CI 1.08-1.30 respectively), increases in neoplasm in the post restriction period (OR 1.31, 95% CI 1.04-1.64) and increases for congenital conditions in the restriction period (OR 2.56, 95% CI 1.39-4.71). Cardiovascular and congenital conditions had increased rates of priority 1 patients in the restriction and post restriction periods. There was a decrease in endocrine and metabolic disease retrievals in the restriction period (OR 0.72, 95% CI 0.53-0.98). There were lower odds during the post-restriction period for retrievals of the respiratory system (OR 0.78, 95% CI 0.67-0.93), and disease of the skin (OR 0.78, 95% CI 0.6-1.0). Distribution between the 2019 and 2020 time periods differed ($P < 0.05$), with the lockdown period resulting in a significant reduction in activity.

Conclusion: The lockdown period resulted in increased AR rates of circulatory and congenital conditions.

Keywords: aeromedical; coronavirus; pandemic; rural and remote.

© 2020 Royal Australasian College of Physicians.

Comment in

- Hospital in the air: Royal Flying Doctor Service retrievals and challenges before, during and after, Australia's COVID-19 lockdown in 2020.
  Ting J. Ting J. Intern Med J. 2020 Dec;50(12):1449-1451. doi: 10.1111/imj.15109. Intern Med J. 2020. PMID: 33354879 No abstract available.

- 27 references

Supplementary info

Publication types, MeSH terms Expand

Publication types

- Observational Study

MeSH terms

- Adult
- Aged
- Air Ambulances*
- Australia / epidemiology
- COVID-19 / epidemiology*
- Communicable Disease Control / methods
- Communicable Disease Control / trends*
- Female
- Humans
- Male
- Middle Aged
- Patient Transfer / trends*
- Quarantine / trends*
- Retrospective Studies

Full text links

WILEY Full Text Article Wiley Free PMC article

Proceed to details

Cite

Share

☐ 879

Observational Study

Arch Cardiovasc Dis

. 2021 May;114(5):415-425.

doi: 10.1016/j.acvd.2021.04.003. Epub 2021 May 24.

History of heart failure in patients with coronavirus disease 2019: Insights from a French registry

Vassili Panagides [1], Flavien Vincent [2], Orianne Weizman [3], Melchior Jonveaux [4], Antonin Trimaille [5], Thibaut Pommier [6], Joffrey Cellier [7], Laura Geneste [8], Wassima Marsou [9], Antoine Deney [10], Sabir Attou [11], Thomas Delmotte [12], Charles Fauvel [13], Nacim Ezzouhairi [14], Benjamin Perin [15], Cyril Zakine [16], Thomas Levasseur [17], Iris Ma [7], Diane Chavignier [18], Nathalie Noirclerc [19], Arthur Darmon [20], Marine Mevelec [19], Clément Karsenty [10], Baptiste Duceau [4], Willy Sutter [4], Delphine Mika [21], Théo Pezel [22], Victor Waldmann [4], Julien Ternacle [23], Ariel Cohen [24], Guillaume Bonnet [25], Critical COVID-19 France Investigators
Affiliations Expand

Affiliations

- [1] Aix-Marseille Université, Intensive Care Unit, Hôpital Nord, AP-HM, 13015 Marseille, France.
- [2] Centre Hospitalier Universitaire de Lille, Institut Cœur Poumon, Cardiology, Department of Interventional Cardiology for Coronary, Valves and Structural Heart Diseases, INSERM U1011, Institut Pasteur de Lille, EGID, Université de Lille, 59800 Lille, France.
- [3] Institut Lorrain du Cœur et des Vaisseaux, CHU de Nancy, 54500 Vandœuvre-les-Nancy, France; Université de Paris, Paris Cardiovascular Research Centre (PARCC), INSERM, UMR-S970, 75015 Paris, France.
- [4] Département de Cardiologie, Expert Valve Center, Assistance Publique-Hôpitaux de Paris, Hôpital Henri Mondor, 94010 Créteil, France.
- [5] Nouvel Hôpital Civil, Centre Hospitalier Régional Universitaire de Strasbourg, 67000 Strasbourg, France.
- [6] Centre Hospitalier Universitaire de Dijon, 21000 Dijon, France.
- [7] Hôpital Européen Georges-Pompidou, Université de Paris, 75015 Paris, France.
- [8] Centre Hospitalier Universitaire d'Amiens-Picardie, 80000 Amiens, France.
- [9] GCS-Groupement des Hôpitaux de l'Institut Catholique de Lille, Faculté de Médecine et de Maïeutique, Université Catholique de Lille, 59800 Lille, France.
- [10] Centre Hospitalier Universitaire de Toulouse, 31400 Toulouse, France.
- [11] Centre Hospitalier Universitaire de Caen-Normandie, 14000 Caen, France.
- [12] Centre Hospitalier Universitaire de Reims, 51100 Reims, France.
- [13] Rouen University Hospital, FHU REMOD-VHF, 76000 Rouen, France.
- [14] University of Bordeaux, Hôpital Cardiologique Haut-Lévêque, Centre Hospitalier Universitaire de Bordeaux, Pessac, France.
- [15] Institut Lorrain du Cœur et des Vaisseaux, CHU de Nancy, 54500 Vandœuvre-les-Nancy, France.
- [16] Clinique Saint-Gatien, 37540 Saint-Cyr-sur-Loire, France.
- [17] Centre Hospitalier Intercommunal Fréjus-Saint-Raphaël, 83600 Fréjus, France.
- [18] Centre Hospitalier Régional d'Orléans, 45100 Orléans, France.
- [19] Centre Hospitalier Annecy Genevois, 74370 Épagny-Metz-Tessy, France.
- [20] Hôpital Bichat-Claude-Bernard, AP-HP, Université de Paris, 75018 Paris, France.

- [21] Université Paris-Saclay, INSERM, UMR-S 1180, 92296 Châtenay-Malabry, France.
- [22] Hôpital Lariboisière, AP-HP, University of Paris, 75010 Paris, France.
- [23] University of Bordeaux, Hôpital Cardiologique Haut-Lévêque, Centre Hospitalier Universitaire de Bordeaux, Pessac, France; Institut Universitaire de Cardiologie et de Pneumologie de Québec, Université Laval/Québec Heart and Lung Institute, Laval University, Québec G1V 4G5, Canada.
- [24] Hôpital Saint-Antoine, 75012 Paris, France. Electronic address: ariel.cohen@aphp.fr.
- [25] Université de Paris, Paris Cardiovascular Research Centre (PARCC), INSERM, UMR-S970, 75015 Paris, France; University of Bordeaux, Hôpital Cardiologique Haut-Lévêque, Centre Hospitalier Universitaire de Bordeaux, Pessac, France.

- PMID: **34099379**
- PMCID: PMC8141712
- DOI: 10.1016/j.acvd.2021.04.003

Free PMC article
Observational Study

History of heart failure in patients with coronavirus disease 2019: Insights from a French registry

Vassili Panagides et al. Arch Cardiovasc Dis. 2021 May.
Free PMC article
Show details
Arch Cardiovasc Dis
. 2021 May;114(5):415-425.
doi: 10.1016/j.acvd.2021.04.003. Epub 2021 May 24.

Authors

Vassili Panagides [1], Flavien Vincent [2], Orianne Weizman [3], Melchior Jonveaux [4], Antonin Trimaille [5], Thibaut Pommier [6], Joffrey Cellier [7], Laura Geneste [8], Wassima Marsou [9], Antoine Deney [10], Sabir Attou [11], Thomas Delmotte [12], Charles Fauvel [13], Nacim Ezzouhairi [14], Benjamin Perin [15], Cyril Zakine [16], Thomas Levasseur [17], Iris Ma [7], Diane Chavignier [18], Nathalie Noirclerc [19], Arthur Darmon [20], Marine Mevelec [19], Clément Karsenty [10], Baptiste Duceau [4], Willy Sutter [4], Delphine Mika [21], Théo Pezel [22], Victor Waldmann [4], Julien Ternacle [23], Ariel Cohen [24], Guillaume Bonnet [25], Critical COVID-19 France Investigators

Affiliations

- [1] Aix-Marseille Université, Intensive Care Unit, Hôpital Nord, AP-HM, 13015 Marseille, France.
- [2] Centre Hospitalier Universitaire de Lille, Institut Cœur Poumon, Cardiology, Department of Interventional Cardiology for Coronary, Valves and Structural Heart Diseases, INSERM U1011, Institut Pasteur de Lille, EGID, Université de Lille, 59800 Lille, France.

- [3] Institut Lorrain du Cœur et des Vaisseaux, CHU de Nancy, 54500 Vandœuvre-les-Nancy, France; Université de Paris, Paris Cardiovascular Research Centre (PARCC), INSERM, UMR-S970, 75015 Paris, France.
- [4] Département de Cardiologie, Expert Valve Center, Assistance Publique-Hôpitaux de Paris, Hôpital Henri Mondor, 94010 Créteil, France.
- [5] Nouvel Hôpital Civil, Centre Hospitalier Régional Universitaire de Strasbourg, 67000 Strasbourg, France.
- [6] Centre Hospitalier Universitaire de Dijon, 21000 Dijon, France.
- [7] Hôpital Européen Georges-Pompidou, Université de Paris, 75015 Paris, France.
- [8] Centre Hospitalier Universitaire d'Amiens-Picardie, 80000 Amiens, France.
- [9] GCS-Groupement des Hôpitaux de l'Institut Catholique de Lille, Faculté de Médecine et de Maïeutique, Université Catholique de Lille, 59800 Lille, France.
- [10] Centre Hospitalier Universitaire de Toulouse, 31400 Toulouse, France.
- [11] Centre Hospitalier Universitaire de Caen-Normandie, 14000 Caen, France.
- [12] Centre Hospitalier Universitaire de Reims, 51100 Reims, France.
- [13] Rouen University Hospital, FHU REMOD-VHF, 76000 Rouen, France.
- [14] University of Bordeaux, Hôpital Cardiologique Haut-Lévêque, Centre Hospitalier Universitaire de Bordeaux, Pessac, France.
- [15] Institut Lorrain du Cœur et des Vaisseaux, CHU de Nancy, 54500 Vandœuvre-les-Nancy, France.
- [16] Clinique Saint-Gatien, 37540 Saint-Cyr-sur-Loire, France.
- [17] Centre Hospitalier Intercommunal Fréjus-Saint-Raphaël, 83600 Fréjus, France.
- [18] Centre Hospitalier Régional d'Orléans, 45100 Orléans, France.
- [19] Centre Hospitalier Annecy Genevois, 74370 Épagny-Metz-Tessy, France.
- [20] Hôpital Bichat-Claude-Bernard, AP-HP, Université de Paris, 75018 Paris, France.
- [21] Université Paris-Saclay, INSERM, UMR-S 1180, 92296 Châtenay-Malabry, France.
- [22] Hôpital Lariboisière, AP-HP, University of Paris, 75010 Paris, France.
- [23] University of Bordeaux, Hôpital Cardiologique Haut-Lévêque, Centre Hospitalier Universitaire de Bordeaux, Pessac, France; Institut Universitaire de Cardiologie et de Pneumologie de Québec, Université Laval/Québec Heart and Lung Institute, Laval University, Québec G1V 4G5, Canada.
- [24] Hôpital Saint-Antoine, 75012 Paris, France. Electronic address: ariel.cohen@aphp.fr.
- [25] Université de Paris, Paris Cardiovascular Research Centre (PARCC), INSERM, UMR-S970, 75015 Paris, France; University of Bordeaux, Hôpital Cardiologique Haut-Lévêque, Centre Hospitalier Universitaire de Bordeaux, Pessac, France.

- PMID: **34099379**
- PMCID: PMC8141712
- DOI: 10.1016/j.acvd.2021.04.003

Abstract
in English, French

Background: Although cardiovascular comorbidities seem to be strongly associated with worse outcomes in patients with coronavirus disease 2019 (COVID-19), data regarding patients with preexisting heart failure are limited.

Aims: To investigate the incidence, characteristics and clinical outcomes of patients with COVID-19 with a history of heart failure with preserved or reduced ejection fraction.

Methods: We performed an observational multicentre study including all patients hospitalized for COVID-19 across 24 centres in France from 26 February to 20 April 2020. The primary endpoint was a composite of in-hospital death or need for orotracheal intubation.

Results: Overall, 2809 patients (mean age 66.4±16.9years) were included. Three hundred and seventeen patients (11.2%) had a history of heart failure; among them, 49.2% had heart failure with reduced ejection fraction and 50.8% had heart failure with preserved ejection fraction. COVID-19 severity at admission, defined by a quick sequential organ failure assessment score>1, was similar in patients with versus without a history of heart failure. Before and after adjustment for age, male sex, cardiovascular comorbidities and quick sequential organ failure assessment score, history of heart failure was associated with the primary endpoint (hazard ratio [HR]: 1.41, 95% confidence interval [CI]: 1.06-1.90; P=0.02). This result seemed to be mainly driven by a history of heart failure with preserved ejection fraction (HR: 1.61, 95% CI: 1.13-2.27; P=0.01) rather than heart failure with reduced ejection fraction (HR: 1.19, 95% CI: 0.79-1.81; P=0.41).

Conclusions: History of heart failure in patients with COVID-19 was associated with a higher risk of in-hospital death or orotracheal intubation. These findings suggest that patients with a history of heart failure, particularly heart failure with preserved ejection fraction, should be considered at high risk of clinical deterioration.

Contexte: Les antécédents cardiovasculaires semblent associés à un risque de complications accru chez les patients atteints de la COVID-19. Cependant, peu de données sont actuellement disponibles concernant les patients ayant une insuffisance cardiaque connue.

Objectifs: Définir l'incidence, les caractéristiques et le pronostic clinique des patients atteints de la COVID-19 avec un antécédent d'insuffisance cardiaque à fraction d'éjection préservée ou réduite.

Méthodes: Via une étude observationnelle rétrospective et multicentrique, tous les patients hospitalisés du 26 février au 20 avril 2020 pour une infection à SARS-CoV-2 parmi 24 centres français ont été inclus. Le critère de jugement principal était un critère composite comprenant la mortalité intra-hospitalière ou la nécessité d'une intubation orotrachéale.

Résultats: Au total, 2809 patients (66,4 ± 16,9 ans) ont été inclus. Trois cent dix-sept patients (11,2 %) avaient un antécédent d'insuffisance cardiaque. Parmi ces derniers, 49,2 % avait une fraction d'éjection réduite et 50,8 % une fraction d'éjection préservée. La sévérité de l'infection à l'admission, définie par un score qSOFA > 1, était similaire entre les patients avec ou sans antécédent d'insuffisance cardiaque. Avec ou sans ajustement sur l'âge, le genre masculin, les comorbidités cardiaques et le score qSOFA, l'existence préalable d'une insuffisance cardiaque était associée de manière significative au critère de jugement principal (HR : 1,41, IC95 % : 1,06 –1,90 ; *p* = 0,02). Ce résultat est principalement imputable aux patients ayant un antécédent d'insuffisance cardiaque à fraction d'éjection préservée (HR : 1,61, IC95 % : 1,13–2,27 ; *p* = 0,01) par rapport à ceux ayant une fraction d'éjection réduite (HR : 1,19, IC95 % : 0,79–1,81 ; *p* = 0,41).

Conclusions: L'existence d'une insuffisance cardiaque préalable chez les patients présentant une infection COVID-19 est associée à un risque accru de mortalité ou d'intubation orotrachéale. Ces résultats suggèrent que ces patients, particulièrement ceux à fraction d'éjection préservée, doivent être considérés comme à haut risque de dégradation clinique et surveillés de manière rapprochée.

Keywords: COVID-19; Facteurs de risque; Femme; HFpEF; HFrEF; Heart failure; RAAS inhibitors; Résultats; SARS-COV 2.

Copyright © 2021. Published by Elsevier Masson SAS.

- 37 references
- 2 figures

Supplementary info

Publication types, MeSH terms Expand

Publication types

- Multicenter Study
- Observational Study

MeSH terms

- Aged
- COVID-19 / blood
- COVID-19 / epidemiology*
- Comorbidity
- Confounding Factors, Epidemiologic
- Female
- France / epidemiology
- Heart Failure / blood
- Heart Failure / epidemiology*
- Heart Failure / physiopathology
- Hospital Mortality
- Humans
- Incidence
- Intubation, Intratracheal / statistics & numerical data
- Kaplan-Meier Estimate
- Male
- Middle Aged
- Procedures and Techniques Utilization
- Registries / statistics & numerical data*
- Retrospective Studies
- Risk Factors
- SARS-CoV-2*
- Stroke Volume
- Treatment Outcome

Full text links

ELSEVIER FULL-TEXT ARTICLE Elsevier Science Free PMC article
Proceed to details
Cite
Share
880

Observational Study

Mediators Inflamm
. 2021 Mar 18;2021:8812304.
doi: 10.1155/2021/8812304. eCollection 2021.

Hyperglycemia and Correlated High Levels of Inflammation Have a Positive Relationship with the Severity of Coronavirus Disease 2019

Wen Zhang [1, 2], Chuanwei Li [2, 3, 4], Yu Xu [1, 3, 5], Binfeng He [1], Mingdong Hu [1, 2, 3], Guoqiang Cao [2, 3, 6], Li Li [2, 3, 6], Shuang Wu [3], Xia Wang [1], Chun Zhang [1], Jianping Zhao [7], Jungang Xie [7], Zihui Xu [8], Qi Li [1, 2, 3], Guansong Wang [1]
Affiliations Expand

Affiliations

- [1] Institute of Respiratory Diseases, Department of Pulmonary and Critical Care Medicine, Xinqiao Hospital, Third Military Medical University, Chongqing 400037, China.
- [2] Infection Division, Wuhan Huoshenshan Hospital, Wuhan 430030, China.
- [3] Infection Division, Wuhan Jinyintan Hospital, Wuhan 430030, China.
- [4] Department of Cardiology, Daping Hospital, Third Military Medical University, Chongqing 400037, China.
- [5] Department of Critical Care Medicine, Wuhan Huoshenshan Hospital, Wuhan 430030, China.
- [6] Department of Pulmonary and Critical Care Medicine, Daping Hospital, Third Military Medical University, Chongqing 400037, China.
- [7] Department of Pulmonary and Critical Care Medicine, Tongji Hospital, Tongji Medical College, Huazhong University of Science and Technology, Wuhan 430030, China.
- [8] Department of Traditional Chinese Medicine, Xinqiao Hospital, Third Military Medical University, Chongqing 400037, China.

- PMID: **33814982**
- PMCID: PMC7977979
- DOI: 10.1155/2021/8812304

Free PMC article
Observational Study

Hyperglycemia and Correlated High Levels of Inflammation Have a Positive Relationship with the Severity of Coronavirus Disease 2019

Wen Zhang et al. Mediators Inflamm. 2021.
Free PMC article
Show details
Mediators Inflamm
. 2021 Mar 18;2021:8812304.
doi: 10.1155/2021/8812304. eCollection 2021.

Authors

Wen Zhang [1, 2], Chuanwei Li [2, 3, 4], Yu Xu [1, 3, 5], Binfeng He [1], Mingdong Hu [1, 2, 3], Guoqiang Cao [2, 3, 6], Li Li [2, 3, 6], Shuang Wu [3], Xia Wang [1], Chun Zhang [1], Jianping Zhao [7], Jungang Xie [7], Zihui Xu [8], Qi Li [1, 2, 3], Guansong Wang [1]

Affiliations

- [1] Institute of Respiratory Diseases, Department of Pulmonary and Critical Care Medicine, Xinqiao Hospital, Third Military Medical University, Chongqing 400037, China.
- [2] Infection Division, Wuhan Huoshenshan Hospital, Wuhan 430030, China.
- [3] Infection Division, Wuhan Jinyintan Hospital, Wuhan 430030, China.
- [4] Department of Cardiology, Daping Hospital, Third Military Medical University, Chongqing 400037, China.
- [5] Department of Critical Care Medicine, Wuhan Huoshenshan Hospital, Wuhan 430030, China.
- [6] Department of Pulmonary and Critical Care Medicine, Daping Hospital, Third Military Medical University, Chongqing 400037, China.
- [7] Department of Pulmonary and Critical Care Medicine, Tongji Hospital, Tongji Medical College, Huazhong University of Science and Technology, Wuhan 430030, China.
- [8] Department of Traditional Chinese Medicine, Xinqiao Hospital, Third Military Medical University, Chongqing 400037, China.

- PMID: **33814982**
- PMCID: PMC7977979
- DOI: 10.1155/2021/8812304

Abstract

Objective: Coronavirus disease 2019 (COVID-19) is a considerable global public health threat. This study sought to investigate whether blood glucose (BG) levels or comorbid diabetes are associated with inflammatory status and disease severity in patients with COVID-19.

Methods: In this retrospective cohort study, the clinical and biochemical characteristics of COVID-19 patients with or without diabetes were compared. The relationship among severity of COVID-19, inflammatory status, and diabetes or hyperglycemia was analyzed. The severity of

COVID-19 in all patients was determined according to the diagnostic and treatment guidelines issued by the Chinese National Health Committee (7th edition).

Results: Four hundred and sixty-one patients were enrolled in our study, and 71.58% of patients with diabetes and 13.03% of patients without diabetes had hyperglycemia. Compared with patients without diabetes ($n = 366$), patients with diabetes ($n = 95$) had a higher leucocyte count, neutrophil count, neutrophil to lymphocyte ratio (NLR), and erythrocyte sedimentation rate (ESR). There was no association between severity of COVID-19 and known diabetes adjusted for age, sex, body mass index (BMI), known hypertension, and coronary heart disease. The leucocyte count, NLR, and C-reactive protein (CRP) level increased with increasing BG level. Hyperglycemia was an independent predictor of critical (OR 4.00, 95% CI 1.72-9.30) or severe (OR 3.55, 95% CI 1.47-8.58) COVID-19, and of increased inflammatory levels (high leucocyte count (OR 4.26, 95% CI 1.65-10.97), NLR (OR 2.76, 95% CI 1.24-6.10), and CRP level (OR 2.49, 95% CI 1.19-5.23)), after adjustment for age, sex, BMI, severity of illness, and known diabetes.

Conclusion: Hyperglycemia was positively correlated with higher inflammation levels and more severe illness, and it is a risk factor for the increased severity of COVID-19. The initial measurement of plasma glucose levels after hospitalization may help identify a subset of patients who are predisposed to a worse clinical course.

Copyright © 2021 Wen Zhang et al.

Conflict of interest statement

No authors declare any conflicts of interest.

- 27 references
- 1 figure

Supplementary info

Publication types, MeSH terms, Substances Expand

Publication types

- Multicenter Study
- Observational Study

MeSH terms

- Aged
- Blood Glucose / metabolism
- Blood Sedimentation
- C-Reactive Protein / metabolism
- COVID-19 / blood*
- COVID-19 / complications*
- COVID-19 / epidemiology
- China / epidemiology

- Diabetes Complications / blood
- Female
- Humans
- Hyperglycemia / blood*
- Hyperglycemia / complications*
- Inflammation / blood*
- Inflammation / complications*
- Leukocyte Count
- Male
- Middle Aged
- Pandemics
- Retrospective Studies
- Risk Factors
- SARS-CoV-2*
- Severity of Illness Index

Substances

- Blood Glucose
- C-Reactive Protein

Full text links

Hindawi FREE Full Text Article Hindawi Limited Free PMC article
Proceed to details
Cite
Share
881
Observational Study
J Neurol
. 2022 Feb;269(2):603-608.
doi: 10.1007/s00415-021-10734-z. Epub 2021 Jul 31.

Delays in thrombolysis during COVID-19 are associated with worse neurological outcomes: the Society of Vascular and Interventional Neurology Multicenter Collaboration

Dinesh V Jillella [1], Fadi Nahab [1], Thanh N Nguyen [2], Mohamad Abdalkader [3], David S Liebeskind [4], Nirav Vora [5], Vivek Rai [5], Diogo C Haussen [6], Raul G Nogueira [6], Shashvat Desai [7] [8], Ashutosh P Jadhav [7] [8], Alexandra L Czap [9], Alicia M Zha [9], Italo Linfante

[10], Ameer E Hassan [11], Darko Quispe-Orozco [12], Santiago Ortega-Gutierrez [12], Priyank Khandelwal [13], Pratit Patel [13], Osama Zaidat [14], Tudor G Jovin [15], Scott Kamen [16], James E Siegler [17]

Affiliations Expand

Affiliations

- [1] Department of Neurology, Emory University School of Medicine, Atlanta, GA, 30322, USA.
- [2] Interventional Neurology and Neuroradiology, Boston Medical Center, Boston, MA, 02118, USA.
- [3] Department of Neurology, Boston Medical Center, Boston, MA, 02118, USA.
- [4] Department of Neurology, Ronald Reagan University of California at Los Angeles, Los Angeles, CA, 90095, USA.
- [5] OhioHealth Neuroscience Center, Riverside Methodist Hospital, Columbus, OH, 43214, USA.
- [6] Marcus Stroke and Neuroscience Center, Grady Memorial Hospital, Atlanta, GA, 30303, USA.
- [7] University of Pittsburgh Medical Center Mercy Hospital, Pittsburgh, PA, 15219, USA.
- [8] University of Pittsburgh Medical Center Presbyterian Medical Center, Pittsburgh, PA, 15213, USA.
- [9] Department of Neurology, University of Texas McGovern Medical School, Houston, TX, 77030, USA.
- [10] Department of Interventional Neuroradiology and Endovascular Neurosurgery, Baptist Health South Florida, Coral Gables, FL, 33146, USA.
- [11] Department of Neurology, University of Texas Rio Grande Valley, Valley Baptist Medical Center, Harlingen, TX, 78550, USA.
- [12] Department of Neurology, Neurosurgery and Radiology, University of Iowa Hospitals and Clinics, Iowa City, IA, 52242, USA.
- [13] Department of Endovascular Neurological Surgery and Neurology, Robert Wood Johnson University Hospital, New Brunswick, NJ, 08901, USA.
- [14] Department of Neurology, Mercy Health St. Vincent Hospital, Toledo, OH, 43608, USA.
- [15] Cooper Neurological Institute, Cooper University Hospital, 3 Cooper Plaza, Suite 320, Camden, NJ, 08103, USA.
- [16] Cooper Medical School of Rowan University, Camden, NJ, 08103, USA.
- [17] Cooper Neurological Institute, Cooper University Hospital, 3 Cooper Plaza, Suite 320, Camden, NJ, 08103, USA. siegler-james@cooperhealth.edu.

- PMID: **34333701**
- PMCID: PMC8325534
- DOI: 10.1007/s00415-021-10734-z

Free PMC article
Observational Study

Delays in thrombolysis during COVID-19 are associated with worse neurological outcomes:

the Society of Vascular and Interventional Neurology Multicenter Collaboration

Dinesh V Jillella et al. J Neurol. 2022 Feb.
Free PMC article
Show details
J Neurol
. 2022 Feb;269(2):603-608.
doi: 10.1007/s00415-021-10734-z. Epub 2021 Jul 31.

Authors

Dinesh V Jillella [1], Fadi Nahab [1], Thanh N Nguyen [2], Mohamad Abdalkader [3], David S Liebeskind [4], Nirav Vora [5], Vivek Rai [5], Diogo C Haussen [6], Raul G Nogueira [6], Shashvat Desai [7] [8], Ashutosh P Jadhav [7] [8], Alexandra L Czap [9], Alicia M Zha [9], Italo Linfante [10], Ameer E Hassan [11], Darko Quispe-Orozco [12], Santiago Ortega-Gutierrez [12], Priyank Khandelwal [13], Pratit Patel [13], Osama Zaidat [14], Tudor G Jovin [15], Scott Kamen [16], James E Siegler [17]

Affiliations

- [1] Department of Neurology, Emory University School of Medicine, Atlanta, GA, 30322, USA.
- [2] Interventional Neurology and Neuroradiology, Boston Medical Center, Boston, MA, 02118, USA.
- [3] Department of Neurology, Boston Medical Center, Boston, MA, 02118, USA.
- [4] Department of Neurology, Ronald Reagan University of California at Los Angeles, Los Angeles, CA, 90095, USA.
- [5] OhioHealth Neuroscience Center, Riverside Methodist Hospital, Columbus, OH, 43214, USA.
- [6] Marcus Stroke and Neuroscience Center, Grady Memorial Hospital, Atlanta, GA, 30303, USA.
- [7] University of Pittsburgh Medical Center Mercy Hospital, Pittsburgh, PA, 15219, USA.
- [8] University of Pittsburgh Medical Center Presbyterian Medical Center, Pittsburgh, PA, 15213, USA.
- [9] Department of Neurology, University of Texas McGovern Medical School, Houston, TX, 77030, USA.
- [10] Department of Interventional Neuroradiology and Endovascular Neurosurgery, Baptist Health South Florida, Coral Gables, FL, 33146, USA.
- [11] Department of Neurology, University of Texas Rio Grande Valley, Valley Baptist Medical Center, Harlingen, TX, 78550, USA.
- [12] Department of Neurology, Neurosurgery and Radiology, University of Iowa Hospitals and Clinics, Iowa City, IA, 52242, USA.
- [13] Department of Endovascular Neurological Surgery and Neurology, Robert Wood Johnson University Hospital, New Brunswick, NJ, 08901, USA.
- [14] Department of Neurology, Mercy Health St. Vincent Hospital, Toledo, OH, 43608, USA.

- [15] Cooper Neurological Institute, Cooper University Hospital, 3 Cooper Plaza, Suite 320, Camden, NJ, 08103, USA.
- [16] Cooper Medical School of Rowan University, Camden, NJ, 08103, USA.
- [17] Cooper Neurological Institute, Cooper University Hospital, 3 Cooper Plaza, Suite 320, Camden, NJ, 08103, USA. siegler-james@cooperhealth.edu.

- PMID: **34333701**
- PMCID: PMC8325534
- DOI: 10.1007/s00415-021-10734-z

Abstract

Introduction: We have demonstrated in a multicenter cohort that the COVID-19 pandemic has led to a delay in intravenous thrombolysis (IVT) among stroke patients. Whether this delay contributes to meaningful short-term outcome differences in these patients warranted further exploration.

Methods: We conducted a nested observational cohort study of adult acute ischemic stroke patients receiving IVT from 9 comprehensive stroke centers across 7 U.S states. Patients admitted prior to the COVID-19 pandemic (1/1/2019-02/29/2020) were compared to patients admitted during the early pandemic (3/1/2020-7/31/2020). Multivariable logistic regression was used to estimate the effect of IVT delay on discharge to hospice or death, with treatment delay on admission during COVID-19 included as an interaction term.

Results: Of the 676 thrombolysed patients, the median age was 70 (IQR 58-81) years, 313 were female (46.3%), and the median NIHSS was 8 (IQR 4-16). Longer treatment delays were observed during COVID-19 (median 46 vs 38 min, $p = 0.01$) and were associated with higher in-hospital death/hospice discharge irrespective of admission period (OR per hour 1.08, 95% CI 1.01-1.17, $p = 0.03$). This effect was strengthened after multivariable adjustment (aOR 1.15, 95% CI 1.07-1.24, $p < 0.001$). There was no interaction of treatment delay on admission during COVID-19 ($p_{interaction} = 0.65$). Every one-hour delay in IVT was also associated with 7% lower odds of being discharged to home or acute inpatient rehabilitation facility (aOR 0.93, 95% CI 0.89-0.97, $p < 0.001$).

Conclusion: Treatment delays observed during the COVID-19 pandemic led to greater early mortality and hospice care, with a lower probability of discharge to home/rehabilitation facility. There was no effect modification of treatment delay on admission during the pandemic, indicating that treatment delay at any time contributes similarly to these short-term outcomes.

Keywords: COVID-19; Mortality; Outcomes; Stroke; Thrombolysis; Treatment delay.

© 2021. Springer-Verlag GmbH Germany, part of Springer Nature.

Conflict of interest statement

RGN reports consulting fees for advisory roles with Anaconda, Biogen, Cerenovus, Genentech, Imperative Care, Medtronic, Phenox, Prolong Pharmaceuticals, Stryker Neurovascular and stock options for advisory roles with Astrocyte, Brainomix, Cerebrotech, Ceretrieve, Corindus Vascular Robotics, Vesalio, Viz-AI, and Perfuze. No other authors report any competing financial interests.

- 13 references

Supplementary info

Publication types, MeSH terms Expand

Publication types

- Multicenter Study
- Observational Study

MeSH terms

- Adult
- Aged
- Brain Ischemia* / complications
- Brain Ischemia* / drug therapy
- Brain Ischemia* / epidemiology
- COVID-19*
- Female
- Hospital Mortality
- Humans
- Neurology*
- Pandemics
- Retrospective Studies
- SARS-CoV-2
- Stroke* / complications
- Stroke* / drug therapy
- Stroke* / epidemiology
- Thrombolytic Therapy
- Treatment Outcome

Full text links

SpringerLink FULL-TEXT ARTICLE Springer Free PMC article
Proceed to details
Cite
Share
☐ 882
Observational Study
J Antimicrob Chemother
. 2021 Feb 11;76(3):796-803.
doi: 10.1093/jac/dkaa475.

An observational cohort study of bacterial co-infection and implications for empirical antibiotic therapy in patients presenting with COVID-19 to hospitals in North West London

Liyang Wang [1], Amit K Amin [1, 2], Priya Khanna [1, 2], Adnan Aali [1], Alastair McGregor [1, 2, 3], Paul Bassett [4], Guduru Gopal Rao [1, 2]
Affiliations Expand

Affiliations

- [1] Department of Microbiology, London North West University Healthcare NHS Trust, London HA1 3UJ, UK.
- [2] Faculty of Medicine, Imperial College London, London SW7 2BU, UK.
- [3] Department of Infectious Diseases, London North West University Healthcare NHS Trust, London HA1 3UJ, UK.
- [4] Statsconsultancy Ltd, 40 Longwood Lane, Amersham HP7 9EN, UK.

- PMID: **33185241**
- PMCID: PMC7717240
- DOI: 10.1093/jac/dkaa475

Free PMC article
Observational Study

An observational cohort study of bacterial co-infection and implications for empirical antibiotic therapy in patients presenting with COVID-19 to hospitals in North West London

Liyang Wang et al. J Antimicrob Chemother. 2021.
Free PMC article
Show details
J Antimicrob Chemother
. 2021 Feb 11;76(3):796-803.
doi: 10.1093/jac/dkaa475.

Authors

Liyang Wang [1], Amit K Amin [1, 2], Priya Khanna [1, 2], Adnan Aali [1], Alastair McGregor [1, 2, 3], Paul Bassett [4], Guduru Gopal Rao [1, 2]

Affiliations

- [1] Department of Microbiology, London North West University Healthcare NHS Trust, London HA1 3UJ, UK.
- [2] Faculty of Medicine, Imperial College London, London SW7 2BU, UK.
- [3] Department of Infectious Diseases, London North West University Healthcare NHS Trust, London HA1 3UJ, UK.
- [4] Statsconsultancy Ltd, 40 Longwood Lane, Amersham HP7 9EN, UK.

- PMID: **33185241**
- PMCID: PMC7717240
- DOI: 10.1093/jac/dkaa475

Abstract

Objectives: To describe the prevalence and nature of bacterial co-infections in COVID-19 patients within 48 hours of hospital admission and assess the appropriateness of empirical antibiotic treatment they received.

Methods: In this retrospective observational cohort study, we included all adult non-pregnant patients who were admitted to two acute hospitals in North West London in March and April 2020 and confirmed to have COVID-19 infection within 2 days of admission. Results of microbiological specimens taken within 48 hours of admission were reviewed and their clinical significance was assessed. Empirical antibiotic treatment of representative patients was reviewed. Patient age, gender, co-morbidities, inflammatory markers at admission, admission to ICU and 30 day all-cause in-hospital mortality were collected and compared between patients with and without bacterial co-infections.

Results: Of the 1396 COVID-19 patients included, 37 patients (2.7%) had clinically important bacterial co-infection within 48 hours of admission. The majority of patients (36/37 in those with co-infection and 98/100 in selected patients without co-infection) received empirical antibiotic treatment. There was no significant difference in age, gender, pre-existing illnesses, ICU admission or 30 day all-cause mortality in those with and without bacterial co-infection. However, white cell count, neutrophil count and CRP on admission were significantly higher in patients with bacterial co-infections.

Conclusions: We found that bacterial co-infection was infrequent in hospitalized COVID-19 patients within 48 hours of admission. These results suggest that empirical antimicrobial treatment may not be necessary in all patients presenting with COVID-19 infection, although the decision could be guided by high inflammatory markers.

© The Author(s) 2020. Published by Oxford University Press on behalf of the British Society for Antimicrobial Chemotherapy. All rights reserved. For permissions, please email: journals.permissions@oup.com.

- 28 references

Supplementary info

Publication types, MeSH terms, Substances Expand

Publication types

- Multicenter Study
- Observational Study
- Research Support, Non-U.S. Gov't

MeSH terms

- Adult
- Aged
- Aged, 80 and over
- Anti-Bacterial Agents / therapeutic use*
- Bacterial Infections / diagnosis
- Bacterial Infections / drug therapy*
- Bacterial Infections / epidemiology
- COVID-19 / diagnosis
- COVID-19 / drug therapy*
- COVID-19 / epidemiology
- Cohort Studies
- Coinfection / diagnosis
- Coinfection / drug therapy*
- Coinfection / epidemiology
- Comorbidity
- Empirical Research*
- Female
- Humans
- London / epidemiology
- Male
- Middle Aged
- Retrospective Studies
- Young Adult

Substances

- Anti-Bacterial Agents

Full text links

OXFORD ACADEMIC Silverchair Information Systems Free PMC article
Proceed to details
Cite
Share

☐ 883
Observational Study
J Med Virol
. 2021 Jul;93(7):4399-4404.
doi: 10.1002/jmv.26980. Epub 2021 Apr 6.

Viral community acquired pneumonia at the emergency department: Report from the pre COVID-19 age

Ornella Spagnolello [1] [2], Alessandra Pierangeli [3], Maria Civita Cedrone [2], Valentina Di Biagio [2], Massimo Gentile [3], Annalisa Leonardi [2], Camilla Valeriano [2], Giuseppe Pietro Innocenti [1], Letizia Santinelli [1], Cristian Borrazzo [1], Alessandro Russo [4], Giuseppe Oliveto [3], Agnese Viscido [3], Massimo Ciccozzi [5], Giuliano Bertazzoni [2], Gabriella d'Ettorre [1], Giancarlo Ceccarelli [1]
Affiliations Expand

Affiliations

- [1] Department of Public Health and Infectious Diseases, University of Rome Sapienza, Rome, Italy.
- [2] Emergency Department, University of Rome Sapienza, Rome, Italy.
- [3] Laboratory of Virology, Department of Molecular Medicine, University of Rome Sapienza, Rome, Italy.
- [4] Department of Public Health and Infectious Diseases, University of Pisa, Pisa, Italy.
- [5] Unit of Medical Statistics and Molecular Epidemiology, University Campus Bio-Medico of Rome, Rome, Italy.

- PMID: **33783850**
- PMCID: PMC8250557
- DOI: 10.1002/jmv.26980

Free PMC article
Observational Study

Viral community acquired pneumonia at the emergency department: Report from the pre COVID-19 age

Ornella Spagnolello et al. J Med Virol. 2021 Jul.
Free PMC article
Show details
J Med Virol

. 2021 Jul;93(7):4399-4404.
doi: 10.1002/jmv.26980. Epub 2021 Apr 6.

Authors

Ornella Spagnolello [1] [2], Alessandra Pierangeli [3], Maria Civita Cedrone [2], Valentina Di Biagio [2], Massimo Gentile [3], Annalisa Leonardi [2], Camilla Valeriano [2], Giuseppe Pietro Innocenti [1], Letizia Santinelli [1], Cristian Borrazzo [1], Alessandro Russo [4], Giuseppe Oliveto [3], Agnese Viscido [3], Massimo Ciccozzi [5], Giuliano Bertazzoni [2], Gabriella d'Ettorre [1], Giancarlo Ceccarelli [1]

Affiliations

- [1] Department of Public Health and Infectious Diseases, University of Rome Sapienza, Rome, Italy.
- [2] Emergency Department, University of Rome Sapienza, Rome, Italy.
- [3] Laboratory of Virology, Department of Molecular Medicine, University of Rome Sapienza, Rome, Italy.
- [4] Department of Public Health and Infectious Diseases, University of Pisa, Pisa, Italy.
- [5] Unit of Medical Statistics and Molecular Epidemiology, University Campus Bio-Medico of Rome, Rome, Italy.

- PMID: **33783850**
- PMCID: PMC8250557
- DOI: 10.1002/jmv.26980

Abstract

The role of viruses in community acquired pneumonia (CAP) has been largely underestimated in the pre-coronavirus disease 2019 age. However, during flu seasonal early identification of viral infection in CAP is crucial to guide treatment and in-hospital management. Though recommended, the routine use of nasopharyngeal swab (NPS) to detect viral infection has been poorly scaled-up, especially in the emergency department (ED). This study sought to assess the prevalence and associated clinical outcomes of viral infections in patients with CAP during peak flu season. In this retrospective, observational study adults presenting at the ED of our hospital (Rome, Italy) with CAP from January 15th to February 22th, 2019 were enrolled. Each patient was tested on admission with Influenza rapid test and real time multiplex assay. Seventy five consecutive patients were enrolled. 30.7% (n = 23) tested positive for viral infection. Of these, 52.1% (n = 12) were H1N1/FluA. 10 patients had multiple virus co-infections. CAP with viral infection did not differ for any demographic, clinic and laboratory features by the exception of CCI and CURB-65. All intra-ED deaths and mechanical ventilations were recorded among CAP with viral infection. Testing only patients with CURB-65 score ≥2, 10 out of 12 cases of H1N1/FluA would have been detected saving up to 40% tests. Viral infection occurred in one-third of CAP during flu seasonal peak 2019. Since not otherwise distinguishable, NPS is so far the only reliable mean to identify CAP with viral infection. Testing only patients with moderate/severe CAP significantly minimize the number of tests.

Keywords: community acquired pneumonia; emergency department; public health; viral infection.

© 2021 The Authors. Journal of Medical Virology published by Wiley Periodicals LLC.

Conflict of interest statement

The authors declare that there are no conflict of interests.

- 22 references
- 3 figures

Supplementary info

Publication types, MeSH terms Expand

Publication types

- Observational Study

MeSH terms

- Aged
- COVID-19 / epidemiology
- Coinfection / virology
- Community-Acquired Infections / epidemiology*
- Emergency Service, Hospital / statistics & numerical data
- Female
- Humans
- Influenza A Virus, H1N1 Subtype / isolation & purification
- Influenza, Human / epidemiology
- Italy / epidemiology
- Male
- Pneumonia / epidemiology*
- Pneumonia / virology*
- Prevalence
- Retrospective Studies
- SARS-CoV-2 / isolation & purification

Full text links

WILEY Full Text Article Wiley Free PMC article
Proceed to details
Cite
Share
884
Observational Study
Echocardiography
. 2020 Nov;37(11):1838-1843.

doi: 10.1111/echo.14849. Epub 2020 Sep 15.

Application of lung ultrasonography in critically ill patients with COVID-19

Shuo Li [1], Ya-Li Qu [2], Mu-Qin Tu [2], Li-Yan Guo [2], Qi-Li Zhang [1], Chao-Yang Lv [1], Rui-Jun Guo [1]
Affiliations Expand

Affiliations

- [1] Department of Ultrasound Medicine, Beijing Chaoyang Hospital, Capital Medical University, Beijing, China.
- [2] Department of Ultrasound Medicine, Wuhan Jin Yin-Tan Hospital, Wuhan, China.

- PMID: **32931069**
- DOI: 10.1111/echo.14849

Observational Study

Application of lung ultrasonography in critically ill patients with COVID-19

Shuo Li et al. Echocardiography. 2020 Nov.
Show details
Echocardiography
. 2020 Nov;37(11):1838-1843.
doi: 10.1111/echo.14849. Epub 2020 Sep 15.

Authors

Shuo Li [1], Ya-Li Qu [2], Mu-Qin Tu [2], Li-Yan Guo [2], Qi-Li Zhang [1], Chao-Yang Lv [1], Rui-Jun Guo [1]

Affiliations

- [1] Department of Ultrasound Medicine, Beijing Chaoyang Hospital, Capital Medical University, Beijing, China.
- [2] Department of Ultrasound Medicine, Wuhan Jin Yin-Tan Hospital, Wuhan, China.

- PMID: **32931069**
- DOI: 10.1111/echo.14849

Abstract

Purpose: Lung ultrasonography (LU) is useful to assess lung lesions and variations at bedside. To investigate the results of LU in severe and critical patients with coronavirus disease 2019 (COVID-19), we performed a single-institution study to evaluate the related lung lesions and variations, and prophylactic strategies, in a large referral and treatment center.

Methods: We included 91 adult patients with severe and critical COVID-19, namely 62 males and 29 females, with an average age of 59 ± 11 years, who underwent LU. We collected the following patient information: sex, age, days in hospital, and days in ICU. In the ultrasound examinations, we recorded the presence of discrete B lines, confluent B lines, consolidation, pleural thickening, pleural effusion, and pneumothorax (PTX).

Results: Among the 91 severe and critical patients, 59 cases had scattered B lines, 56 cases had confluent B lines, 58 cases had alveolar-interstitial syndrome (AIS), 48 cases had lung consolidation, six cases had pleural thickening, 39 cases had pleural effusion (average depth of the pleural effusion: 1.0 ± 1.5 cm), and 20 patients developed PTX. In the Cox multivariate analysis, there were significant differences in age, hospitalization days, ICU days, and lung consolidation.

Conclusion: Lung ultrasonography performed at the bedside can detect lung diseases, such as B lines, PTX, pulmonary edema, lung consolidation, pleural effusion, and variations of these findings. Our findings support the use of LU and measurements for estimating factors, and monitoring response to therapy in severe and critical COVID-19 patients.

Keywords: lung consolidation; lung ultrasonography; severe and critical COVID-19.

© 2020 The Authors. Echocardiography published by Wiley Periodicals LLC.

- 15 references

Supplementary info

Publication types, MeSH terms Expand

Publication types

- Observational Study

MeSH terms

- COVID-19 / complications*
- China
- Critical Care / methods*
- Critical Illness
- Female
- Humans
- Lung / diagnostic imaging
- Lung Diseases / diagnostic imaging*
- Lung Diseases / etiology*
- Male

- Middle Aged
- Retrospective Studies
- Ultrasonography / methods*

Full text links

Wiley Full Text Article Wiley
Proceed to details
Cite
Share
885
Observational Study

Crit Care Med
. 2021 Jan 1;49(1):e31-e40.
doi: 10.1097/CCM.0000000000004748.

Unexpectedly High Frequency of Enterococcal Bloodstream Infections in Coronavirus Disease 2019 Patients Admitted to an Italian ICU: An Observational Study

Cecilia Bonazzetti [1, 2], Valentina Morena [1, 2], Andrea Giacomelli [1, 2], Letizia Oreni [1], Giacomo Casalini [1, 2], Laura Rina Galimberti [1], Matteo Bolis [1, 2], Matteo Rimoldi [3], Elisabetta Ballone [4], Riccardo Colombo [4], Anna Lisa Ridolfo [1], Spinello Antinori [1, 2]
Affiliations Expand

Affiliations

- [1] Department of Infectious Diseases, ASST Fatebenefratelli-Sacco, Luigi Sacco University Hospital, Milan, Italy.
- [2] Luigi Sacco Department of Biomedical and Clinical Sciences, University of Milan, Milan, Italy.
- [3] Technical and Rehabilitation Nursing Hospital Service, Epidemiological Office, SITRA, ASST Fatebenefratelli-Sacco, Luigi Sacco Hospital, Milan, Italy.
- [4] Department of Anesthesiology and Intensive Care, ASST Fatebenefratelli-Sacco, Luigi Sacco Hospital, Milan, Italy.

- PMID: **33122577**
- PMCID: PMC7737701
- DOI: 10.1097/CCM.0000000000004748

Free PMC article
Observational Study

Unexpectedly High Frequency of Enterococcal Bloodstream Infections in Coronavirus Disease 2019 Patients Admitted to an Italian ICU: An Observational Study

Cecilia Bonazzetti et al. Crit Care Med. 2021.
Free PMC article
Show details
Crit Care Med
. 2021 Jan 1;49(1):e31-e40.
doi: 10.1097/CCM.0000000000004748.

Authors

Cecilia Bonazzetti [1 2], Valentina Morena [1 2], Andrea Giacomelli [1 2], Letizia Oreni [1], Giacomo Casalini [1 2], Laura Rina Galimberti [1], Matteo Bolis [1 2], Matteo Rimoldi [3], Elisabetta Ballone [4], Riccardo Colombo [4], Anna Lisa Ridolfo [1], Spinello Antinori [1 2]

Affiliations

- [1] Department of Infectious Diseases, ASST Fatebenefratelli-Sacco, Luigi Sacco University Hospital, Milan, Italy.
- [2] Luigi Sacco Department of Biomedical and Clinical Sciences, University of Milan, Milan, Italy.
- [3] Technical and Rehabilitation Nursing Hospital Service, Epidemiological Office, SITRA, ASST Fatebenefratelli-Sacco, Luigi Sacco Hospital, Milan, Italy.
- [4] Department of Anesthesiology and Intensive Care, ASST Fatebenefratelli-Sacco, Luigi Sacco Hospital, Milan, Italy.

- PMID: **33122577**
- PMCID: PMC7737701
- DOI: 10.1097/CCM.0000000000004748

Abstract

Objectives: We aimed to assess the frequency of ICU-acquired bloodstream infections in coronavirus disease 2019 patients.

Design: Retrospective observational study.

Setting: The emergency expansion of an ICU from eight general beds to 30 coronavirus disease 2019 beds.

Participants: Patients with coronavirus disease 2019 admitted to the ICU of Luigi Sacco Hospital (Milan, Italy) for greater than or equal to 48 hours between February 21, 2020, and April 30, 2020.

Interventions: None.

Measurements and main results: The frequency of bloodstream infections per 1,000 days of ICU stay was calculated in 89 coronavirus disease 2019 patients, and the cumulative probability of bloodstream infection was estimated using death and ICU discharge as competing events. Sixty patients (67.4%) experienced at least one of the 93 recorded episodes of bloodstream infection, a frequency of 87 per 1,000 days of ICU stay (95% CI, 67-112).The patients who experienced a bloodstream infection had a higher Sequential Organ Failure Assessment score upon ICU admission (9.5; interquartile range, 8-12 vs 8, interquartile range, 5-10; $p = 0.042$), a longer median ICU stay (15 d; interquartile range, 11-23 vs 8, interquartile range, 5-12; $p < 0.001$), and more frequently required invasive mechanical ventilation (98.3% vs 82.8%; $p = 0.013$) than those who did not. The median time from ICU admission to the first bloodstream infection episode was 10 days. Gram-positive bacteria accounted for 74 episodes (79.6%), with Enterococcus species being the most prevalent (53 episodes, 55.8%). Thirty-two isolates (27.3%) showed multidrug resistance.

Conclusions: Coronavirus disease 2019 seemed to increase the frequency of bloodstream infections (particularly Enterococcus-related bloodstream infection) after ICU admission. This may have been due to enteric involvement in patients with severe coronavirus disease 2019 and/or limitations in controlling the patient-to-patient transmission of infectious agents in extremely challenging circumstances.

Copyright © 2020 by the Society of Critical Care Medicine and Wolters Kluwer Health, Inc. All Rights Reserved.

Conflict of interest statement

Dr. Giacomelli received funding from consultancy fees from Mylan and educational support from Gilead. Dr. Antinori has received support for research activities from Pfizer and Merck Sharp & Dome. The remaining authors have disclosed that they do not have any potential conflicts of interest.

- 31 references
- 3 figures

Supplementary info

Publication types, MeSH terms Expand

Publication types

- Observational Study

MeSH terms

- Adult
- Aged
- COVID-19 / epidemiology
- COVID-19 / microbiology*
- Critical Illness
- Enterococcus / isolation & purification*

- Female
- Gram-Positive Bacterial Infections / epidemiology
- Gram-Positive Bacterial Infections / microbiology*
- Humans
- Intensive Care Units
- Italy
- Length of Stay / statistics & numerical data*
- Male
- Middle Aged
- Retrospective Studies
- Sepsis / epidemiology
- Sepsis / microbiology*
- Treatment Outcome

Full text links

Wolters Kluwer Wolters Kluwer Free PMC article
Proceed to details
Cite
Share
886
Observational Study
J Am Heart Assoc
. 2020 Oct 20;9(19):e017126.
doi: 10.1161/JAHA.120.017126. Epub 2020 Sep 9.

Who Has Seen Patients With ST-Segment-Elevation Myocardial Infarction? First Results From Italian Real-World Coronavirus Disease 2019

Valeria Cammalleri [1], Saverio Muscoli [1], Daniela Benedetto [1], Giuseppe Stifano [1], Massimiliano Macrini [1], Alessio Di Landro [1], Marco Di Luozzo [1], Massimo Marchei [1], Enrica Giuliana Mariano [1], Linda Cota [1], Domenico Sergi [1], Andrea Bezzeccheri [1], Michela Bonanni [1], Martino Baluci [1], Pasquale De Vico [2], Francesco Romeo [1]
Affiliations Expand

Affiliations

- [1] Department of Cardiovascular Disease Tor Vergata University Rome Italy.
- [2] Department of Anesthesia Tor Vergata University Rome Italy.

- PMID: **32901560**
- PMCID: PMC7792389
- DOI: 10.1161/JAHA.120.017126

Free PMC article
Observational Study

Who Has Seen Patients With ST-Segment-Elevation Myocardial Infarction? First Results From Italian Real-World Coronavirus Disease 2019

Valeria Cammalleri et al. J Am Heart Assoc. 2020.
Free PMC article
Show details
J Am Heart Assoc
. 2020 Oct 20;9(19):e017126.
doi: 10.1161/JAHA.120.017126. Epub 2020 Sep 9.

Authors

Valeria Cammalleri [1], Saverio Muscoli [1], Daniela Benedetto [1], Giuseppe Stifano [1], Massimiliano Macrini [1], Alessio Di Landro [1], Marco Di Luozzo [1], Massimo Marchei [1], Enrica Giuliana Mariano [1], Linda Cota [1], Domenico Sergi [1], Andrea Bezzeccheri [1], Michela Bonanni [1], Martino Baluci [1], Pasquale De Vico [2], Francesco Romeo [1]

Affiliations

- [1] Department of Cardiovascular Disease Tor Vergata University Rome Italy.
- [2] Department of Anesthesia Tor Vergata University Rome Italy.

- PMID: **32901560**
- PMCID: PMC7792389
- DOI: 10.1161/JAHA.120.017126

Abstract

Background After the coronavirus disease 2019 outbreak, social isolation measures were introduced to contain infection. Although there is currently a slowing down of the infection, a reduction of hospitalizations, especially for myocardial infarction, was observed. The aim of our study is to evaluate the impact of the infectious disease on ST-segment-elevation myocardial infarction (STEMI) care during the coronavirus disease 2019 pandemic, through the analysis of recent cases of patients who underwent percutaneous coronary intervention. Methods and Results Consecutive patients affected by STEMI from March 1 to 31, 2020, during social restrictions of Italian government, were collected and compared with patients with STEMI treated during March 2019. During March 2020, we observed a 63% reduction of patients with STEMI who were

admitted to our catheterization laboratory, when compared with the same period of 2019 (13 versus 35 patients). Changes in all time components of STEMI care were notably observed, particularly for longer median time in symptom-to-first medical contact, spoke-to-hub, and the cumulative symptom-to-wire delay. Procedural data and in-hospital outcomes were similar between the 2 groups, whereas the length of hospitalization was longer in patients of 2020. In this group, we also observed higher levels of cardiac biomarkers and a worse left ventricular ejection fraction at baseline and discharge. Conclusions The coronavirus disease 2019 outbreak induced a reduction of hospital access for STEMI with an increase in treatment delay, longer hospitalization, higher levels of cardiac biomarkers, and worse left ventricular function.

Keywords: acute coronary syndrome; complications; coronavirus; interstitial pneumonia; percutaneous coronary intervention.

Conflict of interest statement

None.

- 15 references
- 1 figure

Supplementary info

Publication types, MeSH terms Expand

Publication types

- Observational Study

MeSH terms

- Aged
- Betacoronavirus*
- COVID-19
- Comorbidity
- Coronavirus Infections / epidemiology*
- Echocardiography, Doppler, Color
- Electrocardiography
- Female
- Follow-Up Studies
- Heart Ventricles / diagnostic imaging
- Heart Ventricles / physiopathology*
- Hospital Mortality / trends
- Hospitalization / trends
- Humans
- Incidence
- Italy / epidemiology

- Male
- Middle Aged
- Pandemics
- Percutaneous Coronary Intervention / methods
- Pneumonia, Viral / epidemiology*
- Prognosis
- Retrospective Studies
- SARS-CoV-2
- ST Elevation Myocardial Infarction / epidemiology*
- ST Elevation Myocardial Infarction / physiopathology
- ST Elevation Myocardial Infarction / surgery
- Stroke Volume / physiology
- Survival Rate / trends

Full text links

OPEN ACCESS Atypon Free PMC article
Proceed to details
Cite
Share
887
Observational Study
Viruses
. 2021 Apr 26;13(5):758.
doi: 10.3390/v13050758.

D-Dimer Level and Neutrophils Count as Predictive and Prognostic Factors of Pulmonary Embolism in Severe Non-ICU COVID-19 Patients

Benjamin Thoreau [1] [2], Joris Galland [3], Maxime Delrue [4], Marie Neuwirth [4], Alain Stepanian [4], Anthony Chauvin [5], Azeddine Dellal [6], Olivier Nallet [7], Melanie Roriz [8], Mathilde Devaux [9], Jonathan London [10], Gonzague Martin-Lecamp [11], Antoine Froissart [12], Nouara Arab [12], Bertrand Ferron [13], Marie-Helene Groff [14], Viviane Queyrel [15], Christine Lorut [16], Lucile Regard [16], Emilie Berthoux [17], Guillaume Bayer [18], Chloe Comarmond [19], Bertrand Lioger [20], Arsène Mekinian [21], Tali-Anne Szwebel [1], Thomas Sené [22], Blanca Amador-Borrero [3], Olivier Mangin [3], Pierre O Sellier [23], Virginie Siguret [4], Stéphane Mouly [3], Jean-Philippe Kevorkian [24], Lariboisière Covid Group, Dominique Vodovar [25] [26], Damien Sene [3]
Affiliations Expand

Affiliations

- [1] Department of Internal Medicine, National Referral Center for Rare Systemic Autoimmune Diseases, Cochin Hospital, AP-HP, University of Paris, CEDEX 14, 75679 Paris, France.
- [2] INSERM U1016, Cochin Institute, Paris, University of Paris, CNRS UMR 8104, 75014 Paris, France.
- [3] Department of Internal Medicine, Lariboisière Hospital, Assistance Publique-Hôpitaux de Paris, (AP-HP) Université de Paris, 75010 Paris, France.
- [4] Haemostasis Laboratory, Lariboisière Hospital, AP-HP, University of Paris, 75010 Paris, France.
- [5] Emergency Department, Lariboisière Hospital, AP-HP, University of Paris, 75010 Paris, France.
- [6] Department of Rheumatology and Internal Medicine, Le Raincy-Montfermeil Hospital, 93370 Montfermeil, France.
- [7] Department of Cardiology, Le Raincy-Montfermeil Hospital, 93370 Montfermeil, France.
- [8] Department of Internal Medicine, Hospital Center of Agen, 47923 Agen, France.
- [9] Department of Internal Medicine, Hospital Center of Poissy-Saint Germain, 78300 Saint Germain en Laye, France.
- [10] Department of Internal Medicine, Diaconesses Croix Saint-Simon Hospital, 75012 Paris, France.
- [11] Department of Internal Medicine, Hospital Center of Pau, 64000 Pau, France.
- [12] Department of Internal Medicine, Intermunicipal Hospital Center of Créteil, 94000 Créteil, France.
- [13] Department of Internal Medicine, Hospital Center of Sens, 89100 Sens, France.
- [14] Department of Internal Medicine, Hospital Center of Nord-Mayenne, 53100 Mayenne, France.
- [15] Department of Rheumatology, University Hospital of Nice, 06000 Nice, France.
- [16] Department of Pneumology, Cochin Hospital, AP-HP, Université de Paris, 75014 Paris, France.
- [17] Department of Internal Medicine, Saint Luc-Saint Joseph Hospital, 69007 Lyon, France.
- [18] Department of Internal Medicine, Claude Galien Hospital, 91480 Quincy sous Senart, France.
- [19] Department of Internal Medicine, Pitié-Salpétrière Hospital, AP-HP, Sorbonne University, 75013 Paris, France.
- [20] Department of Internal Medicine, Simone Veil Hospital, 41000 Blois, France.
- [21] Department of Internal Medicine, Saint Antoine Hospital, APHP, 75012 Paris, France.
- [22] Department of Internal Medicine, Fondation Rothschild, 75019 Paris, France.
- [23] Department of Infectious Disease, Lariboisière Hospital, APHP, 75010 Paris, France.
- [24] Department of Endocrinology, Lariboisière Hospital, APHP, 75010 Paris, France.
- [25] Centre Anti-Poison, Fernand Widal Hospital, AP-HP, University of Paris, 75010 Paris, France.
- [26] INSERM UMRS 1144, 75006 Paris, France.

- PMID: **33926038**
- PMCID: PMC8146364
- DOI: 10.3390/v13050758

Free PMC article

Observational Study

D-Dimer Level and Neutrophils Count as Predictive and Prognostic Factors of Pulmonary Embolism in Severe Non-ICU COVID-19 Patients

Benjamin Thoreau et al. Viruses. 2021.
Free PMC article
Show details
Viruses
. 2021 Apr 26;13(5):758.
doi: 10.3390/v13050758.

Authors

Benjamin Thoreau [1, 2], Joris Galland [3], Maxime Delrue [4], Marie Neuwirth [4], Alain Stepanian [4], Anthony Chauvin [5], Azeddine Dellal [6], Olivier Nallet [7], Melanie Roriz [8], Mathilde Devaux [9], Jonathan London [10], Gonzague Martin-Lecamp [11], Antoine Froissart [12], Nouara Arab [12], Bertrand Ferron [13], Marie-Helene Groff [14], Viviane Queyrel [15], Christine Lorut [16], Lucile Regard [16], Emilie Berthoux [17], Guillaume Bayer [18], Chloe Comarmond [19], Bertrand Lioger [20], Arsène Mekinian [21], Tali-Anne Szwebel [1], Thomas Sené [22], Blanca Amador-Borrero [3], Olivier Mangin [3], Pierre O Sellier [23], Virginie Siguret [4], Stéphane Mouly [3], Jean-Philippe Kevorkian [24], Lariboisière Covid Group, Dominique Vodovar [25, 26], Damien Sene [3]

Affiliations

- [1] Department of Internal Medicine, National Referral Center for Rare Systemic Autoimmune Diseases, Cochin Hospital, AP-HP, University of Paris, CEDEX 14, 75679 Paris, France.
- [2] INSERM U1016, Cochin Institute, Paris, University of Paris, CNRS UMR 8104, 75014 Paris, France.
- [3] Department of Internal Medicine, Lariboisière Hospital, Assistance Publique-Hôpitaux de Paris, (AP-HP) Université de Paris, 75010 Paris, France.
- [4] Haemostasis Laboratory, Lariboisière Hospital, AP-HP, University of Paris, 75010 Paris, France.
- [5] Emergency Department, Lariboisière Hospital, AP-HP, University of Paris, 75010 Paris, France.
- [6] Department of Rheumatology and Internal Medicine, Le Raincy-Montfermeil Hospital, 93370 Montfermeil, France.
- [7] Department of Cardiology, Le Raincy-Montfermeil Hospital, 93370 Montfermeil, France.
- [8] Department of Internal Medicine, Hospital Center of Agen, 47923 Agen, France.
- [9] Department of Internal Medicine, Hospital Center of Poissy-Saint Germain, 78300 Saint Germain en Laye, France.

- [10] Department of Internal Medicine, Diaconesses Croix Saint-Simon Hospital, 75012 Paris, France.
- [11] Department of Internal Medicine, Hospital Center of Pau, 64000 Pau, France.
- [12] Department of Internal Medicine, Intermunicipal Hospital Center of Créteil, 94000 Créteil, France.
- [13] Department of Internal Medicine, Hospital Center of Sens, 89100 Sens, France.
- [14] Department of Internal Medicine, Hospital Center of Nord-Mayenne, 53100 Mayenne, France.
- [15] Department of Rheumatology, University Hospital of Nice, 06000 Nice, France.
- [16] Department of Pneumology, Cochin Hospital, AP-HP, Université de Paris, 75014 Paris, France.
- [17] Department of Internal Medicine, Saint Luc-Saint Joseph Hospital, 69007 Lyon, France.
- [18] Department of Internal Medicine, Claude Galien Hospital, 91480 Quincy sous Senart, France.
- [19] Department of Internal Medicine, Pitié-Salpétrière Hospital, AP-HP, Sorbonne University, 75013 Paris, France.
- [20] Department of Internal Medicine, Simone Veil Hospital, 41000 Blois, France.
- [21] Department of Internal Medicine, Saint Antoine Hospital, APHP, 75012 Paris, France.
- [22] Department of Internal Medicine, Fondation Rothschild, 75019 Paris, France.
- [23] Department of Infectious Disease, Lariboisière Hospital, APHP, 75010 Paris, France.
- [24] Department of Endocrinology, Lariboisière Hospital, APHP, 75010 Paris, France.
- [25] Centre Anti-Poison, Fernand Widal Hospital, AP-HP, University of Paris, 75010 Paris, France.
- [26] INSERM UMRS 1144, 75006 Paris, France.

- PMID: **33926038**
- PMCID: PMC8146364
- DOI: 10.3390/v13050758

Abstract

The incidence of pulmonary embolism (PE) is high during severe Coronavirus Disease 2019 (COVID-19). We aimed to identify predictive and prognostic factors of PE in non-ICU hospitalized COVID-19 patients. In the retrospective multicenter observational CLOTVID cohort, we enrolled patients with confirmed RT-PCR COVID-19 who were hospitalized in a medicine ward and also underwent a CT pulmonary angiography for a PE suspicion. Baseline data, laboratory biomarkers, treatments, and outcomes were collected. Predictive and prognostics factors of PE were identified by using logistic multivariate and by Cox regression models, respectively. A total of 174 patients were enrolled, among whom 86 (median [IQR] age of 66 years [55-77]) had post-admission PE suspicion, with 30/86 (34.9%) PE being confirmed. PE occurrence was independently associated with the lack of long-term anticoagulation or thromboprophylaxis (OR [95%CI], 72.3 [3.6-4384.8]) D-dimers ≥ 2000 ng/mL (26.3 [4.1-537.8]) and neutrophils ≥ 7.0 G/L (5.8 [1.4-29.5]). The presence of these two biomarkers was associated with a higher risk of PE ($p = 0.0002$) and death or ICU transfer (HR [95%CI], 12.9 [2.5-67.8], $p < 0.01$). In hospitalized non-ICU severe COVID-19 patients with clinical PE suspicion, the lack of anticoagulation, D-dimers ≥ 2000 ng/mL, neutrophils ≥ 7.0 G/L, and these two biomarkers combined might be useful predictive markers of PE and prognosis, respectively.

Keywords: COVID-19; D-dimer; ICU transfer; anticoagulation; mortality; neutrophil; predictive factor; prognostic; pulmonary embolism.

Conflict of interest statement

The authors certify that they have no affiliation with or involvement in any organization or entity with any financial interest or non-financial interest in the materials discussed in this manuscript.

- 50 references
- 2 figures

Supplementary info

Publication types, MeSH terms, Substances Expand

Publication types

- Observational Study

MeSH terms

- Aged
- COVID-19 / blood
- COVID-19 / pathology*
- Computed Tomography Angiography
- Female
- Fibrin Fibrinogen Degradation Products / metabolism*
- Humans
- Logistic Models
- Male
- Middle Aged
- Neutrophils / pathology*
- Prognosis
- Pulmonary Embolism / blood
- Pulmonary Embolism / pathology
- Pulmonary Embolism / virology*
- Retrospective Studies
- Risk Factors
- SARS-CoV-2 / genetics
- Venous Thromboembolism / blood
- Venous Thromboembolism / pathology
- Venous Thromboembolism / virology

Substances

- Fibrin Fibrinogen Degradation Products
- fibrin fragment D

Full text links

FULL TEXT OPEN ACCESS MDPI Multidisciplinary Digital Publishing Institute (MDPI) Free PMC article

Proceed to details

Cite

Share

☐ 888

Observational Study

Kidney Blood Press Res

. 2020;45(4):612-622.

doi: 10.1159/000509517. Epub 2020 Jul 24.

Acute Kidney Injury in Patients with the Coronavirus Disease 2019: A Multicenter Study

Xiaoyang Cui [1], Xin Yu [1], Xiaojing Wu [1], Linna Huang [1], Ye Tian [1], Xu Huang [1], Zeyu Zhang [1], Zhenshun Cheng [2], Qiang Guo [3], Yi Zhang [1], Ying Cai [1], Qingyuan Zhan [4]

Affiliations Expand

Affiliations

- [1] Department of Pulmonary and Critical Care Medicine, Center of Respiratory Medicine, National Clinical Research Center for Respiratory Diseases, Institute of Respiratory Medicine, China-Japan Friendship Hospital, Beijing, China.
- [2] Department of Pulmonary and Critical Care Medicine, Zhongnan Hospital of Wuhan University, Wuhan, China.
- [3] Department of Critical Care Medicine, The First Affiliated Hospital of Soochow University, Suzhou, China.
- [4] Department of Pulmonary and Critical Care Medicine, Center of Respiratory Medicine, National Clinical Research Center for Respiratory Diseases, Institute of Respiratory Medicine, China-Japan Friendship Hospital, Beijing, China, drzhanqy@163.com.

- PMID: **32712607**
- PMCID: PMC7445371
- DOI: 10.1159/000509517

Free PMC article

Observational Study

Acute Kidney Injury in Patients with the Coronavirus Disease 2019: A Multicenter Study

Xiaoyang Cui et al. Kidney Blood Press Res. 2020.
Free PMC article
Show details
Kidney Blood Press Res
. 2020;45(4):612-622.
doi: 10.1159/000509517. Epub 2020 Jul 24.

Authors

Xiaoyang Cui [1], Xin Yu [1], Xiaojing Wu [1], Linna Huang [1], Ye Tian [1], Xu Huang [1], Zeyu Zhang [1], Zhenshun Cheng [2], Qiang Guo [3], Yi Zhang [1], Ying Cai [1], Qingyuan Zhan [4]

Affiliations

- [1] Department of Pulmonary and Critical Care Medicine, Center of Respiratory Medicine, National Clinical Research Center for Respiratory Diseases, Institute of Respiratory Medicine, China-Japan Friendship Hospital, Beijing, China.
- [2] Department of Pulmonary and Critical Care Medicine, Zhongnan Hospital of Wuhan University, Wuhan, China.
- [3] Department of Critical Care Medicine, The First Affiliated Hospital of Soochow University, Suzhou, China.
- [4] Department of Pulmonary and Critical Care Medicine, Center of Respiratory Medicine, National Clinical Research Center for Respiratory Diseases, Institute of Respiratory Medicine, China-Japan Friendship Hospital, Beijing, China, drzhanqy@163.com.

- PMID: **32712607**
- PMCID: PMC7445371
- DOI: 10.1159/000509517

Abstract

Introduction: Severe acute respiratory viral infections are frequency accompanied by multiple organ dysfunction, including acute kidney injury (AKI). In December 2019, the coronavirus disease 2019 (COVID-19) outbreak began in Wuhan, Hubei Province, China, and rapidly spread worldwide. While diffuse alveolar damage and acute respiratory failure are the main features of COVID-19, other organs may be involved, and the incidence of AKI is not well described. We assessed the incidence and clinical characteristics of AKI in patients with laboratory-confirmed COVID-19 and its effects on clinical outcomes.

Methods: We conducted a multicenter, retrospective, observational study of patients with COVID-19 admitted to two general hospitals in Wuhan from 5 January 2020 to 21 March 2020. Demographic data and information on organ dysfunction were collected daily. AKI was defined

according to the KDIGO clinical practice guidelines. Early and late AKI were defined as AKI occurring within 72 h after admission or after 72 h, respectively.

Results: Of the 116 patients, AKI developed in 21 (18.1%) patients. Among them, early and late AKI were found in 13 (11.2%) and 8 (6.9%) patients, respectively. Compared with patients without AKI, patients with AKI had more severe organ dysfunction, as indicated by a higher level of disease severity status, higher sequential organ failure assessment (SOFA) score on admission, an increased prevalence of shock, and a higher level of respiratory support. Patients with AKI had a higher SOFA score on admission (4.5 ± 2.1 vs. 2.8 ± 1.4, OR 1.498, 95% CI 1.047-2.143) and greater hospital mortality (57.1% vs. 12.6%, OR 3.998, 95% CI 1.088-14.613) than patients without AKI in both the univariate and multivariate analyses. Patients with late AKI, but not those with early AKI, had a significantly prolonged length of stay (19.6 vs. 9.6 days, p = 0.015).

Conclusion: Our findings show that admission SOFA score was an independent risk factor for AKI in COVID-19 patients, and patients with AKI had higher in-hospital mortality. Moreover, AKI development after 72 h of admission was related to prolonged hospitalization time.

Keywords: Acute kidney injury; Coronavirus disease 2019; Extrapulmonary complications.

© 2020 The Author(s) Published by S. Karger AG, Basel.

Conflict of interest statement

The authors have no conflicts of interest to declare.

- 24 references
- 2 figures

Supplementary info

Publication types, MeSH terms Expand

Publication types

- Multicenter Study
- Observational Study

MeSH terms

- Acute Kidney Injury / etiology*
- Acute Kidney Injury / mortality
- Acute Kidney Injury / therapy*
- Adult
- Aged
- Aged, 80 and over
- COVID-19
- China / epidemiology
- Coronavirus Infections / complications*

- Coronavirus Infections / mortality
- Coronavirus Infections / therapy
- Disease Progression
- Female
- Hospital Mortality
- Hospitals, General
- Humans
- Incidence
- Kidney Function Tests
- Length of Stay
- Male
- Middle Aged
- Multiple Organ Failure / etiology
- Multiple Organ Failure / therapy
- Pandemics
- Pneumonia, Viral / complications*
- Pneumonia, Viral / mortality
- Pneumonia, Viral / therapy
- Practice Guidelines as Topic
- Retrospective Studies
- Treatment Outcome
- Water-Electrolyte Balance

Full text links

Karger Free Final Version S. Karger AG, Basel, Switzerland Free PMC article

Proceed to details

Cite

Share

☐ 889

Observational Study

Blood
. 2022 Jan 6;139(1):134-137.
doi: 10.1182/blood.2021014232.

Antibody response after 2 and 3 doses of SARS-CoV-2 mRNA vaccine in allogeneic hematopoietic cell transplant recipients

Alexis Maillard [1], Rabah Redjoul [2], Marion Klemencie [3], Hélène Labussière Wallet [4], Amandine Le Bourgeois [5], Maud D'Aveni [6], Anne Huynh [7], Ana Berceanu [8], Tony

Marchand [9], Sylvain Chantepie [10], Carmen Botella Garcia [11], Michael Loschi [12], Magalie Joris [13], Cristina Castilla-Llorente [14], Anne Thiebaut-Bertrand [15], Sylvie François [3], Mathieu Leclerc [2], Patrice Chevallier [5], Stephanie Nguyen [1]
Affiliations Expand

Affiliations

- [1] Department of Clinical Hematology, Hôpitaux Universitaires Pitié Salpêtrière, Paris, France.
- [2] Department of Clinical Hematology, Assistance Publique des Hôpitaux de Paris (APHP), Hôpital Henri Mondor, Créteil, France.
- [3] Department of Clinical Hematology, Angers University Hospital, Angers, France.
- [4] Department of Clinical Hematology, Centre Hospitalier Lyon Sud, Hospices Civils de Lyon, Lyon, France.
- [5] Department of Clinical Hematology, Nantes University Hospital, Nantes, France.
- [6] Department of Hematology, Nancy Hospital, Nancy, France.
- [7] Department of Hematology, Institut Universitaire du Cancer Toulouse-Oncopole, Service de greffe de cellules souches hematopoïétiques, Toulouse, France.
- [8] Department of Clinical Hematology, Besançon University Hospital, Besançon, France.
- [9] Department of Clinical Hematology, Centre Hospitalier Universitaire (CHU) de Rennes, Rennes, France.
- [10] Institut d'Hématologie de Basse Normandie, Centre Hospitalier Universitaire de Caen, Caen.
- [11] Department of Hematology and Cell Therapy, CHU de Bordeaux, Bordeaux, France.
- [12] Department of Hematology, CHU de Nice, Université Cote d'Azur, Nice, France.
- [13] Department of Hematology, CHU Amiens, Amiens, France.
- [14] Institut de Cancérologie Gustave Roussy, Villejuif, France.
- [15] Department of Clinical Hematology, CHU de Grenoble, Grenoble, France; and.

- PMID: **34818411**
- PMCID: PMC8616709
- DOI: 10.1182/blood.2021014232

Free PMC article
Observational Study

Antibody response after 2 and 3 doses of SARS-CoV-2 mRNA vaccine in allogeneic hematopoietic cell transplant recipients

Alexis Maillard et al. Blood. 2022.
Free PMC article
Show details
Blood
. 2022 Jan 6;139(1):134-137.
doi: 10.1182/blood.2021014232.

Authors

Alexis Maillard [1], Rabah Redjoul [2], Marion Klemencie [3], Hélène Labussière Wallet [4], Amandine Le Bourgeois [5], Maud D'Aveni [6], Anne Huynh [7], Ana Berceanu [8], Tony Marchand [9], Sylvain Chantepie [10], Carmen Botella Garcia [11], Michael Loschi [12], Magalie Joris [13], Cristina Castilla-Llorente [14], Anne Thiebaut-Bertrand [15], Sylvie François [3], Mathieu Leclerc [2], Patrice Chevallier [5], Stephanie Nguyen [1]

Affiliations

- [1] Department of Clinical Hematology, Hôpitaux Universitaires Pitié Salpêtrière, Paris, France.
- [2] Department of Clinical Hematology, Assistance Publique des Hôpitaux de Paris (APHP), Hôpital Henri Mondor, Créteil, France.
- [3] Department of Clinical Hematology, Angers University Hospital, Angers, France.
- [4] Department of Clinical Hematology, Centre Hospitalier Lyon Sud, Hospices Civils de Lyon, Lyon, France.
- [5] Department of Clinical Hematology, Nantes University Hospital, Nantes, France.
- [6] Department of Hematology, Nancy Hospital, Nancy, France.
- [7] Department of Hematology, Institut Universitaire du Cancer Toulouse-Oncopole, Service de greffe de cellules souches hematopoïétiques, Toulouse, France.
- [8] Department of Clinical Hematology, Besançon University Hospital, Besançon, France.
- [9] Department of Clinical Hematology, Centre Hospitalier Universitaire (CHU) de Rennes, Rennes, France.
- [10] Institut d'Hématologie de Basse Normandie, Centre Hospitalier Universitaire de Caen, Caen.
- [11] Department of Hematology and Cell Therapy, CHU de Bordeaux, Bordeaux, France.
- [12] Department of Hematology, CHU de Nice, Université Cote d'Azur, Nice, France.
- [13] Department of Hematology, CHU Amiens, Amiens, France.
- [14] Institut de Cancérologie Gustave Roussy, Villejuif, France.
- [15] Department of Clinical Hematology, CHU de Grenoble, Grenoble, France; and.

- PMID: **34818411**
- PMCID: PMC8616709
- DOI: 10.1182/blood.2021014232

No abstract available

- 19 references
- 2 figures

Supplementary info

Publication types, MeSH terms, Substances Expand

Publication types

- Letter

- Observational Study

MeSH terms

- 2019-nCoV Vaccine mRNA-1273 / administration & dosage*
- Aged
- Allografts
- Antibodies, Viral / biosynthesis*
- Antibodies, Viral / blood
- BNT162 Vaccine / administration & dosage*
- COVID-19 / immunology*
- COVID-19 / prevention & control*
- Dose-Response Relationship, Immunologic
- Female
- Hematopoietic Stem Cell Transplantation / adverse effects*
- Humans
- Immunization, Secondary
- Immunocompromised Host / immunology
- Immunogenicity, Vaccine
- Male
- Middle Aged
- Multivariate Analysis
- Retrospective Studies
- SARS-CoV-2 / immunology*

Substances

- Antibodies, Viral
- 2019-nCoV Vaccine mRNA-1273
- BNT162 Vaccine

Full text links

blood Silverchair Information Systems Free PMC article
Proceed to details
Cite
Share
890
Observational Study
Thromb Res
. 2020 Dec;196:209-212.
doi: 10.1016/j.thromres.2020.09.004. Epub 2020 Sep 3.

Coagulation parameters and venous thromboembolism in patients with and without COVID-19 admitted to the Emergency Department for acute respiratory insufficiency

Roberto Pizzi [1], Giancarlo Gini [2], Lucia Caiano [1], Beniamino Castelli [1], Noga Dotan [1], Federica Magni [1], Alexandra Virano [1], Andrea Roveda [2], Lorenza Bertù [3], Walter Ageno [4]
Affiliations Expand

Affiliations

- [1] Department of Emergency Medicine, Ospedale di Circolo, University of Insubria, Varese, Italy; Department of Medicine and Surgery, University of Insubria, Varese, Italy.
- [2] Department of Emergency Medicine, Ospedale di Circolo, University of Insubria, Varese, Italy.
- [3] Department of Medicine and Surgery, University of Insubria, Varese, Italy.
- [4] Department of Emergency Medicine, Ospedale di Circolo, University of Insubria, Varese, Italy; Department of Medicine and Surgery, University of Insubria, Varese, Italy. Electronic address: walter.ageno@uninsubria.it.

- PMID: **32911392**
- PMCID: PMC7468277
- DOI: 10.1016/j.thromres.2020.09.004

Free PMC article
Observational Study

Coagulation parameters and venous thromboembolism in patients with and without COVID-19 admitted to the Emergency Department for acute respiratory insufficiency

Roberto Pizzi et al. Thromb Res. 2020 Dec.
Free PMC article
Show details
Thromb Res
. 2020 Dec;196:209-212.
doi: 10.1016/j.thromres.2020.09.004. Epub 2020 Sep 3.

Authors

Roberto Pizzi [1], Giancarlo Gini [2], Lucia Caiano [1], Beniamino Castelli [1], Noga Dotan [1], Federica Magni [1], Alexandra Virano [1], Andrea Roveda [2], Lorenza Bertù [3], Walter Ageno [4]

Affiliations

- [1] Department of Emergency Medicine, Ospedale di Circolo, University of Insubria, Varese, Italy; Department of Medicine and Surgery, University of Insubria, Varese, Italy.
- [2] Department of Emergency Medicine, Ospedale di Circolo, University of Insubria, Varese, Italy.
- [3] Department of Medicine and Surgery, University of Insubria, Varese, Italy.
- [4] Department of Emergency Medicine, Ospedale di Circolo, University of Insubria, Varese, Italy; Department of Medicine and Surgery, University of Insubria, Varese, Italy. Electronic address: walter.ageno@uninsubria.it.

- PMID: **32911392**
- PMCID: PMC7468277
- DOI: 10.1016/j.thromres.2020.09.004

Abstract

Background: In the recent outbreak of COVID-19 pandemic, increased D-dimer levels and high rates of venous thromboembolic events were reported. We aimed to compare coagulation parameters on admission between COVID-19 patients and non-COVID-19 patients with acute respiratory insufficiency and to describe VTE diagnosed at entry.

Methods: In this single-centre, observational retrospective study consecutive patients admitted for fever and acute respiratory failure were included. Patients underwent laboratory tests, arterial blood gas, chest X-ray, point of care ultrasound (POCUS), limited compression ultrasonography of the lower limbs (L-CUS), chest CT-scan if necessary, and swab test for COVID-19.

Results: Of 324 patients, 50% had COVID-19. COVID19 patients had significantly lower mean white blood cells, neutrophils, platelet count, and pCT values, and significantly higher CRP, LDH, and ferritin levels than non-COVID19 patients. D-dimer was increased in 86.5% COVID19 patients and in 84.9% non-COVID19 patients; mean values were similar (2185 ng/mL and 2814 ng/mL, respectively, p = n.s.). After multivariate analysis, results were unchanged (Odds Ratio 1.00 95%CI: 0.99-1.00, p = 0.21). PT and aPTT values were also similar between the two groups, fibrinogen levels were higher in COVID19 than in non-COVID19 patients (684 and 496 mg/dL, respectively, p < 0.0001). Five patients had asymptomatic proximal deep vein thrombosis detected by L-CUS (3 COVID19) and 2 patients had symptomatic pulmonary embolism (both non-COVID19).

Conclusions: D-dimer levels were similarly increased in patients with and without SARS-CoV 2 related disease. There were few cases of asymptomatic deep vein thrombosis or symptomatic pulmonary embolism at first day of admission, similarly distributed between COVID19 patients and non-COVID19 patients.

Keywords: COVID-19; Coagulation; D-dimer; Venous thromboembolism.

Copyright © 2020. Published by Elsevier Ltd.

Conflict of interest statement

None.

- 12 references

Supplementary info

Publication types, MeSH terms, Substances Expand

Publication types

- Comparative Study
- Observational Study

MeSH terms

- Aged
- Aged, 80 and over
- Biomarkers / blood
- Blood Coagulation*
- COVID-19 / blood
- COVID-19 / complications*
- COVID-19 / diagnosis
- Emergency Service, Hospital*
- Female
- Fibrin Fibrinogen Degradation Products / analysis
- Humans
- Male
- Middle Aged
- Patient Admission*
- Prognosis
- Pulmonary Embolism / blood
- Pulmonary Embolism / diagnosis
- Pulmonary Embolism / etiology*
- Respiratory Insufficiency / diagnosis
- Respiratory Insufficiency / etiology*
- Retrospective Studies
- Risk Factors
- Up-Regulation
- Venous Thromboembolism / blood
- Venous Thromboembolism / diagnosis
- Venous Thromboembolism / etiology*

- Venous Thrombosis / blood
- Venous Thrombosis / diagnosis
- Venous Thrombosis / etiology*

Substances

- Biomarkers
- Fibrin Fibrinogen Degradation Products
- fibrin fragment D

Full text links

ELSEVIER FULL-TEXT ARTICLE Elsevier Science Free PMC article
Proceed to details
Cite
Share
891
Observational Study
Eur J Neurol
. 2020 Nov;27(11):2322-2328.
doi: 10.1111/ene.14444. Epub 2020 Aug 24.

Assessment of neurological manifestations in hospitalized patients with COVID-19

M Luigetti [1,2], R Iorio [1,2], A R Bentivoglio [1,2], L Tricoli [1,2], V Riso [1,2], J Marotta [1,2], C Piano [1,2], G Primiano [1,2], L Zileri Del Verme [3], M R Lo Monaco [4], P Calabresi [1,2], GEMELLI AGAINST COVID-19 group
Collaborators, Affiliations Expand

- PMID: **32681611**
- PMCID: PMC7405467
- DOI: 10.1111/ene.14444

Free PMC article
Observational Study

Assessment of neurological manifestations in hospitalized patients with COVID-19

M Luigetti et al. Eur J Neurol. 2020 Nov.
Free PMC article
Show details

Eur J Neurol

. 2020 Nov;27(11):2322-2328.
doi: 10.1111/ene.14444. Epub 2020 Aug 24.

- PMID: **32681611**
- PMCID: PMC7405467
- DOI: 10.1111/ene.14444

Abstract

Background and purpose: The objective of this study was to assess the neurological manifestations in a series of consecutive severe acute respiratory syndrome coronavirus 2 (SARS-CoV-2)-positive patients, comparing their frequency with a population hospitalized in the same period for flu/respiratory symptoms, finally not related to SARS-CoV-2.

Methods: Patients with flu/respiratory symptoms admitted to Fondazione Policlinico Gemelli hospital from 14 March 2020 to 20 April 2020 were retrospectively enrolled. The frequency of neurological manifestations of patients with SARS-CoV-2 infection was compared with a control group.

Results: In all, 213 patients were found to be positive for SARS-CoV-2, after reverse transcriptase polymerase chain reaction on nasal or throat swabs, whilst 218 patients were found to be negative and were used as a control group. Regarding central nervous system manifestations, in SARS-CoV-2-positive patients a higher frequency of headache, hyposmia and encephalopathy always related to systemic conditions (fever or hypoxia) was observed. Furthermore, muscular involvement was more frequent in SARS-CoV-2 infection.

Conclusions: Patients with COVID-19 commonly have neurological manifestations but only hyposmia and muscle involvement seem more frequent compared with other flu diseases.

Keywords: COVID-19; SARS-CoV-2; muscle; neurological disorders; precision medicine.

© 2020 European Academy of Neurology.

- 20 references

Supplementary info

Publication types, MeSH terms Expand

Publication types

- Observational Study

MeSH terms

- Adult
- Aged
- Anosmia / epidemiology
- Anosmia / etiology

- Brain Diseases / epidemiology
- Brain Diseases / etiology
- COVID-19 / complications*
- COVID-19 / epidemiology
- Female
- Headache / epidemiology
- Headache / etiology
- Hospitalization
- Humans
- Influenza, Human / complications
- Influenza, Human / epidemiology
- Male
- Middle Aged
- Nervous System Diseases / epidemiology
- Nervous System Diseases / etiology*
- Neuromuscular Diseases / epidemiology
- Neuromuscular Diseases / etiology
- Patients
- Retrospective Studies

Full text links

WILEY Full Text Article Wiley Free PMC article

Proceed to details

Cite

Share

892

Observational Study

J Pediatr Endocrinol Metab

. 2020 Nov 5;33(12):1601-1603.

doi: 10.1515/jpem-2020-0481. Print 2020 Dec 16.

Severity in pediatric type 1 diabetes mellitus debut during the COVID-19 pandemic

María Güemes [1, 2], Pilar Storch-de-Gracia [3], Sara Vinagre Enriquez [3], Álvaro Martín-Rivada [1, 2], Anthony González Brabin [4], Jesús Argente [1, 2, 5, 6, 7]

Affiliations Expand

Affiliations

- [1] Endocrinology Department, Hospital Infantil Universitario Niño Jesús, Madrid, Spain.

- [2] La Princesa Research Institute, Madrid, Spain.
- [3] Emergency Department, Hospital Infantil Universitario Niño Jesús, Madrid, Spain.
- [4] Intensive Care Unit, Hospital Infantil Universitario Niño Jesús, Madrid, Spain.
- [5] Department of Pediatrics, Universidad Autónoma de Madrid, Madrid, Spain.
- [6] Centro de Investigación Biomédica en Red de Fisiopatología de la Obesidad y Nutrición (CIBEROBN), Instituto de Salud Carlos III, Madrid, Spain.
- [7] IMDEA, Food Institute, CEIUAM+CSI, Madrid, Spain.

- PMID: **33151178**
- DOI: 10.1515/jpem-2020-0481

Observational Study

Severity in pediatric type 1 diabetes mellitus debut during the COVID-19 pandemic

María Güemes et al. J Pediatr Endocrinol Metab. 2020.
Show details
J Pediatr Endocrinol Metab
. 2020 Nov 5;33(12):1601-1603.
doi: 10.1515/jpem-2020-0481. Print 2020 Dec 16.

Authors

María Güemes [1] [2], Pilar Storch-de-Gracia [3], Sara Vinagre Enriquez [3], Álvaro Martín-Rivada [1] [2], Anthony González Brabin [4], Jesús Argente [1] [2] [5] [6] [7]

Affiliations

- [1] Endocrinology Department, Hospital Infantil Universitario Niño Jesús, Madrid, Spain.
- [2] La Princesa Research Institute, Madrid, Spain.
- [3] Emergency Department, Hospital Infantil Universitario Niño Jesús, Madrid, Spain.
- [4] Intensive Care Unit, Hospital Infantil Universitario Niño Jesús, Madrid, Spain.
- [5] Department of Pediatrics, Universidad Autónoma de Madrid, Madrid, Spain.
- [6] Centro de Investigación Biomédica en Red de Fisiopatología de la Obesidad y Nutrición (CIBEROBN), Instituto de Salud Carlos III, Madrid, Spain.
- [7] IMDEA, Food Institute, CEIUAM+CSI, Madrid, Spain.

- PMID: **33151178**
- DOI: 10.1515/jpem-2020-0481

No abstract available

Keywords: type 1 diabetes mellitus; COVID-19; children.

- 8 references

Supplementary info

Publication types, MeSH terms Expand

Publication types

- Letter
- Observational Study

MeSH terms

- Adolescent
- COVID-19 / epidemiology*
- Child
- Diabetes Mellitus, Type 1 / diagnosis
- Diabetes Mellitus, Type 1 / epidemiology*
- Diabetes Mellitus, Type 1 / therapy
- Diabetic Ketoacidosis / diagnosis
- Diabetic Ketoacidosis / epidemiology
- Diabetic Ketoacidosis / therapy
- Emergency Service, Hospital / statistics & numerical data
- Humans
- Pandemics
- Retrospective Studies
- SARS-CoV-2*
- Spain / epidemiology

Full text links

De Gruyter

Proceed to details

Cite

Share

☐ 893

Observational Study

J Cardiothorac Vasc Anesth

. 2021 Feb;35(2):389-397.

doi: 10.1053/j.jvca.2020.08.067. Epub 2020 Sep 4.

Anticoagulation Management in Severe Coronavirus Disease 2019 Patients on Extracorporeal Membrane Oxygenation

Zhen Guo [1], Lin Sun [1], Bailing Li [2], Rui Tian [3], Xiaolin Zhang [4], Zhongwei Zhang [5], Sean P Clifford [6], Yuan Liu [7], Jiapeng Huang [8], Xin Li [9]
Affiliations Expand

Affiliations

- [1] Department of Cardiovascular Surgery, Shanghai Chest Hospital, Shanghai Jiao Tong University, Shanghai, China.
- [2] Department of Cardiovascular Surgery, Shanghai Changhai Hospital, The Second Military Medical University, Shanghai, China.
- [3] Department of Emergency and Critical Care, Shanghai General Hospital, Shanghai Jiao Tong University, Shanghai, China.
- [4] Department of Intensive Care Unit, Shanghai Public Health Clinical Center, Fudan University, Shanghai, China.
- [5] Department of Intensive Care Unit, Fudan University Shanghai Cancer Center, Shanghai, China.
- [6] Department of Anesthesiology and Perioperative Medicine, University of Louisville, Louisville, KY.
- [7] Department of Statistics Center, Shanghai Chest Hospital, Shanghai Jiao Tong University, Shanghai, China.
- [8] Department of Anesthesiology and Perioperative Medicine, University of Louisville, Louisville, KY. Electronic address: jiapeng.huang@louisville.edu.
- [9] Department of Cardiovascular Surgery. Zhongshan Hospital, Fudan University, Shanghai, China. Electronic address: li.xin3@zs-hospital.sh.cn.

- PMID: **32994131**
- PMCID: PMC7473345
- DOI: 10.1053/j.jvca.2020.08.067

Free PMC article
Observational Study

Anticoagulation Management in Severe Coronavirus Disease 2019 Patients on Extracorporeal Membrane Oxygenation

Zhen Guo et al. J Cardiothorac Vasc Anesth. 2021 Feb.
Free PMC article
Show details
J Cardiothorac Vasc Anesth

. 2021 Feb;35(2):389-397.
doi: 10.1053/j.jvca.2020.08.067. Epub 2020 Sep 4.

Authors

Zhen Guo [1], Lin Sun [1], Bailing Li [2], Rui Tian [3], Xiaolin Zhang [4], Zhongwei Zhang [5], Sean P Clifford [6], Yuan Liu [7], Jiapeng Huang [8], Xin Li [9]

Affiliations

- [1] Department of Cardiovascular Surgery, Shanghai Chest Hospital, Shanghai Jiao Tong University, Shanghai, China.
- [2] Department of Cardiovascular Surgery, Shanghai Changhai Hospital, The Second Military Medical University, Shanghai, China.
- [3] Department of Emergency and Critical Care, Shanghai General Hospital, Shanghai Jiao Tong University, Shanghai, China.
- [4] Department of Intensive Care Unit, Shanghai Public Health Clinical Center, Fudan University, Shanghai, China.
- [5] Department of Intensive Care Unit, Fudan University Shanghai Cancer Center, Shanghai, China.
- [6] Department of Anesthesiology and Perioperative Medicine, University of Louisville, Louisville, KY.
- [7] Department of Statistics Center, Shanghai Chest Hospital, Shanghai Jiao Tong University, Shanghai, China.
- [8] Department of Anesthesiology and Perioperative Medicine, University of Louisville, Louisville, KY. Electronic address: jiapeng.huang@louisville.edu.
- [9] Department of Cardiovascular Surgery. Zhongshan Hospital, Fudan University, Shanghai, China. Electronic address: li.xin3@zs-hospital.sh.cn.

- PMID: **32994131**
- PMCID: PMC7473345
- DOI: 10.1053/j.jvca.2020.08.067

Abstract

Objective: To explore special coagulation characteristics and anticoagulation management in extracorporeal membrane oxygenation (ECMO)-assisted patients with coronavirus disease 2019 (COVID-19).

Design: Single-center, retrospective observation of a series of patients.

Participants: Laboratory-confirmed severe COVID-19 patients who received venovenous ECMO support from January 20-May 20, 2020.

Interventions: This study analyzed the anticoagulation management and monitoring strategies, bleeding complications, and thrombotic events during ECMO support.

Measurements and main results: Eight of 667 confirmed COVID-19 patients received venovenous ECMO and had an elevated D-dimer level before and during ECMO support. An ECMO circuit pack (oxygenator and tubing) was replaced a total of 13 times in all 8 patients, and coagulation-related complications included oxygenator thrombosis (7/8), tracheal hemorrhage

(5/8), oronasal hemorrhage (3/8), thoracic hemorrhage (3/8), bleeding at puncture sites (4/8), and cannulation site hemorrhage (2/8).

Conclusions: Hypercoagulability and secondary hyperfibrinolysis during ECMO support in COVID-19 patients are common and possibly increase the propensity for thrombotic events and failure of the oxygenator. Currently, there is not enough evidence to support a more aggressive anticoagulation strategy.

Keywords: COVID-19; ECMO; SARS-CoV-2; anticoagulation; coronavirus; extracorporeal membrane oxygenation; severe acute respiratory syndrome coronavirus 2; thrombosis.

Copyright © 2020 Elsevier Inc. All rights reserved.

- 41 references
- 3 figures

Supplementary info

Publication types, MeSH terms, Substances Expand

Publication types

- Observational Study

MeSH terms

- Adult
- Aged
- Aged, 80 and over
- Anticoagulants / administration & dosage
- Anticoagulants / therapeutic use*
- COVID-19 / complications
- COVID-19 / diagnostic imaging
- COVID-19 / therapy*
- Critical Care
- Extracorporeal Membrane Oxygenation* / adverse effects
- Female
- Fibrin Fibrinogen Degradation Products / analysis
- Hemorrhage / chemically induced
- Hemorrhage / epidemiology
- Humans
- Male
- Middle Aged
- Monitoring, Physiologic
- Respiratory Insufficiency / diagnostic imaging
- Respiratory Insufficiency / etiology

- Respiratory Insufficiency / therapy
- Retrospective Studies
- Thrombosis / epidemiology
- Tomography, X-Ray Computed
- Trachea / injuries

Substances

- Anticoagulants
- Fibrin Fibrinogen Degradation Products
- fibrin fragment D

Full text links

ELSEVIER FULL-TEXT ARTICLE Elsevier Science Free PMC article

Proceed to details

Cite

Share

894

Observational Study

Infect Dis Now

. 2021 Aug;51(5):424-428.

doi: 10.1016/j.idnow.2021.06.303. Epub 2021 Jun 19.

SARS Cov2 outbreak management on a landing helicopter dock: An observational retrospective study

Johan Schmitt [1], Emmanuel Genet [2], Marc Danguy Des Deserts [3], Sophie Chauvet-Atin [4], Pierre-Julien Cungi [4], Philippe Aries [3], Christophe Giacardi [3], Sophie Vellieux [5]

Affiliations Expand

Affiliations

- [1] Intensive Care Unit, Military Teaching Hospital Clermont Tonnerre, rue Colonel Fourrier, 29200 Brest, France. Electronic address: schmitt.johan83@gmail.com.
- [2] Medical Unit, 2(e) Military Legion Corps, Nimes, France.
- [3] Intensive Care Unit, Military Teaching Hospital Clermont Tonnerre, rue Colonel Fourrier, 29200 Brest, France.
- [4] Medical Unit, International Medical Centre, Abidjan, Ivory Coast.
- [5] Medical Unit, Land Helicopter Dock Dixmude, Toulon, France.

- PMID: **34157429**

- PMCID: PMC8214318
- DOI: 10.1016/j.idnow.2021.06.303

Free PMC article
Observational Study

SARS Cov2 outbreak management on a landing helicopter dock: An observational retrospective study

Johan Schmitt et al. Infect Dis Now. 2021 Aug.
Free PMC article
Show details
Infect Dis Now
. 2021 Aug;51(5):424-428.
doi: 10.1016/j.idnow.2021.06.303. Epub 2021 Jun 19.

Authors

Johan Schmitt [1], Emmanuel Genet [2], Marc Danguy Des Deserts [3], Sophie Chauvet-Atin [4], Pierre-Julien Cungi [4], Philippe Aries [3], Christophe Giacardi [3], Sophie Vellieux [5]

Affiliations

- [1] Intensive Care Unit, Military Teaching Hospital Clermont Tonnerre, rue Colonel Fourrier, 29200 Brest, France. Electronic address: schmitt.johan83@gmail.com.
- [2] Medical Unit, 2(e) Military Legion Corps, Nimes, France.
- [3] Intensive Care Unit, Military Teaching Hospital Clermont Tonnerre, rue Colonel Fourrier, 29200 Brest, France.
- [4] Medical Unit, International Medical Centre, Abidjan, Ivory Coast.
- [5] Medical Unit, Land Helicopter Dock Dixmude, Toulon, France.

- PMID: **34157429**
- PMCID: PMC8214318
- DOI: 10.1016/j.idnow.2021.06.303

Abstract

Objectives: Even though SARS Cov2 outbreak management has been well-described, scant information is available in military settings. We aimed to describe a SARS Cov2 outbreak and its management on the Dixmude, a French landing helicopter dock.

Patients and methods: We performed an observational retrospective and monocentric study in a ship. Role 1 was reinforced by additional roles 1 and 2. Our analysis included all infected crew personnel. We described demographic data, outbreak course, and biological samples including Covid-19 diagnosis. All infected patients were monitored for 10 days.

Results: Between February 16th 2021 and March 12th 2021, 54 patients (10% of the entire crew) were included. No patient had previously been vaccinated against SARS Cov2. The global mission was maintained. The crew members were healthy, male, and young (median age 28 years) with no medical history. Ranks of every status were concerned. Covid-19 disease was mainly diagnosed by real-time reverse-transcriptase polymerase chain reaction (rt-PCR). Thirty-two patients (59%) were symptomatic, four (8%) were presymptomatic and 18 (33%) remained asymptomatic.

Conclusions: The present work describes specific SARS Cov2 outbreak management in an austere military environment. Early individual and global measures were set and implemented on board.

Keywords: Austere environment; Covid-19; Landing helicopter dock; SARS Cov2.

Copyright © 2021 Elsevier Masson SAS. All rights reserved.

- 12 references
- 2 figures

Supplementary info

Publication types, MeSH terms Expand

Publication types

- Observational Study

MeSH terms

- Adult
- Aircraft
- COVID-19 / epidemiology*
- COVID-19 / therapy
- Disease Outbreaks*
- Female
- France / epidemiology
- Humans
- Male
- Military Facilities
- Retrospective Studies
- Young Adult

Full text links

ELSEVIER FULL-TEXT ARTICLE Elsevier Science Free PMC article

Proceed to details

Cite

Share

☐ 895

Observational Study

J Infect Public Health

. 2021 Mar;14(3):365-370.

doi: 10.1016/j.jiph.2020.12.017. Epub 2020 Dec 29.

Comparing ICU admission rates of mild/moderate COVID-19 patients treated with hydroxychloroquine, favipiravir, and hydroxychloroquine plus favipiravir

Rahmet Guner [1], Imran Hasanoglu [2], Bircan Kayaaslan [3], Adalet Aypak [4], Esragul Akinci [5], Hurrem Bodur [6], Fatma Eser [7], Ayse Kaya Kalem [8], Orhan Kucuksahin [9], Ihsan Ates [10], Aliye Bastug [11], Yasemin Tezer Tekce [12], Zeynep Bilgic [13], Fahriye Melis Gursoy [14], Hatice Nisa Akca [15], Seval Izdes [16], Deniz Erdem [17], Emra Asfuroglu [18], Habibe Hezer [19], Hatice Kilic [20], Musa Cıvak [21], Sibel Aydogan [22], Turan Buzgan [23]

Affiliations Expand

Affiliations

- [1] Department of Infectious Disease and Clinical Microbiology, Ankara Yildirim Beyazit University, Ankara City Hospital, Ankara, Turkey. Electronic address: rahmetguner@yahoo.com.
- [2] Department of Infectious Disease and Clinical Microbiology, Ankara Yildirim Beyazit University, Ankara City Hospital, Ankara, Turkey. Electronic address: imran.solak@gmail.com.
- [3] Department of Infectious Disease and Clinical Microbiology, Ankara Yildirim Beyazit University, Ankara City Hospital, Ankara, Turkey. Electronic address: drbican@gmail.com.
- [4] Department of Infectious Disease and Clinical Microbiology, Ankara City Hospital, Ankara, Turkey. Electronic address: aadalet@yahoo.com.
- [5] Department of Infectious Disease and Clinical Microbiology, University of Health Sciences, Ankara City Hospital, Ankara, Turkey. Electronic address: esragulakinci@gmail.com.
- [6] Department of Infectious Disease and Clinical Microbiology, University of Health Sciences, Ankara City Hospital, Ankara, Turkey. Electronic address: hurrembodur@gmail.com.
- [7] Department of Infectious Disease and Clinical Microbiology, Ankara Yildirim Beyazit University, Ankara City Hospital, Ankara, Turkey. Electronic address: fatmacivelekeser@hotmail.com.
- [8] Department of Infectious Disease and Clinical Microbiology, Ankara Yildirim Beyazit University, Ankara City Hospital, Ankara, Turkey. Electronic address: dr.aysekaya09@hotmail.com.
- [9] Department of Rheumatology, Ankara Yildirim Beyazit University, Ankara City Hospital, Ankara, Turkey. Electronic address: orhankcs@gmail.com.

- [10] Department of Internal Medicine, Ankara City Hospital, Ankara, Turkey. Electronic address: dr.ihsanates@hotmail.com.
- [11] Department of Infectious Disease and Clinical Microbiology, University of Health Sciences, Ankara City Hospital, Ankara, Turkey. Electronic address: dr.aliye@yahoo.com.
- [12] Department of Infectious Disease and Clinical Microbiology, Ankara City Hospital, Ankara, Turkey. Electronic address: ayasmintezer@gmail.com.
- [13] Department of Infectious Disease and Clinical Microbiology, Ankara City Hospital, Ankara, Turkey. Electronic address: zeynepunsal3860@gmail.com.
- [14] Department of Infectious Disease and Clinical Microbiology, Ankara City Hospital, Ankara, Turkey. Electronic address: mlsgursoy@gmail.com.
- [15] Department of Infectious Disease and Clinical Microbiology, Ankara City Hospital, Ankara, Turkey. Electronic address: htcnskc@gmail.com.
- [16] Department of Anaesthesiology and Reanimation, Ankara Yildirim Beyazit University, Ankara City Hospital, Ankara, Turkey. Electronic address: sevalizdes@yahoo.com.
- [17] Department of Anaesthesiology and Reanimation, Ankara City Hospital, Ankara, Turkey. Electronic address: dh2erdem@gmail.com.
- [18] Department of Internal Medicine, Ankara City Hospital, Ankara, Turkey. Electronic address: emra.kalkan@hotmail.com.
- [19] Department of Pulmonary Diseases, Ankara City Hospital, Ankara, Turkey. Electronic address: hoflaz@yahoo.com.
- [20] Department of Pulmonary Diseases, Ankara Yildirim Beyazit University, Ankara City Hospital, Ankara, Turkey. Electronic address: drhaticeb@yahoo.com.
- [21] Department of Internal Medicine, Ankara City Hospital, Ankara, Turkey. Electronic address: drhgk@hotmail.com.
- [22] Department of Virology, Ankara City Hospital, Ankara, Turkey. Electronic address: drsaydogan72@gmail.com.
- [23] Department of Infectious Disease and Clinical Microbiology, Ankara Yildirim Beyazit University, Ankara City Hospital, Ankara, Turkey. Electronic address: turanbuzgan@yahoo.com.

- PMID: **33647553**
- PMCID: PMC7771901
- DOI: 10.1016/j.jiph.2020.12.017

Free PMC article
Observational Study

Comparing ICU admission rates of mild/moderate COVID-19 patients treated with hydroxychloroquine, favipiravir, and hydroxychloroquine plus favipiravir

Rahmet Guner et al. J Infect Public Health. 2021 Mar.
Free PMC article

Show details

J Infect Public Health

. 2021 Mar;14(3):365-370.
doi: 10.1016/j.jiph.2020.12.017. Epub 2020 Dec 29.

Authors

Rahmet Guner[1], Imran Hasanoglu[2], Bircan Kayaaslan[3], Adalet Aypak[4], Esragul Akinci[5], Hurrem Bodur[6], Fatma Eser[7], Ayse Kaya Kalem[8], Orhan Kucuksahin[9], Ihsan Ates[10], Aliye Bastug[11], Yasemin Tezer Tekce[12], Zeynep Bilgic[13], Fahriye Melis Gursoy[14], Hatice Nisa Akca[15], Seval Izdes[16], Deniz Erdem[17], Emra Asfuroglu[18], Habibe Hezer[19], Hatice Kilic[20], Musa Cıvak[21], Sibel Aydogan[22], Turan Buzgan[23]

Affiliations

- [1] Department of Infectious Disease and Clinical Microbiology, Ankara Yildirim Beyazit University, Ankara City Hospital, Ankara, Turkey. Electronic address: rahmetguner@yahoo.com.
- [2] Department of Infectious Disease and Clinical Microbiology, Ankara Yildirim Beyazit University, Ankara City Hospital, Ankara, Turkey. Electronic address: imran.solak@gmail.com.
- [3] Department of Infectious Disease and Clinical Microbiology, Ankara Yildirim Beyazit University, Ankara City Hospital, Ankara, Turkey. Electronic address: drbican@gmail.com.
- [4] Department of Infectious Disease and Clinical Microbiology, Ankara City Hospital, Ankara, Turkey. Electronic address: aadalet@yahoo.com.
- [5] Department of Infectious Disease and Clinical Microbiology, University of Health Sciences, Ankara City Hospital, Ankara, Turkey. Electronic address: esragulakinci@gmail.com.
- [6] Department of Infectious Disease and Clinical Microbiology, University of Health Sciences, Ankara City Hospital, Ankara, Turkey. Electronic address: hurrembodur@gmail.com.
- [7] Department of Infectious Disease and Clinical Microbiology, Ankara Yildirim Beyazit University, Ankara City Hospital, Ankara, Turkey. Electronic address: fatmacivelekeser@hotmail.com.
- [8] Department of Infectious Disease and Clinical Microbiology, Ankara Yildirim Beyazit University, Ankara City Hospital, Ankara, Turkey. Electronic address: dr.aysekaya09@hotmail.com.
- [9] Department of Rheumatology, Ankara Yildirim Beyazit University, Ankara City Hospital, Ankara, Turkey. Electronic address: orhankcs@gmail.com.
- [10] Department of Internal Medicine, Ankara City Hospital, Ankara, Turkey. Electronic address: dr.ihsanates@hotmail.com.
- [11] Department of Infectious Disease and Clinical Microbiology, University of Health Sciences, Ankara City Hospital, Ankara, Turkey. Electronic address: dr.aliye@yahoo.com.
- [12] Department of Infectious Disease and Clinical Microbiology, Ankara City Hospital, Ankara, Turkey. Electronic address: ayasmintezer@gmail.com.
- [13] Department of Infectious Disease and Clinical Microbiology, Ankara City Hospital, Ankara, Turkey. Electronic address: zeynepunsal3860@gmail.com.
- [14] Department of Infectious Disease and Clinical Microbiology, Ankara City Hospital, Ankara, Turkey. Electronic address: mlsgursoy@gmail.com.
- [15] Department of Infectious Disease and Clinical Microbiology, Ankara City Hospital, Ankara, Turkey. Electronic address: htcnskc@gmail.com.

- [16] Department of Anaesthesiology and Reanimation, Ankara Yildirim Beyazit University, Ankara City Hospital, Ankara, Turkey. Electronic address: sevalizdes@yahoo.com.
- [17] Department of Anaesthesiology and Reanimation, Ankara City Hospital, Ankara, Turkey. Electronic address: dh2erdem@gmail.com.
- [18] Department of Internal Medicine, Ankara City Hospital, Ankara, Turkey. Electronic address: emra.kalkan@hotmail.com.
- [19] Department of Pulmonary Diseases, Ankara City Hospital, Ankara, Turkey. Electronic address: hoflaz@yahoo.com.
- [20] Department of Pulmonary Diseases, Ankara Yildirim Beyazit University, Ankara City Hospital, Ankara, Turkey. Electronic address: drhaticeb@yahoo.com.
- [21] Department of Internal Medicine, Ankara City Hospital, Ankara, Turkey. Electronic address: drhgk@hotmail.com.
- [22] Department of Virology, Ankara City Hospital, Ankara, Turkey. Electronic address: drsaydogan72@gmail.com.
- [23] Department of Infectious Disease and Clinical Microbiology, Ankara Yildirim Beyazit University, Ankara City Hospital, Ankara, Turkey. Electronic address: turanbuzgan@yahoo.com.

- PMID: **33647553**
- PMCID: PMC7771901
- DOI: 10.1016/j.jiph.2020.12.017

Abstract

Background: In this study, we aimed to compare the intensive care unit (ICU) admission rate of hospitalized mild/moderate COVID-19 patients treated with hydroxychloroquine (HCQ), favipiravir, and HCQ plus favipiravir.

Methods: Single center retrospective designed observational study conducted in Ankara City Hospital. Patients who were hospitalized between March 15, 2020 and June 1, 2020 in COVID-19 inpatient clinics with laboratory confirmed diagnosis of COVID-19 were included in the study. An inverse probability of treatment weighting (IPTW) for multiple treatment groups approach was used to balance the differences in several variables on admission.

Results: Among 2441 patients hospitalized with diagnosis of COVID-19 during the study period, 824 were eligible for the analysis. Median age of patients was 42 (18-93 years). Among all, 347 (43.2%) of the patients had mild disease, 470 (56.8%) had pneumonia. Propensity scores ranged from 0.1841 to 0.9381 in the HCQ group, from 0.03643 to 0.29885 in the favipiravir group, and from 0.03542 to 0.56184 in the HCQ plus favipiravir group. After IPTW for multiple treatment groups was applied, all the covariates in the planned propensity score had weighted standardized effect sizes below 10% which were ranged from 0.005 to 0.092. Multivariate analysis of treatment effect (adjusted effect of treatment) was indicated that there is no statistically significant difference between HCQ, favipiravir, and HCQ plus favipiravir treatment. After using combination of SMOTE and Bootstrap resampling approach, we found no statistically significant difference between HCQ and HCQ plus favipiravir groups in terms of ICU admission. However, compared with the HCQ group, ICU admission rate was statistically significantly higher in the favipiravir group. We obtained the similar results after the sensitivity analysis.

Conclusions: HCQ with or without favipiravir treatment is associated with reduced risk of ICU admission compared to favipiravir alone in mild to moderate COVID-19 adult patients.

Keywords: COVID-19; Favipiravir; Hydroxychloroquine; ICU; Treatment.

Copyright © 2021 The Authors. Published by Elsevier Ltd.. All rights reserved.

Comment in

- Letter to the editor: The effect of hydroxychloroquine on COVID-19.
  Demir C, Demir AU. Demir C, et al. J Infect Public Health. 2022 Jan;15(1):68. doi: 10.1016/j.jiph.2021.12.006. Epub 2021 Dec 10. J Infect Public Health. 2022. PMID: 34922225 Free PMC article. No abstract available.

- 26 references

Supplementary info

Publication types, MeSH terms, Substances, Supplementary concepts Expand

Publication types

- Observational Study

MeSH terms

- Adult
- Aged
- Aged, 80 and over
- Amides* / therapeutic use
- Antiviral Agents* / therapeutic use
- COVID-19 / drug therapy*
- Drug Therapy, Combination
- Female
- Humans
- Hydroxychloroquine* / therapeutic use
- Intensive Care Units / statistics & numerical data*
- Male
- Middle Aged
- Pyrazines* / therapeutic use
- Retrospective Studies
- Treatment Outcome
- Young Adult

Substances

- Amides
- Antiviral Agents

- Pyrazines
- Hydroxychloroquine
- favipiravir

Supplementary concepts

- COVID-19 drug treatment

Full text links

ELSEVIER OPEN ACCESS Elsevier Science Free PMC article

Proceed to details

Cite

Share

☐ 896

Observational Study

J Immunol
. 2021 Feb 1;206(3):599-606.
doi: 10.4049/jimmunol.2000981. Epub 2020 Dec 9.

Repurposed Tocilizumab in Patients with Severe COVID-19

Jianbo Tian [1], Ming Zhang [1], Meng Jin [2], Fengqin Zhang [3], Qian Chu [3], Xiaoyang Wang [1], Can Chen [1], Huihui Yue [3], Li Zhang [4], Ronghui Du [5], Dong Zhao [2], Zhaofu Zeng [2], Yang Zhao [2], Kui Liu [3], Mengmei Wang [2], Ke Hu [6], Xiaoping Miao [7], Huilan Zhang [8]

Affiliations Expand

Affiliations

- [1] Department of Epidemiology and Biostatistics, Key Laboratory for Environment and Health, School of Public Health, Tongji Medical College, Huazhong University of Sciences and Technology, Wuhan 430030, China.
- [2] Department of Respiratory and Critical Care Medicine, Renmin Hospital of Wuhan University, Wuhan 430060, China.
- [3] Department of Respiratory and Critical Care Medicine, Tongji Hospital, Tongji Medical College, Huazhong University of Science and Technology, Wuhan 430030, China.
- [4] Department of Oncology, Tongji Hospital, Tongji Medical College, Huazhong University of Science and Technology, Wuhan 430030, China; and.
- [5] Department of Respiratory and Critical Care Medicine, Wuhan Pulmonary Hospital, Wuhan 430030, China.
- [6] Department of Respiratory and Critical Care Medicine, Renmin Hospital of Wuhan University, Wuhan 430060, China; huke-rmhospital@163.com miaoxp@hust.edu.cn huilanz_76@163.com.

- [7] Department of Epidemiology and Biostatistics, Key Laboratory for Environment and Health, School of Public Health, Tongji Medical College, Huazhong University of Sciences and Technology, Wuhan 430030, China; huke-rmhospital@163.com miaoxp@hust.edu.cn huilanz_76@163.com.
- [8] Department of Respiratory and Critical Care Medicine, Tongji Hospital, Tongji Medical College, Huazhong University of Science and Technology, Wuhan 430030, China; huke-rmhospital@163.com miaoxp@hust.edu.cn huilanz_76@163.com.

- PMID: **33298617**
- PMCID: PMC7812057
- DOI: 10.4049/jimmunol.2000981

Free PMC article
Observational Study

Repurposed Tocilizumab in Patients with Severe COVID-19

Jianbo Tian et al. J Immunol. 2021.
Free PMC article
Show details
J Immunol
. 2021 Feb 1;206(3):599-606.
doi: 10.4049/jimmunol.2000981. Epub 2020 Dec 9.

Authors

Jianbo Tian [1], Ming Zhang [1], Meng Jin [2], Fengqin Zhang [3], Qian Chu [3], Xiaoyang Wang [1], Can Chen [1], Huihui Yue [3], Li Zhang [4], Ronghui Du [5], Dong Zhao [2], Zhaofu Zeng [2], Yang Zhao [2], Kui Liu [3], Mengmei Wang [2], Ke Hu [6], Xiaoping Miao [7], Huilan Zhang [8]

Affiliations

- [1] Department of Epidemiology and Biostatistics, Key Laboratory for Environment and Health, School of Public Health, Tongji Medical College, Huazhong University of Sciences and Technology, Wuhan 430030, China.
- [2] Department of Respiratory and Critical Care Medicine, Renmin Hospital of Wuhan University, Wuhan 430060, China.
- [3] Department of Respiratory and Critical Care Medicine, Tongji Hospital, Tongji Medical College, Huazhong University of Science and Technology, Wuhan 430030, China.
- [4] Department of Oncology, Tongji Hospital, Tongji Medical College, Huazhong University of Science and Technology, Wuhan 430030, China; and.
- [5] Department of Respiratory and Critical Care Medicine, Wuhan Pulmonary Hospital, Wuhan 430030, China.
- [6] Department of Respiratory and Critical Care Medicine, Renmin Hospital of Wuhan University, Wuhan 430060, China; huke-rmhospital@163.com miaoxp@hust.edu.cn huilanz_76@163.com.

- [7] Department of Epidemiology and Biostatistics, Key Laboratory for Environment and Health, School of Public Health, Tongji Medical College, Huazhong University of Sciences and Technology, Wuhan 430030, China; huke-rmhospital@163.com miaoxp@hust.edu.cn huilanz_76@163.com.
- [8] Department of Respiratory and Critical Care Medicine, Tongji Hospital, Tongji Medical College, Huazhong University of Science and Technology, Wuhan 430030, China; huke-rmhospital@163.com miaoxp@hust.edu.cn huilanz_76@163.com.

- PMID: **33298617**
- PMCID: PMC7812057
- DOI: 10.4049/jimmunol.2000981

Abstract

The coronavirus disease 2019 (COVID-19) has caused a global pandemic, resulting in considerable morbidity and mortality. Tocilizumab, an inhibitor of IL-6, has been widely repurposed as a treatment of severely ill patients without robust evidence supporting its use. In this study, we aimed to systematically describe the effectiveness of treatment and prevention of the cytokine storms in COVID-19 patients with tocilizumab. In this multicentered retrospective and observational cohort study, 65 patients with COVID-19 receiving tocilizumab and 130 not receiving tocilizumab were propensity score matched at a ratio of 2:1 based on age, sex, and comorbidities from January 20, 2020 to March 18, 2020 in Wuhan, China. After adjusting for confounding, the detected risk for in-hospital death was lower in the tocilizumab group versus nontocilizumab group (hazard ratio = 0.47; 95% confidence interval = 0.25-0.90; $p = 0.023$). Moreover, use of tocilizumab was associated with a lower risk of acute respiratory distress syndrome (odds ratio = 0.23; 95% confidence interval = 0.11-0.45; $p < 0.0001$). Furthermore, patients had heightened inflammation and more dysregulated immune cells before treatment, which might aggravate disease progression. After tocilizumab administration, abnormally elevated IL-6, C-reactive protein, fibrinogen, and activated partial thromboplastin time decreased. Tocilizumab may be of value in prolonging survival in patients with severe COVID-19, which provided a novel strategy for COVID-19-induced cytokine release syndrome. Our findings could inform bedside decisions until data from randomized, controlled clinical trials become available.

Copyright © 2021 by The American Association of Immunologists, Inc.

Conflict of interest statement

The authors have no financial conflicts of interest.

- 2 figures

Supplementary info

Publication types, MeSH terms, Substances Expand

Publication types

- Multicenter Study
- Observational Study

MeSH terms

- Aged
- Antibodies, Monoclonal, Humanized / therapeutic use*
- COVID-19 / complications*
- COVID-19 / drug therapy*
- COVID-19 / immunology
- Cohort Studies
- Cytokine Release Syndrome / complications*
- Cytokine Release Syndrome / drug therapy*
- Cytokine Release Syndrome / immunology
- Drug Repositioning*
- Female
- Humans
- Interleukin-6 / immunology
- Male
- Middle Aged
- Respiratory Distress Syndrome / complications*
- Respiratory Distress Syndrome / drug therapy*
- Respiratory Distress Syndrome / immunology
- Retrospective Studies
- SARS-CoV-2
- Severity of Illness Index

Substances

- Antibodies, Monoclonal, Humanized
- Interleukin-6
- tocilizumab

Full text links

HighWire Free PMC article
Proceed to details
Cite
Share
897
Observational Study
Blood Cells Mol Dis
. 2021 Mar;87:102525.
doi: 10.1016/j.bcmd.2020.102525. Epub 2020 Dec 8.

Effect of age, comorbidity and remission status on outcome of COVID-19 in patients with hematological malignancies

Pronamee Borah [1], Sumeet Mirgh [2], Sanjeev Kumar Sharma [3], Sachin Bansal [4], Ashish Dixit [5], Tuphan Kanti Dolai [6], Sweta Lunkad [7], Naveen Gupta [8], Gurmeet Singh [9], Aditi Jain [10], Divya Bansal [11], Dharma Choudhary [3], Vipin Khandelwal [3], Divya Doval [3], Meet Kumar [4], Rahul Bhargava [4], Amrita Chakrabarti [4], Mallikarjun Kalashetty [5], Amit Rauthan [5], Bilal Kazi [6], Prakas Kumar Mandal [6], Preethi Jeyaraman [1], Rahul Naithani [12], AIIMS Hematology Alumni Group
Affiliations Expand

Affiliations

- [1] Max Superspecialty Hospital, Saket, New Delhi, India.
- [2] Tata Memorial Centre, ACTREC, Mumbai, India.
- [3] BLK Superspeciality Hospital, New Delhi, India.
- [4] Fortis Memorial Hospital, Gurugram, India.
- [5] Manipal Hospital, Bengaluru, India.
- [6] NRS Medical College and Hospital, Kolkata, India.
- [7] Avinash Cancer Clinic, Pune, India.
- [8] Mahatma Gandhi Medical College and Hospital, Jaipur, India.
- [9] Jawahar Lal Nehru Hospital & Research Centre, Bhilai, India.
- [10] Safdarjung Hospital, New Delhi, India.
- [11] Manipal Hospital, New Delhi, India.
- [12] Max Superspecialty Hospital, Saket, New Delhi, India. Electronic address: dr_rahul6@hotmail.com.

- PMID: **33338697**
- PMCID: PMC7723067
- DOI: 10.1016/j.bcmd.2020.102525

Free PMC article
Observational Study

Effect of age, comorbidity and remission status on outcome of COVID-19 in patients with hematological malignancies

Pronamee Borah et al. Blood Cells Mol Dis. 2021 Mar.
Free PMC article
Show details
Blood Cells Mol Dis

. 2021 Mar;87:102525.
doi: 10.1016/j.bcmd.2020.102525. Epub 2020 Dec 8.

Authors

Pronamee Borah [1], Sumeet Mirgh [2], Sanjeev Kumar Sharma [3], Sachin Bansal [4], Ashish Dixit [5], Tuphan Kanti Dolai [6], Sweta Lunkad [7], Naveen Gupta [8], Gurmeet Singh [9], Aditi Jain [10], Divya Bansal [11], Dharma Choudhary [3], Vipin Khandelwal [3], Divya Doval [3], Meet Kumar [4], Rahul Bhargava [4], Amrita Chakrabarti [4], Mallikarjun Kalashetty [5], Amit Rauthan [5], Bilal Kazi [6], Prakas Kumar Mandal [6], Preethi Jeyaraman [1], Rahul Naithani [12], AIIMS Hematology Alumni Group

Affiliations

- [1] Max Superspecialty Hospital, Saket, New Delhi, India.
- [2] Tata Memorial Centre, ACTREC, Mumbai, India.
- [3] BLK Superspeciality Hospital, New Delhi, India.
- [4] Fortis Memorial Hospital, Gurugram, India.
- [5] Manipal Hospital, Bengaluru, India.
- [6] NRS Medical College and Hospital, Kolkata, India.
- [7] Avinash Cancer Clinic, Pune, India.
- [8] Mahatma Gandhi Medical College and Hospital, Jaipur, India.
- [9] Jawahar Lal Nehru Hospital & Research Centre, Bhilai, India.
- [10] Safdarjung Hospital, New Delhi, India.
- [11] Manipal Hospital, New Delhi, India.
- [12] Max Superspecialty Hospital, Saket, New Delhi, India. Electronic address: dr_rahul6@hotmail.com.

- PMID: **33338697**
- PMCID: PMC7723067
- DOI: 10.1016/j.bcmd.2020.102525

Abstract

Background: There is scarcity of data on outcome of COVID-19 in patients with hematological malignancies. Primary objective of study was to analyse the 14-day and 28-day mortality. Secondary objectives were to correlate age, comorbidities and remission status with outcome.

Methods: Retrospective multicentre observational study conducted in 11 centres across India. Total 130 patients with hematological malignancies and COVID-19 were enrolled.

Results: Fever and cough were commonest presentation. Eleven percent patients were incidentally detected. Median age of our cohort was 49.5 years. Most of our patients had a lymphoid malignancy (n = 91). One-half patients (52%) had mild infection, while moderate and severe infections contributed to one-fourth each. Sixty seven patients (52%) needed oxygen For treatment of COVID-19 infection, half(n = 66) received antivirals. Median time to RT-PCR COVID-19 negativity was 17 days (7-49 days). Nearly three-fourth (n = 95) of our patients were on anticancer treatment at time of infection, of which nearly two-third (n = 59;64%) had a delay in chemotherapy. Overall, 20% (n = 26) patients succumbed. 14-day survival and 28-day survival for

whole cohort was 85.4% and 80%, respectively. One patient succumbed outside the study period on day 39. Importantly, death rate at 1 month was 50% and 60% in relapse/refractory and severe disease cohorts, respectively. Elderly patients(age ≥ 60) ($p = 0.009$), and severe COVID-19 infection ($p = 0.000$) had a poor 14-day survival. The 28-day survival was significantly better for patients in remission ($p = 0.04$), non-severe infection ($p = 0.00$), and age < 60 years ($p = 0.05$).

Conclusions: Elderly patients with hematological malignancy and severe covid-19 have worst outcomes specially when disease is not in remission.

Keywords: COVID-19; Comorbidity; Hematology; Remission; Survival.

Copyright © 2020 Elsevier Inc. All rights reserved.

Conflict of interest statement

None.

- 30 references
- 1 figure

Supplementary info

Publication types, MeSH terms Expand

Publication types

- Multicenter Study
- Observational Study

MeSH terms

- Adolescent
- Adult
- Age Factors
- Aged
- Aged, 80 and over
- COVID-19 / epidemiology*
- COVID-19 / therapy
- Child
- Child, Preschool
- Comorbidity
- Female
- Hematologic Neoplasms / epidemiology*
- Hematologic Neoplasms / therapy
- Humans
- India / epidemiology

- Male
- Middle Aged
- Remission Induction
- Retrospective Studies
- Survival Analysis
- Treatment Outcome
- Young Adult

Full text links

ELSEVIER FULL-TEXT ARTICLE Elsevier Science Free PMC article
Proceed to details
Cite
Share
☐ 898
Observational Study
Prev Med
. 2021 Oct;151:106586.
doi: 10.1016/j.ypmed.2021.106586.

Measuring the impact of the COVID-19 pandemic on organized cancer screening and diagnostic follow-up care in Ontario, Canada: A provincial, population-based study

Meghan J Walker [1], Olivia Meggetto [2], Julia Gao [2], Gabriela Espino-Hernández [2], Nathaniel Jembere [2], Caroline A Bravo [2], Michelle Rey [2], Usman Aslam [2], Amanda J Sheppard [3], Aisha K Lofters [4], Martin C Tammemägi [5], Jill Tinmouth [6], Rachel Kupets [7], Anna M Chiarelli [3], Linda Rabeneck [8]
Affiliations Expand

Affiliations

- [1] Ontario Health (Cancer Care Ontario), Toronto, Ontario, Canada; Dalla Lana School of Public Health, University of Toronto, Toronto, Ontario, Canada. Electronic address: meghan.walker@ontariohealth.ca.
- [2] Ontario Health (Cancer Care Ontario), Toronto, Ontario, Canada.
- [3] Ontario Health (Cancer Care Ontario), Toronto, Ontario, Canada; Dalla Lana School of Public Health, University of Toronto, Toronto, Ontario, Canada.
- [4] Ontario Health (Cancer Care Ontario), Toronto, Ontario, Canada; Dalla Lana School of Public Health, University of Toronto, Toronto, Ontario, Canada; Peter Gilgan Centre for Women's Cancers, Women's College Hospital, Toronto, Ontairo, Canada; IC/ES, Toronto,

Ontario, Canada; Department of Family & Community Medicine, University of Toronto, Toronto, Canada.

- [5] Ontario Health (Cancer Care Ontario), Toronto, Ontario, Canada; Brock University, St. Catharines, Ontario, Canada.
- [6] Ontario Health (Cancer Care Ontario), Toronto, Ontario, Canada; Dalla Lana School of Public Health, University of Toronto, Toronto, Ontario, Canada; IC/ES, Toronto, Ontario, Canada; Department of Medicine, Sunnybrook Health Sciences Centre, Toronto, Ontario, Canada; Department of Medicine, University of Toronto, Toronto, Ontario, Canada.
- [7] Ontario Health (Cancer Care Ontario), Toronto, Ontario, Canada; Division of Gynecologic Oncology, Odette Cancer Centre, Sunnybrook Health Sciences Centre, Toronto, Ontario, Canada; Department of Obstetrics and Gynecology, University of Toronto, Toronto, Ontario, Canada.
- [8] Ontario Health (Cancer Care Ontario), Toronto, Ontario, Canada; Dalla Lana School of Public Health, University of Toronto, Toronto, Ontario, Canada; IC/ES, Toronto, Ontario, Canada; Department of Medicine, University of Toronto, Toronto, Ontario, Canada.

- PMID: **34217413**
- DOI: 10.1016/j.ypmed.2021.106586

Observational Study

Measuring the impact of the COVID-19 pandemic on organized cancer screening and diagnostic follow-up care in Ontario, Canada: A provincial, population-based study

Meghan J Walker et al. Prev Med. 2021 Oct.

Show details

Prev Med

. 2021 Oct;151:106586.
doi: 10.1016/j.ypmed.2021.106586.

Authors

Meghan J Walker [1], Olivia Meggetto [2], Julia Gao [2], Gabriela Espino-Hernández [2], Nathaniel Jembere [2], Caroline A Bravo [2], Michelle Rey [2], Usman Aslam [2], Amanda J Sheppard [3], Aisha K Lofters [4], Martin C Tammemägi [5], Jill Tinmouth [6], Rachel Kupets [7], Anna M Chiarelli [3], Linda Rabeneck [8]

Affiliations

- [1] Ontario Health (Cancer Care Ontario), Toronto, Ontario, Canada; Dalla Lana School of Public Health, University of Toronto, Toronto, Ontario, Canada. Electronic address: meghan.walker@ontariohealth.ca.
- [2] Ontario Health (Cancer Care Ontario), Toronto, Ontario, Canada.

- [3] Ontario Health (Cancer Care Ontario), Toronto, Ontario, Canada; Dalla Lana School of Public Health, University of Toronto, Toronto, Ontario, Canada.
- [4] Ontario Health (Cancer Care Ontario), Toronto, Ontario, Canada; Dalla Lana School of Public Health, University of Toronto, Toronto, Ontario, Canada; Peter Gilgan Centre for Women's Cancers, Women's College Hospital, Toronto, Ontairo, Canada; IC/ES, Toronto, Ontario, Canada; Department of Family & Community Medicine, University of Toronto, Toronto, Canada.
- [5] Ontario Health (Cancer Care Ontario), Toronto, Ontario, Canada; Brock University, St. Catharines, Ontario, Canada.
- [6] Ontario Health (Cancer Care Ontario), Toronto, Ontario, Canada; Dalla Lana School of Public Health, University of Toronto, Toronto, Ontario, Canada; IC/ES, Toronto, Ontario, Canada; Department of Medicine, Sunnybrook Health Sciences Centre, Toronto, Ontario, Canada; Department of Medicine, University of Toronto, Toronto, Ontario, Canada.
- [7] Ontario Health (Cancer Care Ontario), Toronto, Ontario, Canada; Division of Gynecologic Oncology, Odette Cancer Centre, Sunnybrook Health Sciences Centre, Toronto, Ontario, Canada; Department of Obstetrics and Gynecology, University of Toronto, Toronto, Ontario, Canada.
- [8] Ontario Health (Cancer Care Ontario), Toronto, Ontario, Canada; Dalla Lana School of Public Health, University of Toronto, Toronto, Ontario, Canada; IC/ES, Toronto, Ontario, Canada; Department of Medicine, University of Toronto, Toronto, Ontario, Canada.

- PMID: **34217413**
- DOI: 10.1016/j.ypmed.2021.106586

Abstract

It is essential to quantify the impacts of the COVID-19 pandemic on cancer screening, including for vulnerable sub-populations, to inform the development of evidence-based, targeted pandemic recovery strategies. We undertook a population-based retrospective observational study in Ontario, Canada to assess the impact of the pandemic on organized cancer screening and diagnostic services, and assess whether patterns of cancer screening service use and diagnostic delay differ across population sub-groups during the pandemic. Provincial health databases were used to identify age-eligible individuals who participated in one or more of Ontario's breast, cervical, colorectal, and lung cancer screening programs from January 1, 2019-December 31, 2020. Ontario's screening programs delivered 951,000 (-41%) fewer screening tests in 2020 than in 2019 and volumes for most programs remained more than 20% below historical levels by the end of 2020. A smaller percentage of cervical screening participants were older (50-59 and 60-69 years) during the pandemic when compared with 2019. Individuals in the oldest age groups and in lower-income neighborhoods were significantly more likely to experience diagnostic delay following an abnormal breast, cervical, or colorectal cancer screening test during the pandemic, and individuals with a high probability of living on a First Nation reserve were significantly more likely to experience diagnostic delay following an abnormal fecal test. Ongoing monitoring and management of backlogs must continue. Further evaluation is required to identify populations for whom access to cancer screening and diagnostic care has been disproportionately impacted and quantify impacts of these service disruptions on cancer incidence, stage, and mortality. This information is critical to pandemic recovery efforts that are aimed at achieving equitable and timely access to cancer screening-related care.

Keywords: COVID-19; Cancer screening; Diagnostic assessment; Disparity.

Copyright © 2021. Published by Elsevier Inc.

Supplementary info

Publication types, MeSH terms Expand

Publication types

- Observational Study

MeSH terms

- Aftercare
- COVID-19*
- Delayed Diagnosis
- Early Detection of Cancer
- Female
- Humans
- Lung Neoplasms*
- Ontario
- Pandemics
- SARS-CoV-2
- Uterine Cervical Neoplasms*

Full text links

ELSEVIER FULL-TEXT ARTICLE Elsevier Science
Proceed to details
Cite
Share
☐ 899
Observational Study
J Am Med Dir Assoc
. 2020 Nov;21(11):1539-1545.
doi: 10.1016/j.jamda.2020.09.004. Epub 2020 Sep 9.

COVID-19 In-Hospital Mortality and Use of Renin-Angiotensin System Blockers in Geriatrics Patients

Bastien Genet [1], Jean-Sébastien Vidal [2], Adrien Cohen [2], Clémence Boully [2], Maëlle Beunardeau [2], Louise Marine Harlé [2], Anna Gonçalves [2], Yasmina Boudali [2], Intza Hernandorena [2], Henri Bailly [2], Hermine Lenoir [2], Matthieu Piccoli [2], Anne Chahwakilian [2], Léna Kermanach [2], Laura de Jong [3], Emmanuelle Duron [4], Xavier Girerd [5], Olivier Hanon [6]

Affiliations Expand

Affiliations

- [1] Assistance Publique-Hôpitaux de Paris, Hôpitaux Universitaires Paris Centre, Hôpital Broca, Service de gériatrie, F-75013, Paris, France.
- [2] Assistance Publique-Hôpitaux de Paris, Hôpitaux Universitaires Paris Centre, Hôpital Broca, Service de gériatrie, F-75013, Paris, France; EA 4468, Université de Paris, F-75013, Paris, France.
- [3] Centre Hospitalier Sainte-Anne, GHU Paris Psychiatrie & Neurosciences, F-75014, Paris, France.
- [4] Assistance Publique-Hôpitaux de Paris, Hôpital Paul Brousse, Service de gériatrie, F-94804, Villejuif, France; Université Paris-Sud XI, F-94270, Le Kremlin-Bicêtre, France.
- [5] Fondation de recherche sur l'hypertension artérielle (FRHTA), F-75013, Paris, France; Assistance Publique-Hôpitaux de Paris, Hôpital Pitié Salpêtrière, Sorbonne Université, F-75013, Paris, France.
- [6] Assistance Publique-Hôpitaux de Paris, Hôpitaux Universitaires Paris Centre, Hôpital Broca, Service de gériatrie, F-75013, Paris, France; EA 4468, Université de Paris, F-75013, Paris, France. Electronic address: olivier.hanon@brc.aphp.fr.

- PMID: **33138935**
- PMCID: PMC7480334
- DOI: 10.1016/j.jamda.2020.09.004

Free PMC article
Observational Study

COVID-19 In-Hospital Mortality and Use of Renin-Angiotensin System Blockers in Geriatrics Patients

Bastien Genet et al. J Am Med Dir Assoc. 2020 Nov.
Free PMC article
Show details
J Am Med Dir Assoc
. 2020 Nov;21(11):1539-1545.
doi: 10.1016/j.jamda.2020.09.004. Epub 2020 Sep 9.

Authors

Bastien Genet [1], Jean-Sébastien Vidal [2], Adrien Cohen [2], Clémence Boully [2], Maëlle Beunardeau [2], Louise Marine Harlé [2], Anna Gonçalves [2], Yasmina Boudali [2], Intza Hernandorena [2], Henri Bailly [2], Hermine Lenoir [2], Matthieu Piccoli [2], Anne Chahwakilian [2], Léna Kermanach [2], Laura de Jong [3], Emmanuelle Duron [4], Xavier Girerd [5], Olivier Hanon [6]

Affiliations

- [1] Assistance Publique-Hôpitaux de Paris, Hôpitaux Universitaires Paris Centre, Hôpital Broca, Service de gériatrie, F-75013, Paris, France.
- [2] Assistance Publique-Hôpitaux de Paris, Hôpitaux Universitaires Paris Centre, Hôpital Broca, Service de gériatrie, F-75013, Paris, France; EA 4468, Université de Paris, F-75013, Paris, France.
- [3] Centre Hospitalier Sainte-Anne, GHU Paris Psychiatrie & Neurosciences, F-75014, Paris, France.
- [4] Assistance Publique-Hôpitaux de Paris, Hôpital Paul Brousse, Service de gériatrie, F-94804, Villejuif, France; Université Paris-Sud XI, F-94270, Le Kremlin-Bicêtre, France.
- [5] Fondation de recherche sur l'hypertension artérielle (FRHTA), F-75013, Paris, France; Assistance Publique-Hôpitaux de Paris, Hôpital Pitié Salpêtrière, Sorbonne Université, F-75013, Paris, France.
- [6] Assistance Publique-Hôpitaux de Paris, Hôpitaux Universitaires Paris Centre, Hôpital Broca, Service de gériatrie, F-75013, Paris, France; EA 4468, Université de Paris, F-75013, Paris, France. Electronic address: olivier.hanon@brc.aphp.fr.

- PMID: **33138935**
- PMCID: PMC7480334
- DOI: 10.1016/j.jamda.2020.09.004

Abstract

Objective: The role of treatment with renin-angiotensin-aldosterone system blockers at the onset of COVID-19 infection is not known in the geriatric population. The aim of this study was to assess the relationship between angiotensin receptor blockers (ARBs) and angiotensin-converting enzyme inhibitor (ACEI) use and in-hospital mortality in geriatric patients hospitalized for COVID-19.

Design: This observational retrospective study was conducted in a French geriatric department. Patients were included between March 17 and April 18, 2020.

Setting and participants: All consecutive 201 patients hospitalized for COVID-19 (confirmed by reverse-transcriptase polymerase chain reaction methods) were included. All nondeceased patients had 30 days of follow-up and no patient was lost to follow-up.

Methods: Demographic, clinical, and biological data and medications were collected. In-hospital mortality of patients treated or not by ACEI/ARB was analyzed using multivariate Cox models.

Results: Mean age of the population was 86.3 (8.0) years, 62.7% of patients were institutionalized, 88.6% had dementia, and 53.5% had severe disability (activities of daily living [ADL] score <2). Sixty-three patients were treated with ACEI/ARB and 138 were not. Mean follow-up was 23.4 (10.0) days, 66 (33.8%) patients died after an average of 10.0 days (6.0). Lower mortality rate was observed in patients treated with ACEI/ARB compared with patients not treated with ARB or ACEI (22.2% [14] vs 37.7% [52], hazard ratio [HR] 0.54; 95% confidence interval 0.30-0.97; P = .03). In a multivariate Cox regression model including age, sex, ADL score, Charlson index, renal function, dyspnea, C-reactive protein, and white blood cell count, use of ACEI/ARB was significantly associated with lower in-hospital mortality (HR 0.52 (0.27-0.99), P = .048).

Conclusion and implications: In very old subjects hospitalized in geriatric settings for COVID-19, mortality was significantly lower in subjects treated with ARB or ACEI before the onset of

infection. The continuation of ACEI/ARB therapy should be encouraged during periods of coronavirus outbreak in older subjects.

Keywords: COVID-19; angiotensin receptor blockers; angiotensin-converting enzyme inhibitor; geriatrics; in-hospital mortality; renin-angiotensin-aldosterone system blockers.

Copyright © 2020 AMDA – The Society for Post-Acute and Long-Term Care Medicine. Published by Elsevier Inc. All rights reserved.

- 39 references
- 2 figures

Supplementary info

Publication types, MeSH terms, Substances Expand

Publication types

- Observational Study

MeSH terms

- Aged
- Aged, 80 and over
- Angiotensin-Converting Enzyme Inhibitors / therapeutic use*
- Betacoronavirus
- COVID-19
- Coronavirus Infections / mortality*
- Female
- France / epidemiology
- Geriatric Nursing
- Hospital Mortality / trends*
- Humans
- Male
- Pandemics
- Pneumonia, Viral / mortality*
- Retrospective Studies
- SARS-CoV-2

Substances

- Angiotensin-Converting Enzyme Inhibitors

Full text links

Elsevier Science Free PMC article

Proceed to details

Cite

Share

☐ 900

Observational Study

Philos Ethics Humanit Med

. 2022 Mar 16;17(1):5.

doi: 10.1186/s13010-022-00117-1.

Operation of a triage committee for advanced life support during the COVID-19 pandemic

Benjamín Herreros [1] [2], Rafael Ruiz de Luna [3], Natalia de la Calle [3], Diego Gayoso [4], Paula Martínez [4], Karmele Olaciregui Dague [5], Gregorio Palacios [4] [6]

Affiliations Expand

Affiliations

- [1] Internal Medicine Unit, Alcorcón Foundation University Hospital, Alcorcón, Spain. benjaminherreros@gmail.com.
- [2] Francisco Vallés Institute of Clinical Ethics, European University, Madrid, Spain. benjaminherreros@gmail.com.
- [3] Intensive Medicine Unit, Alcorcón Foundation University Hospital, Madrid, Spain.
- [4] Internal Medicine Unit, Alcorcón Foundation University Hospital, Alcorcón, Spain.
- [5] Epileptology Department, University Hospital Bonn, Bonn, Germany.
- [6] Francisco Vallés Institute of Clinical Ethics, European University, Madrid, Spain.

- PMID: **35292071**
- PMCID: PMC8923824
- DOI: 10.1186/s13010-022-00117-1

Free PMC article

Observational Study

Operation of a triage committee for advanced life support during the COVID-19 pandemic

Benjamín Herreros et al. Philos Ethics Humanit Med. 2022.

Free PMC article

Show details

Philos Ethics Humanit Med

. 2022 Mar 16;17(1):5.

doi: 10.1186/s13010-022-00117-1.

Authors

Benjamín Herreros [1, 2], Rafael Ruiz de Luna [3], Natalia de la Calle [3], Diego Gayoso [4], Paula Martínez [4], Karmele Olaciregui Dague [5], Gregorio Palacios [4, 6]

Affiliations

- [1] Internal Medicine Unit, Alcorcón Foundation University Hospital, Alcorcón, Spain. benjaminherreros@gmail.com.
- [2] Francisco Vallés Institute of Clinical Ethics, European University, Madrid, Spain. benjaminherreros@gmail.com.
- [3] Intensive Medicine Unit, Alcorcón Foundation University Hospital, Madrid, Spain.
- [4] Internal Medicine Unit, Alcorcón Foundation University Hospital, Alcorcón, Spain.
- [5] Epileptology Department, University Hospital Bonn, Bonn, Germany.
- [6] Francisco Vallés Institute of Clinical Ethics, European University, Madrid, Spain.

- PMID: **35292071**
- PMCID: PMC8923824
- DOI: 10.1186/s13010-022-00117-1

Abstract

Background: During the first weeks of March 2020 in Spain, the cases of severe respiratory failure progressively increased, generating an imbalance between the clinical needs for advanced life support (ALS) measures and the effective availability of ALS resources. To address this problem, the creation of triage committees (TC) was proposed, whose main function is to select the best candidates to receive ALS. The main objective of our study is to describe the clinical characteristics of the patients evaluated by the TC of the Alcorcón Foundation University Hospital (AFUH) during the first wave of SARS CoV-2. Other objectives are to determine if there are differences between the patients considered candidates / not candidates for ALS and to analyze the functioning of the TC.

Methods: Retrospective observational study of all patients assessed by the AFUH TC.

Results: There were 19 meetings, in which 181 patients were evaluated, 65.4% male and with a mean age of 70.1 years. 31% had some degree of functional dependence, the Barthel median was 100 and Charlson 4. 58.5% were not considered a candidate for ALS at that time. The patients considered candidates to receive ALS were younger (72 vs 66; $p < 0.001$), had less comorbidity (Charlson 4 vs 3; $p < 0.001$) and had a better previous functional situation. A median of 5 physicians participated in each meeting and, after being assessed by the TC, 13.6% received ALS: 29.3% of those considered candidates for ALS and 2% of the non-candidates.

Conclusions: The patients evaluated by the TC had a mean age of 70 years, high comorbidity and almost a third had some degree of functional dependence. More than half were not considered candidates for ALS at that time, these patients being older, with more comorbidity and a worse previous functional situation. TC decisions, based on objective clinical criteria, were almost always respected. Public institutions must get involved in triage procedures, which should and in our opinion must include the creation of TC in health centers. The implementation of Anticipated Decision programs (ADP) would help enable patients affected by triage decisions to participate in them.

Keywords: COVID-19; Decision-making; Ethics Committees/Consultation; Triage.

© 2022. The Author(s).

Conflict of interest statement

The authors declare that they have no conflict of interest related to this article.

- 31 references

Supplementary info

Publication types, MeSH terms Expand

Publication types

- Observational Study
- Research Support, Non-U.S. Gov't

MeSH terms

- Advanced Cardiac Life Support
- Aged
- COVID-19*
- Female
- Humans
- Male
- Pandemics
- Retrospective Studies
- Triage*

Full text links

Read free full text at BMC BioMed Central Free PMC article

Proceed to details

Cite

Share

☐ 901

Observational Study

Hematol Oncol Stem Cell Ther

. 2021 Dec;14(4):311-317.
doi: 10.1016/j.hemonc.2020.12.001. Epub 2020 Dec 24.

COVID-19-positive cancer patients undergoing active anticancer treatment: An analysis of clinical features and outcomes

Jamshed Ali [1], Kashif Sajjad [2], Amer Rehman Farooqi [2], Muhammad Tahir Aziz [3], Ayesha Rahat [4], Sarah Khan [4]
Affiliations Expand

Affiliations

- [1] Department of Medical Oncology, Shaukat Khanum Memorial Cancer Hospital and Research Centre (SKMCH&RC), Peshawar, Pakistan. Electronic address: jamshedali@skm.org.pk.
- [2] Department of Internal Medicine, Shaukat Khanum Memorial Cancer Hospital and Research Centre (SKMCH&RC), Peshawar, Pakistan.
- [3] Department of Pharmacy, Shaukat Khanum Memorial Cancer Hospital and Research Centre (SKMCH&RC), Peshawar, Pakistan.
- [4] Department of Medical Oncology, Shaukat Khanum Memorial Cancer Hospital and Research Centre (SKMCH&RC), Peshawar, Pakistan.

- PMID: **33387453**
- PMCID: PMC7759333
- DOI: 10.1016/j.hemonc.2020.12.001

Free PMC article
Observational Study

COVID-19-positive cancer patients undergoing active anticancer treatment: An analysis of clinical features and outcomes

Jamshed Ali et al. Hematol Oncol Stem Cell Ther. 2021 Dec.
Free PMC article
Show details
Hematol Oncol Stem Cell Ther
. 2021 Dec;14(4):311-317.
doi: 10.1016/j.hemonc.2020.12.001. Epub 2020 Dec 24.

Authors

Jamshed Ali [1], Kashif Sajjad [2], Amer Rehman Farooqi [2], Muhammad Tahir Aziz [3], Ayesha Rahat [4], Sarah Khan [4]

Affiliations

- [1] Department of Medical Oncology, Shaukat Khanum Memorial Cancer Hospital and Research Centre (SKMCH&RC), Peshawar, Pakistan. Electronic address: jamshedali@skm.org.pk.
- [2] Department of Internal Medicine, Shaukat Khanum Memorial Cancer Hospital and Research Centre (SKMCH&RC), Peshawar, Pakistan.
- [3] Department of Pharmacy, Shaukat Khanum Memorial Cancer Hospital and Research Centre (SKMCH&RC), Peshawar, Pakistan.
- [4] Department of Medical Oncology, Shaukat Khanum Memorial Cancer Hospital and Research Centre (SKMCH&RC), Peshawar, Pakistan.

- PMID: **33387453**
- PMCID: PMC7759333
- DOI: 10.1016/j.hemonc.2020.12.001

Abstract

Background: Cancer patients, particularly those on active anticancer treatment, are reportedly at a high risk of severe coronavirus disease 2019 (COVID-19) infection and death. This study aimed to describe the clinical characteristics and outcomes of patients diagnosed with COVID-19 whilst on anticancer treatment in a developing country.

Methods: This is a retrospective observational study of all adult cancer patients at Shaukat Khanum Memorial Cancer Hospital and Research Centre, Pakistan, from March 15, 2020 to July 10, 2020, diagnosed with COVID-19 within 4 weeks of receiving anticancer treatment, where a purposive sampling was performed. Cancer patients who did not receive anticancer treatment and clinical or radiological diagnosis of COVID-19 without a positive reverse transcription-polymerase chain reaction (RT-PCR) test were excluded. The primary endpoint was all-cause mortality after 30 days of COVID-19 test. Data was analyzed with SPSS version 23 (SPSS Inc., Chicago, IL, USA). Categorical parameters were computed using chi-square test, keeping p value < 0.05 as significant.

Results: A total of 201 cancer patients with COVID-19 were analyzed. The median age of patients was 45 (18-78) years. Mild symptoms were present in 162 (80.6%) patients, whereas severe symptoms were present in 39 (19.4%) patients. The risk of death was statistically significant ($p < .05$) amongst patients with age greater than 50 years, metastatic disease, and ongoing palliative anticancer treatment. Anticancer treatment (chemotherapy, radiotherapy, hormonal therapy, targeted therapy, and surgery) received within preceding 4 weeks had no statistically significant ($p > .05$) impact on mortality.

Conclusions: In cancer patients with COVID-19, mortality appears to be principally driven by age, advanced stage of the disease, and palliative intent of cancer treatment. We did not identify evidence that cancer patients on chemotherapy are at significant risk of mortality from COVID-19 correlating to those not on chemotherapy.

Keywords: COVID-19; Cancer; Outcome; Pakistan.

Copyright © 2020 King Faisal Specialist Hospital & Research Centre. Published by Elsevier Ltd. All rights reserved.

Conflict of interest statement

Declaration of Competing Interest The authors declare that they have no known competing financial interests or personal relationships that could have appeared to influence the work reported in this paper.

- 14 references
- 2 figures

Supplementary info

Publication types, MeSH terms Expand

Publication types

- Observational Study

MeSH terms

- Adolescent
- Adult
- Aged
- COVID-19* / complications
- COVID-19* / mortality
- Humans
- Middle Aged
- Neoplasms* / drug therapy
- Pakistan
- Pandemics
- Retrospective Studies
- Young Adult

Full text links

ELSEVIER OPEN ACCESS Elsevier Science Free PMC article

Proceed to details

Cite

Share

☐ 902

Case Reports

J Pediatr

. 2020 Aug;223:199-203.e1.
doi: 10.1016/j.jpeds.2020.05.007. Epub 2020 May 13.

Severe Coronavirus Disease-2019 in Children and Young Adults in the Washington, DC, Metropolitan Region

Roberta L DeBiasi [1], Xiaoyan Song [2], Meghan Delaney [3], Michael Bell [4], Karen Smith [5], Jay Pershad [6], Emily Ansusinha [7], Andrea Hahn [8], Rana Hamdy [8], Nada Harik [8], Benjamin Hanisch [8], Barbara Jantausch [8], Adeline Koay [8], Robin Steinhorn [9], Kurt Newman [10], David Wessel [4]

Affiliations Expand

Affiliations

- [1] Division of Pediatric Infectious Diseases, Children's National Hospital, Washington, DC; Department of Pediatrics, The George Washington University School of Medicine and Health Sciences, Washington, DC; Department of Microbiology, Immunology and Tropical Medicine, The George Washington University School of Medicine and Health Sciences, Washington, DC. Electronic address: rdebiasi@childrensnational.org.
- [2] Department of Pediatrics, The George Washington University School of Medicine and Health Sciences, Washington, DC; Division of Infection Control and Epidemiology, Children's National Hospital, Washington, DC.
- [3] Department of Pediatrics, The George Washington University School of Medicine and Health Sciences, Washington, DC; Division of Laboratory Medicine and Pathology, Children's National Hospital, Washington, DC.
- [4] Department of Pediatrics, The George Washington University School of Medicine and Health Sciences, Washington, DC; Division of Critical Care Medicine, Children's National Hospital, Washington, DC.
- [5] Department of Pediatrics, The George Washington University School of Medicine and Health Sciences, Washington, DC; Division of Hospitalist Medicine, Children's National Hospital, Washington, DC.
- [6] Department of Pediatrics, The George Washington University School of Medicine and Health Sciences, Washington, DC; Division of Emergency Medicine, Children's National Hospital, Washington, DC.
- [7] Division of Pediatric Infectious Diseases, Children's National Hospital, Washington, DC.
- [8] Division of Pediatric Infectious Diseases, Children's National Hospital, Washington, DC; Department of Pediatrics, The George Washington University School of Medicine and Health Sciences, Washington, DC.
- [9] Department of Pediatrics, The George Washington University School of Medicine and Health Sciences, Washington, DC; Division of Neonatology, Children's National Hospital, Washington, DC.
- [10] Department of Pediatrics, The George Washington University School of Medicine and Health Sciences, Washington, DC; Chief Executive Officer, Division of Pediatric Surgery, Children's National Hospital, Washington, DC.

- PMID: **32405091**
- PMCID: PMC7217783
- DOI: 10.1016/j.jpeds.2020.05.007

Free PMC article
Case Reports

Severe Coronavirus Disease-2019 in Children and Young Adults in the Washington, DC, Metropolitan Region

Roberta L DeBiasi et al. J Pediatr. 2020 Aug.
Free PMC article
Show details
J Pediatr
. 2020 Aug;223:199-203.e1.
doi: 10.1016/j.jpeds.2020.05.007. Epub 2020 May 13.

Authors

Roberta L DeBiasi[1], Xiaoyan Song[2], Meghan Delaney[3], Michael Bell[4], Karen Smith[5], Jay Pershad[6], Emily Ansusinha[7], Andrea Hahn[8], Rana Hamdy[8], Nada Harik[8], Benjamin Hanisch[8], Barbara Jantausch[8], Adeline Koay[8], Robin Steinhorn[9], Kurt Newman[10], David Wessel[4]

Affiliations

- [1] Division of Pediatric Infectious Diseases, Children's National Hospital, Washington, DC; Department of Pediatrics, The George Washington University School of Medicine and Health Sciences, Washington, DC; Department of Microbiology, Immunology and Tropical Medicine, The George Washington University School of Medicine and Health Sciences, Washington, DC. Electronic address: rdebiasi@childrensnational.org.
- [2] Department of Pediatrics, The George Washington University School of Medicine and Health Sciences, Washington, DC; Division of Infection Control and Epidemiology, Children's National Hospital, Washington, DC.
- [3] Department of Pediatrics, The George Washington University School of Medicine and Health Sciences, Washington, DC; Division of Laboratory Medicine and Pathology, Children's National Hospital, Washington, DC.
- [4] Department of Pediatrics, The George Washington University School of Medicine and Health Sciences, Washington, DC; Division of Critical Care Medicine, Children's National Hospital, Washington, DC.
- [5] Department of Pediatrics, The George Washington University School of Medicine and Health Sciences, Washington, DC; Division of Hospitalist Medicine, Children's National Hospital, Washington, DC.
- [6] Department of Pediatrics, The George Washington University School of Medicine and Health Sciences, Washington, DC; Division of Emergency Medicine, Children's National Hospital, Washington, DC.
- [7] Division of Pediatric Infectious Diseases, Children's National Hospital, Washington, DC.
- [8] Division of Pediatric Infectious Diseases, Children's National Hospital, Washington, DC; Department of Pediatrics, The George Washington University School of Medicine and Health Sciences, Washington, DC.

- [9] Department of Pediatrics, The George Washington University School of Medicine and Health Sciences, Washington, DC; Division of Neonatology, Children's National Hospital, Washington, DC.
- [10] Department of Pediatrics, The George Washington University School of Medicine and Health Sciences, Washington, DC; Chief Executive Officer, Division of Pediatric Surgery, Children's National Hospital, Washington, DC.

- PMID: **32405091**
- PMCID: PMC7217783
- DOI: 10.1016/j.jpeds.2020.05.007

Abstract

Despite worldwide spread of severe acute respiratory syndrome coronavirus-2, few publications have reported the potential for severe disease in the pediatric population. We report 177 infected children and young adults, including 44 hospitalized and 9 critically ill patients, with a comparison of patient characteristics between infected hospitalized and nonhospitalized cohorts, as well as critically ill and noncritically ill cohorts. Children <1 year and adolescents and young adults >15 years of age were over-represented among hospitalized patients (P = .07). Adolescents and young adults were over-represented among the critically ill cohort (P = .02).

Keywords: COVID-19; SARS-CoV-2; critical care; hospitalization; pediatric.

Copyright © 2020 Elsevier Inc. All rights reserved.

Comment in

- Reply.
  DeBiasi RL. DeBiasi RL. J Pediatr. 2020 Oct;225:280-281. doi: 10.1016/j.jpeds.2020.06.062. Epub 2020 Jun 23. J Pediatr. 2020. PMID: 32589999 Free PMC article. No abstract available.
- Severe coronavirus disease 2019 in children and young adults.
  Wilkes M, Issa R, Aluf A, Beliard K, Yau M, Rapaport R, Ebekozien O. Wilkes M, et al. J Pediatr. 2020 Oct;225:280. doi: 10.1016/j.jpeds.2020.06.061. Epub 2020 Jun 24. J Pediatr. 2020. PMID: 32590002 Free PMC article. No abstract available.

- 9 references
- 1 figure

Supplementary info

Publication types, MeSH terms, Supplementary concepts Expand

Publication types

- Case Reports
- Comparative Study
- Observational Study

MeSH terms

- Adolescent
- Age Distribution
- Asthma / epidemiology
- Betacoronavirus*
- COVID-19
- Child
- Child, Preschool
- Cohort Studies
- Comorbidity
- Coronavirus Infections / diagnosis
- Coronavirus Infections / epidemiology*
- Cough / virology
- Critical Illness
- District of Columbia / epidemiology
- Dyspnea / virology
- Female
- Fever / virology
- Hospitalization*
- Humans
- Infant
- Infant, Newborn
- Male
- Mucocutaneous Lymph Node Syndrome / complications
- Pandemics
- Pharyngitis / virology
- Pneumonia, Viral / diagnosis
- Pneumonia, Viral / epidemiology*
- Respiration, Artificial / statistics & numerical data
- Retrospective Studies
- SARS-CoV-2
- Systemic Inflammatory Response Syndrome / virology
- Young Adult

Supplementary concepts

- pediatric multisystem inflammatory disease, COVID-19 related

Full text links

ELSEVIER FULL-TEXT ARTICLE Elsevier Science Free PMC article

Proceed to details

Cite

Share

☐ 903

Observational Study

Kidney Blood Press Res

. 2021;46(1):126-134.

doi: 10.1159/000512535. Epub 2021 Jan 27.

Clinical Features of Asymptomatic SARS-CoV-2 Infection in Hemodialysis Patients

Maria Soledad Pizarro-Sánchez [1], Alejandro Avello [2], Sebastian Mas-Fontao [3], Teresa Stock da Cunha [2], Elena Goma-Garcés [2], Mónica Pereira [4], Alberto Ortíz [2] [5], Emilio González-Parra [6] [7]

Affiliations Expand

Affiliations

- [1] Nephrology and Hypertension, Rey Juan Carlos Hospital, Madrid, Spain.
- [2] Nephrology and Hypertension, UH-Fundacion Jimenez Diaz UAM, Madrid, Spain.
- [3] Renal Pathology and Diabetes Laboratory, IIS-FJD/CIBERDEM, Madrid, Spain.
- [4] Iñígo Álvarez de Toledo Renal Foundation (FRIAT), Madrid, Spain.
- [5] Autónoma University of Madrid, Madrid, Spain.
- [6] Nephrology and Hypertension, UH-Fundacion Jimenez Diaz UAM, Madrid, Spain, egonzalezpa@senefro.org.
- [7] Autónoma University of Madrid, Madrid, Spain, egonzalezpa@senefro.org.

- PMID: **33503627**
- PMCID: PMC7900451
- DOI: 10.1159/000512535

Free PMC article

Observational Study

Clinical Features of Asymptomatic SARS-CoV-2 Infection in Hemodialysis Patients

Maria Soledad Pizarro-Sánchez et al. Kidney Blood Press Res. 2021.

Free PMC article

Show details

Kidney Blood Press Res

. 2021;46(1):126-134.

doi: 10.1159/000512535. Epub 2021 Jan 27.

Authors

Maria Soledad Pizarro-Sánchez [1], Alejandro Avello [2], Sebastian Mas-Fontao [3], Teresa Stock da Cunha [2], Elena Goma-Garcés [2], Mónica Pereira [4], Alberto Ortíz [2] [5], Emilio González-Parra [6] [7]

Affiliations

- [1] Nephrology and Hypertension, Rey Juan Carlos Hospital, Madrid, Spain.
- [2] Nephrology and Hypertension, UH-Fundacion Jimenez Diaz UAM, Madrid, Spain.
- [3] Renal Pathology and Diabetes Laboratory, IIS-FJD/CIBERDEM, Madrid, Spain.
- [4] Iñígo Álvarez de Toledo Renal Foundation (FRIAT), Madrid, Spain.
- [5] Autónoma University of Madrid, Madrid, Spain.
- [6] Nephrology and Hypertension, UH-Fundacion Jimenez Diaz UAM, Madrid, Spain, egonzalezpa@senefro.org.
- [7] Autónoma University of Madrid, Madrid, Spain, egonzalezpa@senefro.org.

- PMID: **33503627**
- PMCID: PMC7900451
- DOI: 10.1159/000512535

Abstract

Background: CKD is a risk factor for severe COVID-19. However, the clinical spectrum of COVID-19 in hemodialysis patients is still poorly characterized.

Objective: To analyze the clinical spectrum of COVID-19 on hemodialysis patients.

Method: A retrospective observational study was conducted on 66 hemodialysis patients. Nasopharyngeal swab PCR and serology for SARS-CoV-2, blood analysis, chest radiography, treatment, and outcomes were assessed.

Results: COVID-19 was diagnosed in 50 patients: 38 (76%) were PCR-positive and 12 (24%) were PCR-negative but developed anti-SARS-CoV-2 antibodies. By contrast, 17% of PCR-positive patients failed to develop detectable antibodies against SARS-CoV-2. Among PCR-positive patients, 5/38 (13%) were asymptomatic, while among PCR-negative patients 7/12 (58%) were asymptomatic ($p = 0.005$) for a total of 12/50 (24%) asymptomatic patients. No other differences were found between PCR-positive and PCR-negative patients. No differences in potential predisposing factors were found between asymptomatic and symptomatic patients except for a lower use of ACE inhibitors among asymptomatic patients. Asymptomatic patients had laboratory evidence of milder disease such as higher lymphocyte counts and oxygen saturation and lower troponin I and interleukin-6 levels than symptomatic patients. Overall mortality was 7/50 (14%) and occurred only in symptomatic PCR-positive patients in whom mortality was 7/33 (21%).

Conclusions: Asymptomatic SARS-CoV-2 infection is common in hemodialysis patients, especially among patients with initial negative PCR that later seroconvert. Thus COVID-19 mortality in hemodialysis patients may be lower than previously estimated based on PCR tests alone.

Keywords: Asymptomatic; COVID-19; Chronic kidney disease; Hemodialysis; Mortality.

© 2021 The Author(s) Published by S. Karger AG, Basel.

Conflict of interest statement

The authors have no conflicts of interest to declare.

- 28 references
- 1 figure

Supplementary info

Publication types, MeSH terms Expand

Publication types

- Observational Study

MeSH terms

- Aged
- Aged, 80 and over
- Asymptomatic Diseases / epidemiology*
- COVID-19 / blood
- COVID-19 / diagnosis*
- COVID-19 / epidemiology*
- Female
- Humans
- Male
- Middle Aged
- Renal Dialysis / trends*
- Renal Insufficiency, Chronic / blood
- Renal Insufficiency, Chronic / diagnosis*
- Renal Insufficiency, Chronic / epidemiology*
- Retrospective Studies

Full text links

Karger Free Final Version S. Karger AG, Basel, Switzerland Free PMC article

Proceed to details

Cite

Share

☐ 904

Observational Study

J Thromb Thrombolysis

. 2021 Jan;51(1):33-39.

doi: 10.1007/s11239-020-02181-w.

DVT incidence and risk factors in critically ill patients with COVID-19

Shujing Chen [1], Dingyu Zhang [2], Tianqi Zheng [1], Yongfu Yu [3 4], Jinjun Jiang [5]
Affiliations Expand

Affiliations

- [1] Department of Pulmonary and Critical Care Medicine, Zhongshan Hospital, Fudan University, Shanghai, 200032, China.
- [2] Department of Tuberculosis and Respiratory Disease, Jinyintan Hospital, Wuhan, China.
- [3] Department of Biostatistics, School of Public Health, and The Key Laboratory of Public Health Safety of Ministry of Education, Fudan University, Shanghai, China. yoyu@clin.au.dk.
- [4] Department of Clinical Epidemiology, Aarhus University, Aarhus, Denmark. yoyu@clin.au.dk.
- [5] Department of Pulmonary and Critical Care Medicine, Zhongshan Hospital, Fudan University, Shanghai, 200032, China. jinjundoc@163.com.

- PMID: **32607652**
- PMCID: PMC7324310
- DOI: 10.1007/s11239-020-02181-w

Free PMC article
Observational Study

DVT incidence and risk factors in critically ill patients with COVID-19

Shujing Chen et al. J Thromb Thrombolysis. 2021 Jan.
Free PMC article
Show details
J Thromb Thrombolysis
. 2021 Jan;51(1):33-39.
doi: 10.1007/s11239-020-02181-w.

Authors

Shujing Chen [1], Dingyu Zhang [2], Tianqi Zheng [1], Yongfu Yu [3 4], Jinjun Jiang [5]

Affiliations

- [1] Department of Pulmonary and Critical Care Medicine, Zhongshan Hospital, Fudan University, Shanghai, 200032, China.

- [2] Department of Tuberculosis and Respiratory Disease, Jinyintan Hospital, Wuhan, China.
- [3] Department of Biostatistics, School of Public Health, and The Key Laboratory of Public Health Safety of Ministry of Education, Fudan University, Shanghai, China. yoyu@clin.au.dk.
- [4] Department of Clinical Epidemiology, Aarhus University, Aarhus, Denmark. yoyu@clin.au.dk.
- [5] Department of Pulmonary and Critical Care Medicine, Zhongshan Hospital, Fudan University, Shanghai, 200032, China. jinjundoc@163.com.

- PMID: **32607652**
- PMCID: PMC7324310
- DOI: 10.1007/s11239-020-02181-w

Abstract

Few data are available on the incidence of deep vein thrombosis (DVT) in critically ill COVID-19 with thrombosis prophylaxis. This study retrospectively included 88 patients in the ICU with critically ill COVID-19 at Jinyintan Hospital in Wuhan, China. All patients underwent compression ultrasonography for identifying DVT. Firth logistic regression was used to examine the association of DVT with sex, age, hypoalbuminemia, D-dimer, and SOFA score. The median (interquartile range [IQR]) age and SOFA score of 88 patients were 63 (55-71) years old and 5 (4-6), respectively. Despite all patients receiving guideline-recommended low-molecular-weight heparin (LMWH) thromboprophylaxis, the incidence of DVT was 46% (95% CI 35-56%). Proximal DVT was recognized in 9% (95% CI 3-15%) of the patients, while 46% (95% CI 35-56%) of patients had distal DVT. All of the proximal DVT combined with distal DVT. Risk factors of DVT extension occurred in all distal DVT patients. As Padua score ≥ 4 or IMPROVE score ≥ 2, 53% and 46% of patients had DVT, respectively. Mortality was higher in patients with acute DVT (30%) compared with non-DVT (17%), but did not reach statistical significance. Hypoalbuminemia (odds ratio [OR], 0.17; 95% CI 0.06-0.05, P = 0.001), higher SOFA score (OR per IQR, 2.07; 95% CI 1.38-3.39, P = 0.001), and elevated D-dimer (OR per IQR, 1.04; 95% CI 1.03-1.84, P = 0.029) were significant DVT risk factors in multivariable analyses. High incidence of DVT was identified in patients with critically ill COVID-19, despite the use of guideline-recommended pharmacologic thromboprophylaxis. The presence of hypoalbuminemia, higher SOFA score, and elevated D-dimer were significantly independent risk factors of DVT. More effective VTE prevention and management strategies may need to be addressed.

Keywords: Coronavirus; D-dimer; Deep vein thrombosis; Hypoalbuminemia; SOFA score.

Conflict of interest statement

The authors have no conflict of interest to disclose.

- 23 references
- 1 figure

Supplementary info

Publication types, MeSH terms, Substances, Grant support Expand

Publication types

- Observational Study

MeSH terms

- Age Factors
- Anticoagulants / administration & dosage
- COVID-19* / blood
- COVID-19* / complications
- COVID-19* / diagnosis
- COVID-19* / therapy
- Chemoprevention* / methods
- Chemoprevention* / statistics & numerical data
- China / epidemiology
- Critical Illness
- Female
- Fibrin Fibrinogen Degradation Products / analysis*
- Heparin, Low-Molecular-Weight / administration & dosage*
- Humans
- Hypoalbuminemia* / diagnosis
- Hypoalbuminemia* / etiology
- Male
- Middle Aged
- Organ Dysfunction Scores
- Risk Assessment
- Risk Factors
- SARS-CoV-2 / isolation & purification
- Sex Factors
- Venous Thrombosis* / blood
- Venous Thrombosis* / diagnosis
- Venous Thrombosis* / drug therapy
- Venous Thrombosis* / etiology

Substances

- Anticoagulants
- Fibrin Fibrinogen Degradation Products
- Heparin, Low-Molecular-Weight
- fibrin fragment D

Grant support

- 81900038/National Natural Science Foundation of China
- 81870062/National Natural Science Foundation of China

Full text links

SpringerLink FULL-TEXT ARTICLE Springer Free PMC article
Proceed to details
Cite
Share
☐ 905

Observational Study

Diabetes Obes Metab
. 2020 Aug;22(8):1443-1454.
doi: 10.1111/dom.14086. Epub 2020 Jun 17.

The clinical characteristics and outcomes of patients with diabetes and secondary hyperglycaemia with coronavirus disease 2019: A single-centre, retrospective, observational study in Wuhan

Yang Zhang [1], Haichao Li [2], Jian Zhang [1], Yedi Cao [1], Xue Zhao [1], Nan Yu [1], Ying Gao [1], Jing Ma [2], Hong Zhang [2], Junqing Zhang [1], Xiaohui Guo [1], Xinmin Liu [3]
Affiliations Expand

Affiliations

- [1] Endocrinology Department, Peking University First Hospital, Beijing, China.
- [2] Respiratory and Critical Care Medicine Department, Peking University First Hospital, Beijing, China.
- [3] Geriatrics Department, Peking University First Hospital, Beijing, China.

- PMID: **32406594**
- PMCID: PMC7273002
- DOI: 10.1111/dom.14086

Free PMC article
Observational Study

The clinical characteristics and outcomes of patients with diabetes and secondary hyperglycaemia with coronavirus disease 2019: A single-centre, retrospective, observational study in Wuhan

Yang Zhang et al. Diabetes Obes Metab. 2020 Aug.
Free PMC article
Show details
Diabetes Obes Metab
. 2020 Aug;22(8):1443-1454.
doi: 10.1111/dom.14086. Epub 2020 Jun 17.

Authors

Yang Zhang [1], Haichao Li [2], Jian Zhang [1], Yedi Cao [1], Xue Zhao [1], Nan Yu [1], Ying Gao [1], Jing Ma [2], Hong Zhang [2], Junqing Zhang [1], Xiaohui Guo [1], Xinmin Liu [3]

Affiliations

- [1] Endocrinology Department, Peking University First Hospital, Beijing, China.
- [2] Respiratory and Critical Care Medicine Department, Peking University First Hospital, Beijing, China.
- [3] Geriatrics Department, Peking University First Hospital, Beijing, China.

- PMID: **32406594**
- PMCID: PMC7273002
- DOI: 10.1111/dom.14086

Abstract

Aim: To explore whether coronavirus disease 2019 (COVID-19) patients with diabetes and secondary hyperglycaemia have different clinical characteristics and prognoses than those without significantly abnormal glucose metabolism.

Materials and methods: We retrospectively analysed 166 COVID-19 patients at Tongji Hospital (Wuhan) from 8 February to 21 March 2020. Clinical characteristics and outcomes (as of 4 April 2020) were compared among control (group 1), secondary hyperglycaemia (group 2: no diabetes history, fasting plasma glucose levels of ≥7.0 mmol/L once and HbA1c values <6.5%) and patients with diabetes (group 3).

Results: Compared with group 1, groups 2 and 3 had higher rates of leukocytosis, neutrophilia, lymphocytopenia, eosinopenia and levels of hypersensitive C-reactive protein, ferritin and d-dimer ($P < .05$ for all). Group 2 patients had higher levels of lactate dehydrogenase, prevalence of liver dysfunction and increased interleukin-8 (IL-8) than those in group 1, and a higher prevalence of increased IL-8 was found in group 2 than in group 3 ($P < .05$ for all). The proportions of critical

patients in groups 2 and 3 were significantly higher compared with group 1 (38.1%, 32.8% vs. 9.5%, P < .05 for both). Groups 2 and 3 had significantly longer hospital stays than group 1, which was nearly 1 week longer. The composite outcomes risks were 5.47 (1.56-19.82) and 2.61 (0.86-7.88) times greater in groups 2 and 3 than in group 1.

Conclusions: Hyperglycaemia in both diabetes and secondary hyperglycaemia patients with COVID-19 may indicate poor prognoses. There were differences between patients with secondary hyperglycaemia and those with diabetes. We recommend that clinicians pay more attention to the blood glucose status of COVID-19 patients, even those not diagnosed with diabetes before admission.

Keywords: cohort study, type 2 diabetes.

© 2020 John Wiley & Sons Ltd.

- 31 references

Supplementary info

Publication types, MeSH terms, Substances, Grant support Expand

Publication types

- Observational Study
- Research Support, Non-U.S. Gov't

MeSH terms

- Adult
- Aged
- Betacoronavirus*
- Blood Glucose / analysis
- COVID-19
- China / epidemiology
- Coronavirus Infections / blood*
- Coronavirus Infections / complications
- Coronavirus Infections / mortality
- Diabetes Mellitus / blood
- Diabetes Mellitus / mortality
- Diabetes Mellitus / virology*
- Female
- Glycated Hemoglobin A / analysis
- Hospitalization / statistics & numerical data
- Humans
- Hyperglycemia / blood
- Hyperglycemia / mortality

- Hyperglycemia / virology*
- Male
- Middle Aged
- Pandemics
- Pneumonia, Viral / blood*
- Pneumonia, Viral / complications
- Pneumonia, Viral / mortality
- Prognosis
- Retrospective Studies
- SARS-CoV-2

Substances

- Blood Glucose
- Glycated Hemoglobin A
- hemoglobin A1c protein, human

Grant support

- This work was supported by the Youth Clinical Research Project of Peking University First Hospital (2018CR01)./International

Full text links

Wiley Free PMC article
Proceed to details
Cite
Share
☐ 906
Observational Study
Br J Anaesth
. 2021 Jan;126(1):48-55.
doi: 10.1016/j.bja.2020.09.042. Epub 2020 Oct 10.

Prone positioning for patients intubated for severe acute respiratory distress syndrome (ARDS) secondary to COVID-19: a retrospective observational cohort study

Tyler T Weiss [1], Flor Cerda [2], J Brady Scott [3], Ramandeep Kaur [1], Sarah Sungurlu [4], Sara H Mirza [5], Amnah A Alolaiwat [6], Ramandeep Kaur [6], Ashley E Augustynovich [6], Jie Li [7]

Affiliations Expand

Affiliations

- [1] Department of Respiratory Care, Rush University Medical Center, Chicago, IL, USA.
- [2] Department of Nursing, Medical Intensive Care Unit, Rush University Medical Center, Chicago, IL, USA.
- [3] Department of Respiratory Care, Rush University Medical Center, Chicago, IL, USA; Department of Cardiopulmonary Sciences, Division of Respiratory Care, Rush University, Chicago, IL, USA.
- [4] Department of Pulmonary and Critical Care, Rush University Medical Center, Chicago, IL, USA.
- [5] Department of Respiratory Care, Rush University Medical Center, Chicago, IL, USA; Department of Pulmonary and Critical Care, Rush University Medical Center, Chicago, IL, USA.
- [6] Department of Cardiopulmonary Sciences, Division of Respiratory Care, Rush University, Chicago, IL, USA.
- [7] Department of Respiratory Care, Rush University Medical Center, Chicago, IL, USA; Department of Cardiopulmonary Sciences, Division of Respiratory Care, Rush University, Chicago, IL, USA. Electronic address: Jie_Li@rush.edu.

- PMID: **33158500**
- PMCID: PMC7547633
- DOI: 10.1016/j.bja.2020.09.042

Free PMC article
Observational Study

Prone positioning for patients intubated for severe acute respiratory distress syndrome (ARDS) secondary to COVID-19: a retrospective observational cohort study

Tyler T Weiss et al. Br J Anaesth. 2021 Jan.
Free PMC article
Show details
Br J Anaesth
. 2021 Jan;126(1):48-55.
doi: 10.1016/j.bja.2020.09.042. Epub 2020 Oct 10.

Authors

Tyler T Weiss [1], Flor Cerda [2], J Brady Scott [3], Ramandeep Kaur [1], Sarah Sungurlu [4], Sara H Mirza [5], Amnah A Alolaiwat [6], Ramandeep Kaur [6], Ashley E Augustynovich [6], Jie Li [7]

Affiliations

- [1] Department of Respiratory Care, Rush University Medical Center, Chicago, IL, USA.
- [2] Department of Nursing, Medical Intensive Care Unit, Rush University Medical Center, Chicago, IL, USA.
- [3] Department of Respiratory Care, Rush University Medical Center, Chicago, IL, USA; Department of Cardiopulmonary Sciences, Division of Respiratory Care, Rush University, Chicago, IL, USA.
- [4] Department of Pulmonary and Critical Care, Rush University Medical Center, Chicago, IL, USA.
- [5] Department of Respiratory Care, Rush University Medical Center, Chicago, IL, USA; Department of Pulmonary and Critical Care, Rush University Medical Center, Chicago, IL, USA.
- [6] Department of Cardiopulmonary Sciences, Division of Respiratory Care, Rush University, Chicago, IL, USA.
- [7] Department of Respiratory Care, Rush University Medical Center, Chicago, IL, USA; Department of Cardiopulmonary Sciences, Division of Respiratory Care, Rush University, Chicago, IL, USA. Electronic address: Jie_Li@rush.edu.

- PMID: **33158500**
- PMCID: PMC7547633
- DOI: 10.1016/j.bja.2020.09.042

Abstract

Background: The role of repeated prone positioning in intubated subjects with acute respiratory distress syndrome caused by COVID-19 remains unclear.

Methods: We conducted a retrospective observational cohort study of critically ill intubated patients with COVID-19 who were placed in the prone position between March 18, 2020 and March 31, 2020. Exclusion criteria were pregnancy, reintubation, and previous prone positioning at a referring hospital. Patients were followed up until hospital discharge. The primary outcome was oxygenation assessed by partial pressure of oxygen/fraction of inspired oxygen ratio (Pao_2/Fio_2) ratio. A positive response to proning was defined as an increase in Pao_2/Fio_2 ratio ≥20%. Treatment failure of prone positioning was defined as death or requirement for extracorporeal membrane oxygenation (ECMO).

Results: Forty-two subjects (29 males; age: 59 [52-69] yr) were eligible for analysis. Nine subjects were placed in the prone position only once, with 25 requiring prone positioning on three or more occasions. A total of 31/42 (74%) subjects survived to discharge, with five requiring ECMO; 11/42 (26%) subjects died. After the first prone positioning session, Pao_2/Fio_2 (mean (standard deviation)) ratio increased from 17.9 kPa (7.2) to 28.2 kPa (12.2) ($P<0.01$). After the initial prone positioning session, subjects who were discharged from hospital were more likely to have an improvement in Pao_2/Fio_2 ratio ≥20%, compared with those requiring ECMO or who died.

Conclusion: Patients with COVID-19 acute respiratory distress syndrome frequently responded to initial prone positioning with improved oxygenation. Subsequent prone positioning in subjects discharged from hospital was associated with greater improvements in oxygenation.

Keywords: COVID-19; acute respiratory distress syndrome (ARDS); mechanical ventilation; oxygenation; prone positioning.

Copyright © 2020 British Journal of Anaesthesia. Published by Elsevier Ltd. All rights reserved.

Conflict of interest statement

Declarations of interest JBS discloses a relationship with Ventec Life Systems and Teleflex. JL discloses research support from Fisher & Paykel Healthcare and Rice Foundation outside the submitted work. All other authors declare that they have no conflicts of interest.

Comment in

- Characterising the pulmonary response to prone positioning. Comment on Br J Anaesth 2021; 126: 48-55.
  Chad T. Chad T. Br J Anaesth. 2021 May;126(5):e191-e192. doi: 10.1016/j.bja.2021.02.008. Epub 2021 Feb 18. Br J Anaesth. 2021. PMID: 33722373 Free PMC article. No abstract available.

- 31 references
- 2 figures

Supplementary info

Publication types, MeSH terms Expand

Publication types

- Observational Study

MeSH terms

- Aged
- COVID-19 / complications
- COVID-19 / physiopathology
- COVID-19 / therapy*
- Cohort Studies
- Female
- Follow-Up Studies
- Humans
- Intubation, Intratracheal / methods*
- Male
- Middle Aged
- Prone Position / physiology*
- Respiratory Distress Syndrome / etiology
- Respiratory Distress Syndrome / physiopathology

- Respiratory Distress Syndrome / therapy*
- Respiratory Mechanics / physiology*
- Retrospective Studies

Full text links

ELSEVIER OPEN ACCESS Elsevier Science Free PMC article
Proceed to details
Cite
Share
907
Observational Study
Neurologia (Engl Ed)
. Nov-Dec 2020;35(9):621-627.
doi: 10.1016/j.nrl.2020.07.014. Epub 2020 Aug 4.

Neurological complications in critical patients with COVID-19

[Article in English, Spanish]

M J Abenza-Abildúa [1], M T Ramírez-Prieto [2], R Moreno-Zabaleta [2], N Arenas-Valls [2], M A Salvador-Maya [2], C Algarra-Lucas [3], B Rojo Moreno-Arrones [2], B Sánchez-Cordón [4], J Ojeda-Ruíz de Luna [3], C Jimeno-Montero [3], F J Navacerrada-Barrero [3], C Borrue-Fernández [3], E Malmierca-Corral [5], P Ruíz-Seco [5], P González-Ruano [5], I Palmí-Cortés [3], J Fernández-Travieso [3], M Mata-Álvarez de Santullano [3], M L Almarcha-Menargues [3], G Gutierrez-Gutierrez [3], J A Palacios-Castaño [6], R Alonso-Esteban [7], N Gonzalo-García [8], C Pérez-López [9]
Affiliations Expand

Affiliations

- [1] Sección de Neurología, Hospital Universitario Infanta Sofía, Madrid, España. Electronic address: mjose.abenza@salud.madrid.org.
- [2] Sección de Neumología, Hospital Universitario Infanta Sofía, Madrid, España.
- [3] Sección de Neurología, Hospital Universitario Infanta Sofía, Madrid, España.
- [4] Sección de Neurorradiología, Hospital Universitario Infanta Sofía, Madrid, España.
- [5] Sección de Enfermedades Infecciosas, Hospital Universitario Infanta Sofía, Madrid, España.
- [6] Servicio de Psiquiatría, Hospital Universitario Infanta Sofía, Madrid, España.
- [7] Hospitalización a Domicilio, Hospital Universitario Infanta Sofía, Madrid, España.
- [8] Servicio de Rehabilitación, Hospital Universitario Infanta Sofía, Madrid, España.
- [9] Servicio de Neurocirugía, Complejo Universitario La Paz-Cantoblanco-CarlosIII, Madrid, España.

- PMID: **32912745**
- PMCID: PMC7402100

- DOI: 10.1016/j.nrl.2020.07.014

Free PMC article
Observational Study

Neurological complications in critical patients with COVID-19

[Article in English, Spanish]
M J Abenza-Abildúa et al. Neurologia (Engl Ed). Nov-Dec 2020.
Free PMC article
Show details
Neurologia (Engl Ed)
. Nov-Dec 2020;35(9):621-627.
doi: 10.1016/j.nrl.2020.07.014. Epub 2020 Aug 4.

Authors

M J Abenza-Abildúa [1], M T Ramírez-Prieto [2], R Moreno-Zabaleta [2], N Arenas-Valls [2], M A Salvador-Maya [2], C Algarra-Lucas [3], B Rojo Moreno-Arrones [2], B Sánchez-Cordón [4], J Ojeda-Ruíz de Luna [3], C Jimeno-Montero [3], F J Navacerrada-Barrero [3], C Borrue-Fernández [3], E Malmierca-Corral [5], P Ruíz-Seco [5], P González-Ruano [5], I Palmí-Cortés [3], J Fernández-Travieso [3], M Mata-Álvarez de Santullano [3], M L Almarcha-Menargues [3], G Gutierrez-Gutierrez [3], J A Palacios-Castaño [6], R Alonso-Esteban [7], N Gonzalo-García [8], C Pérez-López [9]

Affiliations

- [1] Sección de Neurología, Hospital Universitario Infanta Sofía, Madrid, España. Electronic address: mjose.abenza@salud.madrid.org.
- [2] Sección de Neumología, Hospital Universitario Infanta Sofía, Madrid, España.
- [3] Sección de Neurología, Hospital Universitario Infanta Sofía, Madrid, España.
- [4] Sección de Neurorradiología, Hospital Universitario Infanta Sofía, Madrid, España.
- [5] Sección de Enfermedades Infecciosas, Hospital Universitario Infanta Sofía, Madrid, España.
- [6] Servicio de Psiquiatría, Hospital Universitario Infanta Sofía, Madrid, España.
- [7] Hospitalización a Domicilio, Hospital Universitario Infanta Sofía, Madrid, España.
- [8] Servicio de Rehabilitación, Hospital Universitario Infanta Sofía, Madrid, España.
- [9] Servicio de Neurocirugía, Complejo Universitario La Paz-Cantoblanco-CarlosIII, Madrid, España.

- PMID: **32912745**
- PMCID: PMC7402100
- DOI: 10.1016/j.nrl.2020.07.014

Abstract

Introduction: We analysed the neurological complications of patients with severe SARS-CoV-2 infection who required intensive care unit (ICU) admission.

Patients and methods: We conducted a retrospective, observational, descriptive study of consecutive patients admitted to the ICU due to severe respiratory symptoms secondary to SARS-CoV-2 infection between 1 April and 1 June 2020.

Results: We included 30 patients with neurological symptoms; 21 were men (72.40%), and mean age (standard deviation [SD]) was 57.41 years (11.61). The mean duration of ICU stay was 18.83 days (14.33). The neurological conditions recorded were acute confusional syndrome in 28 patients (93.33%), neuromuscular disease in 15 (50%), headache in 5 (16.66%), cerebrovascular disease in 4 (13.33%), and encephalopathies/encephalitis in 4 (13.33%). CSF analysis results were normal in 6 patients (20%). Brain MRI or head CT showed alterations in 20 patients (66.6%). EEG was performed in all patients (100%), with 8 (26.66%) showing abnormal findings. In 5 of the 15 patients with clinical myopathy, diagnosis was confirmed with electroneuromyography. We found a correlation between older age and duration of ICU stay (P=.002; 95%CI: 4.032-6.022; OR: 3,594).

Conclusions: Severe COVID-19 mainly affects men, as observed in other series. Half of our patients presented acute myopathy, and almost all patients left the ICU with acute confusional syndrome, which fully resolved; no correlation was found with EEG or neuroimaging findings. Older age is associated with longer ICU stay.

Keywords: Acute myopathy; COVID-19; Critical patient; Encefalopatías; Encephalopathy; Miopatía aguda; Paciente crítico; SARS-CoV-2.

Copyright © 2020 Sociedad Española de Neurología. Publicado por Elsevier España, S.L.U. All rights reserved.

- 38 references
- 2 figures

Supplementary info

Publication types, MeSH terms Expand

Publication types

- Observational Study

MeSH terms

- Acute Disease
- Adult
- Age Factors
- Aged
- Betacoronavirus*
- COVID-19
- Cerebral Hemorrhage / diagnostic imaging

- Cerebral Hemorrhage / epidemiology
- Cerebral Hemorrhage / etiology
- Confusion / epidemiology
- Confusion / etiology
- Coronavirus Infections / complications*
- Coronavirus Infections / epidemiology
- Critical Care
- Critical Illness*
- Female
- Humans
- Length of Stay / statistics & numerical data
- Magnetic Resonance Imaging
- Male
- Middle Aged
- Muscular Diseases / epidemiology
- Muscular Diseases / etiology*
- Nervous System Diseases / epidemiology
- Nervous System Diseases / etiology*
- Neuroimaging
- Pandemics*
- Pneumonia, Viral / complications*
- Pneumonia, Viral / epidemiology
- Retrospective Studies
- SARS-CoV-2
- Spain / epidemiology

Full text links

ELSEVIER OPEN ACCESS Elsevier Science Free PMC article

Proceed to details

Cite

Share

☐ 908

Observational Study

Emerg Infect Dis

. 2020 Aug;26(8):1679-1685.

doi: 10.3201/eid2608.201776. Epub 2020 May 14.

Characteristics and Outcomes of Coronavirus Disease Patients under Nonsurge Conditions, Northern California, USA, March-April 2020

Jessica Ferguson, Joelle I Rosser, Orlando Quintero, Jake Scott, Aruna Subramanian, Mohammad Gumma, Angela Rogers, Shanthi Kappagoda

- PMID: **32407284**
- PMCID: PMC7392471
- DOI: 10.3201/eid2608.201776

Free PMC article
Observational Study

Characteristics and Outcomes of Coronavirus Disease Patients under Nonsurge Conditions, Northern California, USA, March-April 2020

Jessica Ferguson et al. Emerg Infect Dis. 2020 Aug.
Free PMC article
Show details
Emerg Infect Dis
. 2020 Aug;26(8):1679-1685.
doi: 10.3201/eid2608.201776. Epub 2020 May 14.

Authors

Jessica Ferguson, Joelle I Rosser, Orlando Quintero, Jake Scott, Aruna Subramanian, Mohammad Gumma, Angela Rogers, Shanthi Kappagoda

- PMID: **32407284**
- PMCID: PMC7392471
- DOI: 10.3201/eid2608.201776

Abstract

Limited data are available on the clinical presentation and outcomes of coronavirus disease (COVID-19) patients in the United States hospitalized under normal-caseload or nonsurge conditions. We retrospectively studied 72 consecutive adult patients hospitalized with COVID-19 in 2 hospitals in the San Francisco Bay area, California, USA, during March 13-April 11, 2020. The death rate for all hospitalized COVID-19 patients was 8.3%, and median length of hospitalization was 7.5 days. Of the 21 (29% of total) intensive care unit patients, 3 (14.3% died); median length of intensive care unit stay was 12 days. Of the 72 patients, 43 (59.7%) had underlying cardiovascular disease and 19 (26.4%) had underlying pulmonary disease. In this

study, death rates were lower than those reported from regions of the United States experiencing a high volume of COVID-19 patients.

Keywords: COVID-19; California; Coronavirus diseases; ICU; SARS-CoV-2; United States; coronavirus disease; hospitalization; mortality; outcomes; pneumonia; respiratory diseases; severe acute respiratory syndrome coronavirus 2; viruses; zoonoses.

- 25 references

Supplementary info

Publication types, MeSH terms, Substances, Grant support Expand

Publication types

- Observational Study
- Research Support, N.I.H., Extramural

MeSH terms

- Adenosine Monophosphate / analogs & derivatives
- Adenosine Monophosphate / therapeutic use
- Aged
- Aged, 80 and over
- Alanine / analogs & derivatives
- Alanine / therapeutic use
- Asthma / epidemiology
- Asthma / physiopathology
- Azithromycin / therapeutic use
- Betacoronavirus / pathogenicity*
- COVID-19
- COVID-19 Testing
- California / epidemiology
- Clinical Laboratory Techniques / methods
- Comorbidity
- Coronavirus Infections / diagnosis
- Coronavirus Infections / drug therapy
- Coronavirus Infections / epidemiology*
- Coronavirus Infections / mortality
- Diabetes Mellitus / epidemiology*
- Diabetes Mellitus / physiopathology
- Female
- Humans
- Hyperlipidemias / epidemiology*

- Hyperlipidemias / physiopathology
- Hypertension / epidemiology*
- Hypertension / physiopathology
- Intensive Care Units
- Length of Stay
- Male
- Pandemics*
- Pneumonia, Viral / diagnosis
- Pneumonia, Viral / drug therapy
- Pneumonia, Viral / epidemiology*
- Pneumonia, Viral / mortality
- Pulmonary Disease, Chronic Obstructive / epidemiology
- Pulmonary Disease, Chronic Obstructive / physiopathology
- Retrospective Studies
- Reverse Transcriptase Polymerase Chain Reaction
- SARS-CoV-2
- Severity of Illness Index
- Survival Analysis
- Tomography, X-Ray Computed

Substances

- remdesivir
- Adenosine Monophosphate
- Azithromycin
- Alanine

Grant support

- T32 AI052073/AI/NIAID NIH HHS/United States

Full text links

EMERGING INFECTIOUS DISEASES CDC-NCEZID Free PMC article
Proceed to details
Cite
Share
☐ 909
Observational Study
Sci Rep
. 2021 Aug 16;11(1):16529.
doi: 10.1038/s41598-021-96134-9.

First trimester pregnancy outcomes in a large IVF center from the Lombardy County (Italy) during the peak COVID-19 pandemic

P E Levi- Setti [1, 2], F Cirillo [3], V Immediata [3], E Morenghi [4], V Canevisio [3], C Ronchetti [3], A Baggiani [3], E Albani [3], P Patrizio [5]
Affiliations Expand

Affiliations

- [1] Department of Gynecology, Division of Gynecology and Reproductive Medicine, Fertility Center, Humanitas Research Hospital, IRCCS, Via Manzoni 56, 20089, Rozzano, Milan, Italy. paolo.levi_setti@humanitas.it.
- [2] Department of Biomedical Sciences, Humanitas University, Via Rita Levi Montalcini 4, 20090, Pieve Emanuele, Milan, Italy. paolo.levi_setti@humanitas.it.
- [3] Department of Gynecology, Division of Gynecology and Reproductive Medicine, Fertility Center, Humanitas Research Hospital, IRCCS, Via Manzoni 56, 20089, Rozzano, Milan, Italy.
- [4] Biostatistics Unit, Humanitas Research Hospital, IRCCS, Rozzano, Milan, Italy.
- [5] Department of Obstetrics, Gynecology and Reproductive Sciences, School of Medicine, Yale University, New Haven, CT, USA.

- PMID: **34400730**
- PMCID: PMC8368203
- DOI: 10.1038/s41598-021-96134-9

Free PMC article
Observational Study

First trimester pregnancy outcomes in a large IVF center from the Lombardy County (Italy) during the peak COVID-19 pandemic

P E Levi- Setti et al. Sci Rep. 2021.
Free PMC article
Show details
Sci Rep
. 2021 Aug 16;11(1):16529.
doi: 10.1038/s41598-021-96134-9.

Authors

P E Levi- Setti [1, 2], F Cirillo [3], V Immediata [3], E Morenghi [4], V Canevisio [3], C Ronchetti [3], A Baggiani [3], E Albani [3], P Patrizio [5]

Affiliations

- [1] Department of Gynecology, Division of Gynecology and Reproductive Medicine, Fertility Center, Humanitas Research Hospital, IRCCS, Via Manzoni 56, 20089, Rozzano, Milan, Italy. paolo.levi_setti@humanitas.it.
- [2] Department of Biomedical Sciences, Humanitas University, Via Rita Levi Montalcini 4, 20090, Pieve Emanuele, Milan, Italy. paolo.levi_setti@humanitas.it.
- [3] Department of Gynecology, Division of Gynecology and Reproductive Medicine, Fertility Center, Humanitas Research Hospital, IRCCS, Via Manzoni 56, 20089, Rozzano, Milan, Italy.
- [4] Biostatistics Unit, Humanitas Research Hospital, IRCCS, Rozzano, Milan, Italy.
- [5] Department of Obstetrics, Gynecology and Reproductive Sciences, School of Medicine, Yale University, New Haven, CT, USA.

- PMID: **34400730**
- PMCID: PMC8368203
- DOI: 10.1038/s41598-021-96134-9

Abstract

At the beginning of 2020, the Italian Lombardy region was hit by an "epidemic tsunami" which was, at that point in time, one of the worst pandemics ever. At that moment the effects of SARS-COV 2 were still unknown. To evaluate whether the pandemic has influenced ART (Assisted Reproduction Techniques) outcomes in an asymptomatic infertile population treated at one of the major COVID-19 epicentres during the weeks immediately preceding lockdown. All ART procedures performed during two time periods were compared: November 1st, 2018 to February 28th, 2019 (non-COVID-19 risk) and November 1st, 2019 to February 29th, 2020 (COVID-19 risk). In total 1749 fresh cycles (883 non-COVID-19 risk and 866 COVID-19 risk) and1166 embryos and 63 oocytes warming cycles (538 and 37 during non-COVID and 628 and 26 during COVID-19 risk, respectively) were analysed. Clinical pregnancies per cycle were not different: 370 (25.38%) in non-COVID versus 415 (27.30%) ($p = 0.237$) during COVID-19 risk. There were no differences in biochemical pregnancy rates 52 (3.57%) versus 38 (2.50%) ($p = 0.089$) nor in ectopic pregnancies 4 (1.08%) versus 3 (0.72%) ($p = 0.594$), spontaneous miscarriages 84 (22.70%) versus 103 (24.82%) $p = 0.487$, nor in intrauterine ongoing pregnancies 282 (76.22%) versus 309 (74.46%) $p = 0.569$. A multivariate analysis investigating differences in spontaneous miscarriage rate showed no differences between the two timeframes. Our results support no differences in asymptomatic infertile couples' ART outcomes between the pre COVID and COVID-19 periods in one of the earliest and most severe pandemic areas.

© 2021. The Author(s).

Conflict of interest statement

The authors declare no competing interests.

- 43 references

Supplementary info

Publication types, MeSH terms Expand

Publication types

- Comparative Study
- Observational Study

MeSH terms

- Abortion, Spontaneous / epidemiology*
- Adult
- Asymptomatic Infections / epidemiology
- COVID-19 / complications*
- COVID-19 / epidemiology
- COVID-19 / prevention & control
- Communicable Disease Control / standards
- Female
- Humans
- Infertility / therapy*
- Italy / epidemiology
- Male
- Pandemics
- Pregnancy
- Pregnancy Rate*
- Pregnancy Trimester, First
- Reproductive Techniques, Assisted / standards
- Reproductive Techniques, Assisted / statistics & numerical data*
- Retrospective Studies
- Treatment Outcome

Full text links

npg nature publishing group Nature Publishing Group Free PMC article

Proceed to details

Cite

Share

☐ 910

Ann Vasc Surg

. 2022 Mar;80:104-112.
doi: 10.1016/j.avsg.2021.10.021. Epub 2021 Nov 12.

The Impact of the COVID-19 Pandemic on the Workload, Case Mix and hospital Resources at a Tertiary Vascular Unit

Mustafa Musajee [1], Lukla Biasi [2], Narayanan Thulasidasan [2], Meryl Green [2], Federica Francia [2], Martin Arissol [3], Alpa Lakhani [3], Stephen Thomas [3], Sanjay Patel [2], Hany Zayed [2]
Affiliations Expand

Affiliations

- [1] Department of Vascular Surgery, St Thomas Hospital, Guys and St Thomas NHS Foundation trust, London, United Kingdom. Electronic address: mustafa.musajee@gstt.nhs.uk.
- [2] Department of Vascular Surgery, St Thomas Hospital, Guys and St Thomas NHS Foundation trust, London, United Kingdom.
- [3] Diabetic Foot Care, Guys and St. Thomas NHS Foundation Trust, London, United Kingdom.

- PMID: **34775023**
- PMCID: PMC8585553
- DOI: 10.1016/j.avsg.2021.10.021

Free PMC article

The Impact of the COVID-19 Pandemic on the Workload, Case Mix and hospital Resources at a Tertiary Vascular Unit

Mustafa Musajee et al. Ann Vasc Surg. 2022 Mar.
Free PMC article
Show details
Ann Vasc Surg
. 2022 Mar;80:104-112.
doi: 10.1016/j.avsg.2021.10.021. Epub 2021 Nov 12.

Authors

Mustafa Musajee [1], Lukla Biasi [2], Narayanan Thulasidasan [2], Meryl Green [2], Federica Francia [2], Martin Arissol [3], Alpa Lakhani [3], Stephen Thomas [3], Sanjay Patel [2], Hany Zayed [2]

Affiliations

- [1] Department of Vascular Surgery, St Thomas Hospital, Guys and St Thomas NHS Foundation trust, London, United Kingdom. Electronic address: mustafa.musajee@gstt.nhs.uk.
- [2] Department of Vascular Surgery, St Thomas Hospital, Guys and St Thomas NHS Foundation trust, London, United Kingdom.
- [3] Diabetic Foot Care, Guys and St. Thomas NHS Foundation Trust, London, United Kingdom.

- PMID: **34775023**
- PMCID: PMC8585553
- DOI: 10.1016/j.avsg.2021.10.021

Abstract

Background: The aim of this study was to examine the COVID-19 pandemic and its associated impact on the provision of vascular services, and the pattern of presentation and practice in a tertiary referral vascular unit.

Methods: This is a retrospective observational study from a prospectively maintained data-base comparing two time frames, Period 1(15th March-30th May 2019-P1) and Period 2(15th March-30th May 2020-P2)All the patients who presented for a vascular review in the 2 timeframes were included. Metrics of service and patient care episodes were collected and compared including, the number of emergency referrals, patient encounters, consultations, emergency admissions and interventions. Impact on key hospital resources such as critical care and imaging facilities during the two time periods were also examined.

Results: There was an absolute reduction of 44% in the number of patients who required urgent or emergency treatment from P1 to P2 (141 vs 79). We noted a non-significant trend towards an increase in the proportion of patients presenting with Chronic Limb Threatening Ischaemia (CLTI) Rutherford 5&6 (P=0.09) as well as a reduction in the proportion of admissions related to Aortic Aneurysm (P=0.21). There was a significant absolute reduction of 77% in all vascular interventions from P1 to P2 with the greatest reductions noted in Carotid (P=0.02), Deep Venous (P=0.003) and Aortic interventions (P=0.016). The number of lower limb interventions also decreased though there was a significant increase as a relative proportion of all vascular interventions in P2 (P=0.001). There was an absolute reduction in the number of scans performed for vascular pathology; Duplex scans reduced by 86%(P<0.002), CT scans by 68%(P<0.003) and MRIs by 74%(P<0.009).

Conclusion: We report a decrease in urgent and emergency vascular presentations, admissions and interventions. The reduction in patients presenting with lower limb pathology was not as significant as other vascular conditions, resulting in a significant rise in interventions for CLTI and DFI as a proportion of all vascular interventions. These observations will help guide the provision of vascular services during future pandemics.

Copyright © 2021 Elsevier Inc. All rights reserved.

- 26 references

Supplementary info

MeSH terms Expand

MeSH terms

- Ambulatory Care / statistics & numerical data
- COVID-19 / complications
- COVID-19 / epidemiology*
- COVID-19 / therapy
- Critical Care / statistics & numerical data
- Facilities and Services Utilization
- Hospital Units / statistics & numerical data*
- Hospitalization / statistics & numerical data*
- Humans
- Magnetic Resonance Imaging / statistics & numerical data
- Practice Patterns, Physicians' / statistics & numerical data
- Tertiary Healthcare / statistics & numerical data*
- Tomography, X-Ray Computed / statistics & numerical data
- United Kingdom
- Vascular Surgical Procedures / statistics & numerical data*
- Workload / statistics & numerical data*

Full text links

ELSEVIER FULL-TEXT ARTICLE Elsevier Science Free PMC article

Proceed to details

Cite

Share

911

Observational Study

Crit Care Med

. 2021 Jul 1;49(7):e663-e672.

doi: 10.1097/CCM.0000000000004971.

A Comparison of Thrombosis and Hemorrhage Rates in Patients With Severe Respiratory Failure Due to Coronavirus Disease 2019 and Influenza Requiring Extracorporeal Membrane Oxygenation

Andrew J Doyle [1], Beverley J Hunt [1], Barnaby Sanderson [2], Joseph Zhang [2], Sze M Mak [3], Guilia Benedetti [3], Karen A Breen [1], Luigi Camporota [2], Nicholas A Barrett [2] [4], Andrew Retter [2]

Affiliations Expand

Affiliations

- [1] Centre for Thrombosis and Haemostasis, Department of Haematology, Guy's & St Thomas' NHS Foundation Trust, London, United Kingdom.
- [2] Department of Critical Care, Guy's & St Thomas' NHS Foundation Trust, London, United Kingdom.
- [3] Department of Radiology, Guy's & St Thomas' NHS Foundation Trust, London, United Kingdom.
- [4] Centre from Human & Applied Physiological Sciences, King's College London, London, United Kingdom.

- PMID: **33861545**
- DOI: 10.1097/CCM.0000000000004971

Observational Study

A Comparison of Thrombosis and Hemorrhage Rates in Patients With Severe Respiratory Failure Due to Coronavirus Disease 2019 and Influenza Requiring Extracorporeal Membrane Oxygenation

Andrew J Doyle et al. Crit Care Med. 2021.

Show details

Crit Care Med

. 2021 Jul 1;49(7):e663-e672.

doi: 10.1097/CCM.0000000000004971.

Authors

Andrew J Doyle [1], Beverley J Hunt [1], Barnaby Sanderson [2], Joseph Zhang [2], Sze M Mak [3], Guilia Benedetti [3], Karen A Breen [1], Luigi Camporota [2], Nicholas A Barrett [2] [4], Andrew Retter [2]

Affiliations

- [1] Centre for Thrombosis and Haemostasis, Department of Haematology, Guy's & St Thomas' NHS Foundation Trust, London, United Kingdom.
- [2] Department of Critical Care, Guy's & St Thomas' NHS Foundation Trust, London, United Kingdom.
- [3] Department of Radiology, Guy's & St Thomas' NHS Foundation Trust, London, United Kingdom.

- [4] Centre from Human & Applied Physiological Sciences, King's College London, London, United Kingdom.

- PMID: **33861545**
- DOI: 10.1097/CCM.0000000000004971

Abstract

Objectives: Extracorporeal membrane oxygenation is a lifesaving therapy for patients with severe acute respiratory distress syndrome refractory to conventional mechanical ventilation. It is frequently complicated by both thrombosis and hemorrhage. A markedly prothrombotic state associated with high rates of venous thromboembolism has been described in patients with severe acute respiratory syndrome coronavirus 2 (coronavirus disease 2019) infection. These rates have currently not been described during extracorporeal membrane oxygenation in comparison to other viral pneumonias.

Design: Retrospective observational study.

Setting: Single high-volume tertiary critical care department at a university hospital.

Patients: Patients 16 years old or greater receiving venovenous extracorporeal membrane oxygenation between March 1, 2020, and May 31, 2020, with coronavirus disease 2019 were compared with a cohort of patients with influenza pneumonia between June 1, 2012, and May 31, 2020.

Interventions: None.

Measurements and main results: The rates of venous thromboembolism and hemorrhage were compared in patients with coronavirus disease 2019 against a historic population of patients with influenza pneumonia who required extracorporeal membrane oxygenation. There were 51 patients who received extracorporeal membrane oxygenation due to coronavirus disease 2019 and 80 patients with influenza. At cannulation for extracorporeal membrane oxygenation, 37% of patients with coronavirus disease 2019 compared with 8% of patients with influenza had filling defects on CT pulmonary angiography ($p = 0.0001$). Catheter-associated deep vein thrombosis shown on ultrasound Doppler after decannulation was present in 53% with coronavirus disease 2019 versus 25% with influenza ($p = 0.01$). The rates of intracranial hemorrhage at the time of cannulation were 16% with coronavirus disease 2019 and 14% with influenza ($p = 0.8$). Elevated d-dimer levels were seen in both conditions and were significantly higher in those with pulmonary thromboembolism than those without in coronavirus disease 2019 ($p = 0.02$). Fibrinogen and C-reactive protein levels were significantly higher in those with coronavirus disease 2019 than influenza ($p < 0.01$).

Conclusions: Significant rates of pulmonary thromboembolism and of catheter-associated deep vein thrombosis were seen in both viral infections but were greater in those requiring the use of extracorporeal membrane oxygenation in coronavirus disease 2019 than for influenza.

Copyright © 2021 by the Society of Critical Care Medicine and Wolters Kluwer Health, Inc. All Rights Reserved.

Conflict of interest statement

Dr. Hunt disclosed that she is the Medical Director of Thrombosis UK. Dr. Barrett received research and educational support from Maquet and ALung Incorporated. The remaining authors have disclosed that they do not have any potential conflicts of interest.

Comment in

- The Janus Face of Coronavirus Disease 2019-Associated Coagulopathy.
  Stahl K, Seeliger B, Hofmaenner DA, Doebler M, Bode C, David S. Stahl K, et al. Crit Care Med. 2021 Oct 1;49(10):e1049-e1050. doi: 10.1097/CCM.0000000000005128. Crit Care Med. 2021. PMID: 34048369 Free PMC article. No abstract available.
- The authors reply.
  Doyle AJ, Retter A. Doyle AJ, et al. Crit Care Med. 2021 Oct 1;49(10):e1050-e1051. doi: 10.1097/CCM.0000000000005199. Crit Care Med. 2021. PMID: 34166291 Free PMC article. No abstract available.

- 36 references

Supplementary info

Publication types, MeSH terms, Substances Expand

Publication types

- Comparative Study
- Observational Study
- Research Support, Non-U.S. Gov't

MeSH terms

- Adult
- C-Reactive Protein / metabolism
- COVID-19 / therapy*
- Computed Tomography Angiography
- Extracorporeal Membrane Oxygenation*
- Female
- Fibrin Fibrinogen Degradation Products / metabolism
- Fibrinogen / metabolism
- Humans
- Influenza A Virus, H1N1 Subtype
- Influenza A virus
- Influenza B virus
- Influenza, Human / therapy*
- Intracranial Hemorrhages / complications*
- London / epidemiology
- Male

- Middle Aged
- Pulmonary Embolism / complications*
- Retrospective Studies
- SARS-CoV-2
- State Medicine
- Tertiary Care Centers
- Ultrasonography, Doppler
- Venous Thromboembolism / complications*
- Venous Thrombosis / complications*

Substances

- Fibrin Fibrinogen Degradation Products
- fibrin fragment D
- Fibrinogen
- C-Reactive Protein

Full text links

Wolters Kluwer Wolters Kluwer
Proceed to details
Cite
Share
☐ 912
Observational Study
J Thorac Cardiovasc Surg
. 2021 May;161(5):1605-1614.e4.
doi: 10.1016/j.jtcvs.2020.11.074. Epub 2020 Dec 1.

Impact of early Coronavirus Disease 2019 pandemic on pediatric cardiac surgery in China

Guocheng Shi [1], Jihong Huang [1], Mingan Pi [2], Xinxin Chen [3], Xiaofeng Li [4], Yiqun Ding [5], Hao Zhang [6], National Association of Pediatric Cardiology and Cardiac Surgery Working Group
Collaborators, Affiliations Expand

Collaborators

- **National Association of Pediatric Cardiology and Cardiac Surgery Working Group**:

Dongyu Xiao [7], Guojin Huang [8], Teng Ming [8], Peng Huang [7], Caixia Liu [9], Qifeng Zhao [10], Hai Tian [11], Renwei Chen [12], Jianjun Guo [13], Jian Tang [14], WeinMin Chen [15], Huiwen Chen [1]

Affiliations

- [1] Department of Cardiothoracic Surgery, Heart Center and Shanghai Institution of Pediatric Congenital Heart Diseases, Shanghai Children's Medical Center, National Children's Medical Center, Shanghai Jiao Tong University School of Medicine, Shanghai, China; Clinical Research Center, Shanghai Children's Medical Center, National Children's Medical Center, Shanghai Jiao Tong University School of Medicine, Shanghai, China.
- [2] Department of Cardiothoracic Surgery, Wuhan Children's Hospital, Wuhan, Hubei, China.
- [3] Department of Cardiothoracic Surgery, Guangzhou Woman and Children's Hospital, Guangzhou, Guangdong, China.
- [4] Department of Cardiac Surgery, Beijing Children's Hospital, National Center for Children Health, Beijing, China.
- [5] Department of Cardiothoracic Surgery, Shenzhen Children's Hospital, Shenzhen, Guangdong, China. Electronic address: yiqun.ding@139.com.
- [6] Department of Cardiothoracic Surgery, Heart Center and Shanghai Institution of Pediatric Congenital Heart Diseases, Shanghai Children's Medical Center, National Children's Medical Center, Shanghai Jiao Tong University School of Medicine, Shanghai, China. Electronic address: drzhanghao@yahoo.com.
- [7] Department of Cardiothoracic Surgery, Hunan Children's Hospital, Changsha, China.
- [8] Department of Cardiothoracic Surgery, Jiangxi Children's Hospital, Nanchang, Jiangxi, China.
- [9] Department of Cardiothoracic Surgery, Shanxi Children's Hospital, Taiyuan, Shanxi, China.
- [10] Department of Cardiothoracic Surgery, Yuying Children's Hospital, Wenzhou Medical University, Wenzhou, Zhejiang, China.
- [11] Department of Cardiac Surgery, 2nd Affiliated Hospital, Harbin Medical University, Harbin, Heilongjiang, China.
- [12] Heart Center, Hainan Women and Children Medical Center, Haikou, Hainan, China.
- [13] Department of Cardiothoracic Surgery, Xi'an Children's Hospital, Xi'an, Shaanxi, China.
- [14] Department of Cardiac Surgery, Yan'an Hospital, Kunming, Yunnan, China.
- [15] Department of Cardiothoracic Surgery, Heart Center and Shanghai Institution of Pediatric Congenital Heart Diseases, Shanghai Children's Medical Center, National Children's Medical Center, Shanghai Jiao Tong University School of Medicine, Shanghai, China.

- PMID: **33419537**
- PMCID: PMC7704339
- DOI: 10.1016/j.jtcvs.2020.11.074

Free PMC article
Observational Study

Impact of early Coronavirus Disease 2019 pandemic on pediatric cardiac surgery in China

Guocheng Shi et al. J Thorac Cardiovasc Surg. 2021 May.
Free PMC article
Show details
J Thorac Cardiovasc Surg
. 2021 May;161(5):1605-1614.e4.
doi: 10.1016/j.jtcvs.2020.11.074. Epub 2020 Dec 1.

Authors

Guocheng Shi [1], Jihong Huang [1], Mingan Pi [2], Xinxin Chen [3], Xiaofeng Li [4], Yiqun Ding [5], Hao Zhang [6], National Association of Pediatric Cardiology and Cardiac Surgery Working Group

Collaborators

- **National Association of Pediatric Cardiology and Cardiac Surgery Working Group**: Dongyu Xiao [7], Guojin Huang [8], Teng Ming [8], Peng Huang [7], Caixia Liu [9], Qifeng Zhao [10], Hai Tian [11], Renwei Chen [12], Jianjun Guo [13], Jian Tang [14], WeinMin Chen [15], Huiwen Chen [1]

Affiliations

- [1] Department of Cardiothoracic Surgery, Heart Center and Shanghai Institution of Pediatric Congenital Heart Diseases, Shanghai Children's Medical Center, National Children's Medical Center, Shanghai Jiao Tong University School of Medicine, Shanghai, China; Clinical Research Center, Shanghai Children's Medical Center, National Children's Medical Center, Shanghai Jiao Tong University School of Medicine, Shanghai, China.
- [2] Department of Cardiothoracic Surgery, Wuhan Children's Hospital, Wuhan, Hubei, China.
- [3] Department of Cardiothoracic Surgery, Guangzhou Woman and Children's Hospital, Guangzhou, Guangdong, China.
- [4] Department of Cardiac Surgery, Beijing Children's Hospital, National Center for Children Health, Beijing, China.
- [5] Department of Cardiothoracic Surgery, Shenzhen Children's Hospital, Shenzhen, Guangdong, China. Electronic address: yiqun.ding@139.com.
- [6] Department of Cardiothoracic Surgery, Heart Center and Shanghai Institution of Pediatric Congenital Heart Diseases, Shanghai Children's Medical Center, National Children's Medical Center, Shanghai Jiao Tong University School of Medicine, Shanghai, China. Electronic address: drzhanghao@yahoo.com.
- [7] Department of Cardiothoracic Surgery, Hunan Children's Hospital, Changsha, China.
- [8] Department of Cardiothoracic Surgery, Jiangxi Children's Hospital, Nanchang, Jiangxi, China.

- [9] Department of Cardiothoracic Surgery, Shanxi Children's Hospital, Taiyuan, Shanxi, China.
- [10] Department of Cardiothoracic Surgery, Yuying Children's Hospital, Wenzhou Medical University, Wenzhou, Zhejiang, China.
- [11] Department of Cardiac Surgery, 2nd Affiliated Hospital, Harbin Medical University, Harbin, Heilongjiang, China.
- [12] Heart Center, Hainan Women and Children Medical Center, Haikou, Hainan, China.
- [13] Department of Cardiothoracic Surgery, Xi'an Children's Hospital, Xi'an, Shaanxi, China.
- [14] Department of Cardiac Surgery, Yan'an Hospital, Kunming, Yunnan, China.
- [15] Department of Cardiothoracic Surgery, Heart Center and Shanghai Institution of Pediatric Congenital Heart Diseases, Shanghai Children's Medical Center, National Children's Medical Center, Shanghai Jiao Tong University School of Medicine, Shanghai, China.

- PMID: **33419537**
- PMCID: PMC7704339
- DOI: 10.1016/j.jtcvs.2020.11.074

Abstract

Objective: This study aimed to provide an insight into the impact of the early outbreak of the novel Coronavirus Disease 2019 on the care management for patients with congenital heart disease.

Methods: This study respectively enrolled a cohort of surgical patients who underwent surgery in 2018 (group I), 2019 (group II), and 2020 (group III) and a cohort of follow-up patients who had follow-up in 2017 (group A), 2018 (group B), and 2019 (group C) in 13 children hospitals.

Results: During the Coronavirus Disease 2019 era, there was a significant decrease in total surgical volume and a change in case mix in terms of an increase in the proportion of emergency operations. Decrease in migration scale index was correlated to the decrease in both surgical volume (r = 0.64, P = .02) and outpatient visit volume (r = 0.61, P = .03). There was a significantly higher proportion of patients who had follow-up through the internet or phone in group C (26.4% vs 9.6% in group B and 8.9% in group A; P < .0001). There was no statistical difference in death or rehospitalization among the 3 follow-up groups (P = .49). There was higher parents' anxiety score (P < .0001) and more use of telemedicine (P = .004) in group C compared with groups A and B.

Conclusions: The Coronavirus Disease 2019 pandemic has resulted in a considerable decrease in total surgical volume and a change of case mix, which seems to be related to the strict traffic ban. Follow-up through the online medical service appears to be an effective alternative to the conventional method.

Keywords: COVID-19; congenital heart disease; follow-up; surgery.

Copyright © 2020 The American Association for Thoracic Surgery. Published by Elsevier Inc. All rights reserved.

Comment in

- Commentary: Are these times still unprecedented?

Chen JM. Chen JM. J Thorac Cardiovasc Surg. 2021 May;161(5):1615-1616. doi: 10.1016/j.jtcvs.2020.11.088. Epub 2020 Nov 28. J Thorac Cardiovasc Surg. 2021. PMID: 33353747 No abstract available.

- Commentary: Lights and shadows of pediatric cardiac surgery in China during the coronavirus disease 2019 pandemic.
  Luciani GB. Luciani GB. J Thorac Cardiovasc Surg. 2021 May;161(5):1616-1617. doi: 10.1016/j.jtcvs.2020.11.089. Epub 2020 Nov 28. J Thorac Cardiovasc Surg. 2021. PMID: 33353749 No abstract available.

- 34 references
- 8 figures

Supplementary info

Publication types, MeSH terms Expand

Publication types

- Multicenter Study
- Observational Study
- Research Support, Non-U.S. Gov't

MeSH terms

- COVID-19 / epidemiology*
- Cardiac Surgical Procedures / statistics & numerical data*
- Child
- China / epidemiology
- Disease Outbreaks / statistics & numerical data
- Heart Defects, Congenital / epidemiology
- Heart Defects, Congenital / surgery*
- Humans
- Pandemics*
- Patient Care Management / statistics & numerical data
- Retrospective Studies
- SARS-CoV-2
- Thoracic Surgery / statistics & numerical data

Full text links

ELSEVIER FULL-TEXT ARTICLE Elsevier Science Free PMC article
Proceed to details
Cite
Share
☐ 913

Observational Study

Medicina (Kaunas)
. 2021 Sep 4;57(9):931.
doi: 10.3390/medicina57090931.

Aspirin Is Related to Worse Clinical Outcomes of COVID-19

Isaac Kim [1], Siyeong Yoon [2], Minsup Kim [3], Hyunil Lee [4], Sinhyung Park [5], Wonsang Kim [3], Soonchul Lee [2]

Affiliations Expand

Affiliations

- [1] Department of General Surgery, CHA Bundang Medical Center, CHA University School of Medicine, Seongnam 13488, Korea.
- [2] Department of Orthopedic Surgery, CHA Bundang Medical Center, CHA University School of Medicine, Seongnam 13488, Korea.
- [3] inCerebro Drug Discovery Institute, Seoul Technopark, Seoul 01811, Korea.
- [4] Department of Orthopedic Surgery, Ilsan Paik Hospital, Inje University, Goyang 10380, Korea.
- [5] Department of Orthopedic Surgery, Bucheon Hospital, Soonchunhyang University, Bucheon 14584, Korea.

- PMID: **34577854**
- PMCID: PMC8465059
- DOI: 10.3390/medicina57090931

Free PMC article
Observational Study

Aspirin Is Related to Worse Clinical Outcomes of COVID-19

Isaac Kim et al. Medicina (Kaunas). 2021.
Free PMC article
Show details

Medicina (Kaunas)
. 2021 Sep 4;57(9):931.
doi: 10.3390/medicina57090931.

Authors

Isaac Kim [1], Siyeong Yoon [2], Minsup Kim [3], Hyunil Lee [4], Sinhyung Park [5], Wonsang Kim [3], Soonchul Lee [2]

Affiliations

- [1] Department of General Surgery, CHA Bundang Medical Center, CHA University School of Medicine, Seongnam 13488, Korea.
- [2] Department of Orthopedic Surgery, CHA Bundang Medical Center, CHA University School of Medicine, Seongnam 13488, Korea.
- [3] inCerebro Drug Discovery Institute, Seoul Technopark, Seoul 01811, Korea.
- [4] Department of Orthopedic Surgery, Ilsan Paik Hospital, Inje University, Goyang 10380, Korea.
- [5] Department of Orthopedic Surgery, Bucheon Hospital, Soonchunhyang University, Bucheon 14584, Korea.

- PMID: **34577854**
- PMCID: PMC8465059
- DOI: 10.3390/medicina57090931

Abstract

Backgroundand Objectives: Aspirin is used globally to reduce pain and inflammation; however, its effect in patients with coronavirus disease (COVID-19) is not fully investigated and remains controversial. We evaluated the association between aspirin and COVID-19 outcomes using nationwide data from the Korean National Health Insurance System. *Materials and Methods:* This was a retrospective observational cohort study that included 22,660 eligible patients who underwent COVID-19 testing in South Korea between 1 January-31 July 2020. We identified all aspirin users prescribed aspirin within two weeks before or after the index date. The primary outcome was positivity for the COVID-19 test, and secondary outcomes included conventional oxygen therapy, intensive care unit, mechanical ventilation, or death. We applied the propensity score matching method to reduce the possible bias originating from the differences in patients' baseline characteristics. *Results:* Of those eligible, 662 patients were prescribed aspirin. Among them, 136 patients were on aspirin within two weeks before diagnosis and 526 patients were on aspirin after diagnosis. The COVID-19 test positivity rate was not significantly different according to aspirin use. Aspirin use before COVID-19 was related to an increased death rate and aspirin use after COVID-19 was related to a higher risk of the conventional oxygen therapy. *Conclusion:* Aspirin use was associated with adverse effects in COVID-19 patients. Further studies for mechanisms are needed.

Keywords: COVID-19; aspirin; outcome.

Conflict of interest statement

The authors declare no conflict of interest.

- 29 references
- 2 figures

Supplementary info

Publication types, MeSH terms, Substances, Grant support Expand

Publication types

- Observational Study

MeSH terms

- Aspirin* / adverse effects
- COVID-19 Testing
- COVID-19*
- Cohort Studies
- Humans
- SARS-CoV-2

Substances

- Aspirin

Grant support

- No.2020R1G1A1099728/National Research Foundation of Korea

Full text links

FULL TEXT OPEN ACCESS MDPI Multidisciplinary Digital Publishing Institute (MDPI) Free PMC article
Proceed to details
Cite
Share
☐ 914
Observational Study
Semergen
. 2021 Apr;47(3):181-188.
doi: 10.1016/j.semerg.2021.01.004. Epub 2021 Mar 8.

[Evaluation of social distancing measures on the transmissibility of COVID-19 in rural areas. Retrospective logitudinal study of posible cases]

[Article in Spanish]
E Peña-Galo [1], J M Turón-Alcaine [2], E Gracia-Carrasco [3], J Alcedo-González [4]
Affiliations Expand

Affiliations

- [1] Instituto de Investigación Sanitaria (IIS) Aragón, Zaragoza, España; Equipo de Atención Primaria, Servicio Aragonés de la Salud, Calanda, Teruel, España. Electronic address: empenna@salud.aragon.es.
- [2] Equipo de Atención Primaria, Servicio Aragonés de la Salud, Andorra, Teruel, España; Equipo de dirección, Sector Sanitario de Alcañiz, Alcañiz, Teruel, España.
- [3] Equipo de Atención Primaria, Servicio Aragonés de la Salud, Andorra, Teruel, España.
- [4] Instituto de Investigación Sanitaria (IIS) Aragón, Zaragoza, España; Servicio de Aparato Digestivo, Hospital Universitario Miguel Servet, Zaragoza, España.

- PMID: **33812795**
- PMCID: PMC7938792
- DOI: 10.1016/j.semerg.2021.01.004

Free PMC article
Observational Study

[Evaluation of social distancing measures on the transmissibility of COVID-19 in rural areas. Retrospective logitudinal study of posible cases]

[Article in Spanish]
E Peña-Galo et al. Semergen. 2021 Apr.
Free PMC article

Show details

Semergen
. 2021 Apr;47(3):181-188.
doi: 10.1016/j.semerg.2021.01.004. Epub 2021 Mar 8.

Authors

E Peña-Galo [1], J M Turón-Alcaine [2], E Gracia-Carrasco [3], J Alcedo-González [4]

Affiliations

- [1] Instituto de Investigación Sanitaria (IIS) Aragón, Zaragoza, España; Equipo de Atención Primaria, Servicio Aragonés de la Salud, Calanda, Teruel, España. Electronic address: empenna@salud.aragon.es.
- [2] Equipo de Atención Primaria, Servicio Aragonés de la Salud, Andorra, Teruel, España; Equipo de dirección, Sector Sanitario de Alcañiz, Alcañiz, Teruel, España.
- [3] Equipo de Atención Primaria, Servicio Aragonés de la Salud, Andorra, Teruel, España.
- [4] Instituto de Investigación Sanitaria (IIS) Aragón, Zaragoza, España; Servicio de Aparato Digestivo, Hospital Universitario Miguel Servet, Zaragoza, España.

- PMID: **33812795**
- PMCID: PMC7938792
- DOI: 10.1016/j.semerg.2021.01.004

Abstract
in English, Spanish

Aim: To evaluate the social distance effect on the daily frequency of possible SARS-CoV-2 cases in Primary Care, in relation to the predictive model Kermack-McKendrick.

Methods: Longitudinal retrospective study in 2 rural populations of Aragon (13,579h). A time series evaluation with a t-Student analysis was carry on, during the first 70 days of the pandemic. A simple Kermack-McKendrick predictive model was compared with the possible COVID-19 cases. Complementary ANOVA analysis to assess the before-after number of daily cases, follow-up days and days from symptoms onset to first contact with Primary Health Care.

Results: Three hundred and fifty-nine cases were detected (53.4% women; 70.7% under 60). Primary Care followed 95.3% of cases. The number of cases during the first social distancing strategies was higher in comparison with the model (P=.004, P=.006 and P=.004) with a media of decreases of 6.7 possible cases by series. In relation to the lockdown period the model and cases are close (P=.608 and P=.093), with an average decrease of 1.8 cases per series. During post-containment, the number of cases per day (P<.001) and days of follow-up (P<.001) increased.

Conclusions: Social distancing and containment measures were effective in reducing the number of possible COVID-19 cases in rural areas. Primary Care followed most of the cases.

Objetivo: Evaluar el efecto del confinamiento domiciliario en la frecuencia de casos posibles por SARS-CoV-2 en Atención Primaria, respecto a la esperada según el modelo predictivo de Kermack-McKendrick.

Métodos: Estudio longitudinal retrospectivo en 2 poblaciones rurales de Aragón (13.579 h), con evaluación de series temporales, durante los primeros 70 días de la pandemia, incluyendo el inicio de un período con medidas de distanciamiento social que alcanzó el confinamiento domiciliario. Se comparó un modelo predictivo simple Kermack-McKendrick con los casos COVID-19 posibles observados en población no pediátrica, con el estadístico t de Student. Se realizó un análisis complementario ANOVA para evaluar el antes-después del número de casos diario, los días de seguimiento y los días desde el inicio de síntomas hasta el primer contacto con Atención Primaria.

Resultados: Se detectaron 359 casos (53,4% mujeres; 70,7% menores de 60 años). El 95,3% fue seguido por Atención Primaria. El número de casos durante las series correspondientes a las primeras medidas de distanciamiento social fue superior al modelo (p = 0,004, p = 0,006 y p = 0,004), pero con una diminución media de 6,7 casos posibles por serie. Durante el confinamiento domiciliario el modelo y los casos se aproximaron (p = 0,608 y p = 0,093), con una disminución media de 1,8 casos por serie. Durante el posconfinamiento se incrementaron el número de casos por día (p < 0,001) y los días de seguimiento (p < 0,001).

Conclusiones: Las medidas de distanciamiento social y confinamiento fueron efectivas en disminuir el número de casos posibles por COVID-19 en el medio rural. Atención Primaria dio seguimiento a la mayoría de los casos posibles.

Keywords: COVID-19; Distanciamiento social; Evaluación; Evaluation; Rural; SARS-CoV-2; Social distancing.

Copyright © 2021 Sociedad Española de Médicos de Atención Primaria (SEMERGEN). Publicado por Elsevier España, S.L.U. All rights reserved.

- 28 references
- 4 figures

Supplementary info

Publication types, MeSH terms Expand

Publication types

- Observational Study

MeSH terms

- Adolescent
- Adult
- Aged
- Aged, 80 and over
- COVID-19 / epidemiology
- COVID-19 / prevention & control*
- COVID-19 / transmission
- Female
- Follow-Up Studies
- Humans
- Longitudinal Studies
- Male
- Middle Aged
- Physical Distancing*
- Primary Health Care
- Retrospective Studies
- Rural Health / statistics & numerical data*
- Spain / epidemiology
- Young Adult

Full text links

ELSEVIER FULL-TEXT ARTICLE Elsevier Science Free PMC article

Proceed to details

Cite

Share

☐ 915

Observational Study

Saudi Med J
. 2022 Jan;43(1):67-74.
doi: 10.15537/smj.2022.43.1.20210694.

Impacts and effects of COVID-19 infection in pregnancy

Amala Sunder [1], Bessy Varghese [1], Basma Darwish [1], Noor Shaikho [1], Mooza Rashid [1]
Affiliations Expand

Affiliation

- [1] From the Department of Obstetrics and Gynaecology, Bahrain Defence Force Hospital, Riffa, Bahrain.

- PMID: **35022286**
- DOI: 10.15537/smj.2022.43.1.20210694

Free article
Observational Study

Impacts and effects of COVID-19 infection in pregnancy

Amala Sunder et al. Saudi Med J. 2022 Jan.
Free article
Show details
Saudi Med J
. 2022 Jan;43(1):67-74.
doi: 10.15537/smj.2022.43.1.20210694.

Authors

Amala Sunder [1], Bessy Varghese [1], Basma Darwish [1], Noor Shaikho [1], Mooza Rashid [1]

Affiliation

- [1] From the Department of Obstetrics and Gynaecology, Bahrain Defence Force Hospital, Riffa, Bahrain.

- PMID: **35022286**
- DOI: 10.15537/smj.2022.43.1.20210694

Abstract

Objectives: To explore the trimester wise significance of the primary outcome in pregnant women during coronavirus disease-19 (COVID-19) pandemic.

Methods: Retrospective observational study of pregnant women who were infected with COVID-19 from April 2020 until March 2021 at Bahrain Defense Force Hospital, Riffa, Bahrain. The study focused on the effects in relation to gestational age (GA), association with variables, severity, and treatment. A p-value of ≤ 0.05 was considered significant.

Results: During the study period, 74 COVID-19 cases were identified from the recorded 2944 pregnant women. The mean GA at diagnosis was 33.5±12.2 weeks, and the mean GA at birth was 38.4±1.8 weeks. Analysis of the obstetric complications revealed fetal growth restriction (FGR) had a p-value of <0.001. According to the trimester wise analysis, between the gestational period at diagnosis and the outcome of pregnancy, significant p-value of <0.01 was found in miscarriage. There were no significant associations found in GA at diagnosis and delivery, complications in relation to maternal age and body mass index, and no maternal morbidities or mortalities.

Conclusion: In our study, FGR and miscarriage were the identified complications. However, the maternal and neonatal end result of COVID-19 was satisfactory.

Keywords: fetal growth restriction; gestational age; miscarriage; mortality; stillborn.

Copyright: © Saudi Medical Journal.

Supplementary info

Publication types, MeSH terms Expand

Publication types

- Observational Study

MeSH terms

- COVID-19*
- Female
- Humans
- Infant, Newborn
- Pandemics
- Pregnancy
- Pregnancy Complications, Infectious* / epidemiology
- Pregnancy Outcome
- SARS-CoV-2

Full text links

SAUDI MED J FREE FULL TEXT Saudi Medical Journal

Proceed to details

Cite

Share

☐ 916

Observational Study

Cardiovasc Revasc Med

. 2022 Jan;34:80-85.

doi: 10.1016/j.carrev.2021.01.026. Epub 2021 Jan 26.

Initial Single-Center ST-Segment Elevation Myocardial Infarction Experience in New York Before and During the COVID-19 Pandemic

Giorgio A Medranda [1], Kunal Brahmbhatt [2], Basem Alawneh [2], Kevin P Marzo [2], Richard K Schwartz [2], Stephen J Green [3]

Affiliations Expand

Affiliations

- [1] Section of Interventional Cardiology, MedStar Washington Hospital Center, Washington, DC, United States of America.
- [2] Division of Cardiology, NYU Winthrop Hospital, Mineola, NY, United States of America.
- [3] Division of Cardiology, NYU Winthrop Hospital, Mineola, NY, United States of America. Electronic address: stephen.green@nyulangone.org.

- PMID: **33526393**
- PMCID: PMC7837613
- DOI: 10.1016/j.carrev.2021.01.026

Free PMC article

Observational Study

Initial Single-Center ST-Segment Elevation Myocardial Infarction Experience in New York Before and During the COVID-19 Pandemic

Giorgio A Medranda et al. Cardiovasc Revasc Med. 2022 Jan.

Free PMC article

Show details

Cardiovasc Revasc Med

. 2022 Jan;34:80-85.

doi: 10.1016/j.carrev.2021.01.026. Epub 2021 Jan 26.

Authors

Giorgio A Medranda [1], Kunal Brahmbhatt [2], Basem Alawneh [2], Kevin P Marzo [2], Richard K Schwartz [2], Stephen J Green [3]

Affiliations

- [1] Section of Interventional Cardiology, MedStar Washington Hospital Center, Washington, DC, United States of America.
- [2] Division of Cardiology, NYU Winthrop Hospital, Mineola, NY, United States of America.
- [3] Division of Cardiology, NYU Winthrop Hospital, Mineola, NY, United States of America. Electronic address: stephen.green@nyulangone.org.

- PMID: **33526393**
- PMCID: PMC7837613
- DOI: 10.1016/j.carrev.2021.01.026

Abstract

Background/purpose: Severe acute respiratory syndrome coronavirus 2 (SARS-CoV-2) has emerged as a highly contagious and lethal virus, devastating healthcare systems throughout the world. Following a period of stability, the coronavirus disease 2019 (COVID-19) pandemic appears to be re-intensifying globally. As the virus continues to evolve, so does our understanding of its implications on ST-segment elevation myocardial infarction (STEMI). We sought to describe a single center STEMI experience at one of the epicenters during the COVID-19 pandemic.

Methods/materials: We conducted a retrospective, observational study comparing STEMI patients during the pandemic period (March 1 to August 31, 2020) to those with STEMI during the pre-pandemic period (March 1 to August 31, 2019) at NYU Langone Hospital - Long Island, a tertiary-care center in Nassau County, New York. Additionally, we describe our subset of COVID-19 patients with STEMI during the pandemic.

Results: The acute myocardial infarction (AMI) team was activated for 183 patients during both periods. There were a similar number of AMI team activations during the pandemic period (n = 93) compared to the pre-pandemic period (n = 90). Baseline characteristics did not differ during both periods; however, infection control measures and additional investigation were required to clarify the diagnosis during the pandemic, resulting in a signal toward longer door-to-balloon times (95.9 min vs. 74.4 min, p = 0.0587). We observed similar inpatient length of stay (LOS) (3.6 days vs. 5.0 days, p = 0.0901) and mortality (13.2% vs. 9.2%, p = 0.5876). There were 6 COVID-19-positive patients who presented with STEMI, of whom 4 were emergently taken to the cardiac catheterization laboratory with successful percutaneous coronary intervention (PCI) performed in 3 patients. The 2 patients who were not offered primary PCI expired, as both were treated medically, one with thrombolytics.

Conclusions: Our single-center study, in New York, at one of the epicenters of the pandemic, demonstrated a similar number of AMI team activations, mimicking the seasonal variability seen in 2019, but with a signal toward longer door-to-balloon time. Despite this, inpatient LOS and mortality remained similar.

Keywords: COVID-19; STEMI.

Copyright © 2021 Elsevier Inc. All rights reserved.

- 25 references
- 2 figures

Supplementary info

Publication types, MeSH terms Expand

Publication types

- Observational Study

MeSH terms

- COVID-19*
- Humans
- Myocardial Infarction* / diagnosis
- Myocardial Infarction* / epidemiology
- Myocardial Infarction* / therapy
- New York / epidemiology
- Pandemics
- Percutaneous Coronary Intervention*
- Retrospective Studies
- SARS-CoV-2
- ST Elevation Myocardial Infarction* / diagnostic imaging
- ST Elevation Myocardial Infarction* / epidemiology

Full text links

ELSEVIER FULL-TEXT ARTICLE Elsevier Science Free PMC article
Proceed to details
Cite
Share
917
Observational Study
World J Emerg Surg
. 2021 Mar 8;16(1):9.
doi: 10.1186/s13017-021-00354-3.

Serum ferritin levels in inflammation: a retrospective comparative analysis between

COVID-19 and emergency surgical non-COVID-19 patients

Filippo Banchini [1], Gaetano Maria Cattaneo [2], Patrizio Capelli [2]
Affiliations Expand

Affiliations

- [1] Department of General Surgery, Guglielmo da Saliceto Hospital, Piacenza, Italy. filippobanchini@virgilio.it.
- [2] Department of General Surgery, Guglielmo da Saliceto Hospital, Piacenza, Italy.

- PMID: **33685484**
- PMCID: PMC7938265
- DOI: 10.1186/s13017-021-00354-3

Free PMC article
Observational Study

Serum ferritin levels in inflammation: a retrospective comparative analysis between COVID-19 and emergency surgical non-COVID-19 patients

Filippo Banchini et al. World J Emerg Surg. 2021.
Free PMC article
Show details
World J Emerg Surg
. 2021 Mar 8;16(1):9.
doi: 10.1186/s13017-021-00354-3.

Authors

Filippo Banchini [1], Gaetano Maria Cattaneo [2], Patrizio Capelli [2]

Affiliations

- [1] Department of General Surgery, Guglielmo da Saliceto Hospital, Piacenza, Italy. filippobanchini@virgilio.it.
- [2] Department of General Surgery, Guglielmo da Saliceto Hospital, Piacenza, Italy.

- PMID: **33685484**
- PMCID: PMC7938265
- DOI: 10.1186/s13017-021-00354-3

Abstract

Background: SARS-CoV-2 infection has spread worldwide, and the pathogenic mechanism is still under investigation. The presence of a huge inflammatory response, defined as "cytokine storm," is being studied in order to understand what might be the prognostic factors implicated in the progression of the infection, with ferritin being one of such markers. The role of ferritin as a marker of inflammation is already known, and whether it changes differently between COVID and non-COVID patients still remains unclear. The aim of this retrospective analysis is to understand whether the inflammatory process in these two types is different.

Methods: In this retrospective analysis, we compared 17 patients affected by SARS-CoV-2, who had been admitted between February and April 2020 (group A) along with 30 patients admitted for acute surgical disease with SARS-CoV-2 negative swab (group B). A further subgroup of Covid negative patients with leukocytosis was compared to group A.

Results: In group A, the median (interquartile range) serum ferritin was 674 (1284) ng/mL, and it was double the cutoff (300 ng/mL) in 9 out of 17 (52%). The median (IQR) value of ferritin level in the total blood samples of group B was 231, and in the subgroup with leucocytosis, 149 (145). Group A showed a significantly higher ferritin median level compared to the entire group B (two-tailed Mann-Whitney test, $p < 0.0001$) as well as to the subgroup with leucocytosis ($p < 0.0014$).

Conclusions: The role of iron metabolism appears to be directly involved in COVID infection. On the other hand, in the acute inflammation of patients admitted for surgery, and probably in other common phlogistic processes, iron modifications appear to be self-limited. However, our finding suggests the use of ferritin as a marker for COVID infection.

Keywords: COVID; Ferritin; Hepcidin; Iron; SARS-CoV-2; Sepsi; Surgery; Transferrin.

Conflict of interest statement

The authors declare that they have no competing interests.

- 31 references
- 6 figures

Supplementary info

Publication types, MeSH terms, Substances Expand

Publication types

- Comparative Study
- Observational Study

MeSH terms

- Adult
- Aged
- Aged, 80 and over

- Biomarkers / blood
- COVID-19 / blood
- COVID-19 / diagnosis*
- COVID-19 / physiopathology*
- COVID-19 / surgery
- COVID-19 Testing / methods*
- Case-Control Studies
- Emergencies
- Female
- Ferritins / blood*
- Humans
- Inflammation / blood
- Inflammation / diagnosis*
- Inflammation / virology
- Male
- Middle Aged
- Retrospective Studies
- Surgical Procedures, Operative

Substances

- Biomarkers
- Ferritins

Full text links

Read free full text at BMC BioMed Central Free PMC article

Proceed to details

Cite

Share

☐ 918

Observational Study

Int J Med Sci

. 2021 Jan 11;18(5):1198-1206.

doi: 10.7150/ijms.50039. eCollection 2021.

Risk factors for mortality of critically ill patients with COVID-19 receiving invasive ventilation

Ye Tu [1], Ping Yang [1], Yaqun Zhou [1], Xiaoyan Wen [2], Qinqin Li [1], Jing Zhou [1], Jingjing Wang [1], Jinqian Hu [1], Nannan He [1], Kai Wang [3], Chaolong Wang [3], Xuebi Tian [1], Ailin Luo [1], Feng Gao [1]
Affiliations Expand

Affiliations

- [1] Department of Anesthesiology, Tongji Hospital, Tongji Medical College, Huazhong University of Science and Technology, Wuhan, China.
- [2] Department of Clinical Laboratory, Tongji Hospital, Tongji Medical College, Huazhong University of Science and Technology, Wuhan, China.
- [3] Department of Epidemiology and Biostatistics, School of Public Health, Tongji Medical College, Huazhong University of Science and Technology, Wuhan, China.

- PMID: **33526981**
- PMCID: PMC7847616
- DOI: 10.7150/ijms.50039

Free PMC article
Observational Study

Risk factors for mortality of critically ill patients with COVID-19 receiving invasive ventilation

Ye Tu et al. Int J Med Sci. 2021.
Free PMC article
Show details
Int J Med Sci
. 2021 Jan 11;18(5):1198-1206.
doi: 10.7150/ijms.50039. eCollection 2021.

Authors

Ye Tu [1], Ping Yang [1], Yaqun Zhou [1], Xiaoyan Wen [2], Qinqin Li [1], Jing Zhou [1], Jingjing Wang [1], Jinqian Hu [1], Nannan He [1], Kai Wang [3], Chaolong Wang [3], Xuebi Tian [1], Ailin Luo [1], Feng Gao [1]

Affiliations

- [1] Department of Anesthesiology, Tongji Hospital, Tongji Medical College, Huazhong University of Science and Technology, Wuhan, China.
- [2] Department of Clinical Laboratory, Tongji Hospital, Tongji Medical College, Huazhong University of Science and Technology, Wuhan, China.
- [3] Department of Epidemiology and Biostatistics, School of Public Health, Tongji Medical College, Huazhong University of Science and Technology, Wuhan, China.

- PMID: **33526981**
- PMCID: PMC7847616
- DOI: 10.7150/ijms.50039

Abstract

Rationale: Early invasive ventilation may improve outcomes for critically ill patients with COVID-19. The objective of this study is to explore risk factors for 28-day mortality of COVID-19 patients receiving invasive ventilation. **Methods:** 74 consecutive adult invasively ventilated COVID-19 patients were included in this retrospective study. The demographic and clinical data were compared between survivors and non-survivors, and Cox regression analysis was used to explore risk factors for 28-day mortality. The primary outcome was 28-day mortality after initiation of invasive ventilation. Secondary outcome was the time from admission to intubation. **Results:** Of 74 patients with COVID-19, the median age was 68.0 years, 53 (71.6%) were male, 47 (63.5%) had comorbidities with hypertension, and diabetes commonly presented. The most frequent symptoms were fever and dyspnea. The median time from hospital admission to intubation was similar in survivors and non-survivors (6.5 days vs. 5.0 days). The 28-day mortality was 81.1%. High Sequential Organ Failure Assessment (SOFA) score (hazard ratio [HR], 1.54; 95% confidence interval [CI], 1.23-1.92; $p < 0.001$) and longer time from hospital admission to intubation (HR, 2.41; 95% CI, 1.15-5.07; $p = 0.020$) were associated with 28-day mortality in invasively ventilated COVID-19 patients. **Conclusions:** The mortality of invasively ventilated COVID-19 patients was particularly striking. Patients with high SOFA score and receiving delayed invasive ventilation were at high risk of mortality.

Keywords: COVID-19; critically ill; invasive ventilation; mortality; risk factor.

© The author(s).

Conflict of interest statement

Competing Interests: The authors have declared that no competing interest exists.

- 32 references
- 1 figure

Supplementary info

Publication types, MeSH terms Expand

Publication types

- Observational Study

MeSH terms

- Adult
- Aged
- Aged, 80 and over
- COVID-19 / mortality*

- COVID-19 / therapy
- China / epidemiology
- Critical Illness / mortality*
- Female
- Humans
- Male
- Middle Aged
- Respiration, Artificial / mortality*
- Retrospective Studies
- Risk Factors

Full text links

Ivyspring International Publisher Free PMC article
Proceed to details
Cite
Share
919
Observational Study
J Trauma Acute Care Surg
. 2021 Apr 1;90(4):708-713.
doi: 10.1097/TA.0000000000003061.

"Safer at home": The effect of the COVID-19 lockdown on epidemiology, resource utilization, and outcomes at a large urban trauma center

Hiroto Chiba [1], Meghan Lewis, Elizabeth R Benjamin, Dominik A Jakob, Panayiotis Liasidis, Monica D Wong, Sixta Navarrete, Robert Carreon, Demetrios Demetriades
Affiliations Expand

Affiliation

- [1] From the Division of Trauma and Surgical Critical Care, Department of Surgery, Los Angeles County + University of Southern California Medical Center, University of Southern California, Los Angeles, California.

- PMID: **33347094**
- PMCID: PMC7996058
- DOI: 10.1097/TA.0000000000003061

Free PMC article

Observational Study

"Safer at home": The effect of the COVID-19 lockdown on epidemiology, resource utilization, and outcomes at a large urban trauma center

Hiroto Chiba et al. J Trauma Acute Care Surg. 2021.
Free PMC article
Show details
J Trauma Acute Care Surg
. 2021 Apr 1;90(4):708-713.
doi: 10.1097/TA.0000000000003061.

Authors

Hiroto Chiba [1], Meghan Lewis, Elizabeth R Benjamin, Dominik A Jakob, Panayiotis Liasidis, Monica D Wong, Sixta Navarrete, Robert Carreon, Demetrios Demetriades

Affiliation

- [1] From the Division of Trauma and Surgical Critical Care, Department of Surgery, Los Angeles County + University of Southern California Medical Center, University of Southern California, Los Angeles, California.

- PMID: **33347094**
- PMCID: PMC7996058
- DOI: 10.1097/TA.0000000000003061

Abstract

Background: The COVID-19 pandemic has affected the entire global health care system. In California, because of a high burden of cases, a lockdown order was announced on March 19, 2020. This study investigated the impact of the lockdown on the epidemiology and outcomes of trauma admissions at the largest trauma center in Los Angeles.

Methods: A retrospective study comparing epidemiological and clinical characteristics and outcomes of trauma admissions during the lockdown period (March 20, 2020, to June 30, 2020) to a similar period in the previous year (March 20, 2019, to June 30, 2019) was performed. Data collection included demographics, mechanism of injury, prehospital transportation, substance use, injury severity, resource utilization, and outcomes.

Findings: There were 1,202 admissions during the lockdown period in 2020 and 1,143 during the same calendar period in 2019. Following the lockdown, there was a reduction in the automobile versus pedestrian admissions by 42.5%, motorcycle injuries by 38.7%, and bicycle accidents by 28.4% but no significant effect on the number of motor vehicle accident admissions. There was an increase in ground level falls by 32.5%, especially in the elderly group. The absolute number of

gunshot wounds increased by 6.2% and knife injuries by 39.3%. Suicides increased by 38.5%. Positive testing for substance use increased by 20.9%. During the lockdown, patients suffered less severe trauma, with Injury Severity Score of <9 ($p < 0.001$), as well as less severe head ($p = 0.001$) and severe chest trauma ($p < 0.001$). Trauma deaths were reduced by 27.9%, and the crude overall mortality was significantly lower during the lockdown period (4.1% vs. 5.9%, $p = 0.046$). Intensive care unit admission rates, mechanical ventilation, and intensive care unit length of stay were all reduced.

Conclusion: The COVID-19 lockdown in 2020 had a significant effect on the epidemiology, clinical characteristics, and critical care resource utilization of trauma admissions in a large academic trauma center. These findings may help in planning and optimization of hospital resources during the pandemic.

Level of evidence: Epidemiological study, level III; Retrospective observational, level III.

Copyright © 2020 Wolters Kluwer Health, Inc. All rights reserved.

- 15 references

Supplementary info

Publication types, MeSH terms Expand

Publication types

- Observational Study

MeSH terms

- Accidental Falls / statistics & numerical data
- Accidental Injuries / epidemiology*
- Accidents, Traffic / statistics & numerical data*
- Adult
- Aged
- COVID-19* / epidemiology
- COVID-19* / prevention & control
- Communicable Disease Control / methods*
- Female
- Humans
- Intensive Care Units / statistics & numerical data
- Los Angeles / epidemiology
- Male
- Mortality
- Patient Acceptance of Health Care / statistics & numerical data*
- Physical Abuse / statistics & numerical data*
- Retrospective Studies

- SARS-CoV-2
- Trauma Centers / statistics & numerical data
- Trauma Severity Indices
- Wounds, Gunshot / epidemiology

Full text links

Wolters Kluwer Wolters Kluwer Free PMC article
Proceed to details
Cite
Share
920
Observational Study
Clin Med (Lond)
. 2020 Nov;20(6):e222-e228.
doi: 10.7861/clinmed.2020-0423. Epub 2020 Sep 10.

Coronavirus in the elderly: a late lockdown UK cohort

Andrew D Kerr [1], Sybil Rl Stacpoole [2]
Affiliations Expand

Affiliations

- [1] North West Anglia NHS Foundation Trust.
- [2] North West Anglia NHS Foundation Trust sybilstacpoole@nhs.net.

- PMID: **32912964**
- PMCID: PMC7687331
- DOI: 10.7861/clinmed.2020-0423

Free PMC article
Observational Study

Coronavirus in the elderly: a late lockdown UK cohort

Andrew D Kerr et al. Clin Med (Lond). 2020 Nov.
Free PMC article
Show details
Clin Med (Lond)
. 2020 Nov;20(6):e222-e228.
doi: 10.7861/clinmed.2020-0423. Epub 2020 Sep 10.

Authors

Andrew D Kerr [1], Sybil Rl Stacpoole [2]

Affiliations

- [1] North West Anglia NHS Foundation Trust.
- [2] North West Anglia NHS Foundation Trust sybilstacpoole@nhs.net.

- PMID: **32912964**
- PMCID: PMC7687331
- DOI: 10.7861/clinmed.2020-0423

Abstract

Objective: To identify the source of ongoing coronavirus disease 2019 (COVID-19) infections after 4 weeks of lockdown and to characterise the presentation of COVID-19 in the elderly, who represent the highest risk group.

Design: Retrospective observational cohort study of 115 patients at one acute district general hospital with a catchment population of approximately 500,000 people, during weeks 5 and 6 of the UK lockdown.

Results: More than 2 in 3 of the overall cohort had had contacts with the health and social care system prior to diagnosis. This figure rose to 85% in those 70 years and over. In the older cohort, the most common reasons for presentation were shortness of breath or falls, and 1 in 3 had neither cough nor fever.

Conclusion: COVID-19 can present differently in the elderly, overlapping with many common presentations, so focusing testing on those with a cough or fever will miss at least 1 in 3 cases in those over the age of 70. A high degree of vigilance, suspicion and repeated testing is required if streaming into high and low risk areas is to succeed, allowing safe restarting of services such as elective surgery and cancer care.

Keywords: COVID-19; SARS-CoV-2; elderly.

© 2020 Royal College of Physicians 2020. All rights reserved.

- 3 figures

Supplementary info

Publication types, MeSH terms Expand

Publication types

- Observational Study

MeSH terms

- Aged
- Aged, 80 and over
- Betacoronavirus
- COVID-19
- Comorbidity
- Coronavirus Infections* / diagnosis
- Coronavirus Infections* / epidemiology
- Coronavirus Infections* / mortality
- Coronavirus Infections* / therapy
- Female
- Humans
- Male
- Middle Aged
- Pandemics*
- Pneumonia, Viral* / diagnosis
- Pneumonia, Viral* / epidemiology
- Pneumonia, Viral* / mortality
- Pneumonia, Viral* / therapy
- Retrospective Studies
- SARS-CoV-2
- Treatment Outcome
- United Kingdom / epidemiology

Full text links

Full text ClinMed HighWire Free PMC article
Proceed to details
Cite
Share
☐ 921
Observational Study
PLoS One
. 2021 Aug 24;16(8):e0254379.
doi: 10.1371/journal.pone.0254379. eCollection 2021.

Don't close the book on tocilizumab for the treatment of severe COVID-19 pneumonia-the jury is still out: The Kuwait experience

Yousef Al-Shamali [1] [2], Yaser M Ali [3], Rawan A Al-Shamali [4], Maryam Al-Melahi [5], Farah R Al-Shammari [6], Ahmad Alsaber [7], Wasl Al-Adsani [8]

Affiliations Expand

Affiliations

- [1] Department of Gastroenterology, Vancouver General Hospital, Vancouver, Canada.
- [2] Department of Gastroenterology, Jaber Al Sabah Hospital, Kuwait City, Kuwait.
- [3] Department of Rheumatology, Mubarak Al Kabeer Hospital, Jabriya, Kuwait.
- [4] Department of Ophthalmology, Kuwait Institute for Medical Specialization, Al Bahar Hospital, Kuwait.
- [5] Department of Internal Medicine, Amiri Hospital, Kuwait City, Kuwait.
- [6] Department of Internal Medicine, Jahra Hospital, Kuwait City, Kuwait.
- [7] Department of Mathematics and Statistics, University of Strathclyde, Glasgow, United Kingdom.
- [8] Department of Internal Medicine, Department of Infectious Diseases, Mubarak Al-Kabeer Hospital, Kuwait City, Kuwait.

- PMID: **34428204**
- PMCID: PMC8384154
- DOI: 10.1371/journal.pone.0254379

Free PMC article
Observational Study

Don't close the book on tocilizumab for the treatment of severe COVID-19 pneumonia-the jury is still out: The Kuwait experience

Yousef Al-Shamali et al. PLoS One. 2021.
Free PMC article
Show details
PLoS One
. 2021 Aug 24;16(8):e0254379.
doi: 10.1371/journal.pone.0254379. eCollection 2021.

Authors

Yousef Al-Shamali [1] [2], Yaser M Ali [3], Rawan A Al-Shamali [4], Maryam Al-Melahi [5], Farah R Al-Shammari [6], Ahmad Alsaber [7], Wasl Al-Adsani [8]

Affiliations

- [1] Department of Gastroenterology, Vancouver General Hospital, Vancouver, Canada.
- [2] Department of Gastroenterology, Jaber Al Sabah Hospital, Kuwait City, Kuwait.
- [3] Department of Rheumatology, Mubarak Al Kabeer Hospital, Jabriya, Kuwait.
- [4] Department of Ophthalmology, Kuwait Institute for Medical Specialization, Al Bahar Hospital, Kuwait.

- [5] Department of Internal Medicine, Amiri Hospital, Kuwait City, Kuwait.
- [6] Department of Internal Medicine, Jahra Hospital, Kuwait City, Kuwait.
- [7] Department of Mathematics and Statistics, University of Strathclyde, Glasgow, United Kingdom.
- [8] Department of Internal Medicine, Department of Infectious Diseases, Mubarak Al-Kabeer Hospital, Kuwait City, Kuwait.

- PMID: **34428204**
- PMCID: PMC8384154
- DOI: 10.1371/journal.pone.0254379

Abstract

Purpose: This cross-sectional observational study aims to report preliminary data from the first experience using tocilizumab for patients with severe acute respiratory syndrome coronavirus-2 (SARS-CoV-2) infection in three of Kuwait's largest public hospitals City.

Patients and methods: This chart review study examined the benefits of tocilizumab treatment among 127 patients diagnosed with severe coronavirus disease of 2019 (COVID-19) pneumonia.

Results: 90 of 127 patients (71%) survived. Mortality was highest in the elderly with multiple medical conditions.

Conclusion: Despite the small sample size and retrospective nature of the work, our findings are consistent with recent studies suggesting tocilizumab administration in patients presenting with severe COVID pneumonia with associated hyperinflammatory features conferred mortality benefit.

Conflict of interest statement

The authors declare that they have no known competing financial interests or personal relationships that could have appeared to influence the work reported in this paper.

- 38 references

Supplementary info

Publication types, MeSH terms, Substances, Supplementary concepts, Grant support Expand

Publication types

- Observational Study

MeSH terms

- Aged
- Antibodies, Monoclonal, Humanized / therapeutic use*
- COVID-19 / drug therapy*
- Cross-Sectional Studies

- Female
- Humans
- Kuwait / epidemiology
- Male
- Middle Aged
- Pneumonia / drug therapy*
- Retrospective Studies

Substances

- Antibodies, Monoclonal, Humanized
- tocilizumab

Supplementary concepts

- COVID-19 drug treatment

Grant support

The authors received no specific funding for this work.

Full text links

OPEN ACCESS TO FULL TEXT PLOS ONE Public Library of Science Free PMC article
Proceed to details
Cite
Share
☐ 922
Observational Study
BMC Geriatr
. 2021 Oct 29;21(1):610.
doi: 10.1186/s12877-021-02565-4.

Characteristics, hospital referrals and 60-day mortality of older patients living in nursing homes with COVID-19 assessed by a liaison geriatric team during the first wave: a research article

Lorena García-Cabrera [1], Noelia Pérez-Abascal [2], Beatriz Montero-Errasquín [2], Lourdes Rexach Cano [3], Jesús Mateos-Nozal [2], Alfonso Cruz-Jentoft [2]

Affiliations Expand

Affiliations

- [1] Unidad de Cuidados Paliativos, Hospital Universitario Ramón y Cajal (IRYCIS), Carretera de Colmenar km 9,1, 28034, Madrid, Spain. lgcabrera@salud.madrid.org.
- [2] Servicio de Geriatría. Hospital Universitario Ramón y Cajal (IRYCIS), Madrid, Spain.
- [3] Unidad de Cuidados Paliativos, Hospital Universitario Ramón y Cajal (IRYCIS), Carretera de Colmenar km 9,1, 28034, Madrid, Spain.

- PMID: **34715807**
- PMCID: PMC8553905
- DOI: 10.1186/s12877-021-02565-4

Free PMC article
Observational Study

Characteristics, hospital referrals and 60-day mortality of older patients living in nursing homes with COVID-19 assessed by a liaison geriatric team during the first wave: a research article

Lorena García-Cabrera et al. BMC Geriatr. 2021.
Free PMC article
Show details
BMC Geriatr
. 2021 Oct 29;21(1):610.
doi: 10.1186/s12877-021-02565-4.

Authors

Lorena García-Cabrera [1], Noelia Pérez-Abascal [2], Beatriz Montero-Errasquín [2], Lourdes Rexach Cano [3], Jesús Mateos-Nozal [2], Alfonso Cruz-Jentoft [2]

Affiliations

- [1] Unidad de Cuidados Paliativos, Hospital Universitario Ramón y Cajal (IRYCIS), Carretera de Colmenar km 9,1, 28034, Madrid, Spain. lgcabrera@salud.madrid.org.
- [2] Servicio de Geriatría. Hospital Universitario Ramón y Cajal (IRYCIS), Madrid, Spain.
- [3] Unidad de Cuidados Paliativos, Hospital Universitario Ramón y Cajal (IRYCIS), Carretera de Colmenar km 9,1, 28034, Madrid, Spain.

- PMID: **34715807**
- PMCID: PMC8553905

• DOI: 10.1186/s12877-021-02565-4

Abstract

Background: The infection by SARS-CoV-2 (COVID-19) has been especially serious in older patients. The aim of this study is to describe baseline and clinical characteristics, hospital referrals, 60-day mortality, factors associated with hospital referrals and mortality in older patients living in nursing homes (NH) with suspected COVID-19.

Methods: A retrospective observational study was performed during March and April 2020 of institutionalized patients assessed by a liaison geriatric hospital-based team. Were collected all older patients living in 31 nursing homes of a public hospital catchment area assessed by a liaison geriatric team due to the suspicion of COVID-19 during the first wave, when the hospital system was collapsed. Sociodemographic variables, comprehensive geriatric assessment, clinical characteristics, treatment received including care setting, and 60-days mortality were recorded from electronic medical records. A logistic regression analysis was performed to analyze the factors associated with mortality.

Results: 419 patients were included in the study (median age 89 years old, 71.6 % women, 63.7 % with moderate-severe dependence, and 43.8 % with advanced dementia). 31.1 % were referred to the emergency department in the first assessment, with a higher rate of hospital referral in those with better functional and mental status. COVID-19 atypical symptoms like functional decline, delirium, or eating disorders were frequent. 36.9% had died in the 60 days following the first call. According to multivariate logistic regression age (p 0.010), Barthel index <60 (p 0.002), presence of tachypnea (p 0.021), fever (p 0.006) and the use of ceftriaxone (p 0.004) were associated with mortality. No mortality differences were found between those referred to the hospital or cared at the nursing home.

Conclusions and implications: 31% of the nursing home patients assessed by a liaison geriatric hospital-based team for COVID-19 were referred to the hospital, being more frequently referred those with a better functional and cognitive situation. The 60-days mortality rate due to COVID-19 was 36.8% and was associated with older age, functional dependence, the presence of tachypnea and fever, and the use of ceftriaxone. Geriatric comprehensive assessment and coordination between NH and the hospital geriatric department teams were crucial.

Keywords: COVID-19; Liaison geriatric team; Mortality; Nursing homes.

© 2021. The Author(s).

Conflict of interest statement

There is no conflict of interest in any of the authors of the manuscript.

• 25 references

Supplementary info

Publication types, MeSH terms Expand

Publication types

- Observational Study

MeSH terms

- Aged
- Aged, 80 and over
- COVID-19*
- Female
- Geriatric Assessment
- Humans
- Male
- Nursing Homes
- Referral and Consultation
- SARS-CoV-2

Full text links

Read free full text at BMC BioMed Central Free PMC article
Proceed to details
Cite
Share
☐ 923
Observational Study
Diagn Interv Imaging
. 2021 Dec;102(12):717-725.
doi: 10.1016/j.diii.2021.06.007. Epub 2021 Jul 13.

Association between coronary artery calcifications and 6-month mortality in hospitalized patients with COVID-19

Elie Mousseaux [1], Antoine Fayol [2], Nicolas Danchin [3], Gilles Soulat [4], Etienne Charpentier [5], Marine Livrozet [6], Jean-Baptiste Carves [6], Victoria Tea [7], Fares Ben Salem [5], Chekrallah Chamandi [7], Jean-Sébastien Hulot [2], Etienne Puymirat [8]
Affiliations Expand

Affiliations

- [1] Université de Paris, Faculté de Médecine, 75006 Paris, France; Department of Radiology, Assistance Publique-Hôpitaux des Hôpitaux de Paris, Hôpital Européen Georges-Pompidou, 75015 Paris, France; Institut National de la Santé et de la Recherche Médicale, PARCC, UMR970, 75015 Paris, France. Electronic address: elie.mousseaux@aphp.fr.

- [2] Université de Paris, Faculté de Médecine, 75006 Paris, France; Institut National de la Santé et de la Recherche Médicale, PARCC, UMR970, 75015 Paris, France; CIC1418 and DMU CARTE, Assistance Publique-Hôpitaux de Paris, Hôpital Européen Georges-Pompidou, 75015 Paris, France.
- [3] Université de Paris, Faculté de Médecine, 75006 Paris, France; Department of Cardiology, Assistance Publique-Hôpitaux de Paris, Hôpital Européen Georges-Pompidou, 75015 Paris, France.
- [4] Université de Paris, Faculté de Médecine, 75006 Paris, France; Department of Radiology, Assistance Publique-Hôpitaux des Hôpitaux de Paris, Hôpital Européen Georges-Pompidou, 75015 Paris, France; Institut National de la Santé et de la Recherche Médicale, PARCC, UMR970, 75015 Paris, France.
- [5] Université de Paris, Faculté de Médecine, 75006 Paris, France; Department of Radiology, Assistance Publique-Hôpitaux des Hôpitaux de Paris, Hôpital Européen Georges-Pompidou, 75015 Paris, France.
- [6] Université de Paris, Faculté de Médecine, 75006 Paris, France; CIC1418 and DMU CARTE, Assistance Publique-Hôpitaux de Paris, Hôpital Européen Georges-Pompidou, 75015 Paris, France.
- [7] Université de Paris, Faculté de Médecine, 75006 Paris, France; Institut National de la Santé et de la Recherche Médicale, PARCC, UMR970, 75015 Paris, France.
- [8] Université de Paris, Faculté de Médecine, 75006 Paris, France; Institut National de la Santé et de la Recherche Médicale, PARCC, UMR970, 75015 Paris, France; Department of Cardiology, Assistance Publique-Hôpitaux de Paris, Hôpital Européen Georges-Pompidou, 75015 Paris, France.

- PMID: **34312110**
- PMCID: PMC8275480
- DOI: 10.1016/j.diii.2021.06.007

Free PMC article
Observational Study

Association between coronary artery calcifications and 6-month mortality in hospitalized patients with COVID-19

Elie Mousseaux et al. Diagn Interv Imaging. 2021 Dec.
Free PMC article
Show details
Diagn Interv Imaging
. 2021 Dec;102(12):717-725.
doi: 10.1016/j.diii.2021.06.007. Epub 2021 Jul 13.

Authors

Elie Mousseaux [1], Antoine Fayol [2], Nicolas Danchin [3], Gilles Soulat [4], Etienne Charpentier [5], Marine Livrozet [6], Jean-Baptiste Carves [6], Victoria Tea [7], Fares Ben Salem [5], Chekrallah Chamandi [7], Jean-Sébastien Hulot [2], Etienne Puymirat [8]

Affiliations

- [1] Université de Paris, Faculté de Médecine, 75006 Paris, France; Department of Radiology, Assistance Publique-Hôpitaux des Hôpitaux de Paris, Hôpital Européen Georges-Pompidou, 75015 Paris, France; Institut National de la Santé et de la Recherche Médicale, PARCC, UMR970, 75015 Paris, France. Electronic address: elie.mousseaux@aphp.fr.
- [2] Université de Paris, Faculté de Médecine, 75006 Paris, France; Institut National de la Santé et de la Recherche Médicale, PARCC, UMR970, 75015 Paris, France; CIC1418 and DMU CARTE, Assistance Publique-Hôpitaux de Paris, Hôpital Européen Georges-Pompidou, 75015 Paris, France.
- [3] Université de Paris, Faculté de Médecine, 75006 Paris, France; Department of Cardiology, Assistance Publique-Hôpitaux de Paris, Hôpital Européen Georges-Pompidou, 75015 Paris, France.
- [4] Université de Paris, Faculté de Médecine, 75006 Paris, France; Department of Radiology, Assistance Publique-Hôpitaux des Hôpitaux de Paris, Hôpital Européen Georges-Pompidou, 75015 Paris, France; Institut National de la Santé et de la Recherche Médicale, PARCC, UMR970, 75015 Paris, France.
- [5] Université de Paris, Faculté de Médecine, 75006 Paris, France; Department of Radiology, Assistance Publique-Hôpitaux des Hôpitaux de Paris, Hôpital Européen Georges-Pompidou, 75015 Paris, France.
- [6] Université de Paris, Faculté de Médecine, 75006 Paris, France; CIC1418 and DMU CARTE, Assistance Publique-Hôpitaux de Paris, Hôpital Européen Georges-Pompidou, 75015 Paris, France.
- [7] Université de Paris, Faculté de Médecine, 75006 Paris, France; Institut National de la Santé et de la Recherche Médicale, PARCC, UMR970, 75015 Paris, France.
- [8] Université de Paris, Faculté de Médecine, 75006 Paris, France; Institut National de la Santé et de la Recherche Médicale, PARCC, UMR970, 75015 Paris, France; Department of Cardiology, Assistance Publique-Hôpitaux de Paris, Hôpital Européen Georges-Pompidou, 75015 Paris, France.

- PMID: **34312110**
- PMCID: PMC8275480
- DOI: 10.1016/j.diii.2021.06.007

Abstract

Purpose: The purpose of this study was to evaluate the association between coronary artery calcium (CAC) visual score and 6-month mortality in patients with coronavirus disease 2019 (COVID-19).

Material and methods: A single-center prospective observational cohort was conducted in 169 COVID-19 consecutive hospitalized patients between March 13 and April 1, 2020, and follow-up for 6-months. A four-level visual CAC scoring was assessed by analyzing images obtained after the first routine non-ECG-gated CT performed to detect COVID-19 pneumonia.

Results: Among 169 confirmed COVID-19 patients (118 men, 51 women; mean age, 65.6 ± 18.8 [SD] years; age range: 30-95 years) 63 (37%) presented with either moderate (n = 26, 15.3%) or heavy (n = 37, 21.8%) CAC detected by CT and 20 (11.8%) had history of cardiovascular disease requiring specific preventive treatment. At six months, mortality rate (45/169; 26.6%) increased with magnitude of CAC and was 7/64 (10.9%), 11/42 (26.2%), 10/26 (38.5%), 17/37 (45.9%) for no-CAC, mild-CAC, moderate-CAC and heavy-CAC groups, respectively (P = 0.001). Compared

to the no CAC group, risk of death increased after adjustment with magnitude of CAC (HR: 2.23, 95% CI: 0.73-6.87, P = 0.16; HR: 2.78, 95% CI: 0.85-9.07, P0.09; HR: 5.38, 95% CI: 1.57-18.40, P = 0.007; in mild CAC, moderate and heavy CAC groups, respectively). In patients without previous coronary artery disease (154/169; 91%), mortality increased from 10.9% to 45.8% (P = 0.001) according to the magnitude of CAC categories. After adjustment, presence of moderate or heavy CAC was associated with higher mortality (HR: 2.26, 95% CI: 1.09-4.69, P = 0.03).

Conclusion: By using non-ECG-gated CT during the initial pulmonary assessment of COVID-19, heavy CAC is independently associated with 6-month mortality in patients hospitalized for severe COVID-19 pneumonia.

Keywords: COVID-19; Computed tomography; Coronary artery calcification; Myocardial injury; Outcomes.

Copyright © 2021. Published by Elsevier Masson SAS.

- 27 references
- 4 figures

Supplementary info

Publication types, MeSH terms Expand

Publication types

- Observational Study

MeSH terms

- Adult
- Aged
- Aged, 80 and over
- COVID-19*
- Coronary Angiography
- Coronary Artery Disease* / diagnostic imaging
- Female
- Humans
- Male
- Middle Aged
- Predictive Value of Tests
- Retrospective Studies
- Risk Assessment
- Risk Factors
- SARS-CoV-2
- Vascular Calcification* / diagnostic imaging

Full text links

Elsevier Science Free PMC article
Proceed to details
Cite
Share
924
Observational Study
BMJ Open Diabetes Res Care
. 2020 Apr;8(1):e001343.
doi: 10.1136/bmjdrc-2020-001343.

Clinical characteristics and outcomes of patients with severe covid-19 with diabetes

Yongli Yan [1], Yan Yang [1], Fen Wang [1], Huihui Ren [1], Shujun Zhang [1], Xiaoli Shi [1], Xuefeng Yu [1], Kun Dong [2]
Affiliations Expand

Affiliations

- [1] Endocrinology, Tongji Hospital of Tongji Medical College of Huazhong University of Science and Technology, Wuhan, Hubei, China.
- [2] Endocrinology, Tongji Hospital of Tongji Medical College of Huazhong University of Science and Technology, Wuhan, Hubei, China kundong2019@hotmail.com.

- PMID: **32345579**
- PMCID: PMC7222577
- DOI: 10.1136/bmjdrc-2020-001343

Free PMC article
Observational Study

Clinical characteristics and outcomes of patients with severe covid-19 with diabetes

Yongli Yan et al. BMJ Open Diabetes Res Care. 2020 Apr.
Free PMC article
Show details
BMJ Open Diabetes Res Care
. 2020 Apr;8(1):e001343.
doi: 10.1136/bmjdrc-2020-001343.

Authors

Yongli Yan [1], Yan Yang [1], Fen Wang [1], Huihui Ren [1], Shujun Zhang [1], Xiaoli Shi [1], Xuefeng Yu [1], Kun Dong [2]

Affiliations

- [1] Endocrinology, Tongji Hospital of Tongji Medical College of Huazhong University of Science and Technology, Wuhan, Hubei, China.
- [2] Endocrinology, Tongji Hospital of Tongji Medical College of Huazhong University of Science and Technology, Wuhan, Hubei, China kundong2019@hotmail.com.

- PMID: **32345579**
- PMCID: PMC7222577
- DOI: 10.1136/bmjdrc-2020-001343

Abstract

Objective: This study explores the clinical characteristics of patients with diabetes with severe covid-19, and the association of diabetes with survival duration in patients with severe covid-19.

Research design and methods: In this single-center, retrospective, observational study, the clinical and laboratory characteristics of 193 patients with severe covid-19 were collected. 48 patients with severe covid-19 had diabetes, and 145 patients (ie, the controls) did not have diabetes. A severe case was defined as including at least one of the following criteria: (1) Respiratory rate >30/min. (2) Oxygen saturation ≤93%. (3) $PaO_2/FiO_2 \leq 300$ mm Hg. (4) Patients, either with shock or respiratory failure, requiring mechanical ventilation, or combined with other organ failure, requiring admission to intensive care unit (ICU).

Results: Of 193 patients with severe covid-19, 48 (24.9%) had diabetes. Compared with patients with severe covid-19 without diabetes, patients with diabetes were older, susceptible to receiving mechanical ventilation and admission to ICU, and had higher mortality. In addition, patients with severe covid-19 with diabetes had higher levels of leukocyte count, neutrophil count, high-sensitivity C reaction protein, procalcitonin, ferritin, interleukin (IL) 2 receptor, IL-6, IL-8, tumor necrosis factor α, D-dimer, fibrinogen, lactic dehydrogenase and N-terminal pro-brain natriuretic peptide. Among patients with severe covid-19 with diabetes, more non-survivors were men (30 (76.9%) *vs* 9 (23.1%)). Non-survivors had severe inflammatory response, and cardiac, hepatic, renal and coagulation impairment. Finally, the Kaplan-Meier survival curve showed a trend towards poorer survival in patients with severe covid-19 with diabetes than patients without diabetes. The HR was 1.53 (95% CI 1.02 to 2.30; p=0.041) after adjustment for age, sex, hypertension, cardiovascular disease and cerebrovascular disease by Cox regression. The median survival durations from hospital admission in patients with severe covid-19 with and without diabetes were 10 days and 18 days, respectively.

Conclusion: The mortality rate in patients with severe covid-19 with diabetes is considerable. Diabetes may lead to an increase in the risk of death.

Keywords: adult diabetes.

© Author(s) (or their employer(s)) 2020. Re-use permitted under CC BY-NC. No commercial re-use. See rights and permissions. Published by BMJ.

Conflict of interest statement

Competing interests: None declared.

Comment in

- Diabetes and covid-19: a global health challenge.
  Shenoy A, Ismaily M, Bajaj M. Shenoy A, et al. BMJ Open Diabetes Res Care. 2020 Apr;8 (1):e001450. doi: 10.1136/bmjdrc-2020-001450. BMJ Open Diabetes Res Care. 2020. PMID: 32345580 Free PMC article. No abstract available.

- 32 references
- 1 figure

Supplementary info

Publication types, MeSH terms Expand

Publication types

- Observational Study
- Research Support, Non-U.S. Gov't

MeSH terms

- Adult
- Aged
- Betacoronavirus
- COVID-19
- China
- Coronavirus Infections / complications*
- Coronavirus Infections / mortality*
- Diabetes Mellitus / virology*
- Female
- Humans
- Inflammation
- Kaplan-Meier Estimate
- Male
- Middle Aged
- Pandemics
- Pneumonia, Viral / complications*
- Pneumonia, Viral / mortality*
- Respiration, Artificial
- Retrospective Studies
- Risk Factors
- SARS-CoV-2

Full text links

BMJ Free Full Text HighWire Free PMC article
Proceed to details
Cite
Share
925
Observational Study
BMJ Open Respir Res
. 2020 Nov;7(1):e000731.
doi: 10.1136/bmjresp-2020-000731.

Characterisation and outcomes of ARDS secondary to pneumonia in patients with and without SARS-CoV-2: a single-centre experience

Rahul Y Mahida [1], Minesh Chotalia [2 3], Joseph Alderman [2 3], Chhaya Patel [4], Amber Hayden [5], Ruchi Desai [5], Emily Beesley [5], Louise E Crowley [2], Marina Soltan [2], Mansoor Bangash [2 3], Dhruv Parekh [2 3], Jaimin Patel [2 3], David R Thickett [2]
Affiliations Expand

Affiliations

- [1] Birmingham Acute Care Research Group, Institute of Inflammation and Ageing, University of Birmingham, Birmingham, UK r.mahida@bham.ac.uk.
- [2] Birmingham Acute Care Research Group, Institute of Inflammation and Ageing, University of Birmingham, Birmingham, UK.
- [3] Department of Anaesthesia and Critical Care, University Hospitals Birmingham NHS Foundation Trust, Birmingham, Birmingham, UK.
- [4] School of Medical Sciences, The University of Manchester, Manchester, Manchester, UK.
- [5] School of Medical and Dental Sciences, University of Birmingham, Birmingham, Birmingham, UK.

- PMID: **33257441**
- PMCID: PMC7705425
- DOI: 10.1136/bmjresp-2020-000731

Free PMC article
Observational Study

Characterisation and outcomes of ARDS secondary to pneumonia in patients with and

without SARS-CoV-2: a single-centre experience

Rahul Y Mahida et al. BMJ Open Respir Res. 2020 Nov.
Free PMC article
Show details
BMJ Open Respir Res
. 2020 Nov;7(1):e000731.
doi: 10.1136/bmjresp-2020-000731.

Authors

Rahul Y Mahida [1], Minesh Chotalia [2] [3], Joseph Alderman [2] [3], Chhaya Patel [4], Amber Hayden [5], Ruchi Desai [5], Emily Beesley [5], Louise E Crowley [2], Marina Soltan [2], Mansoor Bangash [2] [3], Dhruv Parekh [2] [3], Jaimin Patel [2] [3], David R Thickett [2]

Affiliations

- [1] Birmingham Acute Care Research Group, Institute of Inflammation and Ageing, University of Birmingham, Birmingham, UK r.mahida@bham.ac.uk.
- [2] Birmingham Acute Care Research Group, Institute of Inflammation and Ageing, University of Birmingham, Birmingham, UK.
- [3] Department of Anaesthesia and Critical Care, University Hospitals Birmingham NHS Foundation Trust, Birmingham, Birmingham, UK.
- [4] School of Medical Sciences, The University of Manchester, Manchester, Manchester, UK.
- [5] School of Medical and Dental Sciences, University of Birmingham, Birmingham, Birmingham, UK.

- PMID: **33257441**
- PMCID: PMC7705425
- DOI: 10.1136/bmjresp-2020-000731

Abstract

Introduction: Acute respiratory distress syndrome (ARDS) is the major cause of mortality in patients with SARS-CoV-2 pneumonia. It appears that development of 'cytokine storm' in patients with SARS-CoV-2 pneumonia precipitates progression to ARDS. However, severity scores on admission do not predict severity or mortality in patients with SARS-CoV-2 pneumonia. Our objective was to determine whether patients with SARS-CoV-2 ARDS are clinically distinct, therefore requiring alternative management strategies, compared with other patients with ARDS. We report a single-centre retrospective study comparing the characteristics and outcomes of patients with ARDS with and without SARS-CoV-2.

Methods: Two intensive care unit (ICU) cohorts of patients at the Queen Elizabeth Hospital Birmingham were analysed: SARS-CoV-2 patients admitted between 11 March and 21 April 2020 and all patients with community-acquired pneumonia (CAP) from bacterial or viral infection who developed ARDS between 1 January 2017 and 1 November 2019. All data were routinely collected on the hospital's electronic patient records.

Results: A greater proportion of SARS-CoV-2 patients were from an Asian ethnic group (p=0.002). SARS-CoV-2 patients had lower circulating leucocytes, neutrophils and monocytes (p<0.0001), but higher CRP (p=0.016) on ICU admission. SARS-CoV-2 patients required a longer duration of mechanical ventilation (p=0.01), but had lower vasopressor requirements (p=0.016).

Discussion: The clinical syndromes and respiratory mechanics of SARS-CoV-2 and CAP-ARDS are broadly similar. However, SARS-CoV-2 patients initially have a lower requirement for vasopressor support, fewer circulating leukocytes and require prolonged ventilation support. Further studies are required to determine whether the dysregulated inflammation observed in SARS-CoV-2 ARDS may contribute to the increased duration of respiratory failure.

Keywords: ARDS; pneumonia; viral infection.

© Author(s) (or their employer(s)) 2020. Re-use permitted under CC BY. Published by BMJ.

Conflict of interest statement

Competing interests: None declared.

- 23 references
- 1 figure

Supplementary info

Publication types, MeSH terms, Substances, Grant support Expand

Publication types

- Observational Study
- Research Support, Non-U.S. Gov't

MeSH terms

- C-Reactive Protein / metabolism
- COVID-19 / complications*
- Cohort Studies
- Critical Care / methods*
- Ethnicity / statistics & numerical data
- Female
- Humans
- Leukocytes / metabolism
- Male
- Middle Aged
- Monocytes / metabolism
- Neutrophils / metabolism
- Patient Outcome Assessment*
- Respiration, Artificial / statistics & numerical data

- Respiratory Distress Syndrome / blood*
- Respiratory Distress Syndrome / etiology*
- Respiratory Distress Syndrome / therapy
- Respiratory Mechanics
- Retrospective Studies
- SARS-CoV-2
- Time
- United Kingdom
- Vasoconstrictor Agents / therapeutic use

Substances

- Vasoconstrictor Agents
- C-Reactive Protein

Grant support

- MR/N021185/1/MRC_/Medical Research Council/United Kingdom
- MR/S002782/1/MRC_/Medical Research Council/United Kingdom

Full text links

BMJ Free Full Text HighWire Free PMC article
Proceed to details
Cite
Share
926
Observational Study
Br J Anaesth
. 2021 Jul;127(1):e35-e37.
doi: 10.1016/j.bja.2021.04.001. Epub 2021 Apr 12.

Effectiveness of anaesthesia ventilator use for mechanical ventilation in critically ill patients during the COVID-19 pandemic

Aurélie Gouel-Cheron [1], Yoann Elmaleh [2], Camille Couffignal [3], Elie Kantor [2], Simon Meslin [4], Anaïs Caillard [5], Arthur Salome [6], Sophie Hamada [4], Bernard Cholley [4], Alexandre Mebazaa [7], Dan Longrois [8], Jean-Louis Bourgain [6], Valérie Billard [6], Frédérique Servin [2], Philippe Montravers [9]
Affiliations Expand

Affiliations

- [1] Anaesthesiology and Critical Care Medicine Department, DMU PARABOL, Bichat Hospital, AP-HP, Paris, France; Antibody in Therapy and Pathology, Pasteur Institute, UMR 1222 INSERM, Paris, France; Biostatistics Research Branch, Division of Clinical Research, National Institute of Allergy and Infectious Diseases, National Institutes of Health, Bethesda, MD, USA. Electronic address: aurelie.gouel@aphp.fr.
- [2] Anaesthesiology and Critical Care Medicine Department, DMU PARABOL, Bichat Hospital, AP-HP, Paris, France.
- [3] Clinical Research, Biostatistics and Epidemiology Department, Bichat-Claude Bernard Hospital, AP-HP, Université de Paris, Paris, France; INSERM CIC-EC 1425, Bichat Hospital, AP-HP, Université de Paris, Paris, France.
- [4] Anaesthesiology and Critical Care Medicine Department, Hôpital Européen Georges Pompidou, APHP, Paris, France.
- [5] Anaesthesiology and Critical Care Medicine Department, DMU PARABOL, Lariboisiere Hospital, APHP, Paris, France; INSERM UMR-S 942, Paris, France.
- [6] Department of Anaesthesia, Gustave Roussy, Villejuif, France.
- [7] Université de Paris, FHU PROMICE, Paris, France; Anaesthesiology and Critical Care Medicine Department, DMU PARABOL, Lariboisiere Hospital, APHP, Paris, France; INSERM UMR-S 942, Paris, France.
- [8] Anaesthesiology and Critical Care Medicine Department, DMU PARABOL, Bichat Hospital, AP-HP, Paris, France; Université de Paris, FHU PROMICE, Paris, France; Anaesthesiology and Critical Care Medicine Department, DMU PARABOL, Louis Mourier Hospital, APHP, Colombes, France; INSERM 1148, Paris, France.
- [9] Anaesthesiology and Critical Care Medicine Department, DMU PARABOL, Bichat Hospital, AP-HP, Paris, France; Université de Paris, FHU PROMICE, Paris, France; INSERM UMR 1152, ANR-10-LABX-17, Paris, France.

- PMID: **33934889**
- PMCID: PMC8041185
- DOI: 10.1016/j.bja.2021.04.001

Free PMC article
Observational Study

Effectiveness of anaesthesia ventilator use for mechanical ventilation in critically ill patients during the COVID-19 pandemic

Aurélie Gouel-Cheron et al. Br J Anaesth. 2021 Jul.
Free PMC article

Show details

Br J Anaesth
. 2021 Jul;127(1):e35-e37.
doi: 10.1016/j.bja.2021.04.001. Epub 2021 Apr 12.

Authors

Aurélie Gouel-Cheron [1], Yoann Elmaleh [2], Camille Couffignal [3], Elie Kantor [2], Simon Meslin [4], Anaïs Caillard [5], Arthur Salome [6], Sophie Hamada [4], Bernard Cholley [4], Alexandre Mebazaa [7], Dan Longrois [8], Jean-Louis Bourgain [6], Valérie Billard [6], Frédérique Servin [2], Philippe Montravers [9]

Affiliations

- [1] Anaesthesiology and Critical Care Medicine Department, DMU PARABOL, Bichat Hospital, AP-HP, Paris, France; Antibody in Therapy and Pathology, Pasteur Institute, UMR 1222 INSERM, Paris, France; Biostatistics Research Branch, Division of Clinical Research, National Institute of Allergy and Infectious Diseases, National Institutes of Health, Bethesda, MD, USA. Electronic address: aurelie.gouel@aphp.fr.
- [2] Anaesthesiology and Critical Care Medicine Department, DMU PARABOL, Bichat Hospital, AP-HP, Paris, France.
- [3] Clinical Research, Biostatistics and Epidemiology Department, Bichat-Claude Bernard Hospital, AP-HP, Université de Paris, Paris, France; INSERM CIC-EC 1425, Bichat Hospital, AP-HP, Université de Paris, Paris, France.
- [4] Anaesthesiology and Critical Care Medicine Department, Hôpital Européen Georges Pompidou, APHP, Paris, France.
- [5] Anaesthesiology and Critical Care Medicine Department, DMU PARABOL, Lariboisiere Hospital, APHP, Paris, France; INSERM UMR-S 942, Paris, France.
- [6] Department of Anaesthesia, Gustave Roussy, Villejuif, France.
- [7] Université de Paris, FHU PROMICE, Paris, France; Anaesthesiology and Critical Care Medicine Department, DMU PARABOL, Lariboisiere Hospital, APHP, Paris, France; INSERM UMR-S 942, Paris, France.
- [8] Anaesthesiology and Critical Care Medicine Department, DMU PARABOL, Bichat Hospital, AP-HP, Paris, France; Université de Paris, FHU PROMICE, Paris, France; Anaesthesiology and Critical Care Medicine Department, DMU PARABOL, Louis Mourier Hospital, APHP, Colombes, France; INSERM 1148, Paris, France.
- [9] Anaesthesiology and Critical Care Medicine Department, DMU PARABOL, Bichat Hospital, AP-HP, Paris, France; Université de Paris, FHU PROMICE, Paris, France; INSERM UMR 1152, ANR-10-LABX-17, Paris, France.

- PMID: **33934889**
- PMCID: PMC8041185
- DOI: 10.1016/j.bja.2021.04.001

No abstract available

Keywords: COVID-19; acute respiratory distress syndrome; anaesthesia ventilator; heat and moisture exchange filter; mechanical ventilation.

Comment in

- Use of anaesthesia machines for mechanical ventilation and sedation in patients with COVID-19 ARDS.
  Hanidziar D. Hanidziar D. Br J Anaesth. 2021 Aug;127(2):e72-e73. doi: 10.1016/j.bja.2021.05.002. Epub 2021 May 10. Br J Anaesth. 2021. PMID: 34045065 Free PMC article. No abstract available.

- 13 references

Supplementary info

Publication types, MeSH terms Expand

Publication types

- Letter
- Multicenter Study
- Observational Study

MeSH terms

- Aged
- COVID-19 / epidemiology
- COVID-19 / therapy*
- Cohort Studies
- Critical Illness / epidemiology
- Critical Illness / therapy*
- Female
- Humans
- Male
- Middle Aged
- Respiration, Artificial / methods*
- Retrospective Studies
- Treatment Outcome
- Ventilators, Mechanical / supply & distribution*

Full text links

ELSEVIER FULL-TEXT ARTICLE Elsevier Science Free PMC article
Proceed to details
Cite
Share
☐ 927
HIV Res Clin Pract
. 2021 Jun;22(3):63-70.
Epub 2021 Jul 25.

Impact of the COVID-19 pandemic situation on HIV care in Liège, Belgium

Majdouline El Moussaoui [1], Nicolas Lambert [2], Nathalie Maes [3], Karine Fombellida [1], Dolores Vaira [4], Michel Moutschen [1 4], Gilles Darcis [1]
Affiliations Expand

Affiliations

- [1] Department of Infectious Diseases and General Internal Medicine, Liège University Hospital, Liège, Belgium.
- [2] Department of Neurology, Liège University Hospital, Liège, Belgium.
- [3] Department of Biostatistics and Medico-Economic Information, Liège University Hospital, Liège, Belgium.
- [4] AIDS Reference Laboratory, Liège University, Liège, Belgium.

- PMID: **34308800**

Impact of the COVID-19 pandemic situation on HIV care in Liège, Belgium

Majdouline El Moussaoui et al. HIV Res Clin Pract. 2021 Jun.
Show details
HIV Res Clin Pract
. 2021 Jun;22(3):63-70.
Epub 2021 Jul 25.

Authors

Majdouline El Moussaoui [1], Nicolas Lambert [2], Nathalie Maes [3], Karine Fombellida [1], Dolores Vaira [4], Michel Moutschen [1 4], Gilles Darcis [1]

Affiliations

- [1] Department of Infectious Diseases and General Internal Medicine, Liège University Hospital, Liège, Belgium.
- [2] Department of Neurology, Liège University Hospital, Liège, Belgium.
- [3] Department of Biostatistics and Medico-Economic Information, Liège University Hospital, Liège, Belgium.
- [4] AIDS Reference Laboratory, Liège University, Liège, Belgium.

- PMID: **34308800**

Abstract

Background: Background: The COVID-19 pandemic and associated containment measures dramatically affected the health care systems including the screening of human immunodeficiency virus and the management people living with HIV around the world by making the access to preventive care services and specific medical monitoring more difficult.

Objective: Objective: To study the impact of the COVID-19 pandemic on the holistic care of people living with HIV in Liège (Belgium).

Methods: Methods: In this retrospective observational study conducted in Liège University Hospital, we compared the out-patient follow-up of HIV-infected individuals as well as the number of new HIV diagnoses between 2019 and 2020 and between the different waves of the COVID-19 pandemic in 2020.

Results: Results: In 2020, when compared to 2019, we observed a significant decrease in the number of new HIV diagnoses, especially during the first wave of the pandemic, and in the number of consultations undertaken by sexual health services, psychologists and specialists in infectious diseases at our HIV clinic. We also observed a decrease in the number of viral load assays and blood CD4 + T-cells count analyses performed, although we found less patients with HIV plasma viral load above 400 copies per mL in 2020. Finally, we noted a significant reduction in terms of screening of our HIV-infected patients for hepatitis C, syphilis, colorectal and anal cancers and hypercholesterolemia.

Conclusions: Conclusions: Our experience exhibits the deleterious impact of the COVID-19 pandemic on the HIV care and the need to implement new strategies to guarantee its continuum.

Keywords: Europe; HIV care continuum; HIV epidemiology; HIV prevention; health system; sexually transmitted infections/diseases.

Supplementary info

Publication types, MeSH terms Expand

Publication types

- Research Support, Non-U.S. Gov't

MeSH terms

- Ambulatory Care / statistics & numerical data
- Belgium / epidemiology
- CD4 Lymphocyte Count / statistics & numerical data
- COVID-19 / epidemiology*
- COVID-19 / prevention & control
- Coinfection / diagnosis
- HIV Infections / diagnosis*
- HIV Infections / drug therapy*
- HIV Infections / epidemiology
- HIV Infections / prevention & control

- HIV Long-Term Survivors / psychology
- HIV Long-Term Survivors / statistics & numerical data
- Humans
- Mass Screening / statistics & numerical data
- Referral and Consultation / statistics & numerical data
- Retrospective Studies
- SARS-CoV-2
- Time-to-Treatment / statistics & numerical data
- Viral Load / statistics & numerical data

Proceed to details
Cite
Share
☐ 928
Kidney Int
. 2020 Dec;98(6):1549-1558.
doi: 10.1016/j.kint.2020.08.005. Epub 2020 Aug 24.

An initial report from the French SOT COVID Registry suggests high mortality due to COVID-19 in recipients of kidney transplants

Sophie Caillard [1], Dany Anglicheau [2], Marie Matignon [3], Antoine Durrbach [3], Clarisse Greze [4], Luc Frimat [5], Olivier Thaunat [6], Tristan Legris [7], Valerie Moal [7], Pierre Francois Westeel [8], Nassim Kamar [9], Philippe Gatault [10], Renaud Snanoudj [11], Antoine Sicard [12], Dominique Bertrand [13], Charlotte Colosio [14], Lionel Couzi [15], Jonathan M Chemouny [16], Christophe Masset [17], Gilles Blancho [17], Jamal Bamoulid [18], Agnes Duveau [19], Nicolas Bouvier [20], Nathalie Chavarot [2], Philippe Grimbert [3], Bruno Moulin [21], Yannick Le Meur [22], Marc Hazzan [23], French SOT COVID Registry
Collaborators, Affiliations Expand

Collaborators

- **French SOT COVID Registry**: Sophie Caillard, Bruno Moulin, Samira Fafi-Kremer, Marc Hazzan, Dany Anglicheau, Alexandre Hertig, Jérôme Tourret, Benoit Barrou, Emmanuel Morelon, Olivier Thaunat, Lionel Couzi, Pierre Merville, Valérie Moal, Tristan Legris, Pierre-François Westeel, Maïté Jaureguy, Luc Frimat, Didier Ducloux, Jamal Bamoulid, Dominique Bertrand, Michel Tsimaratos, Florentine Garaix-Gilardo, Jérôme Dumortier, Sacha Mussot, Antoine Roux, Laurent Sebbag, Yannick Le Meur, Gilles Blancho, Christophe Masset, Nassim Kamar, Hélène Francois, Eric Rondeau, Nicolas Bouvier, Christiane Mousson, Matthias Buchler, Philippe Gatault, Jean-François Augusto, Agnès Duveau, Cécile Vigneau, Marie-Christine Morin, Jonathan Chemouny, Leonard

Golbin, Philippe Grimbert, Marie Matignon, Antoine Durrbach, Clarisse Greze, Renaud Snanoudj, Charlotte Colosio, Betoul Schvartz, Paolo Malvezzi, Christophe Mariat, Antoine Thierry, Moglie Le Quintrec, Antoine Sicard, Jean Philippe Rerolle, Anne-Élisabeth Heng, Cyril Garrouste, Henri Vacher Coponat, Éric Epailly, Olivier Brugiere, Sébastien Dharancy, Éphrem Salame, Faouzi Saliba

Affiliations

- [1] Department of Nephrology and Transplantation, Strasbourg University Hospital, Strasbourg, France; INSERM, IRM UMR-S 1109, University of Strasbourg, Strasbourg, France. Electronic address: Sophie.caillard@chru-strasbourg.fr.
- [2] Department of Nephrology and Transplantation, Necker University Hospital - APHP, Paris, France; INEM INSERM U 1151- CNRS UMR 8253, Paris University, Paris, France.
- [3] Department of Nephrology and Renal Transplantation, Henri-Mondor/Albert-Chenevier Hospital, AP-HP, Créteil, France; IFRNT, INSERM U 955, University of Paris-Est-Créteil, Créteil, France.
- [4] Department of Nephrology and Transplantation, Bichat hospital, Paris, France.
- [5] Department of Nephrology and Transplantation, CHRU-Nancy, Vandoeuvre, France; INSERM CIC-EC CIE6, University of Lorraine, Nancy, France.
- [6] Department of Transplantation, Nephrology and Clinical Immunology, Edouard Herriot Hospital, Hospices civils de Lyon, Lyon, France; CIRI, INSERM U1111, University Claude Bernard Lyon 1, Lyon, France.
- [7] Department of Nephrology and Transplantation, Marseille University Hospital, Conception hospital, Aix Marseille University, Marseille, France.
- [8] Department of Nephrology and Transplantation, University of Amiens, Amiens, France.
- [9] Department of Nephrology and Transplantation, University of Toulouse, Toulouse, France.
- [10] Department of Nephrology and Transplantation, University of Tours, Tours, France.
- [11] Department of Nephrology and Transplantation, Bicêtre Hospital, Le Kremlin-Bicêtre, France.
- [12] Department of Nephrology Dialysis and Transplantation, Pasteur 2 Hospital, Nice University Hospital, Nice, France; Unité de Recherche Clinique Côte d'Azur (UR2CA), University of Côte d'Azur, Nice, France.
- [13] Department of Nephrology and Transplantation, University of Rouen, Rouen, France.
- [14] Department of Nephrology and Transplantation, University of Reims, Reims, France.
- [15] Department of Nephrology Dialysis, Transplantation and Apheresis, Bordeaux Pellegrin University Hospital, Bordeaux, France; Research Unit ImmunoConcEpT CNRS 5164, University of Bordeaux, Bordeaux, France.
- [16] Inserm UMR_S 1085, EHESP, University of Rennes, Rennes, France.
- [17] Department of Nephrology and Transplantation, Nantes University Hospital, Nantes, France.
- [18] Department of Nephrology and Transplantation, University of Besançon, Besançon, France.
- [19] Department of Nephrology and Transplantation, University of Angers, Angers, France.
- [20] Department of Nephrology and Transplantation, University of Caen, Caen, France.
- [21] Department of Nephrology and Transplantation, Strasbourg University Hospital, Strasbourg, France; INSERM, IRM UMR-S 1109, University of Strasbourg, Strasbourg, France.

- [22] Department of Nephrology and Transplantation, Brest University Hospital, Brest, France; Inserm UMR1227, University of Brest, Labex IGO, Brest, France.
- [23] Department of Nephrology and Transplantation, University of Lille, Lille, France.

- PMID: **32853631**
- PMCID: PMC7444636
- DOI: 10.1016/j.kint.2020.08.005

Free PMC article

An initial report from the French SOT COVID Registry suggests high mortality due to COVID-19 in recipients of kidney transplants

Sophie Caillard et al. Kidney Int. 2020 Dec.
Free PMC article
Show details
Kidney Int
. 2020 Dec;98(6):1549-1558.
doi: 10.1016/j.kint.2020.08.005. Epub 2020 Aug 24.

Authors

Sophie Caillard [1], Dany Anglicheau [2], Marie Matignon [3], Antoine Durrbach [3], Clarisse Greze [4], Luc Frimat [5], Olivier Thaunat [6], Tristan Legris [7], Valerie Moal [7], Pierre Francois Westeel [8], Nassim Kamar [9], Philippe Gatault [10], Renaud Snanoudj [11], Antoine Sicard [12], Dominique Bertrand [13], Charlotte Colosio [14], Lionel Couzi [15], Jonathan M Chemouny [16], Christophe Masset [17], Gilles Blancho [17], Jamal Bamoulid [18], Agnes Duveau [19], Nicolas Bouvier [20], Nathalie Chavarot [2], Philippe Grimbert [3], Bruno Moulin [21], Yannick Le Meur [22], Marc Hazzan [23], French SOT COVID Registry

Collaborators

- **French SOT COVID Registry**:
  Sophie Caillard, Bruno Moulin, Samira Fafi-Kremer, Marc Hazzan, Dany Anglicheau, Alexandre Hertig, Jérôme Tourret, Benoit Barrou, Emmanuel Morelon, Olivier Thaunat, Lionel Couzi, Pierre Merville, Valérie Moal, Tristan Legris, Pierre-François Westeel, Maïté Jaureguy, Luc Frimat, Didier Ducloux, Jamal Bamoulid, Dominique Bertrand, Michel Tsimaratos, Florentine Garaix-Gilardo, Jérôme Dumortier, Sacha Mussot, Antoine Roux, Laurent Sebbag, Yannick Le Meur, Gilles Blancho, Christophe Masset, Nassim Kamar, Hélène Francois, Eric Rondeau, Nicolas Bouvier, Christiane Mousson, Matthias Buchler, Philippe Gatault, Jean-François Augusto, Agnès Duveau, Cécile Vigneau, Marie-Christine Morin, Jonathan Chemouny, Leonard Golbin, Philippe Grimbert, Marie Matignon, Antoine Durrbach, Clarisse Greze, Renaud Snanoudj, Charlotte Colosio, Betoul Schvartz, Paolo Malvezzi, Christophe Mariat, Antoine

Thierry, Moglie Le Quintrec, Antoine Sicard, Jean Philippe Rerolle, Anne-Élisabeth Heng, Cyril Garrouste, Henri Vacher Coponat, Éric Epailly, Olivier Brugiere, Sébastien Dharancy, Éphrem Salame, Faouzi Saliba

Affiliations

- [1] Department of Nephrology and Transplantation, Strasbourg University Hospital, Strasbourg, France; INSERM, IRM UMR-S 1109, University of Strasbourg, Strasbourg, France. Electronic address: Sophie.caillard@chru-strasbourg.fr.
- [2] Department of Nephrology and Transplantation, Necker University Hospital - APHP, Paris, France; INEM INSERM U 1151- CNRS UMR 8253, Paris University, Paris, France.
- [3] Department of Nephrology and Renal Transplantation, Henri-Mondor/Albert-Chenevier Hospital, AP-HP, Créteil, France; IFRNT, INSERM U 955, University of Paris-Est-Créteil, Créteil, France.
- [4] Department of Nephrology and Transplantation, Bichat hospital, Paris, France.
- [5] Department of Nephrology and Transplantation, CHRU-Nancy, Vandoeuvre, France; INSERM CIC-EC CIE6, University of Lorraine, Nancy, France.
- [6] Department of Transplantation, Nephrology and Clinical Immunology, Edouard Herriot Hospital, Hospices civils de Lyon, Lyon, France; CIRI, INSERM U1111, University Claude Bernard Lyon 1, Lyon, France.
- [7] Department of Nephrology and Transplantation, Marseille University Hospital, Conception hospital, Aix Marseille University, Marseille, France.
- [8] Department of Nephrology and Transplantation, University of Amiens, Amiens, France.
- [9] Department of Nephrology and Transplantation, University of Toulouse, Toulouse, France.
- [10] Department of Nephrology and Transplantation, University of Tours, Tours, France.
- [11] Department of Nephrology and Transplantation, Bicêtre Hospital, Le Kremlin-Bicêtre, France.
- [12] Department of Nephrology Dialysis and Transplantation, Pasteur 2 Hospital, Nice University Hospital, Nice, France; Unité de Recherche Clinique Côte d'Azur (UR2CA), University of Côte d'Azur, Nice, France.
- [13] Department of Nephrology and Transplantation, University of Rouen, Rouen, France.
- [14] Department of Nephrology and Transplantation, University of Reims, Reims, France.
- [15] Department of Nephrology Dialysis, Transplantation and Apheresis, Bordeaux Pellegrin University Hospital, Bordeaux, France; Research Unit ImmunoConcEpT CNRS 5164, University of Bordeaux, Bordeaux, France.
- [16] Inserm UMR_S 1085, EHESP, University of Rennes, Rennes, France.
- [17] Department of Nephrology and Transplantation, Nantes University Hospital, Nantes, France.
- [18] Department of Nephrology and Transplantation, University of Besançon, Besançon, France.
- [19] Department of Nephrology and Transplantation, University of Angers, Angers, France.
- [20] Department of Nephrology and Transplantation, University of Caen, Caen, France.
- [21] Department of Nephrology and Transplantation, Strasbourg University Hospital, Strasbourg, France; INSERM, IRM UMR-S 1109, University of Strasbourg, Strasbourg, France.
- [22] Department of Nephrology and Transplantation, Brest University Hospital, Brest, France; Inserm UMR1227, University of Brest, Labex IGO, Brest, France.
- [23] Department of Nephrology and Transplantation, University of Lille, Lille, France.

- PMID: **32853631**
- PMCID: PMC7444636
- DOI: 10.1016/j.kint.2020.08.005

Abstract

Notwithstanding the ongoing coronavirus disease-2019 (Covid-19) pandemic, information on its clinical presentation and prognosis in recipients of a kidney transplant remain scanty. The aim of this registry-based observational study was to explore characteristics and clinical outcomes of recipients of kidney transplants included in the French nationwide Registry of Solid Organ Transplant Recipients with Covid-19. Covid-19 was diagnosed in symptomatic patients who had a positive PCR assay for SARS-CoV-2 or having typical lung lesions on imaging. Clinical and laboratory characteristics, management of immunosuppression, treatment for Covid-19, and clinical outcomes (hospitalization, admission to intensive care unit, mechanical ventilation, or death) were recorded. Risk factors for severe disease or death were determined. Of the 279 patients, 243 were admitted to hospital and 36 were managed at home. The median age of hospitalized patients was 61.6 years; most had comorbidities (hypertension, 90.1%; overweight, 63.8%; diabetes, 41.3%; cardiovascular disease, 36.2%). Fever, cough, dyspnea, and diarrhea were the most common symptoms on admission. Laboratory findings revealed mild inflammation frequently accompanied by lymphopenia. Immunosuppressive drugs were generally withdrawn (calcineurin inhibitors: 28.7%; antimetabolites: 70.8%). Treatment was mainly based on hydroxychloroquine (24.7%), antiviral drugs (7.8%), and tocilizumab (5.3%). Severe Covid-19 occurred in 106 patients (46%). Forty-three hospitalized patients died (30-day mortality 22.8%). Multivariable analysis identified overweight, fever, and dyspnea as independent risk factors for severe disease, whereas age over 60 years, cardiovascular disease, and dyspnea were independently associated with mortality. Thus, Covid-19 in recipients of kidney transplants portends a high mortality rate. Proper management of immunosuppression and tailored treatment of this population remain challenging.

Keywords: COVID-19; immunosuppression; kidney transplantation; mortality; prognosis.

Copyright © 2020 International Society of Nephrology. Published by Elsevier Inc. All rights reserved.

Comment in

- The authors reply.
  Hazzan M, Caillard S. Hazzan M, et al. Kidney Int. 2020 Dec;98(6):1618-1619. doi: 10.1016/j.kint.2020.09.018. Epub 2020 Oct 13. Kidney Int. 2020. PMID: 33065130 Free PMC article. No abstract available.
- Registry reports in COVID-19 patients: juggling with big data, poor data, and no data.
  Søfteland JM, Karason K, Magnusson J, Schult A, Felldin M, Friman V, Oltean M. Søfteland JM, et al. Kidney Int. 2020 Dec;98(6):1618. doi: 10.1016/j.kint.2020.09.017. Epub 2020 Oct 13. Kidney Int. 2020. PMID: 33065132 Free PMC article. No abstract available.

- 36 references
- 4 figures

Supplementary info

MeSH terms Expand

MeSH terms

- Adult
- Aged
- Aged, 80 and over
- COVID-19 / complications
- COVID-19 / mortality*
- COVID-19 / therapy
- Deprescriptions
- Female
- France / epidemiology
- Humans
- Immunosuppression Therapy
- Kidney Transplantation / mortality*
- Male
- Middle Aged
- Pandemics / statistics & numerical data
- Postoperative Complications / mortality*
- Postoperative Complications / virology
- Registries*
- Retrospective Studies
- Risk Factors
- Young Adult

Full text links

ELSEVIER FULL-TEXT ARTICLE Elsevier Science Free PMC article
Proceed to details
Cite
Share
929
Observational Study
J Intensive Care Med
. 2021 Jun;36(6):655-663.
doi: 10.1177/0885066621995386. Epub 2021 Mar 8.

Pulmonary Hemodynamics and Ventilation in Patients With COVID-19-Related Respiratory Failure and ARDS

André Becker [1, 2], Frederik Seiler [1, 2], Ralf M Muellenbach [3], Guy Danziger [1, 2], Maren Kamphorst [1, 2], Christopher Lotz [4], PACovid Study Group; Robert Bals [1, 2], Philipp M Lepper [1, 2]

Affiliations Expand

Affiliations

- [1] Department of Internal Medicine V-Pneumology, Allergology and Critical Care Medicine, 39072University Hospital of Saarland and Saarland University, Homburg/Saar, Germany.
- [2] Interdisciplinary COVID-19-Center, 39072University Hospital of Saarland, Saarland University, Homburg/Saar, Germany.
- [3] Department of Anaesthesiology and Critical Care, Campus Kassel of the University of Southampton, Kassel, Germany.
- [4] Department of Anaesthesiology and Critical Care Medicine, 9190University of Würzburg, Würzburg, Germany.

- PMID: **33678052**
- DOI: 10.1177/0885066621995386

Observational Study

Pulmonary Hemodynamics and Ventilation in Patients With COVID-19-Related Respiratory Failure and ARDS

André Becker et al. J Intensive Care Med. 2021 Jun.

Show details

J Intensive Care Med

. 2021 Jun;36(6):655-663.
doi: 10.1177/0885066621995386. Epub 2021 Mar 8.

Authors

André Becker [1, 2], Frederik Seiler [1, 2], Ralf M Muellenbach [3], Guy Danziger [1, 2], Maren Kamphorst [1, 2], Christopher Lotz [4], PACovid Study Group; Robert Bals [1, 2], Philipp M Lepper [1, 2]

Affiliations

- [1] Department of Internal Medicine V-Pneumology, Allergology and Critical Care Medicine, 39072University Hospital of Saarland and Saarland University, Homburg/Saar, Germany.
- [2] Interdisciplinary COVID-19-Center, 39072University Hospital of Saarland, Saarland University, Homburg/Saar, Germany.
- [3] Department of Anaesthesiology and Critical Care, Campus Kassel of the University of Southampton, Kassel, Germany.

- [4] Department of Anaesthesiology and Critical Care Medicine, 9190University of Würzburg, Würzburg, Germany.

- PMID: **33678052**
- DOI: 10.1177/0885066621995386

Abstract

Background: It has been suggested that COVID-19-associated severe respiratory failure (CARDS) might differ from usual acute respiratory distress syndrome (ARDS) due to failing autoregulation of pulmonary vessels and higher shunt. We sought to investigate pulmonary hemodynamics and ventilation properties in patients with CARDS compared to patients with ARDS of pulmonary origin.

Methods: This was a retrospective analysis of prospectively collected data from consecutive adults with laboratory-confirmed severe acute respiratory syndrome coronavirus 2 patients treated in our ICU in 04/2020 and a comparison of the data to matched controls with ARDS due to respiratory infections treated in our ICU from 01/2014 to 08/2019 for whom pulmonary artery catheter data were available.

Results: CARDS patients (n = 10) had ventilation characteristics similar to those of ARDS (n = 10) patients. Nevertheless, mechanical power applied by ventilation was significantly higher in CARDS patients (23.4 ± 8.9 J/min) than in ARDS (15.9 ± 4.3 J/min; $P < 0.05$). COVID-19 patients had similar pulmonary artery pressure but significantly lower pulmonary vascular resistance, as cardiac output was higher in CARDS vs. ARDS patients ($P < 0.05$). Shunt fraction and dead space were similar in CARDS compared to ARDS ($P > 0.05$) and were correlated with hypoxemia in both groups. The arteriovenous pCO_2 difference (ΔpCO_2) was elevated (CARDS 5.5 ± 2.8 mmHg vs. ARDS 4.7 ± 1.1 mmHg; $P > 0.05$), as was the $P_{(v-a)}CO_2/C_{(a-v)}O_2$ ratio (CARDS mean 2.2 ± 1.5 vs. ARDS 1.7 ± 0.8; $P > 0.05$).

Conclusions: Respiratory failure in COVID-19 patients seems to differ only slightly from ARDS regarding ventilation characteristics and pulmonary hemodynamics. Our data indicate microcirculatory dysfunction. More data need to be collected to assure these findings and gain more pathophysiological insights into COVID-19 and respiratory failure.

Keywords: COVID-19; acute respiratory distress syndrome; mechanical ventilation; novel coronavirus disease 2019; pulmonary artery catheter.

Supplementary info

Publication types, MeSH terms Expand

Publication types

- Observational Study

MeSH terms

- Aged
- Aged, 80 and over

- COVID-19 / complications*
- COVID-19 / physiopathology*
- COVID-19 / therapy
- Cardiac Output / physiology*
- Female
- Humans
- Male
- Middle Aged
- Pulmonary Artery
- Respiration, Artificial*
- Respiratory Insufficiency / physiopathology*
- Respiratory Insufficiency / therapy
- Respiratory Insufficiency / virology
- Retrospective Studies
- Vascular Resistance / physiology*

Full text links

SAGE journals Atypon
Proceed to details
Cite
Share
930
Observational Study
Aging (Albany NY)
. 2020 Aug 24;12(16):15946-15953.
doi: 10.18632/aging.103745. Epub 2020 Aug 24.

Clinical course and characteristics of patients with coronavirus disease 2019 in Wuhan, China: a single-centered, retrospective, observational study

Yanfang Liu [1], Lina Liu [1], Ye Wang [1], Xinyang Du [1], Hong Ma [1], Jing Yao [1]
Affiliations Expand

Affiliation

- [1] Cancer Center, Union Hospital, Tongji Medical College, Huazhong University of Science and Technology, Wuhan 430022, People's Republic of China.

- PMID: **32833671**

- PMCID: PMC7485711
- DOI: 10.18632/aging.103745

Free PMC article
Observational Study

Clinical course and characteristics of patients with coronavirus disease 2019 in Wuhan, China: a single-centered, retrospective, observational study

Yanfang Liu et al. Aging (Albany NY). 2020.
Free PMC article
Show details
Aging (Albany NY)
. 2020 Aug 24;12(16):15946-15953.
doi: 10.18632/aging.103745. Epub 2020 Aug 24.

Authors

Yanfang Liu [1], Lina Liu [1], Ye Wang [1], Xinyang Du [1], Hong Ma [1], Jing Yao [1]

Affiliation

- [1] Cancer Center, Union Hospital, Tongji Medical College, Huazhong University of Science and Technology, Wuhan 430022, People's Republic of China.

- PMID: **32833671**
- PMCID: PMC7485711
- DOI: 10.18632/aging.103745

Abstract

Background: Severe acute respiratory syndrome coronavirus 2(SARS-CoV-2) is the virus responsible for the coronavirus disease 2019(COVID-19) pandemic. Despite the extensive studies aiming to understand the pathology of COVID-19, the clinicopathological characteristics and risk factors associated with COVID-19 remain mostly unclear. In this study, we assessed the clinical course and features of COVID-19 patients.

Findings: There were 59 patients (54.1%) that had no fever. One-hundred(91.7%) patients required oxygen therapy, which improved percutaneous oxygen saturation (SpO_2). Seventy-two (66.1%) patients aged over 60; these patients were more likely to develop respiratory symptoms. Only 13(11.9%) patients were positive for anti-SARS-CoV-2 antibodies, SARS-CoV-2 nucleic acid, and computed tomography (CT) findings. We found significant differences in age, respiratory symptoms, and heart rates between patients with and without underlying conditions.

Conclusions: Our findings suggest that oxygen plays an important role in the treatment of COVID-19 patients and that age and underlying diseases are significant risk factors for COVID-19. Most COVID-19 patients have no fever, and CT provides higher detection rates than antibody- and nucleic acid-based detection methods.

Methods: We analyzed data from 109 confirmed COVID-19 cases. We compared the clinicopathological characteristic of patients stratified according to age and underlying diseases, as well as assessed the detection rates of different diagnostic methods.

Keywords: COVID-19; SARS-CoV-2; chronic diseases; infection; risk factors.

Conflict of interest statement

CONFLICTS OF INTEREST: The authors declare no conflicts of interest.

- 20 references
- 1 figure

Supplementary info

Publication types, MeSH terms Expand

Publication types

- Observational Study
- Research Support, Non-U.S. Gov't

MeSH terms

- Age Factors
- Aged
- Betacoronavirus / isolation & purification*
- Blood Gas Monitoring, Transcutaneous / methods
- COVID-19
- COVID-19 Testing
- China / epidemiology
- Clinical Laboratory Techniques / methods*
- Comorbidity
- Coronavirus Infections* / diagnosis
- Coronavirus Infections* / epidemiology
- Coronavirus Infections* / physiopathology
- Coronavirus Infections* / therapy
- Female
- Humans
- Male
- Middle Aged

- Oxygen Inhalation Therapy / methods*
- Pandemics*
- Pneumonia, Viral* / diagnosis
- Pneumonia, Viral* / epidemiology
- Pneumonia, Viral* / physiopathology
- Pneumonia, Viral* / therapy
- Retrospective Studies
- Risk Assessment
- Risk Factors
- SARS-CoV-2
- Symptom Assessment / methods

Full text links

Impact Journals, LLC Free PMC article
Proceed to details
Cite
Share
931
Observational Study
Medicine (Baltimore)
. 2020 Nov 13;99(46):e23257.
doi: 10.1097/MD.0000000000023257.

Sputum characteristics and airway clearance methods in patients with severe COVID-19

Yu Wang [1], Meng Zhang [2], Yan Yu [2], Tao Han [2], Ji Zhou [3], Liqing Bi [1]
Affiliations Expand

Affiliations

- [1] Department of Neurosurgery Intensive Care Unit.
- [2] Department of Critical Care Medicine.
- [3] Department of Respiratory and Critical Care Medicine, The First Affiliated Hospital, Nanjing Medical University, Nanjing, PR China.

- PMID: **33181718**
- PMCID: PMC7668462
- DOI: 10.1097/MD.0000000000023257

Free PMC article
Observational Study

Sputum characteristics and airway clearance methods in patients with severe COVID-19

Yu Wang et al. Medicine (Baltimore). 2020.
Free PMC article
Show details
Medicine (Baltimore)
. 2020 Nov 13;99(46):e23257.
doi: 10.1097/MD.0000000000023257.

Authors

Yu Wang [1], Meng Zhang [2], Yan Yu [2], Tao Han [2], Ji Zhou [3], Liqing Bi [1]

Affiliations

- [1] Department of Neurosurgery Intensive Care Unit.
- [2] Department of Critical Care Medicine.
- [3] Department of Respiratory and Critical Care Medicine, The First Affiliated Hospital, Nanjing Medical University, Nanjing, PR China.

- PMID: **33181718**
- PMCID: PMC7668462
- DOI: 10.1097/MD.0000000000023257

Abstract

Critically ill patients with coronavirus disease 2019 (COVID-19) have a high case fatality rate. Hence, controlling the disease progression of severely ill COVID-19 patients to avoid the development of severe-to-critical COVID-19 is the most important target of COVID-19 treatment. The latest autopsy results of COVID-19 patients have shown the presence of viscous secretions in the airways. However, no studies are available that specifically describe and analyze the sputum characteristics and the effects of various sputum drainage methods on the prognosis of COVID-19 patients. In our study, we found that elderly COVID-19 patients were more susceptible to progression to critical illness (P = .024) and were likely to have accompanying lymphopenia (P = .035) or increased neutrophil counts (P = .019). We observed that there was a higher proportion of patients with Grade 3 sticky sputum in the critically ill group than in the noncritically ill group (P = .026), suggesting that changes in sputum characteristics may be one of the early warning signs of critical COVID-19. In addition, we found that the application rates of large doses of ambroxol (P = .043) and prone-position drainage (P = .037) were relatively high in COVID-19 patients with good prognoses, suggesting that the early application of large doses of expectorant drugs and prone-position drainage in COVID-19 patients may avoid progression to critical illness and improve the prognosis.

Conflict of interest statement

The authors report no conflicts of interest.

- 20 references
- 1 figure

Supplementary info

Publication types, MeSH terms, Substances Expand

Publication types

- Observational Study

MeSH terms

- Adult
- Age Factors
- Aged
- Aged, 80 and over
- Airway Management / methods*
- Betacoronavirus
- COVID-19
- Coronavirus Infections / mortality
- Coronavirus Infections / pathology*
- Critical Illness*
- Expectorants / administration & dosage
- Female
- Humans
- Male
- Middle Aged
- Neutrophils / metabolism
- Pandemics
- Pneumonia, Viral / mortality
- Pneumonia, Viral / pathology*
- Prone Position
- Retrospective Studies
- SARS-CoV-2
- Severity of Illness Index
- Sputum / chemistry*

Substances

- Expectorants

Full text links

Wolters Kluwer Free PMC article
Proceed to details
Cite
Share
932
Observational Study

J Intensive Care Med
. 2021 Jun;36(6):719-725.
doi: 10.1177/0885066621989920. Epub 2021 Feb 3.

Comparative Analysis of Intravenous Pumps Relocation for Critically Ill Isolated COVID-19 Patients From Bedside to Outside the Patient Room

Ami Shah [1] [2], Jiashan Xu [1], Seana Friedman [2] [3], John D Puskas [2], Himani V Bhatt [4], Mekeleya Yimen [2]
Affiliations Expand

Affiliations

- [1] Department of Pharmacy, 22527Mount Sinai Morningside Medical Center, New York, NY, USA.
- [2] Department of Cardiothoracic Surgery and Critical Care, 22527Mount Sinai Morningside Medical Center, New York, NY, USA.
- [3] Department of Nursing, 22527Mount Sinai Morningside Medical Center, New York, NY, USA.
- [4] Department of Anesthesiology and Perioperative Medicine, 22527Mount Sinai Morningside Medical Center, New York, NY, USA.

- PMID: **33530822**
- DOI: 10.1177/0885066621989920

Observational Study

Comparative Analysis of Intravenous Pumps Relocation for Critically Ill Isolated COVID-19 Patients From Bedside to Outside the Patient Room

Ami Shah et al. J Intensive Care Med. 2021 Jun.
Show details
J Intensive Care Med
. 2021 Jun;36(6):719-725.
doi: 10.1177/0885066621989920. Epub 2021 Feb 3.

Authors

Ami Shah [1, 2], Jiashan Xu [1], Seana Friedman [2, 3], John D Puskas [2], Himani V Bhatt [4], Mekeleya Yimen [2]

Affiliations

- [1] Department of Pharmacy, 22527Mount Sinai Morningside Medical Center, New York, NY, USA.
- [2] Department of Cardiothoracic Surgery and Critical Care, 22527Mount Sinai Morningside Medical Center, New York, NY, USA.
- [3] Department of Nursing, 22527Mount Sinai Morningside Medical Center, New York, NY, USA.
- [4] Department of Anesthesiology and Perioperative Medicine, 22527Mount Sinai Morningside Medical Center, New York, NY, USA.

- PMID: **33530822**
- DOI: 10.1177/0885066621989920

Abstract

Objectives: To quantify the impact of IV pump relocation for COVID-19 patients from the bedside to outside the patient room on nurse exposure to COVID-19 and conservation of PPE.

Design: Original Article.

Setting: Intensive care units at a single-center teaching hospital.

Patients: Critically ill COVID-19 patients under contact and special droplet precautions.

Interventions: Relocation of intravenous pumps for COVID-19 patients from bedside to outside the patient room using extension tubing.

Measurements and main results: The primary objective of the study was to measure the impact of this strategy on COVID-19 exposure, utilizing the number of nurse entries into the patient room as a surrogate endpoint, and extrapolation of this data to determine the reduction or PPE usage.

Secondary endpoints included incidence of extravasation, hyperglycemia, hypotension, and diagnosis of CLABSI/bacteremia. A statistically significant reduction in the primary endpoint of the study was observed as room entries prior to pump relocation averaged 15.36 (± 4.10) as opposed to an average of 7.92 (± 2.19) following pump relocation ($p < 0.0001$). In both pre- and post-pump relocation groups, there was no incidence of extravasation or CLABSI. No significant differences were noted in number of patients experiencing hyperglycemia, hypotensive episodes, or bacteremia.

Conclusions: There was a significant decrease in COVID-19 exposure based on the number of nurse entries following the relocation of intravenous pumps from inside to outside of the patient room. These results may be cautiously extrapolated to suggest a decrease in personal protective equipment utilization. Future prospective, randomized controlled trials investigating the impact of this strategy are required.

Keywords: COVID-19; coronavirus; infusion pumps; personal protective equipment (PPE); relocation.

Supplementary info

Publication types, MeSH terms Expand

Publication types

- Comparative Study
- Observational Study

MeSH terms

- Aged
- COVID-19 / prevention & control*
- COVID-19 / transmission
- Critical Care*
- Female
- Humans
- Infection Control*
- Infectious Disease Transmission, Patient-to-Professional / prevention & control*
- Infusion Pumps*
- Male
- Middle Aged
- Patients' Rooms*
- Personal Protective Equipment
- Retrospective Studies

Full text links

SAGE journals Atypon

Proceed to details

Cite

Share

933

Observational Study

Crit Care

. 2021 Jan 11;25(1):25.

doi: 10.1186/s13054-021-03460-5.

Ventilator-associated pneumonia in critically ill patients with COVID-19

Mailis Maes [#] [1], Ellen Higginson [#] [1], Joana Pereira-Dias [#] [1], Martin D Curran [2], Surendra Parmar [2], Fahad Khokhar [1], Delphine Cuchet-Lourenço [3], Janine Lux [3], Sapna Sharma-Hajela [4], Benjamin Ravenhill [4], Islam Hamed [4], Laura Heales [4], Razeen Mahroof [4], Amelia Soderholm [1], Sally Forrest [1], Sushmita Sridhar [1] [5], Nicholas M Brown [2], Stephen Baker [1], Vilas Navapurkar [4], Gordon Dougan [1], Josefin Bartholdson Scott [1], Andrew Conway Morris [6] [7]

Affiliations Expand

Affiliations

- [1] Department of Medicine, Cambridge Institute of Therapeutic Immunology and Infectious Disease (CITIID), University of Cambridge, Cambridge, UK.
- [2] Public Health England, Clinical Microbiology and Public Health Laboratory, Addenbrooke's Hospital, Cambridge, UK.
- [3] Division of Anaesthesia, Department of Medicine, University of Cambridge, Level 4, Addenbrooke's Hospital, Hills Road, Cambridge, CB2 0QQ, UK.
- [4] John Farman ICU, Addenbrookes Hospital, Cambridge, UK.
- [5] Wellcome Sanger Institute, Hinxton, UK.
- [6] Division of Anaesthesia, Department of Medicine, University of Cambridge, Level 4, Addenbrooke's Hospital, Hills Road, Cambridge, CB2 0QQ, UK. ac926@cam.ac.uk.
- [7] John Farman ICU, Addenbrookes Hospital, Cambridge, UK. ac926@cam.ac.uk.

[#] Contributed equally.

- PMID: **33430915**
- PMCID: PMC7797892
- DOI: 10.1186/s13054-021-03460-5

Free PMC article

Observational Study

Ventilator-associated pneumonia in critically ill patients with COVID-19

Mailis Maes et al. Crit Care. 2021.
Free PMC article
Show details
Crit Care
. 2021 Jan 11;25(1):25.
doi: 10.1186/s13054-021-03460-5.

Authors

Mailis Maes [# 1], Ellen Higginson [# 1], Joana Pereira-Dias [# 1], Martin D Curran [2], Surendra Parmar [2], Fahad Khokhar [1], Delphine Cuchet-Lourenço [3], Janine Lux [3], Sapna Sharma-Hajela [4], Benjamin Ravenhill [4], Islam Hamed [4], Laura Heales [4], Razeen Mahroof [4], Amelia Soderholm [1], Sally Forrest [1], Sushmita Sridhar [1] [5], Nicholas M Brown [2], Stephen Baker [1], Vilas Navapurkar [4], Gordon Dougan [1], Josefin Bartholdson Scott [1], Andrew Conway Morris [6] [7]

Affiliations

- [1] Department of Medicine, Cambridge Institute of Therapeutic Immunology and Infectious Disease (CITIID), University of Cambridge, Cambridge, UK.
- [2] Public Health England, Clinical Microbiology and Public Health Laboratory, Addenbrooke's Hospital, Cambridge, UK.
- [3] Division of Anaesthesia, Department of Medicine, University of Cambridge, Level 4, Addenbrooke's Hospital, Hills Road, Cambridge, CB2 0QQ, UK.
- [4] John Farman ICU, Addenbrookes Hospital, Cambridge, UK.
- [5] Wellcome Sanger Institute, Hinxton, UK.
- [6] Division of Anaesthesia, Department of Medicine, University of Cambridge, Level 4, Addenbrooke's Hospital, Hills Road, Cambridge, CB2 0QQ, UK. ac926@cam.ac.uk.
- [7] John Farman ICU, Addenbrookes Hospital, Cambridge, UK. ac926@cam.ac.uk.

[#] Contributed equally.

- PMID: **33430915**
- PMCID: PMC7797892
- DOI: 10.1186/s13054-021-03460-5

Erratum in

- Correction to: Ventilator-associated pneumonia in critically ill patients with COVID-19. Maes M, Higginson E, Pereira-Dias J, Curran MD, Parmar S, Khokhar F, Cuchet-Lourenço D, Lux J, Sharma-Hajela S, Ravenhill B, Hamed I, Heales L, Mahroof R, Soderholm A, Forrest S, Sridhar S, Brown NM, Baker S, Navapurkar V, Dougan G, Scott JB, Morris AC. Maes M, et al. Crit Care. 2021 Apr 6;25(1):130. doi: 10.1186/s13054-021-03560-2. Crit Care. 2021. PMID: 33823901 Free PMC article. No abstract available.

Abstract

Background: Pandemic COVID-19 caused by the coronavirus SARS-CoV-2 has a high incidence of patients with severe acute respiratory syndrome (SARS). Many of these patients require admission to an intensive care unit (ICU) for invasive ventilation and are at significant risk of developing a secondary, ventilator-associated pneumonia (VAP).

Objectives: To study the incidence of VAP and bacterial lung microbiome composition of ventilated COVID-19 and non-COVID-19 patients.

Methods: In this retrospective observational study, we compared the incidence of VAP and secondary infections using a combination of microbial culture and a TaqMan multi-pathogen array. In addition, we determined the lung microbiome composition using 16S RNA analysis in a subset of samples. The study involved 81 COVID-19 and 144 non-COVID-19 patients receiving invasive ventilation in a single University teaching hospital between March 15th 2020 and August 30th 2020.

Results: COVID-19 patients were significantly more likely to develop VAP than patients without COVID (Cox proportional hazard ratio 2.01 95% CI 1.14-3.54, p = 0.0015) with an incidence density of 28/1000 ventilator days versus 13/1000 for patients without COVID (p = 0.009). Although the distribution of organisms causing VAP was similar between the two groups, and the pulmonary microbiome was similar, we identified 3 cases of invasive aspergillosis amongst the patients with COVID-19 but none in the non-COVID-19 cohort. Herpesvirade activation was also numerically more frequent amongst patients with COVID-19.

Conclusion: COVID-19 is associated with an increased risk of VAP, which is not fully explained by the prolonged duration of ventilation. The pulmonary dysbiosis caused by COVID-19, and the causative organisms of secondary pneumonia observed are similar to that seen in critically ill patients ventilated for other reasons.

Keywords: COVID-19; Critical care; Molecular diagnostics; Nosocomial infections; SARS-CoV-2; Ventilator-associated pneumonia.

Conflict of interest statement

The authors have declared that no competing interests exist.

- 40 references
- 4 figures

Supplementary info

Publication types, MeSH terms, Grant support Expand

Publication types

- Observational Study
- Research Support, Non-U.S. Gov't

MeSH terms

- Aged
- COVID-19 / diagnosis
- COVID-19 / epidemiology*
- COVID-19 / therapy*
- Critical Illness / epidemiology*
- Critical Illness / therapy*
- Female
- Humans
- Intensive Care Units / trends
- Male
- Middle Aged
- Pneumonia, Ventilator-Associated / diagnosis
- Pneumonia, Ventilator-Associated / epidemiology*
- Retrospective Studies

Grant support

- WT_/Wellcome Trust/United Kingdom
- 205214/Z/16/Z/WT_/Wellcome Trust/United Kingdom
- Cambridge BRC grant/National Institute for Health Research/International
- WT 2055214/Z/16/Z/WT_/Wellcome Trust/United Kingdom

Full text links

Read free full text at BMC BioMed Central Free PMC article
Proceed to details
Cite
Share
☐ 934
Observational Study
Nutrients
. 2021 Jul 26;13(8):2559.
doi: 10.3390/nu13082559.

Mortality in Hemodialysis Patients with COVID-19, the Effect of Paricalcitol or Calcimimetics

María Dolores Arenas Jimenez [1, 2], Emilio González-Parra [3], Marta Riera [1], Abraham Rincón Bello [4], Ana López-Herradón [4], Higini Cao [1], Sara Hurtado [4], Silvia Collado [1], Laura Ribera

[4], Francesc Barbosa [1], Fabiola Dapena [5], Vicent Torregrosa [6], José-Jesús Broseta [5], Carlos Soto Montañez [6], Juan F Navarro-González [7] [8] [9], Rosa Ramos [4], Jordi Bover [10], Xavier Nogués-Solan [11], Marta Crespo [1], Adriana S Dusso [12] [13], Julio Pascual [1]
Affiliations Expand

Affiliations

- [1] Department of Nephrology, Hospital del Mar, IMIM Hospital del Mar Medical Research Institute, RD16/0009/0013 (ISCIII FEDER REDinREN), 08003 Barcelona, Spain.
- [2] Fundación Renal Iñigo Alvarez de Toledo, 28003 Madrid, Spain.
- [3] Fundación Jimenez Díaz, 28040 Madrid, Spain.
- [4] Fresenius Medical Care, Dirección Médica FMC, 28760 Madrid, Spain.
- [5] Department of Nephrology, Consorci Sanitari Alt Penedes Garraf, 08800 Barcelona, Spain.
- [6] Department of Nephrology and Kidney Transplantation, Hospital Clinic, 08036 Barcelona, Spain.
- [7] Research Division and Department of Nephrology, Hospital Nuestra Señora de la Candelaria, 38010 Santa Cruz de Tenerife, Spain.
- [8] Instituto de Tecnologías Biomédicas, Universidad de La Laguna, 38010 Tenerife, Spain.
- [9] Red de Investigación Renal (REDINREN-RD16/0009/0022), Instituto de Salud Carlos III, 28029 Madrid, Spain.
- [10] Department of Nephrology, Hospital Can Ruti, 08916 Barcelona, Spain.
- [11] Department of Internal Medicine, Hospital del Mar, Institut Mar for Medical Research, CIBERFES, 08003 Barcelona, Spain.
- [12] Bone and Mineral Research Unit, Instituto de Investigaciones Sanitarias del Principado de Asturias, 33011 Oviedo, Spain.
- [13] Department of Internal Medicine, Division of Endocrinology, Metabolism and Lipid Research, Washington University School of Medicine, St. Louis, MO 63110, USA.

- PMID: **34444716**
- PMCID: PMC8401800
- DOI: 10.3390/nu13082559

Free PMC article
Observational Study

Mortality in Hemodialysis Patients with COVID-19, the Effect of Paricalcitol or Calcimimetics

María Dolores Arenas Jimenez et al. Nutrients. 2021.
Free PMC article
Show details
Nutrients
. 2021 Jul 26;13(8):2559.
doi: 10.3390/nu13082559.

Authors

María Dolores Arenas Jimenez [1, 2], Emilio González-Parra [3], Marta Riera [1], Abraham Rincón Bello [4], Ana López-Herradón [4], Higini Cao [1], Sara Hurtado [4], Silvia Collado [1], Laura Ribera [4], Francesc Barbosa [1], Fabiola Dapena [5], Vicent Torregrosa [6], José-Jesús Broseta [5], Carlos Soto Montañez [6], Juan F Navarro-González [7, 8, 9], Rosa Ramos [4], Jordi Bover [10], Xavier Nogués-Solan [11], Marta Crespo [1], Adriana S Dusso [12, 13], Julio Pascual [1]

Affiliations

- [1] Department of Nephrology, Hospital del Mar, IMIM Hospital del Mar Medical Research Institute, RD16/0009/0013 (ISCIII FEDER REDinREN), 08003 Barcelona, Spain.
- [2] Fundación Renal Iñigo Alvarez de Toledo, 28003 Madrid, Spain.
- [3] Fundación Jimenez Díaz, 28040 Madrid, Spain.
- [4] Fresenius Medical Care, Dirección Médica FMC, 28760 Madrid, Spain.
- [5] Department of Nephrology, Consorci Sanitari Alt Penedes Garraf, 08800 Barcelona, Spain.
- [6] Department of Nephrology and Kidney Transplantation, Hospital Clinic, 08036 Barcelona, Spain.
- [7] Research Division and Department of Nephrology, Hospital Nuestra Señora de la Candelaria, 38010 Santa Cruz de Tenerife, Spain.
- [8] Instituto de Tecnologías Biomédicas, Universidad de La Laguna, 38010 Tenerife, Spain.
- [9] Red de Investigación Renal (REDINREN-RD16/0009/0022), Instituto de Salud Carlos III, 28029 Madrid, Spain.
- [10] Department of Nephrology, Hospital Can Ruti, 08916 Barcelona, Spain.
- [11] Department of Internal Medicine, Hospital del Mar, Institut Mar for Medical Research, CIBERFES, 08003 Barcelona, Spain.
- [12] Bone and Mineral Research Unit, Instituto de Investigaciones Sanitarias del Principado de Asturias, 33011 Oviedo, Spain.
- [13] Department of Internal Medicine, Division of Endocrinology, Metabolism and Lipid Research, Washington University School of Medicine, St. Louis, MO 63110, USA.

- PMID: **34444716**
- PMCID: PMC8401800
- DOI: 10.3390/nu13082559

Abstract

Background: In COVID-19 patients, low serum vitamin D (VD) levels have been associated with severe acute respiratory failure and poor prognosis. In regular hemodialysis (HD) patients, there is VD deficiency and markedly reduced calcitriol levels, which may predispose them to worse outcomes of COVID-19 infection. Some hemodialysis patients receive treatment with drugs for secondary hyperparathyroidism, which have well known pleiotropic effects beyond mineral metabolism. The aim of this study was to evaluate the impact of VD status and the administration of active vitamin D medications, used to treat secondary hyperparathyroidism, on survival in a cohort of COVID-19 positive HD patients.

Methods: A cross-sectional retrospective observational study was conducted from 12 March to 21 May 2020 in 288 HD patients with positive PCR for SARS-CoV2. Patients were from 52 different centers in Spain.

Results: The percent of HD patients with COVID-19 was 6.1% (288 out of 4743). Mortality rate was 28.4% (81/285). Three patients were lost to follow-up. Serum 25(OH)D (calcidiol) level was 17.1 [10.6-27.5] ng/mL and was not significantly associated to mortality (OR 0.99 (0.97-1.01), p = 0.4). Patients receiving active vitamin D medications (16/94 (17%) vs. 65/191(34%), $p = 0.003$), including calcimimetics (4/49 (8.2%) vs. 77/236 (32.6%), $p = 0.001$), paricalcitol or calcimimetics (19/117 (16.2%) vs. 62/168 (36.9%); $p < 0.001$), and also those on both paricalcitol and calcimimetics, to treat secondary hyperparathyroidism (SHPTH) (1/26 (3.8%) vs. 80/259 (30.9%), $p < 0.001$) showed a lower mortality rate than patients receiving no treatment with either drug. Multivariate Cox regression analysis confirmed this increased survival.

Conclusions: Our findings suggest that the use of paricalcitol, calcimimetics or the combination of both, seem to be associated with the improvement of survival in HD patients with COVID-19. No correlation was found between serum VD levels and prognosis or outcomes in HD patients with COVID-19. Prospective studies and clinical trials are needed to support these findings.

Keywords: COVID-19; SARS-CoV-2; calcifediol; calcitriol; survival; vitamin D.

Conflict of interest statement

The authors declare no conflict of interest except M.D.A.J. and J.B. who declare receipt of advisory and/or lecture fees from Amgen, Sanofi-Genzyme, Shire, and Vifor-Fresenius-Pharma.

- 54 references
- 1 figure

Supplementary info

Publication types, MeSH terms, Substances Expand

Publication types

- Multicenter Study
- Observational Study

MeSH terms

- Aged
- Aged, 80 and over
- COVID-19 / blood
- COVID-19 / mortality*
- Calcifediol / blood
- Calcitriol / administration & dosage*
- Calcium / blood
- Cross-Sectional Studies

- Ergocalciferols / administration & dosage*
- Female
- Humans
- Hyperparathyroidism, Secondary / blood
- Hyperparathyroidism, Secondary / drug therapy
- Male
- Renal Dialysis / mortality*
- Retrospective Studies
- SARS-CoV-2 / isolation & purification
- Survival Analysis
- Vitamin D / blood
- Vitamin D Deficiency / blood
- Vitamin D Deficiency / drug therapy
- Vitamin D Deficiency / mortality
- Vitamin D Deficiency / virology

Substances

- Ergocalciferols
- Vitamin D
- paricalcitol
- Calcitriol
- Calcifediol
- Calcium

Full text links

FULL TEXT OPEN ACCESS MDPI Multidisciplinary Digital Publishing Institute (MDPI) Free PMC article
Proceed to details
Cite
Share
☐ 935
Observational Study
Med J Malaysia
. 2020 Sep;75(5):582-584.

COVID-19 detected from targeted contact tracing, attempting to see the pattern in random happenings: early lessons in Malaysia

B H Ng [1], Y L B Andrea [2], N A Nuratiqah [1], A H Faisal [1], C I Soo [1], K Najma [3], P Periyasamy [3]
Affiliations Expand

Affiliations

- [1] Universiti Kebangsaan Malaysia Medical Centre, Faculty of Medicine, Department of Internal Medicine, Respiratory Unit, Kuala Lumpur, Malaysia.
- [2] Universiti Kebangsaan Malaysia Medical Centre, Faculty of Medicine, Department of Internal Medicine, Respiratory Unit, Kuala Lumpur, Malaysia. andreaban@gmail.com.
- [3] Universiti Kebangsaan Malaysia Medical Centre, Faculty of Medicine, Department of Internal Medicine, Infectious Disease Unit, Kuala Lumpur, Malaysia.

- PMID: **32918430**

Free article
Observational Study

COVID-19 detected from targeted contact tracing, attempting to see the pattern in random happenings: early lessons in Malaysia

B H Ng et al. Med J Malaysia. 2020 Sep.
Free article
Show details
Med J Malaysia
. 2020 Sep;75(5):582-584.

Authors

B H Ng [1], Y L B Andrea [2], N A Nuratiqah [1], A H Faisal [1], C I Soo [1], K Najma [3], P Periyasamy [3]

Affiliations

- [1] Universiti Kebangsaan Malaysia Medical Centre, Faculty of Medicine, Department of Internal Medicine, Respiratory Unit, Kuala Lumpur, Malaysia.
- [2] Universiti Kebangsaan Malaysia Medical Centre, Faculty of Medicine, Department of Internal Medicine, Respiratory Unit, Kuala Lumpur, Malaysia. andreaban@gmail.com.
- [3] Universiti Kebangsaan Malaysia Medical Centre, Faculty of Medicine, Department of Internal Medicine, Infectious Disease Unit, Kuala Lumpur, Malaysia.

- PMID: **32918430**

Abstract

The world feels strange as we face what is for most of us our first ever pandemic. The number of newly diagnosed cases rises daily in many parts of the world, and we are faced with the reality that there are still many things to learn about this new disease. We share here our experience of treating our first 199 COVID-19 patients in the Hospital Canselor Tuanku Muhriz, Pusat Perubatan Universiti Kebangsaan Malaysia (PPUKM).

Supplementary info

Publication types, MeSH terms Expand

Publication types

- Observational Study
- Research Support, Non-U.S. Gov't

MeSH terms

- Adolescent
- Adult
- Aged
- Aged, 80 and over
- Betacoronavirus*
- COVID-19
- Child
- Contact Tracing
- Coronavirus Infections / complications
- Coronavirus Infections / diagnosis*
- Coronavirus Infections / therapy*
- Female
- Humans
- Malaysia
- Male
- Middle Aged
- Pandemics
- Pneumonia, Viral / complications
- Pneumonia, Viral / diagnosis*
- Pneumonia, Viral / therapy*
- Retrospective Studies
- Risk Factors
- SARS-CoV-2
- Symptom Assessment
- Virus Shedding
- Young Adult

Full text links

Med J Malaysia Malaysian Medical Association
Proceed to details
Cite
Share
936
Observational Study

Anaesthesia
. 2021 Mar;76(3):320-326.
doi: 10.1111/anae.15293. Epub 2020 Oct 16.

Renal impairment and its impact on clinical outcomes in patients who are critically ill with COVID-19: a multicentre observational study

M Gasparini [1], S Khan [2], J M Patel [3], D Parekh [3], M N Bangash [3], R Stümpfle [4], A Shah [5], B Baharlo [4], S Soni [6], Collaborators
Collaborators, Affiliations Expand

Collaborators

- **Collaborators**:
S Brett, R Broomhead, P Patel, U Waheed, M Templeton, M Chotalia, J Alderman, E Beesley

Affiliations

- [1] Surgery, Cancer and Cardiovascular Division, Imperial College Healthcare NHS Trust, London, UK.
- [2] Medicine and Integrated Care Division, Imperial College Healthcare NHS Trust, London, UK.
- [3] Department of Critical Care Medicine, University Hospital Birmingham, Birmingham, UK.
- [4] Centre for Peri-operative Medicine and Critical Care Research, Imperial College Healthcare NHS Trust, London, UK.
- [5] University of Oxford, Oxford, UK.
- [6] Division of Anaesthetics, Pain Medicine and Intensive Care, Imperial College London, London, UK.

- PMID: **33948938**
- DOI: 10.1111/anae.15293

Free article
Observational Study

Renal impairment and its impact on clinical outcomes in patients who are critically ill with COVID-19: a multicentre observational study

M Gasparini et al. Anaesthesia. 2021 Mar.
Free article
Show details
Anaesthesia
. 2021 Mar;76(3):320-326.
doi: 10.1111/anae.15293. Epub 2020 Oct 16.

Authors

M Gasparini [1], S Khan [2], J M Patel [3], D Parekh [3], M N Bangash [3], R Stümpfle [4], A Shah [5], B Baharlo [4], S Soni [6], Collaborators

Collaborators

- **Collaborators**: S Brett, R Broomhead, P Patel, U Waheed, M Templeton, M Chotalia, J Alderman, E Beesley

Affiliations

- [1] Surgery, Cancer and Cardiovascular Division, Imperial College Healthcare NHS Trust, London, UK.
- [2] Medicine and Integrated Care Division, Imperial College Healthcare NHS Trust, London, UK.
- [3] Department of Critical Care Medicine, University Hospital Birmingham, Birmingham, UK.
- [4] Centre for Peri-operative Medicine and Critical Care Research, Imperial College Healthcare NHS Trust, London, UK.
- [5] University of Oxford, Oxford, UK.
- [6] Division of Anaesthetics, Pain Medicine and Intensive Care, Imperial College London, London, UK.

- PMID: **33948938**
- DOI: 10.1111/anae.15293

Abstract

Renal impairment is common in patients who are critically ill with coronavirus disease-19 (COVID-19). We examined the association between acute and chronic kidney disease with clinical outcomes in 372 patients with coronavirus disease-19 admitted to four regional intensive care units between 10 March 2020 and 31 July 2020. A total of 216 (58%) patients presented with COVID-19 and renal impairment. Acute kidney injury and/or chronic kidney disease was associated with greater in-hospital mortality compared with patients with preserved renal function

(107/216 patients (50%) (95%CI 44-57) vs. 32/156 (21%) (95%CI 15-28), respectively; p < 0.001, relative risk 2.4 (95%CI 1.7-3.4)). Mortality was greatest in patients with renal transplants (6/7 patients (86%) (95%CI 47-100)). Mortality rates increased in patients with worsening renal injury according to the Kidney Disease: Improving Global Outcomes classification: stage 0 mortality 33/157 patients (21%) (95%CI 15-28) vs. stages 1-3 mortality 91/186 patients (49%) (95%CI 42-56); p < 0.001, relative risk 2.3 (95%CI 1.7-3.3). Survivors were less likely to require renal replacement therapy compared with non-survivors (57/233 patients (24%) vs. 64/139 patients (46%), respectively; p < 0.001, relative risk 1.9 (95%CI 1.4-2.5)). One-fifth of survivors who required renal replacement therapy acutely in intensive care continued to require renal support following discharge. Our data demonstrate that renal impairment in patients admitted to intensive care with COVID-19 is common and is associated with a high mortality and requirement for on-going renal support after discharge from critical care. Our findings have important implications for future pandemic planning in this patient cohort.

Keywords: COVID-19; acute kidney disease; chronic kidney disease; critical illness.

© 2020 The Authors. Anaesthesia published by John Wiley & Sons Ltd on behalf of Association of Anaesthetists.

- 33 references

Supplementary info

Publication types, MeSH terms Expand

Publication types

- Multicenter Study
- Observational Study

MeSH terms

- Acute Kidney Injury / mortality*
- Adult
- Aged
- Aged, 80 and over
- COVID-19 / mortality*
- Causality
- Cohort Studies
- Comorbidity
- Critical Illness
- England / epidemiology
- Female
- Hospital Mortality
- Humans
- Male
- Middle Aged

- Retrospective Studies
- Risk Assessment
- SARS-CoV-2
- Severity of Illness Index
- Young Adult

Full text links

WILEY Free Full Text Wiley
Proceed to details
Cite
Share
☐ 937

Observational Study
S D Med
. 2020 Jul;73(7):312-317.

Critical Illness Due to Covid-19: A Description of the Surge in a Single Center in Sioux Falls

Fady Jamous [1, 2], Natasha Meyer [1], Dana Buus [1], Huthayfa Ateeli [1], Kari Taggart [3, 4], Travis Hanson [1, 5], Mohammed Alzoubaidi [1, 2], Jawad Nazir [6, 2], Joe Devasahayam [1, 2]
Affiliations Expand

Affiliations

- [1] Avera Medical Group - Pulmonary, Critical Care and Sleep Medicine, Sioux Falls, South Dakota.
- [2] University of South Dakota Sanford School of Medicine, Sioux Falls, South Dakota.
- [3] Department of Pharmacy Practice, College of Pharmacy and Allied Health Professions South Dakota State University.
- [4] Avera McKennan Hospital and University Health Center, Sioux Falls, South Dakota.
- [5] Avera McKennan ICU, Sioux Falls, South Dakota.
- [6] Avera Medical Group - Infectious Disease, Sioux Falls, South Dakota.

- PMID: **32805781**

Observational Study

Critical Illness Due to Covid-19: A Description of the Surge in a Single Center in Sioux Falls

Fady Jamous et al. S D Med. 2020 Jul.
Show details
S D Med
. 2020 Jul;73(7):312-317.

Authors

Fady Jamous [1 2], Natasha Meyer [1], Dana Buus [1], Huthayfa Ateeli [1], Kari Taggart [3 4], Travis Hanson [1 5], Mohammed Alzoubaidi [1 2], Jawad Nazir [6 2], Joe Devasahayam [1 2]

Affiliations

- [1] Avera Medical Group - Pulmonary, Critical Care and Sleep Medicine, Sioux Falls, South Dakota.
- [2] University of South Dakota Sanford School of Medicine, Sioux Falls, South Dakota.
- [3] Department of Pharmacy Practice, College of Pharmacy and Allied Health Professions South Dakota State University.
- [4] Avera McKennan Hospital and University Health Center, Sioux Falls, South Dakota.
- [5] Avera McKennan ICU, Sioux Falls, South Dakota.
- [6] Avera Medical Group - Infectious Disease, Sioux Falls, South Dakota.

- PMID: **32805781**

Abstract

Background: We aim to describe the basic demographics, clinical course and outcomes of critically ill patients with Covid-19 admitted to Avera McKennan Hospital and University Health Center Intensive Care Unit (ICU) between March 20 and May 4, 2020.

Methods: In this single centered, retrospective, observational study, we enrolled 37 critically ill adults with COVID-19 pneumonia admitted to the (ICU) between March 20 and May 4, 2020. Demographic data, admitting symptoms, laboratory values, co-morbidities, treatments and clinical outcomes were collected. Data was compared between survivors and non-survivors. We aim to describe our data and report the 28-day mortality as of June 1, 2020.

Results: Of 154 patients admitted with COVID-19 pneumonia during our study period, 37 (24 percent) were critically ill and required an ICU stay. The mean age was 58 years and 76 percent were men. Of these 37 patients, 28 (78 percent) had a chronic illness (diabetes in 43 percent, hypertension in 47 percent). In addition, 54 percent were associated with a local meat packing plant. Most common presenting symptoms were dyspnea (92 percent), cough (70 percent) and fever (68 percent). The mean PaO2/ FiO2 ratio was 143 (67-362). Significant lab findings include the following: 54 percent of patients had lymphocytopenia, the mean ferritin was 850 ng/mL (10-3528), the mean D-Dimer was 4.09 FEU ug/mL and the mean IL-6 was 96.5 pg/mL. At 28 days,

24 percent (nine) had died. Twenty-five (68 percent) patients required mechanical ventilation, with 10 (27 percent) of those patients requiring initiation of neuromuscular blocking agents for ventilator compliance. Of those four (40 percent) did not survive. In addition, 20 patients (54 percent) were proned. Pneumomediastinum or pneumothorax occurred in five of the 37 (14 percent). Renal replacement therapy was required in 6 of the 37 patients, 4 of whom (66 percent) died. Steroids were used in 70 percent of patients, tocilizumab in 59 percent, and hydroxychloroquine in 27 percent. All patients received antibiotics. Convalescent plasma became available for our 5th patient. A total of 29 (78 percent) received convalescent plasma, (86 percent of survivors and 56 percent non-survivors). Median ICU length of stay was 11 days for both survivors (1-49) and non-survivors (1-21). There were no differences in age, body mass index (BMI), or initial PaO2/FiO2 (P/F) among those two groups. Non-survivors (nine) included the two immune compromised patients in our cohort, two patients with pre-existing DNR/DNI status, and one death within two hours of admit. Compared with survivors, more of the non-survivors received vasopressors (78 percent vs 46 percent), dialysis (44 percent vs 7 percent) and hydroxychloroquine (44 percent vs 21 percent). The first 5 patients treated in the ICU did not survive. One month after the initial case was reported in South Dakota, our ICU experienced a six-week surge. At its highest, COVID-19-related census reached 63 percent of the ICU capacity (15/24).

Conclusion: Mortality of critically ill patients with COVID-19 is high. Multi-organ, advanced and prolonged critical care resources are needed. Interpretation of our data is limited by a higher mortality of the earlier members of the cohort, a change in therapeutic practice over time and institution of social distancing.

Copyright© South Dakota State Medical Association.

Supplementary info

Publication types, MeSH terms Expand

Publication types

- Observational Study

MeSH terms

- Betacoronavirus
- COVID-19
- Comorbidity
- Coronavirus Infections / diagnosis*
- Coronavirus Infections / mortality*
- Critical Illness*
- Female
- Humans
- Male
- Meat-Packing Industry
- Middle Aged
- Pandemics

- Pneumonia, Viral / diagnosis*
- Pneumonia, Viral / mortality*
- Retrospective Studies
- SARS-CoV-2
- South Dakota / epidemiology

Proceed to details

Cite

Share

938

Observational Study

Psychiatr Q

. 2021 Dec;92(4):1341-1359.

doi: 10.1007/s11126-021-09907-w. Epub 2021 Mar 26.

Urgent Psychiatric Consultations at Mental Health Center during COVID-19 Pandemic: Retrospective Observational Study

Rosaria Di Lorenzo [1], Gianluca Fiore [2], Alessandra Bruno [3], Margherita Pinelli [2], Davide Bertani [2], Patrizia Falcone [4], Donatella Marrama [5], Fabrizio Starace [6], Paola Ferri [7]

Affiliations Expand

Affiliations

- [1] Psychiatric Intensive Treatment Facility, Mental Health and Drug Abuse Department of AUSL-Modena, Via Paul Harris, 175, 41122, Modena, Italy. saradilorenzo1@alice.it.
- [2] University of Modena and Reggio Emilia, Via del Pozzo, 71, 41124, Modena, Italy.
- [3] School of Nursing, University of Modena and Reggio Emilia, Via del Pozzo, 71, 41124, Modena, Italy.
- [4] Psychiatric Intensive Treatment Facility, Mental Health and Drug Abuse Department of AUSL-Modena, Via Paul Harris, 175, 41122, Modena, Italy.
- [5] Mental Health and Drug Abuse Department of AUSL-Modena, Via Paul Harris, 175, 41122, Modena, Italy.
- [6] Mental Health and Drug Abuse Department of AUSL-Modena, Italian Society of Epidemiological Psychiatry (SIEP), Viale L.A. Muratori 201, 41124 Modena, Italy.
- [7] Department of Biomedical, Metabolic and Neural Sciences, Via G. Campi, 287, 41125, Modena, Italy.

- PMID: **33772425**
- PMCID: PMC7997653
- DOI: 10.1007/s11126-021-09907-w

Free PMC article

Observational Study

Urgent Psychiatric Consultations at Mental Health Center during COVID-19 Pandemic: Retrospective Observational Study

Rosaria Di Lorenzo et al. Psychiatr Q. 2021 Dec.
Free PMC article
Show details
Psychiatr Q
. 2021 Dec;92(4):1341-1359.
doi: 10.1007/s11126-021-09907-w. Epub 2021 Mar 26.

Authors

Rosaria Di Lorenzo [1], Gianluca Fiore [2], Alessandra Bruno [3], Margherita Pinelli [2], Davide Bertani [2], Patrizia Falcone [4], Donatella Marrama [5], Fabrizio Starace [6], Paola Ferri [7]

Affiliations

- [1] Psychiatric Intensive Treatment Facility, Mental Health and Drug Abuse Department of AUSL-Modena, Via Paul Harris, 175, 41122, Modena, Italy. saradilorenzo1@alice.it.
- [2] University of Modena and Reggio Emilia, Via del Pozzo, 71, 41124, Modena, Italy.
- [3] School of Nursing, University of Modena and Reggio Emilia, Via del Pozzo, 71, 41124, Modena, Italy.
- [4] Psychiatric Intensive Treatment Facility, Mental Health and Drug Abuse Department of AUSL-Modena, Via Paul Harris, 175, 41122, Modena, Italy.
- [5] Mental Health and Drug Abuse Department of AUSL-Modena, Via Paul Harris, 175, 41122, Modena, Italy.
- [6] Mental Health and Drug Abuse Department of AUSL-Modena, Italian Society of Epidemiological Psychiatry (SIEP), Viale L.A. Muratori 201, 41124 Modena, Italy.
- [7] Department of Biomedical, Metabolic and Neural Sciences, Via G. Campi, 287, 41125, Modena, Italy.

- PMID: **33772425**
- PMCID: PMC7997653
- DOI: 10.1007/s11126-021-09907-w

Abstract

The coronavirus pandemic and related social distancing measures have brought about dramatic changes in people's lives. In particular, health workers have been forced to change their activities both for the different needs of patients and for preventive measures against the spread of the virus. This study is aimed at comparing the urgent psychiatric consultations (UPC) performed at the outpatient Mental Health Center (MHC) of Modena during the coronavirus outbreak period, from 1 March to 31 August 2020, with the same period in 2019. We retrospectively collected in a database the demographic and clinical characteristics of patients who required UPC in the MHC during the 6-month observation periods in both 2019 and 2020. Data were statistically analyzed. We analyzed 656 urgent psychiatric consultations in 2019 and 811 in 2020, requested by 425

patients in 2019 and 488 in 2020, respectively. In the pandemic period, we observed an increase in the total and daily number of UPC which were more frequently required by patients in care at local outpatient services in comparison with the previous period. During 2020, an increased number of UPC was carried out remotely and the outcome was more frequently represented by discharge at home, avoiding hospitalization as much as possible. In the course of the coronavirus pandemic, MHC had to face an increased demand for clinical activity especially from the most clinically and socially vulnerable patients, who more frequently required UPC in outpatient psychiatric services.

Keywords: COVID-19; Coronavirus pandemic; Mental Health Center; Urgent psychiatric consultations.

© 2021. The Author(s), under exclusive licence to Springer Science+Business Media, LLC, part of Springer Nature.

Conflict of interest statement

All authors declare that they have no conflicts of interest.

- 45 references

Supplementary info

Publication types, MeSH terms Expand

Publication types

- Observational Study

MeSH terms

- Adult
- Ambulatory Care Facilities
- COVID-19* / epidemiology
- COVID-19* / psychology
- Emergency Services, Psychiatric* / statistics & numerical data
- Female
- Humans
- Italy / epidemiology
- Male
- Mental Disorders* / therapy
- Middle Aged
- Pandemics*
- Referral and Consultation* / statistics & numerical data
- Retrospective Studies

Full text links

SpringerLink FULL-TEXT ARTICLE Springer Free PMC article

Proceed to details

Cite

Share

☐ 939

Observational Study

Clin Exp Nephrol

. 2021 Apr;25(4):401-409.

doi: 10.1007/s10157-020-01996-4. Epub 2021 Jan 4.

Hypokalemia in Patients with COVID-19

Gaetano Alfano [1] [2] [3], Annachiara Ferrari [4], Francesco Fontana [5], Rossella Perrone [4], Giacomo Mori [5], Elisabetta Ascione [5], Riccardo Magistroni [4] [5], Giulia Venturi [6], Simone Pederzoli [7], Gianluca Margiotta [7], Marilina Romeo [7], Francesca Piccinini [7], Giacomo Franceschi [8], Sara Volpi [8], Matteo Faltoni [8], Giacomo Ciusa [8], Erica Bacca [8], Marco Tutone [8], Alessandro Raimondi [8], Marianna Menozzi [8], Erica Franceschini [8], Gianluca Cuomo [8], Gabriella Orlando [8], Antonella Santoro [8], Margherita Di Gaetano [8], Cinzia Puzzolante [8], Federica Carli [8], Andrea Bedini [8], Jovana Milic [9] [8], Marianna Meschiari [8], Cristina Mussini [8], Gianni Cappelli [4] [5], Giovanni Guaraldi [8], Modena Covid-19 Working Group (MoCo19)

Collaborators, Affiliations Expand

Collaborators

- **Modena Covid-19 Working Group (MoCo19)**: Cristina Mussini, Giovanni Guaraldi, Erica Bacca, Andrea Bedini, Vanni Borghi, Giulia Burastero, Federica Carli, Giacomo Ciusa, Luca Corradi, Gianluca Cuomo, Margherita Di Gaetano, Giovanni Dolci, Matteo Faltoni, Riccardo Fantini, Giacomo Franceschi, Erica Franceschini, Vittorio Iadisernia, Damiano Larné, Marianna Menozzi, Marianna Meschiari, Jovana Milic, Gabriella Orlando, Francesco Pellegrino, Alessandro Raimondi, Carlotta Rogati, Antonella Santoro, Roberto Tonelli, Marco Tutone, Sara Volpi, Dina Yaacoub, Gianni Cappelli, Riccardo Magistroni, Gaetano Alfano, Francesco Fontana, Ballestri Marco, Giacomo Mori, Roberto Pulizzi, Elisabetta Ascione, Marco Leonelli, Francesca Facchini, Francesca Damiano, Massimo Girardis, Alberto Andreotti, Emanuela Biagioni, Filippo Bondi, Stefano Busani, Giovanni Chierego, Marzia Scotti, Lucia Serio Andrea Cossarizza, Caterina Bellinazzi, Rebecca Borella, Sara De Biasi, Anna De Gaetano, Lucia Fidanza, Lara Gibellini, Anna Iannone, Domenico Lo Tartaro, Marco Mattioli, Milena Nasi, Annamaria Paolini, Marcello Pinti

Affiliations

- [1] Surgical, Medical and Dental Department of Morphological Sciences, Section of Nephrology, University of Modena and Reggio Emilia, via del Pozzo 71, 41124, Modena, Italy. gaetano.alfano@unimore.it.

- [2] Nephrology, Dialysis and Transplant Unit, University Hospital of Modena, Modena, Italy. gaetano.alfano@unimore.it.
- [3] Clinical and Experimental Medicine Ph.D. Program, University of Modena and Reggio Emilia, Modena, Italy. gaetano.alfano@unimore.it.
- [4] Surgical, Medical and Dental Department of Morphological Sciences, Section of Nephrology, University of Modena and Reggio Emilia, via del Pozzo 71, 41124, Modena, Italy.
- [5] Nephrology, Dialysis and Transplant Unit, University Hospital of Modena, Modena, Italy.
- [6] Department of Biomedical, Metabolic and Neural Sciences, Section of Clinical Neurosciences, University of Modena and Reggio Emilia, Modena, Italy.
- [7] Unit of Endocrinology, Department of Biomedical, Metabolic and Neural Sciences, University of Modena and Reggio Emilia,, Modena, Italy.
- [8] Clinic of Infectious Diseases, University Hospital of Modena, Modena, Italy.
- [9] Clinical and Experimental Medicine Ph.D. Program, University of Modena and Reggio Emilia, Modena, Italy.

- PMID: **33398605**
- PMCID: PMC7781399
- DOI: 10.1007/s10157-020-01996-4

Free PMC article
Observational Study

Hypokalemia in Patients with COVID-19

Gaetano Alfano et al. Clin Exp Nephrol. 2021 Apr.
Free PMC article

Show details

Clin Exp Nephrol
. 2021 Apr;25(4):401-409.
doi: 10.1007/s10157-020-01996-4. Epub 2021 Jan 4.

Authors

Gaetano Alfano [1] [2] [3], Annachiara Ferrari [4], Francesco Fontana [5], Rossella Perrone [4], Giacomo Mori [5], Elisabetta Ascione [5], Riccardo Magistroni [4] [5], Giulia Venturi [6], Simone Pederzoli [7], Gianluca Margiotta [7], Marilina Romeo [7], Francesca Piccinini [7], Giacomo Franceschi [8], Sara Volpi [8], Matteo Faltoni [8], Giacomo Ciusa [8], Erica Bacca [8], Marco Tutone [8], Alessandro Raimondi [8], Marianna Menozzi [8], Erica Franceschini [8], Gianluca Cuomo [8], Gabriella Orlando [8], Antonella Santoro [8], Margherita Di Gaetano [8], Cinzia Puzzolante [8], Federica Carli [8], Andrea Bedini [8], Jovana Milic [9] [8], Marianna Meschiari [8], Cristina Mussini [8], Gianni Cappelli [4] [5], Giovanni Guaraldi [8], Modena Covid-19 Working Group (MoCo19)

Collaborators

- **Modena Covid-19 Working Group (MoCo19)**: Cristina Mussini, Giovanni Guaraldi, Erica Bacca, Andrea Bedini, Vanni Borghi, Giulia Burastero, Federica Carli, Giacomo Ciusa, Luca Corradi, Gianluca Cuomo, Margherita Di

Gaetano, Giovanni Dolci, Matteo Faltoni, Riccardo Fantini, Giacomo Franceschi, Erica Franceschini, Vittorio Iadisernia, Damiano Larné, Marianna Menozzi, Marianna Meschiari, Jovana Milic, Gabriella Orlando, Francesco Pellegrino, Alessandro Raimondi, Carlotta Rogati, Antonella Santoro, Roberto Tonelli, Marco Tutone, Sara Volpi, Dina Yaacoub, Gianni Cappelli, Riccardo Magistroni, Gaetano Alfano, Francesco Fontana, Ballestri Marco, Giacomo Mori, Roberto Pulizzi, Elisabetta Ascione, Marco Leonelli, Francesca Facchini, Francesca Damiano, Massimo Girardis, Alberto Andreotti, Emanuela Biagioni, Filippo Bondi, Stefano Busani, Giovanni Chierego, Marzia Scotti, Lucia Serio Andrea Cossarizza, Caterina Bellinazzi, Rebecca Borella, Sara De Biasi, Anna De Gaetano, Lucia Fidanza, Lara Gibellini, Anna Iannone, Domenico Lo Tartaro, Marco Mattioli, Milena Nasi, Annamaria Paolini, Marcello Pinti

Affiliations

- [1] Surgical, Medical and Dental Department of Morphological Sciences, Section of Nephrology, University of Modena and Reggio Emilia, via del Pozzo 71, 41124, Modena, Italy. gaetano.alfano@unimore.it.
- [2] Nephrology, Dialysis and Transplant Unit, University Hospital of Modena, Modena, Italy. gaetano.alfano@unimore.it.
- [3] Clinical and Experimental Medicine Ph.D. Program, University of Modena and Reggio Emilia, Modena, Italy. gaetano.alfano@unimore.it.
- [4] Surgical, Medical and Dental Department of Morphological Sciences, Section of Nephrology, University of Modena and Reggio Emilia, via del Pozzo 71, 41124, Modena, Italy.
- [5] Nephrology, Dialysis and Transplant Unit, University Hospital of Modena, Modena, Italy.
- [6] Department of Biomedical, Metabolic and Neural Sciences, Section of Clinical Neurosciences, University of Modena and Reggio Emilia, Modena, Italy.
- [7] Unit of Endocrinology, Department of Biomedical, Metabolic and Neural Sciences, University of Modena and Reggio Emilia,, Modena, Italy.
- [8] Clinic of Infectious Diseases, University Hospital of Modena, Modena, Italy.
- [9] Clinical and Experimental Medicine Ph.D. Program, University of Modena and Reggio Emilia, Modena, Italy.

- PMID: **33398605**
- PMCID: PMC7781399
- DOI: 10.1007/s10157-020-01996-4

Abstract

Background: Patients with COVID-19 experience multiple clinical conditions that may cause electrolyte imbalances. Hypokalemia is a concerning electrolyte disorder closely associated with severe complications. This study aimed to estimate prevalence, risk factors and outcome of hypokalemia in a cohort of patients with confirmed COVID-19.

Methods: A retrospective analysis was conducted on 290 non-ICU admitted patients with COVID-19 at the tertiary teaching hospital of Modena, Italy, from February 16 to April 14, 2020.

Results: Hypokalemia was detected in 119 out of 290 patients (41%) during hospitalization. Mean serum potassium was 3.1 ± 0.1 meq/L. The majority of patients (90.7%) patients experienced only a mild decrease in serum potassium level (3-3.4 mEq/L). Hypokalemia was associated with hypocalcemia, which was detected in 50% of subjects. Urine potassium-to-creatinine ratio,

measured in a small number of patients (n = 45; 36.1%), revealed an increase of urinary potassium excretion in most cases (95.5%). Risk factors for hypokalemia were female sex (odds ratio (OR) 2.44; 95% CI 1.36-4.37; P 0.003) and diuretic therapy (OR 1.94, 95% CI 1.08-3.48; P 0.027). Hypokalemia, adjusted for sex, age and SOFA score, was not associated with ICU transfer (OR 0.52; 95% CI 0.228-1.212; P = 0.131), in-hospital mortality (OR, 0.47; 95% CI 0.170-1.324; P = 0.154) and composite outcome of ICU transfer or in-hospital mortality (OR 0.48; 95% CI 0.222-1.047; P = 0.065) in our cohort of patients.

Conclusions: Hypokalemia was a frequent disorder in subjects with COVID-19. Female sex and diuretic therapy were identified as risk factors for low serum potassium levels. Hypokalemia was unrelated to ICU transfer and death in this cohort of patients.

Keywords: COVID; Coronavirus; Electrolytes; Hypokalemia; Magnesium; Potassium.

Conflict of interest statement

The authors have no conflicts of interest to declare.

- 29 references
- 1 figure

Supplementary info

Publication types, MeSH terms, Substances Expand

Publication types

- Observational Study

MeSH terms

- Aged
- Aged, 80 and over
- COVID-19 / complications*
- Diuretics / adverse effects
- Female
- Hospital Mortality
- Humans
- Hypokalemia / drug therapy
- Hypokalemia / epidemiology
- Hypokalemia / etiology*
- Male
- Middle Aged
- Potassium / blood
- Potassium / urine
- Prevalence
- Retrospective Studies

- Risk Factors
- SARS-CoV-2*

Substances

- Diuretics
- Potassium

Full text links

SpringerLink FULL-TEXT ARTICLE Springer Free PMC article
Proceed to details
Cite
Share
940
Observational Study
J Orthop Surg Res
. 2021 Feb 2;16(1):103.
doi: 10.1186/s13018-021-02217-8.

Experience of clinical screening for COVID-19 among patients undergoing elective orthopedic surgeries: an alternative proposal

Edwarth Soler [1], Sammy Nicolás Farah [2], Valeria P Bustos [3], Sofía Elizabeth Muñoz Medina [4], Jairo Fernando Gómez [5], Ernesto Martinez Lema [6], Carlos Álvarez Moreno [7]
Affiliations Expand

Affiliations

- [1] Department of Orthopedics, Keralty Group, Clínica Universitaria Colombia, Bogotá, Colombia. edsp33@yahoo.com.
- [2] Department of Orthopedics, Keralty Group, Fundación Universitaria Sanitas, Bogotá, Colombia.
- [3] Faculty of Medicine, Pontificia Universidad Javeriana, Bogotá, Colombia.
- [4] Department of Epidemiology, Keralty Group, Fundación Universitaria Sanitas, Bogotá, Colombia.
- [5] Department of Orthopedics, Keralty Group, Clínica Universitaria Colombia, Bogotá, Colombia.
- [6] Department of Orthopedics, Clínica Reina Sofia, Keralty Group, Bogotá, Colombia.
- [7] Department of Infectious Diseases, Sanitas-Keralty Group, Bogotá, Colombia.

- PMID: **33531037**
- PMCID: PMC7851635

- DOI: 10.1186/s13018-021-02217-8

Free PMC article
Observational Study

Experience of clinical screening for COVID-19 among patients undergoing elective orthopedic surgeries: an alternative proposal

Edwarth Soler et al. J Orthop Surg Res. 2021.
Free PMC article
Show details
J Orthop Surg Res
. 2021 Feb 2;16(1):103.
doi: 10.1186/s13018-021-02217-8.

Authors

Edwarth Soler [1], Sammy Nicolás Farah [2], Valeria P Bustos [3], Sofía Elizabeth Muñoz Medina [4], Jairo Fernando Gómez [5], Ernesto Martinez Lema [6], Carlos Álvarez Moreno [7]

Affiliations

- [1] Department of Orthopedics, Keralty Group, Clínica Universitaria Colombia, Bogotá, Colombia. edsp33@yahoo.com.
- [2] Department of Orthopedics, Keralty Group, Fundación Universitaria Sanitas, Bogotá, Colombia.
- [3] Faculty of Medicine, Pontificia Universidad Javeriana, Bogotá, Colombia.
- [4] Department of Epidemiology, Keralty Group, Fundación Universitaria Sanitas, Bogotá, Colombia.
- [5] Department of Orthopedics, Keralty Group, Clínica Universitaria Colombia, Bogotá, Colombia.
- [6] Department of Orthopedics, Clínica Reina Sofia, Keralty Group, Bogotá, Colombia.
- [7] Department of Infectious Diseases, Sanitas-Keralty Group, Bogotá, Colombia.

- PMID: **33531037**
- PMCID: PMC7851635
- DOI: 10.1186/s13018-021-02217-8

Abstract

Background: The coronavirus disease 2019 (COVID-19) pandemic is the largest global event in recent times, with millions of infected people and hundreds of thousands of deaths worldwide. Colombia has also been affected by the pandemic, including by the cancellation of medically necessary surgical procedures that were categorized as nonessential. The objective of this study was to show the results of the program implemented in two institutions in Bogotá, Colombia, in April 2020 to support the performance of elective essential and nonessential low- and medium-

complexity orthopedic surgeries during the mitigation phase of the COVID-19 pandemic, which involved a presurgical clinical protocol without serological or molecular testing.

Methods: This was a multicenter, observational, retrospective, descriptive study of a cohort of patients who underwent elective orthopedic surgery at two institutions in the city of Bogota, Colombia, in April 2020. We implemented a preoperative clinical protocol that did not involve serological or molecular tests; the protocol consisted of a physical examination, a survey of symptoms and contact with confirmed or suspected cases, and presurgical isolation. We recorded the types of surgeries, the patients' scores on the medically necessary, time-sensitive (MeNTs) scale, the presence of signs, symptoms, and mortality associated with COVID-19 developed after the operation.

Results: A total of 179 patients underwent orthopedic surgery. The average age was 47 years (Shapiro-Wilk, P = 0.021), and the range was between 18 and 81 years. There was a female predominance (61.5%). With regard to the types of surgeries, 86 (48%) were knee operations, 42 (23.5%) were hand surgeries, 34 (19%) were shoulder surgeries, and 17 (9.5%) were foot and ankle surgeries. The average MeNTs score was 44.6 points. During the 2 weeks after surgery, four patients were suspected of having COVID-19 because they developed at least two symptoms associated with the disease. The incidence of COVID-19 in the postoperative period was 2.3%. Two (1.1%) of these four patients visited an emergency department where RT-PCR tests were performed, and they tested negative for severe acute respiratory syndrome coronavirus 2 (SARS-CoV-2). No patients died or were hospitalized for symptoms of COVID-19.

Conclusion: Through the implementation of a presurgical clinical protocol consisting of a physical examination; a clinical survey inquiring about signs, symptoms, and epidemiological contact with suspected or confirmed cases; and presurgical isolation but not involving the performance of molecular or serological diagnostic tests, positive results were obtained with regard to the performance of low- and medium-complexity elective orthopedic surgeries in an early stage of the COVID-19 pandemic.

Level of evidence: IV.

Keywords: Coronavirus; Orthopedic procedures; Outpatient surgical procedures.

Conflict of interest statement

The authors declare that they have no competing interests.

- 21 references
- 1 figure

Supplementary info

Publication types, MeSH terms Expand

Publication types

- Multicenter Study
- Observational Study

MeSH terms

- Adolescent
- Adult
- Aged
- Aged, 80 and over
- COVID-19 / diagnosis*
- COVID-19 / epidemiology
- Cohort Studies
- Elective Surgical Procedures / methods*
- Elective Surgical Procedures / standards
- Female
- Humans
- Male
- Middle Aged
- Orthopedic Procedures / methods*
- Orthopedic Procedures / standards
- Preoperative Care / methods*
- Preoperative Care / standards
- Retrospective Studies
- Surveys and Questionnaires*
- Young Adult

Full text links

Read free full text at BMC BioMed Central Free PMC article
Proceed to details
Cite
Share
☐ 941
Observational Study
Lancet
. 2020 Jun 6;395(10239):1771-1778.
doi: 10.1016/S0140-6736(20)31103-X. Epub 2020 May 13.

An outbreak of severe Kawasaki-like disease at the Italian epicentre of the SARS-CoV-2 epidemic: an observational cohort study

Lucio Verdoni [1], Angelo Mazza [1], Annalisa Gervasoni [1], Laura Martelli [1], Maurizio Ruggeri [1], Matteo Ciuffreda [2], Ezio Bonanomi [3], Lorenzo D'Antiga [4]

Affiliations Expand

Affiliations

- [1] Paediatric Department, Hospital Papa Giovanni XXIII, Bergamo, Italy.
- [2] Paediatric Cardiology, Hospital Papa Giovanni XXIII, Bergamo, Italy.
- [3] Paediatric Intensive Care Unit, Hospital Papa Giovanni XXIII, Bergamo, Italy.
- [4] Paediatric Department, Hospital Papa Giovanni XXIII, Bergamo, Italy. Electronic address: ldantiga@asst-pg23.it.

- PMID: **32410760**
- PMCID: PMC7220177
- DOI: 10.1016/S0140-6736(20)31103-X

Free PMC article
Observational Study

An outbreak of severe Kawasaki-like disease at the Italian epicentre of the SARS-CoV-2 epidemic: an observational cohort study

Lucio Verdoni et al. Lancet. 2020.
Free PMC article
Show details
Lancet
. 2020 Jun 6;395(10239):1771-1778.
doi: 10.1016/S0140-6736(20)31103-X. Epub 2020 May 13.

Authors

Lucio Verdoni [1], Angelo Mazza [1], Annalisa Gervasoni [1], Laura Martelli [1], Maurizio Ruggeri [1], Matteo Ciuffreda [2], Ezio Bonanomi [3], Lorenzo D'Antiga [4]

Affiliations

- [1] Paediatric Department, Hospital Papa Giovanni XXIII, Bergamo, Italy.
- [2] Paediatric Cardiology, Hospital Papa Giovanni XXIII, Bergamo, Italy.
- [3] Paediatric Intensive Care Unit, Hospital Papa Giovanni XXIII, Bergamo, Italy.
- [4] Paediatric Department, Hospital Papa Giovanni XXIII, Bergamo, Italy. Electronic address: ldantiga@asst-pg23.it.

- PMID: **32410760**
- PMCID: PMC7220177
- DOI: 10.1016/S0140-6736(20)31103-X

Abstract

Background: The Bergamo province, which is extensively affected by the severe acute respiratory syndrome coronavirus 2 (SARS-CoV-2) epidemic, is a natural observatory of virus manifestations in the general population. In the past month we recorded an outbreak of Kawasaki disease; we aimed to evaluate incidence and features of patients with Kawasaki-like disease diagnosed during the SARS-CoV-2 epidemic.

Methods: All patients diagnosed with a Kawasaki-like disease at our centre in the past 5 years were divided according to symptomatic presentation before (group 1) or after (group 2) the beginning of the SARS-CoV-2 epidemic. Kawasaki- like presentations were managed as Kawasaki disease according to the American Heart Association indications. Kawasaki disease shock syndrome (KDSS) was defined by presence of circulatory dysfunction, and macrophage activation syndrome (MAS) by the Paediatric Rheumatology International Trials Organisation criteria. Current or previous infection was sought by reverse-transcriptase quantitative PCR in nasopharyngeal and oropharyngeal swabs, and by serological qualitative test detecting SARS-CoV-2 IgM and IgG, respectively.

Findings: Group 1 comprised 19 patients (seven boys, 12 girls; aged 3·0 years [SD 2·5]) diagnosed between Jan 1, 2015, and Feb 17, 2020. Group 2 included ten patients (seven boys, three girls; aged 7·5 years [SD 3·5]) diagnosed between Feb 18 and April 20, 2020; eight of ten were positive for IgG or IgM, or both. The two groups differed in disease incidence (group 1 vs group 2, 0·3 vs ten per month), mean age (3·0 vs 7·5 years), cardiac involvement (two of 19 vs six of ten), KDSS (zero of 19 vs five of ten), MAS (zero of 19 vs five of ten), and need for adjunctive steroid treatment (three of 19 vs eight of ten; all $p<0·01$).

Interpretation: In the past month we found a 30-fold increased incidence of Kawasaki-like disease. Children diagnosed after the SARS-CoV-2 epidemic began showed evidence of immune response to the virus, were older, had a higher rate of cardiac involvement, and features of MAS. The SARS-CoV-2 epidemic was associated with high incidence of a severe form of Kawasaki disease. A similar outbreak of Kawasaki-like disease is expected in countries involved in the SARS-CoV-2 epidemic.

Funding: None.

Copyright © 2020 Elsevier Ltd. All rights reserved.

Comment in

- Autoimmune and inflammatory diseases following COVID-19.
  Galeotti C, Bayry J. Galeotti C, et al. Nat Rev Rheumatol. 2020 Aug;16(8):413-414. doi: 10.1038/s41584-020-0448-7. Nat Rev Rheumatol. 2020. PMID: 32499548 Free PMC article.
- Cytokine Profile in an Adolescent With Pediatric Multisystem Inflammatory Syndrome Temporally Related to COVID-19.
  Buonsenso D, Di Sante G, Sali M; CURE COVID-19 Study Group. Buonsenso D, et al. Pediatr Infect Dis J. 2020 Aug;39(8):e213-e215. doi: 10.1097/INF.0000000000002802. Pediatr Infect Dis J. 2020. PMID: 32677813 No abstract available.
- Association between COVID-19 and Kawasaki-like disease in children is a topic that needs further investigation.
  Moradi S, Radgoodarzi M. Moradi S, et al. Evid Based Nurs. 2021 Apr;24(2):35. doi: 10.1136/ebnurs-2020-103319. Epub 2020 Dec 1. Evid Based Nurs. 2021. PMID: 33262169 No abstract available.

- COVID-19-induced Kawasaki disease.
  Carvalho T. Carvalho T. Nat Med. 2020 Dec;26(12):1807. doi: 10.1038/s41591-020-01163-y. Nat Med. 2020. PMID: 33288939 No abstract available.

- 37 references
- 1 figure

Supplementary info

Publication types, MeSH terms Expand

Publication types

- Observational Study

MeSH terms

- Betacoronavirus
- COVID-19
- Child
- Child, Preschool
- Coronavirus Infections / epidemiology*
- Disease Outbreaks
- Female
- Humans
- Italy
- Male
- Mucocutaneous Lymph Node Syndrome / epidemiology*
- Pandemics
- Pneumonia, Viral / epidemiology*
- Retrospective Studies
- SARS-CoV-2

Full text links

THE LANCET FULL-TEXT ARTICLE Elsevier Science Free PMC article
Proceed to details
Cite
Share
942
Observational Study
Med Mal Infect
. 2020 Aug;50(5):436-439.
doi: 10.1016/j.medmal.2020.04.006. Epub 2020 Apr 17.

Features of anosmia in COVID-19

T Klopfenstein [1], N J Kadiane-Oussou [2], L Toko [2], P-Y Royer [2], Q Lepiller [3], V Gendrin [2], S Zayet [4]

Affiliations Expand

Affiliations

- [1] Infectious Diseases Department, Nord Franche-Comté Hospital, 90400 Trévenans, France. Electronic address: timothee.klopfenstein@hnfc.fr.
- [2] Infectious Diseases Department, Nord Franche-Comté Hospital, 90400 Trévenans, France.
- [3] Virology Department, Centre-Hospitalier Universitaire de Besançon, 25000 Besançon, France.
- [4] Infectious Diseases Department, Nord Franche-Comté Hospital, 90400 Trévenans, France. Electronic address: souhail.zayet@gmail.com.

- PMID: **32305563**
- PMCID: PMC7162775
- DOI: 10.1016/j.medmal.2020.04.006

Free PMC article
Observational Study

Features of anosmia in COVID-19

T Klopfenstein et al. Med Mal Infect. 2020 Aug.
Free PMC article
Show details
Med Mal Infect
. 2020 Aug;50(5):436-439.
doi: 10.1016/j.medmal.2020.04.006. Epub 2020 Apr 17.

Authors

T Klopfenstein [1], N J Kadiane-Oussou [2], L Toko [2], P-Y Royer [2], Q Lepiller [3], V Gendrin [2], S Zayet [4]

Affiliations

- [1] Infectious Diseases Department, Nord Franche-Comté Hospital, 90400 Trévenans, France. Electronic address: timothee.klopfenstein@hnfc.fr.
- [2] Infectious Diseases Department, Nord Franche-Comté Hospital, 90400 Trévenans, France.
- [3] Virology Department, Centre-Hospitalier Universitaire de Besançon, 25000 Besançon, France.
- [4] Infectious Diseases Department, Nord Franche-Comté Hospital, 90400 Trévenans, France. Electronic address: souhail.zayet@gmail.com.

- PMID: **32305563**
- PMCID: PMC7162775
- DOI: 10.1016/j.medmal.2020.04.006

Abstract

Background: Medical publications about anosmia with COVID-19 are scarce. We aimed to describe the prevalence and features of anosmia in COVID-19 patients.

Methods: We retrospectively included COVID-19 patients with anosmia between March 1st and March 17th, 2020. We used SARS-CoV-2 real time PCR in respiratory samples to confirm the cases.

Results: Fifty-four of 114 patients (47%) with confirmed COVID-19 reported anosmia. Mean age of the 54 patients was 47 (±16) years; 67% were females and 37% were hospitalised. The median Charlson comorbidity index was 0.70 (±1.6 [0-7]). Forty-six patients (85%) had dysgeusia and 28% presented with pneumonia. Anosmia began 4.4 (±1.9 [1-8]) days after infection onset. The mean duration of anosmia was 8.9 (±6.3 [1-21]) days and 98% of patients recovered within 28 days.

Conclusions: Anosmia was present in half of our European COVID-19 patients and was often associated with dysgeusia.

Keywords: Anosmia; COVID-19; Dysgeusia.

Copyright © 2020 Elsevier Masson SAS. All rights reserved.

- 19 references
- 1 figure

Supplementary info

Publication types, MeSH terms Expand

Publication types

- Observational Study

MeSH terms

- Adult
- Aged
- Betacoronavirus / physiology
- COVID-19
- Cohort Studies
- Comorbidity
- Coronavirus Infections / complications*
- Coronavirus Infections / epidemiology*

- Coronavirus Infections / pathology
- Coronavirus Infections / therapy
- Female
- France / epidemiology
- Humans
- Male
- Middle Aged
- Olfaction Disorders / epidemiology*
- Olfaction Disorders / therapy
- Olfaction Disorders / virology*
- Oxygen Inhalation Therapy
- Pandemics
- Pneumonia / epidemiology
- Pneumonia / physiopathology
- Pneumonia / therapy
- Pneumonia / virology
- Pneumonia, Viral / complications*
- Pneumonia, Viral / epidemiology*
- Pneumonia, Viral / pathology
- Pneumonia, Viral / therapy
- Prevalence
- Respiratory Function Tests
- Retrospective Studies
- SARS-CoV-2
- Severity of Illness Index
- Time Factors

Full text links

ELSEVIER FULL-TEXT ARTICLE Elsevier Science Free PMC article
Proceed to details
Cite
Share
☐ 943
Observational Study
Diabetes Metab Res Rev
. 2021 Sep;37(6):e3404.
doi: 10.1002/dmrr.3404. Epub 2020 Oct 5.

Reduction of hypoglycaemia, lifestyle modifications and psychological distress

[during lockdown following SARS-CoV-2 outbreak in type 1 diabetes]

Irene Caruso [1], Sergio Di Molfetta [1], Francesca Guarini [1], Fiorella Giordano [1], Angelo Cignarelli [1], Annalisa Natalicchio [1], Sebastio Perrini [1], Anna Leonardini [1], Francesco Giorgino [1], Luigi Laviola [1]
Affiliations Expand

Affiliation

- [1] Department of Emergency and Organ Transplantation, Section of Internal Medicine, Endocrinology, Andrology and Metabolic Diseases, University of Bari Aldo Moro, Bari, Italy.

- PMID: **32918324**
- DOI: 10.1002/dmrr.3404

Observational Study

Reduction of hypoglycaemia, lifestyle modifications and psychological distress during lockdown following SARS-CoV-2 outbreak in type 1 diabetes

Irene Caruso et al. Diabetes Metab Res Rev. 2021 Sep.
Show details
Diabetes Metab Res Rev
. 2021 Sep;37(6):e3404.
doi: 10.1002/dmrr.3404. Epub 2020 Oct 5.

Authors

Irene Caruso [1], Sergio Di Molfetta [1], Francesca Guarini [1], Fiorella Giordano [1], Angelo Cignarelli [1], Annalisa Natalicchio [1], Sebastio Perrini [1], Anna Leonardini [1], Francesco Giorgino [1], Luigi Laviola [1]

Affiliation

- [1] Department of Emergency and Organ Transplantation, Section of Internal Medicine, Endocrinology, Andrology and Metabolic Diseases, University of Bari Aldo Moro, Bari, Italy.

- PMID: **32918324**
- DOI: 10.1002/dmrr.3404

Abstract

Aims: To assess changes in glucose metrics and their association with psychological distress and lifestyle changes in patients with type 1 diabetes (T1D) using flash glucose monitoring (FGM) during lockdown following severe acute respiratory syndrome coronavirus 2 outbreak.

Materials and methods: Single-centre, observational, retrospective study enrolling T1D patients who attended a remote visit on April 2020 at the Endocrinology division of the University Hospital Policlinico Consorziale, Bari, Italy. Lockdown-related changes in physical activity level and dietary habits were assessed on a semi-quantitative basis. Changes in general well-being were assessed by the General Health Questionnaire-12 items with a binary scoring system. Glucose metrics were obtained from the Libreview platform for the first 2 weeks of February 2020 (T0) and the last 2 weeks before the phone visit (T1).

Results: Out of 84 patients assessed for eligibility, 48 had sufficient FGM data to be included in the analysis. FGM data analysis revealed significant reductions in coefficient of variation, number of hypoglycaemic events, and time below range, while no changes were found in time in range, time above range, mean sensor glucose, and glucose management indicator. Moreover, the frequency of sweets consumption was inversely related to the occurrence of hypoglycaemic events during lockdown.

Conclusions: Lockdown-related lifestyle changes, albeit unhealthy, may lead to reduction in FGM-derived measures of hypoglycaemia and glycaemic variability in patients with T1D.

Keywords: COVID-19; FGM; hypoglycaemia; lifestyle; lockdown; type 1 diabetes.

© 2020 John Wiley & Sons Ltd.

- 25 references

Supplementary info

Publication types, MeSH terms Expand

Publication types

- Observational Study

MeSH terms

- Adolescent
- Adult
- Behavior Therapy / statistics & numerical data
- Blood Glucose Self-Monitoring
- COVID-19 / epidemiology
- COVID-19 / prevention & control*
- Communicable Disease Control*
- Diabetes Mellitus, Type 1* / blood

- Diabetes Mellitus, Type 1* / epidemiology
- Diabetes Mellitus, Type 1* / psychology
- Diabetes Mellitus, Type 1* / therapy
- Disease Outbreaks
- Female
- Humans
- Hypoglycemia / blood
- Hypoglycemia / epidemiology*
- Italy / epidemiology
- Life Style
- Male
- Middle Aged
- Pandemics
- Patient Compliance / psychology
- Patient Compliance / statistics & numerical data
- Psychological Distress
- Quarantine / statistics & numerical data
- Remote Consultation
- Retrospective Studies
- SARS-CoV-2
- Stress, Psychological / epidemiology*
- Stress, Psychological / etiology
- Young Adult

Full text links

WILEY Full Text Article Wiley

Proceed to details

Cite

Share

☐ 944

Observational Study

Crit Care

. 2021 Aug 19;25(1):298.
doi: 10.1186/s13054-021-03728-w.

Inhaled liposomal amphotericin-B as a prophylactic treatment for COVID-19-associated pulmonary aspergillosis/aspergillus tracheobronchitis

Sofie Van Ackerbroeck [1], Lynn Rutsaert [1 2], Ella Roelant [3 4], Kathleen Dillen [5], Joost Wauters [6], Niels Van Regenmortel [7 8]
Affiliations Expand

Affiliations

- [1] Department of Intensive Care Medicine, Ziekenhuis Netwerk Antwerpen Campus Stuivenberg, Lange Beeldekensstraat 267, 2060, Antwerp, Belgium.
- [2] Department of Haematology, Gasthuiszusters Antwerpen, Campus St. Augustinus, Oosterveldlaan 24, 2610, Wilrijk, Belgium.
- [3] Center for Statistics, StatUa, University of Antwerp, Prinsstraat 13, 2000, Antwerp, Belgium.
- [4] Clinical Trial Center (CTC), CRC Antwerp, Antwerp University Hospital, University of Antwerp, Drie Eikenstraat 655, 2650, Edegem, Belgium.
- [5] Department of Pharmacy, Ziekenhuis Netwerk Antwerpen Campus Stuivenberg, Lange Beeldekensstraat 267, 2060, Antwerp, Belgium.
- [6] Medical Intensive Care Unit, Department of General Internal Medicine, University Hospitals Leuven, Leuven, Belgium.
- [7] Department of Intensive Care Medicine, Ziekenhuis Netwerk Antwerpen Campus Stuivenberg, Lange Beeldekensstraat 267, 2060, Antwerp, Belgium. niels.vanregenmortel@zna.be.
- [8] Department of Intensive Care Medicine, Antwerp University Hospital, Drie Eikenstraat 655, 2650, Edegem, Antwerp, Belgium. niels.vanregenmortel@zna.be.

- PMID: **34412686**
- PMCID: PMC8374123
- DOI: 10.1186/s13054-021-03728-w

Free PMC article
Observational Study

Inhaled liposomal amphotericin-B as a prophylactic treatment for COVID-19-associated pulmonary aspergillosis/aspergillus tracheobronchitis

Sofie Van Ackerbroeck et al. Crit Care. 2021.

Free PMC article

Show details

Crit Care

. 2021 Aug 19;25(1):298.

doi: 10.1186/s13054-021-03728-w.

Authors

Sofie Van Ackerbroeck [1], Lynn Rutsaert [1 2], Ella Roelant [3 4], Kathleen Dillen [5], Joost Wauters [6], Niels Van Regenmortel [7 8]

Affiliations

- [1] Department of Intensive Care Medicine, Ziekenhuis Netwerk Antwerpen Campus Stuivenberg, Lange Beeldekensstraat 267, 2060, Antwerp, Belgium.
- [2] Department of Haematology, Gasthuiszusters Antwerpen, Campus St. Augustinus, Oosterveldlaan 24, 2610, Wilrijk, Belgium.
- [3] Center for Statistics, StatUa, University of Antwerp, Prinsstraat 13, 2000, Antwerp, Belgium.
- [4] Clinical Trial Center (CTC), CRC Antwerp, Antwerp University Hospital, University of Antwerp, Drie Eikenstraat 655, 2650, Edegem, Belgium.
- [5] Department of Pharmacy, Ziekenhuis Netwerk Antwerpen Campus Stuivenberg, Lange Beeldekensstraat 267, 2060, Antwerp, Belgium.
- [6] Medical Intensive Care Unit, Department of General Internal Medicine, University Hospitals Leuven, Leuven, Belgium.
- [7] Department of Intensive Care Medicine, Ziekenhuis Netwerk Antwerpen Campus Stuivenberg, Lange Beeldekensstraat 267, 2060, Antwerp, Belgium. niels.vanregenmortel@zna.be.
- [8] Department of Intensive Care Medicine, Antwerp University Hospital, Drie Eikenstraat 655, 2650, Edegem, Antwerp, Belgium. niels.vanregenmortel@zna.be.

- PMID: **34412686**
- PMCID: PMC8374123
- DOI: 10.1186/s13054-021-03728-w

No abstract available

Conflict of interest statement

The authors declare that they have no conflict of interest.

- 6 references
- 1 figure

Supplementary info

Publication types, MeSH terms, Substances Expand

Publication types

- Letter
- Observational Study

MeSH terms

- Administration, Inhalation
- Aged
- Amphotericin B / administration & dosage
- Amphotericin B / pharmacology*
- Amphotericin B / therapeutic use
- Aspergillosis / drug therapy*
- Aspergillosis / epidemiology
- Aspergillosis / prevention & control*
- Belgium / epidemiology
- COVID-19 / complications*
- COVID-19 / epidemiology
- Cohort Studies
- Female
- Humans
- Male
- Middle Aged
- Retrospective Studies

Substances

- liposomal amphotericin B
- Amphotericin B

Full text links

Read free full text at BMC BioMed Central Free PMC article
Proceed to details
Cite
Share
945
Observational Study
Transfus Apher Sci
. 2021 Aug;60(4):103160.
doi: 10.1016/j.transci.2021.103160. Epub 2021 May 26.

Use of face mask by blood donors during the COVID-19 pandemic: Impact on donor hemoglobin concentration: A bane or a boon

Rasika Setia [1], Mitu Dogra [2], Anil Handoo [3], Ramesh Yadav [4], Gokhula Prasath Thangavel [5], Amena Ebadur Rahman [6]
Affiliations Expand

Affiliations

- [1] Department of Transfusion Medicine, BLK Superspecialty Hospital, India. Electronic address: rasika_setia@yahoo.co.in.
- [2] Department of Transfusion Medicine, BLK Superspecialty Hospital, India. Electronic address: drmithu25@yahoo.in.
- [3] Department of Hematology, BLK Superspecialty Hospital, India. Electronic address: ahhemat@gmail.com.
- [4] Department of Transfusion Medicine, BLK Superspecialty Hospital, India. Electronic address: ramesh.yadav@blkhospital.com.
- [5] Department of Transfusion Medicine, BLK Superspecialty Hospital, India. Electronic address: gokhula.prasath@blkhospital.com.
- [6] Department of Transfusion Medicine, BLK Superspecialty Hospital, India. Electronic address: amena.rahman@blkhospital.com.

- PMID: **34217601**
- PMCID: PMC8152240
- DOI: 10.1016/j.transci.2021.103160

Free PMC article
Observational Study

Use of face mask by blood donors during the COVID-19 pandemic: Impact on donor hemoglobin concentration: A bane or a boon

Rasika Setia et al. Transfus Apher Sci. 2021 Aug.
Free PMC article
Show details
Transfus Apher Sci
. 2021 Aug;60(4):103160.
doi: 10.1016/j.transci.2021.103160. Epub 2021 May 26.

Authors

Rasika Setia [1], Mitu Dogra [2], Anil Handoo [3], Ramesh Yadav [4], Gokhula Prasath Thangavel [5], Amena Ebadur Rahman [6]

Affiliations

- [1] Department of Transfusion Medicine, BLK Superspecialty Hospital, India. Electronic address: rasika_setia@yahoo.co.in.
- [2] Department of Transfusion Medicine, BLK Superspecialty Hospital, India. Electronic address: drmithu25@yahoo.in.
- [3] Department of Hematology, BLK Superspecialty Hospital, India. Electronic address: ahhemat@gmail.com.
- [4] Department of Transfusion Medicine, BLK Superspecialty Hospital, India. Electronic address: ramesh.yadav@blkhospital.com.
- [5] Department of Transfusion Medicine, BLK Superspecialty Hospital, India. Electronic address: gokhula.prasath@blkhospital.com.
- [6] Department of Transfusion Medicine, BLK Superspecialty Hospital, India. Electronic address: amena.rahman@blkhospital.com.

- PMID: **34217601**
- PMCID: PMC8152240
- DOI: 10.1016/j.transci.2021.103160

Abstract

Background: COVID-19 virus has caused the world's deadliest pandemic. Early April 2020, the Delhi Government made it compulsory for people to wear face masks while going outdoors to curb disease spread. Prolonged use of surgical masks during the pandemic has been reported to cause many adverse effects. Intermittent hypoxia has been shown to activate erythropoietin (EPO leading to increased hemoglobin mass.

Aim: To analyze whether face mask induced intermittent hypoxia has any effect on the hemoglobin levels of healthy blood donors.

Materials and methods: We retrospectively analyzed donor data from 1st July 2019-31st December 2020 for hemoglobin distribution across hemoglobin ranges and donor deferral on basis of hemoglobin. Study population was divided into two cohorts Group 1- (1st July 2019-31 st March 2020): before implementation of mandatory face masks Group 2- (1st April 2020-31 st December 2020): after implementation of mandatory face masks RESULTS: Mean Hb of blood donors in Group 2 (15.01 ± 1.1 g/dl) was higher than Group1 (14.49 ± 1.15 g/dl), ($p < 0.0001$). 47.1 % group2 donors had Hb of 16.1-18 g/dl compared to group1 (38.4 %). 52.9 % group 2 donors had Hb between 12.5-15 g/dl compared to 61.6 % Group 1 ($p < 0.05$). Deferral due to anemia was lesser in group 2 compared to group 1 ($p < 0.00001$). Group 2 had significantly higher deferral due to high Hb (>18 gm/dl) was than Group 1 ($p = 0.0039$).

Conclusion: This study including 19504 blood donors spanning over one and a half year shows that prolonged use of face mask by blood donors may lead to intermittent hypoxia and consequent increase in hemoglobin mass.

Keywords: COVID-19; Donor hemoglobin; Erythropoietin; Face mask; Intermittent hypoxia; Prolonged use.

Copyright © 2021 Elsevier Ltd. All rights reserved.

Conflict of interest statement

All authors declare that they do not have any conflict of interest that could inappropriately influence the present study.

- 20 references
- 1 figure

Supplementary info

Publication types, MeSH terms, Substances Expand

Publication types

- Comparative Study
- Observational Study

MeSH terms

- Adolescent
- Adult
- Aged
- Blood Donors*
- COVID-19 / prevention & control*
- Cross-Sectional Studies
- Donor Selection / standards
- Erythropoietin / physiology*
- Female
- Hemoglobins / analysis*
- Hemoglobins / biosynthesis
- Humans
- Hypoxia / blood
- Hypoxia / etiology*
- Male
- Masks / adverse effects*
- Middle Aged
- Pandemics*
- Retrospective Studies
- SARS-CoV-2*
- Young Adult

Substances

- EPO protein, human
- Hemoglobins
- Erythropoietin

Full text links

Elsevier Science Free PMC article
Proceed to details
Cite
Share
946
Observational Study
J Antimicrob Chemother
. 2020 Nov 1;75(11):3359-3365.
doi: 10.1093/jac/dkaa321.

Effectiveness of remdesivir in patients with COVID-19 under mechanical ventilation in an Italian ICU

Zeno Pasquini [1, 2], Roberto Montalti [3], Chiara Temperoni [1], Benedetta Canovari [1], Mauro Mancini [4], Michele Tempesta [5], Daniela Pimpini [5], Nicoletta Zallocco [4], Francesco Barchiesi [1, 2]

Affiliations Expand

Affiliations

- [1] Malattie Infettive, Azienda Ospedaliera Ospedali Riuniti Marche Nord, Pesaro, Italy.
- [2] Dipartimento di Scienze Biomediche e Sanità Pubblica, Università Politecnica delle Marche, Ancona, Italy.
- [3] Unità di Chirurgia Epato-bilio-pancreatica, Mininvasiva e Robotica, Dipartimento di Sanità Pubblica, Università Federico II, Napoli, Italy.
- [4] Farmacia Ospedaliera, Azienda Ospedaliera Ospedali Riuniti Marche Nord, Pesaro, Italy.
- [5] Anestesia e Rianimazione, Azienda Ospedaliera Ospedali Riuniti Marche Nord, Pesaro, Italy.

- PMID: **32829390**
- PMCID: PMC7499641
- DOI: 10.1093/jac/dkaa321

Free PMC article
Observational Study

Effectiveness of remdesivir in patients with COVID-19 under mechanical ventilation in an Italian ICU

Zeno Pasquini et al. J Antimicrob Chemother. 2020.
Free PMC article
Show details
J Antimicrob Chemother
. 2020 Nov 1;75(11):3359-3365.
doi: 10.1093/jac/dkaa321.

Authors

Zeno Pasquini [1, 2], Roberto Montalti [3], Chiara Temperoni [1], Benedetta Canovari [1], Mauro Mancini [4], Michele Tempesta [5], Daniela Pimpini [5], Nicoletta Zallocco [4], Francesco Barchiesi [1, 2]

Affiliations

- [1] Malattie Infettive, Azienda Ospedaliera Ospedali Riuniti Marche Nord, Pesaro, Italy.
- [2] Dipartimento di Scienze Biomediche e Sanità Pubblica, Università Politecnica delle Marche, Ancona, Italy.
- [3] Unità di Chirurgia Epato-bilio-pancreatica, Mininvasiva e Robotica, Dipartimento di Sanità Pubblica, Università Federico II, Napoli, Italy.
- [4] Farmacia Ospedaliera, Azienda Ospedaliera Ospedali Riuniti Marche Nord, Pesaro, Italy.
- [5] Anestesia e Rianimazione, Azienda Ospedaliera Ospedali Riuniti Marche Nord, Pesaro, Italy.

- PMID: **32829390**
- PMCID: PMC7499641
- DOI: 10.1093/jac/dkaa321

Abstract

Background: Remdesivir is a prodrug with in vitro activity against severe acute respiratory syndrome coronavirus-2 (SARS-CoV-2). Its clinical efficacy in patients with COVID-19 under mechanical ventilation remains to be evaluated.

Methods: This study includes patients under mechanical ventilation with confirmed SARS-CoV-2 infection admitted to the ICU of Pesaro hospital between 29 February and 20 March 2020. During this period, remdesivir was provided on a compassionate use basis. Clinical characteristics and outcome of patients treated with remdesivir were collected retrospectively and compared with those of patients hospitalized in the same time period.

Results: A total of 51 patients were considered, of which 25 were treated with remdesivir. The median (IQR) age was 67 (59-75.5) years, 92% were men and symptom onset was 10 (8-12) days before admission to ICU. At baseline, there was no significant difference in demographic

characteristics, comorbidities and laboratory values between patients treated and not treated with remdesivir. Median follow-up was 52 (46-57) days. Kaplan-Meier curves showed significantly lower mortality among patients who had been treated with remdesivir (56% versus 92%, P < 0.001). Cox regression analysis showed that the Charlson Comorbidity Index was the only factor that had a significant association with higher mortality (OR 1.184; 95% CI 1.027-1.365; P = 0.020), while the use of remdesivir was associated with better survival (OR 3.506; 95% CI 1.768-6.954; P < 0.001).

Conclusions: In this study the mortality rate of patients with COVID-19 under mechanical ventilation is confirmed to be high. The use of remdesivir was associated with a significant beneficial effect on survival.

© The Author(s) 2020. Published by Oxford University Press on behalf of the British Society for Antimicrobial Chemotherapy. All rights reserved. For permissions, please email: journals.permissions@oup.com.

Comment in

- Comment on: Effectiveness of remdesivir in patients with COVID-19 under mechanical ventilation in an Italian ICU.
  Bonazzetti C, Milazzo L, Giacomelli A, Oreni L, Colombo R, Ridolfo AL, Antinori S. Bonazzetti C, et al. J Antimicrob Chemother. 2021 May 12;76(6):1650-1651. doi: 10.1093/jac/dkab033. J Antimicrob Chemother. 2021. PMID: 33561203 Free PMC article. No abstract available.

- 32 references
- 2 figures

Supplementary info

Publication types, MeSH terms, Substances Expand

Publication types

- Observational Study

MeSH terms

- Adenosine Monophosphate / analogs & derivatives*
- Adenosine Monophosphate / therapeutic use
- Aged
- Alanine / analogs & derivatives*
- Alanine / therapeutic use
- Antiviral Agents / therapeutic use*
- Betacoronavirus*
- COVID-19
- Coronavirus Infections / diagnosis
- Coronavirus Infections / mortality*

- Coronavirus Infections / therapy
- Female
- Follow-Up Studies
- Humans
- Intensive Care Units* / trends
- Italy / epidemiology
- Male
- Middle Aged
- Pandemics
- Pneumonia, Viral / diagnosis
- Pneumonia, Viral / mortality*
- Pneumonia, Viral / therapy
- Respiration, Artificial / mortality*
- Respiration, Artificial / trends
- Retrospective Studies
- SARS-CoV-2
- Treatment Outcome

Substances

- Antiviral Agents
- remdesivir
- Adenosine Monophosphate
- Alanine

Full text links

OXFORD ACADEMIC Silverchair Information Systems Free PMC article
Proceed to details
Cite
Share
947
Observational Study
J Am Heart Assoc
. 2021 Mar 16;10(6):e018477.
doi: 10.1161/JAHA.120.018477. Epub 2020 Oct 30.

Troponin and Other Biomarker Levels and Outcomes Among Patients Hospitalized With

COVID-19: Derivation and Validation of the HA $_2$ T $_2$ COVID-19 Mortality Risk Score

Kevin K Manocha [1], Jared Kirzner [1], Xiaohan Ying [1], Ilhwan Yeo [2], Bradley Peltzer [1], Bryan Ang [1], Han A Li [1], Bruce B Lerman [1], Monika M Safford [3], Parag Goyal [1], Jim W Cheung [1]
Affiliations Expand

Affiliations

- [1] Department of Medicine Division of Cardiology Weill Cornell Medicine - New York Presbyterian Hospital New York NY.
- [2] Department of Medicine Division of Cardiology New York Presbyterian Hospital-Queens Flushing NY.
- [3] Division of General Internal Medicine Department of Medicine Weill Cornell Medicine - New York Presbyterian Hospital New York NY.

- PMID: **33121304**
- PMCID: PMC8174190
- DOI: 10.1161/JAHA.120.018477

Free PMC article
Observational Study

Troponin and Other Biomarker Levels and Outcomes Among Patients Hospitalized With COVID-19: Derivation and Validation of the HA $_2$ T $_2$ COVID-19 Mortality Risk Score

Kevin K Manocha et al. J Am Heart Assoc. 2021.
Free PMC article
Show details
J Am Heart Assoc
. 2021 Mar 16;10(6):e018477.
doi: 10.1161/JAHA.120.018477. Epub 2020 Oct 30.

Authors

Kevin K Manocha [1], Jared Kirzner [1], Xiaohan Ying [1], Ilhwan Yeo [2], Bradley Peltzer [1], Bryan Ang [1], Han A Li [1], Bruce B Lerman [1], Monika M Safford [3], Parag Goyal [1], Jim W Cheung [1]

Affiliations

- [1] Department of Medicine Division of Cardiology Weill Cornell Medicine - New York Presbyterian Hospital New York NY.

- [2] Department of Medicine Division of Cardiology New York Presbyterian Hospital-Queens Flushing NY.
- [3] Division of General Internal Medicine Department of Medicine Weill Cornell Medicine - New York Presbyterian Hospital New York NY.

- PMID: **33121304**
- PMCID: PMC8174190
- DOI: 10.1161/JAHA.120.018477

Abstract

Background The independent prognostic value of troponin and other biomarker elevation among patients with coronavirus disease 2019 (COVID-19) are unclear. We sought to characterize biomarker levels in patients hospitalized with COVID-19 and develop and validate a mortality risk score. Methods and Results An observational cohort study of 1053 patients with COVID-19 was conducted. Patients with all of the following biomarkers measured-troponin-I, B-type natriuretic peptide, C-reactive protein, ferritin, and d-dimer (n=446) -were identified. Maximum levels for each biomarker were recorded. The primary end point was 30-day in-hospital mortality. Multivariable logistic regression was used to construct a mortality risk score. Validation of the risk score was performed using an independent patient cohort (n=440). Mean age of patients was 65.0±15.2 years and 65.3% were men. Overall, 444 (99.6%) had elevation of any biomarker. Among tested biomarkers, troponin-I ≥0.34 ng/mL was the only independent predictor of 30-day mortality (adjusted odds ratio, 4.38; P<0.001). Patients with a mortality score using hypoxia on presentation, age, and troponin-I elevation, age (HA_2T_2) ≥3 had a 30-day mortality of 43.7% while those with a score <3 had mortality of 5.9%. Area under the receiver operating characteristic curve of the HA_2T_2 score was 0.834 for the derivation cohort and 0.784 for the validation cohort. Conclusions Elevated troponin and other biomarker levels are commonly seen in patients hospitalized with COVID-19. High troponin levels are a potent predictor of 30-day in-hospital mortality. A simple risk score can stratify patients at risk for COVID-19-associated mortality.

Keywords: COVID-19; biomarkers; mortality; troponin.

Conflict of interest statement

Dr Cheung has received consulting fees from Abbott, Biosense Webster, Biotronik, and Boston Scientific and fellowship grant support from Abbott, Biosense Webster, Biotronik, Boston Scientific, and Medtronic. Dr Safford has received research grant support from Amgen. The remaining authors have no disclosures to report.

- 21 references
- 5 figures

Supplementary info

Publication types, MeSH terms, Substances, Grant support Expand

Publication types

- Observational Study
- Research Support, Non-U.S. Gov't

- Validation Study

MeSH terms

- Aged
- Aged, 80 and over
- Biomarkers / blood
- C-Reactive Protein / analysis
- COVID-19 / blood
- COVID-19 / diagnosis*
- COVID-19 / mortality
- Cardiovascular Diseases / blood
- Cardiovascular Diseases / diagnosis*
- Cardiovascular Diseases / mortality
- Female
- Ferritins / blood
- Fibrin Fibrinogen Degradation Products / analysis
- Health Status Indicators*
- Hospital Mortality
- Hospitalization*
- Humans
- Male
- Middle Aged
- Natriuretic Peptide, Brain / blood
- Predictive Value of Tests
- Prognosis
- Reproducibility of Results
- Retrospective Studies
- Risk Assessment
- Risk Factors
- Time Factors
- Troponin I / blood*
- Up-Regulation

Substances

- Biomarkers
- Fibrin Fibrinogen Degradation Products
- Troponin I
- fibrin fragment D
- Natriuretic Peptide, Brain
- C-Reactive Protein

- Ferritins

Grant support

- UL1 TR000457/TR/NCATS NIH HHS/United States

Full text links

OPEN ACCESS Atypon Free PMC article
Proceed to details
Cite
Share
948
Observational Study
J Thromb Thrombolysis
. 2021 Jul;52(1):95-104.
doi: 10.1007/s11239-020-02340-z. Epub 2020 Nov 16.

Clinical features of patients with acute coronary syndrome during the COVID-19 pandemic

Kensuke Matsushita [1 2], Sebastien Hess [1], Benjamin Marchandot [1], Chisato Sato [1 2], Dinh Phi Truong [1 3], Ngoc Thanh Kim [1 3], Anne Weiss [4], Laurence Jesel [1 2], Patrick Ohlmann [1], Olivier Morel [5 6]
Affiliations Expand

Affiliations

- [1] Pôle d'Activité Médico-Chirurgicale Cardio-Vasculaire, Nouvel Hôpital Civil, Centre Hospitalier Universitaire, Université de Strasbourg, 1 place de l'Hôpital, 67091, Strasbourg cedex, France.
- [2] UMR1260 INSERM, Nanomédecine Régénérative, Faculté de Pharmacie, Université de Strasbourg, Illkirch, France.
- [3] Vietnam National Heart Institute, Bach Mai Hospital, Hanoi, Vietnam.
- [4] Centre Hospitalier Universitaire, SAMU 67, Strasbourg, France.
- [5] Pôle d'Activité Médico-Chirurgicale Cardio-Vasculaire, Nouvel Hôpital Civil, Centre Hospitalier Universitaire, Université de Strasbourg, 1 place de l'Hôpital, 67091, Strasbourg cedex, France. olivier.morel@chru-strasbourg.fr.
- [6] UMR1260 INSERM, Nanomédecine Régénérative, Faculté de Pharmacie, Université de Strasbourg, Illkirch, France. olivier.morel@chru-strasbourg.fr.

- PMID: **33200333**
- PMCID: PMC7668406
- DOI: 10.1007/s11239-020-02340-z

Free PMC article
Observational Study

Clinical features of patients with acute coronary syndrome during the COVID-19 pandemic

Kensuke Matsushita et al. J Thromb Thrombolysis. 2021 Jul.
Free PMC article
Show details
J Thromb Thrombolysis
. 2021 Jul;52(1):95-104.
doi: 10.1007/s11239-020-02340-z. Epub 2020 Nov 16.

Authors

Kensuke Matsushita [1, 2], Sebastien Hess [1], Benjamin Marchandot [1], Chisato Sato [1, 2], Dinh Phi Truong [1, 3], Ngoc Thanh Kim [1, 3], Anne Weiss [4], Laurence Jesel [1, 2], Patrick Ohlmann [1], Olivier Morel [5, 6]

Affiliations

- [1] Pôle d'Activité Médico-Chirurgicale Cardio-Vasculaire, Nouvel Hôpital Civil, Centre Hospitalier Universitaire, Université de Strasbourg, 1 place de l'Hôpital, 67091, Strasbourg cedex, France.
- [2] UMR1260 INSERM, Nanomédecine Régénérative, Faculté de Pharmacie, Université de Strasbourg, Illkirch, France.
- [3] Vietnam National Heart Institute, Bach Mai Hospital, Hanoi, Vietnam.
- [4] Centre Hospitalier Universitaire, SAMU 67, Strasbourg, France.
- [5] Pôle d'Activité Médico-Chirurgicale Cardio-Vasculaire, Nouvel Hôpital Civil, Centre Hospitalier Universitaire, Université de Strasbourg, 1 place de l'Hôpital, 67091, Strasbourg cedex, France. olivier.morel@chru-strasbourg.fr.
- [6] UMR1260 INSERM, Nanomédecine Régénérative, Faculté de Pharmacie, Université de Strasbourg, Illkirch, France. olivier.morel@chru-strasbourg.fr.

- PMID: **33200333**
- PMCID: PMC7668406
- DOI: 10.1007/s11239-020-02340-z

Abstract

Although a reduction in hospital admissions of acute coronary syndromes (ACS) patients has been observed globally during the coronavirus disease 2019 (COVID-19) pandemic, clinical features of those patients have not been fully investigated. The aim of the present analysis is to investigate the incidence, clinical presentation, and outcomes of patients with ACS during the COVID-19 pandemic. We performed a retrospective analysis of consecutive patients who were admitted for ACS at our institution between March 1 and April 20, 2020 and compared with the equivalent

period in 2019. Admissions for acute myocardial infarction (AMI) reduced by 39.5% in 2020 compared with the equivalent period in 2019. Owing to the emergency medical services (EMS) of our region, all time components of ST-elevated myocardial infarction care were similar during the COVID-19 outbreak as compared with the previous year's dataset. Among the 106 ACS patients in 2020, 7 patients tested positive for COVID-19. Higher incidence of type 2 myocardial infarction (29% vs. 4%, p = 0.0497) and elevated D-dimer levels (5650 µg/l [interquartile range (IQR) 1905-13,625 µg/l] vs. 400 µg/l [IQR 270-1050 µg/l], p = 0.02) were observed in COVID-19 patients. In sum, a significant reduction in admission for AMI was observed during the COVID-19 pandemic. COVID-19 patients were characterized by elevated D-dimer levels on admission, reflecting enhanced COVID-19 related thrombogenicity. The prehospital evaluation by EMS may have played an important role for the timely revascularization for STEMI patients.

Keywords: Acute coronary syndrome; Catheterization laboratory; Coronavirus disease 2019; Percutaneous coronary intervention.

© 2020. Springer Science+Business Media, LLC, part of Springer Nature.

Conflict of interest statement

The authors declare that they have no conflict of interest.

- 24 references
- 3 figures

Supplementary info

Publication types, MeSH terms, Substances Expand

Publication types

- Comparative Study
- Observational Study

MeSH terms

- Acute Coronary Syndrome / diagnosis
- Acute Coronary Syndrome / epidemiology
- Acute Coronary Syndrome / therapy*
- Aged
- Aged, 80 and over
- Angina, Unstable / diagnosis
- Angina, Unstable / epidemiology
- Angina, Unstable / therapy*
- Biomarkers / blood
- COVID-19 / diagnosis
- COVID-19 / epidemiology
- COVID-19 / therapy*

- Emergency Medical Services
- Female
- Fibrin Fibrinogen Degradation Products / analysis
- Humans
- Incidence
- Male
- Middle Aged
- Non-ST Elevated Myocardial Infarction / diagnosis
- Non-ST Elevated Myocardial Infarction / epidemiology
- Non-ST Elevated Myocardial Infarction / therapy*
- Patient Admission
- Percutaneous Coronary Intervention*
- Retrospective Studies
- ST Elevation Myocardial Infarction / diagnosis
- ST Elevation Myocardial Infarction / epidemiology
- ST Elevation Myocardial Infarction / therapy*
- Time Factors
- Time-to-Treatment
- Treatment Outcome

Substances

- Biomarkers
- Fibrin Fibrinogen Degradation Products
- fibrin fragment D

Full text links

SpringerLink FULL-TEXT ARTICLE Springer Free PMC article
Proceed to details
Cite
Share
☐ 949
J Diabetes Res
. 2021 Jul 3;2021:5515902.
doi: 10.1155/2021/5515902. eCollection 2021.

Diabetes Management Delivery and Pregnancy Outcomes in Women with Gestational Diabetes Mellitus during the First

Wave of the 2020 COVID-19 Pandemic: A Single-Reference Center Report

Magdalena Wilk [1, 2], Paulina Surowiec [1, 2], Bartłomiej Matejko [1, 2], Albert Wróbel [3], Joanna Zięba-Parkitny [2], Katarzyna Cyganek [1, 2], Hubert Huras [2, 4], Maciej T Małecki [1, 2]
Affiliations Expand

Affiliations

- [1] Department of Metabolic Diseases, Jagiellonian University Medical College, Krakow, Poland.
- [2] University Hospital, Krakow, Poland.
- [3] Students' Scientific Group, Department of Metabolic Diseases, Jagiellonian University Medical College, Krakow, Poland.
- [4] Department of Obstetrics and Perinatology, Jagiellonian University Medical College, Krakow, Poland.

- PMID: **34307689**
- PMCID: PMC8279845
- DOI: 10.1155/2021/5515902

Free PMC article

Diabetes Management Delivery and Pregnancy Outcomes in Women with Gestational Diabetes Mellitus during the First Wave of the 2020 COVID-19 Pandemic: A Single-Reference Center Report

Magdalena Wilk et al. J Diabetes Res. 2021.
Free PMC article
Show details
J Diabetes Res
. 2021 Jul 3;2021:5515902.
doi: 10.1155/2021/5515902. eCollection 2021.

Authors

Magdalena Wilk [1, 2], Paulina Surowiec [1, 2], Bartłomiej Matejko [1, 2], Albert Wróbel [3], Joanna Zięba-Parkitny [2], Katarzyna Cyganek [1, 2], Hubert Huras [2, 4], Maciej T Małecki [1, 2]

Affiliations

- [1] Department of Metabolic Diseases, Jagiellonian University Medical College, Krakow, Poland.
- [2] University Hospital, Krakow, Poland.
- [3] Students' Scientific Group, Department of Metabolic Diseases, Jagiellonian University Medical College, Krakow, Poland.
- [4] Department of Obstetrics and Perinatology, Jagiellonian University Medical College, Krakow, Poland.

- PMID: **34307689**
- PMCID: PMC8279845
- DOI: 10.1155/2021/5515902

Abstract

Objectives: The COVID-19 pandemic has forced a rapid adaptation of healthcare services to secure care for many patient groups. This includes women with gestational diabetes mellitus (GDM). We evaluated the impacts of the first COVID-19 wave on parameters such as the GDM treatment, glycemic control, and pregnancy outcomes.

Methods: In this retrospective study from a reference diabetes center (Krakow, Poland), we compared patient data from two different time periods: the first wave of the COVID-19 pandemic (March 2020-June 2020) and the preceding five months (October 2019-February 2020). Data was collected from the medical records and telephone surveys.

Results: We included 155 consecutive women (group N1 = 73 and group N2 = 82 from the COVID-19 pandemic period and non-COVID-19 period, respectively). During the COVID-19 pandemic, almost half of all GDM women (N1 = 36, 49.3%) used telemedicine as a method of contacting their diabetic specialists while this tool was not utilized in the earlier period. Moreover, these patients reported difficulties in performing blood glucose self-control more often (N1 = 20, 27.4%, vs N2 = 7, 8.5%; $p \leq 0.01$) and spent less time on diabetes education than the control group on average (N1 = 39, 53.4%, vs N2 = 9, 9.8% below 2 hours of training; $p \leq 0.01$). Most analyzed glycemic parameters and pregnancy outcomes were similar. Differences were found with respect to the incidence of prolonged labor (N1 = 12, 16.4%, vs N2 = 3, 3.7%; $p \leq 0.01$) and preeclampsia (N1 = 0 vs N2 = 7, 8.5%; $p = 0.01$).

Conclusion: In this single-center observational study, the first wave of the COVID-19 pandemic did not seem to have a negative impact on pregnancy outcomes in GDM women, despite the difficulties in diabetes management delivery.

Copyright © 2021 Magdalena Wilk et al.

Conflict of interest statement

The authors declare no conflict of interest.

- 33 references

Supplementary info

MeSH terms, Substances Expand

MeSH terms

- Adult
- Blood Glucose / metabolism
- COVID-19 / epidemiology*
- Diabetes, Gestational / blood
- Diabetes, Gestational / therapy*
- Disease Management
- Female
- Humans
- Infant, Newborn
- Male
- Pandemics*
- Poland / epidemiology
- Pregnancy
- Pregnancy Outcome
- Retrospective Studies
- SARS-CoV-2*
- Telemedicine

Substances

- Blood Glucose

Full text links

Hindawi Limited Free PMC article

Proceed to details

Cite

Share

☐ 950

Clinical Trial

Crit Care Med

. 2021 Feb 1;49(2):e191-e198.
doi: 10.1097/CCM.0000000000004711.

Almitrine Infusion in Severe Acute Respiratory Syndrome Coronavirus 2-Induced Acute Respiratory Distress

Syndrome: A Single-Center Observational Study

Morgan Caplan [1], Julien Goutay [1], Anne Bignon [2], Emmanuelle Jaillette [1], Raphael Favory [3], Daniel Mathieu [1], Erika Parmentier-Decrucq [1], Julien Poissy [4], Thibault Duburcq [1], Lille Intensive Care COVID-19 Group
Affiliations Expand

Affiliations

- [1] Pôle de Réanimation, CHU Lille, University of Lille, Lille, France.
- [2] Réanimation Chirurgicale, CHU Lille, University of Lille, Lille, France.
- [3] Pôle de réanimation, Lille Inflammation Research International Center (LIRIC), CHU Lille, Inserm U995, University of Lille, Lille, France.
- [4] Pôle de réanimation, CNRS, UMR 8576 - Unité de Glycobiologie Structurale et Fonctionnelle, CHU Lille, Inserm U1285, University of Lille, Lille, France.

- PMID: **33093279**
- DOI: 10.1097/CCM.0000000000004711

Clinical Trial

Almitrine Infusion in Severe Acute Respiratory Syndrome Coronavirus 2-Induced Acute Respiratory Distress Syndrome: A Single-Center Observational Study

Morgan Caplan et al. Crit Care Med. 2021.
Show details
Crit Care Med
. 2021 Feb 1;49(2):e191-e198.
doi: 10.1097/CCM.0000000000004711.

Authors

Morgan Caplan [1], Julien Goutay [1], Anne Bignon [2], Emmanuelle Jaillette [1], Raphael Favory [3], Daniel Mathieu [1], Erika Parmentier-Decrucq [1], Julien Poissy [4], Thibault Duburcq [1], Lille Intensive Care COVID-19 Group

Affiliations

- [1] Pôle de Réanimation, CHU Lille, University of Lille, Lille, France.

- [2] Réanimation Chirurgicale, CHU Lille, University of Lille, Lille, France.
- [3] Pôle de réanimation, Lille Inflammation Research International Center (LIRIC), CHU Lille, Inserm U995, University of Lille, Lille, France.
- [4] Pôle de réanimation, CNRS, UMR 8576 - Unité de Glycobiologie Structurale et Fonctionnelle, CHU Lille, Inserm U1285, University of Lille, Lille, France.

- PMID: **33093279**
- DOI: 10.1097/CCM.0000000000004711

Abstract

Objectives: Treating acute respiratory failure in patients with coronavirus disease 2019 is challenging due to the lack of knowledge of the underlying pathophysiology. Hypoxemia may be explained in part by the loss of hypoxic pulmonary vasoconstriction. The present study assessed the effect of almitrine, a selective pulmonary vasoconstrictor, on arterial oxygenation in severe acute respiratory syndrome coronavirus 2-induced acute respiratory distress syndrome.

Design: Single-center retrospective observational study.

Setting: ICU of Lille Teaching Hospital, France, from February 27, 2020, to April 14, 2020.

Patients: Patients with coronavirus disease 2019 pneumonia confirmed by positive reverse transcriptase-polymerase chain reaction for severe acute respiratory syndrome-coronavirus 2 and acute respiratory distress syndrome according to Berlin definition. Data focused on clinicobiological features, ventilator settings, therapeutics, outcomes, and almitrine-related adverse events.

Interventions: Almitrine was considered in patients with severe hypoxemia (Pao2/Fio2 ratio < 150 mm Hg) in addition to the recommended therapies, at an hourly IV delivery of 10 µg/kg/min. Comparative blood gases were done before starting almitrine trial and immediately after the end of the infusion. A positive response to almitrine was defined by an increase of Pao2/Fio2 ratio greater than or equal to 20% at the end of the infusion.

Measurements and main results: A total of 169 patients were enrolled. Thirty-two patients with acute respiratory distress syndrome received an almitrine infusion trial. In most cases, almitrine was infused in combination with inhaled nitric oxide (75%). Twenty-one patients (66%) were responders. The median Pao2/Fio2 ratio improvement was 39% (9-93%) and differs significantly between the responders and nonresponders (67% [39-131%] vs 6% [9-16%], respectively; $p < 0.0001$). The 28-day mortality rates were 47.6% and 63.6% ($p = 0.39$) for the responders and nonresponders, respectively. Hemodynamic parameters remained similar before and after the trial, not suggesting acute cor pulmonale.

Conclusions: Almitrine infusion improved oxygenation in severe acute respiratory syndrome coronavirus 2-induced acute respiratory distress syndrome without adverse effects. In a multistep clinical approach to manage severe hypoxemia in this population, almitrine could be an interesting therapeutic option to counteract the loss of hypoxic pulmonary vasoconstriction and redistribute blood flow away from shunting zones.

Copyright © 2020 by the Society of Critical Care Medicine and Wolters Kluwer Health, Inc. All Rights Reserved.

Conflict of interest statement

This work was supported by the French government through the Programme Investissement d'Avenir (I-SITE ULNE/ANR-16-IDEX-0004 ULNE) managed by the Agence Nationale de la Recherche ("PHYSIO COVID" and "PREDICT" projects) The authors have disclosed that they do not have any potential conflicts of interest.

Comment in

- Coronavirus Disease 2019 Acute Respiratory Failure: Almitrine Drug Resuscitation or Resuscitating Patients by Almitrine?
  Payen D. Payen D. Crit Care Med. 2021 Feb 1;49(2):387-389. doi: 10.1097/CCM.0000000000004765. Crit Care Med. 2021. PMID: 33186137 No abstract available.

- 19 references

Supplementary info

Publication types, MeSH terms, Substances Expand

Publication types

- Clinical Trial
- Observational Study

MeSH terms

- Almitrine / therapeutic use*
- COVID-19 / complications
- COVID-19 / drug therapy*
- Critical Care / methods
- Dose-Response Relationship, Drug
- Female
- Humans
- Infusions, Intravenous
- Male
- Middle Aged
- Pulmonary Gas Exchange / drug effects
- Respiratory Distress Syndrome / drug therapy*
- Respiratory Distress Syndrome / etiology
- Respiratory System Agents / therapeutic use*
- Retrospective Studies

Substances

- Respiratory System Agents
- Almitrine

Full text links

Wolters Kluwer Wolters Kluwer
Proceed to details
Cite
Share

951
Observational Study
Lancet Respir Med
. 2021 Feb;9(2):139-148.
doi: 10.1016/S2213-2600(20)30459-8. Epub 2020 Oct 23.

Ventilation management and clinical outcomes in invasively ventilated patients with COVID-19 (PRoVENT-COVID): a national, multicentre, observational cohort study

Michela Botta [1], Anissa M Tsonas [1], Janesh Pillay [2], Leonoor S Boers [1], Anna Geke Algera [1], Lieuwe D J Bos [1], Dave A Dongelmans [1], Marcus W Hollmann [3], Janneke Horn [1], Alexander P J Vlaar [1], Marcus J Schultz [4], Ary Serpa Neto [5], Frederique Paulus [6], PRoVENT-COVID Collaborative Group
Collaborators, Affiliations Expand

Collaborators

- **PRoVENT-COVID Collaborative Group**: Jesse P van Akkeren, Anna Geke Algera, Cheetel K Algoe, Rombout B van Amstel, Onno L Baur, Pablo van de Berg, Alida E van den Berg, Dennis C J J Bergmans, Dido I van den Bersselaar, Freke A Bertens, Alexander J G H Bindels, Milou M de Boer, Sylvia den Boer, Leonoor S Boers, Margriet Bogerd, Lieuwe D J Bos, Michela Botta, Jennifer S Breel, Hendrik de Bruin, Sanne de Bruin, Caro L Bruna, Laura A Buiteman-Kruizinga, Olaf L Cremer, Rogier M Determann, Willem Dieperink, Dave A Dongelmans, Hildegard S Franke, Michal S Galek-Aldridge, Mart J de Graaff, Laura A Hagens, Jasper J Haringman, Sebastiaan T van der Heide, Pim L J van der Heiden, Nanon F L Heijnen, Stephan J P Hiel, Lotte L Hoeijmakers, Liselotte Hol, Markus W Hollmann, Marga E Hoogendoorn, Janneke Horn, Robrecht van der Horst, Evy L K Ie, Dimitri P Ivanov, Nicole Juffermans, Eline Kho, Eline S de Klerk, Ankie W M M Koopman-van

Gemert, Matty Koopmans, Songul Kucukcelebi, Michael A Kuiper, Dylan W de Lange, Niels van Mourik, Sunny G L H Nijbroek, Marisa Onrust, Evelien A N Oostdijk, Frederique Paulus, Charlotte J Pennartz, Janesh Pillay, Luigi Pisani, Ilse M Purmer, Thijs C D Rettig, Jan-Paul Roozeman, Michiel T U Schuijt, Marcus J Schultz, Ary Serpa Neto, Mengalvio E Sleeswijk, Marry R Smit, Peter E Spronk, Willemke Stilma, Aart C Strang, Anissa M Tsonas, Pieter R Tuinman, Christel M A Valk, Felicia L Veen-Schra, Lars I Veldhuis, Patricia van Velzen, Ward H van der Ven, Alexander P J Vlaar, Peter van Vliet, Peter H J van der Voort, Louis van Welie, Henrico J F T Wesselink, Hermien H van der Wier-Lubbers, Bas van Wijk, Tineke Winters, Wing Yi Wong, Arthur R H van Zanten

Affiliations

- [1] Department of Intensive Care, Amsterdam University Medical Centers location Academic Medical Center, Amsterdam, Netherlands.
- [2] Department of Intensive Care, Amsterdam University Medical Centers location Academic Medical Center, Amsterdam, Netherlands; University Medical Center Groningen, Groningen, The Netherlands.
- [3] Department of Anaesthesiology, Amsterdam University Medical Centers location Academic Medical Center, Amsterdam, Netherlands.
- [4] Department of Intensive Care, Amsterdam University Medical Centers location Academic Medical Center, Amsterdam, Netherlands; Mahidol-Oxford Tropical Medicine Research Unit, Mahidol University, Bangkok, Thailand; Nuffield Department of Medicine, University of Oxford, Oxford, UK. Electronic address: marcus.j.schultz@gmail.com.
- [5] Department of Intensive Care, Amsterdam University Medical Centers location Academic Medical Center, Amsterdam, Netherlands; Department of Critical Care Medicine, Hospital Israelita Albert Einstein, Sao Paulo, Brazil; Austin Hospital and University of Melbourne, Melbourne, VIC, Australia.
- [6] Department of Intensive Care, Amsterdam University Medical Centers location Academic Medical Center, Amsterdam, Netherlands; ACHIEVE, Centre of Applied Research, Amsterdam University of Applied Sciences, Faculty of Health, Amsterdam, Netherlands.

- PMID: **33169671**
- PMCID: PMC7584441
- DOI: 10.1016/S2213-2600(20)30459-8

Free PMC article
Observational Study

Ventilation management and clinical outcomes in invasively ventilated patients with COVID-19 (PRoVENT-COVID): a national, multicentre, observational cohort study

Michela Botta et al. Lancet Respir Med. 2021 Feb.
Free PMC article

Show details

Lancet Respir Med
. 2021 Feb;9(2):139-148.
doi: 10.1016/S2213-2600(20)30459-8. Epub 2020 Oct 23.

Authors

Michela Botta [1], Anissa M Tsonas [1], Janesh Pillay [2], Leonoor S Boers [1], Anna Geke Algera [1], Lieuwe D J Bos [1], Dave A Dongelmans [1], Marcus W Hollmann [3], Janneke Horn [1], Alexander P J Vlaar [1], Marcus J Schultz [4], Ary Serpa Neto [5], Frederique Paulus [6], PRoVENT-COVID Collaborative Group

Collaborators

- **PRoVENT-COVID Collaborative Group**: Jesse P van Akkeren, Anna Geke Algera, Cheetel K Algoe, Rombout B van Amstel, Onno L Baur, Pablo van de Berg, Alida E van den Berg, Dennis C J J Bergmans, Dido I van den Bersselaar, Freke A Bertens, Alexander J G H Bindels, Milou M de Boer, Sylvia den Boer, Leonoor S Boers, Margriet Bogerd, Lieuwe D J Bos, Michela Botta, Jennifer S Breel, Hendrik de Bruin, Sanne de Bruin, Caro L Bruna, Laura A Buiteman-Kruizinga, Olaf L Cremer, Rogier M Determann, Willem Dieperink, Dave A Dongelmans, Hildegard S Franke, Michal S Galek-Aldridge, Mart J de Graaff, Laura A Hagens, Jasper J Haringman, Sebastiaan T van der Heide, Pim L J van der Heiden, Nanon F L Heijnen, Stephan J P Hiel, Lotte L Hoeijmakers, Liselotte Hol, Markus W Hollmann, Marga E Hoogendoorn, Janneke Horn, Robrecht van der Horst, Evy L K Ie, Dimitri P Ivanov, Nicole Juffermans, Eline Kho, Eline S de Klerk, Ankie W M M Koopman-van Gemert, Matty Koopmans, Songul Kucukcelebi, Michael A Kuiper, Dylan W de Lange, Niels van Mourik, Sunny G L H Nijbroek, Marisa Onrust, Evelien A N Oostdijk, Frederique Paulus, Charlotte J Pennartz, Janesh Pillay, Luigi Pisani, Ilse M Purmer, Thijs C D Rettig, Jan-Paul Roozeman, Michiel T U Schuijt, Marcus J Schultz, Ary Serpa Neto, Mengalvio E Sleeswijk, Marry R Smit, Peter E Spronk, Willemke Stilma, Aart C Strang, Anissa M Tsonas, Pieter R Tuinman, Christel M A Valk, Felicia L Veen-Schra, Lars I Veldhuis, Patricia van Velzen, Ward H van der Ven, Alexander P J Vlaar, Peter van Vliet, Peter H J van der Voort, Louis van Welie, Henrico J F T Wesselink, Hermien H van der Wier-Lubbers, Bas van Wijk, Tineke Winters, Wing Yi Wong, Arthur R H van Zanten

Affiliations

- [1] Department of Intensive Care, Amsterdam University Medical Centers location Academic Medical Center, Amsterdam, Netherlands.
- [2] Department of Intensive Care, Amsterdam University Medical Centers location Academic Medical Center, Amsterdam, Netherlands; University Medical Center Groningen, Groningen, The Netherlands.
- [3] Department of Anaesthesiology, Amsterdam University Medical Centers location Academic Medical Center, Amsterdam, Netherlands.
- [4] Department of Intensive Care, Amsterdam University Medical Centers location Academic Medical Center, Amsterdam, Netherlands; Mahidol-Oxford Tropical Medicine Research Unit, Mahidol University, Bangkok, Thailand; Nuffield Department of Medicine, University of Oxford, Oxford, UK. Electronic address: marcus.j.schultz@gmail.com.

- [5] Department of Intensive Care, Amsterdam University Medical Centers location Academic Medical Center, Amsterdam, Netherlands; Department of Critical Care Medicine, Hospital Israelita Albert Einstein, Sao Paulo, Brazil; Austin Hospital and University of Melbourne, Melbourne, VIC, Australia.
- [6] Department of Intensive Care, Amsterdam University Medical Centers location Academic Medical Center, Amsterdam, Netherlands; ACHIEVE, Centre of Applied Research, Amsterdam University of Applied Sciences, Faculty of Health, Amsterdam, Netherlands.

- PMID: **33169671**
- PMCID: PMC7584441
- DOI: 10.1016/S2213-2600(20)30459-8

Abstract

Background: Little is known about the practice of ventilation management in patients with COVID-19. We aimed to describe the practice of ventilation management and to establish outcomes in invasively ventilated patients with COVID-19 in a single country during the first month of the outbreak.

Methods: PRoVENT-COVID is a national, multicentre, retrospective observational study done at 18 intensive care units (ICUs) in the Netherlands. Consecutive patients aged at least 18 years were eligible for participation if they had received invasive ventilation for COVID-19 at a participating ICU during the first month of the national outbreak in the Netherlands. The primary outcome was a combination of ventilator variables and parameters over the first 4 calendar days of ventilation: tidal volume, positive end-expiratory pressure (PEEP), respiratory system compliance, and driving pressure. Secondary outcomes included the use of adjunctive treatments for refractory hypoxaemia and ICU complications. Patient-centred outcomes were ventilator-free days at day 28, duration of ventilation, duration of ICU and hospital stay, and mortality. PRoVENT-COVID is registered at ClinicalTrials.gov (NCT04346342).

Findings: Between March 1 and April 1, 2020, 553 patients were included in the study. Median tidal volume was 6·3 mL/kg predicted bodyweight (IQR 5·7-7·1), PEEP was 14·0 cm H_2O (IQR 11·0-15·0), and driving pressure was 14·0 cm H_2O (11·2-16·0). Median respiratory system compliance was 31·9 mL/cm H_2O (26·0-39·9). Of the adjunctive treatments for refractory hypoxaemia, prone positioning was most often used in the first 4 days of ventilation (283 [53%] of 530 patients). The median number of ventilator-free days at day 28 was 0 (IQR 0-15); 186 (35%) of 530 patients had died by day 28. Predictors of 28-day mortality were gender, age, tidal volume, respiratory system compliance, arterial pH, and heart rate on the first day of invasive ventilation.

Interpretation: In patients with COVID-19 who were invasively ventilated during the first month of the outbreak in the Netherlands, lung-protective ventilation with low tidal volume and low driving pressure was broadly applied and prone positioning was often used. The applied PEEP varied widely, despite an invariably low respiratory system compliance. The findings of this national study provide a basis for new hypotheses and sample size calculations for future trials of invasive ventilation for COVID-19. These data could also help in the interpretation of findings from other studies of ventilation practice and outcomes in invasively ventilated patients with COVID-19.

Funding: Amsterdam University Medical Centers, location Academic Medical Center.

Copyright © 2021 Elsevier Ltd. All rights reserved.

Comment in

- Instrumental dead space in ventilator management - Authors' reply.
  Schultz MJ, Bos LDJ, Paulus F, Neto AS. Schultz MJ, et al. Lancet Respir Med. 2021 Mar;9(3):e23. doi: 10.1016/S2213-2600(21)00015-1. Epub 2021 Jan 29. Lancet Respir Med. 2021. PMID: 33524317 No abstract available.
- Instrumental dead space in ventilator management.
  Lellouche F, Grieco DL, Maggiore SM, Antonelli M. Lellouche F, et al. Lancet Respir Med. 2021 Mar;9(3):e22. doi: 10.1016/S2213-2600(21)00024-2. Epub 2021 Jan 29. Lancet Respir Med. 2021. PMID: 33524318 No abstract available.
- [Focus ventilation, oxygen therapy and weaning : Intensive medical care studies from 2020/2021].
  Fiedler MO, Reuß CJ, Bernhard M, Beynon C, Hecker A, Jungk C, Nusshag C, Michalski D, Brenner T, Weigand MA, Dietrich M. Fiedler MO, et al. Anaesthesist. 2021 Nov;70 (11):967-976. doi: 10.1007/s00101-021-00979-8. Epub 2021 Oct 6. Anaesthesist. 2021. PMID: 34613457 Free PMC article. German. No abstract available.

- 34 references
- 3 figures

Supplementary info

Publication types, MeSH terms, Associated data Expand

Publication types

- Multicenter Study
- Observational Study
- Research Support, Non-U.S. Gov't

MeSH terms

- Aged
- COVID-19 / therapy*
- Cohort Studies
- Female
- Humans
- Male
- Middle Aged
- Netherlands
- Respiration, Artificial*
- Retrospective Studies
- Treatment Outcome

Associated data

- ClinicalTrials.gov/NCT04346342

Full text links

ELSEVIER FULL-TEXT ARTICLE Elsevier Science Free PMC article

Proceed to details

Cite

Share

☐ 952

Observational Study

PLoS One
. 2020 Aug 21;15(8):e0237960.
doi: 10.1371/journal.pone.0237960. eCollection 2020.

Prognostic factors in Spanish COVID-19 patients: A case series from Barcelona

Antoni Sisó-Almirall [1 2 3], Belchin Kostov [1 2], Minerva Mas-Heredia [1 3], Sergi Vilanova-Rotllan [1 3], Ethel Sequeira-Aymar [1 4], Mireia Sans-Corrales [1 2 5], Elisenda Sant-Arderiu [1 4], Laia Cayuelas-Redondo [1 2 4], Angela Martínez-Pérez [1 4], Noemí García-Plana [1 5], August Anguita-Guimet [1 3], Jaume Benavent-Àreu [1 2]

Affiliations Expand

Affiliations

- [1] Consorci d'Atenció Primària de Salut Barcelona Esquerra (CAPSBE), Barcelona, Spain.
- [2] Primary Healthcare Transversal Research Group, Institut d'Investigacions Biomèdiques August Pi i Sunyer (IDIBAPS), Barcelona, Spain.
- [3] Primary Care Centre Les Corts, Barcelona, Spain.
- [4] Primary Care Centre Casanova, Barcelona, Spain.
- [5] Primary Care Centre Comte Borrell, Barcelona, Spain.

- PMID: **32822413**
- PMCID: PMC7444503
- DOI: 10.1371/journal.pone.0237960

Free PMC article

Observational Study

Prognostic factors in Spanish COVID-19 patients: A case series from Barcelona

Antoni Sisó-Almirall et al. PLoS One. 2020.

Free PMC article

Show details

PLoS One

. 2020 Aug 21;15(8):e0237960.
doi: 10.1371/journal.pone.0237960. eCollection 2020.

Authors

Antoni Sisó-Almirall [1 2 3], Belchin Kostov [1 2], Minerva Mas-Heredia [1 3], Sergi Vilanova-Rotllan [1 3], Ethel Sequeira-Aymar [1 4], Mireia Sans-Corrales [1 2 5], Elisenda Sant-Arderiu [1 4], Laia Cayuelas-Redondo [1 2 4], Angela Martínez-Pérez [1 4], Noemí García-Plana [1 5], August Anguita-Guimet [1 3], Jaume Benavent-Àreu [1 2]

Affiliations

- [1] Consorci d'Atenció Primària de Salut Barcelona Esquerra (CAPSBE), Barcelona, Spain.
- [2] Primary Healthcare Transversal Research Group, Institut d'Investigacions Biomèdiques August Pi i Sunyer (IDIBAPS), Barcelona, Spain.
- [3] Primary Care Centre Les Corts, Barcelona, Spain.
- [4] Primary Care Centre Casanova, Barcelona, Spain.
- [5] Primary Care Centre Comte Borrell, Barcelona, Spain.

- PMID: **32822413**
- PMCID: PMC7444503
- DOI: 10.1371/journal.pone.0237960

Abstract

Background: In addition to the lack of COVID-19 diagnostic tests for the whole Spanish population, the current strategy is to identify the disease early to limit contagion in the community.

Aim: To determine clinical factors of a poor prognosis in patients with COVID-19 infection.

Design and setting: Descriptive, observational, retrospective study in three primary healthcare centres with an assigned population of 100,000.

Method: Examination of the medical records of patients with COVID-19 infections confirmed by polymerase chain reaction. Logistic multivariate regression models adjusted for age and sex were constructed to analyse independent predictive factors associated with death, ICU admission and hospitalization.

Results: We included 322 patients (mean age 56.7 years, 50% female, 115 (35.7%) aged ≥ 65 years): 123 (38.2) were health workers (doctors, nurses, auxiliaries). Predictors of ICU admission or death were greater age (OR = 1.05; 95%CI = 1.03 to 1.07), male sex (OR = 2.94; 95%CI = 1.55 to 5.82), autoimmune disease (OR = 2.82; 95%CI = 1.00 to 7.84), bilateral pulmonary infiltrates (OR = 2.86; 95%CI = 1.41 to 6.13), elevated lactate-dehydrogenase (OR = 2.85; 95%CI = 1.28 to 6.90), elevated D-dimer (OR = 2.85; 95%CI = 1.22 to 6.98) and elevated C-reactive protein (OR = 2.38; 95%CI = 1.22 to 4.68). Myalgia or arthralgia (OR = 0.31; 95%CI = 0.12 to 0.70) was protective factor against ICU admission and death. Predictors of hospitalization were chills (OR = 5.66; 95%CI = 1.68 to 23.49), fever (OR = 3.33; 95%CI = 1.89 to 5.96), dyspnoea (OR = 2.92; 95%CI = 1.62 to 5.42), depression (OR = 6.06; 95%CI = 1.54 to 40.42), lymphopenia (OR = 3.48; 95%CI = 1.67 to 7.40) and elevated C-reactive protein (OR = 3.27; 95%CI = 1.59 to 7.18).

Anosmia (OR = 0.42; 95%CI = 0.19 to 0.90) was the only significant protective factor for hospitalization after adjusting for age and sex.

Conclusion: Determining the clinical, biological and radiological characteristics of patients with suspected COVID-19 infection will be key to early treatment and isolation and the tracing of contacts.

Conflict of interest statement

The authors have declared that no competing interests exist.

- 28 references
- 2 figures

Supplementary info

Publication types, MeSH terms, Grant support Expand

Publication types

- Multicenter Study
- Observational Study

MeSH terms

- Adolescent
- Adult
- Age Factors
- Aged
- Aged, 80 and over
- Betacoronavirus / genetics*
- COVID-19
- Coronavirus Infections / diagnosis*
- Coronavirus Infections / epidemiology*
- Coronavirus Infections / mortality
- Coronavirus Infections / virology
- Female
- Hospitalization
- Humans
- Intensive Care Units
- Male
- Middle Aged
- Mortality
- Pandemics
- Pneumonia, Viral / diagnosis*

- Pneumonia, Viral / epidemiology*
- Pneumonia, Viral / mortality
- Pneumonia, Viral / virology
- Polymerase Chain Reaction
- Prognosis
- Protective Factors
- Retrospective Studies
- Risk Factors
- SARS-CoV-2
- Sex Factors
- Spain / epidemiology
- Young Adult

Grant support

The author(s) received no specific funding for this work.

Full text links

OPEN ACCESS TO FULL TEXT PLOS ONE Public Library of Science Free PMC article
Proceed to details
Cite
Share
953
Observational Study
Lab Med
. 2021 Mar 15;52(2):141-145.
doi: 10.1093/labmed/lmaa105.

Absolute Lymphocytes, Ferritin, C-Reactive Protein, and Lactate Dehydrogenase Predict Early Invasive Ventilation in Patients With COVID-19

Salvador Payán-Pernía [1], Lucía Gómez Pérez [1], Ángel F Remacha Sevilla [1], Jordi Sierra Gil [1], Silvana Novelli Canales [1]
Affiliations Expand

Affiliation

- [1] Haematology Department, Hospital de la Santa Creu i Sant Pau, Sant Pau Research Institute, Universitat Autònoma de Barcelona, Barcelona, Spain.

- PMID: **33336243**
- PMCID: PMC7798986
- DOI: 10.1093/labmed/lmaa105

Free PMC article
Observational Study

Absolute Lymphocytes, Ferritin, C-Reactive Protein, and Lactate Dehydrogenase Predict Early Invasive Ventilation in Patients With COVID-19

Salvador Payán-Pernía et al. Lab Med. 2021.
Free PMC article
Show details
Lab Med
. 2021 Mar 15;52(2):141-145.
doi: 10.1093/labmed/lmaa105.

Authors

Salvador Payán-Pernía [1], Lucía Gómez Pérez [1], Ángel F Remacha Sevilla [1], Jordi Sierra Gil [1], Silvana Novelli Canales [1]

Affiliation

- [1] Haematology Department, Hospital de la Santa Creu i Sant Pau, Sant Pau Research Institute, Universitat Autònoma de Barcelona, Barcelona, Spain.

- PMID: **33336243**
- PMCID: PMC7798986
- DOI: 10.1093/labmed/lmaa105

Abstract

Objective: Early detection of patients with COVID-19 who will need mechanical invasive ventilation (MIV) may aid in delivering proper care and optimizing the use of limited resources.

Methods: In this single-center retrospective observational study, we aimed to identify simple laboratory parameters that in combination with ferritin (a surrogate marker of severe inflammation) may help predict early (first 48 hours) MIV. A total of 160 patients with COVID-19 in whom serum ferritin, absolute lymphocyte count (ALC), platelet count, C-reactive protein (CRP), and lactate dehydrogenase (LDH) had been analyzed at admission were included.

Results: We found that ferritin, LDH, ALC, and CRP predicted with 88% accuracy the probability of early MIV. Results indicated that LDH showed the greater area under the curve

(AUC), with a value of 89.1%. Using the AUC, we established cutoff values for clinical application. Finally, we developed a classification tree based on LDH for its clinical use.

Conclusion: Ferritin, LDH, ALC, and CRP predict with 88% accuracy the probability of early MIV.

Keywords: COVID-19; biomarkers; ferritin; hematology; lactate dehydrogenase; mechanical ventilation.

© American Society for Clinical Pathology 2020. All rights reserved. For permissions, please e-mail: journals.permissions@oup.com.

- 16 references
- 1 figure

Supplementary info

Publication types, MeSH terms, Substances Expand

Publication types

- Observational Study

MeSH terms

- Adult
- Aged
- Biomarkers / blood
- C-Reactive Protein / metabolism*
- COVID-19 / blood*
- COVID-19 / therapy
- Female
- Ferritins / blood*
- Humans
- L-Lactate Dehydrogenase / blood*
- Logistic Models
- Lymphocyte Count
- Male
- Middle Aged
- Respiration, Artificial / statistics & numerical data*
- Retrospective Studies
- Young Adult

Substances

- Biomarkers

- C-Reactive Protein
- Ferritins
- L-Lactate Dehydrogenase

Full text links

OXFORD ACADEMIC Silverchair Information Systems Free PMC article
Proceed to details
Cite
Share
954
Observational Study
Medicine (Baltimore)
. 2020 Oct 23;99(43):e22635.
doi: 10.1097/MD.0000000000022635.

Analysis of dynamic disturbance in blood coagulation function of patients with Coronavirus Disease 2019: A retrospective observational study

Nian Chen [1], Yuwen Li [2], Haozhi Fan [3], Anran Tian [1], Hui Yuan [1], Zhengyi Jiang [4], Yunxi Yu [5], Lili Ruan [6], Pingping Hu [1], Ming Yue [1], Jun Li [1], Chuanlong Zhu [1]
Affiliations Expand

Affiliations

- [1] Department of Infectious Disease.
- [2] Department of Pediatrics.
- [3] Department of Information, the First Affiliated Hospital of Nanjing Medical University, Nanjing.
- [4] State Key Laboratory for Diagnosis and Treatment of Infectious Diseases, Collaborative Innovation Center for Diagnosis and Treatment of Infectious Diseases, The First Affiliated Hospital, College of Medicine, Zhejiang University, Hangzhou.
- [5] Emergency department, Huangshi Hospital of Traditional Chinese Medicine.
- [6] Department of Anesthesiology, The Fifth Hospital of Huangshi, Huangshi, Hubei Province, China.

- PMID: **33120752**
- PMCID: PMC7581139
- DOI: 10.1097/MD.0000000000022635

Free PMC article
Observational Study

Analysis of dynamic disturbance in blood coagulation function of patients with Coronavirus Disease 2019: A retrospective observational study

Nian Chen et al. Medicine (Baltimore). 2020.
Free PMC article
Show details
Medicine (Baltimore)
. 2020 Oct 23;99(43):e22635.
doi: 10.1097/MD.0000000000022635.

Authors

Nian Chen [1], Yuwen Li [2], Haozhi Fan [3], Anran Tian [1], Hui Yuan [1], Zhengyi Jiang [4], Yunxi Yu [5], Lili Ruan [6], Pingping Hu [1], Ming Yue [1], Jun Li [1], Chuanlong Zhu [1]

Affiliations

- [1] Department of Infectious Disease.
- [2] Department of Pediatrics.
- [3] Department of Information, the First Affiliated Hospital of Nanjing Medical University, Nanjing.
- [4] State Key Laboratory for Diagnosis and Treatment of Infectious Diseases, Collaborative Innovation Center for Diagnosis and Treatment of Infectious Diseases, The First Affiliated Hospital, College of Medicine, Zhejiang University, Hangzhou.
- [5] Emergency department, Huangshi Hospital of Traditional Chinese Medicine.
- [6] Department of Anesthesiology, The Fifth Hospital of Huangshi, Huangshi, Hubei Province, China.

- PMID: **33120752**
- PMCID: PMC7581139
- DOI: 10.1097/MD.0000000000022635

Abstract

Coronavirus Disease 2019 (COVID-19) has became a major problem affecting global health security.To assess the differences and dynamic changes of blood coagulation function in COVID-19 patients with different severity.A total of 261 COVID-19 patients from January 24 to March 25, 2020 in Huangshi, Hubei Province were enrolled.We designed a retrospective observational study. Clinical information, including age, blood routine and blood coagulation function, were collected. According to the Diagnosis and Treatment Guidelines for COVID-19 (seventh version) that issued by the National Health Committee of the People's Republic of China, patients were divided into 3 subgroups: 186 ordinary, 45 severe and 30 critical ones. We compared the differences in blood coagulation factors among groups.Average age in critical group (71.47 ± 11.48 years) was the oldest of 3 subgroups. At admission, statistically differences could be

observed among ordinary, severe and critical patients in D-dimer (0.18 ± 0.33, 0.63 ± 1.13 and 1.16 ± 1.58 mg/L), fibrinogen/fibrin degradation products (FDP) (3.11 ± 5.30, 9.82 ± 23.91 and 21.94 ± 40.98 μg/ml), platelet [(169 ± 62.85), (188 ± 71.56) and (117 ± 38.31) × 10/L)] and lymphocyte count [(1.18 ± 0.46), (0.82 ± 0.35) and (0.75 ± 0.39) × 10/L)], respectively (P < .05). During hospitalization, the peak values of coagulation and valley values of blood routine were monitored. There were significant differences among ordinary, severe and critical patients in D-dimer (0.26 ± 0.46, 1.39 ± 1.51 and 2.89 ± 1.68 mg/L), FDP (3.29 ± 5.52, 23.68 ± 39.07 and 56.11 ± 49.94 μg/ml), platelet [(164 ± 55.53), (171 ± 69.96) and (84 ± 57.80) × 10/L)] and lymphocyte count [(1.10 ± 0.46), (0.65 ± 0.35) and (0.55 ± 0.31) × 10/L)], respectively (P < .001). D-dimer and FDP in the course of disease in severe/critical groups showed a first upward and then downward trend.We concluded that coagulation function indexes such as D-dimer and FDP could be served as markers to estimate COVID-19 patients condition. Close monitoring of coagulation function may be helpful for early diagnosis of severe patients and guidance of treatments.

Conflict of interest statement

The authors have no conflicts of interests to disclose.

- 24 references
- 1 figure

Supplementary info

Publication types, MeSH terms Expand

Publication types

- Observational Study

MeSH terms

- Adult
- Aged
- Aged, 80 and over
- Betacoronavirus*
- Blood Coagulation Disorders / diagnosis
- Blood Coagulation Disorders / physiopathology
- Blood Coagulation Disorders / virology*
- Blood Coagulation Tests
- COVID-19
- Coronavirus Infections / complications*
- Coronavirus Infections / diagnosis
- Coronavirus Infections / physiopathology
- Female
- Humans
- Male

- Middle Aged
- Pandemics
- Pneumonia, Viral / complications*
- Pneumonia, Viral / diagnosis
- Pneumonia, Viral / physiopathology
- Retrospective Studies
- SARS-CoV-2
- Severity of Illness Index

Full text links

Wolters Kluwer Wolters Kluwer Free PMC article
Proceed to details
Cite
Share
955
Observational Study
Diabetes Res Clin Pract
. 2020 Oct;168:108374.
doi: 10.1016/j.diabres.2020.108374. Epub 2020 Aug 15.

Newly-diagnosed diabetes and admission hyperglycemia predict COVID-19 severity by aggravating respiratory deterioration

Gian Paolo Fadini [1], Mario Luca Morieri [2], Federico Boscari [2], Paola Fioretto [2], Alberto Maran [2], Luca Busetto [2], Benedetta Maria Bonora [2], Elisa Selmin [2], Gaetano Arcidiacono [2], Silvia Pinelli [2], Filippo Farnia [2], Daniele Falaguasta [2], Lucia Russo [2], Giacomo Voltan [2], Sara Mazzocut [2], Giorgia Costantini [2], Francesca Ghirardini [2], Silvia Tresso [2], Anna Maria Cattelan [3], Andrea Vianello [4], Angelo Avogaro [2], Roberto Vettor [2]
Affiliations Expand

Affiliations

- [1] Department of Medicine, University of Padova, Italy. Electronic address: gianpaolo.fadini@unipd.it.
- [2] Department of Medicine, University of Padova, Italy.
- [3] Unit of Infectious Disease, University Hospital of Padova, Italy.
- [4] Department of Cardiothoracic Vascular Sciences and Public Health, University of Padova, Italy.

- PMID: **32805345**
- PMCID: PMC7428425
- DOI: 10.1016/j.diabres.2020.108374

Free PMC article
Observational Study

Newly-diagnosed diabetes and admission hyperglycemia predict COVID-19 severity by aggravating respiratory deterioration

Gian Paolo Fadini et al. Diabetes Res Clin Pract. 2020 Oct.
Free PMC article
Show details
Diabetes Res Clin Pract
. 2020 Oct;168:108374.
doi: 10.1016/j.diabres.2020.108374. Epub 2020 Aug 15.

Authors

Gian Paolo Fadini [1], Mario Luca Morieri [2], Federico Boscari [2], Paola Fioretto [2], Alberto Maran [2], Luca Busetto [2], Benedetta Maria Bonora [2], Elisa Selmin [2], Gaetano Arcidiacono [2], Silvia Pinelli [2], Filippo Farnia [2], Daniele Falaguasta [2], Lucia Russo [2], Giacomo Voltan [2], Sara Mazzocut [2], Giorgia Costantini [2], Francesca Ghirardini [2], Silvia Tresso [2], Anna Maria Cattelan [3], Andrea Vianello [4], Angelo Avogaro [2], Roberto Vettor [2]

Affiliations

- [1] Department of Medicine, University of Padova, Italy. Electronic address: gianpaolo.fadini@unipd.it.
- [2] Department of Medicine, University of Padova, Italy.
- [3] Unit of Infectious Disease, University Hospital of Padova, Italy.
- [4] Department of Cardiothoracic Vascular Sciences and Public Health, University of Padova, Italy.

- PMID: **32805345**
- PMCID: PMC7428425
- DOI: 10.1016/j.diabres.2020.108374

Abstract

Aims: We investigated whether pre-existing diabetes, newly-diagnosed diabetes, and admission hyperglycemia were associated with COVID-19 severity independently from confounders.

Methods: We retrospectively analyzed data on patients with COVID-19 hospitalized between February and April 2020 in an outbreak hospital in North-East Italy. Pre-existing diabetes was defined by self-reported history, electronic medical records, or ongoing medications. Newly-diagnosed diabetes was defined by HbA1c and fasting glucose. The primary outcome was a composite of ICU admission or death.

Results: 413 subjects were included, 107 of whom (25.6%) had diabetes, including 21 newly-diagnosed. Patients with diabetes were older and had greater comorbidity burden. The primary outcome occurred in 37.4% of patients with diabetes compared to 20.3% in those without (RR 1.85; 95%C.I. 1.33-2.57; $p < 0.001$). The association was stronger for newly-diagnosed compared to pre-existing diabetes (RR 3.06 vs 1.55; $p = 0.004$). Higher glucose level at admission was associated with COVID-19 severity, with a stronger association among patients without as compared to those with pre-existing diabetes (interaction $p < 0.001$). Admission glucose was correlated with most clinical severity indexes and its association with adverse outcome was mostly mediated by a worse respiratory function.

Conclusion: Newly-diagnosed diabetes and admission hyperglycemia are powerful predictors of COVID-19 severity due to rapid respiratory deterioration.

Keywords: Mediation; Metabolism; Observational; Prediction; SARS-CoV-2; Survival.

Copyright © 2020 Elsevier B.V. All rights reserved.

Conflict of interest statement

Declaration of Competing Interest The authors declare that they have no known competing financial interests or personal relationships that could have appeared to influence the work reported in this paper.

Comment in

- Inpatient use of glucocorticoids may mediate the detrimental effect of new-onset hyperglycemia on COVID-19 severity.
  Li G. Li G. Diabetes Res Clin Pract. 2020 Oct;168:108441. doi: 10.1016/j.diabres.2020.108441. Epub 2020 Sep 12. Diabetes Res Clin Pract. 2020. PMID: 32926957 Free PMC article. No abstract available.

- 32 references
- 3 figures

Supplementary info

Publication types, MeSH terms, Substances Expand

Publication types

- Observational Study

MeSH terms

- Age of Onset
- Aged
- Aged, 80 and over
- Betacoronavirus / physiology
- Blood Glucose / analysis

- Blood Glucose / metabolism
- COVID-19
- Comorbidity
- Coronavirus Infections / complications
- Coronavirus Infections / diagnosis*
- Coronavirus Infections / epidemiology
- Coronavirus Infections / therapy
- Diabetes Complications / blood
- Diabetes Complications / diagnosis*
- Diabetes Complications / epidemiology
- Diabetes Complications / pathology
- Diabetes Mellitus / blood
- Diabetes Mellitus / diagnosis*
- Diabetes Mellitus / epidemiology
- Diabetes Mellitus / therapy
- Female
- Humans
- Hyperglycemia / complications*
- Hyperglycemia / diagnosis*
- Hyperglycemia / epidemiology
- Hyperglycemia / therapy
- Italy / epidemiology
- Male
- Middle Aged
- Pandemics
- Patient Admission*
- Pneumonia, Viral / complications
- Pneumonia, Viral / diagnosis*
- Pneumonia, Viral / epidemiology
- Pneumonia, Viral / therapy
- Prognosis
- Retrospective Studies
- SARS-CoV-2
- Severity of Illness Index
- Treatment Outcome

Substances

- Blood Glucose

Full text links

ELSEVIER FULL-TEXT ARTICLE Elsevier Science Free PMC article
Proceed to details
Cite
Share
☐ 956
Observational Study
Am J Emerg Med
. 2021 Oct;48:1-11.
doi: 10.1016/j.ajem.2021.03.087. Epub 2021 Apr 2.

The impact of Covid-19 on patients with suspected cancer: An analysis of ED presentation and referrals to a quick diagnosis unit

Xavier Bosch [1], Aina Capdevila [2], Ignacio Grafia [2], Andrea Ladino [2], Pedro J Moreno [2], Alfonso López-Soto [2]
Affiliations Expand

Affiliations

- [1] Department of Internal Medicine, Hospital Clínic, Institut d'Investigacions Biomèdiques August Pi i Sunyer (IDIBAPS), University of Barcelona, 08036 Barcelona, Spain. Electronic address: xavbosch@clinic.cat.
- [2] Department of Internal Medicine, Hospital Clínic, Institut d'Investigacions Biomèdiques August Pi i Sunyer (IDIBAPS), University of Barcelona, 08036 Barcelona, Spain.

- PMID: **33836386**
- PMCID: PMC8016540
- DOI: 10.1016/j.ajem.2021.03.087

Free PMC article
Observational Study

The impact of Covid-19 on patients with suspected cancer: An analysis of ED presentation and referrals to a quick diagnosis unit

Xavier Bosch et al. Am J Emerg Med. 2021 Oct.
Free PMC article

Show details

Am J Emerg Med

. 2021 Oct;48:1-11.
doi: 10.1016/j.ajem.2021.03.087. Epub 2021 Apr 2.

Authors

Xavier Bosch [1], Aina Capdevila [2], Ignacio Grafia [2], Andrea Ladino [2], Pedro J Moreno [2], Alfonso López-Soto [2]

Affiliations

- [1] Department of Internal Medicine, Hospital Clínic, Institut d'Investigacions Biomèdiques August Pi i Sunyer (IDIBAPS), University of Barcelona, 08036 Barcelona, Spain. Electronic address: xavbosch@clinic.cat.
- [2] Department of Internal Medicine, Hospital Clínic, Institut d'Investigacions Biomèdiques August Pi i Sunyer (IDIBAPS), University of Barcelona, 08036 Barcelona, Spain.

- PMID: **33836386**
- PMCID: PMC8016540
- DOI: 10.1016/j.ajem.2021.03.087

Abstract

Purpose: Patients evaluated in the emergency department (ED) who have concerning symptoms suggestive of a cancer diagnosis are mostly referred to the quick diagnosis unit of our tertiary hospital. This study analyzed the impact of the Covid-19 pandemic on the volume, disease patterns, and accessibility to essential investigations of patients with suspected cancer referred by the ED to this unit.

Methods: Trends in referrals were analyzed from January 1 to July 8, 2020 and the corresponding dates of 2019. Only non-Covid-19 conditions were evaluated. Three time-based cohorts were defined: prepandemic (January 1-February 19), pandemic (February 19-April 22), and postpandemic (April 22-July 8). Along with descriptive statistics, linear regression was used to test for time trends with weekly referrals as the dependent variable.

Results: There were 384, 193, and 450 patients referred during the prepandemic, pandemic, and postpandemic periods, respectively. Following an increasing rate, referrals decreased to unprecedented levels in the pandemic period (average weekly slope: -2.1 cases), then increasing again until near normalization. Waiting times to most diagnostic procedures including radiology, endoscopic, nuclear medicine, and biopsy/cytology during the pandemic period were significantly delayed and time-to-diagnosis was considerably longer (19.72 ± 10.37 days vs. 8.33 ± 3.94 days in prepandemic and 13.49 ± 6.45 days in postpandemic period; $P < 0.001$ in both). Compared to other cohorts, pandemic cohort patients were more likely to have unintentional weight loss and fever of unknown origin as referral indications while anemia and lymphadenopathy were less common. Patients from the pandemic cohort had a significantly lower rate of malignancies and higher of benign gastrointestinal disorders (40.93% vs. 19.53% and 20.89% in prepandemic and postpandemic periods, respectively; $P < 0.001$ in both), most notably irritable bowel disease, and of mental and behavioral disorders (15.54% vs. 3.39% and 6.00% in prepandemic and postpandemic periods, respectively; $P < 0.001$ in both).

Conclusions: As our hospital switched its traditional care to one focused on Covid-19 patients, recognized indicators of healthcare quality of quick diagnosis units were severely disrupted. The clinical patterns of presentation and diagnosis of the pandemic period suggested that mass media-generated mental and behavioral responses with distressing symptoms played a significant role in most of these patients.

Keywords: Covid-19; Hospital ambulatory medicine; Pandemic; Quick diagnosis units; Suspected cancer.

Copyright © 2021 Elsevier Inc. All rights reserved.

Conflict of interest statement

Declaration of Competing Interest All authors have nothing to declare on conflict of interest.

- 43 references
- 4 figures

Supplementary info

Publication types, MeSH terms Expand

Publication types

- Observational Study

MeSH terms

- Adolescent
- Adult
- Aged
- Aged, 80 and over
- COVID-19 / prevention & control*
- Delayed Diagnosis / trends
- Diagnosis, Differential
- Emergency Service, Hospital / organization & administration
- Emergency Service, Hospital / trends*
- Female
- Follow-Up Studies
- Health Services Accessibility / trends*
- Humans
- Linear Models
- Male
- Middle Aged
- Neoplasms / diagnosis*
- Observer Variation

- Quick Diagnosis Units / trends*
- Referral and Consultation / trends*
- Reproducibility of Results
- Retrospective Studies
- Spain
- Tertiary Care Centers
- Young Adult

Full text links

ELSEVIER OPEN ACCESS Elsevier Science Free PMC article

Proceed to details

Cite

Share

☐ 957

Observational Study

Clin Microbiol Infect

. 2020 Nov;26(11):1525-1536.

doi: 10.1016/j.cmi.2020.07.024. Epub 2020 Aug 4.

Characteristics and predictors of death among 4035 consecutively hospitalized patients with COVID-19 in Spain

Juan Berenguer [1], Pablo Ryan [2], Jesús Rodríguez-Baño [3], Inmaculada Jarrín [4], Jordi Carratalà [5], Jerónimo Pachón [6], María Yllescas [7], José Ramón Arriba [8], COVID-19@Spain Study Group; Fundación SEIMC-GESIDA; Hospital General Universitario Gregorio Marañón; Hospital Universitario La Paz; Hospital Infanta Leonor; Complejo Hospitalario Virgen de la Salud; Hospital Universitario Rafael Méndez; Hospital Universitario de Cruces; Hospital de Melilla; Hospital San Eloy de Barakaldo; Hospital Universitario Central de Asturias; Hospital General Universitario de Alicante; Hospital Virgen de la Victoria; Hospital Universitario Puerto Real; EOXI Pontevedra e Salnés; Hospital de Figueres; Hospital Sant Jaume de Calella; Hospital del Mar; Hospital Virgen de la Arrixaca; Hospital de Can Misses; Hospital de Sagunto; Hospital Clínico San Cecilio; Hospital Universitario Príncipe de Asturias; Parc Sanitari Sant Joan de Déu; Hospital Nuestra Señora de Gracia; HC Marbella Internacional Hospital; Hospital La Princesa; Hospital Josep Trueta; Hospital Dos de Maig; Hospital Arnau de Vilanova-Lliria; Hospital General Universitario de Elche; Hospital Clínico Universitario de Valencia; Complejo Asistencial de Ávila; Hospital Comarcal de Alcañiz; Hospital Universitario Marqués de Valdecilla; Hospital Quiron-Salud de Torrevieja; Hospital Universitario Miguel Servet; SCIAS, Hospital de Barcelona; Fundación Hospital Universitario Alcorcón; Hospital Álvaro Cunqueiro; Complejo Asistencial Universitario de Salamanca; Hospital Universitario Severo Ochoa; Hospital CIMA-Sanitas; Hospital HLA Inmaculada; Hospital Universitario Rio Hortega; Hospital de Guadalajara; Hospital Universitario Infanta Sofía; Hospital Comarcal de Blanes; Hospital Universitari de Tarragona Joan XXIII; Hospital Universitario Basurto; Hospital Universitario de Canarias; Hospital Universitario de Gran Canaria Dr Negrín; Hospital Son

Espases; Hospital Universitario de Móstoles; Complejo Hospitalario Universitario A Coruña; Hospital Costa del Sol; Hospital Clínico Universitario Lozano Blesa; Hospital Mutua de Terrassa; Hospital de la Plana; Hospital Virgen de la Concha–Complejo Asistencial de Zamora; Complejo Hospitalario Universitario Insular Materno-Infantil; Hospital de la Marina Baixa; Hospital Universitario Virgen Macarena; Hospital Universitari de Bellvitge; Hospital Universitario y Politécnico la Fe; Hospital Universitario del Vinalopó; Hospital de Sabadell (Parc Tauli); Hospital Clinic de Barcelona; Hospital Universitario de la Ribera; Fundación Jiménez Díaz; Hospital Clínico Universitario de Valladolid; Hospital Clínico San Carlos; Hospital Santa Creu i Sant Pau; Clínica Universitaria de Navarra–Campus Madrid; Hospital Son Llatzer; Hospital General de la Defensa Gómez Ulla; Hospital Universitario de Álava; Hospital Santos Reyes; Hospital Dr José Molina Orosa; Hospital Vall d'Hebrón; Hospital Universitario Rey Juan Carlos; Complejo Hospitalario Universitario Santa Lucía; Hospital Santa Bárbara; Complejo Hospitalario Universitario de Ferrol; Hospital de l'Esperit Sant; Hospital Universitario los Arcos del Mar Menor; Hospital HLA Universitario Moncloa; Hospital Virgen del Puerto; Hospital Marina Salud de Dénia; Hospital Universitario de Jerez; Hospital Reina Sofía de Tudela; Hospital Clínico Universitario de Santiago de Compostela; Hospital Universitario del Henares; Hospital Universitario Lucus Augusti; Hospital de Donostia; Hospital de Urduliz Alfredo Espinosa; Hospital de Mendaro; Hospital Juan Ramón Jiménez; Hospital de Tortosa Virgen de la Cinta; Hospital Riotinto; Hospital Vega Baja; Hospital Puerta de Hierro; Hospital Universitario de Getafe; Hospital General de la Palma; Hospital El Bierzo; Fundación Hospital de Calahorra; Hospital Alto Deba; Hospital Universitario San Juan de Alicante; Hospital de Guadarrama; Hospital Universitario de Jaén; Hospital de Mataró; Hospital de Palamós; Hospital Universitario de Valme; Clínica Universitaria de Navarra–Campus Navarra; Hospital Clínica Benidorm; Hospital Doce de Octubre; Hospital Universitario Virgen del Rocío; Hospital Universitario Ramón y Cajal; Hospital Universitario San Pedro; Hospital Quirón A Coruña; HM Sanchinarro; Hospital Francesc de Borja; Complejo Hospitalario Universitario Nuestra Señora de La Candelaria; Hospital Universitario HM Montepríncipe; Hospital Universitario HM Puerta del Sur; Hospital Universitario HM Torrelodones; Hospital Universitario HM Madrid; Hospital Don Benito-Villanueva de la Serena; Hospital de Viladecans; Centro Nacional de Epidemiología
Collaborators, Affiliations Expand

- PMID: **32758659**
- PMCID: PMC7399713
- DOI: 10.1016/j.cmi.2020.07.024

Free PMC article
Observational Study

Characteristics and predictors of death among 4035 consecutively hospitalized patients with COVID-19 in Spain

Juan Berenguer et al. Clin Microbiol Infect. 2020 Nov.
Free PMC article
Show details
Clin Microbiol Infect
. 2020 Nov;26(11):1525-1536.
doi: 10.1016/j.cmi.2020.07.024. Epub 2020 Aug 4.

- PMID: **32758659**
- PMCID: PMC7399713
- DOI: 10.1016/j.cmi.2020.07.024

Abstract

Objectives: To analyse the characteristics and predictors of death in hospitalized patients with coronavirus disease 2019 (COVID-19) in Spain.

Methods: A retrospective observational study was performed of the first consecutive patients hospitalized with COVID-19 confirmed by real-time PCR assay in 127 Spanish centres until 17 March 2020. The follow-up censoring date was 17 April 2020. We collected demographic, clinical, laboratory, treatment and complications data. The primary endpoint was all-cause mortality. Univariable and multivariable Cox regression analyses were performed to identify factors associated with death.

Results: Of the 4035 patients, male subjects accounted for 2433 (61.0%) of 3987, the median age was 70 years and 2539 (73.8%) of 3439 had one or more comorbidity. The most common symptoms were a history of fever, cough, malaise and dyspnoea. During hospitalization, 1255 (31.5%) of 3979 patients developed acute respiratory distress syndrome, 736 (18.5%) of 3988 were admitted to intensive care units and 619 (15.5%) of 3992 underwent mechanical ventilation. Virus- or host-targeted medications included lopinavir/ritonavir (2820/4005, 70.4%), hydroxychloroquine (2618/3995, 65.5%), interferon beta (1153/3950, 29.2%), corticosteroids (1109/3965, 28.0%) and tocilizumab (373/3951, 9.4%). Overall, 1131 (28%) of 4035 patients died. Mortality increased with age (85.6% occurring in older than 65 years). Seventeen factors were independently associated with an increased hazard of death, the strongest among them including advanced age, liver cirrhosis, low age-adjusted oxygen saturation, higher concentrations of C-reactive protein and lower estimated glomerular filtration rate.

Conclusions: Our findings provide comprehensive information about characteristics and complications of severe COVID-19, and may help clinicians identify patients at a higher risk of death.

Keywords: COVID-19; Coronavirus; Pneumonia; Respiratory distress syndrome; SARS-CoV-2.

Copyright © 2020 European Society of Clinical Microbiology and Infectious Diseases. Published by Elsevier Ltd. All rights reserved.

Comment in

- The association of dementia with COVID-19 mortality: Evidence based on adjusted effect estimates.
  Yang H, Liang X, Hou H, Xu J, Shi L, Wang Y. Yang H, et al. J Infect. 2021 May;82(5):e6-e10. doi: 10.1016/j.jinf.2021.02.013. Epub 2021 Feb 12. J Infect. 2021. PMID: 33582205 Free PMC article. No abstract available.

- 30 references
- 4 figures

Supplementary info

Publication types, MeSH terms, Substances, Supplementary concepts Expand

Publication types

- Observational Study

MeSH terms

- Aged
- Aged, 80 and over
- Antiviral Agents / therapeutic use
- Betacoronavirus / isolation & purification
- COVID-19
- Cause of Death
- Coronavirus Infections / diagnosis
- Coronavirus Infections / drug therapy
- Coronavirus Infections / mortality*
- Coronavirus Infections / therapy
- Female
- Hospital Mortality
- Hospitalization
- Humans
- Male
- Middle Aged
- Pandemics
- Pneumonia, Viral / diagnosis
- Pneumonia, Viral / mortality*
- Pneumonia, Viral / therapy
- Retrospective Studies
- Risk Factors
- SARS-CoV-2
- Spain / epidemiology

Substances

- Antiviral Agents

Supplementary concepts

- COVID-19 drug treatment

Full text links

ELSEVIER OPEN ACCESS Elsevier Science Free PMC article

Proceed to details
Cite
Share
958
Observational Study
Med Clin (Barc)
. 2021 Apr 23;156(8):386-389.
doi: 10.1016/j.medcli.2020.11.036. Epub 2020 Dec 26.

Early prone positioning therapy for patients with mild COVID-19 disease

[Article in English, Spanish]
Xiaoyi Liu [1], Hui Liu [2], Qing Lan [3], Xiangde Zheng [3], Jun Duan [4], Fanwei Zeng [5]
Affiliations Expand

Affiliations

- [1] Department of Critical Care Medicine, The Central Hospital of Dazhou, Dazhou, Sichuan, PR China. Electronic address: 756904123@qq.com.
- [2] Ophthalmology, The Central Hospital of Dazhou, Dazhou, Sichuan, PR China.
- [3] Department of Critical Care Medicine, The Central Hospital of Dazhou, Dazhou, Sichuan, PR China.
- [4] Department of Respiratory and Critical Care Medicine, The First Affiliated Hospital of Chongqing Medical University, Chongqing, PR China.
- [5] Orthopedics, The Central Hospital of Dazhou, Dazhou, Sichuan, PR China. Electronic address: 1054628507@qq.com.

- PMID: **33478810**
- PMCID: PMC7834567
- DOI: 10.1016/j.medcli.2020.11.036

Free PMC article
Observational Study

Early prone positioning therapy for patients with mild COVID-19 disease

[Article in English, Spanish]
Xiaoyi Liu et al. Med Clin (Barc). 2021.
Free PMC article
Show details
Med Clin (Barc)
. 2021 Apr 23;156(8):386-389.
doi: 10.1016/j.medcli.2020.11.036. Epub 2020 Dec 26.

Authors

Xiaoyi Liu [1], Hui Liu [2], Qing Lan [3], Xiangde Zheng [3], Jun Duan [4], Fanwei Zeng [5]

Affiliations

- [1] Department of Critical Care Medicine, The Central Hospital of Dazhou, Dazhou, Sichuan, PR China. Electronic address: 756904123@qq.com.
- [2] Ophthalmology, The Central Hospital of Dazhou, Dazhou, Sichuan, PR China.
- [3] Department of Critical Care Medicine, The Central Hospital of Dazhou, Dazhou, Sichuan, PR China.
- [4] Department of Respiratory and Critical Care Medicine, The First Affiliated Hospital of Chongqing Medical University, Chongqing, PR China.
- [5] Orthopedics, The Central Hospital of Dazhou, Dazhou, Sichuan, PR China. Electronic address: 1054628507@qq.com.

- PMID: **33478810**
- PMCID: PMC7834567
- DOI: 10.1016/j.medcli.2020.11.036

Abstract
in English, Spanish

Objective: In December 2019, Wuhan, China, experienced an outbreak of coronavirus disease 2019 (COVID-19). Some patients admitted to our hospital were treated with early prone positioning (PP). Here, we analyzed its clinical significance.

Methods: This was a retrospective observational study. We defined the early PP group as mild COVID-19 patients who were placed into a prone position within 24h of admission; others served as the control group. We recorded basic data and outcomes of early PP and compared the results to those of controls.

Results: After 1 day of treatment, oxygenation was greater in the early PP group than in the control group (P/F: 421.6±39.74 vs. 382.1±38.84mmHg [1mmHg=0.133kPa], p<0.01). And early PP group spent less total time in prone position (11.1±4.17 vs. 16.9±5.20 days, p<0.01), and required shorter hospitalization duration (12.2±4.49 vs. 23.2±4.83 days, p<0.001).

Conclusions: Early PP treatment can improve hypoxia and shorten the prone position time and hospitalization duration in mild COVID-19 patients. It is a potential clinically applicable intervention.

Objetivo: En diciembre de 2019, Wuhan, China, experimentó un brote de enfermedad por coronavirus 2019 (COVID-19). Algunos pacientes ingresados en nuestro hospital fueron tratados con posicionamiento temprano en decúbito prono (PP). En este estudio analizamos su significación clínica.

Métodos: Estudio retrospectivo observacional en el que definimos el PP temprano como aquellos pacientes con COVID-19 que fueron posicionados en decúbito prono dentro de las 24 horas siguientes a su ingreso, sirviendo el resto de los pacientes como grupo control. Registramos los datos básicos y los resultados de PP temprano, comparando dichos resultados con los de los controles.

Resultados: Tras un día de tratamiento, la oxigenación fue más alta en el grupo PP temprano que en el grupo control (P/F: 421,6 ± 39,74 vs. 382,1 ± 38,84 mmHg [1 mmHg = 0,133 kPa], p < 0,01). El grupo PP temprano pasó menor tiempo total en posición de decúbito prono (11,1 ± 4,17 vs. 16,9 ± 5,20 días, p < 0,01), y requirió menor tiempo de hospitalización (12,2 ± 4,49 vs. 23,2 ± 4,83 días, p < 0,001).

Conclusiones: El tratamiento de PP temprano puede mejorar la hipoxia y reducir el tiempo de posición en decúbito prono en pacientes con COVID-19 leve. Se trata de una intervención potencialmente aplicable desde el punto de vista clínico.

Keywords: ARDS; COVID-19; Hipoxemia; Hypoxemia; Posición en decúbito prono; Prone position; SARS-CoV-2; SDRA.

Copyright © 2020 Elsevier España, S.L.U. All rights reserved.

- 10 references
- 2 figures

Supplementary info

Publication types, MeSH terms Expand

Publication types

- Observational Study

MeSH terms

- Adult
- COVID-19 / therapy*
- China / epidemiology
- Female
- Humans
- Male
- Middle Aged
- Patient Positioning*
- Prone Position*

Full text links

Ediciones Doyma, S.L. Free PMC article
Proceed to details
Cite
Share
☐ 959
Case Reports
Rev Esp Anestesiol Reanim (Engl Ed)

. Jun-Jul 2021;68(6):346-352.
doi: 10.1016/j.redar.2020.10.006. Epub 2021 Apr 12.

Utility of preoperative polymerase chain reaction testing during SARS-CoV-2 pandemic: The challenge of evolving incidence

[Article in English, Spanish]

M de la Matta[1], J M Delgado-Sánchez[2], G M Gutiérrez[3], J L López Romero[4], M Del Mar Martínez Gómez[4], A Domínguez Blanco[4]
Affiliations Expand

Affiliations

- [1] Servicio de Anestesiología y Reanimación, Hospital Universitario Virgen del Rocío, Sevilla, España. Electronic address: mdlmattam@hotmail.com.
- [2] Departamento de Matemática Aplicada I, Escuela Técnica Superior de Arquitectura, Universidad de Sevilla, Sevilla, España.
- [3] Unidad de Enfermedades Infecciosas, Microbiología y Medicina Preventiva, Hospital Universitario Virgen del Rocío, Sevilla, España.
- [4] Servicio de Anestesiología y Reanimación, Hospital Universitario Virgen del Rocío, Sevilla, España.

- PMID: **33858679**
- PMCID: PMC8041274
- DOI: 10.1016/j.redar.2020.10.006

Free PMC article
Case Reports

Utility of preoperative polymerase chain reaction testing during SARS-CoV-2 pandemic: The challenge of evolving incidence

[Article in English, Spanish]
M de la Matta et al. Rev Esp Anestesiol Reanim (Engl Ed). Jun-Jul 2021.
Free PMC article
Show details
Rev Esp Anestesiol Reanim (Engl Ed)
. Jun-Jul 2021;68(6):346-352.

doi: 10.1016/j.redar.2020.10.006. Epub 2021 Apr 12.

Authors

M de la Matta [1], J M Delgado-Sánchez [2], G M Gutiérrez [3], J L López Romero [4], M Del Mar Martínez Gómez [4], A Domínguez Blanco [4]

Affiliations

- [1] Servicio de Anestesiología y Reanimación, Hospital Universitario Virgen del Rocío, Sevilla, España. Electronic address: mdlmattam@hotmail.com.
- [2] Departamento de Matemática Aplicada I, Escuela Técnica Superior de Arquitectura, Universidad de Sevilla, Sevilla, España.
- [3] Unidad de Enfermedades Infecciosas, Microbiología y Medicina Preventiva, Hospital Universitario Virgen del Rocío, Sevilla, España.
- [4] Servicio de Anestesiología y Reanimación, Hospital Universitario Virgen del Rocío, Sevilla, España.

- PMID: **33858679**
- PMCID: PMC8041274
- DOI: 10.1016/j.redar.2020.10.006

Abstract

Introduction: Due to its high transmissibility, measures aimed at reducing the spread of SARS CoV2 have become mandatory. Different organizations have recommended performing polymerase chain reaction tests (PCR) as part of the preoperative screening of surgical patients. We aimed to determine the performance of PCR testing to detect asymptomatic carriers.

Methods: Observational study carried out at a tertiary care center. We compared the results of preoperative real-time reverse-transcription-PCR test (RT-PCR) performed on a cohort of patients pending surgery with the results we would have expected assuming the epidemiological data released by government offices.

Results: We registered no positives in the 2,722 preoperative RT-PCR tests performed in our health care area between epidemiological Weeks 18 to 21, meaning a cumulative incidence trending to zero. Assuming public epidemiological data, the probabilistic projection of potential asymptomatic individuals ranged from 0.27*10e -4 (according to official data of new cases diagnosed by PCR) to 4.69*10e -4 if we assumed cases confirmed by IgG test in our province. Assuming a RT-PCR sensitivity of 95%, to obtain a positive result we should perform 38,461 and 2,028 tests respectively.

Conclusions: In scenarios of very low prevalence and despite high sensitivity scores, indiscriminate preoperative RT-PCR screening is of a questionable effectiveness for detecting asymptomatic carriers. Our findings evidence the difficulty of establishing reliable predictive models for the episodic and rapidly evolving incidence of infections such as has characterized the SARS CoV2 pandemic.

Keywords: Asymptomatic patient; COVID-19; Coronavirus; Paciente asintomático; Periodo preoperatorio; Preoperative Period; Prevalence; Prevalencia; SARS; Sensibilidad y especificidad; Sensitivity and Specificity.

Copyright © 2020 Sociedad Española de Anestesiología, Reanimación y Terapéutica del Dolor. Publicado por Elsevier España, S.L.U. All rights reserved.

- 18 references
- 3 figures

Supplementary info

Publication types, MeSH terms Expand

Publication types

- Case Reports
- Observational Study

MeSH terms

- Asymptomatic Infections / epidemiology*
- COVID-19 / diagnosis*
- COVID-19 / epidemiology*
- COVID-19 Nucleic Acid Testing / methods*
- COVID-19 Nucleic Acid Testing / statistics & numerical data
- Humans
- Incidence
- Pandemics*
- Preoperative Care*
- Retrospective Studies
- Sensitivity and Specificity

Full text links

ELSEVIER FULL-TEXT ARTICLE Elsevier Science Free PMC article
Proceed to details
Cite
Share
☐ 960
J Infect
. 2020 Jul;81(1):e51-e60.
doi: 10.1016/j.jinf.2020.04.012. Epub 2020 Apr 18.

Suppressed T cell-mediated immunity in patients with COVID-19: A clinical retrospective study in Wuhan, China

Bo Xu [1], Cun-Yu Fan [1], An-Lu Wang [2], Yi-Long Zou [1], Yi-Han Yu [1], Cong He [1], Wen-Guang Xia [3], Ji-Xian Zhang [4], Qing Miao [5]
Affiliations Expand

Affiliations

- [1] Hubei Provincial Hospital of Traditional Chinese & Western Medicine, Wuhan 430015, China.
- [2] Xiyuan Hospital, China Academy of Chinese Medical Sciences, No. 1 Xiyuan Playground, Haidian District, Beijing 100091, China.
- [3] Hubei Provincial Hospital of Traditional Chinese & Western Medicine, Wuhan 430015, China. Electronic address: hbszxyjhyy11@163.com.
- [4] Hubei Provincial Hospital of Traditional Chinese & Western Medicine, Wuhan 430015, China. Electronic address: jxzhang1607@163.com.
- [5] Xiyuan Hospital, China Academy of Chinese Medical Sciences, No. 1 Xiyuan Playground, Haidian District, Beijing 100091, China. Electronic address: miaoqing55@sina.com.

- PMID: **32315725**
- PMCID: PMC7166040
- DOI: 10.1016/j.jinf.2020.04.012

Free PMC article

Suppressed T cell-mediated immunity in patients with COVID-19: A clinical retrospective study in Wuhan, China

Bo Xu et al. J Infect. 2020 Jul.
Free PMC article
Show details
J Infect
. 2020 Jul;81(1):e51-e60.
doi: 10.1016/j.jinf.2020.04.012. Epub 2020 Apr 18.

Authors

Bo Xu [1], Cun-Yu Fan [1], An-Lu Wang [2], Yi-Long Zou [1], Yi-Han Yu [1], Cong He [1], Wen-Guang Xia [3], Ji-Xian Zhang [4], Qing Miao [5]

Affiliations

- [1] Hubei Provincial Hospital of Traditional Chinese & Western Medicine, Wuhan 430015, China.
- [2] Xiyuan Hospital, China Academy of Chinese Medical Sciences, No. 1 Xiyuan Playground, Haidian District, Beijing 100091, China.
- [3] Hubei Provincial Hospital of Traditional Chinese & Western Medicine, Wuhan 430015, China. Electronic address: hbszxyjhyy11@163.com.
- [4] Hubei Provincial Hospital of Traditional Chinese & Western Medicine, Wuhan 430015, China. Electronic address: jxzhang1607@163.com.
- [5] Xiyuan Hospital, China Academy of Chinese Medical Sciences, No. 1 Xiyuan Playground, Haidian District, Beijing 100091, China. Electronic address: miaoqing55@sina.com.

- PMID: **32315725**
- PMCID: PMC7166040
- DOI: 10.1016/j.jinf.2020.04.012

Abstract

Importance: An ongoing outbreak of COVID-19 has exhibited significant threats around the world. We found a significant decrease of T lymphocyte subsets and an increase of inflammatory cytokines of hospitalized patients with COVID-19 in clinical practice.

Methods: We conducted a retrospective, single-center observational study of in-hospital adult patients with confirmed COVID-19 in Hubei Provincial Hospital of traditional Chinese and Western medicine (Wuhan, China) by Mar 1, 2020. Demographic, clinical, laboratory information, especially T lymphocyte subsets and inflammatory cytokines were reported. For patients who died or discharge from hospital, the associations of T lymphocyte subsets on admission were evaluated by univariate logistic regression with odds ratios (ORs) and 95% confidence intervals (CIs), warning values to predict in-hospital death were assessed by Receiver Operator Characteristic (ROC) curves.

Results: A total of 187 patients were enrolled in our study from Dec 26, 2019 to Mar 1, 2020, of whom 145 were survivors (discharge = 117) or non-survivors (in-hospital death ==28). All patients exhibited a significant drop of T lymphocyte subsets counts with remarkably increasing concentrations of SAA, CRP, IL-6, and IL-10 compared to normal values. The median concentrations of SAA and CRP in critically-ill patients were nearly 4- and 10-fold than those of mild-ill patients, respectively. As the severity of COVID-19 getting worse, the counts of T lymphocyte drop lower.28 patients died in hospital, the median lymphocyte, CD3+ T-cell, CD4+ T-cell, CD8+ T-cell and B-cell were significantly lower than other patients. Lower counts (/uL) of T lymphocyte subsets lymphocyte (<500), CD3+T-cell (<200), CD4+ T-cell (<100), CD8+ T-cell (<100) and B-cell (<50) were associated with higher risks of in-hospital death of CIVID-19. The warning values to predict in-hospital death of lymphocyte, CD3+ T-cell, CD4+ T-cell, CD8+ T-cell, and B-cell were 559, 235, 104, 85 and 82, respectively.

Conclusion: We find a significant decrease of T lymphocyte subset is positively correlated with in-hospital death and severity of illness. The decreased levels of T lymphocyte subsets reported in our study were similar with SARS but not common among other virus infection, which may be possible biomarkers for early diagnosis of COVID-19. Our findings may shed light on early warning of high risks of mortality and help early intervention and treatment of COVID-19.

Keywords: COVID-19; Immunity; Retrospective study; T cell subsets.

Copyright © 2020. Published by Elsevier Ltd.

Conflict of interest statement

Declaration of Competing Interest We declare no competing interests.

Comment in

- COVID-19 patients display distinct SARS-CoV-2 specific T-cell responses according to disease severity.
  Kroemer M, Spehner L, Vettoretti L, Bouard A, Eberst G, Pili Floury S, Capellier G, Lepiller Q, Orillard E, Mansi L, Clairet AL, Westeel V, Limat S, Dubois M, Malinowski L, Bohard L, Borg C, Chirouze C, Bouiller K. Kroemer M, et al. J Infect. 2021 Feb;82(2):282-327. doi: 10.1016/j.jinf.2020.08.036. Epub 2020 Aug 25. J Infect. 2021. PMID: 32853599 Free PMC article.

- 24 references
- 4 figures

Supplementary info

Publication types, MeSH terms Expand

Publication types

- Research Support, Non-U.S. Gov't

MeSH terms

- Adult
- Aged
- Betacoronavirus*
- COVID-19
- China / epidemiology
- Coronavirus Infections / epidemiology*
- Coronavirus Infections / immunology*
- Female
- Humans
- Immunity, Cellular*
- Lymphocyte Count
- Male
- Middle Aged
- Pandemics
- Pneumonia, Viral / epidemiology*
- Pneumonia, Viral / immunology*
- Retrospective Studies

- SARS-CoV-2
- T-Lymphocyte Subsets

Full text links

ELSEVIER FULL-TEXT ARTICLE Elsevier Science Free PMC article

Proceed to details

Cite

Share

☐ 961

Clinical Trial

J Clin Invest

. 2020 Dec 1;130(12):6417-6428.

doi: 10.1172/JCI140617.

Corticosteroid treatment in severe COVID-19 patients with acute respiratory distress syndrome

Jiao Liu [1 2], Sheng Zhang [1], Xuan Dong [3], Zhongyi Li [4], Qianghong Xu [5], Huibin Feng [6], Jing Cai [7], Sisi Huang [2], Jun Guo [8], Lidi Zhang [2], Yizhu Chen [2], Wei Zhu [9], Hangxiang Du [1], Yongan Liu [1], Tao Wang [1], Limin Chen [1], Zhenliang Wen [2], Djillali Annane [10], Jieming Qu [11], Dechang Chen [1 2]

Affiliations Expand

Affiliations

- [1] Department of Critical Care Medicine, Ruijin Hospital, Shanghai Jiao Tong University School of Medicine, Shanghai, China.
- [2] Department of Critical Care Medicine, Ruijin Hospital North, Shanghai Jiao Tong University School of Medicine, Shanghai, China.
- [3] Tuberculosis and Respiratory Department, Wuhan Jinyin-tan Hospital, Wuhan, China.
- [4] Department of Critical Care Medicine, Wuhan No. 9 Hospital, Wuhan, China.
- [5] Department of Critical Care Medicine, Zhejiang Hospital, Hangzhou, China.
- [6] Intensive Care Unit, Huangshi Central Hospital, Affiliated Hospital of Hubei Polytechnic University, Edong Healthcare Group, Huangshi, China.
- [7] Department of Critical Care Medicine, Second Affiliated Hospital of Zhejiang University Medical College, Hangzhou, China.
- [8] Intensive Care Unit, Huazhong University of Science and Technology Union Jiangbei Hospital, Wuhan, China.
- [9] Intensive Care Unit, Tianyou Hospital Affiliated to Wuhan University of Science and Technology, Wuhan, China.
- [10] FHU SEPSIS (Saclay and Paris Seine Nord Endeavour to PerSonalize Interventions for Sepsis), RHU RECORDS (Rapi'd rEcognition of CORticosteroiD resistant or sensitive Sepsis), Department of Intensive Care, Hôpital Raymond Poincaré (APHP), Laboratory of

Infection and Inflammation - U1173, School of Medicine Simone Veil, University Versailles Saint Quentin - University Paris Saclay, INSERM, Garches, France.
- [11] Department of Pulmonary and Critical Care Medicine, Ruijin Hospital, Shanghai Jiao Tong University School of Medicine, Shanghai, China.

- PMID: **33141117**
- PMCID: PMC7685724
- DOI: 10.1172/JCI140617

Free PMC article
Clinical Trial

Corticosteroid treatment in severe COVID-19 patients with acute respiratory distress syndrome

Jiao Liu et al. J Clin Invest. 2020.
Free PMC article
Show details
J Clin Invest
. 2020 Dec 1;130(12):6417-6428.
doi: 10.1172/JCI140617.

Authors

Jiao Liu [1, 2], Sheng Zhang [1], Xuan Dong [3], Zhongyi Li [4], Qianghong Xu [5], Huibin Feng [6], Jing Cai [7], Sisi Huang [2], Jun Guo [8], Lidi Zhang [2], Yizhu Chen [2], Wei Zhu [9], Hangxiang Du [1], Yongan Liu [1], Tao Wang [1], Limin Chen [1], Zhenliang Wen [2], Djillali Annane [10], Jieming Qu [11], Dechang Chen [1, 2]

Affiliations

- [1] Department of Critical Care Medicine, Ruijin Hospital, Shanghai Jiao Tong University School of Medicine, Shanghai, China.
- [2] Department of Critical Care Medicine, Ruijin Hospital North, Shanghai Jiao Tong University School of Medicine, Shanghai, China.
- [3] Tuberculosis and Respiratory Department, Wuhan Jinyin-tan Hospital, Wuhan, China.
- [4] Department of Critical Care Medicine, Wuhan No. 9 Hospital, Wuhan, China.
- [5] Department of Critical Care Medicine, Zhejiang Hospital, Hangzhou, China.
- [6] Intensive Care Unit, Huangshi Central Hospital, Affiliated Hospital of Hubei Polytechnic University, Edong Healthcare Group, Huangshi, China.
- [7] Department of Critical Care Medicine, Second Affiliated Hospital of Zhejiang University Medical College, Hangzhou, China.
- [8] Intensive Care Unit, Huazhong University of Science and Technology Union Jiangbei Hospital, Wuhan, China.
- [9] Intensive Care Unit, Tianyou Hospital Affiliated to Wuhan University of Science and Technology, Wuhan, China.

- [10] FHU SEPSIS (Saclay and Paris Seine Nord Endeavour to PerSonalize Interventions for Sepsis), RHU RECORDS (Rapi'd rEcognition of CORticosteroiD resistant or sensitive Sepsis), Department of Intensive Care, Hôpital Raymond Poincaré (APHP), Laboratory of Infection and Inflammation - U1173, School of Medicine Simone Veil, University Versailles Saint Quentin - University Paris Saclay, INSERM, Garches, France.
- [11] Department of Pulmonary and Critical Care Medicine, Ruijin Hospital, Shanghai Jiao Tong University School of Medicine, Shanghai, China.

- PMID: **33141117**
- PMCID: PMC7685724
- DOI: 10.1172/JCI140617

Abstract

BACKGROUNDCorticosteroids are widely used in patients with COVID 19, although their benefit-to-risk ratio remains controversial.METHODSPatients with severe COVID-19-related acute respiratory distress syndrome (ARDS) were included from December 29, 2019 to March 16, 2020 in 5 tertiary Chinese hospitals. Cox proportional hazards and competing risks analyses were conducted to analyze the impact of corticosteroids on mortality and SARS-CoV-2 RNA clearance, respectively. We performed a propensity score (PS) matching analysis to control confounding factors.RESULTSOf 774 eligible patients, 409 patients received corticosteroids, with a median time from hospitalization to starting corticosteroids of 1.0 day (IQR 0.0-3.0 days) . As compared with usual care, treatment with corticosteroids was associated with increased rate of myocardial (15.6% vs. 10.4%, P = 0.041) and liver injury (18.3% vs. 9.9%, P = 0.001), of shock (22.0% vs. 12.6%, P < 0.001), of need for mechanical ventilation (38.1% vs. 19.5%, P < 0.001), and increased rate of 28-day all-cause mortality (44.3% vs. 31.0%, P < 0.001). After PS matching, corticosteroid therapy was associated with 28-day mortality (adjusted HR 1.46, 95% CI 1.01-2.13, P = 0.045). High dose (>200 mg) and early initiation (≤3 days from hospitalization) of corticosteroid therapy were associated with a higher 28-day mortality rate. Corticosteroid use was also associated with a delay in SARS-CoV-2 coronavirus RNA clearance in the competing risk analysis (subhazard ratio 1.59, 95% CI 1.17-2.15, P = 0.003).CONCLUSIONAdministration of corticosteroids in severe COVID-19-related ARDS is associated with increased 28-day mortality and delayed SARS-CoV-2 coronavirus RNA clearance after adjustment for time-varying confounders.FUNDINGNone.

Keywords: COVID-19; Respiration.

Conflict of interest statement

Conflict of interest: The authors have declared that no conflict of interest exists.

Comment in

- Corticosteroids, COVID-19 pneumonia, and acute respiratory distress syndrome.
  Matthay MA, Wick KD. Matthay MA, et al. J Clin Invest. 2020 Dec 1;130(12):6218-6221. doi: 10.1172/JCI143331. J Clin Invest. 2020. PMID: 32976118 Free PMC article.

- 5 figures

Supplementary info

Publication types, MeSH terms, Substances Expand

Publication types

- Clinical Trial
- Multicenter Study
- Observational Study

MeSH terms

- Adrenal Cortex Hormones / administration & dosage*
- Adrenal Cortex Hormones / adverse effects*
- Aged
- COVID-19 / complications
- COVID-19 / drug therapy*
- COVID-19 / mortality*
- Disease-Free Survival
- Female
- Humans
- Male
- Middle Aged
- Respiratory Distress Syndrome / drug therapy*
- Respiratory Distress Syndrome / etiology
- Respiratory Distress Syndrome / mortality*
- Retrospective Studies
- Severity of Illness Index
- Survival Rate

Substances

- Adrenal Cortex Hormones

Full text links

VIEW ARTICLE FULL TEXT American Society for Clinical Investigation Free PMC article
Proceed to details
Cite
Share
☐ 962
Observational Study
Int J Cardiol
. 2021 Feb 1;324:249-254.
doi: 10.1016/j.ijcard.2020.09.062. Epub 2020 Sep 25.

The role of anti-hypertensive treatment, comorbidities and early introduction of LMWH in the setting of COVID-19: A retrospective, observational study in Northern Italy

Antonio Desai [1], Giuseppe Voza [2], Silvia Paiardi [2], Francesca Ilaria Teofilo [2], Giuseppe Caltagirone [3], Marta Ripoll Pons [2], Monia Aloise [2], Maria Kogan [2], Tobia Tommasini [4], Victor Savevski [4], Giulio Stefanini [5], Claudio Angelini [6], Michele Ciccarelli [7], Salvatore Badalamenti [6], Ana Lleo De Nalda [8], Alessio Aghemo [8], Maurizio Cecconi [9], Filippo Martinelli Boneschi [10], Antonio Voza [2], Humanitas COVID-19 task force

Affiliations Expand

Affiliations

- [1] Emergency Department, Humanitas Clinical and Research Center, IRCCS, Milan, Italy; Department of Biomedical Sciences, Humanitas University, Pieve Emanuele, Italy. Electronic address: desaiantonio@gmail.com.
- [2] Emergency Department, Humanitas Clinical and Research Center, IRCCS, Milan, Italy.
- [3] Emergency Department, Humanitas Clinical and Research Center, IRCCS, Milan, Italy; Department of Biomedical Sciences, Humanitas University, Pieve Emanuele, Italy.
- [4] Artificial Intelligence Center, Humanitas Clinical and Research Center, IRCCS, Milan, Italy.
- [5] Cardiology Department, Humanitas Clinical and Research Center, IRCCS, Milan, Italy.
- [6] Humanitas Clinical and Research Center-Nephrology Unit, Rozzano, Milan, Italy.
- [7] Pneumology Department, Humanitas Clinical and Research Center, Rozzano, Milan, Italy.
- [8] Department of Biomedical Sciences, Humanitas University, Pieve Emanuele, Italy; Department of Internal Medicine and Hepatology, Humanitas University, Humanitas Clinical and Research Center, IRCCS, Rozzano, Milan, Italy.
- [9] Department of Biomedical Sciences, Humanitas University, Pieve Emanuele, Italy; Humanitas Clinical and Research Center, Department and Anaesthesia and Intensive Care, Rozzano, Milan, Italy.
- [10] Dino Ferrari Centre, Neuroscience Section, Department of Pathophysiology and Transplantation (DEPT), University of Milan, Milan, Italy; Neurology Unit and MS Centre, Fondazione IRCCS Ca' Granda Ospedale Maggiore Policlinico, Milan, Italy.

- PMID: **32980434**
- PMCID: PMC7516574
- DOI: 10.1016/j.ijcard.2020.09.062

Free PMC article
Observational Study

The role of anti-hypertensive treatment, comorbidities and early introduction of LMWH in the setting of COVID-19: A retrospective, observational study in Northern Italy

Antonio Desai et al. Int J Cardiol. 2021.
Free PMC article
Show details
Int J Cardiol
. 2021 Feb 1;324:249-254.
doi: 10.1016/j.ijcard.2020.09.062. Epub 2020 Sep 25.

Authors

Antonio Desai [1], Giuseppe Voza [2], Silvia Paiardi [2], Francesca Ilaria Teofilo [2], Giuseppe Caltagirone [3], Marta Ripoll Pons [2], Monia Aloise [2], Maria Kogan [2], Tobia Tommasini [4], Victor Savevski [4], Giulio Stefanini [5], Claudio Angelini [6], Michele Ciccarelli [7], Salvatore Badalamenti [6], Ana Lleo De Nalda [8], Alessio Aghemo [8], Maurizio Cecconi [9], Filippo Martinelli Boneschi [10], Antonio Voza [2], Humanitas COVID-19 task force

Affiliations

- [1] Emergency Department, Humanitas Clinical and Research Center, IRCCS, Milan, Italy; Department of Biomedical Sciences, Humanitas University, Pieve Emanuele, Italy. Electronic address: desaiantonio@gmail.com.
- [2] Emergency Department, Humanitas Clinical and Research Center, IRCCS, Milan, Italy.
- [3] Emergency Department, Humanitas Clinical and Research Center, IRCCS, Milan, Italy; Department of Biomedical Sciences, Humanitas University, Pieve Emanuele, Italy.
- [4] Artificial Intelligence Center, Humanitas Clinical and Research Center, IRCCS, Milan, Italy.
- [5] Cardiology Department, Humanitas Clinical and Research Center, IRCCS, Milan, Italy.
- [6] Humanitas Clinical and Research Center-Nephrology Unit, Rozzano, Milan, Italy.
- [7] Pneumology Department, Humanitas Clinical and Research Center, Rozzano, Milan, Italy.
- [8] Department of Biomedical Sciences, Humanitas University, Pieve Emanuele, Italy; Department of Internal Medicine and Hepatology, Humanitas University, Humanitas Clinical and Research Center, IRCCS, Rozzano, Milan, Italy.
- [9] Department of Biomedical Sciences, Humanitas University, Pieve Emanuele, Italy; Humanitas Clinical and Research Center, Department and Anaesthesia and Intensive Care, Rozzano, Milan, Italy.
- [10] Dino Ferrari Centre, Neuroscience Section, Department of Pathophysiology and Transplantation (DEPT), University of Milan, Milan, Italy; Neurology Unit and MS Centre, Fondazione IRCCS Ca' Granda Ospedale Maggiore Policlinico, Milan, Italy.

- PMID: **32980434**
- PMCID: PMC7516574
- DOI: 10.1016/j.ijcard.2020.09.062

Abstract

Background: There is a great deal of debate about the role of cardiovascular comorbidities and the chronic use of antihypertensive agents (such as ACE-I and ARBs) on mortality on COVID-19 patients. Of note, ACE2 is responsible for the host cell entry of the virus.

Methods: We extracted data on 575 consecutive patients with laboratory-confirmed SARS-CoV-2 infection admitted to the Emergency Department (ED) of Humanitas Center, between February 21 and April 14, 2020. The aim of the study was to evaluate the role of chronic treatment with ACE-I or ARBs and other clinical predictors on in-hospital mortality in a cohort of COVID-19 patients.

Results: Multivariate analysis showed that a chronic intake of ACE-I was associated with a trend in reduction of mortality (OR: 0.53; 95% CI: 0.27-1.03; $p = 0.06$), differently from a chronic intake of ARB (OR: 1.1; 95% CI: 0.5-2.8; $p=0.8$). Increased age (ORs ranging from 3.4 to 25.2 and to 39.5 for 60-70, 70-80 and >80 years vs <60) and cardiovascular comorbidities (OR: 1.90; 95% CI: 1.1-3.3; $p = 0.02$) were confirmed as important risk factors for COVID-19 mortality. Timely treatment with low-molecular-weight heparin (LMWH) in ED was found to be protective (OR: 0.36; 95% CI: 0.21-0.62; $p < 0.0001$).

Conclusions: This study can contribute to understand the reasons behind the high mortality rate of patients in Lombardy, a region which accounts for >50% of total Italian deaths. Based on our findings, we support that daily intake of antihypertensive medications in the setting of COVID-19 should not be discontinued and that a timely LMWH administration in ED has shown to decrease in-hospital mortality.

Keywords: ACE-I; ARBs; Cardiovascular; Epidemiology; Hypertension; RAAS.

Copyright © 2020 Elsevier B.V. All rights reserved.

Conflict of interest statement

Declaration of Competing Interest None.

- 34 references
- 2 figures

Supplementary info

Publication types, MeSH terms, Substances Expand

Publication types

- Observational Study

MeSH terms

- Adult
- Aged
- Aged, 80 and over
- Anticoagulants / administration & dosage*
- Antihypertensive Agents / administration & dosage*
- COVID-19 / diagnosis
- COVID-19 / drug therapy*
- COVID-19 / mortality*
- Comorbidity
- Female
- Heparin, Low-Molecular-Weight / administration & dosage*
- Hospital Mortality / trends*
- Humans
- Italy / epidemiology
- Male
- Middle Aged
- Mortality / trends
- Retrospective Studies
- Time-to-Treatment / trends
- Treatment Outcome

Substances

- Anticoagulants
- Antihypertensive Agents
- Heparin, Low-Molecular-Weight

Full text links

ELSEVIER FULL-TEXT ARTICLE Elsevier Science Free PMC article
Proceed to details
Cite
Share
☐ 963
Observational Study
Emerg Med Australas
. 2022 Feb;34(1):52-57.
doi: 10.1111/1742-6723.13835. Epub 2021 Aug 8.

Impact of lockdowns on critical care service demand in a metropolitan hospital in Melbourne, Australia

Sing Chee Tan [1, 2], Anthony Cross [1, 3], Angajendra Ghosh [1, 4]
Affiliations Expand

Affiliations

- [1] Department of Intensive Care Medicine, Northern Health, Melbourne, Victoria, Australia.
- [2] Centre for Digital Transformation of Health, The University of Melbourne, Melbourne, Victoria, Australia.
- [3] Centre for Integrated Critical Care, The University of Melbourne, Melbourne, Victoria, Australia.
- [4] Department of Medical Education, The University of Melbourne, Melbourne, Victoria, Australia.

- PMID: **34369078**
- DOI: 10.1111/1742-6723.13835

Observational Study

Impact of lockdowns on critical care service demand in a metropolitan hospital in Melbourne, Australia

Sing Chee Tan et al. Emerg Med Australas. 2022 Feb.
Show details
Emerg Med Australas
. 2022 Feb;34(1):52-57.
doi: 10.1111/1742-6723.13835. Epub 2021 Aug 8.

Authors

Sing Chee Tan [1, 2], Anthony Cross [1, 3], Angajendra Ghosh [1, 4]

Affiliations

- [1] Department of Intensive Care Medicine, Northern Health, Melbourne, Victoria, Australia.
- [2] Centre for Digital Transformation of Health, The University of Melbourne, Melbourne, Victoria, Australia.
- [3] Centre for Integrated Critical Care, The University of Melbourne, Melbourne, Victoria, Australia.

- [4] Department of Medical Education, The University of Melbourne, Melbourne, Victoria, Australia.

- PMID: **34369078**
- DOI: 10.1111/1742-6723.13835

Abstract

Objective: There is a growing recognition of the impact of lockdowns on non-COVID-19 demand for critical care services. While a reduction in demand has been postulated, there remains a paucity of quantitative data on the extent and nature of this reduction. The present study aims to quantify the impact of lockdown on critical care services, namely ED, intensive care unit (ICU), medical emergency team (MET) and emergency theatre (ET) demand, during the lockdown in Victoria, Australia.

Methods: This is a single-centred, retrospective observational study on critical service demand, comparing activity levels during the lockdown (31 March to 27 October 2020) with the matched time period from 1 year prior.

Results: There was a reduction in presentations to ED (27.2%), MET calls (27.4%), ICU patient episodes (14.5%) and ET bookings (5.8%). There was an unexpected increase in ICU admissions for metabolic diagnoses, comprising drug overdoses and diabetic ketoacidosis, and a reduction in respiratory ICU admissions. There was a reduction across all ED triage categories, which included triage 1 and 2 patients, indicating a reduction even in life-threatening and emergency presentations.

Conclusion: Lockdowns lead to a significant reduction in ICU, MET call and ED demand, and to a lesser extent ET demand. This pattern should be considered in surge capacity and workforce redeployment planning. There are also impacts on public health epidemiology, with potential adverse consequences on mental health and chronic disease management. Further research on the impact of lockdowns on long-term disease outcomes is needed.

Keywords: critical care; emergency medicine; epidemiology; public health; specialties; surgical.

© 2021 Australasian College for Emergency Medicine.

- 24 references

Supplementary info

Publication types, MeSH terms Expand

Publication types

- Observational Study

MeSH terms

- Critical Care*
- Emergency Service, Hospital*

- Hospitals, Urban
- Humans
- Retrospective Studies
- Victoria

Full text links

Wiley Full Text Article Wiley
Proceed to details
Cite
Share
964
Observational Study
Crit Care
. 2021 Dec 13;25(1):423.
doi: 10.1186/s13054-021-03846-5.

Lung histopathologic clusters in severe COVID-19: a link between clinical picture and tissue damage

Maddalena Alessandra Wu [#] [1], Gianluca Lopez [#] [2] [3], Manuela Nebuloni [3] [4], Davide Ottolina [5], Jonathan Montomoli [6], Luca Carsana [3], Tommaso Fossali [5], Antonio Castelli [5], Roberto Rech [5], Chiara Cogliati [1] [4], Emanuele Catena [5], Riccardo Colombo [7]
Affiliations Expand

Affiliations

- [1] Division of Internal Medicine, ASST Fatebenefratelli Sacco, Milan, Italy.
- [2] School of Pathology, University of Milan, Milan, Italy.
- [3] Pathology Unit, ASST Fatebenefratelli Sacco, Milan, Italy.
- [4] Department of Biomedical and Clinical Sciences, University of Milan, Milan, Italy.
- [5] Division of Anesthesiology and Intensive Care, ASST Fatebenefratelli Sacco, Milan, Italy.
- [6] Division of Anesthesiology and Intensive Care, Ospedale Degli Infermi, Rimini, Italy.
- [7] Division of Anesthesiology and Intensive Care, ASST Fatebenefratelli Sacco, Milan, Italy. riccardo.colombo@unimi.it.

[#] Contributed equally.

- PMID: **34903264**
- PMCID: PMC8667540
- DOI: 10.1186/s13054-021-03846-5

Free PMC article
Observational Study

Lung histopathologic clusters in severe COVID-19: a link between clinical picture and tissue damage

Maddalena Alessandra Wu et al. Crit Care. 2021.
Free PMC article
Show details
Crit Care
. 2021 Dec 13;25(1):423.
doi: 10.1186/s13054-021-03846-5.

Authors

Maddalena Alessandra Wu [#] [1], Gianluca Lopez [#] [2] [3], Manuela Nebuloni [3] [4], Davide Ottolina [5], Jonathan Montomoli [6], Luca Carsana [3], Tommaso Fossali [5], Antonio Castelli [5], Roberto Rech [5], Chiara Cogliati [1] [4], Emanuele Catena [5], Riccardo Colombo [7]

Affiliations

- [1] Division of Internal Medicine, ASST Fatebenefratelli Sacco, Milan, Italy.
- [2] School of Pathology, University of Milan, Milan, Italy.
- [3] Pathology Unit, ASST Fatebenefratelli Sacco, Milan, Italy.
- [4] Department of Biomedical and Clinical Sciences, University of Milan, Milan, Italy.
- [5] Division of Anesthesiology and Intensive Care, ASST Fatebenefratelli Sacco, Milan, Italy.
- [6] Division of Anesthesiology and Intensive Care, Ospedale Degli Infermi, Rimini, Italy.
- [7] Division of Anesthesiology and Intensive Care, ASST Fatebenefratelli Sacco, Milan, Italy. riccardo.colombo@unimi.it.

[#] Contributed equally.

- PMID: **34903264**
- PMCID: PMC8667540
- DOI: 10.1186/s13054-021-03846-5

Abstract

Background: Autoptic pulmonary findings have been described in severe COVID-19 patients, but evidence regarding the correlation between clinical picture and lung histopathologic patterns is still weak.

Methods: This was a retrospective cohort observational study conducted at the referral center for infectious diseases in northern Italy. Full lung autoptic findings and clinical data of patients who died from COVID-19 were analyzed. Lung histopathologic patterns were scored according to the extent of tissue damage. To consider coexisting histopathologic patterns, hierarchical clustering of histopathologic findings was applied.

Results: Whole pulmonary examination was available in 75 out of 92 full autopsies. Forty-eight hospitalized patients (64%), 44 from ICU and four from the medical ward, had complete clinical data. The histopathologic patterns had a time-dependent distribution with considerable overlap among patterns. Duration of positive-pressure ventilation ($p < 0.0001$), mean positive end-expiratory pressure (PEEP) ($p = 0.007$), worst serum albumin ($p = 0.017$), interleukin 6 ($p = 0.047$), and kidney SOFA ($p = 0.001$) differed among histopathologic clusters. The amount of PEEP for long-lasting ventilatory treatment was associated with the cluster showing the largest areas of early and late proliferative diffuse alveolar damage. No pharmacologic interventions or comorbidities affected the lung histopathology.

Conclusions: Our study draws a comprehensive link between the clinical and pulmonary histopathologic findings in a large cohort of COVID-19 patients. These results highlight that the positive end-expiratory pressures and the duration of the ventilatory treatment correlate with lung histopathologic patterns, providing new clues to the knowledge of the pathophysiology of severe SARS-CoV-2 pneumonia.

Keywords: COVID-19; Histology; Lung injury; Pneumonia; Positive-pressure ventilation.

© 2021. The Author(s).

Conflict of interest statement

The authors have no competing interests to declare.

- 30 references
- 7 figures

Supplementary info

Publication types, MeSH terms Expand

Publication types

- Observational Study

MeSH terms

- Autopsy
- COVID-19*
- Humans
- Lung* / pathology
- Patient Acuity
- Retrospective Studies

Full text links

Read free full text at BMC BioMed Central Free PMC article
Proceed to details

Cite
Share
☐ 965
Observational Study
Lancet Infect Dis
. 2020 Jun;20(6):689-696.
doi: 10.1016/S1473-3099(20)30198-5. Epub 2020 Mar 25.

Clinical and epidemiological features of 36 children with coronavirus disease 2019 (COVID-19) in Zhejiang, China: an observational cohort study

Haiyan Qiu [1], Junhua Wu [1], Liang Hong [2], Yunling Luo [2], Qifa Song [3], Dong Chen [4]
Affiliations Expand

Affiliations

- [1] Department of Pediatrics, Ningbo Women and Children's Hospital, Ningbo, Zhejiang, China.
- [2] Department of Infectious Diseases, The Third Affiliated Hospital of Wenzhou Medical University, Wenzhou, Zhejiang, China.
- [3] Department of Microbiology, Ningbo Municipal Centre for Disease Control and Prevention, Ningbo, Zhejiang, China. Electronic address: songqf@nbcdc.org.cn.
- [4] Department of Infectious Diseases, Wenzhou Central Hospital and Sixth People's Hospital of Wenzhou, Wenzhou, Zhejiang, China. Electronic address: chendong_wz@126.com.

- PMID: **32220650**
- PMCID: PMC7158906
- DOI: 10.1016/S1473-3099(20)30198-5

Free PMC article
Observational Study

Clinical and epidemiological features of 36 children with coronavirus disease 2019 (COVID-19) in Zhejiang, China: an observational cohort study

Haiyan Qiu et al. Lancet Infect Dis. 2020 Jun.
Free PMC article
Show details

Lancet Infect Dis

. 2020 Jun;20(6):689-696.
doi: 10.1016/S1473-3099(20)30198-5. Epub 2020 Mar 25.

Authors

Haiyan Qiu [1], Junhua Wu [1], Liang Hong [2], Yunling Luo [2], Qifa Song [3], Dong Chen [4]

Affiliations

- [1] Department of Pediatrics, Ningbo Women and Children's Hospital, Ningbo, Zhejiang, China.
- [2] Department of Infectious Diseases, The Third Affiliated Hospital of Wenzhou Medical University, Wenzhou, Zhejiang, China.
- [3] Department of Microbiology, Ningbo Municipal Centre for Disease Control and Prevention, Ningbo, Zhejiang, China. Electronic address: songqf@nbcdc.org.cn.
- [4] Department of Infectious Diseases, Wenzhou Central Hospital and Sixth People's Hospital of Wenzhou, Wenzhou, Zhejiang, China. Electronic address: chendong_wz@126.com.

- PMID: **32220650**
- PMCID: PMC7158906
- DOI: 10.1016/S1473-3099(20)30198-5

Abstract

Background: Since December, 2019, an outbreak of coronavirus disease 2019 (COVID-19) has spread globally. Little is known about the epidemiological and clinical features of paediatric patients with COVID-19.

Methods: We retrospectively retrieved data for paediatric patients (aged 0-16 years) with confirmed COVID-19 from electronic medical records in three hospitals in Zhejiang, China. We recorded patients' epidemiological and clinical features.

Findings: From Jan 17 to March 1, 2020, 36 children (mean age 8·3 [SD 3·5] years) were identified to be infected with severe acute respiratory syndrome coronavirus 2. The route of transmission was by close contact with family members (32 [89%]) or a history of exposure to the epidemic area (12 [33%]); eight (22%) patients had both exposures. 19 (53%) patients had moderate clinical type with pneumonia; 17 (47%) had mild clinical type and either were asymptomatic (ten [28%]) or had acute upper respiratory symptoms (seven [19%]). Common symptoms on admission were fever (13 [36%]) and dry cough (seven [19%]). Of those with fever, four (11%) had a body temperature of 38·5°C or higher, and nine (25%) had a body temperature of 37·5-38·5°C. Typical abnormal laboratory findings were elevated creatine kinase MB (11 [31%]), decreased lymphocytes (11 [31%]), leucopenia (seven [19%]), and elevated procalcitonin (six [17%]). Besides radiographic presentations, variables that were associated significantly with severity of COVID-19 were decreased lymphocytes, elevated body temperature, and high levels of procalcitonin, D-dimer, and creatine kinase MB. All children received interferon alfa by aerosolisation twice a day, 14 (39%) received lopinavir-ritonavir syrup twice a day, and six (17%) needed oxygen inhalation. Mean time in hospital was 14 (SD 3) days. By Feb 28, 2020, all patients were cured.

Interpretation: Although all paediatric patients in our cohort had mild or moderate type of COVID-19, the large proportion of asymptomatic children indicates the difficulty in identifying paediatric patients who do not have clear epidemiological information, leading to a dangerous situation in community-acquired infections.

Funding: Ningbo Clinical Research Center for Children's Health and Diseases, Ningbo Reproductive Medicine Centre, and Key Scientific and Technological Innovation Projects of Wenzhou.

Copyright © 2020 Elsevier Ltd. All rights reserved.

Comment in

- Toward a clinically based classification of disease severity for paediatric COVID-19 - Authors' reply.
  Chen D, Tang F, Lu S, Song Q. Chen D, et al. Lancet Infect Dis. 2021 Jan;21(1):22-23. doi: 10.1016/S1473-3099(20)30397-2. Epub 2020 May 15. Lancet Infect Dis. 2021. PMID: 32422200 Free PMC article. No abstract available.
- Toward a clinically based classification of disease severity for paediatric COVID-19.
  Buonsenso D, Parri N, De Rose C, Valentini P; Gemelli-pediatric COVID-19 team. Buonsenso D, et al. Lancet Infect Dis. 2021 Jan;21(1):22. doi: 10.1016/S1473-3099(20)30396-0. Epub 2020 May 15. Lancet Infect Dis. 2021. PMID: 32422205 Free PMC article. No abstract available.
- Children are protected against SARS-CoV-2 infection.
  Dimeglio C, Mansuy JM, Charpentier S, Claudet I, Izopet J. Dimeglio C, et al. J Clin Virol. 2020 Jul;128:104451. doi: 10.1016/j.jcv.2020.104451. Epub 2020 May 20. J Clin Virol. 2020. PMID: 32454427 Free PMC article. No abstract available.

- 23 references
- 2 figures

Supplementary info

Publication types, MeSH terms Expand

Publication types

- Observational Study
- Research Support, Non-U.S. Gov't

MeSH terms

- Adolescent
- Asymptomatic Infections
- Betacoronavirus
- COVID-19
- Child
- Child, Preschool
- China / epidemiology

- Coronavirus Infections / epidemiology*
- Coronavirus Infections / physiopathology*
- Coronavirus Infections / therapy
- Cough / etiology
- Female
- Fever / etiology
- Humans
- Infant
- Male
- Pandemics
- Pneumonia, Viral / epidemiology*
- Pneumonia, Viral / physiopathology*
- Pneumonia, Viral / therapy
- Retrospective Studies
- SARS-CoV-2

Full text links

THE LANCET FULL-TEXT ARTICLE Elsevier Science Free PMC article

Proceed to details

Cite

Share

☐ 966

Observational Study

Arch Pediatr

. 2021 Jul;28(5):374-380.

doi: 10.1016/j.arcped.2021.04.004. Epub 2021 Apr 24.

Clinical characteristics of COVID-19 infection in polyhandicapped persons in France

M-C Rousseau [1], M Hully [2], M Milh [3], D Juzeau [4], B Pollez [5], S Peudenier [6], N Bahi Buisson [7], V Gautheron [8], French Polyhandicap (PLH), COVID Observatory Group; B Chabrol [3], T Billette de Villemeur [9]

Affiliations Expand

Affiliations

- [1] Service polyhandicap adultes, Hôpital San Salvadour (Assistance Publique Hôpitaux de Paris), BP 30 080, 83407 Hyères cedex, France. Electronic address: marie-christine.rousseau@aphp.fr.
- [2] MD, Services de Neurologie et Rééducation Pédiatriques, Hôpital Necker Enfants Malades, APHP, 75015 Paris, France.

- [3] Service de Neuropédiatrie, Hôpital d'Enfants CHU Timone, 13005 Marseille, France.
- [4] Santé Publique, Co Fondatrice du réseau NeurodeV, 59000 Lille, France.
- [5] APEI Lille, 59000 Lille, France.
- [6] CRDI, Hôpital Morvan, CHRU Brest, 29200 Brest, France.
- [7] Pediatric Neurology, Necker Enfants Malades University Hospital, Université de Paris, 75015 Paris, France; Institut Imagine, INSERM U 1163, Université de Paris, Paris, France.
- [8] Physical and Rehabilitation Medicine, Bellevue University Hospital, 42100 Saint-Étienne, France.
- [9] Service de Neuropédiatrie, Pathologie du développement, hôpital Trousseau-La Roche Guyon, 95780 La Roche-Guyon, France.

- PMID: **33994267**
- PMCID: PMC8064873
- DOI: 10.1016/j.arcped.2021.04.004

Free PMC article
Observational Study

Clinical characteristics of COVID-19 infection in polyhandicapped persons in France

M-C Rousseau et al. Arch Pediatr. 2021 Jul.
Free PMC article

Show details

Arch Pediatr
. 2021 Jul;28(5):374-380.
doi: 10.1016/j.arcped.2021.04.004. Epub 2021 Apr 24.

Authors

M-C Rousseau [1], M Hully [2], M Milh [3], D Juzeau [4], B Pollez [5], S Peudenier [6], N Bahi Buisson [7], V Gautheron [8], French Polyhandicap (PLH), COVID Observatory Group; B Chabrol [3], T Billette de Villemeur [9]

Affiliations

- [1] Service polyhandicap adultes, Hôpital San Salvadour (Assistance Publique Hôpitaux de Paris), BP 30 080, 83407 Hyères cedex, France. Electronic address: marie-christine.rousseau@aphp.fr.
- [2] MD, Services de Neurologie et Rééducation Pédiatriques, Hôpital Necker Enfants Malades, APHP, 75015 Paris, France.
- [3] Service de Neuropédiatrie, Hôpital d'Enfants CHU Timone, 13005 Marseille, France.
- [4] Santé Publique, Co Fondatrice du réseau NeurodeV, 59000 Lille, France.
- [5] APEI Lille, 59000 Lille, France.
- [6] CRDI, Hôpital Morvan, CHRU Brest, 29200 Brest, France.
- [7] Pediatric Neurology, Necker Enfants Malades University Hospital, Université de Paris, 75015 Paris, France; Institut Imagine, INSERM U 1163, Université de Paris, Paris, France.

- [8] Physical and Rehabilitation Medicine, Bellevue University Hospital, 42100 Saint-Étienne, France.
- [9] Service de Neuropédiatrie, Pathologie du développement, hôpital Trousseau-La Roche Guyon, 95780 La Roche-Guyon, France.

- PMID: **33994267**
- PMCID: PMC8064873
- DOI: 10.1016/j.arcped.2021.04.004

Abstract

Aim: Little is known about the clinical profile of COVID-19 infection in polyhandicapped persons. This study aimed to describe the characteristics of this infection among individuals with polyhandicap.

Method: This was a retrospective observational study. Polyhandicap was defined by the combination of motor deficiency, profound mental retardation, and age at onset of cerebral lesion younger than 6 years. A positive COVID-19 status was considered for patients with a positive COVID-19 laboratory test result, or patients presenting with compatible symptoms and living in an institution or at home with other patients or relatives who had laboratory-confirmed COVID-19 infection. Data collection included sociodemographic data, clinical and paraclinical characteristics, as well as the management and treatment for COVID-19 infection.

Results: We collected 98 cases, with a sex ratio of 0.98 and a mean age of 38.5 years (3 months to 73 years). COVID-19 infection was paucisymptomatic in 46% of patients, 20.6% of patients presented with dyspnea, while the most frequent extra-respiratory symptoms were digestive (26.5%) and neurological changes (24.5%); 18 patients required hospital admission, four adults died. The mean duration of infection was longer for adults than for children, and the proportion of taste and smell disorders was higher in older patients.

Conclusion: These findings suggest that PLH persons often develop paucisymptomatic forms of COVID-19 infection, although they may also experience severe outcomes, including death. Clinicians should be aware that COVID-19 symptoms in PLH persons are often extra-respiratory signs, mostly digestive and neurologic, which may help in the earlier identification of COVID-19 infection in this particular population of patients.

Keywords: COVID-19; Clinical characteristics; Death; Polyhandicap.

Copyright © 2021 French Society of Pediatrics. Published by Elsevier Masson SAS. All rights reserved.

- 22 references

Supplementary info

Publication types, MeSH terms Expand

Publication types

- Observational Study

MeSH terms

- Adolescent
- Adult
- Aged
- COVID-19 / complications*
- COVID-19 / diagnosis*
- Child
- Child, Preschool
- Female
- France
- Humans
- Infant
- Intellectual Disability / complications*
- Male
- Middle Aged
- Motor Disorders / complications*
- Retrospective Studies
- Young Adult

Full text links

ELSEVIER FULL-TEXT ARTICLE Elsevier Science Free PMC article

Proceed to details

Cite

Share

967

Observational Study

PLoS One
. 2020 Dec 22;15(12):e0238552.
doi: 10.1371/journal.pone.0238552. eCollection 2020.

Obesity and smoking as risk factors for invasive mechanical ventilation in COVID-19: A retrospective, observational cohort study

Ana C Monteiro [1], Rajat Suri [1], Iheanacho O Emeruwa [1], Robert J Stretch [1], Roxana Y Cortes-Lopez [1], Alexander Sherman [1], Catherine C Lindsay [2], Jennifer A Fulcher [3], David Goodman-Meza [3], Anil Sapru [4], Russell G Buhr [1], Steven Y Chang [1], Tisha Wang [1], Nida Qadir [1]
Affiliations Expand

Affiliations

- [1] Division of Pulmonary and Critical Care, Department of Medicine, UCLA Medical Center, Los Angeles, CA, United States of America.
- [2] Department of Medicine, UCLA Medical Center, Los Angeles, CA, United States of America.
- [3] Division of Infectious Disease, Department of Medicine, UCLA Medical Center, Los Angeles, CA, United States of America.
- [4] Division of Critical Care, Department of Pediatrics, UCLA Medical Center, Los Angeles, CA, United States of America.

- PMID: **33351817**
- PMCID: PMC7755188
- DOI: 10.1371/journal.pone.0238552

Free PMC article
Observational Study

Obesity and smoking as risk factors for invasive mechanical ventilation in COVID-19: A retrospective, observational cohort study

Ana C Monteiro et al. PLoS One. 2020.
Free PMC article

Show details

PLoS One

. 2020 Dec 22;15(12):e0238552.
doi: 10.1371/journal.pone.0238552. eCollection 2020.

Authors

Ana C Monteiro [1], Rajat Suri [1], Iheanacho O Emeruwa [1], Robert J Stretch [1], Roxana Y Cortes-Lopez [1], Alexander Sherman [1], Catherine C Lindsay [2], Jennifer A Fulcher [3], David Goodman-Meza [3], Anil Sapru [4], Russell G Buhr [1], Steven Y Chang [1], Tisha Wang [1], Nida Qadir [1]

Affiliations

- [1] Division of Pulmonary and Critical Care, Department of Medicine, UCLA Medical Center, Los Angeles, CA, United States of America.
- [2] Department of Medicine, UCLA Medical Center, Los Angeles, CA, United States of America.
- [3] Division of Infectious Disease, Department of Medicine, UCLA Medical Center, Los Angeles, CA, United States of America.
- [4] Division of Critical Care, Department of Pediatrics, UCLA Medical Center, Los Angeles, CA, United States of America.

- PMID: **33351817**

- PMCID: PMC7755188
- DOI: 10.1371/journal.pone.0238552

Abstract

Purpose: To describe the trajectory of respiratory failure in COVID-19 and explore factors associated with risk of invasive mechanical ventilation (IMV).

Materials and methods: A retrospective, observational cohort study of 112 inpatient adults diagnosed with COVID-19 between March 12 and April 16, 2020. Data were manually extracted from electronic medical records. Multivariable and Univariable regression were used to evaluate association between baseline characteristics, initial serum markers and the outcome of IMV.

Results: Our cohort had median age of 61 (IQR 45-74) and was 66% male. In-hospital mortality was 6% (7/112). ICU mortality was 12.8% (6/47), and 18% (5/28) for those requiring IMV. Obesity (OR 5.82, CI 1.74-19.48), former (OR 8.06, CI 1.51-43.06) and current smoking status (OR 10.33, CI 1.43-74.67) were associated with IMV after adjusting for age, sex, and high prevalence comorbidities by multivariable analysis. Initial absolute lymphocyte count (OR 0.33, CI 0.11-0.96), procalcitonin (OR 1.27, CI 1.02-1.57), IL-6 (OR 1.17, CI 1.03-1.33), ferritin (OR 1.05, CI 1.005-1.11), LDH (OR 1.57, 95% CI 1.13-2.17) and CRP (OR 1.13, CI 1.06-1.21), were associated with IMV by univariate analysis.

Conclusions: Obesity, smoking history, and elevated inflammatory markers were associated with increased need for IMV in patients with COVID-19.

Conflict of interest statement

I have read the journal's policy and the authors of this manuscript have the following competing interests: SYC consults for PureTech on their deupirfenidone in COVID study. This does not alter our adherence to PLOS ONE policies on sharing data and materials.

Update of

- Obesity and Smoking as Risk Factors for Invasive Mechanical Ventilation in COVID-19: a Retrospective, Observational Cohort Study.
  Monteiro AC, Suri R, Emeruwa IO, Stretch RJ, Cortes-Lopez RY, Sherman A, Lindsay CC, Fulcher JA, Goodman-Meza D, Sapru A, Buhr RG, Chang S, Wang T, Qadir N. Monteiro AC, et al. medRxiv. 2020 Aug 14:2020.08.12.20173849. doi: 10.1101/2020.08.12.20173849. Preprint. medRxiv. 2020. PMID: 32817959 Free PMC article. Updated.

- 37 references
- 2 figures

Supplementary info

Publication types, MeSH terms, Substances, Grant support Expand

Publication types

- Observational Study

- Research Support, N.I.H., Extramural

MeSH terms

- Aged
- C-Reactive Protein
- COVID-19 / blood
- COVID-19 / complications
- COVID-19 / epidemiology*
- COVID-19 / virology
- Cohort Studies
- Female
- Ferritins / blood
- Hospital Mortality
- Humans
- Intensive Care Units
- Interleukin-6 / blood
- L-Lactate Dehydrogenase / blood
- Lymphocyte Count
- Male
- Middle Aged
- Obesity / blood
- Obesity / complications
- Obesity / epidemiology*
- Obesity / virology
- Procalcitonin / blood
- Respiration, Artificial*
- Respiratory Insufficiency / blood
- Respiratory Insufficiency / complications
- Respiratory Insufficiency / epidemiology*
- Respiratory Insufficiency / virology
- Retrospective Studies
- Risk Factors
- SARS-CoV-2 / pathogenicity
- Smoking / adverse effects

Substances

- Interleukin-6
- Procalcitonin
- C-Reactive Protein
- Ferritins

- L-Lactate Dehydrogenase

Grant support

- UL1 TR001881/TR/NCATS NIH HHS/United States
- T32 5T32HL072752-1/NH/NIH HHS/United States
- K08 DA048163/DA/NIDA NIH HHS/United States
- T32 HL072752/HL/NHLBI NIH HHS/United States
- KL2 TR001882/TR/NCATS NIH HHS/United States
- TL1 TR001883/TR/NCATS NIH HHS/United States

Show all 6 grants

Full text links

OPEN ACCESS TO FULL TEXT PLOS ONE Public Library of Science Free PMC article

Proceed to details

Cite

Share

☐ 968

Observational Study

Emerg Med J

. 2021 Jun;38(6):439-445.

doi: 10.1136/emermed-2020-209992. Epub 2021 Apr 13.

Emergency department attendances during the COVID-19 pandemic: a retrospective analysis of attendances following Irish governmental pandemic measures

Ryan Taylor Sless [1], Nathaniel Edward Hayward [2], Paul MacDaragh Ryan [2], Conor Deasy [3], Kantikiran Dasari [3]

Affiliations Expand

Affiliations

- [1] School of Medicine, University College Cork, Cork City, Cork, Ireland ryan.sless@mail.utoronto.ca.
- [2] School of Medicine, University College Cork, Cork City, Cork, Ireland.
- [3] Emergency Medicine, Cork University Hospital, Cork City, Cork, Ireland.

- PMID: **33849939**
- PMCID: PMC8050875
- DOI: 10.1136/emermed-2020-209992

Free PMC article
Observational Study

Emergency department attendances during the COVID-19 pandemic: a retrospective analysis of attendances following Irish governmental pandemic measures

Ryan Taylor Sless et al. Emerg Med J. 2021 Jun.
Free PMC article
Show details
Emerg Med J
. 2021 Jun;38(6):439-445.
doi: 10.1136/emermed-2020-209992. Epub 2021 Apr 13.

Authors

Ryan Taylor Sless [1], Nathaniel Edward Hayward [2], Paul MacDaragh Ryan [2], Conor Deasy [3], Kantikiran Dasari [3]

Affiliations

- [1] School of Medicine, University College Cork, Cork City, Cork, Ireland ryan.sless@mail.utoronto.ca.
- [2] School of Medicine, University College Cork, Cork City, Cork, Ireland.
- [3] Emergency Medicine, Cork University Hospital, Cork City, Cork, Ireland.

- PMID: **33849939**
- PMCID: PMC8050875
- DOI: 10.1136/emermed-2020-209992

Abstract

Background: COVID-19 has resulted in the death of over 1 million people to date. Following government-implemented regulations, there has been concern over the apparent decline in emergency department (ED) attendances and the resultant health legacy. Therefore, we aimed to characterise the attendances to an Irish tertiary hospital ED following the implementation of these regulations during the COVID-19 pandemic.

Methods: This retrospective observational study investigated all attendances to the Cork University Hospital ED from 15 February to 11 April in 2020 and 2017-2019. Attendances were stratified into four periods: Before COVID (BC) (15 February to 5 March), After COVID (AC) (6 March to 12 March), Educational Closure (EC) (13 March to 27 March) and Stay Home (SH) (28 March to 11 April), as per government regulations. Triage presentations of abdominal pain, shortness of breath, chest pain, headache and trauma were examined. Data were analysed by independent t-tests and χ^2 analysis.

Results: There were 8261 attendances to the ED in the 2020 time period compared with a mean of 10 389 attendances during the corresponding periods in 2017-2019. There was a significant decrease in daily attendances in 2020 compared with 2017-2019 in the AC (142 vs 188, p=0.02), EC (122 vs 184, p<0.001) and SH (121 vs 181, p<0.001) periods, including significant decreases in abdominal pain (AC: 9 vs 22, EC: 10 vs 19, SH: 11 vs 18, p<0.001), chest pain (AC: 9 vs 15, EC: 8 vs 15, SH: 9 vs 15, p<0.01), headache (AC: 5 vs 11, EC: 4 vs 9, SH: 4 vs 9, p<0.01) and trauma (AC: 3 vs 5, EC: 2 vs 6, SH: 3 vs 5, p<0.01).

Conclusion: Our findings suggest that the combination of government-imposed restrictions and perceived risk of attending an ED during a pandemic may contribute to reduced attendances. Public confidence in EDs is necessary to reduce collateral damage caused by failure to seek medical attention during a pandemic; adequate infrastructure to allow social distancing and isolation capacity in EDs is a necessity.

Keywords: acute medicine-other; emergency departments; epidemiology; infectious diseases; viral.

© Author(s) (or their employer(s)) 2021. No commercial re-use. See rights and permissions. Published by BMJ.

Conflict of interest statement

Competing interests: None declared.

- 19 references
- 4 figures

Supplementary info

Publication types, MeSH terms Expand

Publication types

- Observational Study

MeSH terms

- Adult
- COVID-19 / epidemiology*
- COVID-19 / prevention & control
- COVID-19 / therapy
- Communicable Disease Control* / methods
- Emergency Service, Hospital / statistics & numerical data*
- Female
- Government Regulation
- Humans
- Ireland / epidemiology
- Male

- Patient Acceptance of Health Care / statistics & numerical data*
- Retrospective Studies

Full text links

BMJ Full Text HighWire Free PMC article

Proceed to details

Cite

Share

☐ 969

Observational Study

Eur J Clin Invest

. 2021 Jan;51(1):e13404.

doi: 10.1111/eci.13404. Epub 2020 Sep 25.

Prognostic implications of neutrophil-lymphocyte ratio in COVID-19

Sara Jimeno [1] [2] [3], Paula S Ventura [3] [4], Jose M Castellano [5], Salvador I García-Adasme [3] [6], Mario Miranda [3] [7], Paula Touza [1] [3], Isabel Lllana [1] [3], Alejandro López-Escobar [1] [2] [3]

Affiliations Expand

Affiliations

- [1] Pediatrics Department, Hospital Universitario HM Puerta del Sur, Móstoles, Madrid, Spain.
- [2] Facultad de Medicina, Universidad CEU San Pablo, Madrid, Spain.
- [3] Fundación de Investigación HM Hospitales, Madrid, Spain.
- [4] Pediatrics Department, Hospital Universitario HM Nens, Barcelona, Spain.
- [5] Cardiology Department, Facultad de Medicina, Centro Nacional de Investigaciones Cardiovasculares, Hospital Universitario HM Montepríncipe, Grupo HM Hospitales, Fundación de Investigación HM Hospitales, Universidad CEU San Pablo, Instituto de Salud Carlos III, Madrid, Spain.
- [6] Escuela de Enfermería, Universidad CEU San Pablo, Madrid, Spain.
- [7] Internal Medicine Department, Hospital Universitario HM Puerta del Sur, Móstoles, Madrid, Spain.

- PMID: **32918295**
- DOI: 10.1111/eci.13404

Observational Study

Prognostic implications of neutrophil-lymphocyte ratio in COVID-19

Sara Jimeno et al. Eur J Clin Invest. 2021 Jan.
Show details
Eur J Clin Invest
. 2021 Jan;51(1):e13404.
doi: 10.1111/eci.13404. Epub 2020 Sep 25.

Authors

Sara Jimeno [1 2 3], Paula S Ventura [3 4], Jose M Castellano [5], Salvador I García-Adasme [3 6], Mario Miranda [3 7], Paula Touza [1 3], Isabel Lllana [1 3], Alejandro López-Escobar [1 2 3]

Affiliations

- [1] Pediatrics Department, Hospital Universitario HM Puerta del Sur, Móstoles, Madrid, Spain.
- [2] Facultad de Medicina, Universidad CEU San Pablo, Madrid, Spain.
- [3] Fundación de Investigación HM Hospitales, Madrid, Spain.
- [4] Pediatrics Department, Hospital Universitario HM Nens, Barcelona, Spain.
- [5] Cardiology Department, Facultad de Medicina, Centro Nacional de Investigaciones Cardiovasculares, Hospital Universitario HM Montepríncipe, Grupo HM Hospitales, Fundación de Investigación HM Hospitales, Universidad CEU San Pablo, Instituto de Salud Carlos III, Madrid, Spain.
- [6] Escuela de Enfermería, Universidad CEU San Pablo, Madrid, Spain.
- [7] Internal Medicine Department, Hospital Universitario HM Puerta del Sur, Móstoles, Madrid, Spain.

- PMID: **32918295**
- DOI: 10.1111/eci.13404

Abstract

Background: The clinical presentation of COVID-19 ranges from a mild, self-limiting disease, to multiple organ failure and death. Most severe COVID-19 cases present low lymphocytes counts and high leukocytes counts, and accumulated evidence suggests that in a subgroup of patients presenting severe COVID-19, there may be a hyperinflammatory response driving a severe hypercytokinaemia which may be, at least in part, signalling the presence of an underlying endothelial dysfunction. In this context, available data suggest a prognostic role of neutrophil-lymphocyte ratio (NLR) in various inflammatory diseases and oncological processes. Following this rationale, we hypothesized that NLR, as a marker of endothelial dysfunction, may be useful in identifying patients with a poor prognosis in hospitalized COVID-19 cases.

Design: A retrospective observational study performed at Hospital Universitario HM Puerta del Sur, Madrid, Spain, which included 119 patients with COVID-19 from 1 March to 31 March 2020. Patients were categorized according to WHO R&D Expert Group.

Results: Forty-five (12.1%) patients experienced severe acute respiratory failure requiring respiratory support. Forty-seven (12.6%) patients died. Those with worse outcomes were older (P = .002) and presented significantly higher NLR at admission (P = .001), greater increase in Peak NLR (P < .001) and higher increasing speed of NLR (P = .003) compared with follow-up patients. In a multivariable logistic regression, age, cardiovascular disease and C-reactive protein at admission and Peak NLR were significantly associated with death.

Conclusions: NLR is an easily measurable, available, cost-effective and reliable parameter, which continuous monitoring could be useful for the diagnosis and treatment of COVID-19.

Keywords: COVID-19; Neutrophil-lymphocyte ratio; endothelial dysfunction; hyperinflammatory response.

© 2020 Stichting European Society for Clinical Investigation Journal Foundation. Published by John Wiley & Sons Ltd.

- 35 references

Supplementary info

Publication types, MeSH terms, Substances Expand

Publication types

- Observational Study

MeSH terms

- Age Factors
- Aged
- Aged, 80 and over
- C-Reactive Protein / immunology
- COVID-19 / blood*
- COVID-19 / immunology
- COVID-19 / mortality
- Cardiovascular Diseases / epidemiology
- Comorbidity
- Diabetes Mellitus / epidemiology
- Endothelium, Vascular / physiopathology
- Female
- Hospital Mortality*
- Humans
- Hypertension / epidemiology
- L-Lactate Dehydrogenase / blood
- Leukocyte Count
- Leukocytosis / blood*

- Leukocytosis / immunology
- Logistic Models
- Lymphocyte Count
- Lymphocytes*
- Lymphopenia / blood*
- Lymphopenia / immunology
- Male
- Middle Aged
- Multivariate Analysis
- Neutrophils*
- Prognosis
- Pulmonary Disease, Chronic Obstructive / epidemiology
- Retrospective Studies
- SARS-CoV-2
- Severity of Illness Index
- Spain / epidemiology

Substances

- C-Reactive Protein
- L-Lactate Dehydrogenase

Full text links

WILEY Full Text Article Wiley

Proceed to details

Cite

Share

☐ 970

Observational Study

Rev Bras Ter Intensiva

. Jan-Mar 2021;33(1):68-74.

doi: 10.5935/0103-507X.20210006.

Changes in the management and clinical outcomes of critically ill patients without COVID-19 during the pandemic

[Article in Portuguese, English]

Iván Alfredo Huespe [1] [2], Agustina Marco [1], Eduardo Prado [1], Indalecio Carboni Bisso [1], Pablo Coria [1], Nicolas Gemelli [1], Eduardo San Román [1], Marcos José Las Heras [1]

Affiliations Expand

Affiliations

- [1] Unidade de Terapia Intensiva, Hospital Italiano de Buenos Aires - Buenos Aires, Argentina.
- [2] Instituto de Medicina Translacional e Engenharia Biomédica, Hospital Italiano de Buenos Aires, Instituto Universitario Hospital Italiano, Consejo Nacional de Investigaciones Científicas y Técnicas - Buenos Aires, Argentina.

- PMID: **33886854**
- PMCID: PMC8075343
- DOI: 10.5935/0103-507X.20210006

Free PMC article
Observational Study

Changes in the management and clinical outcomes of critically ill patients without COVID-19 during the pandemic

[Article in Portuguese, English]
Iván Alfredo Huespe et al. Rev Bras Ter Intensiva. Jan-Mar 2021.
Free PMC article
Show details

Rev Bras Ter Intensiva
. Jan-Mar 2021;33(1):68-74.
doi: 10.5935/0103-507X.20210006.

Authors

Iván Alfredo Huespe [1, 2], Agustina Marco [1], Eduardo Prado [1], Indalecio Carboni Bisso [1], Pablo Coria [1], Nicolas Gemelli [1], Eduardo San Román [1], Marcos José Las Heras [1]

Affiliations

- [1] Unidade de Terapia Intensiva, Hospital Italiano de Buenos Aires - Buenos Aires, Argentina.
- [2] Instituto de Medicina Translacional e Engenharia Biomédica, Hospital Italiano de Buenos Aires, Instituto Universitario Hospital Italiano, Consejo Nacional de Investigaciones Científicas y Técnicas - Buenos Aires, Argentina.

- PMID: **33886854**
- PMCID: PMC8075343
- DOI: 10.5935/0103-507X.20210006

Abstract

in English, Portuguese, Portuguese

Objective: To analyze whether changes in medical care due to the application of COVID-19 protocols affected clinical outcomes in patients without COVID-19 during the pandemic.

Methods: This was a retrospective, observational cohort study carried out in a thirty-eight-bed surgical and medical intensive care unit of a high complexity private hospital. Patients with respiratory failure admitted to the intensive care unit during March and April 2020 and the same months in 2019 were selected. We compared interventions and outcomes of patients without COVID-19 during the pandemic with patients admitted in 2019. The main variables analyzed were intensive care unit respiratory management, number of chest tomography scans and bronchoalveolar lavages, intensive care unit complications, and status at hospital discharge.

Results: In 2020, a significant reduction in the use of a high-flow nasal cannula was observed: 14 (42%) in 2019 compared to 1 (3%) in 2020. Additionally, in 2020, a significant increase was observed in the number of patients under mechanical ventilation admitted to the intensive care unit from the emergency department, 23 (69%) compared to 11 (31%) in 2019. Nevertheless, the number of patients with mechanical ventilation after 5 days of admission was similar in both years: 24 (69%) in 2019 and 26 (79%) in 2020.

Conclusion: Intensive care unit protocols based on international recommendations for the COVID-19 pandemic have produced a change in non-COVID-19 patient management. We observed a reduction in the use of a high-flow nasal cannula and an increased number of tracheal intubations in the emergency department. However, no changes in the percentage of intubated patients in the intensive care unit, the number of mechanical ventilation days or the length of stay in intensive care unit.

Objetivo: Analisar se as modificações na atenção médica em razão da aplicação dos protocolos para COVID-19 afetaram os desfechos clínicos de pacientes sem a doença durante a pandemia.

Métodos: Este foi um estudo observacional de coorte retrospectiva conduzido em uma unidade de terapia intensiva clínica e cirúrgica com 38 leitos, localizada em hospital privado de alta complexidade na cidade de Buenos Aires, Argentina, e envolveu os pacientes com insuficiência respiratória admitidos à unidade de terapia intensiva no período compreendido entre março e abril de 2020 em comparação com o mesmo período no ano de 2019. Compararam-se as intervenções e os desfechos dos pacientes sem COVID-19 tratados durante a pandemia em 2020 e os pacientes admitidos em 2019. As principais variáveis avaliadas foram os cuidados respiratórios na unidade de terapia intensiva, o número de exames de tomografia computadorizada do tórax e lavados broncoalveolares, complicações na unidade de terapia intensiva e condições quando da alta hospitalar.

Resultados: Observou-se, em 2020, uma redução significante do uso de cânula nasal de alto fluxo: 14 (42%), em 2019, em comparação com 1 (3%), em 2020. Além disso, em 2020, observou-se aumento significante no número de pacientes sob ventilação mecânica admitidos à unidade de terapia intensiva a partir do pronto-socorro, de 23 (69%) em comparação com 11 (31%) em 2019. Contudo, o número de pacientes com ventilação mecânica 5 dias após a admissão foi semelhante em ambos os anos: 24 (69%), em 2019, e 26 (79%) em 2020.

Conclusão: Os protocolos para unidades de terapia intensiva com base em recomendações internacionais para a pandemia de COVID-19 modificaram o manejo de pacientes sem COVID-19. Observamos redução do uso da cânula nasal de alto fluxo e aumento no número de intubações traqueais no pronto-socorro. Entretanto, não se identificaram alterações na percentagem de

pacientes intubados na unidade de terapia intensiva, número de dias sob ventilação mecânica ou número de dias na unidade de terapia intensiva.

Objetivo: Analisar se as modificações na atenção médica em razão da aplicação dos protocolos para COVID-19 afetaram os desfechos clínicos de pacientes sem a doença durante a pandemia.

Métodos: Este foi um estudo observacional de coorte retrospectiva conduzido em uma unidade de terapia intensiva clínica e cirúrgica com 38 leitos, localizada em hospital privado de alta complexidade na cidade de Buenos Aires, Argentina, e envolveu os pacientes com insuficiência respiratória admitidos à unidade de terapia intensiva no período compreendido entre março e abril de 2020 em comparação com o mesmo período no ano de 2019. Compararam-se as intervenções e os desfechos dos pacientes sem COVID-19 tratados durante a pandemia em 2020 e os pacientes admitidos em 2019. As principais variáveis avaliadas foram os cuidados respiratórios na unidade de terapia intensiva, o número de exames de tomografia computadorizada do tórax e lavados broncoalveolares, complicações na unidade de terapia intensiva e condições quando da alta hospitalar.

Resultados: Observou-se, em 2020, uma redução significante do uso de cânula nasal de alto fluxo: 14 (42%), em 2019, em comparação com 1 (3%), em 2020. Além disso, em 2020, observou-se aumento significante no número de pacientes sob ventilação mecânica admitidos à unidade de terapia intensiva a partir do pronto-socorro, de 23 (69%) em comparação com 11 (31%) em 2019. Contudo, o número de pacientes com ventilação mecânica 5 dias após a admissão foi semelhante em ambos os anos: 24 (69%), em 2019, e 26 (79%) em 2020.

Conclusão: Os protocolos para unidades de terapia intensiva com base em recomendações internacionais para a pandemia de COVID-19 modificaram o manejo de pacientes sem COVID-19. Observamos redução do uso da cânula nasal de alto fluxo e aumento no número de intubações traqueais no pronto-socorro. Entretanto, não se identificaram alterações na percentagem de pacientes intubados na unidade de terapia intensiva, número de dias sob ventilação mecânica ou número de dias na unidade de terapia intensiva.

Conflict of interest statement

Conflicts of interest: None.

- 15 references
- 1 figure

Supplementary info

Publication types, MeSH terms Expand

Publication types

- Observational Study

MeSH terms

- Aged
- Argentina / epidemiology

- Bronchoalveolar Lavage / statistics & numerical data
- COVID-19 / epidemiology*
- Critical Illness / therapy*
- Disease Management*
- Female
- Health Care Surveys
- Humans
- Intensive Care Units
- Intubation, Intratracheal / statistics & numerical data
- Male
- Middle Aged
- Pandemics*
- Respiration, Artificial / statistics & numerical data
- Retrospective Studies
- Treatment Outcome

Full text links

free full text available at SciELO.org Scientific Electronic Library Online Free PMC article

Proceed to details

Cite

Share

971

Observational Study

J Intern Med

. 2021 Aug;290(2):470-472.

doi: 10.1111/joim.13292. Epub 2021 Apr 23.

Cholestatic liver injury in COVID-19 is a rare and distinct entity and is associated with increased mortality

B L Da [1], K Suchman [2], N Roth [1], A Rizvi [3], M Vincent [4], A J Trindade [2], D Bernstein [1], S K Satapathy [1], Northwell COVID-19 Research Consortium

Affiliations Expand

Affiliations

- [1] From the, Division of Hepatology, North Shore University Hospital, Donald and Barbara Zucker School of Medicine at Hofstra/Northwell, Northwell Health System, Manhasset, NY, USA.

- [2] Department of Internal Medicine, Donald and Barbara Zucker School of Medicine at Hofstra/Northwell, Northwell Health System, Manhasset, NY, USA.
- [3] Division of Gastroenterology, Long Island Jewish Medical Center, Donald and Barbara Zucker School of Medicine at Hofstra/Northwell, Northwell Health System, New Hyde Park, NY, USA.
- [4] Donald and Barbara Zucker School of Medicine at Hofstra/Northwell, Hempstead, NY, USA.

- PMID: **33786906**
- PMCID: PMC8250628
- DOI: 10.1111/joim.13292

Free PMC article
Observational Study

Cholestatic liver injury in COVID-19 is a rare and distinct entity and is associated with increased mortality

B L Da et al. J Intern Med. 2021 Aug.
Free PMC article
Show details
J Intern Med
. 2021 Aug;290(2):470-472.
doi: 10.1111/joim.13292. Epub 2021 Apr 23.

Authors

B L Da [1], K Suchman [2], N Roth [1], A Rizvi [3], M Vincent [4], A J Trindade [2], D Bernstein [1], S K Satapathy [1], Northwell COVID-19 Research Consortium

Affiliations

- [1] From the, Division of Hepatology, North Shore University Hospital, Donald and Barbara Zucker School of Medicine at Hofstra/Northwell, Northwell Health System, Manhasset, NY, USA.
- [2] Department of Internal Medicine, Donald and Barbara Zucker School of Medicine at Hofstra/Northwell, Northwell Health System, Manhasset, NY, USA.
- [3] Division of Gastroenterology, Long Island Jewish Medical Center, Donald and Barbara Zucker School of Medicine at Hofstra/Northwell, Northwell Health System, New Hyde Park, NY, USA.
- [4] Donald and Barbara Zucker School of Medicine at Hofstra/Northwell, Hempstead, NY, USA.

- PMID: **33786906**
- PMCID: PMC8250628
- DOI: 10.1111/joim.13292

No abstract available

Keywords: COVID-19; SARS-CoV2; alkaline phosphatase; cholestasis; mortality.

Conflict of interest statement

The authors have no relevant conflicts of interest.

- 9 references
- 1 figure

Supplementary info

Publication types, MeSH terms, Substances Expand

Publication types

- Letter
- Multicenter Study
- Observational Study

MeSH terms

- Alkaline Phosphatase / blood
- COVID-19 / complications*
- COVID-19 / mortality
- Case-Control Studies
- Cholestasis / virology*
- Hospital Mortality
- Humans
- Liver Diseases / etiology*
- Liver Function Tests
- New York
- Respiration, Artificial
- Retrospective Studies

Substances

- Alkaline Phosphatase

Full text links

WILEY Full Text Article Wiley Free PMC article

Proceed to details

Cite

Share

972

Observational Study

J Nephrol

. 2022 Jan;35(1):99-111.
doi: 10.1007/s40620-021-01100-3. Epub 2021 Jun 25.

Acute kidney injury (AKI) in patients with Covid-19 infection is associated with ventilatory management with elevated positive end-expiratory pressure (PEEP)

Davide Ottolina [#] [1], Luca Zazzeron [#] [2], Letizia Trevisi [3], Andrea Agarossi [4], Riccardo Colombo [4], Tommaso Fossali [4], Mattia Passeri [4], Beatrice Borghi [4], Elisabetta Ballone [4], Roberto Rech [4], Antonio Castelli [4], Emanuele Catena [4], Manuela Nebuloni [5] [6], Maurizio Gallieni [5] [7]
Affiliations Expand

Affiliations

- [1] Department of Anesthesia and Critical Care, "Luigi Sacco" Hospital, ASST Fatebenefratelli-Sacco, Via G.B. Grassi, 74, 20157, Milan, Italy. ottolina.davide@asst-fbf-sacco.it.
- [2] Department of Anesthesia, Critical Care, and Pain Medicine, Massachusetts General Hospital, Boston, MA, USA.
- [3] Department of Global Health and Social Medicine, Harvard Medical School, Boston, MA, USA.
- [4] Department of Anesthesia and Critical Care, "Luigi Sacco" Hospital, ASST Fatebenefratelli-Sacco, Via G.B. Grassi, 74, 20157, Milan, Italy.
- [5] 'L. Sacco' Department of Biomedical and Clinical Sciences, Università degli Studi di Milano, Milan, Italy.
- [6] Pathology Unit, "L. Sacco" Hospital, ASST Fatebenefratelli-Sacco, Milan, Italy.
- [7] Nephrology and Dialysis Unit, "L. Sacco" Hospital, ASST Fatebenefratelli-Sacco, Milan, Italy.

[#] Contributed equally.

- PMID: **34170508**
- PMCID: PMC8226340
- DOI: 10.1007/s40620-021-01100-3

Free PMC article
Observational Study

Acute kidney injury (AKI) in patients with Covid-19 infection is associated with ventilatory management with elevated positive end-expiratory pressure (PEEP)

Davide Ottolina et al. J Nephrol. 2022 Jan.
Free PMC article
Show details
J Nephrol
. 2022 Jan;35(1):99-111.
doi: 10.1007/s40620-021-01100-3. Epub 2021 Jun 25.

Authors

Davide Ottolina [#] [1], Luca Zazzeron [#] [2], Letizia Trevisi [3], Andrea Agarossi [4], Riccardo Colombo [4], Tommaso Fossali [4], Mattia Passeri [4], Beatrice Borghi [4], Elisabetta Ballone [4], Roberto Rech [4], Antonio Castelli [4], Emanuele Catena [4], Manuela Nebuloni [5] [6], Maurizio Gallieni [5] [7]

Affiliations

- [1] Department of Anesthesia and Critical Care, "Luigi Sacco" Hospital, ASST Fatebenefratelli-Sacco, Via G.B. Grassi, 74, 20157, Milan, Italy. ottolina.davide@asst-fbf-sacco.it.
- [2] Department of Anesthesia, Critical Care, and Pain Medicine, Massachusetts General Hospital, Boston, MA, USA.
- [3] Department of Global Health and Social Medicine, Harvard Medical School, Boston, MA, USA.
- [4] Department of Anesthesia and Critical Care, "Luigi Sacco" Hospital, ASST Fatebenefratelli-Sacco, Via G.B. Grassi, 74, 20157, Milan, Italy.
- [5] 'L. Sacco' Department of Biomedical and Clinical Sciences, Università degli Studi di Milano, Milan, Italy.
- [6] Pathology Unit, "L. Sacco" Hospital, ASST Fatebenefratelli-Sacco, Milan, Italy.
- [7] Nephrology and Dialysis Unit, "L. Sacco" Hospital, ASST Fatebenefratelli-Sacco, Milan, Italy.

[#] Contributed equally.

- PMID: **34170508**
- PMCID: PMC8226340
- DOI: 10.1007/s40620-021-01100-3

Abstract

Background: Acute kidney injury (AKI) in Covid-19 patients admitted to the intensive care unit (ICU) is common, and its severity may be associated with unfavorable outcomes. Severe Covid-19 fulfills the diagnostic criteria for acute respiratory distress syndrome (ARDS); however, it is

unclear whether there is any relationship between ventilatory management and AKI development in Covid-19 ICU patients.

Purpose: To describe the clinical course and outcomes of Covid-19 ICU patients, focusing on ventilatory management and factors associated with AKI development.

Methods: Single-center, retrospective observational study, which assessed AKI incidence in Covid-19 ICU patients divided by positive end expiratory pressure (PEEP) tertiles, with median levels of 9.6 (low), 12.0 (medium), and 14.7 cmH_2O (high-PEEP).

Results: Overall mortality was 51.5%. AKI (KDIGO stage 2 or 3) occurred in 38% of 101 patients. Among the AKI patients, 19 (53%) required continuous renal replacement therapy (CRRT). In AKI patients, mortality was significantly higher versus non-AKI (81% vs. 33%, $p < 0.0001$). The incidence of AKI in low-, medium-, or high-PEEP patients were 16%, 38%, and 59%, respectively ($p = 0.002$). In a multivariate analysis, high-PEEP patients showed a higher risk of developing AKI than low-PEEP patients (OR = 4.96 [1.1-21.9] 95% CI $p < 0.05$). ICU mortality rate was higher in high-PEEP patients, compared to medium-PEEP or low-PEEP patients (69% vs. 44% and 42%, respectively; $p = 0.057$).

Conclusion: The use of high PEEP in Covid-19 ICU patients is associated with a fivefold higher risk of AKI, leading to higher mortality. The cause and effect relationship needs further analysis.

Keywords: AKI; ARDS; Covid-19; Intensive care; PEEP.

© 2021. The Author(s).

Conflict of interest statement

All authors have nothing to disclose.

- 29 references
- 2 figures

Supplementary info

Publication types, MeSH terms Expand

Publication types

- Observational Study

MeSH terms

- Acute Kidney Injury* / diagnosis
- Acute Kidney Injury* / epidemiology
- Acute Kidney Injury* / therapy
- COVID-19*
- Humans
- Intensive Care Units

- Positive-Pressure Respiration / adverse effects
- SARS-CoV-2

Full text links

SpringerLink FULL-TEXT ARTICLE Springer Free PMC article

Proceed to details

Cite

Share

973

Endocr Res

. Feb-May 2021;46(2):45-50.

doi: 10.1080/07435800.2020.1856865. Epub 2020 Dec 4.

Insulin Use, Diabetes Control, and Outcomes in Patients with COVID-19

Shayan Riahi [1], Lorenna Rodrigues Silva Sombra [1], Kevin Bryan Lo [1], Shireen R Chacko [1], Alvaro Goncalves Mendes Neto [1], Zurab Azmaiparashvili [1], Gabriel Patarroyo-Aponte [1] [2] [3], Janani Rangaswami [1] [2], Catherine Anastasopoulou [1] [4]

Affiliations Expand

Affiliations

- [1] Department of Medicine, Einstein Medical Center Philadelphia, PA, Philadelphia, USA.
- [2] Department of Medicine, Sidney Kimmel College of Thomas Jefferson University, Philadelphia, PA, USA.
- [3] Pulmonary, Critical Care and Sleep Medicine, Einstein Medical Center Philadelphia.
- [4] Department of Endocrinology, Einstein Medical Center Philadelphia.

- PMID: **33275067**
- DOI: 10.1080/07435800.2020.1856865

Insulin Use, Diabetes Control, and Outcomes in Patients with COVID-19

Shayan Riahi et al. Endocr Res. Feb-May 2021.

Show details

Endocr Res

. Feb-May 2021;46(2):45-50.

doi: 10.1080/07435800.2020.1856865. Epub 2020 Dec 4.

Authors

Shayan Riahi [1], Lorenna Rodrigues Silva Sombra [1], Kevin Bryan Lo [1], Shireen R Chacko [1], Alvaro Goncalves Mendes Neto [1], Zurab Azmaiparashvili [1], Gabriel Patarroyo-Aponte [1] [2] [3], Janani Rangaswami [1] [2], Catherine Anastasopoulou [1] [4]

Affiliations

- [1] Department of Medicine, Einstein Medical Center Philadelphia, PA, Philadelphia, USA.
- [2] Department of Medicine, Sidney Kimmel College of Thomas Jefferson University, Philadelphia, PA, USA.
- [3] Pulmonary, Critical Care and Sleep Medicine, Einstein Medical Center Philadelphia.
- [4] Department of Endocrinology, Einstein Medical Center Philadelphia.

- PMID: **33275067**
- DOI: 10.1080/07435800.2020.1856865

Abstract

Background: The novel coronavirus (SARS CoV-2) has caused significant morbidity and mortality in patients with diabetes. However, the effects of diabetes control including insulin use remain uncertain in terms of clinical outcomes of patients with COVID-19.**Methods**: In this single-center, retrospective observational study, all adult patients admitted to Einstein Medical Center, Philadelphia, from March 1 through April 24, 2020 with a diagnosis of COVID-19 and diabetes were included. Demographic, clinical and laboratory data, insulin dose at home and at the hospital, other anti-hyperglycemic agents use, and outcomes were obtained. Multivariate logistic regression was used to evaluate the factors associated with diabetes control and mortality.**Results**: Patients who used insulin at home had higher mortality compared to those who did not (35% vs 18% $p = .015$), this was true even after adjustment for demographics, comorbidities and a1c OR 2.65 95% CI (1.23-5.71) $p = .013$. However, the mean a1c and the median home requirements of insulin did not significantly differ among patients who died compared to the ones that survived. Patients who died had significantly higher inpatient insulin requirements (highest day insulin requirement recorded in units during hospitalization) 36 (11-86) vs 21 (8-52) $p = .043$ despite similar baseline a1c and steroid doses received. After adjusting for demographics, comorbidities and a1c, peak insulin requirements remained significantly associated with inpatient mortality OR 1.022 95% CI (1.00-1.04) $p = .044$.**Conclusion**: Among diabetic patients infected with COVID-19, insulin therapy at home was significantly independently associated with increased mortality. Peak daily inpatient insulin requirements was also independently associated with increased inpatient mortality.

Keywords: COVID-19; diabetes; mortality; novel coronavirus; outcomes.

Comment in

- Insulin Treatment May Not Be Associated with Increased Mortality in Patients with COVID-19 and Concurrent Diabetes.
  Kow CS, Hasan SS. Kow CS, et al. Endocr Res. 2021 Feb-May;46(2):51-52. doi: 10.1080/07435800.2021.1892748. Epub 2021 Feb 26. Endocr Res. 2021. PMID: 33635726
- Insulin Use and Poor COVID-19 Outcomes among Diabetes Patients: Association Not Necessarily Causation.
  Riahi S, Lo KB, Anastasopoulou C, Rangaswami J. Riahi S, et al. Endocr Res. 2021 Feb-May;46(2):53-54. doi: 10.1080/07435800.2021.1894821. Epub 2021 Mar 8. Endocr Res. 2021. PMID: 33684317

Supplementary info

MeSH terms, Substances Expand

MeSH terms

- Aged
- Blood Glucose / analysis
- COVID-19 / blood
- COVID-19 / complications*
- COVID-19 / mortality
- Diabetes Mellitus, Type 1 / blood
- Diabetes Mellitus, Type 1 / complications*
- Diabetes Mellitus, Type 1 / drug therapy
- Diabetes Mellitus, Type 1 / mortality
- Diabetes Mellitus, Type 2 / blood
- Diabetes Mellitus, Type 2 / complications*
- Diabetes Mellitus, Type 2 / drug therapy
- Diabetes Mellitus, Type 2 / mortality
- Female
- Glycated Hemoglobin A / analysis
- Glycemic Control*
- Hospital Mortality
- Hospitalization
- Humans
- Hypoglycemic Agents / administration & dosage
- Hypoglycemic Agents / therapeutic use*
- Insulin / administration & dosage
- Insulin / therapeutic use*
- Male
- Middle Aged
- Retrospective Studies
- Risk Factors
- Survival Rate

Substances

- Blood Glucose
- Glycated Hemoglobin A
- Hypoglycemic Agents
- Insulin
- hemoglobin A1c protein, human

Full text links

View full text Taylor & Francis
Proceed to details
Cite
Share
☐ 974
Observational Study
Res Social Adm Pharm
. 2021 Jan;17(1):1853-1858.
doi: 10.1016/j.sapharm.2020.06.005. Epub 2020 Jun 6.

On-ward participation of clinical pharmacists in a Chinese intensive care unit for patients with COVID-19: A retrospective, observational study

Rongrong Wang [1], Limin Kong [1], Qiang Xu [1], Ping Yang [1], Xiaojuan Wang [1], Na Chen [1], Lu Li [1], Saiping Jiang [2], Xiaoyang Lu [3]
Affiliations Expand

Affiliations

- [1] Department of Pharmacy, The First Affiliated Hospital, College of Medicine, Zhejiang University, Hangzhou, China.
- [2] Department of Pharmacy, The First Affiliated Hospital, College of Medicine, Zhejiang University, Hangzhou, China. Electronic address: j5145@zju.edu.cn.
- [3] Department of Pharmacy, The First Affiliated Hospital, College of Medicine, Zhejiang University, Hangzhou, China. Electronic address: luxiaoyang@zju.edu.cn.

- PMID: **33317764**
- PMCID: PMC7832950
- DOI: 10.1016/j.sapharm.2020.06.005

Free PMC article
Observational Study

On-ward participation of clinical pharmacists in a Chinese intensive care unit for patients with COVID-19: A retrospective, observational study

Rongrong Wang et al. Res Social Adm Pharm. 2021 Jan.
Free PMC article
Show details
Res Social Adm Pharm
. 2021 Jan;17(1):1853-1858.
doi: 10.1016/j.sapharm.2020.06.005. Epub 2020 Jun 6.

Authors

Rongrong Wang [1], Limin Kong [1], Qiang Xu [1], Ping Yang [1], Xiaojuan Wang [1], Na Chen [1], Lu Li [1], Saiping Jiang [2], Xiaoyang Lu [3]

Affiliations

- [1] Department of Pharmacy, The First Affiliated Hospital, College of Medicine, Zhejiang University, Hangzhou, China.
- [2] Department of Pharmacy, The First Affiliated Hospital, College of Medicine, Zhejiang University, Hangzhou, China. Electronic address: j5145@zju.edu.cn.
- [3] Department of Pharmacy, The First Affiliated Hospital, College of Medicine, Zhejiang University, Hangzhou, China. Electronic address: luxiaoyang@zju.edu.cn.

- PMID: **33317764**
- PMCID: PMC7832950
- DOI: 10.1016/j.sapharm.2020.06.005

Abstract

Background: The practical experiences of active pharmacists involved in managing critically ill patients with coronavirus disease 2019 (COVID-19) have been rarely reported.

Objective: This work aimed to share professional experiences on medication optimization and provide a feasible reference for the pharmaceutical care of critically ill patients with COVID-19.

Methods: This study was conducted in a COVID-19-designated hospital in China. A group of dedicated clinical pharmacists participated in multidisciplinary rounds to optimize the treatments for critically ill patients with COVID-19. Consensus on medication recommendations was reached by a multidisciplinary team through bi-daily discussion. Related drug, classification, cause, and adjustment content for recommendations were recorded and reviewed.

Results: A total of 111 medication recommendations were supplied for 22 out of 33 (56.7%) critically ill patients from 1 February 2020 to 18 March 2020, and 106 (95.5%) of these were accepted. Among these recommendations, 64 (67.7%), 32 (28.8%), and 15 (13.5%) were related to

antibiotics and antifungals, antiviral agents, and other drugs, respectively. Recommendation types significantly differed for different anti-infectives ($p < 0.05$). For antibiotics and antifungals, treatment effectiveness accounted for 60.9% of recommendation types, with 15 (38.5%) cases related to untreated infections. For antiviral agents, adverse drug events were the most common recommendation types (84.4%), with 20 (74.1%) cases related to liver function dysfunction. Discontinuation of suspected antiviral agents (66.7%) was usually recommended after the occurrence of adverse events that may progress and bring poor outcomes.

Conclusion: Forceful and extensive on-ward participation is recommended for clinical pharmacists in managing critically ill patients. Our experiences highlight the need for special attention toward untreated infections and adverse events related to antiviral agents.

Keywords: Coronavirus disease 2019; Intensive care unit; Medication recommendation; Pharmacists.

Copyright © 2020 Elsevier Inc. All rights reserved.

Conflict of interest statement

All authors declared that they have no financial relationships with any organizations or people that might influence or bias the content of the paper within 3 years of beginning of the work; no other relationships or activities that could appear to have influenced the submitted work.

- 30 references
- 2 figures

Supplementary info

Publication types, MeSH terms, Substances, Supplementary concepts Expand

Publication types

- Observational Study

MeSH terms

- Adult
- Aged
- Aged, 80 and over
- Antiviral Agents / administration & dosage
- Antiviral Agents / adverse effects
- COVID-19 / drug therapy
- COVID-19 / therapy*
- China
- Critical Illness
- Female
- Humans
- Intensive Care Units*

- Male
- Middle Aged
- Patient Care Team / organization & administration
- Pharmacists / organization & administration*
- Pharmacy Service, Hospital / organization & administration*
- Professional Role
- Retrospective Studies

Substances

- Antiviral Agents

Supplementary concepts

- COVID-19 drug treatment

Full text links

ELSEVIER FULL-TEXT ARTICLE Elsevier Science Free PMC article

Proceed to details

Cite

Share

975

Observational Study

Farm Hosp

. 2021 Aug 2;45(5):253-257.

Experience in the use of remdesivir in patients with SARS-CoV-2 pneumonia

Anna Murgadella-Sancho [1], Berta Gracia-García [2], José Loureiro-Amigo [3], Ana Coloma-Conde [4], Laura Losa-López [5], Ana Puebla-Villaescusa [6]

Affiliations Expand

Affiliations

- [1] Department of Pharmacy, Moisès Broggi Hospital (Consorci Sanitari Integral), Sant Joan Despí (Barcelona). Spain.. anna.murgadellasancho@sanitatintegral.org.
- [2] Department of Pharmacy, Moisès Broggi Hospital (Consorci Sanitari Integral), Sant Joan Despí (Barcelona). Spain.. berta.gracia@sanitatintegral.org.
- [3] Department of Internal Medicine, Hospital Moisès Broggi (Consorci Sanitari Integral), Sant Joan Despí (Barcelona). Spain.. jose.loureiro@sanitatintegral.org.
- [4] Department of Internal Medicine, Hospital Moisès Broggi (Consorci Sanitari Integral), Sant Joan Despí (Barcelona). Spain.. ana.coloma@sanitatintegral.org.

- [5] Department of Pharmacy, Moisès Broggi Hospital (Consorci Sanitari Integral), Sant Joan Despí (Barcelona). Spain.. laura.losa@sanitatintegral.org.
- [6] Department of Pharmacy, Moisès Broggi Hospital (Consorci Sanitari Integral), Sant Joan Despí (Barcelona). Spain.. ana.puebla@sanitatintegral.org.

- PMID: **34806585**

Free article
Observational Study

Experience in the use of remdesivir in patients with SARS-CoV-2 pneumonia

Anna Murgadella-Sancho et al. Farm Hosp. 2021.
Free article
Show details
Farm Hosp
. 2021 Aug 2;45(5):253-257.

Authors

Anna Murgadella-Sancho [1], Berta Gracia-García [2], José Loureiro-Amigo [3], Ana Coloma-Conde [4], Laura Losa-López [5], Ana Puebla-Villaescusa [6]

Affiliations

- [1] Department of Pharmacy, Moisès Broggi Hospital (Consorci Sanitari Integral), Sant Joan Despí (Barcelona). Spain.. anna.murgadellasancho@sanitatintegral.org.
- [2] Department of Pharmacy, Moisès Broggi Hospital (Consorci Sanitari Integral), Sant Joan Despí (Barcelona). Spain.. berta.gracia@sanitatintegral.org.
- [3] Department of Internal Medicine, Hospital Moisès Broggi (Consorci Sanitari Integral), Sant Joan Despí (Barcelona). Spain.. jose.loureiro@sanitatintegral.org.
- [4] Department of Internal Medicine, Hospital Moisès Broggi (Consorci Sanitari Integral), Sant Joan Despí (Barcelona). Spain.. ana.coloma@sanitatintegral.org.
- [5] Department of Pharmacy, Moisès Broggi Hospital (Consorci Sanitari Integral), Sant Joan Despí (Barcelona). Spain.. laura.losa@sanitatintegral.org.
- [6] Department of Pharmacy, Moisès Broggi Hospital (Consorci Sanitari Integral), Sant Joan Despí (Barcelona). Spain.. ana.puebla@sanitatintegral.org.

- PMID: **34806585**

Abstract
in English, Spanish

Objective: To describe the effectiveness and safety of remdesivir in patients with SARS-CoV-2 pneumonia in real-world clinical practice conditions.

Method: Retrospective observational study that included all adults with SARS-CoV-2 pneumonia admitted at the Moisès Broggi Hospital and treated with remdesivir between July 1st and November 7th, 2020. Efficacy outcomes were time to recovery, 28-day mortality, length of hospital stay, and the need of mechanical ventilation after treatment. The main safetyrelated endpoint was elevation of transaminases after treatment.

Results: A total of 111 patients were included of whom 97 (87.4%) were receiving low-flow oxygen therapy. Median time to recovery was 9 days [6-14]. Seven patients (6.3%) died at 28 days' follow-up. Median length of hospital stay was 12 days [9-22] and 15 patients (13.5%) needed mechanical ventilation after treatment with remdesivir. Severe hypertransaminasemia was observed in 4 patients (4%).

Conclusions: Clinical outcomes of patients with SARS-CoV-2 pneumonia on low-flow oxygen therapy treated with remdesivir were similar to those published in clinical trials, both in terms of time to recovery and 28-day mortality.

Objetivo: Describir la efectividad y seguridad de remdesivir en pacientes con neumonía por SARS-CoV-2 en condiciones de práctica clínica real.Método: Estudio observacional retrospectivo que incluyó a todos los pacientes tratados con remdesivir en el Hospital Moisès Broggi entre el 1 de julio y el 7 de noviembre de 2020. Como variables de efectividad se registraron el tiempo hasta la recuperación, la mortalidad a los 28 días, la estancia hospitalaria y la proporción de pacientes que requirió ventilación mecánica invasiva tras el tratamiento. Como variable de seguridad se registró la alteración en las transaminasas tras el tratamiento.Resultados: Se incluyeron 111 pacientes, 97 (87,4%) con oxigenoterapia de bajo flujo. El tiempo hasta la recuperación fue de 9 días [6-14] de mediana y 7 pacientes (6,3%) habían fallecido a los 28 días de seguimiento. La estancia hospitalaria fue de 12 días [9-22] de mediana. Un total de 15 pacientes (13,5%) requirió ventilación mecánica invasiva tras el tratamiento y 4 pacientes (4%) presentaron una alteración grave de las transaminasas.Conclusiones: El tratamiento con remdesivir en la práctica clínica habitual presenta resultados similares a los publicados en los ensayos clínicos en el subgrupo de pacientes con oxigenoterapia de bajo flujo, tanto en el tiempo hasta la recuperación como en la mortalidad a los 28 días.

Copyright AULA MEDICA EDICIONES 2014. Published by AULA MEDICA. All rights reserved.

Supplementary info

Publication types, MeSH terms, Substances, Supplementary concepts Expand

Publication types

- Observational Study

MeSH terms

- Adenosine Monophosphate / analogs & derivatives
- Adult
- Alanine / analogs & derivatives
- Antiviral Agents / therapeutic use
- COVID-19* / drug therapy

- Humans
- SARS-CoV-2*
- Treatment Outcome

Substances

- Antiviral Agents
- remdesivir
- Adenosine Monophosphate
- Alanine

Supplementary concepts

- COVID-19 drug treatment

Full text links

Aula Medica
Proceed to details
Cite
Share
976
Observational Study
Epidemiol Infect
. 2020 Aug 4;148:e168.
doi: 10.1017/S0950268820001727.

Development and validation of prognosis model of mortality risk in patients with COVID-19

Xuedi Ma [1], Michael Ng [2], Shuang Xu [3], Zhouming Xu [1] [2], Hui Qiu [4], Yuwei Liu [1], Jiayou Lyu [1], Jiwen You [1], Peng Zhao [1], Shihao Wang [1], Yunfei Tang [1], Hao Cui [5], Changxiao Yu [5], Feng Wang [6] [7] [8], Fei Shao [5] [9], Peng Sun [3], Ziren Tang [5] [9]
Affiliations Expand

Affiliations

- [1] AI Research Division, A.I. Phoenix Technology Co., Ltd, Hong Kong, China.
- [2] Research Division for Mathematical and Statistical Science, University of Hong Kong, Hong Kong, China.
- [3] Department of Emergency Medicine, Union Hospital, Tongji Medical College, Huazhong University of Science and Technology, Wuhan, China.

- [4] Department of Emergency Surgery, The west campus of Union Hospital, Tongji Medical College, Huazhong University of Science and Technology, Wuhan, China.
- [5] Department of Emergency Medicine, Beijing Chaoyang Hospital, Capital Medical University, Beijing, China.
- [6] Department of Respiratory and Critical Care Medicine, Beijing Chaoyang Hospital, Capital Medical University, Beijing, China.
- [7] Beijing Institute of Respiratory Medicine, Beijing Engineering Research Center for Diagnosis and Treatment of Respiratory and Critical Care Medicine, Beijing Chaoyang Hospital, Capital Medical University, Beijing, China.
- [8] Beijing Key Laboratory of Respiratory and Pulmonary Circulation Disorders, Beijing, China.
- [9] Beijing Key Laboratory of Cardiopulmonary Cerebral Resuscitation, Beijing, China.

- PMID: **32746957**
- PMCID: PMC7426607
- DOI: 10.1017/S0950268820001727

Free PMC article
Observational Study

Development and validation of prognosis model of mortality risk in patients with COVID-19

Xuedi Ma et al. Epidemiol Infect. 2020.
Free PMC article
Show details
Epidemiol Infect

. 2020 Aug 4;148:e168.
doi: 10.1017/S0950268820001727.

Authors

Xuedi Ma [1], Michael Ng [2], Shuang Xu [3], Zhouming Xu [1] [2], Hui Qiu [4], Yuwei Liu [1], Jiayou Lyu [1], Jiwen You [1], Peng Zhao [1], Shihao Wang [1], Yunfei Tang [1], Hao Cui [5], Changxiao Yu [5], Feng Wang [6] [7] [8], Fei Shao [5] [9], Peng Sun [3], Ziren Tang [5] [9]

Affiliations

- [1] AI Research Division, A.I. Phoenix Technology Co., Ltd, Hong Kong, China.
- [2] Research Division for Mathematical and Statistical Science, University of Hong Kong, Hong Kong, China.
- [3] Department of Emergency Medicine, Union Hospital, Tongji Medical College, Huazhong University of Science and Technology, Wuhan, China.
- [4] Department of Emergency Surgery, The west campus of Union Hospital, Tongji Medical College, Huazhong University of Science and Technology, Wuhan, China.

- [5] Department of Emergency Medicine, Beijing Chaoyang Hospital, Capital Medical University, Beijing, China.
- [6] Department of Respiratory and Critical Care Medicine, Beijing Chaoyang Hospital, Capital Medical University, Beijing, China.
- [7] Beijing Institute of Respiratory Medicine, Beijing Engineering Research Center for Diagnosis and Treatment of Respiratory and Critical Care Medicine, Beijing Chaoyang Hospital, Capital Medical University, Beijing, China.
- [8] Beijing Key Laboratory of Respiratory and Pulmonary Circulation Disorders, Beijing, China.
- [9] Beijing Key Laboratory of Cardiopulmonary Cerebral Resuscitation, Beijing, China.

- PMID: **32746957**
- PMCID: PMC7426607
- DOI: 10.1017/S0950268820001727

Abstract

This study aimed to identify clinical features for prognosing mortality risk using machine-learning methods in patients with coronavirus disease 2019 (COVID-19). A retrospective study of the inpatients with COVID-19 admitted from 15 January to 15 March 2020 in Wuhan is reported. The data of symptoms, comorbidity, demographic, vital sign, CT scans results and laboratory test results on admission were collected. Machine-learning methods (Random Forest and XGboost) were used to rank clinical features for mortality risk. Multivariate logistic regression models were applied to identify clinical features with statistical significance. The predictors of mortality were lactate dehydrogenase (LDH), C-reactive protein (CRP) and age based on 500 bootstrapped samples. A multivariate logistic regression model was formed to predict mortality 292 in-sample patients with area under the receiver operating characteristics (AUROC) of 0.9521, which was better than CURB-65 (AUROC of 0.8501) and the machine-learning-based model (AUROC of 0.4530). An out-sample data set of 13 patients was further tested to show our model (AUROC of 0.6061) was also better than CURB-65 (AUROC of 0.4608) and the machine-learning-based model (AUROC of 0.2292). LDH, CRP and age can be used to identify severe patients with COVID-19 on hospital admission.

Keywords: COVID-19; Random Forest; machine-learning methods; mortality risk; prognosis.

Conflict of interest statement

None.

- 32 references
- 2 figures

Supplementary info

Publication types, MeSH terms Expand

Publication types

- Observational Study
- Research Support, Non-U.S. Gov't

MeSH terms

- Adolescent
- Adult
- Aged
- COVID-19
- China / epidemiology
- Coronavirus Infections / mortality*
- Coronavirus Infections / therapy*
- Female
- Hospitalization
- Humans
- Logistic Models*
- Machine Learning*
- Male
- Middle Aged
- Pandemics
- Pneumonia, Viral / mortality*
- Pneumonia, Viral / therapy*
- Prognosis
- ROC Curve
- Reproducibility of Results
- Retrospective Studies
- Risk Assessment / methods
- Young Adult

Full text links

Cambridge University Press Free PMC article

Proceed to details

Cite

Share

977

Observational Study

Resuscitation

. 2020 Jun;151:18-23.

doi: 10.1016/j.resuscitation.2020.04.005. Epub 2020 Apr 10.

In-hospital cardiac arrest outcomes among patients with COVID-19 pneumonia in Wuhan, China

Fei Shao[1], Shuang Xu[2], Xuedi Ma[3], Zhouming Xu[3], Jiayou Lyu[3], Michael Ng[4], Hao Cui[5], Changxiao Yu[5], Qing Zhang[6], Peng Sun[7], Ziren Tang[8]
Affiliations Expand

Affiliations

- [1] Beijing Key Laboratory of Cardiopulmonary Cerebral Resuscitation, Beijing Chaoyang Hospital, Beijing, China; Department of Emergency Medicine, Beijing Chaoyang Hospital, Capital Medical University, Beijing, China.
- [2] Department of Emergency Medicine, Union Hospital, Tongji Medical College, Huazhong University of Science and Technology, Wuhan, China.
- [3] AI Research Division, A.I. Phoenix Technology Co., Ltd, Hong Kong, China.
- [4] Research Division for Mathematical and Statistical Science, University of Hong Kong, Hong Kong, China.
- [5] Department of Emergency Medicine, Beijing Chaoyang Hospital, Capital Medical University, Beijing, China.
- [6] Departments of Anaesthesiology, Union Hospital, Tongji Medical College, Huazhong University of Science and Technology, Wuhan, China.
- [7] Department of Emergency Medicine, Union Hospital, Tongji Medical College, Huazhong University of Science and Technology, Wuhan, China. Electronic address: simple1111@hust.edu.cn.
- [8] Beijing Key Laboratory of Cardiopulmonary Cerebral Resuscitation, Beijing Chaoyang Hospital, Beijing, China; Department of Emergency Medicine, Beijing Chaoyang Hospital, Capital Medical University, Beijing, China. Electronic address: tangziren1970@163.com.

- PMID: **32283117**
- PMCID: PMC7151543
- DOI: 10.1016/j.resuscitation.2020.04.005

Free PMC article
Observational Study

In-hospital cardiac arrest outcomes among patients with COVID-19 pneumonia in Wuhan, China

Fei Shao et al. Resuscitation. 2020 Jun.
Free PMC article
Show details
Resuscitation

. 2020 Jun;151:18-23.
doi: 10.1016/j.resuscitation.2020.04.005. Epub 2020 Apr 10.

Authors

Fei Shao [1], Shuang Xu [2], Xuedi Ma [3], Zhouming Xu [3], Jiayou Lyu [3], Michael Ng [4], Hao Cui [5], Changxiao Yu [5], Qing Zhang [6], Peng Sun [7], Ziren Tang [8]

Affiliations

- [1] Beijing Key Laboratory of Cardiopulmonary Cerebral Resuscitation, Beijing Chaoyang Hospital, Beijing, China; Department of Emergency Medicine, Beijing Chaoyang Hospital, Capital Medical University, Beijing, China.
- [2] Department of Emergency Medicine, Union Hospital, Tongji Medical College, Huazhong University of Science and Technology, Wuhan, China.
- [3] AI Research Division, A.I. Phoenix Technology Co., Ltd, Hong Kong, China.
- [4] Research Division for Mathematical and Statistical Science, University of Hong Kong, Hong Kong, China.
- [5] Department of Emergency Medicine, Beijing Chaoyang Hospital, Capital Medical University, Beijing, China.
- [6] Departments of Anaesthesiology, Union Hospital, Tongji Medical College, Huazhong University of Science and Technology, Wuhan, China.
- [7] Department of Emergency Medicine, Union Hospital, Tongji Medical College, Huazhong University of Science and Technology, Wuhan, China. Electronic address: simple1111@hust.edu.cn.
- [8] Beijing Key Laboratory of Cardiopulmonary Cerebral Resuscitation, Beijing Chaoyang Hospital, Beijing, China; Department of Emergency Medicine, Beijing Chaoyang Hospital, Capital Medical University, Beijing, China. Electronic address: tangziren1970@163.com.

- PMID: **32283117**
- PMCID: PMC7151543
- DOI: 10.1016/j.resuscitation.2020.04.005

Abstract

Objective: To describe the characteristics and outcomes of patients with severe COVID-19 and in-hospital cardiac arrest (IHCA) in Wuhan, China.

Methods: The outcomes of patients with severe COVID-19 pneumonia after IHCA over a 40-day period were retrospectively evaluated. Between January 15 and February 25, 2020, data for all cardiopulmonary resuscitation (CPR) attempts for IHCA that occurred in a tertiary teaching hospital in Wuhan, China were collected according to the Utstein style. The primary outcome was restoration of spontaneous circulation (ROSC), and the secondary outcomes were 30-day survival, and neurological outcome.

Results: Data from 136 patients showed 119 (87.5%) patients had a respiratory cause for their cardiac arrest, and 113 (83.1%) were resuscitated in a general ward. The initial rhythm was asystole in 89.7%, pulseless electrical activity (PEA) in 4.4%, and shockable in 5.9%. Most patients with IHCA were monitored (93.4%) and in most resuscitation (89%) was initiated <1 min. The average length of hospital stay was 7 days and the time from illness onset to hospital admission was 10 days. The most frequent comorbidity was hypertension (30.2%), and the most

frequent symptom was shortness of breath (75%). Of the patients receiving CPR, ROSC was achieved in 18 (13.2%) patients, 4 (2.9%) patients survived for at least 30 days, and one patient achieved a favourable neurological outcome at 30 days. Cardiac arrest location and initial rhythm were associated with better outcomes.

Conclusion: Survival of patients with severe COVID-19 pneumonia who had an in-hospital cardiac arrest was poor in Wuhan.

Keywords: COVID-19; Cardiopulmonary resuscitation; In-hospital cardiac arrest; ROSC; Survival.

Copyright © 2020 Elsevier B.V. All rights reserved.

Comment in

- Dilemmas in resuscitation of COVID-19 patients based on current evidence.
  Szarpak L, Ruetzler K, Dabrowski M, Nadolny K, Ladny JR, Smereka J, Jaguszewski M, Filipiak KJ. Szarpak L, et al. Cardiol J. 2020;27(3):327-328. doi: 10.5603/CJ.a2020.0066. Epub 2020 May 18. Cardiol J. 2020. PMID: 32419130 Free PMC article. No abstract available.
- Cardiopulmonary resuscitation of inpatients with severe COVID-19 pneumonia: The Wuhan experience.
  Shao F, Sun P, Tang Z. Shao F, et al. Resuscitation. 2020 Jul;152:95-96. doi: 10.1016/j.resuscitation.2020.05.009. Epub 2020 May 11. Resuscitation. 2020. PMID: 32437782 Free PMC article. No abstract available.

- 15 references
- 1 figure

Supplementary info

Publication types, MeSH terms Expand

Publication types

- Observational Study
- Research Support, Non-U.S. Gov't

MeSH terms

- Adult
- Aged
- Aged, 80 and over
- Betacoronavirus*
- COVID-19
- Cardiopulmonary Resuscitation / methods*
- China
- Cohort Studies

- Coronavirus Infections / complications*
- Female
- Heart Arrest / etiology
- Heart Arrest / mortality*
- Heart Arrest / therapy
- Hospital Mortality*
- Humans
- Male
- Middle Aged
- Pandemics
- Pneumonia, Viral / complications
- Pneumonia, Viral / etiology
- Pneumonia, Viral / mortality
- Pneumonia, Viral / therapy*
- Prognosis
- Retrospective Studies
- Risk Assessment
- SARS-CoV-2
- Survival Analysis
- Treatment Outcome

Full text links

ELSEVIER FULL-TEXT ARTICLE Elsevier Science Free PMC article

Proceed to details

Cite

Share

978

Blood

. 2020 Jul 2;136(1):144-147.

doi: 10.1182/blood.2020006941.

Impact of anticoagulation prior to COVID-19 infection: a propensity score-matched cohort study

Douglas Tremblay [1], Maaike van Gerwen [2] [3], Mathilda Alsen [2], Santiago Thibaud [1], Alaina Kessler [1], Sangeetha Venugopal [1], Iman Makki [1], Qian Qin [1], Sirish Dharmapuri [1], Tomi Jun [1], Sheena Bhalla [1], Shana Berwick [1], Jonathan Feld [1], John Mascarenhas [1], Kevin Troy [1], Caroline Cromwell [1], Andrew Dunn [4], William K Oh [1], Leonard Naymagon [1]

Affiliations Expand

Affiliations

- [1] Division of Hematology and Medical Oncology, Tisch Cancer Institute.
- [2] Department of Otolaryngology-Head and Neck Surgery.
- [3] Institute for Translational Epidemiology, and.
- [4] Division of Hospital Medicine, Department of Medicine, Icahn School of Medicine at Mount Sinai, New York, NY.

- PMID: **32462179**
- PMCID: PMC7332896
- DOI: 10.1182/blood.2020006941

Free PMC article

Impact of anticoagulation prior to COVID-19 infection: a propensity score-matched cohort study

Douglas Tremblay et al. Blood. 2020.
Free PMC article
Show details

Blood
. 2020 Jul 2;136(1):144-147.
doi: 10.1182/blood.2020006941.

Authors

Douglas Tremblay [1], Maaike van Gerwen [2] [3], Mathilda Alsen [2], Santiago Thibaud [1], Alaina Kessler [1], Sangeetha Venugopal [1], Iman Makki [1], Qian Qin [1], Sirish Dharmapuri [1], Tomi Jun [1], Sheena Bhalla [1], Shana Berwick [1], Jonathan Feld [1], John Mascarenhas [1], Kevin Troy [1], Caroline Cromwell [1], Andrew Dunn [4], William K Oh [1], Leonard Naymagon [1]

Affiliations

- [1] Division of Hematology and Medical Oncology, Tisch Cancer Institute.
- [2] Department of Otolaryngology-Head and Neck Surgery.
- [3] Institute for Translational Epidemiology, and.
- [4] Division of Hospital Medicine, Department of Medicine, Icahn School of Medicine at Mount Sinai, New York, NY.

- PMID: **32462179**
- PMCID: PMC7332896
- DOI: 10.1182/blood.2020006941

Abstract

Tremblay and colleagues asked whether patients receiving either routine anticoagulation or antiplatelet therapy for existing conditions prior to becoming ill with COVID-19 have different outcomes from patients receiving neither therapy. After matching for existing comorbidities, this retrospective observational study finds no evidence for an effect of prediagnosis anticoagulation on mortality.

- 20 references
- 1 figure

Supplementary info

Publication types, MeSH terms, Substances Expand

Publication types

- Letter

MeSH terms

- Adult
- Aged
- Anticoagulants / therapeutic use*
- Betacoronavirus / drug effects
- COVID-19
- Coronavirus Infections / drug therapy
- Coronavirus Infections / epidemiology*
- Female
- Humans
- Male
- Middle Aged
- Pandemics
- Platelet Aggregation Inhibitors / therapeutic use*
- Pneumonia, Viral / drug therapy
- Pneumonia, Viral / epidemiology*
- Propensity Score
- Protective Agents / therapeutic use*
- Protective Factors
- Retrospective Studies
- SARS-CoV-2
- Survival Analysis

Substances

- Anticoagulants

- Platelet Aggregation Inhibitors
- Protective Agents

Full text links

blood Silverchair Information Systems Free PMC article
Proceed to details
Cite
Share
☐ 979
PLoS One

. 2021 Nov 29;16(11):e0260318.
doi: 10.1371/journal.pone.0260318. eCollection 2021.

Admission criteria in critically ill COVID-19 patients: A physiology-based approach

Samuele Ceruti [1], Andrea Glotta [1], Maira Biggiogero [2], Pier Andrea Maida [2], Martino Marzano [3], Patrizia Urso [4], Giovanni Bona [2], Christian Garzoni [3], Zsolt Molnar [5] [6]
Affiliations Expand

Affiliations

- [1] Department of Critical Care, Clinica Luganese Moncucco, Lugano, Switzerland.
- [2] Clinical Research Unit, Clinica Luganese Moncucco, Lugano, Switzerland.
- [3] Internal Medicine Service, Clinica Luganese Moncucco, Lugano, Switzerland.
- [4] Radiotherapy Service, Clinica Luganese Moncucco, Lugano, Switzerland.
- [5] Department of Anaesthesiology and Intensive Therapy, Semmelweis University, Budapest, Hungary.
- [6] Department of Anaesthesiology and Intensive Therapy, Poznan University of Medical Sciences, Poznan, Poland.

- PMID: **34843531**
- PMCID: PMC8629252
- DOI: 10.1371/journal.pone.0260318

Free PMC article

Admission criteria in critically ill COVID-19 patients: A physiology-based approach

Samuele Ceruti et al. PLoS One. 2021.
Free PMC article
Show details

PLoS One
. 2021 Nov 29;16(11):e0260318.
doi: 10.1371/journal.pone.0260318. eCollection 2021.

Authors

Samuele Ceruti [1], Andrea Glotta [1], Maira Biggiogero [2], Pier Andrea Maida [2], Martino Marzano [3], Patrizia Urso [4], Giovanni Bona [2], Christian Garzoni [3], Zsolt Molnar [5] [6]

Affiliations

- [1] Department of Critical Care, Clinica Luganese Moncucco, Lugano, Switzerland.
- [2] Clinical Research Unit, Clinica Luganese Moncucco, Lugano, Switzerland.
- [3] Internal Medicine Service, Clinica Luganese Moncucco, Lugano, Switzerland.
- [4] Radiotherapy Service, Clinica Luganese Moncucco, Lugano, Switzerland.
- [5] Department of Anaesthesiology and Intensive Therapy, Semmelweis University, Budapest, Hungary.
- [6] Department of Anaesthesiology and Intensive Therapy, Poznan University of Medical Sciences, Poznan, Poland.

- PMID: **34843531**
- PMCID: PMC8629252
- DOI: 10.1371/journal.pone.0260318

Abstract

Introduction: The COVID-19 pandemic required careful management of intensive care unit (ICU) admissions, to reduce ICU overload while facing limitations in resources. We implemented a standardized, physiology-based, ICU admission criteria and analyzed the mortality rate of patients refused from the ICU.

Materials and methods: In this retrospective observational study, COVID-19 patients proposed for ICU admission were consecutively analyzed; Do-Not-Resuscitate patients were excluded. Patients presenting an oxygen peripheral saturation (SpO2) lower than 85% and/or dyspnea and/or mental confusion resulted eligible for ICU admission; patients not presenting these criteria remained in the ward with an intensive monitoring protocol. Primary outcome was both groups' survival rate. Secondary outcome was a sub analysis correlating SpO2 cutoff with ICU admission.

Results: From March 2020 to January 2021, 1623 patients were admitted to our Center; 208 DNR patients were excluded; 97 patients were evaluated. The ICU-admitted group ($n = 63$) mortality rate resulted 15.9% at 28 days and 27% at 40 days; the ICU-refused group ($n = 34$) mortality rate resulted 0% at both intervals ($p < 0.001$). With a SpO2 cut-off of 85%, a significant correlation was found ($p = 0.009$), but with a 92% a cut-off there was no correlation with ICU admission ($p = 0.26$). A similar correlation was also found with dyspnea ($p = 0.0002$).

Conclusion: In COVID-19 patients, standardized ICU admission criteria appeared to safely reduce ICU overload. In the absence of dyspnea and/or confusion, a SpO2 cutoff up to 85% for ICU admission was not burdened by negative outcomes. In a pandemic context, the SpO2 cutoff of 92%, as a threshold for ICU admission, needs critical re-evaluation.

Conflict of interest statement

The authors have declared that no competing interests exist.

- 30 references
- 3 figures

Supplementary info

MeSH terms, Grant support Expand

MeSH terms

- Adult
- Aged
- Aged, 80 and over
- COVID-19 / complications
- COVID-19 / epidemiology*
- COVID-19 / physiopathology*
- Critical Illness*
- Female
- Hospitalization*
- Humans
- Hypoxia / complications
- Intensive Care Units
- Male
- Middle Aged
- Partial Pressure
- Referral and Consultation
- Survival Rate

Grant support

The authors received no specific funding for this work.

Full text links

OPEN ACCESS TO FULL TEXT PLOS ONE Public Library of Science Free PMC article
Proceed to details
Cite
Share
☐ 980
J Burn Care Res
. 2021 Aug 4;42(4):794-800.
doi: 10.1093/jbcr/iraa217.

Burns and COVID-19-Initial Experience and Challenges

Shalabh Kumar [1], Rakesh Kain [1], Amrita More [1], Srushti Sheth [1], Praveen Kumar Arumugam [1]
Affiliations Expand

Affiliation

- [1] Department of Burns, Plastic and Maxillofacial Surgery, Vardhman Mahavir Medical College and Safdarjung Hospital, Delhi, India.

- PMID: **33367625**
- PMCID: PMC7799032
- DOI: 10.1093/jbcr/iraa217

Free PMC article

Burns and COVID-19-Initial Experience and Challenges

Shalabh Kumar et al. J Burn Care Res. 2021.
Free PMC article
Show details
J Burn Care Res
. 2021 Aug 4;42(4):794-800.
doi: 10.1093/jbcr/iraa217.

Authors

Shalabh Kumar [1], Rakesh Kain [1], Amrita More [1], Srushti Sheth [1], Praveen Kumar Arumugam [1]

Affiliation

- [1] Department of Burns, Plastic and Maxillofacial Surgery, Vardhman Mahavir Medical College and Safdarjung Hospital, Delhi, India.

- PMID: **33367625**
- PMCID: PMC7799032
- DOI: 10.1093/jbcr/iraa217

Abstract

The COVID-19 pandemic has brought with it many challenges in the field of healthcare around the world. Managing burn patients has its own challenges as they require a long duration of care and are more susceptible to infection. We conducted a retrospective observational study from January 30 to July 15, 2020 at our center to study the epidemiology of burns treated & patients

and healthcare workers affected by COVID-19 during this period. The number of burn admissions showed a 42.6% reduction as compared to last year. A total of 17 patients (3.67%) and 29 health care workers (8.68%) tested positive for COVID-19 in the burns department. Our strategy underwent changes based on the changing dynamics of COVID-19 and changes in government and institutional policies. We have described the various challenges we faced in managing burns during this time. We found that effective screening of patients and healthcare workers, proper segregation of negative and positive/ suspect population and a low threshold for COVID-19 testing were essential to mitigate transmission of infection.

© The Author(s) 2020. Published by Oxford University Press on behalf of the American Burn Association. All rights reserved. For permissions, please e-mail: journals.permissions@oup.com.

Supplementary info

MeSH terms Expand

MeSH terms

- Burn Units / organization & administration*
- Burns / epidemiology
- Burns / therapy*
- COVID-19 / epidemiology*
- COVID-19 / therapy
- Humans
- India
- Infection Control / organization & administration*
- Personal Protective Equipment / statistics & numerical data
- Retrospective Studies

Full text links

OXFORD ACADEMIC Silverchair Information Systems Free PMC article
Proceed to details
Cite
Share
☐ 981
Observational Study
Crit Care Med
. 2021 Feb 1;49(2):209-214.
doi: 10.1097/CCM.0000000000004747.

Improving Survival of Critical Care Patients With Coronavirus Disease 2019 in England: A National Cohort Study, March to June 2020

John M Dennis [1], Andrew P McGovern [1], Sebastian J Vollmer [2 3], Bilal A Mateen [3 4]
Affiliations Expand

Affiliations

- [1] Institute of Biomedical & Clinical Science, University of Exeter Medical School, Exeter, United Kingdom.
- [2] The Alan Turing Institute, London, United Kingdom.
- [3] Department of Statistics, University of Warwick, Coventry, United Kingdom.
- [4] King's College Hospital NHS Foundation Trust, Denmark Hill, London, United Kingdom.

- PMID: **33105150**
- PMCID: PMC7803441
- DOI: 10.1097/CCM.0000000000004747

Free PMC article
Observational Study

Improving Survival of Critical Care Patients With Coronavirus Disease 2019 in England: A National Cohort Study, March to June 2020

John M Dennis et al. Crit Care Med. 2021.
Free PMC article
Show details
Crit Care Med
. 2021 Feb 1;49(2):209-214.
doi: 10.1097/CCM.0000000000004747.

Authors

John M Dennis [1], Andrew P McGovern [1], Sebastian J Vollmer [2 3], Bilal A Mateen [3 4]

Affiliations

- [1] Institute of Biomedical & Clinical Science, University of Exeter Medical School, Exeter, United Kingdom.
- [2] The Alan Turing Institute, London, United Kingdom.
- [3] Department of Statistics, University of Warwick, Coventry, United Kingdom.
- [4] King's College Hospital NHS Foundation Trust, Denmark Hill, London, United Kingdom.

- PMID: **33105150**
- PMCID: PMC7803441
- DOI: 10.1097/CCM.0000000000004747

Abstract

Objectives: To measure temporal trends in survival over time in people with severe coronavirus disease 2019 requiring critical care (high dependency unit or ICU) management, and to assess whether temporal variation in mortality was explained by changes in patient demographics and comorbidity burden over time.

Design: Retrospective observational cohort; based on data reported to the COVID-19 Hospitalisation in England Surveillance System. The primary outcome was in-hospital 30-day all-cause mortality. Unadjusted survival was estimated by calendar week of admission, and Cox proportional hazards models were used to estimate adjusted survival, controlling for age, sex, ethnicity, major comorbidities, and geographical region.

Setting: One hundred eight English critical care units.

Patients: All adult (18 yr +) coronavirus disease 2019 specific critical care admissions between March 1, 2020, and June 27, 2020.

Interventions: Not applicable.

Measurements and main results: Twenty-one thousand eighty-two critical care patients (high dependency unit n = 15,367; ICU n = 5,715) were included. Unadjusted survival at 30 days was lowest for people admitted in late March in both high dependency unit (71.6% survival) and ICU (58.0% survival). By the end of June, survival had improved to 92.7% in high dependency unit and 80.4% in ICU. Improvements in survival remained after adjustment for patient characteristics (age, sex, ethnicity, and major comorbidities) and geographical region.

Conclusions: There has been a substantial improvement in survival amongst people admitted to critical care with coronavirus disease 2019 in England, with markedly higher survival rates in people admitted in May and June compared with those admitted in March and April. Our analysis suggests this improvement is not due to temporal changes in the age, sex, ethnicity, or major comorbidity burden of admitted patients.

Copyright © 2020 The Author(s). Published by Wolters Kluwer Health, Inc. on behalf of the Society of Critical Care Medicine and Wolters Kluwer Health, Inc.

Comment in

- Survival From Severe Coronavirus Disease 2019: Is It Changing?
  Prescott HC, Levy MM. Prescott HC, et al. Crit Care Med. 2021 Feb 1;49(2):351-353. doi: 10.1097/CCM.0000000000004753. Crit Care Med. 2021. PMID: 33186138 No abstract available.
- Critical care survival rates in COVID-19 patients improved as the first wave of the pandemic developed.
  Bateson ML, McPeake JM. Bateson ML, et al. Evid Based Nurs. 2022 Jan;25(1):13. doi: 10.1136/ebnurs-2020-103370. Epub 2021 May 10. Evid Based Nurs. 2022. PMID: 33972290 No abstract available.

- 11 references

- 2 figures

Supplementary info

Publication types, MeSH terms Expand

Publication types

- Observational Study
- Research Support, Non-U.S. Gov't

MeSH terms

- Adult
- Aged
- COVID-19 / mortality*
- COVID-19 / therapy
- Cohort Studies
- Critical Care / statistics & numerical data*
- Critical Illness / mortality*
- Critical Illness / therapy
- England
- Female
- Humans
- Intensive Care Units
- Male
- Middle Aged
- Retrospective Studies
- Risk Assessment
- Risk Factors
- Survival Rate
- Survivors / statistics & numerical data*

Full text links

Wolters Kluwer Wolters Kluwer Free PMC article
Proceed to details
Cite
Share
☐ 982
Observational Study
J Clin Lab Anal
. 2020 Oct;34(10):e23562.

doi: 10.1002/jcla.23562. Epub 2020 Sep 6.

Predictive criteria of severe cases in COVID-19 patients of early stage: A retrospective observational study

Jinrui Gao [1], Xiu Huang [1], Haibo Gu [1], Lingyun Lou [1], Zhihao Xu [1]
Affiliations Expand

Affiliation

- [1] Department of Respiratory and Critical Care Medicine, Fourth Affiliated Hospital of Zhejiang University School of Medicine, Yiwu, China.

- PMID: **32893398**
- PMCID: PMC7595922
- DOI: 10.1002/jcla.23562

Free PMC article
Observational Study

Predictive criteria of severe cases in COVID-19 patients of early stage: A retrospective observational study

Jinrui Gao et al. J Clin Lab Anal. 2020 Oct.
Free PMC article
Show details
J Clin Lab Anal
. 2020 Oct;34(10):e23562.
doi: 10.1002/jcla.23562. Epub 2020 Sep 6.

Authors

Jinrui Gao [1], Xiu Huang [1], Haibo Gu [1], Lingyun Lou [1], Zhihao Xu [1]

Affiliation

- [1] Department of Respiratory and Critical Care Medicine, Fourth Affiliated Hospital of Zhejiang University School of Medicine, Yiwu, China.

- PMID: **32893398**
- PMCID: PMC7595922
- DOI: 10.1002/jcla.23562

Abstract

Background: Patients with coronavirus disease 2019 (COVID-19) often suffer sudden deterioration of disease around 1-2 weeks after onset. Once the disease progressed to severe phase, clinical prognosis of patients will significantly deteriorate.

Methods: This was a multicenter retrospective study on patients of all adult inpatients (≥18 years old) from Tianyou Hospital (Wuhan, China) and the Fourth Affiliated Hospital, Zhejiang University School of Medicine. All 139 patients had laboratory-confirmed COVID-19 in their early stage, which is defined as within 7 days of clinical symptoms. Univariate and multivariate logistic regression models were used to determine the predictive factors in the early detection of patients who may subsequently develop into severe cases.

Results: Multivariable logistic regression analysis showed that the higher level of hypersensitivity C-reactive protein (OR = 4.77, 95% CI:1.92-11.87, P = .001), elevated alanine aminotransferase (OR = 6.87, 95%CI:1.56-30.21, P = .011), and chronic comorbidities (OR = 11.48, 95% CI:4.44-29.66, P < .001) are the determining risk factors for the progression into severe pneumonia in COVID-19 patients.

Conclusion: Early COVID-19 patients with chronic comorbidities, elevated hs-CRP or elevated ALT are significantly more likely to develop severe pneumonia as the disease progresses. These risk factors may facilitate the early diagnosis of critical patients in clinical practice.

Keywords: COVID-19; SARS-CoV-2; cytokine storm; laboratory parameter; risk factors.

© 2020 The Authors. Journal of Clinical Laboratory Analysis published by Wiley Periodicals LLC.

Conflict of interest statement

There were no conflicts of interest to this work.

- 35 references
- 1 figure

Supplementary info

Publication types, MeSH terms, Grant support Expand

Publication types

- Multicenter Study
- Observational Study

MeSH terms

- Adult
- Aged
- Betacoronavirus

- COVID-19
- China
- Coronavirus Infections* / diagnosis
- Coronavirus Infections* / epidemiology
- Coronavirus Infections* / physiopathology
- Coronavirus Infections* / therapy
- Critical Illness
- Cytokine Release Syndrome
- Early Diagnosis
- Female
- Humans
- Male
- Middle Aged
- Pandemics*
- Pneumonia, Viral* / diagnosis
- Pneumonia, Viral* / epidemiology
- Pneumonia, Viral* / physiopathology
- Pneumonia, Viral* / therapy
- Predictive Value of Tests
- Retrospective Studies
- Risk Factors
- SARS-CoV-2

Grant support

- 2020XG-31/Jinhua special scientific research fund for COVID-19 prevention and control
- 2020XGZX069/Zhejiang University special scientific research fund for COVID-19 prevention and control

Full text links

WILEY Full Text Article Wiley Free PMC article

Proceed to details

Cite

Share

983

Case Reports

Diabetes Res Clin Pract

. 2020 Aug;166:108279.

doi: 10.1016/j.diabres.2020.108279. Epub 2020 Jun 25.

Clinical characteristics and outcome in patients with combined diabetic ketoacidosis and hyperosmolar hyperglycemic state associated with COVID-19: A retrospective, hospital-based observational case series

Kok Hoe Chan [1], Divya Thimmareddygari [2], Amr Ramahi [2], Liana Atallah [2], Nicholas G Baranetsky [3], Jihad Slim [4]
Affiliations Expand

Affiliations

- [1] Department of Medical Education, Saint Michael's Medical Centre, New York Medical College, NJ, United States. Electronic address: kchan2@primehealthcare.com.
- [2] Department of Medical Education, Saint Michael's Medical Centre, New York Medical College, NJ, United States.
- [3] Department of Medical Education, Saint Michael's Medical Centre, New York Medical College, NJ, United States; Department of Medicine, Saint Michael's Medical Centre, New York Medical College, NJ, United States.
- [4] Department of Medical Education, Saint Michael's Medical Centre, New York Medical College, NJ, United States; Department of Infectious Disease, Saint Michael's Medical Centre, New York Medical College, NJ, United States.

- PMID: **32592843**
- PMCID: PMC7314685
- DOI: 10.1016/j.diabres.2020.108279

Free PMC article
Case Reports

Clinical characteristics and outcome in patients with combined diabetic ketoacidosis and hyperosmolar hyperglycemic state associated with COVID-19: A retrospective, hospital-based observational case series

Kok Hoe Chan et al. Diabetes Res Clin Pract. 2020 Aug.
Free PMC article
Show details
Diabetes Res Clin Pract
. 2020 Aug;166:108279.

doi: 10.1016/j.diabres.2020.108279. Epub 2020 Jun 25.

Authors

Kok Hoe Chan [1], Divya Thimmareddygari [2], Amr Ramahi [2], Liana Atallah [2], Nicholas G Baranetsky [3], Jihad Slim [4]

Affiliations

- [1] Department of Medical Education, Saint Michael's Medical Centre, New York Medical College, NJ, United States. Electronic address: kchan2@primehealthcare.com.
- [2] Department of Medical Education, Saint Michael's Medical Centre, New York Medical College, NJ, United States.
- [3] Department of Medical Education, Saint Michael's Medical Centre, New York Medical College, NJ, United States; Department of Medicine, Saint Michael's Medical Centre, New York Medical College, NJ, United States.
- [4] Department of Medical Education, Saint Michael's Medical Centre, New York Medical College, NJ, United States; Department of Infectious Disease, Saint Michael's Medical Centre, New York Medical College, NJ, United States.

- PMID: **32592843**
- PMCID: PMC7314685
- DOI: 10.1016/j.diabres.2020.108279

Abstract

Aim: One of the risk factors for poor outcome with SARS-CoV-2 infection is diabetes mellitus; diabetic ketoacidosis (DKA) and hyperosmolar hyperglycemic state (HHS) are the most serious complications of diabetes mellitus. We aimed to explore the clinical characteristics and outcomes of COVID-19 patients presenting with combined DKA/HHS to our institution.

Methods: A retrospective, hospital based observation case series was performed on patients with SARS-CoV-2 admitted to Intensive Care Unit between 3/20/2020 and 4/20/2020. Inclusion criteria were: (1) Blood Glucose >250 mg/dL; (2) Serum bicarbonate <18 mmol/L; (3) Anion Gap >10; (4) serum pH <7.3; (5) ketonemia or ketonuria; (6) effective/calculated plasma osmolality >304 mOsm/kg and (7) positive SARS-CoV-2 RT-PCR.

Results: We reported 6 patients who presented during this period with combined DKA/HHS. Their median age was 50 years, all males, three Hispanic, and three African American. Hispanic patients, had more severe acidosis, and multiple comorbidities, with a higher mortality. The striking feature was that combined DKA/HHS was the initial presentation for COVID-19 for most of the cases.

Discussion: Our observational retrospective case series shows that diabetic patients are at risk of developing combined DKA/ HHS associated with COVID-19 and a substantial mortality. To our knowledge, we are first to report the clinical characteristics and outcome in this group of patients.

Keywords: COVID-19; Diabetic ketoacidosis; Hyperosmolar hyperglycemic state; SARS-CoV-2.

Copyright © 2020 Elsevier B.V. All rights reserved.

Conflict of interest statement

Declaration of Competing Interest The authors declare that they have no known competing financial interests or personal relationships that could have appeared to influence the work reported in this paper.

- 16 references
- 1 figure

Supplementary info

Publication types, MeSH terms, Substances Expand

Publication types

- Case Reports
- Observational Study

MeSH terms

- Adult
- Betacoronavirus / isolation & purification*
- Blood Glucose
- COVID-19
- Coronavirus Infections / complications
- Coronavirus Infections / epidemiology
- Coronavirus Infections / mortality*
- Coronavirus Infections / virology
- Diabetes Mellitus / mortality*
- Diabetes Mellitus / physiopathology
- Diabetes Mellitus / virology
- Diabetic Ketoacidosis / etiology
- Diabetic Ketoacidosis / mortality*
- Diabetic Ketoacidosis / pathology
- Female
- Hospitalization
- Humans
- Hyperglycemic Hyperosmolar Nonketotic Coma / etiology
- Hyperglycemic Hyperosmolar Nonketotic Coma / mortality*
- Hyperglycemic Hyperosmolar Nonketotic Coma / pathology
- Male
- Middle Aged
- Pandemics
- Pneumonia, Viral / complications

- Pneumonia, Viral / epidemiology
- Pneumonia, Viral / mortality*
- Pneumonia, Viral / virology
- Prognosis
- Retrospective Studies
- Risk Factors
- SARS-CoV-2
- Survival Rate
- Young Adult

Substances

- Blood Glucose

Full text links

ELSEVIER FULL-TEXT ARTICLE Elsevier Science Free PMC article
Proceed to details
Cite
Share
984
Observational Study
Turk Kardiyol Dern Ars
. 2021 Jun;49(4):286-292.
doi: 10.5543/tkda.2021.87750.

Comparison of hypertension prevalence and the use of renin-angiotensin-aldosterone system blockers in hospitalized patients with COVID-19 and non-COVID-19 viral pneumonia

Selçuk Görmez [1], Ceyda Erel Kırışoğlu [2], Mehmet Erkan Ekicibaşı [3], Aleks Değirmencioğlu [4], Ashok Paudel [3], Gökçe Akan [5], Fatmahan Atalar [6], Nevin Sarıgüzel [7], Burak Pamukçu [8]
Affiliations Expand

Affiliations

- [1] Department of Cardiology, Acıbadem Mehmet Ali Aydınlar University School of Medicine, İstanbul, Turkey.

- [2] Department of Chest Diseases and Tuberculosis, Faculty of Medicine, Acibadem Mehmet Ali Aydinlar University, Istanbul, Turkey.
- [3] Department of Cardiology, Acibadem Altunizade Hospital, Acibadem Healthcare Group, Istanbul, Turkey.
- [4] Department of Cardiology, Acibadem Maslak Hospital, Acibadem Healthcare Group, Istanbul, Turkey.
- [5] Muhimbili University of Health and Allied Sciences, Department of Biochemistry, MUHAS Genetics Laboratory, Dar es Salaam, Tanzania.
- [6] Department of Family Health, Child Health Institute, Istanbul University, Istanbul, Turkey.
- [7] Department of Infectious Diseases, Acibadem Kadikoy - Kozyatagi Hospitals, Acibadem Healthcare Group, Istanbul, Turkey.
- [8] Department of First and Emergency Aid, Vocational School of Health Services, Acibadem Mehmet Ali Aydinlar University, Istanbul, Turkey.

- PMID: **34106062**
- DOI: 10.5543/tkda.2021.87750

Free article
Observational Study

Comparison of hypertension prevalence and the use of renin-angiotensin-aldosterone system blockers in hospitalized patients with COVID-19 and non-COVID-19 viral pneumonia

Selçuk Görmez et al. Turk Kardiyol Dern Ars. 2021 Jun.
Free article
Show details
Turk Kardiyol Dern Ars
. 2021 Jun;49(4):286-292.
doi: 10.5543/tkda.2021.87750.

Authors

Selçuk Görmez [1], Ceyda Erel Kırışoğlu [2], Mehmet Erkan Ekicibaşı [3], Aleks Değirmencioğlu [4], Ashok Paudel [3], Gökçe Akan [5], Fatmahan Atalar [6], Nevin Sarıgüzel [7], Burak Pamukçu [8]

Affiliations

- [1] Department of Cardiology, Acıbadem Mehmet Ali Aydınlar University School of Medicine, İstanbul, Turkey.
- [2] Department of Chest Diseases and Tuberculosis, Faculty of Medicine, Acibadem Mehmet Ali Aydinlar University, Istanbul, Turkey.

- [3] Department of Cardiology, Acibadem Altunizade Hospital, Acibadem Healthcare Group, Istanbul, Turkey.
- [4] Department of Cardiology, Acibadem Maslak Hospital, Acibadem Healthcare Group, Istanbul, Turkey.
- [5] Muhimbili University of Health and Allied Sciences, Department of Biochemistry, MUHAS Genetics Laboratory, Dar es Salaam, Tanzania.
- [6] Department of Family Health, Child Health Institute, Istanbul University, Istanbul, Turkey.
- [7] Department of Infectious Diseases, Acibadem Kadikoy - Kozyatagi Hospitals, Acibadem Healthcare Group, Istanbul, Turkey.
- [8] Department of First and Emergency Aid, Vocational School of Health Services, Acibadem Mehmet Ali Aydinlar University, Istanbul, Turkey.

- PMID: **34106062**
- DOI: 10.5543/tkda.2021.87750

Abstract

Objective: To compare the prevalence of hypertension and pre-existing use of renin-angiotensin-aldosterone system blockers in patients with coronavirus disease (COVID-19) and non-COVID-19 viral pneumonias.

Methods: Real-time polymerase chain reaction confirmed COVID-19 and non-COVID-19 pneumonia patients were retrospectively analyzed. The presence of hypertension, coronary artery disease (CAD), and pre-existing use of angiotensin-converting enzyme inhibitors (ACEIs) and angiotensin receptor blockers (ARBs) were compared between the groups.

Results: A total of 103 COVID-19 and 91 non-COVID-19 hospitalized viral pneumonia patients were enrolled. Hypertension and CAD were more common in patients with non-COVID-19 viral pneumonia than in patients with COVID-19 (39.6% vs 22.3%, respectively, p=0.012 and 24.2% vs 4.9%, respectively, p<0.001). In our study, 2.9% and 6.8% of patients with COVID-19 were on ACEIs and ARBs, respectively, whereas 13.2% and 19.8% of patients with non-COVID-19 viral pneumonia were on ACEIs and ARBs, respectively (p=0.009 and p=0.013). Neutrophil-to-lymphocyte ratio (p<0.001) was prominent in patients with non-COVID-19 viral pneumonia compared with patients with COVID-19.

Conclusion: Our study results indicate that hypertension and CAD are more common among patients with non-COVID-19 viral pneumonia than patients with COVID-19. The prevalence of ACEIs and ARBs use was not higher in patients with COVID-19. Our results support that the use of ACEIs and ARBs do not play a specific role in patients with COVID-19.

Supplementary info

Publication types, MeSH terms, Substances Expand

Publication types

- Multicenter Study
- Observational Study

MeSH terms

- Adult
- Angiotensin Receptor Antagonists / therapeutic use*
- Angiotensin-Converting Enzyme Inhibitors / therapeutic use*
- COVID-19* / complications
- COVID-19* / epidemiology
- Coronary Artery Disease / complications
- Coronary Artery Disease / epidemiology
- Female
- Humans
- Hypertension* / complications
- Hypertension* / drug therapy
- Hypertension* / epidemiology
- Male
- Middle Aged
- Pneumonia, Viral / complications
- Pneumonia, Viral / epidemiology
- Prevalence
- Retrospective Studies

Substances

- Angiotensin Receptor Antagonists
- Angiotensin-Converting Enzyme Inhibitors

Full text links

Turk Kardiyol Dern Ars OPEN ACCESS Aves Yayincilik
Proceed to details
Cite
Share
☐ 985
Observational Study
Catheter Cardiovasc Interv
. 2022 Feb;99(2):391-396.
doi: 10.1002/ccd.30056. Epub 2021 Dec 30.

Impact of COVID-19 pandemic on the management of nonculprit lesions in patients presenting with ST-elevation myocardial

[infarction: Outcomes from the pan-London heart attack centers]

Ozan M Demir [1], Callum D Little [2], Richard Jabbour [3], Haseeb Rahman [3], Max Sayers [4], Asrar Ahmed [5], Michelle J Connolly [6], Ritesh Kanyal [7], Philip MacCarthy [7], Simon J Wilson [6], Miles Dalby [5], Ajay Jain [4], Iqbal Malik [3], Roby Rakhit [2], Divaka Perera [1]
Affiliations Expand

Affiliations

- [1] NIHR Biomedical Research Centre and British Heart Foundation Centre of Excellence, School of Cardiovascular Medicine and Sciences, King's College London, London, UK.
- [2] Department of Cardiology, Royal Free London NHS Foundation Trust, London, UK.
- [3] Department of Cardiology, Imperial College Healthcare NHS Foundation Trust, London, UK.
- [4] Department of Cardiology, Barts Health NHS Trust, London, UK.
- [5] Department of Cardiology, Royal Brompton and Harefield NHS Foundation Trust, London, UK.
- [6] Department of Cardiology, St George's University Hospitals NHS Foundation Trust, London, UK.
- [7] Department of Cardiology, King's College Hospital NHS Foundation Trust, London, UK.

- PMID: **34967091**
- DOI: 10.1002/ccd.30056

Observational Study

Impact of COVID-19 pandemic on the management of nonculprit lesions in patients presenting with ST-elevation myocardial infarction: Outcomes from the pan-London heart attack centers

Ozan M Demir et al. Catheter Cardiovasc Interv. 2022 Feb.
Show details
Catheter Cardiovasc Interv
. 2022 Feb;99(2):391-396.
doi: 10.1002/ccd.30056. Epub 2021 Dec 30.

Authors

Ozan M Demir [1], Callum D Little [2], Richard Jabbour [3], Haseeb Rahman [3], Max Sayers [4], Asrar Ahmed [5], Michelle J Connolly [6], Ritesh Kanyal [7], Philip MacCarthy [7], Simon J Wilson [6], Miles Dalby [5], Ajay Jain [4], Iqbal Malik [3], Roby Rakhit [2], Divaka Perera [1]

Affiliations

- [1] NIHR Biomedical Research Centre and British Heart Foundation Centre of Excellence, School of Cardiovascular Medicine and Sciences, King's College London, London, UK.
- [2] Department of Cardiology, Royal Free London NHS Foundation Trust, London, UK.
- [3] Department of Cardiology, Imperial College Healthcare NHS Foundation Trust, London, UK.
- [4] Department of Cardiology, Barts Health NHS Trust, London, UK.
- [5] Department of Cardiology, Royal Brompton and Harefield NHS Foundation Trust, London, UK.
- [6] Department of Cardiology, St George's University Hospitals NHS Foundation Trust, London, UK.
- [7] Department of Cardiology, King's College Hospital NHS Foundation Trust, London, UK.

- PMID: **34967091**
- DOI: 10.1002/ccd.30056

Abstract

Background: The impact of COVID-19 on the diagnosis and management of nonculprit lesions remains unclear.

Objectives: This study sought to evaluate the management and outcomes of patients with nonculprit lesions during the COVID-19 pandemic.

Methods: We conducted a retrospective observational analysis of consecutive primary percutaneous coronary intervention (PPCI) pathway activations across the heart attack center network in London, UK. Data from the study period in 2020 were compared with prepandemic data in 2019. The primary outcome was the rate of nonculprit lesion percutaneous coronary intervention (PCI) and secondary outcomes included major adverse cardiovascular events.

Results: A total of 788 patients undergoing PPCI were identified, 209 (60%) in 2020 cohort and 263 (60%) in 2019 cohort had nonculprit lesions ($p = .89$). There was less functional assessment of the significance of nonculprit lesions in the 2020 cohort compared to 2019 cohort; in 8% 2020 cohort versus 15% 2019 cohort ($p = .01$). There was no difference in rates of PCI for nonculprit disease in the 2019 and 2020 cohorts (31% vs 30%, $p = .11$). Patients in 2020 cohort underwent nonculprit lesion PCI sooner than the 2019 cohort ($p < .001$). At 6 months there was higher rates of unplanned revascularization (4% vs. 2%, $p = .05$) and repeat myocardial infarction (4% vs. 1%, $p = .02$) in the 2019 cohort compared to 2020 cohort.

Conclusion: Changes to clinical practice during the COVID-19 pandemic were associated with reduced rates of unplanned revascularization and myocardial infarction at 6-months follow-up, and despite the pandemic, there was no difference in mortality, suggesting that it is not only safe but maybe more efficacious.

Keywords: COVID-19; FFR; STEMI; bystander; coronary physiology; nonculprit.

© 2021 The Authors. Catheterization and Cardiovascular Interventions published by Wiley Periodicals LLC.

- 15 references

Supplementary info

Publication types, MeSH terms, Grant support Expand

Publication types

- Observational Study
- Research Support, Non-U.S. Gov't

MeSH terms

- COVID-19*
- Humans
- London / epidemiology
- Myocardial Infarction* / etiology
- Pandemics
- Percutaneous Coronary Intervention* / adverse effects
- Retrospective Studies
- SARS-CoV-2
- ST Elevation Myocardial Infarction* / diagnostic imaging
- ST Elevation Myocardial Infarction* / etiology
- ST Elevation Myocardial Infarction* / therapy
- Treatment Outcome

Grant support

- PG/19/9/34228/BHF_/British Heart Foundation/United Kingdom

Full text links

WILEY Full Text Article Wiley

Proceed to details

Cite

Share

☐ 986

Observational Study

Mol Med

. 2021 Oct 18;27(1):129.

doi: 10.1186/s10020-021-00390-4.

CXCL10 levels at hospital admission predict COVID-19 outcome: hierarchical assessment of 53 putative inflammatory biomarkers in an observational study

Nicola I Lorè[#] [1] [2], Rebecca De Lorenzo[#] [3] [4], Paola M V Rancoita[5], Federica Cugnata[5], Alessandra Agresti[6], Francesco Benedetti[4] [7], Marco E Bianchi[4] [6], Chiara Bonini[3] [4], Annalisa Capobianco[3], Caterina Conte[3] [4], Angelo Corti[4] [8], Roberto Furlan[7], Paola Mantegani[3] [9], Norma Maugeri[3] [4], Clara Sciorati[3], Fabio Saliu[3] [9], Laura Silvestri[6], Cristina Tresoldi[10], Bio Angels for COVID-BioB Study Group; Fabio Ciceri[4] [10], Patrizia Rovere-Querini[#] [3] [4], Clelia Di Serio[#] [4] [5] [11], Daniela M Cirillo[#] [3] [9], Angelo A Manfredi[#] [3] [4]
Collaborators, Affiliations Expand

Collaborators

- **Bio Angels for COVID-BioB Study Group**: Nicola Farina, Luigi De Filippo, Marco Battista, Domenico Grosso, Francesca Gorgoni, Carlo Di Biase, Alessio Grazioli Moretti, Lucio Granata, Filippo Bonaldi, Giulia Bettinelli, Elena Delmastro, Damiano Salvato, Giulia Magni, Monica Avino, Paolo Betti, Romina Bucci, Iulia Dumoa, Simona Bossolasco, Federica Morselli

Affiliations

- [1] Division of Immunology, Transplantation and Infectious Diseases, IRCCS San Raffaele Scientific Institute, Via Olgettina 60, 20132, Milano, Italy. lore.nicolaivan@hsr.it.
- [2] Emerging Bacterial Pathogens Unit, IRCCS San Raffaele Scientific Institute, Milano, Italy. lore.nicolaivan@hsr.it.
- [3] Division of Immunology, Transplantation and Infectious Diseases, IRCCS San Raffaele Scientific Institute, Via Olgettina 60, 20132, Milano, Italy.
- [4] Vita-Salute San Raffaele University, Milano, Italy.
- [5] University Centre for Statistics in the Biomedical Sciences (CUSSB), Vita-Salute San Raffaele University, Milan, Italy.
- [6] Division of Genetics and Cell Biology, IRCCS San Raffaele Scientific Institute, Milano, Italy.
- [7] Division of Neuroscience, IRCCS San Raffaele Scientific Institute, Milano, Italy.
- [8] Division of Experimental Oncology, IRCCS San Raffaele Scientific Institute, Milano, Italy.
- [9] Emerging Bacterial Pathogens Unit, IRCCS San Raffaele Scientific Institute, Milano, Italy.
- [10] Hematology and Bone Marrow Transplant, IRCCS San Raffaele Scientific Institute, Milano, Italy.
- [11] Faculty of Biomedical Sciences, Swiss University, Lugano, Switzerland.

[#] Contributed equally.

- PMID: **34663207**
- PMCID: PMC8521494
- DOI: 10.1186/s10020-021-00390-4

Free PMC article
Observational Study

CXCL10 levels at hospital admission predict COVID-19 outcome: hierarchical assessment of 53 putative inflammatory biomarkers in an observational study

Nicola I Lorè et al. Mol Med. 2021.
Free PMC article
Show details
Mol Med
. 2021 Oct 18;27(1):129.
doi: 10.1186/s10020-021-00390-4.

Authors

Nicola I Lorè [#] [1] [2], Rebecca De Lorenzo [#] [3] [4], Paola M V Rancoita [5], Federica Cugnata [5], Alessandra Agresti [6], Francesco Benedetti [4] [7], Marco E Bianchi [4] [6], Chiara Bonini [3] [4], Annalisa Capobianco [3], Caterina Conte [3] [4], Angelo Corti [4] [8], Roberto Furlan [7], Paola Mantegani [3] [9], Norma Maugeri [3] [4], Clara Sciorati [3], Fabio Saliu [3] [9], Laura Silvestri [6], Cristina Tresoldi [10], Bio Angels for COVID-BioB Study Group; Fabio Ciceri [4] [10], Patrizia Rovere-Querini [#] [3] [4], Clelia Di Serio [#] [4] [5] [11], Daniela M Cirillo [#] [3] [9], Angelo A Manfredi [#] [3] [4]

Collaborators

- **Bio Angels for COVID-BioB Study Group**: Nicola Farina, Luigi De Filippo, Marco Battista, Domenico Grosso, Francesca Gorgoni, Carlo Di Biase, Alessio Grazioli Moretti, Lucio Granata, Filippo Bonaldi, Giulia Bettinelli, Elena Delmastro, Damiano Salvato, Giulia Magni, Monica Avino, Paolo Betti, Romina Bucci, Iulia Dumoa, Simona Bossolasco, Federica Morselli

Affiliations

- [1] Division of Immunology, Transplantation and Infectious Diseases, IRCCS San Raffaele Scientific Institute, Via Olgettina 60, 20132, Milano, Italy. lore.nicolaivan@hsr.it.
- [2] Emerging Bacterial Pathogens Unit, IRCCS San Raffaele Scientific Institute, Milano, Italy. lore.nicolaivan@hsr.it.
- [3] Division of Immunology, Transplantation and Infectious Diseases, IRCCS San Raffaele Scientific Institute, Via Olgettina 60, 20132, Milano, Italy.
- [4] Vita-Salute San Raffaele University, Milano, Italy.

- [5] University Centre for Statistics in the Biomedical Sciences (CUSSB), Vita-Salute San Raffaele University, Milan, Italy.
- [6] Division of Genetics and Cell Biology, IRCCS San Raffaele Scientific Institute, Milano, Italy.
- [7] Division of Neuroscience, IRCCS San Raffaele Scientific Institute, Milano, Italy.
- [8] Division of Experimental Oncology, IRCCS San Raffaele Scientific Institute, Milano, Italy.
- [9] Emerging Bacterial Pathogens Unit, IRCCS San Raffaele Scientific Institute, Milano, Italy.
- [10] Hematology and Bone Marrow Transplant, IRCCS San Raffaele Scientific Institute, Milano, Italy.
- [11] Faculty of Biomedical Sciences, Swiss University, Lugano, Switzerland.

[#] Contributed equally.

- PMID: **34663207**
- PMCID: PMC8521494
- DOI: 10.1186/s10020-021-00390-4

Abstract

Background: Host inflammation contributes to determine whether SARS-CoV-2 infection causes mild or life-threatening disease. Tools are needed for early risk assessment.

Methods: We studied in 111 COVID-19 patients prospectively followed at a single reference Hospital fifty-three potential biomarkers including alarmins, cytokines, adipocytokines and growth factors, humoral innate immune and neuroendocrine molecules and regulators of iron metabolism. Biomarkers at hospital admission together with age, degree of hypoxia, neutrophil to lymphocyte ratio (NLR), lactate dehydrogenase (LDH), C-reactive protein (CRP) and creatinine were analysed within a data-driven approach to classify patients with respect to survival and ICU outcomes. Classification and regression tree (CART) models were used to identify prognostic biomarkers.

Results: Among the fifty-three potential biomarkers, the classification tree analysis selected CXCL10 at hospital admission, in combination with NLR and time from onset, as the best predictor of ICU transfer (AUC [95% CI] = 0.8374 [0.6233-0.8435]), while it was selected alone to predict death (AUC [95% CI] = 0.7334 [0.7547-0.9201]). CXCL10 concentration abated in COVID-19 survivors after healing and discharge from the hospital.

Conclusions: CXCL10 results from a data-driven analysis, that accounts for presence of confounding factors, as the most robust predictive biomarker of patient outcome in COVID-19.

Keywords: Biomarkers; COVID-19 severity predictors; CXCL10; Decision tree.

© 2021. The Author(s).

Conflict of interest statement

The authors declare that they have no competing interests.

- 42 references
- 6 figures

Supplementary info

Publication types, MeSH terms, Substances, Grant support Expand

Publication types

- Observational Study
- Research Support, Non-U.S. Gov't

MeSH terms

- Biomarkers / blood
- C-Reactive Protein / metabolism
- COVID-19 / blood
- COVID-19 / diagnosis*
- COVID-19 / immunology
- COVID-19 / mortality
- Chemokine CXCL10 / blood*
- Comorbidity
- Coronary Artery Disease / blood
- Coronary Artery Disease / diagnosis*
- Coronary Artery Disease / immunology
- Coronary Artery Disease / mortality
- Creatine / blood
- Diabetes Mellitus / blood
- Diabetes Mellitus / diagnosis*
- Diabetes Mellitus / immunology
- Diabetes Mellitus / mortality
- Female
- Hospitalization
- Humans
- Hypertension / blood
- Hypertension / diagnosis*
- Hypertension / immunology
- Hypertension / mortality
- Immunity, Humoral
- Immunity, Innate
- Inflammation
- Intensive Care Units
- L-Lactate Dehydrogenase / blood
- Leukocyte Count
- Lymphocytes / immunology

- Lymphocytes / pathology
- Male
- Middle Aged
- Neutrophils / immunology
- Neutrophils / pathology
- Prognosis
- Prospective Studies
- Retrospective Studies
- SARS-CoV-2
- Severity of Illness Index
- Survival Analysis

Substances

- Biomarkers
- CXCL10 protein, human
- Chemokine CXCL10
- C-Reactive Protein
- L-Lactate Dehydrogenase
- Creatine

Grant support

- COVID-2020-12371617/Ministero della Salute

Full text links

Read free full text at BMC BioMed Central Free PMC article
Proceed to details
Cite
Share
☐ 987
Observational Study
Acta Orthop
. 2020 Dec;91(6):639-643.
doi: 10.1080/17453674.2020.1816617. Epub 2020 Sep 8.

Delayed surgery versus nonoperative treatment for hip fractures in post-COVID-19 arena: a retrospective study of 145 patients

Bobin Mi [1], Lang Chen [1], Dake Tong [2], Adriana C Panayi [3], Fang Ji [4], Junfei Guo [5], Zhiyong Ou [5], Yingze Zhang [5], Yuan Xiong [1], Guohui Liu [1]
Affiliations Expand

Affiliations

- [1] Department of Orthopedics, Union Hospital, Tongji Medical College, Huazhong University of Science and Technology, Wuhan, China.
- [2] Department of Orthopedics, Shanghai Ninth People's Hospital, Shanghai Jiaotong University School of Medicine, Shanghai, China.
- [3] Department of Plastic Surgery, Brigham and Women's Hospital, Harvard Medical College, Boston, USA.
- [4] Department of Orthopedics, Changhai Hospital, Shanghai, China.
- [5] Department of Orthopaedic Surgery, Third Hospital of Hebei Medical University, Shijiazhuang, China.

- PMID: **32896189**
- PMCID: PMC8023940
- DOI: 10.1080/17453674.2020.1816617

Free PMC article
Observational Study

Delayed surgery versus nonoperative treatment for hip fractures in post-COVID-19 arena: a retrospective study of 145 patients

Bobin Mi et al. Acta Orthop. 2020 Dec.
Free PMC article
Show details
Acta Orthop
. 2020 Dec;91(6):639-643.
doi: 10.1080/17453674.2020.1816617. Epub 2020 Sep 8.

Authors

Bobin Mi [1], Lang Chen [1], Dake Tong [2], Adriana C Panayi [3], Fang Ji [4], Junfei Guo [5], Zhiyong Ou [5], Yingze Zhang [5], Yuan Xiong [1], Guohui Liu [1]

Affiliations

- [1] Department of Orthopedics, Union Hospital, Tongji Medical College, Huazhong University of Science and Technology, Wuhan, China.
- [2] Department of Orthopedics, Shanghai Ninth People's Hospital, Shanghai Jiaotong University School of Medicine, Shanghai, China.

- [3] Department of Plastic Surgery, Brigham and Women's Hospital, Harvard Medical College, Boston, USA.
- [4] Department of Orthopedics, Changhai Hospital, Shanghai, China.
- [5] Department of Orthopaedic Surgery, Third Hospital of Hebei Medical University, Shijiazhuang, China.

- PMID: **32896189**
- PMCID: PMC8023940
- DOI: 10.1080/17453674.2020.1816617

Abstract

Background and purpose - Following the outbreak of COVID-19 in December 2019, in China, many hip fracture patients were unable to gain timely admission and surgery. We assessed whether delayed surgery improves hip joint function and reduces major complications better than nonoperative therapy. Patients and methods - In this retrospective observational study, we collected data from 24 different hospitals from January 1, 2020, to July 20, 2020. 145 patients with hip fractures aged 65 years or older were eligible. Clinical data was extracted from electronic medical records. The primary outcomes were visual analogue scale (VAS) score and Harris Hip Score. Major complications, including deep venous thrombosis (DVT) and pneumonia within 1 month and 3 months, were collected for further analysis. Results - Of the 145 hip fracture patients 108 (median age 72; 70 females) received delayed surgery and 37 (median age 74; 20 females) received nonoperative therapy. The median time from hip fracture injury to surgery was 33 days (IQR 24-48) in the delayed surgery group. Hypertension, in about half of the patients in both groups, and cerebral infarction, in around a quarter of patients in both groups, were the most common comorbidities. Both VAS score and Harris Hip Score were superior in the delayed surgery group. At the 3-month follow-up, the median VAS score was 1 in the delayed surgery group and 2.5 in the nonoperative group ($p < 0.001$). Also, the percentage of complications was higher in the nonoperative group ($p = 0.004$ for DVT, $p < 0.001$ for pulmonary infection). Interpretation - In hip fracture patients, delayed surgery compared with nonoperative therapy significantly improved hip function and reduced various major complications.

Comment in

- Delayed surgery versus nonoperative treatment for hip fractures in post-COVID-19 situation.
  Wiwanitkit V, Liu G. Wiwanitkit V, et al. Acta Orthop. 2020 Dec;91(6):803. doi: 10.1080/17453674.2020.1831242. Epub 2020 Oct 13. Acta Orthop. 2020. PMID: 33047626
  Free PMC article. No abstract available.

- 12 references
- 2 figures

Supplementary info

Publication types, MeSH terms Expand

Publication types

- Multicenter Study

- Observational Study

MeSH terms

- Aged
- COVID-19 / epidemiology
- COVID-19 / prevention & control
- Cerebral Infarction* / epidemiology
- Cerebral Infarction* / etiology
- Cerebral Infarction* / prevention & control
- China / epidemiology
- Conservative Treatment* / adverse effects
- Conservative Treatment* / methods
- Conservative Treatment* / statistics & numerical data
- Electronic Health Records / statistics & numerical data
- Female
- Fracture Fixation* / adverse effects
- Fracture Fixation* / methods
- Fracture Fixation* / statistics & numerical data
- Hip Fractures* / epidemiology
- Hip Fractures* / therapy
- Humans
- Hypertension* / epidemiology
- Hypertension* / etiology
- Hypertension* / prevention & control
- Male
- Outcome and Process Assessment, Health Care
- Postoperative Complications* / epidemiology
- Postoperative Complications* / prevention & control
- SARS-CoV-2
- Time-to-Treatment / statistics & numerical data*

Full text links

Free PMC article
Proceed to details
Cite
Share
☐ 988
Observational Study
Anatol J Cardiol
. 2021 Mar;25(3):184-190.
doi: 10.14744/AnatolJCardiol.2020.79138.

Effect of triple antimicrobial therapy on electrocardiography parameters in patients with mild-to-moderate coronavirus disease 2019

Burcu Uğurlu Ilgın [1], İrem Müge Akbulut Koyuncu [2], Emrullah Kızıltunç [3]
Affiliations Expand

Affiliations

- [1] Department of Cardiology, Gazi Mustafa Kemal State Hospital; Ankara-Turkey.
- [2] Department of Cardiology, Faculty of Medicine, Ankara University; Ankara-Turkey.
- [3] Department of Cardiology, Faculty of Medicine, Gazi University; Ankara-Turkey.

- PMID: **33690133**
- PMCID: PMC8114734
- DOI: 10.14744/AnatolJCardiol.2020.79138

Free PMC article
Observational Study

Effect of triple antimicrobial therapy on electrocardiography parameters in patients with mild-to-moderate coronavirus disease 2019

Burcu Uğurlu Ilgın et al. Anatol J Cardiol. 2021 Mar.
Free PMC article
Show details
Anatol J Cardiol
. 2021 Mar;25(3):184-190.
doi: 10.14744/AnatolJCardiol.2020.79138.

Authors

Burcu Uğurlu Ilgın [1], İrem Müge Akbulut Koyuncu [2], Emrullah Kızıltunç [3]

Affiliations

- [1] Department of Cardiology, Gazi Mustafa Kemal State Hospital; Ankara-Turkey.
- [2] Department of Cardiology, Faculty of Medicine, Ankara University; Ankara-Turkey.
- [3] Department of Cardiology, Faculty of Medicine, Gazi University; Ankara-Turkey.

- PMID: **33690133**
- PMCID: PMC8114734
- DOI: 10.14744/AnatolJCardiol.2020.79138

Abstract

Objective: The effects of treatment of coronavirus disease 2019 (COVID-19) with a triple combination composed of hydroxychloroquine, an an-tiviral, and an antibiotic on electrocardiography (ECG) parameters in patients with mild-to-moderate symptoms are not wholly understood. We aimed to explore the changes in ECG parameters after treatment with triple combination therapy in patients with mild-to-moderate symptomatic COVID-19.

Methods: This retrospective, single-center case series analyzed 91 patients with mild-to-moderate symptomatic COVID-19 at Ankara Gazi Mus-tafa Kemal State Hospital of Ankara City, Turkey, from April 1, 2020, to April 30, 2020. Forty-three patients were treated with hydroxychloroquine+oseltamivir+azithromycin (Group 1) and 48 patients were treated with hydroxychloroquine+oseltamivir+levofloxacin (Group 2). Heart rate, P wave duration, P wave dispersion, PR interval, QRS duration, corrected QT interval (QTc), QTc dispersion (QTD), delta QTc, Tp-e, Tp-e dispersion, and Tp-e/QTc ratio were all calculated from the baseline and posttreatment 12-lead ECG recordings.

Results: The QTc, QRS duration, Tp-e, PR interval, and P wave duration were significantly increased after treatment ($p<0.001$; $p<0.001$; $p<0.001$; $p=0.001$; $p=0.001$). The posttreatment C-reactive protein level was significantly lower than at baseline in Group 1 ($p=0.014$). At admission, 30% of patients had QT prolongation, and 4.3% of them had a QT duration >500 ms. Both Group 1 and Group 2 showed significant prolongation of the QTc interval (Group 1; $p<0.001$ vs. Group 2; $p<0.001$), QRS duration (Group 1; $p=0.006$ vs. Group 2; $p=0.014$), Tp-e (Group 1; $p=0.036$ vs. Group 2; $p<0.001$), and PR interval (Group 1; $p=0.002$ vs. Group2; $p=0.05$). The QTD was significantly decreased in Group 1 ($p<0.001$). None of the patients experienced any overt ventricular arrhythmia.

Conclusion: To the best of our knowledge, this study is the first to investigate QT prolongation in a population of COVID-19 patients treated with triple combination therapy. We found that there was a significant decrease in the QTD after the treatment in patients who were taking triple therapy including azithromycin.

Conflict of interest statement

Conflict of interest: None declared.

- 1 figure

Supplementary info

Publication types, MeSH terms, Substances Expand

Publication types

- Observational Study

MeSH terms

- Adolescent
- Adult
- Aged
- Anti-Infective Agents / administration & dosage
- Anti-Infective Agents / adverse effects
- Antiviral Agents / administration & dosage
- Antiviral Agents / adverse effects
- COVID-19 / drug therapy*
- COVID-19 / pathology
- Drug Therapy, Combination
- Electrocardiography
- Female
- Humans
- Hydroxychloroquine / administration & dosage
- Hydroxychloroquine / adverse effects
- Levofloxacin / administration & dosage
- Levofloxacin / adverse effects
- Long QT Syndrome / chemically induced*
- Long QT Syndrome / physiopathology
- Male
- Middle Aged
- Oseltamivir / administration & dosage
- Oseltamivir / adverse effects
- Retrospective Studies
- SARS-CoV-2*
- Severity of Illness Index
- Treatment Outcome
- Young Adult

Substances

- Anti-Infective Agents
- Antiviral Agents
- Oseltamivir
- Hydroxychloroquine
- Levofloxacin

Full text links

Free FULLTEXT at www.anatoljcardiol.com Kare Publishing Free PMC article

Proceed to details
Cite
Share
☐ 989
Observational Study
Ann Emerg Med
. 2020 Oct;76(4):442-453.
doi: 10.1016/j.annemergmed.2020.07.022. Epub 2020 Jul 21.

Development and Validation of the Quick COVID-19 Severity Index: A Prognostic Tool for Early Clinical Decompensation

Adrian D Haimovich [1], Neal G Ravindra [2], Stoytcho Stoytchev [1], H Patrick Young [3], Francis P Wilson [4], David van Dijk [2], Wade L Schulz [5], R Andrew Taylor [6]
Affiliations Expand

Affiliations

- [1] Department of Emergency Medicine, Yale University School of Medicine, New Haven, CT.
- [2] Department of Internal Medicine, Section of Cardiovascular Medicine, Yale University School of Medicine, New Haven, CT; Department of Computer Science, Yale University, New Haven, CT.
- [3] Department of Internal Medicine, Yale University School of Medicine, New Haven, CT; Center for Outcomes Research and Evaluation, Yale New Haven Hospital, New Haven, CT.
- [4] Department of Internal Medicine, Yale University School of Medicine, New Haven, CT; Clinical and Translational Research Accelerator, Department of Medicine, Yale University School of Medicine, New Haven, CT.
- [5] Center for Medical Informatics, Yale University School of Medicine, New Haven, CT; Department of Laboratory Medicine, Yale University School of Medicine, New Haven, CT; Center for Outcomes Research and Evaluation, Yale New Haven Hospital, New Haven, CT.
- [6] Department of Emergency Medicine, Yale University School of Medicine, New Haven, CT; Center for Medical Informatics, Yale University School of Medicine, New Haven, CT. Electronic address: richard.taylor@yale.edu.

- PMID: **33012378**
- PMCID: PMC7373004
- DOI: 10.1016/j.annemergmed.2020.07.022

Free PMC article
Observational Study

Development and Validation of the Quick COVID-19 Severity Index: A Prognostic Tool for Early Clinical Decompensation

Adrian D Haimovich et al. Ann Emerg Med. 2020 Oct.
Free PMC article
Show details
Ann Emerg Med
. 2020 Oct;76(4):442-453.
doi: 10.1016/j.annemergmed.2020.07.022. Epub 2020 Jul 21.

Authors

Adrian D Haimovich [1], Neal G Ravindra [2], Stoytcho Stoytchev [1], H Patrick Young [3], Francis P Wilson [4], David van Dijk [2], Wade L Schulz [5], R Andrew Taylor [6]

Affiliations

- [1] Department of Emergency Medicine, Yale University School of Medicine, New Haven, CT.
- [2] Department of Internal Medicine, Section of Cardiovascular Medicine, Yale University School of Medicine, New Haven, CT; Department of Computer Science, Yale University, New Haven, CT.
- [3] Department of Internal Medicine, Yale University School of Medicine, New Haven, CT; Center for Outcomes Research and Evaluation, Yale New Haven Hospital, New Haven, CT.
- [4] Department of Internal Medicine, Yale University School of Medicine, New Haven, CT; Clinical and Translational Research Accelerator, Department of Medicine, Yale University School of Medicine, New Haven, CT.
- [5] Center for Medical Informatics, Yale University School of Medicine, New Haven, CT; Department of Laboratory Medicine, Yale University School of Medicine, New Haven, CT; Center for Outcomes Research and Evaluation, Yale New Haven Hospital, New Haven, CT.
- [6] Department of Emergency Medicine, Yale University School of Medicine, New Haven, CT; Center for Medical Informatics, Yale University School of Medicine, New Haven, CT. Electronic address: richard.taylor@yale.edu.

- PMID: **33012378**
- PMCID: PMC7373004
- DOI: 10.1016/j.annemergmed.2020.07.022

Abstract

Study objective: The goal of this study is to create a predictive, interpretable model of early hospital respiratory failure among emergency department (ED) patients admitted with coronavirus disease 2019 (COVID-19).

Methods: This was an observational, retrospective, cohort study from a 9-ED health system of admitted adult patients with severe acute respiratory syndrome coronavirus 2 (COVID-19) and an

oxygen requirement less than or equal to 6 L/min. We sought to predict respiratory failure within 24 hours of admission as defined by oxygen requirement of greater than 10 L/min by low-flow device, high-flow device, noninvasive or invasive ventilation, or death. Predictive models were compared with the Elixhauser Comorbidity Index, quick Sequential [Sepsis-related] Organ Failure Assessment, and the CURB-65 pneumonia severity score.

Results: During the study period, from March 1 to April 27, 2020, 1,792 patients were admitted with COVID-19, 620 (35%) of whom had respiratory failure in the ED. Of the remaining 1,172 admitted patients, 144 (12.3%) met the composite endpoint within the first 24 hours of hospitalization. On the independent test cohort, both a novel bedside scoring system, the quick COVID-19 Severity Index (area under receiver operating characteristic curve mean 0.81 [95% confidence interval {CI} 0.73 to 0.89]), and a machine-learning model, the COVID-19 Severity Index (mean 0.76 [95% CI 0.65 to 0.86]), outperformed the Elixhauser mortality index (mean 0.61 [95% CI 0.51 to 0.70]), CURB-65 (0.50 [95% CI 0.40 to 0.60]), and quick Sequential [Sepsis-related] Organ Failure Assessment (0.59 [95% CI 0.50 to 0.68]). A low quick COVID-19 Severity Index score was associated with a less than 5% risk of respiratory decompensation in the validation cohort.

Conclusion: A significant proportion of admitted COVID-19 patients progress to respiratory failure within 24 hours of admission. These events are accurately predicted with bedside respiratory examination findings within a simple scoring system.

Copyright © 2020 American College of Emergency Physicians. Published by Elsevier Inc. All rights reserved.

Comment in

- In adults hospitalized with COVID-19, the quick COVID-19 Severity Index predicted 24-h respiratory decompensation.
  Dunne C, Lang E. Dunne C, et al. Ann Intern Med. 2021 Feb;174(2):JC23. doi: 10.7326/ACPJ202102160-023. Epub 2021 Feb 2. Ann Intern Med. 2021. PMID: 33524288

- 46 references
- 4 figures

Supplementary info

Publication types, MeSH terms, Grant support Expand

Publication types

- Observational Study
- Research Support, N.I.H., Extramural
- Research Support, Non-U.S. Gov't
- Validation Study

MeSH terms

- Adolescent
- Adult

- Aged
- Betacoronavirus
- COVID-19
- COVID-19 Testing
- Clinical Laboratory Techniques
- Coronavirus Infections / complications*
- Coronavirus Infections / diagnosis*
- Coronavirus Infections / therapy
- Emergency Service, Hospital*
- Female
- Humans
- Male
- Middle Aged
- Oxygen Inhalation Therapy
- Pandemics
- Pneumonia, Viral / complications*
- Pneumonia, Viral / diagnosis*
- Pneumonia, Viral / therapy
- Respiratory Insufficiency / therapy
- Respiratory Insufficiency / virology*
- Retrospective Studies
- Risk Assessment / methods
- SARS-CoV-2
- Severity of Illness Index*
- Young Adult

Grant support

- P30 DK079310/DK/NIDDK NIH HHS/United States
- R01 DK113191/DK/NIDDK NIH HHS/United States
- UL1 TR001863/TR/NCATS NIH HHS/United States

Full text links

ELSEVIER FULL-TEXT ARTICLE Elsevier Science Free PMC article
Proceed to details
Cite
Share
☐ 990
Reumatismo
. 2021 Jan 18;72(4):189-196.
doi: 10.4081/reumatismo.2020.1333.

Vitamin D and disease severity in coronavirus disease 19 (COVID-19)

G Adami [1], A Giollo [2], A Fassio [3], C Benini [4], E Bertoldo [5], F Bertoldo [6], G Orsolini [7], L Idolazzi [8], O Viapiana [9], S Giannini [10], G Passeri [11], E Tacconelli [12], C Micheletto [13], D Gatti [14], M Rossini [15]

Affiliations Expand

Affiliations

- [1] Rheumatology Unit, University of Verona. adami.g@yahoo.com.
- [2] Rheumatology Unit, University of Verona. alessandrogiollo@gmail.com.
- [3] Rheumatology Unit, University of Verona. angelo.fassio@yahoo.it.
- [4] Rheumatology Unit, University of Verona. camilla.benini@yahoo.it.
- [5] Rheumatology Unit, University of Verona. eugenia.bertoldo@gmail.com.
- [6] Internal Medicine Unit, University of Verona. francesco.bertoldo@univr.it.
- [7] Rheumatology Unit, University of Verona. giovanniorsolini@gmail.com.
- [8] Rheumatology Unit, University of Verona. luca.idolazzi@univr.it.
- [9] Rheumatology Unit, University of Verona. ombretta.viapiana@univr.it.
- [10] Internal Medicine Unit, University of Padua. sandro.giannini@unipd.it.
- [11] Internal Medicine Unit, University of Parma. giovanni.passeri@unipr.it.
- [12] Infectious Diseases Unit, University of Verona. evelina.tacconelli@univr.it.
- [13] Pulmonary Unit, University of Verona. claudio.micheletto@univr.it.
- [14] Rheumatology Unit, University of Verona. davide.gatti@univr.it.
- [15] Rheumatology Unit, University of Verona. maurizio.rossini@univr.it.

- PMID: **33677945**
- DOI: 10.4081/reumatismo.2020.1333

Free article

Vitamin D and disease severity in coronavirus disease 19 (COVID-19)

G Adami et al. Reumatismo. 2021.
Free article
Show details
Reumatismo
. 2021 Jan 18;72(4):189-196.
doi: 10.4081/reumatismo.2020.1333.

Authors

G Adami [1], A Giollo [2], A Fassio [3], C Benini [4], E Bertoldo [5], F Bertoldo [6], G Orsolini [7], L Idolazzi [8], O Viapiana [9], S Giannini [10], G Passeri [11], E Tacconelli [12], C Micheletto [13], D Gatti [14], M Rossini [15]

Affiliations

- [1] Rheumatology Unit, University of Verona. adami.g@yahoo.com.
- [2] Rheumatology Unit, University of Verona. alessandrogiollo@gmail.com.
- [3] Rheumatology Unit, University of Verona. angelo.fassio@yahoo.it.
- [4] Rheumatology Unit, University of Verona. camilla.benini@yahoo.it.
- [5] Rheumatology Unit, University of Verona. eugenia.bertoldo@gmail.com.
- [6] Internal Medicine Unit, University of Verona. francesco.bertoldo@univr.it.
- [7] Rheumatology Unit, University of Verona. giovanniorsolini@gmail.com.
- [8] Rheumatology Unit, University of Verona. luca.idolazzi@univr.it.
- [9] Rheumatology Unit, University of Verona. ombretta.viapiana@univr.it.
- [10] Internal Medicine Unit, University of Padua. sandro.giannini@unipd.it.
- [11] Internal Medicine Unit, University of Parma. giovanni.passeri@unipr.it.
- [12] Infectious Diseases Unit, University of Verona. evelina.tacconelli@univr.it.
- [13] Pulmonary Unit, University of Verona. claudio.micheletto@univr.it.
- [14] Rheumatology Unit, University of Verona. davide.gatti@univr.it.
- [15] Rheumatology Unit, University of Verona. maurizio.rossini@univr.it.

- PMID: **33677945**
- DOI: 10.4081/reumatismo.2020.1333

Abstract

The role of 25-OH-vitamin D in the assessment of coronavirus disease 19 (COVID-19) has not been investigated. We sought to investigate the prevalence of 25-OH-vitamin D deficiency among COVID-19 patients, and to determine the associations between 25-OH-vitamin D status and the severity of the disease. We have conducted a retrospective observational study of COVID-19 patients admitted to the University of Verona Hospital Trust. Demographic, clinical and biochemical parameters were collected at hospital admission, and serum 25-OH-vitamin D levels were measured. The following outcomes were assessed: arterial partial oxygen pressure (PaO2); C-reactive protein (CRP); length of hospitalization; requirement of oxygen therapy; non-invasive ventilation (NIV); mechanical ventilation; and death. Among 61 patients enrolled, 72.1% was 25-OH-vitamin D deficient (<20 ng/mL) and 57.4% had 25-OHvitamin D <15 ng/mL. Patients with arterial PaO2 <60 mmHg had significantly lower mean 25-OH-vitamin D levels compared to patients with PaO2 ≥60 mmHg (13.3 ng/mL vs 20.4 ng/mL respectively, p=0.03). Vitamin D deficiency was associated with 3-fold higher risk of having arterial pO2 <60 mmHg. 25-OH-vitamin D deficiency was associated with increased CRP and dyspnea. 25-OH-vitamin D deficiency was associated with more severe systemic inflammatory response and respiratory failure in COVID-19 patients.

Supplementary info

MeSH terms, Substances Expand

MeSH terms

- Adult
- Aged
- Aged, 80 and over
- C-Reactive Protein / analysis
- COVID-19 / blood*
- COVID-19 / epidemiology
- Comorbidity
- Disease Susceptibility
- Dyspnea / etiology
- Female
- Fibrinogen / analysis
- Humans
- Italy / epidemiology
- Length of Stay / statistics & numerical data
- Male
- Middle Aged
- Oxygen / blood
- Partial Pressure
- Prevalence
- Respiration, Artificial / statistics & numerical data
- Retrospective Studies
- Severity of Illness Index
- Vitamin D / blood*
- Vitamin D Deficiency / blood
- Vitamin D Deficiency / epidemiology

Substances

- Vitamin D
- Fibrinogen
- C-Reactive Protein
- Oxygen

Full text links

REUMATISMO Pagepress Publications
Proceed to details
Cite
Share
☐ 991

Observational Study

Crit Care Med
. 2020 Aug;48(8):e657-e665.
doi: 10.1097/CCM.0000000000004411.

Acute Physiology and Chronic Health Evaluation II Score as a Predictor of Hospital Mortality in Patients of Coronavirus Disease 2019

Xiaojing Zou [1], Shusheng Li [1], Minghao Fang [1], Ming Hu [2], Yi Bian [1], Jianmin Ling [1], Shanshan Yu [1], Liang Jing [1], Donghui Li [1], Jiao Huang [3]
Affiliations Expand

Affiliations

- [1] Department of Emergency, Tongji Hospital, Tongji Medical College, Huazhong University of Science and Technology, Wuhan, China.
- [2] Department of Intensive Care Unit, Wuhan Tuberculosis Control Institute, Wuhan, China.
- [3] Department of Epidemiology and Biostatistics, State Key Laboratory of Environmental Health (Incubating), School of Public Health, Tongji Medical College, Huazhong University of Science and Technology, Wuhan, China.

- PMID: **32697506**
- PMCID: PMC7217128
- DOI: 10.1097/CCM.0000000000004411

Free PMC article
Observational Study

Acute Physiology and Chronic Health Evaluation II Score as a Predictor of Hospital Mortality in Patients of Coronavirus Disease 2019

Xiaojing Zou et al. Crit Care Med. 2020 Aug.
Free PMC article
Show details

Crit Care Med
. 2020 Aug;48(8):e657-e665.
doi: 10.1097/CCM.0000000000004411.

Authors

Xiaojing Zou [1], Shusheng Li [1], Minghao Fang [1], Ming Hu [2], Yi Bian [1], Jianmin Ling [1], Shanshan Yu [1], Liang Jing [1], Donghui Li [1], Jiao Huang [3]

Affiliations

- [1] Department of Emergency, Tongji Hospital, Tongji Medical College, Huazhong University of Science and Technology, Wuhan, China.
- [2] Department of Intensive Care Unit, Wuhan Tuberculosis Control Institute, Wuhan, China.
- [3] Department of Epidemiology and Biostatistics, State Key Laboratory of Environmental Health (Incubating), School of Public Health, Tongji Medical College, Huazhong University of Science and Technology, Wuhan, China.

- PMID: **32697506**
- PMCID: PMC7217128
- DOI: 10.1097/CCM.0000000000004411

Abstract

Objectives: Coronavirus disease 2019 has emerged as a major global health threat with a great number of deaths in China. We aimed to assess the association between Acute Physiology and Chronic Health Evaluation II score and hospital mortality in patients with coronavirus disease 2019, and to compare the predictive ability of Acute Physiology and Chronic Health Evaluation II score, with Sequential Organ Failure Assessment score and Confusion, Urea, Respiratory rate, Blood pressure, Age 65 (CURB65) score.

Design: Retrospective observational cohort.

Setting: Tongji Hospital in Wuhan, China.

Subjects: Confirmed patients with coronavirus disease 2019 hospitalized in the ICU of Tongji hospital from January 10, 2020, to February 10, 2020.

Interventions: None.

Measurements and main results: Of 178 potentially eligible patients with symptoms of coronavirus disease 2019, 23 patients (12.92%) were diagnosed as suspected cases, and one patient (0.56%) suffered from cardiac arrest immediately after admission. Ultimately, 154 patients were enrolled in the analysis and 52 patients (33.77%) died. Mean Acute Physiology and Chronic Health Evaluation II score (23.23 ± 6.05) was much higher in deaths compared with the mean Acute Physiology and Chronic Health Evaluation II score of 10.87 ± 4.40 in survivors ($p < 0.001$). Acute Physiology and Chronic Health Evaluation II score was independently associated with hospital mortality (adjusted hazard ratio, 1.07; 95% CI, 1.01-1.13). In predicting hospital mortality, Acute Physiology and Chronic Health Evaluation II score demonstrated better discriminative ability (area under the curve, 0.966; 95% CI, 0.942-0.990) than Sequential Organ Failure Assessment score (area under the curve, 0.867; 95% CI, 0.808-0.926) and CURB65 score (area under the curve, 0.844; 95% CI, 0.784-0.905). Based on the cut-off value of 17, Acute Physiology and Chronic Health Evaluation II score could predict the death of patients with coronavirus disease 2019 with a sensitivity of 96.15% and a specificity of 86.27%. Kaplan-Meier analysis showed that the survivor probability of patients with coronavirus disease 2019 with Acute Physiology and Chronic Health Evaluation II score less than 17 was notably higher than that of

patients with Acute Physiology and Chronic Health Evaluation II score greater than or equal to 17 ($p < 0.001$).

Conclusions: Acute Physiology and Chronic Health Evaluation II score was an effective clinical tool to predict hospital mortality in patients with coronavirus disease 2019 compared with Sequential Organ Failure Assessment score and CURB65 score. Acute Physiology and Chronic Health Evaluation II score greater than or equal to 17 serves as an early warning indicator of death and may provide guidance to make further clinical decisions.

Conflict of interest statement

The authors have disclosed that they do not have any potential conflicts of interest.

Comment in

- Analysis of Critical Care Severity of Illness Scoring Systems in Patients With Coronavirus Disease 2019: A Retrospective Analysis of Three U.K. ICUs.
  Stephens JR, Stümpfle R, Patel P, Brett S, Broomhead R, Baharlo B, Soni S. Stephens JR, et al. Crit Care Med. 2021 Jan 1;49(1):e105-e107. doi: 10.1097/CCM.0000000000004674. Crit Care Med. 2021. PMID: 32991357 Free PMC article. No abstract available.
- The authors reply.
  Zou X, Huang J. Zou X, et al. Crit Care Med. 2021 Jan 1;49(1):e108. doi: 10.1097/CCM.0000000000004722. Crit Care Med. 2021. PMID: 33337751 No abstract available.

- 28 references
- 2 figures

Supplementary info

Publication types, MeSH terms Expand

Publication types

- Observational Study

MeSH terms

- APACHE
- Adult
- Aged
- COVID-19
- Causality
- Cause of Death*
- China / epidemiology
- Coronavirus Infections / diagnosis*
- Coronavirus Infections / epidemiology*
- Coronavirus Infections / therapy

- Female
- Hospital Mortality*
- Hospitalization / statistics & numerical data
- Hospitals, Urban
- Humans
- Intensive Care Units / statistics & numerical data
- Logistic Models
- Male
- Middle Aged
- Organ Dysfunction Scores
- Pandemics
- Pneumonia, Viral / diagnosis*
- Pneumonia, Viral / epidemiology*
- Pneumonia, Viral / therapy
- Predictive Value of Tests
- Proportional Hazards Models
- ROC Curve
- Retrospective Studies
- Severe Acute Respiratory Syndrome / diagnosis
- Severe Acute Respiratory Syndrome / mortality*
- Severe Acute Respiratory Syndrome / therapy
- Survivors / statistics & numerical data

Full text links

Wolters Kluwer Wolters Kluwer Free PMC article
Proceed to details
Cite
Share
☐ 992
Observational Study
J Am Geriatr Soc
. 2021 Jan;69(1):37-43.
doi: 10.1111/jgs.16956. Epub 2020 Nov 30.

Predicting In-Hospital Mortality in COVID-19 Older Patients with Specifically Developed Scores

Marcello Covino [1], Giuseppe De Matteis [2], Maria Livia Burzo [2], Andrea Russo [3], Evelina Forte [1], Annamaria Carnicelli [1], Andrea Piccioni [1], Benedetta Simeoni [1], Antonio Gasbarrini [4] [5], Francesco Franceschi [1] [5], Claudio Sandroni [6] [5], GEMELLI AGAINST COVID-19 Group
Affiliations Expand

Affiliations

- [1] Emergency Department, Fondazione Policlinico Universitario A. Gemelli IRCCS, Rome, Italy.
- [2] Department of Internal Medicine, Fondazione Policlinico Universitario A. Gemelli IRCCS, Rome, Italy.
- [3] Geriatrics Department, Fondazione Policlinico Universitario A. Gemelli IRCCS, Rome, Italy.
- [4] Department of Internal Medicine and Gastroenterology, Fondazione Policlinico Universitario A. Gemelli IRCCS, Rome, Italy.
- [5] Università Cattolica del Sacro Cuore, Rome, Italy.
- [6] Department of Anesthesiology and Intensive Care Medicine, Fondazione Policlinico Universitario A. Gemelli IRCCS, Rome, Italy.

- PMID: **33197278**
- PMCID: PMC7753731
- DOI: 10.1111/jgs.16956

Free PMC article
Observational Study

Predicting In-Hospital Mortality in COVID-19 Older Patients with Specifically Developed Scores

Marcello Covino et al. J Am Geriatr Soc. 2021 Jan.
Free PMC article
Show details
J Am Geriatr Soc
. 2021 Jan;69(1):37-43.
doi: 10.1111/jgs.16956. Epub 2020 Nov 30.

Authors

Marcello Covino [1], Giuseppe De Matteis [2], Maria Livia Burzo [2], Andrea Russo [3], Evelina Forte [1], Annamaria Carnicelli [1], Andrea Piccioni [1], Benedetta Simeoni [1], Antonio Gasbarrini [4] [5], Francesco Franceschi [1] [5], Claudio Sandroni [6] [5], GEMELLI AGAINST COVID-19 Group

Affiliations

- [1] Emergency Department, Fondazione Policlinico Universitario A. Gemelli IRCCS, Rome, Italy.
- [2] Department of Internal Medicine, Fondazione Policlinico Universitario A. Gemelli IRCCS, Rome, Italy.
- [3] Geriatrics Department, Fondazione Policlinico Universitario A. Gemelli IRCCS, Rome, Italy.
- [4] Department of Internal Medicine and Gastroenterology, Fondazione Policlinico Universitario A. Gemelli IRCCS, Rome, Italy.
- [5] Università Cattolica del Sacro Cuore, Rome, Italy.
- [6] Department of Anesthesiology and Intensive Care Medicine, Fondazione Policlinico Universitario A. Gemelli IRCCS, Rome, Italy.

- PMID: **33197278**
- PMCID: PMC7753731
- DOI: 10.1111/jgs.16956

Abstract

Background/objectives: Several scoring systems have been specifically developed for risk stratification in COVID-19 patients.

Design: We compared, in a cohort of confirmed COVID-19 older patients, three specifically developed scores with a previously established early warning score. Main endpoint was all causes in-hospital death.

Setting: This is a single-center, retrospective observational study, conducted in the Emergency Department (ED) of an urban teaching hospital, referral center for COVID-19.

Participants: We reviewed the clinical records of the confirmed COVID-19 patients aged 60 years or more consecutively admitted to our ED over a 6-week period (March 1st to April 15th, 2020). A total of 210 patients, aged between 60 and 98 years were included in the study cohort.

Measurements: International Severe Acute Respiratory Infection Consortium Clinical Characterization Protocol-Coronavirus Clinical Characterization Consortium (ISARIC-4C) score, COVID-GRAM Critical Illness Risk Score (COVID-GRAM), quick COVID-19 Severity Index (qCSI), National Early Warning Score (NEWS).

Results: Median age was 74 (67-82) and 133 (63.3%) were males. Globally, 42 patients (20.0%) deceased. All the score evaluated showed a fairly good predictive value with respect to in-hospital death. The ISARIC-4C score had the highest area under ROC curve (AUROC) 0.799 (0.738-0.851), followed by the COVID-GRAM 0.785 (0.723-0.838), NEWS 0.764 (0.700-0.819), and qCSI 0.749 (0.685-0.806). However, these differences were not statistical significant.

Conclusion: Among the evaluated scores, the ISARIC-4C and the COVID-GRAM, calculated at ED admission, had the best performance, although the qCSI had similar efficacy by evaluating only three items. However, the NEWS, already widely validated in clinical practice, had a similar performance and could be appropriate for older patients with COVID-19.

Keywords: COVID-19; COVID-GRAM; ISARIC-4C; NEWS; qCSI.

© 2020 The American Geriatrics Society.

Conflict of interest statement

All authors declared no conflict of interests for this paper.

Comment in

- Comment on: Predicting In-Hospital Mortality in COVID-19 Older Patients with Specifically Developed Scores.
  Odille G, Girard N, Sanchez S, Lelarge S, Mignot A, Putot S, Larosa F, Vovelle J, Nuss V, Da Silva S, Barben J, Manckoundia P, Putot A. Odille G, et al. J Am Geriatr Soc. 2021 Apr;69(4):884-886. doi: 10.1111/jgs.17030. Epub 2021 Jan 22. J Am Geriatr Soc. 2021. PMID: 33426643 Free PMC article.

- 27 references
- 1 figure

Supplementary info

Publication types, MeSH terms Expand

Publication types

- Observational Study

MeSH terms

- Aged
- COVID-19 / mortality*
- COVID-19 / therapy
- Cohort Studies
- Critical Illness / mortality*
- Critical Illness / therapy
- Emergency Service, Hospital
- Female
- Hospital Mortality*
- Humans
- Italy
- Male
- Middle Aged
- Retrospective Studies
- Severity of Illness Index*

Full text links

WILEY Full Text Article Wiley Free PMC article

Proceed to details

Cite

Share

☐ 993

Observational Study

Bone Joint J

. 2021 Apr;103-B(4):681-688.

doi: 10.1302/0301-620X.103B.BJJ-2020-1776.R1. Epub 2021 Feb 16.

The rate of COVID-19 and associated mortality after elective hip and knee arthroplasty prior to cessation of elective services in UK

Nicholas D Clement [1] [2], Andrew J Hall [1], Nardeen Kader [2], IMPACT Restart Collaboration; Benjamin Ollivere [3], Sam Oussedik [4], Deiary F Kader [2], David J Deehan [5], Andrew D Duckworth [1] [6]

Affiliations Expand

Affiliations

- [1] Department of Orthopaedics, Royal Infirmary of Edinburgh, Edinburgh, UK.
- [2] South West London Elective Orthopaedic Centre, Epsom, UK.
- [3] Division of Rheumatology, Orthopaedics and Dermatology, Nottingham University, Nottingham, UK.
- [4] Department of Orthopaedics, University College London Hospitals, London, UK.
- [5] Department of Orthopaedics, Freeman Hospital, Newcastle, UK.
- [6] Usher Institute, University of Edinburgh, Edinburgh, UK.

- PMID: **33591211**
- DOI: 10.1302/0301-620X.103B.BJJ-2020-1776.R1

Free article

Observational Study

The rate of COVID-19 and associated mortality after elective hip and knee arthroplasty prior to cessation of elective services in UK

Nicholas D Clement et al. Bone Joint J. 2021 Apr.

Free article

Show details

Bone Joint J
. 2021 Apr;103-B(4):681-688.
doi: 10.1302/0301-620X.103B.BJJ-2020-1776.R1. Epub 2021 Feb 16.

Authors

Nicholas D Clement [1] [2], Andrew J Hall [1], Nardeen Kader [2], IMPACT Restart Collaboration; Benjamin Ollivere [3], Sam Oussedik [4], Deiary F Kader [2], David J Deehan [5], Andrew D Duckworth [1] [6]

Affiliations

- [1] Department of Orthopaedics, Royal Infirmary of Edinburgh, Edinburgh, UK.
- [2] South West London Elective Orthopaedic Centre, Epsom, UK.
- [3] Division of Rheumatology, Orthopaedics and Dermatology, Nottingham University, Nottingham, UK.
- [4] Department of Orthopaedics, University College London Hospitals, London, UK.
- [5] Department of Orthopaedics, Freeman Hospital, Newcastle, UK.
- [6] Usher Institute, University of Edinburgh, Edinburgh, UK.

- PMID: **33591211**
- DOI: 10.1302/0301-620X.103B.BJJ-2020-1776.R1

Abstract

Aims: The primary aim was to assess the rate of postoperative COVID-19 following hip and knee arthroplasty performed in March 2020 in the UK. The secondary aims were to assess whether there were clinical factors associated with COVID-19 status, the mortality rate of patients with COVID-19, and the rate of potential COVID-19 in patients not presenting to healthcare services.

Methods: A multicentre retrospective study was conducted of patients undergoing hip or knee arthroplasty during the first wave of the COVID-19 pandemic (1 March 2020 to 31 March 2020) with a minimum of 60 days follow-up. Patient demographics, American Society of Anesthesiologists grade, procedure type, primary or revision, length of stay (LOS), COVID-19 test status, and postoperative mortality were recorded. A subgroup of patients (n = 211) who had not presented to healthcare services after discharge were contacted and questioned as to whether they had symptoms of COVID-19.

Results: Five (0.5%) of 1,073 patients who underwent hip or knee arthroplasty tested positive for SARS-CoV-2 postoperatively. When adjusting for confounding factors, increasing LOS (p = 0.022) was the only significant factor associated with developing COVID-19 following surgery and a stay greater than three days was a reliable predictor with an area under the curve of 81% (p = 0.018). There were three (0.3%) deaths in the study cohort and the overall mortality rate attributable to COVID-19 was 0.09% (n = 1/1,073), with one (20%) of the five patients with COVID-19 dying postoperatively. Of the 211 patients contacted, two had symptoms within two to 14 days postoperatively with a positive predictive value of 31% and it was therefore estimated that one patient may have had undiagnosed COVID-19.

Conclusion: The rate of postoperative COVID-19 was 0.5% and may have been as high as 1% when accounting for those patients not presenting to healthcare services, which was similar to the estimated population prevalence during the study period. The overall mortality rate secondary to COVID-19 was low (0.09%), however the mortality rate for those patients developing COVID-19 was 20%. Cite this article: *Bone Joint J* 2021;103-B(4):681-688.

Keywords: Arthroplasty; COVID-19; Elective; Hip; Knee; Mortality.

Supplementary info

Publication types, MeSH terms Expand

Publication types

- Multicenter Study
- Observational Study

MeSH terms

- Adult
- Aged
- Aged, 80 and over
- Arthroplasty, Replacement, Hip*
- Arthroplasty, Replacement, Knee*
- COVID-19 / diagnosis
- COVID-19 / epidemiology*
- COVID-19 / etiology
- COVID-19 / prevention & control
- COVID-19 Testing
- Clinical Audit
- Elective Surgical Procedures*
- Female
- Follow-Up Studies
- Health Services Accessibility
- Humans
- Male
- Middle Aged
- Postoperative Complications / diagnosis
- Postoperative Complications / epidemiology*
- Postoperative Complications / etiology
- Retrospective Studies
- Risk Factors
- Sensitivity and Specificity
- Survival Analysis

- United Kingdom / epidemiology

Full text links

The Bone & Joint Journal Full Text Available Atypon THE UNIVERSITY of EDINBURGH Edinburgh Research Explorer, University of Edinburgh
Proceed to details
Cite
Share
994
Observational Study
Rev Esp Cardiol (Engl Ed)
. 2020 Dec;73(12):985-993.
doi: 10.1016/j.rec.2020.07.009. Epub 2020 Jul 31.

Risk factors for in-hospital mortality in patients with acute myocardial infarction during the COVID-19 outbreak

[Article in English, Spanish]
Jorge Solano-López [1], José Luis Zamorano [2], Ana Pardo Sanz [1], Ignacio Amat-Santos [3], Fernando Sarnago [4], Enrique Gutiérrez Ibañes [5], Juan Sanchis [6], Juan Ramón Rey Blas [7], Joan Antoni Gómez-Hospital [8], Sandra Santos Martínez [9], Nicolás Manuel Maneiro-Melón [10], Roberto Mateos Gaitán [11], Jessika González D'Gregorio [12], Luisa Salido [2], José L Mestre [1], Marcelo Sanmartín [2], Ángel Sánchez-Recalde [13]
Affiliations Expand

Affiliations

- [1] Departamento de Cardiología, Hospital Universitario Ramón y Cajal, Madrid, Spain.
- [2] Departamento de Cardiología, Hospital Universitario Ramón y Cajal, Madrid, Spain; Centro de Investigación Biomédica en Red de Enfermedades Cardiovasculares (CIBERCV), Madrid, Spain.
- [3] Centro de Investigación Biomédica en Red de Enfermedades Cardiovasculares (CIBERCV), Madrid, Spain; Departamento de Cardiología, Hospital Clínico Universitario de Valladolid, Instituto de Ciencias del Corazón (ICICOR), Valladolid, Spain.
- [4] Centro de Investigación Biomédica en Red de Enfermedades Cardiovasculares (CIBERCV), Madrid, Spain; Departamento de Cardiología, Hospital Universitario 12 de Octubre, Madrid, Spain.
- [5] Centro de Investigación Biomédica en Red de Enfermedades Cardiovasculares (CIBERCV), Madrid, Spain; Departamento de Cardiología, Hospital General Universitario Gregorio Marañón, Instituto de Investigación Sanitaria Gregorio Marañón, Madrid, Spain.
- [6] Centro de Investigación Biomédica en Red de Enfermedades Cardiovasculares (CIBERCV), Madrid, Spain; Departamento de Cardiología Intervencionista, Hospital Clínic i Universitari de València - Instituto de Investigación Sanitaria INCLIVA, Valencia, Spain.

- [7] Centro de Investigación Biomédica en Red de Enfermedades Cardiovasculares (CIBERCV), Madrid, Spain; Departamento de Cardiología, Hospital Universitario La Paz, Madrid, Spain.
- [8] Centro de Investigación Biomédica en Red de Enfermedades Cardiovasculares (CIBERCV), Madrid, Spain; Departamento de Cardiología, Hospital Universitario Bellvitge, L'Hospitalet de Llobregat, Barcelona, Spain.
- [9] Departamento de Cardiología, Hospital Clínico Universitario de Valladolid, Instituto de Ciencias del Corazón (ICICOR), Valladolid, Spain.
- [10] Departamento de Cardiología, Hospital Universitario 12 de Octubre, Madrid, Spain.
- [11] Departamento de Cardiología, Hospital General Universitario Gregorio Marañón, Instituto de Investigación Sanitaria Gregorio Marañón, Madrid, Spain.
- [12] Departamento de Cardiología Intervencionista, Hospital Clínic i Universitari de València - Instituto de Investigación Sanitaria INCLIVA, Valencia, Spain.
- [13] Departamento de Cardiología, Hospital Universitario Ramón y Cajal, Madrid, Spain; Centro de Investigación Biomédica en Red de Enfermedades Cardiovasculares (CIBERCV), Madrid, Spain. Electronic address: asrecalde@hotmail.com.

- PMID: **32839121**
- PMCID: PMC7832619
- DOI: 10.1016/j.rec.2020.07.009

Free PMC article
Observational Study

Risk factors for in-hospital mortality in patients with acute myocardial infarction during the COVID-19 outbreak

[Article in English, Spanish]
Jorge Solano-López et al. Rev Esp Cardiol (Engl Ed). 2020 Dec.
Free PMC article

Show details

Rev Esp Cardiol (Engl Ed)
. 2020 Dec;73(12):985-993.
doi: 10.1016/j.rec.2020.07.009. Epub 2020 Jul 31.

Authors

Jorge Solano-López [1], José Luis Zamorano [2], Ana Pardo Sanz [1], Ignacio Amat-Santos [3], Fernando Sarnago [4], Enrique Gutiérrez Ibañes [5], Juan Sanchis [6], Juan Ramón Rey Blas [7], Joan Antoni Gómez-Hospital [8], Sandra Santos Martínez [9], Nicolás Manuel Maneiro-Melón [10], Roberto Mateos Gaitán [11], Jessika González D'Gregorio [12], Luisa Salido [2], José L Mestre [1], Marcelo Sanmartín [2], Ángel Sánchez-Recalde [13]

Affiliations

- [1] Departamento de Cardiología, Hospital Universitario Ramón y Cajal, Madrid, Spain.

- [2] Departamento de Cardiología, Hospital Universitario Ramón y Cajal, Madrid, Spain; Centro de Investigación Biomédica en Red de Enfermedades Cardiovasculares (CIBERCV), Madrid, Spain.
- [3] Centro de Investigación Biomédica en Red de Enfermedades Cardiovasculares (CIBERCV), Madrid, Spain; Departamento de Cardiología, Hospital Clínico Universitario de Valladolid, Instituto de Ciencias del Corazón (ICICOR), Valladolid, Spain.
- [4] Centro de Investigación Biomédica en Red de Enfermedades Cardiovasculares (CIBERCV), Madrid, Spain; Departamento de Cardiología, Hospital Universitario 12 de Octubre, Madrid, Spain.
- [5] Centro de Investigación Biomédica en Red de Enfermedades Cardiovasculares (CIBERCV), Madrid, Spain; Departamento de Cardiología, Hospital General Universitario Gregorio Marañón, Instituto de Investigación Sanitaria Gregorio Marañón, Madrid, Spain.
- [6] Centro de Investigación Biomédica en Red de Enfermedades Cardiovasculares (CIBERCV), Madrid, Spain; Departamento de Cardiología Intervencionista, Hospital Clínic i Universitari de València - Instituto de Investigación Sanitaria INCLIVA, Valencia, Spain.
- [7] Centro de Investigación Biomédica en Red de Enfermedades Cardiovasculares (CIBERCV), Madrid, Spain; Departamento de Cardiología, Hospital Universitario La Paz, Madrid, Spain.
- [8] Centro de Investigación Biomédica en Red de Enfermedades Cardiovasculares (CIBERCV), Madrid, Spain; Departamento de Cardiología, Hospital Universitario Bellvitge, L'Hospitalet de Llobregat, Barcelona, Spain.
- [9] Departamento de Cardiología, Hospital Clínico Universitario de Valladolid, Instituto de Ciencias del Corazón (ICICOR), Valladolid, Spain.
- [10] Departamento de Cardiología, Hospital Universitario 12 de Octubre, Madrid, Spain.
- [11] Departamento de Cardiología, Hospital General Universitario Gregorio Marañón, Instituto de Investigación Sanitaria Gregorio Marañón, Madrid, Spain.
- [12] Departamento de Cardiología Intervencionista, Hospital Clínic i Universitari de València - Instituto de Investigación Sanitaria INCLIVA, Valencia, Spain.
- [13] Departamento de Cardiología, Hospital Universitario Ramón y Cajal, Madrid, Spain; Centro de Investigación Biomédica en Red de Enfermedades Cardiovasculares (CIBERCV), Madrid, Spain. Electronic address: asrecalde@hotmail.com.

- PMID: **32839121**
- PMCID: PMC7832619
- DOI: 10.1016/j.rec.2020.07.009

Abstract
in English, Spanish

Introduction and objectives: Despite advances in treatment, patients with acute myocardial infarction (AMI) still exhibit unfavorable short- and long-term prognoses. In addition, there is scant evidence about the clinical outcomes of patients with AMI and coronavirus disease 2019 (COVID-19). The objective of this study was to describe the clinical presentation, complications, and risk factors for mortality in patients admitted for AMI during the COVID-19 pandemic.

Methods: This prospective, multicenter, cohort study included all consecutive patients with AMI who underwent coronary angiography in a 30-day period corresponding chronologically with the COVID-19 outbreak (March 15 to April 15, 2020). Clinical presentations and outcomes were compared between COVID-19 and non-COVID-19 patients. The effect of COVID-19 on mortality was assessed by propensity score matching and with a multivariate logistic regression model.

Results: In total, 187 patients were admitted for AMI, 111 with ST-segment elevation AMI and 76 with non-ST-segment elevation AMI. Of these, 32 (17%) were diagnosed with COVID-19. GRACE score, Killip-Kimball classification, and several inflammatory markers were significantly higher in COVID-19-positive patients. Total and cardiovascular mortality were also significantly higher in COVID-19-positive patients (25% vs 3.8% [P <.001] and 15.2% vs 1.8% [P=.001], respectively). GRACE score> 140 (OR, 23.45; 95%CI, 2.52-62.51; P=.005) and COVID-19 (OR, 6.61; 95%CI, 1.82-24.43; P=.02) were independent predictors of in-hospital death.

Conclusions: During this pandemic, a high GRACE score and COVID-19 were independent risk factors associated with higher in-hospital mortality.

Introducción y objetivos: A pesar de los avances en el tratamiento del infarto agudo de miocardio (IAM), este sigue presentando un pronóstico desfavorable. Hay poca evidencia acerca de la evolución de los pacientes con IAM y la enfermedad por coronavirus 2019 (COVID-19). El objetivo del estudio es describir la presentación clínica, las complicaciones y los factores predictores de mortalidad hospitalaria en pacientes con IAM durante el brote de COVID-19 en España.

Métodos: Se realizó un estudio de cohortes, prospectivo y multicéntrico de todos los pacientes consecutivos con IAM en tratamiento invasivo durante el brote de COVID-19 (15 de marzo a 15 de abril de 2020). Se compararon las características clínicas de los pacientes positivos para COVID-19 con las de los negativos, y se evaluó el efecto de la COVID-19 en la mortalidad mediante emparejamiento por puntuación de propensión y regresión logística.

Resultados: Se incluyó a 187 pacientes con IAM: 111 con elevación del segmento ST y 76 sin elevación. De ellos, 32 (17%) resultaron positivos para COVID-19. Las puntuaciones GRACE y Killip-Kimball y varios marcadores inflamatorios resultaron significativamente mayores en los pacientes con COVID-19. La mortalidad total y cardiovascular fueron significativamente mayores en los pacientes con COVID-19 (el 25 frente al 3,8%; p < 0,001; y el 15,2 frente al 1,8%; p = 0,001). La puntuación GRACE > 140 (OR = 23,45; IC95%, 2,52-62,51; p = 0,005) y la COVID-19 (OR = 6,61; IC95%, 1,82-24,43; p = 0,02) resultaron factores independientes de mortalidad hospitalaria.

Conclusiones: Durante el brote epidémico, la puntuación GRACE elevada y la COVID-19 fueron los factores independientes de mortalidad hospitalaria en los pacientes con IAM.

Keywords: Acute coronary syndrome; COVID-19; Infarto de miocardio; Mortalidad; Mortality; Myocardial infarction; Síndrome coronario agudo.

Copyright © 2020 Sociedad Española de Cardiología. Published by Elsevier España, S.L.U. All rights reserved.

- 37 references
- 3 figures

Supplementary info

Publication types, MeSH terms Expand

Publication types

- Multicenter Study

- Observational Study

MeSH terms

- Aged
- COVID-19 / epidemiology*
- Comorbidity
- Female
- Follow-Up Studies
- Hospital Mortality / trends
- Humans
- Male
- Middle Aged
- Myocardial Infarction / mortality*
- Pandemics
- Prospective Studies
- Retrospective Studies
- Risk Assessment / methods*
- Risk Factors
- SARS-CoV-2*
- Spain / epidemiology

Full text links

FULL TEXT AT REVISTA ESPAÑOLA DE CARDIOLOGIA Ediciones Doyma, S.L. Free PMC article
Proceed to details
Cite
Share
995

Psychiatry Res
. 2021 Apr;298:113776.
doi: 10.1016/j.psychres.2021.113776. Epub 2021 Feb 2.

Epidemiology, Infection Prevention, Testing Data, and Clinical Outcomes of COVID-19 on Five Inpatient Psychiatric Units in a large Academic Medical Center

Luming Li [1], Scott C Roberts [2], William Kulp [3], Angelina Wing [4], Todd Barnes [5], Nicole Colandrea [6], Beth Klink [7], Frank Fortunati [8], Richard Martinello [9]

Affiliations Expand

Affiliations

- [1] Department of Psychiatry, Yale University, New Haven, CT, United States. Electronic address: Luming.li@yale.edu.
- [2] Infectious Disease, Yale New Haven Hospital, New Haven, CT, United States. Electronic address: scott.c.roberts@yale.edu.
- [3] Department of Psychiatry, Yale University, New Haven, CT, United States. Electronic address: William.kulp@yale.edu.
- [4] Yale New Haven Health, Yale New Haven Hospital, New Haven, CT, United States. Electronic address: Angelina.Wing@YNHH.ORG.
- [5] Yale New Haven Health, Yale New Haven Hospital, New Haven, CT, United States. Electronic address: todd.barnes@ynhh.org.
- [6] Yale New Haven Health, Yale New Haven Hospital, New Haven, CT, United States. Electronic address: nicole.colandrea@ynhh.org.
- [7] Yale New Haven Health, Yale New Haven Hospital, New Haven, CT, United States. Electronic address: beth.klink@ynhh.org.
- [8] Department of Psychiatry, Yale University, New Haven, CT, United States. Electronic address: frank.fortunati@yale.edu.
- [9] Infectious Disease, Yale New Haven Hospital, New Haven, CT, United States. Electronic address: richard.martinello@yale.edu.

- PMID: **33571800**
- PMCID: PMC7987366
- DOI: 10.1016/j.psychres.2021.113776

Free PMC article

Epidemiology, Infection Prevention, Testing Data, and Clinical Outcomes of COVID-19 on Five Inpatient Psychiatric Units in a large Academic Medical Center

Luming Li et al. Psychiatry Res. 2021 Apr.
Free PMC article
Show details
Psychiatry Res
. 2021 Apr;298:113776.
doi: 10.1016/j.psychres.2021.113776. Epub 2021 Feb 2.

Authors

Luming Li [1], Scott C Roberts [2], William Kulp [3], Angelina Wing [4], Todd Barnes [5], Nicole Colandrea [6], Beth Klink [7], Frank Fortunati [8], Richard Martinello [9]

Affiliations

- [1] Department of Psychiatry, Yale University, New Haven, CT, United States. Electronic address: Luming.li@yale.edu.
- [2] Infectious Disease, Yale New Haven Hospital, New Haven, CT, United States. Electronic address: scott.c.roberts@yale.edu.
- [3] Department of Psychiatry, Yale University, New Haven, CT, United States. Electronic address: William.kulp@yale.edu.
- [4] Yale New Haven Health, Yale New Haven Hospital, New Haven, CT, United States. Electronic address: Angelina.Wing@YNHH.ORG.
- [5] Yale New Haven Health, Yale New Haven Hospital, New Haven, CT, United States. Electronic address: todd.barnes@ynhh.org.
- [6] Yale New Haven Health, Yale New Haven Hospital, New Haven, CT, United States. Electronic address: nicole.colandrea@ynhh.org.
- [7] Yale New Haven Health, Yale New Haven Hospital, New Haven, CT, United States. Electronic address: beth.klink@ynhh.org.
- [8] Department of Psychiatry, Yale University, New Haven, CT, United States. Electronic address: frank.fortunati@yale.edu.
- [9] Infectious Disease, Yale New Haven Hospital, New Haven, CT, United States. Electronic address: richard.martinello@yale.edu.

- PMID: **33571800**
- PMCID: PMC7987366
- DOI: 10.1016/j.psychres.2021.113776

Abstract

Inpatient psychiatric facilities can face significant challenges in containing infectious outbreaks during the COVID-19 pandemic. The main objective of this study was to characterize the epidemiology, testing data, and containment protocols of COVID-19 in a large academic medical center during the height of the COVID-19 outbreak. A retrospective cohort analysis was conducted on hospitalized individuals on five inpatient psychiatric units from March 1st to July 8th, 2020. Demographic data collected include age, race, gender, ethnicity, diagnosis, and admission status (one or multiple admissions). In addition, a Gantt chart was used to assess outbreak data and timelines for one unit. Testing data was collected for patients admitted to inpatient psychiatric units, emergency room visits, and employees. 964 individuals were hospitalized psychiatrically. The study population included ethnically diverse patients with various mental illnesses. We also describe infection prevention strategies, screening, and triage protocols utilized to safely continue patient flow during and beyond the study period with a low patient and employee infection rate. In summary, our study suggests that early implementation of triage, screening, extensive testing, and unit-specific interventions can help prevent and contain the spread of COVID-19 in inpatient psychiatric units and help facilitate safe delivery of care during a pandemic.

Keywords: COVID-19; infection prevention; inpatient psychiatry; observational study.

Copyright © 2021. Published by Elsevier B.V.

Conflict of interest statement

Dr. Richard Martinello served on a scientific advisory panel for Genentech related to baloxavir. None of the other authors have disclosures to report. The authors declare that they have no competing interests to declare.

- 25 references
- 1 figure

Supplementary info

MeSH terms, Grant support Expand

MeSH terms

- Academic Medical Centers* / standards
- Academic Medical Centers* / statistics & numerical data
- Adult
- COVID-19* / diagnosis
- COVID-19* / epidemiology
- COVID-19* / prevention & control
- Female
- Humans
- Inpatients
- Male
- Mental Disorders* / epidemiology
- Mental Disorders* / therapy
- Middle Aged
- Psychiatric Department, Hospital* / standards
- Psychiatric Department, Hospital* / statistics & numerical data
- Retrospective Studies
- Triage* / standards
- Triage* / statistics & numerical data

Grant support

- UL1 TR001863/TR/NCATS NIH HHS/United States

Full text links

ELSEVIER FULL-TEXT ARTICLE Elsevier Science Free PMC article

Proceed to details

Cite

Share

996

Observational Study

Cell Commun Signal

. 2021 Jul 8;19(1):73.
doi: 10.1186/s12964-021-00754-7.

Single-cell analysis reveals cell communication triggered by macrophages associated with the reduction and exhaustion of CD8 $^{+}$ T cells in COVID-19

Lei He[# 1], Quan Zhang[# 2], Yue Zhang[1], Yixian Fan[3], Fahu Yuan[4], Songming Li[5]
Affiliations Expand

Affiliations

- [1] Department of Blood Transfusion, Tongji Hospital, Tongji Medical College, Huazhong University of Science and Technology, Wuhan, 430030, China.
- [2] Department of Laboratory Medicine, Hubei Provincial Hospital of Integrated Chinese & Western Medicine, Wuhan, 430015, China.
- [3] Department of Physiology, School of Basic Medicine, Tongji Medical College, Huazhong University of Science and Technology, Wuhan, 430030, China.
- [4] School of Medicine, Jianghan University, Wuhan, 430056, China.
- [5] Department of Respiration, Hubei Provincial Hospital of Integrated Chinese & Western Medicine, No. 11, Linjiao Lake Road, Jianghan District, Wuhan, 430015, China. Lsmn6666@sohu.com.

[#] Contributed equally.

- PMID: **34238338**
- PMCID: PMC8264994
- DOI: 10.1186/s12964-021-00754-7

Free PMC article
Observational Study

Single-cell analysis reveals cell communication triggered by macrophages associated with the reduction and exhaustion of CD8 $^{+}$ T cells in COVID-19

Lei He et al. Cell Commun Signal. 2021.
Free PMC article
Show details
Cell Commun Signal

. 2021 Jul 8;19(1):73.
doi: 10.1186/s12964-021-00754-7.

Authors

Lei He[#1], Quan Zhang[#2], Yue Zhang[1], Yixian Fan[3], Fahu Yuan[4], Songming Li[5]

Affiliations

- [1] Department of Blood Transfusion, Tongji Hospital, Tongji Medical College, Huazhong University of Science and Technology, Wuhan, 430030, China.
- [2] Department of Laboratory Medicine, Hubei Provincial Hospital of Integrated Chinese & Western Medicine, Wuhan, 430015, China.
- [3] Department of Physiology, School of Basic Medicine, Tongji Medical College, Huazhong University of Science and Technology, Wuhan, 430030, China.
- [4] School of Medicine, Jianghan University, Wuhan, 430056, China.
- [5] Department of Respiration, Hubei Provincial Hospital of Integrated Chinese & Western Medicine, No. 11, Linjiao Lake Road, Jianghan District, Wuhan, 430015, China. Lsmn6666@sohu.com.

[#] Contributed equally.

- PMID: **34238338**
- PMCID: PMC8264994
- DOI: 10.1186/s12964-021-00754-7

Abstract

Background: The coronavirus disease 2019 (COVID-19) outbreak caused by severe acute respiratory syndrome coronavirus 2 (SARS-Cov-2) has become an ongoing pandemic. Understanding the respiratory immune microenvironment which is composed of multiple cell types, together with cell communication based on ligand-receptor interactions is important for developing vaccines, probing COVID-19 pathogenesis, and improving pandemic control measures.

Methods: A total of 102 consecutive hospitalized patients with confirmed COVID-19 were enrolled in this study. Clinical information, routine laboratory tests, and flow cytometry analysis data with different conditions were collected and assessed for predictive value in COVID-19 patients. Next, we analyzed public single-cell RNA-sequencing (scRNA-seq) data from bronchoalveolar lavage fluid, which offers the closest available view of immune cell heterogeneity as encountered in patients with varying severity of COVID-19. A weighting algorithm was used to calculate ligand-receptor interactions, revealing the communication potentially associated with outcomes across cell types. Finally, serum cytokines including IL6, IL1β, IL10, CXCL10, TNFα, GALECTIN-1, and IGF1 derived from patients were measured.

Results: Of the 102 COVID-19 patients, 42 cases (41.2%) were categorized as severe. Multivariate logistic regression analysis demonstrated that AST, D-dimer, BUN, and WBC were considered as independent risk factors for the severity of COVID-19. T cell numbers including total T cells, $CD4^+$ and $CD8^+$ T cells in the severe disease group were significantly lower than those in the moderate disease group. The risk model containing the above mentioned inflammatory damage parameters, and the counts of T cells, with AUROCs ranged from 0.78 to

0.87. To investigate the molecular mechanism at the cellular level, we analyzed the published scRNA-seq data and found that macrophages displayed specific functional diversity after SARS-Cov-2 infection, and the metabolic pathway activities in the identified macrophage subtypes were influenced by hypoxia status. Importantly, we described ligand-receptor interactions that are related to COVID-19 serverity involving macrophages and T cell subsets by communication analysis.

Conclusions: Our study showed that macrophages driving ligand-receptor crosstalk contributed to the reduction and exhaustion of $CD8^+$ T cells. The identified crucial cytokine panel, including IL6, IL1β, IL10, CXCL10, IGF1, and GALECTIN-1, may offer the selective targets to improve the efficacy of COVID-19 therapy.

Trial registration: This is a retrospective observational study without a trial registration number. Video Abstract.

Keywords: COVID-19; Macrophage; SARS-CoV-2; Single cell RNA-sequencing; T cell.

Conflict of interest statement

The authors declare that they have no competing interests.

- 47 references
- 8 figures

Supplementary info

Publication types, MeSH terms, Substances Expand

Publication types

- Observational Study
- Research Support, Non-U.S. Gov't

MeSH terms

- Aged
- Bronchoalveolar Lavage Fluid / immunology
- CD8-Positive T-Lymphocytes / immunology
- CD8-Positive T-Lymphocytes / pathology
- COVID-19 / epidemiology
- COVID-19 / immunology*
- COVID-19 / pathology*
- COVID-19 / physiopathology
- Cell Communication*
- China / epidemiology
- Cytokines / blood
- Cytokines / immunology

- Female
- Humans
- Macrophages / immunology*
- Macrophages / pathology
- Male
- Middle Aged
- Receptors, Cytokine
- Retrospective Studies
- Sequence Analysis, RNA
- Severity of Illness Index
- Single-Cell Analysis*

Substances

- Cytokines
- Receptors, Cytokine

Full text links

Read free full text at BMC BioMed Central Free PMC article

Proceed to details

Cite

Share

☐ 997

Observational Study

Br J Anaesth

. 2020 Jul;125(1):e28-e37.

doi: 10.1016/j.bja.2020.03.026. Epub 2020 Apr 10.

Emergency tracheal intubation in 202 patients with COVID-19 in Wuhan, China: lessons learnt and international expert recommendations

Wenlong Yao [1], Tingting Wang [2], Bailin Jiang [3], Feng Gao [1], Li Wang [2], Hongbo Zheng [1], Weimin Xiao [2], Shanglong Yao [2], Wei Mei [1], Xiangdong Chen [4], Ailin Luo [5], Liang Sun [3], Tim Cook [6], Elizabeth Behringer [7], Johannes M Huitink [8], David T Wong [9], Meghan Lane-Fall [10], Alistair F McNarry [11], Barry McGuire [12], Andrew Higgs [13], Amit Shah [14], Anil Patel [15], Mingzhang Zuo [16], Wuhua Ma [17], Zhanggang Xue [18], Li-Ming Zhang [19], Wenxian Li [20], Yong Wang [17], Carin Hagberg [21], Ellen P O'Sullivan [22], Lee A Fleisher [10], Huafeng Wei [23], collaborators

Collaborators, Affiliations Expand

Collaborators

- **collaborators**:
  Zhiyong Peng [24], Hansheng Liang [25], Koji Nishikawa [26]

Affiliations

- [1] Department of Anesthesiology, Tongji Hospital, Tongji Medical College, Huazhong University of Science and Technology, Wuhan, China.
- [2] Department of Anesthesiology, Union Hospital, Tongji Medical College, Huazhong University of Science and Technology, Wuhan, China.
- [3] Department of Anesthesiology and Critical Care, University of Pennsylvania, Philadelphia, PA, USA; Department of Anesthesiology, Peking University People's Hospital, Beijing, China.
- [4] Department of Anesthesiology, Union Hospital, Tongji Medical College, Huazhong University of Science and Technology, Wuhan, China. Electronic address: Xiangdongchen2013@163.com.
- [5] Department of Anesthesiology, Tongji Hospital, Tongji Medical College, Huazhong University of Science and Technology, Wuhan, China. Electronic address: alluo@tjh.tjmu.edu.cn.
- [6] Department of Anaesthesia and Intensive Care Medicine, Royal United Hospital, Bath, UK.
- [7] Division of Cardiovascular Surgery and Critical Care, Kaiser Permanente Los Angeles Medical Center, Los Angeles, CA, USA.
- [8] Airway Management Academy, Amsterdam, the Netherlands.
- [9] Department of Anaesthesia, Toronto Western Hospital, University Health Network, University of Toronto, Toronto, ON, Canada.
- [10] Department of Anesthesiology and Critical Care, University of Pennsylvania, Philadelphia, PA, USA.
- [11] Department of Anaesthesia, Western General Hospital, Edinburgh, UK.
- [12] Department of Anaesthesia, Ninewells Hospital, Dundee, UK.
- [13] Department of Anaesthesia and Intensive Care Medicine, Warrington and Halton Hospitals, Warrington, UK.
- [14] Department of Anesthesiology, Kailash Cancer Hospital and Research Center, Muni Seva Ashram, Goraj, India.
- [15] Department of Anaesthesiology, Royal National Throat, Nose and Ear Hospital, London, UK.
- [16] Department of Anesthesiology, Beijing Hospital, National Center of Gerontology, Institute of Geriatric Medicine, Chinese Academy of Medical Sciences, Beijing, China.
- [17] Department of Anesthesiology, First Affiliated Hospital of Guangzhou University of Traditional Chinese Medicine, Guangzhou, China.
- [18] Department of Anesthesiology, Zhongshan Hospital, Fudan University, Shanghai, China.
- [19] Department of Anesthesiology and Perioperative Medicine, University of Pittsburgh Medical Center (UPMC) and University of Pittsburgh School of Medicine, Pittsburgh, PA, USA.
- [20] Department of Anesthesiology, Eye, Ear, Nose and Throat Hospital of Fudan University, Shanghai, China.

- [21] Department of Anesthesiology and Perioperative Medicine, University of Texas MD Anderson Cancer Center, Houston, TX, USA.
- [22] Department of Anaesthesia and Intensive Care Medicine, St James's Hospital, Dublin, Ireland.
- [23] Department of Anesthesiology and Critical Care, University of Pennsylvania, Philadelphia, PA, USA. Electronic address: huafeng.wei@pennmedicine.upenn.edu.
- [24] Department of Critical Care Medicine, Zhongnan Hospital, Wuhan University, Wuhan, China.
- [25] Department of Anesthesiology, Peking University People's Hospital, Beijing, China.
- [26] Department of Anesthesiology and Operating Room, General Sagami Kosei Hospital, Kanagawa, Japan.

- PMID: **32312571**
- PMCID: PMC7151238
- DOI: 10.1016/j.bja.2020.03.026

Free PMC article
Observational Study

Emergency tracheal intubation in 202 patients with COVID-19 in Wuhan, China: lessons learnt and international expert recommendations

Wenlong Yao et al. Br J Anaesth. 2020 Jul.
Free PMC article
Show details
Br J Anaesth
. 2020 Jul;125(1):e28-e37.
doi: 10.1016/j.bja.2020.03.026. Epub 2020 Apr 10.

Authors

Wenlong Yao [1], Tingting Wang [2], Bailin Jiang [3], Feng Gao [1], Li Wang [2], Hongbo Zheng [1], Weimin Xiao [2], Shanglong Yao [2], Wei Mei [1], Xiangdong Chen [4], Ailin Luo [5], Liang Sun [3], Tim Cook [6], Elizabeth Behringer [7], Johannes M Huitink [8], David T Wong [9], Meghan Lane-Fall [10], Alistair F McNarry [11], Barry McGuire [12], Andrew Higgs [13], Amit Shah [14], Anil Patel [15], Mingzhang Zuo [16], Wuhua Ma [17], Zhanggang Xue [18], Li-Ming Zhang [19], Wenxian Li [20], Yong Wang [17], Carin Hagberg [21], Ellen P O'Sullivan [22], Lee A Fleisher [10], Huafeng Wei [23], collaborators

Collaborators

- **collaborators**: Zhiyong Peng [24], Hansheng Liang [25], Koji Nishikawa [26]

Affiliations

- [1] Department of Anesthesiology, Tongji Hospital, Tongji Medical College, Huazhong University of Science and Technology, Wuhan, China.
- [2] Department of Anesthesiology, Union Hospital, Tongji Medical College, Huazhong University of Science and Technology, Wuhan, China.
- [3] Department of Anesthesiology and Critical Care, University of Pennsylvania, Philadelphia, PA, USA; Department of Anesthesiology, Peking University People's Hospital, Beijing, China.
- [4] Department of Anesthesiology, Union Hospital, Tongji Medical College, Huazhong University of Science and Technology, Wuhan, China. Electronic address: Xiangdongchen2013@163.com.
- [5] Department of Anesthesiology, Tongji Hospital, Tongji Medical College, Huazhong University of Science and Technology, Wuhan, China. Electronic address: alluo@tjh.tjmu.edu.cn.
- [6] Department of Anaesthesia and Intensive Care Medicine, Royal United Hospital, Bath, UK.
- [7] Division of Cardiovascular Surgery and Critical Care, Kaiser Permanente Los Angeles Medical Center, Los Angeles, CA, USA.
- [8] Airway Management Academy, Amsterdam, the Netherlands.
- [9] Department of Anaesthesia, Toronto Western Hospital, University Health Network, University of Toronto, Toronto, ON, Canada.
- [10] Department of Anesthesiology and Critical Care, University of Pennsylvania, Philadelphia, PA, USA.
- [11] Department of Anaesthesia, Western General Hospital, Edinburgh, UK.
- [12] Department of Anaesthesia, Ninewells Hospital, Dundee, UK.
- [13] Department of Anaesthesia and Intensive Care Medicine, Warrington and Halton Hospitals, Warrington, UK.
- [14] Department of Anesthesiology, Kailash Cancer Hospital and Research Center, Muni Seva Ashram, Goraj, India.
- [15] Department of Anaesthesiology, Royal National Throat, Nose and Ear Hospital, London, UK.
- [16] Department of Anesthesiology, Beijing Hospital, National Center of Gerontology, Institute of Geriatric Medicine, Chinese Academy of Medical Sciences, Beijing, China.
- [17] Department of Anesthesiology, First Affiliated Hospital of Guangzhou University of Traditional Chinese Medicine, Guangzhou, China.
- [18] Department of Anesthesiology, Zhongshan Hospital, Fudan University, Shanghai, China.
- [19] Department of Anesthesiology and Perioperative Medicine, University of Pittsburgh Medical Center (UPMC) and University of Pittsburgh School of Medicine, Pittsburgh, PA, USA.
- [20] Department of Anesthesiology, Eye, Ear, Nose and Throat Hospital of Fudan University, Shanghai, China.
- [21] Department of Anesthesiology and Perioperative Medicine, University of Texas MD Anderson Cancer Center, Houston, TX, USA.
- [22] Department of Anaesthesia and Intensive Care Medicine, St James's Hospital, Dublin, Ireland.
- [23] Department of Anesthesiology and Critical Care, University of Pennsylvania, Philadelphia, PA, USA. Electronic address: huafeng.wei@pennmedicine.upenn.edu.

- [24] Department of Critical Care Medicine, Zhongnan Hospital, Wuhan University, Wuhan, China.
- [25] Department of Anesthesiology, Peking University People's Hospital, Beijing, China.
- [26] Department of Anesthesiology and Operating Room, General Sagami Kosei Hospital, Kanagawa, Japan.

- PMID: **32312571**
- PMCID: PMC7151238
- DOI: 10.1016/j.bja.2020.03.026

Abstract

Tracheal intubation in coronavirus disease 2019 (COVID-19) patients creates a risk to physiologically compromised patients and to attending healthcare providers. Clinical information on airway management and expert recommendations in these patients are urgently needed. By analysing a two-centre retrospective observational case series from Wuhan, China, a panel of international airway management experts discussed the results and formulated consensus recommendations for the management of tracheal intubation in COVID-19 patients. Of 202 COVID-19 patients undergoing emergency tracheal intubation, most were males (n=136; 67.3%) and aged 65 yr or more (n=128; 63.4%). Most patients (n=152; 75.2%) were hypoxaemic (Sao_2 <90%) before intubation. Personal protective equipment was worn by all intubating healthcare workers. Rapid sequence induction (RSI) or modified RSI was used with an intubation success rate of 89.1% on the first attempt and 100% overall. Hypoxaemia (Sao_2 <90%) was common during intubation (n=148; 73.3%). Hypotension (arterial pressure <90/60 mm Hg) occurred in 36 (17.8%) patients during and 45 (22.3%) after intubation with cardiac arrest in four (2.0%). Pneumothorax occurred in 12 (5.9%) patients and death within 24 h in 21 (10.4%). Up to 14 days post-procedure, there was no evidence of cross infection in the anaesthesiologists who intubated the COVID-19 patients. Based on clinical information and expert recommendation, we propose detailed planning, strategy, and methods for tracheal intubation in COVID-19 patients.

Keywords: ARDS; COVID-19; airway management; consensus recommendations; critical care; infection prevention and control; pneumonia; respiratory failure; tracheal intubation.

Copyright © 2020 The Author(s). Published by Elsevier Ltd.. All rights reserved.

Comment in

- Emergency tracheal intubation in patients with COVID-19: is it any different? Comment on Br J Anaesth 2020; 125: e28-e37.
  Sethi R, Sethi S. Sethi R, et al. Br J Anaesth. 2020 Sep;125(3):e286-e288. doi: 10.1016/j.bja.2020.05.045. Epub 2020 Jun 3. Br J Anaesth. 2020. PMID: 32536442 Free PMC article. No abstract available.

- 58 references
- 2 figures

Supplementary info

Publication types, MeSH terms Expand

Publication types

- Multicenter Study
- Observational Study

MeSH terms

- Aged
- Betacoronavirus*
- COVID-19
- China
- Coronavirus Infections / complications
- Coronavirus Infections / prevention & control
- Coronavirus Infections / therapy*
- Female
- Humans
- Hypotension / etiology
- Hypoxia / etiology
- Intubation, Intratracheal / methods*
- Male
- Pandemics / prevention & control
- Personal Protective Equipment*
- Pneumonia, Viral / complications
- Pneumonia, Viral / prevention & control
- Pneumonia, Viral / therapy*
- Pneumothorax / etiology
- Practice Guidelines as Topic
- Retrospective Studies
- SARS-CoV-2

Full text links

ELSEVIER OPEN ACCESS Elsevier Science Free PMC article

Proceed to details

Cite

Share

☐ 998

Observational Study

Thromb Res

. 2020 Dec;196:318-321.
doi: 10.1016/j.thromres.2020.09.022. Epub 2020 Sep 17.

D-dimer cut-off points and risk of venous thromboembolism in adult hospitalized patients with COVID-19

Justin J Choi [1], Graham T Wehmeyer [2], Han A Li [2], Mark N Alshak [2], Musarrat Nahid [3], Mangala Rajan [3], Bethina Liu [2], Emma M Schatoff [4], Rahmi Elahjji [2], Youmna Abdelghany [3], Debra D'Angelo [5], Daniel Crossman [6], Arthur T Evans [6], Peter Steel [7], Laura C Pinheiro [3], Parag Goyal [6], Monika M Safford [6], Gregory Mints [6], Maria T DeSancho [6]
Affiliations Expand

Affiliations

- [1] Department of Medicine, Weill Cornell Medicine, New York, NY, United States of America; New York Presbyterian Hospital, New York, NY, United States of America. Electronic address: juc9107@med.cornell.edu.
- [2] MD Program, Weill Cornell Medicine, New York, NY, United States of America.
- [3] Department of Medicine, Weill Cornell Medicine, New York, NY, United States of America.
- [4] MD Program, Weill Cornell Medicine, New York, NY, United States of America; Weill Cornell/Rockefeller/Sloan Kettering Tri-Institutional MD-PhD Program, New York, NY, United States of America.
- [5] Department of Population Health Sciences, Weill Cornell Medicine, New York, NY, United States of America.
- [6] Department of Medicine, Weill Cornell Medicine, New York, NY, United States of America; New York Presbyterian Hospital, New York, NY, United States of America.
- [7] New York Presbyterian Hospital, New York, NY, United States of America; Department of Emergency Medicine, Weill Cornell Medicine, New York, NY, United States of America.

- PMID: **32977130**
- PMCID: PMC7495176
- DOI: 10.1016/j.thromres.2020.09.022

Free PMC article
Observational Study

D-dimer cut-off points and risk of venous thromboembolism in adult hospitalized patients with COVID-19

Justin J Choi et al. Thromb Res. 2020 Dec.
Free PMC article
Show details
Thromb Res

. 2020 Dec;196:318-321.
doi: 10.1016/j.thromres.2020.09.022. Epub 2020 Sep 17.

Authors

Justin J Choi [1], Graham T Wehmeyer [2], Han A Li [2], Mark N Alshak [2], Musarrat Nahid [3], Mangala Rajan [3], Bethina Liu [2], Emma M Schatoff [4], Rahmi Elahjji [2], Youmna Abdelghany [3], Debra D'Angelo [5], Daniel Crossman [6], Arthur T Evans [6], Peter Steel [7], Laura C Pinheiro [3], Parag Goyal [6], Monika M Safford [6], Gregory Mints [6], Maria T DeSancho [6]

Affiliations

- [1] Department of Medicine, Weill Cornell Medicine, New York, NY, United States of America; New York Presbyterian Hospital, New York, NY, United States of America. Electronic address: juc9107@med.cornell.edu.
- [2] MD Program, Weill Cornell Medicine, New York, NY, United States of America.
- [3] Department of Medicine, Weill Cornell Medicine, New York, NY, United States of America.
- [4] MD Program, Weill Cornell Medicine, New York, NY, United States of America; Weill Cornell/Rockefeller/Sloan Kettering Tri-Institutional MD-PhD Program, New York, NY, United States of America.
- [5] Department of Population Health Sciences, Weill Cornell Medicine, New York, NY, United States of America.
- [6] Department of Medicine, Weill Cornell Medicine, New York, NY, United States of America; New York Presbyterian Hospital, New York, NY, United States of America.
- [7] New York Presbyterian Hospital, New York, NY, United States of America; Department of Emergency Medicine, Weill Cornell Medicine, New York, NY, United States of America.

- PMID: **32977130**
- PMCID: PMC7495176
- DOI: 10.1016/j.thromres.2020.09.022

No abstract available

Keywords: Coronavirus disease 2019; D-dimer; Deep vein thrombosis; Pulmonary embolism; Venous thromboembolism.

Conflict of interest statement

Dr. Choi has received research support from Allergan and Roche Diagnostics in a topic unrelated to this manuscript. Dr. Safford has received research support from Amgen in a topic unrelated to this manuscript. Dr. DeSancho has served as an advisor to Apellis Pharmaceuticals, Bio Products Laboratory, Sanofi Genzyme unrelated to this manuscript.

Comment in

- D-dimer measurement in COVID-19: Silver bullet or clinical distraction?
  Lippi G, Favaloro EJ. Lippi G, et al. Thromb Res. 2020 Dec;196:635-637. doi: 10.1016/j.thromres.2020.09.040. Epub 2020 Oct 12. Thromb Res. 2020. PMID: 33066999 Free PMC article. No abstract available.

- 10 references
- 1 figure

Supplementary info

Publication types, MeSH terms, Substances, Grant support Expand

Publication types

- Letter
- Observational Study
- Research Support, N.I.H., Extramural
- Research Support, Non-U.S. Gov't

MeSH terms

- Aged
- Biomarkers / blood
- COVID-19 / blood
- COVID-19 / complications*
- COVID-19 / diagnosis
- Female
- Fibrin Fibrinogen Degradation Products / analysis*
- Hospitalization
- Humans
- Male
- Middle Aged
- Predictive Value of Tests
- Pulmonary Embolism / blood
- Pulmonary Embolism / diagnosis*
- Pulmonary Embolism / etiology
- Reproducibility of Results
- Retrospective Studies
- Risk Factors
- Up-Regulation
- Venous Thromboembolism / blood
- Venous Thromboembolism / diagnosis*
- Venous Thromboembolism / etiology
- Venous Thrombosis / blood
- Venous Thrombosis / diagnosis*
- Venous Thrombosis / etiology

Substances

- Biomarkers
- Fibrin Fibrinogen Degradation Products
- fibrin fragment D

Grant support

- F31 CA224800/CA/NCI NIH HHS/United States
- UL1 TR002384/TR/NCATS NIH HHS/United States
- KL2 TR002385/TR/NCATS NIH HHS/United States
- T32 GM007739/GM/NIGMS NIH HHS/United States
- UL1 TR000457/TR/NCATS NIH HHS/United States

Full text links

ELSEVIER FULL-TEXT ARTICLE Elsevier Science Free PMC article

Proceed to details

Cite

Share

☐ 999

Observational Study

Cell Host Microbe

. 2020 Sep 9;28(3):455-464.e2.

doi: 10.1016/j.chom.2020.07.005. Epub 2020 Jul 18.

Retrospective Multicenter Cohort Study Shows Early Interferon Therapy Is Associated with Favorable Clinical Responses in COVID-19 Patients

Nan Wang [1], Yan Zhan [2], Linyu Zhu [3], Zhibing Hou [4], Feng Liu [5], Pinhong Song [5], Feng Qiu [5], Xiaolin Wang [6], Xiafei Zou [6], Deyun Wan [7], Xiaosong Qian [7], Shanshan Wang [8], Yabi Guo [8], Hao Yu [8], Miao Cui [9], Gangling Tong [10], Yunsheng Xu [11], Zhihua Zheng [12], Yingying Lu [13], Peng Hong [14]

Affiliations Expand

Affiliations

- [1] Department of Research, Sun Yat-sen University (SYSU) Seventh Hospital, Shenzhen, Guangdong 518107, China.
- [2] Department of Rehabilitation Medicine, Xiangyang Central Hospital, Xiangyang, Hubei 441021, China; College of Medicine, Hubei University of Arts and Science, Xiangyang, Hubei 441053, China.

- [3] Department of Dermatology, SYSU Seventh Hospital, Shenzhen, Guangdong 518107, China.
- [4] Division of Quality Control, Xiangyang Central Blood Station, Xiangyang, Hubei 441000, China.
- [5] Department of Infectious Diseases, Fever Clinic, Suizhou Zengdu Hospital, Suizhou, Hubei 441300, China.
- [6] Intensive Care Unit, Suizhou Zengdu Hospital, Suizhou, Hubei 441300, China.
- [7] Department of Respiratory Medicine, Suizhou Zengdu Hospital, Suizhou, Hubei 441300, China.
- [8] Department of Rehabilitation Medicine, Xiangyang Central Hospital, Xiangyang, Hubei 441021, China.
- [9] Department of Pathology, Mount Sinai St. Luke's Roosevelt Hospital Center, New York, NY 10025, USA.
- [10] Department of Oncology, Peking University Shenzhen Hospital, Shenzhen, Guangdong 518036, China.
- [11] Department of Research, Sun Yat-sen University (SYSU) Seventh Hospital, Shenzhen, Guangdong 518107, China; Department of Dermatology, SYSU Seventh Hospital, Shenzhen, Guangdong 518107, China.
- [12] Department of Nephrology, Center of Nephrology and Urology, SYSU Seventh Hospital, Shenzhen, Guandgong, 518107, China.
- [13] Department of Research, Sun Yat-sen University (SYSU) Seventh Hospital, Shenzhen, Guangdong 518107, China; Department of Biomedical Science, City University of Hong Kong, Kowloon, Hong Kong SAR, China.
- [14] Department of Research, Sun Yat-sen University (SYSU) Seventh Hospital, Shenzhen, Guangdong 518107, China; Division of Research and Development, US Department of Veterans Affairs New York Harbor Healthcare System, Brooklyn, NY 11209, USA; Department of Cell Biology, State University of New York Downstate Health Sciences University, Brooklyn, NY 11203, USA. Electronic address: peng.hong@downstate.edu.

- PMID: **32707096**
- PMCID: PMC7368656
- DOI: 10.1016/j.chom.2020.07.005

Free PMC article
Observational Study

Retrospective Multicenter Cohort Study Shows Early Interferon Therapy Is Associated with Favorable Clinical Responses in COVID-19 Patients

Nan Wang et al. Cell Host Microbe. 2020.
Free PMC article

Show details

Cell Host Microbe

. 2020 Sep 9;28(3):455-464.e2.

doi: 10.1016/j.chom.2020.07.005. Epub 2020 Jul 18.

Authors

Nan Wang [1], Yan Zhan [2], Linyu Zhu [3], Zhibing Hou [4], Feng Liu [5], Pinhong Song [5], Feng Qiu [5], Xiaolin Wang [6], Xiafei Zou [6], Deyun Wan [7], Xiaosong Qian [7], Shanshan Wang [8], Yabi Guo [8], Hao Yu [8], Miao Cui [9], Gangling Tong [10], Yunsheng Xu [11], Zhihua Zheng [12], Yingying Lu [13], Peng Hong [14]

Affiliations

- [1] Department of Research, Sun Yat-sen University (SYSU) Seventh Hospital, Shenzhen, Guangdong 518107, China.
- [2] Department of Rehabilitation Medicine, Xiangyang Central Hospital, Xiangyang, Hubei 441021, China; College of Medicine, Hubei University of Arts and Science, Xiangyang, Hubei 441053, China.
- [3] Department of Dermatology, SYSU Seventh Hospital, Shenzhen, Guangdong 518107, China.
- [4] Division of Quality Control, Xiangyang Central Blood Station, Xiangyang, Hubei 441000, China.
- [5] Department of Infectious Diseases, Fever Clinic, Suizhou Zengdu Hospital, Suizhou, Hubei 441300, China.
- [6] Intensive Care Unit, Suizhou Zengdu Hospital, Suizhou, Hubei 441300, China.
- [7] Department of Respiratory Medicine, Suizhou Zengdu Hospital, Suizhou, Hubei 441300, China.
- [8] Department of Rehabilitation Medicine, Xiangyang Central Hospital, Xiangyang, Hubei 441021, China.
- [9] Department of Pathology, Mount Sinai St. Luke's Roosevelt Hospital Center, New York, NY 10025, USA.
- [10] Department of Oncology, Peking University Shenzhen Hospital, Shenzhen, Guangdong 518036, China.
- [11] Department of Research, Sun Yat-sen University (SYSU) Seventh Hospital, Shenzhen, Guangdong 518107, China; Department of Dermatology, SYSU Seventh Hospital, Shenzhen, Guangdong 518107, China.
- [12] Department of Nephrology, Center of Nephrology and Urology, SYSU Seventh Hospital, Shenzhen, Guandgong, 518107, China.
- [13] Department of Research, Sun Yat-sen University (SYSU) Seventh Hospital, Shenzhen, Guangdong 518107, China; Department of Biomedical Science, City University of Hong Kong, Kowloon, Hong Kong SAR, China.
- [14] Department of Research, Sun Yat-sen University (SYSU) Seventh Hospital, Shenzhen, Guangdong 518107, China; Division of Research and Development, US Department of Veterans Affairs New York Harbor Healthcare System, Brooklyn, NY 11209, USA; Department of Cell Biology, State University of New York Downstate Health Sciences University, Brooklyn, NY 11203, USA. Electronic address: peng.hong@downstate.edu.

- PMID: **32707096**
- PMCID: PMC7368656
- DOI: 10.1016/j.chom.2020.07.005

Abstract

Interferons (IFNs) are widely used in treating coronavirus disease 2019 (COVID-19) patients. However, a recent report of ACE2, the host factor mediating SARS-Cov-2 infection, identifying it as interferon-stimulated raised considerable safety concern. To examine the association between the use and timing of IFN-α2b and clinical outcomes, we analyzed in a retrospective multicenter cohort study of 446 COVID-19 patients in Hubei, China. Regression models estimated that early administration (≤5 days after admission) of IFN-α2b was associated with reduced in-hospital mortality in comparison with no admission of IFN-α2b, whereas late administration of IFN-α2b was associated with increased mortality. Among survivors, early IFN-α2b was not associated with hospital discharge or computed tomography (CT) scan improvement, whereas late IFN-α2b was associated with delayed recovery. Additionally, early IFN-α2b and umifenovir alone or together were associated with reduced mortality and accelerated recovery in comparison with treatment with lopinavir/ritonavir (LPV/r) alone. We concluded that administration of IFN-α2b during the early stage of COVID-19 could induce favorable clinical responses.

Keywords: RNA virus; anti-retroviral agents; anti-viral immunity; cytokine storm syndrome; infectious disease; respiratory medicine; viral infection.

Copyright © 2020 Elsevier Inc. All rights reserved.

Conflict of interest statement

Declaration of Interests The authors declare no competing interests.

- 22 references
- 4 figures

Supplementary info

Publication types, MeSH terms, Substances, Supplementary concepts, Grant support Expand

Publication types

- Multicenter Study
- Observational Study
- Research Support, Non-U.S. Gov't
- Research Support, U.S. Gov't, Non-P.H.S.

MeSH terms

- Adolescent
- Adult
- Aged
- Aged, 80 and over
- Antiviral Agents / administration & dosage*
- Antiviral Agents / therapeutic use
- Betacoronavirus*

- COVID-19
- Child
- China / epidemiology
- Cohort Studies
- Coronavirus Infections / drug therapy*
- Coronavirus Infections / epidemiology
- Coronavirus Infections / mortality
- Drug Therapy, Combination
- Female
- Hospital Mortality
- Host Microbial Interactions / drug effects
- Humans
- Indoles / administration & dosage
- Interferon alpha-2
- Interferon-alpha / administration & dosage
- Interferon-alpha / therapeutic use*
- Length of Stay
- Lopinavir / administration & dosage
- Male
- Middle Aged
- Pandemics
- Pneumonia, Viral / drug therapy*
- Pneumonia, Viral / epidemiology
- Pneumonia, Viral / mortality
- Retrospective Studies
- Ritonavir / administration & dosage
- SARS-CoV-2
- Treatment Outcome
- Young Adult

Substances

- Antiviral Agents
- Indoles
- Interferon alpha-2
- Interferon-alpha
- Interferon-alpha2b
- Lopinavir
- umifenovir
- Ritonavir

Supplementary concepts

- COVID-19 drug treatment

Grant support

- I01 BX001353/BX/BLRD VA/United States

Full text links

Elsevier Science Free PMC article
Proceed to details
Cite
Share
1,000
Observational Study
Clin Exp Nephrol
. 2022 Jan;26(1):54-58.
doi: 10.1007/s10157-021-02118-4. Epub 2021 Jul 29.

The effect of a single dose of BNT162b2 vaccine on the incidence of severe COVID-19 infection in patients on chronic hemodialysis: a single-centre study

Gabor Cserep [1], David Morrow [1], Karen Latchford [2], Rachael Jesset [2], Agnes Dosa [3], Dimitrios Kirmizis [4]
Affiliations Expand

Affiliations

- [1] East Suffolk & North Essex NHS Foundation Trust, Colchester General Hospital, Turner Rd., Colchester, CO4 5JL, UK.
- [2] Diaverum Dialysis Unit, Colchester , Essex, UK.
- [3] Semmelweis University Medical School, Budapest, Hungary.
- [4] East Suffolk & North Essex NHS Foundation Trust, Colchester General Hospital, Turner Rd., Colchester, CO4 5JL, UK. dkirmizis@yahoo.co.uk.

- PMID: **34324086**
- PMCID: PMC8319692
- DOI: 10.1007/s10157-021-02118-4

Free PMC article
Observational Study

The effect of a single dose of BNT162b2 vaccine on the incidence of severe COVID-19 infection in patients on chronic hemodialysis: a single-centre study

Gabor Cserep et al. Clin Exp Nephrol. 2022 Jan.
Free PMC article
Show details
Clin Exp Nephrol
. 2022 Jan;26(1):54-58.
doi: 10.1007/s10157-021-02118-4. Epub 2021 Jul 29.

Authors

Gabor Cserep [1], David Morrow [1], Karen Latchford [2], Rachael Jesset [2], Agnes Dosa [3], Dimitrios Kirmizis [4]

Affiliations

- [1] East Suffolk & North Essex NHS Foundation Trust, Colchester General Hospital, Turner Rd., Colchester, CO4 5JL, UK.
- [2] Diaverum Dialysis Unit, Colchester , Essex, UK.
- [3] Semmelweis University Medical School, Budapest, Hungary.
- [4] East Suffolk & North Essex NHS Foundation Trust, Colchester General Hospital, Turner Rd., Colchester, CO4 5JL, UK. dkirmizis@yahoo.co.uk.

- PMID: **34324086**
- PMCID: PMC8319692
- DOI: 10.1007/s10157-021-02118-4

Abstract

Introduction: In this single-centre retrospective observational study, the 8-week safety and the efficiency of a single dose of BNT162b2 vaccine was studied in 83 HD patients.

Methods: All clinically stable adult ESRD patients on chronic HD for at least 4 weeks were screened for participation in the study. Exclusion criteria for enrollment in the study included a medical history of COVID-19 infection within the last 12 weeks or delivery of both vaccine doses less than 8 weeks apart from each other. The same patients during the 8-week period that preceded the vaccination served as controls of themselves. The vaccine was administered intramuscularly in the deltoid muscle, on a dialysis day, at least 30 min either pre- or post-dialysis. The primary end-point of the study was severe COVID-19 infection, and/or death due to COVID-19 pneumonitis. Furthermore, all vaccinated patients were scrutinized for any local or systemic reactions within the first 7 days post-vaccination.

Results: Amongst 113 adult HD patients in our Unit, in total 83 patients had the first 30 μg dose of the BNT162b2 vaccine and were considered eligible to be included in the study. The 8-week survival rate was 91% for the controls and 100% for the vaccine group. No life-threatening allergic reaction or other side-effect was observed post-vaccination.

Conclusion: The BNT162b2 vaccine can be safely used in HD patients and seems to offer significant protection against the infection even after the first vaccine dose.

Keywords: BNT162b2 vaccine; COVID-19; Hemodialysis.

© 2021. Japanese Society of Nephrology.

Conflict of interest statement

The author declare no conflict of interest.

- 9 references
- 2 figures

Supplementary info

Publication types, MeSH terms, Substances Expand

Publication types

- Observational Study

MeSH terms

- Adult
- Aged
- Aged, 80 and over
- BNT162 Vaccine / administration & dosage*
- BNT162 Vaccine / adverse effects
- COVID-19 / diagnosis
- COVID-19 / epidemiology
- COVID-19 / prevention & control*
- England / epidemiology
- Female
- Humans
- Immunization Schedule
- Incidence
- Injections, Intramuscular
- Kidney Failure, Chronic / diagnosis
- Kidney Failure, Chronic / epidemiology
- Kidney Failure, Chronic / therapy*

- Male
- Middle Aged
- Patient Safety
- Renal Dialysis*
- Retrospective Studies
- Risk Assessment
- Severity of Illness Index
- Time Factors
- Treatment Outcome
- Young Adult

Substances

- BNT162 Vaccine

Full text links

SpringerLink FULL-TEXT ARTICLE Springer Free PMC article
Proceed to details
Cite
Share
1,388 results
Show more results
[x]
Cite
Copy
Download .nbib
Format: NLM
[x]
Share

-
-

Permalink
Copy
first first First previous previous Prev
Page
5
of 7
Next next next Last last last
Send To

- Clipboard

- Email
- Save
- My Bibliography
- Collections
- Citation Manager

[x]

- Article type
- Species
- Language
- Sex
- Journal
- Age

- ☐ Address
- ☐ Autobiography
- ☐ Bibliography
- ☐ Biography
- ☐ Case Reports
- ☐ Classical Article
- ☐ Clinical Conference
- ☐ Clinical Study
- ☐ Clinical Trial Protocol
- ☐ Clinical Trial, Phase I
- ☐ Clinical Trial, Phase II
- ☐ Clinical Trial, Phase III
- ☐ Clinical Trial, Phase IV
- ☐ Clinical Trial, Veterinary
- ☐ Comment
- ☐ Comparative Study
- ☐ Congress
- ☐ Consensus Development Conference
- ☐ Consensus Development Conference, NIH
- ☐ Controlled Clinical Trial
- ☐ Corrected and Republished Article
- ☐ Dataset
- ☐ Dictionary
- ☐ Directory
- ☐ Duplicate Publication
- ☐ Editorial
- ☐ Electronic Supplementary Materials
- ☐ English Abstract
- ☐ Evaluation Study
- ☐ Festschrift
- ☐ Government Publication
- ☐ Guideline
- ☐ Historical Article
- ☐ Interactive Tutorial
- ☐ Interview

- ☐ Introductory Journal Article
- ☐ Lecture
- ☐ Legal Case
- ☐ Legislation
- ☐ Letter
- ☐ Multicenter Study
- ☐ News
- ☐ Newspaper Article
- ☐ Observational Study
- ☐ Observational Study, Veterinary
- ☐ Overall
- ☐ Patient Education Handout
- ☐ Periodical Index
- ☐ Personal Narrative
- ☐ Portrait
- ☐ Practice Guideline
- ☐ Pragmatic Clinical Trial
- ☐ Preprint
- ☐ Published Erratum
- ☐ Research Support, American Recovery and Reinvestment Act
- ☐ Research Support, N.I.H., Extramural
- ☐ Research Support, N.I.H., Intramural
- ☐ Research Support, Non-U.S. Gov't
- ☐ Research Support, U.S. Gov't, Non-P.H.S.
- ☐ Research Support, U.S. Gov't, P.H.S.
- ☐ Research Support, U.S. Gov't
- ☐ Retracted Publication
- ☐ Retraction of Publication
- ☐ Scientific Integrity Review
- ☐ Technical Report
- ☐ Twin Study
- ☐ Validation Study
- ☐ Video-Audio Media
- ☐ Webcast

- ☐ Humans
- ☐ Other Animals

- ☐ Afrikaans
- ☐ Albanian
- ☐ Arabic
- ☐ Armenian
- ☐ Azerbaijani
- ☐ Bosnian
- ☐ Bulgarian
- ☐ Catalan
- ☐ Chinese
- ☐ Croatian
- ☐ Czech

- ☐ Danish
- ☐ Dutch
- ☐ English
- ☐ Esperanto
- ☐ Estonian
- ☐ Finnish
- ☐ French
- ☐ Georgian
- ☐ German
- ☐ Greek, Modern
- ☐ Hebrew
- ☐ Hindi
- ☐ Hungarian
- ☐ Icelandic
- ☐ Indonesian
- ☐ Italian
- ☐ Japanese
- ☐ Kinyarwanda
- ☐ Korean
- ☐ Latin
- ☐ Latvian
- ☐ Lithuanian
- ☐ Macedonian
- ☐ Malay
- ☐ Malayalam
- ☐ Maori
- ☐ Multiple Languages
- ☐ Norwegian
- ☐ Persian
- ☐ Polish
- ☐ Portuguese
- ☐ Pushto
- ☐ Romanian
- ☐ Russian
- ☐ Sanskrit
- ☐ Scottish gaelic
- ☐ Serbian
- ☐ Slovak
- ☐ Slovenian
- ☐ Spanish
- ☐ Swedish
- ☐ Thai
- ☐ Turkish
- ☐ Ukrainian
- ☐ Undetermined
- ☐ Vietnamese
- ☐ Welsh

- ☐ Female
- ☐ Male

- ☐ MEDLINE

- ☐ Child: birth-18 years
- ☐ Newborn: birth-1 month
- ☐ Infant: birth-23 months
- ☐ Infant: 1-23 months
- ☐ Preschool Child: 2-5 years
- ☐ Child: 6-12 years
- ☐ Adolescent: 13-18 years
- ☐ Adult: 19+ years
- ☐ Young Adult: 19-24 years
- ☐ Adult: 19-44 years
- ☐ Middle Aged + Aged: 45+ years
- ☐ Middle Aged: 45-64 years
- ☐ Aged: 65+ years
- ☐ 80 and over: 80+ years

Cancel Show
Close dialog
Back to Top
Jump to page Close dialog
6 of 7
Jump

NCBI Literature Resources

MeSH PMC Bookshelf Disclaimer

Follow NCBI

[Connect with NLM]

-
-
-

National Library of Medicine
8600 Rockville Pike
Bethesda, MD 20894

Web Policies
FOIA
HHS Vulnerability Disclosure

Help
Accessibility
Careers

- NLM
- NIH
- HHS
- USA.gov

ERREUR p
du site :
Domaine
