# Supplementary material for: COVID-19 treatment of hospital patients worldwide at the onset of the pandemic in 2020: a systematic review
Source: BMC Infect Dis. 2025 Dec 17;26:107. doi: 10.1186/s12879-025-12368-2 (PMC12822144; doi:10.1186/s12879-025-12368-2)
Supplement: Supplementary file 4 — Supplementary Material 4 [file 12879_2025_12368_MOESM4_ESM.zip › 12879_2025_12368_MOESM4_ESM/Search Pubmed 2022 03 28 retrospective observational study hospital treatment covid 601-800.pdf]

Skip to main page content

COVID-19 Information

Public health information (CDC)

Research information (NIH)

SARS-CoV-2 data (NCBI)

Prevention and treatment information (HHS)

Español

Close

Account

Logged in as:
username

- Dashboard
- Publications
- Account settings
- Log out

Access keys NCBI Homepage MyNCBI Homepage Main Content Main Navigation

Search Page

pubmed

Search: retrospective observation Search
Advanced Create alert Create RSS Clipboard
User Guide
Filters 0 Timeline
Sorted by: Best match
Sorted by: Best match Display options
Save Email
Send to

Save citations to file

Selection: All results on this page

Format: Summary (text)
Create file Cancel

# Email citations

Subject: retrospective observational study hospital treatm - PubMed
To: antoine.bosquet@lmr.apl
Selection: All results on this page
Format: Summary
MeSH and other data
Send email Cancel

Send citations to clipboard

Selection: All results on this page
Send Cancel

Add to Collections

Selection: All results on this page

- Create a new collection
- Add to an existing collection

Name your collection:
Name must be less than 100 characters
Choose a collection:
Unable to load your collection due to an error
Please try again
Add Cancel

Add to My Bibliography

Selection: All results on this page

- My Bibliography

Unable to load your delegates due to an error
Please try again
Add Cancel

Create a file for external citation management software

Selection: All results on this page
Create file Cancel

Your saved search

Name of saved search: retrospective observation

Search terms: retrospective observational study

Test search terms

Would you like email updates of new search results?

Saved Search Alert Radio Buttons

- Yes
- No

Email: antoine.bosquet@lmr.aphp.fr (change)

Frequency: Monthly

Which day? The first Sunday

Which day? Sunday

Report format: Summary

Send at most: 5 items

Send even when there aren't any new results

Optional text in email:

Save Cancel

Your RSS Feed

Name of RSS Feed: retrospective observation

Number of items displayed: 15

Create RSS Cancel

RSS Link Your RSS Feed Link Copy

My NCBI Filters

- All (1,388)
- Assistance Publique Hopitaux de Paris (0)
- clinical trial (17)
- Review (1)

Show Fewer

Results by year Expand/collapse timeline

Reset

Table representation of search results timeline featuring number of search results per year.

Year	Number of Results
2020	548
2021	893
2022	147

Text availability

- Abstract
- Free full text
- Full text

Article attribute

- Associated data

Article type

- Books and Documents
- Clinical Trial
- Meta-Analysis
- Randomized Controlled Trial
- Review
- Systematic Review

Publication date

- 1 year
- 5 years
- 10 years
- Custom Range

Additional filters Reset all filters

Search Results

clear all
1,388 results

first first previous previous

Page
4
of 7

next next last last

Use COVID-19 filters from PubMed Clinical Queries to refine your search

- Treatment
- Mechanism
- Transmission
- More filters

See more SARS-CoV-2 literature, sequence, and clinical content from NCBI

Results by year Expand/collapse timeline

Reset

Filters applied: . *Clear all* Select search result to email or save

Page 4

☐ 601

Observational Study

Anaesth Crit Care Pain Med

. 2020 Dec;39(6):740-741.

doi: 10.1016/j.accpm.2020.10.001. Epub 2020 Oct 10.

Three-month quality of life in survivors of ARDS due to COVID-19: A preliminary report from a French academic centre

Arnaud Valent [1], Emmanuel Dudoignon [2], Quentin Ressaire [2], François Dépret [3], Benoît Plaud [2]

Affiliations Expand

Affiliations

- [1] Department of Anaesthesiology and Critical Care, Saint-Louis Hospital, DMU Parabol, AP-HP Nord & University of Paris, 75010 Paris, France; UMR-S 942 MASCOT, Inserm, 75010 Paris, France. Electronic address: arnaud.valent@gmail.com.
- [2] Department of Anaesthesiology and Critical Care, Saint-Louis Hospital, DMU Parabol, AP-HP Nord & University of Paris, 75010 Paris, France; UMR-S 942 MASCOT, Inserm, 75010 Paris, France.
- [3] Department of Anaesthesiology and Critical Care, Saint-Louis Hospital, DMU Parabol, AP-HP Nord & University of Paris, 75010 Paris, France; UMR-S 942 MASCOT, Inserm, 75010 Paris, France; INI-CRCT, 54000 Nancy, France.

- PMID: **33049394**
- PMCID: PMC7547571
- DOI: 10.1016/j.accpm.2020.10.001

Free PMC article

Observational Study

Three-month quality of life in survivors of ARDS due to COVID-19: A preliminary report from a French academic centre

Arnaud Valent et al. Anaesth Crit Care Pain Med. 2020 Dec.
Free PMC article
Show details
Anaesth Crit Care Pain Med
. 2020 Dec;39(6):740-741.
doi: 10.1016/j.accpm.2020.10.001. Epub 2020 Oct 10.

Authors

Arnaud Valent [1], Emmanuel Dudoignon [2], Quentin Ressaire [2], François Dépret [3], Benoît Plaud [2]

Affiliations

- [1] Department of Anaesthesiology and Critical Care, Saint-Louis Hospital, DMU Parabol, AP-HP Nord & University of Paris, 75010 Paris, France; UMR-S 942 MASCOT, Inserm, 75010 Paris, France. Electronic address: arnaud.valent@gmail.com.
- [2] Department of Anaesthesiology and Critical Care, Saint-Louis Hospital, DMU Parabol, AP-HP Nord & University of Paris, 75010 Paris, France; UMR-S 942 MASCOT, Inserm, 75010 Paris, France.
- [3] Department of Anaesthesiology and Critical Care, Saint-Louis Hospital, DMU Parabol, AP-HP Nord & University of Paris, 75010 Paris, France; UMR-S 942 MASCOT, Inserm, 75010 Paris, France; INI-CRCT, 54000 Nancy, France.

- PMID: **33049394**
- PMCID: PMC7547571
- DOI: 10.1016/j.accpm.2020.10.001

No abstract available

Keywords: COVID-19; Post-intensive care syndrome; Quality of life.

Comment in

- Fatigue as long-term consequence of ARDS in COVID-19 patients.
  Zuin M, Rigatelli G, Zuliani G, Roncon L. Zuin M, et al. Anaesth Crit Care Pain Med. 2021 Feb;40(1):100787. doi: 10.1016/j.accpm.2020.10.016. Epub 2020 Nov 26. Anaesth Crit Care Pain Med. 2021. PMID: 33249175 Free PMC article. No abstract available.

- 5 references

Supplementary info

Publication types, MeSH terms Expand

Publication types

- Letter
- Observational Study

MeSH terms

- Academies and Institutes
- Aged
- COVID-19 / complications*
- COVID-19 / epidemiology
- Critical Care / statistics & numerical data
- Critical Illness
- Female
- France / epidemiology
- Humans
- Male
- Middle Aged
- Pain / epidemiology
- Pandemics
- Quality of Life*
- Respiratory Distress Syndrome / etiology
- Respiratory Distress Syndrome / psychology*
- Retrospective Studies
- SARS-CoV-2*
- Survivors / psychology*
- Time Factors

Full text links

ELSEVIER FULL-TEXT ARTICLE Elsevier Science Free PMC article
Proceed to details
Cite
Share
☐ 602
Observational Study
Ophthalmol Retina
. 2021 Dec;5(12):1245-1253.
doi: 10.1016/j.oret.2021.02.005. Epub 2021 Feb 19.

Patterns and Characteristics of a Clinical Implementation of a Self-Monitoring

Program for Retina Diseases during the COVID-19 Pandemic

Kelvin Yi Chong Teo [1], Lucas M Bachmann [2], Dawn Sim [3], Shu Yen Lee [4], Anna Tan [4], Tien Y Wong [4], Chui Ming Gemmy Cheung [4], Gavin Siew Wei Tan [4]
Affiliations Expand

Affiliations

- [1] Singapore Eye Research Institute, Singapore National Eye Centre, Singapore, Republic of Singapore; Duke-NUS Medical School, National University of Singapore, Singapore, Republic of Singapore. Electronic address: kelvin.teo.y.c@singhealth.com.sg.
- [2] Oculocare Medical AG, Zurich, Switzerland.
- [3] NIHR Biomedical Research Centre Biomedical Centre, Moorfields Eye Hospital NHS Foundation Trust and UCL Institute of Ophthalmology, London, United Kingdom; Institute of Ophthalmology, University College of London, London, United Kingdom.
- [4] Singapore Eye Research Institute, Singapore National Eye Centre, Singapore, Republic of Singapore; Duke-NUS Medical School, National University of Singapore, Singapore, Republic of Singapore.

- PMID: **33610833**
- PMCID: PMC8160297
- DOI: 10.1016/j.oret.2021.02.005

Free PMC article
Observational Study

Patterns and Characteristics of a Clinical Implementation of a Self-Monitoring Program for Retina Diseases during the COVID-19 Pandemic

Kelvin Yi Chong Teo et al. Ophthalmol Retina. 2021 Dec.
Free PMC article
Show details
Ophthalmol Retina
. 2021 Dec;5(12):1245-1253.
doi: 10.1016/j.oret.2021.02.005. Epub 2021 Feb 19.

Authors

Kelvin Yi Chong Teo [1], Lucas M Bachmann [2], Dawn Sim [3], Shu Yen Lee [4], Anna Tan [4], Tien Y Wong [4], Chui Ming Gemmy Cheung [4], Gavin Siew Wei Tan [4]

Affiliations

- [1] Singapore Eye Research Institute, Singapore National Eye Centre, Singapore, Republic of Singapore; Duke-NUS Medical School, National University of Singapore, Singapore, Republic of Singapore. Electronic address: kelvin.teo.y.c@singhealth.com.sg.
- [2] Oculocare Medical AG, Zurich, Switzerland.
- [3] NIHR Biomedical Research Centre Biomedical Centre, Moorfields Eye Hospital NHS Foundation Trust and UCL Institute of Ophthalmology, London, United Kingdom; Institute of Ophthalmology, University College of London, London, United Kingdom.
- [4] Singapore Eye Research Institute, Singapore National Eye Centre, Singapore, Republic of Singapore; Duke-NUS Medical School, National University of Singapore, Singapore, Republic of Singapore.

- PMID: **33610833**
- PMCID: PMC8160297
- DOI: 10.1016/j.oret.2021.02.005

Abstract

Purpose: We describe the large-scale self-initiated recruitment of patients to a self-monitoring initiative for macular pathologic features during the coronavirus disease 2019 (COVID-19) pandemic.

Design: Observational study with retrospective analysis.

Participants: A total of 2272 patients from the Singapore National Eye Centre (SNEC) whose visits were rescheduled over lockdown (April 13-June 1, 2020) were offered participation in a self-monitoring initiative administered by SNEC with the Alleye application (Switzerland) as the testing instrument.

Methods: This was an observational study with retrospective analysis. Demographics and characteristics were compared between those who signed up and those who did not. Similar comparisons were made between patients who complied with the initiative versus those who did not. Outcomes were tracked for 6 months starting from the commencement of lockdown.

Main outcome measures: Participation and compliance rates and characteristics of patients who were more likely to participate and comply with the initiative.

Results: Seven hundred thirty-two patients (32%) participated in this self-monitoring initiative. Those who participated were younger (62 years of age vs. 68 years of age; $P < 0.001$), men, and living with family. Patients not receiving treatment and those with poorer vision in the worse-seeing eye were more likely to participate. When grouped according to diagnosis, the proportion who participated was highest for diabetic macular edema (52%), nonneovascular age-related macular degeneration (AMD; 42%), diabetic retinopathy (35%), retinal vein occlusions (18%), and neovascular AMD (15%; $P < 0.001$). Testing compliance rate was 43% (315/732). Patients who complied with the initiative were older, were receiving treatment, and had poorer vision in the worse-seeing eye. Trigger events occurred in 33 patients, with 5 patients having clinically verified disease progression (1.6%).

Conclusions: We provide clinical data on characteristics of patients with stable retinal diseases who were offered, participated in, and complied with a self-monitoring program. The lower participation rate compared with standardized clinical studies reflects the difficulties in

implementation for such initiatives in clinical settings. Despite this, self-monitoring continues to show promise in relieving clinic resources, suggesting the feasibility of scaling such programs beyond the COVID-19 pandemic.

Keywords: COVID-19; Digital; Mobile; Retina; Self-monitoring.

Copyright © 2021 American Academy of Ophthalmology. Published by Elsevier Inc. All rights reserved.

- 19 references
- 4 figures

Supplementary info

Publication types, MeSH terms Expand

Publication types

- Observational Study
- Research Support, Non-U.S. Gov't

MeSH terms

- Aged
- COVID-19 / epidemiology*
- Female
- Health Plan Implementation
- Humans
- Male
- Middle Aged
- Monitoring, Physiologic / methods*
- Patient Compliance
- Patient Participation
- Retinal Diseases / diagnosis*
- Retinal Diseases / physiopathology
- Retrospective Studies
- SARS-CoV-2*
- Self Care / methods*
- Singapore / epidemiology

Full text links

ELSEVIER FULL-TEXT ARTICLE Elsevier Science Free PMC article

Proceed to details

Cite

Share

603

Observational Study

Medicine (Baltimore)

. 2021 Sep 17;100(37):e27281.

doi: 10.1097/MD.0000000000027281.

Demographic profile and outcome of patients admitted to a COVID dedicated hospital in Bangladesh during the second wave

Mohammad Iqbal Hossain [1], Sultana Parvin [1], Md Samiul Islam [2], Mohammad Jane Alam [1], Subrata Podder [1], Raj Datta [1], Touhidul Karim Majumdar [1], Mir Jakib Hossain [1], Faruque Ahmed [1]

Affiliations Expand

Affiliations

- [1] Department of Medical Gastroenterology, Sheikh Russel National Gastroliver Institute & Hospital, Mohakhali, Dhaka, Bangladesh.
- [2] National Institute of Traumatology & Orthopedic Rehabilitation (NITOR), Dhaka, Bangladesh.

- PMID: **34664887**
- PMCID: PMC8448023
- DOI: 10.1097/MD.0000000000027281

Free PMC article

Observational Study

Demographic profile and outcome of patients admitted to a COVID dedicated hospital in Bangladesh during the second wave

Mohammad Iqbal Hossain et al. Medicine (Baltimore). 2021.

Free PMC article

Show details

Medicine (Baltimore)

. 2021 Sep 17;100(37):e27281.

doi: 10.1097/MD.0000000000027281.

Authors

Mohammad Iqbal Hossain [1], Sultana Parvin [1], Md Samiul Islam [2], Mohammad Jane Alam [1], Subrata Podder [1], Raj Datta [1], Touhidul Karim Majumdar [1], Mir Jakib Hossain [1], Faruque Ahmed [1]

Affiliations

- [1] Department of Medical Gastroenterology, Sheikh Russel National Gastroliver Institute & Hospital, Mohakhali, Dhaka, Bangladesh.
- [2] National Institute of Traumatology & Orthopedic Rehabilitation (NITOR), Dhaka, Bangladesh.

- PMID: **34664887**
- PMCID: PMC8448023
- DOI: 10.1097/MD.0000000000027281

Abstract

In December 2019, with pneumonia-like clinical manifestations, a new severe acute respiratory syndrome coronavirus 2 emerged and quickly escalated into a pandemic. Since the first case detected in early March of last year, 8668 have died with an infection mortality rate of 1.52%, as of March 20, 2021. Bangladesh has been struck by the 2nd wave from mid-march 2021. As data on the second wave are sparse, the present study observed the demographic profile, symptoms, and outcomes of Coronavirus Disease 2019 (COVID-19) patients during this wave.The study was conducted at Sheikh Russel National Gastroliver Institute on 486 admitted cases during the 2nd wave of COVID-19 in Bangladesh (March 24-April 24, 2021) using a cross-sectional study design and a convenient sampling technique.Out of 486 cases, 306 (62.9%) were male, and 180 were female, with a mean age of 53.47 ± 13.86. The majority of patients (32.5%) were between the ages of 51 and 60. While fever and cough being the predominant symptoms (>70% cases), the most common co-morbidities were hypertension (41.4) and diabetes mellitus (39.4). Intensive care unit utilization rate was 25%, and a half of the patients had 51% to 70% tomographic lung involvement with an overall mortality rate of 19.3%. Older age, chronic renal disease, percentage of lung involvement, and intensive care unit necessity were important mortality determinants.The present study gives an insight into the demographic profiles and outcomes of admitted patients with COVID-19 during the second wave at a covid dedicated hospital in Bangladesh.

Copyright © 2021 the Author(s). Published by Wolters Kluwer Health, Inc.

Conflict of interest statement

The authors have no funding and conflicts of interest to disclose.

- 24 references
- 1 figure

Supplementary info

Publication types, MeSH terms Expand

Publication types

- Observational Study

MeSH terms

- Adult
- Aged
- Bangladesh / epidemiology
- COVID-19 / complications*
- COVID-19 / epidemiology
- COVID-19 / mortality
- Cross-Sectional Studies
- Demography / statistics & numerical data*
- Female
- Hospitalization / statistics & numerical data
- Humans
- Male
- Middle Aged
- Outcome Assessment, Health Care / methods
- Outcome Assessment, Health Care / statistics & numerical data*
- Retrospective Studies

Full text links

Wolters Kluwer Wolters Kluwer Free PMC article
Proceed to details
Cite
Share
☐ 604
Observational Study
Am J Emerg Med
. 2021 Nov;49:110-113.
doi: 10.1016/j.ajem.2021.05.066. Epub 2021 Jun 1.

Keep your distance! Measuring staff physical distancing during the Sars-Cov-2 pandemic using a real-time locating system

Lindsey Barrick [1], Kevin M Overmann [2], Jonathan LaBare [3], Danny T Y Wu [4]
Affiliations Expand

Affiliations

- [1] Division of Emergency Medicine, Cincinnati Children's Hospital Medical Center, USA; Department of Pediatrics, College of Medicine, University of Cincinnati, USA. Electronic address: Lindsey.barrick@cchmc.org.
- [2] Division of Emergency Medicine, Cincinnati Children's Hospital Medical Center, USA; Department of Pediatrics, College of Medicine, University of Cincinnati, USA. Electronic address: Kevin.overmann@cchmc.org.
- [3] Department of Information Services, Cincinnati Children's Hospital Medical Center, USA. Electronic address: jonathan.labare@cchmc.org.
- [4] Department of Biomedical Informatics, College of Medicine, University of Cincinnati, USA; Department of Pediatrics, College of Medicine, University of Cincinnati, USA. Electronic address: wutz@ucmail.uc.edu.

- PMID: **34098329**
- PMCID: PMC8168304
- DOI: 10.1016/j.ajem.2021.05.066

Free PMC article
Observational Study

Keep your distance! Measuring staff physical distancing during the Sars-Cov-2 pandemic using a real-time locating system

Lindsey Barrick et al. Am J Emerg Med. 2021 Nov.
Free PMC article
Show details
Am J Emerg Med
. 2021 Nov;49:110-113.
doi: 10.1016/j.ajem.2021.05.066. Epub 2021 Jun 1.

Authors

Lindsey Barrick [1], Kevin M Overmann [2], Jonathan LaBare [3], Danny T Y Wu [4]

Affiliations

- [1] Division of Emergency Medicine, Cincinnati Children's Hospital Medical Center, USA; Department of Pediatrics, College of Medicine, University of Cincinnati, USA. Electronic address: Lindsey.barrick@cchmc.org.
- [2] Division of Emergency Medicine, Cincinnati Children's Hospital Medical Center, USA; Department of Pediatrics, College of Medicine, University of Cincinnati, USA. Electronic address: Kevin.overmann@cchmc.org.
- [3] Department of Information Services, Cincinnati Children's Hospital Medical Center, USA. Electronic address: jonathan.labare@cchmc.org.
- [4] Department of Biomedical Informatics, College of Medicine, University of Cincinnati, USA; Department of Pediatrics, College of Medicine, University of Cincinnati, USA. Electronic address: wutz@ucmail.uc.edu.

- PMID: **34098329**
- PMCID: [PMC8168304]
- DOI: [10.1016/j.ajem.2021.05.066]

Abstract

Introduction: Staff-to-staff transmission of SARS-CoV-2 poses a significant risk to the Emergency Department (ED) workforce. We measured close (<6 ft), prolonged (>10 min) staff interactions in a busy pediatric Emergency Department in common work areas over time as the pandemic unfolded, measuring the effectiveness of interventions meant to discourage such close contact.

Methods: We used a Real-Time Locating System to measure staff groupings in crowded common work areas lasting ten or more minutes. We compared the number of these interactions pre-pandemic with those occurring early and then later in the pandemic, as distancing interventions were suggested and then formalized. Nearly all healthcare workers in the ED were included, and the duration of interactions over time were evaluated as well.

Results and conclusions: This study included a total of 12,386 pairs of staff-to-staff encounters over three time periods including just prior to the pandemic, early in the pandemic response, and later in the steady-state pandemic response. Pairs of staff averaged 0.89 high-risk interactions hourly prior to the pandemic, and this continued early in the pandemic with informal recommendations (0.80 high-risk pairs hourly). High-risk staff encounters fell significantly to 0.47 interactions per hour in the steady-state pandemic with formal distancing guidelines in place and decreased patient and staffing volumes. The duration of these encounters remained stable, near 16 min. Close contact between healthcare staff workers did significantly decrease with formal distancing guidelines, though some high-risk interactions remained, warranting additive protective measures such as universal masking.

Keywords: Coronavirus; Covid-19; Employee health; RTLS; Real-time locating system.

Copyright © 2021 Elsevier Inc. All rights reserved.

Conflict of interest statement

Declaration of Competing Interest None.

- 8 references
- 1 figure

Supplementary info

Publication types, MeSH terms Expand

Publication types

- Observational Study

MeSH terms

- COVID-19 / epidemiology*
- COVID-19 / prevention & control
- Computer Systems*
- Contact Tracing*
- Emergency Service, Hospital
- Health Personnel
- Humans
- Longitudinal Studies
- Ohio
- Physical Distancing*
- Retrospective Studies
- SARS-CoV-2

Full text links

ELSEVIER FULL-TEXT ARTICLE Elsevier Science Free PMC article
Proceed to details
Cite
Share
605
Observational Study
Pulmonology
. Jan-Feb 2022;28(1):13-17.
doi: 10.1016/j.pulmoe.2021.04.003. Epub 2021 May 7.

COVID-19 Pneumonia and ROX index: Time to set a new threshold for patients admitted outside the ICU

María Laura Vega [1], Roberto Dongilli [2], Gustavo Olaizola [3], Nicolás Colaianni [4], Mauro Castro Sayat [4], Lara Pisani [5], Micaela Romagnoli [6], Greta Spoladore [7], Irene Prediletto [5], Guillermo Montiel [8], Stefano Nava [9]
Affiliations Expand

Affiliations

- [1] Non-Invasive Respiratory Support Unit, Hospital Juan A. Fernández, Ciudad Autónoma de Buenos Aires. Argentina; IRCCS Azienda Ospedaliera-Universitaria, Division of Respiratory and Critical Care SantOrsola Hospital, Bologna Italia.
- [2] Division of Respiratory Diseases with intermediate respiratory intensive care units, Central Hospital of Bolzano, Bolzano, Italy.
- [3] Unidad Asistencial Cesar Milstein. Ciudad Autónoma de Buenos Aires. Argentina.

- [4] Non-Invasive Respiratory Support Unit, Hospital Juan A. Fernández, Ciudad Autónoma de Buenos Aires. Argentina; Intensive Care Unit, Clínica Zabala, Ciudad Autónoma de Buenos Aires. Argentina.
- [5] IRCCS Azienda Ospedaliera-Universitaria, Division of Respiratory and Critical Care SantOrsol Hospital, Bologna Italia. Alma Mater Studiorum University of Bologna Department of Clinical, Integrated and Experimental Medicine (DIMES), Bologna, Italy.
- [6] Pulmonology Unit, Santa Maria di Ca' Foncello Hospital, Treviso, Italy.
- [7] Division of Infectious Diseases, Central Hospital of Bolzano, Bolzano, Italy.
- [8] Non-Invasive Respiratory Support Unit, Hospital Juan A. Fernández, Ciudad Autónoma de Buenos Aires. Argentina.
- [9] IRCCS Azienda Ospedaliera-Universitaria, Division of Respiratory and Critical Care SantOrsol Hospital, Bologna Italia. Alma Mater Studiorum University of Bologna Department of Clinical, Integrated and Experimental Medicine (DIMES), Bologna, Italy. Electronic address: stefano.nava@unibo.it.

- PMID: **34049831**
- PMCID: PMC8103151
- DOI: 10.1016/j.pulmoe.2021.04.003

Free PMC article
Observational Study

COVID-19 Pneumonia and ROX index: Time to set a new threshold for patients admitted outside the ICU

María Laura Vega et al. Pulmonology. Jan-Feb 2022.
Free PMC article

Show details

Pulmonology
. Jan-Feb 2022;28(1):13-17.
doi: 10.1016/j.pulmoe.2021.04.003. Epub 2021 May 7.

Authors

María Laura Vega [1], Roberto Dongilli [2], Gustavo Olaizola [3], Nicolás Colaianni [4], Mauro Castro Sayat [4], Lara Pisani [5], Micaela Romagnoli [6], Greta Spoladore [7], Irene Prediletto [5], Guillermo Montiel [8], Stefano Nava [9]

Affiliations

- [1] Non-Invasive Respiratory Support Unit, Hospital Juan A. Fernández, Ciudad Autónoma de Buenos Aires. Argentina; IRCCS Azienda Ospedaliera-Universitaria, Division of Respiratory and Critical Care SantOrsola Hospital, Bologna Italia.
- [2] Division of Respiratory Diseases with intermediate respiratory intensive care units, Central Hospital of Bolzano, Bolzano, Italy.
- [3] Unidad Asistencial Cesar Milstein. Ciudad Autónoma de Buenos Aires. Argentina.

- [4] Non-Invasive Respiratory Support Unit, Hospital Juan A. Fernández, Ciudad Autónoma de Buenos Aires. Argentina; Intensive Care Unit, Clínica Zabala, Ciudad Autónoma de Buenos Aires. Argentina.
- [5] IRCCS Azienda Ospedaliera-Universitaria, Division of Respiratory and Critical Care SantOrsol Hospital, Bologna Italia. Alma Mater Studiorum University of Bologna Department of Clinical, Integrated and Experimental Medicine (DIMES), Bologna, Italy.
- [6] Pulmonology Unit, Santa Maria di Ca' Foncello Hospital, Treviso, Italy.
- [7] Division of Infectious Diseases, Central Hospital of Bolzano, Bolzano, Italy.
- [8] Non-Invasive Respiratory Support Unit, Hospital Juan A. Fernández, Ciudad Autónoma de Buenos Aires. Argentina.
- [9] IRCCS Azienda Ospedaliera-Universitaria, Division of Respiratory and Critical Care SantOrsol Hospital, Bologna Italia. Alma Mater Studiorum University of Bologna Department of Clinical, Integrated and Experimental Medicine (DIMES), Bologna, Italy. Electronic address: stefano.nava@unibo.it.

- PMID: **34049831**
- PMCID: PMC8103151
- DOI: 10.1016/j.pulmoe.2021.04.003

Abstract

High flow nasal cannula (HFNC) is used to treat acute hypoxemic respiratory failure (AHRF) even outside the ICU and the ROX index (pulse oximetry/fraction of inspired oxygen/respiratory rate) may predict HFNC failure.

Objective: The purpose of this investigation was therefore to verify whether the ROX index is an accurate predictor of HFNC failure for COVID-19 patients treated outside the intensive care unit (ICU) and to evaluate the validity of the previously suggested threshold.

Design: Multicenter study. Retrospective observational analysis of prospectively collected data.

Setting: 3 centres specialized in non-invasive respiratory support (Buenos Aires, Argentina; Bolzano and Treviso, Italy). Patients treated outside the ICU were analysed MEASUREMENTS: The variables to calculate the ROX index were collected during the first day of therapy at 2, 6, 12 and 24 hours and then recorded every 24 hours. HFNC failure was defined as escalation of respiratory support to invasive mechanical ventilation (IMV) or death.

Main results: A total of 35 (29%) patients failed HFNC and required intubation. ROC analysis identified the 12-hour ROX index as the best predictor of intubation with an AUC of 0.7916[CI 95% 0.6905-0.8927] and the best threshold to be 5.99[Specificity 96% Sensitivity 62%]. In the survival analysis, a ROX value <5.99 was associated with an increased risk of failure (p = 0008 log - rank test). The threshold of 4,9 identified by Roca as the best predictor in non-COVID patients, was not able to discriminate between success and failure (p = 0.4 log-rank test) in our patients.

Conclusions: ROX index may be useful in guiding the clinicians in their decision to intubate patients, especially in patients with moderate ARF, treated therefore outside the ICU. Indeed, it also demonstrates a different threshold value than reported for non-COVID patients, possibly related to the different mechanisms of hypoxia.

Keywords: AHRF; Acute hypoxemic respiratory failure; HFNC; High flow nasal cannula; Non invasive respiratory support; ROX index.

Copyright © 2021 Sociedade Portuguesa de Pneumologia. Published by Elsevier España, S.L.U. All rights reserved.

Comment in

- ROX monitoring in critical COVID-19 patients treated with high flow oxygen: A real added value compared to the respiratory rate?
  Garnier M, Blez D. Garnier M, et al. Pulmonology. 2021 Sep-Oct;27(5):474. doi: 10.1016/j.pulmoe.2021.06.011. Epub 2021 Jul 9. Pulmonology. 2021. PMID: 34312119
  Free PMC article. No abstract available.

- 14 references
- 1 figure

Supplementary info

Publication types, MeSH terms Expand

Publication types

- Multicenter Study
- Observational Study

MeSH terms

- COVID-19*
- Humans
- Intensive Care Units
- Intubation, Intratracheal
- Noninvasive Ventilation* / methods
- Oximetry
- Oxygen Inhalation Therapy / methods*
- Respiratory Insufficiency / therapy*
- Respiratory Rate / physiology
- Retrospective Studies
- SARS-CoV-2

Full text links

ELSEVIER OPEN ACCESS Elsevier Science Free PMC article

Proceed to details

Cite

Share

☐ 606

Observational Study

Rev Esp Enferm Dig
. 2022 Feb;114(2):118-119.
doi: 10.17235/reed.2021.8320/2021.

Switch to subcutaneous infliximab during the SARS-CoV-2 pandemic: preliminary results

Federico Argüelles-Arias [1], Paula Fernández Álvarez [2], Luisa Castro Laria [3], Belén Maldonado Pérez [2], María Belvis Jiménez [3], Vicente Merino-Bohórquez [4], Ángel Caunedo Álvarez [2], Miguel Ángel Calleja Hernández [4]
Affiliations Expand

Affiliations

- [1] Faculty of Medicine, Universidad de Sevilla. Hospital Universitario Virgen Macarena.
- [2] Digestive Diseases, Hospital Universitario Virgen Macarena, España.
- [3] Digestive Diseases, Hospital Universitario Virgen Macarena.
- [4] Pharmacology, Hospital Universitario Virgen Macarena.

- PMID: **34517718**
- DOI: 10.17235/reed.2021.8320/2021

Free article
Observational Study

Switch to subcutaneous infliximab during the SARS-CoV-2 pandemic: preliminary results

Federico Argüelles-Arias et al. Rev Esp Enferm Dig. 2022 Feb.
Free article
Show details
Rev Esp Enferm Dig
. 2022 Feb;114(2):118-119.
doi: 10.17235/reed.2021.8320/2021.

Authors

Federico Argüelles-Arias [1], Paula Fernández Álvarez [2], Luisa Castro Laria [3], Belén Maldonado Pérez [2], María Belvis Jiménez [3], Vicente Merino-Bohórquez [4], Ángel Caunedo Álvarez [2], Miguel Ángel Calleja Hernández [4]

Affiliations

- [1] Faculty of Medicine, Universidad de Sevilla. Hospital Universitario Virgen Macarena.
- [2] Digestive Diseases, Hospital Universitario Virgen Macarena, España.

- [3] Digestive Diseases, Hospital Universitario Virgen Macarena.
- [4] Pharmacology, Hospital Universitario Virgen Macarena.

- PMID: **34517718**
- DOI: 10.17235/reed.2021.8320/2021

Abstract

A new subcutaneous formulation of the infliximab biosimilar CT-P13 has recently been developed for the treatment of inflammatory bowel disease (IBD), providing response rates similar to intravenous treatment. The use of this new formulation was requested, in an effort to limit patient attendance at intravenous infusion centers and to maintain biological treatment during the COVID-19 pandemic. The objective of this observational, retrospective and descriptive study was to assess CT-P13 efficacy and safety after switching from intravenous to a subcutaneous formulation in patients with IBD receiving maintenance therapy. This article shows preliminary results after six months of follow-up.

Supplementary info

Publication types, MeSH terms, Substances Expand

Publication types

- Letter
- Observational Study

MeSH terms

- Biosimilar Pharmaceuticals* / therapeutic use
- COVID-19*
- Drug Substitution / methods
- Gastrointestinal Agents / therapeutic use
- Humans
- Inflammatory Bowel Diseases* / drug therapy
- Infliximab / therapeutic use
- Pandemics
- Prospective Studies
- Retrospective Studies
- SARS-CoV-2
- Treatment Outcome

Substances

- Biosimilar Pharmaceuticals
- Gastrointestinal Agents

- Infliximab

Full text links

REED.es Free full text Aran ediciones, S.L.
Proceed to details
Cite
Share
607
Observational Study
Ann Surg
. 2022 Jan 1;275(1):31-36.
doi: 10.1097/SLA.0000000000005176.

COVID-19 Vaccination Associated With Reduced Postoperative SARS-CoV-2 Infection and Morbidity

Nikhil K Prasad [1, 2], Rachel Lake [1, 2], Brian R Englum [1], Douglas J Turner [1, 2], Tariq Siddiqui [2], Minerva Mayorga-Carlin [1, 2], John D Sorkin [3, 4], Brajesh K Lal [1, 2]
Affiliations Expand

Affiliations

- [1] Department of Surgery, University of Maryland School of Medicine, Baltimore, MD.
- [2] Surgery Service, Veterans Affairs Medical Centre, Baltimore, MD.
- [3] Geriatrics Research, Education, and Clinical Center, Veterans Affairs Medical Centre, Baltimore, MD.
- [4] Department of Medicine, University of Maryland School of Medicine, Baltimore, MD.

- PMID: **34417362**
- PMCID: PMC8678152
- DOI: 10.1097/SLA.0000000000005176

Free PMC article
Observational Study

COVID-19 Vaccination Associated With Reduced Postoperative SARS-CoV-2 Infection and Morbidity

Nikhil K Prasad et al. Ann Surg. 2022.
Free PMC article

Show details

Ann Surg
. 2022 Jan 1;275(1):31-36.
doi: 10.1097/SLA.0000000000005176.

Authors

Nikhil K Prasad [1, 2], Rachel Lake [1, 2], Brian R Englum [1], Douglas J Turner [1, 2], Tariq Siddiqui [2], Minerva Mayorga-Carlin [1, 2], John D Sorkin [3, 4], Brajesh K Lal [1, 2]

Affiliations

- [1] Department of Surgery, University of Maryland School of Medicine, Baltimore, MD.
- [2] Surgery Service, Veterans Affairs Medical Centre, Baltimore, MD.
- [3] Geriatrics Research, Education, and Clinical Center, Veterans Affairs Medical Centre, Baltimore, MD.
- [4] Department of Medicine, University of Maryland School of Medicine, Baltimore, MD.

- PMID: **34417362**
- PMCID: PMC8678152
- DOI: 10.1097/SLA.0000000000005176

Abstract

Objective: The purpose of this study was to determine the effect of COVID-19 vaccination on postoperative mortality, pulmonary and thrombotic complications, readmissions and hospital lengths of stay among patients undergoing surgery in the United States.

Background: While vaccination prevents COVID-19, little is known about its impact on postoperative complications.

Methods: This is a nationwide observational cohort study of all 1,255 Veterans Affairs facilities nationwide. We compared patients undergoing surgery at least 2 weeks after their second dose of the Pfizer BioNTech or Moderna vaccines, to contemporary propensity score matched controls. Primary endpoints were 30-day mortality and postoperative COVID-19 infection. Secondary endpoints were pulmonary or thrombotic complications, readmissions, and hospital lengths of stay.

Results: 30,681 patients met inclusion criteria. After matching, there were 3,104 in the vaccination group (1,903 received the Pfizer BioNTech, and 1,201 received the Moderna vaccine) and 7,438 controls. Full COVID-19 vaccination was associated with lower rates of postoperative 30-day COVID-19 infection (Incidence Rate Ratio and 95% confidence intervals, 0.09 [0.01,0.44]), pulmonary complications (0.54 [0.39, 0.72]), thrombotic complications (0.68 [0.46, 0.99]) and decreased hospital lengths of stay (0.78 [0.69, 0.89]). Complications were also low in vaccinated patients who tested COVID-19 positive before surgery but events were too few to detect a significant difference compared to controls.

Conclusion: COVID-19 vaccination is associated with lower rates of postoperative morbidity. The benefit is most pronounced among individuals who have never had a COVID-19 infection before surgery.

Copyright © 2021 Wolters Kluwer Health, Inc. All rights reserved.

Conflict of interest statement

The authors report no conflicts of interest.

- 27 references
- 1 figure

Supplementary info

Publication types, MeSH terms, Substances, Grant support Expand

Publication types

- Multicenter Study
- Observational Study
- Research Support, N.I.H., Extramural
- Research Support, N.I.H., Intramural
- Research Support, Non-U.S. Gov't
- Research Support, U.S. Gov't, Non-P.H.S.

MeSH terms

- Adult
- Aged
- Aged, 80 and over
- COVID-19 / epidemiology
- COVID-19 / prevention & control*
- COVID-19 Vaccines*
- Female
- Humans
- Length of Stay / statistics & numerical data
- Male
- Matched-Pair Analysis
- Middle Aged
- Patient Readmission / statistics & numerical data
- Poisson Distribution
- Postoperative Complications / epidemiology
- Postoperative Complications / prevention & control*
- Propensity Score
- Regression Analysis
- Retrospective Studies
- Treatment Outcome

Substances

- COVID-19 Vaccines

Grant support

- Z01 AG000513/ImNIH/Intramural NIH HHS/United States
- U01 NS080168/NS/NINDS NIH HHS/United States
- P30 AG028747/AG/NIA NIH HHS/United States
- P30 DK072488/DK/NIDDK NIH HHS/United States
- I01 RX000995/RX/RRD VA/United States

- R01 NS097876/NS/NINDS NIH HHS/United States
- I01 CX001621/CX/CSRD VA/United States
- T32 AG000262/AG/NIA NIH HHS/United States

Show all 8 grants

Full text links

Wolters Kluwer Wolters Kluwer Free PMC article
Proceed to details
Cite
Share
608
Eur J Clin Microbiol Infect Dis
. 2021 Sep;40(9):1963-1974.
doi: 10.1007/s10096-021-04260-z. Epub 2021 May 6.

Sex-specific impact of severe obesity in the outcomes of hospitalized patients with COVID-19: a large retrospective study from the Bronx, New York

Arcelia Guerson-Gil [1 2 3], Leonidas Palaiodimos [4 5], Andrei Assa [4 6], Dimitris Karamanis [7], Damianos Kokkinidis [4 5], Natalia Chamorro-Pareja [4 5], Preeti Kishore [4 5], Jason M Leider [4 5], Lawrence J Brandt [4 8 6]
Affiliations Expand

Affiliations

- [1] Albert Einstein College of Medicine, Bronx, NY, USA. aguerson@montefiore.org.
- [2] Division of Gastroenterology, Montefiore Medical Center, 111 East 210th Street, Bronx, NY, 10467, USA. aguerson@montefiore.org.

- [3] Department of Medicine, Jacobi Medical Center, Bronx, NY, USA. aguerson@montefiore.org.
- [4] Albert Einstein College of Medicine, Bronx, NY, USA.
- [5] Department of Medicine, Jacobi Medical Center, Bronx, NY, USA.
- [6] Department of Medicine, Montefiore Medical Center, Bronx, NY, USA.
- [7] Department of Economics, University of Piraeus, Attica, Greece.
- [8] Division of Gastroenterology, Montefiore Medical Center, 111 East 210th Street, Bronx, NY, 10467, USA.

- PMID: **33956286**
- PMCID: PMC8101338
- DOI: 10.1007/s10096-021-04260-z

Free PMC article

Sex-specific impact of severe obesity in the outcomes of hospitalized patients with COVID-19: a large retrospective study from the Bronx, New York

Arcelia Guerson-Gil et al. Eur J Clin Microbiol Infect Dis. 2021 Sep.
Free PMC article
Show details
Eur J Clin Microbiol Infect Dis
. 2021 Sep;40(9):1963-1974.
doi: 10.1007/s10096-021-04260-z. Epub 2021 May 6.

Authors

Arcelia Guerson-Gil [1 2 3], Leonidas Palaiodimos [4 5], Andrei Assa [4 6], Dimitris Karamanis [7], Damianos Kokkinidis [4 5], Natalia Chamorro-Pareja [4 5], Preeti Kishore [4 5], Jason M Leider [4 5], Lawrence J Brandt [4 8 6]

Affiliations

- [1] Albert Einstein College of Medicine, Bronx, NY, USA. aguerson@montefiore.org.
- [2] Division of Gastroenterology, Montefiore Medical Center, 111 East 210th Street, Bronx, NY, 10467, USA. aguerson@montefiore.org.
- [3] Department of Medicine, Jacobi Medical Center, Bronx, NY, USA. aguerson@montefiore.org.
- [4] Albert Einstein College of Medicine, Bronx, NY, USA.
- [5] Department of Medicine, Jacobi Medical Center, Bronx, NY, USA.
- [6] Department of Medicine, Montefiore Medical Center, Bronx, NY, USA.
- [7] Department of Economics, University of Piraeus, Attica, Greece.

- [8] Division of Gastroenterology, Montefiore Medical Center, 111 East 210th Street, Bronx, NY, 10467, USA.

- PMID: **33956286**
- PMCID: PMC8101338
- DOI: 10.1007/s10096-021-04260-z

Abstract

It has been demonstrated that obesity is an independent risk factor for worse outcomes in patients with COVID-19. Our objectives were to investigate which classes of obesity are associated with higher in-hospital mortality and to assess the association between obesity and systemic inflammation. This was a retrospective study which included consecutive hospitalized patients with COVID-19 in a tertiary center. Three thousand five hundred thirty patients were included in this analysis (female sex: 1579, median age: 65 years). The median body mass index (BMI) was 28.8 kg/m^2. In the overall cohort, a J-shaped association between BMI and in-hospital mortality was depicted. In the subgroup of men, BMI 35-39.9 kg/m^2 and BMI ≥40 kg/m^2 were found to have significant association with higher in-hospital mortality, while only BMI ≥40 kg/m^2 was found significant in the subgroup of women. No significant association between BMI and IL-6 was noted. Obesity classes II and III in men and obesity class III in women were independently associated with higher in-hospital mortality in patients with COVID-19. The male population with severe obesity was the one that mainly drove this association. No significant association between BMI and IL-6 was noted.

Keywords: COVID-19; IL-6; Inflammation; Mortality; Novel coronavirus; Obesity; Observational study; Risk factor; SARS-CoV-2.

© 2021. The Author(s), under exclusive licence to Springer-Verlag GmbH Germany, part of Springer Nature.

Conflict of interest statement

The authors declare no competing interests.

- 66 references
- 2 figures

Supplementary info

MeSH terms Expand

MeSH terms

- Aged
- Aged, 80 and over
- Body Mass Index
- COVID-19 / complications
- COVID-19 / epidemiology
- COVID-19 / mortality

- COVID-19 / therapy*
- Female
- Hospital Mortality
- Hospitalization
- Humans
- Male
- Middle Aged
- New York City / epidemiology
- Obesity, Morbid / complications
- Obesity, Morbid / epidemiology
- Obesity, Morbid / mortality
- Obesity, Morbid / therapy*
- Retrospective Studies
- Sex Factors
- Treatment Outcome

Full text links

SpringerLink FULL-TEXT ARTICLE Springer Free PMC article
Proceed to details
Cite
Share
☐ 609
Observational Study
J Pediatr
. 2020 Sep;224:30-36.
doi: 10.1016/j.jpeds.2020.06.041. Epub 2020 Jun 18.

A Comparison Between Chinese Children Infected with Coronavirus Disease-2019 and with Severe Acute Respiratory Syndrome 2003

Xiaoli Xiong [1], Gilbert T Chua [2], Shuiqing Chi [3], Mike Yat Wah Kwan [4], Wilfred Hing Sang Wong [2], Aifen Zhou [5], Chi Chiu Shek [4], Keith T S Tung [2], Huan Qin [6], Rosa S Wong [2], Xue Li [7], Peng Chen [8], Shuai Li [3], Celine S Chui [7], Winnie W Y Tso [2], Marco H K Ho [2], Ian C K Wong [9], Godfrey C F Chan [2], Yu Lung Lau [2], Kenneth K Y Wong [10], Patrick H Y Chung [10], Hui Li [11], Paul K H Tam [12], Shao-Tao Tang [3], Patrick Ip [13]
Affiliations Expand

Affiliations

- [1] Department of Integrated Chinese and Western Medicine, Wuhan Children's Hospital (Wuhan Maternal and Child Healthcare Hospital), Tongji Medical College, Huazhong University of Science and Technology, Wuhan, China.
- [2] Department of Pediatrics and Adolescent Medicine, The University of Hong Kong, Hong Kong SAR, China.
- [3] Department of Pediatric Surgery, Union Hospital, Tongji Medical College, Huazhong University of Science and Technology, Wuhan, China.
- [4] Department of Pediatrics and Adolescent Medicine, Princess Margaret Hospital, Hong Kong SAR, China.
- [5] Department of Maternal Healthcare, Wuhan Children's Hospital (Wuhan Maternal and Child Healthcare Hospital), Tongji Medical College, Huazhong University of Science and Technology, Wuhan, China.
- [6] Institute of Maternal and Child Health, Wuhan Children's Hospital (Wuhan Maternal and Child Healthcare Hospital), Tongji Medical College, Huazhong University of Science and Technology, Wuhan.
- [7] Department of Pediatrics and Adolescent Medicine, The University of Hong Kong, Hong Kong SAR, China; Center for Safe Medication Practice and Research, Department of Pharmacology and Pharmacy, The University of Hong Kong, Hong Kong SAR, China.
- [8] Department of Respiratory Medicine, Wuhan Children's Hospital (Wuhan Maternal and Child Healthcare Hospital), Tongji Medical College, Huazhong University of Science and Technology, Wuhan, China.
- [9] Center for Safe Medication Practice and Research, Department of Pharmacology and Pharmacy, The University of Hong Kong, Hong Kong SAR, China; Research Department of Practice and Policy, UCL School of Pharmacy, University College, London, UK.
- [10] Division of Pediatric Surgery, Department of Surgery, The University of Hong Kong, Hong Kong SAR, China.
- [11] Department of Hematology, Wuhan Children's Hospital (Wuhan Maternal and Child Healthcare Hospital), Tongji Medical College, Huazhong University of Science and Technology, Wuhan, China.
- [12] Division of Pediatric Surgery, Department of Surgery, The University of Hong Kong, Hong Kong SAR, China; Dr. Li Dak Sum Research Center, The University of Hong Kong-Karolinska Institute Collaboration in Regenerative Medicine, The University of Hong Kong, China.
- [13] Department of Pediatrics and Adolescent Medicine, The University of Hong Kong, Hong Kong SAR, China. Electronic address: patricip@hku.hk.

- PMID: **32565097**
- PMCID: PMC7301144
- DOI: 10.1016/j.jpeds.2020.06.041

Free PMC article
Observational Study

A Comparison Between Chinese Children Infected with Coronavirus Disease-2019 and

with Severe Acute Respiratory Syndrome 2003

Xiaoli Xiong et al. J Pediatr. 2020 Sep.
Free PMC article
Show details
J Pediatr
. 2020 Sep;224:30-36.
doi: 10.1016/j.jpeds.2020.06.041. Epub 2020 Jun 18.

Authors

Xiaoli Xiong [1], Gilbert T Chua [2], Shuiqing Chi [3], Mike Yat Wah Kwan [4], Wilfred Hing Sang Wong [2], Aifen Zhou [5], Chi Chiu Shek [4], Keith T S Tung [2], Huan Qin [6], Rosa S Wong [2], Xue Li [7], Peng Chen [8], Shuai Li [3], Celine S Chui [7], Winnie W Y Tso [2], Marco H K Ho [2], Ian C K Wong [9], Godfrey C F Chan [2], Yu Lung Lau [2], Kenneth K Y Wong [10], Patrick H Y Chung [10], Hui Li [11], Paul K H Tam [12], Shao-Tao Tang [3], Patrick Ip [13]

Affiliations

- [1] Department of Integrated Chinese and Western Medicine, Wuhan Children's Hospital (Wuhan Maternal and Child Healthcare Hospital), Tongji Medical College, Huazhong University of Science and Technology, Wuhan, China.
- [2] Department of Pediatrics and Adolescent Medicine, The University of Hong Kong, Hong Kong SAR, China.
- [3] Department of Pediatric Surgery, Union Hospital, Tongji Medical College, Huazhong University of Science and Technology, Wuhan, China.
- [4] Department of Pediatrics and Adolescent Medicine, Princess Margaret Hospital, Hong Kong SAR, China.
- [5] Department of Maternal Healthcare, Wuhan Children's Hospital (Wuhan Maternal and Child Healthcare Hospital), Tongji Medical College, Huazhong University of Science and Technology, Wuhan, China.
- [6] Institute of Maternal and Child Health, Wuhan Children's Hospital (Wuhan Maternal and Child Healthcare Hospital), Tongji Medical College, Huazhong University of Science and Technology, Wuhan.
- [7] Department of Pediatrics and Adolescent Medicine, The University of Hong Kong, Hong Kong SAR, China; Center for Safe Medication Practice and Research, Department of Pharmacology and Pharmacy, The University of Hong Kong, Hong Kong SAR, China.
- [8] Department of Respiratory Medicine, Wuhan Children's Hospital (Wuhan Maternal and Child Healthcare Hospital), Tongji Medical College, Huazhong University of Science and Technology, Wuhan, China.
- [9] Center for Safe Medication Practice and Research, Department of Pharmacology and Pharmacy, The University of Hong Kong, Hong Kong SAR, China; Research Department of Practice and Policy, UCL School of Pharmacy, University College, London, UK.
- [10] Division of Pediatric Surgery, Department of Surgery, The University of Hong Kong, Hong Kong SAR, China.

- [11] Department of Hematology, Wuhan Children's Hospital (Wuhan Maternal and Child Healthcare Hospital), Tongji Medical College, Huazhong University of Science and Technology, Wuhan, China.
- [12] Division of Pediatric Surgery, Department of Surgery, The University of Hong Kong, Hong Kong SAR, China; Dr. Li Dak Sum Research Center, The University of Hong Kong-Karolinska Institute Collaboration in Regenerative Medicine, The University of Hong Kong, China.
- [13] Department of Pediatrics and Adolescent Medicine, The University of Hong Kong, Hong Kong SAR, China. Electronic address: patricip@hku.hk.

- PMID: **32565097**
- PMCID: PMC7301144
- DOI: 10.1016/j.jpeds.2020.06.041

Abstract

Objectives: To compare the clinical and laboratory features of severe acute respiratory syndrome 2003 (SARS) and coronavirus disease 2019 (COVID-19) in 2 Chinese pediatric cohorts, given that the causative pathogens and are biologically similar.

Study design: This is a cross-sectional study reviewing pediatric patients with SARS (n = 43) and COVID-19 (n = 244) who were admitted to the Princess Margaret Hospital in Hong Kong and Wuhan Children's Hospital in Wuhan, respectively. Demographics, hospital length of stay, and clinical and laboratory features were compared.

Results: Overall, 97.7% of patients with SARS and 85.2% of patients with COVID-19 had epidemiologic associations with known cases. Significantly more patients with SARS developed fever, chills, myalgia, malaise, coryza, sore throat, sputum production, nausea, headache, and dizziness than patients with COVID-19. No patients with SARS were asymptomatic at the time of admission, whereas 29.1% and 20.9% of patients with COVID-19 were asymptomatic on admission and throughout their hospital stay, respectively. More patients with SARS required oxygen supplementation than patients with COVID-19 (18.6 vs 4.7%; $P = .004$). Only 1.6% of patients with COVID-19 and 2.3% of patients with SARS required mechanical ventilation. Leukopenia (37.2% vs 18.6%; $P = .008$), lymphopenia (95.4% vs 32.6%; $P < .01$), and thrombocytopenia (41.9% vs 3.8%; $P < .001$) were significantly more common in patients with SARS than in patients with COVID-19. The duration between positive and negative nasopharyngeal aspirate and the length in hospital stay were similar in patients with COVID-19, regardless of whether they were asymptomatic or symptomatic, suggesting a similar duration of viral shedding.

Conclusions: Children with COVID-19 were less symptomatic and had more favorable hematologic findings than children with SARS.

Keywords: COVID-19; Chinese; SARS; children.

Copyright © 2020 Elsevier Inc. All rights reserved.

- 34 references
- 2 figures

Supplementary info

Publication types, MeSH terms Expand

Publication types

- Comparative Study
- Observational Study

MeSH terms

- Adolescent
- Asymptomatic Infections
- Betacoronavirus
- COVID-19
- Child
- Child, Preschool
- China / epidemiology
- Coronavirus Infections / diagnosis
- Coronavirus Infections / epidemiology*
- Cross-Sectional Studies
- Female
- Hong Kong
- Hospitalization
- Humans
- Infant
- Length of Stay
- Male
- Pandemics
- Pneumonia, Viral / diagnosis
- Pneumonia, Viral / epidemiology*
- Retrospective Studies
- SARS Virus
- SARS-CoV-2
- Severe Acute Respiratory Syndrome / diagnosis
- Severe Acute Respiratory Syndrome / epidemiology*

Full text links

ELSEVIER FULL-TEXT ARTICLE Elsevier Science Free PMC article

Proceed to details

Cite

Share

☐ 610

Observational Study
Diabetes Metab Syndr
. Jan-Feb 2021;15(1):33-38.
doi: 10.1016/j.dsx.2020.12.014. Epub 2020 Dec 5.

Predictors of adverse in-hospital outcome and recovery in patients with diabetes mellitus and COVID-19 pneumonia in Iraq

Hussein Nafakhi [1], Mohammed Alareedh [2], Karrar Al-Buthabhak [3], Foaad Shaghee [4], Ahmed Nafakhi [5], Samet Kasim [6]
Affiliations Expand

Affiliations

- [1] Internal Medicine Department, Medicine College, University of Kufa, Najaf, Iraq. Electronic address: husseinaf.alnaffakh@uokufa.edu.iq.
- [2] Internal Medicine Department, Medicine College, University of Kufa, Najaf, Iraq. Electronic address: mohammed.alareedh@uokufa.edu.iq.
- [3] Internal Medicine Department, Medicine College, University of Kufa, Najaf, Iraq. Electronic address: kararm.zwain@uokufa.edu.iq.
- [4] Internal Medicine Department, Jabir Ibn Hayyan Medical University Faculty of Medicine, Kufa, Iraq. Electronic address: f.fertossy@jmu.edu.iq.
- [5] Research Unit, Najaf Health Bureau, Ministry of Health, Iraq. Electronic address: nafakh06@gmail.com.
- [6] Internal Medicine Department, Medicine College, University of Kufa, Najaf, Iraq. Electronic address: samete.almoula@uokufa.edu.iq.

- PMID: **33296788**
- PMCID: PMC7832757
- DOI: 10.1016/j.dsx.2020.12.014

Free PMC article
Observational Study

Predictors of adverse in-hospital outcome and recovery in patients with diabetes mellitus and COVID-19 pneumonia in Iraq

Hussein Nafakhi et al. Diabetes Metab Syndr. Jan-Feb 2021.
Free PMC article
Show details
Diabetes Metab Syndr
. Jan-Feb 2021;15(1):33-38.

doi: 10.1016/j.dsx.2020.12.014. Epub 2020 Dec 5.

Authors

Hussein Nafakhi [1], Mohammed Alareedh [2], Karrar Al-Buthabhak [3], Foaad Shaghee [4], Ahmed Nafakhi [5], Samet Kasim [6]

Affiliations

- [1] Internal Medicine Department, Medicine College, University of Kufa, Najaf, Iraq. Electronic address: husseinaf.alnaffakh@uokufa.edu.iq.
- [2] Internal Medicine Department, Medicine College, University of Kufa, Najaf, Iraq. Electronic address: mohammed.alareedh@uokufa.edu.iq.
- [3] Internal Medicine Department, Medicine College, University of Kufa, Najaf, Iraq. Electronic address: kararm.zwain@uokufa.edu.iq.
- [4] Internal Medicine Department, Jabir Ibn Hayyan Medical University Faculty of Medicine, Kufa, Iraq. Electronic address: f.fertossy@jmu.edu.iq.
- [5] Research Unit, Najaf Health Bureau, Ministry of Health, Iraq. Electronic address: nafakh06@gmail.com.
- [6] Internal Medicine Department, Medicine College, University of Kufa, Najaf, Iraq. Electronic address: samete.almoula@uokufa.edu.iq.

- PMID: **33296788**
- PMCID: PMC7832757
- DOI: 10.1016/j.dsx.2020.12.014

Abstract

Background and aims: There is limited data about the prognosis and impact of COVID-19 pneumonia on patients with diabetes mellitus (DM). We aimed to assess blood indices, ECG markers of sudden death and malignant arrhythmias on admission, and diabetes lowering drugs as possible predictors of adverse in-hospital outcome and COVID-19 pneumonia recovery status.

Methods: A retrospective study included patients with newly diagnosed COVID-19 pneumonia from August 20, to October 5, 2020.

Results: A total of 192 patients with COVID-19 pneumonia were included in the present study, of whom 67 patients had DM. Low lymphocytes % [0.4(0.1-0.9), P = .011] and QTc interval prolongation [0.4(0.1-0.8), P = .022] were associated with increased length of ICU stay. On the other hand, metformin use [0.3(0.2-4), P = .032] and DPP-4 inhibitors use [0.3(0.2-3), P = .040] were associated with decreased length of ICU stay. QTc interval prolongation [0.4(0.1-0.9), P = .017] was associated with increased length of hospital stay, while using metformin [0.4(0.2-3), P = .022] was associated with decreased length of hospital stay. Low lymphocytes % [0.5(0.4-1.6), P = .001], insulin use [0.4(0.3-5), P = .003], and old age [0.5(0.1-2.3), P = .025] were associated with extensive lung injury. The risk for in-hospital death was associated with high neutrophil% [1 (1-1.4), P = .045], while metformin use was associated with decreased risk for in-hospital death [0.1(0.1-0.6), P = .025]. Insulin use [0.3(0.2-4), P = .013] was associated with partial recovery following acute COVID pneumonia.

Conclusions: Metformin and DPP-4 inhibitors use were associated with favorable in-hospital outcomes, while insulin use was associated with extensive lung injury and post-acute COVID-19 pneumonia partial recovery.

Keywords: COVID-19; Diabetes mellitus; Outcome; Pneumonia; Predictor.

Copyright © 2020 Diabetes India. Published by Elsevier Ltd. All rights reserved.

Conflict of interest statement

Declaration of competing interest The authors declare that they have no conflict of interest.

- 25 references

Supplementary info

Publication types, MeSH terms, Substances Expand

Publication types

- Observational Study

MeSH terms

- Adult
- Aged
- COVID-19 / diagnosis*
- COVID-19 / drug therapy
- COVID-19 / epidemiology*
- Diabetes Mellitus / diagnosis*
- Diabetes Mellitus / drug therapy
- Diabetes Mellitus / epidemiology*
- Dipeptidyl-Peptidase IV Inhibitors / pharmacology
- Dipeptidyl-Peptidase IV Inhibitors / therapeutic use
- Hospitalization / trends*
- Humans
- Hypoglycemic Agents / pharmacology
- Hypoglycemic Agents / therapeutic use
- Iraq
- Metformin / pharmacology
- Metformin / therapeutic use
- Middle Aged
- Prognosis
- Recovery of Function / drug effects
- Recovery of Function / physiology*

- Retrospective Studies
- Treatment Outcome

Substances

- Dipeptidyl-Peptidase IV Inhibitors
- Hypoglycemic Agents
- Metformin

Full text links

ELSEVIER FULL-TEXT ARTICLE Elsevier Science Free PMC article
Proceed to details
Cite
Share
611
Observational Study
J Investig Med
. 2021 Jun;69(5):962-969.
doi: 10.1136/jim-2021-001810. Epub 2021 Apr 13.

Hemogram as marker of in-hospital mortality in COVID-19

Alejandro López-Escobar [1, 2, 3], Rodrigo Madurga [3, 4], José María Castellano [2, 3, 5], Santiago Ruiz de Aguiar [3, 6], Sara Velázquez [3, 7, 8], Marina Bucar [3, 9], Sara Jimeno [10, 2, 3], Paula Sol Ventura [3, 11]
Affiliations Expand

Affiliations

- [1] Pediatrics Department, HM Hospitales, Madrid, Spain alopezescobar@hmhospitales.com.
- [2] Faculty of Medicine, Universidad San Pablo CEU, Madrid, Spain.
- [3] Fundación de Investigación, HM Hospitales, Madrid, Spain.
- [4] Faculty of Experimental Sciences, Universidad Francisco de Vitoria, Pozuelo de Alarcon, Comunidad de Madrid, Spain.
- [5] Cardiology Department, Hospital Universitario HM Montepríncipe, HM Hospitales, Madrid, Spain.
- [6] Medical Management, HM Hospitales, Madrid, Spain.
- [7] Anaesthesia Department, HM Hospitales, Madrid, Spain.
- [8] Anaesthesia Department, Hospital Universitario Santa Cristina, Madrid, Spain.
- [9] Internal Medicine Department, HM Hospitales, Madrid, Spain.
- [10] Pediatrics Department, HM Hospitales, Madrid, Spain.

- [11] Pediatrics Department Hospital Universitario HM Nens, HM Hospitales, Barcelona, Madrid, Spain.

- PMID: **33849952**
- PMCID: PMC8050870
- DOI: 10.1136/jim-2021-001810

Free PMC article
Observational Study

Hemogram as marker of in-hospital mortality in COVID-19

Alejandro López-Escobar et al. J Investig Med. 2021 Jun.
Free PMC article

Show details

J Investig Med

. 2021 Jun;69(5):962-969.
doi: 10.1136/jim-2021-001810. Epub 2021 Apr 13.

Authors

Alejandro López-Escobar [1 2 3], Rodrigo Madurga [3 4], José María Castellano [2 3 5], Santiago Ruiz de Aguiar [3 6], Sara Velázquez [3 7 8], Marina Bucar [3 9], Sara Jimeno [10 2 3], Paula Sol Ventura [3 11]

Affiliations

- [1] Pediatrics Department, HM Hospitales, Madrid, Spain alopezescobar@hmhospitales.com.
- [2] Faculty of Medicine, Universidad San Pablo CEU, Madrid, Spain.
- [3] Fundación de Investigación, HM Hospitales, Madrid, Spain.
- [4] Faculty of Experimental Sciences, Universidad Francisco de Vitoria, Pozuelo de Alarcon, Comunidad de Madrid, Spain.
- [5] Cardiology Department, Hospital Universitario HM Montepríncipe, HM Hospitales, Madrid, Spain.
- [6] Medical Management, HM Hospitales, Madrid, Spain.
- [7] Anaesthesia Department, HM Hospitales, Madrid, Spain.
- [8] Anaesthesia Department, Hospital Universitario Santa Cristina, Madrid, Spain.
- [9] Internal Medicine Department, HM Hospitales, Madrid, Spain.
- [10] Pediatrics Department, HM Hospitales, Madrid, Spain.
- [11] Pediatrics Department Hospital Universitario HM Nens, HM Hospitales, Barcelona, Madrid, Spain.

- PMID: **33849952**
- PMCID: PMC8050870
- DOI: 10.1136/jim-2021-001810

Abstract

The clinical impact of COVID-19 disease calls for the identification of routine variables to identify patients at increased risk of death. Current understanding of moderate-to-severe COVID-19 pathophysiology points toward an underlying cytokine release driving a hyperinflammatory and procoagulant state. In this scenario, white blood cells and platelets play a direct role as effectors of such inflammation and thrombotic response. We investigate whether hemogram-derived ratios such as neutrophil-to-lymphocyte ratio (NLR), platelet-to-lymphocyte ratio and the systemic immune-inflammation index may help to identify patients at risk of fatal outcomes. Activated platelets and neutrophils may be playing a decisive role during the thromboinflammatory phase of COVID-19 so, in addition, we introduce and validate a novel marker, the neutrophil-to-platelet ratio (NPR).Two thousand and eighty-eight hospitalized patients with COVID-19 admitted at any of the hospitals of HM Hospitales group in Spain, from March 1 to June 10, 2020, were categorized according to the primary outcome of in-hospital death.Baseline values, as well as the rate of increase of the four ratios analyzed were significantly higher at hospital admission in patients who died than in those who were discharged ($p<0.0001$). In multivariable logistic regression models, NLR (OR 1.05; 95% CI 1.02 to 1.08, $p=0.00035$) and NPR (OR 1.23; 95% CI 1.12 to 1.36, $p<0.0001$) were significantly and independently associated with in-hospital mortality.According to our results, hemogram-derived ratios obtained at hospital admission, as well as the rate of change during hospitalization, may easily detect, primarily using NLR and the novel NPR, patients with COVID-19 at high risk of in-hospital mortality.

Keywords: COVID-19; blood platelets; critical care; neutrophils.

© American Federation for Medical Research 2021. No commercial re-use. See rights and permissions. Published by BMJ.

Conflict of interest statement

Competing interests: None declared.

- 26 references
- 1 figure

Supplementary info

Publication types, MeSH terms, Substances Expand

Publication types

- Multicenter Study
- Observational Study

MeSH terms

- Aged
- Aged, 80 and over
- Biomarkers / blood
- Blood Cell Count*

- COVID-19 / blood*
- COVID-19 / mortality*
- Female
- Hospital Mortality
- Hospitalization*
- Humans
- Logistic Models
- Male
- Middle Aged
- Neutrophils
- Predictive Value of Tests
- Retrospective Studies
- Spain

Substances

- Biomarkers

Full text links

BMJ Full Text HighWire Free PMC article
Proceed to details
Cite
Share
612
Observational Study
Sci Rep
. 2020 Nov 30;10(1):20834.
doi: 10.1038/s41598-020-77641-7.

Clinical characteristics and predictors of mortality associated with COVID-19 in elderly patients from a long-term care facility

Enrico Maria Trecarichi [1], Maria Mazzitelli [2], Francesca Serapide [2], Maria Chiara Pelle [2], Bruno Tassone [2], Eugenio Arrighi [2], Graziella Perri [2], Paolo Fusco [2], Vincenzo Scaglione [2], Chiara Davoli [2], Rosaria Lionello [2], Valentina La Gamba [2], Giuseppina Marrazzo [3], Maria Teresa Busceti [3], Amerigo Giudice [4], Marco Ricchio [2], Anna Cancelliere [2], Elena Lio [2], Giada Procopio [2], Francesco Saverio Costanzo [5] [6], Daniela Patrizia Foti [7], Giovanni Matera [8], Carlo Torti [2], IDTM UMG COVID-19 Group
Collaborators, Affiliations Expand

Collaborators

- **IDTM UMG COVID-19 Group**:
  Domenico Laganà, Maria Petullà, Bernardo Bertucci, Angela Quirino, Giorgio Settimo Barreca, Aida Giancotti, Luigia Gallo, Angelo Lamberti, Maria Carla Liberto, Nadia Marascio, Adele Emanuela De Francesco

Affiliations

- [1] Infectious and Tropical Disease Unit, Department of Medical and Surgical Sciences, "Magna Graecia" University of Catanzaro, Viale Europa, 88100, Catanzaro, Italy. em.trecarichi@unicz.it.
- [2] Infectious and Tropical Disease Unit, Department of Medical and Surgical Sciences, "Magna Graecia" University of Catanzaro, Viale Europa, 88100, Catanzaro, Italy.
- [3] Respiratory Unit, "Mater Domini" Teaching Hospital, Catanzaro, Italy.
- [4] Department of Health Sciences, "Magna Graecia" University of Catanzaro, Catanzaro, Italy.
- [5] Department of Experimental and Clinical Medicine, "Magna Graecia" University of Catanzaro, Catanzaro, Italy.
- [6] Center of Interdepartmental Services (CIS), "Magna Graecia" University of Catanzaro, Catanzaro, Italy.
- [7] Clinical Pathology Unit, Department of Health Sciences, "Magna Graecia" University of Catanzaro, Catanzaro, Italy.
- [8] Department of Health Sciences, Institute of Microbiology, "Magna Graecia" University of Catanzaro, Catanzaro, Italy.

- PMID: **33257703**
- PMCID: PMC7705720
- DOI: 10.1038/s41598-020-77641-7

Free PMC article
Observational Study

Clinical characteristics and predictors of mortality associated with COVID-19 in elderly patients from a long-term care facility

Enrico Maria Trecarichi et al. Sci Rep. 2020.
Free PMC article
Show details
Sci Rep
. 2020 Nov 30;10(1):20834.
doi: 10.1038/s41598-020-77641-7.

Authors

Enrico Maria Trecarichi [1], Maria Mazzitelli [2], Francesca Serapide [2], Maria Chiara Pelle [2], Bruno Tassone [2], Eugenio Arrighi [2], Graziella Perri [2], Paolo Fusco [2], Vincenzo Scaglione [2], Chiara Davoli [2], Rosaria Lionello [2], Valentina La Gamba [2], Giuseppina Marrazzo [3], Maria Teresa Busceti [3], Amerigo Giudice [4], Marco Ricchio [2], Anna Cancelliere [2], Elena Lio [2], Giada Procopio [2], Francesco Saverio Costanzo [5] [6], Daniela Patrizia Foti [7], Giovanni Matera [8], Carlo Torti [2], IDTM UMG COVID-19 Group

Collaborators

- **IDTM UMG COVID-19 Group**:
Domenico Laganà, Maria Petullà, Bernardo Bertucci, Angela Quirino, Giorgio Settimo Barreca, Aida Giancotti, Luigia Gallo, Angelo Lamberti, Maria Carla Liberto, Nadia Marascio, Adele Emanuela De Francesco

Affiliations

- [1] Infectious and Tropical Disease Unit, Department of Medical and Surgical Sciences, "Magna Graecia" University of Catanzaro, Viale Europa, 88100, Catanzaro, Italy. em.trecarichi@unicz.it.
- [2] Infectious and Tropical Disease Unit, Department of Medical and Surgical Sciences, "Magna Graecia" University of Catanzaro, Viale Europa, 88100, Catanzaro, Italy.
- [3] Respiratory Unit, "Mater Domini" Teaching Hospital, Catanzaro, Italy.
- [4] Department of Health Sciences, "Magna Graecia" University of Catanzaro, Catanzaro, Italy.
- [5] Department of Experimental and Clinical Medicine, "Magna Graecia" University of Catanzaro, Catanzaro, Italy.
- [6] Center of Interdepartmental Services (CIS), "Magna Graecia" University of Catanzaro, Catanzaro, Italy.
- [7] Clinical Pathology Unit, Department of Health Sciences, "Magna Graecia" University of Catanzaro, Catanzaro, Italy.
- [8] Department of Health Sciences, Institute of Microbiology, "Magna Graecia" University of Catanzaro, Catanzaro, Italy.

- PMID: **33257703**
- PMCID: PMC7705720
- DOI: 10.1038/s41598-020-77641-7

Abstract

Since December 2019, coronavirus disease 2019 (COVID-19) pandemic has spread from China all over the world and many COVID-19 outbreaks have been reported in long-term care facilities (LCTF). However, data on clinical characteristics and prognostic factors in such settings are scarce. We conducted a retrospective, observational cohort study to assess clinical characteristics and baseline predictors of mortality of COVID-19 patients hospitalized after an outbreak of SARS-CoV-2 infection in a LTCF. A total of 50 patients were included. Mean age was 80 years (SD, 12 years), and 24/50 (57.1%) patients were males. The overall in-hospital mortality rate was 32%. At Cox regression analysis, significant predictors of in-hospital mortality were: hypernatremia (HR 9.12), lymphocyte count < 1000 cells/μL (HR 7.45), cardiovascular diseases other than hypertension (HR 6.41), and higher levels of serum interleukin-6 (IL-6, pg/mL) (HR 1.005). Our study shows a high in-hospital mortality rate in a cohort of elderly patients with

COVID-19 and hypernatremia, lymphopenia, CVD other than hypertension, and higher IL-6 serum levels were identified as independent predictors of in-hospital mortality. Given the small population size as major limitation of our study, further investigations are necessary to better understand and confirm our findings in elderly patients.

Conflict of interest statement

The authors declare no competing interests.

- 30 references

Supplementary info

Publication types, MeSH terms, Substances Expand

Publication types

- Observational Study

MeSH terms

- Aged
- Aged, 80 and over
- COVID-19 / diagnosis*
- COVID-19 / mortality*
- Cardiovascular Diseases / complications
- China / epidemiology
- Cytokine Release Syndrome / pathology
- Female
- Hospital Mortality*
- Hospitalization
- Humans
- Hypernatremia / complications
- Interleukin-6 / blood
- Long-Term Care / statistics & numerical data*
- Lymphopenia / complications
- Male
- Nursing Homes
- Risk Factors
- SARS-CoV-2

Substances

- IL6 protein, human

- Interleukin-6

Full text links

Nature Publishing Group Free PMC article

Proceed to details

Cite

Share

613

Observational Study

Dig Liver Dis

. 2022 Jan;54(1):10-18.

doi: 10.1016/j.dld.2021.09.017. Epub 2021 Oct 4.

Effect of lockdown on digestive system cancer care amongst older patients during the first wave of COVID-19: The CADIGCOVAGE multicentre cohort study

Thomas Aparicio [1], Richard Layese [2], François Hemery [3], Christophe Tournigand [4], Elena Paillaud [5], Nicola De Angelis [6], Laurent Quero [7], Nathalie Ganne [8], Fredéric Prat [9], Atanas Pachev [10], Gilles Galula [11], Marc-Antoine Benderra [11], Florence Canouï-Poitrine [2], Clinical Data Warehouse of Greater Paris University Hospitals / Inserm COVID-19 research collaboration and Cancer AP-HP Group

Affiliations Expand

Affiliations

- [1] AP-HP, Gastroenterology and Digestive Oncology Department, Saint Louis Hospital, 1 avenue Claude Vellefaux, Paris F-75010, France; Université de Paris, Paris F-75010, France. Electronic address: thomas.aparicio@aphp.fr.
- [2] AP-HP, Public Health and Clinical Research Department, Henri-Mondor Hospital, Créteil F-94010, France; INSERM, IMRB U955, CEpiA Team, University Paris-Est Créteil, Créteil F-94000, France.
- [3] AP-HP, Medical Information Department, Henri-Mondor Hospital, Créteil F-94010, France.
- [4] AP-HP, Medical Oncology Department, Henri-Mondor Hospital, Créteil F-94010, France.
- [5] INSERM, IMRB U955, CEpiA Team, University Paris-Est Créteil, Créteil F-94000, France; AP-HP, Georges Pompidou Hospital, Geriatric Department, Paris Cancer Institute CARPEM, Paris F-75015, France.
- [6] AP-HP, Henri-Mondor Hospital, Digestive Surgery, Créteil F-94010, France.
- [7] AP-HP, Radiotherapy Department, Saint Louis Hospital, Paris F-75010, France; Université de Paris, Paris F-75010, France.
- [8] AP-HP, Hepatology Department, Avicenne Hospital, Bobigny F-93000, France.

- [9] AP-HP, Endoscopy Department, Beaujon Hospital, Clichy F-92110, France; Université de Paris, Paris F-75010, France.
- [10] AP-HP, Radiology Department, Saint Louis Hospital, Paris F-75010, France.
- [11] AP-HP, Medical Oncology, Tenon Hospital, Paris F-75020, France.

- PMID: **34654679**
- PMCID: PMC8487788
- DOI: 10.1016/j.dld.2021.09.017

Free PMC article
Observational Study

Effect of lockdown on digestive system cancer care amongst older patients during the first wave of COVID-19: The CADIGCOVAGE multicentre cohort study

Thomas Aparicio et al. Dig Liver Dis. 2022 Jan.
Free PMC article
Show details
Dig Liver Dis
. 2022 Jan;54(1):10-18.
doi: 10.1016/j.dld.2021.09.017. Epub 2021 Oct 4.

Authors

Thomas Aparicio [1], Richard Layese [2], François Hemery [3], Christophe Tournigand [4], Elena Paillaud [5], Nicola De Angelis [6], Laurent Quero [7], Nathalie Ganne [8], Fredéric Prat [9], Atanas Pachev [10], Gilles Galula [11], Marc-Antoine Benderra [11], Florence Canouï-Poitrine [2], Clinical Data Warehouse of Greater Paris University Hospitals / Inserm COVID-19 research collaboration and Cancer AP-HP Group

Affiliations

- [1] AP-HP, Gastroenterology and Digestive Oncology Department, Saint Louis Hospital, 1 avenue Claude Vellefaux, Paris F-75010, France; Université de Paris, Paris F-75010, France. Electronic address: thomas.aparicio@aphp.fr.
- [2] AP-HP, Public Health and Clinical Research Department, Henri-Mondor Hospital, Créteil F-94010, France; INSERM, IMRB U955, CEpiA Team, University Paris-Est Créteil, Créteil F-94000, France.
- [3] AP-HP, Medical Information Department, Henri-Mondor Hospital, Créteil F-94010, France.
- [4] AP-HP, Medical Oncology Department, Henri-Mondor Hospital, Créteil F-94010, France.
- [5] INSERM, IMRB U955, CEpiA Team, University Paris-Est Créteil, Créteil F-94000, France; AP-HP, Georges Pompidou Hospital, Geriatric Department, Paris Cancer Institute CARPEM, Paris F-75015, France.

- [6] AP-HP, Henri-Mondor Hospital, Digestive Surgery, Créteil F-94010, France.
- [7] AP-HP, Radiotherapy Department, Saint Louis Hospital, Paris F-75010, France; Université de Paris, Paris F-75010, France.
- [8] AP-HP, Hepatology Department, Avicenne Hospital, Bobigny F-93000, France.
- [9] AP-HP, Endoscopy Department, Beaujon Hospital, Clichy F-92110, France; Université de Paris, Paris F-75010, France.
- [10] AP-HP, Radiology Department, Saint Louis Hospital, Paris F-75010, France.
- [11] AP-HP, Medical Oncology, Tenon Hospital, Paris F-75020, France.

- PMID: **34654679**
- PMCID: PMC8487788
- DOI: 10.1016/j.dld.2021.09.017

Abstract

Background: The coronavirus disease 2019 (COVID-19) pandemic has had a dramatic impact on cancer diagnosis and treatment. Most patients newly diagnosed with digestive system cancer are aged 65 and over.

Methods: We performed a retrospective, observational, multicentre cohort study based on prospectively collected electronic health records. All adults aged 65 or over and having been newly treated for a digestive system cancer between January 2018 until August 2020 were enroled.

Results: Data on 7882 patients were analysed. The first COVID-19 lockdown period led to a 42.4% decrease in newly treated digestive system cancers, and the post-lockdown period was associated with a 17% decrease. The decrease in newly treated digestive system cancer did not differ as a function of age, sex, comorbidities, primary tumour site, and disease stage. The proportion of patients admitted to an emergency department increased during the lockdown period. We do not observe a higher 3-month mortality rate in 2020, relative to the corresponding calendar periods in 2018 and 2019.

Conclusion: To avoid a decrease in newly treated cancers during future lockdown periods, access to healthcare will have to be modified. Although 3-month mortality did not increase in any of the patient subgroups, the 2020 cohort must be followed up for long-term mortality.

Keywords: COVID-19; Digestive cancer; Lockdown; Older patients; Public health.

Copyright © 2021 The Author(s). Published by Elsevier Ltd.. All rights reserved.

Conflict of interest statement

Conflict of interest None declared.

- 24 references
- 2 figures

Supplementary info

Publication types, MeSH terms Expand

Publication types

- Multicenter Study
- Observational Study

MeSH terms

- Aged
- Aged, 80 and over
- COVID-19 / epidemiology*
- Communicable Disease Control
- Digestive System Neoplasms / epidemiology*
- Digestive System Neoplasms / therapy*
- Female
- Health Services Accessibility*
- Humans
- Male
- Pandemics
- Paris / epidemiology
- Retrospective Studies
- SARS-CoV-2

Full text links

ELSEVIER OPEN ACCESS Elsevier Science Free PMC article

Proceed to details

Cite

Share

☐ 614

Observational Study

J Am Acad Dermatol

. 2020 Jul;83(1):285-287.

doi: 10.1016/j.jaad.2020.04.085. Epub 2020 Apr 21.

Risk of hospitalization and death from COVID-19 infection in patients with chronic plaque psoriasis receiving a biologic treatment and renal transplant recipients in maintenance immunosuppressive treatment

Paolo Gisondi [1], Gianluigi Zaza [2], Micol Del Giglio [3], Mattia Rossi [2], Valentina Iacono [2], Giampiero Girolomoni [3]
Affiliations Expand

Affiliations

- [1] Section of Dermatology and Venereology, Department of Medicine, University of Verona, Verona, Italy. Electronic address: paolo.gisondi@univr.it.
- [2] Section of Nephrology, Department of Medicine, University of Verona, Verona, Italy.
- [3] Section of Dermatology and Venereology, Department of Medicine, University of Verona, Verona, Italy.

- PMID: **32330632**
- PMCID: PMC7194926
- DOI: 10.1016/j.jaad.2020.04.085

Free PMC article
Observational Study

Risk of hospitalization and death from COVID-19 infection in patients with chronic plaque psoriasis receiving a biologic treatment and renal transplant recipients in maintenance immunosuppressive treatment

Paolo Gisondi et al. J Am Acad Dermatol. 2020 Jul.
Free PMC article
Show details
J Am Acad Dermatol
. 2020 Jul;83(1):285-287.
doi: 10.1016/j.jaad.2020.04.085. Epub 2020 Apr 21.

Authors

Paolo Gisondi [1], Gianluigi Zaza [2], Micol Del Giglio [3], Mattia Rossi [2], Valentina Iacono [2], Giampiero Girolomoni [3]

Affiliations

- [1] Section of Dermatology and Venereology, Department of Medicine, University of Verona, Verona, Italy. Electronic address: paolo.gisondi@univr.it.
- [2] Section of Nephrology, Department of Medicine, University of Verona, Verona, Italy.
- [3] Section of Dermatology and Venereology, Department of Medicine, University of Verona, Verona, Italy.

- PMID: **32330632**
- PMCID: PMC7194926
- DOI: 10.1016/j.jaad.2020.04.085

No abstract available

Comment in

- Immunosuppressive therapies for alopecia areata during COVID-19: A cross-sectional survey study.
  Flanagan KE, Pathoulas JT, Walker CJ, Pupo Wiss IM, Ellison A, Mesinkovska NA, Senna MM. Flanagan KE, et al. Dermatol Ther. 2021 Mar;34(2):e14762. doi: 10.1111/dth.14762. Epub 2021 Jan 16. Dermatol Ther. 2021. PMID: 33404180 Free PMC article. No abstract available.

- 5 references

Supplementary info

Publication types, MeSH terms, Substances Expand

Publication types

- Observational Study

MeSH terms

- Adult
- Aged
- Betacoronavirus / immunology*
- Betacoronavirus / isolation & purification
- Biological Products / adverse effects
- COVID-19
- Coronavirus Infections / immunology
- Coronavirus Infections / mortality*
- Coronavirus Infections / therapy
- Coronavirus Infections / virology
- Dermatologic Agents / adverse effects
- Electronic Health Records / statistics & numerical data
- Female
- Hospitalization / statistics & numerical data*
- Hospitals, University / statistics & numerical data
- Humans
- Immunosuppressive Agents / adverse effects*
- Italy / epidemiology
- Kidney Transplantation / adverse effects

- Male
- Middle Aged
- Pandemics
- Pneumonia, Viral / immunology
- Pneumonia, Viral / mortality*
- Pneumonia, Viral / therapy
- Pneumonia, Viral / virology
- Psoriasis / drug therapy*
- Psoriasis / immunology
- Psoriasis / mortality
- Retrospective Studies
- Risk Assessment
- SARS-CoV-2
- Transplant Recipients / statistics & numerical data*

Substances

- Biological Products
- Dermatologic Agents
- Immunosuppressive Agents

Full text links

ELSEVIER FULL-TEXT ARTICLE Elsevier Science Free PMC article
Proceed to details
Cite
Share
615
Observational Study
Respir Care
. 2021 Jun;66(6):897-908.
doi: 10.4187/respcare.08319. Epub 2021 Jan 14.

Clinical Characteristics, Respiratory Mechanics, and Outcomes in Critically Ill Individuals With COVID-19 Infection in an Underserved Urban Population

Siddique Chaudhary [1], Sadia Benzaquen [2 3 4], Jessica G Woo [5 6], Jack Rubinstein [7], Atul Matta [2], Jeri Albano [4], Robert De Joy 3rd [4], Kevin Bryan Lo [4], Gabriel Patarroyo-Aponte [2 3 4]
Affiliations Expand

Affiliations

- [1] Division of Pulmonary and Critical Care and Sleep Medicine, Einstein Medical Center, Philadelphia, Pennsylvania. chaudhsi@einstein.edu.
- [2] Division of Pulmonary and Critical Care and Sleep Medicine, Einstein Medical Center, Philadelphia, Pennsylvania.
- [3] Department of Medicine, Einstein Medical Center, Philadelphia, Pennsylvania.
- [4] Sidney Kimmel College, Thomas Jefferson University, Philadelphia, Pennsylvania.
- [5] Department of Pediatrics, University of Cincinnati College of Medicine, Cincinnati, Ohio.
- [6] Division of Biostatistics and Epidemiology, Cincinnati Children's Hospital Medical Center, Cincinnati, Ohio.
- [7] Department of Internal Medicine, University of Cincinnati College of Medicine, Cincinnati, Ohio.

- PMID: **33446510**
- DOI: 10.4187/respcare.08319

Observational Study

Clinical Characteristics, Respiratory Mechanics, and Outcomes in Critically Ill Individuals With COVID-19 Infection in an Underserved Urban Population

Siddique Chaudhary et al. Respir Care. 2021 Jun.

Show details

Respir Care

. 2021 Jun;66(6):897-908.

doi: 10.4187/respcare.08319. Epub 2021 Jan 14.

Authors

Siddique Chaudhary [1], Sadia Benzaquen [2 3 4], Jessica G Woo [5 6], Jack Rubinstein [7], Atul Matta [2], Jeri Albano [4], Robert De Joy 3rd [4], Kevin Bryan Lo [4], Gabriel Patarroyo-Aponte [2 3 4]

Affiliations

- [1] Division of Pulmonary and Critical Care and Sleep Medicine, Einstein Medical Center, Philadelphia, Pennsylvania. chaudhsi@einstein.edu.
- [2] Division of Pulmonary and Critical Care and Sleep Medicine, Einstein Medical Center, Philadelphia, Pennsylvania.
- [3] Department of Medicine, Einstein Medical Center, Philadelphia, Pennsylvania.
- [4] Sidney Kimmel College, Thomas Jefferson University, Philadelphia, Pennsylvania.
- [5] Department of Pediatrics, University of Cincinnati College of Medicine, Cincinnati, Ohio.

- [6] Division of Biostatistics and Epidemiology, Cincinnati Children's Hospital Medical Center, Cincinnati, Ohio.
- [7] Department of Internal Medicine, University of Cincinnati College of Medicine, Cincinnati, Ohio.

- PMID: **33446510**
- DOI: 10.4187/respcare.08319

Abstract

Background: The COVID-19 outbreak in the United States has disproportionately affected Black individuals, but little is known about the factors that underlie this observation. Herein, we describe these associations with mortality in a largely minority underserved population.

Methods: This single-center retrospective observational study included all adult subjects with laboratory-confirmed SARS-Cov-2 treated in our ICU between March 15 and May 10, 2020.

Results: 128 critically ill adult subjects were included in the study (median age 68 y [interquartile range 61-76], 45% female, and 64% Black); 124 (97%) required intubation. Eighty (63%) subjects died during their in-patient stay, which did not differ by race/ethnicity. Compared with other racial/ethnic groups, Blacks had a greater proportion of women (52% vs 30%, $P = .02$) and subjects with hypertension (91% vs 78%, $P = .035$). Asthma ($P = .03$) was associated with lower in-patient death, primarily among Black subjects ($P = .02$). Among Black subjects, increased age (odds ratio 1.06 [95% CI 1.05-1.22] per year), positive fluid balance (odds ratio 1.06 [95% CI 1.01-1.11] per 100 mL), and treatment with tocilizumab (odds ratio 25.0 [95% CI 3.5-180]) were independently associated with in-patient death, while higher platelets (odds ratio 0.65 [95% CI 0.47-0.89] per 50×10^3/mL) and treatment with intermediate dose anticoagulants (odds ratio 0.08 [95% CI 0.02-0.43]) were protective. Among other race/ethnic groups, higher total bilirubin (odds ratio 1.75 [95% CI 0.94-3.25] per 0.2 mg/dL) and higher maximum lactate (odds ratio 1.43 [95% CI 0.96-2.13] per mmol/L) were marginally associated with increased death, while tocilizumab treatment was marginally protective (odds ratio 0.24 [95% CI 0.05-1.25]). During first 72 h of ventilation, those who died had less increase in [Formula: see text] ($P = .046$) and less reduction in PEEP ($P = .01$) and [Formula: see text] requirement ($P = .002$); these patterns did not differ by race/ethnicity.

Conclusions: Black and other race/ethnicity subjects had similar mortality rates due to COVID-19 but differed in factors that were associated with increased risk of death. In both groups, subjects who died were older, had a positive fluid balance, and less improvement in [Formula: see text], PEEP, and [Formula: see text] requirement on ventilation.

Keywords: COVID-19; coronavirus; outcomes.

Copyright © 2021 by Daedalus Enterprises.

Conflict of interest statement

The authors have disclosed no conflicts of interest.

Comment in

- COVID-19 Outcomes in Underserved Populations: Reaping What One Sows.

Hatipoğlu U. Hatipoğlu U. Respir Care. 2021 Jun;66(6):1041-1043. doi: 10.4187/respcare.09135. Respir Care. 2021. PMID: 34039763 No abstract available.

Supplementary info

Publication types, MeSH terms Expand

Publication types

- Observational Study

MeSH terms

- Adult
- Aged
- COVID-19*
- Critical Illness
- Female
- Humans
- Male
- Respiratory Mechanics
- Retrospective Studies
- SARS-CoV-2
- United States / epidemiology
- Urban Population
- Vulnerable Populations

Full text links

Final Version Respir Care HighWire
Proceed to details
Cite
Share
☐ 616
Observational Study
J Am Soc Echocardiogr
. 2021 Aug;34(8):831-838.
doi: 10.1016/j.echo.2021.03.010. Epub 2021 Apr 1.

Determining Which Hospitalized Coronavirus Disease 2019 Patients Require Urgent Echocardiography

Neal Yuan [1], Stephanie Wu [2], Florian Rader [2], Robert J Siegel [2]
Affiliations Expand

Affiliations

- [1] Smidt Heart Institute, Cedars-Sinai Medical Center, Los Angeles, California. Electronic address: neal.yuan@cshs.org.
- [2] Smidt Heart Institute, Cedars-Sinai Medical Center, Los Angeles, California.

- PMID: **33812952**
- PMCID: PMC8012271
- DOI: 10.1016/j.echo.2021.03.010

Free PMC article
Observational Study

Determining Which Hospitalized Coronavirus Disease 2019 Patients Require Urgent Echocardiography

Neal Yuan et al. J Am Soc Echocardiogr. 2021 Aug.
Free PMC article
Show details
J Am Soc Echocardiogr
. 2021 Aug;34(8):831-838.
doi: 10.1016/j.echo.2021.03.010. Epub 2021 Apr 1.

Authors

Neal Yuan [1], Stephanie Wu [2], Florian Rader [2], Robert J Siegel [2]

Affiliations

- [1] Smidt Heart Institute, Cedars-Sinai Medical Center, Los Angeles, California. Electronic address: neal.yuan@cshs.org.
- [2] Smidt Heart Institute, Cedars-Sinai Medical Center, Los Angeles, California.

- PMID: **33812952**
- PMCID: PMC8012271
- DOI: 10.1016/j.echo.2021.03.010

Abstract

Background: Patients hospitalized with coronavirus disease 2019 (COVID-19) often have abnormal findings on transthoracic echocardiography (TTE). However, although not all abnormalities on TTE result in changes in clinical management, performing TTE in recently infected patients increases disease transmission risks. It remains unknown whether common biomarker tests, such as troponin and B-type natriuretic peptide (BNP), can help distinguish in which patients with COVID-19 TTE may be safely delayed until infection risks subside.

Methods: Using electronic health records data and chart review, the authors retrospectively studied all patients hospitalized with COVID-19 in a multisite health care system from March 1, 2020, to January 15, 2021, who underwent TTE within 14 days of their first positive COVID-19 result and had BNP and troponin measured before or within 7 days of TTE. The primary outcome was the presence of one or more urgent echocardiographic findings, defined as left ventricular ejection fraction ≤ 35%, wall motion score index ≥ 1.5, moderate or greater right ventricular dysfunction, moderate or greater pericardial effusion, intracardiac thrombus, pulmonary artery systolic pressure > 50 mm Hg, or at least moderate to severe valvular disease. Stepwise logistic regression was conducted to determine biomarkers and comorbidities associated with the outcome. The performance of a rule for classifying TTE using troponin and BNP was evaluated.

Results: Four hundred thirty-four hospitalized and 151 intensive care unit patients with COVID-19 were included. Urgent findings on TTE were present in 105 patients (24.2%). Troponin and BNP were abnormal in 311 (71.7%). Heart failure (odds ratio, 5.41; 95% CI, 2.61-11.68), troponin > 0.04 ng/mL (odds ratio, 4.40; 95% CI, 2.05-10.05), and BNP > 100 pg/mL (odds ratio, 5.85; 95% CI, 2.35-16.09) remained significant predictors of urgent findings on TTE after stepwise selection. No urgent findings on TTE were seen in 95.1% of all patients and in 91.3% of intensive care unit patients with normal troponin and BNP.

Conclusions: Troponin and BNP were highly associated with urgent echocardiographic findings and may be used in triaging algorithms for determining in which patients TTE can be safely delayed until after their peak infectious window has passed.

Keywords: B-type natriuretic peptide; COVID-19; Risk stratification; Safety; Transthoracic echocardiography; Troponin.

Copyright © 2021 American Society of Echocardiography. Published by Elsevier Inc. All rights reserved.

- 33 references
- 3 figures

Supplementary info

Publication types, MeSH terms, Grant support Expand

Publication types

- Multicenter Study
- Observational Study
- Research Support, N.I.H., Extramural

MeSH terms

- Aged
- COVID-19 / epidemiology*
- Comorbidity
- Critical Care / methods*
- Echocardiography / methods*
- Emergencies*
- Female
- Heart Diseases / diagnosis*
- Heart Diseases / epidemiology
- Heart Diseases / physiopathology
- Humans
- Inpatients*
- Male
- Middle Aged
- Retrospective Studies
- SARS-CoV-2
- Stroke Volume / physiology
- Ventricular Function, Left / physiology

Grant support

- T32 HL116273/HL/NHLBI NIH HHS/United States

Full text links

ELSEVIER FULL-TEXT ARTICLE Elsevier Science Free PMC article
Proceed to details
Cite
Share
☐ 617
Observational Study
Emerg Med J
. 2021 Sep;38(9):679-684.
doi: 10.1136/emermed-2020-210409. Epub 2021 Jul 14.

Impact of the COVID-19 pandemic on emergency medical service response to out-of-hospital cardiac arrests in Taiwan: a retrospective observational study

Jiun-Hao Yu [#] [1] [2], Chien-Yu Liu [#] [2] [3], Wei-Kung Chen [2] [3], Shao-Hua Yu [2] [3], Fen-Wei Huang [3], Ming-Tzu Yang [3], Chih-Yu Chen [4], Hong-Mo Shih [5] [3] [6]
Affiliations Expand

Affiliations

- [1] Department of Emergency Medicine, China Medical University Hospital, Hsinchu, Taiwan.
- [2] College of Medicine, China Medical University, Taichung, Taiwan.
- [3] Department of Emergency Medicine, China Medical University Hospital, Taichung, Taiwan.
- [4] Department of Industrial Engineering and Enterprise Information, Tunghai University, Taichung, Taiwan.
- [5] College of Medicine, China Medical University, Taichung, Taiwan homoe042002@hotmail.com.
- [6] Department of Public Health, China Medical University, Taichung, Taiwan.

[#] Contributed equally.

- PMID: **34261763**
- PMCID: PMC8282423
- DOI: 10.1136/emermed-2020-210409

Free PMC article
Observational Study

Impact of the COVID-19 pandemic on emergency medical service response to out-of-hospital cardiac arrests in Taiwan: a retrospective observational study

Jiun-Hao Yu et al. Emerg Med J. 2021 Sep.
Free PMC article
Show details
Emerg Med J
. 2021 Sep;38(9):679-684.
doi: 10.1136/emermed-2020-210409. Epub 2021 Jul 14.

Authors

Jiun-Hao Yu [#, 1, 2], Chien-Yu Liu [#, 2, 3], Wei-Kung Chen [2, 3], Shao-Hua Yu [2, 3], Fen-Wei Huang [3], Ming-Tzu Yang [3], Chih-Yu Chen [4], Hong-Mo Shih [5, 3, 6]

Affiliations

- [1] Department of Emergency Medicine, China Medical University Hospital, Hsinchu, Taiwan.
- [2] College of Medicine, China Medical University, Taichung, Taiwan.
- [3] Department of Emergency Medicine, China Medical University Hospital, Taichung, Taiwan.
- [4] Department of Industrial Engineering and Enterprise Information, Tunghai University, Taichung, Taiwan.
- [5] College of Medicine, China Medical University, Taichung, Taiwan homoe042002@hotmail.com.
- [6] Department of Public Health, China Medical University, Taichung, Taiwan.

[#] Contributed equally.

- PMID: **34261763**
- PMCID: PMC8282423
- DOI: 10.1136/emermed-2020-210409

Abstract

Background: Emergency medical service (EMS) personnel have high COVID-19 risk during resuscitation. The resuscitation protocol for patients with out-of-hospital cardiac arrest (OHCA) was modified in response to the COVID-19 pandemic. However, how the adjustments in the EMS system affected patients with OHCA remains unclear.

Methods: We analysed data from the Taichung OHCA registry system. We compared OHCA outcomes and rescue records for 622 cases during the COVID-19 outbreak period (1 February to 30 April 2020) with those recorded for 570 cases during the same period in 2019.

Results: The two periods did not differ significantly with respect to patient age, patient sex, the presence of witnesses or OHCA location. Bystander cardiopulmonary resuscitation and defibrillation with automated external defibrillators were more common in 2020 (52.81% vs 65.76%, p<0.001%, and 23.51% vs 31.67%, p=0.001, respectively). The EMS response time was longer during the COVID-19 pandemic (445.8±210.2 s in 2020 vs 389.7±201.8 s in 2019, p<0.001). The rate of prehospital return of spontaneous circulation was lower in 2020 (6.49% vs 2.57%, p=0.001); 2019 and 2020 had similar rates of survival discharge (5.96% vs 4.98%). However, significantly fewer cases had favourable neurological function in 2020 (4.21% vs 2.09%, p=0.035).

Conclusion: EMS response time for patients with OHCA was prolonged during the COVID-19 pandemic. Early advanced life support by EMS personnel remains crucial for patients with OHCA.

Keywords: SARS; cardiac arrest; despatch; infectious diseases; prehospital care; resuscitation.

© Author(s) (or their employer(s)) 2021. Re-use permitted under CC BY-NC. No commercial re-use. See rights and permissions. Published by BMJ.

Conflict of interest statement

Competing interests: None declared.

- 28 references
- 3 figures

Supplementary info

Publication types, MeSH terms Expand

Publication types

- Observational Study

MeSH terms

- Adult
- Aged
- Aged, 80 and over
- COVID-19 / complications
- COVID-19 / epidemiology
- COVID-19 / transmission*
- COVID-19 / virology
- Cardiopulmonary Resuscitation / standards
- Cardiopulmonary Resuscitation / statistics & numerical data*
- Emergency Medical Services / standards
- Emergency Medical Services / statistics & numerical data*
- Emergency Medical Technicians / standards
- Emergency Medical Technicians / statistics & numerical data
- Female
- Humans
- Infectious Disease Transmission, Patient-to-Professional / prevention & control*
- Male
- Middle Aged
- Out-of-Hospital Cardiac Arrest / complications
- Out-of-Hospital Cardiac Arrest / epidemiology
- Out-of-Hospital Cardiac Arrest / therapy*
- Pandemics / prevention & control
- Practice Guidelines as Topic
- Registries / statistics & numerical data

- Retrospective Studies
- SARS-CoV-2 / pathogenicity
- Taiwan / epidemiology
- Time-to-Treatment / standards
- Time-to-Treatment / statistics & numerical data
- Young Adult

Full text links

BMJ Open Access Full Text HighWire Free PMC article
Proceed to details
Cite
Share
618
Observational Study
J Cardiovasc Med (Hagerstown)
. 2021 May 1;22(5):344-349.
doi: 10.2459/JCM.0000000000001153.

COVID-19 impact on ST-elevation myocardial infarction incidence rate in a Italian STEMI network: a U-shaped curve phenomenon

Enrico Fabris [1], Riccardo Bessi [1], Annamaria De Bellis [1], Caterina Gregorio [2], Alberto Peratoner [3], Gerardina Lardieri [4], Franco Cominotto [3], Giancarlo Vitrella [1], Serana Rakar [1], Andrea Perkan [1], Gianfranco Sinagra [1]
Affiliations Expand

Affiliations

- [1] Cardiovascular Department.
- [2] Biostatistics Unit, Department of Medical Sciences.
- [3] Emergency Department, University of Trieste, Trieste.
- [4] Division of Cardiology, Gorizia - Monfalcone Hospital, ASUGI, Gorizia, Italy.

- PMID: **33399345**
- DOI: 10.2459/JCM.0000000000001153

Observational Study

COVID-19 impact on ST-elevation myocardial infarction incidence rate in a Italian STEMI network: a U-shaped curve phenomenon

Enrico Fabris et al. J Cardiovasc Med (Hagerstown). 2021.
Show details
J Cardiovasc Med (Hagerstown)
. 2021 May 1;22(5):344-349.
doi: 10.2459/JCM.0000000000001153.

Authors

Enrico Fabris [1], Riccardo Bessi [1], Annamaria De Bellis [1], Caterina Gregorio [2], Alberto Peratoner [3], Gerardina Lardieri [4], Franco Cominotto [3], Giancarlo Vitrella [1], Serana Rakar [1], Andrea Perkan [1], Gianfranco Sinagra [1]

Affiliations

- [1] Cardiovascular Department.
- [2] Biostatistics Unit, Department of Medical Sciences.
- [3] Emergency Department, University of Trieste, Trieste.
- [4] Division of Cardiology, Gorizia - Monfalcone Hospital, ASUGI, Gorizia, Italy.

- PMID: **33399345**
- DOI: 10.2459/JCM.0000000000001153

Abstract

Background: Public health emergencies such as the COVID-19 outbreak may impact on the incidence rate of ST-elevation myocardial infarction (STEMI) in severely affected areas. However, this phenomenon demands attention also in areas where media and patients were focused on the COVID-19 pandemic, but the healthcare system was not overwhelmed by the huge number of COVID-19 patients.

Methods and results: In this observational study, we compared the incidence rate of all consecutive STEMI patients admitted at the University Hospital of Trieste, Italy, during March and April 2020 with the same 2 months of the previous 5 years (2015-2019). Patient characteristics were compared between 2020 and 2019.The incidence rate of STEMI admission in March-April 2020 was lower than those in March-April 2015-2019, 36 vs. 56 cases per 100 000 inhabitants/year [relative risk (RR) 0.65, 95% confidence interval (95% CI) 0.42-0.96, $P = 0.045$]. Considering that the incidence rates were constant in the past years ($P = 0.24$), the turnaround in 2020 is most likely due to the COVID-19 outbreak. Interestingly, this reduction was a dynamic phenomenon with a U-shaped curve during the 2-month period. System-of-care times were similar between 2020 and 2019; however in 2020, patients presented more frequently signs of heart failure compared to 2019 (Killip class ≥2 in 68% vs. 29%, $P = 0.003$).

Conclusion: During the COVID-19 outbreak, we observed a marked reduction in the STEMI incidence rate. This U-shaped phenomenon demands attention because a potential cause for the decrease in STEMI incidence may include the avoidance of medical care. Public campaigns aiming to increase awareness of ischemic symptoms may be needed during community outbreak.

Copyright © 2021 Italian Federation of Cardiology - I.F.C. All rights reserved.

- 20 references

Supplementary info

Publication types, MeSH terms Expand

Publication types

- Observational Study

MeSH terms

- Aged
- COVID-19 / epidemiology*
- Communicable Disease Control
- Emergency Service, Hospital / statistics & numerical data
- Facilities and Services Utilization
- Female
- Hospitalization / statistics & numerical data
- Humans
- Incidence
- Italy
- Male
- Middle Aged
- Retrospective Studies
- ST Elevation Myocardial Infarction / diagnosis
- ST Elevation Myocardial Infarction / epidemiology*
- ST Elevation Myocardial Infarction / therapy
- Time-to-Treatment / statistics & numerical data

Full text links

Wolters Kluwer Wolters Kluwer
Proceed to details
Cite
Share
☐ 619
Observational Study

Ann Biomed Eng
. 2020 Dec;48(12):3003-3013.
doi: 10.1007/s10439-020-02648-0. Epub 2020 Oct 19.

Effects of the Lower Airway Secretions on Airway Opening Pressures and Suction Pressures in Critically Ill COVID-19 Patients: A Computational Simulation

Zhenglong Chen [1], Ming Zhong [2], Li Jiang [3], Nanshan Chen [4], Shengjin Tu [4], Yuan Wei [4], Ling Sang [5], Xia Zheng [6], Chunyuan Zhang [7], Jiale Tao [8], Linhong Deng [9], Yuanlin Song [10]
Affiliations Expand

Affiliations

- [1] School of Medical Instrumentation, Shanghai University of Medicine & Health Sciences, 257 Tianxiong Road, Shanghai, 201318, China.
- [2] Department of Intensive Care Medicine, Zhongshan Hospital, Fudan University, 180 Fenglin Road, Shanghai, 200032, China. zhong_ming@fudan.edu.cn.
- [3] Department of Critical Care Medicine, Xuanwu Hospital, Capital Medical University, 45 Changchun Street, Xicheng District, Beijing, 100053, China.
- [4] Department of Respiratory and Critical Care Medicine, Wuhan Jinyintan Hospital, 1 Yintan Road, Dongxihu District, Wuhan, 430023, China.
- [5] Department of Critical Care Medicine, GuangZhou Institute of Respiratory Health, The First Affiliated Hospital of GuangZhou Medical University, 151 Yanjiangxi Road, Guangzhou, 510120, China.
- [6] Department of Critical Care Medicine, The First Affiliated Hospital of Zhejiang University, Hangzhou, 310003, Zhejiang, China.
- [7] NMPA Key Laboratory for Respiratory and Anaesthetic Equipment, 1 Jinyinhua Road, Shanghai, 201321, China.
- [8] Department of Intensive Care Medicine, Zhongshan Hospital, Fudan University, 180 Fenglin Road, Shanghai, 200032, China.
- [9] Changzhou Key Laboratory of Respiratory Medical Engineering, Institute of Biomedical Engineering and Health Sciences, Changzhou University, Changzhou, 213164, Jiangsu, China.
- [10] Department of Pulmonary Medicine, Zhongshan Hospital, Fudan University, 180 Fenglin Road, Xuhui District, Shanghai, 200032, China.

- PMID: **33078367**
- PMCID: PMC7571532
- DOI: 10.1007/s10439-020-02648-0

Free PMC article
Observational Study

Effects of the Lower Airway Secretions on Airway Opening Pressures and Suction Pressures in Critically Ill COVID-19 Patients: A Computational Simulation

Zhenglong Chen et al. Ann Biomed Eng. 2020 Dec.
Free PMC article
Show details
Ann Biomed Eng
. 2020 Dec;48(12):3003-3013.
doi: 10.1007/s10439-020-02648-0. Epub 2020 Oct 19.

Authors

Zhenglong Chen [1], Ming Zhong [2], Li Jiang [3], Nanshan Chen [4], Shengjin Tu [4], Yuan Wei [4], Ling Sang [5], Xia Zheng [6], Chunyuan Zhang [7], Jiale Tao [8], Linhong Deng [9], Yuanlin Song [10]

Affiliations

- [1] School of Medical Instrumentation, Shanghai University of Medicine & Health Sciences, 257 Tianxiong Road, Shanghai, 201318, China.
- [2] Department of Intensive Care Medicine, Zhongshan Hospital, Fudan University, 180 Fenglin Road, Shanghai, 200032, China. zhong_ming@fudan.edu.cn.
- [3] Department of Critical Care Medicine, Xuanwu Hospital, Capital Medical University, 45 Changchun Street, Xicheng District, Beijing, 100053, China.
- [4] Department of Respiratory and Critical Care Medicine, Wuhan Jinyintan Hospital, 1 Yintan Road, Dongxihu District, Wuhan, 430023, China.
- [5] Department of Critical Care Medicine, GuangZhou Institute of Respiratory Health, The First Affiliated Hospital of GuangZhou Medical University, 151 Yanjiangxi Road, Guangzhou, 510120, China.
- [6] Department of Critical Care Medicine, The First Affiliated Hospital of Zhejiang University, Hangzhou, 310003, Zhejiang, China.
- [7] NMPA Key Laboratory for Respiratory and Anaesthetic Equipment, 1 Jinyinhua Road, Shanghai, 201321, China.
- [8] Department of Intensive Care Medicine, Zhongshan Hospital, Fudan University, 180 Fenglin Road, Shanghai, 200032, China.
- [9] Changzhou Key Laboratory of Respiratory Medical Engineering, Institute of Biomedical Engineering and Health Sciences, Changzhou University, Changzhou, 213164, Jiangsu, China.
- [10] Department of Pulmonary Medicine, Zhongshan Hospital, Fudan University, 180 Fenglin Road, Xuhui District, Shanghai, 200032, China.

- PMID: **33078367**
- PMCID: PMC7571532
- DOI: 10.1007/s10439-020-02648-0

Abstract

In patients with critically ill COVID-19 pneumonia, lower airways are filled with plenty of highly viscous exudates or mucus, leading to airway occlusion. The estimation of airway opening pressures and effective mucus clearance are therefore two issues that clinicians are most concerned about during mechanical ventilation. In this study we retrospectively analyzed respiratory data from 24 critically ill patients with COVID-19 who received invasive mechanical ventilation and recruitment maneuver at Jinyintan Hospital in Wuhan, China. Among 24 patients, the mean inspiratory plateau pressure was 52.4 ± 4.4 cmH_2O (mean ± [SD]). Particularly, the capnograms presented an upward slope during the expiratory plateau, indicting the existence of airway obstruction. A computational model of airway opening was subsequently introduced to investigate possible fluid dynamic mechanisms for the extraordinarily high inspiratory plateau pressures among these patients. Our simulation results showed that the predicted airway opening pressures could be as high as 40-50 cmH_2O and the suction pressure could exceed 20 kPa as the surface tension and viscosity of secretion simulants markedly increased, likely causing the closures of the distal airways. We concluded that, in some critically ill patients with COVID-19, limiting plateau pressure to 30 cmH_2O may not guarantee the opening of airways due to the presence of highly viscous lower airway secretions, not to mention spontaneous inspiratory efforts. Active airway humidification and effective expectorant drugs are therefore strongly recommended during airway management.

Keywords: Airway mucus; Airway opening pressure; Coronavirus disease 2019; Endotracheal suctioning; Respiratory mechanics.

- 33 references
- 7 figures

Supplementary info

Publication types, MeSH terms, Grant support Expand

Publication types

- Observational Study

MeSH terms

- Adult
- Aged
- Air Pressure
- COVID-19 / physiopathology*
- COVID-19 / therapy
- Computer Simulation*
- Critical Illness
- Female
- Humans
- Lung / physiopathology*

- Male
- Middle Aged
- Models, Biological*
- Pulmonary Gas Exchange*
- Respiratory Mechanics*
- Retrospective Studies
- SARS-CoV-2*

Grant support

- 81971807/National Natural Science Foundation of China
- 11532003/National Natural Science Foundation of China
- 31670950/National Natural Science Foundation of China
- 82041003/National Natural Science Foundation of China

Full text links

Springer Free PMC article
Proceed to details
Cite
Share
☐ 620
Observational Study
JMIR Public Health Surveill
. 2021 Jan 8;7(1):e21327.
doi: 10.2196/21327.

Implementation of Telemedicine in a Tertiary Hospital-Based Ambulatory Practice in Detroit During the COVID-19 Pandemic: Observational Study

Alpana Garg [1], Sachin Goyal [1], Rohit Thati [2], Neelima Thati [1]
Affiliations Expand

Affiliations

- [1] Wayne State University, Detroit, MI, United States.
- [2] Georgia State University, Atlanta, GA, United States.

- PMID: **33400680**
- PMCID: PMC7801131
- DOI: 10.2196/21327

Free PMC article
Observational Study

Implementation of Telemedicine in a Tertiary Hospital-Based Ambulatory Practice in Detroit During the COVID-19 Pandemic: Observational Study

Alpana Garg et al. JMIR Public Health Surveill. 2021.
Free PMC article
Show details
JMIR Public Health Surveill
. 2021 Jan 8;7(1):e21327.
doi: 10.2196/21327.

Authors

Alpana Garg [1], Sachin Goyal [1], Rohit Thati [2], Neelima Thati [1]

Affiliations

- [1] Wayne State University, Detroit, MI, United States.
- [2] Georgia State University, Atlanta, GA, United States.

- PMID: **33400680**
- PMCID: PMC7801131
- DOI: 10.2196/21327

Abstract

Background: The COVID-19 pandemic, caused by SARS-CoV-2, has forced the health care delivery structure to change rapidly. The pandemic has further widened the disparities in health care and exposed vulnerable populations. Health care services caring for such populations must not only continue to operate but create innovative methods of care delivery without compromising safety. We present our experience of incorporating telemedicine in our university hospital-based outpatient clinic in one of the worst-hit areas in the world.

Objective: Our goal is to assess the adoption of a telemedicine service in the first month of its implementation in outpatient practice during the COVID-19 pandemic. We also want to assess the need for transitioning to telemedicine, the benefits and challenges in doing so, and ongoing solutions during the initial phase of the implementation of telemedicine services for our patients.

Methods: We conducted a prospective review of clinic operations data from the first month of a telemedicine rollout in the outpatient adult ambulatory clinic from April 1, 2020, to April 30, 2020. A telemedicine visit was defined as synchronous audio-video communication between the provider and patient for clinical care longer than 5 minutes or if the video visit converted to a telephone visit after 5 minutes due to technical problems. We recorded the number of telemedicine

visits scheduled, visits completed, and the time for each visit. We also noted the most frequent billing codes used based on the time spent in the patient care and the number of clinical tasks (eg, activity suggested through diagnosis or procedural code) that were addressed remotely by the physicians.

Results: During the study period, we had 110 telemedicine visits scheduled, of which 94 (85.4%) visits were completed. The average duration of the video visit was 35 minutes, with the most prolonged visit lasting 120 minutes. Of 94 patients, 24 (25.54%) patients were recently discharged from the hospital, and 70 (74.46%) patients were seen for urgent care needs. There was a 50% increase from the baseline in the number of clinical tasks that were addressed by the physicians during the pandemic.

Conclusions: There was a high acceptance of telemedicine services by the patients, which was evident by a high show rate during the COVID-19 pandemic in Detroit. With limited staffing, restricted outpatient work hours, a shortage of providers, and increased outpatient needs, telemedicine was successfully implemented in our practice.

Keywords: COVID-19; Detroit; ambulatory care; internal medicine; pandemic; primary care; telehealth; telemedicine.

©Alpana Garg, Sachin Goyal, Rohit Thati, Neelima Thati. Originally published in JMIR Public Health and Surveillance (http://publichealth.jmir.org), 08.01.2021.

Conflict of interest statement

Conflicts of Interest: None declared.

- 25 references
- 4 figures

Supplementary info

Publication types, MeSH terms Expand

Publication types

- Observational Study

MeSH terms

- Ambulatory Care / methods
- COVID-19 / complications
- COVID-19 / transmission
- Humans
- Michigan
- Pandemics / prevention & control*
- Pandemics / statistics & numerical data
- Patient Satisfaction
- Prospective Studies

- Retrospective Studies
- Telemedicine / methods*
- Telemedicine / trends
- Tertiary Care Centers / organization & administration
- Tertiary Care Centers / trends

Full text links

JMIR Publications Free PMC article
Proceed to details
Cite
Share
621
Observational Study
Int J Clin Oncol
. 2021 Mar;26(3):485-493.
doi: 10.1007/s10147-020-01837-0. Epub 2020 Nov 23.

Characteristics and outcomes of coronavirus disease 2019 (COVID-19) patients with cancer: a single-center retrospective observational study in Tokyo, Japan

Shohei Nakamura [1], Yusuke Kanemasa [2], Yuya Atsuta [3], Sho Fujiwara [4], Masaru Tanaka [4], Kazuaki Fukushima [4], Taiichiro Kobayashi [4], Tatsu Shimoyama [1], Yasushi Omuro [1], Noritaka Sekiya [5] [6], Akifumi Imamura [4]
Affiliations Expand

Affiliations

- [1] Department of Medical Oncology, Tokyo Metropolitan Cancer and Infectious Diseases Center Komagome Hospital, 3-18-22 Honkomagome, Bunkyo-ku, Tokyo, Japan.
- [2] Department of Medical Oncology, Tokyo Metropolitan Cancer and Infectious Diseases Center Komagome Hospital, 3-18-22 Honkomagome, Bunkyo-ku, Tokyo, Japan. y-kanemasa@cick.jp.
- [3] Division of Hematology, Tokyo Metropolitan Cancer and Infectious Diseases Center Komagome Hospital, Tokyo, Japan.
- [4] Department of Infectious Diseases, Tokyo Metropolitan Cancer and Infectious Diseases Center Komagome Hospital, Tokyo, Japan.
- [5] Department of Infection Prevention and Control, Tokyo Metropolitan Cancer and Infectious Diseases Center Komagome Hospital, Tokyo, Japan.
- [6] Department of Clinical Laboratory, Tokyo Metropolitan Cancer and Infectious Diseases Center Komagome Hospital, Tokyo, Japan.

- PMID: **33225396**
- PMCID: PMC7680712
- DOI: 10.1007/s10147-020-01837-0

Free PMC article
Observational Study

Characteristics and outcomes of coronavirus disease 2019 (COVID-19) patients with cancer: a single-center retrospective observational study in Tokyo, Japan

Shohei Nakamura et al. Int J Clin Oncol. 2021 Mar.
Free PMC article
Show details
Int J Clin Oncol
. 2021 Mar;26(3):485-493.
doi: 10.1007/s10147-020-01837-0. Epub 2020 Nov 23.

Authors

Shohei Nakamura [1], Yusuke Kanemasa [2], Yuya Atsuta [3], Sho Fujiwara [4], Masaru Tanaka [4], Kazuaki Fukushima [4], Taiichiro Kobayashi [4], Tatsu Shimoyama [1], Yasushi Omuro [1], Noritaka Sekiya [5] [6], Akifumi Imamura [4]

Affiliations

- [1] Department of Medical Oncology, Tokyo Metropolitan Cancer and Infectious Diseases Center Komagome Hospital, 3-18-22 Honkomagome, Bunkyo-ku, Tokyo, Japan.
- [2] Department of Medical Oncology, Tokyo Metropolitan Cancer and Infectious Diseases Center Komagome Hospital, 3-18-22 Honkomagome, Bunkyo-ku, Tokyo, Japan. y-kanemasa@cick.jp.
- [3] Division of Hematology, Tokyo Metropolitan Cancer and Infectious Diseases Center Komagome Hospital, Tokyo, Japan.
- [4] Department of Infectious Diseases, Tokyo Metropolitan Cancer and Infectious Diseases Center Komagome Hospital, Tokyo, Japan.
- [5] Department of Infection Prevention and Control, Tokyo Metropolitan Cancer and Infectious Diseases Center Komagome Hospital, Tokyo, Japan.
- [6] Department of Clinical Laboratory, Tokyo Metropolitan Cancer and Infectious Diseases Center Komagome Hospital, Tokyo, Japan.

- PMID: **33225396**
- PMCID: PMC7680712
- DOI: 10.1007/s10147-020-01837-0

Abstract

Background: Although severe acute respiratory syndrome coronavirus 2 (SARS-CoV-2) has caused an international outbreak of coronavirus disease 2019 (COVID-19), data on the clinical characteristics of COVID-19 patients with cancer are limited. This study aimed to evaluate the clinical characteristics and outcomes including mortality and viral shedding period in COVID-19 patients with cancer in Japan.

Methods: We retrospectively analyzed 32 patients with a history of cancer who were referred to our hospital between January 31, 2020 and May 25, 2020. We evaluated the association between clinical outcomes and potential prognostic factors using univariate analyses.

Results: The median age was 74.5 (range 24-90) years and 22 patients (69%) were men. A total of 11 patients (34%) died. Our analyses demonstrated that the mortality was significantly associated with lymphocyte count, albumin, lactate dehydrogenase, serum ferritin, and C-reactive protein on admission. The median period between illness onset and the first effective negative SARS-CoV-2 PCR result was 22 days (interquartile range 18-25) in survivors. Of four patients with hematological malignancy who developed COVID-19 within the rest period of chemotherapy, three died and the other patient, who received bendamustine plus rituximab therapy, had the longest duration of viral shedding (56 days).

Conclusion: Our study suggested that the risk factors for mortality previously reported in general COVID-19 patients, including lymphocytopenia, were also effective in cancer patients. Patients who received cytotoxic chemotherapy recently or were treated with chemotherapy, which can lead to lymphocyte reduction, had poor prognosis and prolonged periods of viral shedding.

Keywords: COVID-19; Cancer; Chemotherapy; Japan; Lymphocytopenia.

Conflict of interest statement

No author has any conflict of interest.

- 35 references
- 2 figures

Supplementary info

Publication types, MeSH terms Expand

Publication types

- Observational Study

MeSH terms

- Adult
- Aged
- Aged, 80 and over
- COVID-19*

- China
- Humans
- Japan / epidemiology
- Male
- Middle Aged
- Neoplasms* / drug therapy
- Neoplasms* / epidemiology
- Retrospective Studies
- Risk Factors
- SARS-CoV-2
- Tokyo
- Young Adult

Full text links

SpringerLink FULL-TEXT ARTICLE Springer Free PMC article
Proceed to details
Cite
Share
622
Observational Study
Int J Epidemiol
. 2021 Jan 23;49(6):1940-1950.
doi: 10.1093/ije/dyaa180.

Epidemiological characteristics of patients with severe COVID-19 infection in Wuhan, China: evidence from a retrospective observational study

Fang Wang [1], Jinhong Cao [1], Yong Yu [2], Jianbo Ding [3], Ehab S Eshak [4] [5], Keyang Liu [4] [6], Sumaira Mubarik [1], Fang Shi [1], Haoyu Wen [1], Zixin Zeng [1], Jianjun Bai [1], Chuanhua Yu [1] [7]
Affiliations Expand

Affiliations

- [1] Department of Epidemiology and Biostatistics, School of Health Sciences, Wuhan University, Wuhan, China.
- [2] School of Public Health and Management, Hubei University of Medicine, Shiyan, China.
- [3] YEBIO Bioengineering Co., Ltd. of Qingdao, Qingdao, China.
- [4] Public Health, Department of Social Medicine, Osaka University Graduate School of Medicine, Osaka, Japan.

- [5] Public Health and Community Medicine, Faculty of Medicine, Minia University, Minia, Egypt.
- [6] Department of Epidemiology and Biostatistics, School of Public Health, Peking University, Beijing, China.
- [7] Global Health Institute, Wuhan University, Wuhan, China.

- PMID: **33150437**
- PMCID: PMC7665537
- DOI: 10.1093/ije/dyaa180

Free PMC article
Observational Study

Epidemiological characteristics of patients with severe COVID-19 infection in Wuhan, China: evidence from a retrospective observational study

Fang Wang et al. Int J Epidemiol. 2021.
Free PMC article
Show details
Int J Epidemiol
. 2021 Jan 23;49(6):1940-1950.
doi: 10.1093/ije/dyaa180.

Authors

Fang Wang [1], Jinhong Cao [1], Yong Yu [2], Jianbo Ding [3], Ehab S Eshak [4] [5], Keyang Liu [4] [6], Sumaira Mubarik [1], Fang Shi [1], Haoyu Wen [1], Zixin Zeng [1], Jianjun Bai [1], Chuanhua Yu [1] [7]

Affiliations

- [1] Department of Epidemiology and Biostatistics, School of Health Sciences, Wuhan University, Wuhan, China.
- [2] School of Public Health and Management, Hubei University of Medicine, Shiyan, China.
- [3] YEBIO Bioengineering Co., Ltd. of Qingdao, Qingdao, China.
- [4] Public Health, Department of Social Medicine, Osaka University Graduate School of Medicine, Osaka, Japan.
- [5] Public Health and Community Medicine, Faculty of Medicine, Minia University, Minia, Egypt.
- [6] Department of Epidemiology and Biostatistics, School of Public Health, Peking University, Beijing, China.
- [7] Global Health Institute, Wuhan University, Wuhan, China.

- PMID: **33150437**
- PMCID: PMC7665537

- DOI: 10.1093/ije/dyaa180

Erratum in

- Corrigendum to: Epidemiological characteristics of patients with severe COVID-19 infection in Wuhan, China: evidence from a retrospective, observational study.
  Wang F, Cao J, Yu Y, Ding J, Eshak ES, Liu K, Mubarik S, Shi F, Wen H, Zeng Z, Bai J, Yu C. Wang F, et al. Int J Epidemiol. 2021 May 17;50(2):700. doi: 10.1093/ije/dyab004. Int J Epidemiol. 2021. PMID: 33537778 Free PMC article. No abstract available.

Abstract

Background: The new coronavirus (COVID-19) rapidly resulted in a pandemic. We report the characteristics of patients with severe or critical severe acute respiratory syndrome coronavirus 2 (SARS-CoV-2) infection in Wuhan city, China, and the risk factors related to infection severity and death.

Methods: We extracted the demographic and clinical data of 7283 patients with severe COVID-19 infection from designated Wuhan hospitals as of 25 February 2020. Factors associated with COVID-19 critical illness and mortality were analysed using logistic- and Cox-regression analyses.

Results: We studied 6269 patients with severe COVID-19 illness and 1014 critically ill patients. The median (IQR) age was 64 (53-71) years; 51.2% were male, 38.9% were retirees and 7.4% had self-reported histories of chronic disease. Up to the end of the study, 1180 patients (16.2%) recovered and were discharged, 649 (8.9%) died and the remainder were still receiving treatment. The number of daily confirmed critical cases peaked between 23 January and 1 February 2020. Patients with advanced age [odds ratio (OR), 1.03; 95% confidence intervals (CIs), 1.03-1.04], male sex (OR, 1.57; 95% CI, 1.33-1.86) and pre-existing diabetes (OR, 2.11), hypertension (OR, 2.72), cardiovascular disease (OR, 2.15) or respiratory disease (OR, 3.50) were more likely to be critically ill. Compared with those who recovered and were discharged, patients who died were older [hazard ratio (HR), 1.04; 95% CI, 1.03-1.05], more likely to be male (HR, 1.74; 95% CI, 1.44-2.11) and more likely to have hypertension (HR, 5.58), cardiovascular disease (HR, 1.83) or diabetes (HR, 1.67).

Conclusion: Advanced age, male sex and a history of chronic disease were associated with COVID-19 critical illness and death. Identifying these risk factors could help in the clinical monitoring of susceptible populations.

Keywords: COVID-19; Wuhan; clinical characteristics; oxygen therapy; pneumonia; risk factors; severity.

© The Author(s) 2020. Published by Oxford University Press on behalf of the International Epidemiological Association.

- 36 references
- 2 figures

Supplementary info

Publication types, MeSH terms Expand

Publication types

- Observational Study
- Research Support, Non-U.S. Gov't

MeSH terms

- Aged
- COVID-19 / mortality*
- Cardiovascular Diseases / epidemiology*
- China / epidemiology
- Comorbidity
- Diabetes Mellitus, Type 2 / epidemiology*
- Female
- Humans
- Hypertension / epidemiology*
- Intensive Care Units / statistics & numerical data
- Male
- Middle Aged
- Pandemics
- Real-Time Polymerase Chain Reaction
- Respiratory Tract Diseases / epidemiology*
- Retrospective Studies
- SARS-CoV-2 / isolation & purification*
- Treatment Outcome

Full text links

OXFORD ACADEMIC Silverchair Information Systems Free PMC article
Proceed to details
Cite
Share
☐ 623
Observational Study
AJNR Am J Neuroradiol
. 2020 Nov;41(11):2009-2011.
doi: 10.3174/ajnr.A6728. Epub 2020 Aug 27.

A Double-Edged Sword: Neurologic Complications and Mortality in Extracorporeal Membrane Oxygenation

Therapy for COVID-19-Related Severe Acute Respiratory Distress Syndrome at a Tertiary Care Center

J Masur [1], C W Freeman [2], S Mohan [2]
Affiliations Expand

Affiliations

- [1] Department of Radiology (J.M., C.W.F., S.M.), Hospital of the University of Pennsylvania, Philadelphia, Pennsylvania jonmasur@gmail.com.
- [2] Department of Radiology (J.M., C.W.F., S.M.), Hospital of the University of Pennsylvania, Philadelphia, Pennsylvania.

- PMID: **32855187**
- PMCID: PMC7658835
- DOI: 10.3174/ajnr.A6728

Free PMC article
Observational Study

A Double-Edged Sword: Neurologic Complications and Mortality in Extracorporeal Membrane Oxygenation Therapy for COVID-19-Related Severe Acute Respiratory Distress Syndrome at a Tertiary Care Center

J Masur et al. AJNR Am J Neuroradiol. 2020 Nov.
Free PMC article
Show details
AJNR Am J Neuroradiol
. 2020 Nov;41(11):2009-2011.
doi: 10.3174/ajnr.A6728. Epub 2020 Aug 27.

Authors

J Masur [1], C W Freeman [2], S Mohan [2]

Affiliations

- [1] Department of Radiology (J.M., C.W.F., S.M.), Hospital of the University of Pennsylvania, Philadelphia, Pennsylvania jonmasur@gmail.com.
- [2] Department of Radiology (J.M., C.W.F., S.M.), Hospital of the University of Pennsylvania, Philadelphia, Pennsylvania.

- PMID: **32855187**
- PMCID: PMC7658835
- DOI: 10.3174/ajnr.A6728

Abstract

In this clinical case series, we report our experience to date with neurologic complications of extracorporeal membrane oxygenation therapy for COVID-19 Acute Respiratory Distress Syndrome. We have found an unexpectedly increased rate of complications as demonstrated by neuroimaging compared with meta-analysis data in extracorporeal membrane oxygenation therapy for all Acute Respiratory Distress Syndrome etiologies over the past few decades and compared with the most recent baseline data describing the incidence of neurologic complication in all patients with COVID-19. For our 12-patient cohort, there was a rate of intracranial hemorrhage of 41.7%. Representative cases and images of devastating intracranial hemorrhage are presented. We hypothesize that the interplay between hematologic changes inherent to extracorporeal membrane oxygenation and inflammatory and coagulopathic changes that have begun to be elucidated as part of the COVID-19 disease process are responsible. Continued analysis of extracorporeal membrane oxygenation therapy in this disease paradigm is warranted.

© 2020 by American Journal of Neuroradiology.

- 2 figures

Supplementary info

Publication types, MeSH terms, Grant support Expand

Publication types

- Observational Study

MeSH terms

- Aged
- Betacoronavirus
- COVID-19
- Cohort Studies
- Coronavirus Infections / complications*
- Extracorporeal Membrane Oxygenation / adverse effects*
- Female
- Humans
- Intracranial Hemorrhages / epidemiology

- Intracranial Hemorrhages / etiology
- Male
- Middle Aged
- Pandemics
- Pneumonia, Viral / complications*
- Respiratory Distress Syndrome / therapy*
- Respiratory Distress Syndrome / virology*
- Retrospective Studies
- SARS-CoV-2
- Tertiary Care Centers

Grant support

- T32 EB004311/EB/NIBIB NIH HHS/United States

Full text links

Final Version FREE Am J Neuroradiol HighWire Free PMC article
Proceed to details
Cite
Share
☐ 624
Observational Study
Obesity (Silver Spring)
. 2021 May;29(5):870-878.
doi: 10.1002/oby.23142. Epub 2021 Mar 31.

Obesity and Critical Illness in COVID-19: Respiratory Pathophysiology

Molly Wolf[#,1,2], Jehan Alladina[#,1,2], Allison Navarrete-Welton[1], Benjamin Shoults[1], Kelsey Brait[1], David Ziehr[1,2], Atul Malhotra[3], C Corey Hardin[1,2], Kathryn A Hibbert[1,2]
Affiliations Expand

Affiliations

- [1] Division of Pulmonary and Critical Care Medicine, Massachusetts General Hospital, Boston, Massachusetts, USA.
- [2] Harvard Medical School, Boston, Massachusetts, USA.
- [3] Division of Pulmonary and Critical Care Medicine, University of California San Diego, San Diego, California, USA.

[#] Contributed equally.

- PMID: **33533193**
- PMCID: PMC8014725
- DOI: 10.1002/oby.23142

Free PMC article
Observational Study

Obesity and Critical Illness in COVID-19: Respiratory Pathophysiology

Molly Wolf et al. Obesity (Silver Spring). 2021 May.
Free PMC article
Show details
Obesity (Silver Spring)
. 2021 May;29(5):870-878.
doi: 10.1002/oby.23142. Epub 2021 Mar 31.

Authors

Molly Wolf[#,1,2], Jehan Alladina[#,1,2], Allison Navarrete-Welton[1], Benjamin Shoults[1], Kelsey Brait[1], David Ziehr[1,2], Atul Malhotra[3], C Corey Hardin[1,2], Kathryn A Hibbert[1,2]

Affiliations

- [1] Division of Pulmonary and Critical Care Medicine, Massachusetts General Hospital, Boston, Massachusetts, USA.
- [2] Harvard Medical School, Boston, Massachusetts, USA.
- [3] Division of Pulmonary and Critical Care Medicine, University of California San Diego, San Diego, California, USA.

[#] Contributed equally.

- PMID: **33533193**
- PMCID: PMC8014725
- DOI: 10.1002/oby.23142

Abstract

Objective: Recent cohort studies have identified obesity as a risk factor for poor outcomes in coronavirus disease 2019 (COVID-19). To further explore the relationship between obesity and critical illness in COVID-19, the association of BMI with baseline demographic and intensive care unit (ICU) parameters, laboratory values, and outcomes in a critically ill patient cohort was examined.

Methods: In this retrospective study, the first 277 consecutive patients admitted to Massachusetts General Hospital ICUs with laboratory-confirmed COVID-19 were examined. BMI class, initial ICU laboratory values, physiologic characteristics including gas exchange and ventilatory

mechanics, and ICU interventions as clinically available were measured. Mortality, length of ICU admission, and duration of mechanical ventilation were also measured.

Results: There was no difference found in respiratory system compliance or oxygenation between patients with and without obesity. Patients without obesity had higher initial ferritin and D-dimer levels than patients with obesity. Standard acute respiratory distress syndrome management, including prone ventilation, was equally distributed between BMI groups. There was no difference found in outcomes between BMI groups, including 30- and 60-day mortality and duration of mechanical ventilation.

Conclusions: In this cohort of critically ill patients with COVID-19, obesity was not associated with meaningful differences in respiratory physiology, inflammatory profile, or clinical outcomes.

© 2021 The Obesity Society.

Conflict of interest statement

The authors report no conflicts of interest, including relevant financial interests, activities, relationships, or affiliations. AM reports income from Merck and LivaNova related to medical education and reports that ResMed provided a philanthropic donation to UC San Diego. ANW has received funding from the Olympus Corporation and CRICO Risk Management Foundation for research unrelated to this project.

- 45 references
- 2 figures

Supplementary info

Publication types, MeSH terms, Grant support Expand

Publication types

- Observational Study
- Research Support, N.I.H., Extramural
- Research Support, Non-U.S. Gov't

MeSH terms

- Aged
- Body Mass Index*
- COVID-19 / complications*
- COVID-19 / epidemiology
- Critical Illness
- Female
- Hospitalization
- Humans
- Intensive Care Units
- Male

- Massachusetts
- Middle Aged
- Obesity / complications*
- Respiration, Artificial
- Retrospective Studies
- Risk Factors

Grant support

- T32 HL134632/HL/NHLBI NIH HHS/United States
- UL1 TR002541/TR/NCATS NIH HHS/United States
- K24 HL132105/HL/NHLBI NIH HHS/United States
- R01 HL085188/HL/NHLBI NIH HHS/United States
- T32 HL116275/HL/NHLBI NIH HHS/United States

- R01 HL148436/HL/NHLBI NIH HHS/United States
- RO1 HL148436/Atul Malhotra
- R01 AG063925/AG/NIA NIH HHS/United States
- T32HL116275/David Ziehr

Show all 9 grants

Full text links

WILEY Full Text Article Wiley Free PMC article

Proceed to details

Cite

Share

625

Observational Study

J Med Virol

. 2020 Nov;92(11):2616-2622.

doi: 10.1002/jmv.26082. Epub 2020 Jun 9.

Correlation between the variables collected at admission and progression to severe cases during hospitalization among patients with COVID-19 in Chongqing

Jun Duan [1], Xiaohui Wang [1], Jing Chi [1], Hong Chen [1], Linfu Bai [1], Qianfang Hu [1], Xiaoli Han [1], Wenhui Hu [1], Linxiao Zhu [1], Xue Wang [1], You Li [1], Chenmei Zhou [1], Huaming Mou [2], Xiaofeng Yan [3], Shuliang Guo [1]

Affiliations Expand

Affiliations

- [1] Department of Respiratory and Critical Care Medicine, The First Affiliated Hospital of Chongqing Medical University, Chongqing, China.
- [2] Chongqing Three Gorges Central Hospital, Chongqing, China.
- [3] Chongqing Public Health Medical Center, Chongqing, China.

- PMID: **32470186**
- PMCID: PMC7283752
- DOI: 10.1002/jmv.26082

Free PMC article
Observational Study

Correlation between the variables collected at admission and progression to severe cases during hospitalization among patients with COVID-19 in Chongqing

Jun Duan et al. J Med Virol. 2020 Nov.
Free PMC article

Show details

J Med Virol

. 2020 Nov;92(11):2616-2622.
doi: 10.1002/jmv.26082. Epub 2020 Jun 9.

Authors

Jun Duan [1], Xiaohui Wang [1], Jing Chi [1], Hong Chen [1], Linfu Bai [1], Qianfang Hu [1], Xiaoli Han [1], Wenhui Hu [1], Linxiao Zhu [1], Xue Wang [1], You Li [1], Chenmei Zhou [1], Huaming Mou [2], Xiaofeng Yan [3], Shuliang Guo [1]

Affiliations

- [1] Department of Respiratory and Critical Care Medicine, The First Affiliated Hospital of Chongqing Medical University, Chongqing, China.
- [2] Chongqing Three Gorges Central Hospital, Chongqing, China.
- [3] Chongqing Public Health Medical Center, Chongqing, China.

- PMID: **32470186**
- PMCID: PMC7283752
- DOI: 10.1002/jmv.26082

Abstract

Mortality is high among severe patients with 2019 novel coronavirus-infected disease (COVID-19). Early prediction of progression to severe cases is needed. We retrospectively collected patients with COVID-19 in two hospital of Chongqing from 1st January to 29th February 2020. At admission, we collected the demographics and laboratory tests to predict whether the patient would progress to severe cases in hospitalization. Severe case was confirmed when one of the following criteria occurred: (a) dyspnea, respiratory rate ≥30 breaths/min, (b) blood oxygen saturation ≤93%, and (c) PaO_2 /FiO_2 ≤ 300 mm Hg. At admission, 348 mild cases were enrolled in this study. Of them, 20 (5.7%) patients progressed to severe cases after median 4.0 days (interquartile range: 2.3-6.0). Pulmonary inflammation index, platelet counts, sodium, C-reactive protein, prealbumin, and $PaCO_2$ showed good distinguishing power to predict progression to severe cases (each area under the curve of receiver operating characteristics [AUC] ≥ 0.8). Age, heart rate, chlorine, alanine aminotransferase, aspartate aminotransferase, procalcitonin, creatine kinase, pH, CD3 counts, and CD4 counts showed moderate distinguishing power (each AUC between 0.7-0.8). And potassium, creatinine, temperature, and D-dimer showed mild distinguishing power (each AUC between 0.6-0.7). In addition, higher C-reactive protein was associated with shorter time to progress to severe cases (r = -0.62). Several easily obtained variables at admission are associated with progression to severe cases during hospitalization. These variables provide a reference for the medical staffs when they manage the patients with COVID-19.

Keywords: coronavirus; critical care; pneumonia; predictors.

© 2020 Wiley Periodicals LLC.

Conflict of interest statement

The authors declare that there no conflict of interests.

- 19 references
- 2 figures

Supplementary info

Publication types, MeSH terms, Substances, Grant support Expand

Publication types

- Observational Study
- Research Support, Non-U.S. Gov't

MeSH terms

- Adult
- Aged
- C-Reactive Protein / analysis
- COVID-19 / diagnosis*
- COVID-19 / mortality
- China / epidemiology
- Comorbidity

- Disease Progression
- Female
- Hospitalization / statistics & numerical data*
- Humans
- Male
- Middle Aged
- Platelet Count / statistics & numerical data
- ROC Curve
- Retrospective Studies
- Risk Factors
- Severity of Illness Index*

Substances

- C-Reactive Protein

Grant support

- Chongqing Science and Technology Bureau "new crown pneumonia epidemic emergency science and technology special" the fourth batch of projects/International
- Chongqing Education Board "new coronavirus infection and prevention" emergency scientific research project/International

Full text links

WILEY Full Text Article Wiley Free PMC article
Proceed to details
Cite
Share
626
Rheumatol Int
. 2021 Dec;41(12):2167-2175.
doi: 10.1007/s00296-021-05003-1. Epub 2021 Sep 27.

Rehabilitative management of post-acute COVID-19: clinical pictures and outcomes

Tuba Güler [1], Fatma Gül Yurdakul [2], Filiz Acar Sivas [2], Zeynep Kiliç [2], Emre Adigüzel [2], Evren Yaşar [2], Hatice Bodur [2]
Affiliations Expand

Affiliations

- [1] Ankara City Hospital, Physical Medicine and Rehabilitation Hospital, Ankara, Turkey. tubakulu@yahoo.com.
- [2] Ankara City Hospital, Physical Medicine and Rehabilitation Hospital, Ankara, Turkey.

- PMID: **34580754**
- DOI: 10.1007/s00296-021-05003-1

Rehabilitative management of post-acute COVID-19: clinical pictures and outcomes

Tuba Güler et al. Rheumatol Int. 2021 Dec.

Show details

Rheumatol Int

. 2021 Dec;41(12):2167-2175.
doi: 10.1007/s00296-021-05003-1. Epub 2021 Sep 27.

Authors

Tuba Güler [1], Fatma Gül Yurdakul [2], Filiz Acar Sivas [2], Zeynep Kiliç [2], Emre Adigüzel [2], Evren Yaşar [2], Hatice Bodur [2]

Affiliations

- [1] Ankara City Hospital, Physical Medicine and Rehabilitation Hospital, Ankara, Turkey. tubakulu@yahoo.com.
- [2] Ankara City Hospital, Physical Medicine and Rehabilitation Hospital, Ankara, Turkey.

- PMID: **34580754**
- DOI: 10.1007/s00296-021-05003-1

Abstract

This study aimed to detect patients' characteristics who suffered severe and critical COVID-19 pneumonia admitted to the post-acute COVID-19 rehabilitation clinic in Ankara City Hospital, Physical Medicine and Rehabilitation Hospital and to share our experiences and outcomes of rehabilitation programmes applied. This study was designed as a single-centre, retrospective, observational study. Severe and critical COVID-19 patients, admitted to the post-acute COVID-19 rehabilitation clinic, were included in patient-based rehabilitation programmes, targeting neuromuscular and respiratory recovery. Functional status, oxygen (O_2) requirement and daily living activities were assessed before and after rehabilitation. Eighty-five patients, of which 74% were male, were analysed, with the mean age of 58.27 ± 11.13 and mean body mass index of 25.29 ± 4.81 kg/m^2. The most prevalent comorbidities were hypertension (49.4%) and diabetes mellitus (34.1%). Of the 85 patients, 84 received antiviral drugs, 81 low-molecular-weight heparin, 71 corticosteroids, 11 anakinra, 4 tocilizumab, 16 intravenous immunoglobulin and 6 plasmapheresis. 78.8% of the patients were admitted to the intensive care unit, with a mean length of stay of 19.41 ± 18.99 days, while those who needed O_2 support with mechanic ventilation was 36.1%. Neurological complications, including Guillain-Barré syndrome, critical illness-related myopathy/neuropathy, cerebrovascular disease and steroid myopathy, were observed in 39

patients. On initial functional statuses, 55.3% were bedridden, 22.4% in wheelchair level and 20% mobilised with O_2 support. After rehabilitation, these ratios were 2.4%, 4.7% and 8.2%, respectively. During admission, 71 (83.5%) patients required O_2 support, but decreased to 7 (8.2%) post-rehabilitation. Barthel Index improved statistically from 44.82 ± 27.31 to 88.47 ± 17.56. Patient-based modulated rehabilitation programmes are highly effective in severe and critical COVID-19 complications, providing satisfactory well-being in daily living activities.

Keywords: COVID-19; Inpatient rehabilitation; Neurological manifestations; Patient outcome; Rehabilitation.

© 2021. The Author(s), under exclusive licence to Springer-Verlag GmbH Germany, part of Springer Nature.

- 32 references

Supplementary info

MeSH terms Expand

MeSH terms

- Aged
- COVID-19 / epidemiology
- COVID-19 / rehabilitation*
- Comorbidity
- Exercise Therapy / methods*
- Female
- Humans
- Male
- Middle Aged
- Pandemics
- Rehabilitation Centers / organization & administration*
- Rehabilitation Centers / statistics & numerical data
- Retrospective Studies
- SARS-CoV-2
- Treatment Outcome
- Turkey / epidemiology

Full text links

SpringerLink FULL-TEXT ARTICLE Springer
Proceed to details
Cite
Share
☐ 627
Observational Study

CNS Neurosci Ther

. 2021 Oct;27(10):1127-1135.
doi: 10.1111/cns.13687. Epub 2021 Jun 16.

Encephalopathy at admission predicts adverse outcomes in patients with SARS-CoV-2 infection

Lei Tang [1, 2], Shixin Liu [1, 2], Yanhe Xiao [2], Thi My Linh Tran [3], Ji Whae Choi [3], Jing Wu [4], Kasey Halsey [3], Raymond Y Huang [5], Jerrold Boxerman [3], Sohil H Patel [6], David Kung [7], Renyu Liu [8], Michael D Feldman [9], Daniel D Danoski [9], Wei-Hua Liao [10], Scott E Kasner [11], Tao Liu [12], Bo Xiao [1], Paul J Zhang [9], Michael Reznik [13], Harrison X Bai [3], Li Yang [14]
Affiliations Expand

Affiliations

- [1] Department of Neurology, Xiangya Hospital, Central South University, Changsha, China.
- [2] Xiangya School of Medicine, Central South University, Changsha, China.
- [3] Department of Diagnostic Imaging, Warren Alpert Medical School of Brown University, Providence, RI, USA.
- [4] Department of Radiology, The Second Xiangya Hospital, Central South University, Changsha, China.
- [5] Department of Radiology, Brigham and Women's Hospital, Boston, MA, USA.
- [6] Department of Radiology, University of Virginia, Charlottesville, VA, USA.
- [7] Department of Neurosurgery, Hospital of the University of Pennsylvania, Philadelphia, PA, USA.
- [8] Department of Anaesthesiology and critical care medicine, Hospital of the University of Pennsylvania, Philadelphia, PA, USA.
- [9] Department of Pathology and Laboratory Medicine, Hospital of the University of Pennsylvania, Philadelphia, PA, USA.
- [10] Department of Radiology, Xiangya Hospital, Central South University, Changsha, China.
- [11] Department of Neurology, Hospital of the University of Pennsylvania, Philadelphia, PA, USA.
- [12] Department of Biostatistics and Public Health, Brown University, Providence, RI, USA.
- [13] Department of Neurology, Warren Alpert Medical School of Brown University, Providence, RI, USA.
- [14] Department of Neurology, The Second Xiangya Hospital, Central South University, Changsha, China.

- PMID: **34132473**
- PMCID: PMC8444722
- DOI: 10.1111/cns.13687

Free PMC article
Observational Study

Encephalopathy at admission predicts adverse outcomes in patients with SARS-CoV-2 infection

Lei Tang et al. CNS Neurosci Ther. 2021 Oct.
Free PMC article
Show details
CNS Neurosci Ther
. 2021 Oct;27(10):1127-1135.
doi: 10.1111/cns.13687. Epub 2021 Jun 16.

Authors

Lei Tang [1, 2], Shixin Liu [1, 2], Yanhe Xiao [2], Thi My Linh Tran [3], Ji Whae Choi [3], Jing Wu [4], Kasey Halsey [3], Raymond Y Huang [5], Jerrold Boxerman [3], Sohil H Patel [6], David Kung [7], Renyu Liu [8], Michael D Feldman [9], Daniel D Danoski [9], Wei-Hua Liao [10], Scott E Kasner [11], Tao Liu [12], Bo Xiao [1], Paul J Zhang [9], Michael Reznik [13], Harrison X Bai [3], Li Yang [14]

Affiliations

- [1] Department of Neurology, Xiangya Hospital, Central South University, Changsha, China.
- [2] Xiangya School of Medicine, Central South University, Changsha, China.
- [3] Department of Diagnostic Imaging, Warren Alpert Medical School of Brown University, Providence, RI, USA.
- [4] Department of Radiology, The Second Xiangya Hospital, Central South University, Changsha, China.
- [5] Department of Radiology, Brigham and Women's Hospital, Boston, MA, USA.
- [6] Department of Radiology, University of Virginia, Charlottesville, VA, USA.
- [7] Department of Neurosurgery, Hospital of the University of Pennsylvania, Philadelphia, PA, USA.
- [8] Department of Anaesthesiology and critical care medicine, Hospital of the University of Pennsylvania, Philadelphia, PA, USA.
- [9] Department of Pathology and Laboratory Medicine, Hospital of the University of Pennsylvania, Philadelphia, PA, USA.
- [10] Department of Radiology, Xiangya Hospital, Central South University, Changsha, China.
- [11] Department of Neurology, Hospital of the University of Pennsylvania, Philadelphia, PA, USA.
- [12] Department of Biostatistics and Public Health, Brown University, Providence, RI, USA.
- [13] Department of Neurology, Warren Alpert Medical School of Brown University, Providence, RI, USA.
- [14] Department of Neurology, The Second Xiangya Hospital, Central South University, Changsha, China.

- PMID: **34132473**
- PMCID: PMC8444722
- DOI: 10.1111/cns.13687

Abstract

Aims: To determine if neurologic symptoms at admission can predict adverse outcomes in patients with severe acute respiratory syndrome coronavirus 2 (SARS-CoV-2).

Methods: Electronic medical records of 1053 consecutively hospitalized patients with laboratory-confirmed infection of SARS-CoV-2 from one large medical center in the USA were retrospectively analyzed. Univariable and multivariable Cox regression analyses were performed with the calculation of areas under the curve (AUC) and concordance index (C-index). Patients were stratified into subgroups based on the presence of encephalopathy and its severity using survival statistics. In sensitivity analyses, patients with mild/moderate and severe encephalopathy (defined as coma) were separately considered.

Results: Of 1053 patients (mean age 52.4 years, 48.0% men [n = 505]), 35.1% (n = 370) had neurologic manifestations at admission, including 10.3% (n = 108) with encephalopathy. Encephalopathy was an independent predictor for death (hazard ratio [HR] 2.617, 95% confidence interval [CI] 1.481-4.625) in multivariable Cox regression. The addition of encephalopathy to multivariable models comprising other predictors for adverse outcomes increased AUCs (mortality: 0.84-0.86, ventilation/ intensive care unit [ICU]: 0.76-0.78) and C-index (mortality: 0.78 to 0.81, ventilation/ICU: 0.85-0.86). In sensitivity analyses, risk stratification survival curves for mortality and ventilation/ICU based on severe encephalopathy (n = 15) versus mild/moderate encephalopathy (n = 93) versus no encephalopathy (n = 945) at admission were discriminative ($p < 0.001$).

Conclusions: Encephalopathy at admission predicts later progression to death in SARS-CoV-2 infection, which may have important implications for risk stratification in clinical practice.

Keywords: COVID-19; SARS-CoV-2; encephalopathy; neurologic symptoms.

© 2021 The Authors. CNS Neuroscience & Therapeutics Published by John Wiley & Sons Ltd.

Conflict of interest statement

The authors declare that they have no conflict of interest.

- 43 references
- 4 figures

Supplementary info

Publication types, MeSH terms, Grant support Expand

Publication types

- Multicenter Study
- Observational Study
- Research Support, N.I.H., Extramural
- Research Support, Non-U.S. Gov't

MeSH terms

- Adult
- Aged
- Brain Diseases / diagnosis*
- Brain Diseases / mortality*
- Brain Diseases / therapy
- COVID-19 / diagnosis*
- COVID-19 / mortality*
- COVID-19 / therapy
- Cohort Studies
- Female
- Humans
- Male
- Middle Aged
- Patient Admission / trends*
- Predictive Value of Tests
- Retrospective Studies

Grant support

- R03CA249554/National Cancer Institute (NCI) of the National Institutes of Health under Award
- 2018JJ3709/Hunan Natural Science Foundation under Award
- GR399196/Brown University COVID-19 seed grant
- Research Scholar Grant by RSNA Research & Education Foundation
- 8181101287/National Natural Science Foundation of China grant under Award

- 81971696/National Natural Science Foundation of China grant under Award
- Amazon Web Service for the COVID-19 Diagnostic Development Initiative
- GR399196/Hunan Natural Science Foundation under Award

Show all 8 grants

Full text links

WILEY Full Text Article Wiley Free PMC article

Proceed to details

Cite

Share

628

Observational Study

Otolaryngol Head Neck Surg

. 2020 Jul;163(1):170-178.

doi: 10.1177/0194599820929640. Epub 2020 May 19.

Factors Associated With Intubation and Prolonged Intubation in Hospitalized Patients With COVID-19

Kevin Hur [1], Caroline P E Price [1], Elizabeth L Gray [2], Reeti K Gulati [1], Matthew Maksimoski [1], Samuel D Racette [1], Alexander L Schneider [1], Ashoke R Khanwalkar [1]
Affiliations Expand

Affiliations

- [1] Department of Otolaryngology-Head and Neck Surgery, Feinberg School of Medicine, Northwestern University, Chicago, Illinois, USA.
- [2] Biostatistics Collaboration Center, Feinberg School of Medicine, Northwestern University, Chicago, Illinois, USA.

- PMID: **32423368**
- PMCID: PMC7240317
- DOI: 10.1177/0194599820929640

Free PMC article
Observational Study

Factors Associated With Intubation and Prolonged Intubation in Hospitalized Patients With COVID-19

Kevin Hur et al. Otolaryngol Head Neck Surg. 2020 Jul.
Free PMC article
Show details
Otolaryngol Head Neck Surg
. 2020 Jul;163(1):170-178.
doi: 10.1177/0194599820929640. Epub 2020 May 19.

Authors

Kevin Hur [1], Caroline P E Price [1], Elizabeth L Gray [2], Reeti K Gulati [1], Matthew Maksimoski [1], Samuel D Racette [1], Alexander L Schneider [1], Ashoke R Khanwalkar [1]

Affiliations

- [1] Department of Otolaryngology-Head and Neck Surgery, Feinberg School of Medicine, Northwestern University, Chicago, Illinois, USA.
- [2] Biostatistics Collaboration Center, Feinberg School of Medicine, Northwestern University, Chicago, Illinois, USA.

- PMID: **32423368**
- PMCID: PMC7240317
- DOI: 10.1177/0194599820929640

Erratum in

- Corrigendum to "Factors Associated With Intubation and Prolonged Intubation in Hospitalized Patients With COVID-19".
  [No authors listed] [No authors listed] Otolaryngol Head Neck Surg. 2020 Jul;163(1):NP1. doi: 10.1177/0194599820938340. Otolaryngol Head Neck Surg. 2020. PMID: 32609073 Free PMC article. No abstract available.

Abstract

Objective: To identify risk factors associated with intubation and time to extubation in hospitalized patients with coronavirus disease 2019 (COVID-19).

Study design: Retrospective observational study.

Setting: Ten hospitals in the Chicago metropolitan area.

Subjects and methods: Patients with laboratory-confirmed COVID-19 admitted between March 1 and April 8, 2020, were included. We evaluated sociodemographic and clinical characteristics associated with intubation and prolonged intubation for acute respiratory failure secondary to COVID-19 infection.

Results: Of the 486 hospitalized patients included in the study, the median age was 59 years (interquartile range, 47-69); 271 (55.8%) were male; and the median body mass index was 30.6 (interquartile range, 26.5-35.6). During the hospitalization, 138 (28.4%) patients were intubated; 78 (56.5%) were eventually extubated; 21 (15.2%) died; and 39 (28.3%) remained intubated at a mean ± SD follow-up of 19.6 ± 6.7 days. Intubated patients had a significantly higher median age (65 vs 57 years, $P < .001$) and rate of diabetes (56 [40.6%] vs 104 [29.9%], $P = .031$) as compared with nonintubated patients. Multivariable logistic regression analysis identified age, sex, respiratory rate, oxygen saturation, history of diabetes, and shortness of breath as factors predictive of intubation. Age and body mass index were the only factors independently associated with time to extubation.

Conclusion: In addition to clinical signs of respiratory distress, patients with COVID-19 who are older, male, or diabetic are at higher risk of requiring intubation. Among intubated patients, older and more obese patients are at higher risk for prolonged intubation. Otolaryngologists consulted for airway management should consider these factors in their decision making.

Keywords: COVID-19; SARS-CoV-2; adults; coronavirus; hospitalized; intubation; mechanical ventilator; tracheostomy.

- 31 references
- 3 figures

Supplementary info

Publication types, MeSH terms, Grant support Expand

Publication types

- Multicenter Study
- Observational Study
- Research Support, N.I.H., Extramural

MeSH terms

- Aged
- Betacoronavirus*
- COVID-19
- Coronavirus Infections / complications*
- Coronavirus Infections / epidemiology
- Dyspnea / etiology
- Dyspnea / therapy*
- Female
- Follow-Up Studies
- Humans
- Inpatients*
- Intubation, Intratracheal / methods*
- Male
- Middle Aged
- Pandemics
- Pneumonia, Viral / complications*
- Pneumonia, Viral / epidemiology
- Respiration, Artificial / methods*
- Retrospective Studies
- Risk Factors
- SARS-CoV-2
- Time Factors

Grant support

- UL1 TR001422/TR/NCATS NIH HHS/United States

Full text links

SAGE journals Open access full text Atypon Free PMC article

Proceed to details

Cite

Share

☐ 629

Observational Study

Eur Heart J
. 2020 Jul 1;41(32):3058-3068.
doi: 10.1093/eurheartj/ehaa500.

Pulmonary embolism in COVID-19 patients: a French multicentre cohort study

Charles Fauvel [1], Orianne Weizman [2, 3], Antonin Trimaille [4], Delphine Mika [5], Thibaut Pommier [6], Nathalie Pace [2], Amine Douair [7], Eva Barbin [8], Antoine Fraix [2], Océane Bouchot [7], Othmane Benmansour [8], Guillaume Godeau [9], Yasmine Mecheri [8], Romane Lebourdon [10], Cédric Yvorel [11], Michael Massin [2], Tiphaine Leblon [12], Chaima Chabbi [8], Erwan Cugney [2], Léa Benabou [10], Matthieu Aubry [13], Camille Chan [10], Ines Boufoula [8], Clement Barnaud [8], Léa Bothorel [8], Baptiste Duceau [3], Willy Sutter [3], Victor Waldmann [3, 14], Guillaume Bonnet [3, 14], Ariel Cohen [15], Théo Pezel [16], Critical Covid-19 France Investigators
Affiliations Expand

Affiliations

- [1] Rouen University Hospital, FHU REMOD-VHF, F-76000 Rouen, France.
- [2] Centre Hospitalier Régional Universitaire de Nancy, 54511 Vandoeuvre-Les-Nancy, France.
- [3] Université de Paris, PARCC, INSERM, 75015 Paris, France.
- [4] Nouvel Hôpital Civil, Centre Hospitalier Régional Universitaire de Strasbourg, 67000 Strasbourg, France.
- [5] Université Paris-Saclay, Inserm, UMR-S 1180, 92296 Chatenay-Malabry, France.
- [6] Centre hospitalier Universitaire de Dijon, 21000 Dijon, France.
- [7] Centre Hospitalier Annecy Genevois, 74370 Epagny Metz-Tessy, France.
- [8] Centre Hospitalier Régionnal de Orléans, 45100 Orléans, France.
- [9] Institut Cardiovasculaire Paris Sud, 91300 Massy, France.
- [10] Centre Hospitalier Universitaire de Bordeaux, 33076 Bordeaux, France.
- [11] Centre Hospitalier Universitaire de Saint-Etienne, 42270 Saint-Priest-en-Jarez, France.
- [12] Centre Hospitalier Universitaire de Lille, Université Catholique de Lille, 59000 Lille, France.
- [13] Hospices Civils de Lyon, Centre Hospitalier Universitaire, 69003 Lyon, France.
- [14] Hôpital Européen Georges Pompidou, Université de Paris, 75015 Paris, France.
- [15] Saint Antoine Hospital, 75012 Paris, France.
- [16] Lariboisiere hospital, APHP, University of Paris, 75010 Paris, France.

- PMID: **32656565**
- PMCID: PMC7528952
- DOI: 10.1093/eurheartj/ehaa500

Free PMC article
Observational Study

Pulmonary embolism in COVID-19 patients: a French multicentre cohort study

Charles Fauvel et al. Eur Heart J. 2020.
Free PMC article
Show details
Eur Heart J
. 2020 Jul 1;41(32):3058-3068.
doi: 10.1093/eurheartj/ehaa500.

Authors

Charles Fauvel [1], Orianne Weizman [2 3], Antonin Trimaille [4], Delphine Mika [5], Thibaut Pommier [6], Nathalie Pace [2], Amine Douair [7], Eva Barbin [8], Antoine Fraix [2], Océane Bouchot [7], Othmane Benmansour [8], Guillaume Godeau [9], Yasmine Mecheri [8], Romane Lebourdon [10], Cédric Yvorel [11], Michael Massin [2], Tiphaine Leblon [12], Chaima Chabbi [8], Erwan Cugney [2], Léa Benabou [10], Matthieu Aubry [13], Camille Chan [10], Ines Boufoula [8], Clement Barnaud [8], Léa Bothorel [8], Baptiste Duceau [3], Willy Sutter [3], Victor Waldmann [3 14], Guillaume Bonnet [3 14], Ariel Cohen [15], Théo Pezel [16], Critical Covid-19 France Investigators

Affiliations

- [1] Rouen University Hospital, FHU REMOD-VHF, F-76000 Rouen, France.
- [2] Centre Hospitalier Régional Universitaire de Nancy, 54511 Vandoeuvre-Les-Nancy, France.
- [3] Université de Paris, PARCC, INSERM, 75015 Paris, France.
- [4] Nouvel Hôpital Civil, Centre Hospitalier Régional Universitaire de Strasbourg, 67000 Strasbourg, France.
- [5] Université Paris-Saclay, Inserm, UMR-S 1180, 92296 Chatenay-Malabry, France.
- [6] Centre hospitalier Universitaire de Dijon, 21000 Dijon, France.
- [7] Centre Hospitalier Annecy Genevois, 74370 Epagny Metz-Tessy, France.
- [8] Centre Hospitalier Régionnal de Orléans, 45100 Orléans, France.
- [9] Institut Cardiovasculaire Paris Sud, 91300 Massy, France.
- [10] Centre Hospitalier Universitaire de Bordeaux, 33076 Bordeaux, France.
- [11] Centre Hospitalier Universitaire de Saint-Etienne, 42270 Saint-Priest-en-Jarez, France.
- [12] Centre Hospitalier Universitaire de Lille, Université Catholique de Lille, 59000 Lille, France.
- [13] Hospices Civils de Lyon, Centre Hospitalier Universitaire, 69003 Lyon, France.
- [14] Hôpital Européen Georges Pompidou, Université de Paris, 75015 Paris, France.
- [15] Saint Antoine Hospital, 75012 Paris, France.
- [16] Lariboisiere hospital, APHP, University of Paris, 75010 Paris, France.

- PMID: **32656565**
- PMCID: PMC7528952
- DOI: 10.1093/eurheartj/ehaa500

Abstract

Aims: While pulmonary embolism (PE) appears to be a major issue in COVID-19, data remain sparse. We aimed to describe the risk factors and baseline characteristics of patients with PE in a cohort of COVID-19 patients.

Methods and results: In a retrospective multicentre observational study, we included consecutive patients hospitalized for COVID-19. Patients without computed tomography pulmonary angiography (CTPA)-proven PE diagnosis and those who were directly admitted to an intensive care unit (ICU) were excluded. Among 1240 patients (58.1% men, mean age 64 ± 17 years), 103 (8.3%) patients had PE confirmed by CTPA. The ICU transfer and mechanical ventilation were significantly higher in the PE group (for both $P < 0.001$). In an univariable analysis, traditional venous thrombo-embolic risk factors were not associated with PE ($P > 0.05$), while patients under therapeutic dose anticoagulation before hospitalization or prophylactic dose anticoagulation introduced during hospitalization had lower PE occurrence [odds ratio (OR) 0.40, 95% confidence interval (CI) 0.14-0.91, $P = 0.04$; and OR 0.11, 95% CI 0.06-0.18, $P < 0.001$, respectively]. In a multivariable analysis, the following variables, also statistically significant in univariable analysis, were associated with PE: male gender (OR 1.03, 95% CI 1.003-1.069, $P = 0.04$), anticoagulation with a prophylactic dose (OR 0.83, 95% CI 0.79-0.85, $P < 0.001$) or a therapeutic dose (OR 0.87, 95% CI 0.82-0.92, $P < 0.001$), C-reactive protein (OR 1.03, 95% CI 1.01-1.04, $P = 0.001$), and time from symptom onset to hospitalization (OR 1.02, 95% CI 1.006-1.038, $P = 0.002$).

Conclusion: PE risk factors in the COVID-19 context do not include traditional thrombo-embolic risk factors but rather independent clinical and biological findings at admission, including a major contribution to inflammation.

Keywords: COVID-19; Computed tomography angiography; Intensive care unit; Pulmonary embolism; Risk factors.

Published on behalf of the European Society of Cardiology. All rights reserved. © The Author(s) 2020. For permissions, please email: journals.permissions@oup.com.

Comment in

- COVID-19 and pulmonary embolism: an unwanted alliance.
  Torbicki A. Torbicki A. Eur Heart J. 2020 Jul 1;41(32):3069-3071. doi: 10.1093/eurheartj/ehaa553. Eur Heart J. 2020. PMID: 32656564 Free PMC article. No abstract available.
- Understanding COVID-19: in the end it is the endothelium-what else?
  Lüscher TF. Lüscher TF. Eur Heart J. 2020 Aug 21;41(32):3023-3027. doi: 10.1093/eurheartj/ehaa706. Eur Heart J. 2020. PMID: 33216863 Free PMC article. No abstract available.

- 26 references
- 4 figures

Supplementary info

Publication types, MeSH terms Expand

Publication types

- Multicenter Study
- Observational Study

MeSH terms

- Betacoronavirus*
- COVID-19
- Computed Tomography Angiography / methods
- Coronavirus Infections / complications*
- Coronavirus Infections / epidemiology
- Female
- France / epidemiology
- Hospitalization / trends*
- Humans
- Incidence
- Male
- Middle Aged
- Pandemics*
- Pneumonia, Viral / complications*
- Pneumonia, Viral / epidemiology
- Pulmonary Embolism / diagnosis
- Pulmonary Embolism / epidemiology
- Pulmonary Embolism / etiology*
- Retrospective Studies
- Risk Factors
- SARS-CoV-2
- Survival Rate / trends

Full text links

OXFORD ACADEMIC Silverchair Information Systems Free PMC article
Proceed to details
Cite
Share
☐ 630
Observational Study
Medicine (Baltimore)
. 2021 May 14;100(19):e25913.
doi: 10.1097/MD.0000000000025913.

Differences in clinical characteristics and liver injury between suspected and confirmed COVID-19 patients in Jingzhou, Hubei Province of China

Qing Zhang [1, 2, 3], Jie Li [1], Yan Zhang [1], Jie Gao [1], Peixue Wang [1], Minghua Ai [1], Wen Ding [1], Xiaoping Tan [1]
Affiliations Expand

Affiliations

- [1] Department of Gastroenterology, First Hospital of Yangtze University.
- [2] Clinical Medical College, Yangtze University, Jingzhou, Hubei Province.
- [3] Department of Gastroenterology, Zhongnan Hospital of Wuhan University, Wuhan, China.

- PMID: **34106656**
- PMCID: PMC8133163
- DOI: 10.1097/MD.0000000000025913

Free PMC article
Observational Study

Differences in clinical characteristics and liver injury between suspected and confirmed COVID-19 patients in Jingzhou, Hubei Province of China

Qing Zhang et al. Medicine (Baltimore). 2021.
Free PMC article
Show details
Medicine (Baltimore)
. 2021 May 14;100(19):e25913.
doi: 10.1097/MD.0000000000025913.

Authors

Qing Zhang [1, 2, 3], Jie Li [1], Yan Zhang [1], Jie Gao [1], Peixue Wang [1], Minghua Ai [1], Wen Ding [1], Xiaoping Tan [1]

Affiliations

- [1] Department of Gastroenterology, First Hospital of Yangtze University.

- [2] Clinical Medical College, Yangtze University, Jingzhou, Hubei Province.
- [3] Department of Gastroenterology, Zhongnan Hospital of Wuhan University, Wuhan, China.

- PMID: **34106656**
- PMCID: PMC8133163
- DOI: 10.1097/MD.0000000000025913

Abstract

To evaluate the clinical characteristics and liver injury in coronavirus disease 2019 (COVID-19) patients, and analyze the differences between suspected and confirmed COVID-19 patients, this retrospective study was performed on 157 COVID-19 patients and 93 suspected patients who were ultimately excluded from COVID-19 (control patients). Differences in clinical characteristics and liver injury between suspected and confirmed COVID-19 patients were analyzed. Age, male sex, fever, chest tightness and dyspnea were related to the severity of COVID-19. C-reactive protein (CRP) and D-dimer may be predictors of the severity of COVID-19. Computed tomography (CT) played an important role in the screening of COVID-19 and the evaluation of disease severity. Multiple factors may cause liver injury in COVID-19 patients. Severe acute respiratory syndrome coronavirus 2 (SARS-CoV-2) may be more likely to cause liver injury than common respiratory infectious diseases. Age, temperature (T), white blood cell (WBC), lymphocytes (LY), hematocrit (HCT), CRP, and finger pulse oxygen saturation (SpO2) may correlate with liver function impairment and may predict the occurrence and severity of liver function impairment. Some therapeutic drugs (like glucocorticoid) may be involved in the liver function impairment of COVID-19 patients. Most liver function indices improved significantly after active treatment. Although COVID-19 and other common respiratory infectious diseases share some clinical characteristics, COVID-19 has its own characteristics.

Copyright © 2021 the Author(s). Published by Wolters Kluwer Health, Inc.

Conflict of interest statement

The authors have no conflicts of interests to disclose.

- 38 references
- 5 figures

Supplementary info

Publication types, MeSH terms, Substances Expand

Publication types

- Observational Study

MeSH terms

- Adult
- Age Factors
- Aged

- COVID-19 / complications*
- COVID-19 / diagnostic imaging
- COVID-19 / physiopathology*
- China / epidemiology
- Comorbidity
- Female
- Hematologic Tests
- Humans
- Liver Diseases / diagnostic imaging
- Liver Diseases / etiology*
- Liver Diseases / physiopathology*
- Liver Function Tests
- Male
- Middle Aged
- Oxygen / blood
- Retrospective Studies
- SARS-CoV-2
- Severity of Illness Index
- Sex Factors
- Tomography, X-Ray Computed

Substances

- Oxygen

Full text links

Wolters Kluwer Wolters Kluwer Free PMC article
Proceed to details
Cite
Share
☐ 631
Observational Study
Medicine (Baltimore)
. 2021 Jul 9;100(27):e26526.
doi: 10.1097/MD.0000000000026526.

Smartphone based alerting of first responders during the corona virus disease-19 pandemic: An observational study

Julian Ganter [1], Domagoj Damjanovic [1] [2], Georg Trummer [1] [2] [3], Hans-Jörg Busch [3] [4], Klemens Baldas [5], Mike Hänsel [6], Michael Patrick Müller [2] [3] [5]
Affiliations Expand

Affiliations

- [1] Department of Cardiovascular Surgery, University Heart Center Freiburg, Faculty of Medicine.
- [2] ERC Research NET, European Resuscitation Council, Niel, Belgium.
- [3] German Resuscitation Council (GRC), Ulm, Germany.
- [4] Department of Emergency Medicine, Faculty of Medicine, University Hospital of Freiburg, University of Freiburg.
- [5] Department of Anaesthesiology, Intensive Care, and Emergency Medicine, St. Josef's Hospital, Freiburg.
- [6] Carl Gustav Carus Faculty of Medicine, Carus Teaching Center, Technische Universität Dresden, Dresden, Germany.

- PMID: **34232186**
- PMCID: PMC8270573
- DOI: 10.1097/MD.0000000000026526

Free PMC article
Observational Study

Smartphone based alerting of first responders during the corona virus disease-19 pandemic: An observational study

Julian Ganter et al. Medicine (Baltimore). 2021.
Free PMC article
Show details
Medicine (Baltimore)
. 2021 Jul 9;100(27):e26526.
doi: 10.1097/MD.0000000000026526.

Authors

Julian Ganter [1], Domagoj Damjanovic [1] [2], Georg Trummer [1] [2] [3], Hans-Jörg Busch [3] [4], Klemens Baldas [5], Mike Hänsel [6], Michael Patrick Müller [2] [3] [5]

Affiliations

- [1] Department of Cardiovascular Surgery, University Heart Center Freiburg, Faculty of Medicine.
- [2] ERC Research NET, European Resuscitation Council, Niel, Belgium.
- [3] German Resuscitation Council (GRC), Ulm, Germany.

- [4] Department of Emergency Medicine, Faculty of Medicine, University Hospital of Freiburg, University of Freiburg.
- [5] Department of Anaesthesiology, Intensive Care, and Emergency Medicine, St. Josef's Hospital, Freiburg.
- [6] Carl Gustav Carus Faculty of Medicine, Carus Teaching Center, Technische Universität Dresden, Dresden, Germany.

- PMID: **34232186**
- PMCID: PMC8270573
- DOI: 10.1097/MD.0000000000026526

Abstract

Smartphone alerting systems (SAS) for first responders potentially shorten the resuscitation-free interval of patients with acute cardiac arrest. During the corona virus disease-19 (COVID-19) pandemic, many systems are suspended due to potential risks for the responders.Objective of the study was to establish a concept for SAS during the COVID-19 pandemic and to evaluate whether a SAS can safely be operated in pandemic conditions.A SAS had been implemented in Freiburg (Germany) in 2018 alerting nearby registered first responders in case of emergencies with suspected cardiac arrest. Due to the pandemic, SAS was stopped in March 2020. A concept for a safe restart was elaborated with provision of a set with ventilation bag/mask, airway filter, and personal protective equipment (PPE) for every volunteer. A standard operating procedure was elaborated following the COVID-19 guidelines of the European Resuscitation Council.Willingness of the participants to respond alarms during the pandemic was investigated using an online survey. The response rates of first responders were monitored before and after deactivation, and during the second wave of the pandemic.The system was restarted in May 2020. The willingness to respond to alarms was lower during the pandemic without PPE. It remained lower than before the pandemic when the volunteers had been equipped with PPE, but the alarm response rate remained at approximately 50% during the second wave of the pandemic.When volunteers are equipped with PPE, the operation of a SAS does not need to be paused, and the willingness to respond remains high among first responders.

Copyright © 2021 the Author(s). Published by Wolters Kluwer Health, Inc.

Conflict of interest statement

The authors have no conflicts of interest to disclose.

- 14 references
- 1 figure

Supplementary info

Publication types, MeSH terms Expand

Publication types

- Observational Study

MeSH terms

- COVID-19 / epidemiology*
- Emergency Responders
- Germany / epidemiology
- Humans
- Infectious Disease Transmission, Patient-to-Professional / prevention & control*
- Pandemics*
- Personal Protective Equipment*
- Retrospective Studies
- SARS-CoV-2
- Smartphone*

Full text links

Wolters Kluwer Free PMC article
Proceed to details
Cite
Share
☐ 632
Observational Study
J Hum Hypertens
. 2021 Jul;35(7):588-597.
doi: 10.1038/s41371-020-00405-3. Epub 2020 Aug 24.

Association between renin-angiotensin-aldosterone system inhibitor treatment, neutrophil-lymphocyte ratio, D-Dimer and clinical severity of COVID-19 in hospitalized patients: a multicenter, observational study

Selcuk Gormez [1], Erkan Ekicibasi [2], Aleks Degirmencioglu [3], Ashok Paudel [4], Refik Erdim [5], Hilal Kurtoglu Gumusel [6], Elif Eroglu [7], Ibrahim Halil Tanboga [8] [9] [10], Sinan Dagdelen [7], Nevin Sariguzel [11], Ceyda Erel Kirisoglu [12], Burak Pamukcu [13]
Affiliations Expand

Affiliations

- [1] Department of Cardiology, Faculty of Medicine, Acibadem Kadikoy Hospital, Acibadem Mehmet Ali Aydinlar University, Istanbul, Turkey.

- [2] Department of Cardiology, Vocational School of Health Services, Acibadem Altunizade Hospital, Acibadem Mehmet Ali Aydinlar University, Istanbul, Turkey.
- [3] Department of Cardiology, Faculty of Medicine, Acibadem Maslak Hospital, Acibadem Mehmet Ali Aydinlar University, Istanbul, Turkey.
- [4] Department of Cardiology, Acibadem Altunizade Hospital, Istanbul, Turkey.
- [5] Department of Cardiology, Vocational School of Health Services, Acibadem Kadikoy Hospital, Acibadem Mehmet Ali Aydinlar University, Istanbul, Turkey.
- [6] Department of Cardiology, Acibadem Kadikoy Hospital, Istanbul, Turkey.
- [7] Department of Cardiology, Faculty of Medicine, Acibadem Altunizade Hospital, Acibadem Mehmet Ali Aydinlar University, Istanbul, Turkey.
- [8] Department of Cardiology, Nisantasi University, Istanbul, Turkey.
- [9] Department of Biostatistics, Medical School, Ataturk University, Erzurum, Turkey.
- [10] Department of Cardiology, Hisar Intercontinental Hospital, Istanbul, Turkey.
- [11] Department of Infectious Diseases, Acibadem Kadikoy & Kozyatagi Hospitals, Istanbul, Turkey.
- [12] Department of Chest Diseases and Tuberculosis, Faculty of Medicine, Acibadem Kozyatagi Hospital, Acibadem Mehmet Ali Aydinlar University, Istanbul, Turkey.
- [13] Department of First and Emergency Aid, Department of Cardiology, Acibadem Kozyatagi Hospital, Vocational School of Health Services, Acibadem Mehmet Ali Aydinlar University, Istanbul, Turkey. burak.pamukcu@acibadem.edu.tr.

- PMID: **32839534**
- PMCID: PMC7444679
- DOI: 10.1038/s41371-020-00405-3

Free PMC article
Observational Study

Association between renin-angiotensin-aldosterone system inhibitor treatment, neutrophil-lymphocyte ratio, D-Dimer and clinical severity of COVID-19 in hospitalized patients: a multicenter, observational study

Selcuk Gormez et al. J Hum Hypertens. 2021 Jul.
Free PMC article
Show details
J Hum Hypertens

. 2021 Jul;35(7):588-597.
doi: 10.1038/s41371-020-00405-3. Epub 2020 Aug 24.

Authors

Selcuk Gormez [1], Erkan Ekicibasi [2], Aleks Degirmencioglu [3], Ashok Paudel [4], Refik Erdim [5], Hilal Kurtoglu Gumusel [6], Elif Eroglu [7], Ibrahim Halil Tanboga [8] [9] [10], Sinan Dagdelen [7], Nevin Sariguzel [11], Ceyda Erel Kirisoglu [12], Burak Pamukcu [13]

Affiliations

- [1] Department of Cardiology, Faculty of Medicine, Acibadem Kadikoy Hospital, Acibadem Mehmet Ali Aydinlar University, Istanbul, Turkey.
- [2] Department of Cardiology, Vocational School of Health Services, Acibadem Altunizade Hospital, Acibadem Mehmet Ali Aydinlar University, Istanbul, Turkey.
- [3] Department of Cardiology, Faculty of Medicine, Acibadem Maslak Hospital, Acibadem Mehmet Ali Aydinlar University, Istanbul, Turkey.
- [4] Department of Cardiology, Acibadem Altunizade Hospital, Istanbul, Turkey.
- [5] Department of Cardiology, Vocational School of Health Services, Acibadem Kadikoy Hospital, Acibadem Mehmet Ali Aydinlar University, Istanbul, Turkey.
- [6] Department of Cardiology, Acibadem Kadikoy Hospital, Istanbul, Turkey.
- [7] Department of Cardiology, Faculty of Medicine, Acibadem Altunizade Hospital, Acibadem Mehmet Ali Aydinlar University, Istanbul, Turkey.
- [8] Department of Cardiology, Nisantasi University, Istanbul, Turkey.
- [9] Department of Biostatistics, Medical School, Ataturk University, Erzurum, Turkey.
- [10] Department of Cardiology, Hisar Intercontinental Hospital, Istanbul, Turkey.
- [11] Department of Infectious Diseases, Acibadem Kadikoy & Kozyatagi Hospitals, Istanbul, Turkey.
- [12] Department of Chest Diseases and Tuberculosis, Faculty of Medicine, Acibadem Kozyatagi Hospital, Acibadem Mehmet Ali Aydinlar University, Istanbul, Turkey.
- [13] Department of First and Emergency Aid, Department of Cardiology, Acibadem Kozyatagi Hospital, Vocational School of Health Services, Acibadem Mehmet Ali Aydinlar University, Istanbul, Turkey. burak.pamukcu@acibadem.edu.tr.

- PMID: **32839534**
- PMCID: PMC7444679
- DOI: 10.1038/s41371-020-00405-3

Abstract

The aim of this study was to investigate the possible relationship between worse clinical outcomes and the use of angiotensin-converting enzyme inhibitors (ACEIs) or angiotensin receptor blockers (ARBs) in hospitalized COVID-19 patients. A total of 247 adult patients (154 males, 93 females; mean age: 51.3 ± 14.2 years) hospitalized for COVID-19 as confirmed by polymerase chain reaction (PCR) were retrospectively reviewed. Demographic and clinical characteristics and laboratory parameters were analyzed using various statistical modeling. Primary outcomes were defined as the need for intensive care unit (ICU), mechanical ventilation, or occurrence of death. Of the patients, 48 were treated in the ICU with a high flow oxygen/noninvasive mechanical ventilation (NIMV, n = 12) or mechanical ventilation (n = 36). Median length of ICU stay was 13 (range, 7-18) days. Mortality was seen in four of the ICU patients. Other patients were followed in the COVID-19 services for a median of 7 days. There was no significant correlation between the primary outcomes and use of ACEIs/ARBs (frequentist OR = 0.82, 95% confidence interval (CI) 0.29-2.34, p = 0.715 and Bayesian posterior median OR = 0.80, 95% CI 0.31-2.02) and presence of hypertension (frequentist OR = 1.23, 95% CI 0.52-2.92, p = 0.631 and Bayesian posterior median OR = 1.25, 95% CI 0.58-2.60). Neutrophil-to-lymphocyte ratio (NLR) and D-dimer levels

were strongly associated with primary outcomes. In conclusion, the presence of hypertension and use of ACEIs/ARBs were not significantly associated with poor primary clinical outcomes; however, NLR and D-dimer levels were strong predictors of clinical worsening.

© 2020. The Author(s), under exclusive licence to Springer Nature Limited.

Conflict of interest statement

The authors declare that they have no conflict of interest.

- 41 references
- 5 figures

Supplementary info

Publication types, MeSH terms, Substances Expand

Publication types

- Multicenter Study
- Observational Study

MeSH terms

- Adult
- Aged
- Aldosterone / adverse effects
- Aldosterone / therapeutic use
- Angiotensin Receptor Antagonists / adverse effects
- Angiotensin Receptor Antagonists / therapeutic use*
- Angiotensin-Converting Enzyme Inhibitors / adverse effects
- Angiotensin-Converting Enzyme Inhibitors / therapeutic use*
- COVID-19 / diagnosis*
- COVID-19 Nucleic Acid Testing
- Female
- Fibrin Fibrinogen Degradation Products / analysis
- Humans
- Hypertension / diagnosis
- Hypertension / drug therapy*
- Lymphocytes
- Male
- Middle Aged
- Neutrophils
- Polymerase Chain Reaction
- Renin-Angiotensin System

- Retrospective Studies
- SARS-CoV-2 / genetics
- SARS-CoV-2 / isolation & purification*

Substances

- Angiotensin Receptor Antagonists
- Angiotensin-Converting Enzyme Inhibitors
- Fibrin Fibrinogen Degradation Products
- fibrin fragment D
- Aldosterone

Full text links

Nature Publishing Group Free PMC article
Proceed to details
Cite
Share
633
Observational Study
PLoS One
. 2022 Jan 14;17(1):e0261958.
doi: 10.1371/journal.pone.0261958. eCollection 2022.

COVID-19-associated acute kidney injury patients treated with renal replacement therapy in the intensive care unit: A multicenter study in São Paulo, Brazil

Farid Samaan [1 2 3 4], Elisa Carneiro de Paula [5], Fabrizzio Batista Guimarães de Lima Souza [6], Luiz Fernando Cardoso Mendes [6], Paula Regina Gan Rossi [6], Rafaela Andrade Penalva Freitas [7], Fernando Takahashi Nakagawa [7], Alexandre Toledo Maciel [8], Sylvia Aranha [8], Eduardo Osawa [8], Henrique Pinheiro Konigsfeld [9 10], Riberto Garcia da Silva [9 10], Ricardo Barbosa Cintra de Souza [9 10], Saurus Mayer Coutinho [9 10], Tales Dantas Vieira [1 11], Karina De Bonis Thomaz [1], Elias Marcos Silva Flato [12], Renata Cristina da Silva [12], Lucas Vicente Andrade [13], Muna Badaoui [2 14], Eduardo Pogetti Badaoui [2 14], Miguel Ângelo Goes [1 3], Sergio Henrique do Amaral [1 15], Karlla Cunha [1 15], Inês Marin Muniz [1], Jacqueline Siqueira Sampaio [1 16], Marcelino de Souza Durão Junior [3 17], Dirce M Trevisan Zanetta [18], Emmanuel A Burdmann [2]
Affiliations Expand

Affiliations

- [1] Department of High Complexity Patients, Grupo NotreDame Intermédica, São Paulo, Brazil.
- [2] Nephrology Division, University of São Paulo Medical School, São Paulo, Brazil.
- [3] Nephrology Division, Federal University of São Paulo, São Paulo, Brazil.
- [4] Planning and Evaluation Group, São Paulo State Health Department, São Paulo, Brazil.
- [5] Nephrology Division, Leforte Liberdade Hospital, São Paulo, Brazil.
- [6] Medical Board, Municipal Hospital Vereador José Storopolli, São Paulo, Brazil.
- [7] Internal medicine, Dante Pazzanese Institute of Cardiology, São Paulo, Brazil.
- [8] Imed Research Group, São Camilo Pompeia Hospital, São Paulo, Brazil.
- [9] Nephrology Division, Santa Cruz Hospital, São Paulo, Brazil.
- [10] Nephrology Division, Sepaco Hospital, São Paulo, Brazil.
- [11] Nephrology Division, Cruzeiro do Sul Hospital, Osasco, São Paulo, Brazil.
- [12] Nephrology Division, São Francisco Hospital, Cotia, São Paulo, Brazil.
- [13] Department of General Surgery, Ipiranga Hospital Care Management Unit, São Paulo, Brazil.
- [14] Nephrology Division, Vila Nova Brasilândia Hospital, São Paulo, Brazil.
- [15] Medical Board, Bosque da Saúde Hospital, São Paulo, Brazil.
- [16] Medical Board, Intermédica Guarulhos Hospital, Guarulhos, São Paulo, Brazil.
- [17] Nephrology Division, São Paulo Hospital, São Paulo, Brazil.
- [18] School of Public Health, University of São Paulo, São Paulo, Brazil.

- PMID: **35030179**
- PMCID: PMC8759670
- DOI: 10.1371/journal.pone.0261958

Free PMC article
Observational Study

COVID-19-associated acute kidney injury patients treated with renal replacement therapy in the intensive care unit: A multicenter study in São Paulo, Brazil

Farid Samaan et al. PLoS One. 2022.
Free PMC article
Show details
PLoS One
. 2022 Jan 14;17(1):e0261958.
doi: 10.1371/journal.pone.0261958. eCollection 2022.

Authors

Farid Samaan[1 2 3 4], Elisa Carneiro de Paula[5], Fabrizzio Batista Guimarães de Lima Souza[6], Luiz Fernando Cardoso Mendes[6], Paula Regina Gan Rossi[6], Rafaela Andrade Penalva Freitas[7], Fernando Takahashi Nakagawa[7], Alexandre Toledo Maciel[8], Sylvia Aranha[8], Eduardo Osawa[8], Henrique Pinheiro Konigsfeld[9 10], Riberto Garcia da Silva[9 10], Ricardo Barbosa Cintra de Souza[9 10], Saurus Mayer Coutinho[9 10], Tales Dantas Vieira[1 11], Karina De Bonis Thomaz[1], Elias Marcos Silva Flato[12], Renata Cristina da Silva[12], Lucas Vicente Andrade[13], Muna Badaoui[2 14], Eduardo Pogetti Badaoui[2 14], Miguel Ângelo Goes[1 3], Sergio Henrique do Amaral[1 15], Karlla Cunha[1 15], Inês Marin Muniz[1], Jacqueline Siqueira Sampaio[1 16], Marcelino de Souza Durão Junior[3 17], Dirce M Trevisan Zanetta[18], Emmanuel A Burdmann[2]

Affiliations

- [1] Department of High Complexity Patients, Grupo NotreDame Intermédica, São Paulo, Brazil.
- [2] Nephrology Division, University of São Paulo Medical School, São Paulo, Brazil.
- [3] Nephrology Division, Federal University of São Paulo, São Paulo, Brazil.
- [4] Planning and Evaluation Group, São Paulo State Health Department, São Paulo, Brazil.
- [5] Nephrology Division, Leforte Liberdade Hospital, São Paulo, Brazil.
- [6] Medical Board, Municipal Hospital Vereador José Storopolli, São Paulo, Brazil.
- [7] Internal medicine, Dante Pazzanese Institute of Cardiology, São Paulo, Brazil.
- [8] Imed Research Group, São Camilo Pompeia Hospital, São Paulo, Brazil.
- [9] Nephrology Division, Santa Cruz Hospital, São Paulo, Brazil.
- [10] Nephrology Division, Sepaco Hospital, São Paulo, Brazil.
- [11] Nephrology Division, Cruzeiro do Sul Hospital, Osasco, São Paulo, Brazil.
- [12] Nephrology Division, São Francisco Hospital, Cotia, São Paulo, Brazil.
- [13] Department of General Surgery, Ipiranga Hospital Care Management Unit, São Paulo, Brazil.
- [14] Nephrology Division, Vila Nova Brasilândia Hospital, São Paulo, Brazil.
- [15] Medical Board, Bosque da Saúde Hospital, São Paulo, Brazil.
- [16] Medical Board, Intermédica Guarulhos Hospital, Guarulhos, São Paulo, Brazil.
- [17] Nephrology Division, São Paulo Hospital, São Paulo, Brazil.
- [18] School of Public Health, University of São Paulo, São Paulo, Brazil.

- PMID: **35030179**
- PMCID: PMC8759670
- DOI: 10.1371/journal.pone.0261958

Abstract

Introduction: Multicenter studies involving patients with acute kidney injury (AKI) associated with the disease caused by the new coronavirus (COVID-19) and treated with renal replacement therapy (RRT) in developing countries are scarce. The objectives of this study were to evaluate the demographic profile, clinical picture, risk factors for mortality, and outcomes of critically ill patients with AKI requiring dialysis (AKI-RRT) and with COVID-19 in the megalopolis of São Paulo, Brazil.

Methods: This multicenter, retrospective, observational study was conducted in the intensive care units of 13 public and private hospitals in the metropolitan region of the municipality of São Paulo. Patients hospitalized in an intensive care unit, aged ≥ 18 years, and treated with RRT due to COVID-19-associated AKI were included.

Results: The study group consisted of 375 patients (age 64.1 years, 68.8% male). Most (62.1%) had two or more comorbidities: 68.8%, arterial hypertension; 45.3%, diabetes; 36.3%, anemia; 30.9%, obesity; 18.7%, chronic kidney disease; 15.7%, coronary artery disease; 10.4%, heart failure; and 8.5%, chronic obstructive pulmonary disease. Death occurred in 72.5% of the study population (272 patients). Among the 103 survivors, 22.3% (23 patients) were discharged on RRT. In a multiple regression analysis, the independent factors associated with death were the number of organ dysfunctions at admission and RRT efficiency.

Conclusion: AKI-RRT associated with COVID-19 occurred in patients with an elevated burden of comorbidities and was associated with high mortality (72.5%). The number of organ dysfunctions during hospitalization and RRT efficiency were independent factors associated with mortality. A meaningful portion of survivors was discharged while dependent on RRT (22.3%).

Conflict of interest statement

The authors have declared that no competing interests exist.

- 35 references
- 1 figure

Supplementary info

Publication types, MeSH terms, Grant support Expand

Publication types

- Multicenter Study
- Observational Study
- Research Support, Non-U.S. Gov't

MeSH terms

- Acute Kidney Injury / complications*
- Acute Kidney Injury / epidemiology
- Acute Kidney Injury / mortality
- Acute Kidney Injury / therapy
- Aged
- Brazil / epidemiology
- COVID-19 / complications*
- COVID-19 / epidemiology
- COVID-19 / mortality
- COVID-19 / therapy

- Critical Illness / epidemiology
- Critical Illness / mortality
- Critical Illness / therapy
- Female
- Hospital Mortality
- Humans
- Intensive Care Units
- Male
- Middle Aged
- Renal Replacement Therapy
- Retrospective Studies
- Risk Factors
- SARS-CoV-2 / isolation & purification

Grant support

Grupo NotreDame Intermédica, Brazil, has provided the financial support for the English review of the manuscript. The funders had no role in study design, data collection and analysis, decision to publish, or preparation of the manuscript.

Full text links

OPEN ACCESS TO FULL TEXT PLOS ONE Public Library of Science Free PMC article

Proceed to details

Cite

Share

634

Observational Study

J Assoc Physicians India

. 2021 Aug;69(8):11-12.

The Pattern of Post-viral Arthritis in COVID Pandemic State: An Experience of Tertiary Care Centre

Prakruthi Jaladhar [1], Chandrashekara S [2], Manasa Salanke [3], Devaraj Kori [4]

Affiliations Expand

Affiliations

- [1] Fellow in Rheumatology and Immunology, Bengaluru, Karnataka.
- [2] Managing Director, Chan Re Rheumatology and Immunology Center and Research, Bengaluru, Karnataka.

- [3] Data Scientist,Chan Re Rheumatology and Immunology Center and Research, Bengaluru, Karnataka.
- [4] Resident, Chan Re Rheumatology and Immunology Center and Research, Bengaluru, Karnataka.

- PMID: **34472811**

Observational Study

The Pattern of Post-viral Arthritis in COVID Pandemic State: An Experience of Tertiary Care Centre

Prakruthi Jaladhar et al. J Assoc Physicians India. 2021 Aug.

Show details

J Assoc Physicians India

. 2021 Aug;69(8):11-12.

Authors

Prakruthi Jaladhar [1], Chandrashekara S [2], Manasa Salanke [3], Devaraj Kori [4]

Affiliations

- [1] Fellow in Rheumatology and Immunology, Bengaluru, Karnataka.
- [2] Managing Director, Chan Re Rheumatology and Immunology Center and Research, Bengaluru, Karnataka.
- [3] Data Scientist,Chan Re Rheumatology and Immunology Center and Research, Bengaluru, Karnataka.
- [4] Resident, Chan Re Rheumatology and Immunology Center and Research, Bengaluru, Karnataka.

- PMID: **34472811**

Abstract

Background: Acute onset polyarthritis is a common presentation in rheumatology outpatient consultations, which include both post-infectious arthritis and autoimmune rheumatic diseases (AIRDs). COVID pandemic has added to the list of infectious agents that could result in arthritis.

Materials and methods: The retrospective observational study was conducted at a tertiary care centre. The study included patients who presented with clinical suspicion of post-infectious arthritis between July-September 2019 and 2020. The study was extended for another 2 months to include patients who presented between October-November 2020. The patients were categorized into post-viral arthritis, post-COVID arthritis, chikungunya arthritis and AIRDs. The demographics, comorbidities, clinical presentation, examination findings and laboratory parameters and the response to treatment for each participant were collected and assessed.

Results: In the year 2019 and 2020 (July-Sep), the corresponding number of patients analyzed were 20 and 33. The mean duration of presentation was 1.53 (±3.10) weeks. Chikungunya arthritis was noted in 10% of patients in 2019, while it was 15.15% in 2020. Other post-viral arthritis was identified in 65% and 66.67% of patients in 2019 and 2020 respectively. In the second part of the study, 65.68% of patients were classified as post-viral arthritis, including chikungunya arthritis, post-COVID arthritis and other post-viral arthritis. Around 27% were categorized as AIRDs. Rheumatoid factor negativity and anti-nuclear antibody negativity were found to be significant (P 0.0) in categorizing the patients into post-viral arthritis group, while presence of urinary symptoms (P 0.0) classified the patients into reactive arthritis.

Conclusion: The study revealed that the presence of chikungunya arthritis across the two years was comparable. Post-COVID arthritis needs to be considered as a potential differential in post-infectious arthritis. There are no identifiable characteristics (clinical or a simple routine laboratory parameter) that could differentiate the causes of post-infectious arthritis from AIRDs.

© Journal of the Association of Physicians of India 2011.

Supplementary info

Publication types, MeSH terms Expand

Publication types

- Observational Study

MeSH terms

- Arthritis, Infectious* / diagnosis
- Arthritis, Infectious* / epidemiology
- COVID-19*
- Humans
- Pandemics
- SARS-CoV-2
- Tertiary Care Centers

Proceed to details
Cite
Share
☐ 635
Observational Study
J Neurol Neurosurg Psychiatry
. 2021 Jul;92(7):751-756.
doi: 10.1136/jnnp-2020-324837. Epub 2020 Nov 6.

Guillain-Barré syndrome and COVID-19: an observational multicentre study from two Italian hotspot regions

Massimiliano Filosto [1], Stefano Cotti Piccinelli [2], Stefano Gazzina [3], Camillo Foresti [4], Barbara Frigeni [4], Maria Cristina Servalli [4], Maria Sessa [4], Giuseppe Cosentino [5], Enrico Marchioni [6], Sabrina Ravaglia [5], Chiara Briani [7], Francesca Castellani [7], Gabriella Zara [7], Francesca Bianchi [8], Ubaldo Del Carro [8], Raffaella Fazio [8], Massimo Filippi [8], Eugenio Magni [9], Giuseppe Natalini [10], Francesco Palmerini [9], Anna Maria Perotti [9], Andrea Bellomo [11], Maurizio Osio [12], Giuseppe Scopelliti [11], Marinella Carpo [13], Andrea Rasera [14], Giovanna Squintani [14], Pietro Emiliano Doneddu [15], Valeria Bertasi [16], Maria Sofia Cotelli [16], Laura Bertolasi [17], Gian Maria Fabrizi [17], Sergio Ferrari [17], Federico Ranieri [17], Francesca Caprioli [18], Elena Grappa [19], Laura Broglio [3], Giovanni De Maria [3], Ugo Leggio [3], Loris Poli [20], Frank Rasulo [21], Nicola Latronico [21], Eduardo Nobile-Orazio [15], Alessandro Padovani [# 2], Antonino Uncini [# 22]

Affiliations Expand

Affiliations

- [1] Department of Clinical and Experimental Sciences, University of Brescia; Unit of Neurology, ASST Spedali Civili; NeMO-Brescia Clinical Center for Neuromuscular Diseases, Brescia, Italy massimiliano.filosto@unibs.it.
- [2] Department of Clinical and Experimental Sciences, University of Brescia; Unit of Neurology, ASST Spedali Civili, Brescia, Italy.
- [3] Unit of Neurophysiopathology, ASST Spedali Civili, Brescia, Italy.
- [4] Unit of Neurology and Neurophysiology, ASST PG23, Bergamo, Italy.
- [5] IRCCS Mondino Foundation, Department of Brain and Behavioral Sciences, University of Pavia, Pavia, Italy.
- [6] IRCCS Mondino Foundation, Neurooncology and Neuroinflammation Unit, Pavia, Italy.
- [7] Neurology Unit, Azienda Ospedale-Università di Padova, Padova, Italy.
- [8] Neurology and Neurophysiology Unit, IRCCS San Raffaele Scientific Institute, Vita Salute SanRaffaele University, Milano, Italy.
- [9] Unit of Neurology, Fondazione Poliambulanza, Brescia, Italy.
- [10] Unit of Intensive Care and Anesthesiology, Fondazione Poliambulanza, Brescia, Italy.
- [11] "Luigi Sacco" Department of Biomedical and Clinical Sciences, University of Milano, Milano, Italy.
- [12] Unit of Neurology, ASST Fatebenefratelli Sacco, Milano, Italy.
- [13] Unit of Neurology, ASST Bergamo Ovest, Treviglio, Italy.
- [14] Unit of Neurology, AOUI Verona, Verona, Italy.
- [15] Department of Neurology, Neuromuscular and Neuroimmunology Service, IRCCS Humanitas Clinical and Research Institute; Department of Medical Biotechnology and Translational Medicine,Milan University, Milano, Italy.
- [16] Unit of Neurology, ASST Valcamonica, Esine (Bs), Italy.
- [17] Neurology Unit, Department of Neuroscience, Biomedicine and Movement Sciences, University of Verona, Verona, Italy.

- [18] Unit of Neurology, ASST Cremona, Cremona, Italy.
- [19] Intensive Care Unit, ASST Cremona, Cremona, Italy.
- [20] Unit of Neurology, ASST Spedali Civili, Brescia, Italy.
- [21] Department of Anesthesia, Critical Care and Emergency, ASST Spedali Civili;Department of Medical and Surgical Specialties, Radiological Sciences and Public Health,University of Brescia, Brescia, Italy.
- [22] Department of Neuroscience, Imaging and Clinical Sciences, University "G. d'Annunzio", Chieti-Pescara, Italy.

[#] Contributed equally.

- PMID: **33158914**
- PMCID: PMC7650204
- DOI: 10.1136/jnnp-2020-324837

Free PMC article
Observational Study

Guillain-Barré syndrome and COVID-19: an observational multicentre study from two Italian hotspot regions

Massimiliano Filosto et al. J Neurol Neurosurg Psychiatry. 2021 Jul.
Free PMC article
Show details
J Neurol Neurosurg Psychiatry
. 2021 Jul;92(7):751-756.
doi: 10.1136/jnnp-2020-324837. Epub 2020 Nov 6.

Authors

Massimiliano Filosto [1], Stefano Cotti Piccinelli [2], Stefano Gazzina [3], Camillo Foresti [4], Barbara Frigeni [4], Maria Cristina Servalli [4], Maria Sessa [4], Giuseppe Cosentino [5], Enrico Marchioni [6], Sabrina Ravaglia [5], Chiara Briani [7], Francesca Castellani [7], Gabriella Zara [7], Francesca Bianchi [8], Ubaldo Del Carro [8], Raffaella Fazio [8], Massimo Filippi [8], Eugenio Magni [9], Giuseppe Natalini [10], Francesco Palmerini [9], Anna Maria Perotti [9], Andrea Bellomo [11], Maurizio Osio [12], Giuseppe Scopelliti [11], Marinella Carpo [13], Andrea Rasera [14], Giovanna Squintani [14], Pietro Emiliano Doneddu [15], Valeria Bertasi [16], Maria Sofia Cotelli [16], Laura Bertolasi [17], Gian Maria Fabrizi [17], Sergio Ferrari [17], Federico Ranieri [17], Francesca Caprioli [18], Elena Grappa [19], Laura Broglio [3], Giovanni De Maria [3], Ugo Leggio [3], Loris Poli [20], Frank Rasulo [21], Nicola Latronico [21], Eduardo Nobile-Orazio [15], Alessandro Padovani [# 2], Antonino Uncini [# 22]

Affiliations

- [1] Department of Clinical and Experimental Sciences, University of Brescia; Unit of Neurology, ASST Spedali Civili; NeMO-Brescia Clinical Center for Neuromuscular Diseases, Brescia, Italy massimiliano.filosto@unibs.it.
- [2] Department of Clinical and Experimental Sciences, University of Brescia; Unit of Neurology, ASST Spedali Civili, Brescia, Italy.
- [3] Unit of Neurophysiopathology, ASST Spedali Civili, Brescia, Italy.
- [4] Unit of Neurology and Neurophysiology, ASST PG23, Bergamo, Italy.
- [5] IRCCS Mondino Foundation, Department of Brain and Behavioral Sciences, University of Pavia, Pavia, Italy.
- [6] IRCCS Mondino Foundation, Neurooncology and Neuroinflammation Unit, Pavia, Italy.
- [7] Neurology Unit, Azienda Ospedale-Università di Padova, Padova, Italy.
- [8] Neurology and Neurophysiology Unit, IRCCS San Raffaele Scientific Institute, Vita Salute SanRaffaele University, Milano, Italy.
- [9] Unit of Neurology, Fondazione Poliambulanza, Brescia, Italy.
- [10] Unit of Intensive Care and Anesthesiology, Fondazione Poliambulanza, Brescia, Italy.
- [11] "Luigi Sacco" Department of Biomedical and Clinical Sciences, University of Milano, Milano, Italy.
- [12] Unit of Neurology, ASST Fatebenefratelli Sacco, Milano, Italy.
- [13] Unit of Neurology, ASST Bergamo Ovest, Treviglio, Italy.
- [14] Unit of Neurology, AOUI Verona, Verona, Italy.
- [15] Department of Neurology, Neuromuscular and Neuroimmunology Service, IRCCS Humanitas Clinical and Research Institute; Department of Medical Biotechnology and Translational Medicine,Milan University, Milano, Italy.
- [16] Unit of Neurology, ASST Valcamonica, Esine (Bs), Italy.
- [17] Neurology Unit, Department of Neuroscience, Biomedicine and Movement Sciences, University of Verona, Verona, Italy.
- [18] Unit of Neurology, ASST Cremona, Cremona, Italy.
- [19] Intensive Care Unit, ASST Cremona, Cremona, Italy.
- [20] Unit of Neurology, ASST Spedali Civili, Brescia, Italy.
- [21] Department of Anesthesia, Critical Care and Emergency, ASST Spedali Civili;Department of Medical and Surgical Specialties, Radiological Sciences and Public Health,University of Brescia, Brescia, Italy.
- [22] Department of Neuroscience, Imaging and Clinical Sciences, University "G. d'Annunzio", Chieti-Pescara, Italy.

[#] Contributed equally.

- PMID: **33158914**
- PMCID: PMC7650204
- DOI: 10.1136/jnnp-2020-324837

Abstract

Objective: Single cases and small series of Guillain-Barré syndrome (GBS) have been reported during the SARS-CoV-2 outbreak worldwide. We evaluated incidence and clinical features of GBS in a cohort of patients from two regions of northern Italy with the highest number of patients with COVID-19.

Methods: GBS cases diagnosed in 12 referral hospitals from Lombardy and Veneto in March and April 2020 were retrospectively collected. As a control population, GBS diagnosed in March and April 2019 in the same hospitals were considered.

Results: Incidence of GBS in March and April 2020 was 0.202/100 000/month (estimated rate 2.43/100 000/year) vs 0.077/100 000/month (estimated rate 0.93/100 000/year) in the same months of 2019 with a 2.6-fold increase. Estimated incidence of GBS in COVID-19-positive patients was 47.9/100 000 and in the COVID-19-positive hospitalised patients was 236/100 000. COVID-19-positive patients with GBS, when compared with COVID-19-negative subjects, showed lower MRC sum score (26.3±18.3 vs 41.4±14.8, p=0.006), higher frequency of demyelinating subtype (76.6% vs 35.3%, p=0.011), more frequent low blood pressure (50% vs 11.8%, p=0.017) and higher rate of admission to intensive care unit (66.6% vs 17.6%, p=0.002).

Conclusions: This study shows an increased incidence of GBS during the COVID-19 outbreak in northern Italy, supporting a pathogenic link. COVID-19-associated GBS is predominantly demyelinating and seems to be more severe than non-COVID-19 GBS, although it is likely that in some patients the systemic impairment due to COVID-19 might have contributed to the severity of the whole clinical picture.

© Author(s) (or their employer(s)) 2021. No commercial re-use. See rights and permissions. Published by BMJ.

Conflict of interest statement

Competing interests: None declared.

- 35 references

Supplementary info

Publication types, MeSH terms Expand

Publication types

- Multicenter Study
- Observational Study
- Research Support, Non-U.S. Gov't

MeSH terms

- Adult
- Aged
- COVID-19 / complications*
- COVID-19 / diagnosis
- COVID-19 / therapy
- Female
- Guillain-Barre Syndrome / diagnosis
- Guillain-Barre Syndrome / epidemiology*

- Guillain-Barre Syndrome / therapy
- Hospitalization
- Humans
- Incidence
- Italy
- Male
- Middle Aged
- Referral and Consultation
- Retrospective Studies

Full text links

BMJ Full Text HighWire Free PMC article
Proceed to details
Cite
Share
636
Multicenter Study
Open Heart
. 2021 Nov;8(2):e001833.
doi: 10.1136/openhrt-2021-001833.

Prevalence of cardiac pathology and relation to mortality in a multiethnic population hospitalised with COVID-19

Gabriel Bioh[1], Christina Botrous[1], Emma Howard[1], Ashish Patel[1], Reinette Hampson[1], Roxy Senior[2 3 4]
Affiliations Expand

Affiliations

- [1] Department of Cardiology, Northwick Park Hospital, Harrow, London, UK.
- [2] Department of Cardiology, Northwick Park Hospital, Harrow, London, UK roxysenior@cardiac-research.org.
- [3] Royal Brompton Hospital, London, UK.
- [4] National Heart and Lung Institute, Imperial College London, London, UK.

- PMID: **34782369**
- PMCID: PMC8593271
- DOI: 10.1136/openhrt-2021-001833

Free PMC article
Multicenter Study

Prevalence of cardiac pathology and relation to mortality in a multiethnic population hospitalised with COVID-19

Gabriel Bioh et al. Open Heart. 2021 Nov.
Free PMC article
Show details
Open Heart
. 2021 Nov;8(2):e001833.
doi: 10.1136/openhrt-2021-001833.

Authors

Gabriel Bioh [1], Christina Botrous [1], Emma Howard [1], Ashish Patel [1], Reinette Hampson [1], Roxy Senior [2] [3] [4]

Affiliations

- [1] Department of Cardiology, Northwick Park Hospital, Harrow, London, UK.
- [2] Department of Cardiology, Northwick Park Hospital, Harrow, London, UK roxysenior@cardiac-research.org.
- [3] Royal Brompton Hospital, London, UK.
- [4] National Heart and Lung Institute, Imperial College London, London, UK.

- PMID: **34782369**
- PMCID: PMC8593271
- DOI: 10.1136/openhrt-2021-001833

Abstract

Objective: To determine the prevalence of cardiac abnormalities and their relationship to markers of myocardial injury and mortality in patients admitted to hospital with COVID-19.

Methods: A retrospective and prospective observational study of inpatients referred for transthoracic echocardiography for suspected cardiac pathology due to COVID-19 within a London NHS Trust. Echocardiograms were performed to assess left ventricular (LV), right ventricular (RV) and pulmonary variables along with collection of patient demographics, comorbid conditions, blood biomarkers and outcomes.

Result: In the predominant non-white (72%) population, RV dysfunction was the primary cardiac abnormality noted in 50% of patients, with RV fractional area change <35% being the most common marker of this RV dysfunction. By comparison, LV systolic dysfunction occurred in 18% of patients. RV dysfunction was associated with LV systolic dysfunction and the presence of a D-shaped LV throughout the cardiac cycle (marker of significant pulmonary artery hypertension). LV systolic dysfunction (p=0.002, HR 3.82, 95% CI 1.624 to 8.982), pulmonary valve acceleration time (p=0.024, HR 0.98, 95% CI 0.964 to 0.997)-marker of increased pulmonary vascular resistance, age (p=0.047, HR 1.027, 95% CI 1.000 to 1.055) and an episode of

tachycardia measured from admission to time of echo (p=0.004, HR 6.183, 95% CI 1.772 to 21.575) were independently associated with mortality.

Conclusions: In this predominantly non-white population hospitalised with COVID-19, the most common cardiac pathology was RV dysfunction which is associated with both LV systolic dysfunction and elevated pulmonary artery pressure. The latter two, not RV dysfunction, were associated with mortality.

Keywords: COVID-19; echocardiography; epidemiology.

© Author(s) (or their employer(s)) 2021. Re-use permitted under CC BY-NC. No commercial re-use. See rights and permissions. Published by BMJ.

Conflict of interest statement

Competing interests: Professor Senior was given speaker fees by Bracco, Milan, Italy, Lantheus Medical Imaging, Boston, Massachusetts and Philips Healthcare, Eindhoven, Holland.

- 16 references
- 3 figures

Supplementary info

Publication types, MeSH terms Expand

Publication types

- Multicenter Study
- Research Support, Non-U.S. Gov't

MeSH terms

- COVID-19 / ethnology*
- Comorbidity
- Cross-Sectional Studies
- Echocardiography, Doppler
- Ethnicity*
- Heart Diseases / diagnosis
- Heart Diseases / ethnology*
- Heart Ventricles / diagnostic imaging*
- Hospitalization / trends
- Humans
- Pandemics
- Population Surveillance*
- Prevalence
- Quebec / epidemiology
- Retrospective Studies

- Survival Rate / trends

Full text links

BMJ Open Access Full Text HighWire Free PMC article
Proceed to details
Cite
Share
637
Observational Study
Anaesth Crit Care Pain Med
. 2020 Oct;39(5):579-580.
doi: 10.1016/j.accpm.2020.07.012. Epub 2020 Jul 28.

Outcome of non-invasive ventilation in COVID-19 critically ill patients: A Retrospective observational Study

Ahmed Mukhtar [1], Ahmed Lotfy [2], Ahmed Hasanin [2], Islam El-Hefnawy [3], Akram El Adawy [2]
Affiliations Expand

Affiliations

- [1] Anaesthesia Department, Cairo University, Cairo, Egypt. Electronic address: Ahmed.Mukhtar@kasralainy.edu.eg.
- [2] Anaesthesia Department, Cairo University, Cairo, Egypt.
- [3] Radiology Department Cairo University, Cairo, Egypt.

- PMID: **32736030**
- PMCID: PMC7386290
- DOI: 10.1016/j.accpm.2020.07.012

Free PMC article
Observational Study

Outcome of non-invasive ventilation in COVID-19 critically ill patients: A Retrospective observational Study

Ahmed Mukhtar et al. Anaesth Crit Care Pain Med. 2020 Oct.
Free PMC article
Show details

Anaesth Crit Care Pain Med
. 2020 Oct;39(5):579-580.
doi: 10.1016/j.accpm.2020.07.012. Epub 2020 Jul 28.

Authors

Ahmed Mukhtar [1], Ahmed Lotfy [2], Ahmed Hasanin [2], Islam El-Hefnawy [3], Akram El Adawy [2]

Affiliations

- [1] Anaesthesia Department, Cairo University, Cairo, Egypt. Electronic address: Ahmed.Mukhtar@kasralainy.edu.eg.
- [2] Anaesthesia Department, Cairo University, Cairo, Egypt.
- [3] Radiology Department Cairo University, Cairo, Egypt.

- PMID: **32736030**
- PMCID: PMC7386290
- DOI: 10.1016/j.accpm.2020.07.012

No abstract available

Comment in

- Non-invasive ventilation for acute respiratory failure (in COVID-19 patients): the non-ending story?
  James A, Verdonk F, Bougle A, Constantin JM. James A, et al. Anaesth Crit Care Pain Med. 2020 Oct;39(5):549-550. doi: 10.1016/j.accpm.2020.08.004. Epub 2020 Aug 27. Anaesth Crit Care Pain Med. 2020. PMID: 32860987 Free PMC article. No abstract available.
- Our insight about Mukhtar et al.'s outcome of non-invasive ventilation in COVID-19 critically ill patients.
  Karim HMR, Esquinas AM. Karim HMR, et al. Anaesth Crit Care Pain Med. 2021 Feb;40(1):100781. doi: 10.1016/j.accpm.2020.09.012. Epub 2020 Nov 13. Anaesth Crit Care Pain Med. 2021. PMID: 33197639 Free PMC article. No abstract available.

- 5 references

Supplementary info

Publication types, MeSH terms, Supplementary concepts Expand

Publication types

- Letter
- Observational Study
- Research Support, Non-U.S. Gov't

MeSH terms

- Adult
- Aged
- Algorithms
- Betacoronavirus*
- COVID-19
- Comorbidity
- Coronavirus Infections / drug therapy
- Coronavirus Infections / mortality
- Coronavirus Infections / therapy*
- Critical Illness / therapy
- Female
- Hospital Mortality
- Humans
- Male
- Middle Aged
- Noninvasive Ventilation*
- Oxygen Inhalation Therapy
- Pandemics*
- Pneumonia, Viral / mortality
- Pneumonia, Viral / therapy*
- Retrospective Studies
- SARS-CoV-2
- Treatment Outcome

Supplementary concepts

- COVID-19 drug treatment

Full text links

ELSEVIER FULL-TEXT ARTICLE Elsevier Science Free PMC article

Proceed to details

Cite

Share

☐ 638

Observational Study

Anesth Analg

. 2021 Aug 1;133(2):483-490.

doi: 10.1213/ANE.0000000000005606.

Anesthetic Complications Associated With Severe Acute Respiratory Syndrome Coronavirus 2 in Pediatric Patients

Rita Saynhalath [1, 2], Gijo Alex [1], Proshad N Efune [1, 2], Peter Szmuk [1, 2], Hong Zhu [2, 3], Ethan L Sanford [1, 2]
Affiliations Expand

Affiliations

- [1] From the Division of Pediatric Anesthesia, Department of Anesthesiology and Pain Management, University of Texas Southwestern, Dallas, Texas and Children's Health, Dallas, Texas.
- [2] Outcomes Research Consortium, Cleveland, Ohio.
- [3] Department of Populations and Data Sciences & Harold C. Simmons Comprehensive Cancer Center, University of Texas Southwestern, Dallas, Texas.

- PMID: **33886516**
- DOI: 10.1213/ANE.0000000000005606

Observational Study

Anesthetic Complications Associated With Severe Acute Respiratory Syndrome Coronavirus 2 in Pediatric Patients

Rita Saynhalath et al. Anesth Analg. 2021.
Show details
Anesth Analg
. 2021 Aug 1;133(2):483-490.
doi: 10.1213/ANE.0000000000005606.

Authors

Rita Saynhalath [1, 2], Gijo Alex [1], Proshad N Efune [1, 2], Peter Szmuk [1, 2], Hong Zhu [2, 3], Ethan L Sanford [1, 2]

Affiliations

- [1] From the Division of Pediatric Anesthesia, Department of Anesthesiology and Pain Management, University of Texas Southwestern, Dallas, Texas and Children's Health, Dallas, Texas.
- [2] Outcomes Research Consortium, Cleveland, Ohio.

- [3] Department of Populations and Data Sciences & Harold C. Simmons Comprehensive Cancer Center, University of Texas Southwestern, Dallas, Texas.

- PMID: **33886516**
- DOI: 10.1213/ANE.0000000000005606

Abstract

Background: Coronavirus disease 2019 (COVID-19) is associated with high perioperative morbidity and mortality among adults. The incidence and severity of anesthetic complications in children with severe acute respiratory syndrome coronavirus 2 (SARS-CoV-2) is unknown. We hypothesized that there would be an increased incidence of intra- and postoperative complications in children with SARS-CoV-2 infection as compared to those with negative testing.

Methods: We conducted a retrospective cohort study analyzing complications for children <18 years of age who underwent anesthesia between April 28 and September 30, 2020 at a large, academic pediatric hospital. Each child with a positive SARS-CoV-2 test within the prior 10 days was matched to a patient with a negative SARS-CoV-2 test based on American Society of Anesthesiologists (ASA) physical status, age, gender, and procedure. Children who were intubated before the procedure, underwent organ transplant surgery, or had severe COVID-19 were excluded. The primary outcome was the risk difference of a composite of intra- or postoperative respiratory complications in children positive for SARS-CoV-2 compared to those with negative testing. Secondarily, we used logistic regression to determine the odds ratio for respiratory complications before and after adjustment using propensity scores weighting to adjust for possible confounders. Other secondary outcomes included neurologic, cardiovascular, hematologic, and renal complications, unanticipated postoperative admission to the intensive care unit, length of hospital stay, and mortality.

Results: During the study period, 9812 general anesthetics that had a preoperative SARS-CoV-2 test were identified. Sixty encounters occurred in patients who had positive SARS-CoV-2 testing preoperatively and 51 were included for analysis. The matched controls cohort included 99 encounters. A positive SARS-CoV-2 test was associated with a higher incidence of respiratory complications (11.8% vs 1.0%; risk difference 10.8%, 95% confidence interval [CI], 1.6-19.8; P = .003). After adjustment, the odds ratio for respiratory complications was 14.37 (95% CI, 1.59-130.39; P = .02) for SARS-CoV-2-positive children as compared to controls. There was no occurrence of acute respiratory distress syndrome, postoperative pneumonia, or perioperative mortality in either group.

Conclusions: Pediatric patients with nonsevere SARS-CoV-2 infection had higher rates of perianesthetic respiratory complications than matched controls with negative testing. However, severe morbidity was rare and there were no mortalities. The incidence of complications was similar to previously published rates of perianesthetic complications in the setting of an upper respiratory tract infection. This risk persisted after adjustment for preoperative upper respiratory symptoms, suggesting an increased risk in symptomatic or asymptomatic SARS-CoV-2 infection.

Copyright © 2020 International Anesthesia Research Society.

Conflict of interest statement

The authors declare no conflicts of interest.

- 17 references

Supplementary info

Publication types, MeSH terms Expand

Publication types

- Observational Study

MeSH terms

- Adolescent
- Age Factors
- Anesthesia / adverse effects*
- COVID-19 / diagnosis
- COVID-19 / epidemiology*
- COVID-19 / mortality
- Child
- Child, Preschool
- Female
- Hospital Mortality
- Humans
- Incidence
- Intensive Care Units, Pediatric
- Intraoperative Complications / diagnosis
- Intraoperative Complications / epidemiology*
- Intraoperative Complications / mortality
- Intraoperative Complications / therapy
- Length of Stay
- Male
- Patient Admission
- Postoperative Complications / diagnosis
- Postoperative Complications / epidemiology*
- Postoperative Complications / mortality
- Postoperative Complications / therapy
- Prognosis
- Retrospective Studies
- Risk Assessment
- Risk Factors
- Texas / epidemiology
- Time Factors

Full text links

Wolters Kluwer Wolters Kluwer
Proceed to details
Cite
Share
639
Pharmacol Rep
. 2020 Dec;72(6):1529-1537.
doi: 10.1007/s43440-020-00186-z. Epub 2020 Nov 9.

The effect of tocilizumab on cytokine release syndrome in COVID-19 patients

Carmen de Cáceres [1], Rodrigo Martínez [2], Pablo Bachiller [2], Laura Marín [3], José Manuel García [4]
Affiliations Expand

Affiliations

- [1] Pharmacy Department, Hospital General de Segovia, C/Luis Erik Clavería Neurólogo S/N, 40002, Segovia, Spain. carmendcv@hotmail.com.
- [2] Internal Medicine Department, Hospital General de Segovia, C/Luis Erik Clavería Neurólogo S/N, 40002, Segovia, Spain.
- [3] Pharmacy Department, Hospital General de Segovia, C/Luis Erik Clavería Neurólogo S/N, 40002, Segovia, Spain.
- [4] Análisis Estadísticos 3Datos SL, Salamanca, Spain.

- PMID: **33165762**
- PMCID: PMC7650573
- DOI: 10.1007/s43440-020-00186-z

Free PMC article

The effect of tocilizumab on cytokine release syndrome in COVID-19 patients

Carmen de Cáceres et al. Pharmacol Rep. 2020 Dec.
Free PMC article
Show details
Pharmacol Rep
. 2020 Dec;72(6):1529-1537.
doi: 10.1007/s43440-020-00186-z. Epub 2020 Nov 9.

Authors

Carmen de Cáceres [1], Rodrigo Martínez [2], Pablo Bachiller [2], Laura Marín [3], José Manuel García [4]

Affiliations

- [1] Pharmacy Department, Hospital General de Segovia, C/Luis Erik Clavería Neurólogo S/N, 40002, Segovia, Spain. carmendcv@hotmail.com.
- [2] Internal Medicine Department, Hospital General de Segovia, C/Luis Erik Clavería Neurólogo S/N, 40002, Segovia, Spain.
- [3] Pharmacy Department, Hospital General de Segovia, C/Luis Erik Clavería Neurólogo S/N, 40002, Segovia, Spain.
- [4] Análisis Estadísticos 3Datos SL, Salamanca, Spain.

- PMID: **33165762**
- PMCID: PMC7650573
- DOI: 10.1007/s43440-020-00186-z

Abstract

Background: This study was aimed to assess the efficacy and safety of tocilizumab (TCZ) and to investigate the factors related to the progress and mortality of patients with a secondary cytokine release syndrome caused by SARS-CoV-2.

Methods: A retrospective descriptive observational study of hospitalised patients with a positive polymerase chain reaction (PCR) result for SARS-CoV-2 and whose clinical evolution required the administration of one or more doses of TCZ was conducted. Demographic variables, clinical evolution, radiologic progress and analytical parameters were analysed on days 1, 3 and 5 after administration the first dose of TCZ.

Results: A total of 75 patients with a clinical history of Accurate Respiratory Distress Syndrome (ARDS) were analysed, among whom, 19 had mild ARDS (25.3%), 37 moderate ARDS (49.4%) and 19 severe ARDS (25.3%). Lymphocytopenia and high levels of PCR, D-Dimer and IL-6 were observed in almost all the patients (91.8%). Treatment with TCZ was associated with a reduction of lymphocytopenia, C-reactive protein (CRP) levels, severe ARDS cases and fever. Although a better evolution of PaO_2/FiO_2 was observed in patients who received two or more doses of TCZ (38/75), there was an increase in their mortality (47.4%) and ICU admission (86.8%). The 30-day mortality rate was 30.7% (20.5-42.4% CI) being hypertension, high initial D-dimer levels and ICU admission the only predictive factors found.

Conclusion: Based on our results, treatment with TCZ was associated with a fever, swelling and ventilator support improvement. However, there is no evidence that the administration of two or more doses of TCZ was related to a mortality decrease.

Keywords: Cytokine release syndrome; IL-6; SARS-CoV-2; Tocilizumab.

Conflict of interest statement

The authors declare that they have no conflict of interest.

- 46 references

Supplementary info

MeSH terms, Substances Expand

MeSH terms

- Adult
- Aged
- Aged, 80 and over
- Antibodies, Monoclonal, Humanized / adverse effects
- Antibodies, Monoclonal, Humanized / therapeutic use*
- COVID-19 / complications*
- COVID-19 / drug therapy
- COVID-19 / mortality
- Cytokine Release Syndrome / drug therapy*
- Cytokine Release Syndrome / mortality
- Female
- Humans
- Intensive Care Units / statistics & numerical data*
- Male
- Middle Aged
- Polymerase Chain Reaction
- Respiration, Artificial / statistics & numerical data
- Retrospective Studies
- SARS-CoV-2 / isolation & purification
- Severity of Illness Index
- Treatment Outcome
- Young Adult

Substances

- Antibodies, Monoclonal, Humanized
- tocilizumab

Full text links

Free PMC article
Proceed to details
Cite
Share
☐ 640
Observational Study

J Perinat Med
. 2020 Nov 26;48(9):981-984.
doi: 10.1515/jpm-2020-0236.

Universal screening for SARS-CoV-2 before labor admission during Covid-19 pandemic in Madrid

Ignacio Herraiz [1], Dolores Folgueira [2], Cecilia Villalaín [1], Laura Forcén [1], Rafael Delgado [2], Alberto Galindo [1]
Affiliations Expand

Affiliations

- [1] Fetal Medicine Unit - Maternal and Child Health and Development Network (Red SAMID-RD12/0026/0016), Department of Obstetrics and Gynecology, Hospital Universitario 12 de Octubre, Instituto de Investigación Hospital 12 de Octubre (imas12), Universidad Complutense de Madrid, Madrid, Spain.
- [2] Department of Clinical Microbiology, Hospital Universitario 12 de Octubre, Instituto de Investigación Hospital 12 de Octubre (imas12), Universidad Complutense de Madrid, Madrid, Spain.

- PMID: **32681783**
- DOI: 10.1515/jpm-2020-0236

Observational Study

Universal screening for SARS-CoV-2 before labor admission during Covid-19 pandemic in Madrid

Ignacio Herraiz et al. J Perinat Med. 2020.
Show details

J Perinat Med
. 2020 Nov 26;48(9):981-984.
doi: 10.1515/jpm-2020-0236.

Authors

Ignacio Herraiz [1], Dolores Folgueira [2], Cecilia Villalaín [1], Laura Forcén [1], Rafael Delgado [2], Alberto Galindo [1]

Affiliations

- [1] Fetal Medicine Unit - Maternal and Child Health and Development Network (Red SAMID-RD12/0026/0016), Department of Obstetrics and Gynecology, Hospital Universitario 12 de Octubre, Instituto de Investigación Hospital 12 de Octubre (imas12), Universidad Complutense de Madrid, Madrid, Spain.
- [2] Department of Clinical Microbiology, Hospital Universitario 12 de Octubre, Instituto de Investigación Hospital 12 de Octubre (imas12), Universidad Complutense de Madrid, Madrid, Spain.

- PMID: **32681783**
- DOI: 10.1515/jpm-2020-0236

Abstract

Objectives Asymptomatic women admitted to labor may act as silent spreaders of COVID-19. Therefore, universal screening at admission has been proposed. The objective of the study was to evaluate the performance of universal screening for SARS-CoV-2 using quantitative reverse transcription polymerase-chain-reaction (qRT-PCR) tests in women admitted to labor. Methods Observational retrospective study of a cohort of pregnant women admitted to labor and delivery between April 8 and May 2, 2020 in a large maternity in Madrid. SARS-CoV-2 screening with qRT-PCR from combined nasopharyngeal and oropharyngeal swabs was carried out systematically. Screening performance was described. Results We attended 212 deliveries. Nine cases with COVID-19 diagnosis before admission were excluded. In the remaining 203 women, seven referred COVID-19-related symptoms but only one had a positive qRT-PCR. Among the 194 asymptomatic women, only one case (0.5%) was positive. Conclusions The percentage of positive tests in asymptomatic women admitted to delivery was only 0.5% during the post-peak period.

Keywords: COVID-19; SARS-CoV-2; admission; pregnancy; screening.

- 8 references

Supplementary info

Publication types, MeSH terms Expand

Publication types

- Observational Study

MeSH terms

- Asymptomatic Infections / epidemiology
- Betacoronavirus* / genetics
- Betacoronavirus* / isolation & purification
- COVID-19
- COVID-19 Testing
- Clinical Laboratory Techniques*
- Cohort Studies

- Coronavirus Infections / diagnosis*
- Coronavirus Infections / epidemiology
- Delivery, Obstetric
- Female
- Hospitalization
- Humans
- Infant, Newborn
- Labor, Obstetric*
- Mass Screening*
- Pandemics
- Pneumonia, Viral / diagnosis*
- Pneumonia, Viral / epidemiology
- Pregnancy
- Pregnancy Complications, Infectious / diagnosis
- Retrospective Studies
- Reverse Transcriptase Polymerase Chain Reaction
- SARS-CoV-2
- Spain / epidemiology

Full text links

De Gruyter
Proceed to details
Cite
Share
☐ 641
Observational Study
Pediatr Blood Cancer
. 2021 Dec;68(12):e29223.
doi: 10.1002/pbc.29223. Epub 2021 Jul 20.

SARS-CoV-2 in children with cancer in Brazil: Results of a multicenter national registry

Mariana Cristina M Corso [1], Victor J Soares [2], Anna Maria P Amorim [3], Rosana Cipolotti [4] [5], Isis Maria Q Magalhães [6], Mecneide M Lins [7], Silvana Forsait [8], Luciana N Silva [9], Ana Virginia L de Sousa [10], Nádia G Alves [11], Seila I do Prado [12], Klerize Anecely S Silva [13], Edna K Carbone [14], Melina Brumatti [15], Pablo Santiago [16], Kaline Maria M O Pereira [7], Fabianne A M C Carlesse [10], Marina G Aguiar [11], Rebeca F Marques [17], Ciliana Rechenmacher [1] [17], Liane E Daudt [1] [17] [18], Mariana B Michalowski [1] [17] [19]

Affiliations Expand

Affiliations

- [1] Departamento de Pediatria, Universidade Federal do Rio Grande do Sul, Porto Alegre, Brazil.
- [2] Faculdade de Medicina, Universidade Federal do Rio Grande do Sul, Porto Alegre, Brazil.
- [3] Hospital Oncológico Infantil Octávio Lobo, Belém, Brazil.
- [4] Universidade Federal de Sergipe, São Cristóvão, Brazil.
- [5] Centro de Oncologia de Sergipe Dr. Oswaldo Leite, Aracaju, Brazil.
- [6] Hospital da Criança de Brasília José Alencar, Brasília, Brazil.
- [7] Instituto de Medicina Integral Professor Fernando Figueira, Recife, Brazil.
- [8] Instituto de Tratamento do Câncer Infantil - ITACI, São Paulo, Brazil.
- [9] Hospital Martagão Gesteira, Salvador, Brazil.
- [10] Universidade Federal de São Paulo/Instituto de Oncologia Pediátrica - GRAACC, São Paulo, Brazil.
- [11] Hospital Infantil Albert Sabin, Fortaleza, Brazil.
- [12] Hospital de Câncer de Barretos - Fundação Pio XII, Barretos, Brazil.
- [13] Hospital Criança Conceição, Porto Alegre, Brazil.
- [14] Hospital Pequeno Príncipe, Curitiba, Brazil.
- [15] Hospital Israelita Albert Einstein, São Paulo, Brazil.
- [16] Associação Hospitalar Beneficente São Vicente de Paulo, Passo Fundo, Brazil.
- [17] Laboratório de Pediatria Translacional, Serviço de Pesquisa Experimental, Hospital de Clínicas de Porto Alegre, Porto Alegre, Brazil.
- [18] Unidade de Hematologia e Transplante de Medula Óssea Pediátrica, Serviço de Hematologia Clínica, Hospital de Clínicas de Porto Alegre, Porto Alegre, Brazil.
- [19] Serviço de Oncologia Pediátrica, Hospital de Clínicas de Porto Alegre, Porto Alegre, Brazil.

- PMID: **34288386**
- PMCID: PMC8441618
- DOI: 10.1002/pbc.29223

Free PMC article
Observational Study

SARS-CoV-2 in children with cancer in Brazil: Results of a multicenter national registry

Mariana Cristina M Corso et al. Pediatr Blood Cancer. 2021 Dec.
Free PMC article
Show details
Pediatr Blood Cancer
. 2021 Dec;68(12):e29223.
doi: 10.1002/pbc.29223. Epub 2021 Jul 20.

Authors

Mariana Cristina M Corso [1], Victor J Soares [2], Anna Maria P Amorim [3], Rosana Cipolotti [4] [5], Isis Maria Q Magalhães [6], Mecneide M Lins [7], Silvana Forsait [8], Luciana N Silva [9], Ana Virginia L de Sousa [10], Nádia G Alves [11], Seila I do Prado [12], Klerize Anecely S Silva [13], Edna K Carbone [14], Melina Brumatti [15], Pablo Santiago [16], Kaline Maria M O Pereira [7], Fabianne A M C Carlesse [10], Marina G Aguiar [11], Rebeca F Marques [17], Ciliana Rechenmacher [1] [17], Liane E Daudt [1] [17] [18], Mariana B Michalowski [1] [17] [19]

Affiliations

- [1] Departamento de Pediatria, Universidade Federal do Rio Grande do Sul, Porto Alegre, Brazil.
- [2] Faculdade de Medicina, Universidade Federal do Rio Grande do Sul, Porto Alegre, Brazil.
- [3] Hospital Oncológico Infantil Octávio Lobo, Belém, Brazil.
- [4] Universidade Federal de Sergipe, São Cristóvão, Brazil.
- [5] Centro de Oncologia de Sergipe Dr. Oswaldo Leite, Aracaju, Brazil.
- [6] Hospital da Criança de Brasília José Alencar, Brasília, Brazil.
- [7] Instituto de Medicina Integral Professor Fernando Figueira, Recife, Brazil.
- [8] Instituto de Tratamento do Câncer Infantil - ITACI, São Paulo, Brazil.
- [9] Hospital Martagão Gesteira, Salvador, Brazil.
- [10] Universidade Federal de São Paulo/Instituto de Oncologia Pediátrica - GRAACC, São Paulo, Brazil.
- [11] Hospital Infantil Albert Sabin, Fortaleza, Brazil.
- [12] Hospital de Câncer de Barretos - Fundação Pio XII, Barretos, Brazil.
- [13] Hospital Criança Conceição, Porto Alegre, Brazil.
- [14] Hospital Pequeno Príncipe, Curitiba, Brazil.
- [15] Hospital Israelita Albert Einstein, São Paulo, Brazil.
- [16] Associação Hospitalar Beneficente São Vicente de Paulo, Passo Fundo, Brazil.
- [17] Laboratório de Pediatria Translacional, Serviço de Pesquisa Experimental, Hospital de Clínicas de Porto Alegre, Porto Alegre, Brazil.
- [18] Unidade de Hematologia e Transplante de Medula Óssea Pediátrica, Serviço de Hematologia Clínica, Hospital de Clínicas de Porto Alegre, Porto Alegre, Brazil.
- [19] Serviço de Oncologia Pediátrica, Hospital de Clínicas de Porto Alegre, Porto Alegre, Brazil.

- PMID: **34288386**
- PMCID: PMC8441618
- DOI: 10.1002/pbc.29223

Abstract

Background: Strategies to mitigate the impact of COVID-19 in special populations are complex and challenging. Few studies have addressed the impact of COVID-19 on pediatric patients with cancer in low- and middle-income countries.

Methods: Multicenter observational cohort study with prospective records and retrospective analyses starting in April 2020 in 21 pediatric oncology centers distributed throughout Brazil.

Participants: Patients under 18 years of age who are infected by the SARS-CoV-2 virus (confirmed diagnosis through reverse transcriptase-polymerase chain reaction [RT-PCR]) while under treatment at pediatric oncology centers. The variables of interest included clinical symptoms, diagnostic and therapeutic measures. The repercussions of SARS-CoV-2 infection on cancer treatment and general prognosis were monitored.

Results: One hundred seventy-nine patients were included (median age 6 [4-13] years, 58% male). Of these, 55.9% had acute leukemia and 34.1% had solid tumors. The presence of SARS-CoV-2 was diagnosed by RT-PCR. Various laboratory markers were analyzed, but showed no correlation with outcome. Children with low or high BMI for age had lower overall survival (71.4% and 82.6%, respectively) than those with age-appropriate BMI (92.7%) ($p = .007$). The severity of presentation at diagnosis was significantly associated with outcome ($p < .001$). Overall mortality in the presence of infection was 12.3% (n = 22).

Conclusion: In children with cancer and COVID-19, lower BMI was associated with worse prognosis. The mortality in this group of patients (12.3%) was significantly higher than that described in the pediatric population overall (~1%).

Keywords: BMI; COVID-19; SARS-CoV-2; cancer; pediatric oncology.

© 2021 Wiley Periodicals LLC.

Conflict of interest statement

The authors declare that there is no conflict of interest.

- 27 references
- 2 figures

Supplementary info

Publication types, MeSH terms Expand

Publication types

- Multicenter Study
- Observational Study

MeSH terms

- Adolescent
- Body Mass Index
- Brazil / epidemiology
- COVID-19 / complications*
- COVID-19 / epidemiology
- Child
- Child, Preschool
- Female

- Humans
- Male
- Neoplasms / complications*
- Neoplasms / epidemiology
- Registries
- Retrospective Studies
- Risk Factors
- SARS-CoV-2 / isolation & purification
- Survival Analysis

Full text links

WILEY Full Text Article Wiley Free PMC article

Proceed to details

Cite

Share

642

Observational Study

QJM

. 2021 May 19;114(3):182-189.
doi: 10.1093/qjmed/hcab029.

Should COVID-19 patients >75 years be Ventilated? An Outcome Study

H Raheja [1], N Chukwuka [2], C Agarwal [1], D Sharma [2], A Munoz-Martinez [2], J Fogel [3], M Khalid [1], A T Hashmi [1], S Ehrlich [2], M A Waheed [2], S Siddiqui [1], B A de Brito Gomes [2], A Aslam [2], C J Merino Gualan [4], I Aftab [2], A Tiwari [2], S Singh [2], K Pouching [2], N Somal [2], J Shani [1], G Rojas-Marte [1] [5]

Affiliations Expand

Affiliations

- [1] Department of Cardiology, Maimonides Medical Center, 4802 Tenth Avenue, Brooklyn, NY 11219, USA.
- [2] Department of Internal Medicine, Maimonides Medical Center, 475 Seaview Avenue, Staten Island NY 10305, USA.
- [3] Department of Business Management, Brooklyn College, Brooklyn, NY, USA.
- [4] Department of Volunteer and Student Services, Maimonides Medical Center, Brooklyn, NY, USA.
- [5] Department of Cardiology, Donald and Barbara Zucker School of Medicine at Hofstra/Northwell, Staten Island, NY, USA.

- PMID: **33580251**

- PMCID: PMC7928642
- DOI: 10.1093/qjmed/hcab029

Free PMC article
Observational Study

Should COVID-19 patients >75 years be Ventilated? An Outcome Study

H Raheja et al. QJM. 2021.
Free PMC article
Show details
QJM
. 2021 May 19;114(3):182-189.
doi: 10.1093/qjmed/hcab029.

Authors

H Raheja[1], N Chukwuka[2], C Agarwal[1], D Sharma[2], A Munoz-Martinez[2], J Fogel[3], M Khalid[1], A T Hashmi[1], S Ehrlich[2], M A Waheed[2], S Siddiqui[1], B A de Brito Gomes[2], A Aslam[2], C J Merino Gualan[4], I Aftab[2], A Tiwari[2], S Singh[2], K Pouching[2], N Somal[2], J Shani[1], G Rojas-Marte[1 5]

Affiliations

- [1] Department of Cardiology, Maimonides Medical Center, 4802 Tenth Avenue, Brooklyn, NY 11219, USA.
- [2] Department of Internal Medicine, Maimonides Medical Center, 475 Seaview Avenue, Staten Island NY 10305, USA.
- [3] Department of Business Management, Brooklyn College, Brooklyn, NY, USA.
- [4] Department of Volunteer and Student Services, Maimonides Medical Center, Brooklyn, NY, USA.
- [5] Department of Cardiology, Donald and Barbara Zucker School of Medicine at Hofstra/Northwell, Staten Island, NY, USA.

- PMID: **33580251**
- PMCID: PMC7928642
- DOI: 10.1093/qjmed/hcab029

Abstract

Background: Elderly patients with COVID-19 disease are at increased risk for adverse outcomes. Current data regarding disease characteristics and outcomes in this population are limited.

Aim: To delineate the adverse factors associated with outcomes of COVID-19 patients ≥75 years of age.

Design: Retrospective cohort study.

Methods: Patients were classified into mild/moderate, severe/very severe and critical disease (intubated) based on oxygen requirements. The primary outcome was in-hospital mortality.

Results: A total of 355 patients aged ≥75 years hospitalized with COVID-19 between 19 March and 25 April 2020 were included.Mean age was 84.3 years. One-third of the patients developed critical disease. Mean length of stay was 7.10 days. Vasopressors were required in 27%, with the highest frequency in the critical disease group (74.1%). Overall mortality was 57.2%, with a significant difference between severity groups (mild/moderate disease: 17.4%, severe/very severe disease: 71.3%, critical disease: 94.9%, $P < 0.001$).Increased age, dementia, and severe/very severe and critical disease groups were independently associated with increased odds for mortality while diarrhea was associated with decreased odds for mortality (OR: 0.12, 95% CI: 0.02-0.60, $P < 0.05$). None of the cardiovascular comorbidities were significantly associated with mortality.

Conclusion: Age and dementia are associated with increased odds for mortality in patients ≥75 years of age hospitalized with COVID-19. Those who require intubation have the greatest odds for mortality. Diarrhea as a presenting symptom was associated with lower odds for mortality.

© The Author(s) 2021. Published by Oxford University Press on behalf of the Association of Physicians.

Comment in

- Ventilating the elderly with Covid-19.
  Aujayeb A. Aujayeb A. QJM. 2021 Dec 20;114(10):761. doi: 10.1093/qjmed/hcab201. QJM. 2021. PMID: 34273175 Free PMC article. No abstract available.

- 26 references

Supplementary info

Publication types, MeSH terms Expand

Publication types

- Observational Study

MeSH terms

- Age Factors
- Aged
- Aged, 80 and over
- COVID-19 / epidemiology
- COVID-19 / mortality
- COVID-19 / therapy*
- Decision Making*
- Female
- Hospital Mortality
- Humans

- Male
- New York City / epidemiology
- Pneumonia, Viral / epidemiology
- Pneumonia, Viral / mortality
- Pneumonia, Viral / therapy*
- Pneumonia, Viral / virology
- Respiration, Artificial*
- Retrospective Studies
- Risk Factors
- SARS-CoV-2
- Severity of Illness Index

Full text links

Free PMC article
Proceed to details
Cite
Share
643
Clinics (Sao Paulo)
. 2020;75:e2294.
doi: 10.6061/clinics/2020/e2294. Epub 2020 Aug 26.

Characteristics and outcomes of patients with COVID-19 admitted to the ICU in a university hospital in São Paulo, Brazil - study protocol

Juliana C Ferreira [1], Yeh-Li Ho [2], Bruno A M P Besen [3], Luiz M S Malbuisson [4], Leandro U Taniguchi [3], Pedro V Mendes [3], Eduardo L V Costa [1], Marcelo Park [3], Renato Daltro-Oliveira [5], Roberta M L Roepke [5], João M Silva Jr [4], Maria José C Carmona [4], Carlos Roberto Ribeiro Carvalho [1], EPICCoV Study Group; Adriana Hirota, Alberto Kendy Kanasiro, Alessandra Crescenzi, Amanda Coelho Fernandes, Anna Miethke-Morais, Arthur Petrillo Bellintani, Artur Ribeiro Canasiro, Bárbara Vieira Carneiro, Beatriz Keiko Zanbon, Bernardo Pinheiro De Senna Nogueira Batista, Bianca Ruiz Nicolao, Bruno Adler Maccagnan Pinheiro Besen, Bruno Biselli, Bruno Rocha De Macedo, Caio Machado Gomes De Toledo, Carlos Eduardo Pompilio, Carlos Roberto Ribeiro De Carvalho, Caroline Gomes Mol, Cassio Stipanich, Caue Gasparotto Bueno, Cibele Garzillo, Clarice Tanaka, Daniel Neves Forte, Daniel Joelsons, Daniele Robira, Eduardo Leite Vieira Costa, Elson Mendes Da Silva Júnior, Fabiane Aliotti Regalio, Gabriela Cardoso Segura, Gustavo Brasil Marcelino, Giulia Sefrin Louro, Yeh-Li Ho, Isabela Argollo Ferreira, Jeison de Oliveira Gois, Joao Manoel Da Silva Junior, Jose Otto Reusing Junior, Julia Fray Ribeiro, Juliana Carvalho Ferreira, Karine Vusberg Galleti, Katia Regina Silva, Larissa Padrao Isensee, Larissa dos Santos Oliveira, Leandro Utino Taniguchi, Leila Suemi Letaif, Lígia Trombetta Lima, Lucas Yongsoo Park, Lucas Chaves Netto, Luciana

Cassimiro Nobrega, Luciana Haddad, Ludhmila Hajjar, Luiz Marcelo Malbouisson, Manuela Cristina Adsuara Pandolfi, Marcelo Park, Maria José Carvalho Carmona, Maria Castilho Prandini H De Andrade, Mariana Moreira Santos, Matheus Pereira Bateloche, Mayra Akimi Suiama, Mayron Faria de Oliveira, Mayson Laercio Sousa, Michelle Louvaes, Natassja Huemer, Pedro Mendes, Paulo Ricardo Gessolo Lins, Pedro Gaspar Dos Santos, Pedro Ferreira Paiva Moreira, Renata Mello Guazzelli, Renato Batista Dos Reis, Renato Daltro De Oliveira, Roberta Muriel Longo Roepke, Rodolpho Augusto De Moura Pedro, Rodrigo Kondo, Samia Zahi Rached, Sergio Roberto Silveira Da Fonseca, Thais Sousa Borges, Thalissa Ferreira, Vilson Cobello Junior, Vivian Vieira Tenório Sales, Willaby Serafim Cassa Ferreira
Affiliations Expand

Affiliations

- [1] Divisao de Pneumologia, Instituto do Coracao (InCor), Hospital das Clinicas HCFMUSP, Faculdade de Medicina, Universidade de Sao Paulo, Sao Paulo, SP, BR.
- [2] Hospital das Clinicas HCFMUSP, Faculdade de Medicina, Divisao de Molestias Infecciosas, Universidade de Sao Paulo, Sao Paulo, SP, BR.
- [3] UTI Clinica, Disciplina de Emergencias Clinicas, Departamento de Clinica Medica, Hospital das Clinicas HCFMUSP, Faculdade de Medicina, Universidade de Sao Paulo, Sao Paulo, SP, BR.
- [4] Divisao de Anestesia, Hospital das Clinicas HCFMUSP, Faculdade de Medicina, Universidade de Sao Paulo, Sao Paulo, SP, BR.
- [5] Unidade de Terapia Intensiva, AC Camargo Cancer Center, Sao Paulo, SP, BR.

- PMID: **32876113**
- PMCID: PMC7442378
- DOI: 10.6061/clinics/2020/e2294

Free PMC article

Characteristics and outcomes of patients with COVID-19 admitted to the ICU in a university hospital in São Paulo, Brazil - study protocol

Juliana C Ferreira et al. Clinics (Sao Paulo). 2020.
Free PMC article
Show details
Clinics (Sao Paulo)
. 2020;75:e2294.
doi: 10.6061/clinics/2020/e2294. Epub 2020 Aug 26.

Authors

Juliana C Ferreira [1], Yeh-Li Ho [2], Bruno A M P Besen [3], Luiz M S Malbuisson [4], Leandro U Taniguchi [3], Pedro V Mendes [3], Eduardo L V Costa [1], Marcelo Park [3], Renato Daltro-Oliveira

[5], Roberta M L Roepke [5], João M Silva Jr [4], Maria José C Carmona [4], Carlos Roberto Ribeiro Carvalho [1], EPICCoV Study Group; Adriana Hirota, Alberto Kendy Kanasiro, Alessandra Crescenzi, Amanda Coelho Fernandes, Anna Miethke-Morais, Arthur Petrillo Bellintani, Artur Ribeiro Canasiro, Bárbara Vieira Carneiro, Beatriz Keiko Zanbon, Bernardo Pinheiro De Senna Nogueira Batista, Bianca Ruiz Nicolao, Bruno Adler Maccagnan Pinheiro Besen, Bruno Biselli, Bruno Rocha De Macedo, Caio Machado Gomes De Toledo, Carlos Eduardo Pompilio, Carlos Roberto Ribeiro De Carvalho, Caroline Gomes Mol, Cassio Stipanich, Caue Gasparotto Bueno, Cibele Garzillo, Clarice Tanaka, Daniel Neves Forte, Daniel Joelsons, Daniele Robira, Eduardo Leite Vieira Costa, Elson Mendes Da Silva Júnior, Fabiane Aliotti Regalio, Gabriela Cardoso Segura, Gustavo Brasil Marcelino, Giulia Sefrin Louro, Yeh-Li Ho, Isabela Argollo Ferreira, Jeison de Oliveira Gois, Joao Manoel Da Silva Junior, Jose Otto Reusing Junior, Julia Fray Ribeiro, Juliana Carvalho Ferreira, Karine Vusberg Galleti, Katia Regina Silva, Larissa Padrao Isensee, Larissa dos Santos Oliveira, Leandro Utino Taniguchi, Leila Suemi Letaif, Lígia Trombetta Lima, Lucas Yongsoo Park, Lucas Chaves Netto, Luciana Cassimiro Nobrega, Luciana Haddad, Ludhmila Hajjar, Luiz Marcelo Malbouisson, Manuela Cristina Adsuara Pandolfi, Marcelo Park, Maria José Carvalho Carmona, Maria Castilho Prandini H De Andrade, Mariana Moreira Santos, Matheus Pereira Bateloche, Mayra Akimi Suiama, Mayron Faria de Oliveira, Mayson Laercio Sousa, Michelle Louvaes, Natassja Huemer, Pedro Mendes, Paulo Ricardo Gessolo Lins, Pedro Gaspar Dos Santos, Pedro Ferreira Paiva Moreira, Renata Mello Guazzelli, Renato Batista Dos Reis, Renato Daltro De Oliveira, Roberta Muriel Longo Roepke, Rodolpho Augusto De Moura Pedro, Rodrigo Kondo, Samia Zahi Rached, Sergio Roberto Silveira Da Fonseca, Thais Sousa Borges, Thalissa Ferreira, Vilson Cobello Junior, Vivian Vieira Tenório Sales, Willaby Serafim Cassa Ferreira

Affiliations

- [1] Divisao de Pneumologia, Instituto do Coracao (InCor), Hospital das Clinicas HCFMUSP, Faculdade de Medicina, Universidade de Sao Paulo, Sao Paulo, SP, BR.
- [2] Hospital das Clinicas HCFMUSP, Faculdade de Medicina, Divisao de Molestias Infecciosas, Universidade de Sao Paulo, Sao Paulo, SP, BR.
- [3] UTI Clinica, Disciplina de Emergencias Clinicas, Departamento de Clinica Medica, Hospital das Clinicas HCFMUSP, Faculdade de Medicina, Universidade de Sao Paulo, Sao Paulo, SP, BR.
- [4] Divisao de Anestesia, Hospital das Clinicas HCFMUSP, Faculdade de Medicina, Universidade de Sao Paulo, Sao Paulo, SP, BR.
- [5] Unidade de Terapia Intensiva, AC Camargo Cancer Center, Sao Paulo, SP, BR.

- PMID: **32876113**
- PMCID: PMC7442378
- DOI: 10.6061/clinics/2020/e2294

Abstract

Objectives: We designed a cohort study to describe characteristics and outcomes of patients with coronavirus disease (COVID-19) admitted to the intensive care unit (ICU) in the largest public hospital in Sao Paulo, Brazil, as Latin America becomes the epicenter of the pandemic.

Methods: This is the protocol for a study being conducted at an academic hospital in Brazil with 300 adult ICU beds dedicated to COVID-19 patients. We will include adult patients admitted to the ICU with suspected or confirmed COVID-19 during the study period. The main outcome is ICU survival at 28 days. Data will be collected prospectively and retrospectively by trained

investigators from the hospital's electronic medical records, using an electronic data capture tool. We will collect data on demographics, comorbidities, severity of disease, and laboratorial test results at admission. Information on the need for advanced life support and ventilator parameters will be collected during ICU stay. Patients will be followed up for 28 days in the ICU and 60 days in the hospital. We will plot Kaplan-Meier curves to estimate ICU and hospital survival and perform survival analysis using the Cox proportional hazards model to identify the main risk factors for mortality. ClinicalTrials.gov: NCT04378582.

Results: We expect to include a large sample of patients with COVID-19 admitted to the ICU and to be able to provide data on admission characteristics, use of advanced life support, ICU survival at 28 days, and hospital survival at 60 days.

Conclusions: This study will provide epidemiological data about critically ill patients with COVID-19 in Brazil, which could inform health policy and resource allocation in low- and middle-income countries.

Conflict of interest statement

No potential conflict of interest was reported.

- 29 references
- 2 figures

Supplementary info

MeSH terms, Associated data Expand

MeSH terms

- Betacoronavirus
- Brazil
- COVID-19
- Cohort Studies
- Coronavirus Infections / diagnosis*
- Coronavirus Infections / mortality*
- Coronavirus Infections / therapy*
- Hospital Mortality
- Hospitals, University
- Humans
- Intensive Care Units
- Observational Studies as Topic
- Pandemics
- Pneumonia, Viral / diagnosis*
- Pneumonia, Viral / mortality*
- Pneumonia, Viral / therapy*
- Research Design
- SARS-CoV-2

Associated data

- ClinicalTrials.gov/NCT04378582

Full text links

Free PMC article
Proceed to details
Cite
Share
☐ 644
Observational Study
Am J Emerg Med
. 2021 Aug;46:276-281.
doi: 10.1016/j.ajem.2020.07.071. Epub 2020 Jul 29.

Use of high-flow nasal cannula and noninvasive ventilation in patients with COVID-19: A multicenter observational study

Jun Duan [1], Baixu Chen [2], Xiaoyi Liu [3], Weiwei Shu [4], Wei Zhao [5], Ji Li [6], Yishi Li [7], Yueling Hong [7], Longfang Pan [7], Ke Wang [8]
Affiliations Expand

Affiliations

- [1] Department of Respiratory and Critical Care Medicine, The First Affiliated Hospital of Chongqing Medical University, Chongqing, China. Electronic address: duanjun412589@163.com.
- [2] Department of Critical Care Medicine, West China hospital of Sichuan University, Chengdu, Sichuan, China.
- [3] Department of Critical Care Medicine, The Central Hospital of Dazhou, Dazhou, Shichuan, China.
- [4] Department of Critical Care Medicine, Yongchuan Hospital of Chongqing Medical University, Yongchuan, Chongqing, China.
- [5] Department of Oncology, The Second Affiliated Hospital of Chongqing Medical University, Chongqing, China.
- [6] Department of Thoracic Surgery, Chongqing Public Health Medical Center, Chongqing, China.
- [7] Department of Respiratory and Critical Care Medicine, The First Affiliated Hospital of Chongqing Medical University, Chongqing, China.
- [8] Department of Respiratory and Critical Care Medicine, The Second Affiliated Hospital of Chongqing Medical University, Chongqing, China. Electronic address: 568638714@qq.com.

- PMID: **33046296**

- PMCID: PMC7388754
- DOI: 10.1016/j.ajem.2020.07.071

Free PMC article
Observational Study

Use of high-flow nasal cannula and noninvasive ventilation in patients with COVID-19: A multicenter observational study

Jun Duan et al. Am J Emerg Med. 2021 Aug.
Free PMC article

Show details

Am J Emerg Med
. 2021 Aug;46:276-281.
doi: 10.1016/j.ajem.2020.07.071. Epub 2020 Jul 29.

Authors

Jun Duan [1], Baixu Chen [2], Xiaoyi Liu [3], Weiwei Shu [4], Wei Zhao [5], Ji Li [6], Yishi Li [7], Yueling Hong [7], Longfang Pan [7], Ke Wang [8]

Affiliations

- [1] Department of Respiratory and Critical Care Medicine, The First Affiliated Hospital of Chongqing Medical University, Chongqing, China. Electronic address: duanjun412589@163.com.
- [2] Department of Critical Care Medicine, West China hospital of Sichuan University, Chengdu, Sichuan, China.
- [3] Department of Critical Care Medicine, The Central Hospital of Dazhou, Dazhou, Shichuan, China.
- [4] Department of Critical Care Medicine, Yongchuan Hospital of Chongqing Medical University, Yongchuan, Chongqing, China.
- [5] Department of Oncology, The Second Affiliated Hospital of Chongqing Medical University, Chongqing, China.
- [6] Department of Thoracic Surgery, Chongqing Public Health Medical Center, Chongqing, China.
- [7] Department of Respiratory and Critical Care Medicine, The First Affiliated Hospital of Chongqing Medical University, Chongqing, China.
- [8] Department of Respiratory and Critical Care Medicine, The Second Affiliated Hospital of Chongqing Medical University, Chongqing, China. Electronic address: 568638714@qq.com.

- PMID: **33046296**
- PMCID: PMC7388754
- DOI: 10.1016/j.ajem.2020.07.071

Abstract

Background: The use of high-flow nasal cannula (HFNC) and noninvasive ventilation (NIV) in patients with COVID-19 is debated.

Methods: This study was performed in four hospitals of China from January to March 2020. We retrospectively enrolled 23 and 13 COVID-19 patients who used HFNC and NIV as first-line therapy, respectively.

Results: Among the 23 patients who used HFNC as first-line therapy, 10 experienced HFNC failure and used NIV as rescue therapy. Among the 13 patients who used NIV as first-line therapy, one (8%) used HFNC as rescue therapy due to NIV intolerance. The duration of HFNC + NIV (median 7.1, IQR: 3.5-12.2 vs. 7.3, IQR: 5.3-10.0 days), intubation rate (17% vs. 15%) and mortality (4% vs. 8%) did not differ between patients who used HFNC and NIV as first-line therapy. In total cohorts, 6 (17%) patients received intubation. Time from initiation of HFNC or NIV to intubation was 8.4 days (IQR: 4.4-18.5). And the time from initiation of HFNC or NIV to termination in patients without intubation was 7.1 days (IQR: 3.9-10.3). Among all the patients, C-reactive protein was independently associated with intubation (OR = 1.04, 95% CI: 1.01-1.07). In addition, no medical staff got nosocomial infection who participated in HFNC and NIV management.

Conclusions: In critically ill patients with COVID-19 who used HFNC and NIV as first-line therapy, the duration of HFNC + NIV, intubation rate and mortality did not differ between two groups. And no medical staff got nosocomial infection during this study.

Keywords: COVID-19; High-flow nasal cannula; Intubation; Noninvasive ventilation.

Copyright © 2020 Elsevier Inc. All rights reserved.

Conflict of interest statement

Declaration of Competing Interest We declare that we have no competing interests.

- 30 references
- 2 figures

Supplementary info

Publication types, MeSH terms Expand

Publication types

- Multicenter Study
- Observational Study

MeSH terms

- Aged
- COVID-19 / epidemiology
- COVID-19 / therapy*

- Cannula / statistics & numerical data*
- China / epidemiology
- Equipment Design
- Female
- Humans
- Male
- Middle Aged
- Noninvasive Ventilation / instrumentation*
- Oxygen Inhalation Therapy / instrumentation*
- Pandemics
- SARS-CoV-2

Full text links

ELSEVIER FULL-TEXT ARTICLE Elsevier Science Free PMC article
Proceed to details
Cite
Share
645
Observational Study
Int J Stroke
. 2021 Jul;16(5):573-584.
doi: 10.1177/1747493021991652. Epub 2021 Mar 29.

Global impact of COVID-19 on stroke care

Raul G Nogueira [1], Mohamad Abdalkader [2], Muhammed M Qureshi [3], Michael R Frankel [1], Ossama Yassin Mansour [4], Hiroshi Yamagami [5], Zhongming Qiu [6], Mehdi Farhoudi [7], James E Siegler [8], Shadi Yaghi [9], Eytan Raz [10], Nobuyuki Sakai [11], Nobuyuki Ohara [12], Michel Piotin [13], Laura Mechtouff [14], Omer Eker [15], Vanessa Chalumeau [16], Timothy J Kleinig [17], Raoul Pop [18], Jianmin Liu [19], Hugh S Winters [20], Xianjin Shang [21], Alejandro Rodriguez Vasquez [22], Jordi Blasco [23], Juan F Arenillas [24], Mario Martinez-Galdamez [25], Alex Brehm [26], Marios-Nikos Psychogios [26], Pedro Lylyk [27], Diogo C Haussen [1], Alhamza R Al-Bayati [1], Mahmoud H Mohammaden [1], Luísa Fonseca [28], M Luís Silva [29], Francisco Montalverne [30], Leonardo Renieri [31], Salvatore Mangiafico [31], Urs Fischer [32], Jan Gralla [33], Donald Frei [34], Chandril Chugh [35], Brijesh P Mehta [36], Simon Nagel [37], Markus Mohlenbruch [38], Santiago Ortega-Gutierrez [39], Mudassir Farooqui [39], Ameer E Hassan [40], Allan Taylor [41], Bertrand Lapergue [42], Arturo Consoli [43], Bruce Cv Campbell [44], Malveeka Sharma [45], Melanie Walker [46], Noel Van Horn [47], Jens Fiehler [47], Huy Thang Nguyen [48], Quoc T Nguyen [48], Daisuke Watanabe [49], Hao Zhang [50], Huynh V Le [51], Viet Q Nguyen [51], Ruchir Shah [52], Thomas Devlin [52], Priyank Khandelwal [53], Italo Linfante [54], Wazim Izzath [55], Pablo M Lavados [56], Veronica V Olavarría [56], Gisele Sampaio Silva [57], Anna Verena de Carvalho Sousa [58], Jawad Kirmani [59], Martin Bendszus [38], Tatsuo Amano [60], Ryoo Yamamoto [61], Ryosuke Doijiri [62], Naoki Tokuda [63], Takehiro Yamada [64], Tadashi Terasaki [65], Yukako Yazawa [66], Jane G Morris

[67], Emma Griffin [68], John Thornton [68], Pascale Lavoie [69], Charles Matouk [70], Michael D Hill [71], Andrew M Demchuk [71], Monika Killer-Oberpfalzer [72], Fadi Nahab [73], Dorothea Altschul [74], Anna Ramos-Pachón [75], Natalia Pérez de la Ossa [75], Raghid Kikano [76], William Boisseau [77], Gregory Walker [78], Steve M Cordina [79], Ajit Puri [80], Anna Luisa Kuhn [80], Dheeraj Gandhi [81], Pankajavalli Ramakrishnan [82], Roberta Novakovic-White [83], Alex Chebl [84], Odysseas Kargiotis [85], Alexandra Czap [86], Alicia Zha [86], Hesham E Masoud [87], Carlos Lopez [87], David Ozretic [88], Fawaz Al-Mufti [89], Wenjie Zie [90], Zhenhui Duan [91], Zhengzhou Yuan [92], Wenguo Huang [93], Yonggang Hao [94], Jun Luo [95], Vladimir Kalousek [96], Romain Bourcier [97], Romain Guile [97], Steven Hetts [98], Hosam M Al-Jehani [99], Adel AlHazzani [100], Elyar Sadeghi-Hokmabadi [7], Mohamed Teleb [101], Jeremy Payne [101], Jin Soo Lee [102], Ji Man Hong [102], Sung-Il Sohn [103], Yang-Ha Hwang [104], Dong Hoon Shin [105], Hong Gee Roh [106], Randy Edgell [107], Rakesh Khatri [108], Ainsley Smith [109], Amer Malik [110], David Liebeskind [111], Nabeel Herial [112], Pascal Jabbour [112], Pedro Magalhaes [113], Atilla Ozcan Ozdemir [114], Ozlem Aykac [114], Takeshi Uwatoko [115], Tomohisa Dembo [116], Hisao Shimizu [117], Yuri Sugiura [118], Fumio Miyashita [119], Hiroki Fukuda [120], Kosuke Miyake [121], Junsuke Shimbo [122], Yusuke Sugimura [123], Andre Beer-Furlan [124], Krishna Joshi [124], Luciana Catanese [125], Daniel Giansante Abud [126], Octavio Giansante Neto [127], Masoud Mehrpour [128], Amal Al Hashmi [129], Mahar Saqqur [130], Abdulrahman Mostafa [131], Johanna T Fifi [132], Syed Hussain [133], Seby John [133], Rishi Gupta [134], Rotem Sivan-Hoffmann [135], Anna Reznik [135], Achmad Fidaus Sani [136], Serdar Geyik [137], Eşref Akıl [137], Anchalee Churojana [138], Abdoreza Ghoreishi [139], Mohammad Saadatnia [140], Ehsan Sharifipour [141], Alice Ma [142], Ken Faulder [142], Teddy Wu [143], Lester Leung [144], Adel Malek [145], Barbara Voetsch [146], Ajay Wakhloo [147], Rodrigo Rivera [148], Danny Moises Barrientos Iman [149], Aleksandra Pikula [150], Vasileios-Arsenios Lioutas [146], Gotz Thomalla [151], Lee Birnbaum [152], Paolo Machi [153], Gianmarco Bernava [153], Mollie McDermott [154], Dawn Kleindorfer [154], Ken Wong [155], Mary S Patterson [156], Jose Antonio Fiorot Jr [157], Vikram Huded [158], William Mack [159], Matthew Tenser [159], Clifford Eskey [160], Sumeet Multani [161], Michael Kelly [162], Vallabh Janardhan [163], Oriana Cornett [164], Varsha Singh [164], Yuichi Murayama [165], Maxim Mokin [166], Pengfei Yang [19], Xiaoxi Zhang [19], Congguo Yin [50], Hongxing Han [167], Ya Peng [168], Wenhuo Chen [169], Roberto Crosa [170], Michel Eli Frudit [171], Jeyaraj D Pandian [172], Anirudh Kulkarni [172], Yoshiki Yagita [173], Yohei Takenobu [174], Yuji Matsumaru [175], Satoshi Yamada [176], Ryuhei Kono [177], Takuya Kanamaru [178], Hidekazu Yamazaki [83], Manabu Sakaguchi [179], Kenichi Todo [180], Nobuaki Yamamoto [181], Kazutaka Sonoda [182], Tomoko Yoshida [183], Hiroyuki Hashimoto [184], Ichiro Nakahara [185], Elena Cora [186], David Volders [186], Celina Ducroux [187], Ashkan Shoamanesh [188], Johanna Ospel [189], Artem Kaliaev [190], Saima Ahmed [191], Umair Rashid [191], Leticia C Rebello [192], Vitor Mendes Pereira [150], Robert Fahed [78], Michael Chen [124], Sunil A Sheth [86], Lina Palaiodimou [193], Georgios Tsivgoulis [193], Ronil Chandra [194], Feliks Koyfman [195], Thomas Leung [196], Houman Khosravani [197], Sushrut Dharmadhikari [198], Giovanni Frisullo [199], Paolo Calabresi [199], Alexander Tsiskaridze [200], Nino Lobjanidze [200], Mikayel Grigoryan [201], Anna Czlonkowska [202], Diana Aguiar de Sousa [203], Jelle Demeestere [204], Conrad Liang [205], Navdeep Sangha [206], Helmi L Lutsep [207], Óscar Ayo-Martín [208], Antonio Cruz-Culebras [209], Anh D Tran [51], Chang Y Young [210], Charlotte Cordonnier [211], Francois Caparros [211], Maria Alonso De Lecinana [212], Blanca Fuentes [212], Dileep Yavagal [110], Tudor Jovin [109], Laurent Spelle [16], Jacques Moret [16], Pooja Khatri [213], Osama Zaidat [214], Jean Raymond [77], Sheila Martins [215], Thanh Nguyen [216]

Affiliations Expand

Affiliations

- [1] Neurology, Grady Memorial Hospital, Emory University, Atlanta, Georgia, USA.
- [2] Radiology, Boston Medical Center, Boston University School of Medicine, Boston, USA.
- [3] Radiology, Radiation Oncology, Boston Medical Center, Boston University School of Medicine, Boston, USA.
- [4] Neurology Department, Stroke and Neurointervention Division, Alexandria University Hospital, Alexandria University, Egypt.
- [5] Stroke Neurology, National Hospital Organization, Osaka National Hospital, Japan.
- [6] Neurology, Xinqiao Hospital of the Army Medical University, Chongqing, China.
- [7] Tabriz University, Iran.
- [8] Neurology, Cooper Neurological Institute, Cooper University Hospital, Camden, New Jersey, USA.
- [9] Neurology, Radiology, New York University School of Medicine, New York, USA.
- [10] Radiology, Neurology, New York University School of Medicine, New York, USA.
- [11] Neurosurgery, Kobe City Medical Center General Hospital, Kobe, Japan.
- [12] Neurology, Kobe City Medical Center General Hospital, Kobe, Japan.
- [13] Fondation Ophtalmologique Adolphe de Rothschild, France.
- [14] Neurologie, Hospices Civils de Lyon, France.
- [15] Neuroradiologie, Hospices Civils de Lyon, France.
- [16] Hôpital Bicetre, Paris, France.
- [17] Royal Adelaide Hospital, Australia.
- [18] Hôpitaux Universitaires de Strasbourg, France.
- [19] Changhai Hospital, Shanghai, China.
- [20] Royal Prince Alfred Hospital, Sydney, Australia.
- [21] Yijishan Hospital of Wannan Medical College, China.
- [22] Neurology, Hospital Clinic de Barcelona, Spain.
- [23] Interventional Neuroradiology, Hospital Clinic de Barcelona, Spain.
- [24] Neurology, Hospital Clínico Universitario, Valladolid, Spain.
- [25] Interventional Neuroradiology, Hospital Clínico Universitario, Valladolid, Spain.
- [26] University Hospital Basel, Switzerland.
- [27] Clínica Sagrada Familia, Buenos Aires, Argentina.
- [28] Stroke, Centro Hospitalar Universitário de São João, Portugal.
- [29] Neuroradiology, Centro Hospitalar Universitário de São João, Portugal.
- [30] Hospital Geral de Fortaleza, Brazil.
- [31] Careggi University Hospital, Florence, Italy.
- [32] Neurology, University Hospital Bern, Switzerland.
- [33] Interventional Neuroradiology, University Hospital Bern, Switzerland.
- [34] Swedish Medical Center, USA.
- [35] MAX Superspecialty Hospital, India.
- [36] Memorial Neuroscience Institute, Florida.
- [37] Neurology, University Hospital Heidelberg, Germany.
- [38] Neuroradiology, University Hospital Heidelberg, Germany.
- [39] Neurology, University of Iowa, USA.
- [40] Neurosciences, Valley Baptist Medical Center, Harlingen, Texas, USA.
- [41] Neurosurgery, University of Cape Town, South Africa.

- [42] Neurology, Hôpital Foch, France.
- [43] Interventional Neuroradiology, Hôpital Foch, France.
- [44] Royal Melbourne Hospital, Melbourne, Australia.
- [45] Neurology, University of Washington, Seattle, USA.
- [46] Neurosurgery, University of Washington, Seattle, USA.
- [47] Interventional Neuroradiology, Universitätsklinikum Hamburg-Eppendorf, Germany.
- [48] People's 115 Hospital, Vietnam.
- [49] IMS Tokyo-Katsushika General Hospital, Japan.
- [50] Affiliated Hangzhou First People's Hospital, China.
- [51] Hue Central Hospital, Vietnam.
- [52] Erlanger Medical Center, USA.
- [53] Rutgers University, USA.
- [54] Miami Cardiac and Vascular Institute, USA.
- [55] Nottingham University Hospitals, United Kingdom.
- [56] Clínica Alemana, Universidad del Desarrollo, Chile.
- [57] Universidade Federal de Sao Paulo Hospital Israelita Albert Einstein, Brazil.
- [58] Hospital Israelita Albert Einstein, Brazil.
- [59] Hackensack Meridian Health, New Jersey, USA.
- [60] Kyorin University, Japan.
- [61] Yokohama Brain and Spine Center, Japan.
- [62] Iwate Prefectural Central Hospital, Japan.
- [63] Japanese Red Cross Kyoto Daiichi Hospital, Japan.
- [64] Kyoto Second Red Cross Hospital, Japan.
- [65] Japanese Red Cross Kumamoto Hospital, Japan.
- [66] Kohnan Hospital, Japan.
- [67] Neurology, Maine Medical Center, USA.
- [68] Beaumont Hospital, Dublin, Ireland.
- [69] Hopital Enfant Jesus, Quebec City, Canada.
- [70] Yale New Haven Hospital, USA.
- [71] Neurology, University of Calgary, Canada.
- [72] University Hospital Salzburg, Austria.
- [73] Emory University School of Medicine, USA.
- [74] Valley Hospital, New Jersey, USA.
- [75] University Hospital Germans Trias i Pujol, Barcelona, Spain.
- [76] Lau Medical Center, Beirut, Lebanon.
- [77] CHU Montreal, Canada.
- [78] University of Ottawa, Canada.
- [79] University of South Alabama, USA.
- [80] University of Massachusetts Medical Center, USA.
- [81] University of Maryland, USA.
- [82] Riverside Regional Medical Center, Virginia, USA.
- [83] UT Southwestern, Dallas, Texas, USA.
- [84] Henry Ford Health System, Detroit, USA.
- [85] Metropolitan Hospital, Piraeus, Greece.
- [86] UTHealth McGovern Medical School, Houston, USA.
- [87] SUNY Upstate Medical University Hospital, USA.

- [88] University Hospital Centre Zagreb, Croatia.
- [89] Westchester Medical Center, USA.
- [90] Xinqiao Hospital of the Army Medical University, China.
- [91] Wuhan No.1 Hospital, China.
- [92] Affiliated Hospital of Southwest Medical University, China.
- [93] Maoming Traditional Chinese Medicine Hospital, China.
- [94] Shaw Shaw Hospital, China.
- [95] Mianyang 404 Hospital, China.
- [96] University Clinical Hospital Center Sestre Milosrdnice, Croatia.
- [97] CHU Nantes, France.
- [98] University of California San Francisco, USA.
- [99] King Fahad Hospital of the University, Saudi Arabia.
- [100] King Saud University, Saudi Arabia.
- [101] Banner Desert Medical Center, USA.
- [102] Ajou University Hospital, Korea.
- [103] Kyemyung University, Korea.
- [104] Kyungpook National University Hospital, Korea.
- [105] Gachon University Gil Hospital, Korea.
- [106] Konkuk University Hospital, Korea.
- [107] St. Louis University, USA.
- [108] Texas Tech University, USA.
- [109] Cooper University Hospital, USA.
- [110] University of Miami, USA.
- [111] UCLA, Los Angeles, USA.
- [112] Thomas Jefferson University Hospital, USA.
- [113] Hospital Sao Jose, Brazil.
- [114] Eskisehir Osmangazi University, Turkey.
- [115] Saga-ken Medical Centre Koseikan, Japan.
- [116] Saitama Medical Center, Japan.
- [117] Nara City Hospital, Japan.
- [118] Toyonaka Municipal Hospital, Japan.
- [119] Kagoshima City Hospital, Japan.
- [120] Japanese Red Cross Matsue Hospital, Japan.
- [121] Shiroyama Hospital, Japan.
- [122] Niigata City General Hospital, Japan.
- [123] Sugimura Hospital, Kumamoto, Japan.
- [124] Rush University Medical Center, USA.
- [125] Neurology, McMaster University, Canada.
- [126] Interventional Neuroradiology, Ribeirão Preto Medical School, Brazil.
- [127] Neurosciences, Ribeirão Preto Medical School, Brazil.
- [128] Shahid Beheshti University, Iran.
- [129] Khoula Hospital, Ministry of Health, Oman.
- [130] Hamad Medical Corporation, Qatar.
- [131] Alexandria University Hospital, Egypt Hamad Medical Corporation, Qatar.
- [132] Mount Sinai Health System, New York, USA.
- [133] Cleveland Clinic Abu Dhabi, UAE.

- [134] WellStar Health, Marietta, Georgia, USA.
- [135] Rambam Health Care, Israel.
- [136] General Hospital Dr. Soetomo, Indonesia.
- [137] Istanbul Aydın University, Turkey.
- [138] Siriraj Hospital, Thailand.
- [139] Zanjan University, Iran.
- [140] Isfahan University, Iran.
- [141] Qom University, Iran.
- [142] Royal North Shore Hospital, Australia.
- [143] Christchurch Hospital, Christchurch , New Zealand.
- [144] Neurology, Tufts Medical Center, USA.
- [145] Neurosurgery, Tufts Medical Center, USA.
- [146] Neurology, Beth Israel Lahey Health, USA.
- [147] Interventional Neuroradiology, Beth Israel Lahey Health, USA.
- [148] Neuroradiology, Instituto de Neurocirugia Dr. Asengo, Chile.
- [149] National Institute of Neurological Sciences of Lima, Peru.
- [150] University of Toronto, Canada.
- [151] Neurology, Universitätsklinikum Hamburg-Eppendorf, Germany.
- [152] University of Texas San Antonio, USA.
- [153] University Hospitals of Geneva, Switzerland.
- [154] University of Michigan, USA.
- [155] Royal London Hospital, United Kingdom.
- [156] Bon Secours Mercy Health, USA.
- [157] Hospital-Estadual Central, Brazil.
- [158] NH Mazumdar Shaw Medical Center, India.
- [159] University of Southern California, USA.
- [160] Dartmouth Hitchcock Medical Center, Lebanon, New Hampshire, USA.
- [161] Neurology, Bayhealth Medical Center, Delaware, USA.
- [162] Neurosurgery, University of Saskatchewan, Canada.
- [163] Medical City Plano Texas, USA.
- [164] St. Joseph's University Medical Center, USA.
- [165] Jikei University School of Medicine, Japan.
- [166] University of South Florida, USA.
- [167] Linyi City People Hospital, China.
- [168] First People's Hospital, China.
- [169] Zhangzhou Municipal Hospital, China.
- [170] Centro Endovascular Neurológico Médica, Uruguay.
- [171] Universidade Federal de Sao Paulo, Brazil.
- [172] Christian Medical College, India.
- [173] Kawasaki Medical School, Japan.
- [174] Osaka Red Cross Hospital, Japan.
- [175] University of Tsukuba, Japan.
- [176] Saiseikai Central Hospital, Japan.
- [177] Kinikyochuo Hospital, Japan.
- [178] NTT Medical Center, Japan.
- [179] Osaka General Medical Center, Japan.

- [180] Osaka University Graduate School of Medicine, Japan.
- [181] Tokushima University Graduate School of Biomedical Sciences, Japan.
- [182] Saiseikai Fukuoka General Hospital, Japan.
- [183] Tane General Hospital, Japan.
- [184] Osaka Rosai Hospital, Japan.
- [185] Fujita Health University School of Medicine, Japan.
- [186] Dalhousie University, Nova Scotia, Canada.
- [187] CHU Montreal, Montreal, Canada.
- [188] McMaster University, Canada.
- [189] University of Calgary, Canada.
- [190] Radiology, Boston Medical Center, USA.
- [191] Lahore General Hospital, Pakistan.
- [192] Hospital Brasilia, Brazil.
- [193] National & Kapodistrian University of Athens, Greece.
- [194] Monash Medical Center, Australia.
- [195] New York-Presbyterian Queens, USA.
- [196] Prince of Wales Hospital, Hong Kong.
- [197] Sunnybrook Health Sciences Centre, Canada.
- [198] Baptist Health, Arkansas, USA.
- [199] Fondazione Policlinico Universitario A.Gemelli, Italy.
- [200] Ivane Javakhishvili Tbilisi State University, Georgia.
- [201] Adventist Health Glendale, USA.
- [202] Institute Psychiatry and Neurology, Poland.
- [203] Hospital de Santa Maria, Portugal.
- [204] Leuven University Hospital, Belgium.
- [205] Neurointerventional Radiology, Kaiser Permanente, California, USA.
- [206] Neurology, Kaiser Permanente, California, USA.
- [207] Oregon University, USA.
- [208] Complejo Hospitalario Universitario de Albacete, Spain.
- [209] Hospital Universitario Ramon y Cajal, Unidad de Ictus, Spain.
- [210] Asan Medical Center, Korea.
- [211] CHU de Lille, France.
- [212] La Paz University Hospital, Madrid, Spain.
- [213] University of Cincinnati, USA.
- [214] Bon Secours Mercy Health, Toledo, Ohio, USA.
- [215] Hospital de Clínicas de Porto Alegre, Brazil.
- [216] Radiology, Neurology, Boston Medical Center, USA.

- PMID: **33459583**
- PMCID: PMC8010375
- DOI: 10.1177/1747493021991652

Free PMC article
Observational Study

Global impact of COVID-19 on stroke care

Raul G Nogueira et al. Int J Stroke. 2021 Jul.
Free PMC article

Show details

Int J Stroke
. 2021 Jul;16(5):573-584.
doi: 10.1177/1747493021991652. Epub 2021 Mar 29.

Authors

Raul G Nogueira[1], Mohamad Abdalkader[2], Muhammed M Qureshi[3], Michael R Frankel[1], Ossama Yassin Mansour[4], Hiroshi Yamagami[5], Zhongming Qiu[6], Mehdi Farhoudi[7], James E Siegler[8], Shadi Yaghi[9], Eytan Raz[10], Nobuyuki Sakai[11], Nobuyuki Ohara[12], Michel Piotin[13], Laura Mechtouff[14], Omer Eker[15], Vanessa Chalumeau[16], Timothy J Kleinig[17], Raoul Pop[18], Jianmin Liu[19], Hugh S Winters[20], Xianjin Shang[21], Alejandro Rodriguez Vasquez[22], Jordi Blasco[23], Juan F Arenillas[24], Mario Martinez-Galdamez[25], Alex Brehm[26], Marios-Nikos Psychogios[26], Pedro Lylyk[27], Diogo C Haussen[1], Alhamza R Al-Bayati[1], Mahmoud H Mohammaden[1], Luísa Fonseca[28], M Luís Silva[29], Francisco Montalverne[30], Leonardo Renieri[31], Salvatore Mangiafico[31], Urs Fischer[32], Jan Gralla[33], Donald Frei[34], Chandril Chugh[35], Brijesh P Mehta[36], Simon Nagel[37], Markus Mohlenbruch[38], Santiago Ortega-Gutierrez[39], Mudassir Farooqui[39], Ameer E Hassan[40], Allan Taylor[41], Bertrand Lapergue[42], Arturo Consoli[43], Bruce Cv Campbell[44], Malveeka Sharma[45], Melanie Walker[46], Noel Van Horn[47], Jens Fiehler[47], Huy Thang Nguyen[48], Quoc T Nguyen[48], Daisuke Watanabe[49], Hao Zhang[50], Huynh V Le[51], Viet Q Nguyen[51], Ruchir Shah[52], Thomas Devlin[52], Priyank Khandelwal[53], Italo Linfante[54], Wazim Izzath[55], Pablo M Lavados[56], Veronica V Olavarría[56], Gisele Sampaio Silva[57], Anna Verena de Carvalho Sousa[58], Jawad Kirmani[59], Martin Bendszus[38], Tatsuo Amano[60], Ryoo Yamamoto[61], Ryosuke Doijiri[62], Naoki Tokuda[63], Takehiro Yamada[64], Tadashi Terasaki[65], Yukako Yazawa[66], Jane G Morris[67], Emma Griffin[68], John Thornton[68], Pascale Lavoie[69], Charles Matouk[70], Michael D Hill[71], Andrew M Demchuk[71], Monika Killer-Oberpfalzer[72], Fadi Nahab[73], Dorothea Altschul[74], Anna Ramos-Pachón[75], Natalia Pérez de la Ossa[75], Raghid Kikano[76], William Boisseau[77], Gregory Walker[78], Steve M Cordina[79], Ajit Puri[80], Anna Luisa Kuhn[80], Dheeraj Gandhi[81], Pankajavalli Ramakrishnan[82], Roberta Novakovic-White[83], Alex Chebl[84], Odysseas Kargiotis[85], Alexandra Czap[86], Alicia Zha[86], Hesham E Masoud[87], Carlos Lopez[87], David Ozretic[88], Fawaz Al-Mufti[89], Wenjie Zie[90], Zhenhui Duan[91], Zhengzhou Yuan[92], Wenguo Huang[93], Yonggang Hao[94], Jun Luo[95], Vladimir Kalousek[96], Romain Bourcier[97], Romain Guile[97], Steven Hetts[98], Hosam M Al-Jehani[99], Adel AlHazzani[100], Elyar Sadeghi-Hokmabadi[7], Mohamed Teleb[101], Jeremy Payne[101], Jin Soo Lee[102], Ji Man Hong[102], Sung-Il Sohn[103], Yang-Ha Hwang[104], Dong Hoon Shin[105], Hong Gee Roh[106], Randy Edgell[107], Rakesh Khatri[108], Ainsley Smith[109], Amer Malik[110], David Liebeskind[111], Nabeel Herial[112], Pascal Jabbour[112], Pedro Magalhaes[113], Atilla Ozcan Ozdemir[114], Ozlem Aykac[114], Takeshi Uwatoko[115], Tomohisa Dembo[116], Hisao Shimizu[117], Yuri Sugiura[118], Fumio Miyashita[119], Hiroki Fukuda[120], Kosuke Miyake[121], Junsuke Shimbo[122], Yusuke Sugimura[123], Andre Beer-Furlan[124], Krishna Joshi[124], Luciana Catanese[125], Daniel Giansante Abud[126], Octavio Giansante Neto[127], Masoud Mehrpour[128], Amal Al Hashmi[129], Mahar Saqqur

[130], Abdulrahman Mostafa [131], Johanna T Fifi [132], Syed Hussain [133], Seby John [133], Rishi Gupta [134], Rotem Sivan-Hoffmann [135], Anna Reznik [135], Achmad Fidaus Sani [136], Serdar Geyik [137], Eşref Akıl [137], Anchalee Churojana [138], Abdoreza Ghoreishi [139], Mohammad Saadatnia [140], Ehsan Sharifipour [141], Alice Ma [142], Ken Faulder [142], Teddy Wu [143], Lester Leung [144], Adel Malek [145], Barbara Voetsch [146], Ajay Wakhloo [147], Rodrigo Rivera [148], Danny Moises Barrientos Iman [149], Aleksandra Pikula [150], Vasileios-Arsenios Lioutas [146], Gotz Thomalla [151], Lee Birnbaum [152], Paolo Machi [153], Gianmarco Bernava [153], Mollie McDermott [154], Dawn Kleindorfer [154], Ken Wong [155], Mary S Patterson [156], Jose Antonio Fiorot Jr [157], Vikram Huded [158], William Mack [159], Matthew Tenser [159], Clifford Eskey [160], Sumeet Multani [161], Michael Kelly [162], Vallabh Janardhan [163], Oriana Cornett [164], Varsha Singh [164], Yuichi Murayama [165], Maxim Mokin [166], Pengfei Yang [19], Xiaoxi Zhang [19], Congguo Yin [50], Hongxing Han [167], Ya Peng [168], Wenhuo Chen [169], Roberto Crosa [170], Michel Eli Frudit [171], Jeyaraj D Pandian [172], Anirudh Kulkarni [172], Yoshiki Yagita [173], Yohei Takenobu [174], Yuji Matsumaru [175], Satoshi Yamada [176], Ryuhei Kono [177], Takuya Kanamaru [178], Hidekazu Yamazaki [83], Manabu Sakaguchi [179], Kenichi Todo [180], Nobuaki Yamamoto [181], Kazutaka Sonoda [182], Tomoko Yoshida [183], Hiroyuki Hashimoto [184], Ichiro Nakahara [185], Elena Cora [186], David Volders [186], Celina Ducroux [187], Ashkan Shoamanesh [188], Johanna Ospel [189], Artem Kaliaev [190], Saima Ahmed [191], Umair Rashid [191], Leticia C Rebello [192], Vitor Mendes Pereira [150], Robert Fahed [78], Michael Chen [124], Sunil A Sheth [86], Lina Palaiodimou [193], Georgios Tsivgoulis [193], Ronil Chandra [194], Feliks Koyfman [195], Thomas Leung [196], Houman Khosravani [197], Sushrut Dharmadhikari [198], Giovanni Frisullo [199], Paolo Calabresi [199], Alexander Tsiskaridze [200], Nino Lobjanidze [200], Mikayel Grigoryan [201], Anna Czlonkowska [202], Diana Aguiar de Sousa [203], Jelle Demeestere [204], Conrad Liang [205], Navdeep Sangha [206], Helmi L Lutsep [207], Óscar Ayo-Martín [208], Antonio Cruz-Culebras [209], Anh D Tran [51], Chang Y Young [210], Charlotte Cordonnier [211], Francois Caparros [211], Maria Alonso De Lecinana [212], Blanca Fuentes [212], Dileep Yavagal [110], Tudor Jovin [109], Laurent Spelle [16], Jacques Moret [16], Pooja Khatri [213], Osama Zaidat [214], Jean Raymond [77], Sheila Martins [215], Thanh Nguyen [216]

Affiliations

- [1] Neurology, Grady Memorial Hospital, Emory University, Atlanta, Georgia, USA.
- [2] Radiology, Boston Medical Center, Boston University School of Medicine, Boston, USA.
- [3] Radiology, Radiation Oncology, Boston Medical Center, Boston University School of Medicine, Boston, USA.
- [4] Neurology Department, Stroke and Neurointervention Division, Alexandria University Hospital, Alexandria University, Egypt.
- [5] Stroke Neurology, National Hospital Organization, Osaka National Hospital, Japan.
- [6] Neurology, Xinqiao Hospital of the Army Medical University, Chongqing, China.
- [7] Tabriz University, Iran.
- [8] Neurology, Cooper Neurological Institute, Cooper University Hospital, Camden, New Jersey, USA.
- [9] Neurology, Radiology, New York University School of Medicine, New York, USA.
- [10] Radiology, Neurology, New York University School of Medicine, New York, USA.
- [11] Neurosurgery, Kobe City Medical Center General Hospital, Kobe, Japan.
- [12] Neurology, Kobe City Medical Center General Hospital, Kobe, Japan.
- [13] Fondation Ophtalmologique Adolphe de Rothschild, France.

- [14] Neurologie, Hospices Civils de Lyon, France.
- [15] Neuroradiologie, Hospices Civils de Lyon, France.
- [16] Hôpital Bicetre, Paris, France.
- [17] Royal Adelaide Hospital, Australia.
- [18] Hôpitaux Universitaires de Strasbourg, France.
- [19] Changhai Hospital, Shanghai, China.
- [20] Royal Prince Alfred Hospital, Sydney, Australia.
- [21] Yijishan Hospital of Wannan Medical College, China.
- [22] Neurology, Hospital Clinic de Barcelona, Spain.
- [23] Interventional Neuroradiology, Hospital Clinic de Barcelona, Spain.
- [24] Neurology, Hospital Clínico Universitario, Valladolid, Spain.
- [25] Interventional Neuroradiology, Hospital Clínico Universitario, Valladolid, Spain.
- [26] University Hospital Basel, Switzerland.
- [27] Clínica Sagrada Familia, Buenos Aires, Argentina.
- [28] Stroke, Centro Hospitalar Universitário de São João, Portugal.
- [29] Neuroradiology, Centro Hospitalar Universitário de São João, Portugal.
- [30] Hospital Geral de Fortaleza, Brazil.
- [31] Careggi University Hospital, Florence, Italy.
- [32] Neurology, University Hospital Bern, Switzerland.
- [33] Interventional Neuroradiology, University Hospital Bern, Switzerland.
- [34] Swedish Medical Center, USA.
- [35] MAX Superspecialty Hospital, India.
- [36] Memorial Neuroscience Institute, Florida.
- [37] Neurology, University Hospital Heidelberg, Germany.
- [38] Neuroradiology, University Hospital Heidelberg, Germany.
- [39] Neurology, University of Iowa, USA.
- [40] Neurosciences, Valley Baptist Medical Center, Harlingen, Texas, USA.
- [41] Neurosurgery, University of Cape Town, South Africa.
- [42] Neurology, Hôpital Foch, France.
- [43] Interventional Neuroradiology, Hôpital Foch, France.
- [44] Royal Melbourne Hospital, Melbourne, Australia.
- [45] Neurology, University of Washington, Seattle, USA.
- [46] Neurosurgery, University of Washington, Seattle, USA.
- [47] Interventional Neuroradiology, Universitätsklinikum Hamburg-Eppendorf, Germany.
- [48] People's 115 Hospital, Vietnam.
- [49] IMS Tokyo-Katsushika General Hospital, Japan.
- [50] Affiliated Hangzhou First People's Hospital, China.
- [51] Hue Central Hospital, Vietnam.
- [52] Erlanger Medical Center, USA.
- [53] Rutgers University, USA.
- [54] Miami Cardiac and Vascular Institute, USA.
- [55] Nottingham University Hospitals, United Kingdom.
- [56] Clínica Alemana, Universidad del Desarrollo, Chile.
- [57] Universidade Federal de Sao Paulo Hospital Israelita Albert Einstein, Brazil.
- [58] Hospital Israelita Albert Einstein, Brazil.
- [59] Hackensack Meridian Health, New Jersey, USA.

- [60] Kyorin University, Japan.
- [61] Yokohama Brain and Spine Center, Japan.
- [62] Iwate Prefectural Central Hospital, Japan.
- [63] Japanese Red Cross Kyoto Daiichi Hospital, Japan.
- [64] Kyoto Second Red Cross Hospital, Japan.
- [65] Japanese Red Cross Kumamoto Hospital, Japan.
- [66] Kohnan Hospital, Japan.
- [67] Neurology, Maine Medical Center, USA.
- [68] Beaumont Hospital, Dublin, Ireland.
- [69] Hopital Enfant Jesus, Quebec City, Canada.
- [70] Yale New Haven Hospital, USA.
- [71] Neurology, University of Calgary, Canada.
- [72] University Hospital Salzburg, Austria.
- [73] Emory University School of Medicine, USA.
- [74] Valley Hospital, New Jersey, USA.
- [75] University Hospital Germans Trias i Pujol, Barcelona, Spain.
- [76] Lau Medical Center, Beirut, Lebanon.
- [77] CHU Montreal, Canada.
- [78] University of Ottawa, Canada.
- [79] University of South Alabama, USA.
- [80] University of Massachusetts Medical Center, USA.
- [81] University of Maryland, USA.
- [82] Riverside Regional Medical Center, Virginia, USA.
- [83] UT Southwestern, Dallas, Texas, USA.
- [84] Henry Ford Health System, Detroit, USA.
- [85] Metropolitan Hospital, Piraeus, Greece.
- [86] UTHealth McGovern Medical School, Houston, USA.
- [87] SUNY Upstate Medical University Hospital, USA.
- [88] University Hospital Centre Zagreb, Croatia.
- [89] Westchester Medical Center, USA.
- [90] Xinqiao Hospital of the Army Medical University, China.
- [91] Wuhan No.1 Hospital, China.
- [92] Affiliated Hospital of Southwest Medical University, China.
- [93] Maoming Traditional Chinese Medicine Hospital, China.
- [94] Shaw Shaw Hospital, China.
- [95] Mianyang 404 Hospital, China.
- [96] University Clinical Hospital Center Sestre Milosrdnice, Croatia.
- [97] CHU Nantes, France.
- [98] University of California San Francisco, USA.
- [99] King Fahad Hospital of the University, Saudi Arabia.
- [100] King Saud University, Saudi Arabia.
- [101] Banner Desert Medical Center, USA.
- [102] Ajou University Hospital, Korea.
- [103] Kyemyung University, Korea.
- [104] Kyungpook National University Hospital, Korea.
- [105] Gachon University Gil Hospital, Korea.

- [106] Konkuk University Hospital, Korea.
- [107] St. Louis University, USA.
- [108] Texas Tech University, USA.
- [109] Cooper University Hospital, USA.
- [110] University of Miami, USA.
- [111] UCLA, Los Angeles, USA.
- [112] Thomas Jefferson University Hospital, USA.
- [113] Hospital Sao Jose, Brazil.
- [114] Eskisehir Osmangazi University, Turkey.
- [115] Saga-ken Medical Centre Koseikan, Japan.
- [116] Saitama Medical Center, Japan.
- [117] Nara City Hospital, Japan.
- [118] Toyonaka Municipal Hospital, Japan.
- [119] Kagoshima City Hospital, Japan.
- [120] Japanese Red Cross Matsue Hospital, Japan.
- [121] Shiroyama Hospital, Japan.
- [122] Niigata City General Hospital, Japan.
- [123] Sugimura Hospital, Kumamoto, Japan.
- [124] Rush University Medical Center, USA.
- [125] Neurology, McMaster University, Canada.
- [126] Interventional Neuroradiology, Ribeirão Preto Medical School, Brazil.
- [127] Neurosciences, Ribeirão Preto Medical School, Brazil.
- [128] Shahid Beheshti University, Iran.
- [129] Khoula Hospital, Ministry of Health, Oman.
- [130] Hamad Medical Corporation, Qatar.
- [131] Alexandria University Hospital, Egypt Hamad Medical Corporation, Qatar.
- [132] Mount Sinai Health System, New York, USA.
- [133] Cleveland Clinic Abu Dhabi, UAE.
- [134] WellStar Health, Marietta, Georgia, USA.
- [135] Rambam Health Care, Israel.
- [136] General Hospital Dr. Soetomo, Indonesia.
- [137] Istanbul Aydın University, Turkey.
- [138] Siriraj Hospital, Thailand.
- [139] Zanjan University, Iran.
- [140] Isfahan University, Iran.
- [141] Qom University, Iran.
- [142] Royal North Shore Hospital, Australia.
- [143] Christchurch Hospital, Christchurch , New Zealand.
- [144] Neurology, Tufts Medical Center, USA.
- [145] Neurosurgery, Tufts Medical Center, USA.
- [146] Neurology, Beth Israel Lahey Health, USA.
- [147] Interventional Neuroradiology, Beth Israel Lahey Health, USA.
- [148] Neuroradiology, Instituto de Neurocirugia Dr. Asengo, Chile.
- [149] National Institute of Neurological Sciences of Lima, Peru.
- [150] University of Toronto, Canada.
- [151] Neurology, Universitätsklinikum Hamburg-Eppendorf, Germany.

- [152] University of Texas San Antonio, USA.
- [153] University Hospitals of Geneva, Switzerland.
- [154] University of Michigan, USA.
- [155] Royal London Hospital, United Kingdom.
- [156] Bon Secours Mercy Health, USA.
- [157] Hospital-Estadual Central, Brazil.
- [158] NH Mazumdar Shaw Medical Center, India.
- [159] University of Southern California, USA.
- [160] Dartmouth Hitchcock Medical Center, Lebanon, New Hampshire, USA.
- [161] Neurology, Bayhealth Medical Center, Delaware, USA.
- [162] Neurosurgery, University of Saskatchewan, Canada.
- [163] Medical City Plano Texas, USA.
- [164] St. Joseph's University Medical Center, USA.
- [165] Jikei University School of Medicine, Japan.
- [166] University of South Florida, USA.
- [167] Linyi City People Hospital, China.
- [168] First People's Hospital, China.
- [169] Zhangzhou Municipal Hospital, China.
- [170] Centro Endovascular Neurológico Médica, Uruguay.
- [171] Universidade Federal de Sao Paulo, Brazil.
- [172] Christian Medical College, India.
- [173] Kawasaki Medical School, Japan.
- [174] Osaka Red Cross Hospital, Japan.
- [175] University of Tsukuba, Japan.
- [176] Saiseikai Central Hospital, Japan.
- [177] Kinikyochuo Hospital, Japan.
- [178] NTT Medical Center, Japan.
- [179] Osaka General Medical Center, Japan.
- [180] Osaka University Graduate School of Medicine, Japan.
- [181] Tokushima University Graduate School of Biomedical Sciences, Japan.
- [182] Saiseikai Fukuoka General Hospital, Japan.
- [183] Tane General Hospital, Japan.
- [184] Osaka Rosai Hospital, Japan.
- [185] Fujita Health University School of Medicine, Japan.
- [186] Dalhousie University, Nova Scotia, Canada.
- [187] CHU Montreal, Montreal, Canada.
- [188] McMaster University, Canada.
- [189] University of Calgary, Canada.
- [190] Radiology, Boston Medical Center, USA.
- [191] Lahore General Hospital, Pakistan.
- [192] Hospital Brasilia, Brazil.
- [193] National & Kapodistrian University of Athens, Greece.
- [194] Monash Medical Center, Australia.
- [195] New York-Presbyterian Queens, USA.
- [196] Prince of Wales Hospital, Hong Kong.
- [197] Sunnybrook Health Sciences Centre, Canada.

- [198] Baptist Health, Arkansas, USA.
- [199] Fondazione Policlinico Universitario A.Gemelli, Italy.
- [200] Ivane Javakhishvili Tbilisi State University, Georgia.
- [201] Adventist Health Glendale, USA.
- [202] Institute Psychiatry and Neurology, Poland.
- [203] Hospital de Santa Maria, Portugal.
- [204] Leuven University Hospital, Belgium.
- [205] Neurointerventional Radiology, Kaiser Permanente, California, USA.
- [206] Neurology, Kaiser Permanente, California, USA.
- [207] Oregon University, USA.
- [208] Complejo Hospitalario Universitario de Albacete, Spain.
- [209] Hospital Universitario Ramon y Cajal, Unidad de Ictus, Spain.
- [210] Asan Medical Center, Korea.
- [211] CHU de Lille, France.
- [212] La Paz University Hospital, Madrid, Spain.
- [213] University of Cincinnati, USA.
- [214] Bon Secours Mercy Health, Toledo, Ohio, USA.
- [215] Hospital de Clínicas de Porto Alegre, Brazil.
- [216] Radiology, Neurology, Boston Medical Center, USA.

- PMID: **33459583**
- PMCID: PMC8010375
- DOI: 10.1177/1747493021991652

Abstract

Background: The COVID-19 pandemic led to profound changes in the organization of health care systems worldwide.

Aims: We sought to measure the global impact of the COVID-19 pandemic on the volumes for mechanical thrombectomy, stroke, and intracranial hemorrhage hospitalizations over a three-month period at the height of the pandemic (1 March-31 May 2020) compared with two control three-month periods (immediately preceding and one year prior).

Methods: Retrospective, observational, international study, across 6 continents, 40 countries, and 187 comprehensive stroke centers. The diagnoses were identified by their ICD-10 codes and/or classifications in stroke databases at participating centers.

Results: The hospitalization volumes for any stroke, intracranial hemorrhage, and mechanical thrombectomy were 26,699, 4002, and 5191 in the three months immediately before versus 21,576, 3540, and 4533 during the first three pandemic months, representing declines of 19.2% (95%CI, -19.7 to -18.7), 11.5% (95%CI, -12.6 to -10.6), and 12.7% (95%CI, -13.6 to -11.8), respectively. The decreases were noted across centers with high, mid, and low COVID-19 hospitalization burden, and also across high, mid, and low volume stroke/mechanical thrombectomy centers. High-volume COVID-19 centers (-20.5%) had greater declines in mechanical thrombectomy volumes than mid- (-10.1%) and low-volume (-8.7%) centers ($p < 0.0001$). There was a 1.5% stroke rate across 54,366 COVID-19 hospitalizations. SARS-CoV-2 infection was noted in 3.9% (784/20,250) of all stroke admissions.

Conclusion: The COVID-19 pandemic was associated with a global decline in the volume of overall stroke hospitalizations, mechanical thrombectomy procedures, and intracranial hemorrhage admission volumes. Despite geographic variations, these volume reductions were observed regardless of COVID-19 hospitalization burden and pre-pandemic stroke/mechanical thrombectomy volumes.

Keywords: COVID-19; acute ischemic stroke; epidemiology; intracranial hemorrhage; mechanical thrombectomy; stroke care.

Conflict of interest statement

Declaration of conflicting interests: The author(s) declared no potential conflicts of interest with respect to the research, authorship, and/or publication of this article.

- 29 references
- 2 figures

Supplementary info

Publication types, MeSH terms, Grant support Expand

Publication types

- Multicenter Study
- Observational Study

MeSH terms

- COVID-19*
- Cross-Sectional Studies
- Global Health*
- Hospitalization / trends*
- Hospitals, High-Volume / trends
- Hospitals, Low-Volume / trends
- Humans
- Intracranial Hemorrhages / diagnosis
- Intracranial Hemorrhages / epidemiology
- Intracranial Hemorrhages / therapy*
- Registries
- Retrospective Studies
- Stroke / diagnosis
- Stroke / epidemiology
- Stroke / therapy*
- Thrombectomy / trends*
- Time Factors

Grant support

- UL1 TR001863/TR/NCATS NIH HHS/United States

Full text links

SAGE journals Open access full text Atypon Free PMC article
Proceed to details
Cite
Share
646
Observational Study
Neurol Sci
. 2021 Apr;42(4):1237-1245.
doi: 10.1007/s10072-021-05045-0. Epub 2021 Jan 15.

Effect of the COVID-19 pandemic and the lockdown measures on the local stroke network

Valerio Brunetti [1], Aldobrando Broccolini [1 2], Pietro Caliandro [1], Riccardo Di Iorio [1], Mauro Monforte [1], Roberta Morosetti [1], Carla Piano [1], Fabio Pilato [1], Simone Bellavia [2], Jessica Marotta [2], Irene Scala [2], Alessandro Pedicelli [2 3], Mariano Alberto Pennisi [2 4], Anselmo Caricato [2 4], Cinzia Roberti [5], Maria Concetta Altavista [5], Alessandro Valenza [6], Marisa Distefano [6], Emanuela Cecconi [6], Martina Fanella [7], Sabina Roncacci [7], Miriam Tasillo [7], Paolo Calabresi [1 2], Giovanni Frisullo [8 9 10], Giacomo Della Marca [1 2]
Affiliations Expand

Affiliations

- [1] Dipartimento Scienze dell'Invecchiamento, Neurologiche, Ortopediche e della Testa-Collo, Fondazione Policlinico Universitario A. Gemelli IRCCS - UOC Neurologia, Rome, Italy.
- [2] Università Cattolica del Sacro Cuore, Largo Francesco Vito, 1, 00168, Rome, Italy.
- [3] Dipartimento Diagnostica per Immagini, Radioterapia, Oncologia ed Ematologia, Fondazione Policlinico Universitario A. Gemelli IRCCS - UOC Radiologia e Neuroradiologia, Rome, Italy.
- [4] Dipartimento Scienze dell'emergenza, anestesiologiche e della rianimazione, Fondazione Policlinico Universitario A. Gemelli IRCCS - UOC Anestesia, Rianimazione, Terapia Intensiva e Tossicologia Clinica, Rome, Italy.
- [5] ASL Roma 1, San Filippo Neri Hospital - UOC Neurologia, Rome, Italy.
- [6] Ospedale Belcolle - UOC Neurologia, Viterbo, Italy.
- [7] Unità di Trattamento Neurovascolare (UTN) - Stroke Unit, Ospedale S. Camillo de Lellis, Rieti, Italy.

- [8] Dipartimento Scienze dell'Invecchiamento, Neurologiche, Ortopediche e della Testa-Collo, Fondazione Policlinico Universitario A. Gemelli IRCCS - UOC Neurologia, Rome, Italy. giovanni.frisullo@policlinicogemelli.it.
- [9] Institute of Neurology, Catholic University, Rome, Italy. giovanni.frisullo@policlinicogemelli.it.
- [10] Policlinico Universitario "A. Gemelli", Largo A. Gemelli, 8, 00168, Rome, Italy. giovanni.frisullo@policlinicogemelli.it.

- PMID: **33452656**
- PMCID: PMC7810284
- DOI: 10.1007/s10072-021-05045-0

Free PMC article
Observational Study

Effect of the COVID-19 pandemic and the lockdown measures on the local stroke network

Valerio Brunetti et al. Neurol Sci. 2021 Apr.
Free PMC article
Show details
Neurol Sci

. 2021 Apr;42(4):1237-1245.
doi: 10.1007/s10072-021-05045-0. Epub 2021 Jan 15.

Authors

Valerio Brunetti [1], Aldobrando Broccolini [1] [2], Pietro Caliandro [1], Riccardo Di Iorio [1], Mauro Monforte [1], Roberta Morosetti [1], Carla Piano [1], Fabio Pilato [1], Simone Bellavia [2], Jessica Marotta [2], Irene Scala [2], Alessandro Pedicelli [2] [3], Mariano Alberto Pennisi [2] [4], Anselmo Caricato [2] [4], Cinzia Roberti [5], Maria Concetta Altavista [5], Alessandro Valenza [6], Marisa Distefano [6], Emanuela Cecconi [6], Martina Fanella [7], Sabina Roncacci [7], Miriam Tasillo [7], Paolo Calabresi [1] [2], Giovanni Frisullo [8] [9] [10], Giacomo Della Marca [1] [2]

Affiliations

- [1] Dipartimento Scienze dell'Invecchiamento, Neurologiche, Ortopediche e della Testa-Collo, Fondazione Policlinico Universitario A. Gemelli IRCCS - UOC Neurologia, Rome, Italy.
- [2] Università Cattolica del Sacro Cuore, Largo Francesco Vito, 1, 00168, Rome, Italy.
- [3] Dipartimento Diagnostica per Immagini, Radioterapia, Oncologia ed Ematologia, Fondazione Policlinico Universitario A. Gemelli IRCCS - UOC Radiologia e Neuroradiologia, Rome, Italy.
- [4] Dipartimento Scienze dell'emergenza, anestesiologiche e della rianimazione, Fondazione Policlinico Universitario A. Gemelli IRCCS - UOC Anestesia, Rianimazione, Terapia Intensiva e Tossicologia Clinica, Rome, Italy.

- [5] ASL Roma 1, San Filippo Neri Hospital - UOC Neurologia, Rome, Italy.
- [6] Ospedale Belcolle - UOC Neurologia, Viterbo, Italy.
- [7] Unità di Trattamento Neurovascolare (UTN) - Stroke Unit, Ospedale S. Camillo de Lellis, Rieti, Italy.
- [8] Dipartimento Scienze dell'Invecchiamento, Neurologiche, Ortopediche e della Testa-Collo, Fondazione Policlinico Universitario A. Gemelli IRCCS - UOC Neurologia, Rome, Italy. giovanni.frisullo@policlinicogemelli.it.
- [9] Institute of Neurology, Catholic University, Rome, Italy. giovanni.frisullo@policlinicogemelli.it.
- [10] Policlinico Universitario "A. Gemelli", Largo A. Gemelli, 8, 00168, Rome, Italy. giovanni.frisullo@policlinicogemelli.it.

- PMID: **33452656**
- PMCID: PMC7810284
- DOI: 10.1007/s10072-021-05045-0

Abstract

Introduction: The COVID-19 outbreak highly impacted the acute ischemic stroke care management. The primary end point of the study was to evaluate the impact of the COVID-19 outbreak and the following lockdown measures on our hub-and-spoke network; the secondary end point was to evaluate if the impact of the COVID-19 outbreak was different in hub-and-spoke centers.

Methods: This was a retrospective multicenter observational study conducted at the Stroke Units of Policlinico Gemelli, Ospedale San Filippo Neri, Ospedale di Belcolle, and Ospedale San Camillo de Lellis. We collected clinical reports of all consecutive patients admitted with diagnosis of acute ischemic stroke or transient ischemic attack (TIA) during the phase 1 of the lockdown period (11 March 2020-4 May 2020). As controls, we used all consecutive patients admitted for acute ischemic stroke or TIA in the same period of the previous year.

Results: A total of 156 and 142 clinical reports were collected in 2019 and 2020, respectively. During the COVID-19 outbreak, we observed a reduction of number of thrombolysis, a reduction of the length of hospitalization, and an increase of pneumonia. Regarding performance indicators, we observed an increase in onset-to-door time and in door-to-groin time. We did not observe any statistically significant interaction between year (2019 vs 2020) and facility of admission (hub vs spoke) on all variables analyzed.

Discussion: Our observational study, involving hub-and-spoke stroke network of a wide regional area, indicates that the COVID-19 outbreak impacted on the acute stroke management. This impact was equally observed in hub as well as in spoke centers.

Keywords: COVID-19; Cerebrovascular disease; Hub; Spoke; Stroke.

Conflict of interest statement

The authors declare that they have no conflict of interest.

- 20 references
- 4 figures

Supplementary info

Publication types, MeSH terms Expand

Publication types

- Multicenter Study
- Observational Study

MeSH terms

- Aged
- Aged, 80 and over
- COVID-19*
- Female
- Hospitalization / statistics & numerical data
- Humans
- Ischemic Attack, Transient / epidemiology
- Ischemic Attack, Transient / therapy
- Ischemic Stroke / epidemiology
- Ischemic Stroke / therapy
- Italy / epidemiology
- Length of Stay
- Male
- Middle Aged
- Pandemics*
- Pneumonia / epidemiology
- Quarantine*
- Retrospective Studies
- Stroke / therapy*
- Thrombolytic Therapy / statistics & numerical data

Full text links

SpringerLink FULL-TEXT ARTICLE Springer Free PMC article
Proceed to details
Cite
Share
☐ 647
Observational Study
JACC Heart Fail
. 2021 Jan;9(1):52-61.
doi: 10.1016/j.jchf.2020.10.009. Epub 2020 Oct 29.

COVID-19 in Heart Transplant Recipients: A Multicenter Analysis of the Northern Italian Outbreak

Tomaso Bottio [1], Lorenzo Bagozzi [2], Alessandro Fiocco [2], Matteo Nadali [2], Raphael Caraffa [2], Olimpia Bifulco [2], Matteo Ponzoni [2], Carlo Maria Lombardi [3], Marco Metra [3], Claudio Francesco Russo [4], Maria Frigerio [5], Gabriella Masciocco [5], Luciano Potena [6], Antonio Loforte [6], Davide Pacini [6], Giuseppe Faggian [7], Francesco Onorati [7], Sandro Sponga [8], Ugolino Livi [8], Attilio Iacovoni [9], Amedeo Terzi [9], Michele Senni [9], Mauro Rinaldi [10], Massimo Boffini [10], Matteo Marro [10], Vjola Jorgji [11], Massimiliano Carrozzini [4], Gino Gerosa [2]
Affiliations Expand

Affiliations

- [1] Cardiac Surgery Unit, Department of Cardiac, Thoracic, Vascular Sciences and Public Health, University of Padua, Padua, Italy. Electronic address: tbottio@gmail.com.
- [2] Cardiac Surgery Unit, Department of Cardiac, Thoracic, Vascular Sciences and Public Health, University of Padua, Padua, Italy.
- [3] Cardiology, Department of Medical and Surgical Specialties, Radiological Sciences and Public Health University, Cardiothoracic Department, Spedali Civili of Brescia, Brescia, Italy.
- [4] Cardiac Surgery Unit, Cardiac Thoracic and Vascular Department, Niguarda Hospital, Milan, Italy.
- [5] Transplant Cardiology Unit, Cardiac Thoracic and Vascular Department, Niguarda Hospital, Milan, Italy.
- [6] Cardiac, Thoracic, Vascular and Transplant Department, Policlinico S. Orsola-Malpighi, University of Bologna, Bologna, Italy.
- [7] Division of Cardiac Surgery, Department of Surgery, University of Verona, Verona, Italy.
- [8] Cardiac Surgery Department, University of Udine, Udine, Italy.
- [9] Cardiovascular Department, ASST Papa Giovanni XXIII, Bergamo, Italy.
- [10] Division of Cardiac Surgery, Department of Surgical Sciences, Città della Salute e della Scienza, University of Turin, Turin, Italy.
- [11] Hacohen Lab, Massachusetts General Hospital, Boston, Massachusetts, USA.

- PMID: **33309578**
- PMCID: PMC7604081
- DOI: 10.1016/j.jchf.2020.10.009

Free PMC article
Observational Study

COVID-19 in Heart Transplant Recipients: A Multicenter Analysis of the Northern Italian Outbreak

Tomaso Bottio et al. JACC Heart Fail. 2021 Jan.
Free PMC article
Show details
JACC Heart Fail
. 2021 Jan;9(1):52-61.
doi: 10.1016/j.jchf.2020.10.009. Epub 2020 Oct 29.

Authors

Tomaso Bottio [1], Lorenzo Bagozzi [2], Alessandro Fiocco [2], Matteo Nadali [2], Raphael Caraffa [2], Olimpia Bifulco [2], Matteo Ponzoni [2], Carlo Maria Lombardi [3], Marco Metra [3], Claudio Francesco Russo [4], Maria Frigerio [5], Gabriella Masciocco [5], Luciano Potena [6], Antonio Loforte [6], Davide Pacini [6], Giuseppe Faggian [7], Francesco Onorati [7], Sandro Sponga [8], Ugolino Livi [8], Attilio Iacovoni [9], Amedeo Terzi [9], Michele Senni [9], Mauro Rinaldi [10], Massimo Boffini [10], Matteo Marro [10], Vjola Jorgji [11], Massimiliano Carrozzini [4], Gino Gerosa [2]

Affiliations

- [1] Cardiac Surgery Unit, Department of Cardiac, Thoracic, Vascular Sciences and Public Health, University of Padua, Padua, Italy. Electronic address: tbottio@gmail.com.
- [2] Cardiac Surgery Unit, Department of Cardiac, Thoracic, Vascular Sciences and Public Health, University of Padua, Padua, Italy.
- [3] Cardiology, Department of Medical and Surgical Specialties, Radiological Sciences and Public Health University, Cardiothoracic Department, Spedali Civili of Brescia, Brescia, Italy.
- [4] Cardiac Surgery Unit, Cardiac Thoracic and Vascular Department, Niguarda Hospital, Milan, Italy.
- [5] Transplant Cardiology Unit, Cardiac Thoracic and Vascular Department, Niguarda Hospital, Milan, Italy.
- [6] Cardiac, Thoracic, Vascular and Transplant Department, Policlinico S. Orsola-Malpighi, University of Bologna, Bologna, Italy.
- [7] Division of Cardiac Surgery, Department of Surgery, University of Verona, Verona, Italy.
- [8] Cardiac Surgery Department, University of Udine, Udine, Italy.
- [9] Cardiovascular Department, ASST Papa Giovanni XXIII, Bergamo, Italy.
- [10] Division of Cardiac Surgery, Department of Surgical Sciences, Città della Salute e della Scienza, University of Turin, Turin, Italy.
- [11] Hacohen Lab, Massachusetts General Hospital, Boston, Massachusetts, USA.

- PMID: **33309578**
- PMCID: PMC7604081
- DOI: 10.1016/j.jchf.2020.10.009

Abstract

Objectives: The aim of this study was to assess the clinical course and outcomes of all heart transplant recipients affected by coronavirus disease-2019 (COVID-19) who were followed at the leading heart transplant centers of Northern Italy.

Background: The worldwide severe acute respiratory syndrome-coronavirus-2 (SARS-CoV-2) pandemic has created unprecedented challenges for public health, demanding exceptional efforts for the successful management and treatment of affected patients. Heart transplant patients represent a unique cohort of chronically immunosuppressed subjects in which SARS-CoV-2 may stimulate an unpredictable clinical course of infection.

Methods: Since February 2020, we enrolled all 47 cases (79% male) in a first cohort of patients, with a mean age of 61.8 ± 14.5 years, who tested positive for SARS-CoV-2, out of 2,676 heart transplant recipients alive before the onset of the COVID-19 pandemic at 7 heart transplant centers in Northern Italy.

Results: To date, 38 patients required hospitalization while 9 remained self-home quarantined and 14 died. Compared to the general population, prevalence (18 vs. 7 cases per 1,000) and related case fatality rate (29.7% vs. 15.4%) in heart transplant recipients were doubled. Univariable analysis showed older age ($p = 0.002$), diabetes mellitus ($p = 0.040$), extracardiac arteriopathy ($p = 0.040$), previous PCI ($p = 0.040$), CAV score ($p = 0.039$), lower GFR ($p = 0.004$), and higher NYHA functional classes ($p = 0.023$) were all significantly associated with in-hospital mortality. During the follow-up two patients died and a third patient has prolonged viral-shedding alternating positive and negative swabs. Since July 1st, 2020, we had 6 new patients who tested positive for SARS-CoV-2, 5 patients asymptomatic were self-quarantined, while 1 is still hospitalized for pneumonia. A standard therapy was maintained for all, except for the hospitalized patient.

Conclusions: The prevalence and mortality of SARS-CoV-2 should spur clinicians to immediately refer heart transplant recipients suspected as having SARS-CoV2 infection to centers specializing in the care of this vulnerable population.

Keywords: COVID-19 and heart transplant recipients; SARS-CoV-2; heart transplantation; immunosuppressive therapy.

Copyright © 2021 American College of Cardiology Foundation. Published by Elsevier Inc. All rights reserved.

Conflict of interest statement

Author Disclosures The authors have reported that they have no relationships relevant to the contents of this paper to disclose.

Comment in

- Courage in the Face of Catastrophe: COVID-19 in Heart Transplant Recipients Northern Italian Registry.
  Costanzo MR. Costanzo MR. JACC Heart Fail. 2021 Jan;9(1):62-64. doi: 10.1016/j.jchf.2020.11.004. JACC Heart Fail. 2021. PMID: 33384063 No abstract available.

- 14 references
- 2 figures

Supplementary info

Publication types, MeSH terms Expand

Publication types

- Multicenter Study
- Observational Study

MeSH terms

- Aged
- COVID-19 / epidemiology*
- Comorbidity
- Female
- Follow-Up Studies
- Heart Failure / epidemiology
- Heart Failure / surgery*
- Heart Transplantation*
- Hospital Mortality / trends
- Humans
- Italy / epidemiology
- Male
- Middle Aged
- Pandemics*
- Retrospective Studies
- SARS-CoV-2
- Transplant Recipients*

Full text links

ELSEVIER OPEN ACCESS Elsevier Science Free PMC article

Proceed to details

Cite

Share

☐ 648

Observational Study

Open Heart

. 2021 Oct;8(2):e001785.
doi: 10.1136/openhrt-2021-001785.

Combined anticoagulant and antiplatelet therapy is associated with an improved outcome in hospitalised patients with COVID-19: a propensity matched cohort study

Kamal Matli [1, 2], Nibal Chamoun [3], Aya Fares [#, 2], Victor Zibara [#, 4], Soad Al-Osta [#, 4], Rabih Nasrallah [#, 2], Pascale Salameh [5, 6], Jacques Mokhbat [2, 4], Georges Ghanem [7, 4]
Affiliations Expand

Affiliations

- [1] Cardiology, Lebanese American University Medical Center-Rizk Hospital, Beirut, Lebanon.
- [2] Internal Medicine, Lebanese American University Medical Center-Rizk Hospital, Beirut, Lebanon.
- [3] Department of Pharmacy Practice, Lebanese American University School of Pharmacy, Byblos, Lebanon.
- [4] Lebanese American University School of Medicine, Byblos, Lebanon.
- [5] Lebanese University Faculty of Pharmacy, Hadath, Lebanon.
- [6] University of Nicosia Medical School, Nicosia, Cyprus.
- [7] Cardiology, Lebanese American University Medical Center-Rizk Hospital, Beirut, Lebanon gghanemresearch@gmail.com.

[#] Contributed equally.

- PMID: **34611018**
- PMCID: PMC8493601
- DOI: 10.1136/openhrt-2021-001785

Free PMC article
Observational Study

Combined anticoagulant and antiplatelet therapy is associated with an improved outcome in hospitalised patients with COVID-19: a propensity matched cohort study

Kamal Matli et al. Open Heart. 2021 Oct.
Free PMC article
Show details
Open Heart
. 2021 Oct;8(2):e001785.

doi: 10.1136/openhrt-2021-001785.

Authors

Kamal Matli [1, 2], Nibal Chamoun [3], Aya Fares [#, 2], Victor Zibara [#, 4], Soad Al-Osta [#, 4], Rabih Nasrallah [#, 2], Pascale Salameh [5, 6], Jacques Mokhbat [2, 4], Georges Ghanem [7, 4]

Affiliations

- [1] Cardiology, Lebanese American University Medical Center-Rizk Hospital, Beirut, Lebanon.
- [2] Internal Medicine, Lebanese American University Medical Center-Rizk Hospital, Beirut, Lebanon.
- [3] Department of Pharmacy Practice, Lebanese American University School of Pharmacy, Byblos, Lebanon.
- [4] Lebanese American University School of Medicine, Byblos, Lebanon.
- [5] Lebanese University Faculty of Pharmacy, Hadath, Lebanon.
- [6] University of Nicosia Medical School, Nicosia, Cyprus.
- [7] Cardiology, Lebanese American University Medical Center-Rizk Hospital, Beirut, Lebanon gghanemresearch@gmail.com.

[#] Contributed equally.

- PMID: **34611018**
- PMCID: PMC8493601
- DOI: 10.1136/openhrt-2021-001785

Abstract

Background: COVID-19 is a respiratory disease that results in a prothrombotic state manifesting as thrombotic, microthrombotic and thromboembolic events. As a result, several antithrombotic modalities have been implicated in the treatment of this disease. This study aimed to identify if therapeutic anticoagulation (TAC) or concurrent use of antiplatelet and anticoagulants was associated with an improved outcome in this patient population.

Methods: A retrospective observational cohort study of adult patients admitted to a single university hospital for COVID-19 infection was performed. The primary outcome was a composite of in-hospital mortality, intensive care unit (ICU) admission or the need for mechanical ventilation. The secondary outcomes were each of the components of the primary outcome, in-hospital mortality, ICU admission, or the need for mechanical ventilation.

Results: 242 patients were included in the study and divided into four subgroups: Therapeutic anticoagulation (TAC), prophylactic anticoagulation+antiplatelet (PACAP), TAC+antiplatelet (TACAP) and prophylactic anticoagulation (PAC) which was the reference for comparison. Multivariable Cox regression analysis and propensity matching were done and showed when compared with PAC, TACAP and TAC were associated with less in-hospital all-cause mortality with an adjusted HR (aHR) of 0.113 (95% CI 0.028 to 0.449) and 0.126 (95% CI 0.028 to 0.528), respectively. The number needed to treat in both subgroups was 11. Furthermore, PACAP was associated with a reduced risk of invasive mechanical ventilation with an aHR of 0.07 (95% CI 0.014 to 0.351). However, the was no statistically significant difference in the occurrence of major

or minor bleeds, ICU admission or the composite outcome of in-hospital mortality, ICU admission or the need for mechanical ventilation.

Conclusion: The use of combined anticoagulant and antiplatelet agents or TAC alone in hospitalised patients with COVID-19 was associated with a better outcome in comparison to PAC alone without an increase in the risk of major and minor bleeds. Sufficiently powered randomised controlled trials are needed to further evaluate the safety and efficacy of combining antiplatelet and anticoagulants agents or using TAC in the management of patients with COVID-19 infection.

Keywords: COVID-19; clinical; microvascular angina; pharmacology.

© Author(s) (or their employer(s)) 2021. Re-use permitted under CC BY-NC. No commercial re-use. See rights and permissions. Published by BMJ.

Conflict of interest statement

Competing interests: None declared.

- 42 references
- 2 figures

Supplementary info

Publication types, MeSH terms, Substances Expand

Publication types

- Observational Study

MeSH terms

- Adult
- Aged
- Aged, 80 and over
- Anticoagulants / therapeutic use*
- Blood Coagulation / drug effects
- COVID-19 / blood
- COVID-19 / complications
- COVID-19 / mortality
- COVID-19 / therapy*
- Female
- Hospital Mortality
- Humans
- Inpatients
- Intensive Care Units
- Male
- Middle Aged

- Platelet Aggregation Inhibitors / therapeutic use*
- Retrospective Studies
- SARS-CoV-2
- Survival Analysis
- Thromboembolism / drug therapy
- Thromboembolism / physiopathology
- Thrombosis / drug therapy
- Thrombosis / physiopathology
- Treatment Outcome

Substances

- Anticoagulants
- Platelet Aggregation Inhibitors

Full text links

BMJ Open Access Full Text HighWire Free PMC article
Proceed to details
Cite
Share
☐ 649
Observational Study
AIDS Res Hum Retroviruses
. 2021 Apr;37(4):283-291.
doi: 10.1089/AID.2020.0305.

Darunavir/Cobicistat Is Associated with Negative Outcomes in HIV-Negative Patients with Severe COVID-19 Pneumonia

Jovana Milic [1, 2], Alessio Novella [3], Marianna Meschiari [4], Marianna Menozzi [4], Antonella Santoro [4], Andrea Bedini [4], Gianluca Cuomo [4], Erica Franceschini [4], Margherita Digaetano [4], Federica Carli [4], Giacomo Ciusa [4], Sara Volpi [4], Erica Bacca [4], Giacomo Franceschi [4], Dina Yaacoub [4], Carlotta Rogati [4], Marco Tutone [4], Giulia Burastero [4], Matteo Faltoni [4], Vittorio Iadisernia [4], Giovanni Dolci [4], Andrea Cossarizza [5], Cristina Mussini [1, 4], Luca Pasina [3], Giovanni Guaraldi [1, 4]
Affiliations Expand

Affiliations

- [1] Department of Surgical, Medical, Dental, and Morphological Sciences, University of Modena and Reggio Emilia, Modena, Italy.
- [2] Clinical and Experimental Medicine PhD Program, University of Modena and Reggio Emilia, Modena, Italy.
- [3] Pharmacotherapy and Appropriateness of Drug Prescription Unit, Istituto di Ricerche Farmacologiche Mario Negri IRCCS, Milan, Italy.
- [4] Infectious Diseases Unit, Azienda Ospedaliero-Universitaria di Modena, Modena, Italy.
- [5] Department of Medical and Surgical Sciences for Children and Adults, University of Modena and Reggio Emilia, Modena, Italy.

- PMID: **33619997**
- DOI: 10.1089/AID.2020.0305

Observational Study

Darunavir/Cobicistat Is Associated with Negative Outcomes in HIV-Negative Patients with Severe COVID-19 Pneumonia

Jovana Milic et al. AIDS Res Hum Retroviruses. 2021 Apr.
Show details
AIDS Res Hum Retroviruses
. 2021 Apr;37(4):283-291.
doi: 10.1089/AID.2020.0305.

Authors

Jovana Milic [1 2], Alessio Novella [3], Marianna Meschiari [4], Marianna Menozzi [4], Antonella Santoro [4], Andrea Bedini [4], Gianluca Cuomo [4], Erica Franceschini [4], Margherita Digaetano [4], Federica Carli [4], Giacomo Ciusa [4], Sara Volpi [4], Erica Bacca [4], Giacomo Franceschi [4], Dina Yaacoub [4], Carlotta Rogati [4], Marco Tutone [4], Giulia Burastero [4], Matteo Faltoni [4], Vittorio Iadisernia [4], Giovanni Dolci [4], Andrea Cossarizza [5], Cristina Mussini [1 4], Luca Pasina [3], Giovanni Guaraldi [1 4]

Affiliations

- [1] Department of Surgical, Medical, Dental, and Morphological Sciences, University of Modena and Reggio Emilia, Modena, Italy.
- [2] Clinical and Experimental Medicine PhD Program, University of Modena and Reggio Emilia, Modena, Italy.
- [3] Pharmacotherapy and Appropriateness of Drug Prescription Unit, Istituto di Ricerche Farmacologiche Mario Negri IRCCS, Milan, Italy.
- [4] Infectious Diseases Unit, Azienda Ospedaliero-Universitaria di Modena, Modena, Italy.
- [5] Department of Medical and Surgical Sciences for Children and Adults, University of Modena and Reggio Emilia, Modena, Italy.

- PMID: **33619997**
- DOI: 10.1089/AID.2020.0305

Abstract

The aim of this study was to evaluate both positive outcomes, including reduction of respiratory support aid and duration of hospital stay, and negative ones, including mortality and a composite of invasive mechanical ventilation or death, in patients with coronavirus disease 2019 (COVID-19) pneumonia treated with or without oral darunavir/cobicistat (DRV/c, 800/150 mg/day) used in different treatment durations. The secondary objective was to evaluate the percentage of patients treated with DRV/c who were exposed to potentially severe drug-drug interactions (DDIs) and died during hospitalization. This observational retrospective study was conducted in consecutive patients with COVID-19 pneumonia admitted to a tertiary care hospital in Modena, Italy. Kaplan-Meier survival curves and Cox proportional hazards regression were used to compare patients receiving standard of care with or without DRV/c. Adjustment for key confounders was applied. Two hundred seventy-three patients (115 on DRV/c) were included, 75.8% males, mean age was 64.6 (±13.2) years. Clinical improvement was similar between the groups, depicted by respiratory aid switch ($p > .05$). The same was observed for duration of hospital stay [13.2 (±8.9) for DRV/c vs. 13.4 (±7.2) days for no-DRV/c, $p = .9$]. Patients on DRV/c had higher rates of mortality (25.2% vs. 10.1%, $p < .0001$. The rate of composite outcome of mechanical ventilation and death was higher in the DRV/c group (37.4% vs. 25.3%, $p = .03$). Multiple serious DDI associated with DRV/c were observed in the 19 patients who died. DRV/c should not be recommended as a treatment option for COVID-19 pneumonia outside clinical trials.

Keywords: COVID-19; SARS-CoV-2; darunavir/cobicistat; negative outcomes.

Supplementary info

Publication types, MeSH terms, Substances Expand

Publication types

- Observational Study

MeSH terms

- Adult
- Anti-HIV Agents / adverse effects
- Anti-HIV Agents / therapeutic use*
- COVID-19 / drug therapy*
- COVID-19 / mortality
- COVID-19 / virology
- Cobicistat / adverse effects
- Cobicistat / therapeutic use*
- Darunavir / adverse effects
- Darunavir / therapeutic use*
- Drug Combinations

- Female
- Humans
- Male
- Middle Aged
- Retrospective Studies
- SARS-CoV-2 / isolation & purification

Substances

- Anti-HIV Agents
- Drug Combinations
- cobicistat mixture with darunavir
- Cobicistat
- Darunavir

Full text links

Mary Ann Liebert Atypon
Proceed to details
Cite
Share
☐ 650
Observational Study
Medicine (Baltimore)
. 2021 Mar 12;100(10):e24893.
doi: 10.1097/MD.0000000000024893.

Time on previous renal replacement therapy is associated with worse outcomes of COVID-19 in a regional cohort of kidney transplant and dialysis patients

Luigi Villa [1], Thilo Krüger [2], Claudia Seikrit [3], Anja S Mühlfeld [3], Uta Kunter [3], Cornelius Werner [4], Michael Kleines [5], Maximilian Schulze-Hagen [6], Michael Dreher [7], Alexander Kersten [8], Nikolaus Marx [8], Jürgen Floege [3], Thomas Rauen [3], Gerald S Braun [3]
Affiliations Expand

Affiliations

- [1] Diaverum Dialysis Center, Erkelenz and Heinsberg.
- [2] DaVita Dialysis Center, Geilenkirchen.

- [3] Department of Nephrology and Rheumatology.
- [4] Department of Neurology.
- [5] Division of Virology, Center of laboratory diagnostics.
- [6] Department of Radiology.
- [7] Department of Pneumology and Intensive Care Medicine.
- [8] Department of Cardiology and Intensive Care Medicine, RWTH University Hospital Aachen, Germany.

- PMID: **33725847**
- PMCID: PMC7969209
- DOI: 10.1097/MD.0000000000024893

Free PMC article
Observational Study

Time on previous renal replacement therapy is associated with worse outcomes of COVID-19 in a regional cohort of kidney transplant and dialysis patients

Luigi Villa et al. Medicine (Baltimore). 2021.
Free PMC article
Show details
Medicine (Baltimore)
. 2021 Mar 12;100(10):e24893.
doi: 10.1097/MD.0000000000024893.

Authors

Luigi Villa [1], Thilo Krüger [2], Claudia Seikrit [3], Anja S Mühlfeld [3], Uta Kunter [3], Cornelius Werner [4], Michael Kleines [5], Maximilian Schulze-Hagen [6], Michael Dreher [7], Alexander Kersten [8], Nikolaus Marx [8], Jürgen Floege [3], Thomas Rauen [3], Gerald S Braun [3]

Affiliations

- [1] Diaverum Dialysis Center, Erkelenz and Heinsberg.
- [2] DaVita Dialysis Center, Geilenkirchen.
- [3] Department of Nephrology and Rheumatology.
- [4] Department of Neurology.
- [5] Division of Virology, Center of laboratory diagnostics.
- [6] Department of Radiology.
- [7] Department of Pneumology and Intensive Care Medicine.
- [8] Department of Cardiology and Intensive Care Medicine, RWTH University Hospital Aachen, Germany.

- PMID: **33725847**
- PMCID: PMC7969209
- DOI: 10.1097/MD.0000000000024893

Abstract

Chronic renal replacement therapy by either a kidney transplant (KTX) or hemodialysis (HD) predisposes patients to an increased risk for adverse outcomes of COVID-19. However, details on this interaction remain incomplete. To provide further characterization, we undertook a retrospective observational cohort analysis of the majority of the hemodialysis and renal transplant population affected by the first regional outbreak of severe acute respiratory distress syndrome coronavirus 2 (SARS-CoV-2) in Germany. In a region of 250,000 inhabitants we identified a total of 21 cases with SARS-CoV-2 among 100 KTX and 260 HD patients, that is, 7 KTX with COVID-19, 14 HD with COVID-19, and 3 HD with asymptomatic carrier status. As a first observation, KTX recipients exhibited trends for a higher mortality (43 vs 18%) and a higher proportion of acute respiratory distress syndrome (ARDS) (57 vs 27%) when compared to their HD counterparts. As a novel finding, development of ARDS was significantly associated with the time spent on previous renal replacement therapy (RRT), defined as the composite of dialysis time and time on the transplant (non-ARDS 4.3 vs ARDS 10.6 years, P = .016). Multivariate logistic regression analysis showed an OR of 1.7 per year of RRT. The association remained robust when analysis was confined to KTX patients (5.1 vs 13.2 years, P = .002) or when correlating the time spent on a renal transplant alone (P = .038). Similarly, longer RRT correlated with death vs survival (P = .0002). In conclusion our data suggest renal replacement vintage as a novel risk factor for COVID-19-associated ARDS and death. The findings should be validated by larger cohorts.

Copyright © 2021 the Author(s). Published by Wolters Kluwer Health, Inc.

Conflict of interest statement

The authors have no funding and conflicts of interests to disclose.

- 44 references
- 3 figures

Supplementary info

Publication types, MeSH terms Expand

Publication types

- Observational Study

MeSH terms

- Aged
- Aged, 80 and over
- COVID-19 / epidemiology*
- COVID-19 / mortality

- Female
- Germany / epidemiology
- Humans
- Kidney Failure, Chronic / epidemiology*
- Kidney Failure, Chronic / therapy
- Kidney Transplantation / mortality*
- Logistic Models
- Male
- Middle Aged
- Renal Dialysis / statistics & numerical data*
- Retrospective Studies
- Risk Factors
- SARS-CoV-2

Full text links

Wolters Kluwer Wolters Kluwer Free PMC article
Proceed to details
Cite
Share
651
Observational Study
Actas Urol Esp (Engl Ed)
. 2020 Dec;44(10):659-664.
doi: 10.1016/j.acuro.2020.09.003. Epub 2020 Sep 17.

Impact of the COVID-19 pandemic on the surgical activity of Pediatric Urology: analysis of postoperative complications according to the Clavien-Dindo classification

[Article in English, Spanish]
L Merino-Mateo [1], C Tordable Ojeda [2], D Cabezalí Barbancho [2], A Gómez Fraile [2]
Affiliations Expand

Affiliations

- [1] Servicio de Cirugía Pediátrica. Hospital Universitario 12 de Octubre, Madrid, España. Electronic address: lara.merino@salud.madrid.org.
- [2] Servicio de Cirugía Pediátrica. Hospital Universitario 12 de Octubre, Madrid, España.

- PMID: **33069488**

- PMCID: PMC7498256
- DOI: 10.1016/j.acuro.2020.09.003

Free PMC article
Observational Study

Impact of the COVID-19 pandemic on the surgical activity of Pediatric Urology: analysis of postoperative complications according to the Clavien-Dindo classification

[Article in English, Spanish]
L Merino-Mateo et al. Actas Urol Esp (Engl Ed). 2020 Dec.
Free PMC article
Show details
Actas Urol Esp (Engl Ed)
. 2020 Dec;44(10):659-664.
doi: 10.1016/j.acuro.2020.09.003. Epub 2020 Sep 17.

Authors

L Merino-Mateo [1], C Tordable Ojeda [2], D Cabezalí Barbancho [2], A Gómez Fraile [2]

Affiliations

- [1] Servicio de Cirugía Pediátrica. Hospital Universitario 12 de Octubre, Madrid, España. Electronic address: lara.merino@salud.madrid.org.
- [2] Servicio de Cirugía Pediátrica. Hospital Universitario 12 de Octubre, Madrid, España.

- PMID: **33069488**
- PMCID: PMC7498256
- DOI: 10.1016/j.acuro.2020.09.003

Abstract

Introduction and objective: The coronavirus disease 2019 (COVID-19) has caused a pandemic of global impact that forced social-political measures to be taken, such as the declaration of the state of alarm in Spain. At the same time, the reorganization of the pediatric medical-surgical activities and infrastructures was carried out, with the consequent suspension of the non-urgent surgical activity of Pediatric Urology. We analyzed the impact of the COVID-19 pandemic on surgical activity in a Pediatric Urology division, as well as surgical complications according to the Clavien-Dindo classification.

Materials and methods: A systematic review of epidemiological, clinical and surgical data was carried out, including complications and readmissions of all patients operated on in the division of Pediatric Urology within the duration of the state of alarm. Five time periods have been created according to the de-escalation phases.

Results: Forty-nine surgical procedures were carried out on 45 patients (8 prior to the implementation of the de-escalation phases). High priority pathologies were the most frequent in the first phases, being the ureteropelvic junction (UPJ) obstruction the most prevalent. Four complications were recorded (8.8%), none of them were respiratory.

Conclusions: The EAU recommendations for the resumption of surgical activity have allowed a correct, safe and gradual transition to the routine surgical activity in Pediatric Urology. The Clavien-Dindo classification is useful and valid for application in this division. No respiratory complications have been reported that could be attributable to the pandemic situation.

Keywords: Actividad quirúrgica; COVID-19; Complicaciones quirúrgicas; Pediatric urology; Surgical activity; Surgical complications; Urología pediátrica.

Copyright © 2020 AEU. Publicado por Elsevier España, S.L.U. All rights reserved.

- 10 references
- 2 figures

Supplementary info

Publication types, MeSH terms Expand

Publication types

- Observational Study

MeSH terms

- COVID-19 / epidemiology*
- Child
- Child, Preschool
- Female
- Humans
- Infant
- Male
- Pandemics*
- Patient Readmission / statistics & numerical data
- Postoperative Complications / classification
- Postoperative Complications / epidemiology*
- Prostatic Neoplasms / surgery
- Prune Belly Syndrome / surgery
- Retrospective Studies
- Rhabdomyosarcoma, Embryonal / surgery
- SARS-CoV-2*
- Urinary Bladder Neoplasms / surgery
- Urologic Surgical Procedures / adverse effects*

- Urologic Surgical Procedures / statistics & numerical data
- Urology Department, Hospital

Full text links

ELSEVIER FULL-TEXT ARTICLE Elsevier Science Free PMC article
Proceed to details
Cite
Share
☐ 652
Observational Study
Clin Breast Cancer
. 2021 Feb;21(1):e128-e135.
doi: 10.1016/j.clbc.2020.10.006. Epub 2020 Oct 24.

Breast Cancer Management During COVID-19 Pandemic in Madrid: Surgical Strategy

Juana María Brenes Sánchez [1], Amanda López Picado [2], María Eugenia Olivares Crespo [3], José Ángel García Sáenz [4], Rosa María De La Plata Merlo [3], María Herrera De La Muela [3]
Affiliations Expand

Affiliations

- [1] Breast Cancer Unit, Obstetrics and Gynecology, Women's Health Institute, Hospital Clínico San Carlos, Madrid, Spain. Electronic address: jbrenes@ucm.es.
- [2] Clinical Trials and Research Unit, IdISSC, Hospital Clínico San Carlos, Madrid, Spain.
- [3] Breast Cancer Unit, Obstetrics and Gynecology, Women's Health Institute, Hospital Clínico San Carlos, Madrid, Spain.
- [4] Breast Cancer Unit, Oncology Department, Hospital Clínico San Carlos, Madrid, Spain.

- PMID: **33223393**
- PMCID: PMC7585494
- DOI: 10.1016/j.clbc.2020.10.006

Free PMC article
Observational Study

Breast Cancer Management During COVID-19 Pandemic in Madrid: Surgical Strategy

Juana María Brenes Sánchez et al. Clin Breast Cancer. 2021 Feb.
Free PMC article
Show details

Clin Breast Cancer

. 2021 Feb;21(1):e128-e135.
doi: 10.1016/j.clbc.2020.10.006. Epub 2020 Oct 24.

Authors

Juana María Brenes Sánchez [1], Amanda López Picado [2], María Eugenia Olivares Crespo [3], José Ángel García Sáenz [4], Rosa María De La Plata Merlo [3], María Herrera De La Muela [3]

Affiliations

- [1] Breast Cancer Unit, Obstetrics and Gynecology, Women's Health Institute, Hospital Clínico San Carlos, Madrid, Spain. Electronic address: jbrenes@ucm.es.
- [2] Clinical Trials and Research Unit, IdISSC, Hospital Clínico San Carlos, Madrid, Spain.
- [3] Breast Cancer Unit, Obstetrics and Gynecology, Women's Health Institute, Hospital Clínico San Carlos, Madrid, Spain.
- [4] Breast Cancer Unit, Oncology Department, Hospital Clínico San Carlos, Madrid, Spain.

- PMID: **33223393**
- PMCID: PMC7585494
- DOI: 10.1016/j.clbc.2020.10.006

Abstract

Background: From the first case of SARS-CoV-2 infection in Wuhan (China), the infection spread all around the world causing a pandemic of coronavirus disease-2019 (COVID-19). Spain has been one of the most severely affected countries, and Madrid has reported a high number of cases and deaths. We discuss our strategies for optimal breast cancer management during COVID-19 pandemic.

Patients and methods: This was a retrospective observational study at Clínico San Carlos Hospital to analyze the management of patients with breast cancer during the pandemic outbreak and the surgical strategy after the pandemic outbreak. We created a practical and dynamic tool based on a "traffic light" system for prioritizing surgical time. Every patient was contacted by telephone with a preoperative COVID-19 protocol. After surgical procedures, patient satisfaction was assessed using the European Organisation for Research and Treatment of Cancer in-patient satisfaction with cancer care questionnaire (EORTC IN-PATSAT32).

Results: Patients with breast cancer actively treated with surgical procedures were put on a waiting list and received systemic therapy. Telemedicine was used to evaluate any side effects and to avoid unnecessary hospital visits. Surgery was only considered after the pandemic outbreak, and then, only those procedures designed to minimize surgical complications and, therefore, reduce hospital stay. We also measured patients' satisfaction with medical and nursing scales that resulted in a "very good" evaluation tending to "excellent".

Conclusion: It is necessary to adapt management of oncology treatment and surgical strategy to optimize resources during the COVID-19 pandemic. Patients' perception of care quality and the degree of patients' satisfaction with health services has potential relevance in the absence of outcome data.

Keywords: Breast cancer; Quality care; SARS-CoV-2; Surgical strategy; Systemic therapy.

Copyright © 2020 Elsevier Inc. All rights reserved.

- 22 references
- 4 figures

Supplementary info

Publication types, MeSH terms Expand

Publication types

- Observational Study

MeSH terms

- Breast Neoplasms / pathology
- Breast Neoplasms / surgery
- Breast Neoplasms / therapy*
- COVID-19 / epidemiology*
- COVID-19 / prevention & control
- Disease Management
- Female
- Humans
- Medical Oncology / organization & administration
- Medical Oncology / standards
- Medical Oncology / statistics & numerical data
- Patient Satisfaction / statistics & numerical data
- Retrospective Studies
- SARS-CoV-2
- Spain / epidemiology
- Telemedicine
- Waiting Lists

Full text links

ELSEVIER FULL-TEXT ARTICLE Elsevier Science Free PMC article
Proceed to details
Cite
Share
☐ 653
Observational Study
Br J Cancer
. 2021 Feb;124(4):754-759.
doi: 10.1038/s41416-020-01181-0. Epub 2020 Dec 10.

Severity of COVID-19 in children with cancer: Report from the United Kingdom Paediatric Coronavirus Cancer Monitoring Project

Gerard C Millen [1, 2], Roland Arnold [3], Jean-Baptiste Cazier [4], Helen Curley [3], Richard G Feltbower [5], Ashley Gamble [6], Adam W Glaser [5, 7], Richard G Grundy [6, 8], Lennard Y W Lee [9], Martin G McCabe [10, 11], Robert S Phillips [12, 13], Charles A Stiller [11], Csilla Várnai [3, 14], Pamela R Kearns [15, 16]

Affiliations Expand

Affiliations

- [1] Cancer Research UK Clinical Trials Unit, Institute of Cancer and Genomic Sciences, College of Medical and Dental Sciences, University of Birmingham, Birmingham, B15 2TT, UK. g.c.millen@bham.ac.uk.
- [2] Department of Paediatric Oncology, Birmingham Children's Hospital, Steelhouse Lane, Birmingham, B4 6NH, UK. g.c.millen@bham.ac.uk.
- [3] Institute of Cancer and Genomic Sciences, College of Medical and Dental Sciences, University of Birmingham, Birmingham, B15 2TT, UK.
- [4] Centre for Computational Biology, University of Birmingham, Edgbaston, Birmingham, UK.
- [5] Leeds Institute for Data Analytics (LIDA), School of Medicine, University of Leeds, Leeds, LS2 9JT, UK.
- [6] Children's Cancer and Leukaemia Group (CCLG), Leicester, LE1 7GB, UK.
- [7] Professor of Paediatric Oncology and Late Effects Medicine, Leeds Institute of Medical Research, University of Leeds, Leeds, LS2 9JT, UK.
- [8] Children's Brain Tumour Research Centre, School of Medicine, The University of Nottingham, Nottingham, NG7 2UH, UK.
- [9] Institute of Cancer and Genomic Sciences, University of Birmingham, Edgbaston, Birmingham, UK.
- [10] Division of Cancer Sciences, University of Manchester, Manchester Academic Health Science Centre, Manchester, M13 9PL, UK.
- [11] National Cancer Registration and Analysis Service, Public Health England, London, SE1 8UG, UK.
- [12] Centre for Reviews and Dissemination, University of York, York, UK.
- [13] Department of Paediatric Oncology, Leeds Children's Hospital, Leeds, UK.
- [14] Centre for Computational Biology, University of Birmingham, Birmingham, B15 2TT, UK.
- [15] Department of Paediatric Oncology, Birmingham Children's Hospital, Steelhouse Lane, Birmingham, B4 6NH, UK.
- [16] Cancer Research UK Clinical Trials Unit, NIHR Birmingham Biomedical Research Centre, Institute of Cancer and Genomic Sciences, College of Medical and Dental Sciences, University of Birmingham, Birmingham, B15 2TT, UK.

- PMID: **33299130**
- PMCID: PMC7884399
- DOI: 10.1038/s41416-020-01181-0

Free PMC article
Observational Study

Severity of COVID-19 in children with cancer: Report from the United Kingdom Paediatric Coronavirus Cancer Monitoring Project

Gerard C Millen et al. Br J Cancer. 2021 Feb.
Free PMC article
Show details
Br J Cancer
. 2021 Feb;124(4):754-759.
doi: 10.1038/s41416-020-01181-0. Epub 2020 Dec 10.

Authors

Gerard C Millen [1] [2], Roland Arnold [3], Jean-Baptiste Cazier [4], Helen Curley [3], Richard G Feltbower [5], Ashley Gamble [6], Adam W Glaser [5] [7], Richard G Grundy [6] [8], Lennard Y W Lee [9], Martin G McCabe [10] [11], Robert S Phillips [12] [13], Charles A Stiller [11], Csilla Várnai [3] [14], Pamela R Kearns [15] [16]

Affiliations

- [1] Cancer Research UK Clinical Trials Unit, Institute of Cancer and Genomic Sciences, College of Medical and Dental Sciences, University of Birmingham, Birmingham, B15 2TT, UK. g.c.millen@bham.ac.uk.
- [2] Department of Paediatric Oncology, Birmingham Children's Hospital, Steelhouse Lane, Birmingham, B4 6NH, UK. g.c.millen@bham.ac.uk.
- [3] Institute of Cancer and Genomic Sciences, College of Medical and Dental Sciences, University of Birmingham, Birmingham, B15 2TT, UK.
- [4] Centre for Computational Biology, University of Birmingham, Edgbaston, Birmingham, UK.
- [5] Leeds Institute for Data Analytics (LIDA), School of Medicine, University of Leeds, Leeds, LS2 9JT, UK.
- [6] Children's Cancer and Leukaemia Group (CCLG), Leicester, LE1 7GB, UK.
- [7] Professor of Paediatric Oncology and Late Effects Medicine, Leeds Institute of Medical Research, University of Leeds, Leeds, LS2 9JT, UK.
- [8] Children's Brain Tumour Research Centre, School of Medicine, The University of Nottingham, Nottingham, NG7 2UH, UK.
- [9] Institute of Cancer and Genomic Sciences, University of Birmingham, Edgbaston, Birmingham, UK.

- [10] Division of Cancer Sciences, University of Manchester, Manchester Academic Health Science Centre, Manchester, M13 9PL, UK.
- [11] National Cancer Registration and Analysis Service, Public Health England, London, SE1 8UG, UK.
- [12] Centre for Reviews and Dissemination, University of York, York, UK.
- [13] Department of Paediatric Oncology, Leeds Children's Hospital, Leeds, UK.
- [14] Centre for Computational Biology, University of Birmingham, Birmingham, B15 2TT, UK.
- [15] Department of Paediatric Oncology, Birmingham Children's Hospital, Steelhouse Lane, Birmingham, B4 6NH, UK.
- [16] Cancer Research UK Clinical Trials Unit, NIHR Birmingham Biomedical Research Centre, Institute of Cancer and Genomic Sciences, College of Medical and Dental Sciences, University of Birmingham, Birmingham, B15 2TT, UK.

- PMID: **33299130**
- PMCID: PMC7884399
- DOI: 10.1038/s41416-020-01181-0

Abstract

Background: Children with cancer are frequently immunocompromised. While children are generally thought to be at less risk of severe SARS-CoV-2 infection than adults, comprehensive population-based evidence for the risk in children with cancer is unavailable. We aimed to produce evidence of the incidence and outcomes from SARS-CoV-2 in children with cancer attending all hospitals treating this population across the UK.

Methods: Retrospective and prospective observational study of all children in the UK under 16 diagnosed with cancer through data collection from all hospitals providing cancer care to this population. Eligible patients tested positive for SARS-CoV-2 on reverse transcription polymerase chain reaction (RT-PCR). The primary end-point was death, discharge or end of active care for COVID-19 for those remaining in hospital.

Results: Between 12 March 2020 and 31 July 2020, 54 cases were identified: 15 (28%) were asymptomatic, 34 (63%) had mild infections and 5 (10%) moderate, severe or critical infections. No patients died and only three patients required intensive care support due to COVID-19. Estimated incidence of hospital identified SARS-CoV-2 infection in children with cancer under 16 was 3%.

Conclusions: Children with cancer with SARS-CoV-2 infection do not appear at increased risk of severe infection compared to the general paediatric population. This is reassuring and supports the continued delivery of standard treatment.

Conflict of interest statement

The authors declare no competing interests.

- 31 references
- 2 figures

Supplementary info

Publication types, MeSH terms, Substances, Grant support Expand

Publication types

- Multicenter Study
- Observational Study

MeSH terms

- Adolescent
- COVID-19 / epidemiology*
- COVID-19 / mortality
- Carrier State / epidemiology*
- Child
- Child, Preschool
- Female
- Humans
- Incidence
- Infant
- Male
- Mortality
- Neoplasms / mortality
- Neoplasms / virology*
- Prospective Studies
- RNA, Viral / genetics
- Retrospective Studies
- SARS-CoV-2 / genetics*
- Severity of Illness Index
- United Kingdom / epidemiology

Substances

- RNA, Viral

Grant support

- G0800472/MRC_/Medical Research Council/United Kingdom
- PDF-2014-07-072/DH_/Department of Health/United Kingdom

Full text links

npg nature publishing group Nature Publishing Group Free PMC article

Proceed to details

Cite
Share
654
Observational Study
Clin Cardiol
. 2021 Jul;44(7):963-970.
doi: 10.1002/clc.23628. Epub 2021 May 11.

The prognostic significance of electrocardiography findings in patients with coronavirus disease 2019: A retrospective study

Deyan Yang [1], Jing Li [2], Peng Gao [1], Taibo Chen [1], Zhongwei Cheng [1], Kangan Cheng [1], Hua Deng [1], Quan Fang [1], Chunfeng Yi [2], Hongru Fan [2], Yonghong Wu [2], Liwei Li [2], Yong Fang [2], Guowei Tian [2], Wan Pan [2], Fan Zhang [2]
Affiliations Expand

Affiliations

- [1] Department of Cardiology, Peking Union Medical College Hospital, Chinese Academy of Medical Sciences & Peking Union Medical College, Beijing, China.
- [2] Department of Cardiology, Intervention Cardiology Center, Wuhan No.1 Hospital, No.215 Zhongshan Avenue, QiaoKou District, Wuhan, China.

- PMID: **33973673**
- PMCID: PMC8237010
- DOI: 10.1002/clc.23628

Free PMC article
Observational Study

The prognostic significance of electrocardiography findings in patients with coronavirus disease 2019: A retrospective study

Deyan Yang et al. Clin Cardiol. 2021 Jul.
Free PMC article
Show details
Clin Cardiol
. 2021 Jul;44(7):963-970.

doi: 10.1002/clc.23628. Epub 2021 May 11.

Authors

Deyan Yang [1], Jing Li [2], Peng Gao [1], Taibo Chen [1], Zhongwei Cheng [1], Kangan Cheng [1], Hua Deng [1], Quan Fang [1], Chunfeng Yi [2], Hongru Fan [2], Yonghong Wu [2], Liwei Li [2], Yong Fang [2], Guowei Tian [2], Wan Pan [2], Fan Zhang [2]

Affiliations

- [1] Department of Cardiology, Peking Union Medical College Hospital, Chinese Academy of Medical Sciences & Peking Union Medical College, Beijing, China.
- [2] Department of Cardiology, Intervention Cardiology Center, Wuhan No.1 Hospital, No.215 Zhongshan Avenue, QiaoKou District, Wuhan, China.

- PMID: **33973673**
- PMCID: PMC8237010
- DOI: 10.1002/clc.23628

Abstract

Background: Coronavirus disease 2019 (COVID-19) has reached a pandemic level. Cardiac injury is not uncommon among COVID-19 patients. We sought to describe the electrocardiographic characteristics and to identify the prognostic significance of electrocardiography (ECG) findings of patients with COVID-19.

Hypothesis: ECG abnormality was associated with higher risk of death.

Methods: Consecutive patients with laboratory-confirmed COVID-19 and definite in-hospital outcome were retrospectively included. Demographic characteristics and clinical data were extracted from medical record. Initial ECGs at admission or during hospitalization were reviewed. A point-based scoring system of abnormal ECG findings was formed, in which 1 point each was assigned for the presence of axis deviation, arrhythmias, atrioventricular block, conduction tissue disease, QTc interval prolongation, pathological Q wave, ST-segment change, and T-wave change. The association between abnormal ECG scores and in-hospital mortality was assessed in multivariable Cox regression models.

Results: A total of 306 patients (mean 62.84 ± 14.69 years old, 48.0% male) were included. T-wave change (31.7%), QTc interval prolongation (30.1%), and arrhythmias (16.3%) were three most common found ECG abnormalities. 30 (9.80%) patients died during hospitalization. Abnormal ECG scores were significantly higher among non-survivors (median 2 points vs 1 point, $p < 0.001$). The risk of in-hospital death increased by a factor of 1.478 (HR 1.478, 95% CI 1.131-1.933, $p = 0.004$) after adjusted by age, comorbidities, cardiac injury and treatments.

Conclusions: ECG abnormality was common in patients admitted for COVID-19 and was associated with adverse in-hospital outcome. In-hospital mortality risk increased with increasing abnormal ECG scores.

Keywords: cardiac injury; coronavirus; electrocardiography; outcome.

© 2021 The Authors. Clinical Cardiology published by Wiley Periodicals LLC.

Conflict of interest statement

All authors declare no conflicts of interest that might be relevant to the contents of this manuscript.

- 15 references
- 3 figures

Supplementary info

Publication types, MeSH terms Expand

Publication types

- Observational Study

MeSH terms

- COVID-19 / complications*
- COVID-19 / mortality
- Cardiovascular Diseases / diagnosis*
- Cardiovascular Diseases / mortality
- China / epidemiology
- Electrocardiography*
- Female
- Hospital Mortality
- Humans
- Male
- Middle Aged
- Pandemics
- Pneumonia, Viral / complications*
- Pneumonia, Viral / mortality
- Pneumonia, Viral / virology
- Prognosis
- Retrospective Studies
- Risk Factors
- SARS-CoV-2

Full text links

WILEY Free Full Text Wiley Free PMC article

Proceed to details

Cite

Share

☐ 655
Case Reports

Pediatr Nephrol
. 2021 Jan;36(1):163-169.
doi: 10.1007/s00467-020-04715-z. Epub 2020 Aug 26.

Be aware of acute kidney injury in critically ill children with COVID-19

Xiaowen Wang [1], Xingfeng Chen [2], Feng Tang [3], Wanjun Luo [4], Jian Fang [1], Chang Qi [1], Hua Sun [5], Han Xiao [6], Xuehua Peng [7], Jianbo Shao [8] [9] [10]
Affiliations Expand

Affiliations

- [1] Department of Nephrology, Wuhan Children's Hospital (Wuhan Maternal and Child Healthcare Hospital), Tongji Medical College, Huazhong University of Science & Technology, Wuhan, 430000, China.
- [2] Intensive Care Unit, Wuhan Children's Hospital (Wuhan Maternal and Child Healthcare Hospital), Tongji Medical College, Huazhong University of Science & Technology, Wuhan, 430000, China.
- [3] Department of Laboratory Medicine, Wuhan Children's Hospital (Wuhan Maternal and Child Healthcare Hospital), Tongji Medical College, Huazhong University of Science & Technology, Wuhan, 430000, China.
- [4] Hospital Acquired Infection Control Department, Wuhan Children's Hospital (Wuhan Maternal and Child Healthcare Hospital), Tongji Medical College, Huazhong University of Science & Technology, Wuhan, 430000, China.
- [5] Department of Nephrology, Boston Children's Hospital, Harvard Medical School, Boston, MA, 02115, USA.
- [6] Institute of Maternal and Child Health, Wuhan Children's Hospital (Wuhan Maternal and Child Healthcare Hospital), Tongji Medical College, Huazhong University of Science & Technology, Wuhan, 430000, China.
- [7] Medical Imaging Center, Wuhan Children's Hospital (Wuhan Maternal and Child Healthcare Hospital), Tongji Medical College, Huazhong University of Science & Technology, Wuhan, 430000, China.
- [8] Intensive Care Unit, Wuhan Children's Hospital (Wuhan Maternal and Child Healthcare Hospital), Tongji Medical College, Huazhong University of Science & Technology, Wuhan, 430000, China. shaojb2002@sina.com.
- [9] Medical Imaging Center, Wuhan Children's Hospital (Wuhan Maternal and Child Healthcare Hospital), Tongji Medical College, Huazhong University of Science & Technology, Wuhan, 430000, China. shaojb2002@sina.com.
- [10] Institute of Maternal and Child Health, Wuhan Children's Hospital (Wuhan Maternal and Child Healthcare Hospital), 100# Hongkong Road, Wuhan, 430000, China. shaojb2002@sina.com.

- PMID: **32844290**
- PMCID: PMC7447530
- DOI: 10.1007/s00467-020-04715-z

Free PMC article
Case Reports

Be aware of acute kidney injury in critically ill children with COVID-19

Xiaowen Wang et al. Pediatr Nephrol. 2021 Jan.
Free PMC article
Show details
Pediatr Nephrol
. 2021 Jan;36(1):163-169.
doi: 10.1007/s00467-020-04715-z. Epub 2020 Aug 26.

Authors

Xiaowen Wang [1], Xingfeng Chen [2], Feng Tang [3], Wanjun Luo [4], Jian Fang [1], Chang Qi [1], Hua Sun [5], Han Xiao [6], Xuehua Peng [7], Jianbo Shao [8] [9] [10]

Affiliations

- [1] Department of Nephrology, Wuhan Children's Hospital (Wuhan Maternal and Child Healthcare Hospital), Tongji Medical College, Huazhong University of Science & Technology, Wuhan, 430000, China.
- [2] Intensive Care Unit, Wuhan Children's Hospital (Wuhan Maternal and Child Healthcare Hospital), Tongji Medical College, Huazhong University of Science & Technology, Wuhan, 430000, China.
- [3] Department of Laboratory Medicine, Wuhan Children's Hospital (Wuhan Maternal and Child Healthcare Hospital), Tongji Medical College, Huazhong University of Science & Technology, Wuhan, 430000, China.
- [4] Hospital Acquired Infection Control Department, Wuhan Children's Hospital (Wuhan Maternal and Child Healthcare Hospital), Tongji Medical College, Huazhong University of Science & Technology, Wuhan, 430000, China.
- [5] Department of Nephrology, Boston Children's Hospital, Harvard Medical School, Boston, MA, 02115, USA.
- [6] Institute of Maternal and Child Health, Wuhan Children's Hospital (Wuhan Maternal and Child Healthcare Hospital), Tongji Medical College, Huazhong University of Science & Technology, Wuhan, 430000, China.
- [7] Medical Imaging Center, Wuhan Children's Hospital (Wuhan Maternal and Child Healthcare Hospital), Tongji Medical College, Huazhong University of Science & Technology, Wuhan, 430000, China.
- [8] Intensive Care Unit, Wuhan Children's Hospital (Wuhan Maternal and Child Healthcare Hospital), Tongji Medical College, Huazhong University of Science & Technology, Wuhan, 430000, China. shaojb2002@sina.com.
- [9] Medical Imaging Center, Wuhan Children's Hospital (Wuhan Maternal and Child Healthcare Hospital), Tongji Medical College, Huazhong University of Science & Technology, Wuhan, 430000, China. shaojb2002@sina.com.

- [10] Institute of Maternal and Child Health, Wuhan Children's Hospital (Wuhan Maternal and Child Healthcare Hospital), 100# Hongkong Road, Wuhan, 430000, China. shaojb2002@sina.com.

- PMID: **32844290**
- PMCID: PMC7447530
- DOI: 10.1007/s00467-020-04715-z

Abstract

Background: Acute kidney injury (AKI) is a common complication of critically ill adult patients with COVID-19. However, currently, no studies investigate kidney impairment in children with COVID-19. We investigated incidence and treatment of AKI in pediatric patients with COVID-19 in Wuhan Children's Hospital during the early stages of the COVID-19 pandemic and discuss possible mechanisms of AKI related to SARS-CoV-2 infection.

Methods: By extracting data from electronic medical records, we conducted a retrospective observational study of kidney involvement in confirmed pediatric COVID-19 cases in Wuhan Children's Hospital during the coronavirus outbreak, from January 24 to March 20, 2020. Clinical presentations, clinical courses, laboratory findings, and medical interventions are described below.

Results: Among 238 confirmed COVID-19 cases, only three were critically ill and needed intensive care unit (ICU) admission. All three developed AKI, but AKI was not detected in any non-critically ill patients outside the ICU. Two of the three patients with AKI had prodromal gastrointestinal symptoms. Significantly elevated interleukin-6 (IL-6) levels and complement activation were observed in these patients with AKI. The three patients with AKI were treated with plasma exchange (PE) and continuous kidney replacement therapy (CKRT), resulting in one complete recovery, one partial recovery, and one mortality due to critical illness.

Conclusions: Critically ill children with COVID-19 may develop AKI, especially following prodromal gastrointestinal symptoms. An inflammatory storm and complement-mediated injury may underlie AKI development in children with COVID-19. Our study supports implantation of PE and CKRT in management of critically ill patients with AKI.

Keywords: AKI; CKRT; COVID-19; Plasmapheresis.

Conflict of interest statement

The authors declare that they have no conflicts of interest.

- 34 references
- 3 figures

Supplementary info

Publication types, MeSH terms, Grant support Expand

Publication types

- Case Reports
- Observational Study

- Research Support, Non-U.S. Gov't

MeSH terms

- Acute Kidney Injury / diagnostic imaging
- Acute Kidney Injury / etiology*
- Acute Kidney Injury / therapy
- COVID-19 / complications*
- COVID-19 / diagnostic imaging
- Child
- Critical Illness / therapy
- Cytokine Release Syndrome / etiology
- Fatal Outcome
- Female
- Humans
- Infant
- Male
- Pandemics
- Plasma Exchange
- Retrospective Studies
- SARS-CoV-2
- Treatment Outcome

Grant support

- 2016CFC728/Natural Science Foundation of Hubei Province/International
- WX16A07/Key Projects of Scientific Research Program of Wuhan Health Commission me (CN)/International
- WJ2019M011/Healthy Commission of Hubei Province/International
- WHCDCIRB-K-2020007/Centers For Disease Control And Prevention of Wuhan/International

Full text links

SpringerLink FULL-TEXT ARTICLE Springer Free PMC article
Proceed to details
Cite
Share
☐ 656
Observational Study
Medicine (Baltimore)
. 2021 Aug 13;100(32):e26847.
doi: 10.1097/MD.0000000000026847.

Comparison of emergency department utilization trends between the COVID-19 pandemic and control period

Soo Kang [1], Tae Kyu Ahn [1], Young Ho Seo [1], Young Ju Suh [2], Jin Hui Paik [1]
Affiliations Expand

Affiliations

- [1] Department of Emergency Medicine, Inha University School of Medicine, Incheon, Republic of Korea.
- [2] Department of Biomedical Sciences, Inha University School of Medicine, Incheon, Republic of Korea.

- PMID: **34397892**
- PMCID: PMC8360451
- DOI: 10.1097/MD.0000000000026847

Free PMC article
Observational Study

Comparison of emergency department utilization trends between the COVID-19 pandemic and control period

Soo Kang et al. Medicine (Baltimore). 2021.
Free PMC article
Show details
Medicine (Baltimore)
. 2021 Aug 13;100(32):e26847.
doi: 10.1097/MD.0000000000026847.

Authors

Soo Kang [1], Tae Kyu Ahn [1], Young Ho Seo [1], Young Ju Suh [2], Jin Hui Paik [1]

Affiliations

- [1] Department of Emergency Medicine, Inha University School of Medicine, Incheon, Republic of Korea.
- [2] Department of Biomedical Sciences, Inha University School of Medicine, Incheon, Republic of Korea.

- PMID: **34397892**

- PMCID: PMC8360451
- DOI: 10.1097/MD.0000000000026847

Abstract

Infectious disease pandemics has a great impact on the use of medical facilities. The purpose of this study was to analyze the effects of coronavirus disease 2019 (COVID-19) on the use of emergency medical facilities in the Republic of Korea. This single-center, retrospective observational study was conducted in a tertiary teaching hospital located in Incheon Metropolitan City, Republic of Korea. We set the pandemic period as February 19, 2020 to April 18, 2020, and the control period was set to the same period in 2018 and 2019. All consecutive patients who visited the emergency department (ED) during the study period were included. Patients were divided into 3 groups according to age (pediatric patients, younger adult patients and older adult patients). The total number, demographics, clinical data, and diagnostic codes of ED patients were analyzed. The total number of ED patients in the pandemic period was lower than that in the control period, which was particularly pronounced for pediatric patients. The proportion of patients who used the 119 ambulances increased in all 3 groups (P = .002, P < .001, and P = .001), whereas the proportion of patients who visited on foot was decreased (P = .006, P < .001, and P = .027). In terms of diagnostic codes, a significant decrease was observed in the proportion of certain infectious or parasitic diseases (A00-B99), and respiratory diseases (J00-J99) in the pediatric and younger adult patient groups (P < .001 and P < .001, respectively). The COVID-19 pandemic reduced the number of ED patients; however, the proportion of patients using ambulances increased. In particular, the proportion of patients with diagnostic codes for infectious and respiratory diseases significantly decreased during the pandemic period.

Copyright © 2021 the Author(s). Published by Wolters Kluwer Health, Inc.

Conflict of interest statement

The authors have no potential conflicts of interest to disclose.

- 35 references
- 1 figure

Supplementary info

Publication types, MeSH terms, Grant support Expand

Publication types

- Observational Study

MeSH terms

- Adolescent
- Adult
- Aged
- Aged, 80 and over
- COVID-19 / prevention & control*

- COVID-19 / transmission
- Child
- Child, Preschool
- Emergency Service, Hospital / organization & administration
- Emergency Service, Hospital / statistics & numerical data*
- Female
- Hospitals, University / organization & administration
- Hospitals, University / statistics & numerical data
- Humans
- Infant
- Male
- Middle Aged
- Patient Acceptance of Health Care / statistics & numerical data*
- Republic of Korea
- Retrospective Studies

Grant support

- no/Inha University (KR)

Full text links

Wolters Kluwer Wolters Kluwer Free PMC article
Proceed to details
Cite
Share
☐ 657
Observational Study
Clin Pharmacol Ther
. 2021 Dec;110(6):1498-1511.
doi: 10.1002/cpt.2317. Epub 2021 Jul 2.

Association Between FIASMAs and Reduced Risk of Intubation or Death in Individuals Hospitalized for Severe COVID-19: An Observational Multicenter Study

Nicolas Hoertel [1], Marina Sánchez-Rico [1] [2], Erich Gulbins [3], Johannes Kornhuber [4], Alexander Carpinteiro [3] [5], Eric J Lenze [6], Angela M Reiersen [6], Miriam Abellán [1], Pedro de la Muela [1] [2], Raphaël Vernet [7], Carlos Blanco [8], Céline Cougoule [9], Nathanaël Beeker [10], Antoine Neuraz [11] [12], Philip Gorwood [13], Jesús M Alvarado [2], Pierre Meneton [14], Frédéric Limosin [1], AP-HP /

Université de Paris / INSERM COVID-19 research collaboration, AP-HP COVID CDR Initiative, "Entrepôt de Données de Santé" AP-HP Consortium
Collaborators, Affiliations Expand

Collaborators

- **AP-HP / Université de Paris / INSERM COVID-19 research collaboration, AP-HP COVID CDR Initiative, "Entrepôt de Données de Santé" AP-HP Consortium**: Pierre-Yves Ancel, Alain Bauchet, Nathanaël Beeker, Vincent Benoit, Mélodie Bernaux, Ali Bellamine, Romain Bey, Aurélie Bourmaud, Stéphane Breant, Anita Burgun, Fabrice Carrat, Charlotte Caucheteux, Julien Champ, Sylvie Cormont, Christel Daniel, Julien Dubiel, Catherine Ducloas, Loic Esteve, Marie Frank, Nicolas Garcelon, Alexandre Gramfort, Nicolas Griffon, Olivier Grisel, Martin Guilbaud, Claire Hassen-Khodja, François Hemery, Martin Hilka, Anne Sophie Jannot, Jerome Lambert, Richard Layese, Judith Leblanc, Léo Lebouter, Guillaume Lemaitre, Damien Leprovost, Ivan Lerner, Kankoe Levi Sallah, Aurélien Maire, Marie-France Mamzer, Patricia Martel, Arthur Mensch, Thomas Moreau, Antoine Neuraz, Nina Orlova, Nicolas Paris, Bastien Rance, Hélène Ravera, Antoine Rozes, Elisa Salamanca, Arnaud Sandrin, Patricia Serre, Xavier Tannier, Jean-Marc Treluyer, Damien van Gysel, Gaël Varoquaux, Jill Jen Vie, Maxime Wack, Perceval Wajsburt, Demian Wassermann, Eric Zapletal

Affiliations

- [1] Assistance Publique-Hopitaux de Paris, DMU Psychiatrie et Addictologie, Hôpital Corentin-Celton, Institut de Psychiatrie et Neurosciences de Paris (IPNP), INSERM, UMR_S1266, Université de Paris, Paris, France.
- [2] Department of Psychobiology and Behavioural Sciences Methods, Faculty of Psychology, Universidad Complutense de Madrid, Pozuelo de Alarcón, Madrid, Spain.
- [3] Institute for Molecular Biology, University Medicine Essen, University of Duisburg-Essen, Essen, Germany.
- [4] Department of Psychiatry and Psychotherapy, University Hospital, Friedrich-Alexander-University of Erlangen-Nuremberg, Erlangen, Germany.
- [5] Department of Hematology and Stem Cell Transplantation, University Hospital Essen, University of Duisburg-Essen, Essen, Germany.
- [6] Department of Psychiatry, Washington University in St. Louis School of Medicine, St. Louis, Missouri, USA.
- [7] Medical Informatics, Biostatistics and Public Health Department, Assistance Publique-Hopitaux de Paris, Centre-Université de Paris, Hôpital Européen Georges Pompidou, Paris, France.
- [8] Division of Epidemiology, Services, and Prevention Research, National Institute on Drug Abuse, North Bethesda, Maryland, USA.
- [9] Institut de Pharmacologie et de Biologie Structurale, IPBS, Université de Toulouse, Toulouse, France.
- [10] Unité de Recherche clinique, Hopital Cochin, Assistance Publique-Hopitaux de Paris, Paris, France.
- [11] INSERM, UMR_S 1138, Cordeliers Research Center, Université de Paris, Paris, France.
- [12] Department of Medical Informatics, Necker-Enfants Malades Hospital, Assistance Publique-Hopitaux de Paris, Centre-Université de Paris, Paris, France.

- [13] INSERM, U1266 (Institute of Psychiatry and Neuroscience of Paris), Université de Paris, Paris, France.
- [14] INSERM U1142 LIMICS, UMRS 1142, Sorbonne Universities, UPMC University of Paris 06, University of Paris 13, Paris, France.

- PMID: **34050932**
- PMCID: PMC8239599
- DOI: 10.1002/cpt.2317

Free PMC article
Observational Study

Association Between FIASMAs and Reduced Risk of Intubation or Death in Individuals Hospitalized for Severe COVID-19: An Observational Multicenter Study

Nicolas Hoertel et al. Clin Pharmacol Ther. 2021 Dec.
Free PMC article
Show details
Clin Pharmacol Ther
. 2021 Dec;110(6):1498-1511.
doi: 10.1002/cpt.2317. Epub 2021 Jul 2.

Authors

Nicolas Hoertel [1], Marina Sánchez-Rico [1] [2], Erich Gulbins [3], Johannes Kornhuber [4], Alexander Carpinteiro [3] [5], Eric J Lenze [6], Angela M Reiersen [6], Miriam Abellán [1], Pedro de la Muela [1] [2], Raphaël Vernet [7], Carlos Blanco [8], Céline Cougoule [9], Nathanaël Beeker [10], Antoine Neuraz [11] [12], Philip Gorwood [13], Jesús M Alvarado [2], Pierre Meneton [14], Frédéric Limosin [1], AP-HP / Université de Paris / INSERM COVID-19 research collaboration, AP-HP COVID CDR Initiative, "Entrepôt de Données de Santé" AP-HP Consortium

Collaborators

- **AP-HP / Université de Paris / INSERM COVID-19 research collaboration, AP-HP COVID CDR Initiative, "Entrepôt de Données de Santé" AP-HP Consortium**: Pierre-Yves Ancel, Alain Bauchet, Nathanaël Beeker, Vincent Benoit, Mélodie Bernaux, Ali Bellamine, Romain Bey, Aurélie Bourmaud, Stéphane Breant, Anita Burgun, Fabrice Carrat, Charlotte Caucheteux, Julien Champ, Sylvie Cormont, Christel Daniel, Julien Dubiel, Catherine Ducloas, Loic Esteve, Marie Frank, Nicolas Garcelon, Alexandre Gramfort, Nicolas Griffon, Olivier Grisel, Martin Guilbaud, Claire Hassen-Khodja, François Hemery, Martin Hilka, Anne Sophie Jannot, Jerome Lambert, Richard Layese, Judith Leblanc, Léo Lebouter, Guillaume Lemaitre, Damien Leprovost, Ivan Lerner, Kankoe Levi Sallah, Aurélien Maire, Marie-France Mamzer, Patricia Martel, Arthur Mensch, Thomas Moreau, Antoine Neuraz, Nina Orlova, Nicolas Paris, Bastien Rance, Hélène Ravera, Antoine Rozes, Elisa

Salamanca, Arnaud Sandrin, Patricia Serre, Xavier Tannier, Jean-Marc Treluyer, Damien van Gysel, Gaël Varoquaux, Jill Jen Vie, Maxime Wack, Perceval Wajsburt, Demian Wassermann, Eric Zapletal

Affiliations

- [1] Assistance Publique-Hopitaux de Paris, DMU Psychiatrie et Addictologie, Hôpital Corentin-Celton, Institut de Psychiatrie et Neurosciences de Paris (IPNP), INSERM, UMR_S1266, Université de Paris, Paris, France.
- [2] Department of Psychobiology and Behavioural Sciences Methods, Faculty of Psychology, Universidad Complutense de Madrid, Pozuelo de Alarcón, Madrid, Spain.
- [3] Institute for Molecular Biology, University Medicine Essen, University of Duisburg-Essen, Essen, Germany.
- [4] Department of Psychiatry and Psychotherapy, University Hospital, Friedrich-Alexander-University of Erlangen-Nuremberg, Erlangen, Germany.
- [5] Department of Hematology and Stem Cell Transplantation, University Hospital Essen, University of Duisburg-Essen, Essen, Germany.
- [6] Department of Psychiatry, Washington University in St. Louis School of Medicine, St. Louis, Missouri, USA.
- [7] Medical Informatics, Biostatistics and Public Health Department, Assistance Publique-Hopitaux de Paris, Centre-Université de Paris, Hôpital Européen Georges Pompidou, Paris, France.
- [8] Division of Epidemiology, Services, and Prevention Research, National Institute on Drug Abuse, North Bethesda, Maryland, USA.
- [9] Institut de Pharmacologie et de Biologie Structurale, IPBS, Université de Toulouse, Toulouse, France.
- [10] Unité de Recherche clinique, Hopital Cochin, Assistance Publique-Hopitaux de Paris, Paris, France.
- [11] INSERM, UMR_S 1138, Cordeliers Research Center, Université de Paris, Paris, France.
- [12] Department of Medical Informatics, Necker-Enfants Malades Hospital, Assistance Publique-Hopitaux de Paris, Centre-Université de Paris, Paris, France.
- [13] INSERM, U1266 (Institute of Psychiatry and Neuroscience of Paris), Université de Paris, Paris, France.
- [14] INSERM U1142 LIMICS, UMRS 1142, Sorbonne Universities, UPMC University of Paris 06, University of Paris 13, Paris, France.

- PMID: **34050932**
- PMCID: PMC8239599
- DOI: 10.1002/cpt.2317

Abstract

Several medications commonly used for a number of medical conditions share a property of functional inhibition of acid sphingomyelinase (ASM), or FIASMA. Preclinical and clinical evidence suggest that the ASM/ceramide system may be central to severe acute respiratory syndrome-coronavirus 2 (SARS-CoV-2) infection. We examined the potential usefulness of FIASMA use among patients hospitalized for severe coronavirus disease 2019 (COVID-19) in an observational multicenter study conducted at Greater Paris University hospitals. Of 2,846 adult patients hospitalized for severe COVID-19, 277 (9.7%) were taking an FIASMA medication at the time of their hospital admission. The primary end point was a composite of intubation and/or

death. We compared this end point between patients taking vs. not taking an FIASMA medication in time-to-event analyses adjusted for sociodemographic characteristics and medical comorbidities. The primary analysis was a Cox regression model with inverse probability weighting (IPW). Over a mean follow-up of 9.2 days (SD = 12.5), the primary end point occurred in 104 patients (37.5%) receiving an FIASMA medication, and 1,060 patients (41.4%) who did not. Despite being significantly and substantially associated with older age and greater medical severity, FIASMA medication use was significantly associated with reduced likelihood of intubation or death in both crude (hazard ratio (HR) = 0.71, 95% confidence interval (CI) = 0.58-0.87, P < 0.001) and primary IPW (HR = 0.58, 95%CI = 0.46-0.72, P < 0.001) analyses. This association remained significant in multiple sensitivity analyses and was not specific to one particular FIASMA class or medication. These results show the potential importance of the ASM/ceramide system in COVID-19 and support the continuation of FIASMA medications in these patients. Double-blind controlled randomized clinical trials of these medications for COVID-19 are needed.

© 2021 The Authors. Clinical Pharmacology & Therapeutics published by Wiley Periodicals LLC on behalf of American Society for Clinical Pharmacology and Therapeutics.

Comment in

- Compatibility of FIASMA Pharmacokinetics With Study End Points?
  Le Corre P, Loas G. Le Corre P, et al. Clin Pharmacol Ther. 2022 Feb;111(2):353. doi: 10.1002/cpt.2442. Epub 2021 Oct 31. Clin Pharmacol Ther. 2022. PMID: 34719015 No abstract available.

- 49 references

Supplementary info

Publication types, MeSH terms, Substances Expand

Publication types

- Multicenter Study
- Observational Study

MeSH terms

- Adolescent
- Adult
- Aged
- Aged, 80 and over
- COVID-19 / drug therapy
- COVID-19 / enzymology*
- COVID-19 / mortality*
- COVID-19 Testing / trends
- Cohort Studies
- Enzyme Inhibitors / pharmacology

- Enzyme Inhibitors / therapeutic use
- Female
- Hospitalization / trends*
- Humans
- Intubation, Intratracheal / mortality*
- Intubation, Intratracheal / trends*
- Male
- Middle Aged
- Mortality / trends
- Retrospective Studies
- Sphingomyelin Phosphodiesterase / antagonists & inhibitors*
- Sphingomyelin Phosphodiesterase / metabolism
- Young Adult

Substances

- Enzyme Inhibitors
- SMPD1 protein, human
- Sphingomyelin Phosphodiesterase

Full text links

WILEY Full Text Article Wiley Free PMC article
Proceed to details
Cite
Share
☐ 658
Observational Study
Intensive Crit Care Nurs
. 2021 Feb;62:102967.
doi: 10.1016/j.iccn.2020.102967. Epub 2020 Oct 28.

Impact of COVID-19 on nursing time in intensive care units in Belgium

Arnaud Bruyneel [1], Maria-Cécillia Gallani [2], Jérôme Tack [3], Alain d'Hondt [4], Sebastien Canipel [5], Stéphane Franck [6], Pascal Reper [7], Magali Pirson [8]
Affiliations Expand

Affiliations

- [1] Soins intensifs - Centre Hospitalier Universitaire Tivoli, Belgium; SIZ Nursing, A Society of Intensive Care Nurses, Belgium; Health Economics, Hospital Management and Nursing

Research Dept, School of Public Health, Université Libre de Bruxelles, Belgium. Electronic address: arnaudbruyneel8@gmail.com.

- [2] Université de Laval, Canada.
- [3] SIZ Nursing, A Society of Intensive Care Nurses, Belgium; Soins intensifs - Cliniques Universitaire de Bruxelles - Hôpital Erasme, Belgium.
- [4] Soins intensifs - Centre Hospitalier Universitaire Ambroise Paré, Belgium.
- [5] SIZ Nursing, A Society of Intensive Care Nurses, Belgium; Soins intensifs - Centre Hospitalier Universitaire Ambroise Paré, Belgium.
- [6] Soins intensifs - Centre Hospitalier Universitaire Tivoli, Belgium.
- [7] Soins intensifs - Centre Hospitalier de la Haute Senne, le Tilleriau, Belgium.
- [8] Health Economics, Hospital Management and Nursing Research Dept, School of Public Health, Université Libre de Bruxelles, Belgium.

- PMID: **33162312**
- PMCID: PMC7598359
- DOI: 10.1016/j.iccn.2020.102967

Free PMC article
Observational Study

Impact of COVID-19 on nursing time in intensive care units in Belgium

Arnaud Bruyneel et al. Intensive Crit Care Nurs. 2021 Feb.
Free PMC article
Show details
Intensive Crit Care Nurs
. 2021 Feb;62:102967.
doi: 10.1016/j.iccn.2020.102967. Epub 2020 Oct 28.

Authors

Arnaud Bruyneel [1], Maria-Cécillia Gallani [2], Jérôme Tack [3], Alain d'Hondt [4], Sebastien Canipel [5], Stéphane Franck [6], Pascal Reper [7], Magali Pirson [8]

Affiliations

- [1] Soins intensifs - Centre Hospitalier Universitaire Tivoli, Belgium; SIZ Nursing, A Society of Intensive Care Nurses, Belgium; Health Economics, Hospital Management and Nursing Research Dept, School of Public Health, Université Libre de Bruxelles, Belgium. Electronic address: arnaudbruyneel8@gmail.com.
- [2] Université de Laval, Canada.
- [3] SIZ Nursing, A Society of Intensive Care Nurses, Belgium; Soins intensifs - Cliniques Universitaire de Bruxelles - Hôpital Erasme, Belgium.
- [4] Soins intensifs - Centre Hospitalier Universitaire Ambroise Paré, Belgium.
- [5] SIZ Nursing, A Society of Intensive Care Nurses, Belgium; Soins intensifs - Centre Hospitalier Universitaire Ambroise Paré, Belgium.

- [6] Soins intensifs - Centre Hospitalier Universitaire Tivoli, Belgium.
- [7] Soins intensifs - Centre Hospitalier de la Haute Senne, le Tilleriau, Belgium.
- [8] Health Economics, Hospital Management and Nursing Research Dept, School of Public Health, Université Libre de Bruxelles, Belgium.

- PMID: **33162312**
- PMCID: PMC7598359
- DOI: 10.1016/j.iccn.2020.102967

Abstract

Introduction: The COVID-19 pandemic has had a significant impact on nursing practice in intensive care unit and consequently, on workload.

Objective: To assess the nurse-patient ratio required by COVID-19 patients and to identify the factors that influence nursing in this context.

Design: This study was a retrospective observational study that evaluated the ratio using the Nursing Activities Score (NAS).

Setting: Three Belgian French-speaking hospitals, including five ICUs. Patients included COVID-19 and non-COVID-19 patients.

Measurements and main results: The study included 95 COVID-19 patients and 1604 non-COVID-19 patients (control group) resulting in 905 and 5453 NAS measures, respectively. The NAS was significantly higher among the COVID-19 patients than in the control group ($p = <0.0001$). In the COVID-19 group, these higher scores were also observed per shift and uniformly across the three hospitals. COVID-19 patients required more time in the activities of monitoring and titration ($\chi2 = 457.60$, $p = <0.0001$), mobilisation ($\chi2 = 161.21$, $p = <0.0001$), and hygiene ($\chi2 = 557.77$, $p = <0.0001$). Factors influencing nursing time measured by NAS in the COVID-19 patients were age <65 years old ($p = 0.23$), the use of continuous venovenous hemofiltration ($p = 0.002$), a high APACHE II score ($p = 0.006$) and patient death ($p = 0.002$). A COVID-19 diagnosis was independently associated with an increase in nursing time (OR = 4.8, 95% CI:3.6-6.4).

Conclusions: Patients hospitalised in the ICU due to COVID-19 require significantly more nursing time and need an average ratio of almost 1:1.

Keywords: Coronavirus; Intensive care unit; Nursing activities score; Workload.

Copyright © 2020 Elsevier Ltd. All rights reserved.

Conflict of interest statement

Declaration of Competing Interest The authors declare that they have no known competing financial interests or personal relationships that could have appeared to influence the work reported in this paper.

- 38 references
- 1 figure

Supplementary info

Publication types, MeSH terms Expand

Publication types

- Observational Study

MeSH terms

- APACHE
- Age Factors
- Aged
- Aged, 80 and over
- Belgium
- COVID-19 / nursing*
- Continuous Renal Replacement Therapy / nursing
- Critical Care Nursing*
- Female
- Humans
- Hygiene
- Intensive Care Units
- Male
- Middle Aged
- Mortality
- Moving and Lifting Patients / nursing
- Nurses
- Nursing Care / statistics & numerical data
- Patient Positioning / nursing
- Postoperative Care / nursing*
- Respiration, Artificial / nursing
- Respiratory Insufficiency / nursing*
- Retrospective Studies
- SARS-CoV-2
- Sepsis / nursing*
- Shock, Cardiogenic / nursing*
- Time Factors
- Workload*

Full text links

ELSEVIER FULL-TEXT ARTICLE Elsevier Science Free PMC article

Proceed to details

Cite

Share

☐ 659

Observational Study

Neurol Neurochir Pol

. 2022;56(1):61-67.

doi: 10.5603/PJNNS.a2021.0054. Epub 2021 Aug 4.

COVID-19 pandemic year in a sample of Polish myasthenia gravis patients: an observational study

Łukasz Rzepiński [1] [2], Monika Zawadka-Kunikowska [3]

Affiliations Expand

Affiliations

- [1] Department of Neurology, 10th Military Research Hospital and Polyclinic, Bydgoszcz, Poland. luk.rzepinski@gmail.com.
- [2] Sanitas - Neurology Outpatient Clinic, Bydgoszcz, Poland. luk.rzepinski@gmail.com.
- [3] Department of Exercise Physiology and Functional Anatomy, Ludwik Rydygier Collegium Medicum in Bydgoszcz Nicolaus Copernicus University in Torun, Bydgoszcz, Poland.

- PMID: **34346049**
- DOI: 10.5603/PJNNS.a2021.0054

Free article

Observational Study

COVID-19 pandemic year in a sample of Polish myasthenia gravis patients: an observational study

Łukasz Rzepiński et al. Neurol Neurochir Pol. 2022.

Free article

Show details

Neurol Neurochir Pol

. 2022;56(1):61-67.

doi: 10.5603/PJNNS.a2021.0054. Epub 2021 Aug 4.

Authors

Łukasz Rzepiński [1] [2], Monika Zawadka-Kunikowska [3]

Affiliations

- [1] Department of Neurology, 10th Military Research Hospital and Polyclinic, Bydgoszcz, Poland. luk.rzepinski@gmail.com.
- [2] Sanitas - Neurology Outpatient Clinic, Bydgoszcz, Poland. luk.rzepinski@gmail.com.
- [3] Department of Exercise Physiology and Functional Anatomy, Ludwik Rydygier Collegium Medicum in Bydgoszcz Nicolaus Copernicus University in Torun, Bydgoszcz, Poland.

- PMID: **34346049**
- DOI: 10.5603/PJNNS.a2021.0054

Abstract

Aim of the study: Coronavirus disease 2019 (COVID-19) incidence, mortality, recovery and hospitalisation rates vary in different countries. This study aimed to present the clinical characteristics of a sample of unvaccinated Polish myasthenia gravis (MG) patients during the first year of the COVID-19 pandemic, taking into account the number of MG exacerbations, a detailed description of the severe acute respiratory syndrome coronavirus 2 (SARS-CoV-2) infection course, and the need to modify immunosuppressive therapies. Clinical rationale for the study: To assess the impact of the first COVID-19 pandemic year on MG course in a sample of unvaccinated patients.

Materials and methods: A retrospective observational study involving 30 unvaccinated Polish MG patients consulted in a neurological outpatient clinic on 11-31 March, 2020 (baseline) and 11-31 March, 2021 (endpoint).

Results: During the period of evaluation, exacerbation of MG requiring hospitalisation was reported in 11 patients. Among them, four were treated with intravenous immunoglobulin and another six required plasma exchange. In the study group, COVID-19 was identified in 10 patients. Of them, seven experienced a mild course of SARSCoV-2 infection with spontaneous recovery. In the remaining three patients, both MG exacerbation and SARS-CoV-2 infection were reported. These patients experienced MG exacerbation in the preceding month or concurrently with COVID-19 and were aged over 50 years. Due to the SARS-CoV-2 infection, they required antibiotic and oxygen therapy, and hospitalisation was necessary in the case of two obese patients. None of the patients died due to COVID-19, and nor did any require discontinuation of immunosuppressive therapies during the study period. In total, 12 patients in the study group experienced neither MG exacerbation nor SARS-CoV-2 infection.

Conclusions: In the presented sample of Polish MG patients, favourable outcomes of COVID-19 were observed. Further studies are needed to evaluate the reliable course of COVID-19 taking into account international differences, the types of treatment applied, and the ratio of vaccinated to unvaccinated MG patients.

Keywords: COVID-19; SARS-CoV-2; immunosuppression; myasthenia gravis; pandemic.

Supplementary info

Publication types, MeSH terms Expand

Publication types

- Observational Study

MeSH terms

- Aged
- COVID-19* / epidemiology
- Humans
- Myasthenia Gravis* / epidemiology
- Myasthenia Gravis* / therapy
- Pandemics
- Poland / epidemiology
- SARS-CoV-2

Full text links

Via Medica Medical Publishers

Proceed to details

Cite

Share

660

Observational Study

Respir Med

. 2020 Sep;171:106084.

doi: 10.1016/j.rmed.2020.106084. Epub 2020 Jul 4.

Asthma prevalence in patients with SARS-CoV-2 infection detected by RT-PCR not requiring hospitalization

Eduardo Garcia-Pachon [1], Lucia Zamora-Molina [2], Maria J Soler-Sempere [2], Carlos Baeza-Martinez [2], Justo Grau-Delgado [2], Vicente Canto-Reig [3], Antonio Ramon-Sanchez [3], Isabel Padilla-Navas [2], Montserrat Ruiz-Garcia [4], Nieves Gonzalo-Jimenez [4]

Affiliations Expand

Affiliations

- [1] Section of Respiratory Medicine, Hospital General Universitario de Elche, Alicante, Spain; Department of Clinical Medicine, Universidad Miguel Hernandez de Elche, Alicante, Spain. Electronic address: eduardo.garciap@umh.es.
- [2] Section of Respiratory Medicine, Hospital General Universitario de Elche, Alicante, Spain.

- [3] Allergy Unit, Hospital General Universitario de Elche, Alicante, Spain.
- [4] Section of Microbiology, Hospital General Universitario de Elche, Alicante, Spain.

- PMID: **32658837**
- PMCID: PMC7334641
- DOI: 10.1016/j.rmed.2020.106084

Free PMC article
Observational Study

Asthma prevalence in patients with SARS-CoV-2 infection detected by RT-PCR not requiring hospitalization

Eduardo Garcia-Pachon et al. Respir Med. 2020 Sep.
Free PMC article

Show details

Respir Med
. 2020 Sep;171:106084.
doi: 10.1016/j.rmed.2020.106084. Epub 2020 Jul 4.

Authors

Eduardo Garcia-Pachon [1], Lucia Zamora-Molina [2], Maria J Soler-Sempere [2], Carlos Baeza-Martinez [2], Justo Grau-Delgado [2], Vicente Canto-Reig [3], Antonio Ramon-Sanchez [3], Isabel Padilla-Navas [2], Montserrat Ruiz-Garcia [4], Nieves Gonzalo-Jimenez [4]

Affiliations

- [1] Section of Respiratory Medicine, Hospital General Universitario de Elche, Alicante, Spain; Department of Clinical Medicine, Universidad Miguel Hernandez de Elche, Alicante, Spain. Electronic address: eduardo.garciap@umh.es.
- [2] Section of Respiratory Medicine, Hospital General Universitario de Elche, Alicante, Spain.

- [3] Allergy Unit, Hospital General Universitario de Elche, Alicante, Spain.
- [4] Section of Microbiology, Hospital General Universitario de Elche, Alicante, Spain.

- PMID: **32658837**
- PMCID: PMC7334641
- DOI: 10.1016/j.rmed.2020.106084

Abstract

Introduction: The prevalence of asthma in patients hospitalized with SARS-CoV-2 has been studied and varies widely in the different series. However, the prevalence in SARS-infected patients not requiring hospitalization is not known. The objective of this study was to analyze the

presence of asthma in a consecutive series of patients who tested positive in the RT-PCR assay for SARS-CoV-2 and did not require hospital admission.

Methods and results: A total of 218 patients (58% of those who tested positive) did not require hospitalization; they had a median age of 45 years (IQR 34-57) and 57% were female. Six patients (2.8%) had a previous diagnosis of asthma. Only one patient developed a mild aggravation of asthma symptoms associated with SARS-CoV-2 infection.

Conclusions: Few patients with asthma were infected by SARS-CoV-2, and this infection was not a significant cause of asthma exacerbation.

Keywords: Asthma; COVID-19; Coronavirus; Prevalence; Risk factors.

Copyright © 2020. Published by Elsevier Ltd.

Conflict of interest statement

The authors declare that they have no known competing financial interests or personal relationships that could have appeared to influence the work reported in this paper.

- 15 references

Supplementary info

Publication types, MeSH terms, Substances Expand

Publication types

- Observational Study

MeSH terms

- Anti-Asthmatic Agents / therapeutic use
- Asthma* / diagnosis
- Asthma* / epidemiology
- Asthma* / therapy
- Asthma* / virology
- Betacoronavirus / isolation & purification*
- COVID-19
- COVID-19 Testing
- Clinical Laboratory Techniques / methods*
- Comorbidity
- Coronavirus Infections* / diagnosis
- Coronavirus Infections* / epidemiology
- Coronavirus Infections* / physiopathology
- Female
- Hospitalization / statistics & numerical data

- Humans
- Male
- Middle Aged
- Pandemics*
- Pneumonia, Viral* / diagnosis
- Pneumonia, Viral* / epidemiology
- Pneumonia, Viral* / physiopathology
- Prevalence
- Retrospective Studies
- Risk Assessment / methods
- Risk Factors
- SARS-CoV-2
- Spain / epidemiology
- Symptom Assessment / methods

Substances

- Anti-Asthmatic Agents

Full text links

ELSEVIER FULL-TEXT ARTICLE Elsevier Science Free PMC article
Proceed to details
Cite
Share
☐ 661
Observational Study
Cardiovasc Drugs Ther
. 2022 Feb;36(1):113-120.
doi: 10.1007/s10557-020-07133-3. Epub 2021 Jan 4.

The Association of Low Molecular Weight Heparin Use and In-hospital Mortality Among Patients Hospitalized with COVID-19

Lan Shen[# 1], Lin Qiu[# 2], Dong Liu[# 2], Li Wang[# 3], Hengye Huang[4], Heng Ge[5], Ying Xiao[2], Yi Liu[2], Jingjin Jin[2], Xiulan Liu[2], Dao Wen Wang[6], Eric D Peterson[7], Ben He[8], Ning Zhou[9]
Affiliations Expand

Affiliations

- [1] Department of Cardiology, Shanghai Chest Hospital, Shanghai Jiaotong University, Huaihai Road, Shanghai, 200030, China.
- [2] Department of Pharmacy, Tongji Hospital, Tongji Medical College, Huazhong University of Science and Technology, 1095 Jiefang Ave., Wuhan, 430030, China.
- [3] Department of Geriatrics, Shanghai Renji Hospital, Shanghai Jiaotong University, School of Medicine, Shanghai, 200127, China.
- [4] School of Public Health, Shanghai Jiaotong University, School of Medicine, Shanghai, 200025, China.
- [5] Department of Cardiology, Shanghai Renji Hospital, Shanghai Jiaotong University, School of Medicine, Shanghai, 200127, China.
- [6] Division of Cardiology, Department of Internal Medicine, Tongji Hospital, Tongji Medical College, Huazhong University of Science and Technology, 1095 Jiefang Ave., Wuhan, 430030, China.
- [7] Duke Clinical Research Institute, 300 W Morgan St, Durham, NC, 27701, USA.
- [8] Department of Cardiology, Shanghai Chest Hospital, Shanghai Jiaotong University, Huaihai Road, Shanghai, 200030, China. heben241@126.com.
- [9] Division of Cardiology, Department of Internal Medicine, Tongji Hospital, Tongji Medical College, Huazhong University of Science and Technology, 1095 Jiefang Ave., Wuhan, 430030, China. zhouning@tjh.tjmu.edu.cn.

[#] Contributed equally.

- PMID: **33394360**
- PMCID: PMC7779648
- DOI: 10.1007/s10557-020-07133-3

Free PMC article
Observational Study

The Association of Low Molecular Weight Heparin Use and In-hospital Mortality Among Patients Hospitalized with COVID-19

Lan Shen et al. Cardiovasc Drugs Ther. 2022 Feb.
Free PMC article
Show details
Cardiovasc Drugs Ther
. 2022 Feb;36(1):113-120.
doi: 10.1007/s10557-020-07133-3. Epub 2021 Jan 4.

Authors

Lan Shen [# 1], Lin Qiu [# 2], Dong Liu [# 2], Li Wang [# 3], Hengye Huang [4], Heng Ge [5], Ying Xiao [2], Yi Liu [2], Jingjin Jin [2], Xiulan Liu [2], Dao Wen Wang [6], Eric D Peterson [7], Ben He [8], Ning Zhou [9]

Affiliations

- [1] Department of Cardiology, Shanghai Chest Hospital, Shanghai Jiaotong University, Huaihai Road, Shanghai, 200030, China.
- [2] Department of Pharmacy, Tongji Hospital, Tongji Medical College, Huazhong University of Science and Technology, 1095 Jiefang Ave., Wuhan, 430030, China.
- [3] Department of Geriatrics, Shanghai Renji Hospital, Shanghai Jiaotong University, School of Medicine, Shanghai, 200127, China.
- [4] School of Public Health, Shanghai Jiaotong University, School of Medicine, Shanghai, 200025, China.
- [5] Department of Cardiology, Shanghai Renji Hospital, Shanghai Jiaotong University, School of Medicine, Shanghai, 200127, China.
- [6] Division of Cardiology, Department of Internal Medicine, Tongji Hospital, Tongji Medical College, Huazhong University of Science and Technology, 1095 Jiefang Ave., Wuhan, 430030, China.
- [7] Duke Clinical Research Institute, 300 W Morgan St, Durham, NC, 27701, USA.
- [8] Department of Cardiology, Shanghai Chest Hospital, Shanghai Jiaotong University, Huaihai Road, Shanghai, 200030, China. heben241@126.com.
- [9] Division of Cardiology, Department of Internal Medicine, Tongji Hospital, Tongji Medical College, Huazhong University of Science and Technology, 1095 Jiefang Ave., Wuhan, 430030, China. zhouning@tjh.tjmu.edu.cn.

[#] Contributed equally.

- PMID: **33394360**
- PMCID: PMC7779648
- DOI: 10.1007/s10557-020-07133-3

Abstract

Purpose: To determine the association between low molecular weight heparin (LMWH) use and mortality in hospitalized COVID-19 patients.

Methods: We conducted a retrospective study of patients consecutively enrolled from two major academic hospitals exclusively for COVID-19 in Wuhan, China, from January 26, 2020, to March 26, 2020. The primary outcome was adjusted in-hospital mortality in the LMWH group compared with the non-LMWH group using the propensity score.

Results: Overall, 525 patients with COVID-19 enrolled with a median age of 64 years (IQR 19), and 49.33% men. Among these, 120 (22.86%) were treated with LMWH. Compared with the non-LMWH group, the LMWH group was more likely to be older and male; had a history of hypertension, diabetes, coronary heart disease (CHD), or stroke; and had more severe COVID-19 parameters such as higher inflammatory cytokines or D-dimer. Compared with non-LMWH group, LMWH group had a higher unadjusted in-hospital mortality rate (21.70% vs. 11.10%; $p = 0.004$), but a lower adjusted mortality risk (adjusted odds ratio [OR], 0.20; 95% CI, 0.09-0.46). A propensity score-weighting analysis demonstrated similar findings (adjusted OR, 0.18; 95% CI, 0.10-0.30). Subgroup analysis showed a significant survival benefit among those who were severely (adjusted OR, 0.07; 95% CI, 0.02-0.23) and critically ill (adjusted OR, 0.32; 95% CI, 0.15-0.65), as well as among the elderly patients' age > 65, IL-6 > 10 times upper limit level, and D-dimer > 5 times upper limit level.

Conclusions: Among hospitalized COVID-19 patients, LMWH use was associated with lower all-cause in-hospital mortality than non-LMWH users. The survival benefit was particularly significant among more severely ill patients.

Keywords: COVID-19; In-hospital mortality; LMWH.

© 2021. Springer Science+Business Media, LLC, part of Springer Nature.

Conflict of interest statement

The authors declare that they have no conflict of interest.

- 28 references
- 2 figures

Supplementary info

Publication types, MeSH terms, Substances, Supplementary concepts, Grant support Expand

Publication types

- Multicenter Study
- Observational Study

MeSH terms

- Aged
- Aged, 80 and over
- Anticoagulants / adverse effects
- Anticoagulants / therapeutic use*
- Blood Coagulation / drug effects
- COVID-19 / diagnosis
- COVID-19 / drug therapy*
- COVID-19 / mortality
- China / epidemiology
- Comorbidity
- Female
- Hemorrhage / chemically induced
- Heparin, Low-Molecular-Weight / adverse effects
- Heparin, Low-Molecular-Weight / therapeutic use*
- Hospital Mortality
- Hospitalization*
- Humans
- Male
- Middle Aged

- Retrospective Studies
- Risk Assessment
- Risk Factors
- Severity of Illness Index
- Time Factors
- Treatment Outcome

Substances

- Anticoagulants
- Heparin, Low-Molecular-Weight

Supplementary concepts

- COVID-19 drug treatment

Grant support

- 81900308/National Natural Science Foundation of China
- 81570261/National Natural Science Foundation of China
- DLY201512/School of Medicine, Shanghai Jiao Tong University

Full text links

Springer Free PMC article
Proceed to details
Cite
Share
662
Observational Study
J Immunol
. 2021 Apr 1;206(7):1569-1575.
doi: 10.4049/jimmunol.2001126. Epub 2021 Feb 5.

IL-1 Receptor Antagonist Anakinra in the Treatment of COVID-19 Acute Respiratory Distress Syndrome: A Retrospective, Observational Study

Marco Franzetti [1], Andrea Forastieri [2], Noemi Borsa [3], Alessandro Pandolfo [4], Chiara Molteni [4], Luca Borghesi [4], Silvia Pontiggia [4], Giulia Evasi [2], Lorenzo Guiotto [2], Mattia Erba [2], Ugo Pozzetti [5], Anna Ronchetti [5], Letizia Valsecchi [4], Gioacchino Castaldo [4], Ernesto Longoni

[4], Daniele Colombo [3], Marco Soncini [5], Stefano Crespi [6], Stefano Maggiolini [7], Davide Guzzon [8], Stefania Piconi [4]
Affiliations Expand

Affiliations

- [1] Infectious Diseases Unit, Alessandro Manzoni Hospital, 23900 Lecco, Italy; m.franzetti@asst-lecco.it.
- [2] Intensive Care Unit, Alessandro Manzoni Hospital, 23900 Lecco, Italy.
- [3] Respiratory Unit, Scientific Institute for Research, Hospitalization, and Healthcare, Italian National Institutes of Health and Sciences on Ageing, c/o San Leopoldo Mandic Hospital, 23807 Merate, Italy.
- [4] Infectious Diseases Unit, Alessandro Manzoni Hospital, 23900 Lecco, Italy.
- [5] Medicine Department, Alessandro Manzoni Hospital, 23900 Lecco, Italy.
- [6] Medicine Department, San Leopoldo Mandic Hospital, 23807 Merate, Italy.
- [7] Cardiology Department, San Leopoldo Mandic Hospital, 23807 Merate, Italy; and.
- [8] Intensive Care Unit, San Leopoldo Mandic Hospital, 23807 Merate, Italy.

- PMID: **33547169**
- PMCID: PMC7980530
- DOI: 10.4049/jimmunol.2001126

Free PMC article
Observational Study

IL-1 Receptor Antagonist Anakinra in the Treatment of COVID-19 Acute Respiratory Distress Syndrome: A Retrospective, Observational Study

Marco Franzetti et al. J Immunol. 2021.
Free PMC article
Show details
J Immunol
. 2021 Apr 1;206(7):1569-1575.
doi: 10.4049/jimmunol.2001126. Epub 2021 Feb 5.

Authors

Marco Franzetti [1], Andrea Forastieri [2], Noemi Borsa [3], Alessandro Pandolfo [4], Chiara Molteni [4], Luca Borghesi [4], Silvia Pontiggia [4], Giulia Evasi [2], Lorenzo Guiotto [2], Mattia Erba [2], Ugo Pozzetti [5], Anna Ronchetti [5], Letizia Valsecchi [4], Gioacchino Castaldo [4], Ernesto Longoni [4], Daniele Colombo [3], Marco Soncini [5], Stefano Crespi [6], Stefano Maggiolini [7], Davide Guzzon [8], Stefania Piconi [4]

Affiliations

- [1] Infectious Diseases Unit, Alessandro Manzoni Hospital, 23900 Lecco, Italy; m.franzetti@asst-lecco.it.
- [2] Intensive Care Unit, Alessandro Manzoni Hospital, 23900 Lecco, Italy.
- [3] Respiratory Unit, Scientific Institute for Research, Hospitalization, and Healthcare, Italian National Institutes of Health and Sciences on Ageing, c/o San Leopoldo Mandic Hospital, 23807 Merate, Italy.
- [4] Infectious Diseases Unit, Alessandro Manzoni Hospital, 23900 Lecco, Italy.
- [5] Medicine Department, Alessandro Manzoni Hospital, 23900 Lecco, Italy.
- [6] Medicine Department, San Leopoldo Mandic Hospital, 23807 Merate, Italy.
- [7] Cardiology Department, San Leopoldo Mandic Hospital, 23807 Merate, Italy; and.
- [8] Intensive Care Unit, San Leopoldo Mandic Hospital, 23807 Merate, Italy.

- PMID: **33547169**
- PMCID: PMC7980530
- DOI: 10.4049/jimmunol.2001126

Abstract

The IL-1 receptor antagonist, anakinra, may represent a therapeutic option for acute respiratory distress syndrome (ARDS) associated with coronavirus disease 2019 (COVID-19). In this study, COVID-19 ARDS patients admitted to the Azienda Socio Sanitaria Territoriale of Lecco, Italy, between March 5th to April 15th, 2020, and who had received anakinra off-label were retrospectively evaluated and compared with a cohort of matched controls who did not receive immunomodulatory treatment. The primary end point was survival at day 28. The population consisted of 112 patients (56 treated with anakinra and 56 controls). Survival at day 28 was obtained in 69 patients (61.6%) and was significantly higher in anakinra-treated patients than in the controls (75.0 versus 48.2%, $p = 0.007$). When stratified by continuous positive airway pressure support at baseline, anakinra-treated patients' survival was also significant compared with the controls ($p = 0.008$). Univariate analysis identified anakinra usage (odds ratio, 3.2; 95% confidence interval, 1.47-7.17) as a significant survival predictor. This was not supported by multivariate modeling. The rate of infectious-related adverse events was similar between groups. In conclusion, anakinra improved overall survival and invasive ventilation-free survival and was well tolerated in patients with ARDS associated with COVID-19.

Copyright © 2021 by The American Association of Immunologists, Inc.

Conflict of interest statement

The authors have no financial conflicts of interest.

- 2 figures

Supplementary info

Publication types, MeSH terms, Substances Expand

Publication types

- Observational Study

MeSH terms

- Aged
- COVID-19* / immunology
- COVID-19* / mortality
- COVID-19* / therapy
- Disease-Free Survival
- Female
- Humans
- Interleukin 1 Receptor Antagonist Protein / administration & dosage*
- Interleukin 1 Receptor Antagonist Protein / antagonists & inhibitors
- Interleukin 1 Receptor Antagonist Protein / immunology
- Male
- Middle Aged
- Respiration, Artificial*
- Retrospective Studies
- SARS-CoV-2 / immunology*
- Severe Acute Respiratory Syndrome* / immunology
- Severe Acute Respiratory Syndrome* / mortality
- Severe Acute Respiratory Syndrome* / therapy
- Severe Acute Respiratory Syndrome* / virology
- Survival Rate

Substances

- IL1RN protein, human
- Interleukin 1 Receptor Antagonist Protein

Full text links

Final Version J Immunol HighWire Free PMC article

Proceed to details

Cite

Share

663

Observational Study

Am J Emerg Med

. 2021 Dec;50:156-159.

doi: 10.1016/j.ajem.2021.07.058. Epub 2021 Jul 31.

Influence of Pennsylvania liquor store closures during the COVID-19 pandemic on alcohol withdrawal consultations

Alexandra M Amaducci [1], Ali R Yazdanyar [2], Derek J Fikse [2], Jasdip Kaur [2], Andrew L Koons [2], Gillian A Beauchamp [2], Robert D Cannon [2], Ryan M Surmaitis [2], Matthew D Cook [2], Kenneth D Katz [2]
Affiliations Expand

Affiliations

- [1] Lehigh Valley Health Network, USF Morsani College of Medicine, Allentown, PA, United States of America. Electronic address: Alexandra.Amaducci@lvhn.org.
- [2] Lehigh Valley Health Network, USF Morsani College of Medicine, Allentown, PA, United States of America.

- PMID: **34365065**
- PMCID: PMC8631575
- DOI: 10.1016/j.ajem.2021.07.058

Free PMC article
Observational Study

Influence of Pennsylvania liquor store closures during the COVID-19 pandemic on alcohol withdrawal consultations

Alexandra M Amaducci et al. Am J Emerg Med. 2021 Dec.
Free PMC article
Show details
Am J Emerg Med
. 2021 Dec;50:156-159.
doi: 10.1016/j.ajem.2021.07.058. Epub 2021 Jul 31.

Authors

Alexandra M Amaducci [1], Ali R Yazdanyar [2], Derek J Fikse [2], Jasdip Kaur [2], Andrew L Koons [2], Gillian A Beauchamp [2], Robert D Cannon [2], Ryan M Surmaitis [2], Matthew D Cook [2], Kenneth D Katz [2]

Affiliations

- [1] Lehigh Valley Health Network, USF Morsani College of Medicine, Allentown, PA, United States of America. Electronic address: Alexandra.Amaducci@lvhn.org.

- [2] Lehigh Valley Health Network, USF Morsani College of Medicine, Allentown, PA, United States of America.

- PMID: **34365065**
- PMCID: PMC8631575
- DOI: 10.1016/j.ajem.2021.07.058

Abstract

Introduction: Alcohol withdrawal syndrome (AWS) is a serious consequence of alcohol use disorder (AUD). Due to the current COVID-19 pandemic there was a closure of Pennsylvania (PA) liquor stores on March 17, 2020.

Methods: This is a retrospective, observational study of AWS patients presenting to a tertiary care hospital. We used descriptive statistics for continuous and categorical variables and compared AWS consults placed to the medical toxicology service for six months preceding liquor store closure to those placed between March 17, 2020 and August 31, 2020. We compared this to consults placed to the medical toxicology service placed from October 1, 2019 through March 16, 2020. Charts were identified based on consults placed to the medical toxicology service, and alcohol withdrawal was determined via chart review by a medical toxicologist. This study did not require IRB approval. We evaluated Emergency Department (ED) length of stay (LOS), weekly and monthly consultation rate, rate of admission and ED recidivism, both pre- and post-liquor store closure.

Results: A total of 324 AWS consults were placed during the ten month period. 142 (43.8%) and 182 (56.2%) consults were pre- and post-liquor store closure. The number of consults was not statistically significant comparing these two time frames. There was no significant difference by patient age, gender, or race or by weekly or monthly consultation rate when comparing pre- and post-liquor store periods. The median ED LOS was 7 h (95% Confidence Interval (CI) Larson et al. (2012), Pollard et al. (2020) [5, 11]) and did not significantly differ between pre- and post-liquor store periods (p = 0.78). 92.9% of AWS patients required admission without significant difference between the pre- and post-liquor store closure periods (94.4% vs. 91.8%, p = 0.36). There was a significant increase in the number of AWS patients requiring a return ED visit (Odds Ratio 2.49; 95% CI [1.38, 4.49]) post closure.

Conclusion: There were nearly 2.5 times greater odds of ED recidivism among post-liquor store closure AWS patients compared with pre-closure AWS patients.

Keywords: Alcohol use disorder; Alcohol withdrawal; COVID-19; Toxicology.

Copyright © 2021 Elsevier Inc. All rights reserved.

Conflict of interest statement

Declaration of Competing Interest None.

- 11 references
- 1 figure

Supplementary info

Publication types, MeSH terms Expand

Publication types

- Observational Study

MeSH terms

- Adult
- Alcoholic Beverages*
- Alcoholism / diagnosis
- Alcoholism / epidemiology*
- Alcoholism / therapy
- COVID-19 / epidemiology
- COVID-19 / prevention & control*
- COVID-19 / transmission
- Communicable Disease Control / organization & administration*
- Emergency Service, Hospital / statistics & numerical data
- Facilities and Services Utilization
- Female
- Hospitalization / statistics & numerical data
- Humans
- Male
- Middle Aged
- Pennsylvania / epidemiology
- Referral and Consultation / statistics & numerical data*
- Retrospective Studies
- Substance Withdrawal Syndrome / diagnosis
- Substance Withdrawal Syndrome / epidemiology*
- Substance Withdrawal Syndrome / therapy
- Young Adult

Full text links

ELSEVIER FULL-TEXT ARTICLE Elsevier Science Free PMC article
Proceed to details
Cite
Share
☐ 664
Observational Study
J Palliat Care
. 2022 Jan;37(1):18-25.
doi: 10.1177/08258597211036579. Epub 2021 Aug 17.

Place of Death of Geriatric Population in Turkey: A 7-Year Observational Study

Serdar Ceylan [1], Merve Guner Oytun [1], Arzu Okyar Bas [1], Zeynep Kahyaoglu [1], Burcu B Dogu [1], Mustafa Cankurtaran [1], Meltem G Halil [1]
Affiliations Expand

Affiliation

- [1] Division of Geriatrics, Department of Internal Medicine, Faculty of Medicine, 64005Hacettepe University, Ankara, Turkey.

- PMID: **34402330**
- DOI: 10.1177/08258597211036579

Observational Study

Place of Death of Geriatric Population in Turkey: A 7-Year Observational Study

Serdar Ceylan et al. J Palliat Care. 2022 Jan.
Show details
J Palliat Care
. 2022 Jan;37(1):18-25.
doi: 10.1177/08258597211036579. Epub 2021 Aug 17.

Authors

Serdar Ceylan [1], Merve Guner Oytun [1], Arzu Okyar Bas [1], Zeynep Kahyaoglu [1], Burcu B Dogu [1], Mustafa Cankurtaran [1], Meltem G Halil [1]

Affiliation

- [1] Division of Geriatrics, Department of Internal Medicine, Faculty of Medicine, 64005Hacettepe University, Ankara, Turkey.

- PMID: **34402330**
- DOI: 10.1177/08258597211036579

Abstract

End-of-life care has become an important public health issue in recent years. Place of death is a major component of end-of-life care. Despite attempts to improve end-of-life care, there has not been published any data about place of deaths in Turkey. **Aim:** This retrospective, cross-sectional study investigates the place of death and trends over the years in geriatric age groups in Turkey. **Methods:** Patients who were admitted to geriatric outpatient clinic of a university hospital during

a 7-year period were included. Place and date of death information were received from the death notification system and recorded as hospital or out-of-hospital death. Demographic and clinical data were collected from the hospital information system. Deaths occurring after March 1, 2020 were not included to eliminate the effect of coronavirus disease-2019 pandemic. **Results:** A total of 4025 (20.7%) patients were determined to be dead. Approximately three-quarters of deaths (73.0%) occurred in hospital. The number of deaths reported from nursing homes was only 13 (3.0%). Patients with dementia less frequently died in hospital, however, it was not statistically significant (12.4% vs 14.7%, $P = .05$). The prevalence of death in hospital was significantly higher in patients with chronic renal failure (3.1% vs 1.7%, $P = .02$). The presence of comorbid conditions such as heart failure, cerebrovascular disease, Parkinson's disease, chronic obstructive pulmonary disease/asthma, and cancer did not affect the place of death ($P = .24$, .21, .24, .51, and .18). Out-of-hospital mortality increased with advanced age ($P < .001$). No significant difference was found in the place of death over the years ($P = .41$). **Conclusion:** To the best of our knowledge, this is the first study examining the place of death in Turkey, an aging country. Our results may help to establish policies about end-of-life care in elderly people to improve quality of life by using resources effectively.

Keywords: Turkey; aging country; nursing home; palliative care; place of death.

Supplementary info

Publication types, MeSH terms Expand

Publication types

- Observational Study

MeSH terms

- Aged
- COVID-19*
- Cross-Sectional Studies
- Humans
- Quality of Life
- Retrospective Studies
- SARS-CoV-2
- Terminal Care*
- Turkey / epidemiology

Full text links

SAGE journals Atypon
Proceed to details
Cite
Share
☐ 665
Clinical Trial

PLoS One
. 2021 Aug 19;16(8):e0256331.
doi: 10.1371/journal.pone.0256331. eCollection 2021.

Sociodemographic, laboratory, image data and predictors of gravity risk in patients with COVID-19

Víctor de Oliveira Costa [1] [2], Eveline Montessi Nicolini [3], Bruna Malaquias Arguelles da Costa [4], Victor Hugo Perches Ferreira [5], Ana Julia Rodrigues Tonisi [5], Nathália Munck Machado [6], Marcos de Assis Moura [7], Jorge Montessi [3], Lincoln Eduardo Villela Vieira de Castro Ferreira [8], Rogério Leal Campos [9], Patricia Moreira Costa [10], Maria Antônia Campos [10]
Affiliations Expand

Affiliations

- [1] Medicine, Faculdade de Ciências Médicas e da Saúde de Juiz de Fora - Suprema, Juiz de Fora, Minas Gerais, Brazil.
- [2] Physics, Universidade Federal de Juiz de Fora - UFJF, Juiz de Fora, Minas Gerais, Brazil.
- [3] Department of Toracic Surgery, Hospital Monte Sinai, Juiz de Fora, Minas Gerais, Brazil.
- [4] Nursing, Faculdade de Ciências Médicas e da Saúde de Juiz de Fora - Suprema, Juiz de Fora, Minas Gerais, Brazil.
- [5] Medicine, Universidade Federal de Juiz de Fora, Juiz de Fora, Minas Gerais, Brazil.
- [6] Department of Population Health, University of Kansas Medical Center, Kansas, United States of America.
- [7] Department of Infectology, Faculdade de Ciências Médicas e da Saúde de Juiz de Fora - Suprema and Universidade Federal de Juiz de Fora (UFJF), Juiz de Fora, Minas Gerais, Brazil.
- [8] Digestive Endoscopy Unit, Hospital Monte Sinai and Hospital Universitário - UFJF, Juiz de Fora, Minas Gerais, Brazil.
- [9] Department of Emergency, Hospital Monte Sinai, Juiz de Fora, Minas Gerais, Brazil.
- [10] Department of Intensive Care, Hospital Monte Sinai, Juiz de Fora, Minas Gerais, Brazil.

- PMID: **34411145**
- PMCID: PMC8375972
- DOI: 10.1371/journal.pone.0256331

Free PMC article
Clinical Trial

Sociodemographic, laboratory, image data and predictors of gravity risk in patients with COVID-19

Víctor de Oliveira Costa et al. PLoS One. 2021.
Free PMC article
Show details
PLoS One
. 2021 Aug 19;16(8):e0256331.
doi: 10.1371/journal.pone.0256331. eCollection 2021.

Authors

Víctor de Oliveira Costa [1] [2], Eveline Montessi Nicolini [3], Bruna Malaquias Arguelles da Costa [4], Victor Hugo Perches Ferreira [5], Ana Julia Rodrigues Tonisi [5], Nathália Munck Machado [6], Marcos de Assis Moura [7], Jorge Montessi [3], Lincoln Eduardo Villela Vieira de Castro Ferreira [8], Rogério Leal Campos [9], Patricia Moreira Costa [10], Maria Antônia Campos [10]

Affiliations

- [1] Medicine, Faculdade de Ciências Médicas e da Saúde de Juiz de Fora - Suprema, Juiz de Fora, Minas Gerais, Brazil.
- [2] Physics, Universidade Federal de Juiz de Fora - UFJF, Juiz de Fora, Minas Gerais, Brazil.
- [3] Department of Toracic Surgery, Hospital Monte Sinai, Juiz de Fora, Minas Gerais, Brazil.
- [4] Nursing, Faculdade de Ciências Médicas e da Saúde de Juiz de Fora - Suprema, Juiz de Fora, Minas Gerais, Brazil.
- [5] Medicine, Universidade Federal de Juiz de Fora, Juiz de Fora, Minas Gerais, Brazil.
- [6] Department of Population Health, University of Kansas Medical Center, Kansas, United States of America.
- [7] Department of Infectology, Faculdade de Ciências Médicas e da Saúde de Juiz de Fora - Suprema and Universidade Federal de Juiz de Fora (UFJF), Juiz de Fora, Minas Gerais, Brazil.
- [8] Digestive Endoscopy Unit, Hospital Monte Sinai and Hospital Universitário - UFJF, Juiz de Fora, Minas Gerais, Brazil.
- [9] Department of Emergency, Hospital Monte Sinai, Juiz de Fora, Minas Gerais, Brazil.
- [10] Department of Intensive Care, Hospital Monte Sinai, Juiz de Fora, Minas Gerais, Brazil.

- PMID: **34411145**
- PMCID: PMC8375972
- DOI: 10.1371/journal.pone.0256331

Abstract

Introduction: The effects, severity, and prognosis of COVID-19 infections do not follow a linear pattern in different locations, but change according to the epidemiological data and social issues in each region.

Aims: The purpose of the current study is to provide the clinical and epidemiological standard of the population affected by COVID-19 in the city of Juiz de Fora, MG to better understand the disease and its risk factors, in order to enable more appropriate conduct for patients.

Methods: A retrospective observational study was carried out from March to August of 2020, with 266 participants admitted to the emergency department of the Instituto de Clínicas e Cirurgia de Juiz de Fora-Hospital Monte Sinai. Data were tabulated, analyzed, and classified according to the outcome using an ordinal regression model.

Results: Among the 266 admitted patients, the most common findings were ground-glass opacifications on chest CT (78.8%), cough (75.6%), fever (58.4%), and rhinorrhea (34.5%). There were greater severity and greater need for hospitalization and admission to the ICU in patients who were male, tachypneic at the time of admission, with older age, and with underlying diseases.

Conclusion: Collected data allowed for a better understanding of the disease, its severity criteria, and its pattern of affection in Juiz de Fora, MG. More studies based on the analysis of the behavior of COVID-19 in different regions must be carried out, to improve treatment and support to local populations.

Conflict of interest statement

The authors have declared that no competing interests exist.

- 46 references
- 2 figures

Supplementary info

Publication types, MeSH terms, Supplementary concepts, Grant support Expand

Publication types

- Clinical Trial
- Observational Study

MeSH terms

- Adolescent
- Adult
- Age Factors
- Aged
- Brazil / epidemiology
- COVID-19 / diagnostic imaging
- COVID-19 / drug therapy*
- COVID-19 / epidemiology
- Child
- Child, Preschool
- Emergency Service, Hospital

- Female
- Humans
- Infant
- Infant, Newborn
- Male
- Middle Aged
- Retrospective Studies
- SARS-CoV-2*
- Severity of Illness Index
- Sex Factors
- Tomography, X-Ray Computed*

Supplementary concepts

- COVID-19 drug treatment

Grant support

The author(s) received no specific funding for this work.

Full text links

OPEN ACCESS TO FULL TEXT PLOS ONE Public Library of Science Free PMC article

Proceed to details

Cite

Share

☐ 666

Observational Study

BMC Infect Dis
. 2021 Mar 5;21(1):241.
doi: 10.1186/s12879-021-05912-3.

Tocilizumab improves survival in severe COVID-19 pneumonia with persistent hypoxia: a retrospective cohort study with follow-up from Mumbai, India

Yojana Gokhale [1], Rakshita Mehta [2], Uday Kulkarni [3], Nitin Karnik [2], Sushant Gokhale [2], Uma Sundar [2], Swati Chavan [2], Akshay Kor [2], Sonal Thakur [2], Trupti Trivedi [2], Naveen Kumar [2], Sujata Baveja [2], Aniket Wadal [2], Shaonak Kolte [2], Aukshan Deolankar [2], Sangeeta Pednekar [2], Lalana Kalekar [2], Rupal Padiyar [2], Charulata Londhe [2], Pramod Darole [2], Sujata Pol

[2], Seema Bansode Gokhe [2], Namita Padwal [2], Dharmendra Pandey [2], Dhirendra Yadav [2], Anagha Joshi [2], Harshal Badgujar [2], Mayuri Trivedi [2], Priyanshu Shah [2], Prerna Bhavsar [2]
Affiliations Expand

Affiliations

- [1] Lokmanya Tilak Municipal Medical College, Sion Mumbai, 400022, India. yojana1962@gmail.com.
- [2] Lokmanya Tilak Municipal Medical College, Sion Mumbai, 400022, India.
- [3] Christian Medical College, Vellore, Tamil Nadu, India.

- PMID: **33673818**
- PMCID: PMC7934984
- DOI: 10.1186/s12879-021-05912-3

Free PMC article
Observational Study

Tocilizumab improves survival in severe COVID-19 pneumonia with persistent hypoxia: a retrospective cohort study with follow-up from Mumbai, India

Yojana Gokhale et al. BMC Infect Dis. 2021.
Free PMC article
Show details
BMC Infect Dis
. 2021 Mar 5;21(1):241.
doi: 10.1186/s12879-021-05912-3.

Authors

Yojana Gokhale [1], Rakshita Mehta [2], Uday Kulkarni [3], Nitin Karnik [2], Sushant Gokhale [2], Uma Sundar [2], Swati Chavan [2], Akshay Kor [2], Sonal Thakur [2], Trupti Trivedi [2], Naveen Kumar [2], Sujata Baveja [2], Aniket Wadal [2], Shaonak Kolte [2], Aukshan Deolankar [2], Sangeeta Pednekar [2], Lalana Kalekar [2], Rupal Padiyar [2], Charulata Londhe [2], Pramod Darole [2], Sujata Pol [2], Seema Bansode Gokhe [2], Namita Padwal [2], Dharmendra Pandey [2], Dhirendra Yadav [2], Anagha Joshi [2], Harshal Badgujar [2], Mayuri Trivedi [2], Priyanshu Shah [2], Prerna Bhavsar [2]

Affiliations

- [1] Lokmanya Tilak Municipal Medical College, Sion Mumbai, 400022, India. yojana1962@gmail.com.
- [2] Lokmanya Tilak Municipal Medical College, Sion Mumbai, 400022, India.
- [3] Christian Medical College, Vellore, Tamil Nadu, India.

- PMID: **33673818**
- PMCID: PMC7934984
- DOI: 10.1186/s12879-021-05912-3

Abstract

Background: Cytokine storm triggered by Severe Coronavirus Disease 2019 (COVID-19) is associated with high mortality. With high Interleukin -6 (IL-6) levels reported in COVID-19 related deaths in China, IL-6 is considered to be the key player in COVID-19 cytokine storm. Tocilizumab, a monoclonal antibody against IL-6 receptor, is used on compassionate grounds for treatment of COVID-19 cytokine storm. The aim of this study was to assess effect of tocilizumab on mortality due to COVID-19 cytokine storm.

Method: This retrospective, observational study included patients of severe COVID-19 pneumonia with persistent hypoxia (defined as saturation 94% or less on supplemental Oxygen of 15 L per minute through non-rebreathing mask or PaO2/FiO2 ratio of less than 200) who were admitted to a tertiary care center in Mumbai, India, between 31st March to 5th July 2020. In addition to standard care, single Inj. Tocilizumab 400 mg was given intravenously to 151 consecutive COVID-19 patients with persistent hypoxia, from 13th May to 5th July 2020. These 151 patients were retrospectively analysed and compared with historic controls, ie consecutive COVID-19 patients with persistent hypoxia, defined as stated above (N = 118, from our first COVID-19 admission on 31st March to 12th May 2020 i.e., till tocilizumab was available in hospital). Univariate and multivariate Cox regression analysis was performed for identifying predictors of survival. Statistical analysis was performed using IBM SPSS version 26.

Results: Out of 269 (151 in tocilizumab group and 118 historic controls) patients studied from 31st March to 5th July 2020, median survival in the tocilizumab group was significantly longer than in the control group; 18 days (95% CI, 11.3 to 24.7) versus 9 days (95% CI, 5.7 to 12.3); log rank p 0.007. On multivariate Cox regression analysis, independent predictors of survival were use of tocilizumab (HR 0.621, 95% CI 0.427-0.903, P 0.013) and higher oxygen saturation.

Conclusion: Tocilizumab may improve survival in severe COVID-19 pneumonia with persistent hypoxia. Randomised controlled trials on use of tocilizumab as rescue therapy in patients of severe COVID-19 pneumonia with hypoxia (PaO2/FiO2 less than 200) due to hyperinflammatory state, are warranted.

Keywords: CO-RADS; CT-severity score; Cytokine storm; Hyperinflammatory syndrome; IL-6; Interlukin-6.

Conflict of interest statement

Authors have ‘No competing interests’ to declare.

- 32 references
- 1 figure

Supplementary info

Publication types, MeSH terms, Substances Expand

Publication types

- Observational Study

MeSH terms

- Antibodies, Monoclonal, Humanized / administration & dosage*
- COVID-19* / epidemiology
- COVID-19* / immunology
- COVID-19* / physiopathology
- COVID-19* / therapy
- Compassionate Use Trials / statistics & numerical data
- Cytokine Release Syndrome* / etiology
- Cytokine Release Syndrome* / immunology
- Cytokine Release Syndrome* / therapy
- Female
- Humans
- Hypoxia* / etiology
- Hypoxia* / therapy
- India / epidemiology
- Interleukin-6 / antagonists & inhibitors*
- Interleukin-6 / immunology
- Male
- Middle Aged
- Pneumonia, Viral* / blood
- Pneumonia, Viral* / etiology
- Pneumonia, Viral* / mortality
- Pneumonia, Viral* / therapy
- Respiration, Artificial / methods
- Retrospective Studies
- SARS-CoV-2 / isolation & purification
- Severity of Illness Index
- Survival Analysis
- Treatment Outcome

Substances

- Antibodies, Monoclonal, Humanized
- Interleukin-6
- tocilizumab

Full text links

Read free full text at BMC BioMed Central Free PMC article

Proceed to details

Cite

Share

667

Observational Study

Medicine (Baltimore)

. 2021 May 14;100(19):e25923.

doi: 10.1097/MD.0000000000025923.

Sarilumab (IL-6R antagonist) in critically ill patients with cytokine release syndrome by SARS-CoV2

Hèctor Corominas [1], Ivan Castellví [1], César Diaz-Torné [1], Laia Matas [2], David de la Rosa [3], Maria Antònia Mangues [4], Patricia Moya [1], Virginia Pomar [5], Natividad Benito [5], Ester Moga [6], Nerea Hernandez-de Sosa [2], Jordi Casademont [2], Pere Domingo [5]

Affiliations Expand

Affiliations

- [1] Servei de Reumatologia.
- [2] Servei de Medicina Interna.
- [3] Servei de Pneumologia.
- [4] Servei de Farmàcia Hospitalària.
- [5] Unitat de Malalties Infeccioses.
- [6] Servei Immunologia, Hospital de la Santa Creu i Sant Pau, Barcelona, Universitat Autònoma de Barcelona (UAB), Barcelona, Catalonia, Spain.

- PMID: **34106658**
- PMCID: PMC8133253
- DOI: 10.1097/MD.0000000000025923

Free PMC article

Observational Study

Sarilumab (IL-6R antagonist) in critically ill patients with cytokine release syndrome by SARS-CoV2

Hèctor Corominas et al. Medicine (Baltimore). 2021.

Free PMC article

Show details

Medicine (Baltimore)

. 2021 May 14;100(19):e25923.
doi: 10.1097/MD.0000000000025923.

Authors

Hèctor Corominas [1], Ivan Castellví [1], César Diaz-Torné [1], Laia Matas [2], David de la Rosa [3], Maria Antònia Mangues [4], Patricia Moya [1], Virginia Pomar [5], Natividad Benito [5], Ester Moga [6], Nerea Hernandez-de Sosa [2], Jordi Casademont [2], Pere Domingo [5]

Affiliations

- [1] Servei de Reumatologia.
- [2] Servei de Medicina Interna.
- [3] Servei de Pneumologia.
- [4] Servei de Farmàcia Hospitalària.
- [5] Unitat de Malalties Infeccioses.
- [6] Servei Immunologia, Hospital de la Santa Creu i Sant Pau, Barcelona, Universitat Autònoma de Barcelona (UAB), Barcelona, Catalonia, Spain.

- PMID: **34106658**
- PMCID: PMC8133253
- DOI: 10.1097/MD.0000000000025923

Abstract

Blocking IL-6 pathways with sarilumab, a fully human anti-IL-6R antagonist may potentially curb the inflammatory storm of SARS-CoV2. In the present emergency scenario, we used "off-label" sarilumab in 5 elderly patients in life-threatening condition not candidates to further active measures. We suggest that sarilumab can modulate severe COVID-19-associated Cytokine Release Syndrome.

Copyright © 2021 the Author(s). Published by Wolters Kluwer Health, Inc.

Conflict of interest statement

The authors have no conflicts of interests to disclose.

- 17 references

Supplementary info

Publication types, MeSH terms, Substances, Supplementary concepts Expand

Publication types

- Observational Study

MeSH terms

- Aged
- Anti-Infective Agents / therapeutic use
- Antibodies, Monoclonal, Humanized / therapeutic use*
- Azithromycin / therapeutic use
- COVID-19 / drug therapy*
- COVID-19 / physiopathology
- Comorbidity
- Critical Illness
- Cytokine Release Syndrome / drug therapy*
- Cytokine Release Syndrome / physiopathology
- Humans
- Hydroxychloroquine / therapeutic use
- Interleukin-6 / antagonists & inhibitors*
- Male
- Middle Aged
- RNA, Viral
- Respiration, Artificial / statistics & numerical data
- Retrospective Studies
- SARS-CoV-2

Substances

- Anti-Infective Agents
- Antibodies, Monoclonal, Humanized
- Interleukin-6
- RNA, Viral
- Hydroxychloroquine
- Azithromycin
- sarilumab

Supplementary concepts

- COVID-19 drug treatment

Full text links

Wolters Kluwer Wolters Kluwer Free PMC article

Proceed to details

Cite

Share

☐ 668
Observational Study
J Interv Card Electrophysiol
. 2020 Dec;59(3):485-493.
doi: 10.1007/s10840-020-00896-7. Epub 2020 Oct 31.

T-wave inversion as a manifestation of COVID-19 infection: a case series

Jorge Romero [1], Isabella Alviz [1], Michael Parides [1], Juan Carlos Diaz [1], David Briceno [1], Mohamed Gabr [1], Maria Gamero [1], Kavisha Patel [1], Eric D Braunstein [1], Sutopa Purkayastha [1], Dalvert Polanco [1], Carolina R Valencia [1], Domenico Della Rocca [2], Alejandro Velasco [1], Ruike Yang [1], Nicola Tarantino [1], Xiao-Dong Zhang [1], Sanghamitra Mohanty [2], Juan Bello [1], Andrea Natale [2], Ulrich P Jorde [1], Mario Garcia [1], Luigi Di Biase [3] [4]
Affiliations Expand

Affiliations

- [1] Cardiac Arrhythmia Center, Division of Cardiology, Department of Medicine, Montefiore-Einstein Center for Heart and Vascular Care, Bronx, NY, USA.
- [2] Texas Cardiac Arrhythmia Institute, St. David's Medical Center, Austin, TX, USA.
- [3] Cardiac Arrhythmia Center, Division of Cardiology, Department of Medicine, Montefiore-Einstein Center for Heart and Vascular Care, Bronx, NY, USA. dibbia@gmail.com.
- [4] Texas Cardiac Arrhythmia Institute, St. David's Medical Center, Austin, TX, USA. dibbia@gmail.com.

- PMID: **33128658**
- PMCID: PMC7602831
- DOI: 10.1007/s10840-020-00896-7

Free PMC article
Observational Study

T-wave inversion as a manifestation of COVID-19 infection: a case series

Jorge Romero et al. J Interv Card Electrophysiol. 2020 Dec.
Free PMC article
Show details
J Interv Card Electrophysiol
. 2020 Dec;59(3):485-493.
doi: 10.1007/s10840-020-00896-7. Epub 2020 Oct 31.

Authors

Jorge Romero [1], Isabella Alviz [1], Michael Parides [1], Juan Carlos Diaz [1], David Briceno [1], Mohamed Gabr [1], Maria Gamero [1], Kavisha Patel [1], Eric D Braunstein [1], Sutopa Purkayastha [1], Dalvert Polanco [1], Carolina R Valencia [1], Domenico Della Rocca [2], Alejandro Velasco [1], Ruike Yang [1], Nicola Tarantino [1], Xiao-Dong Zhang [1], Sanghamitra Mohanty [2], Juan Bello [1], Andrea Natale [2], Ulrich P Jorde [1], Mario Garcia [1], Luigi Di Biase [3] [4]

Affiliations

- [1] Cardiac Arrhythmia Center, Division of Cardiology, Department of Medicine, Montefiore-Einstein Center for Heart and Vascular Care, Bronx, NY, USA.
- [2] Texas Cardiac Arrhythmia Institute, St. David's Medical Center, Austin, TX, USA.
- [3] Cardiac Arrhythmia Center, Division of Cardiology, Department of Medicine, Montefiore-Einstein Center for Heart and Vascular Care, Bronx, NY, USA. dibbia@gmail.com.
- [4] Texas Cardiac Arrhythmia Institute, St. David's Medical Center, Austin, TX, USA. dibbia@gmail.com.

- PMID: **33128658**
- PMCID: PMC7602831
- DOI: 10.1007/s10840-020-00896-7

Abstract

Purpose: Cardiac involvement with COVID-19 infection has become evident by elevated troponin, cardiac arrhythmias, ST segment elevation, myocarditis, fulminant heart failure, and sudden cardiac death. We aimed to describe the association of COVID-19 and T-wave inversion (TWI) in a large case series.

Methods: We conducted an observational, retrospective study of confirmed COVID-19 cases with at least one electrocardiogram (ECG) in a large hospital in New York City (March 23, 2020-April 23, 2020). Patients with new TWI or pseudonormalization were further analyzed. Mortality and the need for invasive mechanical ventilation were the main outcomes.

Results: A total of 3225 patients were screened; 195 (6%) were selected for further analysis: 181 with TWI and 14 with T-wave pseudonormalization. Mean age was 66 ± 7 years; 51% were male. TWI were more commonly noted in the lateral (71%), followed by anterior (64%), inferior (57%), and septal (26%) leads. A total of 44 patients (23%) had elevated troponin. A total of 50 patients died (26%). Mortality rates of 35%, and 52% were observed in patients with diffuse TWI, and elevated troponin, respectively. Mortality rate of 80% was observed in patients with both elevated troponin and diffuse TWI. Additionally, 30% of the entire cohort and 58% of patients with elevated troponin required invasive mechanical ventilation.

Conclusion: Our study demonstrates that new TWI is a relatively common finding in COVID-19 patients. Importantly, our findings suggest that new TWI or T-wave pseudonormalization, particularly with elevated troponin, was associated with higher rates of mechanical ventilation and in-hospital mortality.

Keywords: COVID 19; ECG abnormality; Mortality; T-wave inversion.

Conflict of interest statement

Dr. Di Biase is a consultant for Biosense Webster, Stereotaxis, Boston Scientific, and Abbott; has received speaker honoraria/travel from Biosense Webster, Abbott, Boston Scientific, Medtronic, Atricure, Pfizer, and Biotronik. The remaining authors report no conflicts of interest.

Comment in

- Prolonged QT predicts prognosis in COVID-19.
  Akhtar Z, Gallagher MM, Yap YG, Leung LWM, Elbatran AI, Madden B, Ewasiuk V, Gregory L, Breathnach A, Chen Z, Fluck DS, Sharma S. Akhtar Z, et al. Pacing Clin Electrophysiol. 2021 May;44(5):875-882. doi: 10.1111/pace.14232. Epub 2021 Apr 13. Pacing Clin Electrophysiol. 2021. PMID: 33792080 Free PMC article.

- 30 references
- 3 figures

Supplementary info

Publication types, MeSH terms, Substances Expand

Publication types

- Observational Study

MeSH terms

- Aged
- COVID-19 / epidemiology
- COVID-19 / mortality
- COVID-19 / physiopathology*
- Electrocardiography
- Female
- Humans
- Male
- New York City / epidemiology
- Respiration, Artificial
- Retrospective Studies
- SARS-CoV-2
- Troponin / blood
- Ventricular Function*

Substances

- Troponin

Full text links

Springer Free PMC article
Proceed to details
Cite
Share
669
Observational Study
Medicine (Baltimore)
. 2021 Jan 29;100(4):e24524.
doi: 10.1097/MD.0000000000024524.

Co-infection in patients with hypoxemic pneumonia due to COVID-19 in Reunion Island

Nicolas Allou [1, 2], Kevin Larsen [3], Arthur Dubernet [4], Nicolas Traversier [5], Laurie Masse [3], Emilie Foch [3], Léa Bruneau [6, 7], Adrien Maillot [6], Michel André [3], Marie Lagrange-Xelot [8], Jérôme Allyn [1, 2], Vincent Thomas [5], Nathalie Coolen-Allou [3]
Affiliations Expand

Affiliations

- [1] Réanimation polyvalente.
- [2] Département d'Informatique Clinique.
- [3] Pneumologie.
- [4] Médecine Interne.
- [5] Microbiologie, Centre Hospitalier Universitaire Felix Guyon Allée des Topazes, Saint Denis.
- [6] INSERM CIC 1410 Clinical and Epidemiology, University Hospital, Saint Pierre.
- [7] Department of Public health and research support, Methodological Support and Biostatistics Unit, University Hospital, Saint Denis, Reunion Island.
- [8] Service des Maladies Infectieuses, Centre Hospitalier Universitaire Felix Guyon Allée des Topazes, Saint Denis, France.

- PMID: **33530280**
- PMCID: PMC7850706
- DOI: 10.1097/MD.0000000000024524

Free PMC article
Observational Study

Co-infection in patients with hypoxemic pneumonia due to COVID-19 in Reunion Island

Nicolas Allou et al. Medicine (Baltimore). 2021.
Free PMC article
Show details
Medicine (Baltimore)
. 2021 Jan 29;100(4):e24524.
doi: 10.1097/MD.0000000000024524.

Authors

Nicolas Allou [1, 2], Kevin Larsen [3], Arthur Dubernet [4], Nicolas Traversier [5], Laurie Masse [3], Emilie Foch [3], Léa Bruneau [6, 7], Adrien Maillot [6], Michel André [3], Marie Lagrange-Xelot [8], Jérôme Allyn [1, 2], Vincent Thomas [5], Nathalie Coolen-Allou [3]

Affiliations

- [1] Réanimation polyvalente.
- [2] Département d'Informatique Clinique.
- [3] Pneumologie.
- [4] Médecine Interne.
- [5] Microbiologie, Centre Hospitalier Universitaire Felix Guyon Allée des Topazes, Saint Denis.
- [6] INSERM CIC 1410 Clinical and Epidemiology, University Hospital, Saint Pierre.
- [7] Department of Public health and research support, Methodological Support and Biostatistics Unit, University Hospital, Saint Denis, Reunion Island.
- [8] Service des Maladies Infectieuses, Centre Hospitalier Universitaire Felix Guyon Allée des Topazes, Saint Denis, France.

- PMID: **33530280**
- PMCID: PMC7850706
- DOI: 10.1097/MD.0000000000024524

Abstract

This study aimed to evaluate the incidence of co-infection with different types of pathogens in patients with hypoxemic pneumonia due to coronavirus disease 2019 (COVID-19) in Reunion Island.This observational study using a prospectively collected database of hypoxemic pneumonia due to COVID-19 cases was conducted at Félix Guyon University Hospital in Reunion Island, France.Between 18 March 2020 and 15 April 2020, 156 patients were admitted to our hospital for COVID-19. A total of 36 patients had hypoxemic pneumonia (23.1%) due to COVID-19. Thirty of these cases (83.3%) were imported by travelers returning mainly from metropolitan France and Spain. Patients were screened for co-infection with other pathogens at admission: 31 (86.1%) by multiplex polymerase chain reaction (PCR) and 16 (44.4%) by cytobacteriological examination of

sputum culture. Five patients (13.9%) were found to have co-infection: 1 with influenza virus A H1N1 (pdm09) associated with Branhamella catarrhalis, 1 with Streptococcus pneumoniae associated with Haemophilus influenzae, 1 with Human Coronavirus 229E, 1 with Rhinovirus, and 1 with methicillin-susceptible Staphylococcus aureus. Patients with co-infection had higher D-dimer levels than those without co-infection (1.36 [1.34-2.36] µg/mL vs 0.63 [0.51-1.12] µg/mL, P = .05).The incidence of co-infection in our cohort was higher than expected (13.9%). Three co-infections (with influenza virus A(H1N1) pdm09, Streptococcus pneumoniae, and Staphylococcus aureus) required specific treatment. Patients with hypoxemic pneumonia due to COVID-19 should be screened for co-infection using respiratory cultures or multiplex PCR. Whilst our study has a number of limitations, the results from our study suggest that in the absence of screening, patients should be commenced on treatment for co-infection in the presence of an elevated D-dimer.

Copyright © 2021 the Author(s). Published by Wolters Kluwer Health, Inc.

Conflict of interest statement

The authors declare that they have no competing interests.

- 24 references

Supplementary info

Publication types, MeSH terms Expand

Publication types

- Observational Study

MeSH terms

- Adult
- COVID-19 / epidemiology*
- Coinfection / epidemiology*
- Female
- France / epidemiology
- Humans
- Hypoxia
- Male
- Middle Aged
- Pneumonia / epidemiology*
- Pneumonia / microbiology*
- Retrospective Studies
- SARS-CoV-2

Full text links

Wolters Kluwer Wolters Kluwer Free PMC article
Proceed to details
Cite
Share
670
Observational Study
J Stroke Cerebrovasc Dis
. 2020 Aug;29(8):104953.
doi: 10.1016/j.jstrokecerebrovasdis.2020.104953. Epub 2020 May 14.

Falling stroke rates during COVID-19 pandemic at a comprehensive stroke center

J E Siegler [1], M E Heslin [2], L Thau [3], A Smith [4], T G Jovin [5]
Affiliations Expand

Affiliations

- [1] Cooper Neurological Institute, Cooper University Hospital, 3 Cooper Plaza, Suite 320, Camden, NJ 08103, United States. Electronic address: siegler-james@cooperhealth.edu.
- [2] Cooper Medical School of Rowan University, Camden, NJ 08103, United States. Electronic address: heslinm6@rowan.edu.
- [3] Cooper Medical School of Rowan University, Camden, NJ 08103, United States. Electronic address: thaula85@rowan.edu.
- [4] Cooper Neurological Institute, Cooper University Hospital, 3 Cooper Plaza, Suite 320, Camden, NJ 08103, United States. Electronic address: smith-ainsley@cooperhealth.edu.
- [5] Cooper Neurological Institute, Cooper University Hospital, 3 Cooper Plaza, Suite 320, Camden, NJ 08103, United States. Electronic address: jovin-tudor@cooperhealth.edu.

- PMID: **32689621**
- PMCID: PMC7221408
- DOI: 10.1016/j.jstrokecerebrovasdis.2020.104953

Free PMC article
Observational Study

Falling stroke rates during COVID-19 pandemic at a comprehensive stroke center

J E Siegler et al. J Stroke Cerebrovasc Dis. 2020 Aug.
Free PMC article
Show details
J Stroke Cerebrovasc Dis
. 2020 Aug;29(8):104953.
doi: 10.1016/j.jstrokecerebrovasdis.2020.104953. Epub 2020 May 14.

Authors

J E Siegler [1], M E Heslin [2], L Thau [3], A Smith [4], T G Jovin [5]

Affiliations

- [1] Cooper Neurological Institute, Cooper University Hospital, 3 Cooper Plaza, Suite 320, Camden, NJ 08103, United States. Electronic address: siegler-james@cooperhealth.edu.
- [2] Cooper Medical School of Rowan University, Camden, NJ 08103, United States. Electronic address: heslinm6@rowan.edu.
- [3] Cooper Medical School of Rowan University, Camden, NJ 08103, United States. Electronic address: thaula85@rowan.edu.
- [4] Cooper Neurological Institute, Cooper University Hospital, 3 Cooper Plaza, Suite 320, Camden, NJ 08103, United States. Electronic address: smith-ainsley@cooperhealth.edu.
- [5] Cooper Neurological Institute, Cooper University Hospital, 3 Cooper Plaza, Suite 320, Camden, NJ 08103, United States. Electronic address: jovin-tudor@cooperhealth.edu.

- PMID: **32689621**
- PMCID: PMC7221408
- DOI: 10.1016/j.jstrokecerebrovasdis.2020.104953

Abstract

Introduction: Although there is evidence to suggest a high rate of cerebrovascular complications in patients with SARS-CoV-2 infection, anecdotal reports indicate a falling rate of new ischemic stroke diagnoses. We conducted an exploratory single-center analysis to estimate the change in number of new stroke diagnoses in our region, and evaluate the proximate reasons for this change during the COVID-19 pandemic at a tertiary care center in New Jersey.

Patients and methods: A Comprehensive Stroke Center prospective cohort was retrospectively analyzed for the number of stroke admissions, demographic features, and short-term outcomes 5 months prior to 3/1/2020 (pre-COVID-19), and in the 6 weeks that followed (COVID-19 period). The primary outcome was the number of new acute stroke diagnoses before and during the COVID-19 period, as well as the potential reasons for a decline in the number of new diagnoses.

Results: Of the 328 included patients, 53 (16%) presented in the COVID-19 period. There was a mean fall of 38% in new stroke diagnoses (mean 1.13/day [SD 1.07] from 1.82/day [SD 1.38], $p<0.01$), which was related to a 59% decline in the number of daily transfers from referral centers ($p<0.01$), 25% fewer telestroke consultations ($p=0.08$), and 55% fewer patients presenting directly to our institution by private vehicle ($p<0.01$) and 29% fewer patients through emergency services ($p=0.09$). There was no significant change in the monthly number of strokes due to large vessel occlusion (LVO), however the proportion of new LVOs nearly doubled in the COVID-19 period (38% vs. 21%, $p=0.01$).

Conclusions: The observations at our tertiary care center corroborate anecdotal reports that the number of new stroke diagnoses is falling, which seems related to a smaller proportion of patients seeking healthcare services for milder symptoms. These preliminary data warrant validation in larger, multi-center studies.

Keywords: COVID-19; Coronavirus; Epidemiology; Incidence; Ischemic Stroke.

Copyright © 2020 Elsevier Inc. All rights reserved.

- 31 references

Supplementary info

Publication types, MeSH terms Expand

Publication types

- Observational Study

MeSH terms

- Aged
- Aged, 80 and over
- Betacoronavirus / pathogenicity
- COVID-19
- Coronavirus Infections / diagnosis
- Coronavirus Infections / epidemiology*
- Coronavirus Infections / virology
- Emergency Medical Services
- Female
- Humans
- Incidence
- Male
- Middle Aged
- New Jersey / epidemiology
- Pandemics
- Patient Acceptance of Health Care
- Patient Transfer
- Pneumonia, Viral / diagnosis
- Pneumonia, Viral / epidemiology*
- Pneumonia, Viral / virology
- Remote Consultation
- Retrospective Studies
- Risk Factors
- SARS-CoV-2
- Stroke / diagnosis
- Stroke / epidemiology*
- Stroke / therapy
- Stroke / virology
- Tertiary Care Centers*
- Time Factors

Full text links

ELSEVIER FULL-TEXT ARTICLE Elsevier Science Free PMC article

Proceed to details

Cite

Share

☐ 671

Observational Study

Eur J Clin Invest

. 2020 Dec;50(12):e13387.

doi: 10.1111/eci.13387. Epub 2020 Sep 17.

Clinical characteristics and prognosis of hospitalized COVID-19 patients with incident sustained tachyarrhythmias: A multicenter observational study

Vincenzo Russo [1], Marco Di Maio [2], Filiberto Fausto Mottola [1], Gianpiero Pagnano [3], Emilio Attena [4], Nicoletta Verde [1], Pierpaolo Di Micco [5], Angelo Silverio [6], Fernando Scudiero [7], Luigi Nunziata [8], Nunzia Fele [3], Antonello D'Andrea [9], Guido Parodi [10], Stefano Albani [11], Paolo Scacciatella [11], Gerardo Nigro [1], Sergio Severino [3]

Affiliations Expand

Affiliations

- [1] Department of Translational Medical Sciences, University of Campania 'Luigi Vanvitelli'-Monaldi Hospital, Naples, Italy.
- [2] Cardiology Division, "Maria S.S. Addolorata" Hospital, ASL Salerno, Salerno, Italy.
- [3] Cardiology Unit, Cotugno Hospital, Naples, Italy.
- [4] Medicine Unit, Division of Cardiology, San Giuliano Hospital, Naples, Italy.
- [5] Fatebenefratelli Hospital of Naples, Naples, Italy.
- [6] Cardiology Division, Cardiovascular and Thoracic Department, University Hospital "San Giovanni di Dio e Ruggi d' Aragona", Salerno, Italy.
- [7] Cardiology Unit, Health Authority Bergamo East, Italy.
- [8] Cardiology Unit, Boscotrecase Hospital, Naples, Italy.
- [9] Division of Cardiology and Intensive Care Unit, Umberto I Hospital, Nocera Inferiore, Italy.
- [10] Clinical and Interventional Cardiology, Sassari University Hospital, Sassary, Italy.
- [11] Cardiology Department, Aosta Valley Health Authority, Aosta, Italy.

- PMID: **32813877**
- PMCID: PMC7460920
- DOI: 10.1111/eci.13387

Free PMC article
Observational Study

Clinical characteristics and prognosis of hospitalized COVID-19 patients with incident sustained tachyarrhythmias: A multicenter observational study

Vincenzo Russo et al. Eur J Clin Invest. 2020 Dec.
Free PMC article
Show details
Eur J Clin Invest
. 2020 Dec;50(12):e13387.
doi: 10.1111/eci.13387. Epub 2020 Sep 17.

Authors

Vincenzo Russo [1], Marco Di Maio [2], Filiberto Fausto Mottola [1], Gianpiero Pagnano [3], Emilio Attena [4], Nicoletta Verde [1], Pierpaolo Di Micco [5], Angelo Silverio [6], Fernando Scudiero [7], Luigi Nunziata [8], Nunzia Fele [3], Antonello D'Andrea [9], Guido Parodi [10], Stefano Albani [11], Paolo Scacciatella [11], Gerardo Nigro [1], Sergio Severino [3]

Affiliations

- [1] Department of Translational Medical Sciences, University of Campania 'Luigi Vanvitelli'- Monaldi Hospital, Naples, Italy.
- [2] Cardiology Division, "Maria S.S. Addolorata" Hospital, ASL Salerno, Salerno, Italy.
- [3] Cardiology Unit, Cotugno Hospital, Naples, Italy.
- [4] Medicine Unit, Division of Cardiology, San Giuliano Hospital, Naples, Italy.
- [5] Fatebenefratelli Hospital of Naples, Naples, Italy.
- [6] Cardiology Division, Cardiovascular and Thoracic Department, University Hospital "San Giovanni di Dio e Ruggi d' Aragona", Salerno, Italy.
- [7] Cardiology Unit, Health Authority Bergamo East, Italy.
- [8] Cardiology Unit, Boscotrecase Hospital, Naples, Italy.
- [9] Division of Cardiology and Intensive Care Unit, Umberto I Hospital, Nocera Inferiore, Italy.
- [10] Clinical and Interventional Cardiology, Sassari University Hospital, Sassary, Italy.
- [11] Cardiology Department, Aosta Valley Health Authority, Aosta, Italy.

- PMID: **32813877**
- PMCID: PMC7460920
- DOI: 10.1111/eci.13387

Abstract

Introduction: Little is still known about the prognostic impact of incident arrhythmias in hospitalized patients with COVID-19. The aim of this study was to evaluate the incidence and predictors of sustained tachyarrhythmias in hospitalized patients with COVID-19, and their potential association with disease severity and in-hospital mortality.

Materials and methods: This was a retrospective multicenter observation study including consecutive patients with laboratory confirmed COVID-19 admitted to emergency department of ten Italian Hospitals from 15 February to 15 March 2020. The prevalence and the type of incident sustained arrhythmias have been collected. The correlation between the most prevalent arrhythmias and both baseline characteristics and the development of ARDS and in-hospital mortality has been evaluated.

Results: 414 hospitalized patients with COVID-19 (66.9 ± 15.0 years, 61.1% male) were included in the present study. During a median follow-up of 28 days (IQR: 12-45), the most frequent incident sustained arrhythmia was AF (N: 71; 17.1%), of which 50 (12.1%) were new-onset and 21 (5.1%) were recurrent, followed by VT (N: 14, 3.4%) and supraventricular arrhythmias (N: 5, 1.2%). Incident AF, both new-onset and recurrent, did not affect the risk of severe adverse events including ARDS and death during hospitalization; in contrast, incident VT significantly increased the risk of in-hospital mortality (RR: 2.55; P: .003).

Conclusions: AF is the more frequent incident tachyarrhythmia; however, it not seems associated to ARDS development and death. On the other hand, incident VT is a not frequent but independent predictor of in-hospital mortality among hospitalized COVID-19 patients.

Keywords: Atrial Fibrillation; Covid-19; Covid-19 and arrhythmia; Covid-19 and cardiovascular System; Covid-19 and cardiovascular complications; SARS-CoV-2; SARSCoV-2 and ventricular arrhythmias.

© 2020 Stichting European Society for Clinical Investigation Journal Foundation. Published by John Wiley & Sons Ltd.

- 17 references

Supplementary info

Publication types, MeSH terms Expand

Publication types

- Multicenter Study
- Observational Study

MeSH terms

- Age Factors
- Aged
- Aged, 80 and over
- Arrhythmias, Cardiac / epidemiology
- Atrial Fibrillation / epidemiology*
- COVID-19 / mortality*

- COVID-19 / physiopathology
- Female
- Hospital Mortality*
- Hospitalization
- Humans
- Incidence
- Italy / epidemiology
- Male
- Middle Aged
- Multivariate Analysis
- Prognosis
- Recurrence
- Renal Insufficiency, Chronic / epidemiology
- Respiratory Distress Syndrome / epidemiology*
- Respiratory Distress Syndrome / physiopathology
- Retrospective Studies
- Severity of Illness Index
- Tachycardia, Supraventricular / epidemiology*
- Tachycardia, Ventricular / epidemiology*

Full text links

WILEY Full Text Article Wiley Free PMC article

Proceed to details

Cite

Share

☐ 672

Observational Study

Monaldi Arch Chest Dis

. 2020 Nov 9;90(4).

doi: 10.4081/monaldi.2020.1568.

Characteristics of COVID-19 at a non-COVID tertiary pulmonary care centre in Delhi, India

Nitin Goel [1], Sonam Spalgais [2], Parul Mrigpuri [3], Madhu Khanna [4], Balakrishnan Menon [5], Raj Kumar [6]

Affiliations Expand

Affiliations

- [1] Department of Pulmonary Medicine, Vallabhbhai Patel Chest Institute, University of Delhi, New Delhi. drnitingoel@gmail.com.
- [2] Department of Pulmonary Medicine, Vallabhbhai Patel Chest Institute, University of Delhi, New Delhi. sosolrs@gmail.com.
- [3] Department of Pulmonary Medicine, Vallabhbhai Patel Chest Institute, University of Delhi, New Delhi. parul_57mrigpuri@yahoo.com.
- [4] Virology Unit, Department of Microbiology, Vallabhbhai Patel Chest Institute, University of Delhi, New Delhi. madhukhanna@hotmail.com.
- [5] Department of Pulmonary Medicine, Vallabhbhai Patel Chest Institute, University of Delhi, New Delhi. balakmenon@yahoo.co.in.
- [6] Department of Pulmonary Medicine, Vallabhbhai Patel Chest Institute, University of Delhi, New Delhi. rajkumarvpci@gmail.com.

- PMID: **33169599**
- DOI: 10.4081/monaldi.2020.1568

Free article
Observational Study

Characteristics of COVID-19 at a non-COVID tertiary pulmonary care centre in Delhi, India

Nitin Goel et al. Monaldi Arch Chest Dis. 2020.
Free article
Show details
Monaldi Arch Chest Dis
. 2020 Nov 9;90(4).
doi: 10.4081/monaldi.2020.1568.

Authors

Nitin Goel [1], Sonam Spalgais [2], Parul Mrigpuri [3], Madhu Khanna [4], Balakrishnan Menon [5], Raj Kumar [6]

Affiliations

- [1] Department of Pulmonary Medicine, Vallabhbhai Patel Chest Institute, University of Delhi, New Delhi. drnitingoel@gmail.com.
- [2] Department of Pulmonary Medicine, Vallabhbhai Patel Chest Institute, University of Delhi, New Delhi. sosolrs@gmail.com.
- [3] Department of Pulmonary Medicine, Vallabhbhai Patel Chest Institute, University of Delhi, New Delhi. parul_57mrigpuri@yahoo.com.
- [4] Virology Unit, Department of Microbiology, Vallabhbhai Patel Chest Institute, University of Delhi, New Delhi. madhukhanna@hotmail.com.
- [5] Department of Pulmonary Medicine, Vallabhbhai Patel Chest Institute, University of Delhi, New Delhi. balakmenon@yahoo.co.in.

- [6] Department of Pulmonary Medicine, Vallabhbhai Patel Chest Institute, University of Delhi, New Delhi. rajkumarvpci@gmail.com.

- PMID: **33169599**
- DOI: 10.4081/monaldi.2020.1568

Abstract

The pandemic of COVID-19 has emerged as a serious health crisis globally and India too has been extensively affected with 604,641 active cases reported, till date. The present study focuses on the demographic, clinical and laboratory profile of such patients from a tertiary level non-COVID respiratory care hospital. This is a retrospective observational study. Seventy-seven sick patients fulfilling COVID suspect criteria were admitted to the isolation area. Their RT-PCR test was done from the designated laboratory and 35 of them were confirmed to be COVID-19 patients. The detailed demographic, clinical and laboratory profile of these COVID-19 patients was studied. The mean age was 46±17 years with male predominance (57%). Majority of the cases (83%) were symptomatic. The most common symptom was cough (66%) followed by breathlessness and fever. Nineteen (54.3%) patients had one or the other co-morbidity and 16 (45.7%) had chronic lung diseases as one of the comorbidities. Nearly half of the patients (51%) required supplementary oxygen on presentation. Two patients were put on invasive mechanical ventilation while 4 patients required non-invasive ventilation before being shifted to the COVID hospital. Hence, it can be concluded that COVID-19 in patients of chronic respiratory diseases manifests with higher prevalence of symptoms and also higher severity of disease. Further, the symptomatology of COVID-19 closely mimics the acute exacerbation of chronic lung diseases, so cautious screening and testing should be done, especially at the pulmonary department.

Supplementary info

Publication types, MeSH terms Expand

Publication types

- Observational Study

MeSH terms

- Adult
- Asthma / epidemiology
- Betacoronavirus
- Bronchiectasis / epidemiology
- COVID-19
- Chronic Disease
- Comorbidity
- Coronavirus Infections / epidemiology
- Coronavirus Infections / physiopathology*
- Coronavirus Infections / therapy
- Cough / physiopathology

- Diabetes Mellitus / epidemiology*
- Dyspnea / physiopathology
- Female
- Fever / physiopathology
- Hospitals, Special*
- Humans
- Hypertension / epidemiology*
- Hypoxia / physiopathology*
- India / epidemiology
- Lung Diseases / epidemiology*
- Lung Diseases, Interstitial / epidemiology
- Male
- Middle Aged
- Noninvasive Ventilation
- Oxygen Inhalation Therapy
- Pandemics
- Pneumonia, Viral / epidemiology
- Pneumonia, Viral / physiopathology*
- Pneumonia, Viral / therapy
- Pulmonary Disease, Chronic Obstructive / epidemiology
- Pulmonary Medicine*
- Respiration, Artificial
- Retrospective Studies
- SARS-CoV-2
- Tertiary Care Centers*

Full text links

Monaldi Arch Chest Dis Pagepress Publications

Proceed to details

Cite

Share

☐ 673

Multicenter Study

Am J Health Syst Pharm

. 2021 Mar 18;78(7):568-577.

doi: 10.1093/ajhp/zxaa426.

Multicenter point prevalence evaluation of the utilization and safety of drug therapies for

COVID-19 at the onset of the pandemic timeline in the United States

Nathaniel J Rhodes [1 2 3], Atheer Dairem [1], William J Moore [1], Anooj Shah [1 2], Michael J Postelnick [1], Melissa E Badowski [4], Sarah M Michienzi [4], Jaime L Borkowski [5], Radhika S Polisetty [2 6], Karen Fong [7], Emily S Spivak [8], James R Beardsley [9 10], Cory M Hale [11], Andrea M Pallotta [12], Pavithra Srinivas [12], Lucas T Schulz [13]
Affiliations Expand

Affiliations

- [1] Department of Pharmacy, Northwestern Memorial Hospital, Chicago, IL, USA.
- [2] Department of Pharmacy Practice, Midwestern University Chicago College of Pharmacy, Downers Grove, IL, USA.
- [3] Pharmacometrics Center of Excellence, Midwestern University Chicago College of Pharmacy, Downers Grove, IL, USA.
- [4] Department of Pharmacy Practice, Section of Infectious Diseases Pharmacotherapy, University of Illinois at Chicago, College of Pharmacy, Chicago, IL, USA.
- [5] Department of Pharmacy, Northwestern Medicine Delnor Hospital, Geneva, IL, USA.
- [6] Department of Pharmacy, Northwestern Medicine Central DuPage Hospital, Winfield, IL, USA.
- [7] Department of Pharmacy, University of Utah Health.
- [8] Department of Medicine, University of Utah School of Medicine.
- [9] Department of Pharmacy, Wake Forest Baptist Health, Winston-Salem, NC, USA.
- [10] Wake Forest School of Medicine, Winston-Salem, NC, USA.
- [11] Department of Pharmacy, Penn State Health Milton S. Hershey Medical Center, Hershey, PA, USA.
- [12] Department of Pharmacy, Cleveland Clinic Hospital, Cleveland, OH, USA.
- [13] Department of Pharmacy, University of Wisconsin Health, Madison WI, USA.

- PMID: **33537767**
- PMCID: PMC7929420
- DOI: 10.1093/ajhp/zxaa426

Free PMC article
Multicenter Study

Multicenter point prevalence evaluation of the utilization and safety of drug therapies for COVID-19 at the onset of the pandemic timeline in the United States

Nathaniel J Rhodes et al. Am J Health Syst Pharm. 2021.

Free PMC article

Show details

Am J Health Syst Pharm

. 2021 Mar 18;78(7):568-577.

doi: 10.1093/ajhp/zxaa426.

Authors

Nathaniel J Rhodes [1] [2] [3], Atheer Dairem [1], William J Moore [1], Anooj Shah [1] [2], Michael J Postelnick [1], Melissa E Badowski [4], Sarah M Michienzi [4], Jaime L Borkowski [5], Radhika S Polisetty [2] [6], Karen Fong [7], Emily S Spivak [8], James R Beardsley [9] [10], Cory M Hale [11], Andrea M Pallotta [12], Pavithra Srinivas [12], Lucas T Schulz [13]

Affiliations

- [1] Department of Pharmacy, Northwestern Memorial Hospital, Chicago, IL, USA.
- [2] Department of Pharmacy Practice, Midwestern University Chicago College of Pharmacy, Downers Grove, IL, USA.
- [3] Pharmacometrics Center of Excellence, Midwestern University Chicago College of Pharmacy, Downers Grove, IL, USA.
- [4] Department of Pharmacy Practice, Section of Infectious Diseases Pharmacotherapy, University of Illinois at Chicago, College of Pharmacy, Chicago, IL, USA.
- [5] Department of Pharmacy, Northwestern Medicine Delnor Hospital, Geneva, IL, USA.
- [6] Department of Pharmacy, Northwestern Medicine Central DuPage Hospital, Winfield, IL, USA.
- [7] Department of Pharmacy, University of Utah Health.
- [8] Department of Medicine, University of Utah School of Medicine.
- [9] Department of Pharmacy, Wake Forest Baptist Health, Winston-Salem, NC, USA.
- [10] Wake Forest School of Medicine, Winston-Salem, NC, USA.
- [11] Department of Pharmacy, Penn State Health Milton S. Hershey Medical Center, Hershey, PA, USA.
- [12] Department of Pharmacy, Cleveland Clinic Hospital, Cleveland, OH, USA.
- [13] Department of Pharmacy, University of Wisconsin Health, Madison WI, USA.

- PMID: **33537767**
- PMCID: PMC7929420
- DOI: 10.1093/ajhp/zxaa426

Abstract

Key points: In a multicenter point-prevalence study, we found that the rate of supportive care was high; among those receiving COVID-19 drug therapies, adverse reactions occurred in 12% of patients.

Purpose: There are currently no FDA-approved medications for the treatment of coronavirus disease 2019 (COVID-19). At the onset of the pandemic, off-label medication use was supported by limited or no clinical data. We sought to characterize experimental COVID-19 therapies and identify safety signals during this period.

Methods: We conducted a noninterventional, multicenter, point prevalence study of patients hospitalized with suspected/confirmed COVID-19. Clinical and treatment characteristics within a 24-hour window were evaluated in a random sample of up to 30 patients per site. The primary objective was to describe COVID-19-targeted therapies. The secondary objective was to describe adverse drug reactions (ADRs).

Results: A total of 352 patients treated for COVID-19 at 15 US hospitals From April 18 to May 8, 2020, were included in the study. Most patients were treated at academic medical centers (53.4%) or community hospitals (42.6%). Sixty-seven patients (19%) were receiving drug therapy in addition to supportive care. Drug therapies used included hydroxychloroquine (69%), remdesivir (10%), and interleukin-6 antagonists (9%). Five patients (7.5%) were receiving combination therapy. The rate of use of COVID-19-directed drug therapy was higher in patients with vs patients without a history of asthma (14.9% vs 7%, P = 0.037) and in patients enrolled in clinical trials (26.9% vs 3.2%, P < 0.001). Among those receiving drug therapy, 8 patients (12%) experienced an ADR, and ADRs were recognized at a higher rate in patients enrolled in clinical trials (62.5% vs 22%; odds ratio, 5.9; P = 0.028).

Conclusion: While we observed high rates of supportive care for patients with COVID-19, we also found that ADRs were common among patients receiving drug therapy, including those enrolled in clinical trials. Comprehensive systems are needed to identify and mitigate ADRs associated with experimental COVID-19 treatments.

Keywords: COVID-19; SARS-CoV-2; adverse drug reaction; medication safety; observational study; supportive care.

© American Society of Health-System Pharmacists 2021. All rights reserved. For permissions, please e-mail: journals.permissions@oup.com.

Update of

- Multicenter point-prevalence evaluation of the utilization and safety of drug therapies for COVID-19.
  Rhodes NJ, Dairem A, Moore W, Shah A, Postelnick MJ, Badowski ME, Michienzi SM, Borkowski JL, Polisetty RS, Fong K, Spivek ES, Beardsley JR, Hale CM, Pallotta AM, Srinivas P, Schulz LT. Rhodes NJ, et al. medRxiv. 2020 Jun 5:2020.06.03.20121558. doi: 10.1101/2020.06.03.20121558. Preprint. medRxiv. 2020. PMID: 32577687 Free PMC article. Updated.

- 35 references
- 1 figure

Supplementary info

Publication types, MeSH terms, Substances Expand

Publication types

- Multicenter Study

MeSH terms

- Adolescent
- Adult
- Aged
- Aged, 80 and over
- Antiviral Agents / adverse effects
- Antiviral Agents / therapeutic use
- COVID-19 / drug therapy*
- Child
- Child, Preschool
- Drug Therapy, Combination / adverse effects
- Drug Therapy, Combination / statistics & numerical data*
- Drug-Related Side Effects and Adverse Reactions / epidemiology*
- Female
- Humans
- Hydroxychloroquine / adverse effects
- Hydroxychloroquine / therapeutic use
- Infant
- Infant, Newborn
- Male
- Middle Aged
- Pandemics
- Prevalence
- Retrospective Studies
- SARS-CoV-2*
- United States / epidemiology
- Young Adult

Substances

- Antiviral Agents
- Hydroxychloroquine

Full text links

OXFORD ACADEMIC Silverchair Information Systems Free PMC article
Proceed to details
Cite
Share
674
Observational Study
Infection
. 2020 Aug;48(4):543-551.
doi: 10.1007/s15010-020-01432-5. Epub 2020 Apr 28.

Clinical characteristics of 145 patients with corona virus disease 2019 (COVID-19) in Taizhou, Zhejiang, China

Qingqing Chen [1], Zhencang Zheng [1], Chao Zhang [1], Xijiang Zhang [2], Huijuan Wu [3], Jingdong Wang [1], Shuwei Wang [1], Cheng Zheng [4]
Affiliations Expand

Affiliations

- [1] Department of Critical Care Medicine, Taizhou Enze Medical Center (Group) Enze Hospital, Taizhou, 318050, Zhejiang, China.
- [2] Department of Critical Care Medicine, Taizhou Municipal Hospital, Taizhou, 318000, Zhejiang, China.
- [3] Department of Critical Care Medicine, Taizhou Enze Medical Center (Group) Luqiao Hospital, Taizhou, 318050, Zhejiang, China.
- [4] Department of Critical Care Medicine, Taizhou Municipal Hospital, Taizhou, 318000, Zhejiang, China. dr.zhengcheng@foxmail.com.

- PMID: **32342479**
- PMCID: PMC7186187
- DOI: 10.1007/s15010-020-01432-5

Free PMC article
Observational Study

Clinical characteristics of 145 patients with corona virus disease 2019 (COVID-19) in Taizhou, Zhejiang, China

Qingqing Chen et al. Infection. 2020 Aug.
Free PMC article
Show details
Infection
. 2020 Aug;48(4):543-551.
doi: 10.1007/s15010-020-01432-5. Epub 2020 Apr 28.

Authors

Qingqing Chen [1], Zhencang Zheng [1], Chao Zhang [1], Xijiang Zhang [2], Huijuan Wu [3], Jingdong Wang [1], Shuwei Wang [1], Cheng Zheng [4]

Affiliations

- [1] Department of Critical Care Medicine, Taizhou Enze Medical Center (Group) Enze Hospital, Taizhou, 318050, Zhejiang, China.
- [2] Department of Critical Care Medicine, Taizhou Municipal Hospital, Taizhou, 318000, Zhejiang, China.
- [3] Department of Critical Care Medicine, Taizhou Enze Medical Center (Group) Luqiao Hospital, Taizhou, 318050, Zhejiang, China.
- [4] Department of Critical Care Medicine, Taizhou Municipal Hospital, Taizhou, 318000, Zhejiang, China. dr.zhengcheng@foxmail.com.

- PMID: **32342479**
- PMCID: PMC7186187
- DOI: 10.1007/s15010-020-01432-5

Abstract

Objective: The aim of this study was to investigate the clinical characteristics of Corona Virus Disease 2019 in Taizhou, China.

Methods: A single center retrospective observational study was performed between Jan 1, 2020 and Mar 11, 2020 at Taizhou Public Health Medical Center, Zhejiang, China. All patients with confirmed Corona Virus Disease 2019 were enrolled, and their clinical data were gathered by reviewing electronic medical records. Outcomes of severely ill patients and non-severely ill patients were compared.

Results: Of 145 hospitalized patients with COVID-19, the average age was 47.5 years old (standard deviation, 14.6) and 54.5% were men. Hypertension was the most common comorbidity (15.2%), followed by diabetes mellitus (9.7%). Common symptoms included dry cough (81.4%), fever (75.2%), anorexia (42.8%), fatigue (40.7%), chest tightness (32.4%), diarrhea (26.9%) and dizziness (20%). According to imaging examination, 79.3% patients showed bilateral pneumonia, 18.6% showed unilateral pneumonia, 61.4% showed ground-glass opacity, and 2.1% showed no abnormal result. Compared with non-severely ill patients, severely ill patients were older (mean, years, 52.8 vs. 45.3, $p < 0.01$), had a higher proportion of diabetes mellitus (16.3% vs. 6.9%, $p = 0.08$), had a higher body mass index (mean, 24.78 vs. 23.20, $p = 0.02$) and were more likely to have fever (90.7% vs. 68.6%, $p = 0.01$), anorexia (60.5% vs. 35.3%, $p = 0.01$), chest tightness (60.5% vs.20.6%, $p < 0.01$) and dyspnea (7.0% vs. 0%, $p = 0.03$). Of the 43 severely ill patients, 6 (14%) received high-flow nasal cannula oxygen therapy, and 1 (2.3%) received invasive mechanical ventilation.

Conclusions: Older patients or patients with comorbidities such as obesity or diabetes mellitus were more likely to have severe condition. Treatments of COVID-19 is still experimental and more clinical trials are needed.

Keywords: COVID-19; Clinical characteristics; Corona virus disease 2019; Epidemiology; Outcomes; SARS-CoV-2; Treatment.

Conflict of interest statement

The authors declare that they have no conflict interests.

- 25 references
- 2 figures

Supplementary info

Publication types, MeSH terms, Grant support Expand

Publication types

- Observational Study

MeSH terms

- Adult
- Betacoronavirus
- COVID-19
- China / epidemiology
- Comorbidity
- Coronavirus Infections / physiopathology*
- Coronavirus Infections / therapy*
- Diabetes Complications
- Diabetes Mellitus
- Electronic Health Records
- Female
- Humans
- Hypertension / complications
- Male
- Middle Aged
- Oxygen Inhalation Therapy
- Pandemics
- Pneumonia, Viral / physiopathology*
- Pneumonia, Viral / therapy*
- Respiration, Artificial
- Retrospective Studies
- SARS-CoV-2

Grant support

- No. 2019KY781/Medical Health Science and Technology Project of Zhejiang Provincial Health Commission

Full text links

SpringerLink FULL-TEXT ARTICLE Springer Free PMC article
Proceed to details
Cite

Share

☐ 675

Observational Study

J Hosp Med
. 2021 Nov;16(11):659-666.
doi: 10.12788/jhm.3717.

Association of Healthcare Access With Intensive Care Unit Utilization and Mortality in Patients of Hispanic Ethnicity Hospitalized With COVID-19

Ferdinand Velasco [1], Donghan M Yang [2], Minzhe Zhang [2], Tanna Nelson [1], Thomas Sheffield [2], Tony Keller [1], Yiqing Wang [2], Clark Walker [1], Chaitanya Katterapalli [1], Kelli Zimmerman [1], Andrew Masica [1], Christoph U Lehmann [3], Yang Xie [2], John W Hollingsworth [4] [5]
Affiliations Expand

Affiliations

- [1] Texas Health Resources, Arlington, Texas.
- [2] Quantitative Biomedical Research Center, Department of Population and Data Sciences, University of Texas Southwestern Medical Center, Dallas, Texas.
- [3] Clinical Informatics Center, University of Texas Southwestern Medical Center, Dallas, Texas.
- [4] Texas Health Harris Methodist Hospital, Fort Worth, Texas.
- [5] Texas Christian University School of Medicine, Fort Worth, Texas.

- PMID: **34730508**
- PMCID: **PMC8577697** (available on 2022-11-01)
- DOI: 10.12788/jhm.3717

Observational Study

Association of Healthcare Access With Intensive Care Unit Utilization and Mortality in Patients of Hispanic Ethnicity Hospitalized With COVID-19

Ferdinand Velasco et al. J Hosp Med. 2021 Nov.

Show details

J Hosp Med

. 2021 Nov;16(11):659-666.
doi: 10.12788/jhm.3717.

Authors

Ferdinand Velasco [1], Donghan M Yang [2], Minzhe Zhang [2], Tanna Nelson [1], Thomas Sheffield [2], Tony Keller [1], Yiqing Wang [2], Clark Walker [1], Chaitanya Katterapalli [1], Kelli Zimmerman [1], Andrew Masica [1], Christoph U Lehmann [3], Yang Xie [2], John W Hollingsworth [4] [5]

Affiliations

- [1] Texas Health Resources, Arlington, Texas.
- [2] Quantitative Biomedical Research Center, Department of Population and Data Sciences, University of Texas Southwestern Medical Center, Dallas, Texas.
- [3] Clinical Informatics Center, University of Texas Southwestern Medical Center, Dallas, Texas.
- [4] Texas Health Harris Methodist Hospital, Fort Worth, Texas.
- [5] Texas Christian University School of Medicine, Fort Worth, Texas.

- PMID: **34730508**
- PMCID: **PMC8577697** (available on 2022-11-01)
- DOI: 10.12788/jhm.3717

Abstract

Background: Racial and ethnic minority groups in the United States experience a disproportionate burden of COVID-19 deaths.

Objective: To evaluate whether outcome differences between Hispanic and non-Hispanic COVID-19 hospitalized patients exist and, if so, to identify the main malleable contributing factors.

Design, setting, participants: Retrospective, cross-sectional, observational study of 6097 adult COVID-19 patients hospitalized within a single large healthcare system from March to November 2020.

Exposures: Self-reported ethnicity and primary language.

Main outcomes and measures: Clinical outcomes included intensive care unit (ICU) utilization and in-hospital death. We used age-adjusted odds ratios (OR) and multivariable analysis to evaluate the associations between ethnicity/language groups and outcomes.

Results: 32.1% of patients were Hispanic, 38.6% of whom reported a non-English primary language. Hispanic patients were less likely to be insured, have a primary care provider, and have accessed the healthcare system prior to the COVID-19 admission. After adjusting for age, Hispanic inpatients experienced higher ICU utilization (non-English-speaking: OR, 1.75; 95% CI, 1.47-2.08; English-speaking: OR, 1.13; 95% CI, 0.95-1.33) and higher mortality (non-English-speaking: OR, 1.43; 95% CI, 1.10-1.86; English-speaking: OR, 1.53; 95% CI, 1.19-1.98) compared to non-Hispanic inpatients. There were no observed treatment disparities among ethnic groups. After adjusting for age, Hispanic inpatients had elevated disease severity at admission (non-English-speaking: OR, 2.27; 95% CI, 1.89-2.72; English-speaking: OR, 1.33; 95% CI, 1.10-

1.61). In multivariable analysis, the associations between ethnicity/language and clinical outcomes decreased after considering baseline disease severity ($P < .001$).

Conclusion: The associations between ethnicity and clinical outcomes can be explained by elevated disease severity at admission and limited access to healthcare for Hispanic patients, especially non-English-speaking Hispanics.

Supplementary info

Publication types, MeSH terms, Grant support Expand

Publication types

- Observational Study
- Research Support, N.I.H., Extramural

MeSH terms

- Adult
- COVID-19*
- Cross-Sectional Studies
- Ethnicity*
- Health Services Accessibility
- Hispanic or Latino
- Hospital Mortality
- Humans
- Intensive Care Units
- Minority Groups
- Retrospective Studies
- SARS-CoV-2
- United States / epidemiology

Grant support

- R35 GM136375/GM/NIGMS NIH HHS/United States
- T32 CA124334/CA/NCI NIH HHS/United States
- UL1 TR003163/TR/NCATS NIH HHS/United States

Full text links

Journal of Hospital Medicine Frontline Medical Communications Inc

Proceed to details

Cite

Share

☐ 676

Observational Study

J Am Soc Nephrol
. 2022 Jan;33(1):49-57.
doi: 10.1681/ASN.2021060778. Epub 2021 Nov 17.

Real-World Effectiveness and Immunogenicity of BNT162b2 and mRNA-1273 SARS-CoV-2 Vaccines in Patients on Hemodialysis

Scott Sibbel [1], Katherine McKeon [1], Jiacong Luo [1], Karl Wendt [1], Adam G Walker [1], Tara Kelley [1], Rachael Lazar [2], Meredith L Zywno [2], Jeffrey J Connaire [1], Francesca Tentori [1], Amy Young [1], Steven M Brunelli [3]
Affiliations Expand

Affiliations

- [1] DaVita Clinical Research, Minneapolis, Minnesota.
- [2] DaVita Inc., Denver, Colorado.
- [3] DaVita Clinical Research, Minneapolis, Minnesota Steven.Brunelli@DaVita.com.

- PMID: **34789546**
- PMCID: **PMC8763185** (available on 2023-01-01)
- DOI: 10.1681/ASN.2021060778

Observational Study

Real-World Effectiveness and Immunogenicity of BNT162b2 and mRNA-1273 SARS-CoV-2 Vaccines in Patients on Hemodialysis

Scott Sibbel et al. J Am Soc Nephrol. 2022 Jan.

Show details

J Am Soc Nephrol
. 2022 Jan;33(1):49-57.
doi: 10.1681/ASN.2021060778. Epub 2021 Nov 17.

Authors

Scott Sibbel [1], Katherine McKeon [1], Jiacong Luo [1], Karl Wendt [1], Adam G Walker [1], Tara Kelley [1], Rachael Lazar [2], Meredith L Zywno [2], Jeffrey J Connaire [1], Francesca Tentori [1], Amy Young [1], Steven M Brunelli [3]

Affiliations

- [1] DaVita Clinical Research, Minneapolis, Minnesota.
- [2] DaVita Inc., Denver, Colorado.
- [3] DaVita Clinical Research, Minneapolis, Minnesota Steven.Brunelli@DaVita.com.

- PMID: **34789546**
- PMCID: **PMC8763185** (available on 2023-01-01)
- DOI: 10.1681/ASN.2021060778

Abstract

Background: Patients on hemodialysis have an elevated risk for COVID-19 but were not included in efficacy trials of SARS-CoV-2 vaccines.

Methods: We conducted a retrospective, observational study to estimate the real-world effectiveness and immunogenicity of two mRNA SARS-CoV-2 vaccines in a large, representative population of adult hemodialysis patients in the United States. In separate, parallel analyses, patients who began a vaccination series with BNT162b2 or mRNA-1273 in January and February 2021 were matched with unvaccinated patients and risk for outcomes were compared for days 1-21, 22-42, and ≥43 after first dose. In a subset of consented patients, blood samples were collected approximately 28 days after the second dose and anti-SARS-CoV-2 immunoglobulin G was measured.

Results: A total of 12,169 patients received the BNT162b2 vaccine (matched with 44,377 unvaccinated controls); 23,037 patients received the mRNA-1273 vaccine (matched with 63,243 unvaccinated controls). Compared with controls, vaccinated patients' risk of being diagnosed with COVID-19 postvaccination became progressively lower during the study period (hazard ratio and 95% confidence interval for BNT162b2 was 0.21 [0.13, 0.35] and for mRNA-1273 was 0.27 [0.17, 0.42] for days ≥43). After a COVID-19 diagnosis, vaccinated patients were significantly less likely than unvaccinated patients to be hospitalized (for BNT162b2, 28.0% versus 43.4%; for mRNA-1273, 37.2% versus 45.6%) and significantly less likely to die (for BNT162b2, 4.0% versus 12.1%; for mRNA-1273, 5.6% versus 14.5%). Antibodies were detected in 98.1% (309/315) and 96.0% (308/321) of BNT162b2 and mRNA-1273 patients, respectively.

Conclusions: In patients on hemodialysis, vaccination with BNT162b2 or mRNA-1273 was associated with a lower risk of COVID-19 diagnosis and lower risk of hospitalization or death among those diagnosed with COVID-19. SARS-CoV-2 antibodies were detected in nearly all patients after vaccination. These findings support the use of these vaccines in this population.

Keywords: BNT162 vaccine; COVID-19; ESRD; RNA; SARS-CoV-2; coronavirus disease 2019; dialysis; end stage kidney disease; end-stage renal disease; messenger; vaccine.

Copyright © 2022 by the American Society of Nephrology.

Supplementary info

Publication types, MeSH terms, Substances Expand

Publication types

- Multicenter Study
- Observational Study

MeSH terms

- 2019-nCoV Vaccine mRNA-1273 / administration & dosage*
- 2019-nCoV Vaccine mRNA-1273 / immunology*
- Aged
- Aged, 80 and over
- Antibodies, Viral / blood
- BNT162 Vaccine / administration & dosage*
- BNT162 Vaccine / immunology*
- COVID-19 / immunology*
- COVID-19 / prevention & control*
- Dose-Response Relationship, Immunologic
- Female
- Humans
- Male
- Middle Aged
- Prospective Studies
- Renal Dialysis / adverse effects*
- Retrospective Studies
- Risk Factors
- SARS-CoV-2 / immunology*
- Treatment Outcome

Substances

- Antibodies, Viral
- 2019-nCoV Vaccine mRNA-1273
- BNT162 Vaccine

Full text links

Final Version J Am Soc Nephrol HighWire

Proceed to details

Cite

Share

☐ 677
Observational Study
Eur J Intern Med
. 2021 May;87:29-35.
doi: 10.1016/j.ejim.2021.01.017. Epub 2021 Jan 31.

Assessing the impact of COVID-19 on the health of geriatric patients: The European GeroCovid Observational Study

Caterina Trevisan [1], Susanna Del Signore [2], Stefano Fumagalli [3], Pietro Gareri [4], Alba Malara [5], Enrico Mossello [3], Stefano Volpato [6], Fabio Monzani [7], Alessandra Coin [8], Giuseppe Bellelli [9], Gianluca Zia [2], Anette Hylen Ranhoff [10], Raffaele Antonelli Incalzi [11], GeroCovid Working Group
Affiliations Expand

Affiliations

- [1] Geriatrics Unit and the GeroCovid Working Group, Department of Medicine (DIMED), University of Padua, Italy. Electronic address: caterina.trevisan.5@studenti.unipd.it.
- [2] Bluecompanion Ltd, London, UK.
- [3] Geriatric Intensive Care Unit, Department of Experimental and Clinical Medicine, University of Florence, Italy.
- [4] Center for Cognitive Disorders and Dementia - Catanzaro Lido, ASP Catanzaro, Italy.
- [5] Scientific Committee of National Association of Third Age Residences (ANASTE) Calabria, Lamezia Terme (Catanzaro), Italy.
- [6] Department of Medical Sciences, University of Ferrara, Ferrara, Italy.
- [7] Geriatrics Unit, Department of Clinical and Experimental Medicine, University of Pisa, Pisa, Italy.
- [8] Geriatrics Unit and the GeroCovid Working Group, Department of Medicine (DIMED), University of Padua, Italy.
- [9] School of Medicine and Surgery, Acute Geriatric Unit, University of Milano-Bicocca, San Gerardo Hospital, Monza, Italy.
- [10] Department of Clinical Science, University of Bergen, Norway and Diakonhjemmet Hospital, Oslo, Norway.
- [11] Geriatrics Unit, Department of Medicine, Campus Bio-Medico University and Teaching Hospital, Rome, Italy.

- PMID: **33573885**
- PMCID: PMC7847394
- DOI: 10.1016/j.ejim.2021.01.017

Free PMC article
Observational Study

Assessing the impact of COVID-19 on the health of geriatric patients: The European GeroCovid Observational Study

Caterina Trevisan et al. Eur J Intern Med. 2021 May.
Free PMC article
Show details
Eur J Intern Med
. 2021 May;87:29-35.
doi: 10.1016/j.ejim.2021.01.017. Epub 2021 Jan 31.

Authors

Caterina Trevisan [1], Susanna Del Signore [2], Stefano Fumagalli [3], Pietro Gareri [4], Alba Malara [5], Enrico Mossello [3], Stefano Volpato [6], Fabio Monzani [7], Alessandra Coin [8], Giuseppe Bellelli [9], Gianluca Zia [2], Anette Hylen Ranhoff [10], Raffaele Antonelli Incalzi [11], GeroCovid Working Group

Affiliations

- [1] Geriatrics Unit and the GeroCovid Working Group, Department of Medicine (DIMED), University of Padua, Italy. Electronic address: caterina.trevisan.5@studenti.unipd.it.
- [2] Bluecompanion Ltd, London, UK.
- [3] Geriatric Intensive Care Unit, Department of Experimental and Clinical Medicine, University of Florence, Italy.
- [4] Center for Cognitive Disorders and Dementia - Catanzaro Lido, ASP Catanzaro, Italy.
- [5] Scientific Committee of National Association of Third Age Residences (ANASTE) Calabria, Lamezia Terme (Catanzaro), Italy.
- [6] Department of Medical Sciences, University of Ferrara, Ferrara, Italy.
- [7] Geriatrics Unit, Department of Clinical and Experimental Medicine, University of Pisa, Pisa, Italy.
- [8] Geriatrics Unit and the GeroCovid Working Group, Department of Medicine (DIMED), University of Padua, Italy.
- [9] School of Medicine and Surgery, Acute Geriatric Unit, University of Milano-Bicocca, San Gerardo Hospital, Monza, Italy.
- [10] Department of Clinical Science, University of Bergen, Norway and Diakonhjemmet Hospital, Oslo, Norway.
- [11] Geriatrics Unit, Department of Medicine, Campus Bio-Medico University and Teaching Hospital, Rome, Italy.

- PMID: **33573885**
- PMCID: PMC7847394
- DOI: 10.1016/j.ejim.2021.01.017

Abstract

Background: Despite the growing evidence on COVID-19, there are still many gaps in the understanding of this disease, especially in individuals in advanced age. We describe the study protocol of GeroCovid Observational, a multi-purpose, multi-setting and multicenter initiative that aims at investigating: risk factors, clinical presentation and outcomes of individuals affected by COVID-19 in acute and residential care settings; best strategies to prevent infection in long-term care facilities; and, impact of the pandemic on neuropsychologic, functional and physical health, and on medical management in outpatients and home care patients at risk of COVID-19, with a special focus on individuals with dementia.

Methods: GeroCovid involves individuals aged ≥60 years, at risk of or affected by COVID-19, prospectively or retrospectively observed since March 1st, 2020. Data are collected in multiple investigational sites across Italy, Spain and Norway, and recorded in a de-identified clinical e-Registry. A common framework was adapted to different care settings: acute wards, long-term care facilities, geriatric outpatient and home care, and outpatient memory clinics.

Results: At September 16th, 2020, 66 investigational sites obtained their Ethical Committee approval and 1618 cases (mean age 80.6 [SD=9.0] years; 45% men) have been recorded in the e-Registry. The average inclusion rate since the study start on April 25th, 2020, is 11.2 patients/day. New cases enrollment will ended on December 31st , 2020, and the clinical follow-up will end on June 30th, 2021.

Conclusion: GeroCovid will explore relevant aspects of COVID-19 in adults aged ≥60 years with high-quality and comprehensive data, which will help to optimize COVID-19 prevention and management, with practical implications for ongoing and possible future pandemics.

Trial registration: NCT04379440 (clinicaltrial.gov).

Keywords: COVID-19; Health Services for the Aged; Inpatients; Nursing Homes; Observational Study; Outpatients.

Copyright © 2021. Published by Elsevier B.V.

Conflict of interest statement

None.

- 51 references
- 2 figures

Supplementary info

Publication types, MeSH terms, Associated data Expand

Publication types

- Multicenter Study
- Observational Study

MeSH terms

- Adult
- Aged
- Aged, 80 and over
- COVID-19*
- Female
- Humans
- Italy / epidemiology
- Male
- Middle Aged
- Norway
- Retrospective Studies
- SARS-CoV-2
- Spain / epidemiology
- Treatment Outcome

Associated data

- ClinicalTrials.gov/NCT04379440

Full text links

ELSEVIER FULL-TEXT ARTICLE Elsevier Science Free PMC article

Proceed to details

Cite

Share

678

Observational Study

J Med Virol

. 2020 Oct;92(10):2055-2066.

doi: 10.1002/jmv.25966. Epub 2020 Jun 12.

Clinical features of patients with coronavirus disease 2019 from a designated hospital in Beijing, China

Lijun Sun [1], Lijun Shen [2], Junfen Fan [3], Fengjun Gu [4], Mei Hu [5], Yiran An [6], Qing Zhou [6], Huahao Fan [7], Jingfeng Bi [1]

Affiliations Expand

Affiliations

- [1] Research Center for Clinical and Translational Medicine, Beijing 302 Hospital/The Fifth Medical Center of PLA General Hospital, Beijing, China.

- [2] The First Liver Cirrhosis Diagnosis and Treatment Center, Beijing 302 Hospital/The Fifth Medical Center of Chinese PLA General Hospital, Beijing, China.
- [3] Institute of Cerebrovascular Disease Research and Department of Neurology, Xuanwu Hospital of Capital Medical University, Beijing, China.
- [4] Medical Information Center Department, Beijing 302 Hospital/The Fifth Medical Center of Chinese PLA General Hospital, Beijing, China.
- [5] Library, Beijing 302 Hospital/The Fifth Medical Center of Chinese PLA General Hospital, Beijing, China.
- [6] The Medical Center of Clinical Laboratory, Beijing 302 Hospital/the Fifth Medical Center of Chinese PLA General Hospital, Beijing, China.
- [7] College of Life Science and Technology, Beijing University of Chemical Technology, Beijing, China.

- PMID: **32369208**
- PMCID: PMC7267635
- DOI: 10.1002/jmv.25966

Free PMC article
Observational Study

Clinical features of patients with coronavirus disease 2019 from a designated hospital in Beijing, China

Lijun Sun et al. J Med Virol. 2020 Oct.
Free PMC article
Show details
J Med Virol
. 2020 Oct;92(10):2055-2066.
doi: 10.1002/jmv.25966. Epub 2020 Jun 12.

Authors

Lijun Sun [1], Lijun Shen [2], Junfen Fan [3], Fengjun Gu [4], Mei Hu [5], Yiran An [6], Qing Zhou [6], Huahao Fan [7], Jingfeng Bi [1]

Affiliations

- [1] Research Center for Clinical and Translational Medicine, Beijing 302 Hospital/The Fifth Medical Center of PLA General Hospital, Beijing, China.
- [2] The First Liver Cirrhosis Diagnosis and Treatment Center, Beijing 302 Hospital/The Fifth Medical Center of Chinese PLA General Hospital, Beijing, China.
- [3] Institute of Cerebrovascular Disease Research and Department of Neurology, Xuanwu Hospital of Capital Medical University, Beijing, China.
- [4] Medical Information Center Department, Beijing 302 Hospital/The Fifth Medical Center of Chinese PLA General Hospital, Beijing, China.

- [5] Library, Beijing 302 Hospital/The Fifth Medical Center of Chinese PLA General Hospital, Beijing, China.
- [6] The Medical Center of Clinical Laboratory, Beijing 302 Hospital/the Fifth Medical Center of Chinese PLA General Hospital, Beijing, China.
- [7] College of Life Science and Technology, Beijing University of Chemical Technology, Beijing, China.

- PMID: **32369208**
- PMCID: PMC7267635
- DOI: 10.1002/jmv.25966

Abstract

Clinical and laboratory data on patients with coronavirus disease 2019 (COVID-19) in Beijing, China, remain extremely limited. In this study, we summarized the clinical characteristics of patients with COVID-19 from a designated hospital in Beijing. In total, 55 patients with laboratory-confirmed SARS-CoV-2 infection in Beijing 302 Hospital were enrolled in this study. Demographic data, symptoms, comorbidities, laboratory values, treatments, and clinical outcomes were all collected and retrospectively analyzed. A total of 15 (27.3%) patients had severe symptoms, the mean age was 44.0 years (interquartile range [IQR], 34.0-56.0), and the median incubation period was 7.5 days (IQR, 5.0-11.8). A total of 26 (47.3%) patients had exposure history in Wuhan of less than 2 weeks, whereas 20 (36.4%) patients were associated with familial clusters. Also, eighteen (32.7%) patients had underlying comorbidities including hypertension. The most common symptom of illness was fever (45; 81.8%); 51 (92.7%) patients had abnormal findings on chest computed tomography. Laboratory findings showed that neutrophil count, percentage of lymphocyte, percentage of eosinophil, eosinophil count, erythrocyte sedimentation rate, albumin, and serum ferritin are potential risk factors for patients with a poor prognosis. A total of 26 patients (47.3%) were still hospitalized, whereas 29 (52.7%) patients had been discharged. Compared with patients in Wuhan, China, the symptoms of patients in Beijing are relatively mild. Older age, more comorbidities, and more abnormal prominent laboratory markers were associated with a severe condition. On the basis of antiviral drugs, it is observed that antibiotics treatment, appropriate dosage of corticosteroid, and gamma globulin therapy significantly improve patients' outcomes. Early identification and timely medical treatment are important to reduce the severity of patients with COVID-19.

Keywords: SARS-CoV-2; clinical features; coronavirus disease 2019; designated Hospital in Beijing.

© 2020 Wiley Periodicals LLC.

- 34 references

Supplementary info

Publication types, MeSH terms, Substances, Grant support Expand

Publication types

- Observational Study
- Research Support, Non-U.S. Gov't

MeSH terms

- Adrenal Cortex Hormones / therapeutic use
- Adult
- Age Factors
- Antiviral Agents / therapeutic use
- COVID-19 / diagnostic imaging
- COVID-19 / physiopathology*
- COVID-19 / therapy
- COVID-19 / virology
- China
- Comorbidity
- Coronary Disease / diagnostic imaging
- Coronary Disease / physiopathology*
- Coronary Disease / therapy
- Coronary Disease / virology
- Diabetes Mellitus / diagnostic imaging
- Diabetes Mellitus / physiopathology*
- Diabetes Mellitus / therapy
- Diabetes Mellitus / virology
- Eosinophils / pathology
- Eosinophils / virology
- Female
- Ferritins / blood
- Fever / physiopathology
- Hospitalization
- Hospitals
- Humans
- Hypertension / diagnostic imaging
- Hypertension / physiopathology*
- Hypertension / therapy
- Hypertension / virology
- Immunoglobulins, Intravenous / therapeutic use
- Infectious Disease Incubation Period
- Kidney Failure, Chronic / diagnostic imaging
- Kidney Failure, Chronic / physiopathology*
- Kidney Failure, Chronic / therapy
- Kidney Failure, Chronic / virology
- Leukocyte Count
- Lymphocytes / pathology
- Lymphocytes / virology

- Male
- Middle Aged
- Neutrophils / pathology
- Neutrophils / virology
- Retrospective Studies
- Risk Factors
- SARS-CoV-2 / drug effects
- SARS-CoV-2 / pathogenicity
- Severity of Illness Index
- Tomography, X-Ray Computed

Substances

- Adrenal Cortex Hormones
- Antiviral Agents
- Immunoglobulins, Intravenous
- Ferritins

Grant support

- BUCTZY2012/Fundamental Research Funds for Central Universities/International
- 2018ZX09711003-004-003/National Key Research and Development Plan of China/International
- 2019M65072/China Postdoctoral Science Foundation/International

Full text links

WILEY Full Text Article Wiley Free PMC article

Proceed to details

Cite

Share

☐ 679

Observational Study

PLoS One

. 2021 Apr 8;16(4):e0249036.

doi: 10.1371/journal.pone.0249036. eCollection 2021.

Similar incidence of coronavirus disease 2019 (COVID-19) in patients with rheumatic diseases with and without hydroxychloroquine therapy

Juan Macías [1], Paz González-Moreno [2], Esther Sánchez-García [3], Ramón Morillo-Verdugo [4], José J Pérez-Venegas [2], Ana Pinilla [1], MªMar Macho [1], MªVictoria Martínez [1], Alejandro González-Serna [1], Anaïs Corma [1], Luis M Real [1], Juan A Pineda [1]
Affiliations Expand

Affiliations

- [1] Infectious Diseases and Microbiology Unit, Hospital Universitario Virgen de Valme, Seville, Spain.
- [2] Rheumatology Unit, Hospital Universitario Virgen Macarena, Seville, Spain.
- [3] Internal Medicine Service, Hospital Universitario Virgen de Valme, Seville, Spain.
- [4] Pharmacy, Hospital Universitario Virgen de Valme, Seville, Spain.

- PMID: **33831011**
- PMCID: PMC8031374
- DOI: 10.1371/journal.pone.0249036

Free PMC article
Observational Study

Similar incidence of coronavirus disease 2019 (COVID-19) in patients with rheumatic diseases with and without hydroxychloroquine therapy

Juan Macías et al. PLoS One. 2021.
Free PMC article
Show details
PLoS One
. 2021 Apr 8;16(4):e0249036.
doi: 10.1371/journal.pone.0249036. eCollection 2021.

Authors

Juan Macías [1], Paz González-Moreno [2], Esther Sánchez-García [3], Ramón Morillo-Verdugo [4], José J Pérez-Venegas [2], Ana Pinilla [1], MªMar Macho [1], MªVictoria Martínez [1], Alejandro González-Serna [1], Anaïs Corma [1], Luis M Real [1], Juan A Pineda [1]

Affiliations

- [1] Infectious Diseases and Microbiology Unit, Hospital Universitario Virgen de Valme, Seville, Spain.
- [2] Rheumatology Unit, Hospital Universitario Virgen Macarena, Seville, Spain.
- [3] Internal Medicine Service, Hospital Universitario Virgen de Valme, Seville, Spain.
- [4] Pharmacy, Hospital Universitario Virgen de Valme, Seville, Spain.

- PMID: **33831011**
- PMCID: PMC8031374
- DOI: 10.1371/journal.pone.0249036

Abstract

Background: Hydroxychloroquine is not efficacious as post-exposure prophylaxis against coronavirus disease 2019 (COVID-19). It is not known whether as pre-exposure prophylaxis it may prevent COVID-19.

Objective: To compare the incidence of COVID-19 in Spanish patients with autoimmune rheumatic diseases treated with and without hydroxychloroquine.

Patients and methods: Retrospective electronic record review, from February 27th to June 21st, 2020, of patients with autoimmune inflammatory diseases followed at two academic tertiary care hospitals in Seville, Spain. The cumulative incidence of confirmed COVID-19, by PCR or serology, was compared between patients with and without hydroxychloroquine as part of their treatment of autoimmune inflammatory diseases.

Results: Among 722 included patients, 290 (40%) were receiving hydroxychloroquine. During the seventeen-week study period, 10 (3.4% [95% CI: 1.7%-6.7%] cases of COVID-19 were registered among patients with hydroxychloroquine and 13 (3.0% [1.6%-5.1%]) ($p = 0.565$) in those without hydroxychloroquine. COVID-19 was diagnosed by PCR in four (1.4%, 95% CI 0.38%-3.5%) subject with hydroxychloroquine and six (1.4%, 95% CI 0.5%-3.0%) without hydroxychloroquine ($p = 0.697$). Three patients on hydroxychloroquine and four patients without hydroxychloroquine were admitted to the hospital, none of them required to be transferred to the intensive care unit and no patient died during the episode.

Conclusions: The incidence and severity of COVID-19 among patients with autoimmune rheumatic diseases with and without hydroxychloroquine was not significantly different.

Conflict of interest statement

The authors have declared that no competing interests exist.

- 16 references

Supplementary info

Publication types, MeSH terms, Substances, Grant support Expand

Publication types

- Comparative Study
- Multicenter Study
- Observational Study
- Research Support, Non-U.S. Gov't

MeSH terms

- Aged
- COVID-19* / epidemiology
- COVID-19* / prevention & control
- Cross-Sectional Studies
- Female
- Humans
- Hydroxychloroquine / administration & dosage*
- Incidence
- Male
- Middle Aged
- Post-Exposure Prophylaxis*
- Pre-Exposure Prophylaxis*
- Retrospective Studies
- Rheumatic Diseases / drug therapy*
- Rheumatic Diseases / epidemiology
- Risk Factors
- SARS-CoV-2*
- Spain / epidemiology

Substances

- Hydroxychloroquine

Grant support

This study was supported by Instituto de Salud Carlos III in the form of an intensification grant (Grant No. Programa-I3SNS) awarded to JAP, and by the Ministry of Science, Innovation and Universities of Spain in the form of a Miguel Servet Research Contract (CP18/00146) awarded to AG-S. The funders had no role in study design, data collection and analysis, decision to publish, or preparation of the manuscript.

Full text links

OPEN ACCESS TO FULL TEXT PLOS ONE Public Library of Science Free PMC article

Proceed to details

Cite

Share

☐ 680

Observational Study

Med Glas (Zenica)

. 2021 Aug 1;18(2):378-383.

doi: 10.17392/1394-21.

Clinical characteristics, comorbidities and mortality in critically ill mechanically ventilated patients with Covid-19: a retrospective observational study

Adisa Šabanović Adilović [1], Nermina Rizvanović [1], Mirza Kovačević [1], Harun Adilović [2]
Affiliations Expand

Affiliations

- [1] Department of Anaesthesiology and Intensive Care Unit, Cantonal Hospital Zenica, Zenica, Bosnia and Herzegovina.
- [2] Department of Internal Medicine, Cantonal Hospital Zenica, Zenica, Bosnia and Herzegovina.

- PMID: **34308616**
- DOI: 10.17392/1394-21

Free article
Observational Study

Clinical characteristics, comorbidities and mortality in critically ill mechanically ventilated patients with Covid-19: a retrospective observational study

Adisa Šabanović Adilović et al. Med Glas (Zenica). 2021.
Free article
Show details
Med Glas (Zenica)
. 2021 Aug 1;18(2):378-383.
doi: 10.17392/1394-21.

Authors

Adisa Šabanović Adilović [1], Nermina Rizvanović [1], Mirza Kovačević [1], Harun Adilović [2]

Affiliations

- [1] Department of Anaesthesiology and Intensive Care Unit, Cantonal Hospital Zenica, Zenica, Bosnia and Herzegovina.

- [2] Department of Internal Medicine, Cantonal Hospital Zenica, Zenica, Bosnia and Herzegovina.

- PMID: **34308616**
- DOI: 10.17392/1394-21

Abstract

Aim To analyse demographic data, clinical symptoms and signs, laboratory data and comorbidities in patients with COVID-19 pneumonia admitted to the intensive care unit (ICU), mechanically ventilated with fatal outcome. Methods Medical records of 92 patients were retrospectively analysed. Demographic data, clinical symptoms and comorbidities were collected on the day of hospital admission. Clinical signs and laboratory data were collected on the day of hospital admission (T1), on the day of starting non-invasive ventilation (T2), and on the day of starting invasive ventilation (T3). Results Average age of the patients was 60.05 years. Patients over 50 years of age, 71 (77.1%) (p=0.000), and males, 62 (67.4%; p=0.001) were predominant. The most common patient symptoms were exhaustion, myalgia, dyspnoea and cough. Hyperthermia was recorded on the day of hospital admission. Tachycardia, hyperglycaemia, hypoxemia were recorded at all observed study times. The most common comorbidity was hypertension arterialis with a very strong correlation with fatal outcome, followed by diabetes mellitus and chronic heart disease that were moderately correlated with fatal outcome. Conclusion The treatment of COVID-19 patients in ICU with mechanical ventilation has a high failure rate. Demographic data, clinical symptoms and signs as well as accompanying comorbidities can be a significant component in making decisions about diagnostic-therapeutic procedures.

Keywords: comorbidity; fatal outcome; intensive care unit; invasive ventilation.

Copyright© by the Medical Assotiation of Zenica-Doboj Canton.

Supplementary info

Publication types, MeSH terms Expand

Publication types

- Observational Study

MeSH terms

- Aged
- COVID-19* / therapy
- Comorbidity*
- Critical Illness*
- Female
- Humans
- Male
- Middle Aged
- Respiration, Artificial*

- Retrospective Studies

Full text links

FREE FULL TEXT
MEDICINSKI GLASNIK Medical Association of Zenica-Doboj Canton, Bosnia and Herzegovina
Proceed to details
Cite
Share
681

Observational Study
J Pediatr
. 2020 Nov;226:55-63.e2.
doi: 10.1016/j.jpeds.2020.07.039. Epub 2020 Jul 16.

Clinical Manifestations and Outcomes of Critically Ill Children and Adolescents with Coronavirus Disease 2019 in New York City

Kim R Derespina [1], Shubhi Kaushik [2], Anna Plichta [1], Edward E Conway Jr [3], Asher Bercow [3], Jaeun Choi [4], Ruth Eisenberg [4], Jennifer Gillen [2], Anita I Sen [5], Claire M Hennigan [6], Lillian M Zerihun [7], Sule Doymaz [8], Michael A Keenaghan [9], Stephanie Jarrin [10], Franscene Oulds [11], Manoj Gupta [12], Louisdon Pierre [13], Melissa Grageda [14], H Michael Ushay [1], Vinay M Nadkarni [15], Michael S D Agus [16], Shivanand S Medar [17]
Affiliations Expand

Affiliations

- [1] Division of Critical Care Medicine, Department of Pediatrics, Children's Hospital at Montefiore, Bronx, NY.
- [2] Department of Pediatrics, Division of Critical Care Medicine, Kravis Children's Hospital at Mount Sinai, New York, NY.
- [3] Department of Pediatrics, Division of Critical Care Medicine, Jacobi Medical Center, Bronx, NY.
- [4] Department of Epidemiology and Population Health, Albert Einstein College of Medicine, Bronx, NY.
- [5] Department of Pediatrics, Division of Critical Care Medicine, Columbia University Medical Center, New York, NY.
- [6] Department of Pediatrics, New York-Presbyterian Morgan Stanley Children's Hospital, New York, NY.
- [7] Columbia University Vagelos College of Physicians and Surgeons, New York, NY.
- [8] Department of Pediatrics, Division of Critical Care Medicine, State University of New York Downstate Health Sciences University, Brooklyn, NY.
- [9] Department of Pediatrics, Division of Critical Care Medicine, Kings County Medical Center, Brooklyn, NY; Department of Pediatrics, St George's University, Grenada, WI.

- [10] Department of Pediatrics, Division of Critical Care Medicine, Kings County Medical Center, Brooklyn, NY; Department of Pediatrics, State University of New York Downstate, Brooklyn, NY.
- [11] Department of Pediatrics, Division of Critical Care Medicine, Lincoln Medical and Mental Health Center, Bronx, NY.
- [12] Department of Pediatrics, Division of Critical Care Medicine, Lincoln Medical and Mental Health Center, Bronx, NY; Division of Cardiology, Department of Pediatrics, Children's Hospital at Montefiore, Bronx, NY.
- [13] Department of Pediatrics, Division of Critical Care Medicine, The Brooklyn Hospital Center, Brooklyn, NY.
- [14] Department of Pediatrics, Division of Critical Care Medicine, Richmond University Medical Center, Staten Island, NY.
- [15] Division of Critical Care Medicine, Children's Hospital of Philadelphia, Philadelphia, PA.
- [16] Division of Medical Critical Care, Boston Children's Hospital, Boston, MA.
- [17] Division of Critical Care Medicine, Department of Pediatrics, Children's Hospital at Montefiore, Bronx, NY; Division of Cardiology, Department of Pediatrics, Children's Hospital at Montefiore, Bronx, NY. Electronic address: smedar@montefiore.org.

- PMID: **32681989**
- PMCID: PMC7363610
- DOI: 10.1016/j.jpeds.2020.07.039

Free PMC article
Observational Study

Clinical Manifestations and Outcomes of Critically Ill Children and Adolescents with Coronavirus Disease 2019 in New York City

Kim R Derespina et al. J Pediatr. 2020 Nov.
Free PMC article
Show details
J Pediatr
. 2020 Nov;226:55-63.e2.
doi: 10.1016/j.jpeds.2020.07.039. Epub 2020 Jul 16.

Authors

Kim R Derespina [1], Shubhi Kaushik [2], Anna Plichta [1], Edward E Conway Jr [3], Asher Bercow [3], Jaeun Choi [4], Ruth Eisenberg [4], Jennifer Gillen [2], Anita I Sen [5], Claire M Hennigan [6], Lillian M Zerihun [7], Sule Doymaz [8], Michael A Keenaghan [9], Stephanie Jarrin [10], Franscene Oulds [11], Manoj Gupta [12], Louisdon Pierre [13], Melissa Grageda [14], H Michael Ushay [1], Vinay M Nadkarni [15], Michael S D Agus [16], Shivanand S Medar [17]

Affiliations

- [1] Division of Critical Care Medicine, Department of Pediatrics, Children's Hospital at Montefiore, Bronx, NY.
- [2] Department of Pediatrics, Division of Critical Care Medicine, Kravis Children's Hospital at Mount Sinai, New York, NY.
- [3] Department of Pediatrics, Division of Critical Care Medicine, Jacobi Medical Center, Bronx, NY.
- [4] Department of Epidemiology and Population Health, Albert Einstein College of Medicine, Bronx, NY.
- [5] Department of Pediatrics, Division of Critical Care Medicine, Columbia University Medical Center, New York, NY.
- [6] Department of Pediatrics, New York-Presbyterian Morgan Stanley Children's Hospital, New York, NY.
- [7] Columbia University Vagelos College of Physicians and Surgeons, New York, NY.
- [8] Department of Pediatrics, Division of Critical Care Medicine, State University of New York Downstate Health Sciences University, Brooklyn, NY.
- [9] Department of Pediatrics, Division of Critical Care Medicine, Kings County Medical Center, Brooklyn, NY; Department of Pediatrics, St George's University, Grenada, WI.
- [10] Department of Pediatrics, Division of Critical Care Medicine, Kings County Medical Center, Brooklyn, NY; Department of Pediatrics, State University of New York Downstate, Brooklyn, NY.
- [11] Department of Pediatrics, Division of Critical Care Medicine, Lincoln Medical and Mental Health Center, Bronx, NY.
- [12] Department of Pediatrics, Division of Critical Care Medicine, Lincoln Medical and Mental Health Center, Bronx, NY; Division of Cardiology, Department of Pediatrics, Children's Hospital at Montefiore, Bronx, NY.
- [13] Department of Pediatrics, Division of Critical Care Medicine, The Brooklyn Hospital Center, Brooklyn, NY.
- [14] Department of Pediatrics, Division of Critical Care Medicine, Richmond University Medical Center, Staten Island, NY.
- [15] Division of Critical Care Medicine, Children's Hospital of Philadelphia, Philadelphia, PA.
- [16] Division of Medical Critical Care, Boston Children's Hospital, Boston, MA.
- [17] Division of Critical Care Medicine, Department of Pediatrics, Children's Hospital at Montefiore, Bronx, NY; Division of Cardiology, Department of Pediatrics, Children's Hospital at Montefiore, Bronx, NY. Electronic address: smedar@montefiore.org.

- PMID: **32681989**
- PMCID: PMC7363610
- DOI: 10.1016/j.jpeds.2020.07.039

Abstract

Objectives: To describe the clinical manifestations and outcomes of critically ill children with coronavirus disease-19 (COVID-19) in New York City.

Study design: Retrospective observational study of children 1 month to 21 years admitted March 14 to May 2, 2020, to 9 New York City pediatric intensive care units (PICUs) with severe acute respiratory syndrome coronavirus 2 infection.

Results: Of 70 children admitted to PICUs, median age was 15 (IQR 9, 19) years; 61.4% male; 38.6% Hispanic; 32.9% black; and 74.3% with comorbidities. Fever (72.9%) and cough (71.4%)

were the common presenting symptoms. Twelve patients (17%) met severe sepsis criteria; 14 (20%) required vasopressor support; 21 (30%) developed acute respiratory distress syndrome (ARDS); 9 (12.9%) met acute kidney injury criteria; 1 (1.4%) required renal-replacement therapy, and 2 (2.8%) had cardiac arrest. For treatment, 27 (38.6%) patients received hydroxychloroquine; 13 (18.6%) remdesivir; 23 (32.9%) corticosteroids; 3 (4.3%) tocilizumab; and 1 (1.4%) anakinra; no patient was given immunoglobulin or convalescent plasma. Forty-nine (70%) patients required respiratory support: 14 (20.0%) noninvasive mechanical ventilation, 20 (28.6%) invasive mechanical ventilation (IMV), 7 (10%) prone position, 2 (2.8%) inhaled nitric oxide, and 1 (1.4%) extracorporeal membrane oxygenation. Nine (45%) of the 20 patients requiring IMV were extubated by day 14 with median IMV duration of 218 (IQR 79, 310.4) hours. Presence of ARDS was significantly associated with duration of PICU and hospital stay, and lower probability of PICU and hospital discharge at hospital day 14 ($P < .05$ for all).

Conclusions: Critically ill children with COVID-19 predominantly are adolescents, have comorbidities, and require some form of respiratory support. The presence of ARDS is significantly associated with prolonged PICU and hospital stay.

Keywords: pediatric ARDS; pediatric respiratory failure; pediatric viral sepsis.

Copyright © 2020 Elsevier Inc. All rights reserved.

Comment in

- Reply.
  Derespina KR, Kaushik S, Ushay HM, Medar SS. Derespina KR, et al. J Pediatr. 2021 Apr;231:301-302. doi: 10.1016/j.jpeds.2020.11.046. Epub 2020 Nov 26. J Pediatr. 2021. PMID: 33249006 Free PMC article. No abstract available.
- Pediatric acute respiratory distress syndrome associated with respiratory viruses.
  Baird JS. Baird JS. J Pediatr. 2021 Apr;231:300-301. doi: 10.1016/j.jpeds.2020.11.045. Epub 2020 Nov 27. J Pediatr. 2021. PMID: 33253729 Free PMC article. No abstract available.

- 28 references
- 3 figures

Supplementary info

Publication types, MeSH terms, Substances Expand

Publication types

- Observational Study

MeSH terms

- Adolescent
- Antiviral Agents / therapeutic use
- COVID-19 / diagnosis*
- COVID-19 / epidemiology
- COVID-19 / therapy

- Child
- Child, Preschool
- Combined Modality Therapy
- Comorbidity
- Critical Care / methods
- Critical Illness
- Female
- Follow-Up Studies
- Humans
- Infant
- Length of Stay / statistics & numerical data
- Male
- New York City / epidemiology
- Respiratory Therapy / methods
- Retrospective Studies
- Treatment Outcome
- Young Adult

Substances

- Antiviral Agents

Full text links

ELSEVIER FULL-TEXT ARTICLE Elsevier Science Free PMC article
Proceed to details
Cite
Share
☐ 682
Observational Study
Clin Orthop Relat Res
. 2021 May 1;479(5):1158-1166.
doi: 10.1097/CORR.0000000000001568.

What Proportion of Patients with Bone and Soft Tissue Tumors Contracted Coronavirus-19 and Died From Surgical Procedures During the Initial Period of the COVID-19 Pandemic? Results From the Multicenter

British Orthopaedic Oncology Society Observational Study

Raja Bhaskara Rajasekaran [1], Robert U Ashford [2], Thomas D A Cosker [1], Jonathan D Stevenson [3], Lee Jeys [3], Rob Pollock [4], Kenneth S Rankin [5], Paul Cool [6], James T Patton [7], Duncan Whitwell [1], Christopher L M H Gibbons [1], Andrew Carr [8], British Orthopaedic Oncology Society Collaborative Group*
Collaborators, Affiliations Expand

Collaborators

- **British Orthopaedic Oncology Society Collaborative Group***: Adesegun Abudu, Will Aston, Corey D Chan, Scott Evans, Sanjay Gupta, Sanjeev Kotecha, Danielle Maes, Ashish Mahendra, Guy Morris, Michael Parry, Muhammad Sarmad, Muhammad Sarmad Tamimy, Sofia Thoma, Roger Tillman

Affiliations

- [1] R. B. Rajasekaran, T. D. A. Cosker, D. Whitwell, C. L. M. H. Gibbons, Oxford Bone and Soft Tissue Tumour Service, Nuffield Department of Orthopaedics, Rheumatology and Musculoskeletal Science, University of Oxford, Oxford, UK
- [2] R. U. Ashford, East Midlands Sarcoma Service, Nottingham University Hospitals, Nottinghamshire, UK
- [3] J. D. Stevenson, L. Jeys, Royal Orthopaedic Hospital, Birmingham, UK
- [4] R. Pollock, Royal National Orthopaedic Hospital, Middlesex, UK
- [5] K. S. Rankin, North of England Bone and Soft Tissue Tumour Service, Newcastle upon Tyne University Hospitals, Newcastle upon Tyne, UK
- [6] P. Cool, The Greater Manchester and Oswestry Sarcoma Service, The Robert Jones & Agnes Hunt Orthopaedic Hospital, Oswestry, UK
- [7] J. T. Patton, Scottish Sarcoma Network, Edinburgh, UK
- [8] A. Carr, Botnar Research Centre, University of Oxford, Oxford, UK

- PMID: **33196585**
- PMCID: PMC8051862
- DOI: 10.1097/CORR.0000000000001568

Free PMC article
Observational Study

What Proportion of Patients with Bone and Soft Tissue Tumors Contracted Coronavirus-19 and Died From Surgical Procedures During the Initial Period of the COVID-19

Pandemic? Results From the Multicenter British Orthopaedic Oncology Society Observational Study

Raja Bhaskara Rajasekaran et al. Clin Orthop Relat Res. 2021.
Free PMC article
Show details
Clin Orthop Relat Res
. 2021 May 1;479(5):1158-1166.
doi: 10.1097/CORR.0000000000001568.

Authors

Raja Bhaskara Rajasekaran [1], Robert U Ashford [2], Thomas D A Cosker [1], Jonathan D Stevenson [3], Lee Jeys [3], Rob Pollock [4], Kenneth S Rankin [5], Paul Cool [6], James T Patton [7], Duncan Whitwell [1], Christopher L M H Gibbons [1], Andrew Carr [8], British Orthopaedic Oncology Society Collaborative Group*

Collaborators

- **British Orthopaedic Oncology Society Collaborative Group***: Adesegun Abudu, Will Aston, Corey D Chan, Scott Evans, Sanjay Gupta, Sanjeev Kotecha, Danielle Maes, Ashish Mahendra, Guy Morris, Michael Parry, Muhammad Sarmad, Muhammad Sarmad Tamimy, Sofia Thoma, Roger Tillman

Affiliations

- [1] R. B. Rajasekaran, T. D. A. Cosker, D. Whitwell, C. L. M. H. Gibbons, Oxford Bone and Soft Tissue Tumour Service, Nuffield Department of Orthopaedics, Rheumatology and Musculoskeletal Science, University of Oxford, Oxford, UK
- [2] R. U. Ashford, East Midlands Sarcoma Service, Nottingham University Hospitals, Nottinghamshire, UK
- [3] J. D. Stevenson, L. Jeys, Royal Orthopaedic Hospital, Birmingham, UK
- [4] R. Pollock, Royal National Orthopaedic Hospital, Middlesex, UK
- [5] K. S. Rankin, North of England Bone and Soft Tissue Tumour Service, Newcastle upon Tyne University Hospitals, Newcastle upon Tyne, UK
- [6] P. Cool, The Greater Manchester and Oswestry Sarcoma Service, The Robert Jones & Agnes Hunt Orthopaedic Hospital, Oswestry, UK
- [7] J. T. Patton, Scottish Sarcoma Network, Edinburgh, UK
- [8] A. Carr, Botnar Research Centre, University of Oxford, Oxford, UK

- PMID: **33196585**
- PMCID: PMC8051862
- DOI: 10.1097/CORR.0000000000001568

Abstract

Background: Delivering uninterrupted cancer treatment to patients with musculoskeletal tumors has been essential during the rapidly evolving coronavirus 2019 (COVID-19) pandemic, as delays in management can be detrimental. Currently, the risk of contracting COVID-19 in hospitals when admitted for surgery and the susceptibility due to adjuvant therapies and associated mortality due to COVID-19 is unknown, but knowledge of these potential risks would help treating clinicians provide appropriate cancer care.

Questions/purposes: (1) What is the risk of hospital-acquired COVID-19 in patients with musculoskeletal tumors admitted for surgery during the initial period of the pandemic? (2) What is the associated mortality in patients with musculoskeletal tumors who have contracted COVID-19? (3) Are patients with musculoskeletal tumors who have had neoadjuvant therapy (chemotherapy or radiation) preoperatively at an increased risk of contracting COVID-19? (4) Is a higher American Society of Anesthesiologists (ASA) grade in patients with musculoskeletal tumors associated with an increased risk of contracting COVID-19 when admitted to the hospital for surgery?

Methods: This retrospective, observational study analyzed patients with musculoskeletal tumors who underwent surgery in one of eight specialist centers in the United Kingdom, which included the five designated cancer centers in England, one specialist soft tissue sarcoma center, and two centers from Scotland between March 12, 2020 and May 20, 2020. A total of 347 patients were included, with a median (range) age of 53 years (10 to 94); 60% (207 of 347) were men, and the median ASA grade was II (I to IV). These patients had a median hospital stay of 8 days (0 to 53). Eighteen percent (61 of 347) of patients had received neoadjuvant therapy (8% [27] chemotherapy, 8% [28] radiation, 2% [6] chemotherapy and radiation) preoperatively. The decision to undergo surgery was made in adherence with United Kingdom National Health Service and national orthopaedic oncology guidelines, but specific data with regard to the number of patients within each category are not known. Fifty-nine percent (204 of 347) were negative in PCR testing done 48 hours before the surgical procedure; the remaining 41% (143 of 347) were treated before preoperative PCR testing was made mandatory, but these patients were asymptomatic. All patients were followed for 30 days postoperatively, and none were lost to follow-up during that period. The primary outcome of the study was contracting COVID-19 in the hospital after admission. The secondary outcome was associated mortality after contracting COVID-19 within 30 days of the surgical procedure. In addition, we assessed whether there is any association between ASA grade or neoadjuvant treatment and the chances of contracting COVID-19 in the hospital. Electronic patient record system and simple descriptive statistics were used to analyze both outcomes.

Results: Four percent (12 of 347) of patients contracted COVID-19 in the hospital, and 1% (4 of 347) of patients died because of COVID-19-related complications. Patients with musculoskeletal tumors who contracted COVID-19 had increased mortality compared with patients who were asymptomatic or tested negative (odds ratio 55.33 [95% CI 10.60 to 289.01]; $p < 0.001$).With the numbers we had, we could not show that adjuvant therapy had any association with contracting COVID-19 while in the hospital (OR 0.94 [95% CI 0.20 to 4.38]; $p = 0.93$). Increased ASA grade was associated with an increased likelihood of contracting COVID-19 (OR 58 [95% CI 5 to 626]; $p < 0.001$).

Conclusion: Our results show that surgeons must be mindful and inform patients that those with musculoskeletal tumors are at risk of contracting COVID-19 while admitted to the hospital and some may succumb to it. Hospital administrators and governmental agencies should be aware that operations on patients with lower ASA grade appear to have lower risk and should consider restructuring service delivery to ensure that procedures are performed in designated COVID-19-restricted sites. These measures may reduce the likelihood of patients contracting the virus in the

hospital, although we cannot confirm a benefit from this study. Future studies should seek to identify factors influencing these outcomes and also compare surgical complications in those patients with and without COVID-19.

Level of evidence: Level III, therapeutic study.

Copyright © 2020 by the Association of Bone and Joint Surgeons.

Conflict of interest statement

All ICMJE Conflict of Interest Forms for authors and Clinical Orthopaedics and Related Research® editors and board members are on file with the publication and can be viewed on request. Each author certifies that neither he or she, nor any member of his or her immediate family, has funding or commercial associations (consultancies, stock ownership, equity interest, patent/licensing arrangements, etc.) that might pose a conflict of interest in connection with the submitted article.

Comment in

- CORR Insights®: What Proportion of Patients with Bone and Soft Tissue Tumors Contracted Coronavirus-19 and Died From Surgical Procedures During the Initial Period of the COVID-19 Pandemic? Results From the Multicenter British Orthopaedic Oncology Society Observational Study.
  Temple HT. Temple HT. Clin Orthop Relat Res. 2021 May 1;479(5):1167-1169. doi: 10.1097/CORR.0000000000001682. Clin Orthop Relat Res. 2021. PMID: 33704095 Free PMC article. No abstract available.

- 22 references
- 2 figures

Supplementary info

Publication types, MeSH terms Expand

Publication types

- Multicenter Study
- Observational Study

MeSH terms

- Adolescent
- Adult
- Aged
- Aged, 80 and over
- Bone Neoplasms / mortality
- Bone Neoplasms / therapy*
- COVID-19 / complications*

- COVID-19 / mortality
- Child
- Cross Infection / complications*
- Cross Infection / mortality
- Female
- Hospital Mortality
- Humans
- Male
- Middle Aged
- Neoadjuvant Therapy
- Pandemics
- Retrospective Studies
- Risk Factors
- SARS-CoV-2
- Soft Tissue Neoplasms / mortality
- Soft Tissue Neoplasms / therapy*
- United Kingdom / epidemiology
- Young Adult

Full text links

Wolters Kluwer Wolters Kluwer Free PMC article
Proceed to details
Cite
Share
☐ 683
Observational Study
Int J Gynaecol Obstet
. 2020 Oct;151(1):33-38.
doi: 10.1002/ijgo.13296. Epub 2020 Aug 11.

Performing gynecologic cancer surgery during the COVID-19 pandemic in Turkey: A multicenter retrospective observational study

Polat Dursun [1], Haluk Dervisoglu [2], Mine Daggez [3], Taner Turan [4], Fatih Kiliç [4], Özlem M Tekin [4], Işin Üreyen [5], Tayfun Toptaş [5], Gökhan Demirayak [6], Ayşe B Önder [6], Çetin Çelik [7], Denizhan Bayramoğlu [7], Ahmet B Guzel [8], Dagistan T Arioz [9], Filiz Bilir [9], Isa A Ozdemir [10], Tolga Tasci [11], Alper Karalok [12], Ateş Karateke [13]
Affiliations Expand

Affiliations

- [1] Private Gynecologic Oncology, Ankara, Turkey.
- [2] Gynecologic Oncology Division, Department of Obstetrics and Gynecology, Dr. Abdurrahman Yurtaslan Ankara Oncological Education and Research Hospital, Ankara, Turkey.
- [3] Gynecologic Oncology Division, Department of Obstetrics and Gynecology, Erciyes University Faculty of Medicine, Kayseri, Turkey.
- [4] Gynecologic Oncology Division, Department of Obstetrics and Gynecology, Ankara City Hospital, Ankara, Turkey.
- [5] Gynecologic Oncology Division, Department of Obstetrics and Gynecology, University of Health Sciences Antalya Education and Research Hospital, Antalya, Turkey.
- [6] Gynecologic Oncology Division, Department of Obstetrics and Gynecology, Bakirkoy Dr. Sadi Konuk Training & Research Hospital, Istanbul, Turkey.
- [7] Gynecologic Oncology Division, Department of Obstetrics and Gynecology, Selcuk University Faculty of Medicine, Konya, Turkey.
- [8] Gynecologic Oncology Division, Department of Obstetrics and Gynecology, Cukurova University Faculty of Medicine, Adana, Turkey.
- [9] Gynecologic Oncology Division, Department of Obstetrics and Gynecology, Afyonkarahisar Health Sciences University Medical School, Afyonkarahisar, Turkey.
- [10] Gynecologic Oncology Division, Department of Obstetrics and Gynecology, Medipol University, Istanbul, Turkey.
- [11] Gynecologic Oncology Division, Department of Obstetrics and Gynecology, Bahçeşehir University Medical Park Goztepe Hospital, Istanbul, Turkey.
- [12] Gynecologic Oncology Division, Department of Obstetrics and Gynecology, Liv Hospital Ulus, Istanbul, Turkey.
- [13] Gynecologic Oncology Division, Department of Obstetrics and Gynecology, Medeniyet University, Istanbul, Turkey.

- PMID: **32623717**
- DOI: 10.1002/ijgo.13296

Observational Study

Performing gynecologic cancer surgery during the COVID-19 pandemic in Turkey: A multicenter retrospective observational study

Polat Dursun et al. Int J Gynaecol Obstet. 2020 Oct.

Show details

Int J Gynaecol Obstet

. 2020 Oct;151(1):33-38.

doi: 10.1002/ijgo.13296. Epub 2020 Aug 11.

Authors

Polat Dursun [1], Haluk Dervisoglu [2], Mine Daggez [3], Taner Turan [4], Fatih Kiliç [4], Özlem M Tekin [4], Işin Üreyen [5], Tayfun Toptaş [5], Gökhan Demirayak [6], Ayşe B Önder [6], Çetin Çelik [7], Denizhan Bayramoğlu [7], Ahmet B Guzel [8], Dagistan T Arioz [9], Filiz Bilir [9], Isa A Ozdemir [10], Tolga Tasci [11], Alper Karalok [12], Ateş Karateke [13]

Affiliations

- [1] Private Gynecologic Oncology, Ankara, Turkey.
- [2] Gynecologic Oncology Division, Department of Obstetrics and Gynecology, Dr. Abdurrahman Yurtaslan Ankara Oncological Education and Research Hospital, Ankara, Turkey.
- [3] Gynecologic Oncology Division, Department of Obstetrics and Gynecology, Erciyes University Faculty of Medicine, Kayseri, Turkey.
- [4] Gynecologic Oncology Division, Department of Obstetrics and Gynecology, Ankara City Hospital, Ankara, Turkey.
- [5] Gynecologic Oncology Division, Department of Obstetrics and Gynecology, University of Health Sciences Antalya Education and Research Hospital, Antalya, Turkey.
- [6] Gynecologic Oncology Division, Department of Obstetrics and Gynecology, Bakirkoy Dr. Sadi Konuk Training & Research Hospital, Istanbul, Turkey.
- [7] Gynecologic Oncology Division, Department of Obstetrics and Gynecology, Selcuk University Faculty of Medicine, Konya, Turkey.
- [8] Gynecologic Oncology Division, Department of Obstetrics and Gynecology, Cukurova University Faculty of Medicine, Adana, Turkey.
- [9] Gynecologic Oncology Division, Department of Obstetrics and Gynecology, Afyonkarahisar Health Sciences University Medical School, Afyonkarahisar, Turkey.
- [10] Gynecologic Oncology Division, Department of Obstetrics and Gynecology, Medipol University, Istanbul, Turkey.
- [11] Gynecologic Oncology Division, Department of Obstetrics and Gynecology, Bahçeşehir University Medical Park Goztepe Hospital, Istanbul, Turkey.
- [12] Gynecologic Oncology Division, Department of Obstetrics and Gynecology, Liv Hospital Ulus, Istanbul, Turkey.
- [13] Gynecologic Oncology Division, Department of Obstetrics and Gynecology, Medeniyet University, Istanbul, Turkey.

- PMID: **32623717**
- DOI: 10.1002/ijgo.13296

Abstract

Objective: To report the perioperative outcomes of 200 patients with gynecologic cancer who underwent surgery during the Novel Coronavirus Disease (COVID-19) pandemic and the safety of surgical approach.

Methods: Data of patients operated between March 10 and May 20, 2020, were collected retrospectively. Data were statistically analyzed using IBM Statistical Package for the Social Sciences (SPSS) Statistics for Windows v. SP21.0.

Results: Data of 200 patients were included. Their mean age was 56 years. Of the patients, 54% (n=108), 27.5% (n=55), 12.5% (n=25), and 2% (n=4) were diagnosed as having endometrial, ovarian, cervical, and vulvar cancer, respectively. Of them, 98% underwent non-emergent surgery.

A minimally invasive surgical approach was used in 18%. Stage 1 cancer was found in 68% of patients. Surgeons reported COVID-related changes in 10% of the cases. The rate of postoperative complications was 12%. Only two patients had cough and suspected pneumonic lesions on thoracic computed tomography postoperatively, but neither was positive for COVID-19 on polymerase chain reaction testing.

Conclusion: Based on the present findings, it is thought that gynecologic cancer surgery should continue during the COVID-19 pandemic while adhering to the measures. Postponement or non-surgical management should only be considered in patients with documented infection. Gynecologic cancer surgery should continue during the COVID-19 pandemic while adhering to measures. Only 1% of patients developed COVID-19-related symptoms during the postoperative follow-up period.

Keywords: COVID-19; Gynecologic surgical procedures; Lymph node excision; Ovarian neoplasms; Severe acute respiratory syndrome coronavirus 2; Uterine cervical neoplasms; Uterine neoplasms; Vulvar neoplasms.

© 2020 International Federation of Gynecology and Obstetrics.

- 25 references

Supplementary info

Publication types, MeSH terms Expand

Publication types

- Multicenter Study
- Observational Study

MeSH terms

- Adult
- COVID-19 / epidemiology*
- COVID-19 / surgery
- Female
- Genital Neoplasms, Female / pathology
- Gynecologic Surgical Procedures / statistics & numerical data*
- Humans
- Middle Aged
- Postoperative Complications / epidemiology*
- Retrospective Studies
- SARS-CoV-2
- Turkey
- Urogenital Neoplasms / epidemiology*
- Urogenital Neoplasms / surgery*

Full text links

WILEY Full Text Article Wiley
Proceed to details
Cite
Share
684
Observational Study
J Infect

. 2021 Jan;82(1):e22-e24.
doi: 10.1016/j.jinf.2020.08.045. Epub 2020 Sep 1.

Early short-course corticosteroids and furosemide combination to treat non-critically ill COVID-19 patients: An observational cohort study

Jean-Philippe Kevorkian [1], Jean-Pierre Riveline [2], Claire Vandiedonck [3], Diane Girard [3], Joris Galland [4], Florine Féron [2], Jean-François Gautier [2], Bruno Mégarbane [5]
Affiliations Expand

Affiliations

- [1] Department of Diabetes and Endocrinology, Lariboisière Hospital, Assistance Publique-Hôpitaux de Paris, Université de Paris, INSERM UMRS-1138, 2, Rue Ambroise Paré, 75010, Paris, France. Electronic address: jean-philippe.kevorkian@aphp.fr.
- [2] Department of Diabetes and Endocrinology, Lariboisière Hospital, Assistance Publique-Hôpitaux de Paris, Université de Paris, INSERM UMRS-1138, 2, Rue Ambroise Paré, 75010, Paris, France.
- [3] Centre de Recherche des Cordeliers, INSERM, Université de Paris, IMMEDIAB Laboratory, F-75006, Paris, France.
- [4] Department of Internal Medicine, Lariboisière Hospital, Assistance Publique-Hôpitaux de Paris, Université de Paris, Paris, France.
- [5] Department of Medical and Toxicological Critical Care, Lariboisière Hospital, Assistance Publique-Hôpitaux de Paris, Université de Paris, INSERM UMRS-1144, 2, Rue Ambroise Paré, 75010, Paris, France. Electronic address: bruno.megarbane@lrb.aphp.fr.

- PMID: **32888976**
- PMCID: PMC7462464
- DOI: 10.1016/j.jinf.2020.08.045

Free PMC article
Observational Study

Early short-course corticosteroids and furosemide combination to treat non-critically ill COVID-19 patients: An observational cohort study

Jean-Philippe Kevorkian et al. J Infect. 2021 Jan.
Free PMC article
Show details
J Infect
. 2021 Jan;82(1):e22-e24.
doi: 10.1016/j.jinf.2020.08.045. Epub 2020 Sep 1.

Authors

Jean-Philippe Kevorkian [1], Jean-Pierre Riveline [2], Claire Vandiedonck [3], Diane Girard [3], Joris Galland [4], Florine Féron [2], Jean-François Gautier [2], Bruno Mégarbane [5]

Affiliations

- [1] Department of Diabetes and Endocrinology, Lariboisière Hospital, Assistance Publique-Hôpitaux de Paris, Université de Paris, INSERM UMRS-1138, 2, Rue Ambroise Paré, 75010, Paris, France. Electronic address: jean-philippe.kevorkian@aphp.fr.
- [2] Department of Diabetes and Endocrinology, Lariboisière Hospital, Assistance Publique-Hôpitaux de Paris, Université de Paris, INSERM UMRS-1138, 2, Rue Ambroise Paré, 75010, Paris, France.
- [3] Centre de Recherche des Cordeliers, INSERM, Université de Paris, IMMEDIAB Laboratory, F-75006, Paris, France.
- [4] Department of Internal Medicine, Lariboisière Hospital, Assistance Publique-Hôpitaux de Paris, Université de Paris, Paris, France.
- [5] Department of Medical and Toxicological Critical Care, Lariboisière Hospital, Assistance Publique-Hôpitaux de Paris, Université de Paris, INSERM UMRS-1144, 2, Rue Ambroise Paré, 75010, Paris, France. Electronic address: bruno.megarbane@lrb.aphp.fr.

- PMID: **32888976**
- PMCID: PMC7462464
- DOI: 10.1016/j.jinf.2020.08.045

No abstract available

Conflict of interest statement

Declaration of Competing Interest The authors declare that they have no competing interests.

- 10 references
- 1 figure

Supplementary info

Publication types, MeSH terms, Substances Expand

Publication types

- Letter
- Observational Study

MeSH terms

- Adrenal Cortex Hormones / therapeutic use*
- Aged
- Aged, 80 and over
- Anti-Inflammatory Agents / therapeutic use*
- COVID-19 / drug therapy*
- Cohort Studies
- Comorbidity
- Critical Illness / therapy
- Dexamethasone / therapeutic use
- Drug Therapy, Combination
- Female
- Furosemide / therapeutic use*
- Humans
- Male
- Methylprednisolone / therapeutic use
- Middle Aged
- Respiratory Distress Syndrome / drug therapy
- Respiratory Distress Syndrome / prevention & control
- Retrospective Studies
- SARS-CoV-2 / drug effects*
- Sodium Potassium Chloride Symporter Inhibitors / therapeutic use*

Substances

- Adrenal Cortex Hormones
- Anti-Inflammatory Agents
- Sodium Potassium Chloride Symporter Inhibitors
- Furosemide
- Dexamethasone
- Methylprednisolone

Full text links

ELSEVIER FULL-TEXT ARTICLE Elsevier Science Free PMC article

Proceed to details

Cite

Share

685

Observational Study

Turk J Med Sci

. 2021 Aug 30;51(4):1665-1674.

doi: 10.3906/sag-2101-89.

Risk factors associated with mortality in ıntensive care COVID-19 patients: the importance of chest CT score and intubation timing as risk factors

Vecihe Bayrak [1], Nurcan Şentürk Durukan [2], Ferhan Demirer Aydemir [1], Begüm Ergan [3], N. Sinem Gezer [4], Oya Özlem Eren Kutsoylu [5], A. Necati Gökmen [6], Yusuf Savran [7]

Affiliations Expand

Affiliations

- [1] Department of Internal Medicine, Intensive Care Unit, Faculty of Medicine, Dokuz Eylül University, İzmir, Turkey
- [2] Department of Public Health, Faculty of Medicine, Dokuz Eylül University, İzmir, Turkey
- [3] Department of Chest Diseases, Intensive Care Unit, Faculty of Medicine, Dokuz Eylül University, İzmir, Turkey
- [4] Department of Radiology, Faculty of Medicine, Dokuz Eylül University, İzmir, Turkey
- [5] Department of Infectious Diseases, Faculty of Medicine, Dokuz Eylül University, İzmir, Turkey
- [6] Department of Anesthesiology and reanimation, Intensive Care Unit, Faculty of Medicine, Dokuz Eylül University, İzmir, Turkey
- [7] Department of Internal Medicine, International Medicana Hospital, İzmir, Turkey

- PMID: **33957728**
- PMCID: PMC8576336
- DOI: 10.3906/sag-2101-89

Free PMC article

Observational Study

Risk factors associated with mortality in ıntensive care COVID-19 patients: the importance of chest CT score and intubation timing as risk factors

Vecihe Bayrak et al. Turk J Med Sci. 2021.
Free PMC article

Show details

Turk J Med Sci
. 2021 Aug 30;51(4):1665-1674.
doi: 10.3906/sag-2101-89.

Authors

Vecihe Bayrak [1], Nurcan Şentürk Durukan [2], Ferhan Demirer Aydemir [1], Begüm Ergan [3], N. Sinem Gezer [4], Oya Özlem Eren Kutsoylu [5], A. Necati Gökmen [6], Yusuf Savran [7]

Affiliations

- [1] Department of Internal Medicine, Intensive Care Unit, Faculty of Medicine, Dokuz Eylül University, İzmir, Turkey
- [2] Department of Public Health, Faculty of Medicine, Dokuz Eylül University, İzmir, Turkey
- [3] Department of Chest Diseases, Intensive Care Unit, Faculty of Medicine, Dokuz Eylül University, İzmir, Turkey
- [4] Department of Radiology, Faculty of Medicine, Dokuz Eylül University, İzmir, Turkey
- [5] Department of Infectious Diseases, Faculty of Medicine, Dokuz Eylül University, İzmir, Turkey
- [6] Department of Anesthesiology and reanimation, Intensive Care Unit, Faculty of Medicine, Dokuz Eylül University, İzmir, Turkey
- [7] Department of Internal Medicine, International Medicana Hospital, İzmir, Turkey

- PMID: **33957728**
- PMCID: PMC8576336
- DOI: 10.3906/sag-2101-89

Abstract

Background/aim: Coronavirus disease 2019 (COVID-19) is a disease with a high rate of progression to critical illness. However, the predictors of mortality in critically ill patients admitted to the intensive care unit (ICU) are not yet well understood. In this study, we aimed to investigate the risk factors associated with ICU mortality in our hospital.

Materials and methods: In this single-centered retrospective study, we enrolled 86 critically ill adult patients with COVID-19 admitted to ICU of Dokuz Eylül University Hospital (İzmir, Turkey) between 18 March 2020 and 31 October 2020. Data on demographic information, preexisting comorbidities, treatments, the laboratory findings at ICU admission, and clinical

outcomes were collected. The chest computerized tomography (CT) of the patients were evaluated specifically for COVID-19 and CT score was calculated. Data of the survivors and nonsurvivors were compared with survival analysis to identify risk factors of mortality in the ICU.

Results: The mean age of the patients was 71.1 ± 14.1 years. The patients were predominantly male. The most common comorbidity in patients was hypertension. ICU mortality was 62.8%. Being over 60 years old, CT score > 15, acute physiology and chronic health evaluation (APACHE) II score ≥ 15, having dementia, treatment without favipiravir, base excess in blood gas analysis ≤ –2.0, WBC > 10,000/mm3, D-dimer > 1.6 μg/mL, troponin > 24 ng/L, Na ≥ 145 mmol/L were considered to link with ICU mortality according to Kaplan–Meier curves (log-rank test, $p < 0.05$). The APACHE II score (HR: 1.055, 95% CI: 1.021–1.090) and chest CT score (HR: 2.411, 95% CI:1.193–4.875) were associated with ICU mortality in the cox proportional-hazard regression model adjusted for age, dementia, favipiravir treatment and troponin. However, no difference was found between survivors and nonsurvivors in terms of intubation timing.

Conclusions: COVID-19 patients have a high ICU admission and mortality rate. Studies in the ICU are also crucial in this respect. In our study, we investigated the ICU mortality risk factors of COVID-19 patients. We determined a predictive mortality model consisting of APACHE II score and chest CT score. It was thought that this feasible and practical model would assist in making clinical decisions.

Keywords: APACHE II; COVID-19; CT score; ICU mortality; intubation timing.

This work is licensed under a Creative Commons Attribution 4.0 International License.

Conflict of interest statement

CONFLICT OF INTEREST:

none declared

- 32 references
- 2 figures

Supplementary info

Publication types, MeSH terms Expand

Publication types

- Observational Study

MeSH terms

- Adolescent
- Adult
- Aged
- Aged, 80 and over
- COVID-19 / diagnostic imaging*
- COVID-19 / mortality*

- Critical Care / methods*
- Female
- Hospital Mortality*
- Humans
- Intensive Care Units
- Intubation, Intratracheal / methods*
- Intubation, Intratracheal / statistics & numerical data
- Lung / diagnostic imaging
- Male
- Middle Aged
- Retrospective Studies
- Risk Factors
- SARS-CoV-2
- Survival Analysis
- Time Factors
- Tomography, X-Ray Computed / methods*
- Turkey / epidemiology
- Young Adult

Full text links

TUBITAK OPEN ACCESS TUBITAK Free PMC article
Proceed to details
Cite
Share
☐ 686
Clinical Trial
J Clin Invest
. 2021 Oct 1;131(19):e151418.
doi: 10.1172/JCI151418.

In-hospital use of ACE inhibitors/angiotensin receptor blockers associates with COVID-19 outcomes in African American patients

Shilong Li[1], Rangaprasad Sarangarajan[2], Tomi Jun[1], Yu-Han Kao[1], Zichen Wang[1], Ke Hao[1], Emilio Schadt[1], Michael A Kiebish[2], Elder Granger[2], Niven R Narain[2], Rong Chen[1 3], Eric E Schadt[1 3], Li Li[1 3]
Affiliations Expand

Affiliations

- [1] Sema4, Stamford, Connecticut, USA.
- [2] BERG, Framingham, Massachusetts, USA.
- [3] Department of Genetics and Genomic Sciences, the Icahn Institute for Genomics and Multiscale Biology, Icahn School of Medicine at Mount Sinai, New York, New York, USA.

- PMID: **34411004**
- PMCID: PMC8483748
- DOI: 10.1172/JCI151418

Free PMC article
Clinical Trial

In-hospital use of ACE inhibitors/angiotensin receptor blockers associates with COVID-19 outcomes in African American patients

Shilong Li et al. J Clin Invest. 2021.
Free PMC article
Show details
J Clin Invest
. 2021 Oct 1;131(19):e151418.
doi: 10.1172/JCI151418.

Authors

Shilong Li [1], Rangaprasad Sarangarajan [2], Tomi Jun [1], Yu-Han Kao [1], Zichen Wang [1], Ke Hao [1], Emilio Schadt [1], Michael A Kiebish [2], Elder Granger [2], Niven R Narain [2], Rong Chen [1] [3], Eric E Schadt [1] [3], Li Li [1] [3]

Affiliations

- [1] Sema4, Stamford, Connecticut, USA.
- [2] BERG, Framingham, Massachusetts, USA.
- [3] Department of Genetics and Genomic Sciences, the Icahn Institute for Genomics and Multiscale Biology, Icahn School of Medicine at Mount Sinai, New York, New York, USA.

- PMID: **34411004**
- PMCID: PMC8483748
- DOI: 10.1172/JCI151418

Abstract

BACKGROUNDThe angiotensin-converting enzyme (ACE) D allele is more prevalent among African Americans compared with other races and ethnicities and has previously been associated with severe coronavirus disease 2019 (COVID-19) pathogenesis through excessive ACE1 activity. ACE inhibitors/angiotensin receptor blockers (ACE-I/ARB) may counteract this mechanism, but their association with COVID-19 outcomes has not been specifically tested in the African

American population.METHODSWe identified 6218 patients who were admitted into Mount Sinai hospitals with COVID-19 between February 24 and May 31, 2020, in New York City. We evaluated whether the outpatient and in-hospital use of ACE-I/ARB is associated with COVID-19 in-hospital mortality in an African American compared with non-African American population.RESULTSOf the 6218 patients with COVID-19, 1138 (18.3%) were ACE-I/ARB users. In a multivariate logistic regression model, ACE-I/ARB use was independently associated with a reduced risk of in-hospital mortality in the entire population (OR, 0.655; 95% CI, 0.505-0.850; P = 0.001), African American population (OR, 0.44; 95% CI, 0.249-0.779; P = 0.005), and non-African American population (OR, 0.748, 95% CI, 0.553-1.012, P = 0.06). In the African American population, in-hospital use of ACE-I/ARB was associated with improved mortality (OR, 0.378; 95% CI, 0.188-0.766; P = 0.006), whereas outpatient use was not (OR, 0.889; 95% CI, 0.375-2.158; P = 0.812). When analyzing each medication class separately, ARB in-hospital use was significantly associated with reduced in-hospital mortality in the African American population (OR, 0.196; 95% CI, 0.074-0.516; P = 0.001), whereas ACE-I use was not associated with impact on mortality in any population.CONCLUSIONIn-hospital use of ARB was associated with a significant reduction in in-hospital mortality among COVID-19-positive African American patients.FUNDINGNone.

Keywords: COVID-19; Cardiovascular disease; Drug therapy; Epidemiology.

Conflict of interest statement

Conflict of interest: The authors have declared that no conflict of interest exists.

Comment in

- Concerns about the interpretation of subgroup analysis.
  Albuquerque AM, Santolia CB, Verma A. Albuquerque AM, et al. J Clin Invest. 2022 Jan 18;132(2):e155991. doi: 10.1172/JCI155991. J Clin Invest. 2022. PMID: 34855622 Free PMC article. No abstract available.

- 2 figures

Supplementary info

Publication types, MeSH terms, Substances Expand

Publication types

- Clinical Trial
- Comparative Study
- Multicenter Study
- Observational Study

MeSH terms

- African Americans*
- Aged
- Angiotensin Receptor Antagonists / administration & dosage*

- Angiotensin-Converting Enzyme Inhibitors / administration & dosage*
- COVID-19* / drug therapy
- COVID-19* / ethnology
- COVID-19* / metabolism
- COVID-19* / mortality
- Disease-Free Survival
- Female
- Hospital Mortality / ethnology*
- Humans
- Male
- Middle Aged
- Peptidyl-Dipeptidase A / metabolism
- Retrospective Studies
- SARS-CoV-2 / metabolism*
- Survival Rate

Substances

- Angiotensin Receptor Antagonists
- Angiotensin-Converting Enzyme Inhibitors
- ACE protein, human
- Peptidyl-Dipeptidase A

Full text links

VIEW ARTICLE FULL TEXT American Society for Clinical Investigation Free PMC article

Proceed to details

Cite

Share

☐ 687

Observational Study

Medicine (Baltimore)
. 2021 Sep 3;100(35):e27166.
doi: 10.1097/MD.0000000000027166.

Coronavirus disease 2019 pandemic, restriction, and orthopedic trauma: Retrospective observational study

Serdar Toy [1], Oktay Polat, Hakan Özbay
Affiliations Expand

Affiliation

- [1] Department of Orthopedics and Traumatology, Ağri Training and Research Hospital, Ağri, Turkey.

- PMID: **34477174**
- PMCID: PMC8415988
- DOI: 10.1097/MD.0000000000027166

Free PMC article
Observational Study

Coronavirus disease 2019 pandemic, restriction, and orthopedic trauma: Retrospective observational study

Serdar Toy et al. Medicine (Baltimore). 2021.
Free PMC article
Show details
Medicine (Baltimore)
. 2021 Sep 3;100(35):e27166.
doi: 10.1097/MD.0000000000027166.

Authors

Serdar Toy [1], Oktay Polat, Hakan Özbay

Affiliation

- [1] Department of Orthopedics and Traumatology, Ağri Training and Research Hospital, Ağri, Turkey.

- PMID: **34477174**
- PMCID: PMC8415988
- DOI: 10.1097/MD.0000000000027166

Abstract

In 2019, the Coronavirus disease 2019 (Covid-19) was reported in Wuhan, China. Governments in various countries had taken many safeguards. This study investigated the incidence of orthopedic trauma in a rural region epidemiologically and guided source distribution and medical professionals to sustain healthcare systems.Between December 2019 and August 2020, 1651 patients admitted to orthopedics and traumatology clinics with trauma were evaluated in this study. Patients were grouped into 3 groups: pre-covid, restriction, and permitted groups. Age, sex, and fracture types of patients were recorded.The number of patients in the pre-covid period was 629 (38.1%), those were 334 (20.2%) in the restriction period, and 688 (41.7%) patients were admitted in the permitted period. A total of 1203 (72.9%) patients with upper extremity fractures,

383 (23.2%) patients with lower extremity fractures, and 65 (3.9%) patients with axial skeleton and pelvic ring fractures were included in the study. The lowest rates were found in the restriction period when all fractures were evaluated according to the admission periods. There were significant differences between admission dates and the fractures (P < .001).In this study, a decrease in orthopedic trauma rates was observed by half in the restriction period compared with the other 2 periods. Public health precautions had led to a reduction in the incidence of orthopedic trauma in all age groups.

Copyright © 2021 the Author(s). Published by Wolters Kluwer Health, Inc.

Conflict of interest statement

The authors have no funding and conflicts of interest to disclose.

- 14 references
- 2 figures

Supplementary info

Publication types, MeSH terms Expand

Publication types

- Observational Study

MeSH terms

- Adolescent
- Adult
- Aged
- Aged, 80 and over
- COVID-19 / epidemiology
- COVID-19 / prevention & control
- Child
- Child, Preschool
- Communicable Disease Control*
- Female
- Fractures, Bone / epidemiology*
- Humans
- Incidence
- Infant
- Infant, Newborn
- Male
- Middle Aged
- Pandemics
- Retrospective Studies

- SARS-CoV-2
- Turkey / epidemiology
- Young Adult

Full text links

Wolters Kluwer Wolters Kluwer Free PMC article
Proceed to details
Cite
Share
☐ 688
Observational Study
Am J Emerg Med
. 2021 Apr;42:203-210.
doi: 10.1016/j.ajem.2020.11.029. Epub 2020 Nov 19.

Decreased hospital admissions through emergency departments during the COVID-19 pandemic

Sara Nourazari [1], Samuel R Davis [2], Rachel Granovsky [2], Randolph Austin [2], Dean J Straff [3], Joshua W Joseph [4], Leon D Sanchez [4]
Affiliations Expand

Affiliations

- [1] Department of Health Care Administration, California State University, Long Beach, CA, USA. Electronic address: sara.nourazari@csulb.edu.
- [2] LogixHealth, Inc., Bedford, MA, USA.
- [3] Department of Emergency Medicine, White Plains Hospital, White Plains, NY, USA.
- [4] Department of Emergency Medicine, Beth Israel Deaconess Medical Center, Harvard Medical School, Boston, MA, USA.

- PMID: **33279331**
- PMCID: PMC7676321
- DOI: 10.1016/j.ajem.2020.11.029

Free PMC article
Observational Study

Decreased hospital admissions through emergency departments during the COVID-19 pandemic

Sara Nourazari et al. Am J Emerg Med. 2021 Apr.
Free PMC article
Show details
Am J Emerg Med
. 2021 Apr;42:203-210.
doi: 10.1016/j.ajem.2020.11.029. Epub 2020 Nov 19.

Authors

Sara Nourazari [1], Samuel R Davis [2], Rachel Granovsky [2], Randolph Austin [2], Dean J Straff [3], Joshua W Joseph [4], Leon D Sanchez [4]

Affiliations

- [1] Department of Health Care Administration, California State University, Long Beach, CA, USA. Electronic address: sara.nourazari@csulb.edu.
- [2] LogixHealth, Inc., Bedford, MA, USA.
- [3] Department of Emergency Medicine, White Plains Hospital, White Plains, NY, USA.
- [4] Department of Emergency Medicine, Beth Israel Deaconess Medical Center, Harvard Medical School, Boston, MA, USA.

- PMID: **33279331**
- PMCID: PMC7676321
- DOI: 10.1016/j.ajem.2020.11.029

Abstract

Study objective: Emergency Department (ED) visits decreased significantly in the United States during the COVID-19 pandemic. A troubling proportion of this decrease was among patients who typically would have been admitted to the hospital, suggesting substantial deferment of care. We sought to describe and characterize the impact of COVID-19 on hospital admissions through EDs, with a specific focus on diagnosis group, age, gender, and insurance coverage.

Methods: We conducted a retrospective, observational study of aggregated third-party, anonymized ED patient data. This data included 501,369 patient visits from twelve EDs in Massachusetts from 1/1/2019-9/9/2019, and 1/1/2020-9/8/2020. We analyzed the total arrivals and hospital admissions and calculated confidence intervals for the change in admissions for each characteristic. We then developed a Poisson regression model to estimate the relative contribution of each characteristic to the decrease in admissions after the statewide lockdown, corresponding to weeks 11 through 36 (3/11/2020-9/8/2020).

Results: We observed a 32% decrease in admissions during weeks 11 to 36 in 2020, with significant decreases in admissions for chronic respiratory conditions and non-orthopedic needs.

Decreases were particularly acute among women and children, as well as patients with Medicare or without insurance. The most common diagnosis during this time was SARS-CoV-2.

Conclusion: Our findings demonstrate decreased hospital admissions through EDs during the pandemic and suggest that several patient populations may have deferred necessary care. Further research is needed to determine the clinical and operational consequences of this delay.

Keywords: COVID-19; Care deferment; Emergency department; Hospital admissions.

Copyright © 2020 The Authors. Published by Elsevier Inc. All rights reserved.

Comment in

- Non-COVID-19 admissions to the emergency department during the pandemic second wave in Italy: What is changed from the first wave?
  Mantica G, Riccardi N, Terrone C, Gratarola A. Mantica G, et al. Am J Emerg Med. 2021 Jul;45:625-626. doi: 10.1016/j.ajem.2020.11.046. Epub 2020 Nov 26. Am J Emerg Med. 2021. PMID: 33303298 Free PMC article. No abstract available.

- 27 references
- 3 figures

Supplementary info

Publication types, MeSH terms Expand

Publication types

- Observational Study

MeSH terms

- Adolescent
- Adult
- Aged
- Aged, 80 and over
- COVID-19 / epidemiology*
- Child
- Child, Preschool
- Diagnosis-Related Groups / statistics & numerical data
- Emergency Service, Hospital / statistics & numerical data*
- Facilities and Services Utilization
- Female
- Humans
- Infant
- Infant, Newborn
- Male

- Massachusetts
- Middle Aged
- Patient Admission / statistics & numerical data*
- Retrospective Studies
- Socioeconomic Factors
- Young Adult

Full text links

ELSEVIER OPEN ACCESS Elsevier Science Free PMC article
Proceed to details
Cite
Share
☐ 689
Observational Study
Anesth Analg
. 2021 Apr 1;132(4):930-941.
doi: 10.1213/ANE.0000000000005292.

Aspirin Use Is Associated With Decreased Mechanical Ventilation, Intensive Care Unit Admission, and In-Hospital Mortality in Hospitalized Patients With Coronavirus Disease 2019

Jonathan H Chow [1], Ashish K Khanna [2 3], Shravan Kethireddy [4], David Yamane [5], Andrea Levine [6], Amanda M Jackson [7], Michael T McCurdy [6], Ali Tabatabai [6 8], Gagan Kumar [4], Paul Park [9], Ivy Benjenk [1], Jay Menaker [1 2 3 4 5 6 7 8 9 10 11 12 13 14 15 16 17], Nayab Ahmed [11], Evan Glidewell [12], Elizabeth Presutto [9], Shannon Cain [13], Naeha Haridasa [1], Wesley Field [11], Jacob G Fowler [12], Duy Trinh [9], Kathleen N Johnson [12], Aman Kaur [11], Amanda Lee [9], Kyle Sebastian [12], Allison Ulrich [9], Salvador Peña [12], Ross Carpenter [9], Shruti Sudhakar [9], Pushpinder Uppal [9], Benjamin T Fedeles [9], Aaron Sachs [9], Layth Dahbour [9], William Teeter [8 14], Kenichi Tanaka [17], Samuel M Galvagno [8], Daniel L Herr [8], Thomas M Scalea [8 10], Michael A Mazzeffi [15]
Affiliations Expand

Affiliations

- [1] From the Department of Anesthesiology and Critical Care Medicine, George Washington University School of Medicine, Washington, DC.
- [2] Section on Critical Care Medicine, Department of Anesthesiology, Wake Forest School of Medicine, Winston-Salem, North Carolina.

- [3] Outcomes Research Consortium, Cleveland, Ohio.
- [4] Division of Pulmonary and Critical Care, Department of Medicine, Northeast Georgia Health System, Gainesville, Georgia.
- [5] Departments of Emergency Medicine, Anesthesiology, and Critical Care Medicine, George Washington University School of Medicine, Washington, DC.
- [6] Division of Pulmonary and Critical Care, Department of Medicine, University of Maryland School of Medicine, Baltimore, Maryland.
- [7] Division of Gynecologic Oncology, Department of Obstetrics & Gynecology, Walter Reed National Military Medical Center, Bethesda, Maryland.
- [8] Program in Trauma, R Adams Cowley Shock Trauma Center, Baltimore, Maryland.
- [9] Department of Anesthesiology, University of Maryland School of Medicine, Baltimore, Maryland.
- [10] Department of Surgery, University of Maryland School of Medicine, Baltimore, Maryland.
- [11] Department of Medicine, Northeast Georgia Health System, Gainesville, Georgia.
- [12] Department of Anesthesiology, Wake Forest School of Medicine, Winston-Salem, North Carolina.
- [13] Department of Emergency Medicine, George Washington University School of Medicine, Washington, DC.
- [14] Department of Emergency Medicine, University of Maryland School of Medicine, Baltimore, Maryland.
- [15] Division of Cardiothoracic Anesthesiology, Department of Anesthesiology, University of Maryland School of Medicine, Baltimore, Maryland.
- [16] Department of Surgery, University of California San Francisco, San Francisco, California.
- [17] Department of Anesthesiology, The University of Oklahoma School of Medicine, Oklahoma City, Oklahoma.

- PMID: **33093359**
- DOI: [10.1213/ANE.0000000000005292](https://doi.org/10.1213/ANE.0000000000005292)

Observational Study

Aspirin Use Is Associated With Decreased Mechanical Ventilation, Intensive Care Unit Admission, and In-Hospital Mortality in Hospitalized Patients With Coronavirus Disease 2019

Jonathan H Chow et al. Anesth Analg. 2021.

Show details

Anesth Analg
. 2021 Apr 1;132(4):930-941.
doi: 10.1213/ANE.0000000000005292.

Authors

Jonathan H Chow [1], Ashish K Khanna [2] [3], Shravan Kethireddy [4], David Yamane [5], Andrea Levine [6], Amanda M Jackson [7], Michael T McCurdy [6], Ali Tabatabai [6] [8], Gagan Kumar [4], Paul Park [9], Ivy Benjenk [1], Jay Menaker [1] [2] [3] [4] [5] [6] [7] [8] [9] [10] [11] [12] [13] [14] [15] [16] [17], Nayab Ahmed [11], Evan Glidewell [12], Elizabeth Presutto [9], Shannon Cain [13], Naeha Haridasa [1], Wesley Field [11], Jacob G Fowler [12], Duy Trinh [9], Kathleen N Johnson [12], Aman Kaur [11], Amanda Lee [9], Kyle Sebastian [12], Allison Ulrich [9], Salvador Peña [12], Ross Carpenter [9], Shruti Sudhakar [9], Pushpinder Uppal [9], Benjamin T Fedeles [9], Aaron Sachs [9], Layth Dahbour [9], William Teeter [8] [14], Kenichi Tanaka [17], Samuel M Galvagno [8], Daniel L Herr [8], Thomas M Scalea [8] [10], Michael A Mazzeffi [15]

Affiliations

- [1] From the Department of Anesthesiology and Critical Care Medicine, George Washington University School of Medicine, Washington, DC.
- [2] Section on Critical Care Medicine, Department of Anesthesiology, Wake Forest School of Medicine, Winston-Salem, North Carolina.
- [3] Outcomes Research Consortium, Cleveland, Ohio.
- [4] Division of Pulmonary and Critical Care, Department of Medicine, Northeast Georgia Health System, Gainesville, Georgia.
- [5] Departments of Emergency Medicine, Anesthesiology, and Critical Care Medicine, George Washington University School of Medicine, Washington, DC.
- [6] Division of Pulmonary and Critical Care, Department of Medicine, University of Maryland School of Medicine, Baltimore, Maryland.
- [7] Division of Gynecologic Oncology, Department of Obstetrics & Gynecology, Walter Reed National Military Medical Center, Bethesda, Maryland.
- [8] Program in Trauma, R Adams Cowley Shock Trauma Center, Baltimore, Maryland.
- [9] Department of Anesthesiology, University of Maryland School of Medicine, Baltimore, Maryland.
- [10] Department of Surgery, University of Maryland School of Medicine, Baltimore, Maryland.
- [11] Department of Medicine, Northeast Georgia Health System, Gainesville, Georgia.
- [12] Department of Anesthesiology, Wake Forest School of Medicine, Winston-Salem, North Carolina.
- [13] Department of Emergency Medicine, George Washington University School of Medicine, Washington, DC.
- [14] Department of Emergency Medicine, University of Maryland School of Medicine, Baltimore, Maryland.
- [15] Division of Cardiothoracic Anesthesiology, Department of Anesthesiology, University of Maryland School of Medicine, Baltimore, Maryland.
- [16] Department of Surgery, University of California San Francisco, San Francisco, California.
- [17] Department of Anesthesiology, The University of Oklahoma School of Medicine, Oklahoma City, Oklahoma.

- PMID: **33093359**
- DOI: 10.1213/ANE.0000000000005292

Abstract

Background: Coronavirus disease-2019 (COVID-19) is associated with hypercoagulability and increased thrombotic risk in critically ill patients. To our knowledge, no studies have evaluated whether aspirin use is associated with reduced risk of mechanical ventilation, intensive care unit (ICU) admission, and in-hospital mortality.

Methods: A retrospective, observational cohort study of adult patients admitted with COVID-19 to multiple hospitals in the United States between March 2020 and July 2020 was performed. The primary outcome was the need for mechanical ventilation. Secondary outcomes were ICU admission and in-hospital mortality. Adjusted hazard ratios (HRs) for study outcomes were calculated using Cox-proportional hazards models after adjustment for the effects of demographics and comorbid conditions.

Results: Four hundred twelve patients were included in the study. Three hundred fourteen patients (76.3%) did not receive aspirin, while 98 patients (23.7%) received aspirin within 24 hours of admission or 7 days before admission. Aspirin use had a crude association with less mechanical ventilation (35.7% aspirin versus 48.4% nonaspirin, P = .03) and ICU admission (38.8% aspirin versus 51.0% nonaspirin, P = .04), but no crude association with in-hospital mortality (26.5% aspirin versus 23.2% nonaspirin, P = .51). After adjusting for 8 confounding variables, aspirin use was independently associated with decreased risk of mechanical ventilation (adjusted HR, 0.56, 95% confidence interval [CI], 0.37-0.85, P = .007), ICU admission (adjusted HR, 0.57, 95% CI, 0.38-0.85, P = .005), and in-hospital mortality (adjusted HR, 0.53, 95% CI, 0.31-0.90, P = .02). There were no differences in major bleeding (P = .69) or overt thrombosis (P = .82) between aspirin users and nonaspirin users.

Conclusions: Aspirin use may be associated with improved outcomes in hospitalized COVID-19 patients. However, a sufficiently powered randomized controlled trial is needed to assess whether a causal relationship exists between aspirin use and reduced lung injury and mortality in COVID-19 patients.

Copyright © 2020 International Anesthesia Research Society.

Conflict of interest statement

Conflicts of Interest: See Disclosures at the end of the article.

Comment in

- Is Aspirin Effective in Preventing Intensive Care Unit Admission in Patients With Coronavirus Disease 2019 Pneumonia?
  Giorgi-Pierfranceschi M. Giorgi-Pierfranceschi M. Anesth Analg. 2021 May 1;132(5):e89-e90. doi: 10.1213/ANE.0000000000005401. Anesth Analg. 2021. PMID: 33369927 No abstract available.
- Aspirin in Coronavirus Disease 2019-Related Acute Respiratory Distress Syndrome: An Old, Low-Cost Therapy With a Strong Rationale.
  Coppola S, Chiumello D. Coppola S, et al. Anesth Analg. 2021 Apr 1;132(4):927-929. doi: 10.1213/ANE.0000000000005408. Anesth Analg. 2021. PMID: 33481405 No abstract available.
- In Response.

Chow JH, Mazzeffi MA. Chow JH, et al. Anesth Analg. 2021 May 1;132(5):e90. doi: 10.1213/ANE.0000000000005402. Anesth Analg. 2021. PMID: 33858007 No abstract available.

- Aspirin Use, Mechanical Ventilation, and Inhospital Mortality in Coronavirus Disease 2019: Are We Missing Something?
  Senthilnathan M, Ravi R, Sivakumar RK, Majella MG, Chidambaram V. Senthilnathan M, et al. Anesth Analg. 2021 Aug 1;133(2):e31-e33. doi: 10.1213/ANE.0000000000005621. Anesth Analg. 2021. PMID: 34257214 No abstract available.
- Is Aspirin the True Protective Therapy in Coronavirus Disease 2019 Patients?
  Mourad JJ, Suhl J. Mourad JJ, et al. Anesth Analg. 2021 Sep 1;133(3):e41. doi: 10.1213/ANE.0000000000005635. Anesth Analg. 2021. PMID: 34403399 No abstract available.

- 38 references

Supplementary info

Publication types, MeSH terms, Substances Expand

Publication types

- Multicenter Study
- Observational Study

MeSH terms

- Adult
- Aged
- Aspirin / therapeutic use*
- COVID-19 / diagnosis
- COVID-19 / mortality
- COVID-19 / therapy*
- Female
- Fibrinolytic Agents / therapeutic use*
- Hospital Mortality
- Humans
- Intensive Care Units*
- Male
- Middle Aged
- Patient Admission*
- Platelet Aggregation Inhibitors / therapeutic use*
- Registries
- Respiration, Artificial*
- Retrospective Studies
- Risk Assessment

- Risk Factors
- Time Factors
- Treatment Outcome
- United States

Substances

- Fibrinolytic Agents
- Platelet Aggregation Inhibitors
- Aspirin

Full text links

Wolters Kluwer Wolters Kluwer
Proceed to details
Cite
Share
690
Observational Study
Eur J Neurol
. 2020 Dec;27(12):2491-2498.
doi: 10.1111/ene.14467. Epub 2020 Sep 4.

Impact of COVID-19 outbreak in reperfusion therapies of acute ischaemic stroke in northwest Spain

H Tejada Meza [1,2,3], Á Lambea Gil [1,3], A Sancho Saldaña [1,3], M Martínez-Zabaleta [4], E Garmendia Lopetegui [5], E López-Cancio Martínez [6], M Castañón Apilánez [6], M Herrera Isasi [7], J Marta Enguita [7], B Gómez-Vicente [8], J F Arenillas [8,9], N Arenaza Basterrechea [10], J J Timiraos Fernández [10], J Sánchez Herrero [11], J L Maciñeiras Montero [11], M Castellanos Rodrigo [12], D Fernández-Coud [12], I Casado Menéndez [13], M T Temprano Fernández [13], M Freijo [14], A Luna [14], E J Palacio Portilla [15], Y Jiménez López [15], E Rodríguez-Castro [16], M Rodríguez-Yáñez [16], J Tejada García [17], I Beltrán Rodríguez [7], F Julián-Villaverde [18], M P Moreno García [18], J M Trejo Gabriel-Galán [19], A Echavarría Iñiguez [19], C Pérez Lázaro [20], M P Navarro Pérez [20], J Marta Moreno [1,3], NORDICTUS Investigators
Affiliations Expand

Affiliations

- [1] Stroke Unit, Department of Neurology, Hospital Universitario Miguel Servet, Zaragoza, Spain.

- [2] Interventional Neuroradiology Unit, Department of Radiology, Hospital Universitario Miguel Servet, Zaragoza, Spain.
- [3] Instituto de Investigación Sanitaria de Aragón (IISAragón), Zaragoza, Spain.
- [4] Department of Neurology, Donostia University Hospital, San Sebastián, Spain.
- [5] Department of Neuroradiology, Donostia University Hospital, San Sebastián, Spain.
- [6] Department of Neurology, Hospital Universitario Central de Asturias, Oviedo, Spain.
- [7] Department of Neurology, Complejo Hospitalario de Navarra, Pamplona, Spain.
- [8] Department of Neurology, Hospital Clínico Universitario de Valladolid, Valladolid, Spain.
- [9] Neurovascular Research Laboratory, Instituto de Biología y Genética Molecular, Universidad de Valladolid - Consejo Superior de Investigaciones Científicas, Madrid, Spain.
- [10] Stroke Unit, Department of Neurology, Hospital Universitario de Araba, Vitoria, Spain.
- [11] Department of Neurology, Complejo Hospitalario Universitario de Vigo, Vigo, Spain.
- [12] Department of Neurology, Complexo Hospitalario Universitario A Coruña / A Coruña Biomedical Research Insitute, A Coruña, Spain.
- [13] Stroke Unit, Department of Neurology, Hospital Universitario de Cabueñes, Gijón, Spain.
- [14] Neurovascular Department Biocruces Bizkaia Health Research Institute, Osakidetza, Hospital Universitario Cruces, Barakaldo, Spain.
- [15] Department of Neurology, Hospital Universitario Marqués de Valdecilla, Santander, Spain.
- [16] Department of Neurology, Complejo Hospitalario Universitario de Santiago, Santiago de Compostela, Spain.
- [17] Department of Neurology, Complejo Asistencial Universitario de León, León, Spain.
- [18] Department of Neurology, Hospital San Pedro, Logroño, Spain.
- [19] Department of Neurology, Complejo Asistencial Universitario de Burgos, Burgos, Spain.
- [20] Department of Neurology, Hospital Clínico Lozano Blesa, Zaragoza, Spain.

- PMID: **32761981**
- PMCID: PMC7436392
- DOI: 10.1111/ene.14467

Free PMC article
Observational Study

Impact of COVID-19 outbreak in reperfusion therapies of acute ischaemic stroke in northwest Spain

H Tejada Meza et al. Eur J Neurol. 2020 Dec.
Free PMC article
Show details
Eur J Neurol
. 2020 Dec;27(12):2491-2498.
doi: 10.1111/ene.14467. Epub 2020 Sep 4.

Authors

H Tejada Meza [1, 2, 3], Á Lambea Gil [1, 3], A Sancho Saldaña [1, 3], M Martínez-Zabaleta [4], E Garmendia Lopetegui [5], E López-Cancio Martínez [6], M Castañón Apilánez [6], M Herrera Isasi [7], J Marta Enguita [7], B Gómez-Vicente [8], J F Arenillas [8, 9], N Arenaza Basterrechea [10], J J Timiraos Fernández [10], J Sánchez Herrero [11], J L Maciñeiras Montero [11], M Castellanos Rodrigo [12], D Fernández-Coud [12], I Casado Menéndez [13], M T Temprano Fernández [13], M Freijo [14], A Luna [14], E J Palacio Portilla [15], Y Jiménez López [15], E Rodríguez-Castro [16], M Rodríguez-Yáñez [16], J Tejada García [17], I Beltrán Rodríguez [7], F Julián-Villaverde [18], M P Moreno García [18], J M Trejo Gabriel-Galán [19], A Echavarría Iñiguez [19], C Pérez Lázaro [20], M P Navarro Pérez [20], J Marta Moreno [1, 3], NORDICTUS Investigators

Affiliations

- [1] Stroke Unit, Department of Neurology, Hospital Universitario Miguel Servet, Zaragoza, Spain.
- [2] Interventional Neuroradiology Unit, Department of Radiology, Hospital Universitario Miguel Servet, Zaragoza, Spain.
- [3] Instituto de Investigación Sanitaria de Aragón (IISAragón), Zaragoza, Spain.
- [4] Department of Neurology, Donostia University Hospital, San Sebastián, Spain.
- [5] Department of Neuroradiology, Donostia University Hospital, San Sebastián, Spain.
- [6] Department of Neurology, Hospital Universitario Central de Asturias, Oviedo, Spain.
- [7] Department of Neurology, Complejo Hospitalario de Navarra, Pamplona, Spain.
- [8] Department of Neurology, Hospital Clínico Universitario de Valladolid, Valladolid, Spain.
- [9] Neurovascular Research Laboratory, Instituto de Biología y Genética Molecular, Universidad de Valladolid - Consejo Superior de Investigaciones Científicas, Madrid, Spain.
- [10] Stroke Unit, Department of Neurology, Hospital Universitario de Araba, Vitoria, Spain.
- [11] Department of Neurology, Complejo Hospitalario Universitario de Vigo, Vigo, Spain.
- [12] Department of Neurology, Complexo Hospitalario Universitario A Coruña / A Coruña Biomedical Research Insitute, A Coruña, Spain.
- [13] Stroke Unit, Department of Neurology, Hospital Universitario de Cabueñes, Gijón, Spain.
- [14] Neurovascular Department Biocruces Bizkaia Health Research Institute, Osakidetza, Hospital Universitario Cruces, Barakaldo, Spain.
- [15] Department of Neurology, Hospital Universitario Marqués de Valdecilla, Santander, Spain.
- [16] Department of Neurology, Complejo Hospitalario Universitario de Santiago, Santiago de Compostela, Spain.
- [17] Department of Neurology, Complejo Asistencial Universitario de León, León, Spain.
- [18] Department of Neurology, Hospital San Pedro, Logroño, Spain.
- [19] Department of Neurology, Complejo Asistencial Universitario de Burgos, Burgos, Spain.
- [20] Department of Neurology, Hospital Clínico Lozano Blesa, Zaragoza, Spain.

- PMID: **32761981**
- PMCID: PMC7436392
- DOI: 10.1111/ene.14467

Abstract

Background and purpose: Spain has been one of the countries more heavily stricken by SARS-CoV-2, which has had huge implications for stroke care. The aim was to analyse the impact of the COVID-19 epidemic outbreak on reperfusion therapies for acute ischaemic stroke in the northwest of Spain.

Methods: This was a Spanish multicentre retrospective observational study based on data from tertiary hospitals of the NORDICTUS network. All patients receiving reperfusion therapy for ischaemic stroke between 30 December 2019 and 3 May 2020 were recorded, and their baseline, clinical and radiological characteristics, extra- and intra-hospital times of action, Code Stroke activation pathway, COVID-19 status, reperfusion rate, and short-term outcome before and after the setting of the emergency state were analysed.

Results: A total of 796 patients received reperfusion therapies for ischaemic stroke. There was a decrease in the number of patients treated per week (46.5 patients per week vs. 39.0 patients per week, P = 0.043) and a delay in out-of-hospital (95.0 vs. 110.0 min, P = 0.001) and door-to-needle times (51.0 vs. 55.0, P = 0.038). Patients receiving endovascular therapy obtained less successful reperfusion rates (92.9% vs. 86.6%, P = 0.016). COVID-19 patients had more in-hospital mortality.

Conclusion: A decrease in the number of patients benefiting from reperfusion therapies was found, with a delay in out-of-hospital and door-to-needle times and worse reperfusion rates in northwest Spain. COVID-19 patients had more in-hospital mortality.

Keywords: COVID-19; Spain; acute stroke therapy; cerebral infarction; ischaemic stroke; thrombectomy; thrombolysis.

© 2020 European Academy of Neurology.

- 19 references

Supplementary info

Publication types, MeSH terms Expand

Publication types

- Multicenter Study
- Observational Study

MeSH terms

- Adult
- Aged
- Aged, 80 and over
- COVID-19*
- Emergency Medical Services / statistics & numerical data
- Endovascular Procedures / statistics & numerical data

- Female
- Humans
- Ischemic Stroke / epidemiology
- Ischemic Stroke / therapy*
- Length of Stay
- Male
- Middle Aged
- Pandemics*
- Patient Admission / statistics & numerical data
- Registries
- Reperfusion*
- Retrospective Studies
- Spain / epidemiology
- Thrombolytic Therapy / statistics & numerical data
- Treatment Outcome

Full text links

WILEY Full Text Article Wiley Free PMC article

Proceed to details

Cite

Share

☐ 691

Observational Study

Int J Antimicrob Agents

. 2020 Nov;56(5):106136.

doi: 10.1016/j.ijantimicag.2020.106136. Epub 2020 Aug 8.

Effects of Hydroxychloroquine on Covid-19 in Intensive Care Unit Patients: Preliminary Results

Alexandre Lopez [1], Gary Duclos [2], Bruno Pastene [2], Karine Bezulier [2], Romain Guilhaumou [3], Caroline Solas [4], Laurent Zieleskiewicz [5], Marc Leone [2]

Affiliations Expand

Affiliations

- [1] Aix-Marseille University, Assistance Publique Hôpitaux de Marseille, Department of Anesthesiology and Intensive Care, Hôpital Nord, Marseille, 13015, France. Electronic address: alexandre.lopez@ap-hm.fr.

- [2] Aix-Marseille University, Assistance Publique Hôpitaux de Marseille, Department of Anesthesiology and Intensive Care, Hôpital Nord, Marseille, 13015, France.
- [3] Service de Pharmacologie Clinique et Pharmacovigilance, APHM, Institut de neurosciences des systèmes, Inserm UMR 1106, Université d'Aix-Marseille, France.
- [4] Aix-Marseille University, APHM, UMR "Emergence des Pathologies Virales" Inserm 1207 IRD 190, Laboratoire de Pharmacocinétique et Toxicologie, Hôpital La Timone, 13005 Marseille, France.
- [5] Aix-Marseille University, Assistance Publique Hôpitaux de Marseille, Department of Anesthesiology and Intensive Care, Hôpital Nord, Marseille, 13015, France; Center for Cardiovascular and Nutrition Research (C2VN) Aix Marseille Université, INSERM, INRA, Marseille, 13005, France.

- PMID: **32777263**
- PMCID: PMC7413851
- DOI: 10.1016/j.ijantimicag.2020.106136

Free PMC article
Observational Study

Effects of Hydroxychloroquine on Covid-19 in Intensive Care Unit Patients: Preliminary Results

Alexandre Lopez et al. Int J Antimicrob Agents. 2020 Nov.
Free PMC article
Show details
Int J Antimicrob Agents
. 2020 Nov;56(5):106136.
doi: 10.1016/j.ijantimicag.2020.106136. Epub 2020 Aug 8.

Authors

Alexandre Lopez [1], Gary Duclos [2], Bruno Pastene [2], Karine Bezulier [2], Romain Guilhaumou [3], Caroline Solas [4], Laurent Zieleskiewicz [5], Marc Leone [2]

Affiliations

- [1] Aix-Marseille University, Assistance Publique Hôpitaux de Marseille, Department of Anesthesiology and Intensive Care, Hôpital Nord, Marseille, 13015, France. Electronic address: alexandre.lopez@ap-hm.fr.
- [2] Aix-Marseille University, Assistance Publique Hôpitaux de Marseille, Department of Anesthesiology and Intensive Care, Hôpital Nord, Marseille, 13015, France.
- [3] Service de Pharmacologie Clinique et Pharmacovigilance, APHM, Institut de neurosciences des systèmes, Inserm UMR 1106, Université d'Aix-Marseille, France.
- [4] Aix-Marseille University, APHM, UMR "Emergence des Pathologies Virales" Inserm 1207 IRD 190, Laboratoire de Pharmacocinétique et Toxicologie, Hôpital La Timone, 13005 Marseille, France.

- [5] Aix-Marseille University, Assistance Publique Hôpitaux de Marseille, Department of Anesthesiology and Intensive Care, Hôpital Nord, Marseille, 13015, France; Center for Cardiovascular and Nutrition Research (C2VN) Aix Marseille Université, INSERM, INRA, Marseille, 13005, France.

- PMID: **32777263**
- PMCID: PMC7413851
- DOI: 10.1016/j.ijantimicag.2020.106136

Abstract

During the Covid-19 pandemic, many intensive care unit (ICU) patients received hydroxychloroquine. The primary objective of this study was to assess the effects of hydroxychloroquine according to its plasma concentration in ICU patients. A single-center retrospective study was performed from March to April 2020 in an ICU of a university hospital. All patients admitted to the ICU with confirmed Covid-19 pneumonia and treated with hydroxychloroquine were included. The study compared 17 patients in whom the hydroxychloroquine plasma concentration was in the therapeutic target (on-target) and 12 patients in whom the plasma concentration was below the target (off-target). The follow-up of patients was 15 days. No association was found between hydroxychloroquine plasma concentration and viral load evolution (P = 0.77). There was no significant difference between the two groups for duration of mechanical ventilation, length of ICU stay, in-hospital mortality, and 15-days mortality. These findings indicate that hydroxychloroquine administration for Covid-19 patients hospitalized in ICU is not associated with improved outcomes. Larger multicenter studies are needed to confirm these results.

Keywords: Covid-19 pneumonia; Hydroxychloroquine; Intensive care unit.

Copyright © 2020 Elsevier Ltd. All rights reserved.

- 15 references
- 2 figures

Supplementary info

Publication types, MeSH terms, Substances, Supplementary concepts Expand

Publication types

- Observational Study

MeSH terms

- Anti-Inflammatory Agents / adverse effects
- Anti-Inflammatory Agents / blood
- Anti-Inflammatory Agents / therapeutic use*
- Betacoronavirus / drug effects*
- COVID-19
- Coronavirus Infections / drug therapy*

- Critical Care
- Female
- Hospital Mortality
- Humans
- Hydroxychloroquine / adverse effects
- Hydroxychloroquine / blood
- Hydroxychloroquine / therapeutic use*
- Intensive Care Units
- Length of Stay / statistics & numerical data
- Male
- Middle Aged
- Pandemics
- Pneumonia, Viral / drug therapy*
- Preliminary Data
- Retrospective Studies
- SARS-CoV-2
- Viral Load / drug effects

Substances

- Anti-Inflammatory Agents
- Hydroxychloroquine

Supplementary concepts

- COVID-19 drug treatment

Full text links

ELSEVIER FULL-TEXT ARTICLE Elsevier Science Free PMC article
Proceed to details
Cite
Share
☐ 692
Observational Study
Open Heart
. 2020 Nov;7(2):e001428.
doi: 10.1136/openhrt-2020-001428.

Coronary artery disease in patients hospitalised with Coronavirus disease 2019 (COVID-19) infection

Marco Loffi [#] [1], Raffaele Piccolo [#] [2], Valentina Regazzoni [3], Giuseppe Di Tano [3], Luigi Moschini [3], Debora Robba [3], Filippo Quinzani [3], Giovanni Esposito [4], Anna Franzone [2], Gian Battista Danzi [3]
Affiliations Expand

Affiliations

- [1] Division of Cardiology, Hospital of Cremona, Cremona, Italy loffi.marco@gmail.com.
- [2] University of Naples Federico II Department of Advanced Biomedical Sciences, Napoli, Campania, Italy.
- [3] Division of Cardiology, Hospital of Cremona, Cremona, Italy.
- [4] Department of Advanced Biomedical Sciences, University of Naples Federico II Department of Advanced Biomedical Sciences, Napoli, Campania, Italy.

[#] Contributed equally.

- PMID: **33229434**
- PMCID: PMC7684763
- DOI: 10.1136/openhrt-2020-001428

Free PMC article
Observational Study

Coronary artery disease in patients hospitalised with Coronavirus disease 2019 (COVID-19) infection

Marco Loffi et al. Open Heart. 2020 Nov.
Free PMC article
Show details
Open Heart
. 2020 Nov;7(2):e001428.
doi: 10.1136/openhrt-2020-001428.

Authors

Marco Loffi [#] [1], Raffaele Piccolo [#] [2], Valentina Regazzoni [3], Giuseppe Di Tano [3], Luigi Moschini [3], Debora Robba [3], Filippo Quinzani [3], Giovanni Esposito [4], Anna Franzone [2], Gian Battista Danzi [3]

Affiliations

- [1] Division of Cardiology, Hospital of Cremona, Cremona, Italy loffi.marco@gmail.com.
- [2] University of Naples Federico II Department of Advanced Biomedical Sciences, Napoli, Campania, Italy.
- [3] Division of Cardiology, Hospital of Cremona, Cremona, Italy.
- [4] Department of Advanced Biomedical Sciences, University of Naples Federico II Department of Advanced Biomedical Sciences, Napoli, Campania, Italy.

[#] Contributed equally.

- PMID: **33229434**
- PMCID: PMC7684763
- DOI: 10.1136/openhrt-2020-001428

Abstract

Objective: Among patients with Coronavirus disease 2019 (COVID-19), coronary artery disease (CAD) has been identified as a high-risk condition. We aimed to assess the clinical outcomes and mortality among patients with COVID-19 according to CAD status.

Methods: We retrospectively analysed data from patients with COVID-19 admitted to the Cremona Hospital (Lombardy region, Italy) between February and March 2020. The primary outcome was all-cause mortality. CAD was defined as a history of prior myocardial infarction (MI), prior percutaneous coronary intervention (PCI), prior coronary artery bypass grafting (CABG) or CAD that was being medically treated.

Results: Of 1252 consecutive patients with COVID-19, 124 (9.9%) had concomitant CAD. Patients with CAD were older and had a higher prevalence of comorbidities compared with those without CAD. Although patients with CAD had a higher risk of all-cause mortality than patients without CAD (HR 3.01, 95% CI 2.27 to 3.99), this difference was no longer significant in the adjusted model (HR 1.14, 95% CI 0.79 to 1.63). Results were consistent among patients with prior MI (adjusted HR (aHR) 0.87, 95% CI 0.54 to 1.41), prior PCI (aHR 1.10, 95% CI 0.75 to 1.62), prior CABG (aHR 0.91, 95% CI 0.45 to 1.82), or CAD medically treated (aHR 0.84, 95% CI 0.29 to 2.44). Multivariable analysis showed that age (aHR per 5 year increase 1.62, 95% CI 1.53 to 1.72) and female sex (aHR 0.63, 95% CI 0.49 to 0.82) were the only two independent correlates of mortality.

Conclusion: Patients with COVID-19 and CAD have an exceedingly higher risk of mortality, which is mainly attributable to the burden of comorbidities rather than to a direct effect of CAD per se.

Keywords: atherosclerosis; coronary artery disease; risk stratification.

© Author(s) (or their employer(s)) 2020. Re-use permitted under CC BY-NC. No commercial re-use. See rights and permissions. Published by BMJ.

Conflict of interest statement

Competing interests: None declared.

- 15 references

- 3 figures

Supplementary info

Publication types, MeSH terms Expand

Publication types

- Observational Study

MeSH terms

- Age Factors
- Aged
- Aged, 80 and over
- COVID-19 / diagnosis
- COVID-19 / mortality*
- COVID-19 / therapy
- Cause of Death
- Comorbidity
- Coronary Artery Disease / diagnostic imaging
- Coronary Artery Disease / mortality*
- Coronary Artery Disease / therapy
- Female
- Hospital Mortality
- Hospitalization*
- Humans
- Italy / epidemiology
- Male
- Middle Aged
- Prevalence
- Prognosis
- Retrospective Studies
- Risk Assessment
- Risk Factors
- Time Factors

Full text links

HighWire Free PMC article

Proceed to details

Cite

Share

☐ 693
Observational Study
J Cardiothorac Vasc Anesth
. 2022 Feb;36(2):529-533.
doi: 10.1053/j.jvca.2021.04.037. Epub 2021 May 3.

Trend and Pattern of 100 Acute Respiratory Distress Syndrome Patients Referred for Venovenous Extracorporeal Membrane Oxygenation Treatment in a National Referral Center in North Italy During the Last Decade

Marina Pieri [1], Francesco Vettorello [2], Maria Grazia Calabrò [2], Alberto Zangrillo [3], Anna Mara Scandroglio [2]
Affiliations Expand

Affiliations

- [1] Department of Anesthesia and Intensive Care, IRCCS San Raffaele Scientific Institute, Milan, Italy. Electronic address: pieri.marina@hsr.it.
- [2] Department of Anesthesia and Intensive Care, IRCCS San Raffaele Scientific Institute, Milan, Italy.
- [3] Department of Anesthesia and Intensive Care, IRCCS San Raffaele Scientific Institute, Milan, Italy; Vita-Salute San Raffaele University, Milan, Italy.

- PMID: **34088553**
- DOI: 10.1053/j.jvca.2021.04.037

Observational Study

Trend and Pattern of 100 Acute Respiratory Distress Syndrome Patients Referred for Venovenous Extracorporeal Membrane Oxygenation Treatment in a National Referral Center in North Italy During the Last Decade

Marina Pieri et al. J Cardiothorac Vasc Anesth. 2022 Feb.

Show details
J Cardiothorac Vasc Anesth
. 2022 Feb;36(2):529-533.
doi: 10.1053/j.jvca.2021.04.037. Epub 2021 May 3.

Authors

Marina Pieri [1], Francesco Vettorello [2], Maria Grazia Calabrò [2], Alberto Zangrillo [3], Anna Mara Scandroglio [2]

Affiliations

- [1] Department of Anesthesia and Intensive Care, IRCCS San Raffaele Scientific Institute, Milan, Italy. Electronic address: pieri.marina@hsr.it.
- [2] Department of Anesthesia and Intensive Care, IRCCS San Raffaele Scientific Institute, Milan, Italy.
- [3] Department of Anesthesia and Intensive Care, IRCCS San Raffaele Scientific Institute, Milan, Italy; Vita-Salute San Raffaele University, Milan, Italy.

- PMID: **34088553**
- DOI: 10.1053/j.jvca.2021.04.037

Abstract

Objective: Current evidence supports centralization of patients with refractory acute respiratory distress syndrome (ARDS) to institutions with a high level of expertise and with extracorporeal membrane oxygenation (ECMO) capabilities. The aim of this study was to analyze and report the data of transferred refractory ARDS patients managed with venovenous (VV) ECMO at a national referral center over the last 11 years.

Design: Observational study.

Setting: Referral center in Italy.

Participants: The study comprised 100 patients treated from May 2009-November 2020.

Interventions: None.

Measurements and main results: The mean age was 54 ± 14 years, and 65% of patients were male. Patients were treated throughout the year, with seasonal peaks in the winter months. The majority of patients were referred from hospitals within the Lombardia region (81%), mainly from the city of Milan and surrounding area (36% of the total). The most common etiology of refractory ARDS was H1N1 influenza A (42 patients [42%]), followed by bacterial pneumonia (35 patients [35%]), and severe acute respiratory syndrome due to Sars-CoV-2 infection (five patients [5%]). All patients were severely hypoxic at the time of VV ECMO treatment. No transport-related complication was recorded. The most common configuration used in the authors' clinical practice was a bicaval dual-lumen configuration (61 patients [61%]), followed by a femoro-jugular configuration (38 patients [38%]). The intensive care unit survival rate was 55%.

Conclusions: Referral to a specialized center for VV ECMO treatment should be considered expeditiously in case of refractory ARDS, which often is lethal. Transport of patients with an

unstable condition, although challenging, is feasible, and centralization of patient care is associated with good outcomes.

Keywords: VV ECMO; acute respiratory distress syndrome; intensive care unit; mortality; referral; venovenous extracorporeal membrane oxygenation.

Copyright © 2021 Elsevier Inc. All rights reserved.

Supplementary info

Publication types, MeSH terms Expand

Publication types

- Observational Study

MeSH terms

- Adult
- Aged
- COVID-19*
- Extracorporeal Membrane Oxygenation*
- Humans
- Influenza A Virus, H1N1 Subtype*
- Male
- Middle Aged
- Referral and Consultation
- Respiratory Distress Syndrome* / diagnosis
- Respiratory Distress Syndrome* / epidemiology
- Respiratory Distress Syndrome* / therapy
- Retrospective Studies
- SARS-CoV-2

Full text links

ELSEVIER FULL-TEXT ARTICLE Elsevier Science
Proceed to details
Cite
Share
☐ 694
Observational Study
BMJ Open
. 2021 Dec 17;11(12):e052019.
doi: 10.1136/bmjopen-2021-052019.

Background incidence rates of hospitalisations and emergency department visits for thromboembolic and coagulation disorders in Ontario, Canada for COVID-19 vaccine safety assessment: a population-based retrospective observational study

Sharifa Nasreen [1] [2], Andrew J Calzavara [2], Maria E Sundaram [1] [2], Shannon E MacDonald [3] [4], Christiaan H Righolt [5], Menaka Pai [6], Thalia S Field [7], Lily W Zhou [7] [8], Sarah E Wilson [1] [9], Jeffrey C Kwong [10] [2]
Affiliations Expand

Affiliations

- [1] Dalla Lana School of Public Health, University of Toronto, Toronto, Ontario, Canada.
- [2] ICES, Toronto, Ontario, Canada.
- [3] Faculty of Nursing, University of Alberta, Edmonton, Alberta, Canada.
- [4] School of Public Health, University of Alberta, Edmonton, Alberta, Canada.
- [5] Vaccine and Drug Evaluation Centre, Department of Community Health Sciences, University of Manitoba, Winnipeg, Manitoba, Canada.
- [6] Department of Medicine, McMaster University, Hamilton, Ontario, Canada.
- [7] Division of Neurology, The University of British Columbia, Vancouver, Columbia, Canada.
- [8] Stanford Stroke Center, Palo Alto, California, USA.
- [9] Immunization and vaccine-preventable diseases, Public Health Ontario, Toronto, Ontario, Canada.
- [10] Dalla Lana School of Public Health, University of Toronto, Toronto, Ontario, Canada jeff.kwong@utoronto.ca.

- PMID: **34921078**
- PMCID: PMC8685534
- DOI: 10.1136/bmjopen-2021-052019

Free PMC article
Observational Study

Background incidence rates of hospitalisations and emergency department visits for thromboembolic and coagulation disorders in Ontario, Canada for COVID-19

vaccine safety assessment: a population-based retrospective observational study

Sharifa Nasreen et al. BMJ Open. 2021.
Free PMC article
Show details
BMJ Open
. 2021 Dec 17;11(12):e052019.
doi: 10.1136/bmjopen-2021-052019.

Authors

Sharifa Nasreen [1, 2], Andrew J Calzavara [2], Maria E Sundaram [1, 2], Shannon E MacDonald [3, 4], Christiaan H Righolt [5], Menaka Pai [6], Thalia S Field [7], Lily W Zhou [7, 8], Sarah E Wilson [1, 9], Jeffrey C Kwong [10, 2]

Affiliations

- [1] Dalla Lana School of Public Health, University of Toronto, Toronto, Ontario, Canada.
- [2] ICES, Toronto, Ontario, Canada.
- [3] Faculty of Nursing, University of Alberta, Edmonton, Alberta, Canada.
- [4] School of Public Health, University of Alberta, Edmonton, Alberta, Canada.
- [5] Vaccine and Drug Evaluation Centre, Department of Community Health Sciences, University of Manitoba, Winnipeg, Manitoba, Canada.
- [6] Department of Medicine, McMaster University, Hamilton, Ontario, Canada.
- [7] Division of Neurology, The University of British Columbia, Vancouver, Columbia, Canada.
- [8] Stanford Stroke Center, Palo Alto, California, USA.
- [9] Immunization and vaccine-preventable diseases, Public Health Ontario, Toronto, Ontario, Canada.
- [10] Dalla Lana School of Public Health, University of Toronto, Toronto, Ontario, Canada jeff.kwong@utoronto.ca.

- PMID: **34921078**
- PMCID: PMC8685534
- DOI: 10.1136/bmjopen-2021-052019

Abstract

Objective: The objective of this study was to estimate background rates of selected thromboembolic and coagulation disorders in Ontario, Canada.

Design: Population-based retrospective observational study using linked health administrative databases. Records of hospitalisations and emergency department visits were searched to identify cases using International Statistical Classification of Diseases and Related Health Problems, 10th Revision, Canada diagnostic codes.

Participants: All Ontario residents.

Primary outcome measures: Incidence rates of ischaemic stroke, intracerebral haemorrhage, subarachnoid haemorrhage, deep vein thrombosis, pulmonary embolism, idiopathic thrombocytopaenia, disseminated intravascular coagulation and cerebral venous thrombosis during five prepandemic years (2015-2019) and 2020.

Results: The average annual population was 14 million with 51% female. The mean annual rates per 100 000 population during 2015-2019 were 127.1 (95% CI 126.2 to 127.9) for ischaemic stroke, 22.0 (95% CI 21.6 to 22.3) for intracerebral haemorrhage, 9.4 (95% CI 9.2 to 9.7) for subarachnoid haemorrhage, 86.8 (95% CI 86.1 to 87.5) for deep vein thrombosis, 63.7 (95% CI 63.1 to 64.3) for pulmonary embolism, 6.1 (95% CI 5.9 to 6.3) for idiopathic thrombocytopaenia, 1.6 (95% CI 1.5 to 1.7) for disseminated intravascular coagulation, and 1.5 (95% CI 1.4 to 1.6) for cerebral venous thrombosis. Rates were lower in 2020 than during the prepandemic years for ischaemic stroke, deep vein thrombosis and idiopathic thrombocytopaenia. Rates were generally consistent over time, except for pulmonary embolism, which increased from 57.1 to 68.5 per 100 000 between 2015 and 2019. Rates were higher for females than males for subarachnoid haemorrhage, pulmonary embolism and cerebral venous thrombosis, and vice versa for ischaemic stroke and intracerebral haemorrhage. Rates increased with age for most of these conditions, but idiopathic thrombocytopaenia demonstrated a bimodal distribution with incidence peaks at 0-19 years and ≥60 years.

Conclusions: Our estimated background rates help contextualise observed events of these potential adverse events of special interest and to detect potential safety signals related to COVID-19 vaccines.

Keywords: COVID-19; adverse events; stroke; thromboembolism.

© Author(s) (or their employer(s)) 2021. Re-use permitted under CC BY-NC. No commercial re-use. See rights and permissions. Published by BMJ.

Conflict of interest statement

Competing interests: ‘Yes, there are competing interests for one or more authors and I have provided a Competing Interests statement in my manuscript and in the box below’

- 40 references

Supplementary info

Publication types, MeSH terms, Substances Expand

Publication types

- Observational Study
- Research Support, Non-U.S. Gov't

MeSH terms

- Adolescent

- Adult
- Brain Ischemia*
- COVID-19 Vaccines
- COVID-19*
- Child
- Child, Preschool
- Disseminated Intravascular Coagulation*
- Emergency Service, Hospital
- Female
- Hospitalization
- Humans
- Incidence
- Infant
- Infant, Newborn
- Male
- Ontario / epidemiology
- Pulmonary Embolism* / epidemiology
- SARS-CoV-2
- Stroke* / epidemiology
- Young Adult

Substances

- COVID-19 Vaccines

Full text links

BMJ Free Full Text HighWire Free PMC article

Proceed to details

Cite

Share

☐ 695

Observational Study

Int J Immunopathol Pharmacol

. Jan-Dec 2021;35:20587384211059675.

doi: 10.1177/20587384211059675.

Early use of canakinumab to prevent mechanical ventilation in select COVID-19

patients: A retrospective, observational analysis

Antonio Mastroianni [1], Sonia Greco [1], Luciana Chidichimo [1], Filippo Urso [2], Francesca Greco [3], Maria V Mauro [3], Valeria Vangeli [1]
Affiliations Expand

Affiliations

- [1] Infectious Diseases Unit, "Annunziata" Hospital, 220599Azienda Ospedaliera di Cosenza, Cosenza, Italy.
- [2] Hospital Pharmacy, "Annunziata" Hospital, 220599Azienda Ospedaliera di Cosenza, Cosenza, Italy.
- [3] Microbiology Unit, "Annunziata" Hospital, 220599Azienda Ospedaliera di Cosenza, Cosenza, Italy.

- PMID: **34928722**
- PMCID: PMC8725043
- DOI: 10.1177/20587384211059675

Free PMC article
Observational Study

Early use of canakinumab to prevent mechanical ventilation in select COVID-19 patients: A retrospective, observational analysis

Antonio Mastroianni et al. Int J Immunopathol Pharmacol. Jan-Dec 2021.
Free PMC article
Show details
Int J Immunopathol Pharmacol
. Jan-Dec 2021;35:20587384211059675.
doi: 10.1177/20587384211059675.

Authors

Antonio Mastroianni [1], Sonia Greco [1], Luciana Chidichimo [1], Filippo Urso [2], Francesca Greco [3], Maria V Mauro [3], Valeria Vangeli [1]

Affiliations

- [1] Infectious Diseases Unit, "Annunziata" Hospital, 220599Azienda Ospedaliera di Cosenza, Cosenza, Italy.

- [2] Hospital Pharmacy, "Annunziata" Hospital, 220599Azienda Ospedaliera di Cosenza, Cosenza, Italy.
- [3] Microbiology Unit, "Annunziata" Hospital, 220599Azienda Ospedaliera di Cosenza, Cosenza, Italy.

- PMID: **34928722**
- PMCID: PMC8725043
- DOI: 10.1177/20587384211059675

Abstract

Introduction: The fully-human monoclonal anti-interleukin (IL)-1β antibody canakinumab may inhibit the production of inflammatory mediators in patients with coronavirus disease 2019 (COVID-19) and the hyperinflammatory response potentially leading to acute respiratory distress syndrome.

Objectives: The goal of our retrospective, observational analysis was to evaluate the safety and efficacy of subcutaneous (s.c.) canakinumab in combination with our standard of care (SOC) treatment of selected patients with COVID-19 with respiratory failure and elevated reactive pro-inflammatory markers.

Methods: Eight participants received two doses of s.c. canakinumab 150 mg (or 2 mg/kg for participants weighing ≤40 kg) in addition to SOC. 12 patients received only SOC treatment.

Results: Canakinumab treatment reduced the need for mechanical ventilation and reduced proinflammatory markers, resulting in an amelioration of the final outcome, with respect to the control group who received SOC alone. The treatment was safe and well tolerated; no adverse events were reported.

Conclusion: The use of canakinumab (300 mg, s.c.) in the early stage of COVID-19 with mild-to-moderate respiratory failure was superior to SOC at preventing clinical deterioration and may warrant further investigation as a treatment option for patients with COVID-19 who experience a hyperinflammatory response in the early stage of the disease.

Keywords: COVID-19; SARS-CoV-2; biomarkers; canakinumab; case series; interleukin-1β; mechanical ventilation.

Conflict of interest statement

Declaration of conflicting interests: The author(s) declared no potential conflicts of interest with respect to the research, authorship, and/or publication of this article.

- 20 references

Supplementary info

Publication types, MeSH terms, Substances Expand

Publication types

- Observational Study

MeSH terms

- Antibodies, Monoclonal, Humanized* / administration & dosage
- Antibodies, Monoclonal, Humanized* / adverse effects
- Antibodies, Monoclonal, Humanized* / immunology
- Biomarkers / blood
- COVID-19* / complications
- COVID-19* / epidemiology
- COVID-19* / immunology
- COVID-19* / therapy
- Dose-Response Relationship, Drug
- Female
- Humans
- Inflammation Mediators / blood
- Interleukin-1beta* / antagonists & inhibitors
- Interleukin-1beta* / immunology
- Italy / epidemiology
- Male
- Middle Aged
- Monitoring, Immunologic / methods
- Outcome and Process Assessment, Health Care
- Patient Selection
- Respiration, Artificial* / methods
- Respiration, Artificial* / statistics & numerical data
- Retrospective Studies
- SARS-CoV-2
- Time-to-Treatment

Substances

- Antibodies, Monoclonal, Humanized
- Biomarkers
- IL1B protein, human
- Inflammation Mediators
- Interleukin-1beta
- canakinumab

Full text links

SAGE journals Open access full text Atypon Free PMC article

Proceed to details

Cite

Share

☐ 696

Observational Study

Intern Med

. 2021 Dec 1;60(23):3693-3700.

doi: 10.2169/internalmedicine.8220-21. Epub 2021 Sep 25.

Impact of the COVID-19 Pandemic on ST-elevation Myocardial Infarction from a Single-center Experience in Tokyo

Yukihiro Watanabe [1, 2], Hideki Miyachi [1], Kosuke Mozawa [1], Kenta Yamada [1], Eiichiro Oka [1], Reiko Shiomura [1], Yoichiro Sugizaki [1], Junya Matsuda [1], Jun Nakata [1], Shuhei Tara [2], Yukichi Tokita [2], Yu-Ki Iwasaki [2], Takeshi Yamamoto [1], Hitoshi Takano [2], Wataru Shimizu [1, 2]

Affiliations Expand

Affiliations

- [1] Division of Cardiovascular Intensive Care, Nippon Medical School Hospital, Japan.
- [2] Department of Cardiovascular Medicine, Nippon Medical School, Japan.

- PMID: **34565777**
- PMCID: PMC8710378
- DOI: 10.2169/internalmedicine.8220-21

Free PMC article

Observational Study

Impact of the COVID-19 Pandemic on ST-elevation Myocardial Infarction from a Single-center Experience in Tokyo

Yukihiro Watanabe et al. Intern Med. 2021.

Free PMC article

Show details

Intern Med

. 2021 Dec 1;60(23):3693-3700.

doi: 10.2169/internalmedicine.8220-21. Epub 2021 Sep 25.

Authors

Yukihiro Watanabe [1 2], Hideki Miyachi [1], Kosuke Mozawa [1], Kenta Yamada [1], Eiichiro Oka [1], Reiko Shiomura [1], Yoichiro Sugizaki [1], Junya Matsuda [1], Jun Nakata [1], Shuhei Tara [2], Yukichi Tokita [2], Yu-Ki Iwasaki [2], Takeshi Yamamoto [1], Hitoshi Takano [2], Wataru Shimizu [1 2]

Affiliations

- [1] Division of Cardiovascular Intensive Care, Nippon Medical School Hospital, Japan.
- [2] Department of Cardiovascular Medicine, Nippon Medical School, Japan.

- PMID: **34565777**
- PMCID: PMC8710378
- DOI: 10.2169/internalmedicine.8220-21

Abstract

Objective The coronavirus disease 2019 (COVID-19) pandemic has had a significant impact on global healthcare systems. Some studies have reported the negative impact of COVID-19 on ST-elevation myocardial infarction (STEMI) patients; however, the impact in Japan remains unclear. This study investigated the impact of the COVID-19 pandemic on STEMI patients admitted to an academic tertiary-care center in Tokyo, Japan. Methods In this retrospective, observational, cohort study, we included 398 consecutive patients who were admitted to our institute from January 1, 2018, to March 10, 2021, and compared the incidence of hospitalization, clinical characteristics, time course, management, and outcomes before and after March 11, 2020, the date when the World Health Organization declared COVID-19 a pandemic. Results There was a 10.7% reduction in hospitalization of STEMI patients during the COVID-19 pandemic compared with that in the previous year (117 vs. 131 cases). During the COVID-19 pandemic, the incidence of late presentation was significantly higher (26.5% vs. 12.1%, p<0.001), and the onset-to-door [241 (IQR: 70-926) vs. 128 (IQR: 66-493) minutes, p=0.028] and door-to-balloon [72 (IQR: 61-128) vs. 60 (IQR: 43-90) min, p<0.001] times were significantly longer than in the previous year. Furthermore, the in-hospital mortality was higher, but the difference was not significant (9.4% vs. 5.0%, p=0.098). Conclusion The COVID-19 pandemic significantly impacted STEMI patients in Tokyo and resulted in a slight decrease in hospitalization, a significant increase in late presentation and treatment delays, and a slight but nonsignificant increase in mortality. In the COVID-19 era, the acute management system for STEMI in Japan must be reviewed.

Keywords: COVID-19; ST-elevation myocardial infarction; acute coronary syndrome; acute myocardial infarction; coronavirus disease; percutaneous coronary intervention.

Conflict of interest statement

The authors state that they have no Conflict of Interest (COI).

- 24 references
- 3 figures

Supplementary info

Publication types, MeSH terms Expand

Publication types

- Observational Study

MeSH terms

- COVID-19*
- Cohort Studies
- Humans
- Pandemics
- Percutaneous Coronary Intervention*
- Retrospective Studies
- SARS-CoV-2
- ST Elevation Myocardial Infarction* / diagnosis
- ST Elevation Myocardial Infarction* / epidemiology
- Tokyo / epidemiology

Full text links

J-STAGE, Japan Science and Technology Information Aggregator, Electronic
Free PMC article
Proceed to details
Cite
Share
697
Observational Study
J Rehabil Med
. 2020 Sep 4;52(9):jrm00095.
doi: 10.2340/16501977-2731.

Remote rehabilitation for patients with COVID-19

Tomoko Sakai [1], Chisato Hoshino, Reiko Yamaguchi, Masanobu Hirao, Rui Nakahara, Atsushi Okawa
Affiliations Expand

Affiliation

- [1] Department of Rehabilitation Medicine, Tokyo Medical and Dental University, Tokyo, Japan. E-mail: t_sakai.orth@tmd.ac.jp.

- PMID: **32871014**
- DOI: 10.2340/16501977-2731

Free article
Observational Study

Remote rehabilitation for patients with COVID-19

Tomoko Sakai et al. J Rehabil Med. 2020.
Free article
Show details
J Rehabil Med
. 2020 Sep 4;52(9):jrm00095.
doi: 10.2340/16501977-2731.

Authors

Tomoko Sakai [1], Chisato Hoshino, Reiko Yamaguchi, Masanobu Hirao, Rui Nakahara, Atsushi Okawa

Affiliation

- [1] Department of Rehabilitation Medicine, Tokyo Medical and Dental University, Tokyo, Japan. E-mail: t_sakai.orth@tmd.ac.jp.

- PMID: **32871014**
- DOI: 10.2340/16501977-2731

Abstract

Objective: To describe the effectiveness and risk management of remote rehabilitation for coronavirus disease (COVID-19) patients.

Design: Single-centre, retrospective, observational study.

Patients: COVID-19 patients undergoing rehabilitation (24 April to 24 May 2020).

Methods: All COVID-19 inpatients undergoing rehabilitation in the general ward were assessed. Data were collected on age, sex, physical ability, rehabilitation modality (remote/direct), need for intubation or extracorporeal membrane oxygenation, degree of pneumonia, oxygen therapy from the start of rehabilitation, D-dimer and C-reactive protein levels, and rehabilitation-related complications. Activities of daily living were measured using the Barthel Index.

Results: Out of a total of 43 patients, 14 were initially provided with remote rehabilitation and 29 with direct rehabilitation. Four patients were switched from direct to remote rehabilitation during the study, thus at the end of the study there were 18 in the remote rehabilitation group and 25 in the direct rehabilitation group. Patients in remote rehabilitation were significantly younger than those in direct rehabilitation. Of 12 patients who required intubation, 3 were given remote rehabilitation. One extracorporeal membrane oxygenation survivor underwent direct rehabilitation. All patients on remote rehabilitation were discharged home or to a hotel. Twelve

out of 29 patients were transferred to a rehabilitation hospital due to delayed recovery of activities of daily living. No serious adverse events occurred.

Conclusion: Effective and safe remote rehabilitation was performed in 41.9% of COVID-19 patients in this study, which resulted in improved rehabilitation in COVID-19 zones.

Keywords: coronavirus; early ambulation; infection; pulmonary embolism; rehabilitation; risk management.

Supplementary info

Publication types, MeSH terms Expand

Publication types

- Observational Study

MeSH terms

- Adult
- Aged
- Aged, 80 and over
- Betacoronavirus*
- COVID-19
- Coronavirus Infections / rehabilitation*
- Female
- Humans
- Japan
- Male
- Middle Aged
- Pandemics
- Pneumonia, Viral / rehabilitation*
- Retrospective Studies
- Risk Management
- SARS-CoV-2
- Telerehabilitation / methods*
- Treatment Outcome

Full text links

MJS MEDICAL JOURNALS SWEDEN FULL TEXT Medical Journals

Proceed to details

Cite

Share

☐ 698
Observational Study
J Trauma Acute Care Surg
. 2021 Jul 1;91(1):241-246.
doi: 10.1097/TA.0000000000003202.

Do surgical emergencies stay at home? Observations from the first United States Coronavirus epicenter

Caroline T Dong [1], Anna Liveris, Erin R Lewis, Smita Mascharak, Edward Chao, Srinivas H Reddy, Sheldon H Teperman, John McNelis, Melvin E Stone Jr
Affiliations Expand

Affiliation

- [1] From the Department of Surgery (C.T.D., A.L., E.R.L., S.M., E.C., S.H.R., S.H.T., J.M.), Jacobi Medical Center, Bronx; Kings County Hospital Center (M.E.S.J.), Brooklyn; and Albert Einstein College of Medicine (C.T.D., A.L., E.R.L., S.M., E.C., S.H.R., S.H.T., J.M., M.E.S.J.), Bronx, New York.

- PMID: **34144567**
- PMCID: PMC8218982
- DOI: 10.1097/TA.0000000000003202

Free PMC article
Observational Study

Do surgical emergencies stay at home? Observations from the first United States Coronavirus epicenter

Caroline T Dong et al. J Trauma Acute Care Surg. 2021.
Free PMC article
Show details
J Trauma Acute Care Surg
. 2021 Jul 1;91(1):241-246.
doi: 10.1097/TA.0000000000003202.

Authors

Caroline T Dong [1], Anna Liveris, Erin R Lewis, Smita Mascharak, Edward Chao, Srinivas H Reddy, Sheldon H Teperman, John McNelis, Melvin E Stone Jr

Affiliation

- [1] From the Department of Surgery (C.T.D., A.L., E.R.L., S.M., E.C., S.H.R., S.H.T., J.M.), Jacobi Medical Center, Bronx; Kings County Hospital Center (M.E.S.J.), Brooklyn; and Albert Einstein College of Medicine (C.T.D., A.L., E.R.L., S.M., E.C., S.H.R., S.H.T., J.M., M.E.S.J.), Bronx, New York.

- PMID: **34144567**
- PMCID: PMC8218982
- DOI: 10.1097/TA.0000000000003202

Abstract

Background: During the coronavirus disease 2019 pandemic, New York instituted a statewide stay-at-home mandate to lower viral transmission. While public health guidelines advised continued provision of timely care for patients, disruption of safety-net health care and public fear have been proposed to be related to indirect deaths because of delays in presentation. We hypothesized that admissions for emergency general surgery (EGS) diagnoses would decrease during the pandemic and that mortality for these patients would increase.

Methods: A multicenter observational study comparing EGS admissions from January to May 2020 to 2018 and 2019 across 11 NYC hospitals in the largest public health care system in the United States was performed. Emergency general surgery diagnoses were defined using International Classification Diseases, Tenth Revision, codes and grouped into seven common diagnosis categories: appendicitis, cholecystitis, small/large bowel, peptic ulcer disease, groin hernia, ventral hernia, and necrotizing soft tissue infection. Baseline demographics were compared including age, race/ethnicity, and payor status. Outcomes included coronavirus disease (COVID) status and mortality.

Results: A total of 1,376 patients were admitted for EGS diagnoses from January to May 2020, a decrease compared with both 2018 (1,789) and 2019 (1,668) ($p < 0.0001$). This drop was most notable after the stay-at-home mandate (March 22, 2020; week 12). From March to May 2020, 3.3%, 19.2%, and 6.0% of EGS admissions were incidentally COVID positive, respectively. Mortality increased in March to May 2020 compared with 2019 (2.2% vs. 0.7%); this difference was statistically significant between April 2020 and April 2019 (4.1% vs. 0.9%, $p = 0.045$).

Conclusion: Supporting our hypothesis, the coronavirus disease 2019 pandemic and subsequent stay-at-home mandate resulted in decreased EGS admissions between March and May 2020 compared with prior years. During this time, there was also a statistically significant increase in mortality, which peaked at the height of COVID infection rates in our population.

Level of evidence: Epidemiological, level IV.

Copyright © 2021 Wolters Kluwer Health, Inc. All rights reserved.

Comment in

- Separate and unequal: Pandemic-related disparities in operating room access.
  Russo RM, Jurkovich GJ. Russo RM, et al. J Trauma Acute Care Surg. 2021 Oct 1;91 (4):e120-e121. doi: 10.1097/TA.0000000000003354. J Trauma Acute Care Surg. 2021. PMID: 34252064 Free PMC article. No abstract available.
- Response to "Separate and unequal: Pandemic-related disparities in operating room access".

Dong CT, Liveris A, Stone ME Jr. Dong CT, et al. J Trauma Acute Care Surg. 2021 Oct 1;91(4):e121. doi: 10.1097/TA.0000000000003353. J Trauma Acute Care Surg. 2021. PMID: 34252065 Free PMC article. No abstract available.

- 20 references
- 3 figures

Supplementary info

Publication types, MeSH terms Expand

Publication types

- Multicenter Study
- Observational Study

MeSH terms

- Acute Disease / mortality
- Acute Disease / therapy
- Adolescent
- Adult
- Aged
- Aged, 80 and over
- Appendicitis / diagnosis
- Appendicitis / mortality
- Appendicitis / surgery
- COVID-19 / diagnosis
- COVID-19 / epidemiology
- COVID-19 / prevention & control*
- COVID-19 / transmission
- Cholecystitis / diagnosis
- Cholecystitis / mortality
- Cholecystitis / surgery
- Emergencies / epidemiology*
- Emergency Service, Hospital
- Hernia, Inguinal / diagnosis
- Hernia, Inguinal / mortality
- Hernia, Inguinal / surgery
- Hernia, Ventral / diagnosis
- Hernia, Ventral / mortality
- Hernia, Ventral / surgery
- Hospital Mortality / trends*
- Humans

- Male
- Middle Aged
- Necrosis / diagnosis
- Necrosis / mortality
- Necrosis / surgery
- New York / epidemiology
- Pandemics / prevention & control
- Patient Admission / statistics & numerical data*
- Patient Admission / trends
- Peptic Ulcer / diagnosis
- Peptic Ulcer / mortality
- Peptic Ulcer / surgery
- Retrospective Studies
- SARS-CoV-2 / isolation & purification
- Soft Tissue Infections / diagnosis
- Soft Tissue Infections / mortality
- Soft Tissue Infections / surgery
- Time-to-Treatment / statistics & numerical data
- Time-to-Treatment / trends
- Young Adult

Full text links

Wolters Kluwer Wolters Kluwer Free PMC article
Proceed to details
Cite
Share
☐ 699
Observational Study
An Pediatr (Engl Ed)
. 2020 Nov;93(5):323-333.
doi: 10.1016/j.anpedi.2020.07.025. Epub 2020 Aug 31.

[Clinical spectrum and risk factors for complicated disease course in children admitted with SARS-CoV-2 infection]

[Article in Spanish]
Pilar Storch-de-Gracia [1], Inés Leoz-Gordillo [2], David Andina [3], Patricia Flores [4], Enrique Villalobos [4], Silvia Escalada-Pellitero [3], Raquel Jiménez [4]
Affiliations Expand

Affiliations

- [1] Servicio de Urgencias. Hospital Infantil Universitario Niño Jesús, Madrid, España. Electronic address: pilar.storchdegracia@salud.madrid.org.
- [2] Unidad de Cuidados Intensivos. Hospital Infantil Universitario Niño Jesús, Madrid, España.
- [3] Servicio de Urgencias. Hospital Infantil Universitario Niño Jesús, Madrid, España.
- [4] Servicio de Pediatría. Hospital Infantil Universitario Niño Jesús, Madrid, España.

- PMID: **32950434**
- PMCID: PMC7457908
- DOI: 10.1016/j.anpedi.2020.07.025

Free PMC article
Observational Study

[Clinical spectrum and risk factors for complicated disease course in children admitted with SARS-CoV-2 infection]

[Article in Spanish]
Pilar Storch-de-Gracia et al. An Pediatr (Engl Ed). 2020 Nov.
Free PMC article
Show details
An Pediatr (Engl Ed)
. 2020 Nov;93(5):323-333.
doi: 10.1016/j.anpedi.2020.07.025. Epub 2020 Aug 31.

Authors

Pilar Storch-de-Gracia [1], Inés Leoz-Gordillo [2], David Andina [3], Patricia Flores [4], Enrique Villalobos [4], Silvia Escalada-Pellitero [3], Raquel Jiménez [4]

Affiliations

- [1] Servicio de Urgencias. Hospital Infantil Universitario Niño Jesús, Madrid, España. Electronic address: pilar.storchdegracia@salud.madrid.org.
- [2] Unidad de Cuidados Intensivos. Hospital Infantil Universitario Niño Jesús, Madrid, España.
- [3] Servicio de Urgencias. Hospital Infantil Universitario Niño Jesús, Madrid, España.
- [4] Servicio de Pediatría. Hospital Infantil Universitario Niño Jesús, Madrid, España.

- PMID: **32950434**
- PMCID: PMC7457908
- DOI: 10.1016/j.anpedi.2020.07.025

Abstract

in English, Spanish

Introduction: At this time there are still major questions about the characteristics of disease caused by the new coronavirus (COVID-19) in children as well as factors associated with the development of severe forms of the disease.

Study design: Retrospective study including patients under 18 years of age admitted with SARS-CoV-2 infection from March 1 to April 30, 2020. Infection was confirmed by realtime reverse transcriptase-polymerase chain reaction (RT-PCR) or antibody testing. We describe the epidemiological and clinical data, laboratory and imaging findings, as well as treatment and outcome in these patients. In light of these findings, patients were classified into two severity groups and then compared.

Results: Thirty-nine children were included, with a median age of 9 years (range 12 days-16 years); 23 were boys. Cases with uncomplicated disease course (24) mostly presented to the emergency department (ED) with fever and/or respiratory symptoms without significant alterations in laboratory findings. Of the 15 children with a complicated course, 12 developed shock. In addition to fever, they frequently presented altered appearance, extreme tachycardia, abdominal pain, vomiting, diarrhea, rash, and/or conjunctival hyperemia. They also showed greater lymphopenia ($p = 0.001$), elevated neutrophil/lymphocyte ratio ($p = 0.001$), C-reactive protein ($p < 0.001$), procalcitonin ($p = 0.001$), D-dimer ($p < 0.001$), and ferritin ($p < 0.001$).

Conclusions: SARS-CoV-2 infection in admitted children presents with great clinical variability. When provided supportive care, patients with predominant respiratory symptoms without altered laboratory-test results generally have an uncomplicated course. Patients with complicated disease present mainly with fever and abdominal and/or mucocutaneous symptoms. Most develop shock. Elevation of inflammatory markers may allow for early detection and the final outcome is good.

Introducción: En este momento existen todavía grandes interrogantes acerca de las características de enfermedad causada por el nuevo coronavirus (COVID-19) en los niños, así como acerca de los factores asociados al desarrollo de formas graves de la enfermedad.

Métodos: Estudio retrospectivo que incluye pacientes menores de 18 años ingresados debido a infección por SARS-CoV-2. La infección fue confirmada por la reacción en cadena de la transcriptasa inversa-polimerasa (RT-PCR) en tiempo real o por serología. Describimos los datos epidemiológicos y clínicos, los hallazgos de laboratorio y de imágenes, así como el tratamiento y la evolución de estos pacientes. Los pacientes se clasificaron en dos grupos de gravedad y luego se compararon.

Se incluyeron 39 niños, con una mediana de edad de nueve años (rango 12 días-16 años); 23 eran varones. Los casos con evolución no complicada (24) se presentaron en su mayoría con fiebre y/o síntomas respiratorios sin alteraciones significativas en los hallazgos de laboratorio. De los 15 niños con enfermedad complicada, 12 desarrollaron *shock*. Además de la fiebre, frecuentemente presentaban alteraciones de la apariencia, taquicardia extrema, dolor abdominal, vómitos, diarrea, erupción cutánea y/o hiperemia conjuntival. También mostraron mayor linfopenia ($p = 0{,}001$), elevación de la proporción neutrófilos/linfocitos ($p = 0{,}001$), proteína C reactiva ($p < 0{,}001$), procalcitonina ($p = 0{,}001$), dímero D ($p < 0{,}001$) y ferritina ($p < 0{,}001$).

Conclusiones: La infección por SARS-CoV-2 en niños ingresados se presenta con una gran variabilidad clínica. Cuando se les proporciona tratamiento de soporte, los pacientes con síntomas respiratorios que no tienen alteración de las pruebas de laboratorio, generalmente tienen una enfermedad no complicada. Los pacientes con enfermedad complicada se presentan principalmente con fiebre y síntomas abdominales y/o mucocutáneos, la mayoría desarrollan un

shock. La elevación de los marcadores inflamatorios puede permitir una detección temprana y el pronóstico final es bueno.

Keywords: COVID-19; Coronavirus; Neumonía; Pneumonia; Shock.

Copyright © 2020 Asociación Española de Pediatría. Publicado por Elsevier España, S.L.U. All rights reserved.

- 35 references
- 1 figure

Supplementary info

Publication types, MeSH terms Expand

Publication types

- Observational Study

MeSH terms

- Adolescent
- Betacoronavirus* / isolation & purification
- COVID-19
- COVID-19 Testing
- Child
- Child, Preschool
- Clinical Laboratory Techniques
- Coronavirus Infections / complications*
- Coronavirus Infections / diagnosis*
- Coronavirus Infections / epidemiology
- Coronavirus Infections / therapy
- Female
- Hospitalization*
- Humans
- Infant
- Infant, Newborn
- Male
- Pandemics
- Pneumonia, Viral / complications*
- Pneumonia, Viral / diagnosis*
- Pneumonia, Viral / epidemiology
- Pneumonia, Viral / therapy
- Prognosis
- Retrospective Studies

- Risk Factors
- SARS-CoV-2
- Severity of Illness Index
- Spain / epidemiology

Full text links

ELSEVIER OPEN ACCESS Elsevier Science Free PMC article
Proceed to details
Cite
Share
700
Observational Study
Medicine (Baltimore)
. 2021 May 7;100(18):e25832.
doi: 10.1097/MD.0000000000025832.

Off-label tocilizumab and adjuvant iron chelator effectiveness in a group of severe COVID-19 pneumonia patients: A single center experience

Victoria Birlutiu [1], Rares Mircea Birlutiu [2], Liana Chicea [3]
Affiliations Expand

Affiliations

- [1] Lucian Blaga University of Sibiu, Faculty of Medicine Sibiu, Academic Emergency Hospital Sibiu - Infectious Diseases Clinic, Sibiu.
- [2] Lucian Blaga University of Sibiu, Faculty of Medicine Sibiu, FOISOR Clinical Hospital of Orthopedics, Traumatology, and Osteoarticular TB Bucharest.
- [3] Lucian Blaga University of Sibiu, Faculty of Medicine Sibiu, Academic Emergency Hospital Sibiu - Internal Medicine Clinic, Sibiu, Romania.

- PMID: **33950993**
- PMCID: PMC8104262
- DOI: 10.1097/MD.0000000000025832

Free PMC article
Observational Study

Off-label tocilizumab and adjuvant iron chelator effectiveness in a group of severe COVID-19 pneumonia patients: A single center experience

Victoria Birlutiu et al. Medicine (Baltimore). 2021.
Free PMC article
Show details
Medicine (Baltimore)
. 2021 May 7;100(18):e25832.
doi: 10.1097/MD.0000000000025832.

Authors

Victoria Birlutiu [1], Rares Mircea Birlutiu [2], Liana Chicea [3]

Affiliations

- [1] Lucian Blaga University of Sibiu, Faculty of Medicine Sibiu, Academic Emergency Hospital Sibiu - Infectious Diseases Clinic, Sibiu.
- [2] Lucian Blaga University of Sibiu, Faculty of Medicine Sibiu, FOISOR Clinical Hospital of Orthopedics, Traumatology, and Osteoarticular TB Bucharest.
- [3] Lucian Blaga University of Sibiu, Faculty of Medicine Sibiu, Academic Emergency Hospital Sibiu - Internal Medicine Clinic, Sibiu, Romania.

- PMID: **33950993**
- PMCID: PMC8104262
- DOI: 10.1097/MD.0000000000025832

Abstract

Tocilizumab (TCZ), a monoclonal recombinant antibody against IL-6 receptor, is currently used in managing the cytokine release syndrome (CRS) that occurred in coronavirus disease 2019 (COVID-19) selected cases. The primary objective of our study was to establish the effectiveness of TCZ in patients with severe or critical severe acute respiratory syndrome coronavirus-2 (SARS-CoV-2) pneumonia.We retrospectively analyzed 25 consecutive patients, admitted in the Academic Emergency Hospital Sibiu, Romania from April 1, 2020 until May 25, 2020, all with confirmed SARS-CoV-2 infection and severe pneumonia. All patients were treated off-label with TCZ, beside their standard care. Adjuvant iron chelator was associated in 11 patients.Six female and 19 male patients admitted in our hospital all with confirmed SARS-CoV-2 infection and severe pneumonia as defined by Chinese Centers for Disease Control and Prevention were enrolled in this study. Seventeen of the 25 enrolled patients (68%) were seriously ill requiring noninvasive ventilation or oxygen mask, and 8 cases (32%) were critically ill requiring invasive mechanical ventilation. All patients received TCZ, and also received hydroxychloroquine, and lopinavir/ritonavir 200/50 mg for 10 days. Adjuvant iron chelator (deferasirox - marketed as Exjade) was associated in 11 patients who had ferritin serum levels above 1000 ng/mL. No side

effects were encountered during infusions or after TCZ. We observed a rapid increase in arterial oxygen saturation for 20 of the 25 cases (80%) with a favorable evolution toward healing. Survivors were younger than 60 years old (80%), had less comorbidities (10% no comorbidities, 70% with 1 or 2 comorbidities), lower serum ferritin levels (30% under 1000 ng/mL), and 50% had no serum glucose elevation. Our patients with CRS had no response to corticosteroid therapy. Five out of the 25 patients had an unfavorable evolution to death. The off-label use of TCZ in patients with severe or critically ill form of SARS-CoV-2 infection had good results in our study.Off-label use of TCZ in severe and critical cases of COVID-19 pneumonia is effective in managing the "cytokine storm." Better outcomes were noted in younger patients. Associated adjuvant iron chelators may contribute to a good outcome and needs to be confirmed in larger studies.

Copyright © 2021 the Author(s). Published by Wolters Kluwer Health, Inc.

Conflict of interest statement

The authors declare no conflicts of interest.

- 35 references
- 3 figures

Supplementary info

Publication types, MeSH terms, Substances, Supplementary concepts, Grant support Expand

Publication types

- Observational Study

MeSH terms

- Adult
- Aged
- Aged, 80 and over
- Antibodies, Monoclonal, Humanized / therapeutic use*
- COVID-19 / drug therapy*
- Comorbidity
- Cytokine Release Syndrome / drug therapy*
- Deferasirox / therapeutic use*
- Drug Therapy, Combination
- Female
- Humans
- Iron Chelating Agents / therapeutic use*
- Male
- Middle Aged
- Off-Label Use

- Pneumonia, Viral / drug therapy*
- Pneumonia, Viral / virology
- Respiration, Artificial
- Retrospective Studies
- Romania
- SARS-CoV-2

Substances

- Antibodies, Monoclonal, Humanized
- Iron Chelating Agents
- tocilizumab
- Deferasirox

Supplementary concepts

- COVID-19 drug treatment

Grant support

- LBUS-IRG-2020-06/Lucian Blaga University of Sibiu & Hasso Plattner Foundation

Full text links

Wolters Kluwer Wolters Kluwer Free PMC article
Proceed to details
Cite
Share
☐ 701
Observational Study
Acta Biomed
. 2021 Nov 3;92(5):e2021365.
doi: 10.23750/abm.v92i5.11417.

COVID-19 respiratory support outside the ICU's doors. An observational study for a new operative strategy

Elena Bignami [1], Valentina Bellini [2], Giada Maspero [3], Barbara Pifferi [4], Leonardo Fortunati Rossi [5], Andrea Ticinesi [6], Michelangelo Craca [7], Tiziana Meschi [8], Marco Baciarello [9]
Affiliations Expand

Affiliations

- [1] Anesthesiology, Critical Care and Pain Medicine Division, Department of Medicine and Surgery, University of Parma, Viale Gramsci 14, 43126 Parma, Italy. elenagiovanna.bignami@unipr.it.
- [2] Anesthesiology, Critical Care and Pain Medicine Division, Department of Medicine and Surgery, University of Parma, Viale Gramsci 14, 43126 Parma, Italy. bellini.vnt@gmail.com.
- [3] Anesthesiology, Critical Care and Pain Medicine Division, Department of Medicine and Surgery, University of Parma, Viale Gramsci 14, 43126 Parma, Italy. gmaspero@ao.pr.it.
- [4] Anesthesiology, Critical Care and Pain Medicine Division, Department of Medicine and Surgery, University of Parma, Viale Gramsci 14, 43126 Parma, Italy. barbara.pifferi@gmail.com.
- [5] Anesthesiology, Critical Care and Pain Medicine Division, Department of Medicine and Surgery, University of Parma, Viale Gramsci 14, 43126 Parma, Italy. leonardo.fortunati@gmail.com.
- [6] Department of Medicine and Surgery Univeristy of Parma. aticinesi@ao.pr.it.
- [7] Anesthesiology, Critical Care and Pain Medicine Division, Department of Medicine and Surgery, University of Parma, Viale Gramsci 14, 43126 Parma, Italy. michecraca@gmail.com.
- [8] Department of Medicine and Surgery Univeristy of Parma. tiziana.meschi@unipr.it.
- [9] Anesthesiology, Critical Care and Pain Medicine Division, Department of Medicine and Surgery, University of Parma, Viale Gramsci 14, 43126 Parma, Italy. marco.baciarello@unipr.it.

- PMID: **34738575**
- PMCID: PMC8689321
- DOI: 10.23750/abm.v92i5.11417

Free PMC article
Observational Study

COVID-19 respiratory support outside the ICU's doors. An observational study for a new operative strategy

Elena Bignami et al. Acta Biomed. 2021.
Free PMC article

Show details

Acta Biomed
. 2021 Nov 3;92(5):e2021365.
doi: 10.23750/abm.v92i5.11417.

Authors

Elena Bignami [1], Valentina Bellini [2], Giada Maspero [3], Barbara Pifferi [4], Leonardo Fortunati Rossi [5], Andrea Ticinesi [6], Michelangelo Craca [7], Tiziana Meschi [8], Marco Baciarello [9]

Affiliations

- [1] Anesthesiology, Critical Care and Pain Medicine Division, Department of Medicine and Surgery, University of Parma, Viale Gramsci 14, 43126 Parma, Italy. elenagiovanna.bignami@unipr.it.
- [2] Anesthesiology, Critical Care and Pain Medicine Division, Department of Medicine and Surgery, University of Parma, Viale Gramsci 14, 43126 Parma, Italy. bellini.vnt@gmail.com.
- [3] Anesthesiology, Critical Care and Pain Medicine Division, Department of Medicine and Surgery, University of Parma, Viale Gramsci 14, 43126 Parma, Italy. gmaspero@ao.pr.it.
- [4] Anesthesiology, Critical Care and Pain Medicine Division, Department of Medicine and Surgery, University of Parma, Viale Gramsci 14, 43126 Parma, Italy. barbara.pifferi@gmail.com.
- [5] Anesthesiology, Critical Care and Pain Medicine Division, Department of Medicine and Surgery, University of Parma, Viale Gramsci 14, 43126 Parma, Italy. leonardo.fortunati@gmail.com.
- [6] Department of Medicine and Surgery Univeristy of Parma. aticinesi@ao.pr.it.
- [7] Anesthesiology, Critical Care and Pain Medicine Division, Department of Medicine and Surgery, University of Parma, Viale Gramsci 14, 43126 Parma, Italy. michecraca@gmail.com.
- [8] Department of Medicine and Surgery Univeristy of Parma. tiziana.meschi@unipr.it.
- [9] Anesthesiology, Critical Care and Pain Medicine Division, Department of Medicine and Surgery, University of Parma, Viale Gramsci 14, 43126 Parma, Italy. marco.baciarello@unipr.it.

- PMID: **34738575**
- PMCID: PMC8689321
- DOI: 10.23750/abm.v92i5.11417

Abstract

Background and aim: During the first wave of the Severe Acute Respiratory Syndrome CoronaVirus 2 (SARS-CoV-2) pandemic, we faced a massive clinical and organizational challenge having to manage critically ill patients outside the Intensive Care Unit (ICU). This was due to the significant imbalance between ICU bed availability and the number of patients presenting Acute Hypoxemic Respiratory Failure caused by SARS-CoV-2-related interstitial pneumonia. We therefore needed to perform Non-Invasive Ventilation (NIV) in non-intensive wards to assist these patients and relieve pressure on the ICUs and subsequently implemented a new organizational and clinical model. This study was aimed at evaluating its effectiveness and feasibility.

Methods: We recorded the anamnestic, clinical and biochemical data of patients undergoing non-invasive mechanical ventilation while hospitalized in non-intensive CoronaVirus Disease 19 (COVID-19) wards. Data were registered on admission, during anesthesiologist counseling, and when NIV was started and suspended. We retrospectively registered the available results from routine arterial blood gas and laboratory analyses for each time point.

Results: We retrospectively enrolled 231 patients. Based on our criteria, we identified 46 patients as NIV responders, representing 19.9% of the general study population and 29.3% of the patients that spent their entire hospital stay in non-ICU wards. Overall mortality was 56.2%, with no significant differences between patients in non-intensive wards (57.3%) and those later admitted

to the ICU (54%) Conclusions: NIV is safe and manageable in an emergency situation and could become part of an integrated clinical and organizational model.

Conflict of interest statement

Each author declares that he or she has no commercial associations (e.g. consultancies, stock ownership, equity interest, patent/licensing arrangement etc.) that might pose a conflict of interest in connection with the submitted article

- 37 references
- 4 figures

Supplementary info

Publication types, MeSH terms Expand

Publication types

- Observational Study

MeSH terms

- COVID-19*
- Humans
- Intensive Care Units
- Noninvasive Ventilation*
- Pandemics
- Respiration, Artificial
- Respiratory Insufficiency* / therapy
- SARS-CoV-2

Full text links

Free PMC article
Proceed to details
Cite
Share
☐ 702
Observational Study
J Am Heart Assoc
. 2021 Jun 15;10(12):e018451.
doi: 10.1161/JAHA.120.018451. Epub 2021 Jun 5.

Chronic Cardio-Metabolic Disease Increases the Risk of Worse Outcomes Among

Hospitalized Patients With COVID-19: A Multicenter, Retrospective, and Real-World Study

Qijian Chen [1], Lingling Wang [2] [3], Chang Li [4], Weihua Hu [5], Yameng Fan [6], Zaishu Chen [7], Longlong Wu [8], Zhanjin Lu [2], Jianfang Ye [2], Shiyan Chen [2], Junlu Tong [2], Liemin Ruan [9], Jin Mei [9] [10], Hongyun Lu [11]
Affiliations Expand

Affiliations

- [1] Department of Emergency the Fifth Hospital in Wuhan Wuhan Hubei China.
- [2] Department of Endocrinology & Metabolism the Fifth Affiliated Hospital of Sun Yat-sen University Zhuhai Guangdong China.
- [3] Department of Gerontology the Fifth Affiliated Hospital of Sun Yat-sen University Zhuhai Guangdong China.
- [4] Department of Cardiology Hubei No. 3 People's Hospital of Jianghan University Wuhan Hubei China.
- [5] Department of Respiratory Medicine the First Hospital of Jingzhou Clinical Medical College Yangtze University Jingzhou Hubei China.
- [6] School of Health Sciences Wuhan University Wuhan Hubei China.
- [7] People's Hospital of Jiayu County Xianning Hubei China.
- [8] People's Hospital of Nanzhang County Nanzhang Hubei China.
- [9] Central Laboratory Ningbo First Hospital of Zhejiang University Ningbo Zhejiang China.
- [10] Anatomy Department Wenzhou Medical UniversityWenzhou University Town Ningbo Zhejiang China.
- [11] Department of Endocrinology & Metabolism Zhuhai Hospital Affiliated with Jinan UniversityZhuhai People's Hospital Zhuhai Guangdong China.

- PMID: **34096317**
- PMCID: PMC8477891
- DOI: 10.1161/JAHA.120.018451

Free PMC article
Observational Study

Chronic Cardio-Metabolic Disease Increases the Risk of Worse Outcomes Among Hospitalized Patients With COVID-19: A Multicenter, Retrospective, and Real-World Study

Qijian Chen et al. J Am Heart Assoc. 2021.
Free PMC article
Show details
J Am Heart Assoc
. 2021 Jun 15;10(12):e018451.
doi: 10.1161/JAHA.120.018451. Epub 2021 Jun 5.

Authors

Qijian Chen [1], Lingling Wang [2] [3], Chang Li [4], Weihua Hu [5], Yameng Fan [6], Zaishu Chen [7], Longlong Wu [8], Zhanjin Lu [2], Jianfang Ye [2], Shiyan Chen [2], Junlu Tong [2], Liemin Ruan [9], Jin Mei [9] [10], Hongyun Lu [11]

Affiliations

- [1] Department of Emergency the Fifth Hospital in Wuhan Wuhan Hubei China.
- [2] Department of Endocrinology & Metabolism the Fifth Affiliated Hospital of Sun Yat-sen University Zhuhai Guangdong China.
- [3] Department of Gerontology the Fifth Affiliated Hospital of Sun Yat-sen University Zhuhai Guangdong China.
- [4] Department of Cardiology Hubei No. 3 People's Hospital of Jianghan University Wuhan Hubei China.
- [5] Department of Respiratory Medicine the First Hospital of Jingzhou Clinical Medical College Yangtze University Jingzhou Hubei China.
- [6] School of Health Sciences Wuhan University Wuhan Hubei China.
- [7] People's Hospital of Jiayu County Xianning Hubei China.
- [8] People's Hospital of Nanzhang County Nanzhang Hubei China.
- [9] Central Laboratory Ningbo First Hospital of Zhejiang University Ningbo Zhejiang China.
- [10] Anatomy Department Wenzhou Medical UniversityWenzhou University Town Ningbo Zhejiang China.
- [11] Department of Endocrinology & Metabolism Zhuhai Hospital Affiliated with Jinan UniversityZhuhai People's Hospital Zhuhai Guangdong China.

- PMID: **34096317**
- PMCID: PMC8477891
- DOI: 10.1161/JAHA.120.018451

Abstract

Background Although chronic cardio-metabolic disease is a common comorbidity among patients with COVID-19, its effects on the clinical characteristics and outcome are not well known. Methods and Results This study aimed to explore the association between underlying cardio-metabolic disease and mortality with COVID-19 among hospitalized patients. This multicenter, retrospective, and real-world study was conducted from January 22, 2020 to March 25, 2020 in China. Data between patients with and without 5 main cardio-metabolic diseases including hypertension, diabetes mellitus, coronary heart disease, cerebrovascular disease, and hyperlipidemia were compared. A total of 1303 hospitalized patients were included in the final analysis. Of them, 520 patients (39.9%) had cardio-metabolic disease. Compared with patients without cardio-metabolic disease, more patients with cardio-metabolic disease had COVID-related

complications including acute respiratory distress syndrome (9.81% versus 3.32%; *P*<0.001), acute kidney injury (4.23% versus 1.40%; *P*=0.001), secondary infection (13.9% versus 9.8%; *P*=0.026), hypoproteinemia (12.1% versus 5.75%; *P*<0.001), and coagulopathy (19.4% versus 10.3%; *P*<0.001), had higher incidences of the severe type of COVID-19 (32.9% versus 16.7%; *P*<0.001), more were admitted to the intensive care unit (11.7% versus 7.92%; *P*=0.021), and required mechanical ventilation (9.8% versus 4.3%; *P*<0.001). When the number of the patients' cardio-metabolic diseases was 0, 1, and >2, the mortality was 4.2%, 11.1%, and 19.8%, respectively. The multivariable-adjusted hazard ratio of mortality among patients with cardio-metabolic disease was 1.80 (95% CI, 1.17-2.77). Conclusions Cardio-metabolic disease was a common condition among hospitalized patients with COVID-19, and it was associated with higher risks of in-hospital mortality.

Keywords: COVID-19; SARS-CoV-2; cardio-metabolic disease; complications; in-hospital mortality; risk factors.

Conflict of interest statement

None.

- 28 references
- 3 figures

Supplementary info

Publication types, MeSH terms Expand

Publication types

- Multicenter Study
- Observational Study
- Research Support, Non-U.S. Gov't

MeSH terms

- Adult
- Aged
- COVID-19 / complications*
- COVID-19 / diagnosis
- COVID-19 / mortality
- COVID-19 / therapy
- China
- Chronic Disease
- Comorbidity
- Disease Progression
- Female
- Hospital Mortality
- Hospitalization*

- Humans
- Incidence
- Male
- Metabolic Syndrome / complications*
- Metabolic Syndrome / diagnosis
- Metabolic Syndrome / mortality
- Metabolic Syndrome / therapy
- Middle Aged
- Prognosis
- Retrospective Studies
- Risk Assessment
- Risk Factors
- Severity of Illness Index
- Time Factors

Full text links

OPEN ACCESS Atypon Free PMC article
Proceed to details
Cite
Share
703
Observational Study
Int J Cardiol
. 2020 Jul 15;311:116-121.
doi: 10.1016/j.ijcard.2020.03.087. Epub 2020 Apr 8.

Suspected myocardial injury in patients with COVID-19: Evidence from front-line clinical observation in Wuhan, China

Qing Deng [1], Bo Hu [1], Yao Zhang [1], Hao Wang [1], Xiaoyang Zhou [2], Wei Hu [1], Yuting Cheng [1], Jie Yan [2], Haiqin Ping [2], Qing Zhou [3]
Affiliations Expand

Affiliations

- [1] Echo Lab, Department of Ultrasound Imaging, Renmin Hospital of Wuhan University, Wuhan 430060, China.
- [2] Department of Cardiology, Renmin Hospital of Wuhan University, Wuhan 430060, China.
- [3] Echo Lab, Department of Ultrasound Imaging, Renmin Hospital of Wuhan University, Wuhan 430060, China. Electronic address: qingzhou.wh.edu@hotmail.com.

- PMID: **32291207**
- PMCID: PMC7141178
- DOI: 10.1016/j.ijcard.2020.03.087

Free PMC article
Observational Study

Suspected myocardial injury in patients with COVID-19: Evidence from front-line clinical observation in Wuhan, China

Qing Deng et al. Int J Cardiol. 2020.
Free PMC article
Show details
Int J Cardiol
. 2020 Jul 15;311:116-121.
doi: 10.1016/j.ijcard.2020.03.087. Epub 2020 Apr 8.

Authors

Qing Deng [1], Bo Hu [1], Yao Zhang [1], Hao Wang [1], Xiaoyang Zhou [2], Wei Hu [1], Yuting Cheng [1], Jie Yan [2], Haiqin Ping [2], Qing Zhou [3]

Affiliations

- [1] Echo Lab, Department of Ultrasound Imaging, Renmin Hospital of Wuhan University, Wuhan 430060, China.
- [2] Department of Cardiology, Renmin Hospital of Wuhan University, Wuhan 430060, China.
- [3] Echo Lab, Department of Ultrasound Imaging, Renmin Hospital of Wuhan University, Wuhan 430060, China. Electronic address: qingzhou.wh.edu@hotmail.com.

- PMID: **32291207**
- PMCID: PMC7141178
- DOI: 10.1016/j.ijcard.2020.03.087

Abstract

Background: A novel coronavirus disease (COVID-19) in Wuhan has caused an outbreak and become a major public health issue in China and great concern from international community. Myocarditis and myocardial injury were suspected and may even be considered as one of the leading causes for death of COVID-19 patients. Therefore, we focused on the condition of the heart, and sought to provide firsthand evidence for whether myocarditis and myocardial injury were caused by COVID-19.

Methods: We enrolled patients with confirmed diagnosis of COVID-19 retrospectively and collected heart-related clinical data, mainly including cardiac imaging findings, laboratory results

and clinical outcomes. Serial tests of cardiac markers were traced for the analysis of potential myocardial injury/myocarditis.

Results: 112 COVID-19 patients were enrolled in our study. There was evidence of myocardial injury in COVID-19 patients and 14 (12.5%) patients had presented abnormalities similar to myocarditis. Most of patients had normal levels of troponin at admission, that in 42 (37.5%) patients increased during hospitalization, especially in those that died. Troponin levels were significantly increased in the week preceding the death. 15 (13.4%) patients have presented signs of pulmonary hypertension. Typical signs of myocarditis were absent on echocardiography and electrocardiogram.

Conclusions: The clinical evidence in our study suggested that myocardial injury is more likely related to systemic consequences rather than direct damage by the 2019 novel coronavirus. The elevation in cardiac markers was probably due to secondary and systemic consequences and can be considered as the warning sign for recent adverse clinical outcomes of the patients.

Keywords: COVID-19; Cardiac marker; Myocardial injury; Myocarditis; Novel coronavirus.

Copyright © 2020 Elsevier B.V. All rights reserved.

Conflict of interest statement

Declaration of competing interest The authors report no relationships that could be construed as a conflict of interest.

Comment in

- SARS-CoV-2 inflames the heart. The importance of awareness of myocardial injury in COVID-19 patients.
  Ammirati E, Wang DW. Ammirati E, et al. Int J Cardiol. 2020 Jul 15;311:122-123. doi: 10.1016/j.ijcard.2020.03.086. Epub 2020 Apr 6. Int J Cardiol. 2020. PMID: 32276774 Free PMC article. No abstract available.
- Cardiac involvement of COVID-19: Looking forward to novel discoveries and clinically valuable evidence.
  Hu B, Deng Q, Zhou Q. Hu B, et al. Int J Cardiol. 2020 Sep 1;314:95. doi: 10.1016/j.ijcard.2020.05.049. Int J Cardiol. 2020. PMID: 32560765 Free PMC article. No abstract available.
- COVID-19 myocardial injury: We have much more to discover.
  Vrachatis DA, Giotaki SG, Giannopoulos G. Vrachatis DA, et al. Int J Cardiol. 2020 Sep 1;314:96. doi: 10.1016/j.ijcard.2020.05.003. Int J Cardiol. 2020. PMID: 32560766 Free PMC article. No abstract available.

- 25 references
- 1 figure

Supplementary info

Publication types, MeSH terms, Substances Expand

Publication types

- Observational Study

- Research Support, Non-U.S. Gov't

MeSH terms

- Adult
- Biomarkers / blood
- COVID-19
- Cause of Death*
- China
- Cohort Studies
- Comorbidity
- Coronary Angiography / methods
- Coronavirus Infections / diagnosis*
- Coronavirus Infections / epidemiology*
- Echocardiography, Doppler / methods
- Female
- Hospitalization / statistics & numerical data*
- Hospitals, University
- Humans
- Male
- Middle Aged
- Myocardial Infarction / diagnosis
- Myocardial Infarction / epidemiology*
- Myocardial Infarction / therapy
- Myocarditis / diagnosis
- Myocarditis / epidemiology*
- Myocarditis / therapy
- Pandemics
- Pneumonia, Viral / diagnosis*
- Pneumonia, Viral / epidemiology*
- Prognosis
- Retrospective Studies
- Risk Assessment
- Severity of Illness Index
- Survival Analysis
- Treatment Outcome

Substances

- Biomarkers

Full text links

ELSEVIER FULL-TEXT ARTICLE Elsevier Science Free PMC article

Proceed to details

Cite

Share

☐ 704

Observational Study

PLoS One

. 2021 Sep 14;16(9):e0256903.
doi: 10.1371/journal.pone.0256903. eCollection 2021.

Lack of effect on in-hospital mortality of drugs used during COVID-19 pandemic: Findings of the retrospective multicenter COVOCA study

Pia Clara Pafundi [1], Raffaele Galiero [1], Vittorio Simeon [2], Luca Rinaldi [1], Alessandro Perrella [3], Erica Vetrano [1], Alfredo Caturano [1], Maria Alfano [1], Domenico Beccia [1], Riccardo Nevola [1] [4], Raffaele Marfella [1], Celestino Sardu [1], Carmine Coppola [5], Ferdinando Scarano [5], Paolo Maggi [6], Pellegrino De Lucia Sposito [7], Laura Vocciante [8], Carolina Rescigno [9], Costanza Sbreglia [10], Fiorentino Fraganza [11], Roberto Parrella [12], Annamaria Romano [13], Giosuele Calabria [14], Benedetto Polverino [15], Antonio Pagano [16], Carolina Bologna [17], Maria Amitrano [18], Vincenzo Esposito [19], Nicola Coppola [20], Nicola Maturo [21], Luigi Elio Adinolfi [1], Paolo Chiodini [2], Ferdinando Carlo Sasso [1], COVOCA Study Group

Affiliations Expand

Affiliations

- [1] Department of Advanced Medical and Surgical Sciences, University of Campania "Luigi Vanvitelli", Naples, Italy.
- [2] Medical Statistics Unit, Department of Physical and Mental Health and Preventive Medicine, University of Campania "Luigi Vanvitelli", Naples, Italy.
- [3] Task Force Covid-19 Regione Campania, Italy.
- [4] Internal Medicine, Sant'Ottone Frangipane Hospital, ASL Avellino, Ariano Irpino (AV), Italy.
- [5] COVID Center "S. Anna e SS. Madonna della Neve" Hospital, Boscotrecase, Italy.
- [6] U.O.C. Infectious and Tropical diseases, S. Anna e S. Sebastiano Hospital, Caserta, Italy.
- [7] Covid Center - Maddaloni Hospital, Maddaloni, Italy.
- [8] General Medicine Unit, Loreto Mare Hospital, Naples, Italy.
- [9] U.O.C. Infectious Diseases and Neurology, Cotugno Hospital, Naples, Italy.
- [10] U.O.C. Infectious Diseases of the Elderly, Cotugno Hospital, Naples, Italy.
- [11] U.O.C. Anestesia and Intensive Care Unit, Cotugno Hospital, Naples, Italy.

- [12] U.O.C. Respiratory Infectious Diseases, Cotugno Hospital, Naples, Italy.
- [13] U.O.C. Pneumology, Moscati Hospital, Avellino, Italy.
- [14] IXth Division of Infectious Diseases and Interventional Ultrasound, Cotugno Hospital, Naples, Italy.
- [15] "Giovanni da Procida" Hospital, Salerno, Italy.
- [16] Emergency and Acceptance Unit, "Santa Maria delle Grazie" Hospital, Pozzuoli, Italy.
- [17] Internal Medicine Unit, Ospedale Del Mare, Naples, Italy.
- [18] U.O.C. Internal Medicine - Moscati Hospital, Avellino, Italy.
- [19] IVth Division of Immunodeficiency and Gender Infectious Diseases, Cotugno Hospital, Naples, Italy.
- [20] Department of Mental Health and Public Medicine, Centro COVID A.O.U. Vanvitelli, Naples, Italy.
- [21] U.O.S.D. Infectious Diseases Emergency and Acceptance, Cotugno Hospital, Naples, Italy.

- PMID: **34520465**
- PMCID: PMC8439483
- DOI: 10.1371/journal.pone.0256903

Free PMC article
Observational Study

Lack of effect on in-hospital mortality of drugs used during COVID-19 pandemic: Findings of the retrospective multicenter COVOCA study

Pia Clara Pafundi et al. PLoS One. 2021.
Free PMC article
Show details
PLoS One

. 2021 Sep 14;16(9):e0256903.
doi: 10.1371/journal.pone.0256903. eCollection 2021.

Authors

Pia Clara Pafundi [1], Raffaele Galiero [1], Vittorio Simeon [2], Luca Rinaldi [1], Alessandro Perrella [3], Erica Vetrano [1], Alfredo Caturano [1], Maria Alfano [1], Domenico Beccia [1], Riccardo Nevola [1] [4], Raffaele Marfella [1], Celestino Sardu [1], Carmine Coppola [5], Ferdinando Scarano [5], Paolo Maggi [6], Pellegrino De Lucia Sposito [7], Laura Vocciante [8], Carolina Rescigno [9], Costanza Sbreglia [10], Fiorentino Fraganza [11], Roberto Parrella [12], Annamaria Romano [13], Giosuele Calabria [14], Benedetto Polverino [15], Antonio Pagano [16], Carolina Bologna [17], Maria Amitrano [18], Vincenzo Esposito [19], Nicola Coppola [20], Nicola Maturo [21], Luigi Elio Adinolfi [1], Paolo Chiodini [2], Ferdinando Carlo Sasso [1], COVOCA Study Group

Affiliations

- [1] Department of Advanced Medical and Surgical Sciences, University of Campania "Luigi Vanvitelli", Naples, Italy.
- [2] Medical Statistics Unit, Department of Physical and Mental Health and Preventive Medicine, University of Campania "Luigi Vanvitelli", Naples, Italy.
- [3] Task Force Covid-19 Regione Campania, Italy.
- [4] Internal Medicine, Sant'Ottone Frangipane Hospital, ASL Avellino, Ariano Irpino (AV), Italy.
- [5] COVID Center "S. Anna e SS. Madonna della Neve" Hospital, Boscotrecase, Italy.
- [6] U.O.C. Infectious and Tropical diseases, S. Anna e S. Sebastiano Hospital, Caserta, Italy.
- [7] Covid Center - Maddaloni Hospital, Maddaloni, Italy.
- [8] General Medicine Unit, Loreto Mare Hospital, Naples, Italy.
- [9] U.O.C. Infectious Diseases and Neurology, Cotugno Hospital, Naples, Italy.
- [10] U.O.C. Infectious Diseases of the Elderly, Cotugno Hospital, Naples, Italy.
- [11] U.O.C. Anestesia and Intensive Care Unit, Cotugno Hospital, Naples, Italy.
- [12] U.O.C. Respiratory Infectious Diseases, Cotugno Hospital, Naples, Italy.
- [13] U.O.C. Pneumology, Moscati Hospital, Avellino, Italy.
- [14] IXth Division of Infectious Diseases and Interventional Ultrasound, Cotugno Hospital, Naples, Italy.
- [15] "Giovanni da Procida" Hospital, Salerno, Italy.
- [16] Emergency and Acceptance Unit, "Santa Maria delle Grazie" Hospital, Pozzuoli, Italy.
- [17] Internal Medicine Unit, Ospedale Del Mare, Naples, Italy.
- [18] U.O.C. Internal Medicine - Moscati Hospital, Avellino, Italy.
- [19] IVth Division of Immunodeficiency and Gender Infectious Diseases, Cotugno Hospital, Naples, Italy.
- [20] Department of Mental Health and Public Medicine, Centro COVID A.O.U. Vanvitelli, Naples, Italy.
- [21] U.O.S.D. Infectious Diseases Emergency and Acceptance, Cotugno Hospital, Naples, Italy.

- PMID: **34520465**
- PMCID: PMC8439483
- DOI: 10.1371/journal.pone.0256903

Abstract

Introduction: During COVID-19 pandemic, the use of several drugs has represented the worldwide clinical practice. However, though the current increase of knowledge about the disease, there is still no effective treatment for the usage of drugs. Thus, we retrospectively assessed use and effects of therapeutic regimens in hospitalized patients on in-hospital mortality.

Methods: COVOCA is a retrospective observational cohort study on 18 COVID centres throughout Campania Region Hospitals. We included adult patients with confirmed SARS-CoV-2 infection, discharged/dead between March/June 2020.

Results: 618 patients were included, with an overall in-hospital cumulative mortality incidence of 23.1%. Most prescribed early treatments were antivirals (72%), antibiotics (65%) and hydroxychloroquine/anticoagulants (≈50%). Tocilizumab, indeed, was largely prescribed late

during hospitalization. Multivariable models, with a cut-off at day 2 for early COVID-19 therapy administration, did not disclose any significant association of a single drug administration on the clinical outcome.

Discussion: COVOCA represents the first multicenter database in Campania region. None drug class used during the pandemic significantly modified the outcome, regardless of therapy beginning, both overall and net of those already in non-invasive ventilation (NIV)/ orotracheal intubation (OTI) at hospitalization. Our cumulative incidence of mortality seems lower than other described during the same period, particularly in Northern Italy.

Conflict of interest statement

The authors have declared that no competing interests exist.

- 27 references
- 2 figures

Supplementary info

Publication types, MeSH terms, Substances, Grant support Expand

Publication types

- Multicenter Study
- Observational Study

MeSH terms

- Aged
- Antiviral Agents / therapeutic use*
- COVID-19 / drug therapy*
- COVID-19 / epidemiology
- COVID-19 / mortality*
- Female
- Hospital Mortality
- Humans
- Italy / epidemiology
- Male
- Middle Aged
- Pandemics
- Respiratory Therapy
- Retrospective Studies

Substances

- Antiviral Agents

Grant support

The funders had no role in study design, data collection and analysis, decision to publish, or preparation of the manuscript. The authors received no specific funding for this work.

Full text links

OPEN ACCESS TO FULL TEXT PLOS ONE Public Library of Science Free PMC article
Proceed to details
Cite
Share
705
Observational Study
West J Emerg Med
. 2021 Sep 2;22(5):1060-1066.
doi: 10.5811/westjem.2021.5.49968.

Impact of COVID-19 and Shelter in Place on Volume and Type of Traumatic Injuries

James Murrett[1], Emily Fu[1], Zoe Maher[2], Crystal Bae[1], Wayne A Satz[1], Kraftin E Schreyer[1]
Affiliations Expand

Affiliations

- [1] Temple University Hospital, Department of Emergency Medicine, Philadelphia, Pennsylvania.
- [2] Temple University Hospital, Department of Surgery, Philadelphia, Pennsylvania.

- PMID: **34546881**
- PMCID: PMC8463046
- DOI: 10.5811/westjem.2021.5.49968

Free PMC article
Observational Study

Impact of COVID-19 and Shelter in Place on Volume and Type of Traumatic Injuries

James Murrett et al. West J Emerg Med. 2021.
Free PMC article
Show details
West J Emerg Med
. 2021 Sep 2;22(5):1060-1066.
doi: 10.5811/westjem.2021.5.49968.

Authors

James Murrett[1], Emily Fu[1], Zoe Maher[2], Crystal Bae[1], Wayne A Satz[1], Kraftin E Schreyer[1]

Affiliations

- [1] Temple University Hospital, Department of Emergency Medicine, Philadelphia, Pennsylvania.
- [2] Temple University Hospital, Department of Surgery, Philadelphia, Pennsylvania.

- PMID: **34546881**
- PMCID: PMC8463046
- DOI: 10.5811/westjem.2021.5.49968

Abstract

Introduction: Very little is known about the effects of the novel coronavirus (COVID-19) pandemic and its associated social distancing practices on trauma presentations to the emergency department (ED). This study aims to assess the impact of a city-wide stay at home order on the volume, type, and outcomes of traumatic injuries at urban EDs.

Methods: The study was a retrospective chart review of all patients who presented to the ED of an urban Level I Trauma Center and its urban community affiliate in the time period during the 30 days before the institution of city-wide shelter-in-place (preSIP) order and 60 days after the shelter-in-place (SIP) order and the date-matched time periods in the preceding year. Volume and mechanism of traumatic injuries were compared using paired T-tests.

Results: There was a significant decrease in overall ED volume. The volume of certain blunt trauma presentations (motor vehicle collisions) during the first 60 days of SIP compared to the same period from the year prior also significantly decreased. Importantly, the volume of penetrating injuries, including gunshot wounds and stab wounds, did not differ for the preSIP and SIP periods when compared to the prior year. The mortality of traumatic injuries was also unchanged during the SIP comparison period.

Conclusion: While there were significant decreases in visits to the ED and overall trauma volume, penetrating trauma, including gun violence, and other severe traumatic injuries remain a public health crisis that affects urban communities despite social distancing recommendations enacted during the COVID-19 pandemic.

Conflict of interest statement

Conflicts of Interest: By the WestJEM article submission agreement, all authors are required to disclose all affiliations, funding sources and financial or management relationships that could be perceived as potential sources of bias. No author has professional or financial relationships with any companies that are relevant to this study. There are no conflicts of interest or sources of funding to declare.

- 35 references
- 1 figure

Supplementary info

Publication types, MeSH terms Expand

Publication types

- Observational Study

MeSH terms

- COVID-19 / epidemiology
- COVID-19 / psychology*
- Emergency Service, Hospital / statistics & numerical data*
- Emergency Service, Hospital / trends
- Humans
- Pandemics / prevention & control*
- Quarantine*
- Retrospective Studies
- SARS-CoV-2
- Trauma Centers / statistics & numerical data*
- Urban Population
- Wounds and Injuries / epidemiology*

Full text links

Free PMC article
Proceed to details
Cite
Share
☐ 706
Observational Study
Am J Emerg Med
. 2021 Dec;50:22-26.
doi: 10.1016/j.ajem.2021.07.010. Epub 2021 Jul 9.

Patterns of Emergency Department visits for acute and chronic diseases during the two pandemic waves in Italy

Davide Golinelli [1], Francesca Campinoti [2], Francesco Sanmarchi [3], Simona Rosa [4], Michelle Beleffi [5], Gabriele Farina [6], Andrea Tampieri [7], Maria Pia Fantini [8], Fabrizio Giostra [9], Luca Santi [10]

Affiliations Expand

Affiliations

- [1] Department of Biomedical and Neuromotor Sciences (DIBINEM), Alma Mater Studiorum - University of Bologna, Via San Giacomo 12, 40126 Bologna, Italy. Electronic address: davide.golinelli@unibo.it.
- [2] Department of Emergency, Unità operativa di Medicina d'urgenza e Pronto Soccorso, Policlinico S. Orsola-Malpighi. Via Giuseppe Massarenti, 9, 40138 Bologna, Italy. Electronic address: campinotifrancesca@gmail.com.
- [3] Ospedale S. Maria della Scaletta, Dipartimento di Emergenza-urgenza e Accettazione, Unità Operativa di Pronto Soccorso e Medicina d'Urgenza, Viale Amendola, 2, 40026 Imola, Italy. Electronic address: francesco.sanmarchi@gmail.com.
- [4] Department of Biomedical and Neuromotor Sciences (DIBINEM), Alma Mater Studiorum - University of Bologna, Via San Giacomo 12, 40126 Bologna, Italy. Electronic address: simona.rosa@unibo.it.
- [5] Department of Emergency, Unità operativa di Medicina d'urgenza e Pronto Soccorso, Policlinico S. Orsola-Malpighi. Via Giuseppe Massarenti, 9, 40138 Bologna, Italy.
- [6] Department of Emergency, Unità operativa di Medicina d'urgenza e Pronto Soccorso, Policlinico S. Orsola-Malpighi. Via Giuseppe Massarenti, 9, 40138 Bologna, Italy. Electronic address: gabriele.farina@aosp.bo.it.
- [7] Ospedale S. Maria della Scaletta, Dipartimento di Emergenza-urgenza e Accettazione, Unità Operativa di Pronto Soccorso e Medicina d'Urgenza, Viale Amendola, 2, 40026 Imola, Italy. Electronic address: a.tampieri@ausl.imola.bo.it.
- [8] Department of Biomedical and Neuromotor Sciences (DIBINEM), Alma Mater Studiorum - University of Bologna, Via San Giacomo 12, 40126 Bologna, Italy. Electronic address: mariapia.fantini@unibo.it.
- [9] Department of Emergency, Unità operativa di Medicina d'urgenza e Pronto Soccorso, Policlinico S. Orsola-Malpighi. Via Giuseppe Massarenti, 9, 40138 Bologna, Italy. Electronic address: fabrizio.giostra@aosp.bo.it.
- [10] Department of Emergency, Unità operativa di Medicina d'urgenza e Pronto Soccorso, Policlinico S. Orsola-Malpighi. Via Giuseppe Massarenti, 9, 40138 Bologna, Italy. Electronic address: luca.santi@aosp.bo.it.

- PMID: **34271231**
- DOI: 10.1016/j.ajem.2021.07.010

Observational Study

Patterns of Emergency Department visits for acute and chronic diseases during the two pandemic waves in Italy

Davide Golinelli et al. Am J Emerg Med. 2021 Dec.
Show details
Am J Emerg Med
. 2021 Dec;50:22-26.

doi: 10.1016/j.ajem.2021.07.010. Epub 2021 Jul 9.

Authors

Davide Golinelli [1], Francesca Campinoti [2], Francesco Sanmarchi [3], Simona Rosa [4], Michelle Beleffi [5], Gabriele Farina [6], Andrea Tampieri [7], Maria Pia Fantini [8], Fabrizio Giostra [9], Luca Santi [10]

Affiliations

- [1] Department of Biomedical and Neuromotor Sciences (DIBINEM), Alma Mater Studiorum - University of Bologna, Via San Giacomo 12, 40126 Bologna, Italy. Electronic address: davide.golinelli@unibo.it.
- [2] Department of Emergency, Unità operativa di Medicina d'urgenza e Pronto Soccorso, Policlinico S. Orsola-Malpighi. Via Giuseppe Massarenti, 9, 40138 Bologna, Italy. Electronic address: campinotifrancesca@gmail.com.
- [3] Ospedale S. Maria della Scaletta, Dipartimento di Emergenza-urgenza e Accettazione, Unità Operativa di Pronto Soccorso e Medicina d'Urgenza, Viale Amendola, 2, 40026 Imola, Italy. Electronic address: francesco.sanmarchi@gmail.com.
- [4] Department of Biomedical and Neuromotor Sciences (DIBINEM), Alma Mater Studiorum - University of Bologna, Via San Giacomo 12, 40126 Bologna, Italy. Electronic address: simona.rosa@unibo.it.
- [5] Department of Emergency, Unità operativa di Medicina d'urgenza e Pronto Soccorso, Policlinico S. Orsola-Malpighi. Via Giuseppe Massarenti, 9, 40138 Bologna, Italy.
- [6] Department of Emergency, Unità operativa di Medicina d'urgenza e Pronto Soccorso, Policlinico S. Orsola-Malpighi. Via Giuseppe Massarenti, 9, 40138 Bologna, Italy. Electronic address: gabriele.farina@aosp.bo.it.
- [7] Ospedale S. Maria della Scaletta, Dipartimento di Emergenza-urgenza e Accettazione, Unità Operativa di Pronto Soccorso e Medicina d'Urgenza, Viale Amendola, 2, 40026 Imola, Italy. Electronic address: a.tampieri@ausl.imola.bo.it.
- [8] Department of Biomedical and Neuromotor Sciences (DIBINEM), Alma Mater Studiorum - University of Bologna, Via San Giacomo 12, 40126 Bologna, Italy. Electronic address: mariapia.fantini@unibo.it.
- [9] Department of Emergency, Unità operativa di Medicina d'urgenza e Pronto Soccorso, Policlinico S. Orsola-Malpighi. Via Giuseppe Massarenti, 9, 40138 Bologna, Italy. Electronic address: fabrizio.giostra@aosp.bo.it.
- [10] Department of Emergency, Unità operativa di Medicina d'urgenza e Pronto Soccorso, Policlinico S. Orsola-Malpighi. Via Giuseppe Massarenti, 9, 40138 Bologna, Italy. Electronic address: luca.santi@aosp.bo.it.

- PMID: **34271231**
- DOI: 10.1016/j.ajem.2021.07.010

Abstract

Background: Evidence is lacking about the impact of subsequent COVID-19 pandemic waves on Emergency Departments (ED). We analyzed the differences in patterns of ED visits in Italy during the two pandemic waves, focusing on changes in accesses for acute and chronic diseases.

Methods: We conducted a retrospective study using data from a metropolitan area in northern Italy that includes twelve ED. We analyzed weekly trends in non-COVID-19 ED visits during the first (FW) and second wave (SW) of the pandemic. Incidence rate ratios (IRRs) of triage codes, patient destination, and cause-specific ED visits in the FW and SW of the year 2020 vs. 2019 were estimated using Poisson regression models.

Main findings: We found a significant decrease of ED visits by triage code, which was more marked for low priority codes and during the FW. We found an increased share of hospitalizations compared to home discharges both in the FW and in the SW. ED visits for acute and chronic conditions decreased during the FW, ranging, from -70% for injuries (IRR = 0.2862, $p < 0.001$) to -50% and - 60% for ischemic heart disease and heart failure.

Conclusions: The two pandemic waves led to a selection of patients with higher and more urgent needs of acute hospital care. These findings should lead to investigate how to improve systems' capacity to manage changes in population needs.

Keywords: Acute diseases; COVID-19; Chronic diseases; ED visits; Emergency departments; Pandemic.

Copyright © 2021 Elsevier Inc. All rights reserved.

Supplementary info

Publication types, MeSH terms Expand

Publication types

- Observational Study

MeSH terms

- Acute Disease
- Adolescent
- Adult
- Aged
- COVID-19 / epidemiology*
- Child
- Child, Preschool
- Chronic Disease
- Cross-Sectional Studies
- Emergency Service, Hospital / statistics & numerical data*
- Facilities and Services Utilization
- Female
- Hospitalization / statistics & numerical data
- Humans
- Infant
- Infant, Newborn

- Italy / epidemiology
- Male
- Middle Aged
- Retrospective Studies
- Young Adult

Full text links

ELSEVIER FULL-TEXT ARTICLE Elsevier Science

Proceed to details

Cite

Share

707

Observational Study

Can J Cardiol

. 2020 Jul;36(7):1152-1155.

doi: 10.1016/j.cjca.2020.05.023. Epub 2020 May 22.

Decrease and Delay in Hospitalization for Acute Coronary Syndromes During the 2020 SARS-CoV-2 Pandemic

Gioel Gabrio Secco [1], Chiara Zocchi [2], Rosario Parisi [3], Annalisa Roveta [4], Francesca Mirabella [3], Matteo Vercellino [5], Gianfranco Pistis [5], Maurizio Reale [5], Silvia Maggio [5], Andrea Audo [6], Daniela Kozel [4], Giacomo Centini [4], Antonio Maconi [4], Carlo Di Mario [2]

Affiliations Expand

Affiliations

- [1] Department of Cardiology, Interventional Cardiology and Cardiac Surgery, Azienda Ospedaliera SS, Antonio e Biagio e Cesare Arrigo, Alessandria, Italy; Department of Cardiology, Azienda Ospedaliera SS Antonio e Biagio e Cesare Arrigo, Alessandria, Italy. Electronic address: gioel.gabrio.secco@gmail.com.
- [2] Structural Interventional Cardiology, Azienda Ospedaliero-Universitaria Careggi, Florence, Italy.
- [3] Department of Cardiology, Azienda Ospedaliera Ospedali Riuniti Marche Nord, Pesaro, Italy.
- [4] IRFI, Azienda Ospedaliera SS Antonio e Biagio e Cesare Arrigo, Alessandria, Italy.
- [5] Department of Cardiology, Azienda Ospedaliera SS Antonio e Biagio e Cesare Arrigo, Alessandria, Italy.
- [6] Department of Cardiology, Interventional Cardiology and Cardiac Surgery, Azienda Ospedaliera SS, Antonio e Biagio e Cesare Arrigo, Alessandria, Italy.

- PMID: **32447060**
- PMCID: PMC7242185

- DOI: 10.1016/j.cjca.2020.05.023

Free PMC article
Observational Study

Decrease and Delay in Hospitalization for Acute Coronary Syndromes During the 2020 SARS-CoV-2 Pandemic

Gioel Gabrio Secco et al. Can J Cardiol. 2020 Jul.
Free PMC article
Show details
Can J Cardiol
. 2020 Jul;36(7):1152-1155.
doi: 10.1016/j.cjca.2020.05.023. Epub 2020 May 22.

Authors

Gioel Gabrio Secco [1], Chiara Zocchi [2], Rosario Parisi [3], Annalisa Roveta [4], Francesca Mirabella [3], Matteo Vercellino [5], Gianfranco Pistis [5], Maurizio Reale [5], Silvia Maggio [5], Andrea Audo [6], Daniela Kozel [4], Giacomo Centini [4], Antonio Maconi [4], Carlo Di Mario [2]

Affiliations

- [1] Department of Cardiology, Interventional Cardiology and Cardiac Surgery, Azienda Ospedaliera SS, Antonio e Biagio e Cesare Arrigo, Alessandria, Italy; Department of Cardiology, Azienda Ospedaliera SS Antonio e Biagio e Cesare Arrigo, Alessandria, Italy. Electronic address: gioel.gabrio.secco@gmail.com.
- [2] Structural Interventional Cardiology, Azienda Ospedaliero-Universitaria Careggi, Florence, Italy.
- [3] Department of Cardiology, Azienda Ospedaliera Ospedali Riuniti Marche Nord, Pesaro, Italy.
- [4] IRFI, Azienda Ospedaliera SS Antonio e Biagio e Cesare Arrigo, Alessandria, Italy.
- [5] Department of Cardiology, Azienda Ospedaliera SS Antonio e Biagio e Cesare Arrigo, Alessandria, Italy.
- [6] Department of Cardiology, Interventional Cardiology and Cardiac Surgery, Azienda Ospedaliera SS, Antonio e Biagio e Cesare Arrigo, Alessandria, Italy.

- PMID: **32447060**
- PMCID: PMC7242185
- DOI: 10.1016/j.cjca.2020.05.023

Abstract
in English, French

The diffusion of severe acute respiratory syndrome coronavirus-2 (SARS-CoV-2) forced the Italian population to restrictive measures that modified patients' responses to non-SARS-CoV-2 medical conditions. We evaluated all patients with acute coronary syndromes admitted in 3 high-volume hospitals during the first month of SARS-CoV-2 Italian-outbreak and compared them with patients with ACS admitted during the same period 1 year before. Hospitalization for ACS decreased from 162 patients in 2019 to 84 patients in 2020. In 2020, both door-to-balloon and symptoms-to-percutaneous coronary intervention were longer, and admission levels of high-sensitive cardiac troponin I were higher. They had a lower discharged residual left-ventricular function and an increased predicted late cardiovascular mortality based on their Global Registry of Acute Coronary Events (GRACE) scores.

La propagation du coronavirus 2 du syndrome respiratoire aigu sévère (SRAS-CoV-2) a obligé la population italienne à prendre des mesures contraignantes qui ont modifié la réaction des patients face aux affections médicales non liées au SRAS-CoV-2. Nous avons évalué tous les patients atteints de syndromes coronariens aigus (SCA) admis dans 3 hôpitaux à fort volume d'activité au cours du premier mois de l'épidémie italienne de SRAS-CoV-2 et les avons comparés aux patients atteints de SCA admis au cours de la même période un an auparavant. L'hospitalisation pour un SCA a été réduite de 162 patients en 2019 à 84 patients en 2020. En 2020, le délai porte cardio-ballon et le délai symptômes-intervention coronarienne percutanée étaient plus longs, et les niveaux de troponine I cardiaque de haute sensibilité mesurés à l'admission étaient plus élevés. Les patients avaient une fonction ventriculaire gauche résiduelle altérée et une mortalité cardiovasculaire tardive plus élevée que prévu d'après leur score comparé au registre mondial des syndromes coronariens aigus (GRACE).

Copyright © 2020 Canadian Cardiovascular Society. Published by Elsevier Inc. All rights reserved.

- 5 references
- 1 figure

Supplementary info

Publication types, MeSH terms Expand

Publication types

- Comparative Study
- Multicenter Study
- Observational Study

MeSH terms

- Acute Coronary Syndrome / diagnosis
- Acute Coronary Syndrome / mortality
- Acute Coronary Syndrome / therapy*
- Adult
- Aged
- COVID-19
- Cohort Studies

- Coronavirus Infections / epidemiology*
- Disease Outbreaks / statistics & numerical data
- Female
- Hospital Mortality / trends
- Hospitalization / statistics & numerical data
- Hospitals, High-Volume
- Humans
- Italy / epidemiology
- Male
- Middle Aged
- Pandemics / statistics & numerical data*
- Percutaneous Coronary Intervention / methods
- Percutaneous Coronary Intervention / statistics & numerical data*
- Pneumonia, Viral / epidemiology*
- Registries*
- Retrospective Studies
- Risk Assessment
- Statistics, Nonparametric
- Survival Rate
- Time-to-Treatment / statistics & numerical data*
- Treatment Outcome

Full text links

ELSEVIER FULL-TEXT ARTICLE Elsevier Science Free PMC article

Proceed to details

Cite

Share

☐ 708

Observational Study

J Emerg Nurs

. 2021 Nov;47(6):948-954.

doi: 10.1016/j.jen.2021.03.013. Epub 2021 Mar 29.

A Retrospective Analysis of the Impact of the Coronavirus Disease 2019 Pandemic on Health Care Workers in a Tertiary Hospital in Turkey

Berkant Öztürk, Sema Öztürk, Ahmet Çağlar, İlker Kaçer, Muhammet Hacımustafaoğlu, Kemal Öztürk

- PMID: **34294455**
- PMCID: PMC8006193
- DOI: 10.1016/j.jen.2021.03.013

Free PMC article
Observational Study

A Retrospective Analysis of the Impact of the Coronavirus Disease 2019 Pandemic on Health Care Workers in a Tertiary Hospital in Turkey

Berkant Öztürk et al. J Emerg Nurs. 2021 Nov.
Free PMC article
Show details
J Emerg Nurs
. 2021 Nov;47(6):948-954.
doi: 10.1016/j.jen.2021.03.013. Epub 2021 Mar 29.

Authors

Berkant Öztürk, Sema Öztürk, Ahmet Çağlar, İlker Kaçer, Muhammet Hacımustafaoğlu, Kemal Öztürk

- PMID: **34294455**
- PMCID: PMC8006193
- DOI: 10.1016/j.jen.2021.03.013

Abstract

Introduction: Several vaccines have been developed and approved for use against severe acute respiratory syndrome coronavirus-2; however, the use of personal protective equipment remains important owing to the lack of effective specific treatment and whole community immunity. Hydroxychloroquine sulfate was a treatment option in the early days of the pandemic; however, it was subsequently removed owing to a lack of evidence as an effective treatment. We aimed to evaluate the testing and infection characteristics of coronavirus disease 2019 among health care personnel and determine the effectiveness of prophylactic hydroxychloroquine sulfate use to prevent transmission.

Methods: This retrospective observational study was conducted between May 1 and September 30, 2020. The health care personnel included in the study were physicians, nurses, and paraprofessional support personnel. The health records of health care personnel who had been tested for severe acute respiratory syndrome coronavirus-2 using polymerase chain reaction were retrospectively analyzed.

Results: In total, 508 health care personnel were included in the study. A total of 152 (29.9%) health care personnel were diagnosed with coronavirus disease 2019. The positive polymerase

chain reaction rate was 80.3% (n = 122). A comparison of infected and uninfected health care personnel showed a difference in age and occupation and no difference in sex, working area, and prophylactic hydroxychloroquine sulfate use.

Discussion: Protective measures in low-risk areas of our hospital require improvements. All health care personnel should be trained on personal protective equipment use. There was no evidence to support the effectiveness of prophylactic hydroxychloroquine sulfate against severe acute respiratory syndrome coronavirus-2 transmission.

Keywords: Coronavirus disease 2019; Health care personnel; Hydroxychloroquine sulfate; Personal protective equipment; Severe acute respiratory syndrome coronavirus 2.

Copyright © 2021 Emergency Nurses Association. Published by Elsevier Inc. All rights reserved.

- 41 references

Supplementary info

Publication types, MeSH terms Expand

Publication types

- Observational Study

MeSH terms

- COVID-19 Testing
- COVID-19* / epidemiology
- COVID-19* / prevention & control
- COVID-19* / transmission
- Humans
- Infectious Disease Transmission, Patient-to-Professional
- Pandemics*
- Personal Protective Equipment
- Personnel, Hospital* / statistics & numerical data
- Retrospective Studies
- Tertiary Care Centers
- Turkey / epidemiology

Full text links

ELSEVIER FULL-TEXT ARTICLE Elsevier Science Free PMC article

Proceed to details

Cite

Share

☐ 709

Observational Study
Public Health
. 2020 Oct;187:115-119.
doi: 10.1016/j.puhe.2020.08.007. Epub 2020 Aug 18.

Hospital attendance and admission trends for cardiac diseases during the COVID-19 outbreak and lockdown in Greece

E Oikonomou [1], K Aznaouridis [2], J Barbetseas [3], G Charalambous [4], I Gastouniotis [4], V Fotopoulos [5], K-P Gkini [6], A Katsivas [7], G Koudounis [8], P Koudounis [8], M Koutouzis [6], D Lamprinos [5], E Lazaris [6], E Lazaris [6], G Lazaros [2], G Marinos [5], N Platogiannis [9], D Platogiannis [9], G Siasos [10], D Terentes-Printzios [2], A Theodoropoulou [4], P Theofilis [2], K Toutouzas [2], S Tsalamandris [2], I Tsiafoutis [7], M Vavouranakis [2], G Vogiatzi [11], T Zografos [12], E Baka [4], D Tousoulis [2], C Vlachopoulos [2]
Affiliations Expand

Affiliations

- [1] 1st Cardiology Clinic, 'Hippokration' General Hospital, National and Kapodistrian University of Athens, School of Medicine, Athens, Greece; Emergency Department, Hippokration' General Hospital, Athens, Greece. Electronic address: boikono@gmail.com.
- [2] 1st Cardiology Clinic, 'Hippokration' General Hospital, National and Kapodistrian University of Athens, School of Medicine, Athens, Greece.
- [3] Department of Cardiology, Laiko General Hospital, Athens, Greece.
- [4] Emergency Department, Hippokration' General Hospital, Athens, Greece.
- [5] Emergency Department, Laiko General Hospital, Athens, Greece.
- [6] Athens Red Cross Hospital, 2nd Department of Cardiology, Athens, Greece.
- [7] Athens Red Cross Hospital, 1st Department of Cardiology, Athens, Greece.
- [8] General Hospital of Kalamata, Department of Cardiology, Kalamata, Greece.
- [9] General Hospital of Trikala, Department of Cardiology, Trikala, Greece.
- [10] 1st Cardiology Clinic, 'Hippokration' General Hospital, National and Kapodistrian University of Athens, School of Medicine, Athens, Greece; Cardiovascular Division, Brigham and Women's Hospital, Harvard Medical School, Boston, MA, USA.
- [11] 1st Cardiology Clinic, 'Hippokration' General Hospital, National and Kapodistrian University of Athens, School of Medicine, Athens, Greece; Emergency Department, Laiko General Hospital, Athens, Greece.
- [12] 1st Cardiology Clinic, 'Hippokration' General Hospital, National and Kapodistrian University of Athens, School of Medicine, Athens, Greece; Athens Red Cross Hospital, 1st Department of Cardiology, Athens, Greece.

- PMID: **32949881**
- PMCID: PMC7434308
- DOI: 10.1016/j.puhe.2020.08.007

Free PMC article

Observational Study

Hospital attendance and admission trends for cardiac diseases during the COVID-19 outbreak and lockdown in Greece

E Oikonomou et al. Public Health. 2020 Oct.
Free PMC article
Show details
Public Health
. 2020 Oct;187:115-119.
doi: 10.1016/j.puhe.2020.08.007. Epub 2020 Aug 18.

Authors

E Oikonomou [1], K Aznaouridis [2], J Barbetseas [3], G Charalambous [4], I Gastouniotis [4], V Fotopoulos [5], K-P Gkini [6], A Katsivas [7], G Koudounis [8], P Koudounis [8], M Koutouzis [6], D Lamprinos [5], E Lazaris [6], E Lazaris [6], G Lazaros [2], G Marinos [5], N Platogiannis [9], D Platogiannis [9], G Siasos [10], D Terentes-Printzios [2], A Theodoropoulou [4], P Theofilis [2], K Toutouzas [2], S Tsalamandris [2], I Tsiafoutis [7], M Vavouranakis [2], G Vogiatzi [11], T Zografos [12], E Baka [4], D Tousoulis [2], C Vlachopoulos [2]

Affiliations

- [1] 1st Cardiology Clinic, 'Hippokration' General Hospital, National and Kapodistrian University of Athens, School of Medicine, Athens, Greece; Emergency Department, Hippokration' General Hospital, Athens, Greece. Electronic address: boikono@gmail.com.
- [2] 1st Cardiology Clinic, 'Hippokration' General Hospital, National and Kapodistrian University of Athens, School of Medicine, Athens, Greece.
- [3] Department of Cardiology, Laiko General Hospital, Athens, Greece.
- [4] Emergency Department, Hippokration' General Hospital, Athens, Greece.
- [5] Emergency Department, Laiko General Hospital, Athens, Greece.
- [6] Athens Red Cross Hospital, 2nd Department of Cardiology, Athens, Greece.
- [7] Athens Red Cross Hospital, 1st Department of Cardiology, Athens, Greece.
- [8] General Hospital of Kalamata, Department of Cardiology, Kalamata, Greece.
- [9] General Hospital of Trikala, Department of Cardiology, Trikala, Greece.
- [10] 1st Cardiology Clinic, 'Hippokration' General Hospital, National and Kapodistrian University of Athens, School of Medicine, Athens, Greece; Cardiovascular Division, Brigham and Women's Hospital, Harvard Medical School, Boston, MA, USA.
- [11] 1st Cardiology Clinic, 'Hippokration' General Hospital, National and Kapodistrian University of Athens, School of Medicine, Athens, Greece; Emergency Department, Laiko General Hospital, Athens, Greece.
- [12] 1st Cardiology Clinic, 'Hippokration' General Hospital, National and Kapodistrian University of Athens, School of Medicine, Athens, Greece; Athens Red Cross Hospital, 1st Department of Cardiology, Athens, Greece.

- PMID: **32949881**
- PMCID: PMC7434308
- DOI: 10.1016/j.puhe.2020.08.007

Abstract

Objectives: The coronavirus disease 2019 (COVID-19) outbreak, along with implementation of lockdown and strict public movement restrictions, in Greece has affected hospital visits and admissions. We aimed to investigate trends of cardiac disease admissions during the outbreak of the pandemic and possible associations with the applied restrictive measures.

Study design: This is a retrospective observational study.

Methods: Data for 4970 patients admitted via the cardiology emergency department (ED) across 3 large-volume urban hospitals in Athens and 2 regional/rural hospitals from February 3, 2020, up to April 12 were recorded. Data from the equivalent (for the COVID-19 outbreak) time period of 2019 and from the postlockdown time period were also collected.

Results: A falling trend of cardiology ED visits and hospital admissions was observed starting from the week when the restrictive measures due to COVID-19 were implemented. Compared with the pre-COVID-19 outbreak time period, acute coronary syndrome (ACS) [145 (29/week) vs. 60 (12/week), -59%, P < 0.001], ST elevation myocardial infarction [46 (9.2/week) vs. 21 (4.2/week), -54%, P = 0.002], and non-ST elevation ACS [99 cases (19.8/week) vs. 39 (7.8/week), -60% P < 0.001] were reduced at the COVID-19 outbreak time period. Reductions were also noted for heart failure worsening and arrhythmias. The ED visits in the postlockdown period were significantly higher than in the COVID-19 outbreak time period (1511 vs 660; P < 0.05).

Conclusion: Our data show significant drops in cardiology visits and admissions during the COVID-19 outbreak time period. Whether this results from restrictive measures or depicts a true reduction of cardiac disease cases warrants further investigation.

Keywords: Acute coronary syndromes; COVID-19; Emergency department; SARS-CoV-2.

Copyright © 2020 The Royal Society for Public Health. Published by Elsevier Ltd. All rights reserved.

- 6 references
- 1 figure

Supplementary info

Publication types, MeSH terms Expand

Publication types

- Observational Study

MeSH terms

- Adult

- Aged
- Aged, 80 and over
- COVID-19
- Coronavirus Infections / epidemiology*
- Coronavirus Infections / prevention & control
- Emergency Service, Hospital / trends*
- Female
- Greece / epidemiology
- Heart Diseases / therapy*
- Hospitalization / trends*
- Humans
- Male
- Middle Aged
- Pandemics / prevention & control
- Pneumonia, Viral / epidemiology*
- Pneumonia, Viral / prevention & control
- Quarantine / legislation & jurisprudence*
- Retrospective Studies

Full text links

ELSEVIER FULL-TEXT ARTICLE Elsevier Science Free PMC article
Proceed to details
Cite
Share
☐ 710
Observational Study
Mycoses
. 2021 Nov;64(11):1366-1377.
doi: 10.1111/myc.13351. Epub 2021 Jul 31.

Increased incidence of rhino-orbital mucormycosis in an educational therapeutic hospital during the COVID-19 pandemic in western Iran: An observational study

Manouchehr Avatef Fazeli [1], Leila Rezaei [2], Etrat Javadirad [3], Khosro Iranfar [1], Abbas Khosravi [2], Javad Amini Saman [4], Pardis Poursabbagh [1], Mohammad Rasoul Ghadami [1] [5], Mohammad Mehdi Parandin [2], Amrollah Dehghani [1], Touraj Ahmadi Jouybari [6], Behzad Mahdavian [7], Nastaran Eivazi [1], Sohbat Rezaei [1], Alireza Rezaei [1], Bashir Emami [8], Mohadeseh Haqgou [1], Arezoo Bozorgomid [9], Babak Sayad [7] [9]

Affiliations Expand

Affiliations

- [1] Department of Otorhinolaryngology, School of Medicine, Kermanshah University of Medical Sciences, Kermanshah, Iran.
- [2] Department of Ophthalmology, School of Medicine, Kermanshah University of Medical Sciences, Kermanshah, Iran.
- [3] Department of Pathology, School of Medicine, Kermanshah University of Medical Sciences, Kermanshah, Iran.
- [4] Department of Anesthesiology, School of Medicine, Kermanshah University of Medical Sciences, Kermanshah, Iran.
- [5] Sleep Disorders Research Center, Kermanshah University of Medical Sciences, Kermanshah, Iran.
- [6] Department of Interna, Medicine, School of Medicine, Kermanshah University of Medical Sciences, Kermanshah, Iran.
- [7] Department of Infectious Disease, School of Medicine, Kermanshah University of Medical Sciences, Kermanshah, Iran.
- [8] Imam Khomeini and Mohamad Kermanshahi Clinical Research Development Unit, Kermanshah University of Medical Sciences, Kermanshah, Iran.
- [9] Infectious Diseases Research Center, Health Institute, Kermanshah University of Medical Sciences, Kermanshah, Iran.

- PMID: **34252988**
- PMCID: PMC8447086
- DOI: 10.1111/myc.13351

Free PMC article
Observational Study

Increased incidence of rhino-orbital mucormycosis in an educational therapeutic hospital during the COVID-19 pandemic in western Iran: An observational study

Manouchehr Avatef Fazeli et al. Mycoses. 2021 Nov.
Free PMC article
Show details
Mycoses

. 2021 Nov;64(11):1366-1377.
doi: 10.1111/myc.13351. Epub 2021 Jul 31.

Authors

Manouchehr Avatef Fazeli [1], Leila Rezaei [2], Etrat Javadirad [3], Khosro Iranfar [1], Abbas Khosravi [2], Javad Amini Saman [4], Pardis Poursabbagh [1], Mohammad Rasoul Ghadami [1]

[5], Mohammad Mehdi Parandin [2], Amrollah Dehghani [1], Touraj Ahmadi Jouybari [6], Behzad Mahdavian [7], Nastaran Eivazi [1], Sohbat Rezaei [1], Alireza Rezaei [1], Bashir Emami [8], Mohadeseh Haqgou [1], Arezoo Bozorgomid [9], Babak Sayad [7 9]

Affiliations

- [1] Department of Otorhinolaryngology, School of Medicine, Kermanshah University of Medical Sciences, Kermanshah, Iran.
- [2] Department of Ophthalmology, School of Medicine, Kermanshah University of Medical Sciences, Kermanshah, Iran.
- [3] Department of Pathology, School of Medicine, Kermanshah University of Medical Sciences, Kermanshah, Iran.
- [4] Department of Anesthesiology, School of Medicine, Kermanshah University of Medical Sciences, Kermanshah, Iran.
- [5] Sleep Disorders Research Center, Kermanshah University of Medical Sciences, Kermanshah, Iran.
- [6] Department of Interna, Medicine, School of Medicine, Kermanshah University of Medical Sciences, Kermanshah, Iran.
- [7] Department of Infectious Disease, School of Medicine, Kermanshah University of Medical Sciences, Kermanshah, Iran.
- [8] Imam Khomeini and Mohamad Kermanshahi Clinical Research Development Unit, Kermanshah University of Medical Sciences, Kermanshah, Iran.
- [9] Infectious Diseases Research Center, Health Institute, Kermanshah University of Medical Sciences, Kermanshah, Iran.

- PMID: **34252988**
- PMCID: PMC8447086
- DOI: 10.1111/myc.13351

Abstract

Background: COVID-19 patients, especially the patients requiring hospitalisation, have a high risk of several complications such as opportunistic bacterial and fungal infections. Mucormycosis is a rare and opportunistic fungal infection that mainly affects diabetic and immunocompromised patients. An increase has been observed in the number of rhino-orbital mucormycosis in patients with COVID-19 admitted to Imam Khomeini Hospital, Kermanshah, Iran, since October 2020. This is a report of the frequency, risk factors, clinical manifestations, treatment and prognosis of COVID-19 associated with mucormycosis infection.

Methods: The medical records of COVID-19 patients with rhino-orbital mucormycosis who were diagnosed in an educational therapeutic hospital in Kermanshah, west of Iran were surveyed. Several parameters were analysed including demographic, clinical, therapeutic and laboratory characteristics.

Results: Twelve patients with COVID-19-associated rhino-orbital mucormycosis were identified from 12 October to 18 November 2020. All cases reported as proven mucormycosis had a history of hospitalisation due to COVID-19. Comorbidities mainly included diabetes mellitus (83.33%) and hypertension (58.33%). Seventy-five per cent of patients received corticosteroids for COVID-19 treatment. The sites of involvement were rhino-sino-orbital (83%) and rhino-sino (17%).

Amphotericin B/liposomal amphotericin B alone or in combination with surgical debridement or orbital exenteration was used as the first-line therapy. The overall mortality rate was 66.7% (8/12).

Conclusions: We found a high incidence of mucormycosis among COVID-19 patients. Diabetes mellitus and corticosteroid use were the dominant predisposing factor of mucormycosis. Mucormycosis is a life-threatening and opportunistic infection; therefore, physicians should know the signs and symptoms of the disease so that a timely diagnosis and therapy can be performed.

Keywords: COVID-19; Iran; SARS-CoV-2; co-infection; mucormycosis.

© 2021 Wiley-VCH GmbH.

Conflict of interest statement

The authors declare that they have no conflict of interest.

- 33 references
- 2 figures

Supplementary info

Publication types, MeSH terms Expand

Publication types

- Observational Study

MeSH terms

- Aged
- Aged, 80 and over
- COVID-19 / complications*
- COVID-19 / epidemiology
- Female
- Hospitals, Teaching
- Humans
- Incidence
- Iran / epidemiology
- Male
- Middle Aged
- Mucormycosis / complications
- Mucormycosis / diagnostic imaging
- Mucormycosis / epidemiology*
- Orbital Diseases / complications
- Orbital Diseases / diagnostic imaging
- Orbital Diseases / epidemiology*

- Orbital Diseases / microbiology*
- Retrospective Studies
- Rhinitis / complications
- Rhinitis / diagnostic imaging
- Rhinitis / epidemiology*
- Rhinitis / microbiology*

Full text links

WILEY Full Text Article Wiley Free PMC article
Proceed to details
Cite
Share
☐ 711
Observational Study
J Chin Med Assoc
. 2021 Apr 1;84(4):423-427.
doi: 10.1097/JCMA.0000000000000503.

Impact of screening COVID-19 on orthopedic trauma patients at the emergency department: A consecutive series from a level I trauma center

Hsuan-Hsiao Ma [1, 2], Shang-Wen Tsai [1, 2], Cheng-Fong Chen [1, 2], Po-Kuei Wu [1, 2], Chao-Ming Chen [1, 2], Chao-Ching Chiang [1, 2], Wei-Ming Chen [1, 2]
Affiliations Expand

Affiliations

- [1] Department of Orthopaedics and Traumatology, Taipei Veterans General Hospital, Taipei, Taiwan ROC.
- [2] Department of Orthopaedics, School of Medicine, National Yang Ming Chiao Tung University, Taipei, Taiwan, ROC.

- PMID: **33595990**
- DOI: 10.1097/JCMA.0000000000000503

Observational Study

Impact of screening COVID-19 on orthopedic trauma patients at the emergency

department: A consecutive series from a level I trauma center

Hsuan-Hsiao Ma et al. J Chin Med Assoc. 2021.
Show details
J Chin Med Assoc
. 2021 Apr 1;84(4):423-427.
doi: 10.1097/JCMA.0000000000000503.

Authors

Hsuan-Hsiao Ma [1 2], Shang-Wen Tsai [1 2], Cheng-Fong Chen [1 2], Po-Kuei Wu [1 2], Chao-Ming Chen [1 2], Chao-Ching Chiang [1 2], Wei-Ming Chen [1 2]

Affiliations

- [1] Department of Orthopaedics and Traumatology, Taipei Veterans General Hospital, Taipei, Taiwan ROC.
- [2] Department of Orthopaedics, School of Medicine, National Yang Ming Chiao Tung University, Taipei, Taiwan, ROC.

- PMID: **33595990**
- DOI: 10.1097/JCMA.0000000000000503

Abstract

Background: Coronavirus disease 2019 (COVID-19) posed a major threat to the clinical practice of orthopedic surgeons, especially in the emergency department. We aim to present: (1) the criteria established by the Surgery Management Committee of Taipei Veterans General Hospital in response to COVID-19 and (2) the impact of COVID-19 screening on orthopedic trauma patients in the emergency department.

Methods: From April 1 to April 30, 2020, all orthopedic trauma patients in the emergency department were screened for COVID-19 if they fulfilled any of the following: (1) travel from abroad within 14 days, (2) high-risk occupation, (3) contact or cluster history with a COVID-19-positive patient, and (4) any associated symptom, including fever up to 38°C, cough, sore throat, rhinorrhea, loss of taste or smell, muscle soreness, malaise, or shortness of breath. We recorded details on the injury, fever, management, and associated outcomes.

Results: Of the 163 orthopedic trauma patients presenting to the emergency department, 24 were screened for COVID-19; of these, 22 received surgery. Sixty-two patients received surgery without screening for COVID-19. Fever was the most common reason to screen for COVID-19 (N = 20; 83.3%). No patients were COVID-19 positive. Screened patients had a significantly longer mean interval from presentation to the emergency department to surgery (2.7 ± 2.5 vs. 1.5 ± 0.8 days, p = 0.037). Of the 20 patients screened because of fever, the focus was not identified in 12 (60.0%) patients. The other eight had urinary tract infection (N = 6; 27.2%), septic hip (N = 1; 4.6%), and concomitant pneumonia and urinary tract infection (N = 1; 4.6%). The mean duration of fever and hospital stay was 4.3 ± 4.6 and 8.7 ± 4.9 days, respectively. There were no thromboembolic events, surgical complications, or in-hospital mortality.

Conclusion: We developed safe and reliable screening criteria for this COVID-19 pandemic. The delay in surgery was reasonable and did not adversely affect in-patient outcomes.

Copyright © 2021, the Chinese Medical Association.

Conflict of interest statement

Conflicts of interest: The authors declare that they have no conflicts of interest related to the subject matter or materials discussed in this article.

- 21 references

Supplementary info

Publication types, MeSH terms Expand

Publication types

- Observational Study

MeSH terms

- Adult
- Aged
- COVID-19 / diagnosis*
- Child
- Emergency Service, Hospital / statistics & numerical data*
- Female
- Fever
- Fractures, Bone*
- Humans
- Length of Stay
- Male
- Middle Aged
- Orthopedics*
- Pandemics
- Retrospective Studies
- Taiwan
- Trauma Centers / statistics & numerical data*
- Young Adult

Full text links

Wolters Kluwer Wolters Kluwer
Proceed to details

Cite
Share
712
Observational Study
J Am Heart Assoc
. 2020 Nov 17;9(22):e017364.
doi: 10.1161/JAHA.120.017364. Epub 2020 Oct 7.

Antecedent Administration of Angiotensin-Converting Enzyme Inhibitors or Angiotensin II Receptor Antagonists and Survival After Hospitalization for COVID-19 Syndrome

Alberto Palazzuoli [1], Massimo Mancone [2], Gaetano M De Ferrari [3], Giovanni Forleo [4], Gioel G Secco [5], Gaetano M Ruocco [6], Fabrizio D'Ascenzo [3], Silvia Monticone [7], Anita Paggi [8], Marco Vicenzi [9], Anna G Palazzo [10], Maurizio Landolina [11], Erika Taravelli [11], Guido Tavazzi [12], Francesco Blasi [13], Fabio Infusino [2], Francesco Fedele [2], Francesco G De Rosa [10], Michael Emmett [14], Jeffrey M Schussler [14] [15] [16], Kristen M Tecson [14] [15] [16], Peter A McCullough [14] [15] [16]

Affiliations Expand

Affiliations

- [1] Cardiovascular Diseases Unit Department of Medical Sciences AOUS Le Scotte Hospital University of Siena Italy.
- [2] Department of Clinical Internal, Anesthesiological and Cardiovascular Sciences Sapienza, University of Rome Italy.
- [3] Cardiology Department of Medical Science, University of Turin Città della Salute e Della Scienza Le Molinette Hospital Torino Torino Italy.
- [4] Section Head Electrophysiology and Cardiac Pacing Azienda Ospedaliera - Polo Universitario - "Luigi Sacco" Milano Italy.
- [5] Interventional Cardiology and Cardiac Surgery Unit Azienda Ospedaliera SS Antonio e Biagio e Cesare Arrigo Alessandria Italy.
- [6] Cardiology Division Regina Montis Regalis Hospital Mondovì Cuneo Italy.
- [7] Division of Internal Medicine Department of Medical Sciences University of Turin Italy.
- [8] Interventional Cardiology Department of Internal Medicine ASSST Nord Milano E Bassini Hospital Cisanello Balsamo Milan Italy.
- [9] Cardiovascular Disease Unit Department of Internal Medicine Fondazione IRCCS Ca' Granda Ospedale Maggiore Policlinico University of Milano Italy.
- [10] Infectious Disease Department of Medical Sciences AOU Città Della Salute e Della Scienza University of Torino Italy.
- [11] Cardiology Division Ospedale Maggiore of Crema Italy.
- [12] Intensive Care Fondation IRCCS Policlinico San Matteo University of Pavia Italy.

- [13] Respiratory Unit and Adult Cystic Fibrosis Center Department of Internal Medicine Fondazione IRCCS Ca' Granda Ospedale Maggiore Policlinico University of Milano Italy.
- [14] Baylor University Medical Center Dallas TX.
- [15] Baylor Scott and White Heart and Vascular Hospital Dallas TX.
- [16] Baylor Heart and Vascular Institute Dallas TX.

- PMID: **33023356**
- PMCID: PMC7763715
- DOI: 10.1161/JAHA.120.017364

Free PMC article
Observational Study

Antecedent Administration of Angiotensin-Converting Enzyme Inhibitors or Angiotensin II Receptor Antagonists and Survival After Hospitalization for COVID-19 Syndrome

Alberto Palazzuoli et al. J Am Heart Assoc. 2020.
Free PMC article
Show details
J Am Heart Assoc
. 2020 Nov 17;9(22):e017364.
doi: 10.1161/JAHA.120.017364. Epub 2020 Oct 7.

Authors

Alberto Palazzuoli [1], Massimo Mancone [2], Gaetano M De Ferrari [3], Giovanni Forleo [4], Gioel G Secco [5], Gaetano M Ruocco [6], Fabrizio D'Ascenzo [3], Silvia Monticone [7], Anita Paggi [8], Marco Vicenzi [9], Anna G Palazzo [10], Maurizio Landolina [11], Erika Taravelli [11], Guido Tavazzi [12], Francesco Blasi [13], Fabio Infusino [2], Francesco Fedele [2], Francesco G De Rosa [10], Michael Emmett [14], Jeffrey M Schussler [14] [15] [16], Kristen M Tecson [14] [15] [16], Peter A McCullough [14] [15] [16]

Affiliations

- [1] Cardiovascular Diseases Unit Department of Medical Sciences AOUS Le Scotte Hospital University of Siena Italy.
- [2] Department of Clinical Internal, Anesthesiological and Cardiovascular Sciences Sapienza, University of Rome Italy.
- [3] Cardiology Department of Medical Science, University of Turin Città della Salute e Della Scienza Le Molinette Hospital Torino Torino Italy.
- [4] Section Head Electrophysiology and Cardiac Pacing Azienda Ospedaliera - Polo Universitario - "Luigi Sacco" Milano Italy.

- [5] Interventional Cardiology and Cardiac Surgery Unit Azienda Ospedaliera SS Antonio e Biagio e Cesare Arrigo Alessandria Italy.
- [6] Cardiology Division Regina Montis Regalis Hospital Mondovì Cuneo Italy.
- [7] Division of Internal Medicine Department of Medical Sciences University of Turin Italy.
- [8] Interventional Cardiology Department of Internal Medicine ASSST Nord Milano E Bassini Hospital Cisanello Balsamo Milan Italy.
- [9] Cardiovascular Disease Unit Department of Internal Medicine Fondazione IRCCS Ca' Granda Ospedale Maggiore Policlinico University of Milano Italy.
- [10] Infectious Disease Department of Medical Sciences AOU Città Della Salute e Della Scienza University of Torino Italy.
- [11] Cardiology Division Ospedale Maggiore of Crema Italy.
- [12] Intensive Care Fondation IRCCS Policlinico San Matteo University of Pavia Italy.
- [13] Respiratory Unit and Adult Cystic Fibrosis Center Department of Internal Medicine Fondazione IRCCS Ca' Granda Ospedale Maggiore Policlinico University of Milano Italy.
- [14] Baylor University Medical Center Dallas TX.
- [15] Baylor Scott and White Heart and Vascular Hospital Dallas TX.
- [16] Baylor Heart and Vascular Institute Dallas TX.

- PMID: **33023356**
- PMCID: PMC7763715
- DOI: 10.1161/JAHA.120.017364

Abstract

Background Severe acute respiratory syndrome coronavirus 2 (SARS-CoV-2) utilizes the angiotensin-converting enzyme-2 (ACE-2) receptor to enter human cells. Angiotensin-converting enzyme inhibitors (ACEI) and angiotensin II receptor antagonists (ARB) are associated with ACE-2 upregulation. We hypothesized that antecedent use of ACEI/ARB may be associated with mortality in coronavirus disease 2019 (COVID-19). Methods and Results We used the Coracle registry, which contains data of patients hospitalized with COVID-19 in 4 regions of Italy, and restricted analyses to those ≥50 years of age. The primary outcome was in-hospital mortality. Among these 781 patients, 133 (17.0%) used an ARB and 171 (21.9%) used an ACEI. While neither sex nor smoking status differed by user groups, patients on ACEI/ARB were older and more likely to have hypertension, diabetes mellitus, and congestive heart failure. The overall mortality rate was 15.1% (118/781) and increased with age (P_{Trend}<0.0001). The crude odds ratios (ORs) for death for ACEI users and ARB users were 0.98, 95% CI, 0.60-1.60, *P*=0.9333, and 1.13, 95% CI, 0.67-1.91, *P*=0.6385, respectively. After adjusting for age, hypertension, diabetes mellitus, and congestive heart failure, antecedent ACEI administration was associated with reduced mortality (OR, 0.55; 95% CI, 0.31-0.98, *P*=0.0436); a similar, but weaker trend was observed for ARB administration (OR, 0.58; 95% CI, 0.32-1.07, *P*=0.0796). Conclusions In those aged ≥50 years hospitalized with COVID-19, antecedent use of ACEI was independently associated with reduced risk of inpatient death. Our findings suggest a protective role of renin-angiotensin-aldosterone system inhibition in patients with high cardiovascular risk affected by COVID-19.

Keywords: COVID-19; SARS-CoV-2; angiotensin-converting enzyme inhibitor; angiotensin-converting enzyme-2; hospitalization; mortality; renin-angiotensin converting enzyme inhibitor.

Conflict of interest statement

None.

- 19 references
- 3 figures

Supplementary info

Publication types, MeSH terms, Substances Expand

Publication types

- Multicenter Study
- Observational Study
- Research Support, Non-U.S. Gov't

MeSH terms

- Age Factors
- Aged
- Aged, 80 and over
- Angiotensin Receptor Antagonists / therapeutic use*
- Angiotensin-Converting Enzyme Inhibitors / therapeutic use*
- COVID-19 / diagnosis
- COVID-19 / mortality
- COVID-19 / therapy*
- Female
- Hospital Mortality
- Hospitalization*
- Humans
- Italy
- Male
- Middle Aged
- Protective Factors
- Registries
- Retrospective Studies
- Risk Assessment
- Risk Factors
- Time Factors
- Treatment Outcome

Substances

- Angiotensin Receptor Antagonists

- Angiotensin-Converting Enzyme Inhibitors

Full text links

OPEN ACCESS Atypon Free PMC article
Proceed to details
Cite
Share
713
Observational Study
Sci Rep
. 2021 Jun 16;11(1):12716.
doi: 10.1038/s41598-021-92236-6.

Monocyte Distribution Width (MDW) as novel inflammatory marker with prognostic significance in COVID-19 patients

Giovanni Riva [# 1], Sara Castellano [# 2], Vincenzo Nasillo [# 3], Anna Maria Ottomano [3], Giuliano Bergonzini [3], Ambra Paolini [4], Beatrice Lusenti [3], Jovana Milić [5], Sara De Biasi [2], Lara Gibellini [2], Andrea Cossarizza [2], Stefano Busani [6], Massimo Girardis [6], Giovanni Guaraldi [7], Cristina Mussini [7], Rossella Manfredini [8], Mario Luppi [4], Enrico Tagliafico [# 3 2], Tommaso Trenti [# 3]
Affiliations Expand

Affiliations

- [1] Diagnostic Hematology and Clinical Genomics Laboratory, Department of Laboratory Medicine and Pathology, AUSL/AOU Policlinico, Via del Pozzo 71, 41124, Modena, Italy. g.riva@ausl.mo.it.
- [2] Department of Medical and Surgical Sciences, University of Modena and Reggio Emilia, Modena, Italy.
- [3] Diagnostic Hematology and Clinical Genomics Laboratory, Department of Laboratory Medicine and Pathology, AUSL/AOU Policlinico, Via del Pozzo 71, 41124, Modena, Italy.
- [4] Department of Medical and Surgical Sciences, University of Modena and Reggio Emilia, Hematology Unit, AOU Policlinico, Modena, Italy.
- [5] Department of Surgical, Medical, Dental and Morphological Sciences, University of Modena and Reggio Emilia, Modena, Italy.
- [6] Department of Anesthesia and Intensive Care, University of Modena and Reggio Emilia, Intensive Care Unit, AOU Policlinico, Modena, Italy.
- [7] Department of Surgical, Medical, Dental and Morphological Sciences, University of Modena and Reggio Emilia, Infectious Diseases Clinics, AOU Policlinico, Modena, Italy.
- [8] Department of Life Sciences, University of Modena and Reggio Emilia, Centre for Regenerative Medicine "Stefano Ferrari", Modena, Italy.

[#] Contributed equally.

- PMID: **34135448**
- PMCID: PMC8209163
- DOI: 10.1038/s41598-021-92236-6

Free PMC article
Observational Study

Monocyte Distribution Width (MDW) as novel inflammatory marker with prognostic significance in COVID-19 patients

Giovanni Riva et al. Sci Rep. 2021.
Free PMC article

Show details

Sci Rep

. 2021 Jun 16;11(1):12716.
doi: 10.1038/s41598-021-92236-6.

Authors

Giovanni Riva [# 1], Sara Castellano [# 2], Vincenzo Nasillo [# 3], Anna Maria Ottomano [3], Giuliano Bergonzini [3], Ambra Paolini [4], Beatrice Lusenti [3], Jovana Milić [5], Sara De Biasi [2], Lara Gibellini [2], Andrea Cossarizza [2], Stefano Busani [6], Massimo Girardis [6], Giovanni Guaraldi [7], Cristina Mussini [7], Rossella Manfredini [8], Mario Luppi [4], Enrico Tagliafico [# 3 2], Tommaso Trenti [# 3]

Affiliations

- [1] Diagnostic Hematology and Clinical Genomics Laboratory, Department of Laboratory Medicine and Pathology, AUSL/AOU Policlinico, Via del Pozzo 71, 41124, Modena, Italy. g.riva@ausl.mo.it.
- [2] Department of Medical and Surgical Sciences, University of Modena and Reggio Emilia, Modena, Italy.
- [3] Diagnostic Hematology and Clinical Genomics Laboratory, Department of Laboratory Medicine and Pathology, AUSL/AOU Policlinico, Via del Pozzo 71, 41124, Modena, Italy.
- [4] Department of Medical and Surgical Sciences, University of Modena and Reggio Emilia, Hematology Unit, AOU Policlinico, Modena, Italy.
- [5] Department of Surgical, Medical, Dental and Morphological Sciences, University of Modena and Reggio Emilia, Modena, Italy.
- [6] Department of Anesthesia and Intensive Care, University of Modena and Reggio Emilia, Intensive Care Unit, AOU Policlinico, Modena, Italy.
- [7] Department of Surgical, Medical, Dental and Morphological Sciences, University of Modena and Reggio Emilia, Infectious Diseases Clinics, AOU Policlinico, Modena, Italy.
- [8] Department of Life Sciences, University of Modena and Reggio Emilia, Centre for Regenerative Medicine "Stefano Ferrari", Modena, Italy.

[#] Contributed equally.

- PMID: **34135448**
- PMCID: PMC8209163
- DOI: 10.1038/s41598-021-92236-6

Abstract

Monocyte Distribution Width (MDW), a new cytometric parameter correlating with cytomorphologic changes occurring upon massive monocyte activation, has recently emerged as promising early biomarker of sepsis. Similar to sepsis, monocyte/macrophage subsets are considered key mediators of the life-threatening hyper-inflammatory disorder characterizing severe COVID-19. In this study, we longitudinally analyzed MDW values in a cohort of 87 COVID-19 patients consecutively admitted to our hospital, showing significant correlations between MDW and common inflammatory markers, namely CRP ($p < 0.001$), fibrinogen ($p < 0.001$) and ferritin ($p < 0.01$). Moreover, high MDW values resulted to be prognostically associated with fatal outcome in COVID-19 patients (AUC = 0.76, 95% CI: 0.66-0.87, sensitivity 0.75, specificity 0.70, MDW threshold 26.4; RR = 4.91, 95% CI: 1.73-13.96; OR = 7.14, 95% CI: 2.06-24.71). This pilot study shows that MDW can be useful in the monitoring of COVID-19 patients, as this innovative hematologic biomarker is: (1) easy to obtain, (2) directly related to the activation state of a fundamental inflammatory cell subset (i.e. monocytes, pivotal in both cytokine storm and sepsis immunopathogenesis), (3) well correlated with clinical severity of COVID-19-associated inflammatory disorder, and, in turn, (4) endowed with relevant prognostic significance. Additional studies are needed to define further the clinical impact of MDW testing in the management of COVID-19 patients.

Conflict of interest statement

The authors declare no competing interests.

- 34 references
- 3 figures

Supplementary info

Publication types, MeSH terms, Substances Expand

Publication types

- Observational Study

MeSH terms

- Adolescent
- Adult
- Aged
- Aged, 80 and over
- Biomarkers / blood

- C-Reactive Protein / analysis
- COVID-19 / blood*
- COVID-19 / epidemiology
- COVID-19 / virology
- Cell Size*
- Female
- Ferritins / blood
- Fibrinogen / analysis
- Humans
- Inflammation / blood
- Italy / epidemiology
- Longitudinal Studies
- Male
- Middle Aged
- Monocytes / pathology*
- Patient Admission
- Pilot Projects
- Prognosis
- Retrospective Studies
- SARS-CoV-2*
- Sensitivity and Specificity
- Severity of Illness Index*
- Young Adult

Substances

- Biomarkers
- Fibrinogen
- C-Reactive Protein
- Ferritins

Full text links

npg nature publishing group Nature Publishing Group Free PMC article
Proceed to details
Cite
Share
☐ 714
Observational Study
Cardiovasc Res
. 2021 Dec 17;117(14):2807-2820.
doi: 10.1093/cvr/cvab308.

Low-molecular-weight heparin use in coronavirus disease 2019 is associated with curtailed viral persistence: a retrospective multicentre observational study

David Pereyra [1, 2], Stefan Heber [3], Waltraud C Schrottmaier [1], Jonas Santol [2], Anita Pirabe [1], Anna Schmuckenschlager [1], Kerstin Kammerer [1], Daphni Ammon [2], Thomas Sorz [2], Fabian Fritsch [2], Hubert Hayden [4], Erich Pawelka [5], Philipp Krüger [1, 2], Benedikt Rumpf [2, 5], Marianna T Traugott [5], Pia Glaser [6], Christa Firbas [7], Christian Schörgenhofer [7], Tamara Seitz [5], Mario Karolyi [5], Ingrid Pabinger [6], Christine Brostjan [4], Patrick Starlinger [2], Günter Weiss [8], Rosa Bellmann-Weiler [8], Helmut J F Salzer [9], Bernd Jilma [7], Alexander Zoufaly [5], Alice Assinger [1]

Affiliations Expand

Affiliations

- [1] Department of Vascular Biology and Thrombosis Research, Center of Physiology and Pharmacology, Medical University of Vienna, Schwarzspanierstraße 17, 1090 Vienna, Austria.
- [2] Division of Visceral Surgery, Department of Surgery, Medical University of Vienna, General Hospital Vienna, Vienna, Austria.
- [3] Institute of Physiology, Centre of Physiology and Pharmacology, Medical University of Vienna, Vienna, Austria.
- [4] Division of Vascular Surgery, Department of Surgery, Medical University of Vienna, General Hospital Vienna, Vienna, Austria.
- [5] Department of Medicine IV, Kaiser Franz Josef Hospital, Vienna, Austria.
- [6] Department of Medicine I, Medical University of Vienna, General Hospital Vienna, Vienna, Austria.
- [7] Department of Clinical Pharmacology, Medical University of Vienna, General Hospital Vienna, Vienna, Austria.
- [8] Department of Internal Medicine II, Medical University of Innsbruck, Innsbruck, Austria.
- [9] Department of Pulmonology, Kepler University Hospital, Johannes Kepler University, Linz, Austria.

- PMID: **34609480**
- PMCID: PMC8500043
- DOI: 10.1093/cvr/cvab308

Free PMC article
Observational Study

Low-molecular-weight heparin use in coronavirus disease 2019 is associated with

curtailed viral persistence: a retrospective multicentre observational study

David Pereyra et al. Cardiovasc Res. 2021.
Free PMC article
Show details
Cardiovasc Res
. 2021 Dec 17;117(14):2807-2820.
doi: 10.1093/cvr/cvab308.

Authors

David Pereyra [1 2], Stefan Heber [3], Waltraud C Schrottmaier [1], Jonas Santol [2], Anita Pirabe [1], Anna Schmuckenschlager [1], Kerstin Kammerer [1], Daphni Ammon [2], Thomas Sorz [2], Fabian Fritsch [2], Hubert Hayden [4], Erich Pawelka [5], Philipp Krüger [1 2], Benedikt Rumpf [2 5], Marianna T Traugott [5], Pia Glaser [6], Christa Firbas [7], Christian Schörgenhofer [7], Tamara Seitz [5], Mario Karolyi [5], Ingrid Pabinger [6], Christine Brostjan [4], Patrick Starlinger [2], Günter Weiss [8], Rosa Bellmann-Weiler [8], Helmut J F Salzer [9], Bernd Jilma [7], Alexander Zoufaly [5], Alice Assinger [1]

Affiliations

- [1] Department of Vascular Biology and Thrombosis Research, Center of Physiology and Pharmacology, Medical University of Vienna, Schwarzspanierstraße 17, 1090 Vienna, Austria.
- [2] Division of Visceral Surgery, Department of Surgery, Medical University of Vienna, General Hospital Vienna, Vienna, Austria.
- [3] Institute of Physiology, Centre of Physiology and Pharmacology, Medical University of Vienna, Vienna, Austria.
- [4] Division of Vascular Surgery, Department of Surgery, Medical University of Vienna, General Hospital Vienna, Vienna, Austria.
- [5] Department of Medicine IV, Kaiser Franz Josef Hospital, Vienna, Austria.
- [6] Department of Medicine I, Medical University of Vienna, General Hospital Vienna, Vienna, Austria.
- [7] Department of Clinical Pharmacology, Medical University of Vienna, General Hospital Vienna, Vienna, Austria.
- [8] Department of Internal Medicine II, Medical University of Innsbruck, Innsbruck, Austria.
- [9] Department of Pulmonology, Kepler University Hospital, Johannes Kepler University, Linz, Austria.

- PMID: **34609480**
- PMCID: PMC8500043
- DOI: 10.1093/cvr/cvab308

Abstract

Aims: Anticoagulation was associated with improved survival of hospitalized coronavirus disease 2019 (COVID-19) patients in large-scale studies. Yet, the development of COVID-19-associated coagulopathy (CAC) and the mechanism responsible for improved survival of anticoagulated patients with COVID-19 remain largely elusive. This investigation aimed to explore the effects of anticoagulation and low-molecular-weight heparin (LMWH) in particular on patient outcome, CAC development, thromboinflammation, cell death, and viral persistence.

Methods and results: Data of 586 hospitalized COVID-19 patients from three different regions of Austria were evaluated retrospectively. Of these, 419 (71.5%) patients received LMWH and 62 (10.5%) received non-vitamin-K oral anticoagulants (NOACs) during hospitalization. Plasma was collected at different time points in a subset of 106 patients in order to evaluate markers of thromboinflammation (H3Cit-DNA) and the cell death marker cell-free DNA (cfDNA). Use of LMWH was associated with improved survival upon multivariable Cox regression (hazard ratio = 0.561, 95% confidence interval: 0.348-0.906). Interestingly, neither LMWH nor NOAC was associated with attenuation of D-dimer increase over time, or thromboinflammation. In contrast, anticoagulation was associated with a decrease in cfDNA during hospitalization, and curtailed viral persistence was observed in patients using LMWH leading to a 4-day reduction of virus positivity upon quantitative polymerase chain reaction [13 (interquartile range: 6-24) vs. 9 (interquartile range: 5-16) days, P = 0.009].

Conclusion: Time courses of haemostatic and thromboinflammatory biomarkers were similar in patients with and without LMWH, indicating either no effects of LMWH on haemostasis or that LMWH reduced hypercoagulability to levels of patients without LMWH. Nonetheless, anticoagulation with LMWH was associated with reduced mortality, improved markers of cell death, and curtailed viral persistence, indicating potential beneficial effects of LMWH beyond haemostasis, which encourages use of LMWH in COVID-19 patients without contraindications.

Keywords: Anticoagulation; COVID-19-associated coagulopathy; Low-molecular-weight heparin; SARS-CoV-2 viral persistence; Thromboinflammation.

© The Author(s) 2021. Published by Oxford University Press on behalf of the European Society of Cardiology.

- 29 references
- 4 figures

Supplementary info

Publication types, MeSH terms, Substances, Grant support Expand

Publication types

- Multicenter Study
- Observational Study
- Research Support, Non-U.S. Gov't

MeSH terms

- Aged
- Anticoagulants / pharmacology

- Anticoagulants / therapeutic use*
- Austria / epidemiology
- Biomarkers / blood
- COVID-19 / blood
- COVID-19 / complications
- COVID-19 / drug therapy*
- COVID-19 / mortality
- Female
- Hemostasis
- Heparin, Low-Molecular-Weight / pharmacology
- Heparin, Low-Molecular-Weight / therapeutic use*
- Humans
- Male
- Middle Aged
- Predictive Value of Tests
- Retrospective Studies
- SARS-CoV-2 / drug effects
- Thromboinflammation / prevention & control
- Thromboinflammation / virology*

Substances

- Anticoagulants
- Biomarkers
- Heparin, Low-Molecular-Weight

Grant support

- Austrian Federal Ministry of Education, Science and Research
- COVID024/Medical-Scientific Fund of the Mayor of Vienna
- P32064/Austrian Science Fund

Full text links

OXFORD ACADEMIC Silverchair Information Systems Free PMC article
Proceed to details
Cite
Share
715
Observational Study
J Perioper Pract
. 2021 Dec;31(12):446-453.
doi: 10.1177/17504589211026067. Epub 2021 Aug 13.

Characteristics and clinical outcomes of hip fracture patients during the first lockdown of COVID-19, lessons learnt: A retrospective cohort analysis

Karthikeyan P Iyengar [1], Mohammed Khatir [1], Muyed Kamal Awadalla Mohamed [1], Ankur D Kariya [2], Vijay Kumar Jain [3], Chetan Sangani [1], Riad F Adam [1], Khushroo Suraliwala [1]
Affiliations Expand

Affiliations

- [1] Southport and Ormskirk NHS Trust, Southport, UK.
- [2] Wrightington, Wigan and Leigh NHS Trust, Wigan, UK.
- [3] Atal Bihari Vajpayee Institute of Medical Sciences, Dr Ram Manohar Lohia Hospital, New Delhi, India.

- PMID: **34388942**
- DOI: 10.1177/17504589211026067

Observational Study

Characteristics and clinical outcomes of hip fracture patients during the first lockdown of COVID-19, lessons learnt: A retrospective cohort analysis

Karthikeyan P Iyengar et al. J Perioper Pract. 2021 Dec.
Show details
J Perioper Pract
. 2021 Dec;31(12):446-453.
doi: 10.1177/17504589211026067. Epub 2021 Aug 13.

Authors

Karthikeyan P Iyengar [1], Mohammed Khatir [1], Muyed Kamal Awadalla Mohamed [1], Ankur D Kariya [2], Vijay Kumar Jain [3], Chetan Sangani [1], Riad F Adam [1], Khushroo Suraliwala [1]

Affiliations

- [1] Southport and Ormskirk NHS Trust, Southport, UK.
- [2] Wrightington, Wigan and Leigh NHS Trust, Wigan, UK.

- [3] Atal Bihari Vajpayee Institute of Medical Sciences, Dr Ram Manohar Lohia Hospital, New Delhi, India.

- PMID: **34388942**
- DOI: 10.1177/17504589211026067

Abstract

Background: The management of hip fracture patients has been challenging across the UK in the wake of emergency coronavirus disease 2019 guidelines.

Aims: This retrospective, observational cohort study analyses the impact of the first lockdown during the early part of the coronavirus disease 2019 pandemic on the management of hip fracture patients at a district general hospital in the UK.

Methods: Comparative analysis to assess hip fracture patients treated at this Trust between 1 April to 31 May 2019 and 1 April to 31 May 2020 was undertaken. The primary outcome measures appraised were 30 and 60-day mortality and the secondary outcome measure included time to surgery.

Results: There was a higher 30 and 60-day mortality rate in the first lockdown period at 8.1% and 13.5%, respectively, compared to 1.96% and 5.88% in 2019. A significantly lower proportion of hip fracture patients at 59.46% were operated within the 36h target time frame during the first lockdown.

Conclusion: In our Trust, hip fractures were treated as obligatory injuries. However, the mortality was higher in the 2020 cohort with a significant reduction in patients achieving the recommended '36 hours' time to surgery target and accruement of Best Practice Tariff. Enhanced infection control strategies have prepared us for the future.

Keywords: COVID-19; Coronavirus; Elderly; Hip fracture; Mortality; National Hip Fracture Database; Pandemics; The National Institute for Health and Care Excellence.

Supplementary info

Publication types, MeSH terms Expand

Publication types

- Observational Study

MeSH terms

- COVID-19*
- Cohort Studies
- Hip Fractures* / epidemiology
- Hip Fractures* / surgery
- Humans
- Infection Control

- Retrospective Studies
- SARS-CoV-2

Full text links

SAGE journals Atypon
Proceed to details
Cite
Share
716
Observational Study
BMJ Open
. 2021 Nov 18;11(11):e053281.
doi: 10.1136/bmjopen-2021-053281.

Stratification of the risk of developing severe or lethal Covid-19 using a new score from a large Italian population: a population-based cohort study

Giovanni Corrao [1 2], Federico Rea [3 2], Flavia Carle [2 4], Salvatore Scondotto [2 5], Alessandra Allotta [5], Vito Lepore [6], Antonio D'Ettorre [6], Cinzia Tanzarella [6], Patrizia Vittori [7], Sabrina Abena [7], Marica Iommi [4], Liana Spazzafumo [2 8], Michele Ercolanoni [9], Roberto Blaco [9], Simona Carbone [10], Cristina Giordani [10], Dario Manfellotto [11], Massimo Galli [12], Giuseppe Mancia [13 14], 'Monitoring and Assessing care Pathways (MAP)' working group of the Italian Ministry of Health
Collaborators, Affiliations Expand

Collaborators

- **'Monitoring and Assessing care Pathways (MAP)' working group of the Italian Ministry of Health**:
  Donata Bellentani, Simona Carbone, Carla Ceccolini, Angela De Feo, Cristina Giordani, Rosanna Mariniello, Modesta Visca, Natalia Magliocchetti, Giovanna Romano, Antonio Lora, Paola Pisanti, Rinaldo Zanini, Flavia Carle, Marica Iommi, Edlira Skrami, Anna Cantarutti, Giovanni Corrao, Matteo Monzio Compagnoni, Pietro Pugni, Federico Rea, Marina Davoli, Mirko Di Martino, Adele Lallo, Patrizia Vittori, Giuliana Vuillermin, Alfonso Bernardo, Anna Frusciante, Laura Belotti, Rossana De Palma, Andrea Di Lenarda, Marisa Prezza, Danilo Fusco, Chiara Marinacci, Olivia Leoni, Liana Spazzafumo, Simone Pizzi, Lolita Gallo, Ettore Attolini, Vito Lepore, Salvatore Scondotto, Giovanni De Luca, Paolo Francesconi, Carla Rizzuti, Francesco Avossa, Silvia Vigna, Letizia Dondi, Nello Martini, Antonella Pedrini, Carlo Piccinni, Mimma Cosentino, Maria Grazia Marvulli, Aldo Maggioni

Affiliations

- [1] Department of Statistics and Quantitative Methods, University of Milan-Bicocca, Milan, Italy giovanni.corrao@unimib.it.
- [2] National Centre for Healthcare Research and Pharmacoepidemiology, University of Milano-Bicocca, Milan, Italy.
- [3] Department of Statistics and Quantitative Methods, University of Milan-Bicocca, Milan, Italy.
- [4] Center of Epidemiology and Biostatistics, Polytechnic University of Marche, Ancona, Italy.
- [5] Department of Health Services and Epidemiological Observatory, Regional Health Authority of Sicily, Palermo, Italy.
- [6] Regional Health Agency of Puglia, Bari, Italy.
- [7] Regional Health Authority, Aosta, Italy.
- [8] Regional Health Agency of Marche, Ancona, Italy.
- [9] Regional Welfare Service, Milan, Italy.
- [10] Department of Health Planning, Italian Health Ministry, Rome, Italy.
- [11] Department of Internal Medicine, Hospital Fatebenefratelli, Rome, Italy.
- [12] Institute of Tropical and Infectious Diseases, University of Milan L Sacco Hospital, Milan, Italy.
- [13] University of Milano-Bicocca, Milan, Italy.
- [14] Policlinico di Monza, Monza, Italy.

- PMID: **34794995**
- PMCID: PMC8602929
- DOI: 10.1136/bmjopen-2021-053281

Free PMC article
Observational Study

Stratification of the risk of developing severe or lethal Covid-19 using a new score from a large Italian population: a population-based cohort study

Giovanni Corrao et al. BMJ Open. 2021.
Free PMC article
Show details
BMJ Open

. 2021 Nov 18;11(11):e053281.
doi: 10.1136/bmjopen-2021-053281.

Authors

Giovanni Corrao [1, 2], Federico Rea [3, 2], Flavia Carle [2, 4], Salvatore Scondotto [2, 5], Alessandra Allotta [5], Vito Lepore [6], Antonio D'Ettorre [6], Cinzia Tanzarella [6], Patrizia Vittori [7], Sabrina Abena [7], Marica Iommi [4], Liana Spazzafumo [2, 8], Michele Ercolanoni [9], Roberto Blaco [9], Simona Carbone [10], Cristina Giordani [10], Dario Manfellotto [11], Massimo Galli [12], Giuseppe Mancia [13, 14], 'Monitoring and Assessing care Pathways (MAP)' working group of the Italian Ministry of Health

Collaborators

- **'Monitoring and Assessing care Pathways (MAP)' working group of the Italian Ministry of Health**:
  Donata Bellentani, Simona Carbone, Carla Ceccolini, Angela De Feo, Cristina Giordani, Rosanna Mariniello, Modesta Visca, Natalia Magliocchetti, Giovanna Romano, Antonio Lora, Paola Pisanti, Rinaldo Zanini, Flavia Carle, Marica Iommi, Edlira Skrami, Anna Cantarutti, Giovanni Corrao, Matteo Monzio Compagnoni, Pietro Pugni, Federico Rea, Marina Davoli, Mirko Di Martino, Adele Lallo, Patrizia Vittori, Giuliana Vuillermin, Alfonso Bernardo, Anna Frusciante, Laura Belotti, Rossana De Palma, Andrea Di Lenarda, Marisa Prezza, Danilo Fusco, Chiara Marinacci, Olivia Leoni, Liana Spazzafumo, Simone Pizzi, Lolita Gallo, Ettore Attolini, Vito Lepore, Salvatore Scondotto, Giovanni De Luca, Paolo Francesconi, Carla Rizzuti, Francesco Avossa, Silvia Vigna, Letizia Dondi, Nello Martini, Antonella Pedrini, Carlo Piccinni, Mimma Cosentino, Maria Grazia Marvulli, Aldo Maggioni

Affiliations

- [1] Department of Statistics and Quantitative Methods, University of Milan-Bicocca, Milan, Italy giovanni.corrao@unimib.it.
- [2] National Centre for Healthcare Research and Pharmacoepidemiology, University of Milano-Bicocca, Milan, Italy.
- [3] Department of Statistics and Quantitative Methods, University of Milan-Bicocca, Milan, Italy.
- [4] Center of Epidemiology and Biostatistics, Polytechnic University of Marche, Ancona, Italy.
- [5] Department of Health Services and Epidemiological Observatory, Regional Health Authority of Sicily, Palermo, Italy.
- [6] Regional Health Agency of Puglia, Bari, Italy.
- [7] Regional Health Authority, Aosta, Italy.
- [8] Regional Health Agency of Marche, Ancona, Italy.
- [9] Regional Welfare Service, Milan, Italy.
- [10] Department of Health Planning, Italian Health Ministry, Rome, Italy.
- [11] Department of Internal Medicine, Hospital Fatebenefratelli, Rome, Italy.
- [12] Institute of Tropical and Infectious Diseases, University of Milan L Sacco Hospital, Milan, Italy.
- [13] University of Milano-Bicocca, Milan, Italy.
- [14] Policlinico di Monza, Monza, Italy.

- PMID: **34794995**
- PMCID: PMC8602929
- DOI: 10.1136/bmjopen-2021-053281

Abstract

Objectives: To develop a population-based risk stratification model (COVID-19 Vulnerability Score) for predicting severe/fatal clinical manifestations of SARS-CoV-2 infection, using the multiple source information provided by the healthcare utilisation databases of the Italian National Health Service.

Design: Retrospective observational cohort study.

Setting: Population-based study using the healthcare utilisation database from five Italian regions.

Participants: Beneficiaries of the National Health Service, aged 18-79 years, who had the residentship in the five participating regions. Residents in a nursing home were not included. The model was built from the 7 655 502 residents of Lombardy region.

Main outcome measure: The score included gender, age and 29 conditions/diseases selected from a list of 61 conditions which independently predicted the primary outcome, that is, severe (intensive care unit admission) or fatal manifestation of COVID-19 experienced during the first epidemic wave (until June 2020). The score performance was validated by applying the model to several validation sets, that is, Lombardy population (second epidemic wave), and the other four Italian regions (entire 2020) for a total of about 15.4 million individuals and 7031 outcomes. Predictive performance was assessed by discrimination (areas under the receiver operating characteristic curve) and calibration (plot of observed vs predicted outcomes).

Results: We observed a clear positive trend towards increasing outcome incidence as the score increased. The areas under the receiver operating characteristic curve of the COVID-19 Vulnerability Score ranged from 0.85 to 0.88, which compared favourably with the areas of generic scores such as the Charlson Comorbidity Score (0.60). A remarkable performance of the score on the calibration of observed and predicted outcome probability was also observed.

Conclusions: A score based on data used for public health management accurately predicted the occurrence of severe/fatal manifestations of COVID-19. Use of this score may help health decision-makers to more accurately identify high-risk citizens who need early preventive or treatment interventions.

Keywords: COVID-19; health policy; public health.

© Author(s) (or their employer(s)) 2021. Re-use permitted under CC BY-NC. No commercial re-use. See rights and permissions. Published by BMJ.

Conflict of interest statement

Competing interests: GC received research support from the European Community (EC), the Italian Agency of Drug (AIFA), the Italian Ministry of Education, University and Research (MIUR), and the Italian Health Ministry. He took part in a variety of projects that were funded by pharmaceutical companies (ie, Novartis, GSK, Roche, AMGEN and BMS). He also received honoraria as member of Advisory Board from Roche. GM received honoraria for participation as speaker/chairman in national/international meetings from Boehringer Ingelheim, Ferrer, Medtronic, Menarini, Merck Serono, Recordati and Servier.

- 30 references
- 3 figures

Supplementary info

Publication types, MeSH terms Expand

Publication types

- Observational Study
- Research Support, Non-U.S. Gov't

MeSH terms

- Adult
- COVID-19*
- Cohort Studies
- Humans
- Italy / epidemiology
- Retrospective Studies
- SARS-CoV-2
- State Medicine

Full text links

BMJ Free Full Text HighWire Free PMC article

Proceed to details

Cite

Share

717

Observational Study

PLoS One

. 2021 Jun 18;16(6):e0252919.

doi: 10.1371/journal.pone.0252919. eCollection 2021.

Resource utilization and outcomes in emergency general surgery during the COVID19 pandemic: An observational cost analysis

Amelia J Hessheimer [1], Marta Trapero-Bertran [2], Alex Borin [3], Eugenia Butori [3 4], Anna Curell [4], Arlena Sofía Espinoza [3 4], Joaquín Jensen [3], Víctor Turrado [4], Xavier Morales [4], Antonio María de Lacy [4], Constantino Fondevila [1]

Affiliations Expand

Affiliations

- [1] General & Digestive Surgery, Institut de Malaties Digestives i Metabòliques (ICMDM), Hospital Clínic Barcelona, IDIBAPS, CIBERehd, University of Barcelona, Barcelona, Spain.
- [2] Basic Sciences Department, University Institute for Patient Care, Universitat Internacional de Catalunya Barcelona, Barcelona, Spain.
- [3] General & Digestive Surgery, ICMDM, Hospital Clínic Barcelona, Barcelona, Spain.
- [4] Gastrointestinal Surgery, ICMDM, Hospital Clínic Barcelona, Barcelona, Spain.

- PMID: **34143802**
- PMCID: PMC8213049
- DOI: 10.1371/journal.pone.0252919

Free PMC article
Observational Study

Resource utilization and outcomes in emergency general surgery during the COVID19 pandemic: An observational cost analysis

Amelia J Hessheimer et al. PLoS One. 2021.
Free PMC article

Show details

PLoS One

. 2021 Jun 18;16(6):e0252919.
doi: 10.1371/journal.pone.0252919. eCollection 2021.

Authors

Amelia J Hessheimer [1], Marta Trapero-Bertran [2], Alex Borin [3], Eugenia Butori [3] [4], Anna Curell [4], Arlena Sofía Espinoza [3] [4], Joaquín Jensen [3], Víctor Turrado [4], Xavier Morales [4], Antonio María de Lacy [4], Constantino Fondevila [1]

Affiliations

- [1] General & Digestive Surgery, Institut de Malaties Digestives i Metabòliques (ICMDM), Hospital Clínic Barcelona, IDIBAPS, CIBERehd, University of Barcelona, Barcelona, Spain.
- [2] Basic Sciences Department, University Institute for Patient Care, Universitat Internacional de Catalunya Barcelona, Barcelona, Spain.
- [3] General & Digestive Surgery, ICMDM, Hospital Clínic Barcelona, Barcelona, Spain.
- [4] Gastrointestinal Surgery, ICMDM, Hospital Clínic Barcelona, Barcelona, Spain.

- PMID: **34143802**

- PMCID: PMC8213049
- DOI: 10.1371/journal.pone.0252919

Abstract

Background: Over the course of the COVID19 pandemic, global healthcare delivery has declined. Surgery is one of the most resource-intensive area of medicine; loss of surgical care has had untold health and economic consequences. Herein, we evaluate resource utilization, outcomes, and healthcare costs associated with unplanned surgery admissions during the height of the pandemic in 2020 versus the same period in 2019.

Methods: Retrospective analysis on patients ≥18 years admitted from the emergency department to General & Digestive and Gastrointestinal Surgery Services between February and May 2019 and 2020 at our center; clinical outcomes and unadjusted and adjusted per-person healthcare costs were analyzed.

Results: Consults and admissions to surgery declined between February and May 2020 by 37% and 19%, respectively, relative to the same period in 2019, with even greater relative decline during late March and early April. Time between onset of symptoms to diagnosis increased from 2±3 days 2019 to 5±22 days 2020 ($P = 0.01$). Overall hospital stay was two days less in 2020 ($P = 0.19$). Complications (Comprehensive Complication Index 10.3±23.7 2019 vs. 13.9±25.5 2020, $P = 0.10$) and mortality rates (3% vs. 4%, respectively, $P = 0.58$) did not vary. Mean unadjusted per-person costs for patients in the 2019 and 2020 cohorts were 5,886.72€±12,576.33€ and 5,287.62±7,220.16€, respectively ($P = 0.43$). Following multivariate analysis, costs remained similar (4,656.89€±390.53€ 2019 vs. 4,938.54±406.55€ 2020, $P = 0.28$).

Conclusions: Healthcare delivery and spending for unplanned general surgery admissions declined considerably due to COVID19. These results provide a small yet relevant illustration of clinical and economic ramifications of this healthcare crisis.

Conflict of interest statement

Amelia J. Hessheimer and Constantino Fondevila have received consultancy fees from Guanguong Shunde Innovative Design Institute, Guangdong, China, and research funding from Instituto de Salud Carlos III. This does not alter their adherence to PLOS ONE policies on sharing data and materials. The remainder of authors have no disclosures.

- 25 references
- 2 figures

Supplementary info

Publication types, MeSH terms, Grant support Expand

Publication types

- Observational Study

MeSH terms

- Adult
- Aged
- Aged, 80 and over
- COVID-19 / epidemiology*
- Emergency Service, Hospital / economics*
- Female
- Health Care Costs / trends*
- Hospitalization / economics*
- Humans
- Length of Stay
- Male
- Middle Aged
- Retrospective Studies
- Surgery Department, Hospital / economics*
- Young Adult

Grant support

The authors received no specific funding for this work.

Full text links

OPEN ACCESS TO FULL TEXT PLOS ONE Public Library of Science Free PMC article
Proceed to details
Cite
Share
☐ 718
Observational Study
J Pain Symptom Manage
. 2020 Aug;60(2):e75-e78.
doi: 10.1016/j.jpainsymman.2020.04.031. Epub 2020 May 6.

A Dual-Center Observational Review of Hospital-Based Palliative Care in Patients Dying With COVID-19

Jennifer Turner [1], Luke Eliot Hodgson [2], Todd Leckie [3], Lisa Eade [4], Suzanne Ford-Dunn [5]
Affiliations Expand

Affiliations

- [1] ST5 Geriatric Medicine, Worthing Hospital, Western Sussex Hospitals NHS Foundation Trust, Worthing, UK.
- [2] Intensive Care Department, Worthing Hospital Western Sussex Hospitals NHS Foundation Trust, Worthing, UK; Faculty of Health & Medical Sciences, University of Surrey, Guildford, UK.
- [3] ST3 Anaesthetics, Intensive Care Department, Worthing Hospital, Western Sussex Hospitals NHS Foundation Trust, Worthing, UK.
- [4] Palliative Care, St Richards Hospital, Western Sussex Hospitals NHS Foundation Trust, Chichester, UK.
- [5] Palliative Medicine, Worthing Hospital, Western Sussex Hospitals NHS Foundation Trust, Worthing, UK; St Barnabas House Hospice, Worthing, UK. Electronic address: suzanne.ford-dunn@nhs.net.

- PMID: **32387139**
- PMCID: PMC7200379
- DOI: 10.1016/j.jpainsymman.2020.04.031

Free PMC article
Observational Study

A Dual-Center Observational Review of Hospital-Based Palliative Care in Patients Dying With COVID-19

Jennifer Turner et al. J Pain Symptom Manage. 2020 Aug.
Free PMC article

Show details

J Pain Symptom Manage
. 2020 Aug;60(2):e75-e78.
doi: 10.1016/j.jpainsymman.2020.04.031. Epub 2020 May 6.

Authors

Jennifer Turner [1], Luke Eliot Hodgson [2], Todd Leckie [3], Lisa Eade [4], Suzanne Ford-Dunn [5]

Affiliations

- [1] ST5 Geriatric Medicine, Worthing Hospital, Western Sussex Hospitals NHS Foundation Trust, Worthing, UK.
- [2] Intensive Care Department, Worthing Hospital Western Sussex Hospitals NHS Foundation Trust, Worthing, UK; Faculty of Health & Medical Sciences, University of Surrey, Guildford, UK.
- [3] ST3 Anaesthetics, Intensive Care Department, Worthing Hospital, Western Sussex Hospitals NHS Foundation Trust, Worthing, UK.
- [4] Palliative Care, St Richards Hospital, Western Sussex Hospitals NHS Foundation Trust, Chichester, UK.

- [5] Palliative Medicine, Worthing Hospital, Western Sussex Hospitals NHS Foundation Trust, Worthing, UK; St Barnabas House Hospice, Worthing, UK. Electronic address: suzanne.ford-dunn@nhs.net.

- PMID: **32387139**
- PMCID: PMC7200379
- DOI: 10.1016/j.jpainsymman.2020.04.031

Abstract

The current coronavirus disease 2019 (COVID-19) pandemic has put significant strain on all aspects of health care delivery, including palliative care services. Given the high mortality from this disease, particularly in the more vulnerable members of society, it is important to examine how best to deliver a high standard of end-of-life care during this crisis. This case series collected data from two acute hospitals examining the management of patients diagnosed with COVID-19 who subsequently died (n = 36) and compared this with national and local end-of-life audit data for all other deaths. Our results demonstrated a shorter dying phase (38.25 hours vs. 74 hours) and higher rates of syringe driver use (72% vs. 33% in local audits), although with similar average mediation doses. Of note was the significant heterogeneity in the phenotype of deterioration in the dying phase, two distinct patterns emerged, with one group demonstrating severe illness with a short interval between symptom onset and death and another group presenting with a more protracted deterioration. This brief report suggests a spectrum of mode of dying. Overall, the cohort reflects previously described experiences, with increased frailty (median Clinical Frailty Scale score of 5) and extensive comorbidity burden. This brief report provides clinicians with a contemporaneous overview of our experience, knowledge, and pattern recognition when caring for people with COVID-19 and highlights the value of proactive identification of patients and risk of deterioration and palliation.

Keywords: COVID-19; Palliative care; end-of-life care; frailty; symptom management.

Copyright © 2020 American Academy of Hospice and Palliative Medicine. All rights reserved.

- 11 references

Supplementary info

Publication types, MeSH terms Expand

Publication types

- Multicenter Study
- Observational Study

MeSH terms

- Aged
- Aged, 80 and over
- COVID-19
- Comorbidity

- Coronavirus Infections / mortality*
- Coronavirus Infections / therapy*
- Disease Management
- Disease Progression
- Frailty
- Hospitalization*
- Humans
- Middle Aged
- Palliative Care*
- Pandemics
- Pneumonia, Viral / mortality*
- Pneumonia, Viral / therapy*
- Retrospective Studies
- Syringes
- Terminal Care*
- Time Factors

Full text links

ELSEVIER FULL-TEXT ARTICLE Elsevier Science Free PMC article
Proceed to details
Cite
Share
719
Observational Study
Neurocrit Care
. 2021 Jun;34(3):739-747.
doi: 10.1007/s12028-021-01202-7. Epub 2021 Feb 23.

Intracerebral Hemorrhage in COVID-19 Patients with Pulmonary Failure: A Propensity Score-Matched Registry Study

Corinna N Lang [1, 2], Johanna S Dettinger [3, 4], Michael Berchtold-Herz [5], Stefan Utzolino [6], Xavier Bemtgen [3, 4], Viviane Zotzmann [3, 4], Bonaventura Schmid [7], Paul M Biever [3, 4], Christoph Bode [3, 4], Katharina Müller-Peltzer [8], Daniel Duerschmied [3, 4], Tobias Wengenmayer [3, 4], Wolf-Dirk Niesen [#, 9], Dawid L Staudacher [#, 3, 4]
Affiliations Expand

Affiliations

- [1] Department of Cardiology and Angiology I, Faculty of Medicine, Heart Center Freiburg University, University of Freiburg, Hugstetter Str. 55, 79106, Freiburg, Germany. corinna.nadine.lang@uniklinik-freiburg.de.
- [2] Department of Medicine III (Interdisciplinary Medical Intensive Care), Medical Center, Faculty of Medicine, University of Freiburg, Freiburg, Germany. corinna.nadine.lang@uniklinik-freiburg.de.
- [3] Department of Cardiology and Angiology I, Faculty of Medicine, Heart Center Freiburg University, University of Freiburg, Hugstetter Str. 55, 79106, Freiburg, Germany.
- [4] Department of Medicine III (Interdisciplinary Medical Intensive Care), Medical Center, Faculty of Medicine, University of Freiburg, Freiburg, Germany.
- [5] Department of Cardiovascular Surgery, Faculty of Medicine, Heart Center Freiburg University, University of Freiburg, Freiburg, Germany.
- [6] Department of General and Visceral Surgery, Medical Center, University of Freiburg, Freiburg, Germany.
- [7] Department of Emergency Medicine, Faculty of Medicine, University of Freiburg, Freiburg, Germany.
- [8] Department of Radiology, Faculty of Medicine, University of Freiburg, Freiburg, Germany.
- [9] Department of Neurology, Faculty of Medicine, University of Freiburg, Freiburg, Germany.

[#] Contributed equally.

- PMID: **33619668**
- PMCID: PMC7899797
- DOI: 10.1007/s12028-021-01202-7

Free PMC article
Observational Study

Intracerebral Hemorrhage in COVID-19 Patients with Pulmonary Failure: A Propensity Score-Matched Registry Study

Corinna N Lang et al. Neurocrit Care. 2021 Jun.
Free PMC article
Show details
Neurocrit Care
. 2021 Jun;34(3):739-747.
doi: 10.1007/s12028-021-01202-7. Epub 2021 Feb 23.

Authors

Corinna N Lang [1] [2], Johanna S Dettinger [3] [4], Michael Berchtold-Herz [5], Stefan Utzolino [6], Xavier Bemtgen [3] [4], Viviane Zotzmann [3] [4], Bonaventura Schmid [7], Paul M Biever [3] [4], Christoph Bode [3] [4], Katharina Müller-Peltzer [8], Daniel Duerschmied [3] [4], Tobias Wengenmayer [3] [4], Wolf-Dirk Niesen [#] [9], Dawid L Staudacher [#] [3] [4]

Affiliations

- [1] Department of Cardiology and Angiology I, Faculty of Medicine, Heart Center Freiburg University, University of Freiburg, Hugstetter Str. 55, 79106, Freiburg, Germany. corinna.nadine.lang@uniklinik-freiburg.de.
- [2] Department of Medicine III (Interdisciplinary Medical Intensive Care), Medical Center, Faculty of Medicine, University of Freiburg, Freiburg, Germany. corinna.nadine.lang@uniklinik-freiburg.de.
- [3] Department of Cardiology and Angiology I, Faculty of Medicine, Heart Center Freiburg University, University of Freiburg, Hugstetter Str. 55, 79106, Freiburg, Germany.
- [4] Department of Medicine III (Interdisciplinary Medical Intensive Care), Medical Center, Faculty of Medicine, University of Freiburg, Freiburg, Germany.
- [5] Department of Cardiovascular Surgery, Faculty of Medicine, Heart Center Freiburg University, University of Freiburg, Freiburg, Germany.
- [6] Department of General and Visceral Surgery, Medical Center, University of Freiburg, Freiburg, Germany.
- [7] Department of Emergency Medicine, Faculty of Medicine, University of Freiburg, Freiburg, Germany.
- [8] Department of Radiology, Faculty of Medicine, University of Freiburg, Freiburg, Germany.
- [9] Department of Neurology, Faculty of Medicine, University of Freiburg, Freiburg, Germany.

[#] Contributed equally.

- PMID: **33619668**
- PMCID: PMC7899797
- DOI: 10.1007/s12028-021-01202-7

Erratum in

- Correction to: Intracerebral Hemorrhage in COVID-19 Patients with Pulmonary Failure: A Propensity Score-Matched Registry Study.
  Lang CN, Dettinger JS, Berchtold-Herz M, Utzolino S, Bemtgen X, Zotzmann V, Schmid B, Biever PM, Bode C, Müller-Peltzer K, Duerschmied D, Wengenmayer T, Niesen WD, Staudacher DL. Lang CN, et al. Neurocrit Care. 2021 Jun;34(3):1112. doi: 10.1007/s12028-021-01225-0. Neurocrit Care. 2021. PMID: 33846902 Free PMC article. No abstract available.

Abstract

Background: Hypercoagulability in Coronavirus Disease 2019 (COVID-19) causes deep vein thrombosis and pulmonary embolism necessitating systemic anticoagulation. Case reports of intracerebral hemorrhages in ventilated COVID-19 patients warrant precaution. It is unclear, however, if COVID-19 patients with acute respiratory distress syndrome (ARDS) with or without veno-venous extracorporeal membrane oxygenation therapy (VV-ECMO) have more intracerebral hemorrhages (ICH) compared to other ARDS patients.

Methods: We conducted a retrospective observational single-center study enrolling all patients with ARDS from 01/2018 to 05/2020. PCR-positive SARS-CoV-2 patients with ARDS were

allocated to the COVID-19 group. Propensity score matching was performed for age, VV-ECMO, and bleeding risk.

Results: A total of 163 patients with moderate or severe ARDS were identified, 47 (28.8%) in the COVID-19 group, and 116 (71.2%) in the non-COVID-19 group. In 63/163 cases (38.7%), VV-ECMO therapy was required. The ICU survival was 52.8%. COVID-19 patients were older, more often male, and exhibited a lower SOFA score, but the groups showed similar rates of VV-ECMO therapy. Treatments with antiplatelet agents (p = 0.043) and therapeutic anticoagulation (p = 0.028) were significantly more frequent in the COVID-19 patients. ICH was detected in 22 patients (13.5%) with no statistical difference between the groups (11.2 vs. 19.1% without and with SARS-CoV-2, respectively, p = 0.21). Propensity score matching confirmed similar rates of ICH in both groups (12.8 vs. 19.1% without and with SARS-CoV-2, respectively, p = 0.57), thus leveling out possible confounders.

Conclusions: Intracerebral hemorrhage was detected in every tenth patient with ARDS. Despite statistically higher rates of antiplatelet therapy and therapeutic anticoagulation in COVID-19 patients, we found a similar rate of ICH in patients with ARDS due to COVID-19 compared to other causes of ARDS.

Keywords: ARDS; COVID-19; Intracerebral hemorrhage.

Conflict of interest statement

The authors do hereby declare no conflict of interests.

- 31 references
- 2 figures

Supplementary info

Publication types, MeSH terms Expand

Publication types

- Observational Study
- Research Support, Non-U.S. Gov't

MeSH terms

- Adult
- Aged
- Aged, 80 and over
- COVID-19 / complications*
- COVID-19 / mortality
- COVID-19 / therapy
- Cerebral Hemorrhage / epidemiology*
- Cerebral Hemorrhage / therapy
- Cerebral Hemorrhage / virology*

- Critical Care
- Extracorporeal Membrane Oxygenation
- Female
- Germany
- Humans
- Length of Stay
- Male
- Middle Aged
- Propensity Score
- Registries
- Respiration, Artificial
- Respiratory Distress Syndrome / mortality
- Respiratory Distress Syndrome / therapy
- Respiratory Distress Syndrome / virology*
- Retrospective Studies
- Risk Factors
- Survival Rate
- Young Adult

Full text links

SpringerLink FULL-TEXT ARTICLE Springer Free PMC article
Proceed to details
Cite
Share
720

Nutr Metab Cardiovasc Dis
. 2021 Feb 8;31(2):396-398.
doi: 10.1016/j.numecd.2020.09.028. Epub 2020 Oct 1.

Are diabetes and its medications risk factors for the development of COVID-19? Data from a population-based study in Sicily

G Antonio Silverii [1], Matteo Monami [2], Achille Cernigliaro [3], Enrica Vigneri [4], Valentina Guarnotta [4], Salvatore Scondotto [3], Vincenza A Allotta [3], Michela Conti [4], Carla Giordano [4], Edoardo Mannucci [2]
Affiliations Expand

Affiliations

- [1] Experimental and Clinical Biomedical Sciences "Mario Serio" Department, University of Florence, Italy. Electronic address: antonio.silverii@gmail.com.
- [2] Experimental and Clinical Biomedical Sciences "Mario Serio" Department, University of Florence, Italy.
- [3] Health Activities and Epidemiologic Observatory Division, Health Department, Sicily Region, Italy.
- [4] Health Promotion, Department of Health Promotion, Mother and Child Care, Internal Medicine and Medical Specialties "G. D'Alessandro", PROMISE, University of Palermo, Italy.

- PMID: **33223405**
- PMCID: PMC7528967
- DOI: 10.1016/j.numecd.2020.09.028

Free PMC article

Are diabetes and its medications risk factors for the development of COVID-19? Data from a population-based study in Sicily

G Antonio Silverii et al. Nutr Metab Cardiovasc Dis. 2021.
Free PMC article
Show details
Nutr Metab Cardiovasc Dis
. 2021 Feb 8;31(2):396-398.
doi: 10.1016/j.numecd.2020.09.028. Epub 2020 Oct 1.

Authors

G Antonio Silverii [1], Matteo Monami [2], Achille Cernigliaro [3], Enrica Vigneri [4], Valentina Guarnotta [4], Salvatore Scondotto [3], Vincenza A Allotta [3], Michela Conti [4], Carla Giordano [4], Edoardo Mannucci [2]

Affiliations

- [1] Experimental and Clinical Biomedical Sciences "Mario Serio" Department, University of Florence, Italy. Electronic address: antonio.silverii@gmail.com.
- [2] Experimental and Clinical Biomedical Sciences "Mario Serio" Department, University of Florence, Italy.
- [3] Health Activities and Epidemiologic Observatory Division, Health Department, Sicily Region, Italy.
- [4] Health Promotion, Department of Health Promotion, Mother and Child Care, Internal Medicine and Medical Specialties "G. D'Alessandro", PROMISE, University of Palermo, Italy.

- PMID: **33223405**
- PMCID: PMC7528967

• DOI: 10.1016/j.numecd.2020.09.028

Abstract

Background and aims: Diabetes mellitus (DM) has been associated with higher incidence of severe cases of COVID-19 in hospitalized patients, but it is unknown whether DM is a risk factor for the overall COVID-19 incidence. The aim of present study was to investigate whether there is an association of DM with COVID-19 prevalence and case fatality, and between different DM medications and risk for COVID-19 infection and death.

Methods and results: retrospective observational study on all SARS-CoV-2 positive (SARS-CoV-2^{+}) cases and deaths in Sicily up to 2020, May 14th. No difference in COVID-19 prevalence was found between people with and without DM (RR 0.92 [0.79-1.09]). Case fatality was significantly higher in SARS-CoV-2^{+} with DM (RR 4.5 [3.55-5.71]). No diabetes medication was associated with differences in risk for SARS-Cov2 infection.

Conclusions: in Sicily, DM was not a risk factor for COVID-19 infection, whereas it was associated with a higher case fatality.

Keywords: COVID-19 case fatality; COVID-19 incidence; Diabetes mellitus.

Copyright © 2020 The Italian Diabetes Society, the Italian Society for the Study of Atherosclerosis, the Italian Society of Human Nutrition and the Department of Clinical Medicine and Surgery, Federico II University. Published by Elsevier B.V. All rights reserved.

- 11 references

Supplementary info

MeSH terms, Substances Expand

MeSH terms

- Adolescent
- Adult
- Aged
- Aged, 80 and over
- COVID-19 / epidemiology*
- COVID-19 / mortality
- Child
- Child, Preschool
- Diabetes Mellitus / drug therapy
- Diabetes Mellitus / epidemiology*
- Female
- Humans
- Hypoglycemic Agents / adverse effects
- Hypoglycemic Agents / therapeutic use

- Incidence
- Infant
- Male
- Middle Aged
- Prevalence
- Retrospective Studies
- Risk Factors
- SARS-CoV-2
- Sicily / epidemiology
- Young Adult

Substances

- Hypoglycemic Agents

Full text links

ELSEVIER FULL-TEXT ARTICLE Elsevier Science Free PMC article

Proceed to details

Cite

Share

☐ 721

Observational Study

J Nutr Health Aging
. 2021;25(2):189-196.
doi: 10.1007/s12603-020-1479-0.

Impact of Serum 25(OH) Vitamin D Level on Mortality in Patients with COVID-19 in Turkey

S Karahan [1], F Katkat
Affiliations Expand

Affiliation

- [1] Serkan Karahan, Bagcilar Egitim ve Arastirma Hastanesi, Turkey, drserkankarahan@gmail.com.

- PMID: **33491033**
- PMCID: PMC7533663
- DOI: 10.1007/s12603-020-1479-0

Free PMC article

Observational Study

Impact of Serum 25(OH) Vitamin D Level on Mortality in Patients with COVID-19 in Turkey

S Karahan et al. J Nutr Health Aging. 2021.
Free PMC article
Show details
J Nutr Health Aging
. 2021;25(2):189-196.
doi: 10.1007/s12603-020-1479-0.

Authors

S Karahan [1], F Katkat

Affiliation

- [1] Serkan Karahan, Bagcilar Egitim ve Arastirma Hastanesi, Turkey, drserkankarahan@gmail.com.

- PMID: **33491033**
- PMCID: PMC7533663
- DOI: 10.1007/s12603-020-1479-0

Abstract

Background: Because of the lack of sufficient data, we aimed to investigate the role of serum 25 (OH) vitamin D level on COVID severity and related mortality.

Methods: This was a retrospective observational study. Data, including sociodemographic features, clinical characteristics, and laboratory data, and 25(OH) vitamin D levels were recorded for each study participant. Patients were stratified into different vitamin D groups; Normal (Serum 25(OH) vitamin D level >30 ng/mL), Vitamin D insufficiency (21-29 ng/mL), and deficiency (<20 ng/mL). The severity of COVID was classified according to the Chinese Clinical Guideline for classification of COVID-19 severity. Mortality data were determined for participants. Univariate and multivariate Logistic regression analysis was performed to determine independent predictors of in-hospital mortality.

Results: Overall, 149 COVID-19 patients (females 45.6%, mean age 63.5 ± 15.3 (range 24-90 years) years) were included. Forty-seven patients (31.5%) had moderate COVID-19, whereas 102 patients (68.5%) had severe-critical COVID-19. The mean 25(OH) vitamin D level was 15.2 ± 10.3 ng/mL. Thirty-four (22.8%) and 103 (69.1%) patients had vitamin D insufficiency and deficiency, respectively. Mean serum 25(OH) vitamin D level was significantly lower in patients with severe-critical COVID-19 compared with moderate COVID-19 (10.1 ± 6.2 vs. 26.3 ± 8.4 ng/mL, respectively, p<0.001). Vitamin D insufficiency was present in 93.1% of the patients with severe-critical COVID-19. Multivariate logistic regression analysis revealed that only lymphocyte

count, white blood cell count, serum albumin and, 25(OH) vitamin D level were independent predictors of mortality.

Conclusion: Serum 25(OH) vitamin D was independently associated with mortality in COVID-19 patients.

Keywords: COVID-19; mortality; prognosis; vitamin D.

Conflict of interest statement

The authors declare that they have no conflict of interest.

Comment in

- Vitamin D deficiency as a predictor of severity in patients with COVID-19 infection.
  Teama MAEM, Abdelhakam DA, Elmohamadi MA, Badr FM. Teama MAEM, et al. Sci Prog. 2021 Jul-Sep;104(3):368504211036854. doi: 10.1177/00368504211036854. Sci Prog. 2021. PMID: 34347528

- 31 references
- 3 figures

Supplementary info

Publication types, MeSH terms, Substances Expand

Publication types

- Observational Study

MeSH terms

- Adult
- Aged
- Aged, 80 and over
- COVID-19 / blood*
- COVID-19 / drug therapy
- COVID-19 / mortality*
- COVID-19 / virology
- Female
- Humans
- Logistic Models
- Male
- Middle Aged
- Pandemics
- Retrospective Studies
- SARS-CoV-2 / isolation & purification

- Turkey / epidemiology
- Vitamin D / analogs & derivatives*
- Vitamin D / blood
- Vitamin D Deficiency / blood*
- Vitamin D Deficiency / mortality
- Vitamin D Deficiency / virology
- Young Adult

Substances

- Vitamin D
- 25-hydroxyvitamin D

Full text links

SpringerLink FULL-TEXT ARTICLE Springer Free PMC article
Proceed to details
Cite
Share
722
Observational Study
Am J Emerg Med
. 2022 Jan;51:262-266.
doi: 10.1016/j.ajem.2021.11.008. Epub 2021 Nov 8.

Impact of COVID-19 lockdown on self-harm and violence among patients presenting to the emergency department

Suhrith Bhattaram [1], Varsha S Shinde [2], Ishan Lamba [3], Rebecca S Gladwin [1], Kadainti Venkata Subbaraya Sharma [4]
Affiliations Expand

Affiliations

- [1] Department of Emergency Medicine, Dr DY Patil Medical College, Pimpri, Pune, Maharashtra 411018, India.
- [2] Department of Emergency Medicine, Dr DY Patil Medical College, Pimpri, Pune, Maharashtra 411018, India. Electronic address: varsha.shinde@dpu.edu.in.
- [3] Department of Emergency Medicine, Dr DY Patil Medical College, Pimpri, Pune, Maharashtra 411018, India. Electronic address: drishanlamba@yahoo.com.
- [4] Sivadurga Residency, HIG 216&217, Kukatapally, Hyderabad 500072, India.

- PMID: **34781152**
- PMCID: PMC8574082
- DOI: 10.1016/j.ajem.2021.11.008

Free PMC article
Observational Study

Impact of COVID-19 lockdown on self-harm and violence among patients presenting to the emergency department

Suhrith Bhattaram et al. Am J Emerg Med. 2022 Jan.
Free PMC article
Show details
Am J Emerg Med
. 2022 Jan;51:262-266.
doi: 10.1016/j.ajem.2021.11.008. Epub 2021 Nov 8.

Authors

Suhrith Bhattaram [1], Varsha S Shinde [2], Ishan Lamba [3], Rebecca S Gladwin [1], Kadainti Venkata Subbaraya Sharma [4]

Affiliations

- [1] Department of Emergency Medicine, Dr DY Patil Medical College, Pimpri, Pune, Maharashtra 411018, India.
- [2] Department of Emergency Medicine, Dr DY Patil Medical College, Pimpri, Pune, Maharashtra 411018, India. Electronic address: varsha.shinde@dpu.edu.in.
- [3] Department of Emergency Medicine, Dr DY Patil Medical College, Pimpri, Pune, Maharashtra 411018, India. Electronic address: drishanlamba@yahoo.com.
- [4] Sivadurga Residency, HIG 216&217, Kukatapally, Hyderabad 500072, India.

- PMID: **34781152**
- PMCID: PMC8574082
- DOI: 10.1016/j.ajem.2021.11.008

Abstract

Importance: Considering the resurgence of COVID19 and the rapid spread of new and deadlier strains across the globe understanding the incidence and pattern of violence and self harm tendencies during this period might help in formulating better contingency plans for future lockdowns. A deeper look at the available data shows that there is a significant dearth of research into self-harm & violence during the COVID-19 pandemic.

Objective: To identify the incidence and sociodemographic characteristics of self-harm and violence during the COVID19 lockdown and compare with a control group from the previous year.

Design: A cross-sectional retrospective observational study.

Setting: Tertiary care teaching hospital.

Participants: All patients presenting to the emergency department (ED) with self harm and violence during the COVID-19 lockdown period between March 24-June 30, 2020 and March 24-June 30, 2019.

Exposure: The COVID-19 lockdown period.

Main outcome (s) and measure (s): The hypothesis being tested was formulated before the study. The null hypothesis tested was a decline in number of self-harm and violence cases during the lockdown.

Results: A total of 828 patients were analysed over both the time periods, out of which 30% (248) were females while 70% (580) were males. Increases in self-harm and violence were 12.71% and 95.32% respectively per 1000 ED admissions. A significant correlation was found between the COVID-19 lockdown and the increased incidence (X2 (1, N = 828) = 9.2, $p < .05$). An increase of violence by known individuals and between partners was seen. Intimate partner violence also increased to 7%. X2 (3, N = 662) = 21.03, $p < .05$. In the self harm dataset an increase in mortality, ICU admissions and decision to leave against medical advice was noted (X2 (4, N = 166) = 24.49, $p < .05$). Increase in the use of alcohol prior to acts of self harm and violence was noted.

Conclusions: Increase in the incidence of cases of self-harm and violence reported to the ED was noted during the lockdown period. Upgradation of health-care and law enforcement infrastructure maybe needed to deal with similar circumstances in a more efficient manner.

Trial registration: N/A.

Keywords: COVID-19; COVID-19 in India; COVID-19 lockdown self harm in India; Self harm; Violence.

Copyright © 2021 Elsevier Inc. All rights reserved.

Conflict of interest statement

Declaration of Competing Interest There is no conflict of interest. A separate disclosure in accordance with the journal guidelines is provided for the same.

- 27 references
- 2 figures

Supplementary info

Publication types, MeSH terms Expand

Publication types

- Observational Study

MeSH terms

- Adult
- COVID-19 / epidemiology*
- Communicable Disease Control
- Cross-Sectional Studies
- Emergency Service, Hospital / statistics & numerical data
- Female
- Hospitalization
- Humans
- Incidence
- India / epidemiology
- Intimate Partner Violence
- Male
- Middle Aged
- Pandemics
- Retrospective Studies
- Self-Injurious Behavior / epidemiology*
- Violence / statistics & numerical data*
- Young Adult

Full text links

ELSEVIER FULL-TEXT ARTICLE Elsevier Science Free PMC article
Proceed to details
Cite
Share
☐ 723
Observational Study
Isr Med Assoc J
. 2021 Aug;23(8):475-478.

Incidence and Mortality Rate of Hip Fractures in Different Age Groups during the First Wave of the COVID-19 Pandemic

Ilia Prosso [1], Nugzar Oren [1], Gilad Livshits [1], Dror Lakstein [1] [2]
Affiliations Expand

Affiliations

- [1] Department of Orthopedic Surgery, Wolfson Medical Center, Holon, Israel.
- [2] Sackler Faculty of Medicine, Tel Aviv University, Tel Aviv, Israel.

- PMID: **34392620**

Free article
Observational Study

Incidence and Mortality Rate of Hip Fractures in Different Age Groups during the First Wave of the COVID-19 Pandemic

Ilia Prosso et al. Isr Med Assoc J. 2021 Aug.
Free article
Show details
Isr Med Assoc J
. 2021 Aug;23(8):475-478.

Authors

Ilia Prosso [1], Nugzar Oren [1], Gilad Livshits [1], Dror Lakstein [1, 2]

Affiliations

- [1] Department of Orthopedic Surgery, Wolfson Medical Center, Holon, Israel.
- [2] Sackler Faculty of Medicine, Tel Aviv University, Tel Aviv, Israel.

- PMID: **34392620**

Abstract

Background: The coronavirus disease-2019 (COVID-19) pandemic had enormous impact on many aspects of our society, including huge medical, social, and economic challenges.

Objectives: To evaluate the impact of the first wave of the COVID-19 pandemic and the related movement restrictions on the incidence of hip fractures in different age groups.

Methods: This single center retrospective observational study included all patients over 60 years old admitted to our hospital with the diagnosis of hip fracture during March and April 2020. Exclusion criteria were periprosthetic or pathologic fractures and multitrauma. We collected the same data on all patients with hip fractures admitted during March and April of 2018 and 2019.

Results: Mean patient age increased from 81.7 to 85.0 years. Only two of 49 patients tested positive for COVID-19. The data show a decrease of 38% in fracture load, but a striking decrease of 85% and 59% among sexagenarians and septuagenarian, respectively. There was no decrease among nonagenarians. Early mortality, both at 30 days and 90 days, was twice as common during

the pandemic. However, stratification by age group demonstrated that the risks of early mortality were the same as previous years. Mean waiting time for surgery decreased from 27.5 to 18.9 hours. Patient discharge to home over a rehabilitation facility increased from 9% to 17.

Conclusions: The COVID-19 pandemic affected the epidemiology of hip fractures in the elderly. The incidence of fractures and age distribution were significantly different from other years. Discharge destinations were also affected. The management of hip fracture patients was not compromised.

Supplementary info

Publication types, MeSH terms Expand

Publication types

- Observational Study

MeSH terms

- Age Factors
- Aged
- Aged, 80 and over
- COVID-19* / epidemiology
- COVID-19* / prevention & control
- Female
- Hip Fractures* / mortality
- Hip Fractures* / rehabilitation
- Hip Fractures* / therapy
- Humans
- Incidence
- Infection Control / methods
- Israel / epidemiology
- Male
- Middle Aged
- Mortality
- Outcome and Process Assessment, Health Care
- Patient Care Management* / methods
- Patient Care Management* / trends
- Retrospective Studies
- Risk Assessment / methods
- Risk Assessment / statistics & numerical data
- SARS-CoV-2 / isolation & purification

Full text links

Israel Medical Association
Proceed to details
Cite
Share
☐ 724
Observational Study
Monaldi Arch Chest Dis
. 2021 Mar 5;91(2).
doi: 10.4081/monaldi.2021.1561.

Role of awake prone positioning in patients with moderate-to-severe COVID-19: an experience from a developing country

Iffat Khanum [1], Fatima Samar [2], Yousuf Fatimah [3], Awan Safia [4], Aziz Adil [5], Habib Kiren [6], Nosheen Nasir [7], Mahmood Faisal [8], Jamil Bushra [9]
Affiliations Expand

Affiliations

- [1] Department of Medicine, Section of Infectious Diseases, The Aga Khan University Hospital, Karachi. iffat.khanum.ik@gmail.com.
- [2] Department of Medicine, Section of Infectious Diseases, The Aga Khan University Hospital, Karachi. samar.fatima@aku.edu.
- [3] Department of Medicine, Section of Infectious Diseases, The Aga Khan University Hospital, Karachi. fatimasireen@gmail.com.
- [4] Department of Medicine, Section of Infectious Diseases, The Aga Khan University Hospital, Karachi. safia.awan@aku.edu.
- [5] Department of Medicine, Section of Infectious Diseases, The Aga Khan University Hospital, Karachi. adil.aziz@aku.edu.
- [6] Department of Medicine, Section of Infectious Diseases, The Aga Khan University Hospital, Karachi. kiren.habib@aku.edu.
- [7] . nosheen.nasir@aku.edu.
- [8] Department of Medicine, Section of Infectious Diseases, The Aga Khan University Hospital, Karachi. faisal.mahmood@aku.edu.
- [9] Department of Medicine, Section of Infectious Diseases, The Aga Khan University Hospital, Karachi. bushra.jamil@aku.edu.

- PMID: **33666067**
- DOI: 10.4081/monaldi.2021.1561

Free article
Observational Study

Role of awake prone positioning in patients with moderate-to-severe COVID-19: an experience from a developing country

Iffat Khanum et al. Monaldi Arch Chest Dis. 2021.
Free article
Show details
Monaldi Arch Chest Dis
. 2021 Mar 5;91(2).
doi: 10.4081/monaldi.2021.1561.

Authors

Iffat Khanum [1], Fatima Samar [2], Yousuf Fatimah [3], Awan Safia [4], Aziz Adil [5], Habib Kiren [6], Nosheen Nasir [7], Mahmood Faisal [8], Jamil Bushra [9]

Affiliations

- [1] Department of Medicine, Section of Infectious Diseases, The Aga Khan University Hospital, Karachi. iffat.khanum.ik@gmail.com.
- [2] Department of Medicine, Section of Infectious Diseases, The Aga Khan University Hospital, Karachi. samar.fatima@aku.edu.
- [3] Department of Medicine, Section of Infectious Diseases, The Aga Khan University Hospital, Karachi. fatimasireen@gmail.com.
- [4] Department of Medicine, Section of Infectious Diseases, The Aga Khan University Hospital, Karachi. safia.awan@aku.edu.
- [5] Department of Medicine, Section of Infectious Diseases, The Aga Khan University Hospital, Karachi. adil.aziz@aku.edu.
- [6] Department of Medicine, Section of Infectious Diseases, The Aga Khan University Hospital, Karachi. kiren.habib@aku.edu.
- [7] . nosheen.nasir@aku.edu.
- [8] Department of Medicine, Section of Infectious Diseases, The Aga Khan University Hospital, Karachi. faisal.mahmood@aku.edu.
- [9] Department of Medicine, Section of Infectious Diseases, The Aga Khan University Hospital, Karachi. bushra.jamil@aku.edu.

- PMID: **33666067**
- DOI: 10.4081/monaldi.2021.1561

Abstract

There is limited evidence on the efficacy of awake prone positioning (PP) in non-ventilated patients with COVID-19 who have hypoxemia. We, therefore, aim to describe our experience with the use of early proning in awake, non-intubated patients with confirmed COVID-19. In our retrospective observational study, 23 patients with confirmed positive PCR test results for Severe Acute respiratory Syndrome Coronavirus-2 (SARS-CoV-2) and hypoxemia that required oxygen

therapy with or without non-invasive ventilation were treated with PP. Patients were classified into mild, moderate and severe COVID-19 disease. There were no targeted number of hours for proning per day and patients were kept in prone position according to their tolerance. The primary outcome measure was the avoidance of intubation and secondary outcomes were in-hospital mortality, length of hospital stays and complications related to PP. The mean (standard deviation) age of our cohort was 54.5 (11.7) years, and the majority were males (21/23, 91.3%). Sixty-one per cent (14/23) of the patients were suffering from severe disease and 82.6% (19/23) had bilateral lung involvement with interstitial infiltrates. Majority of the patients were prone positioned for a median of 6 days (IQR 4 - 8). Only one patient required transfer to ICU for mechanical ventilation and subsequently died due to severe ARDS. All 22 patients showed progressive improvement in oxygen requirement and PF ratio, mostly after 3-5 days of proning. The mean length of hospital stay was 12 days. All patients, except one, were discharged in stable conditions, on room air or on a minimal oxygen requirement of 1-2 liters. No major complication of PP was recorded. Awake prone positioning is a valuable and safe therapeutic adjunct that can be applied in patients with moderate-to-severe COVID-19. It can also be included in the home-based management protocols of COVID-19 to improve patient outcomes and mitigate the burden on health care facilities.

Supplementary info

Publication types, MeSH terms Expand

Publication types

- Observational Study

MeSH terms

- Adult
- COVID-19 / diagnosis
- COVID-19 / mortality
- COVID-19 / therapy*
- Developing Countries*
- Female
- Hospital Mortality
- Humans
- Male
- Middle Aged
- Oxygen Inhalation Therapy
- Pakistan
- Patient Positioning*
- Prone Position*
- Respiration, Artificial
- Retrospective Studies
- Treatment Outcome
- Wakefulness

Full text links

Monaldi Arch Chest Dis Pagepress Publications

Proceed to details

Cite

Share

☐ 725

Meta-Analysis

Pharmacotherapy
. 2021 Nov;41(11):884-906.
doi: 10.1002/phar.2627. Epub 2021 Oct 1.

Beneficial and harmful outcomes of tocilizumab in severe COVID-19: A systematic review and meta-analysis

Manuel Rubio-Rivas [1], Carlos G Forero [2], José María Mora-Luján [1], Abelardo Montero [1], Francesc Formiga [1], Narcís A Homs [1], Joan Albà-Albalate [1], Laura Sánchez [1], Jordi Rello [2] [3] [4], Xavier Corbella [1] [2]

Affiliations Expand

Affiliations

- [1] Department of Internal Medicine, Bellvitge University Hospital, Bellvitge Biomedical Research Institute-IDIBELL, University of Barcelona, Barcelona, Spain.
- [2] School of Medicine, Universitat Internacional de Catalunya, Barcelona, Spain.
- [3] Centro de Investigación Biomédica en Red (CIBERES), Instituto de Salud Carlos III, Madrid, Spain.
- [4] CRIPS, Vall d'Hebrón Institute of Research, Barcelona, Spain.

- PMID: **34558742**
- PMCID: PMC8661749
- DOI: 10.1002/phar.2627

Free PMC article

Meta-Analysis

Beneficial and harmful outcomes of tocilizumab in severe COVID-19: A systematic review and meta-analysis

Manuel Rubio-Rivas et al. Pharmacotherapy. 2021 Nov.

Free PMC article

Show details

Pharmacotherapy
. 2021 Nov;41(11):884-906.
doi: 10.1002/phar.2627. Epub 2021 Oct 1.

Authors

Manuel Rubio-Rivas [1], Carlos G Forero [2], José María Mora-Luján [1], Abelardo Montero [1], Francesc Formiga [1], Narcís A Homs [1], Joan Albà-Albalate [1], Laura Sánchez [1], Jordi Rello [2] [3] [4], Xavier Corbella [1] [2]

Affiliations

- [1] Department of Internal Medicine, Bellvitge University Hospital, Bellvitge Biomedical Research Institute-IDIBELL, University of Barcelona, Barcelona, Spain.
- [2] School of Medicine, Universitat Internacional de Catalunya, Barcelona, Spain.
- [3] Centro de Investigación Biomédica en Red (CIBERES), Instituto de Salud Carlos III, Madrid, Spain.
- [4] CRIPS, Vall d'Hebrón Institute of Research, Barcelona, Spain.

- PMID: **34558742**
- PMCID: PMC8661749
- DOI: 10.1002/phar.2627

Abstract

Introduction: The results of studies of tocilizumab (TCZ) in COVID-19 are contradictory. Our study aims to update medical evidence from controlled observational studies and randomized clinical trials (RCTs) on the use of TCZ in hospitalized patients with COVID-19.

Methods: We searched the following databases from January 1, 2020 to April 13, 2021 (date of the last search): MEDLINE database through the PubMed search engine and Scopus, using the terms ("COVID-19" [Supplementary Concept]) AND "tocilizumab" [Supplementary Concept]).

Results: Sixty four studies were included in the present study: 54 were controlled observational studies (50 retrospective and 4 prospective) and 10 were RCTs. The overall results provided data from 20,616 hospitalized patients with COVID-19: 7668 patients received TCZ in addition to standard of care (SOC) (including 1915 patients admitted to intensive care units (ICU) with reported mortality) and 12,948 patients only receiving SOC (including 4410 patients admitted to the ICU with reported mortality). After applying the random-effects model, the hospital-wide (including ICU) pooled mortality odds ratio (OR) of patients with COVID-19 treated with TCZ was 0.73 (95% confidence interval (CI) = 0.56-0.93). The pooled hospital-wide mortality OR was 1.25 (95% CI = 0.74-2.18) in patients admitted at conventional wards versus 0.66 (95% CI = 0.59-0.76) in patients admitted to the ICU. The pooled OR of hospital-wide mortality (including ICU) of COVID-19 patients treated with TCZ plus corticosteroids (CS) was 0.67 (95% CI = 0.54-0.84). The pooled in-hospital mortality OR was 0.71 (95% CI = 0.35-1.42) when TCZ was early administered (≤10 days from symptom onset) versus 0.83 (95% CI 0.48-1.45) for late administration (>10 days from symptom onset). The meta-analysis did not find significantly higher risk for secondary infections in COVID-19 patients treated with TCZ.

Conclusions: TCZ prevented mortality in patients hospitalized for COVID-19. This benefit was seen to a greater extent in patients receiving concomitant CS and when TCZ administration occurred within the first 10 days after symptom onset.

Keywords: COVID-19; SARS-CoV-2; coronavirus; meta-analysis; systematic review; tocilizumab.

© 2021 Pharmacotherapy Publications, Inc.

Conflict of interest statement

Jordi Rello has received consultancy honoraria from Roche. All other authors declare no conflicts of interest.

- 132 references
- 2 figures

Supplementary info

Publication types, MeSH terms, Substances, Supplementary concepts Expand

Publication types

- Meta-Analysis
- Systematic Review

MeSH terms

- Adrenal Cortex Hormones
- Antibodies, Monoclonal, Humanized* / adverse effects
- Antibodies, Monoclonal, Humanized* / therapeutic use
- COVID-19* / drug therapy
- Humans
- Observational Studies as Topic
- Randomized Controlled Trials as Topic

Substances

- Adrenal Cortex Hormones
- Antibodies, Monoclonal, Humanized
- tocilizumab

Supplementary concepts

- COVID-19 drug treatment

Full text links

Wiley Full Text Article Wiley Free PMC article

Proceed to details

Cite

Share

726

Observational Study

J Cardiovasc Med (Hagerstown)

. 2021 Nov 1;22(11):857-859.

doi: 10.2459/JCM.0000000000001156.

Cardiac pacing procedures during coronavirus disease 2019 lockdown in Southern Italy: insights from Campania Region

Vincenzo Russo [1], Pia Clara Pafundi [2], Antonio Rapacciuolo [3], Marcello de Divitiis [4], Mario Volpicelli [5], Antonio Ruocco [6], Anna Rago [7], Carlo Uran [8], Felice Nappi [9], Emilio Attena [10], Raffaele Chianese [11], Francesca Esposito [12], Giuseppe Del Giorno [13], Antonello D'Andrea [14], Valentino Ducceschi [4], Giovanni Russo [11], Ernesto Ammendola [7], Angelo Carbone [13], Gregorio Covino [5], Gianluca Manzo [14], Gianna Maria Montella [15], Gerardo Nigro [1], Antonio D'Onofrio [15]

Affiliations Expand

Affiliations

- [1] Chair of Cardiology, Department of Medical Translational Sciences, University of Campania 'Luigi Vanvitelli' - Monaldi Hospital, Naples.
- [2] Department of Advanced Medical and Surgical Sciences, University of Campania 'Luigi Vanvitelli'.
- [3] Division of Cardiology, Department of Advanced Biomedical Sciences, University of Naples Federico II.
- [4] Division of Cardiology, Pellegrini Hospital.
- [5] Division of Cardiology, San Giovanni Bosco Hospital, Health Authority Naples.
- [6] Interventional Cardiology and Cardiological Care Unit, Cardarelli Hospital.
- [7] Cardiology Unit, AORN dei Colli, Naples.
- [8] Cardiology Unit, San Giuseppe and Melorio Hospital, Santa Maria Capua Vetere, Caserta.
- [9] Division of Cardiology, Moscati Hospital, Avellino.
- [10] Division of Cardiology, San Giuliano Hospital, Giugliano in Campania, Health Authority Naples 2 North.
- [11] Division of Cardiology, San Leonardo Hospital, Castellammare di Stabia, Health Authority Naples 3 South, Naples.

- [12] Division of Cardiology, San Giovanni di Dio e Ruggi d'Aragona, Salerno, Italy.
- [13] Cardiology Unit, Maria SS Addolorata, Eboli.
- [14] Cardiology Unit, Umberto I Hospital, Nocera Inferiore.
- [15] Division of Cardiology, Monaldi Hospital, Naples, Italy.

- PMID: **33399343**
- DOI: 10.2459/JCM.0000000000001156

Observational Study

Cardiac pacing procedures during coronavirus disease 2019 lockdown in Southern Italy: insights from Campania Region

Vincenzo Russo et al. J Cardiovasc Med (Hagerstown). 2021.

Show details

J Cardiovasc Med (Hagerstown)

. 2021 Nov 1;22(11):857-859.
doi: 10.2459/JCM.0000000000001156.

Authors

Vincenzo Russo [1], Pia Clara Pafundi [2], Antonio Rapacciuolo [3], Marcello de Divitiis [4], Mario Volpicelli [5], Antonio Ruocco [6], Anna Rago [7], Carlo Uran [8], Felice Nappi [9], Emilio Attena [10], Raffaele Chianese [11], Francesca Esposito [12], Giuseppe Del Giorno [13], Antonello D'Andrea [14], Valentino Ducceschi [4], Giovanni Russo [11], Ernesto Ammendola [7], Angelo Carbone [13], Gregorio Covino [5], Gianluca Manzo [14], Gianna Maria Montella [15], Gerardo Nigro [1], Antonio D'Onofrio [15]

Affiliations

- [1] Chair of Cardiology, Department of Medical Translational Sciences, University of Campania 'Luigi Vanvitelli' - Monaldi Hospital, Naples.
- [2] Department of Advanced Medical and Surgical Sciences, University of Campania 'Luigi Vanvitelli'.
- [3] Division of Cardiology, Department of Advanced Biomedical Sciences, University of Naples Federico II.
- [4] Division of Cardiology, Pellegrini Hospital.
- [5] Division of Cardiology, San Giovanni Bosco Hospital, Health Authority Naples.
- [6] Interventional Cardiology and Cardiological Care Unit, Cardarelli Hospital.
- [7] Cardiology Unit, AORN dei Colli, Naples.
- [8] Cardiology Unit, San Giuseppe and Melorio Hospital, Santa Maria Capua Vetere, Caserta.
- [9] Division of Cardiology, Moscati Hospital, Avellino.

- [10] Division of Cardiology, San Giuliano Hospital, Giugliano in Campania, Health Authority Naples 2 North.
- [11] Division of Cardiology, San Leonardo Hospital, Castellammare di Stabia, Health Authority Naples 3 South, Naples.
- [12] Division of Cardiology, San Giovanni di Dio e Ruggi d'Aragona, Salerno, Italy.
- [13] Cardiology Unit, Maria SS Addolorata, Eboli.
- [14] Cardiology Unit, Umberto I Hospital, Nocera Inferiore.
- [15] Division of Cardiology, Monaldi Hospital, Naples, Italy.

- PMID: **33399343**
- DOI: 10.2459/JCM.0000000000001156

No abstract available

- 12 references

Supplementary info

Publication types, MeSH terms Expand

Publication types

- Multicenter Study
- Observational Study

MeSH terms

- Aged
- Arrhythmias, Cardiac* / epidemiology
- Arrhythmias, Cardiac* / therapy
- COVID-19* / epidemiology
- COVID-19* / prevention & control
- Cardiac Pacing, Artificial* / methods
- Cardiac Pacing, Artificial* / statistics & numerical data
- Cardiology Service, Hospital / organization & administration*
- Female
- Humans
- Infection Control / methods
- Infection Control / organization & administration
- Italy / epidemiology
- Male
- Organizational Innovation
- Outcome and Process Assessment, Health Care
- Retrospective Studies
- SARS-CoV-2

Full text links

Wolters Kluwer Wolters Kluwer
Proceed to details
Cite
Share
☐ 727
Observational Study
An Pediatr (Engl Ed)
. 2020 Aug;93(2):118-122.
doi: 10.1016/j.anpedi.2020.04.022. Epub 2020 May 11.

[Influence of the coronavirus 2 (SARS-Cov-2) pandemic on acute appendicitis]

[Article in Spanish]
María Velayos [1], Antonio Jesús Muñoz-Serrano [2], Karla Estefanía-Fernández [2], Ma Carmen Sarmiento Caldas [2], Lucas Moratilla Lapeña [2], Manuel López-Santamaría [2], Juan Carlos López-Gutiérrez [2]
Affiliations Expand

Affiliations

- [1] Servicio de Cirugía Pediátrica. Hospital Universitario La Paz, Madrid, España. Electronic address: mariavelayos@icloud.com.
- [2] Servicio de Cirugía Pediátrica. Hospital Universitario La Paz, Madrid, España.

- PMID: **32493604**
- PMCID: PMC7211733
- DOI: 10.1016/j.anpedi.2020.04.022

Free PMC article
Observational Study

[Influence of the coronavirus 2 (SARS-Cov-2) pandemic on acute appendicitis]

[Article in Spanish]
María Velayos et al. An Pediatr (Engl Ed). 2020 Aug.
Free PMC article
Show details
An Pediatr (Engl Ed)
. 2020 Aug;93(2):118-122.
doi: 10.1016/j.anpedi.2020.04.022. Epub 2020 May 11.

Authors

María Velayos [1], Antonio Jesús Muñoz-Serrano [2], Karla Estefanía-Fernández [2], Ma Carmen Sarmiento Caldas [2], Lucas Moratilla Lapeña [2], Manuel López-Santamaría [2], Juan Carlos López-Gutiérrez [2]

Affiliations

- [1] Servicio de Cirugía Pediátrica. Hospital Universitario La Paz, Madrid, España. Electronic address: mariavelayos@icloud.com.
- [2] Servicio de Cirugía Pediátrica. Hospital Universitario La Paz, Madrid, España.

- PMID: **32493604**
- PMCID: PMC7211733
- DOI: 10.1016/j.anpedi.2020.04.022

Abstract
in English, Spanish

Introduction: Acute appendicitis (AA) is the most common abdominal surgical emergency. No specific studies have been found that evaluate the impact of the coronavirus 2 (SARS-Cov-2) pandemic on AA and its surgical management. An analysis was made on the influence of this new pathology on the clinical course of AA.

Material and methods: Retrospective observational study was conducted on patients operated on for AA from January to April 2020. They were classified according to the time of the appendectomy, before the declaration of the state of alarm (pre-COVID-19), and after its declaration (post-COVID-19) in Spain, one the most affected countries in the world. An evaluation was made of demographic variables, duration of symptoms, type of appendicitis, surgical time, hospital stay, and postoperative complications.

Results: The study included 66 patients (41 pre-COVID-19; 25 post-COVID-19) with mean age of 10.7 ± 3 and 9.3 ± 3.1; P = .073, respectively. Fever was found in a higher number of post-COVID-19 patients (52 vs. 19.5%; P = 0.013), as well as a higher CRP (72.7 ± 96.2 vs. 31.3 ± 36.2 mg/dL; P = 0.042). This group presented with a higher proportion of complicated appendicitis when compared to pre-COVID-19 (32 vs. 7.3%; P = 0.015). The mean hospital stay was longer in the post-COVID-19 group (5.6 ± 5.9 vs. 3.2 ± 4.3 days; P = 0.041). No differences were found in the time of onset of symptoms or surgical time.

Conclusions: The SARS-Cov-2 pandemic influenced the time of diagnosis of appendicitis, as well as its course, and mean hospital stay. Peritonitis was more frequently seen. As a result of the significant circumstances, delaying diagnosis and treatment of AA during SARS-Cov-2 pandemic, inappropriate management of this common surgical disorder has been noticed.

Introducción: La apendicitis aguda (AA) es la urgencia quirúrgica abdominal más frecuente. No encontramos estudios específicos que evalúen el impacto de la pandemia causada por el coronavirus 2 (SARS-Cov-2) sobre la AA y su tratamiento quirúrgico. Analizamos la influencia de esta nueva patología sobre la AA.

Material y métodos: Estudio observacional retrospectivo en pacientes intervenidos por AA desde enero hasta abril de 2020. Fueron clasificados según el momento de la apendicectomía, antes de la

declaración del estado de alarma (pre-COVID-19) y después de la declaración del estado de alarma (post-COVID-19) en España. Se evaluaron variables demográficas, duración de la sintomatología, tipo de apendicitis, tiempo quirúrgico, estancia hospitalaria y complicaciones postoperatorias.

Resultados: Se incluyeron 66 pacientes (41 pre-COVID-19; 25 post-COVID-19 con edad media de 10,7 ± 3 y 9,33 ± 1; p = 0,073, respectivamente). La fiebre se encontró en un mayor número de pacientes post-COVID19 (52 vs. 19,5%; p = 0,013), así como una PCR más elevada (72,7 ± 96,2 vs. 31,3 ± 36,2 mg/dL; p = 0,042). Este grupo presentó una mayor proporción de apendicitis complicada al compararle con el pre-COVID-19 (32 vs. 7,3%; p = 0,015). La estancia media hospitalaria fue mayor en el grupo post-COVID-19 (5,6 ± 5,9 vs. 3,3 ± 4,3 días; p = 0,041). No se encontraron diferencias en el tiempo de evolución de los síntomas ni en el tiempo quirúrgico.

Conclusiones: La pandemia por SARS-Cov-2 influye en el momento de diagnóstico de la apendicitis, así como en su grado de evolución y estancia hospitalaria. La peritonitis fue lo más frecuentemente observado. Son necesarias una sospecha y una orientación clínica más tempranas para evitar un manejo inadecuado de este trastorno quirúrgico común.

Keywords: Acute appendicitis; Apendicectomía; Apendicitis aguda; Appendectomy; Complicaciones postquirúrgicas; Paciente pediátrico; Paediatric patient; Post-surgical complications; SARS-Cov-2.

Copyright © 2020 Asociación Española de Pediatría. Publicado por Elsevier España, S.L.U. All rights reserved.

- 14 references
- 1 figure

Supplementary info

Publication types, MeSH terms Expand

Publication types

- Observational Study

MeSH terms

- Adolescent
- Appendectomy / methods*
- Appendicitis / surgery*
- COVID-19
- Child
- Coronavirus Infections / epidemiology*
- Female
- Humans
- Length of Stay
- Male

- Pandemics
- Peritonitis / epidemiology
- Pneumonia, Viral / epidemiology*
- Postoperative Complications / epidemiology*
- Retrospective Studies
- Spain / epidemiology

Full text links

ELSEVIER OPEN ACCESS Elsevier Science Free PMC article
Proceed to details
Cite
Share
728
Observational Study
Daru
. 2020 Dec;28(2):507-516.
doi: 10.1007/s40199-020-00353-w. Epub 2020 Jun 19.

The clinical value of two combination regimens in the Management of Patients Suffering from Covid-19 pneumonia: a single centered, retrospective, observational study

Ensieh Vahedi [1], Mostafa Ghanei [2], Ali Ghazvini [1], Hossein Azadi [1], Morteza Izadi [3], Yunes Panahi [4], Saeid Fathi [5], Mahmood Salesi [1], Seyed Hassan Saadat [6], Amir Hossein Ghazale [7], Mohammad Rezapour [7], Abolfazl Mozafari [8], Nahid Zand [9], Mohammadreza Raesi Parsaei [7], Mohammad Hossein Ranjkesh [7], Ramezan Jafari [10], Fatemeh Movaseghi [8], Enayat Darabi [11]
Affiliations Expand

Affiliations

- [1] Chemical Injuries Research Center, Systems Biology and Poisoning Institute, Baqiyatallah University of Medical Sciences, Tehran, Iran.
- [2] Chemical Injuries Research Center, Systems Biology and Poisoning Institute, Baqiyatallah University of Medical Sciences, Tehran, Iran. mghaneister@gmail.com.
- [3] Health Research Center, Baqiyatallah University of Medical Sciences, Tehran, Iran.
- [4] Faculty of pharmacy, pharmacotherapy department, Baqiyatallah University of Medical Sciences, Tehran, Iran.
- [5] University of Tehran, Tehran, Iran.
- [6] Behavioral sciences research center, Lifestyle institute, Baqiatallah University of Medical Sciences, Tehran, Iran.
- [7] Student Research committee, Baqiyatallah University of medical Sciences, Tehran, Iran.

- [8] Department of Medical Science, Qom Branch, Islamic Azad University, Qom, Iran.
- [9] Department of Internal Medicine, Qom university of medical sciences, Qom, Iran.
- [10] Department of Radiology and Chemical Injury Research Center, Baqiyatallah University of Medical Sciences, Tehran, Iran.
- [11] School of Public Health, Tehran University of Medical Sciences, Tehran, Iran.

- PMID: **32562159**
- PMCID: PMC7303568
- DOI: 10.1007/s40199-020-00353-w

Free PMC article
Observational Study

The clinical value of two combination regimens in the Management of Patients Suffering from Covid-19 pneumonia: a single centered, retrospective, observational study

Ensieh Vahedi et al. Daru. 2020 Dec.
Free PMC article
Show details
Daru
. 2020 Dec;28(2):507-516.
doi: 10.1007/s40199-020-00353-w. Epub 2020 Jun 19.

Authors

Ensieh Vahedi [1], Mostafa Ghanei [2], Ali Ghazvini [1], Hossein Azadi [1], Morteza Izadi [3], Yunes Panahi [4], Saeid Fathi [5], Mahmood Salesi [1], Seyed Hassan Saadat [6], Amir Hossein Ghazale [7], Mohammad Rezapour [7], Abolfazl Mozafari [8], Nahid Zand [9], Mohammadreza Raesi Parsaei [7], Mohammad Hossein Ranjkesh [7], Ramezan Jafari [10], Fatemeh Movaseghi [8], Enayat Darabi [11]

Affiliations

- [1] Chemical Injuries Research Center, Systems Biology and Poisoning Institute, Baqiyatallah University of Medical Sciences, Tehran, Iran.
- [2] Chemical Injuries Research Center, Systems Biology and Poisoning Institute, Baqiyatallah University of Medical Sciences, Tehran, Iran. mghaneister@gmail.com.
- [3] Health Research Center, Baqiyatallah University of Medical Sciences, Tehran, Iran.
- [4] Faculty of pharmacy, pharmacotherapy department, Baqiyatallah University of Medical Sciences, Tehran, Iran.
- [5] University of Tehran, Tehran, Iran.
- [6] Behavioral sciences research center, Lifestyle institute, Baqiatallah University of Medical Sciences, Tehran, Iran.
- [7] Student Research committee, Baqiyatallah University of medical Sciences, Tehran, Iran.

- [8] Department of Medical Science, Qom Branch, Islamic Azad University, Qom, Iran.
- [9] Department of Internal Medicine, Qom university of medical sciences, Qom, Iran.
- [10] Department of Radiology and Chemical Injury Research Center, Baqiyatallah University of Medical Sciences, Tehran, Iran.
- [11] School of Public Health, Tehran University of Medical Sciences, Tehran, Iran.

- PMID: **32562159**
- PMCID: PMC7303568
- DOI: 10.1007/s40199-020-00353-w

Abstract

Background: There is no identified pharmacological therapy for COVID-19 patients, where potential therapeutic strategies are underway to determine effective therapy under such unprecedented pandemic. Therefore, combination therapies may have the potential of alleviating the patient's outcome. This study aimed at comparing the efficacy of two different combination regimens in improving outcomes of patients infected by novel coronavirus (COVID-19).

Methods: This is a single centered, retrospective, observational study of 60 laboratory-confirmed COVID-19 positive inpatients (≥18 years old) at two wards of the Baqiyatallah Hospital, Tehran, Iran. Patient's data including clinical and laboratory parameters were recorded. According to the drug regimen, the patients were divided into two groups; group I who received regimen I consisting azithromycin, prednisolone, naproxen, and lopinavir/ritonavir and group II who received regimen II including meropenem, levofloxacin, vancomycin, hydroxychloroquine, and oseltamivir.

Results: The oxygen saturation (SpO_2) and temperature were positively changed in patients receiving regimen I compared to regimen II ($P = 0.013$ and $P = 0.012$, respectively). The serum level of C-reactive protein (CRP) changed positively in group I ($P < 0.001$). Although there was a significant difference in platelets between both groups (75.44 vs 51.62, $P < 0.001$), their change did not clinically differ between two groups. The findings indicated a significant difference of the average length of stay in hospitals (ALOS) between two groups, where the patients under regimen I showed a shorter ALOS (6.97 vs 9.93, $P = 0.001$).

Conclusion: This study revealed the beneficial effect of the short-term use of low-dose prednisolone in combination with azithromycin, naproxen and lopinavir/ritonavir (regimen I), in decreasing ALOS compared to regimen II. Since there is still lack of evidence for safety of this regimen, further investigation in our ongoing follow-up to deal with COVID-19 pneumonia is underway. Graphical abstract.

Keywords: Combination therapy; Coronavirus disease 2019; Length of stay in hospitals (ALOS).

Conflict of interest statement

The authors declare no conflict of interest.

- 45 references
- 4 figures

Supplementary info

Publication types, MeSH terms, Substances, Supplementary concepts Expand

Publication types

- Comparative Study
- Observational Study

MeSH terms

- Adult
- Aged
- Azithromycin / administration & dosage
- COVID-19 / complications
- COVID-19 / drug therapy*
- Drug Combinations
- Drug Therapy, Combination
- Female
- Hospitalization / statistics & numerical data*
- Humans
- Hydroxychloroquine / administration & dosage
- Iran
- Length of Stay
- Levofloxacin / administration & dosage
- Lopinavir / administration & dosage
- Male
- Meropenem / administration & dosage
- Middle Aged
- Naproxen / administration & dosage
- Oseltamivir / administration & dosage
- Pneumonia, Viral / drug therapy*
- Pneumonia, Viral / virology
- Prednisolone / administration & dosage
- Retrospective Studies
- Ritonavir / administration & dosage
- Treatment Outcome
- Vancomycin / administration & dosage

Substances

- Drug Combinations
- lopinavir-ritonavir drug combination
- Oseltamivir
- Lopinavir
- Hydroxychloroquine

- Naproxen
- Levofloxacin
- Vancomycin
- Azithromycin
- Prednisolone
- Meropenem
- Ritonavir

Supplementary concepts

- COVID-19 drug treatment

Full text links

Free PMC article
Proceed to details
Cite
Share
729
Observational Study
PLoS One
. 2020 Nov 12;15(11):e0242127.
doi: 10.1371/journal.pone.0242127. eCollection 2020.

COVID-19 in-hospital mortality and mode of death in a dynamic and non-restricted tertiary care model in Germany

Siegbert Rieg [1], Maja von Cube [2], Johannes Kalbhenn [3], Stefan Utzolino [4], Katharina Pernice [5], Lena Bechet [1], Johanna Baur [6] [7], Corinna N Lang [6] [7], Dirk Wagner [1], Martin Wolkewitz [2], Winfried V Kern [1], Paul Biever [6] [7], COVID UKF Study Group
Affiliations Expand

Affiliations

- [1] Division of Infectious Diseases, Department of Medicine II, Medical Center-University of Freiburg, Faculty of Medicine, University of Freiburg, Freiburg, Germany.
- [2] Institute of Medical Biometry and Statistics, Faculty of Medicine and Medical Center, University of Freiburg, Freiburg, Germany.
- [3] Department of Anesthesiology and Intensive Care Medicine, Medical Center-University of Freiburg, Faculty of Medicine, University of Freiburg, Freiburg, Germany.
- [4] Department of General and Visceral Surgery, Medical Center-University of Freiburg, Faculty of Medicine, University of Freiburg, Freiburg, Germany.

- [5] Department of Cardiovascular Surgery, Heart Center, Medical Center-University of Freiburg, Faculty of Medicine, University of Freiburg, Freiburg, Germany.
- [6] Department of Medicine III (Interdisciplinary Medical Intensive Care), Medical Center-University of Freiburg, Faculty of Medicine, University of Freiburg, Freiburg, Germany.
- [7] Department of Cardiology and Angiology I, Heart Center Freiburg University, Medical Center-University of Freiburg, Faculty of Medicine, University of Freiburg, Freiburg, Germany.

- PMID: **33180830**
- PMCID: PMC7660518
- DOI: 10.1371/journal.pone.0242127

Free PMC article
Observational Study

COVID-19 in-hospital mortality and mode of death in a dynamic and non-restricted tertiary care model in Germany

Siegbert Rieg et al. PLoS One. 2020.
Free PMC article

Show details

PLoS One
. 2020 Nov 12;15(11):e0242127.
doi: 10.1371/journal.pone.0242127. eCollection 2020.

Authors

Siegbert Rieg [1], Maja von Cube [2], Johannes Kalbhenn [3], Stefan Utzolino [4], Katharina Pernice [5], Lena Bechet [1], Johanna Baur [6, 7], Corinna N Lang [6, 7], Dirk Wagner [1], Martin Wolkewitz [2], Winfried V Kern [1], Paul Biever [6, 7], COVID UKF Study Group

Affiliations

- [1] Division of Infectious Diseases, Department of Medicine II, Medical Center-University of Freiburg, Faculty of Medicine, University of Freiburg, Freiburg, Germany.
- [2] Institute of Medical Biometry and Statistics, Faculty of Medicine and Medical Center, University of Freiburg, Freiburg, Germany.
- [3] Department of Anesthesiology and Intensive Care Medicine, Medical Center-University of Freiburg, Faculty of Medicine, University of Freiburg, Freiburg, Germany.
- [4] Department of General and Visceral Surgery, Medical Center-University of Freiburg, Faculty of Medicine, University of Freiburg, Freiburg, Germany.
- [5] Department of Cardiovascular Surgery, Heart Center, Medical Center-University of Freiburg, Faculty of Medicine, University of Freiburg, Freiburg, Germany.
- [6] Department of Medicine III (Interdisciplinary Medical Intensive Care), Medical Center-University of Freiburg, Faculty of Medicine, University of Freiburg, Freiburg, Germany.

- [7] Department of Cardiology and Angiology I, Heart Center Freiburg University, Medical Center-University of Freiburg, Faculty of Medicine, University of Freiburg, Freiburg, Germany.

- PMID: **33180830**
- PMCID: PMC7660518
- DOI: 10.1371/journal.pone.0242127

Abstract

Background: Reported mortality of hospitalised Coronavirus Disease-2019 (COVID-19) patients varies substantially, particularly in critically ill patients. So far COVID-19 in-hospital mortality and modes of death under state of the art care have not been systematically studied.

Methods: This retrospective observational monocenter cohort study was performed after implementation of a non-restricted, dynamic tertiary care model at the University Medical Center Freiburg, an experienced acute respiratory distress syndrome (ARDS) and extracorporeal membrane-oxygenation (ECMO) referral center. All hospitalised patients with PCR-confirmed SARS-CoV-2 infection were included. The primary endpoint was in-hospital mortality, secondary endpoints included major complications and modes of death. A multistate analysis and a Cox regression analysis for competing risk models were performed. Modes of death were determined by two independent reviewers.

Results: Between February 25, and May 8, 213 patients were included in the analysis. The median age was 65 years, 129 patients (61%) were male. 70 patients (33%) were admitted to the intensive care unit (ICU), of which 57 patients (81%) received mechanical ventilation and 23 patients (33%) ECMO support. Using multistate methodology, the estimated probability to die within 90 days after COVID-19 onset was 24% in the whole cohort. If the levels of care at time of study entry were accounted for, the probabilities to die were 16% if the patient was initially on a regular ward, 47% if in the intensive care unit (ICU) and 57% if mechanical ventilation was required at study entry. Age ≥65 years and male sex were predictors for in-hospital death. Predominant complications-as judged by two independent reviewers-determining modes of death were multi-organ failure, septic shock and thromboembolic and hemorrhagic complications.

Conclusion: In a dynamic care model COVID-19-related in-hospital mortality remained very high. In the absence of potent antiviral agents, strategies to alleviate or prevent the identified complications should be investigated. In this context, multistate analyses enable comparison of models-of-care and treatment strategies and allow estimation and allocation of health care resources.

Conflict of interest statement

The authors have declared that no competing interests exist.

- 26 references
- 2 figures

Supplementary info

Publication types, MeSH terms, Grant support Expand

Publication types

- Observational Study
- Research Support, Non-U.S. Gov't

MeSH terms

- Aged
- Aged, 80 and over
- Betacoronavirus
- COVID-19
- Coronavirus Infections / mortality*
- Extracorporeal Membrane Oxygenation
- Female
- Germany / epidemiology
- Hospital Mortality*
- Humans
- Intensive Care Units
- Male
- Middle Aged
- Models, Statistical
- Pandemics
- Pneumonia, Viral / mortality*
- Respiration, Artificial
- Retrospective Studies
- SARS-CoV-2
- Tertiary Healthcare

Grant support

MVC was funded by the EQUIP programme of the Faculty of Medicine, University of Freiburg (https://www.med.uni-freiburg.de/de/forschung/karrierewege/equip/equip). The funders had no role in study design, data collection and analysis, decision to publish, or preparation of the manuscript.

Full text links

OPEN ACCESS TO FULL TEXT PLOS ONE Public Library of Science Free PMC article

Proceed to details

Cite

Share

☐ 730

Observational Study

BMC Infect Dis
. 2020 Dec 1;20(1):910.
doi: 10.1186/s12879-020-05637-9.

Specific dynamic variations in the peripheral blood lymphocyte subsets in COVID-19 and severe influenza A patients: a retrospective observational study

Fang Qian [1], Guiju Gao [1], Yangzi Song [1], Yanli Xu [1], Aibin Wang [1], Sa Wang [1], Yiwei Hao [2], Meiling Chen [2], Xiaoyang Ma [3], Tianwei Zhao [1], Xiaodi Guo [4], Zhihai Chen [5], Fujie Zhang [6]

Affiliations Expand

Affiliations

- [1] Clinical and Research Center of Infectious Diseases, Beijing Ditan Hospital, Capital Medical University, No.8, Jing shun Dong jie, Chaoyang, 100015, District Beijing, China.
- [2] Department of Medical Records and Statistics, Beijing Ditan Hospital, Capital Medical University, Beijing, China.
- [3] Department of Neurology, Beijing Ditan Hospital, Capital Medical University, Beijing, China.
- [4] Department of Oncology, Beijing Ditan Hospital, Capital Medical University, Beijing, China.
- [5] Clinical and Research Center of Infectious Diseases, Beijing Ditan Hospital, Capital Medical University, No.8, Jing shun Dong jie, Chaoyang, 100015, District Beijing, China. chenzhihai0001@126.com.
- [6] Clinical and Research Center of Infectious Diseases, Beijing Ditan Hospital, Capital Medical University, No.8, Jing shun Dong jie, Chaoyang, 100015, District Beijing, China. treatment@chinaaids.cn.

- PMID: **33261583**
- PMCID: PMC7705851
- DOI: 10.1186/s12879-020-05637-9

Free PMC article
Observational Study

Specific dynamic variations in the peripheral blood lymphocyte subsets in COVID-19 and severe influenza A patients: a retrospective observational study

Fang Qian et al. BMC Infect Dis. 2020.
Free PMC article
Show details
BMC Infect Dis
. 2020 Dec 1;20(1):910.
doi: 10.1186/s12879-020-05637-9.

Authors

Fang Qian [1], Guiju Gao [1], Yangzi Song [1], Yanli Xu [1], Aibin Wang [1], Sa Wang [1], Yiwei Hao [2], Meiling Chen [2], Xiaoyang Ma [3], Tianwei Zhao [1], Xiaodi Guo [4], Zhihai Chen [5], Fujie Zhang [6]

Affiliations

- [1] Clinical and Research Center of Infectious Diseases, Beijing Ditan Hospital, Capital Medical University, No.8, Jing shun Dong jie, Chaoyang, 100015, District Beijing, China.
- [2] Department of Medical Records and Statistics, Beijing Ditan Hospital, Capital Medical University, Beijing, China.
- [3] Department of Neurology, Beijing Ditan Hospital, Capital Medical University, Beijing, China.
- [4] Department of Oncology, Beijing Ditan Hospital, Capital Medical University, Beijing, China.
- [5] Clinical and Research Center of Infectious Diseases, Beijing Ditan Hospital, Capital Medical University, No.8, Jing shun Dong jie, Chaoyang, 100015, District Beijing, China. chenzhihai0001@126.com.
- [6] Clinical and Research Center of Infectious Diseases, Beijing Ditan Hospital, Capital Medical University, No.8, Jing shun Dong jie, Chaoyang, 100015, District Beijing, China. treatment@chinaaids.cn.

- PMID: **33261583**
- PMCID: PMC7705851
- DOI: 10.1186/s12879-020-05637-9

Abstract

Background: Both COVID-19 and influenza A contribute to increased mortality among the elderly and those with existing comorbidities. Changes in the underlying immune mechanisms determine patient prognosis. This study aimed to analyze the role of lymphocyte subsets in the immunopathogenesisof COVID-19 and severe influenza A, and examined the clinical significance of their alterations in the prognosis and recovery duration.

Methods: By retrospectively reviewing of patients in four groups (healthy controls, severe influenza A, non-severe COVID-19 and severe COVID-19) who were admitted to Ditan hospital between 2018 to 2020, we performed flow cytometric analysis and compared the absolute counts of leukocytes, lymphocytes, and lymphocyte subsets of the patients at different time points (weeks 1-4).

Results: We reviewed the patients' data of 94 healthy blood donors, 80 Non-severe-COVID-19, 19 Severe-COVID-19 and 37 severe influenza A. We found total lymphocytes (0.81×10^9/L vs 1.74×10^9/L, P = 0.001; 0.87×10^9/L vs 1.74×10^9/L, P < 0.0001, respectively) and lymphocyte subsets (T cells, $CD4^+$ and $CD8^+$ T cell subsets) of severe COVID-19 and severe influenza A patients to be significantly lower than those of healthy donors at early infection stages. Further, significant dynamic variations were observed at different time points (weeks 1-4).

Conclusions: Our study suggests the plausible role of lymphocyte subsets in disease progression, which in turn affects prognosis and recovery duration in patients with severe COVID-19 and influenza A.

Keywords: Coronavirus disease-19 (COVID-19); Lymphocyte; Severe influenza A; T cells subsets.

Conflict of interest statement

The authors declare that they have no competing interests.

- 28 references
- 2 figures

Supplementary info

Publication types, MeSH terms Expand

Publication types

- Observational Study

MeSH terms

- Adult
- Aged
- Beijing / epidemiology
- CD4-Positive T-Lymphocytes / immunology*
- CD8-Positive T-Lymphocytes / immunology*
- COVID-19 / diagnosis
- COVID-19 / epidemiology
- COVID-19 / immunology*
- COVID-19 / virology
- Comorbidity
- Cross-Sectional Studies

- Disease Progression
- Female
- Flow Cytometry
- Humans
- Influenza A virus / genetics*
- Influenza, Human / epidemiology
- Influenza, Human / immunology*
- Influenza, Human / virology
- Lymphocyte Count
- Male
- Middle Aged
- Prognosis
- Retrospective Studies
- SARS-CoV-2 / genetics*
- Severity of Illness Index*

Full text links

Read free full text at BMC BioMed Central Free PMC article
Proceed to details
Cite
Share
731
Meta-Analysis
F1000Res
. 2021 Feb 4;10:73.
doi: 10.12688/f1000research.45046.1. eCollection 2021.

Optimal use of tocilizumab for severe and critical COVID-19: a systematic review and meta-analysis

Cahyo Wibisono Nugroho [1 2], Satriyo Dwi Suryantoro [1 2], Yuliasih Yuliasih [1], Alfian Nur Rosyid [2 3], Tri Pudy Asmarawati [1 2], Lucky Andrianto [2 4], Herley Windo Setiawan [2 3], Bagus Aulia Mahdi [1], Choirina Windradi [1], Esthiningrum Dewi Agustin [5], Jonny Karunia Fajar [6]
Affiliations Expand

Affiliations

- [1] Department of Internal Medicine, Faculty of Medicine, Airlangga University, Surabaya, East Java, 60132, Indonesia.
- [2] Universitas Airlangga Hospital, Surabaya, East Java, 60115, Indonesia.

- [3] Department of Pulmonology and Respiratory Medicine, Faculty of Medicine, Airlangga University, Surabaya, East Java, 60132, Indonesia.
- [4] Department of Anesthesiology and Reanimation, Faculty of Medicine, Airlangga University, Surabaya, East Java, 60132, Indonesia.
- [5] Faculty of Medicine, Airlangga University, Surabaya, East Java, 60132, Indonesia.
- [6] Department of Internal Medicine, Faculty of Medicine, Brawijaya University, Malang, East Java, 65145, Indonesia.

- PMID: **33763201**
- PMCID: PMC7953915
- DOI: 10.12688/f1000research.45046.1

Free PMC article
Meta-Analysis

Optimal use of tocilizumab for severe and critical COVID-19: a systematic review and meta-analysis

Cahyo Wibisono Nugroho et al. F1000Res. 2021.
Free PMC article
Show details
F1000Res
. 2021 Feb 4;10:73.
doi: 10.12688/f1000research.45046.1. eCollection 2021.

Authors

Cahyo Wibisono Nugroho [1] [2], Satriyo Dwi Suryantoro [1] [2], Yuliasih Yuliasih [1], Alfian Nur Rosyid [2] [3], Tri Pudy Asmarawati [1] [2], Lucky Andrianto [2] [4], Herley Windo Setiawan [2] [3], Bagus Aulia Mahdi [1], Choirina Windradi [1], Esthiningrum Dewi Agustin [5], Jonny Karunia Fajar [6]

Affiliations

- [1] Department of Internal Medicine, Faculty of Medicine, Airlangga University, Surabaya, East Java, 60132, Indonesia.
- [2] Universitas Airlangga Hospital, Surabaya, East Java, 60115, Indonesia.
- [3] Department of Pulmonology and Respiratory Medicine, Faculty of Medicine, Airlangga University, Surabaya, East Java, 60132, Indonesia.
- [4] Department of Anesthesiology and Reanimation, Faculty of Medicine, Airlangga University, Surabaya, East Java, 60132, Indonesia.
- [5] Faculty of Medicine, Airlangga University, Surabaya, East Java, 60132, Indonesia.
- [6] Department of Internal Medicine, Faculty of Medicine, Brawijaya University, Malang, East Java, 65145, Indonesia.

- PMID: **33763201**
- PMCID: PMC7953915

- DOI: 10.12688/f1000research.45046.1

Abstract

Background: Several studies have revealed the potential use of tocilizumab in treating COVID-19 since no therapy has yet been approved for COVID-19 pneumonia. Tocilizumab may provide clinical benefits for cytokine release syndrome in COVID-19 patients. **Methods:** We searched for relevant studies in PubMed, Embase, Medline, and Cochrane published from March to October 2020 to evaluate optimal use and baseline criteria for administration of tocilizumab in severe and critically ill COVID-19 patients. Research involving patients with confirmed SARS-CoV-2 infection, treated with tocilizumab and compared with the standard of care (SOC) was included in this study. We conducted a systematic review to find data about the risks and benefits of tocilizumab and outcomes from different baseline criteria for administration of tocilizumab as a treatment for severe and critically ill COVID-19 patients. **Results:** A total of 26 studies, consisting of 23 retrospective studies, one prospective study, and two randomised controlled trials with 2112 patients enrolled in the tocilizumab group and 6160 patients in the SOC group, were included in this meta-analysis. Compared to the SOC, tocilizumab showed benefits for all-cause mortality events and a shorter time until death after first intervention but showed no difference in hospital length of stay. Upon subgroup analysis, tocilizumab showed fewer all-cause mortality events when CRP level ≥100 mg/L, P/F ratio 200-300 mmHg, and P/F ratio <200 mmHg. However, tocilizumab showed a longer length of stay when CRP <100 mg/L than the SOC. **Conclusion:** This meta-analysis demonstrated that tocilizumab has a positive effect on all-cause mortality. It should be cautiously administrated for optimal results and tailored to the patient's eligibility criteria.

Keywords: COVID-19; Severe; critically ill; tocilizumab.

Copyright: © 2021 Nugroho CW et al.

Conflict of interest statement

No competing interests were disclosed.

- 50 references
- 3 figures

Supplementary info

Publication types, MeSH terms, Substances, Supplementary concepts, Associated data, Grant support Expand

Publication types

- Meta-Analysis
- Systematic Review

MeSH terms

- Antibodies, Monoclonal, Humanized / therapeutic use*

- COVID-19 / drug therapy*
- Humans
- Multicenter Studies as Topic
- Observational Studies as Topic
- Randomized Controlled Trials as Topic

Substances

- Antibodies, Monoclonal, Humanized
- tocilizumab

Supplementary concepts

- COVID-19 drug treatment

Associated data

- figshare/10.6084/m9.figshare.13655894.v1

Grant support

The author(s) declared that no grants were involved in supporting this work.

Full text links

F1000Research FREE FULL TEXT F1000 Research Ltd Free PMC article

Proceed to details

Cite

Share

☐ 732

Observational Study

Eur J Clin Microbiol Infect Dis

. 2021 Apr;40(4):761-769.

doi: 10.1007/s10096-020-04078-1. Epub 2020 Oct 20.

High versus standard doses of corticosteroids in severe COVID-19: a retrospective cohort study

Enric Monreal [1], Susana Sainz de la Maza [2], Elena Natera-Villalba [2], Álvaro Beltrán-Corbellini [2], Fernando Rodríguez-Jorge [2], Jose Ignacio Fernández-Velasco [3], Paulette Walo-Delgado [3], Alfonso Muriel [4], Javier Zamora [4], Araceli Alonso-Canovas [2], Jesús Fortún [5], Luis Manzano

[6], Beatriz Montero-Errasquín [7], Lucienne Costa-Frossard [2], Jaime Masjuan [2], Luisa María Villar [3], COVID-HRC group
Collaborators, Affiliations Expand

Collaborators

- **COVID-HRC group**:
L Máiz-Carro, E M Sánchez-García, F Hidalgo, A R Domínguez, J A Pérez-Molina, O Sánchez-Sánchez, B Comeche, B Monge-Maillo, E Barbero, I Barbolla-Díaz, L Aranzábal Orgaz, J Cobo, I Rayo, C Fernández-Golfín, E González, L M Rincón-Díaz, R Ron, B Mateos-Muñoz, E Navas, J Moreno, J Norman, S Serrano, C Quereda Rodríguez-Navarro, A Vallés, S Herrera, J Mateos Del Nozal, M A Moreno-Cobo, F Gioia, M C Concejo-Badorrey, E Y Ortiz Barraza, A Moreno, S Chamorro, J L Casado, C Almonacid, R Nieto, S Diz, E Moreno, M Conde, J M Hermida, M López, J L Chico-García, Á Beltrán-Corbellini, E Rodríguez de Santiago, C G Rita, I Iturrieta-Zuazo, A De Andrés, M Espiño, M Vázquez, M Fernández Lucas, J Martínez-Sanz, N García-Barragán, J Buisán, R Toledano, P Pérez-Torre, M C Matute-Lozano, J L López-Sendón, G García-Ribas, Í Corral, L M Villar

Affiliations

- [1] Department of Neurology, Hospital Universitario Ramón y Cajal, Universidad de Alcalá, IRYCIS, Colmenar Viejo, km 9,100, 28034, Madrid, Spain. enricmonreal@outlook.com.
- [2] Department of Neurology, Hospital Universitario Ramón y Cajal, Universidad de Alcalá, IRYCIS, Colmenar Viejo, km 9,100, 28034, Madrid, Spain.
- [3] Department of Immunology, Hospital Universitario Ramón y Cajal, Universidad de Alcalá, IRYCIS, Madrid, Spain.
- [4] Biostatistics Unit, Hospital Universitario Ramón y Cajal, Universidad de Alcalá, IRYCIS, CIBERESP, Madrid, Spain.
- [5] Department of Infectious Diseases, Hospital Universitario Ramón y Cajal, Universidad de Alcalá, IRYCIS, Madrid, Spain.
- [6] Department of Internal Medicine, Hospital Universitario Ramón y Cajal, Universidad de Alcalá, IRYCIS, Madrid, Spain.
- [7] Department of Geriatrics, Hospital Universitario Ramón y Cajal, Universidad de Alcalá, IRYCIS, Madrid, Spain.

- PMID: **33083917**
- PMCID: PMC7575217
- DOI: 10.1007/s10096-020-04078-1

Free PMC article
Observational Study

High versus standard doses of corticosteroids in severe COVID-19: a retrospective cohort study

Enric Monreal et al. Eur J Clin Microbiol Infect Dis. 2021 Apr.

Free PMC article
Show details
Eur J Clin Microbiol Infect Dis
. 2021 Apr;40(4):761-769.
doi: 10.1007/s10096-020-04078-1. Epub 2020 Oct 20.

Authors

Enric Monreal [1], Susana Sainz de la Maza [2], Elena Natera-Villalba [2], Álvaro Beltrán-Corbellini [2], Fernando Rodríguez-Jorge [2], Jose Ignacio Fernández-Velasco [3], Paulette Walo-Delgado [3], Alfonso Muriel [4], Javier Zamora [4], Araceli Alonso-Canovas [2], Jesús Fortún [5], Luis Manzano [6], Beatriz Montero-Errasquín [7], Lucienne Costa-Frossard [2], Jaime Masjuan [2], Luisa María Villar [3], COVID-HRC group

Collaborators

- **COVID-HRC group**:
  L Máiz-Carro, E M Sánchez-García, F Hidalgo, A R Domínguez, J A Pérez-Molina, O Sánchez-Sánchez, B Comeche, B Monge-Maillo, E Barbero, I Barbolla-Díaz, L Aranzábal Orgaz, J Cobo, I Rayo, C Fernández-Golfín, E González, L M Rincón-Díaz, R Ron, B Mateos-Muñoz, E Navas, J Moreno, J Norman, S Serrano, C Quereda Rodríguez-Navarro, A Vallés, S Herrera, J Mateos Del Nozal, M A Moreno-Cobo, F Gioia, M C Concejo-Badorrey, E Y Ortiz Barraza, A Moreno, S Chamorro, J L Casado, C Almonacid, R Nieto, S Diz, E Moreno, M Conde, J M Hermida, M López, J L Chico-García, Á Beltrán-Corbellini, E Rodríguez de Santiago, C G Rita, I Iturrieta-Zuazo, A De Andrés, M Espiño, M Vázquez, M Fernández Lucas, J Martínez-Sanz, N García-Barragán, J Buisán, R Toledano, P Pérez-Torre, M C Matute-Lozano, J L López-Sendón, G García-Ribas, Í Corral, L M Villar

Affiliations

- [1] Department of Neurology, Hospital Universitario Ramón y Cajal, Universidad de Alcalá, IRYCIS, Colmenar Viejo, km 9,100, 28034, Madrid, Spain. enricmonreal@outlook.com.
- [2] Department of Neurology, Hospital Universitario Ramón y Cajal, Universidad de Alcalá, IRYCIS, Colmenar Viejo, km 9,100, 28034, Madrid, Spain.
- [3] Department of Immunology, Hospital Universitario Ramón y Cajal, Universidad de Alcalá, IRYCIS, Madrid, Spain.
- [4] Biostatistics Unit, Hospital Universitario Ramón y Cajal, Universidad de Alcalá, IRYCIS, CIBERESP, Madrid, Spain.
- [5] Department of Infectious Diseases, Hospital Universitario Ramón y Cajal, Universidad de Alcalá, IRYCIS, Madrid, Spain.
- [6] Department of Internal Medicine, Hospital Universitario Ramón y Cajal, Universidad de Alcalá, IRYCIS, Madrid, Spain.
- [7] Department of Geriatrics, Hospital Universitario Ramón y Cajal, Universidad de Alcalá, IRYCIS, Madrid, Spain.

- PMID: **33083917**
- PMCID: PMC7575217
- DOI: 10.1007/s10096-020-04078-1

Abstract

Despite the increasing evidence of the benefit of corticosteroids for the treatment of moderate-severe coronavirus disease 2019 (COVID-19) patients, no data are available about the potential role of high doses of steroids for these patients. We evaluated the mortality, the risk of need for mechanical ventilation (MV), or death and the risk of developing a severe acute respiratory distress syndrome (ARDS) between high (HD) and standard doses (SD) among patients with a severe COVID-19. All consecutive confirmed COVID-19 patients admitted to a single center were selected, including those treated with steroids and an ARDS. Patients were allocated to the HD (≥ 250 mg/day of methylprednisolone) of corticosteroids or the SD (≤ 1.5 mg/kg/day of methylprednisolone) at discretion of treating physician. Five hundred seventy-three patients were included: 428 (74.7%) men, with a median (IQR) age of 64 (54-73) years. In the HD group, a worse baseline respiratory situation was observed and male gender, older age, and comorbidities were significantly more common. After adjusting by baseline characteristics, HDs were associated with a higher mortality than SD (adjusted OR 2.46, 95% CI 1.59-3.81, $p < 0.001$) and with an increased risk of needing MV or death (adjusted OR 2.35, $p = 0.001$). Conversely, the risk of developing a severe ARDS was similar between groups. Interaction analysis showed that HD increased mortality exclusively in elderly patients. Our real-world experience advises against exceeding 1-1.5 mg/kg/day of corticosteroids for severe COVID-19 with an ARDS, especially in older subjects. This reinforces the rationale of modulating rather than suppressing immune responses in these patients.

Keywords: COVID-19; Corticosteroids; SARS-CoV-2; Severe acute respiratory syndrome coronavirus 2.

Conflict of interest statement

The authors declare that they have no conflict of interest.

- 45 references
- 2 figures

Supplementary info

Publication types, MeSH terms, Substances, Supplementary concepts Expand

Publication types

- Comparative Study
- Observational Study

MeSH terms

- Adult
- Age Factors
- Aged
- Aged, 80 and over
- COVID-19 / complications

- COVID-19 / drug therapy*
- COVID-19 / mortality
- Cohort Studies
- Dose-Response Relationship, Drug
- Female
- Glucocorticoids / administration & dosage*
- Humans
- Logistic Models
- Male
- Methylprednisolone / administration & dosage*
- Middle Aged
- Respiration, Artificial / statistics & numerical data*
- Respiratory Distress Syndrome / epidemiology*
- Respiratory Distress Syndrome / etiology
- Retrospective Studies
- SARS-CoV-2
- Severity of Illness Index

Substances

- Glucocorticoids
- Methylprednisolone

Supplementary concepts

- COVID-19 drug treatment

Full text links

SpringerLink FULL-TEXT ARTICLE Springer Free PMC article
Proceed to details
Cite
Share
☐ 733
Observational Study
Int J Environ Res Public Health
. 2021 May 31;18(11):5911.
doi: 10.3390/ijerph18115911.

Characteristics and Outcomes of Pediatric COVID-19 Patients in Osaka, Japan

Yusuke Katayama [1], Ling Zha [2], Tetsuhisa Kitamura [2], Atsushi Hirayama [3], Taro Takeuchi [2], Kenta Tanaka [2], Sho Komukai [4], Takeshi Shimazu [1], Tomotaka Sobue [2], On Behalf Of The Covid-Epidemiology Research Group Of Osaka University
Affiliations Expand

Affiliations

- [1] Department of Traumatology and Acute Critical Medicine, Osaka University Graduate School of Medicine, Suita, Osaka 565-0871, Japan.
- [2] Division of Environmental Medicine and Population Sciences, Department of Social and Environmental Medicine, Graduate School of Medicine, Osaka University, Suita 565-0871, Japan.
- [3] Division of Public Health, Department of Social Medicine, Osaka University Graduate School of Medicine, Suita, Osaka 565-0871, Japan.
- [4] Division of Biomedical Statistics, Department of Integrated Medicine, Graduate School of Medicine, Osaka University, Suita, Osaka 565-0871, Japan.

- PMID: **34072919**
- PMCID: PMC8198974
- DOI: 10.3390/ijerph18115911

Free PMC article
Observational Study

Characteristics and Outcomes of Pediatric COVID-19 Patients in Osaka, Japan

Yusuke Katayama et al. Int J Environ Res Public Health. 2021.
Free PMC article
Show details
Int J Environ Res Public Health
. 2021 May 31;18(11):5911.
doi: 10.3390/ijerph18115911.

Authors

Yusuke Katayama [1], Ling Zha [2], Tetsuhisa Kitamura [2], Atsushi Hirayama [3], Taro Takeuchi [2], Kenta Tanaka [2], Sho Komukai [4], Takeshi Shimazu [1], Tomotaka Sobue [2], On Behalf Of The Covid-Epidemiology Research Group Of Osaka University

Affiliations

- [1] Department of Traumatology and Acute Critical Medicine, Osaka University Graduate School of Medicine, Suita, Osaka 565-0871, Japan.
- [2] Division of Environmental Medicine and Population Sciences, Department of Social and Environmental Medicine, Graduate School of Medicine, Osaka University, Suita 565-0871, Japan.

- [3] Division of Public Health, Department of Social Medicine, Osaka University Graduate School of Medicine, Suita, Osaka 565-0871, Japan.
- [4] Division of Biomedical Statistics, Department of Integrated Medicine, Graduate School of Medicine, Osaka University, Suita, Osaka 565-0871, Japan.

- PMID: **34072919**
- PMCID: PMC8198974
- DOI: 10.3390/ijerph18115911

Abstract

The epidemiological information on characteristics, in-hospital treatments, and outcomes of the coronavirus disease 2019 (COVID-19) among pediatric patients has not been fully evaluated in Japan. This was a retrospective observational study conducted in the Osaka Prefecture, Japan, and we enrolled laboratory-confirmed COVID-19 patients aged ≤ 19 years old from January to November in 2020. Of 14,846 COVID-19 eligible patients, 1240 pediatric patients (8.4%) were registered during the study period; 329 were children aged 0-9 years (26.5%) and 911 were adolescents aged 10-19 years (73.5%). The majority of the patients exhibited mild symptoms at diagnosis (872, 70.3%), some were asymptomatic (296, 23.9%). Cluster infections occurred in child-care facilities (26, 7.9%) among children and in universities (27, 3.0%) and schools (18, 2.0%) among adolescents. The number of close-contact cases was 260 (69.0%) in children and 459 (50.4%) in adolescents. Sixty of the children (18.2%) and 90 of the adolescents (9.9%) were hospitalized. One patient received mechanical ventilation, and none underwent extracorporeal membrane oxygenation. One patient was admitted to the intensive care unit; there were no deaths. These results are useful for recognizing the clinical course from transmission route to outcomes of this infection in pediatric patients.

Keywords: Japan; SARS-CoV-2; adolescents; children; epidemiology.

Conflict of interest statement

The authors declare no conflict of interest.

- 31 references
- 2 figures

Supplementary info

Publication types, MeSH terms Expand

Publication types

- Observational Study

MeSH terms

- Adolescent
- Adult
- COVID-19*

- Child
- Extracorporeal Membrane Oxygenation*
- Humans
- Japan / epidemiology
- Respiration, Artificial
- SARS-CoV-2
- Young Adult

Full text links

FULL TEXT OPEN ACCESS MDPI Multidisciplinary Digital Publishing Institute (MDPI) Free PMC article
Proceed to details
Cite
Share
☐ 734
Observational Study
Am J Emerg Med
. 2021 Aug;46:525-531.
doi: 10.1016/j.ajem.2020.11.011. Epub 2020 Nov 8.

Clinical value of procalcitonin in critically ill patients infected by SARS-CoV-2

Pedro Garrido [1], Pitter Cueto [2], Conxita Rovira [1], Elisabet Garcia [1], Ana Parra [1], Raquel Enriquez [1], Armando Pinos [1], Manuel Sosa [1], Anna Hernández-Aguilera [3], Immaculada Vallverdú [1]
Affiliations Expand

Affiliations

- [1] Intensive Care Unit, Hospital Universitari Sant Joan, Institut d'Investigació Sanitària Pere Virgili, Universitat Rovira i Virgili, Reus, Spain.
- [2] Intensive Care Unit, Hospital Universitari Sant Joan, Institut d'Investigació Sanitària Pere Virgili, Universitat Rovira i Virgili, Reus, Spain. Electronic address: pfcueto@grupsagessa.com.
- [3] Unitat de Recerca Biomèdica, Hospital Universitari Sant Joan, Institut d'Investigació Sanitària Pere Virgili, Universitat Rovira i Virgili, Reus, Spain.

- PMID: **33221115**
- PMCID: PMC7648886
- DOI: 10.1016/j.ajem.2020.11.011

Free PMC article
Observational Study

Clinical value of procalcitonin in critically ill patients infected by SARS-CoV-2

Pedro Garrido et al. Am J Emerg Med. 2021 Aug.
Free PMC article
Show details
Am J Emerg Med
. 2021 Aug;46:525-531.
doi: 10.1016/j.ajem.2020.11.011. Epub 2020 Nov 8.

Authors

Pedro Garrido [1], Pitter Cueto [2], Conxita Rovira [1], Elisabet Garcia [1], Ana Parra [1], Raquel Enriquez [1], Armando Pinos [1], Manuel Sosa [1], Anna Hernández-Aguilera [3], Immaculada Vallverdú [1]

Affiliations

- [1] Intensive Care Unit, Hospital Universitari Sant Joan, Institut d'Investigació Sanitària Pere Virgili, Universitat Rovira i Virgili, Reus, Spain.
- [2] Intensive Care Unit, Hospital Universitari Sant Joan, Institut d'Investigació Sanitària Pere Virgili, Universitat Rovira i Virgili, Reus, Spain. Electronic address: pfcueto@grupsagessa.com.
- [3] Unitat de Recerca Biomèdica, Hospital Universitari Sant Joan, Institut d'Investigació Sanitària Pere Virgili, Universitat Rovira i Virgili, Reus, Spain.

- PMID: **33221115**
- PMCID: PMC7648886
- DOI: 10.1016/j.ajem.2020.11.011

Abstract

Background Blood procalcitonin (PCT) levels usually increase during infectious diseases and might be helpful to differentiate bacterial from non-bacterial origin. COVID-19 patients could present co-infections at initial presentation in the Emergency Department and nosocomial infections during stay in the ICU. However, the published literature has not established whether PCT changes could aid in the diagnosis of infectious complication during the COVID-19 pandemic. Methods Retrospective, single-center, cohort study, including COVID-19 patients admitted between March and May 2020. The data were prospectively collected for department purposes; laboratory results were collected automatically at admission and during the whole patient admission. Results 56 patients were analyzed (female 32%, male 68%), 35 were admitted to ICU, and 21 received general ward care. 21 ICU patients underwent mechanical ventilation (88%), and 9 died during admission (26%). Non-survivors had higher initial blood PCT levels than survivors at ICU admission (p.

Copyright © 2020 Elsevier Inc. All rights reserved.

Conflict of interest statement

Declaration of Competing Interest The authors have no conflicts of interest to declare.

- 40 references
- 4 figures

Supplementary info

Publication types, MeSH terms, Substances Expand

Publication types

- Observational Study

MeSH terms

- Aged
- Aged, 80 and over
- Biomarkers / blood
- COVID-19 / blood*
- COVID-19 / epidemiology
- Critical Illness*
- Emergency Service, Hospital / standards*
- Female
- Humans
- Intensive Care Units*
- Male
- Middle Aged
- Pandemics
- Patient Admission / trends
- Procalcitonin / blood*
- Retrospective Studies
- Spain / epidemiology

Substances

- Biomarkers
- Procalcitonin

Full text links

ELSEVIER FULL-TEXT ARTICLE Elsevier Science Free PMC article
Proceed to details

Cite
Share
☐ 735
Observational Study
Sleep Med
. 2020 Nov;75:354-360.
doi: 10.1016/j.sleep.2020.08.010. Epub 2020 Aug 17.

Improved night shift schedule related to the mortality of critically ill patients with Corona Virus Disease 2019

Sun Zhang [1], Yuanda Xu [1], Kang Wu [1], Tao Wang [1], Xiaofen Su [1], Qian Han [1], Yin Xi [1], Shitao Zhu [1], Yong Gao [2], Hongbo Wang [2], Yu Hu [2], Chunli Liu [1], Nanshan Zhong [1], Pixin Ran [1], Nuofu Zhang [3]
Affiliations Expand

Affiliations

- [1] State Key Laboratory of Respiratory Disease, National Clinical Research Center for Respiratory Disease, Guangzhou Institute of Respiratory Health, The First Affiliated Hospital of Guangzhou Medical University, Guangzhou, Guangdong, China.
- [2] Union Hospital, Tongji Medical College, Huazhong University of Science and Technology, Wuhan, Hubei, China.
- [3] State Key Laboratory of Respiratory Disease, National Clinical Research Center for Respiratory Disease, Guangzhou Institute of Respiratory Health, The First Affiliated Hospital of Guangzhou Medical University, Guangzhou, Guangdong, China. Electronic address: nfzhanggird@163.com.

- PMID: **32950880**
- PMCID: PMC7429562
- DOI: 10.1016/j.sleep.2020.08.010

Free PMC article
Observational Study

Improved night shift schedule related to the mortality of critically ill patients with Corona Virus Disease 2019

Sun Zhang et al. Sleep Med. 2020 Nov.
Free PMC article
Show details

Sleep Med
. 2020 Nov;75:354-360.
doi: 10.1016/j.sleep.2020.08.010. Epub 2020 Aug 17.

Authors

Sun Zhang [1], Yuanda Xu [1], Kang Wu [1], Tao Wang [1], Xiaofen Su [1], Qian Han [1], Yin Xi [1], Shitao Zhu [1], Yong Gao [2], Hongbo Wang [2], Yu Hu [2], Chunli Liu [1], Nanshan Zhong [1], Pixin Ran [1], Nuofu Zhang [3]

Affiliations

- [1] State Key Laboratory of Respiratory Disease, National Clinical Research Center for Respiratory Disease, Guangzhou Institute of Respiratory Health, The First Affiliated Hospital of Guangzhou Medical University, Guangzhou, Guangdong, China.
- [2] Union Hospital, Tongji Medical College, Huazhong University of Science and Technology, Wuhan, Hubei, China.
- [3] State Key Laboratory of Respiratory Disease, National Clinical Research Center for Respiratory Disease, Guangzhou Institute of Respiratory Health, The First Affiliated Hospital of Guangzhou Medical University, Guangzhou, Guangdong, China. Electronic address: nfzhanggird@163.com.

- PMID: **32950880**
- PMCID: PMC7429562
- DOI: 10.1016/j.sleep.2020.08.010

Abstract

Purpose: To determine the relationship between the improved night shift schedule and the mortality of critically ill patients with Corona Virus Disease 2019 (COVID-19).

Methods: According to the time of the implementation of the new night shift schedule, we divided all patients into two groups: initial period group and recent period group. The clinical electronic medical records, nursing records, laboratory findings, and radiological examinations for all patients with laboratory confirmed Severe Acute Respiratory Syndrome Coronavirus 2 (SARS-CoV-2) infection were reviewed. Cox proportional hazard ratio (HR) models were used to determine the risk factors associated with in hospital death.

Results: A total of 75 patients were included in this study. Initial period group includes 45 patients and recent period group includes 30 patients. The difference in mortality between the two groups was significant, 77.8% and 36.7%, respectively. Leukocytosis at admission and admitted to hospital before the new night shift schedule were associated with increased odds of death.

Conclusions: Shift arrangement of medical staff are associated with the mortality of critically ill patients with COVID-19. The new night shift schedule might improve the continuity of treatment, thereby improving the overall quality of medical work and reducing the mortality of critically ill patients.

Keywords: COVID-19; Mental health; SARS-CoV-2; Shift work; Sleep quality.

Copyright © 2020 Elsevier B.V. All rights reserved.

Conflict of interest statement

The authors declared no potential conflicts of interest with respect to the research, authorship, and/or publication of this article.

The ICMJE Uniform Disclosure Form for Potential Conflicts of Interest associated with this article can be viewed by clicking on the following link: https://doi.org/10.1016/j.sleep.2020.08.010.

- 15 references
- 2 figures

Supplementary info

Publication types, MeSH terms Expand

Publication types

- Observational Study
- Research Support, Non-U.S. Gov't

MeSH terms

- Aged
- COVID-19 / mortality*
- Case-Control Studies
- Comorbidity
- Female
- Hospital Mortality
- Humans
- Male
- Middle Aged
- Proportional Hazards Models
- Quality Improvement
- Retrospective Studies
- SARS-CoV-2
- Shift Work Schedule / statistics & numerical data*

Full text links

ELSEVIER FULL-TEXT ARTICLE Elsevier Science Free PMC article
Proceed to details
Cite
Share
☐ 736

Observational Study

Am J Respir Crit Care Med
. 2020 May 15;201(10):1294-1297.
doi: 10.1164/rccm.202003-0527LE.

Lung Recruitability in COVID-19-associated Acute Respiratory Distress Syndrome: A Single-Center Observational Study

Chun Pan [1 2 3], Lu Chen [4 5 6], Cong Lu [4 5 6], Wei Zhang [7], Jia-An Xia [3], Michael C Sklar [4 5 6], Bin Du [8], Laurent Brochard [4 5 6], Haibo Qiu [1 2 3]
Affiliations Expand

Affiliations

- [1] Zhongda HospitalNanjing, China.
- [2] Southeast UniversityNanjing, China.
- [3] Jinyintan HospitalWuhan, China.
- [4] Keenan Research CentreToronto, Ontario, Canada.
- [5] St. Michael's HospitalToronto, Ontario, Canada.
- [6] University of TorontoToronto, Ontario, Canada.
- [7] PLA 900th Hospital of Joint Service CorpsFuzhou, Chinaand.
- [8] Peking Union Medical College and Chinese Academy of Medical SciencesBeijing, China.

- PMID: **32200645**
- PMCID: PMC7233342
- DOI: 10.1164/rccm.202003-0527LE

Free PMC article
Observational Study

Lung Recruitability in COVID-19-associated Acute Respiratory Distress Syndrome: A Single-Center Observational Study

Chun Pan et al. Am J Respir Crit Care Med. 2020.
Free PMC article
Show details

Am J Respir Crit Care Med
. 2020 May 15;201(10):1294-1297.
doi: 10.1164/rccm.202003-0527LE.

Authors

Chun Pan [1 2 3], Lu Chen [4 5 6], Cong Lu [4 5 6], Wei Zhang [7], Jia-An Xia [3], Michael C Sklar [4 5 6], Bin Du [8], Laurent Brochard [4 5 6], Haibo Qiu [1 2 3]

Affiliations

- [1] Zhongda HospitalNanjing, China.
- [2] Southeast UniversityNanjing, China.
- [3] Jinyintan HospitalWuhan, China.
- [4] Keenan Research CentreToronto, Ontario, Canada.
- [5] St. Michael's HospitalToronto, Ontario, Canada.
- [6] University of TorontoToronto, Ontario, Canada.
- [7] PLA 900th Hospital of Joint Service CorpsFuzhou, Chinaand.
- [8] Peking Union Medical College and Chinese Academy of Medical SciencesBeijing, China.

- PMID: **32200645**
- PMCID: PMC7233342
- DOI: 10.1164/rccm.202003-0527LE

No abstract available

Comment in

- Early application of prone position for management of Covid-19 patients.
  Golestani-Eraghi M, Mahmoodpoor A. Golestani-Eraghi M, et al. J Clin Anesth. 2020 Nov;66:109917. doi: 10.1016/j.jclinane.2020.109917. Epub 2020 May 26. J Clin Anesth. 2020. PMID: 32473503 Free PMC article. No abstract available.
- Reply by Pan *et al.* to Haouzi *et al.*
  Pan C, Chen L, Lu C, Du B, Brochard L, Qiu H. Pan C, et al. Am J Respir Crit Care Med. 2020 Aug 15;202(4):630-631. doi: 10.1164/rccm.202005-2045LE. Am J Respir Crit Care Med. 2020. PMID: 32579031 Free PMC article. No abstract available.

- 7 references
- 1 figure

Supplementary info

Publication types, MeSH terms Expand

Publication types

- Letter
- Observational Study

MeSH terms

- Betacoronavirus

- COVID-19
- Coronavirus Infections* / complications
- Female
- Humans
- Lung / physiopathology*
- Male
- Middle Aged
- Pandemics*
- Pneumonia, Viral* / complications
- Respiration, Artificial
- Respiratory Distress Syndrome / physiopathology
- Respiratory Distress Syndrome / virology*
- Retrospective Studies
- SARS-CoV-2

Full text links

AJRCCM Final Version Atypon Free PMC article
Proceed to details
Cite
Share
737
Observational Study
Rev Neurol
. 2021 Sep 1;73(6):201-209.
doi: 10.33588/rn.7306.2021028.

[Effect of treatment with benzodiazepines on the hospital prognosis of Coronavirus disease 2019]

[Article in Spanish]
E Martínez-Pías [1], D García-Azorín [1], J Trigo-López [1], B Talavera [1], G Valle [1], I Hernández [1], P Simón [1], J F Arenillas-Lara [2] [1] [3]
Affiliations Expand

Affiliations

- [1] Hospital Clínico Universitario de Valladolid, Valladolid, España.
- [2] Universidad de Valladolid, Valladolid, España.
- [3] Universidad de Valladolid-Consejo Superior de Investigaciones Científicas, Madrid, España.

- PMID: **34515333**
- DOI: 10.33588/rn.7306.2021028

Free article
Observational Study

[Effect of treatment with benzodiazepines on the hospital prognosis of Coronavirus disease 2019]

[Article in Spanish]
E Martínez-Pías et al. Rev Neurol. 2021.
Free article
Show details
Rev Neurol

. 2021 Sep 1;73(6):201-209.
doi: 10.33588/rn.7306.2021028.

Authors

E Martínez-Pías [1], D García-Azorín [1], J Trigo-López [1], B Talavera [1], G Valle [1], I Hernández [1], P Simón [1], J F Arenillas-Lara [2] [1] [3]

Affiliations

- [1] Hospital Clínico Universitario de Valladolid, Valladolid, España.
- [2] Universidad de Valladolid, Valladolid, España.
- [3] Universidad de Valladolid-Consejo Superior de Investigaciones Científicas, Madrid, España.

- PMID: **34515333**
- DOI: 10.33588/rn.7306.2021028

Abstract
in English, Spanish

Introduction: The consequences of the use of of benzodiazepines in coronavirus disease 2019 have not yet been studied. We compared the hospital prognosis of patients hospitalized for coronavirus disease 2019 in benzodiazepine users and non-users.

Patients and methods: Observational study with a retrospective cohort design. All consecutive patients admitted with a confirmed diagnosis of coronavirus disease 2019 were included. The patients under chronic treatment with benzodiazepines at the time of admission were studied and compared with non-users. The primary objective was to analyze the mortality of patients who used chronic benzodiazepines at the time of admission and compare them with those who did not use them. The secondary objective was to analyze the risk of severe disease due to coronavirus 2019,

acute respiratory distress syndrome and admission to the Intensive Care Unit in both groups of patients.

Results: We included 576 patients, 138 (24.0%) used benzodiazepines. After adjusting for sex, age, baseline situation and all the different variables between both groups, benzodiazepine users did not show a higher odds of mortality (OR: 1,1, IC 95%: 0,7-1,9, p = 0,682) or higher risk of severe disease due to coronavirus 2019 (OR: 1.2, 95% CI: 0.7-1.8, p = 0.523). They also did not have a higher risk of acute respiratory distress syndrome (OR: 1.2, IC 95%: 0.8-1.9, p = 0.315) or more admission to the Intensive Care Unit (OR: 0.8, 95% CI: 0.4-1.4, p = 0.433).

Conclusion: In our sample, treatment with benzodiazepines at the time of admission was not associated with a worse hospital prognosis in patients with coronavirus disease 2019.

Title: Efecto del tratamiento con benzodiacepinas en el pronóstico hospitalario de la enfermedad por coronavirus 2019.

Introducción. Las consecuencias del consumo de benzodiacepinas en el marco de la la enfermedad por coronavirus 2019 (COVID-19) no se habían estudiado hasta ahora. En el presente estudio se comparó el pronóstico hospitalario de pacientes ingresados por COVID-19 que tomaban benzodiacepinas con el de otros ingresados por idéntico motivo que no las tomaban. Pacientes y métodos. Estudio observacional de cohortes retrospectivo. En el estudio se admitió a todos los pacientes consecutivos ingresados con un diagnóstico confirmado de COVID-19. Se estudió a los pacientes que en el momento del ingreso estaban en tratamiento crónico con benzodiacepinas en comparación con otros que no las tomaban. El objetivo principal fue analizar la mortalidad de dichos pacientes con uso crónico de benzodiacepinas y compararla con la mortalidad de los que no tomaban. El objetivo secundario fue analizar en ambos grupos de pacientes el riesgo de padecer un cuadro grave por COVID-19, el síndrome de dificultad respiratoria aguda o el ingreso en la unidad de cuidados intensivos. Resultados. Se admitieron 576 pacientes, 138 (24,0%) de los cuales tomaban benzodiacepinas. Después del ajuste por sexo, edad, situación inicial y todas las variables diferentes entre ambos grupos, los pacientes que tomaban benzodiacepinas no mostraron una probabilidad mayor de muerte (odds ratio: 1,1; IC 95%: 0,7-1,9; p = 0,682) ni un riesgo más acusado de COVID-19 grave (odds ratio: 1,2; IC 95%: 0,7-1,8; p = 0,523). Tampoco presentaron un riesgo mayor de síndrome de dificultad respiratoria aguda (odds ratio: 1,2; IC 95%: 0,8-1,9; p = 0,315) ni de ingreso en la unidad de cuidados intensivos (odds ratio: 0,8; IC 95%: 0,4-1,4; p = 0,433). Conclusión. En esta muestra de pacientes con COVID-2019, el tratamiento con benzodiacepinas en el momento del ingreso no apareció asociado con un empeoramiento del pronóstico hospitalario.

Supplementary info

Publication types, MeSH terms, Substances Expand

Publication types

- Observational Study

MeSH terms

- Adult
- Aged

- Benzodiazepines / adverse effects
- Benzodiazepines / therapeutic use*
- COVID-19 / mortality*
- Cohort Studies
- Female
- Hospital Mortality
- Hospitalization
- Humans
- Male
- Middle Aged
- Prognosis
- Retrospective Studies
- Severity of Illness Index

Substances

- Benzodiazepines

Full text links

Revista de Neurología Free full text article Viguera Editores, S. L.

Proceed to details

Cite

Share

738

Observational Study

Am J Med
. 2021 Oct;134(10):1247-1251.
doi: 10.1016/j.amjmed.2021.06.008. Epub 2021 Jul 7.

Masking for COVID-19 Is Associated with Decreased Emergency Department Utilization for Non-COVID Viral Illnesses and Respiratory Conditions in Maryland

Zachary D W Dezman [1], Benoit Stryckman [2], Kori S Zachrison [3], Ryan M Conrad [4], David Marcozzi [2], Laura Pimentel [2], Margaret Samuels-Kalow [3], Charles B Cairns [5]
Affiliations Expand

Affiliations

- [1] Department of Emergency Medicine, University of Maryland School of Medicine, Baltimore, Md; Department of Epidemiology and Public Health, University of Maryland School of Medicine, Baltimore, Md. Electronic address: zdezman@som.umaryland.edu.
- [2] Department of Emergency Medicine, University of Maryland School of Medicine, Baltimore, Md.
- [3] Department of Emergency Medicine, Massachusetts General Hospital, Harvard Medical School, Boston, Mass.
- [4] US Food and Drug Administration, Rockville, Md.
- [5] Drexel University, Philadelphia, Penn.

- PMID: **34242620**
- PMCID: PMC8260493
- DOI: 10.1016/j.amjmed.2021.06.008

Free PMC article
Observational Study

Masking for COVID-19 Is Associated with Decreased Emergency Department Utilization for Non-COVID Viral Illnesses and Respiratory Conditions in Maryland

Zachary D W Dezman et al. Am J Med. 2021 Oct.
Free PMC article
Show details
Am J Med
. 2021 Oct;134(10):1247-1251.
doi: 10.1016/j.amjmed.2021.06.008. Epub 2021 Jul 7.

Authors

Zachary D W Dezman [1], Benoit Stryckman [2], Kori S Zachrison [3], Ryan M Conrad [4], David Marcozzi [2], Laura Pimentel [2], Margaret Samuels-Kalow [3], Charles B Cairns [5]

Affiliations

- [1] Department of Emergency Medicine, University of Maryland School of Medicine, Baltimore, Md; Department of Epidemiology and Public Health, University of Maryland School of Medicine, Baltimore, Md. Electronic address: zdezman@som.umaryland.edu.
- [2] Department of Emergency Medicine, University of Maryland School of Medicine, Baltimore, Md.
- [3] Department of Emergency Medicine, Massachusetts General Hospital, Harvard Medical School, Boston, Mass.
- [4] US Food and Drug Administration, Rockville, Md.
- [5] Drexel University, Philadelphia, Penn.

- PMID: **34242620**
- PMCID: PMC8260493
- DOI: 10.1016/j.amjmed.2021.06.008

Abstract

Background: Masking, which is known to decrease the transmission of respiratory viruses, was not widely practiced in the United States until the coronavirus disease 2019 (COVID-19) pandemic. This provides a natural experiment to determine whether the percentage of community masking was associated with decreases in emergency department (ED) visits due to non-COVID viral illnesses (NCVIs) and related respiratory conditions.

Methods: In this observational study of ED encounters in a 11-hospital system in Maryland during 2019-2020, year-on-year ratios for all complaints were calculated to account for "lockdowns" and the global drop in ED visits due to the pandemic. Encounters for specific complaints were identified using the International Classification of Diseases, version 10. Encounters with a positive COVID test were excluded. Linear regression was used to determine the association of publicly available masking data with ED visits for NCVI and exacerbations of asthma and chronic obstructive pulmonary disease (COPD), after adjusting for patient age, sex, and medical history.

Results: There were 285,967 and 252,598 ED visits across the hospital system in 2019 and 2020, respectively. There was a trend toward an association between the year-on-year ratio for all ED visits and the Maryland stay-at-home order (parameter estimate = -0.0804, P = .10). A 10% percent increase in the prevalence of community masking was associated with a 17.0%, 8.8%, and 9.4% decrease in ED visits for NCVI and exacerbations of asthma exacerbations and chronic obstructive pulmonary disease, respectively (P < .001 for all).

Conclusions: Increasing the prevalence of masking is associated with a decrease in ED visits for viral illnesses and exacerbations of asthma and COPD. These findings may be valuable for future public health responses, particularly in future pandemics with respiratory transmission or in severe influenza seasons.

Keywords: COVID; Emergency medicine; Epidemiology; Public health.

Copyright © 2021 Elsevier Inc. All rights reserved.

- 11 references
- 1 figure

Supplementary info

Publication types, MeSH terms Expand

Publication types

- Observational Study
- Research Support, Non-U.S. Gov't

MeSH terms

- COVID-19 / prevention & control*
- Communicable Disease Control*
- Emergency Service, Hospital / statistics & numerical data*
- Female
- Humans
- Male
- Maryland / epidemiology
- Masks*
- Pandemics
- Respiratory Tract Diseases / epidemiology*
- Retrospective Studies
- SARS-CoV-2
- Virus Diseases / epidemiology*

Full text links

ELSEVIER FULL-TEXT ARTICLE Elsevier Science Free PMC article
Proceed to details
Cite
Share
739
Observational Study
Emergencias
. 2021 Aug;33(4):273-281.

Mortality in patients treated for COVID-19 in the emergency department of a tertiary care hospital during the first phase of the pandemic: Derivation of a risk model for emergency departments

[Article in English, Spanish]
Ana García-Martínez [1], Beatriz López-Barbeito [1], Blanca Coll-Vinent [1], Arrate Placer [1], Carme Font [1], Carmen Rosa Vargas [1], Carolina Sánchez [1], Daniela Piñango [1], Elisenda Gómez-Angelats [1], David Curtelin [1], Emilio Salgado [1], Francisco Aya [1], Gemma Martínez-Nadal [1], José Ramón Alonso [1], Julia García-Gozalbes [1], Leticia Fresco [1], Miguel Galicia [1], Milagrosa Perea [1], Miriam Carbó [1], Nerea Iniesta [1], Ona Escoda [1], Rafael Perelló [1], Sandra Cuerpo [1], Vanesa Flores [1], Xavier Alemany [1], Òscar Miró [1], Mª Del Mar Ortega Romero [1], Grupo de Trabajo sobre la atención de la COVID-19 en Urgencias (COVID19-URG)

Affiliations Expand

Affiliation

- [1] Área de Urgencias, Hospital Clinic, Barcelona, España. Universitat de Barcelona, España.

- PMID: **34251140**

Free article
Observational Study

Mortality in patients treated for COVID-19 in the emergency department of a tertiary care hospital during the first phase of the pandemic: Derivation of a risk model for emergency departments

[Article in English, Spanish]
Ana García-Martínez et al. Emergencias. 2021 Aug.
Free article
Show details
Emergencias
. 2021 Aug;33(4):273-281.

Authors

Ana García-Martínez [1], Beatriz López-Barbeito [1], Blanca Coll-Vinent [1], Arrate Placer [1], Carme Font [1], Carmen Rosa Vargas [1], Carolina Sánchez [1], Daniela Piñango [1], Elisenda Gómez-Angelats [1], David Curtelin [1], Emilio Salgado [1], Francisco Aya [1], Gemma Martínez-Nadal [1], José Ramón Alonso [1], Julia García-Gozalbes [1], Leticia Fresco [1], Miguel Galicia [1], Milagrosa Perea [1], Miriam Carbó [1], Nerea Iniesta [1], Ona Escoda [1], Rafael Perelló [1], Sandra Cuerpo [1], Vanesa Flores [1], Xavier Alemany [1], Òscar Miró [1], Mª Del Mar Ortega Romero [1], Grupo de Trabajo sobre la atención de la COVID-19 en Urgencias (COVID19-URG)

Affiliation

- [1] Área de Urgencias, Hospital Clinic, Barcelona, España. Universitat de Barcelona, España.

- PMID: **34251140**

Abstract
in English, Spanish

Objectives: To develop a risk model to predict 30-day mortality after emergency department treatment for COVID-19.

Material and methods: Observational retrospective cohort study including 2511 patients with COVID-19 who came to our emergency department between March 1 and April 30, 2020. We analyzed variables with Kaplan Meier survival and Cox regression analyses.

Results: All-cause mortality was 8% at 30 days. Independent variables associated with higher risk of mortality were age over 50 years, a Barthel index score less than 90, altered mental status, the ratio of arterial oxygen saturation to the fraction of inspired oxygen (SaO2/FIO2), abnormal lung sounds, platelet concentration less than 100 000/mm3, a C-reactive protein concentration of 5 mg/dL or higher, and a glomerular filtration rate less than 45 mL/min. Each independent predictor was assigned 1 point in the score except age, which was assigned 2 points. Risk was distributed in 3 levels: low risk (score of 4 points or less), intermediate risk (5 to 6 points), and high risk (7 points or above). Thirty-day risk of mortality was 1.7% for patients who scored in the low-risk category, 28.2% for patients with an intermediate risk score, and 67.3% for those with a high risk score.

Conclusion: This mortality risk stratification tool for patients with COVID-19 could be useful for managing the course of disease and assigning health care resources in the emergency department.

Objetivo: Derivar un modelo de riesgo para estimar la probabilidad de mortalidad a los 30 días de la visita a urgencias de pacientes con COVID-19.

Metodo: Estudio observacional de cohortes retrospectivo de 2.511 pacientes con COVID-19 atendidos en el servicio de urgencias hospitalario (SUH) del 1 de marzo al 30 de abril de 2020. Se realizó análisis de supervivencia mediante Kaplan Meier y regresión de Cox.

Resultados: La mortalidad por cualquier causa a los 30 días fue de un 8%. Los factores asociados de forma independiente a mayor mortalidad fueron: edad 50 años, índice de Barthel 90 puntos, alteración del nivel de consciencia, índice de SaO2/FIO2 400, auscultación respiratoria anómala, cifra de plaquetas 100.000/mm3, PCR 5 mg/dL y filtrado glomerular 45 mL/min. A estos factores se les asignó una puntuación de 1, excepto a la edad, que se le asignó un valor de 2 puntos. Se dividió el modelo de riesgo en 3 categorías: riesgo bajo (menor o igual a 4 puntos), riesgo intermedio (5-6 puntos) y riesgo alto (igual o superior a 7 puntos). Para los pacientes clasificados como de bajo riesgo la probabilidad de mortalidad a los 30 días fue del 1,7%, en los casos de riesgo intermedio fue del 28,2% y para los de alto riesgo fue del 67,3%.

Conclusiones: Disponer de una herramienta para estratificar el riesgo de mortalidad de los pacientes con COVID-19 que consultan a un SUH podría ser de utilidad para la gestión de los recursos sanitarios disponibles.

Keywords: COVID-19; Emergency department; Indicador de mortalidad; Infección SARS-CoV-2; Mortality; Risk factors; SARS-CoV-2 infection; Urgencias.

Supplementary info

Publication types, MeSH terms, Substances Expand

Publication types

- Observational Study

MeSH terms

- C-Reactive Protein
- COVID-19 / mortality*
- Emergency Service, Hospital / statistics & numerical data*
- Glomerular Filtration Rate
- Humans
- Kaplan-Meier Estimate
- Lung / physiopathology
- Middle Aged
- Models, Theoretical*
- Retrospective Studies
- Risk Factors
- Tertiary Care Centers

Substances

- C-Reactive Protein

Full text links

EMERGENCIAS Grupo Saned
Proceed to details
Cite
Share
☐ 740
Observational Study
Ann Vasc Surg
. 2021 Apr;72:196-204.
doi: 10.1016/j.avsg.2020.12.005. Epub 2020 Dec 31.

Acute Limb Ischemia in a Peruvian Cohort Infected by COVID-19

Juan Bautista Sánchez [1], Juan Diego Cuipal Alcalde [2], Rossi Ramos Isidro [2], Carlos Zúñiga Luna [2], W Samir Cubas [3], André Coaguila Charres [4], José Egoávil Gutiérrez [5], Juan David Ochoa [6], Patricia Fernández Arias [7]
Affiliations Expand

Affiliations

- [1] Vascular and Endovascular Surgery Unit, Cardiac and Vascular Surgery Service, Guillermo Almenara Irigoyen National Hospital, Lima, Peru. Electronic address: juan.bautista.s@upch.pe.
- [2] Vascular and Endovascular Surgery Unit, Cardiac and Vascular Surgery Service, Guillermo Almenara Irigoyen National Hospital, Lima, Peru.
- [3] Vascular and Endovascular Surgery Service, Thoracic and Cardiovascular Surgery Department, Edgardo Rebagliati Martins National Hospital, Lima, Peru.
- [4] Thorax and Cardiovascular Surgery Service, Dos de Mayo National Hospital, Lima, Peru.
- [5] Thorax and Cardiovascular Surgery Service, Cayetano Heredia National Hospital, Lima, Peru.
- [6] Thoracic and Cardiovascular Surgery Department, Hipolito Unanue National Hospital, Lima, Peru.
- [7] Thorax and Cardiovascular Surgery Department, Luis N. Saenz National Police Hospital, Lima, Peru.

- PMID: **33388408**
- PMCID: PMC7836937
- DOI: 10.1016/j.avsg.2020.12.005

Free PMC article
Observational Study

Acute Limb Ischemia in a Peruvian Cohort Infected by COVID-19

Juan Bautista Sánchez et al. Ann Vasc Surg. 2021 Apr.
Free PMC article
Show details
Ann Vasc Surg
. 2021 Apr;72:196-204.
doi: 10.1016/j.avsg.2020.12.005. Epub 2020 Dec 31.

Authors

Juan Bautista Sánchez [1], Juan Diego Cuipal Alcalde [2], Rossi Ramos Isidro [2], Carlos Zúñiga Luna [2], W Samir Cubas [3], André Coaguila Charres [4], José Egoávil Gutiérrez [5], Juan David Ochoa [6], Patricia Fernández Arias [7]

Affiliations

- [1] Vascular and Endovascular Surgery Unit, Cardiac and Vascular Surgery Service, Guillermo Almenara Irigoyen National Hospital, Lima, Peru. Electronic address: juan.bautista.s@upch.pe.
- [2] Vascular and Endovascular Surgery Unit, Cardiac and Vascular Surgery Service, Guillermo Almenara Irigoyen National Hospital, Lima, Peru.
- [3] Vascular and Endovascular Surgery Service, Thoracic and Cardiovascular Surgery Department, Edgardo Rebagliati Martins National Hospital, Lima, Peru.
- [4] Thorax and Cardiovascular Surgery Service, Dos de Mayo National Hospital, Lima, Peru.

- [5] Thorax and Cardiovascular Surgery Service, Cayetano Heredia National Hospital, Lima, Peru.
- [6] Thoracic and Cardiovascular Surgery Department, Hipolito Unanue National Hospital, Lima, Peru.
- [7] Thorax and Cardiovascular Surgery Department, Luis N. Saenz National Police Hospital, Lima, Peru.

- PMID: **33388408**
- PMCID: PMC7836937
- DOI: 10.1016/j.avsg.2020.12.005

Abstract

Background: Coronavirus disease 2019 (COVID-19) infection can cause alterations in the coagulation mechanism conditioning thrombotic phenomena such as acute limb ischemia (ALI) as the only manifestation of the infection. The aim of the study was to describe clinical and surgical characteristics of a group of patients infected with severe acute respiratory syndrome coronavirus 2 who presented ALI in the context of the COVID-19 pandemic at Lima, Peru.

Methods: A multicenter, observational, and retrospective study was performed in six general hospitals, from March to July 2020. The variables considered were the pathological history and associated habits, laboratory tests, the severity of COVID-19 infection and ALI, the anatomic location of the lesion, treatment, evolution, and discharge conditions.

Results: Thirty patients with ALI infected with COVID-19 were evaluated. Their mean age was 60 ± 15 years, the condition being more frequent in men (76.6%). The main comorbidities were arterial hypertension (33.3%), obesity (33.3%), and diabetes mellitus 2 (26.6%). There were 23.3% asymptomatic patients, and their only manifestation was ALI. Rutherford IIA and IIB stage included 93.2% of patients. The most frequent location of the thrombosis was the lower limbs (73.3% vs. 26.6%). Thrombectomy was performed in 76.6% of the patients, and amputation (primary and secondary) was performed in 30% of the patients. The mortality rate was 23.3%, all of it because of acute respiratory distress syndrome.

Conclusions: ALI is a vascular pathology associated with embolic and thrombotic processes. COVID-19 infection can cause severe alterations in coagulation mechanisms, leading some patients to present severe acute arterial complications such as thrombosis, as the only associated manifestation. We report a younger cohort than those described in other studies and with a high frequency of amputations despite adequate surgical treatment.

Copyright © 2021 Elsevier Inc. All rights reserved.

Comment in

- Correspondence.
  Valdivia AR. Valdivia AR. Ann Vasc Surg. 2021 May;73:e5. doi: 10.1016/j.avsg.2021.01.062. Epub 2021 Jan 21. Ann Vasc Surg. 2021. PMID: 33485904 No abstract available.

- 45 references
- 2 figures

Supplementary info

Publication types, MeSH terms Expand

Publication types

- Multicenter Study
- Observational Study

MeSH terms

- Acute Disease
- Amputation
- COVID-19 / complications*
- COVID-19 / epidemiology
- COVID-19 / mortality
- Female
- Humans
- Ischemia / etiology*
- Ischemia / surgery*
- Lower Extremity / blood supply*
- Male
- Middle Aged
- Pandemics
- Peru / epidemiology
- Pneumonia, Viral / epidemiology
- Pneumonia, Viral / mortality
- Pneumonia, Viral / virology
- Retrospective Studies
- SARS-CoV-2
- Thrombectomy

Full text links

ELSEVIER FULL-TEXT ARTICLE Elsevier Science Free PMC article

Proceed to details

Cite

Share

☐ 741

Observational Study

Medicine (Baltimore)

. 2021 Oct 15;100(41):e27400.
doi: 10.1097/MD.0000000000027400.

The clinical characters and prognosis of COVID-19 patients with multiple organ dysfunction

Jianghua Cui [1], Boyun Yuan [2], Yan Li [2], Zhu Li [3], Yadong Yuan [2]
Affiliations Expand

Affiliations

- [1] Department of Intensive Care Medicine, Peking University Shougang Hospital, Beijing, China.
- [2] Department of Respiratory and Critical Care Medicine, Second Hospital of Hebei Medical University, Shijiazhuang Hebei, China.
- [3] Department of Respiratory and Critical Care Medicine, First Hospital of Hebei Medical University, Shijiazhuang Hebei, China.

- PMID: **34731113**
- PMCID: PMC8519259
- DOI: 10.1097/MD.0000000000027400

Free PMC article
Observational Study

The clinical characters and prognosis of COVID-19 patients with multiple organ dysfunction

Jianghua Cui et al. Medicine (Baltimore). 2021.
Free PMC article
Show details
Medicine (Baltimore)
. 2021 Oct 15;100(41):e27400.
doi: 10.1097/MD.0000000000027400.

Authors

Jianghua Cui [1], Boyun Yuan [2], Yan Li [2], Zhu Li [3], Yadong Yuan [2]

Affiliations

- [1] Department of Intensive Care Medicine, Peking University Shougang Hospital, Beijing, China.
- [2] Department of Respiratory and Critical Care Medicine, Second Hospital of Hebei Medical University, Shijiazhuang Hebei, China.

- [3] Department of Respiratory and Critical Care Medicine, First Hospital of Hebei Medical University, Shijiazhuang Hebei, China.

- PMID: **34731113**
- PMCID: PMC8519259
- DOI: 10.1097/MD.0000000000027400

Abstract

To depict the clinical characters and prognosis of coronavirus disease 2019 patients who developed multiple organ dysfunction syndrome (MODS).A cohort consisted of 526 patients, which including 109 patients complicated MODS, was retrospectively analyzed to examine the clinical characteristics and risk factors of MODS.Among the 526 novel coronavirus-infected pneumonia patients, 109 patients developed multiple organ failure, the incidence rate was 20.7%. Among all 109 patients with MODS, 81.7% were over 60 years old, and 63.3% were male. The most common symptoms were fever (79.8%), dyspnea (73.4%), and fatigue (55.0%). Compared with patients non-MODS patients, there were 70 cases of MODS patients with one or more underlying diseases (64.2% vs 41.0%, $P < .001$). Respiratory failure (92.7%), circulatory failure (52.0%), and liver function injury (30.9%) were the most common symptoms within the spectrum of MODS. Invasive ventilator, noninvasive ventilator, and high-flow respiratory support treatment for patients in MODS patients were higher than those in the non-MODS group ($P < .001$). The antiviral therapy and 2 or more antibacterial drug treatments in MODS patients were higher than those in the non-MODS group ($P < .001$). The median hospital stay of all patients was 16 days (interquartile range [IQR], 9-26), of which 20 days (IQR, 11.5-30.5) in the MODS patients, which was approximately 4 days longer than that of non-MODS patients. In addition, our data suggested that lymphocyte counts <1.0 ∗ 109/L, Troponin T > 0.014 ng/mL and lower oxygenation index were risk factors for MODS. In the early stage of hospital admission, higher inflammatory indexes and lactic acid concentration were associated with increased risk of death.MODS often leads to poor prognosis in coronavirus disease 2019. Our data suggested the importance of early identification of MODS. We recommend close monitoring and timely supportive therapy for patients with high risks, stopping the disease progression before it was too late.

Copyright © 2021 the Author(s). Published by Wolters Kluwer Health, Inc.

Conflict of interest statement

The authors have no conflicts of interest to disclose.

- 32 references
- 1 figure

Supplementary info

Publication types, MeSH terms, Grant support Expand

Publication types

- Observational Study

MeSH terms

- Aged
- COVID-19 / epidemiology*
- COVID-19 / physiopathology
- Comorbidity
- Female
- Humans
- Incidence
- Male
- Middle Aged
- Multiple Organ Failure / epidemiology*
- Multiple Organ Failure / etiology
- Multiple Organ Failure / physiopathology
- Pandemics
- Proportional Hazards Models
- Retrospective Studies
- Risk Factors
- SARS-CoV-2

Grant support

- 20277706D/Hebei Province Science and Technology Support Program

Full text links

Wolters Kluwer Free PMC article
Proceed to details
Cite
Share
☐ 742
Observational Study
Medicine (Baltimore)
. 2021 Jan 8;100(1):e24002.
doi: 10.1097/MD.0000000000024002.

Pulmonary thromboembolism in coronavirus disease 2019 patients undergoing thromboprophylaxis

Simone Schiaffino [1], Francesca Giacomazzi [2], Anastassia Esseridou [1], Andrea Cozzi [3], Serena Carriero [4], Daniela P Mazzaccaro [5], Giovanni Nano [5], Giovanni Di Leo [1], Pietro Spagnolo [1], Francesco Sardanelli [1] [3]
Affiliations Expand

Affiliations

- [1] Unit of Radiology.
- [2] Unit of Cardiac Rehabilitation, IRCCS Policlinico San Donato, San Donato Milanese.
- [3] Department of Biomedical Sciences for Health.
- [4] Postgraduate School in Radiodiagnostics, Università degli Studi di Milano, Milano.
- [5] Unit of Vascular Surgery, IRCCS Policlinico San Donato, San Donato Milanese, Italy.

- PMID: **33429763**
- PMCID: PMC7793450
- DOI: 10.1097/MD.0000000000024002

Free PMC article
Observational Study

Pulmonary thromboembolism in coronavirus disease 2019 patients undergoing thromboprophylaxis

Simone Schiaffino et al. Medicine (Baltimore). 2021.
Free PMC article
Show details
Medicine (Baltimore)
. 2021 Jan 8;100(1):e24002.
doi: 10.1097/MD.0000000000024002.

Authors

Simone Schiaffino [1], Francesca Giacomazzi [2], Anastassia Esseridou [1], Andrea Cozzi [3], Serena Carriero [4], Daniela P Mazzaccaro [5], Giovanni Nano [5], Giovanni Di Leo [1], Pietro Spagnolo [1], Francesco Sardanelli [1] [3]

Affiliations

- [1] Unit of Radiology.
- [2] Unit of Cardiac Rehabilitation, IRCCS Policlinico San Donato, San Donato Milanese.
- [3] Department of Biomedical Sciences for Health.
- [4] Postgraduate School in Radiodiagnostics, Università degli Studi di Milano, Milano.
- [5] Unit of Vascular Surgery, IRCCS Policlinico San Donato, San Donato Milanese, Italy.

- PMID: **33429763**

- PMCID: PMC7793450
- DOI: 10.1097/MD.0000000000024002

Abstract

We aimed to investigate the prevalence of pulmonary thromboembolism (PTE) and its association with clinical variables in a cohort of hospitalized coronavirus disease 2019 (COVID-19) patients receiving low-molecular-weight heparin (LMWH) at prophylactic dosage.In this retrospective observational study we included COVID-19 patients receiving prophylactic LMWH from admission but still referred for lower-limbs venous Doppler ultrasound (LL-US) and computed tomography pulmonary angiography (CTPA) for clinical PTE suspicion. A dedicated radiologist reviewed CTPA images to assess PTE presence/extension.From March 1 to April 30, 2020, 45 patients were included (34 men, median age 67 years, interquartile range [IQR] 60-76). Twenty-seven (60%) had PTE signs at CTPA, 17/27 (63%) with bilateral involvement, none with main branch PTE. In 33/45 patients (73%) patients LL-US was performed before CTPA, with 3 patients having superficial vein thrombosis (9%, none with CTPA-confirmed PTE) and 1 patient having deep vein thrombosis (3%, with CTPA-confirmed PTE). Thirty-three patients (73%) had at least one comorbidity, mainly hypertension (23/45, 51%) and cardiovascular disease (15/45, 33%). Before CTPA, 5 patients had high D-dimer (11.21 µg/mL, IQR 9.10-13.02), 19 high fibrinogen (550 mg/dL, IQR 476-590), 26 high interleukin-6 (79 pg/mL, IQR 31-282), and 11 high C-reactive protein (9.60 mg/dL, IQR 6.75-10.65), C-reactive protein being the only laboratory parameter significantly differing between patients with and without PTE (P = .002)High PTE incidence (60%) in COVID-19 hospitalized patients under prophylactic LMWH could substantiate further tailoring of anticoagulation therapy.

Copyright © 2021 the Author(s). Published by Wolters Kluwer Health, Inc.

Conflict of interest statement

F. Giacomazzi, A. Esseridou, A. Cozzi, S. Carriero, D.P. Mazzaccaro, G. Nano, G. Di Leo, and P. Spagnolo all declare that they have no conflict of interest and that they have nothing to disclose. The authors have no conflicts of interest to disclose.

- 36 references
- 2 figures

Supplementary info

Publication types, MeSH terms, Substances Expand

Publication types

- Observational Study

MeSH terms

- Aged
- Anticoagulants / therapeutic use*
- COVID-19 / complications*

- Computed Tomography Angiography
- Female
- Heparin, Low-Molecular-Weight / therapeutic use*
- Hospitalization
- Humans
- Incidence
- Male
- Prevalence
- Pulmonary Embolism / epidemiology*
- Pulmonary Embolism / prevention & control
- Retrospective Studies
- Risk Factors
- Thrombolytic Therapy*
- Ultrasonography, Doppler
- Venous Thromboembolism / diagnostic imaging
- Venous Thromboembolism / prevention & control

Substances

- Anticoagulants
- Heparin, Low-Molecular-Weight

Full text links

Wolters Kluwer Wolters Kluwer Free PMC article
Proceed to details
Cite
Share
☐ 743
Observational Study
Biomed Res Int
. 2021 Mar 1;2021:6695707.
doi: 10.1155/2021/6695707. eCollection 2021.

Impact of Ethnicity and Underlying Comorbidity on COVID-19 Inhospital Mortality: An Observational Study in Abu Dhabi, UAE

Asma Deeb [1], Khulood Khawaja [2], Nida Sakrani [2], Abdulla AlAkhras [3], Ahmed Al Mesabi [4], Ravi Trehan [5], Palat Chirakkara Kumar [6], Zahir Babiker [4], Nico Nagelkerke [7], Emmanuel Fru-Nsutebu [4]
Affiliations Expand

Affiliations

- [1] Division of Paediatric Endocrinology, Sheikh Shakhbout Medical City, Abu Dhabi, UAE.
- [2] Division of Paediatric Rheumatology, Sheikh Shakhbout Medical City, Abu Dhabi, UAE.
- [3] Internal Medicine Department, Sheikh Shakhbout Medical City, Abu Dhabi, UAE.
- [4] Division of Infectious Diseases, Sheikh Shakhbout Medical City, Abu Dhabi, UAE.
- [5] Division of Orthopaedic Surgery, Sheikh Shakhbout Medical City, Abu Dhabi, UAE.
- [6] Division of Neurology, Sheikh Shakhbout Medical City, Abu Dhabi, UAE.
- [7] Public Health Institute, UAE University, Al Ain, UAE.

- PMID: **33708993**
- PMCID: PMC7930915
- DOI: 10.1155/2021/6695707

Free PMC article
Observational Study

Impact of Ethnicity and Underlying Comorbidity on COVID-19 Inhospital Mortality: An Observational Study in Abu Dhabi, UAE

Asma Deeb et al. Biomed Res Int. 2021.
Free PMC article
Show details
Biomed Res Int
. 2021 Mar 1;2021:6695707.
doi: 10.1155/2021/6695707. eCollection 2021.

Authors

Asma Deeb [1], Khulood Khawaja [2], Nida Sakrani [2], Abdulla AlAkhras [3], Ahmed Al Mesabi [4], Ravi Trehan [5], Palat Chirakkara Kumar [6], Zahir Babiker [4], Nico Nagelkerke [7], Emmanuel Fru-Nsutebu [4]

Affiliations

- [1] Division of Paediatric Endocrinology, Sheikh Shakhbout Medical City, Abu Dhabi, UAE.
- [2] Division of Paediatric Rheumatology, Sheikh Shakhbout Medical City, Abu Dhabi, UAE.
- [3] Internal Medicine Department, Sheikh Shakhbout Medical City, Abu Dhabi, UAE.

- [4] Division of Infectious Diseases, Sheikh Shakhbout Medical City, Abu Dhabi, UAE.
- [5] Division of Orthopaedic Surgery, Sheikh Shakhbout Medical City, Abu Dhabi, UAE.
- [6] Division of Neurology, Sheikh Shakhbout Medical City, Abu Dhabi, UAE.
- [7] Public Health Institute, UAE University, Al Ain, UAE.

- PMID: **33708993**
- PMCID: PMC7930915
- DOI: 10.1155/2021/6695707

Abstract

Background: The UAE reported its first cluster of COVID 2019 in a group of returned travellers from Wuhan in January 2020. Various comorbidities are associated with worse disease prognosis. Understanding the impact of ethnicity on the disease outcome is an important public health issue but data from our region is lacking.

Aim: We aim to identify comorbidities among patients hospitalized for COVID-19 that are associated with inhospital death. Also, to assess if ethnicity is correlated with increased risk of death. *Patients and Method*. The study is a single-centre, observational study in Shaikh Shakhbout Medical City, Abu Dhabi. Patients admitted with COVID-19, between 1st of March and the end of May, were enrolled. Records were studied for demography, comorbidity, and ethnicity. Ethnicity was divided into Arabs (Gulf, North Africa, and the Levant), South Asia (India, Pakistan, Bangladesh, Nepal, and Afghanistan), Africans, the Philippines, and others. The study was approved by the Department of Health of Abu Dhabi.

Results: 1075 patients (972 males) were enrolled. There were 24 nationalities under 5 ethnicity groups. Mean (average) age was 51 years (20-81). 101 (9.4%) died with deceased patients being significantly older. Death risk was not significantly influenced by sex. Duration of hospitalization among survivors was 6.2 days (0.2-40.4) with older patients and men staying longer ($P < 0.01$). Comorbidities of diabetes, hypertension, cardiovascular disease, chronic renal disease, liver disease, and malignancy were associated with higher risk of mortality univariate, but only liver disease reached statistical significance after adjustment for age. The highest percentage of death was seen in Arab Levant (21.2) followed by the Asian Afghan (18.8); however, differences among ethnicities did not reach statistical significance ($P = 0.086$).

Conclusion: COVID-19 outcome was worse in older people and those with comorbidities. Men and older patients required longer hospitalization. Ethnicity is not seen to impact the risk of mortality.

Copyright © 2021 Asma Deeb et al.

Conflict of interest statement

None of the authors have any conflict of interest to declare.

- 40 references
- 1 figure

Supplementary info

Publication types, MeSH terms Expand

Publication types

- Observational Study

MeSH terms

- Adult
- Aged
- Aged, 80 and over
- Arabs / statistics & numerical data
- Asia, Southeastern / ethnology
- COVID-19 / epidemiology
- COVID-19 / ethnology*
- COVID-19 / mortality*
- Cardiovascular Diseases / epidemiology
- Comorbidity
- Diabetes Mellitus / epidemiology
- Female
- Hospital Mortality
- Humans
- Length of Stay / statistics & numerical data
- Male
- Middle Aged
- Obesity / epidemiology
- Retrospective Studies
- United Arab Emirates / epidemiology
- Young Adult

Full text links

Hindawi FREE Full Text Article Hindawi Limited Free PMC article

Proceed to details

Cite

Share

☐ 744

Observational Study

J Cardiothorac Vasc Anesth

. 2021 Mar;35(3):846-853.

doi: 10.1053/j.jvca.2020.10.019. Epub 2020 Oct 16.

Myocardial Injury on Admission as a Risk in Critically Ill COVID-19 Patients: A Retrospective in-ICU Study

Hao Qian [1], Peng Gao [1], Ran Tian [1], Xufei Yang [1], Fan Guo [1], Taisheng Li [2], Zhengyin Liu [2], Jinglan Wang [3], Xiang Zhou [4], Yan Qin [5], Long Chang [6], Yanjun Song [1], Xiaowei Yan [1], Wei Wu [7], Shuyang Zhang [8]
Affiliations Expand

Affiliations

- [1] Department of Cardiology, Peking Union Medical College Hospital, Chinese Academy of Medical Sciences and Peking Union Medical College, Beijing, China.
- [2] Department of Infective Disease, Peking Union Medical College Hospital, Chinese Academy of Medical Sciences and Peking Union Medical College, China.
- [3] Department of Pulmonary and Critical Care Medicine, Peking Union Medical College Hospital, Chinese Academy of Medical Sciences and Peking Union Medical College, Beijing, China.
- [4] Department of Intensive Care Medicine, Peking Union Medical College Hospital, Chinese Academy of Medical Sciences and Peking Union Medical College, Beijing, China.
- [5] Department of Nephrology, Peking Union Medical College Hospital, Chinese Academy of Medical Sciences and Peking Union Medical College, Beijing, China.
- [6] Department of Internal Medicine, Peking Union Medical College Hospital, Chinese Academy of Medical Sciences and Peking Union Medical College, Beijing, China.
- [7] Department of Cardiology, Peking Union Medical College Hospital, Chinese Academy of Medical Sciences and Peking Union Medical College, Beijing, China. Electronic address: camsww@126.com.
- [8] Department of Cardiology, Peking Union Medical College Hospital, Chinese Academy of Medical Sciences and Peking Union Medical College, Beijing, China. Electronic address: shuyangzhang103@nrdrs.org.

- PMID: **33162306**
- PMCID: PMC7566673
- DOI: 10.1053/j.jvca.2020.10.019

Free PMC article
Observational Study

Myocardial Injury on Admission as a Risk in Critically Ill COVID-19 Patients: A Retrospective in-ICU Study

Hao Qian et al. J Cardiothorac Vasc Anesth. 2021 Mar.
Free PMC article

Show details

J Cardiothorac Vasc Anesth

. 2021 Mar;35(3):846-853.
doi: 10.1053/j.jvca.2020.10.019. Epub 2020 Oct 16.

Authors

Hao Qian [1], Peng Gao [1], Ran Tian [1], Xufei Yang [1], Fan Guo [1], Taisheng Li [2], Zhengyin Liu [2], Jinglan Wang [3], Xiang Zhou [4], Yan Qin [5], Long Chang [6], Yanjun Song [1], Xiaowei Yan [1], Wei Wu [7], Shuyang Zhang [8]

Affiliations

- [1] Department of Cardiology, Peking Union Medical College Hospital, Chinese Academy of Medical Sciences and Peking Union Medical College, Beijing, China.
- [2] Department of Infective Disease, Peking Union Medical College Hospital, Chinese Academy of Medical Sciences and Peking Union Medical College, China.
- [3] Department of Pulmonary and Critical Care Medicine, Peking Union Medical College Hospital, Chinese Academy of Medical Sciences and Peking Union Medical College, Beijing, China.
- [4] Department of Intensive Care Medicine, Peking Union Medical College Hospital, Chinese Academy of Medical Sciences and Peking Union Medical College, Beijing, China.
- [5] Department of Nephrology, Peking Union Medical College Hospital, Chinese Academy of Medical Sciences and Peking Union Medical College, Beijing, China.
- [6] Department of Internal Medicine, Peking Union Medical College Hospital, Chinese Academy of Medical Sciences and Peking Union Medical College, Beijing, China.
- [7] Department of Cardiology, Peking Union Medical College Hospital, Chinese Academy of Medical Sciences and Peking Union Medical College, Beijing, China. Electronic address: camsww@126.com.
- [8] Department of Cardiology, Peking Union Medical College Hospital, Chinese Academy of Medical Sciences and Peking Union Medical College, Beijing, China. Electronic address: shuyangzhang103@nrdrs.org.

- PMID: **33162306**
- PMCID: PMC7566673
- DOI: 10.1053/j.jvca.2020.10.019

Abstract

Objective: The aim of this study was to investigate the incidence, clinical presentation, cardiovascular (CV) complications, and mortality risk of myocardial injury on admission in critically ill intensive care unit (ICU) inpatients with COVID-19.

Design: A single-center, retrospective, observational study.

Setting: A newly built ICU in Tongji hospital (Sino-French new city campus), Huazhong University of Science and Technology, Wuhan, China.

Participants: Seventy-seven critical COVID-19 patients.

Interventions: Patients were divided into a myocardial injury group and nonmyocardial injury group according to the on-admission levels of high-sensitivity cardiac troponin I.

Measurements and main results: Demographic data, clinical characteristics, laboratory tests, treatment, and clinical outcome were evaluated, stratified by the presence of myocardial injury on admission. Compared with nonmyocardial injury patients, patients with myocardial injury were older (68.4 ± 10.1 v 62.1 ± 13.5 years; p = 0.02), had higher prevalence of underlying CV disease (34.1% v 11.1%; p = 0.02), and in-ICU CV complications (41.5% v 13.9%; p = 0.008), higher Acute Physiology and Chronic Health Evaluation II scores (20.3 ± 7.3 v 14.4 ± 7.4; p = 0.001), and Sequential Organ Failure Assessment scores (7, interquartile range (IQR) 5-10 v 5, IQR 3-6; p < 0.001). Myocardial injury on admission increased the risk of 28-day mortality (hazard ratio [HR], 2.200; 95% confidence interval [CI] 1.29 to 3.74; p = 0.004). Age ≥75 years was another risk factor for mortality (HR, 2.882; 95% CI 1.51-5.50; p = 0.002).

Conclusion: Critically ill patients with COVID-19 had a high risk of CV complications. Myocardial injury on admission may be a common comorbidity and is associated with severity and a high risk of mortality in this population.

Keywords: cardiovascular complication; critically ill; myocardial injury; novel coronavirus disease.

Copyright © 2020 Elsevier Inc. All rights reserved.

Comment in

- COVID-Associated Myocardial Injury: Crossing the Threshold for Mechanical Circulatory Support.
  Ludmir J, Lu S, Dalia A, Shelton KT. Ludmir J, et al. J Cardiothorac Vasc Anesth. 2021 Mar;35(3):854-856. doi: 10.1053/j.jvca.2020.11.038. Epub 2020 Nov 24. J Cardiothorac Vasc Anesth. 2021. PMID: 33358106 Free PMC article. No abstract available.

- 29 references
- 2 figures

Supplementary info

Publication types, MeSH terms Expand

Publication types

- Observational Study

MeSH terms

- Adult
- Aged
- Aged, 80 and over
- COVID-19 / diagnosis
- COVID-19 / mortality*

- COVID-19 / therapy
- Cardiovascular Diseases / diagnosis
- Cardiovascular Diseases / mortality*
- Cardiovascular Diseases / therapy
- Critical Illness / mortality*
- Critical Illness / therapy
- Female
- Humans
- Intensive Care Units / trends*
- Male
- Middle Aged
- Patient Admission / trends*
- Retrospective Studies
- Risk Factors

Full text links

ELSEVIER FULL-TEXT ARTICLE Elsevier Science Free PMC article

Proceed to details

Cite

Share

☐ 745

Influenza Other Respir Viruses

. 2021 Jul;15(4):478-487.

doi: 10.1111/irv.12858. Epub 2021 Apr 7.

Differentiating impacts of non-pharmaceutical interventions on non-coronavirus disease-2019 respiratory viral infections: Hospital-based retrospective observational study in Taiwan

Andrew Po-Liang Chen [1] [2], Isaac Yen-Hao Chu [3], Mei-Lin Yeh [4], Yin-Yin Chen [5] [6], Chia-Lin Lee [7], Hsiao-Hsuan Lin [7], Yu-Jiun Chan [6] [7], Hsin-Pai Chen [1] [8]

Affiliations Expand

Affiliations

- [1] Division of Infectious Diseases, Department of Medicine, Taipei Veterans General Hospital, Taipei, Taiwan.

- [2] Institute of Epidemiology and Preventive Medicine, National Taiwan University, Taipei, Taiwan.
- [3] Department of Public Health, Environments and Society, Faculty of Public Health and Policy, School of Hygiene and Tropical Medicine, London, United Kingdom.
- [4] Nursing Department, Taipei Veterans General Hospital, Taipei, Taiwan.
- [5] Department of Infection Control, Taipei Veterans General Hospital, Taipei, Taiwan.
- [6] Institute of Public Health, School of Medicine, National Yang-Ming University, Taipei, Taiwan.
- [7] Division Microbiology, Department of Pathology and Laboratory Medicine, Taipei Veterans General Hospital, Taipei, Taiwan.
- [8] School of Medicine, National Yang-Ming University, Taipei, Taiwan.

- PMID: **33825310**
- PMCID: PMC8189242
- DOI: 10.1111/irv.12858

Free PMC article

Differentiating impacts of non-pharmaceutical interventions on non-coronavirus disease-2019 respiratory viral infections: Hospital-based retrospective observational study in Taiwan

Andrew Po-Liang Chen et al. Influenza Other Respir Viruses. 2021 Jul.
Free PMC article
Show details
Influenza Other Respir Viruses
. 2021 Jul;15(4):478-487.
doi: 10.1111/irv.12858. Epub 2021 Apr 7.

Authors

Andrew Po-Liang Chen [1, 2], Isaac Yen-Hao Chu [3], Mei-Lin Yeh [4], Yin-Yin Chen [5, 6], Chia-Lin Lee [7], Hsiao-Hsuan Lin [7], Yu-Jiun Chan [6, 7], Hsin-Pai Chen [1, 8]

Affiliations

- [1] Division of Infectious Diseases, Department of Medicine, Taipei Veterans General Hospital, Taipei, Taiwan.
- [2] Institute of Epidemiology and Preventive Medicine, National Taiwan University, Taipei, Taiwan.
- [3] Department of Public Health, Environments and Society, Faculty of Public Health and Policy, School of Hygiene and Tropical Medicine, London, United Kingdom.
- [4] Nursing Department, Taipei Veterans General Hospital, Taipei, Taiwan.

- [5] Department of Infection Control, Taipei Veterans General Hospital, Taipei, Taiwan.
- [6] Institute of Public Health, School of Medicine, National Yang-Ming University, Taipei, Taiwan.
- [7] Division Microbiology, Department of Pathology and Laboratory Medicine, Taipei Veterans General Hospital, Taipei, Taiwan.
- [8] School of Medicine, National Yang-Ming University, Taipei, Taiwan.

- PMID: **33825310**
- PMCID: PMC8189242
- DOI: 10.1111/irv.12858

Abstract

Background: Physical distancing and facemask use are worldwide recognized as effective non-pharmaceutical interventions (NPIs) against the coronavirus disease-2019 (COVID-19). Since January 2020, Taiwan has introduced both NPIs but their effectiveness on non-COVID-19 respiratory viruses (NCRVs) remain underexplored.

Methods: This retrospective observational study examined electronic records at a tertiary hospital in northern Taiwan from pre-COVID (January-December 2019) to post-COVID period (January-May 2020). Patients with respiratory syndromes were tested for both enveloped (eg, influenza virus and seasonal coronavirus) and non-enveloped RVs (eg, enterovirus and rhinovirus) using multiplex reverse transcription polymerase chain reaction assays. Monthly positivity rates of NCRVs among adult and pediatric patients were analyzed with comparison between pre- and post-COVID periods.

Results: A total of 9693 patients underwent 12 127 multiplex RT-PCR tests. The average positivity rate of NCRVs reduced by 11.2% (25.6% to 14.4%) after nationwide PHIs. Despite the COVID-19 pandemic, the most commonly identified enveloped and non-enveloped viruses were influenza virus and enterovirus/rhinovirus, respectively. Observed reduction in NCRV incidence was predominantly contributed by enveloped NCRVs including influenza viruses. We did not observe epidemiological impacts of NPIs on non-enveloped viruses but an increasing trend in enterovirus/rhinovirus test positivity rate among pediatric patients. Our data were validated using Taiwan's national notification database.

Conclusions: Our frontline investigation suggests that the current NPIs in Taiwan might not effectively control the transmission of non-enveloped respiratory viruses, despite their protective effects against influenza and seasonal coronavirus. Health authorities may consider using hydrogen peroxide or chloride-based disinfectants as additional preventative strategies against non-enveloped respiratory viruses in the post-COVID-19 era.

Keywords: COVID-19 pandemic; Influenza virus; enterovirus; enveloped respiratory viruses; infection control; non-enveloped respiratory viruses; non-pharmaceutical intervention; seasonal coronavirus.

© 2021 The Authors. Influenza and Other Respiratory Viruses Published by John Wiley & Sons Ltd.

Conflict of interest statement

No conflict of interest has been declared by the authors.

- 31 references
- 5 figures

Supplementary info

MeSH terms, Substances Expand

MeSH terms

- Adult
- COVID-19 / epidemiology
- COVID-19 / prevention & control
- Child
- Communicable Disease Control / methods*
- Humans
- Masks
- Middle Aged
- Multiplex Polymerase Chain Reaction
- Physical Distancing
- Respiratory Tract Infections / diagnosis
- Respiratory Tract Infections / epidemiology
- Respiratory Tract Infections / prevention & control*
- Retrospective Studies
- SARS-CoV-2
- Species Specificity
- Taiwan / epidemiology
- Tertiary Care Centers
- Viral Envelope Proteins / genetics
- Viral Envelope Proteins / metabolism
- Virus Diseases / diagnosis
- Virus Diseases / epidemiology
- Virus Diseases / prevention & control*
- Viruses / classification
- Viruses / genetics
- Viruses / isolation & purification

Substances

- Viral Envelope Proteins

Full text links

WILEY Free Full Text Wiley Free PMC article

Proceed to details

Cite

Share

☐ 746

Case Reports

Med Sci Monit

. 2021 Nov 25;27:e934267.

doi: 10.12659/MSM.934267.

Use of Monoclonal Antibodies Therapy for Treatment of Mild to Moderate COVID-19 in 4 Patients with Rheumatologic Disorders

Giovanni Franchin [1], Nikhitha Mantri [2], Maleeha Zahid [2], Haozhe Sun [2], Sudharsan R Gongati [2], Diana M Ronderos [2], Snigdha Gadireddy [2], Sridhar Chilimuri [2]

Affiliations Expand

Affiliations

- [1] Division of Rheumatology, Department of Medicine, BronxCare Health System, Affiliated with Icahn School of Medicine at Mount Sinai, Bronx, NY, USA.
- [2] Department of Medicine, BronxCare Health System, Affiliated with Icahn School of Medicine at Mount Sinai, Bronx, NY, USA.

- PMID: **34819488**
- PMCID: PMC8631366
- DOI: 10.12659/MSM.934267

Free PMC article

Case Reports

Use of Monoclonal Antibodies Therapy for Treatment of Mild to Moderate COVID-19 in 4 Patients with Rheumatologic Disorders

Giovanni Franchin et al. Med Sci Monit. 2021.

Free PMC article

Show details

Med Sci Monit

. 2021 Nov 25;27:e934267.

doi: 10.12659/MSM.934267.

Authors

Giovanni Franchin [1], Nikhitha Mantri [2], Maleeha Zahid [2], Haozhe Sun [2], Sudharsan R Gongati [2], Diana M Ronderos [2], Snigdha Gadireddy [2], Sridhar Chilimuri [2]

Affiliations

- [1] Division of Rheumatology, Department of Medicine, BronxCare Health System, Affiliated with Icahn School of Medicine at Mount Sinai, Bronx, NY, USA.
- [2] Department of Medicine, BronxCare Health System, Affiliated with Icahn School of Medicine at Mount Sinai, Bronx, NY, USA.

- PMID: **34819488**
- PMCID: PMC8631366
- DOI: 10.12659/MSM.934267

Abstract

BACKGROUND The use of monoclonal antibodies therapy (MAT) in early mild to moderate Coronavirus disease 2019 (COVID-19) has gained importance in recent times. However, there is limited information on the safety and efficacy of MAT in treating COVID-19 in patients with underlying rheumatologic diseases. Patients with rheumatologic diseases are usually on long-term corticosteroids and immunosuppressive therapy, which increases their risk for progressing to more severe forms of COVID-19. We report a case series of 4 patients with rheumatologic diseases who were treated with MAT for COVID-19. MATERIAL AND METHODS A retrospective observational study was conducted in our institution on patients with underlying rheumatological disorders who received MAT as per the EUA protocol of the FDA. RESULTS Two of the 4 patients were on immunosuppresive therapy at the time of receiving MAT. They recovered from COVID-19 without any adverse outcomes. No flare of underlying rheumatologic disease was noted. CONCLUSIONS MAT was observed to be a safe and effective therapy in 4 patients with rheumatological illnesses and COVID-19 treated at our hospital.

Conflict of interest statement

Conflict of interest: None declared

- 38 references

Supplementary info

Publication types, MeSH terms, Substances Expand

Publication types

- Case Reports

MeSH terms

- Aged
- Antibodies, Monoclonal, Humanized / therapeutic use*

- Arthritis, Rheumatoid / epidemiology*
- COVID-19* / drug therapy
- COVID-19* / epidemiology
- Comorbidity
- Female
- Humans
- Immunotherapy / methods*
- Middle Aged
- SARS-CoV-2 / immunology*
- Treatment Outcome

Substances

- Antibodies, Monoclonal, Humanized

Full text links

International Scientific Literature, Ltd. Free PMC article
Proceed to details
Cite
Share
747
Observational Study
Pancreas
. 2021 Oct 1;50(9):1310-1313.
doi: 10.1097/MPA.0000000000001922.

Analysis of Outcomes in COVID-19 Patients With Varying Degrees of Hyperlipasemia

Petros C Benias [1], Sumant Inamdar [2], Diana Wee [3], Yan Liu [4], Jonathan M Buscaglia [5], Sanjaya K Satapathy, Arvind J Trindade, Northwell COVID-19 Research Consortium
Affiliations Expand

Affiliations

- [1] From the Division of Gastroenterology, Lenox Hill Hospital, Zucker School of Medicine at Hofstra/Northwell, Northwell Health System, New York, NY.
- [2] Division of Gastroenterology, Department of Medicine, University of Arkansas for Medical Sciences, Little Rock, AR.
- [3] Department of Medicine, Long Island Jewish Medical Center, Zucker School of Medicine at Hofstra/Northwell, Northwell Health System, New Hyde Park.
- [4] Feinstein Institutes for Medical Research, Northwell Health, Manhasset.

- [5] Division of Gastroenterology & Hepatology, Renaissance School of Medicine at Stony Brook University, Stony Brook.

- PMID: **34860817**
- DOI: 10.1097/MPA.0000000000001922

Observational Study

Analysis of Outcomes in COVID-19 Patients With Varying Degrees of Hyperlipasemia

Petros C Benias et al. Pancreas. 2021.
Show details
Pancreas
. 2021 Oct 1;50(9):1310-1313.
doi: 10.1097/MPA.0000000000001922.

Authors

Petros C Benias [1], Sumant Inamdar [2], Diana Wee [3], Yan Liu [4], Jonathan M Buscaglia [5], Sanjaya K Satapathy, Arvind J Trindade, Northwell COVID-19 Research Consortium

Affiliations

- [1] From the Division of Gastroenterology, Lenox Hill Hospital, Zucker School of Medicine at Hofstra/Northwell, Northwell Health System, New York, NY.
- [2] Division of Gastroenterology, Department of Medicine, University of Arkansas for Medical Sciences, Little Rock, AR.
- [3] Department of Medicine, Long Island Jewish Medical Center, Zucker School of Medicine at Hofstra/Northwell, Northwell Health System, New Hyde Park.
- [4] Feinstein Institutes for Medical Research, Northwell Health, Manhasset.
- [5] Division of Gastroenterology & Hepatology, Renaissance School of Medicine at Stony Brook University, Stony Brook.

- PMID: **34860817**
- DOI: 10.1097/MPA.0000000000001922

Abstract

Objectives: Coronavirus disease 2019 (COVID-19) patients may have varying degrees of hyperlipasemia. The aim was to compare outcomes among different levels of hyperlipasemia in patients with COVID-19.

Methods: This is a retrospective study examining outcomes among hospitalized COVID-19 patients with a lipase <3× upper limit of normal (ULN), asymptomatic hyperlipasemia (>3× ULN), secondary pancreatitis (typical respiratory COVID-19 symptoms and found to have pancreatitis), and primary pancreatitis (presenting with pancreatitis).

Results: Of 11,883 patients admitted with COVID-19, 1560 patients were included: 1155 patients had normal serum lipase (control group), 270 had elevated lipase <3× ULN, 46 patients had asymptomatic hyperlipasemia with lipase >3× ULN, 57 patients had secondary pancreatitis, and 32 patients had primary pancreatitis. On adjusted multivariate analysis, the elevated lipase <3× ULN and asymptomatic hyperlipasemia groups had worse outcomes with higher mortality (odds ratio [OR], 1.6 [95% confidence interval [CI], 1.2-2.2) and 1.1 [95% CI, 0.5-2.3], respectively), higher need for mechanical ventilation (OR, 2.8 [95% CI, 1.2-2.1] and 2.8 [95% CI, 1.5-5.2], respectively), and longer length of stay (OR, 1.5 [95% CI, 1.1-2.0] and 3.16 [95% CI, 1.5-6.5], respectively).

Conclusions: Patients with COVID-19 with elevated lipase <3× ULN and asymptomatic hyperlipasemia have generally worse outcomes than those with pancreatitis.

Copyright © 2021 Wolters Kluwer Health, Inc. All rights reserved.

Conflict of interest statement

P.C.B. is a consultant for Olympus America, Apollo Medical, FujiFilm, and Boston Scientific. J.M.B. is a consultant and speaker for Abbvie Inc. A.J.T. is a consultant for Olympus America and Pentax Medical. The remaining authors declare no conflict of interest.

- 11 references

Supplementary info

Publication types, MeSH terms, Substances Expand

Publication types

- Comparative Study
- Multicenter Study
- Observational Study

MeSH terms

- Adult
- Aged
- Aged, 80 and over
- Biomarkers / blood
- COVID-19 / blood*
- COVID-19 / diagnosis
- COVID-19 / mortality
- COVID-19 / therapy
- Female
- Hospitalization
- Humans
- Lipase / blood*

- Male
- Middle Aged
- Pancreatitis / blood*
- Pancreatitis / diagnosis
- Pancreatitis / mortality
- Pancreatitis / therapy
- Prognosis
- Retrospective Studies
- Risk Assessment
- Risk Factors
- United States
- Up-Regulation

Substances

- Biomarkers
- Lipase

Full text links

Wolters Kluwer
Proceed to details
Cite
Share
748
Observational Study
J Electrocardiol
. Sep-Oct 2020;62:59-64.
doi: 10.1016/j.jelectrocard.2020.08.008. Epub 2020 Aug 11.

The effect of 5-day course of hydroxychloroquine and azithromycin combination on QT interval in non-ICU COVID19(+) patients

Nijad Bakhshaliyev [1], Mahmut Uluganyan [2], Asim Enhos [2], Erdem Karacop [2], Ramazan Ozdemir [2]
Affiliations Expand

Affiliations

- [1] Bezmialem Vakif University, Department of Cardiology, Turkey. Electronic address: bnijad@bezmialem.edu.tr.
- [2] Bezmialem Vakif University, Department of Cardiology, Turkey.

- PMID: **32827987**
- PMCID: PMC7417268
- DOI: 10.1016/j.jelectrocard.2020.08.008

Free PMC article
Observational Study

The effect of 5-day course of hydroxychloroquine and azithromycin combination on QT interval in non-ICU COVID19(+) patients

Nijad Bakhshaliyev et al. J Electrocardiol. Sep-Oct 2020.
Free PMC article
Show details
J Electrocardiol
. Sep-Oct 2020;62:59-64.
doi: 10.1016/j.jelectrocard.2020.08.008. Epub 2020 Aug 11.

Authors

Nijad Bakhshaliyev [1], Mahmut Uluganyan [2], Asim Enhos [2], Erdem Karacop [2], Ramazan Ozdemir [2]

Affiliations

- [1] Bezmialem Vakif University, Department of Cardiology, Turkey. Electronic address: bnijad@bezmialem.edu.tr.
- [2] Bezmialem Vakif University, Department of Cardiology, Turkey.

- PMID: **32827987**
- PMCID: PMC7417268
- DOI: 10.1016/j.jelectrocard.2020.08.008

Abstract

Background: The combination of Hydroxychloroquine (HCQ) and azithromycin showed effectiveness as a treatment for COVID-19 and is being used widely all around the world. Despite that those drugs are known to cause prolonged QT interval individually there is no study assessing the impact of this combination on electrocardiography (ECG). This study aimed to assess the impact of a 5-day course of HCQ and azithromycin combination on ECG in non-ICU COVID19 (+) patients.

Methods: In this retrospective observational study, we enrolled 109 COVID19(+) patients who required non-ICU hospitalization. All patients received 5-day protocol of HCQ and azithromycin combination. On-treatment ECGs were repeated 3-6 h after the second HCQ loading dose and 48-72 h after the first dose of the combination. ECGs were assessed in terms of rhythm, PR interval, QRS duration, QT and QTc intervals. Baseline and on-treatment ECG findings were compared. Demographic characteristics, laboratory results were recorded. Daily phone call-visit or bed-side visit were performed by attending physician.

Results: Of the 109 patients included in the study, the mean age was 57.3 ± 14.4 years and 48 (44%) were male. Mean baseline PR interval was 158.47 ± 25.10 ms, QRS duration was 94.00 ± 20.55 ms, QTc interval was 435.28 ± 32.78 ms, 415.67 ± 28.51, 412.07 ± 25.65 according to Bazett's, Fridericia's and Framingham Heart Study formulas respectively. ΔPR was -2.94 ± 19.93 ms (p = .55), ΔQRS duration was 5.18 ± 8.94 ms (p = .03). ΔQTc interval was 6.64 ± 9.60 ms (p = .5), 10.67 ± 9.9 ms (p = .19), 14.14 ± 9.68 ms (p = .16) according to Bazett's, Fridericia's and Framingham Heart Study formulas respectively. There were no statistically significant differences between QTc intervals. No ventricular tachycardia, ventricular fibrillation or significant conduction delay was seen during follow-up. There was no death or worsening heart function.

Conclusion: The 5-day course of HCQ- AZM combination did not lead to clinically significant QT prolongation and other conduction delays compared to baseline ECG in non-ICU COVID19 (+) patients.

Keywords: Azithromycin; Coronavirus disease 2019; Electrocardiography; Hydroxychloroquine; QT interval.

Copyright © 2020 Elsevier Inc. All rights reserved.

- 31 references
- 2 figures

Supplementary info

Publication types, MeSH terms, Substances Expand

Publication types

- Observational Study

MeSH terms

- Anti-Bacterial Agents / administration & dosage*
- Azithromycin / administration & dosage*
- COVID-19 / drug therapy*
- Drug Therapy, Combination
- Electrocardiography*
- Enzyme Inhibitors / administration & dosage*
- Female
- Humans
- Hydroxychloroquine / administration & dosage*

- Long QT Syndrome / chemically induced*
- Male
- Middle Aged
- Retrospective Studies
- SARS-CoV-2

Substances

- Anti-Bacterial Agents
- Enzyme Inhibitors
- Hydroxychloroquine
- Azithromycin

Full text links

ELSEVIER FULL-TEXT ARTICLE Elsevier Science Free PMC article
Proceed to details
Cite
Share
749

Observational Study

Ann Vasc Surg
. 2021 Nov;77:71-78.
doi: 10.1016/j.avsg.2021.07.006. Epub 2021 Aug 16.

The effects of COVID-19 pandemic on patients with lower extremity peripheral arterial disease: A near miss disaster

Rafael Trunfio [1], Céline Deslarzes-Dubuis [1], Giacomo Buso [2], Marco Fresa [2], Juliette Brusa [1], Adrian Stefanescu [2], Matthieu Zellweger [1], Jean-Marc Corpataux [1], Sébastien Deglise [1], Lucia Mazzolai [3]
Affiliations Expand

Affiliations

- [1] Vascular Surgery Division, Heart and Vessels Department, Lausanne University Hospital, University of Lausanne, Lausanne, Switzerland.
- [2] Angiology Division, Heart and Vessels Department, Lausanne University Hospital, University of Lausanne, Lausanne, Switzerland.
- [3] Angiology Division, Heart and Vessels Department, Lausanne University Hospital, University of Lausanne, Lausanne, Switzerland. Electronic address: Lucia.Mazzolai@chuv.ch.

- PMID: **34411672**
- PMCID: PMC8366045
- DOI: 10.1016/j.avsg.2021.07.006

Free PMC article
Observational Study

The effects of COVID-19 pandemic on patients with lower extremity peripheral arterial disease: A near miss disaster

Rafael Trunfio et al. Ann Vasc Surg. 2021 Nov.
Free PMC article
Show details
Ann Vasc Surg
. 2021 Nov;77:71-78.
doi: 10.1016/j.avsg.2021.07.006. Epub 2021 Aug 16.

Authors

Rafael Trunfio [1], Céline Deslarzes-Dubuis [1], Giacomo Buso [2], Marco Fresa [2], Juliette Brusa [1], Adrian Stefanescu [2], Matthieu Zellweger [1], Jean-Marc Corpataux [1], Sébastien Deglise [1], Lucia Mazzolai [3]

Affiliations

- [1] Vascular Surgery Division, Heart and Vessels Department, Lausanne University Hospital, University of Lausanne, Lausanne, Switzerland.
- [2] Angiology Division, Heart and Vessels Department, Lausanne University Hospital, University of Lausanne, Lausanne, Switzerland.
- [3] Angiology Division, Heart and Vessels Department, Lausanne University Hospital, University of Lausanne, Lausanne, Switzerland. Electronic address: Lucia.Mazzolai@chuv.ch.

- PMID: **34411672**
- PMCID: PMC8366045
- DOI: 10.1016/j.avsg.2021.07.006

Abstract

Background: The COVID-19 pandemic has led to widespread postponement and cancelation of elective vascular surgeries in Switzerland. The consequences of these decisions are poorly understood.

Patients and methods: In this observational, retrospective, single-center cohort study, we describe the impact of COVID-19 pandemic containment strategies on patients with lower extremity peripheral arterial disease (PAD) referred during the period March 11, to May 11, 2020,

compared to the same time frames in 2018 to 2019. Patients admitted for acute limb ischemia (ALI) or chronic PAD and undergoing urgent or elective vascular surgery or primary amputation were included. Patients' characteristics, indications for admission, and surgical features were analyzed. The occurrence of 30 day outcomes was assessed, including length of stay, rates of major adverse cardiovascular events (MACE) and major adverse limb events (MALE), and procedural and hemodynamic success.

Results: Overall, 166 patients were included. Fewer subjects per 10 day period were operated in 2020 compared to, 2018 to 2019 (6.7 vs. 10.5, respectively; $P < 0.001$). The former had higher rates of chronic obstructive pulmonary disease (COPD) (25% vs. 11.1%; $P = 0.029$), and ASA score (3.13 vs. 2.90; $P = 0.015$). The percentage of patients with ALI in 2020 was about double that of the same period in 2018 to 2019 (47.5% vs. 24.6%; $P = 0.006$). Overall, the types of surgery were similar between 2020 and 2018 to 2019, while palliative care and primary amputations occurred only in 2020 (5 out 40 cases). The rate of post-operative MACE was significantly higher in 2020 (10% vs. 2.4%; $P = 0.037$).

Conclusions: During the first state of emergency for COVID-19 pandemic in 2020, less regular medical follow-up and hindered hospital access could have resulted in more acute and advanced clinical presentations of patients with PAD undergoing surgery. Guidelines are needed to provide appropriate care to this vulnerable population and avoid a large-scale disaster.

Copyright © 2021 The Authors. Published by Elsevier Inc. All rights reserved.

- 29 references
- 1 figure

Supplementary info

Publication types, MeSH terms Expand

Publication types

- Observational Study

MeSH terms

- Aged
- COVID-19 / epidemiology*
- Comorbidity
- Female
- Follow-Up Studies
- Humans
- Male
- Near Miss, Healthcare / methods*
- Pandemics
- Peripheral Arterial Disease / epidemiology*
- Retrospective Studies
- Risk Assessment / methods*

- Risk Factors
- SARS-CoV-2*
- Switzerland / epidemiology

Full text links

Elsevier Science Free PMC article

Proceed to details

Cite

Share

750

Ann Intern Med

. 2022 Feb;175(2):179-190.

doi: 10.7326/M21-2019. Epub 2021 Nov 16.

Comparative Effectiveness of an Automated Text Messaging Service for Monitoring COVID-19 at Home

M Kit Delgado [1], Anna U Morgan [2], David A Asch [3], Ruiying Xiong [4], Austin S Kilaru [5], Kathleen C Lee [6], David Do [7], Ari B Friedman [5], Zachary F Meisel [5], Christopher K Snider [8], Doreen Lam [8], Andrew Parambath [8], Christian Wood [9], Chidinma M Wilson [9], Michael Perez [8], Deena L Chisholm [9], Sheila Kelly [10], Christina J O'Malley [8], Nancy Mannion [11], Ann Marie Huffenberger [11], Susan McGinley [11], Mohan Balachandran [8], Neda Khan [8], Nandita Mitra [12], Krisda H Chaiyachati [13]

Affiliations Expand

Affiliations

- [1] Center for Emergency Care Policy and Research, Department of Emergency Medicine, Department of Biostatistics, Epidemiology, and Informatics, Perelman School of Medicine, and Leonard Davis Institute of Health Economics, University of Pennsylvania, and Center for Health Care Innovation, University of Pennsylvania Health System, Philadelphia, Pennsylvania (M.K.D.).
- [2] Division of General Internal Medicine, Department of Medicine, Perelman School of Medicine and Leonard Davis Institute of Health Economics, University of Pennsylvania, Philadelphia, Pennsylvania (A.U.M.).
- [3] Division of General Internal Medicine, Department of Medicine, Perelman School of Medicine, University of Pennsylvania, Wharton School, and Leonard Davis Institute of Health Economics, University of Pennsylvania, and Center for Health Care Innovation, University of Pennsylvania Health System, Philadelphia, Pennsylvania (D.A.A.).
- [4] Center for Emergency Care Policy and Research, Department of Emergency Medicine, and Division of General Internal Medicine, Department of Medicine, Perelman School of Medicine, and Leonard Davis Institute of Health Economics, University of Pennsylvania, Philadelphia, Pennsylvania (R.X.).

- [5] Center for Emergency Care Policy and Research, Department of Emergency Medicine, Perelman School of Medicine, and Leonard Davis Institute of Health Economics, University of Pennsylvania, Philadelphia, Pennsylvania (A.S.K., A.B.F., Z.F.M.).
- [6] Center for Emergency Care Policy and Research, Department of Emergency Medicine, Perelman School of Medicine, University of Pennsylvania, and Center for Health Care Innovation, University of Pennsylvania Health System, Philadelphia, Pennsylvania (K.C.L.).
- [7] Department of Neurology, Perelman School of Medicine, University of Pennsylvania, and Center for Health Care Innovation, University of Pennsylvania Health System, Philadelphia, Pennsylvania (D.D.).
- [8] Center for Health Care Innovation, University of Pennsylvania Health System, Philadelphia, Pennsylvania (C.K.S., D.L., A.P., M.P., C.J.O., M.B., N.K.).
- [9] Center for Emergency Care Policy and Research, Department of Emergency Medicine, Perelman School of Medicine, University of Pennsylvania, Philadelphia, Pennsylvania (C.W., C.M.W., D.L.C.).
- [10] Division of General Internal Medicine, Department of Medicine, Perelman School of Medicine, University of Pennsylvania, Philadelphia, Pennsylvania (S.K.).
- [11] Center for Connected Care, University of Pennsylvania Health System, Philadelphia, Pennsylvania (N.M., A.M.H., S.M.).
- [12] Department of Biostatistics, Epidemiology, and Informatics, Perelman School of Medicine, and Leonard Davis Institute of Health Economics, University of Pennsylvania, Philadelphia, Pennsylvania (N.M.).
- [13] Division of General Internal Medicine, Department of Medicine, Perelman School of Medicine, Leonard Davis Institute of Health Economics, University of Pennsylvania, and Center for Health Care Innovation and Center for Connected Care, University of Pennsylvania Health System, Philadelphia, Pennsylvania (K.H.C.).

- PMID: **34781715**
- PMCID: PMC8722738
- DOI: 10.7326/M21-2019

Free PMC article

Comparative Effectiveness of an Automated Text Messaging Service for Monitoring COVID-19 at Home

M Kit Delgado et al. Ann Intern Med. 2022 Feb.
Free PMC article

Show details

Ann Intern Med

. 2022 Feb;175(2):179-190.
doi: 10.7326/M21-2019. Epub 2021 Nov 16.

Authors

M Kit Delgado [1], Anna U Morgan [2], David A Asch [3], Ruiying Xiong [4], Austin S Kilaru [5], Kathleen C Lee [6], David Do [7], Ari B Friedman [5], Zachary F Meisel [5], Christopher K Snider [8], Doreen Lam [8], Andrew Parambath [8], Christian Wood [9], Chidinma M Wilson [9], Michael Perez [8], Deena L Chisholm [9], Sheila Kelly [10], Christina J O'Malley [8], Nancy Mannion [11], Ann Marie Huffenberger [11], Susan McGinley [11], Mohan Balachandran [8], Neda Khan [8], Nandita Mitra [12], Krisda H Chaiyachati [13]

Affiliations

- [1] Center for Emergency Care Policy and Research, Department of Emergency Medicine, Department of Biostatistics, Epidemiology, and Informatics, Perelman School of Medicine, and Leonard Davis Institute of Health Economics, University of Pennsylvania, and Center for Health Care Innovation, University of Pennsylvania Health System, Philadelphia, Pennsylvania (M.K.D.).
- [2] Division of General Internal Medicine, Department of Medicine, Perelman School of Medicine and Leonard Davis Institute of Health Economics, University of Pennsylvania, Philadelphia, Pennsylvania (A.U.M.).
- [3] Division of General Internal Medicine, Department of Medicine, Perelman School of Medicine, University of Pennsylvania, Wharton School, and Leonard Davis Institute of Health Economics, University of Pennsylvania, and Center for Health Care Innovation, University of Pennsylvania Health System, Philadelphia, Pennsylvania (D.A.A.).
- [4] Center for Emergency Care Policy and Research, Department of Emergency Medicine, and Division of General Internal Medicine, Department of Medicine, Perelman School of Medicine, and Leonard Davis Institute of Health Economics, University of Pennsylvania, Philadelphia, Pennsylvania (R.X.).
- [5] Center for Emergency Care Policy and Research, Department of Emergency Medicine, Perelman School of Medicine, and Leonard Davis Institute of Health Economics, University of Pennsylvania, Philadelphia, Pennsylvania (A.S.K., A.B.F., Z.F.M.).
- [6] Center for Emergency Care Policy and Research, Department of Emergency Medicine, Perelman School of Medicine, University of Pennsylvania, and Center for Health Care Innovation, University of Pennsylvania Health System, Philadelphia, Pennsylvania (K.C.L.).
- [7] Department of Neurology, Perelman School of Medicine, University of Pennsylvania, and Center for Health Care Innovation, University of Pennsylvania Health System, Philadelphia, Pennsylvania (D.D.).
- [8] Center for Health Care Innovation, University of Pennsylvania Health System, Philadelphia, Pennsylvania (C.K.S., D.L., A.P., M.P., C.J.O., M.B., N.K.).
- [9] Center for Emergency Care Policy and Research, Department of Emergency Medicine, Perelman School of Medicine, University of Pennsylvania, Philadelphia, Pennsylvania (C.W., C.M.W., D.L.C.).
- [10] Division of General Internal Medicine, Department of Medicine, Perelman School of Medicine, University of Pennsylvania, Philadelphia, Pennsylvania (S.K.).
- [11] Center for Connected Care, University of Pennsylvania Health System, Philadelphia, Pennsylvania (N.M., A.M.H., S.M.).
- [12] Department of Biostatistics, Epidemiology, and Informatics, Perelman School of Medicine, and Leonard Davis Institute of Health Economics, University of Pennsylvania, Philadelphia, Pennsylvania (N.M.).
- [13] Division of General Internal Medicine, Department of Medicine, Perelman School of Medicine, Leonard Davis Institute of Health Economics, University of Pennsylvania, and

Center for Health Care Innovation and Center for Connected Care, University of Pennsylvania Health System, Philadelphia, Pennsylvania (K.H.C.).

- PMID: **34781715**
- PMCID: PMC8722738
- DOI: 10.7326/M21-2019

Abstract

Background: Although most patients with SARS-CoV-2 infection can be safely managed at home, the need for hospitalization can arise suddenly.

Objective: To determine whether enrollment in an automated remote monitoring service for community-dwelling adults with COVID-19 at home ("COVID Watch") was associated with improved mortality.

Design: Retrospective cohort analysis.

Setting: Mid-Atlantic academic health system in the United States.

Participants: Outpatients who tested positive for SARS-CoV-2 between 23 March and 30 November 2020.

Intervention: The COVID Watch service consists of twice-daily, automated text message check-ins with an option to report worsening symptoms at any time. All escalations were managed 24 hours a day, 7 days a week by dedicated telemedicine clinicians.

Measurements: Thirty- and 60-day outcomes of patients enrolled in COVID Watch were compared with those of patients who were eligible to enroll but received usual care. The primary outcome was death at 30 days. Secondary outcomes included emergency department (ED) visits and hospitalizations. Treatment effects were estimated with propensity score-weighted risk adjustment models.

Results: A total of 3488 patients enrolled in COVID Watch and 4377 usual care control participants were compared with propensity score weighted models. At 30 days, COVID Watch patients had an odds ratio for death of 0.32 (95% CI, 0.12 to 0.72), with 1.8 fewer deaths per 1000 patients (CI, 0.5 to 3.1) (P = 0.005); at 60 days, the difference was 2.5 fewer deaths per 1000 patients (CI, 0.9 to 4.0) (P = 0.002). Patients in COVID Watch had more telemedicine encounters, ED visits, and hospitalizations and presented to the ED sooner (mean, 1.9 days sooner [CI, 0.9 to 2.9 days]; all $P < 0.001$).

Limitation: Observational study with the potential for unobserved confounding.

Conclusion: Enrollment of outpatients with COVID-19 in an automated remote monitoring service was associated with reduced mortality, potentially explained by more frequent telemedicine encounters and more frequent and earlier presentation to the ED.

Primary funding source: Patient-Centered Outcomes Research Institute.

Conflict of interest statement

Disclosures: Disclosures can be viewed at www.acponline.org/authors/icmje/ConflictOfInterestForms.do?msNum=M21-2019.

Comment in

- Extending a Lifeline to Nonhospitalized Patients With COVID-19 Through Automated Text Messaging.
  Faro JM, Cutrona SL. Faro JM, et al. Ann Intern Med. 2022 Feb;175(2):291-292. doi: 10.7326/M21-4273. Epub 2021 Nov 16. Ann Intern Med. 2022. PMID: 34781713 Free PMC article.

- 55 references
- 2 figures

Supplementary info

MeSH terms Expand

MeSH terms

- Adult
- Aged
- COVID-19 / mortality
- COVID-19 / therapy*
- Comparative Effectiveness Research
- Emergency Service, Hospital
- Female
- Home Care Services
- Hospitalization
- Humans
- Male
- Middle Aged
- Remote Consultation / methods*
- Retrospective Studies
- Text Messaging*
- United States / epidemiology

Full text links

Annals FREE FULL TEXT Atypon Free PMC article
Proceed to details
Cite
Share
☐ 751
Observational Study
Rev Port Cardiol (Engl Ed)
. 2021 Jul;40(7):465-471.
doi: 10.1016/j.repce.2021.07.014.

[Impact of SARS-CoV-2 pandemic on ST-elevation myocardial infarction admissions and outcomes in a Portuguese primary percutaneous coronary intervention center: Preliminary Data]

André Azul Freitas [1], Rui Baptista [2], Valdirene Gonçalves [3], Cátia Ferreira [3], James Milner [3], Carolina Lourenço [3], Susana Costa [3], Fátima Franco [3], Sílvia Monteiro [3], Francisco Gonçalves [3], Lino Gonçalves [4]

Affiliations Expand

Affiliations

- [1] Cardiology Department, Centro Hospitalar e Universitário de Coimbra, Coimbra, Portugal. Electronic address: andre.azulfreitas@gmail.com.
- [2] Faculty of Medicine, University of Coimbra, Coimbra, Portugal; University of Coimbra, Center for Innovative Biomedicine and Biotechnology (CIBB), Coimbra, Portugal; Clinical Academic Center of Coimbra (CACC), Coimbra, Portugal.
- [3] Cardiology Department, Centro Hospitalar e Universitário de Coimbra, Coimbra, Portugal.
- [4] Cardiology Department, Centro Hospitalar e Universitário de Coimbra, Coimbra, Portugal; Faculty of Medicine, University of Coimbra, Coimbra, Portugal; University of Coimbra, Center for Innovative Biomedicine and Biotechnology (CIBB), Coimbra, Portugal; Clinical Academic Center of Coimbra (CACC), Coimbra, Portugal.

- PMID: **34274091**
- PMCID: PMC8278193
- DOI: 10.1016/j.repce.2021.07.014

Free PMC article
Observational Study

Impact of SARS-CoV-2 pandemic on ST-elevation myocardial infarction admissions and outcomes in a Portuguese primary percutaneous coronary intervention center: Preliminary Data

André Azul Freitas et al. Rev Port Cardiol (Engl Ed). 2021 Jul.
Free PMC article
Show details

Rev Port Cardiol (Engl Ed)
. 2021 Jul;40(7):465-471.
doi: 10.1016/j.repce.2021.07.014.

Authors

André Azul Freitas [1], Rui Baptista [2], Valdirene Gonçalves [3], Cátia Ferreira [3], James Milner [3], Carolina Lourenço [3], Susana Costa [3], Fátima Franco [3], Sílvia Monteiro [3], Francisco Gonçalves [3], Lino Gonçalves [4]

Affiliations

- [1] Cardiology Department, Centro Hospitalar e Universitário de Coimbra, Coimbra, Portugal. Electronic address: andre.azulfreitas@gmail.com.
- [2] Faculty of Medicine, University of Coimbra, Coimbra, Portugal; University of Coimbra, Center for Innovative Biomedicine and Biotechnology (CIBB), Coimbra, Portugal; Clinical Academic Center of Coimbra (CACC), Coimbra, Portugal.
- [3] Cardiology Department, Centro Hospitalar e Universitário de Coimbra, Coimbra, Portugal.
- [4] Cardiology Department, Centro Hospitalar e Universitário de Coimbra, Coimbra, Portugal; Faculty of Medicine, University of Coimbra, Coimbra, Portugal; University of Coimbra, Center for Innovative Biomedicine and Biotechnology (CIBB), Coimbra, Portugal; Clinical Academic Center of Coimbra (CACC), Coimbra, Portugal.

- PMID: **34274091**
- PMCID: PMC8278193
- DOI: 10.1016/j.repce.2021.07.014

Abstract
in English, Portuguese

Introduction: Coronavirus disease (COVID-19) has led to significant changes in healthcare systems and its impact on the treatment of cardiovascular conditions, such as ST-elevation myocardial infarction (STEMI), is unknown in countries where the healthcare systems were not saturated, as was the case in Portugal. As such, we aimed to assess the effect on STEMI admissions and outcomes in Portuguese centers.

Methods: We conducted a single-center, observational, retrospective study including all patients admitted to our hospital due to STEMI between the date of the first SARS-CoV-2 case diagnosed in Portugal and the end of the state of emergency (March and April 2020). Patient characteristics and outcomes were assessed and compared with the same period of 2019.

Results: A total of 104 STEMI patients were assessed, 55 in 2019 and 49 in 2020 (-11%). There were no significant differences between groups regarding age (62±12 vs. 65±14 years, p=0.308), gender (84.8% vs. 77.6% males, p=0.295) or comorbidities. In the 2020 group, there was a significant decrease in the proportion of patients transported to the hospital in pre-hospital emergency medical transportation (38.2% vs. 20.4%, p=0.038), an increase in system delay (49 [30-110.25] vs. 140 [90-180] minutes, p=0.019), a higher Killip-Kimball class, with a decrease in class I (74.5% vs. 51%) and an increase in class III (1.8% vs. 8.2%) and IV (5.5% vs. 18.4%) (p=0.038), a greater incidence of vasoactive support (3.7% vs. 26.5%, p=0.001), invasive mechanic ventilation usage (3.6% vs. 14.3%, p=0.056), and an increase in severe left ventricular

dysfunction at hospital discharge (3.6% vs. 16.3%, p=0.03). In-hospital mortality was 14.3% in the 2020 group and 7.3% in the 2019 group p=0.200).

Conclusion: Despite a lack of significant variation in the absolute number of STEMI admissions, there was an increase in STEMI clinical severity and significantly worse outcomes during the SARS-CoV-2 pandemic. An increase in system delay, impaired pre-hospital care and patient fear of in-hospital infection can partially justify these results and should be the target of future actions in further waves of the pandemic.

Introdução: A doença por coronavírus 2019 (COVID-19) originou alterações significativas nos sistemas de saúde e a sua influência no tratamento da patologia cardiovascular, como no caso do enfarte agudo do miocárdio com supradesnivelamento do segmento ST (EAMcSST), é desconhecida em países onde não ocorreu saturação da capacidade dos sistemas de saúde, como é o caso de Portugal. Assim, o nosso objetivo foi determinar o efeito nas admissões por EAMcSST e no seu prognóstico intra-hospitalar na região Centro de Portugal.

Métodos: Realizou-se um estudo unicêntrico, observacional e retrospetivo, incluindo todos os doentes admitidos no nosso hospital por EAMcSST entre a data do primeiro caso de SARS-CoV-2 em Portugal e o término do estado de emergência (março e abril de 2020). Foram avaliadas as características e os resultados dos doentes e foi realizada uma comparação com o período homólogo de 2019.

Resultados: Foram incluídos 104 doentes com EAMcSST, 55 em 2019 e 49 em 2020 (-11%). Não se verificaram diferenças significativas entre os grupos relativamente à idade (62±12 *versus* 65±14 anos, p=0,308), género (84,8% mulheres *versus* 77,6% homens, p=0,295) ou comorbilidades. No grupo de doentes de 2020 verificou-se uma diminuição significativa na proporção de doentes transportados para o hospital pela viatura médica do Instituto Nacional de Emergência Médica (38,2% *versus* 20,4%, p=0,038), um aumento no atraso do sistema de saúde (49 [30-110,25] *versus* 140 [90-180] minutos, p=0,019), uma maior classe Killip-Kimball, com uma redução de doentes em classe I (74,5% *versus* 51%) e um aumento na classe III (1,8% *versus* 8,2%) e IV (5,5% *versus* 18,4%) (p=0,038), uma maior incidência de suporte vasoativo (3,7% *versus* 26,5%, p=0,001), de ventilação mecânica invasiva (3,6% *versus* 14,3%, p=0,056) e um aumento da proporção de doentes com disfunção ventricular esquerda grave na alta hospitalar (3,6% *versus* 16,3%, p=0,03). A mortalidade intra-hospitalar foi de 14,3% no grupo de 2020 e de 7,3% no grupo de 2019 (p=0,200).

Conclusão: Apesar de não se ter verificado uma variação significativa no número de admissões por EAMcSST, existiu um aumento da gravidade, com um prognóstico intra-hospitalar significativamente mais adverso durante a pandemia por SARS-CoV-2. Um aumento no atraso do sistema de saúde, um compromisso nos serviços pré-hospitalares e o receio por parte dos doentes de contraírem uma eventual infeção hospitalar podem justificar parcialmente estes resultados e devem ser planeadas ações para diminuir o seu efeito em novos surtos pandémicos.

Keywords: COVID-19; Coronavírus 2019; EAMcSST; Emergency medical system; SARS-CoV-2; STEMI; Sistema de emergência médica.

Copyright © 2021 Sociedade Portuguesa de Cardiologia. Published by Elsevier España, S.L.U. All rights reserved.

- 23 references
- 3 figures

Supplementary info

Publication types, MeSH terms Expand

Publication types

- Observational Study

MeSH terms

- COVID-19*
- Female
- Humans
- Male
- Pandemics
- Percutaneous Coronary Intervention*
- Portugal / epidemiology
- Preliminary Data
- Retrospective Studies
- SARS-CoV-2
- ST Elevation Myocardial Infarction* / epidemiology

Full text links

ELSEVIER OPEN ACCESS Elsevier Science Free PMC article
Proceed to details
Cite
Share
752
Observational Study
J Cardiovasc Med (Hagerstown)
. 2020 Sep;21(9):722-724.
doi: 10.2459/JCM.0000000000001053.

Impact on hospital admission of ST-elevation myocardial infarction patients during coronavirus disease 2019 pandemic in an Italian Hospital

Ilenia Alessandra Di Liberto [1], Gerlando Pilato [1], Salvatore Geraci [1], Diego Milazzo [1], Giovanni Vaccaro [1], Sergio Buccheri [2], Giuseppe Caramanno [1]
Affiliations Expand

Affiliations

- [1] Department of Interventional Cardiology, San Giovanni Di Dio Hospital, Agrigento, Italy.
- [2] Department of Medical Sciences-Cardiology and Uppsala Clinical Research Center, Uppsala Univerisity, Uppsala, Sweden.

- PMID: **32658006**
- DOI: 10.2459/JCM.0000000000001053

Observational Study

Impact on hospital admission of ST-elevation myocardial infarction patients during coronavirus disease 2019 pandemic in an Italian Hospital

Ilenia Alessandra Di Liberto et al. J Cardiovasc Med (Hagerstown). 2020 Sep.
Show details
J Cardiovasc Med (Hagerstown)
. 2020 Sep;21(9):722-724.
doi: 10.2459/JCM.0000000000001053.

Authors

Ilenia Alessandra Di Liberto [1], Gerlando Pilato [1], Salvatore Geraci [1], Diego Milazzo [1], Giovanni Vaccaro [1], Sergio Buccheri [2], Giuseppe Caramanno [1]

Affiliations

- [1] Department of Interventional Cardiology, San Giovanni Di Dio Hospital, Agrigento, Italy.
- [2] Department of Medical Sciences-Cardiology and Uppsala Clinical Research Center, Uppsala Univerisity, Uppsala, Sweden.

- PMID: **32658006**
- DOI: 10.2459/JCM.0000000000001053

No abstract available

- 5 references

Supplementary info

Publication types, MeSH terms Expand

Publication types

- Comparative Study
- Observational Study

MeSH terms

- Aged
- COVID-19
- Coronavirus Infections*
- Female
- Humans
- Italy
- Male
- Middle Aged
- Pandemics*
- Patient Admission / statistics & numerical data*
- Pneumonia, Viral*
- Retrospective Studies
- ST Elevation Myocardial Infarction*

Full text links

Wolters Kluwer Wolters Kluwer
Proceed to details
Cite
Share

☐ 753

Observational Study

Acta Neurol Scand
. 2022 Jan;145(1):47-52.
doi: 10.1111/ane.13520. Epub 2021 Aug 20.

Systemic thrombolysis in ischaemic stroke patients with COVID-19

Piotr Sobolewski [1] [2], Jacek Antecki [3], Waldemar Brola [2] [4], Małgorzata Fudala [5], Leszek Bieniaszewski [6], Grzegorz Kozera [6]
Affiliations Expand

Affiliations

- [1] Department of Neurology and Stroke Unit in Sandomierz, Jan Kochanowski University, Kielce, Poland.
- [2] Collegium Medicum, Jan Kochanowski University, Kielce, Poland.

- [3] Department of Neurology and Stroke Unit, Regional Hospital in Kielce, Kielce, Poland.
- [4] Department of Neurology and Stroke Unit, Saint Lukas Hospital in Końskie, Końskie, Poland.
- [5] Department of Neurology and Stroke Unit, Skłodowska-Curie Hospital in Skarżysko-Kamienna, Skarżysko-Kamienna, Poland.
- [6] Medical Simulation Center, Medical University of Gdańsk, Gdańsk, Poland.

- PMID: **34415051**
- PMCID: PMC8444791
- DOI: 10.1111/ane.13520

Free PMC article
Observational Study

Systemic thrombolysis in ischaemic stroke patients with COVID-19

Piotr Sobolewski et al. Acta Neurol Scand. 2022 Jan.
Free PMC article

Show details

Acta Neurol Scand
. 2022 Jan;145(1):47-52.
doi: 10.1111/ane.13520. Epub 2021 Aug 20.

Authors

Piotr Sobolewski [1, 2], Jacek Antecki [3], Waldemar Brola [2, 4], Małgorzata Fudala [5], Leszek Bieniaszewski [6], Grzegorz Kozera [6]

Affiliations

- [1] Department of Neurology and Stroke Unit in Sandomierz, Jan Kochanowski University, Kielce, Poland.
- [2] Collegium Medicum, Jan Kochanowski University, Kielce, Poland.
- [3] Department of Neurology and Stroke Unit, Regional Hospital in Kielce, Kielce, Poland.
- [4] Department of Neurology and Stroke Unit, Saint Lukas Hospital in Końskie, Końskie, Poland.
- [5] Department of Neurology and Stroke Unit, Skłodowska-Curie Hospital in Skarżysko-Kamienna, Skarżysko-Kamienna, Poland.
- [6] Medical Simulation Center, Medical University of Gdańsk, Gdańsk, Poland.

- PMID: **34415051**
- PMCID: PMC8444791
- DOI: 10.1111/ane.13520

Abstract

Objective: Intravenous thrombolysis (IVT) with recombinant tissue plasminogen activator is the core medical therapy of acute ischaemic stroke (AIS). COVID-19 infection negatively modifies acute stroke procedures and, due to its pro-coagulative effect, may potentially impact on IVT outcome. Thus, short-term efficacy and safety of IVT were compared in patients with and without evidence of SARS-CoV-2.

Methods: An observational, retrospective study included 70 patients with AIS, including 22 subjects (31%) with evidence of acute COVID-19 infection, consecutively treated with IVT in 4 stroke centres between 15 September and 30 November 2020.

Results: Patients infected with COVID-19 were characterized by higher median of National Institute of Health Stroke Scale (NIHSS) score (11.0 vs. 6.5; $p < .01$) and D-dimers (870 vs. 570; $p = .03$) on admission, higher presence of pneumonia (47.8% vs. 12%; $p < .01$) and lower percentage of 'minor stroke symptoms' (NIHSS 1-5 pts.) (2% vs., 18%; $p < .01$). Hospitalizations were longer in patients with COVID-19 than in those without it (17 vs. 9 days, $p < .01$), but impact of COVID-19 infection on patients' in-hospital mortality or functional status on dismission has been confirmed neither in uni- or multivariate analysis.

Conclusion: SARS-CoV-2 infection prolongs length of stay in hospital after IVT, but does not influence in-hospital outcome.

Keywords: COVID-19; functional outcome; intravenous thrombolysis; ischaemic stroke.

© 2021 John Wiley & Sons A/S. Published by John Wiley & Sons Ltd.

Conflict of interest statement

P. Sobolewski has had lectureship fees covered by Boehringer-Ingelheim, Ever Pharma, Allergan and travel expenses to scientific conferences covered by Boehringer-Ingelheim, Ipsen and Ever Pharma and Angels Initiative. W. Brola has received speaker fees and/or served on Advisory Boards by Bayer, Biogen, Sanofi Genzyme, Merck, Novartis and Roche. He also received support for congress participation, travel and accommodation grants from Biogen, Merck, Roche and Sanofi Genzyme. G. Kozera's research activities have been funded by the Ministry of Science and High Education, Poland, Office of the Governor of Pomerania, Poland; he has received speaker's honoraria from the Boehringer-Ingelheim, Everpharma, Bayer and Phizer and Angels Initiative. L. Bieniaszewski has been funded by the Ministry of Science and High Education, Poland, Poland. J. Antecki and M. Fudala declare that they have no conflict of interest.

- 31 references

Supplementary info

Publication types, MeSH terms, Substances Expand

Publication types

- Observational Study

MeSH terms

- Brain Ischemia* / complications

- Brain Ischemia* / drug therapy
- COVID-19*
- Fibrinolytic Agents / therapeutic use
- Humans
- Ischemic Stroke*
- Retrospective Studies
- SARS-CoV-2
- Stroke* / complications
- Stroke* / drug therapy
- Thrombolytic Therapy
- Tissue Plasminogen Activator / therapeutic use
- Treatment Outcome

Substances

- Fibrinolytic Agents
- Tissue Plasminogen Activator

Full text links

WILEY Full Text Article Wiley Free PMC article
Proceed to details
Cite
Share
☐ 754
Observational Study
Laryngoscope
. 2021 Nov;131(11):E2749-E2754.
doi: 10.1002/lary.29667. Epub 2021 Jun 5.

COVID-19 Cross-Infection Rate After Surgical Procedures: Incidence and Outcome

Bassem Mettias [1], Manish Mair [1], Peter Conboy [1]
Affiliations Expand

Affiliation

- [1] Leicester Royal Infirmary, Infirmary Square, Leicester, LE1 5WW, United Kingdom.

- PMID: **34037248**
- PMCID: PMC8242479
- DOI: 10.1002/lary.29667

Free PMC article
Observational Study

COVID-19 Cross-Infection Rate After Surgical Procedures: Incidence and Outcome

Bassem Mettias et al. Laryngoscope. 2021 Nov.
Free PMC article
Show details
Laryngoscope
. 2021 Nov;131(11):E2749-E2754.
doi: 10.1002/lary.29667. Epub 2021 Jun 5.

Authors

Bassem Mettias [1], Manish Mair [1], Peter Conboy [1]

Affiliation

- [1] Leicester Royal Infirmary, Infirmary Square, Leicester, LE1 5WW, United Kingdom.

- PMID: **34037248**
- PMCID: PMC8242479
- DOI: 10.1002/lary.29667

Abstract

Objectives/hypothesis: Severe acute respiratory syndrome coronavirus 2 (SARSCoV-2) is transmitted by droplet as well as airborne infection. Surgical patients are vulnerable to the infection during their hospital admission. Some surgical procedures are classified as aerosol generating (AGP).

Study design: Retrospective observational study of four specialties associates with known AGP's during the 4 months of the first wave of UK COVID-19 epidermic to identify post-surgical cross-infection with SARSCoV-2 within 14 days of a procedure.

Methods: Retrospective observational study in a tertiary healthcare center of four specialties associates with known AGP's during the 4 months of the first wave of UK COVID-19 epidermic to identify post-surgical cross-infection with SARSCoV-2 within 14 days of a procedure.

Results: There were 3,410 procedures reported during this period. The overall cross-infection rate from tested patients was 1.3% (4 patients), that is, 0.11% of all operations over 4 months. Ear, nose, and throat carried slightly higher rate of infection (0.4%) than gastroenterology (0.08%). The mortality rate was 0.3% (one gastroenterology patient from 304 positive cases) compared to 0% if surgery performed after recovery from SARSCoV-2 and 37.5% when surgery was conducted during the incubation period of the disease. Routine preoperative rapid screening tests and self-isolation are crucial to avoid the risk of cross-infection. Patients with underlying malignancy or receiving chemotherapy were more prone to pulmonary complications and mortality.

Conclusion: The risk of SARS-COV-2 cross-infection after surgical procedure is very low. Preoperative screening and self-isolation together with personal protective measures should be in place to minimize the cross-infection.

Level of evidence: 4 Laryngoscope, 131:E2749-E2754, 2021.

Keywords: SARSCoV-2; mortality; outcome; surgery.

© 2021 The American Laryngological, Rhinological and Otological Society, Inc.

- 11 references
- 3 figures

Supplementary info

Publication types, MeSH terms, Substances Expand

Publication types

- Comparative Study
- Observational Study

MeSH terms

- Aerosols
- Aged
- Aged, 80 and over
- COVID-19 / diagnosis
- COVID-19 / epidemiology
- COVID-19 / transmission*
- COVID-19 / virology
- Cross Infection / epidemiology*
- Cross Infection / prevention & control
- Disease Transmission, Infectious / prevention & control*
- Disease Transmission, Infectious / statistics & numerical data
- Female
- Humans
- Incidence
- Male
- Mass Screening / methods
- Middle Aged
- Mortality / trends
- Outcome Assessment, Health Care
- Particulate Matter / adverse effects
- Patient Isolation / methods

- Personal Protective Equipment / standards
- Preoperative Period
- Retrospective Studies
- Risk Assessment / methods
- SARS-CoV-2 / genetics
- Surgical Procedures, Operative / adverse effects*
- Surgical Procedures, Operative / classification
- Surgical Procedures, Operative / statistics & numerical data
- United Kingdom / epidemiology

Substances

- Aerosols
- Particulate Matter

Full text links

WILEY Full Text Article Wiley Free PMC article
Proceed to details
Cite
Share
755

PLoS One
. 2022 Jan 19;17(1):e0262811.
doi: 10.1371/journal.pone.0262811. eCollection 2022.

Prophylactic versus therapeutic dose anticoagulation effects on survival among critically ill patients with COVID-19

Wouter S Hoogenboom [1], Joyce Q Lu [1], Benjamin Musheyev [2], Lara Borg [2], Rebeca Janowicz [2], Stacey Pamlayne [2], Wei Hou [2], Tim Q Duong [1]
Affiliations Expand

Affiliations

- [1] Albert Einstein College of Medicine and Montefiore Medical Center, Bronx, New York, United States of America.
- [2] Stony Brook University, Renaissance School of Medicine, Stony Brook, New York, United States of America.

- PMID: **35045130**
- PMCID: PMC8769345

- DOI: 10.1371/journal.pone.0262811

Free PMC article

Prophylactic versus therapeutic dose anticoagulation effects on survival among critically ill patients with COVID-19

Wouter S Hoogenboom et al. PLoS One. 2022.
Free PMC article
Show details
PLoS One
. 2022 Jan 19;17(1):e0262811.
doi: 10.1371/journal.pone.0262811. eCollection 2022.

Authors

Wouter S Hoogenboom [1], Joyce Q Lu [1], Benjamin Musheyev [2], Lara Borg [2], Rebeca Janowicz [2], Stacey Pamlayne [2], Wei Hou [2], Tim Q Duong [1]

Affiliations

- [1] Albert Einstein College of Medicine and Montefiore Medical Center, Bronx, New York, United States of America.
- [2] Stony Brook University, Renaissance School of Medicine, Stony Brook, New York, United States of America.

- PMID: **35045130**
- PMCID: PMC8769345
- DOI: 10.1371/journal.pone.0262811

Abstract

Introduction: Although patients with severe COVID-19 are known to be at high risk of developing thrombotic events, the effects of anticoagulation (AC) dose and duration on in-hospital mortality in critically ill patients remain poorly understood and controversial. The goal of this study was to investigate survival of critically ill COVID-19 patients who received prophylactic or therapeutic dose AC and analyze the mortality rate with respect to detailed demographic and clinical characteristics.

Materials and methods: We conducted a retrospective, observational study of critically ill COVID-19 patients admitted to the ICU at Stony Brook University Hospital in New York who received either prophylactic (n = 158) or therapeutic dose AC (n = 153). Primary outcome was in-hospital death assessed by survival analysis and covariate-adjusted Cox proportional hazard model.

Results: For the first 3 weeks of ICU stay, we observed similar survival curves for prophylactic and therapeutic AC groups. However, after 3 or more weeks of ICU stay, the therapeutic AC

group, characterized by high incidence of acute kidney injury (AKI), had markedly higher death incidence rates with 8.6 deaths (95% CI = 6.2-11.9 deaths) per 1,000 person-days and about 5 times higher risk of death (adj. HR = 4.89, 95% CI = 1.71-14.0, p = 0.003) than the prophylactic group (2.4 deaths [95% CI = 0.9-6.3 deaths] per 1,000 person-days). Among therapeutic AC users with prolonged ICU admission, non-survivors were characterized by older males with depressed lymphocyte counts and cardiovascular disease.

Conclusions: Our findings raise the possibility that prolonged use of high dose AC, independent of thrombotic events or clinical background, might be associated with higher risk of in-hospital mortality. Moreover, AKI, age, lymphocyte count, and cardiovascular disease may represent important risk factors that could help identify at-risk patients who require long-term hospitalization with therapeutic dose AC treatment.

Conflict of interest statement

The authors have declared that no competing interests exist.

- 40 references
- 2 figures

Supplementary info

MeSH terms, Substances, Grant support Expand

MeSH terms

- Acute Kidney Injury / complications
- Acute Kidney Injury / diagnosis
- Age Factors
- Aged
- Anticoagulants / adverse effects
- Anticoagulants / therapeutic use*
- COVID-19 / drug therapy
- COVID-19 / mortality
- COVID-19 / pathology*
- COVID-19 / virology
- Cardiovascular Diseases / complications
- Critical Illness
- Female
- Hospital Mortality
- Humans
- Intensive Care Units
- Kaplan-Meier Estimate
- Male
- Middle Aged
- Proportional Hazards Models

- Retrospective Studies
- Risk Factors
- SARS-CoV-2 / isolation & purification
- Sex Factors
- Thrombosis / complications
- Thrombosis / drug therapy*

Substances

- Anticoagulants

Grant support

The author(s) received no specific funding for this work.

Full text links

OPEN ACCESS TO FULL TEXT PLOS ONE Public Library of Science Free PMC article
Proceed to details
Cite
Share
756
Clinical Trial
Nutrients
. 2020 Dec 11;12(12):3799.
doi: 10.3390/nu12123799.

High-Dose Cholecalciferol Booster Therapy is Associated with a Reduced Risk of Mortality in Patients with COVID-19: A Cross-Sectional Multi-Centre Observational Study

Stephanie F Ling [1] [2], Eleanor Broad [1], Rebecca Murphy [1], Joseph M Pappachan [2] [3] [4], Satveer Pardesi-Newton [5], Marie-France Kong [6], Edward B Jude [1] [2] [4]
Affiliations Expand

Affiliations

- [1] Tameside and Glossop Integrated Care NHS Foundation Trust, Fountain Street, Ashton-under-Lyne OL6 9RW, UK.
- [2] The University of Manchester, Oxford Road, Manchester M13 9PL, UK.

- [3] Royal Preston Hospital, Lancashire Teaching Hospitals NHS Foundation Trust, Sharoe Green Lane, Fulwood, Preston PR2 9HT, UK.
- [4] Manchester Metropolitan University, All Saints Building, Oxford Road, Manchester M15 6BH, UK.
- [5] Leicester Royal Infirmary, University Hospitals of Leicester NHS Trust, Infirmary Square, Leicester LE1 5WW, UK.
- [6] Leicester General Hospital, University Hospitals of Leicester NHS Trust, Gwendolen Road, Leicester LE5 4PW, UK.

- PMID: **33322317**
- PMCID: PMC7763301
- DOI: 10.3390/nu12123799

Free PMC article
Clinical Trial

High-Dose Cholecalciferol Booster Therapy is Associated with a Reduced Risk of Mortality in Patients with COVID-19: A Cross-Sectional Multi-Centre Observational Study

Stephanie F Ling et al. Nutrients. 2020.
Free PMC article
Show details
Nutrients
. 2020 Dec 11;12(12):3799.
doi: 10.3390/nu12123799.

Authors

Stephanie F Ling [1, 2], Eleanor Broad [1], Rebecca Murphy [1], Joseph M Pappachan [2, 3, 4], Satveer Pardesi-Newton [5], Marie-France Kong [6], Edward B Jude [1, 2, 4]

Affiliations

- [1] Tameside and Glossop Integrated Care NHS Foundation Trust, Fountain Street, Ashton-under-Lyne OL6 9RW, UK.
- [2] The University of Manchester, Oxford Road, Manchester M13 9PL, UK.
- [3] Royal Preston Hospital, Lancashire Teaching Hospitals NHS Foundation Trust, Sharoe Green Lane, Fulwood, Preston PR2 9HT, UK.
- [4] Manchester Metropolitan University, All Saints Building, Oxford Road, Manchester M15 6BH, UK.
- [5] Leicester Royal Infirmary, University Hospitals of Leicester NHS Trust, Infirmary Square, Leicester LE1 5WW, UK.
- [6] Leicester General Hospital, University Hospitals of Leicester NHS Trust, Gwendolen Road, Leicester LE5 4PW, UK.

- PMID: **33322317**
- PMCID: PMC7763301
- DOI: 10.3390/nu12123799

Abstract

The worldwide pandemic of 2019 novel coronavirus disease (COVID-19) has posed the most substantial and severe public health issue for several generations, and therapeutic options have not yet been optimised. Vitamin D (in its "parent" form, cholecalciferol) has been proposed in the pharmacological management of COVID-19 by various sources. We aimed to determine whether COVID-19 mortality was affected by serum 25-hydroxyvitamin D (25(OH)D) levels, vitamin D status, or cholecalciferol therapy, and to elucidate any other predictors of COVID-19 mortality. Patients hospitalised with COVID-19 were opportunistically recruited from three UK hospitals, and their data were collected retrospectively. Logistic regression was used to determine any relationships between COVID-19 mortality and potential predictors, including 25(OH)D levels and cholecalciferol booster therapy. A total of 986 participants with COVID-19 were studied, of whom 151 (16.0%) received cholecalciferol booster therapy. In the primary cohort of 444 patients, cholecalciferol booster therapy was associated with a reduced risk of COVID-19 mortality, following adjustment for potential confounders (OR_{adj} 0.13, 95% CI 0.05-0.35, $p < 0.001$). This finding was replicated in a validation cohort of 541 patients (OR_{adj} 0.38, 95% CI 0.17-0.84, p = 0.018). In this observational study, treatment with cholecalciferol booster therapy, regardless of baseline serum 25(OH)D levels, appears to be associated with a reduced risk of mortality in acute in-patients admitted with COVID-19. Further work with large population studies needs to be carried out to determine adequate serum 25(OH)D levels, as well as multi-dose clinical trials of cholecalciferol therapy to assess maximum efficacy.

Keywords: 25-hydroxyvitamin D; COVID-19; SARS-CoV-2; cholecalciferol; mortality; novel coronavirus 2019; vitamin D; vitamin D treatment.

Conflict of interest statement

The authors declare no conflict of interest.

- 29 references
- 2 figures

Supplementary info

Publication types, MeSH terms, Substances Expand

Publication types

- Clinical Trial
- Multicenter Study
- Observational Study

MeSH terms

- Aged

- Aged, 80 and over
- COVID-19 / drug therapy*
- COVID-19 / mortality*
- Cholecalciferol / administration & dosage*
- Cross-Sectional Studies
- Female
- Humans
- Male
- Middle Aged
- Risk Factors
- SARS-CoV-2*
- United Kingdom / epidemiology

Substances

- Cholecalciferol

Full text links

FULL TEXT OPEN ACCESS MDPI Multidisciplinary Digital Publishing Institute (MDPI) Free PMC article
Proceed to details
Cite
Share
757
Observational Study
J Surg Res
. 2021 Apr;260:38-45.
doi: 10.1016/j.jss.2020.10.013. Epub 2020 Oct 26.

Short-term Outcomes for Patients and Providers After Elective Tracheostomy in COVID-19-Positive Patients

Patrick Murphy [1], Emma Holler [2], Heidi Lindroth [3], Michelle Laughlin [4], Clark J Simons [4], Erik W Streib [4], Malaz Boustani [5], Damaris Ortiz [6]
Affiliations Expand

Affiliations

- [1] Department of Surgery, Medical College of Wisconsin, Wisconsin.
- [2] Department of Surgery, Trauma and Acute Care Surgery, Eskenazi Health, Indianapolis, Indiana.

- [3] Center for Health Innovation and Implementation Science, Center for Translational Science and Innovation, Indiana University School of Medicine, Indianapolis, Indiana.
- [4] Department of Surgery, Indiana University School of Medicine, Indianapolis, Indiana.
- [5] Center for Health Innovation and Implementation, Indiana University School of Medicine, Indianapolis, Indiana.
- [6] Department of Surgery, Indiana University School of Medicine, Indianapolis, Indiana. Electronic address: damaorti@iu.edu.

- PMID: **33316758**
- PMCID: PMC7587136
- DOI: 10.1016/j.jss.2020.10.013

Free PMC article
Observational Study

Short-term Outcomes for Patients and Providers After Elective Tracheostomy in COVID-19-Positive Patients

Patrick Murphy et al. J Surg Res. 2021 Apr.
Free PMC article
Show details
J Surg Res
. 2021 Apr;260:38-45.
doi: 10.1016/j.jss.2020.10.013. Epub 2020 Oct 26.

Authors

Patrick Murphy [1], Emma Holler [2], Heidi Lindroth [3], Michelle Laughlin [4], Clark J Simons [4], Erik W Streib [4], Malaz Boustani [5], Damaris Ortiz [6]

Affiliations

- [1] Department of Surgery, Medical College of Wisconsin, Wisconsin.
- [2] Department of Surgery, Trauma and Acute Care Surgery, Eskenazi Health, Indianapolis, Indiana.
- [3] Center for Health Innovation and Implementation Science, Center for Translational Science and Innovation, Indiana University School of Medicine, Indianapolis, Indiana.
- [4] Department of Surgery, Indiana University School of Medicine, Indianapolis, Indiana.
- [5] Center for Health Innovation and Implementation, Indiana University School of Medicine, Indianapolis, Indiana.
- [6] Department of Surgery, Indiana University School of Medicine, Indianapolis, Indiana. Electronic address: damaorti@iu.edu.

- PMID: **33316758**
- PMCID: PMC7587136
- DOI: 10.1016/j.jss.2020.10.013

Abstract

Background: Urgent guidance is needed on the safety for providers of percutaneous tracheostomy in patients diagnosed with COVID-19. The objective of the study was to demonstrate that percutaneous dilational tracheostomy (PDT) with a period of apnea in patients requiring prolonged mechanical ventilation due to COVID-19 is safe and can be performed for the usual indications in the intensive care unit.

Methods: This study involves an observational case series at a single-center medical intensive care unit at a level-1 trauma center in patients diagnosed with COVID-19 who were assessed for tracheostomy. Success of a modified technique included direct visualization of tracheal access by bronchoscopy and a blind dilation and tracheostomy insertion during a period of patient apnea to reduce aerosolization. Secondary outcomes include transmission rate of COVID-19 to providers and patient complications.

Results: From April 6th, 2020 to July 21st, 2020, 2030 patients were admitted to the hospital with COVID-19, 615 required intensive care unit care (30.3%), and 254 patients required mechanical ventilation (12.5%). The mortality rate for patients requiring mechanical ventilation was 29%. Eighteen patients were assessed for PDT, and 11 (61%) underwent the procedure. The majority had failed extubation at least once (72.7%), and the median duration of intubation before tracheostomy was 15 d (interquartile range 13-24). The median positive end-expiratory pressure at time of tracheostomy was 10.8. The median partial pressure of oxygen (PaO2)/FiO2 ratio on the day of tracheostomy was 142.8 (interquartile range 104.5-224.4). Two patients had bleeding complications. At 1-week follow-up, eight patients still required ventilator support (73%). At the most recent follow-up, eight patients (73%) have been liberated from the ventilator, one patient (9%) died as a result of respiratory/multiorgan failure, and two were discharged on the ventilator (18%). Average follow-up was 20 d. None of the surgeons performing PDT have symptoms of or have tested positive for COVID-19.

Conclusions: and relevance: PDT for patients with COVID-19 is safe for health care workers and patients despite higher positive end-expiratory pressure requirements and should be performed for the same indications as other causes of respiratory failure.

Keywords: COVID-19; Coronavirus; Critical care; Tracheostomy.

Copyright © 2020 Elsevier Inc. All rights reserved.

- 21 references
- 1 figure

Supplementary info

Publication types, MeSH terms Expand

Publication types

- Observational Study

MeSH terms

- Adult

- Aged
- Airway Extubation / statistics & numerical data
- Bronchoscopy / adverse effects*
- Bronchoscopy / instrumentation
- Bronchoscopy / methods
- Bronchoscopy / standards
- COVID-19 / diagnosis
- COVID-19 / mortality
- COVID-19 / therapy*
- COVID-19 / transmission
- COVID-19 Nucleic Acid Testing / statistics & numerical data
- Female
- Follow-Up Studies
- Hospital Mortality
- Humans
- Infectious Disease Transmission, Patient-to-Professional / prevention & control*
- Infectious Disease Transmission, Patient-to-Professional / statistics & numerical data
- Intensive Care Units / standards
- Intensive Care Units / statistics & numerical data
- Male
- Middle Aged
- Postoperative Complications / epidemiology*
- Postoperative Complications / etiology
- Respiration, Artificial / adverse effects*
- Respiration, Artificial / instrumentation
- Respiration, Artificial / methods
- Respiration, Artificial / statistics & numerical data
- Retrospective Studies
- SARS-CoV-2 / isolation & purification
- Severity of Illness Index
- Time Factors
- Tracheostomy / adverse effects*
- Tracheostomy / instrumentation
- Tracheostomy / methods
- Tracheostomy / standards
- Treatment Outcome

Full text links

ELSEVIER FULL-TEXT ARTICLE Elsevier Science Free PMC article
Proceed to details
Cite

Share

☐ 758

Observational Study

S Afr Med J

. 2020 Dec 4;111(1):52-56.

doi: 10.7196/SAMJ.2021.v111i1.15289.

Locked down: Impact of COVID-19 restrictions on trauma presentations to the emergency department

A Venter [1], C M Lewis, P Saffy, L P Chadinha

Affiliations Expand

Affiliation

- [1] Division of Emergency Medicine, Department of Family Medicine and Primary Care, Faculty of Health Sciences, University of the Witwatersrand, Johannesburg, South Africa. 28aventer@gmail.com.

- PMID: **33404006**
- DOI: 10.7196/SAMJ.2021.v111i1.15289

Free article

Observational Study

Locked down: Impact of COVID-19 restrictions on trauma presentations to the emergency department

A Venter et al. S Afr Med J. 2020.

Free article

Show details

S Afr Med J

. 2020 Dec 4;111(1):52-56.

doi: 10.7196/SAMJ.2021.v111i1.15289.

Authors

A Venter [1], C M Lewis, P Saffy, L P Chadinha

Affiliation

- [1] Division of Emergency Medicine, Department of Family Medicine and Primary Care, Faculty of Health Sciences, University of the Witwatersrand, Johannesburg, South Africa. 28aventer@gmail.com.

- PMID: **33404006**
- DOI: 10.7196/SAMJ.2021.v111i1.15289

Abstract

Background: COVID-19 was recognised as a global pandemic on 11 March 2020. In South Africa (SA), a nationwide lockdown was implemented at midnight on 26 March to prepare for the predicted surge and slow the spread of the virus.

Objectives: To compare the volume and type of presentations of trauma secondary to interpersonal violence and road traffic collisions (RTCs) during two 5-month periods, from February to June 2019 and 2020, in the emergency department (ED) of an academic tertiary hospital in Gauteng Province, SA. In 2020, February - June included the lockdown period.

Methods: An observational retrospective audit of the patient register at the Helen Joseph Hospital ED was conducted, comparing the number of trauma presentations secondary to interpersonal violence (assaults with gunshot wounds, general assaults including mob assaults, assaults with stab wounds) and RTC presentations between February and June 2019 and 2020.

Results: A total of 4 300 trauma presentations secondary to interpersonal violence and RTCs were noted in the 5-month period February - June 2019, as opposed to 3 239 presentations in February - June 2020 (25% decline). A 40% decline in the number of RTCs, from 1 704 in February - June 2019 to 1 026 in the corresponding period for 2020, was noted and found to be statistically significant (p=0.03). Declines in the volume of trauma cases secondary to interpersonal violence and of overall trauma cases were only directional in favour of 2020, but not statistically significant.

Conclusions: The volume of trauma presentations secondary to interpersonal violence and RTCs in the Helen Joseph Hospital ED decreased during the lockdown period. The decline in the volume of RTCs was statistically significant, but declines in the volume of trauma presentations secondary to interpersonal violence and in the volume of overall trauma presentations were not.

Supplementary info

Publication types, MeSH terms Expand

Publication types

- Observational Study

MeSH terms

- Accidents, Traffic / trends*
- COVID-19 / prevention & control*
- Communicable Disease Control*

- Emergency Service, Hospital*
- Humans
- Public Policy
- Retrospective Studies
- SARS-CoV-2
- South Africa / epidemiology
- Violence / trends*
- Wounds and Injuries / epidemiology*
- Wounds, Gunshot / epidemiology
- Wounds, Stab / epidemiology

Full text links

HMPG Health and Medical Publishing Group
Proceed to details
Cite
Share
☐ 759
Observational Study
Int J Surg
. 2020 Nov;83:259-266.
doi: 10.1016/j.ijsu.2020.09.011. Epub 2020 Sep 12.

A single-centre observational cohort study to evaluate volume and severity of emergency general surgery admissions during the COVID-19 pandemic: Is there a "lockdown" effect?

Ross C McLean [1], John Young [1], Aya Musbahi [1], Jing Xian Lee [1], Hena Hidayat [1], Nagi Abdalla [1], Sabyasachi Chowdhury [1], Elizabeth A Baker [1], Kevin Jon Etherson [2]
Affiliations Expand

Affiliations

- [1] Department of Colorectal Surgery, University Hospital of North Tees, Hardwick Road, Stockton-On-Tees, TS19 8PE, UK.
- [2] Department of Colorectal Surgery, University Hospital of North Tees, Hardwick Road, Stockton-On-Tees, TS19 8PE, UK. Electronic address: kevin.etherson@nht.nhs.uk.

- PMID: **32931980**
- PMCID: PMC7486821

• DOI: 10.1016/j.ijsu.2020.09.011

Free PMC article
Observational Study

A single-centre observational cohort study to evaluate volume and severity of emergency general surgery admissions during the COVID-19 pandemic: Is there a "lockdown" effect?

Ross C McLean et al. Int J Surg. 2020 Nov.
Free PMC article
Show details
Int J Surg
. 2020 Nov;83:259-266.
doi: 10.1016/j.ijsu.2020.09.011. Epub 2020 Sep 12.

Authors

Ross C McLean [1], John Young [1], Aya Musbahi [1], Jing Xian Lee [1], Hena Hidayat [1], Nagi Abdalla [1], Sabyasachi Chowdhury [1], Elizabeth A Baker [1], Kevin Jon Etherson [2]

Affiliations

- [1] Department of Colorectal Surgery, University Hospital of North Tees, Hardwick Road, Stockton-On-Tees, TS19 8PE, UK.
- [2] Department of Colorectal Surgery, University Hospital of North Tees, Hardwick Road, Stockton-On-Tees, TS19 8PE, UK. Electronic address: kevin.etherson@nht.nhs.uk.

- PMID: **32931980**
- PMCID: PMC7486821
- DOI: 10.1016/j.ijsu.2020.09.011

Abstract

Introduction: The COVID-19 pandemic has led to changes in NHS surgical service provision, including reduced elective surgical and endoscopic activity, with only essential emergency surgery being undertaken. This, combined with the government-imposed lockdown, may have impacted on patient attendance, severity of surgical disease, and outcomes. The aim of this study was to investigate a possible 'lockdown' effect on the volume and severity of surgical admissions and their outcomes.

Methods: Two separate cohorts of adult emergency general surgery inpatient admissions 30 days immediately before (February 16, 2020 to March 15, 2020), and after UK government advice

(March 16, 2020 to April 15, 2020). Data were collected relating to patient characteristics, severity of disease, clinical outcomes, and compared between these groups.

Results: Following lockdown, a significant reduction in median daily admissions from 7 to 3 per day ($p < 0.001$) was observed. Post-lockdown patients were significantly older, frailer with higher inflammatory indices and rates of acute kidney injury, and also were significantly more likely to present with gastrointestinal cancer, obstruction, and perforation. Patients had significantly higher rates of Clavien-Dindo Grade ≥3 complications ($p = 0.001$), all cause 30-day mortality (8.5% vs. 2.9%, $p = 0.028$), but no significant difference was observed in operative 30-day mortality.

Conclusion: There appears to be a "lockdown" effect on general surgical admissions with a profound impact; fewer surgical admissions, more acutely unwell surgical patients, and an increase in all cause 30-day mortality. Patients should be advised to present promptly with gastrointestinal symptoms, and this should be reinforced for future lockdowns during the pandemic.

Keywords: COVID-19; Emergency surgery; Lockdown effect; Severity; Volume.

Copyright © 2020. Published by Elsevier Ltd.

Conflict of interest statement

Declaration of competing interest Nil.

- 41 references
- 2 figures

Supplementary info

Publication types, MeSH terms Expand

Publication types

- Observational Study

MeSH terms

- Adult
- Aged
- Aged, 80 and over
- COVID-19 / prevention & control*
- Cohort Studies
- Emergencies
- Facilities and Services Utilization / trends*
- Female
- Follow-Up Studies
- General Surgery / trends*
- Hospitalization / trends*

- Humans
- Male
- Middle Aged
- Prospective Studies
- Retrospective Studies
- Severity of Illness Index
- Surgical Procedures, Operative / mortality
- Surgical Procedures, Operative / trends*
- United Kingdom

Full text links

ELSEVIER FULL-TEXT ARTICLE Elsevier Science Free PMC article
Proceed to details
Cite
Share
☐ 760
Observational Study
J Korean Med Sci
. 2021 Jan 18;36(3):e33.
doi: 10.3346/jkms.2021.36.e33.

The Psychological Burden of COVID-19 Stigma: Evaluation of the Mental Health of Isolated Mild Condition COVID-19 Patients

EunKyo Kang [#] [1], Sun Young Lee [#] [1], Min Sun Kim [1] [2], Hyemin Jung [1], Kyae Hyung Kim [1], Kyoung Nam Kim [1], Hye Yoon Park [3], Yu Jin Lee [3], Belong Cho [1] [4], Jee Hoon Sohn [1] [5]
Affiliations Expand

Affiliations

- [1] Public Healthcare Center, Seoul National University Hospital, Seoul, Korea.
- [2] Department of Pediatrics, Seoul National University Hospital, Seoul, Korea.
- [3] Department of Psychiatry, Seoul National University Hospital, Seoul, Korea.
- [4] Department of Family Medicine, Seoul National University Hospital, Seoul National University College of Medicine, Seoul, Korea.
- [5] Department of Psychiatry, Seoul National University Hospital, Seoul, Korea. eliarde@naver.com.

[#] Contributed equally.

- PMID: **33463098**
- PMCID: PMC7813581

- DOI: 10.3346/jkms.2021.36.e33

Free PMC article
Observational Study

The Psychological Burden of COVID-19 Stigma: Evaluation of the Mental Health of Isolated Mild Condition COVID-19 Patients

EunKyo Kang et al. J Korean Med Sci. 2021.
Free PMC article
Show details
J Korean Med Sci
. 2021 Jan 18;36(3):e33.
doi: 10.3346/jkms.2021.36.e33.

Authors

EunKyo Kang [#, 1], Sun Young Lee [#, 1], Min Sun Kim [1, 2], Hyemin Jung [1], Kyae Hyung Kim [1], Kyoung Nam Kim [1], Hye Yoon Park [3], Yu Jin Lee [3], Belong Cho [1, 4], Jee Hoon Sohn [1, 5]

Affiliations

- [1] Public Healthcare Center, Seoul National University Hospital, Seoul, Korea.
- [2] Department of Pediatrics, Seoul National University Hospital, Seoul, Korea.
- [3] Department of Psychiatry, Seoul National University Hospital, Seoul, Korea.
- [4] Department of Family Medicine, Seoul National University Hospital, Seoul National University College of Medicine, Seoul, Korea.
- [5] Department of Psychiatry, Seoul National University Hospital, Seoul, Korea. eliarde@naver.com.

[#] Contributed equally.

- PMID: **33463098**
- PMCID: PMC7813581
- DOI: 10.3346/jkms.2021.36.e33

Abstract

Background: The objective of this article is to assess the mental health issues of the mild condition coronavirus disease 2019 (COVID-19) patients admitted to a community treatment center (CTC) in Korea.

Methods: A total of 107 patients admitted to a CTC were included as the study population, and their mental health problems including depression (patient health questionnaire-9), anxiety (generalized anxiety disorder scale-7), post-traumatic stress disorder (PTSD) (PTSD checklist-5) and somatic symptoms (by patient health questionnaire-15) were evaluated every week during

their stay. The stigma related to COVID-19 infection was evaluated with an adjusted version of the Middle East respiratory syndrome (MERS) stigma scale.

Results: During the first week of isolation, the prevalence of more-than-moderate depression was 24.3%, more-than-moderate anxiety was 14.9%, more-than-moderate somatic symptoms was 36.5% and possible PTSD was 5.6% of total population. For depression and anxiety, previous psychiatric history and stigma of COVID-19 infection were significant risk factors. For PTSD, previous psychiatric history and stigma of COVID-19 infection as well as total duration of isolation were found to be significant risk factors. Prevalence of depression, anxiety and possible PTSD remained similar across the four weeks of observations, though the prevalence of severe depression, increased after four weeks of stay. Somatic symptoms seemed to decrease during their stay.

Conclusion: The results suggest that social mitigation of COVID-19 related stigma, as well as care of patients with pre-existing mental health problems are important mental health measures during this crisis period. It is also important that clinical guidelines and public health policies be well balanced over the protection of the public and those quarantined to minimize the negative psychosocial consequences from isolation of the patients.

Keywords: Anxiety; COVID-19; Depression; Isolation; Stigma.

© 2021 The Korean Academy of Medical Sciences.

Conflict of interest statement

The authors have no potential conflicts of interest to disclose.

- 35 references
- 3 figures

Supplementary info

Publication types, MeSH terms Expand

Publication types

- Observational Study

MeSH terms

- Adult
- Anxiety / epidemiology
- COVID-19 / epidemiology
- COVID-19 / psychology*
- Depression / epidemiology
- Female
- Humans
- Male
- Mental Health*

- Middle Aged
- Pandemics*
- Patient Health Questionnaire
- Patient Isolation / psychology*
- Prevalence
- Quarantine / psychology
- Republic of Korea / epidemiology
- Retrospective Studies
- SARS-CoV-2*
- Social Stigma*
- Stress Disorders, Post-Traumatic / epidemiology

Full text links

JKMS FULL TEXT FREE Journal of Korean Medical Science Korean Academy of Medical Sciences Free PMC article
Proceed to details
Cite
Share
☐ 761
Observational Study
J Med Virol
. 2021 May;93(5):2631-2634.
doi: 10.1002/jmv.26898. Epub 2021 Mar 3.

Potential remdesivir-related transient bradycardia in patients with coronavirus disease 2019 (COVID-19)

Carlo Pallotto [1] [2], Lorenzo R Suardi [1], Andrea Gabbuti [1], Sara Esperti [1], Lorenzo Mecocci [1], Pierluigi Blanc [1]
Affiliations Expand

Affiliations

- [1] Infectious Diseases Unit 1, Santa Maria Annunziata Hospital, Azienda USL Toscana Centro, Florence, Italy.
- [2] Section of Infectious Diseases, Department of Medicine, University of Perugia, Perugia, Italy.

- PMID: **33620107**
- PMCID: PMC8013491
- DOI: 10.1002/jmv.26898

Free PMC article

Observational Study

Potential remdesivir-related transient bradycardia in patients with coronavirus disease 2019 (COVID-19)

Carlo Pallotto et al. J Med Virol. 2021 May.
Free PMC article
Show details
J Med Virol
. 2021 May;93(5):2631-2634.
doi: 10.1002/jmv.26898. Epub 2021 Mar 3.

Authors

Carlo Pallotto [1, 2], Lorenzo R Suardi [1], Andrea Gabbuti [1], Sara Esperti [1], Lorenzo Mecocci [1], Pierluigi Blanc [1]

Affiliations

- [1] Infectious Diseases Unit 1, Santa Maria Annunziata Hospital, Azienda USL Toscana Centro, Florence, Italy.
- [2] Section of Infectious Diseases, Department of Medicine, University of Perugia, Perugia, Italy.

- PMID: **33620107**
- PMCID: PMC8013491
- DOI: 10.1002/jmv.26898

No abstract available

Conflict of interest statement

Carlo Pallotto has received funds for speaking at symposia organized on behalf of Merck, Angelini, and Bristol Myers Squibb.

Comment in

- Remdesivir might induce changes in electrocardiogram beyond bradycardia in patients with coronavirus disease 2019-The pilot study.
  Bistrovic P, Lucijanic M. Bistrovic P, et al. J Med Virol. 2021 Oct;93(10):5724-5725. doi: 10.1002/jmv.27177. Epub 2021 Jul 14. J Med Virol. 2021. PMID: 34232520 Free PMC article. No abstract available.

- 9 references

Supplementary info

Publication types, MeSH terms, Substances, Supplementary concepts Expand

Publication types

- Letter
- Observational Study

MeSH terms

- Adenosine Monophosphate / administration & dosage
- Adenosine Monophosphate / adverse effects
- Adenosine Monophosphate / analogs & derivatives*
- Aged
- Alanine / administration & dosage
- Alanine / adverse effects
- Alanine / analogs & derivatives*
- Bradycardia / epidemiology
- Bradycardia / etiology*
- COVID-19 / drug therapy*
- COVID-19 / epidemiology
- Female
- Humans
- Male
- Middle Aged
- Retrospective Studies
- Risk Factors
- SARS-CoV-2
- Treatment Outcome

Substances

- remdesivir
- Adenosine Monophosphate
- Alanine

Supplementary concepts

- COVID-19 drug treatment

Full text links

WILEY Full Text Article Wiley Free PMC article

Proceed to details
Cite
Share
☐ 762
Observational Study
Clin Immunol
. 2021 Sep;230:108818.
doi: 10.1016/j.clim.2021.108818. Epub 2021 Aug 4.

Guillain-Barré syndrome is infrequent among recipients of the BNT162b2 mRNA COVID-19 vaccine

Miguel García-Grimshaw [1], Anaclara Michel-Chávez [1], Juan Mauricio Vera-Zertuche [2], Javier Andrés Galnares-Olalde [3], Laura E Hernández-Vanegas [3], Melissa Figueroa-Cucurachi [4], Orlando Paredes-Ceballos [4], Gustavo Reyes-Terán [5], Guillermo Carbajal-Sandoval [5], Santa Elizabeth Ceballos-Liceaga [5], Antonio Arauz [3], Sergio Iván Valdés-Ferrer [6]
Affiliations Expand

Affiliations

- [1] Department of Neurology and Psychiatry, Instituto Nacional de Ciencias Médicas y Nutrición Salvador Zubirán, Mexico City, Mexico.
- [2] Department of Endocrinology, Instituto Nacional de Ciencias Médicas y Nutrición Salvador Zubirán, Mexico City, Mexico.
- [3] Department of Neurology, Instituto Nacional de Neurología y Neurocirugía Manuel Velasco Suárez, Mexico City, Mexico.
- [4] Hospital Faro del Mayab, Merida, Yucatán, Mexico.
- [5] Secretaría de Salud, Gobierno de México, Mexico.
- [6] Department of Neurology and Psychiatry, Instituto Nacional de Ciencias Médicas y Nutrición Salvador Zubirán, Mexico City, Mexico; Department of Infectious Diseases, Instituto Nacional de Ciencias Médicas y Nutrición Salvador Zubirán, Mexico City, Mexico; Feinstein Institutes for Medical Research, Manhasset, NY, USA. Electronic address: sergio.valdesf@incmnsz.mx.

- PMID: **34358692**
- PMCID: PMC8332675
- DOI: 10.1016/j.clim.2021.108818

Free PMC article
Observational Study

Guillain-Barré syndrome is infrequent among recipients of the BNT162b2 mRNA COVID-19 vaccine

Miguel García-Grimshaw et al. Clin Immunol. 2021 Sep.
Free PMC article
Show details
Clin Immunol
. 2021 Sep;230:108818.
doi: 10.1016/j.clim.2021.108818. Epub 2021 Aug 4.

Authors

Miguel García-Grimshaw [1], Anaclara Michel-Chávez [1], Juan Mauricio Vera-Zertuche [2], Javier Andrés Galnares-Olalde [3], Laura E Hernández-Vanegas [3], Melissa Figueroa-Cucurachi [4], Orlando Paredes-Ceballos [4], Gustavo Reyes-Terán [5], Guillermo Carbajal-Sandoval [5], Santa Elizabeth Ceballos-Liceaga [5], Antonio Arauz [3], Sergio Iván Valdés-Ferrer [6]

Affiliations

- [1] Department of Neurology and Psychiatry, Instituto Nacional de Ciencias Médicas y Nutrición Salvador Zubirán, Mexico City, Mexico.
- [2] Department of Endocrinology, Instituto Nacional de Ciencias Médicas y Nutrición Salvador Zubirán, Mexico City, Mexico.
- [3] Department of Neurology, Instituto Nacional de Neurología y Neurocirugía Manuel Velasco Suárez, Mexico City, Mexico.
- [4] Hospital Faro del Mayab, Merida, Yucatán, Mexico.
- [5] Secretaría de Salud, Gobierno de México, Mexico.
- [6] Department of Neurology and Psychiatry, Instituto Nacional de Ciencias Médicas y Nutrición Salvador Zubirán, Mexico City, Mexico; Department of Infectious Diseases, Instituto Nacional de Ciencias Médicas y Nutrición Salvador Zubirán, Mexico City, Mexico; Feinstein Institutes for Medical Research, Manhasset, NY, USA. Electronic address: sergio.valdesf@incmnsz.mx.

- PMID: **34358692**
- PMCID: PMC8332675
- DOI: 10.1016/j.clim.2021.108818

Abstract

Vaccines are the most effective strategy to mitigate the global impact of COVID-19. However, vaccine hesitancy is common, particularly among minorities. Guillain-Barré syndrome (GBS) is the most common autoimmune illness of the peripheral nervous system, occurring at an incidence of 1.1/100,000 worldwide. A causal link between mRNA vaccines and GBS has not been previously evaluated. We analyzed a cohort of 3,890,250 Hispanic/Latinx recipients of the BNT162b2 mRNA vaccine (613,780 of whom had already received both doses) for incident GBS occurring within 30 days from vaccine administration. Seven cases of GBS were detected among

first-dose recipients, for an observed incidence of 0.18/100,000 administered doses during the prespecified timeframe of 30 days. No cases were reported after second-dose administration. Our data suggest that, among recipients of the BNT162b2 mRNA vaccine, GBS may occur at the expected community-based rate; however, this should be taken with caution as the current incidence of GBS among the unvaccinated population against COVID-19 is still undetermined. We hope that this preliminary data will increase the public perception of safety toward mRNA-based vaccines and reduce vaccine hesitancy.

Keywords: COVID-19 vaccine; Guillain-Barré syndrome; Vaccine hesitancy; Vaccine safety; mRNA vaccines.

Copyright © 2021 Elsevier Inc. All rights reserved.

- 20 references

Supplementary info

Publication types, MeSH terms, Substances Expand

Publication types

- Observational Study
- Research Support, Non-U.S. Gov't

MeSH terms

- COVID-19 / prevention & control*
- COVID-19 Vaccines / adverse effects*
- COVID-19 Vaccines / immunology*
- Cohort Studies
- Guillain-Barre Syndrome / etiology*
- Humans
- Retrospective Studies
- SARS-CoV-2*

Substances

- COVID-19 Vaccines

Full text links

ELSEVIER FULL-TEXT ARTICLE Elsevier Science Free PMC article
Proceed to details
Cite
Share
☐ 763

Observational Study
J Clin Virol
. 2020 Oct;131:104611.
doi: 10.1016/j.jcv.2020.104611. Epub 2020 Sep 1.

SARS-CoV-2 antibodies, serum inflammatory biomarkers and clinical severity of hospitalized COVID-19 patients

Roberto Gozalbo-Rovira [1], Estela Gimenez [2], Víctor Latorre [3], Clara Francés-Gómez [3], Eliseo Albert [2], Javier Buesa [4], Alberto Marina [5], María Luisa Blasco [6], Jaime Signes-Costa [7], Jesús Rodríguez-Díaz [8], Ron Geller [9], David Navarro [10]
Affiliations Expand

Affiliations

- [1] Department of Microbiology, School of Medicine, University of Valencia, Valencia, Spain.
- [2] Microbiology Service, Clinic University Hospital, INCLIVA Health Research Institute, Valencia, Spain.
- [3] Institute for Integrative Systems Biology (I2SysBio), Universitatde Valencia-CSIC, 46980, Valencia, Spain.
- [4] Department of Microbiology, School of Medicine, University of Valencia, Valencia, Spain; Microbiology Service, Clinic University Hospital, INCLIVA Health Research Institute, Valencia, Spain.
- [5] Department of Genomics and Proteomics, Instituto de Biomedicina de Valencia (IBV-CSIC) and CIBER de Enfermedades Raras (CIBERER-ISCIII), Valencia, Spain; Decoy-SARS-CoV-2 Study Group from the Institute of Biomedicine of Valencia (Group Members List in Acknowledgements Section), Spain.
- [6] Medical Intensive Care Unit, Clinic University Hospital, INCLIVA Health Research Institute, Valencia, Spain.
- [7] Pneumology Service, Clinic University Hospital, INCLIVA Health Research Institute, Valencia, Spain.
- [8] Department of Microbiology, School of Medicine, University of Valencia, Valencia, Spain. Electronic address: jesus.rodriguez@uv.es.
- [9] Institute for Integrative Systems Biology (I2SysBio), Universitatde Valencia-CSIC, 46980, Valencia, Spain. Electronic address: ron.geller@uv.es.
- [10] Department of Microbiology, School of Medicine, University of Valencia, Valencia, Spain; Microbiology Service, Clinic University Hospital, INCLIVA Health Research Institute, Valencia, Spain. Electronic address: david.navarro@uv.es.

- PMID: **32882666**
- PMCID: PMC7459327
- DOI: 10.1016/j.jcv.2020.104611

Free PMC article
Observational Study

SARS-CoV-2 antibodies, serum inflammatory biomarkers and clinical severity of hospitalized COVID-19 patients

Roberto Gozalbo-Rovira et al. J Clin Virol. 2020 Oct.
Free PMC article
Show details
J Clin Virol
. 2020 Oct;131:104611.
doi: 10.1016/j.jcv.2020.104611. Epub 2020 Sep 1.

Authors

Roberto Gozalbo-Rovira [1], Estela Gimenez [2], Víctor Latorre [3], Clara Francés-Gómez [3], Eliseo Albert [2], Javier Buesa [4], Alberto Marina [5], María Luisa Blasco [6], Jaime Signes-Costa [7], Jesús Rodríguez-Díaz [8], Ron Geller [9], David Navarro [10]

Affiliations

- [1] Department of Microbiology, School of Medicine, University of Valencia, Valencia, Spain.
- [2] Microbiology Service, Clinic University Hospital, INCLIVA Health Research Institute, Valencia, Spain.
- [3] Institute for Integrative Systems Biology (I2SysBio), Universitatde Valencia-CSIC, 46980, Valencia, Spain.
- [4] Department of Microbiology, School of Medicine, University of Valencia, Valencia, Spain; Microbiology Service, Clinic University Hospital, INCLIVA Health Research Institute, Valencia, Spain.
- [5] Department of Genomics and Proteomics, Instituto de Biomedicina de Valencia (IBV-CSIC) and CIBER de Enfermedades Raras (CIBERER-ISCIII), Valencia, Spain; Decoy-SARS-CoV-2 Study Group from the Institute of Biomedicine of Valencia (Group Members List in Acknowledgements Section), Spain.
- [6] Medical Intensive Care Unit, Clinic University Hospital, INCLIVA Health Research Institute, Valencia, Spain.
- [7] Pneumology Service, Clinic University Hospital, INCLIVA Health Research Institute, Valencia, Spain.
- [8] Department of Microbiology, School of Medicine, University of Valencia, Valencia, Spain. Electronic address: jesus.rodriguez@uv.es.
- [9] Institute for Integrative Systems Biology (I2SysBio), Universitatde Valencia-CSIC, 46980, Valencia, Spain. Electronic address: ron.geller@uv.es.
- [10] Department of Microbiology, School of Medicine, University of Valencia, Valencia, Spain; Microbiology Service, Clinic University Hospital, INCLIVA Health Research Institute, Valencia, Spain. Electronic address: david.navarro@uv.es.

- PMID: **32882666**
- PMCID: PMC7459327
- DOI: 10.1016/j.jcv.2020.104611

Abstract

Background: The involvement of SARS-CoV-2 antibodies in mediating immunopathogenetic events in COVID-19 patients has been suggested. By using several experimental approaches, we investigated the potential association between SARS-CoV-2 IgGs recognizing the spike (S) protein receptor-binding domain (RBD), neutralizing antibodies (NtAb) targeting S, and COVID-19 severity.

Patients and methods: This unicenter, retrospective, observational study included 51 hospitalized patients (24 at the intensive care unit; ICU). A total of 93 sera from these patients collected at different time points from the onset of symptoms were analyzed. SARS-CoV-2 RBD IgGs were quantitated by ELISA and NtAb50 titers were measured in a GFP reporterbased pseudotyped virus platform. Demographic and clinical data, complete blood counts, as well as serum levels of ferritin, Dimer-D, C reactive protein (CRP), lactose dehydrogenase (LDH), and interleukin-6 (IL-6) were retrieved from clinical charts.

Results: The overall correlation between levels of both antibody measurements was good (Rho = 0.82; P = 0 < 0.001). SARS-CoV-2 RBD IgG and NtAb50 levels in sera collected up to day 30 after the onset of symptoms were comparable between ICU and non-ICU patients (P=>0.1). Four ICU patients died; two of these achieved NtAb50 titers ≥1/160 while the other two exhibited a 1/80 titer. Very weak (Rho=>0.0-<0.2) or weak (Rho=>0.2-<0.4) correlations were observed between anti-RBD IgGs, NtAb50, and serum levels pro-inflammatory biomarkers.

Conclusions: The data presented herein do not support an association between SARS-CoV-2 RBD IgG or NtAb50 levels and COVID-19 severity.

Keywords: COVID-19; Inflammatory biomarkers; Neutralizing antibodies; SARS-CoV-2.

Copyright © 2020 Elsevier B.V. All rights reserved.

Conflict of interest statement

The authors report no declarations of interest.

- 40 references
- 8 figures

Supplementary info

Publication types, MeSH terms, Substances Expand

Publication types

- Observational Study
- Research Support, Non-U.S. Gov't

MeSH terms

- Adult
- Aged

- Antibodies, Neutralizing / blood
- Antibodies, Viral / blood*
- Betacoronavirus
- Binding Sites, Antibody
- Biomarkers / blood
- COVID-19
- Coronavirus Infections / blood*
- Coronavirus Infections / immunology
- Female
- Hospitalization / statistics & numerical data*
- Humans
- Inflammation / blood*
- Inflammation / virology
- Male
- Middle Aged
- Pandemics
- Pneumonia, Viral / blood*
- Pneumonia, Viral / immunology
- Retrospective Studies
- SARS-CoV-2
- Severity of Illness Index
- Spike Glycoprotein, Coronavirus / immunology
- Young Adult

Substances

- Antibodies, Neutralizing
- Antibodies, Viral
- Biomarkers
- Spike Glycoprotein, Coronavirus
- spike protein, SARS-CoV-2

Full text links

ELSEVIER FULL-TEXT ARTICLE Elsevier Science Free PMC article
Proceed to details
Cite
Share
764
Observational Study
Ren Fail
. 2021 Dec;43(1):911-918.
doi: 10.1080/0886022X.2021.1933530.

Outcomes of critically ill patients with acute kidney injury in COVID-19 infection: an observational study

Rodrigo Bezerra [1, 2], Flávio Teles [3], Polyana Bezerra Mendonca [4], Tedla Damte [5], Andrew Likaka [1], Edyniesky Ferrer-Miranda [6], Jones Oliveira de Albuquerque [1, 7], José Luiz de Lima Filho [1, 8]
Affiliations Expand

Affiliations

- [1] Keizo Asami Laboratory of Immunopathology, Federal University of Pernambuco, Recife, Brazil.
- [2] PROCAPE, University of Pernambuco, Recife, Brazil.
- [3] Clinical Medicine Department, Federal University of Alagoas, Maceio, Brazil.
- [4] Federal University of Pernambuco, Recife, Brazil.
- [5] Health department, UNICEF, Lilongwe, Malawi.
- [6] Federal Rural University of Pernambuco, Recife, Brazil.
- [7] Department of Statistics and Informatics, Federal Rural University of Pernambuco, Recife, Brazil.
- [8] Department of Biochemistry, Federal University of Pernambuco, Recife, Brazil.

- PMID: **34057014**
- PMCID: PMC8168780
- DOI: 10.1080/0886022X.2021.1933530

Free PMC article
Observational Study

Outcomes of critically ill patients with acute kidney injury in COVID-19 infection: an observational study

Rodrigo Bezerra et al. Ren Fail. 2021 Dec.
Free PMC article
Show details
Ren Fail
. 2021 Dec;43(1):911-918.
doi: 10.1080/0886022X.2021.1933530.

Authors

Rodrigo Bezerra [1] [2], Flávio Teles [3], Polyana Bezerra Mendonca [4], Tedla Damte [5], Andrew Likaka [1], Edyniesky Ferrer-Miranda [6], Jones Oliveira de Albuquerque [1] [7], José Luiz de Lima Filho [1] [8]

Affiliations

- [1] Keizo Asami Laboratory of Immunopathology, Federal University of Pernambuco, Recife, Brazil.
- [2] PROCAPE, University of Pernambuco, Recife, Brazil.
- [3] Clinical Medicine Department, Federal University of Alagoas, Maceio, Brazil.
- [4] Federal University of Pernambuco, Recife, Brazil.
- [5] Health department, UNICEF, Lilongwe, Malawi.
- [6] Federal Rural University of Pernambuco, Recife, Brazil.
- [7] Department of Statistics and Informatics, Federal Rural University of Pernambuco, Recife, Brazil.
- [8] Department of Biochemistry, Federal University of Pernambuco, Recife, Brazil.

- PMID: **34057014**
- PMCID: PMC8168780
- DOI: 10.1080/0886022X.2021.1933530

Abstract

Background: Early reports indicate that AKI is common during COVID-19 infection. Different mortality rates of AKI due to SARS-CoV-2 have been reported, based on the degree of organic dysfunction and varying from public to private hospitals. However, there is a lack of data about AKI among critically ill patients with COVID-19.

Methods: We conducted a multicenter cohort study of 424 critically ill adults with severe acute respiratory syndrome (SARS) and AKI, both associated with SARS-CoV-2, admitted to six public ICUs in Brazil. We used multivariable logistic regression to identify risk factors for AKI severity and in-hospital mortality.

Results: The average age was 66.42 ± 13.79 years, 90.3% were on mechanical ventilation (MV), 76.6% were at KDIGO stage 3, and 79% underwent hemodialysis. The overall mortality was 90.1%. We found a higher frequency of dialysis (82.7% versus 45.2%), MV (95% versus 47.6%), vasopressors (81.2% versus 35.7%) ($p < 0.001$) and severe AKI (79.3% versus 52.4%; $p = 0.002$) in nonsurvivors. MV, vasopressors, dialysis, sepsis-associated AKI, and death ($p < 0.001$) were more frequent in KDIGO 3. Logistic regression for death demonstrated an association with MV (OR = 8.44; CI 3.43-20.74) and vasopressors (OR = 2.93; CI 1.28-6.71; $p < 0.001$). Severe AKI and dialysis need were not independent risk factors for death. MV (OR = 2.60; CI 1.23-5.45) and vasopressors (OR = 1.95; CI 1.12-3.99) were also independent risk factors for KDIGO 3 ($p <$ 0.001).

Conclusion: Critically ill patients with SARS and AKI due to COVID-19 had high mortality in this cohort. Mortality was largely determined by the need for mechanical ventilation and vasopressors rather than AKI severity.

Keywords: COVID-19; acute kidney injury; critical care; dialysis; mortality risk; renal failure.

Conflict of interest statement

No potential conflict of interest was reported by the author(s).

- 26 references
- 1 figure

Supplementary info

Publication types, MeSH terms Expand

Publication types

- Multicenter Study
- Observational Study
- Video-Audio Media

MeSH terms

- Acute Kidney Injury / mortality
- Acute Kidney Injury / therapy*
- Acute Kidney Injury / virology*
- Aged
- Brazil / epidemiology
- COVID-19 / complications*
- COVID-19 / mortality
- COVID-19 / therapy
- Critical Illness*
- Female
- Hospital Mortality
- Humans
- Intensive Care Units
- Male
- Pneumonia, Viral / mortality
- Pneumonia, Viral / therapy
- Pneumonia, Viral / virology
- Renal Dialysis*
- Respiration, Artificial
- Retrospective Studies
- Risk Factors
- SARS-CoV-2

Full text links

View full text Taylor & Francis Free PMC article
Proceed to details
Cite
Share
765
Observational Study
PLoS One
. 2020 Jul 23;15(7):e0236778.
doi: 10.1371/journal.pone.0236778. eCollection 2020.

Safety, tolerability, and clinical outcomes of hydroxychloroquine for hospitalized patients with coronavirus 2019 disease

Michael J Satlin [1,2], Parag Goyal [1,2], Reed Magleby [2], Grace A Maldarelli [2], Khanh Pham [2], Maiko Kondo [1], Edward J Schenck [1,2], Hanna Rennert [2,3], Lars F Westblade [1,2,3], Justin J Choi [1,2], Monika M Safford [1,2], Roy M Gulick [1,2]
Affiliations Expand

Affiliations

- [1] Department of Medicine, Weill Cornell Medicine, New York, NY, United States of America.
- [2] NewYork-Presbyterian Hospital, Weill Cornell Medical Center, New York, NY, United States of America.
- [3] Department of Pathology and Laboratory Medicine, Weill Cornell Medicine, New York, NY, United States of America.

- PMID: **32701969**
- PMCID: PMC7377460
- DOI: 10.1371/journal.pone.0236778

Free PMC article
Observational Study

Safety, tolerability, and clinical outcomes of hydroxychloroquine for hospitalized patients with coronavirus 2019 disease

Michael J Satlin et al. PLoS One. 2020.
Free PMC article
Show details
PLoS One

. 2020 Jul 23;15(7):e0236778.
doi: 10.1371/journal.pone.0236778. eCollection 2020.

Authors

Michael J Satlin [1 2], Parag Goyal [1 2], Reed Magleby [2], Grace A Maldarelli [2], Khanh Pham [2], Maiko Kondo [1], Edward J Schenck [1 2], Hanna Rennert [2 3], Lars F Westblade [1 2 3], Justin J Choi [1 2], Monika M Safford [1 2], Roy M Gulick [1 2]

Affiliations

- [1] Department of Medicine, Weill Cornell Medicine, New York, NY, United States of America.
- [2] NewYork-Presbyterian Hospital, Weill Cornell Medical Center, New York, NY, United States of America.
- [3] Department of Pathology and Laboratory Medicine, Weill Cornell Medicine, New York, NY, United States of America.

- PMID: **32701969**
- PMCID: PMC7377460
- DOI: 10.1371/journal.pone.0236778

Abstract

Background: Severe acute respiratory coronavirus 2 (SARS-CoV-2) has caused a devastating worldwide pandemic. Hydroxychloroquine (HCQ) has in vitro activity against SARS-CoV-2, but clinical data supporting HCQ for coronavirus disease 2019 (COVID-19) are limited.

Methods: This was a retrospective cohort study of hospitalized patients with COVID-19 who received ≥1 dose of HCQ at two New York City hospitals. We measured incident Grade 3 or 4 blood count and liver test abnormalities, ventricular arrhythmias, and vomiting and diarrhea within 10 days after HCQ initiation, and the proportion of patients who completed HCQ therapy. We also describe changes in Sequential Organ Failure Assessment hypoxia scores between baseline and day 10 after HCQ initiation and in-hospital mortality.

Results: None of the 153 hospitalized patients with COVID-19 who received HCQ developed a sustained ventricular tachyarrhythmia. Incident blood count and liver test abnormalities occurred in <15% of patients and incident vomiting or diarrhea was rare. Eighty-nine percent of patients completed their HCQ course and three patients discontinued therapy because of QT prolongation. Fifty-two percent of patients had improved hypoxia scores 10 days after starting HCQ. Thirty-one percent of patients who were receiving mechanical ventilation at the time of HCQ initiation died during their hospitalization, compared to 18% of patients who were receiving supplemental oxygen but not requiring mechanical ventilation, and 8% of patients who were not requiring supplemental oxygen. Co-administration of azithromycin was not associated with improved outcomes.

Conclusions: HCQ appears to be reasonably safe and tolerable in most hospitalized patients with COVID-19. However, nearly one-half of patients did not improve with this treatment, highlighting the need to evaluate HCQ and alternate therapies in randomized trials.

Conflict of interest statement

The authors have declared that no competing interests exist.

- 31 references
- 2 figures

Supplementary info

Publication types, MeSH terms, Substances, Grant support Expand

Publication types

- Multicenter Study
- Observational Study
- Research Support, N.I.H., Extramural

MeSH terms

- Adult
- Aged
- Azithromycin / therapeutic use
- Betacoronavirus
- COVID-19
- COVID-19 Testing
- Clinical Laboratory Techniques
- Coronavirus Infections / diagnosis
- Coronavirus Infections / drug therapy*
- Female
- Humans
- Hydroxychloroquine / adverse effects
- Hydroxychloroquine / therapeutic use*
- Male
- Middle Aged
- New York City
- Pandemics
- Pneumonia, Viral / drug therapy*
- Retrospective Studies
- SARS-CoV-2
- Treatment Outcome

Substances

- Hydroxychloroquine
- Azithromycin

Grant support

- T32 AI007613/AI/NIAID NIH HHS/United States
- UL1 TR002384/TR/NCATS NIH HHS/United States

Full text links

OPEN ACCESS TO FULL TEXT PLOS ONE Public Library of Science Free PMC article

Proceed to details

Cite

Share

766

Observational Study

Thromb Haemost

. 2022 Jan;122(1):105-112.

doi: 10.1055/a-1503-3875. Epub 2021 May 7.

COVID-19 and Atrial Fibrillation in Older Patients: Does Oral Anticoagulant Therapy Provide a Survival Benefit?-An Insight from the GeroCovid Registry

Stefano Fumagalli [1], Caterina Trevisan [2], Susanna Del Signore [3], Giulia Pelagalli [1], Stefano Volpato [4], Pietro Gareri [5], Enrico Mossello [1], Alba Malara [6], Fabio Monzani [7], Alessandra Coin [2], Giuseppe Bellelli [8], Gianluca Zia [3], Raffaele Antonelli Incalzi [9], GeroCovid Working Group

Affiliations Expand

Affiliations

- [1] Geriatric Intensive Care Unit and Geriatric Arrhythmia Unit, Department of Experimental and Clinical Medicine, University of Florence and AOU Careggi, Florence, Italy.
- [2] Geriatrics Division, Department of Medicine (DIMED), University of Padua, Padua, Italy.
- [3] Bluecompanion ltd, London, United Kingdom.
- [4] Section of Internal and Cardiorespiratory Medicine, Department of Medical Science, University of Ferrara, Ferrara, Italy.
- [5] Center for Cognitive Disorders and Dementia - Catanzaro Lido, ASP Catanzaro, Catanzaro, Italy.
- [6] Scientific Committee of National Association of Third Age Residences (ANASTE) Calabria, Lamezia Terme (Catanzaro), Catanzaro, Italy.

- [7] Geriatrics Unit, Department of Clinical and Experimental Medicine, University of Pisa, Pisa, Italy.
- [8] Acute Geriatric Unit, San Gerardo Hospital, School of Medicine and Surgery, University of Milano-Bicocca, Monza, Italy.
- [9] Unit of Geriatrics, Department of Medicine, Campus Bio Medico University and Teaching Hospital, Rome, Italy.

- PMID: **33962480**
- DOI: 10.1055/a-1503-3875

Observational Study

COVID-19 and Atrial Fibrillation in Older Patients: Does Oral Anticoagulant Therapy Provide a Survival Benefit?-An Insight from the GeroCovid Registry

Stefano Fumagalli et al. Thromb Haemost. 2022 Jan.

Show details

Thromb Haemost

. 2022 Jan;122(1):105-112.
doi: 10.1055/a-1503-3875. Epub 2021 May 7.

Authors

Stefano Fumagalli [1], Caterina Trevisan [2], Susanna Del Signore [3], Giulia Pelagalli [1], Stefano Volpato [4], Pietro Gareri [5], Enrico Mossello [1], Alba Malara [6], Fabio Monzani [7], Alessandra Coin [2], Giuseppe Bellelli [8], Gianluca Zia [3], Raffaele Antonelli Incalzi [9], GeroCovid Working Group

Affiliations

- [1] Geriatric Intensive Care Unit and Geriatric Arrhythmia Unit, Department of Experimental and Clinical Medicine, University of Florence and AOU Careggi, Florence, Italy.
- [2] Geriatrics Division, Department of Medicine (DIMED), University of Padua, Padua, Italy.
- [3] Bluecompanion ltd, London, United Kingdom.
- [4] Section of Internal and Cardiorespiratory Medicine, Department of Medical Science, University of Ferrara, Ferrara, Italy.
- [5] Center for Cognitive Disorders and Dementia - Catanzaro Lido, ASP Catanzaro, Catanzaro, Italy.
- [6] Scientific Committee of National Association of Third Age Residences (ANASTE) Calabria, Lamezia Terme (Catanzaro), Catanzaro, Italy.
- [7] Geriatrics Unit, Department of Clinical and Experimental Medicine, University of Pisa, Pisa, Italy.
- [8] Acute Geriatric Unit, San Gerardo Hospital, School of Medicine and Surgery, University of Milano-Bicocca, Monza, Italy.

- [9] Unit of Geriatrics, Department of Medicine, Campus Bio Medico University and Teaching Hospital, Rome, Italy.

- PMID: **33962480**
- DOI: 10.1055/a-1503-3875

Abstract

Introduction: Atrial fibrillation (AF), the most frequent arrhythmia of older patients, associates with serious thromboembolic complications and high mortality. Coronavirus disease 2019 (COVID-19) severely affects aged subjects, determining an important prothrombotic status. The aim of this study was to evaluate mortality-related factors in older AF patients with COVID-19.

Methods: Between March and June 2020, we enrolled ≥60 year-old in-hospital COVID-19 patients (n = 806) in GeroCovid, a multicenter observational study promoted by the Italian Society of Gerontology and Geriatric Medicine.

Results: The prevalence of AF was 21.8%. In-hospital mortality was higher in the AF group (36.9 vs. 27.5%, $p = 0.015$). At admission, 51.7, 10.2, and 38.1% of AF cases were taking, respectively, oral anticoagulants (OACs), antiplatelet agents, and no antithrombotic therapy. During hospitalization, 51% patients switched to low-molecular-weight heparins. AF patients who survived were younger (81 ± 8 vs. 84 ± 7 years; $p = 0.002$) and had a lower CHA_2DS_2-VASc score (3.9 ± 1.6 vs. 4.4 ± 1.3; $p = 0.02$) than those who died. OAC use before (63.1 vs. 32.3%; $p < 0.001$) and during hospitalization (34.0 vs. 12.7%; $p = 0.002$) was higher among survivors. At multivariable analysis, lower age, higher self-sufficiency, less severe initial COVID-19 presentation, and the use of vitamin K antagonists (odds ratio [OR] = 0.16, 95% confidence interval [CI]: 0.03-0.84) or direct OACs (OR = 0.22, 95% CI: 0.08-0.56) at admission, or the persistence of OAC during hospitalization (OR = 0.05, 95% CI: 0.01-0.24), were associated with a lower chance of in-hospital death.

Conclusion: AF is a prevalent and severe condition in older COVID-19 patients. Advanced age, dependency, and relevant clinical manifestations of disease characterized a worse prognosis. Preadmission and in-hospital anticoagulant therapies were positively associated with survival.

Thieme. All rights reserved.

Conflict of interest statement

None declared.

Supplementary info

Publication types, MeSH terms, Substances Expand

Publication types

- Multicenter Study
- Observational Study

MeSH terms

- Administration, Oral
- Aged
- Aged, 80 and over
- Anticoagulants / administration & dosage
- Anticoagulants / therapeutic use
- Atrial Fibrillation / complications*
- Atrial Fibrillation / drug therapy
- Atrial Fibrillation / mortality
- COVID-19 / complications*
- COVID-19 / drug therapy
- COVID-19 / mortality
- Female
- Hospital Mortality
- Humans
- Italy / epidemiology
- Logistic Models
- Male
- Middle Aged
- Multivariate Analysis
- Odds Ratio
- Prevalence
- Registries
- Retrospective Studies
- Risk Factors
- SARS-CoV-2*

Substances

- Anticoagulants

Full text links

Full text THIEME CONNECT Georg Thieme Verlag Stuttgart, New York

Proceed to details

Cite

Share

☐ 767

Observational Study

Eur J Cardiothorac Surg

. 2020 Oct 1;58(4):738-744.

doi: 10.1093/ejcts/ezaa287.

Clinical features and outcomes of thoracic surgery patients during the COVID-19 pandemic

María Salmerón Jiménez [1], Fátima Hermoso Alarza [1], Ivan Martínez Serna [1], Carmen Marrón Fernández [1], José Carlos Meneses Pardo [1], José Alberto García Salcedo [1], Alejandro Torres Serna [1], Mario Gustavo Manama Gama [1], Oscar Enrique Colmenares Mendoza [1], Vicente Diaz-Hellín Gude [1], Antonio Pablo Gamez García [1]
Affiliations Expand

Affiliation

- [1] Department of Thoracic Surgery, 12 de Octubre University Hospital, Universidad Complutense de Madrid, Madrid, Spain.

- PMID: **32951033**
- PMCID: PMC7543325
- DOI: 10.1093/ejcts/ezaa287

Free PMC article
Observational Study

Clinical features and outcomes of thoracic surgery patients during the COVID-19 pandemic

María Salmerón Jiménez et al. Eur J Cardiothorac Surg. 2020.
Free PMC article
Show details
Eur J Cardiothorac Surg
. 2020 Oct 1;58(4):738-744.
doi: 10.1093/ejcts/ezaa287.

Authors

María Salmerón Jiménez [1], Fátima Hermoso Alarza [1], Ivan Martínez Serna [1], Carmen Marrón Fernández [1], José Carlos Meneses Pardo [1], José Alberto García Salcedo [1], Alejandro Torres Serna [1], Mario Gustavo Manama Gama [1], Oscar Enrique Colmenares Mendoza [1], Vicente Diaz-Hellín Gude [1], Antonio Pablo Gamez García [1]

Affiliation

- [1] Department of Thoracic Surgery, 12 de Octubre University Hospital, Universidad Complutense de Madrid, Madrid, Spain.

- PMID: **32951033**
- PMCID: PMC7543325
- DOI: 10.1093/ejcts/ezaa287

Abstract

Objectives: The goal of this study was to describe the clinical features and outcomes of thoracic surgery patients during the coronavirus disease 2019 (COVID-19) pandemic.

Methods: Thirty-five patients were treated at the 12 de Octubre University Hospital in Madrid between 1 March 2020 and 24 April 2020 during the COVID-19 pandemic. Patient demographics, surgical procedures, complications, COVID-19 symptoms and outcomes were recorded. A protocol was introduced to reduce the risk of operating on patients with COVID-19, including symptom screening, a polymerase chain reaction test for severe acute respiratory syndrome coronavirus 2 and computed tomography scans of the chest. Surgical activity changed significantly during this time, from an initial period of near-normal activity, through an emergency-only period and finally a recovery period when some oncological surgical cases were restarted. Selection criteria for surgical patients are also described.

Results: A total of 34 patients underwent surgery during the pandemic period. We performed 22 lung resections (11 lobectomies and 11 sublobar resections). No hospital deaths were recorded. An elective surgery patient and an emergency surgery patient were diagnosed with COVID-19 (5.88%). The former died within 30 days after surgery.

Conclusions: Severe acute respiratory syndrome coronavirus 2 represents a tremendous limitation for thoracic surgical practice. Preoperative practices to exclude asymptomatic cases infected with the virus allowed us to perform thoracic surgical procedures.

Keywords: Coronavirus disease 2019; Novel coronavirus; Outcomes; Thoracic surgery.

© The Author(s) 2020. Published by Oxford University Press on behalf of the European Association for Cardio-Thoracic Surgery. All rights reserved.

- 28 references
- 3 figures

Supplementary info

Publication types, MeSH terms Expand

Publication types

- Observational Study

MeSH terms

- Adult
- Aged
- Aged, 80 and over
- Betacoronavirus*

- COVID-19
- Clinical Protocols
- Coronavirus Infections / complications
- Coronavirus Infections / diagnosis
- Coronavirus Infections / prevention & control*
- Female
- Health Care Rationing / methods
- Humans
- Infection Control / methods*
- Male
- Middle Aged
- Outcome Assessment, Health Care
- Pandemics / prevention & control*
- Patient Selection
- Perioperative Care / methods*
- Pneumonia, Viral / complications
- Pneumonia, Viral / diagnosis
- Pneumonia, Viral / prevention & control*
- Retrospective Studies
- SARS-CoV-2
- Spain
- Tertiary Care Centers
- Thoracic Surgical Procedures* / mortality

Full text links

OXFORD ACADEMIC Silverchair Information Systems Free PMC article
Proceed to details
Cite
Share
☐ 768
Observational Study
Front Endocrinol (Lausanne)
. 2021 Dec 8;12:791476.
doi: 10.3389/fendo.2021.791476. eCollection 2021.

High Fasting Blood Glucose Level With Unknown Prior History of Diabetes Is Associated With High Risk of Severe Adverse COVID-19 Outcome

Wenjun Wang [1 2 3 4], Zhonglin Chai [5], Mark E Cooper [5], Paul Z Zimmet [5], Hua Guo [6], Junyu Ding [6], Feifei Yang [1 2 3 4], Xu Chen [1 2 3 4], Xixiang Lin [1 2 3 4], Kai Zhang [1], Qin Zhong [1 2 3 4], Zongren Li [1 2 3 4], Peifang Zhang [7], Zhenzhou Wu [7], Xizhou Guan [6], Lei Zhang [5 8 9 10], Kunlun He [1 2 3 4]

Affiliations Expand

Affiliations

- [1] Key Laboratory of Ministry of Industry and Information Technology of Biomedical Engineering and Translational Medicine, Chinese People's Liberation Army (PLA) General Hospital, Beijing, China.
- [2] Translational Medical Research Center, Chinese People's Liberation Army (PLA) General Hospital, Beijing, China.
- [3] Medical Artificial Intelligence Research Center, Chinese People's Liberation Army (PLA) General Hospital, Beijing, China.
- [4] Medical Big Data Center, Chinese People's Liberation Army (PLA) General Hospital, Beijing, China.
- [5] Department of Diabetes, Central Clinical School, Faculty of Medicine, Nursing and Health Sciences, Monash University, Melbourne, VIC, Australia.
- [6] Department of Pulmonary and Critical Care Medicine, Chinese People's Liberation Army (PLA) General Hospital, Beijing, China.
- [7] BioMind Technology, Zhongguancun Medical Engineering Center, Beijing, China.
- [8] China-Australia Joint Research Center for Infectious Diseases, School of Public Health, Xi'an Jiaotong University Health Science Center, Xi'an, China.
- [9] Artificial Intelligence and Modelling in Epidemiology Program, Melbourne Sexual Health Centre, Alfred Health, Melbourne, VIC, Australia.
- [10] Department of Epidemiology and Biostatistics, College of Public Health, Zhengzhou University, Zhengzhou, China.

- PMID: **34956098**
- PMCID: PMC8692378
- DOI: 10.3389/fendo.2021.791476

Free PMC article
Observational Study

High Fasting Blood Glucose Level With Unknown Prior History of Diabetes Is

Associated With High Risk of Severe Adverse COVID-19 Outcome

Wenjun Wang et al. Front Endocrinol (Lausanne). 2021.
Free PMC article
Show details
Front Endocrinol (Lausanne)
. 2021 Dec 8;12:791476.
doi: 10.3389/fendo.2021.791476. eCollection 2021.

Authors

Wenjun Wang [1 2 3 4], Zhonglin Chai [5], Mark E Cooper [5], Paul Z Zimmet [5], Hua Guo [6], Junyu Ding [6], Feifei Yang [1 2 3 4], Xu Chen [1 2 3 4], Xixiang Lin [1 2 3 4], Kai Zhang [1], Qin Zhong [1 2 3 4], Zongren Li [1 2 3 4], Peifang Zhang [7], Zhenzhou Wu [7], Xizhou Guan [6], Lei Zhang [5 8 9 10], Kunlun He [1 2 3 4]

Affiliations

- [1] Key Laboratory of Ministry of Industry and Information Technology of Biomedical Engineering and Translational Medicine, Chinese People's Liberation Army (PLA) General Hospital, Beijing, China.
- [2] Translational Medical Research Center, Chinese People's Liberation Army (PLA) General Hospital, Beijing, China.
- [3] Medical Artificial Intelligence Research Center, Chinese People's Liberation Army (PLA) General Hospital, Beijing, China.
- [4] Medical Big Data Center, Chinese People's Liberation Army (PLA) General Hospital, Beijing, China.
- [5] Department of Diabetes, Central Clinical School, Faculty of Medicine, Nursing and Health Sciences, Monash University, Melbourne, VIC, Australia.
- [6] Department of Pulmonary and Critical Care Medicine, Chinese People's Liberation Army (PLA) General Hospital, Beijing, China.
- [7] BioMind Technology, Zhongguancun Medical Engineering Center, Beijing, China.
- [8] China-Australia Joint Research Center for Infectious Diseases, School of Public Health, Xi'an Jiaotong University Health Science Center, Xi'an, China.
- [9] Artificial Intelligence and Modelling in Epidemiology Program, Melbourne Sexual Health Centre, Alfred Health, Melbourne, VIC, Australia.
- [10] Department of Epidemiology and Biostatistics, College of Public Health, Zhengzhou University, Zhengzhou, China.

- PMID: **34956098**
- PMCID: PMC8692378
- DOI: 10.3389/fendo.2021.791476

Abstract

Background: We aimed to understand how glycaemic levels among COVID-19 patients impact their disease progression and clinical complications.

Methods: We enrolled 2,366 COVID-19 patients from Huoshenshan hospital in Wuhan. We stratified the COVID-19 patients into four subgroups by current fasting blood glucose (FBG) levels and their awareness of prior diabetic status, including patients with FBG<6.1mmol/L with no history of diabetes (group 1), patients with FBG<6.1mmol/L with a history of diabetes diagnosed (group 2), patients with FBG≥6.1mmol/L with no history of diabetes (group 3) and patients with FBG≥6.1mmol/L with a history of diabetes diagnosed (group 4). A multivariate cause-specific Cox proportional hazard model was used to assess the associations between FBG levels or prior diabetic status and clinical adversities in COVID-19 patients.

Results: COVID-19 patients with higher FBG and unknown diabetes in the past (group 3) are more likely to progress to the severe or critical stage than patients in other groups (severe: 38.46% vs 23.46%-30.70%; critical 7.69% vs 0.61%-3.96%). These patients also have the highest abnormal level of inflammatory parameters, complications, and clinical adversities among all four groups (all p<0.05). On day 21 of hospitalisation, group 3 had a significantly higher risk of ICU admission [14.1% (9.6%-18.6%)] than group 4 [7.0% (3.7%-10.3%)], group 2 [4.0% (0.2% -7.8%)] and group 1 [2.1% (1.4%-2.8%)], (P<0.001). Compared with group 1 who had low FBG, group 3 demonstrated 5 times higher risk of ICU admission events during hospitalisation (HR=5.38, 3.46-8.35, P<0.001), while group 4, where the patients had high FBG and prior diabetes diagnosed, also showed a significantly higher risk (HR=1.99, 1.12-3.52, P=0.019), but to a much lesser extent than in group 3.

Conclusion: Our study shows that COVID-19 patients with current high FBG levels but unaware of pre-existing diabetes, or possibly new onset diabetes as a result of COVID-19 infection, have a higher risk of more severe adverse outcomes than those aware of prior diagnosis of diabetes and those with low current FBG levels.

Keywords: COVID-19; FBG levels; complications; diabetes; glycaemia control and treatment.

Copyright © 2021 Wang, Chai, Cooper, Zimmet, Guo, Ding, Yang, Chen, Lin, Zhang, Zhong, Li, Zhang, Wu, Guan, Zhang and He.

Conflict of interest statement

The authors declare that the research was conducted in the absence of any commercial or financial relationships that could be construed as a potential conflict of interest.

- 48 references
- 2 figures

Supplementary info

Publication types, MeSH terms, Substances Expand

Publication types

- Observational Study
- Research Support, Non-U.S. Gov't

MeSH terms

- Adult
- Aged
- Aged, 80 and over
- Blood Glucose / metabolism*
- COVID-19 / blood*
- Fasting / blood
- Female
- Hospitalization
- Humans
- Male
- Middle Aged
- Prognosis
- Retrospective Studies
- Risk Factors

Substances

- Blood Glucose

Full text links

Frontiers Media SA Free PMC article
Proceed to details
Cite
Share
☐ 769
Observational Study
JAMA
. 2020 Jun 23;323(24):2493-2502.
doi: 10.1001/jama.2020.8630.

Association of Treatment With Hydroxychloroquine or Azithromycin With In-Hospital Mortality in Patients With COVID-19 in New York State

Eli S Rosenberg [1], Elizabeth M Dufort [2], Tomoko Udo [1], Larissa A Wilberschied [2], Jessica Kumar [2], James Tesoriero [2], Patti Weinberg [3], James Kirkwood [2], Alison Muse [2], Jack DeHovitz [3] [4], Debra S Blog [2], Brad Hutton [2], David R Holtgrave [1], Howard A Zucker [2]

Affiliations Expand

Affiliations

- [1] University at Albany School of Public Health, State University of New York, Rensselaer.
- [2] New York State Department of Health, Albany.
- [3] IPRO, Lake Success, New York.
- [4] Downstate Health Sciences University, State University of New York, Brooklyn.

- PMID: **32392282**
- PMCID: PMC7215635
- DOI: 10.1001/jama.2020.8630

Free PMC article
Observational Study

Association of Treatment With Hydroxychloroquine or Azithromycin With In-Hospital Mortality in Patients With COVID-19 in New York State

Eli S Rosenberg et al. JAMA. 2020.
Free PMC article
Show details
JAMA
. 2020 Jun 23;323(24):2493-2502.
doi: 10.1001/jama.2020.8630.

Authors

Eli S Rosenberg [1], Elizabeth M Dufort [2], Tomoko Udo [1], Larissa A Wilberschied [2], Jessica Kumar [2], James Tesoriero [2], Patti Weinberg [3], James Kirkwood [2], Alison Muse [2], Jack DeHovitz [3] [4], Debra S Blog [2], Brad Hutton [2], David R Holtgrave [1], Howard A Zucker [2]

Affiliations

- [1] University at Albany School of Public Health, State University of New York, Rensselaer.
- [2] New York State Department of Health, Albany.
- [3] IPRO, Lake Success, New York.
- [4] Downstate Health Sciences University, State University of New York, Brooklyn.

- PMID: **32392282**
- PMCID: PMC7215635
- DOI: 10.1001/jama.2020.8630

Abstract

Importance: Hydroxychloroquine, with or without azithromycin, has been considered as a possible therapeutic agent for patients with coronavirus disease 2019 (COVID-19). However, there are limited data on efficacy and associated adverse events.

Objective: To describe the association between use of hydroxychloroquine, with or without azithromycin, and clinical outcomes among hospital inpatients diagnosed with COVID-19.

Design, setting, and participants: Retrospective multicenter cohort study of patients from a random sample of all admitted patients with laboratory-confirmed COVID-19 in 25 hospitals, representing 88.2% of patients with COVID-19 in the New York metropolitan region. Eligible patients were admitted for at least 24 hours between March 15 and 28, 2020. Medications, preexisting conditions, clinical measures on admission, outcomes, and adverse events were abstracted from medical records. The date of final follow-up was April 24, 2020.

Exposures: Receipt of both hydroxychloroquine and azithromycin, hydroxychloroquine alone, azithromycin alone, or neither.

Main outcomes and measures: Primary outcome was in-hospital mortality. Secondary outcomes were cardiac arrest and abnormal electrocardiogram findings (arrhythmia or QT prolongation).

Results: Among 1438 hospitalized patients with a diagnosis of COVID-19 (858 [59.7%] male, median age, 63 years), those receiving hydroxychloroquine, azithromycin, or both were more likely than those not receiving either drug to have diabetes, respiratory rate >22/min, abnormal chest imaging findings, O2 saturation lower than 90%, and aspartate aminotransferase greater than 40 U/L. Overall in-hospital mortality was 20.3% (95% CI, 18.2%-22.4%). The probability of death for patients receiving hydroxychloroquine + azithromycin was 189/735 (25.7% [95% CI, 22.3%-28.9%]), hydroxychloroquine alone, 54/271 (19.9% [95% CI, 15.2%-24.7%]), azithromycin alone, 21/211 (10.0% [95% CI, 5.9%-14.0%]), and neither drug, 28/221 (12.7% [95% CI, 8.3%-17.1%]). In adjusted Cox proportional hazards models, compared with patients receiving neither drug, there were no significant differences in mortality for patients receiving hydroxychloroquine + azithromycin (HR, 1.35 [95% CI, 0.76-2.40]), hydroxychloroquine alone (HR, 1.08 [95% CI, 0.63-1.85]), or azithromycin alone (HR, 0.56 [95% CI, 0.26-1.21]). In logistic models, compared with patients receiving neither drug cardiac arrest was significantly more likely in patients receiving hydroxychloroquine + azithromycin (adjusted OR, 2.13 [95% CI, 1.12-4.05]), but not hydroxychloroquine alone (adjusted OR, 1.91 [95% CI, 0.96-3.81]) or azithromycin alone (adjusted OR, 0.64 [95% CI, 0.27-1.56]), . In adjusted logistic regression models, there were no significant differences in the relative likelihood of abnormal electrocardiogram findings.

Conclusions and relevance: Among patients hospitalized in metropolitan New York with COVID-19, treatment with hydroxychloroquine, azithromycin, or both, compared with neither treatment, was not significantly associated with differences in in-hospital mortality. However, the interpretation of these findings may be limited by the observational design.

Conflict of interest statement

Conflict of Interest Disclosures: Dr Dufort reported that her spouse has a Gilead Foundation-Focus HIV/HCV testing research grant. No other disclosures were reported.

Comment in

- Impact of Hydroxychloroquine on Antibody Responses to the SARS-CoV-2 Coronavirus.
  de Miranda Santos IKF, Costa CHN. de Miranda Santos IKF, et al. Front Immunol. 2020 Aug 4;11:1739. doi: 10.3389/fimmu.2020.01739. eCollection 2020. Front Immunol. 2020. PMID: 32849619 Free PMC article. No abstract available.
- Dangers of the use of hydroxychloroquine and azithromycin combination in COVID-19 patients.
  Alizargar J. Alizargar J. Travel Med Infect Dis. 2020 Nov-Dec;38:101881. doi: 10.1016/j.tmaid.2020.101881. Epub 2020 Sep 18. Travel Med Infect Dis. 2020. PMID: 32956851 Free PMC article. No abstract available.

- 2 figures

Supplementary info

Publication types, MeSH terms, Substances, Supplementary concepts Expand

Publication types

- Multicenter Study
- Observational Study

MeSH terms

- Adolescent
- Adult
- Aged
- Anti-Infective Agents / adverse effects
- Anti-Infective Agents / therapeutic use*
- Arrhythmias, Cardiac / chemically induced
- Azithromycin / adverse effects
- Azithromycin / therapeutic use*
- Betacoronavirus
- COVID-19
- Coronavirus Infections / drug therapy*
- Coronavirus Infections / mortality
- Drug Therapy, Combination
- Female
- Heart Arrest / etiology
- Hospital Mortality*
- Hospitalization
- Humans
- Hydroxychloroquine / adverse effects
- Hydroxychloroquine / therapeutic use*
- Logistic Models
- Male

- Middle Aged
- New York
- Pandemics
- Pneumonia, Viral / drug therapy*
- Pneumonia, Viral / mortality
- Proportional Hazards Models
- Retrospective Studies
- SARS-CoV-2
- Young Adult

Substances

- Anti-Infective Agents
- Hydroxychloroquine
- Azithromycin

Supplementary concepts

- COVID-19 drug treatment

Full text links

JAMA FULL TEXT Silverchair Information Systems Free PMC article

Proceed to details

Cite

Share

☐ 770

Observational Study

PLoS One

. 2020 Sep 30;15(9):e0240014.

doi: 10.1371/journal.pone.0240014. eCollection 2020.

Safety of bedside surgical tracheostomy during COVID-19 pandemic: A retrospective observational study

Edoardo Picetti [1], Anna Fornaciari [1], Fabio Silvio Taccone [2], Laura Malchiodi [1], Silvia Grossi [1], Filippo Di Lella [3], Maurizio Falcioni [3], Giulia D'Angelo [3], Emanuele Sani [1], Sandra Rossi [1]

Affiliations Expand

Affiliations

- [1] Department of Anesthesia and Intensive Care, Parma University Hospital, Parma, Italy.
- [2] Department of Intensive Care, Erasme Hospital, Université Libre de Bruxelles, Brussels, Belgium.
- [3] Department of Otolaryngology, Parma University Hospital, Parma, Italy.

- PMID: **32997704**
- PMCID: PMC7526872
- DOI: 10.1371/journal.pone.0240014

Free PMC article
Observational Study

Safety of bedside surgical tracheostomy during COVID-19 pandemic: A retrospective observational study

Edoardo Picetti et al. PLoS One. 2020.
Free PMC article
Show details
PLoS One
. 2020 Sep 30;15(9):e0240014.
doi: 10.1371/journal.pone.0240014. eCollection 2020.

Authors

Edoardo Picetti [1], Anna Fornaciari [1], Fabio Silvio Taccone [2], Laura Malchiodi [1], Silvia Grossi [1], Filippo Di Lella [3], Maurizio Falcioni [3], Giulia D'Angelo [3], Emanuele Sani [1], Sandra Rossi [1]

Affiliations

- [1] Department of Anesthesia and Intensive Care, Parma University Hospital, Parma, Italy.
- [2] Department of Intensive Care, Erasme Hospital, Université Libre de Bruxelles, Brussels, Belgium.
- [3] Department of Otolaryngology, Parma University Hospital, Parma, Italy.

- PMID: **32997704**
- PMCID: PMC7526872
- DOI: 10.1371/journal.pone.0240014

Abstract

Data regarding safety of bedside surgical tracheostomy in novel coronavirus 2019 (COVID-19) mechanically ventilated patients admitted to the intensive care unit (ICU) are lacking. We performed this study to assess the safety of bedside surgical tracheostomy in COVID-19 patients admitted to ICU. This retrospective, single-center, cohort observational study (conducted between February, 23 and April, 30, 2020) was performed in our 45-bed dedicated COVID-19 ICU. Inclusion criteria were: a) age over 18 years; b) confirmed diagnosis of COVID-19 infection (with

nasopharyngeal/oropharyngeal swab); c) invasive mechanical ventilation and d) clinical indication for tracheostomy. The objectives of this study were to describe: 1) perioperative complications, 2) perioperative alterations in respiratory gas exchange and 3) occurrence of COVID-19 infection among health-care providers involved into the procedure. A total of 125 COVID-19 patients were admitted to the ICU during the study period. Of those, 66 (53%) underwent tracheostomy. Tracheostomy was performed after a mean of 6.1 (± 2.1) days since ICU admission. Most of tracheostomies (47/66, 71%) were performed by intensivists and the mean time of the procedure was 22 (± 4.4) minutes. No intraprocedural complications was reported. Stoma infection and bleeding were reported in 2 patients and 7 patients, respectively, in the post-procedure period, without significant clinical consequences. The mean PaO2 / FiO2 was significantly lower at the end of tracheostomy (117.6 ± 35.4) then at the beginning (133.4 ± 39.2) or 24 hours before (135.8 ± 51.3) the procedure. However, PaO2/FiO2 progressively increased at 24 hours after tracheostomy (142 ± 50.7). None of the members involved in the tracheotomy procedures developed COVID-19 infection. Bedside surgical tracheostomy appears to be feasible and safe, both for patients and for health care workers, during COVID-19 pandemic in an experienced center.

Conflict of interest statement

NO authors have competing interests

- 26 references
- 1 figure

Supplementary info

Publication types, MeSH terms, Grant support Expand

Publication types

- Observational Study

MeSH terms

- Aged
- Betacoronavirus
- COVID-19
- Coronavirus Infections / prevention & control*
- Coronavirus Infections / transmission
- Female
- Humans
- Infectious Disease Transmission, Patient-to-Professional / prevention & control
- Intensive Care Units
- Male
- Middle Aged
- Pandemics / prevention & control*
- Pneumonia, Viral / prevention & control*

- Pneumonia, Viral / transmission
- Respiration, Artificial
- Retrospective Studies
- SARS-CoV-2
- Safety*
- Tracheostomy*

Grant support

The authors received no specific funding for this work.

Full text links

OPEN ACCESS TO FULL TEXT PLOS ONE Public Library of Science Free PMC article

Proceed to details

Cite

Share

771

Observational Study

J Neurol Sci

. 2021 Jul 15;426:117479.

doi: 10.1016/j.jns.2021.117479. Epub 2021 May 5.

Impact of SARS-CoV-2 infection on acute intracerebral haemorrhage in northern Italy

Davide Sangalli [1], Filippo Martinelli-Boneschi [2], Maurizio Versino [3], Irene Colombo [4], Alfonso Ciccone [5], Simone Beretta [6], Simona Marcheselli [7], Riccardo Altavilla [8], Mauro Roncoroni [9], Sandro Beretta [10], Lorenzo Lorusso [11], Anna Cavallini [12], Alessandro Prelle [13], Donata Guidetti [14], Sara La Gioia [15], Paola Santalucia [16], Carla Zanferrari [17], Giampiero Grampa [18], Elisabetta D'Adda [19], Lorenzo Peverelli [20], Antonio Colombo [21], Andrea Salmaggi [22], SNO-COVID-19 group

Affiliations Expand

Affiliations

- [1] Neurological Department, "Alessandro Manzoni" Hospital, ASST Lecco, Via dell'Eremo 9/11, 23900 Lecco, Italy. Electronic address: d.sangalli@asst-lecco.it.
- [2] IRCCS Fondazione Ca' Granda Ospedale Maggiore Policlinico, Neurology Unit, Via Francesco Sforza 35, 20122 Milan, Italy; Dino Ferrari Center, Department of Pathophysiology and Transplantation, University of Milan, Via Francesco Sforza 35, 20122 Milan, Italy.
- [3] Neurology and Stroke Unit, ASST SetteLaghi, Ospedale di Circolo, DMC, University of Insubria, Varese, Italy.
- [4] Neurology and Stroke Unit, Ospedale di Desio, ASST, Monza, MB, Italy.

- [5] Department of Neurosciences, Carlo Poma Hospital, ASST di Mantova, Mantua, Italy.
- [6] Department of Neurology, San Gerardo Hospital, ASST Monza, University of Milano Bicocca, Monza, NeuroMi (Milan Center for Neuroscience), Milan, Italy.
- [7] Neurologia d'urgenza e Stroke Unit, Humanitas Clinical and Research Center - IRCCS, Rozzano, Milan, Italy.
- [8] Neurology and Stroke Unit, P.O. San Carlo Borromeo, ASST Santi Paolo e Carlo, Milan, Italy.
- [9] Neurology and Stroke Unit, P.O. Saronno, ASST Valle Olona, Varese, Italy.
- [10] Neurology, Vimercate Hospital, ASST Vimercate, Vimercate, MB, Italy.
- [11] Neurological Department, San Leopoldo Mandic Hospital, ASST Lecco, Merate, Italy.
- [12] Neurologia d'Urgenza e Stroke Unit, IRCCS Fondazione Mondino, Pavia, Italy.
- [13] Neurology, ASST Ovest Milanese, Legnano, Italy.
- [14] Neurology Unit, Guglielmo da Saliceto Hospital, Piacenza, Italy.
- [15] Department of neurology, Papa Giovanni XXIII Hospital, Bergamo, Italy.
- [16] Neurology and Stroke Unit, San Giuseppe-Multimedica Hospital, Milan, Italy.
- [17] Neurology and Stroke Unit, PO Vizzolo Predabissi, ASST Melegnano Martesana, Milan, Italy.
- [18] Neurology Unit, S. Anna Hospital, Como, Italy.
- [19] Neurology Unit, Ospedale Maggiore di Crema, ASST Crema, Crema, Italy.
- [20] Neurology, Ospedale Maggiore di Lodi, ASST Lodi, Italy.
- [21] Polo Neurologico Brianteo, Seregno, MB, Italy.
- [22] Neurological Department, "Alessandro Manzoni" Hospital, ASST Lecco, Via dell'Eremo 9/11, 23900 Lecco, Italy.

- PMID: **34004463**
- PMCID: PMC8096523
- DOI: 10.1016/j.jns.2021.117479

Free PMC article
Observational Study

Impact of SARS-CoV-2 infection on acute intracerebral haemorrhage in northern Italy

Davide Sangalli et al. J Neurol Sci. 2021.
Free PMC article
Show details
J Neurol Sci
. 2021 Jul 15;426:117479.
doi: 10.1016/j.jns.2021.117479. Epub 2021 May 5.

Authors

Davide Sangalli [1], Filippo Martinelli-Boneschi [2], Maurizio Versino [3], Irene Colombo [4], Alfonso Ciccone [5], Simone Beretta [6], Simona Marcheselli [7], Riccardo Altavilla [8], Mauro Roncoroni [9], Sandro Beretta [10], Lorenzo Lorusso [11], Anna Cavallini [12], Alessandro Prelle [13], Donata Guidetti [14], Sara La Gioia [15], Paola Santalucia [16], Carla Zanferrari [17], Giampiero Grampa

[18], Elisabetta D'Adda [19], Lorenzo Peverelli [20], Antonio Colombo [21], Andrea Salmaggi [22], SNO-COVID-19 group

Affiliations

- [1] Neurological Department, "Alessandro Manzoni" Hospital, ASST Lecco, Via dell'Eremo 9/11, 23900 Lecco, Italy. Electronic address: d.sangalli@asst-lecco.it.
- [2] IRCCS Fondazione Ca' Granda Ospedale Maggiore Policlinico, Neurology Unit, Via Francesco Sforza 35, 20122 Milan, Italy; Dino Ferrari Center, Department of Pathophysiology and Transplantation, University of Milan, Via Francesco Sforza 35, 20122 Milan, Italy.
- [3] Neurology and Stroke Unit, ASST SetteLaghi, Ospedale di Circolo, DMC, University of Insubria, Varese, Italy.
- [4] Neurology and Stroke Unit, Ospedale di Desio, ASST, Monza, MB, Italy.
- [5] Department of Neurosciences, Carlo Poma Hospital, ASST di Mantova, Mantua, Italy.
- [6] Department of Neurology, San Gerardo Hospital, ASST Monza, University of Milano Bicocca, Monza, NeuroMi (Milan Center for Neuroscience), Milan, Italy.
- [7] Neurologia d'urgenza e Stroke Unit, Humanitas Clinical and Research Center - IRCCS, Rozzano, Milan, Italy.
- [8] Neurology and Stroke Unit, P.O. San Carlo Borromeo, ASST Santi Paolo e Carlo, Milan, Italy.
- [9] Neurology and Stroke Unit, P.O. Saronno, ASST Valle Olona, Varese, Italy.
- [10] Neurology, Vimercate Hospital, ASST Vimercate, Vimercate, MB, Italy.
- [11] Neurological Department, San Leopoldo Mandic Hospital, ASST Lecco, Merate, Italy.
- [12] Neurologia d'Urgenza e Stroke Unit, IRCCS Fondazione Mondino, Pavia, Italy.
- [13] Neurology, ASST Ovest Milanese, Legnano, Italy.
- [14] Neurology Unit, Guglielmo da Saliceto Hospital, Piacenza, Italy.
- [15] Department of neurology, Papa Giovanni XXIII Hospital, Bergamo, Italy.
- [16] Neurology and Stroke Unit, San Giuseppe-Multimedica Hospital, Milan, Italy.
- [17] Neurology and Stroke Unit, PO Vizzolo Predabissi, ASST Melegnano Martesana, Milan, Italy.
- [18] Neurology Unit, S. Anna Hospital, Como, Italy.
- [19] Neurology Unit, Ospedale Maggiore di Crema, ASST Crema, Crema, Italy.
- [20] Neurology, Ospedale Maggiore di Lodi, ASST Lodi, Italy.
- [21] Polo Neurologico Brianteo, Seregno, MB, Italy.
- [22] Neurological Department, "Alessandro Manzoni" Hospital, ASST Lecco, Via dell'Eremo 9/11, 23900 Lecco, Italy.

- PMID: **34004463**
- PMCID: PMC8096523
- DOI: 10.1016/j.jns.2021.117479

Abstract

Introduction: Growing evidence has been published as to the impact of SARS-CoV-2 (Severe acute respiratory syndrome coronavirus 2) on cerebrovascular events over the last few months, with considerable attention paid to ischemic strokes. Conversely, little is known about the clinical course of intracerebral haemorrhage (ICH) and simultaneous SARS-CoV-2 infection.

Method: The Italian Society of Hospital Neurosciences (SNO) promoted a multicentre, retrospective, observational study (SNO-COVID-19), involving 20 Neurological Departments in Northern Italy. Clinical data on patients with acute cerebrovascular diseases, admitted from March 1st to April 30th, 2020, were collected. A comparison was made of the demographical and clinical features of both SARS-CoV-2 positive and negative patients with ICH.

Results: 949 patients were enrolled (average age 73.4 years; 52.7% males); 135 patients had haemorrhagic stroke and 127 (13.4%) had a primary ICH. Only 16 patients with ICH (12.6%) had laboratory confirmed SARS-CoV-2 infection, both symptomatic and asymptomatic. SARS-CoV-2 related pneumonia or respiratory distress (OR 5.4), lobar location (OR 5.0) and previous antiplatelet or anticoagulant treatment (OR 2.9) were the only factors significantly associated with increased mortality in ICH. SARS-CoV-2 infection, regardless of respiratory involvement, led to a non-significantly increased risk of in-hospital death (37.5% vs 23.4%, p = 0.2).

Discussion: ICH patients with COVID-19 did not experience an increase in mortality as striking as ischemic stroke. The inflammatory response and respiratory complications could justify the slight increase of death in ICH. Bleeding sites and previous antiplatelet or anticoagulant treatment were the only other predictors of a worse outcome.

Keywords: COVID-19; Intracerebral haemorrhage; SARS-CoV-2; Stroke.

Copyright © 2021 Elsevier B.V. All rights reserved.

Conflict of interest statement

The authors declare that they have no conflict of interest.

- 19 references
- 2 figures

Supplementary info

Publication types, MeSH terms Expand

Publication types

- Observational Study

MeSH terms

- Aged
- COVID-19*
- Cerebral Hemorrhage / complications
- Cerebral Hemorrhage / epidemiology
- Female
- Hospital Mortality
- Humans
- Italy / epidemiology
- Male

- Retrospective Studies
- SARS-CoV-2*

Full text links

ELSEVIER FULL-TEXT ARTICLE Elsevier Science Free PMC article
Proceed to details
Cite
Share
772
Observational Study

J Am Geriatr Soc
. 2022 Jan;70(1):40-48.
doi: 10.1111/jgs.17463. Epub 2021 Oct 2.

Treatment intensity and mortality among COVID-19 patients with dementia: A retrospective observational study

Amber E Barnato [1] [2], John D Birkmeyer [1] [3], Jonathan S Skinner [1] [4], A James O'Malley [1] [5], Nancy J O Birkmeyer [1]
Affiliations Expand

Affiliations

- [1] The Dartmouth Institute for Health Policy and Clinical Practice, Geisel School of Medicine at Dartmouth, Lebanon, New Hampshire, USA.
- [2] Department of Medicine, Geisel School of Medicine at Dartmouth, Lebanon, New Hampshire, USA.
- [3] Sound Physicians, Tacoma, Washington, USA.
- [4] Department of Economics, Dartmouth College, Hanover, New Hampshire, USA.
- [5] Department of Biomedical Data Science, Geisel School of Medicine at Dartmouth, Lebanon, New Hampshire, USA.

- PMID: **34480354**
- PMCID: **PMC8742761** (available on 2023-01-01)
- DOI: 10.1111/jgs.17463

Observational Study

Treatment intensity and mortality among COVID-19 patients with dementia: A retrospective observational study

Amber E Barnato et al. J Am Geriatr Soc. 2022 Jan.
Show details
J Am Geriatr Soc
. 2022 Jan;70(1):40-48.
doi: 10.1111/jgs.17463. Epub 2021 Oct 2.

Authors

Amber E Barnato [1] [2], John D Birkmeyer [1] [3], Jonathan S Skinner [1] [4], A James O'Malley [1] [5], Nancy J O Birkmeyer [1]

Affiliations

- [1] The Dartmouth Institute for Health Policy and Clinical Practice, Geisel School of Medicine at Dartmouth, Lebanon, New Hampshire, USA.
- [2] Department of Medicine, Geisel School of Medicine at Dartmouth, Lebanon, New Hampshire, USA.
- [3] Sound Physicians, Tacoma, Washington, USA.
- [4] Department of Economics, Dartmouth College, Hanover, New Hampshire, USA.
- [5] Department of Biomedical Data Science, Geisel School of Medicine at Dartmouth, Lebanon, New Hampshire, USA.

- PMID: **34480354**
- PMCID: **PMC8742761** (available on 2023-01-01)
- DOI: 10.1111/jgs.17463

Abstract

Background: We sought to determine whether dementia is associated with treatment intensity and mortality in patients hospitalized with COVID-19.

Methods: This study includes review of the medical records for patients >60 years of age (n = 5394) hospitalized with COVID-19 from 132 community hospitals between March and June 2020. We examined the relationships between dementia and treatment intensity (including intensive care unit [ICU] admission and mechanical ventilation [MV] and care processes that may influence them, including advance care planning [ACP] billing and do-not-resuscitate [DNR] orders) and in-hospital mortality adjusting for age, sex, race/ethnicity, comorbidity, month of hospitalization, and clustering within hospital. We further explored the effect of ACP conversations on the relationship between dementia and outcomes, both at the individual patient level (effect of having ACP) and at the hospital level (effect of being treated at a hospital with low: <10%, medium 10%-20%, or high >20% ACP rates).

Results: Ten percent (n = 522) of the patients had documented dementia. Dementia patients were older (>80 years: 60% vs. 27%, p < 0.0001), had a lower burden of comorbidity (3+ comorbidities: 31% vs. 38%, p = 0.003), were more likely to have ACP (28% vs. 17%, p < 0.0001) and a DNR order (52% vs. 22%, p < 0.0001), had similar rates of ICU admission (26% vs. 28%, p = 0.258), were less likely to receive MV (11% vs. 16%, p = 0.001), and more likely to die (22% vs. 14%, p < 0.0001). Differential treatment intensity among patients with dementia was concentrated in hospitals with low, dementia-biased ACP billing practices (risk-adjusted ICU use: 21% vs. 30%, odds ratio [OR] = 0.6, p = 0.016; risk-adjusted MV use: 6% vs. 16%, OR = 0.3, p < 0.001).

Conclusions: Dementia was associated with lower treatment intensity and higher mortality in patients hospitalized with COVID-19. Differential treatment intensity was concentrated in low ACP billing hospitals suggesting an interplay between provider bias and "preference-sensitive" care for COVID-19.

Keywords: COVID-19; advance care planning; dementia; intensive care; mortality.

© 2021 The American Geriatrics Society.

Conflict of interest statement

CONFLICT OF INTEREST

JDB is an executive at Sound Physicians which provided data for this study. NJB is married to JDB.

Supplementary info

Publication types, MeSH terms, Grant support Expand

Publication types

- Observational Study
- Research Support, N.I.H., Extramural

MeSH terms

- Advance Care Planning / statistics & numerical data
- Aged
- Aged, 80 and over
- COVID-19* / mortality
- COVID-19* / therapy
- Comorbidity
- Dementia / complications*
- Dementia / mortality
- Female
- Hospital Mortality / trends
- Humans

- Intensive Care Units / statistics & numerical data*
- Male
- Respiration, Artificial / statistics & numerical data*
- Resuscitation Orders
- Retrospective Studies

Grant support

- P01 AG019783/AG/NIA NIH HHS/United States
- P01AG019783/AG/NIA NIH HHS/United States
- P01AG019783/AG/NIA NIH HHS/United States

Full text links

Wiley Full Text Article Wiley

Proceed to details

Cite

Share

773

Observational Study

BMC Med
. 2021 Aug 30;19(1):213.
doi: 10.1186/s12916-021-02096-0.

The association between mechanical ventilator compatible bed occupancy and mortality risk in intensive care patients with COVID-19: a national retrospective cohort study

Harrison Wilde [#] [1], Thomas Mellan [#] [2], Iwona Hawryluk [2], John M Dennis [3], Spiros Denaxas [4] [5] [6], Christina Pagel [7], Andrew Duncan [4] [8], Samir Bhatt [2], Seth Flaxman [#] [8], Bilal A Mateen [#] [9] [10] [11], Sebastian J Vollmer [#] [1] [4]

Affiliations Expand

Affiliations

- [1] Department of Statistics, University of Warwick, Coventry, CV4 7AL, UK.
- [2] MRC Centre for Global Infectious Disease Analysis, Abdul Latif Jameel Institute for Disease and Emergency Analytics (J-IDEA), Imperial College London, London, UK.
- [3] Institute of Biomedical & Clinical Science, RILD Building, Royal Devon & Exeter Hospital, University of Exeter Medical School, Barrack Road, Exeter, EX2 5DW, UK.

- [4] The Alan Turing Institute, British Library, 96 Euston Road, London, NW1 2DB, UK.
- [5] Institute of Health Informatics, University College London, 222 Euston Rd, London, London, NW1 2DA, UK.
- [6] Health Data Research UK, Gibbs Building, 215 Euston Road, London, NW1 2BE, UK.
- [7] Clinical Operational Research Unit, University College London, 222 Euston Rd, London, London, NW1 2DA, UK.
- [8] Department of Mathematics, Imperial College, London, London, UK.
- [9] The Alan Turing Institute, British Library, 96 Euston Road, London, NW1 2DB, UK. bilal.mateen@nhs.net.
- [10] Institute of Health Informatics, University College London, 222 Euston Rd, London, London, NW1 2DA, UK. bilal.mateen@nhs.net.
- [11] The Wellcome Trust, Gibbs Building, 215 Euston Road, London, NW1 2BE, UK. bilal.mateen@nhs.net.

[#] Contributed equally.

- PMID: **34461893**
- PMCID: PMC8404408
- DOI: 10.1186/s12916-021-02096-0

Free PMC article
Observational Study

The association between mechanical ventilator compatible bed occupancy and mortality risk in intensive care patients with COVID-19: a national retrospective cohort study

Harrison Wilde et al. BMC Med. 2021.
Free PMC article
Show details
BMC Med
. 2021 Aug 30;19(1):213.
doi: 10.1186/s12916-021-02096-0.

Authors

Harrison Wilde [#, 1], Thomas Mellan [#, 2], Iwona Hawryluk [2], John M Dennis [3], Spiros Denaxas [4, 5, 6], Christina Pagel [7], Andrew Duncan [4, 8], Samir Bhatt [2], Seth Flaxman [#, 8], Bilal A Mateen [#, 9, 10, 11], Sebastian J Vollmer [#, 1, 4]

Affiliations

- [1] Department of Statistics, University of Warwick, Coventry, CV4 7AL, UK.

- [2] MRC Centre for Global Infectious Disease Analysis, Abdul Latif Jameel Institute for Disease and Emergency Analytics (J-IDEA), Imperial College London, London, UK.
- [3] Institute of Biomedical & Clinical Science, RILD Building, Royal Devon & Exeter Hospital, University of Exeter Medical School, Barrack Road, Exeter, EX2 5DW, UK.
- [4] The Alan Turing Institute, British Library, 96 Euston Road, London, NW1 2DB, UK.
- [5] Institute of Health Informatics, University College London, 222 Euston Rd, London, London, NW1 2DA, UK.
- [6] Health Data Research UK, Gibbs Building, 215 Euston Road, London, NW1 2BE, UK.
- [7] Clinical Operational Research Unit, University College London, 222 Euston Rd, London, London, NW1 2DA, UK.
- [8] Department of Mathematics, Imperial College, London, London, UK.
- [9] The Alan Turing Institute, British Library, 96 Euston Road, London, NW1 2DB, UK. bilal.mateen@nhs.net.
- [10] Institute of Health Informatics, University College London, 222 Euston Rd, London, London, NW1 2DA, UK. bilal.mateen@nhs.net.
- [11] The Wellcome Trust, Gibbs Building, 215 Euston Road, London, NW1 2BE, UK. bilal.mateen@nhs.net.

[#] Contributed equally.

- PMID: **34461893**
- PMCID: PMC8404408
- DOI: 10.1186/s12916-021-02096-0

Abstract

Background: The literature paints a complex picture of the association between mortality risk and ICU strain. In this study, we sought to determine if there is an association between mortality risk in intensive care units (ICU) and occupancy of beds compatible with mechanical ventilation, as a proxy for strain.

Methods: A national retrospective observational cohort study of 89 English hospital trusts (i.e. groups of hospitals functioning as single operational units). Seven thousand one hundred thirty-three adults admitted to an ICU in England between 2 April and 1 December, 2020 (inclusive), with presumed or confirmed COVID-19, for whom data was submitted to the national surveillance programme and met study inclusion criteria. A Bayesian hierarchical approach was used to model the association between hospital trust level (mechanical ventilation compatible), bed occupancy, and in-hospital all-cause mortality. Results were adjusted for unit characteristics (pre-pandemic size), individual patient-level demographic characteristics (age, sex, ethnicity, deprivation index, time-to-ICU admission), and recorded chronic comorbidities (obesity, diabetes, respiratory disease, liver disease, heart disease, hypertension, immunosuppression, neurological disease, renal disease).

Results: One hundred thirty-five thousand six hundred patient days were observed, with a mortality rate of 19.4 per 1000 patient days. Adjusting for patient-level factors, mortality was higher for admissions during periods of high occupancy (> 85% occupancy versus the baseline of 45 to 85%) [OR 1.23 (95% posterior credible interval (PCI): 1.08 to 1.39)]. In contrast, mortality was decreased for admissions during periods of low occupancy (< 45% relative to the baseline) [OR 0.83 (95% PCI 0.75 to 0.94)].

Conclusion: Increasing occupancy of beds compatible with mechanical ventilation, a proxy for operational strain, is associated with a higher mortality risk for individuals admitted to ICU. Further research is required to establish if this is a causal relationship or whether it reflects strain on other operational factors such as staff. If causal, the result highlights the importance of strategies to keep ICU occupancy low to mitigate the impact of this type of resource saturation.

Keywords: Coronavirus infection; Critical care; Hospital mortality; Public health surveillance; Quality of healthcare.

© 2021. The Author(s).

Conflict of interest statement

SJV declares funding from IQVIA and Microsoft. BAM is an employee of the Wellcome Trust and holds a Wellcome funded honorary post at University College London for the purposes of carrying out independent research; the views expressed in this manuscript do not necessarily reflect the views or position of the Wellcome Trust. All other authors declare no competing interests.

- 39 references
- 3 figures

Supplementary info

Publication types, MeSH terms, Grant support Expand

Publication types

- Observational Study

MeSH terms

- Adolescent
- Adult
- Aged
- Aged, 80 and over
- Bayes Theorem
- Bed Occupancy / statistics & numerical data*
- COVID-19 / mortality*
- Cause of Death*
- Critical Care / statistics & numerical data*
- Female
- Hospital Mortality*
- Humans
- Intensive Care Units*
- Male
- Middle Aged

- Retrospective Studies
- SARS-CoV-2
- Ventilators, Mechanical*
- Young Adult

Grant support

- MC_PC_20058/MRC_/Medical Research Council/United Kingdom
- MR/R015600/1/MRC_/Medical Research Council/United Kingdom
- MR/V038109/1/MRC_/Medical Research Council/United Kingdom

Full text links

Read free full text at BMC BioMed Central Free PMC article
Proceed to details
Cite
Share
774
Observational Study
Arch Phys Med Rehabil
. 2022 Mar;103(3):418-423.
doi: 10.1016/j.apmr.2021.10.018. Epub 2021 Nov 8.

In a Pandemic That Limits Contact, Can Videoconferencing Enable Interdisciplinary Persistent Pain Services and What Are the Patient's Perspectives?

Jarryd Brown [1], Darren Doherty [2], Andrew P Claus [3], Kelly Gilbert [2], Mandy Nielsen [4]
Affiliations Expand

Affiliations

- [1] Gold Coast University Hospital, Interdisciplinary Persistent Pain Centre, Gold Coast, Queensland, Australia. Electronic address: Jarryd.Brown@health.qld.gov.au.
- [2] Gold Coast University Hospital, Interdisciplinary Persistent Pain Centre, Gold Coast, Queensland, Australia.
- [3] Royal Brisbane and Women's Hospital, Tess Cramond Pain and Research Centre, Brisbane, Australia; School of Health and Rehabilitation Sciences, University of Queensland, Brisbane, Australia.
- [4] The Hopkins Centre, Metro South Health and Griffith University, Brisbane, Australia.

- PMID: **34762855**
- PMCID: PMC8598123

- DOI: 10.1016/j.apmr.2021.10.018

Free PMC article
Observational Study

In a Pandemic That Limits Contact, Can Videoconferencing Enable Interdisciplinary Persistent Pain Services and What Are the Patient's Perspectives?

Jarryd Brown et al. Arch Phys Med Rehabil. 2022 Mar.
Free PMC article
Show details
Arch Phys Med Rehabil
. 2022 Mar;103(3):418-423.
doi: 10.1016/j.apmr.2021.10.018. Epub 2021 Nov 8.

Authors

Jarryd Brown [1], Darren Doherty [2], Andrew P Claus [3], Kelly Gilbert [2], Mandy Nielsen [4]

Affiliations

- [1] Gold Coast University Hospital, Interdisciplinary Persistent Pain Centre, Gold Coast, Queensland, Australia. Electronic address: Jarryd.Brown@health.qld.gov.au.
- [2] Gold Coast University Hospital, Interdisciplinary Persistent Pain Centre, Gold Coast, Queensland, Australia.
- [3] Royal Brisbane and Women's Hospital, Tess Cramond Pain and Research Centre, Brisbane, Australia; School of Health and Rehabilitation Sciences, University of Queensland, Brisbane, Australia.
- [4] The Hopkins Centre, Metro South Health and Griffith University, Brisbane, Australia.

- PMID: **34762855**
- PMCID: PMC8598123
- DOI: 10.1016/j.apmr.2021.10.018

Abstract

Objective: To explore patients' thoughts and satisfaction with using videoconferencing during the COVID-19 pandemic. The current study aimed to gather (1) patient feedback and satisfaction with videoconferencing across all health professions as well as divided into a subgroup for each profession, (2) patient preferences for either videoconference or face-to-face consultations during the pandemic lockdown, and (3) whether patients would consider using videoconferencing once face-to-face appointments were available.

Design: An observational cross-sectional, mixed methods study design.

Setting: Tertiary-level persistent pain center.

Participants: Sixty-five patients aged 18 to 85 years with persistent pain lasting more than 12 months.

Interventions: Not applicable.

Main outcome measures: Data were collected using a patient survey. Descriptive statistics were used to report findings from 5-point Likert scales. Qualitative analysis was guided by content analysis to organize and categorize the open-ended survey response text.

Results: Videoconferencing platform features including audiovisual, usability, and privacy worked well for most patients (≥90%). Two-thirds of those surveyed reported the videoconferencing sessions as equal to face-to-face attendance (68%). In the context of the pandemic, almost as many preferred videoconferencing (65%), whereas 26% preferred face-to-face attendance and 9% were unsure. Preferences for videoconferencing over face-to-face in context to the pandemic varied depending on the health discipline involved: pharmacy (83%), occupational therapy (78%), psychology (61%), pain specialist physician (59%), and physiotherapy (53%). Even outside of a pandemic situation, 80% would consider using videoconferencing in the future. Qualitative analysis on an open-ended question asking patients for any further comments regarding their experience with the videoconference consultation, found 3 main categories: (1) overall satisfaction with videoconferencing, (2) technology qualities and (3) clinical interaction.

Conclusion: In the context of a pandemic, videoconferencing for interdisciplinary persistent pain management services was effective and preferred, and most patients would continue its use into the future. Alternative or mixed modes of support may be needed for the 26% who currently prefer onsite attendance, when that mode of delivery is not available.

Keywords: COVID-19; Patient satisfaction; Rehabilitation; Telehealth; Videoconferencing.

Copyright © 2021 The American Congress of Rehabilitation Medicine. Published by Elsevier Inc. All rights reserved.

- 23 references

Supplementary info

Publication types, MeSH terms Expand

Publication types

- Observational Study

MeSH terms

- COVID-19 / epidemiology*
- Communicable Disease Control / methods*
- Comorbidity
- Cross-Sectional Studies

- Female
- Humans
- Male
- Middle Aged
- Pain / epidemiology*
- Pain Management / methods*
- Pandemics*
- Patient Preference
- Physical Therapy Modalities
- Qualitative Research
- Queensland / epidemiology
- Retrospective Studies
- SARS-CoV-2*
- Telemedicine / methods
- Videoconferencing*

Full text links

ELSEVIER FULL-TEXT ARTICLE Elsevier Science Free PMC article
Proceed to details
Cite
Share
775
Observational Study
Am J Gastroenterol
. 2020 Oct;115(10):1617-1623.
doi: 10.14309/ajg.0000000000000832.

Impact of Famotidine Use on Clinical Outcomes of Hospitalized Patients With COVID-19

Jeffrey F Mather[1], Richard L Seip, Raymond G McKay
Affiliations Expand

Affiliation

- [1] Divisions of Research Administration and Interventional Cardiology, Hartford Hospital, Hartford, Connecticut, USA.

- PMID: **32852338**
- PMCID: PMC7473796
- DOI: 10.14309/ajg.0000000000000832

Free PMC article
Observational Study

Impact of Famotidine Use on Clinical Outcomes of Hospitalized Patients With COVID-19

Jeffrey F Mather et al. Am J Gastroenterol. 2020 Oct.
Free PMC article
Show details
Am J Gastroenterol
. 2020 Oct;115(10):1617-1623.
doi: 10.14309/ajg.0000000000000832.

Authors

Jeffrey F Mather [1], Richard L Seip, Raymond G McKay

Affiliation

- [1] Divisions of Research Administration and Interventional Cardiology, Hartford Hospital, Hartford, Connecticut, USA.

- PMID: **32852338**
- PMCID: PMC7473796
- DOI: 10.14309/ajg.0000000000000832

Abstract

Introduction: To compare outcomes in patients hospitalized with coronavirus (COVID-19) receiving famotidine therapy with those not receiving famotidine.

Methods: Retrospective, propensity-matched observational study of consecutive COVID-19-positive patients between February 24, 2020, and May 13, 2020.

Results: Of 878 patients in the analysis, 83 (9.5%) received famotidine. In comparison to patients not treated with famotidine, patients treated with famotidine were younger (63.5 ± 15.0 vs 67.5 ± 15.8 years, P = 0.021), but did not differ with respect to baseline demographics or preexisting comorbidities. Use of famotidine was associated with a decreased risk of in-hospital mortality (odds ratio 0.37, 95% confidence interval 0.16-0.86, P = 0.021) and combined death or intubation (odds ratio 0.47, 95% confidence interval 0.23-0.96, P = 0.040). Propensity score matching to adjust for age difference between groups did not alter the effect on either outcome. In addition, patients receiving famotidine displayed lower levels of serum markers for severe disease including lower median peak C-reactive protein levels (9.4 vs 12.7 mg/dL, P = 0.002), lower median procalcitonin levels (0.16 vs 0.30 ng/mL, P = 0.004), and a nonsignificant trend to lower median mean ferritin levels (797.5 vs 964.0 ng/mL, P = 0.076). Logistic regression analysis demonstrated that famotidine was an independent predictor of both lower mortality and combined death/intubation, whereas older age, body mass index >30 kg/m, chronic kidney disease, National

Early Warning Score, and higher neutrophil-lymphocyte ratio were all predictors of both adverse outcomes.

Discussion: Famotidine use in hospitalized patients with COVID-19 is associated with a lower risk of mortality, lower risk of combined outcome of mortality and intubation, and lower levels of serum markers for severe disease in hospitalized patients with COVID-19.(Equation is included in full-text article.).

Conflict of interest statement

Guarantor of the article: Jeffrey F. Mather, MS.

Specific author contributions: J.F.M. had full access to all the data in the study and takes responsibility for the integrity of the data and the accuracy of the data analysis. All authors played a part in concept and design. J.F.M. performed data acquisition. All authors had a role in interpretation and drafting the manuscript. J.F.M. and R.L.S. performed statistical analysis. R.G.M. and J.F.M. wrote the manuscript, and all authors gave critical revision of the manuscript for important intellectual content.

Financial support: None to report.

Potential competing interests: None to report.

Comment in

- Association Between Famotidine Use and Clinical Outcomes in Patients With COVID-19: Assessment of Available Evidence.
  Ma T, Wu M. Ma T, et al. Am J Gastroenterol. 2021 Apr;116(4):848-849. doi: 10.14309/ajg.0000000000001008. Am J Gastroenterol. 2021. PMID: 33982973 Free PMC article. No abstract available.
- Response to Ma and Wu.
  Mather J. Mather J. Am J Gastroenterol. 2021 Apr;116(4):849-850. doi: 10.14309/ajg.0000000000001068. Am J Gastroenterol. 2021. PMID: 33982974 No abstract available.
- Famotidine and Hospitalized COVID-19 Patients: Are the Benefits True?
  Amjad W, Malik A. Amjad W, et al. Am J Gastroenterol. 2021 Jul 1;116(7):1561. doi: 10.14309/ajg.0000000000001217. Am J Gastroenterol. 2021. PMID: 34183586 No abstract available.

- 21 references
- 2 figures

Supplementary info

Publication types, MeSH terms, Substances, Supplementary concepts Expand

Publication types

- Comparative Study
- Observational Study

MeSH terms

- Aged
- Aged, 80 and over
- Betacoronavirus / isolation & purification
- Betacoronavirus / pathogenicity
- COVID-19
- Coronavirus Infections / diagnosis
- Coronavirus Infections / drug therapy
- Coronavirus Infections / mortality
- Coronavirus Infections / therapy*
- Coronavirus Infections / virology
- Famotidine / therapeutic use*
- Female
- Humans
- Intubation, Intratracheal / statistics & numerical data*
- Male
- Middle Aged
- Pandemics
- Pneumonia, Viral / diagnosis
- Pneumonia, Viral / mortality
- Pneumonia, Viral / therapy*
- Pneumonia, Viral / virology
- Propensity Score
- Retrospective Studies
- SARS-CoV-2
- Severity of Illness Index
- Tertiary Care Centers / statistics & numerical data
- Treatment Outcome

Substances

- Famotidine

Supplementary concepts

- COVID-19 drug treatment

Full text links

Wolters Kluwer Wolters Kluwer Free PMC article
Proceed to details

Cite

Share

☐ 776

Observational Study

Arch Cardiovasc Dis

. 2021 May;114(5):381-393.
doi: 10.1016/j.acvd.2021.02.003. Epub 2021 Mar 9.

D-dimer at hospital admission for COVID-19 are associated with in-hospital mortality, independent of venous thromboembolism: Insights from a French multicenter cohort study

Richard Chocron [1], Baptiste Duceau [2], Nicolas Gendron [3], Nacim Ezzouhairi [4], Lina Khider [5], Antonin Trimaille [6], Guillaume Goudot [5], Orianne Weizman [7], Jean Marc Alsac [8], Thibault Pommier [9], Olivier Bory [10], Joffrey Cellier [11], Aurélien Philippe [3], Laura Geneste [12], Iannis Ben Abdallah [8], Vassili Panagides [13], Salma El Batti [8], Wassima Marsou [14], Philippe Juvin [10], Antoine Deney [15], Emmanuel Messas [16], Sabir Attou [17], Benjamin Planquette [18], Delphine Mika [19], Pascale Gaussem [20], Charles Fauvel [21], Jean-Luc Diehl [22], Theo Pezel [23], Tristan Mirault [16], Willy Sutter [2], Olivier Sanchez [18], Guillaume Bonnet [2], Ariel Cohen [24], David M Smadja [3], Critical COVID-19 France investigators

Affiliations Expand

Affiliations

- [1] Université de Paris, PARCC, INSERM; Emergency department, Georges-Pompidou European hospital, AP-HP, 75015 Paris, France. Electronic address: richard.chocron@gmail.com.
- [2] Université de Paris, PARCC, INSERM, 75015 Paris, France.
- [3] Université de Paris, Innovative therapies in haemostasis, INSERM; Haematology department and biosurgical research laboratory (Carpentier Foundation), Georges-Pompidou European Hospital, AH-HP, 75015 Paris, France.
- [4] Université de médecine de Bordeaux, Centre hospitalier universitaire de Bordeaux, 33076 Bordeaux, France.
- [5] Université de Paris, Vascular medicine department and biosurgical research laboratory (Carpentier Foundation), Georges-Pompidou European Hospital, AP-HP, 75015 Paris, France.
- [6] Nouvel hôpital civil, Centre hospitalier régional universitaire de Strasbourg, 67000 Strasbourg, France.
- [7] Centre hospitalier régional universitaire de Nancy, 54511 Vandœuvre-Les-Nancy, France.
- [8] Université de Paris, Innovative therapies in haemostasis, INSERM; Vascular surgery department and biosurgical research laboratory (Carpentier Foundation), Georges-Pompidou European Hospital, AH-HP, 75015 Paris, France.

- [9] Centre hospitalier universitaire de Dijon, 21000 Dijon, France.
- [10] Université de Paris, Emergency department, Georges-Pompidou European Hospital, AP-HP, 75015 Paris, France.
- [11] Georges-Pompidou European Hospital, AP-HP, Université de Paris, 75015 Paris, France.
- [12] Centre hospitalier universitaire d'Amiens-Picardie, 80000 Amiens, France.
- [13] Centre hospitalier universitaire de Marseille, 13005 Marseille, France.
- [14] Centre hospitalier universitaire de Lille, Université catholique de Lille, 59000 Lille, France.
- [15] Centre hospitalier universitaire de Toulouse, 31400 Toulouse, France.
- [16] Université de Paris, PARCC, INSERM; Vascular medicine department, Georges-Pompidou European Hospital, AP-HP, 75015 Paris, France.
- [17] Centre hospitalier universitaire de Caen-Normandie, 14000 Caen, France.
- [18] Université de Paris, Innovative therapies in haemostasis, INSERM; Respiratory medicine department and biosurgical research laboratory (Carpentier Foundation), Georges-Pompidou European hospital, AH-HP, 75015 Paris, France.
- [19] Université Paris-Saclay, INSERM, UMR-S 1180, 92296 Chatenay-Malabry, France.
- [20] Université de Paris, Innovative therapies in haemostasis, INSERM; Haematology department, Georges-Pompidou European Hospital, AP-HP, 75015 Paris, France.
- [21] Rouen university hospital, FHU REMOD-VHF, 76000 Rouen, France.
- [22] Université de Paris, Innovative therapies in haemostasis, INSERM; Intensive care medicine department and biosurgical research laboratory (Carpentier Foundation), Georges-Pompidou European Hospital, AH-HP, 75015 Paris, France.
- [23] Lariboisière hospital, AP-HP, Université de Paris, 75010 Paris, France.
- [24] Department of cardiology, Saint-Antoine hospital, AP-HP, 75012 Paris, France.

- PMID: **33846096**
- PMCID: PMC7942155
- DOI: 10.1016/j.acvd.2021.02.003

Free PMC article
Observational Study

D-dimer at hospital admission for COVID-19 are associated with in-hospital mortality, independent of venous thromboembolism: Insights from a French multicenter cohort study

Richard Chocron et al. Arch Cardiovasc Dis. 2021 May.
Free PMC article

Show details

Arch Cardiovasc Dis

. 2021 May;114(5):381-393.
doi: 10.1016/j.acvd.2021.02.003. Epub 2021 Mar 9.

Authors

Richard Chocron [1], Baptiste Duceau [2], Nicolas Gendron [3], Nacim Ezzouhairi [4], Lina Khider [5], Antonin Trimaille [6], Guillaume Goudot [5], Orianne Weizman [7], Jean Marc Alsac [8], Thibault Pommier [9], Olivier Bory [10], Joffrey Cellier [11], Aurélien Philippe [3], Laura Geneste [12], Iannis Ben Abdallah [8], Vassili Panagides [13], Salma El Batti [8], Wassima Marsou [14], Philippe Juvin [10], Antoine Deney [15], Emmanuel Messas [16], Sabir Attou [17], Benjamin Planquette [18], Delphine Mika [19], Pascale Gaussem [20], Charles Fauvel [21], Jean-Luc Diehl [22], Theo Pezel [23], Tristan Mirault [16], Willy Sutter [2], Olivier Sanchez [18], Guillaume Bonnet [2], Ariel Cohen [24], David M Smadja [3], Critical COVID-19 France investigators

Affiliations

- [1] Université de Paris, PARCC, INSERM; Emergency department, Georges-Pompidou European hospital, AP-HP, 75015 Paris, France. Electronic address: richard.chocron@gmail.com.
- [2] Université de Paris, PARCC, INSERM, 75015 Paris, France.
- [3] Université de Paris, Innovative therapies in haemostasis, INSERM; Haematology department and biosurgical research laboratory (Carpentier Foundation), Georges-Pompidou European Hospital, AH-HP, 75015 Paris, France.
- [4] Université de médecine de Bordeaux, Centre hospitalier universitaire de Bordeaux, 33076 Bordeaux, France.
- [5] Université de Paris, Vascular medicine department and biosurgical research laboratory (Carpentier Foundation), Georges-Pompidou European Hospital, AP-HP, 75015 Paris, France.
- [6] Nouvel hôpital civil, Centre hospitalier régional universitaire de Strasbourg, 67000 Strasbourg, France.
- [7] Centre hospitalier régional universitaire de Nancy, 54511 Vandœuvre-Les-Nancy, France.
- [8] Université de Paris, Innovative therapies in haemostasis, INSERM; Vascular surgery department and biosurgical research laboratory (Carpentier Foundation), Georges-Pompidou European Hospital, AH-HP, 75015 Paris, France.
- [9] Centre hospitalier universitaire de Dijon, 21000 Dijon, France.
- [10] Université de Paris, Emergency department, Georges-Pompidou European Hospital, AP-HP, 75015 Paris, France.
- [11] Georges-Pompidou European Hospital, AP-HP, Université de Paris, 75015 Paris, France.
- [12] Centre hospitalier universitaire d'Amiens-Picardie, 80000 Amiens, France.
- [13] Centre hospitalier universitaire de Marseille, 13005 Marseille, France.
- [14] Centre hospitalier universitaire de Lille, Université catholique de Lille, 59000 Lille, France.
- [15] Centre hospitalier universitaire de Toulouse, 31400 Toulouse, France.
- [16] Université de Paris, PARCC, INSERM; Vascular medicine department, Georges-Pompidou European Hospital, AP-HP, 75015 Paris, France.
- [17] Centre hospitalier universitaire de Caen-Normandie, 14000 Caen, France.
- [18] Université de Paris, Innovative therapies in haemostasis, INSERM; Respiratory medicine department and biosurgical research laboratory (Carpentier Foundation), Georges-Pompidou European hospital, AH-HP, 75015 Paris, France.
- [19] Université Paris-Saclay, INSERM, UMR-S 1180, 92296 Chatenay-Malabry, France.

- [20] Université de Paris, Innovative therapies in haemostasis, INSERM; Haematology department, Georges-Pompidou European Hospital, AP-HP, 75015 Paris, France.
- [21] Rouen university hospital, FHU REMOD-VHF, 76000 Rouen, France.
- [22] Université de Paris, Innovative therapies in haemostasis, INSERM; Intensive care medicine department and biosurgical research laboratory (Carpentier Foundation), Georges-Pompidou European Hospital, AH-HP, 75015 Paris, France.
- [23] Lariboisière hospital, AP-HP, Université de Paris, 75010 Paris, France.
- [24] Department of cardiology, Saint-Antoine hospital, AP-HP, 75012 Paris, France.

- PMID: **33846096**
- PMCID: PMC7942155
- DOI: 10.1016/j.acvd.2021.02.003

Abstract
in English, French

Background: Coronavirus disease 2019 (COVID-19) has been associated with coagulation disorders, in particular high concentrations of D-dimer, and increased frequency of venous thromboembolism.

Aim: To explore the association between D-dimer at admission and in-hospital mortality in patients hospitalised for COVID-19, with or without symptomatic venous thromboembolism.

Methods: From 26 February to 20 April 2020, D-dimer concentration at admission and outcomes (in-hospital mortality and venous thromboembolism) of patients hospitalised for COVID-19 in medical wards were retrospectively analysed in a multicenter study in 24 French hospitals.

Results: Among 2878 patients enrolled in the study, 1154 (40.1%) patients had D-dimer measurement at admission. Receiver operating characteristic curve analysis identified a D-dimer concentration>1128ng/mL as the best cut-off value for in-hospital mortality (area under the curve 64.9%, 95% confidence interval [CI] 60-69), with a sensitivity of 71.1% (95% CI 62-78) and a specificity of 55.6% (95% CI 52-58), which did not differ in the subgroup of patients with venous thromboembolism during hospitalisation. Among 545 (47.2%) patients with D-dimer concentration>1128ng/mL at admission, 86 (15.8%) deaths occurred during hospitalisation. After adjustment, in Cox proportional hazards and logistic regression models, D-dimer concentration>1128ng/mL at admission was also associated with a worse prognosis, with an odds ratio of 3.07 (95% CI 2.05-4.69; P<0.001) and an adjusted hazard ratio of 2.11 (95% CI 1.31-3.4; P<0.01).

Conclusions: D-dimer concentration>1128ng/mL is a relevant predictive factor for in-hospital mortality in patients hospitalised for COVID-19 in a medical ward, regardless of the occurrence of venous thromboembolism during hospitalisation.

Contexte: La COVID-19 a été associée à des troubles de la coagulation, en particulier des niveaux élevés de D-dimères, et une fréquence accrue d'évènement thromboembolique veineux (ETEV).

Objectif: Nous avons étudié l'association entre les D-dimères à l'admission au service d'accueil des urgences (SAU) et la mortalité chez les patients hospitalisés pour COVID-19 avec ou sans ETEV symptomatique.

Méthodes: Du 26 février au 20 avril 2020, pour les patients COVID-19 hospitalisés dans un service de médecine, le taux de D-dimères à l'admission et les critères de jugement (décès et

ETEV) ont été rétrospectivement analysés dans une étude multicentrique dans 24 hôpitaux français. Les analyses statistiques comprenaient une courbe ROC, des régressions logistiques et des modèles de Cox. Cette recherche a été réalisée dans le respect de la réglementation sur la recherche.

Résultats: Parmi 2878 patients inclus dans la cohorte, 1154 (40,1 %) patients avaient bénéficié d'un dosage des D-dimères à l'admission au SAU. La courbe ROC a identifié une valeur de D-dimères supérieure à 1128 ng/mL comme valeur seuil optimale pour prédire la mortalité à l'hôpital (AUC 64,9 %; IC à 95 % 60–69) avec une sensibilité de 71,1 % (IC à 95 % 62–78) et une spécificité de 55,6 % (IC à 95 % 52–58). Dans l'analyse de sensibilité, ce seuil était similaire dans le sous-groupe de patients atteints de ETEV pendant l'hospitalisation. Parmi 545 (47.2 %) patients avec un taux de D-dimères > 1128 ng/mL à l'admission, 86 (15,8 %) décès étaient survenus pendant l'hospitalisation. Après ajustement, les modèles de régression logistique et de Cox ont confirmé qu'un taux de D-dimères > 1128 ng/mL à l'admission était associé à un mauvais pronostic avec un OR à 3,07 (IC à 95 % 2,05–4,69 ; $p < 0,001$) et un HR de 2,11 (IC à 95 % 1,31 –3,4 ; $p < 0,01$).

Conclusions: Un taux de D-dimères > 1128 ng/mL est un facteur prédictif pertinent de la mortalité chez les patients COVID-19 avec critère d'hospitalisation, quelle que soit la survenue d' ETEV pendant l'hospitalisation.

Keywords: COVID-19; D-dimer; D-dimères; Deep venous thrombosis; Embolie pulmonaire; Microthrombose; Microvascular thrombosis; Pulmonary embolism; Thrombose veineuse profonde.

Copyright © 2021. Published by Elsevier Masson SAS.

- 32 references
- 3 figures

Supplementary info

Publication types, MeSH terms, Substances Expand

Publication types

- Multicenter Study
- Observational Study

MeSH terms

- Adolescent
- Adult
- Aged
- Aged, 80 and over
- Anticoagulants / therapeutic use
- Area Under Curve
- COVID-19 / blood*
- COVID-19 / complications

- COVID-19 / mortality
- COVID-19 Nucleic Acid Testing
- Child
- Child, Preschool
- Electronic Health Records
- Fibrin Fibrinogen Degradation Products / analysis*
- France / epidemiology
- Hospital Mortality
- Humans
- Infant
- Infant, Newborn
- Kaplan-Meier Estimate
- Male
- Middle Aged
- Patient Admission
- Patients' Rooms
- Prognosis
- Proportional Hazards Models
- ROC Curve
- Retrospective Studies
- Thrombophilia / blood*
- Thrombophilia / drug therapy
- Thrombophilia / etiology
- Venous Thromboembolism / epidemiology
- Young Adult

Substances

- Anticoagulants
- Fibrin Fibrinogen Degradation Products
- fibrin fragment D

Full text links

ELSEVIER FULL-TEXT ARTICLE Elsevier Science Free PMC article
Proceed to details
Cite
Share
☐ 777
Observational Study
J Clin Hypertens (Greenwich)
. 2020 Aug;22(8):1379-1388.
doi: 10.1111/jch.13948. Epub 2020 Jul 25.

Use of distinct anti-hypertensive drugs and risk for COVID-19 among hypertensive people: A population-based cohort study in Southern Catalonia, Spain

Angel Vila-Corcoles [1, 2], Eva Satue-Gracia [1, 2], Olga Ochoa-Gondar [1, 2], Cristina Torrente-Fraga [3], Frederic Gomez-Bertomeu [4], Angel Vila-Rovira [2], Imma Hospital-Guardiola [1], Cinta de Diego-Cabanes [1], Ferran Bejarano-Romero [5], Dolors Rovira-Veciana [6], Josep Basora-Gallisa [7]

Affiliations Expand

Affiliations

- [1] Primary Health Care Service Camp de Tarragona, Institut Catala de la Salut, Tarragona, Spain.
- [2] Unitat de Suport a la Recerca Tarragona-Reus, IDIAP Jordi Gol, Barcelona, Spain.
- [3] Department of Information and Communication Technologies, DAP Camp de Tarragona, Institut Catala de la Salut, Tarragona, Spain.
- [4] Department of Microbiology, Hospital Universitari Joan XXIII, Institut Catala de la Salut, Tarragona, Spain.
- [5] Department of Farmacology, DAP Camp de Tarragona, Institut Catala de la Salut, Tarragona, Spain.
- [6] Department of Laboral Medicine, ACTIVA Mutua, Tarragona, Spain.
- [7] Fundació Institut Universitari per a la recerca a l'Atenció Primària de Salut Jordi Gol i Gurina (IDIAPJGol), Barcelona, Spain.

- PMID: **32710674**
- PMCID: PMC8029672
- DOI: 10.1111/jch.13948

Free PMC article
Observational Study

Use of distinct anti-hypertensive drugs and risk for COVID-19 among hypertensive people: A population-based cohort study in Southern Catalonia, Spain

Angel Vila-Corcoles et al. J Clin Hypertens (Greenwich). 2020 Aug.
Free PMC article
Show details
J Clin Hypertens (Greenwich)

. 2020 Aug;22(8):1379-1388.
doi: 10.1111/jch.13948. Epub 2020 Jul 25.

Authors

Angel Vila-Corcoles [1, 2], Eva Satue-Gracia [1, 2], Olga Ochoa-Gondar [1, 2], Cristina Torrente-Fraga [3], Frederic Gomez-Bertomeu [4], Angel Vila-Rovira [2], Imma Hospital-Guardiola [1], Cinta de Diego-Cabanes [1], Ferran Bejarano-Romero [5], Dolors Rovira-Veciana [6], Josep Basora-Gallisa [7]

Affiliations

- [1] Primary Health Care Service Camp de Tarragona, Institut Catala de la Salut, Tarragona, Spain.
- [2] Unitat de Suport a la Recerca Tarragona-Reus, IDIAP Jordi Gol, Barcelona, Spain.
- [3] Department of Information and Communication Technologies, DAP Camp de Tarragona, Institut Catala de la Salut, Tarragona, Spain.
- [4] Department of Microbiology, Hospital Universitari Joan XXIII, Institut Catala de la Salut, Tarragona, Spain.
- [5] Department of Farmacology, DAP Camp de Tarragona, Institut Catala de la Salut, Tarragona, Spain.
- [6] Department of Laboral Medicine, ACTIVA Mutua, Tarragona, Spain.
- [7] Fundació Institut Universitari per a la recerca a l'Atenció Primària de Salut Jordi Gol i Gurina (IDIAPJGol), Barcelona, Spain.

- PMID: **32710674**
- PMCID: PMC8029672
- DOI: 10.1111/jch.13948

Abstract

The use of some anti-hypertensive drugs in the current COVID-19 pandemic has become controversial. This study investigated possible relationships between anti-hypertensive medications use and COVID-19 infection risk in the ambulatory hypertensive population. This is a population-based retrospective cohort study involving 34 936 hypertensive adults >50 years in Tarragona (Southern Catalonia, Spain) who were retrospectively followed through pandemic period (from 01/03/2020 to 30/04/2020). Two data sets including demographic/clinical characteristics (comorbidities and cardiovascular medications use) and laboratory PCR codes for COVID-19 were linked to construct an anonymized research database. Cox regression was used to calculate multivariable hazard ratios (HRs) and estimate the risk of suffering COVID-19 infection. Across study period, 205 PCR-confirmed COVID-19 cases were observed, which means an overall incidence of 586.8 cases per 100 000 persons-period. In multivariable analyses, only age (HR: 1.03; 95% CI: 1.02-1.05; P < .001) and nursing home residence (HR: 19.60; 95% CI: 13.80-27.84; P < .001) appeared significantly associated with increased risk of COVID-19. Considering anti-hypertensive drugs, receiving diuretics (HR: 1.22; 95% CI: 0.90-1.67; P = .205), calcium channel blockers (HR: 1.29; 95%CI: 0.91-1.82; P = .148), beta-blockers (HR: 0.97; 95% CI: 0.68-1.37; P = .844), and angiotensin-converting enzyme inhibitors (HR: 0.83; 95% CI: 0.61-1.13; P = .238) did not significantly alter the risk of PCR-confirmed COVID-19, whereas receiving angiotensin II receptor blockers was associated with an almost statistically significant reduction risk (HR: 0.67; 95% CI: 0.44-1.01; P = .054). In conclusion, our data support that receiving renin-

angiotensin-aldosterone system inhibitors does not predispose for suffering COVID-19 infection in ambulatory hypertensive people. Conversely, receiving angiotensin II receptor blockers could be related with a reduced risk.

Keywords: COVID-19; SARS-COV-2; angiotensin II receptor blockers; angiotensin-converting enzyme inhibitors; anti-hypertensive medication; hypertension.

©2020 Wiley Periodicals, LLC.

Conflict of interest statement

All authors declared that they have no conflicts of interest.

- 1 figure

Supplementary info

Publication types, MeSH terms, Substances, Grant support Expand

Publication types

- Observational Study
- Research Support, Non-U.S. Gov't

MeSH terms

- Adrenergic beta-Antagonists / adverse effects
- Adrenergic beta-Antagonists / therapeutic use
- Aged
- Aged, 80 and over
- Angiotensin Receptor Antagonists / adverse effects
- Angiotensin Receptor Antagonists / therapeutic use
- Angiotensin-Converting Enzyme Inhibitors / adverse effects
- Angiotensin-Converting Enzyme Inhibitors / therapeutic use
- Antihypertensive Agents / adverse effects*
- Antihypertensive Agents / therapeutic use
- COVID-19 / diagnosis*
- COVID-19 / epidemiology
- COVID-19 / virology
- Calcium Channel Blockers / adverse effects
- Calcium Channel Blockers / therapeutic use
- Case-Control Studies
- Comorbidity
- Diuretics / adverse effects
- Diuretics / therapeutic use

- Female
- Humans
- Hypertension / complications
- Hypertension / drug therapy*
- Incidence
- Male
- Middle Aged
- Outcome Assessment, Health Care
- Renin-Angiotensin System / drug effects*
- Retrospective Studies
- Risk Factors
- SARS-CoV-2 / drug effects*
- SARS-CoV-2 / genetics
- Spain / epidemiology

Substances

- Adrenergic beta-Antagonists
- Angiotensin Receptor Antagonists
- Angiotensin-Converting Enzyme Inhibitors
- Antihypertensive Agents
- Calcium Channel Blockers
- Diuretics

Grant support

- COV20/00852/Instituto de Salud Carlos III/International

Full text links

WILEY Free Full Text Wiley Free PMC article
Proceed to details
Cite
Share
☐ 778
Observational Study
Medicine (Baltimore)
. 2021 Jun 4;100(22):e26253.
doi: 10.1097/MD.0000000000026253.

Personalized ß-lactam dosing in patients with coronavirus disease 2019 (COVID-19) and

pneumonia: A retrospective analysis on pharmacokinetics and pharmacokinetic target attainment

Ute Chiriac [1], Otto R Frey [2], Anka C Roehr [2], Andreas Koeberer [3], Patrick Gronau [3], Thomas Fuchs [4], Jason A Roberts [5] [6] [7], Alexander Brinkmann [3]
Affiliations Expand

Affiliations

- [1] Department of Pharmacy, University Hospital of Heidelberg.
- [2] Department of Pharmacy, Heidenheim General Hospital.
- [3] Department of Anesthesiology and Intensive Care Medicine, Heidenheim General Hospital.
- [4] Department of Anesthesiology and Intensive Care Medicine, Heidenheim General, Heidenheim, Germany.
- [5] University of Queensland Centre for Clinical Research, Faculty of Medicine, The University of Queensland.
- [6] Departments of Pharmacy and Intensive Care Medicine, Royal Brisbane and Women's Hospital, Brisbane, Australia.
- [7] Division of Anaesthesiology Critical Care Emergency and Pain Medicine, Nîmes University Hospital, University of Montpellier, Nîmes France.

- PMID: **34087915**
- PMCID: PMC8183774
- DOI: 10.1097/MD.0000000000026253

Free PMC article
Observational Study

Personalized ß-lactam dosing in patients with coronavirus disease 2019 (COVID-19) and pneumonia: A retrospective analysis on pharmacokinetics and pharmacokinetic target attainment

Ute Chiriac et al. Medicine (Baltimore). 2021.
Free PMC article
Show details
Medicine (Baltimore)
. 2021 Jun 4;100(22):e26253.
doi: 10.1097/MD.0000000000026253.

Authors

Ute Chiriac [1], Otto R Frey [2], Anka C Roehr [2], Andreas Koeberer [3], Patrick Gronau [3], Thomas Fuchs [4], Jason A Roberts [5] [6] [7], Alexander Brinkmann [3]

Affiliations

- [1] Department of Pharmacy, University Hospital of Heidelberg.
- [2] Department of Pharmacy, Heidenheim General Hospital.
- [3] Department of Anesthesiology and Intensive Care Medicine, Heidenheim General Hospital.
- [4] Department of Anesthesiology and Intensive Care Medicine, Heidenheim General, Heidenheim, Germany.
- [5] University of Queensland Centre for Clinical Research, Faculty of Medicine, The University of Queensland.
- [6] Departments of Pharmacy and Intensive Care Medicine, Royal Brisbane and Women's Hospital, Brisbane, Australia.
- [7] Division of Anaesthesiology Critical Care Emergency and Pain Medicine, Nîmes University Hospital, University of Montpellier, Nîmes France.

- PMID: **34087915**
- PMCID: PMC8183774
- DOI: 10.1097/MD.0000000000026253

Abstract

Pathophysiological changes are important risk factors for critically ill patients with pneumonia manifesting sub-therapeutic antibiotic exposures during empirical treatment. The effect of coronavirus disease 2019 (COVID-19) on antibiotic dosing requirements is uncertain. We aimed to determine the effect of COVID-19 on ß-lactam pharmacokinetics (PK) and PK target attainment in critically ill patients with a personalized dosing strategy.Retrospective, single-center analysis of COVID-19 ± critically ill patients with pneumonia (community-acquired pneumonia or hospital-acquired pneumonia) who received continuous infusion of a ß-lactam antibiotic with dosing personalized through dosing software and therapeutic drug monitoring. A therapeutic exposure was defined as serum concentration between (css) 4 to 8 times the EUCAST non-species related breakpoint).Data from 58 patients with pneumonia was analyzed. Nineteen patients were tested COVID-19-positive before the start of the antibiotic therapy for community-acquired pneumonia or hospital-acquired pneumonia. Therapeutic exposure was achieved in 71% of COVID-19 patients (68% considering all patients). All patients demonstrated css above the non-species-related breakpoint. Twenty percent exceeded css above the target range (24% of all patients). The median ß-lactam clearance was 49% compared to ß-lactam clearance in a standard patient without a significant difference regarding antibiotic, time of sampling or present COVID-19 infection. Median daily doses were 50% lower compared to standard bolus dosing.COVID-19 did not significantly affect ß-lactam pharmacokinetics in critically ill patients. Personalized ß-lactam dosing strategies were safe in critically ill patients and lead to high PK target attainment with less resources.

Copyright © 2021 the Author(s). Published by Wolters Kluwer Health, Inc.

Conflict of interest statement

The authors report no conflicts of interest.

- 46 references
- 2 figures

Supplementary info

Publication types, MeSH terms, Substances Expand

Publication types

- Observational Study

MeSH terms

- Aged
- Aged, 80 and over
- Body Mass Index
- COVID-19 / drug therapy*
- Critical Illness
- Dose-Response Relationship, Drug
- Drug Monitoring
- Female
- Humans
- Infusions, Intravenous
- Male
- Metabolic Clearance Rate
- Middle Aged
- Retrospective Studies
- SARS-CoV-2
- Severity of Illness Index
- beta-Lactams / administration & dosage*
- beta-Lactams / economics
- beta-Lactams / pharmacokinetics*
- beta-Lactams / therapeutic use

Substances

- beta-Lactams

Full text links

Wolters Kluwer Wolters Kluwer Free PMC article
Proceed to details
Cite
Share
779
Observational Study
Pan Afr Med J
. 2021 Jun 16;39:134.
doi: 10.11604/pamj.2021.39.134.27627. eCollection 2021.

Clinical characteristics and outcomes for pregnant women diagnosed with COVID-19 disease at the University of Benin Teaching Hospital, Benin City, Nigeria

James Osaikhuwuomwan [1], Michael Ezeanochie [1], Charles Uwagboe [1], Kingsley Ndukwu [1], Sofiat Yusuf [1], Adedapo Ande [1 2]
Affiliations Expand

Affiliations

- [1] Department of Obstetrics and Gynaecology, University of Benin Teaching Hospital, Benin City, Edo State, Nigeria.
- [2] Materno-Foetal Unit, University of Benin Teaching Hospital, Benin City, Edo State, Nigeria.

- PMID: **34527150**
- PMCID: PMC8418185
- DOI: 10.11604/pamj.2021.39.134.27627

Free PMC article
Observational Study

Clinical characteristics and outcomes for pregnant women diagnosed with COVID-19 disease at the University of Benin Teaching Hospital, Benin City, Nigeria

James Osaikhuwuomwan et al. Pan Afr Med J. 2021.
Free PMC article
Show details

Pan Afr Med J
. 2021 Jun 16;39:134.
doi: 10.11604/pamj.2021.39.134.27627. eCollection 2021.

Authors

James Osaikhuwuomwan [1], Michael Ezeanochie [1], Charles Uwagboe [1], Kingsley Ndukwu [1], Sofiat Yusuf [1], Adedapo Ande [1 2]

Affiliations

- [1] Department of Obstetrics and Gynaecology, University of Benin Teaching Hospital, Benin City, Edo State, Nigeria.
- [2] Materno-Foetal Unit, University of Benin Teaching Hospital, Benin City, Edo State, Nigeria.

- PMID: **34527150**
- PMCID: PMC8418185
- DOI: 10.11604/pamj.2021.39.134.27627

Abstract

Introduction: the novel coronavirus disease (COVID-19) pandemic has challenged health systems around the world. This study was designed to describe the socio-demographic characteristics of pregnant women with COVID-19 infection, the common clinical features at presentation and the pregnancy outcome at the University of Benin Teaching Hospital, Edo State, Nigeria.

Methods: a cross-sectional analytical study of all confirmed cases of COVID-19 infection from April to September 2020.

Results: out of 69 suspected cases that were tested, 19 (28.4%) were confirmed with COVID-19 infection. The common presenting complaints were fever (68.4 %), cough (57.9 %), sore throat (31.6%), malaise (42.1%), loss of taste (26.3%), anosmia (21.1%), and difficulty with breathing (10.6%). In terms of treatment outcome, 57.9% delivered while 36.8% recovered with pregnancy on-going, and 1 (5.3%) maternal death. Of the 11 women who delivered, 45.4% had vaginal deliveries and 54.6 % had Caesarean section. The mean birth weight was 3.1kg and most of the neonates (81.8%) had normal Apgar scores at birth. There was 1 perinatal death from prematurity, birth asphyxia, and intrauterine growth restriction. The commonest diagnosed co-morbidity of pregnancy was preeclampsia and it was significantly associated with severe COVID-19 disease requiring oxygen supplementation (P = 0.028).

Conclusion: the clinical symptoms of COVID-19 in pregnancy are similar to those described in the non-pregnant population. It did not seem to worsen the maternal or foetal pregnancy outcome. The occurrence of preeclampsia is significantly associated with severe COVID-19 infection requiring respiratory support.

Keywords: COVID-19; Nigeria; coronavirus; pregnancy.

Copyright: James Osaikhuwuomwan et al.

Conflict of interest statement

The authors declare no competing interests.

- 17 references

Supplementary info

Publication types, MeSH terms, Substances Expand

Publication types

- Observational Study

MeSH terms

- Adult
- COVID-19 / complications*
- COVID-19 / physiopathology
- COVID-19 / therapy
- Cesarean Section / statistics & numerical data
- Cross-Sectional Studies
- Delivery, Obstetric / statistics & numerical data*
- Female
- Hospitals, Teaching
- Humans
- Infant, Newborn
- Maternal Death / statistics & numerical data
- Nigeria
- Oxygen / administration & dosage
- Pre-Eclampsia / epidemiology
- Pregnancy
- Pregnancy Complications, Infectious / virology*
- Pregnancy Outcome*
- Premature Birth / epidemiology
- Retrospective Studies
- Severity of Illness Index
- Young Adult

Substances

- Oxygen

Full text links

PAMJ Full text Pan African Medical Journal Free PMC article

Proceed to details

Cite

Share

780

Observational Study

Kidney Int

. 2020 Dec;98(6):1530-1539.

doi: 10.1016/j.kint.2020.07.030. Epub 2020 Aug 15.

Outcomes of patients with end-stage kidney disease hospitalized with COVID-19

Jia H Ng [1], Jamie S Hirsch [2], Rimda Wanchoo [1], Mala Sachdeva [1], Vipulbhai Sakhiya [1], Susana Hong [1], Kenar D Jhaveri [1], Steven Fishbane [3], Northwell COVID-19 Research Consortium and the Northwell Nephrology COVID-19 Research Consortium

Collaborators, Affiliations Expand

Collaborators

- **Northwell COVID-19 Research Consortium and the Northwell Nephrology COVID-19 Research Consortium**: Mersema Abate, Hugo Paz Andrade, Richard L Barnett, Alessandro Bellucci, Madhu C Bhaskaran, Antonio G Corona, Bessy Suyin Flores Chang, Mark Finger, Steven Fishbane, Michael Gitman, Candice Halinski, Shamir Hasan, Azzour D Hazzan, Susana Hong, Yuriy Khanin, Aireen Kuan, Varun Madireddy, Deepa Malieckal, Abdulrahman Muzib, Gayatri Nair, Jia Hwei Ng, Rushang Parikh, Daniel W Ross, Vipulbhai Sakhiya, Mala Sachdeva, Richard Schwarz, Hitesh H Shah, Purva Sharma, Pravin C Singhal, Nupur N Uppal, Rimda Wanchoo, Jamie S Hirsch, Kenar D Jhaveri, Vinay V Nair

Affiliations

- [1] Division of Kidney Diseases and Hypertension, Department of Medicine, Donald and Barbara Zucker School of Medicine at Hofstra/Northwell, Great Neck, New York, USA.
- [2] Division of Kidney Diseases and Hypertension, Department of Medicine, Donald and Barbara Zucker School of Medicine at Hofstra/Northwell, Great Neck, New York, USA; Institute of Health Innovations and Outcomes Research, Feinstein Institutes for Medical Research, Manhasset, New York, USA; Department of Information Services, Northwell Health, New Hyde Park, New York, USA.
- [3] Division of Kidney Diseases and Hypertension, Department of Medicine, Donald and Barbara Zucker School of Medicine at Hofstra/Northwell, Great Neck, New York, USA. Electronic address: sfishbane@northwell.edu.

- PMID: **32810523**
- PMCID: PMC7428720

• DOI: 10.1016/j.kint.2020.07.030

Free PMC article
Observational Study

Outcomes of patients with end-stage kidney disease hospitalized with COVID-19

Jia H Ng et al. Kidney Int. 2020 Dec.
Free PMC article
Show details
Kidney Int
. 2020 Dec;98(6):1530-1539.
doi: 10.1016/j.kint.2020.07.030. Epub 2020 Aug 15.

Authors

Jia H Ng [1], Jamie S Hirsch [2], Rimda Wanchoo [1], Mala Sachdeva [1], Vipulbhai Sakhiya [1], Susana Hong [1], Kenar D Jhaveri [1], Steven Fishbane [3], Northwell COVID-19 Research Consortium and the Northwell Nephrology COVID-19 Research Consortium

Collaborators

- **Northwell COVID-19 Research Consortium and the Northwell Nephrology COVID-19 Research Consortium**: Mersema Abate, Hugo Paz Andrade, Richard L Barnett, Alessandro Bellucci, Madhu C Bhaskaran, Antonio G Corona, Bessy Suyin Flores Chang, Mark Finger, Steven Fishbane, Michael Gitman, Candice Halinski, Shamir Hasan, Azzour D Hazzan, Susana Hong, Yuriy Khanin, Aireen Kuan, Varun Madireddy, Deepa Malieckal, Abdulrahman Muzib, Gayatri Nair, Jia Hwei Ng, Rushang Parikh, Daniel W Ross, Vipulbhai Sakhiya, Mala Sachdeva, Richard Schwarz, Hitesh H Shah, Purva Sharma, Pravin C Singhal, Nupur N Uppal, Rimda Wanchoo, Jamie S Hirsch, Kenar D Jhaveri, Vinay V Nair

Affiliations

- [1] Division of Kidney Diseases and Hypertension, Department of Medicine, Donald and Barbara Zucker School of Medicine at Hofstra/Northwell, Great Neck, New York, USA.
- [2] Division of Kidney Diseases and Hypertension, Department of Medicine, Donald and Barbara Zucker School of Medicine at Hofstra/Northwell, Great Neck, New York, USA; Institute of Health Innovations and Outcomes Research, Feinstein Institutes for Medical Research, Manhasset, New York, USA; Department of Information Services, Northwell Health, New Hyde Park, New York, USA.
- [3] Division of Kidney Diseases and Hypertension, Department of Medicine, Donald and Barbara Zucker School of Medicine at Hofstra/Northwell, Great Neck, New York, USA. Electronic address: sfishbane@northwell.edu.

- PMID: **32810523**
- PMCID: PMC7428720
- DOI: 10.1016/j.kint.2020.07.030

Abstract

Given the high risk of infection-related mortality, patients with end-stage kidney disease (ESKD) may be at increased risk with COVID-19. To assess this, we compared outcomes of patients with and without ESKD, hospitalized with COVID-19. This was a retrospective study of patients admitted with COVID-19 from 13 New York hospitals from March 1, 2020, to April 27, 2020, and followed through May 27, 2020. We measured primary outcome (in-hospital death), and secondary outcomes (mechanical ventilation and length of stay). Of 10,482 patients with COVID-19, 419 had ESKD. Patients with ESKD were older, had a greater percentage self-identified as Black, and more comorbid conditions. Patients with ESKD had a higher rate of in-hospital death than those without (31.7% vs 25.4%, odds ratio 1.38, 95% confidence interval 1.12 - 1.70). This increase rate remained after adjusting for demographic and comorbid conditions (adjusted odds ratio 1.37, 1.09 - 1.73). The odds of length of stay of seven or more days was higher in the group with compared to the group without ESKD in both the crude and adjusted analysis (1.62, 1.27 - 2.06; vs 1.57, 1.22 - 2.02, respectively). There was no difference in the odds of mechanical ventilation between the groups. Independent risk factors for in-hospital death for patients with ESKD were increased age, being on a ventilator, lymphopenia, blood urea nitrogen and serum ferritin. Black race was associated with a lower risk of death. Thus, among patients hospitalized with COVID-19, those with ESKD had a higher rate of in-hospital death compared to those without ESKD.

Keywords: COVID-19; ESKD; ESRD; dialysis; hemodialysis; peritoneal dialysis.

Copyright © 2020 International Society of Nephrology. Published by Elsevier Inc. All rights reserved.

- 58 references
- 3 figures

Supplementary info

Publication types, MeSH terms, Grant support Expand

Publication types

- Multicenter Study
- Observational Study

MeSH terms

- Adult
- Aged
- Aged, 80 and over
- COVID-19 / complications*
- COVID-19 / mortality
- Female
- Humans
- Inpatients

- Kidney Failure, Chronic / complications*
- Kidney Failure, Chronic / mortality
- Length of Stay / statistics & numerical data
- Male
- Middle Aged
- New York / epidemiology
- Respiration, Artificial / statistics & numerical data
- Retrospective Studies
- Risk Factors

Grant support

- R01 DK118017/DK/NIDDK NIH HHS/United States

Full text links

ELSEVIER FULL-TEXT ARTICLE Elsevier Science Free PMC article
Proceed to details
Cite
Share

☐ 781

Observational Study

J Surg Oncol
. 2020 Dec;122(7):1271-1275.
doi: 10.1002/jso.26193. Epub 2020 Sep 3.

An observational study of the demographic and treatment changes in a tertiary colorectal cancer center during the COVID-19 pandemic

Barath Raj Kumar[1], Diwakar Pandey[1], Jitender Rohila[1], Ashwin deSouza[1], Avanish Saklani[1]
Affiliations Expand

Affiliation

- [1] Division of Colorectal Oncology, Department of Surgical Oncology, Tata Memorial Centre, Homi Bhabha National Institute, Mumbai, India.

- PMID: **32885429**
- DOI: 10.1002/jso.26193

Observational Study

An observational study of the demographic and treatment changes in a tertiary colorectal cancer center during the COVID-19 pandemic

Barath Raj Kumar et al. J Surg Oncol. 2020 Dec.
Show details
J Surg Oncol
. 2020 Dec;122(7):1271-1275.
doi: 10.1002/jso.26193. Epub 2020 Sep 3.

Authors

Barath Raj Kumar [1], Diwakar Pandey [1], Jitender Rohila [1], Ashwin deSouza [1], Avanish Saklani [1]

Affiliation

- [1] Division of Colorectal Oncology, Department of Surgical Oncology, Tata Memorial Centre, Homi Bhabha National Institute, Mumbai, India.

- PMID: **32885429**
- DOI: 10.1002/jso.26193

Abstract

Background and objectives: The COVID-19 pandemic has wreaked havoc in the healthcare infrastructure. While we change our surgical practice, cancer care will take a toll on unprecedented long-term outcomes. We elucidate our experience that has unfolded during this period.

Methods: This study included retrospective data of patients being treated for colorectal cancer and peritoneal surface malignancy between January and May 2020. We compared the treatment changes before and after the national emergency was declared.

Results: There was a 65% decrease in outpatients with a 90% drop in endoscopy procedures. Treatment protocols were changed with a 200% increase in short course radiation in rectal cancer. Colon cancer and anal melanoma were triaged to undergo 'essential' surgery. No robotic or exenteration procedures were performed in April and May. Patients with a low peritoneal cancer index underwent surgery alone. The relative number of emergency surgeries were unchanged.

Conclusion: There is no standard approach to deliver cancer care during the COVID-19 pandemic. Treatment decisions were made based on the state of affairs that COVID-19 had created during that cross-section of time and protocols were redrawn to strike a balance between the risk of death from colorectal cancer and the risk of death from COVID-19 infection.

Keywords: COVID-19; colorectal cancer; peritoneal surface malignancy.

© 2020 Wiley Periodicals LLC.

- 15 references

Supplementary info

Publication types, MeSH terms Expand

Publication types

- Observational Study

MeSH terms

- COVID-19 / epidemiology*
- Colorectal Neoplasms / diagnosis
- Colorectal Neoplasms / epidemiology*
- Colorectal Neoplasms / therapy*
- Humans
- India / epidemiology
- Outpatients / statistics & numerical data
- Pandemics
- Retrospective Studies
- Tertiary Care Centers / statistics & numerical data

Full text links

WILEY Full Text Article Wiley

Proceed to details

Cite

Share

☐ 782

Observational Study

Pediatr Crit Care Med
. 2022 Mar 1;23(3):e145-e152.
doi: 10.1097/PCC.0000000000002850.

Strain Echocardiography and Myocardial Dysfunction in Critically Ill Children With Multisystem Inflammatory Syndrome Unrecognized by Conventional

Echocardiography: A Retrospective Cohort Analysis

Sonali Basu[1], Esther J Kim[1], Matthew P Sharron[1], Ashley Austin[2], Murray M Pollack[1], Ashraf S Harahsheh[3], Niti Dham[3]
Affiliations Expand

Affiliations

- [1] Division of Critical Care Medicine, Department of Pediatrics, Children's National Hospital and George Washington University School of Medicine and Health Sciences, Washington, DC.
- [2] Division of Cardiology, Department of Echocardiography, Children's National Hospital, Washington, DC.
- [3] Division of Cardiology, Department of Pediatrics, Children's National Hospital and George Washington University School of Medicine and Health Sciences, Washington, DC.

- PMID: **34636357**
- PMCID: PMC8887681
- DOI: 10.1097/PCC.0000000000002850

Free PMC article
Observational Study

Strain Echocardiography and Myocardial Dysfunction in Critically Ill Children With Multisystem Inflammatory Syndrome Unrecognized by Conventional Echocardiography: A Retrospective Cohort Analysis

Sonali Basu et al. Pediatr Crit Care Med. 2022.
Free PMC article
Show details
Pediatr Crit Care Med
. 2022 Mar 1;23(3):e145-e152.
doi: 10.1097/PCC.0000000000002850.

Authors

Sonali Basu[1], Esther J Kim[1], Matthew P Sharron[1], Ashley Austin[2], Murray M Pollack[1], Ashraf S Harahsheh[3], Niti Dham[3]

Affiliations

- [1] Division of Critical Care Medicine, Department of Pediatrics, Children's National Hospital and George Washington University School of Medicine and Health Sciences, Washington, DC.
- [2] Division of Cardiology, Department of Echocardiography, Children's National Hospital, Washington, DC.
- [3] Division of Cardiology, Department of Pediatrics, Children's National Hospital and George Washington University School of Medicine and Health Sciences, Washington, DC.

- PMID: **34636357**
- PMCID: PMC8887681
- DOI: 10.1097/PCC.0000000000002850

Abstract

Objectives: Multisystem inflammatory syndrome in children is a newly defined complication of severe acute respiratory syndrome coronavirus 2 infection that can result in cardiogenic shock in the pediatric population. Early detection of cardiac dysfunction is imperative in directing therapy and identifying patients at highest risk for deterioration. This study compares the strengths of conventional and strain echocardiography in identifying cardiac dysfunction in critically ill children with multisystem inflammatory syndrome in children and their association with ICU therapeutic needs and clinical outcomes.

Design: Retrospective, observational cohort study.

Setting: A large, quaternary care PICU.

Patients: Sixty-five pediatric patients admitted to the PICU with the diagnosis of multisystem inflammatory syndrome in children from March 2020 to March 2021.

Interventions: Global longitudinal strain four chamber was measured retrospectively by strain echocardiography and compared with conventional echocardiography. Cardiac dysfunction was defined by left ventricular ejection fraction less than 55% and global longitudinal strain four chamber greater than or equal to -17.2%. Clinical variables examined included cardiac biomarkers, immune therapies, and ICU interventions and outcomes.

Measurements and main results: Twenty-four patients (37%) had abnormal left ventricular ejection fraction and 56 (86%) had abnormal global longitudinal strain four chamber. Between patients with normal and abnormal left ventricular ejection fraction, we failed to identify a difference in cardiac biomarker levels, vasoactive use, respiratory support needs, or ICU length of stay. Global longitudinal strain four chamber was associated with maximum cardiac biomarker levels. Abnormal global longitudinal strain four chamber was associated with greater odds of any vasoactive use (odds ratio, 5.8; 95% CI, 1.3-25.3; z-statistic, 2.3; $p = 0.021$). The number of days of vasoactive infusion was correlated with global longitudinal strain four chamber ($r = 0.400$; 95% CI, 2.4-3.9; $p < 0.001$). Children with abnormal strain had longer ICU length of stay (4.5 d vs 2 d; $p = 0.014$).

Conclusions: Our findings suggest strain echocardiography can detect abnormalities in cardiac function in multisystem inflammatory syndrome in children patients unrecognized by conventional echocardiography. These abnormalities are associated with increased use of intensive

care therapies. Evaluation of these patients with strain echocardiography may better identify those with myocardial dysfunction and need for more intensive therapy.

Copyright © 2021 by the Society of Critical Care Medicine and the World Federation of Pediatric Intensive and Critical Care Societies.

Conflict of interest statement

Dr. Pollack received funding from the National Institutes of Health and Mallinckrodt Pharmaceuticals LLC. The remaining authors have disclosed that they do not have any potential conflicts of interest.

- 33 references
- 1 figure

Supplementary info

Publication types, MeSH terms, Supplementary concepts Expand

Publication types

- Observational Study
- Research Support, N.I.H., Extramural
- Research Support, Non-U.S. Gov't

MeSH terms

- COVID-19* / complications
- COVID-19* / diagnostic imaging
- Child
- Cohort Studies
- Critical Illness / therapy
- Echocardiography
- Humans
- Retrospective Studies
- SARS-CoV-2
- Stroke Volume
- Systemic Inflammatory Response Syndrome
- Ventricular Dysfunction, Left* / diagnostic imaging
- Ventricular Dysfunction, Left* / etiology
- Ventricular Function, Left

Supplementary concepts

- pediatric multisystem inflammatory disease, COVID-19 related

Full text links

Wolters Kluwer Wolters Kluwer Free PMC article
Proceed to details
Cite
Share
783

Observational Study
Gastroenterology
. 2020 Nov;159(5):1979-1981.e3.
doi: 10.1053/j.gastro.2020.07.042. Epub 2020 Jul 25.

Collateral Effect of Coronavirus Disease 2019 Pandemic on Hospitalizations and Clinical Outcomes in Gastrointestinal and Liver Diseases: A Territory-wide Observational Study in Hong Kong

Louis H S Lau [1], Sunny H Wong [2], Terry C F Yip [3], Grace L H Wong [3], Vincent W S Wong [3], Joseph J Y Sung [4]
Affiliations Expand

Affiliations

- [1] Department of Medicine and Therapeutics, Prince of Wales Hospital, Faculty of Medicine, The Chinese University of Hong Kong, Hong Kong, China; Institute of Digestive Diseases, The Chinese University of Hong Kong, Hong Kong, China.
- [2] Department of Medicine and Therapeutics, Prince of Wales Hospital, Faculty of Medicine, The Chinese University of Hong Kong, Hong Kong, China; Institute of Digestive Diseases, The Chinese University of Hong Kong, Hong Kong, China; State Key Laboratory of Digestive Disease, Li Ka Shing Institute of Health Sciences, Faculty of Medicine, The Chinese University of Hong Kong, Hong Kong, China.
- [3] Department of Medicine and Therapeutics, Prince of Wales Hospital, Faculty of Medicine, The Chinese University of Hong Kong, Hong Kong, China; Institute of Digestive Diseases, The Chinese University of Hong Kong, Hong Kong, China; Medical Data Analytic Centre, Department of Medicine and Therapeutics, The Chinese University of Hong Kong, Hong Kong, China.
- [4] Department of Medicine and Therapeutics, Prince of Wales Hospital, Faculty of Medicine, The Chinese University of Hong Kong, Hong Kong, China; Institute of Digestive Diseases, The Chinese University of Hong Kong, Hong Kong, China; State Key Laboratory of Digestive Disease, Li Ka Shing Institute of Health Sciences, Faculty of Medicine, The Chinese University of Hong Kong, Hong Kong, China. Electronic address: jjysung@cuhk.edu.hk.

- PMID: **32721440**
- PMCID: PMC7382332
- DOI: 10.1053/j.gastro.2020.07.042

Free PMC article
Observational Study

Collateral Effect of Coronavirus Disease 2019 Pandemic on Hospitalizations and Clinical Outcomes in Gastrointestinal and Liver Diseases: A Territory-wide Observational Study in Hong Kong

Louis H S Lau et al. Gastroenterology. 2020 Nov.
Free PMC article
Show details
Gastroenterology
. 2020 Nov;159(5):1979-1981.e3.
doi: 10.1053/j.gastro.2020.07.042. Epub 2020 Jul 25.

Authors

Louis H S Lau [1], Sunny H Wong [2], Terry C F Yip [3], Grace L H Wong [3], Vincent W S Wong [3], Joseph J Y Sung [4]

Affiliations

- [1] Department of Medicine and Therapeutics, Prince of Wales Hospital, Faculty of Medicine, The Chinese University of Hong Kong, Hong Kong, China; Institute of Digestive Diseases, The Chinese University of Hong Kong, Hong Kong, China.
- [2] Department of Medicine and Therapeutics, Prince of Wales Hospital, Faculty of Medicine, The Chinese University of Hong Kong, Hong Kong, China; Institute of Digestive Diseases, The Chinese University of Hong Kong, Hong Kong, China; State Key Laboratory of Digestive Disease, Li Ka Shing Institute of Health Sciences, Faculty of Medicine, The Chinese University of Hong Kong, Hong Kong, China.
- [3] Department of Medicine and Therapeutics, Prince of Wales Hospital, Faculty of Medicine, The Chinese University of Hong Kong, Hong Kong, China; Institute of Digestive Diseases, The Chinese University of Hong Kong, Hong Kong, China; Medical Data Analytic Centre, Department of Medicine and Therapeutics, The Chinese University of Hong Kong, Hong Kong, China.
- [4] Department of Medicine and Therapeutics, Prince of Wales Hospital, Faculty of Medicine, The Chinese University of Hong Kong, Hong Kong, China; Institute of Digestive Diseases, The Chinese University of Hong Kong, Hong Kong, China; State Key Laboratory of Digestive Disease, Li Ka Shing Institute of Health Sciences, Faculty of Medicine, The Chinese University of Hong Kong, Hong Kong, China. Electronic address: jjysung@cuhk.edu.hk.

- PMID: **32721440**
- PMCID: PMC7382332
- DOI: 10.1053/j.gastro.2020.07.042

No abstract available

Keywords: COVID-19; Collateral; Gastroenterology; Liver; Pandemic.

- 8 references
- 1 figure

Supplementary info

Publication types, MeSH terms Expand

Publication types

- Observational Study

MeSH terms

- Betacoronavirus / pathogenicity
- COVID-19
- Coronavirus Infections / epidemiology*
- Coronavirus Infections / prevention & control
- Coronavirus Infections / therapy
- Coronavirus Infections / virology
- Delayed Diagnosis
- Endoscopy / statistics & numerical data
- Gastrointestinal Diseases / diagnosis
- Gastrointestinal Diseases / mortality
- Gastrointestinal Diseases / therapy*
- Hong Kong / epidemiology
- Hospital Mortality
- Humans
- Intensive Care Units / statistics & numerical data
- Liver Diseases / diagnosis
- Liver Diseases / mortality
- Liver Diseases / therapy*
- Pandemics / prevention & control
- Patient Admission / statistics & numerical data*
- Pneumonia, Viral / epidemiology*
- Pneumonia, Viral / prevention & control
- Pneumonia, Viral / therapy
- Pneumonia, Viral / virology

- Retrospective Studies
- SARS-CoV-2

Full text links

ELSEVIER FULL-TEXT ARTICLE Elsevier Science Free PMC article

Proceed to details

Cite

Share

784

Observational Study

Biomed Res Int

. 2022 Feb 16;2022:4620037.

doi: 10.1155/2022/4620037. eCollection 2022.

Hematological Findings among COVID-19 Patients Attending King Khalid Hospital at Najran, Kingdom of Saudi Arabia

Ahmed M E Elkhalifa [1] [2], Abozer Y Elderdery [3], Ibrahim Ali Al Bataj [4], Abdelhakam G Tamomh [2], Masaud M Alyami [5], Hussein A Almakrami [6], Mubarak A Alofair [6], Mohammed A Almorish [7], Salem Bashanfer [8], Mohammed I Tabash [9], Hadeil M E Idris [10], Dania Z Ahmed [11], Abdulaziz H Alhamidi [12], Pooi Ling Mok [13]

Affiliations Expand

Affiliations

- [1] College of Health Sciences, Saudi Electronic University, Riyadh, Saudi Arabia.
- [2] Faculty of Medical Laboratory Sciences, University of El Imam El Mahdi, Kosti, Sudan.
- [3] College of Applied Medical Science, Jouf University, Sakaka, Saudi Arabia.
- [4] Healthcare Model, General Directorate of Health Affairs-Najran, Saudi Arabia.
- [5] Dhahran Aljnoob Health Sector-Asser, Saudi Arabia.
- [6] Najran General Directorate of Health Affairs, Saudi Arabia.
- [7] College of Medicine and Health Sciences, Sana'a University, Yemen.
- [8] Centre for Regenerative Medicine, Institute for Regeneration and Repair, University of Edinburgh, Edinburgh, UK.
- [9] Department of Laboratory Medicine, Faculty of Applied Medical Sciences, Al-Azhar University, Gaza, State of Palestine.
- [10] College of Applied Medical Sciences, Shaqra University, Saudi Arabia.
- [11] Hampshire Hospitals NHS Foundation Trust and Health Education, UK.
- [12] Clinical Laboratory Sciences Department, College of Applied Medical Science, King Saud University, Riyadh, Saudi Arabia.
- [13] Department of Biomedical Sciences, Faculty of Medicine and Health Sciences, Universiti Putra Malaysia, 43400 UPM Serdang, Selangor, Malaysia.

- PMID: **35224093**
- PMCID: PMC8865990
- DOI: 10.1155/2022/4620037

Free PMC article
Observational Study

Hematological Findings among COVID-19 Patients Attending King Khalid Hospital at Najran, Kingdom of Saudi Arabia

Ahmed M E Elkhalifa et al. Biomed Res Int. 2022.
Free PMC article
Show details
Biomed Res Int
. 2022 Feb 16;2022:4620037.
doi: 10.1155/2022/4620037. eCollection 2022.

Authors

Ahmed M E Elkhalifa [1] [2], Abozer Y Elderdery [3], Ibrahim Ali Al Bataj [4], Abdelhakam G Tamomh [2], Masaud M Alyami [5], Hussein A Almakrami [6], Mubarak A Alofair [6], Mohammed A Almorish [7], Salem Bashanfer [8], Mohammed I Tabash [9], Hadeil M E Idris [10], Dania Z Ahmed [11], Abdulaziz H Alhamidi [12], Pooi Ling Mok [13]

Affiliations

- [1] College of Health Sciences, Saudi Electronic University, Riyadh, Saudi Arabia.
- [2] Faculty of Medical Laboratory Sciences, University of El Imam El Mahdi, Kosti, Sudan.
- [3] College of Applied Medical Science, Jouf University, Sakaka, Saudi Arabia.
- [4] Healthcare Model, General Directorate of Health Affairs-Najran, Saudi Arabia.
- [5] Dhahran Aljnoob Health Sector-Asser, Saudi Arabia.
- [6] Najran General Directorate of Health Affairs, Saudi Arabia.
- [7] College of Medicine and Health Sciences, Sana'a University, Yemen.
- [8] Centre for Regenerative Medicine, Institute for Regeneration and Repair, University of Edinburgh, Edinburgh, UK.
- [9] Department of Laboratory Medicine, Faculty of Applied Medical Sciences, Al-Azhar University, Gaza, State of Palestine.
- [10] College of Applied Medical Sciences, Shaqra University, Saudi Arabia.
- [11] Hampshire Hospitals NHS Foundation Trust and Health Education, UK.
- [12] Clinical Laboratory Sciences Department, College of Applied Medical Science, King Saud University, Riyadh, Saudi Arabia.
- [13] Department of Biomedical Sciences, Faculty of Medicine and Health Sciences, Universiti Putra Malaysia, 43400 UPM Serdang, Selangor, Malaysia.

- PMID: **35224093**

- PMCID: PMC8865990
- DOI: 10.1155/2022/4620037

Abstract

COVID-19 is a global pandemic viral infection that has affected millions worldwide. Limited data is available on the effect of COVID-19 on hematological parameters in Saudi Arabia. This study is aimed at examining the role of hematological parameters among COVID-19 patients admitted to King Khalid Hospital in Najran, Saudi Arabia. This is a retrospective, hospital-based study of 514 cases who were recruited during August to October 2020. 257 COVID-19 patients formed the study group, and a further 257 negative subjects formed the control group. Anemia was significantly elevated in positive subjects over controls (respectively, 64.2% and 35.8%), with patients 2.5 times more likely to be anemic ($p < 0.01$). Thrombocytopenia was higher in patients over controls (respectively, 62% and 38%), with patients ~1.7 times more likely to be thrombocytopenic ($p < 0.01$). Moreover, leukopenia was significantly higher in patients over controls (respectively, 71% and 29%), with positive subjects ~2.6 times more likely to be leukopenic. Our study results indicate that mild anemia associated with leukopenia may have diagnostic value for COVID-19. Careful assessment of hematological parameters, at baseline and throughout the disease path, will assist physicians in formulating personalized approaches to treatment and promptly offer intensive care to those in greater need.

Copyright © 2022 Ahmed M. E. Elkhalifa et al.

Conflict of interest statement

The authors declare that there is no conflict of interest regarding the publication of this article.

- 36 references

Supplementary info

Publication types, MeSH terms Expand

Publication types

- Observational Study

MeSH terms

- Adult
- Aged
- Anemia / virology
- COVID-19 / blood*
- COVID-19 / complications*
- Female
- Hospitalization
- Humans
- Leukopenia / virology

- Lymphocyte Count
- Male
- Middle Aged
- Retrospective Studies
- Saudi Arabia
- Thrombocytopenia / virology

Full text links

Hindawi Limited Free PMC article
Proceed to details
Cite
Share
785
Observational Study
World J Gastroenterol
. 2022 Feb 7;28(5):570-587.
doi: 10.3748/wjg.v28.i5.570.

Abnormal liver chemistries as a predictor of COVID-19 severity and clinical outcomes in hospitalized patients

Arunkumar Krishnan [1], Laura Prichett [2], Xueting Tao [2], Saleh A Alqahtani [1], James P Hamilton [1], Esteban Mezey [1], Alexandra T Strauss [1], Ahyoung Kim [1], James J Potter [1], Po-Hung Chen [1], Tinsay A Woreta [3]
Affiliations Expand

Affiliations

- [1] Division of Gastroenterology and Hepatology, Johns Hopkins University School of Medicine, Baltimore, MD 21287, United States.
- [2] Department of Pediatrics, Johns Hopkins University School of Medicine, Baltimore, MD 21287, United States.
- [3] Division of Gastroenterology and Hepatology, Johns Hopkins University School of Medicine, Baltimore, MD 21287, United States. tworeta1@jhmi.edu.

- PMID: **35316959**
- PMCID: PMC8905016
- DOI: 10.3748/wjg.v28.i5.570

Free PMC article
Observational Study

Abnormal liver chemistries as a predictor of COVID-19 severity and clinical outcomes in hospitalized patients

Arunkumar Krishnan et al. World J Gastroenterol. 2022.
Free PMC article
Show details
World J Gastroenterol
. 2022 Feb 7;28(5):570-587.
doi: 10.3748/wjg.v28.i5.570.

Authors

Arunkumar Krishnan [1], Laura Prichett [2], Xueting Tao [2], Saleh A Alqahtani [1], James P Hamilton [1], Esteban Mezey [1], Alexandra T Strauss [1], Ahyoung Kim [1], James J Potter [1], Po-Hung Chen [1], Tinsay A Woreta [3]

Affiliations

- [1] Division of Gastroenterology and Hepatology, Johns Hopkins University School of Medicine, Baltimore, MD 21287, United States.
- [2] Department of Pediatrics, Johns Hopkins University School of Medicine, Baltimore, MD 21287, United States.
- [3] Division of Gastroenterology and Hepatology, Johns Hopkins University School of Medicine, Baltimore, MD 21287, United States. tworeta1@jhmi.edu.

- PMID: **35316959**
- PMCID: PMC8905016
- DOI: 10.3748/wjg.v28.i5.570

Abstract

Background: Abnormal liver chemistries are common findings in patients with Coronavirus Disease 2019 (COVID-19). However, the association of these abnormalities with the severity of COVID-19 and clinical outcomes is poorly understood.

Aim: We aimed to assess the prevalence of elevated liver chemistries in hospitalized patients with COVID-19 and compare the serum liver chemistries to predict the severity and in-hospital mortality.

Methods: This retrospective, observational study included 3380 patients with COVID-19 who were hospitalized in the Johns Hopkins Health System (Baltimore, MD, United States). Demographic data, clinical characteristics, laboratory findings, treatment measures, and outcome data were collected. Cox regression modeling was used to explore variables associated with abnormal liver chemistries on admission with disease severity and prognosis.

Results: A total of 2698 (70.4%) had abnormal alanine aminotransferase (ALT) at the time of admission. Other more prevalent abnormal liver chemistries were aspartate aminotransferase

(AST) (44.4%), alkaline phosphatase (ALP) (16.1%), and total bilirubin (T-Bil) (5.9%). Factors associated with liver injury were older age, Asian ethnicity, other race, being overweight, and obesity. Higher ALT, AST, T-Bil, and ALP levels were more commonly associated with disease severity. Multivariable adjusted Cox regression analysis revealed that abnormal AST and T-Bil were associated with the highest mortality risk than other liver injury indicators during hospitalization. Abnormal AST, T-Bil, and ALP were associated with a need for vasopressor drugs, whereas higher levels of AST, T-Bil, and a decreased albumin levels were associated with mechanical ventilation.

Conclusion: Abnormal liver chemistries are common at the time of hospital admission in COVID-19 patients and can be closely related to the patient's severity and prognosis. Elevated liver chemistries, specifically ALT, AST, ALP, and T-Bil levels, can be used to stratify risk and predict the need for advanced therapies in these patients.

Keywords: Alanine aminotransferase; Aspartate aminotransferase; Liver injury; Liver tests; Severe acute respiratory syndrome coronavirus 2; bilirubin.

©The Author(s) 2022. Published by Baishideng Publishing Group Inc. All rights reserved.

Conflict of interest statement

Conflict-of-interest statement: All the Authors have no conflict of interest related to the manuscript.

- 32 references
- 3 figures

Supplementary info

Publication types, MeSH terms, Substances Expand

Publication types

- Observational Study

MeSH terms

- Alanine Transaminase
- Aspartate Aminotransferases
- COVID-19* / therapy
- Humans
- Liver
- Retrospective Studies

Substances

- Aspartate Aminotransferases
- Alanine Transaminase

Full text links

Free BPG Article Baishideng Publishing Group Inc. Free PMC article
Proceed to details
Cite
Share
786

Observational Study
Crit Care
. 2021 May 25;25(1):177.
doi: 10.1186/s13054-021-03588-4.

Relationship between ventilator-associated pneumonia and mortality in COVID-19 patients: a planned ancillary analysis of the coVAPid cohort

Saad Nseir [1, 2], Ignacio Martin-Loeches [3, 4], Pedro Povoa [5, 6], Matthieu Metzelard [7], Damien Du Cheyron [8], Fabien Lambiotte [9], Fabienne Tamion [10], Marie Labruyere [11], Demosthenes Makris [12], Claire Boulle Geronimi [13], Marc Pinetonde Chambrun [14], Martine Nyunga [15], Olivier Pouly [16], Bruno Mégarbane [17], Anastasia Saade [18], Gemma Gomà [19], Eleni Magira [20], Jean-François Llitjos [21], Antoni Torres [22], Iliana Ioannidou [23], Alexandre Pierre [24], Luis Coelho [5], Jean Reignier [25], Denis Garot [26], Louis Kreitmann [27], Jean-Luc Baudel [28], Guillaume Voiriot [29], Damien Contou [30], Alexandra Beurton [31], Pierre Asfar [32], Alexandre Boyer [33], Arnaud W Thille [34], Armand Mekontso-Dessap [35], Vassiliki Tsolaki [12], Christophe Vinsonneau [36], Pierre-Edouard Floch [37], Loïc Le Guennec [38], Adrian Ceccato [39], Antonio Artigas [40], Mathilde Bouchereau [41], Julien Labreuche [42], Alain Duhamel [42], Anahita Rouzé [41, 43], coVAPid study group
Collaborators, Affiliations Expand

Collaborators

- **coVAPid study group**: Raphaël Favory, Sébastien Préau, Mercé Jourdain, Julien Poissy, Piehr Saint Leger, Thierry Van der Linden, Anne Veinstein, Elie Azoulay, Frédéric Pene, Maelle Martin, Keyvan Razazi, Gaëtan Plantefeve, Muriel Fartoukh, Didier Thevenin, Bertrand Guidet, Nicolas Weiss, Achille Kouatchet, Charlotte Salmon, Guillaume Brunin, Safaa Nemlaghi, David Meguerditchian, Laurent Argaud, Sebastian Voicu, Charles-Edouard Luyt, Benjamin Kowalski, Edgar Moglia, Luis Morales, Antonia Koutsoukou, Spyros D Mentzelopoulos, David Nora, Sean Boyd, Julien Maizel, Pierre Cuchet, Quentin Delforge, Jean-Pierre Quenot, Déborah Boyer, Catia Cilloniz

Affiliations

- [1] Médecine Intensive-Réanimation, CHU de Lille, F-59000, Lille, France. s-nseir@chru-lille.fr.
- [2] Inserm U1285, CNRS, UMR 8576-UGSF-Unité de Glycobiologie Structurale et Fonctionnelle, Univ. Lille, Lille, France. s-nseir@chru-lille.fr.
- [3] Department of Intensive Care Medicine, Multidisciplinary Intensive Care Research Organization (MICRO), St. James's Hospital, St. James Street, Dublin 8, Dublin, Eire, Ireland.
- [4] Hospital Clinic, IDIBAPS, Universided de Barcelona, CIBERes, Barcelona, Spain.
- [5] Polyvalent Intensive Care Unit, São Francisco Xavier Hospital, Centro Hospitalar de Lisboa Ocidental, and NOVA Medical School, CHRC, New University of Lisbon, Lisbon, Portugal.
- [6] Center for Clinical Epidemiology and Research Unit of Clinical Epidemiology, OUH Odense University Hospital, Odense, Denmark.
- [7] Medical ICU, Amiens University Hospital, Amiens, France.
- [8] Department of Medical Intensive Care, Caen University Hospital, 14000, Caen, France.
- [9] Service de Réanimation Polyvalente, Centre Hospitalier de Valenciennes, Valenciennes, France.
- [10] Medical Intensive Care Unit, Rouen University Hospital, Normandie Université, UNIROUEN, Inserm U1096, FHU-REMOD-VHF, 76000, Rouen, France.
- [11] Department of Intensive Care, François Mitterrand University Hospital, Dijon, France.
- [12] Intensive Care Unit, University Hospital of Larissa, University of Thessaly, 41110, Biopolis Larissa, Greece.
- [13] Service de Réanimation Et de Soins Intensifs, Centre Hospitalier de Douai, Route de Cambrai, Douai, France.
- [14] Service de Médecine Intensive Réanimation, Institut de Cardiologie, Hôpital Pitié-Salpêtrière, Assistance Publique-Hôpitaux de Paris (APHP), Sorbonne Université, 47-83, Boulevard de L'Hôpital, 75651, Paris Cedex 13, France.
- [15] ICU, Roubaix Hospital, Roubaix, France.
- [16] Médecine Intensive Réanimation, Hôpital Saint Philibert GHICL, Université Catholique, Lille, France.
- [17] Réanimation Médicale Et Toxicologique, Hôpital Lariboisière, Université de Paris, INSERM UMRS-1144, Paris, France.
- [18] Service de Médecine Intensive Et Réanimation, Hôpital Saint-Louis, 1 Avenue Claude Vellefaux, 75010, Paris, France.
- [19] Critical Care Department, Hospital Universitari Parc Taulí, Sabadell, Spain.
- [20] 1St Department of Intensive Care Medicine, National and Kapodistrian University of Athens Medical School, Evaggelismos Hospital, Athens, Greece.
- [21] Medical Intensive Care Unit, Cochin Hospital, AP-HP. Centre, Université de Paris, Paris, France.
- [22] Department of Pulmonology, Hospital Clinic Barcelona, University of Barcelona, IDIBAPS, CIBERES, ICREA, Barcelona, Spain.
- [23] 1St Department of Pulmonary Medicine and Intensive Care Unit, National and Kapodistrian University of Athens, "Sotiria" Chest Hospital, Athens, Greece.
- [24] Réanimation Polyvalente, CH Lens, Lens, France.
- [25] Service de Médecine Intensive Réanimation, CHU de Nantes, Nantes, France.
- [26] Service de Médecine Intensive Réanimation, CHU de Tours, Hôpital Bretonneau, 2 Bd Tonnellé, 37000, Tours, France.
- [27] Service de Médecine Intensive - Réanimation, Hospices Civils de Lyon, Hôpital Edouard Herriot, 5, place d'Arsonval, 69437, Lyon Cedex 03, France.

- [28] Service de Médecine Intensive Réanimation, AP-HP, Hôpital Saint-Antoine, Assistance Publique-Hôpitaux de Paris, 184 rue du Faubourg Saint-Antoine, 75571, Paris Cedex 12, France.
- [29] Sorbonne Université, Assistance Publique-Hôpitaux de Paris, Service de Médecine Intensive Réanimation, Hôpital Tenon, Paris, France.
- [30] Réanimation Polyvalente, CH Victor Dupouy, Argenteuil, France.
- [31] Service de Pneumologie, Médecine Intensive - Réanimation (Département "R3S"), AP-HP, Sorbonne Université, Groupe Hospitalier Universitaire Pitié-Salpêtrière Charles Foix, INSERM, UMRS1158 Neurophysiologie Respiratoire Expérimentale Et Clinique, Paris, France.
- [32] Département de Médecine Intensive-Réanimation, CHU D'Angers, Université D'Angers, 4 rue Larrey, 49933, Angers Cedex 9, France.
- [33] Intensive Care Unit, Pellegrin-Tripode Hospital, University Hospital of Bordeaux, Bordeaux, France.
- [34] CHU de Poitiers, Médecine Intensive Réanimation, CIC 1402 ALIVE, Université de Poitiers, Poitiers, France.
- [35] APHP, CHU Henri Mondor, Service de Médecine Intensive RéanimationUniversité Paris Est-Créteil, Faculté de Santé, Groupe de Recherche Clinique CARMASINSERM U955, Institut Mondor de Recherche Biomédicale, 94010, Créteil, France.
- [36] Service de Médecine Intensive Réanimation, Centre Hospitalier de Béthune, Réseau de Recherche Boréal, 62408, Béthune, France.
- [37] Service de Réanimation, Hôpital Duchenne, Rue Monod, 62200, Boulogne-sur-Mer, France.
- [38] Sorbonne Université, AP-HP, Hôpital de La Pitié-Salpêtrière, Département de Neurologie, Unité de Médecine Intensive Réanimation Neurologique, Paris, France.
- [39] Intensive Care Unit, Hospital Universitari Sagrat Cor, and Ciber de Enfermedades Respiratorias (Ciberes, CB06/06/0028)-Institut D'Investigacions Biomèdiques August Pi I Sunyer (IDIBAPS), Barcelona, Spain.
- [40] Critical Care Center, Corporacion Sanitaria Universitaria Parc Tauli, CIBER Enfermedades Respiratorias, Autonomous University of Barcelona, Parc Tauli 1, 08028, Sabadell, Spain.
- [41] Médecine Intensive-Réanimation, CHU de Lille, F-59000, Lille, France.
- [42] Univ. Lille, CHU Lille, ULR 2694-METRICS: Évaluation Des Technologies de Santé Et Des Pratiques Médicales, 59000, Lille, France.
- [43] Inserm U1285, CNRS, UMR 8576-UGSF-Unité de Glycobiologie Structurale et Fonctionnelle, Univ. Lille, Lille, France.

- PMID: **34034777**
- PMCID: PMC8146175
- DOI: 10.1186/s13054-021-03588-4

Free PMC article
Observational Study

Relationship between ventilator-associated pneumonia and mortality in COVID-19

patients: a planned ancillary analysis of the coVAPid cohort

Saad Nseir et al. Crit Care. 2021.
Free PMC article
Show details
Crit Care
. 2021 May 25;25(1):177.
doi: 10.1186/s13054-021-03588-4.

Authors

Saad Nseir [1] [2], Ignacio Martin-Loeches [3] [4], Pedro Povoa [5] [6], Matthieu Metzelard [7], Damien Du Cheyron [8], Fabien Lambiotte [9], Fabienne Tamion [10], Marie Labruyere [11], Demosthenes Makris [12], Claire Boulle Geronimi [13], Marc Pinetonde Chambrun [14], Martine Nyunga [15], Olivier Pouly [16], Bruno Mégarbane [17], Anastasia Saade [18], Gemma Gomà [19], Eleni Magira [20], Jean-François Llitjos [21], Antoni Torres [22], Iliana Ioannidou [23], Alexandre Pierre [24], Luis Coelho [5], Jean Reignier [25], Denis Garot [26], Louis Kreitmann [27], Jean-Luc Baudel [28], Guillaume Voiriot [29], Damien Contou [30], Alexandra Beurton [31], Pierre Asfar [32], Alexandre Boyer [33], Arnaud W Thille [34], Armand Mekontso-Dessap [35], Vassiliki Tsolaki [12], Christophe Vinsonneau [36], Pierre-Edouard Floch [37], Loïc Le Guennec [38], Adrian Ceccato [39], Antonio Artigas [40], Mathilde Bouchereau [41], Julien Labreuche [42], Alain Duhamel [42], Anahita Rouzé [41] [43], coVAPid study group

Collaborators

- **coVAPid study group**:
Raphaël Favory, Sébastien Préau, Mercé Jourdain, Julien Poissy, Piehr Saint Leger, Thierry Van der Linden, Anne Veinstein, Elie Azoulay, Frédéric Pene, Maelle Martin, Keyvan Razazi, Gaëtan Plantefeve, Muriel Fartoukh, Didier Thevenin, Bertrand Guidet, Nicolas Weiss, Achille Kouatchet, Charlotte Salmon, Guillaume Brunin, Safaa Nemlaghi, David Meguerditchian, Laurent Argaud, Sebastian Voicu, Charles-Edouard Luyt, Benjamin Kowalski, Edgar Moglia, Luis Morales, Antonia Koutsoukou, Spyros D Mentzelopoulos, David Nora, Sean Boyd, Julien Maizel, Pierre Cuchet, Quentin Delforge, Jean-Pierre Quenot, Déborah Boyer, Catia Cilloniz

Affiliations

- [1] Médecine Intensive-Réanimation, CHU de Lille, F-59000, Lille, France. s-nseir@chru-lille.fr.
- [2] Inserm U1285, CNRS, UMR 8576-UGSF-Unité de Glycobiologie Structurale et Fonctionnelle, Univ. Lille, Lille, France. s-nseir@chru-lille.fr.
- [3] Department of Intensive Care Medicine, Multidisciplinary Intensive Care Research Organization (MICRO), St. James's Hospital, St. James Street, Dublin 8, Dublin, Eire, Ireland.
- [4] Hospital Clinic, IDIBAPS, Universided de Barcelona, CIBERes, Barcelona, Spain.

- [5] Polyvalent Intensive Care Unit, São Francisco Xavier Hospital, Centro Hospitalar de Lisboa Ocidental, and NOVA Medical School, CHRC, New University of Lisbon, Lisbon, Portugal.
- [6] Center for Clinical Epidemiology and Research Unit of Clinical Epidemiology, OUH Odense University Hospital, Odense, Denmark.
- [7] Medical ICU, Amiens University Hospital, Amiens, France.
- [8] Department of Medical Intensive Care, Caen University Hospital, 14000, Caen, France.
- [9] Service de Réanimation Polyvalente, Centre Hospitalier de Valenciennes, Valenciennes, France.
- [10] Medical Intensive Care Unit, Rouen University Hospital, Normandie Université, UNIROUEN, Inserm U1096, FHU-REMOD-VHF, 76000, Rouen, France.
- [11] Department of Intensive Care, François Mitterrand University Hospital, Dijon, France.
- [12] Intensive Care Unit, University Hospital of Larissa, University of Thessaly, 41110, Biopolis Larissa, Greece.
- [13] Service de Réanimation Et de Soins Intensifs, Centre Hospitalier de Douai, Route de Cambrai, Douai, France.
- [14] Service de Médecine Intensive Réanimation, Institut de Cardiologie, Hôpital Pitié-Salpêtrière, Assistance Publique-Hôpitaux de Paris (APHP), Sorbonne Université, 47-83, Boulevard de L'Hôpital, 75651, Paris Cedex 13, France.
- [15] ICU, Roubaix Hospital, Roubaix, France.
- [16] Médecine Intensive Réanimation, Hôpital Saint Philibert GHICL, Université Catholique, Lille, France.
- [17] Réanimation Médicale Et Toxicologique, Hôpital Lariboisière, Université de Paris, INSERM UMRS-1144, Paris, France.
- [18] Service de Médecine Intensive Et Réanimation, Hôpital Saint-Louis, 1 Avenue Claude Vellefaux, 75010, Paris, France.
- [19] Critical Care Department, Hospital Universitari Parc Taulí, Sabadell, Spain.
- [20] 1St Department of Intensive Care Medicine, National and Kapodistrian University of Athens Medical School, Evaggelismos Hospital, Athens, Greece.
- [21] Medical Intensive Care Unit, Cochin Hospital, AP-HP. Centre, Université de Paris, Paris, France.
- [22] Department of Pulmonology, Hospital Clinic Barcelona, University of Barcelona, IDIBAPS, CIBERES, ICREA, Barcelona, Spain.
- [23] 1St Department of Pulmonary Medicine and Intensive Care Unit, National and Kapodistrian University of Athens, "Sotiria" Chest Hospital, Athens, Greece.
- [24] Réanimation Polyvalente, CH Lens, Lens, France.
- [25] Service de Médecine Intensive Réanimation, CHU de Nantes, Nantes, France.
- [26] Service de Médecine Intensive Réanimation, CHU de Tours, Hôpital Bretonneau, 2 Bd Tonnellé, 37000, Tours, France.
- [27] Service de Médecine Intensive - Réanimation, Hospices Civils de Lyon, Hôpital Edouard Herriot, 5, place d'Arsonval, 69437, Lyon Cedex 03, France.
- [28] Service de Médecine Intensive Réanimation, AP-HP, Hôpital Saint-Antoine, Assistance Publique-Hôpitaux de Paris, 184 rue du Faubourg Saint-Antoine, 75571, Paris Cedex 12, France.
- [29] Sorbonne Université, Assistance Publique-Hôpitaux de Paris, Service de Médecine Intensive Réanimation, Hôpital Tenon, Paris, France.
- [30] Réanimation Polyvalente, CH Victor Dupouy, Argenteuil, France.
- [31] Service de Pneumologie, Médecine Intensive - Réanimation (Département "R3S"), AP-HP, Sorbonne Université, Groupe Hospitalier Universitaire Pitié-Salpêtrière Charles Foix,

INSERM, UMRS1158 Neurophysiologie Respiratoire Expérimentale Et Clinique, Paris, France.

- [32] Département de Médecine Intensive-Réanimation, CHU D'Angers, Université D'Angers, 4 rue Larrey, 49933, Angers Cedex 9, France.
- [33] Intensive Care Unit, Pellegrin-Tripode Hospital, University Hospital of Bordeaux, Bordeaux, France.
- [34] CHU de Poitiers, Médecine Intensive Réanimation, CIC 1402 ALIVE, Université de Poitiers, Poitiers, France.
- [35] APHP, CHU Henri Mondor, Service de Médecine Intensive RéanimationUniversité Paris Est-Créteil, Faculté de Santé, Groupe de Recherche Clinique CARMASINSERM U955, Institut Mondor de Recherche Biomédicale, 94010, Créteil, France.
- [36] Service de Médecine Intensive Réanimation, Centre Hospitalier de Béthune, Réseau de Recherche Boréal, 62408, Béthune, France.
- [37] Service de Réanimation, Hôpital Duchenne, Rue Monod, 62200, Boulogne-sur-Mer, France.
- [38] Sorbonne Université, AP-HP, Hôpital de La Pitié-Salpêtrière, Département de Neurologie, Unité de Médecine Intensive Réanimation Neurologique, Paris, France.
- [39] Intensive Care Unit, Hospital Universitari Sagrat Cor, and Ciber de Enfermedades Respiratorias (Ciberes, CB06/06/0028)-Institut D'Investigacions Biomèdiques August Pi I Sunyer (IDIBAPS), Barcelona, Spain.
- [40] Critical Care Center, Corporacion Sanitaria Universitaria Parc Tauli, CIBER Enfermedades Respiratorias, Autonomous University of Barcelona, Parc Tauli 1, 08028, Sabadell, Spain.
- [41] Médecine Intensive-Réanimation, CHU de Lille, F-59000, Lille, France.
- [42] Univ. Lille, CHU Lille, ULR 2694-METRICS: Évaluation Des Technologies de Santé Et Des Pratiques Médicales, 59000, Lille, France.
- [43] Inserm U1285, CNRS, UMR 8576-UGSF-Unité de Glycobiologie Structurale et Fonctionnelle, Univ. Lille, Lille, France.

- PMID: **34034777**
- PMCID: PMC8146175
- DOI: 10.1186/s13054-021-03588-4

Erratum in

- Correction to: Relationship between ventilator-associated pneumonia and mortality in COVID-19 patients: a planned ancillary analysis of the coVAPid cohort.
  Nseir S, Martin-Loeches I, Povoa P, Metzelard M, Du Cheyron D, Lambiotte F, Tamion F, Labruyere M, Makris D, Boulle Geronimi C, Pinetonde Chambrun M, Nyunga M, Pouly O, Mégarbane B, Saade A, Gomà G, Magira E, Llitjos JF, Torres A, Ioannidou I, Pierre A, Coelho L, Reignier J, Garot D, Kreitmann L, Baudel JL, Voiriot G, Contou D, Beurton A, Asfar P, Boyer A, Thille AW, Mekontso-Dessap A, Tsolaki V, Vinsonneau C, Floch PE, Le Guennec L, Ceccato A, Artigas A, Bouchereau M, Labreuche J, Duhamel A, Rouzé A; coVAPid study group. Nseir S, et al. Crit Care. 2021 Aug 9;25(1):284. doi: 10.1186/s13054-021-03713-3. Crit Care. 2021. PMID: 34372897 Free PMC article. No abstract available.

Abstract

Background: Patients with SARS-CoV-2 infection are at higher risk for ventilator-associated pneumonia (VAP). No study has evaluated the relationship between VAP and mortality in this population, or compared this relationship between SARS-CoV-2 patients and other populations. The main objective of our study was to determine the relationship between VAP and mortality in SARS-CoV-2 patients.

Methods: Planned ancillary analysis of a multicenter retrospective European cohort. VAP was diagnosed using clinical, radiological and quantitative microbiological criteria. Univariable and multivariable marginal Cox's regression models, with cause-specific hazard for duration of mechanical ventilation and ICU stay, were used to compare outcomes between study groups. Extubation, and ICU discharge alive were considered as events of interest, and mortality as competing event.

Findings: Of 1576 included patients, 568 were SARS-CoV-2 pneumonia, 482 influenza pneumonia, and 526 no evidence of viral infection at ICU admission. VAP was associated with significantly higher risk for 28-day mortality in SARS-CoV-2 (adjusted HR 1.70 (95% CI 1.16-2.47), p = 0.006), and influenza groups (1.75 (1.03-3.02), p = 0.045), but not in the no viral infection group (1.07 (0.64-1.78), p = 0.79). VAP was associated with significantly longer duration of mechanical ventilation in the SARS-CoV-2 group, but not in the influenza or no viral infection groups. VAP was associated with significantly longer duration of ICU stay in the 3 study groups. No significant difference was found in heterogeneity of outcomes related to VAP between the 3 groups, suggesting that the impact of VAP on mortality was not different between study groups.

Interpretation: VAP was associated with significantly increased 28-day mortality rate in SARS-CoV-2 patients. However, SARS-CoV-2 pneumonia, as compared to influenza pneumonia or no viral infection, did not significantly modify the relationship between VAP and 28-day mortality.

Clinical trial registration: The study was registered at ClinicalTrials.gov, number NCT04359693.

Keywords: COVID-19; Mortality; Ventilator-associated pneumonia.

Conflict of interest statement

AR received personal fees from MaatPharma, IML received personal fees from MSD, and Gilead. AA received personal fees from Lilly Foundation, and grants from Grifols and Fischer & Paykel. CEL received personal fees from Bayer, Merck, Aerogen, Biomérieux, ThermoFischer Brahms, and Carmat. SN received personal fees from MSD, Bio Rad, BioMérieux, Gilead, and Pfizer. All other authors declare no competing interests.

- 32 references
- 2 figures

Supplementary info

Publication types, MeSH terms, Associated data Expand

Publication types

- Multicenter Study
- Observational Study

- Research Support, Non-U.S. Gov't

MeSH terms

- Aged
- COVID-19 / mortality*
- COVID-19 / therapy*
- Europe / epidemiology
- Female
- Hospital Mortality
- Humans
- Intensive Care Units
- Length of Stay / statistics & numerical data
- Male
- Middle Aged
- Pneumonia, Ventilator-Associated / epidemiology*
- Respiration, Artificial / statistics & numerical data
- Retrospective Studies

Associated data

- ClinicalTrials.gov/NCT04359693

Full text links

Read free full text at BMC BioMed Central Free PMC article

Proceed to details

Cite

Share

☐ 787

Observational Study

JAMA Otolaryngol Head Neck Surg

. 2021 May 1;147(5):482-484.

doi: 10.1001/jamaoto.2020.5698.

Otolaryngologic Manifestations in Pediatric Inflammatory Multisystem Syndrome Temporally Associated With COVID-19

Ryan C T Cheong [1], Christopher Jephson [1], Claire Frauenfelder [1], Lesley Cavalli [2], Karyn Moshal [3], Colin R Butler [1] [4], Michelle E Wyatt [1]

Affiliations Expand

Affiliations

- [1] Ear, Nose and Throat Department, Great Ormond Street Hospital for Children NHS Trust, London, United Kingdom.
- [2] Speech and Language Therapy Department, Great Ormond Street Hospital for Children NHS Trust, London, United Kingdom.
- [3] Infectious Disease Department, Great Ormond Street Hospital for Children NHS Trust, London, United Kingdom.
- [4] Stem Cell and Regenerative Medicine Division, Institute of Child Health UCL, London, United Kingdom.

- PMID: **33630068**
- PMCID: PMC7907982
- DOI: 10.1001/jamaoto.2020.5698

Free PMC article
Observational Study

Otolaryngologic Manifestations in Pediatric Inflammatory Multisystem Syndrome Temporally Associated With COVID-19

Ryan C T Cheong et al. JAMA Otolaryngol Head Neck Surg. 2021.
Free PMC article

Show details

JAMA Otolaryngol Head Neck Surg
. 2021 May 1;147(5):482-484.
doi: 10.1001/jamaoto.2020.5698.

Authors

Ryan C T Cheong [1], Christopher Jephson [1], Claire Frauenfelder [1], Lesley Cavalli [2], Karyn Moshal [3], Colin R Butler [1] [4], Michelle E Wyatt [1]

Affiliations

- [1] Ear, Nose and Throat Department, Great Ormond Street Hospital for Children NHS Trust, London, United Kingdom.
- [2] Speech and Language Therapy Department, Great Ormond Street Hospital for Children NHS Trust, London, United Kingdom.
- [3] Infectious Disease Department, Great Ormond Street Hospital for Children NHS Trust, London, United Kingdom.
- [4] Stem Cell and Regenerative Medicine Division, Institute of Child Health UCL, London, United Kingdom.

- PMID: **33630068**
- PMCID: PMC7907982

- DOI: 10.1001/jamaoto.2020.5698

Abstract

This cohort study describes the various otolaryngologic manifestations in and rates among patients 18 years or younger with pediatric inflammatory multisystem syndrome temporally associated with severe acute respiratory syndrome coronavirus 2.

Conflict of interest statement

Conflict of Interest Disclosures: None reported.

- 1 figure

Supplementary info

Publication types, MeSH terms, Supplementary concepts Expand

Publication types

- Observational Study

MeSH terms

- Adolescent
- Anosmia / virology
- COVID-19 / complications*
- Child
- Deglutition Disorders / virology
- Dysphonia / virology
- Female
- Humans
- Male
- Otorhinolaryngologic Diseases / virology*
- Retrospective Studies
- SARS-CoV-2
- Systemic Inflammatory Response Syndrome / complications*

Supplementary concepts

- pediatric multisystem inflammatory disease, COVID-19 related

Full text links

FULL TEXT JAMA Otolaryngology–Head & Neck Surgery Silverchair Information Systems Free PMC article
Proceed to details
Cite
Share
788

Observational Study

Indian J Ophthalmol
. 2021 Oct;69(10):2617-2624.
doi: 10.4103/ijo.IJO_2243_21.

Impact of COVID-19 nationwide lockdown on retinoblastoma treatment and outcome: A study of 476 eyes of 326 children

Rolika Bansal [1], Ankita Aishwarya [1], Raksha Rao [2], Marem C Christy [1], Mrittika Sen [1], Harika Regani [1], Poonam Bagai [3], Vijay A Reddy [4], Kaustubh Mulay [1], Pramila Bongoni [1], Santosh G Honavar [1]

Affiliations Expand

Affiliations

- [1] Centre for Sight, Hyderabad, Telangana, India.
- [2] Narayana Nethralaya, Bangalore, Karnataka, India.
- [3] CanKids KidsCan, New Delhi, Delhi, India.
- [4] Apollo Hospitals, Hyderabad, Telangana, India.

- PMID: **34571599**
- PMCID: PMC8597487
- DOI: 10.4103/ijo.IJO_2243_21

Free PMC article
Observational Study

Impact of COVID-19 nationwide lockdown on retinoblastoma treatment and outcome: A study of 476 eyes of 326 children

Rolika Bansal et al. Indian J Ophthalmol. 2021 Oct.
Free PMC article
Show details

Indian J Ophthalmol
. 2021 Oct;69(10):2617-2624.

doi: 10.4103/ijo.IJO_2243_21.

Authors

Rolika Bansal [1], Ankita Aishwarya [1], Raksha Rao [2], Marem C Christy [1], Mrittika Sen [1], Harika Regani [1], Poonam Bagai [3], Vijay A Reddy [4], Kaustubh Mulay [1], Pramila Bongoni [1], Santosh G Honavar [1]

Affiliations

- [1] Centre for Sight, Hyderabad, Telangana, India.
- [2] Narayana Nethralaya, Bangalore, Karnataka, India.
- [3] CanKids KidsCan, New Delhi, Delhi, India.
- [4] Apollo Hospitals, Hyderabad, Telangana, India.

- PMID: **34571599**
- PMCID: PMC8597487
- DOI: 10.4103/ijo.IJO_2243_21

Abstract

Purpose: The novel coronavirus SARS-CoV-2 (COVID-19) and the resultant nationwide lockdown and travel restrictions led to difficulty in providing timely and regular treatment to patients with childhood cancers such as retinoblastoma. This study is aimed at assessing the demography, clinical presentation, treatment strategies, and outcome of treatment defaulters due to the lockdown.

Methods: Cross-sectional, observational study of retinoblastoma patients at a tertiary care ocular oncology center during the first wave of COVID-19 and the resulting nationwide lockdown.

Results: Of the 476 eyes of 326 patients undergoing active management with a median age of 57 months (range: 4-214 months), 205 (63%) patients returned for follow-up after a mean delay of 45.8 ± 24.3 weeks (range: 8-80 weeks) and 121 (37%) were defaulters according to the data analyzed till June 30, 2021. Distance of residence was ≥1000 km for 148 patients (46%). In terms of need for active treatment, the number of emergent cases was 2 (<1%), 11 (3%) were urgent, and 313 (96%) were semi-urgent. International classification groups D (n = 107 eyes, 23%) and E (n = 173 eyes, 36%) were in majority, and 13 eyes (4%) and 4 eyes (1%) were at stages 3 and 4, respectively. Prior to lockdown, 86 eyes (18%) had active tumor, which remained unchanged (n = 26, 30%) or worsened (n = 49, 60%) after failure to follow-up. Vision (47%), eye (92%), and life salvage (98%) were achieved by individualized protocol-based management after the patients returned for further management. Five children succumbed to intracranial extension.

Conclusion: The COVID-19-related nationwide lockdown has deprived retinoblastoma patients of optimal and timely management, leading to prolonged treatment interruptions, delays, permanent default, and death. It is of paramount importance for all the stakeholders to increase awareness, make necessary travel and logistic arrangements, and ensure continuity of care for children with retinoblastoma.

Keywords: COVID-19; SARS-CoV-2; ocular oncology; retinoblastoma; treatment delay.

Conflict of interest statement

None

- 25 references
- 6 figures

Supplementary info

Publication types, MeSH terms Expand

Publication types

- Observational Study

MeSH terms

- COVID-19*
- Child
- Communicable Disease Control
- Cross-Sectional Studies
- Humans
- Retinal Neoplasms* / diagnosis
- Retinal Neoplasms* / epidemiology
- Retinal Neoplasms* / therapy
- Retinoblastoma* / diagnosis
- Retinoblastoma* / epidemiology
- Retinoblastoma* / therapy
- Retrospective Studies
- SARS-CoV-2

Full text links

Free PMC article
Proceed to details
Cite
Share
☐ 789
Observational Study
Clin Transplant
. 2020 Dec;34(12):e14119.
doi: 10.1111/ctr.14119. Epub 2020 Oct 26.

The social and clinical impact of the COVID-19 epidemic on the Strasbourg lung transplant cohort: A single-center retrospective cohort study

Benjamin Renaud-Picard [1 2], Floriane Gallais [3], Marianne Riou [1 2], Eva Chatron [1 2], Tristan Degot [1], Sophie Freudenberger [1], Michele Porzio [1 2], Armelle Schuller [1], Julien Stauder [1], Sandrine Hirschi [1], Romain Kessler [1 2]
Affiliations Expand

Affiliations

- [1] Respiratory Medicine and Strasbourg Lung Transplant Program, Hôpitaux universitaires de Strasbourg, Strasbourg, France.
- [2] Faculty of Medicine, Federation of Translational Medicine (FMTS), Strasbourg, France.
- [3] Laboratory of Virology, Hôpitaux universitaires de Strasbourg, Strasbourg, France.

- PMID: **33048391**
- PMCID: PMC7646014
- DOI: 10.1111/ctr.14119

Free PMC article
Observational Study

The social and clinical impact of the COVID-19 epidemic on the Strasbourg lung transplant cohort: A single-center retrospective cohort study

Benjamin Renaud-Picard et al. Clin Transplant. 2020 Dec.
Free PMC article
Show details
Clin Transplant
. 2020 Dec;34(12):e14119.
doi: 10.1111/ctr.14119. Epub 2020 Oct 26.

Authors

Benjamin Renaud-Picard [1 2], Floriane Gallais [3], Marianne Riou [1 2], Eva Chatron [1 2], Tristan Degot [1], Sophie Freudenberger [1], Michele Porzio [1 2], Armelle Schuller [1], Julien Stauder [1], Sandrine Hirschi [1], Romain Kessler [1 2]

Affiliations

- [1] Respiratory Medicine and Strasbourg Lung Transplant Program, Hôpitaux universitaires de Strasbourg, Strasbourg, France.
- [2] Faculty of Medicine, Federation of Translational Medicine (FMTS), Strasbourg, France.
- [3] Laboratory of Virology, Hôpitaux universitaires de Strasbourg, Strasbourg, France.

- PMID: **33048391**
- PMCID: PMC7646014
- DOI: 10.1111/ctr.14119

Abstract

The clinical and social impacts of the COVID-19 epidemic on lung transplant (LTx) recipients remain poorly known. We aimed to evaluate its social, clinical, and behavioral consequences on the LTx patients followed in Strasbourg university hospital. A questionnaire was used to collect details concerning patients' lifestyles, their protection methods used to avoid COVID-19 contamination, and clinical infection-related information for March 2020. A specific score was created to quantify patients' contacts and the associated risk of infectious contagion. Data were collected from 322 patients (91.2%). A majority reported a higher application than usual of social distancing and barrier measures. 43.8% described infectious-related symptoms and 15.8% needed an anti-infective treatment. There was no difference in symptom onset according to age, native lung disease, diabetes, or obesity. Nineteen patients were tested for COVID-19, and four were diagnosed positive, all with a favorable outcome. The infection risk contact score was higher for symptomatic patients (p: 0.007), those needing extra-medical appointments ($p < .001$), and those receiving anti-infective treatments ($p = .02$). LTx patients reported a careful lifestyle and did not seem at higher risk for COVID-19. Our score showed encouraging preliminary results and could become a useful tool for the usual infection-related follow-up of the LTx patients.

Keywords: COVID-19; lifestyle factors; lung transplantation; protective measures.

© 2020 John Wiley & Sons A/S. Published by John Wiley & Sons Ltd.

Conflict of interest statement

None of the authors has a financial relationship with a commercial entity that has an interest in the subject of the presented manuscript or other conflicts of interest to disclose.

- 20 references
- 3 figures

Supplementary info

Publication types, MeSH terms Expand

Publication types

- Observational Study

MeSH terms

- Adolescent
- Adult
- Aged
- Aged, 80 and over
- COVID-19 / diagnosis
- COVID-19 / epidemiology
- COVID-19 / etiology*
- COVID-19 / prevention & control
- Contact Tracing
- Epidemics
- Female
- France / epidemiology
- Health Behavior*
- Hospitals, University
- Humans
- Life Style
- Lung Transplantation*
- Male
- Middle Aged
- Patient Compliance / psychology
- Patient Compliance / statistics & numerical data
- Physical Distancing
- Postoperative Complications* / diagnosis
- Postoperative Complications* / epidemiology
- Postoperative Complications* / prevention & control
- Postoperative Complications* / psychology
- Retrospective Studies
- Risk Assessment
- Risk Factors
- Social Determinants of Health*
- Transplant Recipients / psychology*
- Young Adult

Full text links

WILEY Full Text Article Wiley Free PMC article

Proceed to details

Cite

Share

☐ 790

Observational Study

Cancer Cell

. 2021 Feb 8;39(2):130-131.

doi: 10.1016/j.ccell.2021.01.002. Epub 2021 Jan 6.

Changes in Treatment Behavior during the COVID-19 Pandemic among Patients at a Cancer Hospital

Yasuyoshi Sato [1], Yu Fujiwara [2], Naoki Fukuda [3], Brian Hayama [4], Yoshinori Ito [5], Shinji Ohno [6], Shunji Takahashi [3], COVID-19 Working Group of The Cancer Institute Hospital of Japanese Foundation for Cancer Research

Affiliations Expand

Affiliations

- [1] Department of Medical Oncology, The Cancer Institute Hospital of Japanese Foundation for Cancer Research, 3-8-31 Ariake, Koto-ku, Tokyo 135-8550, Japan. Electronic address: yasuyoshi.sato@jfcr.or.jp.
- [2] Department of Medical Oncology, The Cancer Institute Hospital of Japanese Foundation for Cancer Research, 3-8-31 Ariake, Koto-ku, Tokyo 135-8550, Japan; Department of Medicine, Icahn School of Medicine at Mount Sinai, Mount Sinai Beth Israel, 281 First Avenue, New York, NY 10003, USA.
- [3] Department of Medical Oncology, The Cancer Institute Hospital of Japanese Foundation for Cancer Research, 3-8-31 Ariake, Koto-ku, Tokyo 135-8550, Japan.
- [4] Department of Infectious Diseases, The Cancer Institute Hospital of Japanese Foundation for Cancer Research, 3-8-31 Ariake, Koto-ku, Tokyo 135-8550, Japan.
- [5] Department of Chemotherapy, The Cancer Institute Hospital of Japanese Foundation for Cancer Research, 3-8-31, Ariake, Koto-ku, Tokyo 1358550, Japan.
- [6] Department of Breast Oncology, The Cancer Institute Hospital of Japanese Foundation for Cancer Research, 3-8-31 Ariake, Koto-ku, Tokyo 135-8550, Japan.

- PMID: **33465338**
- PMCID: PMC7787502
- DOI: 10.1016/j.ccell.2021.01.002

Free PMC article

Observational Study

Changes in Treatment Behavior during the COVID-19 Pandemic among Patients at a Cancer Hospital

Yasuyoshi Sato et al. Cancer Cell. 2021.

Free PMC article

Show details

Cancer Cell

. 2021 Feb 8;39(2):130-131.

doi: 10.1016/j.ccell.2021.01.002. Epub 2021 Jan 6.

Authors

Yasuyoshi Sato [1], Yu Fujiwara [2], Naoki Fukuda [3], Brian Hayama [4], Yoshinori Ito [5], Shinji Ohno [6], Shunji Takahashi [3], COVID-19 Working Group of The Cancer Institute Hospital of Japanese Foundation for Cancer Research

Affiliations

- [1] Department of Medical Oncology, The Cancer Institute Hospital of Japanese Foundation for Cancer Research, 3-8-31 Ariake, Koto-ku, Tokyo 135-8550, Japan. Electronic address: yasuyoshi.sato@jfcr.or.jp.
- [2] Department of Medical Oncology, The Cancer Institute Hospital of Japanese Foundation for Cancer Research, 3-8-31 Ariake, Koto-ku, Tokyo 135-8550, Japan; Department of Medicine, Icahn School of Medicine at Mount Sinai, Mount Sinai Beth Israel, 281 First Avenue, New York, NY 10003, USA.
- [3] Department of Medical Oncology, The Cancer Institute Hospital of Japanese Foundation for Cancer Research, 3-8-31 Ariake, Koto-ku, Tokyo 135-8550, Japan.
- [4] Department of Infectious Diseases, The Cancer Institute Hospital of Japanese Foundation for Cancer Research, 3-8-31 Ariake, Koto-ku, Tokyo 135-8550, Japan.
- [5] Department of Chemotherapy, The Cancer Institute Hospital of Japanese Foundation for Cancer Research, 3-8-31, Ariake, Koto-ku, Tokyo 1358550, Japan.
- [6] Department of Breast Oncology, The Cancer Institute Hospital of Japanese Foundation for Cancer Research, 3-8-31 Ariake, Koto-ku, Tokyo 135-8550, Japan.

- PMID: **33465338**
- PMCID: PMC7787502
- DOI: 10.1016/j.ccell.2021.01.002

No abstract available

Conflict of interest statement

Declaration of Interests Y.S. reports personal fees from ONO Pharmaceutical Co., Ltd.; Bristol-Myers Squibb Company; MSD KK; and TAIHO Pharmaceutical Co., Ltd. outside the submitted work. N.F. reports personal fees from Eisai outside the submitted work. S.O. reports personal fees from Chugai, grants and personal fees from Eisai, grants and personal fees from TAIHO Pharmaceutical Co., personal fees from AstraZeneca, personal fees from Pfizer, personal fees from Eli Lilly, personal fees from Kyowa Kirin, and personal fees from Nippon Kayaku outside the submitted work. S.T. reports grants and personal fees from Bristol- Myers Squibb KK; grants and personal fees from ONO Pharmaceutical Co., Ltd.; grants and personal fees from MSD; grants and personal fees from AstraZeneca; grants and personal fees from Chugai; and grants and personal fees from Bayer outside the submitted work. The other authors report no competing interests to disclose.

- 4 references

Supplementary info

Publication types, MeSH terms Expand

Publication types

- Letter
- Observational Study

MeSH terms

- COVID-19* / epidemiology
- Cancer Care Facilities / statistics & numerical data*
- Humans
- Japan / epidemiology
- Neoplasms* / therapy
- Outpatients / statistics & numerical data
- Retrospective Studies

Full text links

CellPress OPEN ACCESS Elsevier Science Free PMC article

Proceed to details

Cite

Share

☐ 791

Observational Study

Intern Emerg Med

. 2021 Aug;16(5):1141-1152.

doi: 10.1007/s11739-020-02550-6. Epub 2020 Nov 8.

Venous thromboembolism and COVID-19: a single center experience from an academic tertiary referral hospital of Northern Italy

Federica Melazzini [1], Marta Colaneri [2], Federica Fumoso [1], Giulia Freddi [1], Marco Vincenzo Lenti [1], Teresa Chiara Pieri [2], Davide Piloni [3], Patrizia Noris [1], Carla Pieresca [1], Paola Stefania Preti [1], Mariaconcetta Russo [1], Angelo Corsico [3], Guido Tavazzi [4], Fausto Baldanti [5], Antonio Triarico [6], Francesco Mojoli [4], Raffaele Bruno [2], Antonio Di Sabatino [7], San Matteo Pavia COVID-19 Task Force

Collaborators, Affiliations Expand

Collaborators

- **San Matteo Pavia COVID-19 Task Force**: Nicola Aronico, Gaetano Bergamaschi, Giampiera Bertolino, Silvia Codega, Filippo Costanzo, Roberto Cresci, Angela Delliponti, Giuseppe Derosa, Michele Di Stefano, Francesco Falaschi, Carmine Iadarola, Elisabetta Lovati, Pietro Carlo Lucotti, Alessandra Martignoni, Caterina Mengoli, Emanuela Miceli, Amedeo Mugellini, Chiara Muggia, Elisabetta Pagani, Ilaria Palumbo, Alessandro Pecci, Tiziano Perrone, Carmelo Sgarlata, Luisa Siciliani, Andrea Staniscia, Francesca Torello Vjera, Giovanna Achilli, Andrea Agostinelli, Valentina Antoci, Alessia Ballesio, Francesco Banfi, Chiara Barteselli, Irene Benedetti, Federica Borrelli de Andreis, Michele Brattoli, Francesca Calabretta, Ginevra Cambiè, Roberta Canta, Federico Conca, Luigi Coppola, Elisa Maria Cremonte, Gabriele Croce, Virginia Del Rio, Francesco Di Terlizzi, Maria Giovanna Ferrari, Sara Ferrari, Anna Fiengo, Tommaso Forni, Chiara Frigerio, Alessandra Fusco, Margherita Gabba, Matteo Garolfi, Antonella Gentile, Giulia Gori, Giacomo Grandi, Paolo Grimaldi, Alice Lampugnani, Francesco Lapia, Federica Lepore, Gianluca Lettieri, Jacopo Mambella, Chiara Mercanti, Stefania Merli, Francesco Mordà, Alba Nardone, Luca Pace, Lucia Padovini, Alessandro Parodi, Ivan Pellegrino, Lavinia Pitotti, Margherita Reduzzi, Giovanni Rigano, Giovanni Romito, Giorgio Rotola, Umberto Sabatini, Lucia Salvi, Giovanni Santacroce, Jessica Savioli, Simone Soriano, Carmine Spataro, Debora Stefani, Anna Rita Aliberti, Alessandro Amatu, Laura Anfossi, Eric Arisi, Chiara Baldi, Mirko Belliato, Lorenzo Bellini, Alberto Benzi, Germana Bichisao, Antonia Bolongaro, Andrea Bottazzi, Federica Broglia, Giacomo Bruschi, Luca Caneva, Emanuele Capaccio, Valeria Carboni, Fabrizio Cavalloro, Maria Ciceri, Luca Civardi, Maria Paola Delmonte, Elisa Lucia Domenegati, Federica Ferrari, Fiorenza Ferrari, Marta Ferrari, Marinella Fuardo, Maddalena Margherita Gerletti, Simonetta Gualdana, Marcella Ilardi, Claudia Lo Coco, Giuseppe Maggio, Maria Benedetta Mascia, Simonetta Mencherini, Paola Maria Merati, Silvia Mongodi, Anna Maria Mori, Federica Morgante, Thekla Larissa Niebel, Silvano Noli, Anita Orlando, Michele Pagani, Debora Passador, Simona Pellicori, Luciano Perotti, Raffaella Picchioni, Silvia Poma, Marco Pozzi, Emanuela Preti, Roberta Puce, Danila Katia Radolovich, Gianluca Ragni, Filippo Repossi, Francesca Riccardi, Roberto Rizzardi, Giuseppe Rodi, Emanuela Roldi, Giovanni Romito, Cristina Rossi, Giuseppe Sala Gallini, Fabio Sciutti, Debora Sportiello, Giulia Ticozzelli, Federico Visconti, Silvia Zizzi, Alessandro Bagliani, Corrado Belotti, Chiara Bossi, Andrea Colombo, Costanza Natalia Julia Colombo, Luca Cremascoli, Valentino Dammassa, Roberto Discepoli, Maria Adelaide Garlando, Filippo Grandini, Andrea Pellegrini, Cecilia Quaranta, Andrea Stella, Francesco Torresani, Mario Mondelli, Enrico Brunetti, Angela Di Matteo, Elena Seminari, Laura Maiocchi, Valentina Zuccaro, Layla Pagnucco, Bianca Mariani, Serena Ludovisi, Raffaella Lissandrin, Aldo Parisi, Paolo Sacchi, Savino F A Patruno, Giuseppe Michelone, Roberto Gulminetti, Domenico Zanaboni, Stefano Novati, Renato Maserati, Paolo Orsolini, Marco Vecchia, Erika Asperges, Alessandro Di Filippo, Margherita Sambo, Simona Biscarini, Matteo Lupi, Silvia Roda, Ilaria Gallazzi, Michele Sachs, Pietro Valsecchi, Alessandra Ferrari, Bianca Mariani, Matteo Bosio, Alessandro Cascina, Valentina Conio, Rita Di Domenica, Anna Donnetta, Elia Fraolini, Giuseppe Gualtieri, Patrizia Mangiarotti, Francesca Mariani, Federica Meloni, Tiberio Oggionni, Lidia Pasturenzi, Vanessa Ronzoni, Laura Saracino, Giulia Stella, Stefano Tomaselli, Tommaso Abbate, Giulia Accordino, Francesco Bertuccio, Cecilia Burattini, Elisa Cacciatore, Elena Cattaneo, Vittorio Chino, Manuela Coretti, Matteo Della Zoppa, Cristina Infusino, Sara Lettieri, Valeria Maccabruni, Silvia Mancinelli, Claudio Tirelli, Valentina Vertui

Affiliations

- [1] Department of Internal Medicine, San Matteo Hospital Foundation, University of Pavia, Pavia, Italy.
- [2] Department of Infectious Disease, San Matteo Hospital Foundation, University of Pavia, Pavia, Italy.
- [3] Department of Respiratory Disease, San Matteo Hospital Foundation, University of Pavia, Pavia, Italy.
- [4] Department of Intensive Care, San Matteo Hospital Foundation, University of Pavia, Pavia, Italy.
- [5] Molecular Virology Unit, Microbiology and Virology Department, San Matteo Hospital Foundation, University of Pavia, Pavia, Italy.
- [6] Chief Medical Direction, San Matteo Hospital Foundation, Pavia, Italy.
- [7] Department of Internal Medicine, San Matteo Hospital Foundation, University of Pavia, Pavia, Italy. a.disabatino@smatteo.pv.it.

- PMID: **33161478**
- PMCID: PMC7648897
- DOI: 10.1007/s11739-020-02550-6

Free PMC article
Observational Study

Venous thromboembolism and COVID-19: a single center experience from an academic tertiary referral hospital of Northern Italy

Federica Melazzini et al. Intern Emerg Med. 2021 Aug.
Free PMC article
Show details
Intern Emerg Med

. 2021 Aug;16(5):1141-1152.
doi: 10.1007/s11739-020-02550-6. Epub 2020 Nov 8.

Authors

Federica Melazzini [1], Marta Colaneri [2], Federica Fumoso [1], Giulia Freddi [1], Marco Vincenzo Lenti [1], Teresa Chiara Pieri [2], Davide Piloni [3], Patrizia Noris [1], Carla Pieresca [1], Paola Stefania Preti [1], Mariaconcetta Russo [1], Angelo Corsico [3], Guido Tavazzi [4], Fausto Baldanti [5], Antonio Triarico [6], Francesco Mojoli [4], Raffaele Bruno [2], Antonio Di Sabatino [7], San Matteo Pavia COVID-19 Task Force

Collaborators

- **San Matteo Pavia COVID-19 Task Force**:
  Nicola Aronico, Gaetano Bergamaschi, Giampiera Bertolino, Silvia Codega, Filippo Costanzo, Roberto Cresci, Angela Delliponti, Giuseppe Derosa, Michele Di

Stefano, Francesco Falaschi, Carmine Iadarola, Elisabetta Lovati, Pietro Carlo Lucotti, Alessandra Martignoni, Caterina Mengoli, Emanuela Miceli, Amedeo Mugellini, Chiara Muggia, Elisabetta Pagani, Ilaria Palumbo, Alessandro Pecci, Tiziano Perrone, Carmelo Sgarlata, Luisa Siciliani, Andrea Staniscia, Francesca Torello Vjera, Giovanna Achilli, Andrea Agostinelli, Valentina Antoci, Alessia Ballesio, Francesco Banfi, Chiara Barteselli, Irene Benedetti, Federica Borrelli de Andreis, Michele Brattoli, Francesca Calabretta, Ginevra Cambiè, Roberta Canta, Federico Conca, Luigi Coppola, Elisa Maria Cremonte, Gabriele Croce, Virginia Del Rio, Francesco Di Terlizzi, Maria Giovanna Ferrari, Sara Ferrari, Anna Fiengo, Tommaso Forni, Chiara Frigerio, Alessandra Fusco, Margherita Gabba, Matteo Garolfi, Antonella Gentile, Giulia Gori, Giacomo Grandi, Paolo Grimaldi, Alice Lampugnani, Francesco Lapia, Federica Lepore, Gianluca Lettieri, Jacopo Mambella, Chiara Mercanti, Stefania Merli, Francesco Mordà, Alba Nardone, Luca Pace, Lucia Padovini, Alessandro Parodi, Ivan Pellegrino, Lavinia Pitotti, Margherita Reduzzi, Giovanni Rigano, Giovanni Romito, Giorgio Rotola, Umberto Sabatini, Lucia Salvi, Giovanni Santacroce, Jessica Savioli, Simone Soriano, Carmine Spataro, Debora Stefani, Anna Rita Aliberti, Alessandro Amatu, Laura Anfossi, Eric Arisi, Chiara Baldi, Mirko Belliato, Lorenzo Bellini, Alberto Benzi, Germana Bichisao, Antonia Bolongaro, Andrea Bottazzi, Federica Broglia, Giacomo Bruschi, Luca Caneva, Emanuele Capaccio, Valeria Carboni, Fabrizio Cavalloro, Maria Ciceri, Luca Civardi, Maria Paola Delmonte, Elisa Lucia Domenegati, Federica Ferrari, Fiorenza Ferrari, Marta Ferrari, Marinella Fuardo, Maddalena Margherita Gerletti, Simonetta Gualdana, Marcella Ilardi, Claudia Lo Coco, Giuseppe Maggio, Maria Benedetta Mascia, Simonetta Mencherini, Paola Maria Merati, Silvia Mongodi, Anna Maria Mori, Federica Morgante, Thekla Larissa Niebel, Silvano Noli, Anita Orlando, Michele Pagani, Debora Passador, Simona Pellicori, Luciano Perotti, Raffaella Picchioni, Silvia Poma, Marco Pozzi, Emanuela Preti, Roberta Puce, Danila Katia Radolovich, Gianluca Ragni, Filippo Repossi, Francesca Riccardi, Roberto Rizzardi, Giuseppe Rodi, Emanuela Roldi, Giovanni Romito, Cristina Rossi, Giuseppe Sala Gallini, Fabio Sciutti, Debora Sportiello, Giulia Ticozzelli, Federico Visconti, Silvia Zizzi, Alessandro Bagliani, Corrado Belotti, Chiara Bossi, Andrea Colombo, Costanza Natalia Julia Colombo, Luca Cremascoli, Valentino Dammassa, Roberto Discepoli, Maria Adelaide Garlando, Filippo Grandini, Andrea Pellegrini, Cecilia Quaranta, Andrea Stella, Francesco Torresani, Mario Mondelli, Enrico Brunetti, Angela Di Matteo, Elena Seminari, Laura Maiocchi, Valentina Zuccaro, Layla Pagnucco, Bianca Mariani, Serena Ludovisi, Raffaella Lissandrin, Aldo Parisi, Paolo Sacchi, Savino F A Patruno, Giuseppe Michelone, Roberto Gulminetti, Domenico Zanaboni, Stefano Novati, Renato Maserati, Paolo Orsolini, Marco Vecchia, Erika Asperges, Alessandro Di Filippo, Margherita Sambo, Simona Biscarini, Matteo Lupi, Silvia Roda, Ilaria Gallazzi, Michele Sachs, Pietro Valsecchi, Alessandra Ferrari, Bianca Mariani, Matteo Bosio, Alessandro Cascina, Valentina Conio, Rita Di Domenica, Anna Donnetta, Elia Fraolini, Giuseppe Gualtieri, Patrizia Mangiarotti, Francesca Mariani, Federica Meloni, Tiberio Oggionni, Lidia Pasturenzi, Vanessa Ronzoni, Laura Saracino, Giulia Stella, Stefano Tomaselli, Tommaso Abbate, Giulia Accordino, Francesco Bertuccio, Cecilia Burattini, Elisa Cacciatore, Elena Cattaneo, Vittorio Chino, Manuela Coretti, Matteo Della Zoppa, Cristina Infusino, Sara Lettieri, Valeria Maccabruni, Silvia Mancinelli, Claudio Tirelli, Valentina Vertui

Affiliations

- [1] Department of Internal Medicine, San Matteo Hospital Foundation, University of Pavia, Pavia, Italy.

- [2] Department of Infectious Disease, San Matteo Hospital Foundation, University of Pavia, Pavia, Italy.
- [3] Department of Respiratory Disease, San Matteo Hospital Foundation, University of Pavia, Pavia, Italy.
- [4] Department of Intensive Care, San Matteo Hospital Foundation, University of Pavia, Pavia, Italy.
- [5] Molecular Virology Unit, Microbiology and Virology Department, San Matteo Hospital Foundation, University of Pavia, Pavia, Italy.
- [6] Chief Medical Direction, San Matteo Hospital Foundation, Pavia, Italy.
- [7] Department of Internal Medicine, San Matteo Hospital Foundation, University of Pavia, Pavia, Italy. a.disabatino@smatteo.pv.it.

- PMID: **33161478**
- PMCID: PMC7648897
- DOI: 10.1007/s11739-020-02550-6

Erratum in

- Correction to: Venous thromboembolism and COVID-19: a single center experience from an academic tertiary referral hospital of Northern Italy.
  Melazzini F, Colaneri M, Fumoso F, Freddi G, Lenti MV, Pieri TC, Piloni D, Noris P, Pieresca C, Preti PS, Russo M, Corsico A, Tavazzi G, Baldanti F, Triarico A, Mojoli F, Bruno R, Di Sabatino A; San Matteo Pavia COVID-19 Task Force. Melazzini F, et al. Intern Emerg Med. 2021 Aug;16(5):1153. doi: 10.1007/s11739-020-02613-8. Intern Emerg Med. 2021. PMID: 33400165 Free PMC article. No abstract available.

Abstract

Preliminary evidence supports the notion that COVID-19 patients may have an increased susceptibility to develop venous thromboembolism (VTE). However, the magnitude of this association still needs to be defined. Furthermore, clinical predictors of thrombogenesis, and the relationship with the inflammatory status are currently unknown. On this basis, we conducted a retrospective, observational study on 259 consecutive COVID-19 patients admitted to an academic tertiary referral hospital in Northern Italy between March 19th and April 6th, 2020. Records of COVID-19 patients with a definite VTE event were reviewed for demographic information, co-morbidities, risk factors for VTE, laboratory tests, and anticoagulation treatment. Twenty-five cases among 259 COVID-19 patients developed VTE (9.6%), all of them having a Padua score > 4, although being under standard anticoagulation prophylaxis since hospital admission. In the VTE subcohort, we found a significant positive correlation between platelet count (PLT) and either C reactive protein (CRP) ($p < 0.0001$) or lactate dehydrogenase (LDH) ($p = 0.0013$), while a significant inverse correlation was observed between PLT and mean platelet volume ($p < 0.0001$). Platelet-to-lymphocyte ratio significantly correlated with CRP ($p < 0.0001$). The majority of VTE patients was male and younger compared to non-VTE patients ($p = 0.002$ and $p = 0.005$, respectively). No significant difference was found in D-dimer levels between VTE and non VTE patients, while significantly higher levels of LDH ($p = 0.04$) and IL-6 ($p = 0.04$) were observed in VTE patients in comparison to non-VTE patients. In conclusion, our findings showed a quite high prevalence of VTE in COVID-19 patients. Raised inflammatory indexes and increased serum levels of pro-inflammatory cytokines should raise the clinical suspicion of VTE.

Keywords: Anticoagulants; Pulmonary embolism; SARS-CoV-2; Thrombosis.

© 2020. The Author(s).

Conflict of interest statement

The authors declare that they have no conflict of interest.

- 44 references

Supplementary info

Publication types, MeSH terms Expand

Publication types

- Observational Study
- Research Support, Non-U.S. Gov't

MeSH terms

- Academic Medical Centers / organization & administration
- Academic Medical Centers / statistics & numerical data
- Adult
- Aged
- Aged, 80 and over
- COVID-19 / complications*
- COVID-19 / epidemiology
- Female
- Humans
- Italy / epidemiology
- Male
- Middle Aged
- Prevalence
- Retrospective Studies
- Statistics, Nonparametric
- Tertiary Care Centers / organization & administration
- Tertiary Care Centers / statistics & numerical data
- Venous Thromboembolism / epidemiology
- Venous Thromboembolism / etiology*

Full text links

SpringerLink FULL-TEXT ARTICLE Springer Free PMC article
Proceed to details
Cite

Share

☐ 792

Observational Study

J Orthop Sci
. 2021 Jan;26(1):179-181.
doi: 10.1016/j.jos.2020.10.009. Epub 2020 Nov 6.

RT-PCR testing should be performed prior to elective orthopaedic surgery during the COVID-19 pandemic

Tsuyoshi Nakai [1], Haruaki Iwasaki [2], Tomoya Nishikawa [2], Ryo Higuchi [2], Shigeko Nakamura [2], Seizoh Nakata [3]

Affiliations Expand

Affiliations

- [1] Department of Orthopaedic Surgery, Itami City Hospital, Itami, Japan. Electronic address: tsuyoshi223@gmail.com.
- [2] Department of Orthopaedic Surgery, Itami City Hospital, Itami, Japan.
- [3] Administrator, Itami City Hospital, Itami, Japan.

- PMID: **33199087**
- PMCID: PMC7837226
- DOI: 10.1016/j.jos.2020.10.009

Free PMC article

Observational Study

RT-PCR testing should be performed prior to elective orthopaedic surgery during the COVID-19 pandemic

Tsuyoshi Nakai et al. J Orthop Sci. 2021 Jan.

Free PMC article

Show details

J Orthop Sci
. 2021 Jan;26(1):179-181.
doi: 10.1016/j.jos.2020.10.009. Epub 2020 Nov 6.

Authors

Tsuyoshi Nakai [1], Haruaki Iwasaki [2], Tomoya Nishikawa [2], Ryo Higuchi [2], Shigeko Nakamura [2], Seizoh Nakata [3]

Affiliations

- [1] Department of Orthopaedic Surgery, Itami City Hospital, Itami, Japan. Electronic address: tsuyoshi223@gmail.com.
- [2] Department of Orthopaedic Surgery, Itami City Hospital, Itami, Japan.
- [3] Administrator, Itami City Hospital, Itami, Japan.

- PMID: **33199087**
- PMCID: PMC7837226
- DOI: 10.1016/j.jos.2020.10.009

No abstract available

Conflict of interest statement

Declaration of competing interest None.

- 5 references

Supplementary info

Publication types, MeSH terms Expand

Publication types

- Observational Study

MeSH terms

- Adult
- Aged
- Aged, 80 and over
- COVID-19 / diagnosis*
- COVID-19 / epidemiology*
- COVID-19 Nucleic Acid Testing*
- COVID-19 Serological Testing
- Clinical Protocols
- Elective Surgical Procedures*
- Female
- Humans
- Japan
- Male
- Middle Aged

- Orthopedic Procedures*
- Retrospective Studies
- Reverse Transcriptase Polymerase Chain Reaction*

Full text links

ELSEVIER FULL-TEXT ARTICLE Elsevier Science Free PMC article
Proceed to details
Cite
Share
793
Observational Study
Kidney Int
. 2021 Jul;100(1):2-5.
doi: 10.1016/j.kint.2021.04.017. Epub 2021 Apr 28.

Use of peritoneal dialysis for acute kidney injury during the COVID-19 pandemic in New York City: a multicenter observational study

Wei Chen [1], Nina Caplin [2], Osama El Shamy [3], Shuchita Sharma [3], Maryanne Y Sourial [4], Michael J Ross [5], Mina H Sourial [4], Kalyan Prudhvi [4], Ladan Golestaneh [4], Vesh Srivatana [6], Rochelle Dalsan [7], Daniil Shimonov [6], Luis Sanchez-Russo [3], Sara Atallah [3], Jaime Uribarri [3], NYC-PD Consortium
Affiliations Expand

Affiliations

- [1] Division of Nephrology, Albert Einstein College of Medicine, Bronx, New York, USA; Division of Nephrology, Montefiore Medical Center, Bronx, New York, USA; Division of Nephrology, University of Rochester School of Medicine and Dentistry, Rochester, New York, USA. Electronic address: wei.chen@einsteinmed.org.
- [2] Division of Nephrology, New York University Langone Health and New York University Grossman School of Medicine, New York, New York, USA; Department of Medicine, New York City Health and Hospitals/Bellevue, New York, New York, USA.
- [3] Division of Nephrology, Icahn School of Medicine at Mount Sinai, New York, New York, USA.
- [4] Division of Nephrology, Montefiore Medical Center, Bronx, New York, USA.
- [5] Division of Nephrology, Albert Einstein College of Medicine, Bronx, New York, USA; Division of Nephrology, Montefiore Medical Center, Bronx, New York, USA.
- [6] Division of Nephrology and Hypertension, Weill Cornell Medicine, New York, New York, USA; The Rogosin Institute, New York, New York, USA.
- [7] Division of Nephrology, Albert Einstein College of Medicine, Bronx, New York, USA.

- PMID: **33930411**
- PMCID: PMC8079266
- DOI: 10.1016/j.kint.2021.04.017

Free PMC article
Observational Study

Use of peritoneal dialysis for acute kidney injury during the COVID-19 pandemic in New York City: a multicenter observational study

Wei Chen et al. Kidney Int. 2021 Jul.
Free PMC article
Show details
Kidney Int
. 2021 Jul;100(1):2-5.
doi: 10.1016/j.kint.2021.04.017. Epub 2021 Apr 28.

Authors

Wei Chen [1], Nina Caplin [2], Osama El Shamy [3], Shuchita Sharma [3], Maryanne Y Sourial [4], Michael J Ross [5], Mina H Sourial [4], Kalyan Prudhvi [4], Ladan Golestaneh [4], Vesh Srivatana [6], Rochelle Dalsan [7], Daniil Shimonov [6], Luis Sanchez-Russo [3], Sara Atallah [3], Jaime Uribarri [3], NYC-PD Consortium

Affiliations

- [1] Division of Nephrology, Albert Einstein College of Medicine, Bronx, New York, USA; Division of Nephrology, Montefiore Medical Center, Bronx, New York, USA; Division of Nephrology, University of Rochester School of Medicine and Dentistry, Rochester, New York, USA. Electronic address: wei.chen@einsteinmed.org.
- [2] Division of Nephrology, New York University Langone Health and New York University Grossman School of Medicine, New York, New York, USA; Department of Medicine, New York City Health and Hospitals/Bellevue, New York, New York, USA.
- [3] Division of Nephrology, Icahn School of Medicine at Mount Sinai, New York, New York, USA.
- [4] Division of Nephrology, Montefiore Medical Center, Bronx, New York, USA.
- [5] Division of Nephrology, Albert Einstein College of Medicine, Bronx, New York, USA; Division of Nephrology, Montefiore Medical Center, Bronx, New York, USA.
- [6] Division of Nephrology and Hypertension, Weill Cornell Medicine, New York, New York, USA; The Rogosin Institute, New York, New York, USA.
- [7] Division of Nephrology, Albert Einstein College of Medicine, Bronx, New York, USA.

- PMID: **33930411**
- PMCID: PMC8079266

- DOI: 10.1016/j.kint.2021.04.017

Abstract

To demonstrate feasibility of acute peritoneal dialysis (PD) for acute kidney injury during the coronavirus disease 2019 (COVID-19) pandemic, we performed a multicenter, retrospective, observational study of 94 patients who received acute PD in New York City in the spring of 2020. Patient comorbidities, severity of disease, laboratory values, kidney replacement therapy, and patient outcomes were recorded. The mean age was 61 ± 11 years; 34% were women; 94% had confirmed COVID-19; 32% required mechanical ventilation on admission. Compared to the levels prior to initiation of kidney replacement therapy, the mean serum potassium level decreased from 5.1 ± 0.9 to 4.5 ± 0.7 mEq/L on PD day 3 and 4.2 ± 0.6 mEq/L on day 7 ($P < 0.001$ for both); mean serum bicarbonate increased from 20 ± 4 to 21 ± 4 mEq/L on PD day 3 ($P = 0.002$) and 24 ± 4 mEq/L on day 7 ($P < 0.001$). After a median follow-up of 30 days, 46% of patients died and 22% had renal recovery. Male sex and mechanical ventilation on admission were significant predictors of mortality. The rapid implementation of an acute PD program was feasible despite resource constraints and can be lifesaving during crises such as the COVID-19 pandemic.

Keywords: COVID-19; acute kidney injury; acute peritoneal dialysis; kidney replacement therapy; mortality.

Copyright © 2021 International Society of Nephrology. Published by Elsevier Inc. All rights reserved.

- 8 references
- 1 figure

Supplementary info

Publication types, MeSH terms, Grant support Expand

Publication types

- Multicenter Study
- Observational Study
- Research Support, N.I.H., Extramural

MeSH terms

- Acute Kidney Injury* / diagnosis
- Acute Kidney Injury* / epidemiology
- Acute Kidney Injury* / therapy
- Aged
- COVID-19*
- Female
- Humans
- Male

- Middle Aged
- New York City / epidemiology
- Pandemics
- Peritoneal Dialysis* / adverse effects
- Retrospective Studies
- SARS-CoV-2

Grant support

- K23 DK114476/DK/NIDDK NIH HHS/United States
- R01 DK101338/DK/NIDDK NIH HHS/United States
- R01 DK108346/DK/NIDDK NIH HHS/United States
- R18 DK118471/DK/NIDDK NIH HHS/United States

Full text links

ELSEVIER FULL-TEXT ARTICLE Elsevier Science Free PMC article
Proceed to details
Cite
Share
794

Observational Study

BMC Cardiovasc Disord
. 2021 Mar 30;21(1):158.
doi: 10.1186/s12872-021-01963-1.

Coronavirus disease 2019 (COVID-19) and QTc prolongation

Khalid Changal [1], David Paternite [2], Sean Mack [2], Spiro Veria [2], Rehana Bashir [3], Mitra Patel [2], Ronak Soni [4], Muhammad Ali [5], Tanveer Mir [6], Mujeeb Sheikh [7], P Kasi Ramanathan [8]
Affiliations Expand

Affiliations

- [1] Cardiovascular Medicine, University of Toledo, Toledo, OH, USA. khalidchangal@gmail.com.
- [2] University of Toledo College of Medicine and Life Sciences, Toledo, USA.
- [3] Owen's Community College, Toledo, OH, USA.
- [4] Cardiovascular Medicine, University of Toledo, Toledo, OH, USA.
- [5] Internal Medicine, University of Toledo, Toledo, OH, USA.
- [6] Internal Medicine, Detroit Medical Center, Wayne State University, Detroit, MI, USA.
- [7] Department of Cardiovascular Medicine and Interventional Cardiology, Promedica Toledo Hospital, 2109 Hughes Dr, Jobst Tower 3rd, Floor, Toledo, OH, 43606, USA. Smujeeb73@gmail.com.

- [8] Department of Cardiovascular Medicine and Interventional Cardiology, Promedica Toledo Hospital, 2109 Hughes Dr, Jobst Tower 3rd, Floor, Toledo, OH, 43606, USA.

- PMID: **33784966**
- PMCID: PMC8007653
- DOI: 10.1186/s12872-021-01963-1

Free PMC article
Observational Study

Coronavirus disease 2019 (COVID-19) and QTc prolongation

Khalid Changal et al. BMC Cardiovasc Disord. 2021.
Free PMC article
Show details
BMC Cardiovasc Disord
. 2021 Mar 30;21(1):158.
doi: 10.1186/s12872-021-01963-1.

Authors

Khalid Changal [1], David Paternite [2], Sean Mack [2], Spiro Veria [2], Rehana Bashir [3], Mitra Patel [2], Ronak Soni [4], Muhammad Ali [5], Tanveer Mir [6], Mujeeb Sheikh [7], P Kasi Ramanathan [8]

Affiliations

- [1] Cardiovascular Medicine, University of Toledo, Toledo, OH, USA. khalidchangal@gmail.com.
- [2] University of Toledo College of Medicine and Life Sciences, Toledo, USA.
- [3] Owen's Community College, Toledo, OH, USA.
- [4] Cardiovascular Medicine, University of Toledo, Toledo, OH, USA.
- [5] Internal Medicine, University of Toledo, Toledo, OH, USA.
- [6] Internal Medicine, Detroit Medical Center, Wayne State University, Detroit, MI, USA.
- [7] Department of Cardiovascular Medicine and Interventional Cardiology, Promedica Toledo Hospital, 2109 Hughes Dr, Jobst Tower 3rd, Floor, Toledo, OH, 43606, USA. Smujeeb73@gmail.com.
- [8] Department of Cardiovascular Medicine and Interventional Cardiology, Promedica Toledo Hospital, 2109 Hughes Dr, Jobst Tower 3rd, Floor, Toledo, OH, 43606, USA.

- PMID: **33784966**
- PMCID: PMC8007653
- DOI: 10.1186/s12872-021-01963-1

Abstract

Introduction: The cause-and-effect relationship of QTc prolongation in Coronavirus disease 2019 (COVID-19) patients has not been studied well.

Objective: We attempt to better understand the relationship of QTc prolongation in COVID-19 patients in this study.

Methods: This is a retrospective, hospital-based, observational study. All patients with normal baseline QTc interval who were hospitalized with the diagnosis of COVID-19 infection at two hospitals in Ohio, USA were included in this study.

Results: Sixty-nine patients had QTc prolongation, and 210 patients continued to have normal QTc during hospitalization. The baseline QTc intervals were comparable in the two groups. Patients with QTc prolongation were older (mean age 67 vs. 60, P 0.003), more likely to have underlying cardiovascular disease (48% versus 26%, P 0.001), ischemic heart disease (29% versus 17%, P 0.026), congestive heart failure with preserved ejection fraction (16% versus 8%, P 0.042), chronic kidney disease (23% versus 10%, P 0.005), and end-stage renal disease (12% versus 1%, $P < 0.001$). Patients with QTc prolongation were more likely to have received hydroxychloroquine (75% versus 59%, P 0.018), azithromycin (18% vs. 14%, P 0.034), a combination of hydroxychloroquine and azithromycin (29% vs 7%, $P < 0.001$), more than 1 QT prolonging agents (59% vs. 32%, $P < 0.001$). Patients who were on angiotensin-converting enzyme inhibitors (ACEi) were less likely to develop QTc prolongation (11% versus 26%, P 0.014). QTc prolongation was not associated with increased ventricular arrhythmias or mortality.

Conclusion: Older age, ESRD, underlying cardiovascular disease, potential virus mediated cardiac injury, and drugs like hydroxychloroquine/azithromycin, contribute to QTc prolongation in COVID-19 patients. The role of ACEi in preventing QTc prolongation in COVID-19 patients needs to be studied further.

Keywords: COVID-19; Cardiovascular disease; QT prolongation.

Conflict of interest statement

None.

- 34 references

Supplementary info

Publication types, MeSH terms, Supplementary concepts Expand

Publication types

- Observational Study

MeSH terms

- Age Factors
- Aged
- COVID-19 / classification
- COVID-19 / complications

- COVID-19 / drug therapy*
- COVID-19 / epidemiology
- COVID-19 / physiopathology
- COVID-19 / therapy
- Cardiovascular Diseases / epidemiology*
- Comorbidity
- Correlation of Data
- Electrocardiography* / methods
- Electrocardiography* / statistics & numerical data
- Female
- Humans
- Long QT Syndrome* / diagnosis
- Long QT Syndrome* / epidemiology
- Long QT Syndrome* / etiology
- Male
- Middle Aged
- Outcome and Process Assessment, Health Care
- Renal Insufficiency, Chronic / epidemiology*
- Risk Assessment / methods
- SARS-CoV-2 / isolation & purification
- Survival Analysis
- United States / epidemiology

Supplementary concepts

- COVID-19 drug treatment

Full text links

Read free full text at BMC BioMed Central Free PMC article
Proceed to details
Cite
Share
☐ 795
Observational Study
Lancet Respir Med
. 2021 Apr;9(4):397-406.
doi: 10.1016/S2213-2600(20)30579-8. Epub 2021 Feb 15.

Patient factors and temporal trends associated with COVID-19 in-hospital

mortality in England: an observational study using administrative data

Annakan V Navaratnam [1], William K Gray [2], Jamie Day [2], Julia Wendon [3], Tim W R Briggs [4]
Affiliations Expand

Affiliations

- [1] Getting It Right First Time programme, London, UK; University College London Hospitals NHS Foundation Trust, London, UK. Electronic address: annakan.navaratnam@nhs.net.
- [2] Getting It Right First Time programme, London, UK.
- [3] Kings College Hospital NHS Foundation Trust, London, UK.
- [4] Getting It Right First Time programme, London, UK; Royal National Orthopaedic Hospital NHS Trust, London, UK.

- PMID: **33600777**
- PMCID: PMC7906650
- DOI: 10.1016/S2213-2600(20)30579-8

Free PMC article
Observational Study

Patient factors and temporal trends associated with COVID-19 in-hospital mortality in England: an observational study using administrative data

Annakan V Navaratnam et al. Lancet Respir Med. 2021 Apr.
Free PMC article
Show details
Lancet Respir Med
. 2021 Apr;9(4):397-406.
doi: 10.1016/S2213-2600(20)30579-8. Epub 2021 Feb 15.

Authors

Annakan V Navaratnam [1], William K Gray [2], Jamie Day [2], Julia Wendon [3], Tim W R Briggs [4]

Affiliations

- [1] Getting It Right First Time programme, London, UK; University College London Hospitals NHS Foundation Trust, London, UK. Electronic address: annakan.navaratnam@nhs.net.

- [2] Getting It Right First Time programme, London, UK.
- [3] Kings College Hospital NHS Foundation Trust, London, UK.
- [4] Getting It Right First Time programme, London, UK; Royal National Orthopaedic Hospital NHS Trust, London, UK.

- PMID: **33600777**
- PMCID: PMC7906650
- DOI: 10.1016/S2213-2600(20)30579-8

Abstract

Background: Analysis of the effect of COVID-19 on the complete hospital population in England has been lacking. Our aim was to provide a comprehensive account of all hospitalised patients with COVID-19 in England during the early phase of the pandemic and to identify the factors that influenced mortality as the pandemic evolved.

Methods: This was a retrospective exploratory analysis using the Hospital Episode Statistics administrative dataset. All patients aged 18 years or older in England who completed a hospital stay (were discharged alive or died) between March 1 and May 31, 2020, and had a diagnosis of COVID-19 on admission or during their stay were included. In-hospital death was the primary outcome of interest. Multilevel logistic regression was used to model the relationship between death and several covariates: age, sex, deprivation (Index of Multiple Deprivation), ethnicity, frailty (Hospital Frailty Risk Score), presence of comorbidities (Charlson Comorbidity Index items), and date of discharge (whether alive or deceased).

Findings: 91 541 adult patients with COVID-19 were discharged during the study period, among which 28 200 (30·8%) in-hospital deaths occurred. The final multilevel logistic regression model accounted for age, deprivation score, and date of discharge as continuous variables, and sex, ethnicity, and Charlson Comorbidity Index items as categorical variables. In this model, significant predictors of in-hospital death included older age (modelled using restricted cubic splines), male sex (1·457 [1·408-1·509]), greater deprivation (1·002 [1·001-1·003]), Asian (1·211 [1·128-1·299]) or mixed ethnicity (1·317 [1·080-1·605]; vs White ethnicity), and most of the assessed comorbidities, including moderate or severe liver disease (5·433 [4·618-6·392]). Later date of discharge was associated with a lower odds of death (0·977 [0·976-0·978]); adjusted in-hospital mortality improved significantly in a broadly linear fashion, from 52·2% in the first week of March to 16·8% in the last week of May.

Interpretation: Reductions in the adjusted probability of in-hospital mortality for COVID-19 patients over time might reflect the impact of changes in hospital strategy and clinical processes. The reasons for the observed improvements in mortality should be thoroughly investigated to inform the response to future outbreaks. The higher mortality rate reported for certain ethnic minority groups in community-based studies compared with our hospital-based analysis might partly reflect differential infection rates in those at greatest risk, propensity to become severely ill once infected, and health-seeking behaviours.

Funding: None.

Copyright © 2021 Elsevier Ltd. All rights reserved.

Comment in

- Trends in COVID-19-related in-hospital mortality: lessons learned from nationwide samples.
  Madahar P, Wunsch H, Jha P, Slutsky AS, Brodie D. Madahar P, et al. Lancet Respir Med. 2021 Apr;9(4):322-324. doi: 10.1016/S2213-2600(21)00080-1. Epub 2021 Feb 15. Lancet Respir Med. 2021. PMID: 33600776 Free PMC article. No abstract available.

- 34 references
- 3 figures

Supplementary info

Publication types, MeSH terms Expand

Publication types

- Observational Study

MeSH terms

- Adolescent
- Adult
- Age Factors
- Aged
- Aged, 80 and over
- COVID-19 / diagnosis
- COVID-19 / mortality*
- Comorbidity
- Datasets as Topic
- Electronic Health Records / statistics & numerical data
- England / epidemiology
- Female
- Hospital Mortality / trends*
- Humans
- Length of Stay / statistics & numerical data
- Male
- Middle Aged
- Minority Groups / statistics & numerical data*
- Pandemics / statistics & numerical data*
- Patient Acceptance of Health Care / statistics & numerical data*
- Retrospective Studies
- Risk Factors
- Severity of Illness Index
- Sex Factors
- Young Adult

Full text links

ELSEVIER FULL-TEXT ARTICLE Elsevier Science Free PMC article
Proceed to details
Cite
Share
796
Observational Study
Transplant Proc
. 2020 Nov;52(9):2654-2658.
doi: 10.1016/j.transproceed.2020.09.005. Epub 2020 Sep 10.

Spectrum of Coronavirus Disease 2019 Outcomes in Kidney Transplant Recipients: A Single-Center Experience

Erik Lum [1], Suphamai Bunnapradist [1], Ashrit Multani [2], Omer E Beaird [2], Margrit Carlson [2], Pryce Gaynor [2], Camille Kotton [3], Basmah Abdalla [1], Gabriel Danovitch [1], Elizabeth Kendrick [1], Karid Nieves-Borrero [1], Phuong T Pham [1], Julie Yabu [1], Joanna Schaenman [4]
Affiliations Expand

Affiliations

- [1] Division of Nephrology, Department of Medicine, UCLA David Geffen School of Medicine, Los Angeles, California.
- [2] Division of Infectious Diseases, UCLA David Geffen School of Medicine, University of California, Los Angeles, California.
- [3] Transplant and Immunocompromised Host Infectious Diseases, Infectious Diseases Division, Department of Medicine, Massachusetts General Hospital, Harvard Medical School, Boston, Massachusetts.
- [4] Division of Infectious Diseases, UCLA David Geffen School of Medicine, University of California, Los Angeles, California. Electronic address: jschaenman@mednet.ucla.edu.

- PMID: **33041077**
- PMCID: PMC7832798
- DOI: 10.1016/j.transproceed.2020.09.005

Free PMC article
Observational Study

Spectrum of Coronavirus Disease 2019 Outcomes in Kidney Transplant Recipients: A Single-Center Experience

Erik Lum et al. Transplant Proc. 2020 Nov.
Free PMC article
Show details
Transplant Proc
. 2020 Nov;52(9):2654-2658.
doi: 10.1016/j.transproceed.2020.09.005. Epub 2020 Sep 10.

Authors

Erik Lum [1], Suphamai Bunnapradist [1], Ashrit Multani [2], Omer E Beaird [2], Margrit Carlson [2], Pryce Gaynor [2], Camille Kotton [3], Basmah Abdalla [1], Gabriel Danovitch [1], Elizabeth Kendrick [1], Karid Nieves-Borrero [1], Phuong T Pham [1], Julie Yabu [1], Joanna Schaenman [4]

Affiliations

- [1] Division of Nephrology, Department of Medicine, UCLA David Geffen School of Medicine, Los Angeles, California.
- [2] Division of Infectious Diseases, UCLA David Geffen School of Medicine, University of California, Los Angeles, California.
- [3] Transplant and Immunocompromised Host Infectious Diseases, Infectious Diseases Division, Department of Medicine, Massachusetts General Hospital, Harvard Medical School, Boston, Massachusetts.
- [4] Division of Infectious Diseases, UCLA David Geffen School of Medicine, University of California, Los Angeles, California. Electronic address: jschaenman@mednet.ucla.edu.

- PMID: **33041077**
- PMCID: PMC7832798
- DOI: 10.1016/j.transproceed.2020.09.005

Abstract

Purpose: We reviewed the clinical experience of kidney transplant recipients diagnosed with severe acute respiratory syndrome coronavirus 2 infection in order to understand the impact of the current coronavirus disease 2019 (COVID-19) pandemic infection on transplant recipients. Given that early reports from heavily affected areas demonstrated a very high mortality rate amongst kidney transplant recipients, ranging between 30% and 40%, we sought to evaluate outcomes at a center with a high burden of cases but not experiencing acute crisis due to COVID-19.

Procedures: In this single center retrospective observational study, medical records of all kidney transplant recipients at the UCLA Medical Center were reviewed for a diagnosis of COVID-19 by polymerase chain reaction, followed by chart review to determine kidney transplant characteristics and clinical course.

Main findings: A total of 41 kidney transplant recipients were identified with COVID-19 positive polymerase chain reaction. Recipients had been transplanted for a median of 47 months before diagnosis. The large proportion of infected individuals were minorities (Hispanic 65.9%, black 14.6%), on prednisone, tacrolimus, and mycophenolate mofetil (95.1%, 87.8%, and 87.8%, respectively), and had excellent allograft function (median 1.25 mg/dL). The most common presenting symptoms were fever, dyspnea, or cough. Most patients were hospitalized (63.4%); mortality was 9.8% and occurred only in patients in the intensive care unit. The most common treatment was reduction or removal of antimetabolite (77.8%). Approximately 26.9% presented with AKI.

Conclusions: COVID-19 infection in kidney transplant recipients results in a higher rate of hospitalization and mortality than in the general population. In an area with a high number of infections, the mortality rate was lower compared with earlier reports from areas experiencing early surge and strain on the medical system. Minorities were disproportionately affected. Future studies are needed to determine optimal approach to treatment and management of immunosuppression in kidney transplant recipients with COVID-19 infection.

Copyright © 2020 Elsevier Inc. All rights reserved.

- 20 references

Supplementary info

Publication types, MeSH terms, Substances Expand

Publication types

- Observational Study

MeSH terms

- Adult
- Betacoronavirus
- COVID-19
- Coronavirus Infections / immunology*
- Coronavirus Infections / mortality
- Female
- Humans
- Immunocompromised Host*
- Immunosuppressive Agents / therapeutic use
- Kidney Transplantation* / mortality
- Male
- Middle Aged
- Pandemics
- Pneumonia, Viral / immunology*
- Pneumonia, Viral / mortality
- Retrospective Studies

- SARS-CoV-2
- Transplant Recipients*

Substances

- Immunosuppressive Agents

Full text links

ELSEVIER FULL-TEXT ARTICLE Elsevier Science Free PMC article
Proceed to details
Cite
Share
797
Observational Study
Br J Anaesth
. 2021 Mar;126(3):578-589.
doi: 10.1016/j.bja.2020.11.034. Epub 2020 Dec 4.

Clinically applicable approach for predicting mechanical ventilation in patients with COVID-19

Nicholas J Douville [1], Christopher B Douville [2], Graciela Mentz [3], Michael R Mathis [3], Carlo Pancaro [3], Kevin K Tremper [3], Milo Engoren [3]
Affiliations Expand

Affiliations

- [1] Department of Anesthesiology, Michigan Medicine, Ann Arbor, MI, USA; Institute of Healthcare Policy & Innovation, University of Michigan, Ann Arbor, MI, USA. Electronic address: ndouvill@med.umich.edu.
- [2] Ludwig Center for Cancer Genetics and Therapeutics, Johns Hopkins University School of Medicine, Baltimore, MD, USA; Sidney Kimmel Cancer Center, Johns Hopkins University School of Medicine, Baltimore, MD, USA; Sol Goldman Pancreatic Cancer Research Center, Johns Hopkins University School of Medicine, Baltimore, MD, USA.
- [3] Department of Anesthesiology, Michigan Medicine, Ann Arbor, MI, USA.

- PMID: **33454051**
- PMCID: PMC7833820
- DOI: 10.1016/j.bja.2020.11.034

Free PMC article
Observational Study

Clinically applicable approach for predicting mechanical ventilation in patients with COVID-19

Nicholas J Douville et al. Br J Anaesth. 2021 Mar.
Free PMC article
Show details
Br J Anaesth
. 2021 Mar;126(3):578-589.
doi: 10.1016/j.bja.2020.11.034. Epub 2020 Dec 4.

Authors

Nicholas J Douville [1], Christopher B Douville [2], Graciela Mentz [3], Michael R Mathis [3], Carlo Pancaro [3], Kevin K Tremper [3], Milo Engoren [3]

Affiliations

- [1] Department of Anesthesiology, Michigan Medicine, Ann Arbor, MI, USA; Institute of Healthcare Policy & Innovation, University of Michigan, Ann Arbor, MI, USA. Electronic address: ndouvill@med.umich.edu.
- [2] Ludwig Center for Cancer Genetics and Therapeutics, Johns Hopkins University School of Medicine, Baltimore, MD, USA; Sidney Kimmel Cancer Center, Johns Hopkins University School of Medicine, Baltimore, MD, USA; Sol Goldman Pancreatic Cancer Research Center, Johns Hopkins University School of Medicine, Baltimore, MD, USA.
- [3] Department of Anesthesiology, Michigan Medicine, Ann Arbor, MI, USA.

- PMID: **33454051**
- PMCID: PMC7833820
- DOI: 10.1016/j.bja.2020.11.034

Abstract

Background: Patients with coronavirus disease 2019 (COVID-19) requiring mechanical ventilation have high mortality and resource utilisation. The ability to predict which patients may require mechanical ventilation allows increased acuity of care and targeted interventions to potentially mitigate deterioration.

Methods: We included hospitalised patients with COVID-19 in this single-centre retrospective observational study. Our primary outcome was mechanical ventilation or death within 24 h. As clinical decompensation is more recognisable, but less modifiable, as the prediction window shrinks, we also assessed 4, 8, and 48 h prediction windows. Model features included demographic information, laboratory results, comorbidities, medication administration, and vital signs. We created a Random Forest model, and assessed performance using 10-fold cross-validation. The model was compared with models derived from generalised estimating equations using discrimination.

Results: Ninety-three (23%) of 398 patients required mechanical ventilation or died within 14 days of admission. The Random Forest model predicted pending mechanical ventilation with good discrimination (C-statistic=0.858; 95% confidence interval, 0.841-0.874), which is comparable with the discrimination of the generalised estimating equation regression. Vitals sign data including SpO_2/FiO_2 ratio (Random Forest Feature Importance Z-score=8.56), ventilatory frequency (5.97), and heart rate (5.87) had the highest predictive utility. In our highest-risk cohort, the number of patients needed to identify a single new case was 3.2, and for our second quintile it was 5.0.

Conclusion: Machine learning techniques can be leveraged to improve the ability to predict which patients with COVID-19 are likely to require mechanical ventilation, identifying unrecognised bellwethers and providing insight into the constellation of accompanying signs of respiratory failure in COVID-19.

Keywords: COVID-19; critical care medicine; machine learning; mechanical ventilation; predictive models; respiratory failure; respiratory insufficiency.

Copyright © 2020 British Journal of Anaesthesia. Published by Elsevier Ltd. All rights reserved.

- 30 references
- 2 figures

Supplementary info

Publication types, MeSH terms, Grant support Expand

Publication types

- Observational Study
- Research Support, N.I.H., Extramural
- Research Support, Non-U.S. Gov't

MeSH terms

- Aged
- COVID-19 / diagnosis*
- COVID-19 / epidemiology
- COVID-19 / therapy*
- Clinical Decision-Making / methods*
- Female
- Humans
- Machine Learning / trends*
- Male
- Middle Aged
- Predictive Value of Tests
- Respiration, Artificial / trends*
- Retrospective Studies

Grant support

- K01 HL141701/HL/NHLBI NIH HHS/United States

Full text links

ELSEVIER OPEN ACCESS Elsevier Science Free PMC article
Proceed to details
Cite
Share
☐ 798
Observational Study
Acta Anaesthesiol Scand
. 2021 Jul;65(6):761-769.
doi: 10.1111/aas.13819. Epub 2021 Mar 27.

Acute pulmonary hypertension and short-term outcomes in severe Covid-19 patients needing intensive care

Joakim Norderfeldt [1], Andreas Liliequist [2], Claes Frostell [3], Christofer Adding [4], Per Agvald [5], Maria Eriksson [1], Per-Arne Lönnqvist [6]
Affiliations Expand

Affiliations

- [1] Department of Clinical Physiology, Department of Molecular Medicine and Surgery, Karolinska University Hospital, Karolinska Institutet, Stockholm, Sweden.
- [2] Section of Thoracic Anaesthesia and Intensive Care, Perioperative Medicine and Intensive Care, Karolinska University Hospital, Stockholm, Sweden.
- [3] Department of Clinical Sciences, Karolinska Institute, Danderyd Hospital, Stockholm.
- [4] Department of Molecular Medicine and Surgery, Section of Urology, Karolinska Institutet, Stockholm, Sweden.
- [5] Department of Physiology and Pharmacology, Section of Pharmacology, Karolinska Institutet, Stockholm, Sweden.
- [6] Department of Physiology and Pharmacology, Section of Anaesthesiology and Intensive Care, Karolinska Institutet, Stockholm, Sweden.

- PMID: **33728633**
- PMCID: PMC8250592
- DOI: 10.1111/aas.13819

Free PMC article
Observational Study

Acute pulmonary hypertension and short-term outcomes in severe Covid-19 patients needing intensive care

Joakim Norderfeldt et al. Acta Anaesthesiol Scand. 2021 Jul.
Free PMC article
Show details
Acta Anaesthesiol Scand
. 2021 Jul;65(6):761-769.
doi: 10.1111/aas.13819. Epub 2021 Mar 27.

Authors

Joakim Norderfeldt [1], Andreas Liliequist [2], Claes Frostell [3], Christofer Adding [4], Per Agvald [5], Maria Eriksson [1], Per-Arne Lönnqvist [6]

Affiliations

- [1] Department of Clinical Physiology, Department of Molecular Medicine and Surgery, Karolinska University Hospital, Karolinska Institutet, Stockholm, Sweden.
- [2] Section of Thoracic Anaesthesia and Intensive Care, Perioperative Medicine and Intensive Care, Karolinska University Hospital, Stockholm, Sweden.
- [3] Department of Clinical Sciences, Karolinska Institute, Danderyd Hospital, Stockholm.
- [4] Department of Molecular Medicine and Surgery, Section of Urology, Karolinska Institutet, Stockholm, Sweden.
- [5] Department of Physiology and Pharmacology, Section of Pharmacology, Karolinska Institutet, Stockholm, Sweden.
- [6] Department of Physiology and Pharmacology, Section of Anaesthesiology and Intensive Care, Karolinska Institutet, Stockholm, Sweden.

- PMID: **33728633**
- PMCID: PMC8250592
- DOI: 10.1111/aas.13819

Abstract

Introduction: Critically ill Covid-19 pneumonia patients are likely to develop the sequence of acute pulmonary hypertension, right ventricular (RV) strain, and eventually RV failure due to known pathophysiology (endothelial inflammation plus thrombo-embolism) that promotes increased pulmonary vascular resistance and pulmonary artery pressure. This study aimed to investigate the occurrence of acute pulmonary hypertension (aPH) as per established trans-thoracic echocardiography (TTE) criteria in Covid-19 patients receiving intensive care and to explore whether short-term outcomes are affected by the presence of aPH.

Methods: Medical records were reviewed for patients treated in the intensive care units at a tertiary university hospital over a month. The presence of aPH on the TTE was noted, and plasma

NTproBNP and troponin were measured as markers of cardiac failure and myocardial injury, respectively. Follow-up data were collected 21 d after the performance of TTE.

Results: In total, 26 of 67 patients (39%) had an assessed systolic pulmonary artery pressure of > 35 mmHg (group aPH), meeting the TTE definition of aPH. NTproBNP levels (median [range]: 1430 [102-30 300] vs. 470 [45-29 600] ng L^{-1} ; P = .0007), troponin T levels (63 [22-352] vs. 15 [5-407] ng L^{-1} ; P = .0002), and the 21-d mortality rate (46% vs. 7%; P < .001) were substantially higher in patients with aPH compared to patients not meeting aPH criteria.

Conclusion: TTE-defined acute pulmonary hypertension was frequently observed in severely ill Covid-19 patients. Furthermore, aPH was linked to biomarker-defined myocardial injury and cardiac failure, as well as an almost sevenfold increase in 21-d mortality.

Keywords: Covid-19; echocardiography; intensive care; outcome; prevalence; pulmonary hypertension; tricuspid valve regurgitation.

© 2021 The Authors. Acta Anaesthesiologica Scandinavica published by John Wiley & Sons Ltd on behalf of Acta Anaesthesiologica Scandinavica Foundation.

Conflict of interest statement

JN, AL, and ME have no competing interests to declare. CF, and PAL act as advisors to ATTGENO AB, a startup pharmaceutical drug development company (Sweden). CF is also a minor stock owner in ATTGENO AB and directs Claes Frostell Research & Consulting AB (Sweden). PAL is a minor stock owner in ATTGENO AB and directs AnestInvest AB (Sweden). CA and PA are the founders and major owners of ATTGENO AB.

- 19 references
- 3 figures

Supplementary info

Publication types, MeSH terms, Substances, Grant support Expand

Publication types

- Observational Study

MeSH terms

- Acute Disease
- Adult
- Aged
- Biomarkers
- COVID-19 / complications*
- COVID-19 / mortality
- COVID-19 / physiopathology
- COVID-19 / therapy

- Critical Care*
- Echocardiography
- Female
- Fibrin Fibrinogen Degradation Products / analysis
- Follow-Up Studies
- Heart Failure / blood
- Heart Failure / etiology
- Heart Failure / mortality
- Hospital Mortality
- Humans
- Hypertension, Pulmonary / diagnostic imaging
- Hypertension, Pulmonary / epidemiology
- Hypertension, Pulmonary / etiology*
- Intensive Care Units / statistics & numerical data
- Male
- Middle Aged
- Natriuretic Peptide, Brain / blood
- Peptide Fragments / blood
- Procedures and Techniques Utilization
- Respiration, Artificial / statistics & numerical data
- Retrospective Studies
- SARS-CoV-2*
- Sweden
- Tertiary Care Centers / statistics & numerical data
- Treatment Outcome
- Tricuspid Valve Insufficiency / diagnostic imaging
- Tricuspid Valve Insufficiency / etiology
- Troponin T / blood

Substances

- Biomarkers
- Fibrin Fibrinogen Degradation Products
- Peptide Fragments
- Troponin T
- fibrin fragment D
- pro-brain natriuretic peptide (1-76)
- Natriuretic Peptide, Brain

Grant support

- Swedish Research Council (2015-02880)/Vetenskapsrådet

- Unrestricted educational grant from Attgeno AB/Attgeno AB

Full text links

WILEY Full Text Article Wiley Free PMC article

Proceed to details

Cite

Share

☐ 799

Observational Study

Indian J Ophthalmol

. 2021 Mar;69(3):730-733.

doi: 10.4103/ijo.IJO_2807_20.

Profile of patients receiving intravitreal anti-vascular endothelial growth factor injections during COVID-19-related lockdown

Manavi D Sindal [1], Kanika Chhabra [2], Vaibhav Khanna [3]

Affiliations Expand

Affiliations

- [1] Clinical Retina and Training, Vitreoretinal Services, Aravind Eye Hospital and Postgraduate Institute of Ophthalmology, Pondicherry, India.
- [2] Vitreoretinal Services, Aravind Eye Hospital and Postgraduate Institute of Ophthalmology, Pondicherry, India.
- [3] Cornea Services, Aravind Eye Hospital and Postgraduate Institute of Ophthalmology, Pondicherry, India.

- PMID: **33595512**
- PMCID: PMC7942127
- DOI: 10.4103/ijo.IJO_2807_20

Free PMC article

Observational Study

Profile of patients receiving intravitreal anti-vascular endothelial growth factor injections during COVID-19-related lockdown

Manavi D Sindal et al. Indian J Ophthalmol. 2021 Mar.

Free PMC article

Show details

Indian J Ophthalmol
. 2021 Mar;69(3):730-733.
doi: 10.4103/ijo.IJO_2807_20.

Authors

Manavi D Sindal [1], Kanika Chhabra [2], Vaibhav Khanna [3]

Affiliations

- [1] Clinical Retina and Training, Vitreoretinal Services, Aravind Eye Hospital and Postgraduate Institute of Ophthalmology, Pondicherry, India.
- [2] Vitreoretinal Services, Aravind Eye Hospital and Postgraduate Institute of Ophthalmology, Pondicherry, India.
- [3] Cornea Services, Aravind Eye Hospital and Postgraduate Institute of Ophthalmology, Pondicherry, India.

- PMID: **33595512**
- PMCID: PMC7942127
- DOI: 10.4103/ijo.IJO_2807_20

Abstract

Purpose: The aim of this study was to analyze the impact on vision due to delay in presentation of patients requiring intravitreal anti-vascular endothelial growth factor (anti-VEGF) injections, consequent to COVID-19-related travel restrictions.

Methods: Data were collected retrospectively of patients who received anti-VEGF injections during four months of the COVID-19 pandemic. Visual acuities, indication for treatment were noted along with basic demographic characteristics.

Results: Data were analyzed for 303 eyes of 263 patients. The indication for treatment was age-related macular degeneration (AMD) in 60 eyes (19.8%), while 162 eyes (53.5%) had Diabetic Macular Edema, 71 eyes (23.4%) had Retinal Vein Occlusion and 10 eyes (3.3%) had other diagnosis. The visual acuity in the treatment naïve eyes (Group A, n = 168) was significantly worse (P <0.001) than those who presented for retreatment (Group B, n = 135). In Group B, there was a significant decline in vision for the entire cohort (P = 0.009) and those with AMD (P = 0.036). Those in Group B presented at a mean interval of 19.1 ± 10.6 (range, 4-64) weeks for retreatment.

Conclusion: The COVID-19 pandemic has led to a delay in patients receiving anti-VEGF injections. The visual acuity is worse in both treatment naïve as well as those requiring retreatment. This could have long-term impact on vision of patients requiring this vision preserving treatment.

Keywords: Age-related macular degeneration; COVID-19; anti-VEGF; diabetic macular edema; retinal vein occlusion.

Conflict of interest statement

None

- 16 references
- 2 figures

Supplementary info

Publication types, MeSH terms, Substances Expand

Publication types

- Observational Study

MeSH terms

- Adult
- Aged
- Aged, 80 and over
- Angiogenesis Inhibitors / administration & dosage
- Bevacizumab / administration & dosage*
- COVID-19 / epidemiology*
- Comorbidity
- Female
- Follow-Up Studies
- Humans
- Intravitreal Injections
- Male
- Middle Aged
- Quarantine*
- Ranibizumab / administration & dosage*
- Retinal Diseases / drug therapy*
- Retinal Diseases / epidemiology
- Retrospective Studies
- SARS-CoV-2*
- Vascular Endothelial Growth Factor A / antagonists & inhibitors
- Young Adult

Substances

- Angiogenesis Inhibitors
- Vascular Endothelial Growth Factor A
- Bevacizumab
- Ranibizumab

Full text links

Get Free Full Text Wolters Kluwer | Medknow Medknow Publications and Media Pvt Ltd Free PMC article

Proceed to details

Cite

Share

800

Observational Study

Biomarkers

. 2021 Mar;26(2):119-126.

doi: 10.1080/1354750X.2021.1874052. Epub 2021 Jan 18.

Biomarkers and short-term prognosis in COVID-19

Óscar M Peiró [1 2 3], Anna Carrasquer [1 2 3], Raúl Sánchez-Gimenez [1 2 3], Nisha Lal-Trehan [1 2 3], Víctor Del-Moral-Ronda [1 2 3], Gil Bonet [1 2 3], Isabel Fort-Gallifa [4], Ester Picó-Plana [4], Natalia Bastón-Paz [4], Cristina Gutiérrez [4], Alfredo Bardaji [1 2 3]

Affiliations Expand

Affiliations

- [1] Department of Cardiology, Joan XXIII University Hospital, Tarragona, Spain.
- [2] Pere Virgili Health Research Institute (IISPV), Tarragona, Spain.
- [3] Department of Medicine and Surgery, Rovira i Virgili University, Tarragona, Spain.
- [4] Clinical Laboratory, Catalan Institute of Health, Camp de Tarragona-Terres de l'Ebre, Tarragona, Spain.

- PMID: **33426934**
- PMCID: PMC7832452
- DOI: 10.1080/1354750X.2021.1874052

Free PMC article

Observational Study

Biomarkers and short-term prognosis in COVID-19

Óscar M Peiró et al. Biomarkers. 2021 Mar.

Free PMC article

Show details

Biomarkers

. 2021 Mar;26(2):119-126.

doi: 10.1080/1354750X.2021.1874052. Epub 2021 Jan 18.

Authors

Óscar M Peiró [1 2 3], Anna Carrasquer [1 2 3], Raúl Sánchez-Gimenez [1 2 3], Nisha Lal-Trehan [1 2 3], Víctor Del-Moral-Ronda [1 2 3], Gil Bonet [1 2 3], Isabel Fort-Gallifa [4], Ester Picó-Plana [4], Natalia Bastón-Paz [4], Cristina Gutiérrez [4], Alfredo Bardaji [1 2 3]

Affiliations

- [1] Department of Cardiology, Joan XXIII University Hospital, Tarragona, Spain.
- [2] Pere Virgili Health Research Institute (IISPV), Tarragona, Spain.
- [3] Department of Medicine and Surgery, Rovira i Virgili University, Tarragona, Spain.
- [4] Clinical Laboratory, Catalan Institute of Health, Camp de Tarragona-Terres de l'Ebre, Tarragona, Spain.

- PMID: **33426934**
- PMCID: PMC7832452
- DOI: 10.1080/1354750X.2021.1874052

Abstract

Purpose: The aim of our study was to analyse the short-term prognostic value of different biomarkers in patients with COVID-19.

Methods: We included patients admitted to emergency department with COVID-19 and available concentrations of cardiac troponin I (cTnI), D-dimer, C-reactive protein (CRP) and lactate dehydrogenase (LDH). Patients were classified for each biomarker into two groups (low vs. high concentrations) according to their best cut-off point, and 30-day all-cause death was evaluated.

Results: After multivariate adjustment, cTnI ≥21 ng/L, D-dimer ≥1112 ng/mL, CRP ≥10 mg/dL and LDH ≥334 U/L at admission were associated with an increased risk of 30-day all-cause death (hazard ratio (HR) 4.30; 95% CI 1.74-10.58; $p = 0.002$; HR 3.35; 95% CI 1.58-7.13; $p = 0.002$; HR 2.25; 95% CI 1.13-4.50; $p = 0.021$; HR 2.00; 95% CI 1.04-3.84; $p = 0.039$, respectively). The area under the curve for cTnI was 0.825 (95% CI 0.759-0.892) and, in comparison, was significantly better than CRP (0.685; 95% CI 0.600-0.770; $p = 0.009$) and LDH (0.643; 95% CI 0.534-0.753; $p = 0.006$) but non-significantly better than D-dimer (0.756; 95% CI 0.674-0.837; $p = 0.115$).

Conclusions: In patients with COVID-19, increased concentrations of cTnI, D-dimer, CRP and LDH are associated with short-term mortality. Of these, cTnI provides better mortality risk prediction. However, differences with D-dimer were non-significant.

Keywords: Biomarkers; C-reactive protein; COVID-19; D-dimer; lactate dehydrogenase; short-term prognosis; troponin.

Conflict of interest statement

No potential conflict of interest was reported by the author(s).

- 36 references
- 3 figures

Supplementary info

Publication types, MeSH terms, Substances Expand

Publication types

- Observational Study

MeSH terms

- Aged
- Aged, 80 and over
- Biomarkers*
- C-Reactive Protein / analysis
- COVID-19 / diagnosis*
- COVID-19 / mortality
- COVID-19 / pathology
- Cause of Death
- Female
- Fibrin Fibrinogen Degradation Products / analysis
- Humans
- L-Lactate Dehydrogenase / analysis
- Male
- Middle Aged
- Patient Admission
- Predictive Value of Tests
- Prognosis
- ROC Curve
- Retrospective Studies
- Treatment Outcome
- Troponin I / analysis

Substances

- Biomarkers
- Fibrin Fibrinogen Degradation Products
- Troponin I
- fibrin fragment D
- C-Reactive Protein
- L-Lactate Dehydrogenase

Full text links

View full text Taylor & Francis Free PMC article
Proceed to details
Cite
Share
1,388 results
Show more results
[x]
Cite
Copy
Download .nbib
Format: NLM
[x]
Share

-
-

Permalink
Copy
first first First previous previous Prev
Page
4
of 7
Next next next Last last last
Send To

- Clipboard
- Email
- Save
- My Bibliography
- Collections
- Citation Manager

[x]

- Article type
- Species
- Language
- Sex
- Journal
- Age

- ☐ Address
- ☐ Autobiography
- ☐ Bibliography
- ☐ Biography
- ☐ Case Reports

- ☐ Classical Article
- ☐ Clinical Conference
- ☐ Clinical Study
- ☐ Clinical Trial Protocol
- ☐ Clinical Trial, Phase I
- ☐ Clinical Trial, Phase II
- ☐ Clinical Trial, Phase III
- ☐ Clinical Trial, Phase IV
- ☐ Clinical Trial, Veterinary
- ☐ Comment
- ☐ Comparative Study
- ☐ Congress
- ☐ Consensus Development Conference
- ☐ Consensus Development Conference, NIH
- ☐ Controlled Clinical Trial
- ☐ Corrected and Republished Article
- ☐ Dataset
- ☐ Dictionary
- ☐ Directory
- ☐ Duplicate Publication
- ☐ Editorial
- ☐ Electronic Supplementary Materials
- ☐ English Abstract
- ☐ Evaluation Study
- ☐ Festschrift
- ☐ Government Publication
- ☐ Guideline
- ☐ Historical Article
- ☐ Interactive Tutorial
- ☐ Interview
- ☐ Introductory Journal Article
- ☐ Lecture
- ☐ Legal Case
- ☐ Legislation
- ☐ Letter
- ☐ Multicenter Study
- ☐ News
- ☐ Newspaper Article
- ☐ Observational Study
- ☐ Observational Study, Veterinary
- ☐ Overall
- ☐ Patient Education Handout
- ☐ Periodical Index
- ☐ Personal Narrative
- ☐ Portrait
- ☐ Practice Guideline
- ☐ Pragmatic Clinical Trial
- ☐ Preprint

- ☐ Published Erratum
- ☐ Research Support, American Recovery and Reinvestment Act
- ☐ Research Support, N.I.H., Extramural
- ☐ Research Support, N.I.H., Intramural
- ☐ Research Support, Non-U.S. Gov't
- ☐ Research Support, U.S. Gov't, Non-P.H.S.
- ☐ Research Support, U.S. Gov't, P.H.S.
- ☐ Research Support, U.S. Gov't
- ☐ Retracted Publication
- ☐ Retraction of Publication
- ☐ Scientific Integrity Review
- ☐ Technical Report
- ☐ Twin Study
- ☐ Validation Study
- ☐ Video-Audio Media
- ☐ Webcast

- ☐ Humans
- ☐ Other Animals

- ☐ Afrikaans
- ☐ Albanian
- ☐ Arabic
- ☐ Armenian
- ☐ Azerbaijani
- ☐ Bosnian
- ☐ Bulgarian
- ☐ Catalan
- ☐ Chinese
- ☐ Croatian
- ☐ Czech
- ☐ Danish
- ☐ Dutch
- ☐ English
- ☐ Esperanto
- ☐ Estonian
- ☐ Finnish
- ☐ French
- ☐ Georgian
- ☐ German
- ☐ Greek, Modern
- ☐ Hebrew
- ☐ Hindi
- ☐ Hungarian
- ☐ Icelandic
- ☐ Indonesian
- ☐ Italian
- ☐ Japanese
- ☐ Kinyarwanda

- ☐ Korean
- ☐ Latin
- ☐ Latvian
- ☐ Lithuanian
- ☐ Macedonian
- ☐ Malay
- ☐ Malayalam
- ☐ Maori
- ☐ Multiple Languages
- ☐ Norwegian
- ☐ Persian
- ☐ Polish
- ☐ Portuguese
- ☐ Pushto
- ☐ Romanian
- ☐ Russian
- ☐ Sanskrit
- ☐ Scottish gaelic
- ☐ Serbian
- ☐ Slovak
- ☐ Slovenian
- ☐ Spanish
- ☐ Swedish
- ☐ Thai
- ☐ Turkish
- ☐ Ukrainian
- ☐ Undetermined
- ☐ Vietnamese
- ☐ Welsh

- ☐ Female
- ☐ Male

- ☐ MEDLINE

- ☐ Child: birth-18 years
- ☐ Newborn: birth-1 month
- ☐ Infant: birth-23 months
- ☐ Infant: 1-23 months
- ☐ Preschool Child: 2-5 years
- ☐ Child: 6-12 years
- ☐ Adolescent: 13-18 years
- ☐ Adult: 19+ years
- ☐ Young Adult: 19-24 years
- ☐ Adult: 19-44 years
- ☐ Middle Aged + Aged: 45+ years
- ☐ Middle Aged: 45-64 years
- ☐ Aged: 65+ years
- ☐ 80 and over: 80+ years

Cancel Show
Close dialog
Back to Top
Jump to page Close dialog
5 of 7
Jump

NCBI Literature Resources

MeSH PMC Bookshelf Disclaimer

Follow NCBI

Connect with NLM

-
-
-

National Library of Medicine
8600 Rockville Pike
Bethesda, MD 20894

Web Policies
FOIA
HHS Vulnerability Disclosure

[Help](#)
[Accessibility](#)
[Careers](#)

- [NLM](#)
- [NIH](#)
- [HHS](#)
- [USA.gov](#)

ERREUR p
du site :
Domaine
