# Supplementary material for: COVID-19 treatment of hospital patients worldwide at the onset of the pandemic in 2020: a systematic review
Source: BMC Infect Dis. 2025 Dec 17;26:107. doi: 10.1186/s12879-025-12368-2 (PMC12822144; doi:10.1186/s12879-025-12368-2)
Supplement: Supplementary file 4 — Supplementary Material 4 [file 12879_2025_12368_MOESM4_ESM.zip › 12879_2025_12368_MOESM4_ESM/Search Pubmed 2022 03 28 retrospective observational study hospital treatment covid 201-400.pdf]

Skip to main page content

COVID-19 Information

Public health information (CDC)

Research information (NIH)

SARS-CoV-2 data (NCBI)

Prevention and treatment information (HHS)

Español

Close

Account

Logged in as:
username

- Dashboard
- Publications
- Account settings
- Log out

Access keys NCBI Homepage MyNCBI Homepage Main Content Main Navigation

Search Page

pubmed

Search: retrospective observation Search
Advanced Create alert Create RSS Clipboard
User Guide
Filters 0 Timeline
Sorted by: Best match
Sorted by: Best match Display options
Save Email
Send to

Save citations to file

Selection: All results on this page

Format: Summary (text)
Create file Cancel

# Email citations

Subject: retrospective observational study hospital treatm - PubMed
To: antoine.bosquet@lmr.apl
Selection: All results on this page
Format: Summary
MeSH and other data
Send email Cancel

Send citations to clipboard

Selection: All results on this page
Send Cancel

Add to Collections

Selection: All results on this page

- Create a new collection
- Add to an existing collection

Name your collection:
Name must be less than 100 characters
Choose a collection:
Unable to load your collection due to an error
Please try again
Add Cancel

Add to My Bibliography

Selection: All results on this page

- My Bibliography

Unable to load your delegates due to an error
Please try again
Add Cancel

Create a file for external citation management software

Selection: All results on this page
Create file Cancel

Your saved search

Name of saved search: retrospective observation

Search terms: retrospective observational study

Test search terms

Would you like email updates of new search results?

Saved Search Alert Radio Buttons

- Yes
- No

Email: antoine.bosquet@lmr.aphp.fr (change)

Frequency: Monthly

Which day? The first Sunday

Which day? Sunday

Report format: Summary

Send at most: 5 items

Send even when there aren't any new results

Optional text in email:

Save Cancel

Your RSS Feed

Name of RSS Feed: retrospective observation

Number of items displayed: 15

Create RSS Cancel

RSS Link Your RSS Feed Link Copy

My NCBI Filters

- All (1,388)
- Assistance Publique Hopitaux de Paris (0)
- clinical trial (17)
- Review (1)

Show Fewer

Results by year Expand/collapse timeline

Reset

Table representation of search results timeline featuring number of search results per year.

Year	Number of Results
2020	548
2021	893
2022	147

Text availability

- ☐ Abstract
- ☐ Free full text
- ☐ Full text

Article attribute

- ☐ Associated data

Article type

- ☐ Books and Documents
- ☐ Clinical Trial
- ☐ Meta-Analysis
- ☐ Randomized Controlled Trial
- ☐ Review
- ☐ Systematic Review

Publication date

- ○ 1 year
- ○ 5 years
- ○ 10 years
- ○ Custom Range

Additional filters | Reset all filters

Search Results

☐
clear all
1,388 results

first first | previous previous

Page
2
of 7

next next | last last

Use COVID-19 filters from PubMed Clinical Queries to refine your search

- Treatment
- Mechanism
- Transmission
- More filters

See more SARS-CoV-2 literature, sequence, and clinical content from NCBI

Results by year Expand/collapse timeline

Reset

Filters applied: . *Clear all* Select search result to email or save

Page 2

☐ 201

Observational Study

Ann Vasc Surg

. 2021 Jul;74:88-94.

doi: 10.1016/j.avsg.2021.03.003. Epub 2021 Apr 2.

Acute Limb Ischemia in Hospitalized COVID-19 Patients

Ahmet Can Topcu [1], Gozde Ozturk-Altunyurt [2], Dilara Akman [2], Ayse Batirel [2], Recep Demirhan [3]

Affiliations Expand

Affiliations

- [1] Department of Cardiovascular Surgery, University of Health Sciences, Kartal Dr. Lutfi Kirdar City Hospital, Istanbul, Turkey. Electronic address: ahmet.topcu@icloud.com.
- [2] Department of Infectious Diseases and Clinical Microbiology, University of Health Sciences, Kartal Dr. Lutfi Kirdar City Hospital, Istanbul, Turkey.
- [3] Department of Thoracic Surgery, University of Health Sciences, Kartal Dr. Lutfi Kirdar City Hospital, Istanbul, Turkey.

- PMID: **33819591**
- PMCID: PMC8017914
- DOI: 10.1016/j.avsg.2021.03.003

Free PMC article

Observational Study

Acute Limb Ischemia in Hospitalized COVID-19 Patients

Ahmet Can Topcu et al. Ann Vasc Surg. 2021 Jul.
Free PMC article
Show details
Ann Vasc Surg
. 2021 Jul;74:88-94.
doi: 10.1016/j.avsg.2021.03.003. Epub 2021 Apr 2.

Authors

Ahmet Can Topcu [1], Gozde Ozturk-Altunyurt [2], Dilara Akman [2], Ayse Batirel [2], Recep Demirhan [3]

Affiliations

- [1] Department of Cardiovascular Surgery, University of Health Sciences, Kartal Dr. Lutfi Kirdar City Hospital, Istanbul, Turkey. Electronic address: ahmet.topcu@icloud.com.
- [2] Department of Infectious Diseases and Clinical Microbiology, University of Health Sciences, Kartal Dr. Lutfi Kirdar City Hospital, Istanbul, Turkey.
- [3] Department of Thoracic Surgery, University of Health Sciences, Kartal Dr. Lutfi Kirdar City Hospital, Istanbul, Turkey.

- PMID: **33819591**
- PMCID: PMC8017914
- DOI: 10.1016/j.avsg.2021.03.003

Abstract

Background: COVID-19 is a multisystemic disorder. Hematologic and cardiovascular involvement of COVID-19 causes thromboembolic events across multiple organs which mainly manifest as venous thromboembolism, and rarely, peripheral arterial thromboembolic events. In-situ thrombosis of a healthy, non-atherosclerotic native artery is rare, and COVID-19 has been reported to be a cause of this phenomenon. We aimed to report our institutional experience with COVID-19 patients who developed acute limb ischemia (ALI) during hospitalization or after discharge.

Methods: This was a single-center cross-sectional study. Records of all patients ≥18 years of age admitted to a tertiary center with a confirmed diagnosis of COVID-19 infection between September 1 and December 31, 2020 were retrospectively examined. Data regarding patient demographics, co-morbidities and outcomes were collected. Patients were followed-up during index hospitalization and for 30 days postdischarge. Acute limb ischemia was diagnosed by means of duplex ultrasound and computed tomography angiography in the presence of a clinical suspicion.

Results: A total of 681 consecutive patients (38.5% women) were hospitalized with a confirmed diagnosis of COVID-19 during the study period. Median age was 63 years (IQR, 52-74). In-hospital mortality occurred in 94 (13.8%) patients. Ninety (13.2%) patients required intensive care

unit admission at some point of their hospital stay. Six (0.9%) patients (one woman) with a median age of 62 years experienced ALI (IQR, 59-64.3). All patients were receiving low molecular weight heparin when they developed ALI. The median of duration between COVID-19 diagnosis and ALI symptom onset was 13 days (IQR, 11.3-14). Three patients underwent emergent surgical thrombectomy combined with systemic anticoagulation, and 3 received systemic anticoagulation alone. Two patients with ALI did not survive to hospital discharge. Among survivors, 1 patient underwent bilateral major amputations, and another underwent a minor amputation within 1 month of hospital discharge. Symptoms of ALI completely resolved in 2 patients without sequelae.

Conclusions: COVID-19 is a multisystemic disorder with involvement of hematologic and cardiovascular systems. Despite widespread use of thromboprophylaxis, hospitalized patients with COVID-19 are at increased risk of ALI, and subsequent limb loss or even death.

Copyright © 2021. Published by Elsevier Inc.

- Cited by 3 articles
- 25 references
- 3 figures

Supplementary info

Publication types, MeSH terms, Substances Expand

Publication types

- Observational Study

MeSH terms

- Acute Disease
- Aged
- Amputation
- Anticoagulants / therapeutic use
- COVID-19 / complications*
- COVID-19 / diagnosis
- COVID-19 / mortality
- COVID-19 / therapy
- Cross-Sectional Studies
- Female
- Hospital Mortality
- Hospitalization*
- Humans
- Ischemia / diagnostic imaging
- Ischemia / etiology*
- Ischemia / mortality

- Ischemia / therapy
- Limb Salvage
- Male
- Middle Aged
- Patient Discharge
- Peripheral Arterial Disease / diagnostic imaging
- Peripheral Arterial Disease / etiology*
- Peripheral Arterial Disease / mortality
- Peripheral Arterial Disease / therapy
- Retrospective Studies
- Risk Assessment
- Risk Factors
- Thrombectomy
- Time Factors
- Treatment Outcome

Substances

- Anticoagulants

Full text links

ELSEVIER FULL-TEXT ARTICLE Elsevier Science Free PMC article
Proceed to details
Cite
Share
202
Observational Study
Medicine (Baltimore)
. 2021 Mar 19;100(11):e24771.
doi: 10.1097/MD.0000000000024771.

Characteristics and outcomes of patients with COVID-19 in Hainan, South China

Yamei Zheng [1], Yunsuo Gao [2], Biao Wu [3], Linhui Huang [1], Yongxing Chen [1], Xingjun Cai [1]
Affiliations Expand

Affiliations

- [1] Pulmonary and Critical Care Medicine.
- [2] Medical Information Department.

- [3] Disease Control and Prevention Department, Hainan Affiliated Hospital of Hainan Medical University, Hainan, China.

- PMID: **33725944**
- PMCID: PMC7982183
- DOI: 10.1097/MD.0000000000024771

Free PMC article
Observational Study

Characteristics and outcomes of patients with COVID-19 in Hainan, South China

Yamei Zheng et al. Medicine (Baltimore). 2021.
Free PMC article
Show details
Medicine (Baltimore)

. 2021 Mar 19;100(11):e24771.
doi: 10.1097/MD.0000000000024771.

Authors

Yamei Zheng [1], Yunsuo Gao [2], Biao Wu [3], Linhui Huang [1], Yongxing Chen [1], Xingjun Cai [1]

Affiliations

- [1] Pulmonary and Critical Care Medicine.
- [2] Medical Information Department.
- [3] Disease Control and Prevention Department, Hainan Affiliated Hospital of Hainan Medical University, Hainan, China.

- PMID: **33725944**
- PMCID: PMC7982183
- DOI: 10.1097/MD.0000000000024771

Abstract

As an international tourist center, Hainan province includes both imported and local COVID-19 cases. This study aimed to investigate the clinical characteristics and outcomes of COVID-19 patients in Hainan, China.COVID-19 patients hospitalized in Hainan affiliated Hospital of Hainan Medical University in January to March 2020 were retrospectively assessed. Routine blood tests, blood gas analyses, and computed tomography imaging were performed within 24 hours. Virus nucleic acid was detected every other day. The patients were divided into local resident and traveler groups, and differences in clinical data as well as leukocyte, lymphocyte, and neutrophil levels were analyzed.A total of 70 patients aged 51.23 ± 13.54 years were assessed, including 16 local residents and 54 travelers. Of these, 55 cases (78.6%) had fever, 47 (67.1%) had cough and sputum, and 9 (12.9%) had chest dyspnea; 60 and 10 cases were mild/common and severe/critical, respectively. Sex, basic diseases, smoking history and drinking history, Charlson Comorbidity

Index, symptoms, time of onset to admission, clinical severity, white blood cell count, lymphocyte count, neutrophil count, oxygen inhalation, mechanical ventilation, glucocorticoid therapy, treatment, admission to ICU, hospital stay, and mortality were similar between the 2 groups.The warm and humid climate of Hainan does not seem to significantly affect patient features and outcomes from COVID-19. Unnecessary travel to tourist areas should be avoided.

Copyright © 2021 the Author(s). Published by Wolters Kluwer Health, Inc.

Conflict of interest statement

The authors report no conflicts of interest.

- Cited by 1 article
- 31 references
- 1 figure

Supplementary info

Publication types, MeSH terms, Grant support Expand

Publication types

- Observational Study

MeSH terms

- Adult
- Aged
- COVID-19 / diagnosis
- COVID-19 / epidemiology*
- COVID-19 / therapy*
- China / epidemiology
- Cough / epidemiology
- Cough / virology
- Female
- Fever / epidemiology
- Fever / virology
- Hospitalization
- Humans
- Male
- Middle Aged
- Oxygen Inhalation Therapy / methods
- Respiration, Artificial / methods
- Retrospective Studies
- SARS-CoV-2

- Severity of Illness Index
- Tomography, X-Ray Computed
- Travel
- Treatment Outcome

Grant support

- XGZX2020001/2019-ncoṽ Science and Technology Research Project of Hainan Medical University

Full text links

Wolters Kluwer Free PMC article
Proceed to details
Cite
Share
203
Observational Study
West J Emerg Med
. 2021 May 5;22(3):572-579.
doi: 10.5811/westjem.2021.1.50123.

The Impact of COVID-19 on a Large, Canadian Community Emergency Department

Daniel Dongjoo Lee [1, 2], Hyejung Jung [2], Wendy Lou [2], David Rauchwerger [3], Lucas B Chartier [4, 5], Sameer Masood [4, 5], Seyon Sathiaseelan [3, 4, 6], Ahmed Khaled Taher [3, 4, 5]
Affiliations Expand

Affiliations

- [1] Temerty Faculty of Medicine, University of Toronto, Toronto, Ontario, Canada.
- [2] Dalla Lana School of Public Health, University of Toronto, Toronto, Ontario Canada.
- [3] Mackenzie Health, Department of Emergency Medicine, Richmond Hill, Ontario, Canada.
- [4] University of Toronto, Department of Medicine, Division of Emergency Medicine, Toronto, Ontario, Canada.
- [5] University Health Network, Department of Emergency Medicine, Toronto, Ontario, Canada.
- [6] McMaster University, Department of Family Medicine, Hamilton, Ontario, Canada.

- PMID: **34125029**
- PMCID: PMC8202991
- DOI: 10.5811/westjem.2021.1.50123

Free PMC article
Observational Study

The Impact of COVID-19 on a Large, Canadian Community Emergency Department

Daniel Dongjoo Lee et al. West J Emerg Med. 2021.
Free PMC article
Show details
West J Emerg Med
. 2021 May 5;22(3):572-579.
doi: 10.5811/westjem.2021.1.50123.

Authors

Daniel Dongjoo Lee [1] [2], Hyejung Jung [2], Wendy Lou [2], David Rauchwerger [3], Lucas B Chartier [4] [5], Sameer Masood [4] [5], Seyon Sathiaseelan [3] [4] [6], Ahmed Khaled Taher [3] [4] [5]

Affiliations

- [1] Temerty Faculty of Medicine, University of Toronto, Toronto, Ontario, Canada.
- [2] Dalla Lana School of Public Health, University of Toronto, Toronto, Ontario Canada.
- [3] Mackenzie Health, Department of Emergency Medicine, Richmond Hill, Ontario, Canada.
- [4] University of Toronto, Department of Medicine, Division of Emergency Medicine, Toronto, Ontario, Canada.
- [5] University Health Network, Department of Emergency Medicine, Toronto, Ontario, Canada.
- [6] McMaster University, Department of Family Medicine, Hamilton, Ontario, Canada.

- PMID: **34125029**
- PMCID: PMC8202991
- DOI: 10.5811/westjem.2021.1.50123

Abstract

Introduction: As the COVID-19 pandemic unfolded, emergency departments (EDs) across the world braced for surges in volume and demand. However, many EDs experienced decreased demand even for higher acuity illnesses. In this study we sought to examine the change in utilization at a large Canadian community ED, including changes in patient demographics and presentations, as well as structural and administrative changes made in response to the pandemic.

Methods: This retrospective observational study took place in Ontario, Canada, from March 17-June 30, 2020, during province-wide lockdowns in response to COVID-19. We used a control period of March 17-June 30 in 2018-2019. Differences between observed and expected values were calculated for total visits, Canadian Triage and Acuity Scale (CTAS) groups, and age groups

using Fisher's exact test. Length of stay (LOS), physician initial assessment time (PIA), and top primary and admission diagnoses were also examined.

Results: Patient visits fell to 66.3% of expected volume in the exposure period (20,901 vs 31,525, P<0.0001). CTAS-1 (highest acuity) patient volumes dropped to 86.8% of expected (P = 0.1964) while CTAS-5 (lowest acuity) patient volumes dropped to 32.4% of expected (P <0.0001). Youth (0-17), adult (18-64), and senior (65+) visits all decreased to 37.4%, 71.7%, and 72.9% of expected volumes, respectively (P <0.0001). Median PIA and median ED LOS both decreased (1.1 to 0.6 hours and 3.3 to 3.0 hours, respectively). The most common primary diagnosis in both periods was "other chest pain." Viral syndromes were more prevalent in the exposure period. The top admission diagnoses were congestive heart failure in the control period (4.8%) and COVID-19 in the study period (3.5%).

Conclusion: ED utilization changed drastically during COVID-19. Our ED responded with wide stakeholder engagement, spatial reorganization, and human resources changes informed by real-time data. Our experiences can help prepare for potential subsequent "waves" of COVID-19 and future pandemics.

Conflict of interest statement

Conflicts of Interest: By the WestJEM article submission agreement, all authors are required to disclose all affiliations, funding sources and financial or management relationships that could be perceived as potential sources of bias. No author has professional or financial relationships with any companies that are relevant to this study. There are no conflicts of interest or sources of funding to declare.

- Cited by 2 articles
- 25 references
- 1 figure

Supplementary info

Publication types, MeSH terms Expand

Publication types

- Observational Study

MeSH terms

- Adolescent
- Adult
- Aged
- COVID-19 / epidemiology*
- Child
- Child, Preschool
- Emergency Service, Hospital / statistics & numerical data*
- Female

- Humans
- Infant
- Infant, Newborn
- Length of Stay / statistics & numerical data*
- Male
- Middle Aged
- Ontario / epidemiology
- Pandemics
- Retrospective Studies
- SARS-CoV-2
- Young Adult

Full text links

Free PMC article
Proceed to details
Cite
Share
204
Observational Study
Transfusion
. 2020 Sep;60(9):1919-1923.
doi: 10.1111/trf.15947. Epub 2020 Jul 4.

Blood transfusion utilization in hospitalized COVID-19 patients

Christina M Barriteau [1, 2], Patricia Bochey [2], Paul F Lindholm [1, 2], Karyn Hartman [2], Ricardo Sumugod [2], Glenn Ramsey [1, 2]
Affiliations Expand

Affiliations

- [1] Department of Pathology, Feinberg School of Medicine, Northwestern University, Chicago, Illinois, USA.
- [2] Blood Bank, Northwestern Memorial Hospital, Chicago, Illinois, USA.

- PMID: **32583506**
- PMCID: PMC7361376
- DOI: 10.1111/trf.15947

Free PMC article
Observational Study

Blood transfusion utilization in hospitalized COVID-19 patients

Christina M Barriteau et al. Transfusion. 2020 Sep.
Free PMC article
Show details
Transfusion
. 2020 Sep;60(9):1919-1923.
doi: 10.1111/trf.15947. Epub 2020 Jul 4.

Authors

Christina M Barriteau [1 2], Patricia Bochey [2], Paul F Lindholm [1 2], Karyn Hartman [2], Ricardo Sumugod [2], Glenn Ramsey [1 2]

Affiliations

- [1] Department of Pathology, Feinberg School of Medicine, Northwestern University, Chicago, Illinois, USA.
- [2] Blood Bank, Northwestern Memorial Hospital, Chicago, Illinois, USA.

- PMID: **32583506**
- PMCID: PMC7361376
- DOI: 10.1111/trf.15947

Abstract

Background: The acute respiratory illness designated coronavirus disease 2019 (COVID-19) was first reported in Wuhan, China, in December 2019 and caused a worldwide pandemic. Concerns arose about the impact of the COVID-19 pandemic on blood donations and potential significant blood transfusion needs in severely ill COVID-19 patients. Data on blood usage in hospitalized COVID-19 patients are scarce.

Study design and methods: We performed a retrospective observational study of blood component transfusions in the first 4 weeks of COVID-19 ward admissions. The study period began 14 days before the first COVID-19 cohort wards opened in our hospital in March 2020 and ended 28 days afterward. The number of patients and blood components transfused in the COVID-19 wards was tabulated. Transfusion rates of each blood component were compared in COVID-19 wards versus all other inpatient wards.

Results: COVID-19 wards opened with seven suspected patients and after 4 weeks had 305 cumulative COVID-19 admissions. Forty-one of 305 hospitalized COVID-19 patients (13.4%) received transfusions with 11.1% receiving red blood cells (RBCs), 1.6% platelets (PLTs), 1.0% plasma, and 1.0% cryoprecipitate (cryo). COVID-19 wards had significantly lower transfusion rates compared to non-COVID wards for RBCs (0.03 vs 0.08 units/patient-day), PLTs (0.003 vs 0.033), and plasma (0.002 vs 0.018; all $p < 0.0001$). Cryo rates were similar (0.008 vs 0.009, $p = 0.6$).

Conclusions: Hospitalized COVID-19 patients required many fewer blood transfusions than other hospitalized patients. COVID-19 transfusion data will inform planning and preparation of blood resource utilization during the pandemic.

© 2020 AABB.

Conflict of interest statement

The authors have disclosed no conflicts of interest.

- Cited by 15 articles
- 22 references
- 2 figures

Supplementary info

Publication types, MeSH terms Expand

Publication types

- Observational Study

MeSH terms

- Academic Medical Centers / statistics & numerical data
- Appointments and Schedules
- Blood Component Transfusion / statistics & numerical data
- Blood Transfusion / statistics & numerical data*
- COVID-19 / complications
- COVID-19 / therapy*
- Chicago
- Disseminated Intravascular Coagulation / etiology
- Disseminated Intravascular Coagulation / therapy
- Elective Surgical Procedures
- Health Services Needs and Demand / statistics & numerical data
- Hospital Departments
- Hospitals, Urban / statistics & numerical data
- Humans
- Inpatients / statistics & numerical data*
- Procedures and Techniques Utilization
- Retrospective Studies

Full text links

WILEY Full Text Article Wiley Free PMC article

Proceed to details

Cite

Share

☐ 205

Observational Study

Rev Esp Quimioter

. 2021 Feb;34(1):33-43.

doi: 10.37201/req/091.2020. Epub 2020 Dec 15.

COVID-19 and Acute Respiratory Distress Syndrome. Impact of corticosteroid treatment and predictors of poor outcome

P Vidal-Cortés[1], L Del Río-Carbajo, J Nieto-Del Olmo, E Prol-Silva, A I Tizón-Varela, A Rodríguez-Vázquez, P Rodríguez-Rodríguez, M D Díaz-López, P Fernández-Ugidos, M A Pérez-Veloso

Affiliations Expand

Affiliation

- [1] Pablo Vidal-Cortés, Intensive Care Unit. CHU Ourense, Ourense University Hospital. Intensive Care Unit. Ramón Puga 52-54. 32005 Ourense (Spain). pablo.vidal.cortes@sergas.es.

- PMID: **33317261**
- PMCID: PMC7876902
- DOI: 10.37201/req/091.2020

Free PMC article

Observational Study

COVID-19 and Acute Respiratory Distress Syndrome. Impact of corticosteroid treatment and predictors of poor outcome

P Vidal-Cortés et al. Rev Esp Quimioter. 2021 Feb.

Free PMC article

Show details

Rev Esp Quimioter

. 2021 Feb;34(1):33-43.

doi: 10.37201/req/091.2020. Epub 2020 Dec 15.

Authors

P Vidal-Cortés [1], L Del Río-Carbajo, J Nieto-Del Olmo, E Prol-Silva, A I Tizón-Varela, A Rodríguez-Vázquez, P Rodríguez-Rodríguez, M D Díaz-López, P Fernández-Ugidos, M A Pérez-Veloso

Affiliation

- [1] Pablo Vidal-Cortés, Intensive Care Unit. CHU Ourense, Ourense University Hospital. Intensive Care Unit. Ramón Puga 52-54. 32005 Ourense (Spain). pablo.vidal.cortes@sergas.es.

- PMID: **33317261**
- PMCID: PMC7876902
- DOI: 10.37201/req/091.2020

Abstract
in English, Spanish

Objective: To assess the impact of corticosteroids on inflammatory and respiratory parameters of patients with COVID-19 and acute respiratory distress syndrome (ARDS).

Methods: Longitudinal, retrospective, observational study conducted in an ICU of a second level hospital. Adult patients with COVID-19 were included. Baseline characteristics, data on SARS-CoV-2 infection, treatment received, evolution of respiratory and inflammatory parameters, and ICU and hospital stay and mortality were analyzed.

Results: A total of 27 patients were included, 63% men, median age: 68.4 (51.8, 72.2) years. All patients met ARDS criteria and received MV and corticosteroids. After corticosteroids treatment we observed a reduction in the O2 A-a gradient [day 0: 322 (249, 425); day 3: 169 (129.5, 239.5) p<0.001; day 5: 144 (127.5, 228.0) p<0.001; day 7: 192 (120, 261) p=0.002] and an increase in the pO2/FiO2 ratio on days 3 and 5, but not on day 7 [day 0: 129 (100, 168); day 3: 193 (140, 236) p=0.002; day 5: 183 (141, 255) p=0.004; day 7: 170 (116, 251) p=0.057]. CRP also decreased on days 3 and 5 and increased again on day 7 [day 0: 16 (8.6, 24); day 3: 3.4 (1.7, 10.2) p<0.001; day 5: 4.1 (1.4, 10.2) p<0.001; day 7: 13.5 (6.8, 17.3) p=0.063]. Persistence of moderate ARDS on day 7 was related to a greater risk of poor outcome (OR 6.417 [1.091-37.735], p=0.040).

Conclusions: Corticosteroids appears to reduce the inflammation and temporarily improve the oxygenation in COVID-19 and ARDS patients. Persistence of ARDS after 7 days treatment is a predictor of poor outcome.

Objetivos: Evaluar el impacto del tratamiento con corticoides en los parámetros inflamatorios y respiratorios de los pacientes con Síndrome de Dificultad Respiratoria Aguda (SDRA) secundario a COVID-19

Métodos: Estudio longitudinal, retrospectivo, observacional en una UCI de un hospital de segundo nivel. Se incluyeron los pacientes adultos ingresados en UCI por COVID-19. Analizamos características basales, datos de la infección por SARS-CoV-2, tratamiento recibido, evolución de los parámetros respiratorios e inflamatorios y estancia y mortalidad en UCI y hospitalaria.

Resultados: 27 pacientes, 63% hombres, mediana de edad: 68.4 (51.8, 72.2) años. Todos recibieron ventilación mecánica y cumplieron criterios de SDRA. Todos recibieron corticoides. Tras la administración de corticoides observamos una reducción del gradiente A-a de O_2 [día 0: 322 (249, 425); día 3: 169 (129.5, 239.5) p<0.001; día 5: 144 (127.5, 228.0) p<0.001; día 7: 192

(120, 261) p=0.002] y un aumento en la relación pO_2/FiO_2 en los días 3 y 5, pero no al día 7 [día 0: 129 (100, 168); día 3: 193 (140, 236) p=0.002; día 5: 183 (141, 255) p=0.004; día 7: 170 (116, 251) p=0.057]. La PCR descendió a los días 3 y 5 volviendo a subir al día 7 [día 0: 16 (8.6, 24); día 3: 3.4 (1.7, 10.2) p<0.001; día 5: 4.1 (1.4, 10.2) p<0.001; día 7: 13.5 (6.8, 17.3) p=0.063]. La persistencia de SDRA moderado al día 7 se relacionó con un peor pronóstico (OR 6.417 [1.091-37.735], p=0.040)

Conclusión: Los corticosteroides parecen reducir la inflamación y mejorar temporalmente la oxigenación en pacientes con SDRA y COVID-19. La persistencia de SDRA moderado tras 7 días de tratamiento es un predictor de mal pronóstico.

Keywords: ARDS; COVID-19; ICU; corticosteroids; mechanical ventilation.

©The Author 2020. Published by Sociedad Española de Quimioterapia. This article is distributed under the terms of the Creative Commons Attribution-NonCommercial 4.0 International (CC BY-NC 4.0)(https://creativecommons.org/licenses/by-nc/4.0/).

Conflict of interest statement

The authors declare that they have no conflicts of interest

- Cited by 2 articles
- 30 references
- 3 figures

Supplementary info

Publication types, MeSH terms Expand

Publication types

- Observational Study

MeSH terms

- Aged
- COVID-19 / drug therapy*
- COVID-19 / metabolism
- Female
- Humans
- Intensive Care Units
- Longitudinal Studies
- Male
- Middle Aged
- Oxygen Consumption / drug effects*
- Oxygen Consumption / physiology
- Respiration, Artificial

- Respiratory Distress Syndrome / drug therapy*
- Respiratory Distress Syndrome / metabolism
- Retrospective Studies
- SARS-CoV-2*
- Secondary Care Centers
- Spain
- Time Factors
- Treatment Outcome

Full text links

FREE Full Text www.seq.es Sociedad Espanola de Quimioterapia Free PMC article
Proceed to details
Cite
Share
☐ 206
Observational Study
Blood Cells Mol Dis
. 2021 May;88:102548.
doi: 10.1016/j.bcmd.2021.102548. Epub 2021 Feb 18.

Effectiveness of convalescent plasma in Indian patients with COVID-19

Sandeep Budhiraja [1], Arun Dewan [2], Ritesh Aggarwal [3], Omender Singh [4], Deven Juneja [5], Sangeeta Pathak [6], Y P Singh [7], Ajay Gupta [8], Reeta Rai [9], Abhaya Indrayan [10], Vinitaa Jha [11], Rahul Naithani [12]
Affiliations Expand

Affiliations

- [1] Clinical Directorate, Max Healthcare, New Delhi, India; Institute of Internal Medicine, Max Healthcare, New Delhi, India. Electronic address: sbudhiraja@maxhealthcare.com.
- [2] Critical Care, Max Healthcare, New Delhi, India. Electronic address: Arun.Dewan@maxhealthcare.com.
- [3] Critical Care, Max Healthcare, New Delhi, India. Electronic address: Ritesh.Aggarwal@maxhealthcare.com.
- [4] Critical Care, Max Healthcare, New Delhi, India. Electronic address: Omender.Singh@maxhealthcare.com.
- [5] Critical Care, Max Healthcare, New Delhi, India. Electronic address: deven.juneja@maxhealthcare.com.
- [6] Transfusion Medicine, Max Healthcare, New Delhi, India. Electronic address: sangeeta.pathak@maxhealthcare.com.
- [7] Critical Care, Max Healthcare, New Delhi, India. Electronic address: Yogendra.Singh2@maxhealthcare.com.

- [8] Critical Care, Max Healthcare, New Delhi, India. Electronic address: DrAjay.Gupta@maxhealthcare.com.
- [9] Transfusion Medicine, Max Healthcare, New Delhi, India. Electronic address: Reeta.Rai@maxhealthcare.com.
- [10] Division of Bio Statistics, Max Healthcare, New Delhi, India.
- [11] Clinical Directorate, Max Healthcare, New Delhi, India. Electronic address: Vinitaa.jha@maxhealthcare.com.
- [12] Hematology & Bone Marrow Transplant Division, Max Healthcare, New Delhi, India. Electronic address: Rahul.Naithani@maxhealthcare.com.

- PMID: **33621948**
- PMCID: PMC7891064
- DOI: 10.1016/j.bcmd.2021.102548

Free PMC article
Observational Study

Effectiveness of convalescent plasma in Indian patients with COVID-19

Sandeep Budhiraja et al. Blood Cells Mol Dis. 2021 May.
Free PMC article
Show details
Blood Cells Mol Dis
. 2021 May;88:102548.
doi: 10.1016/j.bcmd.2021.102548. Epub 2021 Feb 18.

Authors

Sandeep Budhiraja [1], Arun Dewan [2], Ritesh Aggarwal [3], Omender Singh [4], Deven Juneja [5], Sangeeta Pathak [6], Y P Singh [7], Ajay Gupta [8], Reeta Rai [9], Abhaya Indrayan [10], Vinitaa Jha [11], Rahul Naithani [12]

Affiliations

- [1] Clinical Directorate, Max Healthcare, New Delhi, India; Institute of Internal Medicine, Max Healthcare, New Delhi, India. Electronic address: sbudhiraja@maxhealthcare.com.
- [2] Critical Care, Max Healthcare, New Delhi, India. Electronic address: Arun.Dewan@maxhealthcare.com.
- [3] Critical Care, Max Healthcare, New Delhi, India. Electronic address: Ritesh.Aggarwal@maxhealthcare.com.
- [4] Critical Care, Max Healthcare, New Delhi, India. Electronic address: Omender.Singh@maxhealthcare.com.
- [5] Critical Care, Max Healthcare, New Delhi, India. Electronic address: deven.juneja@maxhealthcare.com.
- [6] Transfusion Medicine, Max Healthcare, New Delhi, India. Electronic address: sangeeta.pathak@maxhealthcare.com.

- [7] Critical Care, Max Healthcare, New Delhi, India. Electronic address: Yogendra.Singh2@maxhealthcare.com.
- [8] Critical Care, Max Healthcare, New Delhi, India. Electronic address: DrAjay.Gupta@maxhealthcare.com.
- [9] Transfusion Medicine, Max Healthcare, New Delhi, India. Electronic address: Reeta.Rai@maxhealthcare.com.
- [10] Division of Bio Statistics, Max Healthcare, New Delhi, India.
- [11] Clinical Directorate, Max Healthcare, New Delhi, India. Electronic address: Vinitaa.jha@maxhealthcare.com.
- [12] Hematology & Bone Marrow Transplant Division, Max Healthcare, New Delhi, India. Electronic address: Rahul.Naithani@maxhealthcare.com.

- PMID: **33621948**
- PMCID: PMC7891064
- DOI: 10.1016/j.bcmd.2021.102548

Abstract

Background: Convalescent plasma (CP) is being used as a treatment option in hospitalized patients with COVID-19. Till date, there is conflicting evidence on efficacy of CP in reducing COVID-19 related mortality.

Objective: To evaluate the effect of CP on 28-day mortality reduction in patients with COVID-19.

Methods: We did a multi-centre, retrospective case control observational study from 1st May 2020 to 31st August 2020. A total of 1079 adult patients with moderate and severe COVID-19 requiring oxygen, were reviewed. Of these, 694 patients were admitted to ICU. Out of these, 333 were given CP along with best supportive care and remaining 361 received best supportive care only.

Results: In the overall group of 1079 patients, mortality in plasma vs no plasma group was statistically not significant (22.4% vs 18.5%; p = 0.125; OR = 1.27, 95% CI: 0.94--1.72). However, in patients with COVID-19 admitted to ICU, mortality was significantly lower in plasma group (25.5% vs 33.2%; p = 0.026; OR = 0.69, 95%CI: 0.50-0.96). This benefit of reduced mortality was most seen in age group 60 to 74 years (26.7% vs 43.0%; p = 0.004; OR = 0.48, 95% CI: 0.29-0.80), driven mostly by females of this age group (23.1% vs 53.5%; p = 0.013; OR = 0.26, 95% CI: 0.09-0.78). Significant difference in mortality was observed in patients with one comorbidity (22.3% vs 36.5%; p = 0.004; OR = 0.50, 95% CI: 0.31-0.80). Moreover, patients on ventilator had significantly lower mortality in the plasma arm (37.2% vs 49.3%; p = 0.009; OR = 0.61, 95% CI: 0.42-0.89); particularly so for patients on invasive mechanical ventilation (63.9% vs 82.9%; p = 0.014; OR = 0.37, 95% CI: 0.16-0.83).

Conclusion: The use of CP was associated with reduced mortality in COVID-19 elderly patients admitted in ICU, above 60 years of age, particularly females, those with comorbidities and especially those who required some form of ventilation.

Keywords: COVID-19; Convalescent plasma therapy; ICU cases; Mortality; Subgroup analysis.

Copyright © 2021 Elsevier Inc. All rights reserved.

- Cited by 14 articles

- 34 references
- 1 figure

Supplementary info

Publication types, MeSH terms, Supplementary concepts Expand

Publication types

- Multicenter Study
- Observational Study

MeSH terms

- Adult
- Age Factors
- Aged
- COVID-19 / epidemiology
- COVID-19 / mortality
- COVID-19 / therapy*
- Case-Control Studies
- Female
- Humans
- Immunization, Passive
- India / epidemiology
- Male
- Middle Aged
- Retrospective Studies
- SARS-CoV-2 / isolation & purification

Supplementary concepts

- COVID-19 serotherapy

Full text links

ELSEVIER FULL-TEXT ARTICLE Elsevier Science Free PMC article
Proceed to details
Cite
Share
☐ 207
Observational Study
Obes Surg
. 2022 Jan;32(1):18-25.

doi: 10.1007/s11695-021-05761-8. Epub 2021 Oct 30.

Bariatric Surgery and COVID-19: What We Have Learned from the Pandemic in Iran: a Retrospective Observational Cohort Study

Gholamreza Moradpour [1], Masoud Amini [1], Nader Moeinvaziri [1], Seyed Vahid Hosseini [1], Shirin Rajabi [1], Cain C T Clark [2], Babak Hosseini [1], Leila Vafa [1], Neda Haghighat [3]
Affiliations Expand

Affiliations

- [1] laparoscopy Research Center, Shiraz University of Medical Sciences, Shiraz, Iran.
- [2] Centre for Intelligent Healthcare, Coventry University, Coventry, CV1 5FB, UK.
- [3] laparoscopy Research Center, Shiraz University of Medical Sciences, Shiraz, Iran. neda.hag@gmail.com.

- PMID: **34716898**
- PMCID: PMC8556836
- DOI: 10.1007/s11695-021-05761-8

Free PMC article
Observational Study

Bariatric Surgery and COVID-19: What We Have Learned from the Pandemic in Iran: a Retrospective Observational Cohort Study

Gholamreza Moradpour et al. Obes Surg. 2022 Jan.
Free PMC article
Show details
Obes Surg
. 2022 Jan;32(1):18-25.
doi: 10.1007/s11695-021-05761-8. Epub 2021 Oct 30.

Authors

Gholamreza Moradpour [1], Masoud Amini [1], Nader Moeinvaziri [1], Seyed Vahid Hosseini [1], Shirin Rajabi [1], Cain C T Clark [2], Babak Hosseini [1], Leila Vafa [1], Neda Haghighat [3]

Affiliations

- [1] laparoscopy Research Center, Shiraz University of Medical Sciences, Shiraz, Iran.
- [2] Centre for Intelligent Healthcare, Coventry University, Coventry, CV1 5FB, UK.

- [3] laparoscopy Research Center, Shiraz University of Medical Sciences, Shiraz, Iran. neda.hag@gmail.com.

- PMID: **34716898**
- PMCID: PMC8556836
- DOI: 10.1007/s11695-021-05761-8

Abstract

Purpose: Little is known about the symptoms of coronavirus disease 2019 (COVID-19) on patients with morbid obesity following bariatric surgery (BS) in Iran. Thus, we sought to investigate the symptoms and effect of COVID-19 in patients with morbid obesity following, or candidates for, BS in Iran.

Materials and methods: In this retrospective observational cohort study, we enrolled 236 morbid obese patients following (surgical group) or candidates (nonsurgical group) for bariatric surgery. Demographics, probable COVID-19 incidence, acute and persistent COVID-19 symptoms, and clinical outcome parameters of bariatric patients and candidates for BS were compared. The incidence of probable COVID-19 was assessed including the clinical definition of probable case, according to World Health Organization criteria.

Results: The incidence of probable COVID-19 among surgical and nonsurgical groups was significantly different (20.6% vs 26.08%, respectively, $p = 0.046$). The probable case of surgical patients had a shorter length of symptoms and hospitalization duration, and a lower proportion of admission in ICUs and hospitals with respect to nonsurgical patients ($p < 0.001$). Surgical patients had a greater prevalence of persistent symptoms including anorexia, food intolerance, and anosmia-hyposmia than nonsurgical patients. Moreover, surgical patients with probable COVID-19 had a significantly higher proportion of diabetic patients than surgical patients without probable COVID-19 (20% vs 9.3%).

Conclusion: These findings highlight the need to evaluate the persistent symptoms of COVID-19 and the importance of nutritional support for at least several weeks after COVID-19 symptom onset. Moreover, it seems that COVID-19 incidence in post-bariatric surgery patients could reduce the effectiveness of bariatric surgery in the resolution of diabetes.

Keywords: Acute symptoms; Bariatric surgery; COVID-19; Long COVID; Obesity; Prolonged symptoms; Weight loss.

© 2021. The Author(s), under exclusive licence to Springer Science+Business Media, LLC, part of Springer Nature.

Conflict of interest statement

The authors declare no competing interests.

- 36 references

Supplementary info

Publication types, MeSH terms Expand

Publication types

- Observational Study

MeSH terms

- Bariatric Surgery*
- COVID-19*
- Humans
- Iran / epidemiology
- Obesity, Morbid* / surgery
- Pandemics
- Retrospective Studies
- SARS-CoV-2

Full text links

SpringerLink FULL-TEXT ARTICLE Springer Free PMC article
Proceed to details
Cite
Share
208
Observational Study
J Vasc Surg Venous Lymphat Disord
. 2021 Jul;9(4):845-852.
doi: 10.1016/j.jvsv.2020.11.004. Epub 2020 Nov 10.

Thrombosis at hospital presentation in patients with and without coronavirus disease 2019

Shari B Brosnahan [1], Nathaniel R Smilowitz [2], Nancy E Amoroso [3], Michael Barfield [4], Jeffery S Berger [2], Ronald Goldenberg [3], Koto Ishida [5], Nina Talmor [6], Jose Torres [5], Shadi Yaghi [5], Eugene Yuriditsky [2], Thomas S Maldonado [4]
Affiliations Expand

Affiliations

- [1] Division of Pulmonary, Critical Care and Sleep Medicine, Department of Medicine, New York University Langone Health, New York, NY. Electronic address: Shari.Brosnahan@nyulangone.org.
- [2] Division of Cardiology, Department of Medicine, New York University Langone Health, New York, NY.

- [3] Division of Pulmonary, Critical Care and Sleep Medicine, Department of Medicine, New York University Langone Health, New York, NY.
- [4] Department of Vascular Surgery, New York University Langone Health, New York, NY.
- [5] Department of Neurology, New York University Langone Health, New York, NY.
- [6] Department of Internal Medicine, New York University Langone Health, New York, NY.

- PMID: **33186750**
- PMCID: PMC7655032
- DOI: 10.1016/j.jvsv.2020.11.004

Free PMC article
Observational Study

Thrombosis at hospital presentation in patients with and without coronavirus disease 2019

Shari B Brosnahan et al. J Vasc Surg Venous Lymphat Disord. 2021 Jul.
Free PMC article

Show details

J Vasc Surg Venous Lymphat Disord
. 2021 Jul;9(4):845-852.
doi: 10.1016/j.jvsv.2020.11.004. Epub 2020 Nov 10.

Authors

Shari B Brosnahan [1], Nathaniel R Smilowitz [2], Nancy E Amoroso [3], Michael Barfield [4], Jeffery S Berger [2], Ronald Goldenberg [3], Koto Ishida [5], Nina Talmor [6], Jose Torres [5], Shadi Yaghi [5], Eugene Yuriditsky [2], Thomas S Maldonado [4]

Affiliations

- [1] Division of Pulmonary, Critical Care and Sleep Medicine, Department of Medicine, New York University Langone Health, New York, NY. Electronic address: Shari.Brosnahan@nyulangone.org.
- [2] Division of Cardiology, Department of Medicine, New York University Langone Health, New York, NY.
- [3] Division of Pulmonary, Critical Care and Sleep Medicine, Department of Medicine, New York University Langone Health, New York, NY.
- [4] Department of Vascular Surgery, New York University Langone Health, New York, NY.
- [5] Department of Neurology, New York University Langone Health, New York, NY.
- [6] Department of Internal Medicine, New York University Langone Health, New York, NY.

- PMID: **33186750**
- PMCID: PMC7655032
- DOI: 10.1016/j.jvsv.2020.11.004

Abstract

Objective: In the present study, we sought to better characterize the patients with coronavirus disease 2019 (COVID-19) most at risk of severe, outpatient thrombosis by defining the patients hospitalized with COVID-19 with arterial or venous thrombosis diagnosed at admission.

Methods: We conducted a single-center, retrospective analysis of COVID-19 patients. We found a shift in the proportions of thrombosis subtypes from 2019 to 2020, with declines in ST-segment myocardial infarction (from 22.0% to 10.1% of thrombotic events) and stroke (from 48.6% to 37.2%) and an increase in venous thromboembolism (from 29.4% to 52.7%). The patients with COVID-19-associated thrombosis were younger (age, 58 years vs 64 years; P = .043) and were less frequently women (31.3% vs 43.9%; P = .16). However, no differences were found in the body mass index or major comorbidities between those with and without COVID-19. COVID-19-associated thrombosis correlated with greater mortality (15.2% vs 4.3%; P = .016). The biometric profile of patients admitted with COVID-19-associated thrombosis compared with regular thrombosis showed significant changes in the complete blood count, liver function test results, D-dimer levels, C-reactive protein, ferritin, and coagulation panels.

Conclusions: Outpatients with COVID-19 who developed thrombosis requiring hospitalization had increased mortality compared with outpatients without COVID-19 who developed thrombosis requiring hospitalization. Given the significantly higher inflammatory marker levels, it is possible this is related to different mechanisms of thrombotic disease in these patients. The inflammation could be a therapeutic target to reduce the risk, or aid in the treatment, of thrombosis. We call for more studies elucidating the role that immunothrombosis might be playing in patients with COVID-19.

Keywords: Biomarkers; COVID-19; Coronavirus; Embolism; Ischemic stroke; Thrombosis.

Copyright © 2020 Society for Vascular Surgery. Published by Elsevier Inc. All rights reserved.

- Cited by 5 articles
- 27 references
- 1 figure

Supplementary info

Publication types, MeSH terms, Substances Expand

Publication types

- Observational Study

MeSH terms

- Aged
- Arteries
- Biomarkers / blood
- COVID-19 / complications*
- Case-Control Studies

- Female
- Hospitalization*
- Humans
- Male
- Middle Aged
- Prognosis
- Retrospective Studies
- SARS-CoV-2
- ST Elevation Myocardial Infarction / diagnosis
- ST Elevation Myocardial Infarction / etiology
- Stroke / diagnosis
- Stroke / etiology
- Thrombosis / diagnosis*
- Thrombosis / etiology
- Venous Thromboembolism / diagnosis
- Venous Thromboembolism / etiology

Substances

- Biomarkers

Full text links

ELSEVIER FULL-TEXT ARTICLE Elsevier Science Free PMC article
Proceed to details
Cite
Share
☐ 209
Observational Study
Acta Anaesthesiol Scand
. 2021 Mar;65(3):351-359.
doi: 10.1111/aas.13726. Epub 2020 Nov 22.

A single-centre, prospective cohort study of COVID-19 patients admitted to ICU for mechanical ventilatory support

Signe Søvik [1, 2], Per M Bådstøløkken [1], Vibecke Sørensen [1], Peder Langeland Myhre [2, 3], Christian Prebensen [2, 4], Torbjørn Omland [2, 3], Jan-Erik Berdal [2, 4]
Affiliations Expand

Affiliations

- [1] Department of Anaesthesia and Intensive Care, Akershus University Hospital, Lørenskog, Norway.
- [2] Institute of Clinical Medicine, Faculty of Medicine, University of Oslo, Oslo, Norway.
- [3] Department of Cardiology, Akershus University Hospital, Lørenskog, Norway.
- [4] Department of Infectious Diseases, Akershus University Hospital, Lørenskog, Norway.

- PMID: **33128800**
- PMCID: PMC7894152
- DOI: 10.1111/aas.13726

Free PMC article
Observational Study

A single-centre, prospective cohort study of COVID-19 patients admitted to ICU for mechanical ventilatory support

Signe Søvik et al. Acta Anaesthesiol Scand. 2021 Mar.
Free PMC article
Show details
Acta Anaesthesiol Scand
. 2021 Mar;65(3):351-359.
doi: 10.1111/aas.13726. Epub 2020 Nov 22.

Authors

Signe Søvik [1, 2], Per M Bådstøløkken [1], Vibecke Sørensen [1], Peder Langeland Myhre [2, 3], Christian Prebensen [2, 4], Torbjørn Omland [2, 3], Jan-Erik Berdal [2, 4]

Affiliations

- [1] Department of Anaesthesia and Intensive Care, Akershus University Hospital, Lørenskog, Norway.
- [2] Institute of Clinical Medicine, Faculty of Medicine, University of Oslo, Oslo, Norway.
- [3] Department of Cardiology, Akershus University Hospital, Lørenskog, Norway.
- [4] Department of Infectious Diseases, Akershus University Hospital, Lørenskog, Norway.

- PMID: **33128800**
- PMCID: PMC7894152
- DOI: 10.1111/aas.13726

Abstract

Background: Mortality rates in COVID-19 patients in need of mechanical ventilation are high, with wide variations between countries. Most studies were retrospective, and results may not be generalizable due to differences in demographics, healthcare organization and surge capacity. We present a cohort of mechanically ventilated COVID-19 patients from a resource-rich, publicly financed healthcare system.

Methods: Prospective study from a tertiary hospital. Consecutive SARS-CoV-2 positive adult patients admitted to the ICU for mechanical ventilation from 10 March 2020 to 04 May 2020 were included. Triage and treatment were protocolized. High-dose dalteparin was adjusted by D-dimer. Demographics, treatments and high-resolution physiological variables were collected. Outcomes were 30-day and hospital mortality. Data are medians (quartiles).

Results: Of the 1484 persons in the hospital catchment area testing positive for SARS-CoV-2, 201 (13.5%) were hospitalized. Thirty-eight (19%) patients were mechanically ventilated, of whom five (13%) died. Of the 163 patients treated with supplemental oxygen, eight (5%) died. In ventilated patients (75% males, age 61 (53-70) years), severe, moderate and mild ARDS was present in 25%, 70% and 5%. Tidal volume ≤8 mL/kg ideal bodyweight was achieved in 34 (94%) patients. Proning and neuromuscular blockers were used in 19 (54%) and 20 (61%) patients. Duration of ventilation was 12 days (8-23). D-dimer peaked at 3.8 mg/L (2.1-5.3), and maximum dalteparin dose was 15 000 IU/24 h (10 000-15 000). Despite organizational changes, a high degree of adherence to treatment protocols was achieved.

Conclusion: In a prospective cohort study of mechanically ventilated COVID-19 patients treated in a resource-rich, publicly financed healthcare system, mortality was considerably lower than previously reported in retrospective studies.

Keywords: COVID-19; SARS-CoV-2; adult respiratory distress syndrome; critical care; mechanical ventilation; thrombosis.

© 2020 The Authors. Acta Anaesthesiologica Scandinavica published by John Wiley & Sons Ltd on behalf of Acta Anaesthesiologica Scandinavica Foundation.

Conflict of interest statement

All authors state that they have no financial or non-financial disclosures.

- Cited by 7 articles
- 27 references
- 2 figures

Supplementary info

Publication types, MeSH terms, Substances Expand

Publication types

- Observational Study

MeSH terms

- Anticoagulants / therapeutic use

- COVID-19 / drug therapy
- COVID-19 / physiopathology
- COVID-19 / therapy*
- Cohort Studies
- Critical Care / methods*
- Dalteparin / therapeutic use
- Female
- Fibrin Fibrinogen Degradation Products
- Humans
- Inpatients / statistics & numerical data
- Intensive Care Units
- Lung / physiopathology
- Male
- Middle Aged
- Prospective Studies
- Respiration, Artificial / methods*
- SARS-CoV-2
- Tertiary Care Centers
- Time
- Treatment Outcome

Substances

- Anticoagulants
- Fibrin Fibrinogen Degradation Products
- fibrin fragment D
- Dalteparin

Full text links

WILEY Full Text Article Wiley Free PMC article

Proceed to details

Cite

Share

☐ 210

Observational Study

Int J Surg

. 2021 Feb;86:32-37.

doi: 10.1016/j.ijsu.2020.12.009. Epub 2021 Jan 16.

Acute appendicitis management during the COVID-19 pandemic: A prospective cohort study from a large UK centre

Ramez Antakia [1], Athanasios Xanthis [2], Fanourios Georgiades [3], Victoria Hudson [2], James Ashcroft [2], Siobhan Rooney [2], Aminder A Singh [2], John R O'Neill [4], Nicola Fearnhead [5], Richard H Hardwick [4], R Justin Davies [5], John M H Bennett [4]
Affiliations Expand

Affiliations

- [1] Division of Digestive Diseases, Addenbrooke's Hospital, Cambridge, UK. Electronic address: ramez.antakia@gmail.com.
- [2] Division of Digestive Diseases, Addenbrooke's Hospital, Cambridge, UK.
- [3] Department of Surgery, University of Cambridge, Cambridge, UK.
- [4] Cambridge Oesophago-Gastric Centre, Addenbrooke's Hospital, Cambridge, UK.
- [5] Cambridge Colorectal Unit, Addenbrooke's Hospital, Cambridge, UK.

- PMID: **33465496**
- PMCID: PMC7985094
- DOI: 10.1016/j.ijsu.2020.12.009

Free PMC article
Observational Study

Acute appendicitis management during the COVID-19 pandemic: A prospective cohort study from a large UK centre

Ramez Antakia et al. Int J Surg. 2021 Feb.
Free PMC article
Show details
Int J Surg
. 2021 Feb;86:32-37.
doi: 10.1016/j.ijsu.2020.12.009. Epub 2021 Jan 16.

Authors

Ramez Antakia [1], Athanasios Xanthis [2], Fanourios Georgiades [3], Victoria Hudson [2], James Ashcroft [2], Siobhan Rooney [2], Aminder A Singh [2], John R O'Neill [4], Nicola Fearnhead [5], Richard H Hardwick [4], R Justin Davies [5], John M H Bennett [4]

Affiliations

- [1] Division of Digestive Diseases, Addenbrooke's Hospital, Cambridge, UK. Electronic address: ramez.antakia@gmail.com.
- [2] Division of Digestive Diseases, Addenbrooke's Hospital, Cambridge, UK.
- [3] Department of Surgery, University of Cambridge, Cambridge, UK.
- [4] Cambridge Oesophago-Gastric Centre, Addenbrooke's Hospital, Cambridge, UK.
- [5] Cambridge Colorectal Unit, Addenbrooke's Hospital, Cambridge, UK.

- PMID: **33465496**
- PMCID: PMC7985094
- DOI: 10.1016/j.ijsu.2020.12.009

Abstract

Background: During the Covid-19 pandemic, non-operative management for acute appendicitis (AA) was implemented in the UK. The aim of this study was to determine the efficacy and outcomes of conservative versus surgical management of AA during the pandemic.

Materials & methods: We conducted an observational study in a tertiary referral centre. Data was collected from all patients (≥16 years) with a diagnosis of AA between November 1, 2019 to March 10, 2020 (pre-COVID period) and March 10, 2020 to July 5, 2020 (COVID period).

Results: A total of 116 patients in the pre-COVID period were included versus 91 in the COVID period. 43.1% (n = 50) of patients pre-COVID were classified as ASA 2 compared to 26.4% (n = 24) during the COVID period (p-value = 0.042). 72.5% (n = 66) of the patients during the COVID period scored as high risk using the Alvarado score compared to 24.1% (n = 28) in the pre-COVID period (p-value<0.001). We observed a significant increase in radiological evaluation, 69.8% versus 87.5% of patients had a CT in the pre-COVID and COVID periods respectively (p-value = 0.008). 94.9% of patients were managed operatively in the pre-COVID period compared to 60.4% in the COVID period (p-value<0.001). We observed more open appendicectomies (37.3% versus 0.9%; p-value<0.001) during the COVID period compared to the pre-COVID period. More abscess formation and free fluid were found intraoperatively in the COVID period (p-value = 0.021 and 0.023 respectively). Re-attendance rate due to appendicitis-related issues was significantly higher in the COVID period (p = 0.027).

Conclusion: Radiological diagnosis of AA was more frequent during the COVID period. More conservative management for AA was employed during the COVID-19 pandemic, and for those managed operatively an open approach was preferred. Intra-operative findings were suggestive of delayed presentation during the COVID period without this affecting the length of hospital stay.

Keywords: Acute appendicitis; Antibiotic therapy; COVID-19; Conservative management; Laparoscopic appendicectomy; Open surgery.

Crown Copyright © 2021. Published by Elsevier Ltd. All rights reserved.

Comment in

- A commentary on "acute appendicitis management during the COVID-19 pandemic: A prospective cohort study from a large UK centre".

Hung CM, Lee HM, Tsai KJ, Yang MC, Chiu CC. Hung CM, et al. Int J Surg. 2021 Mar;87:105902. doi: 10.1016/j.ijsu.2021.105902. Epub 2021 Feb 23. Int J Surg. 2021. PMID: 33636359 Free PMC article. No abstract available.

- Commentary on "Acute appendicitis management during the COVID-19 pandemic: A prospective cohort study from a large UK centre".
  Chiarello MM, Cariati M, Brisinda G. Chiarello MM, et al. Int J Surg. 2021 Apr;88:105914. doi: 10.1016/j.ijsu.2021.105914. Epub 2021 Mar 13. Int J Surg. 2021. PMID: 33727173 Free PMC article. No abstract available.

- Cited by 6 articles
- 25 references

Supplementary info

Publication types, MeSH terms, Substances Expand

Publication types

- Observational Study

MeSH terms

- Acute Disease
- Adolescent
- Adult
- Aged
- Aged, 80 and over
- Anti-Bacterial Agents / therapeutic use
- Appendectomy
- Appendicitis / diagnosis
- Appendicitis / drug therapy*
- Appendicitis / surgery*
- COVID-19* / epidemiology
- Conservative Treatment
- Delayed Diagnosis
- Female
- Humans
- Length of Stay
- Male
- Middle Aged
- Pandemics
- Prospective Studies
- Retrospective Studies
- SARS-CoV-2
- Tertiary Care Centers

- United Kingdom / epidemiology
- Young Adult

Substances

- Anti-Bacterial Agents

Full text links

ELSEVIER FULL-TEXT ARTICLE Elsevier Science Free PMC article
Proceed to details
Cite
Share
211
Observational Study
BMC Ophthalmol
. 2021 Nov 27;21(1):408.
doi: 10.1186/s12886-021-02169-x.

Ocular related emergencies in Spain during the COVID-19 pandemic, a multicenter study

Martín Puzo[1,2], Jorge Sánchez-Monroy[3,4], Carmen A Porcar-Plana[5], Francisco de Asís Bartol-Puyal[3,4,6], Marina Dotti-Boada[7], Pilar Peña-Urbina[8], Jordi Izquierdo-Serra[7], Ana López-Montero[9], Pilar Pérez-García[8], Daniel Bordonaba-Bosque[10], Luis E Pablo[3,4,6], Pilar Calvo[3,4]
Affiliations Expand

Affiliations

- [1] Ophthalmology Department, Miguel Servet University Hospital, Paseo Isabel la Católica 1-3, 50009, Zaragoza, Spain. martinpuzo@hotmail.com.
- [2] Miguel Servet Ophthalmology Research Group (GIMSO), Aragón Institute for Health Research (IIS-Aragón), Zaragoza, Spain. martinpuzo@hotmail.com.
- [3] Ophthalmology Department, Miguel Servet University Hospital, Paseo Isabel la Católica 1-3, 50009, Zaragoza, Spain.
- [4] Miguel Servet Ophthalmology Research Group (GIMSO), Aragón Institute for Health Research (IIS-Aragón), Zaragoza, Spain.
- [5] Ophthalmology Department, Hospital Virgen de los Lirios, Alcoy, Spain.
- [6] University of Zaragoza, Zaragoza, Spain.
- [7] Ophthalmology Department, Hospital Clinic, Barcelona, Spain.
- [8] Ophthalmology Department, Hospital Clínico San Carlos, Madrid, Spain.
- [9] Ophthalmology Department, Hospital Clínico Universitario, Valencia, Spain.
- [10] Instituto Aragonés de Ciencias de la Salud (IACS), Zaragoza, Spain.

- PMID: **34837985**
- PMCID: PMC8626758
- DOI: 10.1186/s12886-021-02169-x

Free PMC article
Observational Study

Ocular related emergencies in Spain during the COVID-19 pandemic, a multicenter study

Martín Puzo et al. BMC Ophthalmol. 2021.
Free PMC article
Show details
BMC Ophthalmol
. 2021 Nov 27;21(1):408.
doi: 10.1186/s12886-021-02169-x.

Authors

Martín Puzo [1, 2], Jorge Sánchez-Monroy [3, 4], Carmen A Porcar-Plana [5], Francisco de Asís Bartol-Puyal [3, 4, 6], Marina Dotti-Boada [7], Pilar Peña-Urbina [8], Jordi Izquierdo-Serra [7], Ana López-Montero [9], Pilar Pérez-García [8], Daniel Bordonaba-Bosque [10], Luis E Pablo [3, 4, 6], Pilar Calvo [3, 4]

Affiliations

- [1] Ophthalmology Department, Miguel Servet University Hospital, Paseo Isabel la Católica 1-3, 50009, Zaragoza, Spain. martinpuzo@hotmail.com.
- [2] Miguel Servet Ophthalmology Research Group (GIMSO), Aragón Institute for Health Research (IIS-Aragón), Zaragoza, Spain. martinpuzo@hotmail.com.
- [3] Ophthalmology Department, Miguel Servet University Hospital, Paseo Isabel la Católica 1-3, 50009, Zaragoza, Spain.
- [4] Miguel Servet Ophthalmology Research Group (GIMSO), Aragón Institute for Health Research (IIS-Aragón), Zaragoza, Spain.
- [5] Ophthalmology Department, Hospital Virgen de los Lirios, Alcoy, Spain.
- [6] University of Zaragoza, Zaragoza, Spain.
- [7] Ophthalmology Department, Hospital Clinic, Barcelona, Spain.
- [8] Ophthalmology Department, Hospital Clínico San Carlos, Madrid, Spain.
- [9] Ophthalmology Department, Hospital Clínico Universitario, Valencia, Spain.
- [10] Instituto Aragonés de Ciencias de la Salud (IACS), Zaragoza, Spain.

- PMID: **34837985**
- PMCID: PMC8626758
- DOI: 10.1186/s12886-021-02169-x

Abstract

Purpose: To evaluate ophthalmological emergencies (OE) during the COVID-19 pandemic comparing them with the same period of the previous year.

Methods: Retrospective observational study of all OE visits in four tertiary hospitals in Spain comparing data from March 16th to April 30th, 2020 (COVID-19 period) and the same period of 2019 (pre-COVID-19 period). Severity of the conditions was assessed following Channa et al. publication. Data on demographics, diagnosis and treatments were collected from Electronic Medical Records.

Results: During lockdown, OE significantly declined by 75.18%, from 7,730 registered in the pre-COVID-19 period to 1,928 attended during the COVID-19 period ($p < 0.001$). In 2019, 23.86% of visits were classified as emergent, 59.50% as non-emergent, and 16.65% could not be determined. In 2020, the percentage of emergent visits increased up to 29.77%, non-emergent visits significantly decreased to 52.92% ($p < 0.001$), and 17.31% of the visits were classified as "could not determine". During the pandemic, people aged between 45 and 65 years old represented the largest attending group (37.89%), compared to 2019, where patients over 65 years were the majority (39.80%). In 2019, most frequent diagnosis was unspecified acute conjunctivitis (11.59%), followed by vitreous degeneration (6.47%), and punctate keratitis (5.86%). During the COVID-19 period, vitreous degeneration was the first cause for consultation (9.28%), followed by unspecified acute conjunctivitis (5.63%) and punctate keratitis (5.85%).

Conclusions: OE visits dropped significantly during the pandemic in Spain (75.18%), although more than half were classified as non-urgent conditions, indicating a lack of understanding of the really emergent ocular pathologies among population.

Keywords: COVID-19; Emergency department; Epidemiology; Ophthalmological emergencies.

© 2021. The Author(s).

Conflict of interest statement

None of the authors have any proprietary interests or conflicts of interest related to this submission.

- 31 references
- 5 figures

Supplementary info

Publication types, MeSH terms Expand

Publication types

- Multicenter Study
- Observational Study

MeSH terms

- Aged
- COVID-19*

- Communicable Disease Control
- Emergencies
- Humans
- Middle Aged
- Pandemics*
- Retrospective Studies
- SARS-CoV-2
- Spain / epidemiology

Full text links

Read free full text at BMC BioMed Central Free PMC article

Proceed to details

Cite

Share

212

Multicenter Study

Transfus Apher Sci

. 2021 Jun;60(3):103075.

doi: 10.1016/j.transci.2021.103075. Epub 2021 Feb 3.

Convalescent plasma therapy for severe Covid-19 in patients with hematological malignancies

Preethi Jeyaraman [1], Narendra Agrawal [2], Rahul Bhargava [3], Divya Bansal [4], Rayaz Ahmed [2], Dinesh Bhurani [2], Sachin Bansal [3], Neha Rastogi [3], Pronamee Borah [1], Rahul Naithani [5], Delhi Hematology Group

Affiliations Expand

Affiliations

- [1] Department of Hematology& Bone Marrow Transplantation, Max Superspecialty Hospital, Saket, New Delhi, 110017, India.
- [2] Rajiv Gandhi Cancer Institute, New Delhi, India.
- [3] Fortis Memorial Hospital, Gurugram, India.
- [4] Manipal Hospital, New Delhi, India.
- [5] Department of Hematology& Bone Marrow Transplantation, Max Superspecialty Hospital, Saket, New Delhi, 110017, India. Electronic address: dr_rahul6@hotmail.com.

- PMID: **33574010**
- PMCID: PMC7857080
- DOI: 10.1016/j.transci.2021.103075

Free PMC article
Multicenter Study

Convalescent plasma therapy for severe Covid-19 in patients with hematological malignancies

Preethi Jeyaraman et al. Transfus Apher Sci. 2021 Jun.
Free PMC article
Show details
Transfus Apher Sci
. 2021 Jun;60(3):103075.
doi: 10.1016/j.transci.2021.103075. Epub 2021 Feb 3.

Authors

Preethi Jeyaraman [1], Narendra Agrawal [2], Rahul Bhargava [3], Divya Bansal [4], Rayaz Ahmed [2], Dinesh Bhurani [2], Sachin Bansal [3], Neha Rastogi [3], Pronamee Borah [1], Rahul Naithani [5], Delhi Hematology Group

Affiliations

- [1] Department of Hematology& Bone Marrow Transplantation, Max Superspecialty Hospital, Saket, New Delhi, 110017, India.
- [2] Rajiv Gandhi Cancer Institute, New Delhi, India.
- [3] Fortis Memorial Hospital, Gurugram, India.
- [4] Manipal Hospital, New Delhi, India.
- [5] Department of Hematology& Bone Marrow Transplantation, Max Superspecialty Hospital, Saket, New Delhi, 110017, India. Electronic address: dr_rahul6@hotmail.com.

- PMID: **33574010**
- PMCID: PMC7857080
- DOI: 10.1016/j.transci.2021.103075

Abstract

Background: Data on convalescent plasma therapy (CPT) in patients of hematological malignancies with severe Covid-19 is scarce.

Objective: To study 14-day mortality in patients who received CPT.

Patients & methods: Retrospective multicentre observational study conducted in 4 centres treating haematological malignancies across Delhi-national capital region. Total 33 haematological malignancies patients with severe Covid-19 who received CPT were analysed.

Results: The median age of the study cohort was 62 years (18-80 years). Twenty one percent patients had 1 comorbidity, 18 % had 2 comorbidities and 6% patients had 3 and 5 comorbidities

each. Twenty four patients were on active therapy. Sixty nine percent of patients required ICU stay. Twenty five patients received plasma therapy within 7 days (early) of diagnosis of Covid-19 infection. Median day of plasma infusion from date of diagnosis of Covid-19 infection was 4 days (range: 2-25 days). Patient who had early initiation of plasma therapy had shorter duration of hospitalisation (12.7 vs 24.3 days, p = 0.000). Overall mortality in the cohort was 45.5%. There was no effect of disease status, active therapy, presence of comorbidity on mortality. There was no difference in the mortality in patients receiving early vs late initiation of plasma therapy or in patients receiving one versus two plasma therapy.

Conclusions: We provide a large series of patients with hematological malignancies and role of CPT in this group.

Keywords: Convalescent plasma; Covid-19; Hematological malignancy; Leukemia; Lymphoma.

Copyright © 2021 Elsevier Ltd. All rights reserved.

Conflict of interest statement

The authors report no declarations of interest.

- Cited by 7 articles
- 24 references

Supplementary info

Publication types, MeSH terms, Supplementary concepts Expand

Publication types

- Multicenter Study

MeSH terms

- Adolescent
- Adult
- Aged
- Aged, 80 and over
- COVID-19 / immunology
- COVID-19 / therapy*
- COVID-19 / virology
- Female
- Hematologic Neoplasms / therapy
- Humans
- Immunization, Passive
- Male
- Middle Aged
- Retrospective Studies

- SARS-CoV-2 / isolation & purification
- Young Adult

Supplementary concepts

- COVID-19 serotherapy

Full text links

ELSEVIER FULL-TEXT ARTICLE Elsevier Science Free PMC article
Proceed to details
Cite
Share
213
Observational Study
Ocul Immunol Inflamm
. 2021 May 19;29(4):743-750.
doi: 10.1080/09273948.2021.1952278. Epub 2021 Aug 31.

Retinopathy and Systemic Disease Morbidity in Severe COVID-19

Jessica G Shantha [1], Sara C Auld [2] [3], Casey Anthony [1], Laura Ward [4], Max W Adelman [4], Cheryl L Maier [5], Kenneth W Price [1], Jesse T Jacob [3] [4], Tolulope Fashina [1], Casey Randleman [1], Lucy T Xu [1], Joshua Barnett [1], Ofer Sadan [6], Prem A Kandiah [6], Jay B Varkey [4], Colleen S Kraft [4] [5], Nadine Rouphael [4], Susanne Linderman [7], Rafi Ahmed [7], Carolyn Drews-Botsch [8], Jesse J Waggoner [4], Max Weinmann [2], David J Murphy [2], Steven Yeh [1] [9], Emory COVID-19 Quality and Clinical Research Collaborative
Affiliations Expand

Affiliations

- [1] Emory Eye Center, Emory University School of Medicine, Atlanta, GA, USA.
- [2] Department of Medicine, Division of Pulmonary, Allergy, Critical Care, and Sleep Medicine, Emory University School of Medicine, Atlanta, GA, USA.
- [3] Department of Epidemiology, Rollins School of Public Health, Emory University, Atlanta, GA, USA.
- [4] Department of Medicine, Division of Infectious Diseases, Emory University School of Medicine, Atlanta, GA, USA.
- [5] Department of Pathology and Laboratory Medicine, Emory University School of Medicine, Atlanta, GA, USA.
- [6] Department of Neurology and Neurosurgery, Division of Neurocritical Care, Emory University School of Medicine, Atlanta, GA, USA.
- [7] Emory Vaccine Center, Emory University School of Medicine, Atlanta, GA, USA.

- [8] Department of Global and Community Health, College of Health and Human Services, George Mason University, Fairfax, VA, USA.
- [9] Truhlsen Eye Institute, University of Nebraska Medical Center, Omaha, NE, USA.

- PMID: **34464544**
- PMCID: **PMC8562588** (available on 2022-08-31)
- DOI: 10.1080/09273948.2021.1952278

Observational Study

Retinopathy and Systemic Disease Morbidity in Severe COVID-19

Jessica G Shantha et al. Ocul Immunol Inflamm. 2021.
Show details
Ocul Immunol Inflamm
. 2021 May 19;29(4):743-750.
doi: 10.1080/09273948.2021.1952278. Epub 2021 Aug 31.

Authors

Jessica G Shantha [1], Sara C Auld [2, 3], Casey Anthony [1], Laura Ward [4], Max W Adelman [4], Cheryl L Maier [5], Kenneth W Price [1], Jesse T Jacob [3, 4], Tolulope Fashina [1], Casey Randleman [1], Lucy T Xu [1], Joshua Barnett [1], Ofer Sadan [6], Prem A Kandiah [6], Jay B Varkey [4], Colleen S Kraft [4, 5], Nadine Rouphael [4], Susanne Linderman [7], Rafi Ahmed [7], Carolyn Drews-Botsch [8], Jesse J Waggoner [4], Max Weinmann [2], David J Murphy [2], Steven Yeh [1, 9], Emory COVID-19 Quality and Clinical Research Collaborative

Affiliations

- [1] Emory Eye Center, Emory University School of Medicine, Atlanta, GA, USA.
- [2] Department of Medicine, Division of Pulmonary, Allergy, Critical Care, and Sleep Medicine, Emory University School of Medicine, Atlanta, GA, USA.
- [3] Department of Epidemiology, Rollins School of Public Health, Emory University, Atlanta, GA, USA.
- [4] Department of Medicine, Division of Infectious Diseases, Emory University School of Medicine, Atlanta, GA, USA.
- [5] Department of Pathology and Laboratory Medicine, Emory University School of Medicine, Atlanta, GA, USA.
- [6] Department of Neurology and Neurosurgery, Division of Neurocritical Care, Emory University School of Medicine, Atlanta, GA, USA.
- [7] Emory Vaccine Center, Emory University School of Medicine, Atlanta, GA, USA.
- [8] Department of Global and Community Health, College of Health and Human Services, George Mason University, Fairfax, VA, USA.
- [9] Truhlsen Eye Institute, University of Nebraska Medical Center, Omaha, NE, USA.

- PMID: **34464544**

- PMCID: **PMC8562588** (available on 2022-08-31)
- DOI: 10.1080/09273948.2021.1952278

Abstract

Purpose: To assess the prevalence of retinopathy and its association with systemic morbidity and laboratory indices of coagulation and inflammatory dysfunction in severe COVID-19.

Design: Retrospective, observational cohort study.

Methods: Adult patients hospitalized with severe COVID-19 who underwent ophthalmic examination from April to July 2020 were reviewed. Retinopathy was defined as one of the following: 1) Retinal hemorrhage; 2) Cotton wool spots; 3) Retinal vascular occlusion. We analyzed medical comorbidities, sequential organ failure assessment (SOFA) scores, clinical outcomes, and laboratory values for their association with retinopathy.

Results: Thirty-seven patients with severe COVID-19 were reviewed, the majority of whom were female (n = 23, 62%), Black (n = 26, 69%), and admitted to the intensive care unit (n = 35, 95%). Fourteen patients had retinopathy (38%) with retinal hemorrhage in 7 (19%), cotton wool spots in 8 (22%), and a branch retinal artery occlusion in 1 (3%) patient. Patients with retinopathy had higher SOFA scores than those without retinopathy (8.0 vs. 5.3, $p = .03$), higher rates of respiratory failure requiring invasive mechanical ventilation and shock requiring vasopressors ($p < .01$). Peak D-dimer levels were 28,971 ng/mL in patients with retinopathy compared to 12,575 ng/mL in those without retinopathy ($p = .03$). Peak CRP was higher in patients with cotton wool spots versus those without cotton wool spots (354 mg/dL vs. 268 mg/dL, $p = .03$). Multivariate logistic regression modeling showed an increased risk of retinopathy with higher peak D-dimers (aOR 1.32, 95% CI 1.01-1.73, $p = .04$) and male sex (aOR 9.6, 95% CI 1.2-75.5, $p = .04$).

Conclusion: Retinopathy in severe COVID-19 was associated with greater systemic disease morbidity involving multiple organs. Given its association with coagulopathy and inflammation, retinopathy may offer insight into disease pathogenesis in patients with severe COVID-19.

Keywords: COVID-19; ophthalmic disease; retinopathy.

Supplementary info

Publication types, MeSH terms, Grant support Expand

Publication types

- Multicenter Study
- Observational Study

MeSH terms

- COVID-19 / diagnosis
- COVID-19 / epidemiology*
- Follow-Up Studies
- Hospitalization / trends

- Morbidity
- Retinal Diseases / epidemiology*
- Retrospective Studies
- SARS-CoV-2*
- Severity of Illness Index
- United States / epidemiology

Grant support

- K23 AI134182/AI/NIAID NIH HHS/United States
- K23 EY030158/EY/NEI NIH HHS/United States
- P30 EY006360/EY/NEI NIH HHS/United States
- R01 EY029594/EY/NEI NIH HHS/United States

Full text links

View full text Taylor & Francis
Proceed to details
Cite
Share
☐ 214
Observational Study
J Cardiothorac Vasc Anesth
. 2021 Jul;35(7):1999-2006.
doi: 10.1053/j.jvca.2021.01.027. Epub 2021 Jan 19.

Six-Month Survival After Extracorporeal Membrane Oxygenation for Severe COVID-19

Fausto Biancari [1], Giovanni Mariscalco [2], Magnus Dalén [3], Nicla Settembre [4], Henryk Welp [5], Andrea Perrotti [6], Karsten Wiebe [5], Enrico Leo [7], Antonio Loforte [8], Sidney Chocron [6], Davide Pacini [8], Tatu Juvonen [9], L Mikael Broman [10], Dario Di Perna [11], Hakeem Yusuff [2], Chris Harvey [2], Nicolas Mongardon [12], Juan P Maureira [13], Bruno Levy [14], Lars Falk [10], Vito G Ruggieri [11], Svante Zipfel [15], Thierry Folliguet [16], Antonio Fiore [16]
Affiliations Expand

Affiliations

- [1] Heart and Lung Center, Helsinki University Hospital, Helsinki, Finland; Research Unit of Surgery, Anesthesia and Critical Care, University of Oulu, Oulu, Finland; Department of Surgery, University of Turku, Turku, Finland. Electronic address: faustobiancari@yahoo.it.
- [2] Department of Intensive Care Medicine and Cardiac Surgery, Glenfield Hospital, University Hospitals of Leicester, Leicester, UK.

- [3] Department of Molecular Medicine and Surgery, Karolinska Institutet, Department of Cardiothoracic Surgery, Karolinska University Hospital, Stockholm, Sweden.
- [4] Department of Vascular and Endovascular Surgery, Nancy University Hospital, University of Lorraine, Nancy, France.
- [5] Department of Cardiothoracic Surgery, Münster University Hospital, Münster, Germany.
- [6] Department of Thoracic and Cardio-Vascular Surgery, University Hospital Jean Minjoz, Besançon, France.
- [7] Division of Vascular Surgery, A. Manzoni Hospital, Lecco, Italy.
- [8] Department of Cardiothoracic, Transplantation and Vascular Surgery, S. Orsola Hospital, University of Bologna, Bologna, Italy.
- [9] Heart and Lung Center, Helsinki University Hospital, Helsinki, Finland; Research Unit of Surgery, Anesthesia and Critical Care, University of Oulu, Oulu, Finland.
- [10] Department of Physiology and Pharmacology, Karolinska Institutet, ECMO Centre Karolinska, Karolinska University Hospital, Stockholm, Sweden.
- [11] Division of Cardiothoracic and Vascular Surgery, Robert Debré University Hospital, Université Reims Champagne-Ardenne, Reims, France.
- [12] Service d'Anesthésie-réanimation Chirurgicale, Hôpital Henri-Mondor, Assistance Publique - Hôpitaux de Paris, Créteil, France.
- [13] Department of Cardiovascular Surgery and Heart Transplantation, Nancy University Hospital, University of Lorraine, Nancy, France.
- [14] Medical Intensive Care Unit Brabois, Nancy University Hospital, University of Lorraine, Nancy, France.
- [15] Heart Center, Hamburg University Hospital, Hamburg, Germany.
- [16] Service de Chirurgie Thoracique et Cardio-vasculaire, Hôpital Henri-Mondor, Assistance Publique - Hôpitaux de Paris, Créteil, France.

- PMID: **33573928**
- PMCID: PMC7816613
- DOI: 10.1053/j.jvca.2021.01.027

Free PMC article
Observational Study

Six-Month Survival After Extracorporeal Membrane Oxygenation for Severe COVID-19

Fausto Biancari et al. J Cardiothorac Vasc Anesth. 2021 Jul.
Free PMC article
Show details
J Cardiothorac Vasc Anesth
. 2021 Jul;35(7):1999-2006.
doi: 10.1053/j.jvca.2021.01.027. Epub 2021 Jan 19.

Authors

Fausto Biancari [1], Giovanni Mariscalco [2], Magnus Dalén [3], Nicla Settembre [4], Henryk Welp [5], Andrea Perrotti [6], Karsten Wiebe [5], Enrico Leo [7], Antonio Loforte [8], Sidney Chocron [6], Davide Pacini [8], Tatu Juvonen [9], L Mikael Broman [10], Dario Di Perna [11], Hakeem Yusuff [2], Chris Harvey [2], Nicolas Mongardon [12], Juan P Maureira [13], Bruno Levy [14], Lars Falk [10], Vito G Ruggieri [11], Svante Zipfel [15], Thierry Folliguet [16], Antonio Fiore [16]

Affiliations

- [1] Heart and Lung Center, Helsinki University Hospital, Helsinki, Finland; Research Unit of Surgery, Anesthesia and Critical Care, University of Oulu, Oulu, Finland; Department of Surgery, University of Turku, Turku, Finland. Electronic address: faustobiancari@yahoo.it.
- [2] Department of Intensive Care Medicine and Cardiac Surgery, Glenfield Hospital, University Hospitals of Leicester, Leicester, UK.
- [3] Department of Molecular Medicine and Surgery, Karolinska Institutet, Department of Cardiothoracic Surgery, Karolinska University Hospital, Stockholm, Sweden.
- [4] Department of Vascular and Endovascular Surgery, Nancy University Hospital, University of Lorraine, Nancy, France.
- [5] Department of Cardiothoracic Surgery, Münster University Hospital, Münster, Germany.
- [6] Department of Thoracic and Cardio-Vascular Surgery, University Hospital Jean Minjoz, Besançon, France.
- [7] Division of Vascular Surgery, A. Manzoni Hospital, Lecco, Italy.
- [8] Department of Cardiothoracic, Transplantation and Vascular Surgery, S. Orsola Hospital, University of Bologna, Bologna, Italy.
- [9] Heart and Lung Center, Helsinki University Hospital, Helsinki, Finland; Research Unit of Surgery, Anesthesia and Critical Care, University of Oulu, Oulu, Finland.
- [10] Department of Physiology and Pharmacology, Karolinska Institutet, ECMO Centre Karolinska, Karolinska University Hospital, Stockholm, Sweden.
- [11] Division of Cardiothoracic and Vascular Surgery, Robert Debré University Hospital, Université Reims Champagne-Ardenne, Reims, France.
- [12] Service d'Anesthésie-réanimation Chirurgicale, Hôpital Henri-Mondor, Assistance Publique - Hôpitaux de Paris, Créteil, France.
- [13] Department of Cardiovascular Surgery and Heart Transplantation, Nancy University Hospital, University of Lorraine, Nancy, France.
- [14] Medical Intensive Care Unit Brabois, Nancy University Hospital, University of Lorraine, Nancy, France.
- [15] Heart Center, Hamburg University Hospital, Hamburg, Germany.
- [16] Service de Chirurgie Thoracique et Cardio-vasculaire, Hôpital Henri-Mondor, Assistance Publique - Hôpitaux de Paris, Créteil, France.

- PMID: **33573928**
- PMCID: PMC7816613
- DOI: 10.1053/j.jvca.2021.01.027

Abstract

Objectives: The authors evaluated the outcome of adult patients with coronavirus disease 2019 (COVID-19)-related acute respiratory distress syndrome (ARDS) requiring the use of extracorporeal membrane oxygenation (ECMO).

Design: Multicenter retrospective, observational study.

Setting: Ten tertiary referral university and community hospitals.

Participants: Patients with confirmed severe COVID-19-related ARDS.

Interventions: Venovenous or venoarterial ECMO.

Measurements and main results: One hundred thirty-two patients (mean age 51.1 ± 9.7 years, female 17.4%) were treated with ECMO for confirmed severe COVID-19-related ARDS. Before ECMO, the mean Sequential Organ Failure Assessment score was 10.1 ± 4.4, mean pH was 7.23 ± 0.09, and mean PaO_2/fraction of inspired oxygen ratio was 77 ± 50 mmHg. Venovenous ECMO was adopted in 122 patients (92.4%) and venoarterial ECMO in ten patients (7.6%) (mean duration, 14.6 ± 11.0 days). Sixty-three (47.7%) patients died on ECMO and 70 (53.0%) during the index hospitalization. Six-month all-cause mortality was 53.0%. Advanced age (per year, hazard ratio [HR] 1.026, 95% CI 1.000-1-052) and low arterial pH (per unit, HR 0.006, 95% CI 0.000-0.083) before ECMO were the only baseline variables associated with increased risk of six-month mortality.

Conclusions: The present findings suggested that about half of adult patients with severe COVID-19-related ARDS can be managed successfully with ECMO with sustained results at six months. Decreased arterial pH before ECMO was associated significantly with early mortality. Therefore, the authors hypothesized that initiation of ECMO therapy before severe metabolic derangements subset may improve survival rates significantly in these patients. These results should be viewed in the light of a strict patient selection policy and may not be replicated in patients with advanced age or multiple comorbidities.

Clinical trial registration: identifier, NCT04383678.

Keywords: ARDS; COVID-19; ECLS; ECMO; acute respiratory distress syndrome; coronavirus disease 2019; extracorporeal membrane oxygenation.

Copyright © 2021 The Authors. Published by Elsevier Inc. All rights reserved.

Comment in

- Long-Term Outcomes Are Important: Extracorporeal Membrane Oxygenation for COVID-19.
  Ortoleva J, Dalia AA. Ortoleva J, et al. J Cardiothorac Vasc Anesth. 2021 Jul;35(7):2007-2008. doi: 10.1053/j.jvca.2021.02.044. Epub 2021 Feb 24. J Cardiothorac Vasc Anesth. 2021. PMID: 33744109 Free PMC article. No abstract available.

- Cited by 17 articles
- 23 references
- 2 figures

Supplementary info

Publication types, MeSH terms, Associated data Expand

Publication types

- Multicenter Study
- Observational Study

MeSH terms

- Adult
- COVID-19*
- Extracorporeal Membrane Oxygenation*
- Female
- Humans
- Middle Aged
- Respiratory Distress Syndrome* / therapy
- Retrospective Studies
- SARS-CoV-2

Associated data

- ClinicalTrials.gov/NCT04383678

Full text links

ELSEVIER OPEN ACCESS Elsevier Science Free PMC article
Proceed to details
Cite
Share
215
Observational Study
Medicine (Baltimore)
. 2021 Feb 26;100(8):e24552.
doi: 10.1097/MD.0000000000024552.

Suspected myocarditis in patients with COVID-19: A multicenter case series

Natascia Laganà [1] [2], Marco Cei [3], Isabella Evangelista [1], Scilla Cerutti [1], Alessandra Colombo [1], Lucia Conte [1], Enricomaria Mormina [4], Giuseppe Rotiroti [1], Antonio Giovanni Versace [2], Cesare Porta [1], Riccardo Capra [1], Valerio Vacirca [1], Josè Vitale [1], Antonino Mazzone [1], Nicola Mumoli [1]
Affiliations Expand

Affiliations

- [1] Department of Internal Medicine, ASST Ovest Milanese, Magenta and Legnano (MI).

- [2] Department of Clinical and Experimental Medicine, Policlinico Universitario "G. Martino", University of Messina, Messina.
- [3] Department of Internal Medicine, Cecina Hospital, Cecina (LI).
- [4] Biomedical and Dental Sciences and of Morphofunctional Imaging, University of Messina, Messina, Italy.

- PMID: **33663062**
- PMCID: PMC7909099
- DOI: 10.1097/MD.0000000000024552

Free PMC article
Observational Study

Suspected myocarditis in patients with COVID-19: A multicenter case series

Natascia Laganà et al. Medicine (Baltimore). 2021.
Free PMC article
Show details
Medicine (Baltimore)
. 2021 Feb 26;100(8):e24552.
doi: 10.1097/MD.0000000000024552.

Authors

Natascia Laganà [1, 2], Marco Cei [3], Isabella Evangelista [1], Scilla Cerutti [1], Alessandra Colombo [1], Lucia Conte [1], Enricomaria Mormina [4], Giuseppe Rotiroti [1], Antonio Giovanni Versace [2], Cesare Porta [1], Riccardo Capra [1], Valerio Vacirca [1], Josè Vitale [1], Antonino Mazzone [1], Nicola Mumoli [1]

Affiliations

- [1] Department of Internal Medicine, ASST Ovest Milanese, Magenta and Legnano (MI).
- [2] Department of Clinical and Experimental Medicine, Policlinico Universitario "G. Martino", University of Messina, Messina.
- [3] Department of Internal Medicine, Cecina Hospital, Cecina (LI).
- [4] Biomedical and Dental Sciences and of Morphofunctional Imaging, University of Messina, Messina, Italy.

- PMID: **33663062**
- PMCID: PMC7909099
- DOI: 10.1097/MD.0000000000024552

Abstract

Although myocarditis can be a severe cardiac complication of COVID-19 patients, few data are available in the literature about the incidence and clinical significance in patients affected by

SARS-CoV-2. This study aims to describe the prevalence and the clinical features of suspected myocarditis in 3 cohorts of patients hospitalized for COVID-19. We retrospectively evaluated all the consecutive patients admitted for COVID-19 without exclusion criteria. Suspect myocarditis was defined according to current guidelines. Age, sex, in-hospital death, length of stay, comorbidities, serum cardiac markers, interleukin-6, electrocardiogram, echocardiogram, and therapy were recorded. Between March 4 to May 20, 2020, 1169 patients with COVID-19 were admitted in 3 Italian Medicine wards. 12 patients (1%) had suspected acute myocarditis; 5 (41.7%) were men, mean age was 76 (SD 11.34; median 78.5 years); length of stay was 38 days on average (SD 8, median value 37.5); 3 (25%) patients died. 8 (66.7%) had a history of cardiac disease; 7 (58.33%) patients had other comorbidities like diabetes, chronic obstructive pulmonary disease, or renal insufficiency. Myocarditis patients had no difference in sex prevalence, rate of death, comorbidities, elevations in serum cardiac markers as compared with patients without myocardial involvement. Otherwise, there was a significantly higher need for oxygen-support and a higher prevalence of cardiac disease in the myocarditis group. Patients with suspected myocarditis were older, had a higher frequency of previous cardiac disease, and significantly more prolonged hospitalization and a lower value of interleukin-6 than other COVID-19 patients. Further studies, specifically designed on this issue, are warranted.

Copyright © 2021 the Author(s). Published by Wolters Kluwer Health, Inc.

Conflict of interest statement

The authors have no funding and conflicts of interest to disclose.

- Cited by 9 articles
- 25 references

Supplementary info

Publication types, MeSH terms, Substances Expand

Publication types

- Multicenter Study
- Observational Study

MeSH terms

- Age Factors
- Aged
- Aged, 80 and over
- COVID-19 / complications*
- COVID-19 / mortality
- COVID-19 / physiopathology
- Comorbidity
- Electrocardiography
- Female
- Hospital Mortality

- Humans
- Interleukin-6 / blood
- Italy / epidemiology
- Length of Stay
- Male
- Middle Aged
- Myocarditis / etiology*
- Myocarditis / physiopathology
- Oxygen Inhalation Therapy
- Retrospective Studies
- SARS-CoV-2
- Sex Factors

Substances

- Interleukin-6

Full text links

Wolters Kluwer Wolters Kluwer Free PMC article
Proceed to details
Cite
Share
216
Observational Study
Neurol Sci
. 2021 Jun;42(6):2167-2172.
doi: 10.1007/s10072-021-05190-6. Epub 2021 Mar 21.

Effects of COVID-19 on the admissions of aneurysmal subarachnoid hemorrhage: the West Greece experience

Andreas Theofanopoulos [1], Dionysia Fermeli [1], Spyros Boulieris [1], George Kalantzis [1], Zinovia Kefalopoulou [2], Vasilios Panagiotopoulos [1], Dimitrios Papadakos [1], Constantine Constantoyannis [3]
Affiliations Expand

Affiliations

- [1] Department of Neurosurgery, University Hospital of Patras, 26504, Rio, Patras, Greece.
- [2] Department of Neurology, University Hospital of Patras, 26504, Rio, Patras, Greece.

- [3] Department of Neurosurgery, University Hospital of Patras, 26504, Rio, Patras, Greece. cconst@upatras.gr.

- PMID: **33745041**
- PMCID: PMC7981384
- DOI: 10.1007/s10072-021-05190-6

Free PMC article
Observational Study

Effects of COVID-19 on the admissions of aneurysmal subarachnoid hemorrhage: the West Greece experience

Andreas Theofanopoulos et al. Neurol Sci. 2021 Jun.
Free PMC article
Show details
Neurol Sci
. 2021 Jun;42(6):2167-2172.
doi: 10.1007/s10072-021-05190-6. Epub 2021 Mar 21.

Authors

Andreas Theofanopoulos [1], Dionysia Fermeli [1], Spyros Boulieris [1], George Kalantzis [1], Zinovia Kefalopoulou [2], Vasilios Panagiotopoulos [1], Dimitrios Papadakos [1], Constantine Constantoyannis [3]

Affiliations

- [1] Department of Neurosurgery, University Hospital of Patras, 26504, Rio, Patras, Greece.
- [2] Department of Neurology, University Hospital of Patras, 26504, Rio, Patras, Greece.
- [3] Department of Neurosurgery, University Hospital of Patras, 26504, Rio, Patras, Greece. cconst@upatras.gr.

- PMID: **33745041**
- PMCID: PMC7981384
- DOI: 10.1007/s10072-021-05190-6

Abstract

Background: Acute subarachnoid hemorrhage (SAH) due to aneurysmal rupture is a devastating vascular disease accounting for 5% of strokes. COVID-19 pandemic resulted in a decrease in elective and emergency admissions in the majority of neurosurgical centers. The main hypothesis was that fear of COVID-19 may have prevented patients with critical medical or surgical emergencies from actively presenting in emergency departments and outpatient clinics.

Methods: We conducted a single-center, retrospective, observational study searching our institutional data regarding the incidence of spontaneous subarachnoid hemorrhage (SAH) and compare the admissions in two different periods: the pre COVID-19 with the COVID-19 period.

Results: The study cohort was comprised of a total of 99 patients. The mean (SD) weekly case rate of patients with SAH was 1.1 (1.1) during the pre-COVID-19 period, compared to 1.7 (1.4) during the COVID-19 period. Analysis revealed that the volume of admitted patients with SAH was 1.5-fold higher during the COVID period compared to the pre-COVID period and this was statistically significant (ExpB = 1.5, CI 95% 1-2.3, p = 0.044). Difference in mortality did not reach any statistical significance between the two periods (p = 0.097), as well as patients' length of stay (p = 0.193).

Conclusions: The presented data cover a more extended time period than so far published reports; it is reasonable that our recent experience may well be demonstrating a general realistic trend of overall increase in aneurysmal rupture rates during lockdown. Hospitalization of patients with SAH cannot afford any reductions in facilities, equipment, or personnel if optimum outcomes are desirable.

Keywords: Coronavirus disease 19 (COVID-19); Subarachnoid hemorrhage (SAH); admission rate; aneurysm.

Conflict of interest statement

The authors declare no competing interests.

- Cited by 1 article
- 20 references
- 1 figure

Supplementary info

Publication types, MeSH terms Expand

Publication types

- Observational Study

MeSH terms

- COVID-19*
- Communicable Disease Control
- Greece / epidemiology
- Hospitalization
- Humans
- Pandemics
- Retrospective Studies
- SARS-CoV-2
- Subarachnoid Hemorrhage* / epidemiology

- Subarachnoid Hemorrhage* / therapy

Full text links

Springer Free PMC article

Proceed to details

Cite

Share

☐ 217

Observational Study

Crit Care

. 2022 Feb 8;26(1):37.

doi: 10.1186/s13054-022-03905-5.

Non-invasive oxygenation support in acutely hypoxemic COVID-19 patients admitted to the ICU: a multicenter observational retrospective study

Pedro David Wendel-Garcia[#] [1], Arantxa Mas [2], Cristina González-Isern [3], Ricard Ferrer [4], Rafael Máñez [5], Joan-Ramon Masclans [6], Elena Sandoval [7], Paula Vera [8], Josep Trenado [9], Rafael Fernández [10], Josep-Maria Sirvent [11], Melcior Martínez [12], Mercedes Ibarz [13], Pau Garro [14], José Luis Lopera [15], María Bodí [16], Joan Carles Yébenes-Reyes [17], Carles Triginer [18], Imma Vallverdú [19], Anna Baró [20], Fernanda Bodí [21], Paula Saludes [22], Mauricio Valencia [23], Ferran Roche-Campo [24], Arturo Huerta [25], Francisco José Cambra [26], Carme Barberà [27], Jorge Echevarria [28], Óscar Peñuelas [29], Jordi Mancebo[#] [30] [31], UCIsCAT study group

Collaborators, Affiliations Expand

Collaborators

- **UCIsCAT study group**:
  R Ferrer, O Roca, X Nuvials, J C Ruiz, E Papiol, R Máñez, V D Gumicio, E Sandoval, G Muñoz, D Toapanta, P Castro, J Osorio, J R Masclans, R Muñoz-Bermúdez, F Parrilla, P Pérez-Teran, J Marin-Corral, A Mas, B Cancio, S Hernández-Marín, M R Koborzan, C A Briones, J Trenado, R Fernández, J M Sirvent, P Sebastian, X Saiz, M Martínez, M Ibarz, P Garro, C Pedrós, E Vendrell, J L Lopera, M Bodí, A Rodríguez, G Moreno, J C Yébenes-Reyes, C Triginer, I Vallverdú, A Baró, M Morales, F Bodí, P Saludes, J-R Cervelló, M Valencia, F Roche-Campo, D Franch-Llasat, A Huerta, P Santigosa, F J Cambra, S Benito, C Barberà, J Echevarría, J Mancebo, P Vera, J-A Santos, J Baldirà, A-J Betbesé, M Izura, I Morán, J-C Suárez, L Zapata, N Rodríguez, M Torrens, A Cordón, C Gomila, M Flores, A Segarra, M Morales, L Mateo, M Martos, C González-Isern

Affiliations

- [1] Institute of Intensive Care Medicine, University Hospital of Zurich, Zurich, Switzerland.
- [2] Intensive Care Department, Hospital de Sant Joan Despí Moisès Broggi, Sant Joan Despí, Spain.
- [3] Medical Technology Department, Hospital de La Santa Creu I Sant Pau, Barcelona, Spain.
- [4] Intensive Care Department/SODIR Research Group, Hospital Universitari General de La Vall d'Hebron, Barcelona, Spain.
- [5] Intensive Care Department, L'Hospitalet de Llobregat, Barcelona, Spain.
- [6] Intensive Care Department, Hospital del Mar, GREPAC Research Group - IMIM, Department Ciències, Experimentals I de La Salut (DCEXS) UPF, Barcelona, Spain.
- [7] Cardiovascular Surgery Department, Hospital Clínic de Barcelona, Barcelona, Spain.
- [8] Intensive Care Department, Hospital de La Santa Creu I Sant Pau, Barcelona, Spain.
- [9] Intensive Care Department, Hospital Mútua de Terrassa, Terrassa, Spain.
- [10] Intensive Care Department, Althaia, Xarxa Assistencial Universitària de Manresa, Manresa, Spain.
- [11] Intensive Care Department, Hospital Universitari Doctor Josep Trueta de Girona, Girona, Spain.
- [12] Intensive Care Department, Hospital General De Cataluña, Sant Cugat del Vallès, Spain.
- [13] Intensive Care Department, Hospital Universitari Sagrat Cor - Grup Quirónsalut, Barcelona, Spain.
- [14] Intensive Care Department, Hospital General de Granollers, Granollers, Spain.
- [15] Intensive Care Department, Hospital General de Vic, Consorci Hospitalari de Vic, Vic, Spain.
- [16] Intensive Care Department, Hospital Universitari de Tarragona Joan XXIII, Tarragona, Spain.
- [17] Intensive Care Department, Hospital de Mataró, Mataró, Spain.
- [18] Intensive Care Department, Hospital d'Igualada, Igualada, Spain.
- [19] Intensive Care Department, Hospital Sant Joan de Reus, Reus, Spain.
- [20] Intensive Care Department, Hospital de Santa Caterina, Salt, Spain.
- [21] Intensive Care Department, Hospital de Sant Pau I Santa Tecla, Tarragona, Spain.
- [22] Intensive Care Department, Hospital HM Delfos, Barcelona, Spain.
- [23] Intensive Care Department, Hospital El Pilar - Grup Quirónsalut, Barcelona, Spain.
- [24] Intensive Care Department, Hospital de Tortosa Verge de La Cinta, Tortosa, Spain.
- [25] Intensive Care Department, Clínica Sagrada Família, Barcelona, Spain.
- [26] Pediatric Intensive Care Department, Hospital Sant Joan de Déu de Barcelona, Esplugues de Llobregat, Spain.
- [27] Intensive Care Department, Hospital Santa Maria, Lleida, Spain.
- [28] Intensive Care Department, Hospital ASEPEYO de Barcelona, Sant Cugat del Vallés, Spain.
- [29] Intensive Care Department Hospital, Universitario de Getafe, CIBER Enfermedades Respiratorias, CIBERES (Spain), Madrid, Spain.
- [30] Intensive Care Department, Hospital de La Santa Creu I Sant Pau, Barcelona, Spain. JMancebo@santpau.cat.
- [31] Institut d, Investigació Biomèdica Sant Pau, ', Servei Medicina Intensiva, Hospital Universitari Sant Pau, Barcelona, Spain. JMancebo@santpau.cat.

[#] Contributed equally.

- PMID: **35135588**

- PMCID: PMC8822661
- DOI: 10.1186/s13054-022-03905-5

Free PMC article
Observational Study

Non-invasive oxygenation support in acutely hypoxemic COVID-19 patients admitted to the ICU: a multicenter observational retrospective study

Pedro David Wendel-Garcia et al. Crit Care. 2022.
Free PMC article
Show details
Crit Care
. 2022 Feb 8;26(1):37.
doi: 10.1186/s13054-022-03905-5.

Authors

Pedro David Wendel-Garcia [#] [1], Arantxa Mas [2], Cristina González-Isern [3], Ricard Ferrer [4], Rafael Máñez [5], Joan-Ramon Masclans [6], Elena Sandoval [7], Paula Vera [8], Josep Trenado [9], Rafael Fernández [10], Josep-Maria Sirvent [11], Melcior Martínez [12], Mercedes Ibarz [13], Pau Garro [14], José Luis Lopera [15], María Bodí [16], Joan Carles Yébenes-Reyes [17], Carles Triginer [18], Imma Vallverdú [19], Anna Baró [20], Fernanda Bodí [21], Paula Saludes [22], Mauricio Valencia [23], Ferran Roche-Campo [24], Arturo Huerta [25], Francisco José Cambra [26], Carme Barberà [27], Jorge Echevarria [28], Óscar Peñuelas [29], Jordi Mancebo [#] [30] [31], UCIsCAT study group

Collaborators

- **UCIsCAT study group**:
  R Ferrer, O Roca, X Nuvials, J C Ruiz, E Papiol, R Máñez, V D Gumicio, E Sandoval, G Muñoz, D Toapanta, P Castro, J Osorio, J R Masclans, R Muñoz-Bermúdez, F Parrilla, P Pérez-Teran, J Marin-Corral, A Mas, B Cancio, S Hernández-Marín, M R Koborzan, C A Briones, J Trenado, R Fernández, J M Sirvent, P Sebastian, X Saiz, M Martínez, M Ibarz, P Garro, C Pedrós, E Vendrell, J L Lopera, M Bodí, A Rodríguez, G Moreno, J C Yébenes-Reyes, C Triginer, I Vallverdú, A Baró, M Morales, F Bodí, P Saludes, J-R Cervelló, M Valencia, F Roche-Campo, D Franch-Llasat, A Huerta, P Santigosa, F J Cambra, S Benito, C Barberà, J Echevarría, J Mancebo, P Vera, J-A Santos, J Baldirà, A-J Betbesé, M Izura, I Morán, J-C Suárez, L Zapata, N Rodríguez, M Torrens, A Cordón, C Gomila, M Flores, A Segarra, M Morales, L Mateo, M Martos, C González-Isern

Affiliations

- [1] Institute of Intensive Care Medicine, University Hospital of Zurich, Zurich, Switzerland.

- [2] Intensive Care Department, Hospital de Sant Joan Despí Moisès Broggi, Sant Joan Despí, Spain.
- [3] Medical Technology Department, Hospital de La Santa Creu I Sant Pau, Barcelona, Spain.
- [4] Intensive Care Department/SODIR Research Group, Hospital Universitari General de La Vall d'Hebron, Barcelona, Spain.
- [5] Intensive Care Department, L'Hospitalet de Llobregat, Barcelona, Spain.
- [6] Intensive Care Department, Hospital del Mar, GREPAC Research Group - IMIM, Department Ciències, Experimentals I de La Salut (DCEXS) UPF, Barcelona, Spain.
- [7] Cardiovascular Surgery Department, Hospital Clínic de Barcelona, Barcelona, Spain.
- [8] Intensive Care Department, Hospital de La Santa Creu I Sant Pau, Barcelona, Spain.
- [9] Intensive Care Department, Hospital Mútua de Terrassa, Terrassa, Spain.
- [10] Intensive Care Department, Althaia, Xarxa Assistencial Universitària de Manresa, Manresa, Spain.
- [11] Intensive Care Department, Hospital Universitari Doctor Josep Trueta de Girona, Girona, Spain.
- [12] Intensive Care Department, Hospital General De Cataluña, Sant Cugat del Vallès, Spain.
- [13] Intensive Care Department, Hospital Universitari Sagrat Cor - Grup Quirónsalut, Barcelona, Spain.
- [14] Intensive Care Department, Hospital General de Granollers, Granollers, Spain.
- [15] Intensive Care Department, Hospital General de Vic, Consorci Hospitalari de Vic, Vic, Spain.
- [16] Intensive Care Department, Hospital Universitari de Tarragona Joan XXIII, Tarragona, Spain.
- [17] Intensive Care Department, Hospital de Mataró, Mataró, Spain.
- [18] Intensive Care Department, Hospital d'Igualada, Igualada, Spain.
- [19] Intensive Care Department, Hospital Sant Joan de Reus, Reus, Spain.
- [20] Intensive Care Department, Hospital de Santa Caterina, Salt, Spain.
- [21] Intensive Care Department, Hospital de Sant Pau I Santa Tecla, Tarragona, Spain.
- [22] Intensive Care Department, Hospital HM Delfos, Barcelona, Spain.
- [23] Intensive Care Department, Hospital El Pilar - Grup Quirónsalut, Barcelona, Spain.
- [24] Intensive Care Department, Hospital de Tortosa Verge de La Cinta, Tortosa, Spain.
- [25] Intensive Care Department, Clínica Sagrada Família, Barcelona, Spain.
- [26] Pediatric Intensive Care Department, Hospital Sant Joan de Déu de Barcelona, Esplugues de Llobregat, Spain.
- [27] Intensive Care Department, Hospital Santa Maria, Lleida, Spain.
- [28] Intensive Care Department, Hospital ASEPEYO de Barcelona, Sant Cugat del Vallés, Spain.
- [29] Intensive Care Department Hospital, Universitario de Getafe, CIBER Enfermedades Respiratorias, CIBERES (Spain), Madrid, Spain.
- [30] Intensive Care Department, Hospital de La Santa Creu I Sant Pau, Barcelona, Spain. JMancebo@santpau.cat.
- [31] Institut d, Investigació Biomèdica Sant Pau, ', Servei Medicina Intensiva, Hospital Universitari Sant Pau, Barcelona, Spain. JMancebo@santpau.cat.

[#] Contributed equally.

- PMID: **35135588**
- PMCID: PMC8822661

- DOI: 10.1186/s13054-022-03905-5

Abstract

Background: Non-invasive oxygenation strategies have a prominent role in the treatment of acute hypoxemic respiratory failure during the coronavirus disease 2019 (COVID-19). While the efficacy of these therapies has been studied in hospitalized patients with COVID-19, the clinical outcomes associated with oxygen masks, high-flow oxygen therapy by nasal cannula and non-invasive mechanical ventilation in critically ill intensive care unit (ICU) patients remain unclear.

Methods: In this retrospective study, we used the best of nine covariate balancing algorithms on all baseline covariates in critically ill COVID-19 patients supported with > 10 L of supplemental oxygen at one of the 26 participating ICUs in Catalonia, Spain, between March 14 and April 15, 2020.

Results: Of the 1093 non-invasively oxygenated patients at ICU admission treated with one of the three stand-alone non-invasive oxygenation strategies, 897 (82%) required endotracheal intubation and 310 (28%) died during the ICU stay. High-flow oxygen therapy by nasal cannula (n = 439) and non-invasive mechanical ventilation (n = 101) were associated with a lower rate of endotracheal intubation (70% and 88%, respectively) than oxygen masks (n = 553 and 91% intubated), $p < 0.001$. Compared to oxygen masks, high-flow oxygen therapy by nasal cannula was associated with lower ICU mortality (hazard ratio 0.75 [95% CI 0.58-0.98), and the hazard ratio for ICU mortality was 1.21 [95% CI 0.80-1.83] for non-invasive mechanical ventilation.

Conclusion: In critically ill COVID-19 ICU patients and, in the absence of conclusive data, high-flow oxygen therapy by nasal cannula may be the approach of choice as the primary non-invasive oxygenation support strategy.

Keywords: Acute hypoxemic respiratory failure; COVID-19; Intensive care; Non-invasive oxygenation.

© 2022. The Author(s).

Conflict of interest statement

P.D Wendel-Garcia has nothing to disclose. A. Mas declares teaching fees from Fundació Parc Tauli, outside the submitted work. C. González-Isern has nothing to disclose. R. Ferrer declares personal fees from MSD, GSK, Alexion, Pfizer, Shionogi, Menarini and Gilead, outside the submitted work. R. Máñez has nothing to disclose. J.R. Masclans declares grants and research support from Fisher&Paykel and fees from Dextro payed to his Institution, outside the submitted work. E. Sandoval, P. Vera, J. Trenado, R. Fernández, J.M. Sirvent, M. Martínez, M. Ibarz, P. Garro, J.L. Lopera, M. Bodí, J.C. Yébenes-Reyes, C. Triginer, I. Vallverdú, A. Baró, F. Bodí, P. Saludes, M. Valencia, F. Roche-Campo, A. Huerta, F.J. Cambra, C. Barberá, J. Echeverria, and O. Peñuelas have nothing to disclose. J. Mancebo declares personal fees from Medtronic and Janssen, a research grant from Covidien/Medtronic and the Canadian Institutes of Health Research and he is consultant for Vyaire, all outside the submitted work.

- 54 references
- 3 figures

Supplementary info

Publication types, MeSH terms Expand

Publication types

- Multicenter Study
- Observational Study

MeSH terms

- COVID-19* / therapy
- Cannula
- Humans
- Intensive Care Units
- Intubation, Intratracheal
- Noninvasive Ventilation*
- Oxygen Inhalation Therapy
- Respiratory Insufficiency* / therapy
- Retrospective Studies
- SARS-CoV-2
- Spain

Full text links

Read free full text at BMC BioMed Central Free PMC article
Proceed to details
Cite
Share
218
Observational Study
Medicine (Baltimore)
. 2021 Apr 9;100(14):e25287.
doi: 10.1097/MD.0000000000025287.

The relationship between nutritional status and the prognosis of COVID-19: A retrospective analysis of 63 patients

Yuhong Li[1], Shijun Tong[2], Xueyuan Hu[3], Yuanjun Wang[2], Ronghua Lv[2], Shaozheng Ai[2], Ming Hou[2], Haining Fan[2], Youlu Shen[4]
Affiliations Expand

Affiliations

- [1] Department of Respiratory, Qinghai University Affiliated Hospital.
- [2] Qinghai University Affiliated Hospital, Xining.
- [3] The Hospital of Traditional Chinese Medicine of XinZhou District, Wuhan.
- [4] Department of Cardiology, Qinghai University Affiliated Hospital, Xining, China.

- PMID: **33832097**
- PMCID: PMC8036093
- DOI: 10.1097/MD.0000000000025287

Free PMC article
Observational Study

The relationship between nutritional status and the prognosis of COVID-19: A retrospective analysis of 63 patients

Yuhong Li et al. Medicine (Baltimore). 2021.
Free PMC article
Show details
Medicine (Baltimore)
. 2021 Apr 9;100(14):e25287.
doi: 10.1097/MD.0000000000025287.

Authors

Yuhong Li [1], Shijun Tong [2], Xueyuan Hu [3], Yuanjun Wang [2], Ronghua Lv [2], Shaozheng Ai [2], Ming Hou [2], Haining Fan [2], Youlu Shen [4]

Affiliations

- [1] Department of Respiratory, Qinghai University Affiliated Hospital.
- [2] Qinghai University Affiliated Hospital, Xining.
- [3] The Hospital of Traditional Chinese Medicine of XinZhou District, Wuhan.
- [4] Department of Cardiology, Qinghai University Affiliated Hospital, Xining, China.

- PMID: **33832097**
- PMCID: PMC8036093
- DOI: 10.1097/MD.0000000000025287

Abstract

It is important for patients to maintain a good nutritional status as a health promotion strategy to improve the immune function and thus the prognosis of coronavirus disease 2019 (COVID-19).The objective of this retrospective study is to analyze the relationships of nutritional status

with inflammation levels, protein reserves, baseline immune status, severity, length of hospital stay, and prognosis of COVID-19 patients.A total of 63 COVID-19 patients hospitalized in the People's Hospital and the Traditional Chinese Medicine Hospital of the Xinzhou District, Wuhan, China, from January 29, 2020 to March 17, 2020. Sixty-three patients were divided into 3 groups according to the guidelines, moderate (n = 22), severe (n = 14), and critical (n = 25), respectively. The differences in the total nutrition risk screening (NRS) score, inflammation level, protein reserve, baseline immune status, length of hospital stay, and prognosis were compared among patients with moderate, severe, and critical COVID-19.Patients with higher NRS scores tend to have more severe COVID-19, higher C-reactive protein and serum procalcitonin levels, higher white blood cell counts, lower lymphocyte counts, and higher mortality rates ($P < .05$).Nutritional status may be an indirect factor of the severity and prognosis of COVID-19.

Copyright © 2021 the Author(s). Published by Wolters Kluwer Health, Inc.

Conflict of interest statement

The authors have no conflicts of interest to disclose.

- Cited by 3 articles
- 10 references
- 4 figures

Supplementary info

Publication types, MeSH terms, Substances, Grant support Expand

Publication types

- Observational Study

MeSH terms

- Adult
- Aged
- Blood Proteins
- Blood Sedimentation
- C-Reactive Protein / analysis
- COVID-19 / physiopathology*
- Female
- Globulins / analysis
- Humans
- Length of Stay / statistics & numerical data
- Leukocyte Count
- Male
- Middle Aged
- Nutritional Status / physiology*
- Procalcitonin / blood

- Prognosis
- Proteins
- Retrospective Studies
- SARS-CoV-2
- Serum Albumin / analysis
- Severity of Illness Index

Substances

- Blood Proteins
- Globulins
- Procalcitonin
- Proteins
- Serum Albumin
- C-Reactive Protein

Grant support

- 2020-SFC-155/Qinghai Special Project for transformation of Scientific and Technological Achievements

Full text links

Wolters Kluwer Wolters Kluwer Free PMC article

Proceed to details

Cite

Share

☐ 219

Observational Study

Sci Rep

. 2021 Apr 30;11(1):9361.

doi: 10.1038/s41598-021-88679-6.

The COVID-19 lab score: an accurate dynamic tool to predict in-hospital outcomes in COVID-19 patients

Pablo Jose Antunez Muiños [1, 2], Diego López Otero [3, 4], Ignacio J Amat-Santos [5], Javier López País [1, 2], Alvaro Aparisi [5], Carla E Cacho Antonio [1, 2], Pablo Catalá [5], Teba González Ferrero [1, 2], Gonzalo Cabezón [5], Oscar Otero García [1, 2], José Francisco Gil [5], Marta Pérez Poza [1, 2], Jordi Candela [5], Gino Rojas [5], Víctor Jiménez Ramos [1, 2], Carlos Veras [5], J Alberto San Román [5], José R González-Juanatey [1, 2]

Affiliations Expand

Affiliations

- [1] Cardiology Department, Hospital Universitario de Santiago de Compostela, Choupana S/N, C.P. 15706, A Coruña, Spain.
- [2] CIBERCV, Madrid, Spain.
- [3] Cardiology Department, Hospital Universitario de Santiago de Compostela, Choupana S/N, C.P. 15706, A Coruña, Spain. birihh@yahoo.es.
- [4] CIBERCV, Madrid, Spain. birihh@yahoo.es.
- [5] Cardiology Department, Hospital Clínico Universitario de Valladolid, Valladolid, Spain.

- PMID: **33931677**
- PMCID: PMC8087839
- DOI: 10.1038/s41598-021-88679-6

Free PMC article
Observational Study

The COVID-19 lab score: an accurate dynamic tool to predict in-hospital outcomes in COVID-19 patients

Pablo Jose Antunez Muiños et al. Sci Rep. 2021.
Free PMC article
Show details
Sci Rep
. 2021 Apr 30;11(1):9361.
doi: 10.1038/s41598-021-88679-6.

Authors

Pablo Jose Antunez Muiños [1, 2], Diego López Otero [3, 4], Ignacio J Amat-Santos [5], Javier López País [1, 2], Alvaro Aparisi [5], Carla E Cacho Antonio [1, 2], Pablo Catalá [5], Teba González Ferrero [1, 2], Gonzalo Cabezón [5], Oscar Otero García [1, 2], José Francisco Gil [5], Marta Pérez Poza [1, 2], Jordi Candela [5], Gino Rojas [5], Víctor Jiménez Ramos [1, 2], Carlos Veras [5], J Alberto San Román [5], José R González-Juanatey [1, 2]

Affiliations

- [1] Cardiology Department, Hospital Universitario de Santiago de Compostela, Choupana S/N, C.P. 15706, A Coruña, Spain.
- [2] CIBERCV, Madrid, Spain.
- [3] Cardiology Department, Hospital Universitario de Santiago de Compostela, Choupana S/N, C.P. 15706, A Coruña, Spain. birihh@yahoo.es.
- [4] CIBERCV, Madrid, Spain. birihh@yahoo.es.

- [5] Cardiology Department, Hospital Clínico Universitario de Valladolid, Valladolid, Spain.

- PMID: **33931677**
- PMCID: PMC8087839
- DOI: 10.1038/s41598-021-88679-6

Abstract

Deterioration is sometimes unexpected in SARS-CoV2 infection. The aim of our study is to establish laboratory predictors of mortality in COVID-19 disease which can help to identify high risk patients. All patients admitted to hospital due to Covid-19 disease were included. Laboratory biomarkers that contributed with significant predictive value for predicting mortality to the clinical model were included. Cut-off points were established, and finally a risk score was built. 893 patients were included. Median age was 68.2 ± 15.2 years. 87(9.7%) were admitted to Intensive Care Unit (ICU) and 72(8.1%) needed mechanical ventilation support. 171(19.1%) patients died. A Covid-19 Lab score ranging from 0 to 30 points was calculated on the basis of a multivariate logistic regression model in order to predict mortality with a weighted score that included haemoglobin, erythrocytes, leukocytes, neutrophils, lymphocytes, creatinine, C-reactive protein, interleukin-6, procalcitonin, lactate dehydrogenase (LDH), and D-dimer. Three groups were established. Low mortality risk group under 12 points, 12 to 18 were included as moderate risk, and high risk group were those with 19 or more points. Low risk group as reference, moderate and high patients showed mortality OR 4.75(CI95% 2.60-8.68) and 23.86(CI 95% 13.61-41.84), respectively. C-statistic was 0-85(0.82-0.88) and Hosmer-Lemeshow p-value 0.63. Covid-19 Lab score can very easily predict mortality in patients at any moment during admission secondary to SARS-CoV2 infection. It is a simple and dynamic score, and it can be very easily replicated. It could help physicians to identify high risk patients to foresee clinical deterioration.

Conflict of interest statement

The authors declare no competing interests.

- Cited by 4 articles
- 21 references
- 3 figures

Supplementary info

Publication types, MeSH terms, Substances Expand

Publication types

- Multicenter Study
- Observational Study

MeSH terms

- Aged
- Biomarkers / analysis
- COVID-19 / diagnosis*

- COVID-19 / mortality
- COVID-19 / pathology
- COVID-19 / therapy
- Female
- Hospitalization
- Humans
- Male
- Multivariate Analysis
- Retrospective Studies
- Risk Assessment
- SARS-CoV-2 / physiology
- Spain / epidemiology
- Treatment Outcome

Substances

- Biomarkers

Full text links

Nature Publishing Group Free PMC article

Proceed to details

Cite

Share

☐ 220

Observational Study

Emerg Med J

. 2021 Oct;38(10):794-797.

doi: 10.1136/emermed-2021-211220. Epub 2021 Aug 6.

Exercise-induced hypoxia among emergency department patients admitted for suspected COVID-19

Peter Davies [1] [2], Timothy Jones [3], Francisca Bartilotti-Matos [3], Tim Crowe [4], Andrew Russell [5], Catie Sykes [3]

Affiliations Expand

Affiliations

- [1] Infectious Diseases, Monklands General Hospital, Airdrie, UK peter.davies@ggc.scot.nhs.uk.

- [2] Microbiology Department, Glasgow Royal Infirmary, Glasgow, UK.
- [3] Infectious Diseases, Monklands General Hospital, Airdrie, UK.
- [4] Respiratory Medicine, Monklands General Hospital, Airdrie, UK.
- [5] Emergency Medicine, Monklands General Hospital, Airdrie, UK.

- PMID: **34362824**
- DOI: 10.1136/emermed-2021-211220

Observational Study

Exercise-induced hypoxia among emergency department patients admitted for suspected COVID-19

Peter Davies et al. Emerg Med J. 2021 Oct.
Show details
Emerg Med J
. 2021 Oct;38(10):794-797.
doi: 10.1136/emermed-2021-211220. Epub 2021 Aug 6.

Authors

Peter Davies [1, 2], Timothy Jones [3], Francisca Bartilotti-Matos [3], Tim Crowe [4], Andrew Russell [5], Catie Sykes [3]

Affiliations

- [1] Infectious Diseases, Monklands General Hospital, Airdrie, UK peter.davies@ggc.scot.nhs.uk.
- [2] Microbiology Department, Glasgow Royal Infirmary, Glasgow, UK.
- [3] Infectious Diseases, Monklands General Hospital, Airdrie, UK.
- [4] Respiratory Medicine, Monklands General Hospital, Airdrie, UK.
- [5] Emergency Medicine, Monklands General Hospital, Airdrie, UK.

- PMID: **34362824**
- DOI: 10.1136/emermed-2021-211220

Abstract

Background: Exercise-induced hypoxia (EIH) has been assessed at ED triage as part of an assessment of COVID-19; however, evidence supporting this practice is incomplete. We assessed the use of a 1-minute sit-to-stand exercise test among ED patients admitted for suspected COVID-19.

Methods: A case note review of all ED patients assessed for suspected COVID-19 between March and May 2020 at Monklands University Hospital was conducted. Demographic characteristics, clinical parameters, baseline blood tests and radiographic findings, hospital length

of stay, intensive care and maximum oxygen requirement were obtained for those admitted. Using logistic regression, the association between EIH at admission triage and COVID-19 diagnosis was explored adjusting for confounding clinical parameters.

Results: Of 127 ED patients admitted for possible COVID-19, 37 were ultimately diagnosed with COVID-19. 36.4% of patients with COVID-19 and EIH had a normal admission chest radiograph. In multivariate analysis, EIH was an independent predictor of COVID-19 (adjusted OR 3.73 (95% CI (1.25 to 11.15)), as were lymphocyte count, self-reported exertional dyspnoea, C-reactive peptide and radiographic changes.

Conclusions: This observational study demonstrates an association between EIH and a COVID-19 diagnosis. Over one-third of patients with COVID-19 and EIH exhibited no radiographic changes. EIH may represent an additional tool to help predict a COVID-19 diagnosis at initial presentation and may assist in triaging need for admission.

Keywords: COVID-19; emergency care systems; emergency departments; infectious diseases; triage.

© Author(s) (or their employer(s)) 2021. No commercial re-use. See rights and permissions. Published by BMJ.

Conflict of interest statement

Competing interests: None declared.

Supplementary info

Publication types, MeSH terms Expand

Publication types

- Observational Study

MeSH terms

- COVID-19*
- Emergency Service, Hospital
- Female
- Humans
- Hypoxia / diagnosis*
- Male
- Medical Records
- Middle Aged
- Patient Admission*
- Retrospective Studies
- SARS-CoV-2*
- State Medicine

- Triage*
- United Kingdom

Full text links

BMJ Full Text HighWire
Proceed to details
Cite
Share
221
Observational Study
Swiss Med Wkly
. 2020 Dec 31;150:w20446.
doi: 10.4414/smw.2020.20446. eCollection 2020 Dec 14.

Early experimental COVID-19 therapies: associations with length of hospital stay, mortality and related costs

Nathalie Vernaz [1], Thomas Agoritsas [2], Alexandra Calmy [3], Angèle Gayet-Ageron [4], Gabriel Gold [5], Arnaud Perrier [6], Fabienne Picard [7], Virginie Prendki [8], Jean-Luc Reny [9], Caroline Samer [10], Jérôme Stirnemann [9], Pauline Vetter [11], Marie-Céline Zanella [12], Dina Zekry [8], Stéphanie Baggio [13]
Affiliations Expand

Affiliations

- [1] Medical Directorate, Finance Directorate, Geneva University Hospitals, Geneva University, Switzerland.
- [2] Division of General Internal Medicine and Division of Clinical Epidemiology, University Hospitals of Geneva, Switzerland / Department of Health Research Methods, Evidence, and Impact, McMaster University, Hamilton, ON, Canada.
- [3] Division of Infectious Diseases, HIV/AIDS Unit, Geneva University Hospitals, Switzerland.
- [4] CRC and Division of Clinical Epidemiology, Department of Health and Community Medicine, University of Geneva and University Hospitals of Geneva, Switzerland.
- [5] Service of Geriatrics, Department of Internal Medicine Rehabilitation and Geriatrics, University Hospitals and University of Geneva, Switzerland.
- [6] Medical Directorate, University of Geneva and University Hospitals of Geneva, Geneva, Switzerland / Department of General Internal Medicine, Geneva University Hospitals, Switzerland.
- [7] Division of Neurology, Department of Clinical Neurosciences, University of Geneva and University Hospitals of Geneva, Switzerland.
- [8] Division of Internal Medicine for the Aged, University of Geneva and University Hospitals of Geneva, Thônex, Switzerland.

- [9] Department of General Internal Medicine, Geneva University Hospitals, Switzerland.
- [10] Division of Clinical Pharmacology and Toxicology, Geneva University Hospitals, Switzerland.
- [11] Geneva Centre for Emerging Viral Diseases, Geneva University Hospitals, Switzerland.
- [12] Division of Infectious Diseases, Geneva University Hospitals, Switzerland.
- [13] Division of Prison Health, University of Geneva and University Hospitals of Geneva, Thônex, Switzerland / Office of Corrections, Department of Justice and Home Affairs of the Canton of Zurich, Zurich, Switzerland.

- PMID: **33382449**
- DOI: 10.4414/smw.2020.20446

Free article
Observational Study

Early experimental COVID-19 therapies: associations with length of hospital stay, mortality and related costs

Nathalie Vernaz et al. Swiss Med Wkly. 2020.
Free article
Show details
Swiss Med Wkly
. 2020 Dec 31;150:w20446.
doi: 10.4414/smw.2020.20446. eCollection 2020 Dec 14.

Authors

Nathalie Vernaz [1], Thomas Agoritsas [2], Alexandra Calmy [3], Angèle Gayet-Ageron [4], Gabriel Gold [5], Arnaud Perrier [6], Fabienne Picard [7], Virginie Prendki [8], Jean-Luc Reny [9], Caroline Samer [10], Jérôme Stirnemann [9], Pauline Vetter [11], Marie-Céline Zanella [12], Dina Zekry [8], Stéphanie Baggio [13]

Affiliations

- [1] Medical Directorate, Finance Directorate, Geneva University Hospitals, Geneva University, Switzerland.
- [2] Division of General Internal Medicine and Division of Clinical Epidemiology, University Hospitals of Geneva, Switzerland / Department of Health Research Methods, Evidence, and Impact, McMaster University, Hamilton, ON, Canada.
- [3] Division of Infectious Diseases, HIV/AIDS Unit, Geneva University Hospitals, Switzerland.
- [4] CRC and Division of Clinical Epidemiology, Department of Health and Community Medicine, University of Geneva and University Hospitals of Geneva, Switzerland.
- [5] Service of Geriatrics, Department of Internal Medicine Rehabilitation and Geriatrics, University Hospitals and University of Geneva, Switzerland.

- [6] Medical Directorate, University of Geneva and University Hospitals of Geneva, Geneva, Switzerland / Department of General Internal Medicine, Geneva University Hospitals, Switzerland.
- [7] Division of Neurology, Department of Clinical Neurosciences, University of Geneva and University Hospitals of Geneva, Switzerland.
- [8] Division of Internal Medicine for the Aged, University of Geneva and University Hospitals of Geneva, Thônex, Switzerland.
- [9] Department of General Internal Medicine, Geneva University Hospitals, Switzerland.
- [10] Division of Clinical Pharmacology and Toxicology, Geneva University Hospitals, Switzerland.
- [11] Geneva Centre for Emerging Viral Diseases, Geneva University Hospitals, Switzerland.
- [12] Division of Infectious Diseases, Geneva University Hospitals, Switzerland.
- [13] Division of Prison Health, University of Geneva and University Hospitals of Geneva, Thônex, Switzerland / Office of Corrections, Department of Justice and Home Affairs of the Canton of Zurich, Zurich, Switzerland.

- PMID: **33382449**
- DOI: 10.4414/smw.2020.20446

Abstract

Aims of the study: Hydroxychloroquine and lopinavir/ritonavir have been used as experimental therapies to treat COVID-19 during the first wave of the pandemic. Randomised controlled trials have recently shown that there are no meaningful benefits of these two therapies in hospitalised patients. Uncertainty remains regarding the potential harmful impact of these therapies as very early treatments and their burden to the health care system. The present study investigated the length of hospital stay (LOS), mortality, and costs of hydroxychloroquine, lopinavir/ritonavir or their combination in comparison with standard of care among patients hospitalised for coronavirus disease 2019 (COVID-19).

Methods: This retrospective observational cohort study took place in the Geneva University Hospitals, Geneva, Switzerland (n = 840) between 26 February and 31 May 2020. Demographics, treatment regimens, comorbidities, the modified National Early Warning Score (mNEWS) on admission, and contraindications to COVID-19 treatment options were assessed. Outcomes included LOS, in-hospital mortality, and drug and LOS costs.

Results: After successful propensity score matching, patients treated with (1) hydroxychloroquine, (2) lopinavir/ritonavir or (3) their combination had on average 3.75 additional hospitalisation days (95% confidence interval [CI] 1.37–6.12, p = 0.002), 1.23 additional hospitalisation days (95% CI −1.24 – 3.51, p = 0.319), and 4.19 additional hospitalisation days (95% CI 1.52–5.31, p <0.001), respectively, compared with patients treated with the standard of care. Neither experimental therapy was significantly associated with mortality. These additional hospital days amounted to 1010.77 additional days for hydroxychloroquine and hydroxychloroquine combined with lopinavir/ritonavir, resulting in an additional cost of US$ 2,492,214 (95%CI US$ 916,839–3,450,619).

Conclusions: Prescribing experimental therapies for COVID-19 was not associated with a reduced LOS and might have increased the pressure put on healthcare systems.

- Cited by 6 articles

Supplementary info

Publication types, MeSH terms, Substances, Supplementary concepts Expand

Publication types

- Observational Study

MeSH terms

- Adolescent
- Adult
- Age Factors
- Aged
- Aged, 80 and over
- Antiviral Agents / administration & dosage
- Antiviral Agents / adverse effects
- Antiviral Agents / therapeutic use*
- COVID-19 / drug therapy*
- COVID-19 / epidemiology*
- COVID-19 / mortality
- Child
- Child, Preschool
- Comorbidity
- Drug Combinations
- Drug Therapy, Combination
- Health Expenditures
- Hospital Mortality / trends
- Humans
- Hydroxychloroquine / administration & dosage
- Hydroxychloroquine / adverse effects
- Hydroxychloroquine / therapeutic use*
- Infant
- Length of Stay / statistics & numerical data
- Lopinavir / administration & dosage
- Lopinavir / adverse effects
- Lopinavir / therapeutic use*
- Middle Aged
- Pandemics
- Retrospective Studies
- Ritonavir / administration & dosage
- Ritonavir / adverse effects

- Ritonavir / therapeutic use*
- SARS-CoV-2
- Severity of Illness Index
- Sex Factors
- Socioeconomic Factors
- Therapies, Investigational / methods
- Young Adult

Substances

- Antiviral Agents
- Drug Combinations
- lopinavir-ritonavir drug combination
- Lopinavir
- Hydroxychloroquine
- Ritonavir

Supplementary concepts

- COVID-19 drug treatment

Full text links

Open access to full text on Swiss Medical Weekly EMH Swiss Medical Publishers Ltd.
Proceed to details
Cite
Share
222
Observational Study
Indian Heart J
. Nov-Dec 2020;72(6):593-598.
doi: 10.1016/j.ihj.2020.10.005. Epub 2020 Nov 4.

Cardiovascular complications and its impact on outcomes in COVID-19

Shekhar Kunal [1], Shashi Mohan Sharma [1], Sohan Kumar Sharma [2], Dinesh Gautam [1], Harnish Bhatia [1], Himanshu Mahla [1], Sandeep Sharma [1], Sudhir Bhandari [3]
Affiliations Expand

Affiliations

- [1] Department of Cardiology, SMS Medical College, Jaipur, Rajasthan, India.
- [2] Department of Cardiology, SMS Medical College, Jaipur, Rajasthan, India. Electronic address: drsohansharma@gmail.com.
- [3] Department of Medicine, SMS Medical College, Jaipur, Rajasthan, India.

- PMID: **33357651**
- PMCID: PMC7609238
- DOI: 10.1016/j.ihj.2020.10.005

Free PMC article
Observational Study

Cardiovascular complications and its impact on outcomes in COVID-19

Shekhar Kunal et al. Indian Heart J. Nov-Dec 2020.
Free PMC article
Show details
Indian Heart J
. Nov-Dec 2020;72(6):593-598.
doi: 10.1016/j.ihj.2020.10.005. Epub 2020 Nov 4.

Authors

Shekhar Kunal [1], Shashi Mohan Sharma [1], Sohan Kumar Sharma [2], Dinesh Gautam [1], Harnish Bhatia [1], Himanshu Mahla [1], Sandeep Sharma [1], Sudhir Bhandari [3]

Affiliations

- [1] Department of Cardiology, SMS Medical College, Jaipur, Rajasthan, India.
- [2] Department of Cardiology, SMS Medical College, Jaipur, Rajasthan, India. Electronic address: drsohansharma@gmail.com.
- [3] Department of Medicine, SMS Medical College, Jaipur, Rajasthan, India.

- PMID: **33357651**
- PMCID: PMC7609238
- DOI: 10.1016/j.ihj.2020.10.005

Abstract

Background: Coronavirus disease 2019 (COVID-19) has led to a widespread morbidity and mortality. Limited data exists regarding the involvement of cardiovascular system in COVID-19 patients. We sought to evaluate the cardiovascular (CV) complications and its impact on outcomes in symptomatic COVID-19 patients.

Methods: This was a single center observational study among symptomatic COVID-19 patients. Data regarding clinical profile, laboratory investigations, CV complications, treatment and outcomes were collected. Cardiac biomarkers and 12 lead electrocardiograms were done in all

while echocardiography was done in those with clinical indications for the same. Corrected QT-interval (QTc) at baseline and maximum value during hospitalization were computed.

Results: Of the 108 patients, majority of them were males with a mean age of 51.2 ± 17.7 years. Hypertension (38%) and diabetes (32.4%) were most prevalent co-morbidities. ECG findings included sinus tachycardia in 18 (16.9%), first degree AV block in 5 (4.6%), VT/VF in 2 (1.8%) and sinus bradycardia in one (0.9%). QTc prolongation was observed in 17.6% subjects. CV complications included acute cardiac injury in 25.9%, heart failure, cardiogenic shock and acute coronary syndrome in 3.7% each, "probable" myocarditis in 2.8% patients. Patients with acute cardiac injury had higher mortality than those without (16/28 [57.1%] vs 14/78 [17.5%]; P < 0.0001). Multivariate logistic regression analysis showed that acute cardiac injury (OR: 11.3), lymphopenia (OR: 4.91), use of inotropic agents (OR: 2.46) and neutrophil-lymphocyte ratio (OR:1.1) were independent predictors of mortality.

Conclusions: CV complications such as acute cardiac injury is common in COVID-19 patients and is associated with worse prognosis.

Keywords: Acute cardiac injury; Acute coronary syndrome; COVID-19; Cardiogenic shock; Heart failure; Myocarditis.

Copyright © 2020 Cardiological Society of India. Published by Elsevier B.V. All rights reserved.

Conflict of interest statement

Declaration of competing interest Authors have no conflict of interest to disclose.

- Cited by 7 articles
- 31 references

Supplementary info

Publication types, MeSH terms Expand

Publication types

- Observational Study

MeSH terms

- COVID-19 / complications*
- COVID-19 / epidemiology
- Cardiovascular Diseases / epidemiology
- Cardiovascular Diseases / etiology*
- Cardiovascular Diseases / therapy
- Comorbidity
- Electrocardiography
- Female
- Hospitalization / trends*

- Humans
- Incidence
- India / epidemiology
- Male
- Middle Aged
- Pandemics
- Prognosis
- Retrospective Studies
- SARS-CoV-2

Full text links

ELSEVIER OPEN ACCESS Elsevier Science Free PMC article
Proceed to details
Cite
Share
223
Observational Study
Klin Monbl Augenheilkd
. 2021 Nov;238(11):1220-1228.
doi: 10.1055/a-1529-6726. Epub 2021 Sep 15.

Effects of the First COVID-19 Lockdown on Ophthalmological Patient Care

Anna Schuh [1], Stefan Kassumeh [1], Valerie Schmelter [1], Lilian Demberg [1], Jakob Siedlecki [1], Andreas Anschütz [1], Thomas Kreutzer [1], Wolfgang J Mayer [1], Thomas Kohnen [2], Mehdi Shajari [1] [2], Siegfried Priglinger [1]
Affiliations Expand

Affiliations

- [1] Augenklinik und Poliklinik, Klinikum der Universität München, LMU München, Germany.
- [2] Augenklinik, Goethe-Universität Frankfurt am Main, Frankfurt am Main, Germany.

- PMID: **34528232**
- DOI: 10.1055/a-1529-6726

Observational Study

Effects of the First COVID-19 Lockdown on Ophthalmological Patient Care

Anna Schuh et al. Klin Monbl Augenheilkd. 2021 Nov.
Show details
Klin Monbl Augenheilkd
. 2021 Nov;238(11):1220-1228.
doi: 10.1055/a-1529-6726. Epub 2021 Sep 15.

Authors

Anna Schuh [1], Stefan Kassumeh [1], Valerie Schmelter [1], Lilian Demberg [1], Jakob Siedlecki [1], Andreas Anschütz [1], Thomas Kreutzer [1], Wolfgang J Mayer [1], Thomas Kohnen [2], Mehdi Shajari [1] [2], Siegfried Priglinger [1]

Affiliations

- [1] Augenklinik und Poliklinik, Klinikum der Universität München, LMU München, Germany.
- [2] Augenklinik, Goethe-Universität Frankfurt am Main, Frankfurt am Main, Germany.

- PMID: **34528232**
- DOI: 10.1055/a-1529-6726

Abstract
in English, German

Purpose: To determine the effect of lockdown on medical care, with the example of ophthalmology.

Methods: Patients in a period during the first lockdown were compared to a non-lockdown period, with a total of 12 259 patients included in an observational study. Changes in different areas (elective, emergency, inpatients, surgeries) and eye care subspecialties were compared. Emergency patients were analyzed according to severity and urgency. Patients showing hints requiring treatment for urgent cardiovascular diseases were determined. Differences in patients who would have suffered severe vision loss without treatment were identified and the QALY (quality-adjusted life years) loss was determined accordingly. A model to prioritize patient visits after the end of lockdown or in future lockdown scenarios was developed. Data were collected at the University Eye Hospital LMU Munich and patient files were reviewed individually by ophthalmologists.

Results: The average patient number decreased by - 59.4% ($p < 0.001$), with a significant loss in all areas (elective, emergency, inpatients, surgeries; $p < 0.001$). There was a decline of - 39.6% for patients at high risk/high severity. Patients with indications of a risk factor of future stroke declined significantly ($p = 0.003$). QALY loss at the university eye hospital was 171, which was estimated to be 3160 - 24 143 for all of Germany. Working up high losses of outpatients during these 8 weeks of projected lockdown in Germany would take 7 - 23 weeks under normal

circumstances, depending on ophthalmologist density. The prioritization model can reduce morbidity by up to 78%.

Conclusion: There was marked loss of emergency cases and patients with chronic diseases. Making up for the losses in examinations and treatments will theoretically take weeks to months. To reduce the risk of morbidity, we recommend a prioritization model for rescheduling and future lockdown scenarios.

Hintergrund: Erfassung der Auswirkungen eines Lockdowns auf die medizinische Versorgung am Beispiel der Augenheilkunde.

Methoden: In einer Beobachtungsstudie wurden Patienten in einem Zeitraum während des ersten Lockdowns mit einem Zeitraum ohne Lockdown verglichen. Hierbei wurden insgesamt 12 259 Patienten eingeschlossen. Es wurden Veränderungen in verschiedenen Bereichen (elektive Fälle, Notfälle, stationäre Fälle, Operationen) und augenärztlichen Subspezialitäten verglichen. Notfallpatienten wurden nach Schweregrad und Dringlichkeit analysiert. Patienten mit ophthalmologischen Erkrankungen, die einen Hinweis auf behandlungsbedürftige, dringende kardiovaskuläre Erkrankungen darstellen, wurden ermittelt. Unterschiede von Patienten, die ohne Behandlung einen schweren Sehverlust erlitten hätten, wurden identifiziert und der QALY-Verlust entsprechend ermittelt. Ein Modell zur Priorisierung von Patientenbesuchen nach dem Ende des Lockdowns oder in zukünftigen Lockdownszenarien wurde entwickelt. Die Daten wurden an der Universitätsaugenklinik LMU München erhoben und die Patientenakten einzeln von Augenärzten geprüft.

Ergebnisse: Die durchschnittliche Patientenzahl sank um − 59,4% ($p < 0,001$) mit einem signifikanten Verlust in allen Bereichen (elektive Fälle, Notfälle, stationäre Fälle, Operationen; $p < 0,001$). Bei Patienten mit hohem Risiko/hohem Schweregrad ergab sich ein Rückgang von − 39,6%. Patienten mit ophthalmologischen Erkrankungen, die einen Hinweis auf ein mögliches erhöhtes Risiko für einen zukünftigen Schlaganfall geben, nahmen signifikant ab ($p = 0,003$). Der QALY-Verlust an der Universitätsaugenklinik betrug 171, geschätzt für Deutschland 3160 – 24 143. Die Aufarbeitung der hohen Verluste an ambulanten Patienten während der 8 Wochen Sperrung würde auf Deutschland hochgerechnet unter normalen Umständen je nach Augenarztdichte 7 – 23 Wochen dauern. Das Priorisierungsmodell kann die Morbidität um bis zu 78% reduzieren.

Fazit: Es kam zu eminenten Verlusten sowohl bei Notfallpatienten als auch bei Patienten mit chronischen Erkrankungen. Das Aufarbeiten der verpassten Untersuchungen und Behandlungen wird theoretisch Wochen bis Monate dauern. Um das Morbiditätsrisiko zu reduzieren, empfehlen wir ein Priorisierungsmodell für die Wiedereinbestellung nach dem Lockdown und künftige Lockdownszenarien.

Thieme. All rights reserved.

Conflict of interest statement

The authors declare that they have no conflict of interest.

Supplementary info

Publication types, MeSH terms Expand

Publication types

- Observational Study

MeSH terms

- COVID-19*
- Communicable Disease Control
- Humans
- Ophthalmology*
- Patient Care
- Retrospective Studies
- SARS-CoV-2

Full text links

Georg Thieme Verlag Stuttgart, New York

Proceed to details

Cite

Share

☐ 224

Observational Study

Rev Inst Med Trop Sao Paulo

. 2022 Feb 25;64:e20.

doi: 10.1590/S1678-9946202264020. eCollection 2022.

Adult patients admitted to a tertiary hospital for COVID-19 and risk factors associated with severity: a retrospective cohort study

Veridiana Baldon Dos Santos [1], Airton Tetelbom Stein [2] [3], Sofia Louise Santin Barilli [1], Andresa Fontoura Garbini [1], Rafaela Charão de Almeida [1], Daniela Dos Reis Carazai [1], Fernanda Costa Dos Santos [1], Raquel Lutkmeier [1], Isadora Helena Greve [1], André Klafke [3], Ketlen Monteiro Mussart [1], Estefania Inez Wittke [3]

Affiliations Expand

Affiliations

- [1] Grupo Hospitalar Conceição, Hospital Nossa Senhora da Conceição, Unidade de Terapia Intensiva Adulto, Porto Alegre, Rio Grande do Sul, Brazil.
- [2] Universidade Federal de Ciências da Saúde de Porto Alegre, Porto Alegre, Rio Grande do Sul, Brazil.

- [3] Grupo Hospitalar Conceição, Programa de Pós-Graduação em Avaliação de Tecnologias para o Sistema Único de Saúde, Porto Alegre, Rio Grande do Sul, Brazil.

- PMID: **35239864**
- PMCID: PMC8901117
- DOI: 10.1590/S1678-9946202264020

Free PMC article
Observational Study

Adult patients admitted to a tertiary hospital for COVID-19 and risk factors associated with severity: a retrospective cohort study

Veridiana Baldon Dos Santos et al. Rev Inst Med Trop Sao Paulo. 2022.
Free PMC article
Show details
Rev Inst Med Trop Sao Paulo
. 2022 Feb 25;64:e20.
doi: 10.1590/S1678-9946202264020. eCollection 2022.

Authors

Veridiana Baldon Dos Santos [1], Airton Tetelbom Stein [2] [3], Sofia Louise Santin Barilli [1], Andresa Fontoura Garbini [1], Rafaela Charão de Almeida [1], Daniela Dos Reis Carazai [1], Fernanda Costa Dos Santos [1], Raquel Lutkmeier [1], Isadora Helena Greve [1], André Klafke [3], Ketlen Monteiro Mussart [1], Estefania Inez Wittke [3]

Affiliations

- [1] Grupo Hospitalar Conceição, Hospital Nossa Senhora da Conceição, Unidade de Terapia Intensiva Adulto, Porto Alegre, Rio Grande do Sul, Brazil.
- [2] Universidade Federal de Ciências da Saúde de Porto Alegre, Porto Alegre, Rio Grande do Sul, Brazil.
- [3] Grupo Hospitalar Conceição, Programa de Pós-Graduação em Avaliação de Tecnologias para o Sistema Único de Saúde, Porto Alegre, Rio Grande do Sul, Brazil.

- PMID: **35239864**
- PMCID: PMC8901117
- DOI: 10.1590/S1678-9946202264020

Abstract

COVID-19 is a disease whose knowledge is still under construction, high transmissibility, with no consensual treatment available to everyone. Therefore, the identification of patients at higher risk of evolving to the critical form of the disease is fundamental. The study aimed to determine risk factors associated with the severity of COVID-19 in adults patients. This is an observational,

retrospective study from a cohort of adult patients with COVID-19 admitted to a public hospital from March to August 2020, whose medical records were evaluated. For the association of possible severity predictors, a Poisson regression was used. The primary outcome was the critical form of the disease (need for admission to the Intensive Care Unit and/or invasive mechanical ventilation). We included 565 patients: mostly men; 55.5% of those who progressed to the critical form of the disease were over sixty years old. Hypertension, diabetes mellitus and obesity were the most frequent comorbidities. There were 39.8% of patients who progressed to the critical form of the disease. The hospital mortality rate was 22.1%, and that of critical patients was 46.7%. The independent factors associated with the severity of the disease were obesity [RR = 1.33 (95% CI 1.07 to 1.66; p = 0.011)], SpO2/FiO2 ratio ≤ 315 [RR = 2.20 (95% CI 1.79 to 2.71; p = 0.000)], C-reactive protein > 100 mg/L [RR = 1.65 (95% CI 1.33 to 2.06; p = 0.000)], and lymphocytes < 1,000/µL [RR = 1.44 (95% CI 1.18 to 1.75; p = 0.000)]. Advanced age and comorbidities were dependent factors strongly associated with the critical form of the disease.

- 40 references

Supplementary info

Publication types, MeSH terms Expand

Publication types

- Observational Study

MeSH terms

- Adult
- COVID-19*
- Cohort Studies
- Hospitalization
- Humans
- Intensive Care Units
- Male
- Middle Aged
- Retrospective Studies
- Risk Factors
- SARS-CoV-2
- Tertiary Care Centers

Full text links

free full text available at SciELO.org Scientific Electronic Library Online Free PMC article

Proceed to details

Cite

Share

☐ 225

Observational Study

Clin Dermatol
. May-Jun 2021;39(3):510-516.
doi: 10.1016/j.clindermatol.2020.11.013. Epub 2020 Nov 28.

"Do-not-resuscitate (DNR)" status determines mortality in patients with COVID-19

Albert Alhatem [1], Odette Spruijt [2], Debra S Heller [1], Ravi J Chokshi [3], Robert A Schwartz [4], W Clark Lambert [5]
Affiliations Expand

Affiliations

- [1] Department of Pathology, Immunology and Laboratory Medicine, Rutgers-New Jersey Medical School, Newark, New Jersey.
- [2] Department of Palliative Medicine, Peter MacCallum Cancer Centre, Melbourne, Australia.
- [3] Department of Surgery, Rutgers-New Jersey Medical School, Newark, New Jersey.
- [4] Department of Pathology, Immunology and Laboratory Medicine, Rutgers-New Jersey Medical School, Newark, New Jersey; Department of Dermatology, Rutgers-New Jersey Medical School, Newark, New Jersey.
- [5] Department of Pathology, Immunology and Laboratory Medicine, Rutgers-New Jersey Medical School, Newark, New Jersey; Department of Dermatology, Rutgers-New Jersey Medical School, Newark, New Jersey. Electronic address: wclambert3129@gmail.com.

- PMID: **34518012**
- PMCID: PMC7698831
- DOI: 10.1016/j.clindermatol.2020.11.013

Free PMC article
Observational Study

"Do-not-resuscitate (DNR)" status determines mortality in patients with COVID-19

Albert Alhatem et al. Clin Dermatol. May-Jun 2021.
Free PMC article
Show details

Clin Dermatol
. May-Jun 2021;39(3):510-516.
doi: 10.1016/j.clindermatol.2020.11.013. Epub 2020 Nov 28.

Authors

Albert Alhatem [1], Odette Spruijt [2], Debra S Heller [1], Ravi J Chokshi [3], Robert A Schwartz [4], W Clark Lambert [5]

Affiliations

- [1] Department of Pathology, Immunology and Laboratory Medicine, Rutgers-New Jersey Medical School, Newark, New Jersey.
- [2] Department of Palliative Medicine, Peter MacCallum Cancer Centre, Melbourne, Australia.
- [3] Department of Surgery, Rutgers-New Jersey Medical School, Newark, New Jersey.
- [4] Department of Pathology, Immunology and Laboratory Medicine, Rutgers-New Jersey Medical School, Newark, New Jersey; Department of Dermatology, Rutgers-New Jersey Medical School, Newark, New Jersey.
- [5] Department of Pathology, Immunology and Laboratory Medicine, Rutgers-New Jersey Medical School, Newark, New Jersey; Department of Dermatology, Rutgers-New Jersey Medical School, Newark, New Jersey. Electronic address: wclambert3129@gmail.com.

- PMID: **34518012**
- PMCID: PMC7698831
- DOI: 10.1016/j.clindermatol.2020.11.013

Abstract

We investigatd the influence of do-not-resuscitate (DNR) status on mortality of hospital inpatients who died of COVID-19. This is a retrospective, observational cohort study of all patients admitted to two New Jersey hospitals between March 15 and May 15, 2020, who had, or developed, COVID-19 (1270 patients). Of these, 640 patients died (570 [89.1%] with and 70 [10.9%] without a DNR order at the time of admission) and 630 survived (180 [28.6%] with and 450 [71.4%] without a DNR order when admitted). Among the 120 patients without COVID-19 who died during this interval, 110 (91.7%) had a DNR order when admitted. Deceased positive severe acute respiratory syndrome coronavirus 2 (SARS-CoV-2) patients were significantly more likely to have a DNR order on admission compared with recovered positive SARS-CoV-2 patients ($P < 0.05$), similar to those who tested negative for SARS-CoV-2. COVID-19 DNR patients had a higher mortality compared with COVID-19 non-DNR patients (log rank $P < 0.001$). DNR patients had a significantly increased hazard ratio of dying (HR 2.2 [1.5-3.2], $P < 0.001$) compared with non-DNR patients, a finding that remained significant in the multivariate model. The risk of death from COVID-19 was significantly influenced by the patients' DNR status.

Copyright © 2020 Elsevier Inc. All rights reserved.

Conflict of interest statement

Conflict of Interest The authors declare that they have no known competing financial interests or personal relationships that could have appeared to influence the work reported in this paper.

- Cited by 1 article
- 10 references
- 3 figures

Supplementary info

Publication types, MeSH terms Expand

Publication types

- Observational Study

MeSH terms

- COVID-19*
- Cohort Studies
- Humans
- Resuscitation Orders*
- Retrospective Studies
- SARS-CoV-2

Full text links

ELSEVIER FULL-TEXT ARTICLE Elsevier Science Free PMC article
Proceed to details
Cite
Share
226
Observational Study
Vaccine
. 2022 Mar 18;40(13):2003-2010.
doi: 10.1016/j.vaccine.2022.02.039. Epub 2022 Feb 19.

Impact of the Sinopharm's BBIBP-CorV vaccine in preventing hospital admissions and death in infected vaccinees: Results from a retrospective study in the emirate of Abu Dhabi, United Arab Emirates (UAE)

Farida Ismail AlHosani [1], Anderson Eduardo Stanciole [2], Bashir Aden [3], Andrey Timoshkin [4], Omar Najim [5], Walid Abbas Zaher [6], Fatima AlSayedsaleh AlDhaheri [7], Shereena Al Mazrouie [8], Tahir Aziz Rizvi [9], Farah Mustafa [10]
Affiliations Expand

Affiliations

- [1] Communicable Disease Sector, Abu Dhabi Public Health Center, Abu Dhabi, United Arab Emirates. Electronic address: falhosani@adphc.gov.ae.
- [2] Health System Financing, Department of Health, Abu Dhabi, United Arab Emirates. Electronic address: astanciole@doh.gov.ae.
- [3] Healthcare Quality Department, Department of Health, Abu Dhabi, United Arab Emirates. Electronic address: baden@doh.gov.ae.
- [4] Statistics and Modelling, Department of Health, Abu Dhabi, United Arab Emirates. Electronic address: atimoshkin@doh.gov.ae.
- [5] Executive Affairs, Department of Health, Abu Dhabi, United Arab Emirates. Electronic address: onajim@doh.gov.ae.
- [6] Group42 Healthcare, Abu Dhabi, United Arab Emirates; College of Medicine and Health Sciences (CMHS), UAE University, United Arab Emirates. Electronic address: Walid.Zaher@g42.ai.
- [7] Health Care Services, Zayed Higher Organization for People of Determination, Abu Dhabi, United Arab Emirates. Electronic address: f.aldhaheri@zho.gov.ae.
- [8] Health Promotion Department, Abu Dhabi Public Health Center, Abu Dhabi, United Arab Emirates. Electronic address: shmazrouie@adphc.gov.ae.
- [9] Department of Microbiology & Immunology, CMHS, UAE University, Al Ain, United Arab Emirates; Zayed Center for Health Sciences, UAE University, Al Ain, United Arab Emirates. Electronic address: tarizvi@uaeu.ac.ae.
- [10] Department of Biochemistry & Molecular Biology, CMHS, UAE University, Al Ain, United Arab Emirates; Zayed Center for Health Sciences, UAE University, Al Ain, United Arab Emirates. Electronic address: fmustafa@uaeu.ac.ae.

- PMID: **35193793**
- PMCID: PMC8857641
- DOI: 10.1016/j.vaccine.2022.02.039

Free PMC article
Observational Study

Impact of the Sinopharm's BBIBP-CorV vaccine in preventing hospital admissions and death in infected vaccinees: Results from a retrospective study in the emirate of Abu Dhabi, United Arab Emirates (UAE)

Farida Ismail AlHosani et al. Vaccine. 2022.
Free PMC article
Show details
Vaccine
. 2022 Mar 18;40(13):2003-2010.
doi: 10.1016/j.vaccine.2022.02.039. Epub 2022 Feb 19.

Authors

Farida Ismail AlHosani [1], Anderson Eduardo Stanciole [2], Bashir Aden [3], Andrey Timoshkin [4], Omar Najim [5], Walid Abbas Zaher [6], Fatima AlSayedsaleh AlDhaheri [7], Shereena Al Mazrouie [8], Tahir Aziz Rizvi [9], Farah Mustafa [10]

Affiliations

- [1] Communicable Disease Sector, Abu Dhabi Public Health Center, Abu Dhabi, United Arab Emirates. Electronic address: falhosani@adphc.gov.ae.
- [2] Health System Financing, Department of Health, Abu Dhabi, United Arab Emirates. Electronic address: astanciole@doh.gov.ae.
- [3] Healthcare Quality Department, Department of Health, Abu Dhabi, United Arab Emirates. Electronic address: baden@doh.gov.ae.
- [4] Statistics and Modelling, Department of Health, Abu Dhabi, United Arab Emirates. Electronic address: atimoshkin@doh.gov.ae.
- [5] Executive Affairs, Department of Health, Abu Dhabi, United Arab Emirates. Electronic address: onajim@doh.gov.ae.
- [6] Group42 Healthcare, Abu Dhabi, United Arab Emirates; College of Medicine and Health Sciences (CMHS), UAE University, United Arab Emirates. Electronic address: Walid.Zaher@g42.ai.
- [7] Health Care Services, Zayed Higher Organization for People of Determination, Abu Dhabi, United Arab Emirates. Electronic address: f.aldhaheri@zho.gov.ae.
- [8] Health Promotion Department, Abu Dhabi Public Health Center, Abu Dhabi, United Arab Emirates. Electronic address: shmazrouie@adphc.gov.ae.
- [9] Department of Microbiology & Immunology, CMHS, UAE University, Al Ain, United Arab Emirates; Zayed Center for Health Sciences, UAE University, Al Ain, United Arab Emirates. Electronic address: tarizvi@uaeu.ac.ae.
- [10] Department of Biochemistry & Molecular Biology, CMHS, UAE University, Al Ain, United Arab Emirates; Zayed Center for Health Sciences, UAE University, Al Ain, United Arab Emirates. Electronic address: fmustafa@uaeu.ac.ae.

- PMID: **35193793**
- PMCID: PMC8857641
- DOI: 10.1016/j.vaccine.2022.02.039

Abstract

Background: This is a community-based, retrospective, observational study conducted to determine effectiveness of the BBIBP-CorV inactivated vaccine in the real-world setting against hospital admissions and death.

Study design: Study participants were selected from 214,940 PCR-positive cases of COVID-19 reported to the Department of Health, Abu Dhabi Emirate, United Arab Emirates (UAE) between September 01, 2020 and May 1, 2021. Of these, 176,640 individuals were included in the study who were aged ≥ 15 years with confirmed COVID-19 positive status who had records linked to their vaccination status. Those with incomplete or missing records were excluded (n = 38,300). Study participants were divided into three groups depending upon their vaccination status: fully vaccinated (two doses), partially vaccinated (single dose), and non-vaccinated. Study outcomes included COVID-19-related admissions to hospital general and critical care wards and death.

Vaccine effectiveness for each outcome was based on the incidence density per 1000 person-years.

Results: The fully-, partially- and non-vaccinated groups included 62,931, 21,768 and 91,941 individuals, respectively. Based on the incidence rate ratios, the vaccine effectiveness in fully vaccinated individuals was 80%, 92%, and 97% in preventing COVID-19-related hospital admissions, critical care admissions, and death, respectively, when compared to the non-vaccinated group. No protection was observed for critical and non-critical care hospital admissions for the partially vaccinated group, while some protection against death was apparent, although statistically insignificant.

Conclusions: In a COVID-19 pandemic, use of the Sinopharm BBIBP-CorV inactivated vaccine is effective in preventing severe disease and death in a two-dose regimen. Lack of protection with the single dose may be explained by insufficient seroconversion and/or neutralizing antibody responses, behavioral factors (i.e., false sense of protection), and/or other biological factors (emergence of variants, possibility of reinfection, duration of vaccine protection, etc.).

Keywords: COVID-19; Inactivated viral vaccine; Real-world vaccine effectiveness study; SARS-CoV-2; Sinopharm BBIBP-CorV vaccine; United Arab Emirates (UAE).

Copyright © 2022 The Authors. Published by Elsevier Ltd.. All rights reserved.

Conflict of interest statement

Declaration of Competing Interest The authors declare that they have no known competing financial interests or personal relationships that could have appeared to influence the work reported in this paper.

- Cited by 1 article
- 37 references
- 4 figures

Supplementary info

Publication types, MeSH terms, Substances Expand

Publication types

- Observational Study

MeSH terms

- Adolescent
- COVID-19* / epidemiology
- COVID-19* / prevention & control
- Hospitals
- Humans
- Pandemics*
- Retrospective Studies

- SARS-CoV-2
- United Arab Emirates / epidemiology
- Vaccines, Inactivated

Substances

- Vaccines, Inactivated

Full text links

ELSEVIER OPEN ACCESS Elsevier Science Free PMC article
Proceed to details
Cite
Share
227
Case Reports
Transpl Infect Dis
. 2021 Oct;23(5):e13700.
doi: 10.1111/tid.13700. Epub 2021 Aug 16.

COVID-19 in lung transplant recipients: A single-center experience

Sinan Turkkan [1], Muhammet Ali Beyoglu [1], Mehmet Furkan Sahin [1], Alkın Yazicioglu [1], Yasemin Tezer Tekce [2], Erdal Yekeler [1]
Affiliations Expand

Affiliations

- [1] Department of Thoracic Surgery and Lung Transplantation, Ankara City Hospital, University of Health Sciences, Ankara, Turkey.
- [2] Department of Infectious Diseases, Ankara City Hospital, University of Health Sciences, Ankara, Turkey.

- PMID: **34323353**
- PMCID: PMC8420517
- DOI: 10.1111/tid.13700

Free PMC article
Case Reports

COVID-19 in lung transplant recipients: A single-center experience

Sinan Turkkan et al. Transpl Infect Dis. 2021 Oct.
Free PMC article
Show details
Transpl Infect Dis
. 2021 Oct;23(5):e13700.
doi: 10.1111/tid.13700. Epub 2021 Aug 16.

Authors

Sinan Turkkan [1], Muhammet Ali Beyoglu [1], Mehmet Furkan Sahin [1], Alkın Yazicioglu [1], Yasemin Tezer Tekce [2], Erdal Yekeler [1]

Affiliations

- [1] Department of Thoracic Surgery and Lung Transplantation, Ankara City Hospital, University of Health Sciences, Ankara, Turkey.
- [2] Department of Infectious Diseases, Ankara City Hospital, University of Health Sciences, Ankara, Turkey.

- PMID: **34323353**
- PMCID: PMC8420517
- DOI: 10.1111/tid.13700

Abstract

Background: Coronavirus disease 2019 (COVID-19) is a global health problem. However, the course of this disease in immunosuppressed patients remains unknown. This study aimed to describe the course of COVID-19 infection and its effects on lung transplant recipients.

Methods: This was a single-center, retrospective, observational study. The recipients with suspicious symptoms and/or a contact history with infected individuals were diagnosed with COVID-19 by performing a reverse transcription-polymerase chain reaction (RT-PCR) test using samples obtained from the nasopharynx swabs or bronchial lavage. We classified the patients into mild, moderate, and high severity groups according to their clinical conditions. In patients with positive RT-PCR results, cell cycle inhibitor drugs were withdrawn, while steroids were maintained at the same level as in patients without clinical deterioration.

Results: Of the seven recipients diagnosed with COVID-19 infection, one experienced a re-infection. Each recipient had at least one comorbidity. Smell disorder (12.5%), cough/dyspnea (37%), and fever/chills/shivering (37%) were the most frequent symptoms. The mean follow-up time after infection was 108 days. No deaths were recorded due to COVID-19; however, the pulmonary function test values of two recipients were decreased during subsequent follow-ups.

Conclusion: In our small group of transplant recipients with COVID-19, there were two cases of pulmonary function deterioration and a case of re-infection, and no recipient died. It is suggested that steroid therapy should be initiated in the early period in patients with pulmonary opacities.

Keywords: COVID-19; lung transplant; rejection.

© 2021 Wiley Periodicals LLC.

Conflict of interest statement

The authors declare that they have no competing interests.

- Cited by 2 articles
- 20 references
- 1 figure

Supplementary info

Publication types, MeSH terms Expand

Publication types

- Case Reports
- Observational Study

MeSH terms

- COVID-19*
- Humans
- Kidney Transplantation*
- Lung
- Retrospective Studies
- SARS-CoV-2
- Transplant Recipients

Full text links

WILEY Full Text Article Wiley Free PMC article
Proceed to details
Cite
Share
☐ 228
Observational Study
Rev Esp Anestesiol Reanim (Engl Ed)
. 2021 Feb;68(2):65-72.
doi: 10.1016/j.redar.2020.10.003. Epub 2020 Oct 21.

Impact of the COVID-19 pandemic on the mortality of the elderly patient with a hip fracture

[Article in English, Spanish]

A Biarnés-Suñé [1], B Solà-Enríquez [2], M Á González Posada [2], J Teixidor-Serra [3], Y García-Sánchez [4], S Manrique Muñóz [2]
Affiliations Expand

Affiliations

- [1] Departamento de Anestesiología y Reanimación, Hospital Universitario Vall d'Hebron, Barcelona, España. Electronic address: abiarnes@vhebron.net.
- [2] Departamento de Anestesiología y Reanimación, Hospital Universitario Vall d'Hebron, Barcelona, España.
- [3] Departamento de Cirugía Ortopédica y Traumatológica, Hospital Universitario Vall d'Hebron, Barcelona, España.
- [4] Departmento de Cirugia Ortopédica y Traumatologia, Vall d'Hebron Institut de Recerca (VHIR), Barcelona, España.

- PMID: **33461768**
- PMCID: PMC7577732
- DOI: 10.1016/j.redar.2020.10.003

Free PMC article
Observational Study

Impact of the COVID-19 pandemic on the mortality of the elderly patient with a hip fracture

[Article in English, Spanish]
A Biarnés-Suñé et al. Rev Esp Anestesiol Reanim (Engl Ed). 2021 Feb.
Free PMC article
Show details

Rev Esp Anestesiol Reanim (Engl Ed)
. 2021 Feb;68(2):65-72.
doi: 10.1016/j.redar.2020.10.003. Epub 2020 Oct 21.

Authors

A Biarnés-Suñé [1], B Solà-Enríquez [2], M Á González Posada [2], J Teixidor-Serra [3], Y García-Sánchez [4], S Manrique Muñóz [2]

Affiliations

- [1] Departamento de Anestesiología y Reanimación, Hospital Universitario Vall d'Hebron, Barcelona, España. Electronic address: abiarnes@vhebron.net.
- [2] Departamento de Anestesiología y Reanimación, Hospital Universitario Vall d'Hebron, Barcelona, España.

- [3] Departamento de Cirugía Ortopédica y Traumatológica, Hospital Universitario Vall d'Hebron, Barcelona, España.
- [4] Departmento de Cirugia Ortopédica y Traumatologia, Vall d'Hebron Institut de Recerca (VHIR), Barcelona, España.

- PMID: **33461768**
- PMCID: PMC7577732
- DOI: 10.1016/j.redar.2020.10.003

Abstract

COVID-19 became a threat to the public health system, compromising the health of the population. Patients with hip fractures, due to their age and comorbidity, were high-risk patients in this pandemic. The purpose of this study was to observe how the pandemic affected the management of hip fractures in elderly patients.

Methods: This is a descriptive, retrospective study of all patients over the age of 65 diagnosed with a hip fracture that came to the emergency room of Vall d'Hebron University Hospital in the COVID-19 pandemic period, from the 11th of March to the 24th of April 2020. They were followed up during their hospital stay and 30 days after the fracture.

Results: A total of 63 patients were included, 18 (28.6%) of whom had a positive RT-qPCR for COVID-19. Four could not be operated on due to the severity of the disease they presented with upon admission, dying a few days afterwards. Three of these patients had COVID-19. The 83.3% of the patients with positive RT-qPCR presented respiratory symptoms during their hospitalization. The length of hospital stays of patients with a positive RT-qPCR (18.25±8.99 days) was longer than that of patients that were RT-qPCR negative (10.9±4.52 days) (P=.01). In-hospital mortality in operated patients was 20% in patients with a positive RT-qPCR, compared with 2.3% in the group of patients who tested negative (P=.018). Mortality at 30 days was 40% in the group with positive RT-qPCR vs 6.8% in patients not infected by SARS-CoV-2 (P=.002).

Conclusion: SARS-CoV-2 infection in elderly patients with hip fractures increases both the length of hospital stay, as well as in-hospital and 30-day mortality.

Keywords: COVID-19; Fractura de cadera; Hip fracture; Mortalidad; Mortality; Perioperative; Perioperatorio.

Copyright © 2020 Sociedad Española de Anestesiología, Reanimación y Terapéutica del Dolor. Publicado por Elsevier España, S.L.U. All rights reserved.

- Cited by 1 article
- 21 references
- 1 figure

Supplementary info

Publication types, MeSH terms Expand

Publication types

- Observational Study

MeSH terms

- Aged
- Aged, 80 and over
- COVID-19 / epidemiology
- COVID-19 / mortality*
- COVID-19 Nucleic Acid Testing / statistics & numerical data
- Female
- Hip Fractures / mortality*
- Hip Fractures / surgery
- Hospital Mortality*
- Humans
- Length of Stay / statistics & numerical data
- Male
- Pandemics*
- Prevalence
- Retrospective Studies
- SARS-CoV-2*
- Sex Factors
- Spain / epidemiology

Full text links

ELSEVIER FULL-TEXT ARTICLE Elsevier Science Free PMC article

Proceed to details

Cite

Share

☐ 229

Observational Study

Int J Stroke

. 2020 Oct;15(7):755-762.

doi: 10.1177/1747493020938301. Epub 2020 Jun 26.

Impact of COVID-19 outbreak on ischemic stroke admissions and in-hospital mortality in North-West Spain

Herbert Tejada Meza [1 2 3], Álvaro Lambea Gil [1 3], Agustín Sancho Saldaña [1 3], Maite Martínez-Zabaleta [4], Patricia de la Riva Juez [4], Elena López-Cancio Martínez [5], María Castañón Apilánez [5], María Herrera Isasi [6], Juan Marta Enguita [6], Mercedes de Lera Alfonso [7], Juan F Arenillas [7 8], Jon Segurola Olaizola [9], Juan José Timiraos Fernández [9], Joaquín Sánchez [10], Mar Castellanos-Rodrigo [11], Alexia Roel [11], Ignacio Casado Menéndez [12], Mar Freijo

[13], Alain Luna Rodriguez [13], Enrique Palacio Portilla [14], Yésica Jiménez López [14], Emilio Rodríguez Castro [15], Susana Arias Rivas [15], Javier Tejada García [16], Iria Beltrán Rodríguez [16], Francisco Julián-Villaverde [17], Maria Pilar Moreno García [17], José María Trejo-Gabriel-Galán [18], Ana Echavarría Iñiguez [18], Carlos Tejero Juste [19], Cristina Pérez Lázaro [19], Javier Marta Moreno [1 3], On Behalf Of The Nordictus Investigators
Affiliations Expand

Affiliations

- [1] Stroke Unit, Department of Neurology, 16488Hospital Universitario Miguel Servet, Zaragoza, Spain.
- [2] Interventional Neuroradiology Unit, Department of Radiology, 16488Hospital Universitario Miguel Servet, Zaragoza, Spain.
- [3] Instituto de Investigación Sanitaria de Aragón (IISAragón), Zaragoza, Spain.
- [4] Department of Neurology, Hospital Universitario Donostia-Donostia Ospitalea, San Sebastián, Spain.
- [5] Department of Neurology, 16474Hospital Universitario Central de Asturias, Oviedo, Spain.
- [6] Department of Neurology, 83011Complejo Hospitalario de Navarra, Pamplona, Spain.
- [7] Department of Neurology, Hospital Clínico Universitario de Valladolid, Valladolid, Spain.
- [8] Neurovascular Research Laboratory, Instituto de Biología y Genética Molecular, Universidad de Valladolid - Consejo Superior de Investigaciones Científicas, Madrid, Spain.
- [9] Stroke Unit, Department of Neurology, Hospital Universitario de Araba, Vitoria, Spain.
- [10] Department of Neurology, Complejo Hospitalario Universitario de Vigo, Vigo, Spain.
- [11] A Coruña Biomedical Research Institute, Department of Neurology, Complexo Hospitalario Universitario A Coruña, A Coruña, Spain.
- [12] Department of Neurology, Hospital Universitario de Cabueñes, Gijón, Spain.
- [13] Neurovascular group, Biocruces Bizkaia Health Research Institute, Osakidetza, Department of Neurology, Hospital Universitario Cruces, Barakaldo, Spain.
- [14] Department of Neurology, Hospital Universitario Marqués de Valdecilla, Santander, Spain.
- [15] Department of Neurology, Complejo Hospitalario Universitario de Santiago, Santiago de Compostela, Spain.
- [16] Department of Neurology, Complejo Asistencial Universitario de León, León, Spain.
- [17] Department of Neurology, 118003Hospital San Pedro, La Rioja, Spain.
- [18] Department of Neurology, Complejo Asistencial Universitario de Burgos, Burgos, Spain.
- [19] Department of Neurology, Hospital Clínico Lozano Blesa, Zaragoza, Spain.

- PMID: **32525468**
- PMCID: PMC7322514
- DOI: 10.1177/1747493020938301

Free PMC article
Observational Study

Impact of COVID-19 outbreak on ischemic stroke admissions and in-hospital mortality in North-West Spain

Herbert Tejada Meza et al. Int J Stroke. 2020 Oct.
Free PMC article
Show details
Int J Stroke
. 2020 Oct;15(7):755-762.
doi: 10.1177/1747493020938301. Epub 2020 Jun 26.

Authors

Herbert Tejada Meza [1, 2, 3], Álvaro Lambea Gil [1, 3], Agustín Sancho Saldaña [1, 3], Maite Martínez-Zabaleta [4], Patricia de la Riva Juez [4], Elena López-Cancio Martínez [5], María Castañón Apilánez [5], María Herrera Isasi [6], Juan Marta Enguita [6], Mercedes de Lera Alfonso [7], Juan F Arenillas [7, 8], Jon Segurola Olaizola [9], Juan José Timiraos Fernández [9], Joaquín Sánchez [10], Mar Castellanos-Rodrigo [11], Alexia Roel [11], Ignacio Casado Menéndez [12], Mar Freijo [13], Alain Luna Rodriguez [13], Enrique Palacio Portilla [14], Yésica Jiménez López [14], Emilio Rodríguez Castro [15], Susana Arias Rivas [15], Javier Tejada García [16], Iria Beltrán Rodríguez [16], Francisco Julián-Villaverde [17], Maria Pilar Moreno García [17], José María Trejo-Gabriel-Galán [18], Ana Echavarría Iñiguez [18], Carlos Tejero Juste [19], Cristina Pérez Lázaro [19], Javier Marta Moreno [1, 3], On Behalf Of The Nordictus Investigators

Affiliations

- [1] Stroke Unit, Department of Neurology, 16488Hospital Universitario Miguel Servet, Zaragoza, Spain.
- [2] Interventional Neuroradiology Unit, Department of Radiology, 16488Hospital Universitario Miguel Servet, Zaragoza, Spain.
- [3] Instituto de Investigación Sanitaria de Aragón (IISAragón), Zaragoza, Spain.
- [4] Department of Neurology, Hospital Universitario Donostia-Donostia Ospitalea, San Sebastián, Spain.
- [5] Department of Neurology, 16474Hospital Universitario Central de Asturias, Oviedo, Spain.
- [6] Department of Neurology, 83011Complejo Hospitalario de Navarra, Pamplona, Spain.
- [7] Department of Neurology, Hospital Clínico Universitario de Valladolid, Valladolid, Spain.
- [8] Neurovascular Research Laboratory, Instituto de Biología y Genética Molecular, Universidad de Valladolid - Consejo Superior de Investigaciones Científicas, Madrid, Spain.
- [9] Stroke Unit, Department of Neurology, Hospital Universitario de Araba, Vitoria, Spain.
- [10] Department of Neurology, Complejo Hospitalario Universitario de Vigo, Vigo, Spain.
- [11] A Coruña Biomedical Research Institute, Department of Neurology, Complexo Hospitalario Universitario A Coruña, A Coruña, Spain.
- [12] Department of Neurology, Hospital Universitario de Cabueñes, Gijón, Spain.

- [13] Neurovascular group, Biocruces Bizkaia Health Research Institute, Osakidetza, Department of Neurology, Hospital Universitario Cruces, Barakaldo, Spain.
- [14] Department of Neurology, Hospital Universitario Marqués de Valdecilla, Santander, Spain.
- [15] Department of Neurology, Complejo Hospitalario Universitario de Santiago, Santiago de Compostela, Spain.
- [16] Department of Neurology, Complejo Asistencial Universitario de León, León, Spain.
- [17] Department of Neurology, 118003Hospital San Pedro, La Rioja, Spain.
- [18] Department of Neurology, Complejo Asistencial Universitario de Burgos, Burgos, Spain.
- [19] Department of Neurology, Hospital Clínico Lozano Blesa, Zaragoza, Spain.

- PMID: **32525468**
- PMCID: PMC7322514
- DOI: 10.1177/1747493020938301

Abstract

Background and purpose: Spain has been one of the countries heavily stricken by COVID-19. But this epidemic has not affected all regions equally. We analyzed the impact of the COVID-19 pandemic on hospital stroke admissions and in-hospital mortality in tertiary referral hospitals from North-West Spain.

Methods: Spanish multicenter retrospective observational study based on data from tertiary hospitals of the NORDICTUS network. We recorded the number of patients admitted for ischemic stroke between 30 December 2019 and 3 May 2020, the number of IVT and EVT procedures, and in-hospital mortality.

Results: In the study period, 2737 patients were admitted with ischemic stroke. There was a decrease in the weekly mean admitted patients during the pandemic (124 vs. 173, $p<0.001$). In-hospital mortality of stroke patients increased significantly (9.9% vs. 6.5%, $p = 0.003$), but there were no differences in the proportion of IVT (17.3% vs. 16.1%, $p = 0.405$) or EVT (22% vs. 23%, $p = 0.504$).

Conclusion: We found a decrease in the number of ischemic stroke admissions and an increase in in-hospital mortality during the COVID-19 epidemic in this large study from North-West Spain. There were regional changes within the network, not fully explained by the severity of the pandemic in different regions.

Keywords: COVID-19; Spain; Stroke; ischemic stroke; mortality; stroke care.

- Cited by 35 articles
- 15 references
- 2 figures

Supplementary info

Publication types, MeSH terms Expand

Publication types

- Multicenter Study
- Observational Study

MeSH terms

- Aged
- Betacoronavirus*
- Brain Ischemia / diagnosis
- Brain Ischemia / mortality*
- Brain Ischemia / therapy
- COVID-19
- Coronavirus Infections / epidemiology*
- Female
- Hospital Mortality
- Hospitalization / statistics & numerical data*
- Humans
- Male
- Pandemics
- Pneumonia, Viral / epidemiology*
- Reperfusion
- Retrospective Studies
- SARS-CoV-2
- Spain
- Stroke / diagnosis
- Stroke / mortality*
- Stroke / therapy
- Tertiary Care Centers

Full text links

SAGE journals Open access full text Atypon Free PMC article

Proceed to details

Cite

Share

☐ 230

Observational Study

Am J Emerg Med

. 2021 Nov;49:378-384.

doi: 10.1016/j.ajem.2021.06.045. Epub 2021 Jun 22.

[Evaluating the effect of SARS-Cov-2 infection on prognosis and mortality in patients with acute pancreatitis]

Rezan Karaali [1], Firdes Topal [2]
Affiliations Expand

Affiliations

- [1] İzmir Katip Çelebi Univetsity Atatürk Training And Research Hospital, Emergency Department, Karabağlar, İzmir, Turkey. Electronic address: rezantahtaci@hotmail.com.
- [2] İzmir Katip Çelebi Univetsity Atatürk Training And Research Hospital, Gastroenterology Department, Karabağlar, İzmir, Turkey.

- PMID: **34246968**
- PMCID: PMC8216870
- DOI: 10.1016/j.ajem.2021.06.045

Free PMC article
Observational Study

Evaluating the effect of SARS-Cov-2 infection on prognosis and mortality in patients with acute pancreatitis

Rezan Karaali et al. Am J Emerg Med. 2021 Nov.
Free PMC article
Show details
Am J Emerg Med
. 2021 Nov;49:378-384.
doi: 10.1016/j.ajem.2021.06.045. Epub 2021 Jun 22.

Authors

Rezan Karaali [1], Firdes Topal [2]

Affiliations

- [1] İzmir Katip Çelebi Univetsity Atatürk Training And Research Hospital, Emergency Department, Karabağlar, İzmir, Turkey. Electronic address: rezantahtaci@hotmail.com.
- [2] İzmir Katip Çelebi Univetsity Atatürk Training And Research Hospital, Gastroenterology Department, Karabağlar, İzmir, Turkey.

- PMID: **34246968**

- PMCID: PMC8216870
- DOI: 10.1016/j.ajem.2021.06.045

Abstract

Introduction: Acute pancreatitis (AP) is the leading cause of hospitalization among gastrointestinal disorders. The aim of our study is to compare the results between AP patients with and without COVID-19, and to reveal the effects of COVID-19 on the course, intensive care needs and mortality of AP patients.

Material methods: This was a single-center, retrospective and observational study. Patients over 18 years of age, who were diagnosed with AP during the current pandemic. According to the RT-PCR test result, patients were divided into two groups: COVID-19 positive and COVID-19 negative. Gender, age, laboratory parameters, intensive care unit admission, length of hospital stay, severity and mortality of AP were compared between these two groups.

Results: We reviewed 562 patients presenting to the emergency department who were diagnosed with acute pancreatitis between 10.03.2020 and 31.12.2020 and included 189 patients in our study. Positive patients need for intensive care (7.23%) were higher compared to negative patients (0.94%). 32.53% of positive patients and 14.15% of negative patients had severe AP ($p < 0.03$). We established that being COVID-19 positive, CCI scores of ≥5, presence of COVID-19 compatible pneumonia on CT and BISAP scores had an effect on mortality ($p < 0,05$).

Conclusion: The severity and mortality of AP increase in patients with both AP and COVID-19. This rate increases even more in the presence of COVID-19-associated pneumonia. We believe that new strategies should be developed for the follow-up and treatment of patients with both these conditions.

Keywords: Acute pancreatitits; COVID-19; Emergency.

Copyright © 2021 Elsevier Inc. All rights reserved.

- Cited by 1 article
- 41 references

Supplementary info

Publication types, MeSH terms Expand

Publication types

- Observational Study

MeSH terms

- Adult
- Aged
- COVID-19 / diagnosis*
- COVID-19 / epidemiology*

- Emergency Service, Hospital
- Female
- Humans
- Intensive Care Units
- Length of Stay / statistics & numerical data
- Logistic Models
- Male
- Middle Aged
- Pancreatitis / diagnosis*
- Pancreatitis / mortality*
- Pancreatitis / physiopathology
- Prognosis
- Retrospective Studies
- SARS-CoV-2 / isolation & purification
- Severity of Illness Index
- Turkey / epidemiology

Full text links

ELSEVIER FULL-TEXT ARTICLE Elsevier Science Free PMC article
Proceed to details
Cite
Share
231
Observational Study
BMC Infect Dis
. 2021 Dec 20;21(1):1271.
doi: 10.1186/s12879-021-06970-3.

Six-month follow-up of functional status in discharged patients with coronavirus disease 2019

Hou-Wei Du[#,1,2], Shuang-Fang Fang[#,1,2], Sang-Ru Wu[#,1,2], Xiao-Ling Chen[#,3], Jun-Nian Chen[4], Yi-Xian Zhang[5], Hua-Yao Huang[5], Han-Han Lei[1,2], Rong-Hua Chen[1,2], Xiao-Bin Pan[6], Xiao-Qing Li[7], Pin-Cang Xia[7], Zhen-Yang Zheng[1,2], Ling-Luo[1,2], Hai-Long Lin[8], Li-Min Chen[9], Nan Liu[10,11], Fujian Medical Team Support Wuhan for COVID19
Affiliations Expand

Affiliations

- [1] Department of Neurology, Fujian Medical University Union Hospital, 29 Xinquan Road, Gulou District, Fuzhou, 35000, China.
- [2] Institute of Clinical Neurology, Fujian Medical University, Fuzhou, China.
- [3] Department of Infectious Disease, Fujian Medical University Union Hospital, Fuzhou, China.
- [4] Department of Critical Care Medicine, Fujian Medical University Union Hospital, Fuzhou, China.
- [5] Department of Rehabilitation, Fujian Medical University Union Hospital, Fuzhou, China.
- [6] Department of Critical Care Medicine, Fujian Provincial Hospital South Branch, Fuzhou, China.
- [7] Fujian Center for Disease Control and Prevention, Fuzhou, China.
- [8] Department of Radiology, Fujian Medical University Union Hospital, Fuzhou, China.
- [9] Department of Respiratory Medicine, Fujian Medical University Union Hospital, Fuzhou, China.
- [10] Department of Neurology, Fujian Medical University Union Hospital, 29 Xinquan Road, Gulou District, Fuzhou, 35000, China. xieheliunan1984@fjmu.edu.cn.
- [11] Department of Rehabilitation, Fujian Medical University Union Hospital, Fuzhou, China. xieheliunan1984@fjmu.edu.cn.

[#] Contributed equally.

- PMID: **34930161**
- PMCID: PMC8686090
- DOI: 10.1186/s12879-021-06970-3

Free PMC article
Observational Study

Six-month follow-up of functional status in discharged patients with coronavirus disease 2019

Hou-Wei Du et al. BMC Infect Dis. 2021.
Free PMC article
Show details
BMC Infect Dis
. 2021 Dec 20;21(1):1271.
doi: 10.1186/s12879-021-06970-3.

Authors

Hou-Wei Du [#, 1, 2], Shuang-Fang Fang [#, 1, 2], Sang-Ru Wu [#, 1, 2], Xiao-Ling Chen [#, 3], Jun-Nian Chen [4], Yi-Xian Zhang [5], Hua-Yao Huang [5], Han-Han Lei [1, 2], Rong-Hua Chen [1, 2], Xiao-Bin Pan [6], Xiao-Qing Li [7], Pin-Cang Xia [7], Zhen-Yang Zheng [1, 2], Ling-Luo [1, 2], Hai-Long Lin [8], Li-Min Chen [9], Nan Liu [10, 11], Fujian Medical Team Support Wuhan for COVID19

Affiliations

- [1] Department of Neurology, Fujian Medical University Union Hospital, 29 Xinquan Road, Gulou District, Fuzhou, 35000, China.
- [2] Institute of Clinical Neurology, Fujian Medical University, Fuzhou, China.
- [3] Department of Infectious Disease, Fujian Medical University Union Hospital, Fuzhou, China.
- [4] Department of Critical Care Medicine, Fujian Medical University Union Hospital, Fuzhou, China.
- [5] Department of Rehabilitation, Fujian Medical University Union Hospital, Fuzhou, China.
- [6] Department of Critical Care Medicine, Fujian Provincial Hospital South Branch, Fuzhou, China.
- [7] Fujian Center for Disease Control and Prevention, Fuzhou, China.
- [8] Department of Radiology, Fujian Medical University Union Hospital, Fuzhou, China.
- [9] Department of Respiratory Medicine, Fujian Medical University Union Hospital, Fuzhou, China.
- [10] Department of Neurology, Fujian Medical University Union Hospital, 29 Xinquan Road, Gulou District, Fuzhou, 35000, China. xieheliunan1984@fjmu.edu.cn.
- [11] Department of Rehabilitation, Fujian Medical University Union Hospital, Fuzhou, China. xieheliunan1984@fjmu.edu.cn.

[#] Contributed equally.

- PMID: **34930161**
- PMCID: PMC8686090
- DOI: 10.1186/s12879-021-06970-3

Abstract

Background: The long-term functional outcome of discharged patients with coronavirus disease 2019 (COVID-19) remains unresolved. We aimed to describe a 6-month follow-up of functional status of COVID-19 survivors.

Methods: We reviewed the data of COVID-19 patients who had been consecutively admitted to the Tumor Center of Union Hospital (Wuhan, China) between 15 February and 14 March 2020. We quantified a 6-month functional outcome reflecting symptoms and disability in COVID-19 survivors using a post-COVID-19 functional status scale ranging from 0 to 4 (PCFS). We examined the risk factors for the incomplete functional status defined as a PCFS > 0 at a 6-month follow-up after discharge.

Results: We included a total of 95 COVID-19 survivors with a median age of 62 (IQR 53-69) who had a complete functional status (PCFS grade 0) at baseline in this retrospective observational study. At 6-month follow-up, 67 (70.5%) patients had a complete functional outcome (grade 0), 9 (9.5%) had a negligible limited function (grade 1), 12 (12.6%) had a mild limited function (grade 2), 7 (7.4%) had moderate limited function (grade 3). Univariable logistic regression analysis showed a significant association between the onset symptoms of muscle or joint pain and an increased risk of incomplete function (unadjusted OR 4.06, 95% CI 1.33-12.37). This association remained after adjustment for age and admission delay (adjusted OR 3.39, 95% CI 1.06-10.81, p = 0.039).

Conclusions: A small proportion of discharged COVID-19 patients may have an incomplete functional outcome at a 6-month follow-up; intervention strategies are required.

Keywords: Coronavirus disease 2019; Follow-up; Functional outcome.

© 2021. The Author(s).

Conflict of interest statement

None.

- Cited by 1 article
- 35 references
- 1 figure

Supplementary info

Publication types, MeSH terms, Grant support Expand

Publication types

- Observational Study

MeSH terms

- COVID-19*
- Follow-Up Studies
- Functional Status
- Humans
- Patient Discharge*
- SARS-CoV-2

Grant support

- 2019Y9099/Fujian Science and Technology Innovation Joint Fund Project
- 2016B014/Fujian Provincial Natural and Science Innovation Project
- JAT190183/the Young Teacher's Foundation of Fujian Provincial Department of Education

Full text links

Read free full text at BMC BioMed Central Free PMC article

Proceed to details

Cite

Share

☐ 232

Observational Study

Int J Infect Dis

. 2021 Jul;108:347-352.
doi: 10.1016/j.ijid.2021.05.073. Epub 2021 Jun 1.

Clinical and epidemiological characteristics of children with COVID-19 in Negeri Sembilan, Malaysia

David Chun-Ern Ng [1], Kah Kee Tan [2], Ling Chin [1], Marlindawati Mohd Ali [3], Ming Lee Lee [1], Fatin Mahirah Mahmood [4], Mohammad Faid Abd Rashid [5], Harlina Abdul Rashid [5], Erwin Jiayuan Khoo [6]
Affiliations Expand

Affiliations

- [1] Department of Paediatrics, Hospital Tuanku Ja'afar Seremban, Malaysia.
- [2] Department of Paediatrics, Perdana University-Royal College of Surgeons in Ireland School of Medicine, Seremban, Malaysia.
- [3] Microbiology Unit, Department of Pathology, Hospital Tuanku Ja'afar Seremban, Malaysia.
- [4] Hospital Rembau, Chembong, Malaysia.
- [5] Negeri Sembilan State Health Department, Seremban, Malaysia.
- [6] Department of Paediatrics, International Medical University, Seremban, Malaysia. Electronic address: jiayuan_khoo@imu.edu.my.

- PMID: **34087485**
- PMCID: PMC8168297
- DOI: 10.1016/j.ijid.2021.05.073

Free PMC article
Observational Study

Clinical and epidemiological characteristics of children with COVID-19 in Negeri Sembilan, Malaysia

David Chun-Ern Ng et al. Int J Infect Dis. 2021 Jul.
Free PMC article
Show details
Int J Infect Dis
. 2021 Jul;108:347-352.
doi: 10.1016/j.ijid.2021.05.073. Epub 2021 Jun 1.

Authors

David Chun-Ern Ng [1], Kah Kee Tan [2], Ling Chin [1], Marlindawati Mohd Ali [3], Ming Lee Lee [1], Fatin Mahirah Mahmood [4], Mohammad Faid Abd Rashid [5], Harlina Abdul Rashid [5], Erwin Jiayuan Khoo [6]

Affiliations

- [1] Department of Paediatrics, Hospital Tuanku Ja'afar Seremban, Malaysia.
- [2] Department of Paediatrics, Perdana University-Royal College of Surgeons in Ireland School of Medicine, Seremban, Malaysia.
- [3] Microbiology Unit, Department of Pathology, Hospital Tuanku Ja'afar Seremban, Malaysia.
- [4] Hospital Rembau, Chembong, Malaysia.
- [5] Negeri Sembilan State Health Department, Seremban, Malaysia.
- [6] Department of Paediatrics, International Medical University, Seremban, Malaysia. Electronic address: jiayuan_khoo@imu.edu.my.

- PMID: **34087485**
- PMCID: PMC8168297
- DOI: 10.1016/j.ijid.2021.05.073

Abstract

Objectives: To describe the clinical and epidemiological characteristics of children with coronavirus disease 2019 (COVID-19) in the state of Negeri Sembilan, Malaysia in the setting of mandatory hospital isolation and quarantine for all confirmed cases.

Methods: A multi-centre, retrospective observational study was performed among children aged ≤12 years with laboratory-proven COVID-19 between 1 February and 31 December 2020.

Results: In total, 261 children (48.7% males, 51.3% females) were included in this study. The median age was 6 years [interquartile range (IQR) 3-10 years]. One hundred and fifty-one children (57.9%) were asymptomatic on presentation. Among the symptomatic cases, fever was the most common presenting symptom. Two hundred and forty-one (92.3%) cases were close contacts of infected household or extended family members. Twenty-one (8.4%) cases had abnormal radiological findings. All cases were discharged alive without requiring supplemental oxygen therapy or any specific treatment during hospitalization. The median duration of hospitalization was 7 days (IQR 6-10 days). One (2.1%) of the uninfected guardians accompanying a child in quarantine tested positive for severe acute respiratory syndrome coronavirus-2 (SARS-CoV-2) upon discharge.

Conclusions: COVID-19 in children was associated with mild symptoms and a good prognosis. Familial clustering was an important epidemiologic feature in the outbreak in Negeri Sembilan. The risk of transmission of SARS-CoV-2 from children to guardians in hospital isolation was minimal despite close proximity.

Keywords: Clinical features; Epidemiology; Paediatric COVID-19; SARS-CoV-2.

Copyright © 2021 The Author(s). Published by Elsevier Ltd.. All rights reserved.

- Cited by 4 articles
- 25 references

- 1 figure

Supplementary info

Publication types, MeSH terms Expand

Publication types

- Observational Study

MeSH terms

- COVID-19*
- Child
- Child, Preschool
- Disease Outbreaks
- Female
- Humans
- Malaysia / epidemiology
- Male
- Quarantine
- Retrospective Studies
- SARS-CoV-2

Full text links

ELSEVIER OPEN ACCESS Elsevier Science Free PMC article

Proceed to details

Cite

Share

☐ 233

Observational Study

Immunol Lett

. 2020 Dec;228:122-128.

doi: 10.1016/j.imlet.2020.10.009. Epub 2020 Nov 5.

Pulmonary vascular improvement in severe COVID-19 patients treated with tocilizumab

Lorenzo Salvati [1], Mariaelena Occhipinti [1], Leonardo Gori [1], Luca Ciani [1], Alessio Mazzoni [1], Laura Maggi [1], Manuela Capone [1], Paola Parronchi [2], Francesco Liotta [2], Vittorio Miele [3], Francesco Annunziato [4], Federico Lavorini [5], Lorenzo Cosmi [6]

Affiliations Expand

Affiliations

- [1] Department of Experimental and Clinical Medicine, University of Florence, Florence, Italy.
- [2] Department of Experimental and Clinical Medicine, University of Florence, Florence, Italy; Immunology and Cell Therapy Unit, Careggi University Hospital, Florence, Italy.
- [3] Department of Emergency Radiology, Careggi University Hospital, Florence, Italy.
- [4] Department of Experimental and Clinical Medicine, University of Florence, Florence, Italy; Flow Cytometry Diagnostic Center and Immunotherapy (CDCI), Careggi University Hospital, Florence, Italy.
- [5] Department of Experimental and Clinical Medicine, University of Florence, Florence, Italy; Pneumology and Intensive Care Unit, Careggi University Hospital, Florence, Italy.
- [6] Department of Experimental and Clinical Medicine, University of Florence, Florence, Italy; Immunology and Cell Therapy Unit, Careggi University Hospital, Florence, Italy. Electronic address: lorenzo.cosmi@unifi.it.

- PMID: **33161002**
- PMCID: PMC7644186
- DOI: 10.1016/j.imlet.2020.10.009

Free PMC article
Observational Study

Pulmonary vascular improvement in severe COVID-19 patients treated with tocilizumab

Lorenzo Salvati et al. Immunol Lett. 2020 Dec.
Free PMC article
Show details
Immunol Lett
. 2020 Dec;228:122-128.
doi: 10.1016/j.imlet.2020.10.009. Epub 2020 Nov 5.

Authors

Lorenzo Salvati [1], Mariaelena Occhipinti [1], Leonardo Gori [1], Luca Ciani [1], Alessio Mazzoni [1], Laura Maggi [1], Manuela Capone [1], Paola Parronchi [2], Francesco Liotta [2], Vittorio Miele [3], Francesco Annunziato [4], Federico Lavorini [5], Lorenzo Cosmi [6]

Affiliations

- [1] Department of Experimental and Clinical Medicine, University of Florence, Florence, Italy.
- [2] Department of Experimental and Clinical Medicine, University of Florence, Florence, Italy; Immunology and Cell Therapy Unit, Careggi University Hospital, Florence, Italy.
- [3] Department of Emergency Radiology, Careggi University Hospital, Florence, Italy.

- [4] Department of Experimental and Clinical Medicine, University of Florence, Florence, Italy; Flow Cytometry Diagnostic Center and Immunotherapy (CDCI), Careggi University Hospital, Florence, Italy.
- [5] Department of Experimental and Clinical Medicine, University of Florence, Florence, Italy; Pneumology and Intensive Care Unit, Careggi University Hospital, Florence, Italy.
- [6] Department of Experimental and Clinical Medicine, University of Florence, Florence, Italy; Immunology and Cell Therapy Unit, Careggi University Hospital, Florence, Italy. Electronic address: lorenzo.cosmi@unifi.it.

- PMID: **33161002**
- PMCID: PMC7644186
- DOI: 10.1016/j.imlet.2020.10.009

Abstract

As of October 2020 management of Coronavirus disease 2019 (COVID-19) is based on supportive care and off-label or compassionate-use therapies. On March 2020 tocilizumab - an anti-IL-6 receptor monoclonal antibody - was suggested as immunomodulatory treatment in severe COVID-19 because hyperinflammatory syndrome occurs in many patients similarly to the cytokine release syndrome that develops after CAR-T cell therapy. In our retrospective observational study, 20 severe COVID-19 patients requiring intensive care were treated with tocilizumab in addition to standard-of-care therapy (SOC) and compared with 13 COVID-19 patients receiving only SOC. Clinical respiratory status, inflammatory markers and vascular radiologic score improved after one week from tocilizumab administration. On the contrary, these parameters were stable or worsened in patients receiving only SOC. Despite major study limitations, improvement of alveolar-arterial oxygen gradient as well as vascular radiologic score after one week may account for improved pulmonary vascular perfusion and could explain the more rapid recovery of COVID-19 patients receiving tocilizumab compared to controls.

Keywords: Alveolar-arterial oxygen gradient; Chest-X ray; IL-6; Severe COVID-19; Tocilizumab; Vascular score.

Copyright © 2020 European Federation of Immunological Societies. Published by Elsevier B.V. All rights reserved.

Conflict of interest statement

The authors declare no competing interest.

- Cited by 7 articles
- 40 references
- 3 figures

Supplementary info

Publication types, MeSH terms, Substances, Supplementary concepts Expand

Publication types

- Observational Study

MeSH terms

- Aged
- Aged, 80 and over
- Antibodies, Monoclonal, Humanized / therapeutic use*
- Biomarkers / blood
- COVID-19 / drug therapy*
- COVID-19 / pathology
- Combined Modality Therapy
- Critical Care
- Female
- Humans
- Male
- Middle Aged
- Receptors, Interleukin-6 / antagonists & inhibitors
- Respiration / drug effects*
- Retrospective Studies
- SARS-CoV-2
- Time Factors
- Treatment Outcome

Substances

- Antibodies, Monoclonal, Humanized
- Biomarkers
- Receptors, Interleukin-6
- tocilizumab

Supplementary concepts

- COVID-19 drug treatment

Full text links

ELSEVIER FULL-TEXT ARTICLE Elsevier Science Free PMC article
Proceed to details
Cite
Share
☐ 234
Observational Study
PLoS One
. 2020 Jul 23;15(7):e0236240.
doi: 10.1371/journal.pone.0236240. eCollection 2020.

Pre-existing traits associated with Covid-19 illness severity

Joseph E Ebinger [1 2], Natalie Achamallah [3 4], Hongwei Ji [5 6], Brian L Claggett [6], Nancy Sun [1 2], Patrick Botting [1 2], Trevor-Trung Nguyen [1 2], Eric Luong [1 2], Elizabeth H Kim [1 2], Eunice Park [7], Yunxian Liu [1 2], Ryan Rosenberry [1 2], Yuri Matusov [3 4], Steven Zhao [3 4], Isabel Pedraza [3 4], Tanzira Zaman [3 4], Michael Thompson [7], Koen Raedschelders [1 8], Anders H Berg [9], Jonathan D Grein [3 10], Paul W Noble [3 11], Sumeet S Chugh [1 2], C Noel Bairey Merz [1 2 12], Eduardo Marbán [2], Jennifer E Van Eyk [1 8 12], Scott D Solomon [6], Christine M Albert [1 2], Peter Chen [3 4 11], Susan Cheng [1 2 12]

Affiliations Expand

Affiliations

- [1] Department of Cardiology, Cedars-Sinai Medical Center, Los Angeles, California, United States of America.
- [2] Smidt Heart Institute, Cedars-Sinai Medical Center, Los Angeles, California, United States of America.
- [3] Department of Medicine, Cedars-Sinai Medical Center, Los Angeles, California, United States of America.
- [4] Division of Pulmonary and Critical Care Medicine, Cedars-Sinai Medical Center, Los Angeles, California, United States of America.
- [5] Shanghai Tenth People's Hospital, Tongji University, Shanghai, China.
- [6] Cardiovascular Division, Brigham and Women's Hospital, Boston, Massachusetts, United States of America.
- [7] Enterprise Information Systems Data Intelligence Team, Cedars-Sinai Medical Center, Los Angeles, California, United States of America.
- [8] Advanced Clinical Biosystems Institute, Cedars-Sinai Medical Center, Los Angeles, California, United States of America.
- [9] Department of Pathology and Laboratory Medicine, Cedars-Sinai Medical Center, Los Angeles, California, United States of America.
- [10] Department of Epidemiology, Cedars-Sinai Medical Center, Los Angeles, California, United States of America.
- [11] Women's Guild Lung Institute, Cedars-Sinai Medical Center, Los Angeles, California, United States of America.
- [12] Barbra Streisand Women's Heart Center, Cedars-Sinai Medical Center, Los Angeles, California, United States of America.

- PMID: **32702044**
- PMCID: PMC7377468
- DOI: 10.1371/journal.pone.0236240

Free PMC article
Observational Study

Pre-existing traits associated with Covid-19 illness severity

Joseph E Ebinger et al. PLoS One. 2020.
Free PMC article
Show details
PLoS One
. 2020 Jul 23;15(7):e0236240.
doi: 10.1371/journal.pone.0236240. eCollection 2020.

Authors

Joseph E Ebinger [1 2], Natalie Achamallah [3 4], Hongwei Ji [5 6], Brian L Claggett [6], Nancy Sun [1 2], Patrick Botting [1 2], Trevor-Trung Nguyen [1 2], Eric Luong [1 2], Elizabeth H Kim [1 2], Eunice Park [7], Yunxian Liu [1 2], Ryan Rosenberry [1 2], Yuri Matusov [3 4], Steven Zhao [3 4], Isabel Pedraza [3 4], Tanzira Zaman [3 4], Michael Thompson [7], Koen Raedschelders [1 8], Anders H Berg [9], Jonathan D Grein [3 10], Paul W Noble [3 11], Sumeet S Chugh [1 2], C Noel Bairey Merz [1 2 12], Eduardo Marbán [2], Jennifer E Van Eyk [1 8 12], Scott D Solomon [6], Christine M Albert [1 2], Peter Chen [3 4 11], Susan Cheng [1 2 12]

Affiliations

- [1] Department of Cardiology, Cedars-Sinai Medical Center, Los Angeles, California, United States of America.
- [2] Smidt Heart Institute, Cedars-Sinai Medical Center, Los Angeles, California, United States of America.
- [3] Department of Medicine, Cedars-Sinai Medical Center, Los Angeles, California, United States of America.
- [4] Division of Pulmonary and Critical Care Medicine, Cedars-Sinai Medical Center, Los Angeles, California, United States of America.
- [5] Shanghai Tenth People's Hospital, Tongji University, Shanghai, China.
- [6] Cardiovascular Division, Brigham and Women's Hospital, Boston, Massachusetts, United States of America.
- [7] Enterprise Information Systems Data Intelligence Team, Cedars-Sinai Medical Center, Los Angeles, California, United States of America.
- [8] Advanced Clinical Biosystems Institute, Cedars-Sinai Medical Center, Los Angeles, California, United States of America.
- [9] Department of Pathology and Laboratory Medicine, Cedars-Sinai Medical Center, Los Angeles, California, United States of America.
- [10] Department of Epidemiology, Cedars-Sinai Medical Center, Los Angeles, California, United States of America.
- [11] Women's Guild Lung Institute, Cedars-Sinai Medical Center, Los Angeles, California, United States of America.
- [12] Barbra Streisand Women's Heart Center, Cedars-Sinai Medical Center, Los Angeles, California, United States of America.

- PMID: **32702044**

- PMCID: PMC7377468
- DOI: 10.1371/journal.pone.0236240

Abstract

Importance: Certain individuals, when infected by SARS-CoV-2, tend to develop the more severe forms of Covid-19 illness for reasons that remain unclear.

Objective: To determine the demographic and clinical characteristics associated with increased severity of Covid-19 infection.

Design: Retrospective observational study. We curated data from the electronic health record, and used multivariable logistic regression to examine the association of pre-existing traits with a Covid-19 illness severity defined by level of required care: need for hospital admission, need for intensive care, and need for intubation.

Setting: A large, multihospital healthcare system in Southern California.

Participants: All patients with confirmed Covid-19 infection (N = 442).

Results: Of all patients studied, 48% required hospitalization, 17% required intensive care, and 12% required intubation. In multivariable-adjusted analyses, patients requiring a higher levels of care were more likely to be older (OR 1.5 per 10 years, P<0.001), male (OR 2.0, P = 0.001), African American (OR 2.1, P = 0.011), obese (OR 2.0, P = 0.021), with diabetes mellitus (OR 1.8, P = 0.037), and with a higher comorbidity index (OR 1.8 per SD, P<0.001). Several clinical associations were more pronounced in younger compared to older patients (Pinteraction<0.05). Of all hospitalized patients, males required higher levels of care (OR 2.5, P = 0.003) irrespective of age, race, or morbidity profile.

Conclusions and relevance: In our healthcare system, greater Covid-19 illness severity is seen in patients who are older, male, African American, obese, with diabetes, and with greater overall comorbidity burden. Certain comorbidities paradoxically augment risk to a greater extent in younger patients. In hospitalized patients, male sex is the main determinant of needing more intensive care. Further investigation is needed to understand the mechanisms underlying these findings.

Conflict of interest statement

The authors have declared that no competing interests exist.

- Cited by 59 articles
- 48 references
- 4 figures

Supplementary info

Publication types, MeSH terms, Grant support Expand

Publication types

- Observational Study

- Research Support, Non-U.S. Gov't

MeSH terms

- Adolescent
- Adult
- African Americans
- Age Factors
- Aged
- Aged, 80 and over
- Betacoronavirus
- COVID-19
- Child
- Comorbidity
- Coronavirus Infections / epidemiology*
- Critical Care / statistics & numerical data*
- Diabetes Mellitus
- Female
- Hospitalization / statistics & numerical data*
- Humans
- Los Angeles / epidemiology
- Male
- Middle Aged
- Obesity
- Pandemics
- Pneumonia, Viral / epidemiology*
- Retrospective Studies
- Risk Factors
- SARS-CoV-2
- Young Adult

Grant support

This work was supported in part by the Erika J. Glazer Family Foundation (JEE; JEVE; CNBM; SC). The funder had no role in study design, data collection and analysis, decision to publish, or preparation of the manuscript. There was no additional external funding received for this study.

Full text links

OPEN ACCESS TO FULL TEXT PLOS ONE Public Library of Science Free PMC article

Proceed to details

Cite

Share

☐ 235
Observational Study
Therapie
. Jul-Aug 2021;76(4):285-295.
doi: 10.1016/j.therap.2021.01.056. Epub 2021 Jan 28.

Pharmacokinetics and pharmacodynamics of hydroxychloroquine in hospitalized patients with COVID-19

Noël Zahr [1], Saik Urien [2], Benoit Llopis [3], Valérie Pourcher [4], Olivier Paccoud [4], Alexandre Bleibtreu [4], Julien Mayaux [5], Estelle Gandjbakhch [6], Guillaume Hekimian [7], Alain Combes [7], Olivier Benveniste [8], David Saadoun [8], Yves Allenbach [8], Bruno Pinna [3], Patrice Cacoub [8], Christian Funck-Brentano [3], Joe-Elie Salem [3]
Affiliations Expand

Affiliations

- [1] AP-HP, Sorbonne Université, Pitié-Salpêtrière Hospital, Department of Pharmacology and Clinical Investigation Center, INSERM, CIC-1901, Sorbonne Université, Faculty of Medicine, 75013 Paris, France. Electronic address: noel.zahr@aphp.fr.
- [2] AP-HP, Université de Paris, INSERM, Cochin Hospital, Department of Pediatric and Perinatal Pharmacology, 75014 Paris, France.
- [3] AP-HP, Sorbonne Université, Pitié-Salpêtrière Hospital, Department of Pharmacology and Clinical Investigation Center, INSERM, CIC-1901, Sorbonne Université, Faculty of Medicine, 75013 Paris, France.
- [4] AP-HP, Sorbonne Université, INSERM 1136, Institut Pierre-Louis d'Épidémiologie et de Santé Publique, Pitié-Salpêtrière Hospital, Service de Maladies Infectieuses et Tropicales, 75013 Paris, France.
- [5] AP-HP, Sorbonne Université, Service de Pneumologie, Médecine intensive - Réanimation (Département "R3S"), Groupe Hospitalier Universitaire Pitié-Salpêtrière-Charles-Foix, 75013 Paris, France.
- [6] AP-HP, Sorbonne Université, Service de Cardiologie, Groupe Hospitalier Universitaire Pitié-Salpêtrière-Charles-Foix, 75013 Paris, France.
- [7] AP-HP, Sorbonne Université, Médecine intensive-Réanimation Médicale Groupe Hospitalier Universitaire Pitié-Salpêtrière-Charles-Foix, 75013 Paris, France.
- [8] AP-HP, Sorbonne Université, Pitié-Salpêtrière Hospital, Department of Internal Medicine and Clinical Immunology, Centre de Référence des Maladies Auto-Immunes et Systémiques Rares, 75013 Paris, France.

- PMID: **33558079**
- PMCID: PMC7842207
- DOI: 10.1016/j.therap.2021.01.056

Free PMC article
Observational Study

Pharmacokinetics and pharmacodynamics of hydroxychloroquine in hospitalized patients with COVID-19

Noël Zahr et al. Therapie. Jul-Aug 2021.
Free PMC article
Show details
Therapie
. Jul-Aug 2021;76(4):285-295.
doi: 10.1016/j.therap.2021.01.056. Epub 2021 Jan 28.

Authors

Noël Zahr [1], Saik Urien [2], Benoit Llopis [3], Valérie Pourcher [4], Olivier Paccoud [4], Alexandre Bleibtreu [4], Julien Mayaux [5], Estelle Gandjbakhch [6], Guillaume Hekimian [7], Alain Combes [7], Olivier Benveniste [8], David Saadoun [8], Yves Allenbach [8], Bruno Pinna [3], Patrice Cacoub [8], Christian Funck-Brentano [3], Joe-Elie Salem [3]

Affiliations

- [1] AP-HP, Sorbonne Université, Pitié-Salpêtrière Hospital, Department of Pharmacology and Clinical Investigation Center, INSERM, CIC-1901, Sorbonne Université, Faculty of Medicine, 75013 Paris, France. Electronic address: noel.zahr@aphp.fr.
- [2] AP-HP, Université de Paris, INSERM, Cochin Hospital, Department of Pediatric and Perinatal Pharmacology, 75014 Paris, France.
- [3] AP-HP, Sorbonne Université, Pitié-Salpêtrière Hospital, Department of Pharmacology and Clinical Investigation Center, INSERM, CIC-1901, Sorbonne Université, Faculty of Medicine, 75013 Paris, France.
- [4] AP-HP, Sorbonne Université, INSERM 1136, Institut Pierre-Louis d'Épidémiologie et de Santé Publique, Pitié-Salpêtrière Hospital, Service de Maladies Infectieuses et Tropicales, 75013 Paris, France.
- [5] AP-HP, Sorbonne Université, Service de Pneumologie, Médecine intensive - Réanimation (Département "R3S"), Groupe Hospitalier Universitaire Pitié-Salpêtrière-Charles-Foix, 75013 Paris, France.
- [6] AP-HP, Sorbonne Université, Service de Cardiologie, Groupe Hospitalier Universitaire Pitié-Salpêtrière-Charles-Foix, 75013 Paris, France.
- [7] AP-HP, Sorbonne Université, Médecine intensive-Réanimation Médicale Groupe Hospitalier Universitaire Pitié-Salpêtrière-Charles-Foix, 75013 Paris, France.
- [8] AP-HP, Sorbonne Université, Pitié-Salpêtrière Hospital, Department of Internal Medicine and Clinical Immunology, Centre de Référence des Maladies Auto-Immunes et Systémiques Rares, 75013 Paris, France.

- PMID: **33558079**
- PMCID: PMC7842207
- DOI: 10.1016/j.therap.2021.01.056

Abstract

Background: Hydroxychloroquine (HCQ) dosage required to reach circulating levels that inhibit SARS-Cov-2 are extrapolated from pharmacokinetic data in non-COVID-19 patients.

Methods: We performed a population-pharmacokinetic analysis from 104 consecutive COVID-19 hospitalized patients (31 in intensive care units, 73 in medical wards, n=149 samples). Plasma HCQ concentration were measured using high performance liquid chromatography with fluorometric detection. Modelling used Monolix-2019R2.

Results: HCQ doses ranged from 200 to 800mg/day administered for 1 to 11days and median HCQ plasma concentration was 151ng/mL. Among the tested covariates, only bodyweight influenced elimination oral clearance (CL) and apparent volume of distribution (Vd). CL/F (F for unknown bioavailability) and Vd/F (relative standard-error, %) estimates were 45.9L/h (21.2) and 6690L (16.1). The derived elimination half-life (t1/2) was 102h. These parameters in COVID-19 differed from those reported in patients with lupus, where CL/F, Vd/F and t1/2 are reported to be 68L/h, 2440 L and 19.5h, respectively. Within 72h of HCQ initiation, only 16/104 (15.4%) COVID-19 patients had HCQ plasma levels above the in vitro half maximal effective concentration of HCQ against SARS-CoV-2 (240ng/mL). HCQ did not influence inflammation status (assessed by C-reactive protein) or SARS-CoV-2 viral clearance (assessed by real-time reverse transcription-PCR nasopharyngeal swabs).

Conclusion: The interindividual variability of HCQ pharmacokinetic parameters in severe COVID-19 patients was important and differed from that previously reported in non-COVID-19 patients. Loading doses of 1600mg HCQ followed by 600mg daily doses are needed to reach concentrations relevant to SARS-CoV-2 inhibition within 72hours in≥60% (95% confidence interval: 49.5-69.0%) of COVID-19 patients.

Keywords: COVID-19; Hydroxychloroquine; Pharmacodynamics; Pharmacokinetics.

Copyright © 2021 Société française de pharmacologie et de thérapeutique. Published by Elsevier Masson SAS. All rights reserved.

- Cited by 3 articles
- 42 references
- 6 figures

Supplementary info

Publication types, MeSH terms, Substances, Supplementary concepts Expand

Publication types

- Observational Study

MeSH terms

- Adult
- COVID-19 / drug therapy*
- COVID-19 / metabolism*

- Female
- Hospitalization / statistics & numerical data*
- Humans
- Hydroxychloroquine / pharmacokinetics*
- Male
- Middle Aged
- Retrospective Studies
- SARS-CoV-2

Substances

- Hydroxychloroquine

Supplementary concepts

- COVID-19 drug treatment

Full text links

Free PMC article
Proceed to details
Cite
Share
☐ 236
PLoS One
. 2021 Mar 22;16(3):e0248995.
doi: 10.1371/journal.pone.0248995. eCollection 2021.

Non-COVID-19 patients in times of pandemic: Emergency department visits, hospitalizations and cause-specific mortality in Northern Italy

Luca Santi [1], Davide Golinelli [2], Andrea Tampieri [3], Gabriele Farina [1], Manfredi Greco [2], Simona Rosa [2], Michelle Beleffi [4], Bianca Biavati [4], Francesca Campinoti [4], Stefania Guerrini [1], Rodolfo Ferrari [3], Paola Rucci [2], Maria Pia Fantini [2], Fabrizio Giostra [1]
Affiliations Expand

Affiliations

- [1] Department of Emergency, Medicina d'Urgenza e Pronto Soccorso, Policlinico S. Orsola-Malpighi, Bologna, Italy.

- [2] Department of Biomedical and Neuromotor Sciences (DIBINEM), Alma Mater Studiorum - University of Bologna, Bologna, Italy.
- [3] Department of Emergency, Medicina d'Urgenza e Pronto Soccorso. Ospedale S. Maria della Scaletta, Imola, Italy.
- [4] Emergency Medicine Specialization School, Alma Mater Studiorum - Università di Bologna, Bologna, Italy.

- PMID: **33750990**
- PMCID: PMC7984614
- DOI: 10.1371/journal.pone.0248995

Free PMC article

Non-COVID-19 patients in times of pandemic: Emergency department visits, hospitalizations and cause-specific mortality in Northern Italy

Luca Santi et al. PLoS One. 2021.
Free PMC article

Show details

PLoS One
. 2021 Mar 22;16(3):e0248995.
doi: 10.1371/journal.pone.0248995. eCollection 2021.

Authors

Luca Santi [1], Davide Golinelli [2], Andrea Tampieri [3], Gabriele Farina [1], Manfredi Greco [2], Simona Rosa [2], Michelle Beleffi [4], Bianca Biavati [4], Francesca Campinoti [4], Stefania Guerrini [1], Rodolfo Ferrari [3], Paola Rucci [2], Maria Pia Fantini [2], Fabrizio Giostra [1]

Affiliations

- [1] Department of Emergency, Medicina d'Urgenza e Pronto Soccorso, Policlinico S. Orsola-Malpighi, Bologna, Italy.
- [2] Department of Biomedical and Neuromotor Sciences (DIBINEM), Alma Mater Studiorum - University of Bologna, Bologna, Italy.
- [3] Department of Emergency, Medicina d'Urgenza e Pronto Soccorso. Ospedale S. Maria della Scaletta, Imola, Italy.
- [4] Emergency Medicine Specialization School, Alma Mater Studiorum - Università di Bologna, Bologna, Italy.

- PMID: **33750990**
- PMCID: PMC7984614
- DOI: 10.1371/journal.pone.0248995

Abstract

The COVID-19 pandemic forced healthcare services organization to adjust to mutating healthcare needs. Not exhaustive data are available on the consequences of this on non-COVID-19 patients. The aim of this study was to assess the impact of the pandemic on non-COVID-19 patients living in a one-million inhabitants' area in Northern Italy (Bologna Metropolitan Area-BMA), analyzing time trends of Emergency Department (ED) visits, hospitalizations and mortality. We conducted a retrospective observational study using data extracted from BMA healthcare informative systems. Weekly trends of ED visits, hospitalizations, in- and out-of-hospital, all-cause and cause-specific mortality between December 1st, 2019 to May 31st, 2020, were compared with those of the same period of the previous year. Non-COVID-19 ED visits and hospitalizations showed a stable trend until the first Italian case of COVID-19 has been recorded, on February 19th, 2020, when they dropped simultaneously. The reduction of ED visits was observed in all age groups and across all severity and diagnosis groups. In the lockdown period a significant increase was found in overall out-of-hospital mortality (43.2%) and cause-specific out-of-hospital mortality related to neoplasms (76.7%), endocrine, nutritional and metabolic (79.5%) as well as cardiovascular (32.7%) diseases. The pandemic caused a sudden drop of ED visits and hospitalizations of non-COVID-19 patients during the lockdown period, and a concurrent increase in out-of-hospital mortality mainly driven by deaths for neoplasms, cardiovascular and endocrine diseases. As recurrencies of the COVID-19 pandemic are underway, the scenario described in this study might be useful to understand both the population reaction and the healthcare system response at the early phases of the pandemic in terms of reduced demand of care and systems capability in intercepting it.

Conflict of interest statement

The authors have declared that no competing interests exist.

- Cited by 23 articles
- 34 references
- 3 figures

Supplementary info

MeSH terms, Grant support Expand

MeSH terms

- COVID-19 / epidemiology
- COVID-19 / pathology
- COVID-19 / virology
- Cardiovascular Diseases / mortality
- Cardiovascular Diseases / pathology
- Cause of Death*
- Emergency Service, Hospital / statistics & numerical data*
- Hospitalization / statistics & numerical data*
- Humans
- Italy / epidemiology
- Metabolic Diseases / mortality

- Metabolic Diseases / pathology
- Neoplasms / mortality
- Neoplasms / pathology
- Pandemics
- Quarantine
- Retrospective Studies
- SARS-CoV-2 / isolation & purification

Grant support

The authors received no specific funding for this work.

Full text links

OPEN ACCESS TO FULL TEXT PLOS ONE Public Library of Science Free PMC article

Proceed to details

Cite

Share

237

Observational Study

J Neurol Sci

. 2020 Dec 15;419:117163.

doi: 10.1016/j.jns.2020.117163. Epub 2020 Oct 1.

Anosmia is associated with lower in-hospital mortality in COVID-19

Blanca Talavera [1], David García-Azorín [2], Enrique Martínez-Pías [1], Javier Trigo [1], Isabel Hernández-Pérez [1], Gonzalo Valle-Peñacoba [1], Paula Simón-Campo [1], Mercedes de Lera [1], Alba Chavarría-Miranda [1], Cristina López-Sanz [1], María Gutiérrez-Sánchez [1], Elena Martínez-Velasco [1], María Pedraza [1], Álvaro Sierra [1], Beatriz Gómez-Vicente [1], Ángel Guerrero [3], Juan Francisco Arenillas [4]

Affiliations Expand

Affiliations

- [1] Department of Neurology, Hospital Clínico Universitario de Valladolid, Av Ramón y Cajal 3, 47003 Valladolid, Spain.
- [2] Department of Neurology, Hospital Clínico Universitario de Valladolid, Av Ramón y Cajal 3, 47003 Valladolid, Spain. Electronic address: dgazorin@ucm.es.
- [3] Department of Neurology, Hospital Clínico Universitario de Valladolid, Av Ramón y Cajal 3, 47003 Valladolid, Spain; Department of Medicine, University of Valladolid, Av Ramón y Cajal 7, 47005 Valladolid, Spain.

- [4] Department of Neurology, Hospital Clínico Universitario de Valladolid, Av Ramón y Cajal 3, 47003 Valladolid, Spain; Department of Medicine, University of Valladolid, Av Ramón y Cajal 7, 47005 Valladolid, Spain; Neurovascular Research Laboratory, Instituto de Biología y Genética Molecular, Universidad de Valladolid, Sanz y Fores St 3, 47003, Valladolid, Consejo Superior de Investigaciones Científicas, Madrid, Spain.

- PMID: **33035870**
- PMCID: PMC7527278
- DOI: 10.1016/j.jns.2020.117163

Free PMC article
Observational Study

Anosmia is associated with lower in-hospital mortality in COVID-19

Blanca Talavera et al. J Neurol Sci. 2020.
Free PMC article

Show details

J Neurol Sci

. 2020 Dec 15;419:117163.
doi: 10.1016/j.jns.2020.117163. Epub 2020 Oct 1.

Authors

Blanca Talavera [1], David García-Azorín [2], Enrique Martínez-Pías [1], Javier Trigo [1], Isabel Hernández-Pérez [1], Gonzalo Valle-Peñacoba [1], Paula Simón-Campo [1], Mercedes de Lera [1], Alba Chavarría-Miranda [1], Cristina López-Sanz [1], María Gutiérrez-Sánchez [1], Elena Martínez-Velasco [1], María Pedraza [1], Álvaro Sierra [1], Beatriz Gómez-Vicente [1], Ángel Guerrero [3], Juan Francisco Arenillas [4]

Affiliations

- [1] Department of Neurology, Hospital Clínico Universitario de Valladolid, Av Ramón y Cajal 3, 47003 Valladolid, Spain.
- [2] Department of Neurology, Hospital Clínico Universitario de Valladolid, Av Ramón y Cajal 3, 47003 Valladolid, Spain. Electronic address: dgazorin@ucm.es.
- [3] Department of Neurology, Hospital Clínico Universitario de Valladolid, Av Ramón y Cajal 3, 47003 Valladolid, Spain; Department of Medicine, University of Valladolid, Av Ramón y Cajal 7, 47005 Valladolid, Spain.
- [4] Department of Neurology, Hospital Clínico Universitario de Valladolid, Av Ramón y Cajal 3, 47003 Valladolid, Spain; Department of Medicine, University of Valladolid, Av Ramón y Cajal 7, 47005 Valladolid, Spain; Neurovascular Research Laboratory, Instituto de Biología y Genética Molecular, Universidad de Valladolid, Sanz y Fores St 3, 47003, Valladolid, Consejo Superior de Investigaciones Científicas, Madrid, Spain.

- PMID: **33035870**
- PMCID: PMC7527278

• DOI: 10.1016/j.jns.2020.117163

Abstract

Background: Anosmia is common in Coronavirus disease 2019, but its impact on prognosis is unknown. We analysed whether anosmia predicts in-hospital mortality; and if patients with anosmia have a different clinical presentation, inflammatory response, or disease severity.

Methods: Retrospective cohort study including all consecutive hospitalized patients with confirmed Covid-19 from March 8th to April 11th, 2020. We determined all-cause mortality and need of intensive care unit (ICU) admission. We registered the first and worst laboratory parameters. Statistical analysis was done by multivariate logistic and linear regression.

Results: We included 576 patients, 43.3% female, and aged 67.2 years in mean. Anosmia was present in 146 (25.3%) patients. Patients with anosmia were more frequently females, younger and less disabled and had less frequently hypertension, diabetes, smoking habit, cardiac and neurological comorbidities. Anosmia was independently associated with lower mortality (OR: 0.180, 95% CI: 0.069-0.472) and ICU admission (OR: 0.438, 95% CI: 0.229-0.838, p = 0.013). In the multivariate analysis, patients with anosmia had a higher frequency of cough (OR: 1.96, 95% CI: 1.18-3.28), headache (OR: 2.58, 95% CI: 1.66-4.03), and myalgia (OR: 1.74, 95% CI: 1.12-2.71). They had higher adjusted values of hemoglobin (+0.87, 95% CI: 0.40-1.34), lymphocytes (+849.24, 95% CI: 157.45-1541.04), glomerular filtration rate (+6.42, 95% CI: 2.14-10.71), and lower D-dimer (-4886.52, 95% CI: -8655.29-(-1117.75)), and C-reactive protein (-24.92, 95% CI: -47.35-(-2.48)).

Conclusions: Hospitalized Covid-19 patients with anosmia had a lower adjusted mortality rate and less severe course of the disease. This could be related to a distinct clinical presentation and a different inflammatory response.

Keywords: Anosmia; COVID-19; Clinical presentation; Mortality; Prognosis.

Copyright © 2020 Elsevier B.V. All rights reserved.

Conflict of interest statement

None.

- Cited by 27 articles
- 37 references
- 1 figure

Supplementary info

Publication types, MeSH terms, Supplementary concepts Expand

Publication types

- Observational Study

MeSH terms

- Aged
- Anosmia / etiology*
- COVID-19 / complications
- COVID-19 / diagnosis
- COVID-19 / drug therapy
- COVID-19 / mortality*
- COVID-19 / therapy
- COVID-19 Testing
- Comorbidity
- Female
- Hospital Mortality
- Hospitalization
- Humans
- Intensive Care Units / statistics & numerical data
- Male
- Middle Aged
- Regression Analysis
- Retrospective Studies

Supplementary concepts

- COVID-19 drug treatment

Full text links

ELSEVIER FULL-TEXT ARTICLE Elsevier Science Free PMC article
Proceed to details
Cite
Share
☐ 238
Observational Study
Viruses
. 2021 Oct 25;13(11):2151.
doi: 10.3390/v13112151.

COVEVOL: Natural Evolution at 6 Months of COVID-19

Louise Messin [1], Marc Puyraveau [2], Yousri Benabdallah [3], Quentin Lepiller [4], Vincent Gendrin [1], Souheil Zayet [1], Timothée Klopfenstein [1], Lynda Toko [1], Alix Pierron [1], Pierre-Yves Royer [1]
Affiliations Expand

Affiliations

- [1] Infectious Disease Department, Nord Franche-Comté Hospital, 90400 Trevenans, France.
- [2] Clinical Investigation Center, Jean Minjoz University Hospital of Besançon, 25000 Besancon, France.
- [3] Department of Pneumology, Nord Franche-Comté Hospital, 90400 Trevenans, France.
- [4] Virology Department, Jean Minjoz University Hospital of Besançon, 25000 Besancon, France.

- PMID: **34834958**
- PMCID: PMC8619893
- DOI: 10.3390/v13112151

Free PMC article
Observational Study

COVEVOL: Natural Evolution at 6 Months of COVID-19

Louise Messin et al. Viruses. 2021.
Free PMC article
Show details
Viruses
. 2021 Oct 25;13(11):2151.
doi: 10.3390/v13112151.

Authors

Louise Messin [1], Marc Puyraveau [2], Yousri Benabdallah [3], Quentin Lepiller [4], Vincent Gendrin [1], Souheil Zayet [1], Timothée Klopfenstein [1], Lynda Toko [1], Alix Pierron [1], Pierre-Yves Royer [1]

Affiliations

- [1] Infectious Disease Department, Nord Franche-Comté Hospital, 90400 Trevenans, France.
- [2] Clinical Investigation Center, Jean Minjoz University Hospital of Besançon, 25000 Besancon, France.
- [3] Department of Pneumology, Nord Franche-Comté Hospital, 90400 Trevenans, France.
- [4] Virology Department, Jean Minjoz University Hospital of Besançon, 25000 Besancon, France.

- PMID: **34834958**
- PMCID: PMC8619893
- DOI: 10.3390/v13112151

Abstract

Many studies have investigated post-COVID symptoms, but the predictors of symptom persistence remain unknown. The objective was to describe the natural course of the disease at 6 months and to identify possible factors favoring the resurgence or persistence of these symptoms. COVEVOL is a retrospective observational descriptive study of 74 patients. All patients with positive SARS-CoV-2 PCR from March 2020 were included. We compared a group with symptom persistence (PS group) with another group without symptom persistence (no-PS group). Fifty-three out of seventy-four patients (71.62%) described at least one persistent symptom at 6 months of SARS-CoV-2 infection. In the PS group, 56.6% were women and the average age was 54.7 years old [21-89.2] ± 16.9. The main symptoms were asthenia (56.6%, $n = 30$), dyspnea (34%, $n = 18$), anxiety (32.1% $n = 17$), anosmia (24.5%, $n = 13$) and agueusia (15.1% $n = 8$). Ten patients (13.51%) presented a resurgence in symptoms. Patients in the PS group were older ($p = 0.0048$), had a higher BMI ($p = 0.0071$), and were more frequently hospitalized ($p = 0.0359$) compared to the no-PS group. Odynophagia and nasal obstruction were less present in the inaugural symptoms of COVID-19 in the PS group ($p = 0.0202$ and $p = 0.0332$). Persistent post-COVID syndromes are common and identification of contributing factors is necessary for understanding this phenomenon and appropriate management.

Keywords: anosmia; anxiety; asthenia; dyspnea; persistent symptoms; post-COVID-19; risk factors; “long COVID”.

Conflict of interest statement

The authors declare no conflict of interest.

- 52 references
- 2 figures

Supplementary info

Publication types, MeSH terms, Supplementary concepts Expand

Publication types

- Observational Study

MeSH terms

- Adult
- Age Factors
- Aged
- Aged, 80 and over
- Body Mass Index
- COVID-19 / complications*
- COVID-19 / epidemiology
- COVID-19 / physiopathology*
- Chronic Disease
- Female
- Hospitalization

- Humans
- Male
- Middle Aged
- Retrospective Studies
- Young Adult

Supplementary concepts

- post-acute COVID-19 syndrome

Full text links

Multidisciplinary Digital Publishing Institute (MDPI) Free PMC article
Proceed to details
Cite
Share
239
Observational Study
Respirology
. 2021 Feb;26(2):204-205.
doi: 10.1111/resp.13985. Epub 2020 Dec 6.

Comparisons of early and late presentation to hospital in COVID-19 patients

Sarah Williams[#, 1], Natasha Sheard[#, 1], Beth Stuart[2], Hang T T Phan[3, 4], Florina Borca[1, 4], Tom M A Wilkinson[1, 5], Hannah Burke[#, 1, 5], Anna Freeman[#, 1, 5], REACT COVID Investigators
Affiliations Expand

Affiliations

- [1] University Hospital Southampton NHS Foundation Trust, Southampton, UK.
- [2] Southampton Clinical Trials Unit, University Hospital Southampton NHS Foundation Trust, Southampton, UK.
- [3] NIHR Southampton Biomedical Research Centre, University Hospital Southampton NHS Foundation Trust, Southampton, UK.
- [4] Clinical Informatics Research Unit, Faculty of Medicine, University of Southampton, Southampton, UK.
- [5] School of Clinical and Experimental Sciences, Faculty of Medicine, University of Southampton, Southampton, UK.

[#] Contributed equally.

- PMID: **33283433**

- DOI: 10.1111/resp.13985

Free article
Observational Study

Comparisons of early and late presentation to hospital in COVID-19 patients

Sarah Williams et al. Respirology. 2021 Feb.
Free article
Show details
Respirology
. 2021 Feb;26(2):204-205.
doi: 10.1111/resp.13985. Epub 2020 Dec 6.

Authors

Sarah Williams [#,1], Natasha Sheard [#,1], Beth Stuart [2], Hang T T Phan [3,4], Florina Borca [1,4], Tom M A Wilkinson [1,5], Hannah Burke [#,1,5], Anna Freeman [#,1,5], REACT COVID Investigators

Affiliations

- [1] University Hospital Southampton NHS Foundation Trust, Southampton, UK.
- [2] Southampton Clinical Trials Unit, University Hospital Southampton NHS Foundation Trust, Southampton, UK.
- [3] NIHR Southampton Biomedical Research Centre, University Hospital Southampton NHS Foundation Trust, Southampton, UK.
- [4] Clinical Informatics Research Unit, Faculty of Medicine, University of Southampton, Southampton, UK.
- [5] School of Clinical and Experimental Sciences, Faculty of Medicine, University of Southampton, Southampton, UK.

[#] Contributed equally.

- PMID: **33283433**
- DOI: 10.1111/resp.13985

No abstract available

- Cited by 2 articles
- 4 references

Supplementary info

Publication types, MeSH terms Expand

Publication types

- Letter
- Observational Study

MeSH terms

- COVID-19 / complications*
- COVID-19 / diagnostic imaging*
- Hospitals
- Humans
- Patient Acceptance of Health Care
- Prognosis
- Retrospective Studies
- SARS-CoV-2
- Severity of Illness Index
- Time Factors
- Time-to-Treatment*

Full text links

WILEY Free Full Text Wiley

Proceed to details

Cite

Share

☐ 240

Observational Study

Eur Rev Med Pharmacol Sci
. 2021 Apr;25(8):3377-3385.
doi: 10.26355/eurrev_202104_25750.

Mortality and critical conditions in COVID-19 patients at private hospitals: weekend effect?

J González-Gancedo [1], I Morales-Cané, P M Rodríguez-Muñoz, P Hidalgo-Lopezosa, M Del Rocío Valverde-León, M E Fernández-Martínez, F Fabbian, M A Rodríguez-Borrego, P J López-Soto
Affiliations Expand

Affiliation

- [1] Department of Nursing, Instituto Maimónides de Investigación Biomédica de Córdoba (IMIBIC), Córdoba, Spain. n82mocai@uco.es.

- PMID: **33928626**
- DOI: 10.26355/eurrev_202104_25750

Free article
Observational Study

Mortality and critical conditions in COVID-19 patients at private hospitals: weekend effect?

J González-Gancedo et al. Eur Rev Med Pharmacol Sci. 2021 Apr.
Free article
Show details
Eur Rev Med Pharmacol Sci
. 2021 Apr;25(8):3377-3385.
doi: 10.26355/eurrev_202104_25750.

Authors

J González-Gancedo [1], I Morales-Cané, P M Rodríguez-Muñoz, P Hidalgo-Lopezosa, M Del Rocío Valverde-León, M E Fernández-Martínez, F Fabbian, M A Rodríguez-Borrego, P J López-Soto

Affiliation

- [1] Department of Nursing, Instituto Maimónides de Investigación Biomédica de Córdoba (IMIBIC), Córdoba, Spain. n82mocai@uco.es.

- PMID: **33928626**
- DOI: 10.26355/eurrev_202104_25750

Abstract

Objective: The aim of the study was to find factors associated with the mortality of admission to the intensive care unit (ICU) in patients with COVID-19.

Materials and methods: Retrospective observational study with a database of 1987 patients with COVID-19 who had attended the emergency department of a private hospital network between February 2020 and April 2020 were analyzed. Clinical variables and some laboratory parameters were studied. The Charlson and Elixhauser comorbidity indices were calculated. The dependent variables were mortality and admission to the ICU. A descriptive and correlational analysis was performed. Logistic regression models and Kaplan-Meier survival curves were established.

Results: Positive correlations were observed between age, creatinine, and D-dimer levels, as well as with the scores obtained with the Charlson and Elixhauser indices. Differences in the levels of

these parameters were also observed when analyzing variables such as mortality, sex or admission to the ICU. Mortality was associated with high creatinine and D-dimer levels and advanced age. Survival curves indicated longer survival in patients not admitted to the ICU, admitted to the hospital during the week, and in those with lower creatinine and D-dimer levels.

Conclusions: Mortality in Spanish patients with COVID-19 admitted to private hospitals was associated with high creatinine and D-dimer levels and advanced age. Longer survival was obtained on weekdays. This study provides valuable information on the management and nursing care of these patients in order to optimize resources in pandemic situations.

- Cited by 1 article

Supplementary info

Publication types, MeSH terms, Substances Expand

Publication types

- Observational Study

MeSH terms

- After-Hours Care / statistics & numerical data
- Age Factors
- Aged
- Aged, 80 and over
- COVID-19 / metabolism
- COVID-19 / mortality
- COVID-19 / physiopathology*
- Comorbidity
- Creatinine / metabolism*
- Critical Illness
- Emergency Service, Hospital
- Female
- Fibrin Fibrinogen Degradation Products / metabolism*
- Hospital Mortality*
- Hospitalization
- Hospitals, Private
- Humans
- Intensive Care Units / statistics & numerical data*
- Kaplan-Meier Estimate
- Logistic Models
- Male
- Middle Aged
- Retrospective Studies

- SARS-CoV-2
- Spain
- Time Factors

Substances

- Fibrin Fibrinogen Degradation Products
- fibrin fragment D
- Creatinine

Full text links

FREE FULL TEXT European Review for Medical and Pharmacological Sciences
Proceed to details
Cite
Share
241
Observational Study
Diagn Microbiol Infect Dis
. 2021 Nov;101(3):115477.
doi: 10.1016/j.diagmicrobio.2021.115477. Epub 2021 Jul 3.

Bacterial co-infections in COVID-19 pneumonia in a tertiary care hospital: Surfing the first wave

Mario Ruiz-Bastián [1], Iker Falces-Romero [2], Juan Carlos Ramos-Ramos [3], Manuela de Pablos [2], Julio García-Rodríguez [2], SARS-CoV-2 Working Group [2]
Affiliations Expand

Affiliations

- [1] Clinical Microbiology and Parasitology Department, Hospital Universitario La Paz, Madrid, Spain. Electronic address: mario.ruiz.bastian@gmail.com.
- [2] Clinical Microbiology and Parasitology Department, Hospital Universitario La Paz, Madrid, Spain.
- [3] Infectious Disease Unit, Hospital Universitario La Paz, Madrid, Spain.

- PMID: **34358876**
- PMCID: PMC8254382
- DOI: 10.1016/j.diagmicrobio.2021.115477

Free PMC article
Observational Study

Bacterial co-infections in COVID-19 pneumonia in a tertiary care hospital: Surfing the first wave

Mario Ruiz-Bastián et al. Diagn Microbiol Infect Dis. 2021 Nov.
Free PMC article
Show details
Diagn Microbiol Infect Dis
. 2021 Nov;101(3):115477.
doi: 10.1016/j.diagmicrobio.2021.115477. Epub 2021 Jul 3.

Authors

Mario Ruiz-Bastián [1], Iker Falces-Romero [2], Juan Carlos Ramos-Ramos [3], Manuela de Pablos [2], Julio García-Rodríguez [2], SARS-CoV-2 Working Group [2]

Affiliations

- [1] Clinical Microbiology and Parasitology Department, Hospital Universitario La Paz, Madrid, Spain. Electronic address: mario.ruiz.bastian@gmail.com.
- [2] Clinical Microbiology and Parasitology Department, Hospital Universitario La Paz, Madrid, Spain.
- [3] Infectious Disease Unit, Hospital Universitario La Paz, Madrid, Spain.

- PMID: **34358876**
- PMCID: PMC8254382
- DOI: 10.1016/j.diagmicrobio.2021.115477

Abstract

The aim of this study is to review bacterial isolates from respiratory samples of patients with severe COVID-19 disease during the first 2 months of the first wave in our hospital. A single-center retrospective observational study in critically ill adult patients was performed. A total of 1251 respiratory samples from 1195 patients were processed. Samples from 66 patients (5.52%) were determined to be microbiologically significant by a semi-quantitative culture. All patients received broad spectrum antibiotherapy as an empirical treatment. The isolated bacteria were mainly Enterobacterales followed by Staphylococcus aureus and Pseudomonas aeruginosa. Bacterial co-infections in ICU stay could seem not dependent on the virus that has produced the viral pneumonia similarly as with other respiratory viruses such as Influenza virus.

Keywords: COVID-19; Co-infection; Pneumonia; Respiratory samples.

Copyright © 2021. Published by Elsevier Inc.

Conflict of interest statement

Declaration of competing interest None.

- Cited by 1 article
- 16 references

Supplementary info

Publication types, MeSH terms, Substances Expand

Publication types

- Observational Study

MeSH terms

- Aged
- Aged, 80 and over
- Anti-Bacterial Agents / therapeutic use
- COVID-19 / complications*
- Coinfection / diagnosis*
- Cross Infection / drug therapy
- Cross Infection / microbiology
- Female
- Humans
- Intensive Care Units
- Male
- Middle Aged
- Pneumonia, Bacterial / complications*
- Retrospective Studies
- Risk Factors
- SARS-CoV-2
- Tertiary Care Centers*

Substances

- Anti-Bacterial Agents

Full text links

ELSEVIER FULL-TEXT ARTICLE Elsevier Science Free PMC article
Proceed to details
Cite
Share

☐ 242
Observational Study

Eur Geriatr Med
. 2021 Dec;12(6):1169-1180.
doi: 10.1007/s41999-021-00541-0. Epub 2021 Jul 21.

Mortality risk factors in a Spanish cohort of oldest-old patients hospitalized with COVID-19 in an acute geriatric unit: the OCTA-COVID study

Isabel Lozano-Montoya [1, 2], Maribel Quezada-Feijoo [3, 4], Javier Jaramillo-Hidalgo [5, 3], Blanca Garmendia-Prieto [5, 3], Pamela Lisette-Carrillo [5, 3], Francisco J Gómez-Pavón [5, 3]
Affiliations Expand

Affiliations

- [1] Servicio de Geriatría, Hospital Central de la Cruz Roja San José y Santa Adela, C/Reina Victoria, 24, 28003, Madrid, Spain. ilozanom@salud.madrid.org.
- [2] Facultad de Medicina, Universidad Alfonso X el Sabio, Avda. de La Universidad, 1, Villanueva de la Cañada, 28691, Madrid, Spain. ilozanom@salud.madrid.org.
- [3] Facultad de Medicina, Universidad Alfonso X el Sabio, Avda. de La Universidad, 1, Villanueva de la Cañada, 28691, Madrid, Spain.
- [4] Servicio de Cardiología, Hospital Central de la Cruz Roja San José y Santa Adela, C/Reina Victoria, 24, 28003, Madrid, Spain.
- [5] Servicio de Geriatría, Hospital Central de la Cruz Roja San José y Santa Adela, C/Reina Victoria, 24, 28003, Madrid, Spain.

- PMID: **34287813**
- PMCID: PMC8294271
- DOI: 10.1007/s41999-021-00541-0

Free PMC article
Observational Study

Mortality risk factors in a Spanish cohort of oldest-old patients hospitalized with COVID-19 in an acute geriatric unit: the OCTA-COVID study

Isabel Lozano-Montoya et al. Eur Geriatr Med. 2021 Dec.
Free PMC article
Show details
Eur Geriatr Med

. 2021 Dec;12(6):1169-1180.
doi: 10.1007/s41999-021-00541-0. Epub 2021 Jul 21.

Authors

Isabel Lozano-Montoya [1] [2], Maribel Quezada-Feijoo [3] [4], Javier Jaramillo-Hidalgo [5] [3], Blanca Garmendia-Prieto [5] [3], Pamela Lisette-Carrillo [5] [3], Francisco J Gómez-Pavón [5] [3]

Affiliations

- [1] Servicio de Geriatría, Hospital Central de la Cruz Roja San José y Santa Adela, C/Reina Victoria, 24, 28003, Madrid, Spain. ilozanom@salud.madrid.org.
- [2] Facultad de Medicina, Universidad Alfonso X el Sabio, Avda. de La Universidad, 1, Villanueva de la Cañada, 28691, Madrid, Spain. ilozanom@salud.madrid.org.
- [3] Facultad de Medicina, Universidad Alfonso X el Sabio, Avda. de La Universidad, 1, Villanueva de la Cañada, 28691, Madrid, Spain.
- [4] Servicio de Cardiología, Hospital Central de la Cruz Roja San José y Santa Adela, C/Reina Victoria, 24, 28003, Madrid, Spain.
- [5] Servicio de Geriatría, Hospital Central de la Cruz Roja San José y Santa Adela, C/Reina Victoria, 24, 28003, Madrid, Spain.

- PMID: **34287813**
- PMCID: PMC8294271
- DOI: 10.1007/s41999-021-00541-0

Abstract

Purpose: To determine predictors of in-hospital mortality related to COVID-19 in oldest-old patients.

Design: Single-center observational study.

Setting and participants: Patients ≥ 75 years admitted to an Acute Geriatric Unit with COVID-19.

Methods: Data from hospital admission were retrieved from the electronic medical records: demographics, geriatric syndromes (delirium, falls, polypharmacy, functional and cognitive status) co-morbidities, previous treatments, clinical, laboratory, and radiographic characteristics. Cox proportional hazard models were used to evaluate in-hospital mortality.

Results: Three hundred patients were consecutively included (62.7% females, mean age of 86.3 ± 6.6 years). Barthel Index (BI) was < 60 in 127 patients (42.8%) and 126 (42.0%) had Charlson Index CI ≥ 3. Most patients (216; 72.7%) were frail (Clinical Frailty Scale ≥ 5) and 134 patients (45.1%) had dementia of some degree. The overall in-hospital mortality rate was 37%. The following factors were associated with higher in-hospital mortality in a multi-variant analysis: CURB-65 score = 3-5 (HR 7.99, 95% CI 3.55-19.96, p < 0.001), incident delirium (HR 1.72, 1.10-2.70, p = 0.017) and dementia (HR 3.01, 95% CI 1.37-6.705, p = 0.017). Protective factors were concurrent use of angiotensin-converting enzyme inhibitors (HR 0.42, 95% CI 0.25-0.72, p = 0.002) or prescription of hydroxychloroquine (HC 0.37 95% CI 0.22-0.62, p < 0.001) treatment during admission.

Conclusions and implications: Our findings suggest that recognition of geriatric syndromes together with the CURB-65 score may be useful tools to help clinicians establish the prognosis of oldest-old patients admitted to hospital with COVID-19.

Keywords: Covid-19; Mortality; Older adults; Risk factors; SARS-COV-2.

© 2021. European Geriatric Medicine Society.

Conflict of interest statement

The authors have declared no conflict of interest for this article and no financial conflicts.

- Cited by 4 articles
- 45 references
- 2 figures

Supplementary info

Publication types, MeSH terms Expand

Publication types

- Observational Study

MeSH terms

- Aged
- Aged, 80 and over
- COVID-19*
- Female
- Hospitalization
- Humans
- Male
- Retrospective Studies
- Risk Factors
- SARS-CoV-2

Full text links

Free PMC article
Proceed to details
Cite
Share
243
Observational Study
Int Heart J
. 2021 Mar 30;62(2):337-343.

doi: 10.1536/ihj.20-323. Epub 2021 Mar 6.

Hypertension in Patients Hospitalized with COVID-19 in Wuhan, China

Yuan Zhang [1], Tong Sha [1], Feng Wu [2], Hongbin Hu [2], Zhongqing Chen [1], Haijun Li [3], Jiafa Han [3], Wenhong Song [3], Qiaobing Huang [2], Zhenhua Zeng [1]
Affiliations Expand

Affiliations

- [1] Department of Critical Care Medicine, Nanfang Hospital, Southern Medical University.
- [2] Guangdong Provincial Key Laboratory of Shock and Microcirculation, School of Basic Medical Sciences, Southern Medical University.
- [3] Department of Radiology, Hankou Hospital of Wuhan.

- PMID: **33678794**
- DOI: 10.1536/ihj.20-323

Free article
Observational Study

Hypertension in Patients Hospitalized with COVID-19 in Wuhan, China

Yuan Zhang et al. Int Heart J. 2021.
Free article
Show details
Int Heart J
. 2021 Mar 30;62(2):337-343.
doi: 10.1536/ihj.20-323. Epub 2021 Mar 6.

Authors

Yuan Zhang [1], Tong Sha [1], Feng Wu [2], Hongbin Hu [2], Zhongqing Chen [1], Haijun Li [3], Jiafa Han [3], Wenhong Song [3], Qiaobing Huang [2], Zhenhua Zeng [1]

Affiliations

- [1] Department of Critical Care Medicine, Nanfang Hospital, Southern Medical University.
- [2] Guangdong Provincial Key Laboratory of Shock and Microcirculation, School of Basic Medical Sciences, Southern Medical University.
- [3] Department of Radiology, Hankou Hospital of Wuhan.

- PMID: **33678794**
- DOI: 10.1536/ihj.20-323

Abstract

It is unclear whether patients with hypertension are more likely to be infected with severe acute respiratory syndrome coronavirus 2 (SARS-CoV-2) than the general population and whether there is a difference in the severity of coronavirus disease (COVID-19) pneumonia in patients who have taken ACEI/ARB drugs compared with those who have not.This observational study included data from all patients with clinically confirmed COVID-19 admitted to Hankou Hospital, Wuhan, China, between January 5 and March 8, 2020. Data were extracted from clinical and laboratory records. Follow-up was cut off on March 8, 2020.A total of 274 patients, 75 with hypertension and 199 without hypertension, were included in the analysis. Compared with patients without hypertension, patients with hypertension were older and were more likely to have preexisting comorbidities, including chronic renal insufficiency, cardiovascular disease, diabetes mellitus, and cerebrovascular disease. Moreover, patients with hypertension tended to have higher positive rate for SARS-CoV-2 PCR detection. Multivariate logistic regression analysis showed that age (P = 0.005) and gender (P = 0.019) were independent risk factors associated with the severity of pneumonia in patients on admission, whereas ACEI/ARB treatment (P = 0.184) was not.Patients with COVID-19 with hypertension were significantly older and were more likely to have underlying comorbidities, including chronic renal insufficiency, cardiovascular disease, diabetes mellitus, and cerebrovascular disease. ACEI/ARB drugs did not influence the severity of pneumonia in patients with SARS-CoV-2. In future studies, a larger sample size and multi-center clinical data would be needed to support these conclusions.

Keywords: ACEI/ARB; Angiotensin-converting enzyme 2; ICU; Pneumonia.

- Cited by 1 article

Supplementary info

Publication types, MeSH terms, Substances Expand

Publication types

- Observational Study

MeSH terms

- Adult
- Age Factors
- Aged
- Angiotensin Receptor Antagonists / therapeutic use
- Angiotensin-Converting Enzyme Inhibitors / therapeutic use
- Antihypertensive Agents / therapeutic use
- COVID-19 / complications
- COVID-19 / diagnosis
- COVID-19 / epidemiology*
- China
- Female

- Hospitalization*
- Humans
- Hypertension / complications*
- Hypertension / drug therapy*
- Logistic Models
- Male
- Middle Aged
- Retrospective Studies
- Risk Factors
- Sex Factors

Substances

- Angiotensin Receptor Antagonists
- Angiotensin-Converting Enzyme Inhibitors
- Antihypertensive Agents

Full text links

J-STAGE FREE J-STAGE, Japan Science and Technology Information Aggregator, Electronic

Proceed to details

Cite

Share

☐ 244

Observational Study

Trop Med Int Health

. 2021 Apr;26(4):444-452.

doi: 10.1111/tmi.13542. Epub 2021 Jan 12.

Differential diagnoses of severe COVID-19 in tropical areas: the experience of Reunion Island

Pauline Krol [1], Nathalie Coolen-Allou [2], Laura Teysseyre [1], Nicolas Traversier [3], Floryan Beasley [1], Mathilde Nativel [1], Nicolas Allou [1] [4], Jerome Allyn [1] [4]

Affiliations Expand

Affiliations

- [1] Réanimation Polyvalente, Centre Hospitalier Universitaire Félix Guyon, La Réunion, France.

- [2] Service de Pneumologie, Centre Hospitalier Universitaire Félix Guyon, La Réunion, France.
- [3] Service de Virologie, Centre Hospitalier Universitaire Félix Guyon, La Réunion, France.
- [4] Département d'Informatique Clinique, Centre Hospitalier Universitaire Félix Guyon, La Réunion, France.

- PMID: **33354821**
- DOI: 10.1111/tmi.13542

Observational Study

Differential diagnoses of severe COVID-19 in tropical areas: the experience of Reunion Island

Pauline Krol et al. Trop Med Int Health. 2021 Apr.

Show details

Trop Med Int Health

. 2021 Apr;26(4):444-452.
doi: 10.1111/tmi.13542. Epub 2021 Jan 12.

Authors

Pauline Krol [1], Nathalie Coolen-Allou [2], Laura Teysseyre [1], Nicolas Traversier [3], Floryan Beasley [1], Mathilde Nativel [1], Nicolas Allou [1 4], Jerome Allyn [1 4]

Affiliations

- [1] Réanimation Polyvalente, Centre Hospitalier Universitaire Félix Guyon, La Réunion, France.
- [2] Service de Pneumologie, Centre Hospitalier Universitaire Félix Guyon, La Réunion, France.
- [3] Service de Virologie, Centre Hospitalier Universitaire Félix Guyon, La Réunion, France.
- [4] Département d'Informatique Clinique, Centre Hospitalier Universitaire Félix Guyon, La Réunion, France.

- PMID: **33354821**
- DOI: 10.1111/tmi.13542

Abstract

Objective: To identify the differential diagnoses of severe COVID-19 and the distinguishing characteristics of critically ill COVID-19 patients in Reunion Island to help improve the triage and management of patients in this tropical setting.

Methods: This retrospective observational study was conducted from 11 March to 4 May 2020 in the only intensive care unit (ICU) authorised to manage COVID-19 patients in Reunion Island, a

French overseas department located in the Indian Ocean region. All patients with unknown COVID-19 status were tested by polymerase chain reaction (PCR) on ICU admission; those who tested negative were transferred to the COVID-19-free area of the ICU.

Results: Over the study period, 99 patients were admitted to our ICU. A total of 33 patients were hospitalised in the COVID-19 isolation ward, of whom 11 were positive for COVID-19. The main differential diagnoses of severe COVID-19 were as follows: community-acquired pneumonia, dengue, leptospirosis causing intra-alveolar haemorrhage and cardiogenic pulmonary oedema. The median age of COVID-19-positive patients was higher than that of COVID-19-negative patients (71 [58-74] vs. 54 [46-63.5] years, P = 0.045). No distinguishing clinical, biological or radiological characteristics were found between the two groups of patients. All COVID-19-positive patients had recently travelled or been in contact with a recent traveller.

Conclusions: In Reunion Island, dengue and leptospirosis are key differential diagnoses of severe COVID-19, and travel is the only distinguishing characteristic of COVID-19-positive patients. Our findings apply only to the particular context of Reunion Island at this time of the epidemic.

Keywords: COVID-19; dengue; differential diagnoses; travel; tropical area.

© 2020 John Wiley & Sons Ltd.

- 19 references

Supplementary info

Publication types, MeSH terms Expand

Publication types

- Observational Study

MeSH terms

- Aged
- COVID-19 / diagnosis*
- Critical Illness*
- Dengue / diagnosis
- Diagnosis, Differential
- Female
- Humans
- Intensive Care Units*
- Leptospirosis / diagnosis
- Male
- Middle Aged
- Patient Isolation*
- Retrospective Studies
- Reunion / epidemiology

- SARS-CoV-2
- Travel
- Triage*

Full text links

WILEY Full Text Article Wiley
Proceed to details
Cite
Share
245
Observational Study
Laryngoscope
. 2021 Jun;131(6):E1797-E1804.
doi: 10.1002/lary.29391. Epub 2021 Jan 27.

Tracheotomy Outcomes in 64 Ventilated COVID-19 Patients at a High-Volume Center in Bronx, NY

Yasmina Ahmed [1], Angela Cao [1], Arielle Thal [1], Sharan Shah [1], Corin Kinkhabwala [1], David Liao [1], Daniel Li [1], Michael Parides [2], Vikas Mehta [1], Thomas Ow [1], Richard Smith [1], Bradley A Schiff [1]
Affiliations Expand

Affiliations

- [1] Department of Otorhinolaryngology-Head and Neck Surgery, Montefiore Medical Center, Albert Einstein College of Medicine, New York, New York, U.S.A.
- [2] Department of Epidemiology and Population Health, Montefiore Medical Center, Albert Einstein College of Medicine, New York, New York, U.S.A.

- PMID: **33410517**
- DOI: 10.1002/lary.29391

Observational Study

Tracheotomy Outcomes in 64 Ventilated COVID-19 Patients at a High-Volume Center in Bronx, NY

Yasmina Ahmed et al. Laryngoscope. 2021 Jun.

Show details

Laryngoscope
. 2021 Jun;131(6):E1797-E1804.
doi: 10.1002/lary.29391. Epub 2021 Jan 27.

Authors

Yasmina Ahmed [1], Angela Cao [1], Arielle Thal [1], Sharan Shah [1], Corin Kinkhabwala [1], David Liao [1], Daniel Li [1], Michael Parides [2], Vikas Mehta [1], Thomas Ow [1], Richard Smith [1], Bradley A Schiff [1]

Affiliations

- [1] Department of Otorhinolaryngology-Head and Neck Surgery, Montefiore Medical Center, Albert Einstein College of Medicine, New York, New York, U.S.A.
- [2] Department of Epidemiology and Population Health, Montefiore Medical Center, Albert Einstein College of Medicine, New York, New York, U.S.A.

- PMID: **33410517**
- DOI: 10.1002/lary.29391

Abstract

Objectives/hypothesis: The COVID-19 pandemic has resulted in a dramatic increase in the number of patients requiring prolonged mechanical ventilation. Few studies have reported COVID-19 specific tracheotomy outcomes, and the optimal timing and patient selection criteria for tracheotomy remains undetermined. We delineate our outcomes for tracheotomies performed on COVID-19 patients during the peak of the pandemic at a major epicenter in the United States.

Methods: This is a retrospective observational cohort study. Mortality, ventilation liberation rate, complication rate, and decannulation rate were analyzed.

Results: Sixty-four patients with COVID-19 underwent tracheotomy between April 1, 2020 and May 19, 2020 at two tertiary care hospitals in Bronx, New York. The average duration of intubation prior to tracheotomy was 20 days ((interquartile range [IQR] 16.5-26.0). The mortality rate was 33% (n = 21), the ventilation liberation rate was 47% (n = 30), the decannulation rate was 28% (n = 18), and the complication rate was 19% (n = 12). Tracheotomies performed by Otolaryngology were associated with significantly improved survival ($P < .05$) with 60% of patients alive at the conclusion of the study compared to 9%, 12%, and 19% of patients undergoing tracheotomy performed by Critical Care, General Surgery, and Pulmonology, respectively.

Conclusions: So far, this is the second largest study describing tracheotomy outcomes in COVID-19 patients in the United States. Our early outcomes demonstrate successful ventilation liberation and decannulation in COVID-19 patients. Further inquiry is necessary to determine the optimal timing and identification of patient risk factors predictive of improved survival in COVID-19 patients undergoing tracheotomy.

Level of evidence: 4-retrospective cohort study Laryngoscope, 131:E1797-E1804, 2021.

Keywords: Tracheotomy; coronavirus; coronavirus disease 2019; severe acute respiratory syndrome coronavirus 2; tracheostomy outcomes.

© 2021 The American Laryngological, Rhinological and Otological Society, Inc.

Comment in

- In Response to Improving the Safety and Science of COVID-19 Tracheostomy: Challenges and Opportunities.
  Ahmed Y, Cao A, Thal A, Shah S, Mehta V, Ow T, Smith R, Schiff BA. Ahmed Y, et al. Laryngoscope. 2021 Jul;131(7):E2159. doi: 10.1002/lary.29520. Epub 2021 Mar 16. Laryngoscope. 2021. PMID: 33724466 Free PMC article. No abstract available.
- Improving the Safety and Science of COVID-19 Tracheostomy: Challenges and Opportunities.
  Brenner MJ, Rassekh CH, Dulguerov P. Brenner MJ, et al. Laryngoscope. 2021 Jul;131(7):E2160-E2161. doi: 10.1002/lary.29512. Epub 2021 Mar 16. Laryngoscope. 2021. PMID: 33724484 Free PMC article. No abstract available.

- Cited by 10 articles
- 27 references

Supplementary info

Publication types, MeSH terms Expand

Publication types

- Observational Study

MeSH terms

- Aged
- COVID-19 / diagnosis
- COVID-19 / mortality
- COVID-19 / therapy*
- COVID-19 / virology
- COVID-19 Nucleic Acid Testing
- Female
- Hospital Mortality
- Hospitals, High-Volume / statistics & numerical data
- Hospitals, Urban / statistics & numerical data
- Humans
- Intubation, Intratracheal / statistics & numerical data*
- Kaplan-Meier Estimate
- Male
- Middle Aged

- New York / epidemiology
- Pandemics / statistics & numerical data*
- Patient Selection
- Retrospective Studies
- SARS-CoV-2 / genetics
- SARS-CoV-2 / isolation & purification
- Time Factors
- Time-to-Treatment / statistics & numerical data
- Tracheostomy / statistics & numerical data*
- Tracheotomy / statistics & numerical data*
- Treatment Outcome

Full text links

WILEY Full Text Article Wiley
Proceed to details
Cite
Share
246
Observational Study
BMJ Open
. 2021 Dec 22;11(12):e057632.
doi: 10.1136/bmjopen-2021-057632.

Characteristics and outcomes of patients with COVID-19 with and without prevalent hypertension: a multinational cohort study

Carlen Reyes [1], Andrea Pistillo [1], Sergio Fernández-Bertolín [1], Martina Recalde [1 2], Elena Roel [1 2], Diana Puente [1 2], Anthony G Sena [3 4], Clair Blacketer [3 4], Lana Lai [5], Thamir M Alshammari [6], Waheed-Ui-Rahman Ahmed [7 8], Osaid Alser [9], Heba Alghoul [10], Carlos Areia [11], Dalia Dawoud [12 13], Albert Prats-Uribe [14], Neus Valveny [15], Gabriel de Maeztu [16], Luisa Sorlí Redó [2 17 18], Jordi Martinez Roldan [19], Inmaculada Lopez Montesinos [17], Lisa M Schilling [20], Asieh Golozar [21 22], Christian Reich [23], Jose D Posada [24], Nigam Shah [24], Seng Chan You [25], Kristine E Lynch [26 27], Scott L DuVall [26 27], Michael E Matheny [26 27], Fredrik Nyberg [28], Anna Ostropolets [29], George Hripcsak [29 30], Peter R Rijnbeek [4], Marc A Suchard [31], Patrick Ryan [3 29], Kristin Kostka [23 32], Talita Duarte-Salles [33]
Affiliations Expand

Affiliations

- [1] Fundació Institut Universitari per a la recerca a l'Atenció Primària de Salut Jordi Gol i Gurina (IDIAPJGol), Barcelona, Spain.
- [2] Universitat Autonoma de Barcelona, Barcelona, Spain.
- [3] Janssen Research and Development Titusville, Titusville, New Jersey, USA.
- [4] Department of Medical Informatics, Erasmus University Medical Center, Rotterdam, The Netherlands.
- [5] School of Medical Sciences, The University of Manchester, Manchester, UK.
- [6] College of Pharmacy, Riyadh Elm University, Riyadh, Saudi Arabia.
- [7] Nuffield Department of Orthopaedics, Rheumatology and Musculoskeletal Sciences, University of Oxford, Botnar Research Center, Oxford, UK.
- [8] College of Medicine and Health, University of Exeter, St Luke's Campus, Exeter, UK.
- [9] Massachusetts General Hospital, Harvard Medical School, Boston, Massachusetts, USA.
- [10] Faculty of Medicine, Islamic University of Gaza, Gaza, Palestine.
- [11] Nuffield Department of Clinical Neurosciences, University of Oxford, Oxford, UK.
- [12] National Institute for Health and Care Excellence (NICE), London, UK.
- [13] Faculty of Pharmacy, Cairo University, Cairo, Egypt.
- [14] Center for Statistics in Medicine, NDORMS, University of Oxford, Botnar Research Center, Nuffield Orthopaedic Center, Oxford, UK.
- [15] Real-World Evidence, TFS, Barcelona, Spain.
- [16] IOMED, Barcelona, Spain.
- [17] Department of Infectious Diseases, Hospital del Mar, Institut Hospital del Mar d'Investigació Mèdica (IMIM), Barcelona, Spain.
- [18] Universitat Pompeu Fabra, Barcelona, Spain.
- [19] Director of Innovation and Digital Transformation, Hospital del Mar, Barcelona, Spain.
- [20] University of Colorado - Anschutz Medical Campus, Aurora, Colorado, USA.
- [21] Johns Hopkins University Bloomberg School of Public Health, Baltimore, Maryland, USA.
- [22] Regeneron Pharmaceuticals, Tarrytown, NY, USA.
- [23] Real-World Solutions, IQVIA, Cambridge, Massachusetts, USA.
- [24] Stanford University School of Medicine, Stanford, California, USA.
- [25] Department of Preventive Medicine, Yonsei University College of Medicine, Seoul, Korea (the Republic of).
- [26] VA Informatics and Computing Infrastructure, VA Salt Lake City Health Care System, Salt Lake City, Utah, USA.
- [27] Department of Internal Medicine, The University of Utah School of Medicine, Salt Lake City, Utah, USA.
- [28] School of Public Health and Community Medicine, Institute of Medicine, Sahlgrenska Academy, University of Gothenburg, Gothenburg, Sweden.
- [29] Department of Biomedical Informatics, Columbia University Irving Medical Center, New York, New York, USA.
- [30] Medical Informatics Services, New York-Presbyterial Hospital, New York, NY, USA.
- [31] Department of Biostatistics, Fielding School of Publich Health, University of California, Los Angeles, California, USA.
- [32] The OHDSI Center at the Roux Institute, Northeastern University, Portland, ME, USA.
- [33] Fundació Institut Universitari per a la recerca a l'Atenció Primària de Salut Jordi Gol i Gurina (IDIAPJGol), Barcelona, Spain tduarte@idiapjgol.org.

- PMID: **34937726**

- PMCID: PMC8704062
- DOI: 10.1136/bmjopen-2021-057632

Free PMC article
Observational Study

Characteristics and outcomes of patients with COVID-19 with and without prevalent hypertension: a multinational cohort study

Carlen Reyes et al. BMJ Open. 2021.
Free PMC article
Show details
BMJ Open
. 2021 Dec 22;11(12):e057632.
doi: 10.1136/bmjopen-2021-057632.

Authors

Carlen Reyes [1], Andrea Pistillo [1], Sergio Fernández-Bertolín [1], Martina Recalde [1] [2], Elena Roel [1] [2], Diana Puente [1] [2], Anthony G Sena [3] [4], Clair Blacketer [3] [4], Lana Lai [5], Thamir M Alshammari [6], Waheed-Ui-Rahman Ahmed [7] [8], Osaid Alser [9], Heba Alghoul [10], Carlos Areia [11], Dalia Dawoud [12] [13], Albert Prats-Uribe [14], Neus Valveny [15], Gabriel de Maeztu [16], Luisa Sorlí Redó [2] [17] [18], Jordi Martinez Roldan [19], Inmaculada Lopez Montesinos [17], Lisa M Schilling [20], Asieh Golozar [21] [22], Christian Reich [23], Jose D Posada [24], Nigam Shah [24], Seng Chan You [25], Kristine E Lynch [26] [27], Scott L DuVall [26] [27], Michael E Matheny [26] [27], Fredrik Nyberg [28], Anna Ostropolets [29], George Hripcsak [29] [30], Peter R Rijnbeek [4], Marc A Suchard [31], Patrick Ryan [3] [29], Kristin Kostka [23] [32], Talita Duarte-Salles [33]

Affiliations

- [1] Fundació Institut Universitari per a la recerca a l'Atenció Primària de Salut Jordi Gol i Gurina (IDIAPJGol), Barcelona, Spain.
- [2] Universitat Autonoma de Barcelona, Barcelona, Spain.
- [3] Janssen Research and Development Titusville, Titusville, New Jersey, USA.
- [4] Department of Medical Informatics, Erasmus University Medical Center, Rotterdam, The Netherlands.
- [5] School of Medical Sciences, The University of Manchester, Manchester, UK.
- [6] College of Pharmacy, Riyadh Elm University, Riyadh, Saudi Arabia.
- [7] Nuffield Department of Orthopaedics, Rheumatology and Musculoskeletal Sciences, University of Oxford, Botnar Research Center, Oxford, UK.
- [8] College of Medicine and Health, University of Exeter, St Luke's Campus, Exeter, UK.
- [9] Massachusetts General Hospital, Harvard Medical School, Boston, Massachusetts, USA.
- [10] Faculty of Medicine, Islamic University of Gaza, Gaza, Palestine.
- [11] Nuffield Department of Clinical Neurosciences, University of Oxford, Oxford, UK.

- [12] National Institute for Health and Care Excellence (NICE), London, UK.
- [13] Faculty of Pharmacy, Cairo University, Cairo, Egypt.
- [14] Center for Statistics in Medicine, NDORMS, University of Oxford, Botnar Research Center, Nuffield Orthopaedic Center, Oxford, UK.
- [15] Real-World Evidence, TFS, Barcelona, Spain.
- [16] IOMED, Barcelona, Spain.
- [17] Department of Infectious Diseases, Hospital del Mar, Institut Hospital del Mar d'Investigació Mèdica (IMIM), Barcelona, Spain.
- [18] Universitat Pompeu Fabra, Barcelona, Spain.
- [19] Director of Innovation and Digital Transformation, Hospital del Mar, Barcelona, Spain.
- [20] University of Colorado - Anschutz Medical Campus, Aurora, Colorado, USA.
- [21] Johns Hopkins University Bloomberg School of Public Health, Baltimore, Maryland, USA.
- [22] Regeneron Pharmaceuticals, Tarrytown, NY, USA.
- [23] Real-World Solutions, IQVIA, Cambridge, Massachusetts, USA.
- [24] Stanford University School of Medicine, Stanford, California, USA.
- [25] Department of Preventive Medicine, Yonsei University College of Medicine, Seoul, Korea (the Republic of).
- [26] VA Informatics and Computing Infrastructure, VA Salt Lake City Health Care System, Salt Lake City, Utah, USA.
- [27] Department of Internal Medicine, The University of Utah School of Medicine, Salt Lake City, Utah, USA.
- [28] School of Public Health and Community Medicine, Institute of Medicine, Sahlgrenska Academy, University of Gothenburg, Gothenburg, Sweden.
- [29] Department of Biomedical Informatics, Columbia University Irving Medical Center, New York, New York, USA.
- [30] Medical Informatics Services, New York-Presbyterial Hospital, New York, NY, USA.
- [31] Department of Biostatistics, Fielding School of Publich Health, University of California, Los Angeles, California, USA.
- [32] The OHDSI Center at the Roux Institute, Northeastern University, Portland, ME, USA.
- [33] Fundació Institut Universitari per a la recerca a l'Atenció Primària de Salut Jordi Gol i Gurina (IDIAPJGol), Barcelona, Spain tduarte@idiapjgol.org.

- PMID: **34937726**
- PMCID: PMC8704062
- DOI: 10.1136/bmjopen-2021-057632

Abstract

Objective: To characterise patients with and without prevalent hypertension and COVID-19 and to assess adverse outcomes in both inpatients and outpatients.

Design and setting: This is a retrospective cohort study using 15 healthcare databases (primary and secondary electronic healthcare records, insurance and national claims data) from the USA, Europe and South Korea, standardised to the Observational Medical Outcomes Partnership common data model. Data were gathered from 1 March to 31 October 2020.

Participants: Two non-mutually exclusive cohorts were defined: (1) individuals diagnosed with COVID-19 (diagnosed cohort) and (2) individuals hospitalised with COVID-19 (hospitalised

cohort), and stratified by hypertension status. Follow-up was from COVID-19 diagnosis/hospitalisation to death, end of the study period or 30 days.

Outcomes: Demographics, comorbidities and 30-day outcomes (hospitalisation and death for the 'diagnosed' cohort and adverse events and death for the 'hospitalised' cohort) were reported.

Results: We identified 2 851 035 diagnosed and 563 708 hospitalised patients with COVID-19. Hypertension was more prevalent in the latter (ranging across databases from 17.4% (95% CI 17.2 to 17.6) to 61.4% (95% CI 61.0 to 61.8) and from 25.6% (95% CI 24.6 to 26.6) to 85.9% (95% CI 85.2 to 86.6)). Patients in both cohorts with hypertension were predominantly >50 years old and female. Patients with hypertension were frequently diagnosed with obesity, heart disease, dyslipidaemia and diabetes. Compared with patients without hypertension, patients with hypertension in the COVID-19 diagnosed cohort had more hospitalisations (ranging from 1.3% (95% CI 0.4 to 2.2) to 41.1% (95% CI 39.5 to 42.7) vs from 1.4% (95% CI 0.9 to 1.9) to 15.9% (95% CI 14.9 to 16.9)) and increased mortality (ranging from 0.3% (95% CI 0.1 to 0.5) to 18.5% (95% CI 15.7 to 21.3) vs from 0.2% (95% CI 0.2 to 0.2) to 11.8% (95% CI 10.8 to 12.8)). Patients in the COVID-19 hospitalised cohort with hypertension were more likely to have acute respiratory distress syndrome (ranging from 0.1% (95% CI 0.0 to 0.2) to 65.6% (95% CI 62.5 to 68.7) vs from 0.1% (95% CI 0.0 to 0.2) to 54.7% (95% CI 50.5 to 58.9)), arrhythmia (ranging from 0.5% (95% CI 0.3 to 0.7) to 45.8% (95% CI 42.6 to 49.0) vs from 0.4% (95% CI 0.3 to 0.5) to 36.8% (95% CI 32.7 to 40.9)) and increased mortality (ranging from 1.8% (95% CI 0.4 to 3.2) to 25.1% (95% CI 23.0 to 27.2) vs from 0.7% (95% CI 0.5 to 0.9) to 10.9% (95% CI 10.4 to 11.4)) than patients without hypertension.

Conclusions: COVID-19 patients with hypertension were more likely to suffer severe outcomes, hospitalisations and deaths compared with those without hypertension.

Keywords: COVID-19; epidemiology; hypertension.

© Author(s) (or their employer(s)) 2021. Re-use permitted under CC BY. Published by BMJ.

Conflict of interest statement

Competing interests: SLD reports grants from Anolinx. MAS reports grants from US National Institutes of Health, grants from Department of Veterans Affairs, during the conduct of the study; grants from IQVIA, personal fees from Janssen Research and Development, grants from US Food and Drug Administration, personal fees from Private Health Management, outside the submitted work. GH reports grants from NIH, during the conduct of the study; and grants from Janssen Research, outside the submitted work. FN reports being an employee of AstraZeneca until 2019 and holds some AstraZeneca shares, outside the submitted work. KK reports personal fees from the National Institutes of Health, outside the submitted work, and at the time of data analysis and initial drafting of the manuscript. KK was an employee of IQVIA. CRei reports he is an employee of IQVIA. GdM is an employee of IOMED. NV is an employee of TFS. AGS reports personal fees from Janssen R&D, outside the submitted work, and is a full-time employee of Janssen R&D and a Johnson and Johnson shareholder. CB reports personal fees from Janssen R&D, outside the submitted work, and is a full-time employee of Janssen R&D and a Johnson and Johnson shareholder. JDP reports grants from the National Library of Medicine, during the conduct of the study. AG is an employee of Regeneron Pharmaceuticals and reports stocks from Regeneron Pharmaceuticals. PRR reports having received research group grants from Innovative Medicine Initiative and Janssen Research and Development. PR reports being an employee of Janssen Research and Development and a shareholder of Johnson & Johnson. ER, SF-B, NS, LMS, DP, SCY, MR, AP-U, HA, KEL, MEM, AO, CA, CRey, TD-S, TMA, OA, W-U-RA, ILM, JMR,

LSR, DD, LL and AP have nothing to declare. No other relationships or activities could appear to have influenced the submitted work.

- 27 references
- 4 figures

Supplementary info

Publication types, MeSH terms, Grant support Expand

Publication types

- Observational Study
- Research Support, N.I.H., Extramural
- Research Support, Non-U.S. Gov't
- Research Support, U.S. Gov't, Non-P.H.S.

MeSH terms

- COVID-19 Testing
- COVID-19*
- Cohort Studies
- Comorbidity
- Female
- Hospitalization
- Humans
- Hypertension* / epidemiology
- Middle Aged
- Retrospective Studies
- SARS-CoV-2

Grant support

- R01 LM006910/LM/NLM NIH HHS/United States
- RES 13-457/RD/ORD VA/United States

Full text links

BMJ Free Full Text HighWire Free PMC article
Proceed to details
Cite
Share
☐ 247
Observational Study
BMC Infect Dis

. 2021 Sep 17;21(1):969.
doi: 10.1186/s12879-021-06663-x.

What about the others: differential diagnosis of COVID-19 in a German emergency department

David Fistera [1] [2], Annalena Härtl [3], Dirk Pabst [3], Randi Manegold [3], Carola Holzner [3], Christian Taube [4], Sebastian Dolff [5], Benedikt Michael Schaarschmidt [6], Lale Umutlu [6], Clemens Kill [3], Joachim Risse [3]
Affiliations Expand

Affiliations

- [1] Center of Emergency Medicine, University Hospital Essen, Hufelandstrasse 55, 45147, Essen, Germany. David.fistera@uk-essen.de.
- [2] Department of Pulmonary Medicine, University Medicine Essen - Ruhrlandklinik, Essen, Germany. David.fistera@uk-essen.de.
- [3] Center of Emergency Medicine, University Hospital Essen, Hufelandstrasse 55, 45147, Essen, Germany.
- [4] Department of Pulmonary Medicine, University Medicine Essen - Ruhrlandklinik, Essen, Germany.
- [5] Department of Infectious Diseases, West German Center of Infectious Diseases, University Hospital Essen, University Duisburg-Essen, Essen, Germany.
- [6] Department of Diagnostic and Interventional Radiology and Neuroradiology, University Hospital Essen, Essen, Germany.

- PMID: **34535095**
- PMCID: PMC8446739
- DOI: 10.1186/s12879-021-06663-x

Free PMC article
Observational Study

What about the others: differential diagnosis of COVID-19 in a German emergency department

David Fistera et al. BMC Infect Dis. 2021.
Free PMC article
Show details
BMC Infect Dis
. 2021 Sep 17;21(1):969.
doi: 10.1186/s12879-021-06663-x.

Authors

David Fistera [1] [2], Annalena Härtl [3], Dirk Pabst [3], Randi Manegold [3], Carola Holzner [3], Christian Taube [4], Sebastian Dolff [5], Benedikt Michael Schaarschmidt [6], Lale Umutlu [6], Clemens Kill [3], Joachim Risse [3]

Affiliations

- [1] Center of Emergency Medicine, University Hospital Essen, Hufelandstrasse 55, 45147, Essen, Germany. David.fistera@uk-essen.de.
- [2] Department of Pulmonary Medicine, University Medicine Essen - Ruhrlandklinik, Essen, Germany. David.fistera@uk-essen.de.
- [3] Center of Emergency Medicine, University Hospital Essen, Hufelandstrasse 55, 45147, Essen, Germany.
- [4] Department of Pulmonary Medicine, University Medicine Essen - Ruhrlandklinik, Essen, Germany.
- [5] Department of Infectious Diseases, West German Center of Infectious Diseases, University Hospital Essen, University Duisburg-Essen, Essen, Germany.
- [6] Department of Diagnostic and Interventional Radiology and Neuroradiology, University Hospital Essen, Essen, Germany.

- PMID: **34535095**
- PMCID: PMC8446739
- DOI: 10.1186/s12879-021-06663-x

Abstract

Background: The ongoing COVID-19 pandemic remains a major challenge for worldwide health care systems and in particular emergency medicine. An early and safe triage in the emergency department (ED) is especially crucial for proper therapy. Clinical symptoms of COVID-19 comprise those of many common diseases; thus, differential diagnosis remains challenging.

Method: We performed a retrospective study of 314 ED patients presenting with conceivable COVID-19 symptoms during the first wave in Germany. All were tested for COVID-19 with SARS-Cov-2-nasopharyngeal swabs. Forty-seven patients were positive. We analyzed the 267 COVID-19 negative patients for their main diagnosis and compared COVID-19 patients with COVID-19 negative respiratory infections for differences in laboratory parameters, symptoms, and vital signs.

Results: Among the 267 COVID-19 negative patients, 42.7% had respiratory, 14.2% had other infectious, and 11.2% had cardiovascular diseases. Further, 9.0% and 6.7% had oncological and gastroenterological diagnoses, respectively. Compared to COVID-19 negative airway infections, COVID-19 patients showed less dyspnea (OR 0.440; $p = 0.024$) but more dysgeusia (OR 7.631; $p = 0.005$). Their hospital stay was significantly longer (9.0 vs. 5.6 days; $p = 0.014$), and their mortality significantly higher (OR 3.979; $p = 0.014$).

Conclusion: For many common ED diagnoses, COVID-19 should be considered a differential diagnosis. COVID-19 cannot be distinguished from COVID-19 negative respiratory infections by clinical signs, symptoms, or laboratory results. When hospitalization is necessary, the clinical course of COVID-19 airway infections seems to be more severe compared to other respiratory infections.

Trial registration: German Clinical Trial Registry DRKS, DRKS-ID of the study: DRKS00021675 date of registration: May 8th, 2020, retrospectively registered.

Keywords: COVID-19; Clinical symptoms; Differential diagnosis; Emergency department; Respiratory infection; SARS-Cov-2; Triage.

© 2021. The Author(s).

Conflict of interest statement

The authors state they have no competing interests.

- Cited by 1 article
- 23 references
- 2 figures

Supplementary info

Publication types, MeSH terms, Associated data Expand

Publication types

- Observational Study

MeSH terms

- COVID-19*
- Diagnosis, Differential
- Emergency Service, Hospital
- Humans
- Pandemics
- Retrospective Studies
- SARS-CoV-2

Associated data

- DRKS/DRKS00021675

Full text links

Read free full text at BMC BioMed Central Free PMC article

Proceed to details

Cite

Share

☐ 248

Observational Study

J Korean Med Sci

. 2020 Oct 19;35(40):e367.
doi: 10.3346/jkms.2020.35.e367.

Operation and Management of Seoul Metropolitan City Community Treatment Center for Mild Condition COVID-19 Patients

Sun Young Lee [1] [2], Kyoung Jun Song [2] [3], Chun Soo Lim [4], Byeong Gwan Kim [5], Young Jun Chai [6], Jung Kyu Lee [7], Su Hwan Kim [5], Hyouk Jae Lim [2] [8]
Affiliations Expand

Affiliations

- [1] Public Healthcare Center, Seoul National University Hospital, Seoul, Korea.
- [2] Laboratory of Emergency Medical Services, Seoul National University Hospital Biomedical Research Institute, Seoul, Korea.
- [3] Department of Emergency Medicine, Seoul Metropolitan Government-Seoul National University Boramae Medical Center, Seoul, Korea. skciva@gmail.com.
- [4] Division of Nephrology, Department of Internal Medicine, Seoul Metropolitan Government-Seoul National University Boramae Medical Center, Seoul National University College of Medicine, Seoul, Korea.
- [5] Division of Gastroenterology, Department of Internal Medicine, Seoul Metropolitan Government-Seoul National University Boramae Medical Center, Seoul, Korea.
- [6] Department of Surgery, Seoul Metropolitan Government-Seoul National University Boramae Medical Center, Seoul, Korea.
- [7] Division of Pulmonary and Critical Care Medicine, Department of Internal Medicine, Seoul Metropolitan Government-Seoul National University Boramae Medical Center, Seoul, Korea.
- [8] Department of Emergency Medicine, Seoul National University Hospital, Seoul, Korea.

- PMID: **33075858**
- PMCID: PMC7572227
- DOI: 10.3346/jkms.2020.35.e367

Free PMC article
Observational Study

Operation and Management of Seoul Metropolitan City Community Treatment Center for Mild Condition COVID-19 Patients

Sun Young Lee et al. J Korean Med Sci. 2020.
Free PMC article
Show details
J Korean Med Sci
. 2020 Oct 19;35(40):e367.
doi: 10.3346/jkms.2020.35.e367.

Authors

Sun Young Lee [1, 2], Kyoung Jun Song [2, 3], Chun Soo Lim [4], Byeong Gwan Kim [5], Young Jun Chai [6], Jung Kyu Lee [7], Su Hwan Kim [5], Hyouk Jae Lim [2, 8]

Affiliations

- [1] Public Healthcare Center, Seoul National University Hospital, Seoul, Korea.
- [2] Laboratory of Emergency Medical Services, Seoul National University Hospital Biomedical Research Institute, Seoul, Korea.
- [3] Department of Emergency Medicine, Seoul Metropolitan Government-Seoul National University Boramae Medical Center, Seoul, Korea. skciva@gmail.com.
- [4] Division of Nephrology, Department of Internal Medicine, Seoul Metropolitan Government-Seoul National University Boramae Medical Center, Seoul National University College of Medicine, Seoul, Korea.
- [5] Division of Gastroenterology, Department of Internal Medicine, Seoul Metropolitan Government-Seoul National University Boramae Medical Center, Seoul, Korea.
- [6] Department of Surgery, Seoul Metropolitan Government-Seoul National University Boramae Medical Center, Seoul, Korea.
- [7] Division of Pulmonary and Critical Care Medicine, Department of Internal Medicine, Seoul Metropolitan Government-Seoul National University Boramae Medical Center, Seoul, Korea.
- [8] Department of Emergency Medicine, Seoul National University Hospital, Seoul, Korea.

- PMID: **33075858**
- PMCID: PMC7572227
- DOI: 10.3346/jkms.2020.35.e367

Abstract

Background: In response to the disaster of coronavirus disease 2019 (COVID-19) pandemic, Seoul Metropolitan Government (SMG) established a patient facility for mild condition patients other than hospital. This study was conducted to investigate the operation and necessary resources

of a community treatment center (CTC) operated in Seoul, a metropolitan city with a population of 10 million.

Methods: To respond COVID-19 epidemic, the SMG designated 5 municipal hospitals as dedicated COVID-19 hospitals and implemented one CTC cooperated with the Boramae Municipal Hospital for COVID-19 patients in Seoul. As a retrospective cross-sectional observational study, retrospective medical records review was conducted for patients admitted to the Seoul CTC. The admission and discharge route of CTC patients were investigated. The patient characteristics were compared according to route of discharge whether the patient was discharged to home or transferred to hospital. To report the operation of CTC, the daily mean number of tests (reverse transcription polymerase chain reaction and chest X-ray) and consultations by medical staffs were calculated per week. The list of frequent used medications and who used medication most frequently were investigated.

Results: Until May 27 when the Seoul CTC was closed, 26.5% (n = 213) of total 803 COVID-19 patients in Seoul were admitted to the CTC. It was 35.7% (n = 213) of 597 newly diagnosed patients in Seoul during the 11 weeks of operation. The median length of stay was 21 days (interquartile range, 12-29 days). A total of 191 patients (89.7%) were discharged to home after virologic remission and 22 (10.3%) were transferred to hospital for further treatment. Fifty percent of transferred patients were within a week since CTC admission. Daily 2.5-3.6 consultations by doctors or nurses and 0.4-0.9 tests were provided to one patient. The most frequently prescribed medication was symptomatic medication for COVID-19 (cough/sputum and rhinorrhea). The next ranking was psychiatric medication for sleep problem and depression/anxiety, which was prescribed more than digestive drug.

Conclusion: In the time of an infectious disease disaster, a metropolitan city can operate a temporary patient facility such as CTC to make a surge capacity and appropriately allocate scarce medical resource.

Keywords: COVID-19; Community Treatment Center; Disaster; Surge Capacity.

© 2020 The Korean Academy of Medical Sciences.

Conflict of interest statement

The authors have no potential conflicts of interest to disclose.

- Cited by 2 articles
- 36 references
- 2 figures

Supplementary info

Publication types, MeSH terms Expand

Publication types

- Observational Study

MeSH terms

- Adolescent
- Adult
- Aged
- Aged, 80 and over
- Betacoronavirus
- COVID-19
- Child
- Child, Preschool
- Cities
- Coronavirus Infections / epidemiology*
- Coronavirus Infections / therapy*
- Cross-Sectional Studies
- Female
- Hospitalization*
- Hospitals*
- Humans
- Infant
- Infant, Newborn
- Length of Stay
- Male
- Middle Aged
- Pandemics
- Patient Discharge
- Patient Transfer
- Pneumonia, Viral / epidemiology*
- Pneumonia, Viral / therapy*
- Retrospective Studies
- SARS-CoV-2
- Seoul / epidemiology
- Young Adult

Full text links

JKMS FULL TEXT FREE Journal of korean Medical Science Korean Academy of Medical Sciences Free PMC article
Proceed to details
Cite
Share
☐ 249
Observational Study
JAMA Netw Open
. 2021 Jan 4;4(1):e2035699.
doi: 10.1001/jamanetworkopen.2020.35699.

Outcomes of COVID-19 Among Hospitalized Health Care Workers in North America

Jeong Yun Yang [1], Michael D Parkins [2], Andrew Canakis [3], Olga C Aroniadis [4], Dhiraj Yadav [5], Rebekah E Dixon [1], B Joseph Elmunzer [6], Nauzer Forbes [7 8], DMC-19 Study Group and the North American Alliance for the Study of Digestive Manifestations of COVID-19

Affiliations Expand

Affiliations

- [1] Department of Medicine, Icahn School of Medicine at Mount Sinai, New York, New York.
- [2] Division of Infectious Diseases, Department of Medicine, University of Calgary, Calgary, Alberta, Canada.
- [3] Section of Gastroenterology, Department of Medicine, Boston University Medical Center, Boston, Massachusetts.
- [4] Division of Gastroenterology, Stony Brook Hospital, Stony Brook, New York.
- [5] Division of Gastroenterology, Hepatology and Nutrition, University of Pittsburgh Medical Center, Pittsburgh, Pennsylvania.
- [6] Division of Gastroenterology and Hepatology, Medical University of South Carolina, Charleston.
- [7] Division of Gastroenterology and Hepatology, Department of Medicine, University of Calgary, Calgary, Alberta, Canada.
- [8] Department of Community Health Sciences, University of Calgary, Calgary, Alberta, Canada.

- PMID: **33507259**
- PMCID: PMC7844592
- DOI: 10.1001/jamanetworkopen.2020.35699

Free PMC article
Observational Study

Outcomes of COVID-19 Among Hospitalized Health Care Workers in North America

Jeong Yun Yang et al. JAMA Netw Open. 2021.
Free PMC article
Show details
JAMA Netw Open
. 2021 Jan 4;4(1):e2035699.
doi: 10.1001/jamanetworkopen.2020.35699.

Authors

Jeong Yun Yang [1], Michael D Parkins [2], Andrew Canakis [3], Olga C Aroniadis [4], Dhiraj Yadav [5], Rebekah E Dixon [1], B Joseph Elmunzer [6], Nauzer Forbes [7] [8], DMC-19 Study Group and the North American Alliance for the Study of Digestive Manifestations of COVID-19

Affiliations

- [1] Department of Medicine, Icahn School of Medicine at Mount Sinai, New York, New York.
- [2] Division of Infectious Diseases, Department of Medicine, University of Calgary, Calgary, Alberta, Canada.
- [3] Section of Gastroenterology, Department of Medicine, Boston University Medical Center, Boston, Massachusetts.
- [4] Division of Gastroenterology, Stony Brook Hospital, Stony Brook, New York.
- [5] Division of Gastroenterology, Hepatology and Nutrition, University of Pittsburgh Medical Center, Pittsburgh, Pennsylvania.
- [6] Division of Gastroenterology and Hepatology, Medical University of South Carolina, Charleston.
- [7] Division of Gastroenterology and Hepatology, Department of Medicine, University of Calgary, Calgary, Alberta, Canada.
- [8] Department of Community Health Sciences, University of Calgary, Calgary, Alberta, Canada.

- PMID: **33507259**
- PMCID: PMC7844592
- DOI: 10.1001/jamanetworkopen.2020.35699

Abstract

Importance: Although health care workers (HCWs) are at higher risk of acquiring coronavirus disease 2019 (COVID-19), it is unclear whether they are at risk of poorer outcomes.

Objective: To evaluate the association between HCW status and outcomes among patients hospitalized with COVID-19.

Design, setting, and participants: This retrospective, observational cohort study included consecutive adult patients hospitalized with a diagnosis of laboratory-confirmed COVID-19 across 36 North American centers from April 15 to June 5, 2020. Data were collected from 1992 patients. Data were analyzed from September 10 to October 1, 2020.

Exposures: Data on patient baseline characteristics, comorbidities, presenting symptoms, treatments, and outcomes were collected, including HCW status.

Main outcomes and measures: The primary outcome was a requirement for mechanical ventilation or death. Multivariable logistic regression was performed to yield adjusted odds ratios (AORs) and 95% CIs for the association between HCW status and COVID-19-related outcomes in a 3:1 propensity score-matched cohort, adjusting for residual confounding after matching.

Results: In total, 1790 patients were included, comprising 127 HCWs and 1663 non-HCWs. After 3:1 propensity score matching, 122 HCWs were matched to 366 non-HCWs. Women comprised 71 (58.2%) of matched HCWs and 214 (58.5%) of matched non-HCWs. Matched HCWs had a mean (SD) age of 52 (13) years, whereas matched non-HCWs had a mean (SD) age of 57 (17)

years. In the matched cohort, the odds of the primary outcome, mechanical ventilation or death, were not significantly different for HCWs compared with non-HCWs (AOR, 0.60; 95% CI, 0.34-1.04). The HCWs were less likely to require admission to an intensive care unit (AOR, 0.56; 95% CI, 0.34-0.92) and were also less likely to require an admission of 7 days or longer (AOR, 0.53; 95% CI, 0.34-0.83). There were no differences between matched HCWs and non-HCWs in terms of mechanical ventilation (AOR, 0.66; 95% CI, 0.37-1.17), death (AOR, 0.47; 95% CI, 0.18-1.27), or vasopressor requirements (AOR, 0.68; 95% CI, 0.37-1.24).

Conclusions and relevance: In this propensity score-matched multicenter cohort study, HCW status was not associated with poorer outcomes among hospitalized patients with COVID-19 and, in fact, was associated with a shorter length of hospitalization and decreased likelihood of intensive care unit admission. Further research is needed to elucidate the proportion of HCW infections acquired in the workplace and to assess whether HCW type is associated with outcomes.

Conflict of interest statement

Conflict of Interest Disclosures: Dr Forbes reported receiving personal fees from Boston Scientific and from Pentax Medical outside the submitted work. No other disclosures were reported.

- Cited by 3 articles
- 37 references

Supplementary info

Publication types, MeSH terms, Substances, Grant support Expand

Publication types

- Multicenter Study
- Observational Study

MeSH terms

- Adult
- Aged
- COVID-19* / etiology
- COVID-19* / mortality
- COVID-19* / therapy
- Comorbidity
- Female
- Health Personnel*
- Hospitalization*
- Humans
- Intensive Care Units*
- Length of Stay
- Male

- Middle Aged
- North America
- Occupational Exposure*
- Odds Ratio
- Respiration, Artificial
- Retrospective Studies
- SARS-CoV-2
- Severity of Illness Index*
- Vasoconstrictor Agents / therapeutic use
- Workplace

Substances

- Vasoconstrictor Agents

Grant support

- P30 DK123704/DK/NIDDK NIH HHS/United States

Full text links

JAMA Network Open FULL TEXT Silverchair Information Systems Free PMC article
Proceed to details
Cite
Share
250
Observational Study
Foot (Edinb)
. 2021 Mar;46:101772.
doi: 10.1016/j.foot.2020.101772. Epub 2021 Jan 2.

The impact of COVID-19 on foot and ankle surgery in a major trauma centre

Howard Stringer [1], Andrew Molloy [2], Joanne Craven [3], John Moorehead [3], Alasdair Santini [2], Lyndon Mason [2]
Affiliations Expand

Affiliations

- [1] Department of Trauma and Orthopaedics, Liverpool University Hospitals NHS Foundation Trust, UK; School of Medicine, The University of Liverpool, UK. Electronic address: howard.stringer@nhs.net.

- [2] Department of Trauma and Orthopaedics, Liverpool University Hospitals NHS Foundation Trust, UK; Faculty of Health & Life Science, The University of Liverpool, UK.
- [3] Department of Trauma and Orthopaedics, Liverpool University Hospitals NHS Foundation Trust, UK.

- PMID: **33453611**
- PMCID: PMC7836313
- DOI: 10.1016/j.foot.2020.101772

Free PMC article
Observational Study

The impact of COVID-19 on foot and ankle surgery in a major trauma centre

Howard Stringer et al. Foot (Edinb). 2021 Mar.
Free PMC article
Show details
Foot (Edinb)
. 2021 Mar;46:101772.
doi: 10.1016/j.foot.2020.101772. Epub 2021 Jan 2.

Authors

Howard Stringer [1], Andrew Molloy [2], Joanne Craven [3], John Moorehead [3], Alasdair Santini [2], Lyndon Mason [2]

Affiliations

- [1] Department of Trauma and Orthopaedics, Liverpool University Hospitals NHS Foundation Trust, UK; School of Medicine, The University of Liverpool, UK. Electronic address: howard.stringer@nhs.net.
- [2] Department of Trauma and Orthopaedics, Liverpool University Hospitals NHS Foundation Trust, UK; Faculty of Health & Life Science, The University of Liverpool, UK.
- [3] Department of Trauma and Orthopaedics, Liverpool University Hospitals NHS Foundation Trust, UK.

- PMID: **33453611**
- PMCID: PMC7836313
- DOI: 10.1016/j.foot.2020.101772

Abstract

Introduction and aims: COVID-19 has had a significant impact on orthopaedic surgery globally. This paper aims to evaluate the impact of COVID-19 on foot and ankle trauma in a major trauma centre.

Methods: A retrospective observational study of prospectively collected data was performed. All foot and ankle trauma patients over a 33 week period (1st December 2019-16th July 2020) were analysed. All patients with trauma classified by the AO/OTA as occurring at locations 43 and 81-88 were included.

Results: Over the 33 weeks analysed, there was a total of 1661 trauma cases performed; of these, only 230 (13.85%) were foot and ankle trauma cases. As percentage of cases during each period of lockdown, foot and ankle made up 15.20% (147 out of 967) pre-lockdown, 8.81% (17 out of 193) during lockdown and 13.17% (66 out of 501) post lockdown. This difference was statistically significant ($p < .001$). The most significant change in trauma management was the treatment of malleolar fractures. Further analysis showed that during the lockdown period 29 foot and ankle fractures were treated the same and 13 were treated differently, (i.e. 31% of fractures were treated conservatively, when the consultants preferred practice would have been surgical intervention). Of the 13 patients, 3 have had surgical management since lockdown has been eased.

Conclusion: It is evident that the trauma case activity within foot and ankle was significantly reduced during the COVID-19 period. The consequences of change in management were mitigated due to a reduction in case load.

Keywords: COVID-19; Foot and ankle surgery; Trauma.

Crown Copyright © 2021. Published by Elsevier Ltd. All rights reserved.

- Cited by 2 articles
- 25 references
- 3 figures

Supplementary info

Publication types, MeSH terms Expand

Publication types

- Observational Study

MeSH terms

- Ankle Injuries / surgery*
- COVID-19 / epidemiology*
- Foot Injuries / surgery*
- Health Care Rationing*
- Humans
- Pandemics
- Retrospective Studies
- SARS-CoV-2
- Trauma Centers / organization & administration*
- Triage
- United Kingdom / epidemiology

Full text links

ELSEVIER FULL-TEXT ARTICLE Elsevier Science Free PMC article

Proceed to details

Cite

Share

☐ 251

Observational Study

J Cardiothorac Surg

. 2021 Mar 22;16(1):43.

doi: 10.1186/s13019-021-01424-y.

Cardiac surgery outcome during the COVID-19 pandemic: a retrospective review of the early experience in nine UK centres

Julie Sanders [1] [2], Enoch Akowuah [3], Jackie Cooper [4], Bilal H Kirmani [5], Mazyar Kanani [3], Metesh Acharya [6], Reuben Jeganathan [7], George Krasopoulos [8], Dumbor Ngaage [9], Indu Deglurkar [10], Patrick Yiu [11], Simon Kendall [3], Aung Ye Oo [12] [4]

Affiliations Expand

Affiliations

- [1] St Bartholomew's Hospital, Barts Health NHS Trust, London, EC1A 7DN, UK. j.sanders@qmul.ac.uk.
- [2] William Harvey Research Institute, Queen Mary University of London, London, UK. j.sanders@qmul.ac.uk.
- [3] Department of Cardiothoracic Surgery, James Cook University Hospital, South Tees NHS Foundation Trust, Middlesbrough, UK.
- [4] William Harvey Research Institute, Queen Mary University of London, London, UK.
- [5] Department of Cardiothoracic Surgery, Liverpool Heart and Chest Hospital, Liverpool, UK.
- [6] Department of Cardiothoracic Surgery, Glenfield Hospital, University Hospitals Leicester NHS Foundation Trust, Leicester, UK.
- [7] Department of Cardiothoracic Surgery, Royal Victoria Hospital, Belfast, Northern Ireland, UK.
- [8] Department of Cardiothoracic Surgery, Oxford University Hospitals NHS Foundation Trust, Oxford, UK.
- [9] Department of Cardiothoracic Surgery, Castle Hill Hospital, Hull, UK.
- [10] Department of Cardiothoracic Surgery, University Hospital of Wales, Cardiff, Wales, UK.
- [11] Heart and Lung Centre, New Cross Hospital, Wolverhampton, UK.
- [12] St Bartholomew's Hospital, Barts Health NHS Trust, London, EC1A 7DN, UK.

- PMID: **33752706**

- PMCID: PMC7983084
- DOI: 10.1186/s13019-021-01424-y

Free PMC article
Observational Study

Cardiac surgery outcome during the COVID-19 pandemic: a retrospective review of the early experience in nine UK centres

Julie Sanders et al. J Cardiothorac Surg. 2021.
Free PMC article
Show details
J Cardiothorac Surg
. 2021 Mar 22;16(1):43.
doi: 10.1186/s13019-021-01424-y.

Authors

Julie Sanders [1 2], Enoch Akowuah [3], Jackie Cooper [4], Bilal H Kirmani [5], Mazyar Kanani [3], Metesh Acharya [6], Reuben Jeganathan [7], George Krasopoulos [8], Dumbor Ngaage [9], Indu Deglurkar [10], Patrick Yiu [11], Simon Kendall [3], Aung Ye Oo [12 4]

Affiliations

- [1] St Bartholomew's Hospital, Barts Health NHS Trust, London, EC1A 7DN, UK. j.sanders@qmul.ac.uk.
- [2] William Harvey Research Institute, Queen Mary University of London, London, UK. j.sanders@qmul.ac.uk.
- [3] Department of Cardiothoracic Surgery, James Cook University Hospital, South Tees NHS Foundation Trust, Middlesbrough, UK.
- [4] William Harvey Research Institute, Queen Mary University of London, London, UK.
- [5] Department of Cardiothoracic Surgery, Liverpool Heart and Chest Hospital, Liverpool, UK.
- [6] Department of Cardiothoracic Surgery, Glenfield Hospital, University Hospitals Leicester NHS Foundation Trust, Leicester, UK.
- [7] Department of Cardiothoracic Surgery, Royal Victoria Hospital, Belfast, Northern Ireland, UK.
- [8] Department of Cardiothoracic Surgery, Oxford University Hospitals NHS Foundation Trust, Oxford, UK.
- [9] Department of Cardiothoracic Surgery, Castle Hill Hospital, Hull, UK.
- [10] Department of Cardiothoracic Surgery, University Hospital of Wales, Cardiff, Wales, UK.
- [11] Heart and Lung Centre, New Cross Hospital, Wolverhampton, UK.
- [12] St Bartholomew's Hospital, Barts Health NHS Trust, London, EC1A 7DN, UK.

- PMID: **33752706**
- PMCID: PMC7983084
- DOI: 10.1186/s13019-021-01424-y

Abstract

Background: Early studies conclude patients with Covid-19 have a high risk of death, but no studies specifically explore cardiac surgery outcome. We investigate UK cardiac surgery outcomes during the early phase of the Covid-19 pandemic.

Methods: This retrospective observational study included all adult patients undergoing cardiac surgery between 1st March and 30th April 2020 in nine UK centres. Data was obtained and linked locally from the National Institute for Cardiovascular Outcomes Research Adult Cardiac Surgery database, the Intensive Care National Audit and Research Centre database and local electronic systems. The anonymised datasets were analysed by the lead centre. Statistical analysis included descriptive statistics, propensity score matching (PSM), conditional logistic regression and hierarchical quantile regression.

Results: Of 755 included individuals, 53 (7.0%) had Covid-19. Comparing those with and without Covid-19, those with Covid-19 had increased mortality (24.5% v 3.5%, $p < 0.0001$) and longer post-operative stay (11 days v 6 days, $p = 0.001$), both of which remained significant after PSM. Patients with a pre-operative Covid-19 diagnosis recovered in a similar way to non-Covid-19 patients. However, those with a post-operative Covid-19 diagnosis remained in hospital for an additional 5 days (12 days v 7 days, $p = 0.024$) and had a considerably higher mortality rate compared to those with a pre-operative diagnosis (37.1% v 0.0%, $p = 0.005$).

Conclusions: To mitigate against the risks of Covid-19, particularly the post-operative burden, robust and effective pre-surgery diagnosis protocols alongside effective strategies to maintain a Covid-19 free environment are needed. Dedicated cardiac surgery hubs could be valuable in achieving safe and continual delivery of cardiac surgery.

Keywords: Cardiac surgery; Covid-19; Mortality; Outcomes.

Conflict of interest statement

GK is a Consultant for Abbott and Medistim.

The other authors declare that they have no competing interests.

- Cited by 6 articles
- 20 references
- 2 figures

Supplementary info

Publication types, MeSH terms Expand

Publication types

- Multicenter Study
- Observational Study

MeSH terms

- Aged
- COVID-19 / epidemiology*
- COVID-19 Testing
- Cardiac Surgical Procedures / statistics & numerical data*
- Comorbidity
- Female
- Heart Diseases / epidemiology
- Heart Diseases / surgery*
- Humans
- Length of Stay
- Male
- Middle Aged
- Pandemics*
- Propensity Score*
- Retrospective Studies
- SARS-CoV-2
- United Kingdom / epidemiology

Full text links

Read free full text at BMC BioMed Central Free PMC article

Proceed to details

Cite

Share

252

Neuroepidemiology
. 2021;55(5):354-360.
doi: 10.1159/000516753. Epub 2021 Jul 8.

Reduction in Acute Stroke Admissions during the COVID-19 Pandemic: Data from a National Stroke Registry

Carmit Libruder[1], Amit Ram[1], Yael Hershkovitz[1], David Tanne[2,3], Natan M Bornstein[4], Ronen R Leker[5], Anat Horev[6], Hen Hallevi[7,8], Shlomi Peretz[9], David Orion[8,10], Inbar Zucker[1,8]
Affiliations Expand

Affiliations

- [1] Israel Center for Disease Control, Israel Ministry of Health, Ramat Gan, Israel.
- [2] Rambam Health Care Campus, Haifa, Israel.
- [3] Technion Faculty of Medicine, Haifa, Israel.
- [4] Shaare Zedek Medical Center, Jerusalem, Israel.
- [5] Hadassah-Hebrew University Medical Center, Jerusalem, Israel.
- [6] Soroka University Medical Center, Beer-Sheva, Israel.
- [7] Tel Aviv Sourasky Medical Center, Tel Aviv, Israel.
- [8] Sackler Faculty of Medicine, Tel Aviv University, Tel Aviv, Israel.
- [9] Rabin Medical Center, Petach Tikva, Israel.
- [10] Chaim Sheba Medical Center, Tel HaShomer, Ramat Gan, Israel.

- PMID: **34237727**
- PMCID: PMC8339012
- DOI: 10.1159/000516753

Free PMC article

Reduction in Acute Stroke Admissions during the COVID-19 Pandemic: Data from a National Stroke Registry

Carmit Libruder et al. Neuroepidemiology. 2021.
Free PMC article
Show details
Neuroepidemiology
. 2021;55(5):354-360.
doi: 10.1159/000516753. Epub 2021 Jul 8.

Authors

Carmit Libruder [1], Amit Ram [1], Yael Hershkovitz [1], David Tanne [2] [3], Natan M Bornstein [4], Ronen R Leker [5], Anat Horev [6], Hen Hallevi [7] [8], Shlomi Peretz [9], David Orion [8] [10], Inbar Zucker [1] [8]

Affiliations

- [1] Israel Center for Disease Control, Israel Ministry of Health, Ramat Gan, Israel.
- [2] Rambam Health Care Campus, Haifa, Israel.
- [3] Technion Faculty of Medicine, Haifa, Israel.
- [4] Shaare Zedek Medical Center, Jerusalem, Israel.
- [5] Hadassah-Hebrew University Medical Center, Jerusalem, Israel.
- [6] Soroka University Medical Center, Beer-Sheva, Israel.
- [7] Tel Aviv Sourasky Medical Center, Tel Aviv, Israel.

- [8] Sackler Faculty of Medicine, Tel Aviv University, Tel Aviv, Israel.
- [9] Rabin Medical Center, Petach Tikva, Israel.
- [10] Chaim Sheba Medical Center, Tel HaShomer, Ramat Gan, Israel.

- PMID: **34237727**
- PMCID: PMC8339012
- DOI: 10.1159/000516753

Abstract

Introduction: The COVID-19 pandemic overwhelmed health-care systems worldwide, and medical care for other acute diseases was negatively impacted. We aimed to investigate the effect of the COVID-19 outbreak on admission rates and in-hospital care for acute stroke and transient ischemic attack (TIA) in Israel, shortly after the start of the pandemic.

Methods: We conducted a retrospective observational study, based on data reported to the Israeli National Stroke Registry from 7 tertiary hospitals. All hospital admissions for acute stroke or TIA that occurred between January 1 and April 30, 2020 were included. Data were stratified into 2 periods according to the timing of COVID-19 restrictions as follows: (1) "pre-pandemic" - January 1 to March 7, 2020 and (2) "pandemic" - March 8 to April 30, 2020. We compared the weekly counts of hospitalizations between the 2 periods. We further investigated changes in demographic characteristics and in some key parameters of stroke care, including the percentage of reperfusion therapies performed, time from hospital arrival to brain imaging and to thrombolysis, length of hospital stay, and in-hospital mortality.

Results: 2,260 cases were included: 1,469 in the pre-COVID-19 period and 791 in the COVID-19 period. Hospital admissions significantly declined between the 2 periods, by 48% for TIA (rate ratio [RR] = 0.52; 95% CI 0.43-0.64) and by 29% for stroke (RR = 0.71; 95% CI 0.64-0.78). No significant changes were detected in demographic characteristics and in most parameters of stroke management. While the percentage of reperfusion therapies performed remained unchanged, the absolute number of patients treated with reperfusion therapies seemed to decrease. Higher in-hospital mortality was observed only for hemorrhagic stroke.

Conclusion: The marked decrease in admissions for acute stroke and TIA, occurring at a time of a relatively low burden of COVID-19, is of great concern. Public awareness campaigns are needed as patients reluctant to seek urgent stroke care are deprived of lifesaving procedures and secondary prevention treatments.

Keywords: COVID-19; Pandemic; Stroke; Stroke care.

© 2021 S. Karger AG, Basel.

Conflict of interest statement

The authors have no conflicts of interest to declare.

- Cited by 2 articles
- 26 references
- 1 figure

Supplementary info

MeSH terms Expand

MeSH terms

- Aged
- Aged, 80 and over
- COVID-19*
- Female
- Hospitalization / statistics & numerical data*
- Humans
- Male
- Middle Aged
- Pandemics
- Registries
- Retrospective Studies
- Stroke / therapy*

Full text links

S. Karger AG, Basel, Switzerland Free PMC article
Proceed to details
Cite
Share
253
Observational Study
Neurol Neurochir Pol
. 2021;55(2):195-201.
doi: 10.5603/PJNNS.a2021.0011. Epub 2021 Feb 2.

Clinical features of neurological patients with coronavirus 2019: an observational study of one centre

Justyna Zielińska-Turek [1], Anna Jasińska [2], Jolanta Kołakowska [3], Joanna Szadurska [2], Dariusz A Kosior [4, 5], Małgorzata Dorobek [2]
Affiliations Expand

Affiliations

- [1] Department of Neurology, Central Clinical Hospital of the Ministry of Internal Affairs and Administration, Woloska street 137, 02-507 Warsaw, Poland. jzturek@gmail.com.

- [2] Department of Neurology, Central Clinical Hospital of the Ministry of Internal Affairs and Administration, Woloska street 137, 02-507 Warsaw, Poland.
- [3] Department of Cardiac Rehabilitation, Central Clinical Hospital of the Ministry of Internal Affairs and Administration, Woloska Street 137, 02-507 Warsaw, Poland.
- [4] Department of Cardiology and Hypertension, Central Clinical Hospital of the Ministry of Internal Affairs and Administration, Woloska Street 137, 02-507 Warsaw, Poland.
- [5] Mossakowski Medical Research Centre Polish Academy of Sciences, Pawinskiego 5, 02-106 Warsaw, Poland.

- PMID: **33528832**
- DOI: 10.5603/PJNNS.a2021.0011

Observational Study

Clinical features of neurological patients with coronavirus 2019: an observational study of one centre

Justyna Zielińska-Turek et al. Neurol Neurochir Pol. 2021.
Show details
Neurol Neurochir Pol
. 2021;55(2):195-201.
doi: 10.5603/PJNNS.a2021.0011. Epub 2021 Feb 2.

Authors

Justyna Zielińska-Turek [1], Anna Jasińska [2], Jolanta Kołakowska [3], Joanna Szadurska [2], Dariusz A Kosior [4, 5], Małgorzata Dorobek [2]

Affiliations

- [1] Department of Neurology, Central Clinical Hospital of the Ministry of Internal Affairs and Administration, Woloska street 137, 02-507 Warsaw, Poland. jzturek@gmail.com.
- [2] Department of Neurology, Central Clinical Hospital of the Ministry of Internal Affairs and Administration, Woloska street 137, 02-507 Warsaw, Poland.
- [3] Department of Cardiac Rehabilitation, Central Clinical Hospital of the Ministry of Internal Affairs and Administration, Woloska Street 137, 02-507 Warsaw, Poland.
- [4] Department of Cardiology and Hypertension, Central Clinical Hospital of the Ministry of Internal Affairs and Administration, Woloska Street 137, 02-507 Warsaw, Poland.
- [5] Mossakowski Medical Research Centre Polish Academy of Sciences, Pawinskiego 5, 02-106 Warsaw, Poland.

- PMID: **33528832**
- DOI: 10.5603/PJNNS.a2021.0011

Abstract

Background: Since the emergence of coronavirus disease 2019 (COVID-19) caused by SARS-CoV-2 (Severe acute respiratory syndrome coronavirus 2) in Wuhan, China, it has been extensively studied by many scientists. Susceptibility to SARS-CoV-2 infection is shown by people of all ages, especially those with different comorbidities. Our goal was to describe the clinical characteristics, treatment, course, and outcome of COVID-19 in patients with pre-existing neurological disorders.

Method: We retrospectively studied 70 patients with COVID-19 and previous neurological diseases who were treated in the Central Clinical Hospital of the Ministry of the Interior and Administration from 16 March to 15 June 2020. Demographic data, symptoms, image data, laboratory results, treatment methods and results, clinical signs and symptoms of patients hospitalised due to CNS diseases with COVID-19 were collected.

Results: The average age of hospitalised patients was 72, and the majority (63%) were women (44/70). The most common neurological disease was dementia, which was present in almost a third of patients (30.76%), followed by ischaemic stroke (24.61%). Chest imaging showed the presence of interstitial changes in 47% (33) of patients. Laboratory tests revealed increased total blood cells, increased levels of C-reactive protein, procalcitonin, D-dimers, liver indicator markers and IL-6 in the most severely affected patients. The treatment of patients was focused on monitoring their clinical condition, and supporting respiratory inefficiency with passive oxygen therapy and mechanical ventilation. According to the guidelines of the Hospital Therapeutic Committee, pharmacological treatment (Arechin®, Kaletra®) was introduced in cases without contraindications. In patients with moderate COVID-19, antimalarial or antiviral agents were applied (78%). 30% of our observed patients died during the hospitalisation.

Conclusions: We studied a select group of patients (elderly, with comorbidities, and moderate or severe COVID-19 course). Pre-existing neurological disorders were additionally associated with a poorer prognosis and a high fatality rate (30%). Dementia and CNS vascular disorder were the most frequent pre-existing neurological conditions. The neurological symptoms of COVID-19 were various. We observed impaired consciousness, dizziness, headache, nausea, myalgia, psychomotor agitation and slowness, delirium, and psychoses. Further analysis is needed to elucidate the incidence of COVID-19 neurological complications.

Keywords: COVID-19; SARS-CoV-2; coronavirus; dementia; stroke.

Supplementary info

Publication types, MeSH terms Expand

Publication types

- Observational Study

MeSH terms

- Aged
- Brain Ischemia*
- COVID-19*

- China / epidemiology
- Female
- Humans
- Male
- Retrospective Studies
- SARS-CoV-2
- Stroke*

Full text links

Full-text VM Via Medica Medical Publishers
Proceed to details
Cite
Share
254
Observational Study
J Gerontol A Biol Sci Med Sci
. 2021 Feb 25;76(3):456-462.
doi: 10.1093/gerona/glaa181.

Acute Kidney Injury Is Associated With In-hospital Mortality in Older Patients With COVID-19

Qi Yan [1], Peiyuan Zuo [1], Ling Cheng [1], Yuanyuan Li [1], Kaixin Song [1], Yuting Chen [1], Yue Dai [1], Yi Yang [1], Lun Zhou [1], Weiwei Yu [1], Yongsheng Li [2], Min Xie [3], Cuntai Zhang [1], Hongyu Gao [1]
Affiliations Expand

Affiliations

- [1] Department of Geriatrics, Tongji Hospital, Tongji Medical College, Huazhong University of Science and Technology, Wuhan, China.
- [2] Department of Intensive Care Medicine, Tongji Hospital, Tongji Medical College, Huazhong University of Science and Technology, Wuhan, China.
- [3] Department of Pulmonary and Critical Care Medicine, Tongji Hospital, Tongji Medical College, Huazhong University of Science and Technology, Wuhan, China.

- PMID: **32766817**
- PMCID: PMC7454401
- DOI: 10.1093/gerona/glaa181

Free PMC article
Observational Study

Acute Kidney Injury Is Associated With In-hospital Mortality in Older Patients With COVID-19

Qi Yan et al. J Gerontol A Biol Sci Med Sci. 2021.
Free PMC article
Show details
J Gerontol A Biol Sci Med Sci
. 2021 Feb 25;76(3):456-462.
doi: 10.1093/gerona/glaa181.

Authors

Qi Yan [1], Peiyuan Zuo [1], Ling Cheng [1], Yuanyuan Li [1], Kaixin Song [1], Yuting Chen [1], Yue Dai [1], Yi Yang [1], Lun Zhou [1], Weiwei Yu [1], Yongsheng Li [2], Min Xie [3], Cuntai Zhang [1], Hongyu Gao [1]

Affiliations

- [1] Department of Geriatrics, Tongji Hospital, Tongji Medical College, Huazhong University of Science and Technology, Wuhan, China.
- [2] Department of Intensive Care Medicine, Tongji Hospital, Tongji Medical College, Huazhong University of Science and Technology, Wuhan, China.
- [3] Department of Pulmonary and Critical Care Medicine, Tongji Hospital, Tongji Medical College, Huazhong University of Science and Technology, Wuhan, China.

- PMID: **32766817**
- PMCID: PMC7454401
- DOI: 10.1093/gerona/glaa181

Abstract

Background: The epidemic of COVID-19 presents a special threat to older adults. However, information on kidney damage in older patients with COVID-19 is limited. Acute kidney injury (AKI) is common in hospitalized adults and associated with poor prognosis. We sought to explore the association between AKI and mortality in older patients with COVID-19.

Methods: We conducted a retrospective, observational cohort study in a large tertiary care university hospital in Wuhan, China. All consecutive inpatients older than 65 years with COVID-19 were enrolled in this cohort. Demographic data, laboratory values, comorbidities, treatments, and clinical outcomes were all collected. Data were compared between patients with AKI and without AKI. The association between AKI and mortality was analyzed.

Results: Of 1764 in-hospital patients, 882 older adult cases were included in this cohort. The median age was 71 years (interquartile range: 68-77), 440 (49.9%) were men. The most presented comorbidity was cardiovascular diseases (58.2%), followed by diabetes (31.4%). Of 882 older patients, 115 (13%) developed AKI and 128 (14.5%) died. Patients with AKI had higher mortality

than those without AKI (68 [59.1%] vs 60 [7.8%]; p < .001). Multivariable Cox regression analysis showed that increasing odds of in-hospital mortality are associated with higher interleukin-6 on admission, myocardial injury, and AKI.

Conclusions: Acute kidney injury is not an uncommon complication in older patients with COVID-19 but is associated with a high risk of death. Physicians should be aware of the risk of AKI in older patients with COVID-19.

Keywords: Acute kidney injury; Aging kidney; COVID-19; Coronavirus; SARS-CoV-2.

© The Author(s) 2020. Published by Oxford University Press on behalf of The Gerontological Society of America. All rights reserved. For permissions, please e-mail: journals.permissions@oup.com.

- Cited by 15 articles
- 31 references
- 2 figures

Supplementary info

Publication types, MeSH terms Expand

Publication types

- Observational Study
- Research Support, Non-U.S. Gov't

MeSH terms

- Acute Kidney Injury / mortality*
- Aged
- Aged, 80 and over
- COVID-19 / epidemiology
- COVID-19 / mortality*
- China / epidemiology
- Female
- Hospital Mortality*
- Hospitalization
- Humans
- Male
- Pandemics
- Pneumonia, Viral / mortality*
- Pneumonia, Viral / virology
- Prognosis
- Retrospective Studies
- SARS-CoV-2

Full text links

OXFORD ACADEMIC Silverchair Information Systems Free PMC article
Proceed to details
Cite
Share
255
Observational Study

Sao Paulo Med J
. Nov-Dec 2020;138(6):490-497.
doi: 10.1590/1516-3180.2020.00365.R1.08092020.

Clinical characteristics and outcomes among Brazilian patients with severe acute respiratory syndrome coronavirus 2 infection: an observational retrospective study

Israel Júnior Borges do Nascimento [1], Luiz Ricardo Pinto [2], Valéria Alves Fernandes [3], Israel Molina Romero [4], João Antonio de Queiroz Oliveira [5], Milena Soriano Marcolino [6], Maria Fátima Leite [7]
Affiliations Expand

Affiliations

- [1] PharmD. Clinical Pathologist and Medical Research Specialist, University Hospital and School of Medicine, Universidade Federal de Minas Gerais, Belo Horizonte (MG), Brazil; Medical Research Specialist, School of Medicine, Medical College of Wisconsin, Milwaukee, Wisconsin, United States.
- [2] PhD. Full Professor, Department of Production Engineering, School of Engineering, Universidade Federal de Minas Gerais, Belo Horizonte (MG), Brazil.
- [3] MSc, PhD. Researcher, University Hospital and School of Medicine, Universidade Federal de Minas Gerais, Belo Horizonte (MG), Brazil.
- [4] MD, PhD. Medical Researcher, Department of Infectious Diseases, Vall d'Hebron Hospital, Barcelona, Spain; Medical Researcher, Instituto Renê Rachou, Fiocruz Minas, Belo Horizonte (MG), Brazil.
- [5] PharmB, MSc. Doctoral Student, University Hospital and School of Medicine, Universidade Federal de Minas Gerais, Belo Horizonte (MG), Brazil.
- [6] MD, PhD. Full Professor, University Hospital and School of Medicine, Universidade Federal de Minas Gerais, Belo Horizonte (MG), Brazil.
- [7] PharmD, PhD. Full Professor, Department of Physiology and Biophysics, Institute of Biological Sciences, Universidade Federal de Minas Gerais, Belo Horizonte (MG), Brazil.

- PMID: **33263706**
- DOI: 10.1590/1516-3180.2020.00365.R1.08092020

Free article
Observational Study

Clinical characteristics and outcomes among Brazilian patients with severe acute respiratory syndrome coronavirus 2 infection: an observational retrospective study

Israel Júnior Borges do Nascimento et al. Sao Paulo Med J. Nov-Dec 2020.
Free article
Show details
Sao Paulo Med J
. Nov-Dec 2020;138(6):490-497.
doi: 10.1590/1516-3180.2020.00365.R1.08092020.

Authors

Israel Júnior Borges do Nascimento [1], Luiz Ricardo Pinto [2], Valéria Alves Fernandes [3], Israel Molina Romero [4], João Antonio de Queiroz Oliveira [5], Milena Soriano Marcolino [6], Maria Fátima Leite [7]

Affiliations

- [1] PharmD. Clinical Pathologist and Medical Research Specialist, University Hospital and School of Medicine, Universidade Federal de Minas Gerais, Belo Horizonte (MG), Brazil; Medical Research Specialist, School of Medicine, Medical College of Wisconsin, Milwaukee, Wisconsin, United States.
- [2] PhD. Full Professor, Department of Production Engineering, School of Engineering, Universidade Federal de Minas Gerais, Belo Horizonte (MG), Brazil.
- [3] MSc, PhD. Researcher, University Hospital and School of Medicine, Universidade Federal de Minas Gerais, Belo Horizonte (MG), Brazil.
- [4] MD, PhD. Medical Researcher, Department of Infectious Diseases, Vall d'Hebron Hospital, Barcelona, Spain; Medical Researcher, Instituto Renê Rachou, Fiocruz Minas, Belo Horizonte (MG), Brazil.
- [5] PharmB, MSc. Doctoral Student, University Hospital and School of Medicine, Universidade Federal de Minas Gerais, Belo Horizonte (MG), Brazil.
- [6] MD, PhD. Full Professor, University Hospital and School of Medicine, Universidade Federal de Minas Gerais, Belo Horizonte (MG), Brazil.
- [7] PharmD, PhD. Full Professor, Department of Physiology and Biophysics, Institute of Biological Sciences, Universidade Federal de Minas Gerais, Belo Horizonte (MG), Brazil.

- PMID: **33263706**
- DOI: 10.1590/1516-3180.2020.00365.R1.08092020

Abstract

Background: Since February 2020, data on the clinical features of patients infected by severe acute respiratory syndrome coronavirus 2 (SARS-CoV-2) and their clinical evolution have been gathered and intensively discussed, especially in countries with dramatic dissemination of this disease.

Objective: To assess the clinical features of Brazilian patients with SARS-CoV-2 and analyze its local epidemiological features.

Design and setting: Observational retrospective study conducted using data from an official electronic platform for recording confirmed SARS-CoV-2 cases.

Methods: We extracted data from patients based in the state of Pernambuco who were registered on the platform of the Center for Strategic Health Surveillance Information, between February 26 and May 25, 2020. Clinical signs/symptoms, case evolution over time, distribution of confirmed, recovered and fatal cases and relationship between age group and gender were assessed.

Results: We included 28,854 patients who were positive for SARS-CoV-2 (56.13% females), of median age 44.18 years. SARS-CoV-2 infection was most frequent among adults aged 30-39 years. Among cases that progressed to death, the most frequent age range was 70-79 years. Overall, the mortality rate in the cohort was 8.06%; recovery rate, 30.7%; and hospital admission rate (up to the end of follow-up), 17.3%. The average length of time between symptom onset and death was 10.3 days. The most commonly reported symptoms were coughing (42.39%), fever (38.03%) and dyspnea/respiratory distress with oxygen saturation < 95% (30.98%).

Conclusion: Coughing, fever and dyspnea/respiratory distress with oxygen saturation < 95% were the commonest symptoms. The case-fatality rate was 8.06% and the hospitalization rate, 17.3%.

- Cited by 5 articles

Supplementary info

Publication types, MeSH terms Expand

Publication types

- Observational Study
- Research Support, Non-U.S. Gov't

MeSH terms

- Adult
- Aged
- Brazil / epidemiology
- COVID-19 / epidemiology*
- COVID-19 / mortality
- Female
- Fever

- Hospitalization / statistics & numerical data
- Humans
- Male
- Middle Aged
- Retrospective Studies

Full text links

free full text available at SciELO.org Scientific Electronic Library Online

Proceed to details

Cite

Share

256

Observational Study

J Infect Dev Ctries

. 2021 Aug 31;15(8):1094-1103.

doi: 10.3855/jidc.14709.

The outcomes of severe COVID-19 pneumonia managed with supportive care in Palestine: an experience from a developing country

Yousef El-Hamshari [1], Majdi Hamarshi [2], Zaher Nazzal [3], Amna Akkawi [4], Dana Saleh [5], Ibtesam Abdullah [4], Mohammed Najjar [1], Razan Rabi [5], Saad Ruzzeh [1]

Affiliations Expand

Affiliations

- [1] Martyrs Medical Military Hospital, Palestinian Ministry of Health.
- [2] University of Missouri, Kansas City, MO, United States.
- [3] Faculty of Medicine and Health Sciences, An-Najah National University. znazzal@najah.edu.
- [4] Dr.Thabet Thabet Hospital, Palestinian Ministry of Health.
- [5] Rafidia Hospital, Palestinian Ministry of Health.

- PMID: **34516416**
- DOI: 10.3855/jidc.14709

Free article

Observational Study

The outcomes of severe COVID-19 pneumonia managed with supportive care in Palestine: an experience from a developing country

Yousef El-Hamshari et al. J Infect Dev Ctries. 2021.
Free article
Show details
J Infect Dev Ctries
. 2021 Aug 31;15(8):1094-1103.
doi: 10.3855/jidc.14709.

Authors

Yousef El-Hamshari [1], Majdi Hamarshi [2], Zaher Nazzal [3], Amna Akkawi [4], Dana Saleh [5], Ibtesam Abdullah [4], Mohammed Najjar [1], Razan Rabi [5], Saad Ruzzeh [1]

Affiliations

- [1] Martyrs Medical Military Hospital, Palestinian Ministry of Health.
- [2] University of Missouri, Kansas City, MO, United States.
- [3] Faculty of Medicine and Health Sciences, An-Najah National University. znazzal@najah.edu.
- [4] Dr.Thabet Thabet Hospital, Palestinian Ministry of Health.
- [5] Rafidia Hospital, Palestinian Ministry of Health.

- PMID: **34516416**
- DOI: 10.3855/jidc.14709

Abstract

Introduction: About 14% of COVID-19 patients experience severe symptoms and require hospitalization. Managing these patients could be challenging for limited-resource countries, such as Palestine. This study aimed to evaluate hospitalized severe COVID-19 patients' treatment outcomes managed with supportive care and steroids.

Methodology: This was a single-center observational retrospective cohort study that enrolled COVID-19 patients admitted to the "Martyrs medical military complex- COVID Hospital" in Palestine. The managing physicians manually collected data through chart reviews, including patients' characteristics, complications, outcomes, and different management modalities. Continuous and categorical variables between those who were discharged alive and who died were compared using t-test and Chi-squares test, respectively.

Results: Overall, 334 patients were included in this study. Median (IQR) age was 62(11) years, 49.1% were males, and 29.6% were ICU status patients. The median (IQR) PaO2/FiO2 ratio was 76 (67), and 67.6% of these patients had moderate to severe acute respiratory distress syndrome,

and 4.8% of the patients received invasive mechanical ventilation. Most of the patients (78.7%) had at least one comorbidity, and 18.3% developed at least one complication. The overall mortality was 12.3% (95% CI 8.9-16.2%), and the median (IQR) length of hospital stay was 11 (8) days. Age (aOR 1.05, $p = 0.08$), smoking (aOR 4.12, $p = 0.019$), IMV (aOR 27.4, $p < 0.001$) and PaO_2/FiO_2 ratio (aOR 1.03, $p < 0.001$) were found to predict higher mortality.

Conclusions: Supportive care for patients with severe COVID-19 pneumonia in a Palestinian hospital with limited resources was associated with in-hospital mortality of 12.3%.

Keywords: Palestine; Supportive care; mortality rate; risk factors; severe COVID-19; steroids.

Copyright (c) 2021 Yousef El-Hamshari, Majdi Hamarshi, Zaher Nazzal, Amna Akkawi, Dana Saleh, Ibtesam Abdullah, Mohammed Najjar, Razan Rabi, Saad Ruzzeh.

Conflict of interest statement

No Conflict of Interest is declared

Supplementary info

Publication types, MeSH terms Expand

Publication types

- Observational Study

MeSH terms

- Aged
- COVID-19 / epidemiology
- COVID-19 / mortality*
- COVID-19 / therapy*
- Comorbidity
- Critical Care / methods
- Critical Care / standards
- Critical Care / statistics & numerical data
- Developing Countries*
- Female
- Health Resources*
- Hospital Mortality*
- Hospitalization / statistics & numerical data
- Humans
- Intensive Care Units / statistics & numerical data
- Male
- Middle Aged
- Palliative Care / methods

- Palliative Care / standards
- Palliative Care / statistics & numerical data*
- Respiration, Artificial
- Retrospective Studies

Full text links

The Journal of Infection in Developing Countries
Proceed to details
Cite
Share
257
Observational Study
Diabetes Metab Syndr
. Jul-Aug 2021;15(4):102131.
doi: 10.1016/j.dsx.2021.05.004. Epub 2021 May 8.

Impact of nationwide lockdown on cancer care during COVID-19 pandemic: A retrospective analysis from western India

Ananya Pareek [1], Apurva A Patel [2], A Harshavardhan [1], Philip G Kuttikat [1], Shantanu Pendse [1], Aruj Dhyani [1], Satish Sharma [1], Nikesh Agarwal [1], Debajyoti Maji [1], Ramidi G Reddy [1], Yuganshu Gupta [1], Harsha P Panchal [1], Sonia Parikh [1]
Affiliations Expand

Affiliations

- [1] Department of Medical Oncology, The Gujarat Cancer and Research Institute, Ahmedabad, India.
- [2] Department of Medical Oncology, The Gujarat Cancer and Research Institute, Ahmedabad, India. Electronic address: dr.apurvapatel20@gmail.com.

- PMID: **34186357**
- PMCID: PMC8105303
- DOI: 10.1016/j.dsx.2021.05.004

Free PMC article
Observational Study

Impact of nationwide lockdown on cancer care during COVID-19 pandemic: A retrospective analysis from western India

Ananya Pareek et al. Diabetes Metab Syndr. Jul-Aug 2021.
Free PMC article
Show details
Diabetes Metab Syndr
. Jul-Aug 2021;15(4):102131.
doi: 10.1016/j.dsx.2021.05.004. Epub 2021 May 8.

Authors

Ananya Pareek [1], Apurva A Patel [2], A Harshavardhan [1], Philip G Kuttikat [1], Shantanu Pendse [1], Aruj Dhyani [1], Satish Sharma [1], Nikesh Agarwal [1], Debajyoti Maji [1], Ramidi G Reddy [1], Yuganshu Gupta [1], Harsha P Panchal [1], Sonia Parikh [1]

Affiliations

- [1] Department of Medical Oncology, The Gujarat Cancer and Research Institute, Ahmedabad, India.
- [2] Department of Medical Oncology, The Gujarat Cancer and Research Institute, Ahmedabad, India. Electronic address: dr.apurvapatel20@gmail.com.

- PMID: **34186357**
- PMCID: PMC8105303
- DOI: 10.1016/j.dsx.2021.05.004

Abstract

Background and aims: COVID-19 has impacted healthcare system worldwide including cancer case. Aim of this study was to describe the experience of lockdown on cancer care concerning patient's visit and reception of treatment in western India.

Methods: This is a retrospective observational study conducted in patients with cancer attending a tertiary care center pre-lockdown and during lockdown (from January to May 2020). Data related to demographic parameters, type of tumor, type of treatment received and functional status of patients were retrieved from hospital medical records of patients.

Results: Of the 5258 patients included, 4363 visited hospital pre-lockdown (median age, 50 years) and 895 visited during the lockdown period (median age, 47 years). A total of 1168 and 106 patients visiting hospital before and during lockdown, respectively, had comorbidities. Breast cancer (25.6% and 29.7%), head and neck cancer (21.3% and 16.9%) were the most common type of solid tumors; leukemia (58.0% and 73.0%), lymphoma (18.8% and 13.5%) and multiple myeloma (18.6% and 12.2%) were the most common type of hematological malignancies observed in patients visiting pre-lockdown and during lockdown, respectively. Chemotherapy was most commonly received treatment (pre-lockdown, 71.8%; during lockdown, 45.9%). Other therapies reported includes supportive/palliative, targeted, hormonal, and immunotherapy. The

majority of patients who visited the hospital pre-lockdown (68.4%) and during lockdown (62.8%) had 0 or 1 Eastern Cooperative Oncology Group (ECOG) score.

Conclusion: Overall observations highlight a substantial impact of an imposed nationwide lockdown during COVID-19 pandemic on cancer care of patients in terms of reduced patient visits and number of treatments received.

Keywords: Chemotherapy; ECOG score; Oncologists; Telemedicine; Transport.

Copyright © 2021. Published by Elsevier Ltd.

Conflict of interest statement

Declaration of competing interest None.

- Cited by 1 article
- 21 references
- 2 figures

Supplementary info

Publication types, MeSH terms Expand

Publication types

- Observational Study

MeSH terms

- Adolescent
- Adult
- Aged
- Aged, 80 and over
- COVID-19 / complications*
- COVID-19 / transmission
- COVID-19 / virology
- Child
- Child, Preschool
- Female
- Hospitalization / statistics & numerical data*
- Humans
- India / epidemiology
- Male
- Middle Aged
- Neoplasms / epidemiology
- Neoplasms / pathology

- Neoplasms / therapy*
- Neoplasms / virology
- Prognosis
- Quarantine / statistics & numerical data*
- Retrospective Studies
- Risk Factors
- SARS-CoV-2 / isolation & purification*
- Survival Rate
- Young Adult

Full text links

ELSEVIER FULL-TEXT ARTICLE Elsevier Science Free PMC article
Proceed to details
Cite
Share
☐ 258
Sci Rep
. 2021 May 27;11(1):11134.
doi: 10.1038/s41598-021-90610-y.

Urine biomarkers for the prediction of mortality in COVID-19 hospitalized patients

Daniel Morell-Garcia [1, 2], David Ramos-Chavarino [3], Josep M Bauça [3, 4], Paula Argente Del Castillo [3], Maria Antonieta Ballesteros-Vizoso [3], Luis García de Guadiana-Romualdo [5], Cristina Gómez-Cobo [3, 4], J Albert Pou [4, 6], Rocío Amezaga-Menéndez [4, 7], Alberto Alonso-Fernández [4, 8], Isabel Llompart [3, 4], Ana García-Raja [3, 4]
Affiliations Expand

Affiliations

- [1] Laboratory Medicine Department, Hospital Universitari Son Espases, Ctra. Valldemossa 79, mòdul 0-J., 07120, Palma de Mallorca, Spain. dr.morell.uab@gmail.com.
- [2] Institut d'Investigació Sanitària de Les Illes Balears (IdISBa), Palma de Mallorca, Spain. dr.morell.uab@gmail.com.
- [3] Laboratory Medicine Department, Hospital Universitari Son Espases, Ctra. Valldemossa 79, mòdul 0-J., 07120, Palma de Mallorca, Spain.
- [4] Institut d'Investigació Sanitària de Les Illes Balears (IdISBa), Palma de Mallorca, Spain.
- [5] Laboratory Medicine Department, Hospital Universitario Santa Lucía, Cartagena, Spain.
- [6] Internal Medicine Department, Hospital Universitari Son Espases, Palma de Mallorca, Spain.
- [7] Intensive Care Unit, Hospital Universitari Son Espases, Palma de Mallorca, Spain.

- [8] Respiratory Medicine Department, Hospital Universitari Son Espases, Palma de Mallorca, Spain.

- PMID: **34045530**
- PMCID: PMC8159957
- DOI: 10.1038/s41598-021-90610-y

Free PMC article

Urine biomarkers for the prediction of mortality in COVID-19 hospitalized patients

Daniel Morell-Garcia et al. Sci Rep. 2021.
Free PMC article
Show details
Sci Rep
. 2021 May 27;11(1):11134.
doi: 10.1038/s41598-021-90610-y.

Authors

Daniel Morell-Garcia [1, 2], David Ramos-Chavarino [3], Josep M Bauça [3, 4], Paula Argente Del Castillo [3], Maria Antonieta Ballesteros-Vizoso [3], Luis García de Guadiana-Romualdo [5], Cristina Gómez-Cobo [3, 4], J Albert Pou [4, 6], Rocío Amezaga-Menéndez [4, 7], Alberto Alonso-Fernández [4, 8], Isabel Llompart [3, 4], Ana García-Raja [3, 4]

Affiliations

- [1] Laboratory Medicine Department, Hospital Universitari Son Espases, Ctra. Valldemossa 79, mòdul 0-J., 07120, Palma de Mallorca, Spain. dr.morell.uab@gmail.com.
- [2] Institut d'Investigació Sanitària de Les Illes Balears (IdISBa), Palma de Mallorca, Spain. dr.morell.uab@gmail.com.
- [3] Laboratory Medicine Department, Hospital Universitari Son Espases, Ctra. Valldemossa 79, mòdul 0-J., 07120, Palma de Mallorca, Spain.
- [4] Institut d'Investigació Sanitària de Les Illes Balears (IdISBa), Palma de Mallorca, Spain.
- [5] Laboratory Medicine Department, Hospital Universitario Santa Lucía, Cartagena, Spain.
- [6] Internal Medicine Department, Hospital Universitari Son Espases, Palma de Mallorca, Spain.
- [7] Intensive Care Unit, Hospital Universitari Son Espases, Palma de Mallorca, Spain.
- [8] Respiratory Medicine Department, Hospital Universitari Son Espases, Palma de Mallorca, Spain.

- PMID: **34045530**
- PMCID: PMC8159957
- DOI: 10.1038/s41598-021-90610-y

Abstract

Risk factors associated with severity and mortality attributable to COVID-19 have been reported in different cohorts, highlighting the occurrence of acute kidney injury (AKI) in 25% of them. Among other, SARS-CoV-2 targets renal tubular cells and can cause acute renal damage. The aim of the present study was to evaluate the usefulness of urinary parameters in predicting intensive care unit (ICU) admission, mortality and development of AKI in hospitalized patients with COVID-19. Retrospective observational study, in a tertiary care hospital, between March 1st and April 19th, 2020. We recruited adult patients admitted consecutively and positive for SARS-CoV-2. Urinary and serum biomarkers were correlated with clinical outcomes (AKI, ICU admission, hospital discharge and in-hospital mortality) and evaluated using a logistic regression model and ROC curves. A total of 199 COVID-19 hospitalized patients were included. In AKI, the logistic regression model with a highest area under the curve (AUC) was reached by the combination of urine blood and previous chronic kidney disease, with an AUC of 0.676 (95%CI 0.512-0.840; $p = 0.023$); urine specific weight, sodium and albumin in serum, with an AUC of 0.837 (95% CI 0.766-0.909; $p < 0.001$) for ICU admission; and age, urine blood and lactate dehydrogenase levels in serum, with an AUC of 0.923 (95%CI 0.866-0.979; $p < 0.001$) for mortality prediction. For hospitalized patients with COVID-19, renal involvement and early alterations of urinary and serum parameters are useful as prognostic factors of AKI, the need for ICU admission and death.

Conflict of interest statement

The authors declare no competing interests.

- Cited by 4 articles
- 40 references
- 4 figures

Supplementary info

Publication types, MeSH terms, Substances Expand

Publication types

- Research Support, Non-U.S. Gov't

MeSH terms

- Acute Kidney Injury / complications
- Acute Kidney Injury / mortality*
- Acute Kidney Injury / physiopathology
- Acute Kidney Injury / urine*
- Adult
- Aged
- Area Under Curve
- Biomarkers / urine
- COVID-19 / complications

- COVID-19 / mortality*
- COVID-19 / physiopathology
- COVID-19 / urine*
- Critical Care
- Female
- Hospitalization
- Humans
- Logistic Models
- Male
- Middle Aged
- Observational Studies as Topic
- Prognosis
- ROC Curve
- Retrospective Studies
- Risk Factors
- Severity of Illness Index
- Urine / chemistry

Substances

- Biomarkers

Full text links

Nature Publishing Group Free PMC article

Proceed to details

Cite

Share

259

Observational Study

Chin Med J (Engl)

. 2021 Oct 26;135(2):187-193.

doi: 10.1097/CM9.0000000000001798.

Usefulness of the CONUT index upon hospital admission as a potential prognostic indicator of COVID-19 health outcomes

Adrián K Bengelloun [1], Guillermo J Ortega [2], Julio Ancochea [1] [3] [4], Ancor Sanz-Garcia [2], Diego A Rodríguez-Serrano [3], Guillermo Fernández-Jiménez [3], Rosa Girón [1] [3] [4], Elena Ávalos [3], Joan B Soriano [1] [3] [4], J Ignacio de Ulíbarri [3]

Affiliations Expand

Affiliations

- [1] Facultad de Medicina, Universidad Autónoma de Madrid, Madrid, Spain.
- [2] Unidad de Análisis de Datos, Instituto de Investigación Sanitaria del Hospital de la Princesa, Madrid, Spain.
- [3] Servicios de Neumología, Admisión y Documentación Clínica, Cuidados Intensivos y Nutrición y Dietética; Hospital Universitario de La Princesa, Madrid, Spain.
- [4] Centro de Investigación en Red de Enfermedades Respiratorias (CIBERES), Instituto de Salud Carlos III (ISCIII), Madrid, España, Spain.

- PMID: **34711718**
- PMCID: PMC8769140
- DOI: 10.1097/CM9.0000000000001798

Free PMC article
Observational Study

Usefulness of the CONUT index upon hospital admission as a potential prognostic indicator of COVID-19 health outcomes

Adrián K Bengelloun et al. Chin Med J (Engl). 2021.
Free PMC article
Show details
Chin Med J (Engl)
. 2021 Oct 26;135(2):187-193.
doi: 10.1097/CM9.0000000000001798.

Authors

Adrián K Bengelloun [1], Guillermo J Ortega [2], Julio Ancochea [1 3 4], Ancor Sanz-Garcia [2], Diego A Rodríguez-Serrano [3], Guillermo Fernández-Jiménez [3], Rosa Girón [1 3 4], Elena Ávalos [3], Joan B Soriano [1 3 4], J Ignacio de Ulíbarri [3]

Affiliations

- [1] Facultad de Medicina, Universidad Autónoma de Madrid, Madrid, Spain.
- [2] Unidad de Análisis de Datos, Instituto de Investigación Sanitaria del Hospital de la Princesa, Madrid, Spain.
- [3] Servicios de Neumología, Admisión y Documentación Clínica, Cuidados Intensivos y Nutrición y Dietética; Hospital Universitario de La Princesa, Madrid, Spain.
- [4] Centro de Investigación en Red de Enfermedades Respiratorias (CIBERES), Instituto de Salud Carlos III (ISCIII), Madrid, España, Spain.

- PMID: **34711718**

- PMCID: PMC8769140
- DOI: 10.1097/CM9.0000000000001798

Abstract

Background: In-hospital mortality in patients with coronavirus disease 2019 (COVID-19) is high. Simple prognostic indices are needed to identify patients at high-risk of COVID-19 health outcomes. We aimed to determine the usefulness of the CONtrolling NUTritional status (CONUT) index as a potential prognostic indicator of mortality in COVID-19 patients upon hospital admission.

Methods: Our study design is of a retrospective observational study in a large cohort of COVID-19 patients. In addition to descriptive statistics, a Kaplan-Meier mortality analysis and a Cox regression were performed, as well as receiver operating curve (ROC).

Results: From February 5, 2020 to January 21, 2021, there was a total of 2969 admissions for COVID-19 at our hospital, corresponding to 2844 patients. Overall, baseline (within 4 days of admission) CONUT index could be scored for 1627 (57.2%) patients. Patients' age was 67.3 ± 16.5 years and 44.9% were women. The CONUT severity distribution was: 194 (11.9%) normal (0-1); 769 (47.2%) light (2-4); 585 (35.9%) moderate (5-8); and 79 (4.9%) severe (9-12). Mortality of 30 days after admission was 3.1% in patients with normal risk CONUT, 9.0% light, 22.7% moderate, and 40.5% in those with severe CONUT ($P < 0.05$). An increased risk of death associated with a greater baseline CONUT stage was sustained in a multivariable Cox regression model ($P < 0.05$). An increasing baseline CONUT stage was associated with a longer duration of admission, a greater requirement for the use of non-invasive and invasive mechanical ventilation, and other clinical outcomes (all $P < 0.05$). The ROC of CONUT for mortality had an area under the curve (AUC) and 95% confidence interval of 0.711 (0.676-0746).

Conclusion: The CONUT index upon admission is potentially a reliable and independent prognostic indicator of mortality and length of hospitalization in COVID-19 patients.

Copyright © 2022 The Chinese Medical Association, produced by Wolters Kluwer, Inc. under the CC-BY-NC-ND license.

Conflict of interest statement

The authors declare there are no conflicts of interest to report related with this research.

- 23 references
- 4 figures

Supplementary info

Publication types, MeSH terms Expand

Publication types

- Observational Study

MeSH terms

- Aged
- Aged, 80 and over
- COVID-19*
- Female
- Hospitalization
- Hospitals
- Humans
- Middle Aged
- Nutrition Assessment
- Nutritional Status
- Outcome Assessment, Health Care
- Prognosis
- Retrospective Studies
- SARS-CoV-2

Full text links

Wolters Kluwer Free PMC article

Proceed to details

Cite

Share

260

Observational Study

Clin Med (Lond)

. 2021 Nov;21(6):e604-e607.

doi: 10.7861/clinmed.2021-0308. Epub 2021 Oct 12.

Failing the frail: The need to broaden the COVID-19 case definition for geriatric patients

Clare Hunt [1] [2], Flora Olcott [3] [2], George Williams [4], Terrence Chan [3]

Affiliations Expand

Affiliations

- [1] Maidstone and Tunbridge Wells NHS Trust, Tunbridge Wells, UK clare.hunt1@nhs.net.
- [2] joint first authors.
- [3] Lewisham and Greenwich NHS Trust, London, UK.
- [4] Barts Health NHS Trust, London, UK.

- PMID: **34642166**
- PMCID: PMC8806293
- DOI: 10.7861/clinmed.2021-0308

Free PMC article
Observational Study

Failing the frail: The need to broaden the COVID-19 case definition for geriatric patients

Clare Hunt et al. Clin Med (Lond). 2021 Nov.
Free PMC article
Show details
Clin Med (Lond)
. 2021 Nov;21(6):e604-e607.
doi: 10.7861/clinmed.2021-0308. Epub 2021 Oct 12.

Authors

Clare Hunt [1] [2], Flora Olcott [3] [2], George Williams [4], Terrence Chan [3]

Affiliations

- [1] Maidstone and Tunbridge Wells NHS Trust, Tunbridge Wells, UK clare.hunt1@nhs.net.
- [2] joint first authors.
- [3] Lewisham and Greenwich NHS Trust, London, UK.
- [4] Barts Health NHS Trust, London, UK.

- PMID: **34642166**
- PMCID: PMC8806293
- DOI: 10.7861/clinmed.2021-0308

Abstract

The older population has a high mortality with COVID-19 and this cohort often presents atypically with infection. This study compares presenting complaints and observations of older patients with COVID-19 against the established case definition to determine whether the case definition should be broadened to better identify SARS-CoV-2 infection in this age group.This retrospective observational study analysed the presenting complaints and observations of people aged 70 years and over who were admitted to a district general hospital with confirmed SARS-CoV-2 infection from March to May 2020.Out of 225 patients, only 11.5% presented with the trio of cough, fever and breathlessness; 30.2% did not present with any of these symptoms ($p<0.001$). The most frequent atypical complaints were delirium (25%), general malaise (20%) and falls (19%). Only 32.4% recorded a temperature ≥37.6°C on admission while 20.4% were hypothermic with a temperature <36.4°C ($p=0.0003$).A significant proportion of older patients with COVID-19 presented with non-specific symptoms and observations. The high proportion of falls and delirium

emphasises the need for early geriatrician input, awareness of COVID-19 as a differential for confusion in older patients and to include falls in the case definition for COVID-19 in the older population.

Keywords: COVID-19; delirium; falls; geriatric medicine; presenting complaints.

© Royal College of Physicians 2021. All rights reserved.

- 1 figure

Supplementary info

Publication types, MeSH terms Expand

Publication types

- Observational Study

MeSH terms

- Aged
- Aged, 80 and over
- COVID-19*
- Frail Elderly
- Hospitalization
- Humans
- Retrospective Studies
- SARS-CoV-2

Full text links

Full text ClinMed HighWire Free PMC article
Proceed to details
Cite
Share
☐ 261
Observational Study
Infect Dis Now
. 2021 Sep;51(6):518-525.
doi: 10.1016/j.idnow.2021.07.002. Epub 2021 Jul 7.

[Predictors of mortality, ICU hospitalization, and extrapulmonary complications in COVID-19 patients]

M Martinot [1], M Eyriey [2], S Gravier [3], T Bonijoly [3], D Kayser [3], C Ion [3], M Mohseni-Zadeh [3], S Camara [2], J Dubois [2], E Haerrel [2], J Drouaine [2], J Kaiser [4], J C Ongagna [2], A Schieber-Pachart [2], C Kempf [2], Centre Alsace COVID-19 Study Group
Affiliations Expand

Affiliations

- [1] Infectious Diseases Department, Hôpitaux Civils, 39, avenue de la liberté, 68024 Colmar, France. Electronic address: martin.martinot@ch-colmar.fr.
- [2] Clinical Research Department, Hôpitaux Civils de Colmar, Colmar, France.
- [3] Infectious Diseases Department, Hôpitaux Civils, 39, avenue de la liberté, 68024 Colmar, France.
- [4] Clinical Research Department, Hôpitaux Civils de Colmar, Colmar, France; Pharmacy Department, Hôpitaux Civils de Colmar, Colmar, France.

- PMID: **34242842**
- PMCID: PMC8260549
- DOI: 10.1016/j.idnow.2021.07.002

Free PMC article
Observational Study

Predictors of mortality, ICU hospitalization, and extrapulmonary complications in COVID-19 patients

M Martinot et al. Infect Dis Now. 2021 Sep.
Free PMC article
Show details
Infect Dis Now
. 2021 Sep;51(6):518-525.
doi: 10.1016/j.idnow.2021.07.002. Epub 2021 Jul 7.

Authors

M Martinot [1], M Eyriey [2], S Gravier [3], T Bonijoly [3], D Kayser [3], C Ion [3], M Mohseni-Zadeh [3], S Camara [2], J Dubois [2], E Haerrel [2], J Drouaine [2], J Kaiser [4], J C Ongagna [2], A Schieber-Pachart [2], C Kempf [2], Centre Alsace COVID-19 Study Group

Affiliations

- [1] Infectious Diseases Department, Hôpitaux Civils, 39, avenue de la liberté, 68024 Colmar, France. Electronic address: martin.martinot@ch-colmar.fr.
- [2] Clinical Research Department, Hôpitaux Civils de Colmar, Colmar, France.
- [3] Infectious Diseases Department, Hôpitaux Civils, 39, avenue de la liberté, 68024 Colmar, France.
- [4] Clinical Research Department, Hôpitaux Civils de Colmar, Colmar, France; Pharmacy Department, Hôpitaux Civils de Colmar, Colmar, France.

- PMID: **34242842**
- PMCID: PMC8260549
- DOI: 10.1016/j.idnow.2021.07.002

Abstract

Objective: A major coronavirus disease 2019 (COVID-19) outbreak occurred in Northeastern France in spring 2020. This single-center retrospective observational cohort study aimed to compare patients with severe COVID-19 and those with non-severe COVID-19 (survivors vs. non-survivors, ICU patients vs. non-ICU patients) and to describe extrapulmonary complications.

Patients and methods: We included all patients with a confirmed diagnosis of COVID-19 admitted to Colmar Hospital in March 2020.

Results: We examined 600 patients (median age 71.09 years; median body mass index: 26.9 kg/m^2); 57.7% were males, 86.3% had at least one comorbidity, 153 (25.5%) required ICU hospitalization, and 115 (19.1%) died. Baseline independent factors associated with death were older age (>75 vs. ≤75 years), male sex, oxygen supply, chronic neurological, renal, and pulmonary diseases, diabetes, cancer, low platelet and hemoglobin counts, and high levels of C-reactive protein (CRP) and serum creatinine. Factors associated with ICU hospitalization were age <75 years, oxygen supply, chronic pulmonary disease, absence of dementia, and high levels of CRP, hemoglobin, and serum creatinine. Among the 600 patients, 80 (13.3%) had an acute renal injury, 33 (5.5%) had a cardiovascular event, 27 (4.5%) had an acute liver injury, 24 (4%) had venous thromboembolism, eight (1.3%) had a neurological event, five (0.8%) had rhabdomyolysis, and one had acute pancreatitis. Most extrapulmonary complications occurred in ICU patients.

Conclusion: This study highlighted the main risk factors for ICU hospitalization and death caused by severe COVID-19 and the frequency of numerous extrapulmonary complications in France.

Keywords: COVID-19; Extrapulmonary COVID-19; Mortality; Outcome; SARS-CoV-2.

Copyright © 2021 Elsevier Masson SAS. All rights reserved.

- Cited by 2 articles
- 31 references
- 3 figures

Supplementary info

Publication types, MeSH terms Expand

Publication types

- Observational Study

MeSH terms

- Acute Kidney Injury / epidemiology*
- Acute Kidney Injury / etiology
- Acute Lung Injury / epidemiology
- Aged
- Aged, 80 and over
- COVID-19 / complications
- COVID-19 / mortality*
- Cardiovascular Diseases / epidemiology*
- Cardiovascular Diseases / etiology
- Comorbidity
- Female
- France / epidemiology
- Hospital Mortality
- Hospitalization / statistics & numerical data*
- Humans
- Intensive Care Units / statistics & numerical data*
- Male
- Middle Aged
- Nervous System Diseases / epidemiology
- Pancreatitis
- Respiration, Artificial / statistics & numerical data
- Retrospective Studies
- Rhabdomyolysis / epidemiology
- Risk Factors
- SARS-CoV-2
- Severity of Illness Index
- Venous Thromboembolism / epidemiology

Full text links

ELSEVIER FULL-TEXT ARTICLE Elsevier Science Free PMC article

Proceed to details

Cite

Share

☐ 262

Observational Study

Endocr Pract
. 2021 Oct;27(10):1046-1051.
doi: 10.1016/j.eprac.2021.07.008. Epub 2021 Jul 18.

Correlation of Hemoglobin A1C and Outcomes in Patients Hospitalized With COVID-19

Amy J Patel [1], Stanislaw P Klek [1], Virginia Peragallo-Dittko [2], Michael Goldstein [3], Eric Burdge [3], Victoria Nadile [3], Julia Ramadhar [3], Shahidul Islam [2], Gary D Rothberger [4]
Affiliations Expand

Affiliations

- [1] Division of Endocrinology, Diabetes, and Metabolism, New York University Long Island School of Medicine, Mineola, New York.
- [2] Department of Foundations of Medicine, New York University Long Island School of Medicine, Mineola, New York.
- [3] Department of Medicine, New York University Long Island School of Medicine, Mineola, New York.
- [4] Division of Endocrinology, Diabetes, and Metabolism, New York University Long Island School of Medicine, Mineola, New York. Electronic address: Gary.rothberger@nyulangone.org.

- PMID: **34284145**
- PMCID: PMC8286241
- DOI: 10.1016/j.eprac.2021.07.008

Free PMC article
Observational Study

Correlation of Hemoglobin A1C and Outcomes in Patients Hospitalized With COVID-19

Amy J Patel et al. Endocr Pract. 2021 Oct.
Free PMC article
Show details

Endocr Pract
. 2021 Oct;27(10):1046-1051.
doi: 10.1016/j.eprac.2021.07.008. Epub 2021 Jul 18.

Authors

Amy J Patel [1], Stanislaw P Klek [1], Virginia Peragallo-Dittko [2], Michael Goldstein [3], Eric Burdge [3], Victoria Nadile [3], Julia Ramadhar [3], Shahidul Islam [2], Gary D Rothberger [4]

Affiliations

- [1] Division of Endocrinology, Diabetes, and Metabolism, New York University Long Island School of Medicine, Mineola, New York.
- [2] Department of Foundations of Medicine, New York University Long Island School of Medicine, Mineola, New York.
- [3] Department of Medicine, New York University Long Island School of Medicine, Mineola, New York.
- [4] Division of Endocrinology, Diabetes, and Metabolism, New York University Long Island School of Medicine, Mineola, New York. Electronic address: Gary.rothberger@nyulangone.org.

- PMID: **34284145**
- PMCID: PMC8286241
- DOI: 10.1016/j.eprac.2021.07.008

Abstract

Objective: Diabetes is a known risk factor for severe coronavirus disease 2019 (COVID-19). We conducted this study to determine if there is a correlation between hemoglobin A1C (HbA1C) level and poor outcomes in hospitalized patients with diabetes and COVID-19.

Methods: This is a retrospective, single-center, observational study of patients with diabetes (defined by an HbA1C level of ≥6.5% or known medical history of diabetes) who had a confirmed case of COVID-19 and required hospitalization. All patients were admitted to our institution between March 3, 2020, and May 5, 2020. HbA1C results for each patient were divided into quartiles: 5.1% to 6.7% (32-50 mmol/mol), 6.8% to 7.5% (51-58 mmol/mol), 7.6% to 8.9% (60-74 mmol/mol), and >9% (>75 mmol/mol). The primary outcome was in-hospital mortality. Secondary outcomes included admission to an intensive care unit, invasive mechanical ventilation, acute kidney injury, acute thrombosis, and length of hospital stay.

Results: A total of 506 patients were included. The number of deaths within quartiles 1 through 4 were 30 (25%), 37 (27%), 34 (27%), and 24 (19%), respectively. There was no statistical difference in the primary or secondary outcomes among the quartiles, except that acute kidney injury was less frequent in quartile 4.

Conclusion: There was no significant association between HbA1C level and adverse clinical outcomes in patients with diabetes who are hospitalized with COVID-19. HbA1C levels should not be used for risk stratification in these patients.

Keywords: COVID-19; diabetes; hemoglobin A1C; mortality; outcomes.

Copyright © 2021 AACE. Published by Elsevier Inc. All rights reserved.

- 39 references

Supplementary info

Publication types, MeSH terms, Substances Expand

Publication types

- Observational Study

MeSH terms

- COVID-19* / complications
- Diabetes Mellitus* / epidemiology
- Diabetes Mellitus* / virology
- Glycated Hemoglobin A / analysis*
- Hospital Mortality
- Hospitalization
- Humans
- Retrospective Studies

Substances

- Glycated Hemoglobin A

Full text links

ELSEVIER FULL-TEXT ARTICLE Elsevier Science Free PMC article
Proceed to details
Cite
Share
263
Observational Study
Eur J Endocrinol
. 2021 Jul 5;185(2):299-311.
doi: 10.1530/EJE-21-0068.

Sex disparities in COVID-19 outcomes of inpatients with diabetes: insights from the CORONADO study

Blandine Tramunt [1], Sarra Smati [2], Sandrine Coudol [3], Matthieu Wargny [2 3], Matthieu Pichelin [2], Béatrice Guyomarch [4], Abdallah Al-Salameh [5 6], Coralie Amadou [7], Sara Barraud [8 9], Edith Bigot [10], Lyse Bordier [11], Sophie Borot [12], Muriel Bourgeon [13], Olivier Bourron [14], Sybil Charrière [15], Nicolas Chevalier [16], Emmanuel Cosson [17 18], Bruno Fève [19 20], Anna Flaus-Furmaniuk [21], Pierre Fontaine [22], Amandine Galioot [23], Céline Gonfroy-Leymarie [24], Bruno

Guerci [25], Sandrine Lablanche [26], Jean-Daniel Lalau [5] [6], Etienne Larger [27], Adèle Lasbleiz [28] [29], Bruno Laviolle [30], Michel Marre [31], Marion Munch [32], Louis Potier [33] [34], Gaëtan Prevost [35], Eric Renard [36], Yves Reznik [37], Dominique Seret-Bégué [38], Paul Sibilia [39], Philippe Thuillier [40], Bruno Vergès [41], Jean-François Gautier [42] [43], Samy Hadjadj [2], Bertrand Cariou [2], Franck Mauvais-Jarvis [44] [45] [46], Pierre Gourdy [1]

Affiliations Expand

Affiliations

- [1] Department of Diabetology, Metabolic Diseases and Nutrition, Toulouse University Hospital, Institute of Metabolic and Cardiovascular Diseases, UMR1297 INSERM/UPS, Toulouse University, Toulouse, France.
- [2] Nantes University, Nantes University Hospital, CNRS, INSERM, L'Institut du Thorax, Nantes, France.
- [3] CIC-EC 1413, Data Clinic.
- [4] Research Department, Methodology and Biostatistics Platform, Nantes University Hospital, Nantes, France.
- [5] Department of Endocrinology, Diabetes Mellitus and Nutrition, Amiens University Hospital, Amiens, France.
- [6] PériTox=UMR_I 01, University of Picardie Jules Verne, Amiens, France.
- [7] Department of Diabetology, Sud Francilien Hospital Center, Corbeil Essonne, France.
- [8] CRESTIC EA 3804, University of Reims Champagne Ardenne, UFR Sciences Exactes et Naturelles, Moulin de la Housse, Reims, France.
- [9] Department of Endocrinology-Diabetes-Nutrition, Reims University Hospital, Avenue du Général Koenig, Reims, France.
- [10] Department of Biochemistry, Nantes University Hospital, G et R Laënnec Hospital, Bd Jacques Monod, Nantes, France.
- [11] Department of Endocrinology, Bégin Hospital, Saint-Mandé, France.
- [12] Department of Endocrinology, Diabetology and Nutrition, Besançon University Hospital, Besançon, France.
- [13] Department of Endocrinology, Diabetology and Nutrition, Assistance Publique Hôpitaux de Paris, Paris Saclay University, Antoine Béclère Hospital, Clamart, Bicêtre Hospital, Le Kremlin Bicêtre, France.
- [14] Department of Diabetology, Sorbonne University, Assistance Publique Hôpitaux de Paris, La Pitié Salpêtrière-Charles Foix University Hospital, Inserm, UMR_S 1138, Cordeliers Research Center, Paris 06, Institute of Cardiometabolism and Nutrition ICAN, Paris, France.
- [15] Federation of Endocrinology - Louis Pradel Cardiovascular Hospital, Hospices Civils de Lyon, INSERM UMR 1060 Carmen, Claude Bernard Lyon 1 University, Lyon, France.
- [16] University of Côte d'Azur, University Hospital, Inserm U1065, C3M, Nice, France.
- [17] Department of Endocrinology, Diabetology and Nutrition, Assistance Publique Hôpitaux de Paris, Avicenne Hospital, Paris 13 University, Sorbonne Paris Cité, CRNH-IdF, CINFO, Bobigny, France.
- [18] Paris 13 University, Sorbonne Paris Cité, UMR U557 Inserm/U11125 INRAE/CNAM/Paris13 University, Nutritional Epidemiological Research Unit, Bobigny, France.
- [19] Department of Endocrinology, Assistance Publique Hôpitaux de Paris, Saint-Antoine Hospital, Reference Center of Rare Diseases of Insulin Secretion and Insulin Sensitivity (PRISIS), Paris, France.

- [20] Sorbonne University, Inserm UMRS 938, Saint-Antoine Research Center, Paris, France.
- [21] Department of Endocrinology-Diabetology, Felix Guyon Site, University Hospital of la Réunion, Saint-Denis de la Réunion, France.
- [22] Department of Endocrinology, Diabetology and Nutrition, Hospital of Huriez, Lille University Hospital, Lille, France.
- [23] Department of Endocrinology, Diabetology and Nutrition, Bordeaux University Hospital and University of Bordeaux, Bordeaux, France.
- [24] Department of Endocrinology and Diabetology, Hospital of Pontoise, Pontoise, France.
- [25] Lorraine University and Endocrinology, Diabetology, Metabolic Diseases and Nutrition, Nancy University Hospital, Nancy, France.
- [26] Grenoble Alpes University, INSERM U1055, LBFA, Endocrinology, Grenoble Alpes University Hospital, France.
- [27] Department of Diabetology, Cochin Hospital, AP-HP, Paris University, Paris, France.
- [28] Department of Endocrinology, Diabetology and Nutrition, Hospital of la Conception, Assistance Publique-Hôpitaux de Marseille, Marseille, France.
- [29] Aix Marseille University, INSERM, INRA, C2VN, Marseille, France.
- [30] Rennes University, Rennes University Hospital, Inserm, CIC 1414 (Clinical Investigation Center), Rennes, France.
- [31] Ambroise Paré Neuilly-sur-Seine Hospital, Cordeliers Research Center, Paris Diderot University, Paris, France.
- [32] Department of Endocrinology, Diabetology and Nutrition, Strasbourg University Hospitals, Strasbourg, France.
- [33] Department of Endocrinology, Diabetology and Nutrition, Bichat Hospital, Assistance Publique Hôpitaux de Paris, Paris, France.
- [34] Cordeliers Research Center, Inserm, U-1138, Paris University, Paris, France.
- [35] Department of Endocrinology, Diabetes and Metabolic Diseases, Normandie University, UNIROUEN, Rouen University Hospital, Rouen, France.
- [36] Department of Endocrinology, Diabetes, Nutrition, Montpellier University Hospital, INSERM Clinical Investigation Centre, Institute of Functional Genomics, CNRS, INSERM, University of Montpellier, Montpellier, France.
- [37] Department of Endocrinology and Diabetology, University Hospital of Côte de Nacre, Caen Cedex, France.
- [38] Department of Diabetology, Hospital of Gonesse, Gonesse, France.
- [39] Department of Endocrinology, Diabetology and Nutrition, Angers University Hospital, Angers, France.
- [40] Department of Endocrinology, Brest University Hospital, EA 3878 GETBO, Brest, France.
- [41] Department of Endocrinology, Diabetology and Metabolic Diseases, Hospital of Bocage, Dijon, France.
- [42] Department of Diabetology and Endocrinology, Lariboisière Hospital, APHP, Paris, France.
- [43] INSERM UMRS 1138, Paris Diderot-Paris VII University, Sorbonne Paris Cité, Paris, France.
- [44] Section of Endocrinology, John W Deming Department of Medicine, Tulane University School of Medicine, New Orleans, Louisiana, USA.
- [45] Southeast Louisiana Veterans Health Care System Medical Center, New Orleans, Louisiana, USA.
- [46] Tulane Center of Excellence in Sex-Based Biology and Medicine, New Orleans, Louisiana, USA.

- PMID: **34085949**
- DOI: 10.1530/EJE-21-0068

Free article
Observational Study

Sex disparities in COVID-19 outcomes of inpatients with diabetes: insights from the CORONADO study

Blandine Tramunt et al. Eur J Endocrinol. 2021.
Free article
Show details
Eur J Endocrinol
. 2021 Jul 5;185(2):299-311.
doi: 10.1530/EJE-21-0068.

Authors

Blandine Tramunt [1], Sarra Smati [2], Sandrine Coudol [3], Matthieu Wargny [2] [3], Matthieu Pichelin [2], Béatrice Guyomarch [4], Abdallah Al-Salameh [5] [6], Coralie Amadou [7], Sara Barraud [8] [9], Edith Bigot [10], Lyse Bordier [11], Sophie Borot [12], Muriel Bourgeon [13], Olivier Bourron [14], Sybil Charrière [15], Nicolas Chevalier [16], Emmanuel Cosson [17] [18], Bruno Fève [19] [20], Anna Flaus-Furmaniuk [21], Pierre Fontaine [22], Amandine Galioot [23], Céline Gonfroy-Leymarie [24], Bruno Guerci [25], Sandrine Lablanche [26], Jean-Daniel Lalau [5] [6], Etienne Larger [27], Adèle Lasbleiz [28] [29], Bruno Laviolle [30], Michel Marre [31], Marion Munch [32], Louis Potier [33] [34], Gaëtan Prevost [35], Eric Renard [36], Yves Reznik [37], Dominique Seret-Bégué [38], Paul Sibilia [39], Philippe Thuillier [40], Bruno Vergès [41], Jean-François Gautier [42] [43], Samy Hadjadj [2], Bertrand Cariou [2], Franck Mauvais-Jarvis [44] [45] [46], Pierre Gourdy [1]

Affiliations

- [1] Department of Diabetology, Metabolic Diseases and Nutrition, Toulouse University Hospital, Institute of Metabolic and Cardiovascular Diseases, UMR1297 INSERM/UPS, Toulouse University, Toulouse, France.
- [2] Nantes University, Nantes University Hospital, CNRS, INSERM, L'Institut du Thorax, Nantes, France.
- [3] CIC-EC 1413, Data Clinic.
- [4] Research Department, Methodology and Biostatistics Platform, Nantes University Hospital, Nantes, France.
- [5] Department of Endocrinology, Diabetes Mellitus and Nutrition, Amiens University Hospital, Amiens, France.
- [6] PériTox=UMR_I 01, University of Picardie Jules Verne, Amiens, France.
- [7] Department of Diabetology, Sud Francilien Hospital Center, Corbeil Essonne, France.
- [8] CRESTIC EA 3804, University of Reims Champagne Ardenne, UFR Sciences Exactes et Naturelles, Moulin de la Housse, Reims, France.

- [9] Department of Endocrinology-Diabetes-Nutrition, Reims University Hospital, Avenue du Général Koenig, Reims, France.
- [10] Department of Biochemistry, Nantes University Hospital, G et R Laënnec Hospital, Bd Jacques Monod, Nantes, France.
- [11] Department of Endocrinology, Bégin Hospital, Saint-Mandé, France.
- [12] Department of Endocrinology, Diabetology and Nutrition, Besançon University Hospital, Besançon, France.
- [13] Department of Endocrinology, Diabetology and Nutrition, Assistance Publique Hôpitaux de Paris, Paris Saclay University, Antoine Béclère Hospital, Clamart, Bicêtre Hospital, Le Kremlin Bicêtre, France.
- [14] Department of Diabetology, Sorbonne University, Assistance Publique Hôpitaux de Paris, La Pitié Salpêtrière-Charles Foix University Hospital, Inserm, UMR_S 1138, Cordeliers Research Center, Paris 06, Institute of Cardiometabolism and Nutrition ICAN, Paris, France.
- [15] Federation of Endocrinology - Louis Pradel Cardiovascular Hospital, Hospices Civils de Lyon, INSERM UMR 1060 Carmen, Claude Bernard Lyon 1 University, Lyon, France.
- [16] University of Côte d'Azur, University Hospital, Inserm U1065, C3M, Nice, France.
- [17] Department of Endocrinology, Diabetology and Nutrition, Assistance Publique Hôpitaux de Paris, Avicenne Hospital, Paris 13 University, Sorbonne Paris Cité, CRNH-IdF, CINFO, Bobigny, France.
- [18] Paris 13 University, Sorbonne Paris Cité, UMR U557 Inserm/U11125 INRAE/CNAM/Paris13 University, Nutritional Epidemiological Research Unit, Bobigny, France.
- [19] Department of Endocrinology, Assistance Publique Hôpitaux de Paris, Saint-Antoine Hospital, Reference Center of Rare Diseases of Insulin Secretion and Insulin Sensitivity (PRISIS), Paris, France.
- [20] Sorbonne University, Inserm UMRS 938, Saint-Antoine Research Center, Paris, France.
- [21] Department of Endocrinology-Diabetology, Felix Guyon Site, University Hospital of la Réunion, Saint-Denis de la Réunion, France.
- [22] Department of Endocrinology, Diabetology and Nutrition, Hospital of Huriez, Lille University Hospital, Lille, France.
- [23] Department of Endocrinology, Diabetology and Nutrition, Bordeaux University Hospital and University of Bordeaux, Bordeaux, France.
- [24] Department of Endocrinology and Diabetology, Hospital of Pontoise, Pontoise, France.
- [25] Lorraine University and Endocrinology, Diabetology, Metabolic Diseases and Nutrition, Nancy University Hospital, Nancy, France.
- [26] Grenoble Alpes University, INSERM U1055, LBFA, Endocrinology, Grenoble Alpes University Hospital, France.
- [27] Department of Diabetology, Cochin Hospital, AP-HP, Paris University, Paris, France.
- [28] Department of Endocrinology, Diabetology and Nutrition, Hospital of la Conception, Assistance Publique-Hôpitaux de Marseille, Marseille, France.
- [29] Aix Marseille University, INSERM, INRA, C2VN, Marseille, France.
- [30] Rennes University, Rennes University Hospital, Inserm, CIC 1414 (Clinical Investigation Center), Rennes, France.
- [31] Ambroise Paré Neuilly-sur-Seine Hospital, Cordeliers Research Center, Paris Diderot University, Paris, France.
- [32] Department of Endocrinology, Diabetology and Nutrition, Strasbourg University Hospitals, Strasbourg, France.

- [33] Department of Endocrinology, Diabetology and Nutrition, Bichat Hospital, Assistance Publique Hôpitaux de Paris, Paris, France.
- [34] Cordeliers Research Center, Inserm, U-1138, Paris University, Paris, France.
- [35] Department of Endocrinology, Diabetes and Metabolic Diseases, Normandie University, UNIROUEN, Rouen University Hospital, Rouen, France.
- [36] Department of Endocrinology, Diabetes, Nutrition, Montpellier University Hospital, INSERM Clinical Investigation Centre, Institute of Functional Genomics, CNRS, INSERM, University of Montpellier, Montpellier, France.
- [37] Department of Endocrinology and Diabetology, University Hospital of Côte de Nacre, Caen Cedex, France.
- [38] Department of Diabetology, Hospital of Gonesse, Gonesse, France.
- [39] Department of Endocrinology, Diabetology and Nutrition, Angers University Hospital, Angers, France.
- [40] Department of Endocrinology, Brest University Hospital, EA 3878 GETBO, Brest, France.
- [41] Department of Endocrinology, Diabetology and Metabolic Diseases, Hospital of Bocage, Dijon, France.
- [42] Department of Diabetology and Endocrinology, Lariboisière Hospital, APHP, Paris, France.
- [43] INSERM UMRS 1138, Paris Diderot-Paris VII University, Sorbonne Paris Cité, Paris, France.
- [44] Section of Endocrinology, John W Deming Department of Medicine, Tulane University School of Medicine, New Orleans, Louisiana, USA.
- [45] Southeast Louisiana Veterans Health Care System Medical Center, New Orleans, Louisiana, USA.
- [46] Tulane Center of Excellence in Sex-Based Biology and Medicine, New Orleans, Louisiana, USA.

- PMID: **34085949**
- DOI: 10.1530/EJE-21-0068

Abstract

Objective: Male sex is one of the determinants of severe coronavirus diseas-e-2019 (COVID-19). We aimed to characterize sex differences in severe outcomes in adults with diabetes hospitalized for COVID-19.

Methods: We performed a sex-stratified analysis of clinical and biological features and outcomes (i.e. invasive mechanical ventilation (IMV), death, intensive care unit (ICU) admission and home discharge at day 7 (D7) or day 28 (D28)) in 2380 patients with diabetes hospitalized for COVID-19 and included in the nationwide CORONADO observational study (NCT04324736).

Results: The study population was predominantly male (63.5%). After multiple adjustments, female sex was negatively associated with the primary outcome (IMV and/or death, OR: 0.66 (0.49-0.88)), death (OR: 0.49 (0.30-0.79)) and ICU admission (OR: 0.57 (0.43-0.77)) at D7 but only with ICU admission (OR: 0.58 (0.43-0.77)) at D28. Older age and a history of microvascular complications were predictors of death at D28 in both sexes, while chronic obstructive pulmonary disease (COPD) was predictive of death in women only. At admission, C-reactive protein (CRP), aspartate amino transferase (AST) and estimated glomerular filtration rate (eGFR), according to the CKD-EPI formula predicted death in both sexes. Lymphocytopenia was an independent

predictor of death in women only, while thrombocytopenia and elevated plasma glucose concentration were predictors of death in men only.

Conclusions: In patients with diabetes admitted for COVID-19, female sex was associated with lower incidence of early severe outcomes, but did not influence the overall in-hospital mortality, suggesting that diabetes mitigates the female protection from COVID-19 severity. Sex-associated biological determinants may be useful to optimize COVID-19 prevention and management in women and men.

- Cited by 4 articles

Supplementary info

Publication types, MeSH terms, Associated data Expand

Publication types

- Multicenter Study
- Observational Study

MeSH terms

- Aged
- Aged, 80 and over
- COVID-19 / complications
- COVID-19 / diagnosis*
- COVID-19 / epidemiology*
- COVID-19 / therapy
- Diabetes Complications / diagnosis
- Diabetes Complications / epidemiology
- Diabetes Mellitus / diagnosis*
- Diabetes Mellitus / epidemiology*
- Female
- France / epidemiology
- Hospital Mortality
- Hospitalization / statistics & numerical data
- Humans
- Incidence
- Inpatients
- Intensive Care Units / statistics & numerical data
- Male
- Middle Aged
- Prognosis
- Respiration, Artificial / statistics & numerical data

- Retrospective Studies
- Risk Factors
- SARS-CoV-2 / physiology
- Severity of Illness Index
- Sex Characteristics*

Associated data

- ClinicalTrials.gov/NCT04324736

Full text links

bioscientifica FULL TEXT Sheridan PubFactory FREE fulltext HAL HAL archives ouvertes
Proceed to details
Cite
Share
☐ 264
Observational Study
Arch Pediatr
. 2021 Nov;28(8):677-682.
doi: 10.1016/j.arcped.2021.09.014. Epub 2021 Oct 6.

Evaluation of changes in pediatric emergency department utilization during COVID-19 pandemic

Nilden Tuygun [1], Can Demir Karacan [2], Aytaç Göktuğ [2], Ayla Akca Çağlar [2], Aysun Tekeli [2], İlknur Bodur [2], Betül Öztürk [2], Ali Güngör [2], Muhammed Mustafa Güneylioğlu [2], Raziye Merve Yaradılmış [2], Zülfikar Akelma [2]
Affiliations Expand

Affiliations

- [1] Department of Pediatric Emergency Medicine, Dr. Sami Ulus Maternity and Child Health and Diseases Training and Research Hospital, Ankara, Turkey.. Electronic address: nildentuygun@gmail.com.
- [2] Department of Pediatric Emergency Medicine, Dr. Sami Ulus Maternity and Child Health and Diseases Training and Research Hospital, Ankara, Turkey.

- PMID: **34702623**
- PMCID: PMC8492613
- DOI: 10.1016/j.arcped.2021.09.014

Free PMC article
Observational Study

Evaluation of changes in pediatric emergency department utilization during COVID-19 pandemic

Nilden Tuygun et al. Arch Pediatr. 2021 Nov.
Free PMC article
Show details
Arch Pediatr
. 2021 Nov;28(8):677-682.
doi: 10.1016/j.arcped.2021.09.014. Epub 2021 Oct 6.

Authors

Nilden Tuygun [1], Can Demir Karacan [2], Aytaç Göktuğ [2], Ayla Akca Çağlar [2], Aysun Tekeli [2], İlknur Bodur [2], Betül Öztürk [2], Ali Güngör [2], Muhammed Mustafa Güneylioğlu [2], Raziye Merve Yaradılmış [2], Zülfikar Akelma [2]

Affiliations

- [1] Department of Pediatric Emergency Medicine, Dr. Sami Ulus Maternity and Child Health and Diseases Training and Research Hospital, Ankara, Turkey.. Electronic address: nildentuygun@gmail.com.
- [2] Department of Pediatric Emergency Medicine, Dr. Sami Ulus Maternity and Child Health and Diseases Training and Research Hospital, Ankara, Turkey.

- PMID: **34702623**
- PMCID: PMC8492613
- DOI: 10.1016/j.arcped.2021.09.014

Abstract

Background: During the coronavirus disease 2019 (COVID-19) pandemic period, the use of emergency services with pediatric non-COVID patients has decreased considerably. We aimed to examine whether there was a change in the demographic data, triage profile, causes, management, and cost of pediatric emergency department (PED) visits of non-COVID patients during the pandemic period.

Methods: This study was a retrospective, single-center, observational comparative study that was conducted at the PED. Patient records were examined during "the pandemic spring" and the same period of the previous year. Patient demographics, waiting time, and outcome of the PED visit were analyzed in the entire population of children admitted to the PED during the study period, whereas more precise data such as the reason for PED use, duration of symptoms, urgency levels according to the Emergency Severity Index (ESI), final diagnosis, management, and cost of patient care were analyzed in a sample of admitted patients. We used the chi-square test, Fisher's exact test, and Mann-Whitney U test for statistical analyses.

Results: A total of 62,593 PED visits occurred. During the pandemic period, PED visits showed a decrease of 55.8% compared to the previous year. Patients included in the sampling study group

were selected using a systematic random sampling method. The median waiting time during the pandemic period was significantly shorter than the previous year (median 14 min [IQR: 5-32] vs. median 5 min [IQR: 2-16]; p<0.001). The median duration of symptoms was 1 day (1-2) in both groups. Emergency Severity Index (ESI) levels I, II, and III showed a significant increase (27.7% vs. 37.3%) in triage scoring compared to levels IV and V (72.3% vs. 62.7%) during the pandemic period (p<0.001). The median cost per patient during the pandemic period was statistically higher compared to the previous year ($19.57 [19.57-40.50] vs. $25.34 [31.50-52.01]; p<0.001). Overall costs during the pandemic period had a 1.6-fold decline.

Conclusion: We highlighted the changes in an ordinary PED profile during an extraordinary period. A shift in ESI levels in a more emergent direction was observed. While the number of nonurgent patients, especially those with infections, decreased, the rates of surgical cases, acute neurological and heart diseases, home accidents, and poisoning increased relative to the pre-pandemic period.

Keywords: COVID-19; Emergency; Pandemic; Pediatric.

Copyright © 2021. Published by Elsevier Masson SAS.

Conflict of interest statement

Declaration of Competing Interest None.

- 21 references
- 2 figures

Supplementary info

Publication types, MeSH terms Expand

Publication types

- Observational Study

MeSH terms

- COVID-19*
- Child
- Child, Preschool
- Emergency Service, Hospital / statistics & numerical data*
- Female
- Hospitalization
- Hospitals, Pediatric
- Humans
- Infant
- Male
- Pandemics*
- Patient Acceptance of Health Care / statistics & numerical data*

- Retrospective Studies
- Tertiary Care Centers
- Triage
- Turkey

Full text links

ELSEVIER FULL-TEXT ARTICLE Elsevier Science Free PMC article

Proceed to details

Cite

Share

265

Observational Study

Braz J Infect Dis

. Jan-Feb 2022;26(1):101702.

doi: 10.1016/j.bjid.2021.101702. Epub 2021 Dec 21.

Tocilizumab or glucocorticoids treatment for patients with SARS-CoV-2 pneumonia: An observational study

Giovanni Dolci [1], Giulia Cassone [2], Giulia Besutti [3], Romina Corsini [4], Fabio Sampaolesi [4], Valentina Iotti [5], Elena Galli [6], Adalgisa Palermo [6], Matteo Fontana [7], Pamela Mancuso [8], Reggio Emilia COVID-19 Working Group

Affiliations Expand

Affiliations

- [1] Infectious Disease Unit, University of Modena and Reggio Emilia, Modena, Italy. Electronic address: giodolci@hotmail.it.
- [2] Rheumatology Unit, IRCCS Arcispedale Santa Maria Nuova, Azienda Unità Sanitaria Locale-IRCCS di Reggio Emilia, Reggio Emilia, Italy; Clinical and Experimental Medicine PhD Program, University of Modena and Reggio Emilia, Modena, Italy.
- [3] Clinical and Experimental Medicine PhD Program, University of Modena and Reggio Emilia, Modena, Italy; Radiology Unit, Department of Imaging and Laboratory Medicine, Azienda USL-IRCCS di Reggio Emilia, Italy.
- [4] Infectious Disease Unit, Azienda USL-IRCCS di Reggio Emilia, Italy.
- [5] Radiology Unit, Department of Imaging and Laboratory Medicine, Azienda USL-IRCCS di Reggio Emilia, Italy.
- [6] Rheumatology Unit, IRCCS Arcispedale Santa Maria Nuova, Azienda Unità Sanitaria Locale-IRCCS di Reggio Emilia, Reggio Emilia, Italy.
- [7] Pneumology Unit, Azienda USL-IRCCS di Reggio Emilia, Reggio Emilia, Italy.
- [8] Servizio di epidemiologia, Azienda USL-IRCCS di Reggio Emilia, Reggio Emilia, Emilia-Romagna, Italy.

- PMID: **34963560**
- PMCID: PMC8687752
- DOI: 10.1016/j.bjid.2021.101702

Free PMC article
Observational Study

Tocilizumab or glucocorticoids treatment for patients with SARS-CoV-2 pneumonia: An observational study

Giovanni Dolci et al. Braz J Infect Dis. Jan-Feb 2022.
Free PMC article
Show details
Braz J Infect Dis
. Jan-Feb 2022;26(1):101702.
doi: 10.1016/j.bjid.2021.101702. Epub 2021 Dec 21.

Authors

Giovanni Dolci [1], Giulia Cassone [2], Giulia Besutti [3], Romina Corsini [4], Fabio Sampaolesi [4], Valentina Iotti [5], Elena Galli [6], Adalgisa Palermo [6], Matteo Fontana [7], Pamela Mancuso [8], Reggio Emilia COVID-19 Working Group

Affiliations

- [1] Infectious Disease Unit, University of Modena and Reggio Emilia, Modena, Italy. Electronic address: giodolci@hotmail.it.
- [2] Rheumatology Unit, IRCCS Arcispedale Santa Maria Nuova, Azienda Unità Sanitaria Locale-IRCCS di Reggio Emilia, Reggio Emilia, Italy; Clinical and Experimental Medicine PhD Program, University of Modena and Reggio Emilia, Modena, Italy.
- [3] Clinical and Experimental Medicine PhD Program, University of Modena and Reggio Emilia, Modena, Italy; Radiology Unit, Department of Imaging and Laboratory Medicine, Azienda USL-IRCCS di Reggio Emilia, Italy.
- [4] Infectious Disease Unit, Azienda USL-IRCCS di Reggio Emilia, Italy.
- [5] Radiology Unit, Department of Imaging and Laboratory Medicine, Azienda USL-IRCCS di Reggio Emilia, Italy.
- [6] Rheumatology Unit, IRCCS Arcispedale Santa Maria Nuova, Azienda Unità Sanitaria Locale-IRCCS di Reggio Emilia, Reggio Emilia, Italy.
- [7] Pneumology Unit, Azienda USL-IRCCS di Reggio Emilia, Reggio Emilia, Italy.
- [8] Servizio di epidemiologia, Azienda USL-IRCCS di Reggio Emilia, Reggio Emilia, Emilia-Romagna, Italy.

- PMID: **34963560**
- PMCID: PMC8687752
- DOI: 10.1016/j.bjid.2021.101702

Abstract

Objective: To estimate the effect of tocilizumab or glucocorticoids in preventing death and intubation in patients hospitalized with SARS-CoV-2 pneumonia.

Methods: This was a retrospective cohort study enrolling all consecutive patients hospitalized at Reggio Emilia AUSL between February the 11th and April 14th 2020 for severe COVID-19 and treated with tocilizumab or glucocorticoids (at least 80 mg/day of methylprednisolone or equivalent for at least 3 days). The primary outcome was death within 30 days from the start of the considered therapies. The secondary outcome was a composite outcome of death and/or intubation. All patients have been followed-up until May 19th 2020, with a follow-up of at least 30 days for every patient. To reduce confounding due to potential non-comparability of the two groups, those receiving tocilizumab and those receiving glucocorticoids, a propensity score was calculated as the inverse probability weighting of receiving treatment conditional on the baseline covariates.

Results and conclusion: Therapy with tocilizumab alone was associated with a reduction of deaths (OR 0.49, 95% CI 0.21-1.17) and of the composite outcome death/intubation (OR 0.35, 95% CI 0.13-0.90) compared to glucocorticoids alone. Nevertheless, this result should be cautiously interpreted due to a potential prescription bias.

Keywords: COVID-19; Corticosteroids; Glucocorticoids; Methylprednisolone; SARS-CoV-2; Tocilizumab.

Copyright © 2021 Sociedade Brasileira de Infectologia. Published by Elsevier España, S.L.U. All rights reserved.

Conflict of interest statement

Conflicts of interest The authors declare that they have no conflict of interest.

- 22 references

Supplementary info

Publication types, MeSH terms, Substances, Supplementary concepts Expand

Publication types

- Observational Study

MeSH terms

- Antibodies, Monoclonal, Humanized
- COVID-19* / drug therapy
- Glucocorticoids* / therapeutic use
- Humans
- Retrospective Studies
- SARS-CoV-2

- Treatment Outcome

Substances

- Antibodies, Monoclonal, Humanized
- Glucocorticoids
- tocilizumab

Supplementary concepts

- COVID-19 drug treatment

Full text links

ELSEVIER OPEN ACCESS Elsevier Science Free PMC article
Proceed to details
Cite
Share
266
Observational Study
Ann Intern Med
. 2021 Aug;174(8):1101-1109.
doi: 10.7326/M21-1102. Epub 2021 Jun 1.

Patient Characteristics and Costs Associated With COVID-19-Related Medical Care Among Medicare Fee-for-Service Beneficiaries

Yuping Tsai [1], Tara M Vogt [1], Fangjun Zhou [1]
Affiliations Expand

Affiliation

- [1] National Center for Immunization and Respiratory Diseases, Centers for Disease Control and Prevention, Atlanta, Georgia (Y.T., T.M.V., F.Z.).

- PMID: **34058109**
- PMCID: PMC8252832
- DOI: 10.7326/M21-1102

Free PMC article
Observational Study

Patient Characteristics and Costs Associated With COVID-19-Related Medical Care Among Medicare Fee-for-Service Beneficiaries

Yuping Tsai et al. Ann Intern Med. 2021 Aug.
Free PMC article
Show details
Ann Intern Med
. 2021 Aug;174(8):1101-1109.
doi: 10.7326/M21-1102. Epub 2021 Jun 1.

Authors

Yuping Tsai [1], Tara M Vogt [1], Fangjun Zhou [1]

Affiliation

- [1] National Center for Immunization and Respiratory Diseases, Centers for Disease Control and Prevention, Atlanta, Georgia (Y.T., T.M.V., F.Z.).

- PMID: **34058109**
- PMCID: PMC8252832
- DOI: 10.7326/M21-1102

Abstract

Background: New cases of COVID-19 continue to occur daily in the United States, and the need for medical treatments continues to grow. Knowledge of the direct medical costs of COVID-19 treatments is limited.

Objective: To examine the characteristics of older adults with COVID-19 and their costs for COVID-19-related medical care.

Design: Retrospective observational study.

Setting: Medical claims for Medicare fee-for-service (FFS) beneficiaries.

Patients: Medicare FFS beneficiaries aged 65 years or older who had a COVID-19-related medical encounter during April through December 2020.

Measurements: Patient characteristics and direct medical costs of COVID-19-related hospitalizations and outpatient visits.

Results: Among 28.1 million Medicare FFS beneficiaries, 1 181 127 (4.2%) sought COVID-19-related medical care. Among these patients, 23.0% had an inpatient stay and 4.2% died during hospitalization. The majority of the patients were female (57.0%), non-Hispanic White (79.6%), and residents of an urban county (77.2%). Medicare FFS costs for COVID-19-related medical care

were $6.3 billion; 92.6% of costs were for hospitalizations. The mean hospitalization cost was $21 752, and the mean length of stay was 9.2 days; hospitalization cost and length of stay were higher if the patient needed a ventilator ($49 441 and 17.1 days) or died ($32 015 and 11.3 days). The mean cost per outpatient visit was $164. Patients aged 75 years or older were more likely to be hospitalized, but their hospitalizations were associated with lower costs than for younger patients. Male sex and non-White race/ethnicity were associated with higher probability of being hospitalized and higher medical costs.

Limitation: Results are based on Medicare FFS patients.

Conclusion: The COVID-19 pandemic has resulted in substantial disease and economic burden among older Americans, particularly those of non-White race/ethnicity.

Primary funding source: None.

Conflict of interest statement

Disclosures: Authors have reported no disclosures of interest. Forms can be viewed at www.acponline.org/authors/icmje/ConflictOfInterestForms.do?msNum=M21-1102.

Comment in

- Putting Medicare Spending for COVID-19 Into Perspective.
  Blumenthal D, Jacobson GA. Blumenthal D, et al. Ann Intern Med. 2021 Aug;174(8):1169-1170. doi: 10.7326/M21-2187. Epub 2021 Jun 1. Ann Intern Med. 2021. PMID: 34058111 Free PMC article.

- Cited by 9 articles
- 33 references
- 2 figures

Supplementary info

Publication types, MeSH terms Expand

Publication types

- Observational Study

MeSH terms

- Aged
- Aged, 80 and over
- Ambulatory Care / economics*
- COVID-19 / economics*
- Direct Service Costs* / trends
- Fee-for-Service Plans
- Female
- Hospital Costs* / trends

- Hospitalization / economics*
- Humans
- Male
- Medicare / economics*
- Pandemics
- Retrospective Studies
- SARS-CoV-2
- United States

Full text links

Atypon Free PMC article
Proceed to details
Cite
Share
☐ 267
Observational Study
BMC Gastroenterol
. 2022 Jan 11;22(1):19.
doi: 10.1186/s12876-021-02024-9.

Delayed diagnosis and subsequently increased severity of acute appendicitis (compatible with clinical-pathologic grounds) during the COVID-19 pandemic: an observational case-control study

Amitai Bickel [#,1,2], Samer Ganam [#,1], Ibrahim Abu Shakra [1], Inbal Farkash [3], Rola Francis [1], Nour Karra [4], Fahed Merei [1], Isaac Cohen [3], Eli Kakiashvili [5,6]
Affiliations Expand

Affiliations

- [1] Department of Surgery A, Galilee Medical Center, 22100, Nahariya, Israel.
- [2] Faculty of Medicine in the Galilee, Bar-Ilan University, Safed, Israel.
- [3] Department of Pathology, Galilee Medical Center, Nahariya, Israel.
- [4] Department of Internal Medicine E, The Chaim Sheba Medical Centre, Tel-Hashomer, Ramat Gan, Israel.
- [5] Department of Surgery A, Galilee Medical Center, 22100, Nahariya, Israel. elik@gmc.gov.il.
- [6] Faculty of Medicine in the Galilee, Bar-Ilan University, Safed, Israel. elik@gmc.gov.il.

# Contributed equally.

- PMID: **35016616**
- PMCID: PMC8751470
- DOI: 10.1186/s12876-021-02024-9

Free PMC article
Observational Study

Delayed diagnosis and subsequently increased severity of acute appendicitis (compatible with clinical-pathologic grounds) during the COVID-19 pandemic: an observational case-control study

Amitai Bickel et al. BMC Gastroenterol. 2022.
Free PMC article

Show details

BMC Gastroenterol
. 2022 Jan 11;22(1):19.
doi: 10.1186/s12876-021-02024-9.

Authors

Amitai Bickel [#] [1] [2], Samer Ganam [#] [1], Ibrahim Abu Shakra [1], Inbal Farkash [3], Rola Francis [1], Nour Karra [4], Fahed Merei [1], Isaac Cohen [3], Eli Kakiashvili [5] [6]

Affiliations

- [1] Department of Surgery A, Galilee Medical Center, 22100, Nahariya, Israel.
- [2] Faculty of Medicine in the Galilee, Bar-Ilan University, Safed, Israel.
- [3] Department of Pathology, Galilee Medical Center, Nahariya, Israel.
- [4] Department of Internal Medicine E, The Chaim Sheba Medical Centre, Tel-Hashomer, Ramat Gan, Israel.
- [5] Department of Surgery A, Galilee Medical Center, 22100, Nahariya, Israel. elik@gmc.gov.il.
- [6] Faculty of Medicine in the Galilee, Bar-Ilan University, Safed, Israel. elik@gmc.gov.il.

# Contributed equally.

- PMID: **35016616**
- PMCID: PMC8751470
- DOI: 10.1186/s12876-021-02024-9

Abstract

Background: During a global crisis like the current COVID-19 pandemic, delayed admission to hospital in cases of emergent medical illness may lead to serious adverse consequences. We aimed to determine whether such delayed admission affected the severity of an inflammatory process regarding acute appendicitis, and its convalescence.

Methods: In a retrospective observational cohort case-control study, we analyzed the medical data of 60 patients who were emergently and consecutively admitted to our hospital due to acute appendicitis as established by clinical presentation and imaging modalities, during the period of the COVID-19 pandemic (our study group). We matched a statistically control group consisting of 97 patients who were admitted during a previous 12-month period for the same etiology. All underwent laparoscopic appendectomy. The main study parameters included intraoperative findings (validated by histopathology), duration of abdominal pain prior to admission, hospital stay and postoperative convalescence (reflecting the consequences of delay in diagnosis and surgery).

Results: The mean duration of abdominal pain until surgery was significantly longer in the study group. The rate of advanced appendicitis (suppurative and gangrenous appendicitis as well as peri-appendicular abscess) was greater in the study than in the control group (38.3 vs. 21.6%, 23.3 vs. 16.5%, and 5 vs. 1% respectively), as well as mean hospital stay.

Conclusions: A global crisis like the current viral pandemic may significantly affect emergent admissions to hospital (as in case of acute appendicitis), leading to delayed surgical interventions and its consequences.

Keywords: Acute appendicitis; COVID-19; Laparoscopic appendectomy.

© 2021. The Author(s).

Conflict of interest statement

The authors declare that they have no competing interests.

- 31 references

Supplementary info

Publication types, MeSH terms Expand

Publication types

- Observational Study

MeSH terms

- Acute Disease
- Appendectomy
- Appendicitis* / diagnosis
- Appendicitis* / epidemiology

- Appendicitis* / surgery
- COVID-19*
- Case-Control Studies
- Delayed Diagnosis
- Humans
- Laparoscopy*
- Length of Stay
- Pandemics
- Postoperative Complications / epidemiology
- Retrospective Studies
- SARS-CoV-2

Full text links

Read free full text at BMC BioMed Central Free PMC article
Proceed to details
Cite
Share
268
Observational Study
J Neurosurg Anesthesiol
. 2021 Jul 1;33(3):268-272.
doi: 10.1097/ANA.0000000000000748.

Management of Endovascular Treatment for Acute Ischemic Stroke During the COVID-19 Pandemic at a Single Institution in Beijing, China: A Brief Report

Shu Li [1], Min Zeng [1], Jia Dong [1], Muhan Li [1], Xiang Yan [1], Ruowen Li [1], Yuewei Zhang [2], Xiaochuan Huo [3], Zhongrong Miao [3], Shuo Wang [4], Yuming Peng [1], Ruquan Han [1]
Affiliations Expand

Affiliations

- [1] Departments of Anesthesiology.
- [2] Infection Prevention and Control.
- [3] Neuro-Intervention.
- [4] Neurosurgery, Beijing Tiantan Hospital, Capital Medical University, Beijing, China.

- PMID: **33229942**
- PMCID: PMC8195677

- DOI: 10.1097/ANA.0000000000000748

Free PMC article
Observational Study

Management of Endovascular Treatment for Acute Ischemic Stroke During the COVID-19 Pandemic at a Single Institution in Beijing, China: A Brief Report

Shu Li et al. J Neurosurg Anesthesiol. 2021.
Free PMC article
Show details
J Neurosurg Anesthesiol
. 2021 Jul 1;33(3):268-272.
doi: 10.1097/ANA.0000000000000748.

Authors

Shu Li [1], Min Zeng [1], Jia Dong [1], Muhan Li [1], Xiang Yan [1], Ruowen Li [1], Yuewei Zhang [2], Xiaochuan Huo [3], Zhongrong Miao [3], Shuo Wang [4], Yuming Peng [1], Ruquan Han [1]

Affiliations

- [1] Departments of Anesthesiology.
- [2] Infection Prevention and Control.
- [3] Neuro-Intervention.
- [4] Neurosurgery, Beijing Tiantan Hospital, Capital Medical University, Beijing, China.

- PMID: **33229942**
- PMCID: PMC8195677
- DOI: 10.1097/ANA.0000000000000748

Abstract

Background: The coronavirus disease (COVID-19) pandemic is currently a major challenge for health care systems around the world. For a time-sensitive emergency such as acute ischemic stroke (AIS), streamlined workflow times are essential to ensure good clinical outcomes.

Methods: The aim of this single-center, retrospective, observational study was to describe changes in stroke workflow patterns and clinical care during the COVID-19 pandemic. Data from AIS patients undergoing emergent endovascular treatment (EVT) between 23 January and 8 April 2020 were retrospectively collected and compared with data from patients admitted during a similar period in 2019. The primary outcome was difference in time from symptom onset to recanalization. Secondary outcomes included workflow times, clinical management, discharge outcomes, and health-economic data.

Results: In all, 21 AIS patients were admitted for emergent EVT during the 77-day study period, compared with 42 cases in 2019. Median time from symptom onset to recanalization was 132 minutes longer during the pandemic compared with the previous year (672 vs. 540 min, P=0.049). Patients admitted during the pandemic had a higher likelihood of endotracheal intubation (84.6% vs. 42.4%, P<0.05) and a higher incidence of delayed extubation after EVT (69.2% vs. 45.5%, P<0.05). National Institutes of Health Stroke Scale at hospital discharge was similar in the 2 cohorts, whereas neurointensive care unit stay was longer in patients admitted during the pandemic (10 vs. 7 days, P=0.013) and hospitalization costs were higher (123.9 vs. 95.2 thousand Chinese Yuan, P=0.052).

Conclusion: Disruptions to medical services during the COVID-19 pandemic has particularly impacted AIS patients undergoing emergent EVT, resulting in increased workflow times. A structured and multidisciplinary protocol should be implemented to minimize treatment delays and maximize patient outcomes.

Copyright © 2020 Wolters Kluwer Health, Inc. All rights reserved.

Conflict of interest statement

R.H. is a member of the Editorial Board of Journal of Neurosurgical Anesthesiology. The remaining authors have no conflicts of interest to disclose.

- Cited by 1 article
- 19 references
- 1 figure

Supplementary info

Publication types, MeSH terms Expand

Publication types

- Observational Study

MeSH terms

- Acute Disease
- Aged
- Beijing
- COVID-19 / prevention & control*
- Endovascular Procedures / methods*
- Female
- Humans
- Ischemic Stroke / therapy*
- Male
- Middle Aged
- Pandemics
- Retrospective Studies

- SARS-CoV-2
- Time-to-Treatment / statistics & numerical data*
- Treatment Outcome

Full text links

Wolters Kluwer Wolters Kluwer Free PMC article
Proceed to details
Cite
Share
269
Observational Study

Diabetes Res Clin Pract
. 2021 Aug;178:108955.
doi: 10.1016/j.diabres.2021.108955. Epub 2021 Jul 14.

Higher admission activated partial thromboplastin time, neutrophil-lymphocyte ratio, serum sodium, and anticoagulant use predict in-hospital COVID-19 mortality in people with Diabetes: Findings from Two University Hospitals in the U.K

Ahmed Iqbal [1], Marni Greig [2], Muhammad Fahad Arshad [1], Thomas H Julian [3], Sher Ee Tan [3], Jackie Elliott [4]
Affiliations Expand

Affiliations

- [1] Department of Diabetes and Endocrinology, Sheffield Teaching Hospitals, Sheffield, UK; Department of Oncology and Metabolism, The Medical School, The University of Sheffield, Sheffield, UK.
- [2] Department of Diabetes and Endocrinology, Sheffield Teaching Hospitals, Sheffield, UK; Department of Infection, Immunity and Cardiovascular Disease, The Medical School, The University of Sheffield, Sheffield, UK.
- [3] Department of Diabetes and Endocrinology, Sheffield Teaching Hospitals, Sheffield, UK.
- [4] Department of Diabetes and Endocrinology, Sheffield Teaching Hospitals, Sheffield, UK; Department of Oncology and Metabolism, The Medical School, The University of Sheffield, Sheffield, UK. Electronic address: j.elliott@sheffield.ac.uk.

- PMID: **34273452**
- PMCID: PMC8278840

- DOI: 10.1016/j.diabres.2021.108955

Free PMC article
Observational Study

Higher admission activated partial thromboplastin time, neutrophil-lymphocyte ratio, serum sodium, and anticoagulant use predict in-hospital COVID-19 mortality in people with Diabetes: Findings from Two University Hospitals in the U.K

Ahmed Iqbal et al. Diabetes Res Clin Pract. 2021 Aug.
Free PMC article
Show details
Diabetes Res Clin Pract
. 2021 Aug;178:108955.
doi: 10.1016/j.diabres.2021.108955. Epub 2021 Jul 14.

Authors

Ahmed Iqbal [1], Marni Greig [2], Muhammad Fahad Arshad [1], Thomas H Julian [3], Sher Ee Tan [3], Jackie Elliott [4]

Affiliations

- [1] Department of Diabetes and Endocrinology, Sheffield Teaching Hospitals, Sheffield, UK; Department of Oncology and Metabolism, The Medical School, The University of Sheffield, Sheffield, UK.
- [2] Department of Diabetes and Endocrinology, Sheffield Teaching Hospitals, Sheffield, UK; Department of Infection, Immunity and Cardiovascular Disease, The Medical School, The University of Sheffield, Sheffield, UK.
- [3] Department of Diabetes and Endocrinology, Sheffield Teaching Hospitals, Sheffield, UK.
- [4] Department of Diabetes and Endocrinology, Sheffield Teaching Hospitals, Sheffield, UK; Department of Oncology and Metabolism, The Medical School, The University of Sheffield, Sheffield, UK. Electronic address: j.elliott@sheffield.ac.uk.

- PMID: **34273452**
- PMCID: PMC8278840
- DOI: 10.1016/j.diabres.2021.108955

Abstract

Aims: To create and compare survival models from admission laboratory indices in people hospitalized with coronavirus disease 2019 (COVID-19) with and without diabetes.

Methods: Retrospective observational study of patients with COVID-19 with or without diabetes admitted to Sheffield Teaching Hospitals from 29 February to 01 May 2020. Predictive variables for in-hospital mortality from COVID-19 were explored using Cox proportional hazard models.

Results: Out of 505 patients, 156 (30.8%) had diabetes mellitus (DM) of which 143 (91.7%) had type 2 diabetes. There were significantly higher in-hospital COVID-19 deaths in those with DM [DM COVID-19 deaths 54 (34.6%) vs. non-DM COVID-19 deaths 88 (25.2%): $P < 0.05$]. Activated partial thromboplastin time (APPT) > 24 s without anticoagulants (HR 6.38, 95% CI: 1.07-37.87: $P = 0.04$), APTT > 24 s with anticoagulants (HR 24.01, 95% CI: 3.63-159.01: $P < 0.001$), neutrophil-lymphocyte ratio > 8 (HR 6.18, 95% CI: 2.36-16.16: $P < 0.001$), and sodium > 136 mmol/L (HR 3.27, 95% CI: 1.12-9.56: $P = 0.03$) at admission, were only associated with in-hospital COVID-19 mortality for those with diabetes.

Conclusions: At admission, elevated APTT with or without anticoagulants, neutrophil-lymphocyte ratio and serum sodium are unique factors that predict in-hospital COVID-19 mortality in patients with diabetes compared to those without. This novel finding may lead to research into haematological and biochemical mechanisms to understand why those with diabetes are more susceptible to poor outcomes when infected with Covid-19, and contribute to identification of those most at risk when admitted to hospital.

Keywords: Activated partial thromboplastin time (APTT); Anticoagulants; COVID-19; Diabetes mellitus; Mortality; Neutrophil-lymphocyte ratio.

Copyright © 2021 Elsevier B.V. All rights reserved.

Conflict of interest statement

Declaration of Competing Interest The authors declare that they have no known competing financial interests or personal relationships that could have appeared to influence the work reported in this paper.

- Cited by 1 article
- 41 references
- 2 figures

Supplementary info

Publication types, MeSH terms, Substances Expand

Publication types

- Observational Study

MeSH terms

- Adolescent
- Adult

- Aged
- Aged, 80 and over
- Anticoagulants / therapeutic use
- COVID-19* / diagnosis
- COVID-19* / mortality
- Diabetes Mellitus, Type 2* / complications
- Female
- Hospital Mortality*
- Hospitalization
- Hospitals, University
- Humans
- Lymphocytes / cytology
- Male
- Middle Aged
- Neutrophils / cytology
- Partial Thromboplastin Time
- Retrospective Studies
- Risk Factors
- Sodium / blood
- United Kingdom
- Young Adult

Substances

- Anticoagulants
- Sodium

Full text links

ELSEVIER FULL-TEXT ARTICLE Elsevier Science Free PMC article
Proceed to details
Cite
Share
☐ 270
Observational Study
Acta Diabetol
. 2021 Jan;58(1):33-38.
doi: 10.1007/s00592-020-01592-8. Epub 2020 Aug 17.

The relationship between diabetes and clinical outcomes in COVID-19: a single-center retrospective analysis

Tamaryn Fox [#] [1], Kathleen Ruddiman [#] [2], Kevin Bryan Lo [2], Eric Peterson [2], Robert DeJoy 3rd [2], Grace Salacup [2], Jerald Pelayo [2], Ruchika Bhargav [2], Fahad Gul [2], Jeri Albano [2], Zurab Azmaiparashvili [2], Catherine Anastasopoulou [2] [3] [4], Gabriel Patarroyo-Aponte [2] [4] [5]
Affiliations Expand

Affiliations

- [1] Department of Medicine, Einstein Medical Center Philadelphia, 5501 Old York Road, Philadelphia, PA, 19141, USA. FoxTamar@einstein.edu.
- [2] Department of Medicine, Einstein Medical Center Philadelphia, 5501 Old York Road, Philadelphia, PA, 19141, USA.
- [3] Department of Endocrinology, Einstein Medical Center Philadelphia, Philadelphia, USA.
- [4] Sidney Kimmel College of Thomas Jefferson University, Philadelphia, PA, USA.
- [5] Pulmonary, Critical Care and Sleep Medicine, Einstein Medical Center Philadelphia, Philadelphia, USA.

[#] Contributed equally.

- PMID: **32804317**
- PMCID: PMC7429932
- DOI: 10.1007/s00592-020-01592-8

Free PMC article
Observational Study

The relationship between diabetes and clinical outcomes in COVID-19: a single-center retrospective analysis

Tamaryn Fox et al. Acta Diabetol. 2021 Jan.
Free PMC article
Show details
Acta Diabetol
. 2021 Jan;58(1):33-38.
doi: 10.1007/s00592-020-01592-8. Epub 2020 Aug 17.

Authors

Tamaryn Fox [#, 1], Kathleen Ruddiman [#, 2], Kevin Bryan Lo [2], Eric Peterson [2], Robert DeJoy 3rd [2], Grace Salacup [2], Jerald Pelayo [2], Ruchika Bhargav [2], Fahad Gul [2], Jeri Albano [2], Zurab Azmaiparashvili [2], Catherine Anastasopoulou [2, 3, 4], Gabriel Patarroyo-Aponte [2, 4, 5]

Affiliations

- [1] Department of Medicine, Einstein Medical Center Philadelphia, 5501 Old York Road, Philadelphia, PA, 19141, USA. FoxTamar@einstein.edu.
- [2] Department of Medicine, Einstein Medical Center Philadelphia, 5501 Old York Road, Philadelphia, PA, 19141, USA.
- [3] Department of Endocrinology, Einstein Medical Center Philadelphia, Philadelphia, USA.
- [4] Sidney Kimmel College of Thomas Jefferson University, Philadelphia, PA, USA.
- [5] Pulmonary, Critical Care and Sleep Medicine, Einstein Medical Center Philadelphia, Philadelphia, USA.

[#] Contributed equally.

- PMID: **32804317**
- PMCID: PMC7429932
- DOI: 10.1007/s00592-020-01592-8

Abstract

Aims: Coronavirus disease 19 (COVID-19) has become a pandemic. Diabetic patients tend to have poorer outcomes and more severe disease (Kumar et al. in Diabetes Metab Syndr 14(4):535-545, 2020. https://doi.org/10.1016/j.dsx.2020.04.044). However, the vast majority of studies are representative of Asian and Caucasian population and fewer represent an African-American population.

Methods: In this single-center, retrospective observational study, we included all adult patients (> 18 years old) admitted to Einstein Medical Center, Philadelphia, with a diagnosis of COVID-19. Patients were classified according to having a known diagnosis of diabetes mellitus. Demographic and clinical data, comorbidities, outcomes and laboratory findings were obtained.

Results: Our sample included a total of 355 patients. 70% were African-American, and 47% had diabetes. Patients with diabetes had higher peak inflammatory markers like CRP 184 (111-258) versus 142 (65-229) $p = 0.012$ and peak LDH 560 (384-758) versus 499 (324-655) $p = 0.017$. The need for RRT/HD was significantly higher in patients with diabetes (21% vs 11% $p = 0.013$) as well as the need for vasopressors (28% vs 18% $p = 0.023$). Only age was found to be an independent predictor of mortality. We found no significant differences in inpatient mortality $p = 0.856$, need for RRT/HD $p = 0.429$, need for intubation $p = 1.000$ and need for vasopressors $p = 0.471$ in African-Americans with diabetes when compared to non-African-Americans.

Conclusions: Our study demonstrates that patients with COVID-19 and diabetes tend to have more severe disease and poorer clinical outcomes. African-American patients with diabetes did not differ in outcomes or disease severity when compared to non-African-American patients.

Keywords: COVID-19; Diabetes; Mortality; Novel coronavirus; Outcomes.

Conflict of interest statement

None of the authors have any conflicts of interest to disclose.

- Cited by 11 articles
- 20 references
- 1 figure

Supplementary info

Publication types, MeSH terms Expand

Publication types

- Observational Study

MeSH terms

- African Americans / statistics & numerical data*
- Aged
- COVID-19* / mortality
- COVID-19* / therapy
- Comorbidity
- Diabetes Mellitus* / diagnosis
- Diabetes Mellitus* / epidemiology
- Female
- Hospitalization / statistics & numerical data
- Humans
- Male
- Mortality / ethnology
- Outcome and Process Assessment, Health Care
- Philadelphia / epidemiology
- Retrospective Studies
- SARS-CoV-2 / isolation & purification
- Severity of Illness Index

Full text links

SpringerLink FULL-TEXT ARTICLE Springer Free PMC article
Proceed to details
Cite
Share
☐ 271
Observational Study
Pathog Glob Health
. 2021 Jun;115(4):243-249.

doi: 10.1080/20477724.2021.1893485. Epub 2021 Feb 28.

Clinical features and outcomes of hospitalized COVID-19 patients in a low burden region

Mylona Eleni [1], Margellou Evangelia [2], Kranidioti Eleftheria [3], Vlachakos Vasilios [4], Sypsa Vana [5], Sakka Vissaria [6], Balis Evangelos [7], Kalomenidis Ioannis [8]
Affiliations Expand

Affiliations

- [1] 5thDepartment of Internal Medicine and Infectious Diseases, Evangelismos, Athens, Greece.
- [2] 1st Department of Internal Medicine, Evaggelismos Hospital, Athens, Greece.
- [3] 3rd Department of Internal Medicine, Evaggelismos Hospital, Athens, Greece.
- [4] 1st Department of Critical Care and Pulmonary Medicine, National and Kapodistrian University of Athens, Evaggelismos Hospital, Athens, Greece.
- [5] Department of Hygiene, Epidemiology & Medical Statistics, Medical School, National and Kapodistrian University of Athens, Athens, Greece.
- [6] 3rd Department of Internal Medicine and Infectious Diseases, Red Cross Hospital, Athens, Greece.
- [7] Department of Pulmonary Medicine, Evaggelismos Hospital, Athens, Greece.
- [8] 1 Department of Critical Care and Pulmonary Medicine, National and Kapodistrian University of Athens, Evaggelismos Hospital, Athens, Greece.

- PMID: **33645468**
- PMCID: PMC8168732
- DOI: 10.1080/20477724.2021.1893485

Free PMC article
Observational Study

Clinical features and outcomes of hospitalized COVID-19 patients in a low burden region

Mylona Eleni et al. Pathog Glob Health. 2021 Jun.
Free PMC article
Show details
Pathog Glob Health
. 2021 Jun;115(4):243-249.
doi: 10.1080/20477724.2021.1893485. Epub 2021 Feb 28.

Authors

Mylona Eleni [1], Margellou Evangelia [2], Kranidioti Eleftheria [3], Vlachakos Vasilios [4], Sypsa Vana [5], Sakka Vissaria [6], Balis Evangelos [7], Kalomenidis Ioannis [8]

Affiliations

- [1] 5thDepartment of Internal Medicine and Infectious Diseases, Evangelismos, Athens, Greece.
- [2] 1st Department of Internal Medicine, Evaggelismos Hospital, Athens, Greece.
- [3] 3rd Department of Internal Medicine, Evaggelismos Hospital, Athens, Greece.
- [4] 1st Department of Critical Care and Pulmonary Medicine, National and Kapodistrian University of Athens, Evaggelismos Hospital, Athens, Greece.
- [5] Department of Hygiene, Epidemiology & Medical Statistics, Medical School, National and Kapodistrian University of Athens, Athens, Greece.
- [6] 3rd Department of Internal Medicine and Infectious Diseases, Red Cross Hospital, Athens, Greece.
- [7] Department of Pulmonary Medicine, Evaggelismos Hospital, Athens, Greece.
- [8] 1 Department of Critical Care and Pulmonary Medicine, National and Kapodistrian University of Athens, Evaggelismos Hospital, Athens, Greece.

- PMID: **33645468**
- PMCID: PMC8168732
- DOI: 10.1080/20477724.2021.1893485

Abstract

Data on the clinical features and outcomes of COVID-19 patients from countries with low disease burden are rare. Greece, however, presented a low burden of COVID-19 disease during the first pandemic outbreak. This is a retrospective study of COVID-19 hospitalized patients in Greece. Clinical data were extracted from medical records using univariable and multivariable logistic regression analyses to assess the factors associated with Intensive Care Unit (ICU) admission and in-hospital death. Eighty-five patients were included in this study, 49 (57.7%) male with median (25th-75th) age 60 (49-72) years old. Sixty-one (72%) of them had at least one comorbidity with hypertension being the most common (45,6%). More than half (56%) had severe or critical disease, 20% required ICU care (14% received invasive ventilation) and 10.7% died. Solid tumor ($p = 0.021$) and NEWS score ($p = 0.048$), thrombocytopenia ($p = 0.036$) or involvement of all lung fields in chest x-ray ($p = 0.002$) on admission were independent risk factors for ICU admission. Immunosuppression ($p = 0.032$) and thrombocytopenia ($p = 0.049$) were independent predictors of death. Hospitalized COVID-19 patients in a European country with a low burden of the disease, in which hospital capacities had not been overwhelmed, had lower mortality rate compared to those reported for patients hospitalized in regions with a high burden of the disease.

Keywords: COVID-19; SARS-CoV2; mortality; pneumonia; respiratory failure.

Conflict of interest statement

The authors declare no conflict of interest.

- Cited by 2 articles

Supplementary info

Publication types, MeSH terms, Substances Expand

Publication types

- Observational Study

MeSH terms

- Adenosine Monophosphate / administration & dosage
- Adenosine Monophosphate / analogs & derivatives
- Adenosine Monophosphate / therapeutic use
- Adrenal Cortex Hormones
- Adult
- Aged
- Alanine / administration & dosage
- Alanine / analogs & derivatives
- Alanine / therapeutic use
- Anti-Bacterial Agents / administration & dosage
- Anti-Bacterial Agents / therapeutic use
- Antimalarials / administration & dosage
- Antimalarials / therapeutic use
- Antiviral Agents / administration & dosage
- Antiviral Agents / therapeutic use
- Azithromycin / administration & dosage
- Azithromycin / therapeutic use
- COVID-19 / epidemiology
- COVID-19 / pathology*
- COVID-19 / therapy*
- Colchicine / administration & dosage
- Colchicine / therapeutic use
- Drug Therapy, Combination
- Female
- Greece / epidemiology
- Hospitalization
- Humans
- Hydroxychloroquine / administration & dosage
- Hydroxychloroquine / therapeutic use
- Male
- Middle Aged
- Retrospective Studies

- SARS-CoV-2*
- Treatment Outcome
- Young Adult

Substances

- Adrenal Cortex Hormones
- Anti-Bacterial Agents
- Antimalarials
- Antiviral Agents
- remdesivir
- Adenosine Monophosphate
- Hydroxychloroquine
- Azithromycin
- Alanine
- Colchicine

Full text links

View full text Taylor & Francis Free PMC article
Proceed to details
Cite
Share
272
Observational Study
Emerg Radiol
. 2021 Jun;28(3):485-495.
doi: 10.1007/s10140-021-01907-4. Epub 2021 Jan 30.

Effect of the COVID-19 pandemic on CT scans ordered from the emergency department for abdominal complaints

April M Griffith [1], Patrick Ockerse [2], Akram Shaaban [3], Christopher Kelly [2]
Affiliations Expand

Affiliations

- [1] Department of Radiology and Imaging Sciences, University of Utah Health, 30 North 1900 East #1A071, Salt Lake City, UT, 84132-2140, USA. April.Griffith@hsc.utah.edu.
- [2] Department of Surgery, Division of Emergency Medicine, University of Utah Health, Salt Lake City, UT, USA.

- [3] Department of Radiology and Imaging Sciences, University of Utah Health, 30 North 1900 East #1A071, Salt Lake City, UT, 84132-2140, USA.

- PMID: **33517547**
- PMCID: PMC7847299
- DOI: 10.1007/s10140-021-01907-4

Free PMC article
Observational Study

Effect of the COVID-19 pandemic on CT scans ordered from the emergency department for abdominal complaints

April M Griffith et al. Emerg Radiol. 2021 Jun.
Free PMC article
Show details
Emerg Radiol
. 2021 Jun;28(3):485-495.
doi: 10.1007/s10140-021-01907-4. Epub 2021 Jan 30.

Authors

April M Griffith [1], Patrick Ockerse [2], Akram Shaaban [3], Christopher Kelly [2]

Affiliations

- [1] Department of Radiology and Imaging Sciences, University of Utah Health, 30 North 1900 East #1A071, Salt Lake City, UT, 84132-2140, USA. April.Griffith@hsc.utah.edu.
- [2] Department of Surgery, Division of Emergency Medicine, University of Utah Health, Salt Lake City, UT, USA.
- [3] Department of Radiology and Imaging Sciences, University of Utah Health, 30 North 1900 East #1A071, Salt Lake City, UT, 84132-2140, USA.

- PMID: **33517547**
- PMCID: PMC7847299
- DOI: 10.1007/s10140-021-01907-4

Abstract

Purpose: The COVID-19 pandemic has affected healthcare systems and patients alike across the USA. We seek to elucidate changes in abdominal imaging ordered from the emergency department (ED) in a healthcare system undergoing non-surge conditions in April 2020 compared to April 2019.

Methods: We performed a retrospective, observational study comparing patients undergoing CT scans of the abdomen and pelvis ordered from the ED in April 2020 vs. April 2019 at a single healthcare center. Via review of the radiology report and electronic medical record, we determined

the positive or negative status of these scans. We evaluated percentages of positive CT scans and differences in outcomes, including admission rates, interventions, and mortality.

Results: Comparing 2020 to 2019, there was a 31.6% decrease in the number of CT scans performed from the ED. We found a higher percentage of positive CT findings, 58.2% vs. 50.8% (p = 0.025), and increased admission rates, 40.8% vs. 34.1% (p = 0.036). Differences were found in rates of appendicitis, colitis, and cholangitis. No difference was found in ICU admissions, interventions, or in-hospital mortality.

Conclusion: During the COVID-19 pandemic in a region undergoing non-surge conditions, we found increased rates of positive CT scans performed from the ED for abdominal complaints with an increased percentage of hospital admissions compared to a control year. No differences in ICU admissions or rates of procedural intervention were found to suggest higher acuity of pathology on presentation. Our findings suggest appropriately decreased healthcare utilization in our study period, driven by pre-hospital patient self-selection.

Keywords: Abdominal pain; COVID-19; Computed tomography; Emergency department; Healthcare utilization.

Conflict of interest statement

The authors declare that they have no conflict of interest.

- Cited by 3 articles
- 33 references
- 2 figures

Supplementary info

Publication types, MeSH terms Expand

Publication types

- Observational Study

MeSH terms

- Abdomen, Acute / diagnostic imaging*
- Abdomen, Acute / mortality
- Adolescent
- Adult
- Aged
- Aged, 80 and over
- COVID-19 / epidemiology*
- Emergency Service, Hospital*
- Female
- Hospitalization / statistics & numerical data
- Humans

- Male
- Middle Aged
- Pandemics
- Retrospective Studies
- SARS-CoV-2
- Tomography, X-Ray Computed / statistics & numerical data*
- Utah / epidemiology
- Utilization Review

Full text links

SpringerLink FULL-TEXT ARTICLE Springer Free PMC article
Proceed to details
Cite
Share
273
Observational Study
BMC Nephrol
. 2021 Jan 14;22(1):29.
doi: 10.1186/s12882-021-02233-0.

Determinants of mortality in a large group of hemodialysis patients hospitalized for COVID-19

Kenan Turgutalp [1], Savas Ozturk [2], Mustafa Arici [3], Necmi Eren [4], Numan Gorgulu [5], Mahmut Islam [6], Sami Uzun [2], Tamer Sakaci [7], Zeki Aydin [8], Erkan Sengul [9], Bulent Demirelli [10], Yavuz Ayar [11], Mehmet Riza Altiparmak [12], Savas Sipahi [13], Ilay Berke Mentes [14], Tuba Elif Ozler [15], Ebru Gok Oguz [16], Bulent Huddam [17], Ender Hur [18], Rumeyza Kazancioglu [19], Ozkan Gungor [20], Bulent Tokgoz [21], Halil Zeki Tonbul [22], Alaattin Yildiz [23], Siren Sezer [24], Ali Riza Odabas [25], Kenan Ates [26]
Affiliations Expand

Affiliations

- [1] Department of Nephrology, Mersin University Faculty of Medicine, Mersin, Turkey.
- [2] Department of Nephrology, Haseki Training and Research Hospital, Istanbul, Turkey.
- [3] Department of Nephrology, Hacettepe University Faculty of Medicine, Ankara, Turkey. marici@hacettepe.edu.tr.
- [4] Department of Nephrology, Kocaeli University Faculty of Medicine, Kocaeli, Turkey.
- [5] Department of Internal Medicine, Division of Nephrology, University of Health Sciences, Bagcilar Training and Research Hospital, Istanbul, Turkey.
- [6] Department of Nephrology, Zonguldak Ataturk State Hospital, Zonguldak, Turkey.

- [7] Health Sciences University, Sisli Hamidiye Etfal Education and Research Hospital, Nephrology Department, Istanbul, Turkey.
- [8] Department of Nephrology, Darica Farabi Training and Research Hospital, Kocaeli, Turkey.
- [9] Health Science University, Kocaeli Derince Education and Research Hospital, Division of Nephrology, Kocaeli, Turkey.
- [10] Department of Nephrology, University of Health Sciences, Haydarpasa Numune Education and Research Hospital, Istanbul, Turkey.
- [11] Department of Nephrology, Bursa City Hospital, Bursa, Turkey.
- [12] Department of Internal Medicine, Division of Nephrology, Istanbul University-Cerrahpasa Cerrahpasa Faculty of Medicine, Istanbul, Turkey.
- [13] Department of Internal Medicine, Division of Nephrology, Sakarya University Faculty of Medicine, Education and Research Hospital, Sakarya, Turkey.
- [14] Department of Internal Medicine, Division of Nephrology, Marmara University School of Medicine. Marmara University Pendik Education and Research Hospital, Istanbul, Turkey.
- [15] Department of Internal Medicine, Division of Nephrology, Kanuni Sultan Suleyman Research and Training Hospital, Istanbul, Turkey.
- [16] Department of Nephrology, University of Health Sciences, Diskapi Yildirim Beyazit Education and Research Hospital, Ankara, Turkey.
- [17] Departmant of Internal Medicine, Division of Nefrology, Mugla Sitki Kocman University Faculty of Medicine, Education and Research Hospital, Mugla, Turkey.
- [18] Department of Nephrology, Manisa Merkezefendi State Hospital, Manisa, Turkey.
- [19] Department of Internal Medicine, Division of Nephrology, Bezmialem Vakif University, Istanbul, Turkey.
- [20] Department of Nephrology, Kahramanmaras Sutcu Imam University, Faculty of Medicine, Kahramanmaras, Turkey.
- [21] Erciyes University School of Medicine Department of Nephrology, Kayseri, Turkey.
- [22] Department of Internal Medicine, Division of Nephrology, Necmettin Erbakan University, Konya, Turkey.
- [23] Department of Internal Medicine, Division of Nephrology, Istanbul University, Istanbul School of Medicine, Istanbul, Turkey.
- [24] Department of Internal Medicine and Nephrology, Atılım University Faculty of Medicine, Ankara, Turkey.
- [25] Department of Nephrology, University of Health Sciences, Sultan Abdulhamid Han Training and Research Hospital, Istanbul, Turkey.
- [26] Ankara University Faculty of Medicine Department of Nephrology, Ankara, Turkey.

- PMID: **33446135**
- PMCID: PMC7808398
- DOI: 10.1186/s12882-021-02233-0

Free PMC article
Observational Study

Determinants of mortality in a large group of hemodialysis patients hospitalized for COVID-19

Kenan Turgutalp et al. BMC Nephrol. 2021.
Free PMC article
Show details
BMC Nephrol
. 2021 Jan 14;22(1):29.
doi: 10.1186/s12882-021-02233-0.

Authors

Kenan Turgutalp [1], Savas Ozturk [2], Mustafa Arici [3], Necmi Eren [4], Numan Gorgulu [5], Mahmut Islam [6], Sami Uzun [2], Tamer Sakaci [7], Zeki Aydin [8], Erkan Sengul [9], Bulent Demirelli [10], Yavuz Ayar [11], Mehmet Riza Altiparmak [12], Savas Sipahi [13], Ilay Berke Mentes [14], Tuba Elif Ozler [15], Ebru Gok Oguz [16], Bulent Huddam [17], Ender Hur [18], Rumeyza Kazancioglu [19], Ozkan Gungor [20], Bulent Tokgoz [21], Halil Zeki Tonbul [22], Alaattin Yildiz [23], Siren Sezer [24], Ali Riza Odabas [25], Kenan Ates [26]

Affiliations

- [1] Department of Nephrology, Mersin University Faculty of Medicine, Mersin, Turkey.
- [2] Department of Nephrology, Haseki Training and Research Hospital, Istanbul, Turkey.
- [3] Department of Nephrology, Hacettepe University Faculty of Medicine, Ankara, Turkey. marici@hacettepe.edu.tr.
- [4] Department of Nephrology, Kocaeli University Faculty of Medicine, Kocaeli, Turkey.
- [5] Department of Internal Medicine, Division of Nephrology, University of Health Sciences, Bagcilar Training and Research Hospital, Istanbul, Turkey.
- [6] Department of Nephrology, Zonguldak Ataturk State Hospital, Zonguldak, Turkey.
- [7] Health Sciences University, Sisli Hamidiye Etfal Education and Research Hospital, Nephrology Department, Istanbul, Turkey.
- [8] Department of Nephrology, Darica Farabi Training and Research Hospital, Kocaeli, Turkey.
- [9] Health Science University, Kocaeli Derince Education and Research Hospital, Division of Nephrology, Kocaeli, Turkey.
- [10] Department of Nephrology, University of Health Sciences, Haydarpasa Numune Education and Research Hospital, Istanbul, Turkey.
- [11] Department of Nephrology, Bursa City Hospital, Bursa, Turkey.
- [12] Department of Internal Medicine, Division of Nephrology, Istanbul University-Cerrahpasa Cerrahpasa Faculty of Medicine, Istanbul, Turkey.
- [13] Department of Internal Medicine, Division of Nephrology, Sakarya University Faculty of Medicine, Education and Research Hospital, Sakarya, Turkey.
- [14] Department of Internal Medicine, Division of Nephrology, Marmara University School of Medicine. Marmara University Pendik Education and Research Hospital, Istanbul, Turkey.

- [15] Department of Internal Medicine, Division of Nephrology, Kanuni Sultan Suleyman Research and Training Hospital, Istanbul, Turkey.
- [16] Department of Nephrology, University of Health Sciences, Diskapi Yildirim Beyazit Education and Research Hospital, Ankara, Turkey.
- [17] Departmant of Internal Medicine, Division of Nefrology, Mugla Sitki Kocman University Faculty of Medicine, Education and Research Hospital, Mugla, Turkey.
- [18] Department of Nephrology, Manisa Merkezefendi State Hospital, Manisa, Turkey.
- [19] Department of Internal Medicine, Division of Nephrology, Bezmialem Vakif University, Istanbul, Turkey.
- [20] Department of Nephrology, Kahramanmaras Sutcu Imam University, Faculty of Medicine, Kahramanmaras, Turkey.
- [21] Erciyes University School of Medicine Department of Nephrology, Kayseri, Turkey.
- [22] Department of Internal Medicine, Division of Nephrology, Necmettin Erbakan University, Konya, Turkey.
- [23] Department of Internal Medicine, Division of Nephrology, Istanbul University, Istanbul School of Medicine, Istanbul, Turkey.
- [24] Department of Internal Medicine and Nephrology, Atılım University Faculty of Medicine, Ankara, Turkey.
- [25] Department of Nephrology, University of Health Sciences, Sultan Abdulhamid Han Training and Research Hospital, Istanbul, Turkey.
- [26] Ankara University Faculty of Medicine Department of Nephrology, Ankara, Turkey.

- PMID: **33446135**
- PMCID: PMC7808398
- DOI: 10.1186/s12882-021-02233-0

Abstract

Background: Maintenance hemodialysis (MHD) patients are at increased risk for coronavirus disease 2019 (COVID-19). The aim of this study was to describe clinical, laboratory, and radiologic characteristics and determinants of mortality in a large group of MHD patients hospitalized for COVID-19.

Methods: This multicenter, retrospective, observational study collected data from 47 nephrology clinics in Turkey. Baseline clinical, laboratory and radiological characteristics, and COVID-19 treatments during hospitalization, need for intensive care and mechanical ventilation were recorded. The main study outcome was in-hospital mortality and the determinants were analyzed by Cox regression survival analysis.

Results: Of 567 MHD patients, 93 (16.3%) patients died, 134 (23.6%) patients admitted to intensive care unit (ICU) and 91 of the ones in ICU (67.9%) needed mechanical ventilation. Patients who died were older (median age, 66 [57-74] vs. 63 [52-71] years, $p = 0.019$), had more congestive heart failure (34.9% versus 20.7%, $p = 0.004$) and chronic obstructive pulmonary disease (23.6% versus 12.7%, $p = 0.008$) compared to the discharged patients. Most patients (89.6%) had radiological manifestations compatible with COVID-19 pulmonary involvement. Median platelet (166×10^3 per mm^3 versus 192×10^3 per mm^3, $p = 0.011$) and lymphocyte (800 per mm^3 versus 1000 per mm^3, $p < 0.001$) counts and albumin levels (median, 3.2 g/dl versus 3.5 g/dl, $p = 0.001$) on admission were lower in patients who died. Age (HR: 1.022 [95% CI, 1.003-1.041], $p = 0.025$), severe-critical disease clinical presentation at the time of diagnosis (HR: 6.223 [95% CI, 2.168-17.863], $p < 0.001$), presence of congestive heart failure (HR: 2.247 [95%

CI, 1.228-4.111], p = 0.009), ferritin levels on admission (HR; 1.057 [95% CI, 1.006-1.111], p = 0.028), elevation of aspartate aminotransferase (AST) (HR; 3.909 [95% CI, 2.143-7.132], p < 0.001) and low platelet count (< 150 × 10^3 per mm^3) during hospitalization (HR; 1.864 [95% CI, 1.025-3.390], p = 0.041) were risk factors for mortality.

Conclusion: Hospitalized MHD patients with COVID-19 had a high mortality rate. Older age, presence of heart failure, clinical severity of the disease at presentation, ferritin level on admission, decrease in platelet count and increase in AST level during hospitalization may be used to predict the mortality risk of these patients.

Keywords: COVID-19; Clinical findings; Hemodialysis; Mortality; Radiological manifestations.

Conflict of interest statement

The authors declare that they have no competing interests.

- Cited by 15 articles
- 32 references
- 1 figure

Supplementary info

Publication types, MeSH terms Expand

Publication types

- Multicenter Study
- Observational Study
- Research Support, Non-U.S. Gov't

MeSH terms

- Aged
- COVID-19 / complications*
- COVID-19 / diagnostic imaging
- COVID-19 / mortality*
- COVID-19 / therapy
- Critical Care
- Female
- Heart Failure / complications
- Hospital Mortality
- Hospitalization
- Humans
- Kidney Failure, Chronic / complications*
- Kidney Failure, Chronic / therapy*
- Male

- Middle Aged
- Pandemics
- Pulmonary Disease, Chronic Obstructive / complications
- Radiography
- Renal Dialysis*
- Respiration, Artificial
- Retrospective Studies
- Risk Factors
- SARS-CoV-2
- Turkey / epidemiology

Full text links

Read free full text at BMC BioMed Central Free PMC article
Proceed to details
Cite
Share
274
Observational Study
Saudi Med J
. 2021 Jan;42(1):105-109.
doi: 10.15537/smj.2021.1.25572.

Coronavirus Disease 2019 (COVID-19) in pediatric emergency. Presentation and disposition

Roaa S Jamjoom [1]
Affiliations Expand

Affiliation

- [1] Department of Emergency Medicine, Faculty of Medicine King Abdulaziz University, Jeddah, Kingdom of Saudi Arabia. E-mail. rsjamjoom@kau.edu.sa.

- PMID: **33399179**
- PMCID: PMC7989312
- DOI: 10.15537/smj.2021.1.25572

Free PMC article
Observational Study

Coronavirus Disease 2019 (COVID-19) in pediatric emergency. Presentation and disposition

Roaa S Jamjoom. Saudi Med J. 2021 Jan.
Free PMC article
Show details
Saudi Med J
. 2021 Jan;42(1):105-109.
doi: 10.15537/smj.2021.1.25572.

Author

Roaa S Jamjoom [1]

Affiliation

- [1] Department of Emergency Medicine, Faculty of Medicine King Abdulaziz University, Jeddah, Kingdom of Saudi Arabia. E-mail. rsjamjoom@kau.edu.sa.

- PMID: **33399179**
- PMCID: PMC7989312
- DOI: 10.15537/smj.2021.1.25572

Abstract

Objectives: To examine the demographics and common presentations of pediatric coronavirus disease 2019 patients in the emergency department (ED), as well as their contact with positive COVID-19 cases, return visits, and patients' disposition from the ED. Methods: A retrospective chart review of confirmed cases of COVID-19 presenting to the Pediatric ED from March 2020 until June 2020 was conducted.

Results: Fifty-two patients were identified, with a higher frequency of male patients. Forty-four (85%) patients were discharged from the ED, and 8 (15%) required admission. Three patients were admitted to the pediatric intensive care unit and 2 died, resulting in a mortality rate of 3.8%. The most frequent presentations were fever (85%), cough (48%), and diarrhea (23%). Conclusion: In our study, the second most affected system after the respiratory tract was the gastrointestinal tract, which was also the system responsible for the most return visits due to diarrhea. Coronavirus disease 2019 poses clinical and operational challenges given its variable clinical presentations.

- 14 references
- 1 figure

Supplementary info

Publication types, MeSH terms Expand

Publication types

- Observational Study

MeSH terms

- COVID-19 / diagnosis*
- COVID-19 / therapy*
- Child
- Cough / virology
- Critical Care
- Diarrhea / chemically induced
- Emergency Service, Hospital*
- Female
- Fever / virology
- Hospitalization
- Humans
- Male
- Pandemics
- Patient Readmission
- Retrospective Studies
- SARS-CoV-2
- Saudi Arabia

Full text links

Saudi Medical Journal Free PMC article

Proceed to details

Cite

Share

☐ 275

Observational Study

Clin Infect Dis

. 2021 Dec 6;73(11):e4064-e4072.
doi: 10.1093/cid/ciaa791.

Compassionate Use of Hydroxychloroquine in Clinical Practice for Patients With Mild to Severe COVID-19 in a French University Hospital

Olivier Paccoud [1], Florence Tubach [2], Amandine Baptiste [2], Alexandre Bleibtreu [1], David Hajage [2], Gentiane Monsel [1], Gianpiero Tebano [1], David Boutolleau [3] [4], Elise Klement [1], Nagisa Godefroy [1], Romain Palich [1], Oula Itani [1], Antoine Faiçal [1], Marc-Antoine Valantin [1], Roland Tubiana [1], Sonia Burrel [3] [4], Vincent Calvez [3] [4], Eric Caumes [1] [4], Anne-Geneviève Marcelin [3] [4], Valérie Pourcher [1] [4]
Affiliations Expand

Affiliations

- [1] Assistance Publique - Hôpitaux de Paris, Hôpitaux Universitaires Pitié-Salpêtrière Charles Foix, Service de Maladies infectieuses et Tropicales, Paris, France.
- [2] Sorbonne Université, INSERM UMR 1136, Département de Santé Publique, Unité de Recherche Clinique Pitié Salpêtrière - Charles Foix, Centre de Pharmaco-épidémiologie de l'AP-HP, Hôpitaux Universitaires Pitié-Salpêtrière Charles Foix, Paris, France.
- [3] Sorbonne Université, INSERM, Institut Pierre Louis d'Epidémiologie et de Santé Publique, AP-HP, Hôpitaux Universitaires Pitié-Salpêtrière-Charles Foix, laboratoire de virologie, Paris, France.
- [4] Sorbonne Université, INSERM 1136, Institut Pierre Louis d'Epidémiologie et de Santé Publique, Paris, France.

- PMID: **32556143**
- PMCID: PMC7337663
- DOI: 10.1093/cid/ciaa791

Free PMC article
Observational Study

Compassionate Use of Hydroxychloroquine in Clinical Practice for Patients With Mild to Severe COVID-19 in a French University Hospital

Olivier Paccoud et al. Clin Infect Dis. 2021.
Free PMC article
Show details
Clin Infect Dis
. 2021 Dec 6;73(11):e4064-e4072.

doi: 10.1093/cid/ciaa791.

Authors

Olivier Paccoud [1], Florence Tubach [2], Amandine Baptiste [2], Alexandre Bleibtreu [1], David Hajage [2], Gentiane Monsel [1], Gianpiero Tebano [1], David Boutolleau [3 4], Elise Klement [1], Nagisa Godefroy [1], Romain Palich [1], Oula Itani [1], Antoine Faiçal [1], Marc-Antoine Valantin [1], Roland Tubiana [1], Sonia Burrel [3 4], Vincent Calvez [3 4], Eric Caumes [1 4], Anne-Geneviève Marcelin [3 4], Valérie Pourcher [1 4]

Affiliations

- [1] Assistance Publique - Hôpitaux de Paris, Hôpitaux Universitaires Pitié-Salpêtrière Charles Foix, Service de Maladies infectieuses et Tropicales, Paris, France.
- [2] Sorbonne Université, INSERM UMR 1136, Département de Santé Publique, Unité de Recherche Clinique Pitié Salpêtrière - Charles Foix, Centre de Pharmaco-épidémiologie de l'AP-HP, Hôpitaux Universitaires Pitié-Salpêtrière Charles Foix, Paris, France.
- [3] Sorbonne Université, INSERM, Institut Pierre Louis d'Epidémiologie et de Santé Publique, AP-HP, Hôpitaux Universitaires Pitié-Salpêtrière-Charles Foix, laboratoire de virologie, Paris, France.
- [4] Sorbonne Université, INSERM 1136, Institut Pierre Louis d'Epidémiologie et de Santé Publique, Paris, France.

- PMID: **32556143**
- PMCID: PMC7337663
- DOI: 10.1093/cid/ciaa791

Abstract

Background: Data from nonrandomized studies have suggested that hydroxychloroquine could be an effective therapeutic agent against coronavirus disease 2019 (COVID-19).

Methods: We conducted an observational, retrospective cohort study involving hospitalized adult patients with confirmed, mild to severe COVID-19 in a French university hospital. Patients who received hydroxychloroquine (200 mg 3 times daily dosage for 10 days) on a compassionate basis in addition to standard of care (SOC) were compared with patients without contraindications to hydroxychloroquine who received SOC alone. A propensity score-weighted analysis was performed to control for confounders: age, sex, time between symptom onset and admission ≤ 7 days, Charlson comorbidity index, medical history of arterial hypertension, obesity, National Early Warning Score 2 (NEWS2) score at admission, and pneumonia severity. The primary endpoint was time to unfavorable outcome, defined as: death, admission to an intensive care unit, or decision to withdraw or withhold life-sustaining treatments, whichever came first.

Results: Data from 89 patients with laboratory-confirmed COVID-19 were analyzed, 84 of whom were considered in the primary analysis; 38 patients treated with hydroxychloroquine and 46 patients treated with SOC alone. At admission, the mean age of patients was 66 years, the median Charlson comorbidity index was 3, and the median NEWS2 severity score was 3. After propensity score weighting, treatment with hydroxychloroquine was not associated with a significantly reduced risk of unfavorable outcome (hazard ratio, 0.90 [95% confidence interval, .38-2.1], P = .81). Overall survival was not significantly different between the 2 groups (hazard ratio, 0.89 [0.23; 3.47], P = 1).

Conclusion: In hospitalized adults with COVID-19, no significant reduction of the risk of unfavorable outcomes was observed with hydroxychloroquine in comparison to SOC. Unmeasured confounders may have persisted however, despite careful propensity-weighted analysis and the study might be underpowered. Ongoing controlled trials in patients with varying degrees of initial severity on a larger scale will help determine whether there is a place for hydroxychloroquine in the treatment of COVID-19. In hospitalized adults with COVID-19, no significant reduction of the risk of unfavorable outcomes was observed with hydroxychloroquine in comparison to SOC.

Keywords: COVID-19; SARS-CoV-2; hydroxychloroquine.

© The Author(s) 2020. Published by Oxford University Press for the Infectious Diseases Society of America. All rights reserved. For permissions, e-mail: journals.permissions@oup.com.

Comment in

- Reply to Million et al.
  Hajage D, Pourcher V, Paccoud O, Bleibtreu A, Caumes E, Tubach F. Hajage D, et al. Clin Infect Dis. 2021 May 4;72(9):e440-e441. doi: 10.1093/cid/ciaa1122. Clin Infect Dis. 2021. PMID: 32761119 No abstract available.
- Hydroxychloroquine Failure: The End Does Not Justify the Means.
  Million M, Chaudet H, Raoult D. Million M, et al. Clin Infect Dis. 2021 May 4;72(9):e439. doi: 10.1093/cid/ciaa1117. Clin Infect Dis. 2021. PMID: 32761148 No abstract available.

- Cited by 28 articles

Supplementary info

Publication types, MeSH terms, Substances, Supplementary concepts Expand

Publication types

- Observational Study

MeSH terms

- Adult
- Aged
- COVID-19* / drug therapy
- Compassionate Use Trials
- Hospitals, University
- Humans
- Hydroxychloroquine* / therapeutic use
- Retrospective Studies
- SARS-CoV-2
- Treatment Outcome

Substances

- Hydroxychloroquine

Supplementary concepts

- COVID-19 drug treatment

Full text links

OXFORD ACADEMIC Silverchair Information Systems Free PMC article
Proceed to details
Cite
Share
276
Observational Study

Cancer
. 2021 Apr 1;127(7):1057-1067.
doi: 10.1002/cncr.33335. Epub 2020 Dec 9.

Racial disparities in patients with coronavirus disease 2019 infection and gynecologic malignancy

Olivia D Lara [1], Maria J Smith [1], Yuyan Wang [2], Roisin O'Cearbhaill [3], Stephanie V Blank [4], Valentin Kolev [4], Caitlin Carr [4], Anne Knisely [5], Jennifer McEachron [6], Lisa Gabor [7], Eloise Chapman-Davis [8], Justin Jee [2], Julia Fehniger [1], Yi-Chun Lee [6], Sara Isani [7], Mengling Liu [2] [9], Jason D Wright [5], Bhavana Pothuri [1]
Affiliations Expand

Affiliations

- [1] Department of Obstetrics and Gynecology, Division of Gynecologic Oncology, Perlmutter Cancer Center, New York University (NYU) Langone Health, New York, New York.
- [2] Department of Population Health, NYU Langone Health, New York, New York.
- [3] Department of Medical Oncology, Memorial Sloan Kettering Cancer Center, Weill Cornell Medical College, New York, New York.
- [4] Department of Obstetrics, Gynecologic and Reproductive Science, Division of Gynecologic Oncology, Icahn School of Medicine at Mount Sinai, New York, New York.
- [5] Department of Obstetrics and Gynecology, College of Physicians and Surgeons, Columbia University, New York, New York.
- [6] Department of Obstetrics and Gynecology, State University of New York Downstate Medical Center, Brooklyn, New York.

- [7] Department of Obstetrics and Gynecology and Women's Health, Montefiore Medical Center and Albert Einstein College of Medicine, Bronx, New York.
- [8] Department of Obstetrics and Gynecology, Cornell University, New York, New York.
- [9] Department of Environmental Medicine, NYU Langone Health, New York, New York.

- PMID: **33294978**
- PMCID: **PMC8504148** (available on 2022-04-01)
- DOI: 10.1002/cncr.33335

Observational Study

Racial disparities in patients with coronavirus disease 2019 infection and gynecologic malignancy

Olivia D Lara et al. Cancer. 2021.

Show details

Cancer

. 2021 Apr 1;127(7):1057-1067.
doi: 10.1002/cncr.33335. Epub 2020 Dec 9.

Authors

Olivia D Lara [1], Maria J Smith [1], Yuyan Wang [2], Roisin O'Cearbhaill [3], Stephanie V Blank [4], Valentin Kolev [4], Caitlin Carr [4], Anne Knisely [5], Jennifer McEachron [6], Lisa Gabor [7], Eloise Chapman-Davis [8], Justin Jee [2], Julia Fehniger [1], Yi-Chun Lee [6], Sara Isani [7], Mengling Liu [2] [9], Jason D Wright [5], Bhavana Pothuri [1]

Affiliations

- [1] Department of Obstetrics and Gynecology, Division of Gynecologic Oncology, Perlmutter Cancer Center, New York University (NYU) Langone Health, New York, New York.
- [2] Department of Population Health, NYU Langone Health, New York, New York.
- [3] Department of Medical Oncology, Memorial Sloan Kettering Cancer Center, Weill Cornell Medical College, New York, New York.
- [4] Department of Obstetrics, Gynecologic and Reproductive Science, Division of Gynecologic Oncology, Icahn School of Medicine at Mount Sinai, New York, New York.
- [5] Department of Obstetrics and Gynecology, College of Physicians and Surgeons, Columbia University, New York, New York.
- [6] Department of Obstetrics and Gynecology, State University of New York Downstate Medical Center, Brooklyn, New York.
- [7] Department of Obstetrics and Gynecology and Women's Health, Montefiore Medical Center and Albert Einstein College of Medicine, Bronx, New York.
- [8] Department of Obstetrics and Gynecology, Cornell University, New York, New York.
- [9] Department of Environmental Medicine, NYU Langone Health, New York, New York.

- PMID: **33294978**
- PMCID: **PMC8504148** (available on 2022-04-01)
- DOI: 10.1002/cncr.33335

Abstract

Background: Mounting evidence suggests disproportionate coronavirus disease 2019 (COVID-19) hospitalizations and deaths because of racial disparities. The association of race in a cohort of gynecologic oncology patients with severe acute respiratory syndrome-coronavirus 2 infection is unknown.

Methods: Data were abstracted from gynecologic oncology patients with COVID-19 infection among 8 New York City area hospital systems. A multivariable mixed-effects logistic regression model accounting for county clustering was used to analyze COVID-19-related hospitalization and mortality.

Results: Of 193 patients who had gynecologic cancer and COVID-19, 67 (34.7%) were Black, and 126 (65.3%) were non-Black. Black patients were more likely to require hospitalization compared with non-Black patients (71.6% [48 of 67] vs 46.0% [58 of 126]; P = .001). Of 34 (17.6%) patients who died from COVID-19, 14 (41.2%) were Black. Among those who were hospitalized, compared with non-Black patients, Black patients were more likely to: have ≥3 comorbidities (81.1% [30 of 37] vs 59.2% [29 of 49]; P = .05), to reside in Brooklyn (81.0% [17 of 21] vs 44.4% [12 of 27]; P = .02), to live with family (69.4% [25 of 36] vs 41.6% [37 of 89]; P = .009), and to have public insurance (79.6% [39 of 49] vs 53.4% [39 of 73]; P = .006). In multivariable analysis, among patients aged <65 years, Black patients were more likely to require hospitalization compared with non-Black patients (odds ratio, 4.87; 95% CI, 1.82-12.99; P = .002).

Conclusions: Although Black patients represented only one-third of patients with gynecologic cancer, they accounted for disproportionate rates of hospitalization (>45%) and death (>40%) because of COVID-19 infection; younger Black patients had a nearly 5-fold greater risk of hospitalization. Efforts to understand and improve these disparities in COVID-19 outcomes among Black patients are critical.

Keywords: coronavirus disease 2019 (COVID-19); gynecologic cancer; outcomes; racial disparities; severe acute respiratory syndrome-coronavirus 2 (SARS-CoV-2).

© 2020 American Cancer Society.

- Cited by 3 articles

Supplementary info

Publication types, MeSH terms, Grant support Expand

Publication types

- Multicenter Study
- Observational Study
- Research Support, N.I.H., Extramural

MeSH terms

- Adult
- African Americans / statistics & numerical data*
- Aged
- COVID-19 / complications
- COVID-19 / ethnology*
- COVID-19 / virology
- Female
- Genital Neoplasms, Female / complications
- Genital Neoplasms, Female / ethnology*
- Health Status Disparities*
- Hospitalization / statistics & numerical data
- Humans
- Logistic Models
- Middle Aged
- Multivariate Analysis
- New York City
- Retrospective Studies
- Risk Factors
- SARS-CoV-2 / physiology
- Survival Analysis
- Whites / statistics & numerical data*

Grant support

- P30 CA008748/CA/NCI NIH HHS/United States

Full text links

WILEY Full Text Article Wiley
Proceed to details
Cite
Share
☐ 277
Observational Study
Public Health Rep
. 2021 May;136(3):375-383.
doi: 10.1177/0033354921999385. Epub 2021 Mar 5.

COVID-19 in the New York City Jail System: Epidemiology and Health Care Response, March-April 2020

Justin Chan[1], Kelsey Burke[1], Rachael Bedard[1], James Grigg[1], John Winters[1], Colleen Vessell[1], Zachary Rosner[1], Jeffrey Cheng[1], Monica Katyal[1], Patricia Yang[1], Ross MacDonald[1]
Affiliations Expand

Affiliation

- [1] 2012 Correctional Health Services, NYC Health + Hospitals, New York, NY, USA.

- PMID: **33673760**
- PMCID: **PMC8580401** (available on 2022-05-01)
- DOI: 10.1177/0033354921999385

Observational Study

COVID-19 in the New York City Jail System: Epidemiology and Health Care Response, March-April 2020

Justin Chan et al. Public Health Rep. 2021 May.
Show details
Public Health Rep
. 2021 May;136(3):375-383.
doi: 10.1177/0033354921999385. Epub 2021 Mar 5.

Authors

Justin Chan[1], Kelsey Burke[1], Rachael Bedard[1], James Grigg[1], John Winters[1], Colleen Vessell[1], Zachary Rosner[1], Jeffrey Cheng[1], Monica Katyal[1], Patricia Yang[1], Ross MacDonald[1]

Affiliation

- [1] 2012 Correctional Health Services, NYC Health + Hospitals, New York, NY, USA.

- PMID: **33673760**
- PMCID: **PMC8580401** (available on 2022-05-01)
- DOI: 10.1177/0033354921999385

Abstract

Objectives: People detained in correctional facilities are at high risk for infection with severe acute respiratory syndrome coronavirus 2, the virus that causes coronavirus disease 2019 (COVID-19). We described the epidemiology of the COVID-19 outbreak in a large urban jail system, including signs and symptoms at time of testing and risk factors for hospitalization.

Methods: This retrospective observational cohort study included all patients aged ≥18 years who were tested for COVID-19 during March 11-April 28, 2020, while in custody in the New York City jail system (N = 978). We described demographic characteristics and signs and symptoms at the time of testing and performed Cox regression analysis to identify factors associated with hospitalization among those with a positive test result.

Results: Of 978 people tested for COVID-19, 568 received a positive test result. Among symptomatic patients, the most common symptoms among those who received a positive test result were cough (n = 293 of 510, 57%) and objective fever (n = 288 of 510, 56%). Of 257 asymptomatic patients who were tested, 58 (23%) received a positive test result. Forty-five (8%) people who received a positive test result were hospitalized for COVID-19. Older age (aged ≥55 vs 18-34) (adjusted hazard ratio [aHR] = 13.41; 95% CI, 3.80-47.33) and diabetes mellitus (aHR = 1.99; 95% CI, 1.00-3.95) were significantly associated with hospitalization.

Conclusions: A substantial proportion of people tested in New York City jails received a positive test result for COVID-19, including a large proportion of people tested while asymptomatic. During periods of ongoing transmission, asymptomatic screening should complement symptom-driven COVID-19 testing in correctional facilities. Older patients and people with diabetes mellitus should be closely monitored after COVID-19 diagnosis because of their increased risk for hospitalization.

Keywords: COVID-19; SARS-CoV-2; correctional facilities/prisons; incarcerated; jail.

Conflict of interest statement

Declaration of Conflicting Interests: The authors declared no potential conflicts of interest with respect to the research, authorship, and/or publication of this article.

- Cited by 5 articles

Supplementary info

Publication types, MeSH terms Expand

Publication types

- Observational Study

MeSH terms

- Adult
- COVID-19 / diagnosis*
- COVID-19 / epidemiology*

- COVID-19 Testing / statistics & numerical data*
- Cohort Studies
- Female
- Hospitalization / statistics & numerical data*
- Humans
- Jails*
- Male
- Middle Aged
- New York City / epidemiology
- Retrospective Studies
- Risk Factors
- SARS-CoV-2*

Full text links

SAGE journals Atypon
Proceed to details
Cite
Share
278
Observational Study
Rev Esp Cardiol (Engl Ed)
. 2021 Jan;74(1):24-32.
doi: 10.1016/j.rec.2020.08.027. Epub 2020 Oct 15.

Prognostic implications of myocardial injury in patients with and without COVID-19 infection treated in a university hospital

[Article in English, Spanish]
Alfredo Bardají [1], Anna Carrasquer [2], Raúl Sánchez-Giménez [3], Nisha Lal-Trehan [3], Víctor Del-Moral-Ronda [3], Óscar M Peiró [3], Gil Bonet [3], Gislaine Castilho [4], Isabel Fort-Gallifa [5], Clara Benavent [5], Gemma Recio [5], Cristina Gutiérrez [5], Christian Villavicencio [6], Teresa Auguet [7], Carme Boqué [8]
Affiliations Expand

Affiliations

- [1] Servicio de Cardiología, Hospital Universitario de Tarragona Joan XXIII, Tarragona, Spain; Universidad Rovira Virgili, Tarragona, Spain; Institut d'Investigació Sanitària Pere Virgili (IISPV), Tarragona, Spain. Electronic address: abardaji.hj23.ics@gencat.cat.

- [2] Servicio de Cardiología, Hospital Universitario de Tarragona Joan XXIII, Tarragona, Spain; Universidad Rovira Virgili, Tarragona, Spain; Institut d'Investigació Sanitària Pere Virgili (IISPV), Tarragona, Spain.
- [3] Servicio de Cardiología, Hospital Universitario de Tarragona Joan XXIII, Tarragona, Spain.
- [4] Servicio de Cardiología, Hospital Universitario de Tarragona Joan XXIII, Tarragona, Spain; Institut d'Investigació Sanitària Pere Virgili (IISPV), Tarragona, Spain.
- [5] Laboratori Clínic ICS Camp de Tarragona-Terres de l'Ebre, Instituto Catalán de la Salud, Tarragona, Spain.
- [6] Servicio de Cuidados Intensivos, Hospital Universitario de Tarragona Joan XXIII, Tarragona, Spain.
- [7] Universidad Rovira Virgili, Tarragona, Spain; Institut d'Investigació Sanitària Pere Virgili (IISPV), Tarragona, Spain; Servicio de Medicina Interna, Hospital Universitario de Tarragona Joan XXIII, Tarragona, Spain.
- [8] Universidad Rovira Virgili, Tarragona, Spain; Institut d'Investigació Sanitària Pere Virgili (IISPV), Tarragona, Spain; Servicio de Urgencias, Hospital Universitario de Tarragona Joan XXIII, Tarragona, Spain.

- PMID: **33144126**
- PMCID: PMC7561309
- DOI: 10.1016/j.rec.2020.08.027

Free PMC article
Observational Study

Prognostic implications of myocardial injury in patients with and without COVID-19 infection treated in a university hospital

[Article in English, Spanish]
Alfredo Bardají et al. Rev Esp Cardiol (Engl Ed). 2021 Jan.
Free PMC article

Show details

Rev Esp Cardiol (Engl Ed)
. 2021 Jan;74(1):24-32.
doi: 10.1016/j.rec.2020.08.027. Epub 2020 Oct 15.

Authors

Alfredo Bardají [1], Anna Carrasquer [2], Raúl Sánchez-Giménez [3], Nisha Lal-Trehan [3], Víctor Del-Moral-Ronda [3], Óscar M Peiró [3], Gil Bonet [3], Gislaine Castilho [4], Isabel Fort-Gallifa [5], Clara Benavent [5], Gemma Recio [5], Cristina Gutiérrez [5], Christian Villavicencio [6], Teresa Auguet [7], Carme Boqué [8]

Affiliations

- [1] Servicio de Cardiología, Hospital Universitario de Tarragona Joan XXIII, Tarragona, Spain; Universidad Rovira Virgili, Tarragona, Spain; Institut d'Investigació Sanitària Pere Virgili (IISPV), Tarragona, Spain. Electronic address: abardaji.hj23.ics@gencat.cat.
- [2] Servicio de Cardiología, Hospital Universitario de Tarragona Joan XXIII, Tarragona, Spain; Universidad Rovira Virgili, Tarragona, Spain; Institut d'Investigació Sanitària Pere Virgili (IISPV), Tarragona, Spain.
- [3] Servicio de Cardiología, Hospital Universitario de Tarragona Joan XXIII, Tarragona, Spain.
- [4] Servicio de Cardiología, Hospital Universitario de Tarragona Joan XXIII, Tarragona, Spain; Institut d'Investigació Sanitària Pere Virgili (IISPV), Tarragona, Spain.
- [5] Laboratori Clínic ICS Camp de Tarragona-Terres de l'Ebre, Instituto Catalán de la Salud, Tarragona, Spain.
- [6] Servicio de Cuidados Intensivos, Hospital Universitario de Tarragona Joan XXIII, Tarragona, Spain.
- [7] Universidad Rovira Virgili, Tarragona, Spain; Institut d'Investigació Sanitària Pere Virgili (IISPV), Tarragona, Spain; Servicio de Medicina Interna, Hospital Universitario de Tarragona Joan XXIII, Tarragona, Spain.
- [8] Universidad Rovira Virgili, Tarragona, Spain; Institut d'Investigació Sanitària Pere Virgili (IISPV), Tarragona, Spain; Servicio de Urgencias, Hospital Universitario de Tarragona Joan XXIII, Tarragona, Spain.

- PMID: **33144126**
- PMCID: PMC7561309
- DOI: 10.1016/j.rec.2020.08.027

Abstract
in English, Spanish

Introduction and objectives: Cardiac troponin, a marker of myocardial injury, is frequently observed in patients with COVID-19 infection. Our objective was to analyze myocardial injury and its prognostic implications in patients with and without COVID-19 infection treated in the same period of time.

Methods: The present study included patients treated in a university hospital with cardiac troponin I measurements and with suspected COVID-19 infection, confirmed or ruled out by polymerase chain reaction analysis. The impact was analyzed of cardiac troponin I positivity on 30-day mortality.

Results: In total, 433 patients were distributed among the following groups: confirmed COVID-19 (n=186), 22% with myocardial injury (n=41); and ruled out COVID-19 (n=247), 21.5% with myocardial injury (n=52). The confirmed and ruled out COVID-19 groups had a similar age, sex, and cardiovascular history. Mortality was significantly higher in the confirmed COVID-19 group than in the ruled out group (19.9% vs 5.3%, P <.001). In Cox multivariate regression analysis, cardiac troponin I was a predictor of mortality in both groups (confirmed COVID-19 group: HR, 3.54; 95%CI, 1.70-7.34; P=.001; ruled out COVID-19 group: HR, 5.57; 95%CI, 1.70-18.20; P=.004). The predictive model analyzed by ROC curves was similar in the 2 groups (P=.701), with AUCs of 0.808 in the confirmed COVID-19 group (0.750-0.865) and 0.812 in the ruled out COVID-19 group (0.760-0.864).

Conclusions: Myocardial injury is detected in 1 in every 5 patients with confirmed or ruled out COVID-19 and predicts 30-day mortality to a similar extent in both circumstances.

Introducción y objetivos: La elevación de la troponina cardiaca como marcador de daño miocárdico es un predictor pronóstico en pacientes con COVID-19. Sin embargo, se desconoce su rendimiento en pacientes coetáneos con sospecha de COVID-19 pero con prueba de reacción en cadena de la polimerasa negativa.

Métodos: Estudio de cohortes retrospectivo que incluyó a todos los pacientes consecutivos atendidos en un hospital universitario con sospecha de COVID-19, confirmada o descartada mediante prueba de reacción en cadena de la polimerasa, todos ellos con determinaciones de troponina cardiaca I. Se analizó el impacto de la positividad de la troponina cardiaca I en la mortalidad a 30 días.

Resultados: Un total de 433 pacientes quedaron distribuidos en los siguientes grupos: COVID-19 confirmada (n = 186), el 22% de ellos con daño miocárdico (n = 41), y COVID-19 descartada (n = 247), el 21,5% de ellos con daño miocárdico (n = 52). Los grupos de COVID-19 confirmada y descartada tuvieron similares edad, sexo y antecedentes cardiovasculares. La mortalidad en el grupo de COVID-19 confirmada frente al de descartada fue significativamente superior (el 19,9 frente al 5,3%; p < 0,001). En ambos grupos, el daño miocárdico fue predictor de mortalidad en el análisis multivariado de regresión de Cox (grupo de COVID-19 confirmada, HR = 3,54; IC95%, 1,70-7,34; p = 0,001; grupo de COVID-19 descartada, HR = 5,57; IC95%, 1,70-18,20; p = 0,004). El modelo predictivo analizado por curvas ROC fue similar en ambos grupos: COVID-19 confirmada, AUC = 0,808 (0,750-0,865); COVID-19 descartada, AUC = 0,812 (0,760-0,864) (p = 0,701).

Conclusiones: Se detecta daño miocárdico en 1 de cada 5 pacientes con infección por COVID-19 confirmada o descartada. En ambas circunstancias, el daño miocárdico es predictor de mortalidad a 30 días en similar grado.

Keywords: COVID-19; Daño miocárdico; Myocardial damage; Troponin; Troponina.

Copyright © 2020 Sociedad Española de Cardiología. Published by Elsevier España, S.L.U. All rights reserved.

- Cited by 6 articles
- 25 references
- 3 figures

Supplementary info

Publication types, MeSH terms, Substances Expand

Publication types

- Observational Study

MeSH terms

- Aged
- COVID-19 / blood
- COVID-19 / complications
- COVID-19 / diagnosis

- COVID-19 / mortality*
- COVID-19 Nucleic Acid Testing / statistics & numerical data
- Cardiomyopathies / blood
- Cardiomyopathies / mortality*
- Confidence Intervals
- Female
- Hospitalization / statistics & numerical data
- Hospitals, University
- Humans
- Intensive Care Units / statistics & numerical data
- Lung / diagnostic imaging
- Male
- Middle Aged
- Prognosis
- ROC Curve
- Regression Analysis
- Retrospective Studies
- Risk Factors
- SARS-CoV-2*
- Troponin I / blood*

Substances

- Troponin I

Full text links

FULL TEXT AT REVISTA ESPAÑOLA DE CARDIOLOGIA Ediciones Doyma, S.L. Free PMC article

Proceed to details

Cite

Share

279

Observational Study

J Clin Endocrinol Metab
. 2021 Jan 23;106(2):e936-e942.
doi: 10.1210/clinem/dgaa825.

COVID-19 Hospitalization in Adults with Type 1 Diabetes: Results from the T1D Exchange Multicenter Surveillance Study

Grenye O'Malley [1], Osagie Ebekozien [2], Marisa Desimone [3], Catherina T Pinnaro [4], Alissa Roberts [5], Sarit Polsky [6], Nudrat Noor [2], Grazia Aleppo [7], Marina Basina [8], Michael Tansey [4], Devin Steenkamp [9], Francesco Vendrame [10], Ilona Lorincz [11], Priyanka Mathias [12], Shivani Agarwal [12], Lauren Golden [13], Irl B Hirsch [14], Carol J Levy [1]
Affiliations Expand

Affiliations

- [1] Department of Endocrinology, Diabetes and Bone Diseases, Icahn School of Medicine, New York, New York.
- [2] T1D Exchange, Boston, Massachusetts.
- [3] Division of Endocrinology, Diabetes and Metabolism, SUNY Upstate Medical University, Syracuse, New York.
- [4] Stead Family Department of Pediatrics, University of Iowa, Iowa City, Iowa.
- [5] Department of Pediatrics, University of Washington, Seattle, Washington.
- [6] Barbara Davis Center for Diabetes, University of Colorado Anschutz Medical Campus, Aurora, Colorado.
- [7] Feinberg School of Medicine, Northwestern University, Chicago, Illinois.
- [8] Stanford University School of Medicine, Stanford, California.
- [9] Boston University School of Medicine and Boston Medical Center, Boston, Massachusetts.
- [10] Division of Endocrinology, Diabetes, and Metabolism, University of Miami, Miami, Florida.
- [11] Department of Endocrinology, Diabetes, and Metabolism, Hospital of University of Pennsylvania, Philadelphia, Pennsylvania.
- [12] Fleischer Institute for Diabetes and Metabolism, NY-Regional Center for Diabetes and Translational Research, Albert Einstein College of Medicine, New York, New York.
- [13] NYU Langone Medical Center, New York, New York.
- [14] University of Washington School of Medicine, Seattle, Washington.

- PMID: **33165563**
- PMCID: PMC7717244
- DOI: 10.1210/clinem/dgaa825

Free PMC article
Observational Study

COVID-19 Hospitalization in Adults with Type 1 Diabetes: Results from the T1D Exchange Multicenter Surveillance Study

Grenye O'Malley et al. J Clin Endocrinol Metab. 2021.
Free PMC article
Show details
J Clin Endocrinol Metab
. 2021 Jan 23;106(2):e936-e942.

doi: 10.1210/clinem/dgaa825.

Authors

Grenye O'Malley [1], Osagie Ebekozien [2], Marisa Desimone [3], Catherina T Pinnaro [4], Alissa Roberts [5], Sarit Polsky [6], Nudrat Noor [2], Grazia Aleppo [7], Marina Basina [8], Michael Tansey [4], Devin Steenkamp [9], Francesco Vendrame [10], Ilona Lorincz [11], Priyanka Mathias [12], Shivani Agarwal [12], Lauren Golden [13], Irl B Hirsch [14], Carol J Levy [1]

Affiliations

- [1] Department of Endocrinology, Diabetes and Bone Diseases, Icahn School of Medicine, New York, New York.
- [2] T1D Exchange, Boston, Massachusetts.
- [3] Division of Endocrinology, Diabetes and Metabolism, SUNY Upstate Medical University, Syracuse, New York.
- [4] Stead Family Department of Pediatrics, University of Iowa, Iowa City, Iowa.
- [5] Department of Pediatrics, University of Washington, Seattle, Washington.
- [6] Barbara Davis Center for Diabetes, University of Colorado Anschutz Medical Campus, Aurora, Colorado.
- [7] Feinberg School of Medicine, Northwestern University, Chicago, Illinois.
- [8] Stanford University School of Medicine, Stanford, California.
- [9] Boston University School of Medicine and Boston Medical Center, Boston, Massachusetts.
- [10] Division of Endocrinology, Diabetes, and Metabolism, University of Miami, Miami, Florida.
- [11] Department of Endocrinology, Diabetes, and Metabolism, Hospital of University of Pennsylvania, Philadelphia, Pennsylvania.
- [12] Fleischer Institute for Diabetes and Metabolism, NY-Regional Center for Diabetes and Translational Research, Albert Einstein College of Medicine, New York, New York.
- [13] NYU Langone Medical Center, New York, New York.
- [14] University of Washington School of Medicine, Seattle, Washington.

- PMID: **33165563**
- PMCID: PMC7717244
- DOI: 10.1210/clinem/dgaa825

Abstract

Context: Diabetes mellitus is associated with increased COVID-19 morbidity and mortality, but there are few data focusing on outcomes in people with type 1 diabetes.

Objective: The objective of this study was to analyze characteristics of adults with type 1 diabetes for associations with COVID-19 hospitalization.

Design: An observational multisite cross-sectional study was performed. Diabetes care providers answered a 33-item questionnaire regarding demographics, symptoms, and diabetes- and COVID-19-related care and outcomes. Descriptive statistics were used to describe the study population,

and multivariate logistic regression models were used to analyze the relationship between glycated hemoglobin (HbA1c), age, and comorbidities and hospitalization.

Setting: Cases were submitted from 52 US sites between March and August 2020.

Patients or other participants: Adults over the age of 19 with type 1 diabetes and confirmed COVID-19 infection were included.

Interventions: None.

Main outcome measures: Hospitalization for COVID-19 infection.

Results: A total of 113 cases were analyzed. Fifty-eight patients were hospitalized, and 5 patients died. Patients who were hospitalized were more likely to be older, to identify as non-Hispanic Black, to use public insurance, or to have hypertension, and less likely to use continuous glucose monitoring or insulin pumps. Median HbA1c was 8.6% (70 mmol/mol) and was positively associated with hospitalization (odds ratio 1.42, 95% confidence interval 1.18-1.76), which persisted after adjustment for age, sex, race, and obesity.

Conclusions: Baseline glycemic control and access to care are important modifiable risk factors which need to be addressed to optimize care of people with type 1 diabetes during the worldwide COVID-19 pandemic.

Keywords: COVID-19; adult; hospitalization; type 1 diabetes.

© The Author(s) 2020. Published by Oxford University Press on behalf of the Endocrine Society. All rights reserved. For permissions, please e-mail: journals.permissions@oup.com.

- Cited by 7 articles

Supplementary info

Publication types, MeSH terms, Grant support Expand

Publication types

- Multicenter Study
- Observational Study

MeSH terms

- Adult
- Aged
- Aged, 80 and over
- COVID-19 / complications
- COVID-19 / diagnosis
- COVID-19 / epidemiology*
- COVID-19 / therapy*
- Comorbidity

- Cross-Sectional Studies
- Diabetes Mellitus, Type 1 / complications
- Diabetes Mellitus, Type 1 / diagnosis
- Diabetes Mellitus, Type 1 / epidemiology*
- Diabetes Mellitus, Type 1 / therapy*
- Female
- Hospitalization / statistics & numerical data*
- Humans
- Male
- Middle Aged
- Pandemics
- Population Surveillance
- Prognosis
- Retrospective Studies
- SARS-CoV-2 / physiology
- Treatment Outcome
- United States / epidemiology
- Young Adult

Grant support

- K23 DK115896/DK/NIDDK NIH HHS/United States
- P30 DK020541/DK/NIDDK NIH HHS/United States
- P30 DK111022/DK/NIDDK NIH HHS/United States
- T32 DK112751/DK/NIDDK NIH HHS/United States

Full text links

ENDOCRINE SOCIETY Silverchair Information Systems Free PMC article
Proceed to details
Cite
Share
280
Observational Study
J Med Virol
. 2021 Apr;93(4):2270-2280.
doi: 10.1002/jmv.26674. Epub 2020 Nov 22.

Tocilizumab in hospitalized patients with COVID-19: Clinical outcomes, inflammatory marker kinetics, and safety

Joshua A Hill [1 2 3], Manoj P Menon [1 2 3], Shireesha Dhanireddy [1], Mark M Wurfel [1], Margaret Green [1], Rupali Jain [1 4], Jeannie D Chan [1 4], Joanne Huang [1 4], Danika Bethune [5], Cameron Turtle [1 3], Christine Johnston [1 2], Hu Xie [3], Wendy M Leisenring [3], H Nina Kim [1], Guang-Shing Cheng [1 3]
Affiliations Expand

Affiliations

- [1] Department of Medicine, University of Washington, Seattle, Washington, USA.
- [2] Vaccine and Infectious Disease Division, Fred Hutchinson Cancer Research Center, Seattle, Washington, USA.
- [3] Clinical Research Division, Fred Hutchinson Cancer Research Center, Seattle, Washington, USA.
- [4] University of Washington School of Pharmacy, Seattle, Washington, USA.
- [5] University of Washington School of Medicine, Seattle, Washington, USA.

- PMID: **33200828**
- PMCID: PMC7753799
- DOI: 10.1002/jmv.26674

Free PMC article
Observational Study

Tocilizumab in hospitalized patients with COVID-19: Clinical outcomes, inflammatory marker kinetics, and safety

Joshua A Hill et al. J Med Virol. 2021 Apr.
Free PMC article
Show details
J Med Virol
. 2021 Apr;93(4):2270-2280.
doi: 10.1002/jmv.26674. Epub 2020 Nov 22.

Authors

Joshua A Hill [1 2 3], Manoj P Menon [1 2 3], Shireesha Dhanireddy [1], Mark M Wurfel [1], Margaret Green [1], Rupali Jain [1 4], Jeannie D Chan [1 4], Joanne Huang [1 4], Danika Bethune [5], Cameron Turtle [1 3], Christine Johnston [1 2], Hu Xie [3], Wendy M Leisenring [3], H Nina Kim [1], Guang-Shing Cheng [1 3]

Affiliations

- [1] Department of Medicine, University of Washington, Seattle, Washington, USA.
- [2] Vaccine and Infectious Disease Division, Fred Hutchinson Cancer Research Center, Seattle, Washington, USA.

- [3] Clinical Research Division, Fred Hutchinson Cancer Research Center, Seattle, Washington, USA.
- [4] University of Washington School of Pharmacy, Seattle, Washington, USA.
- [5] University of Washington School of Medicine, Seattle, Washington, USA.

- PMID: **33200828**
- PMCID: PMC7753799
- DOI: 10.1002/jmv.26674

Abstract

Coronavirus disease 2019 (COVID-19) due to infection with severe acute respiratory syndrome coronavirus 2 causes substantial morbidity. Tocilizumab, an interleukin-6 receptor antagonist, might improve outcomes by mitigating inflammation. We conducted a retrospective study of patients admitted to the University of Washington Hospital system with COVID-19 and requiring supplemental oxygen. Outcomes included clinical improvement, defined as a two-point reduction in severity on a six-point ordinal scale or discharge, and mortality within 28 days. We used Cox proportional-hazards models with propensity score inverse probability weighting to compare outcomes in patients who did and did not receive tocilizumab. We evaluated 43 patients who received tocilizumab and 45 who did not. Patients receiving tocilizumab were younger with fewer comorbidities but higher baseline oxygen requirements. Tocilizumab treatment was associated with reduced C-reactive protein, fibrinogen, and temperature, but there were no meaningful differences in time to clinical improvement (adjusted hazard ratio [aHR], 0.92; 95% confidence interval [CI], 0.38-2.22) or mortality (aHR, 0.57; 95% CI, 0.21-1.52). A numerically higher proportion of tocilizumab-treated patients had subsequent infections, transaminitis, and cytopenias. Tocilizumab did not improve outcomes in hospitalized patients with COVID-19. However, this study was not powered to detect small differences, and there remains the possibility for a survival benefit.

Keywords: COVID-19; SARS-CoV-2; coronavirus; immunomodulatory; tocilizumab.

© 2020 Wiley Periodicals LLC.

Conflict of interest statement

The authors declare that there are no conflict of interests.

- Cited by 22 articles
- 50 references
- 3 figures

Supplementary info

Publication types, MeSH terms, Substances, Supplementary concepts Expand

Publication types

- Observational Study

MeSH terms

- Aged
- Antibodies, Monoclonal, Humanized / administration & dosage*
- C-Reactive Protein / metabolism
- COVID-19 / drug therapy*
- COVID-19 / metabolism
- COVID-19 / mortality
- COVID-19 / virology
- Female
- Fibrinogen / metabolism
- Hospitalization
- Humans
- Immunomodulation
- Inflammation / drug therapy
- Inflammation Mediators / metabolism
- Male
- Middle Aged
- Receptors, Interleukin-6 / metabolism
- Retrospective Studies
- SARS-CoV-2 / drug effects
- Treatment Outcome

Substances

- Antibodies, Monoclonal, Humanized
- Inflammation Mediators
- Receptors, Interleukin-6
- Fibrinogen
- C-Reactive Protein
- tocilizumab

Supplementary concepts

- COVID-19 drug treatment

Full text links

WILEY Full Text Article Wiley Free PMC article

Proceed to details

Cite

Share

☐ 281

Epidemiol Prev
. Sep-Dec 2020;44(5-6 Suppl 2):323-329.
doi: 10.19191/EP20.5-6.S2.133.

COVID-19 infections in the Friuli Venezia Giulia Region (Northern Italy): a population-based retrospective analysis

Elena Calagnan [1], Michele Gobbato [2], Ivana Burba [2], Stefania Del Zotto [2], Federica Toffolutti [3], Diego Serraino [3], Giuseppe Tonutti [2]
Affiliations Expand

Affiliations

- [1] Azienda regionale di coordinamento per la salute (ARCS), Udine (Italy); elena.clagnan@arcs.sanita.fvg.it.
- [2] Azienda regionale di coordinamento per la salute (ARCS), Udine (Italy).
- [3] Cancer Epidemiology Unit, Centro di riferimento oncologico di Aviano (CRO) IRCCS Aviano (Italy).

- PMID: **33412825**
- DOI: 10.19191/EP20.5-6.S2.133

Free article

COVID-19 infections in the Friuli Venezia Giulia Region (Northern Italy): a population-based retrospective analysis

Elena Calagnan et al. Epidemiol Prev. Sep-Dec 2020.
Free article
Show details

Epidemiol Prev
. Sep-Dec 2020;44(5-6 Suppl 2):323-329.
doi: 10.19191/EP20.5-6.S2.133.

Authors

Elena Calagnan [1], Michele Gobbato [2], Ivana Burba [2], Stefania Del Zotto [2], Federica Toffolutti [3], Diego Serraino [3], Giuseppe Tonutti [2]

Affiliations

- [1] Azienda regionale di coordinamento per la salute (ARCS), Udine (Italy); elena.clagnan@arcs.sanita.fvg.it.
- [2] Azienda regionale di coordinamento per la salute (ARCS), Udine (Italy).
- [3] Cancer Epidemiology Unit, Centro di riferimento oncologico di Aviano (CRO) IRCCS Aviano (Italy).

- PMID: **33412825**
- DOI: 10.19191/EP20.5-6.S2.133

Abstract

Objectives: to study the cumulative incidence, the demographics and health conditions of the population tested for COVID-19, and to map the evolving distribution of individual cases in the population of the Friuli Venezia Giulia Region (North-Eastern Italy).

Design: population-based observational study based on a record linkage procedure of databases included in the electronic health information system of the Friuli Venezia Giulia Region.

Setting and participants: the study group consisted of individuals who resided in the Friuli Venezia Giulia Region and who underwent COVID-19 testing from 01.03 to 24.04.2020. The study group was identified from the laboratory database, which contains all the microbiological testing performed in regional facilities. Tested people were categorized into positive or negative cases, based on test results.

Main outcome measures: probability of being tested for and cumulative incidence of COVID-19.

Results: the cumulative probability of being tested for COVID-19 was 278/10,000 inhabitants, while the cumulative incidence was 22 cases/10,000. Out of 33,853 tested people, 2,744 (8.1%) turned out to be positive for COVID-19. Women were tested more often than men (337 vs 216/10,000), and they showed a higher incidence of infection than men (25 and 19 infected cases/10,000 residents, respectively). Both cumulative incidence and cumulative probability of being tested were higher in the elderly population. About 25% of infected people was hosted in retirement homes and 9% was represented by healthcare workers. Thirty seven percent of positive cases had hypertension, 15% cardiologic diseases, while diabetes and cancer characterized 11.7% and 10% of the infected population, respectively. The geographic distribution of positive cases showed a faster spread of the infection in the city of Trieste, an urban area with the highest regional population density.

Conclusions: the COVID-19 pandemic did not hit the Friuli Venezia Giulia Region as hard as other Northern Italian Regions. In the early phase, as documented in this study, the COVID-19 pandemic particularly affected women and elderly people, especially those living in retirement homes in Trieste.

Keywords: COVID-19; Friuli Venezia Giulia Region; North-Eastern Italy; epidemiology; geography.

- Cited by 2 articles

Supplementary info

MeSH terms, Substances Expand

MeSH terms

- Adolescent
- Adult
- Age Distribution
- Aged
- Aged, 80 and over
- Angiotensin II Type 1 Receptor Blockers / therapeutic use
- Angiotensin Receptor Antagonists / therapeutic use
- COVID-19 / diagnosis
- COVID-19 / epidemiology*
- COVID-19 Testing / statistics & numerical data
- Child
- Child, Preschool
- Comorbidity
- Databases, Factual
- Female
- Geography, Medical
- Homes for the Aged / statistics & numerical data
- Hospitalization / statistics & numerical data
- Humans
- Incidence
- Infant
- Infant, Newborn
- Italy / epidemiology
- Male
- Middle Aged
- Pandemics*
- Procedures and Techniques Utilization
- Retrospective Studies
- SARS-CoV-2*
- Sex Distribution
- Young Adult

Substances

- Angiotensin II Type 1 Receptor Blockers
- Angiotensin Receptor Antagonists

Full text links

Free Full Text Inferenze scarl

Proceed to details

Cite

Share

☐ 282

Observational Study

Am J Emerg Med

. 2021 Dec;50:106-110.

doi: 10.1016/j.ajem.2021.07.049. Epub 2021 Jul 27.

Admission SpO $_2$ and ROX index predict outcome in patients with COVID-19

Ahmed Mukhtar[1], Ashraf Rady[1], Ahmed Hasanin[2], Ahmed Lotfy[1], Akram El Adawy[1], Amr Hussein[1], Islam El-Hefnawy[3], Mohamed Hassan[4], Hanan Mostafa[1]

Affiliations Expand

Affiliations

- [1] Anesthesia and Surgical Intensive Care Department, Cairo University, Cairo, Egypt.
- [2] Anesthesia and Surgical Intensive Care Department, Cairo University, Cairo, Egypt. Electronic address: ahmedmohamedhasanin@gmail.com.
- [3] Radiology Department, Cairo University, Cairo, Egypt.
- [4] Pulmonology Department, Cairo University, Cairo, Egypt.

- PMID: **34332217**
- PMCID: PMC8313790
- DOI: 10.1016/j.ajem.2021.07.049

Free PMC article

Observational Study

Admission SpO $_2$ and ROX index predict outcome in patients with COVID-19

Ahmed Mukhtar et al. Am J Emerg Med. 2021 Dec.

Free PMC article

Show details

Am J Emerg Med

. 2021 Dec;50:106-110.

doi: 10.1016/j.ajem.2021.07.049. Epub 2021 Jul 27.

Authors

Ahmed Mukhtar [1], Ashraf Rady [1], Ahmed Hasanin [2], Ahmed Lotfy [1], Akram El Adawy [1], Amr Hussein [1], Islam El-Hefnawy [3], Mohamed Hassan [4], Hanan Mostafa [1]

Affiliations

- [1] Anesthesia and Surgical Intensive Care Department, Cairo University, Cairo, Egypt.
- [2] Anesthesia and Surgical Intensive Care Department, Cairo University, Cairo, Egypt. Electronic address: ahmedmohamedhasanin@gmail.com.
- [3] Radiology Department, Cairo University, Cairo, Egypt.
- [4] Pulmonology Department, Cairo University, Cairo, Egypt.

- PMID: **34332217**
- PMCID: PMC8313790
- DOI: 10.1016/j.ajem.2021.07.049

Abstract

Background: This study aimed to evaluate the accuracy of pulse oximetry-derived oxygen saturation (SpO_2) on room air, determined at hospital admission, as a predictor for the need for mechanical ventilatory support in patients with Coronavirus Disease-2019 (COVID-19).

Methods: In this retrospective observational study, demographic and clinical details of the patients were obtained during ICU admission. SpO_2 and respiratory rate (RR) on room air were determined within the first 6 h of hospital admission. As all measurements were obtained on room air, we calculated the simplified respiratory rate-oxygenation (ROX) index by dividing the SpO_2 by the RR. Based on the use of any assistance of mechanical ventilator (invasive or noninvasive), patients were divided into mechanical ventilation (MV) group and oxygen therapy group. The accuracy of the SpO_2, CT score, and ROX index to predict the need to MV were determined using the Area under receiver operating curve (AUC).

Results: We included 72 critically ill patients who tested COVID-19-positive. SpO_2 on the room air could predict any MV requirement (AUC [95% confidence interval]: 0.9 [0.8-0.96], sensitivity: 70%, specificity 100%, cut-off value ≤78%, $P < 0.001$). Within the MV group, the use of noninvasive ventilation (NIV) was successful in 37 (74%) patients, whereas 13 patients (26%) required endotracheal intubation. The cut-off ROX value for predicting early NIV failure was ≤1.4, with a sensitivity of 85%, a specificity of 86%, and an AUC of 0.86 (95% confidence interval of 0.73-0.94, $P < 0.0001$).

Conclusions: A baseline SpO_2 ≤78% is an excellent predictor of MV requirement with a positive predictive value of 100%. Moreover, the ROX index measured within the first 6 h of hospital admission is a good indicator of early NIV failure.

Keywords: COVID-19; Mechanical ventilatory support; Peripheral oxygen saturation; ROX index.

Copyright © 2021 Elsevier Inc. All rights reserved.

Conflict of interest statement

None.

- Cited by 4 articles
- 20 references
- 1 figure

Supplementary info

Publication types, MeSH terms Expand

Publication types

- Observational Study

MeSH terms

- Adult
- Aged
- Blood Gas Analysis
- COVID-19 / metabolism*
- COVID-19 / physiopathology
- COVID-19 / therapy*
- Critical Care*
- Diagnostic Tests, Routine
- Female
- Hospitalization
- Humans
- Male
- Middle Aged
- Oxygen Inhalation Therapy
- Oxygen Saturation*
- Predictive Value of Tests
- Respiration, Artificial*
- Respiratory Rate*
- Retrospective Studies
- Treatment Outcome

Full text links

ELSEVIER FULL-TEXT ARTICLE Elsevier Science Free PMC article
Proceed to details
Cite
Share
☐ 283

Observational Study
Diabetes Metab
. 2021 Jul;47(4):101222.
doi: 10.1016/j.diabet.2020.101222. Epub 2020 Dec 31.

Association of diabetes and outcomes in patients with COVID-19: Propensity score-matched analyses from a French retrospective cohort

Willy Sutter [1], Baptiste Duceau [1], Maxime Vignac [1], Guillaume Bonnet [2], Aurélie Carlier [3], Ronan Roussel [4], Antonin Trimaille [5], Thibaut Pommier [6], Pierre Guilleminot [6], Audrey Sagnard [6], Julie Pastier [6], Orianne Weizman [7], Gauthier Giordano [7], Joffrey Cellier [8], Laura Geneste [9], Vassili Panagides [10], Wassima Marsou [11], Antoine Deney [12], Clément Karsenty [12], Sabir Attou [13], Thomas Delmotte [14], Sophie Ribeyrolles [15], Pascale Chemaly [16], Alexandre Gautier [16], Charles Fauvel [17], Corentin Chaumont [18], Delphine Mika [19], Théo Pezel [20], Ariel Cohen [20], Louis Potier [21], Critical COVID-19 France Investigators
Affiliations Expand

Affiliations

- [1] Université de Paris, PARCC, INSERM, 75015 Paris, France.
- [2] Université de Paris, PARCC, INSERM, 75015 Paris, France; Hôpital Européen Georges Pompidou, Université de Paris, 75015 Paris, France.
- [3] Diabetology, Endocrinology and Nutrition Department, Bichat Hospital, APHP, Univeresité de Paris, Paris, France.
- [4] Diabetology, Endocrinology and Nutrition Department, Bichat Hospital, APHP, Univeresité de Paris, Paris, France; Centre de Recherche des Cordeliers, INSERM, Sorbonne Université, Université de Paris, Immediab Team, F-75006 Paris, France.
- [5] Nouvel Hôpital Civil, Centre Hospitalier Régional Universitaire de Strasbourg, 67000 Strasbourg, France.
- [6] Centre Hospitalier Universitaire de Dijon, 21000 Dijon, France.
- [7] Centre Hospitalier Régional Universitaire de Nancy, 54511 Vandoeuvre-Les-Nancy, France.
- [8] Hôpital Européen Georges Pompidou, Université de Paris, 75015 Paris, France.
- [9] Centre Hospitalier Universitaire d'Amiens-Picardie, 80000 Amiens, France.
- [10] Centre Hospitalier Universitaire de Marseille, 13005 Marseille, France.
- [11] GCS-Groupement des Hôpitaux de l'Institut Catholique de Lille, Faculté de Médecine et de Maïeutique, Université Catholique de Lille, Lille, France.
- [12] Centre Hospitalier Universitaire de Toulouse, 31400 Toulouse, France.
- [13] Centre Hospitalier Universitaire de Caen-Normandie, 14000 Caen, France.
- [14] Centre Hospitalier Universitaire de Reims, 51100 Reims, France.
- [15] Institut Mutualiste Montsouris, 75014 Paris, France.
- [16] Institut Cardiovasculaire Paris Sud, 91300 Massy, France.

- [17] Rouen University Hospital, FHU REMOD-VHF, F76000 Rouen, France.
- [18] Université Paris-Saclay, INSERM, UMR-S 1180, 92296 Chatenay-Malabry, France.
- [19] Hôpital Lariboisière, APHP, Université de Paris, 75010 Paris, France.
- [20] Saint-Antoine Hospital, 75012 Paris, France.
- [21] Diabetology, Endocrinology and Nutrition Department, Bichat Hospital, APHP, Univeresité de Paris, Paris, France; Centre de Recherche des Cordeliers, INSERM, Sorbonne Université, Université de Paris, Immediab Team, F-75006 Paris, France. Electronic address: louis.potier@gmail.com.

- PMID: **33388386**
- PMCID: PMC7774010
- DOI: 10.1016/j.diabet.2020.101222

Free PMC article
Observational Study

Association of diabetes and outcomes in patients with COVID-19: Propensity score-matched analyses from a French retrospective cohort

Willy Sutter et al. Diabetes Metab. 2021 Jul.
Free PMC article

Show details

Diabetes Metab
. 2021 Jul;47(4):101222.
doi: 10.1016/j.diabet.2020.101222. Epub 2020 Dec 31.

Authors

Willy Sutter [1], Baptiste Duceau [1], Maxime Vignac [1], Guillaume Bonnet [2], Aurélie Carlier [3], Ronan Roussel [4], Antonin Trimaille [5], Thibaut Pommier [6], Pierre Guilleminot [6], Audrey Sagnard [6], Julie Pastier [6], Orianne Weizman [7], Gauthier Giordano [7], Joffrey Cellier [8], Laura Geneste [9], Vassili Panagides [10], Wassima Marsou [11], Antoine Deney [12], Clément Karsenty [12], Sabir Attou [13], Thomas Delmotte [14], Sophie Ribeyrolles [15], Pascale Chemaly [16], Alexandre Gautier [16], Charles Fauvel [17], Corentin Chaumont [18], Delphine Mika [19], Théo Pezel [20], Ariel Cohen [20], Louis Potier [21], Critical COVID-19 France Investigators

Affiliations

- [1] Université de Paris, PARCC, INSERM, 75015 Paris, France.
- [2] Université de Paris, PARCC, INSERM, 75015 Paris, France; Hôpital Européen Georges Pompidou, Université de Paris, 75015 Paris, France.
- [3] Diabetology, Endocrinology and Nutrition Department, Bichat Hospital, APHP, Univeresité de Paris, Paris, France.

- [4] Diabetology, Endocrinology and Nutrition Department, Bichat Hospital, APHP, Univeresité de Paris, Paris, France; Centre de Recherche des Cordeliers, INSERM, Sorbonne Université, Université de Paris, Immediab Team, F-75006 Paris, France.
- [5] Nouvel Hôpital Civil, Centre Hospitalier Régional Universitaire de Strasbourg, 67000 Strasbourg, France.
- [6] Centre Hospitalier Universitaire de Dijon, 21000 Dijon, France.
- [7] Centre Hospitalier Régional Universitaire de Nancy, 54511 Vandoeuvre-Les-Nancy, France.
- [8] Hôpital Européen Georges Pompidou, Université de Paris, 75015 Paris, France.
- [9] Centre Hospitalier Universitaire d'Amiens-Picardie, 80000 Amiens, France.
- [10] Centre Hospitalier Universitaire de Marseille, 13005 Marseille, France.
- [11] GCS-Groupement des Hôpitaux de l'Institut Catholique de Lille, Faculté de Médecine et de Maïeutique, Université Catholique de Lille, Lille, France.
- [12] Centre Hospitalier Universitaire de Toulouse, 31400 Toulouse, France.
- [13] Centre Hospitalier Universitaire de Caen-Normandie, 14000 Caen, France.
- [14] Centre Hospitalier Universitaire de Reims, 51100 Reims, France.
- [15] Institut Mutualiste Montsouris, 75014 Paris, France.
- [16] Institut Cardiovasculaire Paris Sud, 91300 Massy, France.
- [17] Rouen University Hospital, FHU REMOD-VHF, F76000 Rouen, France.
- [18] Université Paris-Saclay, INSERM, UMR-S 1180, 92296 Chatenay-Malabry, France.
- [19] Hôpital Lariboisière, APHP, Université de Paris, 75010 Paris, France.
- [20] Saint-Antoine Hospital, 75012 Paris, France.
- [21] Diabetology, Endocrinology and Nutrition Department, Bichat Hospital, APHP, Univeresité de Paris, Paris, France; Centre de Recherche des Cordeliers, INSERM, Sorbonne Université, Université de Paris, Immediab Team, F-75006 Paris, France. Electronic address: louis.potier@gmail.com.

- PMID: **33388386**
- PMCID: PMC7774010
- DOI: 10.1016/j.diabet.2020.101222

Abstract

Background: Our study aimed to compare the clinical outcomes of patients with and without diabetes admitted to hospital with COVID-19.

Methods: This retrospective multicentre cohort study comprised 24 tertiary medical centres in France, and included 2851 patients (675 with diabetes) hospitalized for COVID-19 between 26 February and 20 April 2020. A propensity score-matching (PSM) method (1:1 matching including patients' characteristics, medical history, vital statistics and laboratory results) was used to compare patients with and without diabetes (n = 603 per group). The primary outcome was admission to an intensive care unit (ICU) and/or in-hospital death.

Results: After PSM, all baseline characteristics were well balanced between those with and without diabetes: mean age was 71.2 years; 61.8% were male; and mean BMI was 29 kg/m^2. A history of cardiovascular, chronic kidney and chronic obstructive pulmonary diseases were found in 32.8%, 22.1% and 6.4% of participants, respectively. The risk of experiencing the primary outcome was similar in patients with or without diabetes [hazard ratio (HR): 1.16, 95% confidence interval (CI): 0.95-1.41; P = 0.14], and was 1.29 (95% CI: 0.97-1.69) for in-hospital death, 1.26

(95% CI: 0.9-1.72) for death with no transfer to an ICU and 1.14 (95% CI: 0.88-1.47) with transfer to an ICU.

Conclusion: In this retrospective study cohort of patients hospitalized for COVID-19, diabetes was not significantly associated with a higher risk of severe outcomes after PSM.

Trial registration number: NCT04344327.

Keywords: Covid-19; Diabetes; Mortality; Propensity score-matching.

Copyright © 2020. Published by Elsevier Masson SAS.

- Cited by 6 articles
- 26 references
- 2 figures

Supplementary info

Publication types, MeSH terms, Associated data Expand

Publication types

- Observational Study

MeSH terms

- Aged
- Aged, 80 and over
- COVID-19 / epidemiology*
- COVID-19 / mortality
- COVID-19 / physiopathology
- Comorbidity
- Diabetes Mellitus / epidemiology*
- Female
- France / epidemiology
- Hospital Mortality*
- Humans
- Intensive Care Units*
- Length of Stay
- Male
- Middle Aged
- Patient Transfer / statistics & numerical data*
- Propensity Score
- Retrospective Studies
- SARS-CoV-2

Associated data

- ClinicalTrials.gov/NCT04344327

Full text links

ELSEVIER FULL-TEXT ARTICLE Elsevier Science Free PMC article
Proceed to details
Cite
Share
☐ 284
Observational Study
Am J Health Syst Pharm
. 2021 Mar 31;78(8):689-696.
doi: 10.1093/ajhp/zxab056.

Impact of hydroxychloroquine on disease progression and ICU admissions in patients with SARS-CoV-2 infection

Nirvana Awad[1], Daryl S Schiller[1], Magda Fulman[1], Azfar Chak[1]
Affiliations Expand

Affiliation

- [1] Montefiore Nyack Hospital, Nyack, NY, USA.

- PMID: **33599721**
- PMCID: PMC7929454
- DOI: 10.1093/ajhp/zxab056

Free PMC article
Observational Study

Impact of hydroxychloroquine on disease progression and ICU admissions in patients with SARS-CoV-2 infection

Nirvana Awad et al. Am J Health Syst Pharm. 2021.
Free PMC article
Show details
Am J Health Syst Pharm
. 2021 Mar 31;78(8):689-696.

doi: 10.1093/ajhp/zxab056.

Authors

Nirvana Awad [1], Daryl S Schiller [1], Magda Fulman [1], Azfar Chak [1]

Affiliation

- [1] Montefiore Nyack Hospital, Nyack, NY, USA.

- PMID: **33599721**
- PMCID: PMC7929454
- DOI: 10.1093/ajhp/zxab056

Abstract

Purpose: To evaluate whether use of hydroxychloroquine was associated with a reduced likelihood of intensive care unit (ICU) admission in patients with coronavirus disease 2019 (COVID-19) in the early weeks of the pandemic.

Methods: A retrospective, observational cohort study was conducted to determine selected treatment outcomes in 336 patients hospitalized with COVID-19 at an acute care community hospital in the Hudson Valley region of New York from March 20 to April 20, 2020. Eligibility included admission to the hospital, a laboratory-confirmed diagnosis of SARS-CoV-2 infection, and no need for intubation or intensive care at admission. The median (interquartile range) ages of patients who received hydroxychloroquine (n = 188) and those who did not (n = 148) were 68 (58-82) and 64 (51-73) years, respectively. In a multivariable model that included age, gender, obesity, diabetes, and hydroxychloroquine use, patients who received hydroxychloroquine were significantly more likely than those not treated with the drug to be transferred to an ICU (odds ratio, [OR], 8.1; 95% confidence interval [CI]: 3.8-17) and significantly more likely to be intubated (OR, 7.99; 95% CI, 3.76-16.91); these associations were not influenced by disease severity. In-hospital mortality did not differ significantly with disease severity between those who did and those who did not receive hydroxychloroquine.

Conclusion: Hydroxychloroquine use was significantly associated with increased risks of ICU admission and intubation in patients with mild, moderate, and severe symptoms of COVID-19. There were no significant between-group differences in mortality with use vs nonuse of hydroxychloroquine.

Keywords: coronavirus; hydroxychloroquine; infection.

Published by Oxford University Press on behalf of the American Society of Health-System Pharmacists 2021.

- Cited by 2 articles

Supplementary info

Publication types, MeSH terms, Substances Expand

Publication types

- Observational Study

MeSH terms

- Adolescent
- Adult
- Aged
- Aged, 80 and over
- COVID-19 / drug therapy*
- COVID-19 / mortality
- Cohort Studies
- Disease Progression
- Female
- Humans
- Hydroxychloroquine / administration & dosage
- Hydroxychloroquine / therapeutic use*
- Intensive Care Units
- Length of Stay
- Male
- Middle Aged
- New York
- Patient Admission*
- Retrospective Studies
- SARS-CoV-2*
- Survival Analysis
- Young Adult

Substances

- Hydroxychloroquine

Full text links

OXFORD ACADEMIC Silverchair Information Systems Free PMC article

Proceed to details

Cite

Share

☐ 285

Observational Study

Medicine (Baltimore)

. 2021 Oct 15;100(41):e27528.
doi: 10.1097/MD.0000000000027528.

Early viral versus late antibiotic-associated diarrhea in novel coronavirus infection

Roman Maslennikov [1 2 3], Andrey Svistunov [1], Vladimir Ivashkin [1], Anna Ufimtseva [1], Elena Poluektova [1 2], Irina Efremova [1], Anatoly Ulyanin [1], Alexey Okhlobystin [1], Svetlana Kardasheva [1], Anastasia Kurbatova [1], Anna Levshina [1], Diana Grigoriadis [1], Shamil Magomedov [1], Natiya Dzhakhaya [1], Oleg Shifrin [1], Maria Zharkova [1], Elena Yuryeva [1], Nataliya Kokina [1], Manana Shirtladze [1], Olga Kiseleva [1]
Affiliations Expand

Affiliations

- [1] Department of Internal Medicine, Gastroenterology and Hepatology, Sechenov University, Moscow, Russian Federation.
- [2] The Interregional Public Organization "Scientific Community for the Promotion of the Clinical Study of the Human Microbiome," Moscow, Russian Federation.
- [3] Consultative and Diagnostic Center 2 of the Moscow City Health Department, Moscow, Russian Federation.

- PMID: **34731146**
- PMCID: PMC8519250
- DOI: 10.1097/MD.0000000000027528

Free PMC article
Observational Study

Early viral versus late antibiotic-associated diarrhea in novel coronavirus infection

Roman Maslennikov et al. Medicine (Baltimore). 2021.
Free PMC article
Show details
Medicine (Baltimore)
. 2021 Oct 15;100(41):e27528.
doi: 10.1097/MD.0000000000027528.

Authors

Roman Maslennikov [1 2 3], Andrey Svistunov [1], Vladimir Ivashkin [1], Anna Ufimtseva [1], Elena Poluektova [1 2], Irina Efremova [1], Anatoly Ulyanin [1], Alexey Okhlobystin [1], Svetlana Kardasheva [1], Anastasia Kurbatova [1], Anna Levshina [1], Diana Grigoriadis [1], Shamil

Magomedov [1], Natiya Dzhakhaya [1], Oleg Shifrin [1], Maria Zharkova [1], Elena Yuryeva [1], Nataliya Kokina [1], Manana Shirtladze [1], Olga Kiseleva [1]

Affiliations

- [1] Department of Internal Medicine, Gastroenterology and Hepatology, Sechenov University, Moscow, Russian Federation.
- [2] The Interregional Public Organization "Scientific Community for the Promotion of the Clinical Study of the Human Microbiome," Moscow, Russian Federation.
- [3] Consultative and Diagnostic Center 2 of the Moscow City Health Department, Moscow, Russian Federation.

- PMID: **34731146**
- PMCID: PMC8519250
- DOI: 10.1097/MD.0000000000027528

Abstract

Diarrhea is one of the manifestations of the novel coronavirus disease (COVID-19), but it also develops as a complication of massive antibiotic therapy in this disease. This study aimed to compare these types of diarrhea.We included patients with COVID-19 in a cohort study and excluded patients with chronic diarrhea, laxative use, and those who died during the first day of hospitalization.There were 89 (9.3%), 161 (16.7%), and 731 (75.7%) patients with early viral, late antibiotic-associated, and without diarrhea, respectively. Late diarrhea lasted longer (6 [4-10] vs 5 [3-7] days, P < .001) and was more severe. Clostridioides difficile was found in 70.5% of tested patients with late diarrhea and in none with early diarrhea. Presence of late diarrhea was associated with an increased risk of death after 20 days of disease (P = .009; hazard ratio = 4.7). Patients with late diarrhea had a longer hospital stay and total disease duration, and a higher proportion of these patients required intensive care unit admission. Oral amoxicillin/clavulanate (odds ratio [OR] = 2.23), oral clarithromycin (OR = 3.79), and glucocorticoids (OR = 4.41) use was a risk factor for the development of late diarrhea, while ceftriaxone use (OR = 0.35) had a protective effect. Before the development of late diarrhea, decrease in C-reactive protein levels and increase in lymphocyte count stopped but the white blood cell and neutrophil count increased. An increase in neutrophils by >0.6 × 109 cells/L predicted the development of late diarrhea in the coming days (sensitivity 82.0%, specificity 70.8%, area under the curve = 0.791 [0.710-0.872]).Diarrhea in COVID-19 is heterogeneous, and different types of diarrhea require different management.

Copyright © 2021 the Author(s). Published by Wolters Kluwer Health, Inc.

Conflict of interest statement

The authors reprot no conflicts of interest.

- 15 references
- 4 figures

Supplementary info

Publication types, MeSH terms, Substances Expand

Publication types

- Comparative Study
- Observational Study

MeSH terms

- Aged
- Anti-Bacterial Agents / adverse effects*
- COVID-19 / epidemiology*
- Diarrhea / chemically induced*
- Diarrhea / classification
- Diarrhea / epidemiology
- Diarrhea / virology*
- Humans
- Length of Stay
- Middle Aged
- Pandemics
- Retrospective Studies
- Risk Factors
- SARS-CoV-2

Substances

- Anti-Bacterial Agents

Full text links

Wolters Kluwer Wolters Kluwer Free PMC article
Proceed to details
Cite
Share
☐ 286
Observational Study
Clin Nutr ESPEN
. 2021 Aug;44:211-217.
doi: 10.1016/j.clnesp.2021.06.016. Epub 2021 Jun 29.

Energy requirements of long-term ventilated COVID-19 patients with resolved SARS-CoV-2 infection

Janusz von Renesse [1], Simone von Bonin [2], Hanns-Christoph Held [3], Ralph Schneider [2], Adrian M Seifert [1], Lena Seifert [1], Peter Spieth [4], Jürgen Weitz [1], Thilo Welsch [1], Ronny Meisterfeld [5]
Affiliations Expand

Affiliations

- [1] Department of Visceral, Thoracic and Vascular Surgery, University Hospital Carl Gustav Carus, University of Technology Dresden, Dresden, Germany.
- [2] Department of Medicine I, University Hospital Carl Gustav Carus, University of Technology Dresden, Dresden, Germany.
- [3] University Centre of Surgery Intensive Care Unit, University Hospital Carl Gustav Carus, University of Technology Dresden, Dresden, Germany.
- [4] Department of Anesthesiology and Critical Care Medicine, University Hospital Carl Gustav Carus, University of Technology Dresden, Dresden, Germany.
- [5] Department of Visceral, Thoracic and Vascular Surgery, University Hospital Carl Gustav Carus, University of Technology Dresden, Dresden, Germany; University Centre of Surgery Intensive Care Unit, University Hospital Carl Gustav Carus, University of Technology Dresden, Dresden, Germany. Electronic address: ronny.meisterfeld@uniklinikum-dresden.de.

- PMID: **34330468**
- PMCID: PMC8238638
- DOI: 10.1016/j.clnesp.2021.06.016

Free PMC article
Observational Study

Energy requirements of long-term ventilated COVID-19 patients with resolved SARS-CoV-2 infection

Janusz von Renesse et al. Clin Nutr ESPEN. 2021 Aug.
Free PMC article
Show details
Clin Nutr ESPEN
. 2021 Aug;44:211-217.
doi: 10.1016/j.clnesp.2021.06.016. Epub 2021 Jun 29.

Authors

Janusz von Renesse [1], Simone von Bonin [2], Hanns-Christoph Held [3], Ralph Schneider [2], Adrian M Seifert [1], Lena Seifert [1], Peter Spieth [4], Jürgen Weitz [1], Thilo Welsch [1], Ronny Meisterfeld [5]

Affiliations

- [1] Department of Visceral, Thoracic and Vascular Surgery, University Hospital Carl Gustav Carus, University of Technology Dresden, Dresden, Germany.
- [2] Department of Medicine I, University Hospital Carl Gustav Carus, University of Technology Dresden, Dresden, Germany.
- [3] University Centre of Surgery Intensive Care Unit, University Hospital Carl Gustav Carus, University of Technology Dresden, Dresden, Germany.
- [4] Department of Anesthesiology and Critical Care Medicine, University Hospital Carl Gustav Carus, University of Technology Dresden, Dresden, Germany.
- [5] Department of Visceral, Thoracic and Vascular Surgery, University Hospital Carl Gustav Carus, University of Technology Dresden, Dresden, Germany; University Centre of Surgery Intensive Care Unit, University Hospital Carl Gustav Carus, University of Technology Dresden, Dresden, Germany. Electronic address: ronny.meisterfeld@uniklinikum-dresden.de.

- PMID: **34330468**
- PMCID: PMC8238638
- DOI: 10.1016/j.clnesp.2021.06.016

Abstract

Background & aims: Coronavirus disease 2019 (COVID-19) caused by severe acute respiratory syndrome coronavirus 2 (SARS-CoV-2) infection can rapidly progress into acute respiratory distress syndrome accompanied by multi-organ failure requiring invasive mechanical ventilation and critical care treatment. Nutritional therapy is a fundamental pillar in the management of hospitalized patients. It is broadly acknowledged that overfeeding and underfeeding of intensive care unit (ICU) patients are associated with increased morbidity and mortality. This study aimed to assess the energy demands of long-term ventilated COVID-19 patients using indirect calorimetry and to evaluate the applicability of established predictive equations to estimate their energy expenditure.

Methods: We performed a retrospective, single-center study in 26 mechanically ventilated COVID-19 patients with resolved SARS-CoV-2 infection in three independent intensive care units. Resting energy expenditure (REE) was evaluated by repetitive indirect calorimetry (IC) measurements. Simultaneously the performance of 12 predictive equations was examined. Patient's clinical data were retrieved from electronic medical charts. Bland-Altman plots were used to assess agreement between measured and calculated REE.

Results: Mean mREE was 1687 kcal/day and 20.0 kcal relative to actual body weight (ABW) per day (kcal/kg/day). Longitudinal mean mREE did not change significantly over time, although mREE values had a high dispersion (SD of mREE ±487). Obese individuals were found to have significantly increased mREE, but lower energy expenditure relative to their body mass. Calculated REE showed poor agreement with mREE ranging from 33 to 54%.

Conclusion: Resolution of SARS-CoV-2 infection confirmed by negative PCR leads to stabilization of energy demands at an average 20 kcal/kg in ventilated critically ill patients. Due to

high variations in mREE and low agreement with calculated energy expenditure IC remains the gold standard for the guidance of nutritional therapy.

Keywords: COVID-19; Critical care; Energy expenditure; Indirect calorimetry; Predictive equations; SARS-CoV-2.

Copyright © 2021 European Society for Clinical Nutrition and Metabolism. Published by Elsevier Ltd. All rights reserved.

Conflict of interest statement

Declaration of competing interest The authors declare that they have no conflict of interest. This investigator-initiated study was conducted independent of grant funding.

- 28 references
- 2 figures

Supplementary info

Publication types, MeSH terms Expand

Publication types

- Observational Study

MeSH terms

- COVID-19 / physiopathology*
- Calorimetry, Indirect
- Critical Care / methods*
- Critical Illness
- Energy Metabolism / physiology*
- Female
- Humans
- Male
- Middle Aged
- Nutritional Requirements / physiology*
- Respiration, Artificial / methods*
- Retrospective Studies
- SARS-CoV-2
- Time

Full text links

ELSEVIER FULL-TEXT ARTICLE Elsevier Science Free PMC article
Proceed to details

Cite
Share
287
Observational Study
Sci Rep
. 2021 Mar 11;11(1):5803.
doi: 10.1038/s41598-021-85081-0.

Comparison of clinical characteristics and disease outcome of COVID-19 and seasonal influenza

Thomas Theo Brehm [1] [2], Marc van der Meirschen [3], Annette Hennigs [3], Kevin Roedl [4], Dominik Jarczak [4], Dominic Wichmann [4], Daniel Frings [4], Axel Nierhaus [4], Tim Oqueka [5], Walter Fiedler [5], Maximilian Christopeit [6], Christian Kraef [3] [7], Alexander Schultze [8], Marc Lütgehetmann [9] [10], Marylyn M Addo [3] [9], Stefan Schmiedel [3] [9], Stefan Kluge [#] [4], Julian Schulze Zur Wiesch [#] [3] [9]
Affiliations Expand

Affiliations

- [1] I. Department of Internal Medicine, University Medical Center Hamburg-Eppendorf, Martinistraße 52, 20246, Hamburg, Germany. t.brehm@uke.de.
- [2] German Center for Infection Research (DZIF), Partner Site Hamburg-Lübeck-Borstel-Riems, Hamburg, Germany. t.brehm@uke.de.
- [3] I. Department of Internal Medicine, University Medical Center Hamburg-Eppendorf, Martinistraße 52, 20246, Hamburg, Germany.
- [4] Department of Intensive Care Medicine, University Medical Center Hamburg-Eppendorf, Martinistraße 52, 20246, Hamburg, Germany.
- [5] Department of Oncology, Hematology and Bone Marrow Transplantation with Section Pneumology, University Medical Center Hamburg-Eppendorf, Martinistraße 52, 20246, Hamburg, Germany.
- [6] Department of Stem Cell Transplantation, University Medical Center Hamburg-Eppendorf, 20246, Hamburg, Germany.
- [7] CHIP (Centre of Excellence for Health, Immunity and Infections), Department of Infectious Disease, Rigshospitalet, University of Copenhagen, Copenhagen, Denmark.
- [8] Department of Emergency Medicine, University Medical Center Hamburg-Eppendorf, Martinistraße 52, 20246, Hamburg, Germany.
- [9] German Center for Infection Research (DZIF), Partner Site Hamburg-Lübeck-Borstel-Riems, Hamburg, Germany.
- [10] Institute of Medical Microbiology, Virology and Hygiene, University Medical Center Hamburg-Eppendorf, Martinistraße 52, 20246, Hamburg, Germany.

[#] Contributed equally.

- PMID: **33707550**

- PMCID: PMC7970952
- DOI: 10.1038/s41598-021-85081-0

Free PMC article
Observational Study

Comparison of clinical characteristics and disease outcome of COVID-19 and seasonal influenza

Thomas Theo Brehm et al. Sci Rep. 2021.
Free PMC article
Show details
Sci Rep
. 2021 Mar 11;11(1):5803.
doi: 10.1038/s41598-021-85081-0.

Authors

Thomas Theo Brehm [1, 2], Marc van der Meirschen [3], Annette Hennigs [3], Kevin Roedl [4], Dominik Jarczak [4], Dominic Wichmann [4], Daniel Frings [4], Axel Nierhaus [4], Tim Oqueka [5], Walter Fiedler [5], Maximilian Christopeit [6], Christian Kraef [3, 7], Alexander Schultze [8], Marc Lütgehetmann [9, 10], Marylyn M Addo [3, 9], Stefan Schmiedel [3, 9], Stefan Kluge [#, 4], Julian Schulze Zur Wiesch [#, 3, 9]

Affiliations

- [1] I. Department of Internal Medicine, University Medical Center Hamburg-Eppendorf, Martinistraße 52, 20246, Hamburg, Germany. t.brehm@uke.de.
- [2] German Center for Infection Research (DZIF), Partner Site Hamburg-Lübeck-Borstel-Riems, Hamburg, Germany. t.brehm@uke.de.
- [3] I. Department of Internal Medicine, University Medical Center Hamburg-Eppendorf, Martinistraße 52, 20246, Hamburg, Germany.
- [4] Department of Intensive Care Medicine, University Medical Center Hamburg-Eppendorf, Martinistraße 52, 20246, Hamburg, Germany.
- [5] Department of Oncology, Hematology and Bone Marrow Transplantation with Section Pneumology, University Medical Center Hamburg-Eppendorf, Martinistraße 52, 20246, Hamburg, Germany.
- [6] Department of Stem Cell Transplantation, University Medical Center Hamburg-Eppendorf, 20246, Hamburg, Germany.
- [7] CHIP (Centre of Excellence for Health, Immunity and Infections), Department of Infectious Disease, Rigshospitalet, University of Copenhagen, Copenhagen, Denmark.
- [8] Department of Emergency Medicine, University Medical Center Hamburg-Eppendorf, Martinistraße 52, 20246, Hamburg, Germany.
- [9] German Center for Infection Research (DZIF), Partner Site Hamburg-Lübeck-Borstel-Riems, Hamburg, Germany.

- [10] Institute of Medical Microbiology, Virology and Hygiene, University Medical Center Hamburg-Eppendorf, Martinistraße 52, 20246, Hamburg, Germany.

[#] Contributed equally.

- PMID: **33707550**
- PMCID: PMC7970952
- DOI: 10.1038/s41598-021-85081-0

Abstract

While several studies have described the clinical course of patients with coronavirus disease 2019 (COVID-19), direct comparisons with patients with seasonal influenza are scarce. We compared 166 patients with COVID-19 diagnosed between February 27 and June 14, 2020, and 255 patients with seasonal influenza diagnosed during the 2017-18 season at the same hospital to describe common features and differences in clinical characteristics and course of disease. Patients with COVID-19 were younger (median age [IQR], 59 [45-71] vs 66 [52-77]; P < 0001) and had fewer comorbidities at baseline with a lower mean overall age-adjusted Charlson Comorbidity Index (mean [SD], 3.0 [2.6] vs 4.0 [2.7]; P < 0.001) than patients with seasonal influenza. COVID-19 patients had a longer duration of hospitalization (mean [SD], 25.9 days [26.6 days] vs 17.2 days [21.0 days]; P = 0.002), a more frequent need for oxygen therapy (101 [60.8%] vs 103 [40.4%]; P < 0.001) and invasive ventilation (52 [31.3%] vs 32 [12.5%]; P < 0.001) and were more frequently admitted to the intensive care unit (70 [42.2%] vs 51 [20.0%]; P < 0.001) than seasonal influenza patients. Among immunocompromised patients, those in the COVID-19 group had a higher hospital mortality compared to those in the seasonal influenza group (13 [33.3%] vs 8 [11.6%], P = 0.01). In conclusion, we show that COVID-19 patients were younger and had fewer baseline comorbidities than seasonal influenza patients but were at increased risk for severe illness. The high mortality observed in immunocompromised COVID-19 patients emphasizes the importance of protecting these patient groups from SARS-CoV-2 infection.

Conflict of interest statement

Axel Nierhaus declares that he received lecture honoraria and travel reimbursement from Thermo Fisher Scientific and CytoSorbents Europe. The authors declare they have no competing financial interest.

- Cited by 14 articles
- 55 references
- 2 figures

Supplementary info

Publication types, MeSH terms Expand

Publication types

- Comparative Study
- Observational Study
- Research Support, Non-U.S. Gov't

MeSH terms

- Aged
- COVID-19 / epidemiology*
- Comorbidity
- Female
- Germany / epidemiology
- Hospitalization / statistics & numerical data
- Humans
- Immunosuppression Therapy
- Influenza, Human / epidemiology*
- Male
- Middle Aged
- Retrospective Studies
- SARS-CoV-2

Full text links

Nature Publishing Group Free PMC article
Proceed to details
Cite
Share
☐ 288
Observational Study
Int J Artif Organs
. 2022 Feb;45(2):239-242.
doi: 10.1177/0391398821989065. Epub 2021 Jan 28.

Observational study of thrombosis and bleeding in COVID-19 VV ECMO patients

Brianda Ripoll [1], Antonio Rubino [1], Martin Besser [2], Chinmay Patvardhan [1], William Thomas [2], Karen Sheares [3], Hilary Shanahan [1], Bobby Agrawal, Stephen Webb [1], Alain Vuylsteke [1]
Affiliations Expand

Affiliations

- [1] Anaesthesia and Intensive Care, Royal Papworth Hospital, Cambridge, UK.
- [2] Department of Respiratory Medicine, Addenbrooke's Hospital, Cambridge, Cambridgeshire, UK.
- [3] Department of Haematology, Addenbrooke's Hospital Cambridge, UK.

- PMID: **33506708**

• DOI: 10.1177/0391398821989065

Observational Study

Observational study of thrombosis and bleeding in COVID-19 VV ECMO patients

Brianda Ripoll et al. Int J Artif Organs. 2022 Feb.
Show details
Int J Artif Organs
. 2022 Feb;45(2):239-242.
doi: 10.1177/0391398821989065. Epub 2021 Jan 28.

Authors

Brianda Ripoll [1], Antonio Rubino [1], Martin Besser [2], Chinmay Patvardhan [1], William Thomas [2], Karen Sheares [3], Hilary Shanahan [1], Bobby Agrawal, Stephen Webb [1], Alain Vuylsteke [1]

Affiliations

- [1] Anaesthesia and Intensive Care, Royal Papworth Hospital, Cambridge, UK.
- [2] Department of Respiratory Medicine, Addenbrooke's Hospital, Cambridge, Cambridgeshire, UK.
- [3] Department of Haematology, Addenbrooke's Hospital Cambridge, UK.

- PMID: **33506708**
- DOI: 10.1177/0391398821989065

Abstract

Introduction: COVID-19 has been associated with increased risk of thrombosis, heparin resistance and coagulopathy in critically ill patients admitted to intensive care. We report the incidence of thrombotic and bleeding events in a single center cohort of 30 consecutive patients with COVID-19 supported by veno-venous extracorporeal oxygenation (ECMO) and who had a whole body Computed Tomography Scanner (CT) on admission.

Methodology: All patients were initially admitted to other hospitals and later assessed and retrieved by our ECMO team. ECMO was initiated in the referral center and all patients admitted through our CT scan before settling in our intensive care unit. Clinical management was guided by our institutional ECMO guidelines, established since 2011 and applied to at least 40 patients every year.

Results: We diagnosed a thrombotic event in 13 patients on the initial CT scan. Two of these 13 patients subsequently developed further thrombotic complications. Five of those 13 patients had a subsequent clinically significant major bleeding. In addition, two patients presented with isolated intracranial bleeds. Of the 11 patients who did not have baseline thrombotic events, one had a subsequent oropharyngeal hemorrhage. When analyzed by ROC analysis, the area under the curve for % time in intended anticoagulation range did not predict thrombosis or bleeding during the ECMO run (0.36 (95% CI 0.10-0.62); and 0.51 (95% CI 0.25-0.78); respectively).

Conclusion: We observed a high prevalence of VTE and a significant number of hemorrhages in these severely ill patients with COVID-19 requiring veno-venous ECMO support.

Keywords: COVID-19; acute respiratory distress syndrome; extracorporeal membrane oxygenation; heparin resistance; pulmonary embolism.

- Cited by 3 articles

Supplementary info

Publication types, MeSH terms, Substances Expand

Publication types

- Observational Study

MeSH terms

- Anticoagulants / adverse effects
- COVID-19*
- Extracorporeal Membrane Oxygenation* / adverse effects
- Hemorrhage / etiology
- Hemorrhage / therapy
- Humans
- Retrospective Studies
- SARS-CoV-2
- Thrombosis* / epidemiology
- Thrombosis* / etiology

Substances

- Anticoagulants

Full text links

SAGE journals Atypon
Proceed to details
Cite
Share
☐ 289
Observational Study
Medicine (Baltimore)
. 2021 Jul 9;100(27):e26583.
doi: 10.1097/MD.0000000000026583.

Analysis of pediatric emergency department patient volume trends during the COVID-19 pandemic

Matthew Philip Pepper [1], Ernest Leva, Prerna Trivedy, James Luckey, Mark Douglas Baker
Affiliations Expand

Affiliation

- [1] Pediatrics, Rutgers Robert Wood Johnson Medical School, New Brunswick, NJ.

- PMID: **34232205**
- PMCID: PMC8270607
- DOI: 10.1097/MD.0000000000026583

Free PMC article
Observational Study

Analysis of pediatric emergency department patient volume trends during the COVID-19 pandemic

Matthew Philip Pepper et al. Medicine (Baltimore). 2021.
Free PMC article
Show details
Medicine (Baltimore)
. 2021 Jul 9;100(27):e26583.
doi: 10.1097/MD.0000000000026583.

Authors

Matthew Philip Pepper [1], Ernest Leva, Prerna Trivedy, James Luckey, Mark Douglas Baker

Affiliation

- [1] Pediatrics, Rutgers Robert Wood Johnson Medical School, New Brunswick, NJ.

- PMID: **34232205**
- PMCID: PMC8270607
- DOI: 10.1097/MD.0000000000026583

Abstract

During the early period of the COVID-19 pandemic there was a substantial decrease in pediatric emergency department (PED) visitation. The intent of this study is to report PED utilization during the COVID-19 pandemic in an urban pediatric referral center located close to the epicenter in the northeastern US.A retrospective analysis of medical records of patients visiting the PED at Robert Wood Johnson University Hospital (RWJUH) was performed. Data included: daily census, admission rate, Emergency Severity Index, and ICD-10 diagnosis codes for the period of February through July, 2018 to 2020.By the week of March 26th, visits had decreased by 70% compared to the average of the previous 2 years. This census nadir lasted for 6 weeks. At 5 weeks postnadir the average daily census recovered to levels 40% lower than prior year norms and remained at that level during subsequent months. The greatest decreases were seen in low-acuity visits. Visits for behavioral health and fractures decreased by approximately 50% and 70%, respectively, but recovered to prior year norms by June and July of 2020. Visits for asthma exacerbation decreased by as much as 87% and remained at record lows for the remainder of the study period.A substantial and persistent decrease in PED visitation was experienced during the COVID-19 pandemic. Whereas visits for behavioral health and fractures have recovered to prior year norms, visits for asthma exacerbation remain at record lows. Further research is needed to ascertain the causes of these changes, including patient perceptions of the PED.

Copyright © 2021 the Author(s). Published by Wolters Kluwer Health, Inc.

Conflict of interest statement

The authors have no funding and conflicts of interests to disclose.

- Cited by 3 articles
- 22 references
- 6 figures

Supplementary info

Publication types, MeSH terms Expand

Publication types

- Observational Study

MeSH terms

- COVID-19 / epidemiology*
- COVID-19 / therapy
- Child
- Emergency Service, Hospital / statistics & numerical data*
- Female
- Hospitalization / statistics & numerical data*
- Hospitals, Pediatric / statistics & numerical data*
- Humans
- Male
- Pandemics*

- Retrospective Studies
- SARS-CoV-2
- United States / epidemiology

Full text links

Wolters Kluwer Wolters Kluwer Free PMC article
Proceed to details
Cite
Share
290
Observational Study
Swiss Med Wkly
. 2020 Dec 31;150:w20448.
doi: 10.4414/smw.2020.20448. eCollection 2020 Dec 14.

Impact of the COVID-19 pandemic on acute coronary syndromes

Nils Perrin[1], Juan F Iglesias[1], Florian Rey[1], Lamyae Benzakour[2], Murat Cimci[1], Stephane Noble[1], Sophie Degrauwe[1], Elena Tessitore[1], François Mach[1], Marco Roffi[1]
Affiliations Expand

Affiliations

- [1] Cardiology division, Geneva University Hospitals, Geneva, Switzerland.
- [2] Psychiatric department, Geneva University Hospitals, Geneva, Switzerland.

- PMID: **33382905**
- DOI: 10.4414/smw.2020.20448

Free article
Observational Study

Impact of the COVID-19 pandemic on acute coronary syndromes

Nils Perrin et al. Swiss Med Wkly. 2020.
Free article
Show details
Swiss Med Wkly
. 2020 Dec 31;150:w20448.
doi: 10.4414/smw.2020.20448. eCollection 2020 Dec 14.

Authors

Nils Perrin [1], Juan F Iglesias [1], Florian Rey [1], Lamyae Benzakour [2], Murat Cimci [1], Stephane Noble [1], Sophie Degrauwe [1], Elena Tessitore [1], François Mach [1], Marco Roffi [1]

Affiliations

- [1] Cardiology division, Geneva University Hospitals, Geneva, Switzerland.
- [2] Psychiatric department, Geneva University Hospitals, Geneva, Switzerland.

- PMID: **33382905**
- DOI: 10.4414/smw.2020.20448

Abstract

Aim: To assess the impact of the first wave of the COVID-19 pandemic on acute coronary syndromes and on the delay from symptom onset to first medical contact among patients presenting with ST-segment elevation myocardial infarction (STEMI), as well as to investigate whether there were patient-related reasons related to COVID-19 for delaying first medical contact.

Methods and results: All patients undergoing percutaneous coronary intervention (PCI) at the Geneva University Hospitals for acute coronary syndromes (ACS) during the first COVID-19 wave were compared with a control group consisting of all ACS patients who underwent PCI during the same period in 2019 and those treated in the period immediately preceding the pandemic. The primary outcome measure was the difference in the delay from symptom onset to first medical contact in the setting of STEMI between the COVID-19 period and the control period. Secondary outcome measures were the difference in ACS incidence and the impact of the COVID-19 pandemic on patients’ decisions to call the emergency services, assessed using a questionnaire. Delay from symptom onset to first medical contact was longer among patients suffering from STEMI in the COVID-19 period compared with the control period (112 min vs 60 min, p = 0.049). The incidence rate of ACS was lower during the COVID-19 period (incidence rate ratio 0.6, 95% confidence interval [CI] 0.449–0.905). ACS patients delayed their call to the emergency services mainly because of fear of contracting or spreading COVID-19 following hospital admission, as well as of adding burden to the healthcare system.

Conclusion: We observed prolonged delays from symptom onset to first medical contact and a decline in overall ACS incidence during the first wave of the COVID-19 pandemic, with a higher threshold to call for help among ACS patients.

- Cited by 5 articles

Supplementary info

Publication types, MeSH terms, Substances Expand

Publication types

- Observational Study

MeSH terms

- Acute Coronary Syndrome / epidemiology*
- Acute Coronary Syndrome / surgery
- Aged
- COVID-19 / epidemiology*
- Comorbidity
- Female
- Humans
- Length of Stay
- Male
- Middle Aged
- Out-of-Hospital Cardiac Arrest / epidemiology
- Out-of-Hospital Cardiac Arrest / therapy
- Pandemics
- Percutaneous Coronary Intervention / statistics & numerical data
- Retrospective Studies
- SARS-CoV-2
- ST Elevation Myocardial Infarction / epidemiology*
- ST Elevation Myocardial Infarction / therapy*
- Time-to-Treatment / statistics & numerical data*
- Troponin / blood

Substances

- Troponin

Full text links

Open access to full text on Swiss Medical Weekly EMH Swiss Medical Publishers Ltd.
Proceed to details
Cite
Share
☐ 291
Observational Study
Medicine (Baltimore)
. 2021 Sep 17;100(37):e27265.
doi: 10.1097/MD.0000000000027265.

Variation in COVID-19 disease severity at hospital admission over time and across

hospitals: A multi-institution cohort of Michigan hospitals

Max T Wayne [1], Wenjing Weng [2], Megan O'Malley [3], Paul Bozyk [4], Mona M Doshi [5], Scott A Flanders [3], Jakob I McSparron [1], Pratima Sharma [5], Lakshmi Swaminathan [6], Hallie C Prescott [1] [7]

Affiliations Expand

Affiliations

- [1] Division of Pulmonary and Critical Care Medicine, Department of Internal Medicine, University of Michigan, Ann Arbor, MI.
- [2] Michigan Surgical Quality Collaborative, University of Michigan, Ann Arbor, MI.
- [3] Division of Hospital Medicine, Department of Internal Medicine, University of Michigan, Ann Arbor, MI.
- [4] Section of Pulmonary and Critical Care Medicine, Department of Internal Medicine, Beaumont Health, Royal Oak, MI.
- [5] Department of Internal Medicine, University of Michigan, Ann Arbor, MI.
- [6] Division of Hospital Medicine, St. Joseph Mercy Hospital, Ann Arbor, MI.
- [7] VA Center for Clinical Management Research, Ann Arbor, MI.

- PMID: **34664879**
- PMCID: PMC8448065
- DOI: 10.1097/MD.0000000000027265

Free PMC article
Observational Study

Variation in COVID-19 disease severity at hospital admission over time and across hospitals: A multi-institution cohort of Michigan hospitals

Max T Wayne et al. Medicine (Baltimore). 2021.
Free PMC article
Show details
Medicine (Baltimore)
. 2021 Sep 17;100(37):e27265.
doi: 10.1097/MD.0000000000027265.

Authors

Max T Wayne [1], Wenjing Weng [2], Megan O'Malley [3], Paul Bozyk [4], Mona M Doshi [5], Scott A Flanders [3], Jakob I McSparron [1], Pratima Sharma [5], Lakshmi Swaminathan [6], Hallie C Prescott [1] [7]

Affiliations

- [1] Division of Pulmonary and Critical Care Medicine, Department of Internal Medicine, University of Michigan, Ann Arbor, MI.
- [2] Michigan Surgical Quality Collaborative, University of Michigan, Ann Arbor, MI.
- [3] Division of Hospital Medicine, Department of Internal Medicine, University of Michigan, Ann Arbor, MI.
- [4] Section of Pulmonary and Critical Care Medicine, Department of Internal Medicine, Beaumont Health, Royal Oak, MI.
- [5] Department of Internal Medicine, University of Michigan, Ann Arbor, MI.
- [6] Division of Hospital Medicine, St. Joseph Mercy Hospital, Ann Arbor, MI.
- [7] VA Center for Clinical Management Research, Ann Arbor, MI.

- PMID: **34664879**
- PMCID: PMC8448065
- DOI: 10.1097/MD.0000000000027265

Abstract

During the spring 2020 COVID-19 surge, hospitals in Southeast Michigan were overwhelmed, and hospital beds were limited. However, it is unknown whether threshold for hospital admission varied across hospitals or over time.Using a statewide registry, we performed a retrospective cohort study. We identified adult patients hospitalized with COVID-19 in Southeast Michigan (3/1/2020-6/1/2020). We classified disease severity on admission using the World Health Organization (WHO) ordinal scale. Our primary measure of interest was the proportion of patients admitted on room air. We also determined the proportion without acute organ dysfunction on admission or any point during hospitalization. We quantified variation across hospitals and over time by half-month epochs.Among 1315 hospitalizations across 22 hospitals, 57.3% (754/1,315) were admitted on room air, and 26.1% (343/1,315) remained on room air for the duration of hospitalization. Across hospitals, the proportion of COVID-19 hospitalizations admitted on room air varied from 32.3% to 80.0%. Across half-month epochs, the proportion ranged from 49.4% to 69.4% and nadired in early April 2020. Among patients admitted on room air, 75.1% (566/754) had no acute organ dysfunction on admission, and 35.3% (266/754) never developed acute organ dysfunction at any point during hospitalization; there was marked variation in both proportions across hospitals. In-hospital mortality was 13.7% for patients admitted on room air vs 26.3% for patients requiring nasal cannula oxygen.Among patients hospitalized with COVID-19 during the spring 2020 surge in Southeast Michigan, more than half were on room air and a third had no acute organ dysfunction upon admission, but experienced high rates of disease progression and in-hospital mortality.

Copyright © 2021 the Author(s). Published by Wolters Kluwer Health, Inc.

Conflict of interest statement

The authors have no conflicts of interests to disclose.

- 40 references

- 2 figures

Supplementary info

Publication types, MeSH terms, Grant support Expand

Publication types

- Multicenter Study
- Observational Study

MeSH terms

- Aged
- COVID-19 / complications*
- Cohort Studies
- Female
- Hospitalization / statistics & numerical data*
- Humans
- Male
- Michigan
- Middle Aged
- Severity of Illness Index
- Time Factors

Grant support

- no/Blue Cross Blue Shield of Michigan Foundation

Full text links

Wolters Kluwer Wolters Kluwer Free PMC article
Proceed to details
Cite
Share
292
Observational Study
Int J Environ Res Public Health
. 2020 Dec 25;18(1):102.
doi: 10.3390/ijerph18010102.

Cardiovascular Comorbidities and Pharmacological Treatments of COVID-19 Patients Not Requiring Hospitalization

Vincenzo Russo [1], Gaetano Piccinocchi [2], Vincenzo Mandaliti [2], Saverio Annunziata [3], Giovanni Cimmino [1], Emilio Attena [4], Nicola Moio [5], Pierpaolo Di Micco [6], Sergio Severino [4], Roberta Trotta [7], Michele Del Guercio [8]
Affiliations Expand

Affiliations

- [1] Cardiology Unit, Department of Translational Medical Sciences, University of Campania Luigi Vanvitelli-Monaldi Hospital, 80131 Naples, Italy.
- [2] Comegen Primary Care Physicians Cooperative SIMG, Italian Society of Family Medicine, 80125 Naples, Italy.
- [3] KOS Primary Care Physicians Cooperative, 80128 Naples, Italy.
- [4] Cardiology Unit, Cotugno Hospital, 80131 Naples, Italy.
- [5] Cardiology Department, Santa Maria delle Grazie Hospital, 80078 Pozzuoli, Italy.
- [6] Medicine Unit, Fatebenefratelli Hospital, 80131 Naples, Italy.
- [7] Medical Affairs Department-Daiichi Sankyo, 00142 Roma, Italy.
- [8] Angiology Unit, District 24, Health Authority Naples 1, 80131 Naples, Italy.

- PMID: **33375676**
- PMCID: PMC7795623
- DOI: 10.3390/ijerph18010102

Free PMC article
Observational Study

Cardiovascular Comorbidities and Pharmacological Treatments of COVID-19 Patients Not Requiring Hospitalization

Vincenzo Russo et al. Int J Environ Res Public Health. 2020.
Free PMC article
Show details
Int J Environ Res Public Health
. 2020 Dec 25;18(1):102.
doi: 10.3390/ijerph18010102.

Authors

Vincenzo Russo [1], Gaetano Piccinocchi [2], Vincenzo Mandaliti [2], Saverio Annunziata [3], Giovanni Cimmino [1], Emilio Attena [4], Nicola Moio [5], Pierpaolo Di Micco [6], Sergio Severino [4], Roberta Trotta [7], Michele Del Guercio [8]

Affiliations

- [1] Cardiology Unit, Department of Translational Medical Sciences, University of Campania Luigi Vanvitelli-Monaldi Hospital, 80131 Naples, Italy.
- [2] Comegen Primary Care Physicians Cooperative SIMG, Italian Society of Family Medicine, 80125 Naples, Italy.
- [3] KOS Primary Care Physicians Cooperative, 80128 Naples, Italy.
- [4] Cardiology Unit, Cotugno Hospital, 80131 Naples, Italy.
- [5] Cardiology Department, Santa Maria delle Grazie Hospital, 80078 Pozzuoli, Italy.
- [6] Medicine Unit, Fatebenefratelli Hospital, 80131 Naples, Italy.
- [7] Medical Affairs Department-Daiichi Sankyo, 00142 Roma, Italy.
- [8] Angiology Unit, District 24, Health Authority Naples 1, 80131 Naples, Italy.

- PMID: **33375676**
- PMCID: PMC7795623
- DOI: 10.3390/ijerph18010102

Abstract

Introduction: The Coronavirus disease 2019 (COVID-19) outbreak is a whole Earth health emergency related to a highly pathogenic human coronavirus responsible for severe acute respiratory syndrome (SARS-CoV-2). Despite the fact that the majority of infected patients were managed in outpatient settings, little is known about the clinical characteristics of COVID-19 patients not requiring hospitalization. The aim of our study was to describe the clinical comorbidity and the pharmacological therapies of COVID-19 patients managed in outpatient settings.

Materials and methods: We performed an observational, retrospective analysis of laboratory-confirmed COVID-19 patients managed in outpatient settings in Naples, Italy between 9 March and 1 May 2020. Data were sourced from the prospectively maintained Health Search (HS)/Thales database, shared by 128 primary care physicians (PCPs) in Naples, Italy. The clinical features and pharmacological therapies of COVID-19 patients not requiring hospitalization and managed in outpatient settings have been described.

Results: A total of 351 laboratory-confirmed COVID-19 patients (mean age 54 ± 17 years; 193 males) with outpatient management were evaluated. Hypertension was the most prevalent comorbidity (35%). The distribution of cardiovascular comorbidities showed no gender-related differences. A total of 201 patients (57.3%) were treated with at least one experimental drug for COVID-19. Azithromycin, alone (42.78%) or in combination (27.44%), was the most widely used experimental anti-COVID drug in outpatient settings. Low Molecular Weight Heparin and Cortisone were prescribed in 24.87% and 19.4% of the study population, respectively. At multivariate regression model, diabetes (risk ratio (RR): 3.74; 95% CI 1.05 to 13.34; $p = 0.04$) and hypertension (RR: 1.69; 95% CI 1.05 to 2.7; $p = 0.03$) were significantly associated with the experimental anti-COVID drug administration. Moreover, only diabetes (RR: 2.43; 95% CI 1.01 to 5.8; $p = 0.03$) was significantly associated with heparin administration.

Conclusions: Our data show a high prevalence of hypertension, more likely treated with renin-angiotensin-aldosterone system (RASS) inhibitors, among COVID-19 patients not requiring hospitalization. Experimental COVID-19 therapies have been prescribed to COVID-19 patients considered at risk for increased venous thromboembolism based on concomitant comorbidities, in particular diabetes and hypertension.

Keywords: COVID-19; anticoagulation; cardiovascular diseases; experimental drugs; hypertension; low molecular weight heparin; outpatient's setting; risk factors; venous thromboembolism.

Conflict of interest statement

The authors declare no conflict of interest

- Cited by 7 articles
- 27 references

Supplementary info

Publication types, MeSH terms Expand

Publication types

- Observational Study

MeSH terms

- Adult
- Aged
- COVID-19 / complications*
- COVID-19 / drug therapy*
- Cardiovascular Diseases / complications*
- Comorbidity*
- Diabetes Mellitus
- Female
- Hospitalization
- Humans
- Hypertension / complications
- Italy / epidemiology
- Male
- Middle Aged
- Retrospective Studies
- Risk Factors

Full text links

FULL TEXT OPEN ACCESS MDPI Multidisciplinary Digital Publishing Institute (MDPI) Free PMC article
Proceed to details
Cite
Share
293

Observational Study

Prof Inferm
. Jan-Mar 2021;74(1):21-30.
doi: 10.7429/pi.2021.741021.

[COVID 19: Nursing Leadership and innovations on the field to face the pandemic]

[Article in Italian]

Gabriella Costamagna [1], Nicoletta Dasso [2], Giulia Ottonello [3], Milko Zanini [4], Gianluca Catania [5], Loredana Sasso [6], Annamaria Bagnasco [7], Collaboration Group ASO Mauriziano, Turin
Collaborators, Affiliations Expand

Collaborators

- **Collaboration Group ASO Mauriziano, Turin**:
Silvia Bagnato, Angela Delsal, Laura Perretta, Giuseppe Fiumanò, Laura Sandrone

Affiliations

- [1] Direttore SC, Direzione professioni sanitarie (Di.P.Sa), Azienda Ospedaliera Ordine Mauriziano, Torino.
- [2] PhD, MNS, RN, Dipartimento di Scienze della Salute, Università degli Studi di Genova.
- [3] PhD Student, MNS, RN, Dipartimento di Scienze della Salute, Università degli Studi di Genova.
- [4] Ricercatore, PhD, MNS, RN,Dipartimento di Scienze della Salute, Università degli Studi di Genova.
- [5] Ricercatore, PhD, MNS, RN, Dipartimento di Scienze della Salute, Università degli Studi di Genova.
- [6] Full Professor, MEdSc, MSN, RN, FAAN, FFNMRCSI, Dipartimento di Scienze della Salute, Università degli Studi di Genova.
- [7] PhD, RN, Full Professor, Nursing Science,Dipartimento di Scienze della Salute, Università degli Studi di Genova.Corresponding Author: annamaria.bagnasco@unige.it.

- PMID: **34089638**
- DOI: 10.7429/pi.2021.741021

Observational Study

[COVID 19: Nursing Leadership and innovations on the field to face the pandemic]

[Article in Italian]
Gabriella Costamagna et al. Prof Inferm. Jan-Mar 2021.
Show details
Prof Inferm
. Jan-Mar 2021;74(1):21-30.
doi: 10.7429/pi.2021.741021.

Authors

Gabriella Costamagna [1], Nicoletta Dasso [2], Giulia Ottonello [3], Milko Zanini [4], Gianluca Catania [5], Loredana Sasso [6], Annamaria Bagnasco [7], Collaboration Group ASO Mauriziano, Turin

Collaborators

- **Collaboration Group ASO Mauriziano, Turin**: Silvia Bagnato, Angela Delsal, Laura Perretta, Giuseppe Fiumanò, Laura Sandrone

Affiliations

- [1] Direttore SC, Direzione professioni sanitarie (Di.P.Sa), Azienda Ospedaliera Ordine Mauriziano, Torino.
- [2] PhD, MNS, RN, Dipartimento di Scienze della Salute, Università degli Studi di Genova.
- [3] PhD Student, MNS, RN, Dipartimento di Scienze della Salute, Università degli Studi di Genova.
- [4] Ricercatore, PhD, MNS, RN,Dipartimento di Scienze della Salute, Università degli Studi di Genova.
- [5] Ricercatore, PhD, MNS, RN, Dipartimento di Scienze della Salute, Università degli Studi di Genova.
- [6] Full Professor, MEdSc, MSN, RN, FAAN, FFNMRCSI, Dipartimento di Scienze della Salute, Università degli Studi di Genova.
- [7] PhD, RN, Full Professor, Nursing Science,Dipartimento di Scienze della Salute, Università degli Studi di Genova.Corresponding Author: annamaria.bagnasco@unige.it.

- PMID: **34089638**
- DOI: 10.7429/pi.2021.741021

Abstract
in English, Italian

Introduction: In the Italian and European literature there are still few studies describing the impact of the COVID-19 pandemic on the organization of nursing care in hospitals, on the actions taken by nursing leaders to contain it, and on the outcomes of these interventions.

Aim: To describe nursing leaders' experiences with reorganizing healthcare pathways, through management data and personal accounts.

Methods: A retrospective quali-quantitative observational study was conducted at the Mauriziano Hospital in Turin, with 484 beds and over 1,700 workers. Quantitative data were analyzed through descriptive statistical indices and integrated with qualitative data collected through semi-structured telephone interviews. The analysis of the quantitative and qualitative data provided an objective and experiential representation of the implemented interventions.

Results: We described the impact of interventions introduced by the nursing leadership during the pandemic on hospital services. In particular, the reorganization of the emergency department and of the prevention and psychological support services for the hospital's health workers. In addition, specific initiatives to support the discharge of COVID-19 positive patients, and to support and manage contacts with family members both during the hospitalization of their loved ones and following death are described.

Discussion and conclusion: This study contributes to the discussion on some crucial issues: the increasingly clear relationship between adequate staffing and safety of patients and professionals, the importance of a good working environment and a solid leadership, the importance of continuing education for professionals and adequate skill mixes; all highlighted by the pandemic.

Introduzione: Nella letteratura italiana ed europea sono ancora pochi gli studi che descrivono l'impatto della pandemia COVID-19 sull'organizzazione dell'assistenza infermieristica negli ospedali, sulle azioni attuate dai leader infermieristici per contenerla e sui risultati di tali interventi.

Obiettivo: Descrivere le esperienze di riorganizzazione dei percorsi assistenziali da parte dei leader infermieristici, attraverso i dati di gestione e le testimonianze.

Metodi: Studio osservazionale retrospettivo quali-quantitativo, svolto presso l'Ospedale Mauriziano di Torino, 484 posti letto e oltre 1700 operatori. I dati quantitativi sono stati analizzati con indici di statistica descrittiva e sono stati integrati con i dati qualitativi raccolti mediante interviste telefoniche semi strutturate. L'analisi dei dati quanti-qualitativi ha fornito una rappresentazione oggettiva ed esperienziale degli interventi implementati.

Risultati: I dati raccolti descrivono la ricaduta sui servizi degli interventi introdotti dalla leadership infermieristica durante la pandemia; in particolare la riorganizzazione del pronto soccorso e dei servizi di prevenzione e supporto psicologico per sostenere i dipendenti dell'Azienda. Inoltre, sono state descritte alcune iniziative particolari a sostegno delle dimissioni dei pazienti COVID-19 positivi e del sostegno e contatto con i familiari durante la degenza dei propri cari e in seguito al decesso.

Discussione e conclusione: I risultati di questo studio sono un contributo alla discussione su alcuni temi cruciali: il rapporto sempre pi˘ chiaro tra staffing adeguato e sicurezza dei pazienti e dei professionisti, l'importanza delle caratteristiche dell'ambiente di lavoro e di una leadership consolidata, l'importanza della formazione dei professionisti e di skill mix adeguati. Resi più evidenti dal contesto di emergenza pandemica.

Supplementary info

Publication types, MeSH terms Expand

Publication types

- Observational Study

MeSH terms

- COVID-19 / nursing*
- Education, Continuing
- Humans
- Interviews as Topic
- Italy
- Leadership*
- Nurse Administrators / organization & administration
- Nursing Care / organization & administration*
- Nursing Staff, Hospital / organization & administration*
- Patient Discharge
- Retrospective Studies

Proceed to details
Cite
Share
☐ 294
Observational Study
Ann R Coll Surg Engl
. 2021 May;103(5):337-344.
doi: 10.1308/rcsann.2020.7071. Epub 2021 Mar 10.

The effect of the COVID-19 lockdown on the epidemiology of hip fractures in the elderly: a multicentre cohort study

K Malik-Tabassum [1], A Robertson [1], B J Tadros [2], G Chan [3] [4], M Crooks [1], C Buckle [5], B Rogers [5], G Selmon [1], G Arealis [2]
Affiliations Expand

Affiliations

- [1] East Sussex Healthcare NHS Trust, UK.
- [2] East Kent Hospitals University NHS Foundation Trust, UK.
- [3] Western Sussex Hospitals NHS Trust, UK.
- [4] Brighton and Sussex Medical School, UK.
- [5] Brighton and Sussex University Hospitals NHS Trust, UK.

- PMID: **33715420**
- DOI: 10.1308/rcsann.2020.7071

Observational Study

The effect of the COVID-19 lockdown on the epidemiology of hip fractures in the elderly: a multicentre cohort study

K Malik-Tabassum et al. Ann R Coll Surg Engl. 2021 May.

Show details

Ann R Coll Surg Engl

. 2021 May;103(5):337-344.
doi: 10.1308/rcsann.2020.7071. Epub 2021 Mar 10.

Authors

K Malik-Tabassum [1], A Robertson [1], B J Tadros [2], G Chan [3] [4], M Crooks [1], C Buckle [5], B Rogers [5], G Selmon [1], G Arealis [2]

Affiliations

- [1] East Sussex Healthcare NHS Trust, UK.
- [2] East Kent Hospitals University NHS Foundation Trust, UK.
- [3] Western Sussex Hospitals NHS Trust, UK.
- [4] Brighton and Sussex Medical School, UK.
- [5] Brighton and Sussex University Hospitals NHS Trust, UK.

- PMID: **33715420**
- DOI: 10.1308/rcsann.2020.7071

Abstract

Introduction: The COVID-19 pandemic presented extraordinary challenges to the UK healthcare system. This study aimed to assess the impact of the COVID-19 lockdown on the epidemiology, treatment pathways and 30-day mortality rates of hip fractures. Outcomes of COVID-19 positive patients were compared against those who tested negative.

Methods: An observational, retrospective, multicentre study was conducted across six hospitals in the South East of England. Data were retrieved from the National Hip Fracture Database and electronic medical records. Data was collected for the strictest UK lockdown period (period B=23 March 2020-11 May 2020), and the corresponding period in 2019 (period A).

Results: A total of 386 patients were admitted during period A, whereas 381 were admitted during period B. Despite the suspension of the 'Best Practice Tariff' during period B, time to surgery, time to orthogeriatric assessment, and 30-day mortality were similar between period A and B. The length of inpatient stay was significantly shorter during period B (11.5 days vs 17.0 days,

p<0.001). Comparison of COVID-19 positive and negative patients during period B demonstrated that a positive test was associated with a significantly higher rate of 30-day mortality (53.6% vs 6.7%), surgical delay >36h (46.4% vs 30.8%, *p*=0.049), and increased length of inpatient stay (15.8 vs 11.7 days, *p*=0.015).

Conclusions: The COVID-19 lockdown did not alter the epidemiology of hip fractures. A substantially higher mortality rate was observed among patients with a COVID-19 positive test. These findings should be taken into consideration by the healthcare policymakers while formulating contingency plans for a potential 'second wave'.

Keywords: COVID-19; Coronavirus; Proximal femur fractures; SARS-CoV-2; Trauma.

- Cited by 1 article

Supplementary info

Publication types, MeSH terms Expand

Publication types

- Multicenter Study
- Observational Study

MeSH terms

- Aged
- Aged, 80 and over
- Arthroplasty, Replacement, Hip
- Bone Screws
- COVID-19 / complications
- COVID-19 / prevention & control*
- Cohort Studies
- Communicable Disease Control*
- England / epidemiology
- Female
- Fracture Fixation, Internal
- Fracture Fixation, Intramedullary
- Hemiarthroplasty
- Hip Fractures / complications
- Hip Fractures / epidemiology*
- Hip Fractures / surgery
- Humans
- Length of Stay / statistics & numerical data*
- Male
- Mortality*

- Public Policy*
- Reoperation
- SARS-CoV-2
- Time-to-Treatment / statistics & numerical data*

Full text links

annals FULL TEXT Atypon
Proceed to details
Cite
Share
295
Observational Study
Surgeon
. 2021 Oct;19(5):e125-e131.
doi: 10.1016/j.surge.2020.08.015. Epub 2020 Sep 22.

Are there benefits to maintaining Covid-19 pandemic pathways for the long-term? A surgical assessment unit based study

Naomi Simone Laskar[1], Alexander Hunt[2], Dilhara Karunaratne[2], Hannah Brooke-Ball[2], Matthew T V Miller[2]
Affiliations Expand

Affiliations

- [1] General Surgery, Conquest Hospital, East Sussex Healthcare NHS Trust, UK. Electronic address: Naomi.laskar@nhs.net.
- [2] General Surgery, Conquest Hospital, East Sussex Healthcare NHS Trust, UK.

- PMID: **33028491**
- PMCID: PMC7508548
- DOI: 10.1016/j.surge.2020.08.015

Free PMC article
Observational Study

Are there benefits to maintaining Covid-19 pandemic pathways for the long-term? A surgical assessment unit based study

Naomi Simone Laskar et al. Surgeon. 2021 Oct.
Free PMC article
Show details
Surgeon
. 2021 Oct;19(5):e125-e131.
doi: 10.1016/j.surge.2020.08.015. Epub 2020 Sep 22.

Authors

Naomi Simone Laskar[1], Alexander Hunt[2], Dilhara Karunaratne[2], Hannah Brooke-Ball[2], Matthew T V Miller[2]

Affiliations

- [1] General Surgery, Conquest Hospital, East Sussex Healthcare NHS Trust, UK. Electronic address: Naomi.laskar@nhs.net.
- [2] General Surgery, Conquest Hospital, East Sussex Healthcare NHS Trust, UK.

- PMID: **33028491**
- PMCID: PMC7508548
- DOI: 10.1016/j.surge.2020.08.015

Abstract

Background: The Covid-19 pandemic has led to the introduction of conservative non-operative approaches to surgical management favouring community driven care. The aim of this study was to determine the effect of these pathways on patients attending a surgical assessment unit (SAU).

Method: This was a retrospective observational cohort study. We included all consecutive attendances to the SAU in April 2020 (Covid-19 period) and April 2019 (pre-Covid-19). The Covid-19 period saw a shift in clinical practice towards a more conservative approach to the management of acute surgical presentations. The primary outcome measure was 30-day readmission. The secondary outcome measures were length of hospital stay, inpatient investigations undertaken and 30-day mortality.

Results: A total of 451 patients were included. This represented 277 and 174 attendances in pre-Covid-19, and Covid-19 groups respectively. The rates of unplanned 30-day readmission rates in the Covid-19 and pre-Covid-19 periods were 16.7% and 12.6% respectively ($P = 0.232$). There were significantly fewer planned follow-ups in the Covid-19 (36.2%) compared to the pre-Covid-19 group (49.1%; $P < 0.01$; OR 1.7, 95% CI 1.15-2.51). There were no significant differences in length of hospital stay ($P = 0.802$), and 30-day mortality rate ($P = 0.716$; OR 1.9, 95% CI 0.38-9.54) between the two periods.

Conclusion: There were no differences in 30-day readmission rates, length of hospital stay, and 30-day mortality with the changes to pathways. Our findings suggest the resource efficient conservative Covid-19 pathways could potentially continue long-term. However, further multi-centre studies with larger sample sizes and longer follow-up duration will be required to validate our findings.

Keywords: Covid-19; General surgery; Pandemic; Surgical assessment unit.

Crown Copyright © 2020. Published by Elsevier Ltd. All rights reserved.

Conflict of interest statement

Declaration of competing interest None.

- 12 references
- 1 figure

Supplementary info

Publication types, MeSH terms Expand

Publication types

- Observational Study

MeSH terms

- Adult
- Aged
- COVID-19 / complications
- COVID-19 / epidemiology
- COVID-19 / prevention & control*
- Critical Pathways*
- Female
- Hospital Mortality
- Humans
- Length of Stay
- Male
- Middle Aged
- Outcome Assessment, Health Care
- Patient Readmission
- Patient Selection
- Retrospective Studies
- Surgical Procedures, Operative*
- Survival Rate

Full text links

ELSEVIER FULL-TEXT ARTICLE Elsevier Science Free PMC article

Proceed to details

Cite

Share

☐ 296
Observational Study

Physiotherapy
. 2022 Mar;114:47-53.
doi: 10.1016/j.physio.2021.09.001. Epub 2021 Sep 24.

Physiotherapy-assisted prone or modified prone positioning in ward-based patients with COVID-19: a retrospective cohort study

Claudia Tatlow [1], Sophie Heywood [2], Carol Hodgson [3], Georgina Cunningham [4], Matthew Conron [5], Hui Yi Ng [6], Harry Georgiou [7], Gemma Pound [8]
Affiliations Expand

Affiliations

- [1] St Vincent's Hospital Melbourne, Physiotherapy Department, 41 Victoria Parade, Fitzroy 3065, Victoria, Australia. Electronic address: claudia.tatlow@gmail.com.
- [2] St Vincent's Hospital Melbourne, Physiotherapy Department, 41 Victoria Parade, Fitzroy 3065, Victoria, Australia. Electronic address: sophie.heywood@svha.org.au.
- [3] The Alfred Hospital, Physiotherapy Department, 55 Commercial Rd, Melbourne 3004, Victoria, Australia; Australian and New Zealand Intensive Care Research Centre (ANZIC-RC), School of Public Health and Preventive Medicine, Monash University, Victoria, Australia. Electronic address: carol.hodgson@monash.edu.
- [4] St Vincent's Hospital Melbourne, Department of General Medicine, 41 Victoria Parade, Fitzroy 3065, Victoria, Australia. Electronic address: georgina.cunningham@svha.org.au.
- [5] St Vincent's Hospital Melbourne, Department of Respiratory Medicine and Specialty Services, 41 Victoria Parade, Fitzroy 3065, Victoria, Australia. Electronic address: Matthew.CONRON@svha.org.au.
- [6] St Vincent's Hospital Melbourne, Department of General Medicine, 41 Victoria Parade, Fitzroy 3065, Victoria, Australia. Electronic address: huiyi.ng@svha.org.au.
- [7] St Vincent's Hospital Melbourne, Department of Respiratory Medicine and Specialty Services, 41 Victoria Parade, Fitzroy 3065, Victoria, Australia. Electronic address: harry.georgiou@svha.org.au.
- [8] St Vincent's Hospital Melbourne, Physiotherapy Department, 41 Victoria Parade, Fitzroy 3065, Victoria, Australia; Australian and New Zealand Intensive Care Research Centre (ANZIC-RC), School of Public Health and Preventive Medicine, Monash University, Victoria, Australia. Electronic address: gemma.pound@svha.org.au.

- PMID: **35091328**
- PMCID: PMC8462002
- DOI: 10.1016/j.physio.2021.09.001

Free PMC article
Observational Study

Physiotherapy-assisted prone or modified prone positioning in ward-based patients with COVID-19: a retrospective cohort study

Claudia Tatlow et al. Physiotherapy. 2022 Mar.
Free PMC article
Show details
Physiotherapy
. 2022 Mar;114:47-53.
doi: 10.1016/j.physio.2021.09.001. Epub 2021 Sep 24.

Authors

Claudia Tatlow [1], Sophie Heywood [2], Carol Hodgson [3], Georgina Cunningham [4], Matthew Conron [5], Hui Yi Ng [6], Harry Georgiou [7], Gemma Pound [8]

Affiliations

- [1] St Vincent's Hospital Melbourne, Physiotherapy Department, 41 Victoria Parade, Fitzroy 3065, Victoria, Australia. Electronic address: claudia.tatlow@gmail.com.
- [2] St Vincent's Hospital Melbourne, Physiotherapy Department, 41 Victoria Parade, Fitzroy 3065, Victoria, Australia. Electronic address: sophie.heywood@svha.org.au.
- [3] The Alfred Hospital, Physiotherapy Department, 55 Commercial Rd, Melbourne 3004, Victoria, Australia; Australian and New Zealand Intensive Care Research Centre (ANZIC-RC), School of Public Health and Preventive Medicine, Monash University, Victoria, Australia. Electronic address: carol.hodgson@monash.edu.
- [4] St Vincent's Hospital Melbourne, Department of General Medicine, 41 Victoria Parade, Fitzroy 3065, Victoria, Australia. Electronic address: georgina.cunningham@svha.org.au.
- [5] St Vincent's Hospital Melbourne, Department of Respiratory Medicine and Specialty Services, 41 Victoria Parade, Fitzroy 3065, Victoria, Australia. Electronic address: Matthew.CONRON@svha.org.au.
- [6] St Vincent's Hospital Melbourne, Department of General Medicine, 41 Victoria Parade, Fitzroy 3065, Victoria, Australia. Electronic address: huiyi.ng@svha.org.au.
- [7] St Vincent's Hospital Melbourne, Department of Respiratory Medicine and Specialty Services, 41 Victoria Parade, Fitzroy 3065, Victoria, Australia. Electronic address: harry.georgiou@svha.org.au.
- [8] St Vincent's Hospital Melbourne, Physiotherapy Department, 41 Victoria Parade, Fitzroy 3065, Victoria, Australia; Australian and New Zealand Intensive Care Research Centre (ANZIC-RC), School of Public Health and Preventive Medicine, Monash University, Victoria, Australia. Electronic address: gemma.pound@svha.org.au.

- PMID: **35091328**
- PMCID: PMC8462002
- DOI: 10.1016/j.physio.2021.09.001

Abstract

Objectives: To evaluate short-term change in oxygenation and feasibility of physiotherapy-assisted prone or modified prone positioning in awake, ward-based patients with COVID-19.

Design: Retrospective observational cohort study.

Setting: General wards, single-centre tertiary hospital in Australia.

Participants: Patients were included if ≥18 years, had COVID-19, required $FiO_2 \geq 0.28$ or oxygen flow rate ≥4 l/minute and consented to positioning.

Main outcome measures: Feasibility measures included barriers to therapy, assistance required, and comfort. Short-term change in oxygenation (SpO_2) and oxygen requirements before and 15 minutes after positioning.

Results: Thirteen patients, mean age 75 (SD 14) years; median Clinical Frailty Scale score 6 (IQR 4 to 7) participated in 32 sessions of prone or modified prone positioning from a total of 125 ward-based patients admitted with COVID-19 who received physiotherapy intervention. Nine of thirteen patients (69%) required physiotherapy assistance and modified positions were utilised in 8/13 (62%). SpO2 increased in 27/32 sessions, with a mean increase from 90% (SD 5) pre-positioning to 94% (SD 4) (mean difference 4%; 95%CI 3 to 5%) after 15 minutes. Oxygen requirement decreased in 14/32 sessions, with a mean pre-positioning requirement of 8 l/minute (SD 4) to 7 l/minute (SD 4) (mean difference 2 l/minute; 95%CI 1 to 3 l/minute) after 15 minutes. In three sessions oxygen desaturation and discomfort occurred but resolved immediately by returning supine.

Conclusion: Physiotherapy-assisted prone or modified prone positioning may be a feasible option leading to short-term improvements in oxygenation in awake, ward-based patients with hypoxemia due to COVID-19. Further research exploring longerterm health outcomes and safety is required.

Keywords: Acute respiratory failure; COVID-19; Physical therapy modalities; Prone positioning.

Crown Copyright © 2021. Published by Elsevier Ltd. All rights reserved.

- 24 references
- 2 figures

Supplementary info

Publication types, MeSH terms Expand

Publication types

- Observational Study

MeSH terms

- Aged
- COVID-19*
- Humans
- Physical Therapy Modalities

- Prone Position
- Retrospective Studies
- SARS-CoV-2

Full text links

ELSEVIER FULL-TEXT ARTICLE Elsevier Science Free PMC article
Proceed to details
Cite
Share
297
Multicenter Study
Br J Haematol
. 2021 Oct;195(1):85-89.
doi: 10.1111/bjh.17579. Epub 2021 Jun 24.

The clinical course of COVID-19 in pregnant versus non-pregnant women requiring hospitalisation: results from the multicentre UK CA-COVID-19 study

Christina Crossette-Thambiah [1, 2], Phillip Nicolson [3], Indika Rajakaruna [4], Alexander Langridge [5], Zara Sayar [6], Maria R Perelta [7], Sarah Essex [8], Roderick Oakes [9], Philip Mounter [10], Sarah Lewis [11], Tina Dutt [12], Ian Scott [13], Nini Aung [14], Susie Shapiro [15], Mike Laffan [1, 2], Deepa R J Arachchillage [1, 2]
Affiliations Expand

Affiliations

- [1] Centre for Haematology, Imperial College London, London, UK.
- [2] Department of Haematology, Imperial College Healthcare NHS Trust Imperial College London, London, UK.
- [3] Department of Haematology, University Hospitals Birmingham NHS Foundation Trust, Birmingham, UK.
- [4] Department of Computer Science, University of East London, London, UK.
- [5] Department of Haematology, Newcastle Hospitals NHS Foundation Trust, Newcastle Upon Tyne, UK.
- [6] Department of Haematology, Whittington Health NHS Trust, London, UK.
- [7] Department of Haematology, Royal Free London NHS Foundation Trust, London, UK.
- [8] Department of Haematology, South Tees NHS Foundation Trust, Middlesbrough, UK.
- [9] Department of Haematology, North Cumbria University Hospitals NHS Trust, Penrith, UK.

- [10] Department of Haematology, County Durham and Darlington NHS Foundation Trust, Darlington, UK.
- [11] Department of Haematology, Aneurin Bevan University Health Board, Newport, UK.
- [12] Department of Haematology, Royal Liverpool University Hospital, Liverpool, UK.
- [13] Department of Anaesthesia and Critical Care, NHS Grampian, Aberdeen, UK.
- [14] Department of Haematology, North Tees and Hartlepool NHS Foundation Trust, Hartlepool, UK.
- [15] Oxford Haemophilia and Thrombosis Centre, Oxford University Hospitals NHS Foundation Trust, Oxford, UK.

- PMID: **34132386**
- PMCID: PMC8444732
- DOI: 10.1111/bjh.17579

Free PMC article
Multicenter Study

The clinical course of COVID-19 in pregnant versus non-pregnant women requiring hospitalisation: results from the multicentre UK CA-COVID-19 study

Christina Crossette-Thambiah et al. Br J Haematol. 2021 Oct.
Free PMC article

Show details

Br J Haematol
. 2021 Oct;195(1):85-89.
doi: 10.1111/bjh.17579. Epub 2021 Jun 24.

Authors

Christina Crossette-Thambiah [1, 2], Phillip Nicolson [3], Indika Rajakaruna [4], Alexander Langridge [5], Zara Sayar [6], Maria R Perelta [7], Sarah Essex [8], Roderick Oakes [9], Philip Mounter [10], Sarah Lewis [11], Tina Dutt [12], Ian Scott [13], Nini Aung [14], Susie Shapiro [15], Mike Laffan [1, 2], Deepa R J Arachchillage [1, 2]

Affiliations

- [1] Centre for Haematology, Imperial College London, London, UK.
- [2] Department of Haematology, Imperial College Healthcare NHS Trust Imperial College London, London, UK.
- [3] Department of Haematology, University Hospitals Birmingham NHS Foundation Trust, Birmingham, UK.
- [4] Department of Computer Science, University of East London, London, UK.

- [5] Department of Haematology, Newcastle Hospitals NHS Foundation Trust, Newcastle Upon Tyne, UK.
- [6] Department of Haematology, Whittington Health NHS Trust, London, UK.
- [7] Department of Haematology, Royal Free London NHS Foundation Trust, London, UK.
- [8] Department of Haematology, South Tees NHS Foundation Trust, Middlesbrough, UK.
- [9] Department of Haematology, North Cumbria University Hospitals NHS Trust, Penrith, UK.
- [10] Department of Haematology, County Durham and Darlington NHS Foundation Trust, Darlington, UK.
- [11] Department of Haematology, Aneurin Bevan University Health Board, Newport, UK.
- [12] Department of Haematology, Royal Liverpool University Hospital, Liverpool, UK.
- [13] Department of Anaesthesia and Critical Care, NHS Grampian, Aberdeen, UK.
- [14] Department of Haematology, North Tees and Hartlepool NHS Foundation Trust, Hartlepool, UK.
- [15] Oxford Haemophilia and Thrombosis Centre, Oxford University Hospitals NHS Foundation Trust, Oxford, UK.

- PMID: **34132386**
- PMCID: PMC8444732
- DOI: 10.1111/bjh.17579

Abstract

The impact of COVID-19 infection on pregnant women remains relatively unknown but the physiological changes of pregnancy and hypercoagulability of COVID-19 may further increase thrombotic risk. In this retrospective multicentre observational study, we report clinical characteristics and outcomes in 36 pregnant women requiring hospitalisation for COVID-19 compared to a propensity-matched cohort of non-pregnant women. Pregnant women had a lower haemoglobin and higher lymphocyte counts but no differences in other haematological or biochemical parameters on admission compared to non-pregnant women. There was no significant difference in the duration of hospitalisation; median two days (1-77) for pregnant versus eight days (1-49) for non-pregnant women. A higher proportion of non-pregnant women required mechanical ventilation [11/36 (31%) vs 3/36 (8%), P = 0·03] and received thromboprophylaxis with low-molecular-weight heparin (LMWH) within 24 h of admission [25/36 (69%) vs 15 /36 (42%), P = 0·03] compared to pregnant women. One pregnant woman required extracorporeal membrane oxygenation. The rate of thrombosis was similar in both groups (one in each group). No women developed major bleeding or died. Data suggest that although non-pregnant women had a severe clinical course, overall outcomes were not different between women with or without pregnancy. The use of thromboprophylaxis was inconsistent, demonstrating a need for establishing evidence-based guidance for COVID-19 during pregnancy.

Keywords: COVID-19; bleeding; coagulopathy; pregnancy; thrombosis.

© 2021 British Society for Haematology and John Wiley & Sons Ltd.

Conflict of interest statement

PN received research grants from Novartis, Principia and Rigel, unrestricted grants from Sanofi, Chugai and Octapharma, and honoraria from Bayer. SS has also received meeting sponsorship, speaker fees and/or consultancy from Bayer, Pfizer, NovoNordisk, Sobi, Chugai/Roche and Shire/Takeda. ML received speaker fees/consulting/advisory from Leo Pharma, Pfizer, Takeda,

Sobi, Takeda, Pfizer and AstraZeneca. DJA received funding from Bayer plc to set up the multicentre database of the study as an investigator-initiated funding and research funding from Leo Pharma. The remaining authors declare no conflicts of interest.

- Cited by 1 article
- 14 references
- 1 figure

Supplementary info

Publication types, MeSH terms, Grant support Expand

Publication types

- Multicenter Study
- Research Support, Non-U.S. Gov't

MeSH terms

- Adult
- COVID-19 / blood*
- Female
- Hospitalization
- Humans
- Middle Aged
- Pregnancy
- Pregnant Women
- Retrospective Studies
- Thrombosis / drug therapy*
- United Kingdom
- Young Adult

Grant support

- P87339/Bayer Public Limited Company

Full text links

WILEY Full Text Article Wiley Free PMC article

Proceed to details

Cite

Share

☐ 298

Observational Study

Ulus Travma Acil Cerrahi Derg

. 2021 Jan;27(1):22-25.
doi: 10.14744/tjtes.2020.46487.

Comparative analysis of the management of acute appendicitis between the normal period and COVID-19 pandemic

Serhat Meriç [1], Talar Vartanoglu Aktokmakyan [1], Merve Tokocin [1], Yunus Emre Aktimur [1], Nadir Adnan Hacım [1], Osman Bilgin Gülcicek [1]
Affiliations Expand

Affiliation

- [1] Department of General Surgery, İstanbul Bağcılar Training and Research Hospital, İstanbul-Turkey.

- PMID: **33394473**
- DOI: 10.14744/tjtes.2020.46487

Free article
Observational Study

Comparative analysis of the management of acute appendicitis between the normal period and COVID-19 pandemic

Serhat Meriç et al. Ulus Travma Acil Cerrahi Derg. 2021 Jan.
Free article
Show details
Ulus Travma Acil Cerrahi Derg
. 2021 Jan;27(1):22-25.
doi: 10.14744/tjtes.2020.46487.

Authors

Serhat Meriç [1], Talar Vartanoglu Aktokmakyan [1], Merve Tokocin [1], Yunus Emre Aktimur [1], Nadir Adnan Hacım [1], Osman Bilgin Gülcicek [1]

Affiliation

- [1] Department of General Surgery, İstanbul Bağcılar Training and Research Hospital, İstanbul-Turkey.

- PMID: **33394473**

- DOI: 10.14744/tjtes.2020.46487

Abstract

Background: Acute appendicitis is the most common abdominal surgical emergency. There is no adequate information to evaluate the effects of the COVID-19 pandemic on acute appendicitis and its surgical management. The present comparative study reports successful appendectomy and infection control in patients with appendicitis during the COVID-19 pandemic and last year covering the same period.

Methods: This retrospective observational study was conducted in acute appendicitis-treated patients from 13.03.19 to 13.05.19 and from 13.03.20 to 13.05.20, respectively.

Results: This study included 150 patients (110 patients in 2019; 40 patients in 2020 (during COVID-19 pandemic)). The patients were named as Group A (Normal period) and Group B (Pandemic period), respectively. The groups were comparable as there was no significant difference between the mean age, mean BMI, and mean length of stay. There is a significant difference between the comorbidities of Group A and Group B (p=0.033). There was no significant difference between the laboratory and radiological findings of Group A and Group B. There was a significant difference between the perforation number of Group A and Group B (p=0.029). There was no significant difference between the needs of ICU and conversion from laparoscopic to conventional appendectomy of Group A and Group B.

Conclusion: The findings obtained in this study suggest that late admission to the hospital caused complicated cases and made acute appendicitis management more difficult during the pandemic period, which was already a troubling period. During the COVID-19 pandemic, the principles applied to emergency surgery for infected patients should be applied to both suspected and confirmed cases.

- Cited by 3 articles

Supplementary info

Publication types, MeSH terms Expand

Publication types

- Observational Study

MeSH terms

- Acute Disease
- Appendectomy / statistics & numerical data
- Appendicitis* / epidemiology
- Appendicitis* / surgery
- COVID-19*
- Comorbidity
- Hospitalization / statistics & numerical data

- Humans
- Pandemics
- Retrospective Studies
- SARS-CoV-2
- Time-to-Treatment / statistics & numerical data

Full text links

FREE full text article at www.journalagent.com LookUs Bilisim
Proceed to details
Cite
Share
299

Observational Study
BMC Nephrol
. 2021 Feb 27;22(1):73.
doi: 10.1186/s12882-021-02270-9.

Age and multimorbidities as poor prognostic factors for COVID-19 in hemodialysis: a Lebanese national study

Mabel Aoun [1,2], Rabab Khalil [3], Walid Mahfoud [4], Haytham Fatfat [5], Line Bou Khalil [6], Rashad Alameddine [7], Nabil Afiouni [8], Issam Ibrahim [9], Mohamad Hassan [10], Haytham Zarzour [11], Ali Jebai [12], Nina Mourad Khalil [12], Luay Tawil [13], Zeina Mechref [14], Zuhair El Imad [14], Fadia Chamma [15], Ayman Khalil [16], Sandy Zeidan [17], Balsam El Ghoul [17], Georges Dahdah [17], Sarah Mouawad [18,19], Hiba Azar [18,19], Kamal Abou Chahine [20], Siba Kallab [21], Bashir Moawad [21], Ahmad Fawaz [22], Joseph Homsi [23], Carmen Tabaja [24], Maya Delbani [24], Rami Kallab [25], Hassan Hoballah [26], Wahib Haykal [27], Najat Fares [28], Walid Rahal [29], Wael Mroueh [30], Mohammed Youssef [31], Jamale Rizkallah [32], Ziad Sebaaly [32], Antoine Dfouni [33], Norma Ghosn [33], Nagi Nawfal [34], Walid Abou Jaoude [35], Nadine Bassil [35,36], Therese Maroun [35,36], Nabil Bassil [35,36], Chadia Beaini [37], Boutros Haddad [28], Elie Moubarak [38], Houssam Rabah [3], Amer Attieh [9], Serge Finianos [18,19], Dania Chelala [18,19]
Affiliations Expand

Affiliations

- [1] Department of Nephrology, Saint-George Hospital Ajaltoun, Ajaltoun, Lebanon. aounmabel@yahoo.fr.
- [2] Faculty of Medicine, Saint-Joseph University, Beirut, Lebanon. aounmabel@yahoo.fr.
- [3] Makassed General Hospital, Beirut, Lebanon.
- [4] Saydet Zghorta Hospital, Zgharta, Lebanon.
- [5] Mounla Hospital, Tripoli, Lebanon.

- [6] Mount-Lebanon Hospital, Hazmiyeh, Lebanon.
- [7] Orange Nassau Hospital, Tripoli, Lebanon.
- [8] Islamic Hospital, Tripoli, Lebanon.
- [9] Youssef Akkar Hospital, Halba, Lebanon.
- [10] Dar Al-Chifae Hospital, Tripoli, Lebanon.
- [11] Zahraa Hospital, Jnah, Lebanon.
- [12] Bahman Hospital, Beirut, Lebanon.
- [13] Siblin Governmental Hospital, Sebline, Lebanon.
- [14] Ain Wazein Medical Village, Ain Wazein, Lebanon.
- [15] Serhal Hospital, Beirut, Lebanon.
- [16] Iklim Hospital, Mazboud, Lebanon.
- [17] Centre Hospitalier du Nord, Zghorta, Lebanon.
- [18] Faculty of Medicine, Saint-Joseph University, Beirut, Lebanon.
- [19] Hotel-Dieu de France Hospital, Beirut, Lebanon.
- [20] Beqaa Hospital, Zahlé, Lebanon.
- [21] Abou Jaoude Hospital, Jal el Dib, Lebanon.
- [22] Labib Hospital, Sidon, Lebanon.
- [23] Khoury Hospital, Zahle, Lebanon.
- [24] Hammoud Hospital University Medical Center, Sidon, Lebanon.
- [25] FMC Hospital, Mejdlaya, Lebanon.
- [26] Sahel Hospital, Haret Hreik, Lebanon.
- [27] Beit Chabeb Hospital, Beit Chabeb, Lebanon.
- [28] Saint-Georges Orthodoxe Hospital, Beirut, Lebanon.
- [29] Rahal Hospital, Akkar, Lebanon.
- [30] Jabal Amel Hospital, Tyre, Lebanon.
- [31] Dinnieh Hospital, Dinnieh, Lebanon.
- [32] Haroun Hospital, Zalka, Lebanon.
- [33] Centre Hospitalier Universitaire Notre Dame de Secours Hospital, Byblos, Lebanon.
- [34] Sacre-Coeur Hospital, Baabda, Lebanon.
- [35] Middle-East Institute of Health, Bsalim, Lebanon.
- [36] Saint-Joseph Hospital, Dora, Lebanon.
- [37] Bellevue Medical Center, Mansourieh, Lebanon.
- [38] Hayek Hospital, Al Hayek, Lebanon.

- PMID: **33639881**
- PMCID: PMC7912967
- DOI: 10.1186/s12882-021-02270-9

Free PMC article
Observational Study

Age and multimorbidities as poor prognostic factors for COVID-19 in hemodialysis: a Lebanese national study

Mabel Aoun et al. BMC Nephrol. 2021.
Free PMC article
Show details
BMC Nephrol
. 2021 Feb 27;22(1):73.
doi: 10.1186/s12882-021-02270-9.

Authors

Mabel Aoun [1, 2], Rabab Khalil [3], Walid Mahfoud [4], Haytham Fatfat [5], Line Bou Khalil [6], Rashad Alameddine [7], Nabil Afiouni [8], Issam Ibrahim [9], Mohamad Hassan [10], Haytham Zarzour [11], Ali Jebai [12], Nina Mourad Khalil [12], Luay Tawil [13], Zeina Mechref [14], Zuhair El Imad [14], Fadia Chamma [15], Ayman Khalil [16], Sandy Zeidan [17], Balsam El Ghoul [17], Georges Dahdah [17], Sarah Mouawad [18, 19], Hiba Azar [18, 19], Kamal Abou Chahine [20], Siba Kallab [21], Bashir Moawad [21], Ahmad Fawaz [22], Joseph Homsi [23], Carmen Tabaja [24], Maya Delbani [24], Rami Kallab [25], Hassan Hoballah [26], Wahib Haykal [27], Najat Fares [28], Walid Rahal [29], Wael Mroueh [30], Mohammed Youssef [31], Jamale Rizkallah [32], Ziad Sebaaly [32], Antoine Dfouni [33], Norma Ghosn [33], Nagi Nawfal [34], Walid Abou Jaoude [35], Nadine Bassil [35, 36], Therese Maroun [35, 36], Nabil Bassil [35, 36], Chadia Beaini [37], Boutros Haddad [28], Elie Moubarak [38], Houssam Rabah [3], Amer Attieh [9], Serge Finianos [18, 19], Dania Chelala [18, 19]

Affiliations

- [1] Department of Nephrology, Saint-George Hospital Ajaltoun, Ajaltoun, Lebanon. aounmabel@yahoo.fr.
- [2] Faculty of Medicine, Saint-Joseph University, Beirut, Lebanon. aounmabel@yahoo.fr.
- [3] Makassed General Hospital, Beirut, Lebanon.
- [4] Saydet Zghorta Hospital, Zgharta, Lebanon.
- [5] Mounla Hospital, Tripoli, Lebanon.
- [6] Mount-Lebanon Hospital, Hazmiyeh, Lebanon.
- [7] Orange Nassau Hospital, Tripoli, Lebanon.
- [8] Islamic Hospital, Tripoli, Lebanon.
- [9] Youssef Akkar Hospital, Halba, Lebanon.
- [10] Dar Al-Chifae Hospital, Tripoli, Lebanon.
- [11] Zahraa Hospital, Jnah, Lebanon.
- [12] Bahman Hospital, Beirut, Lebanon.
- [13] Siblin Governmental Hospital, Sebline, Lebanon.
- [14] Ain Wazein Medical Village, Ain Wazein, Lebanon.
- [15] Serhal Hospital, Beirut, Lebanon.
- [16] Iklim Hospital, Mazboud, Lebanon.
- [17] Centre Hospitalier du Nord, Zghorta, Lebanon.
- [18] Faculty of Medicine, Saint-Joseph University, Beirut, Lebanon.
- [19] Hotel-Dieu de France Hospital, Beirut, Lebanon.
- [20] Beqaa Hospital, Zahlé, Lebanon.
- [21] Abou Jaoude Hospital, Jal el Dib, Lebanon.
- [22] Labib Hospital, Sidon, Lebanon.
- [23] Khoury Hospital, Zahle, Lebanon.

- [24] Hammoud Hospital University Medical Center, Sidon, Lebanon.
- [25] FMC Hospital, Mejdlaya, Lebanon.
- [26] Sahel Hospital, Haret Hreik, Lebanon.
- [27] Beit Chabeb Hospital, Beit Chabeb, Lebanon.
- [28] Saint-Georges Orthodoxe Hospital, Beirut, Lebanon.
- [29] Rahal Hospital, Akkar, Lebanon.
- [30] Jabal Amel Hospital, Tyre, Lebanon.
- [31] Dinnieh Hospital, Dinnieh, Lebanon.
- [32] Haroun Hospital, Zalka, Lebanon.
- [33] Centre Hospitalier Universitaire Notre Dame de Secours Hospital, Byblos, Lebanon.
- [34] Sacre-Coeur Hospital, Baabda, Lebanon.
- [35] Middle-East Institute of Health, Bsalim, Lebanon.
- [36] Saint-Joseph Hospital, Dora, Lebanon.
- [37] Bellevue Medical Center, Mansourieh, Lebanon.
- [38] Hayek Hospital, Al Hayek, Lebanon.

- PMID: **33639881**
- PMCID: PMC7912967
- DOI: 10.1186/s12882-021-02270-9

Abstract

Background: Hemodialysis patients with COVID-19 have been reported to be at higher risk for death than the general population. Several prognostic factors have been identified in the studies from Asian, European or American countries. This is the first national Lebanese study assessing the factors associated with SARS-CoV-2 mortality in hemodialysis patients.

Methods: This is an observational study that included all chronic hemodialysis patients in Lebanon who were tested positive for SARS-CoV-2 from 31st March to 1st November 2020. Data on demographics, comorbidities, admission to hospital and outcome were collected retrospectively from the patients' medical records. A binary logistic regression analysis was performed to assess risk factors for mortality.

Results: A total of 231 patients were included. Mean age was 61.46 ± 13.99 years with a sex ratio of 128 males to 103 females. Around half of the patients were diabetics, 79.2% presented with fever. A total of 115 patients were admitted to the hospital, 59% of them within the first day of diagnosis. Hypoxia was the major reason for hospitalization. Death rate was 23.8% after a median duration of 6 (IQR, 2 to 10) days. Adjusted regression analysis showed a higher risk for death among older patients (odds ratio = 1.038; 95% confidence interval: 1.013, 1.065), patients with heart failure (odds ratio = 4.42; 95% confidence interval: 2.06, 9.49), coronary artery disease (odds ratio = 3.27; 95% confidence interval: 1.69, 6.30), multimorbidities (odds ratio = 1.593; 95% confidence interval: 1.247, 2.036), fever (odds ratio = 6.66; 95% confidence interval: 1.94, 27.81), CRP above 100 mg/L (odds ratio = 4.76; 95% confidence interval: 1.48, 15.30), and pneumonia (odds ratio = 19.18; 95% confidence interval: 6.47, 56.83).

Conclusions: This national study identified older age, coronary artery disease, heart failure, multimorbidities, fever and pneumonia as risk factors for death in patients with COVID-19 on chronic hemodialysis. The death rate was comparable to other countries and estimated at 23.8%.

Keywords: COVID-19; Hemodialysis; Mortality; Multimorbidities; National study; SARS-CoV-2.

Conflict of interest statement

The authors declare that they have no competing interests.

- Cited by 1 article
- 34 references

Supplementary info

Publication types, MeSH terms Expand

Publication types

- Observational Study

MeSH terms

- Age Factors
- Aged
- COVID-19 / complications
- COVID-19 / mortality*
- Coronary Disease / complications
- Critical Care
- Dementia / complications
- Female
- Fever / complications
- Heart Failure / complications
- Hospitalization
- Humans
- Lebanon / epidemiology
- Male
- Middle Aged
- Multimorbidity*
- Prognosis
- Renal Dialysis*
- Retrospective Studies
- Risk Factors
- SARS-CoV-2
- Stroke / complications

Full text links

Read free full text at BMC BioMed Central Free PMC article
Proceed to details
Cite
Share
300
Observational Study
Korean J Intern Med
. 2021 Jan;36(1):1-10.
doi: 10.3904/kjim.2020.329. Epub 2020 Dec 1.

Clinical implication and risk factor of pneumonia development in mild coronavirus disease 2019 patients

Hyun Woo Lee [1], Seo-Young Yoon [1], Jung-Kyu Lee [1], Tae Yeon Park [1], Deog Kyeom Kim [1], Hee Soon Chung [1], Eun Young Heo [1]
Affiliations Expand

Affiliation

- [1] Division of Respiratory and Critical Care, Department of Internal Medicine, Seoul Metropolitan Government Seoul National University Boramae Medical Center, Seoul, Korea.

- PMID: **32972121**
- PMCID: PMC7820649
- DOI: 10.3904/kjim.2020.329

Free PMC article
Observational Study

Clinical implication and risk factor of pneumonia development in mild coronavirus disease 2019 patients

Hyun Woo Lee et al. Korean J Intern Med. 2021 Jan.
Free PMC article
Show details
Korean J Intern Med
. 2021 Jan;36(1):1-10.
doi: 10.3904/kjim.2020.329. Epub 2020 Dec 1.

Authors

Hyun Woo Lee [1], Seo-Young Yoon [1], Jung-Kyu Lee [1], Tae Yeon Park [1], Deog Kyeom Kim [1], Hee Soon Chung [1], Eun Young Heo [1]

Affiliation

- [1] Division of Respiratory and Critical Care, Department of Internal Medicine, Seoul Metropolitan Government Seoul National University Boramae Medical Center, Seoul, Korea.

- PMID: **32972121**
- PMCID: PMC7820649
- DOI: 10.3904/kjim.2020.329

Abstract

Background/aims: Although a majority of coronavirus disease 2019 (COVID-19) cases were characterized as mild, data assessing the development of pneumonia in mild COVID-19 patients are limited. We aimed to examine the effect of pneumonia development on the clinical course of mild COVID-19 in hospitalized patients.

Methods: A retrospective cohort study was conducted via medical record review between February 25, 2020 and April 11, 2020 at a single center. The impact of pneumonia development on the time to viral clearance in mild COVID-19 patients was evaluated. Risk factors associated with the development of pneumonia were also identified.

Results: Chest radiographs revealed the development of pneumonia in 26.8% of mild COVID-19 patients. The time to pneumonia development was a median of 8.0 days from the onset of symptoms and 3.5 days after hospital admission. A multivariate analysis for predicting pneumonia development identified age ≥ 65 years (odds ratio [OR], 3.15; 95% confidence interval [CI], 1.14 to 8.73), cough (OR, 2.18; 95% CI, 1.29 to 3.68), dyspnea (OR, 3.58; 95% CI, 1.10 to 11.69), and diarrhea (OR, 2.69; 95% CI, 1.51 to 4.78) as significant variables. The time to negative conversion was longer in mild COVID-19 patients who developed pneumonia (23.6 days vs. 18.4 days, $p = 0.003$). In Kaplan-Meier estimation and multivariate Cox regression analyses, newly developed pneumonia was significantly related with delayed time to negative conversion (log-rank test, $p = 0.02$; hazard ratio, 2.90; 95% CI, 1.06 to 7.97).

Conclusion: The development of pneumonia delayed viral clearance in patients with mild COVID-19. Elderly patients or those suffering from diarrhea should be closely monitored, given the increased risk of developing pneumonia.

Keywords: COVID-19; Mortality; Pneumonia; Risk factors; Viruses.

Conflict of interest statement

Conflict of interest

No potential conflict of interest relevant to this article was reported.

- Cited by 4 articles
- 29 references

- 2 figures

Supplementary info

Publication types, MeSH terms Expand

Publication types

- Observational Study

MeSH terms

- Adolescent
- Adult
- COVID-19 / complications
- COVID-19 / diagnosis
- COVID-19 / virology*
- Disease Progression
- Female
- Hospitalization
- Host-Pathogen Interactions
- Humans
- Lung / diagnostic imaging
- Lung / virology*
- Male
- Middle Aged
- Prognosis
- Retrospective Studies
- Risk Assessment
- Risk Factors
- SARS-CoV-2 / pathogenicity*
- Severity of Illness Index
- Time Factors
- Young Adult

Full text links

KJIM FREE FULL TEXT M2PI Free PMC article
Proceed to details
Cite
Share
☐ 301
Observational Study

Surg Obes Relat Dis
. 2021 Oct;17(10):1780-1786.
doi: 10.1016/j.soard.2021.05.029. Epub 2021 Jun 29.

Metabolic surgery may protect against admission for COVID-19 in persons with nonalcoholic fatty liver disease

Christopher J Tignanelli [1], Carolyn T Bramante [2], Nirjhar Dutta [2], Leonardo Tamariz [3], Michael G Usher [2], Sayeed Ikramuddin [4]
Affiliations Expand

Affiliations

- [1] Department of Surgery, University of Minnesota, Division of Surgical Oncology, Minneapolis, Minnesota. Electronic address: bramante@umn.edu.
- [2] Department of Medicine, University of Minnesota, Division of General Internal Medicine, Minneapolis, Minnesota.
- [3] Division of Cardiology, University of Miami, and Miami VA Healthcare Administration, Miami, Florida.
- [4] Department of Surgery, University of Minnesota, Division of Surgical Oncology, Minneapolis, Minnesota.

- PMID: **34326020**
- PMCID: PMC8238640
- DOI: 10.1016/j.soard.2021.05.029

Free PMC article
Observational Study

Metabolic surgery may protect against admission for COVID-19 in persons with nonalcoholic fatty liver disease

Christopher J Tignanelli et al. Surg Obes Relat Dis. 2021 Oct.
Free PMC article
Show details
Surg Obes Relat Dis
. 2021 Oct;17(10):1780-1786.
doi: 10.1016/j.soard.2021.05.029. Epub 2021 Jun 29.

Authors

Christopher J Tignanelli [1], Carolyn T Bramante [2], Nirjhar Dutta [2], Leonardo Tamariz [3], Michael G Usher [2], Sayeed Ikramuddin [4]

Affiliations

- [1] Department of Surgery, University of Minnesota, Division of Surgical Oncology, Minneapolis, Minnesota. Electronic address: bramante@umn.edu.
- [2] Department of Medicine, University of Minnesota, Division of General Internal Medicine, Minneapolis, Minnesota.
- [3] Division of Cardiology, University of Miami, and Miami VA Healthcare Administration, Miami, Florida.
- [4] Department of Surgery, University of Minnesota, Division of Surgical Oncology, Minneapolis, Minnesota.

- PMID: **34326020**
- PMCID: PMC8238640
- DOI: 10.1016/j.soard.2021.05.029

Abstract

Background: SARS-CoV-2 (COVID-19) disease causes significant morbidity and mortality through increased inflammation and thrombosis. Nonalcoholic fatty liver disease (NAFLD) and nonalcoholic steatohepatitis (NASH) are states of chronic inflammation and indicate advanced metabolic disease.

Objective: The purpose of this observational study was to characterize the risk of hospitalization for COVID-19 in patients with NAFLD/NASH and evaluate the mitigating effect of various metabolic treatments.

Setting: Retrospective analysis of electronic medical record data of 26,896 adults from a 12-hospital Midwest healthcare system with a positive COVID-19 polymerase chain reaction (PCR) test from March 1, 2020, to January 26, 2021.

Methods: Variable selection was guided by the least absolute shrinkage and selection operator (LASSO) method, and multiple imputation was used to account for missing data. Multivariable logistic regression and competing risk models were used to assess the odds of being hospitalized within 45 days of a COVID-19 diagnosis. Analysis assessed the risk of hospitalization among patients with a prescription for metformin and statin use within the 3 months prior to the COVID-19 PCR result, history of home glucagon-like peptide 1 receptor agonist (GLP-1 RA) use, and history of metabolic and bariatric surgery (MBS). Interactions were assessed by sex and race.

Results: A history of NAFLD/NASH was associated with increased odds of admission for COVID-19 (odds ratio [OR], 1.88; 95% confidence interval [CI], 1.57-2.26; $P < .001$) and mortality (OR, 1.96; 95% CI, 1.45-2.67; $P < .001$). Each additional year of having NAFLD/NASH was associated with a significant increased risk of being hospitalized for COVID-19 (OR, 1.24; 95% CI, 1.14-1.35; $P < .001$). NAFLD/NASH increased the risk of hospitalization in men, but not women, and increased the risk of hospitalization in all multiracial/multiethnic subgroups. Medication treatments for metabolic syndrome were associated with significantly reduced risk of admission (OR, .81; 95% CI, .67-.99; $P < .001$ for home metformin use; OR, .71; 95% CI, .65-.83; $P < .001$ for home statin use). MBS was associated with a significant decreased risk of admission (OR, .48; 95% CI, .33-.69; $P < .001$).

Conclusions: NAFLD/NASH is a significant risk factor for hospitalization for COVID-19 and appears to account for risk attributed to obesity. Other significant risks include factors associated with socioeconomic status and other co-morbidities, such as history of venous thromboembolism. Treatments for metabolic disease mitigated risks from NAFLD/NASH. More research is needed to confirm the risk associated with visceral adiposity, and patients should be screened for and informed of treatments for metabolic syndrome.

Keywords: COVID-19; fatty liver disease; metabolic and bariatric surgery.

Copyright © 2021. Published by Elsevier Inc.

- 21 references
- 3 figures

Supplementary info

Publication types, MeSH terms, Grant support Expand

Publication types

- Observational Study

MeSH terms

- Adult
- Bariatric Surgery*
- COVID-19 Testing
- COVID-19*
- Hospitalization
- Humans
- Liver
- Male
- Non-alcoholic Fatty Liver Disease*
- Retrospective Studies
- SARS-CoV-2

Grant support

- K12 HS026379/HS/AHRQ HHS/United States
- KL2 TR002492/TR/NCATS NIH HHS/United States
- UL1 TR002494/TR/NCATS NIH HHS/United States

Full text links

ELSEVIER FULL-TEXT ARTICLE Elsevier Science Free PMC article

Proceed to details

Cite

Share

302

Observational Study

Chest

. 2021 Jan;159(1):196-204.
doi: 10.1016/j.chest.2020.08.2114. Epub 2020 Sep 14.

Critically Ill Adults With Coronavirus Disease 2019 in New Orleans and Care With an Evidence-Based Protocol

David R Janz [1], Scott Mackey [2], Nirav Patel [3], Beau P Saccoccia [4], Michelle St Romain [4], Bethany Busack [4], Hayoung Lee [4], Lana Phan [4], Jordan Vaughn [4], David Feinswog [5], Ryan Chan [5], Lauren Auerbach [4], Nicholas Sausen [4], Joseph Grace [5], Marian Sackey [4], Anushka Das [5], Angellica O Gordon [5], Jennifer Schwehm [3], Robin McGoey [3], Kyle I Happel [6], Stephen P Kantrow [6]

Affiliations Expand

Affiliations

- [1] University Medical Center, New Orleans, LA; Section of Pulmonary/Critical Care & Allergy/Immunology, LSU School of Medicine, New Orleans, LA. Electronic address: David.janz@lcmchealth.org.
- [2] Louisiana Children's Medical Center, New Orleans, LA; Section of Emergency Medicine, LSU School of Medicine, New Orleans, LA.
- [3] University Medical Center, New Orleans, LA.
- [4] Section of Emergency Medicine, LSU School of Medicine, New Orleans, LA.
- [5] LSU School of Medicine, New Orleans, LA.
- [6] Section of Pulmonary/Critical Care & Allergy/Immunology, LSU School of Medicine, New Orleans, LA.

- PMID: **32941862**
- PMCID: PMC7487861
- DOI: 10.1016/j.chest.2020.08.2114

Free PMC article

Observational Study

Critically Ill Adults With Coronavirus Disease 2019 in New Orleans and Care With an Evidence-Based Protocol

David R Janz et al. Chest. 2021 Jan.

Free PMC article

Show details

Chest
. 2021 Jan;159(1):196-204.
doi: 10.1016/j.chest.2020.08.2114. Epub 2020 Sep 14.

Authors

David R Janz [1], Scott Mackey [2], Nirav Patel [3], Beau P Saccoccia [4], Michelle St Romain [4], Bethany Busack [4], Hayoung Lee [4], Lana Phan [4], Jordan Vaughn [4], David Feinswog [5], Ryan Chan [5], Lauren Auerbach [4], Nicholas Sausen [4], Joseph Grace [5], Marian Sackey [4], Anushka Das [5], Angellica O Gordon [5], Jennifer Schwehm [3], Robin McGoey [3], Kyle I Happel [6], Stephen P Kantrow [6]

Affiliations

- [1] University Medical Center, New Orleans, LA; Section of Pulmonary/Critical Care & Allergy/Immunology, LSU School of Medicine, New Orleans, LA. Electronic address: David.janz@lcmchealth.org.
- [2] Louisiana Children's Medical Center, New Orleans, LA; Section of Emergency Medicine, LSU School of Medicine, New Orleans, LA.
- [3] University Medical Center, New Orleans, LA.
- [4] Section of Emergency Medicine, LSU School of Medicine, New Orleans, LA.
- [5] LSU School of Medicine, New Orleans, LA.
- [6] Section of Pulmonary/Critical Care & Allergy/Immunology, LSU School of Medicine, New Orleans, LA.

- PMID: **32941862**
- PMCID: PMC7487861
- DOI: 10.1016/j.chest.2020.08.2114

Abstract

Background: Characteristics of critically ill adults with coronavirus disease 2019 (COVID-19) in an academic safety net hospital and the effect of evidence-based practices in these patients are unknown.

Research question: What are the outcomes of critically ill adults with COVID-19 admitted to a network of hospitals in New Orleans, Louisiana, and what is an evidence-based protocol for care associated with improved outcomes?

Study design and methods: In this multi-center, retrospective, observational cohort study of ICUs in four hospitals in New Orleans, Louisiana, we collected data on adults admitted to an ICU and tested for severe acute respiratory syndrome coronavirus 2 (SARS-CoV-2) between March 9, 2020 and April 14, 2020. The exposure of interest was admission to an ICU that implemented an evidence-based protocol for COVID-19 care. The primary outcome was ventilator-free days.

Results: The initial 147 patients admitted to any ICU and tested positive for SARS-CoV-2 constituted the cohort for this study. In the entire network, exposure to an evidence-based protocol was associated with more ventilator-free days (25 days; 0-28) compared with non-protocolized ICUs (0 days; 0-23, P = .005), including in adjusted analyses (P = .02). Twenty patients (37%)

admitted to protocolized ICUs died compared with 51 (56%; P = .02) in non-protocolized ICUs. Among 82 patients admitted to the academic safety net hospital's ICUs, the median number of ventilator-free days was 22 (interquartile range, 0-27) and mortality rate was 39%.

Interpretation: Care of critically ill COVID-19 patients with an evidence-based protocol is associated with increased time alive and free of invasive mechanical ventilation. In-hospital survival occurred in most critically ill adults with COVID-19 admitted to an academic safety net hospital's ICUs despite a high rate of comorbidities.

Keywords: ARDS; COVID-19; critical care.

Copyright © 2020 American College of Chest Physicians. Published by Elsevier Inc. All rights reserved.

Comment in

- Steady As She Goes: Practicing Evidence-Based Critical Care When the Evidence Is Limited.
  Savel RH, Kupfer Y, Shiloh AL. Savel RH, et al. Chest. 2021 Jan;159(1):7-8. doi: 10.1016/j.chest.2020.09.245. Chest. 2021. PMID: 33422231 Free PMC article. No abstract available.

- 24 references
- 3 figures

Supplementary info

Publication types, MeSH terms, Grant support Expand

Publication types

- Multicenter Study
- Observational Study
- Research Support, N.I.H., Extramural

MeSH terms

- Aged
- COVID-19 / therapy*
- Clinical Protocols
- Cohort Studies
- Critical Care / standards*
- Critical Illness
- Evidence-Based Medicine
- Female
- Hospitalization
- Humans

- Male
- Middle Aged
- New Orleans
- Retrospective Studies

Grant support

- U54 GM104940/GM/NIGMS NIH HHS/United States
- UL1 TR000445/TR/NCATS NIH HHS/United States

Full text links

ELSEVIER FULL-TEXT ARTICLE Elsevier Science Free PMC article
Proceed to details
Cite
Share
303
Observational Study
J Vasc Surg Venous Lymphat Disord
. 2021 Jul;9(4):835-844.e4.
doi: 10.1016/j.jvsv.2020.11.006. Epub 2020 Nov 12.

Venous thrombosis, thromboembolism, biomarkers of inflammation, and coagulation in coronavirus disease 2019

Vikas Thondapu [1], Daniel Montes [2], Rachel Rosovsky [3], Anahita Dua [4], Shaunagh McDermott [5], Michael T Lu [1], Brian Ghoshhajra [1], Udo Hoffmann [1], Marie Denise Gerhard-Herman [6], Sandeep Hedgire [7]
Affiliations Expand

Affiliations

- [1] Cardiovascular Imaging Research Center, Division of Cardiovascular Imaging, Department of Radiology, Massachusetts General Hospital, Harvard Medical School, Boston, Mass.
- [2] Division of Neuroradiology, Department of Radiology, Massachusetts General Hospital, Harvard Medical School, Boston, Mass.
- [3] Division of Hematology, Department of Medicine, Massachusetts General Hospital, Harvard Medical School, Boston, Mass.
- [4] Division of Vascular Surgery, Department of Surgery, Massachusetts General Hospital, Harvard Medical School, Boston, Mass.
- [5] Division of Thoracic Imaging, Department of Radiology, Massachusetts General Hospital, Harvard Medical School, Boston, Mass.

- [6] Division of Cardiovascular Medicine, Department of Medicine, Brigham and Women's Hospital, Harvard Medical School, Boston, Mass.
- [7] Cardiovascular Imaging Research Center, Division of Cardiovascular Imaging, Department of Radiology, Massachusetts General Hospital, Harvard Medical School, Boston, Mass. Electronic address: hedgire.sandeep@mgh.harvard.edu.

- PMID: **33188961**
- PMCID: PMC7657877
- DOI: 10.1016/j.jvsv.2020.11.006

Free PMC article
Observational Study

Venous thrombosis, thromboembolism, biomarkers of inflammation, and coagulation in coronavirus disease 2019

Vikas Thondapu et al. J Vasc Surg Venous Lymphat Disord. 2021 Jul.
Free PMC article
Show details
J Vasc Surg Venous Lymphat Disord
. 2021 Jul;9(4):835-844.e4.
doi: 10.1016/j.jvsv.2020.11.006. Epub 2020 Nov 12.

Authors

Vikas Thondapu [1], Daniel Montes [2], Rachel Rosovsky [3], Anahita Dua [4], Shaunagh McDermott [5], Michael T Lu [1], Brian Ghoshhajra [1], Udo Hoffmann [1], Marie Denise Gerhard-Herman [6], Sandeep Hedgire [7]

Affiliations

- [1] Cardiovascular Imaging Research Center, Division of Cardiovascular Imaging, Department of Radiology, Massachusetts General Hospital, Harvard Medical School, Boston, Mass.
- [2] Division of Neuroradiology, Department of Radiology, Massachusetts General Hospital, Harvard Medical School, Boston, Mass.
- [3] Division of Hematology, Department of Medicine, Massachusetts General Hospital, Harvard Medical School, Boston, Mass.
- [4] Division of Vascular Surgery, Department of Surgery, Massachusetts General Hospital, Harvard Medical School, Boston, Mass.
- [5] Division of Thoracic Imaging, Department of Radiology, Massachusetts General Hospital, Harvard Medical School, Boston, Mass.
- [6] Division of Cardiovascular Medicine, Department of Medicine, Brigham and Women's Hospital, Harvard Medical School, Boston, Mass.

- [7] Cardiovascular Imaging Research Center, Division of Cardiovascular Imaging, Department of Radiology, Massachusetts General Hospital, Harvard Medical School, Boston, Mass. Electronic address: hedgire.sandeep@mgh.harvard.edu.

- PMID: **33188961**
- PMCID: PMC7657877
- DOI: 10.1016/j.jvsv.2020.11.006

Abstract

Objective: Coronavirus disease 2019 (COVID-19) is associated with abnormal inflammatory and coagulation markers, potentially mediating thrombotic events. Our objective was to investigate the incidence, time course, laboratory features, and in-hospital outcomes of COVID-19 patients with suspected venous thromboembolism (VTE).

Methods: A retrospective observational cohort study was conducted of patients hospitalized with COVID-19 who had undergone ultrasound imaging for suspected VTE from March 13 to May 18, 2020. The medical records of the included patients were reviewed for D-dimer, fibrinogen, prothrombin time, partial thromboplastin time, platelet count, C-reactive protein (CRP), and high-sensitivity troponin T at admission and at up to seven time points before and after ultrasound examination. The clinical outcomes included superficial venous thrombosis, deep vein thrombosis, pulmonary embolism, intubation, and death. Mixed effects logistic, linear, and Cox proportional hazards methods were used to evaluate the relationships between the laboratory markers and VTE and other in-hospital outcomes.

Results: Of 138 patients who had undergone imaging studies, 44 (31.9%) had evidence of VTE. On univariable analysis, an elevated admission CRP (odds ratio [OR], 1.05; 95% confidence interval [CI], 1.01-1.09; P = .02; per 10-U increase in CRP), platelet count (OR, 1.48; 95% CI, 1.04-2.12; P = .03; per 1000-U increase in platelet count), and male sex (OR, 2.64; 95% CI, 1.19-5.84; P = .02), were associated with VTE. However only male sex remained significant on multivariable analysis (OR, 2.37; 95% CI, 1.01-5.56; P = .048). The independent predictors of death included older age (hazard ratio [HR], 1.04; 95% CI, 1.00-1.07; P = .04), active malignancy (HR, 4.39; 95% CI, 1.39-13.91; P = .01), elevated admission D-dimer (HR, 1.016; 95% CI, 1.003-1.029; P = .02), and evidence of disseminated intravascular coagulation (HR, 4.81; 95% CI, 1.76-13.10; P = .002).

Conclusions: Male sex, elevated CRP, and elevated platelet count at admission were associated with VTE on univariable analysis. However, only male sex remained significant on multivariable analysis. Older age, active malignancy, disseminated intravascular coagulation, and elevated D-dimer at admission were independently associated with death for patients hospitalized with COVID-19.

Keywords: C-reactive protein; COVID-19; D-dimer; Disseminated intravascular coagulation; Intubation; Venous thromboembolism.

Copyright © 2020 Society for Vascular Surgery. Published by Elsevier Inc. All rights reserved.

- Cited by 8 articles
- 35 references
- 2 figures

Supplementary info

Publication types, MeSH terms, Substances Expand

Publication types

- Multicenter Study
- Observational Study

MeSH terms

- Adult
- Aged
- Biomarkers / blood
- C-Reactive Protein / metabolism
- COVID-19 / complications*
- COVID-19 / therapy
- Female
- Humans
- Inflammation / diagnosis
- Inflammation / etiology
- Length of Stay
- Male
- Middle Aged
- Platelet Count
- Pulmonary Embolism / etiology
- Respiration, Artificial
- Retrospective Studies
- Risk Factors
- SARS-CoV-2
- Sex Factors
- Treatment Outcome
- Venous Thromboembolism / diagnosis
- Venous Thromboembolism / etiology*
- Venous Thrombosis / diagnosis
- Venous Thrombosis / etiology*

Substances

- Biomarkers
- C-Reactive Protein

Full text links

ELSEVIER FULL-TEXT ARTICLE Elsevier Science Free PMC article
Proceed to details
Cite
Share
304
Observational Study
Biomed Res Int
. 2021 Aug 6;2021:5822259.
doi: 10.1155/2021/5822259. eCollection 2021.

Neurological Presentations of COVID-19: Characteristic Features in a Case Series of Hospitalized Patients from Abu Dhabi, UAE

Asma Deeb [1], Palat Chirakkara Kumar [2], Nida Sakrani [3], Ravi Kumar Trehan [4], Vijay Ram Papinenei [5]
Affiliations Expand

Affiliations

- [1] Division of Paediatric Endocrinology, Sheikh Shakhbout Medical City, Abu Dhabi, UAE.
- [2] Division of Neurology, Sheikh Shakhbout Medical City, Abu Dhabi, UAE.
- [3] Division of Paediatric Rheumatology, Sheikh Shakhbout Medical City, Abu Dhabi, UAE.
- [4] Division of Orthopaedic Surgery, Sheikh Shakhbout Medical City, Abu Dhabi, UAE.
- [5] Radiology Department, Sheikh Shakhbout Medical City, Abu Dhabi, UAE.

- PMID: **34423037**
- PMCID: PMC8376468
- DOI: 10.1155/2021/5822259

Free PMC article
Observational Study

Neurological Presentations of COVID-19: Characteristic Features in a Case Series of Hospitalized Patients from Abu Dhabi, UAE

Asma Deeb et al. Biomed Res Int. 2021.
Free PMC article
Show details
Biomed Res Int
. 2021 Aug 6;2021:5822259.
doi: 10.1155/2021/5822259. eCollection 2021.

Authors

Asma Deeb [1], Palat Chirakkara Kumar [2], Nida Sakrani [3], Ravi Kumar Trehan [4], Vijay Ram Papinenei [5]

Affiliations

- [1] Division of Paediatric Endocrinology, Sheikh Shakhbout Medical City, Abu Dhabi, UAE.
- [2] Division of Neurology, Sheikh Shakhbout Medical City, Abu Dhabi, UAE.
- [3] Division of Paediatric Rheumatology, Sheikh Shakhbout Medical City, Abu Dhabi, UAE.
- [4] Division of Orthopaedic Surgery, Sheikh Shakhbout Medical City, Abu Dhabi, UAE.
- [5] Radiology Department, Sheikh Shakhbout Medical City, Abu Dhabi, UAE.

- PMID: **34423037**
- PMCID: PMC8376468
- DOI: 10.1155/2021/5822259

Abstract

Background: COVID-19 patients can present with neurological manifestations in the form of headache, dizziness, hyposmia, myalgia, peripheral neuropathy, acute cerebrovascular disease, and encephalopathy. Neurological involvement could be due to virus-induced brain hypoxia, brain infection, or immune reaction. We aim to describe the neurological presentation of COVID-19 patients and study their neuroimaging findings and disease outcome.

Method: The study is a single-centre, retrospective, observational study in Sheikh Shakhbout Medical City (SSMC), Abu Dhabi, UAE. Patients diagnosed with COVID-19 between March and May 2020 who presented with neuropathological features with or without respiratory manifestations of COVID-19 were enrolled. Electronic records were studied for age, sex, duration of hospitalization, detailed neurological presentation, history or documented concomitant fever and respiratory features of COVID-19, inflammatory markers, neuroimaging, progress, and disease outcome.

Results: Thirty-three patients of 10 nationalities presented with neurological manifestations. Mean (range) age was 51.4 (21-86) years. Twenty-four had comorbidities, and 18 had no prior or concomitant respiratory symptoms. Ten patients presented with encephalopathy and exhibited altered behavior/sensorium: 7 presented with myositis, 8 with stroke, and 4 with seizures, and 4 had peripheral and cranial nerve involvement. The mean (average) duration of hospital stay was 11.4 days (1-38) with the longest observed in stroke patients. Fifteen patients (45%) died and 3 (9%) had residual weakness. Serum ferritin, CRP, and procalcitonin were higher in the severe disease group and correlated with risk of death. Twelve of 22 brain images showed abnormalities including haemorrhage, infarcts, small vessel ischemia, and oedema. Risk of death was higher in older age but did not differ based on the underlying neuropathology.

Conclusion: COVID-19 patients who present with neurological involvement have a higher risk of mortality which is aggravated by older age and higher inflammatory markers. The type of neurological pathology does not seem to influence the risk of mortality.

Copyright © 2021 Asma Deeb et al.

Conflict of interest statement

None of the authors have any conflict of interest to declare.

- 30 references
- 5 figures

Supplementary info

Publication types, MeSH terms, Substances Expand

Publication types

- Observational Study

MeSH terms

- Adult
- Aged
- Aged, 80 and over
- Biomarkers / blood
- Brain / diagnostic imaging
- COVID-19 / complications*
- COVID-19 / diagnosis*
- COVID-19 / epidemiology
- Female
- Hospitalization
- Humans
- Inflammation Mediators / blood
- Male
- Middle Aged
- Nervous System Diseases / diagnosis
- Nervous System Diseases / diagnostic imaging
- Nervous System Diseases / etiology*
- Neuroimaging
- Pandemics
- Prognosis
- Respiratory Therapy
- Retrospective Studies
- SARS-CoV-2* / pathogenicity
- United Arab Emirates / epidemiology
- Young Adult

Substances

- Biomarkers
- Inflammation Mediators

Full text links

Hindawi Limited Free PMC article
Proceed to details
Cite
Share
305
Observational Study
Rev Esp Salud Publica
. 2022 Feb 23;96:e202202022.

[Impact of covid-19 vaccination on hospitalization in a third-level hospital]

[Article in Spanish]
Ana Mª Haro Pérez [1, 2], Vega E Benito López [1, 2], Mar Jiménez Rodríguez [1], Irene Ai-Ling García Yu [1], Saray Martín Monteagudo [1]
Affiliations Expand

Affiliations

- [1] Servicio de Medicina Preventiva. Complejo Asistencial Universitario de Salamanca. Gerencia Regional de Salud de Castilla y León (SACYL). Salamanca. España.
- [2] Departamento de Ciencias Biomédicas. Facultad de Medicina. Universidad de Salamanca. Salamanca. España.

- PMID: **35194012**

Free article
Observational Study

[Impact of covid-19 vaccination on hospitalization in a third-level hospital]

[Article in Spanish]
Ana Mª Haro Pérez et al. Rev Esp Salud Publica. 2022.
Free article
Show details
Rev Esp Salud Publica

. 2022 Feb 23;96:e202202022.

Authors

Ana Mª Haro Pérez [1,2], Vega E Benito López [1,2], Mar Jiménez Rodríguez [1], Irene Ai-Ling García Yu [1], Saray Martín Monteagudo [1]

Affiliations

- [1] Servicio de Medicina Preventiva. Complejo Asistencial Universitario de Salamanca. Gerencia Regional de Salud de Castilla y León (SACYL). Salamanca. España.
- [2] Departamento de Ciencias Biomédicas. Facultad de Medicina. Universidad de Salamanca. Salamanca. España.

- PMID: **35194012**

Abstract
in English, Spanish

Objective: This study presents information on the evolution of severe cases of SARS-CoV-2 infection that required hospitalization since the beginning of vaccination in Spain. The objective was to know the impact of vaccination against COVID-19 on the hospitalization of patients with SARS-CoV-2 infection, hospital mortality and readmissions for this cause, and to describe the characteristics of vaccinated patients who required admission.

Methods: A retrospective, observational epidemiological study was conducted of all patients admitted with SARS-CoV-2 infection confirmed by a diagnostic test for active infection (PDIA) in a tertiary hospital, from January 2021 to June 2021. The incidence of admissions was calculated based on the vaccination status of the patients and age groups at different times according to the progress of the strategy of vaccination COVID-19.

Results: Between December 27, 2020 and June 30, 2021, 1,308 patients with positive PDIA were admitted to the University Hospital of Salamanca, of which 1,167 (89.2%) were not vaccinated, 129 (9.9%) had received one dose of vaccine and 12 (0.9%) were fully vaccinated. Of the latter, none were admitted to the ICU and 2 died.

Conclusions: Vaccination against COVID-19 has contributed to the decrease in hospitalizations, since February 2021, of older and institutionalized people. Fully vaccinated people have a lower risk of admission to the ICU and death. These data, together with the information available on recent cases of new SARS-CoV-2 infections in unvaccinated young people, are in favor of achieving high vaccination coverage of the entire population in the shortest possible time.

Objetivo: Este estudio presenta información sobre la evolución de los casos graves de infección SARS-CoV-2 que requirieron hospitalización desde el inicio de la vacunación en España. El objetivo fue conocer el impacto de la vacunación frente a COVID-19 sobre la hospitalización de pacientes con infección SARS-CoV-2, la mortalidad intrahospitalaria y los reingresos por esta causa, y describir las características de los pacientes vacunados que precisaron ingreso.

Metodos: Se realizó un estudio epidemiológico observacional retrospectivo, de todos los pacientes ingresados con infección por SARS-CoV-2 confirmada mediante una prueba de diagnóstico de infección activa (PDIA) en un hospital de tercer nivel, de enero de 2021 a junio de

2021. Se calculó la incidencia de ingresos en función del estado vacunal de los pacientes y grupos de edad en diferentes momentos según el avance de la campaña de vacunación.

Resultados: Entre el 27 de diciembre de 2020 y el 30 de junio de 2021 ingresaron en el Hospital Universitario de Salamanca 1.308 pacientes con PDIA positiva, de los cuales 1.167 (89,2%) no estaban vacunados, 129 (9,9%) habían recibido una dosis de vacuna y 12 (0,9%) estaban completamente vacunados. De estos últimos, ninguno ingresó en UCI y 2 fallecieron.

Conclusiones: La vacunación frente a COVID-19 ha contribuido al descenso de las hospitalizaciones desde el mes de febrero de 2021, sobre todo en personas mayores e institucionalizadas. Las personas completamente vacunadas parecen tener menor riesgo de ingreso en UCI y fallecimiento. Estos datos, junto con la información disponible de los casos recientes de nuevas infecciones por SARS-CoV-2 en personas jóvenes no vacunadas, están a favor de conseguir una cobertura vacunal elevada de toda la población en el menor tiempo posible.

Keywords: COVID-19; Hospitalization; SARS-CoV-2 infection; Severity; Spain; Vaccination.

Supplementary info

Publication types, MeSH terms, Substances Expand

Publication types

- Observational Study

MeSH terms

- Adolescent
- COVID-19 Vaccines*
- COVID-19*
- Hospitalization
- Humans
- Retrospective Studies
- SARS-CoV-2
- Spain / epidemiology
- Tertiary Care Centers
- Vaccination

Substances

- COVID-19 Vaccines

Full text links

Revista Española de Salud Pública FULL TEXT article Revista Espanola de Salud Publica

Proceed to details

Cite
Share
☐ 306

Observational Study
J Gastroenterol Hepatol
. 2021 Nov;36(11):3050-3055.
doi: 10.1111/jgh.15591. Epub 2021 Jul 5.

Lower incidence of COVID-19 in patients with inflammatory bowel disease treated with non-gut selective biologic therapy

Sandro Ardizzone [1], Francesca Ferretti [1], Maria Camilla Monico [1], Anna Maria Carvalhas Gabrielli [1], Stefania Carmagnola [1], Cristina Bezzio [2], Simone Saibeni [2], Matteo Bosani [3], Flavio Caprioli [4] [5], Stefano Mazza [4], Valentina Casini [6], Claudio Camillo Cortelezzi [7], Marco Parravicini [7], Andrea Cassinotti [7], Paola Cosimo [8], Amedeo Indriolo [8], Antonio Di Sabatino [9], Marco Vincenzo Lenti [9], Luca Pastorelli [10], Francesco Conforti [10], Chiara Ricci [11], Piercarlo Sarzi-Puttini [12], Maurizio Vecchi [4] [5], Giovanni Maconi [1]
Affiliations Expand

Affiliations

- [1] Gastroenterology Unit, ASST Fatebenefratelli-Sacco, L. Sacco University Hospital, Department of Biochemical and Clinical Sciences, University of Milan, Milan, Italy.
- [2] Gastroenterology Unit, ASST Rhodense, Rho Hospital, Rho, Italy.
- [3] ASST Ovest Milanese, Legnano Hospital, Legnano, Italy.
- [4] Gastroenterology and Endoscopy Unit, IRCCS Ca' Granda Ospedale Maggiore Policlinico Foundation, Milan, Italy.
- [5] Department of Pathophysiology and Transplantation, University of Milan, Milan, Italy.
- [6] UOC Gastroenterology and Digestive Endoscopy, ASST Bergamo Est, Seriate, Bergamo, Italy.
- [7] ASST Sette Laghi, Gastroenterology and Endoscopy Unit, Circolo Hospital and Macchi Foundation, Varese, Italy.
- [8] Gastroenterology and Endoscopy Unit, Papa Giovanni XXIII Hospital, Bergamo, Italy.
- [9] Department of Internal Medicine, IRCCS San Matteo Hospital Foundation, University of Pavia, Pavia, Italy.
- [10] Gastroenterology Unit, IRCCS Policlinico San Donato Research Hospital, Milan, Italy.
- [11] Gastroenterology Unit, Spedali Civili Hospital, Department of Experimental and Clinical Sciences, University of Brescia, Brescia, Italy.
- [12] Rheumatology Unit, ASST-Fatebenefratelli L. Sacco University Hospital, University of Milan, Milan, Italy.

- PMID: **34159648**
- PMCID: PMC8447454
- DOI: 10.1111/jgh.15591

Free PMC article
Observational Study

Lower incidence of COVID-19 in patients with inflammatory bowel disease treated with non-gut selective biologic therapy

Sandro Ardizzone et al. J Gastroenterol Hepatol. 2021 Nov.
Free PMC article
Show details
J Gastroenterol Hepatol
. 2021 Nov;36(11):3050-3055.
doi: 10.1111/jgh.15591. Epub 2021 Jul 5.

Authors

Sandro Ardizzone [1], Francesca Ferretti [1], Maria Camilla Monico [1], Anna Maria Carvalhas Gabrielli [1], Stefania Carmagnola [1], Cristina Bezzio [2], Simone Saibeni [2], Matteo Bosani [3], Flavio Caprioli [4] [5], Stefano Mazza [4], Valentina Casini [6], Claudio Camillo Cortelezzi [7], Marco Parravicini [7], Andrea Cassinotti [7], Paola Cosimo [8], Amedeo Indriolo [8], Antonio Di Sabatino [9], Marco Vincenzo Lenti [9], Luca Pastorelli [10], Francesco Conforti [10], Chiara Ricci [11], Piercarlo Sarzi-Puttini [12], Maurizio Vecchi [4] [5], Giovanni Maconi [1]

Affiliations

- [1] Gastroenterology Unit, ASST Fatebenefratelli-Sacco, L. Sacco University Hospital, Department of Biochemical and Clinical Sciences, University of Milan, Milan, Italy.
- [2] Gastroenterology Unit, ASST Rhodense, Rho Hospital, Rho, Italy.
- [3] ASST Ovest Milanese, Legnano Hospital, Legnano, Italy.
- [4] Gastroenterology and Endoscopy Unit, IRCCS Ca' Granda Ospedale Maggiore Policlinico Foundation, Milan, Italy.
- [5] Department of Pathophysiology and Transplantation, University of Milan, Milan, Italy.
- [6] UOC Gastroenterology and Digestive Endoscopy, ASST Bergamo Est, Seriate, Bergamo, Italy.
- [7] ASST Sette Laghi, Gastroenterology and Endoscopy Unit, Circolo Hospital and Macchi Foundation, Varese, Italy.
- [8] Gastroenterology and Endoscopy Unit, Papa Giovanni XXIII Hospital, Bergamo, Italy.
- [9] Department of Internal Medicine, IRCCS San Matteo Hospital Foundation, University of Pavia, Pavia, Italy.
- [10] Gastroenterology Unit, IRCCS Policlinico San Donato Research Hospital, Milan, Italy.
- [11] Gastroenterology Unit, Spedali Civili Hospital, Department of Experimental and Clinical Sciences, University of Brescia, Brescia, Italy.
- [12] Rheumatology Unit, ASST-Fatebenefratelli L. Sacco University Hospital, University of Milan, Milan, Italy.

- PMID: **34159648**

- PMCID: PMC8447454
- DOI: 10.1111/jgh.15591

Abstract

Background and aim: Since the outbreak of COVID-19, concerns have been raised as to whether inflammatory bowel disease (IBD) patients under biologic therapy may be more susceptible to the disease. This study aimed to determine the incidence and outcomes of COVID-19 in a large cohort of IBD patients on biologic therapy.

Methods: This observational retrospective multicenter study collected data about COVID-19 in IBD patients on biologic therapy in Italy, between February and May 2020. The main end-points were (i) to assess both the cumulative incidence and clinical outcome of COVID-19, according to different biologic agents and (ii) to compare them with the general population and a cohort IBD patients undergoing non-biologic therapies.

Results: Among 1816 IBD patients, the cumulative incidence of COVID-19 was 3.9 per 1000 (7/1816) with a 57% hospitalization rate and a 29% case-fatality rate. The class of biologic agents was the only risk factor of developing COVID-19 ($P = 0.01$). Non-gut selective agents were associated with a lower incidence of COVID-19 cases, related symptoms, and hospitalization ($P < 0.05$). Compared with the general population of Lombardy, an overall lower incidence of COVID-19 was observed (3.9 vs 8.5 per 1000, $P = 0.03$). Compared with 565 IBD patients on non-biologic therapies, a lower rate of COVID-19 symptoms was observed in our cohort (7.5% vs 18%, $P < 0.001$).

Conclusions: Compared with the general population, IBD patients on biologic therapy are not exposed to a higher risk of COVID-19. Non-gut selective agents are associated with a lower incidence of symptomatic disease, supporting the decision of maintaining the ongoing treatment.

Keywords: Biologic therapy; COVID-19; Inflammatory bowel disease; SARS-CoV-2.

© 2021 Journal of Gastroenterology and Hepatology Foundation and John Wiley & Sons Australia, Ltd.

- Cited by 4 articles
- 29 references

Supplementary info

Publication types, MeSH terms, Substances Expand

Publication types

- Multicenter Study
- Observational Study

MeSH terms

- Adolescent
- Adult

- Aged
- Biological Factors / administration & dosage*
- Biological Therapy / adverse effects*
- COVID-19 / epidemiology*
- Child
- Child, Preschool
- Colitis
- Female
- Humans
- Incidence
- Infant
- Infant, Newborn
- Inflammatory Bowel Diseases / drug therapy*
- Inflammatory Bowel Diseases / epidemiology
- Male
- Middle Aged
- Retrospective Studies
- SARS-CoV-2
- Young Adult

Substances

- Biological Factors

Full text links

WILEY Full Text Article Wiley Free PMC article
Proceed to details
Cite
Share
☐ 307
Observational Study
Ann R Coll Surg Engl
. 2021 Jul;103(7):524-529.
doi: 10.1308/rcsann.2021.0053.

Safety and feasibility of resuming bariatric surgery under the cloud of COVID-19

R Mamidanna [1], A Askari [1], K Patel [1], M T Adil [1], V Jain [1], P Jambulingam [1], D Whitelaw [1], F Rashid [1], A Munasinghe [1], O Al-Taan [1]
Affiliations Expand

Affiliation

- [1] Bedfordshire Hospitals NHS Foundation Trust, UK.

- PMID: **34192498**
- DOI: 10.1308/rcsann.2021.0053

Observational Study

Safety and feasibility of resuming bariatric surgery under the cloud of COVID-19

R Mamidanna et al. Ann R Coll Surg Engl. 2021 Jul.

Show details

Ann R Coll Surg Engl

. 2021 Jul;103(7):524-529.
doi: 10.1308/rcsann.2021.0053.

Authors

R Mamidanna [1], A Askari [1], K Patel [1], M T Adil [1], V Jain [1], P Jambulingam [1], D Whitelaw [1], F Rashid [1], A Munasinghe [1], O Al-Taan [1]

Affiliation

- [1] Bedfordshire Hospitals NHS Foundation Trust, UK.

- PMID: **34192498**
- DOI: 10.1308/rcsann.2021.0053

Abstract

Introduction: Because of the COVID-19 pandemic, numerous bariatric surgical units globally have halted weight loss surgery. Obesity itself has been shown to be a predictor of poor outcome in people infected with the virus. The aim of this study was to report our experience as a high-volume bariatric institution resuming elective weight loss surgery safely amidst emergency admissions of COVID-19-positive patients.

Methods: A standard operating procedure based on national guidance and altered to accommodate local considerations was initiated across the hospital. Data were collected prospectively for 50 consecutive patients undergoing bariatric surgery following recommencement of elective surgery after the first national lockdown in the UK.

Results: Between 28 June and 5 August 2020, a total of 50 patients underwent bariatric surgery of whom 94% were female. Median age was 41 years and median body mass index was 43.8 (interquartile range 40.0-48.8)kg/m^2. Half of the patients (n = 25/50) underwent laparoscopic sleeve gastrectomy and half underwent Roux-en-Y gastric bypass (RYGB). Of these 50 patients, 9 (18%) had revisional bariatric surgery. Overall median length of hospital stay was 1 day, with

96% of the study population being discharged within 24h of surgery. The overall rate of readmission was 6% and one patient (2%) returned to theatre with an obstruction proximal to jejuno-jejunal anastomosis. None of the patients exhibited symptoms or tested positive for COVID-19.

Conclusion: With appropriately implemented measures and precautions, resumption of bariatric surgery during the COVID-19 pandemic appears feasible and safe with no increased risk to patients.

Keywords: Bariatric; Bypass; Metabolic; Obesity; Pandemic; Surgery.

Supplementary info

Publication types, MeSH terms Expand

Publication types

- Observational Study

MeSH terms

- Adult
- Bariatric Surgery / adverse effects*
- Bariatric Surgery / standards
- Bariatric Surgery / statistics & numerical data
- COVID-19 / diagnosis
- COVID-19 / epidemiology
- COVID-19 / prevention & control*
- COVID-19 / transmission
- COVID-19 Testing / standards
- COVID-19 Testing / statistics & numerical data
- Clinical Protocols / standards
- Communicable Disease Control / organization & administration
- Communicable Disease Control / standards
- Elective Surgical Procedures / adverse effects*
- Elective Surgical Procedures / standards
- Elective Surgical Procedures / statistics & numerical data
- Enhanced Recovery After Surgery / standards
- Feasibility Studies
- Female
- Humans
- Length of Stay / statistics & numerical data
- Male
- Middle Aged

- Obesity, Morbid / complications
- Obesity, Morbid / surgery*
- Pandemics / prevention & control
- Patient Readmission / statistics & numerical data
- Postoperative Complications / epidemiology*
- Postoperative Complications / etiology
- Prospective Studies
- Retrospective Studies
- Risk Assessment / statistics & numerical data
- SARS-CoV-2 / isolation & purification
- Surgery Department, Hospital / organization & administration
- Surgery Department, Hospital / standards
- Surgery Department, Hospital / statistics & numerical data

Full text links

annals FULL TEXT Atypon
Proceed to details
Cite
Share
308
Observational Study
Am J Epidemiol
. 2022 Jan 1;191(1):137-146.
doi: 10.1093/aje/kwab252.

Pressure on the Health-Care System and Intensive Care Utilization During the COVID-19 Outbreak in the Lombardy Region of Italy: A Retrospective Observational Study in 43,538 Hospitalized Patients

Filippo Trentini, Valentina Marziano, Giorgio Guzzetta, Marcello Tirani, Danilo Cereda, Piero Poletti, Raffaella Piccarreta, Antonio Barone, Giuseppe Preziosi, Fabio Arduini, Petra Giulia Della Valle, Alberto Zanella, Francesca Grosso, Gabriele Del Castillo, Ambra Castrofino, Giacomo Grasselli, Alessia Melegaro, Alessandra Piatti, Aida Andreassi, Maria Gramegna, Marco Ajelli, Stefano Merler

- PMID: **34652416**
- PMCID: PMC8549288

- DOI: 10.1093/aje/kwab252

Free PMC article
Observational Study

Pressure on the Health-Care System and Intensive Care Utilization During the COVID-19 Outbreak in the Lombardy Region of Italy: A Retrospective Observational Study in 43,538 Hospitalized Patients

Filippo Trentini et al. Am J Epidemiol. 2022.
Free PMC article
Show details
Am J Epidemiol
. 2022 Jan 1;191(1):137-146.
doi: 10.1093/aje/kwab252.

Authors

Filippo Trentini, Valentina Marziano, Giorgio Guzzetta, Marcello Tirani, Danilo Cereda, Piero Poletti, Raffaella Piccarreta, Antonio Barone, Giuseppe Preziosi, Fabio Arduini, Petra Giulia Della Valle, Alberto Zanella, Francesca Grosso, Gabriele Del Castillo, Ambra Castrofino, Giacomo Grasselli, Alessia Melegaro, Alessandra Piatti, Aida Andreassi, Maria Gramegna, Marco Ajelli, Stefano Merler

- PMID: **34652416**
- PMCID: PMC8549288
- DOI: 10.1093/aje/kwab252

Abstract

During the spring of 2020, the coronavirus disease 2019 (COVID-19) epidemic caused an unprecedented demand for intensive-care resources in the Lombardy region of Italy. Using data on 43,538 hospitalized patients admitted between February 21 and July 12, 2020, we evaluated variations in intensive care unit (ICU) admissions and mortality over the course of 3 periods: the early phase of the pandemic (February 21-March 13), the period of highest pressure on the health-care system (March 14-April 25, when numbers of COVID-19 patients exceeded prepandemic ICU bed capacity), and the declining phase (April 26-July 12). Compared with the early phase, patients aged 70 years or more were less often admitted to an ICU during the period of highest pressure on the health-care system (odds ratio (OR) = 0.47, 95% confidence interval (CI): 0.41, 0.54), with longer ICU delays (incidence rate ratio = 1.82, 95% CI: 1.52, 2.18) and lower chances of dying in the ICU (OR = 0.47, 95% CI: 0.34, 0.64). Patients under 56 years of age had more limited changes in the probability of (OR = 0.65, 95% CI: 0.56, 0.76) and delay to (incidence rate

ratio = 1.16, 95% CI: 0.95, 1.42) ICU admission and increased mortality (OR = 1.43, 95% CI: 1.00, 2.07). In the declining phase, all quantities decreased for all age groups. These patterns may suggest that limited health-care resources during the peak phase of the epidemic in Lombardy forced a shift in ICU admission criteria to prioritize patients with higher chances of survival.

Keywords: COVID-19; COVID-19 hospitalization; coronavirus disease 2019; health-care system; hospital admission; intensive care; intensive care unit admission; mortality.

© The Author(s) 2021. Published by Oxford University Press on behalf of the Johns Hopkins Bloomberg School of Public Health. All rights reserved. For permissions, please e-mail: journals.permissions@oup.com.

- Cited by 4 articles

Supplementary info

Publication types, MeSH terms Expand

Publication types

- Observational Study
- Research Support, Non-U.S. Gov't

MeSH terms

- Adult
- Age Factors
- Aged
- Aged, 80 and over
- COVID-19 / epidemiology*
- COVID-19 / mortality
- COVID-19 / therapy*
- Comorbidity
- Delivery of Health Care / statistics & numerical data*
- Humans
- Intensive Care Units / statistics & numerical data*
- Italy / epidemiology
- Middle Aged
- Pandemics
- Retrospective Studies
- Risk Factors
- SARS-CoV-2
- Sex Factors
- Time Factors

Full text links

OXFORD ACADEMIC Silverchair Information Systems Free PMC article
Proceed to details
Cite
Share
309
Observational Study
Nefrologia (Engl Ed)
. May-Jun 2021;41(3):329-336.
doi: 10.1016/j.nefro.2020.09.002. Epub 2020 Nov 5.

COVID-19 incidence and outcomes in a home dialysis unit in Madrid (Spain) at the height of the pandemic

[Article in English, Spanish]
María Maldonado [1], Marta Ossorio [2], Gloria Del Peso [3], Carlos Santos [4], Laura Álvarez [2], Rafael Sánchez-Villanueva [2], Begoña Rivas [5], Cristina Vega [5], Rafael Selgas [6], María A Bajo [6]
Affiliations Expand

Affiliations

- [1] Departamento de Nefrología, Hospital Universitario La Paz, Madrid, España. Electronic address: maria.maldonado@salud.madrid.org.
- [2] Departamento de Nefrología, Hospital Universitario La Paz, Madrid, España; Instituto de Investigación La Paz (IdiPAZ), Madrid, España.
- [3] Departamento de Nefrología, Hospital Universitario La Paz, Madrid, España; Instituto de Investigación La Paz (IdiPAZ), Madrid, España; Red de Investigación Renal (REDINREN), Instituto de Salud Carlos III, Madrid, España.
- [4] Departamento de Nefrología, Hospital Universitario La Paz, Madrid, España.
- [5] Departamento de Nefrología, Hospital Universitario La Paz, Madrid, España; Instituto de Investigación La Paz (IdiPAZ), Madrid, España; Departamento de Nefrología, Universidad Autónoma de Madrid, Madrid, España.
- [6] Departamento de Nefrología, Hospital Universitario La Paz, Madrid, España; Instituto de Investigación La Paz (IdiPAZ), Madrid, España; Red de Investigación Renal (REDINREN), Instituto de Salud Carlos III, Madrid, España; Departamento de Nefrología, Universidad Autónoma de Madrid, Madrid, España.

- PMID: **33248799**
- PMCID: PMC8373627
- DOI: 10.1016/j.nefro.2020.09.002

Free PMC article
Observational Study

COVID-19 incidence and outcomes in a home dialysis unit in Madrid (Spain) at the height of the pandemic

[Article in English, Spanish]
María Maldonado et al. Nefrologia (Engl Ed). May-Jun 2021.
Free PMC article
Show details
Nefrologia (Engl Ed)
. May-Jun 2021;41(3):329-336.
doi: 10.1016/j.nefro.2020.09.002. Epub 2020 Nov 5.

Authors

María Maldonado [1], Marta Ossorio [2], Gloria Del Peso [3], Carlos Santos [4], Laura Álvarez [2], Rafael Sánchez-Villanueva [2], Begoña Rivas [5], Cristina Vega [5], Rafael Selgas [6], María A Bajo [6]

Affiliations

- [1] Departamento de Nefrología, Hospital Universitario La Paz, Madrid, España. Electronic address: maria.maldonado@salud.madrid.org.
- [2] Departamento de Nefrología, Hospital Universitario La Paz, Madrid, España; Instituto de Investigación La Paz (IdiPAZ), Madrid, España.
- [3] Departamento de Nefrología, Hospital Universitario La Paz, Madrid, España; Instituto de Investigación La Paz (IdiPAZ), Madrid, España; Red de Investigación Renal (REDINREN), Instituto de Salud Carlos III, Madrid, España.
- [4] Departamento de Nefrología, Hospital Universitario La Paz, Madrid, España.
- [5] Departamento de Nefrología, Hospital Universitario La Paz, Madrid, España; Instituto de Investigación La Paz (IdiPAZ), Madrid, España; Departamento de Nefrología, Universidad Autónoma de Madrid, Madrid, España.
- [6] Departamento de Nefrología, Hospital Universitario La Paz, Madrid, España; Instituto de Investigación La Paz (IdiPAZ), Madrid, España; Red de Investigación Renal (REDINREN), Instituto de Salud Carlos III, Madrid, España; Departamento de Nefrología, Universidad Autónoma de Madrid, Madrid, España.

- PMID: **33248799**
- PMCID: PMC8373627
- DOI: 10.1016/j.nefro.2020.09.002

Abstract
in English, Spanish

Introduction: The 2019 coronavirus (COVID-19) is a viral infection caused by a new coronavirus that is affecting the entire world. There have been studies of patients on in-center hemodialysis,

but home dialysis population data are scarce. Our objective is to study the incidence and course of COVID-19 in a home dialysis unit (HDU) at the height of the pandemic.

Methods: An observational, retrospective study enrolling all patients diagnosed with COVID-19 from the HDU of Hospital Universitario La Paz (La Paz University Hospital) (Madrid, Spain) between March 10 and May 15, 2020. We collected clinical data from the HDU (57 patients on peritoneal dialysis and 22 patients on home hemodialysis) and compared the clinical characteristics and course of patients with and without COVID-19 infection.

Results: Twelve patients were diagnosed with COVID-19 (9 peritoneal dialysis; 3 home hemodialysis). There were no statistically significant differences in terms of clinical characteristics between patients with COVID-19 and the rest of the unit. The mean age was 62±18.5 years; most were men (75%). All patients but one required hospitalization. Ten patients (83%) were discharged following a mean of 16.4±9.7 days of hospitalization. Two patients were diagnosed while hospitalized for other conditions, and these were the only patients who died. Those who died were older than those who survived.

Conclusion: The incidence of COVID-19 in our HDU in Madrid at the height of the pandemic was high, especially in patients on peritoneal dialysis. No potential benefit for preventing the infection in patients on home dialysis was observed. Advanced age and nosocomial transmission were the main factors linked to a worse prognosis.

Introducción: La enfermedad por coronavirus 2019 (COVID-19) es una infección viral causada por un nuevo coronavirus que está afectando a todo el mundo. Hay estudios previos de pacientes en hemodiálisis en centro (HD), pero hay pocos datos sobre población en diálisis domiciliaria. Nuestro objetivo es estudiar la incidencia y evolución de COVID-19 en una unidad de diálisis domiciliaria (UDD) durante el pico de la pandemia.

Métodos: estudio observacional y retrospectivo que incluye todos los pacientes diagnosticados de COVID-19 de la UDD del Hospital Universitario La Paz (Madrid, España) entre el 10 de marzo y el 15 de mayo de 2020. Se recogieron los datos clínicos de la UDD (57 pacientes en diálisis peritoneal [PD] y 22 pacientes en hemodiálisis domiciliaria [HDD]) y comparamos las características clínicas y la evolución de los pacientes con o sin infección por COVID-19.

Resultados: doce pacientes fueron diagnosticados de COVID-19 (9 DP, 3 HDD). No hubo diferencias estadísticamente significativas entre las características clínicas de los pacientes con COVID-19 y el resto de la unidad. La edad media fue 62 ± 18.5 años; la mayoría eran varones (75%). Todos los pacientes menos uno necesitaron hospitalización. Diez pacientes (83%) fueron dados de alta tras una media de 16,4 ± 9,7 días de hospitalización. Dos pacientes fueron diagnosticados durante su hospitalización por otro motivo y fueron los únicos que fallecieron. Los fallecidos eran de mayor edad que los supervivientes.

Conclusión: La incidencia de COVID-19 en nuestra UDD en Madrid durante el pico de la pandemia fue alto, especialmente en los pacientes en DP, sin observarse un potencial beneficio para prevenir la infección en los pacientes en diálisis domiciliaria. La edad avanzada y la transmisión nosocomial fueron los principales factores relacionados con peor pronóstico.

Keywords: COVID-19; Diálisis domiciliaria; Diálisis peritoneal; España; Hemodiálisis domiciliaria; Home dialysis; Home hemodialysis; Peritoneal dialysis; Spain.

Copyright © 2020 Sociedad Española de Nefrología. Published by Elsevier España, S.L.U. All rights reserved.

- Cited by 2 articles

- 25 references

Supplementary info

Publication types, MeSH terms Expand

Publication types

- Observational Study

MeSH terms

- Adult
- Aged
- Aged, 80 and over
- COVID-19 / epidemiology*
- COVID-19 / mortality
- Female
- Hemodialysis, Home / statistics & numerical data*
- Hospitalization / statistics & numerical data
- Humans
- Incidence
- Male
- Middle Aged
- Pandemics*
- Peritoneal Dialysis / mortality
- Peritoneal Dialysis / statistics & numerical data*
- Retrospective Studies
- Spain / epidemiology

Full text links

FREE Fulltext at nefrologia Ediciones Doyma, S.L. Free PMC article

Proceed to details

Cite

Share

310

Observational Study

PLoS One

. 2021 Aug 5;16(8):e0255427.
doi: 10.1371/journal.pone.0255427. eCollection 2021.

6-month mortality and readmissions of hospitalized COVID-19 patients: A nationwide cohort study of 8,679 patients in Germany

Christian Günster [1], Reinhard Busse [2], Melissa Spoden [1], Tanja Rombey [2], Gerhard Schillinger [1], Wolfgang Hoffmann [3], Steffen Weber-Carstens [4], Andreas Schuppert [5], Christian Karagiannidis [6]

Affiliations Expand

Affiliations

- [1] Research Institute of the Local Health Care Funds, Federal Association of the Local Health Care Funds, Berlin, Germany.
- [2] Department of Health Care Management, Technische Universität Berlin, Berlin, Germany.
- [3] Section Epidemiology of Health Care and Community Health, Institute for Community Medicine, University Medicine Greifswald, Greifswald, Germany.
- [4] Department of Anesthesiology and Operative Intensive Care Medicine, Charité - Universitätsmedizin Berlin, Berlin, Germany.
- [5] Institute for Computational Biomedicine II, University Hospital RWTH Aachen University, Aachen, Germany.
- [6] Department of Pneumology and Critical Care Medicine, Cologne-Merheim Hospital, Kliniken der Stadt Köln, Witten/Herdecke University Hospital, Cologne, Germany.

- PMID: **34351975**
- PMCID: PMC8341502
- DOI: 10.1371/journal.pone.0255427

Free PMC article
Observational Study

6-month mortality and readmissions of hospitalized COVID-19 patients: A nationwide cohort study of 8,679 patients in Germany

Christian Günster et al. PLoS One. 2021.
Free PMC article
Show details
PLoS One

. 2021 Aug 5;16(8):e0255427.
doi: 10.1371/journal.pone.0255427. eCollection 2021.

Authors

Christian Günster [1], Reinhard Busse [2], Melissa Spoden [1], Tanja Rombey [2], Gerhard Schillinger [1], Wolfgang Hoffmann [3], Steffen Weber-Carstens [4], Andreas Schuppert [5], Christian Karagiannidis [6]

Affiliations

- [1] Research Institute of the Local Health Care Funds, Federal Association of the Local Health Care Funds, Berlin, Germany.
- [2] Department of Health Care Management, Technische Universität Berlin, Berlin, Germany.
- [3] Section Epidemiology of Health Care and Community Health, Institute for Community Medicine, University Medicine Greifswald, Greifswald, Germany.
- [4] Department of Anesthesiology and Operative Intensive Care Medicine, Charité - Universitätsmedizin Berlin, Berlin, Germany.
- [5] Institute for Computational Biomedicine II, University Hospital RWTH Aachen University, Aachen, Germany.
- [6] Department of Pneumology and Critical Care Medicine, Cologne-Merheim Hospital, Kliniken der Stadt Köln, Witten/Herdecke University Hospital, Cologne, Germany.

- PMID: **34351975**
- PMCID: PMC8341502
- DOI: 10.1371/journal.pone.0255427

Abstract

Background: COVID-19 frequently necessitates in-patient treatment and in-patient mortality is high. Less is known about the long-term outcomes in terms of mortality and readmissions following in-patient treatment.

Aim: The aim of this paper is to provide a detailed account of hospitalized COVID-19 patients up to 180 days after their initial hospital admission.

Methods: An observational study with claims data from the German Local Health Care Funds of adult patients hospitalized in Germany between February 1 and April 30, 2020, with PCR-confirmed COVID-19 and a related principal diagnosis, for whom 6-month all-cause mortality and readmission rates for 180 days after admission or until death were available. A multivariable logistic regression model identified independent risk factors for 180-day all-cause mortality in this cohort.

Results: Of the 8,679 patients with a median age of 72 years, 2,161 (24.9%) died during the index hospitalization. The 30-day all-cause mortality rate was 23.9% (2,073/8,679), the 90-day rate was 27.9% (2,425/8,679), and the 180-day rate, 29.6% (2,566/8,679). The latter was 52.3% (1,472/2,817) for patients aged ≥80 years 23.6% (1,621/6,865) if not ventilated during index hospitalization, but 53.0% in case of those ventilated invasively (853/1,608). Risk factors for the 180-day all-cause mortality included coagulopathy, BMI ≥ 40, and age, while the female sex was a protective factor beyond a fewer prevalence of comorbidities. Of the 6,235 patients discharged alive, 1,668 were readmitted a total of 2,551 times within 180 days, resulting in an overall readmission rate of 26.8%.

Conclusions: The 180-day follow-up data of hospitalized COVID-19 patients in a nationwide cohort representing almost one-third of the German population show significant long-term, all-cause mortality and readmission rates, especially among patients with coagulopathy, whereas women have a profoundly better and long-lasting clinical outcome compared to men.

Conflict of interest statement

RB reports grants from Berlin University Alliance, during the conduct of the study; grants from Federal Ministry of Research and Education, grants from Federal Ministry of Health, grants from Innovation Fonds of the Federal Joint Committee, grants from World Health Organization, outside the submitted work, AS reports grants from Bayer AG, outside the submitted work. CK reports personal fees from Maquet, personal fees from Xenios, personal fees from Bayer, non-financial support from Speaker of the German register of ICUs, grants from German Ministry of Research and Education, during the conduct of the study. CG, MS, TR, GS, WH, and SWC have nothing to disclose.

- Cited by 6 articles
- 33 references
- 4 figures

Supplementary info

Publication types, MeSH terms, Grant support Expand

Publication types

- Observational Study
- Research Support, Non-U.S. Gov't

MeSH terms

- Adult
- Aged
- Aged, 80 and over
- COVID-19 / epidemiology*
- COVID-19 / mortality*
- Cohort Studies
- Comorbidity
- Female
- Germany / epidemiology
- Hospital Mortality / trends
- Hospitalization / trends
- Humans
- Kaplan-Meier Estimate
- Logistic Models
- Male

- Middle Aged
- Patient Discharge / trends
- Patient Readmission / statistics & numerical data
- Patient Readmission / trends*
- Retrospective Studies
- Risk Factors
- SARS-CoV-2 / pathogenicity
- Time Factors

Grant support

Institutional support and physical resources were provided by the University Witten/ Herdecke and Kliniken der Stadt Köln, the Federal Association of the Local Health Care Funds and the Technical University of Berlin. The latter also received a grant from the Berlin University Alliance (112_PreEP_Corona). Article processing fees were funded by the authors. No funding source had a role in the design or conduct of the study; data collection, management, analysis, or interpretation; or the preparation, review, or approval of the manuscript.

Full text links

OPEN ACCESS TO FULL TEXT PLOS ONE Public Library of Science Free PMC article

Proceed to details

Cite

Share

311

Observational Study

World Neurosurg

. 2021 Oct;154:e118-e129.

doi: 10.1016/j.wneu.2021.06.147. Epub 2021 Jul 6.

Outcomes and Surgical Considerations for Neurosurgical Patients Hospitalized with COVID-19-A Multicenter Case Series

Lina Marenco-Hillembrand [1], Young Erben [2], Paola Suarez-Meade [1], Camila Franco-Mesa [2], Wendy Sherman [3], Benjamin H Eidelman [3], David A Miller [4], Nancy L O'Keefe [5], Bernard R Bendok [6], Robert J Spinner [7], Kaisorn L Chaichana [1], James F Meschia [3], Alfredo Quiñones-Hinojosa [8]

Affiliations Expand

Affiliations

- [1] Department of Neurological Surgery, Mayo Clinic, Jacksonville, Florida.
- [2] Division of Vascular and Endovascular Surgery, Mayo Clinic, Jacksonville, Florida.

- [3] Department of Neurology, Mayo Clinic, Jacksonville, Florida.
- [4] Department of Neurological Surgery, Mayo Clinic, Jacksonville, Florida; Department of Radiology, Mayo Clinic, Jacksonville, Florida.
- [5] Quality Management Services, Mayo Clinic, Jacksonville, Florida.
- [6] Department of Neurological Surgery, Mayo Clinic, Phoenix, Arizona, USA.
- [7] Department of Neurological Surgery, Mayo Clinic, Rochester, Minnesota, USA.
- [8] Department of Neurological Surgery, Mayo Clinic, Jacksonville, Florida. Electronic address: quinones-hinojosa.Alfredo@mayo.edu.

- PMID: **34237448**
- PMCID: PMC8257398
- DOI: 10.1016/j.wneu.2021.06.147

Free PMC article
Observational Study

Outcomes and Surgical Considerations for Neurosurgical Patients Hospitalized with COVID-19-A Multicenter Case Series

Lina Marenco-Hillembrand et al. World Neurosurg. 2021 Oct.
Free PMC article
Show details
World Neurosurg
. 2021 Oct;154:e118-e129.
doi: 10.1016/j.wneu.2021.06.147. Epub 2021 Jul 6.

Authors

Lina Marenco-Hillembrand [1], Young Erben [2], Paola Suarez-Meade [1], Camila Franco-Mesa [2], Wendy Sherman [3], Benjamin H Eidelman [3], David A Miller [4], Nancy L O'Keefe [5], Bernard R Bendok [6], Robert J Spinner [7], Kaisorn L Chaichana [1], James F Meschia [3], Alfredo Quiñones-Hinojosa [8]

Affiliations

- [1] Department of Neurological Surgery, Mayo Clinic, Jacksonville, Florida.
- [2] Division of Vascular and Endovascular Surgery, Mayo Clinic, Jacksonville, Florida.
- [3] Department of Neurology, Mayo Clinic, Jacksonville, Florida.
- [4] Department of Neurological Surgery, Mayo Clinic, Jacksonville, Florida; Department of Radiology, Mayo Clinic, Jacksonville, Florida.
- [5] Quality Management Services, Mayo Clinic, Jacksonville, Florida.
- [6] Department of Neurological Surgery, Mayo Clinic, Phoenix, Arizona, USA.
- [7] Department of Neurological Surgery, Mayo Clinic, Rochester, Minnesota, USA.
- [8] Department of Neurological Surgery, Mayo Clinic, Jacksonville, Florida. Electronic address: quinones-hinojosa.Alfredo@mayo.edu.

- PMID: **34237448**
- PMCID: PMC8257398
- DOI: 10.1016/j.wneu.2021.06.147

Abstract

Objective: Neurosurgical patients are at a higher risk of having a severe course of coronavirus disease 2019 (COVID-19). The objective of this study was to determine morbidity, hospital course, and mortality of neurosurgical patients during the coronavirus disease 2019 (COVID-19) pandemic in a multicenter health care system.

Methods: A retrospective observational study was conducted to identify all hospitalized neurosurgical patients positive for COVID-19 from March 11, 2020 to November 2, 2020 at Mayo Clinic and the Mayo Clinic Health System.

Results: Eleven hospitalized neurosurgical patients (0.68%) were positive for COVID-19. Four patients (36.6%) were men and 7 (63.3%) were women. The mean age was 65.7 years (range, 35-81 years). All patients had comorbidities. The mean length of stay was 13.4 days (range, 4-30 days). Seven patients had a central nervous system malignancy (4 metastases, 1 meningioma, 1 glioblastoma, and 1 schwannoma). Three patients presented with cerebrovascular complications, comprising 2 spontaneous intraparenchymal hemorrhages and 1 ischemic large-vessel stroke. One patient presented with an unstable traumatic spinal burst fracture. Four patients underwent neurosurgical/neuroendovascular interventions. Discharge disposition was to home in 5 patients, rehabilitation facility in 3, and hospice in 3. Five patients had died at follow-up, 3 within 30 days from COVID-19 complications and 2 from progression of their metastatic cancer.

Conclusions: COVID-19 is rare among the inpatient neurosurgical population. In all cases, patients had multiple comorbidities. All symptomatic patients from the respiratory standpoint had complications during their hospitalization. Deaths of 3 patients who died within 30 days of hospitalization were all related to COVID-19 complications. Neurosurgical procedures were performed only if deemed emergent.

Keywords: COVID; Coronavirus; Neurosurgery; Neurosurgical.

Copyright © 2021 Elsevier Inc. All rights reserved.

- 30 references
- 4 figures

Supplementary info

Publication types, MeSH terms Expand

Publication types

- Multicenter Study
- Observational Study

MeSH terms

- Adult
- Aged
- Aged, 80 and over
- COVID-19 / complications*
- COVID-19 / mortality
- Central Nervous System Neoplasms / surgery
- Cerebrovascular Disorders / complications
- Comorbidity
- Female
- Hospitalization
- Humans
- Length of Stay
- Male
- Middle Aged
- Neurosurgical Procedures / methods*
- Neurosurgical Procedures / mortality
- Pandemics
- Patient Readmission / statistics & numerical data
- Postoperative Complications / epidemiology
- Retrospective Studies
- Spinal Fractures / surgery
- Treatment Outcome

Full text links

ELSEVIER FULL-TEXT ARTICLE Elsevier Science Free PMC article

Proceed to details

Cite

Share

312

Observational Study

Bratisl Lek Listy
. 2021;122(8):582-589.
doi: 10.4149/BLL_2021_093.

Impact of proactive low-molecular weight heparin therapy on outcomes in COVID-1

S Gormez, H K Gumusel, E Ekicibasi, A Degirmencioglu, A Paudel, G Akan, F Atalar, R Erdim, E Eroglu, S Dagdelen, N Sariguzel, C E Kirisoglu, B Pamukcu

- PMID: **34282625**
- DOI: 10.4149/BLL_2021_093

Observational Study

Impact of proactive low-molecular weight heparin therapy on outcomes in COVID-1

S Gormez et al. Bratisl Lek Listy. 2021.
Show details
Bratisl Lek Listy
. 2021;122(8):582-589.
doi: 10.4149/BLL_2021_093.

Authors

S Gormez, H K Gumusel, E Ekicibasi, A Degirmencioglu, A Paudel, G Akan, F Atalar, R Erdim, E Eroglu, S Dagdelen, N Sariguzel, C E Kirisoglu, B Pamukcu

- PMID: **34282625**
- DOI: 10.4149/BLL_2021_093

Abstract

Objectives: Low molecular weight heparin (LMWH) may provide beneficial effects on outcomes of COVID-19. We aimed to examine the impact of LMWH treatment on clinical outcomes (duration of hospitalization, admission to intensive care unit, the requirement for mechanical ventilation, and death) of COVID-19 patients with normal D-dimer levels at admission.

Background: Coronavirus disease-2019 (COVID-19) predisposes patients to arterial and venous thrombosis.

Methods: In this retrospective, multicentre and observational study we analysed the data of 308 confirmed COVID-19 patients with normal D-dimer levels at initial admission. After propensity score matching (PSM) patients were grouped; Group 1; patients who received LMWH with D-dimer ≤0.5 mg/L, Group 2; patients who received LMWH after D-dimer levels exceeded 0.5 mg/L, and Group 3; patients who did not receive LMWH.

Results: After PSM, each group comprised 40 patients. The patients in Group1 had the best clinical outcomes compared to the other groups. Group 3 had the worst clinical outcomes ($p<0.005$). The benefit of LMWH increased with early prophylactic therapy especially when started while the D-dimer levels were ≤0.5 mg/L.

Conclusion: Our results strongly suggest that proactive LMWH therapy improves clinical outcomes in hospitalized COVID-19 patients even with normal D-dimer levels (≤ 0.5 mg/L) (Tab. 3, Fig. 2, Ref. 34).

Keywords: COVID-19; SARS-CoV-2; thrombosis; antithrombotic therapy; low-molecular-weight heparin; D-dimer; enoxaparin..

Supplementary info

Publication types, MeSH terms, Substances Expand

Publication types

- Multicenter Study
- Observational Study

MeSH terms

- Anticoagulants
- COVID-19*
- Heparin
- Heparin, Low-Molecular-Weight*
- Humans
- Molecular Weight
- Retrospective Studies
- SARS-CoV-2

Substances

- Anticoagulants
- Heparin, Low-Molecular-Weight
- Heparin

Full text links

AEPress AEPress, s r. o.
Proceed to details
Cite
Share
☐ 313
Observational Study
J Bone Miner Res
. 2021 Nov;36(11):2177-2183.
doi: 10.1002/jbmr.4419. Epub 2021 Aug 22.

The Use of Oral Amino-Bisphosphonates and Coronavirus Disease 2019 (COVID-19) Outcomes

Luca Degli Esposti [1], Valentina Perrone [1], Diego Sangiorgi [1], Margherita Andretta [2], Fausto Bartolini [3], Arturo Cavaliere [4], Andrea Ciaccia [5], Stefania Dell'orco [6], Stefano Grego [7], Sara Salzano [8], Loredana Ubertazzo [8], Adriano Vercellone [9], Davide Gatti [10], Angelo Fassio [10], Ombretta Viapiana [10], Maurizio Rossini [10], Giovanni Adami [10]
Affiliations Expand

Affiliations

- [1] CliCon S.r.l. Health, Economics & Outcomes Research, Bologna, Italy.
- [2] UOC Assistenza Farmaceutica Territoriale, Azienda ULSS 8 Berica, Vicenza, Italy.
- [3] Dipartimento Farmaceutico, USL Umbria 2, Perugia, Italy.
- [4] UOC Farmacia Aziendale, ASL, Viterbo, Italy.
- [5] Dipartimento Farmaceutico, ASL, Foggia, Italy.
- [6] UOC Farmaceutica Territoriale, ASL Roma 6, Rome, Italy.
- [7] Dipartimento Tecnico-Amministrativo, ASL 3 Genovese, Genova, Italy.
- [8] UOC Farmacia Territoriale, ASL Roma 4, Rome, Italy.
- [9] Dipartimento Farmaceutico, ASL Naples 3 Sud, Naples, Italy.
- [10] Rheumatology Unit, University of Verona, Verona, Italy.

- PMID: **34405441**
- PMCID: PMC8420492
- DOI: 10.1002/jbmr.4419

Free PMC article
Observational Study

The Use of Oral Amino-Bisphosphonates and Coronavirus Disease 2019 (COVID-19) Outcomes

Luca Degli Esposti et al. J Bone Miner Res. 2021 Nov.
Free PMC article
Show details
J Bone Miner Res
. 2021 Nov;36(11):2177-2183.
doi: 10.1002/jbmr.4419. Epub 2021 Aug 22.

Authors

Luca Degli Esposti [1], Valentina Perrone [1], Diego Sangiorgi [1], Margherita Andretta [2], Fausto Bartolini [3], Arturo Cavaliere [4], Andrea Ciaccia [5], Stefania Dell'orco [6], Stefano Grego [7], Sara Salzano [8], Loredana Ubertazzo [8], Adriano Vercellone [9], Davide Gatti [10], Angelo Fassio [10], Ombretta Viapiana [10], Maurizio Rossini [10], Giovanni Adami [10]

Affiliations

- [1] CliCon S.r.l. Health, Economics & Outcomes Research, Bologna, Italy.
- [2] UOC Assistenza Farmaceutica Territoriale, Azienda ULSS 8 Berica, Vicenza, Italy.
- [3] Dipartimento Farmaceutico, USL Umbria 2, Perugia, Italy.
- [4] UOC Farmacia Aziendale, ASL, Viterbo, Italy.
- [5] Dipartimento Farmaceutico, ASL, Foggia, Italy.
- [6] UOC Farmaceutica Territoriale, ASL Roma 6, Rome, Italy.
- [7] Dipartimento Tecnico-Amministrativo, ASL 3 Genovese, Genova, Italy.
- [8] UOC Farmacia Territoriale, ASL Roma 4, Rome, Italy.
- [9] Dipartimento Farmaceutico, ASL Naples 3 Sud, Naples, Italy.
- [10] Rheumatology Unit, University of Verona, Verona, Italy.

- PMID: **34405441**
- PMCID: PMC8420492
- DOI: 10.1002/jbmr.4419

Abstract

The determinants of the susceptibility to severe acute respiratory syndrome-coronavirus-2 (SARS-CoV-2) infection and severe coronavirus disease 2019 (COVID-19) manifestations are yet not fully understood. Amino-bisphosphonates (N-BPs) have anti-inflammatory properties and have been shown to reduce the incidence of lower respiratory infections, cardiovascular events, and cancer. We conducted a population-based retrospective observational cohort study with the primary objective of determining if oral N-BPs treatment can play a role in the susceptibility to development of severe COVID-19. Administrative International Classification of Diseases, Ninth Revision, Clinical ModificationI (ICD-9-CM) and anatomical-therapeutic chemical (ATC) code data, representative of Italian population (9% sample of the overall population), were analyzed. Oral N-BPs (mainly alendronate and risedronate) were included in the analysis, zoledronic acid was excluded because of the low number of patients at risk. Incidence of COVID-19 hospitalization was 12.32 (95% confidence interval [CI], 9.61-15.04) and 11.55 (95% CI, 8.91-14.20), of intensive care unit (ICU) utilization because of COVID-19 was 1.25 (95% CI, 0.38-2.11) and 1.42 (95% CI, 0.49-2.36), and of all-cause death was 4.06 (95% CI, 2.50-5.61) and 3.96 (95% CI, 2.41-5.51) for oral N-BPs users and nonusers, respectively. Sensitivity analyses that excluded patients with prevalent vertebral or hip fragility fractures and without concomitant glucocorticoid treatment yielded similar results. In conclusion, we found that the incidence of COVID-19 hospitalization, intensive care unit (ICU) utilization, and COVID-19 potentially related mortality were similar in N-BPs-treated and nontreated subjects. Similar results were found in N-BPs versus other anti-osteoporotic drugs. We provide real-life data on the safety of oral N-BPs in terms of severe COVID-19 risk on a population-based cohort. Our results do not support the hypothesis that oral N-BPs can prevent COVID-19 infection and/or severe COVID-19; however, they do not seem to increase the risk. © 2021 The Authors. Journal of Bone and Mineral Research published by Wiley Periodicals LLC on behalf of American Society for Bone and Mineral Research (ASBMR).

Keywords: BISPHOSPHONATES; COVID-19; INFECTION RISK; OSTEOPOROSIS; SARS-COV-2.

© 2021 The Authors. Journal of Bone and Mineral Research published by Wiley Periodicals LLC on behalf of American Society for Bone and Mineral Research (ASBMR).

- Cited by 1 article
- 23 references

- 4 figures

Supplementary info

Publication types, MeSH terms, Substances, Grant support Expand

Publication types

- Observational Study

MeSH terms

- COVID-19*
- Diphosphonates / therapeutic use
- Humans
- Retrospective Studies
- Risedronic Acid
- SARS-CoV-2

Substances

- Diphosphonates
- Risedronic Acid

Grant support

- Open Access Funding provided by Universita degli Studi di Verona within the CRUI-CARE Agreement. WOA Institution: Universita degli Studi di Verona Blended DEAL: CARE

Full text links

WILEY Full Text Article Wiley Free PMC article

Proceed to details

Cite

Share

☐ 314

Observational Study

J Epidemiol Glob Health

. 2021 Jun;11(2):233-237.

doi: 10.2991/jegh.k.210112.001. Epub 2021 Jan 22.

A Case Series of Severe Hospitalized COVID-19 Patients Treated with Tocilizumab and

Glucocorticoids: A Report from Saudi Arabian Hospital

Salma AlBahrani [1], Jaffar A Al-Tawfiq [2 3 4], Abdulaziz R Alshaer [1], Amal Shilash [1], Khalid Alswefy [1], Razan Salamah Al-Zayer [1], Amr Mohamed Abouelela [1]
Affiliations Expand

Affiliations

- [1] King Fahad Military Medical Complex, Dhahran, Saudi Arabia.
- [2] Infectious Disease Unit, Specialty Internal Medicine, Johns Hopkins Aramco Healthcare, Dhahran, Saudi Arabia.
- [3] Department of Medicine, Indiana University School of Medicine, Indianapolis, IN, USA.
- [4] Department of Medicine, Johns Hopkins University School of Medicine, Baltimore, MD, USA.

- PMID: **33605118**
- PMCID: PMC8242122
- DOI: 10.2991/jegh.k.210112.001

Free PMC article
Observational Study

A Case Series of Severe Hospitalized COVID-19 Patients Treated with Tocilizumab and Glucocorticoids: A Report from Saudi Arabian Hospital

Salma AlBahrani et al. J Epidemiol Glob Health. 2021 Jun.
Free PMC article
Show details
J Epidemiol Glob Health
. 2021 Jun;11(2):233-237.
doi: 10.2991/jegh.k.210112.001. Epub 2021 Jan 22.

Authors

Salma AlBahrani [1], Jaffar A Al-Tawfiq [2 3 4], Abdulaziz R Alshaer [1], Amal Shilash [1], Khalid Alswefy [1], Razan Salamah Al-Zayer [1], Amr Mohamed Abouelela [1]

Affiliations

- [1] King Fahad Military Medical Complex, Dhahran, Saudi Arabia.

- [2] Infectious Disease Unit, Specialty Internal Medicine, Johns Hopkins Aramco Healthcare, Dhahran, Saudi Arabia.
- [3] Department of Medicine, Indiana University School of Medicine, Indianapolis, IN, USA.
- [4] Department of Medicine, Johns Hopkins University School of Medicine, Baltimore, MD, USA.

- PMID: **33605118**
- PMCID: PMC8242122
- DOI: 10.2991/jegh.k.210112.001

Abstract

Background: The clinical spectrum of COVID-19 is variable and ranges from asymptomatic, mildly symptomatic, moderately severe and severe disease. A small proportion might develop severe disease and may have cytokine storm. One of the therapeutic options to treat such cases is Tocilizumab (TCZ). In this study, we present cases of severe COVID-19 treated with TCZ and glucocorticoids and discuss the treatment responses.

Methods: This is a retrospective observational study of severe COVID-19 cases treated with TCZ and glucocorticoids. The case series examined the characteristics and outcome of those patients.

Results: This study included 40 Severe Respiratory Syndrome Coronavirus 2 (SARS-CoV-2) confirmed patients who received TCZ and glucocorticoids. The mean age of the included patients was 57.55 (±Standard deviation 12.86) years. There were 34 (85%) males, 19 (47.5%) were obese (BMI >30), 13 (32.5%) over weight, and five (12.5%) normal weight. The mean days from positive SARS-CoV-2 polymerase chain reaction (PCR) test to admission was 1.641 (±3.2) days. Of the patients, 18 (45%) had diabetes mellitus, 14 (35%) had hypertension. The mean days from hospital admission to ICU was 1.8 (±2.6), 20 (50%) required mechanical ventilation, 39 (97.5%) had received prone position, seven (17.5%) had renal replacement therapy, 13 (32.5%) required inotropes, four (10%) had plasmapheresis, one (2.5%) had intravenous immunoglobulin, all patients received steroid therapy, and the majority 31 (77.5%) did not receive any anti-viral therapy. Of all the patients, six (15%) died, 28 (70%) were discharged and six (15%) were still in hospital.

Conclusion: The overall mortality rate was lower than those cited in meta-analysis. As our understanding of the COVID-19 continues, the approach and therapeutics are also evolving.

Keywords: COVID-19; SARS-COV-2; Tocilizumab; steroid; therapy.

© 2021 The Authors. Published by Atlantis Press International B.V.

Conflict of interest statement

The authors declare they have no conflicts of interest.

- Cited by 5 articles
- 39 references
- 4 figures

Supplementary info

Publication types, MeSH terms, Substances, Supplementary concepts Expand

Publication types

- Observational Study

MeSH terms

- Antibodies, Monoclonal, Humanized / therapeutic use*
- COVID-19 / drug therapy*
- COVID-19 / mortality
- Female
- Glucocorticoids / therapeutic use*
- Hospitals
- Humans
- Male
- Middle Aged
- Retrospective Studies
- SARS-CoV-2
- Saudi Arabia / epidemiology
- Treatment Outcome

Substances

- Antibodies, Monoclonal, Humanized
- Glucocorticoids
- tocilizumab

Supplementary concepts

- COVID-19 drug treatment

Full text links

Atlantis Press Free PMC article

Proceed to details

Cite

Share

☐ 315

Observational Study

J Osteopath Med

. 2021 Dec 16;122(2):111-115.

doi: 10.1515/jom-2021-0182.

Glycemic control is associated with lower odds of mortality and successful extubation in severe COVID-19

Jay M Pescatore[1], Juan Sarmiento[1], Ruben A Hernandez-Acosta[1], Britt Skaathun[2], Nancy Quesada-Rodriguez[3], Katayoun Rezai[4] [5]
Affiliations Expand

Affiliations

- [1] Department of Medicine, John H. Stroger Hospital of Cook County, Chicago, IL, USA.
- [2] Department of Medicine, University of California, San Diego, La Jolla, CA, USA.
- [3] Department of Pulmonary Medicine and Critical Care, John H. Stroger Hospital of Cook County, Chicago, IL, USA.
- [4] Department of Infectious Diseases, John H. Stroger Hospital of Cook County, Chicago, IL, USA.
- [5] Rush University, Chicago, IL, USA.

- PMID: **34908253**
- DOI: 10.1515/jom-2021-0182

Free article
Observational Study

Glycemic control is associated with lower odds of mortality and successful extubation in severe COVID-19

Jay M Pescatore et al. J Osteopath Med. 2021.
Free article
Show details
J Osteopath Med
. 2021 Dec 16;122(2):111-115.
doi: 10.1515/jom-2021-0182.

Authors

Jay M Pescatore[1], Juan Sarmiento[1], Ruben A Hernandez-Acosta[1], Britt Skaathun[2], Nancy Quesada-Rodriguez[3], Katayoun Rezai[4] [5]

Affiliations

- [1] Department of Medicine, John H. Stroger Hospital of Cook County, Chicago, IL, USA.

- [2] Department of Medicine, University of California, San Diego, La Jolla, CA, USA.
- [3] Department of Pulmonary Medicine and Critical Care, John H. Stroger Hospital of Cook County, Chicago, IL, USA.
- [4] Department of Infectious Diseases, John H. Stroger Hospital of Cook County, Chicago, IL, USA.
- [5] Rush University, Chicago, IL, USA.

- PMID: **34908253**
- DOI: 10.1515/jom-2021-0182

Abstract

Context: Corticosteroids, specifically dexamethasone, have become the mainstay of treatment for moderate to severe COVID-19. Although the RECOVERY trial did not report adverse effects of corticosteroids, the METCOVID (Methylprednisolone as Adjunctive Therapy for Patients Hospitalized with COVID-19) study reported a higher blood glucose level in patients receiving methylprednisolone.

Objectives: This study aims to analyze the association between corticosteroids and COVID-19-related outcomes in patients admitted to the medical ICU (MICU) for COVID-19 pneumonia.

Methods: This is an observational study of 141 patients admitted to the MICU between March 18 and June 7, 2020. Data on demographics, laboratory and imaging studies, and clinical course were obtained, including data on corticosteroid use. Bivariate analyses and logistic regression were performed between patient characteristics and mortality and successful extubation.

Results: Of the 141 patients, 86 required mechanical ventilation, 50 received steroids, and 71 died. Regarding demographics, patients had a median age of 58 (interquartile range [IQR] 48, 65), Hispanic (57.4%, n=81), and non-Hispanic Black (37.5%, n=53). The most prevalent comorbidities were hypertension (49.6%, n=70) and diabetes (48.2%, n=68). Lower blood glucose levels on admission (125.5 vs. 148 mg/dL, p=0.025) and lower peak blood glucose levels on corticosteroids (215.5 vs. 361 mg/dL, p=0.0021) were associated with lower prevalence of mortality. Patients who were successfully extubated had a lower admission blood glucose (126.5 vs. 149 mg/dL, p=0.0074) and lower peak blood glucose on corticosteroids (217 vs. 361 mg/dL, p=0.0023).

Conclusions: Lower blood glucose on admission and lower maximum blood glucose on corticosteroids were associated with lower odds of mortality and successful extubation, regardless of preexisting diabetes. Hyperglycemia may be negating any potential benefit of corticosteroid therapy. These findings suggest that glucose control could be a parameter that impacts the outcome of patients receiving corticosteroids for COVID-19 pneumonia.

Keywords: COVID-19; corticosteroids; glycemic control; hyperglycemia.

© 2021 Jay M. Pescatore et al., published by De Gruyter, Berlin/Boston.

- 10 references

Supplementary info

Publication types, MeSH terms Expand

Publication types

- Observational Study

MeSH terms

- Airway Extubation
- COVID-19*
- Glycemic Control
- Humans
- Retrospective Studies
- SARS-CoV-2

Full text links

De Gruyter
Proceed to details
Cite
Share
316
Observational Study
Int J Clin Pract
. 2021 Sep;75(9):e14426.
doi: 10.1111/ijcp.14426. Epub 2021 Jun 16.

Time to hospitalisation, CT pulmonary involvement and in-hospital death in COVID-19 patients in an Emergency Medicine Unit

Luca Marino [1] [2], Marianna Suppa [2], Antonello Rosa [2], Adriana Servello [2], Alessandro Coppola [2], Mariangela Palladino [2], Anna Maria Mazzocchitti [2], Emanuela Bresciani [2], Luigi Petramala [2], Giuliano Bertazzoni [2], Daniele Pastori [2]
Affiliations Expand

Affiliations

- [1] Department of Mechanical and Aerospace Engineering, Sapienza University of Rome, Roma, Italy.
- [2] Emergency Medicine Unit, Department of Clinical, Internal, Anesthesiological and Cardiovascular Sciences, Sapienza University of Rome, Roma, Italy.

- PMID: **34076933**
- PMCID: PMC8236995

• DOI: 10.1111/ijcp.14426

Free PMC article
Observational Study

Time to hospitalisation, CT pulmonary involvement and in-hospital death in COVID-19 patients in an Emergency Medicine Unit

Luca Marino et al. Int J Clin Pract. 2021 Sep.
Free PMC article
Show details
Int J Clin Pract
. 2021 Sep;75(9):e14426.
doi: 10.1111/ijcp.14426. Epub 2021 Jun 16.

Authors

Luca Marino [1, 2], Marianna Suppa [2], Antonello Rosa [2], Adriana Servello [2], Alessandro Coppola [2], Mariangela Palladino [2], Anna Maria Mazzocchitti [2], Emanuela Bresciani [2], Luigi Petramala [2], Giuliano Bertazzoni [2], Daniele Pastori [2]

Affiliations

- [1] Department of Mechanical and Aerospace Engineering, Sapienza University of Rome, Roma, Italy.
- [2] Emergency Medicine Unit, Department of Clinical, Internal, Anesthesiological and Cardiovascular Sciences, Sapienza University of Rome, Roma, Italy.

- PMID: **34076933**
- PMCID: PMC8236995
- DOI: 10.1111/ijcp.14426

Abstract

Background: Patients with coronavirus disease 2019 (COVID-19) are often treated at home given the limited healthcare resources. Many patients may have sudden clinical worsening and may be already compromised at hospitalisation. We investigated the burden of lung involvement according to the time to hospitalisation.

Methods: In this observational cohort study, 55 consecutive COVID-19-related pneumonia patients were admitted to the Emergency Medicine Unit. Groups of lung involvement at computed tomography were classified as follows: 0 (<5%), 1 (5%-25%), 2 (26%-50%), 3 (51%-75%) and 4 (>75%). We also investigated in-hospital death and the predictive value of Yan-XGBoost model and PREDI-CO scores for death.

Results: The median age was 74 years and 34 were men. Time to admission increased from 2 days in group 0 to 8.5-9 days in groups 3 and 4. A progressive increase in LDH, CRP and d-dimer

was found across groups, while a decrease of lymphocytes paO_2 /FiO_2 ratio and SpO_2 was found. Ten (18.2%) patients died during the in-hospital staying. Patients who died were older, with a trend to lower lymphocytes, a higher d-dimer, creatine phosphokinase and troponin T. The Yan-XGBoost model did not accurately predict in-hospital death with an AUC of 0.57 (95% confidence interval [CI] 0.37-0.76), which improved after the addition of the lung involvement groups (AUC 0.68, 95%CI 0.45-0.90). Conversely, a good predictive value was found for the original PREDI-CO score with an AUC of 0.76 (95% CI 0.58-0.93) which remained similar after the addition of the lung involvement (AUC 0.76, 95% CI 0.57-0.94).

Conclusion: We found that delayed hospital admission is associated with higher lung involvement. Hence, our data suggest that patients at risk for more severe disease, such as those with high LDH, CRP and d-dimer, should be promptly referred to hospital care.

© 2021 The Authors. International Journal of Clinical Practice published by John Wiley & Sons Ltd.

Conflict of interest statement

The authors declare no conflict of interest.

- Cited by 1 article
- 30 references
- 1 figure

Supplementary info

Publication types, MeSH terms Expand

Publication types

- Observational Study

MeSH terms

- Aged
- COVID-19*
- Emergency Medicine*
- Hospital Mortality
- Hospitalization
- Humans
- Male
- Retrospective Studies
- SARS-CoV-2
- Tomography, X-Ray Computed

Full text links

WILEY Full Text Article Wiley Free PMC article
Proceed to details
Cite
Share
317
Observational Study
J Trop Pediatr
. 2021 Dec 8;67(6):fmab102.
doi: 10.1093/tropej/fmab102.

Comparison of Clinical Findings in SARS-CoV-2 with Other Respiratory Viruses in Critically Ill Children during the COVID-19 Pandemic

Oktay Perk [1], Serhan Ozcan [1], Serhat Emeksiz [1], Emel Uyar [1], Belgin Gulhan [2]
Affiliations Expand

Affiliations

- [1] Department of Pediatric Intensive Care, Ankara City Hospital, 06800 Ankara, Turkey.
- [2] Department of Pediatric Infectious Disease, Ankara City Hospital, 06800 Ankara, Turkey.

- PMID: **34865169**
- PMCID: PMC8690266
- DOI: 10.1093/tropej/fmab102

Free PMC article
Observational Study

Comparison of Clinical Findings in SARS-CoV-2 with Other Respiratory Viruses in Critically Ill Children during the COVID-19 Pandemic

Oktay Perk et al. J Trop Pediatr. 2021.
Free PMC article
Show details
J Trop Pediatr
. 2021 Dec 8;67(6):fmab102.
doi: 10.1093/tropej/fmab102.

Authors

Oktay Perk [1], Serhan Ozcan [1], Serhat Emeksiz [1], Emel Uyar [1], Belgin Gulhan [2]

Affiliations

- [1] Department of Pediatric Intensive Care, Ankara City Hospital, 06800 Ankara, Turkey.
- [2] Department of Pediatric Infectious Disease, Ankara City Hospital, 06800 Ankara, Turkey.

- PMID: **34865169**
- PMCID: PMC8690266
- DOI: 10.1093/tropej/fmab102

Abstract

Objectives: The aim of this study was to compare the clinical and laboratory findings in SARS-CoV-2 (COVID-19) with those of other respiratory viruses in critically ill children.

Methods: It is a single center retrospective descriptive study conducted in a 32-bed pediatric intensive care unit (PICU). Our study was performed in Ankara City Hospital, Ankara, Turkey, between 1 March 2020, and 1 March 2021. Demographic and clinical characteristics of the patients were collected and we recorded the antibiotic use, antiviral treatments, respiratory and extracorporeal supports, PICU stay and survival rates.

Results: A total of 202 pediatric patients who tested positive for either COVID-19 or for another respiratory virus panel (RVP) were included in the study. Seventy-two patients were COVID-19 positive. The median age of COVID-19 positive patients and RVP positive patients was 97 and 17 months, respectively. Hypoxia was much more common in patients with RVP than in COVID-19 patients. Low oxygen saturation in arterial blood (SaO2), increased oxygen saturation index (OSI) and fraction of inspired oxygen (FiO2) needs were more significant in RVP patients than in COVID-19 patients. Respiratory support therapies, such as high-flow nasal cannula and non-invasive ventilation (NIV), were used more frequently in RVP patients than in COVID-19 patients.

Conclusion: It is important to distinguish between COVID-19 and RVP cases in order to prioritize intensive care needs in these patients. In addition, non-Covid diseases should not be left aside in the pandemic and appropriate care should be provided to them.

Keywords: COVID-19; SARS-CoV-2; children; pediatric intensive care; respiratory airway viruses.

Plain Language Summary

COVID-19 originated in Wuhan, China, at the end of 2019 and has since spread around the world. During the key period of the pandemic from 1 March 2020, to 1 March 2021, the pediatric intensive care unit registered a total of 72 patients testing positive for SARS-CoV-2 and 130 patients positive for RVP on the respiratory virus panel. In this single-center study, we compared the clinical differences and course of the disease in pediatric intensive care patients infected with SARS-CoV-2 with patients diagnosed with respiratory tract viruses during the COVID-19 outbreak. Unlike previous studies, this is the first to compare the clinical manifestations of COVID-19 with other respiratory pathogens requiring intensive care. Respiratory support therapy, such as high-flow nasal cannula (HFNC) and NIV, was prescribed more frequently in RVP

patients than in COVID-19 patients. In our study, low oxygen saturation in the arterial blood (SaO2), increased OSI and increased fraction of inspired oxygen (FiO2) requirements were more significant in RVP patients than in COVID-19 patients. In parallel, the need for mechanical ventilation was higher in RVP patients than in COVID-19 patients. Therefore, we believe that RVP patients should be followed more carefully during this pandemic period.

© The Author(s) [2021]. Published by Oxford University Press. All rights reserved. For permissions, please email: journals.permissions@oup.com.

Supplementary info

Publication types, MeSH terms Expand

Publication types

- Observational Study

MeSH terms

- COVID-19* / diagnosis
- Child
- Child, Preschool
- Critical Illness
- Female
- Humans
- Infant
- Intensive Care Units, Pediatric
- Male
- Oxygen Saturation
- Pandemics
- Respiratory Tract Diseases / diagnosis
- Respiratory Tract Diseases / virology*
- Retrospective Studies
- SARS-CoV-2
- Turkey
- Viruses / classification

Full text links

OXFORD ACADEMIC Silverchair Information Systems Free PMC article
Proceed to details
Cite
Share
☐ 318

Observational Study

Transplant Proc
. Jan-Feb 2022;54(1):22-24.
doi: 10.1016/j.transproceed.2021.09.067. Epub 2021 Nov 12.

Hospital Management and Ambulatory Patient Care After COVID-19 Infection in Kidney Transplant

Magdalena Terán Redondo [1], Claudia Muñoz Martínez [2], Pedro Rosa Guerrero [2], M Luisa Agüera Morales [2], Álvaro Torres De Rueda [2], Alberto Rodríguez Benot [2]
Affiliations Expand

Affiliations

- [1] Nephrology Department, University Hospital of Burgos, Burgos, Spain. Electronic address: mteranredondo@gmail.com.
- [2] Nephrology Department, Reina Sofia University Hospital of Cordoba, Cordoba, Spain.

- PMID: **34963513**
- PMCID: PMC8585592
- DOI: 10.1016/j.transproceed.2021.09.067

Free PMC article
Observational Study

Hospital Management and Ambulatory Patient Care After COVID-19 Infection in Kidney Transplant

Magdalena Terán Redondo et al. Transplant Proc. Jan-Feb 2022.
Free PMC article
Show details

Transplant Proc
. Jan-Feb 2022;54(1):22-24.
doi: 10.1016/j.transproceed.2021.09.067. Epub 2021 Nov 12.

Authors

Magdalena Terán Redondo [1], Claudia Muñoz Martínez [2], Pedro Rosa Guerrero [2], M Luisa Agüera Morales [2], Álvaro Torres De Rueda [2], Alberto Rodríguez Benot [2]

Affiliations

- [1] Nephrology Department, University Hospital of Burgos, Burgos, Spain. Electronic address: mteranredondo@gmail.com.
- [2] Nephrology Department, Reina Sofia University Hospital of Cordoba, Cordoba, Spain.

- PMID: **34963513**
- PMCID: PMC8585592
- DOI: 10.1016/j.transproceed.2021.09.067

Abstract

Background: A large number of registries have been collected for kidney transplant recipients infected with COVID-19.

Methods: From March 2020 to April 2021, our team conducted an observational study, which included all patients who showed a polymerase chain reaction positive for COVID-19. Patients were divided into 2 groups: patients who required ambulatory care and patients who needed hospital admission.

Results: A total of 76 kidney transplant recipients were infected with COVID-19. A total of 33% required hospital admission and 65% received ambulatory treatment; 28% of our patients were asymptomatic and 6.8% died. Immunosuppressive treatment was modified in both study groups, and there were not any acute rejection episodes or changes in the human leukocyte antigen antibodies profile in our patients during our clinical trial.

Conclusions: In our study there was a significant percentage of patients who did not require hospital admission compared with other studies. In addition, we think that the reduction of immunosuppression can be a safe and reliable treatment.

Copyright © 2021 Elsevier Inc. All rights reserved.

- 7 references
- 1 figure

Supplementary info

Publication types, MeSH terms, Substances Expand

Publication types

- Observational Study

MeSH terms

- Ambulatory Care
- COVID-19*
- Hospitals
- Humans
- Immunosuppressive Agents / adverse effects
- Kidney Transplantation* / adverse effects

- Retrospective Studies
- SARS-CoV-2
- Transplant Recipients

Substances

- Immunosuppressive Agents

Full text links

ELSEVIER FULL-TEXT ARTICLE Elsevier Science Free PMC article

Proceed to details

Cite

Share

319

Observational Study

PLoS One

. 2021 Aug 10;16(8):e0256023.

doi: 10.1371/journal.pone.0256023. eCollection 2021.

Characteristics and outcomes of acute kidney injury in hospitalized COVID-19 patients: A multicenter study by the Turkish society of nephrology

Hakki Arikan [1], Savas Ozturk [2], Bulent Tokgoz [3], Belda Dursun [4], Nurhan Seyahi [5], Sinan Trabulus [5], Mahmud Islam [6], Yavuz Ayar [7], Numan Gorgulu [8], Serhat Karadag [2], Mahmut Gok [9], Esra Akcali [10], Feyza Bora [11], Zeki Aydın [12], Eda Altun [13], Elbis Ahbap [14], Mehmet Polat [15], Zeki Soypacacı [16], Ebru Gok Oguz [17], Sumeyra Koyuncu [3], Hulya Colak [18], İdris Sahin [19], Murside Esra Dolarslan [20], Ozant Helvacı [21], Ilhan Kurultak [22], Zehra Eren [23], Hamad Dheir [24], Melike Betul Ogutmen [25], Dilek Guven Taymez [26], Dilek Gibyeli Genek [27], Sultan Ozkurt [28], Elif Ari Bakır [29], Enver Yuksel [30], Tuncay Sahutoglu [31], Ozgur Akin Oto [32], Gulsah Boz [33], Erkan Sengul [34], Ekrem Kara [35], Serhan Tuglular [1]

Affiliations Expand

Affiliations

- [1] Department of Internal Medicine, Division of Nephrology, Marmara University School of Medicine, Istanbul, Turkey.
- [2] Department of Nephrology, Haseki Training and Research Hospital, Istanbul, Turkey.
- [3] Department of Internal Medicine, Division of Nephrology, Erciyes University School of Medicine, Kayseri, Turkey.

- [4] Department of Internal Medicine, Division of Nephrology, Pamukkale University Medical School, Denizli, Turkey.
- [5] Department of Nephrology, Cerrahpasa Medical Faculty, Istanbul University, Istanbul, Turkey.
- [6] Division of Nephrology, Zonguldak Ataturk State Hospital, Zonguldak, Turkey.
- [7] Division of Nephrology, Bursa City Hospital, Faculty of Medicine, University of Health Sciences, Bursa, Turkey.
- [8] Department of Nephrology, Istanbul Bagcilar Training and Research Hospital, University of Health Sciences, Istanbul, Turkey.
- [9] Department of Nephrology, Sultan 2.Abdulhamid Han Training and Research Hospital, Istanbul, Turkey.
- [10] Department of Nephrology, Mersin University Faculty of Medicine, Mersin, Turkey.
- [11] Department of Internal Medicine, Division of Nephrology, Akdeniz University Faculty of Medicine, Antalya, Turkey.
- [12] Department of Nephrology, Kocaeli Darica Farabi Training and Research Hospital, University of Health Sciences, Kocaeli, Turkey.
- [13] Division of Nephrology, Golcuk Necati Celik State Hospital, Kocaeli, Turkey.
- [14] Department of Nephrology, Sisli Hamidiye Etfal Education and Research Hospital, Istanbul, Turkey.
- [15] Division of Nephrology, Nevsehir State Hospital, Nevsehir, Turkey.
- [16] Department of Nephrology, Ataturk Training and Research Hospital, University of Katip Celebi, Izmir, Turkey.
- [17] Department of Nephrology, Diskapi Yildirim Beyazit Education and Research Hospital, University of Health Sciences, Ankara, Turkey.
- [18] Division of Nephrology, Tepecik Education and Research Hospital University of Health Sciences, İzmir, Turkey.
- [19] Department of Internal Medicine, Division of Nephrology, Inonu University Faculty of Medicine, Malatya, Turkey.
- [20] Division of Nephrology, Trabzon Kanuni Education and Research Hospital, University of Health Sciences, Trabzon, Turkey.
- [21] Division of Nephrology, Yenimahalle Research and Training Hospital, Yildirim Beyazit University Faculty of Medicine, Ankara, Turkey.
- [22] Department of Nephrology, Trakya University Faculty of Medicine, Edirne, Turkey.
- [23] Department of Nephrology, Alanya Alaaddin Keykubat University School of Medicine, Antalya, Turkey.
- [24] Department of Internal Medicine, Division of Nephrology, Sakarya University Medical Faculty Education and Research Hospital, Sakarya, Turkey.
- [25] Division of Nephrology, Haydarpasa Numune Education and Research Hospital, University of Health Sciences, Istanbul, Turkey.
- [26] Nephrology and Dialysis Department, Kocaeli State Hospital, Kocaeli, Turkey.
- [27] Department of Nephrology, Faculty of Medicine, Mugla Sitki Kocman University, Mugla, Turkey.
- [28] Department of Nephrology, Faculty of Medicine, Eskisehir Osmangazi University, Eskisehir, Turkey.
- [29] Department of Nephrology, Bahcesehir University Hospital, Istanbul, Turkey.
- [30] Department of Nephrology, Gaziyasargil Training and Research Hospital, University of Health Sciences, Diyarbakir, Turkey.
- [31] Nephrology Unit, Sanliurfa Mehmet Akif Inan Training and Research Hospital, Sanliurfa, Turkey.

- [32] Department of Internal Medicine, Division of Nephrology, Istanbul Medical Faculty, Istanbul University, Istanbul, Turkey.
- [33] Division of Nephrology, Kayseri City Training and Research Hospital, Kayseri, Turkey.
- [34] Division of Nephrology, Kocaeli Derince Education and Research Hospital, University of Health Sciences, Kocaeli, Turkey.
- [35] Department of Internal Medicine, Division of Nephrology, Faculty of Medicine, Recep Tayyip Erdogan University, Rize, Turkey.

- PMID: **34375366**
- PMCID: PMC8354466
- DOI: 10.1371/journal.pone.0256023

Free PMC article
Observational Study

Characteristics and outcomes of acute kidney injury in hospitalized COVID-19 patients: A multicenter study by the Turkish society of nephrology

Hakki Arikan et al. PLoS One. 2021.
Free PMC article

Show details

PLoS One

. 2021 Aug 10;16(8):e0256023.
doi: 10.1371/journal.pone.0256023. eCollection 2021.

Authors

Hakki Arikan [1], Savas Ozturk [2], Bulent Tokgoz [3], Belda Dursun [4], Nurhan Seyahi [5], Sinan Trabulus [5], Mahmud Islam [6], Yavuz Ayar [7], Numan Gorgulu [8], Serhat Karadag [2], Mahmut Gok [9], Esra Akcali [10], Feyza Bora [11], Zeki Aydın [12], Eda Altun [13], Elbis Ahbap [14], Mehmet Polat [15], Zeki Soypacacı [16], Ebru Gok Oguz [17], Sumeyra Koyuncu [3], Hulya Colak [18], İdris Sahin [19], Murside Esra Dolarslan [20], Ozant Helvacı [21], Ilhan Kurultak [22], Zehra Eren [23], Hamad Dheir [24], Melike Betul Ogutmen [25], Dilek Guven Taymez [26], Dilek Gibyeli Genek [27], Sultan Ozkurt [28], Elif Ari Bakır [29], Enver Yuksel [30], Tuncay Sahutoglu [31], Ozgur Akin Oto [32], Gulsah Boz [33], Erkan Sengul [34], Ekrem Kara [35], Serhan Tuglular [1]

Affiliations

- [1] Department of Internal Medicine, Division of Nephrology, Marmara University School of Medicine, Istanbul, Turkey.
- [2] Department of Nephrology, Haseki Training and Research Hospital, Istanbul, Turkey.
- [3] Department of Internal Medicine, Division of Nephrology, Erciyes University School of Medicine, Kayseri, Turkey.

- [4] Department of Internal Medicine, Division of Nephrology, Pamukkale University Medical School, Denizli, Turkey.
- [5] Department of Nephrology, Cerrahpasa Medical Faculty, Istanbul University, Istanbul, Turkey.
- [6] Division of Nephrology, Zonguldak Ataturk State Hospital, Zonguldak, Turkey.
- [7] Division of Nephrology, Bursa City Hospital, Faculty of Medicine, University of Health Sciences, Bursa, Turkey.
- [8] Department of Nephrology, Istanbul Bagcilar Training and Research Hospital, University of Health Sciences, Istanbul, Turkey.
- [9] Department of Nephrology, Sultan 2.Abdulhamid Han Training and Research Hospital, Istanbul, Turkey.
- [10] Department of Nephrology, Mersin University Faculty of Medicine, Mersin, Turkey.
- [11] Department of Internal Medicine, Division of Nephrology, Akdeniz University Faculty of Medicine, Antalya, Turkey.
- [12] Department of Nephrology, Kocaeli Darica Farabi Training and Research Hospital, University of Health Sciences, Kocaeli, Turkey.
- [13] Division of Nephrology, Golcuk Necati Celik State Hospital, Kocaeli, Turkey.
- [14] Department of Nephrology, Sisli Hamidiye Etfal Education and Research Hospital, Istanbul, Turkey.
- [15] Division of Nephrology, Nevsehir State Hospital, Nevsehir, Turkey.
- [16] Department of Nephrology, Ataturk Training and Research Hospital, University of Katip Celebi, Izmir, Turkey.
- [17] Department of Nephrology, Diskapi Yildirim Beyazit Education and Research Hospital, University of Health Sciences, Ankara, Turkey.
- [18] Division of Nephrology, Tepecik Education and Research Hospital University of Health Sciences, İzmir, Turkey.
- [19] Department of Internal Medicine, Division of Nephrology, Inonu University Faculty of Medicine, Malatya, Turkey.
- [20] Division of Nephrology, Trabzon Kanuni Education and Research Hospital, University of Health Sciences, Trabzon, Turkey.
- [21] Division of Nephrology, Yenimahalle Research and Training Hospital, Yildirim Beyazit University Faculty of Medicine, Ankara, Turkey.
- [22] Department of Nephrology, Trakya University Faculty of Medicine, Edirne, Turkey.
- [23] Department of Nephrology, Alanya Alaaddin Keykubat University School of Medicine, Antalya, Turkey.
- [24] Department of Internal Medicine, Division of Nephrology, Sakarya University Medical Faculty Education and Research Hospital, Sakarya, Turkey.
- [25] Division of Nephrology, Haydarpasa Numune Education and Research Hospital, University of Health Sciences, Istanbul, Turkey.
- [26] Nephrology and Dialysis Department, Kocaeli State Hospital, Kocaeli, Turkey.
- [27] Department of Nephrology, Faculty of Medicine, Mugla Sitki Kocman University, Mugla, Turkey.
- [28] Department of Nephrology, Faculty of Medicine, Eskisehir Osmangazi University, Eskisehir, Turkey.
- [29] Department of Nephrology, Bahcesehir University Hospital, Istanbul, Turkey.
- [30] Department of Nephrology, Gaziyasargil Training and Research Hospital, University of Health Sciences, Diyarbakir, Turkey.
- [31] Nephrology Unit, Sanliurfa Mehmet Akif Inan Training and Research Hospital, Sanliurfa, Turkey.

- [32] Department of Internal Medicine, Division of Nephrology, Istanbul Medical Faculty, Istanbul University, Istanbul, Turkey.
- [33] Division of Nephrology, Kayseri City Training and Research Hospital, Kayseri, Turkey.
- [34] Division of Nephrology, Kocaeli Derince Education and Research Hospital, University of Health Sciences, Kocaeli, Turkey.
- [35] Department of Internal Medicine, Division of Nephrology, Faculty of Medicine, Recep Tayyip Erdogan University, Rize, Turkey.

- PMID: **34375366**
- PMCID: PMC8354466
- DOI: 10.1371/journal.pone.0256023

Abstract

Background: Acute kidney injury (AKI) is common in coronavirus disease-2019 (COVID-19) and the severity of AKI is linked to adverse outcomes. In this study, we investigated the factors associated with in-hospital outcomes among hospitalized patients with COVID-19 and AKI.

Methods: In this multicenter retrospective observational study, we evaluated the characteristics and in-hospital renal and patient outcomes of 578 patients with confirmed COVID-19 and AKI. Data were collected from 34 hospitals in Turkey from March 11 to June 30, 2020. AKI definition and staging were based on the Kidney Disease Improving Global Outcomes criteria. Patients with end-stage kidney disease or with a kidney transplant were excluded. Renal outcomes were identified only in discharged patients.

Results: The median age of the patients was 69 years, and 60.9% were males. The most frequent comorbid conditions were hypertension (70.5%), diabetes mellitus (43.8%), and chronic kidney disease (CKD) (37.6%). The proportions of AKI stages 1, 2, and 3 were 54.0%, 24.7%, and 21.3%, respectively. 291 patients (50.3%) were admitted to the intensive care unit. Renal improvement was complete in 81.7% and partial in 17.2% of the patients who were discharged. Renal outcomes were worse in patients with AKI stage 3 or baseline CKD. The overall in-hospital mortality in patients with AKI was 38.9%. In-hospital mortality rate was not different in patients with preexisting non-dialysis CKD compared to patients without CKD (34.4 versus 34.0%, p = 0.924). By multivariate Cox regression analysis, age (hazard ratio [HR] [95% confidence interval (95%CI)]: 1.01 [1.0-1.03], p = 0.035], male gender (HR [95%CI]: 1.47 [1.04-2.09], p = 0.029), diabetes mellitus (HR [95%CI]: 1.51 [1.06-2.17], p = 0.022) and cerebrovascular disease (HR [95%CI]: 1.82 [1.08-3.07], p = 0.023), serum lactate dehydrogenase (greater than two-fold increase) (HR [95%CI]: 1.55 [1.05-2.30], p = 0.027) and AKI stage 2 (HR [95%CI]: 1.98 [1.25-3.14], p = 0.003) and stage 3 (HR [95%CI]: 2.25 [1.44-3.51], p = 0.0001) were independent predictors of in-hospital mortality.

Conclusions: Advanced-stage AKI is associated with extremely high mortality among hospitalized COVID-19 patients. Age, male gender, comorbidities, which are risk factors for mortality in patients with COVID-19 in the general population, are also related to in-hospital mortality in patients with AKI. However, preexisting non-dialysis CKD did not increase in-hospital mortality rate among AKI patients. Renal problems continue in a significant portion of the patients who were discharged.

Conflict of interest statement

The authors have declared that no competing interests exist.

- Cited by 4 articles
- 52 references
- 2 figures

Supplementary info

Publication types, MeSH terms, Substances, Grant support Expand

Publication types

- Multicenter Study
- Observational Study
- Research Support, Non-U.S. Gov't

MeSH terms

- Acute Kidney Injury / etiology
- Acute Kidney Injury / pathology*
- Aged
- COVID-19 / complications
- COVID-19 / mortality
- COVID-19 / pathology*
- COVID-19 / virology
- Comorbidity
- Female
- Hospital Mortality
- Hospitalization
- Humans
- Intensive Care Units
- L-Lactate Dehydrogenase / blood
- Male
- Middle Aged
- Proportional Hazards Models
- Retrospective Studies
- Risk Factors
- SARS-CoV-2 / isolation & purification
- Severity of Illness Index
- Sex Factors
- Turkey

Substances

- L-Lactate Dehydrogenase

Grant support

The study was unconditionally supported by the Turkish Society of Nephrology.

Full text links

OPEN ACCESS TO FULL TEXT PLOS ONE Public Library of Science Free PMC article
Proceed to details
Cite
Share
☐ 320

Observational Study
Expert Rev Respir Med
. 2021 Dec;15(12):1619-1625.
doi: 10.1080/17476348.2021.1960824. Epub 2021 Aug 18.

COVID-19 clinical phenotypes and short-term outcomes: differences between the first and the second wave of pandemic in Italy

Andrea Portacci [1], Giovanna Elisiana Carpagnano [1], Maria Grazia Tummolo [1], Carla Santomasi [1], Lavinia Palma [1], Domenico Fasano [2], Emanuela Resta [3], Madia Lozupone [4], Vincenzo Solfrizzi [2], Francesco Panza [5], Onofrio Resta [1]
Affiliations Expand

Affiliations

- [1] Institute of Respiratory Disease, Department of Basic Medical Science, Neuroscience, and Sense Organs, University of Bari, "Aldo Moro", Bari, Italy.
- [2] Medical Clinic "C. Frugoni" and Geriatric Medicine Unit, University of Bari "Aldo Moro", Bari, Italy.
- [3] Translational Medicine and Health System Management, University of Foggia, Foggia, Italy.
- [4] Neurodegenerative Disease Unit, Department of Basic Medicine, Neuroscience, and Sense Organs, University of Bari Aldo Moro, Bari, Italy.
- [5] Population Health Unit, "Salus in Apulia Study", National Institute of Gastroenterology "Saverio De Bellis", Research Hospital, Castellana Grotte, Bari, Italy.

- PMID: **34311634**
- PMCID: PMC8436419
- DOI: 10.1080/17476348.2021.1960824

Free PMC article
Observational Study

COVID-19 clinical phenotypes and short-term outcomes: differences between the first and the second wave of pandemic in Italy

Andrea Portacci et al. Expert Rev Respir Med. 2021 Dec.
Free PMC article
Show details
Expert Rev Respir Med
. 2021 Dec;15(12):1619-1625.
doi: 10.1080/17476348.2021.1960824. Epub 2021 Aug 18.

Authors

Andrea Portacci [1], Giovanna Elisiana Carpagnano [1], Maria Grazia Tummolo [1], Carla Santomasi [1], Lavinia Palma [1], Domenico Fasano [2], Emanuela Resta [3], Madia Lozupone [4], Vincenzo Solfrizzi [2], Francesco Panza [5], Onofrio Resta [1]

Affiliations

- [1] Institute of Respiratory Disease, Department of Basic Medical Science, Neuroscience, and Sense Organs, University of Bari, "Aldo Moro", Bari, Italy.
- [2] Medical Clinic "C. Frugoni" and Geriatric Medicine Unit, University of Bari "Aldo Moro", Bari, Italy.
- [3] Translational Medicine and Health System Management, University of Foggia, Foggia, Italy.
- [4] Neurodegenerative Disease Unit, Department of Basic Medicine, Neuroscience, and Sense Organs, University of Bari Aldo Moro, Bari, Italy.
- [5] Population Health Unit, "Salus in Apulia Study", National Institute of Gastroenterology "Saverio De Bellis", Research Hospital, Castellana Grotte, Bari, Italy.

- PMID: **34311634**
- PMCID: PMC8436419
- DOI: 10.1080/17476348.2021.1960824

Abstract

Objectives: There are no comparative studies between patients belonging to the first and second waves of the SARS-CoV-2 pandemic, the virus triggering coronavirus disease 2019 (COVID-19). In this retrospective observational study, we analyzed the clinical characteristics and the short-term outcomes of two groups of laboratory-confirmed COVID-19 patients with moderate-to-severe acute respiratory distress syndrome (ARDS) belonging to two different waves of the pandemic. **Methods:** We analyzed 97 consecutive patients from 11 March 2020 to 31 May 2020 and 52 consecutive patients from 28 August 2020 to 15 October 2020. **Results:** Patients belonging to the second wave were younger, had a lower number of concomitant chronic conditions (multimorbidity), and had a milder clinical phenotype. Medical treatments and respiratory support use have changed during the COVID-19 pandemic, based on different laboratory results and disease clinical features. Patients in the second wave had better short-term clinical outcomes, with

lower death rates and more step-down transfers to a general ward. **Conclusion:** The present findings show a clear phenotypic difference in patients hospitalized at different stages of the COVID-19 pandemic in Italy. These results can help to stratify clinical risk and to better tailor medical treatments and respiratory support for patients with ARDS and COVID-19 pneumonia.

Keywords: Critical care; Italy; assisted ventilation; intermediate RICU; pandemic; survival.

- Cited by 1 article
- 30 references

Supplementary info

Publication types, MeSH terms, Grant support Expand

Publication types

- Observational Study

MeSH terms

- COVID-19*
- Hospitalization
- Humans
- Pandemics
- Phenotype
- SARS-CoV-2

Grant support

This paper was not funded.

Full text links

View full text Taylor & Francis Free PMC article

Proceed to details

Cite

Share

☐ 321

Observational Study

J Korean Med Sci

. 2021 Dec 20;36(49):e341.

doi: 10.3346/jkms.2021.36.e341.

Clinical Features of Adult COVID-19 Patients without Risk Factors before and after the

Nationwide SARS-CoV-2 B.1.617.2 (Delta) -variant Outbreak in Korea: Experience from Gyeongsangnam-do

Byung-Han Ryu [1], Sun In Hong [1], Su Jin Lim [2], Younghwa Cho [3], Cheolgu Hwang [4], Hyungseok Kang [5], Si-Ho Kim [6], Yu Mi Wi [6], Kyung-Wook Hong [7], In-Gyu Bae [7], Oh-Hyun Cho [1] [8]

Affiliations Expand

Affiliations

- [1] Department of Internal Medicine, Gyeongsang National University Changwon Hospital, Changwon, Korea.
- [2] Department of Internal Medicine, Gyeongsangnam-do Masan Medical Center, Changwon, Korea.
- [3] Department of Internal Medicine, Korea Labour Welfare Corporation Changwon Hospital, Changwon, Korea.
- [4] Department of Internal Medicine, Busan Medical Center, Busan, Korea.
- [5] Department of Chest Medicine, Masan National Tuberculosis Hospital, Changwon, Korea.
- [6] Department of Internal Medicine, Samsung Changwon Hospital, Sungkyunkwan University School of Medicine, Changwon, Korea.
- [7] Department of Internal Medicine, Gyeongsang National University Hospital, Gyeongsang National University College of Medicine, Jinju, Korea.
- [8] Gyeongsang Institute of Health Sciences, Gyeongsang National University College of Medicine, Jinju, Korea. zenmd@naver.com.

- PMID: **34931500**
- PMCID: PMC8688347
- DOI: 10.3346/jkms.2021.36.e341

Free PMC article
Observational Study

Clinical Features of Adult COVID-19 Patients without Risk Factors before and after the Nationwide SARS-CoV-2 B.1.617.2 (Delta) -variant Outbreak in Korea: Experience from Gyeongsangnam-do

Byung-Han Ryu et al. J Korean Med Sci. 2021.
Free PMC article
Show details

J Korean Med Sci
. 2021 Dec 20;36(49):e341.
doi: 10.3346/jkms.2021.36.e341.

Authors

Byung-Han Ryu [1], Sun In Hong [1], Su Jin Lim [2], Younghwa Cho [3], Cheolgu Hwang [4], Hyungseok Kang [5], Si-Ho Kim [6], Yu Mi Wi [6], Kyung-Wook Hong [7], In-Gyu Bae [7], Oh-Hyun Cho [1] [8]

Affiliations

- [1] Department of Internal Medicine, Gyeongsang National University Changwon Hospital, Changwon, Korea.
- [2] Department of Internal Medicine, Gyeongsangnam-do Masan Medical Center, Changwon, Korea.
- [3] Department of Internal Medicine, Korea Labour Welfare Corporation Changwon Hospital, Changwon, Korea.
- [4] Department of Internal Medicine, Busan Medical Center, Busan, Korea.
- [5] Department of Chest Medicine, Masan National Tuberculosis Hospital, Changwon, Korea.
- [6] Department of Internal Medicine, Samsung Changwon Hospital, Sungkyunkwan University School of Medicine, Changwon, Korea.
- [7] Department of Internal Medicine, Gyeongsang National University Hospital, Gyeongsang National University College of Medicine, Jinju, Korea.
- [8] Gyeongsang Institute of Health Sciences, Gyeongsang National University College of Medicine, Jinju, Korea. zenmd@naver.com.

- PMID: **34931500**
- PMCID: PMC8688347
- DOI: 10.3346/jkms.2021.36.e341

Abstract

Background: Data on severe acute respiratory syndrome-coronavirus-2 (SARS-CoV-2) delta variant virulence are insufficient. We retrospectively compared the clinical features of adult coronavirus disease 2019 (COVID-19) patients without risk factors for severe COVID-19 who entered residential treatment centers (RTCs) before and after the delta variant outbreak.

Methods: We collected medical information from two RTCs in South Korea. On the basis of nationwide delta variant surveillance, we divided the patients into two groups: 1) the delta-minor group (diagnosed from December 2020-June 2021, detection rate < 10%) and 2) the delta-dominant group (diagnosed during August 2021, detection rate > 90%). After propensity-score matching, the incidences of pneumonia, hospital transfer and need for supplemental oxygen were compared between the groups. In addition, risk factors for hospital transfer were analysed.

Results: A total of 1,915 patients were included. The incidence of pneumonia (14.6% vs. 9.2%, $P = 0.009$), all-cause hospital transfer (10.4% vs. 6.3%, $P = 0.020$) and COVID-19-related hospital transfer (7.5% vs. 4.8%, $P = 0.081$) were higher in the delta-dominant group than those in the delta-minor group. In the multivariate analysis, the delta-dominant group was an independent risk

factor for all-cause (adjusted odds ratio [aOR], 1.91; 95% confidence interval [CI], 1.16-3.13; *P* = 0.011) and COVID-19-related hospital transfer (aOR, 1.86; 95% CI, 1.04-3.32; *P* = 0.036).

Conclusion: Hospitalization rates were increased in the adult COVID-19 patients during the delta variant nationwide outbreak. Our results showed that the delta variant may be more virulent than previous lineages.

Keywords: COVID-19; Delta Variant; Hospitalization; South Korea; Virulence.

© 2021 The Korean Academy of Medical Sciences.

Conflict of interest statement

The authors have no potential conflicts of interest to disclose.

- Cited by 1 article
- 14 references
- 1 figure

Supplementary info

Publication types, MeSH terms, Supplementary concepts, Grant support Expand

Publication types

- Observational Study

MeSH terms

- Adult
- COVID-19 / diagnosis*
- COVID-19 / epidemiology*
- Disease Outbreaks
- Female
- Hospitalization*
- Humans
- Incidence
- Male
- Middle Aged
- Odds Ratio
- Republic of Korea / epidemiology
- Retrospective Studies
- Risk Factors
- SARS-CoV-2*
- Time Factors

Supplementary concepts

- SARS-CoV-2 variants

Grant support

- Gyeongsang National University/Korea

Full text links

Korean Academy of Medical Sciences Free PMC article
Proceed to details
Cite
Share
322
Observational Study
Ulus Travma Acil Cerrahi Derg
. 2020 Sep;26(5):685-692.
doi: 10.14744/etd.2020.67927.

The impacts of the COVID-19 outbreak on emergency department visits of surgical patients

Beslen Göksoy [1], Muhammed Tahir Akça [1], Ömer Faruk Inanç [1]
Affiliations Expand

Affiliation

- [1] Department of General Surgery, Şehit Prof. Dr. İlhan Varank Sancaktepe Training and Research Hospital, İstanbul-Turkey.

- PMID: **32946100**
- DOI: 10.14744/etd.2020.67927

Free article
Observational Study

The impacts of the COVID-19 outbreak on emergency department visits of surgical patients

Beslen Göksoy et al. Ulus Travma Acil Cerrahi Derg. 2020 Sep.
Free article
Show details
Ulus Travma Acil Cerrahi Derg
. 2020 Sep;26(5):685-692.
doi: 10.14744/etd.2020.67927.

Authors

Beslen Göksoy [1], Muhammed Tahir Akça [1], Ömer Faruk Inanç [1]

Affiliation

- [1] Department of General Surgery, Şehit Prof. Dr. İlhan Varank Sancaktepe Training and Research Hospital, İstanbul-Turkey.

- PMID: **32946100**
- DOI: 10.14744/etd.2020.67927

Abstract

Background: The novel Coronavirus Disease 2019 (COVID-19) formed the basis for emergency department visits. This study aims to evaluate the effects of the pandemic on emergency department visits of surgical patients.

Methods: The hospital database records of general surgery patients who presented at the emergency department in the period of March 2020-May 2020 (pandemic period) and March 2019-May 2019 (non-pandemic period) were retrospectively analyzed and compared. The primary outcome of this study was the emergency department visit rate of patients requiring a general surgery consultation. Secondary outcomes of this study were patient complaints, diagnosis and treatments, treatment rejection rate, triage category data, the effects of age and gender, and the hospitalization rate.

Results: In this study, 618 patients were included: 265 patients from the pandemic period and 353 patients from the non-pandemic period. The analysis and comparison revealed that during the pandemic period, the presentation rate of female patients was lower than that of male patients (45.5% vs. 55.5%, respectively, p=0.045). The triage category rates of patient visits to the hospital during the pandemic period were higher in yellow and red, and lower in green (p<0.01). The incidence of a surgical pathology finding was higher during the pandemic period (p=0.019). The incidence of diseases related to the gastrointestinal tract was higher during the pandemic period (p=0.011). The rate of open surgery in the pandemic period was higher than that of the non-pandemic period (80.5% vs. 32.7%, respectively; p<0.01). The treatment rejection rate was also higher in the pandemic period compared with the non-pandemic period (20% vs. 3.7%, respectively; p<0.01, r). In addition, the hospitalization period was shorter in the pandemic period (p=0.021).

Conclusion: A 25% reduction in the number of surgical patient visits to the emergency department was observed during the COVID-19 outbreak. The biggest decrease was seen in patients with a green triage code and female patients. It is significant to evaluate the effects of the pandemic on surgical patients visited the emergency department to manage the post-epidemic period and to prepare for possible future epidemic periods.

- Cited by 6 articles

Supplementary info

Publication types, MeSH terms Expand

Publication types

- Observational Study

MeSH terms

- Adult
- Aged
- Anxiety / epidemiology
- Betacoronavirus*
- COVID-19
- Coronavirus Infections / epidemiology*
- Coronavirus Infections / psychology
- Emergency Service, Hospital / statistics & numerical data*
- Female
- Gastrointestinal Diseases / epidemiology
- Gastrointestinal Diseases / surgery
- Hospitalization
- Humans
- Incidence
- Male
- Middle Aged
- Pandemics / statistics & numerical data*
- Pneumonia, Viral / epidemiology*
- Pneumonia, Viral / psychology
- Retrospective Studies
- SARS-CoV-2
- Stress Disorders, Traumatic, Acute / epidemiology
- Tertiary Care Centers / statistics & numerical data
- Turkey / epidemiology

Full text links

FREE full text article at www.journalagent.com LookUs Bilisim
Proceed to details
Cite
Share

☐ 323
Observational Study
Neurol Sci
. 2021 Jan;42(1):15-20.
doi: 10.1007/s10072-020-04775-x. Epub 2020 Oct 6.

Impact of COVID-19 on stroke admissions, treatments, and outcomes at a comprehensive stroke centre in the United Kingdom

Nishita Padmanabhan [1, 2], Indira Natarajan [3], Rachel Gunston [3], Marko Raseta [4], Christine Roffe [3, 5]
Affiliations Expand

Affiliations

- [1] Neurosciences, Royal Stoke University Hospital, Stoke-on-Trent, UK. nishita.padmanabhan@uhnm.nhs.uk.
- [2] Faculty of Medicine and Health Sciences, Keele University, Staffordshire, UK. nishita.padmanabhan@uhnm.nhs.uk.
- [3] Neurosciences, Royal Stoke University Hospital, Stoke-on-Trent, UK.
- [4] Statistics and Mathematical Modelling, Department of Molecular Genetics, Erasmus MC, Rotterdam, Netherlands.
- [5] Faculty of Medicine and Health Sciences, Keele University, Staffordshire, UK.

- PMID: **33021704**
- PMCID: PMC7537376
- DOI: 10.1007/s10072-020-04775-x

Free PMC article
Observational Study

Impact of COVID-19 on stroke admissions, treatments, and outcomes at a comprehensive stroke centre in the United Kingdom

Nishita Padmanabhan et al. Neurol Sci. 2021 Jan.
Free PMC article
Show details
Neurol Sci
. 2021 Jan;42(1):15-20.
doi: 10.1007/s10072-020-04775-x. Epub 2020 Oct 6.

Authors

Nishita Padmanabhan [1, 2], Indira Natarajan [3], Rachel Gunston [3], Marko Raseta [4], Christine Roffe [3, 5]

Affiliations

- [1] Neurosciences, Royal Stoke University Hospital, Stoke-on-Trent, UK. nishita.padmanabhan@uhnm.nhs.uk.
- [2] Faculty of Medicine and Health Sciences, Keele University, Staffordshire, UK. nishita.padmanabhan@uhnm.nhs.uk.
- [3] Neurosciences, Royal Stoke University Hospital, Stoke-on-Trent, UK.
- [4] Statistics and Mathematical Modelling, Department of Molecular Genetics, Erasmus MC, Rotterdam, Netherlands.
- [5] Faculty of Medicine and Health Sciences, Keele University, Staffordshire, UK.

- PMID: **33021704**
- PMCID: PMC7537376
- DOI: 10.1007/s10072-020-04775-x

Abstract

Introduction: The coronavirus disease (COVID-19) pandemic has changed routine clinical practice worldwide with major impacts on the provision of care and treatment for stroke patients.

Methods: This retrospective observational study included all patients admitted to the Royal Stoke University Hospital in Stoke-on-Trent, UK, with a stroke or transient ischaemic attack between March 15th and April 14th, 2020 (COVID). Patient demographics, characteristics of the stroke, treatment details and logistics were compared with patients admitted in the corresponding weeks in the year before (2019).

Results: There was a 39.5% (n = 101 vs n = 167) reduction in admissions in the COVID cohort compared with 2019 with more severe strokes (median National Institutes of Health Stroke Scale (NIHSS) 7 vs 4, p = 0.02), and fewer strokes with no visible acute pathology (21.8 vs 37.1%, p = 0.01) on computed tomography. There was no statistically significant difference in the rates of thrombolysis (10.9 vs 13.2%, p = 0.72) and/or thrombectomy (5.9 vs 4.8%, p = 0.90) and no statistically significant difference in time from stroke onset to arrival at hospital (734 vs 576 min, p = 0.34), door-to-needle time for thrombolysis (54 vs 64 min, p = 0.43) and door-to-thrombectomy time (181 vs 445 min, p = 0.72). Thirty-day mortality was not significantly higher in the COVID year (10.9 vs 8.9%, p = 0.77). None of the 7 stroke patients infected with COVID-19 died.

Conclusions: During the COVID-19 pandemic, the number of stroke admissions fell, and stroke severity increased. There was no statistically significant change in the delivery of thrombolysis and mechanical thrombectomy and no increase in mortality.

Keywords: COVID-19; Infarct; Mortality; Stroke; Thrombectomy; Thrombolysis.

Conflict of interest statement

The authors declare that they have no conflict of interest.

- Cited by 13 articles
- 17 references

Supplementary info

Publication types, MeSH terms Expand

Publication types

- Comparative Study
- Observational Study

MeSH terms

- Aged
- Aged, 80 and over
- COVID-19*
- Female
- Humans
- Ischemic Attack, Transient / drug therapy
- Ischemic Attack, Transient / therapy*
- Male
- Mechanical Thrombolysis / statistics & numerical data*
- Mechanical Thrombolysis / trends
- Middle Aged
- Patient Admission / statistics & numerical data*
- Patient Admission / trends
- Retrospective Studies
- Severity of Illness Index
- Stroke / drug therapy
- Stroke / therapy*
- Tertiary Care Centers / statistics & numerical data*
- Tertiary Care Centers / trends
- Thrombolytic Therapy / statistics & numerical data*
- Thrombolytic Therapy / trends
- United Kingdom

Full text links

SpringerLink FULL-TEXT ARTICLE Springer Free PMC article
Proceed to details
Cite
Share

☐ 324
Observational Study
Hypertension
. 2021 Mar 3;77(3):846-855.
doi: 10.1161/HYPERTENSIONAHA.120.16472. Epub 2020 Dec 16.

Association Between Blood Pressure Control and Coronavirus Disease 2019 Outcomes in 45 418 Symptomatic Patients With Hypertension: An Observational Cohort Study

James P Sheppard [1], Brian D Nicholson [1], Joseph Lee [1], Dylan McGagh [1], Julian Sherlock [1], Constantinos Koshiaris [1], Jason Oke [1], Nicholas R Jones [1], William Hinton [1], Laura Armitage [1], Oliver Van Hecke [1], Sarah Lay-Flurrie [1], Clare R Bankhead [1], Harshana Liyanage [1], John Williams [1], Filipa Ferreira [1], Michael D Feher [1], Andrew J Ashworth [2], Mark P Joy [1], Simon de Lusignan [#] [1], F D Richard Hobbs [#] [1]
Affiliations Expand

Affiliations

- [1] From the Nuffield Department of Primary Care Health Sciences, University of Oxford, United Kingdom (J.P.S., B.D.N., J.L., D.M., J.S., C.K., J.O., N.R.J., W.H., L.A., O.V.H., S.L.-F., C.R.B., H.L., J.W., F.F., M.D.F., M.P.J., S.d.L., F.D.R.H.).
- [2] Bonhard Medical, Bonhard House, Bo'ness, United Kingdom (A.J.A.).

[#] Contributed equally.

- PMID: **33325240**
- PMCID: PMC7884248
- DOI: 10.1161/HYPERTENSIONAHA.120.16472

Free PMC article
Observational Study

Association Between Blood Pressure Control and Coronavirus Disease 2019 Outcomes in 45 418 Symptomatic Patients With Hypertension: An Observational Cohort Study

James P Sheppard et al. Hypertension. 2021.
Free PMC article
Show details
Hypertension
. 2021 Mar 3;77(3):846-855.
doi: 10.1161/HYPERTENSIONAHA.120.16472. Epub 2020 Dec 16.

Authors

James P Sheppard[1], Brian D Nicholson[1], Joseph Lee[1], Dylan McGagh[1], Julian Sherlock[1], Constantinos Koshiaris[1], Jason Oke[1], Nicholas R Jones[1], William Hinton[1], Laura Armitage[1], Oliver Van Hecke[1], Sarah Lay-Flurrie[1], Clare R Bankhead[1], Harshana Liyanage[1], John Williams[1], Filipa Ferreira[1], Michael D Feher[1], Andrew J Ashworth[2], Mark P Joy[1], Simon de Lusignan[#][1], F D Richard Hobbs[#][1]

Affiliations

- [1] From the Nuffield Department of Primary Care Health Sciences, University of Oxford, United Kingdom (J.P.S., B.D.N., J.L., D.M., J.S., C.K., J.O., N.R.J., W.H., L.A., O.V.H., S.L.-F., C.R.B., H.L., J.W., F.F., M.D.F., M.P.J., S.d.L., F.D.R.H.).
- [2] Bonhard Medical, Bonhard House, Bo'ness, United Kingdom (A.J.A.).

[#] Contributed equally.

- PMID: **33325240**
- PMCID: PMC7884248
- DOI: 10.1161/HYPERTENSIONAHA.120.16472

Abstract

Hypertension has been identified as a risk factor for coronavirus disease 2019 (COVID-19) and associated adverse outcomes. This study examined the association between preinfection blood pressure (BP) control and COVID-19 outcomes using data from 460 general practices in England. Eligible patients were adults with hypertension who were tested or diagnosed with COVID-19. BP control was defined by the most recent BP reading within 24 months of the index date (January 1, 2020). BP was defined as controlled (<130/80 mm Hg), raised (130/80-139/89 mm Hg), stage 1 uncontrolled (140/90-159/99 mm Hg), or stage 2 uncontrolled (≥160/100 mm Hg). The primary outcome was death within 28 days of COVID-19 diagnosis. Secondary outcomes were COVID-19 diagnosis and COVID-19-related hospital admission. Multivariable logistic regression was used to

examine the association between BP control and outcomes. Of the 45 418 patients (mean age, 67 years; 44.7% male) included, 11 950 (26.3%) had controlled BP. These patients were older, had more comorbidities, and had been diagnosed with hypertension for longer. A total of 4277 patients (9.4%) were diagnosed with COVID-19 and 877 died within 28 days. Individuals with stage 1 uncontrolled BP had lower odds of COVID-19 death (odds ratio, 0.76 [95% CI, 0.62-0.92]) compared with patients with well-controlled BP. There was no association between BP control and COVID-19 diagnosis or hospitalization. These findings suggest BP control may be associated with worse COVID-19 outcomes, possibly due to these patients having more advanced atherosclerosis and target organ damage. Such patients may need to consider adhering to stricter social distancing, to limit the impact of COVID-19 as future waves of the pandemic occur.

Keywords: COVID-19; blood pressure; electronic health records; mortality; pandemic.

Conflict of interest statement

None.

- Cited by 14 articles
- 28 references
- 3 figures

Supplementary info

Publication types, MeSH terms, Substances, Grant support Expand

Publication types

- Observational Study
- Research Support, Non-U.S. Gov't

MeSH terms

- Adult
- Aged
- Aged, 80 and over
- Antihypertensive Agents / therapeutic use
- Atherosclerosis / epidemiology
- Blood Pressure / drug effects*
- COVID-19 / epidemiology*
- COVID-19 / prevention & control
- Comorbidity
- England / epidemiology
- Ethnicity / statistics & numerical data
- Female
- Follow-Up Studies
- Hospitalization / statistics & numerical data

- Humans
- Hypertension / drug therapy
- Hypertension / epidemiology*
- Logistic Models
- Male
- Middle Aged
- Odds Ratio
- Pandemics*
- Primary Health Care / statistics & numerical data
- Retrospective Studies
- Risk Factors
- SARS-CoV-2*
- Severity of Illness Index
- Survival Analysis
- Treatment Outcome

Substances

- Antihypertensive Agents

Grant support

- 211182/Wellcome Trust/United Kingdom
- NIHR300738/DH_/Department of Health/United Kingdom

Full text links

OPEN ACCESS Atypon Free PMC article
Proceed to details
Cite
Share
☐ 325
Observational Study
Eur J Endocrinol
. 2021 May 28;185(1):137-144.
doi: 10.1530/EJE-20-1447.

Serum sodium alterations in SARS CoV-2 (COVID-19) infection: impact on patient outcome

Andrea Berni [1], Danilo Malandrino [2], Giovanni Corona [3], Mario Maggi [4 5], Gabriele Parenti [4 6], Benedetta Fibbi [4 6], Loredana Poggesi [1 2], Alessandro Bartoloni [2 7], Federico Lavorini [2 8], Andrea Fanelli [9], Giulia Scocchera [9], Carlo Nozzoli [10], Adriano Peris [11], Filippo Pieralli [12], Riccardo Pini [2 13], Andrea Ungar [2 14], Alessandro Peri [4 5 6]
Affiliations Expand

Affiliations

- [1] Internal Medicine Unit 3, Careggi University Hospital, Florence, Italy.
- [2] Department of Experimental and Clinical Medicine, University of Florence, Florence, Italy.
- [3] Endocrinology Unit, Medical Department, Azienda Usl Bologna Maggiore-Bellaria Hospital, Bologna, Italy.
- [4] Endocrinology Unit, Careggi University Hospital, Florence, Italy.
- [5] Department of Experimental and Clinical Biomedical Sciences 'Mario Serio', University of Florence, Florence, Italy.
- [6] Pituitary Diseases and Sodium Alterations Unit, Careggi University Hospital, Florence, Italy.
- [7] Infectious and Tropical Diseases Unit, Careggi University Hospital, Florence, Italy.
- [8] Pneumology and Thoraco Pulmonary Pathophysiology Unit, Careggi University Hospital, Florence, Italy.
- [9] Internal Medicine Unit 2, Careggi University Hospital, Florence, Italy.
- [10] Internal Medicine Unit 1, Careggi University Hospital, Florence, Italy.
- [11] Intensive Care Unit and Regional ECMO Referral Center, Careggi University Hospital, Florence, Italy.
- [12] High Intensity Internal Medicine Unit, Careggi University Hospital, Florence, Italy.
- [13] Department of Internal and Emergency Medicine, Careggi Hospital, Florence, Italy.
- [14] Geriatric-UTIG Unit, Careggi University Hospital, Florence, Italy.

- PMID: **33950864**
- DOI: 10.1530/EJE-20-1447

Observational Study

Serum sodium alterations in SARS CoV-2 (COVID-19) infection: impact on patient outcome

Andrea Berni et al. Eur J Endocrinol. 2021.
Show details
Eur J Endocrinol
. 2021 May 28;185(1):137-144.
doi: 10.1530/EJE-20-1447.

Authors

Andrea Berni [1], Danilo Malandrino [2], Giovanni Corona [3], Mario Maggi [4 5], Gabriele Parenti [4 6], Benedetta Fibbi [4 6], Loredana Poggesi [1 2], Alessandro Bartoloni [2 7], Federico Lavorini [2 8], Andrea Fanelli [9], Giulia Scocchera [9], Carlo Nozzoli [10], Adriano Peris [11], Filippo Pieralli [12], Riccardo Pini [2 13], Andrea Ungar [2 14], Alessandro Peri [4 5 6]

Affiliations

- [1] Internal Medicine Unit 3, Careggi University Hospital, Florence, Italy.
- [2] Department of Experimental and Clinical Medicine, University of Florence, Florence, Italy.
- [3] Endocrinology Unit, Medical Department, Azienda Usl Bologna Maggiore-Bellaria Hospital, Bologna, Italy.
- [4] Endocrinology Unit, Careggi University Hospital, Florence, Italy.
- [5] Department of Experimental and Clinical Biomedical Sciences 'Mario Serio', University of Florence, Florence, Italy.
- [6] Pituitary Diseases and Sodium Alterations Unit, Careggi University Hospital, Florence, Italy.
- [7] Infectious and Tropical Diseases Unit, Careggi University Hospital, Florence, Italy.
- [8] Pneumology and Thoraco Pulmonary Pathophysiology Unit, Careggi University Hospital, Florence, Italy.
- [9] Internal Medicine Unit 2, Careggi University Hospital, Florence, Italy.
- [10] Internal Medicine Unit 1, Careggi University Hospital, Florence, Italy.
- [11] Intensive Care Unit and Regional ECMO Referral Center, Careggi University Hospital, Florence, Italy.
- [12] High Intensity Internal Medicine Unit, Careggi University Hospital, Florence, Italy.
- [13] Department of Internal and Emergency Medicine, Careggi Hospital, Florence, Italy.
- [14] Geriatric-UTIG Unit, Careggi University Hospital, Florence, Italy.

- PMID: **33950864**
- DOI: 10.1530/EJE-20-1447

Abstract

Objective: Hyponatremia is the most common electrolyte disorder in hospitalized patients and occurs in about 30% of patients with pneumonia. Hyponatremia has been associated with a worse outcome in several pathologic conditions The main objective of this study was to determine whether serum sodium alterations may be independent predictors of the outcome of hospitalized COVID-19 patients.

Design and methods: In this observational study, data from 441 laboratory-confirmed COVID-19 patients admitted to a University Hospital were collected. After excluding 61 patients (no serum sodium at admission available, saline solution infusion before sodium assessment, transfer from another hospital), data from 380 patients were analyzed.

Results: 274 (72.1%) patients had normonatremia at admission, 87 (22.9%) patients had hyponatremia and 19 (5%) patients had hypernatremia. We found an inverse correlation between serum sodium and IL-6, whereas a direct correlation between serum sodium and PaO2/FiO2 ratio was observed. Patients with hyponatremia had a higher prevalence of non-invasive ventilation and ICU transfer than those with normonatremia or hypernatremia. Hyponatremia was an independent

predictor of in-hospital mortality (2.7-fold increase vs normonatremia) and each mEq/L of serum sodium reduction was associated with a 14.4% increased risk of death.

Conclusions: These results suggest that serum sodium at admission may be considered as an early prognostic marker of disease severity in hospitalized COVID-19 patients.

- Cited by 7 articles

Supplementary info

Publication types, MeSH terms, Substances Expand

Publication types

- Observational Study

MeSH terms

- Aged
- Aged, 80 and over
- COVID-19 / blood*
- COVID-19 / epidemiology
- COVID-19 / mortality
- Comorbidity
- Critical Care / statistics & numerical data
- Female
- Fluorocarbons / blood
- Hospital Mortality
- Hospitalization / statistics & numerical data
- Humans
- Hydrocarbons, Brominated / blood
- Hypernatremia / epidemiology
- Hyponatremia / epidemiology
- Interleukin-6 / blood
- Male
- Middle Aged
- Respiration, Artificial / statistics & numerical data
- Retrospective Studies
- SARS Virus
- SARS-CoV-2*
- Severity of Illness Index*
- Sodium / blood*

Substances

- Fluorocarbons
- Hydrocarbons, Brominated
- Interleukin-6
- Sodium
- perflubron

Full text links

bioscientifica FULL TEXT Sheridan PubFactory
Proceed to details
Cite
Share
326
Observational Study
Respir Med
. Aug-Sep 2021;185:106495.
doi: 10.1016/j.rmed.2021.106495. Epub 2021 Jun 8.

Symptoms in patients with asthma infected by SARS-CoV-2

Eduardo Garcia-Pachon [1], Sandra Ruiz-Alcaraz [2], Carlos Baeza-Martinez [2], Lucia Zamora-Molina [2], Maria J Soler-Sempere [2], Isabel Padilla-Navas [2], Justo Grau-Delgado [2]
Affiliations Expand

Affiliations

- [1] Section of Respiratory Medicine, Hospital General Universitario de Elche, Elche, Alicante, Spain. Electronic address: egpachon@gmail.com.
- [2] Section of Respiratory Medicine, Hospital General Universitario de Elche, Elche, Alicante, Spain.

- PMID: **34126579**
- PMCID: PMC8184356
- DOI: 10.1016/j.rmed.2021.106495

Free PMC article
Observational Study

Symptoms in patients with asthma infected by SARS-CoV-2

Eduardo Garcia-Pachon et al. Respir Med. Aug-Sep 2021.
Free PMC article
Show details
Respir Med
. Aug-Sep 2021;185:106495.
doi: 10.1016/j.rmed.2021.106495. Epub 2021 Jun 8.

Authors

Eduardo Garcia-Pachon [1], Sandra Ruiz-Alcaraz [2], Carlos Baeza-Martinez [2], Lucia Zamora-Molina [2], Maria J Soler-Sempere [2], Isabel Padilla-Navas [2], Justo Grau-Delgado [2]

Affiliations

- [1] Section of Respiratory Medicine, Hospital General Universitario de Elche, Elche, Alicante, Spain. Electronic address: egpachon@gmail.com.
- [2] Section of Respiratory Medicine, Hospital General Universitario de Elche, Elche, Alicante, Spain.

- PMID: **34126579**
- PMCID: PMC8184356
- DOI: 10.1016/j.rmed.2021.106495

Abstract

Introduction: Organ tropism of SARS-CoV-2 to the respiratory tract could potentially aggravate asthma. The susceptibility of patients with asthma to develop an exacerbation when they are infected with SARS-CoV-2 is unknown. We aimed to investigate the symptoms presented in patients with asthma who became infected with SARS-CoV-2.

Methods and results: All patients over 14 years of age who tested positive for SARS-CoV-2 (by RT-PCR) were included (n = 2995). In patients with asthma (n = 77, 2.6%; 44 females), symptoms, therapy and phenotype were recorded. Seventeen (22%) patients had mild asthma, 55 (71%) moderate and five severe (6%). Twenty-six patients with asthma (34%) were asymptomatic, 34 (44%) developed symptoms but did not require hospital admission, and 17 (22%) were hospitalised. One patient was admitted because of asthma exacerbation without pneumonia or other symptoms. Ten patients (13%) had wheezes (six with pneumonia). Comparison of wheezing between patients with non-T2 asthma and the rest of the patients was statistically significant, ($p < 0.001$).

Conclusions: SARS-CoV-2 infection is not a significant cause of asthma exacerbation, although some patients may present wheezing, especially in cases of pneumonia. The severity of asthma does not seem to be associated with symptoms of the disease.

Keywords: Asthma; Covid-19; Virus.

Copyright © 2021 Elsevier Ltd. All rights reserved.

Conflict of interest statement

The authors declare that they have no known competing financial interests or personal relationships that could have appeared to influence the work reported in this paper.

- Cited by 1 article
- 12 references

Supplementary info

Publication types, MeSH terms Expand

Publication types

- Observational Study

MeSH terms

- Adult
- Asthma / diagnosis*
- Asthma / epidemiology
- COVID-19 / epidemiology*
- Comorbidity
- Female
- Humans
- Male
- Middle Aged
- Retrospective Studies
- SARS-CoV-2*
- Severity of Illness Index

Full text links

ELSEVIER FULL-TEXT ARTICLE Elsevier Science Free PMC article
Proceed to details
Cite
Share
☐ 327
Observational Study
J Med Virol
. 2021 May;93(5):2971-2978.
doi: 10.1002/jmv.26828. Epub 2021 Feb 9.

Clinical characteristics and outcomes of COVID-19 in people living with HIV in Belgium: A multicenter, retrospective cohort

Rakan Nasreddine [1], Eric Florence [2], Michel Moutschen [3], Jean-Cyr Yombi [4], Jean-Christophe Goffard [5], Inge Derdelinckx [6], Patrick Lacor [7], Linos Vandekerckhove [8], Peter Messiaen [9], Stefaan Vandecasteele [10], Marc Delforge [1], Stéphane De Wit [1], Belgian Research on AIDS and HIV Consortium (BREACH)
Affiliations Expand

Affiliations

- [1] Division of Infectious Diseases, Saint-Pierre University Hospital, Université Libre de Bruxelles, Brussels, Belgium.
- [2] Department of Clinical Sciences, Institute of Tropical Medicine, Antwerp, Belgium.
- [3] Department of Infectious Diseases, Liège University Hospital, Université de Liège, Liège, Belgium.
- [4] Department of Internal Medicine and Infectious Diseases, Saint-Luc University Hospital, Université Catholique de Louvain, Brussels, Belgium.
- [5] Department of Internal Medicine, Erasme University Hospital, Université Libre de Bruxelles, Brussels, Belgium.
- [6] Department of Internal Medicine, Leuven University Hospital, Katholieke Universiteit Leuven, Leuven, Belgium.
- [7] Department of Internal Medicine and Infectious Diseases, Brussels University Hospital, Vrije Universiteit Brussel, Brussels, Belgium.
- [8] Department of Internal Medicine and Pediatrics, Ghent University Hospital, Ghent University, Ghent, Belgium.
- [9] Department of Infectious Diseases and Immunity, Jessa Hospital, Hasselt, Belgium.
- [10] Department of Nephrology and Infectious Diseases, General Hospital Sint-Jan Brugge-Oostende AV, Bruges, Belgium.

- PMID: **33506953**
- PMCID: PMC8014531
- DOI: 10.1002/jmv.26828

Free PMC article
Observational Study

Clinical characteristics and outcomes of COVID-19 in people living with HIV in Belgium: A multicenter, retrospective cohort

Rakan Nasreddine et al. J Med Virol. 2021 May.
Free PMC article

Show details

J Med Virol

. 2021 May;93(5):2971-2978.
doi: 10.1002/jmv.26828. Epub 2021 Feb 9.

Authors

Rakan Nasreddine [1], Eric Florence [2], Michel Moutschen [3], Jean-Cyr Yombi [4], Jean-Christophe Goffard [5], Inge Derdelinckx [6], Patrick Lacor [7], Linos Vandekerckhove [8], Peter Messiaen [9], Stefaan Vandecasteele [10], Marc Delforge [1], Stéphane De Wit [1], Belgian Research on AIDS and HIV Consortium (BREACH)

Affiliations

- [1] Division of Infectious Diseases, Saint-Pierre University Hospital, Université Libre de Bruxelles, Brussels, Belgium.
- [2] Department of Clinical Sciences, Institute of Tropical Medicine, Antwerp, Belgium.
- [3] Department of Infectious Diseases, Liège University Hospital, Université de Liège, Liège, Belgium.
- [4] Department of Internal Medicine and Infectious Diseases, Saint-Luc University Hospital, Université Catholique de Louvain, Brussels, Belgium.
- [5] Department of Internal Medicine, Erasme University Hospital, Université Libre de Bruxelles, Brussels, Belgium.
- [6] Department of Internal Medicine, Leuven University Hospital, Katholieke Universiteit Leuven, Leuven, Belgium.
- [7] Department of Internal Medicine and Infectious Diseases, Brussels University Hospital, Vrije Universiteit Brussel, Brussels, Belgium.
- [8] Department of Internal Medicine and Pediatrics, Ghent University Hospital, Ghent University, Ghent, Belgium.
- [9] Department of Infectious Diseases and Immunity, Jessa Hospital, Hasselt, Belgium.
- [10] Department of Nephrology and Infectious Diseases, General Hospital Sint-Jan Brugge-Oostende AV, Bruges, Belgium.

- PMID: **33506953**
- PMCID: PMC8014531
- DOI: 10.1002/jmv.26828

Abstract

The aim of this study was to describe the clinical characteristics and outcomes of coronavirus disease 2019 (COVID-19) among people living with HIV (PLWH) in Belgium. We performed a retrospective multicenter cohort analysis of PLWH with either laboratory-confirmed, radiologically diagnosed, or clinically suspected COVID-19 between February 15, 2020 and May 31, 2020. The primary endpoint was outcome of COVID-19. Secondary endpoints included rate of hospitalization and length of hospital stay and rate of Intensive Care Unit (ICU) admission and mechanical ventilation. One hundred and one patients were included in this study. Patients were categorized as having either laboratory-confirmed (n = 65), radiologically-diagnosed (n = 3), or clinically suspected COVID-19 (n = 33). The median age was 51.3 years (interquartile range [IQR] 41.3-57.3) and 44% were female. Ninety-four percent of patients were virologically

suppressed and 67% had a $CD4^+$ cell count more than or equal to 500 cells/µl. Overall, 46% of patients required hospitalization and the median length of hospital stay was 6 days (IQR 3-15). Age more than or equal to 50 years, Black Sub-Saharan African patients, and being on an integrase strand transfer inhibitor-based regimen were associated with being hospitalized. ICU admission and mechanical ventilation was required for 15% and 10% of all patients respectively. Overall, 9% of patients died while 78 (77%) patients made a full recovery. HIV patients with COVID-19 experienced a high degree of hospitalization despite having elevated $CD4^+$ cell counts and a high rate of virologic suppression. Matched case-control studies are warranted to measure the impact that HIV may have on patients with COVID-19.

Keywords: Belgium; COVID-19; clinical characteristics; outcomes; people living with HIV.

© 2021 Wiley Periodicals LLC.

Conflict of interest statement

The authors declare that there are no conflict of interests.

- Cited by 1 article
- 26 references

Supplementary info

Publication types, MeSH terms Expand

Publication types

- Multicenter Study
- Observational Study

MeSH terms

- Adult
- Belgium / epidemiology
- CD4 Lymphocyte Count
- COVID-19 / diagnosis*
- COVID-19 / epidemiology*
- COVID-19 / therapy
- Case-Control Studies
- Female
- HIV Infections / epidemiology*
- HIV Infections / immunology
- Hospitalization
- Humans
- Male
- Middle Aged

- Retrospective Studies
- Risk Factors
- SARS-CoV-2
- Treatment Outcome

Full text links

WILEY Full Text Article Wiley Free PMC article
Proceed to details
Cite
Share
328

Observational Study
Medicine (Baltimore)
. 2021 Sep 3;100(35):e26752.
doi: 10.1097/MD.0000000000026752.

Assessment of outcomes of elective cancer surgeries in children during coronavirus disease 2019 pandemic: Retrospective cohort study from a tertiary cancer center in India

Sajid S Qureshi [1, 2], Deepak Ramraj [1], Girish Chinnaswamy [2, 3], Badira C Parambil [2, 3], Maya Prasad [2, 3], Nayana Amin [2, 4], Subramaniam Ramanathan [2, 3], Nehal Khanna [2, 5], Siddharth Laskar [2, 5]
Affiliations Expand

Affiliations

- [1] Division of Paediatric Surgical Oncology, Department of Surgical Oncology, Tata Memorial Hospital and Advanced Centre for Training Research and Education in Cancer (ACTREC), Tata Memorial Centre, Mumbai, India.
- [2] Homi Bhabha National Institute (HBNI), Mumbai, India.
- [3] Division of Pediatric Oncology, Department of Medical Oncology, Tata Memorial Hospital and Advanced Centre for Training Research and Education in Cancer (ACTREC), Tata Memorial Centre, Mumbai, India.
- [4] Department of Anaesthesia, Tata Memorial Hospital, and Advanced Centre for Training Research and Education in Cancer (ACTREC), Tata Memorial Centre, Mumbai, India.
- [5] Department of Radiation Oncology, Tata Memorial Hospital and Advanced Centre for Training Research and Education in Cancer (ACTREC), Tata Memorial Centre, Mumbai, India.

- PMID: **34477115**
- PMCID: PMC8415926

- DOI: 10.1097/MD.0000000000026752

Free PMC article
Observational Study

Assessment of outcomes of elective cancer surgeries in children during coronavirus disease 2019 pandemic: Retrospective cohort study from a tertiary cancer center in India

Sajid S Qureshi et al. Medicine (Baltimore). 2021.
Free PMC article
Show details
Medicine (Baltimore)
. 2021 Sep 3;100(35):e26752.
doi: 10.1097/MD.0000000000026752.

Authors

Sajid S Qureshi [1, 2], Deepak Ramraj [1], Girish Chinnaswamy [2, 3], Badira C Parambil [2, 3], Maya Prasad [2, 3], Nayana Amin [2, 4], Subramaniam Ramanathan [2, 3], Nehal Khanna [2, 5], Siddharth Laskar [2, 5]

Affiliations

- [1] Division of Paediatric Surgical Oncology, Department of Surgical Oncology, Tata Memorial Hospital and Advanced Centre for Training Research and Education in Cancer (ACTREC), Tata Memorial Centre, Mumbai, India.
- [2] Homi Bhabha National Institute (HBNI), Mumbai, India.
- [3] Division of Pediatric Oncology, Department of Medical Oncology, Tata Memorial Hospital and Advanced Centre for Training Research and Education in Cancer (ACTREC), Tata Memorial Centre, Mumbai, India.
- [4] Department of Anaesthesia, Tata Memorial Hospital, and Advanced Centre for Training Research and Education in Cancer (ACTREC), Tata Memorial Centre, Mumbai, India.
- [5] Department of Radiation Oncology, Tata Memorial Hospital and Advanced Centre for Training Research and Education in Cancer (ACTREC), Tata Memorial Centre, Mumbai, India.

- PMID: **34477115**
- PMCID: PMC8415926
- DOI: 10.1097/MD.0000000000026752

Abstract

To describe the outcomes of elective cancer surgeries and adverse consequences on the patients and medical staff due to the surgical interventions in children during the Coronavirus Disease

2019 (COVID-19) pandemic.The study included children younger than 15 years who underwent elective cancer surgeries from March 4, 2020 and December 3, 2020.A total of 121 patients (62% male; median age, 3 years) underwent surgery. The surgical procedures included nephrectomies (n = 18), neuroblastoma (n = 26) and soft tissue tumor resections (n = 24) and complex surgical procedures like extended liver resections (n = 2), intra-atrial thrombectomy under cardiopulmonary bypass (n = 2), pancreatoduodenectomy (n = 1), and free microvascular flaps (n = 7). Clavien-Dindo Grade III complications were 5% (n = 6), and there were no postoperative deaths. Preoperative COVID-19 testing was performed in 82% of children, and only 2% showed severe acute respiratory syndrome coronavirus 2 positivity. Postoperatively, 26 children were tested because of specific symptoms and, 6 tested positive for severe acute respiratory syndrome coronavirus 2. Except for a median delay of 23 days in treatment, none of the patients with COVID-19 required critical hospital management. None of the surgical residents or faculty acquired COVID-19, while 4 each medical and support staff were tested positive in the study period.COVID-19 was not a deterrent for continued cancer care, and surgeries could be safely performed adopting universal preventive measures without any added morbidity from COVID-19. Caregivers and centers dealing with childhood cancers can be encouraged to sustain or seek early healthcare.

Copyright © 2021 the Author(s). Published by Wolters Kluwer Health, Inc.

Conflict of interest statement

The authors have no funding and conflicts of interest to disclose.

- 17 references

Supplementary info

Publication types, MeSH terms Expand

Publication types

- Observational Study

MeSH terms

- Adolescent
- COVID-19 / diagnosis
- COVID-19 / epidemiology
- Child
- Child, Preschool
- Elective Surgical Procedures / statistics & numerical data*
- Female
- Humans
- India / epidemiology
- Infant
- Male

- Neoplasms / surgery*
- Pandemics
- Retrospective Studies
- SARS-CoV-2
- Tertiary Care Centers / statistics & numerical data

Full text links

Wolters Kluwer Wolters Kluwer Free PMC article
Proceed to details
Cite
Share
329
Pan Afr Med J
. 2020 Jul 28;35(Suppl 2):125.
doi: 10.11604/pamj.supp.2020.35.2.24977. eCollection 2020.

The changing trend of teleconsultations during COVID-19 era at a tertiary facility in Tanzania

Philip Babatunde Adebayo [1], Ahmed Jusabani [2], Murtaza Mukhtar [3], Ali Akbar Zehri [4]
Affiliations Expand

Affiliations

- [1] Neurology Unit, Department of Medicine, Aga Khan, University, Dar Es Salaam, Tanzania.
- [2] Department of Radiology, Aga Khan Hospital, Dar es Salaam.
- [3] Outpatient Service Department, Aga Khan Hospital, Dar es Salaam.
- [4] Department of Surgery, Aga Khan University, Dar es Salaam.

- PMID: **33282080**
- PMCID: PMC7687499
- DOI: 10.11604/pamj.supp.2020.35.2.24977

Free PMC article

The changing trend of teleconsultations during COVID-19 era at a tertiary facility in Tanzania

Philip Babatunde Adebayo et al. Pan Afr Med J. 2020.
Free PMC article
Show details
Pan Afr Med J
. 2020 Jul 28;35(Suppl 2):125.
doi: 10.11604/pamj.supp.2020.35.2.24977. eCollection 2020.

Authors

Philip Babatunde Adebayo [1], Ahmed Jusabani [2], Murtaza Mukhtar [3], Ali Akbar Zehri [4]

Affiliations

- [1] Neurology Unit, Department of Medicine, Aga Khan, University, Dar Es Salaam, Tanzania.
- [2] Department of Radiology, Aga Khan Hospital, Dar es Salaam.
- [3] Outpatient Service Department, Aga Khan Hospital, Dar es Salaam.
- [4] Department of Surgery, Aga Khan University, Dar es Salaam.

- PMID: **33282080**
- PMCID: PMC7687499
- DOI: 10.11604/pamj.supp.2020.35.2.24977

Abstract

Introduction: the current COVID-19 pandemic has occasioned the increased adoption of telemedicine. This study reports the uptake and trend of a new teleconsultation service in a Tanzanian hospital.

Methods: this is a retrospective observational study that profiled requests for teleconsultations and uptake of the service between April 1, 2020, and June 30, 2020.

Results: two hundred and eighteen telephone inquiries were received over the 3 months. One hundred and sixteen (53.2%) individuals followed through with the teleconsultations. Paediatric (38.8%) and Internal medicine (32.8%) were the subspecialties with the highest number of teleconsultations. In a frame of 3 months, teleconsultation uptake was highest in May and lowest in June.

Conclusion: there was a steady rise and a rapid fall in requests and uptake of teleconsultation services over the period under evaluation. Lack of insurance coverage for teleconsultations was a significant barrier. We propose a re-education and reiteration of the benefits of telemedicine to all stakeholders. This is important for the current era and beyond.

Keywords: COVID-19; Tanzania; health services; teleconsultation; telemedicine.

©Philip Babatunde Adebayo et al.

Conflict of interest statement

The authors declare no competing interests.

- Cited by 1 article
- 9 references
- 1 figure

Supplementary info

MeSH terms Expand

MeSH terms

- COVID-19 / epidemiology
- COVID-19 / therapy*
- Delivery of Health Care / trends*
- Humans
- Remote Consultation / statistics & numerical data*
- Retrospective Studies
- SARS-CoV-2*
- Tanzania / epidemiology
- Tertiary Care Centers*

Full text links

Pan African Medical Journal Free PMC article
Proceed to details
Cite
Share
330
Observational Study
J Infect Public Health
. 2021 Nov;14(11):1668-1670.
doi: 10.1016/j.jiph.2021.09.013. Epub 2021 Sep 23.

Effects of cardiac toxicity of combination therapy with hydroxychloroquine and azithromycin in COVID-19 patients

Usha Yendrapalli [1], Hassoun Ali [2], Jacqueline L Green [3], Jonathan Edwards [4]
Affiliations Expand

Affiliations

- [1] Department of Internal Medicine, Huntsville Hospital, AL, USA. Electronic address: ushachowdhary.y@gmail.com.

- [2] Department of Infectious Disease, Huntsville Hospital, AL, USA. Electronic address: ali_hasoun@yahoo.com.
- [3] Department of Cardiology, Huntsville Hospital, AL, USA.
- [4] Department of Pharmacology, Huntsville Hospital, AL, USA.

- PMID: **34627063**
- PMCID: PMC8459546
- DOI: 10.1016/j.jiph.2021.09.013

Free PMC article
Observational Study

Effects of cardiac toxicity of combination therapy with hydroxychloroquine and azithromycin in COVID-19 patients

Usha Yendrapalli et al. J Infect Public Health. 2021 Nov.
Free PMC article
Show details
J Infect Public Health
. 2021 Nov;14(11):1668-1670.
doi: 10.1016/j.jiph.2021.09.013. Epub 2021 Sep 23.

Authors

Usha Yendrapalli [1], Hassoun Ali [2], Jacqueline L Green [3], Jonathan Edwards [4]

Affiliations

- [1] Department of Internal Medicine, Huntsville Hospital, AL, USA. Electronic address: ushachowdhary.y@gmail.com.
- [2] Department of Infectious Disease, Huntsville Hospital, AL, USA. Electronic address: ali_hasoun@yahoo.com.
- [3] Department of Cardiology, Huntsville Hospital, AL, USA.
- [4] Department of Pharmacology, Huntsville Hospital, AL, USA.

- PMID: **34627063**
- PMCID: PMC8459546
- DOI: 10.1016/j.jiph.2021.09.013

Abstract

Coronavirus disease 2019 (COVID-19), which began in China, caused a global pandemic. Few studies have shown the benefit of hydroxychloroquine (HY) ± azithromycin (AZ) for treating COVID-19. Concerns of QT prolongation and increased risks of torsade's de pointes (TdP) with this combination have been raised since each agent can individually prolong the QT interval. This retrospective, observational study included hospitalized patients treated with HY and AZ from

March 2020 to May 2020 at a large community hospital. Serial assessments of the QT interval were performed. Our aim is to evaluate the safety and characterize the change in QTc interval and arrhythmic events in COVID-19 patients treated with HY/AZ. A total of 21 COVID patients who received at least four days of HY and AZ were included in this study. Mean baseline was QTc 403 ms, mean maximum QTc was 440 ms, mean change in QTc was 36 ms. Only one patient (4.8%) developed prolonged QTc > 500 ms. No patient had a change in QTc of 60 ms or more. No patient developed TdP. Fifteen patients (71.4%) had hypoxia on admission, with only two patients (9.5%) required oxygen of 1-2 L at discharge. 80.9% of patients have been discharged home or inpatient rehabilitation.

Keywords: Azithromycin; COVID-19; Hydroxychloroquine; QT interval; Torsade de pointes.

Copyright © 2021. Published by Elsevier Ltd.

- 13 references
- 1 figure

Supplementary info

Publication types, MeSH terms, Substances, Supplementary concepts Expand

Publication types

- Observational Study

MeSH terms

- Azithromycin / adverse effects
- COVID-19* / drug therapy
- Cardiotoxicity
- Humans
- Hydroxychloroquine* / adverse effects
- Retrospective Studies
- SARS-CoV-2

Substances

- Hydroxychloroquine
- Azithromycin

Supplementary concepts

- COVID-19 drug treatment

Full text links

ELSEVIER OPEN ACCESS Elsevier Science Free PMC article
Proceed to details
Cite
Share
331
Observational Study
J Intern Med
. 2021 Jul;290(1):157-165.
doi: 10.1111/joim.13241. Epub 2021 Feb 5.

Assessment of thirty-day readmission rate, timing, causes and predictors after hospitalization with COVID-19

I Yeo [1, 2], S Baek [3], J Kim [1], H Elshakh [3], A Voronina [3], M S Lou [3], J Vapnik [3], R Kaler [3], X Dai [1], S Goldbarg [1]
Affiliations Expand

Affiliations

- [1] From the, Division of Cardiology, Department of Medicine, New York-Presbyterian Queens, Flushing, NY, USA.
- [2] Graduate School of Biomedical Science, Icahn School of Medicine at Mount Sinai, New York, NY, USA.
- [3] Department of Medicine, New York-Presbyterian Queens, Flushing, NY, USA.

- PMID: **33452824**
- PMCID: PMC8013754
- DOI: 10.1111/joim.13241

Free PMC article
Observational Study

Assessment of thirty-day readmission rate, timing, causes and predictors after hospitalization with COVID-19

I Yeo et al. J Intern Med. 2021 Jul.
Free PMC article
Show details
J Intern Med
. 2021 Jul;290(1):157-165.
doi: 10.1111/joim.13241. Epub 2021 Feb 5.

Authors

I Yeo [1, 2], S Baek [3], J Kim [1], H Elshakh [3], A Voronina [3], M S Lou [3], J Vapnik [3], R Kaler [3], X Dai [1], S Goldbarg [1]

Affiliations

- [1] From the, Division of Cardiology, Department of Medicine, New York-Presbyterian Queens, Flushing, NY, USA.
- [2] Graduate School of Biomedical Science, Icahn School of Medicine at Mount Sinai, New York, NY, USA.
- [3] Department of Medicine, New York-Presbyterian Queens, Flushing, NY, USA.

- PMID: **33452824**
- PMCID: PMC8013754
- DOI: 10.1111/joim.13241

Abstract

Background: There are limited data on the characteristics of 30-day readmission after hospitalization with coronavirus disease 2019 (COVID-19).

Objectives: To examine the rate, timing, causes, predictors and outcomes of 30-day readmission after COVID-19 hospitalization.

Methods: From 13 March to 9 April 2020, all patients hospitalized with COVID-19 and discharged alive were included in this retrospective observational study. Multivariable logistic regression was used to identify the predictors of 30-day readmission, and a restricted cubic spline function was utilized to assess the linearity of the association between continuous predictors and 30-day readmission.

Results: A total of 1062 patients were included in the analysis, with a median follow-up time of 62 days. The mean age of patients was 56.5 years, and 40.5% were women. At the end of the study, a total of 48 (4.5%) patients were readmitted within 30 days of discharge, and a median time to readmission was 5 days. The most common primary diagnosis of 30-day readmission was a hypoxic respiratory failure (68.8%) followed by thromboembolism (12.5%) and sepsis (6.3%). The patients with a peak serum creatinine level of ≥1.29 mg/dL during the index hospitalization, compared to those with a creatinine of <1.29 mg/dL, had 2.4 times increased risk of 30-day readmission (adjusted odds ratio: 2.41; 95% CI: 1.23-4.74). The mortality rate during the readmission was 22.9%.

Conclusion: With 4.5% of the thirty-day readmission rate, COVID-19 survivors were readmitted early after hospital discharge, mainly due to morbidities of COVID-19. One in five readmitted COVID-19 survivors died during their readmission.

Keywords: COVID-19; epidemiology; patient readmission; readmission mortality; readmission predictor.

© 2021 The Association for the Publication of the Journal of Internal Medicine.

Conflict of interest statement

All authors report no potential conflicts of interest to disclose.

- Cited by 17 articles
- 23 references
- 5 figures

Supplementary info

Publication types, MeSH terms Expand

Publication types

- Observational Study

MeSH terms

- COVID-19 / therapy*
- Female
- Hospitalization*
- Humans
- Male
- Middle Aged
- Pandemics
- Patient Readmission / statistics & numerical data*
- Pneumonia, Viral / therapy*
- Pneumonia, Viral / virology
- Prognosis
- Retrospective Studies
- Risk Factors
- SARS-CoV-2
- Time Factors

Full text links

WILEY Full Text Article Wiley Free PMC article

Proceed to details

Cite

Share

332

Observational Study

Am J Manag Care

. 2021 Apr 1;27(4):e135-e136.

doi: 10.37765/ajmc.2021.88623.

Small but nimble: measures taken to face the COVID-19 pandemic in small French military hospitals

Parisian Military Anti–COVID-19 Group

- PMID: **33877781**
- DOI: 10.37765/ajmc.2021.88623

Free article
Observational Study

Small but nimble: measures taken to face the COVID-19 pandemic in small French military hospitals

Parisian Military Anti–COVID-19 Group. Am J Manag Care. 2021.
Free article
Show details
Am J Manag Care
. 2021 Apr 1;27(4):e135-e136.
doi: 10.37765/ajmc.2021.88623.

Author

Parisian Military Anti–COVID-19 Group

- PMID: **33877781**
- DOI: 10.37765/ajmc.2021.88623

Abstract

Objectives: To describe a complete panel of actions of the Service de Santé des Armées (SSA) (ie, French Military Health Service) that together contributed to prevent French health system saturation during the coronavirus disease 2019 (COVID-19) pandemic.

Study design: Observational retrospective study.

Methods: Actions taken by military practitioners in the Parisian military hospitals, which contained 500 beds, to fight COVID-19 were listed and described.

Results: The Parisian military hospitals were fully reorganized to offer 147% more intensive care unit beds and took care of 665 inpatients with COVID-19 while continuing their core mission of war-wounded military care. A strategy to prioritize the use of medicine and medical devices was designed to avoid shortages. Field intensive care unit deployment and airborne collective medical evacuation by the SSA's MoRPHEE system avoided hospital saturation.

Conclusions: Key facets of this achievement were interunit collaboration, esprit de corps, and health workers' adaptability. Small hospitals can provide a coherent answer to the COVID-19 pandemic, as long as they organize and prioritize the patients' care.

Supplementary info

Publication types, MeSH terms Expand

Publication types

- Observational Study

MeSH terms

- COVID-19 / prevention & control*
- France / epidemiology
- Health Personnel / organization & administration
- Hospitals, Military / organization & administration*
- Humans
- Intensive Care Units / organization & administration
- Retrospective Studies
- SARS-CoV-2

Full text links

AJMC FREE FULL TEXT Managed Care & Healthcare Communications, LLC
Proceed to details
Cite
Share
333
Observational Study
Ann Ital Chir
. 2021;92:317-322.

Impact of SARS-Cov-2 pandemic on Emergency General Surgery. A single-center observational study

Alfonso Amendola, Giuseppe Palomba, Maria Gaudiello, Vincenza Paola Dinuzzi, Ester Marra, Ferdinando Fusco, Michele Lanza, Massimo Antropoli, Antonio Brillantino, Federica Mastella, Maurizio Castriconi

- PMID: **34312333**

Observational Study

Impact of SARS-Cov-2 pandemic on Emergency General Surgery. A single-center observational study

Alfonso Amendola et al. Ann Ital Chir. 2021.
Show details
Ann Ital Chir
. 2021;92:317-322.

Authors

Alfonso Amendola, Giuseppe Palomba, Maria Gaudiello, Vincenza Paola Dinuzzi, Ester Marra, Ferdinando Fusco, Michele Lanza, Massimo Antropoli, Antonio Brillantino, Federica Mastella, Maurizio Castriconi

- PMID: **34312333**

Abstract
in English, Italian

Introduction: Several articles have been published on impact COVID-19 infection about reduction of surgical activity. We have focused on the differences between our surgical activity in an Emergency General Surgery department in "Cardarelli" Hospital in Naples.

Methods: This retrospective study compared the patients treated from March 9, 2020 to April 27, 2020 (Italian lockdown time) and the patients treated in the same period of 2019. We recruited 75 patients in Group A (2020) and 165 patients in Group B (2019).

Results: There was a reduction in hospitalizations for non-trauma disease (69 in group A and 122 in group B with p: 0.001), a reduction in transfers from other hospitals (2 patients in group A and 17 in group B with p. 0.04) and a reduction in hospitalizations for trauma disease (6 in group A and 43 in group B with p: 0.001). The severity of the disease in 2020 was greater than in the same period in 2019 and there was a higher rate of high-grade complications CONCLUSION: From data analysis, we conclude that there has been a reduction in hospitalizations and surgical interventions in our emergency surgery department. The patients, however, had a much more severe disease that resulted in a greater number of complications in the peri and post-operative time.

Key words: COVID-19, Lockdown, Emergency Surgery, Severity of disease.

Sono stati pubblicati numerosi articoli sull'impatto dell'infezione da COVID-19 sulla riduzione dell'attività chirurgica. Ci siamo pertanto interessati a confrontare la differenza tra l'attività chirurgica in un dipartimento di Chirurgia generale d'urgenza dell'Ospedale Cardarelli di Napoli in questa evenienza. Si tratta di uno studio retrospettivo comparativo dei pazienti trattati dal 9 Marzo 2020 al 27 Aprile dello stesso anno – nel periodo di lock down in Italia – ed i pazienti trattati nello stesso periodo del 2019. Abbiamo reclutato 75 pazienti nel Gruppo A (2020) e 165

pazienti nel Gruppo B (2019). Abbiamo riscontrato una riduzione dei ricoveri ospedalieri per patologia non traumatica (69 nel Gruppo A e 122 nel Gruppo B, con p. 0.001), una riduzione dei trasferimenti da altri ospedali (2 pazienti nel Gruppo A e 17 nel Gruppo B, con p. 0.04) e una riduzione dei ricoveri ospedalieri per patologia traumatica (6 nel Gruppo A e 43 nel Gruppo B, con p. 0.001). La gravità delle patologie nel 2020 è risultata superioe nel 2020 rispetto allo stesso periodo nel 2019, così come la gravità delle complicanze. Dall'analisi di nostri dati possiamo concludere che c'è stata una riduzione dell'ospedalizzazione e degli interventi chirurgici nel nostro dipartimento. Infine, i pazienti presentavano presentavano patologie di maggiore gravità, con un aumento del numero di complicanze sia peri- che postoperatorie.

Supplementary info

Publication types, MeSH terms Expand

Publication types

- Comparative Study
- Observational Study

MeSH terms

- COVID-19*
- Communicable Disease Control
- Emergency Service, Hospital
- General Surgery
- Hospitalization*
- Humans
- Italy
- Pandemics*
- Retrospective Studies
- SARS-CoV-2*
- Severity of Illness Index
- Surgical Procedures, Operative*
- Workload

Proceed to details

Cite

Share

☐ 334

Observational Study

Pan Afr Med J

. 2021 Mar 8;38:246.

doi: 10.11604/pamj.2021.38.246.28169. eCollection 2021.

Clinical and epidemiological characteristics and outcomes of patients hospitalized for COVID-19 in Douala, Cameroon

David Mekolo [1], Francois Adrien Bokalli [1, 2], Fru McWright Chi [2], Steve Beukou Fonkou [1, 2], Mbachan Maseoli Takere [2], Conrald Metuge Ekukole [2], Jean Moise Bikoy Balomoth [1], Dickson Shey Nsagha [3], Noel Emmanuel Essomba [1], Louis Richard Njock [4], Marcellin Ngowe Ngowe [4]
Affiliations Expand

Affiliations

- [1] Intensive Care and Emergency Department, Laquintinie Hospital, Douala, Cameroon.
- [2] Department of Medicine, Faculty of Health Sciences, University of Buea, Buea Cameroon.
- [3] Department of Public Health and Hygiene, Faculty of Health Sciences, University of Buea, Buea Cameroon.
- [4] Department of Surgery and Specialties, Faculty of Medicine and Pharmaceutical Sciences, University of Douala, Douala, Cameroon.

- PMID: **34104294**
- PMCID: PMC8164425
- DOI: 10.11604/pamj.2021.38.246.28169

Free PMC article
Observational Study

Clinical and epidemiological characteristics and outcomes of patients hospitalized for COVID-19 in Douala, Cameroon

David Mekolo et al. Pan Afr Med J. 2021.
Free PMC article
Show details
Pan Afr Med J
. 2021 Mar 8;38:246.
doi: 10.11604/pamj.2021.38.246.28169. eCollection 2021.

Authors

David Mekolo [1], Francois Adrien Bokalli [1, 2], Fru McWright Chi [2], Steve Beukou Fonkou [1, 2], Mbachan Maseoli Takere [2], Conrald Metuge Ekukole [2], Jean Moise Bikoy Balomoth [1], Dickson Shey Nsagha [3], Noel Emmanuel Essomba [1], Louis Richard Njock [4], Marcellin Ngowe Ngowe [4]

Affiliations

- [1] Intensive Care and Emergency Department, Laquintinie Hospital, Douala, Cameroon.
- [2] Department of Medicine, Faculty of Health Sciences, University of Buea, Buea Cameroon.
- [3] Department of Public Health and Hygiene, Faculty of Health Sciences, University of Buea, Buea Cameroon.
- [4] Department of Surgery and Specialties, Faculty of Medicine and Pharmaceutical Sciences, University of Douala, Douala, Cameroon.

- PMID: **34104294**
- PMCID: PMC8164425
- DOI: 10.11604/pamj.2021.38.246.28169

Abstract

Introduction: the coronavirus disease (COVID-19) is a disease that originated from Wuhan in December 2019. It rapidly spread across the globe causing high mortality especially among the elderly. Africa though not spared has limited studies regarding its effects on its population. We therefore sought to describe the epidemiological and clinical characteristics of COVID-19 in Douala, Cameroon.

Methods: we conducted a single-centre, retrospective, and observational study by reviewing records of patients managed for COVID-19 between the 8th March 2020 and 31st, May 2020. Cases were confirmed by real-time reverse transcriptase - polymerase chain reaction and were analysed for epidemiological, demographic, clinical, and radiological features. Outcomes were either clinical improvement by Day-28 or in-hospital mortality.

Results: we analyzed 282 case files, 192 were males (M: F=2: 1). The mean age was 52 (+/- 15) years. Hypertension and diabetes accounted for 75% of the chronic medical conditions identified. Main presenting complaints were dyspnea, cough, asthenia, and fever (55-60%). Radiographic analysis showed a ground-glass appearance in 85% of cases. Chloroquine/Hydroxychloroquine was the most (91.8%) frequently used drug in management protocols, 35% needed oxygen supplementation while 6 patients were intubated. Severe pneumonia (11.3%) was the commonest complication. They were 91 admissions in the intensive care unit. The average length of hospital stay was 10 (+/- 5) days. The mortality rate was 32%.

Conclusion: our findings are concordant with universally reported data of COVID-19 hospitalised patients. These parameters are essential in designing effective prevention and control programs aimed at reducing the impact of the COVID-19 pandemic particularly in countries with limited resources.

Keywords: COVID-19; Cameroon; clinical characteristics; epidemiology; outcomes.

Copyright: David Mekolo et al.

Conflict of interest statement

The authors declare no competing interests.

- Cited by 1 article
- 31 references
- 2 figures

Supplementary info

Publication types, MeSH terms, Supplementary concepts Expand

Publication types

- Observational Study

MeSH terms

- Adult
- Aged
- COVID-19 / drug therapy
- COVID-19 / epidemiology
- COVID-19 / mortality
- COVID-19 / therapy*
- Cameroon / epidemiology
- Female
- Hospitalization / statistics & numerical data*
- Humans
- Intensive Care Units / statistics & numerical data*
- Length of Stay / statistics & numerical data*
- Male
- Middle Aged
- Real-Time Polymerase Chain Reaction
- Retrospective Studies
- Reverse Transcriptase Polymerase Chain Reaction
- Young Adult

Supplementary concepts

- COVID-19 drug treatment

Full text links

Pan African Medical Journal Free PMC article

Proceed to details

Cite

Share

☐ 335

Observational Study

BMC Infect Dis

. 2021 Jun 10;21(1):549.

doi: 10.1186/s12879-021-06282-6.

[Associated risk factors with disease severity and antiviral drug therapy in patients with COVID-19]

Xiaowei Gong [1], Shiwei Kang [1], Xianfeng Guo [2], Yan Li [1], Haixiang Gao [3], Yadong Yuan [4]
Affiliations Expand

Affiliations

- [1] Department of Respiratory and Critical Care Medicine, The Second Hospital of Hebei Medical University, Shijiazhuang, 050000, China.
- [2] Department of orthopedics, No. 7 Hospital of Wuhan, Wuhan, China.
- [3] Department of Respiratory Medicine, Hebei General Hospital, Shijiazhuang, China.
- [4] Department of Respiratory and Critical Care Medicine, The Second Hospital of Hebei Medical University, Shijiazhuang, 050000, China. yuanyd1108@163.com.

- PMID: **34112084**
- PMCID: PMC8190755
- DOI: 10.1186/s12879-021-06282-6

Free PMC article
Observational Study

Associated risk factors with disease severity and antiviral drug therapy in patients with COVID-19

Xiaowei Gong et al. BMC Infect Dis. 2021.
Free PMC article
Show details
BMC Infect Dis
. 2021 Jun 10;21(1):549.
doi: 10.1186/s12879-021-06282-6.

Authors

Xiaowei Gong [1], Shiwei Kang [1], Xianfeng Guo [2], Yan Li [1], Haixiang Gao [3], Yadong Yuan [4]

Affiliations

- [1] Department of Respiratory and Critical Care Medicine, The Second Hospital of Hebei Medical University, Shijiazhuang, 050000, China.

- [2] Department of orthopedics, No. 7 Hospital of Wuhan, Wuhan, China.
- [3] Department of Respiratory Medicine, Hebei General Hospital, Shijiazhuang, China.
- [4] Department of Respiratory and Critical Care Medicine, The Second Hospital of Hebei Medical University, Shijiazhuang, 050000, China. yuanyd1108@163.com.

- PMID: **34112084**
- PMCID: PMC8190755
- DOI: 10.1186/s12879-021-06282-6

Abstract

Background: Due to the latent onset of novel coronavirus disease 2019 (COVID-19), it is important to identify patients with increased probabilities for disease progression early in order to implement timely medical strategies. This study aimed to identify the factors associated with increased COVID-19 severity and evaluate the current antiviral drugs, especially in severe patients.

Methods: This was a retrospective observational study performed at the No. 7 Hospital of Wuhan (Wuhan, China) with hospitalized patients confirmed with COVID-19 from January 11 to March 13, 2020. Multivariable logistic regression analysis was used to identify the associated factors of severe COVID. Treatments of antivirus drugs were collected and evaluated.

Results: Of the 550 patients, 292 (53.1%) were female and 277 (50.4%) were > 60 years old. The most common symptom was fever (n = 372, 67.7%), followed by dry cough (n = 257, 46.7%), and dyspnea (n = 237, 43.1%), and fatigue (n = 224, 40.7%). Among the severe patients, 20.2% required invasive ventilator support and 18.0% required non-invasive ventilator. The identified risk factors for severe cases were: age ≥ 60 years (odds ratio (OR) =3.02, 95% confidence interval (CI): 1.13-8.08, P = 0.028), D-dimer > 0.243 μg/ml (OR = 2.734, 95%CI: 1.012-7.387, P = 0.047), and low oxygenation index (OR = 0.984, 95%CI: 0.980-0.989, P < 0.001). In severe cases, the benefits (relief of clinical symptoms, clinical outcome, and discharge rate) of arbidol alone was 73.3%, which was better than ribavirin (7/17, 41.2%, P = 0.029).

Conclusions: Age > 60 years, D-dimer > 0.243 μg/ml, and lower oxygenation index were associated with severe COVID-19. Arbidol might provide more clinical benefits in treating patients with severe COVID-19 compared with ribavirin.

Keywords: Antiviral drug; Associated factors; COVID-19; Disease severity; SARS-CoV-2.

Conflict of interest statement

The authors declare that they have no competing interests.

- Cited by 1 article
- 48 references
- 1 figure

Supplementary info

Publication types, MeSH terms, Substances, Grant support Expand

Publication types

- Observational Study

MeSH terms

- Adult
- Aged
- Antiviral Agents / therapeutic use*
- COVID-19 / diagnosis*
- COVID-19 / drug therapy*
- COVID-19 / pathology
- COVID-19 / physiopathology
- China / epidemiology
- Female
- Hospitals
- Humans
- Male
- Middle Aged
- Retrospective Studies
- Risk Factors
- SARS-CoV-2
- Severity of Illness Index

Substances

- Antiviral Agents

Grant support

- 20277706D/the Hebei Province Science and Technology Support Program

Full text links

Read free full text at BMC BioMed Central Free PMC article
Proceed to details
Cite
Share
336
Observational Study
Am J Emerg Med
. 2021 Dec;50:513-517.
doi: 10.1016/j.ajem.2021.08.077. Epub 2021 Sep 2.

Predicting 30 - Day outcomes in emergency department patients discharged with COVID-19

Benjamin McKay [1], Matthew Meyers [2], Leah Rivard [3], Jill Stoltzfus [4], Guhan Rammohan [5], Holly Stankewicz [6]
Affiliations Expand

Affiliations

- [1] Temple/St. Luke's Medical School, 801 Ostrum Street, Bethlehem, PA 18015, United States. Electronic address: tug39112@temple.edu.
- [2] St. Luke's University Health Network, 801 Ostrum Street, Bethlehem, PA 18015, United States. Electronic address: Matthew.meyers@sluhn.org.
- [3] St. Luke's University Health Network, 801 Ostrum Street, Bethlehem, PA 18015, United States. Electronic address: leah.rivard@sluhn.org.
- [4] St. Luke's University Health Network, 801 Ostrum Street, Bethlehem, PA 18015, United States. Electronic address: Jill.stoltzfus@sluhn.org.
- [5] St. Luke's University Health Network, 801 Ostrum Street, Bethlehem, PA 18015, United States. Electronic address: Guhan.rammohan@sluhn.org.
- [6] St. Luke's University Health Network, 801 Ostrum Street, Bethlehem, PA 18015, United States. Electronic address: holly.stankewicz@sluhn.org.

- PMID: **34537576**
- PMCID: PMC8410217
- DOI: 10.1016/j.ajem.2021.08.077

Free PMC article
Observational Study

Predicting 30 - Day outcomes in emergency department patients discharged with COVID-19

Benjamin McKay et al. Am J Emerg Med. 2021 Dec.
Free PMC article
Show details
Am J Emerg Med
. 2021 Dec;50:513-517.
doi: 10.1016/j.ajem.2021.08.077. Epub 2021 Sep 2.

Authors

Benjamin McKay [1], Matthew Meyers [2], Leah Rivard [3], Jill Stoltzfus [4], Guhan Rammohan [5], Holly Stankewicz [6]

Affiliations

- [1] Temple/St. Luke's Medical School, 801 Ostrum Street, Bethlehem, PA 18015, United States. Electronic address: tug39112@temple.edu.
- [2] St. Luke's University Health Network, 801 Ostrum Street, Bethlehem, PA 18015, United States. Electronic address: Matthew.meyers@sluhn.org.
- [3] St. Luke's University Health Network, 801 Ostrum Street, Bethlehem, PA 18015, United States. Electronic address: leah.rivard@sluhn.org.
- [4] St. Luke's University Health Network, 801 Ostrum Street, Bethlehem, PA 18015, United States. Electronic address: Jill.stoltzfus@sluhn.org.
- [5] St. Luke's University Health Network, 801 Ostrum Street, Bethlehem, PA 18015, United States. Electronic address: Guhan.rammohan@sluhn.org.
- [6] St. Luke's University Health Network, 801 Ostrum Street, Bethlehem, PA 18015, United States. Electronic address: holly.stankewicz@sluhn.org.

- PMID: **34537576**
- PMCID: PMC8410217
- DOI: 10.1016/j.ajem.2021.08.077

Abstract

Introduction: Determining disposition for COVID-19 patients can be difficult for emergency medicine clinicians. Previous studies have demonstrated risk factors which predict severe infection and mortality however little is known about which risk factors are associated with failure of outpatient management and subsequent admission for COVID-19 patients.

Methods: We conducted a retrospective observational chart review of patients who had a confirmed positive COVID-19 test collected during an ED visit between March 1, 2020 and October 11, 2020. Patients were divided into two groups based on presence or absence of a subsequent 30-day hospitalization. Clinical and demographic information were collected including chief complaint, triage vital signs and comorbid medical conditions.

Results: 1038 patients were seen and discharged from a network ED with a positive SARS-CoV-2 PCR test. 94 patients (9.1%) were admitted to a hospital within 30 days of the index ED visit while 944 (90.9%) were not admitted to a network hospital within 30 days. Patients that were admitted were more likely to be older (aOR = 1.04 (95% CI 1.03-1.06)), hypoxic (aOR = 2.16 (95% CI 1.14-4.10)) and tachycardic (aOR = 2.13 (95% CI 1.34-3.38)) on initial ED presentation. Preexisting hypertension, diabetes mellitus, coronary artery disease, chronic kidney disease and malignancy were all highly significant risk factors for 30-day hospital admission following initial ED discharge ($p < 0.0001$).

Conclusion: Emergency Department providers should consider age, chief complaint, vital signs and comorbid medical conditions when determining disposition for patients diagnosed with COVID-19.

Copyright © 2021 Elsevier Inc. All rights reserved.

Conflict of interest statement

Declaration of Competing Interest None.

- Cited by 1 article
- 14 references

Supplementary info

Publication types, MeSH terms Expand

Publication types

- Observational Study

MeSH terms

- Adult
- Age Factors
- Aged
- Aged, 80 and over
- COVID-19 / diagnosis
- COVID-19 / mortality
- COVID-19 / therapy*
- Emergency Service, Hospital*
- Female
- Humans
- Male
- Middle Aged
- Outcome Assessment, Health Care
- Patient Discharge
- Patient Readmission*
- Predictive Value of Tests
- Retrospective Studies
- Risk Factors

Full text links

ELSEVIER FULL-TEXT ARTICLE Elsevier Science Free PMC article
Proceed to details
Cite
Share
☐ 337
Observational Study

South Med J
. 2020 Dec;113(12):618-622.
doi: 10.14423/SMJ.0000000000001182.

Characteristics and Outcomes Based on Perceived Illness Severity in SARS-CoV-2

David Snipelisky [1], Rachel Johnson [1], Rajnish Prasad [1], Baqir Lakhani [1], Jeffrey Ellington [1]
Affiliations Expand

Affiliation

- [1] From the Department of Cardiovascular Medicine, Division of Advanced Heart Failure and Transplantation, the Department of Medicine, Division of Internal Medicine, the Department of Cardiovascular Medicine, Department of Critical Care Medicine, and the Department of Medicine, Division of Hospital Medicine, Wellstar Kennestone Medical Center, Marietta, Georgia.

- PMID: **33263129**
- PMCID: PMC8055091
- DOI: 10.14423/SMJ.0000000000001182

Free PMC article
Observational Study

Characteristics and Outcomes Based on Perceived Illness Severity in SARS-CoV-2

David Snipelisky et al. South Med J. 2020 Dec.
Free PMC article
Show details
South Med J
. 2020 Dec;113(12):618-622.
doi: 10.14423/SMJ.0000000000001182.

Authors

David Snipelisky [1], Rachel Johnson [1], Rajnish Prasad [1], Baqir Lakhani [1], Jeffrey Ellington [1]

Affiliation

- [1] From the Department of Cardiovascular Medicine, Division of Advanced Heart Failure and Transplantation, the Department of Medicine, Division of Internal Medicine, the Department of Cardiovascular Medicine, Department of Critical Care Medicine, and the Department of Medicine, Division of Hospital Medicine, Wellstar Kennestone Medical Center, Marietta, Georgia.

- PMID: **33263129**
- PMCID: PMC8055091
- DOI: 10.14423/SMJ.0000000000001182

Abstract

Objectives: The severe acute respiratory syndrome-coronavirus-2 (SARS-CoV-2) epidemic is characterized by a global sense of uncertainty, partly driven by the paucity of real-life clinical data. This study assessed whether admission patient characteristics were associated with need for intensive care unit (ICU) care.

Methods: The observational study included consecutive patients admitted to a large community teaching hospital with a diagnosis of SARS-CoV-2 between March 6, 2020 and March 31, 2020. Comparisons were made based on the need for ICU admission.

Results: A total of 156 patients were admitted, 42 of whom (26.9%) required ICU admission and 114 (73.1%) did not. No difference in age (61.9 years vs 60.5 years, $P = 0.67$), race/ethnicity, or comorbidities were noted, except that patients requiring ICU care had lower serum albumin levels and lymphocyte counts and higher liver function tests, white blood cell count, and absolute neutrophil count on admission. The average time from admission to death was similar (10 days in an ICU subset vs 9.2 days in a non-ICU subset, $P = 0.78$), yet patients necessitating ICU care had longer hospital lengths of stay (10.2 vs 5.1 days, $P = 0.0002$). At the time of data extraction, 15 patients in the ICU had died, 7 were discharged from the hospital, and 20 were still admitted while 5 patients died in the non-ICU cohort with 97 discharged and 12 patients admitted.

Conclusions: This is the largest study assessing clinical differences based on the need for ICU admission in inpatients with SARS-CoV-2. It found few major differences in clinical variables between subsets. Among patients admitted to the ICU, outcomes were generally poor.

Conflict of interest statement

D.S. has received compensation from Pfizer. R.P. has received compensation from Abiomed. The remaining authors did not report any financial relationships or conflicts of interest.

- Cited by 1 article
- 14 references

Supplementary info

Publication types, MeSH terms, Substances Expand

Publication types

- Observational Study

MeSH terms

- Adult
- Age Factors

- Aged
- Alanine Transaminase / blood
- Aspartate Aminotransferases / blood
- COVID-19 / blood*
- COVID-19 / mortality
- COVID-19 / physiopathology
- COVID-19 / therapy
- Electrocardiography
- Ethnicity / statistics & numerical data
- Female
- Hospital Mortality*
- Hospitalization
- Hospitals, Community
- Hospitals, Teaching
- Humans
- Intensive Care Units / statistics & numerical data*
- Length of Stay / statistics & numerical data
- Leukocyte Count
- Lymphocyte Count
- Male
- Middle Aged
- Neutrophils
- Prognosis
- Respiration, Artificial / statistics & numerical data*
- Retrospective Studies
- Serum Albumin / metabolism
- Severity of Illness Index
- Time Factors

Substances

- Serum Albumin
- Aspartate Aminotransferases
- Alanine Transaminase

Full text links

SMJ Full Text Southern Medical Association Free PMC article

Proceed to details

Cite

Share

☐ 338

Observational Study
ESC Heart Fail
. 2021 Apr;8(2):1691-1695.
doi: 10.1002/ehf2.13158. Epub 2021 Jan 6.

Impact of COVID-19 on inpatient referral of acute heart failure: a single-centre experience from the south-west of the UK

Gemina Doolub [1], Chih Wong [1], Lynsey Hewitson [1], Ahmed Mohamed [1], Fraser Todd [1], Laisha Gogola [1], Andrew Skyrme-Jones [1 2], Shahid Aziz [1 2], Eva Sammut [2 3], Amardeep Dastidar [1 2 3]
Affiliations Expand

Affiliations

- [1] North Bristol NHS Trust, Bristol, UK.
- [2] University Hospitals Bristol and Weston NHS Foundation Trust, Bristol, UK.
- [3] University of Bristol, Bristol, UK.

- PMID: **33410281**
- PMCID: PMC8006615
- DOI: 10.1002/ehf2.13158

Free PMC article
Observational Study

Impact of COVID-19 on inpatient referral of acute heart failure: a single-centre experience from the south-west of the UK

Gemina Doolub et al. ESC Heart Fail. 2021 Apr.
Free PMC article
Show details
ESC Heart Fail
. 2021 Apr;8(2):1691-1695.
doi: 10.1002/ehf2.13158. Epub 2021 Jan 6.

Authors

Gemina Doolub [1], Chih Wong [1], Lynsey Hewitson [1], Ahmed Mohamed [1], Fraser Todd [1], Laisha Gogola [1], Andrew Skyrme-Jones [1 2], Shahid Aziz [1 2], Eva Sammut [2 3], Amardeep Dastidar [1 2 3]

Affiliations

- [1] North Bristol NHS Trust, Bristol, UK.
- [2] University Hospitals Bristol and Weston NHS Foundation Trust, Bristol, UK.
- [3] University of Bristol, Bristol, UK.

- PMID: **33410281**
- PMCID: PMC8006615
- DOI: 10.1002/ehf2.13158

Abstract

Aims: Healthcare services worldwide have been significantly impacted by the COVID-19 pandemic. Recent reports have shown a decline in hospitalization for emergency cardiac conditions. The impact of the COVID-19 pandemic on hospitalization and particularly mortality due to acute heart failure has not been thoroughly described.

Methods and results: In this single-centre observational study, we examined referrals to the acute heart failure team over a period of 16 weeks (7 January to 27 April 2020) spanning the ongoing COVID-19 pandemic; 283 patients referred to our acute heart failure services over the study period were included on the basis of typical symptoms, raised BNP, and echocardiogram. There was a substantial but statistically non-significant drop in referrals with 164 referred in the 8 weeks before the first UK death due to COVID-19 on 2 March 2020 (BC), compared with 119 referred after (AC) in the subsequent 8 weeks, representing a 27% reduction overall (P = 0.06). The 30 day case fatality rate was increased from 11% in the BC group compared with 21% in the AC group (risk ratio = 1.9, 95% confidence interval 1.09-3.3). Age, gender, length of stay, left ventricular ejection fraction, and N-terminal pro-brain natriuretic peptide were similar between the groups. Admission creatinine, age, and AC cohort status were found to be univariable predictors of mortality. On multivariate Cox regression analysis, only age (hazard ratio 1.04, P = 0.03) and AC cohort status (hazard ratio 2.1, P = 0.017) remained significant predictors of mortality. On sensitivity analysis, this increased mortality was driven by COVID-19 positive status.

Conclusions: There was a reduction in referral of patients with acute heart failure with significant increase in mortality in the 8 weeks following the first reported UK death due to COVID-19. The observation of increased mortality does not appear related to a change in population in terms of demographics, left ventricular ejection fraction, or N-terminal pro-brain natriuretic peptide. The observed increased mortality appears to be related to the coexistence of COVID19 infection with acute heart failure. The study highlights the need for widespread preventative and shielding measures particularly in this group of patients especially in the light of the second wave. Longer follow-up with inclusion of data from other centres and community heart failure services will be needed.

Keywords: Acute heart failure; COVID-19; Mortality.

© 2021 The Authors. ESC Heart Failure published by John Wiley & Sons Ltd on behalf of the European Society of Cardiology.

Conflict of interest statement

None declared.

- Cited by 13 articles

- 17 references
- 2 figures

Supplementary info

Publication types, MeSH terms Expand

Publication types

- Observational Study

MeSH terms

- Aged
- Aged, 80 and over
- COVID-19 / diagnosis
- COVID-19 / epidemiology*
- COVID-19 / therapy
- Female
- Heart Failure / diagnosis
- Heart Failure / mortality*
- Heart Failure / therapy*
- Hospitalization*
- Humans
- Male
- Referral and Consultation*
- Retrospective Studies
- Stroke Volume
- Survival Rate
- United Kingdom

Full text links

WILEY Free Full Text Wiley Free PMC article

Proceed to details

Cite

Share

☐ 339

Observational Study

Intern Emerg Med

. 2021 Jun;16(4):989-996.

doi: 10.1007/s11739-020-02617-4. Epub 2021 Feb 23.

Clinical risk scores for the early prediction of severe outcomes in patients hospitalized for COVID-19

Walter Ageno [1], Chiara Cogliati [2], Martina Perego [2], Domenico Girelli [3], Ernesto Crisafulli [3], Francesca Pizzolo [3], Oliviero Olivieri [3], Marco Cattaneo [4], Alberto Benetti [4], Elena Corradini [5], Lorenza Bertù [6], Antonello Pietrangelo [5], List of contributors
Collaborators, Affiliations Expand

Collaborators

- **List of contributors**:
  Lucia Maria Caiano, Federica Magni, Elisabetta Tombolini, Chiara Aloise, Francesca Maria Casanova, Benedetta Peroni, Andrea Ricci, Stefania Scarlini, Ivan Silvestri, Matteo Morandi, Sara Pezzato, Francesca Stefani, Virginia Trevisan

Affiliations

- [1] University of Insubria, Varese, Italy. walter.ageno@uninsubria.it.
- [2] Ospedale Sacco, Milan, Italy.
- [3] Università Degli Studi Di Verona, Verona, Italy.
- [4] Ospedale San Paolo E Università Degli Studi Di Milano, Milan, Italy.
- [5] Università Di Modena E Reggio Emilia, Modena, Italy.
- [6] University of Insubria, Varese, Italy.

- PMID: **33620680**
- PMCID: PMC7900378
- DOI: 10.1007/s11739-020-02617-4

Free PMC article
Observational Study

Clinical risk scores for the early prediction of severe outcomes in patients hospitalized for COVID-19

Walter Ageno et al. Intern Emerg Med. 2021 Jun.
Free PMC article
Show details
Intern Emerg Med
. 2021 Jun;16(4):989-996.
doi: 10.1007/s11739-020-02617-4. Epub 2021 Feb 23.

Authors

Walter Ageno [1], Chiara Cogliati [2], Martina Perego [2], Domenico Girelli [3], Ernesto Crisafulli [3], Francesca Pizzolo [3], Oliviero Olivieri [3], Marco Cattaneo [4], Alberto Benetti [4], Elena Corradini [5], Lorenza Bertù [6], Antonello Pietrangelo [5], List of contributors

Collaborators

- **List of contributors**:
  Lucia Maria Caiano, Federica Magni, Elisabetta Tombolini, Chiara Aloise, Francesca Maria Casanova, Benedetta Peroni, Andrea Ricci, Stefania Scarlini, Ivan Silvestri, Matteo Morandi, Sara Pezzato, Francesca Stefani, Virginia Trevisan

Affiliations

- [1] University of Insubria, Varese, Italy. walter.ageno@uninsubria.it.
- [2] Ospedale Sacco, Milan, Italy.
- [3] Università Degli Studi Di Verona, Verona, Italy.
- [4] Ospedale San Paolo E Università Degli Studi Di Milano, Milan, Italy.
- [5] Università Di Modena E Reggio Emilia, Modena, Italy.
- [6] University of Insubria, Varese, Italy.

- PMID: **33620680**
- PMCID: PMC7900378
- DOI: 10.1007/s11739-020-02617-4

Abstract

Coronavirus disease of 2019 (COVID-19) is associated with severe acute respiratory failure. Early identification of high-risk COVID-19 patients is crucial. We aimed to derive and validate a simple score for the prediction of severe outcomes. A retrospective cohort study of patients hospitalized for COVID-19 was carried out by the Italian Society of Internal Medicine. Epidemiological, clinical, laboratory, and treatment variables were collected at hospital admission at five hospitals. Three algorithm selection models were used to construct a predictive risk score: backward Selection, Least Absolute Shrinkage and Selection Operator (LASSO), and Random Forest. Severe outcome was defined as the composite of need for non-invasive ventilation, need for orotracheal intubation, or death. A total of 610 patients were included in the analysis, 313 had a severe outcome. The subset for the derivation analysis included 335 patients, the subset for the validation analysis 275 patients. The LASSO selection identified 6 variables (age, history of coronary heart disease, CRP, AST, D-dimer, and neutrophil/lymphocyte ratio) and resulted in the best performing score with an area under the curve of 0.79 in the derivation cohort and 0.80 in the validation cohort. Using a cut-off of 7 out of 13 points, sensitivity was 0.93, specificity 0.34, positive predictive value 0.59, and negative predictive value 0.82. The proposed score can identify patients at low risk for severe outcome who can be safely managed in a low-intensity setting after hospital admission for COVID-19.

Keywords: COVID-19; Respiratory failure; Risk prediction model; SARS-CoV 2.

Conflict of interest statement

The authors have no conflicts of interest to disclose related to this study.

Comment in

- Clinical risk scores for the early prediction of severe outocomes in patients hospitalized for COVID-19: comment.
  Rossio R, Tettamanti M, Nobili A, Harari S, Mannucci PM, Bandera A, Peyvandi F; COVID-19 network working group. Rossio R, et al. Intern Emerg Med. 2022 Jan;17(1):303-306. doi: 10.1007/s11739-021-02807-8. Epub 2021 Aug 3. Intern Emerg Med. 2022. PMID: 34342788 Free PMC article. No abstract available.

- Cited by 5 articles
- 13 references
- 1 figure

Supplementary info

Publication types, MeSH terms Expand

Publication types

- Multicenter Study
- Observational Study
- Research Support, Non-U.S. Gov't

MeSH terms

- Aged
- COVID-19 / complications
- COVID-19 / epidemiology*
- COVID-19 / therapy*
- Female
- Hospitalization*
- Humans
- Intubation, Intratracheal
- Italy
- Male
- Middle Aged
- Outcome Assessment, Health Care
- Predictive Value of Tests
- ROC Curve
- Respiration, Artificial
- Retrospective Studies
- Risk Assessment
- Risk Factors

- Survival Rate

Full text links

SpringerLink FULL-TEXT ARTICLE Springer Free PMC article

Proceed to details

Cite

Share

340

Observational Study

N Z Med J

. 2021 Jul 9;134(1538):111-119.

COVID-19 and the impact on urology service provision at Capital & Coast District Health Board

Simon Lambracos [1], Lance Yuan [2], Andrew Kennedy-Smith [2]

Affiliations Expand

Affiliations

- [1] General Surgery Department, Poole Hospital NHS Trust, Longfleet Road, Poole BH15 2JB, UK.
- [2] Urology Department, Capital & Coast DHB, Riddiford Street, Newtown, Wellington 6011.

- PMID: **34239150**

Observational Study

COVID-19 and the impact on urology service provision at Capital & Coast District Health Board

Simon Lambracos et al. N Z Med J. 2021.

Show details

N Z Med J

. 2021 Jul 9;134(1538):111-119.

Authors

Simon Lambracos [1], Lance Yuan [2], Andrew Kennedy-Smith [2]

Affiliations

- [1] General Surgery Department, Poole Hospital NHS Trust, Longfleet Road, Poole BH15 2JB, UK.
- [2] Urology Department, Capital & Coast DHB, Riddiford Street, Newtown, Wellington 6011.

- PMID: **34239150**

Abstract

Aim: To determine the impact on the Capital & Coast District Health Board (CCDHB) urology service of the implementation of nationwide healthcare restrictions in response to the COVID-19 pandemic.

Methods: This is an observational retrospective study over a 21 working day period during the implementation of National Hospital Response Framework Alert (NHRFA) level 2. We obtained patient data during this period and a corresponding control period prior to the pandemic. The data was focussed on the volume of operating theatre cases, outpatient consultations, procedural clinic appointments and the estimated avoided outpatient travel.

Results: Total urology admissions decreased by 27% during the 21-day NHRFA level 2 period. However, acute surgical procedures increased by 30% whereas elective surgical procedures decreased by 32%. Outpatient consultations overall decreased by 32% during NHRFA level 2 despite virtual phone consultations increasing by 274%. Procedural clinic appointments decreased by 85%. The virtual platform also saved each patient an estimated 22.7km of average travel.

Conclusion: The data demonstrate the effects of restrictions in response to a crisis and set a precedent for future management in such scenarios. The data also show how service efficiency can be optimised while providing an environmentally friendly alternative for routine clinical practice.

Conflict of interest statement

Nil.

Supplementary info

Publication types, MeSH terms Expand

Publication types

- Observational Study

MeSH terms

- Ambulatory Care / statistics & numerical data*
- COVID-19 / prevention & control*
- Delivery of Health Care / statistics & numerical data*
- Elective Surgical Procedures / statistics & numerical data

- Humans
- New Zealand
- Office Visits / statistics & numerical data
- Patient Admission / statistics & numerical data
- Retrospective Studies
- SARS-CoV-2
- Telemedicine / statistics & numerical data
- Travel / statistics & numerical data
- Urologic Surgical Procedures / statistics & numerical data*
- Urology / statistics & numerical data*

Proceed to details
Cite
Share
☐ 341
Observational Study
Arch Gerontol Geriatr
. Sep-Oct 2021;96:104462.
doi: 10.1016/j.archger.2021.104462. Epub 2021 Jun 16.

Clinical characteristics of 1544 Brazilians aged 60 years and over with laboratory evidence for SARS-CoV-2

Marcelo de Maio Nascimento [1]
Affiliations Expand

Affiliation

- [1] Federal University of Sao Francisco Valley: Universidade Federal do Vale do Sao Francisco, Petrolina, Pernambuco BRAZIL. Electronic address: marcelo.nascimento@univasf.edu.br.

- PMID: **34175730**
- PMCID: PMC8216862
- DOI: 10.1016/j.archger.2021.104462

Free PMC article
Observational Study

Clinical characteristics of 1544 Brazilians aged 60 years and over with laboratory evidence for SARS-CoV-2

Marcelo de Maio Nascimento. Arch Gerontol Geriatr. Sep-Oct 2021.
Free PMC article
Show details
Arch Gerontol Geriatr
. Sep-Oct 2021;96:104462.
doi: 10.1016/j.archger.2021.104462. Epub 2021 Jun 16.

Author

Marcelo de Maio Nascimento [1]

Affiliation

- [1] Federal University of Sao Francisco Valley: Universidade Federal do Vale do Sao Francisco, Petrolina, Pernambuco BRAZIL. Electronic address: marcelo.nascimento@univasf.edu.br.

- PMID: **34175730**
- PMCID: PMC8216862
- DOI: 10.1016/j.archger.2021.104462

Abstract

Introduction: Infection with the new coronavirus responsible for Severe Acute Respiratory Syndrome (SARS-CoV-2) continues to spread worldwide. In Brazil, there are already more than 230 thousand dead, many of these older adults.

Objective: To present the clinical characteristics of older Brazilian adults infected by COVID-19, in the epidemiological weeks (EW) 34-52, and to verify factors responsible for the increased risk of death.

Methods: Retrospective and observational study conducted with secondary publicly available data, provided by the Brazilian Ministry of Health. 1,544 confirmed cases of registered COVID-19 infection were included between August 16 and December 26, 2020, aged 60 or older.

Outcomes: Demographic data, comorbidity, symptoms for disease, clinical information: days of hospitalization, chest X-ray, type of RT-PCR.

Results: 48% of patients admitted to the ICU with evidence for SARS-CoV-2 died. Symptoms and comorbidities related to increased chance of death (OR) were immunodeficiency (188%), kidney disease (166%), neurological disease (103%), dyspnea (86%), pneumopathy (55%), O_2 saturation <95% (53%), respiratory discomfort (49%), age (36%), sore throat (31%), and sex (0.5%). There was a 5% increase in the chance of death for each year of life.

Conclusion: Heart disease and Diabetes mellitus were the most frequent comorbidities, but did not indicate an increased risk of death from SARS-CoV-2 infection. Age, sex, sore throat, dyspnea, respiratory discomfort, O_2 saturation <95%, neurological disease, pneumopathy, immunodeficiency, and kidney disease were significantly associated with risk of death from COVID-19.

Keywords: COVID-19; Clinical characteristics; Comorbidity; Older Adult; SARS-CoV-2.

Copyright © 2021. Published by Elsevier B.V.

Conflict of interest statement

No potential conflict of interest was reported by the author.

- 26 references

Supplementary info

Publication types, MeSH terms Expand

Publication types

- Observational Study

MeSH terms

- Aged
- Brazil / epidemiology
- COVID-19*
- Comorbidity
- Hospitalization
- Humans
- Laboratories
- Middle Aged
- Retrospective Studies
- SARS-CoV-2*

Full text links

ELSEVIER FULL-TEXT ARTICLE Elsevier Science Free PMC article
Proceed to details
Cite
Share
☐ 342
Observational Study
Clin Transl Sci

. 2020 Nov;13(6):1071-1076.
doi: 10.1111/cts.12860. Epub 2020 Oct 13.

Effect of Combination Therapy of Hydroxychloroquine and Azithromycin on Mortality in Patients With COVID-19

Marinella Lauriola [1], Arianna Pani [2], Giovanbattista Ippoliti [3], Andrea Mortara [4], Stefano Milighetti [3], Marjieh Mazen [3], Gianluca Perseghin [3] [5], Daniele Pastori [6], Paolo Grosso [7], Francesco Scaglione [2]
Affiliations Expand

Affiliations

- [1] Infectious Disease Department, Policlinico di Monza, Monza, Italy.
- [2] Department of Oncology and Hemato-Oncology, Università degli Studi di Milano, Milan, Italy.
- [3] Internal Medicine Department, Policlinico di Monza, Monza, Italy.
- [4] Cardiology Department, Policlinico di Monza, Monza, Italy.
- [5] Department of Medicine and Surgery, Università degli Studi di Milano Bicocca, Milan, Italy.
- [6] Department of Clinical, Internal, Anesthesiologic, and Cardiovascular Sciences, Sapienza University of Rome, Rome, Italy.
- [7] Intensive Care Unit, Policlinico di Monza, Monza, Italy.

- PMID: **32926573**
- PMCID: PMC7719367
- DOI: 10.1111/cts.12860

Free PMC article
Observational Study

Effect of Combination Therapy of Hydroxychloroquine and Azithromycin on Mortality in Patients With COVID-19

Marinella Lauriola et al. Clin Transl Sci. 2020 Nov.
Free PMC article
Show details
Clin Transl Sci
. 2020 Nov;13(6):1071-1076.
doi: 10.1111/cts.12860. Epub 2020 Oct 13.

Authors

Marinella Lauriola [1], Arianna Pani [2], Giovanbattista Ippoliti [3], Andrea Mortara [4], Stefano Milighetti [3], Marjieh Mazen [3], Gianluca Perseghin [3] [5], Daniele Pastori [6], Paolo Grosso [7], Francesco Scaglione [2]

Affiliations

- [1] Infectious Disease Department, Policlinico di Monza, Monza, Italy.
- [2] Department of Oncology and Hemato-Oncology, Università degli Studi di Milano, Milan, Italy.
- [3] Internal Medicine Department, Policlinico di Monza, Monza, Italy.
- [4] Cardiology Department, Policlinico di Monza, Monza, Italy.
- [5] Department of Medicine and Surgery, Università degli Studi di Milano Bicocca, Milan, Italy.
- [6] Department of Clinical, Internal, Anesthesiologic, and Cardiovascular Sciences, Sapienza University of Rome, Rome, Italy.
- [7] Intensive Care Unit, Policlinico di Monza, Monza, Italy.

- PMID: **32926573**
- PMCID: PMC7719367
- DOI: 10.1111/cts.12860

Abstract

Conflicting evidence regarding the use of hydroxychloroquine (HCQ) and azithromycin for the treatment of severe acute respiratory syndrome coronavirus 2 (SARS-CoV-2) infection do exist. We performed a retrospective single-center cohort study including 377 consecutive patients admitted for pneumonia related to coronavirus disease 2019 (COVID-19). Of these, 297 were in combination treatment, 17 were on HCQ alone, and 63 did not receive either of these 2 drugs because of contraindications. The primary end point was in-hospital death. Mean age was 71.8 ± 13.4 years and 34.2% were women. We recorded 146 deaths: 35 in no treatment, 7 in HCQ treatment group, and 102 in HCQ + azithromycin treatment group (log rank test for Kaplan-Meier curve $P < 0.001$). At multivariable Cox proportional hazard regression analysis, age (hazard ratio (HR) 1.057, 95% confidence interval (CI) 1.035-1.079, $P < 0.001$), mechanical ventilation/continuous positive airway pressure (HR 2.726, 95% CI 1.823-4.074, $P < 0.001$), and C reactive protein above the median (HR 2.191, 95% CI 1.479-3.246, $P < 0.001$) were directly associated with death, whereas use of HCQ + azithromycin (vs. no treatment; HR 0.265, 95% CI 0.171-0.412, $P < 0.001$) was inversely associated. In this study, we found a reduced in-hospital mortality in patients treated with a combination of HCQ and azithromycin after adjustment for comorbidities. A large randomized trial is necessary to confirm these findings.

© 2020 The Authors. Clinical and Translational Science published by Wiley Periodicals LLC on behalf of the American Society for Clinical Pharmacology and Therapeutics.

Conflict of interest statement

All authors declared no competing interests for this work.

- Cited by 14 articles

- 28 references
- 2 figures

Supplementary info

Publication types, MeSH terms, Substances, Supplementary concepts Expand

Publication types

- Observational Study

MeSH terms

- Aged
- Aged, 80 and over
- Azithromycin / administration & dosage*
- COVID-19 / drug therapy*
- COVID-19 / mortality
- Drug Therapy, Combination
- Female
- Hospital Mortality
- Humans
- Hydroxychloroquine / administration & dosage*
- Male
- Middle Aged
- Retrospective Studies
- SARS-CoV-2*

Substances

- Hydroxychloroquine
- Azithromycin

Supplementary concepts

- COVID-19 drug treatment

Full text links

WILEY Full Text Article Wiley Free PMC article
Proceed to details
Cite
Share

☐ 343

Observational Study

Am J Trop Med Hyg
. 2021 Dec 2;106(1):108-113.
doi: 10.4269/ajtmh.21-0480.

COVID-19 versus Seasonal Influenza: Comparison of Profiles of Older Adults Hospitalized in a Short-Term Geriatric Ward in France

Lidvine Godaert [1], Agnès Cebille [1], Emeline Proye [1], Moustapha Dramé [2, 3]

Affiliations Expand

Affiliations

- [1] Department of Geriatrics, General Hospital of Valenciennes, Valenciennes, France.
- [2] Faculty of Medicine, University of the French West Indies, Fort-de-France, Martinique, France.
- [3] Department of Clinical Research and Innovation, University Hospital of Martinique, Fort-de-France, Martinique, France.

- PMID: **34856536**
- PMCID: PMC8733482
- DOI: 10.4269/ajtmh.21-0480

Free PMC article

Observational Study

COVID-19 versus Seasonal Influenza: Comparison of Profiles of Older Adults Hospitalized in a Short-Term Geriatric Ward in France

Lidvine Godaert et al. Am J Trop Med Hyg. 2021.

Free PMC article

Show details

Am J Trop Med Hyg
. 2021 Dec 2;106(1):108-113.
doi: 10.4269/ajtmh.21-0480.

Authors

Lidvine Godaert [1], Agnès Cebille [1], Emeline Proye [1], Moustapha Dramé [2] [3]

Affiliations

- [1] Department of Geriatrics, General Hospital of Valenciennes, Valenciennes, France.
- [2] Faculty of Medicine, University of the French West Indies, Fort-de-France, Martinique, France.
- [3] Department of Clinical Research and Innovation, University Hospital of Martinique, Fort-de-France, Martinique, France.

- PMID: **34856536**
- PMCID: PMC8733482
- DOI: 10.4269/ajtmh.21-0480

Abstract

The objective was to compare the profile and outcomes of older adults admitted to a geriatric short-stay unit for COVID-19, to those of older adults admitted to the same unit for seasonal influenza infection. This was an observational study performed in a General Hospital in France. Patients ≥ 70 years admitted to a geriatric short-stay unit for COVID-19 between March 18 and November 15, 2020 were included. They were compared with patients of the same age group, admitted to the same geriatric short-stay unit for seasonal influenza infection over the periods January to March 2019 and January to March 2020. Data collection included demographic information, medical history, clinical signs and symptoms, outcomes, and hospital discharge patterns. Descriptive and intergroup comparison analyses were performed. In total, 153 patients were included in the study, 82 in the seasonal influenza group, and 71 in the COVID-19 group. The average age was 87.6 ± 4.8 and 87.6 ± 6.5 years in the COVID-19 and seasonal influenza groups, respectively. There was no difference between groups regarding the Charlson comorbidity index (3.4 ± 3.0 versus 3.4 ± 2.8). The seasonal influenza group more often had fever, cough, sputum, and renal failure, whereas the COVID-19 group more often experienced diarrhea, and death. The COVID-19 group was frequently living in collective housing. The profile at admission of older adults hospitalized for COVID-19 or seasonal influenza infection was similar. Although fever and respiratory signs were less common in the COVID-19 group, these patients experienced more complications (such as renal failure or oxygen therapy requirement) and higher mortality.

- 50 references

Supplementary info

Publication types, MeSH terms Expand

Publication types

- Comparative Study
- Observational Study

MeSH terms

- Aged
- Aged, 80 and over
- COVID-19 / epidemiology*
- COVID-19 / mortality
- Female
- France
- Geriatrics
- Hospital Units
- Hospitalization
- Humans
- Influenza, Human / epidemiology*
- Influenza, Human / mortality
- Longitudinal Studies
- Male
- Retrospective Studies
- Seasons

Full text links

Full Text THE AMERICAN JOURNAL OF TROPICAL MEDICINE AND HYGIENE Sheridan PubFactory Free PMC article

Proceed to details

Cite

Share

☐ 344

Observational Study

Ann Noninvasive Electrocardiol

. 2021 Jul;26(4):e12846.

doi: 10.1111/anec.12846. Epub 2021 May 6.

The impact of hydroxychloroquine-azithromycin combination on Tpeak-to-end and Tpeak-to-end/QT ratio during a short treatment course

Nijad Bakhshaliyev [1], Ramazan Özdemir [1]

Affiliations Expand

Affiliation

- [1] Bezmialem Vakif University, Istanbul, Turkey.

- PMID: **33956361**
- PMCID: PMC8293593
- DOI: 10.1111/anec.12846

Free PMC article
Observational Study

The impact of hydroxychloroquine-azithromycin combination on Tpeak-to-end and Tpeak-to-end/QT ratio during a short treatment course

Nijad Bakhshaliyev et al. Ann Noninvasive Electrocardiol. 2021 Jul.
Free PMC article
Show details
Ann Noninvasive Electrocardiol
. 2021 Jul;26(4):e12846.
doi: 10.1111/anec.12846. Epub 2021 May 6.

Authors

Nijad Bakhshaliyev [1], Ramazan Özdemir [1]

Affiliation

- [1] Bezmialem Vakif University, Istanbul, Turkey.

- PMID: **33956361**
- PMCID: PMC8293593
- DOI: 10.1111/anec.12846

Abstract

Background: Since there was no proven treatment of coronavirus disease 2019 (COVID-19), hydroxychloroquine-azithromycin (HCQ-AZM) combination is being used in different countries as a treatment option. Many controversies exist related to the safety and effectiveness of this combination, and questions about how HCQ-AZM combination affects the ventricular repolarization are still unknown.

Objective: The aim of the study was to show whether the hydroxychloroquine-azithromycin (HCQ-AZM) combination prolonged Tpeak-to-end (TpTe) duration and TpTe/QT interval ratio or not.

Methods: One hundred and twenty-six consequent COVID-19(+) patients meeting the study criteria were enrolled in this study. Baseline ECGs were obtained immediately after

hospitalization and before commencing the HCQ-AZM combination. On-treatment ECG was obtained 24-48 hr after the loading dose of HCQ/AZM. ECG parameters including PR interval, QRS duration, QT interval, QTc interval, TpTe duration, and TpTe/QT interval ratio were assessed. Demographic and laboratory findings were collected from an electronic recording system.

Results: ECGs of 126 COVID-19(+) patients who received HCQ-AZM combination were assessed. Mean baseline QTc (by Fridericia formula), TpTe, and TpTe/QT ratio were 420.0 ± 26.5 ms, 82.43 ± 9.77 ms, and 0.22 ± 0.02, respectively. On-treatment QTc, TpTe and TpTe/QT ratio were 425.7 ± 27.18 ms, 85.17 ± 11.17 ms, and 0.22 ± 0.03, respectively. No statistically significant acute impacts of HCQ-AZM combination on TpTe duration and TpTe/QT interval ratio were observed compared with baseline values. No ventricular tachycardia/fibrillation and the significant conduction delays were seen during in-hospital follow-up.

Conclusion: HCQ-AZM combination increased TpTe duration. However, no significant impact on TpTe/QT interval ratio was observed.

Keywords: Tpeak-to-end; azithromycin; cardiac death; coronavirus disease 2019; electrocardiography; hydroxychloroquine.

© 2021 The Authors. Annals of Noninvasive Electrocardiology published by Wiley Periodicals LLC.

Conflict of interest statement

None.

- 28 references
- 1 figure

Supplementary info

Publication types, MeSH terms, Substances Expand

Publication types

- Observational Study

MeSH terms

- Anti-Bacterial Agents / pharmacology
- Azithromycin / pharmacology*
- COVID-19 / drug therapy*
- Drug Administration Schedule
- Drug Therapy, Combination
- Electrocardiography / drug effects*
- Enzyme Inhibitors / pharmacology
- Female
- Heart Ventricles / drug effects*

- Humans
- Hydroxychloroquine / pharmacology*
- Male
- Middle Aged
- Retrospective Studies
- SARS-CoV-2
- Treatment Outcome

Substances

- Anti-Bacterial Agents
- Enzyme Inhibitors
- Hydroxychloroquine
- Azithromycin

Full text links

WILEY Full Text Article Wiley Free PMC article
Proceed to details
Cite
Share
345
Observational Study
Minerva Med
. 2021 Jun;112(3):329-337.
doi: 10.23736/S0026-4806.21.07134-2. Epub 2021 Jan 19.

Management of patients with severe acute respiratory failure due to SARS-CoV-2 pneumonia with noninvasive ventilatory support outside Intensive Care Unit

Teresa Diaz DE Teran [1], Monica Gonzales Martinez [1], Paolo Banfi [2], Giancarlo Garuti [3], Gianluca Ferraioli [4], Giuseppe Russo [4], Francesco Casu [4], Michela Vivarelli [4], Monica Bonfiglio [4], Alessandro Perazzo [5], Cornelius Barlascini [6], Armando Bauleo [7], Antonello Nicolini [8], Paolo Solidoro [9] [10]
Affiliations Expand

Affiliations

- [1] Unit of Pulmonary Sleep Disorders and Noninvasive Ventilation, Marqués de Valdecilla Hospital, Santander, Spain.

- [2] Unit of Rehabilitation Pulmonology, IRCCS Don Gnocchi Foundation, Milan, Italy.
- [3] Unit of Pulmonology, Santa Maria Bianca Hospital, Mirandola, Modena, Italy.
- [4] COVID Respiratory Intensive Care Unit, General Hospital, Sestri Levante, Genoa, Italy.
- [5] Unit of Respiratory Diseases, General Hospital, Sestri Levante, Genoa, Italy.
- [6] Unit of Hygiene and Health Care Medicine, General Hospital, Sestri Levante, Genoa, Italy.
- [7] Independent Scientific Consultant, Modena, Italy.
- [8] Unit of Respiratory Diseases, General Hospital, Sestri Levante, Genoa, Italy - antonellonicolini@gmail.com.
- [9] Unit of Respiratory Diseases, Department Cardiovascular and Thoracic Diseases, Città della Salute e della Scienza, Turin, Italy.
- [10] Department of Medical Science, University of Turin, Turin, Italy.

- PMID: **33464224**
- DOI: 10.23736/S0026-4806.21.07134-2

Observational Study

Management of patients with severe acute respiratory failure due to SARS-CoV-2 pneumonia with noninvasive ventilatory support outside Intensive Care Unit

Teresa Diaz DE Teran et al. Minerva Med. 2021 Jun.

Show details

Minerva Med

. 2021 Jun;112(3):329-337.
doi: 10.23736/S0026-4806.21.07134-2. Epub 2021 Jan 19.

Authors

Teresa Diaz DE Teran [1], Monica Gonzales Martinez [1], Paolo Banfi [2], Giancarlo Garuti [3], Gianluca Ferraioli [4], Giuseppe Russo [4], Francesco Casu [4], Michela Vivarelli [4], Monica Bonfiglio [4], Alessandro Perazzo [5], Cornelius Barlascini [6], Armando Bauleo [7], Antonello Nicolini [8], Paolo Solidoro [9] [10]

Affiliations

- [1] Unit of Pulmonary Sleep Disorders and Noninvasive Ventilation, Marqués de Valdecilla Hospital, Santander, Spain.
- [2] Unit of Rehabilitation Pulmonology, IRCCS Don Gnocchi Foundation, Milan, Italy.
- [3] Unit of Pulmonology, Santa Maria Bianca Hospital, Mirandola, Modena, Italy.
- [4] COVID Respiratory Intensive Care Unit, General Hospital, Sestri Levante, Genoa, Italy.
- [5] Unit of Respiratory Diseases, General Hospital, Sestri Levante, Genoa, Italy.

- [6] Unit of Hygiene and Health Care Medicine, General Hospital, Sestri Levante, Genoa, Italy.
- [7] Independent Scientific Consultant, Modena, Italy.
- [8] Unit of Respiratory Diseases, General Hospital, Sestri Levante, Genoa, Italy - antonellonicolini@gmail.com.
- [9] Unit of Respiratory Diseases, Department Cardiovascular and Thoracic Diseases, Città della Salute e della Scienza, Turin, Italy.
- [10] Department of Medical Science, University of Turin, Turin, Italy.

- PMID: **33464224**
- DOI: [10.23736/S0026-4806.21.07134-2](10.23736/S0026-4806.21.07134-2)

Abstract

Background: COVID-19 has high mortality rate mainly stemming from acute respiratory distress leading to respiratory failure (ARF). Aim of the study was to evaluate the management of severe ARF due to COVID-19 pneumonia using noninvasive ventilatory support (NIVS), studying safety and effectiveness of NIVS.

Methods: This is a retrospective, multicenter study. Primary outcomes were NIVS failure with intubation rate and hospital mortality. Secondary outcomes were hospital stay and factors related to NIVS failure and mortality. These outcomes were compared with patients intubated and admitted to ICU.

Results: One hundred sixty-two patients were hospitalized because of severe respiratory failure (PaO<inf>2</inf>/FiO<inf>2</inf> ratio <250). One hundred thirty-eight patients were admitted to Respiratory Intermediate Care Unit (RICU) for a NIVS trial. One hundred patients were treated successfully with NIVS (74.5%); 38 failed NIVS trial (27.5%). In-hospital mortality was 23.18% in RICU group and 30.55% in ICU group. Patients with NIVS failure were older, had a lower number of lymphocytes, a higher IL-6, lower PaO<inf>2</inf>, PaC O<inf>2</inf>, PaO<inf>2</inf>/FiO<inf>2</inf> ratio, higher respiratory rate (RR) and heart rate at admission and lower PaO2, and PaO<inf>2</inf>/FiO<inf>2</inf> ratio and higher RR after 1-6 hours. Multivariate analysis identified higher age, C-reactive protein as well as RR after 1-6 hours and PaO<inf>2</inf>/FiO<inf>2</inf> ratio after 1-6 hours as an independent predictor mortality.

Conclusions: NIVS is a safe and effective strategy in the treatment of severe ARF due to COVID-19 related pneumonia, that reduces mortality and length of hospital stay in the carefully selected patients.

Supplementary info

Publication types, MeSH terms Expand

Publication types

- Multicenter Study
- Observational Study

MeSH terms

- Acute Disease
- Age Factors
- Aged
- COVID-19 / complications*
- COVID-19 / drug therapy
- Female
- Heart Rate
- Hospital Mortality
- Humans
- Intensive Care Units / statistics & numerical data
- Length of Stay
- Male
- Middle Aged
- Multivariate Analysis
- Noninvasive Ventilation* / adverse effects
- Noninvasive Ventilation* / methods
- Noninvasive Ventilation* / statistics & numerical data
- Respiratory Insufficiency / mortality
- Respiratory Insufficiency / therapy*
- Respiratory Rate
- Retrospective Studies
- SARS-CoV-2
- Treatment Failure
- Treatment Outcome

Full text links

FULL TEXT article at minervamedica.it Minerva Medica

Proceed to details

Cite

Share

☐ 346

Observational Study

Medicine (Baltimore)

. 2021 Feb 19;100(7):e24720.
doi: 10.1097/MD.0000000000024720.

Management of outpatient with totally implantable venous access Ports during the COVID-19 epidemic

Weipeng Yan [1], Chaoya Zhang, Chenggang Luo, Zilin Li
Affiliations Expand

Affiliation

- [1] Department of Radiology, Hubei Cancer Hospital, Tongji Medical College, Huazhong University of Science and Technology, Wuhan, Hubei Province, China.

- PMID: **33607812**
- PMCID: PMC7899843
- DOI: 10.1097/MD.0000000000024720

Free PMC article
Observational Study

Management of outpatient with totally implantable venous access Ports during the COVID-19 epidemic

Weipeng Yan et al. Medicine (Baltimore). 2021.
Free PMC article
Show details
Medicine (Baltimore)
. 2021 Feb 19;100(7):e24720.
doi: 10.1097/MD.0000000000024720.

Authors

Weipeng Yan [1], Chaoya Zhang, Chenggang Luo, Zilin Li

Affiliation

- [1] Department of Radiology, Hubei Cancer Hospital, Tongji Medical College, Huazhong University of Science and Technology, Wuhan, Hubei Province, China.

- PMID: **33607812**
- PMCID: PMC7899843
- DOI: 10.1097/MD.0000000000024720

Abstract

The purpose of this study was to explore the management experience of outpatient with totally implantable central venous access Ports (TIVAPs, Ports) during the epidemic, including whether the extension of the irrigation interval will affect the incidence of catheter occlusion, the reasons for the port removal rate, and the corresponding protective treatment strategies during the COVID-19 epidemic.We retrospectively analyzed the Ports evaluation and flushing procedure data between February 3, 2020 and April 3, 2020; the cases were divided into the normal group and delayed group according to the critical point of the maintenance interval of 28 days (4 weeks). We compared the incidence of catheter obstruction between the 2 groups, analyzed the causes of catheter removal events in the 2 groups, and proposed corresponding protective treatment recommendations.During the period, 329 cases were included in the study. There was no significant difference in the incidence of catheter obstruction between the 2 groups. There were 15 patients with catheter removal, 8 cases of infection, 5 cases of catheter obstruction, and 1 case of an ectopic catheter, as well as 1 case of an overturned port. During the epidemic, no hospital infections related to the Ports flushing procedure occurred.The interval of Ports flushing procedures for patients without clinical symptoms can be appropriately extended during the COVID-19 epidemic. However, once the local infection symptoms or other sources of discomfort appear, Ports assessment needs to be performed as soon as possible. Take enhanced protected and isolation measures did not increase cross-infection during outpatient's flushing procedure at non-COVID-19-designated diagnosis and treatment hospitals.

Copyright © 2021 the Author(s). Published by Wolters Kluwer Health, Inc.

Conflict of interest statement

The authors have no conflicts of interest to disclose.

- 27 references
- 5 figures

Supplementary info

Publication types, MeSH terms Expand

Publication types

- Observational Study

MeSH terms

- COVID-19 / epidemiology*
- Catheterization, Central Venous*
- Device Removal
- Female
- Humans
- Male
- Middle Aged

- Outpatients*
- Retrospective Studies
- SARS-CoV-2

Full text links

Wolters Kluwer Wolters Kluwer Free PMC article
Proceed to details
Cite
Share
347
Observational Study
PLoS One
. 2020 Dec 31;15(12):e0244777.
doi: 10.1371/journal.pone.0244777. eCollection 2020.

SARS-CoV-2 PCR cycle threshold at hospital admission associated with patient mortality

Jui Choudhuri [1], Jamal Carter [1], Randin Nelson [1], Karin Skalina [1], Marika Osterbur-Badhey [1], Andrew Johnston [2], Doctor Goldstein [1], Monika Paroder [1], James Szymanski [1]
Affiliations Expand

Affiliations

- [1] Department of Pathology, Montefiore Medical Center, Albert Einstein College of Medicine, Bronx, New York, United States of America.
- [2] Department of Medicine, Saint Vincent's Medical Center, Bridgeport, Connecticut, United States of America.

- PMID: **33382805**
- PMCID: PMC7774957
- DOI: 10.1371/journal.pone.0244777

Free PMC article
Observational Study

SARS-CoV-2 PCR cycle threshold at hospital admission associated with patient mortality

Jui Choudhuri et al. PLoS One. 2020.
Free PMC article
Show details
PLoS One

. 2020 Dec 31;15(12):e0244777.
doi: 10.1371/journal.pone.0244777. eCollection 2020.

Authors

Jui Choudhuri [1], Jamal Carter [1], Randin Nelson [1], Karin Skalina [1], Marika Osterbur-Badhey [1], Andrew Johnston [2], Doctor Goldstein [1], Monika Paroder [1], James Szymanski [1]

Affiliations

- [1] Department of Pathology, Montefiore Medical Center, Albert Einstein College of Medicine, Bronx, New York, United States of America.
- [2] Department of Medicine, Saint Vincent's Medical Center, Bridgeport, Connecticut, United States of America.

- PMID: **33382805**
- PMCID: PMC7774957
- DOI: 10.1371/journal.pone.0244777

Abstract

Background: Severe acute respiratory syndrome coronavirus 2 (SARS-CoV-2) cycle threshold (Ct) has been suggested as an approximate measure of initial viral burden. The utility of cycle threshold, at admission, as a predictor of disease severity has not been thoroughly investigated.

Methods and findings: We conducted a retrospective study of SARS-CoV-2 positive, hospitalized patients from 3/26/2020 to 8/5/2020 who had SARS-CoV-2 Ct data within 48 hours of admission (n = 1044). Only patients with complete survival data, discharged (n = 774) or died in hospital (n = 270), were included in our analysis. Laboratory, demographic, and clinical data were extracted from electronic medical records. Multivariable logistic regression was applied to examine the relationship of patient mortality with Ct values while adjusting for established risk factors. Ct was analyzed as continuous variable and subdivided into quartiles to better illustrate its relationship with outcome. Cumulative incidence curves were created to assess whether there was a survival difference in the setting of the competing risks of death versus patient discharge. Mean Ct at admission was higher for survivors (28.6, SD = 5.8) compared to non-survivors (24.8, SD = 6.0, P<0.001). In-hospital mortality significantly differed (p<0.05) by Ct quartile. After adjusting for age, gender, BMI, hypertension and diabetes, increased cycle threshold was associated with decreased odds of in-hospital mortality (0.91, CI 0.89-0.94, p<0.001). Compared to the 4th Quartile, patients with Ct values in the 1st Quartile (Ct <22.9) and 2nd Quartile (Ct 23.0-27.3) had an adjusted odds ratio of in-hospital mortality of 3.8 and 2.6 respectively (p<0.001). The discriminative ability of Ct to predict inpatient mortality was found to be limited, possessing an area under the curve (AUC) of 0.68 (CI 0.63-0.71).

Conclusion: SARS-CoV-2 Ct was found to be an independent predictor of patient mortality. However, further study is needed on how to best clinically utilize such information given the result variation due to specimen quality, phase of disease, and the limited discriminative ability of the test.

Conflict of interest statement

NO authors have competing interests.

- Cited by 12 articles
- 35 references
- 7 figures

Supplementary info

Publication types, MeSH terms, Grant support Expand

Publication types

- Observational Study

MeSH terms

- Adult
- Age Factors
- Aged
- Aged, 80 and over
- COVID-19 / diagnosis
- COVID-19 / mortality*
- COVID-19 / therapy*
- Female
- Hospital Mortality*
- Humans
- Incidence
- Male
- Middle Aged
- Patient Admission
- Patient Discharge
- Retrospective Studies
- Risk Factors
- SARS-CoV-2*
- Sex Factors

Grant support

The authors received no specific funding for this work.

Full text links

OPEN ACCESS TO FULL TEXT PLOS ONE Public Library of Science Free PMC article

Proceed to details

Cite

Share

☐ 348
Observational Study
Diabetes Metab Syndr
. Nov-Dec 2021;15(6):102306.
doi: 10.1016/j.dsx.2021.102306. Epub 2021 Oct 4.

Symptomatic post-vaccination SARS-CoV-2 infections in healthcare workers- A multicenter cohort study

Raju Vaishya [1], Anupam Sibal [2], Arpita Malani [3], Sujoy Kar [4], Hari Prasad K [5], Kiran Sv [6], Sangita Reddy [7], Shobana Kamineni [8], Suneeta Reddy [9], Preetha Reddy [10], Prathap Chandra Reddy [11]
Affilations Expand

Affiliations

- [1] Indraprastha Apollo Hospitals, New Delhi, India. Electronic address: raju_vaishya@apollohospitalsdelhi.com.
- [2] Apollo Hospitals Group, Chennai, India. Electronic address: anupamsibal@apollohospitals.com.
- [3] Indraprastha Apollo Hospitals, New Delhi, India. Electronic address: Malanidr_arpita@apollohospitalsdelhi.com.
- [4] Apollo Hospitals Group, Chennai, India. Electronic address: drsujoy_k@apollohospitals.com.
- [5] Apollo Hospitals Group, Chennai, India. Electronic address: drhariprasad_k@apollohospitals.com.
- [6] Apollo Hospitals Group, Chennai, India. Electronic address: kiran_sv@apollohospitals.com.
- [7] Apollo Hospitals Group, Chennai, India. Electronic address: sangita_reddy@apollohospitals.com.
- [8] Apollo Hospitals Group, Chennai, India. Electronic address: shobana@apollohospitals.com.
- [9] Apollo Hospitals Group, Chennai, India. Electronic address: suneetareddy@apollohospitals.com.
- [10] Apollo Hospitals Group, Chennai, India. Electronic address: preethareddy@apollohospitals.com.
- [11] Apollo Hospitals Group, Chennai, India. Electronic address: chairman@apollohospitals.com.

- PMID: **34619430**
- PMCID: PMC8489275
- DOI: 10.1016/j.dsx.2021.102306

Free PMC article
Observational Study

Symptomatic post-vaccination SARS-CoV-2 infections in healthcare workers- A multicenter cohort study

Raju Vaishya et al. Diabetes Metab Syndr. Nov-Dec 2021.
Free PMC article
Show details
Diabetes Metab Syndr
. Nov-Dec 2021;15(6):102306.
doi: 10.1016/j.dsx.2021.102306. Epub 2021 Oct 4.

Authors

Raju Vaishya [1], Anupam Sibal [2], Arpita Malani [3], Sujoy Kar [4], Hari Prasad K [5], Kiran Sv [6], Sangita Reddy [7], Shobana Kamineni [8], Suneeta Reddy [9], Preetha Reddy [10], Prathap Chandra Reddy [11]

Affiliations

- [1] Indraprastha Apollo Hospitals, New Delhi, India. Electronic address: raju_vaishya@apollohospitalsdelhi.com.
- [2] Apollo Hospitals Group, Chennai, India. Electronic address: anupamsibal@apollohospitals.com.
- [3] Indraprastha Apollo Hospitals, New Delhi, India. Electronic address: Malanidr_arpita@apollohospitalsdelhi.com.
- [4] Apollo Hospitals Group, Chennai, India. Electronic address: drsujoy_k@apollohospitals.com.
- [5] Apollo Hospitals Group, Chennai, India. Electronic address: drhariprasad_k@apollohospitals.com.
- [6] Apollo Hospitals Group, Chennai, India. Electronic address: kiran_sv@apollohospitals.com.
- [7] Apollo Hospitals Group, Chennai, India. Electronic address: sangita_reddy@apollohospitals.com.
- [8] Apollo Hospitals Group, Chennai, India. Electronic address: shobana@apollohospitals.com.
- [9] Apollo Hospitals Group, Chennai, India. Electronic address: suneetareddy@apollohospitals.com.
- [10] Apollo Hospitals Group, Chennai, India. Electronic address: preethareddy@apollohospitals.com.
- [11] Apollo Hospitals Group, Chennai, India. Electronic address: chairman@apollohospitals.com.

- PMID: **34619430**
- PMCID: PMC8489275
- DOI: 10.1016/j.dsx.2021.102306

Abstract

Background and aims: During the COVID-19 vaccination program in India, the healthcare workers were given the first priority. There are concerns regarding the occurrence of breakthrough infections after vaccination. We aimed to investigate the effictiveness of COVID-19 vaccines in preventing and reducing the severity of post-vaccination infections.

Methods: This retrospective test-negative case-control study examined 28342 vaccinated healthcare workers for symptomatic SARS-CoV-2 infections between January 16 to June 15, 2021. They worked at 43 Apollo Group hospitals in 24 Indian cities. These cohorts received either ChAdOx nCOV-19 (Recombinant) or the whole virion inactivated Vero cell vaccines. Various demographic, vaccination related and clinical parameters were evaluated.

Results: Symptomatic symptomatic post-vaccination infections occurred in a small number of vaccinated cohorts (5.07%, $p < 0.001$), and these were predominantly mild and did not result in hospitalization ($p < 0.0001$), or death. Both vaccines provided similar protection, with symptomatic infections in 5.11% and 4.58%, following ChAdOx nCOV-19 (Recombinant) and the whole virion inactivated Vero cell vaccines, respectively ($p < 0.001$). Nursing and Clinical staff and cohorts >50 years contracted more infections ($p < 0.001$). Two-dose vaccination has significantly lower odds of developing symptomatic infection (0.83, 95%CI - 0.72 to 0.97). Maximum infections occurred during the peak of the second COVID-19 wave from mid-April to May 2021 ($p < 0.001$). No significant difference existed in the infection between sex, vaccine type, and the number of vaccine doses received ($p \geq 0.05$).

Conclusion: Symptomatic infections occurred in a small percentage of healthcare workers after COVID vaccination. Vaccination protected them from not only infection but also severe disease.

Keywords: Break through infections; COVID-19; Healthcare workers; SARS-CoV-2; Vaccine.

Copyright © 2021 Diabetes India. Published by Elsevier Ltd. All rights reserved.

Conflict of interest statement

Declaration of competing interest None.

- 35 references
- 2 figures

Supplementary info

Publication types, MeSH terms, Substances Expand

Publication types

- Multicenter Study
- Observational Study

MeSH terms

- Adolescent

- Adult
- Aged
- Aged, 80 and over
- COVID-19 / epidemiology*
- COVID-19 / prevention & control
- COVID-19 / virology
- COVID-19 Vaccines / administration & dosage*
- Case-Control Studies
- Female
- Follow-Up Studies
- Health Personnel / statistics & numerical data*
- Hospitalization / statistics & numerical data*
- Humans
- India / epidemiology
- Male
- Middle Aged
- Prognosis
- Retrospective Studies
- SARS-CoV-2 / isolation & purification*
- Vaccination
- Young Adult

Substances

- COVID-19 Vaccines

Full text links

ELSEVIER FULL-TEXT ARTICLE Elsevier Science Free PMC article
Proceed to details
Cite
Share
☐ 349
Observational Study
PLoS One
. 2020 Aug 12;15(8):e0237558.
doi: 10.1371/journal.pone.0237558. eCollection 2020.

Risk factors for severe illness in hospitalized Covid-19 patients at a regional hospital

Justin J Turcotte [1], Barry R Meisenberg [2], James H MacDonald [1], Nandakumar Menon [1], Marcia B Fowler [1], Michaline West [1], Jane Rhule [3], Sadaf S Qureshi [3], Eileen B MacDonald [2]

Affiliations Expand

Affiliations

- [1] Department of Orthopedics, Anne Arundel Medical Center, Annapolis, MD, United States of America.
- [2] Department of Medicine, Anne Arundel Medical Center, Annapolis, MD, United States of America.
- [3] Anne Arundel Research Institute, Anne Arundel Medical Center, Annapolis, MD, United States of America.

- PMID: **32785285**
- PMCID: PMC7423129
- DOI: 10.1371/journal.pone.0237558

Free PMC article
Observational Study

Risk factors for severe illness in hospitalized Covid-19 patients at a regional hospital

Justin J Turcotte et al. PLoS One. 2020.
Free PMC article
Show details
PLoS One
. 2020 Aug 12;15(8):e0237558.
doi: 10.1371/journal.pone.0237558. eCollection 2020.

Authors

Justin J Turcotte [1], Barry R Meisenberg [2], James H MacDonald [1], Nandakumar Menon [1], Marcia B Fowler [1], Michaline West [1], Jane Rhule [3], Sadaf S Qureshi [3], Eileen B MacDonald [2]

Affiliations

- [1] Department of Orthopedics, Anne Arundel Medical Center, Annapolis, MD, United States of America.
- [2] Department of Medicine, Anne Arundel Medical Center, Annapolis, MD, United States of America.
- [3] Anne Arundel Research Institute, Anne Arundel Medical Center, Annapolis, MD, United States of America.

- PMID: **32785285**
- PMCID: PMC7423129

• DOI: 10.1371/journal.pone.0237558

Abstract

Background: The Covid-19 pandemic threatens to overwhelm scarce clinical resources. Risk factors for severe illness must be identified to make efficient resource allocations.

Objective: To evaluate risk factors for severe illness.

Design: Retrospective, observational case series.

Setting: Single-institution.

Participants: First 117 consecutive patients hospitalized for Covid-19 from March 1 to April 12, 2020.

Exposure: None.

Main outcomes and measures: Intensive care unit admission or death.

Results: In-hospital mortality was 24.8% and average total length of stay was 11.82 days (95% CI: 10.01 to 13.63 days). 30.8% of patients required intensive care unit admission and 29.1% required mechanical ventilation. Multivariate regression identified the amount of supplemental oxygen required at admission (OR: 1.208, 95% CI: 1.011-1.443, p = .037), sputum production (OR: 6.734, 95% CI: 1.630-27.812, p = .008), insulin dependent diabetes mellitus (OR: 11.873, 95% CI: 2.218-63.555, p = .004) and chronic kidney disease (OR: 4.793, 95% CI: 1.528-15.037, p = .007) as significant risk factors for intensive care unit admission or death. Of the 48 patients who were admitted to the intensive care unit or died, this occurred within 3 days of arrival in 42%, within 6 days in 71%, and within 9 days in 88% of patients.

Conclusions: At our regional medical center, patients with Covid-19 had an average length of stay just under 12 days, required ICU care in 31% of cases, and had a 25% mortality rate. Patients with increased sputum production and higher supplemental oxygen requirements at admission, and insulin dependent diabetes or chronic kidney disease may be at increased risk for severe illness. A model for predicting intensive care unit admission or death with excellent discrimination was created that may aid in treatment decisions and resource allocation. Early identification of patients at increased risk for severe illness may lead to improved outcomes in patients hospitalized with Covid-19.

Conflict of interest statement

The authors have declared that no competing interests exist.

- Cited by 16 articles
- 33 references
- 4 figures

Supplementary info

Publication types, MeSH terms, Grant support Expand

Publication types

- Observational Study

MeSH terms

- Aged
- Aged, 80 and over
- Betacoronavirus / genetics*
- COVID-19
- Coronavirus Infections / epidemiology*
- Coronavirus Infections / mortality
- Coronavirus Infections / pathology*
- Coronavirus Infections / virology
- Critical Illness
- Female
- Health Care Rationing
- Hospital Mortality
- Hospitalization*
- Hospitals, Community
- Humans
- Intensive Care Units
- Length of Stay
- Male
- Maryland / epidemiology
- Middle Aged
- Pandemics
- Pneumonia, Viral / epidemiology*
- Pneumonia, Viral / mortality
- Pneumonia, Viral / pathology*
- Pneumonia, Viral / virology
- Respiration, Artificial
- Retrospective Studies
- Reverse Transcriptase Polymerase Chain Reaction
- Risk Factors
- SARS-CoV-2

Grant support

The author(s) received no specific funding for this work.

Full text links

OPEN ACCESS TO FULL TEXT PLOS ONE Public Library of Science Free PMC article
Proceed to details
Cite
Share
350
Observational Study
J Pharm Pharm Sci
. 2021;24:210-219.
doi: 10.18433/jpps31969.

Clinical Outcomes of COVID-19 Patients Treated with Convalescent Plasma or Remdesivir Alone and in Combination at a Community Hospital in California's Central Valley

Regine Padilla [1], Jered Arquiette [1], Yvonne Mai [2], Gurinder Singh [3], Kristine Galang [4], Edward Liang [2]
Affiliations Expand

Affiliations

- [1] San Joaquin General Hospital, French Camp, CA, USA.
- [2] University of the Pacific, Thomas J. Long School of Pharmacy, Stockton CA, USA.
- [3] San Joaquin General Hospital, Department: Internal Medicine, French Camp, CA, USA.
- [4] San Joaquin General Hospital, Department of Internal Medicine, French Camp, CA, USA.

- PMID: **33939951**
- DOI: 10.18433/jpps31969

Free article
Observational Study

Clinical Outcomes of COVID-19 Patients Treated with Convalescent Plasma or Remdesivir Alone and in Combination at a Community Hospital in California's Central Valley

Regine Padilla et al. J Pharm Pharm Sci. 2021.
Free article
Show details
J Pharm Pharm Sci
. 2021;24:210-219.
doi: 10.18433/jpps31969.

Authors

Regine Padilla [1], Jered Arquiette [1], Yvonne Mai [2], Gurinder Singh [3], Kristine Galang [4], Edward Liang [2]

Affiliations

- [1] San Joaquin General Hospital, French Camp, CA, USA.
- [2] University of the Pacific, Thomas J. Long School of Pharmacy, Stockton CA, USA.
- [3] San Joaquin General Hospital, Department: Internal Medicine, French Camp, CA, USA.
- [4] San Joaquin General Hospital, Department of Internal Medicine, French Camp, CA, USA.

- PMID: **33939951**
- DOI: 10.18433/jpps31969

Abstract

Purpose: The purpose of this study was to compare how treatment with convalescent plasma (CP) monotherapy, remdesivir (RDV) monotherapy, and combination therapy (CP + RDV) in patients with COVID-19 affected clinical outcomes.

Methods: Patients with COVID-19 infection who were admitted to the hospital received CP, RDV, or combination of both. Mortality, discharge disposition, hospital length of stay (LOS), intensive care unit (ICU) LOS, and total ventilation days were compared between each treatment group and stratified by ABO blood group. An exploratory analysis identified risk factors for mortality. Adverse effects were also evaluated.

Results: RDV monotherapy showed an increased chance of survival compared to combination therapy or CP monotherapy ($p = 0.052$). There were 15, 3, and 6 deaths in the CP, RDV, and combination therapy groups, respectively. The combination therapy group had the longest median ICU LOS (8, IQR 4.5-15.5, $p = 0.220$) and hospital LOS (11, IQR 7-15.5, $p = 0.175$). Age ($p = 0.036$), initial SOFA score ($p = 0.013$), and intubation ($p = 0.005$) were statistically significant predictors of mortality. Patients with type O blood had decreased ventilation days, ICU LOS, and total LOS. Thirteen treatment-related adverse events occurred.

Conclusion: No significant differences in clinical outcomes were observed between patients treated with RDV, CP, or combination therapy. Elderly patients, those with a high initial SOFA score, and those who require intubation are at increased risk of mortality associated with COVID-19. Blood type did not affect clinical outcomes.

Supplementary info

Publication types, MeSH terms, Substances, Supplementary concepts Expand

Publication types

- Observational Study

MeSH terms

- Adenosine Monophosphate / administration & dosage
- Adenosine Monophosphate / analogs & derivatives*
- Adult
- Aged
- Alanine / administration & dosage
- Alanine / analogs & derivatives*
- Antiviral Agents / administration & dosage*
- COVID-19 / diagnosis
- COVID-19 / epidemiology
- COVID-19 / mortality
- COVID-19 / therapy*
- California / epidemiology
- Combined Modality Therapy / methods
- Female
- Hospitals, Community / trends*
- Humans
- Immunization, Passive / mortality
- Length of Stay / trends
- Male
- Middle Aged
- Retrospective Studies
- Treatment Outcome

Substances

- Antiviral Agents
- remdesivir
- Adenosine Monophosphate
- Alanine

Supplementary concepts

- COVID-19 serotherapy

Full text links

JPPS Canadian Society for Pharmaceutical Sciences
Proceed to details
Cite
Share
351

Observational Study
J Infect Public Health
. 2021 Nov;14(11):1623-1629.
doi: 10.1016/j.jiph.2021.09.014. Epub 2021 Sep 23.

Clinical and biochemical characteristics and outcomes of suspected COVID-19 hospitalized patients: RT-PCR swab positive and negative comparison

Assim A Alfadda [1], Mohammad AlKhowaiter [2], Naif Alotaibi [3], Khalid Alayed [2], Musa Alzahrani [2], Khalifa Binkhamis [4], Khalid Siddiqui [5], Amira Youssef [5], Haifa Altalhi [6], Ibrahim Almaghlouth [7], Mohammed Alarifi [8], Saleh Albanyan [2], Mohammed Faraj Alosaimi [9], Rana Hasanato [10], Arthur Isnani [11], Hafedh Dekhil [11], Mohamed Rafiullah [5]
Affiliations Expand

Affiliations

- [1] Department of Internal Medicine, College of Medicine, and King Khalid University Hospital, King Saud University Medical City, King Saud University, Riyadh, Saudi Arabia; Strategic Center for Diabetes Research, College of Medicine, King Saud University, Riyadh, Saudi Arabia; Obesity Research Center, College of Medicine, King Saud University, Riyadh, Saudi Arabia. Electronic address: aalfadda@ksu.edu.sa.
- [2] Department of Internal Medicine, College of Medicine, and King Khalid University Hospital, King Saud University Medical City, King Saud University, Riyadh, Saudi Arabia.
- [3] Communicable Diseases Department, King Khalid University Hospital, King Saud University Medical City, King Saud University, Riyadh, Saudi Arabia.
- [4] Department of Pathology, College of Medicine, King Saud University, Riyadh, Saudi Arabia; King Saud University Medical City, King Saud University, Riyadh, Saudi Arabia.
- [5] Strategic Center for Diabetes Research, College of Medicine, King Saud University, Riyadh, Saudi Arabia.
- [6] Infection Control Department, King Khalid University Hospital, King Saud University Medical City, King Saud University, Riyadh, Saudi Arabia.
- [7] Rheumatology Unit, Department of Internal Medicine, King Khalid University Hospital, King Saud University Medical City, King Saud University, Riyadh, Saudi Arabia.
- [8] Intensive Care Department, King Khalid University Hospital, King Saud University Medical City, King Saud University, Riyadh, Saudi Arabia.

- [9] Immunology unit, Department of Pediatrics, College of Medicine, and King Khalid University Hospital, King Saud University Medical City, King Saud University, Riyadh, Saudi Arabia.
- [10] Department of Pathology, King Khalid University Hospital, King Saud University Medical City, King Saud University, Riyadh, Saudi Arabia.
- [11] Obesity Research Center, College of Medicine, King Saud University, Riyadh, Saudi Arabia.

- PMID: **34624717**
- PMCID: PMC8457915
- DOI: 10.1016/j.jiph.2021.09.014

Free PMC article
Observational Study

Clinical and biochemical characteristics and outcomes of suspected COVID-19 hospitalized patients: RT-PCR swab positive and negative comparison

Assim A Alfadda et al. J Infect Public Health. 2021 Nov.
Free PMC article
Show details
J Infect Public Health
. 2021 Nov;14(11):1623-1629.
doi: 10.1016/j.jiph.2021.09.014. Epub 2021 Sep 23.

Authors

Assim A Alfadda [1], Mohammad AlKhowaiter [2], Naif Alotaibi [3], Khalid Alayed [2], Musa Alzahrani [2], Khalifa Binkhamis [4], Khalid Siddiqui [5], Amira Youssef [5], Haifa Altalhi [6], Ibrahim Almaghlouth [7], Mohammed Alarifi [8], Saleh Albanyan [2], Mohammed Faraj Alosaimi [9], Rana Hasanato [10], Arthur Isnani [11], Hafedh Dekhil [11], Mohamed Rafiullah [5]

Affiliations

- [1] Department of Internal Medicine, College of Medicine, and King Khalid University Hospital, King Saud University Medical City, King Saud University, Riyadh, Saudi Arabia; Strategic Center for Diabetes Research, College of Medicine, King Saud University, Riyadh, Saudi Arabia; Obesity Research Center, College of Medicine, King Saud University, Riyadh, Saudi Arabia. Electronic address: aalfadda@ksu.edu.sa.
- [2] Department of Internal Medicine, College of Medicine, and King Khalid University Hospital, King Saud University Medical City, King Saud University, Riyadh, Saudi Arabia.
- [3] Communicable Diseases Department, King Khalid University Hospital, King Saud University Medical City, King Saud University, Riyadh, Saudi Arabia.

- [4] Department of Pathology, College of Medicine, King Saud University, Riyadh, Saudi Arabia; King Saud University Medical City, King Saud University, Riyadh, Saudi Arabia.
- [5] Strategic Center for Diabetes Research, College of Medicine, King Saud University, Riyadh, Saudi Arabia.
- [6] Infection Control Department, King Khalid University Hospital, King Saud University Medical City, King Saud University, Riyadh, Saudi Arabia.
- [7] Rheumatology Unit, Department of Internal Medicine, King Khalid University Hospital, King Saud University Medical City, King Saud University, Riyadh, Saudi Arabia.
- [8] Intensive Care Department, King Khalid University Hospital, King Saud University Medical City, King Saud University, Riyadh, Saudi Arabia.
- [9] Immunology unit, Department of Pediatrics, College of Medicine, and King Khalid University Hospital, King Saud University Medical City, King Saud University, Riyadh, Saudi Arabia.
- [10] Department of Pathology, King Khalid University Hospital, King Saud University Medical City, King Saud University, Riyadh, Saudi Arabia.
- [11] Obesity Research Center, College of Medicine, King Saud University, Riyadh, Saudi Arabia.

- PMID: **34624717**
- PMCID: PMC8457915
- DOI: 10.1016/j.jiph.2021.09.014

Abstract

Background: COVID-19 is diagnosed using RT-PCR assays of samples from nasal and oropharyngeal swabs. People with negative RT-PCR often presented with clinical manifestations of COVID-19. The data on such patients are lacking. The present study aims to characterize the patients who were suspected COVID-19 cases and tested negative in RT-PCR compared to patients who had been tested RT-PCR positive.

Methods: This is a retrospective, observational study of adult suspected and confirmed patients of COVID-19 admitted to King Saud University Medical City, Riyadh, Saudi Arabia, from 1st March 2020 until 30th November 2020. Laboratory confirmation is done through nasal/pharyngeal swab specimens, tested positive in RT-PCR assay. Patients with initial negative RT-PCR test results were assessed again within 48-72 h to avoid false-negative results. Patient data were extracted from the electronic medical files of each included patient using a predesigned case report form.

Results: The study included 488 (80.93%) patients with RT-PCR swab results positive, and 115 (19.07%) patients who were negative. Respiratory rate and diastolic blood pressure were higher among the swab-positive cases. More number of swab-negative patients had comorbidities such as coronary heart disease, chronic kidney disease, and carcinoma. Fever, cough, and shortness of breath were reported higher among the swab-positive cases. ALT and AST, and LDH levels were found higher among RT-PCR-positive patients. Serum creatinine, blood urea nitrogen and troponin were more elevated in RT-PCR-negative patients. Antibiotics, anticoagulants, and corticosteroids were used more by swab-positive patients. Significantly higher number of RT-PCR-positive patients required proning, high-flow nasal cannula, non-invasive mechanical ventilation, and invasive mechanical ventilation. Acute cardiac ischemia and death were found to be similar among the patients. However, deaths occurred significantly earlier among the swab-positive cases when compared to the swab-negative group.

Conclusion: Distinctive symptoms and markers of COVID-19 are more frequent among patients who had RT-PCR-positive results.

Keywords: COVID-19; Clinical characteristics; Negative RT-PCR; Suspected COVID-19.

Copyright © 2021. Published by Elsevier Ltd.

- Cited by 1 article
- 24 references
- 3 figures

Supplementary info

Publication types, MeSH terms Expand

Publication types

- Observational Study

MeSH terms

- Adult
- COVID-19*
- Comorbidity
- Hospitalization
- Humans
- Reverse Transcriptase Polymerase Chain Reaction
- SARS-CoV-2

Full text links

ELSEVIER OPEN ACCESS Elsevier Science Free PMC article
Proceed to details
Cite
Share
☐ 352
BMJ Open

. 2021 Nov 16;11(11):e045718.
doi: 10.1136/bmjopen-2020-045718.

Epidemiology, management and the associated burden of mental health illness, atopic and autoimmune conditions, and

common infections in alopecia areata: protocol for an observational study series

Matthew Harries[1], Abby E Macbeth[2], Susan Holmes[3], Andrew R Thompson[4], Wing Sin Chiu[5], William Romero Gallardo[5], Andrew G Messenger[6], Christos Tziotzios[7], Simon de Lusignan[8][9]

Affiliations Expand

Affiliations

- [1] The Dermatology Centre, Salford Royal Hospital, Northern Care Alliance NHS Foundation Trust, Greater Manchester, UK.
- [2] Department of Dermatology, Norfolk and Norwich University Hospitals NHS Foundation Trust, Norwich, UK.
- [3] Alan Lyell Centre for Dermatology, Queen Elizabeth University Hospital, Glasgow, UK.
- [4] South Wales Clinical Psychology Training Programme, Department of Psychology, Cardiff University, Cardiff, UK.
- [5] Pfizer, Tadworth, UK.
- [6] Department of Dermatology, Royal Hallamshire Hospital, Sheffield, UK.
- [7] St John's Institute of Dermatology, King's College London, London, UK.
- [8] Nuffield Department of Primary Care Health Sciences, University of Oxford, Oxford, UK simon.delusignan@phc.ox.ac.uk.
- [9] Department of Clinical and Experimental Medicine, University of Surrey, Guildford, UK.

- PMID: **34785540**
- PMCID: PMC8596050
- DOI: 10.1136/bmjopen-2020-045718

Free PMC article

Epidemiology, management and the associated burden of mental health illness, atopic and autoimmune conditions, and common infections in alopecia areata: protocol for an observational study series

Matthew Harries et al. BMJ Open. 2021.
Free PMC article
Show details
BMJ Open
. 2021 Nov 16;11(11):e045718.
doi: 10.1136/bmjopen-2020-045718.

Authors

Matthew Harries [1], Abby E Macbeth [2], Susan Holmes [3], Andrew R Thompson [4], Wing Sin Chiu [5], William Romero Gallardo [5], Andrew G Messenger [6], Christos Tziotzios [7], Simon de Lusignan [8] [9]

Affiliations

- [1] The Dermatology Centre, Salford Royal Hospital, Northern Care Alliance NHS Foundation Trust, Greater Manchester, UK.
- [2] Department of Dermatology, Norfolk and Norwich University Hospitals NHS Foundation Trust, Norwich, UK.
- [3] Alan Lyell Centre for Dermatology, Queen Elizabeth University Hospital, Glasgow, UK.
- [4] South Wales Clinical Psychology Training Programme, Department of Psychology, Cardiff University, Cardiff, UK.
- [5] Pfizer, Tadworth, UK.
- [6] Department of Dermatology, Royal Hallamshire Hospital, Sheffield, UK.
- [7] St John's Institute of Dermatology, King's College London, London, UK.
- [8] Nuffield Department of Primary Care Health Sciences, University of Oxford, Oxford, UK simon.delusignan@phc.ox.ac.uk.
- [9] Department of Clinical and Experimental Medicine, University of Surrey, Guildford, UK.

- PMID: **34785540**
- PMCID: PMC8596050
- DOI: 10.1136/bmjopen-2020-045718

Abstract

Introduction: Alopecia areata (AA) is a common cause of immune-mediated non-scarring hair loss. Links between AA and common mental health, autoimmune and atopic conditions, and common infections have previously been described but remain incompletely elucidated and contemporary descriptions of the epidemiology of AA in the UK are lacking.

Methods and analysis: Retrospective study series using a large population-based cohort (5.2 million) from the Oxford Royal College of General Practitioners (RCGP) Research and Surveillance Centre (RSC) database, exploring four themes: AA epidemiology, mental health comorbidities, autoimmune/atopic associations and common infections.In the epidemiology theme, we will describe the incidence and point prevalence of AA overall and by age, sex and sociodemographic factors. Healthcare utilisation (primary care visits and secondary care referrals) and treatments for AA will also be assessed. In the mental health theme, we will explore the prevalence and incidence of mental health conditions (anxiety, depressive episodes, recurrent depressive disorder, adjustment disorder, agoraphobia, self-harm and parasuicide) in people with AA compared with matched controls. We will also explore the mental health treatment patterns (medication and psychological interventions), time off work and unemployment rates. Within the autoimmune/atopic associations theme, we will examine the prevalence of atopic (atopic dermatitis, allergic rhinitis, asthma) and autoimmune conditions (Crohn's disease, ulcerative colitis, coeliac disease, type 1 diabetes, Hashimoto's thyroiditis, Graves' disease, rheumatoid arthritis, psoriatic arthritis, ankylosing spondylitis, systemic lupus erythematosus (SLE), polymyalgia rheumatica, Sjögren's syndrome, psoriasis, vitiligo, multiple sclerosis, pernicious anaemia) in people with AA compared with matched controls. We will also estimate the incidence

of new-onset atopic and autoimmune conditions after AA diagnosis. Within the common infections theme, we will examine the incidence of common infections (respiratory tract infection, pneumonia, acute bronchitis, influenza, skin infection, urinary tract infection, genital infections, gastrointestinal infection, herpes simplex, herpes zoster, meningitis, COVID-19) in people with AA compared with matched controls.

Ethics and dissemination: The Health Research Authority decision tool classed this a study of usual practice, ethics approval was not required. Study approval was granted by the RCGP RSC Study Approval Committee. Results will be disseminated through peer-reviewed publications.

Observational study registration number: NCT04239521.

Keywords: dermatology; epidemiology; immunology; infectious diseases; mental health; primary care.

© Author(s) (or their employer(s)) 2021. Re-use permitted under CC BY-NC. No commercial re-use. See rights and permissions. Published by BMJ.

Conflict of interest statement

Competing interests: SdL is Director of RCGP RSC. He has received funding for projects from Eli Lilly, AstraZeneca, GSK, Seqirus and Takeda—all through his universities and none related to this study. WSC and WRG are employees of Pfizer. SH and CT are investigators on the Pfizer-funded Allegro clinical trial.

- Cited by 1 article
- 74 references

Supplementary info

Publication types, MeSH terms, Associated data Expand

Publication types

- Research Support, Non-U.S. Gov't

MeSH terms

- Alopecia Areata* / epidemiology
- Autoimmune Diseases* / epidemiology
- COVID-19*
- Dermatitis, Atopic* / epidemiology
- Humans
- Mental Health
- Observational Studies as Topic
- Retrospective Studies
- SARS-CoV-2

Associated data

- ClinicalTrials.gov/NCT04239521

Full text links

BMJ Free Full Text HighWire Free PMC article

Proceed to details

Cite

Share

353

Observational Study

Am J Trop Med Hyg

. 2021 Aug 9;105(3):718-726.

doi: 10.4269/ajtmh.21-0234.

Role of Prealbumin in Predicting the Prognosis of Severely and Critically Ill COVID-19 Patients

Ningning Cui [1], Haihui Tong [2], Yan Li [1], Yanyan Ge [1], Yuxin Shi [1], Ping Lv [1], Xiaobo Zhao [2], Jianchu Zhang [3], Gui Fu [2], Yanfen Zhou [2], Ke Jiang [4], Nengxing Lin [5], Tao Bai [6], Runming Jin [2], Sheng Wei [7], Xuefeng Yang [1], Xin Li [2]

Affiliations Expand

Affiliations

- [1] 1Department of Nutrition and Food Hygiene, Hubei Key Laboratory of Food Nutrition and Safety, MOE Key Laboratory of Environment and Health, School of Public Health, Tongji Medical College, Huazhong University of Science and Technology, Wuhan, Hubei, P.R. China.
- [2] 2Department of Paediatrics, Union Hospital, Tongji Medical College, Huazhong University of Science and Technology, Wuhan, Hubei Province, P.R. China.
- [3] 3Department of Respiratory and Critical Care Medicine, Union Hospital, Tongji Medical College, Huazhong University of Science and Technology, Wuhan, Hubei Province, P.R. China.
- [4] 4Department of Thoracic Surgery, Union Hospital, Tongji Medical College, Huazhong University of Science and Technology, Wuhan, Hubei Province, P.R. China.
- [5] 5Department of Dermatology, Union Hospital, Tongji Medical College, Huazhong University of Science and Technology, Wuhan, Hubei Province, P.R. China.
- [6] 6Department of Gastroenterology, Union Hospital, Tongji Medical College, Huazhong University of Science and Technology, Wuhan, Hubei Province, P.R. China.
- [7] 7Department of Epidemiology and Biostatistics, Ministry of Education Key Laboratory of Environment and Health, School of Public Health, Tongji Medical College, Huazhong University of Science and Technology, Wuhan, Hubei, China.

- PMID: **34242179**
- PMCID: PMC8592333
- DOI: 10.4269/ajtmh.21-0234

Free PMC article
Observational Study

Role of Prealbumin in Predicting the Prognosis of Severely and Critically Ill COVID-19 Patients

Ningning Cui et al. Am J Trop Med Hyg. 2021.
Free PMC article
Show details
Am J Trop Med Hyg
. 2021 Aug 9;105(3):718-726.
doi: 10.4269/ajtmh.21-0234.

Authors

Ningning Cui [1], Haihui Tong [2], Yan Li [1], Yanyan Ge [1], Yuxin Shi [1], Ping Lv [1], Xiaobo Zhao [2], Jianchu Zhang [3], Gui Fu [2], Yanfen Zhou [2], Ke Jiang [4], Nengxing Lin [5], Tao Bai [6], Runming Jin [2], Sheng Wei [7], Xuefeng Yang [1], Xin Li [2]

Affiliations

- [1] 1Department of Nutrition and Food Hygiene, Hubei Key Laboratory of Food Nutrition and Safety, MOE Key Laboratory of Environment and Health, School of Public Health, Tongji Medical College, Huazhong University of Science and Technology, Wuhan, Hubei, P.R. China.
- [2] 2Department of Paediatrics, Union Hospital, Tongji Medical College, Huazhong University of Science and Technology, Wuhan, Hubei Province, P.R. China.
- [3] 3Department of Respiratory and Critical Care Medicine, Union Hospital, Tongji Medical College, Huazhong University of Science and Technology, Wuhan, Hubei Province, P.R. China.
- [4] 4Department of Thoracic Surgery, Union Hospital, Tongji Medical College, Huazhong University of Science and Technology, Wuhan, Hubei Province, P.R. China.
- [5] 5Department of Dermatology, Union Hospital, Tongji Medical College, Huazhong University of Science and Technology, Wuhan, Hubei Province, P.R. China.
- [6] 6Department of Gastroenterology, Union Hospital, Tongji Medical College, Huazhong University of Science and Technology, Wuhan, Hubei Province, P.R. China.
- [7] 7Department of Epidemiology and Biostatistics, Ministry of Education Key Laboratory of Environment and Health, School of Public Health, Tongji Medical College, Huazhong University of Science and Technology, Wuhan, Hubei, China.

- PMID: **34242179**
- PMCID: PMC8592333

- DOI: 10.4269/ajtmh.21-0234

Abstract

Most critically ill patients experience malnutrition, resulting in a poor prognosis. This study aimed to evaluate the association of prealbumin (PAB) with the prognosis for severely and critically ill coronavirus disease 2019 (COVID-19) patients and explore factors related to this association. Patients with laboratory-confirmed COVID-19 from West Campus of Union Hospital in Wuhan from January 29, 2020 to March 31, 2020 were enrolled in this study. Patients were classified into the PAB1 (150-400 mg/L; N = 183) and PAB2 (< 150 mg/L; N = 225) groups. Data collection was performed using the hospital's electronic medical records system. The predictive value of PAB was evaluated by measuring the area under the receiver-operating characteristic (AUROC) curve. Patients were defined as severely or critically ill based on the Guidance for COVID-19 (7th edition) by the National Health Commission of China. During this analysis, 316 patients had severe cases and 65 had critical cases. A reduced PAB level was associated with a higher risk of mortality and a longer hospital stay. The AUROC curve for the prognosis based on the PAB level was 0.93, with sensitivity of 97.2% and specificity of 77.6%. For severe cases, a lower level of PAB was associated with a higher risk of malnutrition, higher NK cell counts, and lower B lymphocyte counts; these factors were not significant in critical cases. C-reactive protein and nutritional status mediated the association between PAB and prognosis. This retrospective analysis suggests that the PAB level on admission is an indicator of the prognosis for COVID-19.

- 49 references
- 4 figures

Supplementary info

Publication types, MeSH terms, Substances Expand

Publication types

- Observational Study
- Research Support, Non-U.S. Gov't

MeSH terms

- Adult
- Aged
- C-Reactive Protein / analysis
- COVID-19 / blood
- COVID-19 / mortality*
- Critical Illness
- Female
- Humans
- Length of Stay
- Male
- Middle Aged

- Prealbumin / analysis*
- Prognosis
- Retrospective Studies
- SARS-CoV-2*
- Severity of Illness Index

Substances

- Prealbumin
- C-Reactive Protein

Full text links

Full Text THE AMERICAN JOURNAL OF TROPICAL MEDICINE AND HYGIENE Sheridan PubFactory Free PMC article
Proceed to details
Cite
Share
354
Observational Study
PLoS One
. 2021 May 14;16(5):e0251768.
doi: 10.1371/journal.pone.0251768. eCollection 2021.

The impact of chest CT body composition parameters on clinical outcomes in COVID-19 patients

Giulia Besutti [1, 2], Massimo Pellegrini [3, 4], Marta Ottone [5], Michele Cantini [6], Jovana Milic [2, 6], Efrem Bonelli [1, 7], Giovanni Dolci [8], Giulia Cassone [2, 9], Guido Ligabue [10], Lucia Spaggiari [1], Pierpaolo Pattacini [1], Tommaso Fasano [7], Simone Canovi [7], Marco Massari [8], Carlo Salvarani [9], Giovanni Guaraldi [6], Paolo Giorgi Rossi [5], Reggio Emilia COVID-19 Working Group
Affiliations Expand

Affiliations

- [1] Radiology Unit, Azienda USL-IRCCS di Reggio Emilia, Reggio Emilia, Italy.
- [2] Clinical and Experimental Medicine PhD Program, University of Modena and Reggio Emilia, Modena, Italy.
- [3] Clinical Nutrition Unit, Azienda USL-IRCCS di Reggio Emilia, Reggio Emilia, Italy.
- [4] Department of Biomedical, Metabolic and Neural Sciences, University of Modena and Reggio Emilia, Modena, Italy.
- [5] Epidemiology Unit, Azienda USL-IRCCS di Reggio Emilia, Reggio Emilia, Italy.

- [6] Modena HIV Metabolic Clinic, University of Modena and Reggio Emilia, Modena, Italy.
- [7] Department of Diagnostic Imaging and Laboratory Medicine, Clinical Chemistry and Endocrinology Laboratory, Azienda USL-IRCCS di Reggio Emilia, Reggio Emilia, Italy.
- [8] Infectious Disease Unit, Azienda USL-IRCCS di Reggio Emilia, Reggio Emilia, Italy.
- [9] Rheumatology Unit, Azienda USL-IRCCS di Reggio Emilia, Reggio Emilia, Italy.
- [10] Radiology Unit, Azienda Ospedaliero-Universitaria di Modena, University of Modena and Reggio Emilia, Modena, Italy.

- PMID: **33989341**
- PMCID: PMC8121324
- DOI: 10.1371/journal.pone.0251768

Free PMC article
Observational Study

The impact of chest CT body composition parameters on clinical outcomes in COVID-19 patients

Giulia Besutti et al. PLoS One. 2021.
Free PMC article
Show details
PLoS One

. 2021 May 14;16(5):e0251768.
doi: 10.1371/journal.pone.0251768. eCollection 2021.

Authors

Giulia Besutti [1, 2], Massimo Pellegrini [3, 4], Marta Ottone [5], Michele Cantini [6], Jovana Milic [2, 6], Efrem Bonelli [1, 7], Giovanni Dolci [8], Giulia Cassone [2, 9], Guido Ligabue [10], Lucia Spaggiari [1], Pierpaolo Pattacini [1], Tommaso Fasano [7], Simone Canovi [7], Marco Massari [8], Carlo Salvarani [9], Giovanni Guaraldi [6], Paolo Giorgi Rossi [5], Reggio Emilia COVID-19 Working Group

Affiliations

- [1] Radiology Unit, Azienda USL-IRCCS di Reggio Emilia, Reggio Emilia, Italy.
- [2] Clinical and Experimental Medicine PhD Program, University of Modena and Reggio Emilia, Modena, Italy.
- [3] Clinical Nutrition Unit, Azienda USL-IRCCS di Reggio Emilia, Reggio Emilia, Italy.
- [4] Department of Biomedical, Metabolic and Neural Sciences, University of Modena and Reggio Emilia, Modena, Italy.
- [5] Epidemiology Unit, Azienda USL-IRCCS di Reggio Emilia, Reggio Emilia, Italy.
- [6] Modena HIV Metabolic Clinic, University of Modena and Reggio Emilia, Modena, Italy.
- [7] Department of Diagnostic Imaging and Laboratory Medicine, Clinical Chemistry and Endocrinology Laboratory, Azienda USL-IRCCS di Reggio Emilia, Reggio Emilia, Italy.

- [8] Infectious Disease Unit, Azienda USL-IRCCS di Reggio Emilia, Reggio Emilia, Italy.
- [9] Rheumatology Unit, Azienda USL-IRCCS di Reggio Emilia, Reggio Emilia, Italy.
- [10] Radiology Unit, Azienda Ospedaliero-Universitaria di Modena, University of Modena and Reggio Emilia, Modena, Italy.

- PMID: **33989341**
- PMCID: PMC8121324
- DOI: 10.1371/journal.pone.0251768

Abstract

We assessed the impact of chest CT body composition parameters on outcomes and disease severity at hospital presentation of COVID-19 patients, focusing also on the possible mediation of body composition in the relationship between age and death in these patients. Chest CT scans performed at hospital presentation by consecutive COVID-19 patients (02/27/2020-03/13/2020) were retrospectively reviewed to obtain pectoralis muscle density and total, visceral, and intermuscular adipose tissue areas (TAT, VAT, IMAT) at the level of T7-T8 vertebrae. Primary outcomes were: hospitalization, mechanical ventilation (MV) and/or death, death alone. Secondary outcomes were: C-reactive protein (CRP), oxygen saturation (SO2), CT disease extension at hospital presentation. The mediation of body composition in the effect of age on death was explored. Of the 318 patients included in the study (median age 65.7 years, females 37.7%), 205 (64.5%) were hospitalized, 68 (21.4%) needed MV, and 58 (18.2%) died. Increased muscle density was a protective factor while increased TAT, VAT, and IMAT were risk factors for hospitalization and MV/death. All these parameters except TAT had borderline effects on death alone. All parameters were associated with SO2 and extension of lung parenchymal involvement at CT; VAT was associated with CRP. Approximately 3% of the effect of age on death was mediated by decreased muscle density. In conclusion, low muscle quality and ectopic fat accumulation were associated with COVID-19 outcomes, VAT was associated with baseline inflammation. Low muscle quality partly mediated the effect of age on mortality.

Conflict of interest statement

The authors have declared that no competing interests exist.

- Cited by 4 articles
- 46 references
- 3 figures

Supplementary info

Publication types, MeSH terms, Grant support Expand

Publication types

- Observational Study
- Research Support, Non-U.S. Gov't

MeSH terms

- Aged
- Aged, 80 and over
- Body Composition*
- Body Mass Index
- COVID-19 / diagnostic imaging*
- COVID-19 / epidemiology
- COVID-19 / mortality*
- COVID-19 / virology
- Female
- Hospitalization
- Humans
- Intra-Abdominal Fat / diagnostic imaging*
- Italy / epidemiology
- Male
- Mass Chest X-Ray / methods*
- Middle Aged
- Respiration, Artificial
- Retrospective Studies
- Risk Factors
- SARS-CoV-2 / genetics*
- Tomography, X-Ray Computed / methods*
- Treatment Outcome

Grant support

This study is part of a larger project supported by Ministry of Health (Grant number COVID-2020-12371808).

Full text links

OPEN ACCESS TO FULL TEXT PLOS ONE Public Library of Science Free PMC article

Proceed to details

Cite

Share

☐ 355

Observational Study

Clin Microbiol Infect

. 2021 Aug;27(8):1137-1144.

doi: 10.1016/j.cmi.2020.12.010. Epub 2021 Jan 20.

Better prognosis in females with severe COVID-19 pneumonia: possible role of inflammation as potential mediator

Cristina Mussini [1], Alessandro Cozzi-Lepri [2], Marianna Menozzi [3], Marianna Meschiari [3], Erica Franceschini [3], Carlotta Rogati [4], Gianluca Cuomo [3], Andrea Bedini [3], Vittorio Iadisernia [4], Sara Volpi [4], Jovana Milic [5], Roberto Tonelli [6], Lucio Brugioni [7], Antonello Pietrangelo [8], Massimo Girardis [9], Andrea Cossarizza [8], Enrico Clini [10], Giovanni Guaraldi [11], Modena Covid-19 Working Group (MoCo19); Office of information and communication technologies of Policlinico di Modena

Collaborators, Affiliations Expand

Collaborators

- Erica Bacca, Andrea Bedini, Vanni Borghi, Giulia Burastero, Federica Carli, Giacomo Ciusa, Luca Corradi, Margherita Di Gaetano, Matteo Faltoni, Giacomo Franceschi, Gabriella Orlando, Francesco Pellegrino, Cinzia Puzzolante, Alessandro Raimondi, Antonella Santoro, Marco Tutone, Dina Yaacoub, Alberto Andreotti, Emanuela Biagioni, Filippo Bondi, Stefano Busani, Giovanni Chierego, Marzia Scotti, Lucia Serio, Caterina Bellinazzi, Rebecca Borella, Sara De Biasi, Anna De Gaetano, Lucia Fidanza, Lara Gibellini, Anna Iannone, Domenico Lo Tartaro, Marco Mattioli, Annamaria Paolini, Rossella Fogliani, Grazia Righini, Mario Lugli

Affiliations

- [1] Department of Infectious Diseases, Azienda Ospedaliero-Universitaria Policlinico of Modena, Modena, Italy; Department of Surgical, Medical, Dental and Morphological Sciences, University of Modena and Reggio Emilia, Italy. Electronic address: cristina.mussini@unimore.it.
- [2] Centre for Clinical Research, Epidemiology, Modelling and Evaluation (CREME), Institute for Global Health, UCL, London, UK.
- [3] Department of Infectious Diseases, Azienda Ospedaliero-Universitaria Policlinico of Modena, Modena, Italy.
- [4] Department of Surgical, Medical, Dental and Morphological Sciences, University of Modena and Reggio Emilia, Italy.
- [5] Department of Surgical, Medical, Dental and Morphological Sciences, University of Modena and Reggio Emilia, Italy; Clinical and Experimental Medicine PhD Program, University of Modena and Reggio Emilia, Modena, Italy.
- [6] Clinical and Experimental Medicine PhD Program, University of Modena and Reggio Emilia, Modena, Italy; Respiratory Diseases Unit, Azienda Ospedaliero-Universitaria Policlinic o of Modena, Modena, Italy.
- [7] Internal Medicine Department, Azienda Ospedaliero-Universitaria Policlinico of Modena, Modena, Italy.
- [8] Department of Medical and Surgical Sciences for Children and Adults, University of Modena and Reggio Emilia, Italy.

- [9] Department of Surgical, Medical, Dental and Morphological Sciences, University of Modena and Reggio Emilia, Italy; Department of Anaesthesia and Intensive Care Unit, Azienda Ospedaliero-Universitaria Policlinico of Modena, Modena, Italy.
- [10] Respiratory Diseases Unit, Azienda Ospedaliero-Universitaria Policlinic o of Modena, Modena, Italy; Department of Medical and Surgical Sciences for Children and Adults, University of Modena and Reggio Emilia, Italy.
- [11] Department of Infectious Diseases, Azienda Ospedaliero-Universitaria Policlinico of Modena, Modena, Italy; Department of Surgical, Medical, Dental and Morphological Sciences, University of Modena and Reggio Emilia, Italy.

- PMID: **33359539**
- PMCID: PMC7816626
- DOI: 10.1016/j.cmi.2020.12.010

Free PMC article
Observational Study

Better prognosis in females with severe COVID-19 pneumonia: possible role of inflammation as potential mediator

Cristina Mussini et al. Clin Microbiol Infect. 2021 Aug.
Free PMC article

Show details

Clin Microbiol Infect
. 2021 Aug;27(8):1137-1144.
doi: 10.1016/j.cmi.2020.12.010. Epub 2021 Jan 20.

Authors

Cristina Mussini [1], Alessandro Cozzi-Lepri [2], Marianna Menozzi [3], Marianna Meschiari [3], Erica Franceschini [3], Carlotta Rogati [4], Gianluca Cuomo [3], Andrea Bedini [3], Vittorio Iadisernia [4], Sara Volpi [4], Jovana Milic [5], Roberto Tonelli [6], Lucio Brugioni [7], Antonello Pietrangelo [8], Massimo Girardis [9], Andrea Cossarizza [8], Enrico Clini [10], Giovanni Guaraldi [11], Modena Covid-19 Working Group (MoCo19); Office of information and communication technologies of Policlinico di Modena

Collaborators

- Erica Bacca, Andrea Bedini, Vanni Borghi, Giulia Burastero, Federica Carli, Giacomo Ciusa, Luca Corradi, Margherita Di Gaetano, Matteo Faltoni, Giacomo Franceschi, Gabriella Orlando, Francesco Pellegrino, Cinzia Puzzolante, Alessandro Raimondi, Antonella Santoro, Marco Tutone, Dina Yaacoub, Alberto Andreotti, Emanuela Biagioni, Filippo Bondi, Stefano Busani, Giovanni Chierego, Marzia Scotti, Lucia Serio, Caterina Bellinazzi, Rebecca Borella, Sara De Biasi, Anna De Gaetano, Lucia Fidanza, Lara Gibellini, Anna Iannone, Domenico Lo Tartaro, Marco Mattioli, Annamaria Paolini, Rossella Fogliani, Grazia Righini, Mario Lugli

Affiliations

- [1] Department of Infectious Diseases, Azienda Ospedaliero-Universitaria Policlinico of Modena, Modena, Italy; Department of Surgical, Medical, Dental and Morphological Sciences, University of Modena and Reggio Emilia, Italy. Electronic address: cristina.mussini@unimore.it.
- [2] Centre for Clinical Research, Epidemiology, Modelling and Evaluation (CREME), Institute for Global Health, UCL, London, UK.
- [3] Department of Infectious Diseases, Azienda Ospedaliero-Universitaria Policlinico of Modena, Modena, Italy.
- [4] Department of Surgical, Medical, Dental and Morphological Sciences, University of Modena and Reggio Emilia, Italy.
- [5] Department of Surgical, Medical, Dental and Morphological Sciences, University of Modena and Reggio Emilia, Italy; Clinical and Experimental Medicine PhD Program, University of Modena and Reggio Emilia, Modena, Italy.
- [6] Clinical and Experimental Medicine PhD Program, University of Modena and Reggio Emilia, Modena, Italy; Respiratory Diseases Unit, Azienda Ospedaliero-Universitaria Policlinic o of Modena, Modena, Italy.
- [7] Internal Medicine Department, Azienda Ospedaliero-Universitaria Policlinico of Modena, Modena, Italy.
- [8] Department of Medical and Surgical Sciences for Children and Adults, University of Modena and Reggio Emilia, Italy.
- [9] Department of Surgical, Medical, Dental and Morphological Sciences, University of Modena and Reggio Emilia, Italy; Department of Anaesthesia and Intensive Care Unit, Azienda Ospedaliero-Universitaria Policlinico of Modena, Modena, Italy.
- [10] Respiratory Diseases Unit, Azienda Ospedaliero-Universitaria Policlinic o of Modena, Modena, Italy; Department of Medical and Surgical Sciences for Children and Adults, University of Modena and Reggio Emilia, Italy.
- [11] Department of Infectious Diseases, Azienda Ospedaliero-Universitaria Policlinico of Modena, Modena, Italy; Department of Surgical, Medical, Dental and Morphological Sciences, University of Modena and Reggio Emilia, Italy.

- PMID: **33359539**
- PMCID: PMC7816626
- DOI: 10.1016/j.cmi.2020.12.010

Abstract

Objectives: Sex differences in COVID-19 severity and mortality have been described. Key aims of this analysis were to compare the risk of invasive mechanical ventilation (IMV) and mortality by sex and to explore whether variation in specific biomarkers could mediate this difference.

Methods: This was a retrospective, observational cohort study among patients with severe COVID-19 pneumonia. A survival analysis was conducted to compare time to the composite endpoint of IMV or death according to sex. Interaction was formally tested to compare the risk difference by sex in sub-populations. Mediation analysis with a binary endpoint IMV or death (yes/no) by day 28 of follow-up for a number of inflammation/coagulation biomarkers in the context of counterfactual prediction was also conducted.

Results: Among 415 patients, 134 were females (32%) and 281 males (67%), median age 66 years (IQR 54-77). At admission, females showed a significantly less severe clinical and

respiratory profiles with a higher PaO_2/FiO_2 (254 mmHg vs. 191 mmHg; p 0.023). By 28 days from admission, 49.2% (95% CI 39.6-58.9%) of males vs. 31.7% (17.9-45.4%) of females underwent IMV or death (log-rank $p < 0.0001$) and this amounted to a difference in terms of HR of 0.40 (0.26-0.63, p 0.0001). The area under the curve in C-reactive protein (CRP) over the study period appeared to explain 85% of this difference in risk by sex.

Discussion: Our analysis confirms a difference in the risk of COVID-19 clinical progression by sex and provides a hypothesis for potential mechanisms leading to this. Specifically, CRP showed a predominant role to mediate the difference in risk by sex.

Keywords: COVID-19; COVID-19 pneumonia; Inflammation; Mediation; Prognosis; Sex differences.

Copyright © 2020 European Society of Clinical Microbiology and Infectious Diseases. Published by Elsevier Ltd. All rights reserved.

Comment in

- Sex-biased clinical presentation and outcomes from COVID-19.
  Scully EP, Gupta A, Klein SL. Scully EP, et al. Clin Microbiol Infect. 2021 Aug;27 (8):1072-1073. doi: 10.1016/j.cmi.2021.03.027. Epub 2021 Apr 1. Clin Microbiol Infect. 2021. PMID: 33813116 Free PMC article. No abstract available.

- Cited by 10 articles
- 24 references
- 4 figures

Supplementary info

Publication types, MeSH terms Expand

Publication types

- Observational Study

MeSH terms

- Aged
- COVID-19 / diagnosis*
- COVID-19 / epidemiology
- COVID-19 / virology
- Cohort Studies
- Female
- Hospitalization
- Humans
- Inflammation
- Male
- Middle Aged

- Prognosis
- Respiration, Artificial
- Retrospective Studies
- Risk Factors
- SARS-CoV-2 / immunology*
- Sex Factors

Full text links

ELSEVIER FULL-TEXT ARTICLE Elsevier Science Free PMC article
Proceed to details
Cite
Share
356
Observational Study
J Clin Gastroenterol
. 2021 Jan;55(1):84-87.
doi: 10.1097/MCG.0000000000001462.

High Prevalence of Gastrointestinal Manifestations of COVID-19 Infection in Hospitalized Patients With Cancer

Shilpa Grover[1,2], Walker D Redd[3,2], Joyce C Zhou[2], Cheikh Nije[3,2], Danny Wong[3,2], Kelly E Hathorn[1,2], Thomas R McCarty[1,2], Ahmad N Bazarbashi[2], Lin Shen[1,2], Walter W Chan[1,2]
Affiliations Expand

Affiliations

- [1] Departments of Medicine, Division of Gastroenterology.
- [2] Harvard Medical School, Boston, MA.
- [3] Medicine, Brigham and Women's Hospital.

- PMID: **33116066**
- PMCID: PMC7718415
- DOI: 10.1097/MCG.0000000000001462

Free PMC article
Observational Study

High Prevalence of Gastrointestinal Manifestations of COVID-19 Infection in Hospitalized Patients With Cancer

Shilpa Grover et al. J Clin Gastroenterol. 2021 Jan.
Free PMC article
Show details
J Clin Gastroenterol
. 2021 Jan;55(1):84-87.
doi: 10.1097/MCG.0000000000001462.

Authors

Shilpa Grover [1] [2], Walker D Redd [3] [2], Joyce C Zhou [2], Cheikh Nije [3] [2], Danny Wong [3] [2], Kelly E Hathorn [1] [2], Thomas R McCarty [1] [2], Ahmad N Bazarbashi [2], Lin Shen [1] [2], Walter W Chan [1] [2]

Affiliations

- [1] Departments of Medicine, Division of Gastroenterology.
- [2] Harvard Medical School, Boston, MA.
- [3] Medicine, Brigham and Women's Hospital.

- PMID: **33116066**
- PMCID: PMC7718415
- DOI: 10.1097/MCG.0000000000001462

Abstract

Background and aim: Gastrointestinal (GI) symptoms have been reported with SARS-CoV-2 infection, but data on the prevalence and severity of GI symptoms in patients with cancer are limited. We sought to characterize the GI manifestations of coronavirus disease-19 (COVID-19) in oncology patients.

Materials and methods: We performed a multicenter cohort study of adult patients hospitalized with COVID-19 in 9 Massachusetts medical centers and identified those with an active malignancy. We evaluated the prevalence and severity of GI symptoms among hospitalized COVID-19 patients with cancer.

Results: Of 395 hospitalized patients with COVID-19, 36 (9%) had an active malignancy. Of the 36 cancer patients, 23 (63%) reported ≥1 new GI symptom. The most prevalent symptoms were anorexia (12, 52%), diarrhea (9, 39%), and vomiting (8, 35%). GI symptoms were the initial symptom in 4/36 (11%) patients, were the predominant symptom in 5/36 (14%) patients, and were severe in 4/23 (17%) patients. Four of 5 patients with GI symptoms at presentation reported concurrent fever; notably 1 patient had no fever or respiratory symptoms. Twelve (33%) patients had elevations in liver transaminases at presentation; patients with elevated transaminases were more likely to have associated GI symptoms (83% vs. 54%, P=0.04).

Conclusions: Acute GI symptoms associated with COVID-19 are highly prevalent in hospitalized cancer patients and can occur as a presenting symptom without respiratory symptoms. Symptoms are severe in a small subset of patients.

Conflict of interest statement

Disclosures:

Shilpa Grover is a gastroenterology editor (employment) at UpToDate, Wolters Kluwer Inc.

Walker D. Redd has no conflicts to disclose.

Joyce C. Zhou has no conflicts to disclose.

Cheikh Nije has no conflicts to disclose.

Danny Wong has no conflicts to disclose.

Kelly E. Hathorn has no conflicts to disclose.

Thomas R. McCarty has no conflicts to disclose.

Ahmad Najdat Bazarbashi has no conflicts to disclose.

Lin Shen has no conflicts to disclose.

Walter W. Chan has no conflicts to disclose.

- Cited by 3 articles

Supplementary info

Publication types, MeSH terms, Grant support Expand

Publication types

- Multicenter Study
- Observational Study

MeSH terms

- Adolescent
- Adult
- Aged
- Aged, 80 and over
- COVID-19 / complications*
- COVID-19 / diagnosis
- COVID-19 Testing
- Female

- Gastrointestinal Diseases / diagnosis
- Gastrointestinal Diseases / epidemiology
- Gastrointestinal Diseases / virology*
- Hospitalization
- Humans
- Male
- Massachusetts
- Middle Aged
- Neoplasms / complications*
- Prevalence
- Retrospective Studies
- Severity of Illness Index
- Young Adult

Grant support

- T32 DK007533/DK/NIDDK NIH HHS/United States

Full text links

Wolters Kluwer Wolters Kluwer Free PMC article
Proceed to details
Cite
Share
357
Observational Study
Front Med
. 2020 Dec;14(6):760-775.
doi: 10.1007/s11684-020-0803-8. Epub 2020 Sep 14.

Clinical features and the traditional Chinese medicine therapeutic characteristics of 293 COVID-19 inpatient cases

Zixin Shu[1], Yana Zhou[2], Kai Chang[1], Jifen Liu[2], Xiaojun Min[2], Qing Zhang[2], Jing Sun[2], Yajuan Xiong[2], Qunsheng Zou[1], Qiguang Zheng[1], Jinghui Ji[1], Josiah Poon[3] [4], Baoyan Liu[5], Xuezhong Zhou[6], Xiaodong Li[7] [8]
Affiliations Expand

Affiliations

- [1] Institute of Medical Intelligence, School of Computer and Information Technology, Beijing Jiaotong University, Beijing, 100044, China.

- [2] Hubei Provincial Hospital of Traditional Chinese Medicine, Wuhan, 430061, China.
- [3] School of Computer Science, The University of Sydney, Sydney, NSW, 2006, Australia. josiah.poon@sydney.edu.au.
- [4] Analytic and Clinical Cooperative Laboratory for Integrative Medicine, USYD & CUHK, Sydney, NSW, 2006, Australia. josiah.poon@sydney.edu.au.
- [5] China Academy of Chinese Medical Sciences, Beijing, 100700, China. liuby5505@139.com.
- [6] Institute of Medical Intelligence, School of Computer and Information Technology, Beijing Jiaotong University, Beijing, 100044, China. xzzhou@bjtu.edu.cn.
- [7] Hubei Provincial Hospital of Traditional Chinese Medicine, Wuhan, 430061, China. lixiaodong555@126.com.
- [8] Institute of Liver Diseases, Wuhan, 430061, China. lixiaodong555@126.com.

- PMID: **32926319**
- PMCID: PMC7488634
- DOI: 10.1007/s11684-020-0803-8

Free PMC article
Observational Study

Clinical features and the traditional Chinese medicine therapeutic characteristics of 293 COVID-19 inpatient cases

Zixin Shu et al. Front Med. 2020 Dec.
Free PMC article
Show details
Front Med
. 2020 Dec;14(6):760-775.
doi: 10.1007/s11684-020-0803-8. Epub 2020 Sep 14.

Authors

Zixin Shu [1], Yana Zhou [2], Kai Chang [1], Jifen Liu [2], Xiaojun Min [2], Qing Zhang [2], Jing Sun [2], Yajuan Xiong [2], Qunsheng Zou [1], Qiguang Zheng [1], Jinghui Ji [1], Josiah Poon [3] [4], Baoyan Liu [5], Xuezhong Zhou [6], Xiaodong Li [7] [8]

Affiliations

- [1] Institute of Medical Intelligence, School of Computer and Information Technology, Beijing Jiaotong University, Beijing, 100044, China.
- [2] Hubei Provincial Hospital of Traditional Chinese Medicine, Wuhan, 430061, China.
- [3] School of Computer Science, The University of Sydney, Sydney, NSW, 2006, Australia. josiah.poon@sydney.edu.au.
- [4] Analytic and Clinical Cooperative Laboratory for Integrative Medicine, USYD & CUHK, Sydney, NSW, 2006, Australia. josiah.poon@sydney.edu.au.

- [5] China Academy of Chinese Medical Sciences, Beijing, 100700, China. liuby5505@139.com.
- [6] Institute of Medical Intelligence, School of Computer and Information Technology, Beijing Jiaotong University, Beijing, 100044, China. xzzhou@bjtu.edu.cn.
- [7] Hubei Provincial Hospital of Traditional Chinese Medicine, Wuhan, 430061, China. lixiaodong555@126.com.
- [8] Institute of Liver Diseases, Wuhan, 430061, China. lixiaodong555@126.com.

- PMID: **32926319**
- PMCID: PMC7488634
- DOI: 10.1007/s11684-020-0803-8

Abstract

Coronavirus disease 2019 (COVID-19) is now pandemic worldwide and has heavily overloaded hospitals in Wuhan City, China during the time between late January and February. We reported the clinical features and therapeutic characteristics of moderate COVID-19 cases in Wuhan that were treated via the integration of traditional Chinese medicine (TCM) and Western medicine. We collected electronic medical record (EMR) data, which included the full clinical profiles of patients, from a designated TCM hospital in Wuhan. The structured data of symptoms and drugs from admission notes were obtained through an information extraction process. Other key clinical entities were also confirmed and normalized to obtain information on the diagnosis, clinical treatments, laboratory tests, and outcomes of the patients. A total of 293 COVID-19 inpatient cases, including 207 moderate and 86 (29.3%) severe cases, were included in our research. Among these cases, 238 were discharged, 31 were transferred, and 24 (all severe cases) died in the hospital. Our COVID-19 cases involved elderly patients with advanced ages (57 years on average) and high comorbidity rates (61%). Our results reconfirmed several well-recognized risk factors, such as age, gender (male), and comorbidities, as well as provided novel laboratory indications (e.g., cholesterol) and TCM-specific phenotype markers (e.g., dull tongue) that were relevant to COVID-19 infections and prognosis. In addition to antiviral/antibiotics and standard supportive therapies, TCM herbal prescriptions incorporating 290 distinct herbs were used in 273 (93%) cases. The cases that received TCM treatment had lower death rates than those that did not receive TCM treatment (17/273 = 6.2% vs. 7/20= 35%, P = 0.0004 for all cases; 17/77= 22% vs. 7/9= 77.7%, P = 0.002 for severe cases). The TCM herbal prescriptions used for the treatment of COVID-19 infections mainly consisted of Pericarpium Citri Reticulatae, Radix Scutellariae, Rhizoma Pinellia, and their combinations, which reflected the practical TCM principles (e.g., clearing heat and dampening phlegm). Lastly, 59% of the patients received treatment, including antiviral, antibiotics, and Chinese patent medicine, before admission. This situation might have some effects on symptoms, such as fever and dry cough. By using EMR data, we described the clinical features and therapeutic characteristics of 293 COVID-19 cases treated via the integration of TCM herbal prescriptions and Western medicine. Clinical manifestations and treatments before admission and in the hospital were investigated. Our results preliminarily showed the potential effectiveness of TCM herbal prescriptions and their regularities in COVID-19 treatment.

Keywords: COVID-19; clinical features; traditional Chinese medicine.

Conflict of interest statement

Zixin Shu, Yana Zhou, Kai Chang, Jifen Liu, Xiaojun Min, Qing Zhang, Jing Sun, Yajuan Xiong, Qunsheng Zou, Qiguang Zheng, Jinghui Ji, Josiah Poon, Baoyan Liu, Xuezhong Zhou, and Xiaodong Li declare that they have no conflict of interest. This study was approved by the ethics

review board of Hubei Provincial Hospital of Traditional Chinese Medicine (HBZY2020-C01-01). Written consent was waived due to the retrospective nature. This study was reported according to STROBE (The Strengthening the Reporting of Observational Studies in Epidemiology).

- Cited by 15 articles
- 41 references

Supplementary info

Publication types, MeSH terms, Substances, Supplementary concepts Expand

Publication types

- Observational Study

MeSH terms

- Adult
- Aged
- Aged, 80 and over
- COVID-19 / complications
- COVID-19 / drug therapy*
- COVID-19 / mortality
- COVID-19 / therapy*
- China
- Combined Modality Therapy
- Drugs, Chinese Herbal / therapeutic use*
- Female
- Hospitalization
- Humans
- Male
- Medicine, Chinese Traditional*
- Middle Aged
- Retrospective Studies
- Survival Rate
- Treatment Outcome

Substances

- Drugs, Chinese Herbal

Supplementary concepts

- COVID-19 drug treatment

Full text links

Springer Free PMC article
Proceed to details
Cite
Share
358
Case Reports
Med Clin (Barc)
. 2021 Jun 25;156(12):602-605.
doi: 10.1016/j.medcli.2021.01.006. Epub 2021 Jan 28.

Glucocorticoids alone versus tocilizumab alone or glucocorticoids plus tocilizumab in patients with severe SARS-CoV-2 pneumonia and mild inflammation

[Article in English, Spanish]
Ismael Francisco Aomar-Millán [1], Juan Salvatierra [2], Úrsula Torres-Parejo [3], María Nuñez-Nuñez [4], José Hernández-Quero [5], Francisco Anguita-Santos [5]
Affiliations Expand

Affiliations

- [1] Servicio de Medicina Interna, Hospital Universitario San Cecilio, Granada, España. Electronic address: iaomarmillan@hotmail.com.
- [2] Servicio de Reumatología, Hospital Universitario San Cecilio, Granada, España.
- [3] Departamento de Estadística, Universidad de Granada, Granada, España.
- [4] Servicio de Farmacia, Hospital Universitario San Cecilio, Granada, España; Servicio de Enfermedades Infecciosas, Hospital Universitario San Cecilio, Granada, España.
- [5] Servicio de Enfermedades Infecciosas, Hospital Universitario San Cecilio, Granada, España.

- PMID: **33622529**
- PMCID: PMC7843156
- DOI: 10.1016/j.medcli.2021.01.006

Free PMC article
Case Reports

Glucocorticoids alone versus tocilizumab alone or glucocorticoids plus tocilizumab in

patients with severe SARS-CoV-2 pneumonia and mild inflammation

[Article in English, Spanish]
Ismael Francisco Aomar-Millán et al. Med Clin (Barc). 2021.
Free PMC article
Show details
Med Clin (Barc)
. 2021 Jun 25;156(12):602-605.
doi: 10.1016/j.medcli.2021.01.006. Epub 2021 Jan 28.

Authors

Ismael Francisco Aomar-Millán [1], Juan Salvatierra [2], Úrsula Torres-Parejo [3], María Nuñez-Nuñez [4], José Hernández-Quero [5], Francisco Anguita-Santos [5]

Affiliations

- [1] Servicio de Medicina Interna, Hospital Universitario San Cecilio, Granada, España. Electronic address: iaomarmillan@hotmail.com.
- [2] Servicio de Reumatología, Hospital Universitario San Cecilio, Granada, España.
- [3] Departamento de Estadística, Universidad de Granada, Granada, España.
- [4] Servicio de Farmacia, Hospital Universitario San Cecilio, Granada, España; Servicio de Enfermedades Infecciosas, Hospital Universitario San Cecilio, Granada, España.
- [5] Servicio de Enfermedades Infecciosas, Hospital Universitario San Cecilio, Granada, España.

- PMID: **33622529**
- PMCID: PMC7843156
- DOI: 10.1016/j.medcli.2021.01.006

Abstract

Aim: To assess clinical outcomes according to the immunosuppressive treatment administered to patients with severe SARS-CoV-2 pneumonia and moderate inflammation.

Methods: A retrospective observational cohort study involving 142 patients with severe COVID-19 pneumonia and moderate inflammation divided into three treatment groups (pulses of methylprednisolone alone [groupI], tocilizumab alone [groupII] and methylprednisolone plus tocilizumab [groupIII]). The aim was to assess intergroups differences in the clinical course with a 60-day follow-up and related analytical factors.

Results: 14 patients (9,8%) died: 8 (10%) in groupI and 6 (9,5%) in groupsII andIII. 15 (10,6%) were admitted to ICU: 2 (2,5%) from groupI, 4 (28,5%) from groupII and 9 (18,4%) from groupIII. The mean hospital stay was longer in groupII and clinical outcome was not associated with treatment.

Conclusions: Tocilizumab seems to be not associated with better clinical outcomes and should be reserved for clinical trial scenario, since its widespread use may result in higher rate of ICU admission and longer mean hospital stay without differences in mortality rate and potentially adverse events.

Keywords: COVID-19; Cytokine storm syndrome; Inflamación; Inflammation; Methylprednisolone; Metilprednisolona; Síndrome de tormenta de citoquinas; Tocilizumab.

Copyright © 2021 Elsevier España, S.L.U. All rights reserved.

- Cited by 1 article
- 10 references
- 1 figure

Supplementary info

Publication types, MeSH terms, Substances, Supplementary concepts Expand

Publication types

- Case Reports
- Observational Study

MeSH terms

- Antibodies, Monoclonal, Humanized
- COVID-19* / drug therapy
- Glucocorticoids* / therapeutic use
- Humans
- Inflammation
- Retrospective Studies
- SARS-CoV-2
- Treatment Outcome

Substances

- Antibodies, Monoclonal, Humanized
- Glucocorticoids
- tocilizumab

Supplementary concepts

- COVID-19 drug treatment

Full text links

Ediciones Doyma, S.L. Free PMC article
Proceed to details
Cite
Share
359
Observational Study
PM R
. 2022 Feb;14(2):202-209.
doi: 10.1002/pmrj.12645. Epub 2021 Jul 29.

Outcomes of patients with COVID-19 after inpatient rehabilitation

Suzanne L Groah [1, 2], Cynthia T Pham [3], Amanda K Rounds [1, 4], Jennifer J Semel [1]
Affiliations Expand

Affiliations

- [1] MedStar National Rehabilitation Hospital, Washington, District of Columbia, USA.
- [2] MedStar Georgetown University Hospital Department of Rehabilitation Medicine, Washington, District of Columbia, USA.
- [3] Georgetown University School of Medicine, Washington, District of Columbia, USA.
- [4] MedStar Health Research Institute, Hyattsville, Maryland, USA.

- PMID: **34021974**
- PMCID: PMC8242539
- DOI: 10.1002/pmrj.12645

Free PMC article
Observational Study

Outcomes of patients with COVID-19 after inpatient rehabilitation

Suzanne L Groah et al. PM R. 2022 Feb.
Free PMC article
Show details
PM R
. 2022 Feb;14(2):202-209.
doi: 10.1002/pmrj.12645. Epub 2021 Jul 29.

Authors

Suzanne L Groah [1, 2], Cynthia T Pham [3], Amanda K Rounds [1, 4], Jennifer J Semel [1]

Affiliations

- [1] MedStar National Rehabilitation Hospital, Washington, District of Columbia, USA.
- [2] MedStar Georgetown University Hospital Department of Rehabilitation Medicine, Washington, District of Columbia, USA.
- [3] Georgetown University School of Medicine, Washington, District of Columbia, USA.
- [4] MedStar Health Research Institute, Hyattsville, Maryland, USA.

- PMID: **34021974**
- PMCID: PMC8242539
- DOI: 10.1002/pmrj.12645

Abstract

Background: Rehabilitation outcomes of patients with coronavirus disease 2019 (COVID-19) are unknown.

Objective: To describe patients with COVID-19 who are undergoing inpatient rehabilitation and their rehabilitation outcomes.

Design: Retrospective observational cohort study of all inpatients from a rehabilitation hospital between March 1 and September 30, 2020.

Setting: Inpatient rehabilitation hospital.

Patients: Among all inpatients, inclusion criteria are: ≥18 years of age and admission and discharge within the study time frame. The initial search yielded 920 patients; 896 met the inclusion criteria. Subjects were stratified by COVID-19 status and rehabilitation impairment.

Main outcome measures: Data included age, gender, body mass index (BMI), length of stay (LOS), discharge location, and functional ability in self-care and mobility (FA-SC, FA-Mob). One-sample t-tests were used to assess the difference of age, BMI, LOS, FA-SC, FA-Mob, and FA efficiency between COVID-19+ and COVID-19- patients.

Results: COVID-19+ patients were younger (59.4 years vs 62.9 years; $t[894] = -2.05$, $p = .04$) with a higher mean BMI (32 vs 28; $t[894] = 3.51$, $p < .01$) than COVID-19- patients. COVID-19+ patients had equivalent or superior improvements in FA-SC and FA-Mob, functional change efficiency, and LOS relative to COVID-19- patients. When medically complex patients were compared, those with COVID-19 had greater FA-SC and FA-Mob efficiencies than COVID-19- patients. COVID-19+ patients had similar rates of return to the community.

Conclusions: Patients with COVID-19 who meet the admission criteria for inpatient rehabilitation can benefit from inpatient rehabilitation similarly to their non-COVID-19 counterparts with similar rehabilitation-specific diagnoses.

© 2021 American Academy of Physical Medicine and Rehabilitation.

- 23 references

Supplementary info

Publication types, MeSH terms, Grant support Expand

Publication types

- Observational Study

MeSH terms

- Activities of Daily Living
- COVID-19*
- Humans
- Inpatients*
- Length of Stay
- Recovery of Function
- Rehabilitation Centers
- Retrospective Studies
- SARS-CoV-2
- Treatment Outcome

Grant support

- This study did not utilize any funding source

Full text links

WILEY Full Text Article Wiley Free PMC article
Proceed to details
Cite
Share
360
Observational Study
Medicine (Baltimore)
. 2021 Mar 26;100(12):e25083.
doi: 10.1097/MD.0000000000025083.

Combined clinical and imaging features better predict the critical outcomes of patients with SARS-COV-2

Ting Yue [1], Wenli Zhou [1], Jie He [1], Huilin Wang [1], Yongjiu Liu [2], Bing Wang [2], QingQing Zhu [1], Huawei Xia [1], Hongjie Hu [1]
Affiliations Expand

Affiliations

- [1] Department of Radiology, Sir Run Run Shaw Hospital, Zhejiang University School of Medicine, Hangzhou, Zhejiang.
- [2] Department of Radiology, First People's Hospital of Jingmen, Jingmen, Hubei, China.

- PMID: **33761668**
- DOI: 10.1097/MD.0000000000025083

Free article
Observational Study

Combined clinical and imaging features better predict the critical outcomes of patients with SARS-COV-2

Ting Yue et al. Medicine (Baltimore). 2021.
Free article
Show details
Medicine (Baltimore)
. 2021 Mar 26;100(12):e25083.
doi: 10.1097/MD.0000000000025083.

Authors

Ting Yue [1], Wenli Zhou [1], Jie He [1], Huilin Wang [1], Yongjiu Liu [2], Bing Wang [2], QingQing Zhu [1], Huawei Xia [1], Hongjie Hu [1]

Affiliations

- [1] Department of Radiology, Sir Run Run Shaw Hospital, Zhejiang University School of Medicine, Hangzhou, Zhejiang.
- [2] Department of Radiology, First People's Hospital of Jingmen, Jingmen, Hubei, China.

- PMID: **33761668**
- DOI: 10.1097/MD.0000000000025083

Abstract

The purpose of this study was to investigate the predictive value of combined clinical and imaging features, compared with the clinical or radiological risk factors only. Moreover, the expected results aimed to improve the identification of severe acute respiratory syndrome coronavirus-2 (SARS-COV-2) patients who may have critical outcomes.This retrospective study included laboratory-confirmed SARS-COV-2 cases between January 18, 2020, and February 16, 2020. The patients were divided into 2 groups with noncritical illness and critical illness regarding severity status within the hospitalization. Univariable and multivariable logistic regression models were used to explore the risk factors associated with clinical and radiological outcomes in patients with

SARS-COV-2. The ROC curves were performed to compare the prediction performance of different factors.A total of 180 adult patients in this study included 20 critical patients and 160 noncritical patients. In univariate logistic regression analysis, 15 risk factors were significantly associated with critical outcomes. Of importance, C-reactive protein (1.051, 95% confidence interval 1.024-1.078), D-dimer (1.911, 95% CI, 1.050-3.478), and CT score (1.29, 95% CI, 1.053-1.529) on admission were independent risk factors in multivariate analysis. The combined model achieved a better performance in disease severity prediction (P = .05).CRP, D-dimer, and CT score on admission were independent risk factors for critical illness in adults with SARS-COV-2. The combined clinical and radiological model achieved better predictive performance than clinical or radiological factors alone.

Copyright © 2021 the Author(s). Published by Wolters Kluwer Health, Inc.

Conflict of interest statement

The authors have no potential conflicts of interest to this work.

- 25 references

Supplementary info

Publication types, MeSH terms, Substances, Grant support Expand

Publication types

- Observational Study

MeSH terms

- Adult
- Aged
- C-Reactive Protein / analysis
- COVID-19 / epidemiology*
- COVID-19 / physiopathology*
- Diagnostic Techniques and Procedures / statistics & numerical data*
- Female
- Fibrin Fibrinogen Degradation Products / analysis
- Hospitalization / statistics & numerical data
- Humans
- Male
- Middle Aged
- ROC Curve
- Retrospective Studies
- Risk Factors
- SARS-CoV-2
- Severity of Illness Index

- Tomography, X-Ray Computed

Substances

- Fibrin Fibrinogen Degradation Products
- fibrin fragment D
- C-Reactive Protein

Grant support

- 2020XGZX051/Zhejiang University special scientific research fund for COVID-19 prevention and control

Full text links

Wolters Kluwer

Proceed to details

Cite

Share

☐ 361

Observational Study

PLoS One

. 2020 Dec 9;15(12):e0242953.

doi: 10.1371/journal.pone.0242953. eCollection 2020.

Development and external validation of a prognostic tool for COVID-19 critical disease

Daniel S Chow [1], Justin Glavis-Bloom [1], Jennifer E Soun [1], Brent Weinberg [2], Theresa Berens Loveless [3], Xiaohui Xie [4], Simukayi Mutasa [5], Edwin Monuki [6], Jung In Park [7], Daniela Bota [8], Jie Wu [9], Leslie Thompson [9], Bernadette Boden-Albala [10], Saahir Khan [11] [12], Alpesh N Amin [12], Peter D Chang [1] [4]

Affiliations Expand

Affiliations

- [1] Department of Radiological Sciences, University of California, Irvine, California, United States of America.
- [2] Department of Radiological Sciences, Emory University, Atlanta, Georgia, United States of America.
- [3] Department of Biomedical Engineering, University of California, Irvine, California, United States of America.
- [4] Department of Computer Science, University of California, Irvine, California, United States of America.

- [5] Department of Radiological Sciences, Columbia University Medical Center, New York, New York, United States of America.
- [6] Department of Pathology and Laboratory Medicine, University of California, Irvine, California, United States of America.
- [7] Sue and Bill Gross School of Nursing, University of California, Irvine, California, United States of America.
- [8] UCI Center for Clinical Research, University of California, Irvine, California, United States of America.
- [9] School of Biological Sciences, University of California, Irvine, California, United States of America.
- [10] Department of Population Health and Disease Prevention and Department of Epidemiology, University of California, Irvine, California, United States of America.
- [11] Division of Infectious Diseases, University of California, Irvine, California, United States of America.
- [12] Department of Medicine, University of California, Irvine, California, United States of America.

- PMID: **33296357**
- PMCID: PMC7725393
- DOI: 10.1371/journal.pone.0242953

Free PMC article
Observational Study

Development and external validation of a prognostic tool for COVID-19 critical disease

Daniel S Chow et al. PLoS One. 2020.
Free PMC article

Show details

PLoS One

. 2020 Dec 9;15(12):e0242953.
doi: 10.1371/journal.pone.0242953. eCollection 2020.

Authors

Daniel S Chow [1], Justin Glavis-Bloom [1], Jennifer E Soun [1], Brent Weinberg [2], Theresa Berens Loveless [3], Xiaohui Xie [4], Simukayi Mutasa [5], Edwin Monuki [6], Jung In Park [7], Daniela Bota [8], Jie Wu [9], Leslie Thompson [9], Bernadette Boden-Albala [10], Saahir Khan [11] [12], Alpesh N Amin [12], Peter D Chang [1] [4]

Affiliations

- [1] Department of Radiological Sciences, University of California, Irvine, California, United States of America.
- [2] Department of Radiological Sciences, Emory University, Atlanta, Georgia, United States of America.

- [3] Department of Biomedical Engineering, University of California, Irvine, California, United States of America.
- [4] Department of Computer Science, University of California, Irvine, California, United States of America.
- [5] Department of Radiological Sciences, Columbia University Medical Center, New York, New York, United States of America.
- [6] Department of Pathology and Laboratory Medicine, University of California, Irvine, California, United States of America.
- [7] Sue and Bill Gross School of Nursing, University of California, Irvine, California, United States of America.
- [8] UCI Center for Clinical Research, University of California, Irvine, California, United States of America.
- [9] School of Biological Sciences, University of California, Irvine, California, United States of America.
- [10] Department of Population Health and Disease Prevention and Department of Epidemiology, University of California, Irvine, California, United States of America.
- [11] Division of Infectious Diseases, University of California, Irvine, California, United States of America.
- [12] Department of Medicine, University of California, Irvine, California, United States of America.

- PMID: **33296357**
- PMCID: PMC7725393
- DOI: 10.1371/journal.pone.0242953

Abstract

Background: The rapid spread of coronavirus disease 2019 (COVID-19) revealed significant constraints in critical care capacity. In anticipation of subsequent waves, reliable prediction of disease severity is essential for critical care capacity management and may enable earlier targeted interventions to improve patient outcomes. The purpose of this study is to develop and externally validate a prognostic model/clinical tool for predicting COVID-19 critical disease at presentation to medical care.

Methods: This is a retrospective study of a prognostic model for the prediction of COVID-19 critical disease where critical disease was defined as ICU admission, ventilation, and/or death. The derivation cohort was used to develop a multivariable logistic regression model. Covariates included patient comorbidities, presenting vital signs, and laboratory values. Model performance was assessed on the validation cohort by concordance statistics. The model was developed with consecutive patients with COVID-19 who presented to University of California Irvine Medical Center in Orange County, California. External validation was performed with a random sample of patients with COVID-19 at Emory Healthcare in Atlanta, Georgia.

Results: Of a total 3208 patients tested in the derivation cohort, 9% (299/3028) were positive for COVID-19. Clinical data including past medical history and presenting laboratory values were available for 29% (87/299) of patients (median age, 48 years [range, 21-88 years]; 64% [36/55] male). The most common comorbidities included obesity (37%, 31/87), hypertension (37%, 32/87), and diabetes (24%, 24/87). Critical disease was present in 24% (21/87). After backward stepwise selection, the following factors were associated with greatest increased risk of critical disease: number of comorbidities, body mass index, respiratory rate, white blood cell count, % lymphocytes, serum creatinine, lactate dehydrogenase, high sensitivity troponin I, ferritin,

procalcitonin, and C-reactive protein. Of a total of 40 patients in the validation cohort (median age, 60 years [range, 27-88 years]; 55% [22/40] male), critical disease was present in 65% (26/40). Model discrimination in the validation cohort was high (concordance statistic: 0.94, 95% confidence interval 0.87-1.01). A web-based tool was developed to enable clinicians to input patient data and view likelihood of critical disease.

Conclusions and relevance: We present a model which accurately predicted COVID-19 critical disease risk using comorbidities and presenting vital signs and laboratory values, on derivation and validation cohorts from two different institutions. If further validated on additional cohorts of patients, this model/clinical tool may provide useful prognostication of critical care needs.

Conflict of interest statement

Alpesh Amin reported serving as PI or co-I of clinical trials sponsored by NIH/NIAID, NeuroRx Pharma, Pulmotect, Blade Therapeutics, Novartis, Takeda, Humanigen, Eli Lilly, PTC Therapeutics, OctaPharma, Fulcrum Therapeutics, Alexion. He has served as consultant and/or speaker for BMS, Pfizer, BI, Portola, Sunovion, Mylan, Salix, Alexion, AstraZeneca, Novartis, Nabriva, Paratek, Bayer, Tetraphase, Achogen LaJolla, Millenium, Ferring, PeraHealth, HeartRite, Aseptiscope, Sprightly. This does not alter our adherence to PLOS ONE policies on sharing data and materials.

- Cited by 4 articles
- 24 references
- 3 figures

Supplementary info

Publication types, MeSH terms, Grant support Expand

Publication types

- Observational Study
- Research Support, Non-U.S. Gov't
- Validation Study

MeSH terms

- Adult
- Aged
- Aged, 80 and over
- COVID-19* / blood
- COVID-19* / diagnosis
- COVID-19* / diagnostic imaging
- COVID-19* / epidemiology
- Critical Care*
- Female
- Hospitalization*

- Humans
- Male
- Middle Aged
- Models, Biological*
- Prognosis
- Retrospective Studies
- Risk Assessment
- Risk Factors
- SARS-CoV-2*

Grant support

- KL2 TR001416/TR/NCATS NIH HHS/United States
- UL1 TR001414/TR/NCATS NIH HHS/United States

Full text links

OPEN ACCESS TO FULL TEXT PLOS ONE Public Library of Science Free PMC article

Proceed to details

Cite

Share

☐ 362

Observational Study

Sci Rep
. 2020 Dec 9;10(1):21545.
doi: 10.1038/s41598-020-78392-1.

Vital signs assessed in initial clinical encounters predict COVID-19 mortality in an NYC hospital system

Elza Rechtman [1], Paul Curtin [1], Esmeralda Navarro [1], Sharon Nirenberg [2], Megan K Horton [3]

Affiliations Expand

Affiliations

- [1] Department of Environmental Medicine and Public Health, Icahn School of Medicine at Mount Sinai, One Gustave Levy Place, Box 1057, New York, NY, 10029, USA.
- [2] Scientific Computing, Icahn School of Medicine at Mount Sinai, New York, NY, USA.
- [3] Department of Environmental Medicine and Public Health, Icahn School of Medicine at Mount Sinai, One Gustave Levy Place, Box 1057, New York, NY, 10029, USA. megan.horton@mssm.edu.

- PMID: **33298991**

- PMCID: PMC7726000
- DOI: 10.1038/s41598-020-78392-1

Free PMC article
Observational Study

Vital signs assessed in initial clinical encounters predict COVID-19 mortality in an NYC hospital system

Elza Rechtman et al. Sci Rep. 2020.
Free PMC article
Show details
Sci Rep
. 2020 Dec 9;10(1):21545.
doi: 10.1038/s41598-020-78392-1.

Authors

Elza Rechtman [1], Paul Curtin [1], Esmeralda Navarro [1], Sharon Nirenberg [2], Megan K Horton [3]

Affiliations

- [1] Department of Environmental Medicine and Public Health, Icahn School of Medicine at Mount Sinai, One Gustave Levy Place, Box 1057, New York, NY, 10029, USA.
- [2] Scientific Computing, Icahn School of Medicine at Mount Sinai, New York, NY, USA.
- [3] Department of Environmental Medicine and Public Health, Icahn School of Medicine at Mount Sinai, One Gustave Levy Place, Box 1057, New York, NY, 10029, USA. megan.horton@mssm.edu.

- PMID: **33298991**
- PMCID: PMC7726000
- DOI: 10.1038/s41598-020-78392-1

Abstract

Timely and effective clinical decision-making for COVID-19 requires rapid identification of risk factors for disease outcomes. Our objective was to identify characteristics available immediately upon first clinical evaluation related COVID-19 mortality. We conducted a retrospective study of 8770 laboratory-confirmed cases of SARS-CoV-2 from a network of 53 facilities in New-York City. We analysed 3 classes of variables; demographic, clinical, and comorbid factors, in a two-tiered analysis that included traditional regression strategies and machine learning. COVID-19 mortality was 12.7%. Logistic regression identified older age (OR, 1.69 [95% CI 1.66-1.92]), male sex (OR, 1.57 [95% CI 1.30-1.90]), higher BMI (OR, 1.03 [95% CI 1.102-1.05]), higher heart rate (OR, 1.01 [95% CI 1.00-1.01]), higher respiratory rate (OR, 1.05 [95% CI 1.03-1.07]), lower oxygen saturation (OR, 0.94 [95% CI 0.93-0.96]), and chronic kidney disease (OR, 1.53 [95% CI 1.20-1.95]) were associated with COVID-19 mortality. Using gradient-boosting machine learning,

these factors predicted COVID-19 related mortality (AUC = 0.86) following cross-validation in a training set. Immediate, objective and culturally generalizable measures accessible upon clinical presentation are effective predictors of COVID-19 outcome. These findings may inform rapid response strategies to optimize health care delivery in parts of the world who have not yet confronted this epidemic, as well as in those forecasting a possible second outbreak.

Conflict of interest statement

The authors declare no competing interests.

- Cited by 8 articles
- 16 references
- 2 figures

Supplementary info

Publication types, MeSH terms, Grant support Expand

Publication types

- Observational Study
- Research Support, N.I.H., Extramural

MeSH terms

- Aged
- Aged, 80 and over
- COVID-19* / mortality
- COVID-19* / physiopathology
- COVID-19* / therapy
- Female
- Hospitalization*
- Humans
- Machine Learning*
- Male
- Middle Aged
- Models, Biological*
- New York City
- Pandemics*
- Predictive Value of Tests
- Retrospective Studies
- Risk Factors
- SARS-CoV-2*
- Vital Signs*

Grant support

- P30 ES023515/ES/NIEHS NIH HHS/United States
- UL1TR001433/TR/NCATS NIH HHS/United States

Full text links

Nature Publishing Group Free PMC article
Proceed to details
Cite
Share
363
Observational Study
Expert Rev Med Devices
. 2020 Nov;17(11):1207-1210.
doi: 10.1080/17434440.2020.1841632. Epub 2020 Nov 10.

Arrhythmogenic syncope leading to cardiac rhythm management procedures during COVID-19 lockdown

Vincenzo Russo [1], Pia Clara Pafundi [2], Antonio Rapacciuolo [3], Antonello D'Andrea [4], Marcello de Devitiis [5], Mario Volpicelli [6], Antonio Ruocco [7], Gerardo Nigro [1], Antonio D'Onofrio [8]
Affiliations Expand

Affiliations

- [1] Division of Cardiology, Department of Medical Translational Sciences, University of Campania "Luigi Vanvitelli" , Naples, Italy.
- [2] Department of Advanced Medical and Surgical Sciences, University of Campania "Luigi Vanvitelli" , Naples, Italy.
- [3] Division of Cardiology, Department of Advanced Biomedical Sciences, University of Naples Federico II , Naples, Italy.
- [4] Division of Cardiology, Umberto I Hospital, Nocera Inferiore , Salerno, Italy.
- [5] Division of Cardiology, Pellegrini Hospital, Health Authority Naples 1 , Naples, Italy.
- [6] Division of Cardiology, San Giovanni Bosco Hospital, Health Authority Naples 1 , Naples, Italy.
- [7] Interventional Cardiology and Cardiological Care Unit, Cardarelli Hospital , Naples, Italy.
- [8] Division of Cardiology, Monaldi Hospital , Naples, Italy.

- PMID: **33090884**
- DOI: 10.1080/17434440.2020.1841632

Observational Study

Arrhythmogenic syncope leading to cardiac rhythm management procedures during COVID-19 lockdown

Vincenzo Russo et al. Expert Rev Med Devices. 2020 Nov.
Show details
Expert Rev Med Devices
. 2020 Nov;17(11):1207-1210.
doi: 10.1080/17434440.2020.1841632. Epub 2020 Nov 10.

Authors

Vincenzo Russo [1], Pia Clara Pafundi [2], Antonio Rapacciuolo [3], Antonello D'Andrea [4], Marcello de Devitiis [5], Mario Volpicelli [6], Antonio Ruocco [7], Gerardo Nigro [1], Antonio D'Onofrio [8]

Affiliations

- [1] Division of Cardiology, Department of Medical Translational Sciences, University of Campania "Luigi Vanvitelli" , Naples, Italy.
- [2] Department of Advanced Medical and Surgical Sciences, University of Campania "Luigi Vanvitelli" , Naples, Italy.
- [3] Division of Cardiology, Department of Advanced Biomedical Sciences, University of Naples Federico II , Naples, Italy.
- [4] Division of Cardiology, Umberto I Hospital, Nocera Inferiore , Salerno, Italy.
- [5] Division of Cardiology, Pellegrini Hospital, Health Authority Naples 1 , Naples, Italy.
- [6] Division of Cardiology, San Giovanni Bosco Hospital, Health Authority Naples 1 , Naples, Italy.
- [7] Interventional Cardiology and Cardiological Care Unit, Cardarelli Hospital , Naples, Italy.
- [8] Division of Cardiology, Monaldi Hospital , Naples, Italy.

- PMID: **33090884**
- DOI: 10.1080/17434440.2020.1841632

Abstract

Introduction: Following the coronavirus disease (COVID-19) outbreak, the Italian government adopted strict rules of lockdown and social distancing. The aim of our study was to assess admission rate for syncope leading to cardiac rhythm management (CRM) procedures in Campania, the third-most-populous region of Italy, during COVID-19 lockdown. **Methods**: Data were sourced from 14 referral hospitals in Campania from 10th March to 4 May 2020 (lockdown period) and during the same period in 2019. Among consecutive patients hospitalized for CRM procedures during the two observational periods, we retrospectively evaluated those admitted for arrhythmogenic syncope. Admission rate and the type of hospital admission between the two observational periods were compared. **Results**: Among 951 consecutive patients hospitalized for CRM procedures, 204 were admitted for arrhythmogenic syncope leading to CRM procedures. A significant increase in admission was shown in 2020 compared to 2019 (26.4% vs. 18.3%; *P* =

0.003). Moreover, regarding the type of admission to hospitals, attendance at the emergency department (ED) significantly increased (83.5% vs. 56.1%; $P < 0.001$); conversely, a significant decrease in urgent unplanned hospitalizations (6.2% vs. 35.5%; $P < 0.001$) was observed during COVID-19 lockdown. **Conclusions**: The hospitalization for arrhythmogenic syncope leading to CRM procedures increased during COVID-19 lockdown.

Keywords: Arrhythmias; COVID-19 defibrillator; cardiac implanted device; hospitalization; pacemaker; syncope.

- Cited by 3 articles

Supplementary info

Publication types, MeSH terms Expand

Publication types

- Observational Study

MeSH terms

- Aged
- Aged, 80 and over
- Arrhythmias, Cardiac / etiology
- Arrhythmias, Cardiac / therapy*
- COVID-19* / complications
- Communicable Disease Control
- Emergency Service, Hospital
- Hospitalization
- Humans
- Italy / epidemiology
- Retrospective Studies
- SARS-CoV-2
- Syncope

Full text links

View full text Taylor & Francis

Proceed to details

Cite

Share

☐ 364

Observational Study

Scand J Trauma Resusc Emerg Med

. 2021 Mar 22;29(1):51.

doi: 10.1186/s13049-021-00864-8.

Impact of the SARS-COV-2 outbreak on epidemiology and management of major traumain France: a registry-based study (the COVITRAUMA study)

Jean-Denis Moyer [1], Arthur James [2], Clément Gakuba [3], Mathieu Boutonnet [4], Emeline Angles [5], Emmanuel Rozenberg [6], Jean Bardon [7], Thomas Clavier [8], Vincent Legros [9], Marie Werner [10], Quentin Mathais [11], Véronique Ramonda [12], Pierre Le Minh [13], Yann Berthelot [14], Clélia Colas [14], Julien Pottecher [15], Tobias Gauss [13], and the Traumabase Group
Affiliations Expand

Affiliations

- [1] Department of Anesthesiology and Critical Care, Beaujon Hospital, DMU Parabol, AP-HP.Nord, 100 boulevard du General Leclerc, F92110, Clichy, France. Jean-denis.moyer@aphp.fr.
- [2] Department of Anaesthesiology and critical care, Pitié-Salpêtrière Hospital, Sorbonne University, GRC 29, AP-HP, DMU DREAM, Paris, France.
- [3] Department of Anesthesiology and Critical Care Medicine, Caen University Hospital, Avenue de la cote de Nacre, Caen, France.
- [4] Intensive Care Unit, Percy Military Teaching Hospital. 101 avenue Henri Barbusse 92140, Clamart, Val de Grace Academy, place Alphonse Laveran, 75005, Paris, France.
- [5] Department of Anesthesiology and Critical Care, Bordeaux University Hospital, Pellegrin, Bordeaux, France.
- [6] Department of Anesthesiology and Critical Care, Hôpital Européen Georges Pompidou, Paris, France.
- [7] Department of Anesthesiology and Critical Care, Hôpital Henri Mondor, Créteil, France.
- [8] Department of Anesthesiology and Critical Care, Rouen University Hospital, 1 rue de Germont, 76000, Rouen, France.
- [9] Department of Anesthesiology and Critical Care, Hopital Maison Blanche - CHU de Reims, Reims, France.
- [10] Department of Anesthesiology and Critical Care, APH-HP, Bicêtre Hôpitaux Universitaires Paris-Sud, Université Paris Saclay, Le Kremlin Bicêtre, France.
- [11] Department of Anesthesiology and Critical Care, Military Teaching Hospital, Sainte-Anne, Toulon, France.
- [12] Department of Anesthesiology and Critical Care, University Toulouse 3-Paul-Sabatier, University Hospital of Toulouse, Hôpital Pierre-Paul Riquet, CHU Toulouse-Purpan, 31059, Toulouse, France.
- [13] Department of Anesthesiology and Critical Care, Beaujon Hospital, DMU Parabol, AP-HP.Nord, 100 boulevard du General Leclerc, F92110, Clichy, France.
- [14] Capgemini Invent, Insight Driven Enterprise, focused on Data & Artificial Intelligence services, Issy-les-Moulineaux, France.

- [15] Hôpitaux Universitaires de Strasbourg, Pôle d'Anesthésie-Réanimation & Médecine Péri-Opératoire, Service d'Anesthésie-Réanimation & Médecine Péri-Opératoire Hôpital de Hautepierre - Université de Strasbourg, Faculté de Médecine, Fédération de Médecine Translationnelle de Strasbourg (FMTS), UR3072, Strasbourg, France.

- PMID: **33752728**
- PMCID: PMC7983347
- DOI: 10.1186/s13049-021-00864-8

Free PMC article
Observational Study

Impact of the SARS-COV-2 outbreak on epidemiology and management of major traumain France: a registry-based study (the COVITRAUMA study)

Jean-Denis Moyer et al. Scand J Trauma Resusc Emerg Med. 2021.
Free PMC article
Show details
Scand J Trauma Resusc Emerg Med
. 2021 Mar 22;29(1):51.
doi: 10.1186/s13049-021-00864-8.

Authors

Jean-Denis Moyer [1], Arthur James [2], Clément Gakuba [3], Mathieu Boutonnet [4], Emeline Angles [5], Emmanuel Rozenberg [6], Jean Bardon [7], Thomas Clavier [8], Vincent Legros [9], Marie Werner [10], Quentin Mathais [11], Véronique Ramonda [12], Pierre Le Minh [13], Yann Berthelot [14], Clélia Colas [14], Julien Pottecher [15], Tobias Gauss [13], and the Traumabase Group

Affiliations

- [1] Department of Anesthesiology and Critical Care, Beaujon Hospital, DMU Parabol, AP-HP.Nord, 100 boulevard du General Leclerc, F92110, Clichy, France. Jean-denis.moyer@aphp.fr.
- [2] Department of Anaesthesiology and critical care, Pitié-Salpêtrière Hospital, Sorbonne University, GRC 29, AP-HP, DMU DREAM, Paris, France.
- [3] Department of Anesthesiology and Critical Care Medicine, Caen University Hospital, Avenue de la cote de Nacre, Caen, France.
- [4] Intensive Care Unit, Percy Military Teaching Hospital. 101 avenue Henri Barbusse 92140, Clamart, Val de Grace Academy, place Alphonse Laveran, 75005, Paris, France.
- [5] Department of Anesthesiology and Critical Care, Bordeaux University Hospital, Pellegrin, Bordeaux, France.
- [6] Department of Anesthesiology and Critical Care, Hôpital Européen Georges Pompidou, Paris, France.

- [7] Department of Anesthesiology and Critical Care, Hôpital Henri Mondor, Créteil, France.
- [8] Department of Anesthesiology and Critical Care, Rouen University Hospital, 1 rue de Germont, 76000, Rouen, France.
- [9] Department of Anesthesiology and Critical Care, Hopital Maison Blanche - CHU de Reims, Reims, France.
- [10] Department of Anesthesiology and Critical Care, APH-HP, Bicêtre Hôpitaux Universitaires Paris-Sud, Université Paris Saclay, Le Kremlin Bicêtre, France.
- [11] Department of Anesthesiology and Critical Care, Military Teaching Hospital, Sainte-Anne, Toulon, France.
- [12] Department of Anesthesiology and Critical Care, University Toulouse 3-Paul-Sabatier, University Hospital of Toulouse, Hôpital Pierre-Paul Riquet, CHU Toulouse-Purpan, 31059, Toulouse, France.
- [13] Department of Anesthesiology and Critical Care, Beaujon Hospital, DMU Parabol, AP-HP.Nord, 100 boulevard du General Leclerc, F92110, Clichy, France.
- [14] Capgemini Invent, Insight Driven Enterprise, focused on Data & Artificial Intelligence services, Issy-les-Moulineaux, France.
- [15] Hôpitaux Universitaires de Strasbourg, Pôle d'Anesthésie-Réanimation & Médecine Péri-Opératoire, Service d'Anesthésie-Réanimation & Médecine Péri-Opératoire Hôpital de Hautepierre - Université de Strasbourg, Faculté de Médecine, Fédération de Médecine Translationnelle de Strasbourg (FMTS), UR3072, Strasbourg, France.

- PMID: **33752728**
- PMCID: PMC7983347
- DOI: 10.1186/s13049-021-00864-8

Abstract

Background: Emerging evidence suggests that the reallocation of health care resources during the COVID-19 pandemic negatively impacts health care system. This study describes the epidemiology and the outcome of major trauma patients admitted to centers in France during the first wave of the COVID-19 outbreak.

Methods: This retrospective observational study included all consecutive trauma patients aged 15 years and older admitted into 15 centers contributing to the TraumaBase® registry during the first wave of the SARS-CoV-2 pandemic in France. This COVID-19 trauma cohort was compared to historical cohorts (2017-2019).

Results: Over a 4 years-study period, 5762 patients were admitted between the first week of February and mid-June. This cohort was split between patients admitted during the first 2020 pandemic wave in France (pandemic period, 1314 patients) and those admitted during the corresponding period in the three previous years (2017-2019, 4448 patients). Trauma patient demographics changed substantially during the pandemic especially during the lockdown period, with an observed reduction in both the absolute numbers and proportion exposed to road traffic accidents and subsequently admitted to traumacenters (348 annually 2017-2019 [55.4% of trauma admissions] vs 143 [36.8%] in 2020 $p < 0.005$). The in-hospital observed mortality and predicted mortality during the pandemic period were not different compared to the non-pandemic years.

Conclusions: During this first wave of COVID-19 in France, and more specifically during lockdown there was a significant reduction of patients admitted to designated trauma centers. Despite the reallocation and reorganization of medical resources this reduction prevented the

saturation of the trauma rescue chain and has allowed maintaining a high quality of care for trauma patients.

Keywords: COVID-19; France; Trauma; Traumacenter.

Conflict of interest statement

TG reports personal fees from Laboratoire du Biomédicament Français, outside the submitted work

- Cited by 1 article
- 18 references
- 4 figures

Supplementary info

Publication types, MeSH terms Expand

Publication types

- Multicenter Study
- Observational Study

MeSH terms

- Adult
- COVID-19 / epidemiology*
- COVID-19 / therapy
- Communicable Disease Control / organization & administration*
- Delivery of Health Care / methods*
- Disease Management*
- Female
- France / epidemiology
- Hospitalization / trends
- Humans
- Male
- Pandemics / prevention & control*
- Registries*
- Retrospective Studies
- SARS-CoV-2
- Trauma Centers / statistics & numerical data*

Full text links

Read free full text at BMC BioMed Central Free PMC article

Proceed to details
Cite
Share
☐ 365
Observational Study
ASAIO J
. 2021 May 1;67(5):503-510.
doi: 10.1097/MAT.0000000000001403.

The Use of Venovenous Extracorporeal Membrane Oxygenation in COVID-19 Infection: One Region's Comprehensive Experience

Zachary R Bergman [1], Jillian K Wothe [2], Fatima S Alwan [2], Alex Dunn [3], Elizabeth R Lusczek [1], Arianna E Lofrano [4], Kelly M Tointon [5], Melissa Doucette [5], John K Bohman [6], Ramiro Saavedra-Romero [5], Matthew E Prekker [3] [4], Melissa E Brunsvold [1]
Affiliations Expand

Affiliations

- [1] From the Department of Surgery, University of Minnesota, Minneapolis, Minnesota.
- [2] Medical School, University of Minnesota, Minneapolis, Minnesota.
- [3] Department of Emergency Medicine, Hennepin Healthcare, Minneapolis, Minnesota.
- [4] Department of Internal Medicine, Hennepin Healthcare, Minneapolis, Minnesota.
- [5] Department of Critical Care Medicine, Abbott Northwestern Hospital, Minneapolis, Minnesota.
- [6] Department of Anesthesiology and Perioperative Medicine, Mayo Clinic, Rochester, Minnesota.

- PMID: **33492851**
- PMCID: PMC8078021
- DOI: 10.1097/MAT.0000000000001403

Free PMC article
Observational Study

The Use of Venovenous Extracorporeal Membrane Oxygenation in COVID-19 Infection: One Region's Comprehensive Experience

Zachary R Bergman et al. ASAIO J. 2021.
Free PMC article
Show details
ASAIO J
. 2021 May 1;67(5):503-510.
doi: 10.1097/MAT.0000000000001403.

Authors

Zachary R Bergman [1], Jillian K Wothe [2], Fatima S Alwan [2], Alex Dunn [3], Elizabeth R Lusczek [1], Arianna E Lofrano [4], Kelly M Tointon [5], Melissa Doucette [5], John K Bohman [6], Ramiro Saavedra-Romero [5], Matthew E Prekker [3] [4], Melissa E Brunsvold [1]

Affiliations

- [1] From the Department of Surgery, University of Minnesota, Minneapolis, Minnesota.
- [2] Medical School, University of Minnesota, Minneapolis, Minnesota.
- [3] Department of Emergency Medicine, Hennepin Healthcare, Minneapolis, Minnesota.
- [4] Department of Internal Medicine, Hennepin Healthcare, Minneapolis, Minnesota.
- [5] Department of Critical Care Medicine, Abbott Northwestern Hospital, Minneapolis, Minnesota.
- [6] Department of Anesthesiology and Perioperative Medicine, Mayo Clinic, Rochester, Minnesota.

- PMID: **33492851**
- PMCID: PMC8078021
- DOI: 10.1097/MAT.0000000000001403

Abstract

Severe acute respiratory distress syndrome (ARDS) unresponsive to conventional intensive care unit (ICU) management is an accepted indication for venovenous extracorporeal membrane oxygenation (V-V ECMO) support. The frequency with which patients with coronavirus disease 2019 (COVID-19) pneumonia are selected for V-V ECMO has not been described. This was a cohort study including all patients placed on either V-V ECMO or venoarteriovenous ECMO at the four adult ECMO Centers of Excellence. Primary outcomes evaluated were survival to decannulation from the ECMO circuit, survival to discharge, and 60-day survival. Secondary outcomes were hospital length of stay (LOS), ICU LOS, length of ECMO cannulation, and length of intubation. During the study period, which corresponded to the first surge in COVID-19 hospitalizations in Minnesota, 35 patients with ARDS were selected for V-V ECMO support out of 1,849 adult ICU patients with COVID-19 infection in the state (1.9% incidence; 95% CI, 1.3-2.6%). This represents 46 (95% CI, 34-61) expected V-V ECMO patients per 100,000 confirmed positive cases of COVID-19. Twenty-six of the 35 patients (74.3%) supported with V-V ECMO survived to 60-day post-ECMO decannulation. Recent studies have demonstrated ongoing success rescuing patients with severe ARDS in COVID-19 infection. Our data add to the support of ECMO and the consideration for encouraging cooperation among regional ECMO centers to ensure access to this highest level of care. Finally, by evaluating all the patients of a single region, we estimate overall need for this resource intensive intervention based on the overall number of COVID-19 cases and ICU admissions.

Copyright © ASAIO 2021.

Conflict of interest statement

Disclosure: The authors have no conflicts of interest to report.

- Cited by 3 articles
- 25 references
- 3 figures

Supplementary info

Publication types, MeSH terms Expand

Publication types

- Observational Study

MeSH terms

- Adult
- Aged
- COVID-19 / complications
- COVID-19 / therapy*
- Extracorporeal Membrane Oxygenation*
- Female
- Humans
- Male
- Middle Aged
- Respiratory Distress Syndrome / therapy
- Retrospective Studies
- SARS-CoV-2*

Full text links

Wolters Kluwer Wolters Kluwer Free PMC article
Proceed to details
Cite
Share
☐ 366
Observational Study
Int J Qual Health Care
. 2021 Mar 3;33(1):mzab029.
doi: 10.1093/intqhc/mzab029.

Impact of a prolonged COVID-19 lockdown on patterns of admission, mortality and performance indicators in a cardiovascular intensive care unit

Jorge Luis Szarfer [1], Luciana Puente [2], Leandro Bono [2], María Laura Estrella [3], Eugenia Doppler [4], Mariano Napoli Llobera [4], María Patricia Arce [5], Karina Alejandra Borri [6], Mariana Elisa Fiandesio [6], Marta Josefina Ferraris [6], Juan Gagliardi [5]
Affiliations Expand

Affiliations

- [1] Chief of Cardiovascular Intensive Care Unit, HGA Dr. Cosme Argerich, La Pampa 3280, Ciudad Autónoma de Buenos Aires 1428, Argentina.
- [2] Cardiac Intensive Care Unit, HGA Dr. Cosme Argerich, Pi Y Margall 750, Ciudad Autónoma de Buenos Aires C1155AHD, Argentina.
- [3] Chief Resident of Cardiology Division, HGA Dr. Cosme Argerich, Pi Y Margall 750, Ciudad Autónoma de Buenos Aires C1155AHD, Argentina.
- [4] Resident of Cardiology Division, HGA Dr. Cosme Argerich, Pi Y Margall 750, Ciudad Autónoma de Buenos Aires C1155AHD, Argentina.
- [5] Cardiology Division, HGA Dr. Cosme Argerich, Pi Y Margall 750, Ciudad Autónoma de Buenos Aires C1155AHD, Argentina.
- [6] Hospital Pharmacy, HGA Dr. Cosme Argerich, Pi Y Margall 750, Ciudad Autónoma de Buenos Aires C1155AHD, Argentina.

- PMID: **33620065**
- PMCID: PMC7928899
- DOI: 10.1093/intqhc/mzab029

Free PMC article
Observational Study

Impact of a prolonged COVID-19 lockdown on patterns of admission, mortality and performance indicators in a cardiovascular intensive care unit

Jorge Luis Szarfer et al. Int J Qual Health Care. 2021.
Free PMC article
Show details
Int J Qual Health Care
. 2021 Mar 3;33(1):mzab029.

doi: 10.1093/intqhc/mzab029.

Authors

Jorge Luis Szarfer [1], Luciana Puente [2], Leandro Bono [2], María Laura Estrella [3], Eugenia Doppler [4], Mariano Napoli Llobera [4], María Patricia Arce [5], Karina Alejandra Borri [6], Mariana Elisa Fiandesio [6], Marta Josefina Ferraris [6], Juan Gagliardi [5]

Affiliations

- [1] Chief of Cardiovascular Intensive Care Unit, HGA Dr. Cosme Argerich, La Pampa 3280, Ciudad Autónoma de Buenos Aires 1428, Argentina.
- [2] Cardiac Intensive Care Unit, HGA Dr. Cosme Argerich, Pi Y Margall 750, Ciudad Autónoma de Buenos Aires C1155AHD, Argentina.
- [3] Chief Resident of Cardiology Division, HGA Dr. Cosme Argerich, Pi Y Margall 750, Ciudad Autónoma de Buenos Aires C1155AHD, Argentina.
- [4] Resident of Cardiology Division, HGA Dr. Cosme Argerich, Pi Y Margall 750, Ciudad Autónoma de Buenos Aires C1155AHD, Argentina.
- [5] Cardiology Division, HGA Dr. Cosme Argerich, Pi Y Margall 750, Ciudad Autónoma de Buenos Aires C1155AHD, Argentina.
- [6] Hospital Pharmacy, HGA Dr. Cosme Argerich, Pi Y Margall 750, Ciudad Autónoma de Buenos Aires C1155AHD, Argentina.

- PMID: **33620065**
- PMCID: PMC7928899
- DOI: 10.1093/intqhc/mzab029

Abstract

Background: The effects of an early and prolonged lockdown during the coronavirus disease 2019 (COVID-19) pandemic on cardiovascular intensive care units (CICUs) are not well established.

Objectives: This study analyses patterns of admission, mortality and performance indicators in a CICU before and during the Argentine lockdown in the COVID-19 pandemic.

Methods: This is a retrospective observational cross-sectional study of all consecutive patients aged 18 years or more admitted to the cardiac intensive care unit at a high-volume reference hospital in Buenos Aires, Argentina, comparing hospitalization rates, primary causes of admission, inpatient utilization indicators, pharmacy supplies' expenditures and in-hospital mortality between 5 March and 31 July 2020, with two corresponding control periods in 2019 and 2018.

Results: We included 722 female patients [mean age of 61.6 (SD 15.5) years; 237 (32.8%)]. Overall hospitalizations dropped 53.2% (95%CI: 45.3, 61.0%), from 295.5 patients/year over the periods 2018/2019 to 137 patients in 2020. Cardiovascular disease-related admissions dropped 59.9%, while admission for non-cardiac causes doubled its prevalence from 9.6% over the periods 2018/2019 to 22.6% in the study period ($P < 0.001$).In the period 2020, the bed occupancy rate fell from 82.2% to 77.4%, and the bed turnover rate dropped 50% from 7.88 to 3.91 monthly discharges/bed. The average length of stay doubled from 3.26 to 6.75 days, and the turnover interval increased from 3.8 to 8.39 days in 2020.Pharmacy supplies' expenditures per discharge

increased 134% along with a rise in antibiotics usage from 6.5 to 11.4 vials/ampoules per discharge (P < 0.02).Overall mortality increased from 7% (n = 41) to 13.9% (n = 19) (P = 0.008) at the expense of non-cardiac-related admissions (3.6-19.4%, P = 0.01).

Conclusions: This study found a significant reduction in overall and cardiovascular disease-related causes of admission to the cardiac intensive care unit, worse performance indicators and increased in-hospital mortality along the first 5 months of the early and long-lasting COVID-19 lockdown in Argentina. These results highlight the need to foster public awareness concerning the risks of avoiding hospital attendance. Moreover, health systems should follow strict screening protocols to prevent potential biases in the admission of patients with critical conditions unrelated to the COVID-19 pandemic.

Keywords: cardiovascular diseases; equity in health care; intensive care; patient outcomes; public health; quality indicators.

© The Author(s) 2021. Published by Oxford University Press on behalf of International Society for Quality in Health Care. All rights reserved. For permissions, please e-mail: journals.permissions@oup.com.

Supplementary info

Publication types, MeSH terms Expand

Publication types

- Observational Study

MeSH terms

- Adult
- Aged
- Argentina / epidemiology
- Bed Occupancy / statistics & numerical data
- COVID-19 / epidemiology*
- Cardiovascular Diseases / epidemiology*
- Cross-Sectional Studies
- Female
- Health Policy
- Hospital Mortality / trends
- Humans
- Intensive Care Units / statistics & numerical data*
- Length of Stay / statistics & numerical data
- Male
- Middle Aged
- Pandemics
- Patient Admission / statistics & numerical data*

- Pharmacy Service, Hospital / economics
- Pharmacy Service, Hospital / statistics & numerical data
- Quality Indicators, Health Care / statistics & numerical data*
- Retrospective Studies
- SARS-CoV-2

Full text links

OXFORD ACADEMIC Silverchair Information Systems Free PMC article
Proceed to details
Cite
Share
367
Observational Study
Pediatrics
. 2022 Jan 1;149(1):e2021053498.
doi: 10.1542/peds.2021-053498.

Birth Hospital Length of Stay and Rehospitalization During COVID-19

Sara C Handley [1] [2] [3], Kieran Gallagher [4], Amy Breden [4], Eric Lindgren [4], Justin Y Lo [4], Moeun Son [5], Daria Murosko [1], Kevin Dysart [1] [2], Scott A Lorch [1] [2] [3], Jay Greenspan [6] [7], Jennifer F Culhane [5], Heather H Burris [1] [2] [3]
Affiliations Expand

Affiliations

- [1] Division of Neonatology, The Children's Hospital of Philadelphia, Philadelphia, Pennsylvania.
- [2] Department of Pediatrics, Perelman School of Medicine, University of Pennsylvania, Philadelphia, Pennsylvania.
- [3] Leonard Davis Institute of Health Economics, Philadelphia, Pennsylvania.
- [4] Epic Systems, Verona, Wisconsin.
- [5] Yale School of Medicine, Yale University, New Haven, Connecticut.
- [6] Division of Neonatology, Nemours duPont Pediatrics, Philadelphia, Pennsylvania.
- [7] Sidney Kimmel Medical College, Thomas Jefferson University, Philadelphia, Pennsylvania.

- PMID: **34889449**
- DOI: 10.1542/peds.2021-053498

Observational Study

Birth Hospital Length of Stay and Rehospitalization During COVID-19

Sara C Handley et al. Pediatrics. 2022.
Show details
Pediatrics
. 2022 Jan 1;149(1):e2021053498.
doi: 10.1542/peds.2021-053498.

Authors

Sara C Handley [1 2 3], Kieran Gallagher [4], Amy Breden [4], Eric Lindgren [4], Justin Y Lo [4], Moeun Son [5], Daria Murosko [1], Kevin Dysart [1 2], Scott A Lorch [1 2 3], Jay Greenspan [6 7], Jennifer F Culhane [5], Heather H Burris [1 2 3]

Affiliations

- [1] Division of Neonatology, The Children's Hospital of Philadelphia, Philadelphia, Pennsylvania.
- [2] Department of Pediatrics, Perelman School of Medicine, University of Pennsylvania, Philadelphia, Pennsylvania.
- [3] Leonard Davis Institute of Health Economics, Philadelphia, Pennsylvania.
- [4] Epic Systems, Verona, Wisconsin.
- [5] Yale School of Medicine, Yale University, New Haven, Connecticut.
- [6] Division of Neonatology, Nemours duPont Pediatrics, Philadelphia, Pennsylvania.
- [7] Sidney Kimmel Medical College, Thomas Jefferson University, Philadelphia, Pennsylvania.

- PMID: **34889449**
- DOI: 10.1542/peds.2021-053498

Abstract

Objectives: To determine if birth hospitalization length of stay (LOS) and infant rehospitalization changed during the coronavirus disease 2019 (COVID-19) era among healthy, term infants.

Methods: Retrospective cohort study using Epic's Cosmos data from 35 health systems of term infants discharged ≤5 days of birth. Short birth hospitalization LOS (vaginal birth <2 midnights; cesarean birth <3 midnights) and, secondarily, infant rehospitalization ≤7 days after birth hospitalization discharge were compared between the COVID-19 (March 1 to August 31, 2020) and prepandemic eras (March 1 to August 31, 2017, 2018, 2019). Mixed-effects models were used to estimate adjusted odds ratios (aORs) comparing the eras.

Results: Among 202 385 infants (57 110 from the COVID-19 era), short birth hospitalization LOS increased from 28.5% to 43.0% for all births (vaginal: 25.6% to 39.3%, cesarean: 40.1% to 61.0%) during the pandemic and persisted after multivariable adjustment (all: aOR 2.30, 95% confidence interval [CI] 2.25-2.36; vaginal: aOR 2.12, 95% CI 2.06-2.18; cesarean: aOR 3.01, 95% CI 2.87-3.15). Despite shorter LOS, infant rehospitalizations decreased slightly during the

pandemic (1.2% to 1.1%); results were similar in adjusted analysis (all: aOR 0.83, 95% CI 0.76-0.92; vaginal: aOR 0.82, 95% CI 0.74-0.91; cesarean: aOR 0.87, 95% CI 0.69-1.10). There was no change in the proportion of rehospitalization diagnoses between eras.

Conclusions: Short infant LOS was 51% more common in the COVID-19 era, yet infant rehospitalization within a week did not increase. This natural experiment suggests shorter birth hospitalization LOS among family- and clinician-selected, healthy term infants may be safe with respect to infant rehospitalization, although examination of additional outcomes is needed.

Copyright © 2022 by the American Academy of Pediatrics.

Conflict of interest statement

FINANCIAL DISCLOSURE: The authors have indicated they have no financial relationships relevant to this article to disclose.

Supplementary info

Publication types, MeSH terms Expand

Publication types

- Observational Study

MeSH terms

- COVID-19 / prevention & control*
- Female
- Humans
- Infant, Newborn
- Length of Stay / trends*
- Male
- Patient Readmission / trends*
- Practice Patterns, Physicians' / trends*
- Pregnancy
- Retrospective Studies
- Term Birth*
- United States

Full text links

AAP Publications Silverchair Information Systems

Proceed to details

Cite

Share

☐ 368

Observational Study

Acta Biomed

. 2020 Aug 10;91(4):e2020201.
doi: 10.23750/abm.v91i4.10227.

Analysis of the Patients Who Admitted To A Turkish Emergency Department During COVID-19 Pandemic

Ertuğrul Altınbilek [1], Derya Öztürk [2], Ceren Atasoy [3], Miray Özlem [4], Fevzi Yılmaz [5], Cemil Kavalci [6]

Affiliations Expand

Affiliations

- [1] Array. ertugrulaltinbilek@gmail.com.
- [2] University of Health Sciences Sisli Etfal Training and ResearchHospital, Istanbul. drderyaozturk@yahoo.com.
- [3] Istanbul Sisli Hamidiye Etfal Education and Research Hospital, Department of Infectious Diseases, Istanbul, Turkey. drcerenatasoy.i@gmail.com.
- [4] University of Health Sciences, Ankara City Hospital, Department of Emergency Medicine, Ankara, Turkey. dr.mirayozlem@gmail.com.
- [5] Health Sciences University, Antalya Education and Research Hospital; Department of Emergency Medicine, Antalya /Turkey. fevzi_yilmaz2002@yahoo.com.
- [6] University of Health Sciences Diskapi Yildirim Beyazit Education and Research Hospital, Department of Emergency Medicine, Ankara. cemkavalci@yahoo.com.

- PMID: **33525265**
- PMCID: PMC7927554
- DOI: 10.23750/abm.v91i4.10227

Free PMC article
Observational Study

Analysis of the Patients Who Admitted To A Turkish Emergency Department During COVID-19 Pandemic

Ertuğrul Altınbilek et al. Acta Biomed. 2020.
Free PMC article

Show details

Acta Biomed

. 2020 Aug 10;91(4):e2020201.
doi: 10.23750/abm.v91i4.10227.

Authors

Ertuğrul Altınbilek [1], Derya Öztürk [2], Ceren Atasoy [3], Miray Özlem [4], Fevzi Yılmaz [5], Cemil Kavalci [6]

Affiliations

- [1] Array. ertugrulaltinbilek@gmail.com.
- [2] University of Health Sciences Sisli Etfal Training and ResearchHospital, Istanbul. drderyaozturk@yahoo.com.
- [3] Istanbul Sisli Hamidiye Etfal Education and Research Hospital, Department of Infectious Diseases, Istanbul, Turkey. drcerenatasoy.i@gmail.com.
- [4] University of Health Sciences, Ankara City Hospital, Department of Emergency Medicine, Ankara, Turkey. dr.mirayozlem@gmail.com.
- [5] Health Sciences University, Antalya Education and Research Hospital; Department of Emergency Medicine, Antalya /Turkey. fevzi_yilmaz2002@yahoo.com.
- [6] University of Health Sciences Diskapi Yildirim Beyazit Education and Research Hospital, Department of Emergency Medicine, Ankara. cemkavalci@yahoo.com.

- PMID: **33525265**
- PMCID: PMC7927554
- DOI: 10.23750/abm.v91i4.10227

Abstract

Background and aim: In this study, it was aimed to review patients who presented to a Turkish emergency department (ED) with fever and at least one symptom and finding of acute respiratory infection (cough, shortness of breath) in Sisli Hamidiye Etfal Education and Research Hospital Tertiary Medical Care Center during COVID-19 pandemic.

Methods: This retrospective, descriptive, observational study included patients presented between March 10, 2020 and April 25, 2020. The patients were classified into two groups according to RT-PCR test result: RT-PCR (+) and RT-PCR (-). The demographic characteristics and clinical endpoint-related factors were analyzed in the patients.

Results: The study included 840 patients; 461 men (54.9%) and 379 women (45.1%). RT-PCR test was positive in 345 patients (41.0%). The most common comorbidity was hypertension (HT) in 119 patients (34.5%); followed by diabetes mellitus (DM) in 61 patients (18.3%). At time of ED presentation, there was mild clinical manifestation in 72.2%, whereas moderate in 21.7% and severe in 6.1% of patients with positive RT-PCR testing. Of the patients with positive RT-PCR testing, 64 patients (18.6%) were discharged from ED while 255 patients (73.9%) were admitted to COVID clinic and 26 were admitted to COVID intensive care unit (ICU). Of the patients admitted, 299 patients (86.7%) were discharged while 46 patients (13.3%) died due to multi-organ failure (MOF) (50%), acute respiratory distress syndrome (ARDS) (32.6%), acute pulmonary embolism (APE) (10.9%) and acute coronary syndrome (ACS) (6.5%).

Conclusions: The RT-PCR positivity rate seemed lower in our study when compared to literature. In addition, mortality rate was lower in our study when compared to other countries.

Conflict of interest statement

"Each author declares that he or she has no commercial associations (e. g. consultancies, stock ownership, equity interest, patent/licensing arrangement etc.) that might pose a conflict of interest in connection with the submitted article".

- Cited by 1 article
- 22 references
- 2 figures

Supplementary info

Publication types, MeSH terms Expand

Publication types

- Observational Study

MeSH terms

- Adult
- Aged
- COVID-19 / complications
- COVID-19 / diagnosis*
- COVID-19 / epidemiology*
- COVID-19 Nucleic Acid Testing*
- Critical Care*
- Emergency Service, Hospital*
- Female
- Hospitalization*
- Humans
- Male
- Middle Aged
- Retrospective Studies
- Reverse Transcriptase Polymerase Chain Reaction
- Turkey

Full text links

Free PMC article
Proceed to details
Cite
Share
☐ 369
Observational Study
Vaccine
. 2022 Mar 15;40(12):1790-1798.

doi: 10.1016/j.vaccine.2022.02.008. Epub 2022 Feb 8.

Impact of the COVID-19 pandemic on routine immunization coverage in children under 2 years old in Ontario, Canada: A retrospective cohort study

Catherine Ji [1], Pierre-Philippe Piché-Renaud [2], Jemisha Apajee [3], Ellen Stephenson [3], Milena Forte [4], Jeremy N Friedman [5], Michelle Science [2], Stanley Zlotkin [6], Shaun K Morris [7], Karen Tu [8]

Affiliations Expand

Affiliations

- [1] Department of Family and Community Medicine, University of Toronto, 500 University Avenue, 5th Floor, Toronto, Ontario M5G 1V7, Canada; Toronto Western Family Health Team, University Health Network, 440 Bathurst Street, 3rd Floor, Toronto, Ontario M5T 2S6, Canada. Electronic address: catherine.ji@uhn.ca.
- [2] Division of Infectious Diseases, The Hospital for Sick Children, 555 University Avenue, Toronto, Ontario M5G 1X8, Canada; Department of Paediatrics, University of Toronto, 555 University Avenue, Toronto, Ontario, Canada, M5G 1X8.
- [3] Department of Family and Community Medicine, University of Toronto, 500 University Avenue, 5th Floor, Toronto, Ontario M5G 1V7, Canada.
- [4] Department of Family and Community Medicine, University of Toronto, 500 University Avenue, 5th Floor, Toronto, Ontario M5G 1V7, Canada; Mount Sinai Academic Family Health Team, 60 Murray Street, 4th Floor, Toronto, Ontario M5T 3L9, Canada.
- [5] Department of Paediatrics, University of Toronto, 555 University Avenue, Toronto, Ontario, Canada, M5G 1X8; Division of Paediatric Medicine, The Hospital for Sick Children, 555 University Avenue, Toronto, Ontario M5G 1X8, Canada.
- [6] Department of Paediatrics, University of Toronto, 555 University Avenue, Toronto, Ontario, Canada, M5G 1X8; Division of Paediatric Medicine, The Hospital for Sick Children, 555 University Avenue, Toronto, Ontario M5G 1X8, Canada; Centre for Global Child Health and the SickKids Research Institute, Peter Gilgan Centre for Research and Learning, 686 Bay Street, 11th Floor, Suite 11.9805, Toronto, Ontario M5G 0A4, Canada; Dalla Lana School of Public Health, University of Toronto, 155 College Street, Toronto, Ontario M5T 3M7, Canada.
- [7] Division of Infectious Diseases, The Hospital for Sick Children, 555 University Avenue, Toronto, Ontario M5G 1X8, Canada; Department of Paediatrics, University of Toronto, 555 University Avenue, Toronto, Ontario, Canada, M5G 1X8; Centre for Global Child Health and the SickKids Research Institute, Peter Gilgan Centre for Research and Learning, 686 Bay Street, 11th Floor, Suite 11.9805, Toronto, Ontario M5G 0A4, Canada.
- [8] Department of Family and Community Medicine, University of Toronto, 500 University Avenue, 5th Floor, Toronto, Ontario M5G 1V7, Canada; Toronto Western Family Health Team, University Health Network, 440 Bathurst Street, 3rd Floor, Toronto, Ontario M5T 2S6, Canada; North York General Hospital, 4001 Leslie Street, Toronto, Ontario M2K 1E1, Canada.

- PMID: **35164987**
- PMCID: PMC8824235
- DOI: 10.1016/j.vaccine.2022.02.008

Free PMC article
Observational Study

Impact of the COVID-19 pandemic on routine immunization coverage in children under 2 years old in Ontario, Canada: A retrospective cohort study

Catherine Ji et al. Vaccine. 2022.
Free PMC article
Show details
Vaccine
. 2022 Mar 15;40(12):1790-1798.
doi: 10.1016/j.vaccine.2022.02.008. Epub 2022 Feb 8.

Authors

Catherine Ji [1], Pierre-Philippe Piché-Renaud [2], Jemisha Apajee [3], Ellen Stephenson [3], Milena Forte [4], Jeremy N Friedman [5], Michelle Science [2], Stanley Zlotkin [6], Shaun K Morris [7], Karen Tu [8]

Affiliations

- [1] Department of Family and Community Medicine, University of Toronto, 500 University Avenue, 5th Floor, Toronto, Ontario M5G 1V7, Canada; Toronto Western Family Health Team, University Health Network, 440 Bathurst Street, 3rd Floor, Toronto, Ontario M5T 2S6, Canada. Electronic address: catherine.ji@uhn.ca.
- [2] Division of Infectious Diseases, The Hospital for Sick Children, 555 University Avenue, Toronto, Ontario M5G 1X8, Canada; Department of Paediatrics, University of Toronto, 555 University Avenue, Toronto, Ontario, Canada, M5G 1X8.
- [3] Department of Family and Community Medicine, University of Toronto, 500 University Avenue, 5th Floor, Toronto, Ontario M5G 1V7, Canada.
- [4] Department of Family and Community Medicine, University of Toronto, 500 University Avenue, 5th Floor, Toronto, Ontario M5G 1V7, Canada; Mount Sinai Academic Family Health Team, 60 Murray Street, 4th Floor, Toronto, Ontario M5T 3L9, Canada.
- [5] Department of Paediatrics, University of Toronto, 555 University Avenue, Toronto, Ontario, Canada, M5G 1X8; Division of Paediatric Medicine, The Hospital for Sick Children, 555 University Avenue, Toronto, Ontario M5G 1X8, Canada.
- [6] Department of Paediatrics, University of Toronto, 555 University Avenue, Toronto, Ontario, Canada, M5G 1X8; Division of Paediatric Medicine, The Hospital for Sick Children, 555 University Avenue, Toronto, Ontario M5G 1X8, Canada; Centre for Global Child Health and the SickKids Research Institute, Peter Gilgan Centre for Research and

Learning, 686 Bay Street, 11th Floor, Suite 11.9805, Toronto, Ontario M5G 0A4, Canada; Dalla Lana School of Public Health, University of Toronto, 155 College Street, Toronto, Ontario M5T 3M7, Canada.

- [7] Division of Infectious Diseases, The Hospital for Sick Children, 555 University Avenue, Toronto, Ontario M5G 1X8, Canada; Department of Paediatrics, University of Toronto, 555 University Avenue, Toronto, Ontario, Canada, M5G 1X8; Centre for Global Child Health and the SickKids Research Institute, Peter Gilgan Centre for Research and Learning, 686 Bay Street, 11th Floor, Suite 11.9805, Toronto, Ontario M5G 0A4, Canada.
- [8] Department of Family and Community Medicine, University of Toronto, 500 University Avenue, 5th Floor, Toronto, Ontario M5G 1V7, Canada; Toronto Western Family Health Team, University Health Network, 440 Bathurst Street, 3rd Floor, Toronto, Ontario M5T 2S6, Canada; North York General Hospital, 4001 Leslie Street, Toronto, Ontario M2K 1E1, Canada.

- PMID: **35164987**
- PMCID: PMC8824235
- DOI: 10.1016/j.vaccine.2022.02.008

Abstract

Background: The COVID-19 pandemic has caused a disruption in childhood immunization coverage around the world. This study aimed to determine the change in immunization coverage for children under 2 years old in Ontario, Canada, comparing time periods pre-pandemic to during the first year of the pandemic.

Methods: Observational retrospective open cohort study, using primary care electronic medical record data from the University of Toronto Practice-Based Research Network (UTOPIAN) database, from January 2019 to December 2020. Children under 2 years old who had at least 2 visits recorded in UTOPIAN were included. We measured up-to-date (UTD) immunization coverage rates, overall and by type of vaccine (DTaP-IPV-Hib, PCV13, Rota, Men-C-C, MMR, Var), and on-time immunization coverage rates by age milestone (2, 4, 6, 12, 15, 18 months). We compared average coverage rates over 3 periods of time: January 2019-March 2020 (T1); March-July 2020 (T2); and August-December 2020 (T3).

Results: 12,313 children were included. Overall UTD coverage for all children was 71.0% in T1, dropped by 5.7% (95% CI: -6.2, -5.1) in T2, slightly increased in T3 but remained lower than in T1. MMR vaccine UTD coverage slightly decreased in T2 and T3 by approximately 2%. The largest decreases were seen at ages 15-month and 18-month old, with drops in on-time coverage of 14.7% (95% CI: -18.7, -10.6) and 16.4% (95% CI: -20.0, -12.8) respectively during T2. When stratified by sociodemographic characteristics, no specific subgroup of children was found to have been differentially impacted by the pandemic.

Conclusion: Childhood immunization coverage rates for children under 2 years in Ontario decreased significantly during the early period of the COVID-19 pandemic and only partially recovered during the rest of 2020. Public health and educational interventions for providers and parents are needed to ensure adequate catch-up of delayed/missed immunizations to prevent potential outbreaks of vaccine-preventable diseases.

Keywords: COVID-19; Childhood immunization; Family medicine; Health services; Immunization coverage; Pediatrics; Primary care; Retrospective observational study.

Copyright © 2022 The Author(s). Published by Elsevier Ltd.. All rights reserved.

Conflict of interest statement

Declaration of Competing Interest The authors declare that they have no known competing financial interests or personal relationships that could have appeared to influence the work reported in this paper.

- 45 references
- 6 figures

Supplementary info

Publication types, MeSH terms Expand

Publication types

- Observational Study
- Research Support, Non-U.S. Gov't

MeSH terms

- COVID-19* / epidemiology
- COVID-19* / prevention & control
- Cohort Studies
- Humans
- Immunization
- Immunization Programs
- Infant
- Male
- Ontario / epidemiology
- Pandemics*
- Retrospective Studies
- SARS-CoV-2
- Vaccination Coverage

Full text links

ELSEVIER OPEN ACCESS Elsevier Science Free PMC article

Proceed to details

Cite

Share

☐ 370

Observational Study

Metabolism

. 2021 Apr;117:154703.
doi: 10.1016/j.metabol.2021.154703. Epub 2021 Jan 6.

Excess body weight is an independent risk factor for severe forms of COVID-19

Léa Pietri [1], Roch Giorgi [2], Audrey Bégu [3], Manon Lojou [3], Marie Koubi [4], Raphael Cauchois [5], Rachel Grangeot [3], Noémie Dubois [3], Gilles Kaplanski [4], René Valéro [1], Sophie Béliard [6]
Affiliations Expand

Affiliations

- [1] Aix Marseille Univ, APHM, INSERM, INRAE, C2VN, Marseille, France; APHM, Department of Nutrition, Metabolic Diseases, Endocrinology, University Hospital La Conception, Marseille, France.
- [2] Aix Marseille Univ, APHM, INSERM, IRD, SESSTIM, Sciences Economiques & Sociales de la Santé & Traitement de l'Information Médicale, Hop Timone, BioSTIC, Biostatistique et Technologies de l'Information et de la, Communication, Marseille, France.
- [3] APHM, Department of Nutrition, Metabolic Diseases, Endocrinology, University Hospital La Conception, Marseille, France.
- [4] Department of Internal Medicine and Clinical Immunology, La Conception Hospital, APHM, 147 Bd Baille, 13005 Marseille, France.
- [5] Aix Marseille Univ, APHM, INSERM, INRAE, C2VN, Marseille, France; Department of Internal Medicine and Clinical Immunology, La Conception Hospital, APHM, 147 Bd Baille, 13005 Marseille, France.
- [6] Aix Marseille Univ, APHM, INSERM, INRAE, C2VN, Marseille, France; APHM, Department of Nutrition, Metabolic Diseases, Endocrinology, University Hospital La Conception, Marseille, France. Electronic address: sophie.beliard@ap-hm.fr.

- PMID: **33421506**
- PMCID: PMC7834365
- DOI: 10.1016/j.metabol.2021.154703

Free PMC article
Observational Study

Excess body weight is an independent risk factor for severe forms of COVID-19

Léa Pietri et al. Metabolism. 2021 Apr.
Free PMC article
Show details
Metabolism
. 2021 Apr;117:154703.
doi: 10.1016/j.metabol.2021.154703. Epub 2021 Jan 6.

Authors

Léa Pietri [1], Roch Giorgi [2], Audrey Bégu [3], Manon Lojou [3], Marie Koubi [4], Raphael Cauchois [5], Rachel Grangeot [3], Noémie Dubois [3], Gilles Kaplanski [4], René Valéro [1], Sophie Béliard [6]

Affiliations

- [1] Aix Marseille Univ, APHM, INSERM, INRAE, C2VN, Marseille, France; APHM, Department of Nutrition, Metabolic Diseases, Endocrinology, University Hospital La Conception, Marseille, France.
- [2] Aix Marseille Univ, APHM, INSERM, IRD, SESSTIM, Sciences Economiques & Sociales de la Santé & Traitement de l'Information Médicale, Hop Timone, BioSTIC, Biostatistique et Technologies de l'Information et de la, Communication, Marseille, France.
- [3] APHM, Department of Nutrition, Metabolic Diseases, Endocrinology, University Hospital La Conception, Marseille, France.
- [4] Department of Internal Medicine and Clinical Immunology, La Conception Hospital, APHM, 147 Bd Baille, 13005 Marseille, France.
- [5] Aix Marseille Univ, APHM, INSERM, INRAE, C2VN, Marseille, France; Department of Internal Medicine and Clinical Immunology, La Conception Hospital, APHM, 147 Bd Baille, 13005 Marseille, France.
- [6] Aix Marseille Univ, APHM, INSERM, INRAE, C2VN, Marseille, France; APHM, Department of Nutrition, Metabolic Diseases, Endocrinology, University Hospital La Conception, Marseille, France. Electronic address: sophie.beliard@ap-hm.fr.

- PMID: **33421506**
- PMCID: PMC7834365
- DOI: 10.1016/j.metabol.2021.154703

Abstract

Background and aims: Few studies distinguished the independent role of overweight/obesity or their associated-comorbidities in the evolution towards severe forms of COVID-19. Obesity as a unifying risk factor for severe COVID-19 is an emerging hypothesis. The aim of this study was to evaluate whether excessive body weight per se, was a risk factor for developing a severe form of COVID-19.

Patients and methods: We included 131 patients hospitalized for COVID-19 pneumonia in a single center of the internal medicine department in Marseille, France. We recorded anthropometric and metabolic parameters such as fasting glycaemia, insulinemia, HOMA-IR, lipids, and all clinical criteria linked to SARS-CoV-2 infection at the admission. Excess body weight was defined by a BMI ≥ 25 kg/m^2. The occurrence of a serious event was defined as a high-debit oxygen requirement over 6 L/min, admission into the intensive care unit, or death.

Results: Among 113 patients, two thirds (n = 76, 67%) had an excess body weight. The number of serious events was significantly higher in excess body weight patients compared to normal weight patients (respectively 25% vs 8%, p = 0.03) although excess body weight patients were younger (respectively 63.6 vs 70.3 years old, p = 0.01). In multivariate analyses, the excess body weight status was the only predictor for developing a serious event linked to SARS-CoV-2 infection, with an odds ratio at 5.6 (95% CI: 1.30-23.96; p = 0.02), independently of previous obesity associated comorbidities. There was a trend towards a positive association between the BMI (normal weight, overweight and obesity) and the risk of serious events linked to COVID-19, with a marked increase from 8.1% to 20% and 30.6% respectively (p = 0.05).

Conclusion: Excess body weight was significantly associated with severe forms of the disease, independently of its classical associated comorbidities. Physicians and specialists in Public Health must be sensitized to better protect people with an excess body weight against SARS-CoV-2 infection.

Keywords: COVID-19; Excess body weight; Obesity; Obesity comorbidities; Overweight; SARS-CoV-2.

Copyright © 2021 Elsevier Inc. All rights reserved.

Conflict of interest statement

Declaration of competing interest LP, RG, AB, ML, MK, RC, RG, ND, GK, RV and SB have no conflicts of interest linked to this work.

- Cited by 2 articles
- 25 references
- 2 figures

Supplementary info

Publication types, MeSH terms Expand

Publication types

- Observational Study

MeSH terms

- Aged
- Aged, 80 and over
- Body Mass Index
- Body Weight / physiology*
- COVID-19 / diagnosis*
- COVID-19 / epidemiology
- COVID-19 / etiology
- COVID-19 / pathology*
- Comorbidity
- Critical Illness
- Female
- France / epidemiology
- Hospitalization / statistics & numerical data
- Humans
- Male
- Middle Aged
- Obesity / complications

- Obesity / epidemiology
- Overweight / complications
- Overweight / epidemiology
- Prognosis
- Retrospective Studies
- Risk Factors
- SARS-CoV-2 / physiology
- Severity of Illness Index

Full text links

ELSEVIER FULL-TEXT ARTICLE Elsevier Science Free PMC article

Proceed to details

Cite

Share

☐ 371

Observational Study

Vox Sang
. 2021 Oct;116(9):983-989.
doi: 10.1111/vox.13087. Epub 2021 Feb 26.

Transfusion profile, clinical characteristics, comorbidities and outcomes of 3014 hospitalized patients diagnosed with COVID-19 in Brazil

Leandro Felipe Figueiredo Dalmazzo [1], Alyne Ferreira de Almendra Freitas [1], Brunna Eulálio Alves [1], Diogo Kloppel Cardoso [1], Eduardo Ferro de Carvalho [1], Fabiana Akil [1], Fernanda da Cunha Vieira Perini [1], Karina Todeschini Pires [1], Ludmila Coutinho de Aguiar [1], Mara Cabral Moraes [1], Maria Isabel Ayrosa Madeira [1], Pablo Raphael Gomiero Alves [1], Ruth Helena Perdiz Watanabe [1], Silvia Helena da Silva Sá Teixeira [1], Tatiana Covas Pereira [1], Viviani de Lourdes Rosa Pessoa [1], Sérgio Domingos Vieira [1]

Affiliations Expand

Affiliation

- [1] Grupo Gestor de Serviços de Hemoterapia - Grupo GSH, Sao Paulo, Brazil.

- PMID: **33634890**
- PMCID: PMC8014314
- DOI: 10.1111/vox.13087

Free PMC article

Observational Study

Transfusion profile, clinical characteristics, comorbidities and outcomes of 3014 hospitalized patients diagnosed with COVID-19 in Brazil

Leandro Felipe Figueiredo Dalmazzo et al. Vox Sang. 2021 Oct.
Free PMC article
Show details
Vox Sang
. 2021 Oct;116(9):983-989.
doi: 10.1111/vox.13087. Epub 2021 Feb 26.

Authors

Leandro Felipe Figueiredo Dalmazzo [1], Alyne Ferreira de Almendra Freitas [1], Brunna Eulálio Alves [1], Diogo Kloppel Cardoso [1], Eduardo Ferro de Carvalho [1], Fabiana Akil [1], Fernanda da Cunha Vieira Perini [1], Karina Todeschini Pires [1], Ludmila Coutinho de Aguiar [1], Mara Cabral Moraes [1], Maria Isabel Ayrosa Madeira [1], Pablo Raphael Gomiero Alves [1], Ruth Helena Perdiz Watanabe [1], Silvia Helena da Silva Sá Teixeira [1], Tatiana Covas Pereira [1], Viviani de Lourdes Rosa Pessoa [1], Sérgio Domingos Vieira [1]

Affiliation

- [1] Grupo Gestor de Serviços de Hemoterapia - Grupo GSH, Sao Paulo, Brazil.

- PMID: **33634890**
- PMCID: PMC8014314
- DOI: 10.1111/vox.13087

Abstract

Background: The novel coronavirus disease-2019 (COVID-19) caused a sudden and unexpected increase in the number of hospital admissions and deaths worldwide. The impact of social distancing on blood stocks was significant. Data on the use of blood products by patients with COVID-19 are scarce.

Material and methods: A retrospective observational study was conducted by analysing the medical records of 3014 hospitalized COVID-19 patients in 16 Brazilian hospitals. Individual data related to clinical, laboratory and transfusion characteristics and outcomes of these patients were collected. Patients characteristics association with mortality and transfusion need were tested independently by logistic regression models.

Results: Patients mean age was 57·6 years. In 2298 (76·2%) patients, there was an underlying clinical comorbidity. A total of 1657 (55%) patients required admission to intensive care unit

(ICU), and 943 (31%) patients required ventilatory support and orotracheal intubation (OTI). There was a total of 471 (15·6%) deaths among all patients. 325 patients (10·7%) required blood transfusion; 3187 blood products were transfused: 1364 red blood cells in 303 patients, 1092 platelet units in 78 patients, 303 fresh frozen plasma in 49 patients and 423 cryoprecipitates in 21 patients. The mortality among patients who received transfusion was substantially higher than that among the total study population.

Conclusion: Need for transfusion was low in COVID-19 patients, but significantly higher in patients admitted to ICU and in those who needed OTI. Knowledge of the transfusion profile of these patients allows better strategies for maintaining the blood stocks of hospitals during the pandemic.

Keywords: COVID-19; SARS; SARS-COV-2; mortality; transfusion.

© 2021 International Society of Blood Transfusion.

Conflict of interest statement

The authors have no conflict of interest.

- 21 references

Supplementary info

Publication types, MeSH terms Expand

Publication types

- Observational Study

MeSH terms

- Blood Transfusion
- Brazil / epidemiology
- COVID-19*
- Comorbidity
- Hospitalization
- Humans
- Infant, Newborn
- Intensive Care Units
- Respiration, Artificial
- Retrospective Studies
- SARS-CoV-2

Full text links

WILEY Full Text Article Wiley Free PMC article

Proceed to details

Cite

Share

372

Observational Study

Cancer Rep (Hoboken)

. 2021 Oct;4(5):e1388.

doi: 10.1002/cnr2.1388. Epub 2021 May 20.

Rapid real-world data analysis of patients with cancer, with and without COVID-19, across distinct health systems

Clara Hwang [1], Monika A Izano [2], Michael A Thompson [3], Shirish M Gadgeel [1], James L Weese [3], Tom Mikkelsen [1], Andrew Schrag [2], Mahder Teka [2], Sheetal Walters [2], Frank M Wolf [2], Jonathan Hirsch [2], Donna R Rivera [4], Paul G Kluetz [4], Harpreet Singh [4], Thomas D Brown [2]

Affiliations Expand

Affiliations

- [1] Henry Ford Cancer Institute, Henry Ford Health System, Detroit, Michigan, USA.
- [2] Syapse, San Francisco, California, USA.
- [3] Aurora Cancer Care, Advocate Aurora Health, Milwaukee, Wisconsin, USA.
- [4] Oncology Center of Excellence, United States Food and Drug Administration, Silver Spring, Maryland, USA.

- PMID: **34014037**
- PMCID: PMC8209944
- DOI: 10.1002/cnr2.1388

Free PMC article

Observational Study

Rapid real-world data analysis of patients with cancer, with and without COVID-19, across distinct health systems

Clara Hwang et al. Cancer Rep (Hoboken). 2021 Oct.

Free PMC article

Show details

Cancer Rep (Hoboken)

. 2021 Oct;4(5):e1388.

doi: 10.1002/cnr2.1388. Epub 2021 May 20.

Authors

Clara Hwang [1], Monika A Izano [2], Michael A Thompson [3], Shirish M Gadgeel [1], James L Weese [3], Tom Mikkelsen [1], Andrew Schrag [2], Mahder Teka [2], Sheetal Walters [2], Frank M Wolf [2], Jonathan Hirsch [2], Donna R Rivera [4], Paul G Kluetz [4], Harpreet Singh [4], Thomas D Brown [2]

Affiliations

- [1] Henry Ford Cancer Institute, Henry Ford Health System, Detroit, Michigan, USA.
- [2] Syapse, San Francisco, California, USA.
- [3] Aurora Cancer Care, Advocate Aurora Health, Milwaukee, Wisconsin, USA.
- [4] Oncology Center of Excellence, United States Food and Drug Administration, Silver Spring, Maryland, USA.

- PMID: **34014037**
- PMCID: PMC8209944
- DOI: 10.1002/cnr2.1388

Abstract

Background: The understanding of the impact of COVID-19 in patients with cancer is evolving, with need for rapid analysis.

Aims: This study aims to compare the clinical and demographic characteristics of patients with cancer (with and without COVID-19) and characterize the clinical outcomes of patients with COVID-19 and cancer.

Methods and results: Real-world data (RWD) from two health systems were used to identify 146 702 adults diagnosed with cancer between 2015 and 2020; 1267 COVID-19 cases were identified between February 1 and July 30, 2020. Demographic, clinical, and socioeconomic characteristics were extracted. Incidence of all-cause mortality, hospitalizations, and invasive respiratory support was assessed between February 1 and August 14, 2020. Among patients with cancer, patients with COVID-19 were more likely to be Non-Hispanic black (NHB), have active cancer, have comorbidities, and/or live in zip codes with median household income <$30 000. Patients with COVID-19 living in lower-income areas and NHB patients were at greatest risk for hospitalization from pneumonia, fluid and electrolyte disorders, cough, respiratory failure, and acute renal failure and were more likely to receive hydroxychloroquine. All-cause mortality, hospital admission, and invasive respiratory support were more frequent among patients with cancer and COVID-19. Male sex, increasing age, living in zip codes with median household income <$30 000, history of pulmonary circulation disorders, and recent treatment with immune checkpoint inhibitors or chemotherapy were associated with greater odds of all-cause mortality in multivariable logistic regression models.

Conclusion: RWD can be rapidly leveraged to understand urgent healthcare challenges. Patients with cancer are more vulnerable to COVID-19 effects, especially in the setting of active cancer and comorbidities, with additional risk observed in NHB patients and those living in zip codes with median household income <$30 000.

Keywords: cancer risk factors; epidemiology; medical oncology; viral infection.

© 2021 The Authors. Cancer Reports published by Wiley Periodicals LLC. This article has been contributed to by US Government employees and their work is in the public domain in the USA.

Conflict of interest statement

The authors have no conflicts of interest to disclose.

- Cited by 1 article
- 34 references
- 1 figure

Supplementary info

Publication types, MeSH terms Expand

Publication types

- Observational Study

MeSH terms

- Aged
- COVID-19 / diagnosis
- COVID-19 / epidemiology*
- COVID-19 / therapy
- COVID-19 / virology
- Comorbidity
- Data Analysis
- Female
- Hospital Mortality
- Humans
- Male
- Middle Aged
- Neoplasms / complications
- Neoplasms / epidemiology*
- Neoplasms / immunology
- Patient Admission / statistics & numerical data
- Respiration, Artificial
- Retrospective Studies
- Risk Factors
- SARS-CoV-2 / immunology
- Severity of Illness Index
- Social Determinants of Health / statistics & numerical data*

- Socioeconomic Factors*
- United States / epidemiology

Full text links

Wiley Free PMC article
Proceed to details
Cite
Share
373
Observational Study
Palliat Med
. 2020 Oct;34(9):1228-1234.
doi: 10.1177/0269216320940566. Epub 2020 Jul 17.

The Palliative Performance Scale predicts mortality in hospitalized patients with COVID-19

Michele Fiorentino[1], Sri Ram Pentakota[1], Anne C Mosenthal[2], Nina E Glass[1]
Affiliations Expand

Affiliations

- [1] Rutgers New Jersey Medical School, Newark, NJ, USA.
- [2] Tufts University School of Medicine, Boston, MA, USA.

- PMID: **32677509**
- PMCID: PMC7378312
- DOI: 10.1177/0269216320940566

Free PMC article
Observational Study

The Palliative Performance Scale predicts mortality in hospitalized patients with COVID-19

Michele Fiorentino et al. Palliat Med. 2020 Oct.
Free PMC article
Show details
Palliat Med

. 2020 Oct;34(9):1228-1234.
doi: 10.1177/0269216320940566. Epub 2020 Jul 17.

Authors

Michele Fiorentino [1], Sri Ram Pentakota [1], Anne C Mosenthal [2], Nina E Glass [1]

Affiliations

- [1] Rutgers New Jersey Medical School, Newark, NJ, USA.
- [2] Tufts University School of Medicine, Boston, MA, USA.

- PMID: **32677509**
- PMCID: PMC7378312
- DOI: 10.1177/0269216320940566

Abstract

Background: Coronavirus disease 2019 (COVID-19) has a substantial mortality risk with increased rates in the elderly. We hypothesized that age is not sufficient, and that frailty measured by preadmission Palliative Performance Scale would be a predictor of outcomes. Improved ability to identify high-risk patients will improve clinicians' ability to provide appropriate palliative care, including engaging in shared decision-making about life-sustaining therapies.

Aim: To evaluate whether preadmission Palliative Performance Scale predicts mortality in hospitalized patients with COVID-19.

Design: Retrospective observational cohort study of patients admitted with COVID-19. Palliative Performance Scale was calculated from the chart. Using logistic regression, Palliative Performance Scale was assessed as a predictor of mortality controlling for demographics, comorbidities, palliative care measures and socioeconomic status.

Setting/participants: Patients older than 18 years of age admitted with COVID-19 to a single urban public hospital in New Jersey, USA.

Results: Of 443 admitted patients, we determined the Palliative Performance Scale score for 374. Overall mortality was 31% and 81% in intubated patients. In all, 36% (134) of patients had a low Palliative Performance Scale score. Compared with patients with a high score, patients with a low score were more likely to die, have do not intubate orders and be discharged to a facility. Palliative Performance Scale independently predicts mortality (odds ratio 2.89; 95% confidence interval 1.42-5.85).

Conclusions: Preadmission Palliative Performance Scale independently predicts mortality in patients hospitalized with COVID-19. Improved predictors of mortality can help clinicians caring for patients with COVID-19 to discuss prognosis and provide appropriate palliative care including decisions about life-sustaining therapy.

Keywords: Palliative care; coronavirus; frailty; hospital mortality; physical functional performance.

Conflict of interest statement

Declaration of conflicting interests: The author(s) declared no potential conflicts of interest with respect to the research, authorship and/or publication of this article.

- Cited by 6 articles
- 18 references

Supplementary info

Publication types, MeSH terms Expand

Publication types

- Observational Study
- Research Support, Non-U.S. Gov't

MeSH terms

- Adult
- Aged
- Aged, 80 and over
- Betacoronavirus
- COVID-19
- Cohort Studies
- Coronavirus Infections / epidemiology
- Coronavirus Infections / mortality*
- Coronavirus Infections / therapy*
- Female
- Frail Elderly / statistics & numerical data*
- Hospital Mortality*
- Hospitalization / statistics & numerical data*
- Humans
- Male
- Middle Aged
- Mortality
- New Jersey / epidemiology
- Palliative Care / statistics & numerical data*
- Pandemics
- Pneumonia, Viral / epidemiology
- Pneumonia, Viral / mortality*
- Pneumonia, Viral / therapy*
- Prognosis*
- Retrospective Studies
- SARS-CoV-2

- Young Adult

Full text links

SAGE journals Open access full text Atypon Free PMC article
Proceed to details
Cite
Share
374
Observational Study
PLoS One
. 2021 Jul 30;16(7):e0255228.
doi: 10.1371/journal.pone.0255228. eCollection 2021.

Development and validation of a prognostic 40-day mortality risk model among hospitalized patients with COVID-19

Donald A Berry [1, 2], Andrew Ip [3, 4, 5], Brett E Lewis [4, 5], Scott M Berry [1], Nicholas S Berry [1], Mary MrKulic [4], Virginia Gadalla [4], Burcu Sat [4], Kristen Wright [4], Michelle Serna [4], Rashmi Unawane [4], Katerina Trpeski [4], Michael Koropsak [4], Puneet Kaur [4], Zachary Sica [4], Andrew McConnell [4], Urszula Bednarz [4], Michael Marafelias [4], Andre H Goy [4, 5], Andrew L Pecora [4, 5], Ihor S Sawczuk [5, 6], Stuart L Goldberg [3, 4, 5]
Affiliations Expand

Affiliations

- [1] Berry Consultants LLC, Austin, Texas, United States of America.
- [2] M.D. Anderson Cancer Center of the University of Texas, Houston, Texas, United States of America.
- [3] Division of Outcomes and Value Research, John Theurer Cancer Center at Hackensack University Medical Center, Hackensack, New Jersey, United States of America.
- [4] John Theurer Cancer Center at Hackensack University Medical Center, Hackensack, New Jersey, United States of America.
- [5] Hackensack Meridian Health School of Medicine, Nutley, New Jersey, United States of America.
- [6] Hackensack Meridian Health, Edison, New Jersey, United States of America.

- PMID: **34329317**
- PMCID: PMC8323891
- DOI: 10.1371/journal.pone.0255228

Free PMC article
Observational Study

Development and validation of a prognostic 40-day mortality risk model among hospitalized patients with COVID-19

Donald A Berry et al. PLoS One. 2021.
Free PMC article
Show details
PLoS One
. 2021 Jul 30;16(7):e0255228.
doi: 10.1371/journal.pone.0255228. eCollection 2021.

Authors

Donald A Berry [1, 2], Andrew Ip [3, 4, 5], Brett E Lewis [4, 5], Scott M Berry [1], Nicholas S Berry [1], Mary MrKulic [4], Virginia Gadalla [4], Burcu Sat [4], Kristen Wright [4], Michelle Serna [4], Rashmi Unawane [4], Katerina Trpeski [4], Michael Koropsak [4], Puneet Kaur [4], Zachary Sica [4], Andrew McConnell [4], Urszula Bednarz [4], Michael Marafelias [4], Andre H Goy [4, 5], Andrew L Pecora [4, 5], Ihor S Sawczuk [5, 6], Stuart L Goldberg [3, 4, 5]

Affiliations

- [1] Berry Consultants LLC, Austin, Texas, United States of America.
- [2] M.D. Anderson Cancer Center of the University of Texas, Houston, Texas, United States of America.
- [3] Division of Outcomes and Value Research, John Theurer Cancer Center at Hackensack University Medical Center, Hackensack, New Jersey, United States of America.
- [4] John Theurer Cancer Center at Hackensack University Medical Center, Hackensack, New Jersey, United States of America.
- [5] Hackensack Meridian Health School of Medicine, Nutley, New Jersey, United States of America.
- [6] Hackensack Meridian Health, Edison, New Jersey, United States of America.

- PMID: **34329317**
- PMCID: PMC8323891
- DOI: 10.1371/journal.pone.0255228

Abstract

Objectives: The development of a prognostic mortality risk model for hospitalized COVID-19 patients may facilitate patient treatment planning, comparisons of therapeutic strategies, and public health preparations.

Methods: We retrospectively reviewed the electronic health records of patients hospitalized within a 13-hospital New Jersey USA network between March 1, 2020 and April 22, 2020 with positive polymerase chain reaction results for SARS-CoV-2, with follow-up through May 29, 2020. With death or hospital discharge by day 40 as the primary endpoint, we used univariate followed by stepwise multivariate proportional hazard models to develop a risk score on one-half

the data set, validated on the remainder, and converted the risk score into a patient-level predictive probability of 40-day mortality based on the combined dataset.

Results: The study population consisted of 3123 hospitalized COVID-19 patients; median age 63 years; 60% were men; 42% had >3 coexisting conditions. 713 (23%) patients died within 40 days of hospitalization for COVID-19. From 22 potential candidate factors 6 were found to be independent predictors of mortality and were included in the risk score model: age, respiratory rate ≥25/minute upon hospital presentation, oxygenation <94% on hospital presentation, and pre-hospital comorbidities of hypertension, coronary artery disease, or chronic renal disease. The risk score was highly prognostic of mortality in a training set and confirmatory set yielding in the combined dataset a hazard ratio of 1.80 (95% CI, 1.72, 1.87) for one unit increases. Using observed mortality within 20 equally sized bins of risk scores, a predictive model for an individual's 40-day risk of mortality was generated as -14.258 + 13.460*RS + 1.585*(RS-2.524)^2-0.403*(RS-2.524)^3. An online calculator of this 40-day COVID-19 mortality risk score is available at www.HackensackMeridianHealth.org/CovidRS.

Conclusions: A risk score using six variables is able to prognosticate mortality within 40-days of hospitalization for COVID-19.

Trial registration: Clinicaltrials.gov Identifier: NCT04347993.

Conflict of interest statement

The authors declare no conflicts of interest. Authors DAB and SMB are co-owners and NSB is an employee of Berry Consultants, LLC, a company that designs and analyses clinical trials for pharmaceutical and medical device companies, NIH cooperative groups, patient advocacy groups, and international consortia. Berry Consultants received no funding related to this project. This does not alter our adherence to PLOS ONE policies on sharing data and materials.

- Cited by 1 article
- 16 references
- 4 figures

Supplementary info

Publication types, MeSH terms, Associated data, Grant support Expand

Publication types

- Multicenter Study
- Observational Study
- Validation Study

MeSH terms

- Adolescent
- Adult
- Aged
- Aged, 80 and over

- COVID-19 / diagnosis
- COVID-19 / mortality*
- COVID-19 Nucleic Acid Testing
- Child
- Child, Preschool
- Female
- Hospital Mortality*
- Hospitalization*
- Humans
- Infant
- Infant, Newborn
- Male
- Middle Aged
- Models, Biological*
- SARS-CoV-2*
- Time Factors

Associated data

- ClinicalTrials.gov/NCT04347993

Grant support

No funding or salary support for this project. Authors DAB, SMB, and NSB are associated with Berry Consultants, LLC. Their contribution was on their own time and was not funded by Berry Consultants. Nor did these individuals nor Berry Consultants itself provide funding for this project from any other source nor did they nor Berry Consultants receive any funding related to this project from any source.

Full text links

OPEN ACCESS TO FULL TEXT PLOS ONE Public Library of Science Free PMC article
Proceed to details
Cite
Share
375
Observational Study
Eur Ann Otorhinolaryngol Head Neck Dis
. 2021 Dec;138(6):443-449.
doi: 10.1016/j.anorl.2021.03.002. Epub 2021 Mar 4.

Tracheostomies after SARS-CoV-2 intubation, performed by academic

otorhinolaryngologists in the Paris area of France: Preliminary results

S Bartier [1], C La Croix [2], D Evrard [3], R Hervochon [4], O Laccourreye [5], C Gasne [6], A Excoffier [6], L Tanaka [7], B Barry [3], A Coste [8], F Tankere [4], R Kania [9], J Nevoux [7]
Affiliations Expand

Affiliations

- [1] Service ORL, Centre hospitalier intercommunal de Créteil, Université Paris Est, 40, avenue de Verdun, 94000 Créteil, France. Electronic address: sophiebartier@hotmail.fr.
- [2] Service ORL, AP-HP, Hôpital Cochin AP-HP, Université Paris centre, 27, rue du Faubourg-St.-Jacques, 75014 Paris, France.
- [3] Service ORL, Hôpital Bichat, AP-HP, Université Paris centre, 46, rue Henri-Huchard, 75018 Paris, France.
- [4] Service ORL, AP-HP, Hôpital La Pitié-Salpétrière, Université Paris Sorbonne, 47-83, boulevard de l'Hôpital, 75013 Paris, France.
- [5] Service ORL, AP-HP, HEGP, Université Paris Centre, 20-40, rue Leblanc, 75015 Paris, France.
- [6] Service ORL, AP-HP, Hôpital Tenon, AP-HP, Université Paris Sorbonne, 4, rue de la Chine, 75020 Paris, France.
- [7] Service ORL, AP-HP, Hôpital Bicêtre, Université Paris Saclay, 78, rue du Général Leclerc, 94270 Le Kremlin-Bicêtre, France.
- [8] Service ORL, Centre hospitalier intercommunal de Créteil, Université Paris Est, 40, avenue de Verdun, 94000 Créteil, France.
- [9] Service ORL, AP-HP, Hôpital Lariboisière, Université Paris Nord, 2, rue Ambroise-Paré, 75010 Paris, France.

- PMID: **33707069**
- PMCID: PMC7931693
- DOI: 10.1016/j.anorl.2021.03.002

Free PMC article
Observational Study

Tracheostomies after SARS-CoV-2 intubation, performed by academic otorhinolaryngologists in the Paris area of France: Preliminary results

S Bartier et al. Eur Ann Otorhinolaryngol Head Neck Dis. 2021 Dec.
Free PMC article
Show details
Eur Ann Otorhinolaryngol Head Neck Dis

. 2021 Dec;138(6):443-449.
doi: 10.1016/j.anorl.2021.03.002. Epub 2021 Mar 4.

Authors

S Bartier [1], C La Croix [2], D Evrard [3], R Hervochon [4], O Laccourreye [5], C Gasne [6], A Excoffier [6], L Tanaka [7], B Barry [3], A Coste [8], F Tankere [4], R Kania [9], J Nevoux [7]

Affiliations

- [1] Service ORL, Centre hospitalier intercommunal de Créteil, Université Paris Est, 40, avenue de Verdun, 94000 Créteil, France. Electronic address: sophiebartier@hotmail.fr.
- [2] Service ORL, AP-HP, Hôpital Cochin AP-HP, Université Paris centre, 27, rue du Faubourg-St.-Jacques, 75014 Paris, France.
- [3] Service ORL, Hôpital Bichat, AP-HP, Université Paris centre, 46, rue Henri-Huchard, 75018 Paris, France.
- [4] Service ORL, AP-HP, Hôpital La Pitié-Salpétrière, Université Paris Sorbonne, 47-83, boulevard de l'Hôpital, 75013 Paris, France.
- [5] Service ORL, AP-HP, HEGP, Université Paris Centre, 20-40, rue Leblanc, 75015 Paris, France.
- [6] Service ORL, AP-HP, Hôpital Tenon, AP-HP, Université Paris Sorbonne, 4, rue de la Chine, 75020 Paris, France.
- [7] Service ORL, AP-HP, Hôpital Bicêtre, Université Paris Saclay, 78, rue du Général Leclerc, 94270 Le Kremlin-Bicêtre, France.
- [8] Service ORL, Centre hospitalier intercommunal de Créteil, Université Paris Est, 40, avenue de Verdun, 94000 Créteil, France.
- [9] Service ORL, AP-HP, Hôpital Lariboisière, Université Paris Nord, 2, rue Ambroise-Paré, 75010 Paris, France.

- PMID: **33707069**
- PMCID: PMC7931693
- DOI: 10.1016/j.anorl.2021.03.002

Abstract

Objective: To analyse tracheostomies after intubation for SARS-Cov-2 infection performed by otorhinolaryngologists in 7 university hospitals in the Paris area of France during the month March 24 to April 23, 2020.

Material and methods: A multicentre retrospective observational study included 59 consecutive patients. The main goals were to evaluate the number, characteristics and practical conditions of tracheostomies, and the COVID-19 status of the otorhinolaryngologists. Secondary goals were to analyse tracheostomy time, decannulation rate, immediate postoperative complications and laryngotracheal axis status.

Results: Tracheostomy indications were for ventilatory weaning and extubation failure in 86% and 14% of cases, respectively. The technique was surgical, percutaneous or hybrid in 91.5%, 3.4% and 5.1% of cases, respectively. None of the operators developed symptoms consistent with COVID-19. Postoperative complications occurred in 15% of cases, with no significant difference between surgical and percutaneous/hybrid techniques (P=0.33), although no complications

occurred after percutaneous or hybrid tracheostomies. No procedures or complications resulted in death. The decannulation rate was 74.5% with a mean tracheostomy time of 20±12 days. In 55% of the patients evaluated by flexible endoscopy after decannulation, a laryngeal abnormality was found. On univariate analysis, no clinical features had a significant influence on tracheostomy time, decannulation rate or occurrence of laryngeal lesions.

Conclusion: The main findings of the present retrospective study were: absence of contamination of the surgeons, heterogeneity of practices between centres, a high rate of complications and laryngeal lesions whatever the technique, and the specificities of the patients.

Keywords: France; Otorhinolaryngology; SARS-CoV-2; Tracheostomy.

Copyright © 2021 Elsevier Masson SAS. All rights reserved.

- 32 references
- 3 figures

Supplementary info

Publication types, MeSH terms Expand

Publication types

- Observational Study

MeSH terms

- COVID-19*
- Humans
- Intubation, Intratracheal / adverse effects
- Paris
- Retrospective Studies
- SARS-CoV-2
- Surgeons*
- Tracheostomy

Full text links

ELSEVIER FULL-TEXT ARTICLE Elsevier Science Free PMC article

Proceed to details

Cite

Share

☐ 376

Observational Study

Medicine (Baltimore)

. 2021 May 14;100(19):e25917.

doi: 10.1097/MD.0000000000025917.

National early warning score on admission as risk factor for invasive mechanical ventilation in COVID-19 patients: A STROBE-compliant study

Min Cheol Chang [1], Tae Uk Kim [2], Donghwi Park [3]
Affiliations Expand

Affiliations

- [1] Department of Physical Medicine and Rehabilitation, College of Medicine, Yeungnam University, Daegu.
- [2] Department of Physical Medicine and Rehabilitation, College of Medicine, Dankook University, Cheonan.
- [3] Department of Physical Medicine and Rehabilitation, Ulsan University Hospital, University of Ulsan College of Medicine, Ulsan, Republic of Korea.

- PMID: **34106657**
- PMCID: PMC8133259
- DOI: 10.1097/MD.0000000000025917

Free PMC article
Observational Study

National early warning score on admission as risk factor for invasive mechanical ventilation in COVID-19 patients: A STROBE-compliant study

Min Cheol Chang et al. Medicine (Baltimore). 2021.
Free PMC article
Show details
Medicine (Baltimore)
. 2021 May 14;100(19):e25917.
doi: 10.1097/MD.0000000000025917.

Authors

Min Cheol Chang [1], Tae Uk Kim [2], Donghwi Park [3]

Affiliations

- [1] Department of Physical Medicine and Rehabilitation, College of Medicine, Yeungnam University, Daegu.
- [2] Department of Physical Medicine and Rehabilitation, College of Medicine, Dankook University, Cheonan.
- [3] Department of Physical Medicine and Rehabilitation, Ulsan University Hospital, University of Ulsan College of Medicine, Ulsan, Republic of Korea.

- PMID: **34106657**
- PMCID: PMC8133259
- DOI: 10.1097/MD.0000000000025917

Abstract

The coronavirus disease (COVID-19) has become a global pandemic. Invasive mechanical ventilation is recommended for the management of patients with COVID-19 who have severe respiratory symptoms. However, various complications can develop after its use. The efficient and appropriate management of patients requires the identification of factors associated with an aggravation of COVID-19 respiratory symptoms to a degree where invasive mechanical ventilation becomes necessary, thereby enabling clinicians to prevent such ventilation. This retrospective study included 138 inpatients with COVID-19 at a tertiary hospital. We evaluated the differences in the demographic and clinical data between 27 patients who required invasive mechanical ventilation and 111 patients who did not. Multivariate logistic regression analysis indicated that the duration of fever, national early warning score (NEWS), and lactate dehydrogenase (LDH) levels on admission were significantly associated with invasive mechanical ventilation in this cohort. The optimal cut-off values were: fever duration ≥1 day (sensitivity 100.0%, specificity 54.95%), NEWS ≥7 (sensitivity 72.73%, specificity 92.52%), and LDH >810 mg/dL (sensitivity 56.0%, specificity 90.29%). These findings can assist in the early identification of patients who will require invasive mechanical ventilation. Further studies in larger patient populations are recommended to validate our findings.

Copyright © 2021 the Author(s). Published by Wolters Kluwer Health, Inc.

Conflict of interest statement

The authors have no conflicts of interest to disclose.

- 36 references
- 1 figure

Supplementary info

Publication types, MeSH terms, Substances Expand

Publication types

- Observational Study

MeSH terms

- Adult
- Age Factors
- Aged
- Aged, 80 and over
- Antiviral Agents / therapeutic use
- COVID-19 / drug therapy
- COVID-19 / physiopathology*
- Early Warning Score*
- Female
- Fever / physiopathology
- Humans
- Hydroxychloroquine / therapeutic use
- L-Lactate Dehydrogenase / blood
- Logistic Models
- Male
- Middle Aged
- Pandemics
- Real-Time Polymerase Chain Reaction
- Republic of Korea
- Respiration, Artificial / statistics & numerical data*
- Retrospective Studies
- Risk Assessment
- Risk Factors
- SARS-CoV-2
- Sex Factors
- Socioeconomic Factors
- Tertiary Care Centers
- Young Adult

Substances

- Antiviral Agents
- Hydroxychloroquine
- L-Lactate Dehydrogenase

Full text links

Wolters Kluwer Wolters Kluwer Free PMC article

Proceed to details

Cite

Share

☐ 377

Observational Study

Am J Emerg Med
. 2021 Dec;50:437-441.
doi: 10.1016/j.ajem.2021.08.032. Epub 2021 Aug 16.

Impact of early versus late administration of bamlanivimab on readmissions in patients with high-risk COVID-19

James D Melton 3rd [1], Kayla Wilson [2], Fred Blind [1], Andrew Barbera [1], Donna Bhisitkul [3], Shannon Hasara [1], Karen Homa [4], Juliana Karp [1], Hal Escowitz [1], Todd Haber [1], Diana DeGroot [1], Jonathan Anderson [1], Jason DeLeon [1], Jesse De Los Santos [1], Donna Faviere [1], Joanne Fuell [1], Rita Gillespie [1], Jesse Glueck [1], Cliff Reeber [1], David J Rhodes [3], Vashun Rodriguez [1]
Affiliations Expand

Affiliations

- [1] Department of Emergency Medicine, Lakeland Regional Health, Lakeland, FL, USA.
- [2] Department of Emergency Medicine, Lakeland Regional Health, Lakeland, FL, USA. Electronic address: Kayla.Wilson@mylrh.org.
- [3] Department of Pediatric Emergency Medicine, Lakeland Regional Health, Lakeland, FL, USA.
- [4] Department of Research and Sponsored Studies, Lakeland Regional Health, Lakeland, FL, USA.

- PMID: **34487951**
- PMCID: PMC8366036
- DOI: 10.1016/j.ajem.2021.08.032

Free PMC article
Observational Study

Impact of early versus late administration of bamlanivimab on readmissions in patients with high-risk COVID-19

James D Melton 3rd et al. Am J Emerg Med. 2021 Dec.
Free PMC article
Show details
Am J Emerg Med
. 2021 Dec;50:437-441.
doi: 10.1016/j.ajem.2021.08.032. Epub 2021 Aug 16.

Authors

James D Melton 3rd [1], Kayla Wilson [2], Fred Blind [1], Andrew Barbera [1], Donna Bhisitkul [3], Shannon Hasara [1], Karen Homa [4], Juliana Karp [1], Hal Escowitz [1], Todd Haber [1], Diana DeGroot [1], Jonathan Anderson [1], Jason DeLeon [1], Jesse De Los Santos [1], Donna Faviere [1], Joanne Fuell [1], Rita Gillespie [1], Jesse Glueck [1], Cliff Reeber [1], David J Rhodes [3], Vashun Rodriguez [1]

Affiliations

- [1] Department of Emergency Medicine, Lakeland Regional Health, Lakeland, FL, USA.
- [2] Department of Emergency Medicine, Lakeland Regional Health, Lakeland, FL, USA. Electronic address: Kayla.Wilson@mylrh.org.
- [3] Department of Pediatric Emergency Medicine, Lakeland Regional Health, Lakeland, FL, USA.
- [4] Department of Research and Sponsored Studies, Lakeland Regional Health, Lakeland, FL, USA.

- PMID: **34487951**
- PMCID: PMC8366036
- DOI: 10.1016/j.ajem.2021.08.032

Abstract

Background: Recombinant monoclonal antibody therapies have been utilized under emergency use authorization (EUA) for the prevention of clinical decompensation in high-risk COVID-19 positive patients for up to 10 days from symptom onset. The purpose of this study was to determine the impact of the timing of the monoclonal antibody, bamlanivimab, on clinical outcomes in high-risk COVID-19 positive patients.

Methods: This was an IRB-approved, retrospective evaluation of adult patients who received bamlanivimab per EUA criteria in the emergency department (ED). Patients were dichotomized into two groups- 3 days of symptoms or less (early) versus 4 to 10 days (late). The primary outcome was hospitalization for COVID-related illness at 28 days (or treatment failure). Secondary outcomes were COVID-related ED visits at 28 days, hospital and intensive care unit (ICU) length of stay (LOS), and in-hospital mortality at 28 days.

Results: A total of 839 patients were included in the analysis. There was no difference observed in COVID-related hospitalization rates within 28 days between the early and late bamlanivimab administration groups (7.5% vs. 8.2%, p = 0.71). There was no difference in COVID-related ED visits within 28 days with 13% of patients returning to the ED.

Conclusions: In conclusion, there were no differences in the rates of hospitalization at 28 days when bamlanivimab was administered in the first 3 days of illness versus days 4 to 10. Future prospective studies are warranted to expand upon the characteristics of patients that may or may not benefit from monoclonal antibody therapy.

Keywords: Bamlanivimab; COVID-19; High-risk COVID-19; Monoclonal antibody therapy.

Copyright © 2021 Elsevier Inc. All rights reserved.

Conflict of interest statement

Declaration of Competing Interest Nothing to disclose.

- 7 references
- 1 figure

Supplementary info

Publication types, MeSH terms, Substances Expand

Publication types

- Comparative Study
- Observational Study

MeSH terms

- Adolescent
- Adult
- Age Factors
- Aged
- Antibodies, Monoclonal, Humanized / administration & dosage*
- Antibodies, Neutralizing / administration & dosage*
- Antiviral Agents / administration & dosage*
- Body Mass Index
- COVID-19 / diagnosis
- COVID-19 / drug therapy*
- COVID-19 / mortality
- Drug Administration Schedule
- Emergency Service, Hospital
- Female
- Hospital Mortality
- Humans
- Length of Stay
- Male
- Middle Aged
- Patient Readmission*
- Retrospective Studies
- Risk Factors
- Treatment Outcome
- Young Adult

Substances

- Antibodies, Monoclonal, Humanized
- Antibodies, Neutralizing
- Antiviral Agents
- bamlanivimab

Full text links

ELSEVIER FULL-TEXT ARTICLE Elsevier Science Free PMC article
Proceed to details
Cite
Share
378
Observational Study
Malays J Pathol
. 2021 Dec;43(3):375-380.

Procalcitonin as a predictor of severity and mortality in a cohort of patients hospitalised with COVID-19

S Ahmed[1], Z A Ahmed[2], N H Rashid[3], M Mansoor[4], I Siddiqui[1], L Jafri[5]
Affiliations Expand

Affiliations

- [1] Aga Khan University, Department of Pathology and Laboratory Medicine, Section of Chemical Pathology, Stadium Road, Karachi 74800, Pakistan.
- [2] Aga Khan University, Department of Pathology and Laboratory Medicine, Section of Molecular Pathology, Stadium Road, Karachi 74800, Pakistan.
- [3] Aga Khan University, Department of Medicine, Intensive Care Unit, Stadium Road, Karachi 74800, Pakistan.
- [4] Aga Khan University, Stadium Road, Karachi 74800, Pakistan.
- [5] Aga Khan University, Department of Pathology and Laboratory Medicine, Section of Chemical Pathology, Stadium Road, Karachi 74800, Pakistan. ena.jafri@aku.edu.

- PMID: **34958058**

Free article
Observational Study

Procalcitonin as a predictor of severity and mortality in a cohort of patients hospitalised with COVID-19

S Ahmed et al. Malays J Pathol. 2021 Dec.
Free article
Show details
Malays J Pathol
. 2021 Dec;43(3):375-380.

Authors

S Ahmed [1], Z A Ahmed [2], N H Rashid [3], M Mansoor [4], I Siddiqui [1], L Jafri [5]

Affiliations

- [1] Aga Khan University, Department of Pathology and Laboratory Medicine, Section of Chemical Pathology, Stadium Road, Karachi 74800, Pakistan.
- [2] Aga Khan University, Department of Pathology and Laboratory Medicine, Section of Molecular Pathology, Stadium Road, Karachi 74800, Pakistan.
- [3] Aga Khan University, Department of Medicine, Intensive Care Unit, Stadium Road, Karachi 74800, Pakistan.
- [4] Aga Khan University, Stadium Road, Karachi 74800, Pakistan.
- [5] Aga Khan University, Department of Pathology and Laboratory Medicine, Section of Chemical Pathology, Stadium Road, Karachi 74800, Pakistan. ena.jafri@aku.edu.

- PMID: **34958058**

Abstract

Introduction: To evaluate the association of Procalcitonin (PCT) with severity in Coronavirus disease 2019 (COVID-19), hospitalised patients and to test the hypothesis that it is an independent predictor of mortality.

Materials and methods: This study was conducted at Chemical Pathology, Department of Pathology and Laboratory Medicine and Department of Medicine, Aga Khan University (AKU), Karachi Pakistan. Electronic medical records of all in-patients including both genders and all age groups with documented COVID-19 from March to August 2020 were reviewed and recorded on a pre-structured performa. The subjects were divided into two categories severe and non-severe COVID-19; and survivors and non-survivors. Between-group differences were tested using the Chi-square and Mann-Whitney's U-test. The receiver operating characteristic curve was plotted for serum PCT with severity and mortality. A binary logistic regression was used to identify variables independently associated with mortality. The data was analysed using SPSS.

Results: 336 patients were reviewed as declared COVID-19 positive during the study duration, and 136 were included in the final analysis including 101 males and 35 females. A statistically significant difference in PCT was found between severe and non-severe COVID-19 (p

value=0.01); and survivors and nonsurvivors (p value<0.0001). PCT, older age and increased duration of hospital stay were revealed as variables independently associated with mortality. On ROC analysis, an AUC of 0.76 for mortality prediction was generated for PCT.

Conclusion: Baseline serum PCT concentration is a promising predictor of mortality and severity in COVID-19 cases when considered in combination with clinical details and other laboratory tests.

Supplementary info

Publication types, MeSH terms, Substances Expand

Publication types

- Comparative Study
- Observational Study

MeSH terms

- Aged
- Biomarkers / blood
- COVID-19 / blood
- COVID-19 / mortality*
- Calcitonin Gene-Related Peptide
- Female
- Hospitalization
- Humans
- Inpatients
- Male
- Pakistan
- Procalcitonin / blood*
- Prognosis
- Retrospective Studies
- SARS-CoV-2
- Sepsis / complications
- Sepsis / mortality
- Severity of Illness Index

Substances

- Biomarkers
- CALCA protein, human
- Procalcitonin
- Calcitonin Gene-Related Peptide

Full text links

Malays J Pathol College of Pathologists, Academy of Medicine of Malaysia
Proceed to details
Cite
Share
☐ 379
Observational Study
Saudi J Kidney Dis Transpl
. Mar-Apr 2021;32(2):377-386.
doi: 10.4103/1319-2442.335450.

Frequency of deranged renal profile in patients with COVID-19: Tertiary Care Experience from a developing country

Muhammad Sohaib Asghar [1], Muhammad Nadeem Ahsan [2], Mohammed Akram [3], Maira Hassan [3], Uzma Rasheed [3], Syed Muhammad Adnan [4]
Affiliations Expand

Affiliations

- [1] Department of Internal Medicine, Dow University Hospital, Dow University of Health Sciences, Karachi, Pakistan.
- [2] Department of Nephrology, Dow University Hospital, Dow University of Health Sciences, Karachi, Pakistan.
- [3] Department of Internal Medicine, Liaquat National Hospital and Medical College, Karachi, Pakistan.
- [4] Department of Epidemiology and Biostatistics, Dow University Hospital, Dow University of Health Sciences, Karachi, Pakistan.

- PMID: **35017332**
- DOI: 10.4103/1319-2442.335450

Free article
Observational Study

Frequency of deranged renal profile in patients with COVID-19: Tertiary Care Experience from a developing country

Muhammad Sohaib Asghar et al. Saudi J Kidney Dis Transpl. Mar-Apr 2021.
Free article

Show details

Saudi J Kidney Dis Transpl
. Mar-Apr 2021;32(2):377-386.
doi: 10.4103/1319-2442.335450.

Authors

Muhammad Sohaib Asghar [1], Muhammad Nadeem Ahsan [2], Mohammed Akram [3], Maira Hassan [3], Uzma Rasheed [3], Syed Muhammad Adnan [4]

Affiliations

- [1] Department of Internal Medicine, Dow University Hospital, Dow University of Health Sciences, Karachi, Pakistan.
- [2] Department of Nephrology, Dow University Hospital, Dow University of Health Sciences, Karachi, Pakistan.
- [3] Department of Internal Medicine, Liaquat National Hospital and Medical College, Karachi, Pakistan.
- [4] Department of Epidemiology and Biostatistics, Dow University Hospital, Dow University of Health Sciences, Karachi, Pakistan.

- PMID: **35017332**
- DOI: 10.4103/1319-2442.335450

Abstract

Coronavirus disease-2019 (COVID-19) is a global pandemic, also affecting Pakistan with its first case reported on February 26, 2020. Since then, it has been declared a pandemic by the World Health Organization. Our study aimed to evaluate the renal derangements associated with COVID-19 infection in our population. A retrospective, observational study was conducted to include all the admitted patients having COVID-19 positive, and evaluated those for derangements of renal function (n = 362). Out of the 362 patients, 229were admitted in the ward, 133 were in intensive care unit (ICU), 258 of them recovered, while 104 deaths reported. At admission, the renal profile was deranged in almost one-half of ICU admissions and mortalities which increased to two-third during the hospital stay, with around 80% of deaths reported with increased urea and creatinine levels. Among the deceased patients, around one-third of the mortalities developed renal profile derangements during the hospital stay although they were admitted with a normal renal profile. An estimated glomerular filtration rate showed a mean increase of 13.37 mL/min/1.73 m^2 during the hospital stay of surviving patients, while a decline of 19.92 in nonsurviving patients. A hazard ratio of 3.293 (P <0.001) for admitting serum urea and 3.795 (P = 0.009) at discharge and for serum creatinine at 5.392 (P <0.001) on discharge was associated significantly with mortality. Kaplan-Meier plot showed a significant decline in days of survival with deranged urea and creatinine (P <0.001). The deranged renal function in COVID-19 patients is associated with an increased number of ICU admissions as well as mortalities.

Supplementary info

Publication types, MeSH terms, Substances Expand

Publication types

- Observational Study

MeSH terms

- Acute Kidney Injury / etiology*
- Acute Kidney Injury / mortality
- Acute Kidney Injury / therapy
- COVID-19 / complications*
- COVID-19 / mortality
- COVID-19 Nucleic Acid Testing
- Creatinine / blood
- Glomerular Filtration Rate
- Hospital Mortality*
- Humans
- Incidence
- Intensive Care Units / statistics & numerical data*
- Kidney Function Tests
- Pakistan / epidemiology
- Renal Dialysis
- Retrospective Studies
- SARS-CoV-2* / genetics
- SARS-CoV-2* / isolation & purification
- Tertiary Healthcare
- Urea / blood

Substances

- Urea
- Creatinine

Full text links

Medknow Publications and Media Pvt Ltd

Proceed to details

Cite

Share

☐ 380

Observational Study

Rev Esp Cardiol (Engl Ed)

. 2021 Feb;74(2):175-182.
doi: 10.1016/j.rec.2020.05.018. Epub 2020 Jun 5.

Impact of angiotensin-converting enzyme inhibitors and angiotensin receptor blockers on COVID-19 in a western population. CARDIOVID registry

[Article in English, Spanish]

Diego López-Otero [1], Javier López-Pais [2], Carla Eugenia Cacho-Antonio [3], Pablo José Antúnez-Muiños [3], Teba González-Ferrero [3], Marta Pérez-Poza [3], Óscar Otero-García [3], Brais Díaz-Fernández [2], María Bastos-Fernández [2], Noelia Bouzas-Cruz [4], Xoan Carlos Sanmartín-Pena [2], Alfonso Varela-Román [2], Manuel Portela-Romero [5], Luis Valdés-Cuadrado [6], Antonio Pose-Reino [7], José Ramón González-Juanatey [2]
Affiliations Expand

Affiliations

- [1] Servicio de Cardiología, Hospital Clínico Universitario de Santiago de Compostela, Santiago de Compostela, A Coruña, Spain; Centro de Investigación Biomédica en Red Enfermedades Cardiovasculares, CIBERCV, Madrid, Spain; Instituto de Investigación Sanitaria Santiago de Compostela (IDICHUS), Santiago de Compostela, Spain. Electronic address: birihh@yahoo.es.
- [2] Servicio de Cardiología, Hospital Clínico Universitario de Santiago de Compostela, Santiago de Compostela, A Coruña, Spain; Centro de Investigación Biomédica en Red Enfermedades Cardiovasculares, CIBERCV, Madrid, Spain; Instituto de Investigación Sanitaria Santiago de Compostela (IDICHUS), Santiago de Compostela, Spain.
- [3] Servicio de Cardiología, Hospital Clínico Universitario de Santiago de Compostela, Santiago de Compostela, A Coruña, Spain.
- [4] Servicio de Cardiología, Hospital Clínico Universitario de Santiago de Compostela, Santiago de Compostela, A Coruña, Spain; Instituto de Investigación Sanitaria Santiago de Compostela (IDICHUS), Santiago de Compostela, Spain.
- [5] Centro de Salud Concepción Arenal, Santiago de Compostela, A Coruña, Spain.
- [6] Servicio de Neumología, Hospital Clínico Universitario de Santiago de Compostela, Santiago de Compostela, A Coruña, Spain.
- [7] Servicio de Medicina Interna, Hospital Clínico Universitario de Santiago de Compostela, Santiago de Compostela, A Coruña, Spain.

- PMID: **32600991**
- PMCID: PMC7274611
- DOI: 10.1016/j.rec.2020.05.018

Free PMC article
Observational Study

Impact of angiotensin-converting enzyme inhibitors and angiotensin receptor blockers

on COVID-19 in a western population. CARDIOVID registry

[Article in English, Spanish]
Diego López-Otero et al. Rev Esp Cardiol (Engl Ed). 2021 Feb.
Free PMC article
Show details
Rev Esp Cardiol (Engl Ed)
. 2021 Feb;74(2):175-182.
doi: 10.1016/j.rec.2020.05.018. Epub 2020 Jun 5.

Authors

Diego López-Otero [1], Javier López-Pais [2], Carla Eugenia Cacho-Antonio [3], Pablo José Antúnez-Muiños [3], Teba González-Ferrero [3], Marta Pérez-Poza [3], Óscar Otero-García [3], Brais Díaz-Fernández [2], María Bastos-Fernández [2], Noelia Bouzas-Cruz [4], Xoan Carlos Sanmartín-Pena [2], Alfonso Varela-Román [2], Manuel Portela-Romero [5], Luis Valdés-Cuadrado [6], Antonio Pose-Reino [7], José Ramón González-Juanatey [2]

Affiliations

- [1] Servicio de Cardiología, Hospital Clínico Universitario de Santiago de Compostela, Santiago de Compostela, A Coruña, Spain; Centro de Investigación Biomédica en Red Enfermedades Cardiovasculares, CIBERCV, Madrid, Spain; Instituto de Investigación Sanitaria Santiago de Compostela (IDICHUS), Santiago de Compostela, Spain. Electronic address: birihh@yahoo.es.
- [2] Servicio de Cardiología, Hospital Clínico Universitario de Santiago de Compostela, Santiago de Compostela, A Coruña, Spain; Centro de Investigación Biomédica en Red Enfermedades Cardiovasculares, CIBERCV, Madrid, Spain; Instituto de Investigación Sanitaria Santiago de Compostela (IDICHUS), Santiago de Compostela, Spain.
- [3] Servicio de Cardiología, Hospital Clínico Universitario de Santiago de Compostela, Santiago de Compostela, A Coruña, Spain.
- [4] Servicio de Cardiología, Hospital Clínico Universitario de Santiago de Compostela, Santiago de Compostela, A Coruña, Spain; Instituto de Investigación Sanitaria Santiago de Compostela (IDICHUS), Santiago de Compostela, Spain.
- [5] Centro de Salud Concepción Arenal, Santiago de Compostela, A Coruña, Spain.
- [6] Servicio de Neumología, Hospital Clínico Universitario de Santiago de Compostela, Santiago de Compostela, A Coruña, Spain.
- [7] Servicio de Medicina Interna, Hospital Clínico Universitario de Santiago de Compostela, Santiago de Compostela, A Coruña, Spain.

- PMID: **32600991**
- PMCID: PMC7274611
- DOI: 10.1016/j.rec.2020.05.018

Abstract
in English, Spanish

Introduction and objectives: Coronavirus disease (COVID-19) has been designated a global pandemic by the World Health Organization. It is unclear whether previous treatment with angiotensin-converting enzyme inhibitors (ACEI) and angiotensin receptor blockers (ARB) affects the prognosis of COVID-19 patients. The aim of this study was to evaluate the clinical implications of previous treatment with ACEI/ARB on the prognosis of patients with COVID-19 infection.

Methods: Single-center, retrospective, observational cohort study based on all the inhabitants of our health area. Analyses of main outcomes (mortality, heart failure, hospitalization, intensive care unit [ICU] admission, and major acute cardiovascular events [a composite of mortality and heart failure]) were adjusted by multivariate logistic regression and propensity score matching models.

Results: Of the total population, 447 979 inhabitants, 965 patients (0.22%) were diagnosed with COVID-19 infection, and 210 (21.8%) were under ACEI or ARB treatment at the time of diagnosis. Treatment with ACEI/ARB (combined and individually) had no effect on mortality (OR, 0.62; 95%CI, 0.17-2.26; P=.486), heart failure (OR, 1.37; 95%CI, 0.39-4.77; P=.622), hospitalization rate (OR, 0.85; 95%CI, 0.45-1.64; P=.638), ICU admission (OR, 0.87; 95%CI, 0.30-2.50; P=.798), or major acute cardiovascular events (OR, 1.06; 95%CI, 0.39-2.83; P=.915). This neutral effect remained in a subgroup analysis of patients requiring hospitalization.

Conclusions: Previous treatment with ACEI/ARB in patients with COVID-19 had no effect on mortality, heart failure, requirement for hospitalization, or ICU admission. Withdrawal of ACEI/ARB in patients testing positive for COVID-19 would not be justified, in line with current recommendations of scientific societies and government agencies.

Introducción y objetivos: La Organización Mundial de la Salud calificó la enfermedad de coronavirus (COVID-19) como una pandemia global. No está claro si el tratamiento previo con inhibidores de la enzima de conversión de la angiotensina (IECA) y antagonistas de los receptores de angiotensina (ARA-II) tiene un impacto en el pronóstico de los pacientes infectados con COVID-19. El objetivo fue evaluar la implicación clínica del tratamiento previo con IECA/ARA-II en el pronóstico de la COVID-19.

Métodos: Estudio observacional, retrospectivo, unicéntrico, de cohortes basado en todos los habitantes del área de salud. El análisis de los resultados principales (mortalidad, insuficiencia cardiaca, hospitalización, ingreso en la unidad de cuidados intensivos (UCI) y eventos cardiovasculares agudos mayores [un compuesto de mortalidad e insuficiencia cardiaca]), se ajustó mediante modelos de regresión logística multivariada y modelos de coincidencia de puntaje de propensión.

Resultados: De una población total, 447.979 habitantes, 965 pacientes (0,22%), fueron diagnosticados de infección por COVID-19, 210 (21,8%) estaban bajo tratamiento con IECA o ARA-II en el momento del diagnóstico. El tratamiento con IECA/ARA-II (combinado e individualmente) no tuvo efecto sobre la mortalidad (OR = 0,62; IC95%, 0,17-2,26; p = 0,486), insuficiencia cardiaca (OR = 1,37; IC95%, 0,39-4,77; p = 0,622), tasa de hospitalización (OR = 0,85; IC95%, 0,45-1,64; p = 0,638), ingreso en UCI (OR = 0,87; IC95%, 0,30-2,50; p = 0,798) y cardiopatía aguda grave eventos (OR = 1,06; IC95%, 0,39-2,83; p = 0,915). En el análisis del subgrupos de pacientes que requirieron hospitalización, el efecto se mantuvo neutral.

Conclusiones: El tratamiento previo con IECA/ARA-II en pacientes con COVID-19 no tuvo efecto sobre la mortalidad, la aparición de insuficiencia cardíaca, ni en la necesidad de

hospitalización ni ingreso en UCI. La supresión de IECA/ARA-II en pacientes con COVID-19 no estaría justificada en ningún caso, de acuerdo a las recomendaciones actuales de las sociedades científicas y las agencias gubernamentales.

Keywords: Angiotensin receptor blockers; Angiotensin-converting enzyme inhibitors; Antagonistas del receptor de la angiotensina II; COVID-19; Inhibidores de la enzima de conversión de la angiotensina.

Copyright © 2020 Sociedad Española de Cardiología. Published by Elsevier España, S.L.U. All rights reserved.

- Cited by 13 articles
- 28 references
- 3 figures

Supplementary info

Publication types, MeSH terms, Substances, Supplementary concepts Expand

Publication types

- Observational Study

MeSH terms

- Adolescent
- Adult
- Aged
- Angiotensin Receptor Antagonists / therapeutic use*
- Angiotensin-Converting Enzyme Inhibitors / therapeutic use*
- COVID-19 / diagnosis
- COVID-19 / drug therapy*
- COVID-19 / mortality
- Child
- Child, Preschool
- Female
- Heart Failure / epidemiology
- Hospitalization / statistics & numerical data
- Humans
- Infant
- Infant, Newborn
- Male
- Middle Aged
- Prognosis
- Registries

- Retrospective Studies
- Spain
- Young Adult

Substances

- Angiotensin Receptor Antagonists
- Angiotensin-Converting Enzyme Inhibitors

Supplementary concepts

- COVID-19 drug treatment

Full text links

Ediciones Doyma, S.L. Free PMC article
Proceed to details
Cite
Share
381
Observational Study
Eur Arch Otorhinolaryngol
. 2021 Jun;278(6):2107-2114.
doi: 10.1007/s00405-020-06597-1. Epub 2021 Jan 9.

Open versus percutaneous tracheostomy in COVID-19: a multicentre comparison and recommendation for future resource utilisation

Aleix Rovira[#, 1], Stephen Tricklebank[2], Pavol Surda[3], Stephen Whebell[2], Joe Zhang[2], Arun Takhar[3], Elizabeth Yeung[4], Kathleen Fan[4], Imran Ahmed[5], Phillip Hopkins[6], Deborah Dawson[7], Jonathan Ball[7], Ram Kumar[8], Waqas Khaliq[9], Ricard Simo[3], Asit Arora[3]
Affiliations Expand

Affiliations

- [1] Department of Otolaryngology and Head and Neck Surgery, Guy's and St Thomas' NHS Foundation Trust, Great Maze Pond, London, SE1 9RT, UK. aleix.rovira@gstt.nhs.uk.
- [2] Department of Critical Care, Guy's and St Thomas' NHS Foundation Trust, London, UK.
- [3] Department of Otolaryngology and Head and Neck Surgery, Guy's and St Thomas' NHS Foundation Trust, Great Maze Pond, London, SE1 9RT, UK.

- [4] Department of Oral and Maxillofacial Surgery, Kings College Hospital NHS Foundation Trust, London, UK.
- [5] Department of Anaesthesia, Guy's and St Thomas' NHS Foundation Trust, London, UK.
- [6] Department of Critical Care, Kings College Hospital NHS Foundation Trust, London, UK.
- [7] Department of Critical Care, St George's University Hospitals NHS Foundation Trust, London, UK.
- [8] Department of Critical Care, Kingston Hospital NHS Foundation Trust, London, UK.
- [9] Department of Critical Care, Lewisham and Greenwich NHS Trust, London, UK.

[#] Contributed equally.

- PMID: **33420842**
- PMCID: PMC7796696
- DOI: 10.1007/s00405-020-06597-1

Free PMC article
Observational Study

Open versus percutaneous tracheostomy in COVID-19: a multicentre comparison and recommendation for future resource utilisation

Aleix Rovira et al. Eur Arch Otorhinolaryngol. 2021 Jun.
Free PMC article
Show details
Eur Arch Otorhinolaryngol
. 2021 Jun;278(6):2107-2114.
doi: 10.1007/s00405-020-06597-1. Epub 2021 Jan 9.

Authors

Aleix Rovira [#] [1], Stephen Tricklebank [2], Pavol Surda [3], Stephen Whebell [2], Joe Zhang [2], Arun Takhar [3], Elizabeth Yeung [4], Kathleen Fan [4], Imran Ahmed [5], Phillip Hopkins [6], Deborah Dawson [7], Jonathan Ball [7], Ram Kumar [8], Waqas Khaliq [9], Ricard Simo [3], Asit Arora [3]

Affiliations

- [1] Department of Otolaryngology and Head and Neck Surgery, Guy's and St Thomas' NHS Foundation Trust, Great Maze Pond, London, SE1 9RT, UK. aleix.rovira@gstt.nhs.uk.
- [2] Department of Critical Care, Guy's and St Thomas' NHS Foundation Trust, London, UK.
- [3] Department of Otolaryngology and Head and Neck Surgery, Guy's and St Thomas' NHS Foundation Trust, Great Maze Pond, London, SE1 9RT, UK.
- [4] Department of Oral and Maxillofacial Surgery, Kings College Hospital NHS Foundation Trust, London, UK.
- [5] Department of Anaesthesia, Guy's and St Thomas' NHS Foundation Trust, London, UK.

- [6] Department of Critical Care, Kings College Hospital NHS Foundation Trust, London, UK.
- [7] Department of Critical Care, St George's University Hospitals NHS Foundation Trust, London, UK.
- [8] Department of Critical Care, Kingston Hospital NHS Foundation Trust, London, UK.
- [9] Department of Critical Care, Lewisham and Greenwich NHS Trust, London, UK.

[#] Contributed equally.

- PMID: **33420842**
- PMCID: PMC7796696
- DOI: 10.1007/s00405-020-06597-1

Abstract

Purpose: The COVID-19 pandemic placed an unprecedented demand on critical care services for the provision of mechanical ventilation. Tracheostomy formation facilitates liberation from mechanical ventilation with advantages for both the patient and wider critical care resource, and can be performed using both percutaneous dilatational and surgical techniques. We compared outcomes in those patients undergoing percutaneous dilatational tracheostomy to those undergoing surgical tracheostomy and make recommendations for provision of tracheostomy services in any future surge.

Methods: Multicentre multidisciplinary retrospective observational cohort study including 201 patients with COVID-19 pneumonitis admitted to an ICU in one of five NHS Trusts within the South London Adult Critical Care Network who required mechanical ventilation and subsequent tracheostomy.

Results: Percutaneous dilatational tracheostomy was performed in 124 (62%) of patients, and surgical tracheostomy in 77 (38%) of patients. There was no difference between percutaneous dilatational tracheostomy and surgical tracheostomy in either the rate of peri-operative complications (16.9 vs. 22.1%, p = 0.46), median [IQR(range)] time to decannulation [19.0 (15.0-30.2 (5.0-65.0)] vs. 21.0 [15.5-36.0 (5.0-70.0) days] or mortality (13.7% vs. 15.6%, p = 0.84). Of the 172 patients that were alive at follow-up, two remained ventilated and 163 were decannulated.

Conclusion: In patients with COVID-19 pneumonitis that require tracheostomy to facilitate weaning from mechanical ventilation, there was no difference in outcomes between those patients that had percutaneous dilatational tracheostomy compared with those that had surgical tracheostomy. Planning for future surges in COVID-19-related critical care demands should utilise all available resource and expertise.

Keywords: Covid-19; Mechanical ventilation; Percutaneous tracheostomy; Surgical tracheostomy.

Conflict of interest statement

The authors do not have any conflict of interest.

Comment in

- Response to comments to "Open versus percutaneous tracheostomy in COVID-19: a multicentre comparison and recommendationfor future resource utilisation".

Rovira A, Tricklebank S, Surda P, Whebell S, Zhang J, Takhar A, Yeung E, Fan K, Ahmed I, Hopkins P, Dawson D, Ball J, Kumar R, Khaliq W, Simo R, Arora A. Rovira A, et al. Eur Arch Otorhinolaryngol. 2021 Jun;278(6):2165-2166. doi: 10.1007/s00405-021-06775-9. Epub 2021 Apr 8. Eur Arch Otorhinolaryngol. 2021. PMID: 33830366 Free PMC article. No abstract available.

- Cited by 9 articles
- 22 references
- 2 figures

Supplementary info

Publication types, MeSH terms Expand

Publication types

- Multicenter Study
- Observational Study

MeSH terms

- Adult
- COVID-19*
- Humans
- London
- Pandemics
- Respiration, Artificial
- Retrospective Studies
- SARS-CoV-2
- Tracheostomy*

Full text links

SpringerLink FULL-TEXT ARTICLE Springer Free PMC article
Proceed to details
Cite
Share
☐ 382
Observational Study
J Med Virol
. 2021 Apr;93(4):2243-2251.
doi: 10.1002/jmv.26656. Epub 2020 Nov 22.

[Non-severe immunosuppression might be associated with a lower risk of moderate-severe acute respiratory distress syndrome in COVID-19: A pilot study]

Enric Monreal [1], Sainz de la Maza S [1], Pedro Gullón [2], Elena Natera-Villalba [1], Juan Luis Chico-García [1], Álvaro Beltrán-Corbellini [1], Javier Martínez-Sanz [3], Nuria García-Barragán [1], Javier Buisán [1], Rafael Toledano [1], Araceli Alonso-Canovas [1], Paula Pérez-Torre [1], María C Matute-Lozano [1], Jose Luis López-Sendón [1], Guillermo García-Ribas [1], Íñigo Corral [1], Jesús Fortún [3], Beatriz Montero-Errasquín [4], Luis Manzano [5], Luis Máiz-Carro [6], Lucienne Costa-Frossard [1], Jaime Masjuan [1], COVID-HRC Group
Affiliations Expand

Affiliations

- [1] Department of Neurology, Hospital Universitario Ramón y Cajal, Universidad de Alcalá, IRYCIS, Madrid, Spain.
- [2] Public Health and Epidemiology Research Group, Universidad de Alcalá, Madrid, Spain.
- [3] Department of Infectious Diseases, Hospital Universitario Ramón y Cajal, Universidad de Alcalá, IRYCIS, Madrid, Spain.
- [4] Department of Geriatrics, Hospital Universitario Ramón y Cajal, Universidad de Alcalá, IRYCIS, Madrid, Spain.
- [5] Department of Internal Medicine, Hospital Universitario Ramón y Cajal, Universidad de Alcalá, IRYCIS, Madrid, Spain.
- [6] Department of Neumology, Hospital Universitario Ramón y Cajal, Universidad de Alcalá, IRYCIS, Madrid, Spain.

- PMID: **33165922**
- DOI: [10.1002/jmv.26656]

Observational Study

[Non-severe immunosuppression might be associated with a lower risk of moderate-severe acute respiratory distress syndrome in COVID-19: A pilot study]

Enric Monreal et al. J Med Virol. 2021 Apr.
Show details
J Med Virol
. 2021 Apr;93(4):2243-2251.
doi: 10.1002/jmv.26656. Epub 2020 Nov 22.

Authors

Enric Monreal [1], Sainz de la Maza S [1], Pedro Gullón [2], Elena Natera-Villalba [1], Juan Luis Chico-García [1], Álvaro Beltrán-Corbellini [1], Javier Martínez-Sanz [3], Nuria García-Barragán [1], Javier Buisán [1], Rafael Toledano [1], Araceli Alonso-Canovas [1], Paula Pérez-Torre [1], María C Matute-Lozano [1], Jose Luis López-Sendón [1], Guillermo García-Ribas [1], Íñigo Corral [1], Jesús Fortún [3], Beatriz Montero-Errasquín [4], Luis Manzano [5], Luis Máiz-Carro [6], Lucienne Costa-Frossard [1], Jaime Masjuan [1], COVID-HRC Group

Affiliations

- [1] Department of Neurology, Hospital Universitario Ramón y Cajal, Universidad de Alcalá, IRYCIS, Madrid, Spain.
- [2] Public Health and Epidemiology Research Group, Universidad de Alcalá, Madrid, Spain.
- [3] Department of Infectious Diseases, Hospital Universitario Ramón y Cajal, Universidad de Alcalá, IRYCIS, Madrid, Spain.
- [4] Department of Geriatrics, Hospital Universitario Ramón y Cajal, Universidad de Alcalá, IRYCIS, Madrid, Spain.
- [5] Department of Internal Medicine, Hospital Universitario Ramón y Cajal, Universidad de Alcalá, IRYCIS, Madrid, Spain.
- [6] Department of Neumology, Hospital Universitario Ramón y Cajal, Universidad de Alcalá, IRYCIS, Madrid, Spain.

- PMID: **33165922**
- DOI: 10.1002/jmv.26656

Abstract

The role of immunosuppression among coronavirus disease 2019 (COVID-19) patients has not been elucidated and management may be challenging. This observational study included confirmed COVID-19 patients. The primary endpoint was the development of moderate-severe acute respiratory distress syndrome (ARDS). Time to moderate-severe ARDS, the need for mechanical or noninvasive ventilation (MV/NIV), death, and a composite of death or MV/NIV were secondary endpoints. Of 138 patients included, 27 (19.6%) were immunosuppressed (IS) and 95 (68.8%) were male, with a median (IQR) age of 68 (54-78) years. A significantly lower proportion of IS patients (25.9%) compared to non-IS patients (52.3%) developed moderate-severe ARDS, in both unadjusted (0.32; 95% CI, 0.13-0.83; p = .017) and adjusted (aOR, 0.25; 95% CI, 0.08-0.80; p = .019) analyses. After stratifying by pathologies, only IS patients with autoimmune diseases remained significant (aOR 0.25; 95% CI, 0.07-0.98; p = .046). Nonsignificant trends toward a longer time to moderate or severe ARDS, a lower need for MV/NIV, and a lower risk of death or MV/NIV were detected among IS. In our cohort of COVID-19 patients, nonsevere immunosuppression was associated with a lower risk of moderate-severe ARDS, especially among AD. This suggests a potential protective effect from a hypothesized hyper-inflammatory response.

Keywords: COVID-19; acute respiratory distress syndrome; autoimmune diseases; immunosuppression; severe acute respiratory syndrome coronavirus 2.

© 2020 Wiley Periodicals LLC.

- Cited by 4 articles
- 35 references

Supplementary info

Publication types, MeSH terms Expand

Publication types

- Observational Study

MeSH terms

- Aged
- Aged, 80 and over
- COVID-19 / epidemiology
- COVID-19 / immunology*
- COVID-19 / virology
- Cohort Studies
- Coinfection
- Female
- Hospitalization
- Humans
- Immunosuppression Therapy
- Male
- Middle Aged
- Pilot Projects
- Respiratory Distress Syndrome / epidemiology
- Respiratory Distress Syndrome / immunology*
- Respiratory Distress Syndrome / virology
- Retrospective Studies
- SARS-CoV-2 / immunology
- Severity of Illness Index
- Spain / epidemiology

Full text links

WILEY Full Text Article Wiley
Proceed to details
Cite
Share
☐ 383
Observational Study

Biomarkers
. 2021 Jul;26(5):401-409.
doi: 10.1080/1354750X.2021.1921031. Epub 2021 May 24.

Interpretation of myocardial injury subtypes in COVID-19 disease per fourth version of Universal Definition of Myocardial Infarction

Christian Salbach [1], Matthias Mueller-Hennessen [1], Moritz Biener [1], Kiril Stoyanov [1], Michael Preusch [1], Lars Kihm [2], Uta Merle [3], Paul Schnitzler [4], Hugo A Katus [1], Evangelos Giannitsis [1]
Affiliations Expand

Affiliations

- [1] Department of Internal Medicine III, Cardiology, University of Heidelberg, Heidelberg, Germany.
- [2] Department Internal Medicine I, Endocrinology, University of Heidelberg, Heidelberg, Germany.
- [3] Department of Internal Medicine IV, Gastroenterology, University of Heidelberg, Heidelberg, Germany.
- [4] Department for Infectious Diseases, Virology, University of Heidelberg, Heidelberg, Germany.

- PMID: **33998352**
- DOI: 10.1080/1354750X.2021.1921031

Observational Study

Interpretation of myocardial injury subtypes in COVID-19 disease per fourth version of Universal Definition of Myocardial Infarction

Christian Salbach et al. Biomarkers. 2021 Jul.
Show details
Biomarkers
. 2021 Jul;26(5):401-409.
doi: 10.1080/1354750X.2021.1921031. Epub 2021 May 24.

Authors

Christian Salbach [1], Matthias Mueller-Hennessen [1], Moritz Biener [1], Kiril Stoyanov [1], Michael Preusch [1], Lars Kihm [2], Uta Merle [3], Paul Schnitzler [4], Hugo A Katus [1], Evangelos Giannitsis [1]

Affiliations

- [1] Department of Internal Medicine III, Cardiology, University of Heidelberg, Heidelberg, Germany.
- [2] Department Internal Medicine I, Endocrinology, University of Heidelberg, Heidelberg, Germany.
- [3] Department of Internal Medicine IV, Gastroenterology, University of Heidelberg, Heidelberg, Germany.
- [4] Department for Infectious Diseases, Virology, University of Heidelberg, Heidelberg, Germany.

- PMID: **33998352**
- DOI: 10.1080/1354750X.2021.1921031

Abstract

Background: Application of the 4th version of Universal Definition of Myocardial Infarction (UDMI) to characterize rates and prognostic relevance of myocardial injury in COVID-19 disease.

Methods: This retrospective, single-centre observational study enrolled 104 patients hospitalized with SARS-CoV-2 infection. Kaplan-Meier analysis and multivariate Cox regression were used to identify influence of acute or chronic myocardial injury on a composite primary (mortality, incident acute respiratory distress syndrome, incident mechanical ventilation) and secondary endpoint (mortality, incident acute myocardial injury during hospitalization, incident venous thrombosis, pulmonary embolism or stroke).

Results: A total of 27 (26.0%) patients presented with chronic myocardial injury, and 19 (18.3%) with acute myocardial injury. 42 patients(40.4%) developed an incident myocardial injury during hospitalization. The presence of acute or chronic myocardial injury on admission and incident myocardial injury during hospitalization were associated with higher rates of endpoints. Independent predictors for the primary endpoint were higher severity stages according to Siddiqi *et al.* classification system and history of dyslipidaemia. Maximal hs-cTnT and D-dimer concentrations during hospitalization showed an association ($r = 0.61$).

Conclusions: Objective description of myocardial injury according to the 4th UDMI in the current COVID-19 pandemic is crucial in order to discriminate patients with acute myocardial infarction and acute, chronic or incident myocardial injury.

Keywords: COVID-19; Myocardial injury; SARS-CoV-2; Universal Definition of Myocardial Infarction; myocardial infarction.

- Cited by 1 article

Supplementary info

Publication types, MeSH terms, Substances Expand

Publication types

- Observational Study

MeSH terms

- Adult
- Aged
- Aged, 80 and over
- COVID-19 / epidemiology
- COVID-19 / prevention & control*
- COVID-19 / virology
- Female
- Fibrin Fibrinogen Degradation Products / analysis
- Germany / epidemiology
- Heart Injuries / diagnosis*
- Heart Injuries / epidemiology
- Hospitalization / statistics & numerical data
- Humans
- Kaplan-Meier Estimate
- Male
- Middle Aged
- Myocardial Infarction / diagnosis*
- Myocardial Infarction / epidemiology
- Pandemics
- Prevalence
- Prognosis
- Retrospective Studies
- SARS-CoV-2 / isolation & purification*
- SARS-CoV-2 / physiology
- Troponin T / analysis

Substances

- Fibrin Fibrinogen Degradation Products
- Troponin T
- fibrin fragment D

Full text links

View full text Taylor & Francis
Proceed to details
Cite
Share
☐ 384
Observational Study

Virol Sin
. 2020 Dec;35(6):768-775.
doi: 10.1007/s12250-020-00281-8. Epub 2020 Aug 31.

Patients with Prolonged Positivity of SARS-CoV-2 RNA Benefit from Convalescent Plasma Therapy: A Retrospective Study

Yongran Wu[# 1], Ke Hong[# 2], Lianguo Ruan[# 2], Xiaobo Yang[1], Jiancheng Zhang[1], Jiqian Xu[1], Shangwen Pan[1], Lehao Ren[1], Lu Chen[2], Chaolin Huang[3], You Shang[4]
Affiliations Expand

Affiliations

- [1] Department of Critical Care Medicine, Union Hospital, Tongji Medical College, Huazhong University of Science and Technology, Wuhan, 430022, China.
- [2] Research Center for Translational Medicine, Jinyintan Hospital, Wuhan, 430022, China.
- [3] Research Center for Translational Medicine, Jinyintan Hospital, Wuhan, 430022, China. chaolin2020@163.com.
- [4] Department of Critical Care Medicine, Union Hospital, Tongji Medical College, Huazhong University of Science and Technology, Wuhan, 430022, China. you_shanghust@163.com.

[#] Contributed equally.

- PMID: **32865701**
- PMCID: PMC7457444
- DOI: 10.1007/s12250-020-00281-8

Free PMC article
Observational Study

Patients with Prolonged Positivity of SARS-CoV-2 RNA Benefit from Convalescent Plasma Therapy: A Retrospective Study

Yongran Wu et al. Virol Sin. 2020 Dec.
Free PMC article
Show details
Virol Sin
. 2020 Dec;35(6):768-775.
doi: 10.1007/s12250-020-00281-8. Epub 2020 Aug 31.

Authors

Yongran Wu [#] [1], Ke Hong [#] [2], Lianguo Ruan [#] [2], Xiaobo Yang [1], Jiancheng Zhang [1], Jiqian Xu [1], Shangwen Pan [1], Lehao Ren [1], Lu Chen [2], Chaolin Huang [3], You Shang [4]

Affiliations

- [1] Department of Critical Care Medicine, Union Hospital, Tongji Medical College, Huazhong University of Science and Technology, Wuhan, 430022, China.
- [2] Research Center for Translational Medicine, Jinyintan Hospital, Wuhan, 430022, China.
- [3] Research Center for Translational Medicine, Jinyintan Hospital, Wuhan, 430022, China. chaolin2020@163.com.
- [4] Department of Critical Care Medicine, Union Hospital, Tongji Medical College, Huazhong University of Science and Technology, Wuhan, 430022, China. you_shanghust@163.com.

[#] Contributed equally.

- PMID: **32865701**
- PMCID: PMC7457444
- DOI: 10.1007/s12250-020-00281-8

Abstract

Convalescent plasma therapy has been implemented in a few cases of severe coronavirus disease 2019. No report about convalescent plasma therapy in treating patients with prolonged positivity of SARS-CoV-2 RNA has been published. In this study, we conducted a retrospective observational study in 27 patients with prolonged positivity of SARS-CoV-2 RNA, the clinical benefit of convalescent plasma therapy were analyzed. qRT-PCR test of SARS-CoV-2 RNA turned negative (≤ 7 days) in a part of patients (early negative group, n = 15) after therapy, others (late negative group, n = 12) turned negative in more than 7 days. Pulmonary imaging improvement was confirmed in 7 patients in early negative group and 8 in late negative group after CP therapy. Viral load decreased in early negative group compared with late negative group at day 3, 5, 7 after implementing convalescent plasma therapy. Patients in early negative group had a shorter median length of hospital stay. In conclusion, convalescent plasma therapy might help eliminate virus and shorten length of hospital stay in patients with prolonged positivity of SARS-CoV-2 RNA.

Keywords: Convalescent plasma therapy; Coronavirus disease 2019 (COVID-19); Prolonged positivity; SARS-CoV-2.

Conflict of interest statement

The authors declare that they have no conflict of interest.

- Cited by 5 articles
- 37 references
- 2 figures

Supplementary info

Publication types, MeSH terms, Substances, Supplementary concepts Expand

Publication types

- Observational Study

MeSH terms

- Aged
- Antibodies, Viral / blood
- Antibodies, Viral / immunology
- COVID-19 / diagnostic imaging
- COVID-19 / epidemiology
- COVID-19 / immunology
- COVID-19 / therapy*
- China / epidemiology
- Female
- Humans
- Immunization, Passive / methods*
- Length of Stay
- Male
- Middle Aged
- RNA, Viral / blood
- RNA, Viral / immunology*
- Retrospective Studies
- SARS-CoV-2 / genetics
- SARS-CoV-2 / immunology*
- Viral Load

Substances

- Antibodies, Viral
- RNA, Viral

Supplementary concepts

- COVID-19 serotherapy

Full text links

SpringerLink FULL-TEXT ARTICLE Springer Free PMC article
Proceed to details

Cite
Share
385
Observational Study
Medicina (B Aires)
. 2020;80(5):433-438.

[Initial experience in the attention of patients with COVID-19 in a private third-level hospital in Buenos Aires City]

[Article in Spanish]
Laura Pulido [1], Marco A Solís-Aramayo [2], Manuel Ibarrola [1], Marcela Heres [1], Jimena Falco [1], Giselle Tomaszuk [1], Lisandro Churin [1], Patricia Maggio [1], Vanina Martin [1], Marcos Hernandez [1], Sebastián García-Zamora [3], Silvia Quadrelli [1]
Affiliations Expand

Affiliations

- [1] Servicio de Neumonología, Fundación Sanatorio Güemes, Buenos Aires, Argentina.
- [2] Servicio de Neumonología, Fundación Sanatorio Güemes, Buenos Aires, Argentina. E-mail: msolisaramayo@yahoo.com.ar.
- [3] Servicio de Cardiodiagnóstico, Investigaciones Médicas, Buenos Aires, Argentina.

- PMID: **33048785**

Free article
Observational Study

[Initial experience in the attention of patients with COVID-19 in a private third-level hospital in Buenos Aires City]

[Article in Spanish]
Laura Pulido et al. Medicina (B Aires). 2020.
Free article
Show details
Medicina (B Aires)
. 2020;80(5):433-438.

Authors

Laura Pulido [1], Marco A Solís-Aramayo [2], Manuel Ibarrola [1], Marcela Heres [1], Jimena Falco [1], Giselle Tomaszuk [1], Lisandro Churin [1], Patricia Maggio [1], Vanina Martin [1], Marcos Hernandez [1], Sebastián García-Zamora [3], Silvia Quadrelli [1]

Affiliations

- [1] Servicio de Neumonología, Fundación Sanatorio Güemes, Buenos Aires, Argentina.
- [2] Servicio de Neumonología, Fundación Sanatorio Güemes, Buenos Aires, Argentina. E-mail: msolisaramayo@yahoo.com.ar.
- [3] Servicio de Cardiodiagnóstico, Investigaciones Médicas, Buenos Aires, Argentina.

- PMID: **33048785**

Abstract
in English, Spanish

Infection with the SARS coronavirus type 2 (COVID-19) has a variety of presentations, with little data on the evolution of affected patients in Argentina. This is a retrospective and observational study of patients with virological confirmation of coronavirus treated during the months of March to May in a private third-level university hospital in Buenos Aires. O ne hundred and fifty-five adult patients were included, of which 30.3% attended only for a swab; 59.4% were admitted to the hospital and 10.3% were hospitalized at home with daily telephone follow-up. Fifty-four point two percent of participants were women and the median age was 35 years (ICQ 29 to 50). About 59.3% of patients had some risk factor, including age (65 years old or more), underlying chronic disease, were health workers or personnel/residents in a nursing home. The most frequent symptom was fever (75.9%), followed by cough (65.7%), and odyno phagia (48.2%). Globally, 93.5% experienced some symptoms while 17.6% of the participants presented some symptoms but without fever. Chest tomographies were performed to 5 patients. Their chest radiograph was normal or non-diagnostic. Fourteen patients required intensive therapy and 6 of them required mechanical ventilation, 4 of them died. The remaining 2 patients were referred to chronic care centers. No patient with home hospitalization required admission to hospital or died. While this observation is encouraging, it will need to be confirmed with new studies.

La infección por COVID-19 tiene presentaciones variadas, siendo aún escasos los datos de evolución de pacientes afectados en Argentina. Este es un estudio retrospectivo, observacional de pacientes con confirmación virológica de coronavirus atendidos entre marzo y mayo 2020 en un hospital privado universitario de tercer nivel de Buenos Aires. Se incluyeron 155 pacientes adultos de los cuales 47 (30.3%) concurrieron solo para realizarse un hisopado; 92 (59.4%) fueron internados en el hospital y 16 (10.3%) tuvieron internación domiciliaria con seguimiento telefónico diario. El 54.2% fueron mujeres con mediana de edad de 35 años (rango intercuartil [RIC] 29 a 50). El 59.4% (92) tenían algún factor de riesgo, incluyendo edad igual o mayor a 65 años, enfermedad crónica predisponente, eran personal de salud o trabajaban/residían en geriátrico. En los 108 que tuvieron seguimiento, el síntoma más frecuente fue fiebre (75.9%), seguido de tos (65.7%), y odinofagia (48.2%). La odinofagia fue más frecuente en mujeres ($p = 0.035$) y la disnea en hombres ($p = 0.014$). El 93.5% de los participantes (101) experimentaron síntomas, mientras que 17.6% (19) presentó algún síntoma, pero encontrándose afebriles. En 5 participantes a los que se les realizó una tomografía se observó que la radiografía había sido normal o no diagnóstica. Catorce pacientes requirieron terapia intensiva y 6 de ellos necesitaron ventilación mecánica, falleciendo cuatro. Los 2 pacientes restantes fueron derivados a centros de cuidados crónicos. Ningún paciente con internación domiciliaria requirió ser hospitalizado ni falleció. Si bien esta observación resulta alentadora, deberá ser confirmado en nuevos estudios.

Keywords: SARS-CoV-2 infection; coronavirus-19; hospitalization; mortality.

Supplementary info

Publication types, MeSH terms Expand

Publication types

- Observational Study

MeSH terms

- Adult
- Argentina / epidemiology
- Betacoronavirus
- COVID-19
- Coronavirus Infections / epidemiology*
- Female
- Hospitals, Private
- Humans
- Middle Aged
- Pandemics*
- Pneumonia, Viral / epidemiology*
- Retrospective Studies
- SARS-CoV-2

Full text links

Free Full Text medicina Fundacion Revista Medicina (Buenos Aires)
Proceed to details
Cite
Share
☐ 386
Observational Study
Int J Psychiatry Med
. 2021 Jul;56(4):266-277.
doi: 10.1177/0091217420982100. Epub 2020 Dec 15.

The effect of COVID-19 lockdown on the incidence of deliberate self-harm injuries presenting to the emergency room

Nader Henry [1], Sunil Parthiban [1], Azzam Farroha [1]
Affiliations Expand

Affiliation

- [1] University Hospitals Birmingham NHS Foundation Trust, Birmingham, UK.

- PMID: **33322983**
- PMCID: PMC8689101
- DOI: 10.1177/0091217420982100

Free PMC article
Observational Study

The effect of COVID-19 lockdown on the incidence of deliberate self-harm injuries presenting to the emergency room

Nader Henry et al. Int J Psychiatry Med. 2021 Jul.
Free PMC article
Show details
Int J Psychiatry Med
. 2021 Jul;56(4):266-277.
doi: 10.1177/0091217420982100. Epub 2020 Dec 15.

Authors

Nader Henry [1], Sunil Parthiban [1], Azzam Farroha [1]

Affiliation

- [1] University Hospitals Birmingham NHS Foundation Trust, Birmingham, UK.

- PMID: **33322983**
- PMCID: PMC8689101
- DOI: 10.1177/0091217420982100

Abstract

Objective: The World Health Organization declared COVID-19 a pandemic on 11th March 2020. The UK government introduced strict social distancing measures on 23rd March 2020, with the country put into a full lockdown to further halt the spread of the virus.The aims of this article are to ascertain whether there was a rise in the incidence of deliberate self-harm (DSH) presentations to the emergency department at a level one trauma center associated with the introduction of lockdown measures.

Method: An observational study from a level one trauma center was carried out. Retrospective data from 23rd March 2020 to 1st May 2020 was collected and compared to the same time period in 2019. Data was collected from coded electronic patient records.

Results: Total attendances to the Emergency Department (ED) reduced from 2019 to 2020 (5198 and 3059 respectively). There was a significant increase in the total number of self-harm presentations between 2019 and 2020 (103 vs 113, p-value <0.001) as well as paracetamol, NSAID and opiate overdoses, with more cases requiring hospital admission in 2020 vs 2019.

Conclusions: Societal lockdown measures secondary to the COVID-19 pandemic have had a significant effect on the mental health of patients. One way this can be detected is through an increased incidence and severity of deliberate self-harm injuries presenting to the ED. These findings, in conjunction with the available, literature provide valuable implications for community and emergency physicians and psychiatrists for any future wave of disease or pandemic.

Keywords: COVID 19; deliberate self-harm; mental health; trauma.

Conflict of interest statement

Declaration of conflicting interests: The author(s) declared no potential conflicts of interest with respect to the research, authorship, and/or publication of this article.

- Cited by 4 articles
- 39 references

Supplementary info

Publication types, MeSH terms Expand

Publication types

- Observational Study

MeSH terms

- Adult
- COVID-19 / psychology*
- Emergency Service, Hospital
- Hospitalization
- Humans
- Incidence
- Pandemics
- Physical Distancing*
- Quarantine / psychology*
- Quarantine / statistics & numerical data
- Retrospective Studies
- SARS-CoV-2

- Self-Injurious Behavior / epidemiology*
- Self-Injurious Behavior / psychology*
- United Kingdom / epidemiology

Full text links

SAGE journals Atypon Free PMC article
Proceed to details
Cite
Share
387
Observational Study
J Nepal Health Res Counc
. 2021 Sep 6;19(2):396-401.
doi: 10.33314/jnhrc.v19i2.3623.

Characteristics and Outcome of Patients with COVID-19 Undergoing Invasive Mechanical Ventilation for Respiratory Failure in a Tertiary Level Hospital in Nepal

Sachit Sharma [1], Hem Raj Paneru [1], Gentle Sunder Shrestha [1], Pramesh Sunder Shrestha [1], Subhash Prasad Acharya [1]
Affiliations Expand

Affiliation

- [1] Department of Anaesthesiology, Tribhuvan University Teaching Hospital, Maharajgunj, Kathmandu, Nepal.

- PMID: **34601537**
- DOI: 10.33314/jnhrc.v19i2.3623

Observational Study

Characteristics and Outcome of Patients with COVID-19 Undergoing Invasive Mechanical Ventilation for Respiratory Failure in a Tertiary Level Hospital in Nepal

Sachit Sharma et al. J Nepal Health Res Counc. 2021.

Show details

J Nepal Health Res Counc

. 2021 Sep 6;19(2):396-401.
doi: 10.33314/jnhrc.v19i2.3623.

Authors

Sachit Sharma [1], Hem Raj Paneru [1], Gentle Sunder Shrestha [1], Pramesh Sunder Shrestha [1], Subhash Prasad Acharya [1]

Affiliation

- [1] Department of Anaesthesiology, Tribhuvan University Teaching Hospital, Maharajgunj, Kathmandu, Nepal.

- PMID: **34601537**
- DOI: 10.33314/jnhrc.v19i2.3623

Abstract

Background: Corona virus disease 2019 has become a global health issue. The goal of this study was to investigate the characteristics and outcomes of patients with corona virus disease 2019 undergoing invasive mechanical ventilation and identify factors associated with mortality.

Methods: Ninety four consecutive critically ill patients with confirmed corona virus disease 2019 undergoing invasive mechanical ventilation were included in this retrospective, single-center, observational study. The outcome variable was mortality of patients undergoing invasive mechanical ventilation and factors associated with it during intensive care unit stay.

Results: Seventy nine (84%) out of 94 patients with confirmed corona virus disease 2019 who underwent invasive mechanical ventilation didn't survive. Ninety four percent of patients who had Type 2 Diabetes Mellitus did not survive in comparison to 72 percent of patients who didn't have Type 2 Diabetes Mellitus. Similarly, 48 (94.1%) out of 51 patients with a positive C-reactive protein value didn't survive in comparison to 31 (72%) out of 43 patients with a negative C-reactive protein.

Conclusions: The presence of Type 2 Diabetes Mellitus and a positive C-reactive protein value were strongly associated with mortality. Patients with a Sequential organ failure assessment score of more than eight at intensive care unit admission and peak D-dimer level of more than or equal to two during intensive care unit stay didn't show significant association with mortality. These findings need further exploration through larger prospective studies.

Keywords: COVID-19; critically ill patients; invasive mechanical ventilation; Nepal; outcomes..

Supplementary info

Publication types, MeSH terms Expand

Publication types

- Observational Study

MeSH terms

- COVID-19*
- Diabetes Mellitus, Type 2* / therapy
- Humans
- Nepal
- Respiration, Artificial
- Respiratory Insufficiency*
- Retrospective Studies
- SARS-CoV-2
- Tertiary Care Centers

Proceed to details
Cite
Share
☐ 388
Observational Study
Clin Nutr ESPEN
. 2021 Dec;46:206-209.
doi: 10.1016/j.clnesp.2021.10.007. Epub 2021 Oct 20.

Effect of parenteral nutrition in oxygen escalation/de-escalation in SARS-CoV-2 infected patients who are pre-intubation: A multicenter, observational study

Kritika Subramanian [1], Nadia Solomon [2], Robert Faillace [3], Vidya Menon [4], Farbod Raiszadeh [5], Gary Brandeis [6]
Affiliations Expand

Affiliations

- [1] Department of Molecular Imaging and Therapeutics, Weill Cornell Medicine, New York, NY, USA; Department of Microbiology, Immunology and Transplantation, Laboratory of Clinical and Epidemiological Virology, Rega Institute for Medical Research, KU Leuven, Leuven, Belgium.
- [2] Department of Radiology, Yale University, New Haven, CT, USA.
- [3] Department of Medicine, Jacobi Medical Center, Albert Einstein College of Medicine, Bronx, NY, USA.
- [4] Department of Medicine, Lincoln Hospital Center, Weill Cornell Medicine, Bronx, NY, USA.

- [5] Division of Cardiology, Department of Medicine, Harlem Hospital Center, Columbia University, New York, NY, USA.
- [6] Division of Geriatrics, Department of Medicine, Elmhurst Hospital Center, Icahn School of Medicine at Mount Sinai, Elmhurst, NY, USA; Brookdale Department of Geriatrics and Palliative Medicine, Icahn School of Medicine at Mount Sinai, New York, NY, USA. Electronic address: brandeig@nychhc.org.

- PMID: **34857197**
- PMCID: PMC8526114
- DOI: 10.1016/j.clnesp.2021.10.007

Free PMC article
Observational Study

Effect of parenteral nutrition in oxygen escalation/de-escalation in SARS-CoV-2 infected patients who are pre-intubation: A multicenter, observational study

Kritika Subramanian et al. Clin Nutr ESPEN. 2021 Dec.
Free PMC article
Show details
Clin Nutr ESPEN
. 2021 Dec;46:206-209.
doi: 10.1016/j.clnesp.2021.10.007. Epub 2021 Oct 20.

Authors

Kritika Subramanian [1], Nadia Solomon [2], Robert Faillace [3], Vidya Menon [4], Farbod Raiszadeh [5], Gary Brandeis [6]

Affiliations

- [1] Department of Molecular Imaging and Therapeutics, Weill Cornell Medicine, New York, NY, USA; Department of Microbiology, Immunology and Transplantation, Laboratory of Clinical and Epidemiological Virology, Rega Institute for Medical Research, KU Leuven, Leuven, Belgium.
- [2] Department of Radiology, Yale University, New Haven, CT, USA.
- [3] Department of Medicine, Jacobi Medical Center, Albert Einstein College of Medicine, Bronx, NY, USA.
- [4] Department of Medicine, Lincoln Hospital Center, Weill Cornell Medicine, Bronx, NY, USA.
- [5] Division of Cardiology, Department of Medicine, Harlem Hospital Center, Columbia University, New York, NY, USA.
- [6] Division of Geriatrics, Department of Medicine, Elmhurst Hospital Center, Icahn School of Medicine at Mount Sinai, Elmhurst, NY, USA; Brookdale Department of Geriatrics and

Palliative Medicine, Icahn School of Medicine at Mount Sinai, New York, NY, USA. Electronic address: brandeig@nychhc.org.

- PMID: **34857197**
- PMCID: PMC8526114
- DOI: 10.1016/j.clnesp.2021.10.007

Abstract

Background & aims: SARS-CoV-2 infection includes a variety of gastrointestinal manifestations along with the usual viral symptoms of malaise and myalgias. The objective of this study was to determine if intravenous parenteral nutrition (PN) affected the risk of intubation in SARS-CoV-2 patients who were dependent on non-invasive ventilation.

Methods: Retrospective, multicenter case-control study which analyzed oxygen requirements for 1974 adults with SARS-CoV-2, who were admitted to the local public hospital system between March 1 and May 17, 2020. Relevant baseline biomarkers were studied over 5 days. The main outcome was an escalation or de-escalation of oxygen requirements relative to the exposure of PN.

Results: 111 patients received PN while on non-invasive ventilation. Patients who received PN had a significantly lower odds ($p < 0.001$) of oxygen escalation in comparison to their control group counterparts (OR = 0.804, 95% CI 0.720, 0.899) when matched for age, body mass index, Charlson comorbidity index, and gender.

Conclusion: Initiating PN in the setting of non-invasive ventilation of SARS-CoV-2 infected patients was significantly associated with a lower odds of oxygen escalation. PN does not independently exacerbate oxygen requirements in SARS-CoV-2 infected pre-intubated patients.

Keywords: Non-invasive ventilation; Oxygen requirements; Parenteral nutrition; Pre-intubation; SARS-CoV-2.

Copyright © 2021 European Society for Clinical Nutrition and Metabolism. Published by Elsevier Ltd. All rights reserved.

Conflict of interest statement

Declaration of competing interest The authors declare no conflict of interest.

- 20 references

Supplementary info

Publication types, MeSH terms, Substances Expand

Publication types

- Multicenter Study
- Observational Study

MeSH terms

- Adult
- COVID-19*
- Case-Control Studies
- Humans
- Intubation, Intratracheal
- Oxygen
- Parenteral Nutrition
- Retrospective Studies
- SARS-CoV-2*

Substances

- Oxygen

Full text links

ELSEVIER FULL-TEXT ARTICLE Elsevier Science Free PMC article
Proceed to details
Cite
Share
389
Observational Study
CMAJ
. 2021 Oct 25;193(42):E1619-E1625.
doi: 10.1503/cmaj.211248. Epub 2021 Oct 4.

Evaluation of the relative virulence of novel SARS-CoV-2 variants: a retrospective cohort study in Ontario, Canada

David N Fisman [1], Ashleigh R Tuite [2]
Affiliations Expand

Affiliations

- [1] Dalla Lana School of Public Health, University of Toronto, Toronto, Ont. david.fisman@utoronto.ca.
- [2] Dalla Lana School of Public Health, University of Toronto, Toronto, Ont.

- PMID: **34610919**
- PMCID: PMC8562985

- DOI: 10.1503/cmaj.211248

Free PMC article
Observational Study

Evaluation of the relative virulence of novel SARS-CoV-2 variants: a retrospective cohort study in Ontario, Canada

David N Fisman et al. CMAJ. 2021.
Free PMC article
Show details
CMAJ
. 2021 Oct 25;193(42):E1619-E1625.
doi: 10.1503/cmaj.211248. Epub 2021 Oct 4.

Authors

David N Fisman [1], Ashleigh R Tuite [2]

Affiliations

- [1] Dalla Lana School of Public Health, University of Toronto, Toronto, Ont. david.fisman@utoronto.ca.
- [2] Dalla Lana School of Public Health, University of Toronto, Toronto, Ont.

- PMID: **34610919**
- PMCID: PMC8562985
- DOI: 10.1503/cmaj.211248

Abstract

Background: Between February and June 2021, the initial wild-type strains of SARS-CoV-2 were supplanted in Ontario, Canada, by new variants of concern (VOCs), first those with the N501Y mutation (i.e., Alpha/B1.1.17, Beta/B.1.351 and Gamma/P.1 variants) and then the Delta/B.1.617 variant. The increased transmissibility of these VOCs has been documented, but knowledge about their virulence is limited. We used Ontario's COVID-19 case data to evaluate the virulence of these VOCs compared with non-VOC SARS-CoV-2 strains, as measured by risk of hospitalization, intensive care unit (ICU) admission and death.

Methods: We created a retrospective cohort of people in Ontario who tested positive for SARS-CoV-2 and were screened for VOCs, with dates of test report between Feb. 7 and June 27, 2021. We constructed mixed-effect logistic regression models with hospitalization, ICU admission and death as outcome variables. We adjusted models for age, sex, time, vaccination status, comorbidities and pregnancy status. We included health units as random intercepts.

Results: Our cohort included 212 326 people. Compared with non-VOC SARS-CoV-2 strains, the adjusted elevation in risk associated with N501Y-positive variants was 52% (95% confidence

interval [CI] 42%-63%) for hospitalization, 89% (95% CI 67%-117%) for ICU admission and 51% (95% CI 30%-78%) for death. Increased risk with the Delta variant was more pronounced at 108% (95% CI 78%-140%) for hospitalization, 235% (95% CI 160%-331%) for ICU admission and 133% (95% CI 54%-231%) for death.

Interpretation: The increasing virulence of SARS-CoV-2 VOCs will lead to a considerably larger, and more deadly, pandemic than would have occurred in the absence of the emergence of VOCs.

© 2021 CMA Joule Inc. or its licensors.

Conflict of interest statement

Competing interests: David Fisman has served on advisory boards related to influenza and SARS-CoV-2 vaccines for Seqirus, Pfizer, AstraZeneca and Sanofi-Pasteur, and has served as a legal expert on issues related to COVID-19 epidemiology for the Elementary Teachers Federation of Ontario and the Registered Nurses Association of Ontario. He was previously a member of the Ontario COVID-19 Modelling Consensus Table. Ashleigh Tuite serves on the Ontario COVID-19 Modelling Consensus Table.

Comment in

- Countering more virulent SARS-CoV-2 variants will require a smarter pandemic response.
  Patrick K. Patrick K. CMAJ. 2021 Oct 25;193(42):E1633-E1634. doi: 10.1503/cmaj.211656. Epub 2021 Oct 4. CMAJ. 2021. PMID: 34610918 Free PMC article. No abstract available.
- Is it time to reconsider ring vaccination for COVID-19?
  McAlister VC. McAlister VC. CMAJ. 2022 Jan 10;194(1):E19. doi: 10.1503/cmaj.80442. CMAJ. 2022. PMID: 35012951 Free PMC article. No abstract available.

- Cited by 34 articles
- 23 references
- 2 figures

Supplementary info

Publication types, MeSH terms Expand

Publication types

- Observational Study

MeSH terms

- Age Distribution
- COVID-19 / mortality*
- COVID-19 / transmission
- Comorbidity
- Female

- Hospitalization / statistics & numerical data
- Humans
- Intensive Care Units / statistics & numerical data
- Male
- Ontario / epidemiology
- Pandemics
- Pregnancy
- Retrospective Studies
- Risk Assessment
- SARS-CoV-2 / pathogenicity*
- Vaccination Coverage / statistics & numerical data

Full text links

Free full text on cmaj.ca HighWire Free PMC article

Proceed to details

Cite

Share

☐ 390

Observational Study

PLoS One

. 2020 Nov 20;15(11):e0242400.

doi: 10.1371/journal.pone.0242400. eCollection 2020.

C-Reactive protein as a prognostic indicator in hospitalized patients with COVID-19

Milad Sharifpour [1], Srikant Rangaraju [2], Michael Liu [3], Darwish Alabyad [4], Fadi B Nahab [2], Christina M Creel-Bulos [1], Craig S Jabaley [1], Emory COVID-19 Quality & Clinical Research Collaborative

Affiliations Expand

Affiliations

- [1] Department of Anesthesiology and Critical Care, Emory University Hospital, Atlanta, Georgia, United States of America.
- [2] Department of Neurology, Emory University Hospital, Atlanta, Georgia, United States of America.
- [3] Emory University School of Medicine, Atlanta, Georgia, United States of America.
- [4] Morehouse University School of Medicine, Atlanta, Georgia, United States of America.

- PMID: **33216774**
- PMCID: PMC7679150
- DOI: 10.1371/journal.pone.0242400

Free PMC article
Observational Study

C-Reactive protein as a prognostic indicator in hospitalized patients with COVID-19

Milad Sharifpour et al. PLoS One. 2020.
Free PMC article
Show details
PLoS One
. 2020 Nov 20;15(11):e0242400.
doi: 10.1371/journal.pone.0242400. eCollection 2020.

Authors

Milad Sharifpour [1], Srikant Rangaraju [2], Michael Liu [3], Darwish Alabyad [4], Fadi B Nahab [2], Christina M Creel-Bulos [1], Craig S Jabaley [1], Emory COVID-19 Quality & Clinical Research Collaborative

Affiliations

- [1] Department of Anesthesiology and Critical Care, Emory University Hospital, Atlanta, Georgia, United States of America.
- [2] Department of Neurology, Emory University Hospital, Atlanta, Georgia, United States of America.
- [3] Emory University School of Medicine, Atlanta, Georgia, United States of America.
- [4] Morehouse University School of Medicine, Atlanta, Georgia, United States of America.

- PMID: **33216774**
- PMCID: PMC7679150
- DOI: 10.1371/journal.pone.0242400

Abstract

Recent studies have reported that CRP levels are elevated in patients with COVID-19 and may correlate with severity of disease and disease progression. We conducted a retrospective cohort analysis of the medical records of 268 adult patients, who were admitted to one of the six cohorted COVID ICUs across Emory Healthcare System and had at least two CRP values within the first seven days of admission to study the temporal progression of CRP and its association with all-cause in-hospital mortality. The median CRP during hospitalization for the entire cohort was 130 mg/L (IQR 82-191 mg/L), and the median CRP on ICU admission was 169 (IQR 111-234). The hospitalization-wide median CRP was significantly higher amongst the patients who died, compared to those who survived [206 mg/L (157-288 mg/L) vs 114 mg/L (72-160 mg/L), $p<0.001$]. CRP levels increased in a linear fashion during the first week of hospitalization and peaked on day 5. Compared to patients who died, those who survived had lower peak CRP levels and earlier declines. CRP levels were significantly higher in patients who died compared to those who survived ($p<0.001$). Our findings support the utility of daily CRP values in hospitalized

COVID-19 patients and provide early thresholds during hospitalization that may facilitate risk stratification and prognostication.

Conflict of interest statement

No competing interests.

- Cited by 28 articles
- 20 references
- 2 figures

Supplementary info

Publication types, MeSH terms, Substances, Grant support Expand

Publication types

- Observational Study
- Research Support, N.I.H., Extramural
- Research Support, Non-U.S. Gov't

MeSH terms

- Adult
- Aged
- Betacoronavirus
- Biomarkers / analysis
- C-Reactive Protein / analysis*
- COVID-19
- Coronavirus Infections / diagnosis
- Coronavirus Infections / epidemiology*
- Female
- Hospital Mortality*
- Hospitalization
- Humans
- Male
- Middle Aged
- Pandemics
- Pneumonia, Viral / diagnosis
- Pneumonia, Viral / epidemiology*
- Prognosis
- Retrospective Studies
- Risk Factors
- SARS-CoV-2

Substances

- Biomarkers
- C-Reactive Protein

Grant support

- UL1 TR002378/TR/NCATS NIH HHS/United States

Full text links

OPEN ACCESS TO FULL TEXT PLOS ONE Public Library of Science Free PMC article

Proceed to details

Cite

Share

☐ 391

Observational Study

Int J Dermatol
. 2020 Nov;59(11):1425-1426.
doi: 10.1111/ijd.15157. Epub 2020 Sep 4.

COVID-19 and systemic therapies in psoriasis: experience of a tertiary hospital in Madrid

Jon Fulgencio-Barbarin[1], Mario Puerta-Peña[1], Pablo Ortiz-Romero[1], Carmen García-Donoso[1], Raquel Rivera-Diaz[1]

Affiliations Expand

Affiliation

- [1] 12 de Octubre University Hospital, Madrid, Spain.

- PMID: **32886364**
- DOI: 10.1111/ijd.15157

Observational Study

COVID-19 and systemic therapies in psoriasis: experience of a tertiary hospital in Madrid

Jon Fulgencio-Barbarin et al. Int J Dermatol. 2020 Nov.
Show details
Int J Dermatol
. 2020 Nov;59(11):1425-1426.
doi: 10.1111/ijd.15157. Epub 2020 Sep 4.

Authors

Jon Fulgencio-Barbarin [1], Mario Puerta-Peña [1], Pablo Ortiz-Romero [1], Carmen García-Donoso [1], Raquel Rivera-Diaz [1]

Affiliation

- [1] 12 de Octubre University Hospital, Madrid, Spain.

- PMID: **32886364**
- DOI: 10.1111/ijd.15157

No abstract available

- Cited by 3 articles
- 5 references

Supplementary info

Publication types, MeSH terms, Substances Expand

Publication types

- Letter
- Observational Study

MeSH terms

- Adult
- Aged
- COVID-19 / complications*
- Dermatologic Agents / therapeutic use
- Female
- Humans
- Immunosuppressive Agents / therapeutic use
- Male
- Middle Aged
- Psoriasis / complications*
- Psoriasis / drug therapy*
- Retrospective Studies

- SARS-CoV-2
- Spain
- Tertiary Care Centers

Substances

- Dermatologic Agents
- Immunosuppressive Agents

Full text links

WILEY Full Text Article Wiley
Proceed to details
Cite
Share
392
Observational Study
J Prim Care Community Health
. Jan-Dec 2021;12:21501327211017016.
doi: 10.1177/21501327211017016.

Characteristics and Outcomes of COVID-19 Infection from an Urban Ambulatory COVID-19 Clinic-Guidance for Outpatient Clinicians in Triaging Patients

Dweep Barbhaya [1], Stephanie Franco [2], Kejal Gandhi [1], Radhika Arya [1], Rabin Neupane [1], Negar Foroughi [1], Nnenna Oluigbo [1], Dawn Fishbein [1 3], Jennifer Tran [1]
Affiliations Expand

Affiliations

- [1] Medstar Washington Hospital Center, Washington, DC, USA.
- [2] Georgetown University School of Medicine, Washington, DC, USA.
- [3] MedStar Health Research Institute, Washington, DC, USA.

- PMID: **33985374**
- PMCID: PMC8127736
- DOI: 10.1177/21501327211017016

Free PMC article
Observational Study

Characteristics and Outcomes of COVID-19 Infection from an Urban Ambulatory COVID-19 Clinic-Guidance for Outpatient Clinicians in Triaging Patients

Dweep Barbhaya et al. J Prim Care Community Health. Jan-Dec 2021.
Free PMC article
Show details
J Prim Care Community Health
. Jan-Dec 2021;12:21501327211017016.
doi: 10.1177/21501327211017016.

Authors

Dweep Barbhaya [1], Stephanie Franco [2], Kejal Gandhi [1], Radhika Arya [1], Rabin Neupane [1], Negar Foroughi [1], Nnenna Oluigbo [1], Dawn Fishbein [1 3], Jennifer Tran [1]

Affiliations

- [1] Medstar Washington Hospital Center, Washington, DC, USA.
- [2] Georgetown University School of Medicine, Washington, DC, USA.
- [3] MedStar Health Research Institute, Washington, DC, USA.

- PMID: **33985374**
- PMCID: PMC8127736
- DOI: 10.1177/21501327211017016

Abstract

Background: Coronavirus infection (COVID) presents with flu-like symptoms and can cause serious complications. Here, we discuss the presentation and outcomes of COVID in an ambulatory setting along with distribution of positive cases amongst healthcare workers (HCWs).

Method: Patients who visited the COVID clinic between 03/11/2020 and 06/14/2020 were tested based on the CDC guidelines at the time using PCR-detection methods. Medical records were reviewed and captured on a RedCap database. Statistical analysis was performed using both univariate and bivariate analysis using Fischer's exact test with 2-sided *P* values.

Results: Of the 2471 evaluated patients, 846 (34.2%) tested positive for COVID. Mean age of positivity was 43.4 years (SD ± 15.4), 60.1% were female and 49% were Black. 58.7% of people tested had a known exposure, and amongst those with exposure, 57.3% tested positive. Ninety-four patients were hospitalized (11.1%), of which 22 patients (23.4%) required ICU admission and 10 patients died. The overall death rate of patients presenting to clinic was 0.4%, or 1.2% amongst positive patients. Median length of hospital stay was 6 days (range 1-51). Symptoms significantly associated with COVID included: anosmia, fever, change in taste, anorexia, myalgias, cough, chills, and fatigue. Increased risk of COVID occurred with diabetes, whereas individuals with lung disease or malignancy were not associated with increased risk of COVID. Amongst COVID

positive HCWs, the majority were registered nurses (23.4%), most working in general medicine (39.8%) followed by critical care units (14.3%).

Discussion/conclusion: Blacks and females had the highest infection rates. There was a broad range in presentation from those who are very ill and require hospitalization and those who remain ambulatory. The above data could assist health care professionals perform a targeted review of systems and co-morbidities, allowing for appropriate patient triage.

Keywords: COVID-19; ambulatory clinic; healthcare associated infection; high risk population; presenting symptoms.

Conflict of interest statement

Declaration of Conflicting Interests: The author(s) declared the following potential conflicts of interest with respect to the research, authorship, and/or publication of this article: Dr. Dawn Fishbein has served on a Gilead Advisory Board regarding HCV. However, No external funding for this COVID research was received.

- Cited by 2 articles
- 15 references
- 3 figures

Supplementary info

Publication types, MeSH terms, Grant support Expand

Publication types

- Observational Study

MeSH terms

- Adult
- Aged
- Ambulatory Care / statistics & numerical data*
- COVID-19 / diagnosis*
- COVID-19 / epidemiology
- COVID-19 Testing
- Cross Infection
- Female
- Guidelines as Topic*
- Health Personnel / statistics & numerical data*
- Hospitalization
- Humans
- Male
- Middle Aged
- Outpatients / statistics & numerical data*

- Pandemics
- Retrospective Studies
- SARS-CoV-2
- Triage*
- Urban Population

Grant support

- UL1 TR001409/TR/NCATS NIH HHS/United States

Full text links

SAGE journals Open access full text Atypon Free PMC article
Proceed to details
Cite
Share
393
Observational Study
J Anesth
. 2021 Dec;35(6):827-836.
doi: 10.1007/s00540-021-02986-w. Epub 2021 Aug 15.

Peripheral oxygen saturation to inspiratory oxygen fraction ratio-based identification of critically ill coronavirus disease patients for early therapeutic interventions

Masaru Shimizu [1], Satoru Hashimoto [2]
Affiliations Expand

Affiliations

- [1] Department of Anesthesiology, University Hospital, Kyoto Prefectural University of Medicine, 465 Kajiicho, Kamigyo Ward, Kyoto, 602-8566, Japan. masaru@koto.kpu-m.ac.jp.
- [2] Department of Intensive Care, University Hospital, Kyoto Prefectural University of Medicine, 465 Kajiicho, Kamigyo Ward, Kyoto, 602-8566, Japan.

- PMID: **34392404**
- PMCID: PMC8364630
- DOI: 10.1007/s00540-021-02986-w

Free PMC article
Observational Study

Peripheral oxygen saturation to inspiratory oxygen fraction ratio-based identification of critically ill coronavirus disease patients for early therapeutic interventions

Masaru Shimizu et al. J Anesth. 2021 Dec.
Free PMC article
Show details
J Anesth
. 2021 Dec;35(6):827-836.
doi: 10.1007/s00540-021-02986-w. Epub 2021 Aug 15.

Authors

Masaru Shimizu [1], Satoru Hashimoto [2]

Affiliations

- [1] Department of Anesthesiology, University Hospital, Kyoto Prefectural University of Medicine, 465 Kajiicho, Kamigyo Ward, Kyoto, 602-8566, Japan. masaru@koto.kpu-m.ac.jp.
- [2] Department of Intensive Care, University Hospital, Kyoto Prefectural University of Medicine, 465 Kajiicho, Kamigyo Ward, Kyoto, 602-8566, Japan.

- PMID: **34392404**
- PMCID: PMC8364630
- DOI: 10.1007/s00540-021-02986-w

Abstract

Background: Early identification of critically ill coronavirus disease (COVID-19) patients in clinical settings is crucial in reducing the mortality rate. Therefore, this study aimed to determine whether the saturation of peripheral oxygen (SpO_2) to fraction of inspiratory oxygen (FiO_2) ratio (SF ratio) at admission is useful for the early identification of severe COVID-19.

Methods: This single-center, retrospective, observational study conducted at the University Hospital, Kyoto, Japan, included 26 patients diagnosed with COVID-19 between January 24 and May 6, 2020. COVID-19 severity was classified into two groups based on the SF ratio: ≤ 235 (moderate to severe disease: low group) and > 235 (normal to mild disease: high group). The characteristics, laboratory data, and outcomes of the patients were examined retrospectively and compared between the groups.

Results: Of the 26 patients [median age 51.5 years, interquartile range 35.8-67.0], 6 were in the low group (23%) and 20 in the high group (77%). The low group had a higher respiratory rate than the high group ($p < 0.05$). Blood tests immediately after admission showed that the low group had significantly lower albumin ($p < 0.01$), and higher lactate dehydrogenase ($p < 0.01$), C-reactive protein ($p < 0.01$), and D-dimer ($p < 0.01$) levels than the high group. Moreover, all patients

received antiviral agents; four received continuous renal replacement therapy and invasive positive pressure ventilation, one received extracorporeal membrane oxygenation, and two died in the low group.

Conclusion: SF ratio measurement at admission could assist clinicians in the early identification of severe COVID-19, which in turn can lead to early therapeutic interventions.

Keywords: COVID-19; Early identification; SF ratio; Severity; SpO2/FiO2.

© 2021. Japanese Society of Anesthesiologists.

Conflict of interest statement

MS, YK, TY, BO, and SH report no conflict of interest.

- 32 references
- 5 figures

Supplementary info

Publication types, MeSH terms, Substances Expand

Publication types

- Observational Study

MeSH terms

- COVID-19*
- Critical Illness*
- Humans
- Middle Aged
- Oxygen*
- Retrospective Studies
- SARS-CoV-2

Substances

- Oxygen

Full text links

SpringerLink FULL-TEXT ARTICLE Springer Free PMC article
Proceed to details
Cite
Share
☐ 394

Observational Study

Stroke
. 2021 Apr;52(4):1362-1369.
doi: 10.1161/STROKEAHA.120.032312. Epub 2021 Feb 25.

Hospitalizations for Stroke in France During the COVID-19 Pandemic Before, During, and After the National Lockdown

Anne-Sophie Mariet[#][1][2][3], Maurice Giroud[#][4], Eric Benzenine[#][1], Jonathan Cottenet[#][1], Adrien Roussot[#][1], Ludwig Serge Aho-Glélé[#][5], Pascale Tubert-Bitter[#][6], Yannick Béjot[#][4], Catherine Quantin[#][1][2][3][6]

Affiliations Expand

Affiliations

- [1] Biostatistics and Bioinformatics (DIM), University Hospital, Dijon, France (A.-S.M., E.B., J.C., A.R., C.Q.).
- [2] Inserm, CIC 1432, Dijon, France (A.-S.M., C.Q.).
- [3] Dijon University Hospital, Clinical Investigation Center, clinical epidemiology/clinical trials unit, France (A.-S.M., C.Q.).
- [4] Dijon Stroke Registry - EA7460 (Pathophysiology and Epidemiology of Cerebro-Cardio-Vascular Diseases), University of Burgundy - UBFC (M.G., Y.B.), University Hospital of Dijon, Bourgogne, France.
- [5] Department of Epidemiology and Hospital Hygiene (L.S.A.-G.), University Hospital of Dijon, Bourgogne, France.
- [6] Université Paris-Saclay, UVSQ, Université Paris-Sud, Inserm, High-Dimensional Biostatistics for Drug Safety and Genomics, CESP, Villejuif, France (P.T.-B., C.Q.).

[#] Contributed equally.

- PMID: **33626900**
- DOI: 10.1161/STROKEAHA.120.032312

Free article
Observational Study

Hospitalizations for Stroke in France During the COVID-19 Pandemic Before, During, and After the National Lockdown

Anne-Sophie Mariet et al. Stroke. 2021 Apr.
Free article
Show details

Stroke
. 2021 Apr;52(4):1362-1369.
doi: 10.1161/STROKEAHA.120.032312. Epub 2021 Feb 25.

Authors

Anne-Sophie Mariet [#] [1] [2] [3], Maurice Giroud [#] [4], Eric Benzenine [#] [1], Jonathan Cottenet [#] [1], Adrien Roussot [#] [1], Ludwig Serge Aho-Glélé [#] [5], Pascale Tubert-Bitter [#] [6], Yannick Béjot [#] [4], Catherine Quantin [#] [1] [2] [3] [6]

Affiliations

- [1] Biostatistics and Bioinformatics (DIM), University Hospital, Dijon, France (A.-S.M., E.B., J.C., A.R., C.Q.).
- [2] Inserm, CIC 1432, Dijon, France (A.-S.M., C.Q.).
- [3] Dijon University Hospital, Clinical Investigation Center, clinical epidemiology/clinical trials unit, France (A.-S.M., C.Q.).
- [4] Dijon Stroke Registry - EA7460 (Pathophysiology and Epidemiology of Cerebro-Cardio-Vascular Diseases), University of Burgundy - UBFC (M.G., Y.B.), University Hospital of Dijon, Bourgogne, France.
- [5] Department of Epidemiology and Hospital Hygiene (L.S.A.-G.), University Hospital of Dijon, Bourgogne, France.
- [6] Université Paris-Saclay, UVSQ, Université Paris-Sud, Inserm, High-Dimensional Biostatistics for Drug Safety and Genomics, CESP, Villejuif, France (P.T.-B., C.Q.).

[#] Contributed equally.

- PMID: **33626900**
- DOI: 10.1161/STROKEAHA.120.032312

Abstract

Background and purpose: In France, the entire population was put under a total lockdown from March 17 to May 11, 2020 during the peak of the coronavirus disease 2019 (COVID-19) pandemic. Whether the lockdown had consequences on the management of medical emergencies such as stroke and transient ischemic attack (TIA) has yet to be fully evaluated. This article describes hospitalization rates for acute stroke in 2 French regions that experienced contrasting rates of COVID-19 infection, before, during, and after the nationwide lockdown (January to June 2020).

Methods: All patients admitted for acute stroke/TIA into all public and private hospitals of the 2 study regions were included. Data were retrieved from the National Hospitalization Database (PMSI). In the most affected region (Grand-Est), the hospitalization rates observed in April 2020 were compared with the rates in the same period in the least affected region (Occitanie) and in the 3 prior years (2017-2019).

Results: There was a significant decline in hospitalization rates for stroke/TIA within the region most affected by COVID-19 during the month of April 2020 compared with previous years, while no significant change was seen in the least affected region. After lockdown, we observed a fast rebound in the rate of hospitalization for stroke/TIA in the most affected region, contrasting with a

slower rebound in the least affected region. In both regions, patients with COVID-19 stroke more frequently had ischemic stroke, a nonsignificant greater prevalence of diabetes, they were less frequently admitted to stroke units, and mortality was higher than in patients without COVID-19.

Conclusions: Our results demonstrates a significant drop in stroke/TIA hospitalizations and a fast recovery after the end of the French lockdown in the most affected region, while the least affected region saw a nonsignificant drop in stroke/TIA hospitalizations and a slow recovery. These results and recommendations could be used by the health authorities to prepare for future challenges.

Keywords: coronavirus disease 2019; hemorrhagic stroke; hospitalization; incidence; ischemic attack, transient; ischemic stroke.

- Cited by 9 articles

Supplementary info

Publication types, MeSH terms Expand

Publication types

- Comparative Study
- Observational Study
- Research Support, Non-U.S. Gov't

MeSH terms

- Aged
- Aged, 80 and over
- COVID-19 / epidemiology*
- COVID-19 / therapy
- Communicable Disease Control / methods
- Communicable Disease Control / trends*
- Female
- France / epidemiology
- Hospitalization / trends*
- Humans
- Male
- Middle Aged
- Pandemics*
- Retrospective Studies
- Stroke / epidemiology*
- Stroke / therapy

Full text links

FREE FULL TEXT Atypon
Proceed to details
Cite
Share
395
Observational Study
Clin Microbiol Infect
. 2021 Aug;27(8):1190-1192.
doi: 10.1016/j.cmi.2021.05.023. Epub 2021 May 17.

Drug reaction with eosinophilia and systemic symptoms (DRESS) in patients with COVID-19

Giuseppe A Ramirez [1], Emanuel Della-Torre [1], Moreno Tresoldi [2], Paolo Scarpellini [3], Fabio Ciceri [4], Lorenzo Dagna [1], Mona-Rita Yacoub [5]
Affiliations Expand

Affiliations

- [1] Università Vita-Salute San Raffaele, Milan, Italy; Unit of Immunology, Rheumatology, Allergy and Rare Diseases (UniRAR), IRCCS San Raffaele Scientific Institute, Milan, Italy.
- [2] Unit of General Medicine and Advanced Care, IRCCS San Raffaele Scientific Institute, Milan, Italy.
- [3] Unit of Infectious Diseases, IRCCS San Raffaele Scientific Institute, Milan, Italy.
- [4] Università Vita-Salute San Raffaele, Milan, Italy; Unit of Haematology, IRCCS San Raffaele Scientific Institute, Milan, Italy.
- [5] Università Vita-Salute San Raffaele, Milan, Italy; Unit of Immunology, Rheumatology, Allergy and Rare Diseases (UniRAR), IRCCS San Raffaele Scientific Institute, Milan, Italy. Electronic address: yacoub.monarita@hsr.it.

- PMID: **34015530**
- PMCID: PMC8127514
- DOI: 10.1016/j.cmi.2021.05.023

Free PMC article
Observational Study

Drug reaction with eosinophilia and systemic symptoms (DRESS) in patients with COVID-19

Giuseppe A Ramirez et al. Clin Microbiol Infect. 2021 Aug.

Free PMC article

Show details

Clin Microbiol Infect

. 2021 Aug;27(8):1190-1192.

doi: 10.1016/j.cmi.2021.05.023. Epub 2021 May 17.

Authors

Giuseppe A Ramirez [1], Emanuel Della-Torre [1], Moreno Tresoldi [2], Paolo Scarpellini [3], Fabio Ciceri [4], Lorenzo Dagna [1], Mona-Rita Yacoub [5]

Affiliations

- [1] Università Vita-Salute San Raffaele, Milan, Italy; Unit of Immunology, Rheumatology, Allergy and Rare Diseases (UniRAR), IRCCS San Raffaele Scientific Institute, Milan, Italy.
- [2] Unit of General Medicine and Advanced Care, IRCCS San Raffaele Scientific Institute, Milan, Italy.
- [3] Unit of Infectious Diseases, IRCCS San Raffaele Scientific Institute, Milan, Italy.
- [4] Università Vita-Salute San Raffaele, Milan, Italy; Unit of Haematology, IRCCS San Raffaele Scientific Institute, Milan, Italy.
- [5] Università Vita-Salute San Raffaele, Milan, Italy; Unit of Immunology, Rheumatology, Allergy and Rare Diseases (UniRAR), IRCCS San Raffaele Scientific Institute, Milan, Italy. Electronic address: yacoub.monarita@hsr.it.

- PMID: **34015530**
- PMCID: PMC8127514
- DOI: 10.1016/j.cmi.2021.05.023

No abstract available

- Cited by 2 articles
- 5 references

Supplementary info

Publication types, MeSH terms Expand

Publication types

- Letter
- Observational Study

MeSH terms

- Aged
- COVID-19 / blood*
- COVID-19 / complications*
- Comorbidity

- Critical Illness
- Drug Hypersensitivity Syndrome / blood
- Drug Hypersensitivity Syndrome / etiology*
- Drug Hypersensitivity Syndrome / virology
- Eosinophilia / drug therapy*
- Eosinophilia / etiology*
- Female
- Hospitalization / statistics & numerical data
- Humans
- Male
- Middle Aged
- Prospective Studies
- Retrospective Studies
- SARS-CoV-2 / drug effects

Full text links

ELSEVIER FULL-TEXT ARTICLE Elsevier Science Free PMC article
Proceed to details
Cite
Share
396
Observational Study
Eur Radiol
. 2020 Dec;30(12):6770-6778.
doi: 10.1007/s00330-020-07013-2. Epub 2020 Jun 26.

Quantitative chest CT analysis in COVID-19 to predict the need for oxygenation support and intubation

Ezio Lanza [1], Riccardo Muglia [2], Isabella Bolengo [2], Orazio Giuseppe Santonocito [2], Costanza Lisi [3], Giovanni Angelotti [4], Pierandrea Morandini [4], Victor Savevski [4], Letterio Salvatore Politi [5] [3], Luca Balzarini [5]
Affiliations Expand

Affiliations

- [1] Department of Diagnostic and Interventional Radiology, Humanitas Clinical and Research Center - IRCCS, Via A. Manzoni 56, Rozzano, 20089, Milan, Italy. eziolanza@gmail.com.
- [2] Training School in Radiology, Humanitas University, Via Rita Levi Montalcini 4, Pieve Emanuele, 20090, Milan, Italy.

- [3] Department of Biomedical Sciences, Humanitas University, Via Rita Levi Montalcini 4, Pieve Emanuele, 20090, Milan, Italy.
- [4] Artificial Intelligence Center, Humanitas Clinical and Research Center - IRCCS, Via A. Manzoni 56, Rozzano, 20089, Milan, Italy.
- [5] Department of Diagnostic and Interventional Radiology, Humanitas Clinical and Research Center - IRCCS, Via A. Manzoni 56, Rozzano, 20089, Milan, Italy.

- PMID: **32591888**
- PMCID: PMC7317888
- DOI: 10.1007/s00330-020-07013-2

Free PMC article
Observational Study

Quantitative chest CT analysis in COVID-19 to predict the need for oxygenation support and intubation

Ezio Lanza et al. Eur Radiol. 2020 Dec.
Free PMC article
Show details
Eur Radiol
. 2020 Dec;30(12):6770-6778.
doi: 10.1007/s00330-020-07013-2. Epub 2020 Jun 26.

Authors

Ezio Lanza [1], Riccardo Muglia [2], Isabella Bolengo [2], Orazio Giuseppe Santonocito [2], Costanza Lisi [3], Giovanni Angelotti [4], Pierandrea Morandini [4], Victor Savevski [4], Letterio Salvatore Politi [5] [3], Luca Balzarini [5]

Affiliations

- [1] Department of Diagnostic and Interventional Radiology, Humanitas Clinical and Research Center - IRCCS, Via A. Manzoni 56, Rozzano, 20089, Milan, Italy. eziolanza@gmail.com.
- [2] Training School in Radiology, Humanitas University, Via Rita Levi Montalcini 4, Pieve Emanuele, 20090, Milan, Italy.
- [3] Department of Biomedical Sciences, Humanitas University, Via Rita Levi Montalcini 4, Pieve Emanuele, 20090, Milan, Italy.
- [4] Artificial Intelligence Center, Humanitas Clinical and Research Center - IRCCS, Via A. Manzoni 56, Rozzano, 20089, Milan, Italy.
- [5] Department of Diagnostic and Interventional Radiology, Humanitas Clinical and Research Center - IRCCS, Via A. Manzoni 56, Rozzano, 20089, Milan, Italy.

- PMID: **32591888**
- PMCID: PMC7317888
- DOI: 10.1007/s00330-020-07013-2

Abstract

Objective: Lombardy (Italy) was the epicentre of the COVID-19 pandemic in March 2020. The healthcare system suffered from a shortage of ICU beds and oxygenation support devices. In our Institution, most patients received chest CT at admission, only interpreted visually. Given the proven value of quantitative CT analysis (QCT) in the setting of ARDS, we tested QCT as an outcome predictor for COVID-19.

Methods: We performed a single-centre retrospective study on COVID-19 patients hospitalised from January 25, 2020, to April 28, 2020, who received CT at admission prompted by respiratory symptoms such as dyspnea or desaturation. QCT was performed using a semi-automated method (3D Slicer). Lungs were divided by Hounsfield unit intervals. Compromised lung (%CL) volume was the sum of poorly and non-aerated volumes (- 500, 100 HU). We collected patient's clinical data including oxygenation support throughout hospitalisation.

Results: Two hundred twenty-two patients (163 males, median age 66, IQR 54-6) were included; 75% received oxygenation support (20% intubation rate). Compromised lung volume was the most accurate outcome predictor (logistic regression, $p < 0.001$). %CL values in the 6-23% range increased risk of oxygenation support; values above 23% were at risk for intubation. %CL showed a negative correlation with PaO_2/FiO_2 ratio ($p < 0.001$) and was a risk factor for in-hospital mortality ($p < 0.001$).

Conclusions: QCT provides new metrics of COVID-19. The compromised lung volume is accurate in predicting the need for oxygenation support and intubation and is a significant risk factor for in-hospital death. QCT may serve as a tool for the triaging process of COVID-19.

Key points: • Quantitative computer-aided analysis of chest CT (QCT) provides new metrics of COVID-19. • The compromised lung volume measured in the - 500, 100 HU interval predicts oxygenation support and intubation and is a risk factor for in-hospital death. • Compromised lung values in the 6-23% range prompt oxygenation therapy; values above 23% increase the need for intubation.

Keywords: COVID-19; Intubation; Pulmonary ventilation; Tomography, spiral computed.

Conflict of interest statement

The authors of this manuscript declare no relationships with any companies whose products or services may be related to the subject matter of the article.

- Cited by 48 articles
- 37 references
- 4 figures

Supplementary info

Publication types, MeSH terms Expand

Publication types

- Observational Study

MeSH terms

- Betacoronavirus*
- COVID-19
- Coronavirus Infections / diagnosis*
- Coronavirus Infections / epidemiology
- Coronavirus Infections / therapy
- Female
- Hospital Mortality
- Hospitalization
- Humans
- Intubation, Intratracheal / methods*
- Italy / epidemiology
- Lung / diagnostic imaging*
- Male
- Middle Aged
- Oxygen Inhalation Therapy / methods*
- Pandemics
- Pneumonia, Viral / diagnosis*
- Pneumonia, Viral / epidemiology
- Pneumonia, Viral / therapy
- Prognosis
- Retrospective Studies
- SARS-CoV-2
- Tomography, X-Ray Computed / methods*

Full text links

SpringerLink FULL-TEXT ARTICLE Springer Free PMC article
Proceed to details
Cite
Share
☐ 397
Observational Study
Clin Microbiol Infect
. 2021 Dec;27(12):1863.e1-1863.e4.
doi: 10.1016/j.cmi.2021.09.002. Epub 2021 Sep 8.

Outcomes and proportions of pregnant women during the first and consecutive waves

# of coronavirus disease 2019: observational cohort study

Cathrin Kodde [1], Marzia Bonsignore [2], Sven Hohenstein [3], Ralf Kuhlen [4], Andreas Meier-Hellmann [5], Andreas Bollmann [2], Irit Nachtigall [6]
Affiliations Expand

Affiliations

- [1] Department of Pneumology, Lungenklinik Heckeshorn, Helios Klinikum Emil von Behring, Berlin, Germany.
- [2] Centre for Hygiene, Evangelische Kliniken Gelsenkirchen, Gelsenkirchen, Germany.
- [3] Heart Centre Leipzig at University of Leipzig and Leipzig Heart Institute, Leipzig, Germany.
- [4] Helios Health, Berlin, Germany.
- [5] Helios Kliniken, Berlin, Germany.
- [6] Department of Infectious Diseases and Infection Prevention, HELIOS Hospital Emil-von-Behring, Berlin, Germany; Charité - Universitätsmedizin Berlin, Institute of Hygiene and Environmental Medicine, Berlin, Germany. Electronic address: Irit.nachtigall@helios-gesundheit.de.

- PMID: **34508888**
- PMCID: PMC8425671
- DOI: 10.1016/j.cmi.2021.09.002

Free PMC article
Observational Study

Outcomes and proportions of pregnant women during the first and consecutive waves of coronavirus disease 2019: observational cohort study

Cathrin Kodde et al. Clin Microbiol Infect. 2021 Dec.
Free PMC article
Show details
Clin Microbiol Infect
. 2021 Dec;27(12):1863.e1-1863.e4.
doi: 10.1016/j.cmi.2021.09.002. Epub 2021 Sep 8.

Authors

Cathrin Kodde [1], Marzia Bonsignore [2], Sven Hohenstein [3], Ralf Kuhlen [4], Andreas Meier-Hellmann [5], Andreas Bollmann [2], Irit Nachtigall [6]

Affiliations

- [1] Department of Pneumology, Lungenklinik Heckeshorn, Helios Klinikum Emil von Behring, Berlin, Germany.
- [2] Centre for Hygiene, Evangelische Kliniken Gelsenkirchen, Gelsenkirchen, Germany.
- [3] Heart Centre Leipzig at University of Leipzig and Leipzig Heart Institute, Leipzig, Germany.
- [4] Helios Health, Berlin, Germany.
- [5] Helios Kliniken, Berlin, Germany.
- [6] Department of Infectious Diseases and Infection Prevention, HELIOS Hospital Emil-von-Behring, Berlin, Germany; Charité - Universitätsmedizin Berlin, Institute of Hygiene and Environmental Medicine, Berlin, Germany. Electronic address: Irit.nachtigall@helios-gesundheit.de.

- PMID: **34508888**
- PMCID: PMC8425671
- DOI: 10.1016/j.cmi.2021.09.002

Abstract

Objectives: It has been suggested that pregnant women were affected more severely during the late wave, as opposed to the early wave of the coronavirus disease 2019 (COVID-19) pandemic. The aim of our study was to compare the proportion of pregnant women among hospitalized women of childbearing age, their rate of intensive care (ICU) admission, need for mechanical ventilation and mortality during the waves.

Methods: The study is a retrospective analysis of claims data on women of childbearing age (16-49 years) admitted to 76 hospitals with a laboratory-confirmed severe acute respiratory syndrome coronavirus 2 infection. The observation period was divided into first wave (7 March 2020 to 30 September 2020) and second wave (1 October to 17 April 2021). Co-morbidities derived from claims data were summarized in the Elixhauser Co-morbidity Index (ECI).

Results: A total of 1879 women were included, 532 of whom were pregnant. During the second wave, the proportion of pregnant women was higher (29.3% (484/1650) versus 21.0% (48/229), p < 0.01). They were older (mean ± SD 29.1 ± 5.9 years versus 27 ± 6.3 years, p 0.02 in the first wave) and had comparable co-morbidities (ECI mean ± SD 0.3 ± 3.5 versus -0.2 ± 2.0, p 0.30). Of the pregnant women, 6.2% (3/48) were admitted to ICU during the first wave versus 3.3% (16/484) during the second wave (OR 0.51, 95% CI 0.14-1.83, p 0.30), 2.1% (1/48) were ventilated versus 1.2% (6/484, OR 0.60, 95% CI 0.07-5.23, p 0.64). No deaths were observed among the hospitalized pregnant women in either wave.

Conclusions: Proportionally more pregnant women with COVID-19 were hospitalized in the second wave compared with the first wave but no more severe outcomes were registered.

Keywords: Coronavirus disease 2019; Intensive care unit; Mortality; Pregnant women; Second wave.

Copyright © 2021 European Society of Clinical Microbiology and Infectious Diseases. Published by Elsevier Ltd. All rights reserved.

- 14 references
- 1 figure

Supplementary info

Publication types, MeSH terms Expand

Publication types

- Observational Study

MeSH terms

- Adolescent
- Adult
- COVID-19* / epidemiology
- Female
- Hospitalization
- Humans
- Middle Aged
- Pregnancy
- Pregnant Women*
- Retrospective Studies
- Young Adult

Full text links

ELSEVIER FULL-TEXT ARTICLE Elsevier Science Free PMC article

Proceed to details

Cite

Share

398

Observational Study

Ann Emerg Med
. 2021 Nov;78(5):619-627.
doi: 10.1016/j.annemergmed.2021.05.022. Epub 2021 May 29.

Personal Protective Equipment Adherence of Pediatric Resuscitation Team Members During the COVID-19 Pandemic

Emily C Alberto [1], Kathleen H McCarthy [1], Colleen A Hamilton [1], Jacob Shalkevich [1], Zachary P Milestone [1], Rima Izem [2], Jennifer L Fritzeen [1], Ivan Marsic [3], Aleksandra Sarcevic [4], Karen J O'Connell [5], Randall S Burd [6]
Affiliations Expand

Affiliations

- [1] Division of Trauma and Burn Surgery, Children's National Hospital, Washington, DC.
- [2] Division of Biostatistics and Study Methods, Children's National Research Institute, Washington, DC; Department of Pediatrics and the Department of Epidemiology, George Washington University, Washington, DC.
- [3] Department of Electrical and Computer Engineering, Rutgers University, Piscataway, NJ.
- [4] College of Computing and Informatics, Drexel University, Philadelphia, PA.
- [5] Division of Emergency Medicine, Children's National Hospital, Washington, DC.
- [6] Division of Trauma and Burn Surgery, Children's National Hospital, Washington, DC. Electronic address: rburd@childrensnational.org.

- PMID: **34353649**
- PMCID: PMC8164378
- DOI: 10.1016/j.annemergmed.2021.05.022

Free PMC article
Observational Study

Personal Protective Equipment Adherence of Pediatric Resuscitation Team Members During the COVID-19 Pandemic

Emily C Alberto et al. Ann Emerg Med. 2021 Nov.
Free PMC article
Show details
Ann Emerg Med
. 2021 Nov;78(5):619-627.
doi: 10.1016/j.annemergmed.2021.05.022. Epub 2021 May 29.

Authors

Emily C Alberto [1], Kathleen H McCarthy [1], Colleen A Hamilton [1], Jacob Shalkevich [1], Zachary P Milestone [1], Rima Izem [2], Jennifer L Fritzeen [1], Ivan Marsic [3], Aleksandra Sarcevic [4], Karen J O'Connell [5], Randall S Burd [6]

Affiliations

- [1] Division of Trauma and Burn Surgery, Children's National Hospital, Washington, DC.
- [2] Division of Biostatistics and Study Methods, Children's National Research Institute, Washington, DC; Department of Pediatrics and the Department of Epidemiology, George Washington University, Washington, DC.
- [3] Department of Electrical and Computer Engineering, Rutgers University, Piscataway, NJ.
- [4] College of Computing and Informatics, Drexel University, Philadelphia, PA.
- [5] Division of Emergency Medicine, Children's National Hospital, Washington, DC.

- [6] Division of Trauma and Burn Surgery, Children's National Hospital, Washington, DC. Electronic address: rburd@childrensnational.org.

- PMID: **34353649**
- PMCID: PMC8164378
- DOI: 10.1016/j.annemergmed.2021.05.022

Abstract

Study objective: During the COVID-19 pandemic, health care workers have had the highest risk of infection among essential workers. Although personal protective equipment (PPE) use is associated with lower infection rates, appropriate use of PPE has been variable among health care workers, even in settings with COVID-19 patients. We aimed to evaluate the patterns of PPE adherence during emergency department resuscitations that included aerosol-generating procedures.

Methods: We conducted a retrospective, video-based review of pediatric resuscitations involving one or more aerosol-generating procedures during the first 3 months of the COVID-19 pandemic in the United States (March to June 2020). Recommended adherence (complete, inadequate, absent) with 5 PPE items (headwear, eyewear, masks, gowns, gloves) and the duration of potential exposure were evaluated for individuals in the room after aerosol-generating procedure initiation.

Results: Among the 345 health care workers observed during 19 resuscitations, 306 (88.7%) were nonadherent (inadequate or absent adherence) with the recommended use of at least 1 PPE type at some time during the resuscitation, 23 (6.7%) of whom had no PPE. One hundred and forty health care workers (40.6%) altered or removed at least 1 type of PPE during the event. The aggregate time in the resuscitation room for health care workers across all events was 118.7 hours. During this time, providers had either absent or inadequate eyewear for 46.4 hours (39.1%) and absent or inadequate masks for 35.2 hours (29.7%).

Conclusion: Full adherence with recommended PPE use was limited in a setting at increased risk for SARS-CoV-2 virus aerosolization. In addition to ensuring appropriate donning, approaches are needed for ensuring ongoing adherence with PPE recommendations during exposure.

Copyright © 2021 American College of Emergency Physicians. Published by Elsevier Inc. All rights reserved.

- Cited by 1 article
- 31 references
- 3 figures

Supplementary info

Publication types, MeSH terms, Grant support Expand

Publication types

- Observational Study

MeSH terms

- COVID-19 / epidemiology
- COVID-19 / prevention & control*
- COVID-19 / transmission
- Child
- Emergency Service, Hospital / standards*
- Guideline Adherence*
- Hospitals, Pediatric
- Humans
- Infection Control / methods
- Infection Control / standards*
- Pandemics*
- Patient Care Team / standards
- Personal Protective Equipment / standards*
- Practice Guidelines as Topic
- Resuscitation*
- Retrospective Studies
- SARS-CoV-2

Grant support

- UL1 TR001876/TR/NCATS NIH HHS/United States

Full text links

ELSEVIER FULL-TEXT ARTICLE Elsevier Science Free PMC article

Proceed to details

Cite

Share

399

Observational Study

Fam Pract

. 2021 Sep 25;38(5):549-555.

doi: 10.1093/fampra/cmaa130.

Clinical learnings from a virtual primary care program monitoring mild to moderate COVID-19 patients at home

Nicholas Pimlott [1, 2], Payal Agarwal [1], Lisa M McCarthy [1, 3], Miles J Luke [4], Susan Hum [1], Sumeet Gill [1], Ruth Heisey [1, 2]

Affiliations Expand

Affiliations

- [1] Department of Family and Community Medicine, Women's College Hospital, Toronto, ON, Canada.
- [2] Department of Family and Community Medicine, University of Toronto, Toronto, ON, Canada.
- [3] Women's College Research Institute and Pharmacy Services, Women's College Hospital, Toronto, ON, Canada.
- [4] Pharmacy Services, Women's College Hospital, Toronto, ON, Canada.

- PMID: **33340398**
- PMCID: PMC7953959
- DOI: 10.1093/fampra/cmaa130

Free PMC article
Observational Study

Clinical learnings from a virtual primary care program monitoring mild to moderate COVID-19 patients at home

Nicholas Pimlott et al. Fam Pract. 2021.
Free PMC article
Show details
Fam Pract

. 2021 Sep 25;38(5):549-555.
doi: 10.1093/fampra/cmaa130.

Authors

Nicholas Pimlott [1, 2], Payal Agarwal [1], Lisa M McCarthy [1, 3], Miles J Luke [4], Susan Hum [1], Sumeet Gill [1], Ruth Heisey [1, 2]

Affiliations

- [1] Department of Family and Community Medicine, Women's College Hospital, Toronto, ON, Canada.
- [2] Department of Family and Community Medicine, University of Toronto, Toronto, ON, Canada.
- [3] Women's College Research Institute and Pharmacy Services, Women's College Hospital, Toronto, ON, Canada.
- [4] Pharmacy Services, Women's College Hospital, Toronto, ON, Canada.

- PMID: **33340398**
- PMCID: PMC7953959
- DOI: 10.1093/fampra/cmaa130

Abstract

Background: Virtual consults have replaced in-person visits for many home-isolated patients with COVID-19 disease.

Objectives: To describe the natural history, clinical management and outcomes of community-dwelling patients with COVID-19, who received support from a family medicine-led, virtual CovidCare@Home program in Toronto, Ontario, Canada.

Methods: Observational, descriptive study conducted by retrospective chart review of 98 patients enrolled during the first 5 weeks of program implementation (8 April-11 May 2020); 73 patients with laboratory-confirmed COVID-19, with symptom onset ≤ 14 days before initial consult were included for analysis. Patients were classified as mild, moderate or severe based on WHO Criteria.

Results: All patients in the program experienced mild (88%) or moderate (12.3%) disease. No patients were hospitalized or died. Patients were mainly female (70%); with mean age of 43.3 years. Most patients (82.2%) worked in higher risk, healthcare settings. Almost 40% had no medical co-morbidities. Common symptoms were cough (65.8%), fatigue (60.3%), headache (42.5%) and myalgia (39.7%), followed by fever (32.9%), sore throat (21.9%), nasal congestion (21.9%) and rhinorrhea (20.5%). Headache (51%) and anosmia (45.1%) were common among females; fever and breathlessness among males (40.9%). Nine patients (12.3%) experienced worsening of symptoms (mainly respiratory) or exacerbation of co-morbidities, which required care outside the virtual service.

Conclusion: Patients with mild to moderate COVID-19 disease can be managed safely and effectively in a family medicine-led virtual program. Some sex differences in symptoms were observed. Future work should focus on long-term follow up in view of the existence of so-called 'long-haulers'.

Keywords: COVID-19; SARS-CoV-2; family practice; multidisciplinary care; primary health care; telemedicine.

© The Author(s) 2020. Published by Oxford University Press.

- Cited by 3 articles
- 24 references
- 1 figure

Supplementary info

Publication types, MeSH terms, Grant support Expand

Publication types

- Observational Study
- Research Support, Non-U.S. Gov't

MeSH terms

- Adult

- COVID-19 / therapy*
- Female
- Home Care Services / organization & administration*
- Humans
- Male
- Primary Health Care / organization & administration*
- Retrospective Studies
- Risk Factors
- SARS-CoV-2
- Symptom Assessment*
- Telemedicine*

Grant support

- Women's College Hospital

Full text links

OXFORD ACADEMIC Silverchair Information Systems Free PMC article
Proceed to details
Cite
Share
400
Clinical Trial
PLoS One
. 2021 Feb 19;16(2):e0247422.
doi: 10.1371/journal.pone.0247422. eCollection 2021.

Healthcare workers hospitalized due to COVID-19 have no higher risk of death than general population. Data from the Spanish SEMI-COVID-19 Registry

Jesús Díez-Manglano [1], Marta Nataya Solís-Marquínez [2], Andrea Álvarez García [2], Nicolás Alcalá-Rivera [1], Irene Maderuelo Riesco [2], Martín Gericó Aseguinolaza [1], José Luis Beato Pérez [3], Manuel Méndez Bailón [4], Ane-Elbire Labirua-Iturburu Ruiz [5], Miriam García Gómez [6], Carmen Martínez Cilleros [7], Paula María Pesqueira Fontan [8], Lucy Abella Vázquez [9], Julio César Blázquez Encinar [10], Ramon Boixeda [11], Ricardo Gil Sánchez [12], Andrés de la Peña Fernández [13], José Loureiro Amigo [14], Joaquín Escobar Sevilla [15], Marcos Guzmán Garcia [16], María Dolores Martín Escalante [17], Jeffrey Oskar Magallanes Gamboa [18], Ángel Luis Martínez González [19], Carlos Lumbreras Bermejo [20], Juan Miguel Antón Santos [21], SEMI-COVID-19 Network

Affiliations Expand

Affiliations

- [1] Internal Medicine Department, Royo Villanova Hospital, Zaragoza, Spain.
- [2] Internal Medicine Department, San Agustin University Hospital, Avilés, Asturias, Spain.
- [3] Internal Medicine Department, Albacete University Hospital, Albacete, Spain.
- [4] Internal Medicine Department, San Carlos Clinical Hospital, Madrid, Spain.
- [5] Internal Medicine Department, Santa Marina Hospital, Bilbao, Spain.
- [6] Internal Medicine Department, Urduliz Alfredo Espinosa Hospital, Urdúliz, Vizcaya, Spain.
- [7] Internal Medicine Department, HLA Moncloa Hospital, Madrid, Spain.
- [8] Internal Medicine Department, Santiago Clinical Hospital, Santiago de Compostela, A Coruña, Spain.
- [9] Internal Medicine Department, Nuestra Señora Candelaria University Hospital, Santa Cruz de Tenerife, Spain.
- [10] Internal Medicine Department, Torrevieja University Hospital, Torrevieja, Alicante, Spain.
- [11] Internal Medicine Department, Mataró Hospital, Mataró, Barcelona, Spain.
- [12] Internal Medicine Department, La Fe University Hospital, Valencia, Spain.
- [13] Internal Medicine Department, Son Llàtzer University Hospital, Palma de Mallorca, Spain.
- [14] Internal Medicine Department, Moisès Broggi Hospital, Sant Joan Despí, Barcelona, Spain.
- [15] Internal Medicine Department, Virgen de las Nieves University Hospital, Granada, Spain.
- [16] Internal Medicine Department, San Juan de la Cruz Hospital, Úbeda, Jaén, Spain.
- [17] Internal Medicine Department, Costa del Sol Hospital, Marbella, Málaga, Spain.
- [18] Internal Medicine Department, Nuestra Señora del Prado Hospital, Talavera de la Reina, Toledo, Spain.
- [19] Internal Medicine Department, León University Hospital, León, Spain.
- [20] Internal Medicine Department, 12 de Octubre University Hospital, Madrid, Spain.
- [21] Internal Medicine Department, Infanta Cristina University Hospital, Parla, Madrid, Spain.

- PMID: **33606820**
- PMCID: PMC7894924
- DOI: 10.1371/journal.pone.0247422

Free PMC article
Clinical Trial

Healthcare workers hospitalized due to COVID-19 have no higher risk of death than general population. Data from the Spanish SEMI-COVID-19 Registry

Jesús Díez-Manglano et al. PLoS One. 2021.
Free PMC article
Show details
PLoS One
. 2021 Feb 19;16(2):e0247422.
doi: 10.1371/journal.pone.0247422. eCollection 2021.

Authors

Jesús Díez-Manglano [1], Marta Nataya Solís-Marquínez [2], Andrea Álvarez García [2], Nicolás Alcalá-Rivera [1], Irene Maderuelo Riesco [2], Martín Gericó Aseguinolaza [1], José Luis Beato Pérez [3], Manuel Méndez Bailón [4], Ane-Elbire Labirua-Iturburu Ruiz [5], Miriam García Gómez [6], Carmen Martínez Cilleros [7], Paula María Pesqueira Fontan [8], Lucy Abella Vázquez [9], Julio César Blázquez Encinar [10], Ramon Boixeda [11], Ricardo Gil Sánchez [12], Andrés de la Peña Fernández [13], José Loureiro Amigo [14], Joaquín Escobar Sevilla [15], Marcos Guzmán Garcia [16], María Dolores Martín Escalante [17], Jeffrey Oskar Magallanes Gamboa [18], Ángel Luis Martínez González [19], Carlos Lumbreras Bermejo [20], Juan Miguel Antón Santos [21], SEMI-COVID-19 Network

Affiliations

- [1] Internal Medicine Department, Royo Villanova Hospital, Zaragoza, Spain.
- [2] Internal Medicine Department, San Agustin University Hospital, Avilés, Asturias, Spain.
- [3] Internal Medicine Department, Albacete University Hospital, Albacete, Spain.
- [4] Internal Medicine Department, San Carlos Clinical Hospital, Madrid, Spain.
- [5] Internal Medicine Department, Santa Marina Hospital, Bilbao, Spain.
- [6] Internal Medicine Department, Urduliz Alfredo Espinosa Hospital, Urdúliz, Vizcaya, Spain.
- [7] Internal Medicine Department, HLA Moncloa Hospital, Madrid, Spain.
- [8] Internal Medicine Department, Santiago Clinical Hospital, Santiago de Compostela, A Coruña, Spain.
- [9] Internal Medicine Department, Nuestra Señora Candelaria University Hospital, Santa Cruz de Tenerife, Spain.
- [10] Internal Medicine Department, Torrevieja University Hospital, Torrevieja, Alicante, Spain.
- [11] Internal Medicine Department, Mataró Hospital, Mataró, Barcelona, Spain.
- [12] Internal Medicine Department, La Fe University Hospital, Valencia, Spain.
- [13] Internal Medicine Department, Son Llàtzer University Hospital, Palma de Mallorca, Spain.
- [14] Internal Medicine Department, Moisès Broggi Hospital, Sant Joan Despí, Barcelona, Spain.
- [15] Internal Medicine Department, Virgen de las Nieves University Hospital, Granada, Spain.
- [16] Internal Medicine Department, San Juan de la Cruz Hospital, Úbeda, Jaén, Spain.
- [17] Internal Medicine Department, Costa del Sol Hospital, Marbella, Málaga, Spain.
- [18] Internal Medicine Department, Nuestra Señora del Prado Hospital, Talavera de la Reina, Toledo, Spain.
- [19] Internal Medicine Department, León University Hospital, León, Spain.
- [20] Internal Medicine Department, 12 de Octubre University Hospital, Madrid, Spain.

- [21] Internal Medicine Department, Infanta Cristina University Hospital, Parla, Madrid, Spain.

- PMID: **33606820**
- PMCID: PMC7894924
- DOI: 10.1371/journal.pone.0247422

Abstract

Aim: To determine whether healthcare workers (HCW) hospitalized in Spain due to COVID-19 have a worse prognosis than non-healthcare workers (NHCW).

Methods: Observational cohort study based on the SEMI-COVID-19 Registry, a nationwide registry that collects sociodemographic, clinical, laboratory, and treatment data on patients hospitalised with COVID-19 in Spain. Patients aged 20-65 years were selected. A multivariate logistic regression model was performed to identify factors associated with mortality.

Results: As of 22 May 2020, 4393 patients were included, of whom 419 (9.5%) were HCW. Median (interquartile range) age of HCW was 52 (15) years and 62.4% were women. Prevalence of comorbidities and severe radiological findings upon admission were less frequent in HCW. There were no difference in need of respiratory support and admission to intensive care unit, but occurrence of sepsis and in-hospital mortality was lower in HCW (1.7% vs. 3.9%; p = 0.024 and 0.7% vs. 4.8%; p<0.001 respectively). Age, male sex and comorbidity, were independently associated with higher in-hospital mortality and healthcare working with lower mortality (OR 0.211, 95%CI 0.067-0.667, p = 0.008). 30-days survival was higher in HCW (0.968 vs. 0.851 p<0.001).

Conclusions: Hospitalized COVID-19 HCW had fewer comorbidities and a better prognosis than NHCW. Our results suggest that professional exposure to COVID-19 in HCW does not carry more clinical severity nor mortality.

Conflict of interest statement

The authors have declared that no competing interests exist.

- Cited by 2 articles
- 28 references
- 2 figures

Supplementary info

Publication types, MeSH terms, Grant support Expand

Publication types

- Clinical Trial
- Multicenter Study
- Observational Study

MeSH terms

- Adult
- Aged
- COVID-19 / mortality*
- COVID-19 / therapy
- Female
- Health Personnel*
- Hospitalization*
- Humans
- Male
- Middle Aged
- Occupational Exposure / adverse effects*
- Prevalence
- Registries*
- Retrospective Studies
- Risk Factors
- SARS-CoV-2*
- Spain / epidemiology

Grant support

The authors received no specific funding for this work.

Full text links

OPEN ACCESS TO FULL TEXT PLOS ONE Public Library of Science Free PMC article

Proceed to details

Cite

Share

1,388 results

Show more results

[x]

Cite

Copy

Download .nbib

Format: NLM

[x]

Share

-
-

Permalink

Copy

first first First

previous previous Prev

Page
2
of 7

Next next next | Last last last

Send To

- Clipboard
- Email
- Save
- My Bibliography
- Collections
- Citation Manager

[x]

- Article type
- Species
- Language
- Sex
- Journal
- Age

- ☐ Address
- ☐ Autobiography
- ☐ Bibliography
- ☐ Biography
- ☐ Case Reports
- ☐ Classical Article
- ☐ Clinical Conference
- ☐ Clinical Study
- ☐ Clinical Trial Protocol
- ☐ Clinical Trial, Phase I
- ☐ Clinical Trial, Phase II
- ☐ Clinical Trial, Phase III
- ☐ Clinical Trial, Phase IV
- ☐ Clinical Trial, Veterinary
- ☐ Comment
- ☐ Comparative Study
- ☐ Congress
- ☐ Consensus Development Conference
- ☐ Consensus Development Conference, NIH
- ☐ Controlled Clinical Trial
- ☐ Corrected and Republished Article
- ☐ Dataset
- ☐ Dictionary
- ☐ Directory
- ☐ Duplicate Publication
- ☐ Editorial
- ☐ Electronic Supplementary Materials

- ☐ English Abstract
- ☐ Evaluation Study
- ☐ Festschrift
- ☐ Government Publication
- ☐ Guideline
- ☐ Historical Article
- ☐ Interactive Tutorial
- ☐ Interview
- ☐ Introductory Journal Article
- ☐ Lecture
- ☐ Legal Case
- ☐ Legislation
- ☐ Letter
- ☐ Multicenter Study
- ☐ News
- ☐ Newspaper Article
- ☐ Observational Study
- ☐ Observational Study, Veterinary
- ☐ Overall
- ☐ Patient Education Handout
- ☐ Periodical Index
- ☐ Personal Narrative
- ☐ Portrait
- ☐ Practice Guideline
- ☐ Pragmatic Clinical Trial
- ☐ Preprint
- ☐ Published Erratum
- ☐ Research Support, American Recovery and Reinvestment Act
- ☐ Research Support, N.I.H., Extramural
- ☐ Research Support, N.I.H., Intramural
- ☐ Research Support, Non-U.S. Gov't
- ☐ Research Support, U.S. Gov't, Non-P.H.S.
- ☐ Research Support, U.S. Gov't, P.H.S.
- ☐ Research Support, U.S. Gov't
- ☐ Retracted Publication
- ☐ Retraction of Publication
- ☐ Scientific Integrity Review
- ☐ Technical Report
- ☐ Twin Study
- ☐ Validation Study
- ☐ Video-Audio Media
- ☐ Webcast

- ☐ Humans
- ☐ Other Animals

- ☐ Afrikaans
- ☐ Albanian
- ☐ Arabic

- ☐ Armenian
- ☐ Azerbaijani
- ☐ Bosnian
- ☐ Bulgarian
- ☐ Catalan
- ☐ Chinese
- ☐ Croatian
- ☐ Czech
- ☐ Danish
- ☐ Dutch
- ☐ English
- ☐ Esperanto
- ☐ Estonian
- ☐ Finnish
- ☐ French
- ☐ Georgian
- ☐ German
- ☐ Greek, Modern
- ☐ Hebrew
- ☐ Hindi
- ☐ Hungarian
- ☐ Icelandic
- ☐ Indonesian
- ☐ Italian
- ☐ Japanese
- ☐ Kinyarwanda
- ☐ Korean
- ☐ Latin
- ☐ Latvian
- ☐ Lithuanian
- ☐ Macedonian
- ☐ Malay
- ☐ Malayalam
- ☐ Maori
- ☐ Multiple Languages
- ☐ Norwegian
- ☐ Persian
- ☐ Polish
- ☐ Portuguese
- ☐ Pushto
- ☐ Romanian
- ☐ Russian
- ☐ Sanskrit
- ☐ Scottish gaelic
- ☐ Serbian
- ☐ Slovak
- ☐ Slovenian
- ☐ Spanish

- ☐ Swedish
- ☐ Thai
- ☐ Turkish
- ☐ Ukrainian
- ☐ Undetermined
- ☐ Vietnamese
- ☐ Welsh

- ☐ Female
- ☐ Male

- ☐ MEDLINE

- ☐ Child: birth-18 years
- ☐ Newborn: birth-1 month
- ☐ Infant: birth-23 months
- ☐ Infant: 1-23 months
- ☐ Preschool Child: 2-5 years
- ☐ Child: 6-12 years
- ☐ Adolescent: 13-18 years
- ☐ Adult: 19+ years
- ☐ Young Adult: 19-24 years
- ☐ Adult: 19-44 years
- ☐ Middle Aged + Aged: 45+ years
- ☐ Middle Aged: 45-64 years
- ☐ Aged: 65+ years
- ☐ 80 and over: 80+ years

Cancel Show
Close dialog
Back to Top
Jump to page Close dialog
3 of 7
Jump

NCBI Literature Resources

MeSH PMC Bookshelf Disclaimer

Follow NCBI

Connect with NLM

-

-
-

National Library of Medicine
8600 Rockville Pike
Bethesda, MD 20894

Web Policies
FOIA
HHS Vulnerability Disclosure

Help
Accessibility
Careers

- NLM
- NIH
- HHS
- USA.gov

ERREUR p
du site :
Domaine
