# Supplementary material for: COVID-19 treatment of hospital patients worldwide at the onset of the pandemic in 2020: a systematic review
Source: BMC Infect Dis. 2025 Dec 17;26:107. doi: 10.1186/s12879-025-12368-2 (PMC12822144; doi:10.1186/s12879-025-12368-2)
Supplement: Supplementary file 4 — Supplementary Material 4 [file 12879_2025_12368_MOESM4_ESM.zip › 12879_2025_12368_MOESM4_ESM/Search Pubmed 2022 03 28 retrospective observational study hospital treatment covid 1-200.pdf]

Skip to main page content

COVID-19 Information

Public health information (CDC)

Research information (NIH)

SARS-CoV-2 data (NCBI)

Prevention and treatment information (HHS)

Español

Close

Account

Logged in as:
username

- Dashboard
- Publications
- Account settings
- Log out

Access keys NCBI Homepage MyNCBI Homepage Main Content Main Navigation

Search Page

pubmed

Search: retrospective observation Search
Advanced Create alert Create RSS Clipboard
User Guide
Filters 0 Timeline
Sorted by: Best match
Sorted by: Best match Display options
Save Email
Send to

Save citations to file

Selection: All results on this page

Format: Summary (text)

Create file Cancel

# Email citations

Subject: retrospective observational study hospital treatm - PubMed

To: antoine.bosquet@lmr.apl

Selection: All results on this page

Format: Summary

☐ MeSH and other data

Send email Cancel

Send citations to clipboard

Selection: All results on this page

Send Cancel

Add to Collections

Selection: All results on this page

- ○ Create a new collection
- ◉ Add to an existing collection

Name your collection:

Name must be less than 100 characters

Choose a collection:

Unable to load your collection due to an error

Please try again

Add Cancel

Add to My Bibliography

Selection: All results on this page

- ◉ My Bibliography

Unable to load your delegates due to an error

Please try again

Add Cancel

Create a file for external citation management software

Selection: All results on this page

Create file Cancel

Your saved search

Name of saved search: retrospective observation

Search terms: retrospective observational study

Test search terms

Would you like email updates of new search results?

Saved Search Alert Radio Buttons

- Yes
- No

Email: antoine.bosquet@lmr.aphp.fr (change)

Frequency: Monthly

Which day? The first Sunday

Which day? Sunday

Report format: Summary

Send at most: 5 items

Send even when there aren't any new results

Optional text in email:

Save Cancel

Your RSS Feed

Name of RSS Feed: retrospective observation

Number of items displayed: 15

Create RSS Cancel

RSS Link Your RSS Feed Link Copy

My NCBI Filters

- All (1,388)
- Assistance Publique Hopitaux de Paris (0)
- clinical trial (17)
- Review (1)

Show Fewer

Results by year Expand/collapse timeline

Reset

Table representation of search results timeline featuring number of search results per year.

Year	Number of Results
2020	548
2021	893
2022	147

Text availability

- Abstract
- Free full text
- Full text

Article attribute

- Associated data

Article type

- Books and Documents
- Clinical Trial
- Meta-Analysis
- Randomized Controlled Trial
- Review
- Systematic Review

Publication date

- 1 year
- 5 years
- 10 years
- Custom Range

Additional filters Reset all filters

Search Results

clear all

1,388 results

first first previous previous

Page

1

of 7

next next last last

Use COVID-19 filters from PubMed Clinical Queries to refine your search

- Treatment
- Mechanism
- Transmission
- More filters

See more SARS-CoV-2 literature, sequence, and clinical content from NCBI

Results by year Expand/collapse timeline

Reset

Filters applied: . *Clear all* Select search result to email or save

Page 1

☐ 1

Observational Study

Clin Microbiol Infect

. 2021 Jan;27(1):83-88.

doi: 10.1016/j.cmi.2020.07.041. Epub 2020 Jul 31.

Incidence of co-infections and superinfections in hospitalized patients with COVID-19: a retrospective cohort study

Carolina Garcia-Vidal [1], Gemma Sanjuan [2], Estela Moreno-García [2], Pedro Puerta-Alcalde [2], Nicole Garcia-Pouton [2], Mariana Chumbita [2], Mariana Fernandez-Pittol [3], Cristina Pitart [3], Alexy Inciarte [2], Marta Bodro [2], Laura Morata [2], Juan Ambrosioni [2], Ignacio Grafia [2], Fernanda Meira [2], Irene Macaya [2], Celia Cardozo [2], Climent Casals [3], Adrian Tellez [4], Pedro Castro [4], Francesc Marco [3], Felipe García [2], Josep Mensa [2], José Antonio Martínez [2], Alex Soriano [2], COVID-19 Researchers Group

Collaborators, Affiliations Expand

Collaborators

- **COVID-19 Researchers Group**: Verónica Rico [5], Marta Hernández-Meneses [5], Daiana Agüero [5], Berta Torres [5], Ana González [5], Lorena de la Mora [5], Jhon Rojas [5], Laura Linares [5], Berta Fidalgo [5], Natalia Rodriguez [5], David Nicolas [5], Laia Albiach [5], José Muñoz [5], Alex Almuedo [5], Daniel Camprubí [5], Ma Angeles Marcos [5], Daniel Camprubí [5], Catia Cilloniz [5], Sara Fernández [5], Jose M Nicolas [5], Antoni Torres [5]

Affiliations

- [1] Department of Infectious Diseases, Hospital Clinic of Barcelona, IDIBAPS, Barcelona, Spain. Electronic address: cgarciav@clinic.cat.

- [2] Department of Infectious Diseases, Hospital Clinic of Barcelona, IDIBAPS, Barcelona, Spain.
- [3] Department of Microbiology, Hospital Clinic, University of Barcelona, ISGLOBAL, Barcelona, Spain.
- [4] Medical Intensive Care Unit, Hospital Clinic of Barcelona, IDIBAPS, Barcelona, Spain.
- [5] Hospital Clinic-IDIBAPS, University of Barcelona, Barcelona, Spain.

- PMID: **32745596**
- PMCID: PMC7836762
- DOI: 10.1016/j.cmi.2020.07.041

Free PMC article
Observational Study

Incidence of co-infections and superinfections in hospitalized patients with COVID-19: a retrospective cohort study

Carolina Garcia-Vidal et al. Clin Microbiol Infect. 2021 Jan.
Free PMC article

Show details

Clin Microbiol Infect
. 2021 Jan;27(1):83-88.
doi: 10.1016/j.cmi.2020.07.041. Epub 2020 Jul 31.

Authors

Carolina Garcia-Vidal [1], Gemma Sanjuan [2], Estela Moreno-García [2], Pedro Puerta-Alcalde [2], Nicole Garcia-Pouton [2], Mariana Chumbita [2], Mariana Fernandez-Pittol [3], Cristina Pitart [3], Alexy Inciarte [2], Marta Bodro [2], Laura Morata [2], Juan Ambrosioni [2], Ignacio Grafia [2], Fernanda Meira [2], Irene Macaya [2], Celia Cardozo [2], Climent Casals [3], Adrian Tellez [4], Pedro Castro [4], Francesc Marco [3], Felipe García [2], Josep Mensa [2], José Antonio Martínez [2], Alex Soriano [2], COVID-19 Researchers Group

Collaborators

- **COVID-19 Researchers Group**: Verónica Rico [5], Marta Hernández-Meneses [5], Daiana Agüero [5], Berta Torres [5], Ana González [5], Lorena de la Mora [5], Jhon Rojas [5], Laura Linares [5], Berta Fidalgo [5], Natalia Rodriguez [5], David Nicolas [5], Laia Albiach [5], José Muñoz [5], Alex Almuedo [5], Daniel Camprubí [5], Ma Angeles Marcos [5], Daniel Camprubí [5], Catia Cilloniz [5], Sara Fernández [5], Jose M Nicolas [5], Antoni Torres [5]

Affiliations

- [1] Department of Infectious Diseases, Hospital Clinic of Barcelona, IDIBAPS, Barcelona, Spain. Electronic address: cgarciav@clinic.cat.
- [2] Department of Infectious Diseases, Hospital Clinic of Barcelona, IDIBAPS, Barcelona, Spain.
- [3] Department of Microbiology, Hospital Clinic, University of Barcelona, ISGLOBAL, Barcelona, Spain.
- [4] Medical Intensive Care Unit, Hospital Clinic of Barcelona, IDIBAPS, Barcelona, Spain.
- [5] Hospital Clinic-IDIBAPS, University of Barcelona, Barcelona, Spain.

- PMID: **32745596**
- PMCID: PMC7836762
- DOI: 10.1016/j.cmi.2020.07.041

Abstract

Objectives: To describe the burden, epidemiology and outcomes of co-infections and superinfections occurring in hospitalized patients with coronavirus disease 2019 (COVID-19).

Methods: We performed an observational cohort study of all consecutive patients admitted for ≥48 hours to the Hospital Clinic of Barcelona for COVID-19 (28 February to 22 April 2020) who were discharged or dead. We describe demographic, epidemiologic, laboratory and microbiologic results, as well as outcome data retrieved from electronic health records.

Results: Of a total of 989 consecutive patients with COVID-19, 72 (7.2%) had 88 other microbiologically confirmed infections: 74 were bacterial, seven fungal and seven viral. Community-acquired co-infection at COVID-19 diagnosis was uncommon (31/989, 3.1%) and mainly caused by Streptococcus pneumoniae and Staphylococcus aureus. A total of 51 hospital-acquired bacterial superinfections, mostly caused by Pseudomonas aeruginosa and Escherichia coli, were diagnosed in 43 patients (4.7%), with a mean (SD) time from hospital admission to superinfection diagnosis of 10.6 (6.6) days. Overall mortality was 9.8% (97/989). Patients with community-acquired co-infections and hospital-acquired superinfections had worse outcomes.

Conclusions: Co-infection at COVID-19 diagnosis is uncommon. Few patients developed superinfections during hospitalization. These findings are different compared to those of other viral pandemics. As it relates to hospitalized patients with COVID-19, such findings could prove essential in defining the role of empiric antimicrobial therapy or stewardship strategies.

Keywords: COVID-19; Co-infections; Mortality; SARS-CoV-2; Superinfections.

Copyright © 2020 European Society of Clinical Microbiology and Infectious Diseases. Published by Elsevier Ltd. All rights reserved.

- 19 references

Supplementary info

Publication types, MeSH terms, Substances Expand

Publication types

- Observational Study

MeSH terms

- Aged
- Anti-Bacterial Agents / therapeutic use
- Bacterial Infections / epidemiology*
- Bacterial Infections / microbiology
- Bacterial Infections / mortality
- Bacterial Infections / therapy
- Bacterial Typing Techniques
- Blood Culture / methods
- COVID-19 / epidemiology*
- COVID-19 / mortality
- COVID-19 / therapy
- COVID-19 / virology
- Coinfection
- Community-Acquired Infections
- Cross Infection / epidemiology*
- Cross Infection / microbiology
- Cross Infection / mortality
- Cross Infection / therapy
- Female
- Hospitalization
- Hospitals
- Humans
- Incidence
- Male
- Middle Aged
- Mycoses / epidemiology*
- Mycoses / microbiology
- Mycoses / mortality
- Mycoses / therapy
- Retrospective Studies
- SARS-CoV-2 / pathogenicity*
- Spain / epidemiology
- Sputum / microbiology
- Superinfection / epidemiology*
- Superinfection / mortality
- Superinfection / therapy
- Superinfection / virology
- Survival Analysis
- Virus Diseases / epidemiology*

- Virus Diseases / mortality
- Virus Diseases / therapy
- Virus Diseases / virology

Substances

- Anti-Bacterial Agents

Full text links

ELSEVIER OPEN ACCESS Elsevier Science Free PMC article
Proceed to details
Cite
Share
2
Observational Study
BMJ
. 2021 Mar 31;372:n693.
doi: 10.1136/bmj.n693.

Post-covid syndrome in individuals admitted to hospital with covid-19: retrospective cohort study

Daniel Ayoubkhani [1], Kamlesh Khunti [2], Vahé Nafilyan [1], Thomas Maddox [1], Ben Humberstone [1], Ian Diamond [1], Amitava Banerjee [3] [4] [5]
Affiliations Expand

Affiliations

- [1] Office for National Statistics, Government Buildings, Newport, UK.
- [2] Diabetes Research Centre, University of Leicester, Leicester, UK.
- [3] Institute of Health Informatics, University College London, London NW1 2DA, UK ami.banerjee@ucl.ac.uk.
- [4] University College London Hospitals NHS Trust, London, UK.
- [5] Barts Health NHS Trust, Royal London Hospital, London, UK.

- PMID: **33789877**
- PMCID: PMC8010267
- DOI: 10.1136/bmj.n693

Free PMC article
Observational Study

Post-covid syndrome in individuals admitted to hospital with covid-19: retrospective cohort study

Daniel Ayoubkhani et al. BMJ. 2021.
Free PMC article
Show details
BMJ
. 2021 Mar 31;372:n693.
doi: 10.1136/bmj.n693.

Authors

Daniel Ayoubkhani [1], Kamlesh Khunti [2], Vahé Nafilyan [1], Thomas Maddox [1], Ben Humberstone [1], Ian Diamond [1], Amitava Banerjee [3] [4] [5]

Affiliations

- [1] Office for National Statistics, Government Buildings, Newport, UK.
- [2] Diabetes Research Centre, University of Leicester, Leicester, UK.
- [3] Institute of Health Informatics, University College London, London NW1 2DA, UK ami.banerjee@ucl.ac.uk.
- [4] University College London Hospitals NHS Trust, London, UK.
- [5] Barts Health NHS Trust, Royal London Hospital, London, UK.

- PMID: **33789877**
- PMCID: PMC8010267
- DOI: 10.1136/bmj.n693

Abstract

Objective: To quantify rates of organ specific dysfunction in individuals with covid-19 after discharge from hospital compared with a matched control group from the general population.

Design: Retrospective cohort study.

Setting: NHS hospitals in England.

Participants: 47 780 individuals (mean age 65, 55% men) in hospital with covid-19 and discharged alive by 31 August 2020, exactly matched to controls from a pool of about 50 million people in England for personal and clinical characteristics from 10 years of electronic health records.

Main outcome measures: Rates of hospital readmission (or any admission for controls), all cause mortality, and diagnoses of respiratory, cardiovascular, metabolic, kidney, and liver diseases until 30 September 2020. Variations in rate ratios by age, sex, and ethnicity.

Results: Over a mean follow-up of 140 days, nearly a third of individuals who were discharged from hospital after acute covid-19 were readmitted (14 060 of 47 780) and more than 1 in 10 (5875) died after discharge, with these events occurring at rates four and eight times greater, respectively, than in the matched control group. Rates of respiratory disease ($P<0.001$), diabetes ($P<0.001$), and cardiovascular disease ($P<0.001$) were also significantly raised in patients with covid-19, with 770 (95% confidence interval 758 to 783), 127 (122 to 132), and 126 (121 to 131) diagnoses per 1000 person years, respectively. Rate ratios were greater for individuals aged less than 70 than for those aged 70 or older, and in ethnic minority groups compared with the white population, with the largest differences seen for respiratory disease (10.5 (95% confidence interval 9.7 to 11.4) for age less than 70 years *v* 4.6 (4.3 to 4.8) for age ≥70, and 11.4 (9.8 to 13.3) for non-white *v* 5.2 (5.0 to 5.5) for white individuals).

Conclusions: Individuals discharged from hospital after covid-19 had increased rates of multiorgan dysfunction compared with the expected risk in the general population. The increase in risk was not confined to the elderly and was not uniform across ethnicities. The diagnosis, treatment, and prevention of post-covid syndrome requires integrated rather than organ or disease specific approaches, and urgent research is needed to establish the risk factors.

© Author(s) (or their employer(s)) 2019. Re-use permitted under CC BY. No commercial re-use. See rights and permissions. Published by BMJ.

Conflict of interest statement

Competing interests: All authors have completed the ICMJE uniform disclosure form at www.icmje.org/coi_disclosure.pdf and declare: no support from any organisation for the submitted work; no financial relationships with any organisations that might have an interest in the submitted work in the previous three years; KK is chair of the ethnicity subgroup of the Independent Scientific Advisory Group for Emergencies (SAGE), a member of Independent SAGE, a trustee of the South Asian Health Foundation (SAHF), and director of the University of Leicester Centre for Black Minority Ethnic Health; and AB is a trustee of SAHF and has received a research grant unrelated to the current work from AstraZeneca.

Comment in

- Fresh evidence of the scale and scope of long covid.
  Sivan M, Rayner C, Delaney B. Sivan M, et al. BMJ. 2021 Apr 1;373:n853. doi: 10.1136/bmj.n853. BMJ. 2021. PMID: 33795224 No abstract available.
- Acknowledging breathlessness post-covid.
  Hopkinson N. Hopkinson N. BMJ. 2021 May 20;373:n1264. doi: 10.1136/bmj.n1264. BMJ. 2021. PMID: 34016654 No abstract available.
- Developing useful early warning and prognostic scores for COVID-19.
  Coughlan C, Rahman S, Honeyford K, Costelloe CE. Coughlan C, et al. Postgrad Med J. 2021 Aug;97(1150):477-480. doi: 10.1136/postgradmedj-2021-140086. Epub 2021 May 28. Postgrad Med J. 2021. PMID: 34049992 No abstract available.

- 36 references
- 3 figures

Supplementary info

Publication types, MeSH terms Expand

Publication types

- Observational Study

MeSH terms

- Adult
- Aged
- COVID-19 / complications*
- COVID-19 / diagnosis
- COVID-19 / mortality
- COVID-19 / virology
- Cardiovascular Diseases / epidemiology
- Case-Control Studies
- Diabetes Mellitus / epidemiology
- England / epidemiology
- Ethnicity
- Female
- Hospitalization / statistics & numerical data*
- Humans
- Male
- Middle Aged
- Multiple Organ Failure / epidemiology*
- Patient Discharge / statistics & numerical data
- Patient Readmission / statistics & numerical data*
- Respiratory Tract Diseases / epidemiology
- Retrospective Studies
- Risk Factors
- SARS-CoV-2 / genetics
- SARS-CoV-2 / isolation & purification

Full text links

BMJ Open Access Full Text HighWire Free PMC article
Proceed to details
Cite
Share
☐ 3
Comment
Diabetes Care
. 2020 Dec;43(12):2999-3006.
doi: 10.2337/dc20-1521. Epub 2020 Sep 29.

Sitagliptin Treatment at the Time of Hospitalization Was Associated With Reduced Mortality in Patients With Type 2 Diabetes and COVID-19: A Multicenter, Case-Control, Retrospective, Observational Study

Sebastiano Bruno Solerte [1 2], Francesca D'Addio [3], Roberto Trevisan [4], Elisabetta Lovati [5], Antonio Rossi [6], Ida Pastore [6], Marco Dell'Acqua [3 6], Elio Ippolito [3], Cristiana Scaranna [4], Rosalia Bellante [4], Silvia Galliani [4], Alessandro Roberto Dodesini [4], Giuseppe Lepore [4], Francesca Geni [1 2], Roberta Maria Fiorina [3], Emanuele Catena [7], Angelo Corsico [8], Riccardo Colombo [7], Marco Mirani [9], Carlo De Riva [10], Salvatore Endrio Oleandri [11], Reza Abdi [12], Joseph V Bonventre [12], Stefano Rusconi [13 14], Franco Folli [15], Antonio Di Sabatino [5], Gianvincenzo Zuccotti [16 17], Massimo Galli [13 14], Paolo Fiorina [18 6 19]
Affiliations Expand

Affiliations

- [1] Department of Internal Medicine, Geriatric and Diabetology Unit, University of Pavia, Italy.
- [2] School of Geriatrics, University of Pavia Azienda di Servizi alla Persona-Pavia, Pavia, Italy.
- [3] International Center for T1D, Pediatric Clinical Research Center Romeo ed Enrica Invernizzi, Dipartimento di Scienze Biomediche e Cliniche L. Sacco, Università di Milano, Milan, Italy.
- [4] Unità Operativa Complessa Malattie Endocrine 1-Diabetologia, Ospedale Papa Giovanni XXIII Azienda Socio Sanitaria Territoriale-PG XXIII, Bergamo, Italy.
- [5] Internal Medicine Unit, University of Pavia and IRCCS Policlinico San Matteo, Pavia, Italy.
- [6] Division of Endocrinology, Azienda Socio Sanitaria Territoriale Fatebenefratelli Sacco, Milan, Italy.
- [7] Department of Anesthesia and Intensive Care Unit, Azienda Socio Sanitaria Territoriale Fatebenefratelli Sacco, Luigi Sacco Hospital, Università di Milano, Milan, Italy.
- [8] Pneumology Unit, Fondazione IRCCS Policlinico San Matteo and Department of Internal Medicine and Therapeutics, University of Pavia, Pavia, Italy.
- [9] Endocrinology and Diabetology Unit, Humanitas Research Hospital, Rozzano, Milan, Italy.
- [10] Unità Operativa di Malattie Endocrine ULSS3-Ospedale dell'Angelo Mestre, Mestre, Italy.
- [11] Department of Endocrinology and Metabolism, Azienda Sanitaria Locale Città di Torino, Torino, Italy.
- [12] Renal Division, Brigham and Women's Hospital, Boston, MA.

- [13] Department of Biomedical and Clinical Sciences "Luigi Sacco," Univerisità di Milano, Milan, Italy.
- [14] III Division of Infectious Diseases, Azienda Socio Sanitaria Territoriale Fatebenefratelli Sacco, Luigi Sacco Hospital, Milan, Italy.
- [15] Endocrinology and Metabolism, Department of Health Science, Università di Milano, Azienda Socio Sanitaria Territoriale Santi Paolo e Carlo, Milan, Italy.
- [16] Pediatric Clinical Research Center Romeo ed Enrica Invernizzi, Dipartimento di Scienze Biomediche e Cliniche, Università di Milano, Milan, Italy.
- [17] Department of Pediatrics, "V. Buzzi" Children's Hospital, Milan, Italy.
- [18] International Center for T1D, Pediatric Clinical Research Center Romeo ed Enrica Invernizzi, Dipartimento di Scienze Biomediche e Cliniche L. Sacco, Università di Milano, Milan, Italy paolo.fiorina@childrens.harvard.edu.
- [19] Division of Nephrology, Boston Children's Hospital, Harvard Medical School, Boston, MA.

- PMID: **32994187**
- PMCID: PMC7770266
- DOI: 10.2337/dc20-1521

Free PMC article
Comment

Sitagliptin Treatment at the Time of Hospitalization Was Associated With Reduced Mortality in Patients With Type 2 Diabetes and COVID-19: A Multicenter, Case-Control, Retrospective, Observational Study

Sebastiano Bruno Solerte et al. Diabetes Care. 2020 Dec.
Free PMC article
Show details
Diabetes Care
. 2020 Dec;43(12):2999-3006.
doi: 10.2337/dc20-1521. Epub 2020 Sep 29.

Authors

Sebastiano Bruno Solerte [1] [2], Francesca D'Addio [3], Roberto Trevisan [4], Elisabetta Lovati [5], Antonio Rossi [6], Ida Pastore [6], Marco Dell'Acqua [3] [6], Elio Ippolito [3], Cristiana Scaranna [4], Rosalia Bellante [4], Silvia Galliani [4], Alessandro Roberto Dodesini [4], Giuseppe Lepore [4], Francesca Geni [1] [2], Roberta Maria Fiorina [3], Emanuele Catena [7], Angelo Corsico [8], Riccardo Colombo [7], Marco Mirani [9], Carlo De Riva [10], Salvatore Endrio Oleandri [11], Reza Abdi

[12], Joseph V Bonventre [12], Stefano Rusconi [13] [14], Franco Folli [15], Antonio Di Sabatino [5], Gianvincenzo Zuccotti [16] [17], Massimo Galli [13] [14], Paolo Fiorina [18] [6] [19]

Affiliations

- [1] Department of Internal Medicine, Geriatric and Diabetology Unit, University of Pavia, Italy.
- [2] School of Geriatrics, University of Pavia Azienda di Servizi alla Persona-Pavia, Pavia, Italy.
- [3] International Center for T1D, Pediatric Clinical Research Center Romeo ed Enrica Invernizzi, Dipartimento di Scienze Biomediche e Cliniche L. Sacco, Università di Milano, Milan, Italy.
- [4] Unità Operativa Complessa Malattie Endocrine 1-Diabetologia, Ospedale Papa Giovanni XXIII Azienda Socio Sanitaria Territoriale-PG XXIII, Bergamo, Italy.
- [5] Internal Medicine Unit, University of Pavia and IRCCS Policlinico San Matteo, Pavia, Italy.
- [6] Division of Endocrinology, Azienda Socio Sanitaria Territoriale Fatebenefratelli Sacco, Milan, Italy.
- [7] Department of Anesthesia and Intensive Care Unit, Azienda Socio Sanitaria Territoriale Fatebenefratelli Sacco, Luigi Sacco Hospital, Università di Milano, Milan, Italy.
- [8] Pneumology Unit, Fondazione IRCCS Policlinico San Matteo and Department of Internal Medicine and Therapeutics, University of Pavia, Pavia, Italy.
- [9] Endocrinology and Diabetology Unit, Humanitas Research Hospital, Rozzano, Milan, Italy.
- [10] Unità Operativa di Malattie Endocrine ULSS3-Ospedale dell'Angelo Mestre, Mestre, Italy.
- [11] Department of Endocrinology and Metabolism, Azienda Sanitaria Locale Città di Torino, Torino, Italy.
- [12] Renal Division, Brigham and Women's Hospital, Boston, MA.
- [13] Department of Biomedical and Clinical Sciences "Luigi Sacco," Univeristà di Milano, Milan, Italy.
- [14] III Division of Infectious Diseases, Azienda Socio Sanitaria Territoriale Fatebenefratelli Sacco, Luigi Sacco Hospital, Milan, Italy.
- [15] Endocrinology and Metabolism, Department of Health Science, Università di Milano, Azienda Socio Sanitaria Territoriale Santi Paolo e Carlo, Milan, Italy.
- [16] Pediatric Clinical Research Center Romeo ed Enrica Invernizzi, Dipartimento di Scienze Biomediche e Cliniche, Università di Milano, Milan, Italy.
- [17] Department of Pediatrics, "V. Buzzi" Children's Hospital, Milan, Italy.
- [18] International Center for T1D, Pediatric Clinical Research Center Romeo ed Enrica Invernizzi, Dipartimento di Scienze Biomediche e Cliniche L. Sacco, Università di Milano, Milan, Italy paolo.fiorina@childrens.harvard.edu.
- [19] Division of Nephrology, Boston Children's Hospital, Harvard Medical School, Boston, MA.

- PMID: **32994187**
- PMCID: PMC7770266
- DOI: 10.2337/dc20-1521

Abstract

Objective: Poor outcomes have been reported in patients with type 2 diabetes and coronavirus disease 2019 (COVID-19); thus, it is mandatory to explore novel therapeutic approaches for this population.

Research design and methods: In a multicenter, case-control, retrospective, observational study, sitagliptin, an oral and highly selective dipeptidyl peptidase 4 inhibitor, was added to standard of care (e.g., insulin administration) at the time of hospitalization in patients with type 2 diabetes who were hospitalized with COVID-19. Every center also recruited at a 1:1 ratio untreated control subjects matched for age and sex. All patients had pneumonia and exhibited oxygen saturation <95% when breathing ambient air or when receiving oxygen support. The primary end points were discharge from the hospital/death and improvement of clinical outcomes, defined as an increase in at least two points on a seven-category modified ordinal scale. Data were collected retrospectively from patients receiving sitagliptin from 1 March through 30 April 2020.

Results: Of the 338 consecutive patients with type 2 diabetes and COVID-19 admitted in Northern Italy hospitals included in this study, 169 were on sitagliptin, while 169 were on standard of care. Treatment with sitagliptin at the time of hospitalization was associated with reduced mortality (18% vs. 37% of deceased patients; hazard ratio 0.44 [95% CI 0.29-0.66]; P = 0.0001), with an improvement in clinical outcomes (60% vs. 38% of improved patients; P = 0.0001) and with a greater number of hospital discharges (120 vs. 89 of discharged patients; P = 0.0008) compared with patients receiving standard of care, respectively.

Conclusions: In this multicenter, case-control, retrospective, observational study of patients with type 2 diabetes admitted to the hospital for COVID-19, sitagliptin treatment at the time of hospitalization was associated with reduced mortality and improved clinical outcomes as compared with standard-of-care treatment. The effects of sitagliptin in patients with type 2 diabetes and COVID-19 should be confirmed in an ongoing randomized, placebo-controlled trial.

© 2020 by the American Diabetes Association.

Comment in

- Reduced COVID-19 Mortality With Sitagliptin Treatment? Weighing the Dissemination of Potentially Lifesaving Findings Against the Assurance of High Scientific Standards.
  Nauck MA, Meier JJ. Nauck MA, et al. Diabetes Care. 2020 Dec;43(12):2906-2909. doi: 10.2337/dci20-0062. Epub 2020 Oct 8. Diabetes Care. 2020. PMID: 33033068 No abstract available.

Comment on

- Reduced COVID-19 Mortality With Sitagliptin Treatment? Weighing the Dissemination of Potentially Lifesaving Findings Against the Assurance of High Scientific Standards.
  Nauck MA, Meier JJ. Nauck MA, et al. Diabetes Care. 2020 Dec;43(12):2906-2909. doi: 10.2337/dci20-0062. Epub 2020 Oct 8. Diabetes Care. 2020. PMID: 33033068 No abstract available.

- 47 references
- 2 figures

Supplementary info

Publication types, MeSH terms, Substances, Associated data, Grant support Expand

Publication types

- Research Support, Non-U.S. Gov't
- Comment

MeSH terms

- Betacoronavirus
- COVID-19
- Coronavirus Infections*
- Coronavirus*
- Diabetes Mellitus, Type 2* / drug therapy
- Hospitalization
- Humans
- Italy
- Pandemics*
- Pneumonia, Viral*
- Retrospective Studies
- SARS-CoV-2
- Sitagliptin Phosphate / therapeutic use

Substances

- Sitagliptin Phosphate

Associated data

- figshare/10.2337/figshare.12907490

Grant support

- R37 DK039773/DK/NIDDK NIH HHS/United States

Full text links

American Diabetes Association FULL TEXT Silverchair Information Systems Free PMC article
Proceed to details
Cite
Share
☐ 4

Observational Study

Pulm Pharmacol Ther

. 2021 Apr;67:101989.
doi: 10.1016/j.pupt.2021.101989. Epub 2021 Jan 16.

Antihistamines and azithromycin as a treatment for COVID-19 on primary health care - A retrospective observational study in elderly patients

Juan Ignacio Morán Blanco [1], Judith A Alvarenga Bonilla [1], Sakae Homma [2], Kazuo Suzuki [3], Philip Fremont-Smith [4], Karina Villar Gómez de Las Heras [5]

Affiliations Expand

Affiliations

- [1] Servicio de Salud de Castilla-La Mancha (SESCAM), Toledo, Spain; Centro de Salud de Yepes, Av. Santa Reliquia, 26, 45313, Yepes, Toledo, Spain.
- [2] Department of Advanced and Integrated Interstitial Lung Diseases Research, School of Medicine, Toho University, Ota-ku, Tokyo, 143-8540, Japan.
- [3] Asia International Institute of Infectious Disease Control, and Department of Health Protection, Graduate School of Medicine, Teikyo University, Itabashi-ku, Tokyo, 173-8605, Japan.
- [4] Massachusetts Institute of Technology Lincoln Laboratory, Lexington, MA, USA.
- [5] Delegación Provincial de la Consejería de Sanidad. Servicio de Salud Pública, C/ Río Guadalmena, 2, 45007, Toledo, Spain. Electronic address: kvillar@jccm.es.

- PMID: **33465426**
- PMCID: PMC7833340
- DOI: 10.1016/j.pupt.2021.101989

Free PMC article
Observational Study

Antihistamines and azithromycin as a treatment for COVID-19 on primary health care - A retrospective observational study in elderly patients

Juan Ignacio Morán Blanco et al. Pulm Pharmacol Ther. 2021 Apr.
Free PMC article
Show details

Pulm Pharmacol Ther
. 2021 Apr;67:101989.
doi: 10.1016/j.pupt.2021.101989. Epub 2021 Jan 16.

Authors

Juan Ignacio Morán Blanco [1], Judith A Alvarenga Bonilla [1], Sakae Homma [2], Kazuo Suzuki [3], Philip Fremont-Smith [4], Karina Villar Gómez de Las Heras [5]

Affiliations

- [1] Servicio de Salud de Castilla-La Mancha (SESCAM), Toledo, Spain; Centro de Salud de Yepes, Av. Santa Reliquia, 26, 45313, Yepes, Toledo, Spain.
- [2] Department of Advanced and Integrated Interstitial Lung Diseases Research, School of Medicine, Toho University, Ota-ku, Tokyo, 143-8540, Japan.
- [3] Asia International Institute of Infectious Disease Control, and Department of Health Protection, Graduate School of Medicine, Teikyo University, Itabashi-ku, Tokyo, 173-8605, Japan.
- [4] Massachusetts Institute of Technology Lincoln Laboratory, Lexington, MA, USA.
- [5] Delegación Provincial de la Consejería de Sanidad. Servicio de Salud Pública, C/ Río Guadalmena, 2, 45007, Toledo, Spain. Electronic address: kvillar@jccm.es.

- PMID: **33465426**
- PMCID: PMC7833340
- DOI: 10.1016/j.pupt.2021.101989

Abstract

Between March and April 2020, 84 elderly patients with suspected COVID-19 living in two nursing homes of Yepes, Toledo (Spain) were treated early with antihistamines (dexchlorpheniramine, cetirizine or loratadine), adding azithromycin in the 25 symptomatic cases. The outcomes are retrospectively reported. The primary endpoint is the fatality rate of COVID-19. The secondary endpoints are the hospital and ICU admission rates. Endpoints were compared with the official Spanish rates for the elderly. The mean age of our population was 85 and 48% were over 80 years old. No hospital admissions, deaths, nor adverse drug effects were reported in our patient population. By the end of June, 100% of the residents had positive serology for COVID-19. Although clinical trials are needed to determine the efficacy of both drugs in the treatment of COVID-19, this analysis suggests that primary care diagnosis and treatment with antihistamines, plus azithromycin in selected cases, may treat COVID-19 and prevent progression to severe disease in elderly patients.

Keywords: Antihistamines; Azithromycin; Coronavirus; Elderly; SARS-CoV-2.

Copyright © 2021 Elsevier Ltd. All rights reserved.

Conflict of interest statement

The authors declare no conflict of interest.

- 45 references
- 2 figures

Supplementary info

Publication types, MeSH terms, Substances, Supplementary concepts Expand

Publication types

- Observational Study

MeSH terms

- Aged
- Aged, 80 and over
- Azithromycin / therapeutic use*
- COVID-19 / drug therapy*
- COVID-19 / epidemiology
- COVID-19 / virology
- Female
- Histamine Antagonists / therapeutic use*
- Humans
- Male
- Middle Aged
- Primary Health Care
- Retrospective Studies
- SARS-CoV-2 / isolation & purification
- Spain / epidemiology
- Treatment Outcome

Substances

- Histamine Antagonists
- Azithromycin

Supplementary concepts

- COVID-19 drug treatment

Full text links

ELSEVIER FULL-TEXT ARTICLE Elsevier Science Free PMC article
Proceed to details
Cite
Share
☐ 5

Observational Study

Hepatology
. 2020 Oct;72(4):1169-1176.
doi: 10.1002/hep.31487.

Abnormal Liver Tests in COVID-19: A Retrospective Observational Cohort Study of 1,827 Patients in a Major U.S. Hospital Network

Melanie A Hundt [1], Yanhong Deng [2], Maria M Ciarleglio [2], Michael H Nathanson [1] [3], Joseph K Lim [1] [3]
Affiliations Expand

Affiliations

- [1] Department of Medicine, Yale School of Medicine, New Haven, CT.
- [2] Yale Center for Analytical Sciences, Yale School of Public Health, New Haven, CT.
- [3] Yale Liver Center and Section of Digestive Diseases, Yale School of Medicine, New Haven, CT.

- PMID: **32725890**
- DOI: 10.1002/hep.31487

Observational Study

Abnormal Liver Tests in COVID-19: A Retrospective Observational Cohort Study of 1,827 Patients in a Major U.S. Hospital Network

Melanie A Hundt et al. Hepatology. 2020 Oct.
Show details
Hepatology
. 2020 Oct;72(4):1169-1176.
doi: 10.1002/hep.31487.

Authors

Melanie A Hundt [1], Yanhong Deng [2], Maria M Ciarleglio [2], Michael H Nathanson [1] [3], Joseph K Lim [1] [3]

Affiliations

- [1] Department of Medicine, Yale School of Medicine, New Haven, CT.
- [2] Yale Center for Analytical Sciences, Yale School of Public Health, New Haven, CT.
- [3] Yale Liver Center and Section of Digestive Diseases, Yale School of Medicine, New Haven, CT.

- PMID: **32725890**
- DOI: 10.1002/hep.31487

Abstract

Background and aims: The coronavirus-19 disease (COVID-19) pandemic, caused by the severe acute respiratory syndrome coronavirus 2 virus, is associated with significant morbidity and mortality attributable to pneumonia, acute respiratory distress syndrome, and multiorgan failure. Liver injury has been reported as a nonpulmonary manifestation of COVID-19, but characterization of liver test abnormalities and their association with clinical outcomes is incomplete.

Approach and results: We conducted a retrospective cohort study of 1,827 patients with confirmed COVID-19 who were hospitalized within the Yale-New Haven Health System between March 14, 2020 and April 23, 2020. Clinical characteristics, liver tests (aspartate aminotransferase [AST], alanine aminotransferase [ALT], alkaline phosphatase [ALP], total bilirubin [TBIL], and albumin) at three time points (preinfection baseline, admission, and peak hospitalization), and hospitalization outcomes (severe COVID-19, intensive care unit [ICU] admission, mechanical ventilation, and death) were analyzed. Abnormal liver tests were commonly observed in hospitalized patients with COVID-19, both at admission (AST 66.9%, ALT 41.6%, ALP 13.5%, and TBIL 4.3%) and peak hospitalization (AST 83.4%, ALT 61.6%, ALP 22.7%, and TBIL 16.1%). Most patients with abnormal liver tests at admission had minimal elevations 1-2× the upper limit of normal (ULN; AST 63.7%, ALT 63.5%, ALP 80.0%, and TBIL 75.7%). A significant proportion of these patients had abnormal liver tests prehospitalization (AST 25.9%, ALT 38.0%, ALP 56.8%, and TBIL 44.4%). Multivariate analysis revealed an association between abnormal liver tests and severe COVID-19, including ICU admission, mechanical ventilation, and death; associations with age, male sex, body mass index, and diabetes mellitus were also observed. Medications used in COVID-19 treatment (lopinavir/ritonavir, hydroxychloroquine, remdesivir, and tocilizumab) were associated with peak hospitalization liver transaminase elevations >5× ULN.

Conclusions: Abnormal liver tests occur in most hospitalized patients with COVID-19 and may be associated with poorer clinical outcomes.

© 2020 by the American Association for the Study of Liver Diseases.

Comment in

- Letter to the Editor: Serum Albumin in COVID-19: A Good Example in Which Analytical and Clinical Performance of a Laboratory Test Are Strictly Intertwined.
  Pasqualetti S, Aloisio E, Panteghini M. Pasqualetti S, et al. Hepatology. 2021 Nov;74 (5):2905-2907. doi: 10.1002/hep.31791. Epub 2021 Sep 16. Hepatology. 2021. PMID: 33665867 Free PMC article.

- 31 references

Supplementary info

Publication types, MeSH terms, Grant support Expand

Publication types

- Observational Study
- Research Support, N.I.H., Extramural

MeSH terms

- Adolescent
- Adult
- Aged
- Aged, 80 and over
- COVID-19 / physiopathology*
- Child
- Child, Preschool
- Female
- Hospitalization
- Humans
- Infant
- Liver / physiopathology*
- Liver Function Tests
- Male
- Middle Aged
- Retrospective Studies
- SARS-CoV-2*
- Young Adult

Grant support

- P01-DK57751/DK/NIDDK NIH HHS/United States
- P30-DK34989/DK/NIDDK NIH HHS/United States
- R01-DK 114041/DK/NIDDK NIH HHS/United States
- R01-DK112797/DK/NIDDK NIH HHS/United States

Full text links

WILEY Full Text Article Wiley

Proceed to details

Cite

Share

☐ 6

Observational Study

J Am Med Dir Assoc
. 2020 Jul;21(7):928-932.e1.
doi: 10.1016/j.jamda.2020.06.008. Epub 2020 Jun 9.

Frailty and Mortality in Hospitalized Older Adults With COVID-19: Retrospective Observational Study

Robert De Smet [1], Bea Mellaerts [1], Hannelore Vandewinckele [1], Peter Lybeert [1], Eric Frans [2], Sara Ombelet [3], Wim Lemahieu [3], Rolf Symons [4], Erwin Ho [5], Johan Frans [5], Annick Smismans [5], Michaël R Laurent [6]

Affiliations Expand

Affiliations

- [1] Geriatrics Department, Imelda Hospital, Bonheiden, Belgium.
- [2] Pulmonology and Intensive Care Medicine, Imelda Hospital, Bonheiden, Belgium.
- [3] Nephrology and Infectious Diseases, Imelda Hospital, Bonheiden, Belgium.
- [4] Radiology Department, Imelda Hospital, Bonheiden, Belgium.
- [5] Department of Medical Microbiology, Imelda Hospital, Bonheiden, Belgium.
- [6] Geriatrics Department, Imelda Hospital, Bonheiden, Belgium. Electronic address: michael.laurent@imelda.be.

- PMID: **32674821**
- PMCID: PMC7280137
- DOI: 10.1016/j.jamda.2020.06.008

Free PMC article
Observational Study

Frailty and Mortality in Hospitalized Older Adults With COVID-19: Retrospective Observational Study

Robert De Smet et al. J Am Med Dir Assoc. 2020 Jul.
Free PMC article
Show details
J Am Med Dir Assoc
. 2020 Jul;21(7):928-932.e1.
doi: 10.1016/j.jamda.2020.06.008. Epub 2020 Jun 9.

Authors

Robert De Smet [1], Bea Mellaerts [1], Hannelore Vandewinckele [1], Peter Lybeert [1], Eric Frans [2], Sara Ombelet [3], Wim Lemahieu [3], Rolf Symons [4], Erwin Ho [5], Johan Frans [5], Annick Smismans [5], Michaël R Laurent [6]

Affiliations

- [1] Geriatrics Department, Imelda Hospital, Bonheiden, Belgium.
- [2] Pulmonology and Intensive Care Medicine, Imelda Hospital, Bonheiden, Belgium.
- [3] Nephrology and Infectious Diseases, Imelda Hospital, Bonheiden, Belgium.
- [4] Radiology Department, Imelda Hospital, Bonheiden, Belgium.
- [5] Department of Medical Microbiology, Imelda Hospital, Bonheiden, Belgium.
- [6] Geriatrics Department, Imelda Hospital, Bonheiden, Belgium. Electronic address: michael.laurent@imelda.be.

- PMID: **32674821**
- PMCID: PMC7280137
- DOI: 10.1016/j.jamda.2020.06.008

Abstract

Objectives: To determine the association between frailty and short-term mortality in older adults hospitalized for coronavirus disease 2019 (COVID-19).

Design: Retrospective single-center observational study.

Setting and participants: Eighty-one patients with COVID-19 confirmed by reverse-transcriptase polymerase chain reaction (RT-PCR), at the Geriatrics department of a general hospital in Belgium.

Measurements: Frailty was graded according to the Rockwood Clinical Frailty Scale (CFS). Demographic, biochemical, and radiologic variables, comorbidities, symptoms, and treatment were extracted from electronic medical records.

Results: Participants (N = 48 women, 59%) had a median age of 85 years (range 65-97 years) and a median CFS score of 7 (range 2-9); 42 (52%) were long-term care residents. Within 6 weeks, 18 patients died. Mortality was significantly but weakly associated with age (Spearman $r = 0.241$, $P = .03$) and CFS score ($r = 0.282$, $P = .011$), baseline lactate dehydrogenase (LDH; $r = 0.301$, $P = .009$), lymphocyte count ($r = -0.262$, $P = .02$), and RT-PCR cycle threshold (Ct, $r = -0.285$, $P = .015$). Mortality was not associated with long-term care residence, dementia, delirium, or polypharmacy. In multivariable logistic regression analyses, CFS, LDH, and RT-PCR Ct (but not age) remained independently associated with mortality. Both age and frailty had poor specificity to predict survival. A multivariable model combining age, CFS, LDH, and viral load significantly predicted survival.

Conclusions and implications: Although their prognosis is worse, even the oldest and most severely frail patients may benefit from hospitalization for COVID-19, if sufficient resources are available.

Keywords: COVID-19; frailty; hospitalization; older adults; severe acute respiratory syndrome coronavirus 2.

Copyright © 2020 AMDA – The Society for Post-Acute and Long-Term Care Medicine. Published by Elsevier Inc. All rights reserved.

- 30 references
- 2 figures

Supplementary info

Publication types, MeSH terms Expand

Publication types

- Observational Study

MeSH terms

- Aged
- Aged, 80 and over
- Belgium / epidemiology
- COVID-19
- Cohort Studies
- Coronavirus Infections / epidemiology*
- Coronavirus Infections / prevention & control
- Disease Outbreaks / statistics & numerical data*
- Female
- Frail Elderly
- Frailty / mortality*
- Geriatric Assessment
- Hospital Mortality*
- Hospitalization / statistics & numerical data
- Hospitals, General
- Humans
- Incidence
- Male
- Pandemics / prevention & control
- Pandemics / statistics & numerical data*
- Pneumonia, Viral / epidemiology*
- Pneumonia, Viral / prevention & control
- Retrospective Studies

Full text links

ELSEVIER FULL-TEXT ARTICLE Elsevier Science Free PMC article
Proceed to details
Cite
Share
7
Observational Study
BMC Infect Dis
. 2020 Oct 22;20(1):787.
doi: 10.1186/s12879-020-05452-2.

Clinical characteristics of 116 hospitalized patients with COVID-19 in Wuhan, China: a single-centered, retrospective, observational study

Shiqiang Xiong [1], Lin Liu [2], Feng Lin [1], Jinhu Shi [1], Lei Han [3], Huijian Liu [1], Lewei He [1], Qijun Jiang [1], Zeyang Wang [1], Wenbo Fu [1], Zhigang Li [1], Qing Lu [1], Zhinan Chen [1], Shifang Ding [4]
Affiliations Expand

Affiliations

- [1] Department of Cardiology, the General Hospital of Central Theater Command, Wuhan, 430000, China.
- [2] Department of Integrated Traditional Chinese and Western Internal Medicine, the General Hospital of Central Theater Command, Wuhan, 430000, China.
- [3] Division of Medical Management, the General Hospital of Central Theater Command, Wuhan, 430000, China.
- [4] Department of Cardiology, the General Hospital of Central Theater Command, Wuhan, 430000, China. dsfmdwh@163.com.

- PMID: **33092539**
- PMCID: PMC7578439
- DOI: 10.1186/s12879-020-05452-2

Free PMC article
Observational Study

Clinical characteristics of 116 hospitalized patients with COVID-19 in Wuhan, China: a

single-centered, retrospective, observational study

Shiqiang Xiong et al. BMC Infect Dis. 2020.
Free PMC article
Show details
BMC Infect Dis
. 2020 Oct 22;20(1):787.
doi: 10.1186/s12879-020-05452-2.

Authors

Shiqiang Xiong [1], Lin Liu [2], Feng Lin [1], Jinhu Shi [1], Lei Han [3], Huijian Liu [1], Lewei He [1], Qijun Jiang [1], Zeyang Wang [1], Wenbo Fu [1], Zhigang Li [1], Qing Lu [1], Zhinan Chen [1], Shifang Ding [4]

Affiliations

- [1] Department of Cardiology, the General Hospital of Central Theater Command, Wuhan, 430000, China.
- [2] Department of Integrated Traditional Chinese and Western Internal Medicine, the General Hospital of Central Theater Command, Wuhan, 430000, China.
- [3] Division of Medical Management, the General Hospital of Central Theater Command, Wuhan, 430000, China.
- [4] Department of Cardiology, the General Hospital of Central Theater Command, Wuhan, 430000, China. dsfmdwh@163.com.

- PMID: **33092539**
- PMCID: PMC7578439
- DOI: 10.1186/s12879-020-05452-2

Abstract

Background: A cluster of acute respiratory illness, now known as Corona Virus Disease 2019 (COVID-19) caused by 2019 novel coronavirus (SARS-CoV-2), has become a global pandemic. Aged population with cardiovascular diseases are more likely be to infected with SARS-CoV-2 and result in more severe outcomes and elevated case-fatality rate. Meanwhile, cardiovascular diseases have a high prevalence in the middle-aged and elderly population. However, despite of several researches in COVID-19, cardiovascular implications related to it still remains largely unclear. Therefore, a specific analysis in regard to cardiovascular implications of COVID-19 patients is in great need.

Methods: In this single-centered, retrospective, observational study, 116 patients with laboratory-confirmed COVID-19 were enrolled, who admitted to the General Hospital of Central Theater Command (Wuhan, China) from January 20 to March 8, 2020. The demographic data, underlying comorbidities, clinical symptoms and signs, laboratory findings, chest computed tomography, treatment measures, and outcome data were collected from electronic medical records. Data were compared between non-severe and severe cases.

Results: Of 116 hospitalized patients with COVID-19, the median age was 58.5 years (IQR, 47.0-69.0), and 36 (31.0%) were female. Hypertension (45 [38.8%]), diabetes (19 [16.4%]), and coronary heart disease (17 [14.7%]) were the most common coexisting conditions. Common symptoms included fever [99 (85.3%)], dry cough (61 [52.6%]), fatigue (60 [51.7%]), dyspnea (52 [44.8%]), anorexia (50 [43.1%]), and chest discomfort (50 [43.1%]). Local and/or bilateral patchy shadowing were the typical radiological findings on chest computed tomography. Lymphopenia (lymphocyte count, 1.0×10^9/L [IQR, 0.7-1.3]) was observed in 66 patients (56.9%), and elevated lactate dehydrogenase (245.5 U/L [IQR, 194.3-319.8]) in 69 patients (59.5%). Hypokalemia occurred in 24 (20.7%) patients. Compared with non-severe cases, severe cases were older (64.0 years [IQR, 53.0-76.0] vs 56.0 years [IQR, 37.0-64.0]), more likely to have comorbidities (35 [63.6%] vs 24 [39.3%]), and more likely to develop acute cardiac injury (19 [34.5%] vs 4 [6.6%]), acute heart failure (18 [32.7%] vs 3 [4.9%]), and ARDS (20 [36.4%] vs 0 [0%]). During hospitalization, the prevalence of new onset hypertension was significantly higher in severe patients (55.2% vs 19.0%) than in non-severe ones.

Conclusions: In this single-centered, retrospective, observational study, we found that the infection of SARS-CoV-2 was more likely to occur in middle and aged population with cardiovascular comorbidities. Cardiovascular complications, including new onset hypertension and heart injury were common in severe patients with COVID-19. More detailed researches in cardiovascular involvement in COVID-19 are urgently needed to further understand the disease.

Keywords: COVID-19; Cardiovascular disease; Coronary heart disease; Hypertension; SARS-CoV-2.

Conflict of interest statement

The authors declare that they have no competing interests.

- 24 references
- 2 figures

Supplementary info

Publication types, MeSH terms, Grant support Expand

Publication types

- Observational Study

MeSH terms

- Aged
- Betacoronavirus
- COVID-19
- China / epidemiology
- Comorbidity*
- Coronavirus Infections / epidemiology*
- Coronavirus Infections / pathology
- Coronavirus Infections / physiopathology*

- Cough / epidemiology
- Female
- Fever / epidemiology
- Hospitalization / statistics & numerical data*
- Humans
- Lymphopenia / epidemiology
- Lymphopenia / pathology
- Male
- Middle Aged
- Pandemics
- Pneumonia, Viral / epidemiology*
- Pneumonia, Viral / pathology
- Pneumonia, Viral / physiopathology*
- Retrospective Studies
- SARS-CoV-2
- Severe Acute Respiratory Syndrome / epidemiology

Grant support

- 31600942/National Natural Science Foundation of China

Full text links

Read free full text at BMC BioMed Central Free PMC article

Proceed to details

Cite

Share

☐ 8

Observational Study

Clin Microbiol Infect

. 2021 Jan;27(1):105-111.

doi: 10.1016/j.cmi.2020.09.014. Epub 2020 Sep 22.

Efficacy of corticosteroid treatment for hospitalized patients with severe COVID-19: a multicentre study

Michele Bartoletti [1], Lorenzo Marconi [2], Luigia Scudeller [3], Livia Pancaldi [4], Sara Tedeschi [4], Maddalena Giannella [4], Matteo Rinaldi [4], Linda Bussini [4], Ilaria Valentini [5], Anna Filomena Ferravante [5], Antonella Potalivo [6], Elisa Marchionni [2], Giacomo Fornaro [4], Renato Pascale [4], Zeno Pasquini [7], Massimo Puoti [8], Marco Merli [8], Francesco Barchiesi [7], Francesca Volpato

[4], Arianna Rubin [4], Annalisa Saracino [9], Tommaso Tonetti [10], Paolo Gaibani [11], Vito Marco Ranieri [10], Pierluigi Viale [4], Francesco Cristini [2], PREDICO Study Group
Affiliations Expand

Affiliations

- [1] Infectious Diseases Unit, Department of Medical and Surgical Sciences, Alma Mater Studiorum University of Bologna, Italy. Electronic address: m.bartoletti@unibo.it.
- [2] Infectious Diseases Unit, Rimini-Forlì-Cesena Hospitals, Italy.
- [3] Clinical Trials Team, Scientific Direction, IRCCS Ca' Granda Ospedale Maggiore Policlinico di Milano, Italy.
- [4] Infectious Diseases Unit, Department of Medical and Surgical Sciences, Alma Mater Studiorum University of Bologna, Italy.
- [5] Unit of Pneumology, ASL della Romagna, Italy.
- [6] Department of Emergency, Anaesthesia and Intensive Care Section, Infermi Hospital, Rimini, Italy.
- [7] Infectious Diseases Unit, Azienda Ospedaliera Ospedali Riuniti Marche Nord, Pesaro, Italy; Clinica Malattie Infettive, Dipartimento di Scienze Biomediche e Sanità Pubblica, Università Politecnica delle Marche, Azienda Ospedaliera Universitaria, Ospedali Riuniti Umberto I-Lancisi-Salesi, Ancona, Italy.
- [8] Division of Infectious Diseases, ASST Grande Ospedale Metropolitano Niguarda, Milan, Italy.
- [9] Department of Biomedical Sciences and Human Oncology, Infectious Disease Unit, University of Bari, Policlinico di Bari, Bari, Italy.
- [10] Intensive Care Unit, Department of Medical and Surgical Sciences, Italy.
- [11] Centro di riferimento regionale per le emergenze microbiologiche (CRREM), Clinical Microbiology Unit, Department of Experimental, Diagnostic and Specialty Medicine, Policlinico Sant'Orsola, Bologna, Italy.

- PMID: **32971254**
- PMCID: PMC7506332
- DOI: 10.1016/j.cmi.2020.09.014

Free PMC article
Observational Study

Efficacy of corticosteroid treatment for hospitalized patients with severe COVID-19: a multicentre study

Michele Bartoletti et al. Clin Microbiol Infect. 2021 Jan.
Free PMC article
Show details
Clin Microbiol Infect
. 2021 Jan;27(1):105-111.
doi: 10.1016/j.cmi.2020.09.014. Epub 2020 Sep 22.

Authors

Michele Bartoletti [1], Lorenzo Marconi [2], Luigia Scudeller [3], Livia Pancaldi [4], Sara Tedeschi [4], Maddalena Giannella [4], Matteo Rinaldi [4], Linda Bussini [4], Ilaria Valentini [5], Anna Filomena Ferravante [5], Antonella Potalivo [6], Elisa Marchionni [2], Giacomo Fornaro [4], Renato Pascale [4], Zeno Pasquini [7], Massimo Puoti [8], Marco Merli [8], Francesco Barchiesi [7], Francesca Volpato [4], Arianna Rubin [4], Annalisa Saracino [9], Tommaso Tonetti [10], Paolo Gaibani [11], Vito Marco Ranieri [10], Pierluigi Viale [4], Francesco Cristini [2], PREDICO Study Group

Affiliations

- [1] Infectious Diseases Unit, Department of Medical and Surgical Sciences, Alma Mater Studiorum University of Bologna, Italy. Electronic address: m.bartoletti@unibo.it.
- [2] Infectious Diseases Unit, Rimini-Forlì-Cesena Hospitals, Italy.
- [3] Clinical Trials Team, Scientific Direction, IRCCS Ca' Granda Ospedale Maggiore Policlinico di Milano, Italy.
- [4] Infectious Diseases Unit, Department of Medical and Surgical Sciences, Alma Mater Studiorum University of Bologna, Italy.
- [5] Unit of Pneumology, ASL della Romagna, Italy.
- [6] Department of Emergency, Anaesthesia and Intensive Care Section, Infermi Hospital, Rimini, Italy.
- [7] Infectious Diseases Unit, Azienda Ospedaliera Ospedali Riuniti Marche Nord, Pesaro, Italy; Clinica Malattie Infettive, Dipartimento di Scienze Biomediche e Sanità Pubblica, Università Politecnica delle Marche, Azienda Ospedaliera Universitaria, Ospedali Riuniti Umberto I-Lancisi-Salesi, Ancona, Italy.
- [8] Division of Infectious Diseases, ASST Grande Ospedale Metropolitano Niguarda, Milan, Italy.
- [9] Department of Biomedical Sciences and Human Oncology, Infectious Disease Unit, University of Bari, Policlinico di Bari, Bari, Italy.
- [10] Intensive Care Unit, Department of Medical and Surgical Sciences, Italy.
- [11] Centro di riferimento regionale per le emergenze microbiologiche (CRREM), Clinical Microbiology Unit, Department of Experimental, Diagnostic and Specialty Medicine, Policlinico Sant'Orsola, Bologna, Italy.

- PMID: **32971254**
- PMCID: PMC7506332
- DOI: 10.1016/j.cmi.2020.09.014

Abstract

Objective: To assess the efficacy of corticosteroids in patients with coronavirus disease 2019 (COVID-19).

Methods: A multicentre observational study was performed from 22 February through 30 June 2020. We included consecutive adult patients with severe COVID-19, defined as respiratory rate ≥30 breath per minute, oxygen saturation ≤93% on ambient air or arterial partial pressure of oxygen to fraction of inspired oxygen ≤300 mm Hg. We excluded patients being treated with other immunomodulant drugs, receiving low-dose corticosteroids and receiving corticosteroids 72 hours after admission. The primary endpoint was 30-day mortality from hospital admission. The main

exposure variable was corticosteroid therapy at a dose of ≥0.5 mg/kg of prednisone equivalents. It was introduced as binomial covariate in a logistic regression model for the primary endpoint and inverse probability of treatment weighting using the propensity score.

Results: Of 1717 patients with COVID-19 evaluated, 513 were included in the study, and of these, 170 (33%) were treated with corticosteroids. During hospitalization, 166 patients (34%) met the criteria of the primary outcome (60/170, 35% in the corticosteroid group and 106/343, 31% in the noncorticosteroid group). At multivariable analysis corticosteroid treatment was not associated with lower 30-day mortality rate (adjusted odds ratio, 0.59; 95% confidence interval (CI), 0.20-1.74; p 0.33). After inverse probability of treatment weighting, corticosteroids were not associated with lower 30-day mortality (average treatment effect, 0.05; 95% CI, -0.02 to 0.09; p 0.12). However, subgroup analysis revealed that in patients with PO2/FiO2 < 200 mm Hg at admission (135 patients, 52 (38%) treated with corticosteroids), corticosteroid treatment was associated with a lower risk of 30-day mortality (23/52, 44% vs. 45/83, 54%; adjusted odds ratio, 0.20; 95% CI, 0.04-0.90; p 0.036).

Conclusions: The effect of corticosteroid treatment on mortality might be limited to critically ill COVID-19 patients.

Keywords: ARDS; COVID-19; Corticosteroids; Mortality; SARS-CoV-2.

Copyright © 2020 European Society of Clinical Microbiology and Infectious Diseases. Published by Elsevier Ltd. All rights reserved.

- 19 references
- 1 figure

Supplementary info

Publication types, MeSH terms, Substances Expand

Publication types

- Multicenter Study
- Observational Study

MeSH terms

- Adrenal Cortex Hormones / therapeutic use*
- Adult
- Aged
- Antiviral Agents / therapeutic use
- COVID-19 / drug therapy*
- COVID-19 / mortality*
- COVID-19 / pathology
- Critical Illness
- Female
- Heparin, Low-Molecular-Weight / therapeutic use

- Hospital Mortality
- Hospitals
- Humans
- Hydroxychloroquine / therapeutic use
- Italy
- Length of Stay / statistics & numerical data
- Male
- Middle Aged
- Odds Ratio
- Respiratory Distress Syndrome / drug therapy*
- Respiratory Distress Syndrome / mortality*
- Respiratory Distress Syndrome / pathology
- Retrospective Studies
- SARS-CoV-2 / drug effects
- SARS-CoV-2 / pathogenicity*
- Severity of Illness Index
- Survival Analysis
- Treatment Outcome

Substances

- Adrenal Cortex Hormones
- Antiviral Agents
- Heparin, Low-Molecular-Weight
- Hydroxychloroquine

Full text links

ELSEVIER OPEN ACCESS Elsevier Science Free PMC article
Proceed to details
Cite
Share
☐ 9
Observational Study
Respir Care
. 2021 Jun;66(6):909-919.
doi: 10.4187/respcare.08631. Epub 2020 Dec 16.

High-Flow Nasal Cannula Therapy in COVID-19: Using the ROX Index to Predict Success

Abhimanyu Chandel [1], Saloni Patolia [2], A Whitney Brown [3], A Claire Collins [4], Dhwani Sahjwani [5], Vikramjit Khangoora [3], Paula C Cameron [6], Mehul Desai [7], Aditya Kasarabada [6], Jack K Kilcullen [6], Steven D Nathan [3], Christopher S King [3]
Affiliations Expand

Affiliations

- [1] Department of Pulmonary and Critical Care, Walter Reed National Military Medical Center, Bethesda, Maryland. abhimanyu.chandel.mil@mail.mil.
- [2] Virginia Commonwealth University School of Medicine, Richmond, Virginia.
- [3] Department of Advanced Lung Disease and Transplant, Inova Fairfax Hospital, Falls Church, Virginia.
- [4] Advanced Lung Disease Research, Inova Fairfax Hospital, Falls Church, Virginia.
- [5] Department of Pediatrics, Inova Fairfax Hospital, Falls Church, Virginia.
- [6] Respiratory Therapy, Inova Fairfax Hospital, Falls Church, Virginia.
- [7] Medical Critical Care Service, Inova Fairfax Hospital, Falls Church, Virginia.

- PMID: **33328179**
- DOI: 10.4187/respcare.08631

Observational Study

High-Flow Nasal Cannula Therapy in COVID-19: Using the ROX Index to Predict Success

Abhimanyu Chandel et al. Respir Care. 2021 Jun.
Show details
Respir Care
. 2021 Jun;66(6):909-919.
doi: 10.4187/respcare.08631. Epub 2020 Dec 16.

Authors

Abhimanyu Chandel [1], Saloni Patolia [2], A Whitney Brown [3], A Claire Collins [4], Dhwani Sahjwani [5], Vikramjit Khangoora [3], Paula C Cameron [6], Mehul Desai [7], Aditya Kasarabada [6], Jack K Kilcullen [6], Steven D Nathan [3], Christopher S King [3]

Affiliations

- [1] Department of Pulmonary and Critical Care, Walter Reed National Military Medical Center, Bethesda, Maryland. abhimanyu.chandel.mil@mail.mil.
- [2] Virginia Commonwealth University School of Medicine, Richmond, Virginia.
- [3] Department of Advanced Lung Disease and Transplant, Inova Fairfax Hospital, Falls Church, Virginia.
- [4] Advanced Lung Disease Research, Inova Fairfax Hospital, Falls Church, Virginia.
- [5] Department of Pediatrics, Inova Fairfax Hospital, Falls Church, Virginia.
- [6] Respiratory Therapy, Inova Fairfax Hospital, Falls Church, Virginia.
- [7] Medical Critical Care Service, Inova Fairfax Hospital, Falls Church, Virginia.

- PMID: **33328179**
- DOI: 10.4187/respcare.08631

Abstract

Background: Optimal timing of mechanical ventilation in COVID-19 is uncertain. We sought to evaluate outcomes of delayed intubation and examine the ROX index (ie, [[Formula: see text]]/breathing frequency) to predict weaning from high-flow nasal cannula (HFNC) in patients with COVID-19.

Methods: We performed a multicenter, retrospective, observational cohort study of subjects with respiratory failure due to COVID-19 and managed with HFNC. The ROX index was applied to predict HFNC success. Subjects that failed HFNC were divided into early HFNC failure (≤ 48 h of HFNC therapy prior to mechanical ventilation) and late failure (> 48 h). Standard statistical comparisons and regression analyses were used to compare overall hospital mortality and secondary end points, including time-specific mortality, need for extracorporeal membrane oxygenation, and ICU length of stay between early and late failure groups.

Results: 272 subjects with COVID-19 were managed with HFNC. One hundred sixty-four (60.3%) were successfully weaned from HFNC, and 111 (67.7%) of those weaned were managed solely in non-ICU settings. ROX index >3.0 at 2, 6, and 12 hours after initiation of HFNC was 85.3% sensitive for identifying subsequent HFNC success. One hundred eight subjects were intubated for failure of HFNC (61 early failures and 47 late failures). Mortality after HFNC failure was high (45.4%). There was no statistical difference in hospital mortality (39.3% vs 53.2%, $P = .18$) or any of the secondary end points between early and late HFNC failure groups. This remained true even when adjusted for covariates.

Conclusions: In this retrospective review, HFNC was a viable strategy and mechanical ventilation was unecessary in the majority of subjects. In the minority that progressed to mechanical ventilation, duration of HFNC did not differentiate subjects with worse clinical outcomes. The ROX index was sensitive for the identification of subjects successfully weaned from HFNC. Prospective studies in COVID-19 are warranted to confirm these findings and to optimize patient selection for use of HFNC in this disease.

Keywords: COVID-19; SARS-CoV-2; high-flow nasal cannula; hypoxemic respiratory failure; respiratory insufficiency; viral pneumonia.

Copyright © 2021 by Daedalus Enterprises.

Conflict of interest statement

The authors have disclosed no conflicts of interest.

Comment in

- Predicting Success of High-Flow Nasal Cannula in COVID-19.
  Varipapa RJ Jr, Sonti R. Varipapa RJ Jr, et al. Respir Care. 2021 Jun;66(6):1044-1045. doi: 10.4187/respcare.09212. Respir Care. 2021. PMID: 34039764 No abstract available.

Supplementary info

Publication types, MeSH terms Expand

Publication types

- Multicenter Study
- Observational Study

MeSH terms

- COVID-19*
- Cannula
- Humans
- Noninvasive Ventilation*
- Oxygen Inhalation Therapy
- Prospective Studies
- Respiratory Insufficiency* / therapy
- Retrospective Studies
- SARS-CoV-2

Full text links

Final Version Respir Care HighWire
Proceed to details
Cite
Share
☐ 10
Observational Study
Clin Nutr
. 2021 Apr;40(4):2154-2161.
doi: 10.1016/j.clnu.2020.09.040. Epub 2020 Oct 1.

Nutritional risk and therapy for severe and critical COVID-19 patients: A multicenter retrospective observational study

Gang Li [1], Chen-Liang Zhou [2], Yuan-Ming Ba [3], Ye-Ming Wang [4], Bin Song [5], Xiao-Bin Cheng [6], Qiu-Fen Dong [7], Liu-Lin Wang [8], Sha-Sha You [9]
Affiliations Expand

Affiliations

- [1] Department of Critical Care Medicine, Hubei Provincial Hospital of Traditional Chinese Medicine, Wuhan, China; Hubei Province Academy of Traditional Chinese Medicine, Wuhan, China. Electronic address: marty007@163.com.
- [2] Department of Critical Care Medicine, Renmin Hospital of Wuhan University, Wuhan, China. Electronic address: 54665420@qq.com.
- [3] Department of Nephrology, Hubei Provincial Hospital of Traditional Chinese Medicine, Wuhan, China. Electronic address: 1723426138@qq.com.
- [4] Department of Critical Care Medicine, Hubei Provincial Hospital of Integrated Chinese & Western Medicine, Wuhan, China. Electronic address: 804995318@qq.com.
- [5] Department of Critical Care Medicine, Jin Yin-tan Hospital, Wuhan, China. Electronic address: 76499599@qq.com.
- [6] Department of Critical Care Medicine, Hubei Provincial Hospital of Traditional Chinese Medicine, Wuhan, China. Electronic address: baggion2015@163.com.
- [7] Department of Critical Care Medicine, Hubei Provincial Hospital of Traditional Chinese Medicine, Wuhan, China. Electronic address: dongqiufen2011@sina.com.
- [8] Department of Critical Care Medicine, Hubei Provincial Hospital of Traditional Chinese Medicine, Wuhan, China. Electronic address: wangll60@163.com.
- [9] Department of Critical Care Medicine, Hubei Provincial Hospital of Integrated Chinese & Western Medicine, Wuhan, China. Electronic address: 805015396@qq.com.

- PMID: **33077274**
- PMCID: PMC7527833
- DOI: 10.1016/j.clnu.2020.09.040

Free PMC article
Observational Study

Nutritional risk and therapy for severe and critical COVID-19 patients: A multicenter retrospective observational study

Gang Li et al. Clin Nutr. 2021 Apr.
Free PMC article
Show details

Clin Nutr
. 2021 Apr;40(4):2154-2161.
doi: 10.1016/j.clnu.2020.09.040. Epub 2020 Oct 1.

Authors

Gang Li [1], Chen-Liang Zhou [2], Yuan-Ming Ba [3], Ye-Ming Wang [4], Bin Song [5], Xiao-Bin Cheng [6], Qiu-Fen Dong [7], Liu-Lin Wang [8], Sha-Sha You [9]

Affiliations

- [1] Department of Critical Care Medicine, Hubei Provincial Hospital of Traditional Chinese Medicine, Wuhan, China; Hubei Province Academy of Traditional Chinese Medicine, Wuhan, China. Electronic address: marty007@163.com.
- [2] Department of Critical Care Medicine, Renmin Hospital of Wuhan University, Wuhan, China. Electronic address: 54665420@qq.com.
- [3] Department of Nephrology, Hubei Provincial Hospital of Traditional Chinese Medicine, Wuhan, China. Electronic address: 1723426138@qq.com.
- [4] Department of Critical Care Medicine, Hubei Provincial Hospital of Integrated Chinese & Western Medicine, Wuhan, China. Electronic address: 804995318@qq.com.
- [5] Department of Critical Care Medicine, Jin Yin-tan Hospital, Wuhan, China. Electronic address: 76499599@qq.com.
- [6] Department of Critical Care Medicine, Hubei Provincial Hospital of Traditional Chinese Medicine, Wuhan, China. Electronic address: baggion2015@163.com.
- [7] Department of Critical Care Medicine, Hubei Provincial Hospital of Traditional Chinese Medicine, Wuhan, China. Electronic address: dongqiufen2011@sina.com.
- [8] Department of Critical Care Medicine, Hubei Provincial Hospital of Traditional Chinese Medicine, Wuhan, China. Electronic address: wangll60@163.com.
- [9] Department of Critical Care Medicine, Hubei Provincial Hospital of Integrated Chinese & Western Medicine, Wuhan, China. Electronic address: 805015396@qq.com.

- PMID: **33077274**
- PMCID: PMC7527833
- DOI: 10.1016/j.clnu.2020.09.040

Abstract

Objective: To evaluate the nutritional risk and therapy in severe and critical patients with COVID-19.

Methods: A total of 523 patients enrolled from four hospitals in Wuhan, China. The inclusion time was from January 2, 2020 to February 15. Clinical characteristics and laboratory values were obtained from electronic medical records, nursing records, and related examinations.

Results: Of these patients, 211 (40.3%) were admitted to the ICU and 115 deaths (22.0%). Patients admitted to the ICU had lower BMI and plasma protein levels. The median Nutrition risk in critically ill (NUTRIC) score of 211 patients in the ICU was 5 (4, 6) and Nutritional Risk Screening (NRS) score was 5 (3, 6). The ratio of parenteral nutrition (PN) therapy in non-survivors was greater than that in survivors, and the time to start nutrition therapy was later than that in survivors. The NUTRIC score can independently predict the risk of death in the hospital

(OR = 1.197, 95%CI: 1.091-1.445, p = 0.006) and high NRS score patients have a higher risk of poor outcome in the ICU (OR = 1.880, 95%CI: 1.151-3.070, p = 0.012). After adjusted age and sex, for each standard deviation increase in BMI, the risk of in-hospital death was reduced by 13% (HR = 0.871, 95%CI: 0.795-0.955, p = 0.003), and the risk of ICU transfer was reduced by 7% (HR = 0.932, 95%CI:0.885-0.981, p = 0.007). The in-hospital survival time of patients with albumin level ≤35 g/L was significantly decreased (15.9 d, 95% CI: 13.7-16.3, vs 24.2 d, 95% CI: 22.3-29.7, p < 0.001).

Conclusion: Severe and critical patients with COVID-19 have a high risk of malnutrition. Low BMI and protein levels were significantly associated with adverse events. Early nutritional risk screening and therapy for patients with COVID-19 are necessary.

Keywords: COVID-19; NRS score; NUTRIC score; Nutritional risk.

Copyright © 2020 Elsevier Ltd and European Society for Clinical Nutrition and Metabolism. All rights reserved.

- 34 references
- 3 figures

Supplementary info

Publication types, MeSH terms Expand

Publication types

- Multicenter Study
- Observational Study

MeSH terms

- Adult
- Aged
- COVID-19 / epidemiology*
- COVID-19 / mortality
- COVID-19 / therapy*
- China / epidemiology
- Critical Illness / epidemiology*
- Critical Illness / mortality
- Critical Illness / therapy*
- Female
- Hospital Mortality
- Hospitalization
- Humans
- Intensive Care Units
- Kaplan-Meier Estimate
- Male

- Malnutrition / epidemiology*
- Malnutrition / mortality
- Malnutrition / therapy*
- Middle Aged
- Nutrition Assessment
- Nutritional Status
- Nutritional Support*
- Proportional Hazards Models
- Retrospective Studies
- Risk Assessment
- SARS-CoV-2
- Severity of Illness Index
- Time-to-Treatment

Full text links

ELSEVIER FULL-TEXT ARTICLE Elsevier Science Free PMC article
Proceed to details
Cite
Share
☐ 11
Observational Study
JAMA Neurol
. 2020 Jun 1;77(6):683-690.
doi: 10.1001/jamaneurol.2020.1127.

Neurologic Manifestations of Hospitalized Patients With Coronavirus Disease 2019 in Wuhan, China

Ling Mao [1], Huijuan Jin [1], Mengdie Wang [1], Yu Hu [2], Shengcai Chen [1], Quanwei He [1], Jiang Chang [3], Candong Hong [1], Yifan Zhou [1], David Wang [4], Xiaoping Miao [3], Yanan Li [1], Bo Hu [1]

Affiliations Expand

Affiliations

- [1] Department of Neurology, Union Hospital, Tongji Medical College, Huazhong University of Science and Technology, Wuhan, China.
- [2] Department of Hematology, Union Hospital, Tongji Medical College, Huazhong University of Science and Technology, Wuhan, China.

- [3] Department of Epidemiology and Biostatistics, Key Laboratory for Environment and Health, School of Public Health, Tongji Medical College, Huazhong University of Science and Technology, Wuhan, China.
- [4] Neurovascular Division, Department of Neurology, Barrow Neurological Institute, Saint Joseph's Hospital and Medical Center, Phoenix, Arizona.

- PMID: **32275288**
- PMCID: PMC7149362
- DOI: 10.1001/jamaneurol.2020.1127

Free PMC article
Observational Study

Neurologic Manifestations of Hospitalized Patients With Coronavirus Disease 2019 in Wuhan, China

Ling Mao et al. JAMA Neurol. 2020.
Free PMC article
Show details
JAMA Neurol
. 2020 Jun 1;77(6):683-690.
doi: 10.1001/jamaneurol.2020.1127.

Authors

Ling Mao [1], Huijuan Jin [1], Mengdie Wang [1], Yu Hu [2], Shengcai Chen [1], Quanwei He [1], Jiang Chang [3], Candong Hong [1], Yifan Zhou [1], David Wang [4], Xiaoping Miao [3], Yanan Li [1], Bo Hu [1]

Affiliations

- [1] Department of Neurology, Union Hospital, Tongji Medical College, Huazhong University of Science and Technology, Wuhan, China.
- [2] Department of Hematology, Union Hospital, Tongji Medical College, Huazhong University of Science and Technology, Wuhan, China.
- [3] Department of Epidemiology and Biostatistics, Key Laboratory for Environment and Health, School of Public Health, Tongji Medical College, Huazhong University of Science and Technology, Wuhan, China.
- [4] Neurovascular Division, Department of Neurology, Barrow Neurological Institute, Saint Joseph's Hospital and Medical Center, Phoenix, Arizona.

- PMID: **32275288**
- PMCID: PMC7149362
- DOI: 10.1001/jamaneurol.2020.1127

Abstract

Importance: The outbreak of coronavirus disease 2019 (COVID-19) in Wuhan, China, is serious and has the potential to become an epidemic worldwide. Several studies have described typical clinical manifestations including fever, cough, diarrhea, and fatigue. However, to our knowledge, it has not been reported that patients with COVID-19 had any neurologic manifestations.

Objective: To study the neurologic manifestations of patients with COVID-19.

Design, setting, and participants: This is a retrospective, observational case series. Data were collected from January 16, 2020, to February 19, 2020, at 3 designated special care centers for COVID-19 (Main District, West Branch, and Tumor Center) of the Union Hospital of Huazhong University of Science and Technology in Wuhan, China. The study included 214 consecutive hospitalized patients with laboratory-confirmed diagnosis of severe acute respiratory syndrome coronavirus 2 infection.

Main outcomes and measures: Clinical data were extracted from electronic medical records, and data of all neurologic symptoms were checked by 2 trained neurologists. Neurologic manifestations fell into 3 categories: central nervous system manifestations (dizziness, headache, impaired consciousness, acute cerebrovascular disease, ataxia, and seizure), peripheral nervous system manifestations (taste impairment, smell impairment, vision impairment, and nerve pain), and skeletal muscular injury manifestations.

Results: Of 214 patients (mean [SD] age, 52.7 [15.5] years; 87 men [40.7%]) with COVID-19, 126 patients (58.9%) had nonsevere infection and 88 patients (41.1%) had severe infection according to their respiratory status. Overall, 78 patients (36.4%) had neurologic manifestations. Compared with patients with nonsevere infection, patients with severe infection were older, had more underlying disorders, especially hypertension, and showed fewer typical symptoms of COVID-19, such as fever and cough. Patients with more severe infection had neurologic manifestations, such as acute cerebrovascular diseases (5 [5.7%] vs 1 [0.8%]), impaired consciousness (13 [14.8%] vs 3 [2.4%]), and skeletal muscle injury (17 [19.3%] vs 6 [4.8%]).

Conclusions and relevance: Patients with COVID-19 commonly have neurologic manifestations. During the epidemic period of COVID-19, when seeing patients with neurologic manifestations, clinicians should suspect severe acute respiratory syndrome coronavirus 2 infection as a differential diagnosis to avoid delayed diagnosis or misdiagnosis and lose the chance to treat and prevent further transmission.

Conflict of interest statement

Conflict of Interest Disclosures: None reported.

Comment in

- Anosmia and Ageusia: Common Findings in COVID-19 Patients.
  Vaira LA, Salzano G, Deiana G, De Riu G. Vaira LA, et al. Laryngoscope. 2020 Jul;130 (7):1787. doi: 10.1002/lary.28692. Epub 2020 Apr 15. Laryngoscope. 2020. PMID: 32237238 Free PMC article.
- COVID-19: what if the brain had a role in causing the deaths?
  Tassorelli C, Mojoli F, Baldanti F, Bruno R, Benazzo M. Tassorelli C, et al. Eur J Neurol. 2020 Sep;27(9):e41-e42. doi: 10.1111/ene.14275. Epub 2020 May 14. Eur J Neurol. 2020. PMID: 32333819 Free PMC article. No abstract available.

- [Neurological manifestations of SARS-CoV-2 infection].
Orozco-Hernández JP, Marin-Medina DS, Sánchez-Duque JA. Orozco-Hernández JP, et al. Semergen. 2020 Aug;46 Suppl 1:106-108. doi: 10.1016/j.semerg.2020.05.004. Epub 2020 May 11. Semergen. 2020. PMID: 32439269 Free PMC article. Spanish. No abstract available.
- COVID-19 international neurological registries.
Román GC, Reis J, Spencer PS, Buguet A, Öztürk S, Wasay M; World Federation of Neurology Environmental Neurology Specialty Group. Román GC, et al. Lancet Neurol. 2020 Jun;19(6):484-485. doi: 10.1016/S1474-4422(20)30148-4. Epub 2020 May 26. Lancet Neurol. 2020. PMID: 32470417 Free PMC article. No abstract available.
- Guillain-Barré syndrome in a patient with antibodies against SARS-COV-2.
Helbok R, Beer R, Löscher W, Boesch S, Reindl M, Hornung R, Schiefecker AJ, Deisenhammer F, Pfausler B. Helbok R, et al. Eur J Neurol. 2020 Sep;27(9):1754-1756. doi: 10.1111/ene.14388. Eur J Neurol. 2020. PMID: 32531083 Free PMC article.
- Challenges to medical education at a time of physical distancing.
Gill D, Whitehead C, Wondimagegn D. Gill D, et al. Lancet. 2020 Jul 11;396(10244):77-79. doi: 10.1016/S0140-6736(20)31368-4. Epub 2020 Jun 11. Lancet. 2020. PMID: 32534631 Free PMC article. No abstract available.
- Nervous system: subclinical target of SARS-CoV-2 infection.
Mariotto S, Savoldi A, Donadello K, Zanzoni S, Bozzetti S, Carta S, Zivelonghi C, Alberti D, Piraino F, Minuz P, Girelli D, Crisafulli E, Romano S, Marcon D, Marchi G, Gottin L, Polati E, Zanatta P, Monaco S, Tacconelli E, Ferrari S. Mariotto S, et al. J Neurol Neurosurg Psychiatry. 2020 Sep;91(9):1010-1012. doi: 10.1136/jnnp-2020-323881. Epub 2020 Jun 23. J Neurol Neurosurg Psychiatry. 2020. PMID: 32576611 Free PMC article. No abstract available.
- COVID-19 and neurologic manifestations: a still missing link and a call for neurologists.
Spallazzi M, Morelli N, Taga A. Spallazzi M, et al. Neurol Sci. 2020 Aug;41(8):1997-1998. doi: 10.1007/s10072-020-04540-0. Epub 2020 Jun 26. Neurol Sci. 2020. PMID: 32592104 Free PMC article. No abstract available.
- How does COVID-19 affect the brain?
Aamodt AH, Flinstad Harbo H, Eldøen G, Barratt-Due A, Aukrust P. Aamodt AH, et al. Tidsskr Nor Laegeforen. 2020 May 29;140(10). doi: 10.4045/tidsskr.20.0444. Print 2020 Jun 30. Tidsskr Nor Laegeforen. 2020. PMID: 32602331 English, Norwegian. No abstract available.
- Disease of the Year: COVID-19 and Its Neuro-ophthalmic Complications.
Chwalisz BK, Dinkin MJ. Chwalisz BK, et al. J Neuroophthalmol. 2020 Sep;40(3):283-284. doi: 10.1097/WNO.0000000000001046. J Neuroophthalmol. 2020. PMID: 32604246 Free PMC article. No abstract available.
- Headache in a group of SARS-COVID-19 patients: an observational prospectical study.
Coppola A, Tonini MC, Baratelli E, Barillà C, Bassani R, Gonano EF, Grassi F, Guidi I, Lucchelli F, Mantica D, Mattioli M, Perri G, Riggio MG, Ronzoni M, Tiriticco M, Vanotti A; Garbagnate Covid Group. Coppola A, et al. Neurol Sci. 2020 Dec;41(Suppl 2):503-504. doi: 10.1007/s10072-020-04676-z. Neurol Sci. 2020. PMID: 32915343 Free PMC article. No abstract available.

- 1 figure

Supplementary info

Publication types, MeSH terms Expand

Publication types

- Multicenter Study
- Observational Study

MeSH terms

- Adult
- Aged
- Betacoronavirus*
- COVID-19
- China / epidemiology
- Coronavirus Infections / blood
- Coronavirus Infections / diagnosis*
- Coronavirus Infections / epidemiology*
- Female
- Hospitalization / trends*
- Humans
- Male
- Middle Aged
- Nervous System Diseases / blood
- Nervous System Diseases / diagnosis*
- Nervous System Diseases / epidemiology*
- Pandemics
- Pneumonia, Viral / blood
- Pneumonia, Viral / diagnosis*
- Pneumonia, Viral / epidemiology*
- Retrospective Studies
- SARS-CoV-2

Full text links

FULL TEXT JAMA Neurology Silverchair Information Systems Free PMC article
Proceed to details
Cite
Share
☐ 12
Observational Study
Diabetologia
. 2021 Apr;64(4):778-794.
doi: 10.1007/s00125-020-05351-w. Epub 2021 Feb 17.

Predictors of hospital discharge and mortality in patients with diabetes and COVID-19: updated results from the nationwide CORONADO study

Matthieu Wargny [1 2], Louis Potier [3 4], Pierre Gourdy [5 6], Matthieu Pichelin [1], Coralie Amadou [7 8], Pierre-Yves Benhamou [9 10], Jean-Baptiste Bonnet [11], Lyse Bordier [12], Olivier Bourron [13 14 15 16], Claude Chaumeil [17], Nicolas Chevalier [18], Patrice Darmon [19 20], Blandine Delenne [21], Delphine Demarsy [22], Marie Dumas [23], Olivier Dupuy [24], Anna Flaus-Furmaniuk [25], Jean-François Gautier [4 26], Anne-Marie Guedj [27], Nathalie Jeandidier [28], Etienne Larger [29], Jean-Philippe Le Berre [30], Myriam Lungo [31], Nathanaëlle Montanier [32], Philippe Moulin [33], Françoise Plat [34], Vincent Rigalleau [35], René Robert [36], Dominique Seret-Bégué [37], Pierre Sérusclat [38], Sarra Smati [1], Jean-François Thébaut [17], Blandine Tramunt [5 6], Camille Vatier [39 40], Fritz-Line Velayoudom [41], Bruno Vergès [42], Patrice Winiszewski [43], Audrey Zabulon [44], Pierre-Antoine Gourraud [2], Ronan Roussel [3 4], Bertrand Cariou [45], Samy Hadjadj [46], CORONADO investigators

Affiliations Expand

Affiliations

- [1] l'institut du thorax, Inserm, CNRS, UNIV Nantes, CHU Nantes, Nantes, France.
- [2] CHU de Nantes, Inserm, CIC 1413, Pôle Hospitalo-Universitaire 11: Santé Publique, Clinique des Données, Nantes, France.
- [3] Département d'Endocrinologie, Diabétologie et Nutrition, Hôpital Bichat, Assistance Publique Hôpitaux de Paris, Paris, France.
- [4] Centre de Recherche des Cordeliers, Inserm, U-1138, Université de Paris, Paris, France.
- [5] Département d'Endocrinologie, Diabétologie et Nutrition, CHU Toulouse, Toulouse, France.
- [6] Institut des Maladies Métaboliques et Cardiovasculaires, UMR1048 Inserm/UPS, Université de Toulouse, Toulouse, France.
- [7] Département de Diabétologie, Centre Hospitalier Sud Francilien, Corbeil-Essonnes, France.
- [8] Université Paris-Saclay, Paris, France.
- [9] Service Endocrinologie-Diabétologie-Nutrition, CHU Grenoble, Grenoble, France.
- [10] University Grenoble-Alpes, Grenoble, France.
- [11] Département d'Endocrinologie, Diabète, Nutrition et CIC Inserm 1411, CHU de Montpellier, Montpellier, France.
- [12] Département de Diabétologie, H.I.A. Begin, Saint Mandé, France.
- [13] Sorbonne Université, Paris, France.
- [14] Assistance Publique Hôpitaux de Paris, Département de Diabétologie, CHU La Pitié Salpêtrière-Charles Foix, Paris, France.
- [15] Centre de Recherche des Cordeliers, Inserm, U-1138, Paris, France.
- [16] Institute of Cardiometabolism and Nutrition ICAN, Paris, France.
- [17] Fédération Française des Diabétiques (FFD), Paris, France.

- [18] Université Côte d'Azur, CHU, Inserm U1065, C3M, Nice, France.
- [19] Service d'Endocrinologie, Maladies Métaboliques et Nutrition, Hôpital de la Conception, Assistance Publique Hôpitaux de Marseille, Marseille, France.
- [20] C2VN, Inserm, INRA, Aix Marseille Université, Marseille, France.
- [21] Service d'Endocrinologie, Diabétologie et Maladies Métaboliques, Centre Hospitalier d'Aix-en-Provence, Aix-en-Provence, France.
- [22] Service Endocrinologie-Diabétologie, Centre Hospitalier de la Côte Basque, Bayonne, France.
- [23] Service Endocrinologie-Diabétologie, Hôpital Saint Vincent de Paul Lille, Lille, France.
- [24] Service de Diabétologie Endocrinologie, Hôpital Paris Saint-Joseph, Paris, France.
- [25] Service d'Endocrinologie - Diabétologie, Site Felix Guyon, CHU de la Réunion, Saint-Denis de la Réunion, France.
- [26] Service de Diabétologie et d'Endocrinologie, Hôpital Lariboisière, Assistance Publique Hôpitaux de Paris, Paris, France.
- [27] Service des Maladies Métaboliques et Endocriniennes, CHU de Nîmes, Université de Montpellier, Nîmes, France.
- [28] Service d'Endocrinologie, Diabétologie et Nutrition, Hôpitaux Universitaires de Strasbourg, UdS, Strasbourg, France.
- [29] Service de Diabétologie et Immunologie Clinique, Hôpital Cochin, Assistance Publique Hôpitaux de Paris, Centre-Université de Paris, Paris, France.
- [30] Service de Diabétologie et d'Endocrinologie, Médipôle, Villeurbanne, France.
- [31] Service d'Endocrinologie et de Diabétologie, Centre Hospitalier de Bastia, Bastia, France.
- [32] Service d'Endocrinologie, Centre Hospitalier du Forez, Montbrison, France.
- [33] Hôpital Cardiovasculaire Louis Pradel, Hospices Civils de Lyon, Inserm UMR 1060 Carmen, Université Claude Bernard Lyon 1, Lyon, France.
- [34] Service d'Endocrinologie et Maladies Métaboliques, Centre Hospitalier d'Avignon, Avignon, France.
- [35] Endocrinology-Nutrition Department, Centre Hospitalier Universitaire de Bordeaux, Université de Bordeaux, Bordeaux, France.
- [36] Université de Poitiers; CIC Inserm 1402; Médecine Intensive Réanimation, Centre Hospitalier Universitaire de Poitiers, Poitiers, France.
- [37] Service de Diabétologie, Endocrinologie et Nutrition, Centre Hospitalier de Gonesse, Gonesse, France.
- [38] Service d'Endocrinologie, Diabétologie et Maladies Métaboliques, Groupe Hospitalier Mutualiste Les Portes du Sud, Venissieux, France.
- [39] Assistance Publique Hôpitaux de Paris, Saint-Antoine Hospital, Reference Center of Rare Diseases of Insulin Secretion and Insulin Sensitivity (PRISIS), Department of Endocrinology, Paris, France.
- [40] Sorbonne University, Inserm UMRS 938, Saint-Antoine Research Center, Paris, France.
- [41] Service d'Endocrinologie, Diabétologie et Métabolisme, Centre Hospitalier Universitaire de Guadeloupe, Pointe-à-Pitre, France.
- [42] Service Endocrinologie, Diabétologie et Maladies Métaboliques, Hôpital du Bocage, Dijon, France.
- [43] Service d'Endocrinologie, Diabétologie et Nutrition, Hôpital Nord Franche-Comté, Trévenans, France.
- [44] Service d'Endocrinologie et Diabétologie, CHU de Martinique, Fort-de-France, France.
- [45] l'institut du thorax, Inserm, CNRS, UNIV Nantes, CHU Nantes, Nantes, France. bertrand.cariou@univ-nantes.fr.

- [46] l'institut du thorax, Inserm, CNRS, UNIV Nantes, CHU Nantes, Nantes, France. samy.hadjadj@univ-nantes.fr.

- PMID: **33599800**
- PMCID: PMC7890396
- DOI: 10.1007/s00125-020-05351-w

Free PMC article
Observational Study

Predictors of hospital discharge and mortality in patients with diabetes and COVID-19: updated results from the nationwide CORONADO study

Matthieu Wargny et al. Diabetologia. 2021 Apr.
Free PMC article

Show details

Diabetologia
. 2021 Apr;64(4):778-794.
doi: 10.1007/s00125-020-05351-w. Epub 2021 Feb 17.

Authors

Matthieu Wargny [1, 2], Louis Potier [3, 4], Pierre Gourdy [5, 6], Matthieu Pichelin [1], Coralie Amadou [7, 8], Pierre-Yves Benhamou [9, 10], Jean-Baptiste Bonnet [11], Lyse Bordier [12], Olivier Bourron [13, 14, 15, 16], Claude Chaumeil [17], Nicolas Chevalier [18], Patrice Darmon [19, 20], Blandine Delenne [21], Delphine Demarsy [22], Marie Dumas [23], Olivier Dupuy [24], Anna Flaus-Furmaniuk [25], Jean-François Gautier [4, 26], Anne-Marie Guedj [27], Nathalie Jeandidier [28], Etienne Larger [29], Jean-Philippe Le Berre [30], Myriam Lungo [31], Nathanaëlle Montanier [32], Philippe Moulin [33], Françoise Plat [34], Vincent Rigalleau [35], René Robert [36], Dominique Seret-Bégué [37], Pierre Sérusclat [38], Sarra Smati [1], Jean-François Thébaut [17], Blandine Tramunt [5, 6], Camille Vatier [39, 40], Fritz-Line Velayoudom [41], Bruno Vergès [42], Patrice Winiszewski [43], Audrey Zabulon [44], Pierre-Antoine Gourraud [2], Ronan Roussel [3, 4], Bertrand Cariou [45], Samy Hadjadj [46], CORONADO investigators

Affiliations

- [1] l'institut du thorax, Inserm, CNRS, UNIV Nantes, CHU Nantes, Nantes, France.
- [2] CHU de Nantes, Inserm, CIC 1413, Pôle Hospitalo-Universitaire 11: Santé Publique, Clinique des Données, Nantes, France.
- [3] Département d'Endocrinologie, Diabétologie et Nutrition, Hôpital Bichat, Assistance Publique Hôpitaux de Paris, Paris, France.
- [4] Centre de Recherche des Cordeliers, Inserm, U-1138, Université de Paris, Paris, France.

- [5] Département d'Endocrinologie, Diabétologie et Nutrition, CHU Toulouse, Toulouse, France.
- [6] Institut des Maladies Métaboliques et Cardiovasculaires, UMR1048 Inserm/UPS, Université de Toulouse, Toulouse, France.
- [7] Département de Diabétologie, Centre Hospitalier Sud Francilien, Corbeil-Essonnes, France.
- [8] Université Paris-Saclay, Paris, France.
- [9] Service Endocrinologie-Diabétologie-Nutrition, CHU Grenoble, Grenoble, France.
- [10] University Grenoble-Alpes, Grenoble, France.
- [11] Département d'Endocrinologie, Diabète, Nutrition et CIC Inserm 1411, CHU de Montpellier, Montpellier, France.
- [12] Département de Diabétologie, H.I.A. Begin, Saint Mandé, France.
- [13] Sorbonne Université, Paris, France.
- [14] Assistance Publique Hôpitaux de Paris, Département de Diabétologie, CHU La Pitié Salpêtrière-Charles Foix, Paris, France.
- [15] Centre de Recherche des Cordeliers, Inserm, U-1138, Paris, France.
- [16] Institute of Cardiometabolism and Nutrition ICAN, Paris, France.
- [17] Fédération Française des Diabétiques (FFD), Paris, France.
- [18] Université Côte d'Azur, CHU, Inserm U1065, C3M, Nice, France.
- [19] Service d'Endocrinologie, Maladies Métaboliques et Nutrition, Hôpital de la Conception, Assistance Publique Hôpitaux de Marseille, Marseille, France.
- [20] C2VN, Inserm, INRA, Aix Marseille Université, Marseille, France.
- [21] Service d'Endocrinologie, Diabétologie et Maladies Métaboliques, Centre Hospitalier d'Aix-en-Provence, Aix-en-Provence, France.
- [22] Service Endocrinologie-Diabétologie, Centre Hospitalier de la Côte Basque, Bayonne, France.
- [23] Service Endocrinologie-Diabétologie, Hôpital Saint Vincent de Paul Lille, Lille, France.
- [24] Service de Diabétologie Endocrinologie, Hôpital Paris Saint-Joseph, Paris, France.
- [25] Service d'Endocrinologie - Diabétologie, Site Felix Guyon, CHU de la Réunion, Saint-Denis de la Réunion, France.
- [26] Service de Diabétologie et d'Endocrinologie, Hôpital Lariboisière, Assistance Publique Hôpitaux de Paris, Paris, France.
- [27] Service des Maladies Métaboliques et Endocriniennes, CHU de Nîmes, Université de Montpellier, Nîmes, France.
- [28] Service d'Endocrinologie, Diabétologie et Nutrition, Hôpitaux Universitaires de Strasbourg, UdS, Strasbourg, France.
- [29] Service de Diabétologie et Immunologie Clinique, Hôpital Cochin, Assistance Publique Hôpitaux de Paris, Centre-Université de Paris, Paris, France.
- [30] Service de Diabétologie et d'Endocrinologie, Médipôle, Villeurbanne, France.
- [31] Service d'Endocrinologie et de Diabétologie, Centre Hospitalier de Bastia, Bastia, France.
- [32] Service d'Endocrinologie, Centre Hospitalier du Forez, Montbrison, France.
- [33] Hôpital Cardiovasculaire Louis Pradel, Hospices Civils de Lyon, Inserm UMR 1060 Carmen, Université Claude Bernard Lyon 1, Lyon, France.
- [34] Service d'Endocrinologie et Maladies Métaboliques, Centre Hospitalier d'Avignon, Avignon, France.
- [35] Endocrinology-Nutrition Department, Centre Hospitalier Universitaire de Bordeaux, Université de Bordeaux, Bordeaux, France.

- [36] Université de Poitiers; CIC Inserm 1402; Médecine Intensive Réanimation, Centre Hospitalier Universitaire de Poitiers, Poitiers, France.
- [37] Service de Diabétologie, Endocrinologie et Nutrition, Centre Hospitalier de Gonesse, Gonesse, France.
- [38] Service d'Endocrinologie, Diabétologie et Maladies Métaboliques, Groupe Hospitalier Mutualiste Les Portes du Sud, Venissieux, France.
- [39] Assistance Publique Hôpitaux de Paris, Saint-Antoine Hospital, Reference Center of Rare Diseases of Insulin Secretion and Insulin Sensitivity (PRISIS), Department of Endocrinology, Paris, France.
- [40] Sorbonne University, Inserm UMRS 938, Saint-Antoine Research Center, Paris, France.
- [41] Service d'Endocrinologie, Diabétologie et Métabolisme, Centre Hospitalier Universitaire de Guadeloupe, Pointe-à-Pitre, France.
- [42] Service Endocrinologie, Diabétologie et Maladies Métaboliques, Hôpital du Bocage, Dijon, France.
- [43] Service d'Endocrinologie, Diabétologie et Nutrition, Hôpital Nord Franche-Comté, Trévenans, France.
- [44] Service d'Endocrinologie et Diabétologie, CHU de Martinique, Fort-de-France, France.
- [45] l'institut du thorax, Inserm, CNRS, UNIV Nantes, CHU Nantes, Nantes, France. bertrand.cariou@univ-nantes.fr.
- [46] l'institut du thorax, Inserm, CNRS, UNIV Nantes, CHU Nantes, Nantes, France. samy.hadjadj@univ-nantes.fr.

- PMID: **33599800**
- PMCID: PMC7890396
- DOI: 10.1007/s00125-020-05351-w

Abstract

Aims/hypothesis: This is an update of the results from the previous report of the CORONADO (Coronavirus SARS-CoV-2 and Diabetes Outcomes) study, which aims to describe the outcomes and prognostic factors in patients with diabetes hospitalised for coronavirus disease-2019 (COVID-19).

Methods: The CORONADO initiative is a French nationwide multicentre study of patients with diabetes hospitalised for COVID-19 with a 28-day follow-up. The patients were screened after hospital admission from 10 March to 10 April 2020. We mainly focused on hospital discharge and death within 28 days.

Results: We included 2796 participants: 63.7% men, mean age 69.7 ± 13.2 years, median BMI (25th-75th percentile) 28.4 (25.0-32.4) kg/m^2. Microvascular and macrovascular diabetic complications were found in 44.2% and 38.6% of participants, respectively. Within 28 days, 1404 (50.2%; 95% CI 48.3%, 52.1%) were discharged from hospital with a median duration of hospital stay of 9 (5-14) days, while 577 participants died (20.6%; 95% CI 19.2%, 22.2%). In multivariable models, younger age, routine metformin therapy and longer symptom duration on admission were positively associated with discharge. History of microvascular complications, anticoagulant routine therapy, dyspnoea on admission, and higher aspartate aminotransferase, white cell count and C-reactive protein levels were associated with a reduced chance of discharge. Factors associated with death within 28 days mirrored those associated with discharge, and also included routine treatment by insulin and statin as deleterious factors.

Conclusions/interpretation: In patients with diabetes hospitalised for COVID-19, we established prognostic factors for hospital discharge and death that could help clinicians in this pandemic period.

Trial registration: Clinicaltrials.gov identifier: NCT04324736.

Keywords: Admission plasma glucose; COVID-19; Death; Diabetes; Discharge; HbA1c; Home discharge; Mechanical ventilation.

- 32 references
- 2 figures

Supplementary info

Publication types, MeSH terms, Associated data Expand

Publication types

- Multicenter Study
- Observational Study
- Research Support, Non-U.S. Gov't

MeSH terms

- Aged
- Aged, 80 and over
- COVID-19 / complications
- COVID-19 / diagnosis*
- COVID-19 / mortality*
- COVID-19 / therapy
- Diabetes Complications / diagnosis
- Diabetes Complications / mortality
- Diabetes Complications / therapy
- Diabetes Mellitus / diagnosis*
- Diabetes Mellitus / mortality*
- Diabetes Mellitus / therapy
- Female
- Follow-Up Studies
- France / epidemiology
- Hospital Mortality
- Hospitalization / statistics & numerical data
- Humans
- Length of Stay / statistics & numerical data
- Male
- Middle Aged

- Patient Discharge* / statistics & numerical data
- Prognosis
- Respiration, Artificial / statistics & numerical data
- Retrospective Studies
- Risk Factors
- SARS-CoV-2 / physiology

Associated data

- ClinicalTrials.gov/NCT04324736

Full text links

Springer Free PMC article
Proceed to details
Cite
Share
13
Observational Study
Am J Kidney Dis
. 2021 Feb;77(2):204-215.e1.
doi: 10.1053/j.ajkd.2020.09.002. Epub 2020 Sep 19.

Outcomes Among Patients Hospitalized With COVID-19 and Acute Kidney Injury

Jia H Ng [1], Jamie S Hirsch [2], Azzour Hazzan [1], Rimda Wanchoo [1], Hitesh H Shah [1], Deepa A Malieckal [1], Daniel W Ross [1], Purva Sharma [1], Vipulbhai Sakhiya [1], Steven Fishbane [1], Kenar D Jhaveri [3], Northwell Nephrology COVID-19 Research Consortium
Collaborators, Affiliations Expand

Collaborators

- **Northwell Nephrology COVID-19 Research Consortium**: Mersema Abate, Hugo Paz Andrade, Richard L Barnett, Alessandro Bellucci, Madhu C Bhaskaran, Antonio G Corona, Bessy Suyin Flores Chang, Mark Finger, Steven Fishbane, Michael Gitman, Candice Halinski, Shamir Hasan, Azzour D Hazzan, Jamie S Hirsch, Susana Hong, Kenar D Jhaveri, Yuriy Khanin, Aireen Kuan, Varun Madireddy, Deepa Malieckal, Abdulrahman Muzib, Gayatri Nair, Vinay V Nair, Jia Hwei Ng, Rushang Parikh, Daniel W Ross, Vipulbhai Sakhiya, Mala Sachdeva, Richard Schwarz, Hitesh H Shah, Purva Sharma, Pravin C Singhal, Nupur N Uppal, Rimda Wanchoo

Affiliations

- [1] Division of Kidney Diseases and Hypertension, Department of Medicine, Donald and Barbara Zucker School of Medicine at Hofstra/Northwell, Great Neck, NY.
- [2] Division of Kidney Diseases and Hypertension, Department of Medicine, Donald and Barbara Zucker School of Medicine at Hofstra/Northwell, Great Neck, NY; Institute of Health Innovations and Outcomes Research, Feinstein Institutes for Medical Research, Manhasset, NY; Department of Information Services, Northwell Health, New Hyde Park, NY.
- [3] Division of Kidney Diseases and Hypertension, Department of Medicine, Donald and Barbara Zucker School of Medicine at Hofstra/Northwell, Great Neck, NY. Electronic address: kjhaveri@northwell.edu.

- PMID: **32961245**
- PMCID: PMC7833189
- DOI: 10.1053/j.ajkd.2020.09.002

Free PMC article
Observational Study

Outcomes Among Patients Hospitalized With COVID-19 and Acute Kidney Injury

Jia H Ng et al. Am J Kidney Dis. 2021 Feb.
Free PMC article
Show details
Am J Kidney Dis
. 2021 Feb;77(2):204-215.e1.
doi: 10.1053/j.ajkd.2020.09.002. Epub 2020 Sep 19.

Authors

Jia H Ng [1], Jamie S Hirsch [2], Azzour Hazzan [1], Rimda Wanchoo [1], Hitesh H Shah [1], Deepa A Malieckal [1], Daniel W Ross [1], Purva Sharma [1], Vipulbhai Sakhiya [1], Steven Fishbane [1], Kenar D Jhaveri [3], Northwell Nephrology COVID-19 Research Consortium

Collaborators

- **Northwell Nephrology COVID-19 Research Consortium**: Mersema Abate, Hugo Paz Andrade, Richard L Barnett, Alessandro Bellucci, Madhu C Bhaskaran, Antonio G Corona, Bessy Suyin Flores Chang, Mark Finger, Steven Fishbane, Michael Gitman, Candice Halinski, Shamir Hasan, Azzour D Hazzan, Jamie S Hirsch, Susana Hong, Kenar D Jhaveri, Yuriy Khanin, Aireen Kuan, Varun Madireddy, Deepa Malieckal, Abdulrahman Muzib, Gayatri Nair, Vinay V Nair, Jia Hwei Ng, Rushang Parikh, Daniel W Ross, Vipulbhai Sakhiya, Mala Sachdeva, Richard Schwarz, Hitesh H Shah, Purva Sharma, Pravin C Singhal, Nupur N Uppal, Rimda Wanchoo

Affiliations

- [1] Division of Kidney Diseases and Hypertension, Department of Medicine, Donald and Barbara Zucker School of Medicine at Hofstra/Northwell, Great Neck, NY.
- [2] Division of Kidney Diseases and Hypertension, Department of Medicine, Donald and Barbara Zucker School of Medicine at Hofstra/Northwell, Great Neck, NY; Institute of Health Innovations and Outcomes Research, Feinstein Institutes for Medical Research, Manhasset, NY; Department of Information Services, Northwell Health, New Hyde Park, NY.
- [3] Division of Kidney Diseases and Hypertension, Department of Medicine, Donald and Barbara Zucker School of Medicine at Hofstra/Northwell, Great Neck, NY. Electronic address: kjhaveri@northwell.edu.

- PMID: **32961245**
- PMCID: PMC7833189
- DOI: 10.1053/j.ajkd.2020.09.002

Abstract

Rationale & objective: Outcomes of patients hospitalized with coronavirus disease 2019 (COVID-19) and acute kidney injury (AKI) are not well understood. The goal of this study was to investigate the survival and kidney outcomes of these patients.

Study design: Retrospective cohort study.

Setting & participants: Patients (aged≥18 years) hospitalized with COVID-19 at 13 hospitals in metropolitan New York between March 1, 2020, and April 27, 2020, followed up until hospital discharge.

Exposure: AKI.

Outcomes: Primary outcome: in-hospital death.

Secondary outcomes: requiring dialysis at discharge, recovery of kidney function.

Analytical approach: Univariable and multivariable time-to-event analysis and logistic regression.

Results: Among 9,657 patients admitted with COVID-19, the AKI incidence rate was 38.4/1,000 patient-days. Incidence rates of in-hospital death among patients without AKI, with AKI not requiring dialysis (AKI stages 1-3), and with AKI receiving dialysis (AKI 3D) were 10.8, 31.1, and 37.5/1,000 patient-days, respectively. Taking those without AKI as the reference group, we observed greater risks for in-hospital death for patients with AKI 1-3 and AKI 3D (HRs of 5.6 [95% CI, 5.0-6.3] and 11.3 [95% CI, 9.6-13.1], respectively). After adjusting for demographics, comorbid conditions, and illness severity, the risk for death remained higher among those with AKI 1-3 (adjusted HR, 3.4 [95% CI, 3.0-3.9]) and AKI 3D (adjusted HR, 6.4 [95% CI, 5.5-7.6]) compared with those without AKI. Among patients with AKI 1-3 who survived, 74.1% achieved kidney recovery by the time of discharge. Among those with AKI 3D who survived, 30.6% remained on dialysis at discharge, and prehospitalization chronic kidney disease was the only independent risk factor associated with needing dialysis at discharge (adjusted OR, 9.3 [95% CI, 2.3-37.8]).

Limitations: Observational retrospective study, limited to the NY metropolitan area during the peak of the COVID-19 pandemic.

Conclusions: AKI in hospitalized patients with COVID-19 was associated with significant risk for death.

Keywords: AKI-on-CKD; COVID-19 outcomes; Coronavirus disease 2019 (COVID-19); acute kidney injury (AKI); acute renal failure (ARF); death; dialysis; hospitalization; in-hospital mortality; kidney replacement therapy (KRT); recovery; renal prognosis; renal recovery; severe acute respiratory syndrome coronavirus 2 (SARS-CoV-2).

Copyright © 2020 National Kidney Foundation, Inc. Published by Elsevier Inc. All rights reserved.

Comment in

- COVID-19: The Kidneys Tell a Tale.
  Chan L, Hindi J, Nadkarni GN. Chan L, et al. Am J Kidney Dis. 2021 Feb;77(2):175-177. doi: 10.1053/j.ajkd.2020.11.001. Epub 2020 Dec 17. Am J Kidney Dis. 2021. PMID: 33342610 Free PMC article. No abstract available.

- 38 references
- 5 figures

Supplementary info

Publication types, MeSH terms, Grant support Expand

Publication types

- Observational Study
- Research Support, Non-U.S. Gov't

MeSH terms

- Acute Kidney Injury* / diagnosis
- Acute Kidney Injury* / epidemiology
- Acute Kidney Injury* / physiopathology
- Acute Kidney Injury* / therapy
- COVID-19* / diagnosis
- COVID-19* / mortality
- COVID-19* / physiopathology
- COVID-19* / therapy
- Female
- Hospital Mortality*
- Hospitalization / statistics & numerical data*
- Humans
- Incidence
- Kidney Function Tests / methods
- Kidney Function Tests / statistics & numerical data

- Male
- Middle Aged
- New York / epidemiology
- Outcome and Process Assessment, Health Care
- Renal Dialysis* / methods
- Renal Dialysis* / statistics & numerical data
- Retrospective Studies
- Risk Factors
- SARS-CoV-2 / isolation & purification
- Survival Analysis

Grant support

- R01 DK118017/DK/NIDDK NIH HHS/United States

Full text links

ELSEVIER FULL-TEXT ARTICLE Elsevier Science Free PMC article

Proceed to details

Cite

Share

☐ 14

Observational Study

Chest
. 2021 Jan;159(1):108-111.
doi: 10.1016/j.chest.2020.06.082. Epub 2020 Jul 22.

The Minimal Effect of Zinc on the Survival of Hospitalized Patients With COVID-19: An Observational Study

Jasper Seth Yao [1], Joseph Alexander Paguio [1], Edward Christopher Dee [2], Hanna Clementine Tan [3], Achintya Moulick [1], Carmelo Milazzo [1], Jerry Jurado [1], Nicolás Della Penna [4], Leo Anthony Celi [5]

Affiliations Expand

Affiliations

- [1] Hoboken University Medical Center, Hoboken, NJ.
- [2] Harvard Medical School, Boston, MA.
- [3] University of the Philippines College of Medicine, The Philippines.
- [4] Massachusetts Institute of Technology, Cambridge, MA.

- [5] Harvard Medical School, Boston, MA; Massachusetts Institute of Technology, Cambridge, MA. Electronic address: LCeli@mit.edu.

- PMID: **32710890**
- PMCID: PMC7375307
- DOI: 10.1016/j.chest.2020.06.082

Free PMC article
Observational Study

The Minimal Effect of Zinc on the Survival of Hospitalized Patients With COVID-19: An Observational Study

Jasper Seth Yao et al. Chest. 2021 Jan.
Free PMC article

Show details

Chest
. 2021 Jan;159(1):108-111.
doi: 10.1016/j.chest.2020.06.082. Epub 2020 Jul 22.

Authors

Jasper Seth Yao [1], Joseph Alexander Paguio [1], Edward Christopher Dee [2], Hanna Clementine Tan [3], Achintya Moulick [1], Carmelo Milazzo [1], Jerry Jurado [1], Nicolás Della Penna [4], Leo Anthony Celi [5]

Affiliations

- [1] Hoboken University Medical Center, Hoboken, NJ.
- [2] Harvard Medical School, Boston, MA.
- [3] University of the Philippines College of Medicine, The Philippines.
- [4] Massachusetts Institute of Technology, Cambridge, MA.
- [5] Harvard Medical School, Boston, MA; Massachusetts Institute of Technology, Cambridge, MA. Electronic address: LCeli@mit.edu.

- PMID: **32710890**
- PMCID: PMC7375307
- DOI: 10.1016/j.chest.2020.06.082

No abstract available

Comment in

- Zinc and Coronavirus Disease 2019: Causal or Casual Association?

Khurana AK, Karna ST, Hussain A. Khurana AK, et al. Chest. 2021 Jan;159(1):449-450. doi: 10.1016/j.chest.2020.08.2092. Chest. 2021. PMID: 33422222 Free PMC article. No abstract available.

- Response.
  Yao JS, Paguio JA, Dee EC, Tan HC, Moulick A, Milazzo C, Jurado J, Della Penna N, Celi LA. Yao JS, et al. Chest. 2021 Jan;159(1):450-451. doi: 10.1016/j.chest.2020.08.2093. Chest. 2021. PMID: 33422223 Free PMC article. No abstract available.

- 10 references

Supplementary info

Publication types, MeSH terms, Substances, Grant support Expand

Publication types

- Letter
- Observational Study
- Research Support, N.I.H., Extramural

MeSH terms

- Aged
- Aged, 80 and over
- COVID-19 / drug therapy*
- COVID-19 / mortality*
- Female
- Hospitalization
- Humans
- Male
- Middle Aged
- Retrospective Studies
- Survival Rate
- Zinc / therapeutic use*

Substances

- Zinc

Grant support

- R01 EB017205/EB/NIBIB NIH HHS/United States

Full text links

ELSEVIER FULL-TEXT ARTICLE Elsevier Science Free PMC article

Proceed to details

Cite

Share

15

Observational Study

Int J Infect Dis

. 2021 Apr;105:245-251.

doi: 10.1016/j.ijid.2021.02.057. Epub 2021 Feb 17.

Tocilizumab treatment in critically ill patients with COVID-19: A retrospective observational study

Edmund Huang [1], Sharon Isonaka [2], Haoshu Yang [2], Erin Salce [2], Elisa Rosales [2], Stanley C Jordan [3]

Affiliations Expand

Affiliations

- [1] Department of Medicine, Division of Nephrology, Transplant Immunology Laboratory, Transplant Immunotherapy Program, United States.
- [2] Department of Clinical Transformation, Cedars-Sinai Medical Center, Los Angeles, California, United States.
- [3] Department of Medicine, Division of Nephrology, Transplant Immunology Laboratory, Transplant Immunotherapy Program, United States. Electronic address: stan.jordan@cshs.org.

- PMID: **33609773**
- PMCID: PMC7889004
- DOI: 10.1016/j.ijid.2021.02.057

Free PMC article

Observational Study

Tocilizumab treatment in critically ill patients with COVID-19: A retrospective observational study

Edmund Huang et al. Int J Infect Dis. 2021 Apr.

Free PMC article

Show details

Int J Infect Dis

. 2021 Apr;105:245-251.
doi: 10.1016/j.ijid.2021.02.057. Epub 2021 Feb 17.

Authors

Edmund Huang [1], Sharon Isonaka [2], Haoshu Yang [2], Erin Salce [2], Elisa Rosales [2], Stanley C Jordan [3]

Affiliations

- [1] Department of Medicine, Division of Nephrology, Transplant Immunology Laboratory, Transplant Immunotherapy Program, United States.
- [2] Department of Clinical Transformation, Cedars-Sinai Medical Center, Los Angeles, California, United States.
- [3] Department of Medicine, Division of Nephrology, Transplant Immunology Laboratory, Transplant Immunotherapy Program, United States. Electronic address: stan.jordan@cshs.org.

- PMID: **33609773**
- PMCID: PMC7889004
- DOI: 10.1016/j.ijid.2021.02.057

Abstract

Objective: Elevated levels of pro-inflammatory cytokines are observed in severe COVID-19 infections, and cytokine storm is associated with disease severity. Tocilizumab, an interleukin-6 receptor antagonist, is used to treat chimeric antigen receptor T cell-induced cytokine release syndrome and may attenuate the dysregulated immune response in COVID-19. We compared outcomes among tocilizumab-treated and non-tocilizumab-treated critically ill COVID-19 patients.

Design, setting, and participants: This was a retrospective observational study conducted at a tertiary referral center investigating all patients admitted to the intensive care unit for COVID-19 who had a disposition from the hospital because of death or hospital discharge between March 1 and May 18, 2020 ($n = 96$). The percentages of death and secondary infections were compared between patients treated with tocilizumab ($n = 55$) and those who were not ($n = 41$).

Measurements and main results: More tocilizumab-treated patients required mechanical ventilation (44/55, 80%) compared to non-treated patients (15/41, 37%; $P < 0.001$). Of 55 patients treated with tocilizumab, 32 (58%) were on mechanical ventilation at the time of administration, and 12 (22%) progressed to mechanical ventilation after treatment. Of patients treated with tocilizumab requiring mechanical ventilation, 30/44 (68%) were intubated within 1 day of administration. Fewer deaths were observed among tocilizumab-treated patients, both in the overall population (15% vs 37%; $P = 0.02$) and among the subgroup of patients requiring mechanical ventilation (14% vs 60%; $P = 0.001$). Secondary infections were not different between the 2 groups (tocilizumab: 31%, non-tocilizumab: 17%; $P = 0.16$) and were predominantly related to invasive devices, such as urinary and central venous catheters.

Conclusions: Tocilizumab treatment was associated with fewer deaths compared to non-treatment despite predominantly being used in patients with more advanced respiratory disease.

Keywords: Acute respiratory distress syndrome; COVID-19; Cytokine release syndrome; Pneumonia; SARS-CoV2; Tocilizumab.

Copyright © 2021 The Authors. Published by Elsevier Ltd.. All rights reserved.

- 27 references
- 3 figures

Supplementary info

Publication types, MeSH terms, Substances, Supplementary concepts Expand

Publication types

- Observational Study

MeSH terms

- Adult
- Aged
- Aged, 80 and over
- Antibodies, Monoclonal, Humanized / therapeutic use*
- COVID-19 / drug therapy*
- Critical Illness*
- Female
- Humans
- Male
- Middle Aged
- Respiration, Artificial
- Retrospective Studies
- SARS-CoV-2*

Substances

- Antibodies, Monoclonal, Humanized
- tocilizumab

Supplementary concepts

- COVID-19 drug treatment

Full text links

ELSEVIER OPEN ACCESS Elsevier Science Free PMC article

Proceed to details
Cite
Share
☐ 16
Observational Study
Med Sci Monit
. 2021 Dec 30;27:e935379.
doi: 10.12659/MSM.935379.

Retrospective Study of Outcomes and Hospitalization Rates of Patients in Italy with a Confirmed Diagnosis of Early COVID-19 and Treated at Home Within 3 Days or After 3 Days of Symptom Onset with Prescribed and Non-Prescribed Treatments Between November 2020 and August 2021

Serafino Fazio [1], Paolo Bellavite [2], Elisabetta Zanolin [3], Peter A McCullough [4], Sergio Pandolfi [5], Flora Affuso [6]
Affiliations Expand

Affiliations

- [1] Retired Professor of Internal Medicine, Medical School University Federico II, Naples, Italy.
- [2] Physiopathology Chair, Homeopathic Medical School of Verona, Verona, Italy.
- [3] Unit of Epidemiology and Medical Statistics, Department of Diagnostics and Public Health, University of Verona, Verona, Italy.
- [4] Department of Cardiology, Truth for Health Foundation, Tucson, AZ, USA.
- [5] Department of Neurosurgery, Villa Mafalda Clinics, Rome, Italy.
- [6] Independent Researcher, Gallipoli, Italy.

- PMID: **34966165**
- PMCID: PMC8725339
- DOI: 10.12659/MSM.935379

Free PMC article
Observational Study

Retrospective Study of Outcomes and Hospitalization Rates of Patients in Italy with

a Confirmed Diagnosis of Early COVID-19 and Treated at Home Within 3 Days or After 3 Days of Symptom Onset with Prescribed and Non-Prescribed Treatments Between November 2020 and August 2021

Serafino Fazio et al. Med Sci Monit. 2021.
Free PMC article
Show details
Med Sci Monit
. 2021 Dec 30;27:e935379.
doi: 10.12659/MSM.935379.

Authors

Serafino Fazio [1], Paolo Bellavite [2], Elisabetta Zanolin [3], Peter A McCullough [4], Sergio Pandolfi [5], Flora Affuso [6]

Affiliations

- [1] Retired Professor of Internal Medicine, Medical School University Federico II, Naples, Italy.
- [2] Physiopathology Chair, Homeopathic Medical School of Verona, Verona, Italy.
- [3] Unit of Epidemiology and Medical Statistics, Department of Diagnostics and Public Health, University of Verona, Verona, Italy.
- [4] Department of Cardiology, Truth for Health Foundation, Tucson, AZ, USA.
- [5] Department of Neurosurgery, Villa Mafalda Clinics, Rome, Italy.
- [6] Independent Researcher, Gallipoli, Italy.

- PMID: **34966165**
- PMCID: PMC8725339
- DOI: 10.12659/MSM.935379

Abstract

BACKGROUND This retrospective study aimed to investigate outcomes and hospitalization rates in patients with a confirmed diagnosis of early COVID-19 treated at home with prescribed and non-prescribed treatments. MATERIAL AND METHODS The medical records of a cohort of 158 Italian patients with early COVID-19 treated at home were analyzed. Treatments consisted of indomethacin, low-dose aspirin, omeprazole, and a flavonoid-based food supplement, plus azithromycin, low-molecular-weight heparin, and betamethasone as needed. The association of treatment timeliness and of clinical variables with the duration of symptoms and with the risk of hospitalization was evaluated by logistic regression. RESULTS Patients were divided into 2 groups: group 1 (n=85) was treated at the earliest possible time (<72 h from onset of symptoms), and group 2 (n=73) was treated >72 h after the onset of symptoms. Clinical severity at the

beginning of treatment was similar in the 2 groups. In group 1, symptom duration was shorter than in group 2 (median 6.0 days vs 13.0 days, P<0.001) and no hospitalizations occurred, compared with 19.18% hospitalizations in group 2. One patient in group 1 developed chest X-ray alterations and 2 patients experienced an increase in D-dimer levels, compared with 30 and 22 patients, respectively, in group 2. The main factor determining the duration of symptoms and the risk of hospitalization was the delay in starting therapy (P<0.001). CONCLUSIONS This real-world study of patients in the community showed that early diagnosis and early supportive patient management reduced the severity of COVID-19 and reduced the rate of hospitalization.

Conflict of interest statement

Conflict of interest: Paolo Bellavite has a consultancy agreement with Vanda s.r.l. (Frascati, Rome), but he had no role in the treatments. Other authors have no competing interests to declare

- 69 references
- 2 figures

Supplementary info

Publication types, MeSH terms, Substances Expand

Publication types

- Observational Study

MeSH terms

- Aged
- Aged, 80 and over
- Aspirin / therapeutic use
- Betamethasone / therapeutic use
- COVID-19 / diagnosis*
- COVID-19 / drug therapy*
- Cohort Studies
- Dietary Supplements
- Early Diagnosis
- Female
- Flavonoids / therapeutic use
- Follow-Up Studies
- Heparin, Low-Molecular-Weight / therapeutic use
- Hospitalization / statistics & numerical data*
- Humans
- Indomethacin / therapeutic use
- Italy
- Male

- Middle Aged
- Omeprazole / therapeutic use
- Patient Acuity
- Retrospective Studies
- Risk Assessment
- SARS-CoV-2
- Time
- Time-to-Treatment / statistics & numerical data*
- Treatment Outcome

Substances

- Flavonoids
- Heparin, Low-Molecular-Weight
- Betamethasone
- Omeprazole
- Aspirin
- Indomethacin

Full text links

International Scientific Literature, Ltd. Free PMC article

Proceed to details

Cite

Share

17

Observational Study

J Med Microbiol

. 2021 Apr;70(4):001350.

doi: 10.1099/jmm.0.001350.

Co-infection in critically ill patients with COVID-19: an observational cohort study from England

Vadsala Baskaran [1 2 3], Hannah Lawrence [1 2 3], Louise E Lansbury [2], Karmel Webb [2], Shahideh Safavi [3 4], Nurul I Zainuddin [1], Tausif Huq [1], Charlotte Eggleston [1], Jayne Ellis [5], Clare Thakker [5], Bethan Charles [6], Sara Boyd [7 8], Tom Williams [7], Claire Phillips [9], Ethan Redmore [9], Sarah Platt [10], Eve Hamilton [10], Andrew Barr [10], Lucy Venyo [10], Peter Wilson [5], Tom Bewick [11], Priya Daniel [11], Paul Dark [12 6], Adam R Jeans [6], Jamie McCanny [7], Jonathan D Edgeworth [7], Martin J Llewelyn [9], Matthias L Schmid [10], Tricia M McKeever [2 3], Martin Beed [13 14], Wei Shen Lim [1 3]

Affiliations Expand

Affiliations

- [1] Department of Respiratory Medicine, Nottingham University Hospital NHS Trust, Nottingham NG5 1PB, UK.
- [2] Division of Epidemiology and Public Health, School of Medicine, University of Nottingham, Clinical Sciences Building, Nottingham City Hospital Campus, Hucknall Road, Nottingham NG5 1PB, UK.
- [3] NIHR Nottingham Biomedical Research Centre, Queen's Medical Centre, Nottingham NG7 2UH, UK.
- [4] Division of Respiratory Medicine, School of Medicine, University of Nottingham, Queens Medical Centre, Derby Rd, Nottingham NG7 2UH, UK.
- [5] University College London Hospitals NHS Foundation Trust, 250 Euston Rd, London NW1 2PG, UK.
- [6] Salford Royal NHS Foundation Trust, Stott Ln, Salford M6 8HD, UK.
- [7] Guy's and St Thomas' NHS Foundation Trust, Great Maze Pond, London SE1 9RT, UK.
- [8] Antimicrobial Pharmacodynamics and Therapeutics, Department of Molecular and Clinical Pharmacology, University of Liverpool, Liverpool, L69 3GE, UK.
- [9] Brighton and Sussex University Hospitals NHS trust, Eastern Road, Brighton BN2 1ES, UK.
- [10] Newcastle Upon Tyne Hospitals NHS Foundation Trust, Freeman Rd, High Heaton, Newcastle upon Tyne NE7 7DN, UK.
- [11] University Hospitals of Derby and Burton NHS Foundation Trust, Uttoxeter Road, Derby DE22 3NE, UK.
- [12] Division of Infection, Immunity and Respiratory Medicine, NIHR Manchester Biomedical Research Centre, University of Manchester, Manchester, M23 9PT, UK.
- [13] Department of Critical Care, Nottingham University Hospital NHS Trust, Nottingham NG5 1PB, UK.
- [14] Division of Anaesthesia, School of Medicine, University of Nottingham, Queens Medical Centre, Derby Rd, Nottingham NG7 2UH, UK.

- PMID: **33861190**
- PMCID: PMC8289210
- DOI: 10.1099/jmm.0.001350

Free PMC article
Observational Study

Co-infection in critically ill patients with COVID-19: an observational cohort study from England

Vadsala Baskaran et al. J Med Microbiol. 2021 Apr.
Free PMC article
Show details
J Med Microbiol

. 2021 Apr;70(4):001350.
doi: 10.1099/jmm.0.001350.

Authors

Vadsala Baskaran [1 2 3], Hannah Lawrence [1 2 3], Louise E Lansbury [2], Karmel Webb [2], Shahideh Safavi [3 4], Nurul I Zainuddin [1], Tausif Huq [1], Charlotte Eggleston [1], Jayne Ellis [5], Clare Thakker [5], Bethan Charles [6], Sara Boyd [7 8], Tom Williams [7], Claire Phillips [9], Ethan Redmore [9], Sarah Platt [10], Eve Hamilton [10], Andrew Barr [10], Lucy Venyo [10], Peter Wilson [5], Tom Bewick [11], Priya Daniel [11], Paul Dark [12 6], Adam R Jeans [6], Jamie McCanny [7], Jonathan D Edgeworth [7], Martin J Llewelyn [9], Matthias L Schmid [10], Tricia M McKeever [2 3], Martin Beed [13 14], Wei Shen Lim [1 3]

Affiliations

- [1] Department of Respiratory Medicine, Nottingham University Hospital NHS Trust, Nottingham NG5 1PB, UK.
- [2] Division of Epidemiology and Public Health, School of Medicine, University of Nottingham, Clinical Sciences Building, Nottingham City Hospital Campus, Hucknall Road, Nottingham NG5 1PB, UK.
- [3] NIHR Nottingham Biomedical Research Centre, Queen's Medical Centre, Nottingham NG7 2UH, UK.
- [4] Division of Respiratory Medicine, School of Medicine, University of Nottingham, Queens Medical Centre, Derby Rd, Nottingham NG7 2UH, UK.
- [5] University College London Hospitals NHS Foundation Trust, 250 Euston Rd, London NW1 2PG, UK.
- [6] Salford Royal NHS Foundation Trust, Stott Ln, Salford M6 8HD, UK.
- [7] Guy's and St Thomas' NHS Foundation Trust, Great Maze Pond, London SE1 9RT, UK.
- [8] Antimicrobial Pharmacodynamics and Therapeutics, Department of Molecular and Clinical Pharmacology, University of Liverpool, Liverpool, L69 3GE, UK.
- [9] Brighton and Sussex University Hospitals NHS trust, Eastern Road, Brighton BN2 1ES, UK.
- [10] Newcastle Upon Tyne Hospitals NHS Foundation Trust, Freeman Rd, High Heaton, Newcastle upon Tyne NE7 7DN, UK.
- [11] University Hospitals of Derby and Burton NHS Foundation Trust, Uttoxeter Road, Derby DE22 3NE, UK.
- [12] Division of Infection, Immunity and Respiratory Medicine, NIHR Manchester Biomedical Research Centre, University of Manchester, Manchester, M23 9PT, UK.
- [13] Department of Critical Care, Nottingham University Hospital NHS Trust, Nottingham NG5 1PB, UK.
- [14] Division of Anaesthesia, School of Medicine, University of Nottingham, Queens Medical Centre, Derby Rd, Nottingham NG7 2UH, UK.

- PMID: **33861190**
- PMCID: PMC8289210
- DOI: 10.1099/jmm.0.001350

Abstract

Introduction. During previous viral pandemics, reported co-infection rates and implicated pathogens have varied. In the 1918 influenza pandemic, a large proportion of severe illness and death was complicated by bacterial co-infection, predominantly *Streptococcus pneumoniae* and *Staphylococcus aureus*.**Gap statement.** A better understanding of the incidence of co-infection in patients with COVID-19 infection and the pathogens involved is necessary for effective antimicrobial stewardship.**Aim.** To describe the incidence and nature of co-infection in critically ill adults with COVID-19 infection in England.**Methodology.** A retrospective cohort study of adults with COVID-19 admitted to seven intensive care units (ICUs) in England up to 18 May 2020, was performed. Patients with completed ICU stays were included. The proportion and type of organisms were determined at <48 and >48 h following hospital admission, corresponding to community and hospital-acquired co-infections.**Results.** Of 254 patients studied (median age 59 years (IQR 49-69); 64.6 % male), 139 clinically significant organisms were identified from 83 (32.7 %) patients. Bacterial co-infections/ co-colonisation were identified within 48 h of admission in 14 (5.5 %) patients; the commonest pathogens were *Staphylococcus aureus* (four patients) and *Streptococcus pneumoniae* (two patients). The proportion of pathogens detected increased with duration of ICU stay, consisting largely of Gram-negative bacteria, particularly *Klebsiella pneumoniae* and *Escherichia coli*. The co-infection/ co-colonisation rate >48 h after admission was 27/1000 person-days (95 % CI 21.3-34.1). Patients with co-infections/ co-colonisation were more likely to die in ICU (crude OR 1.78,95 % CI 1.03-3.08, *P*=0.04) compared to those without co-infections/ co-colonisation.**Conclusion.** We found limited evidence for community-acquired bacterial co-infection in hospitalised adults with COVID-19, but a high rate of Gram-negative infection acquired during ICU stay.

Keywords: COVID-19; ICU; antibiotic resistance; co-infection.

Conflict of interest statement

Professor Lim reports grants from National Institute for Health Research (NIHR), grants from Pfizer, outside the submitted work. Paul Dark is funded by NIHR Manchester BRC as sub-theme lead in Respiratory Infections.

- 40 references
- 2 figures

Supplementary info

Publication types, MeSH terms Expand

Publication types

- Multicenter Study
- Observational Study

MeSH terms

- Adult
- Aged
- Aged, 80 and over
- Bacteria / classification

- Bacteria / isolation & purification
- Bacterial Infections / epidemiology*
- Bacterial Infections / microbiology
- COVID-19 / epidemiology*
- COVID-19 / microbiology
- Coinfection / epidemiology*
- Coinfection / microbiology
- Critical Illness
- Cross Infection / epidemiology
- Cross Infection / microbiology
- England / epidemiology
- Female
- Hospitalization
- Humans
- Intensive Care Units
- Male
- Middle Aged
- Odds Ratio
- Retrospective Studies
- SARS-CoV-2
- Young Adult

Full text links

MICROBIOLOGY SOCIETY Ingenta plc Free PMC article

Proceed to details

Cite

Share

☐ 18

Meta-Analysis

Mol Psychiatry

. 2021 Sep;26(9):5199-5212.

doi: 10.1038/s41380-021-01021-4. Epub 2021 Feb 4.

Association between antidepressant use and reduced risk of intubation or death in hospitalized patients with COVID-19: results from an observational study

Nicolas Hoertel [1 2 3], Marina Sánchez-Rico [4 5], Raphaël Vernet [6], Nathanaël Beeker [7], Anne-Sophie Jannot [8 6 9], Antoine Neuraz [9 10], Elisa Salamanca [11], Nicolas Paris [12 13], Christel Daniel [12 14], Alexandre Gramfort [15], Guillaume Lemaitre [15], Mélodie Bernaux [16], Ali Bellamine [7], Cédric Lemogne [4 17 8], Guillaume Airagnes [4 17 8], Anita Burgun [9], Frédéric Limosin [4 17 8], AP-HP / Universities / INSERM COVID-19 Research Collaboration and AP-HP COVID CDR Initiative
Affiliations Expand

Affiliations

- [1] AP-HP.Centre-Université de Paris, Hôpital Corentin-Celton, Département Médico-Universitaire de Psychiatrie et Addictologie, 92130, Issy-les-Moulineaux, France. nico.hoertel@yahoo.fr.
- [2] INSERM, Institut de Psychiatrie et Neurosciences de Paris, UMR_S1266, Paris, France. nico.hoertel@yahoo.fr.
- [3] Université de Paris, Faculté de Santé, UFR de Médecine, Paris, France. nico.hoertel@yahoo.fr.
- [4] AP-HP.Centre-Université de Paris, Hôpital Corentin-Celton, Département Médico-Universitaire de Psychiatrie et Addictologie, 92130, Issy-les-Moulineaux, France.
- [5] Department of Psychobiology & Behavioural Sciences Methods, Faculty of Psychology, Universidad Complutense de Madrid, Campus de Somosaguas, Pozuelo de Alarcon, Spain.
- [6] AP-HP.Centre-Université de Paris, Hôpital Européen Georges Pompidou, Medical Informatics, Biostatistics and Public Health Department, F-75015, Paris, France.
- [7] AP-HP.Centre-Université de Paris, Unité de Recherche clinique, Hôpital Cochin, Paris, France.
- [8] Université de Paris, Faculté de Santé, UFR de Médecine, Paris, France.
- [9] INSERM, UMR_S1138, Cordeliers Research Center, Université de Paris, Paris, France.
- [10] AP-HP.Centre-Université de Paris, Necker-Enfants Malades Hospital, Department of Medical Informatics, 75015, Paris, France.
- [11] Banque Nationale de Données Maladies Rares, Campus Picpus, Département WIND (Web Innovation Données), Paris, France.
- [12] AP-HP, DSI-WIND (Web Innovation Données), Paris, France.
- [13] LIMSI, CNRS, Université Paris-Sud and Université Paris-Saclay, F-91405, Orsay, France.
- [14] Sorbonne University, University Paris 13, Sorbonne Paris Cité, INSERM UMR_S1142, F-75012, Paris, France.
- [15] Université Paris-Saclay, INRIA, CEA, Palaiseau, France.
- [16] Direction de la stratégie et de la transformation, AP-HP, Paris, France.
- [17] INSERM, Institut de Psychiatrie et Neurosciences de Paris, UMR_S1266, Paris, France.

- PMID: **33536545**
- DOI: 10.1038/s41380-021-01021-4

Meta-Analysis

Association between antidepressant use and reduced risk of intubation or death in

hospitalized patients with COVID-19: results from an observational study

Nicolas Hoertel et al. Mol Psychiatry. 2021 Sep.
Show details
Mol Psychiatry
. 2021 Sep;26(9):5199-5212.
doi: 10.1038/s41380-021-01021-4. Epub 2021 Feb 4.

Authors

Nicolas Hoertel [1 2 3], Marina Sánchez-Rico [4 5], Raphaël Vernet [6], Nathanaël Beeker [7], Anne-Sophie Jannot [8 6 9], Antoine Neuraz [9 10], Elisa Salamanca [11], Nicolas Paris [12 13], Christel Daniel [12 14], Alexandre Gramfort [15], Guillaume Lemaitre [15], Mélodie Bernaux [16], Ali Bellamine [7], Cédric Lemogne [4 17 8], Guillaume Airagnes [4 17 8], Anita Burgun [9], Frédéric Limosin [4 17 8], AP-HP / Universities / INSERM COVID-19 Research Collaboration and AP-HP COVID CDR Initiative

Affiliations

- [1] AP-HP.Centre-Université de Paris, Hôpital Corentin-Celton, Département Médico-Universitaire de Psychiatrie et Addictologie, 92130, Issy-les-Moulineaux, France. nico.hoertel@yahoo.fr.
- [2] INSERM, Institut de Psychiatrie et Neurosciences de Paris, UMR_S1266, Paris, France. nico.hoertel@yahoo.fr.
- [3] Université de Paris, Faculté de Santé, UFR de Médecine, Paris, France. nico.hoertel@yahoo.fr.
- [4] AP-HP.Centre-Université de Paris, Hôpital Corentin-Celton, Département Médico-Universitaire de Psychiatrie et Addictologie, 92130, Issy-les-Moulineaux, France.
- [5] Department of Psychobiology & Behavioural Sciences Methods, Faculty of Psychology, Universidad Complutense de Madrid, Campus de Somosaguas, Pozuelo de Alarcon, Spain.
- [6] AP-HP.Centre-Université de Paris, Hôpital Européen Georges Pompidou, Medical Informatics, Biostatistics and Public Health Department, F-75015, Paris, France.
- [7] AP-HP.Centre-Université de Paris, Unité de Recherche clinique, Hôpital Cochin, Paris, France.
- [8] Université de Paris, Faculté de Santé, UFR de Médecine, Paris, France.
- [9] INSERM, UMR_S1138, Cordeliers Research Center, Université de Paris, Paris, France.
- [10] AP-HP.Centre-Université de Paris, Necker-Enfants Malades Hospital, Department of Medical Informatics, 75015, Paris, France.
- [11] Banque Nationale de Données Maladies Rares, Campus Picpus, Département WIND (Web Innovation Données), Paris, France.
- [12] AP-HP, DSI-WIND (Web Innovation Données), Paris, France.
- [13] LIMSI, CNRS, Université Paris-Sud and Université Paris-Saclay, F-91405, Orsay, France.
- [14] Sorbonne University, University Paris 13, Sorbonne Paris Cité, INSERM UMR_S1142, F-75012, Paris, France.
- [15] Université Paris-Saclay, INRIA, CEA, Palaiseau, France.

- [16] Direction de la stratégie et de la transformation, AP-HP, Paris, France.
- [17] INSERM, Institut de Psychiatrie et Neurosciences de Paris, UMR_S1266, Paris, France.

- PMID: **33536545**
- DOI: 10.1038/s41380-021-01021-4

Abstract

A prior meta-analysis showed that antidepressant use in major depressive disorder was associated with reduced plasma levels of several pro-inflammatory mediators, which have been associated with severe COVID-19. Recent studies also suggest that several antidepressants may inhibit acid sphingomyelinase activity, which may prevent the infection of epithelial cells with SARS-CoV-2, and that the SSRI fluoxetine may exert in-vitro antiviral effects on SARS-CoV-2. We examined the potential usefulness of antidepressant use in patients hospitalized for COVID-19 in an observational multicenter retrospective cohort study conducted at AP-HP Greater Paris University hospitals. Of 7230 adults hospitalized for COVID-19, 345 patients (4.8%) received an antidepressant within 48 h of hospital admission. The primary endpoint was a composite of intubation or death. We compared this endpoint between patients who received antidepressants and those who did not in time-to-event analyses adjusted for patient characteristics, clinical and biological markers of disease severity, and other psychotropic medications. The primary analysis was a multivariable Cox model with inverse probability weighting. This analysis showed a significant association between antidepressant use and reduced risk of intubation or death (HR, 0.56; 95% CI, 0.43-0.73, $p < 0.001$). This association remained significant in multiple sensitivity analyses. Exploratory analyses suggest that this association was also significant for SSRI and non-SSRI antidepressants, and for fluoxetine, paroxetine, escitalopram, venlafaxine, and mirtazapine (all $p < 0.05$). These results suggest that antidepressant use could be associated with lower risk of death or intubation in patients hospitalized for COVID-19. Double-blind controlled randomized clinical trials of antidepressant medications for COVID-19 are needed.

© 2021. The Author(s), under exclusive licence to Springer Nature Limited part of Springer Nature.

Comment in

- Can antidepressants unlock prescription of rimonabant in the fight against COVID-19?
  Salles J, Briand-Mésange F, Trudel S, Ausseil J, Salles JP, Chap H. Salles J, et al. Mol Psychiatry. 2021 Dec;26(12):7091-7092. doi: 10.1038/s41380-021-01221-y. Epub 2021 Jul 19. Mol Psychiatry. 2021. PMID: 34282263 Free PMC article. No abstract available.
- Diversity of mechanism of action of psychotropic drugs in their anti-COVID-19 properties.
  Stip E, Arnone D, Abdel Aziz K, Javaid SF. Stip E, et al. Mol Psychiatry. 2021 Dec;26 (12):7093-7097. doi: 10.1038/s41380-021-01222-x. Epub 2021 Jul 19. Mol Psychiatry. 2021. PMID: 34282264 Free PMC article. No abstract available.

- 35 references

Supplementary info

Publication types, MeSH terms, Substances Expand

Publication types

- Meta-Analysis

MeSH terms

- Antidepressive Agents / therapeutic use
- COVID-19*
- Depressive Disorder, Major* / drug therapy
- Humans
- Intubation, Intratracheal
- Multicenter Studies as Topic
- Observational Studies as Topic
- Retrospective Studies
- SARS-CoV-2

Substances

- Antidepressive Agents

Full text links

Nature Publishing Group
Proceed to details
Cite
Share
19
Observational Study
Int J Infect Dis
. 2020 Aug;97:396-403.
doi: 10.1016/j.ijid.2020.06.099. Epub 2020 Jul 2.

Treatment with hydroxychloroquine, azithromycin, and combination in patients hospitalized with COVID-19

Samia Arshad [1], Paul Kilgore [2], Zohra S Chaudhry [1], Gordon Jacobsen [3], Dee Dee Wang [4], Kylie Huitsing [1], Indira Brar [1], George J Alangaden [5], Mayur S Ramesh [1], John E McKinnon [1], William O'Neill [4], Marcus Zervos [6], Henry Ford COVID-19 Task Force
Collaborators, Affiliations Expand

Collaborators

- **Henry Ford COVID-19 Task Force**:

Varidhi Nauriyal, Asif Abdul Hamed, Owais Nadeem, Jennifer Swiderek, Amanda Godfrey, Jeffrey Jennings, Jayna Gardner-Gray, Adam M Ackerman, Jonathan Lezotte, Joseph Ruhala, Raef Fadel, Amit Vahia, Smitha Gudipati, Tommy Parraga, Anita Shallal, Gina Maki, Zain Tariq, Geehan Suleyman, Nicholas Yared, Erica Herc, Johnathan Williams, Odaliz Abreu Lanfranco, Pallavi Bhargava, Katherine Reyes

Affiliations

- [1] Infectious Diseases, Henry Ford Hospital, Detroit, MI, United States.
- [2] Eugene Applebaum College of Pharmacy, Wayne State University, Detroit, MI, United States; Wayne State University School of Medicine, Detroit, MI, United States.
- [3] Public Health Sciences, Henry Ford Hospital, Detroit, MI, United States.
- [4] Division of Cardiovascular Disease & Structural Heart, Henry Ford Hospital, Detroit, MI, United States.
- [5] Infectious Diseases, Henry Ford Hospital, Detroit, MI, United States; Wayne State University School of Medicine, Detroit, MI, United States.
- [6] Infectious Diseases, Henry Ford Hospital, Detroit, MI, United States; Wayne State University School of Medicine, Detroit, MI, United States. Electronic address: MZervos1@hfhs.org.

- PMID: **32623082**
- PMCID: PMC7330574
- DOI: 10.1016/j.ijid.2020.06.099

Free PMC article
Observational Study

Treatment with hydroxychloroquine, azithromycin, and combination in patients hospitalized with COVID-19

Samia Arshad et al. Int J Infect Dis. 2020 Aug.
Free PMC article
Show details
Int J Infect Dis
. 2020 Aug;97:396-403.
doi: 10.1016/j.ijid.2020.06.099. Epub 2020 Jul 2.

Authors

Samia Arshad [1], Paul Kilgore [2], Zohra S Chaudhry [1], Gordon Jacobsen [3], Dee Dee Wang [4], Kylie Huitsing [1], Indira Brar [1], George J Alangaden [5], Mayur S Ramesh [1], John E McKinnon [1], William O'Neill [4], Marcus Zervos [6], Henry Ford COVID-19 Task Force

Collaborators

- **Henry Ford COVID-19 Task Force**:

Varidhi Nauriyal, Asif Abdul Hamed, Owais Nadeem, Jennifer Swiderek, Amanda Godfrey, Jeffrey Jennings, Jayna Gardner-Gray, Adam M Ackerman, Jonathan Lezotte, Joseph Ruhala, Raef Fadel, Amit Vahia, Smitha Gudipati, Tommy Parraga, Anita Shallal, Gina Maki, Zain Tariq, Geehan Suleyman, Nicholas Yared, Erica Herc, Johnathan Williams, Odaliz Abreu Lanfranco, Pallavi Bhargava, Katherine Reyes

Affiliations

- [1] Infectious Diseases, Henry Ford Hospital, Detroit, MI, United States.
- [2] Eugene Applebaum College of Pharmacy, Wayne State University, Detroit, MI, United States; Wayne State University School of Medicine, Detroit, MI, United States.
- [3] Public Health Sciences, Henry Ford Hospital, Detroit, MI, United States.
- [4] Division of Cardiovascular Disease & Structural Heart, Henry Ford Hospital, Detroit, MI, United States.
- [5] Infectious Diseases, Henry Ford Hospital, Detroit, MI, United States; Wayne State University School of Medicine, Detroit, MI, United States.
- [6] Infectious Diseases, Henry Ford Hospital, Detroit, MI, United States; Wayne State University School of Medicine, Detroit, MI, United States. Electronic address: MZervos1@hfhs.org.

- PMID: **32623082**
- PMCID: PMC7330574
- DOI: 10.1016/j.ijid.2020.06.099

Abstract

Significance: The United States is in an acceleration phase of the COVID-19 pandemic. Currently there is no known effective therapy or vaccine for treatment of SARS-CoV-2, highlighting urgency around identifying effective therapies.

Objective: The purpose of this study was to evaluate the role of hydroxychloroquine therapy alone and in combination with azithromycin in hospitalized patients positive for COVID-19.

Design: Multi-center retrospective observational study.

Setting: The Henry Ford Health System (HFHS) in Southeast Michigan: large six hospital integrated health system; the largest of hospitals is an 802-bed quaternary academic teaching hospital in urban Detroit, Michigan.

Participants: Consecutive patients hospitalized with a COVID-related admission in the health system from March 10, 2020 to May 2, 2020 were included. Only the first admission was included for patients with multiple admissions. All patients evaluated were 18 years of age and older and were treated as inpatients for at least 48h unless expired within 24h.

Exposure: Receipt of hydroxychloroquine alone, hydroxychloroquine in combination with azithromycin, azithromycin alone, or neither.

Main outcome: The primary outcome was in-hospital mortality.

Results: Of 2,541 patients, with a median total hospitalization time of 6 days (IQR: 4-10 days), median age was 64 years (IQR:53-76 years), 51% male, 56% African American, with median time to follow-up of 28.5 days (IQR:3-53). Overall in-hospital mortality was 18.1% (95% CI:16.6%

-19.7%); by treatment: hydroxychloroquine+azithromycin, 157/783 (20.1% [95% CI: 17.3% -23.0%]), hydroxychloroquine alone, 162/1202 (13.5% [95% CI: 11.6%-15.5%]), azithromycin alone, 33/147 (22.4% [95% CI: 16.0%-30.1%]), and neither drug, 108/409 (26.4% [95% CI: 22.2%-31.0%]). Primary cause of mortality was respiratory failure (88%); no patient had documented torsades de pointes. From Cox regression modeling, predictors of mortality were age>65 years (HR:2.6 [95% CI:1.9-3.3]), white race (HR:1.7 [95% CI:1.4-2.1]), CKD (HR:1.7 [95%CI:1.4-2.1]), reduced O2 saturation level on admission (HR:1.5 [95%CI:1.1-2.1]), and ventilator use during admission (HR: 2.2 [95%CI:1.4-3.3]). Hydroxychloroquine provided a 66% hazard ratio reduction, and hydroxychloroquine+azithromycin 71% compared to neither treatment (p<0.001).

Conclusions and relevance: In this multi-hospital assessment, when controlling for COVID-19 risk factors, treatment with hydroxychloroquine alone and in combination with azithromycin was associated with reduction in COVID-19 associated mortality. Prospective trials are needed to examine this impact.

Keywords: COVID-19; Coronavirus; Hydroxychloroquine; Mortality; SARS-COV-2; Therapy.

Copyright © 2020 The Author(s). Published by Elsevier Ltd.. All rights reserved.

Comment in

- Clarifying the record on hydroxychloroquine for the treatment of patients hospitalized with COVID-19.
  Rosenberg ES, Holtgrave DR, Udo T. Rosenberg ES, et al. Int J Infect Dis. 2020 Oct;99:38-39. doi: 10.1016/j.ijid.2020.07.055. Epub 2020 Jul 29. Int J Infect Dis. 2020. PMID: 32738483 Free PMC article. No abstract available.
- The continued dilemma about the usage of hydroxychloroquine: Respite is in randomized control trials.
  Malviya A. Malviya A. Int J Infect Dis. 2020 Oct;99:310-311. doi: 10.1016/j.ijid.2020.07.054. Epub 2020 Jul 29. Int J Infect Dis. 2020. PMID: 32738490 Free PMC article. No abstract available.
- Effectiveness of hydroxychloroquine in COVID-19 disease: A done and dusted deal?
  d'Arminio Monforte A, Tavelli A, Bai F, Marchetti G, Cozzi-Lepri A. d'Arminio Monforte A, et al. Int J Infect Dis. 2020 Oct;99:75-76. doi: 10.1016/j.ijid.2020.07.056. Epub 2020 Jul 29. Int J Infect Dis. 2020. PMID: 32738491 Free PMC article. No abstract available.
- Problems with the analysis in "Treatment with Hydroxychloroquine, Azithromycin, and Combination in Patients Hospitalized with COVID-19".
  Atkinson JG. Atkinson JG. Int J Infect Dis. 2020 Oct;99:37. doi: 10.1016/j.ijid.2020.07.057. Epub 2020 Jul 30. Int J Infect Dis. 2020. PMID: 32738492 Free PMC article. No abstract available.
- Possible synergistic effects of hydroxychloroquine and steroids in COVID-19, time for a nuanced approach. Comment on Arshad et al.
  Wiseman DM. Wiseman DM. Int J Infect Dis. 2020 Oct;99:344-345. doi: 10.1016/j.ijid.2020.07.064. Epub 2020 Aug 5. Int J Infect Dis. 2020. PMID: 32768694 Free PMC article. No abstract available.
- Hydroxychloroquine in COVID-19: Taking care of statistics to take care of patients.
  Brito-Azevedo A. Brito-Azevedo A. Int J Infect Dis. 2020 Oct;99:324. doi: 10.1016/j.ijid.2020.07.079. Epub 2020 Aug 6. Int J Infect Dis. 2020. PMID: 32768698 Free PMC article. No abstract available.
- Comment on Arshad et al.: Treatment with Hydroxychloroquine, Azithromycin, and Combination in Patients Hospitalized with COVID-19.

Varisco TJ, Johnson ML, Thornton D. Varisco TJ, et al. Int J Infect Dis. 2020 Oct;99:373. doi: 10.1016/j.ijid.2020.07.071. Epub 2020 Aug 6. Int J Infect Dis. 2020. PMID: 32771630
Free PMC article. No abstract available.

- 40 references
- 2 figures

Supplementary info

Publication types, MeSH terms, Substances, Supplementary concepts Expand

Publication types

- Multicenter Study
- Observational Study

MeSH terms

- Aged
- Azithromycin / therapeutic use*
- Betacoronavirus
- COVID-19
- Coronavirus Infections / drug therapy*
- Coronavirus Infections / mortality
- Drug Therapy, Combination
- Female
- Hospital Mortality*
- Hospitalization
- Humans
- Hydroxychloroquine / therapeutic use*
- Inpatients
- Male
- Middle Aged
- Pandemics
- Pneumonia, Viral / drug therapy*
- Pneumonia, Viral / mortality
- Retrospective Studies
- Risk Factors
- SARS-CoV-2

Substances

- Hydroxychloroquine
- Azithromycin

Supplementary concepts

- COVID-19 drug treatment

Full text links

ELSEVIER OPEN ACCESS Elsevier Science Free PMC article
Proceed to details
Cite
Share
20
Observational Study
Lancet Digit Health
. 2021 Mar;3(3):e166-e174.
doi: 10.1016/S2589-7500(20)30316-2. Epub 2021 Feb 8.

A simple nomogram for predicting failure of non-invasive respiratory strategies in adults with COVID-19: a retrospective multicentre study

Ling Liu [1], Jianfeng Xie [1], Wenjuan Wu [2], Hui Chen [3], Shusheng Li [4], Hongli He [5], Yuetian Yu [6], Ming Hu [7], Jinxiu Li [8], Ruiqiang Zheng [9], Xuyan Li [10], Haibo Qiu [1], Zhaohui Tong [10], Bin Du [11], Eddy Fan [12], Yi Yang [13], Arthur S Slutsky [14]
Affiliations Expand

Affiliations

- [1] Jiangsu Provincial Key Laboratory of Critical Care Medicine, Department of Critical Care Medicine, Zhongda Hospital, School of Medicine, Southeast University, Nanjing, China.
- [2] Department of Critical Care Medicine, Wuhan Jinyintan Hospital, Wuhan, China.
- [3] Department of Intensive Care Medicine, The First Affiliated Hospital of Soochow University, Suzhou, China.
- [4] Department of Critical Care Medicine, Tongji Hospital, Tongji Medical College Huazhong University of Science and Technology, Wuhan, China.
- [5] Department of Critical Care Medicine, Sichuan Provincial People's Hospital, University of Electronic Science and Technology of China, Chengdu, China.
- [6] Department of Critical Care Medicine, Ren Ji Hospital, School of Medicine, Shanghai Jiao Tong University, Shanghai, China.
- [7] Department of Infectious Disease, Wuhan Pulmonary Hospital, Wuhan, China.
- [8] Department of Critical Care Medicine, Shenzhen Third People's Hospital, Shenzhen, China.

- [9] Department of Critical Care Medicine, Northern Jiangsu People's Hospital, Clinical Medical School, Yangzhou University, Yangzhou, China.
- [10] Department of Respiratory and Critical Care Medicine, Beijing Institute of Respiratory Medicine, Beijing Chaoyang Hospital, Capital Medical University, Beijing, China.
- [11] Medical Intensive Care Unit, Peking Union Medical College Hospital, Peking Union Medical College and Chinese Academy of Medical Sciences, Beijing, China.
- [12] Interdepartmental Division of Critical Care Medicine, University of Toronto, Toronto, ON, Canada; Division of Respirology and Critical Care Medicine, Toronto General Hospital, Toronto, ON, Canada.
- [13] Jiangsu Provincial Key Laboratory of Critical Care Medicine, Department of Critical Care Medicine, Zhongda Hospital, School of Medicine, Southeast University, Nanjing, China. Electronic address: yiyiyang2004@163.com.
- [14] Interdepartmental Division of Critical Care Medicine, University of Toronto, Toronto, ON, Canada; Keenan Research Center, Li Ka Shing Knowledge Institute, St Michael's Hospital, Toronto, ON, Canada; Department of Medicine, Department of Surgery, and Department of Biomedical Engineering, University of Toronto, Toronto, ON, Canada.

- PMID: **33573999**
- PMCID: PMC7906717
- DOI: 10.1016/S2589-7500(20)30316-2

Free PMC article
Observational Study

A simple nomogram for predicting failure of non-invasive respiratory strategies in adults with COVID-19: a retrospective multicentre study

Ling Liu et al. Lancet Digit Health. 2021 Mar.
Free PMC article
Show details
Lancet Digit Health
. 2021 Mar;3(3):e166-e174.
doi: 10.1016/S2589-7500(20)30316-2. Epub 2021 Feb 8.

Authors

Ling Liu [1], Jianfeng Xie [1], Wenjuan Wu [2], Hui Chen [3], Shusheng Li [4], Hongli He [5], Yuetian Yu [6], Ming Hu [7], Jinxiu Li [8], Ruiqiang Zheng [9], Xuyan Li [10], Haibo Qiu [1], Zhaohui Tong [10], Bin Du [11], Eddy Fan [12], Yi Yang [13], Arthur S Slutsky [14]

Affiliations

- [1] Jiangsu Provincial Key Laboratory of Critical Care Medicine, Department of Critical Care Medicine, Zhongda Hospital, School of Medicine, Southeast University, Nanjing, China.

- [2] Department of Critical Care Medicine, Wuhan Jinyintan Hospital, Wuhan, China.
- [3] Department of Intensive Care Medicine, The First Affiliated Hospital of Soochow University, Suzhou, China.
- [4] Department of Critical Care Medicine, Tongji Hospital, Tongji Medical College Huazhong University of Science and Technology, Wuhan, China.
- [5] Department of Critical Care Medicine, Sichuan Provincial People's Hospital, University of Electronic Science and Technology of China, Chengdu, China.
- [6] Department of Critical Care Medicine, Ren Ji Hospital, School of Medicine, Shanghai Jiao Tong University, Shanghai, China.
- [7] Department of Infectious Disease, Wuhan Pulmonary Hospital, Wuhan, China.
- [8] Department of Critical Care Medicine, Shenzhen Third People's Hospital, Shenzhen, China.
- [9] Department of Critical Care Medicine, Northern Jiangsu People's Hospital, Clinical Medical School, Yangzhou University, Yangzhou, China.
- [10] Department of Respiratory and Critical Care Medicine, Beijing Institute of Respiratory Medicine, Beijing Chaoyang Hospital, Capital Medical University, Beijing, China.
- [11] Medical Intensive Care Unit, Peking Union Medical College Hospital, Peking Union Medical College and Chinese Academy of Medical Sciences, Beijing, China.
- [12] Interdepartmental Division of Critical Care Medicine, University of Toronto, Toronto, ON, Canada; Division of Respirology and Critical Care Medicine, Toronto General Hospital, Toronto, ON, Canada.
- [13] Jiangsu Provincial Key Laboratory of Critical Care Medicine, Department of Critical Care Medicine, Zhongda Hospital, School of Medicine, Southeast University, Nanjing, China. Electronic address: yiyiyang2004@163.com.
- [14] Interdepartmental Division of Critical Care Medicine, University of Toronto, Toronto, ON, Canada; Keenan Research Center, Li Ka Shing Knowledge Institute, St Michael's Hospital, Toronto, ON, Canada; Department of Medicine, Department of Surgery, and Department of Biomedical Engineering, University of Toronto, Toronto, ON, Canada.

- PMID: **33573999**
- PMCID: PMC7906717
- DOI: 10.1016/S2589-7500(20)30316-2

Abstract

Background: Non-invasive respiratory strategies (NIRS) including high-flow nasal cannula (HFNC) and non-invasive ventilation (NIV) have become widely used in patients with COVID-19 who develop acute respiratory failure. However, use of these therapies, if ineffective, might delay initiation of invasive mechanical ventilation (IMV) in some patients. We aimed to determine early predictors of NIRS failure and develop a simple nomogram and online calculator that can identify patients at risk of NIRS failure.

Methods: We did a retrospective, multicentre observational study in 23 hospitals designated for patients with COVID-19 in China. Adult patients (≥18 years) with severe acute respiratory syndrome coronavirus 2 infection and acute respiratory failure receiving NIRS were enrolled. A training cohort of 652 patients (21 hospitals) was used to identify early predictors of NIRS failure, defined as subsequent need for IMV or death within 28 days after intensive care unit admission. A nomogram was developed by multivariable logistic regression and concordance statistics (C-statistics) computed. C-statistics were validated internally by cross-validation in the training cohort, and externally in a validation cohort of 107 patients (two hospitals).

Findings: Patients were enrolled between Jan 1 and Feb 29, 2020. NIV failed in 211 (74%) of 286 patients and HFNC in 204 (56%) of 366 patients in the training cohort. NIV failed in 48 (81%) of 59 patients and HFNC in 26 (54%) of 48 patients in the external validation cohort. Age, number of comorbidities, respiratory rate-oxygenation index (ratio of pulse oximetry oxygen saturation/fraction of inspired oxygen to respiratory rate), Glasgow coma scale score, and use of vasopressors on the first day of NIRS in the training cohort were independent risk factors for NIRS failure. Based on the training dataset, the nomogram had a C-statistic of 0·80 (95% CI 0·74-0·85) for predicting NIV failure, and a C-statistic of 0·85 (0·82-0·89) for predicting HFNC failure. C-statistic values were stable in both internal validation (NIV group mean 0·79 [SD 0·10], HFNC group mean 0·85 [0·07]) and external validation (NIV group value 0·88 [95% CI 0·72-0·96], HFNC group value 0·86 [0·72-0·93]).

Interpretation: We have developed a nomogram and online calculator that can be used to identify patients with COVID-19 who are at risk of NIRS failure. These patients might benefit from early triage and more intensive monitoring.

Funding: Ministry of Science and Technology of the People's Republic of China, Key Research and Development Plan of Jiangsu Province, Chinese Academy of Medical Sciences.

Copyright © 2021 The Author(s). Published by Elsevier Ltd. This is an Open Access article under the CC BY-NC-ND 4.0 license. Published by Elsevier Ltd.. All rights reserved.

Comment in

- A nomogram for use of non-invasive respiratory strategies in COVID-19.
  Hess DR. Hess DR. Lancet Digit Health. 2021 Mar;3(3):e140-e141. doi: 10.1016/S2589-7500(21)00006-6. Epub 2021 Feb 8. Lancet Digit Health. 2021. PMID: 33574000 Free PMC article. No abstract available.

- 33 references
- 2 figures

Supplementary info

Publication types, MeSH terms Expand

Publication types

- Multicenter Study
- Observational Study
- Research Support, Non-U.S. Gov't

MeSH terms

- Adult
- Aged
- COVID-19 / therapy*
- China
- Comorbidity

- Female
- Forecasting
- Humans
- Male
- Medical Records
- Middle Aged
- Nomograms*
- Noninvasive Ventilation*
- Retrospective Studies
- SARS-CoV-2
- Treatment Failure*
- Young Adult

Full text links

ELSEVIER OPEN ACCESS Elsevier Science Free PMC article

Proceed to details

Cite

Share

☐ 21

Observational Study

Infect Dis (Lond)

. 2021 Feb;53(2):102-110.

doi: 10.1080/23744235.2020.1839672. Epub 2020 Oct 24.

Few bacterial co-infections but frequent empiric antibiotic use in the early phase of hospitalized patients with COVID-19: results from a multicentre retrospective cohort study in The Netherlands

Zara Karami [1] [2], Bram T Knoop [1] [2], Anton S M Dofferhoff [3], Marc J T Blaauw [1] [2] [3] [4], Nico A Janssen [1] [2], Marjan van Apeldoorn [5], Angèle P M Kerckhoffs [5], Josephine S van de Maat [1] [2], Jacobien J Hoogerwerf [1] [2], Jaap Ten Oever [1] [2]

Affiliations Expand

Affiliations

- [1] Radboud University Medical Center, Nijmegen, The Netherlands.
- [2] Radboud Center for Infectious diseases, Nijmegen, The Netherlands.
- [3] Canisius Wilhelmina Ziekenhuis, Nijmegen, The Netherlands.

- [4] Bernhoven University, Uden, The Netherlands.
- [5] Jeroen Bosch Ziekenhuis, Den Bosch, The Netherlands.

- PMID: **33103530**
- DOI: 10.1080/23744235.2020.1839672

Observational Study

Few bacterial co-infections but frequent empiric antibiotic use in the early phase of hospitalized patients with COVID-19: results from a multicentre retrospective cohort study in The Netherlands

Zara Karami et al. Infect Dis (Lond). 2021 Feb.

Show details

Infect Dis (Lond)

. 2021 Feb;53(2):102-110.
doi: 10.1080/23744235.2020.1839672. Epub 2020 Oct 24.

Authors

Zara Karami [1, 2], Bram T Knoop [1, 2], Anton S M Dofferhoff [3], Marc J T Blaauw [1, 2, 3, 4], Nico A Janssen [1, 2], Marjan van Apeldoorn [5], Angèle P M Kerckhoffs [5], Josephine S van de Maat [1, 2], Jacobien J Hoogerwerf [1, 2], Jaap Ten Oever [1, 2]

Affiliations

- [1] Radboud University Medical Center, Nijmegen, The Netherlands.
- [2] Radboud Center for Infectious diseases, Nijmegen, The Netherlands.
- [3] Canisius Wilhelmina Ziekenhuis, Nijmegen, The Netherlands.
- [4] Bernhoven University, Uden, The Netherlands.
- [5] Jeroen Bosch Ziekenhuis, Den Bosch, The Netherlands.

- PMID: **33103530**
- DOI: 10.1080/23744235.2020.1839672

Abstract

Background: Knowledge on bacterial co-infections in COVID-19 is crucial to use antibiotics appropriately. Therefore, we aimed to determine the incidence of bacterial co-infections, antibiotic use and application of antimicrobial stewardship principles in hospitalized patients with COVID-19.

Methods: We performed a retrospective observational study in four hospitals (1 university, 2 non-university teaching, 1 non-teaching hospital) in the Netherlands from March to May 2020 including consecutive patients with PCR-confirmed COVID-19. Data on first microbiological investigations obtained at the discretion of the physician and antibiotic use in the first week of hospital admission were collected.

Results: Twelve (1.2%) of the 925 patients included had a documented bacterial co-infection (75.0% pneumonia) within the first week. Microbiological testing was performed in 749 (81%) patients: sputum cultures in 105 (11.4%), blood cultures in 711 (76.9%), pneumococcal urinary antigen testing in 202 (21.8%), and *Legionella* urinary antigen testing in 199 (21.5%) patients, with clear variation between hospitals. On presentation 556 (60.1%; range 33.3-73.4%) patients received antibiotics for a median duration of 2 days (IQR 1-4). Intravenous to oral switch was performed in 41 of 413 (9.9%) patients who received intravenous treatment >48 h. Mean adherence to the local guideline on empiric antibiotic therapy on day 1 was on average 60.3% (range 45.3%-74.7%).

Conclusions: On presentation to the hospital bacterial co-infections are rare, while empiric antibiotic use is abundant. This implies that in patients with COVID-19 empiric antibiotic should be withheld. This has the potential to dramatically reduce the current overuse of antibiotics in the COVID-19 pandemic.

Keywords: COVID-19; SARS-CoV-2; antibiotic use; antimicrobial stewardship; bacterial co-infections; pneumonia.

Supplementary info

Publication types, MeSH terms, Substances Expand

Publication types

- Multicenter Study
- Observational Study

MeSH terms

- Aged
- Anti-Bacterial Agents / administration & dosage*
- Antimicrobial Stewardship
- Bacterial Infections / drug therapy*
- Bacterial Infections / epidemiology*
- Bacterial Infections / microbiology
- Blood Culture
- COVID-19 / epidemiology*
- COVID-19 / virology
- Coinfection
- Drug Administration Routes
- Drug Administration Schedule

- Female
- Guideline Adherence / statistics & numerical data
- Hospitalization
- Humans
- Incidence
- Male
- Middle Aged
- Netherlands / epidemiology
- Pandemics*
- Prescription Drug Overuse / prevention & control
- Prescription Drug Overuse / statistics & numerical data*
- Retrospective Studies
- SARS-CoV-2 / pathogenicity

Substances

- Anti-Bacterial Agents

Full text links

View full text Taylor & Francis
Proceed to details
Cite
Share
22
Observational Study
Virol J
. 2021 May 25;18(1):102.
doi: 10.1186/s12985-021-01577-1.

Benefits of treatment with favipiravir in hospitalized patients for COVID-19: a retrospective observational case-control study

Anıl Ucan [1], Pamir Cerci [2], Serdar Efe [3], Hakan Akgun [4], Ahmet Ozmen [5], Aysel Yagmuroglu [6], Muzaffer Bilgin [7], Deniz Avci [8]
Affiliations Expand

Affiliations

- [1] Department of Internal Medicine, Eskisehir City Hospital, 71 Evler Neighborhood, Çavdarlar Street, 26080, Odunpazarı, Eskişehir, Turkey. anil.ucan@saglik.gov.tr.

- [2] Division of Immunology, Department of Internal Medicine, Eskisehir City Hospital, 71 Evler Neighborhood, Çavdarlar Street, 26080, Odunpazarı, Eskişehir, Turkey.
- [3] Division of Intensive Care, Department of Internal Medicine, Eskisehir City Hospital, 71 Evler Neighborhood, Çavdarlar Street, 26080, Odunpazarı, Eskişehir, Turkey.
- [4] Department of Thoracic Medicine, Eskisehir City Hospital, 71 Evler Neighborhood, Çavdarlar Street, 26080, Odunpazarı, Eskişehir, Turkey.
- [5] Department of Infection Diseases, Eskisehir City Hospital, 71 Evler Neighborhood, Çavdarlar Street, 26080, Odunpazarı, Eskişehir, Turkey.
- [6] Department of Microbiology, Eskisehir City Hospital, 71 Evler Neighborhood, Çavdarlar Street, 26080, Odunpazarı, Eskişehir, Turkey.
- [7] Department of Biostatistics, Faculty of Medicine, Eskisehir Osmangazi University, Meşelik Kampüsü Büyükdere Mah. Prof. Dr. Nabi Avcı Bulvarı No: 4, 26040, Odunpazarı, Eskişehir, Turkey.
- [8] Department of Internal Medicine, Kayseri City Hospital, Muhsin Yazıcıoğlu Bulvarı No: 77, 38080, Kocasinan, Kayseri, Turkey.

- PMID: **34034765**
- PMCID: PMC8148395
- DOI: 10.1186/s12985-021-01577-1

Free PMC article
Observational Study

Benefits of treatment with favipiravir in hospitalized patients for COVID-19: a retrospective observational case-control study

Anıl Ucan et al. Virol J. 2021.
Free PMC article
Show details
Virol J
. 2021 May 25;18(1):102.
doi: 10.1186/s12985-021-01577-1.

Authors

Anıl Ucan [1], Pamir Cerci [2], Serdar Efe [3], Hakan Akgun [4], Ahmet Ozmen [5], Aysel Yagmuroglu [6], Muzaffer Bilgin [7], Deniz Avci [8]

Affiliations

- [1] Department of Internal Medicine, Eskisehir City Hospital, 71 Evler Neighborhood, Çavdarlar Street, 26080, Odunpazarı, Eskişehir, Turkey. anil.ucan@saglik.gov.tr.
- [2] Division of Immunology, Department of Internal Medicine, Eskisehir City Hospital, 71 Evler Neighborhood, Çavdarlar Street, 26080, Odunpazarı, Eskişehir, Turkey.
- [3] Division of Intensive Care, Department of Internal Medicine, Eskisehir City Hospital, 71 Evler Neighborhood, Çavdarlar Street, 26080, Odunpazarı, Eskişehir, Turkey.

- [4] Department of Thoracic Medicine, Eskisehir City Hospital, 71 Evler Neighborhood, Çavdarlar Street, 26080, Odunpazarı, Eskişehir, Turkey.
- [5] Department of Infection Diseases, Eskisehir City Hospital, 71 Evler Neighborhood, Çavdarlar Street, 26080, Odunpazarı, Eskişehir, Turkey.
- [6] Department of Microbiology, Eskisehir City Hospital, 71 Evler Neighborhood, Çavdarlar Street, 26080, Odunpazarı, Eskişehir, Turkey.
- [7] Department of Biostatistics, Faculty of Medicine, Eskisehir Osmangazi University, Meşelik Kampüsü Büyükdere Mah. Prof. Dr. Nabi Avcı Bulvarı No: 4, 26040, Odunpazarı, Eskişehir, Turkey.
- [8] Department of Internal Medicine, Kayseri City Hospital, Muhsin Yazıcıoğlu Bulvarı No: 77, 38080, Kocasinan, Kayseri, Turkey.

- PMID: **34034765**
- PMCID: PMC8148395
- DOI: 10.1186/s12985-021-01577-1

Abstract

Background: Although more than a year past since COVID-19 was defined, there is no specific treatment yet. Since COVID-19 management differs over time, it is hard to determine which therapy is more efficacious. In this study, we aimed to evaluate the efficacy of the regimen with Favipiravir (FPV) and determine if the timing of FPV addition offers any improvement.

Methods: A retrospective observational case-controlled cohort study was performed between March and September 2020, including adults with COVID-19 in a single-center in Turkey. We categorized patients into age-sex matched three groups, group 1 (n = 48) and group 2 (n = 48) included patients treated with the combination of FPV plus Hydroxychloroquine (HQ) early and late, respectively. Group 3 (n = 48) consisted of patients on HQ monotherapy. In Group 2, if the respiratory or clinic condition had not improved sufficiently, FPV was added on or after day 3.

Results: We found that starting FPV early had an impact on PCR negativity and the progression of the disease. 'No progression' was defined as the absence of a new finding in the control radiological examination and the absence of accompanying clinical deterioration. Also, the decrease in C-reactive protein (CRP) was greater in Group 1 than Group 3 ($p < 0.001$). However, we found that early initiation of FPV treatment did not have a positive effect on the estimated survival time.

Conclusions: According to this retrospective study results, we believe that for better clinical outcomes, FPV treatment should be started promptly to enhance antiviral effects and improve clinical outcomes.

Keywords: Antiviral therapy; Favipiravir; Hydroxychloroquine; Pneumonia; Severe acute respiratory syndrome coronavirus 2.

Conflict of interest statement

The authors declare that they have no competing interests.

- 27 references
- 2 figures

Supplementary info

Publication types, MeSH terms, Substances, Supplementary concepts Expand

Publication types

- Observational Study

MeSH terms

- Aged
- Amides / therapeutic use*
- Antiviral Agents / therapeutic use*
- COVID-19 / drug therapy*
- COVID-19 / physiopathology
- COVID-19 / virology
- Case-Control Studies
- Drug Administration Schedule
- Female
- Humans
- Hydroxychloroquine / therapeutic use
- Male
- Middle Aged
- Pyrazines / therapeutic use*
- Retrospective Studies
- SARS-CoV-2 / drug effects*
- SARS-CoV-2 / isolation & purification
- Treatment Outcome

Substances

- Amides
- Antiviral Agents
- Pyrazines
- Hydroxychloroquine
- favipiravir

Supplementary concepts

- COVID-19 drug treatment

Full text links

Read free full text at BMC BioMed Central Free PMC article
Proceed to details
Cite
Share
23

Observational Study

Crit Care
. 2021 Feb 18;25(1):72.
doi: 10.1186/s13054-021-03493-w.

Epidemiology and microbiology of ventilator-associated pneumonia in COVID-19 patients: a multicenter retrospective study in 188 patients in an un-inundated French region

Gauthier Blonz [1], Achille Kouatchet [2], Nicolas Chudeau [3], Emmanuel Pontis [4], Julien Lorber [5], Anthony Lemeur [6], Lucie Planche [7], Jean-Baptiste Lascarrou [8], Gwenhael Colin [9]
Affiliations Expand

Affiliations

- [1] Médecine Intensive Réanimation, Centre Hospitalier Départemental de Vendee, Les Oudairies, 85000, La Roche-Sur-Yon, France.
- [2] Medical Intensive Care Unit, University Hospital of Angers, 4 rue Larrey 49933, Angers, France.
- [3] Médecine Intensive Réanimation, Centre Hospitalier Le Mans, 194 Avenue Rubillard, 72037, Le Mans, France.
- [4] Médecine Intensive Réanimation, Centre Hospitalier de Laval, 33, rue du Haut Roche, Laval, 53015, Canada.
- [5] Médecine Intensive Réanimation, Centre Hospitalier de Saint-Nazaire, 11 Boulevard Georges Charpak, 44600, Saint-Nazaire, France.
- [6] Médecine Intensive Réanimation, Centre Hospitalier de Cholet, 1 Rue de Marengo, 49300, Cholet, France.
- [7] Clinical Research Unit, Centre Hospitalier Départmental de Vendée, Les Oudairies, 85000, La Roche-Sur-Yon, France.
- [8] Médecine Intensive Réanimation, University Hospital of Nantes, 1 Place Alexis-Ricordeau, 44000, Nantes, France.
- [9] Médecine Intensive Réanimation, Centre Hospitalier Départemental de Vendee, Les Oudairies, 85000, La Roche-Sur-Yon, France. gwenhael.colin@chd-vendee.fr.

- PMID: **33602296**
- PMCID: PMC7891465
- DOI: 10.1186/s13054-021-03493-w

Free PMC article

Observational Study

Epidemiology and microbiology of ventilator-associated pneumonia in COVID-19 patients: a multicenter retrospective study in 188 patients in an un-inundated French region

Gauthier Blonz et al. Crit Care. 2021.
Free PMC article
Show details
Crit Care
. 2021 Feb 18;25(1):72.
doi: 10.1186/s13054-021-03493-w.

Authors

Gauthier Blonz [1], Achille Kouatchet [2], Nicolas Chudeau [3], Emmanuel Pontis [4], Julien Lorber [5], Anthony Lemeur [6], Lucie Planche [7], Jean-Baptiste Lascarrou [8], Gwenhael Colin [9]

Affiliations

- [1] Médecine Intensive Réanimation, Centre Hospitalier Départemental de Vendee, Les Oudairies, 85000, La Roche-Sur-Yon, France.
- [2] Medical Intensive Care Unit, University Hospital of Angers, 4 rue Larrey 49933, Angers, France.
- [3] Médecine Intensive Réanimation, Centre Hospitalier Le Mans, 194 Avenue Rubillard, 72037, Le Mans, France.
- [4] Médecine Intensive Réanimation, Centre Hospitalier de Laval, 33, rue du Haut Roche, Laval, 53015, Canada.
- [5] Médecine Intensive Réanimation, Centre Hospitalier de Saint-Nazaire, 11 Boulevard Georges Charpak, 44600, Saint-Nazaire, France.
- [6] Médecine Intensive Réanimation, Centre Hospitalier de Cholet, 1 Rue de Marengo, 49300, Cholet, France.
- [7] Clinical Research Unit, Centre Hospitalier Départmental de Vendée, Les Oudairies, 85000, La Roche-Sur-Yon, France.
- [8] Médecine Intensive Réanimation, University Hospital of Nantes, 1 Place Alexis-Ricordeau, 44000, Nantes, France.
- [9] Médecine Intensive Réanimation, Centre Hospitalier Départemental de Vendee, Les Oudairies, 85000, La Roche-Sur-Yon, France. gwenhael.colin@chd-vendee.fr.

- PMID: **33602296**
- PMCID: PMC7891465
- DOI: 10.1186/s13054-021-03493-w

Abstract

Background: The COVID-19 pandemic is responsible for many hospitalizations in intensive care units (ICU), with widespread use of invasive mechanical ventilation (IMV) which exposes patients to the risk of ventilator-associated pneumonia (VAP). The characteristics of VAP in COVID-19 patients remain unclear.

Methods: We retrospectively collected data on all patients hospitalized for COVID-19 during the first phase of the epidemic in one of the seven ICUs of the Pays-de-Loire region (North-West France) and who were on invasive mechanical ventilation for more than 48 h. We studied the characteristics of VAP in these patients. VAP was diagnosed based on official recommendations, and we included only cases of VAP that were confirmed by a quantitative microbiological culture.

Findings: We analyzed data from 188 patients. Of these patients, 48.9% had VAP and 19.7% experienced multiple episodes. Our study showed an incidence of 39.0 VAP per 1000 days of IMV (until the first VAP episode) and an incidence of 33.7 VAP per 1000 days of IMV (including all 141 episodes of VAP). Multi-microbial VAP accounted for 39.0% of all VAP, and 205 pathogens were identified. Enterobacteria accounted for 49.8% of all the isolated pathogens. Bacteremia was associated in 15 (10.6%) cases of VAP. Pneumonia was complicated by thoracic empyema in five cases (3.5%) and by pulmonary abscess in two cases (1.4%). Males were associated with a higher risk of VAP (sHR 2.24 CI95% [1.18; 4.26] p = 0.013).

Interpretation: Our study showed an unusually high incidence of VAP in patients admitted to the ICU for severe COVID-19, even though our services were not inundated during the first wave of the epidemic. We also noted a significant proportion of enterobacteria. VAP-associated complications (abscess, empyema) were not exceptional.

Registration: As an observational study, this study has not been registered.

Keywords: Acute respiratory distress syndrome; COVID-19; SARS-CoV-2; Ventilator-associated pneumonia.

Conflict of interest statement

The authors declare that they have no competing interests.

- 28 references
- 2 figures

Supplementary info

Publication types, MeSH terms Expand

Publication types

- Multicenter Study
- Observational Study
- Research Support, Non-U.S. Gov't

MeSH terms

- Aged
- COVID-19 / epidemiology
- COVID-19 / therapy*
- Female
- France / epidemiology
- Hospitalization
- Humans
- Intensive Care Units
- Male
- Middle Aged
- Pneumonia, Ventilator-Associated / epidemiology*
- Pneumonia, Ventilator-Associated / microbiology*
- Respiration, Artificial / adverse effects*
- Retrospective Studies

Full text links

Read free full text at BMC BioMed Central Free PMC article
Proceed to details
Cite
Share
24
Observational Study
J Assoc Physicians India
. 2020 Oct;68(10):18-24.

Preliminary Observations and Experiences of Physiotherapy Practice in Acute Care Setup of COVID 19: A Retrospective Observational Study

Mariya P Jiandani [1], Santosh B Salagre [2], Shabana Kazi [3], Saraswati Iyer [4], Poonam Patil [5], Wasim Y Khot [6], Ekta Patil [7], Mashira Sopariwala [7]
Affiliations Expand

Affiliations

- [1] Associate Professor, Department of Physiotherapy, Seth GSMC and KEM Hospital, Mumbai, Maharashtra.

- [2] Professor and In-charge Covid ICU, Department of Medicine, Seth GSMC and KEM Hospital, Mumbai, Maharashtra.
- [3] Junior Physiotherapist, Seth GSMC and KEM Hospital, Mumbai, Maharashtra.
- [4] Prof. and Head, Department of Physiotherapy, Seth GSMC and KEM Hospital, Mumbai, Maharashtra.
- [5] Assistant Physiotherapist, Department of Physiotherapy, Seth GSMC and KEM Hospital, Mumbai, Maharashtra.
- [6] Assistant Professor, Department of Medicine, Seth GSMC and KEM Hospital, Mumbai, Maharashtra.
- [7] Postgraduate Student, Department of Physiotherapy, Seth GSMC and KEM Hospital, Mumbai, Maharashtra.

- PMID: **32978920**

Observational Study

Preliminary Observations and Experiences of Physiotherapy Practice in Acute Care Setup of COVID 19: A Retrospective Observational Study

Mariya P Jiandani et al. J Assoc Physicians India. 2020 Oct.

Show details

J Assoc Physicians India

. 2020 Oct;68(10):18-24.

Authors

Mariya P Jiandani [1], Santosh B Salagre [2], Shabana Kazi [3], Saraswati Iyer [4], Poonam Patil [5], Wasim Y Khot [6], Ekta Patil [7], Mashira Sopariwala [7]

Affiliations

- [1] Associate Professor, Department of Physiotherapy, Seth GSMC and KEM Hospital, Mumbai, Maharashtra.
- [2] Professor and In-charge Covid ICU, Department of Medicine, Seth GSMC and KEM Hospital, Mumbai, Maharashtra.
- [3] Junior Physiotherapist, Seth GSMC and KEM Hospital, Mumbai, Maharashtra.
- [4] Prof. and Head, Department of Physiotherapy, Seth GSMC and KEM Hospital, Mumbai, Maharashtra.
- [5] Assistant Physiotherapist, Department of Physiotherapy, Seth GSMC and KEM Hospital, Mumbai, Maharashtra.
- [6] Assistant Professor, Department of Medicine, Seth GSMC and KEM Hospital, Mumbai, Maharashtra.

- [7] Postgraduate Student, Department of Physiotherapy, Seth GSMC and KEM Hospital, Mumbai, Maharashtra.

- PMID: **32978920**

Abstract

Background: The rapid outbreak of coronavirus disease 2019 (COVID-19), a public health emergency of grave concern, warranted hospital admissions with almost 90,000 cases in June 2020 in city of Mumbai. 3-10% of the patients with moderate to severe involvement required intensive care unit (ICU) admission with respiratory support. Patients admitted in ICU with an acute COVID event present with respiratory dysfunction and are more likely to have critical illness myopathy and neuropathy (CIMN). Physiotherapy services being integral part of non-pharmacological management of any ICU was implemented for patients with COVID 19; a novel viral disease.

Objective: This retrospective study was undertaken to explore the physiotherapy practices that could be implemented in patients admitted with COVID 19 in the ICU and its effect on mobility and oxygen requirement as an outcome.

Methodology: Following ethical permission of institute, the data was extracted from electronic data record sheet in which daily parameters for physiotherapy intervention were recorded. Data from a single ICU and step down unit (SDU) from 5th June to 5th July 2020 was analysed. Records of patients diagnosed with COVID 19 and admitted in ICU or SDU were studied. Those in the age group of 18 to 90 years, of either gender were included. Demographic characteristics, disease severity, oxygen requirement, mobility status, physiotherapy intervention were studied.

Results: 278 record sheets (110 ICU and 168 SDU) were retrospectively analysed for demographics. 44.55% of patients improved with side lying position, 37.27% with prone position and 10.91% with quarter prone position. 4.55% of patient maintained oxygenation in propped up sitting. 2.73% could not be positioned. Chest physiotherapy techniques applied were deep breathing, ACBT, paced breathing and diaphragmatic breathing. Deep intercostal pressure on NIV along with vibrations was given to 12.72% of patients in the ICU. Group therapy sessions were conducted in SDU where 50.59% patients participated. ICU mobility score showed significant improvement on Wilcoxon Signed Ranks test status on day 7 in the ICU (z=-5.99, p=0.00) and SDU (z= 7.676, p=0.00) compared to day 1. Descriptive analysis showed a definitive reduction in oxygen support requirement.

Conclusion: Most common form of physiotherapy interventions in patients with Covid 19 were therapeutic positioning, early mobilization and breathing exercises. Physiotherapy intervention appears promising in facilitating early patient ambulation and discharge. This study shows that it is safe and feasible to provide early physiotherapy treatment techniques in patients with COVID-19 using appropriate measures of infection prevention and cross contamination.

© Journal of the Association of Physicians of India 2011.

Supplementary info

Publication types, MeSH terms Expand

Publication types

- Observational Study

MeSH terms

- Adolescent
- Adult
- Aged
- Aged, 80 and over
- Betacoronavirus
- COVID-19
- Coronavirus Infections*
- Humans
- Intensive Care Units
- Middle Aged
- Pandemics*
- Physical Therapy Modalities
- Pneumonia, Viral*
- Retrospective Studies
- SARS-CoV-2
- Young Adult

Proceed to details
Cite
Share
☐ 25
Observational Study
Clin Infect Dis
. 2020 Nov 5;71(8):1962-1968.
doi: 10.1093/cid/ciaa674.

Predictors for Severe COVID-19 Infection

Ashish Bhargava [1], Elisa Akagi Fukushima [1], Miriam Levine [1], Wei Zhao [1], Farah Tanveer [1], Susanna M Szpunar [1], Louis Saravolatz [1]
Affiliations Expand

Affiliation

- [1] Ascension St John Hospital, Detroit, Michigan, USA.

- PMID: **32472676**
- PMCID: PMC7314166
- DOI: 10.1093/cid/ciaa674

Free PMC article

Observational Study

Predictors for Severe COVID-19 Infection

Ashish Bhargava et al. Clin Infect Dis. 2020.
Free PMC article
Show details
Clin Infect Dis
. 2020 Nov 5;71(8):1962-1968.
doi: 10.1093/cid/ciaa674.

Authors

Ashish Bhargava[1], Elisa Akagi Fukushima[1], Miriam Levine[1], Wei Zhao[1], Farah Tanveer[1], Susanna M Szpunar[1], Louis Saravolatz[1]

Affiliation

- [1] Ascension St John Hospital, Detroit, Michigan, USA.

- PMID: **32472676**
- PMCID: PMC7314166
- DOI: 10.1093/cid/ciaa674

Abstract

Background: COVID-19 is a pandemic disease caused by a novel coronavirus, severe acute respiratory syndrome coronavirus 2 (SARS-CoV-2). Predictors for severe COVID-19 infection have not been well defined. Determination of risk factors for severe infection would enable identifying patients who may benefit from aggressive supportive care and early intervention.

Methods: We conducted a retrospective observational study of 197 patients with confirmed COVID-19 admitted to a tertiary academic medical center.

Results: Of 197 hospitalized patients, the mean (SD) age of the cohort was 60.6 (16.2) years, 103 (52.3%) were male, and 156 (82.1%) were black. Severe COVID-19 infection was noted in 74 (37.6%) patients, requiring intubation. Patients aged above 60 were significantly more likely to have severe infection. Patients with severe infection were significantly more likely to have diabetes, renal disease, and chronic pulmonary disease and had significantly higher white blood cell counts, lower lymphocyte counts, and increased C-reactive protein (CRP) than patients with nonsevere infection. In multivariable logistic regression analysis, risk factors for severe infection included pre-existing renal disease (odds ratio [OR], 7.4; 95% CI, 2.5-22.0), oxygen requirement at hospitalization (OR, 2.9; 95% CI, 1.3-6.7), acute renal injury (OR, 2.7; 95% CI, 1.3-5.6), and CRP on admission (OR, 1.006; 95% CI, 1.001-1.01). Race, age, and socioeconomic status were not independent predictors.

Conclusions: Acute or pre-existing renal disease, supplemental oxygen upon hospitalization, and admission CRP were independent predictors for the development of severe COVID-19. Every 1-unit increase in CRP increased the risk of severe disease by 0.06%.

Keywords: predictors; risk factors; severe COVID-19.

© The Author(s) 2020. Published by Oxford University Press for the Infectious Diseases Society of America. All rights reserved. For permissions, e-mail: journals.permissions@oup.com.

Comment in

- Nasopharyngeal SARS-CoV-2 Load at Hospital Admission as a Predictor of Mortality.
  Alteri C, Cento V, Vecchi M, Colagrossi L, Fanti D, Vismara C, Puoti M, Perno CF; SCoVA Study Group. Alteri C, et al. Clin Infect Dis. 2021 May 18;72(10):1868-1869. doi: 10.1093/cid/ciaa956. Clin Infect Dis. 2021. PMID: 32674129 Free PMC article. No abstract available.
- Reply to Alteri et al.
  Bhargava A, Fukushima EA, Levine M, Zhao W, Tanveer F, Szpunar SM, Saravolatz L. Bhargava A, et al. Clin Infect Dis. 2021 May 18;72(10):1870. doi: 10.1093/cid/ciaa960. Clin Infect Dis. 2021. PMID: 32674132 No abstract available.

Supplementary info

Publication types, MeSH terms Expand

Publication types

- Observational Study

MeSH terms

- Age Factors
- Aged
- Betacoronavirus
- COVID-19
- Comorbidity
- Coronavirus Infections / epidemiology*
- Female
- Hospitalization / statistics & numerical data
- Humans
- Male
- Middle Aged
- Pandemics
- Pneumonia, Viral / epidemiology*
- Retrospective Studies
- Risk Assessment*
- Risk Factors
- SARS-CoV-2
- Severity of Illness Index*

Full text links

OXFORD ACADEMIC Silverchair Information Systems Free PMC article

Proceed to details

Cite

Share

26

Observational Study

Sci Rep

. 2021 Oct 25;11(1):20964.

doi: 10.1038/s41598-021-00243-4.

Treatment for COVID-19-a cohort study from Northern Italy

Lorenzo Guglielmetti [1] [2] [3], Daniela Aschieri [4], Irina Kontsevaya [5] [6] [7], Francesco Calabrese [8], Alessandra Donisi [9], Alberto Faggi [8], Patrizia Ferrante [8] [10], Elisa Fronti [8], Laura Gerna [8], Maria Cristina Leoni [8], Franco Paolillo [8], Giovanna Ratti [8], Alessandro Ruggieri [8], Daria Sacchini [8], Marta Scotti [8], Caterina Valdatta [8], Marco Stabile [11], Gloria Taliani [8] [12] [13], Mauro Codeluppi [8]

Affiliations Expand

Affiliations

- [1] Sorbonne Université, INSERM, U1135, Centre d'Immunologie et des Maladies Infectieuses, Cimi-Paris, équipe 13, Paris, France. lorenzo.guglielmetti@aphp.fr.
- [2] APHP, Groupe Hospitalier Universitaire Sorbonne Université, Hôpital Pitié-Salpêtrière, Centre National de Référence Des Mycobactéries Et de La Résistance Des Mycobactéries Aux Antituberculeux, Paris, France. lorenzo.guglielmetti@aphp.fr.
- [3] Infectious Diseases Unit, Guglielmo da Saliceto Hospital, Piacenza, Italy. lorenzo.guglielmetti@aphp.fr.
- [4] Cardiology Unit, Castel San Giovanni Hospital, Piacenza, Italy.
- [5] Research Center Borstel, Borstel, Germany.
- [6] German Center for Infection Research, Hamburg-Lübeck-Borstel-Riems, Borstel, Germany.
- [7] International Health/Infectious Diseases, University of Lübeck, Lübeck, Germany.
- [8] Infectious Diseases Unit, Guglielmo da Saliceto Hospital, Piacenza, Italy.
- [9] Migration Health Unit, Primary Health Care Department, Guglielmo da Saliceto Hospital, Piacenza, Italy.
- [10] Institute for Cross-Disciplinary Physics and Complex Systems IFISC (UIB-CSIC), Campus Universitat Illes Balears, 07122, Palma de Mallorca, Spain.
- [11] Plastic Surgery Unit, Castel San Giovanni Hospital, Piacenza, Italy.
- [12] Infectious and Tropical Disease Unit, Department of Translational and Precision Medicine, Sapienza University of Rome, Rome, Italy.
- [13] Anti-COVID Task Force of the Italian Civil Protection, Rome, Italy.

- PMID: **34697322**
- PMCID: PMC8545945
- DOI: 10.1038/s41598-021-00243-4

Free PMC article
Observational Study

Treatment for COVID-19-a cohort study from Northern Italy

Lorenzo Guglielmetti et al. Sci Rep. 2021.
Free PMC article
Show details
Sci Rep
. 2021 Oct 25;11(1):20964.
doi: 10.1038/s41598-021-00243-4.

Authors

Lorenzo Guglielmetti [1] [2] [3], Daniela Aschieri [4], Irina Kontsevaya [5] [6] [7], Francesco Calabrese [8], Alessandra Donisi [9], Alberto Faggi [8], Patrizia Ferrante [8] [10], Elisa Fronti [8], Laura Gerna [8], Maria Cristina Leoni [8], Franco Paolillo [8], Giovanna Ratti [8], Alessandro Ruggieri [8], Daria Sacchini [8], Marta Scotti [8], Caterina Valdatta [8], Marco Stabile [11], Gloria Taliani [8] [12] [13], Mauro Codeluppi [8]

Affiliations

- [1] Sorbonne Université, INSERM, U1135, Centre d'Immunologie et des Maladies Infectieuses, Cimi-Paris, équipe 13, Paris, France. lorenzo.guglielmetti@aphp.fr.
- [2] APHP, Groupe Hospitalier Universitaire Sorbonne Université, Hôpital Pitié-Salpêtrière, Centre National de Référence Des Mycobactéries Et de La Résistance Des Mycobactéries Aux Antituberculeux, Paris, France. lorenzo.guglielmetti@aphp.fr.
- [3] Infectious Diseases Unit, Guglielmo da Saliceto Hospital, Piacenza, Italy. lorenzo.guglielmetti@aphp.fr.
- [4] Cardiology Unit, Castel San Giovanni Hospital, Piacenza, Italy.
- [5] Research Center Borstel, Borstel, Germany.
- [6] German Center for Infection Research, Hamburg-Lübeck-Borstel-Riems, Borstel, Germany.
- [7] International Health/Infectious Diseases, University of Lübeck, Lübeck, Germany.
- [8] Infectious Diseases Unit, Guglielmo da Saliceto Hospital, Piacenza, Italy.
- [9] Migration Health Unit, Primary Health Care Department, Guglielmo da Saliceto Hospital, Piacenza, Italy.
- [10] Institute for Cross-Disciplinary Physics and Complex Systems IFISC (UIB-CSIC), Campus Universitat Illes Balears, 07122, Palma de Mallorca, Spain.
- [11] Plastic Surgery Unit, Castel San Giovanni Hospital, Piacenza, Italy.
- [12] Infectious and Tropical Disease Unit, Department of Translational and Precision Medicine, Sapienza University of Rome, Rome, Italy.

- [13] Anti-COVID Task Force of the Italian Civil Protection, Rome, Italy.

- PMID: **34697322**
- PMCID: PMC8545945
- DOI: 10.1038/s41598-021-00243-4

Abstract

Multicentre, retrospective cohort study with multivariable Cox proportional-hazards modelling and survival-time inverse-probability-weighting, evaluating the impact of different treatments on survival of proven COVID-19 patients admitted to two Hospitals in the province of Piacenza, Italy. Use of tocilizumab and of high doses of low molecular weight heparin, but not of antivirals (either alone or in combination), azithromycin, and any corticosteroid, was independently associated with lower mortality. Our results support further clinical evaluation of high doses of low molecular weight heparin and tocilizumab as COVID-19 therapeutics.

© 2021. The Author(s).

Conflict of interest statement

The authors declare no competing interests.

- 10 references

Supplementary info

Publication types, MeSH terms, Substances Expand

Publication types

- Multicenter Study
- Observational Study
- Research Support, Non-U.S. Gov't

MeSH terms

- Adrenal Cortex Hormones / administration & dosage
- Aged
- Antibodies, Monoclonal, Humanized / administration & dosage*
- Antiviral Agents / administration & dosage*
- Azithromycin / administration & dosage
- COVID-19 / drug therapy*
- COVID-19 / epidemiology*
- Female
- Heparin / administration & dosage*
- Hospital Mortality

- Hospitalization
- Humans
- Italy / epidemiology
- Male
- Middle Aged
- Patient Admission
- Probability
- Proportional Hazards Models
- Retrospective Studies
- SARS-CoV-2
- Treatment Outcome

Substances

- Adrenal Cortex Hormones
- Antibodies, Monoclonal, Humanized
- Antiviral Agents
- Azithromycin
- Heparin
- tocilizumab

Full text links

Nature Publishing Group Free PMC article

Proceed to details

Cite

Share

27

Observational Study

JAMA
. 2021 Jun 22;325(24):2457-2465.
doi: 10.1001/jama.2021.7152.

Association Between Vaccination With BNT162b2 and Incidence of Symptomatic and Asymptomatic SARS-CoV-2 Infections Among Health Care Workers

Yoel Angel [1] [2], Avishay Spitzer [2] [3] [4], Oryan Henig [2] [5], Esther Saiag [2] [6], Eli Sprecher [2] [7] [8], Hagit Padova [2] [9], Ronen Ben-Ami [2] [5]
Affiliations Expand

Affiliations

- [1] Department of Physician Affairs, Tel Aviv Sourasky Medical Center, Tel Aviv, Israel.
- [2] Sackler Faculty of Medicine, Tel Aviv University, Tel Aviv, Israel.
- [3] Department of Oncology, Tel Aviv Sourasky Medical Center, Tel Aviv, Israel.
- [4] Department of Molecular Cell Biology, Weizmann Institute of Science, Rehovot, Israel.
- [5] Department of Infectious Diseases and Infection Control, Tel Aviv Sourasky Medical Center, Tel Aviv, Israel.
- [6] Department of Information Systems and Operations, Tel Aviv Sourasky Medical Center, Tel Aviv, Israel.
- [7] Department of Research and Development, Tel Aviv Sourasky Medical Center, Tel Aviv, Israel.
- [8] Department of Dermatology, Tel Aviv Sourasky Medical Center, Tel Aviv, Israel.
- [9] Department of Patient Safety, Tel Aviv Sourasky Medical Center, Tel Aviv, Israel.

- PMID: **33956048**
- PMCID: PMC8220476
- DOI: 10.1001/jama.2021.7152

Free PMC article
Observational Study

Association Between Vaccination With BNT162b2 and Incidence of Symptomatic and Asymptomatic SARS-CoV-2 Infections Among Health Care Workers

Yoel Angel et al. JAMA. 2021.
Free PMC article
Show details
JAMA
. 2021 Jun 22;325(24):2457-2465.
doi: 10.1001/jama.2021.7152.

Authors

Yoel Angel [1, 2], Avishay Spitzer [2, 3, 4], Oryan Henig [2, 5], Esther Saiag [2, 6], Eli Sprecher [2, 7, 8], Hagit Padova [2, 9], Ronen Ben-Ami [2, 5]

Affiliations

- [1] Department of Physician Affairs, Tel Aviv Sourasky Medical Center, Tel Aviv, Israel.
- [2] Sackler Faculty of Medicine, Tel Aviv University, Tel Aviv, Israel.
- [3] Department of Oncology, Tel Aviv Sourasky Medical Center, Tel Aviv, Israel.
- [4] Department of Molecular Cell Biology, Weizmann Institute of Science, Rehovot, Israel.

- [5] Department of Infectious Diseases and Infection Control, Tel Aviv Sourasky Medical Center, Tel Aviv, Israel.
- [6] Department of Information Systems and Operations, Tel Aviv Sourasky Medical Center, Tel Aviv, Israel.
- [7] Department of Research and Development, Tel Aviv Sourasky Medical Center, Tel Aviv, Israel.
- [8] Department of Dermatology, Tel Aviv Sourasky Medical Center, Tel Aviv, Israel.
- [9] Department of Patient Safety, Tel Aviv Sourasky Medical Center, Tel Aviv, Israel.

- PMID: **33956048**
- PMCID: PMC8220476
- DOI: 10.1001/jama.2021.7152

Abstract

Importance: Randomized clinical trials have provided estimates of the effectiveness of the BNT162b2 vaccine against symptomatic SARS-CoV-2 infection, but its effect on asymptomatic infections remains unclear.

Objective: To estimate the association of vaccination with the Pfizer-BioNTech BNT162b2 vaccine with symptomatic and asymptomatic SARS-CoV-2 infections among health care workers.

Design, setting, and participants: This was a single-center, retrospective cohort study conducted at a tertiary medical center in Tel Aviv, Israel. Data were collected on symptomatic and asymptomatic SARS-CoV-2 infections confirmed via polymerase chain reaction (PCR) tests in health care workers undergoing regular screening with nasopharyngeal swabs between December 20, 2020, and February 25, 2021. Logistic regression was used to calculate incidence rate ratios (IRRs) comparing the incidence of infection between fully vaccinated and unvaccinated participants, controlling for demographics and the number of PCR tests performed.

Exposures: Vaccination with the BNT162b2 vaccine vs unvaccinated status was ascertained from the employee health database. Full vaccination was defined as more than 7 days after receipt of the second vaccine dose.

Main outcomes and measures: The primary outcome was the regression-adjusted IRR for symptomatic and asymptomatic SARS-CoV-2 infection of fully vaccinated vs unvaccinated health care workers. The secondary outcomes included IRRs for partially vaccinated health care workers (days 7-28 after first dose) and for those considered as late fully vaccinated (>21 days after second dose).

Results: A total of 6710 health care workers (mean [SD] age, 44.3 [12.5] years; 4465 [66.5%] women) were followed up for a median period of 63 days; 5953 health care workers (88.7%) received at least 1 dose of the BNT162b2 vaccine, 5517 (82.2%) received 2 doses, and 757 (11.3%) were not vaccinated. Vaccination was associated with older age compared with those who were not vaccinated (mean age, 44.8 vs 40.7 years, respectively) and male sex (31.4% vs 17.7%). Symptomatic SARS-CoV-2 infection occurred in 8 fully vaccinated health care workers and 38 unvaccinated health care workers (incidence rate, 4.7 vs 149.8 per 100 000 person-days, respectively, adjusted IRR, 0.03 [95% CI, 0.01-0.06]). Asymptomatic SARS-CoV-2 infection occurred in 19 fully vaccinated health care workers and 17 unvaccinated health care workers (incidence rate, 11.3 vs 67.0 per 100 000 person-days, respectively, adjusted IRR, 0.14 [95% CI, 0.07-0.31]). The results were qualitatively unchanged by the propensity score sensitivity analysis.

Conclusions and relevance: Among health care workers at a single center in Tel Aviv, Israel, receipt of the BNT162b2 vaccine compared with no vaccine was associated with a significantly lower incidence of symptomatic and asymptomatic SARS-CoV-2 infection more than 7 days after the second dose. Findings are limited by the observational design.

Conflict of interest statement

Conflict of Interest Disclosures: Dr Angel reported receiving research grants from Pfizer outside the scope of this work. Dr Spitzer reported being partially supported by the Israeli Council for Higher Education via the Weizmann Data Science Research Center and by a research grant from Madame Olga Klein–Astrachan. Dr Ben-Ami reported receiving consulting fees from Pfizer, Gilead, and Merck Sharp & Dohme outside the scope of this work. No other disclosures were reported.

Comment in

- Technical note: The calculated real world BNT162b2 vaccine efficacy was 88% when accounting for asymptomatic cases.
  Junghans RP. Junghans RP. Hum Vaccin Immunother. 2021 Dec 2;17(12):5133-5134. doi: 10.1080/21645515.2021.1994800. Hum Vaccin Immunother. 2021. PMID: 35213948

- 3 figures

Supplementary info

Publication types, MeSH terms, Substances Expand

Publication types

- Observational Study

MeSH terms

- Adult
- Asymptomatic Infections / epidemiology
- BNT162 Vaccine
- COVID-19 / diagnosis
- COVID-19 / epidemiology*
- COVID-19 / prevention & control
- COVID-19 Vaccines*
- Female
- Health Personnel*
- Humans
- Incidence
- Israel
- Male
- Middle Aged

- Polymerase Chain Reaction
- Propensity Score
- Retrospective Studies
- SARS-CoV-2 / isolation & purification
- Tertiary Care Centers

Substances

- COVID-19 Vaccines
- BNT162 Vaccine

Full text links

JAMA FULL TEXT Silverchair Information Systems Free PMC article
Proceed to details
Cite
Share
28
Observational Study
Hypertension
. 2021 Mar 3;77(3):833-842.
doi: 10.1161/HYPERTENSIONAHA.120.16314. Epub 2021 Jan 11.

Antihypertensive Drugs and COVID-19 Risk: A Cohort Study of 2 Million Hypertensive Patients

Laura Semenzato [#] [1], Jérémie Botton [#] [1], Jérôme Drouin [1], Bérangère Baricault [1], Clémentine Vabre [1], François Cuenot [1], Laetitia Penso [1], Philippe Herlemont [1], Emilie Sbidian [2], Alain Weill [1], Rosemary Dray-Spira [1], Mahmoud Zureik [3]
Affiliations Expand

Affiliations

- [1] EPI-PHARE Scientific Interest Group in Epidemiology of Health Products from the French National Agency for the Safety of Medicines and Health Products, Saint-Denis, France and the French National Health Insurance, Paris, France (L.S., J.B., B.B., C.V., J.D., F.C., L.P., P.H., E.S., A.W., R.D.-S., M.Z.).
- [2] Epidemiology in Dermatology and Evaluation of Therapeutics Research Unit, University Paris-Est Créteil (E.S.).
- [3] University Paris-Saclay, UVSQ, University Paris-Sud, Inserm, Anti-Infective Evasion and Pharmacoepidemiology, CESP, 78180, Montigny le Bretonneux, France (M.Z.).

[#] Contributed equally.

- PMID: **33423528**
- PMCID: PMC7884243
- DOI: 10.1161/HYPERTENSIONAHA.120.16314

Free PMC article
Observational Study

Antihypertensive Drugs and COVID-19 Risk: A Cohort Study of 2 Million Hypertensive Patients

Laura Semenzato et al. Hypertension. 2021.
Free PMC article

Show details

Hypertension

. 2021 Mar 3;77(3):833-842.
doi: 10.1161/HYPERTENSIONAHA.120.16314. Epub 2021 Jan 11.

Authors

Laura Semenzato [#] [1], Jérémie Botton [#] [1], Jérôme Drouin [1], Bérangère Baricault [1], Clémentine Vabre [1], François Cuenot [1], Laetitia Penso [1], Philippe Herlemont [1], Emilie Sbidian [2], Alain Weill [1], Rosemary Dray-Spira [1], Mahmoud Zureik [3]

Affiliations

- [1] EPI-PHARE Scientific Interest Group in Epidemiology of Health Products from the French National Agency for the Safety of Medicines and Health Products, Saint-Denis, France and the French National Health Insurance, Paris, France (L.S., J.B., B.B., C.V., J.D., F.C., L.P., P.H., E.S., A.W., R.D.-S., M.Z.).
- [2] Epidemiology in Dermatology and Evaluation of Therapeutics Research Unit, University Paris-Est Créteil (E.S.).
- [3] University Paris-Saclay, UVSQ, University Paris-Sud, Inserm, Anti-Infective Evasion and Pharmacoepidemiology, CESP, 78180, Montigny le Bretonneux, France (M.Z.).

[#] Contributed equally.

- PMID: **33423528**
- PMCID: PMC7884243
- DOI: 10.1161/HYPERTENSIONAHA.120.16314

Abstract

After initially hypothesizing a positive relationship between use of renin-angiotensin-aldosterone system inhibitors and risk of coronavirus disease 2019 (COVID-19), more recent evidence

suggests negative associations. We examined whether COVID-19 risk differs according to antihypertensive drug class in patients treated by ACE (angiotensin-converting enzyme) inhibitors and angiotensin receptor blockers (ARBs) compared with calcium channel blockers (CCBs). Three exclusive cohorts of prevalent ACE inhibitors, ARB and CCB users, aged 18 to 80 years, from the French National Health Insurance databases were followed from February 15, 2020 to June 7, 2020. We excluded patients with a history of diabetes, known cardiovascular disease, chronic renal failure, or chronic respiratory disease during the previous 5 years, to only consider patients treated for uncomplicated hypertension and to limit indication bias. The primary end point was time to hospitalization for COVID-19. The secondary end point was time to intubation/death during a hospital stay for COVID-19. In a population of almost 2 million hypertensive patients (ACE inhibitors: 566 023; ARB: 958 227; CCB: 358 306) followed for 16 weeks, 2338 were hospitalized and 526 died or were intubated for COVID-19. ACE inhibitors and ARBs were associated with a lower risk of COVID-19 hospitalization compared with CCBs (hazard ratio, 0.74 [95% CI, 0.65-0.83] and 0.84 [0.76-0.93], respectively) and a lower risk of intubation/death. Risks were slightly lower for ACE inhibitor users than for ARB users. This large observational study may suggest a lower COVID-19 risk in hypertensive patients treated over a long period with ACE inhibitors or ARBs compared with CCBs. These results, if confirmed, tend to contradict previous hypotheses and raise new hypotheses.

Keywords: COVID-19; angiotensin receptor blockers; angiotensin-converting enzyme inhibitors; calcium channel blockers; hospitalization; mortality; pharmacoepidemiology.

Conflict of interest statement

None.

Comment in

- Could Renin-Angiotensin System Inhibitors Be Protective From Severe COVID-19?
  Carey RM. Carey RM. Hypertension. 2021 Mar 3;77(3):843-845. doi: 10.1161/HYPERTENSIONAHA.120.16800. Epub 2021 Feb 10. Hypertension. 2021. PMID: 33566689 Free PMC article. No abstract available.

- 32 references
- 3 figures

Supplementary info

Publication types, MeSH terms, Substances Expand

Publication types

- Observational Study

MeSH terms

- Adolescent
- Adult
- Aged

- Aged, 80 and over
- Angiotensin Receptor Antagonists / adverse effects*
- Angiotensin Receptor Antagonists / therapeutic use
- Angiotensin-Converting Enzyme 2 / drug effects*
- Angiotensin-Converting Enzyme Inhibitors / adverse effects*
- Angiotensin-Converting Enzyme Inhibitors / therapeutic use
- Antihypertensive Agents / adverse effects*
- Antihypertensive Agents / therapeutic use
- COVID-19 / epidemiology*
- COVID-19 / etiology
- Calcium Channel Blockers / adverse effects
- Calcium Channel Blockers / therapeutic use
- Comorbidity
- Disease Susceptibility
- Drug Utilization
- Female
- Follow-Up Studies
- France / epidemiology
- Hospital Mortality
- Hospitalization / statistics & numerical data
- Humans
- Hypertension / drug therapy*
- Hypertension / epidemiology
- Intubation, Intratracheal / statistics & numerical data
- Male
- Middle Aged
- Pandemics*
- Receptors, Virus / drug effects*
- Retrospective Studies
- SARS-CoV-2 / physiology*
- Young Adult

Substances

- Angiotensin Receptor Antagonists
- Angiotensin-Converting Enzyme Inhibitors
- Antihypertensive Agents
- Calcium Channel Blockers
- Receptors, Virus
- ACE2 protein, human
- Angiotensin-Converting Enzyme 2

Full text links

OPEN ACCESS Atypon Free PMC article
Proceed to details
Cite
Share
29
Observational Study
Clin Drug Investig
. 2021 Mar;41(3):221-233.
doi: 10.1007/s40261-021-01001-0. Epub 2021 Feb 9.

Observational Study of Chlorpromazine in Hospitalized Patients with COVID-19

Nicolas Hoertel [1, 2, 3], Marina Sánchez-Rico [4, 5], Raphaël Vernet [6], Anne-Sophie Jannot [3, 6, 7], Antoine Neuraz [7, 8], Carlos Blanco [9], Cédric Lemogne [1, 2, 3], Guillaume Airagnes [1, 2, 3], Nicolas Paris [10, 11], Christel Daniel [10, 12], Alexandre Gramfort [13], Guillaume Lemaitre [13], Mélodie Bernaux [14], Ali Bellamine [15], Nathanaël Beeker [15], Frédéric Limosin [1, 2, 3], AP-HP/Universities/INSERM COVID-19 Research Collaboration and AP-HP COVID CDR Initiative
Collaborators, Affiliations Expand

Collaborators

- **AP-HP/Universities/INSERM COVID-19 Research Collaboration and AP-HP COVID CDR Initiative**:
  Pierre-Yves Ancel, Alain Bauchet, Nathanaël Beeker, Vincent Benoit, Mélodie Bernaux, Ali Bellamine, Romain Bey, Aurélie Bourmaud, Stéphane Breant, Anita Burgun, Fabrice Carrat, Charlotte Caucheteux, Julien Champ, Sylvie Cormont, Christel Daniel, Julien Dubiel, Catherine Ducloas, Loic Esteve, Marie Frank, Nicolas Garcelon, Alexandre Gramfort, Nicolas Griffon, Olivier Grisel, Martin Guilbaud, Claire Hassen-Khodja, François Hemery, Martin Hilka, Anne Sophie Jannot, Jerome Lambert, Richard Layese, Judith Leblanc, Léo Lebouter, Guillaume Lemaitre, Damien Leprovost, Ivan Lerner, Kankoe Levi Sallah, Aurélien Maire, Marie-France Mamzer, Patricia Martel, Arthur Mensch, Thomas Moreau, Antoine Neuraz, Nina Orlova, Nicolas Paris, Bastien Rance, Hélène Ravera, Antoine Rozes, Elisa Salamanca, Arnaud Sandrin, Patricia Serre, Xavier Tannier, Jean-Marc Treluyer, Damien van Gysel, Gaël Varoquaux, Jill Jen Vie, Maxime Wack, Perceval Wajsburt, Demian Wassermann, Eric Zapletal

Affiliations

- [1] DMU Psychiatrie et Addictologie, AP-HP. Centre-Université de Paris, Hôpital Corentin-Celton, 4 parvis Corentin Celton, 92130, Issy-les-Moulineaux, France.
- [2] INSERM, Institut de Psychiatrie et Neurosciences de Paris, UMR_S1266, Paris, France.
- [3] Faculté de Santé, UFR de Médecine, Université de Paris, Paris, France.

- [4] DMU Psychiatrie et Addictologie, AP-HP. Centre-Université de Paris, Hôpital Corentin-Celton, 4 parvis Corentin Celton, 92130, Issy-les-Moulineaux, France. marinals@ucm.es.
- [5] Department of Psychobiology and Behavioural Sciences Methods, Faculty of Psychology, Universidad Complutense de Madrid, Campus de Somosaguas, Madrid, Spain. marinals@ucm.es.
- [6] Medical Informatics, Biostatistics and Public Health Department, AP-HP. Centre-Université de Paris, Hôpital Européen Georges Pompidou, 75015, Paris, France.
- [7] INSERM, UMR_S 1138, Cordeliers Research Center, Université de Paris, Paris, France.
- [8] Department of Medical Informatics, AP-HP. Centre-Université de Paris, Necker-Enfants Malades Hospital, 75015, Paris, France.
- [9] National Institute on Drug Abuse, Bethesda, MD, USA.
- [10] AP-HP. Département Web Innovation Données (DSI-WIND), Paris, France.
- [11] LIMSI, CNRS, Université Paris-Sud, Université Paris-Saclay, 91405, Orsay, France.
- [12] Sorbonne University, University Paris 13, Sorbonne Paris Cité, INSERM UMR_S 1142, 75012, Paris, France.
- [13] Université Paris-Saclay, Inria, CEA, Palaiseau, France.
- [14] AP-HP, Direction de la stratégie et de la transformation, Paris, France.
- [15] Unité de Recherche clinique, Hôpital Cochin, AP-HP. Centre-Université de Paris, Paris, France.

- PMID: **33559821**
- PMCID: PMC7871023
- DOI: 10.1007/s40261-021-01001-0

Free PMC article
Observational Study

Observational Study of Chlorpromazine in Hospitalized Patients with COVID-19

Nicolas Hoertel et al. Clin Drug Investig. 2021 Mar.
Free PMC article
Show details
Clin Drug Investig
. 2021 Mar;41(3):221-233.
doi: 10.1007/s40261-021-01001-0. Epub 2021 Feb 9.

Authors

Nicolas Hoertel [1 2 3], Marina Sánchez-Rico [4 5], Raphaël Vernet [6], Anne-Sophie Jannot [3 6 7], Antoine Neuraz [7 8], Carlos Blanco [9], Cédric Lemogne [1 2 3], Guillaume Airagnes [1 2 3], Nicolas Paris [10 11], Christel Daniel [10 12], Alexandre Gramfort [13], Guillaume Lemaitre [13], Mélodie Bernaux [14], Ali Bellamine [15], Nathanaël Beeker [15], Frédéric Limosin [1 2 3], AP-HP/Universities/INSERM COVID-19 Research Collaboration and AP-HP COVID CDR Initiative

Collaborators

- **AP-HP/Universities/INSERM COVID-19 Research Collaboration and AP-HP COVID CDR Initiative**:
  Pierre-Yves Ancel, Alain Bauchet, Nathanaël Beeker, Vincent Benoit, Mélodie Bernaux, Ali Bellamine, Romain Bey, Aurélie Bourmaud, Stéphane Breant, Anita Burgun, Fabrice Carrat, Charlotte Caucheteux, Julien Champ, Sylvie Cormont, Christel Daniel, Julien Dubiel, Catherine Ducloas, Loic Esteve, Marie Frank, Nicolas Garcelon, Alexandre Gramfort, Nicolas Griffon, Olivier Grisel, Martin Guilbaud, Claire Hassen-Khodja, François Hemery, Martin Hilka, Anne Sophie Jannot, Jerome Lambert, Richard Layese, Judith Leblanc, Léo Lebouter, Guillaume Lemaitre, Damien Leprovost, Ivan Lerner, Kankoe Levi Sallah, Aurélien Maire, Marie-France Mamzer, Patricia Martel, Arthur Mensch, Thomas Moreau, Antoine Neuraz, Nina Orlova, Nicolas Paris, Bastien Rance, Hélène Ravera, Antoine Rozes, Elisa Salamanca, Arnaud Sandrin, Patricia Serre, Xavier Tannier, Jean-Marc Treluyer, Damien van Gysel, Gaël Varoquaux, Jill Jen Vie, Maxime Wack, Perceval Wajsburt, Demian Wassermann, Eric Zapletal

Affiliations

- [1] DMU Psychiatrie et Addictologie, AP-HP. Centre-Université de Paris, Hôpital Corentin-Celton, 4 parvis Corentin Celton, 92130, Issy-les-Moulineaux, France.
- [2] INSERM, Institut de Psychiatrie et Neurosciences de Paris, UMR_S1266, Paris, France.
- [3] Faculté de Santé, UFR de Médecine, Université de Paris, Paris, France.
- [4] DMU Psychiatrie et Addictologie, AP-HP. Centre-Université de Paris, Hôpital Corentin-Celton, 4 parvis Corentin Celton, 92130, Issy-les-Moulineaux, France. marinals@ucm.es.
- [5] Department of Psychobiology and Behavioural Sciences Methods, Faculty of Psychology, Universidad Complutense de Madrid, Campus de Somosaguas, Madrid, Spain. marinals@ucm.es.
- [6] Medical Informatics, Biostatistics and Public Health Department, AP-HP. Centre-Université de Paris, Hôpital Européen Georges Pompidou, 75015, Paris, France.
- [7] INSERM, UMR_S 1138, Cordeliers Research Center, Université de Paris, Paris, France.
- [8] Department of Medical Informatics, AP-HP. Centre-Université de Paris, Necker-Enfants Malades Hospital, 75015, Paris, France.
- [9] National Institute on Drug Abuse, Bethesda, MD, USA.
- [10] AP-HP. Département Web Innovation Données (DSI-WIND), Paris, France.
- [11] LIMSI, CNRS, Université Paris-Sud, Université Paris-Saclay, 91405, Orsay, France.
- [12] Sorbonne University, University Paris 13, Sorbonne Paris Cité, INSERM UMR_S 1142, 75012, Paris, France.
- [13] Université Paris-Saclay, Inria, CEA, Palaiseau, France.
- [14] AP-HP, Direction de la stratégie et de la transformation, Paris, France.
- [15] Unité de Recherche clinique, Hôpital Cochin, AP-HP. Centre-Université de Paris, Paris, France.

- PMID: **33559821**
- PMCID: PMC7871023
- DOI: 10.1007/s40261-021-01001-0

Abstract

Introduction: Chlorpromazine has been suggested as being potentially useful in patients with coronavirus disease 2019 (COVID-19) on the grounds of its potential antiviral and anti-inflammatory effects.

Objective: The aim of this study was to examine the association between chlorpromazine use and mortality among adult patients hospitalized for COVID-19.

Methods: We conducted an observational, multicenter, retrospective study at Assistance Publique-Hôpitaux de Paris (AP-HP) Greater Paris University hospitals. Study baseline was defined as the date of first prescription of chlorpromazine during hospitalization for COVID-19. The primary endpoint was death. Among patients who had not been hospitalized in intensive care units (ICUs), we compared this endpoint between those who received chlorpromazine and those who did not, in time-to-event analyses adjusted for patient characteristics, clinical markers of disease severity, and other psychotropic medications. The primary analysis used a Cox regression model with inverse probability weighting. Multiple sensitivity analyses were performed.

Results: Of the 14,340 adult inpatients hospitalized outside ICUs for COVID-19, 55 patients (0.4%) received chlorpromazine. Over a mean follow-up of 14.3 days (standard deviation [SD] 18.2), death occurred in 13 patients (23.6%) who received chlorpromazine and 1289 patients (9.0%) who did not. In the primary analysis, there was no significant association between chlorpromazine use and mortality (hazard ratio [HR] 2.01, 95% confidence interval [CI] 0.75-5.40; p = 0.163). Sensitivity analyses included a Cox regression in a 1:5 ratio matched analytic sample that showed a similar result (HR 1.67, 95% CI 0.91-3.06; p = 0.100) and a multivariable Cox regression that indicated a significant positive association (HR 3.10, 95% CI 1.31-7.34; p = 0.010).

Conclusion: Our results suggest that chlorpromazine prescribed at a mean daily dose of 70.8 mg (SD 65.3) was not associated with reduced mortality.

Conflict of interest statement

Nicolas Hoertel has received personal fees and non-financial support from Lundbeck, outside the submitted work; Cédric Lemogne reports personal fees and non-financial support from Janssen-Cilag, Lundbeck, Otsuka Pharmaceutical, and Boehringer Ingelheim, outside the submitted work; Guillaume Airagnes reports personal fees from Pfizer, Pierre Fabre and Lundbeck, outside the submitted work; and Frédéric Limosin has received speaker and consulting fees from Janssen-Cilag outside the submitted work. Marina Sanchez-Rico, Raphaël Vernet, Anne-Sophie Jannot, Antoine Neuraz, Carlos Blanco, Nicolas Paris, Christel Daniel, Alexandre Gramfort, Guillaume Lemaitre, Mélodie Bernaux, Ali Bellamine, and Nathanaël Beeker declare no competing interests.

- 49 references
- 2 figures

Supplementary info

Publication types, MeSH terms, Substances, Supplementary concepts Expand

Publication types

- Multicenter Study
- Observational Study

MeSH terms

- Adolescent
- Adult
- Aged
- Aged, 80 and over
- COVID-19 / drug therapy*
- Chlorpromazine / therapeutic use*
- Female
- Hospitalization
- Humans
- Male
- Middle Aged
- Proportional Hazards Models
- Retrospective Studies
- SARS-CoV-2*
- Severity of Illness Index
- Young Adult

Substances

- Chlorpromazine

Supplementary concepts

- COVID-19 drug treatment

Full text links

SpringerLink FULL-TEXT ARTICLE Springer Free PMC article
Proceed to details
Cite
Share
☐ 30
Observational Study
BMC Infect Dis
. 2020 Nov 23;20(1):870.
doi: 10.1186/s12879-020-05617-z.

Atypical presentation of COVID-19; an observational retrospective study

Maryam Haghighi-Morad [1], Ilad Alavi Darazam [2], Hooman Bahrami-Moltagh [1], Maryam Amerifar [1], Nasim Zamani [3] [4], Hossein Hassanian-Moghaddam [5] [6]
Affiliations Expand

Affiliations

- [1] Department of Radiology, Loghman-Hakim Hospital, School of Medicine, Shahid Beheshti University of Medical Sciences, Tehran, Iran.
- [2] Department of Infectious Disease and Tropical Medicine, Loghman Hakim Hospital, School of Medicine, Shahid Beheshti University of Medical Sciences, Tehran, Iran.
- [3] Social Determinants of Health Research Center, Shahid Beheshti University of Medical Sciences, Tehran, Iran.
- [4] Department of Clinical Toxicology, Loghman-Hakim Hospital, School of Medicine, Shahid Beheshti University of Medical Sciences, South Karegar Avenue, Tehran, Iran.
- [5] Social Determinants of Health Research Center, Shahid Beheshti University of Medical Sciences, Tehran, Iran. hassanian@sbmu.ac.ir.
- [6] Department of Clinical Toxicology, Loghman-Hakim Hospital, School of Medicine, Shahid Beheshti University of Medical Sciences, South Karegar Avenue, Tehran, Iran. hassanian@sbmu.ac.ir.

- PMID: **33225911**
- PMCID: PMC7681183
- DOI: 10.1186/s12879-020-05617-z

Free PMC article
Observational Study

Atypical presentation of COVID-19; an observational retrospective study

Maryam Haghighi-Morad et al. BMC Infect Dis. 2020.
Free PMC article
Show details
BMC Infect Dis
. 2020 Nov 23;20(1):870.
doi: 10.1186/s12879-020-05617-z.

Authors

Maryam Haghighi-Morad [1], Ilad Alavi Darazam [2], Hooman Bahrami-Moltagh [1], Maryam Amerifar [1], Nasim Zamani [3] [4], Hossein Hassanian-Moghaddam [5] [6]

Affiliations

- [1] Department of Radiology, Loghman-Hakim Hospital, School of Medicine, Shahid Beheshti University of Medical Sciences, Tehran, Iran.

- [2] Department of Infectious Disease and Tropical Medicine, Loghman Hakim Hospital, School of Medicine, Shahid Beheshti University of Medical Sciences, Tehran, Iran.
- [3] Social Determinants of Health Research Center, Shahid Beheshti University of Medical Sciences, Tehran, Iran.
- [4] Department of Clinical Toxicology, Loghman-Hakim Hospital, School of Medicine, Shahid Beheshti University of Medical Sciences, South Karegar Avenue, Tehran, Iran.
- [5] Social Determinants of Health Research Center, Shahid Beheshti University of Medical Sciences, Tehran, Iran. hassanian@sbmu.ac.ir.
- [6] Department of Clinical Toxicology, Loghman-Hakim Hospital, School of Medicine, Shahid Beheshti University of Medical Sciences, South Karegar Avenue, Tehran, Iran. hassanian@sbmu.ac.ir.

- PMID: **33225911**
- PMCID: PMC7681183
- DOI: 10.1186/s12879-020-05617-z

Abstract

Background: COVID-19 infection may present with atypical signs and symptoms and false negative polymerase chain reaction (PCR) tests predisposing healthy people and health care workers to infection. The aim of the current study is to evaluate the features of atypical presentations in COVID-19 infection in a referral center in Tehran, Iran.

Methods: Hospital database of inpatients admitted to Loghman Hakim hospital between February 20th and May 11th, 2020 was reviewed and all patients with final diagnosis of COVID-19 infection were evaluated for their presenting symptoms. Patients with chief complaints of "fever", "dyspnea", and/or "cough" as typical presentations of COVID-19 were excluded and those with other clinical presentations were included.

Results: Nineteen patients were included with a mean age of 51 ± 19 years, of whom, 17 were males (89%). Median [IQR] Glasgow coma scale (GCS) was 14 [13, 15]. Almost 10 had referred with chief complaint of methanol poisoning and overdose on substances of abuse. Only 8 cases (42%) had positive COVID-19 test. Nine (47%) needed invasive mechanical ventilation, of whom, two had positive COVID-19 test results (p = ns). Eight patients (42%) died with three of them having positive PCRs.

Conclusions: In patients referring to emergency departments with chief complaint of poisoning (especially poisonings that can result in dyspnea including substances of abuse and toxic alcohols), gastrointestinal, and constitutional respiratory symptoms, attention should be given not to miss possible cases of COVID-19.

Keywords: Atypical; COVID-19; False negative; Infection; Signs; Symptoms.

Conflict of interest statement

The authors declare that they have no competing interests.

- 20 references

Supplementary info

Publication types, MeSH terms, Substances Expand

Publication types

- Observational Study

MeSH terms

- Adult
- Aged
- Alcoholic Intoxication / complications*
- COVID-19 / complications*
- COVID-19 / epidemiology
- COVID-19 / physiopathology*
- COVID-19 / virology
- Drug Overdose / complications*
- Emergency Service, Hospital
- Female
- Hospitalization
- Humans
- Iran / epidemiology
- Male
- Methanol / poisoning*
- Middle Aged
- Polymerase Chain Reaction
- Respiration, Artificial
- Retrospective Studies
- SARS-CoV-2 / genetics*

Substances

- Methanol

Full text links

Read free full text at BMC BioMed Central Free PMC article
Proceed to details
Cite
Share
☐ 31
Observational Study
J Glob Antimicrob Resist
. 2020 Dec;23:1-3.

doi: 10.1016/j.jgar.2020.08.001. Epub 2020 Aug 20.

A comprehensive strategy for the early treatment of COVID-19 with azithromycin/hydroxychloroquine and/or corticosteroids: Results of a retrospective observational study in the French overseas department of Réunion Island

Arthur Dubernet [1], Kevin Larsen [1], Laurie Masse [1], Jérôme Allyn [2], Emilie Foch [1], Lea Bruneau [3], Adrien Maillot [4], Marie Lagrange-Xelot [5], Vincent Thomas [6], Marie-Christine Jaffar-Bandjee [6], Loraine Gauzere [7], Loic Raffray [7], Karine Borsu [8], Servane Dibernardo [8], Stéphane Renaud [9], Michel André [1], Diane Moreau [1], Julien Jabot [10], Nathalie Coolen-Allou [1], Nicolas Allou [11]

Affiliations Expand

Affiliations

- [1] Pneumologie, Centre Hospitalier Universitaire Felix-Guyon, Allée des Topazes, 97405 Saint Denis, Réunion Island, France.
- [2] Réanimation polyvalente, Centre Hospitalier Universitaire Felix-Guyon, Allée des Topazes, 97405 Saint Denis, Réunion Island, France; Département d'Informatique Clinique, Centre Hospitalier Universitaire Felix-Guyon, Allée des Topazes, 97405 Saint Denis, Réunion Island, France.
- [3] INSERM CIC 1410 Clinical and Epidemiology, University Hospital, Saint Pierre, Réunion Island, France; Department of Public Health and Research Support, Methodological Support and Biostatistics Unit, University Hospital, Saint Denis, Réunion Island, France.
- [4] Department of Public Health and Research Support, Methodological Support and Biostatistics Unit, University Hospital, Saint Denis, Réunion Island, France.
- [5] Service des Maladies Infectieuses, Centre Hospitalier Universitaire Felix-Guyon, Allée des Topazes, 97405 Saint Denis, Réunion Island, France.
- [6] Microbiologie, Centre Hospitalier Universitaire Felix-Guyon, Allée des Topazes, 97405 Saint Denis, Réunion Island, France.
- [7] Médecine Interne, Centre Hospitalier Universitaire Felix-Guyon, Allée des Topazes, 97405 Saint Denis, Réunion Island, France.
- [8] Médecine Polyvalente, Centre Hospitalier Universitaire Felix-Guyon, Allée des Topazes, 97405 Saint Denis, Réunion Island, France.
- [9] Gériatrie, Centre Hospitalier Universitaire Felix-Guyon, Allée des Topazes, 97405 Saint Denis, Réunion Island, France.
- [10] Réanimation polyvalente, Centre Hospitalier Universitaire Felix-Guyon, Allée des Topazes, 97405 Saint Denis, Réunion Island, France.
- [11] Réanimation polyvalente, Centre Hospitalier Universitaire Felix-Guyon, Allée des Topazes, 97405 Saint Denis, Réunion Island, France; Département d'Informatique Clinique,

Centre Hospitalier Universitaire Felix-Guyon, Allée des Topazes, 97405 Saint Denis, Réunion Island, France. Electronic address: nicolas.allou@hotmail.fr.

- PMID: **32828896**
- PMCID: PMC7439827
- DOI: 10.1016/j.jgar.2020.08.001

Free PMC article
Observational Study

A comprehensive strategy for the early treatment of COVID-19 with azithromycin/hydroxychloroquine and/or corticosteroids: Results of a retrospective observational study in the French overseas department of Réunion Island

Arthur Dubernet et al. J Glob Antimicrob Resist. 2020 Dec.
Free PMC article
Show details
J Glob Antimicrob Resist

. 2020 Dec;23:1-3.
doi: 10.1016/j.jgar.2020.08.001. Epub 2020 Aug 20.

Authors

Arthur Dubernet [1], Kevin Larsen [1], Laurie Masse [1], Jérôme Allyn [2], Emilie Foch [1], Lea Bruneau [3], Adrien Maillot [4], Marie Lagrange-Xelot [5], Vincent Thomas [6], Marie-Christine Jaffar-Bandjee [6], Loraine Gauzere [7], Loic Raffray [7], Karine Borsu [8], Servane Dibernardo [8], Stéphane Renaud [9], Michel André [1], Diane Moreau [1], Julien Jabot [10], Nathalie Coolen-Allou [1], Nicolas Allou [11]

Affiliations

- [1] Pneumologie, Centre Hospitalier Universitaire Felix-Guyon, Allée des Topazes, 97405 Saint Denis, Réunion Island, France.
- [2] Réanimation polyvalente, Centre Hospitalier Universitaire Felix-Guyon, Allée des Topazes, 97405 Saint Denis, Réunion Island, France; Département d'Informatique Clinique, Centre Hospitalier Universitaire Felix-Guyon, Allée des Topazes, 97405 Saint Denis, Réunion Island, France.
- [3] INSERM CIC 1410 Clinical and Epidemiology, University Hospital, Saint Pierre, Réunion Island, France; Department of Public Health and Research Support, Methodological Support and Biostatistics Unit, University Hospital, Saint Denis, Réunion Island, France.

- [4] Department of Public Health and Research Support, Methodological Support and Biostatistics Unit, University Hospital, Saint Denis, Réunion Island, France.
- [5] Service des Maladies Infectieuses, Centre Hospitalier Universitaire Felix-Guyon, Allée des Topazes, 97405 Saint Denis, Réunion Island, France.
- [6] Microbiologie, Centre Hospitalier Universitaire Felix-Guyon, Allée des Topazes, 97405 Saint Denis, Réunion Island, France.
- [7] Médecine Interne, Centre Hospitalier Universitaire Felix-Guyon, Allée des Topazes, 97405 Saint Denis, Réunion Island, France.
- [8] Médecine Polyvalente, Centre Hospitalier Universitaire Felix-Guyon, Allée des Topazes, 97405 Saint Denis, Réunion Island, France.
- [9] Gériatrie, Centre Hospitalier Universitaire Felix-Guyon, Allée des Topazes, 97405 Saint Denis, Réunion Island, France.
- [10] Réanimation polyvalente, Centre Hospitalier Universitaire Felix-Guyon, Allée des Topazes, 97405 Saint Denis, Réunion Island, France.
- [11] Réanimation polyvalente, Centre Hospitalier Universitaire Felix-Guyon, Allée des Topazes, 97405 Saint Denis, Réunion Island, France; Département d'Informatique Clinique, Centre Hospitalier Universitaire Felix-Guyon, Allée des Topazes, 97405 Saint Denis, Réunion Island, France. Electronic address: nicolas.allou@hotmail.fr.

- PMID: **32828896**
- PMCID: PMC7439827
- DOI: 10.1016/j.jgar.2020.08.001

Abstract

Background: This study aimed to evaluate the prognosis of COVID-19 patients in Reunion Island, with a particular focus on the management of patients with hypoxemic pneumonia.

Methods: This retrospective observational study was conducted from 11 March to 17 April 2020 at the only hospital authorized to manage patients with COVID-19 in Reunion Island.

Results: Over the study period, 164 out of 398 patients (41.2%) infected with COVID-19 were admitted to Félix Guyon University Hospital. Of these, 36 (22%) developed hypoxemic pneumonia. Patients with hypoxemic pneumonia were aged 66 [56-77] years, 69% were male and 33% had hypertension. Ten patients (27.8%) were hospitalized in intensive care unit (ICU). Hydroxychloroquine/azithromycin treatment was associated with a lower ICU admission rate (P=0.008). None of the 6 patients treated with corticosteroids were hospitalized in ICU (P=0.16). There were no deaths at follow up (minimum 80 days).

Conclusions: Despite the risk profile of COVID-19 patients with severe hypoxemic pneumonia, the mortality rate of the disease in Reunion Island was 0%. This may be due to the care bundle used in our hospital (early hospitalisation, treatment with hydroxychloroquine/azithromycin and/or corticosteroids, non-invasive respiratory support, etc).

Keywords: Azithromycin; COVID-19; Corticosteroids; Hydroxychloroquine; SARS-CoV-2.

Copyright © 2020 The Authors. Published by Elsevier Ltd.. All rights reserved.

- 5 references
- 1 figure

Supplementary info

Publication types, MeSH terms, Substances Expand

Publication types

- Observational Study

MeSH terms

- Adrenal Cortex Hormones / administration & dosage*
- Aged
- Azithromycin / administration & dosage*
- COVID-19 / drug therapy*
- COVID-19 / virology
- Drug Therapy, Combination
- Female
- Hospitalization
- Humans
- Hydroxychloroquine / administration & dosage*
- Intensive Care Units
- Kaplan-Meier Estimate
- Male
- Middle Aged
- Retrospective Studies
- Reunion
- SARS-CoV-2 / isolation & purification

Substances

- Adrenal Cortex Hormones
- Hydroxychloroquine
- Azithromycin

Full text links

ELSEVIER OPEN ACCESS Elsevier Science Free PMC article
Proceed to details
Cite
Share
☐ 32
Observational Study
Medicine (Baltimore)

. 2021 Oct 29;100(43):e27685.
doi: 10.1097/MD.0000000000027685.

Epidemiology of COVID-19 in Jiangxi, China: A retrospective observational study

Yonghai Dong [1], Sheng Ding [1], Jingyu Zhang [2], Yun Liu [3]
Affiliations Expand

Affiliations

- [1] Jiangxi Provincial Center for Disease Control and Prevention, Nanchang, Jiangxi, China.
- [2] Clinical Laboratory, the First Affiliated Hospital of Nanchang University, Nanchang, Jiangxi, China.
- [3] Department of Imaging, Jiangxi Provincial People's Hospital Affiliated to Nanchang University, Nanchang, Jiangxi, China.

- PMID: **34713866**
- PMCID: PMC8556035
- DOI: 10.1097/MD.0000000000027685

Free PMC article
Observational Study

Epidemiology of COVID-19 in Jiangxi, China: A retrospective observational study

Yonghai Dong et al. Medicine (Baltimore). 2021.
Free PMC article
Show details
Medicine (Baltimore)
. 2021 Oct 29;100(43):e27685.
doi: 10.1097/MD.0000000000027685.

Authors

Yonghai Dong [1], Sheng Ding [1], Jingyu Zhang [2], Yun Liu [3]

Affiliations

- [1] Jiangxi Provincial Center for Disease Control and Prevention, Nanchang, Jiangxi, China.
- [2] Clinical Laboratory, the First Affiliated Hospital of Nanchang University, Nanchang, Jiangxi, China.
- [3] Department of Imaging, Jiangxi Provincial People's Hospital Affiliated to Nanchang University, Nanchang, Jiangxi, China.

- PMID: **34713866**
- PMCID: PMC8556035
- DOI: 10.1097/MD.0000000000027685

Abstract

To analyze the epidemiological characteristics of coronavirus disease 2019 (COVID-19) in Jiangxi Province, China, from January 21 to April 9, 2020.COVID-19 epidemic information was obtained from the official websites of the Jiangxi Provincial Health Committee, Hubei Provincial Health Committee, and National Health Commission of the People's Republic of China. ArcGIS 10.0 was used to draw a map of the spatial distribution of the cases.On January 21, 2020, the first COVID-19 confirmed case in Jiangxi was reported. By January 27, COVID-19 had spread rapidly to all cities in Jiangxi. The outbreak peaked on February 3, with a daily incidence of 85 cases. The last indigenous case reported on February 27. From January 21 to April 9, a total of 937 confirmed cases of COVID-19 were reported, with a cumulative incidence of 2.02/100,000. Of those, 936 patients (99.89%) were cured, and 1 (0.11%) died due to COVID-19. The COVID-19 epidemic trend in Jiangxi was basically consistent with the national epidemic trend (except Hubei). Throughout the epidemic prevention and control phase, Jiangxi province has taken targeted prevention and control measures based on the severity of the spread of COVID-19.The COVID-19 epidemic in Jiangxi was widespread and developed rapidly. In less than 1 month, the epidemic situation was effectively controlled, and the epidemic situation shifted to a low-level distribution state. All these proved that the COVID-19 prevention and control strategies and measures adopted by Jiangxi Province were right, positive and effective.

Copyright © 2021 the Author(s). Published by Wolters Kluwer Health, Inc.

Conflict of interest statement

The authors have no conflicts of interests to disclose.

- 37 references
- 5 figures

Supplementary info

Publication types, MeSH terms Expand

Publication types

- Observational Study

MeSH terms

- Adaptation, Psychological
- COVID-19 / epidemiology*
- COVID-19 / prevention & control
- China / epidemiology
- Communicable Disease Control / organization & administration

- Epidemics
- Humans
- Retrospective Studies
- SARS-CoV-2
- Spatio-Temporal Analysis

Full text links

Wolters Kluwer Wolters Kluwer Free PMC article

Proceed to details

Cite

Share

33

Observational Study

J Assoc Physicians India

. 2021 Jan;69(1):19-22.

Role of Mycobacterium w for the Treatment of COVID-19: An Observational Study

Atul Ingale [1], Farah Ingale [2], Brajesh Kunwar [3], Shakeel Ahmed [4], Kumar Salvi [5], Vishwas Chavan [5], Manish Sontakke [6], T Chandrashekhar [7]

Affiliations Expand

Affiliations

- [1] Director, Dept. of Nephrology, Fortis Hiranandani Hospitals, Vashi, Mumbai, Maharashtra.
- [2] Director, Dept. of Internal Medicine, Fortis Hiranandani Hospitals, Vashi, Mumbai, Maharashtra.
- [3] Director, Dept. of Cardiology, Fortis Hiranandani Hospitals, Vashi, Mumbai, Maharashtra.
- [4] Head, Dept. of EM, Fortis Hiranandani Hospitals, Vashi, Mumbai, Maharashtra.
- [5] Consultant, Dept. of Pediatrics, Fortis Hiranandani Hospitals, Vashi, Mumbai, Maharashtra.
- [6] Consultant, Dept. of Orthopedics, Fortis Hiranandani Hospitals, Vashi, Mumbai, Maharashtra.
- [7] Head, Dept. of Critical Care, Fortis Hiranandani Hospitals, Vashi, Mumbai, Maharashtra.

- PMID: **34227770**

Observational Study

Role of Mycobacterium w for the Treatment of COVID-19: An Observational Study

Atul Ingale et al. J Assoc Physicians India. 2021 Jan.
Show details
J Assoc Physicians India
. 2021 Jan;69(1):19-22.

Authors

Atul Ingale [1], Farah Ingale [2], Brajesh Kunwar [3], Shakeel Ahmed [4], Kumar Salvi [5], Vishwas Chavan [5], Manish Sontakke [6], T Chandrashekhar [7]

Affiliations

- [1] Director, Dept. of Nephrology, Fortis Hiranandani Hospitals, Vashi, Mumbai, Maharashtra.
- [2] Director, Dept. of Internal Medicine, Fortis Hiranandani Hospitals, Vashi, Mumbai, Maharashtra.
- [3] Director, Dept. of Cardiology, Fortis Hiranandani Hospitals, Vashi, Mumbai, Maharashtra.
- [4] Head, Dept. of EM, Fortis Hiranandani Hospitals, Vashi, Mumbai, Maharashtra.
- [5] Consultant, Dept. of Pediatrics, Fortis Hiranandani Hospitals, Vashi, Mumbai, Maharashtra.
- [6] Consultant, Dept. of Orthopedics, Fortis Hiranandani Hospitals, Vashi, Mumbai, Maharashtra.
- [7] Head, Dept. of Critical Care, Fortis Hiranandani Hospitals, Vashi, Mumbai, Maharashtra.

- PMID: **34227770**

Abstract

Background: COVID-19 has taken a big toll on the world in terms of morbidity and mortality. The disease may progress in some of the patients leading to trigger of "cytokine storm" which is shown to be associated with adverse outcomes. Heat killed Mycobacterium w (Mw) is a known immunomodulator which is approved for the treatment of gram negative sepsis. This study was carried out to evaluate the role of Mw in the treatment of COVID-19 early in the course of the disease.

Method: In this retrospective observational study, 117 (84 males, 33 females) COVID-19 patients admitted between July 3, 2020 and Aug 26, 2020 in the covid ward of Fortis Hiranandani hospital, Mumbai, were enrolled. Patients were tested COVID-19 positive on RTPCR and were treated with standard of care treatment along with Mw 0.3 ml intradermal injection per day for 3 consecutive days. Patients were evaluated for live discharge as well as changes in the levels of inflammatory markers.

Results: Use of Mw was seen to be associated with rapid recovery in 116/117 patients from COVID-19 who were discharged from the hospital within 10 days. A decrease in the levels of CRP and IL6 was observed after the administration of Mw. This decrease was associated with

improvement in the patients' condition. The use of Mw was seen to be associated with no systemic side effects.

Conclusion: The patients of COVID-19 may deteriorate due to exaggerated production of cytokines which may result in adverse outcomes. Mw used earlier in the disease not only effectively prevents excessive cytokine production but also contribute to rapid recovery. Mw was also found to be safe in use. Larger randomized controlled trials are recommended to assess the role of Mw in COVID-19.

© Journal of the Association of Physicians of India 2011.

Supplementary info

Publication types, MeSH terms Expand

Publication types

- Observational Study

MeSH terms

- COVID-19*
- Female
- Hospitalization
- Humans
- Male
- Mycobacterium*
- Respiration, Artificial
- SARS-CoV-2
- Treatment Outcome

Proceed to details
Cite
Share
☐ 34
Observational Study
Pediatr Cardiol
. 2020 Oct;41(7):1391-1401.
doi: 10.1007/s00246-020-02391-2. Epub 2020 Jun 12.

Paediatric Inflammatory Multisystem Syndrome: Temporally Associated with SARS-CoV-2 (PIMS-TS): Cardiac Features,

Management and Short-Term Outcomes at a UK Tertiary Paediatric Hospital

Tristan Ramcharan [1], Oscar Nolan [1], Chui Yi Lai [1], Nanda Prabhu [1], Raghu Krishnamurthy [2], Alex G Richter [3], Deepthi Jyothish [4], Hari Krishnan Kanthimathinathan [5] [6], Steven B Welch [7], Scott Hackett [7], Eslam Al-Abadi [6] [8], Barnaby R Scholefield [#] [5] [9], Ashish Chikermane [#] [10]
Affiliations Expand

Affiliations

- [1] Department of Cardiology, Birmingham Women's and Children's NHS Foundation Trust, Steelhouse Lane, Birmingham, B4 6NH, UK.
- [2] Department of Paediatrics, Manor Hospital, Walsall, WS2 9PS, UK.
- [3] Institute of Immunology and Immunotherapy, University of Birmingham, Birmingham, B15 2TT, UK.
- [4] Department of Paediatrics, Birmingham Women's and Children's NHS Foundation Trust, Birmingham, B4 6NH, UK.
- [5] Paediatric Intensive Care Unit, Birmingham Women's and Children's NHS Foundation Trust, Birmingham, B4 6NH, UK.
- [6] Birmingham Clinical Trials Unit, University of Birmingham, Birmingham, B15 2TT, UK.
- [7] Department of Paediatrics, Birmingham Chest Clinic and Heartlands Hospital, University Hospitals Birmingham NHS Foundation Trust, Birmingham, B9 5SS, UK.
- [8] Childhood Arthritis and Rheumatic Diseases Unit, Birmingham Women's and Children's NHS Foundation Trust, Birmingham, B4 6NH, UK.
- [9] Birmingham Acute Care Research Group, Institute of Inflammation and Ageing, University of Birmingham, Birmingham, B15 2TT, UK.
- [10] Department of Cardiology, Birmingham Women's and Children's NHS Foundation Trust, Steelhouse Lane, Birmingham, B4 6NH, UK. a.chikermane@nhs.net.

[#] Contributed equally.

- PMID: **32529358**
- PMCID: PMC7289638
- DOI: 10.1007/s00246-020-02391-2

Free PMC article
Observational Study

Paediatric Inflammatory Multisystem Syndrome: Temporally Associated with SARS-CoV-2 (PIMS-TS): Cardiac Features, Management and Short-Term Outcomes at a UK Tertiary Paediatric Hospital

Tristan Ramcharan et al. Pediatr Cardiol. 2020 Oct.
Free PMC article
Show details
Pediatr Cardiol
. 2020 Oct;41(7):1391-1401.
doi: 10.1007/s00246-020-02391-2. Epub 2020 Jun 12.

Authors

Tristan Ramcharan [1], Oscar Nolan [1], Chui Yi Lai [1], Nanda Prabhu [1], Raghu Krishnamurthy [2], Alex G Richter [3], Deepthi Jyothish [4], Hari Krishnan Kanthimathinathan [5] [6], Steven B Welch [7], Scott Hackett [7], Eslam Al-Abadi [6] [8], Barnaby R Scholefield [#] [5] [9], Ashish Chikermane [#] [10]

Affiliations

- [1] Department of Cardiology, Birmingham Women's and Children's NHS Foundation Trust, Steelhouse Lane, Birmingham, B4 6NH, UK.
- [2] Department of Paediatrics, Manor Hospital, Walsall, WS2 9PS, UK.
- [3] Institute of Immunology and Immunotherapy, University of Birmingham, Birmingham, B15 2TT, UK.
- [4] Department of Paediatrics, Birmingham Women's and Children's NHS Foundation Trust, Birmingham, B4 6NH, UK.
- [5] Paediatric Intensive Care Unit, Birmingham Women's and Children's NHS Foundation Trust, Birmingham, B4 6NH, UK.
- [6] Birmingham Clinical Trials Unit, University of Birmingham, Birmingham, B15 2TT, UK.
- [7] Department of Paediatrics, Birmingham Chest Clinic and Heartlands Hospital, University Hospitals Birmingham NHS Foundation Trust, Birmingham, B9 5SS, UK.
- [8] Childhood Arthritis and Rheumatic Diseases Unit, Birmingham Women's and Children's NHS Foundation Trust, Birmingham, B4 6NH, UK.
- [9] Birmingham Acute Care Research Group, Institute of Inflammation and Ageing, University of Birmingham, Birmingham, B15 2TT, UK.
- [10] Department of Cardiology, Birmingham Women's and Children's NHS Foundation Trust, Steelhouse Lane, Birmingham, B4 6NH, UK. a.chikermane@nhs.net.

[#] Contributed equally.

- PMID: **32529358**
- PMCID: PMC7289638
- DOI: 10.1007/s00246-020-02391-2

Abstract

Children were relatively spared during COVID-19 pandemic. However, the recently reported hyperinflammatory syndrome with overlapping features of Kawasaki disease and toxic shock syndrome-"Paediatric Inflammatory Multisystem Syndrome-temporally associated with SARS-CoV-2" (PIMS-TS) has caused concern. We describe cardiac findings and short-term outcomes in children with PIMS-TS at a tertiary children's hospital. Single-center observational study of children with PIMS-TS from 10th April to 9th May 2020. Data on ECG and echocardiogram were retrospectively analyzed along with demographics, clinical features and blood parameters. Fifteen children with median age of 8.8 (IQR 6.4-11.2) years were included, all were from African/Afro-Caribbean, South Asian, Mixed or other minority ethnic groups. All showed raised inflammatory/cardiac markers (CRP, ferritin, Troponin I, CK and pro-BNP). Transient valve regurgitation was present in 10 patients (67%). Left Ventricular ejection fraction was reduced in 12 (80%), fractional shortening in 8 (53%) with resolution in all but 2. Fourteen (93%) had coronary artery abnormalities, with normalization in 6. ECG abnormalities were present in 9 (60%) which normalized in 6 by discharge. Ten (67%) needed inotropes and/or vasopressors. None needed extracorporeal life support. Improvement in cardiac biochemical markers was closely followed by improvement in ECG/echocardiogram. All patients were discharged alive and twelve (80%) have been reviewed since. Our entire cohort with PIMS-TS had cardiac involvement and this degree of involvement is significantly more than other published series and emphasizes the need for specialist cardiac review. We believe that our multi-disciplinary team approach was crucial for the good short-term outcomes.

Keywords: COVID-19; Hyper-inflammatory; Kawasaki; MIS-C; PIMS-TS; SARS-CoV-2.

Conflict of interest statement

There are no disclosures or conflicts of interest.

- 26 references

Supplementary info

Publication types, MeSH terms, Substances, Supplementary concepts, Grant support Expand

Publication types

- Observational Study

MeSH terms

- Betacoronavirus
- COVID-19
- Child
- Coronavirus Infections / complications
- Coronavirus Infections / therapy*
- Echocardiography
- Female

- Heart Diseases / complications*
- Heart Diseases / diagnostic imaging
- Heart Diseases / therapy
- Hospitals, Pediatric*
- Humans
- Immunoglobulins, Intravenous / therapeutic use
- Male
- Mucocutaneous Lymph Node Syndrome / complications
- Pandemics
- Patient Discharge
- Pneumonia, Viral / complications
- Pneumonia, Viral / therapy*
- Retrospective Studies
- SARS-CoV-2
- Stroke Volume
- Systemic Inflammatory Response Syndrome / complications
- Systemic Inflammatory Response Syndrome / therapy*
- Treatment Outcome
- United Kingdom
- Vasoconstrictor Agents / therapeutic use
- Ventricular Function, Left

Substances

- Immunoglobulins, Intravenous
- Vasoconstrictor Agents

Supplementary concepts

- pediatric multisystem inflammatory disease, COVID-19 related

Grant support

- CS-2015-15-016/DH_/Department of Health/United Kingdom

Full text links

SpringerLink FULL-TEXT ARTICLE Springer Free PMC article
Proceed to details
Cite
Share
☐ 35
Observational Study

Eur J Phys Rehabil Med
. 2021 Apr;57(2):208-215.
doi: 10.23736/S1973-9087.21.06674-0. Epub 2021 Feb 4.

Comprehensive rehabilitation treatment for sub-acute COVID-19 patients: an observational study

Federica Bertolucci [1], Laura Sagliocco [1], Martina Tolaini [1], Federico Posteraro [2]
Affiliations Expand

Affiliations

- [1] Department of Rehabilitation, Versilia Hospital, AUSL Toscana Nord Ovest, Lucca, Italy.
- [2] Department of Rehabilitation, Versilia Hospital, AUSL Toscana Nord Ovest, Lucca, Italy - federico.posteraro@uslnordovest.toscana.it.

- PMID: **33541042**
- DOI: 10.23736/S1973-9087.21.06674-0

Free article
Observational Study

Comprehensive rehabilitation treatment for sub-acute COVID-19 patients: an observational study

Federica Bertolucci et al. Eur J Phys Rehabil Med. 2021 Apr.
Free article
Show details

Eur J Phys Rehabil Med
. 2021 Apr;57(2):208-215.
doi: 10.23736/S1973-9087.21.06674-0. Epub 2021 Feb 4.

Authors

Federica Bertolucci [1], Laura Sagliocco [1], Martina Tolaini [1], Federico Posteraro [2]

Affiliations

- [1] Department of Rehabilitation, Versilia Hospital, AUSL Toscana Nord Ovest, Lucca, Italy.
- [2] Department of Rehabilitation, Versilia Hospital, AUSL Toscana Nord Ovest, Lucca, Italy - federico.posteraro@uslnordovest.toscana.it.

- PMID: **33541042**
- DOI: 10.23736/S1973-9087.21.06674-0

Abstract

Background: COVID-19 is a respiratory infection, but it should be considered as a systemic illness with increasing interest on the survivors' sequelae and their management. Considering multi-organ disabilities, a comprehensive rehabilitation provided in sub-acute phase could be considered a suitable setting for these patients.

Aim: The aim of this article was to report the features and rehabilitative outcomes of patients requiring rehabilitation due to disabilities related to severe COVID-19 infection.

Design: Longitudinal Observational Study.

Setting: Department of Rehabilitation in General Hospital.

Population: Patients showing multiple disabilities due to severe COVID-19 infection.

Methods: Thirty-nine consecutive patients were admitted to a rehabilitation ward transferred from ICU or Medical wards. Barthel Index (BI) and Functional Ambulation Categories (FAC) were scored as disabilities measures. Data regarding comorbidity, rehabilitation course, swabs, procedures in acute phase, non-respiratory manifestations, dysphagia, mental confusion, PaO2/FiO2, oxygen supplementation have been collected to admission and discharge. For all patients a comprehensive rehabilitation treatment have been provided.

Results: Functional outcome is good with a statistically significant improvement in BI and FAC scores. Thirty-eight patients were discharged at their home. Mean lenght of stay (LOS) in acute wards was 46 days. Mean LOS in rehabilitation was 20 day. Eleven patients still had tracheostomy at admission, none at discharge and all dysphagic patients recovered a normal oral feeding. The change in PaO2/FiO2 and the reduction of the oxygen supplementation testify a good recovery of pulmonary function.

Conclusions: Our results showed a consistent recovery with little caregiver burden at discharge. Fast relocation from ICU makes beds available which are very valuable during pandemic. Comprehensive rehabilitation treatment provided in sub-acute phase for patients still positive for SARS-CoV-2, would be desirable as it seems to be an effective setting. In this setting a strong medical assistance must be ensured.

Clinical rehabilitation impact: The activation of comprehensive rehabilitation settings able to assist sub-acute patients still positive would be desirable as it could be a very efficient Healthcare Systems answer to the catastrophic pandemic, decompressing acute hospital as well. Furthermore, contagious patients with swabs positivity affected by other kind of disabilities (i.e. Stroke, Femur Fracture) can be treated avoiding to lose the early rehabilitation.

Supplementary info

Publication types, MeSH terms Expand

Publication types

- Observational Study

MeSH terms

- Aged
- COVID-19 / epidemiology
- COVID-19 / rehabilitation*
- Female
- Humans
- Intensive Care Units
- Male
- Pandemics*
- Retrospective Studies
- SARS-CoV-2

Full text links

FREE FULL TEXT article at minervamedica.it Minerva Medica
Proceed to details
Cite
Share
☐ 36
Comparative Study
J Am Soc Nephrol
. 2020 Sep;31(9):2145-2157.
doi: 10.1681/ASN.2020040509. Epub 2020 Jul 15.

AKI in Hospitalized Patients with and without COVID-19: A Comparison Study

Molly Fisher [1], Joel Neugarten [2], Eran Bellin [3], Milagros Yunes [2], Lindsay Stahl [4], Tanya S Johns [2], Matthew K Abramowitz [2], Rebecca Levy [2], Neelja Kumar [2], Michele H Mokrzycki [2], Maria Coco [2], Mary Dominguez [2], Kalyan Prudhvi [2], Ladan Golestaneh [2]
Affiliations Expand

Affiliations

- [1] Division of Nephrology, Department of Medicine, Albert Einstein College of Medicine/Montefiore Medical Center, Bronx, New York mfisher@montefiore.org.
- [2] Division of Nephrology, Department of Medicine, Albert Einstein College of Medicine/Montefiore Medical Center, Bronx, New York.
- [3] Department of Clinical Epidemiology and Population Health, Albert Einstein College of Medicine/Montefiore Medical Center, Bronx, New York.
- [4] Information Technology, Montefiore Medical Center, Bronx, New York.

- PMID: **32669322**

- PMCID: PMC7461660
- DOI: 10.1681/ASN.2020040509

Free PMC article
Comparative Study

AKI in Hospitalized Patients with and without COVID-19: A Comparison Study

Molly Fisher et al. J Am Soc Nephrol. 2020 Sep.
Free PMC article
Show details
J Am Soc Nephrol
. 2020 Sep;31(9):2145-2157.
doi: 10.1681/ASN.2020040509. Epub 2020 Jul 15.

Authors

Molly Fisher [1], Joel Neugarten [2], Eran Bellin [3], Milagros Yunes [2], Lindsay Stahl [4], Tanya S Johns [2], Matthew K Abramowitz [2], Rebecca Levy [2], Neelja Kumar [2], Michele H Mokrzycki [2], Maria Coco [2], Mary Dominguez [2], Kalyan Prudhvi [2], Ladan Golestaneh [2]

Affiliations

- [1] Division of Nephrology, Department of Medicine, Albert Einstein College of Medicine/Montefiore Medical Center, Bronx, New York mfisher@montefiore.org.
- [2] Division of Nephrology, Department of Medicine, Albert Einstein College of Medicine/Montefiore Medical Center, Bronx, New York.
- [3] Department of Clinical Epidemiology and Population Health, Albert Einstein College of Medicine/Montefiore Medical Center, Bronx, New York.
- [4] Information Technology, Montefiore Medical Center, Bronx, New York.

- PMID: **32669322**
- PMCID: PMC7461660
- DOI: 10.1681/ASN.2020040509

Abstract

Background: Reports from centers treating patients with coronavirus disease 2019 (COVID-19) have noted that such patients frequently develop AKI. However, there have been no direct comparisons of AKI in hospitalized patients with and without COVID-19 that would reveal whether there are aspects of AKI risk, course, and outcomes unique to this infection.

Methods: In a retrospective observational study, we evaluated AKI incidence, risk factors, and outcomes for 3345 adults with COVID-19 and 1265 without COVID-19 who were hospitalized in a large New York City health system and compared them with a historical cohort of 9859 individuals hospitalized a year earlier in the same health system. We also developed a model to identify predictors of stage 2 or 3 AKI in our COVID-19.

Results: We found higher AKI incidence among patients with COVID-19 compared with the historical cohort (56.9% versus 25.1%, respectively). Patients with AKI and COVID-19 were more likely than those without COVID-19 to require RRT and were less likely to recover kidney function. Development of AKI was significantly associated with male sex, Black race, and older age (>50 years). Male sex and age >50 years associated with the composite outcome of RRT or mortality, regardless of COVID-19 status. Factors that were predictive of stage 2 or 3 AKI included initial respiratory rate, white blood cell count, neutrophil/lymphocyte ratio, and lactate dehydrogenase level.

Conclusions: Patients hospitalized with COVID-19 had a higher incidence of severe AKI compared with controls. Vital signs at admission and laboratory data may be useful for risk stratification to predict severe AKI. Although male sex, Black race, and older age associated with development of AKI, these associations were not unique to COVID-19.

Keywords: AKI; COVID-19; outcomes; race; risk factors; sex.

Copyright © 2020 by the American Society of Nephrology.

- 4 figures

Supplementary info

Publication types, MeSH terms, Grant support Expand

Publication types

- Comparative Study

MeSH terms

- Acute Kidney Injury / epidemiology*
- Acute Kidney Injury / etiology
- Adult
- Aged
- Aged, 80 and over
- Betacoronavirus*
- COVID-19
- Coronavirus Infections / complications*
- Female
- Hospital Mortality
- Hospitalization*
- Humans
- Incidence
- Intensive Care Units
- Male
- Middle Aged
- Pandemics

- Pneumonia, Viral / complications*
- Prognosis
- Renal Replacement Therapy
- Resource Allocation
- Respiration, Artificial
- Retrospective Studies
- SARS-CoV-2

Grant support

- KL2 TR002558/TR/NCATS NIH HHS/United States
- UL1 TR002556/TR/NCATS NIH HHS/United States

Full text links

Final Version FREE J Am Soc Nephrol HighWire Free PMC article
Proceed to details
Cite
Share
37
Observational Study
J Intensive Care Med
. 2021 Mar;36(3):327-333.
doi: 10.1177/0885066620976525. Epub 2020 Nov 25.

Responsiveness of Inhaled Epoprostenol in Respiratory Failure due to COVID-19

Rajiv Sonti [1], C William Pike [2], Nathan Cobb [1]
Affiliations Expand

Affiliations

- [1] Division of Pulmonary, Critical Care and Sleep Medicine, 12230Georgetown University Medical Center, Washington, DC, USA.
- [2] 12230Georgetown University School of Medicine, Washington, DC, USA.

- PMID: **33234007**
- PMCID: PMC7724253
- DOI: 10.1177/0885066620976525

Free PMC article
Observational Study

Responsiveness of Inhaled Epoprostenol in Respiratory Failure due to COVID-19

Rajiv Sonti et al. J Intensive Care Med. 2021 Mar.
Free PMC article
Show details
J Intensive Care Med
. 2021 Mar;36(3):327-333.
doi: 10.1177/0885066620976525. Epub 2020 Nov 25.

Authors

Rajiv Sonti [1], C William Pike [2], Nathan Cobb [1]

Affiliations

- [1] Division of Pulmonary, Critical Care and Sleep Medicine, 12230Georgetown University Medical Center, Washington, DC, USA.
- [2] 12230Georgetown University School of Medicine, Washington, DC, USA.

- PMID: **33234007**
- PMCID: PMC7724253
- DOI: 10.1177/0885066620976525

Abstract

Background: Inhaled pulmonary vasodilators are used as adjunctive therapies for the treatment of refractory hypoxemia. Available evidence suggest they improve oxygenation in a subset of patients without changing long-term trajectory. Given the differences in respiratory failure due to COVID-19 and "traditional" ARDS, we sought to identify their physiologic impact.

Methods: This is a retrospective observational study of patients mechanically ventilated for COVID-19, from the ICUs of 2 tertiary care centers, who received inhaled epoprostenol (iEpo) for the management of hypoxemia. The primary outcome is change in PaO_2/FiO_2. Additionally, we measured several patient level features to predict iEpo responsiveness (or lack thereof).

Results: Eighty patients with laboratory confirmed SARS-CoV2 received iEpo while mechanically ventilated and had PaO_2/FiO_2 measured before and after. The median PaO_2/FiO_2 prior to receiving iEpo was 92 mmHg and interquartile range (74 - 122). The median change in PaO_2/FiO_2 was 9 mmHg (-9 - 37) corresponding to a 10% improvement (-8 - 41). Fifty-percent (40 / 80) met our a priori definition of a clinically significant improvement in PaO_2/FiO_2 (increase in 10% from the baseline value). Prone position and lower PaO_2/FiO_2 when iEpo was started predicted a more robust response, which held after multivariate adjustment. For proned individuals, improvement in PaO_2/FiO_2 was 14 mmHg (-6 to 45) vs. 3 mmHg (-11 - 20), $p = 0.04$ for supine individuals; for those with severe ARDS ($PaO_2/FiO_2 < 100$, $n = 49$) the median improvement was 16 mmHg (-2 - 46).

Conclusion: Fifty percent of patients have a clinically significant improvement in PaO_2/FiO_2 after the initiation of iEpo. This suggests it is worth trying as a rescue therapy; although generally the

benefit was modest with a wide variability. Those who were prone and had lower PaO_2/FiO_2 were more likely to respond.

Keywords: ARDS; COVID-19; inhaled epoprostenol.

Conflict of interest statement

Declaration of Conflicting Interests: The author(s) declared no potential conflicts of interest with respect to the research, authorship, and/or publication of this article.

- 41 references
- 1 figure

Supplementary info

Publication types, MeSH terms, Substances Expand

Publication types

- Observational Study

MeSH terms

- Administration, Inhalation
- Aged
- COVID-19 / therapy*
- Epoprostenol / therapeutic use*
- Female
- Humans
- Hypoxia / metabolism
- Hypoxia / therapy*
- Male
- Middle Aged
- Oxygen / metabolism
- Partial Pressure
- Patient Positioning
- Prone Position
- Respiration, Artificial*
- Respiratory Insufficiency / therapy*
- Retrospective Studies
- SARS-CoV-2
- Severity of Illness Index
- Tertiary Care Centers
- Treatment Outcome
- Vasodilator Agents / therapeutic use*

Substances

- Vasodilator Agents
- Epoprostenol
- Oxygen

Full text links

SAGE journals Open access full text Atypon Free PMC article
Proceed to details
Cite
Share
38
Observational Study
J Infect Dis
. 2021 Oct 28;224(8):1278-1286.
doi: 10.1093/infdis/jiab377.

Real-World Clinical Outcomes of Bamlanivimab and Casirivimab-Imdevimab Among High-Risk Patients With Mild to Moderate Coronavirus Disease 2019

Ravindra Ganesh [1], Lindsey M Philpot [1], Dennis M Bierle [1], Ryan J Anderson [1], Lori L Arndt [2], Richard F Arndt [2], Tracy L Culbertson [3], Molly J Destro Borgen [1], Sara N Hanson [3], Brian D Kennedy [4], Brian B Kottke [3], Jennifer J Larsen [1], Priya Ramar [1], Jordan K Rosedahl [1], Maria Teresa Seville [5], Leigh L Speicher [6], Sidna M Tulledge-Scheitel [1], Caroline G Wilker [7], Raymund R Razonable [1]
Affiliations Expand

Affiliations

- [1] Mayo Clinic, Rochester, Minnesota, USA.
- [2] Mayo Clinic Health System, Eau Claire, Wisconsin, USA.
- [3] Mayo Clinic Health System, Mankato, Minnesota, USA.
- [4] Mayo Clinic Health System, Lake City, Minnesota, USA.
- [5] Mayo Clinic Arizona, Phoenix, Arizona, USA.
- [6] Mayo Clinic, Jacksonville, Florida, USA.
- [7] Mayo Clinic Health System-Franciscan Healthcare, La Crosse, Wisconsin, USA.

- PMID: **34279629**
- PMCID: PMC8344643
- DOI: 10.1093/infdis/jiab377

Free PMC article
Observational Study

Real-World Clinical Outcomes of Bamlanivimab and Casirivimab-Imdevimab Among High-Risk Patients With Mild to Moderate Coronavirus Disease 2019

Ravindra Ganesh et al. J Infect Dis. 2021.
Free PMC article
Show details
J Infect Dis
. 2021 Oct 28;224(8):1278-1286.
doi: 10.1093/infdis/jiab377.

Authors

Ravindra Ganesh [1], Lindsey M Philpot [1], Dennis M Bierle [1], Ryan J Anderson [1], Lori L Arndt [2], Richard F Arndt [2], Tracy L Culbertson [3], Molly J Destro Borgen [1], Sara N Hanson [3], Brian D Kennedy [4], Brian B Kottke [3], Jennifer J Larsen [1], Priya Ramar [1], Jordan K Rosedahl [1], Maria Teresa Seville [5], Leigh L Speicher [6], Sidna M Tulledge-Scheitel [1], Caroline G Wilker [7], Raymund R Razonable [1]

Affiliations

- [1] Mayo Clinic, Rochester, Minnesota, USA.
- [2] Mayo Clinic Health System, Eau Claire, Wisconsin, USA.
- [3] Mayo Clinic Health System, Mankato, Minnesota, USA.
- [4] Mayo Clinic Health System, Lake City, Minnesota, USA.
- [5] Mayo Clinic Arizona, Phoenix, Arizona, USA.
- [6] Mayo Clinic, Jacksonville, Florida, USA.
- [7] Mayo Clinic Health System-Franciscan Healthcare, La Crosse, Wisconsin, USA.

- PMID: **34279629**
- PMCID: PMC8344643
- DOI: 10.1093/infdis/jiab377

Abstract

Background: Bamlanivimab and casirivimab-imdevimab are authorized for treatment of mild to moderate coronavirus disease 2019 (COVID-19) in high-risk patients. We compared the outcomes of patients who received these therapies to identify factors associated with hospitalization and other clinical outcomes.

Methods: Adult patients who received monoclonal antibody from 19 November 2020 to 11 February 2021 were selected and divided into those who received bamlanivimab (n = 2747) and casirivimab-imdevimab (n = 849). The 28-day all-cause and COVID-19-related hospitalizations were compared between the groups.

Results: The population included 3596 patients; the median age was 62 years, and 50% were female. All had ≥1 medical comorbidity; 55% had multiple comorbidities. All-cause and COVID-19-related hospitalization rates at 28 days were 3.98% and 2.56%, respectively. After adjusting for medical comorbidities, there was no significant difference in all-cause and COVID-19-related hospitalization rates between bamlanivimab and casirivimab-imdevimab (adjusted hazard ratios [95% confidence interval], 1.4 [.9-2.2] and 1.6 [.8-2.7], respectively). Chronic kidney, respiratory and cardiovascular diseases, and immunocompromised status were associated with higher likelihood of hospitalization.

Conclusions: This observational study on the use of bamlanivimab and casirivimab-imdevimab in high-risk patients showed similarly low rates of hospitalization. The number and type of medical comorbidities are associated with hospitalizations after monoclonal antibody treatment.

Keywords: COVID-19; bamlanivimab; casirivimab; hospitalization; outcomes.

© The Author(s) 2021. Published by Oxford University Press for the Infectious Diseases Society of America. All rights reserved. For permissions, e-mail: journals.permissions@oup.com.

Comment in

- Not All Monoclonal Antibodies for Coronavirus Disease 2019 Are Created Equal.
  Stosor V, Angarone MP. Stosor V, et al. J Infect Dis. 2021 Oct 28;224(8):1275-1277. doi: 10.1093/infdis/jiab380. J Infect Dis. 2021. PMID: 34273151 Free PMC article. No abstract available.
- Severe Acute Respiratory Syndrome Coronavirus 2-Specific Monoclonal Antibody for the Treatment of Mild to Moderate Coronavirus Disease 2019 in Cancer Patients: A Single-Center Experience.
  Puing AG, Ho S, Frankel P, Tegtmeier B, Martin A, Ross J, Nanayakkara D, Dickter J, Seto T, Nakamura R, Taplitz R, Dadwal S. Puing AG, et al. J Infect Dis. 2022 Jan 18;225(2):352-354. doi: 10.1093/infdis/jiab406. J Infect Dis. 2022. PMID: 34379763 Free PMC article. No abstract available.

Supplementary info

Publication types, MeSH terms, Substances, Supplementary concepts, Grant support Expand

Publication types

- Observational Study
- Research Support, Non-U.S. Gov't

MeSH terms

- Adolescent
- Adult

- Aged
- Aged, 80 and over
- Antibodies, Monoclonal, Humanized / therapeutic use*
- COVID-19 / diagnosis
- COVID-19 / drug therapy*
- COVID-19 / epidemiology
- Drug Combinations
- Female
- Hospitalization / statistics & numerical data
- Humans
- Male
- Middle Aged
- Multimorbidity
- Retrospective Studies
- Risk Factors
- SARS-CoV-2 / isolation & purification
- Severity of Illness Index
- Treatment Outcome
- Young Adult

Substances

- Antibodies, Monoclonal, Humanized
- Drug Combinations
- casirivimab and imdevimab drug combination
- bamlanivimab

Supplementary concepts

- COVID-19 drug treatment

Grant support

- Mayo Clinic

Full text links

OXFORD ACADEMIC Silverchair Information Systems Free PMC article
Proceed to details
Cite
Share
☐ 39
Observational Study

Diabetes Metab Res Rev
. 2021 Mar;37(3):e3388.
doi: 10.1002/dmrr.3388. Epub 2020 Aug 18.

Characteristics and outcomes of COVID-19 in hospitalized patients with and without diabetes

Abdallah Al-Salameh [1] [2], Jean-Philippe Lanoix [3], Youssef Bennis [4], Claire Andrejak [5], Etienne Brochot [6], Guillaume Deschasse [7], Hervé Dupont [8], Vincent Goeb [9], Maité Jaureguy [10], Sylvie Lion [11], Julien Maizel [12], Julien Moyet [7], Benoit Vaysse [13], Rachel Desailloud [1] [2], Olivier Ganry [14], Jean-Luc Schmit [3], Jean-Daniel Lalau [1] [2]
Affiliations Expand

Affiliations

- [1] Department of Endocrinology, Diabetes Mellitus and Nutrition, Amiens University Hospital, Amiens, France.
- [2] PériTox = UMR_I 01, University of Picardy Jules Verne, Amiens, France.
- [3] Department of Infectious Diseases, Amiens University Hospital, Amiens, France.
- [4] Laboratory of Clinical Pharmacology, Amiens University Hospital, Amiens, France.
- [5] Department of Pulmonary diseases, Amiens University Hospital, Amiens, France.
- [6] Laboratory of Virology, Amiens University Hospital, Amiens, France.
- [7] Department of Geriatrics, Amiens University Hospital, Amiens, France.
- [8] Surgical Intensive Care Unit, Amiens University Hospital, Amiens, France.
- [9] Department of Rheumatology, Amiens University Hospital, Amiens, France.
- [10] Department of Nephrology, Amiens University Hospital, Amiens, France.
- [11] Department of Orthopaedics and Traumatology, Amiens University Hospital, Amiens, France.
- [12] Medical Intensive Care Unit, Amiens University Hospital, Amiens, France.
- [13] Department of Medical Informatics, Amiens University Hospital, Amiens, France.
- [14] Department of Epidemiology, Amiens University Hospital, Amiens, France.

- PMID: **32683744**
- PMCID: PMC7404605
- DOI: 10.1002/dmrr.3388

Free PMC article
Observational Study

Characteristics and outcomes of COVID-19 in hospitalized patients with and without diabetes

Abdallah Al-Salameh et al. Diabetes Metab Res Rev. 2021 Mar.
Free PMC article
Show details
Diabetes Metab Res Rev
. 2021 Mar;37(3):e3388.
doi: 10.1002/dmrr.3388. Epub 2020 Aug 18.

Authors

Abdallah Al-Salameh [1 2], Jean-Philippe Lanoix [3], Youssef Bennis [4], Claire Andrejak [5], Etienne Brochot [6], Guillaume Deschasse [7], Hervé Dupont [8], Vincent Goeb [9], Maité Jaureguy [10], Sylvie Lion [11], Julien Maizel [12], Julien Moyet [7], Benoit Vaysse [13], Rachel Desailloud [1 2], Olivier Ganry [14], Jean-Luc Schmit [3], Jean-Daniel Lalau [1 2]

Affiliations

- [1] Department of Endocrinology, Diabetes Mellitus and Nutrition, Amiens University Hospital, Amiens, France.
- [2] PériTox = UMR_I 01, University of Picardy Jules Verne, Amiens, France.
- [3] Department of Infectious Diseases, Amiens University Hospital, Amiens, France.
- [4] Laboratory of Clinical Pharmacology, Amiens University Hospital, Amiens, France.
- [5] Department of Pulmonary diseases, Amiens University Hospital, Amiens, France.
- [6] Laboratory of Virology, Amiens University Hospital, Amiens, France.
- [7] Department of Geriatrics, Amiens University Hospital, Amiens, France.
- [8] Surgical Intensive Care Unit, Amiens University Hospital, Amiens, France.
- [9] Department of Rheumatology, Amiens University Hospital, Amiens, France.
- [10] Department of Nephrology, Amiens University Hospital, Amiens, France.
- [11] Department of Orthopaedics and Traumatology, Amiens University Hospital, Amiens, France.
- [12] Medical Intensive Care Unit, Amiens University Hospital, Amiens, France.
- [13] Department of Medical Informatics, Amiens University Hospital, Amiens, France.
- [14] Department of Epidemiology, Amiens University Hospital, Amiens, France.

- PMID: **32683744**
- PMCID: PMC7404605
- DOI: 10.1002/dmrr.3388

Abstract

Background: Coronavirus disease 2019 (COVID-19) is a rapidly progressing pandemic, with four million confirmed cases and 280 000 deaths at the time of writing. Some studies have suggested

that diabetes is associated with a greater risk of developing severe forms of COVID-19. The primary objective of the present study was to compare the clinical features and outcomes in hospitalized COVID-19 patients with vs without diabetes.

Methods: All consecutive adult patients admitted to Amiens University Hospital (Amiens, France) with confirmed COVID-19 up until April 21st, 2020, were included. The composite primary endpoint comprised admission to the intensive care unit (ICU) and death. Both components were also analysed separately in a logistic regression analysis and a Cox proportional hazards model.

Results: A total of 433 patients (median age: 72; 238 (55%) men; diabetes: 115 (26.6%)) were included. Most of the deaths occurred in non-ICU units and among older adults. Multivariate analyses showed that diabetes was associated neither with the primary endpoint (odds ratio (OR): 1.12; 95% confidence interval (CI): 0.66-1.90) nor with mortality (hazard ratio: 0.73; 95%CI: 0.40-1.34) but was associated with ICU admission (OR: 2.06; 95%CI 1.09-3.92, P = .027) and a longer length of hospital stay. Age was negatively associated with ICU admission and positively associated with death.

Conclusions: Diabetes was prevalent in a quarter of the patients hospitalized with COVID-19; it was associated with a greater risk of ICU admission but not with a significant elevation in mortality. Further investigation of the relationship between COVID-19 severity and diabetes is warranted.

Keywords: acute respiratory distress syndrome; coronavirus disease 2019 (COVID-19); diabetes; intensive care; mortality; outcome.

© 2020 John Wiley & Sons Ltd.

- 25 references

Supplementary info

Publication types, MeSH terms Expand

Publication types

- Observational Study

MeSH terms

- Aged
- Aged, 80 and over
- Body Mass Index
- COVID-19* / complications
- COVID-19* / diagnosis
- COVID-19* / epidemiology
- COVID-19* / therapy
- Cohort Studies
- Comorbidity

- Diabetes Mellitus / diagnosis*
- Diabetes Mellitus / epidemiology*
- Diabetes Mellitus / mortality
- Diabetes Mellitus / therapy
- Female
- France / epidemiology
- Hospital Mortality
- Hospitalization / statistics & numerical data*
- Humans
- Intensive Care Units / statistics & numerical data*
- Length of Stay / statistics & numerical data
- Male
- Middle Aged
- Pandemics
- Prognosis
- Retrospective Studies
- Risk Factors
- SARS-CoV-2 / physiology
- Severity of Illness Index
- Treatment Outcome

Full text links

WILEY Full Text Article Wiley Free PMC article

Proceed to details

Cite

Share

☐ 40

Observational Study

Nephrol Dial Transplant

. 2020 Dec 4;35(12):2083-2095.

doi: 10.1093/ndt/gfaa271.

Mortality analysis of COVID-19 infection in chronic kidney disease, haemodialysis and renal transplant patients compared with patients without kidney disease: a nationwide analysis from Turkey

Savas Ozturk [1], Kenan Turgutalp [2], Mustafa Arici [3], Ali Riza Odabas [4], Mehmet Riza Altiparmak [5], Zeki Aydin [6], Egemen Cebeci [1], Taner Basturk [7], Zeki Soypacaci [8], Garip Sahin [9], Tuba Elif Ozler [10], Ekrem Kara [11], Hamad Dheir [12], Necmi Eren [13], Gultekin Suleymanlar [14], Mahmud Islam [15], Melike Betul Ogutmen [16], Erkan Sengul [17], Yavuz Ayar [18], Murside Esra Dolarslan [19], Serkan Bakirdogen [20], Seda Safak [21], Ozkan Gungor [22], Idris Sahin [23], Ilay Berke Mentese [24], Ozgur Merhametsiz [25], Ebru Gok Oguz [26], Dilek Gibyeli Genek [27], Nadir Alpay [28], Nimet Aktas [29], Murat Duranay [30], Selma Alagoz [31], Hulya Colak [32], Zelal Adibelli [33], Irem Pembegul [34], Ender Hur [35], Alper Azak [36], Dilek Guven Taymez [37], Erhan Tatar [38], Rumeyza Kazancioglu [39], Aysegul Oruc [40], Enver Yuksel [41], Engin Onan [42], Kultigin Turkmen [43], Nuri Baris Hasbal [44], Ali Gurel [45], Berna Yelken [46], Tuncay Sahutoglu [47], Mahmut Gok [4], Nurhan Seyahi [5], Mustafa Sevinc [7], Sultan Ozkurt [9], Savas Sipahi [48], Sibel Gokcay Bek [13], Feyza Bora [14], Bulent Demirelli [16], Ozgur Akin Oto [21], Orcun Altunoren [22], Serhan Zubeyde Tuglular [24], Mehmet Emin Demir [25], Mehmet Deniz Ayli [26], Bulent Huddam [27], Mehmet Tanrisev [49], Ilter Bozaci [38], Meltem Gursu [39], Betul Bakar [30], Bulent Tokgoz [50], Halil Zeki Tonbul [42], Alaattin Yildiz [21], Siren Sezer [51], Kenan Ates [52]

Affiliations Expand

Affiliations

- [1] Department of Nephrology, University of Health Sciences, Haseki Training and Research Hospital, Istanbul, Turkey.
- [2] Department of Internal Medicine, Division of Nephrology, Faculty of Medicine, Mersin University Training and Research Hospital, Mersin, Turkey.
- [3] Department of Nephrology, Faculty of Medicine, Hacettepe University, Ankara, Turkey.
- [4] Department of Nephrology, University of Health Sciences, Sultan 2.Abdulhamid Han Training and Research Hospital, Istanbul, Turkey.
- [5] Department of Internal Medicine, Division of Nephrology, Istanbul University - Cerrahpasa Cerrahpasa Faculty of Medicine, Istanbul, Turkey.
- [6] Department of Nephrology, Darica Farabi Training and Research Hospital, Kocaeli, Turkey.
- [7] Department of Nephrology, Sisli Hamidiye Etfal Training and Research Hospital, University of Health Sciences, Istanbul, Turkey.
- [8] Department of Internal Medicine, Division of Nephrology, Izmir Katip Celebi University, Atatürk Training and Research Hospital, Izmir, Turkey.
- [9] Department of Internal Medicine, Division of Nephrology, Faculty of Medicine, Eskisehir Osmangazi University, Eskisehir, Turkey.
- [10] Department of Internal Medicine, Division of Nephrology, University of Health Sciences, Kanuni Sultan Suleyman Training and Research Hospital, Istanbul, Turkey.
- [11] Department of Internal Medicine, Division of Nephrology, Faculty of Medicine, Recep Tayyip Erdogan University, Rize, Turkey.
- [12] Department of Internal Medicine, Division of Nephrology, Sakarya University Training and Research Hospital, Sakarya, Turkey.
- [13] Department of Nephrology, Faculty of Medicine, Kocaeli University, Kocaeli, Turkey.
- [14] Department of Internal Medicine, Division of Nephrology, Faculty of Medicine, Akdeniz University, Antalya, Turkey.
- [15] Department of Nephrology, Zonguldak Ataturk State Hospital, Zonguldak, Turkey.
- [16] Department of Nephrology, University of Health Sciences, Haydarpasa Numune Training and Research Hospital, Istanbul, Turkey.

- [17] Department of Nephrology, University of Health Sciences, Kocaeli Derince Training and Research Hospital, Kocaeli, Turkey.
- [18] Department of Nephrology, Bursa City Hospital, Bursa, Turkey.
- [19] Department of Nephrology, University of Health Sciences, Trabzon Kanuni Training and Research Hospital, Trabzon, Turkey.
- [20] Department of Internal Medicine, Division of Nephrology, Canakkale Onsekiz Mart University, Canakkale, Turkey.
- [21] Department of Internal Medicine, Division of Nephrology, Istanbul University Faculty of Medicine, Istanbul, Turkey.
- [22] Department of Nephrology, Kahramanmaras Sutcu Imam University Faculty of Medicine, Kahramanmaras, Turkey.
- [23] Department of Internal Medicine, Division of Nephrology, Inonu University Faculty of Medicine, Turgut Ozal Medical Center, Malatya, Turkey.
- [24] Department of Internal Medicine, Division of Nephrology, Marmara University Faculty of Medicine, Marmara University Pendik Training and Research Hospital, Istanbul, Turkey.
- [25] Department of Internal Medicine, Division of Nephrology, Yeni Yuzyil University Faculty of Medicine, Istanbul, Turkey.
- [26] Department of Nephrology, University of Health Sciences, Diskapi Yildirim Beyazit Training and Research Hospital, Ankara, Turkey.
- [27] Department of Internal Medicine, Division of Nephrology, Mugla Sitki Kocman University Faculty of Medicine, Training and Research Hospital, Mugla, Turkey.
- [28] Department of Internal Medicine, Division of Nephrology, Memorial Hizmet Hospital, Istanbul, Turkey.
- [29] Department of Nephrology, University of Health Sciences, Bursa Higher Specialization Training and Research Hospital, Bursa, Turkey.
- [30] Department of Nephrology, University of Health Sciences, Ankara Training and Research Hospital, Ankara, Turkey.
- [31] Department of Internal Medicine, Division of Nephrology, University of Health Sciences, Bagcilar Training and Research Hospital, Istanbul, Turkey.
- [32] Department of Internal Medicine, Division of Nephrology, University of Health Sciences, Training and Research Hospital, Izmir, Turkey.
- [33] Department of Internal Medicine, Division of Nephrology, Faculty of Medicine, Usak University, Usak, Turkey.
- [34] Department of Nephrology, Malatya Training and Research Hospital, Malatya, Turkey.
- [35] Department of Nephrology, Manisa Merkezefendi State Hospital, Manisa, Turkey.
- [36] Department of Nephrology, Balıkesir Ataturk City Hospital, Balikesir, Turkey.
- [37] Department of Nephrology, Kocaeli State Hospital, Kocaeli, Turkey.
- [38] Department of Nephrology, University of Health Sciences, Izmir Bozyaka Training and Research Hospital, Izmir, Turkey.
- [39] Department of Internal Medicine, Division of Nephrology, Bezmialem Vakif University Faculty of Medicine, Istanbul, Turkey.
- [40] Department of Nephrology, Bursa Uludag University Faculty of Medicine, Bursa, Turkey.
- [41] Department of Nephrology, University of Health Sciences, Diyarbakır Gazi Yasargil Training and Research Hospital, Diyarbakir, Turkey.
- [42] Department of Nephrology, Adana City Training and Research Hospital, Adana, Turkey.
- [43] Department of Nephrology, Necmettin Erbakan University Meram Faculty of Medicine, Konya, Turkey.
- [44] Department of Nephrology, Hakkari State Hospital, Hakkari, Turkey.

- [45] Department of Internal Medicine, Division of Nephrology, Firat University Faculty of Medicine, Elazig, Turkey.
- [46] Department of Transplantation, Koc University Hospital, Istanbul, Turkey.
- [47] Department of Nephrology, University of Health Sciences, Mehmet Akif Inan Training and Research Hospital, Sanliurfa, Turkey.
- [48] Department of Internal Medicine, Division of Nephrology, Sakarya University Faculty of Medicine, Training and Research Hospital, Sakarya, Turkey.
- [49] Department of Internal Medicine, Division of Nephrology, University of Health Sciences, Tepecik Training and Research Hospital, Izmir, Turkey.
- [50] Department of Nephrology, Erciyes University Faculty of Medicine, Kayseri, Turkey.
- [51] Department of Internal Medicine, Division of Nephrology, Atilim University Faculty of Medicine, Istanbul, Turkey.
- [52] Department of Internal Medicine, Division of Nephrology, Ankara University Faculty of Medicine Ankara, Turkey.

- PMID: **33275763**
- PMCID: PMC7716804
- DOI: 10.1093/ndt/gfaa271

Free PMC article
Observational Study

Mortality analysis of COVID-19 infection in chronic kidney disease, haemodialysis and renal transplant patients compared with patients without kidney disease: a nationwide analysis from Turkey

Savas Ozturk et al. Nephrol Dial Transplant. 2020.
Free PMC article

Show details

Nephrol Dial Transplant

. 2020 Dec 4;35(12):2083-2095.
doi: 10.1093/ndt/gfaa271.

Authors

Savas Ozturk [1], Kenan Turgutalp [2], Mustafa Arici [3], Ali Riza Odabas [4], Mehmet Riza Altiparmak [5], Zeki Aydin [6], Egemen Cebeci [1], Taner Basturk [7], Zeki Soypacaci [8], Garip Sahin [9], Tuba Elif Ozler [10], Ekrem Kara [11], Hamad Dheir [12], Necmi Eren [13], Gultekin Suleymanlar [14], Mahmud Islam [15], Melike Betul Ogutmen [16], Erkan Sengul [17], Yavuz Ayar [18], Murside Esra Dolarslan [19], Serkan Bakirdogen [20], Seda Safak [21], Ozkan Gungor [22], Idris Sahin [23], Ilay Berke Mentese [24], Ozgur Merhametsiz [25], Ebru Gok Oguz [26], Dilek Gibyeli Genek [27], Nadir Alpay [28], Nimet Aktas [29], Murat Duranay [30], Selma Alagoz [31], Hulya Colak [32], Zelal Adibelli [33], Irem

Pembegul [34], Ender Hur [35], Alper Azak [36], Dilek Guven Taymez [37], Erhan Tatar [38], Rumeyza Kazancioglu [39], Aysegul Oruc [40], Enver Yuksel [41], Engin Onan [42], Kultigin Turkmen [43], Nuri Baris Hasbal [44], Ali Gurel [45], Berna Yelken [46], Tuncay Sahutoglu [47], Mahmut Gok [4], Nurhan Seyahi [5], Mustafa Sevinc [7], Sultan Ozkurt [9], Savas Sipahi [48], Sibel Gokcay Bek [13], Feyza Bora [14], Bulent Demirelli [16], Ozgur Akin Oto [21], Orcun Altunoren [22], Serhan Zubeyde Tuglular [24], Mehmet Emin Demir [25], Mehmet Deniz Ayli [26], Bulent Huddam [27], Mehmet Tanrisev [49], Ilter Bozaci [38], Meltem Gursu [39], Betul Bakar [30], Bulent Tokgoz [50], Halil Zeki Tonbul [42], Alaattin Yildiz [21], Siren Sezer [51], Kenan Ates [52]

Affiliations

- [1] Department of Nephrology, University of Health Sciences, Haseki Training and Research Hospital, Istanbul, Turkey.
- [2] Department of Internal Medicine, Division of Nephrology, Faculty of Medicine, Mersin University Training and Research Hospital, Mersin, Turkey.
- [3] Department of Nephrology, Faculty of Medicine, Hacettepe University, Ankara, Turkey.
- [4] Department of Nephrology, University of Health Sciences, Sultan 2.Abdulhamid Han Training and Research Hospital, Istanbul, Turkey.
- [5] Department of Internal Medicine, Division of Nephrology, Istanbul University - Cerrahpasa Cerrahpasa Faculty of Medicine, Istanbul, Turkey.
- [6] Department of Nephrology, Darica Farabi Training and Research Hospital, Kocaeli, Turkey.
- [7] Department of Nephrology, Sisli Hamidiye Etfal Training and Research Hospital, University of Health Sciences, Istanbul, Turkey.
- [8] Department of Internal Medicine, Division of Nephrology, Izmir Katip Celebi University, Atatürk Training and Research Hospital, Izmir, Turkey.
- [9] Department of Internal Medicine, Division of Nephrology, Faculty of Medicine, Eskisehir Osmangazi University, Eskisehir, Turkey.
- [10] Department of Internal Medicine, Division of Nephrology, University of Health Sciences, Kanuni Sultan Suleyman Training and Research Hospital, Istanbul, Turkey.
- [11] Department of Internal Medicine, Division of Nephrology, Faculty of Medicine, Recep Tayyip Erdogan University, Rize, Turkey.
- [12] Department of Internal Medicine, Division of Nephrology, Sakarya University Training and Research Hospital, Sakarya, Turkey.
- [13] Department of Nephrology, Faculty of Medicine, Kocaeli University, Kocaeli, Turkey.
- [14] Department of Internal Medicine, Division of Nephrology, Faculty of Medicine, Akdeniz University, Antalya, Turkey.
- [15] Department of Nephrology, Zonguldak Ataturk State Hospital, Zonguldak, Turkey.
- [16] Department of Nephrology, University of Health Sciences, Haydarpasa Numune Training and Research Hospital, Istanbul, Turkey.
- [17] Department of Nephrology, University of Health Sciences, Kocaeli Derince Training and Research Hospital, Kocaeli, Turkey.
- [18] Department of Nephrology, Bursa City Hospital, Bursa, Turkey.
- [19] Department of Nephrology, University of Health Sciences, Trabzon Kanuni Training and Research Hospital, Trabzon, Turkey.
- [20] Department of Internal Medicine, Division of Nephrology, Canakkale Onsekiz Mart University, Canakkale, Turkey.

- [21] Department of Internal Medicine, Division of Nephrology, Istanbul University Faculty of Medicine, Istanbul, Turkey.
- [22] Department of Nephrology, Kahramanmaras Sutcu Imam University Faculty of Medicine, Kahramanmaras, Turkey.
- [23] Department of Internal Medicine, Division of Nephrology, Inonu University Faculty of Medicine, Turgut Ozal Medical Center, Malatya, Turkey.
- [24] Department of Internal Medicine, Division of Nephrology, Marmara University Faculty of Medicine, Marmara University Pendik Training and Research Hospital, Istanbul, Turkey.
- [25] Department of Internal Medicine, Division of Nephrology, Yeni Yuzyil University Faculty of Medicine, Istanbul, Turkey.
- [26] Department of Nephrology, University of Health Sciences, Diskapi Yildirim Beyazit Training and Research Hospital, Ankara, Turkey.
- [27] Department of Internal Medicine, Division of Nephrology, Mugla Sitki Kocman University Faculty of Medicine, Training and Research Hospital, Mugla, Turkey.
- [28] Department of Internal Medicine, Division of Nephrology, Memorial Hizmet Hospital, Istanbul, Turkey.
- [29] Department of Nephrology, University of Health Sciences, Bursa Higher Specialization Training and Research Hospital, Bursa, Turkey.
- [30] Department of Nephrology, University of Health Sciences, Ankara Training and Research Hospital, Ankara, Turkey.
- [31] Department of Internal Medicine, Division of Nephrology, University of Health Sciences, Bagcilar Training and Research Hospital, Istanbul, Turkey.
- [32] Department of Internal Medicine, Division of Nephrology, University of Health Sciences, Training and Research Hospital, Izmir, Turkey.
- [33] Department of Internal Medicine, Division of Nephrology, Faculty of Medicine, Usak University, Usak, Turkey.
- [34] Department of Nephrology, Malatya Training and Research Hospital, Malatya, Turkey.
- [35] Department of Nephrology, Manisa Merkezefendi State Hospital, Manisa, Turkey.
- [36] Department of Nephrology, Balıkesir Ataturk City Hospital, Balikesir, Turkey.
- [37] Department of Nephrology, Kocaeli State Hospital, Kocaeli, Turkey.
- [38] Department of Nephrology, University of Health Sciences, Izmir Bozyaka Training and Research Hospital, Izmir, Turkey.
- [39] Department of Internal Medicine, Division of Nephrology, Bezmialem Vakif University Faculty of Medicine, Istanbul, Turkey.
- [40] Department of Nephrology, Bursa Uludag University Faculty of Medicine, Bursa, Turkey.
- [41] Department of Nephrology, University of Health Sciences, Diyarbakır Gazi Yasargil Training and Research Hospital, Diyarbakir, Turkey.
- [42] Department of Nephrology, Adana City Training and Research Hospital, Adana, Turkey.
- [43] Department of Nephrology, Necmettin Erbakan University Meram Faculty of Medicine, Konya, Turkey.
- [44] Department of Nephrology, Hakkari State Hospital, Hakkari, Turkey.
- [45] Department of Internal Medicine, Division of Nephrology, Firat University Faculty of Medicine, Elazig, Turkey.
- [46] Department of Transplantation, Koc University Hospital, Istanbul, Turkey.
- [47] Department of Nephrology, University of Health Sciences, Mehmet Akif Inan Training and Research Hospital, Sanliurfa, Turkey.
- [48] Department of Internal Medicine, Division of Nephrology, Sakarya University Faculty of Medicine, Training and Research Hospital, Sakarya, Turkey.

- [49] Department of Internal Medicine, Division of Nephrology, University of Health Sciences, Tepecik Training and Research Hospital, Izmir, Turkey.
- [50] Department of Nephrology, Erciyes University Faculty of Medicine, Kayseri, Turkey.
- [51] Department of Internal Medicine, Division of Nephrology, Atilim University Faculty of Medicine, Istanbul, Turkey.
- [52] Department of Internal Medicine, Division of Nephrology, Ankara University Faculty of Medicine Ankara, Turkey.

- PMID: **33275763**
- PMCID: PMC7716804
- DOI: 10.1093/ndt/gfaa271

Abstract

Background: Chronic kidney disease (CKD) and immunosuppression, such as in renal transplantation (RT), stand as one of the established potential risk factors for severe coronavirus disease 2019 (COVID-19). Case morbidity and mortality rates for any type of infection have always been much higher in CKD, haemodialysis (HD) and RT patients than in the general population. A large study comparing COVID-19 outcome in moderate to advanced CKD (Stages 3-5), HD and RT patients with a control group of patients is still lacking.

Methods: We conducted a multicentre, retrospective, observational study, involving hospitalized adult patients with COVID-19 from 47 centres in Turkey. Patients with CKD Stages 3-5, chronic HD and RT were compared with patients who had COVID-19 but no kidney disease. Demographics, comorbidities, medications, laboratory tests, COVID-19 treatments and outcome [in-hospital mortality and combined in-hospital outcome mortality or admission to the intensive care unit (ICU)] were compared.

Results: A total of 1210 patients were included [median age, 61 (quartile 1-quartile 3 48-71) years, female 551 (45.5%)] composed of four groups: control (n = 450), HD (n = 390), RT (n = 81) and CKD (n = 289). The ICU admission rate was 266/1210 (22.0%). A total of 172/1210 (14.2%) patients died. The ICU admission and in-hospital mortality rates in the CKD group [114/289 (39.4%); 95% confidence interval (CI) 33.9-45.2; and 82/289 (28.4%); 95% CI 23.9-34.5)] were significantly higher than the other groups: HD = 99/390 (25.4%; 95% CI 21.3-29.9; $P < 0.001$) and 63/390 (16.2%; 95% CI 13.0-20.4; $P < 0.001$); RT = 17/81 (21.0%; 95% CI 13.2-30.8; $P = 0.002$) and 9/81 (11.1%; 95% CI 5.7-19.5; $P = 0.001$); and control = 36/450 (8.0%; 95% CI 5.8-10.8; $P < 0.001$) and 18/450 (4%; 95% CI 2.5-6.2; $P < 0.001$). Adjusted mortality and adjusted combined outcomes in CKD group and HD groups were significantly higher than the control group [hazard ratio (HR) (95% CI) CKD: 2.88 (1.52-5.44); $P = 0.001$; 2.44 (1.35-4.40); $P = 0.003$; HD: 2.32 (1.21-4.46); $P = 0.011$; 2.25 (1.23-4.12); $P = 0.008$), respectively], but these were not significantly different in the RT from in the control group [HR (95% CI) 1.89 (0.76-4.72); $P = 0.169$; 1.87 (0.81-4.28); $P = 0.138$, respectively].

Conclusions: Hospitalized COVID-19 patients with CKDs, including Stages 3-5 CKD, HD and RT, have significantly higher mortality than patients without kidney disease. Stages 3-5 CKD patients have an in-hospital mortality rate as much as HD patients, which may be in part because of similar age and comorbidity burden. We were unable to assess if RT patients were or were not at increased risk for in-hospital mortality because of the relatively small sample size of the RT patients in this study.

Keywords: COVID-19; haemodialysis; kidney disease; mortality; renal transplantation.

© The Author(s) 2020. Published by Oxford University Press on behalf of ERA-EDTA.

- 36 references
- 2 figures

Supplementary info

Publication types, MeSH terms Expand

Publication types

- Multicenter Study
- Observational Study
- Research Support, Non-U.S. Gov't

MeSH terms

- Adult
- Aged
- COVID-19 / epidemiology*
- Comorbidity
- Female
- Hospital Mortality / trends
- Hospitalization / trends
- Humans
- Kidney Transplantation*
- Male
- Middle Aged
- Renal Dialysis / methods*
- Renal Insufficiency, Chronic / epidemiology*
- Renal Insufficiency, Chronic / therapy
- Retrospective Studies
- Risk Factors
- SARS-CoV-2
- Time Factors
- Turkey / epidemiology

Full text links

OXFORD ACADEMIC Silverchair Information Systems Free PMC article
Proceed to details
Cite
Share

☐ 41
Observational Study
J Diabetes Sci Technol
. 2020 Jul;14(4):813-821.
doi: 10.1177/1932296820924469. Epub 2020 May 9.

Glycemic Characteristics and Clinical Outcomes of COVID-19 Patients Hospitalized in the United States

Bruce Bode [1], Valerie Garrett [2], Jordan Messler [2], Raymie McFarland [2], Jennifer Crowe [2], Robby Booth [2], David C Klonoff [3]
Affiliations Expand

Affiliations

- [1] Atlanta Diabetes Associates, GA, USA.
- [2] Glytec, Inc., Waltham, MA, USA.
- [3] Diabetes Research Institute, Mills-Peninsula Medical Center, San Mateo, CA, USA.

- PMID: **32389027**
- PMCID: PMC7673150
- DOI: 10.1177/1932296820924469

Free PMC article
Observational Study

Glycemic Characteristics and Clinical Outcomes of COVID-19 Patients Hospitalized in the United States

Bruce Bode et al. J Diabetes Sci Technol. 2020 Jul.
Free PMC article
Show details
J Diabetes Sci Technol
. 2020 Jul;14(4):813-821.
doi: 10.1177/1932296820924469. Epub 2020 May 9.

Authors

Bruce Bode [1], Valerie Garrett [2], Jordan Messler [2], Raymie McFarland [2], Jennifer Crowe [2], Robby Booth [2], David C Klonoff [3]

Affiliations

- [1] Atlanta Diabetes Associates, GA, USA.
- [2] Glytec, Inc., Waltham, MA, USA.
- [3] Diabetes Research Institute, Mills-Peninsula Medical Center, San Mateo, CA, USA.

- PMID: **32389027**
- PMCID: PMC7673150
- DOI: 10.1177/1932296820924469

Erratum in

- Corrigendum to Glycemic Characteristics and Clinical Outcomes of COVID-19 Patients Hospitalized in the United States.
  [No authors listed] [No authors listed] J Diabetes Sci Technol. 2020 Jun 10:1932296820932678. doi: 10.1177/1932296820932678. Online ahead of print. J Diabetes Sci Technol. 2020. PMID: 32522034 No abstract available.

Abstract

Introduction: Diabetes has emerged as an important risk factor for severe illness and death from COVID-19. There is a paucity of information on glycemic control among hospitalized COVID-19 patients with diabetes and acute hyperglycemia.

Methods: This retrospective observational study of laboratory-confirmed COVID-19 adults evaluated glycemic and clinical outcomes in patients with and without diabetes and/or acutely uncontrolled hyperglycemia hospitalized March 1 to April 6, 2020. Diabetes was defined as A1C ≥6.5%. Uncontrolled hyperglycemia was defined as ≥2 blood glucoses (BGs) > 180 mg/dL within any 24-hour period. Data were abstracted from Glytec's data warehouse.

Results: Among 1122 patients in 88 U.S. hospitals, 451 patients with diabetes and/or uncontrolled hyperglycemia spent 37.8% of patient days having a mean BG > 180 mg/dL. Among 570 patients who died or were discharged, the mortality rate was 28.8% in 184 diabetes and/or uncontrolled hyperglycemia patients, compared with 6.2% of 386 patients without diabetes or hyperglycemia ($P < .001$). Among the 184 patients with diabetes and/or hyperglycemia who died or were discharged, 40 of 96 uncontrolled hyperglycemia patients (41.7%) died compared with 13 of 88 patients with diabetes (14.8%, $P < .001$). Among 493 discharged survivors, median length of stay (LOS) was longer in 184 patients with diabetes and/or uncontrolled hyperglycemia compared with 386 patients without diabetes or hyperglycemia (5.7 vs 4.3 days, $P < .001$).

Conclusion: Among hospitalized patients with COVID-19, diabetes and/or uncontrolled hyperglycemia occurred frequently. These COVID-19 patients with diabetes and/or uncontrolled hyperglycemia had a longer LOS and markedly higher mortality than patients without diabetes or uncontrolled hyperglycemia. Patients with uncontrolled hyperglycemia had a particularly high mortality rate. We recommend health systems which ensure that inpatient hyperglycemia is safely and effectively treated.

Keywords: COVID-19; Glytec; diabetes; glucose; hospital; hyperglycemia; length of stay; mortality.

Conflict of interest statement

Declaration of Conflicting Interests: The author(s) declared the following potential conflicts of interest with respect to the research, authorship, and/or publication of this article: Bruce Bode is an advisory board member for Glytec and owns stock in Aseko/Glytec; his employer, Atlanta Diabetes Associates, receives grant support from DexCom, Insulet, Lilly, Medtronic, Novo Nordisk, and Sanofi. He is on the speaker bureau and consults with Lilly, Medtronic, Novo Nordisk, and Sanofi. Valerie Garrett, Jordan Messler, Raymie McFarland, Jennifer Crowe, and Robby Booth are employed by Glytec. David Klonoff is a consultant to Abbott, Ascensia, Dexcom, EOFlow, Fractyl, Lifecare, Novo, Roche, and Thirdwayv.

- 4 figures

Supplementary info

Publication types, MeSH terms, Substances Expand

Publication types

- Observational Study

MeSH terms

- Aged
- Betacoronavirus
- Blood Glucose Self-Monitoring / methods
- COVID-19
- Comorbidity
- Coronavirus Infections / complications*
- Coronavirus Infections / epidemiology
- Coronavirus Infections / therapy*
- Data Collection
- Female
- Glycated Hemoglobin A / analysis
- Hospitalization
- Humans
- Hyperglycemia / complications
- Hyperglycemia / epidemiology
- Hyperglycemia / therapy*
- Insulin Infusion Systems
- Length of Stay
- Male
- Middle Aged
- Pandemics
- Patient Discharge
- Pneumonia, Viral / complications*

- Pneumonia, Viral / epidemiology
- Pneumonia, Viral / therapy*
- Respiratory Distress Syndrome / complications
- Retrospective Studies
- Risk Factors
- SARS-CoV-2
- Software
- Treatment Outcome
- United States / epidemiology

Substances

- Glycated Hemoglobin A
- hemoglobin A1c protein, human

Full text links

SAGE journals Open access full text Atypon Free PMC article

Proceed to details

Cite

Share

42

Observational Study

BMJ Open

. 2021 Jul 23;11(7):e044921.

doi: 10.1136/bmjopen-2020-044921.

Variation in COVID-19 characteristics, treatment and outcomes in Michigan: an observational study in 32 hospitals

Vineet Chopra [1], Scott A Flanders [2], Valerie Vaughn [2], Lindsay Petty [3], Tejal Gandhi [3], Jakob Israel McSparron [4], Anurag Malani [5], Megan O'Malley [2], Tae Kim [6], Elizabeth McLaughlin [2], Hallie Prescott [4]

Affiliations Expand

Affiliations

- [1] Division of Hospital Medicine, Department of Internal Medicine, University of Michigan, Ann Arbor, Michigan, USA vineetc@umich.edu.
- [2] Division of Hospital Medicine, Department of Internal Medicine, University of Michigan, Ann Arbor, Michigan, USA.

- [3] Division of Infectious Diseases, Department of Internal Medicine, University of Michigan, Ann Arbor, Michigan, USA.
- [4] Division of Pulmonary and Critical Care, Department of Internal Medicine, University of Michigan, Ann Arbor, Michigan, USA.
- [5] St Joseph Mercy Health System, Ann Arbor, Michigan, USA.
- [6] Department of Orthopedics, University of Michigan, Ann Arbor, Michigan, USA.

- PMID: **34301650**
- PMCID: PMC8313307
- DOI: 10.1136/bmjopen-2020-044921

Free PMC article
Observational Study

Variation in COVID-19 characteristics, treatment and outcomes in Michigan: an observational study in 32 hospitals

Vineet Chopra et al. BMJ Open. 2021.
Free PMC article
Show details
BMJ Open

. 2021 Jul 23;11(7):e044921.
doi: 10.1136/bmjopen-2020-044921.

Authors

Vineet Chopra [1], Scott A Flanders [2], Valerie Vaughn [2], Lindsay Petty [3], Tejal Gandhi [3], Jakob Israel McSparron [4], Anurag Malani [5], Megan O'Malley [2], Tae Kim [6], Elizabeth McLaughlin [2], Hallie Prescott [4]

Affiliations

- [1] Division of Hospital Medicine, Department of Internal Medicine, University of Michigan, Ann Arbor, Michigan, USA vineetc@umich.edu.
- [2] Division of Hospital Medicine, Department of Internal Medicine, University of Michigan, Ann Arbor, Michigan, USA.
- [3] Division of Infectious Diseases, Department of Internal Medicine, University of Michigan, Ann Arbor, Michigan, USA.
- [4] Division of Pulmonary and Critical Care, Department of Internal Medicine, University of Michigan, Ann Arbor, Michigan, USA.
- [5] St Joseph Mercy Health System, Ann Arbor, Michigan, USA.
- [6] Department of Orthopedics, University of Michigan, Ann Arbor, Michigan, USA.

- PMID: **34301650**
- PMCID: PMC8313307
- DOI: 10.1136/bmjopen-2020-044921

Abstract

Objective: To describe patient characteristics, symptoms, patterns of care and outcomes for patients hospitalised with COVID-19 in Michigan.

Design: Multicentre retrospective cohort study.

Setting: 32 acute care hospitals in the state of Michigan.

Participants: Patients discharged (16 March-11 May 2020) with suspected or confirmed COVID-19 were identified. Trained abstractors collected demographic information on all patients and detailed clinical data on a subset of COVID-19-positive patients.

Primary outcome measurements: Patient characteristics, treatment and outcomes including cardiopulmonary resuscitation, mortality and venous thromboembolism within and across hospitals.

Results: Demographic-only data from 1593 COVID-19-positive and 1259 persons under investigation discharges were collected. Among 1024 cases with detailed data, the median age was 63 years; median body mass index was 30.6; and 51.4% were black. Cough, fever and shortness of breath were the top symptoms. 37.2% reported a known COVID-19 contact; 7.0% were healthcare workers; and 16.1% presented from congregated living facilities.During hospitalisation, 232 (22.7%) patients were treated in an intensive care unit (ICU); 558 (54.9%) in a 'cohorted' unit; 161 (15.7%) received mechanical ventilation; and 90 (8.8%) received high-flow nasal cannula. ICU patients more often received hydroxychloroquine (66% vs 46%), corticosteroids (34% vs 18%) and antibiotic therapy (92% vs 71%) than general ward patients ($p<0.05$ for all). Overall, 219 (21.4%) patients died, with in-hospital mortality ranging from 7.9% to 45.7% across hospitals. 73% received at least one COVID-19-specific treatment, ranging from 32% to 96% across sites.Across 14 hospitals, the proportion of patients admitted directly to an ICU ranged from 0% to 43.8%; mechanical ventilation on admission from 0% to 12.8%; mortality from 7.9% to 45.7%. Use of at least one COVID-19-specific therapy varied from 32% to 96.3% across sites.

Conclusions: During the early days of the Michigan outbreak of COVID-19, patient characteristics, treatment and outcomes varied widely within and across hospitals.

Keywords: COVID-19; protocols & guidelines; quality in healthcare.

© Author(s) (or their employer(s)) 2021. Re-use permitted under CC BY-NC. No commercial re-use. See rights and permissions. Published by BMJ.

Conflict of interest statement

Competing interests: None declared.

- 31 references
- 2 figures

Supplementary info

Publication types, MeSH terms Expand

Publication types

- Observational Study

MeSH terms

- COVID-19*
- Hospitals
- Humans
- Intensive Care Units
- Michigan / epidemiology
- Middle Aged
- Retrospective Studies
- SARS-CoV-2
- Treatment Outcome

Full text links

BMJ Free Full Text HighWire Free PMC article
Proceed to details
Cite
Share
43
Observational Study
Ann Intern Med
. 2021 Mar;174(3):316-325.
doi: 10.7326/M20-5206. Epub 2020 Nov 24.

Surviving COVID-19 After Hospital Discharge: Symptom, Functional, and Adverse Outcomes of Home Health Recipients

Kathryn H Bowles [1], Margaret McDonald [2], Yolanda Barrón [2], Erin Kennedy [3], Melissa O'Connor [4], Mark Mikkelsen [5]
Affiliations Expand

Affiliations

- [1] University of Pennsylvania School of Nursing, Philadelphia, Pennsylvania, and Visiting Nurse Service of New York, New York, New York (K.H.B.).
- [2] Visiting Nurse Service of New York, New York, New York (M.M., Y.B.).
- [3] University of Pennsylvania School of Nursing, Philadelphia, Pennsylvania (E.K.).

- [4] Villanova University, Villanova, Pennsylvania (M.O.).
- [5] Perelman School of Medicine at the University of Pennsylvania, Philadelphia, Pennsylvania (M.M.).

- PMID: **33226861**
- PMCID: PMC7707212
- DOI: 10.7326/M20-5206

Free PMC article
Observational Study

Surviving COVID-19 After Hospital Discharge: Symptom, Functional, and Adverse Outcomes of Home Health Recipients

Kathryn H Bowles et al. Ann Intern Med. 2021 Mar.
Free PMC article
Show details
Ann Intern Med
. 2021 Mar;174(3):316-325.
doi: 10.7326/M20-5206. Epub 2020 Nov 24.

Authors

Kathryn H Bowles [1], Margaret McDonald [2], Yolanda Barrón [2], Erin Kennedy [3], Melissa O'Connor [4], Mark Mikkelsen [5]

Affiliations

- [1] University of Pennsylvania School of Nursing, Philadelphia, Pennsylvania, and Visiting Nurse Service of New York, New York, New York (K.H.B.).
- [2] Visiting Nurse Service of New York, New York, New York (M.M., Y.B.).
- [3] University of Pennsylvania School of Nursing, Philadelphia, Pennsylvania (E.K.).
- [4] Villanova University, Villanova, Pennsylvania (M.O.).
- [5] Perelman School of Medicine at the University of Pennsylvania, Philadelphia, Pennsylvania (M.M.).

- PMID: **33226861**
- PMCID: PMC7707212
- DOI: 10.7326/M20-5206

Abstract

Background: Little is known about recovery from coronavirus disease 2019 (COVID-19) after hospital discharge.

Objective: To describe the home health recovery of patients with COVID-19 and risk factors associated with rehospitalization or death.

Design: Retrospective observational cohort.

Setting: New York City.

Participants: 1409 patients with COVID-19 admitted to home health care (HHC) between 1 April and 15 June 2020 after hospitalization.

Measurements: Covariates and outcomes were obtained from the mandated OASIS (Outcome and Assessment Information Set). Cox proportional hazards models were used to estimate the hazard ratio (HR) of risk factors associated with rehospitalization or death.

Results: After an average of 32 days in HHC, 94% of patients were discharged and most achieved statistically significant improvements in symptoms and function. Activity-of-daily-living dependencies decreased from an average of 6 (95% CI, 5.9 to 6.1) to 1.2 (CI, 1.1 to 1.3). Risk for rehospitalization or death was higher for male patients (HR, 1.45 [CI, 1.04 to 2.03]); White patients (HR, 1.74 [CI, 1.22 to 2.47]); and patients with heart failure (HR, 2.12 [CI, 1.41 to 3.19]), diabetes with complications (HR, 1.71 [CI, 1.17 to 2.52]), 2 or more emergency department visits in the past 6 months (HR, 1.78 [CI, 1.21 to 2.62]), pain daily or all the time (HR, 1.46 [CI, 1.05 to 2.05]), cognitive impairment (HR, 1.49 [CI, 1.04 to 2.13]), or functional dependencies (HR, 1.09 [CI, 1.00 to 1.20]). Eleven patients (1%) died, 137 (10%) were rehospitalized, and 23 (2%) remain on service.

Limitations: Care was provided by 1 home health agency. Information on rehospitalization and death after HHC discharge is not available.

Conclusion: Symptom burden and functional dependence were common at the time of HHC admission but improved for most patients. Comorbid conditions of heart failure and diabetes, as well as characteristics present at admission, identified patients at greatest risk for an adverse event.

Primary funding source: No direct funding.

Conflict of interest statement

Disclosures: Disclosures can be viewed at www.acponline.org/authors/icmje/ConflictOfInterestForms.do?msNum=M20-5206.

- 33 references

Supplementary info

Publication types, MeSH terms, Grant support Expand

Publication types

- Observational Study
- Research Support, N.I.H., Extramural
- Research Support, Non-U.S. Gov't

MeSH terms

- Age Factors
- Aged
- Aged, 80 and over
- COVID-19 / complications*
- COVID-19 / mortality
- COVID-19 / therapy*
- Female
- Home Care Services*
- Humans
- Male
- Middle Aged
- New York City / epidemiology
- Outcome Assessment, Health Care
- Patient Discharge*
- Patient Readmission*
- Proportional Hazards Models
- Retrospective Studies
- Risk Factors
- SARS-CoV-2
- Treatment Outcome

Grant support

- T32 NR009356/NR/NINR NIH HHS/United States

Full text links

Atypon Free PMC article
Proceed to details
Cite
Share
☐ 44
Observational Study
Pathog Dis
. 2021 Jan 9;79(1):ftaa064.
doi: 10.1093/femspd/ftaa064.

[SARS-CoV-2 RT-PCR profile in 298 Indian COVID-19 patients: a retrospective observational study]

Bisakh Bhattacharya [1], Rohit Kumar [1], Ved Prakash Meena [1], Manish Soneja [1], Amit Singh [2], Rojaleen Das [2], Ashit Xess [2], Nazneen Arif [2], Saurabh Vig [3], Vandana Rastogi [4], Pavan Tiwari [5], Sushma Bhatnagar [3], Anant Mohan [5], Naveet Wig [1], Lalit Dar [2]
Affiliations Expand

Affiliations

- [1] Department of Medicine, All India Institute of Medical Sciences, Medicine Office, 3rd floor, Teaching block, AIIMS, New Delhi, India.
- [2] Department of Microbiology, Teaching block, 2nd floor, All India Institute of Medical Sciences, New Delhi, India.
- [3] Department of Onco-anesthesia and Palliative Medicine, Dr. B.R.A.I.R.C.H, All India Institute of Medical Sciences, New Delhi, India.
- [4] Department of Biostatistics, All India Institute of Medical Sciences, New Delhi, India.
- [5] Department of Pulmonary Medicine and Sleep Disorders, All India Institute of Medical Sciences, New Delhi, India.

- PMID: **33537743**
- DOI: 10.1093/femspd/ftaa064

Observational Study

SARS-CoV-2 RT-PCR profile in 298 Indian COVID-19 patients: a retrospective observational study

Bisakh Bhattacharya et al. Pathog Dis. 2021.
Show details
Pathog Dis
. 2021 Jan 9;79(1):ftaa064.
doi: 10.1093/femspd/ftaa064.

Authors

Bisakh Bhattacharya [1], Rohit Kumar [1], Ved Prakash Meena [1], Manish Soneja [1], Amit Singh [2], Rojaleen Das [2], Ashit Xess [2], Nazneen Arif [2], Saurabh Vig [3], Vandana Rastogi [4], Pavan Tiwari [5], Sushma Bhatnagar [3], Anant Mohan [5], Naveet Wig [1], Lalit Dar [2]

Affiliations

- [1] Department of Medicine, All India Institute of Medical Sciences, Medicine Office, 3rd floor, Teaching block, AIIMS, New Delhi, India.
- [2] Department of Microbiology, Teaching block, 2nd floor, All India Institute of Medical Sciences, New Delhi, India.
- [3] Department of Onco-anesthesia and Palliative Medicine, Dr. B.R.A.I.R.C.H, All India Institute of Medical Sciences, New Delhi, India.
- [4] Department of Biostatistics, All India Institute of Medical Sciences, New Delhi, India.
- [5] Department of Pulmonary Medicine and Sleep Disorders, All India Institute of Medical Sciences, New Delhi, India.

- PMID: **33537743**
- DOI: 10.1093/femspd/ftaa064

Abstract

Background: despite being in the 5th month of pandemic, knowledge with respect to viral dynamics, infectivity and RT-PCR positivity continues to evolve.

Aim: to analyse the SARS CoV-2 nucleic acid RT-PCR profiles in COVID-19 patients.

Design: it was a retrospective, observational study conducted at COVID facilities under AIIMS, New Delhi.

Methods: patients admitted with laboratory confirmed COVID-19 were eligible for enrolment. Patients with incomplete details, or only single PCR tests were excluded. Data regarding demographic details, comorbidities, treatment received and results of SARS-CoV-2 RT-PCR performed on nasopharyngeal and oropharyngeal swabs, collected at different time points, was retrieved from the hospital records.

Results: a total of 298 patients were included, majority were males (75·8%) with mean age of 39·07 years (0·6-88 years). The mean duration from symptom onset to first positive RT-PCR was 4·7 days (SD 3·67), while that of symptom onset to last positive test was 17·83 days (SD 6·22). Proportions of positive RT-PCR tests were 100%, 49%, 24%, 8·7% and 20·6% in the 1st, 2nd, 3rd, 4th and >4 weeks of illness. A total of 12 symptomatic patients had prolonged positive test results even after 3 weeks of symptom onset. Age > = 60 years was associated with prolonged RT-PCR positivity (statistically significant).

Conclusion: this study showed that the average period of PCR positivity is more than 2 weeks in COVID-19 patients; elderly patients have prolonged duration of RT-PCR positivity and requires further follow up.

Keywords: COVID-19; RT-PCR; SARS-CoV-2; profile.

© The Author(s) 2020. Published by Oxford University Press on behalf of FEMS.

Supplementary info

Publication types, MeSH terms Expand

Publication types

- Observational Study
- Research Support, Non-U.S. Gov't

MeSH terms

- Adolescent
- Adult
- Aged
- Aged, 80 and over
- COVID-19 / epidemiology
- COVID-19 / virology*
- COVID-19 Testing / methods
- Child
- Child, Preschool
- Comorbidity
- Female
- Hospitalization
- Humans
- India / epidemiology
- Infant
- Male
- Middle Aged
- Nasopharynx / virology
- Oropharynx / virology
- Pandemics
- Retrospective Studies
- Reverse Transcriptase Polymerase Chain Reaction / methods
- SARS-CoV-2 / genetics*
- Young Adult

Full text links

OXFORD ACADEMIC Silverchair Information Systems
Proceed to details
Cite
Share
45
Observational Study
Epilepsy Res
. 2021 Aug;174:106650.
doi: 10.1016/j.eplepsyres.2021.106650. Epub 2021 May 5.

Acute symptomatic seizures and COVID-19: Hospital-based study

Eman M Khedr [1], Ahmed Shoyb [2], Mahmoud Mohammaden [3], Mostafa Saber [2]
Affiliations Expand

Affiliations

- [1] Department of Neuropsychiatry, Faculty of Medicine, Assiut University, Egypt. Electronic address: emankhedr99@yahoo.com.
- [2] Department of Neuropsychiatry, Faculty of Medicine, Aswan University, Egypt.
- [3] Department of Neuropsychiatry, Faculty of Medicine, South Valley University, Egypt.

- PMID: **33993018**
- PMCID: PMC8096525
- DOI: 10.1016/j.eplepsyres.2021.106650

Free PMC article
Observational Study

Acute symptomatic seizures and COVID-19: Hospital-based study

Eman M Khedr et al. Epilepsy Res. 2021 Aug.
Free PMC article
Show details
Epilepsy Res
. 2021 Aug;174:106650.
doi: 10.1016/j.eplepsyres.2021.106650. Epub 2021 May 5.

Authors

Eman M Khedr [1], Ahmed Shoyb [2], Mahmoud Mohammaden [3], Mostafa Saber [2]

Affiliations

- [1] Department of Neuropsychiatry, Faculty of Medicine, Assiut University, Egypt. Electronic address: emankhedr99@yahoo.com.
- [2] Department of Neuropsychiatry, Faculty of Medicine, Aswan University, Egypt.
- [3] Department of Neuropsychiatry, Faculty of Medicine, South Valley University, Egypt.

- PMID: **33993018**
- PMCID: PMC8096525
- DOI: 10.1016/j.eplepsyres.2021.106650

Abstract

Background and purpose: Post COVID-19 seizures are relatively rare. The aim of the present study was to estimate the frequency of acute symptomatic seizures among patients with COVID-19 and to discuss possible pathophysiological mechanisms.

Material and methods: Out of 439 cases with COVID-19 that were admitted to Assiut and Aswan University hospitals during the period from 1 June to 10 August 2020, 19 patients (4.3 %) presented with acute symptomatic seizures. Each patient underwent computed tomography (CT) or magnetic resonance imaging (MRI) of the brain and conventional electroencephalography (EEG). Laboratory investigations included: blood gases, complete blood picture, serum D-Dimer, Ferritin, C-reactive protein, renal and liver functions, and coagulation profile.

Results: Of the 19 patients, 3 had new onset seizures without underlying pathology (0.68 % out of the total 439 patients); 2 others (0.46 %) had previously diagnosed controlled epilepsy with breakthrough seizures. The majority of cases (14 patients, 3.19 %) had primary pathology that could explain the occurrence of seizures: 5 suffered a post COVID-19 stroke (3 ischemic and 2 hemorrhagic stroke); 6 patients had COVID-related encephalitis; 2 patients were old ischemic stroke patients; 1 patient had a brain tumor and developed seizures post COVID-19.

Conclusion: acute symptomatic seizure is not a rare complication of post COVID-19 infection. Both new onset seizures and seizures secondary to primary brain insult (post COVID encephalitis or recent stroke) were observed.

Keywords: CNS; COVID-19; Cerebrovascular stroke; Encephalitis; Epilepsy; Seizure.

Copyright © 2021 Elsevier B.V. All rights reserved.

- 27 references
- 1 figure

Supplementary info

Publication types, MeSH terms, Substances Expand

Publication types

- Observational Study

MeSH terms

- Adult
- Aged
- Anticonvulsants / therapeutic use
- Brain / diagnostic imaging
- COVID-19 / epidemiology*
- COVID-19 / physiopathology
- Egypt / epidemiology
- Electroencephalography

- Encephalitis, Viral / epidemiology*
- Epilepsy / drug therapy
- Epilepsy / epidemiology*
- Female
- Hospitalization
- Humans
- Magnetic Resonance Imaging
- Male
- Middle Aged
- Retrospective Studies
- SARS-CoV-2
- Seizures / epidemiology*
- Seizures / physiopathology
- Stroke / diagnostic imaging
- Stroke / epidemiology*
- Tomography, X-Ray Computed

Substances

- Anticonvulsants

Full text links

ELSEVIER FULL-TEXT ARTICLE Elsevier Science Free PMC article

Proceed to details

Cite

Share

☐ 46

Observational Study

BMC Infect Dis

. 2021 Sep 14;21(1):952.

doi: 10.1186/s12879-021-06588-5.

Pneumonia-targeted lopinavir/ritonavir-based treatment for patients with COVID-19: an early-period retrospective single center observational study

Jongkyu Kim [#] [1], Jiwoong Jung [#] [2], Tae Ho Kim [3], Naree Kang [3], Hanzo Choi [4], Dong Hyun Oh [3], Mi Young Ahn [3], Su Hyun Kim [3], Chorom Hahm [3], Young Kyong Lee [5], Keunhong Park [6], Kiho Hong [7], Jae-Phil Choi [8]

Affiliations Expand

Affiliations

- [1] Department of Physical Medicine and Rehabilitation, Seoul Medical Center, Seoul, Republic of Korea.
- [2] Department of Surgery, Seoul Medical Center, Seoul, Republic of Korea.
- [3] Department of Internal Medicine, Seoul Medical Center, Seoul, Republic of Korea.
- [4] Department of Emergency Medicine, Kyunghee University at Gangdong, Seoul, Republic of Korea.
- [5] Department of Radiology, Seoul Medical Center, Seoul, Republic of Korea.
- [6] Department of Emergency Medicine, Seoul Medical Center, Seoul, Republic of Korea.
- [7] Department of Laboratory Medicine, Seoul Medical Center, Seoul, Republic of Korea.
- [8] Department of Internal Medicine, Seoul Medical Center, Seoul, Republic of Korea. dasole@seoulmc.or.kr.

[#] Contributed equally.

- PMID: **34521365**
- PMCID: PMC8439533
- DOI: 10.1186/s12879-021-06588-5

Free PMC article
Observational Study

Pneumonia-targeted lopinavir/ritonavir-based treatment for patients with COVID-19: an early-period retrospective single center observational study

Jongkyu Kim et al. BMC Infect Dis. 2021.
Free PMC article
Show details
BMC Infect Dis
. 2021 Sep 14;21(1):952.
doi: 10.1186/s12879-021-06588-5.

Authors

Jongkyu Kim [#1], Jiwoong Jung [#2], Tae Ho Kim [3], Naree Kang [3], Hanzo Choi [4], Dong Hyun Oh [3], Mi Young Ahn [3], Su Hyun Kim [3], Chorom Hahm [3], Young Kyong Lee [5], Keunhong Park [6], Kiho Hong [7], Jae-Phil Choi [8]

Affiliations

- [1] Department of Physical Medicine and Rehabilitation, Seoul Medical Center, Seoul, Republic of Korea.
- [2] Department of Surgery, Seoul Medical Center, Seoul, Republic of Korea.
- [3] Department of Internal Medicine, Seoul Medical Center, Seoul, Republic of Korea.
- [4] Department of Emergency Medicine, Kyunghee University at Gangdong, Seoul, Republic of Korea.
- [5] Department of Radiology, Seoul Medical Center, Seoul, Republic of Korea.
- [6] Department of Emergency Medicine, Seoul Medical Center, Seoul, Republic of Korea.
- [7] Department of Laboratory Medicine, Seoul Medical Center, Seoul, Republic of Korea.
- [8] Department of Internal Medicine, Seoul Medical Center, Seoul, Republic of Korea. dasole@seoulmc.or.kr.

[#] Contributed equally.

- PMID: **34521365**
- PMCID: PMC8439533
- DOI: 10.1186/s12879-021-06588-5

Abstract

Background: Robust evidenced treatment strategy for Coronavirus disease 2019 (COVID-19) has not been established yet. Early, targeted, comprehensive management approach can be essential.

Methods: A lopinavir/ritonavir (LPV/r)-based antiviral treatment was administered to the patients with computed tomography (CT)-documented pneumonia. Medical records of patients with COVID-19, previously discharged or hospitalized for ≥ 21 days at the Seoul Medical Center from January 29 to April 15, 2020 were reviewed to analyze clinical and virological outcomes. Patients were divided into two groups (PCR-Negative conversion group vs. Non-negative conversion group and requiring oxygen group vs. Non-requiring oxygen group).

Results: In total, 136 patients with a mean age of 41.8 ± 18.2 years were included with median 3-day delay of hospitalization after illness. Thirteen (9.56%) were initially asymptomatic, and 5 (3.67%) were persistently asymptomatic. Eighty-five (62.5%) had CT-documented pneumonia, 94% of whom received LPV/r treatments. A total of 53 patients (38.97%) had negative polymerase chain reaction (PCR) results within 28 days. Eight (9.4%) out of 85 pneumonic patients received oxygen supplementation. Patients with initial lower respiratory symptoms showed significant delay in PCR negative conversion (> 28 days) (odds ratio [OR] 0.166; 95% confidence interval [CI] 0.067-0.477; P < 0.001). However, antiviral treatment for pneumonic patients was significantly related with early conversion within 28 days (OR 3.049; 95% CI 1.128-8.243; P = 0.028). Increasing age increased the likelihood of oxygen supplementation requirement in the pneumonic patient group (OR 1.108; 95% CI 1.021-1.202; P = 0.014).

Conclusions: Early, pneumonia targeted LPV/r-based antiviral therapy resulted in a significantly higher probability of negative conversion of PCR within 28 days compared to symptomatic treatment.

Keywords: Coronavirus disease 2019 (COVID-19); Lopinavir/ritonavir; Severe acute respiratory syndrome coronavirus 2 SARS-CoV-2; Treatment outcome; Virus shedding.

© 2021. The Author(s).

Conflict of interest statement

The authors declare that they have no competing interests.

- 20 references
- 1 figure

Supplementary info

Publication types, MeSH terms, Substances, Supplementary concepts Expand

Publication types

- Observational Study

MeSH terms

- Antiviral Agents / therapeutic use
- COVID-19* / drug therapy
- Drug Combinations
- Humans
- Infant, Newborn
- Lopinavir / therapeutic use
- Pneumonia* / drug therapy
- Retrospective Studies
- Ritonavir / therapeutic use
- SARS-CoV-2

Substances

- Antiviral Agents
- Drug Combinations
- Lopinavir
- Ritonavir

Supplementary concepts

- COVID-19 drug treatment

Full text links

Read free full text at BMC BioMed Central Free PMC article
Proceed to details
Cite

Share

☐ 47

Observational Study

JPEN J Parenter Enteral Nutr

. 2021 Jan;45(1):32-42.

doi: 10.1002/jpen.1953. Epub 2020 Jul 20.

Evaluation of Nutrition Risk and Its Association With Mortality Risk in Severely and Critically Ill COVID-19 Patients

Xiaobo Zhao [1], Yan Li [2], Yanyan Ge [2], Yuxin Shi [2], Ping Lv [2], Jianchu Zhang [3], Gui Fu [1], Yanfen Zhou [1], Ke Jiang [4], Nengxing Lin [5], Tao Bai [6], Runming Jin [1], Yuanjue Wu [7], Xuefeng Yang [2], Xin Li [1]

Affiliations Expand

Affiliations

- [1] Department of Paediatrics, Union Hospital, Tongji Medical College, Huazhong University of Science and Technology, Wuhan, Hubei Province, P. R. China.
- [2] Department of Nutrition and Food Hygiene, Hubei Key Laboratory of Food Nutrition and Safety, MOE Key Laboratory of Environment and Health, School of Public Health, Tongji Medical College, Huazhong University of Science and Technology, Wuhan, Hubei, P. R. China.
- [3] Department of Respiratory and Critical Care Medicine, Union Hospital, Tongji Medical College, Huazhong University of Science and Technology, Wuhan, Hubei Province, P. R. China.
- [4] Department of Thoracic Surgery, Union Hospital, Tongji Medical College, Huazhong University of Science and Technology, Wuhan, Hubei Province, P. R. China.
- [5] Department of Dermatology, Union Hospital, Tongji Medical College, Huazhong University of Science and Technology, Wuhan, Hubei Province, P. R. China.
- [6] Department of Gastroenterology, Union Hospital, Tongji Medical College, Huazhong University of Science and Technology, Wuhan, Hubei Province, P. R. China.
- [7] Department of Clinical Nutrition, Union Hospital, Tongji Medical College, Huazhong University of Science and Technology, Wuhan, Hubei Province, P. R. China.

- PMID: **32613660**
- PMCID: PMC7361906
- DOI: 10.1002/jpen.1953

Free PMC article

Observational Study

Evaluation of Nutrition Risk and Its Association With Mortality Risk in Severely and Critically Ill COVID-19 Patients

Xiaobo Zhao et al. JPEN J Parenter Enteral Nutr. 2021 Jan.
Free PMC article
Show details
JPEN J Parenter Enteral Nutr
. 2021 Jan;45(1):32-42.
doi: 10.1002/jpen.1953. Epub 2020 Jul 20.

Authors

Xiaobo Zhao [1], Yan Li [2], Yanyan Ge [2], Yuxin Shi [2], Ping Lv [2], Jianchu Zhang [3], Gui Fu [1], Yanfen Zhou [1], Ke Jiang [4], Nengxing Lin [5], Tao Bai [6], Runming Jin [1], Yuanjue Wu [7], Xuefeng Yang [2], Xin Li [1]

Affiliations

- [1] Department of Paediatrics, Union Hospital, Tongji Medical College, Huazhong University of Science and Technology, Wuhan, Hubei Province, P. R. China.
- [2] Department of Nutrition and Food Hygiene, Hubei Key Laboratory of Food Nutrition and Safety, MOE Key Laboratory of Environment and Health, School of Public Health, Tongji Medical College, Huazhong University of Science and Technology, Wuhan, Hubei, P. R. China.
- [3] Department of Respiratory and Critical Care Medicine, Union Hospital, Tongji Medical College, Huazhong University of Science and Technology, Wuhan, Hubei Province, P. R. China.
- [4] Department of Thoracic Surgery, Union Hospital, Tongji Medical College, Huazhong University of Science and Technology, Wuhan, Hubei Province, P. R. China.
- [5] Department of Dermatology, Union Hospital, Tongji Medical College, Huazhong University of Science and Technology, Wuhan, Hubei Province, P. R. China.
- [6] Department of Gastroenterology, Union Hospital, Tongji Medical College, Huazhong University of Science and Technology, Wuhan, Hubei Province, P. R. China.
- [7] Department of Clinical Nutrition, Union Hospital, Tongji Medical College, Huazhong University of Science and Technology, Wuhan, Hubei Province, P. R. China.

- PMID: **32613660**
- PMCID: PMC7361906
- DOI: 10.1002/jpen.1953

Abstract

Background: The nutrition status of coronavirus disease 2019 patients is unknown. This study evaluates clinical and nutrition characteristics of severely and critically ill patients infected with severe acute respiratory syndrome coronavirus 2 (SARS-CoV-2) and investigates the relationship between nutrition risk and clinical outcomes.

Methods: A retrospective, observational study was conducted at West Campus of Union Hospital in Wuhan. Patients confirmed with SARS-CoV-2 infection by a nucleic acid-positive test and identified as severely or critically ill were enrolled in this study. Clinical data and outcomes information were collected and nutrition risk was assessed using Nutritional Risk Screening 2002 (NRS).

Results: In total, 413 patients were enrolled in this study, including 346 severely and 67 critically ill patients. Most patients, especially critically ill patients, had significant changes in nutrition-related parameters and inflammatory markers. As for nutrition risk, the critically ill patients had significantly higher proportion of high NRS scores ($P < .001$), which were correlated with inflammatory and nutrition-related markers. Among 342 patients with NRS score ≥3, only 84 (of 342, 25%) received nutrition support. Critically ill patients and those with higher NRS score had a higher risk of mortality and longer stay in hospital. In logistic regression models, 1-unit increase in NRS score was associated with the risk of mortality increasing by 1.23 times (adjusted odds ratio, 2.23; 95% CI, 1.10-4.51; $P = .026$).

Conclusions: Most severely and critically ill patients infected with SARS-CoV-2 are at nutrition risk. The patients with higher nutrition risk have worse outcome and require nutrition therapy.

Keywords: COVID-19; Nutritional Risk Screening 2002; clinical outcomes; inflammatory marker; nutritional status.

© 2020 American Society for Parenteral and Enteral Nutrition.

Comment in

- Nutrition Status Affects COVID-19 Patient Outcomes.
  Berger MM. Berger MM. JPEN J Parenter Enteral Nutr. 2020 Sep;44(7):1166-1167. doi: 10.1002/jpen.1954. Epub 2020 Jul 15. JPEN J Parenter Enteral Nutr. 2020. PMID: 32613691 Free PMC article. No abstract available.

- 39 references

Supplementary info

Publication types, MeSH terms Expand

Publication types

- Observational Study

MeSH terms

- COVID-19 / diagnosis
- COVID-19 / mortality
- COVID-19 / therapy*
- COVID-19 Nucleic Acid Testing
- China / epidemiology
- Critical Care

- Critical Illness*
- Humans
- Nutrition Assessment*
- Nutritional Status*
- Nutritional Support
- Retrospective Studies
- SARS-CoV-2

Full text links

WILEY Full Text Article Wiley Free PMC article
Proceed to details
Cite
Share
48
Observational Study
Hong Kong Med J
. 2021 Feb;27(1):7-17.
doi: 10.12809/hkmj208725. Epub 2020 Aug 27.

Clinical and radiological characteristics of COVID-19: a multicentre, retrospective, observational study

Y Wang [1], S Luo [2], C S Zhou [2], Z Q Wen [3], W Chen [4] [5], W Chen [6], W H Liao [7], J Liu [8], Y Yang [9], J C Shi [10], S D Liu [10], F Xia [2], Z H Yan [5], X Lu [11], T Chen [12], F Yan [11], B Zhang [1], D Y Zhang [9], Z Y Sun [2]
Affiliations Expand

Affiliations

- [1] Department of Radiology, The Affiliated Nanjing Drum Tower Hospital of Nanjing University Medical School, Nanjing, Jiangsu, China.
- [2] Department of Medical Imaging, Jinling Hospital, Medical School of Nanjing University, Nanjing, Jiangsu, China.
- [3] Department of Outpatient, Jinling Hospital, Medical School of Nanjing University, Nanjing, Jiangsu, China.
- [4] Department of Radiology, Jinling Hospital, Southern Medical University, Nanjing, Jiangsu, China.
- [5] Department of Radiology, The Second Affiliated Hospital and Yuying Children's Hospital of Wenzhou Medical University, Wenzhou, Zhejiang, China.
- [6] Department of Medical Imaging, Taihe Hospital, Shiyan, Hubei, China.
- [7] Department of Medical Imaging, Xiangya Hospital of Central South University, Changsha, Hunan, China.

- [8] Department of Medical Imaging, The Second Xiangya Hospital of Central South University, Changsha, Hunan, China.
- [9] Department of Medical Imaging, Wuhan First Hospital, Wuhan, Hubei, China.
- [10] Department of Infectious Disease, Wenzhou Central Hospital, Wenzhou, Zhejiang, China.
- [11] State Key Laboratory of Natural Medicines, Research Center of Biostatistics and Computational Pharmacy, China Pharmaceutical University, Nanjing, Jiangsu, China.
- [12] Medical School of Nanjing University, Nanjing, Jiangsu, China.

- PMID: **32848097**
- DOI: 10.12809/hkmj208725

Free article
Observational Study

Clinical and radiological characteristics of COVID-19: a multicentre, retrospective, observational study

Y Wang et al. Hong Kong Med J. 2021 Feb.
Free article
Show details
Hong Kong Med J
. 2021 Feb;27(1):7-17.
doi: 10.12809/hkmj208725. Epub 2020 Aug 27.

Authors

Y Wang [1], S Luo [2], C S Zhou [2], Z Q Wen [3], W Chen [4] [5], W Chen [6], W H Liao [7], J Liu [8], Y Yang [9], J C Shi [10], S D Liu [10], F Xia [2], Z H Yan [5], X Lu [11], T Chen [12], F Yan [11], B Zhang [1], D Y Zhang [9], Z Y Sun [2]

Affiliations

- [1] Department of Radiology, The Affiliated Nanjing Drum Tower Hospital of Nanjing University Medical School, Nanjing, Jiangsu, China.
- [2] Department of Medical Imaging, Jinling Hospital, Medical School of Nanjing University, Nanjing, Jiangsu, China.
- [3] Department of Outpatient, Jinling Hospital, Medical School of Nanjing University, Nanjing, Jiangsu, China.
- [4] Department of Radiology, Jinling Hospital, Southern Medical University, Nanjing, Jiangsu, China.
- [5] Department of Radiology, The Second Affiliated Hospital and Yuying Children's Hospital of Wenzhou Medical University, Wenzhou, Zhejiang, China.
- [6] Department of Medical Imaging, Taihe Hospital, Shiyan, Hubei, China.
- [7] Department of Medical Imaging, Xiangya Hospital of Central South University, Changsha, Hunan, China.

- [8] Department of Medical Imaging, The Second Xiangya Hospital of Central South University, Changsha, Hunan, China.
- [9] Department of Medical Imaging, Wuhan First Hospital, Wuhan, Hubei, China.
- [10] Department of Infectious Disease, Wenzhou Central Hospital, Wenzhou, Zhejiang, China.
- [11] State Key Laboratory of Natural Medicines, Research Center of Biostatistics and Computational Pharmacy, China Pharmaceutical University, Nanjing, Jiangsu, China.
- [12] Medical School of Nanjing University, Nanjing, Jiangsu, China.

- PMID: **32848097**
- DOI: 10.12809/hkmj208725

Abstract

Background: Multicentre cohort investigations of patients with coronavirus disease 2019 (COVID-19) have been limited. We investigated the clinical and chest computed tomography characteristics of patients with COVID-19 at the peak of the epidemic from multiple centres in China.

Methods: We retrospectively analysed the epidemiologic, clinical, laboratory, and radiological characteristics of 189 patients with confirmed COVID-19 who were admitted to seven hospitals in four Chinese provinces from 18 January 2020 to 3 February 2020.

Results: The mean patient age was 44 years and 52.9% were men; 186/189 had ≥1 co-existing medical condition. Fever, cough, fatigue, myalgia, diarrhoea, and headache were common symptoms at onset; hypertension was the most common co-morbidity. Common clinical signs included dyspnoea, hypoxia, leukopenia, lymphocytopenia, and neutropenia; most lesions exhibited subpleural distribution. The most common radiological manifestation was mixed ground-glass opacity with consolidation (mGGO-C); most patients had grid-like shadows and some showed paving stones. Patients with hypertension, dyspnoea, or hypoxia exhibited more severe lobe involvement and diffusely distributed lesions. Patients in severely affected areas exhibited higher body temperature; more fatigue and dyspnoea; and more manifestations of multiple lesions, lobe involvement, and mGGO-C. During the Wuhan lockdown period, cough, nausea, and dyspnoea were alleviated in patients with newly confirmed COVID-19; lobe involvement was also improved.

Conclusions: Among patients with COVID-19 hospitalised at the peak of the epidemic in China, fever, cough, and dyspnoea were the main symptoms at initial diagnosis, accompanied by lymphocytopenia and hypoxaemia. Patients with severe disease showed more severe lobe involvement and diffuse pulmonary lesion distribution.

Keywords: COVID-19; Dyspnea; Hypertension; Hypoxia; Lymphopenia; Severe acute respiratory syndrome coronavirus 2.

Conflict of interest statement

The authors declare no competing interests.

Supplementary info

Publication types, MeSH terms Expand

Publication types

- Multicenter Study
- Observational Study
- Research Support, Non-U.S. Gov't

MeSH terms

- Adult
- COVID-19 / diagnostic imaging*
- COVID-19 / epidemiology
- China / epidemiology
- Comorbidity
- Female
- Hospitalization
- Humans
- Male
- Pneumonia, Viral / diagnostic imaging*
- Pneumonia, Viral / epidemiology
- Pneumonia, Viral / virology
- Radiography, Thoracic*
- Retrospective Studies
- SARS-CoV-2
- Severity of Illness Index
- Tomography, X-Ray Computed*

Full text links

FREE Full Text Hong Kong Med J Hong Kong Academy of Medicine Press

Proceed to details

Cite

Share

☐ 49

Observational Study

Ann Clin Biochem

. 2021 Mar;58(2):95-101.

doi: 10.1177/0004563220972539. Epub 2020 Nov 20.

Correlating arterial blood gas, acid-base and blood pressure abnormalities with outcomes in COVID-19 intensive care patients

Morne C Bezuidenhout [1], Owen J Wiese [1], Desiree Moodley [2], Elizna Maasdorp [2], Mogamat R Davids [2], Coenraad Fn Koegelenberg [2], Usha Lalla [2], Aye A Khine-Wamono [1], Annalise E Zemlin [1], Brian W Allwood [2]
Affiliations Expand

Affiliations

- [1] National Health Laboratory Service and Stellenbosch University, Tygerberg Hospital, Cape Town, South Africa.
- [2] Faculty of Medicine and Health Sciences, Stellenbosch University, Cape Town, South Africa.

- PMID: **33103442**
- DOI: 10.1177/0004563220972539

Observational Study

Correlating arterial blood gas, acid-base and blood pressure abnormalities with outcomes in COVID-19 intensive care patients

Morne C Bezuidenhout et al. Ann Clin Biochem. 2021 Mar.
Show details
Ann Clin Biochem
. 2021 Mar;58(2):95-101.
doi: 10.1177/0004563220972539. Epub 2020 Nov 20.

Authors

Morne C Bezuidenhout [1], Owen J Wiese [1], Desiree Moodley [2], Elizna Maasdorp [2], Mogamat R Davids [2], Coenraad Fn Koegelenberg [2], Usha Lalla [2], Aye A Khine-Wamono [1], Annalise E Zemlin [1], Brian W Allwood [2]

Affiliations

- [1] National Health Laboratory Service and Stellenbosch University, Tygerberg Hospital, Cape Town, South Africa.
- [2] Faculty of Medicine and Health Sciences, Stellenbosch University, Cape Town, South Africa.

- PMID: **33103442**
- DOI: 10.1177/0004563220972539

Erratum in

- Correction to Bezuidenhout et al. (2021).
  [No authors listed] [No authors listed] Ann Clin Biochem. 2022 Mar;59(2):152. doi: 10.1177/00045632221077327. Ann Clin Biochem. 2022. PMID: 35189722 Free PMC article. No abstract available.

Abstract

Background: During the outbreak of coronavirus disease 2019 (COVID-19), many studies have investigated laboratory biomarkers in management and prognostication of COVID-19 patients, however to date, few have investigated arterial blood gas, acid-base and blood pressure patterns. The aim of the study is to assess the arterial blood gas and acid-base patterns, blood pressure findings and their association with the outcomes of COVID-19 patients admitted to an intensive care unit.

Methods: A single-centre retrospective, observational study in a dedicated COVID-19 intensive care unit in Cape Town, South Africa. Admission arterial blood gas, serum electrolytes, renal function and blood pressure readings performed on COVID-19 patients admitted between 26 March and 2 June 2020 were analysed and compared between survivors and non-survivors.

Results: A total of 56 intensive care unit patients had admission arterial blood gas performed at the time of intensive care unit admission. An alkalaemia (pH > 7.45) was observed in 36 (64.3%) patients. A higher arterial pH (median 7.48 [interquartile range: 7.45-7.51] versus 7.46 [interquartile range: 7.40-7.48], $P = 0.049$) and partial pressure of oxygen in arterial blood (median 7.9 kPa [interquartile range: 7.3-9.6] versus 6.5 kPa [interquartile range: 5.2-7.3], $P = <0.001$) were significantly associated with survival. Survivors also tended to have a higher systolic blood pressure (median: 144 mmHg [interquartile range: 134-152] versus 139 mmHg [interquartile range: 125-142], $P = 0.078$) and higher arterial HCO_3 (median: 28.0 mmol/L [interquartile range: 25.7-28.8] versus 26.3 mmol/L [interquartile range: 24.3-27.9], $P = 0.059$).

Conclusions: The majority of the study population admitted to intensive care unit had an alkalaemia on arterial blood gas. A higher pH and lower partial pressure of oxygen in arterial blood on arterial blood gas analysis were significantly associated with survival.

Keywords: COVID-19; SARS-CoV-2; acid–base; blood gas; critical care.

Supplementary info

Publication types, MeSH terms, Substances Expand

Publication types

- Observational Study

MeSH terms

- Acid-Base Equilibrium*
- Adult
- Biomarkers / blood
- Blood Gas Analysis
- Blood Pressure*
- COVID-19* / blood
- COVID-19* / mortality
- COVID-19* / physiopathology
- COVID-19* / therapy
- Critical Care*
- Female
- Humans
- Intensive Care Units
- Male
- Middle Aged
- Retrospective Studies
- SARS-CoV-2 / metabolism*

Substances

- Biomarkers

Full text links

SAGE journals Atypon
Proceed to details
Cite
Share
☐ 50
Observational Study
Scand J Trauma Resusc Emerg Med
. 2021 Dec 20;29(1):173.
doi: 10.1186/s13049-021-00984-1.

Limitation of life-sustaining treatment and patient involvement in decision-making: a retrospective study of a Danish COVID-19 patient cohort

Hanne Irene Jensen [1] [2] [3], Sevim Ozden [4], Gitte Schultz Kristensen [5], Mihnaz Azizi [6], Siri Aas Smedemark [7], Christian Backer Mogensen [8] [5]

Affiliations Expand

Affiliations

- [1] Department of Anaesthesiology and Intensive Care, Kolding Hospital, University Hospital of Southern Denmark, Sygehusvej 24, 6000, Kolding, Denmark. hanne.irene.jensen@rsyd.dk.
- [2] Department of Anaesthesiology and Intensive Care, Vejle Hospital, University Hospital of Southern Denmark, Vejle, Denmark. hanne.irene.jensen@rsyd.dk.
- [3] Department of Regional Health Research, University of Southern Denmark, Odense, Denmark. hanne.irene.jensen@rsyd.dk.
- [4] Department of Anaesthesiology and Intensive Care, Kolding Hospital, University Hospital of Southern Denmark, Sygehusvej 24, 6000, Kolding, Denmark.
- [5] Department of Emergency, Hospital Sønderjylland, University Hospital of Southern Denmark, Aabenraa, Denmark.
- [6] Emergency Medicine, Hospital of South West Jutland, University Hospital of Southern Denmark, Esbjerg, Denmark.
- [7] Department of Geriatric Medicine, Kolding Hospital, University Hospital of Southern Denmark, Kolding, Denmark.
- [8] Department of Regional Health Research, University of Southern Denmark, Odense, Denmark.

- PMID: **34930420**
- PMCID: PMC8686092
- DOI: 10.1186/s13049-021-00984-1

Free PMC article
Observational Study

Limitation of life-sustaining treatment and patient involvement in decision-making: a retrospective study of a Danish COVID-19 patient cohort

Hanne Irene Jensen et al. Scand J Trauma Resusc Emerg Med. 2021.
Free PMC article
Show details
Scand J Trauma Resusc Emerg Med
. 2021 Dec 20;29(1):173.
doi: 10.1186/s13049-021-00984-1.

Authors

Hanne Irene Jensen [1 2 3], Sevim Ozden [4], Gitte Schultz Kristensen [5], Mihnaz Azizi [6], Siri Aas Smedemark [7], Christian Backer Mogensen [8 5]

Affiliations

- [1] Department of Anaesthesiology and Intensive Care, Kolding Hospital, University Hospital of Southern Denmark, Sygehusvej 24, 6000, Kolding, Denmark. hanne.irene.jensen@rsyd.dk.
- [2] Department of Anaesthesiology and Intensive Care, Vejle Hospital, University Hospital of Southern Denmark, Vejle, Denmark. hanne.irene.jensen@rsyd.dk.
- [3] Department of Regional Health Research, University of Southern Denmark, Odense, Denmark. hanne.irene.jensen@rsyd.dk.
- [4] Department of Anaesthesiology and Intensive Care, Kolding Hospital, University Hospital of Southern Denmark, Sygehusvej 24, 6000, Kolding, Denmark.
- [5] Department of Emergency, Hospital Sønderjylland, University Hospital of Southern Denmark, Aabenraa, Denmark.
- [6] Emergency Medicine, Hospital of South West Jutland, University Hospital of Southern Denmark, Esbjerg, Denmark.
- [7] Department of Geriatric Medicine, Kolding Hospital, University Hospital of Southern Denmark, Kolding, Denmark.
- [8] Department of Regional Health Research, University of Southern Denmark, Odense, Denmark.

- PMID: **34930420**
- PMCID: PMC8686092
- DOI: 10.1186/s13049-021-00984-1

Abstract

Background: The coronavirus (COVID-19) pandemic and the risk of an extensive overload of the healthcare systems have elucidated the need to make decisions on the level of life-sustaining treatment for patients requiring hospitalisation. The purpose of the study was to investigate the proportion and characteristics of COVID-19 patients with limitation of life-sustaining treatment decisions and the degree of patient involvement in the decisions.

Methods: A retrospective observational descriptive study was conducted in three Danish regional hospitals, looking at all patients $\geq$ 18 years of age admitted in 2020 with COVID-19 as the primary diagnosis. Lists of hospitalised patients admitted due to COVID-19 were extracted. The data registration included age, gender, comorbidities, including mental state, body mass index, frailty, recent hospital admissions, COVID-19 life-sustaining treatment, ICU admission, decisions on limitations of life-sustaining treatment before and during current hospitalisation, hospital length of stay, and hospital mortality.

Results: A total of 476 patients were included. For 7% (33/476), a decision about limitation of life-sustaining treatment had been made prior to hospital admission. At the time of admission, one or more limitations of life-sustaining treatment were registered for 16% (75/476) of patients. During the admission, limitation decisions were made for an additional 11 patients, totaling 18% (86/476). For 40% (34/86), the decisions were either made by or discussed with the patient. The decisions not made by patients were made by physicians. For 36% (31/86), no information was disclosed about patient involvement.

Conclusions: Life-sustaining treatment limitation decisions were made for 18% of a COVID-19 patient cohort. Hereof, more than a third of the decisions had been made before hospital admission. Many records lacked information on patient involvement in the decisions.

Keywords: COVID-19; End-of-life; Life-sustaining treatment; Patient involvement; Shared decision-making.

© 2021. The Author(s).

Conflict of interest statement

The authors declare that they have no competing interests.

- 44 references
- 1 figure

Supplementary info

Publication types, MeSH terms Expand

Publication types

- Observational Study

MeSH terms

- COVID-19*
- Denmark / epidemiology
- Humans
- Patient Participation
- Retrospective Studies
- SARS-CoV-2

Full text links

Read free full text at BMC BioMed Central Free PMC article

Proceed to details

Cite

Share

☐ 51

Observational Study

J Endocrinol Invest

. 2021 Apr;44(4):765-771.

doi: 10.1007/s40618-020-01370-x. Epub 2020 Aug 9.

Vitamin D deficiency as a predictor of poor prognosis in patients with acute respiratory failure due to COVID-19

G E Carpagnano [#, 1], V Di Lecce [#, 1], V N Quaranta [2], A Zito [3], E Buonamico [4], E Capozza [1], A Palumbo [1], G Di Gioia [1], V N Valerio [1], O Resta [1]
Affiliations Expand

Affiliations

- [1] Institute of Respiratory Disease, Department of Basic Medical Science, Neuroscience, and Sense Organs, University of Bari "Aldo Moro", piazza Giulio Cesare 11, 70125, Bari, Italy.
- [2] Pneumology Department, "Di Venere" Hospital Bari, Bari, Italy.
- [3] Cardiology Department, "SS Annunziata" Hospital, Taranto, Italy.
- [4] Institute of Respiratory Disease, Department of Basic Medical Science, Neuroscience, and Sense Organs, University of Bari "Aldo Moro", piazza Giulio Cesare 11, 70125, Bari, Italy. enricobuonamico@gmail.com.

[#] Contributed equally.

- PMID: **32772324**
- PMCID: PMC7415009
- DOI: 10.1007/s40618-020-01370-x

Free PMC article
Observational Study

Vitamin D deficiency as a predictor of poor prognosis in patients with acute respiratory failure due to COVID-19

G E Carpagnano et al. J Endocrinol Invest. 2021 Apr.
Free PMC article
Show details
J Endocrinol Invest
. 2021 Apr;44(4):765-771.
doi: 10.1007/s40618-020-01370-x. Epub 2020 Aug 9.

Authors

G E Carpagnano [#, 1], V Di Lecce [#, 1], V N Quaranta [2], A Zito [3], E Buonamico [4], E Capozza [1], A Palumbo [1], G Di Gioia [1], V N Valerio [1], O Resta [1]

Affiliations

- [1] Institute of Respiratory Disease, Department of Basic Medical Science, Neuroscience, and Sense Organs, University of Bari "Aldo Moro", piazza Giulio Cesare 11, 70125, Bari, Italy.
- [2] Pneumology Department, "Di Venere" Hospital Bari, Bari, Italy.
- [3] Cardiology Department, "SS Annunziata" Hospital, Taranto, Italy.
- [4] Institute of Respiratory Disease, Department of Basic Medical Science, Neuroscience, and Sense Organs, University of Bari "Aldo Moro", piazza Giulio Cesare 11, 70125, Bari, Italy. enricobuonamico@gmail.com.

[#] Contributed equally.

- PMID: **32772324**
- PMCID: PMC7415009
- DOI: 10.1007/s40618-020-01370-x

Abstract

Purpose: Hypovitaminosis D is a highly spread condition correlated with increased risk of respiratory tract infections. Nowadays, the world is in the grip of the Coronavirus disease 19 (COVID 19) pandemic. In these patients, cytokine storm is associated with disease severity. In consideration of the role of vitamin D in the immune system, aim of this study was to analyse vitamin D levels in patients with acute respiratory failure due to COVID-19 and to assess any correlations with disease severity and prognosis.

Methods: In this retrospective, observational study, we analysed demographic, clinical and laboratory data of 42 patients with acute respiratory failure due to COVID-19, treated in Respiratory Intermediate Care Unit (RICU) of the Policlinic of Bari from March, 11 to April 30, 2020.

Results: Eighty one percent of patients had hypovitaminosis D. Based on vitamin D levels, the population was stratified into four groups: no hypovitaminosis D, insufficiency, moderate deficiency, and severe deficiency. No differences regarding demographic and clinical characteristics were found. A survival analysis highlighted that, after 10 days of hospitalization, severe vitamin D deficiency patients had a 50% mortality probability, while those with vitamin D $\geq$ 10 ng/mL had a 5% mortality risk ($p = 0.019$).

Conclusions: High prevalence of hypovitaminosis D was found in COVID-19 patients with acute respiratory failure, treated in a RICU. Patients with severe vitamin D deficiency had a significantly higher mortality risk. Severe vitamin D deficiency may be a marker of poor prognosis in these patients, suggesting that adjunctive treatment might improve disease outcomes.

Keywords: Acute respiratory failure; COVID-19; Mortality risk; Vitamin D deficiency.

Conflict of interest statement

All authors disclose no interests related to the present work.

Comment in

- Vitamin D deficiency as a predictor of severity in patients with COVID-19 infection.

Teama MAEM, Abdelhakam DA, Elmohamadi MA, Badr FM. Teama MAEM, et al. Sci Prog. 2021 Jul-Sep;104(3):368504211036854. doi: 10.1177/00368504211036854. Sci Prog. 2021. PMID: 34347528

- 18 references
- 1 figure

Supplementary info

Publication types, MeSH terms, Substances Expand

Publication types

- Observational Study

MeSH terms

- Acute Disease
- Aged
- COVID-19 / epidemiology*
- COVID-19 / immunology
- COVID-19 / mortality*
- Comorbidity
- Cytokine Release Syndrome
- Female
- Hospitalization
- Humans
- Male
- Middle Aged
- Prognosis
- Respiratory Insufficiency / epidemiology*
- Retrospective Studies
- Risk Factors
- SARS-CoV-2
- Severity of Illness Index
- Vitamin D / blood
- Vitamin D Deficiency / epidemiology*
- Vitamin D Deficiency / immunology

Substances

- Vitamin D

Full text links

Springer Free PMC article
Proceed to details
Cite
Share
52
Observational Study
BMJ Open
. 2020 Nov 23;10(11):e042712.
doi: 10.1136/bmjopen-2020-042712.

Retrospective cohort study of admission timing and mortality following COVID-19 infection in England

Ahmed Alaa [1], Zhaozhi Qian [2], Jem Rashbass [3], Jonathan Benger [4], Mihaela van der Schaar [2]
Affiliations Expand

Affiliations

- [1] University of California, Los Angeles, California, USA.
- [2] Centre for Mathematical Sciences, Cambridge University, Cambridge, UK.
- [3] NHS Digital, Leeds, UK.
- [4] NHS Digital, Leeds, UK JBenger@nhs.net.

- PMID: **33234660**
- PMCID: PMC7684820
- DOI: 10.1136/bmjopen-2020-042712

Free PMC article
Observational Study

Retrospective cohort study of admission timing and mortality following COVID-19 infection in England

Ahmed Alaa et al. BMJ Open. 2020.
Free PMC article
Show details
BMJ Open
. 2020 Nov 23;10(11):e042712.
doi: 10.1136/bmjopen-2020-042712.

Authors

Ahmed Alaa [1], Zhaozhi Qian [2], Jem Rashbass [3], Jonathan Benger [4], Mihaela van der Schaar [2]

Affiliations

- [1] University of California, Los Angeles, California, USA.
- [2] Centre for Mathematical Sciences, Cambridge University, Cambridge, UK.
- [3] NHS Digital, Leeds, UK.
- [4] NHS Digital, Leeds, UK JBenger@nhs.net.

- PMID: **33234660**
- PMCID: PMC7684820
- DOI: 10.1136/bmjopen-2020-042712

Abstract

Objectives: We investigated whether the timing of hospital admission is associated with the risk of mortality for patients with COVID-19 in England, and the factors associated with a longer interval between symptom onset and hospital admission.

Design: Retrospective observational cohort study of data collected by the COVID-19 Hospitalisation in England Surveillance System (CHESS). Data were analysed using multivariate regression analysis.

Setting: Acute hospital trusts in England that submit data to CHESS routinely.

Participants: Of 14 150 patients included in CHESS until 13 May 2020, 401 lacked a confirmed diagnosis of COVID-19 and 7666 lacked a recorded date of symptom onset. This left 6083 individuals, of whom 15 were excluded because the time between symptom onset and hospital admission exceeded 3 months. The study cohort therefore comprised 6068 unique individuals.

Main outcome measures: All-cause mortality during the study period.

Results: Timing of hospital admission was an independent predictor of mortality following adjustment for age, sex, comorbidities, ethnicity and obesity. Each additional day between symptom onset and hospital admission was associated with a 1% increase in mortality risk (HR 1.01; $p<0.005$). Healthcare workers were most likely to have an increased interval between symptom onset and hospital admission, as were people from Black, Asian and minority ethnic (BAME) backgrounds, and patients with obesity.

Conclusion: The timing of hospital admission is associated with mortality in patients with COVID-19. Healthcare workers and individuals from a BAME background are at greater risk of later admission, which may contribute to reports of poorer outcomes in these groups. Strategies to identify and admit patients with high-risk and those showing signs of deterioration in a timely way may reduce the consequent mortality from COVID-19, and should be explored.

Keywords: health policy; infectious diseases; public health.

© Author(s) (or their employer(s)) 2020. Re-use permitted under CC BY-NC. No commercial re-use. See rights and permissions. Published by BMJ.

Conflict of interest statement

Competing interests: None declared.

- 19 references
- 4 figures

Supplementary info

Publication types, MeSH terms Expand

Publication types

- Multicenter Study
- Observational Study

MeSH terms

- Aged
- COVID-19 / mortality*
- England / epidemiology
- Female
- Follow-Up Studies
- Hospital Mortality / trends
- Humans
- Male
- Pandemics*
- Patient Admission / trends*
- Retrospective Studies
- Risk Factors
- SARS-CoV-2*
- Survival Rate / trends
- Time Factors

Full text links

BMJ Free Full Text HighWire Free PMC article
Proceed to details
Cite
Share
☐ 53
Observational Study
PLoS One
. 2021 Mar 24;16(3):e0248276.

doi: 10.1371/journal.pone.0248276. eCollection 2021.

Reduced mortality in COVID-19 patients treated with colchicine: Results from a retrospective, observational study

Lucio Manenti [1], Umberto Maggiore [1], Enrico Fiaccadori [1], Tiziana Meschi [2], Anna Degli Antoni [3], Antonio Nouvenne [2], Andrea Ticinesi [2], Nicoletta Cerundolo [2], Beatrice Prati [2], Marco Delsante [1], Ilaria Gandoflini [1], Lorenzo Donghi [3], Micaela Gentile [1], Maria Teresa Farina [1], Vincenzo Oliva [1], Cristina Zambrano [1], Giuseppe Regolisti [4], Alessandra Palmisano [1], Caterina Caminiti [5], Enrico Cocchi [6], Carlo Ferrari [3], Leonardo V Riella [7], Paolo Cravedi [8], Licia Peruzzi [6]
Affiliations Expand

Affiliations

- [1] Dipartimento di Medicina e Chirurgia, Università di Parma e UO Nefrologia, Azienda Ospedaliero-Universitaria di Parma, Parma, Italy.
- [2] Dipartimento Geriatrico-Riabilitativo, Azienda Ospedaliero-Universitaria di Parma, Parma, Italy.
- [3] UO Malattie Infettive ed Epatologia, Azienda Ospedaliero-Universitaria di Parma, Parma, Italy.
- [4] Dipartimento di Medicina e Chirurgia, Università di Parma e UO Clinica e Immunologia Medica, Azienda Ospedaliero-Universitaria di Parma, Parma, Italy.
- [5] UO Ricerca e Innovazione, Azienda Ospedaliera-Universitaria di Parma, Parma, Italy.
- [6] Pediatric Nephrology Unit, Regina Margherita Children's Hospital, Città della Salute e della Scienza di Torino, Turin, Italy.
- [7] Division of Nephrology, Massachusetts General Hospital, Harvard Medical School, Boston, MA, United States of America.
- [8] Renal Division, Department of Medicine, Icahn School of Medicine at Mount Sinai, New York, NY, United States of America.

- PMID: **33760858**
- PMCID: PMC7990208
- DOI: 10.1371/journal.pone.0248276

Free PMC article
Observational Study

Reduced mortality in COVID-19 patients treated with colchicine: Results from a retrospective, observational study

Lucio Manenti et al. PLoS One. 2021.

Free PMC article
Show details
PLoS One
. 2021 Mar 24;16(3):e0248276.
doi: 10.1371/journal.pone.0248276. eCollection 2021.

Authors

Lucio Manenti [1], Umberto Maggiore [1], Enrico Fiaccadori [1], Tiziana Meschi [2], Anna Degli Antoni [3], Antonio Nouvenne [2], Andrea Ticinesi [2], Nicoletta Cerundolo [2], Beatrice Prati [2], Marco Delsante [1], Ilaria Gandoflini [1], Lorenzo Donghi [3], Micaela Gentile [1], Maria Teresa Farina [1], Vincenzo Oliva [1], Cristina Zambrano [1], Giuseppe Regolisti [4], Alessandra Palmisano [1], Caterina Caminiti [5], Enrico Cocchi [6], Carlo Ferrari [3], Leonardo V Riella [7], Paolo Cravedi [8], Licia Peruzzi [6]

Affiliations

- [1] Dipartimento di Medicina e Chirurgia, Università di Parma e UO Nefrologia, Azienda Ospedaliero-Universitaria di Parma, Parma, Italy.
- [2] Dipartimento Geriatrico-Riabilitativo, Azienda Ospedaliero-Universitaria di Parma, Parma, Italy.
- [3] UO Malattie Infettive ed Epatologia, Azienda Ospedaliero-Universitaria di Parma, Parma, Italy.
- [4] Dipartimento di Medicina e Chirurgia, Università di Parma e UO Clinica e Immunologia Medica, Azienda Ospedaliero-Universitaria di Parma, Parma, Italy.
- [5] UO Ricerca e Innovazione, Azienda Ospedaliera-Universitaria di Parma, Parma, Italy.
- [6] Pediatric Nephrology Unit, Regina Margherita Children's Hospital, Città della Salute e della Scienza di Torino, Turin, Italy.
- [7] Division of Nephrology, Massachusetts General Hospital, Harvard Medical School, Boston, MA, United States of America.
- [8] Renal Division, Department of Medicine, Icahn School of Medicine at Mount Sinai, New York, NY, United States of America.

- PMID: **33760858**
- PMCID: PMC7990208
- DOI: 10.1371/journal.pone.0248276

Abstract

Objectives: Effective treatments for coronavirus disease 2019 (COVID-19) are urgently needed. We hypothesized that colchicine, by counteracting proinflammatory pathways implicated in the uncontrolled inflammatory response of COVID-19 patients, reduces pulmonary complications, and improves survival.

Methods: This retrospective study included 71 consecutive COVID-19 patients (hospitalized with pneumonia on CT scan or outpatients) who received colchicine and compared with 70 control patients who did not receive colchicine in two serial time periods at the same institution. We used inverse probability of treatment propensity-score weighting to examine differences in mortality,

clinical improvement (using a 7-point ordinary scale), and inflammatory markers between the two groups.

Results: Amongst the 141 COVID-19 patients (118 [83.7%] hospitalized), 70 (50%) received colchicine. The 21-day crude cumulative mortality was 7.5% in the colchicine group and 28.5% in the control group (P = 0.006; adjusted hazard ratio: 0.24 [95%CI: 0.09 to 0.67]); 21-day clinical improvement occurred in 40.0% of the patients on colchicine and in 26.6% of control patients (adjusted relative improvement rate: 1.80 [95%CI: 1.00 to 3.22]). The strong association between the use of colchicine and reduced mortality was further supported by the diverging linear trends of percent daily change in lymphocyte count (P = 0.018), neutrophil-to-lymphocyte ratio (P = 0.003), and in C-reactive protein levels (P = 0.009). Colchicine was stopped because of transient side effects (diarrhea or skin rashes) in 7% of patients.

Conclusion: In this retrospective cohort study colchicine was associated with reduced mortality and accelerated recovery in COVID-19 patients. This support the rationale for current larger randomized controlled trials testing the safety/efficacy profile of colchicine in COVID-19 patients.

Conflict of interest statement

The authors have declared that no competing interests exist.

- 18 references
- 4 figures

Supplementary info

Publication types, MeSH terms, Substances, Grant support Expand

Publication types

- Observational Study
- Research Support, N.I.H., Extramural

MeSH terms

- Aged
- Aged, 80 and over
- COVID-19 / drug therapy*
- COVID-19 / mortality*
- Colchicine / metabolism
- Colchicine / therapeutic use*
- Female
- Hospitalization
- Humans
- Male
- Middle Aged
- Retrospective Studies

- SARS-CoV-2 / pathogenicity
- Treatment Outcome

Substances

- Colchicine

Grant support

- U01 AI063594/AI/NIAID NIH HHS/United States

Full text links

OPEN ACCESS TO FULL TEXT PLOS ONE Public Library of Science Free PMC article

Proceed to details

Cite

Share

☐ 54

Observational Study

J Med Microbiol

. 2020 Oct;69(10):1228-1234.

doi: 10.1099/jmm.0.001250. Epub 2020 Sep 15.

Zinc sulfate in combination with a zinc ionophore may improve outcomes in hospitalized COVID-19 patients

Philip M Carlucci [1], Tania Ahuja [2], Christopher Petrilli [3] [1], Harish Rajagopalan [3], Simon Jones [4] [5], Joseph Rahimian [1]

Affiliations Expand

Affiliations

- [1] New York University Grossman School of Medicine, Department of Medicine, New York, NY, USA.
- [2] New York University Langone Health, Department of Pharmacy, New York, NY, USA.
- [3] NYU Langone Health, New York, NY, USA.
- [4] Center for Healthcare Innovation and Delivery Science, NYU Langone Health, New York, NY, USA.
- [5] Division of Healthcare Delivery Science, Department of Population Health, NYU Grossman School of Medicine, New York, NY, USA.

- PMID: **32930657**
- PMCID: PMC7660893

• DOI: 10.1099/jmm.0.001250

Free PMC article
Observational Study

Zinc sulfate in combination with a zinc ionophore may improve outcomes in hospitalized COVID-19 patients

Philip M Carlucci et al. J Med Microbiol. 2020 Oct.
Free PMC article
Show details
J Med Microbiol
. 2020 Oct;69(10):1228-1234.
doi: 10.1099/jmm.0.001250. Epub 2020 Sep 15.

Authors

Philip M Carlucci [1], Tania Ahuja [2], Christopher Petrilli [3] [1], Harish Rajagopalan [3], Simon Jones [4] [5], Joseph Rahimian [1]

Affiliations

- [1] New York University Grossman School of Medicine, Department of Medicine, New York, NY, USA.
- [2] New York University Langone Health, Department of Pharmacy, New York, NY, USA.
- [3] NYU Langone Health, New York, NY, USA.
- [4] Center for Healthcare Innovation and Delivery Science, NYU Langone Health, New York, NY, USA.
- [5] Division of Healthcare Delivery Science, Department of Population Health, NYU Grossman School of Medicine, New York, NY, USA.

- PMID: **32930657**
- PMCID: PMC7660893
- DOI: 10.1099/jmm.0.001250

Abstract

Introduction. COVID-19 has rapidly emerged as a pandemic infection that has caused significant mortality and economic losses. Potential therapies and prophylaxis against COVID-19 are urgently needed to combat this novel infection. As a result of *in vitro* evidence suggesting zinc sulphate may be efficacious against COVID-19, our hospitals began using zinc sulphate as add-on therapy to hydroxychloroquine and azithromycin.**Aim.** To compare outcomes among hospitalized COVID-19 patients ordered to receive hydroxychloroquine and azithromycin plus zinc sulphate versus hydroxychloroquine and azithromycin alone.**Methodology.** This was a retrospective observational study. Data was collected from medical records for all patients with admission dates ranging from 2 March 2020 through to 11 April 2020. Initial clinical characteristics on

presentation, medications given during the hospitalization, and hospital outcomes were recorded. The study included patients admitted to any of four acute care NYU Langone Health Hospitals in New York City. Patients included were admitted to the hospital with at least one positive COVID-19 test and had completed their hospitalization. Patients were excluded from the study if they were never admitted to the hospital or if there was an order for other investigational therapies for COVID-19.**Results.** Patients taking zinc sulphate in addition to hydroxychloroquine and azithromycin (*n*=411) and patients taking hydroxychloroquine and azithromycin alone (*n*=521) did not differ in age, race, sex, tobacco use or relevant comorbidities. The addition of zinc sulphate did not impact the length of hospitalization, duration of ventilation or intensive care unit (ICU) duration. In univariate analyses, zinc sulphate increased the frequency of patients being discharged home, and decreased the need for ventilation, admission to the ICU and mortality or transfer to hospice for patients who were never admitted to the ICU. After adjusting for the time at which zinc sulphate was added to our protocol, an increased frequency of being discharged home (OR 1.53, 95 % CI 1.12-2.09) and reduction in mortality or transfer to hospice among patients who did not require ICU level of care remained significant (OR 0.449, 95 % CI 0.271-0.744).**Conclusion.** This study provides the first *in vivo* evidence that zinc sulphate may play a role in therapeutic management for COVID-19.

Keywords: coronavirus; covid 19; hydroxychloroquine; ionophore; mortality; zinc.

Conflict of interest statement

The authors declare that there are no conflicts of interest.

Comment in

- To zinc or not to zinc for COVID-19 prophylaxis or treatment?
  Chiang KC, Gupta A. Chiang KC, et al. J Med Microbiol. 2021 Sep;70(9):001299. doi: 10.1099/jmm.0.001299. J Med Microbiol. 2021. PMID: 34468306 Free PMC article. No abstract available.

- 23 references

Supplementary info

Publication types, MeSH terms, Substances, Supplementary concepts Expand

Publication types

- Comparative Study
- Observational Study

MeSH terms

- Azithromycin / therapeutic use*
- Betacoronavirus / drug effects
- COVID-19
- Cell Membrane Permeability / drug effects
- Coronavirus Infections / drug therapy*

- Drug Therapy, Combination
- Hospitalization
- Humans
- Hydroxychloroquine / therapeutic use*
- Ionophores / therapeutic use
- Length of Stay
- Pandemics
- Pneumonia, Viral / drug therapy*
- Retrospective Studies
- SARS-CoV-2
- Zinc Sulfate / therapeutic use*

Substances

- Ionophores
- Hydroxychloroquine
- Zinc Sulfate
- Azithromycin

Supplementary concepts

- COVID-19 drug treatment

Full text links

MICROBIOLOGY SOCIETY Ingenta plc Free PMC article

Proceed to details

Cite

Share

☐ 55

J Med Internet Res

. 2021 Jul 14;23(7):e28615.

doi: 10.2196/28615.

Emergency Physician Twitter Use in the COVID-19 Pandemic as a Potential Predictor of Impending Surge: Retrospective Observational Study

Colton Margus [1, 2], Natasha Brown [1, 2], Attila J Hertelendy [1, 3], Michelle R Safferman [4, 5], Alexander Hart [1, 2], Gregory R Ciottone [1, 2]
Affiliations Expand

Affiliations

- [1] Division of Disaster Medicine, Department of Emergency Medicine, Beth Israel Deaconess Medical Center, Boston, MA, United States.
- [2] Department of Emergency Medicine, Harvard Medical School, Boston, MA, United States.
- [3] Department of Information Systems and Business Analytics, College of Business, Florida International University, Miami, FL, United States.
- [4] Department of Emergency Medicine, Icahn School of Medicine at Mount Sinai, New York, NY, United States.
- [5] Department of Emergency Medicine, Mount Sinai Morningside-West, New York, NY, United States.

- PMID: **34081612**
- PMCID: PMC8281822
- DOI: 10.2196/28615

Free PMC article

Emergency Physician Twitter Use in the COVID-19 Pandemic as a Potential Predictor of Impending Surge: Retrospective Observational Study

Colton Margus et al. J Med Internet Res. 2021.
Free PMC article
Show details
J Med Internet Res
. 2021 Jul 14;23(7):e28615.
doi: 10.2196/28615.

Authors

Colton Margus [1, 2], Natasha Brown [1, 2], Attila J Hertelendy [1, 3], Michelle R Safferman [4, 5], Alexander Hart [1, 2], Gregory R Ciottone [1, 2]

Affiliations

- [1] Division of Disaster Medicine, Department of Emergency Medicine, Beth Israel Deaconess Medical Center, Boston, MA, United States.
- [2] Department of Emergency Medicine, Harvard Medical School, Boston, MA, United States.

- [3] Department of Information Systems and Business Analytics, College of Business, Florida International University, Miami, FL, United States.
- [4] Department of Emergency Medicine, Icahn School of Medicine at Mount Sinai, New York, NY, United States.
- [5] Department of Emergency Medicine, Mount Sinai Morningside-West, New York, NY, United States.

- PMID: **34081612**
- PMCID: PMC8281822
- DOI: 10.2196/28615

Abstract

Background: The early conversations on social media by emergency physicians offer a window into the ongoing response to the COVID-19 pandemic.

Objective: This retrospective observational study of emergency physician Twitter use details how the health care crisis has influenced emergency physician discourse online and how this discourse may have use as a harbinger of ensuing surge.

Methods: Followers of the three main emergency physician professional organizations were identified using Twitter's application programming interface. They and their followers were included in the study if they identified explicitly as US-based emergency physicians. Statuses, or tweets, were obtained between January 4, 2020, when the new disease was first reported, and December 14, 2020, when vaccination first began. Original tweets underwent sentiment analysis using the previously validated Valence Aware Dictionary and Sentiment Reasoner (VADER) tool as well as topic modeling using latent Dirichlet allocation unsupervised machine learning. Sentiment and topic trends were then correlated with daily change in new COVID-19 cases and inpatient bed utilization.

Results: A total of 3463 emergency physicians produced 334,747 unique English-language tweets during the study period. Out of 3463 participants, 910 (26.3%) stated that they were in training, and 466 of 902 (51.7%) participants who provided their gender identified as men. Overall tweet volume went from a pre-March 2020 mean of 481.9 (SD 72.7) daily tweets to a mean of 1065.5 (SD 257.3) daily tweets thereafter. Parameter and topic number tuning led to 20 tweet topics, with a topic coherence of 0.49. Except for a week in June and 4 days in November, discourse was dominated by the health care system (45,570/334,747, 13.6%). Discussion of pandemic response, epidemiology, and clinical care were jointly found to moderately correlate with COVID-19 hospital bed utilization (Pearson r=0.41), as was the occurrence of "covid," "coronavirus," or "pandemic" in tweet texts (r=0.47). Momentum in COVID-19 tweets, as demonstrated by a sustained crossing of 7- and 28-day moving averages, was found to have occurred on an average of 45.0 (SD 12.7) days before peak COVID-19 hospital bed utilization across the country and in the four most contributory states.

Conclusions: COVID-19 Twitter discussion among emergency physicians correlates with and may precede the rising of hospital burden. This study, therefore, begins to depict the extent to which the ongoing pandemic has affected the field of emergency medicine discourse online and suggests a potential avenue for understanding predictors of surge.

Keywords: COVID-19; COVID-19 pandemic; Twitter; crisis standards of care; disaster medicine; emergency medicine; infodemiology; internet; latent Dirichlet allocation; physician wellness; sentiment analysis; social media; surge capacity; topic modeling.

©Colton Margus, Natasha Brown, Attila J Hertelendy, Michelle R Safferman, Alexander Hart, Gregory R Ciottone. Originally published in the Journal of Medical Internet Research (https://www.jmir.org), 14.07.2021.

Conflict of interest statement

Conflicts of Interest: None declared.

Comment in

- The Unclear Role of the Physician on Social Media During the COVID-19 Pandemic. Comment on "Emergency Physician Twitter Use in the COVID-19 Pandemic as a Potential Predictor of Impending Surge: Retrospective Observational Study".
  MacLeod S, Singh NP, Boyd CJ. MacLeod S, et al. J Med Internet Res. 2022 Mar 2;24 (3):e34870. doi: 10.2196/34870. J Med Internet Res. 2022. PMID: 35120018 Free PMC article. No abstract available.

- 69 references
- 8 figures

Supplementary info

MeSH terms, Substances Expand

MeSH terms

- COVID-19 / diagnosis
- COVID-19 / epidemiology*
- COVID-19 Vaccines / administration & dosage
- Communication*
- Emergency Medicine*
- Forecasting / methods*
- Hospitalization / statistics & numerical data*
- Hospitalization / trends*
- Humans
- Latent Class Analysis
- Longitudinal Studies
- Pandemics
- Physicians*
- Retrospective Studies
- SARS-CoV-2
- Social Media / statistics & numerical data*
- Vaccination / statistics & numerical data

Substances

- COVID-19 Vaccines

Full text links

JMIR Publications Free PMC article

Proceed to details

Cite

Share

56

Observational Study

Sci Rep

. 2021 Jun 21;11(1):12985.

doi: 10.1038/s41598-021-92415-5.

Evaluation of a revised resuscitation protocol for out-of-hospital cardiac arrest patients due to COVID-19 safety protocols: a single-center retrospective study in Japan

Kenji Kandori [1], Yohei Okada [2 3], Wataru Ishii [4], Hiromichi Narumiya [4], Ryoji Iizuka [4]

Affiliations Expand

Affiliations

- [1] Department of Emergency and Critical Care Medicine, Japanese Red Cross Society, Kyoto Daini Hospital, 355-5 Haruobicho Kamigyoku, Kyoto, 602-8026, Japan. knj.kandori@gmail.com.
- [2] Preventive Services, School of Public Health, Kyoto University, Kyoto, Japan.
- [3] Department of Primary Care and Emergency Medicine, Graduate School of Medicine, Kyoto University, Kyoto, Japan.
- [4] Department of Emergency and Critical Care Medicine, Japanese Red Cross Society, Kyoto Daini Hospital, 355-5 Haruobicho Kamigyoku, Kyoto, 602-8026, Japan.

- PMID: **34155299**
- PMCID: PMC8217508
- DOI: 10.1038/s41598-021-92415-5

Free PMC article

Observational Study

Evaluation of a revised resuscitation protocol for out-of-hospital cardiac arrest patients due

to COVID-19 safety protocols: a single-center retrospective study in Japan

Kenji Kandori et al. Sci Rep. 2021.
Free PMC article
Show details
Sci Rep
. 2021 Jun 21;11(1):12985.
doi: 10.1038/s41598-021-92415-5.

Authors

Kenji Kandori [1], Yohei Okada [2] [3], Wataru Ishii [4], Hiromichi Narumiya [4], Ryoji Iizuka [4]

Affiliations

- [1] Department of Emergency and Critical Care Medicine, Japanese Red Cross Society, Kyoto Daini Hospital, 355-5 Haruobicho Kamigyoku, Kyoto, 602-8026, Japan. knj.kandori@gmail.com.
- [2] Preventive Services, School of Public Health, Kyoto University, Kyoto, Japan.
- [3] Department of Primary Care and Emergency Medicine, Graduate School of Medicine, Kyoto University, Kyoto, Japan.
- [4] Department of Emergency and Critical Care Medicine, Japanese Red Cross Society, Kyoto Daini Hospital, 355-5 Haruobicho Kamigyoku, Kyoto, 602-8026, Japan.

- PMID: **34155299**
- PMCID: PMC8217508
- DOI: 10.1038/s41598-021-92415-5

Abstract

This study aimed to determine the association between cardiopulmonary resuscitation (CPR) under the coronavirus 2019 (COVID-19) safety protocols in our hospital and the prognosis of out-of-hospital cardiac arrest (OHCA) patients, in an urban area, where the prevalence of COVID-19 infection is relatively low. This was a single-center, retrospective, observational, cohort study conducted at a tertiary critical care center in Kyoto City, Japan. Adult OHCA patients arriving at our hospital under CPR between January 1, 2019, and December 31, 2020 were included. Our hospital implemented a revised resuscitation protocol for OHCA patients on April 1, 2020 to prevent COVID-19 transmission. This study defined the conventional CPR period as January 1, 2019 to March 31, 2020, and the COVID-19 safety protocol period as April 1, 2020 to December 31, 2020. Throughout the prehospital and in-hospital settings, resuscitation protocols about wearing personal protective equipment and airway management were revised in order to minimize the risk of infection; otherwise, the other resuscitation management had not been changed. The primary outcome was hospitalization survival. The secondary outcomes were return of spontaneous circulation after hospital arrival and 1-month survival after OHCA occurrence. The adjusted odds ratios with 95% confidence intervals (CI) were calculated for outcomes to compare the two study periods, and the multivariable logistic model was used to adjust for potential confounders. The study analyzed 443 patients, with a median age of 76 years (65-85), and

included 261 men (58.9%). The percentage of hospitalization survivors during the entire research period was 16.9% (75/443 patients), with 18.7% (50/267) during the conventional CPR period and 14.2% (25/176) during the COVID-19 safety protocol period. The adjusted odds ratio for hospitalization survival during the COVID-19 safety protocol period was 0.61 (95% CI 0.32-1.18), as compared with conventional CPR. There were no cases of COVID-19 infection among the staff involved in the resuscitation in our hospital. There was no apparent difference in hospitalization survival between the OHCA patients resuscitated under the conventional CPR protocol compared with the current revised protocol for controlling COVID-19 transmission.

Conflict of interest statement

The authors declare no competing interests.

- 38 references
- 1 figure

Supplementary info

Publication types, MeSH terms Expand

Publication types

- Observational Study

MeSH terms

- Adolescent
- Adult
- Aged
- Aged, 80 and over
- COVID-19 / epidemiology*
- COVID-19 / prevention & control*
- COVID-19 / virology
- Cardiopulmonary Resuscitation / methods*
- Emergency Medical Services / methods*
- Female
- Hospitalization
- Humans
- Japan / epidemiology
- Male
- Middle Aged
- Odds Ratio
- Out-of-Hospital Cardiac Arrest / therapy*
- Retrospective Studies
- SARS-CoV-2*

- Treatment Outcome
- Young Adult

Full text links

Nature Publishing Group Free PMC article
Proceed to details
Cite
Share
57
Observational Study
PLoS One
. 2021 Feb 19;16(2):e0245394.
doi: 10.1371/journal.pone.0245394. eCollection 2021.

Features of patients that died for COVID-19 in a hospital in the south of Mexico: A observational cohort study

Jesús Arturo Ruíz-Quiñonez [1], Crystell Guadalupe Guzmán-Priego [2], Germán Alberto Nolasco-Rosales [2], Carlos Alfonso Tovilla-Zarate [3], Oscar Israel Flores-Barrientos [1], Víctor Narváez-Osorio [1], Guadalupe Del Carmen Baeza-Flores [2], Thelma Beatriz Gonzalez-Castro [4], Carlos Ramón López-Brito [1], Carlos Alberto Denis-García [1], Agustín Pérez-García [1], Isela Esther Juárez-Rojop [2]
Affiliations Expand

Affiliations

- [1] Secretaría de Salud, Hospital de Alta Especialidad Dr. Juan Graham Casasús, Villahermosa, Tabasco, México.
- [2] División Académica de Ciencias de la Salud, Universidad Juárez Autónoma de Tabasco, Villahermosa, Tabasco, México.
- [3] División Académica Multidisciplinaria de Comalcalco, Universidad Juárez Autónoma de Tabasco, Comalcalco, Tabasco, México.
- [4] División Académica de Multidisciplinaria de Jalpa de Méndez, Universidad Juárez Autónoma de Tabasco, Jalpa de Méndez, Tabasco, México.

- PMID: **33606711**
- PMCID: PMC7894952
- DOI: 10.1371/journal.pone.0245394

Free PMC article
Observational Study

Features of patients that died for COVID-19 in a hospital in the south of Mexico: A observational cohort study

Jesús Arturo Ruíz-Quiñonez et al. PLoS One. 2021.
Free PMC article
Show details
PLoS One
. 2021 Feb 19;16(2):e0245394.
doi: 10.1371/journal.pone.0245394. eCollection 2021.

Authors

Jesús Arturo Ruíz-Quiñonez [1], Crystell Guadalupe Guzmán-Priego [2], Germán Alberto Nolasco-Rosales [2], Carlos Alfonso Tovilla-Zarate [3], Oscar Israel Flores-Barrientos [1], Víctor Narváez-Osorio [1], Guadalupe Del Carmen Baeza-Flores [2], Thelma Beatriz Gonzalez-Castro [4], Carlos Ramón López-Brito [1], Carlos Alberto Denis-García [1], Agustín Pérez-García [1], Isela Esther Juárez-Rojop [2]

Affiliations

- [1] Secretaría de Salud, Hospital de Alta Especialidad Dr. Juan Graham Casasús, Villahermosa, Tabasco, México.
- [2] División Académica de Ciencias de la Salud, Universidad Juárez Autónoma de Tabasco, Villahermosa, Tabasco, México.
- [3] División Académica Multidisciplinaria de Comalcalco, Universidad Juárez Autónoma de Tabasco, Comalcalco, Tabasco, México.
- [4] División Académica de Multidisciplinaria de Jalpa de Méndez, Universidad Juárez Autónoma de Tabasco, Jalpa de Méndez, Tabasco, México.

- PMID: **33606711**
- PMCID: PMC7894952
- DOI: 10.1371/journal.pone.0245394

Abstract

Background: Due to the wide spread of SARS-CoV2 around the world, the risk of death in individuals with metabolic comorbidities has dangerously increased. Mexico has a high number of infected individuals and deaths by COVID-19 as well as an important burden of metabolic diseases; nevertheless, reports about features of Mexican individuals with COVID-19 are scarce. The aim of this study was to evaluate demographic features, clinical characteristics and the pharmacological treatment of individuals who died by COVID-19 in the south of Mexico.

Methods: We performed an observational study including the information of 185 deceased individuals with confirmed diagnoses of COVID-19. Data were retrieved from medical records. Categorical data were expressed as proportions (%) and numerical data were expressed as mean ±

standard deviation. Comorbidities and overlapping symptoms were plotted as Venn diagrams. Drug clusters were plotted as dendrograms.

Results: The mean age was 59.53 years. There was a male predominance (60.1%). The mean hospital stay was 4.75 ± 4.43 days. The most frequent symptoms were dyspnea (88.77%), fever (71.42%) and dry cough (64.28%). Present comorbidities included diabetes (60.63%), hypertension (59.57%) and obesity (43.61%). The main drugs used for treating COVID-19 were azithromycin (60.6%), hydroxychloroquine (53.0%) and oseltamivir (27.3%).

Conclusions: Mexican individuals who died of COVID-19 had shorter hospital stays, higher frequency of shortness of breath, and higher prevalence of diabetes than individuals from other countries. Also, there was a high frequency of off-label use of drugs for their treatment.

Conflict of interest statement

The authors have declared that no competing interests exist.

- 29 references
- 4 figures

Supplementary info

Publication types, MeSH terms, Substances, Supplementary concepts, Grant support Expand

Publication types

- Observational Study

MeSH terms

- Adult
- Aged
- Azithromycin / administration & dosage*
- COVID-19 / drug therapy*
- COVID-19 / mortality
- COVID-19 / pathology
- Diabetes Mellitus, Type 1* / complications
- Diabetes Mellitus, Type 1* / drug therapy
- Diabetes Mellitus, Type 1* / mortality
- Diabetes Mellitus, Type 1* / pathology
- Female
- Hospital Mortality*
- Hospitals
- Humans
- Hydroxychloroquine / administration & dosage*
- Length of Stay

- Male
- Mexico
- Middle Aged
- Obesity* / complications
- Obesity* / drug therapy
- Obesity* / mortality
- Obesity* / pathology
- Oseltamivir / administration & dosage*
- Retrospective Studies
- SARS-CoV-2*
- Sex Factors

Substances

- Oseltamivir
- Hydroxychloroquine
- Azithromycin

Supplementary concepts

- COVID-19 drug treatment

Grant support

The authors received no specific funding for this work.

Full text links

OPEN ACCESS TO FULL TEXT PLOS ONE Public Library of Science Free PMC article

Proceed to details

Cite

Share

☐ 58

Observational Study

J Med Virol

. 2021 Jul;93(7):4319-4325.

doi: 10.1002/jmv.26925. Epub 2021 Mar 26.

The role of teicoplanin in the treatment of SARS-CoV-2 infection: A retrospective study

in critically ill COVID-19 patients (Tei-COVID study)

Giancarlo Ceccarelli [1, 2], Francesco Alessandri [2, 3], Alessandra Oliva [1, 2], Cristian Borrazzo [1], Serena Dell'Isola [4], Anna Maria Ialungo [4], Elena Rastrelli [4], Massimiliano Pelli [5], Giammarco Raponi [6], Ombretta Turriziani [7], Franco Ruberto [2, 3], Monica Rocco [5], Francesco Pugliese [2, 3], Alessandro Russo [8, 9], Gabriella d'Ettorre [1, 2], Mario Venditti [1, 2]

Affiliations Expand

Affiliations

- [1] Department of Public Health and Infectious Diseases, Sapienza University of Rome, Rome, Italy.
- [2] Covid Division, Azienda Ospedaliero-Universitaria Policlinico Umberto I, Rome, Italy.
- [3] Department of Anesthesiology and Intensive Care, Sapienza University of Rome, Rome, Italy.
- [4] Protect Medicine Division, Belcolle Hospital, Viterbo, Italy.
- [5] Intensive Care Unit, Department of medical and Surgical Science and Traslational Medicine, Sant'Andrea Hospital, Sapienza University of Rome, Rome, Italy.
- [6] Microbiology Unit, Department of Public Health and Infectious Diseases, Sapienza University of Rome, Rome, Italy.
- [7] Virology Unit, Department of Molecular Medicine, Sapienza University of Rome, Rome, Italy.
- [8] Department of Clinical and Experimental Medicine, University of Pisa, Pisa, Italy.
- [9] COVID Unit - Medicine Division, Casilino Hospital, Rome, Italy.

- PMID: **33675235**
- PMCID: PMC8250836
- DOI: 10.1002/jmv.26925

Free PMC article
Observational Study

The role of teicoplanin in the treatment of SARS-CoV-2 infection: A retrospective study in critically ill COVID-19 patients (Tei-COVID study)

Giancarlo Ceccarelli et al. J Med Virol. 2021 Jul.
Free PMC article

Show details

J Med Virol
. 2021 Jul;93(7):4319-4325.
doi: 10.1002/jmv.26925. Epub 2021 Mar 26.

Authors

Giancarlo Ceccarelli [1 2], Francesco Alessandri [2 3], Alessandra Oliva [1 2], Cristian Borrazzo [1], Serena Dell'Isola [4], Anna Maria Ialungo [4], Elena Rastrelli [4], Massimiliano Pelli [5], Giammarco Raponi [6], Ombretta Turriziani [7], Franco Ruberto [2 3], Monica Rocco [5], Francesco Pugliese [2 3], Alessandro Russo [8 9], Gabriella d'Ettorre [1 2], Mario Venditti [1 2]

Affiliations

- [1] Department of Public Health and Infectious Diseases, Sapienza University of Rome, Rome, Italy.
- [2] Covid Division, Azienda Ospedaliero-Universitaria Policlinico Umberto I, Rome, Italy.
- [3] Department of Anesthesiology and Intensive Care, Sapienza University of Rome, Rome, Italy.
- [4] Protect Medicine Division, Belcolle Hospital, Viterbo, Italy.
- [5] Intensive Care Unit, Department of medical and Surgical Science and Traslational Medicine, Sant'Andrea Hospital, Sapienza University of Rome, Rome, Italy.
- [6] Microbiology Unit, Department of Public Health and Infectious Diseases, Sapienza University of Rome, Rome, Italy.
- [7] Virology Unit, Department of Molecular Medicine, Sapienza University of Rome, Rome, Italy.
- [8] Department of Clinical and Experimental Medicine, University of Pisa, Pisa, Italy.
- [9] COVID Unit - Medicine Division, Casilino Hospital, Rome, Italy.

- PMID: **33675235**
- PMCID: PMC8250836
- DOI: 10.1002/jmv.26925

Abstract

Teicoplanin has a potential antiviral activity expressed against severe acute respiratory syndrome coronavirus 2 (SARS-CoV-2) and was suggested as a complementary option to treat coronavirus disease 2019 (COVID-19) patients. In this multicentric, retrospective, observational research the aim was to evaluate the impact of teicoplanin on the course of COVID-19 in critically ill patients. Fifty-five patients with severe COVID-19, hospitalized in the intensive care units (ICUs) and treated with best available therapy were retrospectively analysed. Among them 34 patients were also treated with teicoplanin (Tei-COVID group), while 21 without teicoplanin (control group). Crude in-hospital Day-30 mortality was lower in Tei-COVID group (35.2%) than in control group (42.8%), however not reaching statistical significance ($p = .654$). No statistically significant differences in length of stay in the ICU were observed between Tei-COVID group and control group ($p = .248$). On Day 14 from the ICU hospitalization, viral clearance was achieved in 64.7% patients of Tei-COVID group and 57.1% of control group, without statistical difference. Serum C-reactive protein level was significantly reduced in Tei-COVID group compared to control group, but not other biochemical parameters. Finally, Gram-positive were the causative pathogens for 25% of BSIs in Tei-COVID group and for 70.6% in controls. No side effects related to teicoplanin use were observed. Despite several limitations require further research, in this study the use of teicoplanin is not associated with a significant improvement in outcomes analysed. The antiviral activity of teicoplanin against SARS-CoV-2, previously documented, is probably more effective at early clinical stages.

Keywords: COVID-19; ICU; SARS-CoV-2; Teicoplanin; glycopeptide; intensive care unit; pneumonia.

© 2021 Wiley Periodicals LLC.

Conflict of interest statement

The authors declare that there are no conflict of interests.

- 26 references
- 2 figures

Supplementary info

Publication types, MeSH terms, Substances Expand

Publication types

- Multicenter Study
- Observational Study

MeSH terms

- Aged
- Antiviral Agents / therapeutic use*
- C-Reactive Protein / analysis
- COVID-19 / drug therapy*
- Critical Care / statistics & numerical data
- Critical Illness / therapy
- Female
- Hospital Mortality*
- Humans
- Intensive Care Units
- Length of Stay / statistics & numerical data
- Male
- Middle Aged
- Retrospective Studies
- SARS-CoV-2 / drug effects*
- Teicoplanin / therapeutic use*

Substances

- Antiviral Agents
- Teicoplanin
- C-Reactive Protein

Full text links

Wiley Full Text Article Wiley Free PMC article

Proceed to details

Cite

Share

☐ 59

Observational Study

J Gerontol A Biol Sci Med Sci

. 2021 Feb 25;76(3):e28-e37.

doi: 10.1093/gerona/glaa243.

Clinical Characteristics and Risk Factors for Mortality in Very Old Patients Hospitalized With COVID-19 in Spain

Jose-Manuel Ramos-Rincon [1], Verónica Buonaiuto [2], Michele Ricci [2], Jesica Martín-Carmona [2], Diana Paredes-Ruíz [3], María Calderón-Moreno [4], Manel Rubio-Rivas [5], José-Luis Beato-Pérez [6], Francisco Arnalich-Fernández [7], Daniel Monge-Monge [8], Juan-Antonio Vargas-Núñez [9], Gonzalo Acebes-Repiso [10], Manuel Mendez-Bailon [11], Isabel Perales-Fraile [12], Gema-María García-García [13], Pablo Guisado-Vasco [14], Alaaeldeen Abdelhady-Kishta [15], Maria-de-Los-Reyes Pascual-Pérez [16], Cristina Rodríguez-Fernández-Viagas [17], Adrián Montaño-Martínez [18], Antonio López-Ruiz [19], Maria-Jesus Gonzalez-Juarez [20], Cristina Pérez-García [21], José-Manuel Casas-Rojo [22], Ricardo Gómez-Huelgas [2], SEMI-COVID-19 Network

Affiliations Expand

Affiliations

- [1] Department of Clinical Medicine, Miguel Hernandez University of Elche, Alicante, Spain.
- [2] Internal Medicine Department, Málaga Regional University Hospital, Spain.
- [3] Internal Medicine Department, 12 de Octubre University Hospital, Madrid, Spain.
- [4] Internal Medicine Department, Gregorio Marañon University Hospital, Madrid, Spain.
- [5] Internal Medicine Department, Bellvitge University Hospital, L'Hospitalet de Llobregat (Barcelona), Spain.
- [6] Internal Medicine Department, Albacete University Hospital Complex, Spain.
- [7] Internal Medicine Department, La Paz University Hospital, Madrid, Spain.
- [8] Internal Medicine Department, Segovia Hospital Complex, Spain.
- [9] Internal Medicine Department, Puerta de Hierro University Hospital, Majadahonda, Madrid, Spain.
- [10] Internal Medicine Department, Miguel Servet, Zaragoza Hospital, Spain.
- [11] Internal Medicine Department, Clinico San Carlos Hospital, Madrid, Spain.
- [12] Internal Medicine Department, Infanta Sofia Hospital, S. S. de los Reyes, Madrid, Spain.
- [13] Internal Medicine Department, Badajoz University Hospital Complex, Spain.

- [14] Internal Medicine Department, Quironsalud Madrid University Hospital, Pozuelo de Alarcón, Spain.
- [15] Internal Medicine Department, Nuestra Señora de Sonsoles Hospital, Ávila, Spain.
- [16] Internal Medicine Department, Elda University General Hospital, Alicante, Spain.
- [17] Internal Medicine Department, Puerta del Mar University Hospital, Cádiz, Spain.
- [18] Internal Medicine Department, Montilla Hospital, Córdoba, Spain.
- [19] Internal Medicine Department, Axarquía Hospital, Vélez-Málaga, Málaga, Spain.
- [20] Internal Medicine Department, Virgen del Mar Hospital, Madrid, Spain.
- [21] Internal Medicine Department, Do Salnes Hospital, Vilagarcía de Arousa (Pontevedra), Spain.
- [22] Internal Medicine Department, Infanta Cristina University Hospital, Parla, Madrid, Spain.

- PMID: **33103720**
- PMCID: PMC7797762
- DOI: 10.1093/gerona/glaa243

Free PMC article
Observational Study

Clinical Characteristics and Risk Factors for Mortality in Very Old Patients Hospitalized With COVID-19 in Spain

Jose-Manuel Ramos-Rincon et al. J Gerontol A Biol Sci Med Sci. 2021.
Free PMC article
Show details
J Gerontol A Biol Sci Med Sci
. 2021 Feb 25;76(3):e28-e37.
doi: 10.1093/gerona/glaa243.

Authors

Jose-Manuel Ramos-Rincon [1], Verónica Buonaiuto [2], Michele Ricci [2], Jesica Martín-Carmona [2], Diana Paredes-Ruíz [3], María Calderón-Moreno [4], Manel Rubio-Rivas [5], José-Luis Beato-Pérez [6], Francisco Arnalich-Fernández [7], Daniel Monge-Monge [8], Juan-Antonio Vargas-Núñez [9], Gonzalo Acebes-Repiso [10], Manuel Mendez-Bailon [11], Isabel Perales-Fraile [12], Gema-María García-García [13], Pablo Guisado-Vasco [14], Alaaeldeen Abdelhady-Kishta [15], Maria-de-Los-Reyes Pascual-Pérez [16], Cristina Rodríguez-Fernández-Viagas [17], Adrián Montaño-Martínez [18], Antonio López-Ruiz [19], Maria-Jesus Gonzalez-Juarez [20], Cristina Pérez-García [21], José-Manuel Casas-Rojo [22], Ricardo Gómez-Huelgas [2], SEMI-COVID-19 Network

Affiliations

- [1] Department of Clinical Medicine, Miguel Hernandez University of Elche, Alicante, Spain.
- [2] Internal Medicine Department, Málaga Regional University Hospital, Spain.
- [3] Internal Medicine Department, 12 de Octubre University Hospital, Madrid, Spain.

- [4] Internal Medicine Department, Gregorio Marañon University Hospital, Madrid, Spain.
- [5] Internal Medicine Department, Bellvitge University Hospital, L'Hospitalet de Llobregat (Barcelona), Spain.
- [6] Internal Medicine Department, Albacete University Hospital Complex, Spain.
- [7] Internal Medicine Department, La Paz University Hospital, Madrid, Spain.
- [8] Internal Medicine Department, Segovia Hospital Complex, Spain.
- [9] Internal Medicine Department, Puerta de Hierro University Hospital, Majadahonda, Madrid, Spain.
- [10] Internal Medicine Department, Miguel Servet, Zaragoza Hospital, Spain.
- [11] Internal Medicine Department, Clinico San Carlos Hospital, Madrid, Spain.
- [12] Internal Medicine Department, Infanta Sofía Hospital, S. S. de los Reyes, Madrid, Spain.
- [13] Internal Medicine Department, Badajoz University Hospital Complex, Spain.
- [14] Internal Medicine Department, Quironsalud Madrid University Hospital, Pozuelo de Alarcón, Spain.
- [15] Internal Medicine Department, Nuestra Señora de Sonsoles Hospital, Ávila, Spain.
- [16] Internal Medicine Department, Elda University General Hospital, Alicante, Spain.
- [17] Internal Medicine Department, Puerta del Mar University Hospital, Cádiz, Spain.
- [18] Internal Medicine Department, Montilla Hospital, Córdoba, Spain.
- [19] Internal Medicine Department, Axarquía Hospital, Vélez-Málaga, Málaga, Spain.
- [20] Internal Medicine Department, Virgen del Mar Hospital, Madrid, Spain.
- [21] Internal Medicine Department, Do Salnes Hospital, Vilagarcía de Arousa (Pontevedra), Spain.
- [22] Internal Medicine Department, Infanta Cristina University Hospital, Parla, Madrid, Spain.

- PMID: **33103720**
- PMCID: PMC7797762
- DOI: 10.1093/gerona/glaa243

Abstract

Background: Advanced age is a well-known risk factor for poor prognosis in COVID-19. However, few studies have specifically focused on very old inpatients with COVID-19. This study aims to describe the clinical characteristics of very old inpatients with COVID-19 and identify risk factors for in-hospital mortality at admission.

Methods: We conducted a nationwide, multicenter, retrospective, observational study in patients ≥ 80 years hospitalized with COVID-19 in 150 Spanish hospitals (SEMI-COVID-19) Registry (March 1-May 29, 2020). The primary outcome was in-hospital mortality. A uni- and multivariate logistic regression was performed to assess predictors of mortality at admission.

Results: A total of 2772 consecutive patients (49.4% men, median age 86.3 years) were analyzed. Rates of atherosclerotic cardiovascular disease, diabetes mellitus, dementia, and Barthel Index < 60 were 30.8%, 25.6%, 30.5%, and 21.0%, respectively. The overall case-fatality rate was 46.9% (n: 1301) and increased with age (80-84 years: 41.6%; 85-90 years: 47.3%; 90-94 years: 52.7%; ≥95 years: 54.2%). After analysis, male sex and moderate-to-severe dependence were independently associated with in-hospital mortality; comorbidities were not predictive. At admission, independent risk factors for death were: oxygen saturation < 90%; temperature ≥ 37.8° C; quick sequential organ failure assessment (qSOFA) score ≥ 2; and unilateral-bilateral infiltrates on chest x-rays. Some analytical findings were independent risk factors for death, including estimated glomerular filtration rate < 45 mL/min/1.73 m2; lactate dehydrogenase ≥ 500 U/L;

C-reactive protein ≥ 80 mg/L; neutrophils ≥ 7.5 × 103/μL; lymphocytes < 0.8 × 103/μL; and monocytes < 0.5 × 103/μL.

Conclusions: This first large, multicenter cohort of very old inpatients with COVID-19 shows that age, male sex, and poor preadmission functional status-not comorbidities-are independently associated with in-hospital mortality. Severe COVID-19 at admission is related to poor prognosis.

Keywords: Age ≥ 80; COVID-19; Mortality; Prognostic factors; SARS-CoV-2.

© The Author(s) 2020. Published by Oxford University Press on behalf of The Gerontological Society of America. All rights reserved. For permissions, please e-mail: journals.permissions@oup.com.

- 46 references
- 1 figure

Supplementary info

Publication types, MeSH terms Expand

Publication types

- Multicenter Study
- Observational Study

MeSH terms

- Aged, 80 and over
- COVID-19 / epidemiology
- COVID-19 / mortality*
- COVID-19 / therapy*
- Female
- Hospital Mortality*
- Hospitalization
- Humans
- Male
- Pneumonia, Viral / epidemiology
- Pneumonia, Viral / mortality*
- Pneumonia, Viral / therapy*
- Prognosis
- Retrospective Studies
- Risk Factors
- SARS-CoV-2
- Spain / epidemiology

Full text links

OXFORD ACADEMIC Silverchair Information Systems Free PMC article

Proceed to details

Cite

Share

60

Observational Study

BMC Med

. 2021 May 20;19(1):129.

doi: 10.1186/s12916-021-02003-7.

Sequelae, persistent symptomatology and outcomes after COVID-19 hospitalization: the ANCOHVID multicentre 6-month follow-up study

Álvaro Romero-Duarte [1], Mario Rivera-Izquierdo [2] [3] [4], Inmaculada Guerrero-Fernández de Alba [5] [6], Marina Pérez-Contreras [7], Nicolás Francisco Fernández-Martínez [8] [9], Rafael Ruiz-Montero [8] [9], Álvaro Serrano-Ortiz [8] [9], Rocío Ortiz González-Serna [8] [9], Inmaculada Salcedo-Leal [8] [9], Eladio Jiménez-Mejías [10] [11], Antonio Cárdenas-Cruz [1] [12]

Affiliations Expand

Affiliations

- [1] School of Medicine, University of Granada, Granada, Spain.
- [2] Service of Preventive Medicine and Public Health, Hospital Universitario Clínico San Cecilio, Granada, Spain. mariorivera@ugr.es.
- [3] Department of Preventive Medicine and Public Health, University of Granada, Avda. de la Investigación n°11, 18016, Granada, Spain. mariorivera@ugr.es.
- [4] Instituto de Investigación Biosanitaria, ibs.GRANADA, Granada, Spain. mariorivera@ugr.es.
- [5] Service of Preventive Medicine and Public Health, Hospital Universitario Clínico San Cecilio, Granada, Spain.
- [6] Service of Preventive Medicine and Public Health, Complejo Hospitalario de Jaén, Jaén, Spain.
- [7] Service of Preventive Medicine and Public Health, Hospital Universitario de Puerto Real, Puerto Real, Cádiz, Spain.
- [8] Unidad de Gestión Clínica Interniveles de Prevención, Promoción y Vigilancia de la Salud, Hospital Universitario Reina Sofía, Córdoba, Spain.
- [9] Instituto Maimónides de Investigación Biomédica de Córdoba (Imibic), Córdoba, Spain.
- [10] Department of Preventive Medicine and Public Health, University of Granada, Avda. de la Investigación n°11, 18016, Granada, Spain.

- [11] Chair of Teaching and Research in Family Medicine, SEMERGEN-UGR, University of Granada, Granada, Spain.
- [12] Intensive Care Unit, Hospital de Poniente, El Ejido, Almería, Spain.

- PMID: **34011359**
- PMCID: PMC8134820
- DOI: 10.1186/s12916-021-02003-7

Free PMC article
Observational Study

Sequelae, persistent symptomatology and outcomes after COVID-19 hospitalization: the ANCOHVID multicentre 6-month follow-up study

Álvaro Romero-Duarte et al. BMC Med. 2021.
Free PMC article
Show details
BMC Med
. 2021 May 20;19(1):129.
doi: 10.1186/s12916-021-02003-7.

Authors

Álvaro Romero-Duarte [1], Mario Rivera-Izquierdo [2, 3, 4], Inmaculada Guerrero-Fernández de Alba [5, 6], Marina Pérez-Contreras [7], Nicolás Francisco Fernández-Martínez [8, 9], Rafael Ruiz-Montero [8, 9], Álvaro Serrano-Ortiz [8, 9], Rocío Ortiz González-Serna [8, 9], Inmaculada Salcedo-Leal [8, 9], Eladio Jiménez-Mejías [10, 11], Antonio Cárdenas-Cruz [1, 12]

Affiliations

- [1] School of Medicine, University of Granada, Granada, Spain.
- [2] Service of Preventive Medicine and Public Health, Hospital Universitario Clínico San Cecilio, Granada, Spain. mariorivera@ugr.es.
- [3] Department of Preventive Medicine and Public Health, University of Granada, Avda. de la Investigación n°11, 18016, Granada, Spain. mariorivera@ugr.es.
- [4] Instituto de Investigación Biosanitaria, ibs.GRANADA, Granada, Spain. mariorivera@ugr.es.
- [5] Service of Preventive Medicine and Public Health, Hospital Universitario Clínico San Cecilio, Granada, Spain.
- [6] Service of Preventive Medicine and Public Health, Complejo Hospitalario de Jaén, Jaén, Spain.
- [7] Service of Preventive Medicine and Public Health, Hospital Universitario de Puerto Real, Puerto Real, Cádiz, Spain.

- [8] Unidad de Gestión Clínica Interniveles de Prevención, Promoción y Vigilancia de la Salud, Hospital Universitario Reina Sofía, Córdoba, Spain.
- [9] Instituto Maimónides de Investigación Biomédica de Córdoba (Imibic), Córdoba, Spain.
- [10] Department of Preventive Medicine and Public Health, University of Granada, Avda. de la Investigación n°11, 18016, Granada, Spain.
- [11] Chair of Teaching and Research in Family Medicine, SEMERGEN-UGR, University of Granada, Granada, Spain.
- [12] Intensive Care Unit, Hospital de Poniente, El Ejido, Almería, Spain.

- PMID: **34011359**
- PMCID: PMC8134820
- DOI: 10.1186/s12916-021-02003-7

Abstract

Background: Long-term effects of COVID-19, also called Long COVID, affect more than 10% of patients. The most severe cases (i.e. those requiring hospitalization) present a higher frequency of sequelae, but detailed information on these effects is still lacking. The objective of this study is to identify and quantify the frequency and outcomes associated with the presence of sequelae or persistent symptomatology (SPS) during the 6 months after discharge for COVID-19.

Methods: Retrospective observational 6-month follow-up study conducted in four hospitals of Spain. A cohort of all 969 patients who were hospitalized with PCR-confirmed SARS-CoV-2 from March 1 to April 15, 2020, was included. We collected all the SPS during the 6 months after discharge reported by patients during follow-up from primary care records. Cluster analyses were performed to validate the measures. The main outcome measures were return to the Emergency Services, hospital readmission and post-discharge death. Surviving patients' outcomes were collected through clinical histories and primary care reports. Multiple logistic regression models were applied.

Results: The 797 (82.2%) patients who survived constituted the sample followed, while the rest died from COVID-19. The mean age was 63.0 years, 53.7% of them were men and 509 (63.9%) reported some sequelae during the first 6 months after discharge. These sequelae were very diverse, but the most frequent were respiratory (42.0%), systemic (36.1%), neurological (20.8%), mental health (12.2%) and infectious (7.9%) SPS, with some differences by sex. Women presented higher frequencies of headache and mental health SPS, among others. A total of 160 (20.1%) patients returned to the Emergency Services, 35 (4.4%) required hospital readmission and 8 (1.0%) died during follow-up. The main factors independently associated with the return to Emergency Services were persistent fever, dermatological SPS, arrythmia or palpitations, thoracic pain and pneumonia.

Conclusions: COVID-19 cases requiring hospitalization during the first wave of the pandemic developed a significant range of mid- to long-term SPS. A detailed list of symptoms and outcomes is provided in this multicentre study. Identification of possible factors associated with these SPS could be useful to optimize preventive follow-up strategies in primary care for the coming months of the pandemic.

Keywords: COVID-19; Follow-up; Long COVID; Persistent symptoms; Post-discharge; Primary care; Sequelae.

Conflict of interest statement

The authors declare that they have no competing interests.

- 57 references
- 2 figures

Supplementary info

Publication types, MeSH terms Expand

Publication types

- Multicenter Study
- Observational Study
- Research Support, Non-U.S. Gov't

MeSH terms

- Adult
- Aftercare
- Aged
- Aged, 80 and over
- COVID-19 / complications*
- Cohort Studies
- Disease Progression
- Female
- Follow-Up Studies
- Hospitalization
- Humans
- Longitudinal Studies
- Male
- Middle Aged
- Pandemics
- Patient Discharge
- Patient Readmission
- Retrospective Studies
- SARS-CoV-2
- Spain
- Young Adult

Full text links

Read free full text at BMC BioMed Central Free PMC article
Proceed to details

Cite
Share
☐ 61
Observational Study
RMD Open
. 2021 Jan;7(1):e001439.
doi: 10.1136/rmdopen-2020-001439.

Biological agents for rheumatic diseases in the outbreak of COVID-19: friend or foe?

Cristiana Sieiro Santos [1], Xenia Cásas Férnandez [2], Clara Moriano Morales [3], Elvira Díez Álvarez [3], Carolina Álvarez Castro [3], Alejandra López Robles [3], Trinidad Pérez Sandoval [3]
Affiliations Expand

Affiliations

- [1] Rheumatology, Complejo Asistencial Universitario de León, Leon, Castilla y León, Spain cristysieirosantos@gmail.com.
- [2] Pharmacy, Complejo Asistencial Universitario de León, Leon, Spain.
- [3] Rheumatology, Complejo Asistencial Universitario de León, Leon, Castilla y León, Spain.

- PMID: **33455920**
- PMCID: PMC7813407
- DOI: 10.1136/rmdopen-2020-001439

Free PMC article
Observational Study

Biological agents for rheumatic diseases in the outbreak of COVID-19: friend or foe?

Cristiana Sieiro Santos et al. RMD Open. 2021 Jan.
Free PMC article
Show details
RMD Open
. 2021 Jan;7(1):e001439.
doi: 10.1136/rmdopen-2020-001439.

Authors

Cristiana Sieiro Santos [1], Xenia Cásas Férnandez [2], Clara Moriano Morales [3], Elvira Díez Álvarez [3], Carolina Álvarez Castro [3], Alejandra López Robles [3], Trinidad Pérez Sandoval [3]

Affiliations

- [1] Rheumatology, Complejo Asistencial Universitario de León, Leon, Castilla y León, Spain cristysieirosantos@gmail.com.
- [2] Pharmacy, Complejo Asistencial Universitario de León, Leon, Spain.
- [3] Rheumatology, Complejo Asistencial Universitario de León, Leon, Castilla y León, Spain.

- PMID: **33455920**
- PMCID: PMC7813407
- DOI: 10.1136/rmdopen-2020-001439

Abstract

Background: The recent outbreak of COVID-19 has raised concerns in the rheumatology community about the management of immunosuppressed patients diagnosed with inflammatory rheumatic diseases. It is not clear whether the use of biological agents may suppose a risk or protection against SARS-CoV-2 infection; however, it has been suggested that severe respiratory forms of COVID-19 occur as a result of exacerbated inflammation status and cytokine production. This prompted the use of interleukin 6 (IL-6) (tocilizumab and sarilumab) and IL-1 inhibitors (anakinra) in severe COVID-19 disease and more recently JAK1/2 inhibitor (baricitinib). Therefore, patients with rheumatic diseases provide a great opportunity to learn about the use of biological agents as protective drugs against SARS-CoV-2.

Objectives: To estimate COVID-19 infection rate in patients treated with biological disease-modifying antirheumatic drugs (bDMARDs) for inflammatory rheumatic diseases (RMD), determine the influence of biological agents treatment as risk or protective factors and study the prognosis of patients with rheumatic diseases receiving biological agents compared to the general population in a third-level hospital setting in León, Spain.

Methods: We performed a retrospective observational study including patients seen at our rheumatology department who received bDMARDs for rheumatic diseases between December 1st 2019 and December 1st 2020, and analysed COVID-19 infection rate. All patients who attended our rheumatology outpatient clinic with diagnosis of inflammatory rheumatic disease receiving treatment with biological agents were included. Main variable was the hospital admission related to COVID-19. The covariates were age, sex, comorbidities, biological agent, duration of treatment, mean dose of glucocorticoids and need for intensive care unit . We performed an univariate and multivariate logistic regression models to assess risk factors of COVID-19 infection.

Results: There were a total of 4464 patients with COVID-19 requiring hospitalisation. 40 patients out of a total of 820 patients with rheumatic diseases (4.8%) receiving bDMARDs contracted COVID-19 and 4 required hospital care. Crude incidence rate of COVID-19 requiring hospital care among the general population was 3.6%, and it was 0.89% among the group with underlying rheumatic diseases. 90% of patients receiving bDMARDS with COVID-19 did not require hospitalisation. Out of the 4464 patients, 869 patients died, 2 of which received treatment with biological agents. Patients with rheumatic diseases who tested positive for COVID-19 were older (female: median age 60.8 IQR 46-74; male: median age 61.9 IQR 52-70.3) than those who were negative for COVID-19 (female: median age 58.3 IQR 48-69; male: median age 56.2 IQR 47-66), more likely to have hypertension (45% vs 26%, OR 2.25 (CI 1.18-4.27),p 0.02), cardiovascular disease (23 % vs 9.6%, OR 2.73 (1.25-5.95), p 0.02), be smokers (13% vs 4.6%, OR 2.95 (CI 1.09-7.98), p 0.04), receiving treatment with rituximab (20% vs 8%, 2.28 (CI 1.24-6.32), p 0.02) and a higher dose of glucocorticoids (OR 2.5 (1.3-10.33, p 0.02) and were less likely to be

receiving treatment with IL-6 inhibitors (2.5% vs 14%, OR 0.16, (CI 0.10-0.97, p 0.03). When exploring the effect of the rest of the therapies between groups (affected patients vs unaffected), we found no significant differences in bDMARD proportions. IL-1 inhibitors, IL-6 inhibitors, JAK inhibitors and belimumab-treated patients showed the lowest incidence of COVID-19 among adult patients with rheumatic diseases. We found no differences in sex or rheumatological disease between patients who tested positive for COVID-19 and patients who tested negative.

Conclusions: Overall, the use of biological disease-modifying antirheumatic drugs (bDMARDs) does not associate with severe manifestations of COVID-19. Patients with rheumatic disease diagnosed with COVID-19 were more likely to be receiving a higher dose of glucocorticoids and treatment with rituximab. IL-6 inhibitors may have a protective effect.

Keywords: autoimmunity; biological therapy; epidemiology.

© Author(s) (or their employer(s)) 2021. Re-use permitted under CC BY-NC. No commercial re-use. See rights and permissions. Published by BMJ.

Conflict of interest statement

Competing interests: None declared.

- 16 references

Supplementary info

Publication types, MeSH terms, Substances, Supplementary concepts Expand

Publication types

- Observational Study

MeSH terms

- Aged
- Antibodies, Monoclonal, Humanized / pharmacology
- Antibodies, Monoclonal, Humanized / therapeutic use*
- Antirheumatic Agents / therapeutic use*
- Biological Factors / therapeutic use*
- COVID-19 / drug therapy*
- COVID-19 / epidemiology
- Disease Outbreaks*
- Female
- Glucocorticoids / therapeutic use*
- Humans
- Interleukin-6 / antagonists & inhibitors
- Male
- Middle Aged

- Protective Agents / therapeutic use*
- Retrospective Studies
- Rheumatic Diseases / drug therapy*
- Risk Factors
- Rituximab / therapeutic use*
- SARS-CoV-2 / genetics
- SARS-CoV-2 / immunology
- SARS-CoV-2 / isolation & purification*
- Spain / epidemiology
- Treatment Outcome

Substances

- Antibodies, Monoclonal, Humanized
- Antirheumatic Agents
- Biological Factors
- Glucocorticoids
- IL6 protein, human
- Interleukin-6
- Protective Agents
- Rituximab
- tocilizumab
- sarilumab

Supplementary concepts

- COVID-19 drug treatment

Full text links

BMJ Free Full Text HighWire Free PMC article
Proceed to details
Cite
Share
☐ 62
Observational Study
Mayo Clin Proc
. 2021 Apr;96(4):875-886.
doi: 10.1016/j.mayocp.2021.01.001. Epub 2021 Jan 9.

Vitamin D Status Is Associated With In-Hospital Mortality and Mechanical

Ventilation: A Cohort of COVID-19 Hospitalized Patients

Angeliki M Angelidi [1], Matthew J Belanger [1], Michael K Lorinsky [1], Dimitrios Karamanis [2], Natalia Chamorro-Pareja [3], Jennifer Ognibene [4], Leonidas Palaiodimos [5], Christos S Mantzoros [6]
Affiliations Expand

Affiliations

- [1] Department of Medicine, Beth Israel Deaconess Medical Center/Harvard Medical School, Boston, MA.
- [2] Department of Economics, University of Piraeus, Greece.
- [3] Albert Einstein College of Medicine, Bronx, NY; Department of Medicine, Jacobi Medical Center, Albert Einstein College of Medicine, Bronx, NY.
- [4] Albert Einstein College of Medicine, Bronx, NY.
- [5] Albert Einstein College of Medicine, Bronx, NY; Department of Medicine, Jacobi Medical Center, Albert Einstein College of Medicine, Bronx, NY; Division of Hospital Medicine, Montefiore Medical Center, Albert Einstein College of Medicine, Bronx, NY.
- [6] Department of Medicine, Beth Israel Deaconess Medical Center/Harvard Medical School, Boston, MA; Section of Endocrinology, VA Boston Healthcare System, Harvard Medical School, Boston, MA. Electronic address: mbelang1@bidmc.harvard.edu.

- PMID: **33714594**
- PMCID: PMC7834253
- DOI: 10.1016/j.mayocp.2021.01.001

Free PMC article
Observational Study

Vitamin D Status Is Associated With In-Hospital Mortality and Mechanical Ventilation: A Cohort of COVID-19 Hospitalized Patients

Angeliki M Angelidi et al. Mayo Clin Proc. 2021 Apr.
Free PMC article
Show details
Mayo Clin Proc
. 2021 Apr;96(4):875-886.
doi: 10.1016/j.mayocp.2021.01.001. Epub 2021 Jan 9.

Authors

Angeliki M Angelidi [1], Matthew J Belanger [1], Michael K Lorinsky [1], Dimitrios Karamanis [2], Natalia Chamorro-Pareja [3], Jennifer Ognibene [4], Leonidas Palaiodimos [5], Christos S Mantzoros [6]

Affiliations

- [1] Department of Medicine, Beth Israel Deaconess Medical Center/Harvard Medical School, Boston, MA.
- [2] Department of Economics, University of Piraeus, Greece.
- [3] Albert Einstein College of Medicine, Bronx, NY; Department of Medicine, Jacobi Medical Center, Albert Einstein College of Medicine, Bronx, NY.
- [4] Albert Einstein College of Medicine, Bronx, NY.
- [5] Albert Einstein College of Medicine, Bronx, NY; Department of Medicine, Jacobi Medical Center, Albert Einstein College of Medicine, Bronx, NY; Division of Hospital Medicine, Montefiore Medical Center, Albert Einstein College of Medicine, Bronx, NY.
- [6] Department of Medicine, Beth Israel Deaconess Medical Center/Harvard Medical School, Boston, MA; Section of Endocrinology, VA Boston Healthcare System, Harvard Medical School, Boston, MA. Electronic address: mbelang1@bidmc.harvard.edu.

- PMID: **33714594**
- PMCID: PMC7834253
- DOI: 10.1016/j.mayocp.2021.01.001

Abstract

Objective: To explore the possible associations of serum 25-hydroxyvitamin D [25(OH)D] concentration with coronavirus disease 2019 (COVID-19) in-hospital mortality and need for invasive mechanical ventilation.

Patients and methods: A retrospective, observational, cohort study was conducted at 2 tertiary academic medical centers in Boston and New York. Eligible participants were hospitalized adult patients with laboratory-confirmed COVID-19 between February 1, 2020, and May 15, 2020. Demographic and clinical characteristics, comorbidities, medications, and disease-related outcomes were extracted from electronic medical records.

Results: The final analysis included 144 patients with confirmed COVID-19 (median age, 66 years; 64 [44.4%] male). Overall mortality was 18%, whereas patients with 25(OH)D levels of 30 ng/mL (to convert to nmol/L, multiply by 2.496) and higher had lower rates of mortality compared with those with 25(OH)D levels below 30 ng/mL (9.2% vs 25.3%; P=.02). In the adjusted multivariable analyses, 25(OH)D as a continuous variable was independently significantly associated with lower in-hospital mortality (odds ratio, 0.94; 95% CI, 0.90 to 0.98; P=.007) and need for invasive mechanical ventilation (odds ratio, 0.96; 95% CI, 0.93 to 0.99; P=.01). Similar data were obtained when 25(OH)D was studied as a continuous variable after logarithm transformation and as a dichotomous (<30 ng/mL vs ≥30 ng/mL) or ordinal variable (quintiles) in the multivariable analyses.

Conclusion: Among patients admitted with laboratory-confirmed COVID-19, 25(OH)D levels were inversely associated with in-hospital mortality and the need for invasive mechanical ventilation. Further observational studies are needed to confirm these findings, and randomized clinical trials must be conducted to assess the role of vitamin D administration in improving the morbidity and mortality of COVID-19.

Copyright © 2021 Mayo Foundation for Medical Education and Research. All rights reserved.

- 40 references

Supplementary info

Publication types, MeSH terms, Substances Expand

Publication types

- Observational Study

MeSH terms

- COVID-19* / immunology
- COVID-19* / mortality
- COVID-19* / physiopathology
- COVID-19* / therapy
- Female
- Hospital Mortality
- Hospitalization / statistics & numerical data
- Humans
- Male
- Middle Aged
- New York / epidemiology
- Outcome and Process Assessment, Health Care
- Respiration, Artificial* / methods
- Respiration, Artificial* / statistics & numerical data
- Retrospective Studies
- SARS-CoV-2 / isolation & purification
- Severity of Illness Index
- Tertiary Care Centers / statistics & numerical data
- Vitamin D / analogs & derivatives*
- Vitamin D / blood
- Vitamin D Deficiency* / diagnosis
- Vitamin D Deficiency* / epidemiology
- Vitamin D Deficiency* / immunology
- Vitamin D Deficiency* / therapy

Substances

- Vitamin D
- 25-hydroxyvitamin D

Full text links

ELSEVIER FULL-TEXT ARTICLE Elsevier Science Free PMC article

Proceed to details

Cite

Share

☐ 63

BMJ Open

. 2021 Nov 19;11(11):e054861.

doi: 10.1136/bmjopen-2021-054861.

Retrospective cohort study to evaluate medication use in patients hospitalised with COVID-19 in Scotland: protocol for a national observational study

Tanja Mueller [1] [2], Steven Kerr [3], Stuart McTaggart [4], Amanj Kurdi [5] [2], Eleftheria Vasileiou [3], Annemarie Docherty [3], Kenny Fraser [6], Ting Shi [3], Colin R Simpson [3] [7], Marion Bennie [4] [8], Aziz Sheikh [3] [9]

Affiliations Expand

Affiliations

- [1] Strathclyde Institute of Pharmacy and Biomedical Sciences, University of Strathclyde, Glasgow, UK tanja.muller@strath.ac.uk.
- [2] Public Health Scotland Glasgow Office, Glasgow, UK.
- [3] Usher Institute, The University of Edinburgh, Edinburgh, UK.
- [4] Public Health Scotland, Edinburgh, UK.
- [5] Strathclyde Institute of Pharmacy and Biomedical Sciences, University of Strathclyde, Glasgow, UK.
- [6] Triscribe Limited, Glasgow, UK.
- [7] School of Health, Wellington Faculty of Health, Victoria University of Wellington, Wellington, New Zealand.
- [8] Institute of Pharmacy and Biomedical Sciences, University of Strathclyde, Glasgow, UK.
- [9] BREATHE Hub, HDR UK, Edinburgh, UK.

- PMID: **34799365**
- PMCID: PMC8609490
- DOI: 10.1136/bmjopen-2021-054861

Free PMC article

Retrospective cohort study to evaluate medication use in patients hospitalised with COVID-19 in Scotland: protocol for a national observational study

Tanja Mueller et al. BMJ Open. 2021.
Free PMC article
Show details
BMJ Open
. 2021 Nov 19;11(11):e054861.
doi: 10.1136/bmjopen-2021-054861.

Authors

Tanja Mueller [1, 2], Steven Kerr [3], Stuart McTaggart [4], Amanj Kurdi [5, 2], Eleftheria Vasileiou [3], Annemarie Docherty [3], Kenny Fraser [6], Ting Shi [3], Colin R Simpson [3, 7], Marion Bennie [4, 8], Aziz Sheikh [3, 9]

Affiliations

- [1] Strathclyde Institute of Pharmacy and Biomedical Sciences, University of Strathclyde, Glasgow, UK tanja.muller@strath.ac.uk.
- [2] Public Health Scotland Glasgow Office, Glasgow, UK.
- [3] Usher Institute, The University of Edinburgh, Edinburgh, UK.
- [4] Public Health Scotland, Edinburgh, UK.
- [5] Strathclyde Institute of Pharmacy and Biomedical Sciences, University of Strathclyde, Glasgow, UK.
- [6] Triscribe Limited, Glasgow, UK.
- [7] School of Health, Wellington Faculty of Health, Victoria University of Wellington, Wellington, New Zealand.
- [8] Institute of Pharmacy and Biomedical Sciences, University of Strathclyde, Glasgow, UK.
- [9] BREATHE Hub, HDR UK, Edinburgh, UK.

- PMID: **34799365**
- PMCID: PMC8609490
- DOI: 10.1136/bmjopen-2021-054861

Abstract

Introduction: COVID-19 has caused millions of hospitalisations and deaths globally. A range of vaccines have been developed and are being deployed at scale in the UK to prevent SARS-CoV-2 infection, which have reduced risk of infection and severe COVID-19 outcomes. Those with COVID-19 are now being treated with several repurposed drugs based on evidence emerging from recent clinical trials. However, there is currently limited real-world data available related to the use of these drugs in routine clinical practice. The purpose of this study is to address the prevailing

knowledge gaps regarding the use of dexamethasone, remdesivir and tocilizumab by conducting an exploratory drug utilisation study, aimed at providing in-depth descriptions of patients receiving these drugs as well as the treatment patterns observed in Scotland.

Methods and analysis: Retrospective cohort study, comprising adult patients admitted to hospital with confirmed or suspected COVID-19 across five Scottish Health Boards using data from in-hospital ePrescribing linked to the Early Estimation of Vaccine and Anti-Viral Effectiveness (EAVE II) COVID-19 surveillance platform. The primary outcome will be exposure to the medicines of interest (dexamethasone, remdesivir, tocilizumab), either alone or in combination; exposure will be described in terms of drug(s) of choice; prescribed and administered dose; treatment duration; and any changes in treatment, for example, dose escalation and/or switching to an alternative drug. Analyses will primarily be descriptive in nature.

Ethics and dissemination: Ethical and information governance approvals have been obtained by the National Research Ethics Service Committee, South East Scotland 02 and the Public Benefit and Privacy Panel for Health and Social Care, respectively. Findings from this study will be presented at academic and clinical conferences, and to the funders and other interested parties as appropriate; study findings will also be published in peer-reviewed journals. Publications will be available on the EAVE II website (https://www.ed.ac.uk/usher/eave-ii/key-outputs/our-publications), alongside lay summaries and infographics aimed at the general public. Press releases will also be considered, if appropriate.

Keywords: COVID-19; clinical pharmacology; therapeutics.

© Author(s) (or their employer(s)) 2021. Re-use permitted under CC BY. Published by BMJ.

Conflict of interest statement

Competing interests: AS reports grants from NIHR, grants from MRC, grants from HDR UK, during the conduct of the study. CRS reports funding from NIHR (UK), MRC (UK), CSO (UK), Health Research Council (NZ) and Ministry for Business, Innovation and Employment (NZ) during the conduct of this study. KF is Director of Triscribe, a company providing data quality services and software support. All other authors report no conflicts of interest.

- 41 references

Supplementary info

Publication types, MeSH terms, Substances, Grant support Expand

Publication types

- Research Support, Non-U.S. Gov't

MeSH terms

- Adult
- Antiviral Agents
- COVID-19*
- Humans

- Observational Studies as Topic
- Retrospective Studies
- SARS-CoV-2
- Scotland

Substances

- Antiviral Agents

Grant support

- MC_PC_19075/MRC_/Medical Research Council/United Kingdom
- ERC_/European Research Council/International

Full text links

BMJ Free Full Text HighWire Free PMC article
Proceed to details
Cite
Share
☐ 64
Observational Study
Int J Infect Dis
. 2021 Feb;103:647-653.
doi: 10.1016/j.ijid.2020.09.1475. Epub 2020 Oct 2.

Clinical characteristics of 30 COVID-19 patients with epilepsy: A retrospective study in Wuhan

Minxian Sun [1], Xiaoyun Ruan [2], Yuanyuan Li [2], Pei Wang [1], Shasha Zheng [1], Guiying Shui [1], Li Li [1], Yan Huang [3], Hongmei Zhang [4]
Affiliations Expand

Affiliations

- [1] Department of Endocrinology, The Central Hospital of Wuhan, Tongji Medical College, Huazhong University of Science & Technology, Wuhan, Hubei, 430021, China.
- [2] Department of Pharmacy, The Central Hospital of Wuhan, Tongji Medical College, Huazhong University of Science & Technology, Wuhan, Hubei, 430021, China.
- [3] Department of Bidding, The Central Hospital of Wuhan, Tongji Medical College, Huazhong University of Science & Technology, Wuhan, Hubei, 430021, China. Electronic address: 1700164748@qq.com.

- [4] Department of Endocrinology, The Central Hospital of Wuhan, Tongji Medical College, Huazhong University of Science & Technology, Wuhan, Hubei, 430021, China. Electronic address: zhm7001@163.com.

- PMID: **33017697**
- PMCID: PMC7531277
- DOI: 10.1016/j.ijid.2020.09.1475

Free PMC article
Observational Study

Clinical characteristics of 30 COVID-19 patients with epilepsy: A retrospective study in Wuhan

Minxian Sun et al. Int J Infect Dis. 2021 Feb.
Free PMC article
Show details
Int J Infect Dis
. 2021 Feb;103:647-653.
doi: 10.1016/j.ijid.2020.09.1475. Epub 2020 Oct 2.

Authors

Minxian Sun [1], Xiaoyun Ruan [2], Yuanyuan Li [2], Pei Wang [1], Shasha Zheng [1], Guiying Shui [1], Li Li [1], Yan Huang [3], Hongmei Zhang [4]

Affiliations

- [1] Department of Endocrinology, The Central Hospital of Wuhan, Tongji Medical College, Huazhong University of Science & Technology, Wuhan, Hubei, 430021, China.
- [2] Department of Pharmacy, The Central Hospital of Wuhan, Tongji Medical College, Huazhong University of Science & Technology, Wuhan, Hubei, 430021, China.
- [3] Department of Bidding, The Central Hospital of Wuhan, Tongji Medical College, Huazhong University of Science & Technology, Wuhan, Hubei, 430021, China. Electronic address: 1700164748@qq.com.
- [4] Department of Endocrinology, The Central Hospital of Wuhan, Tongji Medical College, Huazhong University of Science & Technology, Wuhan, Hubei, 430021, China. Electronic address: zhm7001@163.com.

- PMID: **33017697**
- PMCID: PMC7531277
- DOI: 10.1016/j.ijid.2020.09.1475

Abstract

Objective: This study aims to present the clinical characteristics of 30 hospitalized cases with epileptic seizures and coronavirus disease 2019(COVID-19).

Methods: This is a retrospective observational research study. Clinical data were extracted from electronic medical records in 1550 patients with a laboratory-confirmed diagnosis of COVID-19, who were hospitalized in Wuhan Central Hospital, China, from 1 January to 31 April 2020. 30 COVID-19 patients with the diagnosis of epilepsy were enrolled. The clinical characteristics, complications, treatments, and clinical outcomes of 30 cases were collected and analyzed.

Result: Of 30 patients with a diagnosis of epilepsy and COVID-19, 13 patients (43.4%) had new-onset epileptic seizures without an epilepsy history(new-onset seizure group, NS group), ten patients(33.3%) had an epilepsy history with a recurrent epileptic seizure (recurrent seizure group, RS group) and seven patients(23.3%) had an epilepsy history but no seizure during the course of COVID-19 (epilepsy history group, EH group). Patients in the RS group had a larger number of other-neurological-disease histories than those in the NS and EH groups (7/10[70%] VS 1/13 [7.7%] VS 1/7[14.3%]); the difference between the RS group and NS group is significant ($P < 0.05$). Patients in the NE and RS groups suffered more severe/critical COVID-19 infection than patients in the EH group (10/13[76.9%] VS 6/10[60%] VS 1/7[14.3%]); the difference between the NS group and EH group is significant ($P < 0.05$). 36.7% of patients had one to five neurological complications, and 46.4% of patients had 6-10 neurological complications. The complications in patients with seizures (in the RS and NS groups) seem to be more than those without seizures (in the EH group), but it did not reach statistical significance. The proportion of antiepileptic drugs (AEDs) treatment before admission was higher in the EH group than in the RE group(7/7 [100%] VS 2/10 [20%], $P < 0.05$). The mortality of 30 patients with epilepsy and COVID-19 was 36.67%. The mortality of the NS group(38.5%) and the RS group(50%) were a little higher than in the EH group(14.3%). None of the convalescent patients had a recurrent seizure, and there were no more deaths in the 3-month follow-up after discharge.

Conclusions: COVID-19 patients with recurrent epileptic seizures had more underlying neurological diseases than patients who had an epilepsy history but without a seizure. Patients with new-onset and recurrent epileptic seizures suffered more severe/critical COVID-19, which may lead to a worse prognosis. If patients with epilepsy history continue using AEDs during COVID-19 pandemics, the risk of recurrent seizure may be reduced, and a good prognosis for patients with epilepsy history could be expected.

Keywords: COVID-19; Clinical outcomes; Complication; Epilepsy; New-Onset; Recurrent; Seizure.

Copyright © 2020 The Author(s). Published by Elsevier Ltd.. All rights reserved.

- 26 references

Supplementary info

Publication types, MeSH terms, Substances Expand

Publication types

- Observational Study

MeSH terms

- Adult
- Aged
- Anticonvulsants / therapeutic use
- COVID-19 / complications*
- Epilepsy / drug therapy*
- Female
- Humans
- Male
- Middle Aged
- Retrospective Studies
- SARS-CoV-2*

Substances

- Anticonvulsants

Full text links

ELSEVIER OPEN ACCESS Elsevier Science Free PMC article
Proceed to details
Cite
Share
65
Observational Study
BMC Infect Dis
. 2021 Jan 14;21(1):72.
doi: 10.1186/s12879-021-05773-w.

Hydroxychloroquine in the treatment of outpatients with mildly symptomatic COVID-19: a multi-center observational study

Andrew Ip [1] [2] [3], Jaeil Ahn [4], Yizhao Zhou [4], Andre H Goy [5], Eric Hansen [6], Andrew L Pecora [5], Brittany A Sinclaire [5], Urszula Bednarz [5], Michael Marafelias [5], Ihor S Sawczuk [7] [8], Joseph P Underwood 3rd [9], David M Walker [9], Rajiv Prasad [10], Robert L Sweeney [11], Marie G Ponce [11], Samuel La Capra [12], Frank J Cunningham [12], Arthur G Calise [13], Bradley L Pulver [14], Dominic Ruocco [15], Greggory E Mojares [16], Michael P Eagan [17], Kristy L Ziontz [18], Paul Mastrokyriakos [19], Stuart L Goldberg [20]
Affiliations Expand

Affiliations

- [1] Division of Outcomes and Value Research, John Theurer Cancer Center at Hackensack University Medical Center, 92 Second Street, Hackensack, NJ, 07601, USA. Andrew.Ip@hackensackmeridian.org.
- [2] Hackensack Meridian Health, Hackensack, NJ, USA. Andrew.Ip@hackensackmeridian.org.
- [3] Hackensack Meridian School of Medicine at Seton Hall University, Nutley, NJ, USA. Andrew.Ip@hackensackmeridian.org.
- [4] Department of Biostatistics, Bioinformatics, and Biomathematics, Georgetown University, Washington, D.C., USA.
- [5] John Theurer Cancer Center at Hackensack University Medical Center, Hackensack, NJ, USA.
- [6] COTA, Boston, MA, USA.
- [7] Hackensack Meridian Health, Hackensack, NJ, USA.
- [8] Hackensack Meridian School of Medicine at Seton Hall University, Nutley, NJ, USA.
- [9] Hackensack University Medical Center, Hackensack, NJ, USA.
- [10] Bayshore Medical Center, Holmdel, NJ, USA.
- [11] Jersey Shore University Medical Center, Neptune City, NJ, USA.
- [12] JFK Medical Center, Edison, NJ, USA.
- [13] Hackensack Meridian Mountainside Medical Center, Montclair, NJ, USA.
- [14] Ocean Medical Center, Brick, NJ, USA.
- [15] Palisades Medical Center, North Bergen, NJ, USA.
- [16] Pascack Valley Medical Center, Westwood, NJ, USA.
- [17] Raritan Bay Medical Center, Old Bridge, NJ, USA.
- [18] Riverview Medical Center, Red Bank, NJ, USA.
- [19] Southern Ocean Medical Center, Manahawkin, NJ, USA.
- [20] Division of Outcomes and Value Research, John Theurer Cancer Center at Hackensack University Medical Center, 92 Second Street, Hackensack, NJ, 07601, USA.

- PMID: **33446136**
- PMCID: PMC7807228
- DOI: 10.1186/s12879-021-05773-w

Free PMC article
Observational Study

Hydroxychloroquine in the treatment of outpatients with mildly symptomatic COVID-19: a multi-center observational study

Andrew Ip et al. BMC Infect Dis. 2021.
Free PMC article

Show details

BMC Infect Dis

. 2021 Jan 14;21(1):72.

doi: 10.1186/s12879-021-05773-w.

Authors

Andrew Ip [1] [2] [3], Jaeil Ahn [4], Yizhao Zhou [4], Andre H Goy [5], Eric Hansen [6], Andrew L Pecora [5], Brittany A Sinclaire [5], Urszula Bednarz [5], Michael Marafelias [5], Ihor S Sawczuk [7] [8], Joseph P Underwood 3rd [9], David M Walker [9], Rajiv Prasad [10], Robert L Sweeney [11], Marie G Ponce [11], Samuel La Capra [12], Frank J Cunningham [12], Arthur G Calise [13], Bradley L Pulver [14], Dominic Ruocco [15], Greggory E Mojares [16], Michael P Eagan [17], Kristy L Ziontz [18], Paul Mastrokyriakos [19], Stuart L Goldberg [20]

Affiliations

- [1] Division of Outcomes and Value Research, John Theurer Cancer Center at Hackensack University Medical Center, 92 Second Street, Hackensack, NJ, 07601, USA. Andrew.Ip@hackensackmeridian.org.
- [2] Hackensack Meridian Health, Hackensack, NJ, USA. Andrew.Ip@hackensackmeridian.org.
- [3] Hackensack Meridian School of Medicine at Seton Hall University, Nutley, NJ, USA. Andrew.Ip@hackensackmeridian.org.
- [4] Department of Biostatistics, Bioinformatics, and Biomathematics, Georgetown University, Washington, D.C., USA.
- [5] John Theurer Cancer Center at Hackensack University Medical Center, Hackensack, NJ, USA.
- [6] COTA, Boston, MA, USA.
- [7] Hackensack Meridian Health, Hackensack, NJ, USA.
- [8] Hackensack Meridian School of Medicine at Seton Hall University, Nutley, NJ, USA.
- [9] Hackensack University Medical Center, Hackensack, NJ, USA.
- [10] Bayshore Medical Center, Holmdel, NJ, USA.
- [11] Jersey Shore University Medical Center, Neptune City, NJ, USA.
- [12] JFK Medical Center, Edison, NJ, USA.
- [13] Hackensack Meridian Mountainside Medical Center, Montclair, NJ, USA.
- [14] Ocean Medical Center, Brick, NJ, USA.
- [15] Palisades Medical Center, North Bergen, NJ, USA.
- [16] Pascack Valley Medical Center, Westwood, NJ, USA.
- [17] Raritan Bay Medical Center, Old Bridge, NJ, USA.
- [18] Riverview Medical Center, Red Bank, NJ, USA.
- [19] Southern Ocean Medical Center, Manahawkin, NJ, USA.
- [20] Division of Outcomes and Value Research, John Theurer Cancer Center at Hackensack University Medical Center, 92 Second Street, Hackensack, NJ, 07601, USA.

- PMID: **33446136**
- PMCID: PMC7807228
- DOI: 10.1186/s12879-021-05773-w

Abstract

Background: Hydroxychloroquine has not been associated with improved survival among hospitalized COVID-19 patients in the majority of observational studies and similarly was not identified as an effective prophylaxis following exposure in a prospective randomized trial. We aimed to explore the role of hydroxychloroquine therapy in mildly symptomatic patients diagnosed in the outpatient setting.

Methods: We examined the association between outpatient hydroxychloroquine exposure and the subsequent progression of disease among mildly symptomatic non-hospitalized patients with documented SARS-CoV-2 infection. The primary outcome assessed was requirement of hospitalization. Data was obtained from a retrospective review of electronic health records within a New Jersey USA multi-hospital network. We compared outcomes in patients who received hydroxychloroquine with those who did not applying a multivariable logistic model with propensity matching.

Results: Among 1274 outpatients with documented SARS-CoV-2 infection 7.6% were prescribed hydroxychloroquine. In a 1067 patient propensity matched cohort, 21.6% with outpatient exposure to hydroxychloroquine were hospitalized, and 31.4% without exposure were hospitalized. In the primary multivariable logistic regression analysis with propensity matching there was an association between exposure to hydroxychloroquine and a decreased rate of hospitalization from COVID-19 (OR 0.53; 95% CI, 0.29, 0.95). Sensitivity analyses revealed similar associations. QTc prolongation events occurred in 2% of patients prescribed hydroxychloroquine with no reported arrhythmia events among those with data available.

Conclusions: In this retrospective observational study of SARS-CoV-2 infected non-hospitalized patients hydroxychloroquine exposure was associated with a decreased rate of subsequent hospitalization. Additional exploration of hydroxychloroquine in this mildly symptomatic outpatient population is warranted.

Keywords: COVID-19; Hydroxychloroquine; Outpatient.

Conflict of interest statement

Potential conflicts of interest: AHG reports being a study investigator for Genentech-Hoffman La Roche, during the conduct of the study; research funding as study investigator from Acerta, AstraZeneca, Celgene, Kite Pharma, Elsevier's PracticeUpdate Oncology, Gilead, Medscape, MJH Associates, OncLive Peer Exchange, Physicians Education Resource, and Xcenda, outside the submitted work, and research funding as a study investigator for Constellation, Infinity, Infinity Verastem, Janssen, Karyopharm, and Pharmacyclics, outside of the submitted work.

Potential conflicts of interest: EH report consulting for Regional Cancer Care Associates and Hackensack Meridian Health, outside the submitted work.

Potential conflicts of interest: ALP and SLG report having equity ownership in COTA, outside the submitted work.

No conflicts of interest: AI, JA, YZ, BAS, UB, MM, ISS, JPU, DMW, RP, RLS, MGP, SLC, FJC, AGC, BLP, DR, GEM, MPE, KLZ, and PM.

- 38 references
- 2 figures

Supplementary info

Publication types, MeSH terms, Substances Expand

Publication types

- Multicenter Study
- Observational Study

MeSH terms

- Adult
- Aged
- COVID-19 / drug therapy*
- COVID-19 / virology
- Female
- Hospitalization
- Humans
- Hydroxychloroquine / administration & dosage*
- Logistic Models
- Male
- Middle Aged
- New Jersey
- Outpatients / statistics & numerical data
- Retrospective Studies
- SARS-CoV-2 / drug effects
- SARS-CoV-2 / genetics
- SARS-CoV-2 / physiology
- Severity of Illness Index

Substances

- Hydroxychloroquine

Full text links

Read free full text at BMC BioMed Central Free PMC article
Proceed to details
Cite
Share
66
Observational Study
Age Ageing
. 2021 Feb 26;50(2):307-316.
doi: 10.1093/ageing/afaa167.

Comparing associations between frailty and mortality in hospitalised older adults with or without COVID-19 infection: a retrospective observational study using electronic health records

Rhiannon K Owen [1], Simon P Conroy [2], Nicholas Taub [3], Will Jones [4], Daniele Bryden [5], Manish Pareek [6], Christina Faull [1], Keith R Abrams [1], Daniel Davis [7], Jay Banerjee [8]
Affiliations Expand

Affiliations

- [1] Health Sciences, University of Leicester, Leicester, Leicestershire, UK.
- [2] Department of Health Sciences, Centre for Medicine University of Leicester, University of Leicester School of Medicine, Leicester LE1 7HA, UK.
- [3] Department of Health Sciences, University of Leicester, Leicester LE1 6TP, UK.
- [4] Emergency Department, University Hospitals of Leicester NHS Trust, Leicester, UK.
- [5] Critical Care, Sheffield Teaching Hospitals NHS Foundation Trust, Sheffield, UK.
- [6] Cardiovascular Sciences, University of Leicester, Leicester, UK.
- [7] MRC Unit for Lifelong Health and Ageing, University College London, London WC1B 5JU, UK.
- [8] University Hospitals of Leicester NHS Trust, Leicester, UK.

- PMID: **32678866**
- PMCID: PMC7454252
- DOI: 10.1093/ageing/afaa167

Free PMC article
Observational Study

Comparing associations between frailty and mortality in hospitalised older adults with or without COVID-19 infection: a retrospective observational study using electronic health records

Rhiannon K Owen et al. Age Ageing. 2021.
Free PMC article
Show details
Age Ageing

. 2021 Feb 26;50(2):307-316.
doi: 10.1093/ageing/afaa167.

Authors

Rhiannon K Owen [1], Simon P Conroy [2], Nicholas Taub [3], Will Jones [4], Daniele Bryden [5], Manish Pareek [6], Christina Faull [1], Keith R Abrams [1], Daniel Davis [7], Jay Banerjee [8]

Affiliations

- [1] Health Sciences, University of Leicester, Leicester, Leicestershire, UK.
- [2] Department of Health Sciences, Centre for Medicine University of Leicester, University of Leicester School of Medicine, Leicester LE1 7HA, UK.
- [3] Department of Health Sciences, University of Leicester, Leicester LE1 6TP, UK.
- [4] Emergency Department, University Hospitals of Leicester NHS Trust, Leicester, UK.
- [5] Critical Care, Sheffield Teaching Hospitals NHS Foundation Trust, Sheffield, UK.
- [6] Cardiovascular Sciences, University of Leicester, Leicester, UK.
- [7] MRC Unit for Lifelong Health and Ageing, University College London, London WC1B 5JU, UK.
- [8] University Hospitals of Leicester NHS Trust, Leicester, UK.

- PMID: **32678866**
- PMCID: PMC7454252
- DOI: 10.1093/ageing/afaa167

Abstract

Background: The aim of this study was to describe outcomes in hospitalised older people with different levels of frailty and COVID-19 infection.

Methods: We undertook a single-centre, retrospective cohort study examining COVID-19-related mortality using electronic health records, for older people (65 and over) with frailty, hospitalised with or without COVID-19 infection. Baseline covariates included demographics, early warning scores, Charlson Comorbidity Indices and frailty (Clinical Frailty Scale, CFS), linked to COVID-19 status.

Findings: We analysed outcomes on 1,071 patients with COVID-19 test results (285 (27%) were positive for COVID-19). The mean age at ED arrival was 79.7 and 49.4% were female. All-cause mortality (by 30 days) rose from 9 (not frail) to 33% (severely frail) in the COVID-negative cohort but was around 60% for all frailty categories in the COVID-positive cohort. In adjusted analyses, the hazard ratio for death in those with COVID-19 compared to those without COVID-19 was 7.3 (95% CI: 3.00, 18.0) with age, comorbidities and illness severity making small additional contributions.

Interpretation: In this study, frailty measured using the CFS appeared to make little incremental contribution to the hazard of dying in older people hospitalised with COVID-19 infection; illness severity and comorbidity had a modest association with the overall adjusted hazard of death, whereas confirmed COVID-19 infection dominated, with a sevenfold hazard for death.

Keywords: COVID-19; cohort study; frailty; older people.

© The Author(s) 2020. Published by Oxford University Press on behalf of the British Geriatrics Society. All rights reserved. For permissions, please email: journals.permissions@oup.com.

- 34 references
- 3 figures

Supplementary info

Publication types, MeSH terms, Grant support Expand

Publication types

- Observational Study
- Research Support, Non-U.S. Gov't

MeSH terms

- Aged
- COVID-19* / mortality
- COVID-19* / therapy
- Comorbidity
- Early Warning Score
- Electronic Health Records / statistics & numerical data
- Female
- Frail Elderly / statistics & numerical data*
- Frailty* / diagnosis
- Frailty* / epidemiology
- Geriatric Assessment* / methods
- Geriatric Assessment* / statistics & numerical data
- Hospital Mortality*
- Hospitalization / statistics & numerical data
- Humans
- Male
- Proportional Hazards Models
- Retrospective Studies
- SARS-CoV-2 / isolation & purification
- Severity of Illness Index
- United Kingdom / epidemiology

Grant support

- PDF-2015-08-102/DH_/Department of Health/United Kingdom

Full text links

OXFORD ACADEMIC Silverchair Information Systems Free PMC article
Proceed to details
Cite
Share
☐ 67
Observational Study
Respir Med
. 2021 Mar;178:106314.
doi: 10.1016/j.rmed.2021.106314. Epub 2021 Jan 28.

A comparison of characteristics and outcomes of patients with community-acquired and hospital-acquired COVID-19 in the United Kingdom: An observational study

Haaris A Shiwani [1], Muhammad Bilal [2], Muhammad U Shahzad [2], Alson Rodrigues [2], Jehad A Suliman [2], Muhammad Soban [2], Shahzeb Mirza [2], Nicoleta Lotca [2], Mohammed R Ruslan [2], Danyal Memon [3], Muhammad A Arshad [4], Kiran Fatima [5], Asma Kamran [2], Emmanuel E Egom [6], Abdul Aziz [7]
Affiliations Expand

Affiliations

- [1] Royal Lancaster Infirmary, University Hospitals of Morecambe Bay NHS Foundation Trust, Lancaster, LA1 4RP, United Kingdom; Royal Preston Hospital, Lancashire Teaching Hospitals NHS Foundation Trust, Preston, PR2 9HT, United Kingdom. Electronic address: shiwanih@tcd.ie.
- [2] Royal Lancaster Infirmary, University Hospitals of Morecambe Bay NHS Foundation Trust, Lancaster, LA1 4RP, United Kingdom.
- [3] Our Lady of Lourdes Hospital, Drogheda, Louth, Ireland.
- [4] Ameer-ud-Din Medical College, Lahore, Pakistan.
- [5] Khawaja Muhammad Safdar Medical College, Sialkot, Pakistan.
- [6] Egom Clinical & Translational Research Services Ltd., Dartmouth, Canada; Jewish General Hospital and Lady Davis Research Institute, Montreal, Quebec, Canada.
- [7] Royal Liverpool University Hospital, Royal Liverpool and Broadgreen University Hospitals NHS Trust, Liverpool, L7 8XP, United Kingdom.

- PMID: **33550150**
- PMCID: PMC7843030
- DOI: 10.1016/j.rmed.2021.106314

Free PMC article
Observational Study

A comparison of characteristics and outcomes of patients with community-acquired and hospital-acquired COVID-19 in the United Kingdom: An observational study

Haaris A Shiwani et al. Respir Med. 2021 Mar.
Free PMC article
Show details
Respir Med
. 2021 Mar;178:106314.
doi: 10.1016/j.rmed.2021.106314. Epub 2021 Jan 28.

Authors

Haaris A Shiwani [1], Muhammad Bilal [2], Muhammad U Shahzad [2], Alson Rodrigues [2], Jehad A Suliman [2], Muhammad Soban [2], Shahzeb Mirza [2], Nicoleta Lotca [2], Mohammed R Ruslan [2], Danyal Memon [3], Muhammad A Arshad [4], Kiran Fatima [5], Asma Kamran [2], Emmanuel E Egom [6], Abdul Aziz [7]

Affiliations

- [1] Royal Lancaster Infirmary, University Hospitals of Morecambe Bay NHS Foundation Trust, Lancaster, LA1 4RP, United Kingdom; Royal Preston Hospital, Lancashire Teaching Hospitals NHS Foundation Trust, Preston, PR2 9HT, United Kingdom. Electronic address: shiwanih@tcd.ie.
- [2] Royal Lancaster Infirmary, University Hospitals of Morecambe Bay NHS Foundation Trust, Lancaster, LA1 4RP, United Kingdom.
- [3] Our Lady of Lourdes Hospital, Drogheda, Louth, Ireland.
- [4] Ameer-ud-Din Medical College, Lahore, Pakistan.
- [5] Khawaja Muhammad Safdar Medical College, Sialkot, Pakistan.
- [6] Egom Clinical & Translational Research Services Ltd., Dartmouth, Canada; Jewish General Hospital and Lady Davis Research Institute, Montreal, Quebec, Canada.
- [7] Royal Liverpool University Hospital, Royal Liverpool and Broadgreen University Hospitals NHS Trust, Liverpool, L7 8XP, United Kingdom.

- PMID: **33550150**
- PMCID: PMC7843030
- DOI: 10.1016/j.rmed.2021.106314

Abstract

Background and objectives: Reports comparing the characteristics of patients and their clinical outcomes between community-acquired (CA) and hospital-acquired (HA) COVID-19 have not yet been reported in the literature. We aimed to characterise and compare clinical, biochemical and haematological features, in addition to clinical outcomes, between these patients.

Methods: This multi-centre, retrospective, observational study enrolled 488 SARS-CoV-2 positive patients - 339 with CA infection and 149 with HA infection. All patients were admitted to a hospital within the University Hospitals of Morecambe Bay NHS Foundation Trust between March 7th and May 18th' 2020.

Results: The CA cohort comprised of a significantly younger population, median age 75 years, versus 80 years in the HA cohort (P = 0·0002). Significantly less patients in the HA group experienced fever (P = 0·03) and breathlessness (P < 0·0001). Furthermore, significantly more patients had anaemia and hypoalbuminaemia in the HA group, compared to the CA group (P < 0·0001 for both). Hypertension and a lower median BMI were also significantly more pronounced in the HA cohort (P = 0·03 and P = 0·0001, respectively). The mortality rate was not significantly different between the two cohorts (34% in the CA group and 32% in the HA group, P = 0·64). However, the CA group required significantly greater ICU care (10% versus 3% in the HA group, P = 0·009).

Conclusion: Hospital-acquired and community-acquired COVID-19 display similar rates of mortality despite significant differences in baseline characteristics of the respective patient populations. Delineation of community- and hospital-acquired COVID-19 in future studies on COVID-19 may allow for more accurate interpretation of results.

Keywords: COVID-19; Community-acquired; Hospital-acquired; SARS-CoV-2.

Copyright © 2021 Elsevier Ltd. All rights reserved.

Conflict of interest statement

The authors declare that they have no known competing financial interests or personal relationships that could have appeared to influence the work reported in this paper.

The authors declare the following financial interests/personal relationships which may be considered as potential competing interests:

- 24 references
- 2 figures

Supplementary info

Publication types, MeSH terms Expand

Publication types

- Comparative Study
- Observational Study

MeSH terms

- Adolescent
- Adult
- Aged
- Aged, 80 and over

- COVID-19 / complications*
- COVID-19 / diagnosis
- COVID-19 / mortality*
- Community-Acquired Infections / complications*
- Community-Acquired Infections / diagnosis
- Community-Acquired Infections / mortality*
- Cross Infection / complications*
- Cross Infection / diagnosis
- Cross Infection / mortality*
- Female
- Hospital Mortality
- Hospitalization
- Humans
- Male
- Middle Aged
- Retrospective Studies
- Survival Rate
- Symptom Assessment
- United Kingdom
- Young Adult

Full text links

ELSEVIER FULL-TEXT ARTICLE Elsevier Science Free PMC article
Proceed to details
Cite
Share
68
Observational Study
Ear Nose Throat J
. 2021 Apr;100(2_suppl):116S-119S.
doi: 10.1177/0145561320965196. Epub 2020 Oct 9.

The Role of Tracheotomy and Timing of Weaning and Decannulation in Patients Affected by Severe COVID-19

Cecilia Botti [1, 2], Francesca Lusetti [2], Stefano Peroni [3], Tommaso Neri [3], Andrea Castellucci [2], Pierpaolo Salsi [3], Angelo Ghidini [2]
Affiliations Expand

Affiliations

- [1] PhD Program in Clinical and Experimental Medicine, 208968University of Modena and Reggio Emilia, Reggio Emilia, Italy.
- [2] Otolaryngology Unit, 9242Azienda USL-IRCCS, di Reggio Emilia, Italy.
- [3] Intensive Care Unit, 9242Azienda USL-IRCCS, di Reggio Emilia, Italy.

- PMID: **33035129**
- PMCID: PMC7548540
- DOI: 10.1177/0145561320965196

Free PMC article
Observational Study

The Role of Tracheotomy and Timing of Weaning and Decannulation in Patients Affected by Severe COVID-19

Cecilia Botti et al. Ear Nose Throat J. 2021 Apr.
Free PMC article
Show details
Ear Nose Throat J
. 2021 Apr;100(2_suppl):116S-119S.
doi: 10.1177/0145561320965196. Epub 2020 Oct 9.

Authors

Cecilia Botti [1] [2], Francesca Lusetti [2], Stefano Peroni [3], Tommaso Neri [3], Andrea Castellucci [2], Pierpaolo Salsi [3], Angelo Ghidini [2]

Affiliations

- [1] PhD Program in Clinical and Experimental Medicine, 208968University of Modena and Reggio Emilia, Reggio Emilia, Italy.
- [2] Otolaryngology Unit, 9242Azienda USL-IRCCS, di Reggio Emilia, Italy.
- [3] Intensive Care Unit, 9242Azienda USL-IRCCS, di Reggio Emilia, Italy.

- PMID: **33035129**
- PMCID: PMC7548540
- DOI: 10.1177/0145561320965196

Abstract

Objectives: Patients with acute respiratory failure due to coronavirus disease 2019 (COVID-19) have a high likelihood of needing prolonged intubation and may subsequently require tracheotomy. Indications and timing for performing tracheotomy in patients affected by severe COVID-19 pneumonia are still elusive. The aim of this study is to analyze the role of tracheotomy

in the context of this pandemic. Moreover, we report the timing of the procedure and the time needed to complete weaning and decannulation in our center.

Methods: This retrospective, observational cohort study included adults (≥18 years) with severe COVID-19 pneumonia who were admitted to the intensive care unit (ICU) of the tertiary care center of Reggio Emilia (Italy). All patients underwent orotracheal intubation with invasive mechanical ventilation, followed by percutaneous or open surgical tracheotomy. Indications, timing of the procedure, and time needed to complete weaning and decannulation were reported.

Results: Forty-four patients were included in the analysis. Median time from orotracheal intubation to surgery was 7 (range 2-17) days. Fifteen (34.1%) patients died during the follow-up period (median 22 days, range 8-68) after the intubation. Weaning from the ventilator was first attempted on median 25th day (range 13-43) from orotracheal intubation. A median of 35 (range 18-79) days was required to complete weaning. Median duration of ICU stay was 22 (range 10-67) days. Mean decannulation time was 36 (range 10-77) days from surgery.

Conclusions: Since it is not possible to establish an optimal timing for performing tracheotomy, decision-making should be made on case-by-case basis. It should be adapted to the context of the pandemic, taking into account the availability of intensive care resources, potential risks for health care workers, and benefits for the individual patient.

Keywords: COVID; COVID-19; SARS-CoV-2; tracheostomy; tracheotomy; weaning.

Conflict of interest statement

Declaration of Conflicting Interests: The author(s) declared no potential conflicts of interest with respect to the research, authorship, and/or publication of this article.

- 14 references
- 1 figure

Supplementary info

Publication types, MeSH terms Expand

Publication types

- Observational Study

MeSH terms

- Adult
- Aged
- Airway Extubation*
- COVID-19 / complications
- COVID-19 / therapy*
- Disease Progression
- Female
- Hospital Mortality*

- Humans
- Intensive Care Units
- Intubation, Intratracheal
- Italy
- Length of Stay*
- Male
- Middle Aged
- Respiration, Artificial*
- Respiratory Distress Syndrome / etiology
- Respiratory Distress Syndrome / therapy*
- Retrospective Studies
- SARS-CoV-2
- Severity of Illness Index
- Time Factors
- Tracheotomy / methods*
- Ventilator Weaning*

Full text links

SAGE journals Open access full text Atypon Free PMC article
Proceed to details
Cite
Share
☐ 69
Observational Study
J Anesth
. 2021 Apr;35(2):213-221.
doi: 10.1007/s00540-021-02897-w. Epub 2021 Jan 23.

Clinical characteristics and outcomes of critically ill patients with COVID-19 in Kobe, Japan: a single-center, retrospective, observational study

Jiro Ito [1], Ryutaro Seo [2], Daisuke Kawakami [3], Yoshinori Matsuoka [2], Kenjiro Ouchi [3], Suguru Nonami [3], Yusuke Miyoshi [3], Masao Tatebe [3], Takahiro Tsuchida [3], Yoko Asaka [2], Machi Yanai [2], Hiroshi Ueta [3], Takahiro Shimozono [3], Hiroyuki Mima [3], Asako Doi [4], Keisuke Tomii [5], Koichi Ariyoshi [2]
Affiliations Expand

Affiliations

- [1] Department of Anesthesia and Critical Care, Kobe City Medical Center General Hospital, 2-1-1, Minatojima-minamimachi, Chuo-ku, Kobe, Hyogo, 650-0047, Japan. phyandeth69boo@gmail.com.
- [2] Department of Emergency Medicine, Kobe City Medical Center General Hospital, Kobe, Hyogo, Japan.
- [3] Department of Anesthesia and Critical Care, Kobe City Medical Center General Hospital, 2-1-1, Minatojima-minamimachi, Chuo-ku, Kobe, Hyogo, 650-0047, Japan.
- [4] Department of Infectious Diseases, Kobe City Medical Center General Hospital, Kobe, Hyogo, Japan.
- [5] Department of Respiratory Medicine, Kobe City Medical Center General Hospital, Kobe, Hyogo, Japan.

- PMID: **33484361**
- PMCID: PMC7823169
- DOI: 10.1007/s00540-021-02897-w

Free PMC article
Observational Study

Clinical characteristics and outcomes of critically ill patients with COVID-19 in Kobe, Japan: a single-center, retrospective, observational study

Jiro Ito et al. J Anesth. 2021 Apr.
Free PMC article
Show details
J Anesth
. 2021 Apr;35(2):213-221.
doi: 10.1007/s00540-021-02897-w. Epub 2021 Jan 23.

Authors

Jiro Ito [1], Ryutaro Seo [2], Daisuke Kawakami [3], Yoshinori Matsuoka [2], Kenjiro Ouchi [3], Suguru Nonami [3], Yusuke Miyoshi [3], Masao Tatebe [3], Takahiro Tsuchida [3], Yoko Asaka [2], Machi Yanai [2], Hiroshi Ueta [3], Takahiro Shimozono [3], Hiroyuki Mima [3], Asako Doi [4], Keisuke Tomii [5], Koichi Ariyoshi [2]

Affiliations

- [1] Department of Anesthesia and Critical Care, Kobe City Medical Center General Hospital, 2-1-1, Minatojima-minamimachi, Chuo-ku, Kobe, Hyogo, 650-0047, Japan. phyandeth69boo@gmail.com.

- [2] Department of Emergency Medicine, Kobe City Medical Center General Hospital, Kobe, Hyogo, Japan.
- [3] Department of Anesthesia and Critical Care, Kobe City Medical Center General Hospital, 2-1-1, Minatojima-minamimachi, Chuo-ku, Kobe, Hyogo, 650-0047, Japan.
- [4] Department of Infectious Diseases, Kobe City Medical Center General Hospital, Kobe, Hyogo, Japan.
- [5] Department of Respiratory Medicine, Kobe City Medical Center General Hospital, Kobe, Hyogo, Japan.

- PMID: **33484361**
- PMCID: PMC7823169
- DOI: 10.1007/s00540-021-02897-w

Abstract

Purpose: Coronavirus disease 2019 (COVID-19) has placed a great burden on critical care services worldwide. Data regarding critically ill COVID-19 patients and their demand of critical care services outside of initial COVID-19 epicenters are lacking. This study described clinical characteristics and outcomes of critically ill COVID-19 patients and the capacity of a COVID-19-dedicated intensive care unit (ICU) in Kobe, Japan.

Methods: This retrospective observational study included critically ill COVID-19 patients admitted to a 14-bed COVID-19-dedicated ICU in Kobe between March 3, 2020 and June 21, 2020. Clinical and daily ICU occupancy data were obtained from electrical medical records. The last follow-up day was June 28, 2020.

Results: Of 32 patients included, the median hospital follow-up period was 27 (interquartile range 19-50) days. The median age was 68 (57-76) years; 23 (72%) were men and 25 (78%) had at least one comorbidity. Nineteen (59%) patients received invasive mechanical ventilation for a median duration of 14 (8-27) days. Until all patients were discharged from the ICU on June 5, 2020, the median daily ICU occupancy was 50% (36-71%). As of June 28, 2020, six (19%) died during hospitalization. Of 26 (81%) survivors, 23 (72%) were discharged from the hospital and three (9%) remained in the hospital.

Conclusion: During the first months of the outbreak in Kobe, most critically ill patients were men aged ≥ 60 years with at least one comorbidity and on mechanical ventilation; the ICU capacity was not strained, and the case-fatality rate was 19%.

Keywords: Bed occupancy; Coronavirus; Critical care; Mortality; Respiration, artificial; Respiratory distress syndrome, adult; Severe acute respiratory syndrome coronavirus 2.

Conflict of interest statement

The authors declare that they have no conflicts of interest.

- 30 references
- 1 figure

Supplementary info

Publication types, MeSH terms Expand

Publication types

- Observational Study

MeSH terms

- Aged
- COVID-19*
- Critical Illness*
- Humans
- Intensive Care Units
- Japan
- Male
- Respiration, Artificial
- Retrospective Studies
- SARS-CoV-2

Full text links

Springer Free PMC article
Proceed to details
Cite
Share
☐ 70
Observational Study
Clin Med (Lond)
. 2020 Nov;20(6):e229-e233.
doi: 10.7861/clinmed.2020-0483. Epub 2020 Sep 22.

How COVID-19 has changed the unselected medical take: an observational study

Kai Man Alexander Ho [1], Ananthi Anandhakrishnan [2], Arun Mahay [2], Yiwen Soo [2], Laurence B Lovat [3], Andrew P Rochford [2]
Affiliations Expand

Affiliations

- [1] Newham University Hospital, London, UK and University College London, London, UK alexander.ho@ucl.ac.uk.
- [2] Newham University Hospital, London, UK.
- [3] University College London, London, UK.

- PMID: **32962974**
- PMCID: PMC7687303
- DOI: 10.7861/clinmed.2020-0483

Free PMC article
Observational Study

How COVID-19 has changed the unselected medical take: an observational study

Kai Man Alexander Ho et al. Clin Med (Lond). 2020 Nov.
Free PMC article
Show details
Clin Med (Lond)
. 2020 Nov;20(6):e229-e233.
doi: 10.7861/clinmed.2020-0483. Epub 2020 Sep 22.

Authors

Kai Man Alexander Ho [1], Ananthi Anandhakrishnan [2], Arun Mahay [2], Yiwen Soo [2], Laurence B Lovat [3], Andrew P Rochford [2]

Affiliations

- [1] Newham University Hospital, London, UK and University College London, London, UK alexander.ho@ucl.ac.uk.
- [2] Newham University Hospital, London, UK.
- [3] University College London, London, UK.

- PMID: **32962974**
- PMCID: PMC7687303
- DOI: 10.7861/clinmed.2020-0483

Abstract

Introduction: COVID-19 has had a profound effect on the NHS. Little information has been published as to how the unselected medical take has been affected.

Methods: We retrospectively reviewed patients who were referred to general medicine during March 2020. We compared clinical outcomes of patients with and without COVID-19.

Results: 814 patients were included, comprising 777 unique patients. On average, 26 patients were admitted per day. 38% of admitted patients were suspected of COVID-19, with greater numbers of COVID-19 patients in the second half compared to the first half of the month (p<0.001). Logistic regression analyses showed suspected COVID-19 was an independent predictor for inpatient mortality (odds ratio [OR] = 6.09, p<0.001) and 30-day mortality (OR = 4.66, p<0.001).

Conclusions: COVID-19 patients had worse clinical outcomes and increased healthcare use compared to non-COVID-19 patients. Our study highlights some of the challenges in healthcare provision faced during this pandemic.

Keywords: COVID-19; SARS-CoV-2; medical admissions; medical take.

© 2020 Royal College of Physicians 2020. All rights reserved.

- 2 figures

Supplementary info

Publication types, MeSH terms Expand

Publication types

- Observational Study

MeSH terms

- Adult
- Aged
- Aged, 80 and over
- COVID-19
- Coronavirus Infections*
- Female
- Health Services Needs and Demand* / statistics & numerical data
- Health Services Needs and Demand* / trends
- Humans
- Male
- Middle Aged
- Pandemics*
- Patient Admission* / statistics & numerical data
- Patient Admission* / trends
- Pneumonia, Viral*
- Retrospective Studies
- Treatment Outcome
- United Kingdom

Full text links

Full text ClinMed HighWire Free PMC article
Proceed to details
Cite
Share

☐ 71
Observational Study
Ann Clin Microbiol Antimicrob
. 2021 Jan 6;20(1):3.
doi: 10.1186/s12941-020-00412-9.

Clinical characteristics and predictors of mortality in young adults with severe COVID-19: a retrospective observational study

Yanjiao Lu [1], Zhenli Huang [1], Meijia Wang [1], Kun Tang [1], Shanshan Wang [1], Pengfei Gao [2], Jungang Xie [1], Tao Wang [3], Jianping Zhao [4]
Affiliations Expand

Affiliations

- [1] Department of Respiratory and Critical Care Medicine, National Clinical Research Center of Respiratory Disease, Tongji Hospital, Tongji Medical College, Huazhong University of Science and Technology, Wuhan, 430030, China.
- [2] Department of Respiratory and Critical Care Medicine, the First Affiliated Hospital of Henan University of Science and Technology, Luoyang, 471003, China.
- [3] Department of Respiratory and Critical Care Medicine, National Clinical Research Center of Respiratory Disease, Tongji Hospital, Tongji Medical College, Huazhong University of Science and Technology, Wuhan, 430030, China. wt7636@126.com.
- [4] Department of Respiratory and Critical Care Medicine, National Clinical Research Center of Respiratory Disease, Tongji Hospital, Tongji Medical College, Huazhong University of Science and Technology, Wuhan, 430030, China. Zhaojp88@126.com.

- PMID: **33407543**
- PMCID: PMC7787410
- DOI: 10.1186/s12941-020-00412-9

Free PMC article
Observational Study

Clinical characteristics and predictors of mortality in young adults with severe COVID-19: a retrospective observational study

Yanjiao Lu et al. Ann Clin Microbiol Antimicrob. 2021.
Free PMC article
Show details
Ann Clin Microbiol Antimicrob
. 2021 Jan 6;20(1):3.

doi: 10.1186/s12941-020-00412-9.

Authors

Yanjiao Lu [1], Zhenli Huang [1], Meijia Wang [1], Kun Tang [1], Shanshan Wang [1], Pengfei Gao [2], Jungang Xie [1], Tao Wang [3], Jianping Zhao [4]

Affiliations

- [1] Department of Respiratory and Critical Care Medicine, National Clinical Research Center of Respiratory Disease, Tongji Hospital, Tongji Medical College, Huazhong University of Science and Technology, Wuhan, 430030, China.
- [2] Department of Respiratory and Critical Care Medicine, the First Affiliated Hospital of Henan University of Science and Technology, Luoyang, 471003, China.
- [3] Department of Respiratory and Critical Care Medicine, National Clinical Research Center of Respiratory Disease, Tongji Hospital, Tongji Medical College, Huazhong University of Science and Technology, Wuhan, 430030, China. wt7636@126.com.
- [4] Department of Respiratory and Critical Care Medicine, National Clinical Research Center of Respiratory Disease, Tongji Hospital, Tongji Medical College, Huazhong University of Science and Technology, Wuhan, 430030, China. Zhaojp88@126.com.

- PMID: **33407543**
- PMCID: PMC7787410
- DOI: 10.1186/s12941-020-00412-9

Abstract

Background and objective: Little is yet known whether pathogenesis of COVID-19 is different between young and elder patients. Our study aimed to investigate the clinical characteristics and provide predictors of mortality for young adults with severe COVID-19.

Methods: A total of 77 young adults with confirmed severe COVID-19 were recruited retrospectively at Tongji Hospital. Clinical characteristics, laboratory findings, treatment and outcomes were obtained from electronic medical records. The prognostic effects of variables were analyzed using logistic regression model.

Results: In this retrospective cohort, non-survivors showed higher incidence of dyspnea and co-existing laboratory abnormalities, compared with young survivals in severe COVID-19. Multivariate logistic regression analysis showed that lymphopenia, elevated level of d-dimer, hypersensitive cardiac troponin I (hs-CTnI) and high sensitivity C-reactive protein (hs-CRP) were independent predictors of mortality in young adults with severe COVID-19. Further analysis showed that severely young adults with two or more factors abnormalities above would be more prone to death. The similar predictive effect of above four factors had been observed in all-age patients with severe COVID-19.

Conclusion: Lymphopenia, elevated level of d-dimer, hs-CTnI and hs-CRP predicted clinical outcomes of young adults with severe COVID-19.

Keywords: COVID-19; Predictors; SARS-CoV-2; Severe; Young adults.

Conflict of interest statement

The authors declare that they have no competing interests.

- 29 references
- 1 figure

Supplementary info

Publication types, MeSH terms, Grant support Expand

Publication types

- Observational Study

MeSH terms

- COVID-19 / mortality*
- COVID-19 / physiopathology*
- COVID-19 / therapy
- China / epidemiology
- Cohort Studies
- Female
- Humans
- Incidence
- Logistic Models
- Male
- Middle Aged
- Pandemics
- Regression Analysis
- Retrospective Studies
- Risk Factors
- SARS-CoV-2 / isolation & purification
- Treatment Outcome
- Young Adult

Grant support

- 2017ZX10103004/National Major Science and Technology Projects of China
- 2020kfyXGYJ015/Tongji Medical College, Huazhong University of Science and Technology

Full text links

Read free full text at BMC BioMed Central Free PMC article
Proceed to details
Cite
Share
72

Observational Study

Int J Environ Res Public Health
. 2021 Jul 28;18(15):7963.
doi: 10.3390/ijerph18157963.

Impact of COVID-19 on Dental Care during a National Lockdown: A Retrospective Observational Study

Elias Walter [1], Leonard von Bronk [1], Reinhard Hickel [1], Karin Christine Huth [1]
Affiliations Expand

Affiliation

- [1] Department of Conservative Dentistry and Periodontology, University Hospital, LMU, 80336 Munich, Germany.

- PMID: **34360255**
- PMCID: PMC8345748
- DOI: 10.3390/ijerph18157963

Free PMC article
Observational Study

Impact of COVID-19 on Dental Care during a National Lockdown: A Retrospective Observational Study

Elias Walter et al. Int J Environ Res Public Health. 2021.
Free PMC article
Show details

Int J Environ Res Public Health
. 2021 Jul 28;18(15):7963.
doi: 10.3390/ijerph18157963.

Authors

Elias Walter [1], Leonard von Bronk [1], Reinhard Hickel [1], Karin Christine Huth [1]

Affiliation

- [1] Department of Conservative Dentistry and Periodontology, University Hospital, LMU, 80336 Munich, Germany.

- PMID: **34360255**
- PMCID: PMC8345748
- DOI: 10.3390/ijerph18157963

Abstract

The coronavirus disease 19 (COVID-19) has challenged dental health professions. This study analyzes its impact on urgent dental care in the Department of Conservative Dentistry and Periodontology, University Hospital Munich and Bavaria, Germany. Patient numbers without and with positive/suspected COVID-19 infection, their reasons for attendance, and treatments were retrospectively recorded (February-July 2020) and linked to local COVID-19 infection numbers, control measures, and numbers/reasons for closures of private dental practices in Bavaria, Germany. Patient numbers decreased within the urgent care unit and the private dental practices followed by a complete recovery by the end of July. While non-emergency visits dropped to almost zero during the first lockdown, pain-related treatments were administered invariably also in patients with positive/suspected COVID-19 infections. Reasons for practice closures were lack of personal protective equipment (PPE), lack of employees, staff's increased health risks, and infected staff, which accounted for 0.72% (3.6% closures in total). Pain-driven urgent dental care remains a constant necessity even in times of high infection risk, and measures established at the beginning of the pandemic seem to have provided a safe environment for patients as well as oral health care providers. PPE storage is important to ensure patients' treatment under high-risk conditions, and its storage and provision by regulatory units might guarantee a stable and safe oral health care system in the future.

Keywords: COVID-19; lockdown; pandemic; urgent dental care.

Conflict of interest statement

The authors declare no conflict of interest.

- 33 references
- 6 figures

Supplementary info

Publication types, MeSH terms Expand

Publication types

- Observational Study

MeSH terms

- COVID-19*

- Communicable Disease Control
- Dental Care
- Humans
- Retrospective Studies
- SARS-CoV-2

Full text links

FULL TEXT OPEN ACCESS MDPI Multidisciplinary Digital Publishing Institute (MDPI) Free PMC article

Proceed to details

Cite

Share

☐ 73

Observational Study

JAMA Cardiol
. 2020 Sep 1;5(9):1020-1026.
doi: 10.1001/jamacardio.2020.1855.

Association of Use of Angiotensin-Converting Enzyme Inhibitors and Angiotensin II Receptor Blockers With Testing Positive for Coronavirus Disease 2019 (COVID-19)

Neil Mehta [1], Ankur Kalra [2] [3], Amy S Nowacki [4], Scott Anjewierden [1], Zheyi Han [1], Pavan Bhat [2], Andres E Carmona-Rubio [2], Miriam Jacob [2], Gary W Procop [5], Susan Harrington [5], Alex Milinovich [4], Lars G Svensson [6], Lara Jehi [7], James B Young [1] [2], Mina K Chung [2] [8]

Affiliations Expand

Affiliations

- [1] Department of Medicine, Cleveland Clinic Lerner College of Medicine of Case Western Reserve University, Cleveland Clinic, Cleveland, Ohio.
- [2] Heart, Vascular and Thoracic Institute, Department of Cardiovascular Medicine, Cleveland Clinic, Cleveland, Ohio.
- [3] Section of Cardiovascular Research, Heart, Vascular and Thoracic Department, Cleveland Clinic Akron General, Akron, Ohio.
- [4] Lerner Research Institute, Department of Quantitative Health Sciences, Cleveland Clinic, Cleveland, Ohio.
- [5] Pathology and Laboratory Medicine Institute, Department of Laboratory Medicine, Cleveland Clinic, Cleveland, Ohio.
- [6] Heart, Vascular and Thoracic Institute, Department of Thoracic and Cardiovascular Surgery, Cleveland Clinic, Cleveland, Ohio.
- [7] Cleveland Clinic, Cleveland, Ohio.

- [8] Lerner Research Institute, Department of Cardiovascular and Metabolic Sciences, Cleveland Clinic, Cleveland, Ohio.

- PMID: **32936273**
- PMCID: PMC7201375
- DOI: 10.1001/jamacardio.2020.1855

Free PMC article
Observational Study

Association of Use of Angiotensin-Converting Enzyme Inhibitors and Angiotensin II Receptor Blockers With Testing Positive for Coronavirus Disease 2019 (COVID-19)

Neil Mehta et al. JAMA Cardiol. 2020.
Free PMC article
Show details
JAMA Cardiol
. 2020 Sep 1;5(9):1020-1026.
doi: 10.1001/jamacardio.2020.1855.

Authors

Neil Mehta [1], Ankur Kalra [2, 3], Amy S Nowacki [4], Scott Anjewierden [1], Zheyi Han [1], Pavan Bhat [2], Andres E Carmona-Rubio [2], Miriam Jacob [2], Gary W Procop [5], Susan Harrington [5], Alex Milinovich [4], Lars G Svensson [6], Lara Jehi [7], James B Young [1, 2], Mina K Chung [2, 8]

Affiliations

- [1] Department of Medicine, Cleveland Clinic Lerner College of Medicine of Case Western Reserve University, Cleveland Clinic, Cleveland, Ohio.
- [2] Heart, Vascular and Thoracic Institute, Department of Cardiovascular Medicine, Cleveland Clinic, Cleveland, Ohio.
- [3] Section of Cardiovascular Research, Heart, Vascular and Thoracic Department, Cleveland Clinic Akron General, Akron, Ohio.
- [4] Lerner Research Institute, Department of Quantitative Health Sciences, Cleveland Clinic, Cleveland, Ohio.
- [5] Pathology and Laboratory Medicine Institute, Department of Laboratory Medicine, Cleveland Clinic, Cleveland, Ohio.
- [6] Heart, Vascular and Thoracic Institute, Department of Thoracic and Cardiovascular Surgery, Cleveland Clinic, Cleveland, Ohio.
- [7] Cleveland Clinic, Cleveland, Ohio.
- [8] Lerner Research Institute, Department of Cardiovascular and Metabolic Sciences, Cleveland Clinic, Cleveland, Ohio.

- PMID: **32936273**
- PMCID: PMC7201375
- DOI: 10.1001/jamacardio.2020.1855

Abstract

Importance: The role of angiotensin-converting enzyme inhibitors (ACEI) and angiotensin II receptor blockers (ARB) in the setting of the coronavirus disease 2019 (COVID-19) pandemic is hotly debated. There have been recommendations to discontinue these medications, which are essential in the treatment of several chronic disease conditions, while, in the absence of clinical evidence, professional societies have advocated their continued use.

Objective: To study the association between use of ACEIs/ARBs with the likelihood of testing positive for COVID-19 and to study outcome data in subsets of patients taking ACEIs/ARBs who tested positive with severity of clinical outcomes of COVID-19 (eg, hospitalization, intensive care unit admission, and requirement for mechanical ventilation).

Design, setting, and participants: Retrospective cohort study with overlap propensity score weighting was conducted at the Cleveland Clinic Health System in Ohio and Florida. All patients tested for COVID-19 between March 8 and April 12, 2020, were included.

Exposures: History of taking ACEIs or ARBs at the time of COVID-19 testing.

Main outcomes and measures: Results of COVID-19 testing in the entire cohort, number of patients requiring hospitalizations, intensive care unit admissions, and mechanical ventilation among those who tested positive.

Results: A total of 18 472 patients tested for COVID-19. The mean (SD) age was 49 (21) years, 7384 (40%) were male, and 12 725 (69%) were white. Of 18 472 patients who underwent COVID-19 testing, 2285 (12.4%) were taking either ACEIs or ARBs. A positive COVID-19 test result was observed in 1735 of 18 472 patients (9.4%). Among patients who tested positive, 421 (24.3%) were admitted to the hospital, 161 (9.3%) were admitted to an intensive care unit, and 111 (6.4%) required mechanical ventilation. Overlap propensity score weighting showed no significant association of ACEI and/or ARB use with COVID-19 test positivity (overlap propensity score-weighted odds ratio, 0.97; 95% CI, 0.81-1.15).

Conclusions and relevance: This study found no association between ACEI or ARB use and COVID-19 test positivity. These clinical data support current professional society guidelines to not discontinue ACEIs or ARBs in the setting of the COVID-19 pandemic. However, further study in larger numbers of hospitalized patients receiving ACEI and ARB therapy is needed to determine the association with clinical measures of COVID-19 severity.

Conflict of interest statement

Conflict of Interest Disclosures: Dr Milinovich reports royalties for use of REDCap from nPhase during the conduct of the study and grants from Novo Nordisk, Boehringer Ingelheim, Merck, Novartis, Otsuka, and the National Institutes of Health outside the submitted work. No other disclosures were reported.

Comment in

- Overlap Weighting: A Propensity Score Method That Mimics Attributes of a Randomized Clinical Trial.
  Thomas LE, Li F, Pencina MJ. Thomas LE, et al. JAMA. 2020 Jun 16;323(23):2417-2418. doi: 10.1001/jama.2020.7819. JAMA. 2020. PMID: 32369102 No abstract available.
- Understanding Observational Treatment Comparisons in the Setting of Coronavirus Disease 2019 (COVID-19).
  Thomas LE, Bonow RO, Pencina MJ. Thomas LE, et al. JAMA Cardiol. 2020 Sep 1;5 (9):988-990. doi: 10.1001/jamacardio.2020.1874. JAMA Cardiol. 2020. PMID: 32936260 No abstract available.

- 1 figure

Supplementary info

Publication types, MeSH terms, Substances, Grant support Expand

Publication types

- Observational Study
- Research Support, N.I.H., Extramural

MeSH terms

- Adult
- Angiotensin Receptor Antagonists / therapeutic use*
- Angiotensin-Converting Enzyme Inhibitors / therapeutic use*
- Betacoronavirus*
- COVID-19
- COVID-19 Testing
- Clinical Laboratory Techniques
- Coronavirus Infections / diagnosis*
- Coronavirus Infections / epidemiology*
- Critical Care
- Female
- Hospitalization
- Humans
- Hypertension / complications
- Hypertension / drug therapy
- Male
- Middle Aged
- Pandemics
- Pneumonia, Viral / diagnosis*
- Pneumonia, Viral / epidemiology*
- Respiration, Artificial
- Retrospective Studies

- Risk Factors
- SARS-CoV-2

Substances

- Angiotensin Receptor Antagonists
- Angiotensin-Converting Enzyme Inhibitors

Grant support

- UL1 TR002548/TR/NCATS NIH HHS/United States

Full text links

FULL TEXT JAMA Cardiology Silverchair Information Systems Free PMC article

Proceed to details

Cite

Share

74

Observational Study

PLoS One

. 2021 Sep 29;16(9):e0257253.

doi: 10.1371/journal.pone.0257253. eCollection 2021.

Risk factors, predictions, and progression of acute kidney injury in hospitalized COVID-19 patients: An observational retrospective cohort study

Maryam N Naser [1], Rana Al-Ghatam [1], Abdulla H Darwish [1], Manaf M Alqahtani [1], Hajar A Alahmadi [1], Khalifa A Mohamed [1], Nahed K Hasan [1], Nuria S Perez [1]

Affiliations Expand

Affiliation

- [1] Bahrain Defence Force Hospital, Royal Medical Services, Riffa, Kingdom of Bahrain.

- PMID: **34587189**
- PMCID: PMC8480894
- DOI: 10.1371/journal.pone.0257253

Free PMC article

Observational Study

Risk factors, predictions, and progression of acute kidney injury in hospitalized COVID-19 patients: An observational retrospective cohort study

Maryam N Naser et al. PLoS One. 2021.
Free PMC article
Show details
PLoS One
. 2021 Sep 29;16(9):e0257253.
doi: 10.1371/journal.pone.0257253. eCollection 2021.

Authors

Maryam N Naser [1], Rana Al-Ghatam [1], Abdulla H Darwish [1], Manaf M Alqahtani [1], Hajar A Alahmadi [1], Khalifa A Mohamed [1], Nahed K Hasan [1], Nuria S Perez [1]

Affiliation

- [1] Bahrain Defence Force Hospital, Royal Medical Services, Riffa, Kingdom of Bahrain.

- PMID: **34587189**
- PMCID: PMC8480894
- DOI: 10.1371/journal.pone.0257253

Abstract

Objectives: Studies have shown that acute kidney injury (AKI) occurrence post SARS-CoV-2 infection is complex and has a poor prognosis. Therefore, more studies are needed to understand the rate and the predications of AKI involvement among hospitalized COVID-19 patients and AKI's impact on prognosis while under different types of medications.

Patients and methods: This study is a retrospective observational cohort study conducted at Bahrain Defence Force (BDF) Royal Medical Services. Medical records of COVID-19 patients admitted to BDF hospital, treated, and followed up from April 2020 to October 2020 were retrieved. Data were analyzed using univariate and multivariate logistic regression with covariate adjustment, and the odds ratio (OR) and 95% confidence (95% CI) interval were reported.

Results: Among 353 patients admitted with COVID-19, 47.6% developed AKI. Overall, 51.8% of patients with AKI died compared to 2.2% of patients who did not develop AKI (p< 0.001 with OR 48.6 and 95% CI 17.2-136.9). Besides, deaths in patients classified with AKI staging were positively correlated and multivariate regression analysis revealed that moderate to severe hypoalbuminemia (<32 g/L) was independently correlated to death in AKI patients with an OR of 10.99 (CI 95% 4.1-29.3, p<0.001). In addition, 78.2% of the dead patients were on mechanical ventilation. Besides age as a predictor of AKI development, diabetes and hypertension were the major risk factors of AKI development (OR 2.04, p<0.01, and 0.05 for diabetes and hypertension, respectively). Also, two or more comorbidities substantially increased the risk of AKI

development in COVID-19 patients. Furthermore, high levels upon hospital admission of D-Dimer, Troponin I, and ProBNP and low serum albumin were associated with AKI development. Lastly, patients taking ACEI/ARBs had less chance to develop AKI stage II/III with OR of 0.19-0.27 (p<0.05-0.01).

Conclusions: The incidence of AKI in hospitalized COVID-19 patients and the mortality rate among AKI patients were high and correlated with AKI staging. Furthermore, laboratory testing for serum albumin, hypercoagulability and cardiac injury markers maybe indicative for AKI development. Therefore, clinicians should be mandated to perform such tests on admission and follow-up in hospitalized patients.

Conflict of interest statement

The authors have declared that no competing interests exist.

- 29 references
- 3 figures

Supplementary info

Publication types, MeSH terms, Grant support Expand

Publication types

- Observational Study

MeSH terms

- Acute Kidney Injury / epidemiology*
- Acute Kidney Injury / metabolism
- Acute Kidney Injury / physiopathology
- Adult
- Aged
- Bahrain / epidemiology
- COVID-19 / complications*
- COVID-19 / physiopathology
- Cohort Studies
- Comorbidity
- Female
- Hospital Mortality
- Hospitalization / trends
- Hospitals
- Humans
- Incidence
- Intensive Care Units
- Male

- Middle Aged
- Prognosis
- Respiration, Artificial / adverse effects
- Retrospective Studies
- Risk Factors
- SARS-CoV-2 / metabolism
- SARS-CoV-2 / pathogenicity

Grant support

The author(s) received no specific funding for this work.

Full text links

OPEN ACCESS TO FULL TEXT PLOS ONE Public Library of Science Free PMC article

Proceed to details

Cite

Share

☐ 75

Observational Study

Int J Clin Pharmacol Ther

. 2021 May;59(5):378-385.

doi: 10.5414/CP203861.

Lopinavir-ritonavir alone or combined with arbidol in the treatment of 73 hospitalized patients with COVID-19: A pilot retrospective study

Xiu Lan, Chuxiao Shao, Xu Zeng, Zhenbo Wu, Yanyan Xu

- PMID: **33624583**
- DOI: 10.5414/CP203861

Observational Study

Lopinavir-ritonavir alone or combined with arbidol in the treatment of 73 hospitalized patients with COVID-19: A pilot retrospective study

Xiu Lan et al. Int J Clin Pharmacol Ther. 2021 May.
Show details
Int J Clin Pharmacol Ther
. 2021 May;59(5):378-385.
doi: 10.5414/CP203861.

Authors

Xiu Lan, Chuxiao Shao, Xu Zeng, Zhenbo Wu, Yanyan Xu

- PMID: **33624583**
- DOI: 10.5414/CP203861

Abstract

Objectives: This study aimed to evaluate the antiviral efficacy of lopinavir-ritonavir alone or combined with arbidol in the treatment of hospitalized patients with common coronavirus disease-19 (COVID-19).

Materials and methods: In this retrospective observational study, hospitalized COVID-19 patients were identified and divided into two groups based on the antiviral agents during their hospitalization. Patients in group LR were treated with lopinavir-ritonavir 400 mg/100 mg, twice a day, while patients in group LR+Ar were treated with lopinavir-ritonavir 400 mg/100 mg twice a day and arbidol 200 mg three times a day for at least 3 days. Data from these patients were collected from electronic medical record management system.

Results: 73 patients were divided into two groups: group LR (34 cases) and group LR+Ar (39 cases), according to the antiviral agents. The overall cure rate of COVID-19 in group LR+Ar and group LR were 92.3% and 97.1%, respectively, with no significant difference ($p = 0.62$). In a modified intention-to-treat analysis, lopinavir-ritonavir combined with arbidol led to a median time of hospital stay that was shorter by 1.5 days than in group LR (12.5 days vs. 14 days). The percentages of -COVID-19 RNA clearance was 92.3 in group LR and 97.1 in group LR+Ar which was similar to the cure rate. The median time to nucleic acid turning negative = (date of first negative PCR test) - (date of last positive PCR test) was 8.0 days in both groups with no significant difference ($p = 0.59$). Treatment of lopinavir-ritonavir combined with arbidol did not significantly accelerate main symptom improvement and promote the image absorption of pulmonary inflammation.

Conclusion: No benefit was observed in the antiviral effect of lopinavir-ritonavir combined with arbidol compared with lopinavir-ritonavir alone in the hospitalized patients with COVID-19. More clinical observations in COVID-19 patients may help to confirm or exclude the effect of antiviral agents.

Supplementary info

Publication types, MeSH terms, Substances, Supplementary concepts Expand

Publication types

- Observational Study

MeSH terms

- Antiviral Agents / therapeutic use
- COVID-19* / drug therapy
- Drug Combinations
- Humans
- Indoles
- Lopinavir / therapeutic use
- Retrospective Studies
- Ritonavir* / therapeutic use
- SARS-CoV-2

Substances

- Antiviral Agents
- Drug Combinations
- Indoles
- Lopinavir
- umifenovir
- Ritonavir

Supplementary concepts

- COVID-19 drug treatment

Full text links

Dustri-Verlag Dr. Karl Feistle GmbH & Co. KG
Proceed to details
Cite
Share
☐ 76
Observational Study
Sci Rep
. 2021 Jul 26;11(1):15205.

doi: 10.1038/s41598-021-94570-1.

Clinical outcomes of hospitalized COVID-19 patients with renal injury: a multi-hospital observational study from Wuhan

Kehong Chen[#, 1, 2], Yu Lei[#, 3, 4], Yani He[1], Fei Xiao[1], Yan Yu[1, 2], Xiaodong Lai[5, 6], Yang Liu[7, 8], Jiang Wang[9, 10], Huanzi Dai[11, 12]
Affiliations Expand

Affiliations

- [1] Department of Nephrology, Daping Hospital, Army Medical University, Changjiang Branch Road 10, Yu Zhong district, Chongqing, 400042, China.
- [2] Department of Critical Care Medicine, Taikang Tongji Hospital, Wuhan, China.
- [3] Department of Infectious Disease, Huo Shen Shan Hospital, Wuhan, China.
- [4] Department of Anaesthesiology, Daping Hospital, Army Medical University, Chongqing, China.
- [5] Department of Critical Care Medicine, The People's Hospital of Wuhan University, Wuhan, China.
- [6] Department of Critical Care Medicine, The Thirteenth People's Hospital of Chongqing, Chongqing, China.
- [7] Department of Infectious Disease, Taikang Tongji Hospital, Wuhan, China.
- [8] Department of Neurology, The No. 988 Hospital of Joint Logistic Support Force, Zhengzhou, China.
- [9] Department of Infectious Disease, Taikang Tongji Hospital, Wuhan, China. 504641821@qq.com.
- [10] Department of Cardiology, Xinqiao Hospital, Army Medical University, Chongqing, China. 504641821@qq.com.
- [11] Department of Nephrology, Daping Hospital, Army Medical University, Changjiang Branch Road 10, Yu Zhong district, Chongqing, 400042, China. dhz084426@163.com.
- [12] Department of Infectious Disease, Taikang Tongji Hospital, Wuhan, China. dhz084426@163.com.

[#] Contributed equally.

- PMID: **34312430**
- PMCID: PMC8313555
- DOI: 10.1038/s41598-021-94570-1

Free PMC article
Observational Study

Clinical outcomes of hospitalized COVID-19 patients with renal injury: a multi-hospital observational study from Wuhan

Kehong Chen et al. Sci Rep. 2021.
Free PMC article
Show details
Sci Rep
. 2021 Jul 26;11(1):15205.
doi: 10.1038/s41598-021-94570-1.

Authors

Kehong Chen[#,1,2], Yu Lei[#,3,4], Yani He[1], Fei Xiao[1], Yan Yu[1,2], Xiaodong Lai[5,6], Yang Liu[7,8], Jiang Wang[9,10], Huanzi Dai[11,12]

Affiliations

- [1] Department of Nephrology, Daping Hospital, Army Medical University, Changjiang Branch Road 10, Yu Zhong district, Chongqing, 400042, China.
- [2] Department of Critical Care Medicine, Taikang Tongji Hospital, Wuhan, China.
- [3] Department of Infectious Disease, Huo Shen Shan Hospital, Wuhan, China.
- [4] Department of Anaesthesiology, Daping Hospital, Army Medical University, Chongqing, China.
- [5] Department of Critical Care Medicine, The People's Hospital of Wuhan University, Wuhan, China.
- [6] Department of Critical Care Medicine, The Thirteenth People's Hospital of Chongqing, Chongqing, China.
- [7] Department of Infectious Disease, Taikang Tongji Hospital, Wuhan, China.
- [8] Department of Neurology, The No. 988 Hospital of Joint Logistic Support Force, Zhengzhou, China.
- [9] Department of Infectious Disease, Taikang Tongji Hospital, Wuhan, China. 504641821@qq.com.
- [10] Department of Cardiology, Xinqiao Hospital, Army Medical University, Chongqing, China. 504641821@qq.com.
- [11] Department of Nephrology, Daping Hospital, Army Medical University, Changjiang Branch Road 10, Yu Zhong district, Chongqing, 400042, China. dhz084426@163.com.
- [12] Department of Infectious Disease, Taikang Tongji Hospital, Wuhan, China. dhz084426@163.com.

[#] Contributed equally.

- PMID: **34312430**
- PMCID: PMC8313555
- DOI: 10.1038/s41598-021-94570-1

Abstract

Renal injury is common in patients with coronavirus disease 2019 (COVID-19). We aimed to determine the relationship of estimated glomerular filtration rate (eGFR) and acute kidney injury (AKI) with the characteristics, progression, and prognosis of COVID-19 in-patients. We retrospectively reviewed 1851 COVID-19 patients admitted to 3 hospitals in Wuhan, China. Clinical, laboratory, radiological, treatment, complication, and outcome data were analyzed. Patients were stratified according to levels of eGFR (≥ 90 vs. 60-89 vs. < 60 mL/min/1.73 m^2). The risk of reaching the composite endpoint-intensive care unit admission, invasive ventilation, or death-was compared. On admission, 25.5% patients had renal impairment (eGFR < 90 mL/min/1.73 m^2), but only 2.6% patients had chronic kidney disease (CKD). The overall in-hospital AKI incidence was 6.7%. Severe illness and comorbidities (hypertension, diabetes, CKD, and cardiovascular/cerebrovascular diseases) were more common among patients with low eGFR (< 90 mL/min/1.73 m^2). Despite the more frequent use of intensive oxygen therapy, continuous blood purification, and glucocorticoid treatment, the prognosis of these patients was unsatisfactory, with the incidence of the composite endpoint (15.4% vs. 19.6% vs. 54.5%; P = 0.000) and complications (AKI, respiratory failure, cardiac injury, coagulation disorders, sepsis, etc.) increasing with decreasing eGFR. Kaplan-Meier survival analysis revealed that patients with eGFR < 90 mL/min/1.73 m^2 or AKI had significantly escalated risks of reaching the composite endpoint. Multivariate regression analysis showed that renal insufficiency (eGFR < 60 mL/min/1.73 m^2) on admission and in-hospital AKI independently predicted poor prognosis among COVID-19 in-patients. And renal impairment on admission was a greater predictor of poor prognosis in non-elderly patients than that in elderly patients. Early and continuous renal-function monitoring and early AKI diagnosis are necessary to predict and prevent the progression of COVID-19.

© 2021. The Author(s).

Conflict of interest statement

The authors declare no competing interests.

- 43 references
- 3 figures

Supplementary info

Publication types, MeSH terms, Grant support Expand

Publication types

- Multicenter Study
- Observational Study
- Research Support, Non-U.S. Gov't

MeSH terms

- Acute Kidney Injury / complications*

- Acute Kidney Injury / diagnosis
- Acute Kidney Injury / epidemiology
- Acute Kidney Injury / therapy
- Aged
- Aged, 80 and over
- COVID-19 / complications*
- COVID-19 / diagnosis
- COVID-19 / epidemiology
- COVID-19 / therapy
- China / epidemiology
- Disease Management
- Female
- Glomerular Filtration Rate
- Hospitalization
- Hospitals
- Humans
- Kaplan-Meier Estimate
- Male
- Middle Aged
- Prognosis
- Retrospective Studies
- SARS-CoV-2 / isolation & purification

Grant support

- 81770731/National Natural Science Foundation of China
- cstc2020jcyj-msxmX0013/Chongqing Municipal Natural Science Foundation

Full text links

Nature Publishing Group Free PMC article

Proceed to details

Cite

Share

☐ 77

Observational Study

Clin Infect Dis

. 2020 Jul 28;71(15):799-806.

doi: 10.1093/cid/ciaa351.

Factors Associated With Prolonged Viral RNA Shedding in Patients with Coronavirus Disease 2019 (COVID-19)

Kaijin Xu [1], Yanfei Chen [1], Jing Yuan [2], Ping Yi [1], Cheng Ding [1], Wenrui Wu [1], Yongtao Li [1], Qin Ni [1], Rongrong Zou [2], Xiaohe Li [2], Min Xu [1], Ying Zhang [1], Hong Zhao [1], Xuan Zhang [1], Liang Yu [1], Junwei Su [1], Guanjing Lang [1], Jun Liu [1], Xiaoxin Wu [1], Yongzheng Guo [1], Jingjing Tao [1], Ding Shi [1], Ling Yu [1], Qing Cao [1], Bing Ruan [1], Lei Liu [2], Zhaoqin Wang [2], Yan Xu [1], Yingxia Liu [2], Jifang Sheng [1], Lanjuan Li [1]
Affiliations Expand

Affiliations

- [1] State Key Laboratory for Diagnosis and Treatment of Infectious Diseases, National Clinical Research Center for Infectious Diseases, Collaborative Innovation Center for Diagnosis and Treatment of Infectious Diseases, Department of Infectious Diseases, The First Affiliated Hospital, College of Medicine, Zhejiang University, Hangzhou City, China.
- [2] Diagnosis and Treatment of Infectious Diseases Research Laboratory, Shenzhen Third People's Hospital, Shenzhen, China.

- PMID: **32271376**
- PMCID: PMC7184421
- DOI: 10.1093/cid/ciaa351

Free PMC article
Observational Study

Factors Associated With Prolonged Viral RNA Shedding in Patients with Coronavirus Disease 2019 (COVID-19)

Kaijin Xu et al. Clin Infect Dis. 2020.
Free PMC article
Show details
Clin Infect Dis
. 2020 Jul 28;71(15):799-806.
doi: 10.1093/cid/ciaa351.

Authors

Kaijin Xu [1], Yanfei Chen [1], Jing Yuan [2], Ping Yi [1], Cheng Ding [1], Wenrui Wu [1], Yongtao Li [1], Qin Ni [1], Rongrong Zou [2], Xiaohe Li [2], Min Xu [1], Ying Zhang [1], Hong Zhao [1], Xuan Zhang [1], Liang Yu [1], Junwei Su [1], Guanjing Lang [1], Jun Liu [1], Xiaoxin Wu [1], Yongzheng Guo

[1], Jingjing Tao [1], Ding Shi [1], Ling Yu [1], Qing Cao [1], Bing Ruan [1], Lei Liu [2], Zhaoqin Wang [2], Yan Xu [1], Yingxia Liu [2], Jifang Sheng [1], Lanjuan Li [1]

Affiliations

- [1] State Key Laboratory for Diagnosis and Treatment of Infectious Diseases, National Clinical Research Center for Infectious Diseases, Collaborative Innovation Center for Diagnosis and Treatment of Infectious Diseases, Department of Infectious Diseases, The First Affiliated Hospital, College of Medicine, Zhejiang University, Hangzhou City, China.
- [2] Diagnosis and Treatment of Infectious Diseases Research Laboratory, Shenzhen Third People's Hospital, Shenzhen, China.

- PMID: **32271376**
- PMCID: PMC7184421
- DOI: 10.1093/cid/ciaa351

Abstract

Background: An outbreak of coronavirus disease 2019 (COVID-19) is becoming a public health emergency. Data are limited on the duration and host factors related to viral shedding.

Methods: In this retrospective study, risk factors associated with severe acute respiratory syndrome coronavirus 2 (SARS-CoV-2) RNA shedding were evaluated in a cohort of 113 symptomatic patients from 2 hospitals outside Wuhan.

Results: The median (interquartile range) duration of SARS-CoV-2 RNA detection was 17 (13-22) days as measured from illness onset. When comparing patients with early (<15 days) and late (≥15 days after illness onset) viral RNA clearance, prolonged SARS-CoV-2 RNA shedding was associated with male sex (P = .009), old age (P = .033), concomitant hypertension (P = .009), delayed admission to hospital after illness onset (P = .001), severe illness at admission (P = .049), invasive mechanical ventilation (P = .006), and corticosteroid treatment (P = .025). Patients with longer SARS-CoV-2 RNA shedding duration had slower recovery of body temperature (P < .001) and focal absorption on radiograph images (P < .001) than patients with early SARS-CoV-2 RNA clearance. Male sex (OR, 3.24; 95% CI, 1.31-8.02), delayed hospital admission (OR, 1.30; 95% CI, 1.10-1.54), and invasive mechanical ventilation (OR, 9.88; 95% CI, 1.11-88.02) were independent risk factors for prolonged SARS-CoV-2 RNA shedding.

Conclusions: Male sex, delayed admission to hospital after illness onset, and invasive mechanical ventilation were associated with prolonged SARS-CoV-2 RNA shedding. Hospital admission and general treatments should be started as soon as possible in symptomatic COVID-19 patients, especially male patients.

Keywords: COVID-19; SARS-COV-2; coronavirus; risk factors; viral shedding.

© The Author(s) 2020. Published by Oxford University Press for the Infectious Diseases Society of America. All rights reserved. For permissions, e-mail: journals.permissions@oup.com.

Supplementary info

Publication types, MeSH terms, Substances Expand

Publication types

- Multicenter Study
- Observational Study
- Research Support, Non-U.S. Gov't

MeSH terms

- Adult
- Betacoronavirus / isolation & purification*
- Betacoronavirus / pathogenicity
- COVID-19
- China / epidemiology
- Cohort Studies
- Coronavirus Infections / epidemiology
- Coronavirus Infections / virology*
- Disease Progression
- Female
- Hospitalization
- Humans
- Male
- Middle Aged
- Pandemics
- Pneumonia, Viral / epidemiology
- Pneumonia, Viral / virology*
- RNA, Viral / isolation & purification*
- Respiration, Artificial / adverse effects
- Retrospective Studies
- Risk Factors
- SARS-CoV-2
- Sex Factors
- Time Factors
- Time-to-Treatment
- Virus Shedding*

Substances

- RNA, Viral

Full text links

OXFORD ACADEMIC Silverchair Information Systems Free PMC article

Proceed to details
Cite
Share
78
Observational Study
Clin Infect Dis
. 2020 Nov 19;71(16):2276-2278.
doi: 10.1093/cid/ciaa579.

Clinical Features and Outcomes of Patients With Human Immunodeficiency Virus With COVID-19

Cristina Gervasoni [1] [2], Paola Meraviglia [1], Agostino Riva [1], Andrea Giacomelli [1], Letizia Oreni [1], Davide Minisci [1], Chiara Atzori [1], Annalisa Ridolfo [1], Dario Cattaneo [2] [3]
Affiliations Expand

Affiliations

- [1] Department of Infectious Diseases, ASST Fatebenefratelli Sacco University Hospital, Milan, Italy.
- [2] Gestione Ambulatoriale Politerapie (GAP) Outpatient Clinic, ASST Fatebenefratelli Sacco University Hospital, Milan, Italy.
- [3] Unit of Clinical Pharmacology, ASST Fatebenefratelli Sacco University Hospital, Milan, Italy.

- PMID: **32407467**
- PMCID: PMC7239244
- DOI: 10.1093/cid/ciaa579

Free PMC article
Observational Study

Clinical Features and Outcomes of Patients With Human Immunodeficiency Virus With COVID-19

Cristina Gervasoni et al. Clin Infect Dis. 2020.
Free PMC article
Show details
Clin Infect Dis
. 2020 Nov 19;71(16):2276-2278.
doi: 10.1093/cid/ciaa579.

Authors

Cristina Gervasoni [1] [2], Paola Meraviglia [1], Agostino Riva [1], Andrea Giacomelli [1], Letizia Oreni [1], Davide Minisci [1], Chiara Atzori [1], Annalisa Ridolfo [1], Dario Cattaneo [2] [3]

Affiliations

- [1] Department of Infectious Diseases, ASST Fatebenefratelli Sacco University Hospital, Milan, Italy.
- [2] Gestione Ambulatoriale Politerapie (GAP) Outpatient Clinic, ASST Fatebenefratelli Sacco University Hospital, Milan, Italy.
- [3] Unit of Clinical Pharmacology, ASST Fatebenefratelli Sacco University Hospital, Milan, Italy.

- PMID: **32407467**
- PMCID: PMC7239244
- DOI: 10.1093/cid/ciaa579

Abstract

Little is known about the clinical outcomes of patients with human immunodeficiency virus infected with SARS-CoV-2. We describe 47 patients referred to our hospital between 21 February and 16 April 2020 with proven/probable COVID-19, 45 (96%) of whom fully recovered and 2 who died.

Keywords: COVID-19; HIV; SARS-CoV-2; observational study; real-life.

© The Author(s) 2020. Published by Oxford University Press for the Infectious Diseases Society of America. All rights reserved. For permissions, e-mail: journals.permissions@oup.com.

Comment in

- Reply to Childs et al.
  Gervasoni C, Meraviglia P, Cattaneo D. Gervasoni C, et al. Clin Infect Dis. 2020 Nov 5;71 (8):2023. doi: 10.1093/cid/ciaa659. Clin Infect Dis. 2020. PMID: 32459828 No abstract available.
- Hospitalized Patients With COVID-19 and Human Immunodeficiency Virus: A Case Series.
  Childs K, Post FA, Norcross C, Ottaway Z, Hamlyn E, Quinn K, Juniper T, Taylor C. Childs K, et al. Clin Infect Dis. 2020 Nov 5;71(8):2021-2022. doi: 10.1093/cid/ciaa657. Clin Infect Dis. 2020. PMID: 32459833 Free PMC article. No abstract available.

Supplementary info

Publication types, MeSH terms, Substances Expand

Publication types

- Observational Study

MeSH terms

- Adult
- Antiviral Agents / therapeutic use
- COVID-19 / complications*
- COVID-19 / drug therapy
- Female
- HIV Infections / complications*
- Hospitalization
- Humans
- Italy
- Male
- Middle Aged
- Prognosis
- Retrospective Studies
- Risk Factors
- SARS-CoV-2

Substances

- Antiviral Agents

Full text links

OXFORD ACADEMIC Silverchair Information Systems Free PMC article
Proceed to details
Cite
Share
☐ 79
Observational Study
Rev Assoc Med Bras (1992)
. 2021 Jul;67(7):997-1002.
doi: 10.1590/1806-9282.20210433.

The impact of healthcare-associated infections on COVID-19 mortality: a cohort study from a Brazilian public hospital

Bruna Cuoco Provenzano [1], Thiago Bartholo [1], Marcelo Ribeiro-Alves [2], Ana Paula Gomes Dos Santos [1], Thiago Thomaz Mafort [1], Marcos Cesar Santos de Castro [1], Jose Gustavo Pugliese de

Oliveira [1], Leonardo Palermo Bruno [1], Agnaldo José Lopes [1], Claudia Henrique da Costa [1], Rogerio Rufino [1]
Affiliations Expand

Affiliations

- [1] Universidade do Estado do Rio de Janeiro, Department of Pneumology and Tisiology - Rio de Janeiro (RJ), Brazil.
- [2] Fundação Oswaldo Cruz - Rio de Janeiro (RJ), Brazil.

- PMID: **34817513**
- DOI: 10.1590/1806-9282.20210433

Free article
Observational Study

The impact of healthcare-associated infections on COVID-19 mortality: a cohort study from a Brazilian public hospital

Bruna Cuoco Provenzano et al. Rev Assoc Med Bras (1992). 2021 Jul.
Free article
Show details
Rev Assoc Med Bras (1992)
. 2021 Jul;67(7):997-1002.
doi: 10.1590/1806-9282.20210433.

Authors

Bruna Cuoco Provenzano [1], Thiago Bartholo [1], Marcelo Ribeiro-Alves [2], Ana Paula Gomes Dos Santos [1], Thiago Thomaz Mafort [1], Marcos Cesar Santos de Castro [1], Jose Gustavo Pugliese de Oliveira [1], Leonardo Palermo Bruno [1], Agnaldo José Lopes [1], Claudia Henrique da Costa [1], Rogerio Rufino [1]

Affiliations

- [1] Universidade do Estado do Rio de Janeiro, Department of Pneumology and Tisiology - Rio de Janeiro (RJ), Brazil.
- [2] Fundação Oswaldo Cruz - Rio de Janeiro (RJ), Brazil.

- PMID: **34817513**
- DOI: 10.1590/1806-9282.20210433

Abstract

Objective: This study aims to analyze the risk factors for in-hospital mortality in a cohort of patients admitted to a newly adapted intensive care unit in a public hospital in Rio de Janeiro.

Methods: This was an observational, retrospective, and descriptive study. Data were obtained from electronic medical records. Coronavirus disease 2019 (COVID-19) was diagnosed by detecting viral ribonucleic acid using reverse transcription polymerase chain reaction. Factors associated with the risk/protection from death were determined using the odds ratio and adjusted odds ratio.

Results: Fifty-one patients were admitted to the hospital. The median age of the patients was 63 years, 60% were male patients, and 54% were white patients. Sixty-seven percent of the patients were diagnosed with COVID-19. Sepsis at admission increased the chance of in-hospital death by 21 times (adjusted odds ratio=21.06 [0.79-555.2]; p=0.06). The strongest risk factor for death was the development of septic shock during hospitalization (adjusted odds ratio=98.56 [2.75-352.5]; p=0.01), and one in four patients had multidrug-resistant bacteria. Mechanical ventilation, vasopressors, neuromuscular blockers, and sedatives were also the risk factors for in-hospital mortality. The in-hospital mortality rate was 41%, and the mortality rate of patients on mechanical ventilation was 60%. The diagnosis of COVID-19 was not statistically related to the adverse outcomes.

Conclusions: In this cohort, the strongest risk factor for in-hospital death was the development of nosocomial septic shock. Healthcare-associated infections have a significant impact on mortality rates. Therefore, to have a better outcome, it is important to consider not only the availability of beds but also the way healthcare is delivered.

Supplementary info

Publication types, MeSH terms Expand

Publication types

- Observational Study

MeSH terms

- Brazil / epidemiology
- COVID-19*
- Cohort Studies
- Cross Infection*
- Delivery of Health Care
- Hospital Mortality
- Hospitalization
- Hospitals, Public
- Humans
- Intensive Care Units
- Male
- Middle Aged
- Retrospective Studies

- Risk Factors
- SARS-CoV-2

Full text links

free full text available at SciELO.org Scientific Electronic Library Online
Proceed to details
Cite
Share
80
Observational Study
South Med J
. 2021 May;114(5):305-310.
doi: 10.14423/SMJ.0000000000001245.

COVID-19 in a Mississippi Community Hospital

Ijlal Babar [1], Okechukwu Ekenna [1], Maggie Ramsey Clarkson [1], Daralyn Boudreaux [1], William Bennett [1], Randy Roth [1]
Affiliations Expand

Affiliation

- [1] From the Department of Medicine, Pulmonary, and Critical Care, the Department of Medicine and Infectious Disease, the Department of Clinical Research, the Department of Infection Prevention, and the Department of Medicine, Singing River Health System, Ocean Springs, Mississippi.

- PMID: **33942116**
- DOI: 10.14423/SMJ.0000000000001245

Observational Study

COVID-19 in a Mississippi Community Hospital

Ijlal Babar et al. South Med J. 2021 May.
Show details
South Med J
. 2021 May;114(5):305-310.
doi: 10.14423/SMJ.0000000000001245.

Authors

Ijlal Babar [1], Okechukwu Ekenna [1], Maggie Ramsey Clarkson [1], Daralyn Boudreaux [1], William Bennett [1], Randy Roth [1]

Affiliation

- [1] From the Department of Medicine, Pulmonary, and Critical Care, the Department of Medicine and Infectious Disease, the Department of Clinical Research, the Department of Infection Prevention, and the Department of Medicine, Singing River Health System, Ocean Springs, Mississippi.

- PMID: **33942116**
- DOI: 10.14423/SMJ.0000000000001245

Abstract

Objectives: Mississippi recorded the first case of coronavirus disease 2019 (COVID-19) on March 11, 2020. This report describes the initial COVID-19 experience of the single healthcare system serving Jackson County, Mississippi. The intent of this retrospective review of COVID-19 hospitalized patients was to identify any characteristics or interventions amenable to improving care management and clinical outcomes for patients within our community hospital.

Methods: All hospitalized patients 18 years of age and older in our health system with positive tests for COVID-19 (severe acute respiratory syndrome-coronavirus-2 [SARS CoV-2]) by reverse transcriptase-polymerase chain reaction between March 15 and April 10, 2020 are included in this retrospective observational report.

Results: During the study period, 158 patients of the 1384 tested (11.4%) were positive for COVID-19 infection. Of the 158 patients, 41 (26%) were hospitalized, with 17 (41%) admitted to the intensive care unit (ICU). The remaining 24 patients did not require ICU admission. The mean age of the 158 COVID-19-positive patients was 55 years (range 2-103). Obesity was noted in 68% of the hospitalized patients, including 13 (54%) of the non-ICU patients and 15 (88%) of the ICU patients. All 9 deceased patients were obese. Twelve of 17 patients received invasive mechanical ventilation (IMV) and 3 patients received only high-flow nasal cannula oxygen. Only 25% (3 of 12) of the IMV patients were successfully extubated during the study period. The median duration on IMV was 17 days (range 4-35). The mortality in the 158 COVID-19-positive patients was 5.7% (9 of 158). None of the 24 non-ICU patients died. The ICU mortality rate was 53% (9 of 17).

Conclusions: This report describes a community hospital experience with COVID-19. Patient outcome was comparable to that reported at larger centers. Obesity was a major comorbidity and correlated with adverse outcomes. Amidst the initial wave of COVID-19 with high demand for inpatient treatment, it is reassuring that appropriate care can be provided in a community health system.

Supplementary info

Publication types, MeSH terms Expand

Publication types

- Observational Study

MeSH terms

- Adolescent
- Adult
- Aged
- Aged, 80 and over
- COVID-19 / diagnosis
- COVID-19 / epidemiology
- COVID-19 / therapy*
- COVID-19 Nucleic Acid Testing
- Child
- Child, Preschool
- Critical Care / methods*
- Critical Care / organization & administration
- Female
- Hospitals, Community* / organization & administration
- Humans
- Male
- Middle Aged
- Mississippi / epidemiology
- Retrospective Studies
- Treatment Outcome
- Young Adult

Full text links

SMJ Full Text Southern Medical Association
Proceed to details
Cite
Share
81
Observational Study
PLoS One
. 2021 Jun 18;16(6):e0253154.
doi: 10.1371/journal.pone.0253154. eCollection 2021.

COVID-19 in persons aged 70+ in an early affected German district: Risk factors, mortality and post-COVID care needs-A

[retrospective observational study of hospitalized and non-hospitalized patients]

Matthias L Herrmann [1 2 3], Johannes-Martin Hahn [4], Birgit Walter-Frank [5], Desiree M Bollinger [5], Kristina Schmauder [6], Günter Schnauder [6], Michael Bitzer [6], Nisar P Malek [6], Gerhard W Eschweiler [1 2], Siri Göpel [6 7]
Affiliations Expand

Affiliations

- [1] Geriatric Center, University Hospital Tübingen, Tübingen, Germany.
- [2] Department of Psychiatry and Psychotherapy, University Hospital Tübingen, Tübingen, Germany.
- [3] Department of Neurology and Neuroscience, Medical Center-University of Freiburg, Faculty of Medicine, University of Freiburg, Freiburg, Germany.
- [4] Paul-Lechler-Hospital Tübingen, Tübingen, Germany.
- [5] Health Department, Tübingen District Administration, Tübingen, Germany.
- [6] Department of Internal Medicine 1, University Hospital Tübingen, Tübingen, Germany.
- [7] Comprehensive Infectious Disease Center Tübingen, Tübingen, Germany.

- PMID: **34143823**
- PMCID: PMC8213147
- DOI: 10.1371/journal.pone.0253154

Free PMC article
Observational Study

COVID-19 in persons aged 70+ in an early affected German district: Risk factors, mortality and post-COVID care needs-A retrospective observational study of hospitalized and non-hospitalized patients

Matthias L Herrmann et al. PLoS One. 2021.
Free PMC article
Show details
PLoS One
. 2021 Jun 18;16(6):e0253154.
doi: 10.1371/journal.pone.0253154. eCollection 2021.

Authors

Matthias L Herrmann [1,2,3], Johannes-Martin Hahn [4], Birgit Walter-Frank [5], Desiree M Bollinger [5], Kristina Schmauder [6], Günter Schnauder [6], Michael Bitzer [6], Nisar P Malek [6], Gerhard W Eschweiler [1,2], Siri Göpel [6,7]

Affiliations

- [1] Geriatric Center, University Hospital Tübingen, Tübingen, Germany.
- [2] Department of Psychiatry and Psychotherapy, University Hospital Tübingen, Tübingen, Germany.
- [3] Department of Neurology and Neuroscience, Medical Center-University of Freiburg, Faculty of Medicine, University of Freiburg, Freiburg, Germany.
- [4] Paul-Lechler-Hospital Tübingen, Tübingen, Germany.
- [5] Health Department, Tübingen District Administration, Tübingen, Germany.
- [6] Department of Internal Medicine 1, University Hospital Tübingen, Tübingen, Germany.
- [7] Comprehensive Infectious Disease Center Tübingen, Tübingen, Germany.

- PMID: **34143823**
- PMCID: PMC8213147
- DOI: 10.1371/journal.pone.0253154

Abstract

Background: Cohorts of hospitalized COVID-19 patients have been studied in several countries since the beginning of the pandemic. So far, there is no complete survey of older patients in a German district that includes both outpatients and inpatients. In this retrospective observational cohort study, we aimed to investigate risk factors, mortality, and functional outcomes of all patients with COVID-19 aged 70 and older living in the district of Tübingen in the southwest of Germany.

Methods: We retrospectively analysed all 256 patients who tested positive for SARS-CoV-2 in one of the earliest affected German districts during the first wave of the disease from February to April 2020. To ensure inclusion of all infected patients, we analysed reported data from the public health department as well as the results of a comprehensive screening intervention in all nursing homes of the district (n = 1169). Furthermore, we examined clinical data of all hospitalized patients with COVID-19 (n = 109).

Results: The all-cause mortality was 18%. Screening in nursing homes showed a point-prevalence of 4.6%. 39% of residents showed no COVID-specific symptoms according to the official definition at that time. The most important predictors of mortality were the need for inpatient treatment (odds ratio (OR): 3.95 [95%-confidence interval (CI): 2.00-7.86], $p<0.001$) and care needs before infection (non-hospitalized patients: OR: 3.79 [95%-CI: 1.01-14.27], $p = 0.037$, hospitalized patients: OR: 2.89 [95%-CI 1.21-6.92], $p = 0.015$). Newly emerged care needs were a relevant complication of COVID-19: 27% of previously self-sufficient patients who survived the disease were not able to return to their home environment after discharge from the hospital.

Conclusion: Our findings demonstrate the importance of a differentiated view of risk groups and long-term effects within the older population. These findings should be included in the political and social debate during the ongoing pandemic to evaluate the true effect of COVID-19 on healthcare systems and individual functional status.

Conflict of interest statement

The authors have declared that no competing interests exist.

- 23 references
- 3 figures

Supplementary info

Publication types, MeSH terms, Grant support Expand

Publication types

- Observational Study
- Research Support, Non-U.S. Gov't

MeSH terms

- Aged
- Aged, 80 and over
- COVID-19 / epidemiology
- COVID-19 / prevention & control*
- COVID-19 / virology
- Data Collection / methods
- Data Collection / statistics & numerical data
- Female
- Germany / epidemiology
- Hospitalization / statistics & numerical data*
- Humans
- Inpatients / statistics & numerical data*
- Male
- Nursing Homes / statistics & numerical data*
- Outpatients / statistics & numerical data*
- Pandemics
- Prevalence
- Retrospective Studies
- Risk Factors
- SARS-CoV-2 / isolation & purification*
- SARS-CoV-2 / physiology

Grant support

The authors received support from the Open Access Publishing Fund of the University of Tübingen. No additional external funding was received for this study.

Full text links

OPEN ACCESS TO FULL TEXT PLOS ONE Public Library of Science Free PMC article

Proceed to details

Cite

Share

82

Observational Study

Swiss Med Wkly
. 2021 Mar 1;151:w20482.
doi: 10.4414/smw.2021.20482.

Acute kidney injury in patients with COVID-19: a retrospective cohort study from Switzerland

Matthias Diebold [1], Stefan Schaub [1], Emmanuelle Landmann [1], Jürg Steiger [1], Michael Dickenmann [1]

Affiliations Expand

Affiliation

- [1] Clinic for Transplantation Immunology and Nephrology, University Hospital Basel, Switzerland.

- PMID: **33706383**
- DOI: 10.4414/smw.2021.20482

Free article

Observational Study

Acute kidney injury in patients with COVID-19: a retrospective cohort study from Switzerland

Matthias Diebold et al. Swiss Med Wkly. 2021.

Free article

Show details

Swiss Med Wkly
. 2021 Mar 1;151:w20482.
doi: 10.4414/smw.2021.20482.

Authors

Matthias Diebold [1], Stefan Schaub [1], Emmanuelle Landmann [1], Jürg Steiger [1], Michael Dickenmann [1]

Affiliation

- [1] Clinic for Transplantation Immunology and Nephrology, University Hospital Basel, Switzerland.

- PMID: **33706383**
- DOI: 10.4414/smw.2021.20482

Abstract

BACKGROUND: Data about patients in Europe with corona virus disease-2019 (COVID-19) and acute kidney injury (AKI) are scarce. We examined characteristics, presentation and risk factors of AKI in patients hospitalised with COVID-19 in a tertiary hospital in Switzerland. METHODS: We reviewed health records of patients hospitalised with a positive nasopharyngeal polymerase chain reaction test for SARS-CoV2 between 1 February and 30 June 2020, at the University Hospital of Basel. The nadir creatinine of the hospitalisation was used as baseline. AKI was defined according the KDIGO guidelines as a 1.5× increase of baseline creatinine and in-hospital renal recovery as a discharge creatinine <1.25× baseline creatinine. Least absolute shrinkage and selection operator (LASSO) regression was performed to select predictive variables of AKI. Based on this a final model was chosen. RESULTS: Of 188 patients with COVID-19, 41 (22%) developed AKI, and 11 (6%) required renal replacement therapy. AKI developed after a median of 9 days (interquartile range [IQR] 5-12) after the first symptoms and a median of 1 day (IQR 0-5) after hospital admission. The peak AKI stages were stage 1 in 39%, stage 2 in 24% and stage 3 in 37%. A total of 29 (15%) patients were admitted to the intensive care unit and of these 23 (79%) developed AKI. In-hospital renal recovery at discharge was observed in 61% of all AKI episodes. In-hospital mortality was 27% in patients with AKI and 10% in patients without AKI. Age (adjusted odds ratio [aOR] 1.04, 95% confidence interval [CI] 1.01–1.08; p = 0.024), history of chronic kidney disease (aOR 3.47, 95% CI 1.16–10.49;p = 0.026), C-reactive protein levels (aOR 1.09, 95% CI 1.03–1.06; p = 0.002) and creatinine kinase (aOR 1.03, 95% CI 1.01–1.06; p = 0.002) were associated with development of AKI. CONCLUSIONS: AKI is common in hospitalised patients with COVID-19 and more often seen in patients with severe COVID-19 illness. AKI is associated with a high in-hospital mortality.

Supplementary info

Publication types, MeSH terms, Substances Expand

Publication types

- Observational Study

MeSH terms

- Acute Kidney Injury / etiology*

- Acute Kidney Injury / mortality
- Acute Kidney Injury / pathology
- Age Factors
- Aged
- COVID-19 / complications*
- COVID-19 / epidemiology*
- COVID-19 / mortality
- COVID-19 / pathology
- Comorbidity
- Creatinine / blood
- Female
- Humans
- Male
- Middle Aged
- Retrospective Studies
- Risk Factors
- SARS-CoV-2
- Severity of Illness Index
- Sex Factors
- Socioeconomic Factors
- Switzerland
- Tertiary Care Centers
- Time Factors

Substances

- Creatinine

Full text links

Open access to full text on Swiss Medical Weekly EMH Swiss Medical Publishers Ltd.

Proceed to details

Cite

Share

☐ 83

Arch Dis Child

. 2021 Sep;106(9):918-919.
doi: 10.1136/archdischild-2020-320628. Epub 2020 Dec 22.

Association between suicide behaviours in children and adolescents and the COVID-19

lockdown in Paris, France: a retrospective observational study

Maymouna Mourouvaye[#,1], Hugo Bottemanne[#,2,3], Guillaume Bonny[1], Lola Fourcade[1], Francois Angoulvant[4,5], Jérémie F Cohen[6,7], Lisa Ouss[1]
Affiliations Expand

Affiliations

- [1] Department of Child and Adolescent Psychiatry, Necker-Enfants Malades University Hospital, University of Paris, Assistance Publique - Hôpitaux de Paris (AP-HP), Paris, Île-de-France, France.
- [2] Department of Child and Adolescent Psychiatry, Necker-Enfants Malades University Hospital, University of Paris, Assistance Publique - Hôpitaux de Paris (AP-HP), Paris, Île-de-France, France hugo.bottemanne@gmail.com.
- [3] Paris Brain Institute - Institut du Cerveau (ICM), UMR 7225 / UMRS 1127, Sorbonne University / CNRS / INSERM, Paris, France, Paris, France.
- [4] Emergency Department, Necker-Enfants Malades University Hospital, University of Paris, Assistance Publique - Hôpitaux de Paris (AP-HP), Paris, France.
- [5] INSERM, Centre de Recherche des Cordeliers, UMRS 1138, Sorbonne University, University of Paris, Paris, France.
- [6] INSERM UMR 1153, Obstetrical, Perinatal and Pediatric Epidemiology Research Team (EPOPé), Center for Epidemiology and Statistics (CRESS), DHU Risks in Pregnancy, Paris, France.
- [7] Department of General Pediatrics and Pediatric Infectious Diseases, Necker-Enfants Malades University Hospital, University of Paris, Assistance Publique - Hôpitaux de Paris (AP-HP), Paris, France.

[#] Contributed equally.

- PMID: **33355154**
- PMCID: PMC8380898
- DOI: 10.1136/archdischild-2020-320628

Free PMC article

Association between suicide behaviours in children and adolescents and the COVID-19 lockdown in Paris, France: a retrospective observational study

Maymouna Mourouvaye et al. Arch Dis Child. 2021 Sep.
Free PMC article
Show details

Arch Dis Child
. 2021 Sep;106(9):918-919.
doi: 10.1136/archdischild-2020-320628. Epub 2020 Dec 22.

Authors

Maymouna Mourouvaye [#, 1], Hugo Bottemanne [#, 2, 3], Guillaume Bonny [1], Lola Fourcade [1], Francois Angoulvant [4, 5], Jérémie F Cohen [6, 7], Lisa Ouss [1]

Affiliations

- [1] Department of Child and Adolescent Psychiatry, Necker-Enfants Malades University Hospital, University of Paris, Assistance Publique - Hôpitaux de Paris (AP-HP), Paris, Île-de-France, France.
- [2] Department of Child and Adolescent Psychiatry, Necker-Enfants Malades University Hospital, University of Paris, Assistance Publique - Hôpitaux de Paris (AP-HP), Paris, Île-de-France, France hugo.bottemanne@gmail.com.
- [3] Paris Brain Institute - Institut du Cerveau (ICM), UMR 7225 / UMRS 1127, Sorbonne University / CNRS / INSERM, Paris, France, Paris, France.
- [4] Emergency Department, Necker-Enfants Malades University Hospital, University of Paris, Assistance Publique - Hôpitaux de Paris (AP-HP), Paris, France.
- [5] INSERM, Centre de Recherche des Cordeliers, UMRS 1138, Sorbonne University, University of Paris, Paris, France.
- [6] INSERM UMR 1153, Obstetrical, Perinatal and Pediatric Epidemiology Research Team (EPOPé), Center for Epidemiology and Statistics (CRESS), DHU Risks in Pregnancy, Paris, France.
- [7] Department of General Pediatrics and Pediatric Infectious Diseases, Necker-Enfants Malades University Hospital, University of Paris, Assistance Publique - Hôpitaux de Paris (AP-HP), Paris, France.

[#] Contributed equally.

- PMID: **33355154**
- PMCID: PMC8380898
- DOI: 10.1136/archdischild-2020-320628

Erratum in

- Correction: *Association between suicide behaviours in children and adolescents and the COVID-19 lockdown in Paris, France: a retrospective observational study.*
  [No authors listed] [No authors listed] Arch Dis Child. 2021 Nov;106(11):e42. doi: 10.1136/archdischild-2020-320628corr1. Arch Dis Child. 2021. PMID: 34670740 Free PMC article. No abstract available.

Abstract

This retrospective observational study conducted in Necker Hospital for Sick Children, France (January 2018-June 2020) evaluated a potential temporal association between admissions for suicide behaviours in children and adolescents and the national COVID-19 lockdown (March-May 2020). During the study period, 234 patients were admitted for suicide behaviours (28% male;

mean age 13.4 years). Using Poisson regression, we found a significant decrease in the incidence of admissions for suicide behaviour during the lockdown (adjusted incidence rate ratio: 0.46; 95% CI 0.24 to 0.86). This association might result from reduced help-seeking and decreased hospital admission rates during the lockdown, as well as cognitive and environmental factors. Further multicentre studies should be conducted to confirm these findings and investigate whether a compensatory rise in admissions for suicide behaviour occurred in the postlockdown period.

Keywords: COVID-19; adolescent health; child psychiatry; epidemiology.

© Author(s) (or their employer(s)) 2021. No commercial re-use. See rights and permissions. Published by BMJ.

Conflict of interest statement

Competing interests: None declared.

- 6 references

Supplementary info

MeSH terms Expand

MeSH terms

- Adolescent
- Adolescent Behavior*
- COVID-19 / epidemiology*
- COVID-19 / psychology
- Child
- Child Behavior*
- Emergency Service, Hospital / statistics & numerical data*
- Female
- Follow-Up Studies
- Hospitalization / trends
- Humans
- Incidence
- Male
- Paris / epidemiology
- Population Surveillance*
- Quarantine*
- Retrospective Studies
- SARS-CoV-2
- Suicide / statistics & numerical data*

Full text links

BMJ Full Text HighWire Free PMC article
Proceed to details
Cite
Share
84

Observational Study

Med Intensiva (Engl Ed)
. Jan-Feb 2021;45(1):27-34.
doi: 10.1016/j.medin.2020.06.015. Epub 2020 Jul 11.

Acute respiratory distress syndrome due to COVID-19. Clinical and prognostic features from a medical Critical Care Unit in Valencia, Spain

[Article in English, Spanish]

P Ramírez [1], M Gordón [2], M Martín-Cerezuela [2], E Villarreal [2], E Sancho [2], M Padrós [2], J Frasquet [3], G Leyva [2], I Molina [2], M Barrios [2], S Gimeno [2], Á Castellanos [2]
Affiliations Expand

Affiliations

- [1] Critical care department, Hospital Universitario y Politécnico la Fe, Valencia, Spain. Electronic address: Ramirez_pau@gva.es.
- [2] Critical care department, Hospital Universitario y Politécnico la Fe, Valencia, Spain.
- [3] Microbiology Department, Hospital Universitario y Politécnico la Fe, Valencia, Spain.

- PMID: **32919796**
- PMCID: PMC7836701
- DOI: 10.1016/j.medin.2020.06.015

Free PMC article
Observational Study

Acute respiratory distress syndrome due to COVID-19. Clinical and prognostic features from a medical Critical Care Unit in Valencia, Spain

[Article in English, Spanish]
P Ramírez et al. Med Intensiva (Engl Ed). Jan-Feb 2021.

Free PMC article

Show details

Med Intensiva (Engl Ed)
. Jan-Feb 2021;45(1):27-34.
doi: 10.1016/j.medin.2020.06.015. Epub 2020 Jul 11.

Authors

P Ramírez [1], M Gordón [2], M Martín-Cerezuela [2], E Villarreal [2], E Sancho [2], M Padrós [2], J Frasquet [3], G Leyva [2], I Molina [2], M Barrios [2], S Gimeno [2], Á Castellanos [2]

Affiliations

- [1] Critical care department, Hospital Universitario y Politécnico la Fe, Valencia, Spain. Electronic address: Ramirez_pau@gva.es.
- [2] Critical care department, Hospital Universitario y Politécnico la Fe, Valencia, Spain.
- [3] Microbiology Department, Hospital Universitario y Politécnico la Fe, Valencia, Spain.

- PMID: **32919796**
- PMCID: PMC7836701
- DOI: 10.1016/j.medin.2020.06.015

Abstract
in English, Spanish

Objective: Information from critically ill coronavirus disease 2019 (COVID-19) patients is limited and in many cases coming from health systems approaches different from the national public systems existing in most countries in Europe. Besides, patient follow-up remains incomplete in many publications. Our aim is to characterize acute respiratory distress syndrome (ARDS) patients admitted to a medical critical care unit (MCCU) in a referral hospital in Spain.

Design: Retrospective case series of consecutive ARDS COVID-19 patients admitted and treated in our MCCU.

Setting: 36-bed MCCU in referral tertiary hospital.

Patients and participants: SARS-CoV-2 infection confirmed by real-time reverse transcriptase-polymerase chain reaction (RT-PCR) assay of nasal/pharyngeal swabs.

Interventions: None MAIN VARIABLES OF INTEREST: Demographic and clinical data were collected, including data on clinical management, respiratory failure, and patient mortality.

Results: Forty-four ARDS COVID-19 patients were included in the study. Median age was 61.50 (53.25 - 67) years and most of the patients were male (72.7%). Hypertension and dyslipidemia were the most frequent co-morbidities (52.3 and 36.4% respectively). Steroids (1mg/Kg/day) and tocilizumab were administered in almost all patients (95.5%). 77.3% of the patients needed invasive mechanical ventilation for a median of 16 days [11-28]. Prone position ventilation was performed in 33 patients (97%) for a median of 3 sessions [2-5] per patient. Nosocomial infection was diagnosed in 13 patients (29.5%). Tracheostomy was performed in ten patients (29.4%). At study closing all patients had been discharged from the CCU and only two (4.5%) remained in

hospital ward. MCCU length of stay was 18 days [10-27]. Mortality at study closing was 20.5% (n 9); 26.5% among ventilated patients.

Conclusions: The seven-week period in which our MCCU was exclusively dedicated to COVID-19 patients has been challenging. Despite the severity of the patients and the high need for invasive mechanical ventilation, mortality was 20.5%.

Objetivo: La información de pacientes críticos con enfermedad por coronavirus 2019 (COVID-19) es limitada y, en muchos casos, proviene de sistemas de salud diferentes a la organización pública de la mayoría de los países de Europa. Además, el seguimiento del paciente sigue siendo incompleto en muchas publicaciones. Nuestro objetivo es caracterizar a los pacientes con síndrome de distres respiratorio agudo (SDRA) ingresados en una unidad de cuidados críticos médicos (MCCU) en un hospital de referencia en España.

Diseño: Serie retrospectiva de casos de pacientes consecutivos con SDRA por COVID-19 ingresados y tratados en nuestra MCCU.

Lugar: UCC de 36 camas en un hospital terciario de referencia

Pacientes y participantes: Infección por SARS-CoV-2 confirmada por ensayo en tiempo real de la transcriptasa inversa-reacción en cadena de la polimerasa (RT-PCR) de hisopos nasales/faríngeos.

Intervenciones: Ninguna

Principales variables de interés: Se recopilaron datos demográficos y clínicos, incluidos datos sobre manejo clínico, insuficiencia respiratoria y mortalidad del paciente.

Resultados: Cuarenta y cuatro pacientes con SDRA por COVID-19 fueron incluidos en el estudio. La mediana de edad fue de 61.50 (53.25 - 67) años y la mayoría de los pacientes eran hombres (72.7%). La hipertensión y la dislipidemia fueron las comorbilidades más frecuentes (52,3 y 36,4%, respectivamente). Se administraron esteroides (1mg/kg/día) y tocilizumab en casi todos los pacientes (95,5%). El 77,3% de los pacientes necesitaron ventilación mecánica invasiva durante una mediana de 16 días [11-28]. La ventilación en posición prono se realizó en 33 pacientes (97%) con una mediana de 3 sesiones [2-5] por paciente. Se diagnosticó una infección nosocomial en 13 pacientes (29,5%). La traqueotomía se realizó en diez pacientes (29,4%). Al cierre del estudio, todos los pacientes habían sido dados de alta de la MCCU y solo dos permanecían hospitalizados. La estancia en MCCU fue de 18 días [10-27]. La mortalidad al cierre del estudio fue del 20,5% (n 9); 26.5% para pacientes ventilados.

Conclusiones: El período de siete semanas en el que nuestra MCCU se dedicó exclusivamente a pacientes con COVID-19 ha sido un gran desafío. A pesar de la gravedad de los pacientes y la elevada necesidad de ventilación mecánica invasiva, la mortalidad fue del 20,5%.

Keywords: ARDS; COVID-19; Critical care; Cuidados críticos; Neumonía viral; SARS-CoV-2; SDRA; Viral pneumonia.

Copyright © 2020 Elsevier España, S.L.U. y SEMICYUC. All rights reserved.

- 29 references
- 1 figure

Supplementary info

Publication types, MeSH terms, Substances Expand

Publication types

- Observational Study

MeSH terms

- Aged
- Antibodies, Monoclonal, Humanized / therapeutic use
- COVID-19 / complications*
- COVID-19 / epidemiology
- COVID-19 / mortality
- COVID-19 / therapy
- Comorbidity
- Critical Illness
- Cross Infection / epidemiology
- Diabetes Mellitus / epidemiology
- Dyslipidemias / epidemiology
- Female
- Humans
- Hypertension / epidemiology
- Length of Stay
- Male
- Middle Aged
- Prognosis
- Prone Position
- Respiration, Artificial / methods
- Respiration, Artificial / statistics & numerical data
- Respiratory Distress Syndrome / etiology*
- Respiratory Distress Syndrome / mortality
- Retrospective Studies
- SARS-CoV-2*
- Spain / epidemiology
- Steroids / therapeutic use
- Tracheostomy / statistics & numerical data

Substances

- Antibodies, Monoclonal, Humanized
- Steroids
- tocilizumab

Full text links

ELSEVIER FULL-TEXT ARTICLE Elsevier Science Free PMC article

Proceed to details

Cite

Share

☐ 85

Observational Study

Ital J Pediatr

. 2020 Oct 16;46(1):155.

doi: 10.1186/s13052-020-00915-3.

The impact of the COVID-19 pandemic on pediatric operations: a retrospective study of Chinese children

Yi Wei [1, 2], Chengjun Yu [1, 2], Tian Xin Zhao [1, 2], Tao Lin [1, 3], H E Dawei [1, 4], Sheng-de Wu [5, 6, 7], Guang-Hui Wei [2, 3, 4, 8, 9]

Affiliations Expand

Affiliations

- [1] Department of Urology, Children's Hospital of Chongqing Medical University, Room 806, Kejiao Building (NO.6 Building), No.136, 2nd Zhongshan Road, Yuzhong District, Chongqing City, 40014, China.
- [2] Chongqing Key Laboratory of Children Urogenital Development and Tissue Engineering, Chongqing City, China.
- [3] Ministry of Education Key Laboratory of Child Development and Disorders, Room 806, Kejiao Building (NO.6 Building), No.136, 2nd Zhongshan Roadm, Yuzhong District, Chongqing City, 400014, China.
- [4] China International Science and Technology Cooperation base of Child development and Critical Disorders, Chongqing City, China.
- [5] Department of Urology, Children's Hospital of Chongqing Medical University, Room 806, Kejiao Building (NO.6 Building), No.136, 2nd Zhongshan Road, Yuzhong District, Chongqing City, 40014, China. shengdewu@yeah.net.
- [6] Chongqing Key Laboratory of Children Urogenital Development and Tissue Engineering, Chongqing City, China. shengdewu@yeah.net.
- [7] Ministry of Education Key Laboratory of Child Development and Disorders, Room 806, Kejiao Building (NO.6 Building), No.136, 2nd Zhongshan Roadm, Yuzhong District, Chongqing City, 400014, China. shengdewu@yeah.net.
- [8] National Clinical Research Center for Child Health and Disorders, Chongqing City, China.
- [9] Chongqing Key Laboratory of Pediatrics Chongqing, Chongqing City, China.

- PMID: **33066803**
- PMCID: PMC7563908
- DOI: 10.1186/s13052-020-00915-3

Free PMC article
Observational Study

The impact of the COVID-19 pandemic on pediatric operations: a retrospective study of Chinese children

Yi Wei et al. Ital J Pediatr. 2020.
Free PMC article
Show details
Ital J Pediatr
. 2020 Oct 16;46(1):155.
doi: 10.1186/s13052-020-00915-3.

Authors

Yi Wei [1, 2], Chengjun Yu [1, 2], Tian Xin Zhao [1, 2], Tao Lin [1, 3], H E Dawei [1, 4], Sheng-de Wu [5, 6, 7], Guang-Hui Wei [2, 3, 4, 8, 9]

Affiliations

- [1] Department of Urology, Children's Hospital of Chongqing Medical University, Room 806, Kejiao Building (NO.6 Building), No.136, 2nd Zhongshan Road, Yuzhong District, Chongqing City, 40014, China.
- [2] Chongqing Key Laboratory of Children Urogenital Development and Tissue Engineering, Chongqing City, China.
- [3] Ministry of Education Key Laboratory of Child Development and Disorders, Room 806, Kejiao Building (NO.6 Building), No.136, 2nd Zhongshan Roadm, Yuzhong District, Chongqing City, 400014, China.
- [4] China International Science and Technology Cooperation base of Child development and Critical Disorders, Chongqing City, China.
- [5] Department of Urology, Children's Hospital of Chongqing Medical University, Room 806, Kejiao Building (NO.6 Building), No.136, 2nd Zhongshan Road, Yuzhong District, Chongqing City, 40014, China. shengdewu@yeah.net.
- [6] Chongqing Key Laboratory of Children Urogenital Development and Tissue Engineering, Chongqing City, China. shengdewu@yeah.net.
- [7] Ministry of Education Key Laboratory of Child Development and Disorders, Room 806, Kejiao Building (NO.6 Building), No.136, 2nd Zhongshan Roadm, Yuzhong District, Chongqing City, 400014, China. shengdewu@yeah.net.
- [8] National Clinical Research Center for Child Health and Disorders, Chongqing City, China.
- [9] Chongqing Key Laboratory of Pediatrics Chongqing, Chongqing City, China.

- PMID: **33066803**
- PMCID: PMC7563908
- DOI: 10.1186/s13052-020-00915-3

Abstract

Background: The aim of this study was to quantify the impact of coronavirus disease 2019 (COVID-19) on pediatric operations, and establish preoperative, intraoperative, and postoperative protocols to improve the pediatric operations.

Methods: We here compare the number of patients who underwent surgery in Chongqing Medical University Affiliated Children's Hospital during the pandemic (January 23-March 11), after the pandemic (March 12-April 30), after our measures were put in place (May 1-May 21), and the equivalent period in 2019.

Result: During the COVID-19 pandemic, 62.68% fewer patients underwent surgery than during the homologous period of time 1 year earlier ($P < 0.01$). After the COVID-19 pandemic, the number of orchidopexy cases increased significantly from 175.14 to 504.57 per week ($P < 0.01$). The large number of patients that accrued in our hospital may have increased the risk of COVID-19 transmission. In response, hospitals and clinics have made protocols and reorganized healthcare facilities (e.g., performing nucleic acid tests (NAT), adding adequate personal protective equipment (PPE)) from May 1, 2020. After the measures were implemented, the number of operations performed remained stable and comparable to the pre-pandemic period. COVID-19 RNA detection was performed in 5104 cases and there were no new confirmed cases in our hospital.

Conclusion: This outbreak of COVID-19 has affected not only individuals with COVID-19 but also patients seeking surgical operations. Understanding the present situation helps clinicians provide a high level of treatment to all children.

Keywords: COVID-19; Children; China; Nucleic acid test; Operation.

Conflict of interest statement

The authors have no conflict of interest to declare.

- 7 references
- 2 figures

Supplementary info

Publication types, MeSH terms, Grant support Expand

Publication types

- Letter
- Observational Study

MeSH terms

- Betacoronavirus*
- COVID-19
- Child

- China / epidemiology
- Comorbidity
- Coronavirus Infections / epidemiology*
- Hospitals, Pediatric / statistics & numerical data*
- Humans
- Pandemics*
- Pneumonia, Viral / epidemiology*
- Retrospective Studies
- SARS-CoV-2
- Surgical Procedures, Operative / statistics & numerical data*

Grant support

- NO.81873828/National Natural Science Foundation of China
- NO.81771566/National Natural Science Foundation of China

Full text links

Read free full text at BMC BioMed Central Free PMC article
Proceed to details
Cite
Share
☐ 86
Observational Study
Int Immunopharmacol
. 2021 Nov;100:108163.
doi: 10.1016/j.intimp.2021.108163. Epub 2021 Sep 20.

Plasma zinc status and hyperinflammatory syndrome in hospitalized COVID-19 patients: An observational study

Gil Verschelden [1], Maxim Noeparast [2], Maryam Noparast [3], Mathijs Christiaan Goossens [4], Maïlis Lauwers [5], Frédéric Cotton [5], Charlotte Michel [6], Cleo Goyvaerts [7], Maya Hites [8]
Affiliations Expand

Affiliations

- [1] Clinic of Infectious Diseases, Cliniques Universitaires de Bruxelles (CUB), Erasme Hospital, Université Libre de Bruxelles (ULB), 1070 Brussels, Belgium. Electronic address: Gil.Verschelden@erasme.ulb.ac.be.
- [2] Institute of Molecular Oncology, Member of the German Center for Lung Research (DZL), Philipps University, 35043 Marburg, Germany; Fonds Wetenschappelijk Onderzoek

(FWO) - Vlaanderen, Belgium. Electronic address: Maxim.Noeparast@staff.uni-marburg.de.

- [3] Department of Urologic Sciences, University of British Columbia, Vancouver, BC, Canada.
- [4] Vrije Universiteit Brussel, Laarbeeklaan 103, 1090 Brussels, Belgium.
- [5] Department of Clinical Chemistry, Laboratoire Hospitalier Universitaire de Bruxelles (LHUB-ULB), Université Libre de Bruxelles (ULB), Brussels, Belgium.
- [6] Department of Microbiology, Laboratoire Hospitalier Universitaire de Bruxelles (LHUB-ULB), Brussels, Belgium.
- [7] Laboratory for Molecular and Cellular Therapy (LMCT), Vrije Universiteit Brussel (VUB), 1090 Brussels, Belgium.
- [8] Clinic of Infectious Diseases, Cliniques Universitaires de Bruxelles (CUB), Erasme Hospital, Université Libre de Bruxelles (ULB), 1070 Brussels, Belgium.

- PMID: **34583122**
- PMCID: PMC8450071
- DOI: 10.1016/j.intimp.2021.108163

Free PMC article
Observational Study

Plasma zinc status and hyperinflammatory syndrome in hospitalized COVID-19 patients: An observational study

Gil Verschelden et al. Int Immunopharmacol. 2021 Nov.
Free PMC article
Show details
Int Immunopharmacol
. 2021 Nov;100:108163.
doi: 10.1016/j.intimp.2021.108163. Epub 2021 Sep 20.

Authors

Gil Verschelden [1], Maxim Noeparast [2], Maryam Noparast [3], Mathijs Christiaan Goossens [4], Maïlis Lauwers [5], Frédéric Cotton [5], Charlotte Michel [6], Cleo Goyvaerts [7], Maya Hites [8]

Affiliations

- [1] Clinic of Infectious Diseases, Cliniques Universitaires de Bruxelles (CUB), Erasme Hospital, Université Libre de Bruxelles (ULB), 1070 Brussels, Belgium. Electronic address: Gil.Verschelden@erasme.ulb.ac.be.
- [2] Institute of Molecular Oncology, Member of the German Center for Lung Research (DZL), Philipps University, 35043 Marburg, Germany; Fonds Wetenschappelijk Onderzoek (FWO) - Vlaanderen, Belgium. Electronic address: Maxim.Noeparast@staff.uni-marburg.de.

- [3] Department of Urologic Sciences, University of British Columbia, Vancouver, BC, Canada.
- [4] Vrije Universiteit Brussel, Laarbeeklaan 103, 1090 Brussels, Belgium.
- [5] Department of Clinical Chemistry, Laboratoire Hospitalier Universitaire de Bruxelles (LHUB-ULB), Université Libre de Bruxelles (ULB), Brussels, Belgium.
- [6] Department of Microbiology, Laboratoire Hospitalier Universitaire de Bruxelles (LHUB-ULB), Brussels, Belgium.
- [7] Laboratory for Molecular and Cellular Therapy (LMCT), Vrije Universiteit Brussel (VUB), 1090 Brussels, Belgium.
- [8] Clinic of Infectious Diseases, Cliniques Universitaires de Bruxelles (CUB), Erasme Hospital, Université Libre de Bruxelles (ULB), 1070 Brussels, Belgium.

- PMID: **34583122**
- PMCID: PMC8450071
- DOI: 10.1016/j.intimp.2021.108163

Abstract

Zinc deficiency is associated with impaired antiviral response, cytokine releasing syndrome (CRS), and acute respiratory distress syndrome. Notably, similar complications are being observed during severe SARS-CoV-2 infection. We conducted a prospective, single-center, observational study in a tertiary university hospital (CUB-Hôpital Erasme, Brussels) to address the zinc status, the association between the plasma zinc concentration, development of CRS, and the clinical outcomes in PCR-confirmed and hospitalized COVID-19 patients. One hundred and thirty-nine eligible patients were included between May 2020 and November 2020 (median age of 65 years [IQR = 54, 77]). Our cohort's median plasma zinc concentration was 57 µg/dL (interquartile range [IQR] = 45, 67) compared to 74 µg/dL (IQR = 64, 84) in the retrospective non-COVID-19 control group (N = 1513; p < 0.001). Markedly, the absolute majority of COVID-19 patients (96%) were zinc deficient (<80 µg/dL). The median zinc concentration was lower in patients with CRS compared to those without CRS (-5 µg/dL; 95% CI = -10.5, 0.051; p = 0.048). Among the tested outcomes, zinc concentration is significantly correlated with only the length of hospital stay (rho = -0.19; p = 0.022), but not with mortality or morbidity. As such, our findings do not support the role of zinc as a robust prognostic marker among hospitalized COVID-19 patients who in our cohort presented a high prevalence of zinc deficiency. It might be more beneficial to explore the role of zinc as a biomarker for assessing the risk of developing a tissue-damaging CRS and predicting outcomes in patients diagnosed with COVID-19 at the early stage of the disease.

Keywords: COVID-19; Inflammation; SARS-CoV-2; Zinc; cHIS.

Copyright © 2021 Elsevier B.V. All rights reserved.

Conflict of interest statement

The authors declare that they have no known competing financial interests or personal relationships that could have appeared to influence the work reported in this paper.

- 35 references
- 2 figures

Supplementary info

Publication types, MeSH terms, Substances Expand

Publication types

- Observational Study

MeSH terms

- Aged
- COVID-19 / blood
- COVID-19 / complications*
- Cytokine Release Syndrome / blood
- Cytokine Release Syndrome / etiology*
- Female
- Hospitalization
- Humans
- Male
- Middle Aged
- Prospective Studies
- SARS-CoV-2*
- Zinc / blood*
- Zinc / physiology

Substances

- Zinc

Full text links

ELSEVIER FULL-TEXT ARTICLE Elsevier Science Free PMC article
Proceed to details
Cite
Share
87
Observational Study
Crit Care
. 2021 Feb 17;25(1):70.
doi: 10.1186/s13054-021-03504-w.

Surge effects and survival to hospital discharge in critical care patients with

COVID-19 during the early pandemic: a cohort study

Christopher R Dale [1] [2], Rachael W Starcher [3], Shu Ching Chang [4], Ari Robicsek [5], Guilford Parsons [5], Jason D Goldman [6], Andre Vovan [7] [8], David Hotchkin [5], Tyler J Gluckman [4]
Affiliations Expand

Affiliations

- [1] Swedish Health Services, 600 Broadway, Suite 610, Seattle, WA, 98122, USA. Christopher.dale@swedish.org.
- [2] School of Public Health, University of Washington, Seattle, WA, USA. Christopher.dale@swedish.org.
- [3] Providence Portland Medical Center, Portland, OR, USA.
- [4] Center for Cardiovascular Analytics, Research and Data Science (CARDS), Providence Heart Institute, Providence St. Joseph Health, Portland, OR, USA.
- [5] Providence St. Joseph Health, Renton, WA, USA.
- [6] Swedish Health Services, Seattle, WA, USA.
- [7] Providence Health & Services, Portland, OR, USA.
- [8] The Oregon Clinic, Portland, OR, USA.

- PMID: **33596975**
- PMCID: PMC7887411
- DOI: 10.1186/s13054-021-03504-w

Free PMC article
Observational Study

Surge effects and survival to hospital discharge in critical care patients with COVID-19 during the early pandemic: a cohort study

Christopher R Dale et al. Crit Care. 2021.
Free PMC article
Show details
Crit Care
. 2021 Feb 17;25(1):70.
doi: 10.1186/s13054-021-03504-w.

Authors

Christopher R Dale [1] [2], Rachael W Starcher [3], Shu Ching Chang [4], Ari Robicsek [5], Guilford Parsons [5], Jason D Goldman [6], Andre Vovan [7] [8], David Hotchkin [5], Tyler J Gluckman [4]

Affiliations

- [1] Swedish Health Services, 600 Broadway, Suite 610, Seattle, WA, 98122, USA. Christopher.dale@swedish.org.
- [2] School of Public Health, University of Washington, Seattle, WA, USA. Christopher.dale@swedish.org.
- [3] Providence Portland Medical Center, Portland, OR, USA.
- [4] Center for Cardiovascular Analytics, Research and Data Science (CARDS), Providence Heart Institute, Providence St. Joseph Health, Portland, OR, USA.
- [5] Providence St. Joseph Health, Renton, WA, USA.
- [6] Swedish Health Services, Seattle, WA, USA.
- [7] Providence Health & Services, Portland, OR, USA.
- [8] The Oregon Clinic, Portland, OR, USA.

- PMID: **33596975**
- PMCID: PMC7887411
- DOI: 10.1186/s13054-021-03504-w

Abstract

Background: The early months of the COVID-19 pandemic were fraught with much uncertainty and some resource constraint. We assessed the change in survival to hospital discharge over time for intensive care unit patients with COVID-19 during the first 3 months of the pandemic and the presence of any surge effects on patient outcomes.

Methods: Retrospective cohort study using electronic medical record data for all patients with laboratory-confirmed COVID-19 admitted to intensive care units from February 25, 2020, to May 15, 2020, at one of 26 hospitals within an integrated delivery system in the Western USA. Patient demographics, comorbidities, and severity of illness were measured along with medical therapies and hospital outcomes over time. Multivariable logistic regression models were constructed to assess temporal changes in survival to hospital discharge during the study period.

Results: Of 620 patients with COVID-19 admitted to the ICU [mean age 63.5 years (SD 15.7) and 69% male], 403 (65%) survived to hospital discharge and 217 (35%) died in the hospital. Survival to hospital discharge increased over time, from 60.0% in the first 2 weeks of the study period to 67.6% in the last 2 weeks. In a multivariable logistic regression analysis, the risk-adjusted odds of survival to hospital discharge increased over time (biweekly change, adjusted odds ratio [aOR] 1.22, 95% CI 1.04-1.40, P = 0.02). Additionally, an a priori-defined explanatory model showed that after adjusting for both hospital occupancy and percent hospital capacity by COVID-19-positive individuals and persons under investigation (PUI), the temporal trend in risk-adjusted patient survival to hospital discharge remained the same (biweekly change, aOR 1.18, 95% CI 1.00-1.38, P = 0.04). The presence of greater rates of COVID-19 positive/PUI as a percentage of hospital capacity was, however, significantly and inversely associated with survival to hospital discharge (aOR 0.95, 95% CI 0.92-0.98, P < 0.01).

Conclusions: During the early COVID-19 pandemic, risk-adjusted survival to hospital discharge increased over time for critical care patients. An association was also seen between a greater

COVID-19-positive/PUI percentage of hospital capacity and a lower survival rate to hospital discharge.

Keywords: COVID-19; Critical care; Health services; Healthcare delivery; Outcomes; Surge effects.

Conflict of interest statement

No financial or non-financial competing interests exist. All study authors are employees of providence family of healthcare delivery organizations.

- 43 references
- 1 figure

Supplementary info

Publication types, MeSH terms Expand

Publication types

- Observational Study

MeSH terms

- Aged
- COVID-19 / epidemiology*
- COVID-19 / mortality
- COVID-19 / therapy*
- Critical Care
- Female
- Hospital Mortality
- Humans
- Intensive Care Units
- Male
- Middle Aged
- Pandemics*
- Patient Discharge / statistics & numerical data*
- Retrospective Studies
- SARS-CoV-2 / isolation & purification
- Survival Analysis
- United States / epidemiology

Full text links

Read free full text at BMC BioMed Central Free PMC article

Proceed to details

Cite

Share

☐ 88

Observational Study

J Microbiol Biotechnol

. 2021 Mar 28;31(3):380-386.

doi: 10.4014/jmb.2009.09029.

Clinical Effect of Traditional Chinese Medicine Shenhuang Granule in Critically Ill Patients with COVID-19: A Single-Centered, Retrospective, Observational Study

Jun Feng[1], Bangjiang Fang[2], Daixing Zhou[1], Junshuai Wang[1], Dengxiu Zou[1], Gang Yu[1], Yikuan Fen[1], Dan Peng[1], Jifa Hu[3], Daqian Zhan[1]

Affiliations Expand

Affiliations

- [1] Department of Critical Care Medicine, Tongji Hospital, Tongji Medical College, Huazhong University of Science and Technology, Wuhan 430030, P.R. China.
- [2] Department of Emergency Medicine, LongHua Hospital, Shanghai University of Traditional Chinese Medicine, Shanghai 200032, P.R. China.
- [3] Office of Academic Research, Tongji Hospital, Tongji Medical College, Huazhong University of Science and Technology, Wuhan 430030, P.R. China.

- PMID: **33746189**
- DOI: 10.4014/jmb.2009.09029

Free article

Observational Study

Clinical Effect of Traditional Chinese Medicine Shenhuang Granule in Critically Ill Patients with COVID-19: A Single-Centered, Retrospective, Observational Study

Jun Feng et al. J Microbiol Biotechnol. 2021.

Free article

Show details

J Microbiol Biotechnol

. 2021 Mar 28;31(3):380-386.
doi: 10.4014/jmb.2009.09029.

Authors

Jun Feng [1], Bangjiang Fang [2], Daixing Zhou [1], Junshuai Wang [1], Dengxiu Zou [1], Gang Yu [1], Yikuan Fen [1], Dan Peng [1], Jifa Hu [3], Daqian Zhan [1]

Affiliations

- [1] Department of Critical Care Medicine, Tongji Hospital, Tongji Medical College, Huazhong University of Science and Technology, Wuhan 430030, P.R. China.
- [2] Department of Emergency Medicine, LongHua Hospital, Shanghai University of Traditional Chinese Medicine, Shanghai 200032, P.R. China.
- [3] Office of Academic Research, Tongji Hospital, Tongji Medical College, Huazhong University of Science and Technology, Wuhan 430030, P.R. China.

- PMID: **33746189**
- DOI: 10.4014/jmb.2009.09029

Abstract

The coronavirus disease 2019 (COVID-19) pandemic has become a public health emergency of global concern. In China, traditional Chinese medicine has been widely administered to COVID-19 patients without sufficient evidence. To evaluate the efficacy of Shenhuang Granule (SHG) for treating critically ill patients with COVID-19, we included in this study 118 patients who were admitted to the ICU of Tongji Hospital between January 28, 2020 and March 28, 2020. Among these patients, 33 (27.9%) received standard care plus SHG (treatment group) and 85 (72.1%) received standard care alone (control group). Enrolled patients had a median (IQR) age of 68 (57-75) years, and most (79 [67.1%]) were men. At end point of this study, 83 (70.3%) had died in ICU, 29 (24.5%) had been discharged from ICU, and 6 patients (5.2%) were still in ICU. Compared with control group, mortality was significantly lower in treatment group (45.4% vs. 80%, $p < .001$). Patients in treatment group were less likely to develop acute respiratory distress syndrome (ARDS) (12 [36.3%] vs. 54 [63.5%], $p = 0.012$) and cardiac injury (5 [15.1%] vs. 32 [37.6%], $p = 0.026$), and less likely to receive mechanical ventilation (22 [66.7%] vs. 72 [84.7%], $p = 0.028$) than those in control group. The median time from ICU admission to discharge was shorter in treatment group (32 [20-73] days vs. 76 [63-79] days, $p = 0.0074$). These findings suggest that SHG treatment as a complementary therapy might be effective for critically ill adults with COVID-19 and warrant further clinical trials.

Keywords: Coronavirus disease 2019; Shenhuang Granule; inflammation; traditional Chinese medicine.

Supplementary info

Publication types, MeSH terms, Substances Expand

Publication types

- Observational Study

MeSH terms

- Aged
- COVID-19 / drug therapy*
- China
- Critical Illness
- Drugs, Chinese Herbal / therapeutic use*
- Female
- Hospitalization
- Humans
- Male
- Medicine, Chinese Traditional / methods
- Middle Aged
- Pandemics / prevention & control
- Retrospective Studies

Substances

- Drugs, Chinese Herbal

Full text links

The Korean Society for Microbiology and Biotechnology.
Proceed to details
Cite
Share
☐ 89
Observational Study
Clin Neurol Neurosurg
. 2020 Dec;199:106227.
doi: 10.1016/j.clineuro.2020.106227. Epub 2020 Sep 11.

Clinical characteristics and admission patterns of stroke patients during the COVID 19 pandemic: A single center retrospective, observational study from the Abu Dhabi, United Arab Emirates

Seby John [1], Syed Irteza Hussain [2], Bartlomiej Piechowski-Jozwiak [3], Jamil Dibu [4], Praveen Kesav [3], Ahmad Bayrlee [4], Hussam Elkambergy [4], Terrence Lee St John [5], Florian Roser [6], Victoria Ann Mifsud [3]
Affiliations Expand

Affiliations

- [1] Department of Neurology, Neurological Institute, Cleveland Clinic Abu Dhabi; Neurointerventional Surgery, Neurological Institute, Cleveland Clinic Abu Dhabi. Electronic address: Johns5@ClevelandClinicAbuDhabi.ae.
- [2] Department of Neurology, Neurological Institute, Cleveland Clinic Abu Dhabi; Neurointerventional Surgery, Neurological Institute, Cleveland Clinic Abu Dhabi.
- [3] Department of Neurology, Neurological Institute, Cleveland Clinic Abu Dhabi.
- [4] Neurocritical Care Unit, Critical Care Institute, Cleveland Clinic Abu Dhabi.
- [5] Department of Research, Academic Institute, Cleveland Clinic Abu Dhabi.
- [6] Department of Neurosurgery, Neurological Institute, Cleveland Clinic Abu Dhabi.

- PMID: **33011516**
- PMCID: PMC7485577
- DOI: 10.1016/j.clineuro.2020.106227

Free PMC article
Observational Study

Clinical characteristics and admission patterns of stroke patients during the COVID 19 pandemic: A single center retrospective, observational study from the Abu Dhabi, United Arab Emirates

Seby John et al. Clin Neurol Neurosurg. 2020 Dec.
Free PMC article
Show details
Clin Neurol Neurosurg
. 2020 Dec;199:106227.
doi: 10.1016/j.clineuro.2020.106227. Epub 2020 Sep 11.

Authors

Seby John [1], Syed Irteza Hussain [2], Bartlomiej Piechowski-Jozwiak [3], Jamil Dibu [4], Praveen Kesav [3], Ahmad Bayrlee [4], Hussam Elkambergy [4], Terrence Lee St John [5], Florian Roser [6], Victoria Ann Mifsud [3]

Affiliations

- [1] Department of Neurology, Neurological Institute, Cleveland Clinic Abu Dhabi; Neurointerventional Surgery, Neurological Institute, Cleveland Clinic Abu Dhabi. Electronic address: Johns5@ClevelandClinicAbuDhabi.ae.
- [2] Department of Neurology, Neurological Institute, Cleveland Clinic Abu Dhabi; Neurointerventional Surgery, Neurological Institute, Cleveland Clinic Abu Dhabi.
- [3] Department of Neurology, Neurological Institute, Cleveland Clinic Abu Dhabi.
- [4] Neurocritical Care Unit, Critical Care Institute, Cleveland Clinic Abu Dhabi.
- [5] Department of Research, Academic Institute, Cleveland Clinic Abu Dhabi.
- [6] Department of Neurosurgery, Neurological Institute, Cleveland Clinic Abu Dhabi.

- PMID: **33011516**
- PMCID: PMC7485577
- DOI: 10.1016/j.clineuro.2020.106227

Abstract

Objective: To compare ischemic and hemorrhagic stroke patients with COVID-19 to non-COVID-19 controls, and to describe changes in stroke admission patterns during the pandemic.

Methods: This is a single center, retrospective, observational study. All consecutive patients admitted with primary diagnosis of ischemic/ hemorrhagic stroke between March1st -May10th 2020 were included and compared with the same time period in 2019.

Results: There was a 41.9% increase in stroke admissions in 2020 (148 vs 210,P = .001). When comparing all ischemic strokes, higher rate of large vessel occlusion (LVO) (18.3% vs 33.8%,P = .008) and significant delay in initiation of mechanical thrombectomy after hospital arrival (67.75 vs 104.30 minutes,P = .001) was observed in 2020. When comparing all hemorrhagic strokes, there were no differences between the two years. Among 591 COVID-19 admissions, 31 (5.24%) patients with stroke including 19 with ischemic (3.21%) and 12 with hemorrhagic stroke (2.03%) were identified. Patients with COVID-19 and ischemic stroke were significantly younger (58.74 vs 48.11 years,P = .002), predominantly male (68.18% vs 94.74%,P = .016), had lesser vascular risk factors, had more severe clinical presentation (NIHSS 7.01 vs 17.05,P < .001), and higher rate of LVO (23.6% vs. 63.1%,P = .006). There was no difference in the rate of endovascular thrombectomy, but time to groin puncture was significantly longer in COVID-19 patients (83.41 vs 129.50 minutes,P = .003). For hemorrhagic stroke, COVID-19 patients did not differ from non-COVID-19 patients.

Conclusions: Stroke continues to occur during this pandemic and stroke pathways have been affected by the pandemic. Stroke occurs in approximately 5% of patients with COVID-19. COVID-19 associated ischemic stroke occurs in predominantly male patients who are younger, with fewer vascular risk factors, can be more severe, and have higher rates of LVO. Despite an increase in LVO during the pandemic, treatment with mechanical thrombectomy has not increased. COVID-19 associated hemorrhagic stroke does not differ from non-COVID-19 hemorrhagic stroke patients.

Keywords: Admissions; COVID-19; Clinical characteristics; Hemorrhagic; Ischemic; Stroke.

Copyright © 2020. Published by Elsevier B.V.

Conflict of interest statement

None

- 31 references
- 1 figure

Supplementary info

Publication types, MeSH terms Expand

Publication types

- Observational Study

MeSH terms

- Adult
- Aged
- Brain Ischemia / complications
- Brain Ischemia / diagnosis
- Brain Ischemia / therapy
- COVID-19 / complications*
- COVID-19 / mortality
- COVID-19 / therapy
- Endovascular Procedures
- Female
- Hospitalization*
- Humans
- Intracranial Hemorrhages / complications
- Intracranial Hemorrhages / diagnosis
- Intracranial Hemorrhages / therapy
- Male
- Middle Aged
- Retrospective Studies
- Risk Factors
- SARS-CoV-2*
- Stroke / epidemiology*
- Stroke / mortality
- Stroke / therapy*
- Survival Rate
- Thrombectomy
- United Arab Emirates

Full text links

ELSEVIER FULL-TEXT ARTICLE Elsevier Science Free PMC article
Proceed to details
Cite
Share
90
Observational Study
Rev Assoc Med Bras (1992)
. 2021 Nov;67(11):1610-1615.
doi: 10.1590/1806-9282.20210675.

Readmission rates of patients with COVID-19 after hospital discharge

Recep Alanli [1], Murat Bulent Kucukay [1], Kadir Serkan Yalcin [1]
Affiliations Expand

Affiliation

- [1] Lokman Hekim University, Faculty of Medicine, Ankara Hospital, Department of Internal Diseases - Ankara, Turkey.

- PMID: **34909887**
- DOI: 10.1590/1806-9282.20210675

Free article
Observational Study

Readmission rates of patients with COVID-19 after hospital discharge

Recep Alanli et al. Rev Assoc Med Bras (1992). 2021 Nov.
Free article
Show details
Rev Assoc Med Bras (1992)
. 2021 Nov;67(11):1610-1615.
doi: 10.1590/1806-9282.20210675.

Authors

Recep Alanli [1], Murat Bulent Kucukay [1], Kadir Serkan Yalcin [1]

Affiliation

- [1] Lokman Hekim University, Faculty of Medicine, Ankara Hospital, Department of Internal Diseases - Ankara, Turkey.

- PMID: **34909887**
- DOI: 10.1590/1806-9282.20210675

Abstract

Objective: The purpose of this study was to inspect return and readmission reasons and rates of discharged patients with coronavirus disease 2019 (COVID-19).

Methods: This is an observational descriptive retrospective study that was conducted with patients who had confirmed COVID-19 diagnosed with severe respiratory syndrome coronavirus-2 (SARS-CoV-2) polymerase chain reaction (PCR) and hospitalized between April 2020 and June 2021 in a tertiary care university hospital. Patients returning to the hospital after treatment for COVID-19, with symptoms related to COVID-19 within 30 days, were included. Patients under 18 years of age and who were hospitalized in the intensive care unit were excluded.

Results: It was determined that of 369 discharged patients, 87 (23.5%) returned to the hospital, 9 (2.4%) were readmitted, and 1 (0.02%) was deceased within 30 days. The most frequent reasons for returning to the hospital were dyspnea and cough complaints. Existence of pneumonia at first admission, levels of aspartate aminotransferase, lactate dehydrogenase, C-reactive protein, D-dimer, neutrophil counts, lymphocyte counts, and neutrophil-to-lymphocyte count ratios were found to be higher in patients who returned to hospital, compared with the patients who did not return.

Conclusions: Return rate of patients to hospital after discharge with COVID-19 was comparatively high, but readmissions to hospital and mortality rate were low. Comparatively, the higher rate of return to hospital within 30 days of discharge was thought to be resulting from prolonged signs and symptoms related to COVID-19. Since COVID-19 is a new and enigmatic disease and its long-term effects still need to be elucidated, long-term follow-ups of discharged patients will be adequate.

Supplementary info

Publication types, MeSH terms Expand

Publication types

- Observational Study

MeSH terms

- Adolescent
- COVID-19*
- Hospitals
- Humans
- Patient Discharge
- Patient Readmission

- Retrospective Studies
- SARS-CoV-2

Full text links

free full text available at SciELO.org Scientific Electronic Library Online

Proceed to details

Cite

Share

☐ 91

Observational Study

PLoS One
. 2021 Mar 24;16(3):e0248829.
doi: 10.1371/journal.pone.0248829. eCollection 2021.

Baseline clinical characteristics and prognostic factors in hospitalized COVID-19 patients aged ≤ 65 years: A retrospective observational study

Marta Betti [1], Marinella Bertolotti [1], Daniela Ferrante [2], Annalisa Roveta [1], Carolina Pelazza [1], Fabio Giacchero [1], Serena Penpa [1], Costanza Massarino [1], Tatiana Bolgeo [1], Antonella Cassinari [1], Marco Mussa [3], Guido Chichino [3], Antonio Maconi [1]

Affiliations Expand

Affiliations

- [1] Infrastruttura Ricerca Formazione Innovazione, Dipartimento Attività Integrate Ricerca Innovazione, Azienda Ospedaliera "SS Antonio e Biagio e Cesare Arrigo", Alessandria, Italy.
- [2] Unit of Medical Statistics, Department of Translational Medicine, University of "Piemonte Orientale" and Cancer Epidemiology, CPO Piemonte, Novara, Italy.
- [3] Infectious Diseases Unit, Azienda Ospedaliera "SS Antonio e Biagio e Cesare Arrigo", Alessandria, Italy.

- PMID: **33760885**
- PMCID: PMC7990225
- DOI: 10.1371/journal.pone.0248829

Free PMC article

Observational Study

Baseline clinical characteristics and prognostic factors in hospitalized COVID-19 patients aged ≤ 65 years: A retrospective observational study

Marta Betti et al. PLoS One. 2021.
Free PMC article
Show details
PLoS One
. 2021 Mar 24;16(3):e0248829.
doi: 10.1371/journal.pone.0248829. eCollection 2021.

Authors

Marta Betti [1], Marinella Bertolotti [1], Daniela Ferrante [2], Annalisa Roveta [1], Carolina Pelazza [1], Fabio Giacchero [1], Serena Penpa [1], Costanza Massarino [1], Tatiana Bolgeo [1], Antonella Cassinari [1], Marco Mussa [3], Guido Chichino [3], Antonio Maconi [1]

Affiliations

- [1] Infrastruttura Ricerca Formazione Innovazione, Dipartimento Attività Integrate Ricerca Innovazione, Azienda Ospedaliera "SS Antonio e Biagio e Cesare Arrigo", Alessandria, Italy.
- [2] Unit of Medical Statistics, Department of Translational Medicine, University of "Piemonte Orientale" and Cancer Epidemiology, CPO Piemonte, Novara, Italy.
- [3] Infectious Diseases Unit, Azienda Ospedaliera "SS Antonio e Biagio e Cesare Arrigo", Alessandria, Italy.

- PMID: **33760885**
- PMCID: PMC7990225
- DOI: 10.1371/journal.pone.0248829

Abstract

Background: Individual differences in susceptibility to SARS-CoV-2 infection, symptomatology and clinical manifestation of COVID-19 have thus far been observed but little is known about the prognostic factors of young patients.

Methods: A retrospective observational study was conducted on 171 patients aged ≤ 65 years hospitalized in Alessandria's Hospital from 1st March to 30th April 2020 with laboratory confirmed COVID-19. Epidemiological data, symptoms at onset, clinical manifestations, Charlson Comorbidity Index, laboratory parameters, radiological findings and complications were considered. Patients were divided into two groups on the basis of COVID-19 severity. Multivariable logistic regression analysis was used to establish factors associated with the development of a moderate or severe disease.

Findings: A total of 171 patients (89 with mild/moderate disease, 82 with severe/critical disease), of which 61% males and a mean age (± SD) of 53.6 (± 9.7) were included. The multivariable logistic model identified age (50-65 vs 18-49; OR = 3.23 CI95% 1.42-7.37), platelet count (per 100 units of increase OR = 0.61 CI95% 0.42-0.89), c-reactive protein (CPR) (per unit of increase OR = 1.12 CI95% 1.06-1.20) as risk factors for severe or critical disease. The multivariable logistic model showed a good discriminating capacity with a C-index value of 0.76.

Interpretation: Patients aged ≥ 50 years with low platelet count and high CRP are more likely to develop severe or critical illness. These findings might contribute to improved clinical management.

Conflict of interest statement

The authors have declared that no competing interests exist.

- 30 references
- 1 figure

Supplementary info

Publication types, MeSH terms, Substances, Grant support Expand

Publication types

- Observational Study

MeSH terms

- Adult
- C-Reactive Protein / analysis
- COVID-19 / epidemiology*
- COVID-19 / transmission
- Female
- Hospitalization / trends*
- Humans
- Italy / epidemiology
- Male
- Middle Aged
- Platelet Count / trends
- Prognosis
- Retrospective Studies
- Risk Factors
- SARS-CoV-2 / pathogenicity
- Severity of Illness Index*

Substances

- C-Reactive Protein

Grant support

The authors received no specific funding for this work.

Full text links

PLOS ONE Public Library of Science Free PMC article
Proceed to details
Cite
Share
☐ 92
Observational Study
BMC Infect Dis
. 2021 Jan 12;21(1):57.
doi: 10.1186/s12879-020-05741-w.

Clinical characteristics and predictive value of lower CD4 $^{+}$ T cell level in patients with moderate and severe COVID-19: a multicenter retrospective study

Xue-Song Wen [1], Dan Jiang [1], Lei Gao [1], Jian-Zhong Zhou [1], Jun Xiao [2], Xiao-Cheng Cheng [1], Bin He [1], Yue Chen [1], Peng Lei [1], Xiao-Wei Tan [1], Shu Qin [1], Dong-Ying Zhang [3]
Affiliations Expand

Affiliations

- [1] Department of Cardiovascular Medicine, The First Affiliated Hospital of Chongqing Medical University, No 1, Youyi Road, Chongqing, 400016, China.
- [2] Department of Cardiovascular Medicine, Chongqing University Center Hospital, Chongqing, 400014, China.
- [3] Department of Cardiovascular Medicine, The First Affiliated Hospital of Chongqing Medical University, No 1, Youyi Road, Chongqing, 400016, China. zhangdongying@cqmu.edu.cn.

- PMID: **33435865**
- PMCID: PMC7803000
- DOI: 10.1186/s12879-020-05741-w

Free PMC article

Observational Study

Clinical characteristics and predictive value of lower CD4 + T cell level in patients with moderate and severe COVID-19: a multicenter retrospective study

Xue-Song Wen et al. BMC Infect Dis. 2021.
Free PMC article
Show details
BMC Infect Dis
. 2021 Jan 12;21(1):57.
doi: 10.1186/s12879-020-05741-w.

Authors

Xue-Song Wen [1], Dan Jiang [1], Lei Gao [1], Jian-Zhong Zhou [1], Jun Xiao [2], Xiao-Cheng Cheng [1], Bin He [1], Yue Chen [1], Peng Lei [1], Xiao-Wei Tan [1], Shu Qin [1], Dong-Ying Zhang [3]

Affiliations

- [1] Department of Cardiovascular Medicine, The First Affiliated Hospital of Chongqing Medical University, No 1, Youyi Road, Chongqing, 400016, China.
- [2] Department of Cardiovascular Medicine, Chongqing University Center Hospital, Chongqing, 400014, China.
- [3] Department of Cardiovascular Medicine, The First Affiliated Hospital of Chongqing Medical University, No 1, Youyi Road, Chongqing, 400016, China. zhangdongying@cqmu.edu.cn.

- PMID: **33435865**
- PMCID: PMC7803000
- DOI: 10.1186/s12879-020-05741-w

Abstract

Background: In December 2019, coronavirus disease 2019 (COVID-19) caused by severe acute respiratory syndrome coronavirus 2 (SARS-CoV-2) emerged in Wuhan, Hubei, China. Moreover, it has become a global pandemic. This is of great value in describing the clinical symptoms of COVID-19 patients in detail and looking for markers which are significant to predict the prognosis of COVID-19 patients.

Methods: In this multicenter, retrospective study, 476 patients with COVID-19 were enrolled from a consecutive series. After screening, a total of 395 patients were included in this study. All-cause death was the primary endpoint. All patients were followed up from admission till discharge or death.

Results: The main symptoms observed in the study included fever on admission, cough, fatigue, and shortness of breath. The most common comorbidities were hypertension and diabetes mellitus. Patients with lower $CD4^{+}$T cell level were older and more often male compared to those with higher $CD4^{+}$T cell level. Reduced $CD8^{+}$T cell level was an indicator of the severity of COVID-19. Both decreased $CD4^{+}$T [HR:13.659; 95%CI: 3.235-57.671] and $CD8^{+}$T [HR: 10.883; 95%CI: 3.277-36.145] cell levels were associated with in-hospital death in COVID-19 patients, but only the decrease of $CD4^{+}$T cell level was an independent predictor of in-hospital death in COVID-19 patients.

Conclusions: Reductions in lymphocytes and lymphocyte subsets were common in COVID-19 patients, especially in severe cases of COVID-19. It was the $CD8^{+}$T cell level, not the $CD4^{+}$T cell level, that reflected the severity of the patient's disease. Only reduced $CD4^{+}$T cell level was independently associated with increased in-hospital death in COVID-19 patients.

Trial registration: Prognostic Factors of Patients With COVID-19, NCT04292964 . Registered 03 March 2020. Retrospectively registered.

Keywords: CD4+T cells; COVID-19; In-hospital death; SARS-CoV-2.

Conflict of interest statement

All authors declare no conflict of interest.

- 24 references
- 4 figures

Supplementary info

Publication types, MeSH terms, Associated data, Grant support Expand

Publication types

- Multicenter Study
- Observational Study

MeSH terms

- Adult
- Aged
- CD4-Positive T-Lymphocytes / cytology*
- CD8-Positive T-Lymphocytes / cytology
- COVID-19 / blood*
- COVID-19 / diagnosis
- COVID-19 / mortality
- COVID-19 / therapy
- Comorbidity
- Female

- Follow-Up Studies
- Hospitalization
- Humans
- Lymphocyte Count
- Male
- Middle Aged
- Pandemics
- Patient Discharge
- Prognosis
- Retrospective Studies
- SARS-CoV-2 / genetics
- SARS-CoV-2 / immunology*

Associated data

- ClinicalTrials.gov/NCT04292964

Grant support

- 81970203/National Natural Science Foundation of China (CN)
- 81570212/National Natural Science Foundation of China
- 31800976/National Natural Science Foundation of China

Full text links

Read free full text at BMC BioMed Central Free PMC article

Proceed to details

Cite

Share

93

Observational Study

Medicine (Baltimore)

. 2021 Feb 12;100(6):e24544.

doi: 10.1097/MD.0000000000024544.

Effect of low-dose aspirin on mortality and viral duration of the hospitalized adults with COVID-19

Qiang Liu [1], Na Huang [1], Anni Li [1], Yuanhong Zhou [1], Liang Liang [1], Xinyu Song [1], Zhanqiu Yang [2], Xiaolin Zhou [1]

Affiliations Expand

Affiliations

- [1] Department of Infectious Disease, Yichang Central People's Hospital, The First College of Clinical Medical Science, China Three Gorges University, Yichang.
- [2] State Key Laboratory of Virology, Institute of Medical Virology, School of Medicine, Wuhan University, Wuhan, Hubei, China.

- PMID: **33578548**
- PMCID: PMC7886487
- DOI: 10.1097/MD.0000000000024544

Free PMC article
Observational Study

Effect of low-dose aspirin on mortality and viral duration of the hospitalized adults with COVID-19

Qiang Liu et al. Medicine (Baltimore). 2021.
Free PMC article
Show details
Medicine (Baltimore)
. 2021 Feb 12;100(6):e24544.
doi: 10.1097/MD.0000000000024544.

Authors

Qiang Liu [1], Na Huang [1], Anni Li [1], Yuanhong Zhou [1], Liang Liang [1], Xinyu Song [1], Zhanqiu Yang [2], Xiaolin Zhou [1]

Affiliations

- [1] Department of Infectious Disease, Yichang Central People's Hospital, The First College of Clinical Medical Science, China Three Gorges University, Yichang.
- [2] State Key Laboratory of Virology, Institute of Medical Virology, School of Medicine, Wuhan University, Wuhan, Hubei, China.

- PMID: **33578548**
- PMCID: PMC7886487
- DOI: 10.1097/MD.0000000000024544

Abstract

To clarify the effect of aspirin on mortality and viral duration in adults infected with respiratory syndrome coronavirus 2 (SARS-Cov-2).After propensity score-matched (PSM) case-control analyses 24 pairs of patients were enrolled and followed up for 2 months. Both 30-day and 60-day mortality in the aspirin group were significantly lower than that in the non-aspirin group (P = .021

and P = .030, respectively). The viral duration time between the 2 groups was not significantly different (P = .942).Among adults (with hypertension, cardiovascular diseases) infected with SARS-Cov-2, low-dose aspirin medication (100 mg/day) was associated with lower risk of mortality compared with non-aspirin users.

Copyright © 2021 the Author(s). Published by Wolters Kluwer Health, Inc.

Conflict of interest statement

All authors have submitted the ICMJE Form for Disclosure of Potential Conflicts of Interest. The authors have no conflicts of interests to disclose.

- 10 references
- 2 figures

Supplementary info

Publication types, MeSH terms, Substances, Grant support Expand

Publication types

- Observational Study

MeSH terms

- Adult
- Aged
- Aspirin / therapeutic use*
- COVID-19 / complications
- COVID-19 / drug therapy
- COVID-19 / mortality*
- COVID-19 / virology
- China / epidemiology
- Embolism / prevention & control*
- Embolism / virology
- Female
- Fibrinolytic Agents / therapeutic use*
- Humans
- Male
- Middle Aged
- Retrospective Studies
- SARS-CoV-2 / isolation & purification

Substances

- Fibrinolytic Agents
- Aspirin

Grant support

- 81402404/National Nature Science Foundation of China
- 81402404/National Nature Science Foundation of China

Full text links

Wolters Kluwer Wolters Kluwer Free PMC article
Proceed to details
Cite
Share
94
Observational Study
BMJ Qual Saf
. 2022 Jan;31(1):45-53.
doi: 10.1136/bmjqs-2021-013721. Epub 2021 Oct 5.

COVID-19 hospital prevalence as a risk factor for mortality: an observational study of a multistate cohort of 62 hospitals

Mohamad Ghazi Fakih [1], Allison Ottenbacher [2], Baligh Yehia [3], Richard Fogel [3], Collin Miller [2], Angela Winegar [2], Christine Jesser [4], Joseph Cacchione [3]
Affiliations Expand

Affiliations

- [1] Clinical & Network Services, Ascension Health, Saint Louis, Missouri, USA Mohamad.Fakih@ascension.org.
- [2] Ascension Data Science Institute, Ascension Health, Saint Louis, Missouri, USA.
- [3] Clinical & Network Services, Ascension Health, Saint Louis, Missouri, USA.
- [4] Ascension Clinical Research Institute, Ascension Health, Saint Louis, Missouri, USA.

- PMID: **34611041**
- PMCID: PMC8494532
- DOI: 10.1136/bmjqs-2021-013721

Free PMC article
Observational Study

COVID-19 hospital prevalence as a risk factor for mortality: an observational study of a multistate cohort of 62 hospitals

Mohamad Ghazi Fakih et al. BMJ Qual Saf. 2022 Jan.
Free PMC article
Show details
BMJ Qual Saf
. 2022 Jan;31(1):45-53.
doi: 10.1136/bmjqs-2021-013721. Epub 2021 Oct 5.

Authors

Mohamad Ghazi Fakih [1], Allison Ottenbacher [2], Baligh Yehia [3], Richard Fogel [3], Collin Miller [2], Angela Winegar [2], Christine Jesser [4], Joseph Cacchione [3]

Affiliations

- [1] Clinical & Network Services, Ascension Health, Saint Louis, Missouri, USA Mohamad.Fakih@ascension.org.
- [2] Ascension Data Science Institute, Ascension Health, Saint Louis, Missouri, USA.
- [3] Clinical & Network Services, Ascension Health, Saint Louis, Missouri, USA.
- [4] Ascension Clinical Research Institute, Ascension Health, Saint Louis, Missouri, USA.

- PMID: **34611041**
- PMCID: PMC8494532
- DOI: 10.1136/bmjqs-2021-013721

Abstract

Background: The associated mortality with COVID-19 has improved compared with the early pandemic period. The effect of hospital COVID-19 patient prevalence on COVID-19 mortality has not been well studied.

Methods: We analysed data for adults with confirmed SARS-CoV-2 infection admitted to 62 hospitals within a multistate health system over 12 months. Mortality was evaluated based on patient demographic and clinical risk factors, COVID-19 hospital prevalence and calendar time period of the admission, using a generalised linear mixed model with site of care as the random effect.

Results: 38 104 patients with COVID-19 were hospitalised, and during their encounters, the prevalence of COVID-19 averaged 16% of the total hospitalised population. Between March-April 2020 and January-February 2021, COVID-19 mortality declined from 19% to 12% (p<0.001). In the adjusted multivariable analysis, mid and high COVID-19 inpatient prevalence were associated with a 25% and 41% increase in the odds (absolute contribution to probability of death of 2%-3%) of COVID-19 mortality compared with patients with COVID-19 in facilities with low prevalence (<10%), respectively (high prevalence >25%: adjusted OR (AOR) 1.41, 95% CI 1.23 to 1.61; mid-

prevalence (10%-25%): AOR 1.25, 95% CI 1.13 to 1.38). Mid and high COVID-19 prevalence accounted for 76% of patient encounters.

Conclusions: Although inpatient mortality for patients with COVID-19 has sharply declined compared with earlier in the pandemic, higher COVID-19 hospital prevalence remained a common risk factor for COVID-19 mortality. Hospital leaders need to reconsider how we provide support to care for patients in times of increased volume and complexity, such as those experienced during COVID-19 surges.

Keywords: COVID-19; hospital medicine; mortality (standardised mortality ratios).

© Author(s) (or their employer(s)) 2022. Re-use permitted under CC BY-NC. No commercial re-use. See rights and permissions. Published by BMJ.

Conflict of interest statement

Competing interests: None declared.

- 34 references
- 3 figures

Supplementary info

Publication types, MeSH terms Expand

Publication types

- Observational Study

MeSH terms

- Adult
- COVID-19*
- Hospital Mortality
- Hospitalization
- Hospitals
- Humans
- Prevalence
- Retrospective Studies
- Risk Factors
- SARS-CoV-2

Full text links

BMJ Open Access Full Text HighWire Free PMC article

Proceed to details

Cite

Share

☐ 95

Observational Study

BMC Cancer

. 2021 May 20;21(1):578.

doi: 10.1186/s12885-021-08349-8.

Safety of systemic anti-cancer treatment in oncology patients with non-severe COVID-19: a cohort study

C van Marcke[#,1,2], N Honoré[#,1,2], A van der Elst[#,1,2], S Beyaert[2], F Derouane[1,2], C Dumont[1], F Aboubakar Nana[3,4], J F Baurain[1,2], I Borbath[1,2,5], P Collard[3,4], F Cornélis[1,2], A De Cuyper[1,2], F P Duhoux[1,2], B Filleul[1,6], R Galot[1,2], M Gizzi[1,7], F Mazzeo[1,2], T Pieters[3,4], E Seront[1,6], I Sinapi[1,7], M Van den Eynde[1,2,5], N Whenham[1,8], J C Yombi[2,9], A Scohy[10], A van Maanen[11], J P Machiels[12,13]

Affilliations Expand

Affiliations

- [1] Department of Medical Oncology, Institut Roi Albert II, Cliniques universitaires Saint-Luc, Avenue Hippocrate 10, 1200, Brussels, Belgium.
- [2] Institute for Experimental and Clinical Research (IREC, pôle MIRO), Université catholique de Louvain (UCLouvain), Avenue Hippocrate 10, 1200, Brussels, Belgium.
- [3] Department of Pneumology, Institut Roi Albert II, Cliniques universitaires Saint-Luc, Brussels, Belgium.
- [4] Institute for Experimental and Clinical Research (IREC, pôle PNEU), Université catholique de Louvain (UCLouvain), Brussels, Belgium.
- [5] Department of Hepatogastroenterology, Institut Roi Albert II, Cliniques universitaires Saint-Luc, Brussels, Belgium.
- [6] Department of Medical Oncology, Hôpital de Jolimont, Haine-Saint-Paul, Belgium.
- [7] Department of Medical Oncology, Grand Hôpital de Charleroi (GHdC), Charleroi, Belgium.
- [8] Department of Medical Oncology, Clinique Saint-Pierre, Ottignies, Belgium.
- [9] Department of General Internal Medicine, Cliniques universitaires Saint-Luc, Brussels, Belgium.
- [10] Department of Microbiology, Cliniques universitaires Saint-Luc, Brussels, Belgium.
- [11] Statistics unit, Institut Roi Albert II, Cliniques universitaires Saint-Luc, Brussels, Belgium.
- [12] Department of Medical Oncology, Institut Roi Albert II, Cliniques universitaires Saint-Luc, Avenue Hippocrate 10, 1200, Brussels, Belgium. jean-pascal.machiels@uclouvain.be.
- [13] Institute for Experimental and Clinical Research (IREC, pôle MIRO), Université catholique de Louvain (UCLouvain), Avenue Hippocrate 10, 1200, Brussels, Belgium. jean-pascal.machiels@uclouvain.be.

[#] Contributed equally.

- PMID: **34016086**
- PMCID: PMC8134961
- DOI: 10.1186/s12885-021-08349-8

Free PMC article
Observational Study

Safety of systemic anti-cancer treatment in oncology patients with non-severe COVID-19: a cohort study

C van Marcke et al. BMC Cancer. 2021.
Free PMC article
Show details
BMC Cancer
. 2021 May 20;21(1):578.
doi: 10.1186/s12885-021-08349-8.

Authors

C van Marcke[#,1,2], N Honoré[#,1,2], A van der Elst[#,1,2], S Beyaert[2], F Derouane[1,2], C Dumont[1], F Aboubakar Nana[3,4], J F Baurain[1,2], I Borbath[1,2,5], P Collard[3,4], F Cornélis[1,2], A De Cuyper[1,2], F P Duhoux[1,2], B Filleul[1,6], R Galot[1,2], M Gizzi[1,7], F Mazzeo[1,2], T Pieters[3,4], E Seront[1,6], I Sinapi[1,7], M Van den Eynde[1,2,5], N Whenham[1,8], J C Yombi[2,9], A Scohy[10], A van Maanen[11], J P Machiels[12,13]

Affiliations

- [1] Department of Medical Oncology, Institut Roi Albert II, Cliniques universitaires Saint-Luc, Avenue Hippocrate 10, 1200, Brussels, Belgium.
- [2] Institute for Experimental and Clinical Research (IREC, pôle MIRO), Université catholique de Louvain (UCLouvain), Avenue Hippocrate 10, 1200, Brussels, Belgium.
- [3] Department of Pneumology, Institut Roi Albert II, Cliniques universitaires Saint-Luc, Brussels, Belgium.
- [4] Institute for Experimental and Clinical Research (IREC, pôle PNEU), Université catholique de Louvain (UCLouvain), Brussels, Belgium.
- [5] Department of Hepatogastroenterology, Institut Roi Albert II, Cliniques universitaires Saint-Luc, Brussels, Belgium.
- [6] Department of Medical Oncology, Hôpital de Jolimont, Haine-Saint-Paul, Belgium.
- [7] Department of Medical Oncology, Grand Hôpital de Charleroi (GHdC), Charleroi, Belgium.
- [8] Department of Medical Oncology, Clinique Saint-Pierre, Ottignies, Belgium.
- [9] Department of General Internal Medicine, Cliniques universitaires Saint-Luc, Brussels, Belgium.
- [10] Department of Microbiology, Cliniques universitaires Saint-Luc, Brussels, Belgium.

- [11] Statistics unit, Institut Roi Albert II, Cliniques universitaires Saint-Luc, Brussels, Belgium.
- [12] Department of Medical Oncology, Institut Roi Albert II, Cliniques universitaires Saint-Luc, Avenue Hippocrate 10, 1200, Brussels, Belgium. jean-pascal.machiels@uclouvain.be.
- [13] Institute for Experimental and Clinical Research (IREC, pôle MIRO), Université catholique de Louvain (UCLouvain), Avenue Hippocrate 10, 1200, Brussels, Belgium. jean-pascal.machiels@uclouvain.be.

[#] Contributed equally.

- PMID: **34016086**
- PMCID: PMC8134961
- DOI: 10.1186/s12885-021-08349-8

Abstract

Background: The viral pandemic coronavirus disease 2019 (COVID-19) has disrupted cancer patient management around the world. Most reported data relate to incidence, risk factors, and outcome of severe COVID-19. The safety of systemic anti-cancer therapy in oncology patients with non-severe COVID-19 is an important matter in daily practice.

Methods: ONCOSARS-1 was a single-center, academic observational study. Adult patients with solid tumors treated in the oncology day unit with systemic anti-cancer therapy during the initial phase of the COVID-19 pandemic in Belgium were prospectively included. All patients (n = 363) underwent severe acute respiratory syndrome coronavirus-2 (SARS-CoV-2) serological testing after the first peak of the pandemic in Belgium. Additionally, 141 of these patients also had a SARS-CoV-2 RT-PCR test during the pandemic. The main objective was to retrospectively determine the safety of systemic cancer treatment, measured by the rate of adverse events according to the Common Terminology Criteria for Adverse Events, in SARS-CoV-2-positive patients compared with SARS-CoV-2-negative patients.

Results: Twenty-two (6%) of the 363 eligible patients were positive for SARS-CoV-2 by RT-PCR and/or serology. Of these, three required transient oxygen supplementation, but none required admission to the intensive care unit. Hematotoxicity was the only adverse event more frequently observed in SARS-CoV-2 -positive patients than in SARS-CoV-2-negative patients: 73% vs 35% ($P < 0.001$). This association remained significant (odds ratio (OR) 4.1, $P = 0.009$) even after adjusting for performance status and type of systemic treatment. Hematological adverse events led to more treatment delays for the SARS-CoV-2-positive group: 55% vs 20% ($P < 0.001$). Median duration of treatment interruption was similar between the two groups: 14 and 11 days, respectively. Febrile neutropenia, infections unrelated to COVID-19, and bleeding events occurred at a low rate in the SARS-CoV-2-positive patients.

Conclusion: Systemic anti-cancer therapy appeared safe in ambulatory oncology patients treated during the COVID-19 pandemic. There were, however, more treatment delays in the SARS-CoV-2-positive population, mainly due to a higher rate of hematological adverse events.

Keywords: Ambulatory; Non-severe COVID-19; Safety; Systemic anti-cancer treatment.

Conflict of interest statement

The authors declare that they have no competing interests.

- 29 references
- 1 figure

Supplementary info

Publication types, MeSH terms Expand

Publication types

- Observational Study

MeSH terms

- Aged
- Ambulatory Care / statistics & numerical data
- Belgium / epidemiology
- COVID-19 / complications
- COVID-19 / diagnosis*
- COVID-19 / epidemiology*
- Cancer Care Facilities
- Cohort Studies
- Female
- Health Personnel / statistics & numerical data
- Humans
- Male
- Middle Aged
- Neoplasms / epidemiology
- Neoplasms / therapy*
- Risk Factors
- SARS-CoV-2

Full text links

Read free full text at BMC BioMed Central Free PMC article

Proceed to details

Cite

Share

☐ 96

Observational Study

Med Sci Monit

. 2021 Feb 11;27:e926751.
doi: 10.12659/MSM.926751.

Risk Factors of Coronavirus Disease 2019-Related Mortality and Optimal Treatment Regimens: A Retrospective Study

Yuehong Wang [1], Shuang Yao [2], Xiaoling Liu [3], Yinghao Cao [4], Yaling Wang [5], Mao Xie [6]
Affiliations Expand

Affiliations

- [1] Department of Pediatrics, The Fourth Affiliated Hospital, Zhejiang University School of Medicine, Yiwu, Zhejiang, China (mainland).
- [2] Cancer Prevention Office, Hubei Cancer Hospital, Tongji Medical College, Huazhong University of Science and Technology, Wuhan, Hubei, China (mainland).
- [3] Department of Endocrinology, Liyuan Hospital, Tongji Medical College, Huazhong University of Since and Technology, Wuhan, Hubei, China (mainland).
- [4] Department of Gastrointestinal Surgery, Union Hospital, Tongji Medical College, Huazhong University of Science and Technology, Wuhan, Hubei, China (mainland).
- [5] Wuhan Institution for Tuberculosis Control, Wuhan Pulmonary Hospital, Wuhan, Hubei, China (mainland).
- [6] Department of Orthopedics, Union Hospital, Tongji Medical College, Huazhong University of Science and Technology, Wuhan, Hubei, China (mainland).

- PMID: **33571171**
- PMCID: PMC7885294
- DOI: 10.12659/MSM.926751

Free PMC article
Observational Study

Risk Factors of Coronavirus Disease 2019-Related Mortality and Optimal Treatment Regimens: A Retrospective Study

Yuehong Wang et al. Med Sci Monit. 2021.
Free PMC article
Show details
Med Sci Monit
. 2021 Feb 11;27:e926751.
doi: 10.12659/MSM.926751.

Authors

Yuehong Wang [1], Shuang Yao [2], Xiaoling Liu [3], Yinghao Cao [4], Yaling Wang [5], Mao Xie [6]

Affiliations

- [1] Department of Pediatrics, The Fourth Affiliated Hospital, Zhejiang University School of Medicine, Yiwu, Zhejiang, China (mainland).
- [2] Cancer Prevention Office, Hubei Cancer Hospital, Tongji Medical College, Huazhong University of Science and Technology, Wuhan, Hubei, China (mainland).
- [3] Department of Endocrinology, Liyuan Hospital, Tongji Medical College, Huazhong University of Since and Technology, Wuhan, Hubei, China (mainland).
- [4] Department of Gastrointestinal Surgery, Union Hospital, Tongji Medical College, Huazhong University of Science and Technology, Wuhan, Hubei, China (mainland).
- [5] Wuhan Institution for Tuberculosis Control, Wuhan Pulmonary Hospital, Wuhan, Hubei, China (mainland).
- [6] Department of Orthopedics, Union Hospital, Tongji Medical College, Huazhong University of Science and Technology, Wuhan, Hubei, China (mainland).

- PMID: **33571171**
- PMCID: PMC7885294
- DOI: 10.12659/MSM.926751

Abstract

BACKGROUND Coronavirus disease 2019 (COVID-19) is spreading rapidly worldwide, and scientists are trying to find a way to overcome the disease. We explored the risk factors that influence patient outcomes, including treatment regimens, which can provide a reference for further treatment. MATERIAL AND METHODS A retrospective cohort study analysis was performed using data from 97 patients with COVID-19 who visited Wuhan Union Hospital from February 2020 to March 2020. We collected data on demographics, comorbidities, clinical manifestations, laboratory tests, treatment methods, outcomes, and complications. Patients were divided into a recovered group and a deceased group. We compared the differences between the 2 groups and analyzed risk factors influencing the treatment effect. RESULTS Seventy-six patients recovered and 21 died. The average age and body mass index (BMI) of the deceased group were significantly higher than those of the recovered group (69.81±6.80 years vs 60.79±11.28 years, $P<0.001$ and 24.95±3.14 kg/m² vs 23.09±2.97 kg/m², $P=0.014$, respectively). The combination of antiviral drugs and supportive therapy appears to be associated with the lowest mortality ($P<0.05$). Multivariate Cox regression analysis revealed that age, BMI, H-CRP, shock, and acute respiratory distress syndrome (ARDS) were independent risk factors for patients with COVID-19 ($P<0.05$). CONCLUSIONS Elderly patients and those with a high BMI, as well as patients who experience shock and ARDS, may have a higher risk of death from COVID-19. The combination of antiviral drugs and supportive therapy appears to be associated with lower mortality, although further research is needed.

Conflict of interest statement

Conflict of Interest

None.

- 21 references
- 2 figures

Supplementary info

Publication types, MeSH terms, Substances, Supplementary concepts Expand

Publication types

- Observational Study

MeSH terms

- Age Factors
- Aged
- Antiviral Agents / therapeutic use
- COVID-19 / complications
- COVID-19 / drug therapy*
- COVID-19 / mortality*
- COVID-19 / virology
- China / epidemiology
- Drug Therapy, Combination / methods
- Drugs, Chinese Herbal / therapeutic use
- Female
- Hospital Mortality
- Humans
- Kaplan-Meier Estimate
- Male
- Middle Aged
- Prognosis
- Respiratory Distress Syndrome / etiology
- Respiratory Distress Syndrome / mortality*
- Respiratory Distress Syndrome / therapy
- Retrospective Studies
- Risk Factors
- SARS-CoV-2 / isolation & purification
- SARS-CoV-2 / pathogenicity
- Shock / etiology
- Shock / mortality*
- Shock / therapy
- Treatment Outcome
- gamma-Globulins / therapeutic use

Substances

- Antiviral Agents
- Drugs, Chinese Herbal
- gamma-Globulins

Supplementary concepts

- COVID-19 drug treatment

Full text links

International Scientific Literature, Ltd. Free PMC article
Proceed to details
Cite
Share
97
Observational Study
Mayo Clin Proc
. 2021 Jan;96(1):32-39.
doi: 10.1016/j.mayocp.2020.10.003. Epub 2020 Oct 10.

Inverse Relationship of Maximal Exercise Capacity to Hospitalization Secondary to Coronavirus Disease 2019

Clinton A Brawner [1], Jonathan K Ehrman [2], Shane Bole [3], Dennis J Kerrigan [2], Sachin S Parikh [2], Barry K Lewis [2], Ryan M Gindi [2], Courtland Keteyian [4], Khaled Abdul-Nour [2], Steven J Keteyian [2]
Affiliations Expand

Affiliations

- [1] Division of Cardiovascular Medicine, Henry Ford Hospital, Detroit, MI. Electronic address: cbrawne1@hfhs.org.
- [2] Division of Cardiovascular Medicine, Henry Ford Hospital, Detroit, MI.
- [3] Public Health Sciences, Henry Ford Health System, Detroit, MI.
- [4] Population Health, Henry Ford Allegiance Health, Jackson, MI.

- PMID: **33413833**
- PMCID: PMC7547590
- DOI: 10.1016/j.mayocp.2020.10.003

Free PMC article
Observational Study

Inverse Relationship of Maximal Exercise Capacity to Hospitalization Secondary to Coronavirus Disease 2019

Clinton A Brawner et al. Mayo Clin Proc. 2021 Jan.
Free PMC article
Show details
Mayo Clin Proc
. 2021 Jan;96(1):32-39.
doi: 10.1016/j.mayocp.2020.10.003. Epub 2020 Oct 10.

Authors

Clinton A Brawner [1], Jonathan K Ehrman [2], Shane Bole [3], Dennis J Kerrigan [2], Sachin S Parikh [2], Barry K Lewis [2], Ryan M Gindi [2], Courtland Keteyian [4], Khaled Abdul-Nour [2], Steven J Keteyian [2]

Affiliations

- [1] Division of Cardiovascular Medicine, Henry Ford Hospital, Detroit, MI. Electronic address: cbrawne1@hfhs.org.
- [2] Division of Cardiovascular Medicine, Henry Ford Hospital, Detroit, MI.
- [3] Public Health Sciences, Henry Ford Health System, Detroit, MI.
- [4] Population Health, Henry Ford Allegiance Health, Jackson, MI.

- PMID: **33413833**
- PMCID: PMC7547590
- DOI: 10.1016/j.mayocp.2020.10.003

Abstract

Objective: To investigate the relationship between maximal exercise capacity measured before severe acute respiratory syndrome coronavirus 2 (SARS-CoV-2) infection and hospitalization due to coronavirus disease 2019 (COVID-19).

Methods: We identified patients (≥18 years) who completed a clinically indicated exercise stress test between January 1, 2016, and February 29, 2020, and had a test for SARS-CoV-2 (ie, real-time reverse transcriptase polymerase chain reaction test) between February 29, 2020, and May 30, 2020. Maximal exercise capacity was quantified in metabolic equivalents of task (METs). Logistic regression was used to evaluate the likelihood that hospitalization secondary to COVID-19 is related to peak METs, with adjustment for 13 covariates previously identified as associated with higher risk for severe illness from COVID-19.

Results: We identified 246 patients (age, 59±12 years; 42% male; 75% black race) who had an exercise test and tested positive for SARS-CoV-2. Among these, 89 (36%) were hospitalized. Peak METs were significantly lower ($P<.001$) among patients who were hospitalized (6.7±2.8) compared with those not hospitalized (8.0±2.4). Peak METs were inversely associated with the

likelihood of hospitalization in unadjusted (odds ratio, 0.83; 95% CI, 0.74-0.92) and adjusted models (odds ratio, 0.87; 95% CI, 0.76-0.99).

Conclusion: Maximal exercise capacity is independently and inversely associated with the likelihood of hospitalization due to COVID-19. These data further support the important relationship between cardiorespiratory fitness and health outcomes. Future studies are needed to determine whether improving maximal exercise capacity is associated with lower risk of complications due to viral infections, such as COVID-19.

Copyright © 2020 Mayo Foundation for Medical Education and Research. Published by Elsevier Inc. All rights reserved.

Comment in

- Fit Is It in COVID-19, Future Pandemics, and Overall Healthy Living.
  Lavie CJ, Sanchis-Gomar F, Arena R. Lavie CJ, et al. Mayo Clin Proc. 2021 Jan;96(1):7-9. doi: 10.1016/j.mayocp.2020.11.013. Epub 2020 Nov 24. Mayo Clin Proc. 2021. PMID: 33413836 Free PMC article. No abstract available.

- 19 references
- 3 figures

Supplementary info

Publication types, MeSH terms Expand

Publication types

- Observational Study

MeSH terms

- COVID-19 / physiopathology*
- COVID-19 Testing
- Exercise Test
- Exercise Tolerance*
- Female
- Hospitalization / statistics & numerical data*
- Humans
- Male
- Middle Aged
- Pneumonia, Viral / epidemiology
- Pneumonia, Viral / physiopathology*
- Pneumonia, Viral / virology
- Retrospective Studies
- SARS-CoV-2

Full text links

ELSEVIER FULL-TEXT ARTICLE Elsevier Science Free PMC article

Proceed to details

Cite

Share

98

Observational Study

J Crit Care

. 2021 Oct;65:200-204.

doi: 10.1016/j.jcrc.2021.06.014. Epub 2021 Jun 29.

Management of SARS-CoV-2 pneumonia in intensive care unit: An observational retrospective study comparing two bundles

Alexandre Lopez [1], Ines Lakbar [2], Louis Delamarre [2], Aurélien Culver [3], Charlotte Arbelot [3], Gary Duclos [3], Emmanuelle Hammad [3], Bruno Pastene [3], François Antonini [3], Laurent Zieleskiewicz [3], Marc Leone [3]

Affiliations Expand

Affiliations

- [1] Aix-Marseille University, Assistance Publique Hôpitaux de Marseille, Department of Anesthesiology and Intensive Care, Hôpital Nord, Marseille 13015, France. Electronic address: alexandre.lopez@ap-hm.fr.
- [2] Department of Anesthesiology and Intensive Care Unit, Hôpital Nord, Toulouse, France.
- [3] Aix-Marseille University, Assistance Publique Hôpitaux de Marseille, Department of Anesthesiology and Intensive Care, Hôpital Nord, Marseille 13015, France.

- PMID: **34225084**
- PMCID: PMC8238648
- DOI: 10.1016/j.jcrc.2021.06.014

Free PMC article

Observational Study

Management of SARS-CoV-2 pneumonia in intensive care unit: An observational retrospective study comparing two bundles

Alexandre Lopez et al. J Crit Care. 2021 Oct.

Free PMC article

Show details

J Crit Care

. 2021 Oct;65:200-204.
doi: 10.1016/j.jcrc.2021.06.014. Epub 2021 Jun 29.

Authors

Alexandre Lopez [1], Ines Lakbar [2], Louis Delamarre [2], Aurélien Culver [3], Charlotte Arbelot [3], Gary Duclos [3], Emmanuelle Hammad [3], Bruno Pastene [3], François Antonini [3], Laurent Zieleskiewicz [3], Marc Leone [3]

Affiliations

- [1] Aix-Marseille University, Assistance Publique Hôpitaux de Marseille, Department of Anesthesiology and Intensive Care, Hôpital Nord, Marseille 13015, France. Electronic address: alexandre.lopez@ap-hm.fr.
- [2] Department of Anesthesiology and Intensive Care Unit, Hôpital Nord, Toulouse, France.
- [3] Aix-Marseille University, Assistance Publique Hôpitaux de Marseille, Department of Anesthesiology and Intensive Care, Hôpital Nord, Marseille 13015, France.

- PMID: **34225084**
- PMCID: PMC8238648
- DOI: 10.1016/j.jcrc.2021.06.014

Abstract

Purpose: To compare the effects of two therapeutic bundles of management in SARS-CoV2 ICU patients.

Materials and methods: Our retrospective, observational study was performed in a university ICU from March to June 2020 (first wave) and from September 2020 to January 2021 (second wave). In first wave, patients received bundle 1 including early invasive ventilation, hydroxychloroquine, cefotaxime and azithromycin. In second wave, bundle 2 included non-invasive oxygenation support and dexamethasone. The main outcome was in-hospital mortality. Secondary outcomes included ICU and hospital length of stay, ICU supportive therapies, viral clearance and antimicrobial resistance emergence.

Results: 129 patients with SARS-CoV-2 pneumonia were admitted to our ICU. Thirty-five were treated according to bundle 1 and 76 to bundle 2. In-hospital mortality was similar in the two groups (23%, $p = 1$). The hospital ($p = 0.003$) and ICU ($p = 0.01$) length of stay and ventilator-free days at 28 days ($p = 0.03$) were significantly reduced in bundle 2. Increasing age, vasopressor use and PaO_2/FiO_2 ratio < 125 were associated with in-hospital mortality.

Conclusion: Within the limitations of our study, changes in therapeutic bundles for SARS-Cov-2 ICU patients might have no effect on in-hospital mortality but were associated with less exposure to mechanical ventilation and reduced hospital length of stay.

Keywords: Bundle; COVID-19; Dexamethasone; Hydroxychloroquine; Intensive care unit; Treatment.

Copyright © 2021 Elsevier Inc. All rights reserved.

- 21 references
- 2 figures

Supplementary info

Publication types, MeSH terms, Substances Expand

Publication types

- Observational Study

MeSH terms

- COVID-19*
- Humans
- Intensive Care Units
- RNA, Viral
- Respiration, Artificial
- Retrospective Studies
- SARS-CoV-2*

Substances

- RNA, Viral

Full text links

ELSEVIER FULL-TEXT ARTICLE Elsevier Science Free PMC article
Proceed to details
Cite
Share
99
Observational Study
Pol Merkur Lekarski
. 2021 Dec 16;49(294):389-393.

The efficacy of amantadine hydrochloride in the treatment of COVID-19 - a single-center observation study

Wlodzimierz Bodnar [1], Gonzalo Aranda-Abreu [2], Monika Slabon-Willand [3], Sylwia Kotecka [4], Malgorzata Farnik [5], Jaroslaw Bodnar [1]

Affiliations Expand

Affiliations

- [1] General Practice Clinic OPTIMA, Przemyśl, Poland.
- [2] University Veracruzana, Xalapa, Veracruz, MX.
- [3] Emergency Department, Leszek Giec Upper-Silesian Medical Centre of the Silesian Medical University in Katowice, Poland.
- [4] Pharmacy "Wielkopolska", Pharmacy Group Czerniewicz, Poznań, Poland.
- [5] Department of Pulmonology, School of Medicine in Katowice, Medical University of Silesia, Katowice, Poland.

- PMID: **34919079**

Observational Study

The efficacy of amantadine hydrochloride in the treatment of COVID-19 - a single-center observation study

Wlodzimierz Bodnar et al. Pol Merkur Lekarski. 2021.
Show details
Pol Merkur Lekarski
. 2021 Dec 16;49(294):389-393.

Authors

Wlodzimierz Bodnar [1], Gonzalo Aranda-Abreu [2], Monika Slabon-Willand [3], Sylwia Kotecka [4], Malgorzata Farnik [5], Jaroslaw Bodnar [1]

Affiliations

- [1] General Practice Clinic OPTIMA, Przemyśl, Poland.
- [2] University Veracruzana, Xalapa, Veracruz, MX.
- [3] Emergency Department, Leszek Giec Upper-Silesian Medical Centre of the Silesian Medical University in Katowice, Poland.
- [4] Pharmacy "Wielkopolska", Pharmacy Group Czerniewicz, Poznań, Poland.
- [5] Department of Pulmonology, School of Medicine in Katowice, Medical University of Silesia, Katowice, Poland.

- PMID: **34919079**

Abstract

Coronavirus disease 19 (COVID-19) rapidly spread worldwide. The search for effective measures to counter the development and effects of the pandemic includes: identifying the disease pathogen,

introducing methods of reducing its transmission, building the population immunity, and the search for a cure, both among the new and already-known substances with potential antivirus activity such as amantadine hydrochloride.

Aim: The aim of the study was an observational single-center analysis of confirmed COVID-19 cases treated with amantadine in ambulatory settings.

Materials and methods: The 55 patients with confirmed COVID-19 diagnosis were treated in ambulatory settings by amantadine with a treatment schema varied from 200 mg to 500 mg per day. A retrospective analysis was based on symptoms, hospitalization, and number of deaths.

Results: The mean age of the patients was 55.9 years (SD=15), and most patients were male (60%). Despite the majority of patients 64% (n=35) suffering from comorbidities and 53% (n=29) of patients having been diagnosed with pneumonia, none of them died, and only four had required hospitalization in the course of COVID-19. Clinical stabilization was achieved in 91% (n=50) of patients within 48 hours after the first dose of amantadine with further improvement; additionally, all patients experienced remission of COVID-19. In total, 93% (n=51) of patients did not require hospitalization during the treatment.

Conclusions: The data may suggest that amantadine hydrochloride shows efficacy in preventing hospitalization and deaths in patients with COVID-19. At the same time, it emphasizes that daily monitoring of the patient and regular examination are important in the case of SARS-CoV-2 infection dynamics. It may be justified to carry out a prospective, randomized, and double-blinded clinical study with the postulated amantadine scheme.

Keywords: Amantadine hydrochloride; COVID-19; SARS-CoV-2.

© 2021 MEDPRESS.

Supplementary info

Publication types, MeSH terms, Substances Expand

Publication types

- Observational Study

MeSH terms

- Amantadine* / therapeutic use
- COVID-19 Testing
- COVID-19*
- Humans
- Male
- Middle Aged
- Prospective Studies
- Retrospective Studies
- SARS-CoV-2

Substances

- Amantadine

Full text links

MEDPRESS full text PDF MEDPRESS
Proceed to details
Cite
Share
☐ 100
Observational Study
J Med Virol
. 2020 Nov;92(11):2718-2724.
doi: 10.1002/jmv.26147. Epub 2020 Jun 29.

Clinical and epidemiological characteristics of 320 deceased patients with COVID-19 in an Italian Province: A retrospective observational study

Andrea Biagi [1], Luca Rossi [1], Alessandro Malagoli [2], Alessia Zanni [1], Concetta Sticozzi [1], Greta Comastri [1], Stefano Gandolfi [3], Giovanni Quinto Villani [1]
Affiliations Expand

Affiliations

- [1] Division of Cardiology, Department of Cardiovascular and Emergency, Guglielmo da Saliceto Hospital, Piacenza, Italy.
- [2] Division of Cardiology, Department of Nephro-Cardiovascular, "S. Agostino-Estense" Public Hospital, University of Modena and Reggio Emilia, Modena, Italy.
- [3] Health Management, Department of Health Administration, Guglielmo da Saliceto Hospital, ASL Piacenza, Piacenza, Italy.

- PMID: **32515500**
- PMCID: PMC7300458
- DOI: 10.1002/jmv.26147

Free PMC article
Observational Study

Clinical and epidemiological characteristics of 320 deceased patients with COVID-19 in an Italian Province: A retrospective observational study

Andrea Biagi et al. J Med Virol. 2020 Nov.
Free PMC article
Show details
J Med Virol
. 2020 Nov;92(11):2718-2724.
doi: 10.1002/jmv.26147. Epub 2020 Jun 29.

Authors

Andrea Biagi [1], Luca Rossi [1], Alessandro Malagoli [2], Alessia Zanni [1], Concetta Sticozzi [1], Greta Comastri [1], Stefano Gandolfi [3], Giovanni Quinto Villani [1]

Affiliations

- [1] Division of Cardiology, Department of Cardiovascular and Emergency, Guglielmo da Saliceto Hospital, Piacenza, Italy.
- [2] Division of Cardiology, Department of Nephro-Cardiovascular, "S. Agostino-Estense" Public Hospital, University of Modena and Reggio Emilia, Modena, Italy.
- [3] Health Management, Department of Health Administration, Guglielmo da Saliceto Hospital, ASL Piacenza, Piacenza, Italy.

- PMID: **32515500**
- PMCID: PMC7300458
- DOI: 10.1002/jmv.26147

Abstract

Studies have described clinical features of patients with coronavirus disease (COVID-19). However, limited data concerning the clinical characteristics of the Italian deaths are available. We aim to describe the clinical and epidemiological characteristics of 320 deceased from the Italian experience. We retrospectively collected all consecutive non-survivor patients with laboratory-confirmed COVID-19 infection admitted to the Emergency Rooms (ERs) Piacenza Hospital Network during the first month of COVID-19 pandemic in Italy. Clinical history, comorbidities, laboratory findings and treatment were recorded for each patient. A total of 1050 patients with confirmed COVID-19 pneumonia were admitted to the ERs between 24 February and 22 March 2020. Three hundred and twenty (30.5%) patients died with a median age of 78.0 years, 205 (64%) non-survivors were above 65 years old, 230 (71.9%) were male. Non-survivor patients showed frequently several coexisting medical conditions, with hypertension being the most common comorbidity (235 patients, 73.4%). The in-hospital mortality did not change during the progression of the pandemic. In this retrospective Italian study, most of COVID-19 deceased patients were elderly male aged over than 65 years. Hypertension was the most common

coexisting disease. In-hospital mortality was high and showed no variation during the first month of the COVID-19 italian epidemic.

Keywords: COVID-19; SARS-CoV-2; deceased; hypertension.

© 2020 Wiley Periodicals LLC.

- 28 references

Supplementary info

Publication types, MeSH terms Expand

Publication types

- Observational Study

MeSH terms

- Adult
- Aged
- Aged, 80 and over
- COVID-19 / mortality*
- Comorbidity
- Female
- Geography
- Hospital Mortality
- Hospitalization / statistics & numerical data
- Humans
- Hypertension / complications
- Hypertension / epidemiology
- Italy / epidemiology
- Male
- Middle Aged
- Retrospective Studies
- Risk Factors

Full text links

WILEY Full Text Article Wiley Free PMC article

Proceed to details

Cite

Share

☐ 101

Clin Infect Dis
. 2021 May 18;72(10):e558-e565.
doi: 10.1093/cid/ciaa1268.

Patient Characteristics and Outcomes of 11 721 Patients With Coronavirus Disease 2019 (COVID-19) Hospitalized Across the United States

Michael W Fried[1], Julie M Crawford[1], Andrea R Mospan[1], Stephanie E Watkins[1], Breda Munoz[1], Richard C Zink[1], Sherry Elliott[2], Kyle Burleson[1], Charles Landis[3], K Rajender Reddy[4], Robert S Brown[5]
Affiliations Expand

Affiliations

- [1] TARGET PharmaSolutions Inc., Durham, North Carolina, USA.
- [2] Elliott Health Information Pros Inc., Cary, North Carolina, USA.
- [3] Liver Care and Transplantation Services at University of Washington Medical Center, Seattle, Washington, USA.
- [4] Department of Medicine, University of Pennsylvania, Philadelphia, Pennsylvania, USA.
- [5] Department of Medicine, Division of Gastroenterology and Hepatology, Weill Cornell Medicine Center for Liver Disease, New York, New York, USA.

- PMID: **32856034**
- PMCID: PMC7499515
- DOI: 10.1093/cid/ciaa1268

Free PMC article

Patient Characteristics and Outcomes of 11 721 Patients With Coronavirus Disease 2019 (COVID-19) Hospitalized Across the United States

Michael W Fried et al. Clin Infect Dis. 2021.
Free PMC article
Show details
Clin Infect Dis
. 2021 May 18;72(10):e558-e565.
doi: 10.1093/cid/ciaa1268.

Authors

Michael W Fried [1], Julie M Crawford [1], Andrea R Mospan [1], Stephanie E Watkins [1], Breda Munoz [1], Richard C Zink [1], Sherry Elliott [2], Kyle Burleson [1], Charles Landis [3], K Rajender Reddy [4], Robert S Brown [5]

Affiliations

- [1] TARGET PharmaSolutions Inc., Durham, North Carolina, USA.
- [2] Elliott Health Information Pros Inc., Cary, North Carolina, USA.
- [3] Liver Care and Transplantation Services at University of Washington Medical Center, Seattle, Washington, USA.
- [4] Department of Medicine, University of Pennsylvania, Philadelphia, Pennsylvania, USA.
- [5] Department of Medicine, Division of Gastroenterology and Hepatology, Weill Cornell Medicine Center for Liver Disease, New York, New York, USA.

- PMID: **32856034**
- PMCID: PMC7499515
- DOI: 10.1093/cid/ciaa1268

Abstract

Background: As coronavirus disease 2019 (COVID-19) disseminates throughout the United States, a better understanding of the patient characteristics associated with hospitalization, morbidity, and mortality in diverse geographic regions is essential.

Methods: Hospital chargemaster data on adult patients with COVID-19 admitted to 245 hospitals across 38 states between 15 February and 20 April 2020 were assessed. The clinical course from admission, through hospitalization, and to discharge or death was analyzed.

Results: A total of 11 721 patients were included (majority were >60 years of age [59.9%] and male [53.4%]). Comorbidities included hypertension (46.7%), diabetes (27.8%), cardiovascular disease (18.6%), obesity (16.1%), and chronic kidney disease (12.2%). Mechanical ventilation was required by 1967 patients (16.8%). Mortality among hospitalized patients was 21.4% and increased to 70.5% among those on mechanical ventilation. Male sex, older age, obesity, geographic region, and the presence of chronic kidney disease or a preexisting cardiovascular disease were associated with increased odds of mechanical ventilation. All aforementioned risk factors, with the exception of obesity, were associated with increased odds of death (all P values < .001). Many patients received investigational medications for treatment of COVID-19, including 48 patients on remdesivir and 4232 on hydroxychloroquine.

Conclusions: This large observational cohort describes the clinical course and identifies factors associated with the outcomes of hospitalized patients with COVID-19 across the United States. These data can inform strategies to prioritize prevention and treatment for this disease.

Keywords: COVID-19; SARS-CoV-2; hydroxychloroquine; observational study; remdesivir.

© The Author(s) 2020. Published by Oxford University Press for the Infectious Diseases Society of America. All rights reserved. For permissions, e-mail: journals.permissions@oup.com.

Comment in

- Be Careful With Big Data: Reanalysis of Patient Characteristics and Outcomes of 11 721 Patients With Coronavirus Disease 2019 Hospitalized Across the United States.
Roussel Y, Million M, Chabriere E, Lagier JC, Raoult D. Roussel Y, et al. Clin Infect Dis. 2021 Jun 1;72(11):e928. doi: 10.1093/cid/ciaa1618. Clin Infect Dis. 2021. PMID: 33086376 Free PMC article. No abstract available.
- Reply to Roussel et al.
Fried MW, Crawford JM, Mospan AR, Watkins SE, Munoz B, Zink RC, Elliott S, Burleson K, Landis C, Reddy KR, Brown RS. Fried MW, et al. Clin Infect Dis. 2021 Jun 1;72(11):e929. doi: 10.1093/cid/ciaa1621. Clin Infect Dis. 2021. PMID: 33086377 No abstract available.

Supplementary info

Publication types, MeSH terms, Substances, Grant support Expand

Publication types

- Research Support, Non-U.S. Gov't

MeSH terms

- Adult
- Aged
- COVID-19*
- Comorbidity
- Hospitalization
- Humans
- Hydroxychloroquine
- Male
- Respiration, Artificial
- Retrospective Studies
- SARS-CoV-2
- United States / epidemiology

Substances

- Hydroxychloroquine

Grant support

- P30 CA016086/CA/NCI NIH HHS/United States

Full text links

OXFORD ACADEMIC Silverchair Information Systems Free PMC article
Proceed to details
Cite
Share
102

Observational Study

BMC Infect Dis
. 2021 Mar 17;21(1):271.
doi: 10.1186/s12879-021-05957-4.

Characteristics of patients with Coronavirus Disease 2019 (COVID-19) and seasonal influenza at time of hospital admission: a single center comparative study

Pablo Sieber [1], Domenica Flury [1], Sabine Güsewell [2], Werner C Albrich [1], Katia Boggian [1], Céline Gardiol [3], Matthias Schlegel [1], Robert Sieber [4], Pietro Vernazza [1], Philipp Kohler [5]
Affiliations Expand

Affiliations

- [1] Division of Infectious Diseases and Hospital Epidemiology, Cantonal Hospital St. Gallen, Rorschacher Strasse 95, 9007, St. Gallen, Switzerland.
- [2] Clinical Trial Unit, Cantonal Hospital St. Gallen, St. Gallen, Switzerland.
- [3] Federal Office of Public Health, Berne, Switzerland.
- [4] Emergency Department, Cantonal Hospital St. Gallen, St. Gallen, Switzerland.
- [5] Division of Infectious Diseases and Hospital Epidemiology, Cantonal Hospital St. Gallen, Rorschacher Strasse 95, 9007, St. Gallen, Switzerland. philipp.kohler@kssg.ch.

- PMID: **33731019**
- PMCID: PMC7968573
- DOI: 10.1186/s12879-021-05957-4

Free PMC article
Observational Study

Characteristics of patients with Coronavirus Disease 2019 (COVID-19) and seasonal influenza at time of hospital admission: a single center comparative study

Pablo Sieber et al. BMC Infect Dis. 2021.
Free PMC article
Show details
BMC Infect Dis
. 2021 Mar 17;21(1):271.
doi: 10.1186/s12879-021-05957-4.

Authors

Pablo Sieber [1], Domenica Flury [1], Sabine Güsewell [2], Werner C Albrich [1], Katia Boggian [1], Céline Gardiol [3], Matthias Schlegel [1], Robert Sieber [4], Pietro Vernazza [1], Philipp Kohler [5]

Affiliations

- [1] Division of Infectious Diseases and Hospital Epidemiology, Cantonal Hospital St. Gallen, Rorschacher Strasse 95, 9007, St. Gallen, Switzerland.
- [2] Clinical Trial Unit, Cantonal Hospital St. Gallen, St. Gallen, Switzerland.
- [3] Federal Office of Public Health, Berne, Switzerland.
- [4] Emergency Department, Cantonal Hospital St. Gallen, St. Gallen, Switzerland.
- [5] Division of Infectious Diseases and Hospital Epidemiology, Cantonal Hospital St. Gallen, Rorschacher Strasse 95, 9007, St. Gallen, Switzerland. philipp.kohler@kssg.ch.

- PMID: **33731019**
- PMCID: PMC7968573
- DOI: 10.1186/s12879-021-05957-4

Abstract

Background: In the future, co-circulation of severe acute respiratory syndrome coronavirus 2 (SARS-CoV-2) and influenza viruses A/B is likely. From a clinical point of view, differentiation of the two disease entities is crucial for patient management. We therefore aim to detect clinical differences between Coronavirus Disease 2019 (COVID-19) and seasonal influenza patients at time of hospital admission.

Methods: In this single-center observational study, we included all consecutive patients hospitalized for COVID-19 or influenza between November 2019 and May 2020. Data were extracted from a nationwide surveillance program and from electronic health records. COVID-19 and influenza patients were compared in terms of baseline characteristics, clinical presentation and outcome. We used recursive partitioning to generate a classification tree to discriminate COVID-19 from influenza patients.

Results: We included 96 COVID-19 and 96 influenza patients. Median age was 68 vs. 70 years ($p = 0.90$), 72% vs. 56% ($p = 0.024$) were males, and median Charlson Comorbidity Index (CCI) was 1 vs. 2 ($p = 0.027$) in COVID-19 and influenza patients, respectively. Time from symptom onset to hospital admission was longer for COVID-19 (median 7 days, IQR 3-10) than for influenza patients (median 3 days, IQR 2-5, $p < 0.001$). Other variables favoring a diagnosis of COVID-19 in the classification tree were higher systolic blood pressure, lack of productive sputum, and lack of headache. The tree classified 86/192 patients (45%) into two subsets with ≥80% of patients having influenza or COVID-19, respectively. In-hospital mortality was higher for COVID-19 patients (16% vs. 5%, $p = 0.018$).

Conclusion: Discriminating COVID-19 from influenza patients based on clinical presentation is challenging. Time from symptom onset to hospital admission is considerably longer in COVID-19 than in influenza patients and showed the strongest discriminatory power in our classification tree. Although they had fewer comorbidities, in-hospital mortality was higher for COVID-19 patients.

Keywords: COVID-19; Classification tree; Comparative; Differences; Influenza; SARS-CoV-2.

Conflict of interest statement

The authors declare that they have no competing interests.

- 25 references
- 4 figures

Supplementary info

Publication types, MeSH terms Expand

Publication types

- Observational Study

MeSH terms

- Aged
- Aged, 80 and over
- COVID-19 / diagnosis*
- COVID-19 / epidemiology
- Comorbidity
- Diagnosis, Differential
- Female
- Hospital Mortality
- Hospitalization
- Humans
- Influenza, Human / diagnosis*
- Influenza, Human / epidemiology
- Male
- Middle Aged
- Retrospective Studies
- Switzerland

Full text links

Read free full text at BMC BioMed Central Free PMC article
Proceed to details

Cite
Share
☐ 103
Observational Study
PLoS One
. 2021 Sep 2;16(9):e0256977.
doi: 10.1371/journal.pone.0256977. eCollection 2021.

Possibility of deterioration of respiratory status when steroids precede antiviral drugs in patients with COVID-19 pneumonia: A retrospective study

Yu Shionoya [1], Toshibumi Taniguchi [2], Hajime Kasai [1] [3], Noriko Sakuma [1], Shun Imai [1], Kohei Shikano [1], Shin Takayanagi [4], Misuzu Yahaba [2], Taka-Aki Nakada [5], Hidetoshi Igari [2], Seiichiro Sakao [1], Takuji Suzuki [1]
Affiliations Expand

Affiliations

- [1] Department of Respirology, Graduate School of Medicine, Chiba University, Chiba, Japan.
- [2] Department of Infectious Diseases, Chiba University Hospital, Chiba, Japan.
- [3] Health Professional Development Center, Chiba University Hospital, Chiba, Japan.
- [4] Matsudo City General Hospital Department of Infectious Diseases, Matsudo, Japan.
- [5] Department of Emergency and Critical Care Medicine, Chiba University Graduate School of Medicine, Chiba, Japan.

- PMID: **34473766**
- PMCID: PMC8412353
- DOI: 10.1371/journal.pone.0256977

Free PMC article
Observational Study

Possibility of deterioration of respiratory status when steroids precede antiviral drugs in patients with COVID-19 pneumonia: A retrospective study

Yu Shionoya et al. PLoS One. 2021.
Free PMC article

Show details

PLoS One
. 2021 Sep 2;16(9):e0256977.
doi: 10.1371/journal.pone.0256977. eCollection 2021.

Authors

Yu Shionoya [1], Toshibumi Taniguchi [2], Hajime Kasai [1] [3], Noriko Sakuma [1], Shun Imai [1], Kohei Shikano [1], Shin Takayanagi [4], Misuzu Yahaba [2], Taka-Aki Nakada [5], Hidetoshi Igari [2], Seiichiro Sakao [1], Takuji Suzuki [1]

Affiliations

- [1] Department of Respirology, Graduate School of Medicine, Chiba University, Chiba, Japan.
- [2] Department of Infectious Diseases, Chiba University Hospital, Chiba, Japan.
- [3] Health Professional Development Center, Chiba University Hospital, Chiba, Japan.
- [4] Matsudo City General Hospital Department of Infectious Diseases, Matsudo, Japan.
- [5] Department of Emergency and Critical Care Medicine, Chiba University Graduate School of Medicine, Chiba, Japan.

- PMID: **34473766**
- PMCID: PMC8412353
- DOI: 10.1371/journal.pone.0256977

Abstract

Introduction: Coronavirus disease (COVID-19) is caused by severe acute respiratory syndrome coronavirus 2. Although most patients with COVID-19 develop asymptomatic or mild disease, some patients develop severe disease. The effectiveness of various therapeutic agents, including antiviral drugs, steroids, and anti-inflammatories for COVID-19, have been being confirmed. The effect of administering steroids in early disease is unclear. This study therefore aimed to evaluate the effectiveness and risk of exacerbation of steroids administered preceding antiviral drugs in patients with COVID-19 pneumonia.

Methods: This retrospective, single-center, observational study included consecutive patients with COVID-19 between March 2020 and March 2021. Patients were divided into a steroids-first group and antiviral-drugs-first group. Mortality, duration of hospitalization, incidence rate and duration of intensive care unit (ICU) admission, intubation, and extracorporeal membrane oxygenation (ECMO) induction of the two groups were compared.

Results: A total of 258 patients were admitted during the study period. After excluding patients who received symptomatic treatment only, who were taking immunosuppressive drugs, or who were administered antiviral drugs only, 68 patients were included in the analysis, 16 in the steroids-first group and 52 in the antiviral-drugs-first group. The rate of intubation, ICU admission and ECMO induction were significantly higher in the steroids-first group than in the antiviral-drugs-first group (81.3% vs. 33.3, $p<0.001$, 75.0% vs. 29.4%, $p = 0.001$, and 31.3% vs. 7.8%, $p = 0.017$, respectively). Furthermore, patients who received steroids within ten days after starting antiviral drugs had significantly lower rates of ICU admission, intubation, and ECMO induction.

(81.3% vs. 42.9% p = 0.011, 75.0% vs. 37.1% p = 0.012, and 31.3% vs. 8.6% p = 0.039, respectively).

Conclusions: Administering steroids prior to antiviral drugs soon after symptom onset can aggravate disease severity. When administration of steroids is considered soon after symptom onset, it may be safer to initiate antiviral drugs first.

Conflict of interest statement

T.T. has received honorarium for lecture from Gilead Sciences Inc., Janssen Pharmaceuticals Inc., ViiV Healthcare Limited, and MSD Limited, respectively. T.T. is the member of advisory board of Janssen Pharmaceuticals Inc., ViiV Healthcare Limited, and MSD Limited, respectively. This does not alter our adherence to PLOS ONE policies on sharing data and materials.

- 29 references
- 2 figures

Supplementary info

Publication types, MeSH terms, Substances, Grant support Expand

Publication types

- Observational Study

MeSH terms

- Aged
- Antiviral Agents / administration & dosage
- Antiviral Agents / therapeutic use*
- COVID-19 / drug therapy*
- COVID-19 / physiopathology
- COVID-19 / virology
- Dexamethasone / administration & dosage
- Dexamethasone / therapeutic use*
- Drug Administration Schedule
- Female
- Glucocorticoids / administration & dosage
- Glucocorticoids / therapeutic use
- Hospital Mortality
- Hospitalization / statistics & numerical data*
- Humans
- Intensive Care Units / statistics & numerical data
- Kaplan-Meier Estimate
- Male
- Middle Aged

- Respiratory Distress Syndrome / drug therapy*
- Respiratory Distress Syndrome / physiopathology
- Retrospective Studies
- SARS-CoV-2 / drug effects*
- SARS-CoV-2 / physiology
- Severity of Illness Index
- Treatment Outcome

Substances

- Antiviral Agents
- Glucocorticoids
- Dexamethasone

Grant support

The authors received no specific funding for this work.

Full text links

OPEN ACCESS TO FULL TEXT PLOS ONE Public Library of Science Free PMC article

Proceed to details

Cite

Share

☐ 104

Observational Study

Diagnosis (Berl)

. 2021 Jan 19;8(3):327-332.

doi: 10.1515/dx-2020-0114. Print 2021 Aug 26.

Delayed treatment of bacteremia during the COVID-19 pandemic

Taiju Miyagami [1], Yuki Uehara [1] [2], Taku Harada [3] [4], Takashi Watari [5], Taro Shimizu [4], Ayako Nakamura [6], Naoya Ogura [6], Seiko Kushiro [1], Katsutoshi Masuyama [1], Yoshinori Kanai [1], Kwang-Seok Yang [1], Toshio Naito [1]

Affiliations Expand

Affiliations

- [1] Department of General Medicine, Juntendo University Faculty of Medicine, Tokyo, Japan.
- [2] Department of Clinical Laboratory, St Luke's International Hospital, Tokyo, Japan.
- [3] Division of General Medicine, Showa University Koto Toyosu Hospital, Tokyo, Japan.

- [4] Department of Diagnostic and Generalist Medicine, Dokkyo Medical University Hospital, Tochigi, Japan.
- [5] Postgraduate Clinical Training Center, Shimane University Hospital, Izumo City, Shimane, Japan.
- [6] Department of Clinical Laboratory, Juntendo Tokyo Koto Geriatric Medical Center, Tokyo, Japan.

- PMID: **33470952**
- DOI: 10.1515/dx-2020-0114

Observational Study

Delayed treatment of bacteremia during the COVID-19 pandemic

Taiju Miyagami et al. Diagnosis (Berl). 2021.

Show details

Diagnosis (Berl)

. 2021 Jan 19;8(3):327-332.
doi: 10.1515/dx-2020-0114. Print 2021 Aug 26.

Authors

Taiju Miyagami [1], Yuki Uehara [1] [2], Taku Harada [3] [4], Takashi Watari [5], Taro Shimizu [4], Ayako Nakamura [6], Naoya Ogura [6], Seiko Kushiro [1], Katsutoshi Masuyama [1], Yoshinori Kanai [1], Kwang-Seok Yang [1], Toshio Naito [1]

Affiliations

- [1] Department of General Medicine, Juntendo University Faculty of Medicine, Tokyo, Japan.
- [2] Department of Clinical Laboratory, St Luke's International Hospital, Tokyo, Japan.
- [3] Division of General Medicine, Showa University Koto Toyosu Hospital, Tokyo, Japan.
- [4] Department of Diagnostic and Generalist Medicine, Dokkyo Medical University Hospital, Tochigi, Japan.
- [5] Postgraduate Clinical Training Center, Shimane University Hospital, Izumo City, Shimane, Japan.
- [6] Department of Clinical Laboratory, Juntendo Tokyo Koto Geriatric Medical Center, Tokyo, Japan.

- PMID: **33470952**
- DOI: 10.1515/dx-2020-0114

Abstract

Objectives: Coronavirus disease (COVID-19) blindness, that is, the excessive consideration of the disease in diagnosis, has reportedly led to delayed diagnosis of some diseases. We compared several clinical measures between patients admitted for bacteremia during the two months of the

COVID-19 pandemic and those admitted during the same period in 2019. We hypothesized that the pandemic has led to delayed treatment of bacteremia.

Methods: This retrospective observational study compared several measures undertaken for patients who visited the emergency unit in two hospitals between March 1 and May 31, 2020, during the COVID-19 pandemic and whose blood cultures tested positive for bacteremia with those for corresponding patients treated during the same period in 2019. The primary measure was time from consultation to blood culture/antimicrobials.

Results: We included 29 eligible patients from 2020 and 26 from 2019. In 2020, the time from consultation to antimicrobial administration was significantly longer than in 2019 (mean [range], 222 [145-309] min vs. 139 [102-179] min, p=0.002). The frequency of chest computed tomography (CT) was significantly higher in 2020 (96.6 vs. 73.1%, p=0.021). Significant differences were not observed in the time to blood culture or chest CT preceding the blood culture between the two periods.

Conclusions: Our findings suggested that due to the COVID-19 epidemic/pandemic, focusing on the exclusion of its infection using CT scans leads to an overall delay in the diagnosis and treatment of bacteremia. Medical providers must be aware of COVID-19 blindness and evaluate patients objectively based on rational criteria and take appropriate action.

Keywords: COVID-19; COVID-19 blindness; bacteremia.

© 2020 Walter de Gruyter GmbH, Berlin/Boston.

- 21 references

Supplementary info

Publication types, MeSH terms Expand

Publication types

- Observational Study
- Research Support, Non-U.S. Gov't

MeSH terms

- Aged
- Aged, 80 and over
- Bacteremia / diagnosis
- Bacteremia / drug therapy*
- Bacteremia / epidemiology*
- COVID-19 / epidemiology*
- Female
- Humans
- Male
- Pandemics*

- Retrospective Studies
- SARS-CoV-2
- Time-to-Treatment / statistics & numerical data*

Full text links

De Gruyter
Proceed to details
Cite
Share

105

Observational Study

J Glob Health
. 2021 Dec 4;11:05023.
doi: 10.7189/jogh.11.05023. eCollection 2021.

The health and economic impact of constructing temporary field hospitals to meet the COVID-19 pandemic surge: Wuhan Leishenshan Hospital in China as a case study

Yi Cai[#] [1] [2], Yilin Chen[#] [3], Linqi Xiao [2] [4], Sara Khor [3], Tongzu Liu [5] [4], Yong Han [2] [4], Yufeng Yuan [6] [4], Lin Cai [7] [4], Guang Zeng [5] [4], Xinghuan Wang [5] [4]
Affiliations Expand

Affiliations

- [1] Department of Global Health, Wuhan University School of Public Health, Wuhan, PR China.
- [2] Hospital Management Institute, Zhongnan Hospital of Wuhan University, Wuhan, PR China.
- [3] The Comparative Health Outcomes, Policy, and Economics Institute, School of Pharmacy, University of Washington, Seattle, Washington, USA.
- [4] Leishenshan Hospital in Wuhan, Wuhan, PR China.
- [5] Department of Urology, Zhongnan Hospital of Wuhan University, Wuhan, Hubei, PR China.
- [6] Department of Hepatobiliary & Pancreatic Surgery, Zhongnan Hospital of Wuhan University, Wuhan, Hubei, PR China.
- [7] Department of Spine Surgery and Musculoskeletal Tumor, Zhongnan Hospital of Wuhan University, Wuhan, Hubei, PRChina.

[#] Contributed equally.

- PMID: **34912549**

- PMCID: PMC8645218
- DOI: 10.7189/jogh.11.05023

Free PMC article
Observational Study

The health and economic impact of constructing temporary field hospitals to meet the COVID-19 pandemic surge: Wuhan Leishenshan Hospital in China as a case study

Yi Cai et al. J Glob Health. 2021.
Free PMC article
Show details
J Glob Health
. 2021 Dec 4;11:05023.
doi: 10.7189/jogh.11.05023. eCollection 2021.

Authors

Yi Cai [#] [1] [2], Yilin Chen [#] [3], Linqi Xiao [2] [4], Sara Khor [3], Tongzu Liu [5] [4], Yong Han [2] [4], Yufeng Yuan [6] [4], Lin Cai [7] [4], Guang Zeng [5] [4], Xinghuan Wang [5] [4]

Affiliations

- [1] Department of Global Health, Wuhan University School of Public Health, Wuhan, PR China.
- [2] Hospital Management Institute, Zhongnan Hospital of Wuhan University, Wuhan, PR China.
- [3] The Comparative Health Outcomes, Policy, and Economics Institute, School of Pharmacy, University of Washington, Seattle, Washington, USA.
- [4] Leishenshan Hospital in Wuhan, Wuhan, PR China.
- [5] Department of Urology, Zhongnan Hospital of Wuhan University, Wuhan, Hubei, PR China.
- [6] Department of Hepatobiliary & Pancreatic Surgery, Zhongnan Hospital of Wuhan University, Wuhan, Hubei, PR China.
- [7] Department of Spine Surgery and Musculoskeletal Tumor, Zhongnan Hospital of Wuhan University, Wuhan, Hubei, PRChina.

[#] Contributed equally.

- PMID: **34912549**
- PMCID: PMC8645218
- DOI: 10.7189/jogh.11.05023

Abstract

Background: In response to the COVID-19 pandemic, two new temporary hospitals were constructed in record time in Wuhan, China, to help combat the fast-spreading virus in February 2020. Using the experience of one of the hospitals as a case study, we discuss the health and economic implications of this response strategy and its potential application in other countries.

Methods: This retrospective observational study analyzed health resource utilization and clinical outcomes data for 2011 inpatients diagnosed with COVID-19 and admitted to Leishenshan Hospital during its 67 days of operation from February 8th to April 14th, 2020. We used a top-down costing approach to estimate the total cost of treating patients at the Leishenshan Hospital, including capital cost for hospital construction, health personnel costs, and direct health care costs. We used a multivariate generalized linear model to examine risk factors associated with in-hospital deaths.

Results: During the 67 days of hospital operation, 19 medical teams comprising of 933 doctors and 2312 nurses were gradually transferred to Leishenshan Hospital from across China. Of the 2011 admissions, 4.5% used intensive care and 2.0% used ventilators. Overall median length of stay was 19 days, and 21 days for patients in the intensive care unit (ICU). The case fatality rate (CFR) was 2.3% overall, 41.8% in the ICU, and 0.4% in general ward (GW). CFRs were 55% and 50% among patients using non-invasive and invasive ventilators, respectively. The mean total cost and direct health care cost were CNY806 997 (US$114 793) and CNY16 087 (US$2288), respectively. Patients admitted to the ICU had much higher direct health care costs, on average, compared to those in the GW (CNY150 415 vs CNY9720, or US$21 396 vs US$1383). The mean direct health care cost per patient with severe or critical diseases was more than five times higher than those with mild or moderate diseases (CNY45 191 vs CNY8838, or US$6428 vs US$1257). Older age, having comorbidities, and critical disease were associated with higher risks of death from COVID-19. Lower health worker to patient ratio (<2.6) was not associated with in-hospital death.

Conclusion: An adequate health workforce were mobilized and deployed to a new temporary hospital. The Leishenshan Hospital increased access to care during the surge in COVID-19 infections, facilitated timely treatment, and transferred COVID-19 patients between GWs and ICUs within the hospital, all of which are potential contributors to lowering the CFR. Patients in the ICU experienced a much higher CFR and a greater burden of health care cost than those in GW. Our results have important implications for other countries interested in constructing temporary emergency hospitals, such as the need for adequate infrastructure capacities and financial support, centralized strategies to mobilize health workforce and to provide respiratory protective devices, and improvement in access to health care.

Copyright © 2021 by the Journal of Global Health. All rights reserved.

Conflict of interest statement

Competing interests: The authors completed the ICMJER Declaration of Interest Form (available upon request from the corresponding author), and declare no conflicts of interest.

- 37 references
- 5 figures

Supplementary info

Publication types, MeSH terms Expand

Publication types

- Observational Study

MeSH terms

- Aged
- COVID-19*
- Hospital Mortality
- Hospitals
- Humans
- Mobile Health Units
- Pandemics
- SARS-CoV-2

Full text links

LinkOut to related resource International Society of Global Health Free PMC article
Proceed to details
Cite
Share
106
Observational Study
Front Immunol
. 2022 Jan 27;13:834851.
doi: 10.3389/fimmu.2022.834851. eCollection 2022.

Association Between Sex Hormone Levels and Clinical Outcomes in Patients With COVID-19 Admitted to Hospital: An Observational, Retrospective, Cohort Study

Anna Beltrame [1], Pedro Salguero [2], Emanuela Rossi [3], Ana Conesa [4] [5], Lucia Moro [1], Laura Rachele Bettini [6], Eleonora Rizzi [1], Mariella D'Angió [6], Michela Deiana [1], Chiara Piubelli [1], Paola Rebora [3], Silvia Duranti [1], Paolo Bonfanti [7] [8], Ilaria Capua [9], Sonia Tarazona [2], Maria Grazia Valsecchi [3]
Affiliations Expand

Affiliations

- [1] Department of Infectious, Tropical Diseases and Microbiology Istituto di Ricovero e Cura a Carattere Scientifico (I.R.C.C.S). Sacro Cuore Don Calabria Hospital, Negrar di Valpolicella, Italy.
- [2] Department of Applied Statistics, Operations Research and Quality, Universitat Politècnica de València, Valencia, Spain.
- [3] Bicocca Center of Bioinformatics, Biostatistics and Bioimaging, School of Medicine and Surgery, Milano-Bicocca University, Milano, Italy.
- [4] Institute for Integrative Systems Biology, Spanish National Research Council, Paterna, Spain.
- [5] Department of Microbiology and Cell Sciences, University of Florida, Gainesville, FL, United States.
- [6] Pediatric Departement and Centro Tettamanti-European Reference Network PaedCan, EuroBloodNet, MetabERN-University of Milano-Bicocca-Fondazione MONZA e BRIANZA per il BAMBINO e la sua MAMMA (MBBM)-Ospedale, San Gerardo, Monza, Italy.
- [7] School of Medicine and Surgery, Milano-Bicocca University, Milano, Italy.
- [8] Infectious Diseases Unit, Azienda Socio Sanitaria Territoriale (ASST) Monza, San Gerardo Hospital, Monza, Italy.
- [9] One Health Center of Excellence, University of Florida, Gainesville, FL, United States.

- PMID: **35154158**
- PMCID: PMC8829540
- DOI: 10.3389/fimmu.2022.834851

Free PMC article
Observational Study

Association Between Sex Hormone Levels and Clinical Outcomes in Patients With COVID-19 Admitted to Hospital: An Observational, Retrospective, Cohort Study

Anna Beltrame et al. Front Immunol. 2022.
Free PMC article
Show details
Front Immunol
. 2022 Jan 27;13:834851.
doi: 10.3389/fimmu.2022.834851. eCollection 2022.

Authors

Anna Beltrame [1], Pedro Salguero [2], Emanuela Rossi [3], Ana Conesa [4] [5], Lucia Moro [1], Laura Rachele Bettini [6], Eleonora Rizzi [1], Mariella D'Angió [6], Michela Deiana [1], Chiara Piubelli

[1], Paola Rebora [3], Silvia Duranti [1], Paolo Bonfanti [7] [8], Ilaria Capua [9], Sonia Tarazona [2], Maria Grazia Valsecchi [3]

Affiliations

- [1] Department of Infectious, Tropical Diseases and Microbiology Istituto di Ricovero e Cura a Carattere Scientifico (I.R.C.C.S). Sacro Cuore Don Calabria Hospital, Negrar di Valpolicella, Italy.
- [2] Department of Applied Statistics, Operations Research and Quality, Universitat Politècnica de València, Valencia, Spain.
- [3] Bicocca Center of Bioinformatics, Biostatistics and Bioimaging, School of Medicine and Surgery, Milano-Bicocca University, Milano, Italy.
- [4] Institute for Integrative Systems Biology, Spanish National Research Council, Paterna, Spain.
- [5] Department of Microbiology and Cell Sciences, University of Florida, Gainesville, FL, United States.
- [6] Pediatric Departement and Centro Tettamanti-European Reference Network PaedCan, EuroBloodNet, MetabERN-University of Milano-Bicocca-Fondazione MONZA e BRIANZA per il BAMBINO e la sua MAMMA (MBBM)-Ospedale, San Gerardo, Monza, Italy.
- [7] School of Medicine and Surgery, Milano-Bicocca University, Milano, Italy.
- [8] Infectious Diseases Unit, Azienda Socio Sanitaria Territoriale (ASST) Monza, San Gerardo Hospital, Monza, Italy.
- [9] One Health Center of Excellence, University of Florida, Gainesville, FL, United States.

- PMID: **35154158**
- PMCID: PMC8829540
- DOI: 10.3389/fimmu.2022.834851

Abstract

Understanding the cause of sex disparities in COVID-19 outcomes is a major challenge. We investigate sex hormone levels and their association with outcomes in COVID-19 patients, stratified by sex and age. This observational, retrospective, cohort study included 138 patients aged 18 years or older with COVID-19, hospitalized in Italy between February 1 and May 30, 2020. The association between sex hormones (testosterone, estradiol, progesterone, dehydroepiandrosterone) and outcomes (ARDS, severe COVID-19, in-hospital mortality) was explored in 120 patients aged 50 years and over. STROBE checklist was followed. The median age was 73.5 years [IQR 61, 82]; 55.8% were male. In older males, testosterone was lower if ARDS and severe COVID-19 were reported than if not (3.6 *vs.* 5.3 nmol/L, p =0.0378 and 3.7 *vs.* 8.5 nmol/L, p =0.0011, respectively). Deceased males had lower testosterone (2.4 *vs.* 4.8 nmol/L, p =0.0536) and higher estradiol than survivors (40 *vs.* 24 pg/mL, p = 0.0006). Testosterone was negatively associated with ARDS (OR 0.849 [95% CI 0.734, 0.982]), severe COVID-19 (OR 0.691 [95% CI 0.546, 0.874]), and in-hospital mortality (OR 0.742 [95% CI 0.566, 0.972]), regardless of potential confounders, though confirmed only in the regression model on males. Higher estradiol was associated with a higher probability of death (OR 1.051 [95% CI 1.018, 1.084]), confirmed in both sex models. In males, higher testosterone seems to be protective against any considered outcome. Higher estradiol was associated with a higher probability of death in both sexes.

Keywords: ARDS; COVID-19; estradiol; outcome; severity; sex hormones; testosterone.

Copyright © 2022 Beltrame, Salguero, Rossi, Conesa, Moro, Bettini, Rizzi, D'Angió, Deiana, Piubelli, Rebora, Duranti, Bonfanti, Capua, Tarazona and Valsecchi.

Conflict of interest statement

The authors declare that the research was conducted in the absence of any commercial or financial relationships that could be construed as a potential conflict of interest.

- 30 references
- 2 figures

Supplementary info

Publication types, MeSH terms, Substances Expand

Publication types

- Observational Study
- Research Support, Non-U.S. Gov't

MeSH terms

- Aged
- Aged, 80 and over
- COVID-19 / blood*
- Cohort Studies
- Female
- Gonadal Steroid Hormones / blood*
- Hospital Mortality
- Hospitalization
- Humans
- Italy
- Male
- Middle Aged
- Retrospective Studies
- Risk Factors
- SARS-CoV-2
- Sex Characteristics*

Substances

- Gonadal Steroid Hormones

Full text links

Frontiers Media SA Free PMC article
Proceed to details
Cite
Share
107
Observational Study
Minerva Anestesiol
. 2021 Nov;87(11):1209-1216.
doi: 10.23736/S0375-9393.21.15595-6. Epub 2021 Aug 2.

Comparison of renal replacement therapy and renal recovery before and during the COVID-19 pandemic: a single center observational study

Rebecca Ryan [1], Isabel Taylor [1], Chris Laing [2], Mervyn Singer [1 3], Dereck Gondongwe [1], Niall Maccallum [1], Nishkantha Arulkumaran [4 3]
Affiliations Expand

Affiliations

- [1] Intensive Care Unit, University College London Hospital, London, UK.
- [2] Department of Renal Medicine, University College London, Royal Free Hospital, London, UK.
- [3] Division of Medicine, Bloomsbury Institute of Intensive Care Medicine, University College London, London, UK.
- [4] Intensive Care Unit, University College London Hospital, London, UK - nisharulkumaran@doctors.org.uk.

- PMID: **34337912**
- DOI: 10.23736/S0375-9393.21.15595-6

Free article
Observational Study

Comparison of renal replacement therapy and renal recovery before and during the COVID-19 pandemic: a single center observational study

Rebecca Ryan et al. Minerva Anestesiol. 2021 Nov.
Free article
Show details
Minerva Anestesiol
. 2021 Nov;87(11):1209-1216.
doi: 10.23736/S0375-9393.21.15595-6. Epub 2021 Aug 2.

Authors

Rebecca Ryan [1], Isabel Taylor [1], Chris Laing [2], Mervyn Singer [1 3], Dereck Gondongwe [1], Niall Maccallum [1], Nishkantha Arulkumaran [4 3]

Affiliations

- [1] Intensive Care Unit, University College London Hospital, London, UK.
- [2] Department of Renal Medicine, University College London, Royal Free Hospital, London, UK.
- [3] Division of Medicine, Bloomsbury Institute of Intensive Care Medicine, University College London, London, UK.
- [4] Intensive Care Unit, University College London Hospital, London, UK - nisharulkumaran@doctors.org.uk.

- PMID: **34337912**
- DOI: 10.23736/S0375-9393.21.15595-6

Abstract

Background: Our objective was to the describe indications, management, complications and outcomes of renal replacement therapy (RRT) in COVID-19 critically ill patients. To contextualize these findings, comparisons were made against 36 non-COVID-19 consecutive patients requiring RRT on ICU.

Methods: We conducted a retrospective single center observational cohort study of patients requiring acute RRT between 1st March and 30th June 2020. Comparison was made against those receiving RRT in the pre-COVID-19 period from January 2019 to February 2020.

Results: Of 154 COVID-19 patients, 47 (30.5%) received continuous venovenous hemofiltration (CVVHF), all of whom required mechanical ventilation and vasopressor support. The requirement for RRT was related to fluid balance rather than azotemia. Compared to 36 non-COVID-19 patients, those with COVID-19 were younger (P=0.016) with a lower serum creatinine on hospital admission (P=0.049), and lesser degrees of metabolic acidosis (P<0.001) and lactatemia (P<0.001)

before initiation of RRT. In addition, the duration of RRT requirement was longer (P<0.001). Despite lower CVVHF exchange rates with higher serum creatinine levels following RRT initiation in the COVID-19 patients, metabolic abnormalities were corrected. Hospital mortality was 60% among COVID-19 patients requiring RRT, compared to 67% in non-COVID-19 patients (P=0.508), and renal recovery among survivors without pre-existing CKD was similar (P=0.231).

Conclusions: The requirement for RRT in COVID-19 patients was primarily related to fluid balance. Using lower CVVHF exchange rates was effective to correct metabolic abnormalities. Renal recovery occurred in all but one patient by 60 days in the 40% of patients who survived.

Supplementary info

Publication types, MeSH terms Expand

Publication types

- Observational Study

MeSH terms

- Acute Kidney Injury* / epidemiology
- Acute Kidney Injury* / therapy
- COVID-19*
- Critical Illness / therapy
- Humans
- Intensive Care Units
- Pandemics
- Renal Replacement Therapy
- Retrospective Studies
- SARS-CoV-2

Full text links

FREE FULL TEXT article at minervamedica.it Minerva Medica
Proceed to details
Cite
Share
☐ 108
Observational Study
Curr Med Sci
. 2021 Dec;41(6):1096-1104.
doi: 10.1007/s11596-021-2434-y. Epub 2021 Sep 13.

Prolonged SARS-CoV-2 Viral Shedding in Patients with COVID-19 was Associated with Delayed Initiation of Arbidol Treatment and Consulting Doctor Later: A Retrospective Cohort Study

Xin-Liang He [1], Ya-Ya Zhou [1], Wei Fu [1], Yu-E Xue [1], Meng-Yuan Liang [1], Bo-Han Yang [1], Wan-Li Ma [1], Qiong Zhou [1], Long Chen [1], Jian-Chu Zhang [2], Xiao-Rong Wang [3]
Affilations Expand

Affiliations

- [1] Department of Respiratory and Critical Care Medicine, Union Hospital, Tongji Medical College, Huazhong University of Science and Technology, Wuhan, 430022, China.
- [2] Department of Respiratory and Critical Care Medicine, Union Hospital, Tongji Medical College, Huazhong University of Science and Technology, Wuhan, 430022, China. zsn0928@163.com.
- [3] Department of Respiratory and Critical Care Medicine, Union Hospital, Tongji Medical College, Huazhong University of Science and Technology, Wuhan, 430022, China. rong-100@163.com.

- PMID: **34515914**
- PMCID: PMC8436017
- DOI: 10.1007/s11596-021-2434-y

Free PMC article
Observational Study

Prolonged SARS-CoV-2 Viral Shedding in Patients with COVID-19 was Associated with Delayed Initiation of Arbidol Treatment and Consulting Doctor Later: A Retrospective Cohort Study

Xin-Liang He et al. Curr Med Sci. 2021 Dec.
Free PMC article
Show details
Curr Med Sci
. 2021 Dec;41(6):1096-1104.
doi: 10.1007/s11596-021-2434-y. Epub 2021 Sep 13.

Authors

Xin-Liang He [1], Ya-Ya Zhou [1], Wei Fu [1], Yu-E Xue [1], Meng-Yuan Liang [1], Bo-Han Yang [1], Wan-Li Ma [1], Qiong Zhou [1], Long Chen [1], Jian-Chu Zhang [2], Xiao-Rong Wang [3]

Affiliations

- [1] Department of Respiratory and Critical Care Medicine, Union Hospital, Tongji Medical College, Huazhong University of Science and Technology, Wuhan, 430022, China.
- [2] Department of Respiratory and Critical Care Medicine, Union Hospital, Tongji Medical College, Huazhong University of Science and Technology, Wuhan, 430022, China. zsn0928@163.com.
- [3] Department of Respiratory and Critical Care Medicine, Union Hospital, Tongji Medical College, Huazhong University of Science and Technology, Wuhan, 430022, China. rong-100@163.com.

- PMID: **34515914**
- PMCID: PMC8436017
- DOI: 10.1007/s11596-021-2434-y

Abstract

Objective: To study data about SARS-CoV-2 virus shedding and clarify the risk factors for prolonged virus shedding.

Methods: Data were retrospectively collected from adults hospitalized with laboratory-confirmed coronavirus disease-19 (COVID-19) in Wuhan Union Hospital. We compared clinical features among patients with prolonged (a positive SARS-CoV-2 RNA on day 23 after illness onset) and short virus shedding and evaluated risk factors associated with prolonged virus shedding by multivariate regression analysis.

Results: Among 238 patients, the median age was 55.5 years, 57.1% were female, 92.9% (221/238) were administered with arbidol, 58.4% (139/238) were given arbidol in combination with interferon. The median duration of SARS-CoV-2 virus shedding was 23 days (IQR, 17.8-30 days) with a longest one of 51 days. The patients with prolonged virus shedding had higher value of D-dimer (P=0.002), IL-6 (P<0.001), CRP (P=0.005) and more lobes lung lesion (P=0.014) on admission, as well as older age (P=0.017) and more patients with hypertension (P=0.044) than in those the virus shedding less than 23 days. Multivariate regression analysis revealed that prolonged viral shedding was significantly associated with initiation arbidol >8 days after symptom onset [OR: 2.447, 95% CI (1.351-4.431)], ≥3 days from onset of symptoms to first medical visitation [OR: 1.880, 95% CI (1.035-3.416)], illness onset before Jan. 31, 2020 [OR: 3.289, 95% CI (1.474-7.337)]. Arbidol in combination with interferon was also significantly associated with shorter virus shedding [OR: 0.363, 95% CI (0.191-0.690)].

Conclusion: Duration of SARS-CoV-2 virus shedding was long. Early initiation of arbidol and arbidol in combination with interferon as well as consulting doctor timely after illness onset were helpful for SARS-CoV-2 clearance.

Keywords: SARS-CoV-2; antiviral treatment; arbidol; risk factors; viral shedding.

© 2021. Huazhong University of Science and Technology.

- 37 references

Supplementary info

Publication types, MeSH terms, Substances Expand

Publication types

- Observational Study

MeSH terms

- Adult
- Aged
- Antiviral Agents / administration & dosage*
- COVID-19 / drug therapy*
- COVID-19 / epidemiology
- COVID-19 / virology*
- China / epidemiology
- Cohort Studies
- Female
- Hospitalization
- Humans
- Indoles / administration & dosage*
- Interferons / administration & dosage
- Logistic Models
- Male
- Middle Aged
- Multivariate Analysis
- Pandemics
- RNA, Viral / analysis
- Retrospective Studies
- Risk Factors
- SARS-CoV-2* / drug effects
- SARS-CoV-2* / isolation & purification
- Time Factors
- Virus Shedding* / drug effects

Substances

- Antiviral Agents
- Indoles

- RNA, Viral
- Interferons
- umifenovir

Full text links

SpringerLink FULL-TEXT ARTICLE Springer Free PMC article
Proceed to details
Cite
Share
109
Observational Study
PLoS One
. 2020 Dec 10;15(12):e0243700.
doi: 10.1371/journal.pone.0243700. eCollection 2020.

Impact of chronic liver disease upon admission on COVID-19 in-hospital mortality: Findings from COVOCA study

Raffaele Galiero [1], Pia Clara Pafundi [1], Vittorio Simeon [2], Luca Rinaldi [1], Alessandro Perrella [3], Erica Vetrano [1], Alfredo Caturano [1], Maria Alfano [1], Domenico Beccia [1], Riccardo Nevola [1] [4], Raffaele Marfella [1], Celestino Sardu [1], Carmine Coppola [5], Ferdinando Scarano [5], Paolo Maggi [6], Pellegrino De Lucia Sposito [7], Laura Vocciante [8], Carolina Rescigno [9], Costanza Sbreglia [10], Fiorentino Fraganza [11], Roberto Parrella [12], Annamaria Romano [13], Giosuele Calabria [14], Benedetto Polverino [15], Antonio Pagano [16], Carolina Bologna [17], Maria Amitrano [18], Vincenzo Esposito [19], Nicola Coppola [20], Nicola Maturo [21], Luigi Elio Adinolfi [1], Paolo Chiodini [2], Ferdinando Carlo Sasso [1], COVOCA Study Group
Affiliations Expand

Affiliations

- [1] Department of Advanced Medical and Surgical Sciences, University of Campania "Luigi Vanvitelli", Naples, Italy.
- [2] Medical Statistics Unit, Department of Physical and Mental Health and Preventive Medicine, University of Campania "Luigi Vanvitelli", Naples, Italy.
- [3] Task Force Covid-19 Regione Campania, Napoli, Italy.
- [4] Internal Medicine, Sant'Ottone Frangipane Hospital, Ariano Irpino, Italy.
- [5] COVID Center "S. Anna e SS. Madonna della Neve" Hospital, Boscotrecase, Italy.
- [6] U.O.C. Infectious and Tropical Diseases, S. Anna e S. Sebastiano Hospital, Caserta, Italy.
- [7] Covid Center-Maddaloni Hospital, Maddaloni, Italy.
- [8] General Medicine Unit, Loreto Mare Hospital, Naples, Italy.
- [9] U.O.C. Infectious Diseases and Neurology, Cotugno Hospital, Naples, Italy.
- [10] U.O.C. Infectious Diseases of the Elderly, Cotugno Hospital, Naples, Italy.

- [11] U.O.C. Anestesia and Intensive Care Unit, Cotugno Hospital, Naples, Italy.
- [12] U.O.C. Respiratory Infectious Diseases, Cotugno Hospital, Naples, Italy.
- [13] U.O.C. Pneumology, Moscati Hospital, Avellino, Italy.
- [14] IXth Division of Infectious Diseases and Interventional Ultrasound, Cotugno Hospital, Naples, Italy.
- [15] "Giovanni da Procida" Hospital, Salerno, Italy.
- [16] Emergency and Acceptance Unit, "Santa Maria delle Grazie" Hospital, Pozzuoli, Italy.
- [17] Internal Medicine Unit, Ospedale Del Mare, Naples, Italy.
- [18] U.O.C. Internal Medicine-Moscati Hospital, Avellino, Italy.
- [19] IVth Division of Immunodeficiency and Gender Infectious Diseases, Cotugno Hospital, Naples, Italy.
- [20] Department of Mental Health and Public Medicine, Centro COVID A.O.U. Vanvitelli, Naples, Italy.
- [21] U.O.S.D. Infectious Diseases Emergency and Acceptance, Cotugno Hospital, Naples, Italy.

- PMID: **33301529**
- PMCID: PMC7728173
- DOI: 10.1371/journal.pone.0243700

Free PMC article
Observational Study

Impact of chronic liver disease upon admission on COVID-19 in-hospital mortality: Findings from COVOCA study

Raffaele Galiero et al. PLoS One. 2020.
Free PMC article
Show details
PLoS One
. 2020 Dec 10;15(12):e0243700.
doi: 10.1371/journal.pone.0243700. eCollection 2020.

Authors

Raffaele Galiero [1], Pia Clara Pafundi [1], Vittorio Simeon [2], Luca Rinaldi [1], Alessandro Perrella [3], Erica Vetrano [1], Alfredo Caturano [1], Maria Alfano [1], Domenico Beccia [1], Riccardo Nevola [1, 4], Raffaele Marfella [1], Celestino Sardu [1], Carmine Coppola [5], Ferdinando Scarano [5], Paolo Maggi [6], Pellegrino De Lucia Sposito [7], Laura Vocciante [8], Carolina Rescigno [9], Costanza Sbreglia [10], Fiorentino Fraganza [11], Roberto Parrella [12], Annamaria Romano [13], Giosuele Calabria [14], Benedetto Polverino [15], Antonio Pagano [16], Carolina Bologna [17], Maria Amitrano [18], Vincenzo Esposito [19], Nicola Coppola [20], Nicola Maturo [21], Luigi Elio Adinolfi [1], Paolo Chiodini [2], Ferdinando Carlo Sasso [1], COVOCA Study Group

Affiliations

- [1] Department of Advanced Medical and Surgical Sciences, University of Campania "Luigi Vanvitelli", Naples, Italy.
- [2] Medical Statistics Unit, Department of Physical and Mental Health and Preventive Medicine, University of Campania "Luigi Vanvitelli", Naples, Italy.
- [3] Task Force Covid-19 Regione Campania, Napoli, Italy.
- [4] Internal Medicine, Sant'Ottone Frangipane Hospital, Ariano Irpino, Italy.
- [5] COVID Center "S. Anna e SS. Madonna della Neve" Hospital, Boscotrecase, Italy.
- [6] U.O.C. Infectious and Tropical Diseases, S. Anna e S. Sebastiano Hospital, Caserta, Italy.
- [7] Covid Center-Maddaloni Hospital, Maddaloni, Italy.
- [8] General Medicine Unit, Loreto Mare Hospital, Naples, Italy.
- [9] U.O.C. Infectious Diseases and Neurology, Cotugno Hospital, Naples, Italy.
- [10] U.O.C. Infectious Diseases of the Elderly, Cotugno Hospital, Naples, Italy.
- [11] U.O.C. Anestesia and Intensive Care Unit, Cotugno Hospital, Naples, Italy.
- [12] U.O.C. Respiratory Infectious Diseases, Cotugno Hospital, Naples, Italy.
- [13] U.O.C. Pneumology, Moscati Hospital, Avellino, Italy.
- [14] IXth Division of Infectious Diseases and Interventional Ultrasound, Cotugno Hospital, Naples, Italy.
- [15] "Giovanni da Procida" Hospital, Salerno, Italy.
- [16] Emergency and Acceptance Unit, "Santa Maria delle Grazie" Hospital, Pozzuoli, Italy.
- [17] Internal Medicine Unit, Ospedale Del Mare, Naples, Italy.
- [18] U.O.C. Internal Medicine-Moscati Hospital, Avellino, Italy.
- [19] IVth Division of Immunodeficiency and Gender Infectious Diseases, Cotugno Hospital, Naples, Italy.
- [20] Department of Mental Health and Public Medicine, Centro COVID A.O.U. Vanvitelli, Naples, Italy.
- [21] U.O.S.D. Infectious Diseases Emergency and Acceptance, Cotugno Hospital, Naples, Italy.

- PMID: **33301529**
- PMCID: PMC7728173
- DOI: 10.1371/journal.pone.0243700

Abstract

Background: Italy has been the first Western country to be heavily affected by the spread of SARS-COV-2 infection and among the pioneers of the clinical management of pandemic. To improve the outcome, identification of patients at the highest risk seems mandatory.

Objectives: Aim of this study is to identify comorbidities and clinical conditions upon admission associated with in-hospital mortality in several COVID Centers in Campania Region (Italy).

Methods: COVOCA is a multicentre retrospective observational cohort study, which involved 18 COVID Centers throughout Campania Region, Italy. Data were collected from patients who completed their hospitalization between March-June 2020. The endpoint was in-hospital mortality, assessed either from data at discharge or death certificate, whilst all exposure variables were collected at hospital admission.

Results: Among 618 COVID-19 hospitalized patients included in the study, 143 in-hospital mortality events were recorded, with a cumulative incidence of about 23%. At multivariable logistic analysis, male sex (OR 2.63, 95%CI 1.42-4.90; p = 0.001), Chronic Liver Disease (OR 5.88, 95%CI 2.39-14.46; p<0.001) and malignancies (OR 2.62, 95%CI 1.21-5.68; p = 0.015) disclosed an independent association with a poor prognosis, Glasgow Coma Scale (GCS) and Respiratory Severity Scale allowed to identify at higher mortality risk. Sensitivity analysis further enhanced these findings.

Conclusion: Mortality of patients hospitalized for COVID-19 appears strongly affected by both clinical conditions on admission and comorbidities. Originally, we observed a very poor outcome in subjects with a chronic liver disease, alongside with an increase of hepatic damage.

Conflict of interest statement

The authors have declared that no competing interests exist.

- 50 references
- 2 figures

Supplementary info

Publication types, MeSH terms, Grant support Expand

Publication types

- Multicenter Study
- Observational Study

MeSH terms

- Aged
- Aged, 80 and over
- COVID-19 / diagnosis
- COVID-19 / epidemiology*
- COVID-19 / mortality
- Chronic Disease
- Comorbidity
- Female
- Hospital Mortality
- Hospitalization
- Humans
- Italy / epidemiology
- Liver Diseases / diagnosis
- Liver Diseases / epidemiology*
- Liver Diseases / mortality
- Male

- Middle Aged
- Prognosis
- Retrospective Studies
- SARS-CoV-2 / isolation & purification

Grant support

The author(s) received no specific funding for this work.

Full text links

OPEN ACCESS TO FULL TEXT PLOS ONE Public Library of Science Free PMC article

Proceed to details

Cite

Share

☐ 110

Observational Study

J Clin Endocrinol Metab

. 2021 Mar 8;106(3):e1354-e1361.

doi: 10.1210/clinem/dgaa793.

Adrenal Insufficiency at the Time of COVID-19: A Retrospective Study in Patients Referring to a Tertiary Center

Giulia Carosi [1 2], Valentina Morelli [1], Giulia Del Sindaco [1 3], Andreea Liliana Serban [1 2], Arianna Cremaschi [1 3], Sofia Frigerio [1 3], Giulia Rodari [1 3], Eriselda Profka [3], Rita Indirli [1 3], Roberta Mungari [1], Veronica Resi [1], Emanuela Orsi [1], Emanuele Ferrante [1], Alessia Dolci [1], Claudia Giavoli [1 3], Maura Arosio [1 3], Giovanna Mantovani [1 3]

Affiliations Expand

Affiliations

- [1] Endocrinology Unit, Fondazione IRCCS Cà Granda Ospedale Maggiore Policlinico, Milan, Italy.
- [2] Department of Experimental Medicine, Sapienza University of Rome, Rome, Italy.
- [3] Department of Clinical Sciences and Community Health, University of Milan, Italy.

- PMID: **34932807**
- DOI: 10.1210/clinem/dgaa793

Observational Study

Adrenal Insufficiency at the Time of COVID-19: A Retrospective Study in Patients Referring to a Tertiary Center

Giulia Carosi et al. J Clin Endocrinol Metab. 2021.
Show details
J Clin Endocrinol Metab
. 2021 Mar 8;106(3):e1354-e1361.
doi: 10.1210/clinem/dgaa793.

Authors

Giulia Carosi [1 2], Valentina Morelli [1], Giulia Del Sindaco [1 3], Andreea Liliana Serban [1 2], Arianna Cremaschi [1 3], Sofia Frigerio [1 3], Giulia Rodari [1 3], Eriselda Profka [3], Rita Indirli [1 3], Roberta Mungari [1], Veronica Resi [1], Emanuela Orsi [1], Emanuele Ferrante [1], Alessia Dolci [1], Claudia Giavoli [1 3], Maura Arosio [1 3], Giovanna Mantovani [1 3]

Affiliations

- [1] Endocrinology Unit, Fondazione IRCCS Cà Granda Ospedale Maggiore Policlinico, Milan, Italy.
- [2] Department of Experimental Medicine, Sapienza University of Rome, Rome, Italy.
- [3] Department of Clinical Sciences and Community Health, University of Milan, Italy.

- PMID: **34932807**
- DOI: 10.1210/clinem/dgaa793

Abstract

Context: Coronavirus disease 2019 (COVID-19) represents a global health emergency, and infected patients with chronic diseases often present with a severe impairment. Adrenal insufficiency (AI) is supposed to be associated with an increased infection risk, which could trigger an adrenal crisis.

Objective: Our primary aim was to evaluate the incidence of COVID-19 symptoms and complications in AI patients.

Design and setting: We conducted a retrospective case-control study. All patients were on active follow-up and lived in Lombardy, Italy, one of the most affected areas.

Patients: We enrolled 279 patients with primary and secondary AI and 112 controls (patients with benign pituitary lesions without hormonal alterations). All AI patients had been previously trained to modify their replacement therapy on stress doses.

Intervention: By administering a standardized questionnaire by phone, we collected data on COVID-19 suggestive symptoms and consequences.

Results: In February through April 2020, the prevalence of symptomatic patients (complaining at least 1 symptom of viral infection) was similar between the 2 groups (24% in AI and 22.3% in controls, P = 0.79). Highly suggestive COVID-19 symptoms (at least 2 including fever and/or cough) also occurred equally in AI and controls (12.5% in both groups). No patient required hospitalization and no adrenal crisis was reported. Few nasopharyngeal swabs were performed (n = 12), as indicated by sanitary regulations, limiting conclusions on the exact infection rate (2 positive results in AI and none in controls, P = 0.52).

Conclusions: AI patients who are adequately treated and trained seem to display the same incidence of COVID-19-suggestive symptoms and disease severity as controls.

Keywords: COVID-19; adrenal insufficiency; hypopituitarism; infectious diseases.

© The Author(s) 2020. Published by Oxford University Press on behalf of the Endocrine Society.

Supplementary info

Publication types, MeSH terms, Substances Expand

Publication types

- Observational Study

MeSH terms

- Adrenal Insufficiency / complications*
- Adrenal Insufficiency / drug therapy
- Adrenal Insufficiency / immunology
- Adult
- Aged
- COVID-19 / diagnosis
- COVID-19 / epidemiology*
- COVID-19 / immunology
- Case-Control Studies
- Cortisone / administration & dosage
- Female
- Humans
- Hydrocortisone / administration & dosage
- Incidence
- Italy / epidemiology
- Male
- Middle Aged
- Prevalence
- Retrospective Studies
- Risk Factors

- SARS-CoV-2 / isolation & purification
- Severity of Illness Index
- Tertiary Care Centers / statistics & numerical data

Substances

- Cortisone
- Hydrocortisone

Full text links

ENDOCRINE SOCIETY Silverchair Information Systems
Proceed to details
Cite
Share
111
Observational Study

J Clin Endocrinol Metab
. 2021 Mar 8;106(3):e1354-e1361.
doi: 10.1210/clinem/dgaa793.

Adrenal Insufficiency at the Time of COVID-19: A Retrospective Study in Patients Referring to a Tertiary Center

Giulia Carosi [1, 2], Valentina Morelli [1], Giulia Del Sindaco [1, 3], Andreea Liliana Serban [1, 2], Arianna Cremaschi [1, 3], Sofia Frigerio [1, 3], Giulia Rodari [1, 3], Eriselda Profka [3], Rita Indirli [1, 3], Roberta Mungari [1], Veronica Resi [1], Emanuela Orsi [1], Emanuele Ferrante [1], Alessia Dolci [1], Claudia Giavoli [1, 3], Maura Arosio [1, 3], Giovanna Mantovani [1, 3]
Affiliations Expand

Affiliations

- [1] Endocrinology Unit, Fondazione IRCCS Cà Granda Ospedale Maggiore Policlinico, Milan, Italy.
- [2] Department of Experimental Medicine, Sapienza University of Rome, Rome, Italy.
- [3] Department of Clinical Sciences and Community Health, University of Milan, Italy.

- PMID: **33107576**
- PMCID: PMC7665569
- DOI: 10.1210/clinem/dgaa793

Free PMC article
Observational Study

Adrenal Insufficiency at the Time of COVID-19: A Retrospective Study in Patients Referring to a Tertiary Center

Giulia Carosi et al. J Clin Endocrinol Metab. 2021.
Free PMC article
Show details
J Clin Endocrinol Metab
. 2021 Mar 8;106(3):e1354-e1361.
doi: 10.1210/clinem/dgaa793.

Authors

Giulia Carosi [1 2], Valentina Morelli [1], Giulia Del Sindaco [1 3], Andreea Liliana Serban [1 2], Arianna Cremaschi [1 3], Sofia Frigerio [1 3], Giulia Rodari [1 3], Eriselda Profka [3], Rita Indirli [1 3], Roberta Mungari [1], Veronica Resi [1], Emanuela Orsi [1], Emanuele Ferrante [1], Alessia Dolci [1], Claudia Giavoli [1 3], Maura Arosio [1 3], Giovanna Mantovani [1 3]

Affiliations

- [1] Endocrinology Unit, Fondazione IRCCS Cà Granda Ospedale Maggiore Policlinico, Milan, Italy.
- [2] Department of Experimental Medicine, Sapienza University of Rome, Rome, Italy.
- [3] Department of Clinical Sciences and Community Health, University of Milan, Italy.

- PMID: **33107576**
- PMCID: PMC7665569
- DOI: 10.1210/clinem/dgaa793

Abstract

Context: Coronavirus disease 2019 (COVID-19) represents a global health emergency, and infected patients with chronic diseases often present with a severe impairment. Adrenal insufficiency (AI) is supposed to be associated with an increased infection risk, which could trigger an adrenal crisis.

Objective: Our primary aim was to evaluate the incidence of COVID-19 symptoms and complications in AI patients.

Design and setting: We conducted a retrospective case-control study. All patients were on active follow-up and lived in Lombardy, Italy, one of the most affected areas.

Patients: We enrolled 279 patients with primary and secondary AI and 112 controls (patients with benign pituitary lesions without hormonal alterations). All AI patients had been previously trained to modify their replacement therapy on stress doses.

Intervention: By administering a standardized questionnaire by phone, we collected data on COVID-19 suggestive symptoms and consequences.

Results: In February through April 2020, the prevalence of symptomatic patients (complaining at least 1 symptom of viral infection) was similar between the 2 groups (24% in AI and 22.3% in controls, P = 0.79). Highly suggestive COVID-19 symptoms (at least 2 including fever and/or cough) also occurred equally in AI and controls (12.5% in both groups). No patient required hospitalization and no adrenal crisis was reported. Few nasopharyngeal swabs were performed (n = 12), as indicated by sanitary regulations, limiting conclusions on the exact infection rate (2 positive results in AI and none in controls, P = 0.52).

Conclusions: AI patients who are adequately treated and trained seem to display the same incidence of COVID-19-suggestive symptoms and disease severity as controls.

Keywords: COVID-19; adrenal insufficiency; hypopituitarism; infectious diseases.

© The Author(s) 2020. Published by Oxford University Press on behalf of the Endocrine Society.

- 41 references
- 2 figures

Supplementary info

Publication types, MeSH terms Expand

Publication types

- Observational Study

MeSH terms

- Adrenal Insufficiency / complications
- Adrenal Insufficiency / diagnosis
- Adrenal Insufficiency / epidemiology*
- Adrenal Insufficiency / therapy
- Adult
- Aged
- Aged, 80 and over
- COVID-19 / complications*
- COVID-19 / diagnosis
- COVID-19 / epidemiology*
- COVID-19 / therapy
- Case-Control Studies
- Female
- Humans
- Hypopituitarism / complications
- Hypopituitarism / diagnosis
- Hypopituitarism / epidemiology
- Hypopituitarism / therapy

- Incidence
- Italy / epidemiology
- Male
- Middle Aged
- Referral and Consultation / statistics & numerical data
- Retrospective Studies
- Risk Factors
- SARS-CoV-2 / physiology
- Severity of Illness Index
- Tertiary Care Centers / statistics & numerical data
- Young Adult

Full text links

ENDOCRINE SOCIETY Silverchair Information Systems Free PMC article
Proceed to details
Cite
Share
☐ 112
Observational Study
Medicina (Kaunas)
. 2021 Oct 13;57(10):1099.
doi: 10.3390/medicina57101099.

Clostridium Difficile and COVID-19: General Data, Ribotype, Clinical Form, Treatment-Our Experience from the Largest Infectious Diseases Hospital in Western Romania

Adelina Raluca Marinescu [1 2 3], Ruxandra Laza [1 2], Virgil Filaret Musta [1 2], Talida Georgiana Cut [1 2 3 4], Raluca Dumache [4 5], Anca Tudor [6], Mirela Porosnicu [2 3], Voichita Elena Lazureanu [1 2], Monica Licker [7 8]
Affiliations Expand

Affiliations

- [1] Department XIII, Discipline of Infectious Diseases, University of Medicine and Pharmacy "Victor Babes", 300041 Timisoara, Romania.
- [2] Clinical Hospital of Infectious Diseases and Pneumophtisiology "Doctor Victor Babes" Timisoara, 300310 Timisoara, Romania.
- [3] Doctoral School, University of Medicine and Pharmacy "Victor Babes", 300041 Timisoara, Romania.

- [4] Center for Ethics in Human Genetic Identifications, University of Medicine and Pharmacy "Victor Babes", 300041 Timisoara, Romania.
- [5] Department VIII, Discipline of Forensic Medicine, Bioethics, Deontology and Medical Law, University of Medicine and Pharmacy "Victor Babes", 300041 Timisoara, Romania.
- [6] Department III, Discipline of Informatics and Medical Biostatistics, University of Medicine and Pharmacy "Victor Babes", 300041 Timisoara, Romania.
- [7] Department XIV, Discipline of Microbiology, University of Medicine and Pharmacy "Victor Babes", 300041 Timisoara, Romania.
- [8] Multidisciplinary Research Centre on Antimicrobial Resistance, University of Medicine and Pharmacy "Victor Babes", 300041 Timisoara, Romania.

- PMID: **34684136**
- PMCID: PMC8539017
- DOI: 10.3390/medicina57101099

Free PMC article
Observational Study

Clostridium Difficile and COVID-19: General Data, Ribotype, Clinical Form, Treatment-Our Experience from the Largest Infectious Diseases Hospital in Western Romania

Adelina Raluca Marinescu et al. Medicina (Kaunas). 2021.
Free PMC article

Show details

Medicina (Kaunas)
. 2021 Oct 13;57(10):1099.
doi: 10.3390/medicina57101099.

Authors

Adelina Raluca Marinescu [1 2 3], Ruxandra Laza [1 2], Virgil Filaret Musta [1 2], Talida Georgiana Cut [1 2 3 4], Raluca Dumache [4 5], Anca Tudor [6], Mirela Porosnicu [2 3], Voichita Elena Lazureanu [1 2], Monica Licker [7 8]

Affiliations

- [1] Department XIII, Discipline of Infectious Diseases, University of Medicine and Pharmacy "Victor Babes", 300041 Timisoara, Romania.
- [2] Clinical Hospital of Infectious Diseases and Pneumophtisiology "Doctor Victor Babes" Timisoara, 300310 Timisoara, Romania.
- [3] Doctoral School, University of Medicine and Pharmacy "Victor Babes", 300041 Timisoara, Romania.
- [4] Center for Ethics in Human Genetic Identifications, University of Medicine and Pharmacy "Victor Babes", 300041 Timisoara, Romania.

- [5] Department VIII, Discipline of Forensic Medicine, Bioethics, Deontology and Medical Law, University of Medicine and Pharmacy "Victor Babes", 300041 Timisoara, Romania.
- [6] Department III, Discipline of Informatics and Medical Biostatistics, University of Medicine and Pharmacy "Victor Babes", 300041 Timisoara, Romania.
- [7] Department XIV, Discipline of Microbiology, University of Medicine and Pharmacy "Victor Babes", 300041 Timisoara, Romania.
- [8] Multidisciplinary Research Centre on Antimicrobial Resistance, University of Medicine and Pharmacy "Victor Babes", 300041 Timisoara, Romania.

- PMID: **34684136**
- PMCID: PMC8539017
- DOI: 10.3390/medicina57101099

Abstract

Background and Objectives: In Coronavirus Disease 2019 (COVID-19), which is caused by the infection with severe acute respiratory syndrome coronavirus 2 (SARS-CoV-2), the clinical manifestations are primarily related to the pulmonary system. Under 10% of cases also develop gastrointestinal events such as diarrhea, nausea, vomiting and abdominal pain. *Materials and Methods*: We conducted an observational, retrospective study in the Infectious Diseases Clinic of "Victor Babes" Hospital, Timis County, in order to assess the incidence, outcome and risk factors for clostridium difficile infection (CDI) in COVID-19 patients. *Results*: Out of 2065 COVID-19 cases, hospitalized between 1st September 2020 and 30th April 2021, 40 cases of CDI were identified with 32 cases of hospital-onset of CDI and eight cases of community-onset and healthcare-associated CDI. By randomization, polymerase chain reaction ribotyping of Clostridium Difficile was performed in six cases. All the randomized cases tested positive for ribotype 027. The percentage of cases recovered with complications at discharge was higher among COVID-19 patients and CDI ($p = 0.001$). The in-hospital stay, 36 days versus 28 days, was longer among COVID-19 patients and CDI ($p = 0.01$). The presence of previous hospitalization ($p = 0.004$) and administration of antibiotics during the hospital stay, increased the risk of CDI among COVID-19 patients. The mean adjusted CCI at admission was lower among controls ($p = 0.01$). In two cases, exitus was strictly CDI-related, with one case positive for 027 ribotype. *Conclusions*: CDI has complicated the outcome of COVID-19 patients, especially for those with comorbidities or previously exposed to the healthcare system. In the face of the COVID-19 pandemic and the widespread, extensive use of antibiotics, clinicians should remain vigilant for possible CDI and SARS-CoV-2 co-infection.

Keywords: COVID-19 pandemic; antibiotic usage; clostridium difficile infection (CDI); outcome; ribotype; risk factors.

Conflict of interest statement

The authors declare no conflict of interest.

- 36 references

Supplementary info

Publication types, MeSH terms, Substances Expand

Publication types

- Observational Study

MeSH terms

- Anti-Bacterial Agents / therapeutic use
- COVID-19*
- Clostridioides difficile* / genetics
- Communicable Diseases* / drug therapy
- Cross Infection*
- Hospitals
- Humans
- Pandemics
- Retrospective Studies
- Ribotyping
- Romania / epidemiology
- SARS-CoV-2

Substances

- Anti-Bacterial Agents

Full text links

FULL TEXT OPEN ACCESS MDPI Multidisciplinary Digital Publishing Institute (MDPI) Free PMC article

Proceed to details

Cite

Share

☐ 113

Observational Study

BMC Pregnancy Childbirth

. 2021 Jun 4;21(1):416.

doi: 10.1186/s12884-021-03884-5.

Resilience and vulnerability of maternity services in Zimbabwe: a comparative analysis of the effect of Covid-19 and lockdown control measures on maternal and perinatal

outcomes, a single-centre cross-sectional study at Mpilo Central Hospital

Clare Shakespeare [1], Handsome Dube [2], Sikhangezile Moyo [3], Solwayo Ngwenya [2] [4]
Affiliations Expand

Affiliations

- [1] Department of Obstetrics and Gynaecology, Mpilo Central Hospital, Vera Road, Mzilikazi, Bulawayo, Zimbabwe. clare.shakespeare@gmail.com.
- [2] Department of Obstetrics and Gynaecology, Mpilo Central Hospital, Vera Road, Mzilikazi, Bulawayo, Zimbabwe.
- [3] Maternity Department Matron, Mpilo Central Hospital, Vera Road, Mzilikazi, Bulawayo, Zimbabwe.
- [4] National University of Science and Technology, Bulawayo, Zimbabwe.

- PMID: **34088285**
- PMCID: PMC8177257
- DOI: 10.1186/s12884-021-03884-5

Free PMC article
Observational Study

Resilience and vulnerability of maternity services in Zimbabwe: a comparative analysis of the effect of Covid-19 and lockdown control measures on maternal and perinatal outcomes, a single-centre cross-sectional study at Mpilo Central Hospital

Clare Shakespeare et al. BMC Pregnancy Childbirth. 2021.
Free PMC article
Show details
BMC Pregnancy Childbirth
. 2021 Jun 4;21(1):416.
doi: 10.1186/s12884-021-03884-5.

Authors

Clare Shakespeare [1], Handsome Dube [2], Sikhangezile Moyo [3], Solwayo Ngwenya [2] [4]

Affiliations

- [1] Department of Obstetrics and Gynaecology, Mpilo Central Hospital, Vera Road, Mzilikazi, Bulawayo, Zimbabwe. clare.shakespeare@gmail.com.
- [2] Department of Obstetrics and Gynaecology, Mpilo Central Hospital, Vera Road, Mzilikazi, Bulawayo, Zimbabwe.
- [3] Maternity Department Matron, Mpilo Central Hospital, Vera Road, Mzilikazi, Bulawayo, Zimbabwe.
- [4] National University of Science and Technology, Bulawayo, Zimbabwe.

- PMID: **34088285**
- PMCID: PMC8177257
- DOI: 10.1186/s12884-021-03884-5

Abstract

Background: On the 27th of March 2020 the Zimbabwean government declared the Covid-19 pandemic a 'national disaster'. Travel restrictions and emergency regulations have had significant impacts on maternity services, including resource stock-outs, and closure of antenatal clinics during the lockdown period. Estimates of the indirect impact of Covid-19 on maternal and perinatal mortality was expected it to be considerable, but little data was yet available. This study aimed to examine the impact of Covid-19 and lockdown control measures on non-Covid outcomes in a government tertiary level maternity unit in Bulawayo, Zimbabwe, by comparing maternal and perinatal morbidity and mortality before, and after the lockdown was implemented.

Methods: This was a retrospective, observational study, using a cross-sectional design to compare routine monthly maternal and perinatal statistics three months before and after Covid-19 emergency measures were implemented at Mpilo Central Hospital.

Results: Between January-March and April-June 2020, the mean monthly deliveries reduced from 747.3 (SD ± 61.3) in the first quarter of 2020 to 681.0 (SD ± 17.6) during lockdown, but this was not statistically significant, p = 0.20. The Caesarean section rates fell from a mean of 29.8% (SD ± 1.7) versus 28.0% (SD ± 1.7), which was also not statistically significant, p = 0.18. During lockdown, the percentage of women delivering at Mpilo Central Hospital who were booked at the hospital fell from a mean of 41.6% (SD ± 1.1) to 35.8% (SD ± 4.3) which was statistically significant, p = 0.03. There was no significant change, however, in maternal mortality or severe maternal morbidity (such as post-partum haemorrhage (PPH), uterine rupture, and severe preeclampsia/eclampsia), stillbirth rate or special care baby unit admission. There was an increase in the mean total number of early neonatal deaths (ENND) (mean 18.7 (SD ± 2.9) versus 24.0 (SD ± 4.6), but this was not statistically significant, p = 0.32.

Conclusions: Overall, maternity services at Mpilo showed resilience during the lockdown period, with no significant change in maternal and perinatal adverse outcomes, with the same number of man-hours worked before and during the lockdown Maternal and perinatal outcomes should continue to be monitored to assess the impact of Covid-19 and the lockdown measures as the pandemic in Zimbabwe unfolds. Further studies would be beneficial to explore women's experiences and understand how bookings and deliveries at local clinics changed during this time.

Keywords: Covid-19; Healthcare systems; Indirect maternal outcomes; Indirect perinatal outcomes; Pandemic; Resilience; Vulnerability.

Conflict of interest statement

The authors declare that they have no competing interests.

- 21 references
- 3 figures

Supplementary info

Publication types, MeSH terms Expand

Publication types

- Comparative Study
- Observational Study

MeSH terms

- COVID-19 / prevention & control*
- Communicable Disease Control*
- Cross-Sectional Studies
- Female
- Humans
- Maternal Health Services / statistics & numerical data*
- Maternal Health Services / trends
- Maternal Mortality
- Morbidity
- Perinatal Mortality
- Pregnancy
- Pregnancy Outcome
- Retrospective Studies
- SARS-CoV-2
- Tertiary Care Centers / statistics & numerical data*
- Tertiary Care Centers / trends
- Workload / statistics & numerical data
- Zimbabwe / epidemiology

Full text links

Read free full text at BMC BioMed Central Free PMC article

Proceed to details

Cite

Share

☐ 114

Observational Study

J Womens Health (Larchmt)
. 2021 Apr;30(4):492-501.
doi: 10.1089/jwh.2020.8974.

Sex-Based Differences in COVID-19 Outcomes

Astha Tejpal [1], Eugenia Gianos [1, 2], Jane Cerise [3], Jamie S Hirsch [2, 3, 4, 5], Stacey Rosen [2, 6], Nina Kohn [3], Martin Lesser [2, 3], Catherine Weinberg [1, 2], David Majure [2, 6], Sanjaya K Satapathy [2, 7], David Bernstein [2, 7], Matthew A Barish [2, 8], Alex C Spyropoulos [2, 3, 9], Rachel-Maria Brown [1, 2]

Affiliations Expand

Affiliations

- [1] Division of Cardiology, Lenox Hill Hospital, Northwell Health, New York, New York, USA.
- [2] Donald and Barbara Zucker School of Medicine at Hofstra/Northwell, Northwell Health, Hempstead, New York, USA.
- [3] Feinstein Institutes for Medical Research, Northwell Health, Manhasset, New York, USA.
- [4] Department of Nephrology and Hypertension, North Shore University Hospital, Manhasset, New York, USA.
- [5] Department of Information Services, Northwell Health, New Hyde Park, New York, USA.
- [6] Division of Cardiology, North Shore University Medical Center, Manhasset, New York, USA.
- [7] Division of Hepatology, North Shore University Medical Center, Manhasset, New York, USA.
- [8] Department of Radiology, North Shore University Hospital, Northwell Health, Manhasset, New York, USA.
- [9] Department of Internal Medicine, North Shore University Medical Center, Manhasset, New York, USA.

- PMID: **33885345**
- PMCID: **PMC8182657** (available on 2022-04-01)
- DOI: 10.1089/jwh.2020.8974

Observational Study

Sex-Based Differences in COVID-19 Outcomes

Astha Tejpal et al. J Womens Health (Larchmt). 2021 Apr.

Show details

J Womens Health (Larchmt)

. 2021 Apr;30(4):492-501.
doi: 10.1089/jwh.2020.8974.

Authors

Astha Tejpal [1], Eugenia Gianos [1, 2], Jane Cerise [3], Jamie S Hirsch [2, 3, 4, 5], Stacey Rosen [2, 6], Nina Kohn [3], Martin Lesser [2, 3], Catherine Weinberg [1, 2], David Majure [2, 6], Sanjaya K Satapathy [2, 7], David Bernstein [2, 7], Matthew A Barish [2, 8], Alex C Spyropoulos [2, 3, 9], Rachel-Maria Brown [1, 2]

Affiliations

- [1] Division of Cardiology, Lenox Hill Hospital, Northwell Health, New York, New York, USA.
- [2] Donald and Barbara Zucker School of Medicine at Hofstra/Northwell, Northwell Health, Hempstead, New York, USA.
- [3] Feinstein Institutes for Medical Research, Northwell Health, Manhasset, New York, USA.
- [4] Department of Nephrology and Hypertension, North Shore University Hospital, Manhasset, New York, USA.
- [5] Department of Information Services, Northwell Health, New Hyde Park, New York, USA.
- [6] Division of Cardiology, North Shore University Medical Center, Manhasset, New York, USA.
- [7] Division of Hepatology, North Shore University Medical Center, Manhasset, New York, USA.
- [8] Department of Radiology, North Shore University Hospital, Northwell Health, Manhasset, New York, USA.
- [9] Department of Internal Medicine, North Shore University Medical Center, Manhasset, New York, USA.

- PMID: **33885345**
- PMCID: **PMC8182657** (available on 2022-04-01)
- DOI: 10.1089/jwh.2020.8974

Abstract

Background: Smaller studies suggest lower morbidity and mortality associated with coronavirus disease 2019 (COVID-19) in women. Our aim is to assess the impact of female sex on outcomes in a large cohort of patients hospitalized with COVID-19. ***Materials and Methods:*** This is a retrospective observational cohort study of 10,630 adult patients hospitalized with a confirmed COVID-19 polymerase chain reaction between March 1, 2020 and April 27, 2020, with follow-up conducted through June 4, 2020. Logistic regression was used to examine the relationship between sex and the primary outcomes, including length of stay, admission to intensive care unit (ICU), need for mechanical ventilation, pressor requirement, and all-cause mortality as well as major adverse events and in-hospital COVID-19 treatments. ***Results:*** In the multivariable analysis, women had 27% lower odds of in-hospital mortality (odds ratio [OR] = 0.73, 95% confidence interval [CI] 0.66-0.81; $p < 0.001$), 24% lower odds of ICU admission (OR = 0.76, 95% CI 0.69-0.84; $p < 0.001$), 26% lower odds of mechanical ventilation (OR = 0.74, 95% CI 0.66-0.82; $p < 0.001$), and 25% lower odds of vasopressor requirement (OR = 0.75, 95% CI 0.67-0.84; $p < 0.001$). Women had 34% less odds of having acute cardiac injury (OR = 0.66, 95% CI 0.59-0.74; $p < 0.001$; $n = 7,289$), 16% less odds of acute kidney injury (OR = 0.84, 95% CI 0.76-0.92; $p <$

0.001; *n* = 9,840), and 27% less odds of venous thromboembolism (OR = 0.73, 95% CI 0.56-0.96; $p < 0.02$; *c*-statistic 0.85, *n* = 9,407). ***Conclusions:*** Female sex is associated with lower odds of in-hospital outcomes, major adverse events, and all-cause mortality. There may be protective mechanisms inherent to female sex, which explain differences in COVID-19 outcomes.

Keywords: COVID-19; gender; hospital outcomes; intensive care; mortality; sex.

Conflict of interest statement

No competing financial interests exist.

Comment in

- Lessons Learned from Coronavirus Disease 2019 Sex Disparities.
  Suboc T, Gomez JMD, Volgman C, Volgman AS. Suboc T, et al. J Womens Health (Larchmt). 2021 Apr;30(4):453-454. doi: 10.1089/jwh.2021.0110. Epub 2021 Mar 30. J Womens Health (Larchmt). 2021. PMID: 33784474 No abstract available.

Supplementary info

Publication types, MeSH terms, Grant support Expand

Publication types

- Observational Study
- Research Support, N.I.H., Extramural
- Research Support, U.S. Gov't, P.H.S.

MeSH terms

- Adolescent
- Adult
- Aged
- Aged, 80 and over
- COVID-19 / diagnosis
- COVID-19 / epidemiology
- COVID-19 / therapy*
- Cohort Studies
- Female
- Hospital Mortality*
- Hospitalization / statistics & numerical data*
- Humans
- Intensive Care Units / statistics & numerical data*
- Male
- Middle Aged
- New York / epidemiology

- Pandemics
- Retrospective Studies
- SARS-CoV-2
- Sex Distribution
- Sex Factors
- Treatment Outcome
- Young Adult

Grant support

- R24 AG064191/AG/NIA NIH HHS/United States
- R01 LM012836/LM/NLM NIH HHS/United States

Full text links

Atypon
Proceed to details
Cite
Share
115
Observational Study
Br J Clin Pharmacol
. 2021 Oct;87(10):3766-3775.
doi: 10.1111/bcp.14784. Epub 2021 Mar 10.

Dexamethasone use and mortality in hospitalized patients with coronavirus disease 2019: A multicentre retrospective observational study

Nicolas Hoertel[1 2 3], Marina Sánchez-Rico[1 4], Raphaël Vernet[5], Nathanaël Beeker[6], Antoine Neuraz[7 8], Jesús M Alvarado[4], Christel Daniel[9 10], Nicolas Paris[9 11], Alexandre Gramfort[12], Guillaume Lemaitre[12], Elisa Salamanca[13], Mélodie Bernaux[14], Ali Bellamine[15], Anita Burgun[7], Frédéric Limosin[1 2 3], AP-HP/Université de Paris/INSERM Covid-19 research collaboration and AP-HP Covid CDR Initiative
Affiliations Expand

Affiliations

- [1] Département de Psychiatrie, AP-HP.Centre, Hôpital Corentin-Celton, Issy-les-Moulineaux, France.
- [2] Institut de Psychiatrie et Neurosciences de Paris, Université de Paris, INSERM, Paris, France.

- [3] Faculté de Santé, UFR de Médecine, Université de Paris, Paris, France.
- [4] Department of Psychobiology & Behavioural Sciences Methods, Faculty of Psychology, Universidad Complutense de Madrid, Campus de Somosaguas, Pozuelo de Alarcon, Spain.
- [5] Hôpital Européen Georges Pompidou, Medical Informatics, Biostatistics and Public Health Department, AP-HP.Centre-Université de Paris, Paris, France.
- [6] Unité de Recherche clinique, Hopital Cochin, Assistance Publique-Hopitaux de Paris, Paris, France.
- [7] INSERM, UMR_S 1138, Cordeliers Research Center, Université de Paris, France.
- [8] Department of Medical Informatics, Necker-Enfants Malades Hospital, AP-HP. Centre-Université de Paris, Paris, France.
- [9] AP-HP, DSI-WIND (Web Innovation Données), Paris, France.
- [10] Sorbonne University, University Paris 13, Sorbonne Paris Cité, INSERM UMR_S 1142, Paris, France.
- [11] LIMSI, CNRS, Université Paris-Sud, Université Paris-Saclay, Orsay, France.
- [12] Université Paris-Saclay, INRIA, CEA, Palaiseau, France.
- [13] Banque Nationale de Données Maladies Rares (BNDMR), Campus Picpus, Département WIND (Web Innovation Données), Paris, France.
- [14] Direction de la stratégie et de la transformation, AP-HP, Paris, France.
- [15] Unité de Recherche clinique, Hôpital Cochin, AP-HP.Centre-Université de Paris, Paris, France.

- PMID: **33608891**
- PMCID: PMC8013383
- DOI: 10.1111/bcp.14784

Free PMC article
Observational Study

Dexamethasone use and mortality in hospitalized patients with coronavirus disease 2019: A multicentre retrospective observational study

Nicolas Hoertel et al. Br J Clin Pharmacol. 2021 Oct.
Free PMC article
Show details
Br J Clin Pharmacol
. 2021 Oct;87(10):3766-3775.
doi: 10.1111/bcp.14784. Epub 2021 Mar 10.

Authors

Nicolas Hoertel [1] [2] [3], Marina Sánchez-Rico [1] [4], Raphaël Vernet [5], Nathanaël Beeker [6], Antoine Neuraz [7] [8], Jesús M Alvarado [4], Christel Daniel [9] [10], Nicolas Paris [9] [11], Alexandre Gramfort [12], Guillaume Lemaitre [12], Elisa Salamanca [13], Mélodie Bernaux [14], Ali Bellamine

[15], Anita Burgun [7], Frédéric Limosin [1 2 3], AP-HP/Université de Paris/INSERM Covid-19 research collaboration and AP-HP Covid CDR Initiative

Affiliations

- [1] Département de Psychiatrie, AP-HP.Centre, Hôpital Corentin-Celton, Issy-les-Moulineaux, France.
- [2] Institut de Psychiatrie et Neurosciences de Paris, Université de Paris, INSERM, Paris, France.
- [3] Faculté de Santé, UFR de Médecine, Université de Paris, Paris, France.
- [4] Department of Psychobiology & Behavioural Sciences Methods, Faculty of Psychology, Universidad Complutense de Madrid, Campus de Somosaguas, Pozuelo de Alarcon, Spain.
- [5] Hôpital Européen Georges Pompidou, Medical Informatics, Biostatistics and Public Health Department, AP-HP.Centre-Université de Paris, Paris, France.
- [6] Unité de Recherche clinique, Hopital Cochin, Assistance Publique-Hopitaux de Paris, Paris, France.
- [7] INSERM, UMR_S 1138, Cordeliers Research Center, Université de Paris, France.
- [8] Department of Medical Informatics, Necker-Enfants Malades Hospital, AP-HP. Centre-Université de Paris, Paris, France.
- [9] AP-HP, DSI-WIND (Web Innovation Données), Paris, France.
- [10] Sorbonne University, University Paris 13, Sorbonne Paris Cité, INSERM UMR_S 1142, Paris, France.
- [11] LIMSI, CNRS, Université Paris-Sud, Université Paris-Saclay, Orsay, France.
- [12] Université Paris-Saclay, INRIA, CEA, Palaiseau, France.
- [13] Banque Nationale de Données Maladies Rares (BNDMR), Campus Picpus, Département WIND (Web Innovation Données), Paris, France.
- [14] Direction de la stratégie et de la transformation, AP-HP, Paris, France.
- [15] Unité de Recherche clinique, Hôpital Cochin, AP-HP.Centre-Université de Paris, Paris, France.

- PMID: **33608891**
- PMCID: PMC8013383
- DOI: 10.1111/bcp.14784

Abstract

Aims: To examine the association between dexamethasone use and mortality among patients hospitalized for COVID-19.

Methods: We examined the association between dexamethasone use and mortality at AP-HP Greater Paris University hospitals. Study baseline was defined as the date of hospital admission. The primary endpoint was time to death. We compared this endpoint between patients who received dexamethasone and those who did not in time-to-event analyses adjusted for patient characteristics (such as age, sex and comorbidity) and clinical and biological markers of clinical severity of COVID-19, and stratified by the need for respiratory support, i.e. mechanical ventilation or oxygen. The primary analysis was a multivariable Cox regression model.

Results: Of 12 217 adult patients hospitalized with a positive COVID-19 reverse transcriptase-polymerase chain reaction test, 171 (1.4%) received dexamethasone orally or by intravenous perfusion during the visit. Among patients who required respiratory support, the end-point

occurred in 10/63 (15.9%) patients who received dexamethasone and 298/1129 (26.4%) patients who did not. In this group, there was a significant association between dexamethasone use and reduced mortality in the primary analysis (hazard ratio, 0.46; 95% confidence interval 0.22-0.96, P = .039). Among patients who did not require respiratory support, there was no significant association between dexamethasone use and the endpoint.

Conclusions: In this multicentre observational study, dexamethasone use administered either orally or by intravenous injection at a cumulative dose between 60 mg and 150 mg was associated with reduced mortality among patients with COVID-19 requiring respiratory support.

Keywords: COVID-19; SARS-CoV-2; dexamethasone; efficacy; mortality; oxygen; treatment; ventilation.

© 2021 British Pharmacological Society.

Conflict of interest statement

Dr Hoertel has received personal fees and nonfinancial support from Lundbeck, outside the submitted work. Dr Limosin has received speaker and consulting fees from Janssen-Cilag outside the submitted work. Other authors declare no competing interests.

- 24 references
- 4 figures

Supplementary info

Publication types, MeSH terms, Substances, Supplementary concepts Expand

Publication types

- Multicenter Study
- Observational Study

MeSH terms

- Adult
- COVID-19* / drug therapy
- Coronavirus Infections*
- Dexamethasone
- Hospitalization
- Humans
- Retrospective Studies
- SARS-CoV-2

Substances

- Dexamethasone

Supplementary concepts

- COVID-19 drug treatment

Full text links

BJCP Wiley Free PMC article
Proceed to details
Cite
Share
☐ 116
Observational Study
J Korean Med Sci
. 2021 Feb 1;36(5):e44.
doi: 10.3346/jkms.2021.36.e44.

Impact of the COVID-19 Outbreak on Trends in Emergency Department Utilization in Children: a Multicenter Retrospective Observational Study in Seoul Metropolitan Area, Korea

Dong Hyun Choi [1], Jae Yun Jung [2], Dongbum Suh [3], Jea Yeon Choi [4], Se Uk Lee [5], Yoo Jin Choi [6], Young Ho Kwak [1], Do Kyun Kim [1]
Affiliations Expand

Affiliations

- [1] Department of Emergency Medicine, Seoul National University Hospital, Seoul, Korea.
- [2] Department of Emergency Medicine, Seoul National University Hospital, Seoul, Korea. matewoos@snuh.org.
- [3] Department of Emergency Medicine, Seoul National University Bundang Hospital, Seongnam, Korea.
- [4] Department of Emergency Medicine, Gachon University Gil Medical Center, Gachon University College of Medicine, Incheon, Korea.
- [5] Department of Emergency Medicine, Samsung Medical Center, Seoul, Korea.
- [6] Department of Emergency Medicine, Ajou University School of Medicine, Suwon, Korea.

- PMID: **33527786**
- PMCID: PMC7850866
- DOI: 10.3346/jkms.2021.36.e44

Free PMC article

Observational Study

Impact of the COVID-19 Outbreak on Trends in Emergency Department Utilization in Children: a Multicenter Retrospective Observational Study in Seoul Metropolitan Area, Korea

Dong Hyun Choi et al. J Korean Med Sci. 2021.
Free PMC article
Show details
J Korean Med Sci
. 2021 Feb 1;36(5):e44.
doi: 10.3346/jkms.2021.36.e44.

Authors

Dong Hyun Choi [1], Jae Yun Jung [2], Dongbum Suh [3], Jea Yeon Choi [4], Se Uk Lee [5], Yoo Jin Choi [6], Young Ho Kwak [1], Do Kyun Kim [1]

Affiliations

- [1] Department of Emergency Medicine, Seoul National University Hospital, Seoul, Korea.
- [2] Department of Emergency Medicine, Seoul National University Hospital, Seoul, Korea. matewoos@snuh.org.
- [3] Department of Emergency Medicine, Seoul National University Bundang Hospital, Seongnam, Korea.
- [4] Department of Emergency Medicine, Gachon University Gil Medical Center, Gachon University College of Medicine, Incheon, Korea.
- [5] Department of Emergency Medicine, Samsung Medical Center, Seoul, Korea.
- [6] Department of Emergency Medicine, Ajou University School of Medicine, Suwon, Korea.

- PMID: **33527786**
- PMCID: PMC7850866
- DOI: 10.3346/jkms.2021.36.e44

Abstract

Background: Understanding the changes in emergency department (ED) visit patterns during the coronavirus disease 2019 (COVID-19) outbreak is important for effectively operating EDs during the pandemic. We aimed to analyze the changes in pediatric ED visits during the COVID-19 pandemic and examine the relationship between the number of ED visits and the stringency of government social distancing measures.

Methods: This multicenter retrospective study used data of pediatric (age < 18 years) ED visits in Seoul metropolitan area from June 1, 2018, to May 31, 2020. Patient demographics, ED results, and diagnoses were compared during the COVID-19 period and the previous year. To evaluate the effect of the stringency of social distancing measures on the number of ED visits, a Poisson regression model was developed with month, year, and the average monthly Government Response Stringency Index (GRSI) as fixed effects.

Results: In total, 190,732 patients were included. The number of pediatric ED visits during the COVID-19 period was 58.1% lower than in the previous year. There were disproportionate decreases in the numbers of ED visits for children in early childhood (66.5%), low-acuity children (55.2-63.8%), those who did not use an ambulance (59.0%), and those visiting the ED for noninjury complaints (64.9%). The proportion of admissions increased from 11.9% to 16.6%. For every 10-point increase in the GRSI, there was a 15.1% decrease in monthly ED visits.

Conclusion: A striking decrease in pediatric ED visits was observed during the COVID-19 outbreak, the scale which was associated with the stringency of government policies. Changes in the number and characteristics of children visiting the ED should be considered to facilitate the effective operation of EDs during the pandemic.

Keywords: COVID-19; Emergency Department; Korea; Pediatrics.

© 2021 The Korean Academy of Medical Sciences.

Conflict of interest statement

All authors have no potential conflicts of interest to disclose.

- 19 references
- 2 figures

Supplementary info

Publication types, MeSH terms Expand

Publication types

- Multicenter Study
- Observational Study

MeSH terms

- Adolescent
- COVID-19 / epidemiology*
- Child
- Child, Preschool
- Disease Outbreaks
- Emergency Service, Hospital / organization & administration*
- Emergency Service, Hospital / statistics & numerical data*
- Female

- Hospitalization
- Humans
- Infant
- Infant, Newborn
- Male
- Pediatrics / organization & administration*
- Physical Distancing
- Poisson Distribution
- Republic of Korea / epidemiology
- Retrospective Studies
- Seoul / epidemiology
- Tertiary Care Centers

Full text links

Korean Academy of Medical Sciences Free PMC article
Proceed to details
Cite
Share
117
Observational Study
Swiss Med Wkly
. 2021 Jul 28;151:w20547.
doi: 10.4414/smw.2021.20547. eCollection 2021 Jul 19.

Risk factors for severe outcomes for COVID-19 patients hospitalised in Switzerland during the first pandemic wave, February to August 2020: prospective observational cohort study

Filipe Maximiano Sousa [1], Maroussia Roelens [2], Brian Fricker [3], Amaury Thiabaud [2], Anne Iten [4], Alexia Cusini [5], Domenica Flury [6], Michael Buettcher [7], Franziska Zukol [8], Carlo Balmelli [9], Petra Zimmermann [10], Nicolas Troillet [11], Danielle Vuichard-Gysin [12], Peter W Schreiber [13], Sara Bernhard-Stirnemann [14], Sarah Tschudin-Sutter [15], Yvonne Nussbaumer-Ochsner [16], Rami Sommerstein [17], Roman Gaudenz [18], Jonas Marschall [19], Laurence Senn [20], Céline Gardiol [21], Olivia Keiser [2], Gertraud Schüpbach [1], Monica Wymann [21], Beatriz Vidondo [1], Ch-Sur Study Group
Affiliations Expand

Affiliations

- [1] Swiss Federal Office of Public Health, Bern, Switzerland / Veterinary Public Health Institute, University of Bern, Switzerland.
- [2] Institute of Global Health, Faculty of Medicine, University of Geneva, Switzerland.
- [3] Swiss Federal Office of Public Health, Bern, Switzerland / Veterinary Public Health Institute, University of Bern, Switzerlan.
- [4] Service of Prevention and Infection Control, Directorate of Medicine and Quality, University Hospital Geneva, HUG, Geneva, Switzerland.
- [5] Department of Infectious Diseases, Cantonal Hospital Graubuenden, Chur, Switzerland.
- [6] Division of Infectious Diseases and Hospital Epidemiology, Cantonal Hospital St Gallen, Switzerland.
- [7] Paediatric Infectious Diseases, Department of Paediatrics, Children's Hospital, Cantonal Hospital Lucerne, Switzerland.
- [8] Paediatric Infectious Diseases, Department of Paediatrics, Cantonal Hospital Winterthur, Switzerland.
- [9] Infection Control Programme, EOC Hospitals, Ticino, Switzerland.
- [10] Faculty of Science and Medicine, University of Fribourg, Switzerland / Department of Paediatrics, Fribourg Hospital HFR, Fribourg, Switzerland.
- [11] Service of Infectious Diseases, Central Institute, Valais Hospitals, Sion, Switzerland.
- [12] Division of Infectious Diseases and Hospital Hygiene, Thurgau Hospital Group Münsterlingen and Frauenfeld, Switzerland.
- [13] Division of Infectious Diseases and Hospital Epidemiology, University Hospital Zurich and University of Zurich, Switzerland.
- [14] Children's Hospital Aarau, Switzerland.
- [15] Division of Infectious Diseases and Hospital Epidemiology, University Hospital Basel and University of Basel, Switzerland.
- [16] Clinic for Internal Medicine, Cantonal Hospital, Hospitals Schaffhausen, Switzerland.
- [17] Department of Infectious Diseases, Bern University Hospital (Inselspital), Bern, Switzerland / Infectious Diseases and Hospital Hygiene, Hirslanden Central Switzerland, Lucerne, Switzerland.
- [18] Internal Medicine and Infectiology, Cantonal Hospital Nidwalden, Stans, Switzerland.
- [19] Department of Infectious Diseases, Bern University Hospital (Inselspital), Bern, Switzerland.
- [20] Service of Preventive Medicine, Lausanne University Hospital, CHUV, Lausanne, Switzerland.
- [21] Swiss Federal Office of Public Health, Bern, Switzerland.

- PMID: **34324698**
- DOI: 10.4414/smw.2021.20547

Free article
Observational Study

Risk factors for severe outcomes for COVID-19 patients hospitalised in Switzerland during the first pandemic wave, February to August 2020: prospective observational cohort study

Filipe Maximiano Sousa et al. Swiss Med Wkly. 2021.
Free article
Show details
Swiss Med Wkly
. 2021 Jul 28;151:w20547.
doi: 10.4414/smw.2021.20547. eCollection 2021 Jul 19.

Authors

Filipe Maximiano Sousa [1], Maroussia Roelens [2], Brian Fricker [3], Amaury Thiabaud [2], Anne Iten [4], Alexia Cusini [5], Domenica Flury [6], Michael Buettcher [7], Franziska Zukol [8], Carlo Balmelli [9], Petra Zimmermann [10], Nicolas Troillet [11], Danielle Vuichard-Gysin [12], Peter W Schreiber [13], Sara Bernhard-Stirnemann [14], Sarah Tschudin-Sutter [15], Yvonne Nussbaumer-Ochsner [16], Rami Sommerstein [17], Roman Gaudenz [18], Jonas Marschall [19], Laurence Senn [20], Céline Gardiol [21], Olivia Keiser [2], Gertraud Schüpbach [1], Monica Wymann [21], Beatriz Vidondo [1], Ch-Sur Study Group

Affiliations

- [1] Swiss Federal Office of Public Health, Bern, Switzerland / Veterinary Public Health Institute, University of Bern, Switzerland.
- [2] Institute of Global Health, Faculty of Medicine, University of Geneva, Switzerland.
- [3] Swiss Federal Office of Public Health, Bern, Switzerland / Veterinary Public Health Institute, University of Bern, Switzerlan.
- [4] Service of Prevention and Infection Control, Directorate of Medicine and Quality, University Hospital Geneva, HUG, Geneva, Switzerland.
- [5] Department of Infectious Diseases, Cantonal Hospital Graubuenden, Chur, Switzerland.
- [6] Division of Infectious Diseases and Hospital Epidemiology, Cantonal Hospital St Gallen, Switzerland.
- [7] Paediatric Infectious Diseases, Department of Paediatrics, Children's Hospital, Cantonal Hospital Lucerne, Switzerland.
- [8] Paediatric Infectious Diseases, Department of Paediatrics, Cantonal Hospital Winterthur, Switzerland.
- [9] Infection Control Programme, EOC Hospitals, Ticino, Switzerland.
- [10] Faculty of Science and Medicine, University of Fribourg, Switzerland / Department of Paediatrics, Fribourg Hospital HFR, Fribourg, Switzerland.
- [11] Service of Infectious Diseases, Central Institute, Valais Hospitals, Sion, Switzerland.
- [12] Division of Infectious Diseases and Hospital Hygiene, Thurgau Hospital Group Münsterlingen and Frauenfeld, Switzerland.

- [13] Division of Infectious Diseases and Hospital Epidemiology, University Hospital Zurich and University of Zurich, Switzerland.
- [14] Children's Hospital Aarau, Switzerland.
- [15] Division of Infectious Diseases and Hospital Epidemiology, University Hospital Basel and University of Basel, Switzerland.
- [16] Clinic for Internal Medicine, Cantonal Hospital, Hospitals Schaffhausen, Switzerland.
- [17] Department of Infectious Diseases, Bern University Hospital (Inselspital), Bern, Switzerland / Infectious Diseases and Hospital Hygiene, Hirslanden Central Switzerland, Lucerne, Switzerland.
- [18] Internal Medicine and Infectiology, Cantonal Hospital Nidwalden, Stans, Switzerland.
- [19] Department of Infectious Diseases, Bern University Hospital (Inselspital), Bern, Switzerland.
- [20] Service of Preventive Medicine, Lausanne University Hospital, CHUV, Lausanne, Switzerland.
- [21] Swiss Federal Office of Public Health, Bern, Switzerland.

- PMID: **34324698**
- DOI: 10.4414/smw.2021.20547

Abstract

Background: As clinical signs of COVID-19 differ widely among individuals, from mild to severe, the definition of risk groups has important consequences for recommendations to the public, control measures and patient management, and needs to be reviewed regularly.

Aim: The aim of this study was to explore risk factors for in-hospital mortality and intensive care unit (ICU) admission for hospitalised COVID-19 patients during the first epidemic wave in Switzerland, as an example of a country that coped well during the first wave of the pandemic.

Methods: This study included all (n = 3590) adult polymerase chain reaction (PCR)-confirmed hospitalised patients in 17 hospitals from the hospital-based surveillance of COVID-19 (CH-Sur) by 1 September 2020. We calculated univariable and multivariable (adjusted) (1) proportional hazards (Fine and Gray) survival regression models and (2) logistic regression models for in-hospital mortality and admission to ICU, to evaluate the most common comorbidities as potential risk factors.

Results and discussion: We found that old age was the strongest factor for in-hospital mortality after having adjusted for gender and the considered comorbidities (hazard ratio [HR] 2.46, 95% confidence interval [CI] 2.33−2.59 and HR 5.6 95% CI 5.23−6 for ages 65 and 80 years, respectively). In addition, male gender remained an important risk factor in the multivariable models (HR 1.47, 95% CI 1.41−1.53). Of all comorbidities, renal disease, oncological pathologies, chronic respiratory disease, cardiovascular disease (but not hypertension) and dementia were also risk factors for in-hospital mortality. With respect to ICU admission risk, the pattern was different, as patients with higher chances of survival might have been admitted more often to ICU. Male gender (OR 1.91, 95% CI 1.58−2.31), hypertension (OR 1.3, 95% CI 1.07−1.59) and age 55–79 years (OR 1.15, 95% CI 1.06−1.26) are risk factors for ICU admission. Patients aged 80+ years, as well as patients with dementia or with liver disease were admitted less often to ICU.

Conclusion: We conclude that increasing age is the most important risk factor for in-hospital mortality of hospitalised COVID-19 patients in Switzerland, along with male gender and followed

by the presence of comorbidities such as renal diseases, chronic respiratory or cardiovascular disease, oncological malignancies and dementia. Male gender, hypertension and age between 55 and 79 years are, however, risk factors for ICU admission. Mortality and ICU admission need to be considered as separate outcomes when investigating risk factors for pandemic control measures and for hospital resources planning.

Supplementary info

Publication types, MeSH terms Expand

Publication types

- Observational Study

MeSH terms

- Adult
- Aged
- COVID-19* / diagnosis
- COVID-19* / mortality
- Comorbidity
- Hospital Mortality*
- Hospitalization / statistics & numerical data*
- Humans
- Intensive Care Units
- Male
- Middle Aged
- Pandemics*
- Prospective Studies
- Retrospective Studies
- Risk Factors
- SARS-CoV-2
- Switzerland / epidemiology

Full text links

Open access to full text on Swiss Medical Weekly EMH Swiss Medical Publishers Ltd.
Proceed to details
Cite
Share
☐ 118
Observational Study
Aging (Albany NY)
. 2021 Sep 9;13(17):20906-20914.

doi: 10.18632/aging.203503. Epub 2021 Sep 9.

High-dose vitamin C ameliorates cardiac injury in COVID-19 pandemic: a retrospective cohort study

Guozhi Xia [1], Bowen Qin [2], Chaoran Ma [3], Yaowu Zhu [4], Qiangsun Zheng [1]
Affiliations Expand

Affiliations

- [1] Department of Cardiology, The Second Affiliated Hospital of Xi'an Jiaotong University, Xi'an 710004, Shaanxi Province, China.
- [2] National-Local Joint Engineering Research Center of Biodiagnostics and Biotherapy, The Second Affiliated Hospital of Xi'an Jiaotong University, Xi'an 710004, Shaanxi Province, China.
- [3] Department of Nutritional Sciences, Pennsylvania State University, University Park, PA 16802, USA.
- [4] Department of Laboratory Medicine, Tongji Hospital of Huazhong University of Science and Technology, Wuhan 430030, Hubei Province, China.

- PMID: **34499050**
- PMCID: PMC8457586
- DOI: 10.18632/aging.203503

Free PMC article
Observational Study

High-dose vitamin C ameliorates cardiac injury in COVID-19 pandemic: a retrospective cohort study

Guozhi Xia et al. Aging (Albany NY). 2021.
Free PMC article
Show details
Aging (Albany NY)
. 2021 Sep 9;13(17):20906-20914.
doi: 10.18632/aging.203503. Epub 2021 Sep 9.

Authors

Guozhi Xia [1], Bowen Qin [2], Chaoran Ma [3], Yaowu Zhu [4], Qiangsun Zheng [1]

Affiliations

- [1] Department of Cardiology, The Second Affiliated Hospital of Xi'an Jiaotong University, Xi'an 710004, Shaanxi Province, China.
- [2] National-Local Joint Engineering Research Center of Biodiagnostics and Biotherapy, The Second Affiliated Hospital of Xi'an Jiaotong University, Xi'an 710004, Shaanxi Province, China.
- [3] Department of Nutritional Sciences, Pennsylvania State University, University Park, PA 16802, USA.
- [4] Department of Laboratory Medicine, Tongji Hospital of Huazhong University of Science and Technology, Wuhan 430030, Hubei Province, China.

- PMID: **34499050**
- PMCID: PMC8457586
- DOI: 10.18632/aging.203503

Abstract

Background: Cardiac injury is common and associated with poor clinical outcomes in COVID-19. Data are lacking whether high-dose intravenous vitamin C (HIVC) could help to ameliorate myocardial injury in the pandemic.

Methods: The retrospective cohort study included consecutive severe and critically ill COVID-19 patients with cardiac injury receiving symptomatic supportive treatments alone or together with HIVC. Troponin I and inflammatory markers were collected at admission and day 21 during hospitalization from the electronic medical records.

Results: The patients (n = 113) were categorized into the ameliorated cardiac injury (ACI) group (n = 70) and the non-ameliorated cardiac injury (NACI) group (n = 43). Overall, fifty-one (45.1%) patients were administered with HIVC, the percentages of patients with HIVC were higher in the ACI group than those in the NACI group. Logistic regression analysis revealed that HIVC was independently associated with the improvement of myocardial injury. Further analysis showed that inflammatory markers levels significantly decreased at day 21 during hospitalization in patients with HIVC therapy compared to those administered with symptomatic supportive treatments alone. Meanwhile, similar results were also observed regarding changes in inflammatory markers levels from baseline to day 21 during hospitalization in the patients treated with HIVC.

Conclusions: HIVC can ameliorate cardiac injury through alleviating hyperinflammation in severe and critically ill patients with COVID-19.

Keywords: COVID-19; cardiac injury; inflammation; vitamin C.

Conflict of interest statement

CONFLICTS OF INTEREST: The authors declare that they have no conflicts of interest.

- 20 references
- 3 figures

Supplementary info

Publication types, MeSH terms, Substances Expand

Publication types

- Observational Study

MeSH terms

- Aged
- Ascorbic Acid / therapeutic use*
- Biomarkers / blood
- COVID-19 / blood
- COVID-19 / drug therapy*
- COVID-19 / epidemiology*
- Dose-Response Relationship, Drug
- Female
- Heart Injuries / drug therapy*
- Hospitalization
- Humans
- Inflammation / pathology
- Inflammation Mediators / blood
- Male
- Middle Aged
- Pandemics*
- Retrospective Studies
- Troponin I / metabolism

Substances

- Biomarkers
- Inflammation Mediators
- Troponin I
- Ascorbic Acid

Full text links

Impact Journals, LLC Free PMC article

Proceed to details

Cite

Share

☐ 119

Observational Study

J Med Virol
. 2021 Jul;93(7):4265-4272.
doi: 10.1002/jmv.26794. Epub 2021 Apr 12.

A U-shaped association between baseline neutrophil count and COVID-19-related mortality: A retrospective cohort study

Wei Fu [1], Chi Chen [2], Xin-Lin Chen [3], Kun Wang [4], Peiyuan Zuo [5], Yuwei Liu [6], Meng Zhang [4], Xiaofang Zhao [4], Songpu Xie [4], Hao Zhang [4], Yan Geng [7], Chengyun Liu [4]
Affiliations Expand

Affiliations

- [1] Department of Gastroenterology, 925th Hospital of PLA Joint Logistics Support Force, Guiyang, China.
- [2] Department of Immunology and Microbiology, GuiZhou University of Traditional Chinese Medicine, Guiyang, Guizhou, China.
- [3] Department of Geriatrics, Zhongnan Hospital of Wuhan University, Wuhan, China.
- [4] Department of Epidemiology and Biostatistics, Empower U, X&Y solutions Inc., Boston, USA.
- [5] Department of Geriatrics, Union Hospital, Tongji Medical College, Huazhong University of Science and Technology, Wuhan, China.
- [6] Department of Geriatrics, Tongji Hospital, Tongji Medical College, Huazhong University of Science and Technology, Wuhan, China.
- [7] Department of Gastroenterology, 923th Hospital of PLA Joint Logistics Support Force, Nanning, China.

- PMID: **33463721**
- PMCID: PMC8014534
- DOI: 10.1002/jmv.26794

Free PMC article
Observational Study

A U-shaped association between baseline neutrophil count and COVID-19-related mortality: A retrospective cohort study

Wei Fu et al. J Med Virol. 2021 Jul.
Free PMC article
Show details
J Med Virol
. 2021 Jul;93(7):4265-4272.

doi: 10.1002/jmv.26794. Epub 2021 Apr 12.

Authors

Wei Fu [1], Chi Chen [2], Xin-Lin Chen [3], Kun Wang [4], Peiyuan Zuo [5], Yuwei Liu [6], Meng Zhang [4], Xiaofang Zhao [4], Songpu Xie [4], Hao Zhang [4], Yan Geng [7], Chengyun Liu [4]

Affiliations

- [1] Department of Gastroenterology, 925th Hospital of PLA Joint Logistics Support Force, Guiyang, China.
- [2] Department of Immunology and Microbiology, GuiZhou University of Traditional Chinese Medicine, Guiyang, Guizhou, China.
- [3] Department of Geriatrics, Zhongnan Hospital of Wuhan University, Wuhan, China.
- [4] Department of Epidemiology and Biostatistics, Empower U, X&Y solutions Inc., Boston, USA.
- [5] Department of Geriatrics, Union Hospital, Tongji Medical College, Huazhong University of Science and Technology, Wuhan, China.
- [6] Department of Geriatrics, Tongji Hospital, Tongji Medical College, Huazhong University of Science and Technology, Wuhan, China.
- [7] Department of Gastroenterology, 923th Hospital of PLA Joint Logistics Support Force, Nanning, China.

- PMID: **33463721**
- PMCID: PMC8014534
- DOI: 10.1002/jmv.26794

Abstract

Several descriptive studies have reported that higher neutrophil count (NC) may be correlated with poor prognosis in patients with confirmed COVID-19 infection. However, the findings from these studies are limited by methodology and data analysis. This study is a cohort study. We nonselectively and consecutively collected a total of 663 participants in a Chinese hospital from January 7 to February 28. Standardized and two-piecewise Cox regression model were employed to evaluate the association between baseline neutrophil count (bNC), neutrophil count change rate (NCR), and death. bNC had a U-shaped association with death. In the range of 0.1 to $\leq 1.49 \times 10^9$ /L (hazard ratio [HR] = 0.19, 95% confidence interval [CI] = 0.05-0.66) and $>3.55 \times 10^9$ /L of bNC (HR = 2.82, 95% CI = 1.19-6.67), the trends on bNC with mortality were opposite. By recursive algorithm, the bNC at which the risk of the death was lower in the range of >1.49 to $\leq 3.55 \times 10^9$ /L (HR = 13.64, 95% CI = 0.25-74.71). In addition, we find that NCRs (NCR1 and NCR2) are not associated with COVID-19-related deaths. Compared with NCR, bNC has the potential to be used for early risk stratification in patients with COVID-19 infection. The relationship between bNC and mortality was U-shaped. The safe range of bNC was 1.64-4.0 $\times 10^9$ /L. Identifying the correlation may be helpful for early risk stratification and medical decision-making.

Keywords: COVID-19-infection; baseline neutrophil count; change rate; nonlinearity.

© 2021 Wiley Periodicals LLC.

- 21 references

Supplementary info

Publication types, MeSH terms, Grant support Expand

Publication types

- Observational Study
- Research Support, Non-U.S. Gov't

MeSH terms

- COVID-19 / immunology*
- COVID-19 / mortality*
- China
- Female
- Hospitalization / statistics & numerical data
- Humans
- Lymphocyte Count
- Male
- Middle Aged
- Neutrophils / immunology*
- Prognosis
- Retrospective Studies
- Risk
- Risk Factors
- SARS-CoV-2 / immunology*

Grant support

- 81974222/National Natural Science Foundation of China
- 81671386/National Natural Science Foundation of China
- [2020]No.6/Supported by Foundation of Guiyang Municipal Science and Technology Bureau

Full text links

WILEY Full Text Article Wiley Free PMC article
Proceed to details
Cite
Share
☐ 120
Observational Study

Psychiatry Res
. 2021 Aug;302:113999.
doi: 10.1016/j.psychres.2021.113999. Epub 2021 May 13.

Examination of the impact of COVID-19 public health quarantine measures on acute mental health care services: A retrospective observational study

Nadine Nejati [1], Candice Crocker [2], Miroslava Kolajova [3], Jason Morrison [3], Patryk Simon [4], Sanjana Sridharan [3], Philip Tibbo [3]
Affiliations Expand

Affiliations

- [1] Department of Psychiatry, Dalhousie University, Halifax, Nova Scotia, Canada; Department of Mental Health and Addictions, Nova Scotia Health, Nova Scotia, Canada. Electronic address: nadine.nejati@nshealth.ca.
- [2] Department of Psychiatry, Dalhousie University, Halifax, Nova Scotia, Canada; Department of Diagnostic Radiology, Dalhousie University, Halifax, Nova Scotia, Canada.
- [3] Department of Psychiatry, Dalhousie University, Halifax, Nova Scotia, Canada; Department of Mental Health and Addictions, Nova Scotia Health, Nova Scotia, Canada.
- [4] Department of Mental Health and Addictions, Nova Scotia Health, Nova Scotia, Canada.

- PMID: **34038806**
- PMCID: PMC8117541
- DOI: 10.1016/j.psychres.2021.113999

Free PMC article
Observational Study

Examination of the impact of COVID-19 public health quarantine measures on acute mental health care services: A retrospective observational study

Nadine Nejati et al. Psychiatry Res. 2021 Aug.
Free PMC article
Show details
Psychiatry Res
. 2021 Aug;302:113999.
doi: 10.1016/j.psychres.2021.113999. Epub 2021 May 13.

Authors

Nadine Nejati [1], Candice Crocker [2], Miroslava Kolajova [3], Jason Morrison [3], Patryk Simon [4], Sanjana Sridharan [3], Philip Tibbo [3]

Affiliations

- [1] Department of Psychiatry, Dalhousie University, Halifax, Nova Scotia, Canada; Department of Mental Health and Addictions, Nova Scotia Health, Nova Scotia, Canada. Electronic address: nadine.nejati@nshealth.ca.
- [2] Department of Psychiatry, Dalhousie University, Halifax, Nova Scotia, Canada; Department of Diagnostic Radiology, Dalhousie University, Halifax, Nova Scotia, Canada.
- [3] Department of Psychiatry, Dalhousie University, Halifax, Nova Scotia, Canada; Department of Mental Health and Addictions, Nova Scotia Health, Nova Scotia, Canada.
- [4] Department of Mental Health and Addictions, Nova Scotia Health, Nova Scotia, Canada.

- PMID: **34038806**
- PMCID: PMC8117541
- DOI: 10.1016/j.psychres.2021.113999

Abstract

This study assesses for the impact of Covid-19 public health quarantine measures on acute care psychiatric admissions, by comparing admission data from the quarantine period to a comparator period. A chart review was conducted for all admissions to an urban acute care psychiatric centre from Mar 22 - June 5 2020 (quarantine) and January 5 - Mar 21 2020 (comparator). Data was collected on the number of admissions, demographics, patients' psychiatric history, characteristics of admissions, discharge information, patients' substance use and social factors. Data was analyzed using a student's t-test for continuous variables and Chi squared analyses for categorical variables. Results demonstrated 185 admissions during quarantine and 190 during the comparator, with no significant differences in the distribution of admissions across time periods. There was a significantly greater frequency of admissions in the 35-44 age bracket and admissions involving substance use during quarantine. Additionally, admissions during quarantine were significantly shorter, with increased frequency of involuntary status and use of seclusion. The data suggests a vulnerability specific to individuals in their 30-40s during quarantine and demonstrates a need to better understand factors impacting this group. It also suggests that quarantine is associated with changes to substance use, potentiating high acuity illness requiring admission.

Keywords: Inpatients; Mental disorders; Pandemics; Psychotic disorders; Substance related disorders.

Copyright © 2021 The Authors. Published by Elsevier B.V. All rights reserved.

Conflict of interest statement

Dr. Nejati, Dr. Crocker, Dr. Kolajova, Dr. Morrison, Mr. Simon, and Dr. Sridharan have no declarations of interest or financial supports to declare.

Dr. Tibbo has received advisory board honoraria and speaker fees from Janssen Inc., Otsuka and Lundbeck on topics not related to this manuscript.

- 25 references

Supplementary info

Publication types, MeSH terms Expand

Publication types

- Observational Study

MeSH terms

- Adult
- Aged
- COVID-19 / epidemiology
- COVID-19 / prevention & control*
- Female
- Hospitalization / statistics & numerical data*
- Humans
- Male
- Mental Disorders / therapy*
- Mental Health Services / statistics & numerical data*
- Middle Aged
- Nova Scotia / epidemiology
- Public Health / legislation & jurisprudence
- Quarantine / psychology*
- Retrospective Studies
- Young Adult

Full text links

ELSEVIER OPEN ACCESS Elsevier Science Free PMC article

Proceed to details

Cite

Share

☐ 121

Observational Study

PLoS One

. 2021 Jun 9;16(6):e0252388.
doi: 10.1371/journal.pone.0252388. eCollection 2021.

Lack of efficacy of hydroxychloroquine and azithromycin in patients hospitalized for COVID-19 pneumonia: A retrospective study

Anis Saib [1], Walid Amara [1], Pascal Wang [2], Simon Cattan [1], Azeddine Dellal [3], Kais Regaieg [4], Stephane Nahon [5], Olivier Nallet [1], Lee S Nguyen [6]
Affiliations Expand

Affiliations

- [1] Cardiology Department, Groupe Hospitalier Intercommunal Le Raincy-Montfermeil, Montfermeil, France.
- [2] Pneumology Department, Groupe Hospitalier Intercommunal Le Raincy-Montfermeil, Montfermeil, France.
- [3] Rheumatology Department, Groupe Hospitalier Intercommunal Le Raincy-Montfermeil, Montfermeil, France.
- [4] Intensive Care Medicine Department, Groupe Hospitalier Intercommunal Le Raincy-Montfermeil, Montfermeil, France.
- [5] Gastroenterology Department, Groupe Hospitalier Intercommunal Le Raincy-Montfermeil, Montfermeil, France.
- [6] Research & Innovation Department (RICAP), CMC Ambroise Paré, Neuilly-sur-Seine, France.

- PMID: **34106964**
- PMCID: PMC8189518
- DOI: 10.1371/journal.pone.0252388

Free PMC article
Observational Study

Lack of efficacy of hydroxychloroquine and azithromycin in patients hospitalized for COVID-19 pneumonia: A retrospective study

Anis Saib et al. PLoS One. 2021.
Free PMC article
Show details
PLoS One
. 2021 Jun 9;16(6):e0252388.
doi: 10.1371/journal.pone.0252388. eCollection 2021.

Authors

Anis Saib [1], Walid Amara [1], Pascal Wang [2], Simon Cattan [1], Azeddine Dellal [3], Kais Regaieg [4], Stephane Nahon [5], Olivier Nallet [1], Lee S Nguyen [6]

Affiliations

- [1] Cardiology Department, Groupe Hospitalier Intercommunal Le Raincy-Montfermeil, Montfermeil, France.
- [2] Pneumology Department, Groupe Hospitalier Intercommunal Le Raincy-Montfermeil, Montfermeil, France.
- [3] Rheumatology Department, Groupe Hospitalier Intercommunal Le Raincy-Montfermeil, Montfermeil, France.
- [4] Intensive Care Medicine Department, Groupe Hospitalier Intercommunal Le Raincy-Montfermeil, Montfermeil, France.
- [5] Gastroenterology Department, Groupe Hospitalier Intercommunal Le Raincy-Montfermeil, Montfermeil, France.
- [6] Research & Innovation Department (RICAP), CMC Ambroise Paré, Neuilly-sur-Seine, France.

- PMID: **34106964**
- PMCID: PMC8189518
- DOI: 10.1371/journal.pone.0252388

Abstract

Background: Hydroxychloroquine combined with azithromycin (HCQ/AZI) has initially been used against coronavirus disease-2019 (COVID-19). In this retrospective study, we assessed the clinical effects of HCQ/AZI, with a 28-days follow-up.

Methods: In a registry-study which included patients hospitalized for COVID-19 between March 15 and April 2, 2020, we compared patients who received HCQ/AZI to those who did not, regarding a composite outcome of mortality and mechanical ventilation with a 28-days follow-up. QT was monitored for patients treated with HCQ/AZI. Were excluded patients in intensive care units, palliative care and ventilated within 24 hours of admission. Three analyses were performed to adjust for selection bias: propensity score matching, multivariable survival, and inverse probability score weighting (IPSW) analyses.

Results: Overall, 203 patients were included: 60 patients treated by HCQ/AZI and 143 control patients. During the 28-days follow-up, 32 (16.3%) patients presented the primary outcome and 23 (12.3%) patients died. Propensity-score matching identified 52 unique pairs of patients with similar characteristics. In the matched cohort (n = 104), HCQ/AZI was not associated with the primary composite outcome (log-rank p-value = 0.16). In the overall cohort (n = 203), survival and IPSW analyses also found no benefit from HCQ/AZI. In the HCQ/AZI group, 11 (18.3%) patients prolonged QT interval duration, requiring treatment cessation.

Conclusions: HCQ/AZI combination therapy was not associated with lower in-hospital mortality and mechanical ventilation rate, with a 28-days follow-up. In the HCQ/AZI group, 18.3% of patients presented a prolonged QT interval requiring treatment cessation, however, control group was not monitored for this adverse event, making comparison impossible.

Conflict of interest statement

The authors have declared that no competing interests exist.

- 32 references
- 3 figures

Supplementary info

Publication types, MeSH terms, Substances, Grant support Expand

Publication types

- Observational Study

MeSH terms

- Anti-Bacterial Agents / therapeutic use
- Antimalarials / therapeutic use
- Azithromycin / therapeutic use*
- COVID-19 / drug therapy*
- COVID-19 / mortality
- COVID-19 / pathology
- COVID-19 / virology
- Female
- Follow-Up Studies
- Humans
- Hydroxychloroquine / therapeutic use*
- Intensive Care Units
- Male
- Middle Aged
- Respiration, Artificial
- Retrospective Studies
- SARS-CoV-2 / drug effects*
- SARS-CoV-2 / isolation & purification
- Survival Rate
- Treatment Outcome

Substances

- Anti-Bacterial Agents
- Antimalarials
- Hydroxychloroquine
- Azithromycin

Grant support

The author(s) received no specific funding for this work.

Full text links

PLOS ONE Public Library of Science Free PMC article
Proceed to details
Cite
Share
122
Observational Study

J Cardiovasc Pharmacol
. 2021 Jul 1;78(1):e94-e100.
doi: 10.1097/FJC.0000000000001041.

Preadmission Statin Therapy and Clinical Outcome in Hospitalized Patients With COVID-19: An Italian Multicenter Observational Study

Vincenzo Russo [1], Angelo Silverio [2], Fernando Scudiero [3], Emilio Attena [4], Antonello D'Andrea [5], Luigi Nunziata [6], Guido Parodi [7], Dario Celentani [8], Ferdinando Varbella [8], Stefano Albani [9], Giuseppe Musumeci [9], Pierpaolo Di Micco [10], Marco Di Maio [2] [11]
Affiliations Expand

Affiliations

- [1] Division of Cardiology, Department of Translational Medical Sciences, University of Campania "Luigi Vanvitelli"-Monaldi and Cotugno Hospital, Naples, Italy.
- [2] Department of Medicine, Surgery and Dentistry, University of Salerno, Baronissi (Salerno), Italy.
- [3] Cardiology Unit, Health Authority Bergamo East, Seriate (Bergamo), Italy.
- [4] Division of Cardiology, San Giuliano Hospital, Naples, Italy.
- [5] Cardiology and Intensive Care Unit, Umberto I Hospital, Nocera Inferiore, Italy.
- [6] Cardiology Unit, Boscotrecase Hospital, Naples, Italy.
- [7] Clinical and Interventional Cardiology, Sassari University Hospital, Sassary, Italy.
- [8] Cardiology Unit, Rivoli Hospital, Turin, Italy.
- [9] Cardiology Unit, Mauriziano Hospital, Turin, Italy.
- [10] Medicine Unit, Division of Cardiology, Fatebenefratelli Hospital of Naples, Naples, Italy; and.
- [11] Division of Cardiology, Maria SS. Addolorata Hospital, Eboli (Salerno), Italy.

- PMID: **34173802**

- PMCID: PMC8253374
- DOI: 10.1097/FJC.0000000000001041

Free PMC article
Observational Study

Preadmission Statin Therapy and Clinical Outcome in Hospitalized Patients With COVID-19: An Italian Multicenter Observational Study

Vincenzo Russo et al. J Cardiovasc Pharmacol. 2021.
Free PMC article
Show details
J Cardiovasc Pharmacol
. 2021 Jul 1;78(1):e94-e100.
doi: 10.1097/FJC.0000000000001041.

Authors

Vincenzo Russo [1], Angelo Silverio [2], Fernando Scudiero [3], Emilio Attena [4], Antonello D'Andrea [5], Luigi Nunziata [6], Guido Parodi [7], Dario Celentani [8], Ferdinando Varbella [8], Stefano Albani [9], Giuseppe Musumeci [9], Pierpaolo Di Micco [10], Marco Di Maio [2] [11]

Affiliations

- [1] Division of Cardiology, Department of Translational Medical Sciences, University of Campania "Luigi Vanvitelli"-Monaldi and Cotugno Hospital, Naples, Italy.
- [2] Department of Medicine, Surgery and Dentistry, University of Salerno, Baronissi (Salerno), Italy.
- [3] Cardiology Unit, Health Authority Bergamo East, Seriate (Bergamo), Italy.
- [4] Division of Cardiology, San Giuliano Hospital, Naples, Italy.
- [5] Cardiology and Intensive Care Unit, Umberto I Hospital, Nocera Inferiore, Italy.
- [6] Cardiology Unit, Boscotrecase Hospital, Naples, Italy.
- [7] Clinical and Interventional Cardiology, Sassari University Hospital, Sassary, Italy.
- [8] Cardiology Unit, Rivoli Hospital, Turin, Italy.
- [9] Cardiology Unit, Mauriziano Hospital, Turin, Italy.
- [10] Medicine Unit, Division of Cardiology, Fatebenefratelli Hospital of Naples, Naples, Italy; and.
- [11] Division of Cardiology, Maria SS. Addolorata Hospital, Eboli (Salerno), Italy.

- PMID: **34173802**
- PMCID: PMC8253374
- DOI: 10.1097/FJC.0000000000001041

Abstract

Statin therapy has been recently suggested as possible adjuvant treatment to improve the clinical outcome in patients with coronavirus disease 2019 (COVID-19). The aim of this study was to describe the prevalence of preadmission statin therapy in hospitalized patients with COVID-19 and to investigate its potential association with acute distress respiratory syndrome (ARDS) at admission and in-hospital mortality. We retrospectively recruited 467 patients with laboratory-confirmed COVID-19 admitted to the emergency department of 10 Italian hospitals. The study population was divided in 2 groups according to the ARDS diagnosis at admission and in-hospital mortality. A multivariable regression analysis was performed to assess the risk of ARDS at admission and death during hospitalization among patients with COVID-19. A competing risk analysis in patients taking or not statins before admission was also performed. ARDS at admission was reported in 122 cases (26.1%). There was no statistically significant difference for clinical characteristics between patients presenting with and without ARDS. One hundred seven patients (18.5%) died during the hospitalization; they showed increased age (69.6 ± 13.1 vs. 66.1 ± 14.9; P = 0.001), coronary artery disease (23.4% vs. 12.8%; P = 0.012), and chronic kidney disease (20.6% vs. 11.1%; P = 0.018) prevalence; moreover, they presented more frequently ARDS at admission (48.6% vs. 19.4%; P < 0.001). At multivariable regression model, statin therapy was not associated neither with ARDS at admission nor with in-hospital mortality. Preadmission statin therapy does not seem to show a protective effect in severe forms of COVID-19 complicated by ARDS at presentation and rapidly evolving toward death.

Copyright © 2021 Wolters Kluwer Health, Inc. All rights reserved.

Conflict of interest statement

The authors report no conflicts of interest.

- 28 references
- 3 figures

Supplementary info

Publication types, MeSH terms, Substances Expand

Publication types

- Multicenter Study
- Observational Study

MeSH terms

- Aged
- Aged, 80 and over
- COVID-19 / diagnosis
- COVID-19 / mortality
- COVID-19 / therapy*
- Comorbidity

- Disease Progression
- Dyslipidemias / diagnosis
- Dyslipidemias / drug therapy*
- Dyslipidemias / mortality
- Female
- Hospital Mortality
- Hospitalization*
- Humans
- Hydroxymethylglutaryl-CoA Reductase Inhibitors / therapeutic use*
- Italy
- Male
- Middle Aged
- Prognosis
- Retrospective Studies
- Risk Assessment
- Risk Factors
- Time Factors

Substances

- Hydroxymethylglutaryl-CoA Reductase Inhibitors

Full text links

Wolters Kluwer Wolters Kluwer Free PMC article
Proceed to details
Cite
Share
123
Observational Study
J Med Virol
. 2021 May;93(5):2782-2789.
doi: 10.1002/jmv.26617. Epub 2021 Mar 1.

Clinical features and antibody response of patients from a COVID-19 treatment hospital in Wuhan, China

Yong Chen [1, 2], Yuehua Ke [1, 2], Xiong Liu [1, 2], Zhihua Wang [1, 3], Ruizhong Jia [1, 2], Wei Liu [1, 2], Chaojie Yang [1, 2], Leili Jia [1, 2], Yong Wang [1, 2], Li Han [1, 2], Xinyi Xia [1, 4], Sibing Zhang [1, 5], Changjun Wang [1, 2]

Affiliations Expand

Affiliations

- [1] Wuhan Huoshenshan Hospital, Wuhan, China.
- [2] Chinese PLA Center for Disease Control and Prevention, Beijing, China.
- [3] Department of Clinical laboratory, The 907th Hospital of Joint Logistic Armed Force, Nanping, China.
- [4] Research Institute of Laboratory Medicine, Eastern Theater General Hospital, Nanjing, China.
- [5] Executive office, The Fourth Military Medical University, Xi'an, China.

- PMID: **33085103**
- DOI: 10.1002/jmv.26617

Observational Study

Clinical features and antibody response of patients from a COVID-19 treatment hospital in Wuhan, China

Yong Chen et al. J Med Virol. 2021 May.

Show details

J Med Virol

. 2021 May;93(5):2782-2789.
doi: 10.1002/jmv.26617. Epub 2021 Mar 1.

Authors

Yong Chen [1, 2], Yuehua Ke [1, 2], Xiong Liu [1, 2], Zhihua Wang [1, 3], Ruizhong Jia [1, 2], Wei Liu [1, 2], Chaojie Yang [1, 2], Leili Jia [1, 2], Yong Wang [1, 2], Li Han [1, 2], Xinyi Xia [1, 4], Sibing Zhang [1, 5], Changjun Wang [1, 2]

Affiliations

- [1] Wuhan Huoshenshan Hospital, Wuhan, China.
- [2] Chinese PLA Center for Disease Control and Prevention, Beijing, China.
- [3] Department of Clinical laboratory, The 907th Hospital of Joint Logistic Armed Force, Nanping, China.
- [4] Research Institute of Laboratory Medicine, Eastern Theater General Hospital, Nanjing, China.
- [5] Executive office, The Fourth Military Medical University, Xi'an, China.

- PMID: **33085103**
- DOI: 10.1002/jmv.26617

Abstract

Coronavirus disease 2019 (COVID-19) has rapidly evolved into a global pandemic. A total of 1578 patients admitted into a newly built hospital specialized for COVID-19 treatment in Wuhan, China, were enrolled. Clinical features and the levels of severe acute respiratory syndrome coronavirus 2 (SARS-CoV-2) immunoglobulin (Ig)M and IgG were analyzed. In total, 1532 patients (97.2%) were identified as laboratory-confirmed cases. Seventy-seven patients were identified as asymptomatic carriers (n = 64) or SARS-CoV-2 RNA positive before symptom onset (n = 13). The positive rates of SARS-CoV-2 IgM and IgG were 80.4% and 96.8%, respectively. The median of IgM and IgG titers were 37.0A U/ml (interquartile range [IQR]: 13.4-81.1 AU/ml) and 156.9 AU/ml (IQR: 102.8-183.3 AU/ml), respectively. The IgM and IgG levels of asymptomatic patients (median titers, 8.3 AU/ml and 100.3 AU/ml) were much lower than those in symptomatic patients (median titers, 38.0 AU/ml and 158.2 AU/ml). A much lower IgG level was observed in critically ill patients 42-60 days after symptom onset. There were 153 patients with viral RNA shedding after IgG detection. These patients had a higher proportion of critical illness during hospitalization ($p < .001$) and a longer hospital stay ($p < .001$) compared to patients with viral clearance after IgG detection. Coronary heart disease (odds ratio [OR], 1.89 [95% confidence interval [CI], 1.11-3.24]; $p = .020$), and intensive care unit admission (OR, 2.47 [95% CI, 1.31-4.66]; $p = .005$) were independent risk factors associated with viral RNA shedding after IgG detection. Symptomatic patients produced more antibodies than asymptomatic patients. The patients who had SARS-CoV-2 RNA shedding after developing IgG were more likely to be sicker patients.

Keywords: COVID-19; SARS-CoV-2 specific antibody; clinical features; epidemiology; viral shedding.

© 2020 Wiley Periodicals LLC.

- 29 references

Supplementary info

Publication types, MeSH terms, Substances, Supplementary concepts, Grant support Expand

Publication types

- Observational Study
- Research Support, Non-U.S. Gov't

MeSH terms

- Adolescent
- Adult
- Aged
- Antibodies, Viral / immunology*
- Antibody Formation*
- COVID-19 / drug therapy*
- COVID-19 / immunology*

- COVID-19 / physiopathology
- China
- Female
- Hospitalization
- Hospitals
- Humans
- Immunoglobulin G / immunology
- Immunoglobulin M / immunology
- Male
- Middle Aged
- Pandemics
- RNA, Viral
- Retrospective Studies
- Risk Factors
- SARS-CoV-2
- Virus Shedding
- Young Adult

Substances

- Antibodies, Viral
- Immunoglobulin G
- Immunoglobulin M
- RNA, Viral

Supplementary concepts

- COVID-19 drug treatment

Grant support

- National key research and development program 2019YFC1200501
- National Key Program for Infectious Diseases of China 2018ZX10733-402
- COVID-19 Research Program YJGG2020-01 YJGG2020-02
- Beijing Nova Program Z181100006218107
- 31900151/National Natural Science Fundation of China

Full text links

WILEY Full Text Article Wiley

Proceed to details

Cite

Share

124

Multicenter Study
BMJ Open
. 2020 Dec 2;10(12):e041417.
doi: 10.1136/bmjopen-2020-041417.

Design and rationale of the COVID-19 Critical Care Consortium international, multicentre, observational study

Gianluigi Li Bassi [#] [1] [2] [3] [4], Jacky Suen [#] [5] [2], Adrian Gerard Barnett [6], Amanda Corley [5] [2], Jonathan Millar [7], Jonathon Fanning [5] [2] [8], India Lye [5] [2], Sebastiano Colombo [5] [2] [9], Karin Wildi [5] [2], Samantha Livingstone [5] [2], Gabriella Abbate [5], Samuel Hinton [2], Benoit Liquet [10] [11], Sally Shrapnel [10], Heidi Dalton [12], John F Fraser [5] [2] [3] [8], COVID-19 Critical Care Consortium Investigators
Affiliations Expand

Affiliations

- [1] Critical Care Research Group, Prince Charles Hospital, Chermside, Queensland, Australia g.libassi@uq.edu.au.
- [2] Faculty of Medicine, University of Queensland, Brisbane, Queensland, Australia.
- [3] Queensland University of Technology, Brisbane, Queensland, Australia.
- [4] Institut d'Investigacions Biomèdiques August Pi i Sunyer, Barcelona, Spain.
- [5] Critical Care Research Group, Prince Charles Hospital, Chermside, Queensland, Australia.
- [6] Institute of Health and Biomedical Innovation, Queensland University of Technology, Brisbane, Queensland, Australia.
- [7] Roslin Institute, University of Edinburgh, Edinburgh, United Kingdom.
- [8] Critical Care Medicine, UnitingCare Health, Brisbane, Queensland, Australia.
- [9] Department of Pathophysiology and Transplantation, University of Milan, Milan, Italy.
- [10] University of Queensland, Brisbane, Queensland, Australia.
- [11] University of Pau et Pays De L'Adour, Pau, France.
- [12] Inova Fairfax Medical Campus, Falls Church, Virginia, USA.

[#] Contributed equally.

- PMID: **33268426**
- PMCID: PMC7714653
- DOI: 10.1136/bmjopen-2020-041417

Free PMC article
Multicenter Study

Design and rationale of the COVID-19 Critical Care Consortium international, multicentre, observational study

Gianluigi Li Bassi et al. BMJ Open. 2020.
Free PMC article
Show details
BMJ Open
. 2020 Dec 2;10(12):e041417.
doi: 10.1136/bmjopen-2020-041417.

Authors

Gianluigi Li Bassi [#] [1] [2] [3] [4], Jacky Suen [#] [5] [2], Adrian Gerard Barnett [6], Amanda Corley [5] [2], Jonathan Millar [7], Jonathon Fanning [5] [2] [8], India Lye [5] [2], Sebastiano Colombo [5] [2] [9], Karin Wildi [5] [2], Samantha Livingstone [5] [2], Gabriella Abbate [5], Samuel Hinton [2], Benoit Liquet [10] [11], Sally Shrapnel [10], Heidi Dalton [12], John F Fraser [5] [2] [3] [8], COVID-19 Critical Care Consortium Investigators

Affiliations

- [1] Critical Care Research Group, Prince Charles Hospital, Chermside, Queensland, Australia g.libassi@uq.edu.au.
- [2] Faculty of Medicine, University of Queensland, Brisbane, Queensland, Australia.
- [3] Queensland University of Technology, Brisbane, Queensland, Australia.
- [4] Institut d'Investigacions Biomèdiques August Pi i Sunyer, Barcelona, Spain.
- [5] Critical Care Research Group, Prince Charles Hospital, Chermside, Queensland, Australia.
- [6] Institute of Health and Biomedical Innovation, Queensland University of Technology, Brisbane, Queensland, Australia.
- [7] Roslin Institute, University of Edinburgh, Edinburgh, United Kingdom.
- [8] Critical Care Medicine, UnitingCare Health, Brisbane, Queensland, Australia.
- [9] Department of Pathophysiology and Transplantation, University of Milan, Milan, Italy.
- [10] University of Queensland, Brisbane, Queensland, Australia.
- [11] University of Pau et Pays De L'Adour, Pau, France.
- [12] Inova Fairfax Medical Campus, Falls Church, Virginia, USA.

[#] Contributed equally.

- PMID: **33268426**
- PMCID: PMC7714653
- DOI: 10.1136/bmjopen-2020-041417

Abstract

Introduction: There is a paucity of data that can be used to guide the management of critically ill patients with COVID-19. In response, a research and data-sharing collaborative-The COVID-19

Critical Care Consortium-has been assembled to harness the cumulative experience of intensive care units (ICUs) worldwide. The resulting observational study provides a platform to rapidly disseminate detailed data and insights crucial to improving outcomes.

Methods and analysis: This is an international, multicentre, observational study of patients with confirmed or suspected SARS-CoV-2 infection admitted to ICUs. This is an evolving, open-ended study that commenced on 1 January 2020 and currently includes >350 sites in over 48 countries. The study enrols patients at the time of ICU admission and follows them to the time of death, hospital discharge or 28 days post-ICU admission, whichever occurs last. Key data, collected via an electronic case report form devised in collaboration with the International Severe Acute Respiratory and Emerging Infection Consortium/Short Period Incidence Study of Severe Acute Respiratory Illness networks, include: patient demographic data and risk factors, clinical features, severity of illness and respiratory failure, need for non-invasive and/or mechanical ventilation and/or extracorporeal membrane oxygenation and associated complications, as well as data on adjunctive therapies.

Ethics and dissemination: Local principal investigators will ensure that the study adheres to all relevant national regulations, and that the necessary approvals are in place before a site may contribute data. In jurisdictions where a waiver of consent is deemed insufficient, prospective, representative or retrospective consent will be obtained, as appropriate. A web-based dashboard has been developed to provide relevant data and descriptive statistics to international collaborators in real-time. It is anticipated that, following study completion, all de-identified data will be made open access.

Trial registration number: ACTRN12620000421932 (http://anzctr.org.au/ACTRN12620000421932.aspx).

Keywords: epidemiology; intensive & critical care; public health; respiratory infections.

© Author(s) (or their employer(s)) 2020. Re-use permitted under CC BY-NC. No commercial re-use. See rights and permissions. Published by BMJ.

Conflict of interest statement

Competing interests: GLB and JFF received research funds, through their affiliated institution, from Fisher & Paykel for studies related to high-flow oxygen therapy.

- 22 references
- 1 figure

Supplementary info

Publication types, MeSH terms Expand

Publication types

- Clinical Trial Protocol
- Multicenter Study
- Research Support, Non-U.S. Gov't

MeSH terms

- COVID-19 / mortality
- COVID-19 / therapy*
- Evidence-Based Medicine
- Global Health
- Humans
- Intensive Care Units / statistics & numerical data*
- Observational Studies as Topic
- Outcome Assessment, Health Care
- Pandemics
- Pragmatic Clinical Trials as Topic
- Registries*
- SARS-CoV-2

Full text links

BMJ Free Full Text HighWire Free PMC article

Proceed to details

Cite

Share

125

Observational Study

Antimicrob Resist Infect Control

. 2021 Aug 5;10(1):114.

doi: 10.1186/s13756-021-00984-x.

SARS-CoV-2 nosocomial infection acquired in a French university hospital during the 1st wave of the Covid-19 pandemic, a prospective study

A Landoas [1], F Cazzorla [1], M Gallouche [1] [2], S Larrat [3], B Nemoz [3] [4], C Giner [1], M Le Maréchal [5], P Pavese [5], O Epaulard [5], P Morand [3] [4], M-R Mallaret [1] [2], C Landelle [6] [7] [8]

Affiliations Expand

Affiliations

- [1] Infection Control Unit, Grenoble Alpes University Hospital, Grenoble, France.
- [2] Grenoble Alpes University/CNRS, Grenoble INP, MESP TIM-C UMR 5525, Grenoble, France.

- [3] Virology Laboratory, Grenoble Alpes University Hospital, Grenoble, France.
- [4] Grenoble Alpes University/CNRS/CEA, Institut de Biologie Structurale (IBS), HIV and persistent viral infections, Grenoble, France.
- [5] Infectious Diseases Department, Grenoble Alpes University Hospital, Grenoble, France.
- [6] Infection Control Unit, Grenoble Alpes University Hospital, Grenoble, France. caroline.landelle@gmail.com.
- [7] Grenoble Alpes University/CNRS, Grenoble INP, MESP TIM-C UMR 5525, Grenoble, France. caroline.landelle@gmail.com.
- [8] Hospital Hygiene Department, Pavilion E - Grenoble Alpes University Hospital, CS 10217, 38043, Grenoble Cedex 9, France. caroline.landelle@gmail.com.

- PMID: **34353356**
- PMCID: PMC8339707
- DOI: 10.1186/s13756-021-00984-x

Free PMC article
Observational Study

SARS-CoV-2 nosocomial infection acquired in a French university hospital during the 1st wave of the Covid-19 pandemic, a prospective study

A Landoas et al. Antimicrob Resist Infect Control. 2021.
Free PMC article
Show details
Antimicrob Resist Infect Control
. 2021 Aug 5;10(1):114.
doi: 10.1186/s13756-021-00984-x.

Authors

A Landoas [1], F Cazzorla [1], M Gallouche [1, 2], S Larrat [3], B Nemoz [3, 4], C Giner [1], M Le Maréchal [5], P Pavese [5], O Epaulard [5], P Morand [3, 4], M-R Mallaret [1, 2], C Landelle [6, 7, 8]

Affiliations

- [1] Infection Control Unit, Grenoble Alpes University Hospital, Grenoble, France.
- [2] Grenoble Alpes University/CNRS, Grenoble INP, MESP TIM-C UMR 5525, Grenoble, France.
- [3] Virology Laboratory, Grenoble Alpes University Hospital, Grenoble, France.
- [4] Grenoble Alpes University/CNRS/CEA, Institut de Biologie Structurale (IBS), HIV and persistent viral infections, Grenoble, France.
- [5] Infectious Diseases Department, Grenoble Alpes University Hospital, Grenoble, France.
- [6] Infection Control Unit, Grenoble Alpes University Hospital, Grenoble, France. caroline.landelle@gmail.com.

- [7] Grenoble Alpes University/CNRS, Grenoble INP, MESP TIM-C UMR 5525, Grenoble, France. caroline.landelle@gmail.com.
- [8] Hospital Hygiene Department, Pavilion E - Grenoble Alpes University Hospital, CS 10217, 38043, Grenoble Cedex 9, France. caroline.landelle@gmail.com.

- PMID: **34353356**
- PMCID: PMC8339707
- DOI: 10.1186/s13756-021-00984-x

Abstract

Background: In healthcare facilities, nosocomial transmissions of respiratory viruses are a major issue. SARS-CoV-2 is not exempt from nosocomial transmission. Our goals were to describe COVID-19 nosocomial cases during the first pandemic wave among patients in a French university hospital and compliance with hygiene measures.

Methods: We conducted a prospective observational study in Grenoble Alpes University Hospital from 01/03/2020 to 11/05/2020. We included all hospitalised patients with a documented SARS-CoV-2 diagnosis. Nosocomial case was defined by a delay of 5 days between hospitalisation and first symptoms. Hygiene measures were evaluated between 11/05/2020 and 22/05/2020. Lockdown measures were effective in France on 17/03/2020 and ended on 11/05/2020. Systematic wearing of mask was mandatory for all healthcare workers (HCW) and visits were prohibited in our institution from 13/03/2021 and for the duration of the lockdown period.

Results: Among 259 patients included, 14 (5.4%) were considered as nosocomial COVID-19. Median time before symptom onset was 25 days (interquartile range: 12-42). Eleven patients (79%) had risk factors for severe COVID-19. Five died (36%) including 4 deaths attributable to COVID-19. Two clusters were identified. The first cluster had 5 cases including 3 nosocomial acquisitions and no tested HCWs were positive. The second cluster had 3 cases including 2 nosocomial cases and 4 HCWs were positive. Surgical mask wearing and hand hygiene compliance were adequate for 95% and 61% of HCWs, respectively.

Conclusions: The number of nosocomial COVID-19 cases in our hospital was low. Compliance regarding mask wearing, hand hygiene and lockdown measures drastically reduced transmission of the virus. Monitoring of nosocomial COVID-19 cases during the first wave enabled us to determine to what extent the hygiene measures taken were effective and patients protected. Trial registration Study ethics approval was obtained retrospectively on 30 September 2020 (CECIC Rhône-Alpes-Auvergne, Clermont-Ferrand, IRB 5891).

Keywords: COVID-19; Hand hygiene; Healthcare-associated infection; Mask; Outbreak; SARS-CoV-2.

© 2021. The Author(s).

Conflict of interest statement

All authors report no conflicts of interest relevant to this article. An abstract containing partial data was presented at the ESCMID Conference on Coronavirus Disease (ECCVID), 2020, online conference.

- 32 references
- 1 figure

Supplementary info

Publication types, MeSH terms Expand

Publication types

- Observational Study

MeSH terms

- Aged
- Aged, 80 and over
- COVID-19 / diagnosis
- COVID-19 / epidemiology*
- COVID-19 / virology
- COVID-19 Testing / methods
- Cross Infection / epidemiology*
- Cross Infection / virology
- Female
- France / epidemiology
- Hand Hygiene / methods
- Health Personnel
- Hospitals, University / statistics & numerical data
- Humans
- Infection Control / methods
- Male
- Masks / microbiology
- Middle Aged
- Pandemics
- Prospective Studies
- Retrospective Studies
- SARS-CoV-2 / isolation & purification*

Full text links

Read free full text at BMC BioMed Central Free PMC article
Proceed to details
Cite
Share
☐ 126
Observational Study
Eur Rev Med Pharmacol Sci
. 2021 Jan;25(2):1158-1162.

doi: 10.26355/eurrev_202101_24686.

Impact of the COVID-19 pandemic on clinical research in hospitals: observational study in the first epicenter of the epidemic during the general lockdown in France

G Becker [1], T Martin, A N Sabo, F Bertrand, A Hutt, E Ayme-Dietrich, B Michel, L Monassier, B Gourieux
Affiliations Expand

Affiliation

- [1] Pôle Pharmacie-Pharmacologie, Hôpitaux Universitaires de Strasbourg, Strasbourg, France. g.becker@unistra.fr.

- PMID: **33577072**
- DOI: 10.26355/eurrev_202101_24686

Free article
Observational Study

Impact of the COVID-19 pandemic on clinical research in hospitals: observational study in the first epicenter of the epidemic during the general lockdown in France

G Becker et al. Eur Rev Med Pharmacol Sci. 2021 Jan.
Free article
Show details
Eur Rev Med Pharmacol Sci
. 2021 Jan;25(2):1158-1162.
doi: 10.26355/eurrev_202101_24686.

Authors

G Becker [1], T Martin, A N Sabo, F Bertrand, A Hutt, E Ayme-Dietrich, B Michel, L Monassier, B Gourieux

Affiliation

- [1] Pôle Pharmacie-Pharmacologie, Hôpitaux Universitaires de Strasbourg, Strasbourg, France. g.becker@unistra.fr.

- PMID: **33577072**
- DOI: 10.26355/eurrev_202101_24686

Abstract

Objective: The COVID-19 epidemic has had a strong impact on the entire healthcare sector in France with priority being given to research for new therapeutic options for COVID-19. Nevertheless, continuity of care for patients suffering from other diseases represents a crucial challenge, and clinical research is no exception in this respect. This study aims to assess the impact of the strict Covid-19 lockdown on non-Covid-19 clinical research in the French University Hospital of Strasbourg.

Materials and methods: Clinical research activity (non-Covid-19) from the point of view of pharmacy department was estimated and compared to the pre-lockdown period. The impact of lockdown was assessed through five indicators: site initiation visits, the initiation of experimental therapies in non-Covid-19 patients, the delivery of non-Covid-19 investigational medical products, the number of drug shipments to patients' homes, and the number of monitoring or closure visits.

Results: During the study period, the number of site initiation visits decreased by 90%, total inclusions by 72%, and delivery of investigational medical products by 30%. During the lockdown period, 15 treatments were sent to patients' homes. Monitoring activity decreased by 98%.

Conclusions: Although the COVID-19 outbreak has created an incredible momentum in the field of clinical research, research not focused on SaRS-CoV-2 has suffered greatly from this situation. The impact on patients is difficult to estimate but should be further investigated.

Supplementary info

Publication types, MeSH terms Expand

Publication types

- Observational Study

MeSH terms

- Biomedical Research / trends*
- COVID-19 / epidemiology*
- COVID-19 / prevention & control
- COVID-19 / therapy
- Clinical Trials as Topic*
- France / epidemiology
- Hospitals, University / trends*
- Humans
- Pandemics*

- Quarantine / trends*
- Retrospective Studies

Full text links

FREE FULL TEXT European Review for Medical and Pharmacological Sciences
Proceed to details
Cite
Share
127
Observational Study
Respir Med

. 2021 Jan;176:106271.
doi: 10.1016/j.rmed.2020.106271. Epub 2020 Nov 28.

Predictive value of chest CT scoring in COVID-19 patients in Wuhan, China: A retrospective cohort study

Shu Li [1], Shaoyu Liu [1], Ben Wang [2], Qiuyu Li [3], Hua Zhang [4], Lin Zeng [4], Hongxia Ge [1], Qingbian Ma [1], Ning Shen [5]
Affiliations Expand

Affiliations

- [1] Department of Emergency Medicine, Peking University Third Hospital, Beijing, China.
- [2] Department of Orthopedics, Peking University Third Hospital, Beijing, China.
- [3] Department of Pulmonary and Critical Care Medicine, Peking University Third Hospital, Beijing, China.
- [4] Clinical Epidemiology Research Center, Peking University Third Hospital, Beijing, China.
- [5] Department of Pulmonary and Critical Care Medicine, Peking University Third Hospital, Beijing, China. Electronic address: puh3shenning@bjmu.edu.cn.

- PMID: **33296777**
- PMCID: PMC7695948
- DOI: 10.1016/j.rmed.2020.106271

Free PMC article
Observational Study

Predictive value of chest CT scoring in COVID-19 patients in Wuhan, China: A retrospective cohort study

Shu Li et al. Respir Med. 2021 Jan.
Free PMC article
Show details
Respir Med
. 2021 Jan;176:106271.
doi: 10.1016/j.rmed.2020.106271. Epub 2020 Nov 28.

Authors

Shu Li [1], Shaoyu Liu [1], Ben Wang [2], Qiuyu Li [3], Hua Zhang [4], Lin Zeng [4], Hongxia Ge [1], Qingbian Ma [1], Ning Shen [5]

Affiliations

- [1] Department of Emergency Medicine, Peking University Third Hospital, Beijing, China.
- [2] Department of Orthopedics, Peking University Third Hospital, Beijing, China.
- [3] Department of Pulmonary and Critical Care Medicine, Peking University Third Hospital, Beijing, China.
- [4] Clinical Epidemiology Research Center, Peking University Third Hospital, Beijing, China.
- [5] Department of Pulmonary and Critical Care Medicine, Peking University Third Hospital, Beijing, China. Electronic address: puh3shenning@bjmu.edu.cn.

- PMID: **33296777**
- PMCID: PMC7695948
- DOI: 10.1016/j.rmed.2020.106271

Abstract

Background: Computed tomography (CT) findings of COVID-19 patients were demonstrated by cases series and descriptive studies, but quantitative analysis performed by clinical doctors and studies on its predictive value were rarely seen. The aim of the study is to analyze CT score in COVID-19 patients and explore its predictive value.

Materials and methods: We conducted a retrospective cohort study among confirmed COVID -19 patients with available CT images between February 8, 2020 and March 7, 2020. The lung was divided into six zones by the level of tracheal carina and the level of inferior pulmonary vein bilaterally on CT. Ground-glass opacity (GGO), consolidation, crazy-paving pattern and overall lung involvement were rated by Likert scale of 0-4 or binary as 0 or 1. Global severity score for each targeted pattern was calculated as total score of six zones.

Results: There were 53 patients and 137 CT scans included in the study. There were 18(34%) of the patients classified as moderate cases while 35(66%) patients were severe/critical cases. Severe/critical patients had higher CT scores in several types of abnormalities than moderate

patients from the second week to the fourth week post symptom onset. Overall lung involvement score in the second week demonstrated predictive value for severity with a sensitivity of 81.0% and specificity of 69.2%.

Conclusions: Our modified semi-quantitative CT scoring system for COVID-19 patients demonstrated feasibility. Overall lung involvement score on the second week had predictive value for clinical severity and could be indicator for further treatment.

Keywords: COVID-19; Computed tomography; Pneumonia; Prognosis; Severity of illness.

Copyright © 2020 The Authors. Published by Elsevier Ltd.. All rights reserved.

Conflict of interest statement

The authors have disclosed that there is no financial, consultant, institutional, and other relationships that might lead to bias or a conflict of interest.

- 23 references
- 4 figures

Supplementary info

Publication types, MeSH terms Expand

Publication types

- Observational Study
- Research Support, Non-U.S. Gov't

MeSH terms

- Adult
- Aged
- COVID-19 / complications
- COVID-19 / diagnostic imaging*
- COVID-19 / therapy
- China
- Female
- Hospitalization
- Humans
- Lung / diagnostic imaging*
- Male
- Middle Aged
- Predictive Value of Tests
- ROC Curve
- Retrospective Studies

- Severity of Illness Index
- Tomography, X-Ray Computed*

Full text links

ELSEVIER OPEN ACCESS Elsevier Science Free PMC article
Proceed to details
Cite
Share
☐ 128
Observational Study
RMD Open
. 2021 Jan;7(1):e001464.
doi: 10.1136/rmdopen-2020-001464.

Older age, comorbidity, glucocorticoid use and disease activity are risk factors for COVID-19 hospitalisation in patients with inflammatory rheumatic and musculoskeletal diseases

Rebecca Hasseli [1], Ulf Mueller-Ladner [1], Bimba F Hoyer [2], Andreas Krause [3], Hanns-Martin Lorenz [4], Alexander Pfeil [5], Jutta Richter [6], Martin Schäfer [7], Tim Schmeiser [8], Anja Strangfeld [7], Hendrik Schulze-Koops [9], Reinhard E Voll [10], Christof Specker [# 11], Anne Constanze Regierer [# 12]
Affiliations Expand

Affiliations

- [1] Department of Rheumatology and Clinical Immunology, Campus Kerkhoff, Justus-Liebig-University, Giessen, Giessen, Germany.
- [2] Department of Rheumatology and Clinical Immunology, Clinic for Internal Medicine I, University of Schleswig-Holstein at Kiel, Kiel, Germany.
- [3] Department of Rheumatology, Clinical Immunology and Osteology, Immanuel Hospital, Berlin, Germany.
- [4] Department of Rheumatology, University Hospital, Heidelberg, Germany.
- [5] Department of Internal Medicine III, Universitatsklinikum Jena, Jena, Germany.
- [6] Department of Rheumatology and Hiller Research Unit, Medical Faculty, Heinrich-Heine-Universitat Dusseldorf, Dusseldorf, Nordrhein-Westfalen, Germany.
- [7] Epidemiology Unit, German Rheumatism Research Center Berlin, Berlin, Germany.
- [8] Department of Rheumatology and Immunology, Saint Josef Hospital, Wuppertal, Germany.

- [9] Division of Rheumatology and Clinical Immunology, Department of Internal Medicine IV, University of Munich, Munich, Germany.
- [10] Dep. of Rheumatology and Clinical Immunology, University of Freiburg Faculty of Medicine, Freiburg, Germany.
- [11] Department of Rheumatology and Clinical Immunology, Kliniken Essen-Mitte, Essen, Germany.
- [12] Epidemiology Unit, German Rheumatism Research Center Berlin, Berlin, Germany Anne.Regierer@drfz.de.

[#] Contributed equally.

- PMID: **33479021**
- PMCID: PMC7823432
- DOI: 10.1136/rmdopen-2020-001464

Free PMC article
Observational Study

Older age, comorbidity, glucocorticoid use and disease activity are risk factors for COVID-19 hospitalisation in patients with inflammatory rheumatic and musculoskeletal diseases

Rebecca Hasseli et al. RMD Open. 2021 Jan.
Free PMC article
Show details
RMD Open
. 2021 Jan;7(1):e001464.
doi: 10.1136/rmdopen-2020-001464.

Authors

Rebecca Hasseli [1], Ulf Mueller-Ladner [1], Bimba F Hoyer [2], Andreas Krause [3], Hanns-Martin Lorenz [4], Alexander Pfeil [5], Jutta Richter [6], Martin Schäfer [7], Tim Schmeiser [8], Anja Strangfeld [7], Hendrik Schulze-Koops [9], Reinhard E Voll [10], Christof Specker [#] [11], Anne Constanze Regierer [#] [12]

Affiliations

- [1] Department of Rheumatology and Clinical Immunology, Campus Kerkhoff, Justus-Liebig-University, Giessen, Giessen, Germany.
- [2] Department of Rheumatology and Clinical Immunology, Clinic for Internal Medicine I, University of Schleswig-Holstein at Kiel, Kiel, Germany.

- [3] Department of Rheumatology, Clinical Immunology and Osteology, Immanuel Hospital, Berlin, Germany.
- [4] Department of Rheumatology, University Hospital, Heidelberg, Germany.
- [5] Department of Internal Medicine III, Universitatsklinikum Jena, Jena, Germany.
- [6] Department of Rheumatology and Hiller Research Unit, Medical Faculty, Heinrich-Heine-Universitat Dusseldorf, Dusseldorf, Nordrhein-Westfalen, Germany.
- [7] Epidemiology Unit, German Rheumatism Research Center Berlin, Berlin, Germany.
- [8] Department of Rheumatology and Immunology, Saint Josef Hospital, Wuppertal, Germany.
- [9] Division of Rheumatology and Clinical Immunology, Department of Internal Medicine IV, University of Munich, Munich, Germany.
- [10] Dep. of Rheumatology and Clinical Immunology, University of Freiburg Faculty of Medicine, Freiburg, Germany.
- [11] Department of Rheumatology and Clinical Immunology, Kliniken Essen-Mitte, Essen, Germany.
- [12] Epidemiology Unit, German Rheumatism Research Center Berlin, Berlin, Germany Anne.Regierer@drfz.de.

[#] Contributed equally.

- PMID: **33479021**
- PMCID: PMC7823432
- DOI: 10.1136/rmdopen-2020-001464

Abstract

Introduction: Whether patients with inflammatory rheumatic and musculoskeletal diseases (RMD) are at higher risk to develop severe courses of COVID-19 has not been fully elucidated. Aim of this analysis was to describe patients with RMD according to their COVID-19 severity and to identify risk factors for hospitalisation.

Methods: Patients with RMD with PCR confirmed SARS-CoV-2 infection reported to the German COVID-19 registry from 30 March to 1 November 2020 were evaluated. Multivariable logistic regression was used to estimate ORs for hospitalisation due to COVID-19.

Results: Data from 468 patients with RMD with SARS-CoV-2 infection were reported. Most frequent diagnosis was rheumatoid arthritis, RA (48%). 29% of the patients were hospitalised, 5.5% needed ventilation. 19 patients died. Multivariable analysis showed that age >65 years (OR 2.24; 95% CI 1.12 to 4.47), but even more>75 years (OR 3.94; 95% CI 1.86 to 8.32), cardiovascular disease (CVD; OR 3.36; 95% CI 1.5 to 7.55), interstitial lung disease/chronic obstructive pulmonary disease (ILD/COPD) (OR 2.79; 95% CI 1.2 to 6.49), chronic kidney disease (OR 2.96; 95% CI 1.16 to 7.5), moderate/high RMD disease activity (OR 1.96; 95% CI 1.02 to 3.76) and treatment with glucocorticoids (GCs) in dosages >5 mg/day (OR 3.67; 95% CI 1.49 to 9.05) were associated with higher odds of hospitalisation. Spondyloarthritis patients showed a smaller risk of hospitalisation compared with RA (OR 0.46; 95% CI 0.23 to 0.91).

Conclusion: Age was a major risk factor for hospitalisation as well as comorbidities such as CVD, ILD/COPD, chronic kidney disease and current or prior treatment with GCs. Moderate to high RMD disease activity was also an independent risk factor for hospitalisation, underlining the importance of continuing adequate RMD treatment during the pandemic.

Keywords: arthritis; epidemiology; glucocorticoids.

© Author(s) (or their employer(s)) 2021. Re-use permitted under CC BY-NC. No commercial re-use. See rights and permissions. Published by BMJ.

Conflict of interest statement

Competing interests: None declared.

- 32 references
- 1 figure

Supplementary info

Publication types, MeSH terms, Substances Expand

Publication types

- Observational Study
- Research Support, Non-U.S. Gov't

MeSH terms

- Adult
- Age Factors
- Aged
- Aged, 80 and over
- Arthritis, Rheumatoid / complications
- Arthritis, Rheumatoid / drug therapy
- COVID-19 / diagnosis*
- COVID-19 / epidemiology
- COVID-19 / therapy
- COVID-19 / virology
- Case-Control Studies
- Comorbidity
- Female
- Germany / epidemiology
- Glucocorticoids / adverse effects*
- Glucocorticoids / therapeutic use
- Hospitalization / statistics & numerical data
- Humans
- Male
- Middle Aged
- Musculoskeletal Diseases / complications*
- Musculoskeletal Diseases / drug therapy

- Registries
- Respiration, Artificial / methods
- Retrospective Studies
- Rheumatic Diseases / complications*
- Rheumatic Diseases / drug therapy
- Risk Factors
- SARS-CoV-2 / genetics*

Substances

- Glucocorticoids

Full text links

BMJ Free Full Text HighWire Free PMC article
Proceed to details
Cite
Share
129
Observational Study
PLoS One
. 2020 Nov 13;15(11):e0240781.
doi: 10.1371/journal.pone.0240781. eCollection 2020.

Epidemiology, risk factors and clinical course of SARS-CoV-2 infected patients in a Swiss university hospital: An observational retrospective study

Jean Regina [1], Matthaios Papadimitriou-Olivgeris [1, 2], Raphaël Burger [1, 3], Marie-Annick Le Pogam [4], Tapio Niemi [4], Paraskevas Filippidis [1], Jonathan Tschopp [1], Florian Desgranges [1], Benjamin Viala [1], Eleftheria Kampouri [1], Laurence Rochat [1], David Haefliger [1], Mehdi Belkoniene [1, 3], Carlos Fidalgo [1, 3], Antonios Kritikos [1], Katia Jaton [5], Laurence Senn [2], Pierre-Alexandre Bart [3], Jean-Luc Pagani [6], Oriol Manuel [1], Loïc Lhopitallier [1]
Affiliations Expand

Affiliations

- [1] Service of Infectious Diseases, Lausanne University Hospital and University of Lausanne, Lausanne, Switzerland.
- [2] Service of Hospital Preventive Medicine, Lausanne University Hospital and University of Lausanne, Lausanne, Switzerland.

- [3] Service of Internal Medicine, Lausanne University Hospital and University of Lausanne, Lausanne, Switzerland.
- [4] Centre for Primary Care and Public Health (Unisanté), University of Lausanne, Lausanne, Switzerland.
- [5] Institute of Microbiology, Lausanne University Hospital and University of Lausanne, Lausanne, Switzerland.
- [6] Service of Intensive Care, Lausanne University Hospital and University of Lausanne, Lausanne, Switzerland.

- PMID: **33186355**
- PMCID: PMC7665644
- DOI: 10.1371/journal.pone.0240781

Free PMC article
Observational Study

Epidemiology, risk factors and clinical course of SARS-CoV-2 infected patients in a Swiss university hospital: An observational retrospective study

Jean Regina et al. PLoS One. 2020.
Free PMC article
Show details
PLoS One
. 2020 Nov 13;15(11):e0240781.
doi: 10.1371/journal.pone.0240781. eCollection 2020.

Authors

Jean Regina [1], Matthaios Papadimitriou-Olivgeris [1 2], Raphaël Burger [1 3], Marie-Annick Le Pogam [4], Tapio Niemi [4], Paraskevas Filippidis [1], Jonathan Tschopp [1], Florian Desgranges [1], Benjamin Viala [1], Eleftheria Kampouri [1], Laurence Rochat [1], David Haefliger [1], Mehdi Belkoniene [1 3], Carlos Fidalgo [1 3], Antonios Kritikos [1], Katia Jaton [5], Laurence Senn [2], Pierre-Alexandre Bart [3], Jean-Luc Pagani [6], Oriol Manuel [1], Loïc Lhopitallier [1]

Affiliations

- [1] Service of Infectious Diseases, Lausanne University Hospital and University of Lausanne, Lausanne, Switzerland.
- [2] Service of Hospital Preventive Medicine, Lausanne University Hospital and University of Lausanne, Lausanne, Switzerland.
- [3] Service of Internal Medicine, Lausanne University Hospital and University of Lausanne, Lausanne, Switzerland.
- [4] Centre for Primary Care and Public Health (Unisanté), University of Lausanne, Lausanne, Switzerland.

- [5] Institute of Microbiology, Lausanne University Hospital and University of Lausanne, Lausanne, Switzerland.
- [6] Service of Intensive Care, Lausanne University Hospital and University of Lausanne, Lausanne, Switzerland.

- PMID: **33186355**
- PMCID: PMC7665644
- DOI: 10.1371/journal.pone.0240781

Abstract

Background: This study aims to describe the epidemiology of COVID-19 patients in a Swiss university hospital.

Methods: This retrospective observational study included all adult patients hospitalized with a laboratory confirmed SARS-CoV-2 infection from March 1 to March 25, 2020. We extracted data from electronic health records. The primary outcome was the need to mechanical ventilation at day 14. We used multivariate logistic regression to identify risk factors for mechanical ventilation. Follow-up was of at least 14 days.

Results: 145 patients were included in the multivariate model, of whom 36 (24.8%) needed mechanical ventilation at 14 days. The median time from symptoms onset to mechanical ventilation was 9·5 days (IQR 7.00, 12.75). Multivariable regression showed increased odds of mechanical ventilation with age (OR 1.09 per year, 95% CI 1.03-1.16, p = 0.002), in males (OR 6.99, 95% CI 1.68-29.03, p = 0.007), in patients who presented with a qSOFA score ≥2 (OR 7.24, 95% CI 1.64-32.03, p = 0.009), with bilateral infiltrate (OR 18.92, 3.94-98.23, p<0.001) or with a CRP of 40 mg/l or greater (OR 5.44, 1.18-25.25; p = 0.030) on admission. Patients with more than seven days of symptoms on admission had decreased odds of mechanical ventilation (0.087, 95% CI 0.02-0.38, p = 0.001).

Conclusions: This study gives some insight in the epidemiology and clinical course of patients admitted in a European tertiary hospital with SARS-CoV-2 infection. Age, male sex, high qSOFA score, CRP of 40 mg/l or greater and a bilateral radiological infiltrate could help clinicians identify patients at high risk for mechanical ventilation.

Conflict of interest statement

The authors have declared that no competing interests exist.

- 27 references
- 1 figure

Supplementary info

Publication types, MeSH terms, Grant support Expand

Publication types

- Observational Study

MeSH terms

- Adolescent
- Adult
- Aged
- Aged, 80 and over
- Betacoronavirus
- COVID-19
- Coronavirus Infections / epidemiology*
- Electronic Health Records
- Female
- Hospitalization
- Hospitals, University
- Humans
- Logistic Models
- Male
- Middle Aged
- Multivariate Analysis
- Pandemics
- Pneumonia, Viral / epidemiology*
- Respiration, Artificial / statistics & numerical data*
- Retrospective Studies
- Risk Factors
- SARS-CoV-2
- Switzerland
- Tertiary Care Centers
- Young Adult

Grant support

The author(s) received no specific funding for this work.

Full text links

OPEN ACCESS TO FULL TEXT PLOS ONE Public Library of Science Free PMC article

Proceed to details

Cite

Share

☐ 130

Observational Study

J Med Internet Res

. 2021 Feb 10;23(2):e24246.

doi: 10.2196/24246.

A Machine Learning Prediction Model of Respiratory Failure Within 48 Hours of Patient Admission for COVID-19: Model Development and Validation

Siavash Bolourani [1], Max Brenner [1], Ping Wang [1], Thomas McGinn [1], Jamie S Hirsch [1], Douglas Barnaby [# 1], Theodoros P Zanos [# 1], Northwell COVID-19 Research Consortium [2]
Collaborators, Affiliations Expand

Collaborators

- **Northwell COVID-19 Research Consortium**: Matthew Barish, Stuart Cohen, Kevin Coppa, Karina Davidson, Shubham Debnath, Lawrence Lau, Todd Levy, Alexander Makhnevich, Marc Paradis, Viktor Tóth

Affiliations

- [1] Feinstein Institutes for Medical Research, Northwell Health, Manhasset, NY, United States.
- [2] See Acknowledgments, .

[#] Contributed equally.

- PMID: **33476281**
- PMCID: PMC7879728
- DOI: 10.2196/24246

Free PMC article
Observational Study

A Machine Learning Prediction Model of Respiratory Failure Within 48 Hours of Patient Admission for COVID-19: Model Development and Validation

Siavash Bolourani et al. J Med Internet Res. 2021.
Free PMC article
Show details

J Med Internet Res
. 2021 Feb 10;23(2):e24246.
doi: 10.2196/24246.

Authors

Siavash Bolourani [1], Max Brenner [1], Ping Wang [1], Thomas McGinn [1], Jamie S Hirsch [1], Douglas Barnaby [#] [1], Theodoros P Zanos [#] [1], Northwell COVID-19 Research Consortium [2]

Collaborators

- **Northwell COVID-19 Research Consortium**:
  Matthew Barish, Stuart Cohen, Kevin Coppa, Karina Davidson, Shubham Debnath, Lawrence Lau, Todd Levy, Alexander Makhnevich, Marc Paradis, Viktor Tóth

Affiliations

- [1] Feinstein Institutes for Medical Research, Northwell Health, Manhasset, NY, United States.
- [2] See Acknowledgments, .

[#] Contributed equally.

- PMID: **33476281**
- PMCID: PMC7879728
- DOI: 10.2196/24246

Abstract

Background: Predicting early respiratory failure due to COVID-19 can help triage patients to higher levels of care, allocate scarce resources, and reduce morbidity and mortality by appropriately monitoring and treating the patients at greatest risk for deterioration. Given the complexity of COVID-19, machine learning approaches may support clinical decision making for patients with this disease.

Objective: Our objective is to derive a machine learning model that predicts respiratory failure within 48 hours of admission based on data from the emergency department.

Methods: Data were collected from patients with COVID-19 who were admitted to Northwell Health acute care hospitals and were discharged, died, or spent a minimum of 48 hours in the hospital between March 1 and May 11, 2020. Of 11,525 patients, 933 (8.1%) were placed on invasive mechanical ventilation within 48 hours of admission. Variables used by the models included clinical and laboratory data commonly collected in the emergency department. We trained and validated three predictive models (two based on XGBoost and one that used logistic regression) using cross-hospital validation. We compared model performance among all three models as well as an established early warning score (Modified Early Warning Score) using receiver operating characteristic curves, precision-recall curves, and other metrics.

Results: The XGBoost model had the highest mean accuracy (0.919; area under the curve=0.77), outperforming the other two models as well as the Modified Early Warning Score. Important predictor variables included the type of oxygen delivery used in the emergency department, patient age, Emergency Severity Index level, respiratory rate, serum lactate, and demographic characteristics.

Conclusions: The XGBoost model had high predictive accuracy, outperforming other early warning scores. The clinical plausibility and predictive ability of XGBoost suggest that the model could be used to predict 48-hour respiratory failure in admitted patients with COVID-19.

Keywords: COVID-19; artificial intelligence; development; machine learning; model; modeling; pandemic; prognostic; severe acute respiratory syndrome coronavirus 2; validation.

©Siavash Bolourani, Max Brenner, Ping Wang, Thomas McGinn, Jamie S Hirsch, Douglas Barnaby, Theodoros P Zanos, Northwell COVID-19 Research Consortium. Originally published in the Journal of Medical Internet Research (http://www.jmir.org), 10.02.2021.

Conflict of interest statement

Conflicts of Interest: None declared.

- 52 references
- 4 figures

Supplementary info

Publication types, MeSH terms, Grant support Expand

Publication types

- Observational Study
- Validation Study

MeSH terms

- Aged
- COVID-19 / complications
- COVID-19 / physiopathology*
- Clinical Decision Rules
- Early Warning Score
- Emergency Service, Hospital
- Female
- Hospitalization*
- Hospitals
- Humans
- Intubation, Intratracheal / statistics & numerical data*
- Logistic Models
- Machine Learning*
- Male
- Middle Aged
- Patient Admission
- ROC Curve

- Respiration, Artificial / statistics & numerical data*
- Respiratory Insufficiency / epidemiology*
- Respiratory Insufficiency / etiology
- Retrospective Studies
- SARS-CoV-2
- Triage

Grant support

- R01 LM012836/LM/NLM NIH HHS/United States
- R24 AG064191/AG/NIA NIH HHS/United States
- R35 GM118337/GM/NIGMS NIH HHS/United States

Full text links

JMIR Publications Free PMC article

Proceed to details

Cite

Share

131

Observational Study

PLoS One

. 2020 Nov 25;15(11):e0240206.

doi: 10.1371/journal.pone.0240206. eCollection 2020.

Characterisation of 22445 patients attending UK emergency departments with suspected COVID-19 infection: Observational cohort study

Steve Goodacre [1], Ben Thomas [1], Ellen Lee [1], Laura Sutton [1], Amanda Loban [1], Simon Waterhouse [1], Richard Simmonds [1], Katie Biggs [1], Carl Marincowitz [1], Jose Schutter [1], Sarah Connelly [1], Elena Sheldon [1], Jamie Hall [1], Emma Young [1], Andrew Bentley [2], Kirsty Challen [3], Chris Fitzsimmons [4], Tim Harris [5], Fiona Lecky [1], Andrew Lee [1], Ian Maconochie [6], Darren Walter [7]

Affiliations Expand

Affiliations

- [1] School of Health and Related Research (ScHARR), University of Sheffield, Sheffield, United Kingdom.
- [2] Intensive Care, Manchester University NHS Foundation Trust, Wythenshawe Hospital, Manchester, United Kingdom.

- [3] Emergency Department, Lancashire Teaching Hospitals NHS Foundation Trust, Preston, United Kingdom.
- [4] Emergency Department, Sheffield Children's NHS Foundation Trust, Sheffield, United Kingdom.
- [5] Emergency Department, Barts Health NHS Trust, London, United Kingdom.
- [6] Emergency Department, Imperial College Healthcare NHS Trust, London, United Kingdom.
- [7] Emergency Department, Manchester University NHS Foundation Trust, Wythenshawe Hospital, Manchester, United Kingdom.

- PMID: **33237907**
- PMCID: PMC7688143
- DOI: 10.1371/journal.pone.0240206

Free PMC article
Observational Study

Characterisation of 22445 patients attending UK emergency departments with suspected COVID-19 infection: Observational cohort study

Steve Goodacre et al. PLoS One. 2020.
Free PMC article
Show details
PLoS One
. 2020 Nov 25;15(11):e0240206.
doi: 10.1371/journal.pone.0240206. eCollection 2020.

Authors

Steve Goodacre [1], Ben Thomas [1], Ellen Lee [1], Laura Sutton [1], Amanda Loban [1], Simon Waterhouse [1], Richard Simmonds [1], Katie Biggs [1], Carl Marincowitz [1], Jose Schutter [1], Sarah Connelly [1], Elena Sheldon [1], Jamie Hall [1], Emma Young [1], Andrew Bentley [2], Kirsty Challen [3], Chris Fitzsimmons [4], Tim Harris [5], Fiona Lecky [1], Andrew Lee [1], Ian Maconochie [6], Darren Walter [7]

Affiliations

- [1] School of Health and Related Research (ScHARR), University of Sheffield, Sheffield, United Kingdom.
- [2] Intensive Care, Manchester University NHS Foundation Trust, Wythenshawe Hospital, Manchester, United Kingdom.
- [3] Emergency Department, Lancashire Teaching Hospitals NHS Foundation Trust, Preston, United Kingdom.

- [4] Emergency Department, Sheffield Children's NHS Foundation Trust, Sheffield, United Kingdom.
- [5] Emergency Department, Barts Health NHS Trust, London, United Kingdom.
- [6] Emergency Department, Imperial College Healthcare NHS Trust, London, United Kingdom.
- [7] Emergency Department, Manchester University NHS Foundation Trust, Wythenshawe Hospital, Manchester, United Kingdom.

- PMID: **33237907**
- PMCID: PMC7688143
- DOI: 10.1371/journal.pone.0240206

Abstract

Background: Hospital emergency departments play a crucial role in the initial assessment and management of suspected COVID-19 infection. This needs to be guided by studies of people presenting with suspected COVID-19, including those admitted and discharged, and those who do not ultimately have COVID-19 confirmed. We aimed to characterise patients attending emergency departments with suspected COVID-19, including subgroups based on sex, ethnicity and COVID-19 test results.

Methods and findings: We undertook a mixed prospective and retrospective observational cohort study in 70 emergency departments across the United Kingdom (UK). We collected presenting data from 22445 people attending with suspected COVID-19 between 26 March 2020 and 28 May 2020. Outcomes were admission to hospital, COVID-19 result, organ support (respiratory, cardiovascular or renal), and death, by record review at 30 days. Mean age was 58.4 years, 11200 (50.4%) were female and 11034 (49.6%) male. Adults (age >16 years) were acutely unwell (median NEWS2 score of 4), frequently had limited performance status (46.9%) and had high rates of admission (67.1%), COVID-19 positivity (31.2%), organ support (9.8%) and death (15.5%). Children had much lower rates of admission (27.4%), COVID-19 positivity (1.2%), organ support (1.4%) and death (0.3%). Similar numbers of men and women presented to the ED, but men were more likely to be admitted (72.9% v 61.4%), require organ support (12.2% v 7.7%) and die (18.2% v 13.0%). Black or Asian adults tended to be younger than White adults (median age 54, 50 and 67 years), were less likely to have impaired performance status (43.1%, 26.8% and 51.6%), be admitted to hospital (60.8%, 57.3%, 69.6%) or die (11.6%, 11.2%, 16.4%), but were more likely to require organ support (15.9%, 14.3%, 8.9%) or have a positive COVID-19 test (40.8%, 42.1%, 30.0%). Adults admitted with suspected and confirmed COVID-19 had similar age, performance status and comorbidities (except chronic lung disease) to those who did not have COVID-19 confirmed, but were much more likely to need organ support (22.2% v 8.9%) or die (32.1% v 15.5%).

Conclusions: Important differences exist between patient groups presenting to the emergency department with suspected COVID-19. Adults and children differ markedly and require different approaches to emergency triage. Admission and adverse outcome rates among adults suggest that policies to avoid unnecessary ED attendance achieved their aim. Subsequent COVID-19 confirmation confers a worse prognosis and greater need for organ support.

Registration: ISRCTN registry, ISRCTN56149622, http://www.isrctn.com/ISRCTN28342533.

Conflict of interest statement

All authors declare grant funding to their employing institutions from the National Institute for Health Research (NIHR), as outlined under financial disclosure information. SG is Deputy Director of the NIHR Health Technology Assessment (HTA) Programme, which funded the study, and chairs the NIHR HTA commissioning committee. These competing interests do not alter our adherence to PLOS ONE policies on sharing data and materials.

- 23 references
- 3 figures

Supplementary info

Publication types, MeSH terms, Grant support Expand

Publication types

- Observational Study
- Research Support, Non-U.S. Gov't

MeSH terms

- Age Factors
- Aged
- COVID-19 / epidemiology*
- COVID-19 / virology
- Child
- Child, Preschool
- Comorbidity
- Emergency Service, Hospital*
- Female
- Humans
- Infant
- Infant, Newborn
- Male
- Middle Aged
- Pandemics*
- Patient Admission
- Prospective Studies
- Retrospective Studies
- SARS-CoV-2*
- Triage
- United Kingdom / epidemiology

Grant support

- 11/46/07/DH_/Department of Health/United Kingdom

Full text links

OPEN ACCESS TO FULL TEXT PLOS ONE Public Library of Science Free PMC article

Proceed to details

Cite

Share

132

Observational Study

Pulm Pharmacol Ther

. 2021 Aug;69:102007.

doi: 10.1016/j.pupt.2021.102007. Epub 2021 Jun 12.

Inhaled bronchodilators use and clinical course of adult inpatients with Covid-19 pneumonia in Spain: A retrospective cohort study

Elena Villamañán [1], Carmen Sobrino [2], Carlos Carpio [3], Marta Moreno [2], Ana Arancón [2], Catalina Lara [2], Ester Pérez [2], Carlos Jiménez [2], Ester Zamarrón [3], Inmaculada Jiménez-Nácher [2], Alicia Herrero [2], Rodolfo Álvarez-Sala [3]

Affiliations Expand

Affiliations

- [1] Pharmacy Department, La Paz University Hospital, IdiPAZ, Madrid, Spain. Electronic address: evillabueno@telefonica.net.
- [2] Pharmacy Department, La Paz University Hospital, IdiPAZ, Madrid, Spain.
- [3] Pneumology Department, La Paz University Hospital, IdiPAZ, Madrid, Spain.

- PMID: **34129946**
- PMCID: PMC8196225
- DOI: 10.1016/j.pupt.2021.102007

Free PMC article

Observational Study

Inhaled bronchodilators use and clinical course of adult inpatients with Covid-19 pneumonia in Spain: A retrospective cohort study

Elena Villamañán et al. Pulm Pharmacol Ther. 2021 Aug.
Free PMC article
Show details
Pulm Pharmacol Ther
. 2021 Aug;69:102007.
doi: 10.1016/j.pupt.2021.102007. Epub 2021 Jun 12.

Authors

Elena Villamañán [1], Carmen Sobrino [2], Carlos Carpio [3], Marta Moreno [2], Ana Arancón [2], Catalina Lara [2], Ester Pérez [2], Carlos Jiménez [2], Ester Zamarrón [3], Inmaculada Jiménez-Nácher [2], Alicia Herrero [2], Rodolfo Álvarez-Sala [3]

Affiliations

- [1] Pharmacy Department, La Paz University Hospital, IdiPAZ, Madrid, Spain. Electronic address: evillabueno@telefonica.net.
- [2] Pharmacy Department, La Paz University Hospital, IdiPAZ, Madrid, Spain.
- [3] Pneumology Department, La Paz University Hospital, IdiPAZ, Madrid, Spain.

- PMID: **34129946**
- PMCID: PMC8196225
- DOI: 10.1016/j.pupt.2021.102007

Abstract

Background: In the current coronavirus health crisis, inhaled bronchodilators(IB) have been suggested as a possible treatment for patients hospitalized. Patients with evidence of Covid-19 pneumonia worldwide have been prescribed these medications as part of therapy for the disease, an indication for which this medications could be ineffective taken on account the pathophysiology and mechanisms of disease progression.

Objective: The main objective was to evaluate whether there is an association between IB use and length of stay. Primary end points were the number of days that a patient stayed in the hospital and death as a final event in a time to event analysis. Pneumonia severity, oxygen requirement, involved drugs, comorbidity, historical or current respiratory diagnoses and other drugs prescribed to treat coronavirus pneumonia were also evaluated.

Methods: A descriptive, observational, cross-sectional study was performed in this tertiary hospital in Madrid (Spain). Data were obtained regarding patients hospitalized with Covid-19, excluding those who were intubated. The primary and secondary outcomes such as duration of hospitalization and death were compared in patients who received IB with those in patients who did not.

Results: 327 patients were evaluated, mean age was 64.4 ± 15.8 years. Median length of hospitalization stay was 10 days. Of them 292 (89.3%) overcame the disease, the remaining 35 died. Patients who had received IB did not have less mortality rate (odds ratio 0.839; 95% CI: 0.401 to 1.752) and less hospitalization period when compared with patients who did not received IB (odds ratio 1.280; 95% CI: 0.813 to 2.027). There was no significant association between IB use and recovery or death. Hypertension and diabetes were the most common comorbidities. The prevalence of chronic respiratory disease in our cohort was low (21.1%). Anticholinergics were

the IB more frequently prescribed for Covid-19 pneumonia. Better response in patients treated with inhaled corticosteroids was not observed.

Conclusion: Off-label indication of inhaled-bronchodilators for Covid-19 patients are common in admitted patients. Taken on account our results, the use of IB for coronavirus pneumonia apparently is not associated with a significantly patient's improvement. Our study confirms the hypothesis that inhaled bronchodilators do not improve clinical outcomes or reduce the risk of Covid-19 mortality. This could be due to the fact that the virus mainly affects the lung parenchyma and the pulmonary vasculature and probably not the airway. More researches are necessary in order to fill the gap in evidence for this new indication.

Keywords: Covid-19; Inhaled bronchodilators; Pneumonia.

Copyright © 2021 Elsevier Ltd. All rights reserved.

Conflict of interest statement

The authors declare no conflict of interest in this article.

- 27 references
- 3 figures

Supplementary info

Publication types, MeSH terms, Substances Expand

Publication types

- Observational Study

MeSH terms

- Adult
- Bronchodilator Agents*
- COVID-19*
- Cohort Studies
- Cross-Sectional Studies
- Hospitalization
- Humans
- Inpatients
- Middle Aged
- Retrospective Studies
- SARS-CoV-2
- Spain / epidemiology

Substances

- Bronchodilator Agents

Full text links

ELSEVIER FULL-TEXT ARTICLE Elsevier Science Free PMC article

Proceed to details

Cite

Share

☐ 133

BMJ Open
. 2021 Jun 25;11(6):e047561.
doi: 10.1136/bmjopen-2020-047561.

Hypertension is the major predictor of poor outcomes among inpatients with COVID-19 infection in the UK: a retrospective cohort study

Ansu Basu [1 2], Juliana Chizo Agwu [3 4], Nicola Barlow [5], Brian Lee [6]

Affiliations Expand

Affiliations

- [1] Diabetes, Endocrinology and Lipid Metabolism, Sandwell and West Birmingham NHS Trust, Birmingham, UK ansu.basu@nhs.net.
- [2] Institute of Metabolism and Systems Research, University of Birmingham College of Medical and Dental Sciences, Birmingham, UK.
- [3] Paediatrics, Sandwell and West Birmingham NHS Trust, Birmingham, UK.
- [4] Institute of Clinical Sciences, University of Birmingham College of Medical and Dental Sciences, Birmingham, UK.
- [5] Black Country Pathology Services, Sandwell and West Birmingham NHS Trust, Birmingham, UK.
- [6] Diabetes, Endocrinology and Lipid Metabolism, Sandwell and West Birmingham NHS Trust, Birmingham, UK.

- PMID: **34172549**
- PMCID: PMC8238530
- DOI: 10.1136/bmjopen-2020-047561

Free PMC article

Hypertension is the major predictor of poor outcomes among inpatients with COVID-19

infection in the UK: a retrospective cohort study

Ansu Basu et al. BMJ Open. 2021.
Free PMC article
Show details
BMJ Open
. 2021 Jun 25;11(6):e047561.
doi: 10.1136/bmjopen-2020-047561.

Authors

Ansu Basu [1] [2], Juliana Chizo Agwu [3] [4], Nicola Barlow [5], Brian Lee [6]

Affiliations

- [1] Diabetes, Endocrinology and Lipid Metabolism, Sandwell and West Birmingham NHS Trust, Birmingham, UK ansu.basu@nhs.net.
- [2] Institute of Metabolism and Systems Research, University of Birmingham College of Medical and Dental Sciences, Birmingham, UK.
- [3] Paediatrics, Sandwell and West Birmingham NHS Trust, Birmingham, UK.
- [4] Institute of Clinical Sciences, University of Birmingham College of Medical and Dental Sciences, Birmingham, UK.
- [5] Black Country Pathology Services, Sandwell and West Birmingham NHS Trust, Birmingham, UK.
- [6] Diabetes, Endocrinology and Lipid Metabolism, Sandwell and West Birmingham NHS Trust, Birmingham, UK.

- PMID: **34172549**
- PMCID: PMC8238530
- DOI: 10.1136/bmjopen-2020-047561

Abstract

Objective: To assess the impact of diabetes, hypertension and cardiovascular diseases on inpatient mortality from COVID-19, and its relationship to ethnicity and social deprivation.

Design: Retrospective, single-centre observational study SETTING: Birmingham, UK.

Participants: 907 hospitalised patients with laboratory-confirmed COVID-19 from a multi-ethnic community, admitted between 1 March 2020 and 31 May 2020.

Main outcome measures: The primary analysis was an evaluation of cardiovascular conditions and diabetes in relation to ethnicity and social deprivation, with the end-point of inpatient death or death within 30 days of discharge. A multivariable logistic regression model was used to calculate HRs while adjusting for confounders.

Results: 361/907 (39.8%) died in hospital or within 30 days of discharge. The presence of diabetes and hypertension together appears to confer the greatest mortality risk (OR 2.75; 95% CI

1.80 to 4.21; p<0.001) compared with either condition alone. Age >65 years (OR 3.32; 95% CI 2.15 to 5.11), male sex (OR 2.04; 95% CI 1.47 to 2.82), hypertension (OR 1.69; 95% CI 1.10 to 2.61) and cerebrovascular disease (OR 1.87; 95% CI 1.31 to 2.68) were independently associated with increased risk of death. The mortality risk did not differ between the quintiles of deprivation. High-sensitivity troponin I was the best predictor of mortality among biomarkers (OR 4.43; 95% CI 3.10 to 7.10). Angiotensin-receptor blockers (OR 0.57; 95% CI 0.33 to 0.96) and ACE inhibitors (OR 0.65; 95% CI 0.43 to 0.97) were not associated with adverse outcome. The Charlson Index of Comorbidity scores were significantly higher in non-survivors.

Conclusions: The combined prevalence of hypertension and diabetes appears to confer the greatest risk, where diabetes may have a modulating effect. Hypertension and cerebrovascular disease had a significant impact on inpatient mortality. Social deprivation and ethnicity did not have any effect once the patient was in hospital.

Keywords: COVID-19; epidemiology; general diabetes; hypertension.

© Author(s) (or their employer(s)) 2021. Re-use permitted under CC BY-NC. No commercial re-use. See rights and permissions. Published by BMJ.

Conflict of interest statement

Competing interests: None declared.

- 31 references
- 2 figures

Supplementary info

MeSH terms Expand

MeSH terms

- Aged
- COVID-19*
- Comorbidity
- Hospital Mortality
- Hospitalization
- Humans
- Hypertension* / epidemiology
- Inpatients
- Male
- Retrospective Studies
- Risk Factors
- SARS-CoV-2
- United Kingdom / epidemiology

Full text links

BMJ Free Full Text HighWire Free PMC article
Proceed to details
Cite
Share
134
Observational Study
Med Sci Monit
. 2020 Dec 2;26:e928755.
doi: 10.12659/MSM.928755.

A Retrospective Study on the Effects of Convalescent Plasma Therapy in 24 Patients Diagnosed with COVID-19 Pneumonia in February and March 2020 at 2 Centers in Wuhan, China

Shuang Huang [1], Changxin Shen [1], Chengliang Xia [1], Xiaoxing Huang [1], Yourong Fu [1], Li Tian [1]
Affiliations Expand

Affiliation

- [1] Department of Blood Transfusion, Zhongnan Hospital of Wuhan University, Wuhan, Hubei, China (mainland).

- PMID: **33264276**
- PMCID: PMC7720430
- DOI: 10.12659/MSM.928755

Free PMC article
Observational Study

A Retrospective Study on the Effects of Convalescent Plasma Therapy in 24 Patients Diagnosed with COVID-19 Pneumonia in February and March 2020 at 2 Centers in Wuhan, China

Shuang Huang et al. Med Sci Monit. 2020.
Free PMC article

Show details

Med Sci Monit
. 2020 Dec 2;26:e928755.
doi: 10.12659/MSM.928755.

Authors

Shuang Huang [1], Changxin Shen [1], Chengliang Xia [1], Xiaoxing Huang [1], Yourong Fu [1], Li Tian [1]

Affiliation

- [1] Department of Blood Transfusion, Zhongnan Hospital of Wuhan University, Wuhan, Hubei, China (mainland).

- PMID: **33264276**
- PMCID: PMC7720430
- DOI: 10.12659/MSM.928755

Abstract

BACKGROUND This retrospective study aimed to describe the effects of convalescent plasma therapy in 24 patients diagnosed with coronavirus disease 2019 (COVID-19) pneumonia due to severe acute respiratory syndrome coronavirus 2 (SARS-CoV-2) infection during February and March 2020 in Wuhan, China. MATERIAL AND METHODS The confirmation of SARS-CoV-2 infection was made by the reverse transcription-polymerase chain reaction test. We retrospectively analyzed the clinical data and laboratory test reports of patients with severe COVID-19 pneumonia who received a convalescent plasma transfusion. RESULTS A total of 24 patients with COVID-19 pneumonia who were transfused with ABO-compatible convalescent plasma were enrolled in the study. Convalescent plasma transfusion showed an effective clinical outcome in 14 of 24 patients (an effective rate of 58.3%). No patients had an adverse reaction to the transfusion. Compared with before convalescent plasma transfusion, the lymphocyte count after convalescent plasma transfusion increased to a normal level (median: 0.80×10^9/L vs. 1.12×10^9/L, P=0.004). Other laboratory indicators such as white blood cells, high-sensitivity C-reactive protein, procalcitonin, alanine aminotransferase, and aspartate transaminase showed a decreasing trend after transfusion. CONCLUSIONS This retrospective observational clinical study showed that convalescent plasma therapy could have beneficial effects on patient outcomes. Recently, regulatory authorization has been given for the use of convalescent plasma therapy, and clinical guidelines have been developed for the collection and use of convalescent plasma and hyperimmune immunoglobulin in patients with COVID-19.

Conflict of interest statement

Conflict of interest

None.

- 22 references
- 1 figure

Supplementary info

Publication types, MeSH terms, Substances, Supplementary concepts Expand

Publication types

- Multicenter Study
- Observational Study

MeSH terms

- Adult
- Aged
- Aged, 80 and over
- Blood Component Transfusion / methods*
- COVID-19 / diagnosis
- COVID-19 / immunology
- COVID-19 / therapy*
- COVID-19 / virology
- COVID-19 Nucleic Acid Testing
- China
- Female
- Humans
- Immunization, Passive / methods
- Male
- Middle Aged
- RNA, Viral / isolation & purification
- Retrospective Studies
- Reverse Transcriptase Polymerase Chain Reaction
- SARS-CoV-2 / genetics
- SARS-CoV-2 / immunology*
- SARS-CoV-2 / isolation & purification
- Severity of Illness Index
- Treatment Outcome

Substances

- RNA, Viral

Supplementary concepts

- COVID-19 serotherapy

Full text links

International Scientific Literature, Ltd. Free PMC article

Proceed to details

Cite

Share

135

Observational Study

Epidemiol Infect

. 2020 Oct 13;148:e251.

doi: 10.1017/S0950268820002472.

Risk factors for severe disease in patients admitted with COVID-19 to a hospital in London, England: a retrospective cohort study

J W Goodall [1], T A N Reed [2], M Ardissino [1], P Bassett [3], A M Whittington [1], D L Cohen [2], N Vaid [2]

Affiliations Expand

Affiliations

- [1] Department of Infection, Northwick Park Hospital, London North West University Healthcare NHS Trust, London, UK.
- [2] Department of Acute Medicine, Northwick Park Hospital, London North West University Healthcare NHS Trust, London, UK.
- [3] Statsconsultancy Ltd., London, UK.

- PMID: **33046155**
- PMCID: PMC7591271
- DOI: 10.1017/S0950268820002472

Free PMC article

Observational Study

Risk factors for severe disease in patients admitted with COVID-19 to a hospital in London, England: a retrospective cohort study

J W Goodall et al. Epidemiol Infect. 2020.
Free PMC article
Show details
Epidemiol Infect
. 2020 Oct 13;148:e251.
doi: 10.1017/S0950268820002472.

Authors

J W Goodall [1], T A N Reed [2], M Ardissino [1], P Bassett [3], A M Whittington [1], D L Cohen [2], N Vaid [2]

Affiliations

- [1] Department of Infection, Northwick Park Hospital, London North West University Healthcare NHS Trust, London, UK.
- [2] Department of Acute Medicine, Northwick Park Hospital, London North West University Healthcare NHS Trust, London, UK.
- [3] Statsconsultancy Ltd., London, UK.

- PMID: **33046155**
- PMCID: PMC7591271
- DOI: 10.1017/S0950268820002472

Abstract

COVID-19 has caused a major global pandemic and necessitated unprecedented public health restrictions in almost every country. Understanding risk factors for severe disease in hospitalised patients is critical as the pandemic progresses. This observational cohort study aimed to characterise the independent associations between the clinical outcomes of hospitalised patients and their demographics, comorbidities, blood tests and bedside observations. All patients admitted to Northwick Park Hospital, London, UK between 12 March and 15 April 2020 with COVID-19 were retrospectively identified. The primary outcome was death. Associations were explored using Cox proportional hazards modelling. The study included 981 patients. The mortality rate was 36.0%. Age (adjusted hazard ratio (aHR) 1.53), respiratory disease (aHR 1.37), immunosuppression (aHR 2.23), respiratory rate (aHR 1.28), hypoxia (aHR 1.36), Glasgow Coma Scale <15 (aHR 1.92), urea (aHR 2.67), alkaline phosphatase (aHR 2.53), C-reactive protein (aHR 1.15), lactate (aHR 2.67), platelet count (aHR 0.77) and infiltrates on chest radiograph (aHR 1.89) were all associated with mortality. These important data will aid clinical risk stratification and provide direction for further research.

Keywords: COVID-19; hospitalisation; severe disease.

Conflict of interest statement

All authors declare no conflict of interest.

- 33 references
- 3 figures

Supplementary info

Publication types, MeSH terms Expand

Publication types

- Observational Study

MeSH terms

- Aged
- Aged, 80 and over
- Betacoronavirus*
- COVID-19
- Coronavirus Infections / complications
- Coronavirus Infections / epidemiology*
- Coronavirus Infections / mortality
- Female
- Hospitalization
- Humans
- London / epidemiology
- Male
- Middle Aged
- Pandemics
- Pneumonia, Viral / complications
- Pneumonia, Viral / epidemiology*
- Pneumonia, Viral / mortality
- Proportional Hazards Models
- Retrospective Studies
- Risk Factors
- SARS-CoV-2

Full text links

Cambridge University Press Free PMC article
Proceed to details
Cite
Share
136
Observational Study
Int Immunopharmacol
. 2022 Jan;102:108384.
doi: 10.1016/j.intimp.2021.108384. Epub 2021 Nov 20.

Tocilizumab in critically ill COVID-19 patients: An observational study

Muhammad Z Mushtaq [1], Saad B Z Mahmood [1], Aysha Almas [1], Syed Ather Wasti [1], Syed Ahsan Ali [2]
Affiliations Expand

Affiliations

- [1] Department of Medicine, The Aga Khan University Hospital, Stadium Road, Karachi, Pakistan.
- [2] Department of Medicine, The Aga Khan University Hospital, Stadium Road, Karachi, Pakistan. Electronic address: Syed.ahsan@aku.edu.

- PMID: **34838490**
- PMCID: PMC8604692
- DOI: 10.1016/j.intimp.2021.108384

Free PMC article
Observational Study

Tocilizumab in critically ill COVID-19 patients: An observational study

Muhammad Z Mushtaq et al. Int Immunopharmacol. 2022 Jan.
Free PMC article
Show details
Int Immunopharmacol
. 2022 Jan;102:108384.
doi: 10.1016/j.intimp.2021.108384. Epub 2021 Nov 20.

Authors

Muhammad Z Mushtaq [1], Saad B Z Mahmood [1], Aysha Almas [1], Syed Ather Wasti [1], Syed Ahsan Ali [2]

Affiliations

- [1] Department of Medicine, The Aga Khan University Hospital, Stadium Road, Karachi, Pakistan.
- [2] Department of Medicine, The Aga Khan University Hospital, Stadium Road, Karachi, Pakistan. Electronic address: Syed.ahsan@aku.edu.

- PMID: **34838490**
- PMCID: PMC8604692
- DOI: 10.1016/j.intimp.2021.108384

Abstract

Tocilizumab decreases inflammatory response in the cytokine storm which is one of the mechanisms behind the development of ARDS in COVID-19 patients. The objective of our study was to determine response of tocilizumab in patients suffering from COVID-19 by analyzing clinical parameters and inflammatory markers. A single-arm observational retrospective study was conducted from March 15, 2020 to March 15, 2021. Clinical outcomes in terms of mortality, weaning from mechanical ventilator, improvement in laboratory parameters including inflammatory cytokines, and length of hospital stay were documented. Reduction in values of inflammatory markers, and patients discharged home in stable condition were defined as an improvement after tocilizumab administration. A total of 514 patients received tocilizumab, majority of whom were critically sick 333 (64.8%). Out of the total sample 363 (70.6%) patients were discharged home in stable condition. Overall mean length of stay was 11.50 ± 8.4 days. There was significant difference in length of stay of patients who required invasive mechanical ventilation as compared to those who were kept only on supplemental oxygen ($p < 0.05$). Patients who were discharged home showed significant improvement in inflammatory markers and neutrophil to lymphocyte ratio as compared to those who expired ($p < 0.05$). A total of 21 (4.1%) patients had positive blood culture while 57 (11.1%) had positive culture of tracheal aspirate. Hence, tocilizumab is found to be a reasonable therapeutic option for worsening COVID-19 pneumonia by decreasing the need for mechanical ventilation. However, it is associated with adverse events including bacterial and fungal infections.

Keywords: COVID-19; Cytokine release syndrome; SARS-CoV-2; Tocilizumab.

Copyright © 2021. Published by Elsevier B.V.

Conflict of interest statement

The authors declare that they have no known competing financial interests or personal relationships that could have appeared to influence the work reported in this paper.

- 41 references
- 1 figure

Supplementary info

Publication types, MeSH terms, Substances, Supplementary concepts Expand

Publication types

- Observational Study

MeSH terms

- Aged
- Antibodies, Monoclonal, Humanized / administration & dosage*
- Antibodies, Monoclonal, Humanized / adverse effects
- Bacterial Infections / chemically induced
- Bacterial Infections / epidemiology*

- Bacterial Infections / immunology
- COVID-19 / drug therapy*
- COVID-19 / therapy*
- Critical Illness / therapy
- Female
- Humans
- Length of Stay / statistics & numerical data
- Male
- Middle Aged
- Mycoses / chemically induced
- Mycoses / epidemiology*
- Mycoses / immunology
- Pakistan / epidemiology
- Patient Discharge / statistics & numerical data
- Respiration, Artificial / instrumentation
- Respiration, Artificial / statistics & numerical data
- Retrospective Studies
- SARS-CoV-2
- Treatment Outcome

Substances

- Antibodies, Monoclonal, Humanized
- tocilizumab

Supplementary concepts

- COVID-19 drug treatment

Full text links

ELSEVIER FULL-TEXT ARTICLE Elsevier Science Free PMC article
Proceed to details
Cite
Share
☐ 137
Observational Study
Neurol Sci
. 2020 Dec;41(12):3395-3399.
doi: 10.1007/s10072-020-04754-2. Epub 2020 Oct 8.

The stroke mothership model survived during COVID-19 era: an observational single-center study in Emilia-Romagna, Italy

Andrea Zini [1], Michele Romoli [2] [3], Mauro Gentile [1], Ludovica Migliaccio [1], Cosimo Picoco [4], Oscar Dell'Arciprete [4], Luigi Simonetti [5], Federica Naldi [1], Laura Piccolo [1], Giovanni Gordini [4], Francesco Tagliatela [5], Vincenzo Bua [4], Luigi Cirillo [6] [7], Ciro Princiotta [6], Carlo Coniglio [4], Carlo Descovich [8], Pietro Cortelli [9] [10]

Affiliations Expand

Affiliations

- [1] IRCCS Istituto delle Scienze Neurologiche di Bologna, Department of Neurology and Metropolitan Stroke Center, "C.A. Pizzardi" Maggiore Hospital, Largo Nigrisoli 2, 40133, Bologna, Italy.
- [2] IRCCS Istituto delle Scienze Neurologiche di Bologna, Department of Neurology and Metropolitan Stroke Center, "C.A. Pizzardi" Maggiore Hospital, Largo Nigrisoli 2, 40133, Bologna, Italy. michele.romoli@studenti.unipg.it.
- [3] Neurology Clinic, University of Perugia, Perugia, Italy. michele.romoli@studenti.unipg.it.
- [4] Department of Anaesthesia, Intensive Care and Emergency Medical Services, Maggiore Hospital, Bologna, Italy.
- [5] Neuroradiology Unit, Maggiore Hospital, IRCCS Istituto delle Scienze Neurologiche di Bologna, Bologna, Italy.
- [6] Neuroradiology Unit, Bellaria Hospital, IRCCS Istituto delle Scienze Neurologiche di Bologna, Bologna, Italy.
- [7] DIMES, Department of Specialty, Diagnostic and Experimental Medicine, University of Bologna, Bologna, Italy.
- [8] Department of Clinical Governance and Quality, Bologna Local Healthcare Authority, Bologna, Italy.
- [9] Department of Biomedical and Neuromotor Sciences, Alma Mater Studiorum-University of Bologna, Bologna, Italy.
- [10] IRCCS Istituto delle Scienze Neurologiche di Bologna, Bologna, Italy.

- PMID: **33030622**
- PMCID: PMC7541754
- DOI: 10.1007/s10072-020-04754-2

Free PMC article
Observational Study

The stroke mothership model survived during COVID-19 era: an observational single-center study in Emilia-Romagna, Italy

Andrea Zini et al. Neurol Sci. 2020 Dec.
Free PMC article
Show details
Neurol Sci
. 2020 Dec;41(12):3395-3399.
doi: 10.1007/s10072-020-04754-2. Epub 2020 Oct 8.

Authors

Andrea Zini [1], Michele Romoli [2, 3], Mauro Gentile [1], Ludovica Migliaccio [1], Cosimo Picoco [4], Oscar Dell'Arciprete [4], Luigi Simonetti [5], Federica Naldi [1], Laura Piccolo [1], Giovanni Gordini [4], Francesco Tagliatela [5], Vincenzo Bua [4], Luigi Cirillo [6, 7], Ciro Princiotta [6], Carlo Coniglio [4], Carlo Descovich [8], Pietro Cortelli [9, 10]

Affiliations

- [1] IRCCS Istituto delle Scienze Neurologiche di Bologna, Department of Neurology and Metropolitan Stroke Center, "C.A. Pizzardi" Maggiore Hospital, Largo Nigrisoli 2, 40133, Bologna, Italy.
- [2] IRCCS Istituto delle Scienze Neurologiche di Bologna, Department of Neurology and Metropolitan Stroke Center, "C.A. Pizzardi" Maggiore Hospital, Largo Nigrisoli 2, 40133, Bologna, Italy. michele.romoli@studenti.unipg.it.
- [3] Neurology Clinic, University of Perugia, Perugia, Italy. michele.romoli@studenti.unipg.it.
- [4] Department of Anaesthesia, Intensive Care and Emergency Medical Services, Maggiore Hospital, Bologna, Italy.
- [5] Neuroradiology Unit, Maggiore Hospital, IRCCS Istituto delle Scienze Neurologiche di Bologna, Bologna, Italy.
- [6] Neuroradiology Unit, Bellaria Hospital, IRCCS Istituto delle Scienze Neurologiche di Bologna, Bologna, Italy.
- [7] DIMES, Department of Specialty, Diagnostic and Experimental Medicine, University of Bologna, Bologna, Italy.
- [8] Department of Clinical Governance and Quality, Bologna Local Healthcare Authority, Bologna, Italy.
- [9] Department of Biomedical and Neuromotor Sciences, Alma Mater Studiorum-University of Bologna, Bologna, Italy.
- [10] IRCCS Istituto delle Scienze Neurologiche di Bologna, Bologna, Italy.

- PMID: **33030622**
- PMCID: PMC7541754
- DOI: 10.1007/s10072-020-04754-2

Abstract

Introduction: A reduction of the hospitalization and reperfusion treatments was reported during COVID-19 pandemic. However, high variability in results emerged, potentially due to logistic paradigms adopted. Here, we analyze stroke code admissions, hospitalizations, and stroke belt performance for ischemic stroke patients in the metropolitan Bologna region, comparing temporal trends between 2019 and 2020 to define the impact of COVID-19 on the stroke network.

Methods: This retrospective observational study included all people admitted at the Bologna Metropolitan Stroke Center in timeframes 1 March 2019-30 April 2019 (cohort-2019) and 1 March 2020-30 April 2020 (cohort-2020). Diagnosis, treatment strategy, and timing were compared between the two cohorts to define temporal trends.

Results: Overall, 283 patients were admitted to the Stroke Center, with no differences in demographic factors between cohort-2019 and cohort-2020. In cohort-2020, transient ischemic attack (TIA) was significantly less prevalent than 2019 (6.9% vs 14.4%, p = .04). Among 216 ischemic stroke patients, moderate-to-severe stroke was more represented in cohort-2020 (17.8% vs 6.2%, p = .027). Similar proportions of patients underwent reperfusion (45.9% in 2019 vs 53.4% in 2020), although a slight increase in combined treatment was detected (14.4% vs 25.4%, p = .05). Door-to-scan timing was significantly prolonged in 2020 compared with 2019 (28.4 ± 12.6 vs 36.7 ± 14.6, p = .03), although overall timing from stroke to treatment was preserved.

Conclusion: During COVID-19 pandemic, TIA and minor stroke consistently reduced compared to the same timeframe in 2019. Longer stroke-to-call and door-to-scan times, attributable to change in citizen behavior and screening at hospital arrival, did not impact on stroke-to-treatment time. Mothership model might have minimized the effects of the pandemic on the stroke care organization.

Keywords: COVID-19; Epidemiology; Ischemic stroke; Transient ischemic-attack.

Conflict of interest statement

The authors declare that they have no conflict of interest.

- 11 references
- 1 figure

Supplementary info

Publication types, MeSH terms Expand

Publication types

- Observational Study

MeSH terms

- Betacoronavirus
- COVID-19
- Coronavirus Infections*
- Humans
- Italy / epidemiology
- Neurology / trends*
- Pandemics*
- Pneumonia, Viral*
- Prevalence

- Retrospective Studies
- SARS-CoV-2
- Stroke / epidemiology*
- Stroke / therapy*
- Time-to-Treatment / trends

Full text links

SpringerLink FULL-TEXT ARTICLE Springer Free PMC article
Proceed to details
Cite
Share
138
Observational Study
Infect Dis (Lond)
. 2022 Apr;54(4):283-291.
doi: 10.1080/23744235.2021.2013528. Epub 2021 Dec 8.

Convalescent plasma treatment in severely immunosuppressed patients hospitalized with COVID-19: an observational study of 28 cases

Oskar Ljungquist [1] [2], Maria Lundgren [3], Elena Iliachenko [3], Fredrik Månsson [1] [4], Blenda Böttiger [5], Mona Landin-Olsson [4] [6], Christian Wikén [4] [7], Ebba Rosendal [8], Anna K Överby [8], Byström J Wigren [8], Mattias N E Forsell [8], Jens Kjeldsen-Kragh [3], Magnus Rasmussen [4] [7], Fredrik Kahn [4] [7], Karin Holm [4] [7]
Affiliations Expand

Affiliations

- [1] Department of Translational Medicine, Clinical Infection Medicine, Faculty of Medicine, Lund University, Malmö, Sweden.
- [2] Department of Infectious Diseases, Helsingborg Hospital, Helsingborg, Sweden.
- [3] Department of Clinical Immunology and Transfusion Medicine, Office of Medical Services, Lund, Sweden.
- [4] Skåne University Hospital, Malmö, Sweden.
- [5] Department of Clinical Microbiology, University and Regional Laboratories, Lund, Sweden.
- [6] Department of Clinical Science, Division of Internal Medicine, Lund University, Lund, Sweden.
- [7] Department of Clinical Sciences, Division of Infection Medicine, Lund University, Lund, Sweden.
- [8] Department of Clinical Microbiology, Umeå University, Umeå, Sweden.

- PMID: **34878955**
- PMCID: PMC8726003
- DOI: 10.1080/23744235.2021.2013528

Free PMC article
Observational Study

Convalescent plasma treatment in severely immunosuppressed patients hospitalized with COVID-19: an observational study of 28 cases

Oskar Ljungquist et al. Infect Dis (Lond). 2022 Apr.
Free PMC article

Show details

Infect Dis (Lond)
. 2022 Apr;54(4):283-291.
doi: 10.1080/23744235.2021.2013528. Epub 2021 Dec 8.

Authors

Oskar Ljungquist [1] [2], Maria Lundgren [3], Elena Iliachenko [3], Fredrik Månsson [1] [4], Blenda Böttiger [5], Mona Landin-Olsson [4] [6], Christian Wikén [4] [7], Ebba Rosendal [8], Anna K Överby [8], Byström J Wigren [8], Mattias N E Forsell [8], Jens Kjeldsen-Kragh [3], Magnus Rasmussen [4] [7], Fredrik Kahn [4] [7], Karin Holm [4] [7]

Affiliations

- [1] Department of Translational Medicine, Clinical Infection Medicine, Faculty of Medicine, Lund University, Malmö, Sweden.
- [2] Department of Infectious Diseases, Helsingborg Hospital, Helsingborg, Sweden.
- [3] Department of Clinical Immunology and Transfusion Medicine, Office of Medical Services, Lund, Sweden.
- [4] Skåne University Hospital, Malmö, Sweden.
- [5] Department of Clinical Microbiology, University and Regional Laboratories, Lund, Sweden.
- [6] Department of Clinical Science, Division of Internal Medicine, Lund University, Lund, Sweden.
- [7] Department of Clinical Sciences, Division of Infection Medicine, Lund University, Lund, Sweden.
- [8] Department of Clinical Microbiology, Umeå University, Umeå, Sweden.

- PMID: **34878955**
- PMCID: PMC8726003
- DOI: 10.1080/23744235.2021.2013528

Abstract

Background: Immunosuppressed patients are particularly vulnerable to severe infection from the severe acute respiratory syndrome coronavirus 2 (SARS-CoV-2), risking prolonged viremia and symptom duration. In this study we describe clinical and virological treatment outcomes in a heterogeneous group of patients with severe immunosuppression due to various causes suffering from COVID-19 infection, who were all treated with convalescent plasma (CCP) along with standard treatment.

Methods: We performed an observational, retrospective case series between May 2020 to March 2021 at three sites in Skåne, Sweden, with a population of nearly 1.4 million people. All patients hospitalized for COVID-19 who received CCP with the indication severe immunosuppression as defined by the treating physician were included in the study ($n = 28$).

Results: In total, 28 severely immunocompromised patients, half of which previously had been treated with rituximab, who had received in-hospital convalescent plasma treatment of COVID-19 were identified. One week after CCP treatment, 13 of 28 (46%) patients had improved clinically defined as a decrease of at least one point at the WHO-scale. Three patients had increased score points of whom two had died. For 12 patients, the WHO-scale was unchanged.

Conclusion: As one of only few studies on CCP treatment of COVID-19 in hospitalized patients with severe immunosuppression, this study adds descriptive data. The study design prohibits conclusions on safety and efficacy, and the results should be interpreted with caution. Prospective, randomized trials are needed to investigate this further.

Keywords: Antibodies; COVID-19; PCR; SARS-CoV-2; convalescent plasma; immunosuppression; lymphoma; pandemic; rituximab.

Conflict of interest statement

No potential conflict of interest was reported by the authors.

- 34 references
- 1 figure

Supplementary info

Publication types, MeSH terms, Supplementary concepts, Grant support Expand

Publication types

- Observational Study
- Research Support, Non-U.S. Gov't

MeSH terms

- COVID-19* / therapy
- Humans
- Immunization, Passive*

- Immunocompromised Host*
- Prospective Studies
- Retrospective Studies
- SARS-CoV-2
- Sweden

Supplementary concepts

- COVID-19 serotherapy

Grant support

This study was funded by Vetenskapsrådet [#2020-06235] to MNEF, and the Swedish governmental funds for clinical research (ALF).

Full text links

View full text Taylor & Francis Free PMC article
Proceed to details
Cite
Share
☐ 139
Observational Study
J Med Internet Res
. 2020 Aug 11;22(8):e21385.
doi: 10.2196/21385.

Advance Care Planning Among Users of a Patient Portal During the COVID-19 Pandemic: Retrospective Observational Study

Jennifer D Portz [1], Adreanne Brungardt [2], Prajakta Shanbhag [2], Elizabeth W Staton [3], Seuli Bose-Brill [4], Chen-Tan Lin [1], Jean S Kutner [1], Hillary D Lum [2]
Affiliations Expand

Affiliations

- [1] Division of General Internal Medicine, Department of Medicine, University of Colorado School of Medicine, Aurora, CO, United States.
- [2] Division of Geriatric Medicine, Department of Medicine, University of Colorado School of Medicine, Aurora, CO, United States.
- [3] Department of Family Medicine, University of Colorado School of Medicine, Aurora, CO, United States.

- [4] Department of Medicine, The Ohio State University College of Medicine, Columbus, OH, United States.

- PMID: **32716900**
- PMCID: PMC7423389
- DOI: 10.2196/21385

Free PMC article
Observational Study

Advance Care Planning Among Users of a Patient Portal During the COVID-19 Pandemic: Retrospective Observational Study

Jennifer D Portz et al. J Med Internet Res. 2020.
Free PMC article
Show details
J Med Internet Res
. 2020 Aug 11;22(8):e21385.
doi: 10.2196/21385.

Authors

Jennifer D Portz [1], Adreanne Brungardt [2], Prajakta Shanbhag [2], Elizabeth W Staton [3], Seuli Bose-Brill [4], Chen-Tan Lin [1], Jean S Kutner [1], Hillary D Lum [2]

Affiliations

- [1] Division of General Internal Medicine, Department of Medicine, University of Colorado School of Medicine, Aurora, CO, United States.
- [2] Division of Geriatric Medicine, Department of Medicine, University of Colorado School of Medicine, Aurora, CO, United States.
- [3] Department of Family Medicine, University of Colorado School of Medicine, Aurora, CO, United States.
- [4] Department of Medicine, The Ohio State University College of Medicine, Columbus, OH, United States.

- PMID: **32716900**
- PMCID: PMC7423389
- DOI: 10.2196/21385

Abstract

Background: Advance care planning is the process of discussing health care treatment preferences based on patients' personal values, and it often involves the completion of advance directives. In the first months of 2020, a novel coronavirus, severe acute respiratory syndrome coronavirus 2 (SARS-CoV-2), began circulating widely in the American state of Colorado,

leading to widespread diagnosis of coronavirus disease (COVID-19), hospitalizations, and deaths. In this context, the importance of technology-based, non-face-to-face methods to conduct advance care planning via patient portals has increased.

Objective: The aim of this study was to determine the rates of use of a web-based advance care planning tool through a health system-based electronic patient portal both before and in the early months of the COVID-19 pandemic.

Methods: In 2017, we implemented web-based tools through the patient portal of UCHealth's electronic health record (EHR) for patients to learn about advance care planning and complete an electronically signed medical durable power of attorney (MDPOA) to legally appoint a medical decision maker. Patients accessing the portal can complete and submit a legally valid MDPOA, which becomes part of their medical record. We collected data on the patients' date of MDPOA completion, use of advance care planning messaging, age, sex, and geographic location during the early phase of the COVID-19 pandemic (December 29, 2019, to May 30, 2020).

Results: Over a 5-month period that includes the early phase of the COVID-19 pandemic in Colorado, total monthly use of the advance care planning portal tool increased from 418 users in January to 1037 users in April and then decreased slightly to 815 users in May. The number of MDPOA forms submitted per week increased 2.4-fold after the stay-at-home order was issued in Colorado on March 26, 2020 ($P<.001$). The mean age of the advance care planning portal users was 47.7 years (SD 16.1), and 2206/3292 (67.0%) were female. Women were more likely than men to complete an MDPOA, particularly in younger age groups ($P<.001$). The primary use of the advance care planning portal tools was the completion of an MDPOA (3138/3292, 95.3%), compared to sending an electronic message (148/3292, 4.5%). Over 50% of patients who completed an MDPOA did not have a prior agent in the EHR.

Conclusions: Use of a web-based patient portal to complete an MDPOA increased substantially during the first months of the COVID-19 pandemic in Colorado. There was an increase in advance care planning that corresponded with state government shelter-in-place orders as well as public health reports of increased numbers of COVID-19 cases and deaths. Patient portals are an important tool for providing advance care planning resources and documenting medical decision makers during the pandemic to ensure that medical treatment aligns with patient goals and values.

Keywords: COVID-19; advance care planning; advance directives; electronic health records; health system; pandemic; patient portal; planning; web-based tool.

©Jennifer D Portz, Adreanne Brungardt, Prajakta Shanbhag, Elizabeth W Staton, Seuli Bose-Brill, Chen-Tan Lin, Jean S Kutner, Hillary D Lum. Originally published in the Journal of Medical Internet Research (http://www.jmir.org), 11.08.2020.

Conflict of interest statement

Conflicts of Interest: None declared.

- 37 references
- 3 figures

Supplementary info

Publication types, MeSH terms, Grant support Expand

Publication types

- Observational Study
- Research Support, N.I.H., Extramural

MeSH terms

- Adult
- Advance Care Planning
- Betacoronavirus*
- COVID-19
- Coronavirus Infections*
- Electronic Health Records
- Female
- Hospitalization
- Humans
- Male
- Middle Aged
- Pandemics*
- Patient Portals
- Pneumonia, Viral*
- Retrospective Studies
- SARS-CoV-2

Grant support

- K76 AG059934/AG/NIA NIH HHS/United States
- UL1 RR025780/RR/NCRR NIH HHS/United States
- K76 AG054782/AG/NIA NIH HHS/United States

Full text links

JMIR Publications OPEN ACCESS JMIR Publications Free PMC article

Proceed to details

Cite

Share

☐ 140

Observational Study

Int J Antimicrob Agents

. 2021 Dec;58(6):106453.

doi: 10.1016/j.ijantimicag.2021.106453. Epub 2021 Oct 13.

Change in Antimicrobial Use During COVID-19 Pandemic in South Carolina Hospitals: A Multicenter Observational Cohort Study

Hana R Winders [1], Pamela Bailey [2], Joseph Kohn [3], Carmen M Faulkner-Fennell [4], Sara Utley [5], Evan Lantz [6], Lloyd Sarbacker [7], Julie Ann Justo [1], P Brandon Bookstaver [1], Sharon Weissman [8], Hannah Ruegner [9], Majdi N Al-Hasan [8]
Affiliations Expand

Affiliations

- [1] Department of Clinical Pharmacy and Outcomes Sciences, University of South Carolina College of Pharmacy, Columbia, SC, USA; Department of Pharmacy, Prisma Health-Midlands, Columbia, SC, USA.
- [2] University of South Carolina School of Medicine, Columbia, SC, USA; Department of Medicine, Division of Infectious Diseases, Prisma Health-Midlands, Columbia, SC, USA. Electronic address: Pamela.Bailey@uscmed.sc.edu.
- [3] Department of Pharmacy, Prisma Health-Midlands, Columbia, SC, USA.
- [4] Department of Pharmacy, Prisma Health-Upstate, Greenville, SC, USA.
- [5] Department of Pharmacy, Roper St. Francis Healthcare, Charleston, SC, USA.
- [6] Department of Pharmacy, Spartanburg Regional Healthcare System, Spartanburg, SC, USA.
- [7] Department of Pharmacy, Bon Secours St. Francis, Greenville, SC, USA.
- [8] University of South Carolina School of Medicine, Columbia, SC, USA; Department of Medicine, Division of Infectious Diseases, Prisma Health-Midlands, Columbia, SC, USA.
- [9] Department of Clinical Pharmacy and Outcomes Sciences, University of South Carolina College of Pharmacy, Columbia, SC, USA; South Carolina Department of Health and Environmental Control, Columbia, SC, USA.

- PMID: **34655733**
- PMCID: PMC8513515
- DOI: 10.1016/j.ijantimicag.2021.106453

Free PMC article
Observational Study

Change in Antimicrobial Use During COVID-19 Pandemic in South Carolina Hospitals: A Multicenter Observational Cohort Study

Hana R Winders et al. Int J Antimicrob Agents. 2021 Dec.
Free PMC article
Show details
Int J Antimicrob Agents

. 2021 Dec;58(6):106453.
doi: 10.1016/j.ijantimicag.2021.106453. Epub 2021 Oct 13.

Authors

Hana R Winders [1], Pamela Bailey [2], Joseph Kohn [3], Carmen M Faulkner-Fennell [4], Sara Utley [5], Evan Lantz [6], Lloyd Sarbacker [7], Julie Ann Justo [1], P Brandon Bookstaver [1], Sharon Weissman [8], Hannah Ruegner [9], Majdi N Al-Hasan [8]

Affiliations

- [1] Department of Clinical Pharmacy and Outcomes Sciences, University of South Carolina College of Pharmacy, Columbia, SC, USA; Department of Pharmacy, Prisma Health-Midlands, Columbia, SC, USA.
- [2] University of South Carolina School of Medicine, Columbia, SC, USA; Department of Medicine, Division of Infectious Diseases, Prisma Health-Midlands, Columbia, SC, USA. Electronic address: Pamela.Bailey@uscmed.sc.edu.
- [3] Department of Pharmacy, Prisma Health-Midlands, Columbia, SC, USA.
- [4] Department of Pharmacy, Prisma Health-Upstate, Greenville, SC, USA.
- [5] Department of Pharmacy, Roper St. Francis Healthcare, Charleston, SC, USA.
- [6] Department of Pharmacy, Spartanburg Regional Healthcare System, Spartanburg, SC, USA.
- [7] Department of Pharmacy, Bon Secours St. Francis, Greenville, SC, USA.
- [8] University of South Carolina School of Medicine, Columbia, SC, USA; Department of Medicine, Division of Infectious Diseases, Prisma Health-Midlands, Columbia, SC, USA.
- [9] Department of Clinical Pharmacy and Outcomes Sciences, University of South Carolina College of Pharmacy, Columbia, SC, USA; South Carolina Department of Health and Environmental Control, Columbia, SC, USA.

- PMID: **34655733**
- PMCID: PMC8513515
- DOI: 10.1016/j.ijantimicag.2021.106453

Abstract

Objectives: This retrospective cohort study examined the impact of the pandemic on antimicrobial use (AU) in South Carolina hospitals.

Methods: Antimicrobial use in days of therapy (DOT) per 1000 days-present was evaluated in 17 hospitals in South Carolina. Matched-pairs mean difference was used to compare AU during the pandemic (March-June 2020) with that during the same months in 2019 in hospitals that did and did not admit patients with COVID-19.

Results: There was a 6.6% increase in overall AU in the seven hospitals that admitted patients with COVID-19 (from 530.9 to 565.8; mean difference (MD) 34.9 DOT/1000 days-present; 95% CI 4.3, 65.6; P = 0.03). There was no significant change in overall AU in the remaining 10 hospitals that did not admit patients with COVID-19 (MD 6.0 DOT/1000 days-present; 95% CI -55.5, 67.6; P = 0.83). Most of the increase in AU in the seven hospitals that admitted patients with COVID-19 was observed in broad-spectrum antimicrobial agents. A 16.4% increase was observed in agents predominantly used for hospital-onset infections (from 122.3 to 142.5; MD

20.1 DOT/1000 days-present; 95% CI 11.1, 29.1; P = 0.002). There was also a 9.9% increase in the use of anti-methicillin-resistant Staphylococcus aureus (MRSA) agents (from 66.7 to 73.3; MD 6.6 DOT/1000 days-present; 95% CI 2.3, 10.8; P = 0.01).

Conclusion: The COVID-19 pandemic appears to drive overall and broad-spectrum antimicrobial use in South Carolina hospitals admitting patients with COVID-19. Additional antimicrobial stewardship resources are needed to curtail excessive antimicrobial use in hospitals to prevent subsequent increases in antimicrobial resistance and Clostridioides difficile infection rates, given the continuing nature of the pandemic.

Keywords: Antibiotics; Antimicrobial stewardship; Antipseudomonal agents; Novel coronavirus; Vancomycin.

Copyright © 2021 Elsevier Ltd. All rights reserved.

Conflict of interest statement

Declarations of Competing Interests PB, JK, CMF, SU, EL, LS, SW, and MNA: no conflicts HRW: bioMérieux, Speaker's Bureau (nonbranded content); JJ: bioMérieux, Speaker's Bureau (nonbranded content), Merck, Advisory Board; PBB: bioMérieux, Speaker's Bureau (nonbranded content), Kedrion Biopharma Advisory Board, research grant.

- 21 references
- 1 figure

Supplementary info

Publication types, MeSH terms, Substances Expand

Publication types

- Multicenter Study
- Observational Study

MeSH terms

- Anti-Bacterial Agents / therapeutic use*
- Antimicrobial Stewardship
- COVID-19
- Clostridium Infections / drug therapy
- Cross Infection / drug therapy*
- Drug Utilization Review / statistics & numerical data*
- Hospitals
- Humans
- Methicillin-Resistant Staphylococcus aureus / drug effects
- Pandemics*
- Retrospective Studies

- SARS-CoV-2
- South Carolina

Substances

- Anti-Bacterial Agents

Full text links

ELSEVIER FULL-TEXT ARTICLE Elsevier Science Free PMC article
Proceed to details
Cite
Share
141
Observational Study
Elife
. 2020 Nov 26;9:e63195.
doi: 10.7554/eLife.63195.

A novel haemocytometric COVID-19 prognostic score developed and validated in an observational multicentre European hospital-based study

Joachim Linssen [1], Anthony Ermens [2], Marvin Berrevoets [3], Michela Seghezzi [4], Giulia Previtali [4], Simone van der Sar-van der Brugge [2], Henk Russcher [5], Annelies Verbon [5], Judith Gillis [6], Jürgen Riedl [7], Eva de Jongh [7], Jarob Saker [1], Marion Münster [1], Imke Ca Munnix [8], Anthonius Dofferhof [8], Volkher Scharnhorst [9], Heidi Ammerlaan [9], Kathleen Deiteren [10], Stephan Jl Bakker [11], Lucas Joost Van Pelt [11], Yvette Kluiters-de Hingh [3], Mathie Pg Leers [12], Andre J van der Ven [13]
Affiliations Expand

Affiliations

- [1] Sysmex Europe GmbH, Hamburg, Germany.
- [2] Amphia Hospital, Breda, Netherlands.
- [3] Elisabeth-Tweesteden Hospital, Tilburg, Netherlands.
- [4] Hospital Papa Giovanni XXIII, Bergamo, Italy.
- [5] Erasmus MC, University Medical Center, Rotterdam, Netherlands.
- [6] Leiden University Medical Center, Leiden, Netherlands.
- [7] Albert Schweitzer Hospital, Dordrecht, Netherlands.
- [8] Canisius Wilhelmina Hospital, Nijmegen, Netherlands.

- [9] Catharina Hospital, Eindhoven, Netherlands.
- [10] University Hospital Antwerp, Antwerp, Belgium.
- [11] University Medical Center Groningen, University of Groningen, Groningen, Netherlands.
- [12] Zuyderland Medical Center, Sittard-Geleen, Netherlands.
- [13] Radboud University Medical Center, Nijmegen, Netherlands.

- PMID: **33241996**
- PMCID: PMC7732342
- DOI: 10.7554/eLife.63195

Free PMC article
Observational Study

A novel haemocytometric COVID-19 prognostic score developed and validated in an observational multicentre European hospital-based study

Joachim Linssen et al. Elife. 2020.
Free PMC article

Show details

Elife

. 2020 Nov 26;9:e63195.
doi: 10.7554/eLife.63195.

Authors

Joachim Linssen [1], Anthony Ermens [2], Marvin Berrevoets [3], Michela Seghezzi [4], Giulia Previtali [4], Simone van der Sar-van der Brugge [2], Henk Russcher [5], Annelies Verbon [5], Judith Gillis [6], Jürgen Riedl [7], Eva de Jongh [7], Jarob Saker [1], Marion Münster [1], Imke Ca Munnix [8], Anthonius Dofferhof [8], Volkher Scharnhorst [9], Heidi Ammerlaan [9], Kathleen Deiteren [10], Stephan Jl Bakker [11], Lucas Joost Van Pelt [11], Yvette Kluiters-de Hingh [3], Mathie Pg Leers [12], Andre J van der Ven [13]

Affiliations

- [1] Sysmex Europe GmbH, Hamburg, Germany.
- [2] Amphia Hospital, Breda, Netherlands.
- [3] Elisabeth-Tweesteden Hospital, Tilburg, Netherlands.
- [4] Hospital Papa Giovanni XXIII, Bergamo, Italy.
- [5] Erasmus MC, University Medical Center, Rotterdam, Netherlands.
- [6] Leiden University Medical Center, Leiden, Netherlands.
- [7] Albert Schweitzer Hospital, Dordrecht, Netherlands.
- [8] Canisius Wilhelmina Hospital, Nijmegen, Netherlands.
- [9] Catharina Hospital, Eindhoven, Netherlands.

- [10] University Hospital Antwerp, Antwerp, Belgium.
- [11] University Medical Center Groningen, University of Groningen, Groningen, Netherlands.
- [12] Zuyderland Medical Center, Sittard-Geleen, Netherlands.
- [13] Radboud University Medical Center, Nijmegen, Netherlands.

- PMID: **33241996**
- PMCID: PMC7732342
- DOI: 10.7554/eLife.63195

Abstract

COVID-19 induces haemocytometric changes. Complete blood count changes, including new cell activation parameters, from 982 confirmed COVID-19 adult patients from 11 European hospitals were retrospectively analysed for distinctive patterns based on age, gender, clinical severity, symptom duration, and hospital days. The observed haemocytometric patterns formed the basis to develop a multi-haemocytometric-parameter prognostic score to predict, during the first three days after presentation, which patients will recover without ventilation or deteriorate within a two-week timeframe, needing intensive care or with fatal outcome. The prognostic score, with ROC curve AUC at baseline of 0.753 (95% CI 0.723-0.781) increasing to 0.875 (95% CI 0.806-0.926) on day 3, was superior to any individual parameter at distinguishing between clinical severity. Findings were confirmed in a validation cohort. Aim is that the score and haemocytometry results are simultaneously provided by analyser software, enabling wide applicability of the score as haemocytometry is commonly requested in COVID-19 patients.

Keywords: COVID-19; complete blood count; haemocytometry; human; intensive care; medicine; prognostic score.

© 2020, Linssen et al.

Conflict of interest statement

JL, JS, MM is a permanent employee of Sysmex Europe GMBH who provided free of charge study reagents to the study centres. AE, MB, MS, GP, Sv, HR, AV, JG, JR, Ed, IM, AD, VS, HA, KD, SB, LV, YK, ML No competing interests declared, Av has an ad hoc consultancy agreement with Sysmex Europe GMBH who provided free of charge study reagents to the study centres.

- 53 references
- 12 figures

Supplementary info

Publication types, MeSH terms, Grant support Expand

Publication types

- Multicenter Study
- Observational Study

MeSH terms

- Adolescent
- Adult
- Aged
- Aged, 80 and over
- Blood Cell Count / instrumentation
- Blood Cell Count / methods
- Blood Cell Count / statistics & numerical data*
- COVID-19 / blood*
- COVID-19 / epidemiology
- COVID-19 / virology
- Cohort Studies
- Europe
- Female
- Hospitalization / statistics & numerical data*
- Hospitals*
- Humans
- Male
- Middle Aged
- Pandemics
- Prognosis
- Retrospective Studies
- SARS-CoV-2 / physiology
- Young Adult

Grant support

Sysmex Europe GMBH provided free of charge reagents for the study. No monetary payments were made to any of the investigators. Joachim Linssen, Jarob Saker and Marion Münster are full-time employees of Sysmex Europe GMBH and Andre van der Ven has an ad hoc consultancy agreement with Sysmex Europe GMBH.

Full text links

eLife Sciences Publications, Ltd Free PMC article

Proceed to details

Cite

Share

142

Observational Study

J Int Med Res

. 2020 Dec;48(12):300060520979151.
doi: 10.1177/0300060520979151.

Impact of renin-angiotensin system inhibitors use on mortality in severe COVID-19 patients with hypertension: a retrospective observational study

Yanjun Zhong [1], Lishu Zhao [2], Guobao Wu [1], Chunhong Hu [2], Chenfang Wu [1], Min Xu [1], Haiyun Dong [1], Quan Zhang [3], Guyi Wang [1], Bo Yu [1], Jianlei Lv [3], Chao Wu [4] [5], Siye Zhang [1], Chenghui Cao [6], Long Shu [2], Yue Pan [2], Xianling Liu [2], Fang Wu [2]
Affiliations Expand

Affiliations

- [1] Department of Critical Care Medicine, the Second Xiangya Hospital, Central South University, Changsha, Hunan, China.
- [2] Department of Oncology, the Second Xiangya Hospital, Central South University, Changsha, Hunan, China.
- [3] Department of Critical Care Medicine, the First Hospital of Changsha, Hunan, China.
- [4] Department of Metabolism & Endocrinology, the Second Xiangya Hospital, Central South University, Changsha, Hunan, China.
- [5] Key Laboratory of Diabetes Immunology (Central South University), Ministry of Education; National Clinical Research Center for Metabolic Diseases, Changsha, Hunan, China.
- [6] Xiangya School of Medicine, Central South University, Changsha, Hunan, China.

- PMID: **33322988**
- PMCID: PMC7745588
- DOI: 10.1177/0300060520979151

Free PMC article
Observational Study

Impact of renin-angiotensin system inhibitors use on mortality in severe COVID-19 patients with hypertension: a retrospective observational study

Yanjun Zhong et al. J Int Med Res. 2020 Dec.
Free PMC article
Show details
J Int Med Res
. 2020 Dec;48(12):300060520979151.
doi: 10.1177/0300060520979151.

Authors

Yanjun Zhong [1], Lishu Zhao [2], Guobao Wu [1], Chunhong Hu [2], Chenfang Wu [1], Min Xu [1], Haiyun Dong [1], Quan Zhang [3], Guyi Wang [1], Bo Yu [1], Jianlei Lv [3], Chao Wu [4] [5], Siye Zhang [1], Chenghui Cao [6], Long Shu [2], Yue Pan [2], Xianling Liu [2], Fang Wu [2]

Affiliations

- [1] Department of Critical Care Medicine, the Second Xiangya Hospital, Central South University, Changsha, Hunan, China.
- [2] Department of Oncology, the Second Xiangya Hospital, Central South University, Changsha, Hunan, China.
- [3] Department of Critical Care Medicine, the First Hospital of Changsha, Hunan, China.
- [4] Department of Metabolism & Endocrinology, the Second Xiangya Hospital, Central South University, Changsha, Hunan, China.
- [5] Key Laboratory of Diabetes Immunology (Central South University), Ministry of Education; National Clinical Research Center for Metabolic Diseases, Changsha, Hunan, China.
- [6] Xiangya School of Medicine, Central South University, Changsha, Hunan, China.

- PMID: **33322988**
- PMCID: PMC7745588
- DOI: 10.1177/0300060520979151

Abstract

Objective: Association of angiotensin-converting enzyme inhibitors (ACEI) or angiotensin receptor blockers (ARB) use with coronavirus disease 2019 (COVID-19) remains controversial. We aimed to investigate the impact of ACEI/ARB use on all-cause mortality in severe COVID-19 patients with hypertension.

Methods: We enrolled 650 COVID-19 patients from Changsha and Wuhan city between 17 January 2020 and 8 March 2020. Demographic, clinical characteristics, and outcomes were collected. Multivariable analysis and propensity-score matching were performed to assess the impact of ACEI/ARB therapy on mortality.

Results: Among the 650 patients, 126 who had severe COVID-19 concomitant with hypertension were analyzed. The average age was 66 years and 56 (44.4%) were men. There were 37 ACEI/ARB users and 21 in-hospital deaths (mortality rate, 16.7%). Male sex (odds ratio [OR], 5.13; 95% confidence interval [CI], 1.75 to 17.8), but not ACEI/ARB use (OR, 1.09; 95%CI, 0.31 to 3.43), was an independent risk factor for mortality in severe COVID-19 patients with hypertension. After propensity-score matching, 60 severe COVID-19 patients were included and no significant correlation between use of ACEI/ARB and mortality was observed.

Conclusions: There was no significant association of ACEI/ARB use with mortality in severe COVID-19 patients with hypertension. These findings support the continuation of ACEI/ARB therapy for such patients.

Keywords: Angiotensin-converting enzyme inhibitors; angiotensin receptor blockers; coronavirus disease 2019; hypertension; mortality; renin–angiotensin system inhibitors; severe.

Conflict of interest statement

Declaration of conflicting interest: The authors declare that there is no conflict of interest.

- 32 references
- 2 figures

Supplementary info

Publication types, MeSH terms, Substances Expand

Publication types

- Observational Study

MeSH terms

- Aged
- Angiotensin Receptor Antagonists / adverse effects*
- Angiotensin-Converting Enzyme 2 / metabolism
- Angiotensin-Converting Enzyme Inhibitors / adverse effects*
- COVID-19 / complications
- COVID-19 / diagnosis
- COVID-19 / mortality*
- COVID-19 / virology
- Female
- Gene Expression Regulation / drug effects
- Hospital Mortality
- Humans
- Hypertension / complications
- Hypertension / drug therapy*
- Male
- Pandemics
- Renin-Angiotensin System / drug effects
- Retrospective Studies
- Risk Factors
- SARS-CoV-2 / drug effects
- SARS-CoV-2 / pathogenicity
- Severity of Illness Index
- Spike Glycoprotein, Coronavirus / metabolism
- Virus Internalization / drug effects

Substances

- Angiotensin Receptor Antagonists
- Angiotensin-Converting Enzyme Inhibitors
- Spike Glycoprotein, Coronavirus
- spike protein, SARS-CoV-2
- ACE2 protein, human
- Angiotensin-Converting Enzyme 2

Full text links

SAGE journals Open access full text Atypon Free PMC article
Proceed to details
Cite
Share
143
Observational Study
PLoS One
. 2020 Jun 25;15(6):e0235107.
doi: 10.1371/journal.pone.0235107. eCollection 2020.

Sociodemographic, clinical and laboratory factors on admission associated with COVID-19 mortality in hospitalized patients: A retrospective observational study

Mario Rivera-Izquierdo [1 2 3], María Del Carmen Valero-Ubierna [1], Juan Luis R-delAmo [4], Miguel Ángel Fernández-García [1], Silvia Martínez-Diz [1], Arezu Tahery-Mahmoud [5], Marta Rodríguez-Camacho [4], Ana Belén Gámiz-Molina [6], Nicolás Barba-Gyengo [6], Pablo Gámez-Baeza [6], Celia Cabrero-Rodríguez [6], Pedro Antonio Guirado-Ruiz [4], Divina Tatiana Martín-Romero [1], Antonio Jesús Láinez-Ramos-Bossini [7], María Rosa Sánchez-Pérez [8], José Mancera-Romero [8], Miguel García-Martín [2 3 9], Luis Miguel Martín-delosReyes [2 7 9], Virginia Martínez-Ruiz [2 3 9], Pablo Lardelli-Claret [2 3 9], Eladio Jiménez-Mejías [2 3 8 9]
Affiliations Expand

Affiliations

- [1] Preventive Medicine and Public Health Service, Hospital Universitario Clínico San Cecilio, Granada, Spain.
- [2] Department of Preventive Medicine and Public Health, University of Granada, Granada, Spain.
- [3] Instituto Biosanitario de Granada, IBS.Granada, Granada, Spain.

- [4] Neurology Service, Hospital Universitario Clínico San Cecilio, Granada, Spain.
- [5] Psychiatry Service, Hospital Universitario Clínico San Cecilio, Granada, Spain.
- [6] Pulmonology Service, Hospital Universitario Clínico San Cecilio, Granada, Spain.
- [7] Clinical Medicine and Public Heath, Doctorate Program, University of Granada, Granada, Spain.
- [8] SEMERGEN-UGR Chair of Teaching and Research in Family Medicine, University of Granada, Granada, Spain.
- [9] CIBER de Epidemiología y Salud Pública de España, CIBERESP, Madrid, Spain.

- PMID: **32584868**
- PMCID: PMC7316360
- DOI: 10.1371/journal.pone.0235107

Free PMC article
Observational Study

Sociodemographic, clinical and laboratory factors on admission associated with COVID-19 mortality in hospitalized patients: A retrospective observational study

Mario Rivera-Izquierdo et al. PLoS One. 2020.
Free PMC article

Show details

PLoS One

. 2020 Jun 25;15(6):e0235107.
doi: 10.1371/journal.pone.0235107. eCollection 2020.

Authors

Mario Rivera-Izquierdo [1 2 3], María Del Carmen Valero-Ubierna [1], Juan Luis R-delAmo [4], Miguel Ángel Fernández-García [1], Silvia Martínez-Diz [1], Arezu Tahery-Mahmoud [5], Marta Rodríguez-Camacho [4], Ana Belén Gámiz-Molina [6], Nicolás Barba-Gyengo [6], Pablo Gámez-Baeza [6], Celia Cabrero-Rodríguez [6], Pedro Antonio Guirado-Ruiz [4], Divina Tatiana Martín-Romero [1], Antonio Jesús Láinez-Ramos-Bossini [7], María Rosa Sánchez-Pérez [8], José Mancera-Romero [8], Miguel García-Martín [2 3 9], Luis Miguel Martín-delosReyes [2 7 9], Virginia Martínez-Ruiz [2 3 9], Pablo Lardelli-Claret [2 3 9], Eladio Jiménez-Mejías [2 3 8 9]

Affiliations

- [1] Preventive Medicine and Public Health Service, Hospital Universitario Clínico San Cecilio, Granada, Spain.
- [2] Department of Preventive Medicine and Public Health, University of Granada, Granada, Spain.
- [3] Instituto Biosanitario de Granada, IBS.Granada, Granada, Spain.

- [4] Neurology Service, Hospital Universitario Clínico San Cecilio, Granada, Spain.
- [5] Psychiatry Service, Hospital Universitario Clínico San Cecilio, Granada, Spain.
- [6] Pulmonology Service, Hospital Universitario Clínico San Cecilio, Granada, Spain.
- [7] Clinical Medicine and Public Heath, Doctorate Program, University of Granada, Granada, Spain.
- [8] SEMERGEN-UGR Chair of Teaching and Research in Family Medicine, University of Granada, Granada, Spain.
- [9] CIBER de Epidemiología y Salud Pública de España, CIBERESP, Madrid, Spain.

- PMID: **32584868**
- PMCID: PMC7316360
- DOI: 10.1371/journal.pone.0235107

Abstract

Background: To identify and quantify associations between baseline characteristics on hospital admission and mortality in patients with COVID-19 at a tertiary hospital in Spain.

Methods and findings: This retrospective case series included 238 patients hospitalized for COVID-19 at Hospital Universitario Clínico San Cecilio (Granada, Spain) who were discharged or who died. Electronic medical records were reviewed to obtain information on sex, age, personal antecedents, clinical features, findings on physical examination, and laboratory results for each patient. Associations between mortality and baseline characteristics were estimated as hazard ratios (HR) calculated with Cox regression models. Series mortality was 25.6%. Among patients with dependence for basic activities of daily living, 78.7% died, and among patients residing in retirement homes, 80.8% died. The variables most clearly associated with a greater hazard of death were age (3% HR increase per 1-year increase in age; 95%CI 1-6), diabetes mellitus (HR 2.42, 95%CI 1.43-4.09), SatO2/FiO2 ratio (43% HR reduction per 1-point increase; 95%CI 23-57), SOFA score (19% HR increase per 1-point increase, 95%CI 5-34) and CURB-65 score (76% HR increase per 1-point increase, 95%CI 23-143).

Conclusions: The patients residing in retirement homes showed great vulnerability. The main baseline factors that were independently associated with mortality in patients hospitalized for COVID-19 were older age, diabetes mellitus, low SatO2/FiO2 ratio, and high SOFA and CURB-65 scores.

Conflict of interest statement

The authors have declared that no competing interests exist.

- 45 references

Supplementary info

Publication types, MeSH terms, Substances, Grant support Expand

Publication types

- Observational Study
- Research Support, Non-U.S. Gov't

MeSH terms

- Activities of Daily Living
- Adult
- Age Factors*
- Aged
- Aged, 80 and over
- Betacoronavirus
- COVID-19
- Comorbidity
- Coronavirus Infections / mortality*
- Diabetes Mellitus*
- Female
- Hospitalization
- Humans
- Male
- Middle Aged
- Oxygen
- Pandemics
- Pneumonia, Viral / mortality*
- Proportional Hazards Models
- Retrospective Studies
- Risk Factors
- SARS-CoV-2
- Spain / epidemiology

Substances

- Oxygen

Grant support

This work was supported by the SEMERGEN-UGR Chair of Teaching and Research in Family Medicine (Cátedra de Docencia e Investigación en Medicina de Familia SEMERGEN-UGR), University of Granada, Spain to MRI. The funder played no role in the study design, data collection or analysis, and decision to publish.

Full text links

OPEN ACCESS TO FULL TEXT PLOS ONE Public Library of Science Free PMC article

Proceed to details

Cite

Share

☐ 144

Observational Study

BMC Infect Dis

. 2021 Feb 9;21(1):163.

doi: 10.1186/s12879-021-05840-2.

Clinical characteristics and outcomes of critically ill COVID-19 patients in Tokyo: a single-center observational study from the first wave

Aya Banno [1], Toru Hifumi [2], Hiroshi Okamoto [3], Minori Masaki [3], Koichiro Seki [3], Shutaro Isokawa [2], Norio Otani [2], Kuniyoshi Hayashi [4], Shinichi Ishimatsu [2]

Affiliations Expand

Affiliations

- [1] Department of Anesthesia and Intensive Care, St. Luke's International Hospital, 9-1 Akashicho, Chuo-ku, Tokyo, 104-8560, Japan. ayabanno@luke.ac.jp.
- [2] Department of Emergency and Critical Care Medicine, St. Luke's International Hospital, Tokyo, Japan.
- [3] Department of Anesthesia and Intensive Care, St. Luke's International Hospital, 9-1 Akashicho, Chuo-ku, Tokyo, 104-8560, Japan.
- [4] Graduate School of Public Health, St. Luke's International University, Tokyo, Japan.

- PMID: **33563218**
- PMCID: PMC7871305
- DOI: 10.1186/s12879-021-05840-2

Free PMC article

Observational Study

Clinical characteristics and outcomes of critically ill COVID-19 patients in Tokyo: a single-center observational study from the first wave

Aya Banno et al. BMC Infect Dis. 2021.

Free PMC article

Show details

BMC Infect Dis

. 2021 Feb 9;21(1):163.

doi: 10.1186/s12879-021-05840-2.

Authors

Aya Banno [1], Toru Hifumi [2], Hiroshi Okamoto [3], Minori Masaki [3], Koichiro Seki [3], Shutaro Isokawa [2], Norio Otani [2], Kuniyoshi Hayashi [4], Shinichi Ishimatsu [2]

Affiliations

- [1] Department of Anesthesia and Intensive Care, St. Luke's International Hospital, 9-1 Akashicho, Chuo-ku, Tokyo, 104-8560, Japan. ayabanno@luke.ac.jp.
- [2] Department of Emergency and Critical Care Medicine, St. Luke's International Hospital, Tokyo, Japan.
- [3] Department of Anesthesia and Intensive Care, St. Luke's International Hospital, 9-1 Akashicho, Chuo-ku, Tokyo, 104-8560, Japan.
- [4] Graduate School of Public Health, St. Luke's International University, Tokyo, Japan.

- PMID: **33563218**
- PMCID: PMC7871305
- DOI: 10.1186/s12879-021-05840-2

Abstract

Background: Many studies have been published about critically ill coronavirus disease 2019 (COVID-19) during the early phases of the pandemic but the characteristic or survival of critically ill Japanese patients have not yet been investigated. We sought to investigate the characteristics, inflammatory laboratory finding trends, and outcomes among critically ill Japanese patients who were admitted to the intensive care unit (ICU) with the first wave of COVID-19.

Methods: A retrospective observational study was performed in a single institution in the center of Tokyo. Laboratory-confirmed COVID-19 patients admitted to the ICU from March 19 to April 30, 2020 were included. Trends for significant inflammatory laboratory findings were analyzed. In-hospital death, days of mechanical ventilation or oxygen supplementation, days of ICU or hospital stay were followed until May 26, 2020.

Results: Twenty-four patients were included. Median age was 57.5 years, and 79% were male. The neutrophil-to-lymphocyte ratio was elevated to a median of 10.1 on admission and peaked on Day 10 of illness. Seventeen patients were intubated on Day 11 of illness and received mechanical ventilation. One patient underwent extracorporeal membrane oxygenation. The majority (88%) received systemic steroids, including 16 patients who received high dose methylprednisolone (500-1000 mg). Favipiravir was used in 38% of patients. Two patients, including 1 who refused intensive care, died. Eighteen patients were discharged. Median length of ICU and hospital stay for all patients was 6 and 22 days, respectively. Median length of ventilator dependency was 7 days. Four patients underwent a tracheostomy and received prolonged ventilation for more than 21 days. One patient receiving mechanical ventilation died. All survivors discontinued ventilator use.

Conclusions: Mortality was remarkably low in our single institutional study. Three survivors received mechanical ventilation for more than 3 weeks. Trends of clinically significant laboratory markers reflected the clinical course of COVID-19.

Keywords: COVID-19; Coronavirus disease; Favipiravir; ICU; Japan; Mortality; Systemic steroid.

Conflict of interest statement

The authors declare that they have no competing interests.

- 38 references
- 3 figures

Supplementary info

Publication types, MeSH terms, Substances Expand

Publication types

- Observational Study

MeSH terms

- Adult
- Aged
- Aged, 80 and over
- Antiviral Agents / therapeutic use
- C-Reactive Protein / analysis
- COVID-19 / immunology
- COVID-19 / mortality
- COVID-19 / physiopathology*
- COVID-19 / therapy*
- Critical Illness
- Extracorporeal Membrane Oxygenation
- Female
- Fibrin Fibrinogen Degradation Products / analysis
- Hospitalization
- Humans
- Intensive Care Units
- Length of Stay
- Leukocyte Count
- Male
- Methylprednisolone / therapeutic use
- Middle Aged
- Respiration, Artificial
- Retrospective Studies
- Tokyo

Substances

- Antiviral Agents
- Fibrin Fibrinogen Degradation Products
- fibrin fragment D
- C-Reactive Protein
- Methylprednisolone

Full text links

Read free full text at BMC BioMed Central Free PMC article
Proceed to details
Cite
Share
145
Observational Study
Am J Trop Med Hyg
. 2020 Dec 30;104(2):532-536.
doi: 10.4269/ajtmh.20-1356.

Clinical Profile and Treatment of COVID-19 Patients: Experiences from an Ethiopian Treatment Center

Seid Getahun Abdela [1], Seid Hassen Abegaz [2], Wondmagegn Demsiss [3], Koku Sisay Tamirat [4], Saskia van Henten [5], Johan van Griensven [5]
Affiliations Expand

Affiliations

- [1] 1Department of Internal Medicine, College of Medicine and Health Sciences, Wollo University, Dessie, Ethiopia.
- [2] 2Boru Meda Hospital, Dessie, Ethiopia.
- [3] 3Department of Medical Laboratory Science, College of Medicine and Health Sciences, Wollo University, Dessie, Ethiopia.
- [4] 4Institute of Public Health, College of Medicine and Health Sciences, University of Gondar, Gondar, Ethiopia.
- [5] 5Institute of Tropical Medicine, Antwerp, Belgium.

- PMID: **33382029**
- PMCID: PMC7866348
- DOI: 10.4269/ajtmh.20-1356

Free PMC article

Observational Study

Clinical Profile and Treatment of COVID-19 Patients: Experiences from an Ethiopian Treatment Center

Seid Getahun Abdela et al. Am J Trop Med Hyg. 2020.
Free PMC article
Show details
Am J Trop Med Hyg
. 2020 Dec 30;104(2):532-536.
doi: 10.4269/ajtmh.20-1356.

Authors

Seid Getahun Abdela [1], Seid Hassen Abegaz [2], Wondmagegn Demsiss [3], Koku Sisay Tamirat [4], Saskia van Henten [5], Johan van Griensven [5]

Affiliations

- [1] 1Department of Internal Medicine, College of Medicine and Health Sciences, Wollo University, Dessie, Ethiopia.
- [2] 2Boru Meda Hospital, Dessie, Ethiopia.
- [3] 3Department of Medical Laboratory Science, College of Medicine and Health Sciences, Wollo University, Dessie, Ethiopia.
- [4] 4Institute of Public Health, College of Medicine and Health Sciences, University of Gondar, Gondar, Ethiopia.
- [5] 5Institute of Tropical Medicine, Antwerp, Belgium.

- PMID: **33382029**
- PMCID: PMC7866348
- DOI: 10.4269/ajtmh.20-1356

Abstract

COVID-19 is not well studied in Africa. Understanding the clinical profile and management of COVID-19 will help to plan better prevention and treatment strategies taking the local context into consideration. In this study, we described the clinical profile, treatment used, and outcomes of COVID-19 patients in one of the COVID-19 treatment centers of Ethiopia, Boru Meda Hospital. An institution-based retrospective cross-sectional study was carried out using medical records of COVID-19 patients who were admitted to Boru Meda Hospital with a positive reverse transcription (RT)-PCR result from May 9, 2020 to September 20, 2020. All patients with a positive RT-PCR were admitted to the hospital, regardless of symptom and severity status. A total of 279 COVID-19 patients were included in the final analysis. The median age of patients was 28 years (interquartile range 23-40). The majority (69.5%) were male. Around a quarter (n = 73; 26.2%) of the patients were symptomatic, of which cough (n = 49; 67.1%) and fever (n = 32; 43.8%) were common symptoms. Among symptomatic patients, 48 (65.8%) were mild, four

(5.5%) moderate, 12 (16.4%) severe, and nine (12.3%) were critical. The case fatality rate was 2.1%. Hypertension, age older than 25 years, and HIV/AIDS were significantly associated with symptomatic infection. In this study, most of the COVID-19 patients were asymptomatic. However, the proportion of severe and critical patients among those with symptoms was high. More studies are needed to assess the effect of HIV/AIDS on the severity and mortality of COVID-19.

- 14 references
- 1 figure

Supplementary info

Publication types, MeSH terms, Supplementary concepts Expand

Publication types

- Observational Study

MeSH terms

- Adolescent
- Adult
- COVID-19 / drug therapy*
- COVID-19 / epidemiology
- COVID-19 / pathology
- Cough
- Disease Management
- Ethiopia / epidemiology
- Female
- Fever
- Hospitals / statistics & numerical data*
- Humans
- Male
- Middle Aged
- Retrospective Studies
- SARS-CoV-2 / drug effects*
- Young Adult

Supplementary concepts

- COVID-19 drug treatment

Full text links

Full Text Sheridan PubFactory Free PMC article
Proceed to details
Cite
Share
146
Observational Study
Int J Clin Pract
. 2021 Feb;75(2):e13896.
doi: 10.1111/ijcp.13896. Epub 2020 Dec 15.

Hydroxychloroquine/azithromycin treatment, QT interval and ventricular arrhythmias in hospitalised patients with COVID-19

İbrahim Halil Özdemir [1], Bülent Özlek [2], Mehmet Burak Özen [1], Ramazan Gündüz [1], Nurullah Çetin [3], Ali Rıza Bilge [3]
Affiliations Expand

Affiliations

- [1] Department of Cardiology, Manisa City Hospital, Manisa, Turkey.
- [2] Department of Cardiology, Mugla Sitki Kocman University Training and Research Hospital, Mugla, Turkey.
- [3] Department of Cardiology, Faculty of Medicine, Manisa Celal Bayar University, Manisa, Turkey.

- PMID: **33280207**
- PMCID: PMC7883090
- DOI: 10.1111/ijcp.13896

Free PMC article
Observational Study

Hydroxychloroquine/azithromycin treatment, QT interval and ventricular arrhythmias in hospitalised patients with COVID-19

İbrahim Halil Özdemir et al. Int J Clin Pract. 2021 Feb.
Free PMC article
Show details
Int J Clin Pract
. 2021 Feb;75(2):e13896.
doi: 10.1111/ijcp.13896. Epub 2020 Dec 15.

Authors

İbrahim Halil Özdemir [1], Bülent Özlek [2], Mehmet Burak Özen [1], Ramazan Gündüz [1], Nurullah Çetin [3], Ali Rıza Bilge [3]

Affiliations

- [1] Department of Cardiology, Manisa City Hospital, Manisa, Turkey.
- [2] Department of Cardiology, Mugla Sitki Kocman University Training and Research Hospital, Mugla, Turkey.
- [3] Department of Cardiology, Faculty of Medicine, Manisa Celal Bayar University, Manisa, Turkey.

- PMID: **33280207**
- PMCID: PMC7883090
- DOI: 10.1111/ijcp.13896

Abstract

Background: Hydroxychloroquine (HCQ) and azithromycin (AZM) are widely used in off-label treatment of novel coronavirus disease (COVID-19). However, cardiac safety of these drugs is still controversial in COVID-19. Therefore, we aimed to evaluate association of HCQ or HCQ + AZM treatment regimens, corrected QT (QTc) interval and malignant ventricular arrhythmias in hospitalized patients.

Methods: This is a single-center, retrospective and observational study. All data were extracted from the electronic medical records. The initial and post-treatment mean QTc intervals were calculated and compared in patients with HCQ alone or HCQ + AZM therapy. Associated factors with QTc prolongation, the incidence of ventricular arrhythmia during treatment and in-hospital mortality because of ventricular arrhythmias were evaluated.

Results: Our cohort comprised 101 hospitalized COVID-19 patients (mean age of 49.60 ± 18 years, 54.4% men). HCQ + AZM combination therapy group (n = 56) was more likely to have comorbidities. After 5-days treatment, 19 (18.8%) patients had QTc prolongation, and significant increase in the QTc interval was observed in both two groups ($P < .001$). However, HCQ + AZM combination group had significantly higher ΔQTc compared to HCQ group (22.5 ± 18.4 vs 7.5 ± 15.3 ms, $P < .001$). All of 101 patients completed the 5-days treatment without interruption. Also, no malignant ventricular arrhythmia or death secondary to ventricular arrhythmia occurred during the treatment in both groups.

Conclusions: The present study revealed that although HCQ + AZM treatment was independently associated with QTc prolongation, none of patients experienced malignant ventricular arrhythmia or death during treatment. Further prospective studies are needed to determine the exact implications of these drugs on arrhythmias in patients with COVID-19.

© 2020 John Wiley & Sons Ltd.

Conflict of interest statement

The authors declare that they have no conflict of interest.

- 32 references
- 3 figures

Supplementary info

Publication types, MeSH terms, Substances Expand

Publication types

- Observational Study

MeSH terms

- Adult
- Aged
- Anti-Bacterial Agents / therapeutic use*
- Arrhythmias, Cardiac / drug therapy*
- Arrhythmias, Cardiac / epidemiology
- Azithromycin / therapeutic use*
- COVID-19 / drug therapy*
- COVID-19 / epidemiology
- Comorbidity
- Drug Therapy, Combination
- Electrocardiography
- Female
- Humans
- Hydroxychloroquine / therapeutic use*
- Long QT Syndrome / drug therapy*
- Long QT Syndrome / epidemiology
- Male
- Middle Aged
- Retrospective Studies
- SARS-CoV-2

Substances

- Anti-Bacterial Agents
- Hydroxychloroquine
- Azithromycin

Full text links

WILEY Full Text Article Wiley Free PMC article

Proceed to details
Cite
Share
☐ 147
Observational Study
J Med Internet Res
. 2020 Aug 13;22(8):e20108.
doi: 10.2196/20108.

Characteristics and Outcomes of a Sample of Patients With COVID-19 Identified Through Social Media in Wuhan, China: Observational Study

Dong Liu [1], Yuyan Wang [# 2], Juan Wang [# 3], Jue Liu [# 4], Yongjie Yue [# 1], Wenjun Liu [1], Fuhai Zhang [# 5], Ziping Wang [2]
Affiliations Expand

Affiliations

- [1] Renmin University of China, Beijing, China.
- [2] Key Laboratory of Carcinogenesis and Translational Research (Ministry of Education/Beijing), Department of Thoracic Medical Oncology, Peking University Cancer Hospital & Institute, Beijing, China.
- [3] Department of Radiology, Peking University Shougang Hospital, Beijing, China.
- [4] Department of Epidemiology and Biostatistics, School of Public Health, Peking University, Beijing, China.
- [5] School of Education, Hebei Normal University, Shijiazhuang, China.

[#] Contributed equally.

- PMID: **32716901**
- PMCID: PMC7431239
- DOI: 10.2196/20108

Free PMC article
Observational Study

Characteristics and Outcomes of a Sample of Patients With COVID-19 Identified Through Social Media in Wuhan, China: Observational Study

Dong Liu et al. J Med Internet Res. 2020.
Free PMC article
Show details
J Med Internet Res
. 2020 Aug 13;22(8):e20108.
doi: 10.2196/20108.

Authors

Dong Liu [1], Yuyan Wang [# 2], Juan Wang [# 3], Jue Liu [# 4], Yongjie Yue [# 1], Wenjun Liu [1], Fuhai Zhang [# 5], Ziping Wang [2]

Affiliations

- [1] Renmin University of China, Beijing, China.
- [2] Key Laboratory of Carcinogenesis and Translational Research (Ministry of Education/Beijing), Department of Thoracic Medical Oncology, Peking University Cancer Hospital & Institute, Beijing, China.
- [3] Department of Radiology, Peking University Shougang Hospital, Beijing, China.
- [4] Department of Epidemiology and Biostatistics, School of Public Health, Peking University, Beijing, China.
- [5] School of Education, Hebei Normal University, Shijiazhuang, China.

[#] Contributed equally.

- PMID: **32716901**
- PMCID: PMC7431239
- DOI: 10.2196/20108

Abstract

Background: The number of deaths worldwide caused by coronavirus disease (COVID-19) is increasing rapidly. Information about the clinical characteristics of patients with COVID-19 who were not admitted to hospital is limited. Some risk factors of mortality associated with COVID-19 are controversial (eg, smoking). Moreover, the impact of city closure on mortality and admission rates is unknown.

Objective: The aim of this study was to explore the risk factors of mortality associated with COVID-19 infection among a sample of patients in Wuhan whose conditions were reported on social media.

Methods: We enrolled 599 patients with COVID-19 from 67 hospitals in Wuhan in the study; 117 of the participants (19.5%) were not admitted to hospital. The demographic, epidemiological, clinical, and radiological features of the patients were extracted from their social media posts and coded. Telephone follow-up was conducted 1 month later (between March 15 and 23, 2020) to check the clinical outcomes of the patients and acquire other relevant information.

Results: The median age of patients with COVID-19 who died (72 years, IQR 66.5-82.0) was significantly higher than that of patients who recovered (61 years, IQR 53-69, P<.001). We found that lack of admission to hospital (odds ratio [OR] 5.82, 95% CI 3.36-10.1; P<.001), older age (OR 1.08, 95% CI 1.06-1.1; P<.001), diffuse distribution (OR 11.09, 95% CI 0.93-132.9; P=.058), and hypoxemia (odds ratio 2.94, 95% CI 1.32-6.6; P=.009) were associated with increasing odds of death. Smoking was not significantly associated with mortality risk (OR 0.9, 95% CI 0.44-1.85; P=.78).

Conclusions: Older age, diffuse distribution, and hypoxemia are factors that can help clinicians identify patients with COVID-19 who have poor prognosis. Our study suggests that aggregated data from social media can also be comprehensive, immediate, and informative in disease prognosis.

Keywords: COVID-19; clinical characteristic; coronavirus; infectious disease; mortality; outcome; prognosis, China; risk factors; social media; web-based data.

©Dong Liu, Yuyan Wang, Juan Wang, Jue Liu, Yongjie Yue, Wenjun Liu, Fuhai Zhang, Ziping Wang. Originally published in the Journal of Medical Internet Research (http://www.jmir.org), 13.08.2020.

Conflict of interest statement

Conflicts of Interest: None declared.

- 22 references
- 10 figures

Supplementary info

Publication types, MeSH terms Expand

Publication types

- Observational Study
- Research Support, Non-U.S. Gov't

MeSH terms

- Adolescent
- Adult
- Aged
- Aged, 80 and over
- Betacoronavirus*

- COVID-19
- Child
- Child, Preschool
- China
- Coronavirus Infections*
- Female
- Hospitalization
- Humans
- Infant
- Infant, Newborn
- Male
- Middle Aged
- Odds Ratio
- Pandemics*
- Pneumonia, Viral*
- Prognosis
- Retrospective Studies
- Risk Factors
- SARS-CoV-2
- Social Media
- Young Adult

Full text links

JMIR Publications Free PMC article

Proceed to details

Cite

Share

☐ 148

Observational Study

Drug Discov Ther
. 2021 Mar 10;15(1):1-8.
doi: 10.5582/ddt.2020.03068. Epub 2021 Feb 28.

Clinical profile and outcomes of asymptomatic vs. symptomatic travellers diagnosed with COVID-19: An observational study from a coastal town in South India

[Nitin Gupta][1, 2], [Anupa John][3], [Mohammed Safeer Kokkottil][1], [Muralidhar Varma][1], [Shashikiran Umakanth][4], [Kavitha Saravu][1, 2]
Affiliations Expand

Affiliations

- [1] Department of Infectious Diseases, Kasturba Medical College, Manipal Academy of Higher Education, Manipal, Karnataka, India.
- [2] Manipal Center for Infectious Diseases, Prasanna School of Public Health, Manipal Academy of Higher Education, Manipal, Karnataka, India.
- [3] Department of Medicine, Kasturba Medical College, Manipal Academy of Higher Education, Manipal, Karnataka, India.
- [4] Department of Medicine, Dr TMA Pai Hospital, Melaka Manipal Medical College, Manipal Academy of Higher Education, Manipal, Karnataka, India.

- PMID: **33642450**
- DOI: 10.5582/ddt.2020.03068

Free article
Observational Study

Clinical profile and outcomes of asymptomatic vs. symptomatic travellers diagnosed with COVID-19: An observational study from a coastal town in South India

Nitin Gupta et al. Drug Discov Ther. 2021.
Free article
Show details
Drug Discov Ther
. 2021 Mar 10;15(1):1-8.
doi: 10.5582/ddt.2020.03068. Epub 2021 Feb 28.

Authors

[Nitin Gupta][1, 2], [Anupa John][3], [Mohammed Safeer Kokkottil][1], [Muralidhar Varma][1], [Shashikiran Umakanth][4], [Kavitha Saravu][1, 2]

Affiliations

- [1] Department of Infectious Diseases, Kasturba Medical College, Manipal Academy of Higher Education, Manipal, Karnataka, India.
- [2] Manipal Center for Infectious Diseases, Prasanna School of Public Health, Manipal Academy of Higher Education, Manipal, Karnataka, India.
- [3] Department of Medicine, Kasturba Medical College, Manipal Academy of Higher Education, Manipal, Karnataka, India.

- [4] Department of Medicine, Dr TMA Pai Hospital, Melaka Manipal Medical College, Manipal Academy of Higher Education, Manipal, Karnataka, India.

- PMID: **33642450**
- DOI: 10.5582/ddt.2020.03068

Abstract

Despite the high number of coronavirus disease-19 (COVID-19) cases from India, there are few reports from India describing the clinical epidemiology of COVID-19. This study aimed to describe the clinical/epidemiological characteristics and outcomes of asymptomatic vs. symptomatic COVID-19 patients. This was a retrospective chart review of all admitted patients with COVID-19 above 18 years with a history of travel within one month of the admission. The patients were categorized into asymptomatic and symptomatic. The symptomatic patients were further classified into mild, moderate and severe. The demographic profile, risk factors, clinical features, laboratory parameters, treatment details and outcome of all patients were recorded. The clinical and laboratory parameters were compared between symptomatic patients and asymptomatic patients. Of the 127 recruited patients, 75 were asymptomatic. Of the 52 symptomatic patients, 41 patients were classified as a mild illness. The mean age of the patients was 44.5 ± 15 years. A total of 73 patients had one or more risk factors. The male patients were more commonly found to be symptomatic compared to female patients. Neutrophil-lymphocyte ratio, C-reactive protein and lactate dehydrogenase were significantly elevated in symptomatic patients. A total of five individuals required supplemental oxygen therapy, and one of them required mechanical ventilation. All the patients had favourable outcomes. Asymptomatic and mild illness form a significant proportion of positive patients and have excellent outcomes without therapeutic interventions.

Keywords: COVID-19; Pregnancy; Presymptomatic; SARS-CoV-2; asymptomatic; household contact; transmission.

Supplementary info

Publication types, MeSH terms, Substances Expand

Publication types

- Comparative Study
- Observational Study

MeSH terms

- Adult
- Asymptomatic Infections / epidemiology*
- C-Reactive Protein / metabolism
- COVID-19 / blood
- COVID-19 / epidemiology*
- COVID-19 / therapy*
- Communicable Diseases, Imported / blood

- Communicable Diseases, Imported / epidemiology*
- Communicable Diseases, Imported / therapy*
- Communicable Diseases, Imported / virology
- Female
- Hospitalization / statistics & numerical data
- Humans
- India / epidemiology
- L-Lactate Dehydrogenase / blood
- Lymphocyte Count
- Male
- Middle Aged
- Neutrophils / metabolism
- Oxygen Inhalation Therapy
- Prognosis
- Respiration, Artificial
- Retrospective Studies
- Travel-Related Illness
- Young Adult

Substances

- C-Reactive Protein
- L-Lactate Dehydrogenase

Full text links

J-STAGE, Japan Science and Technology Information Aggregator, Electronic
Proceed to details
Cite
Share
149
Observational Study
J Assoc Physicians India
. 2022 Jan;70(1):11-12.

Adjuvant Tocilizumab in the Treatment of Patients with Moderate to Severe COVID-19 Pneumonia: An Observational Study

Minal Shastri [1], Darshankumar Manubhai Raval [2], Vaishnavi M Rathod [3], Dhruti Patel [4]
Affiliations Expand

Affiliations

- [1] Head of Unit, Department of Medicine, Baroda Medical College, SSGH, Vadodara, Gujarat.
- [2] Resident Doctor, Department of Medicine, Baroda Medical College, SSGH, Vadodara, Gujarat.
- [3] Resident Doctor, Department of Medicine, Baroda Medical College, SSGH, Vadodara, Gujarat; Corresponding Author.
- [4] Intern Doctor, GMERS Medical College and Hospital, Vadodara, Gujarat.

- PMID: **35062798**

Observational Study

Adjuvant Tocilizumab in the Treatment of Patients with Moderate to Severe COVID-19 Pneumonia: An Observational Study

Minal Shastri et al. J Assoc Physicians India. 2022 Jan.
Show details
J Assoc Physicians India
. 2022 Jan;70(1):11-12.

Authors

Minal Shastri [1], Darshankumar Manubhai Raval [2], Vaishnavi M Rathod [3], Dhruti Patel [4]

Affiliations

- [1] Head of Unit, Department of Medicine, Baroda Medical College, SSGH, Vadodara, Gujarat.
- [2] Resident Doctor, Department of Medicine, Baroda Medical College, SSGH, Vadodara, Gujarat.
- [3] Resident Doctor, Department of Medicine, Baroda Medical College, SSGH, Vadodara, Gujarat; Corresponding Author.
- [4] Intern Doctor, GMERS Medical College and Hospital, Vadodara, Gujarat.

- PMID: **35062798**

Abstract

Importance: As there is no definitive treatment available for covid-19 pneumonia, with timed administration of tocilizumab as an adjuvant therapy in moderate to severe covid-19 pneumonia, we can reduce mortality due to cytokine storm.

Objective: To determine effectiveness of tocilizumab as an adjuvant therapy in moderate to severe covid-19 pneumonia patients.

Design: Retrospective Observational study from the time period of April 2020 to December 2020.

Setting: This study has been carried out at SSG Hospital, a tertiary care hospital at Vadodara, Gujarat.

Participants: 80 moderate to severe COVID-19 positive patients in the group of age 18-80 years requiring hospitalisation in whom tocilizumab (8mg/kg to a maximum dose of 800mg) was given, were enrolled in the study. The following patient details were collected and studied in relation to the use of tocilizumab- Biodata, Presenting complaints, past history of any illness, Drug history, Vitals and physical examination, Investigations, Treatment. Exposures: 80 patients of moderate to severe COVID-19 pneumonia who were given Injection tocilizumab (8mg/kg to a maximum dose of 800mg) were selected retrospectively. Main outcomes and Measures: We have correlated the outcome in the form of discharged or death with the help of parameters likes: Time to Clinical Improvement, Ventilator-Free Days, Duration of ICU Stay, Time to Clinical Failure, and Time to Hospital Discharge.

Results: Out of the total 80 patients, 29 patients were in the age group of more than 60 years, of which 7 were discharged and 22 died. The other 51 patients were less than 60 years of age, of which 28 patients were discharged and the rest 23 died. (p value 0.007). 63 patients out of the total 80 patients were males of which 31 (49.21%) recovered while 32 (50.79%) of them died. 5 (29.11%) females out of the total 17 female covid positive patients recovered while the other 12 (70.59 %) died. (p value 0.14). Conclusion and Relevance: In our study, we have observed that even after giving tocilizumab mortality was higher in patients above 60 years of age, two or more co-morbidities, SpO2 of less than 85% on the room air, SOFA scoring of more than or equal to 3, radiological involvement of more than 2 zones, higher score (>3) of inflammatory markers, higher level of mode of respiratory support like BiPAP or invasive ventilation. . This shows that the ideal time to give tocilizumab is when the patient is on NRBM support. By this it gives the maximum benefit. There was no difference in outcome in patients of either gender, blood pressure and on admission blood sugar levels, and co-administration of injection Ramdesivir. Significant percentage of patients died who had uncontrolled diabetes mellitus with insignificant p value, so further studies are required to find out the association.

© Journal of the Association of Physicians of India 2011.

Supplementary info

Publication types, MeSH terms, Substances, Supplementary concepts Expand

Publication types

- Observational Study

MeSH terms

- Adolescent
- Adult
- Aged
- Aged, 80 and over
- Antibodies, Monoclonal, Humanized
- COVID-19* / drug therapy

- Female
- Humans
- Male
- Middle Aged
- Oxygen Saturation
- Retrospective Studies
- SARS-CoV-2
- Treatment Outcome
- Young Adult

Substances

- Antibodies, Monoclonal, Humanized
- tocilizumab

Supplementary concepts

- COVID-19 drug treatment

Proceed to details
Cite
Share
☐ 150
Observational Study
J Korean Med Sci
. 2020 Jun 29;35(25):e236.
doi: 10.3346/jkms.2020.35.e236.

The Epidemiological and Clinical Characteristics of 81 Children with COVID-19 in a Pandemic Hospital in Turkey: an Observational Cohort Study

Muhammet Furkan Korkmaz [1], Esra Türe [2], Bayram Ali Dorum [3], Zeliha Banu Kılıç [4]
Affiliations Expand

Affiliations

- [1] Department of Pediatrics, Bursa City Hospital, Bursa, Turkey. korkmazmfurkan@gmail.com.
- [2] Department of Pediatric Emergency, Bursa City Hospital, Bursa, Turkey.
- [3] Department of Neonatology, Bursa City Hospital, Bursa, Turkey.

- [4] Department of Clinical Microbiology, Bursa City Hospital, Bursa, Turkey.

- PMID: **32597047**
- PMCID: PMC7324269
- DOI: 10.3346/jkms.2020.35.e236

Free PMC article
Observational Study

The Epidemiological and Clinical Characteristics of 81 Children with COVID-19 in a Pandemic Hospital in Turkey: an Observational Cohort Study

Muhammet Furkan Korkmaz et al. J Korean Med Sci. 2020.
Free PMC article
Show details
J Korean Med Sci
. 2020 Jun 29;35(25):e236.
doi: 10.3346/jkms.2020.35.e236.

Authors

Muhammet Furkan Korkmaz [1], Esra Türe [2], Bayram Ali Dorum [3], Zeliha Banu Kılıç [4]

Affiliations

- [1] Department of Pediatrics, Bursa City Hospital, Bursa, Turkey. korkmazmfurkan@gmail.com.
- [2] Department of Pediatric Emergency, Bursa City Hospital, Bursa, Turkey.
- [3] Department of Neonatology, Bursa City Hospital, Bursa, Turkey.
- [4] Department of Clinical Microbiology, Bursa City Hospital, Bursa, Turkey.

- PMID: **32597047**
- PMCID: PMC7324269
- DOI: 10.3346/jkms.2020.35.e236

Abstract

Background: Coronavirus disease-2019 (COVID-19) pandemic has affected millions of people throughout the world since December 2019. However, there is a limited amount of data about pediatric patients infected with the disease agent, the severe acute respiratory syndrome coronavirus 2 (SARS-CoV-2).

Methods: The epidemiological, laboratory, radiological, and treatment features of the pediatric patients who were positive for SARS-CoV-2 based on the reverse-transcription polymerase chain reaction (RT-PCR) test, were investigated retrospectively.

Results: The median age of 81 children included in the study was 9.50 years (0-17.75 years). The most frequent symptoms at the time of admission were fever (58%), cough (52%), and fatigue or myalgia (19%). The abnormal laboratory findings in these cases were decreased lymphocytes (2.5%, n = 2), leucopenia (5%, n = 4), and increased lactate dehydrogenase (17.2%, n = 14), C-reactive protein (16%, n = 13), procalcitonin (3.7%, n = 3), and D-dimer (12.3%, n = 10). Three (4%) patients had consolidation in chest computed tomography, and three (4%) had ground-glass opacities. None of the patients needed intensive care except for the newborns. The median time to turn SARS-CoV-2 negative in the RT-PCR test was 5 (3-10) days. The median length of hospital stay was 5 (4-10) days. The time to turn SARS-CoV-2 negative in the RT-PCR test and the length of hospital stay were significantly longer for those aged five years or younger than others (P = 0.037, P = 0.01).

Conclusion: Compared to adults, COVID-19 is milder and more distinctive in children. As a result, more conservative approaches might be preferred in children for the diagnostic, clinical, and even therapeutic applications.

Keywords: COVID-19; Children; Clinical Symptoms; Coronavirus; Newborn; SARS-CoV-2.

© 2020 The Korean Academy of Medical Sciences.

Conflict of interest statement

The authors have no potential conflicts of interest to disclose.

- 28 references
- 1 figure

Supplementary info

Publication types, MeSH terms Expand

Publication types

- Observational Study

MeSH terms

- Adolescent
- Betacoronavirus
- Blood Chemical Analysis
- COVID-19
- Child
- Child, Preschool
- Coronavirus Infections / diagnosis
- Coronavirus Infections / epidemiology*

- Coronavirus Infections / pathology*
- Female
- Hospitalization
- Humans
- Infant
- Infant, Newborn
- Length of Stay
- Lung / pathology
- Male
- Pandemics
- Pneumonia, Viral / diagnosis
- Pneumonia, Viral / epidemiology*
- Pneumonia, Viral / pathology*
- Polymerase Chain Reaction
- Retrospective Studies
- SARS-CoV-2
- Turkey / epidemiology

Full text links

JKMS FULL TEXT FREE Journal of Korean Medical Science Korean Academy of Medical Sciences Free PMC article

Proceed to details

Cite

Share

151

Observational Study

Medicine (Baltimore)

. 2020 Nov 6;99(45):e22971.

doi: 10.1097/MD.0000000000022971.

Risk factors associated with disease aggravation among 126 hospitalized patients with COVID-19 in different places in China: A retrospective observational study

Shuai Shao [1 2 3], Zhiling Zhao [1 2 3], Feng Wang [1 2 3], Dandan Chang [4], Yong Liu [5], Shi Liu [6], Xiaoguang Xu [7], Xuyan Li [1 2 3], Chunguo Jiang [1 2 3], Ziren Tang [8]

Affiliations Expand

Affiliations

- [1] Department of Respiratory and Critical Care Medicine, Beijing Chaoyang Hospital, Capital Medical University.
- [2] Beijing Institute of Respiratory Medicine.
- [3] Beijing Engineering Research Center for Diagnosis and Treatment of Respiratory and Critical Care Medicine.
- [4] Biomedical Engineering School, Beijing Key Laboratory of Fundamental Research on Biomechanics in Clinical Application, Capital Medical University, Beijing.
- [5] Department of Respiratory and Critical Care Medicine, Zhoukou Central Hospital, Zhoukou, Henan Province.
- [6] Department of Gastroenterology, Union Hospital, Tongji Medical College, Huazhong University of Science and Technology, Wuhan, Hubei Province.
- [7] Department of Respiratory and Critical Care Medicine, Affiliated Hospital of Jilin Medical University, Jilin.
- [8] Department of Emergency Medicine, Beijing Chaoyang Hospital, Capital Medical University, Beijing, China.

- PMID: **33157938**
- PMCID: PMC7647556
- DOI: 10.1097/MD.0000000000022971

Free PMC article
Observational Study

Risk factors associated with disease aggravation among 126 hospitalized patients with COVID-19 in different places in China: A retrospective observational study

Shuai Shao et al. Medicine (Baltimore). 2020.
Free PMC article
Show details
Medicine (Baltimore)
. 2020 Nov 6;99(45):e22971.
doi: 10.1097/MD.0000000000022971.

Authors

Shuai Shao [1 2 3], Zhiling Zhao [1 2 3], Feng Wang [1 2 3], Dandan Chang [4], Yong Liu [5], Shi Liu [6], Xiaoguang Xu [7], Xuyan Li [1 2 3], Chunguo Jiang [1 2 3], Ziren Tang [8]

Affiliations

- [1] Department of Respiratory and Critical Care Medicine, Beijing Chaoyang Hospital, Capital Medical University.
- [2] Beijing Institute of Respiratory Medicine.

- [3] Beijing Engineering Research Center for Diagnosis and Treatment of Respiratory and Critical Care Medicine.
- [4] Biomedical Engineering School, Beijing Key Laboratory of Fundamental Research on Biomechanics in Clinical Application, Capital Medical University, Beijing.
- [5] Department of Respiratory and Critical Care Medicine, Zhoukou Central Hospital, Zhoukou, Henan Province.
- [6] Department of Gastroenterology, Union Hospital, Tongji Medical College, Huazhong University of Science and Technology, Wuhan, Hubei Province.
- [7] Department of Respiratory and Critical Care Medicine, Affiliated Hospital of Jilin Medical University, Jilin.
- [8] Department of Emergency Medicine, Beijing Chaoyang Hospital, Capital Medical University, Beijing, China.

- PMID: **33157938**
- PMCID: PMC7647556
- DOI: 10.1097/MD.0000000000022971

Abstract

Coronavirus disease 2019 (COVID-19) has rapidly spread on a global scale. Therefore, it is urgent to identify risk factors that could be associated with severe type of COVID-19 from common type.For this retrospective study, we recruited patients with COVID-19 in Wuhan and Zhoukou. Patients were classified into a severe group and common group based on guidelines after admission. Clinical manifestations and laboratory tests were compared, and univariate binary logistic regression and multivariate regression analyses were applied to assess potential risk factors.A total of 126 patients were recruited from January 23 to March 23, 2020. Ninety cases were identified as the common type and 36 as the severe type. The average age in the severe group was significantly older than that in the common group ($P = .008$). Patients with severe COVID-19 exhibited higher proportions of dyspnea ($P = .001$), weakness ($P = .023$), and diarrhea ($P = .046$). Moreover, there were more patients with hypertension ($P = .01$) or coinfection ($P = .001$) in the severe group than in the common group. Additionally, severe COVID-19 was associated with increased neutrophil counts ($P < .001$), C-reactive protein ($P < .001$), procalcitonin ($P = .024$) and decreased lymphocyte counts ($P = .001$), hemoglobin ($P < .001$), total protein (TP) ($P < .001$), and albumin (ALB) ($P < .001$). Based on logistic regression analysis, dyspnea ($P < .001$), TP ($P = .042$), and ALB ($P = .003$) were independent risk factors for severe disease.Patients with lower TP, ALB, and dyspnea should be carefully monitored, and early intervention should be implemented to prevent the development of severe disease.

Conflict of interest statement

The authors have no conflicts of interest to disclose.

- 49 references
- 2 figures

Supplementary info

Publication types, MeSH terms, Substances Expand

Publication types

- Multicenter Study
- Observational Study

MeSH terms

- Adult
- Aged
- Aged, 80 and over
- Betacoronavirus
- Blood Proteins / analysis
- C-Reactive Protein / analysis
- COVID-19
- China
- Coronavirus Infections / diagnosis*
- Disease Progression*
- Dyspnea / virology
- Female
- Hospitalization*
- Humans
- Male
- Middle Aged
- Pandemics
- Pneumonia, Viral / diagnosis*
- Retrospective Studies
- Risk Factors
- SARS-CoV-2
- Serum Albumin, Human / analysis
- Young Adult

Substances

- Blood Proteins
- C-Reactive Protein
- Serum Albumin, Human

Full text links

Wolters Kluwer Wolters Kluwer Free PMC article
Proceed to details
Cite

Share

☐ 152

Observational Study

Geriatr Gerontol Int

. 2021 Jan;21(1):60-65.

doi: 10.1111/ggi.14102. Epub 2020 Dec 2.

COVID-19 in older adults: What are the differences with younger patients?

Ana B Gómez-Belda [1], Mar Fernández-Garcés [1], Elisabeth Mateo-Sanchis [1], Manuel Madrazo [1], Mar Carmona [1], Laura Piles-Roger [1], Arturo Artero [1] [2]

Affiliations Expand

Affiliations

- [1] Department of Internal Medicine, Dr. Peset University Hospital, Valencia, Spain.
- [2] University of Valencia, Valencia, Spain.

- PMID: **33264816**
- PMCID: PMC7753273
- DOI: 10.1111/ggi.14102

Free PMC article

Observational Study

COVID-19 in older adults: What are the differences with younger patients?

Ana B Gómez-Belda et al. Geriatr Gerontol Int. 2021 Jan.

Free PMC article

Show details

Geriatr Gerontol Int

. 2021 Jan;21(1):60-65.

doi: 10.1111/ggi.14102. Epub 2020 Dec 2.

Authors

Ana B Gómez-Belda [1], Mar Fernández-Garcés [1], Elisabeth Mateo-Sanchis [1], Manuel Madrazo [1], Mar Carmona [1], Laura Piles-Roger [1], Arturo Artero [1] [2]

Affiliations

- [1] Department of Internal Medicine, Dr. Peset University Hospital, Valencia, Spain.
- [2] University of Valencia, Valencia, Spain.

- PMID: **33264816**
- PMCID: PMC7753273
- DOI: 10.1111/ggi.14102

Abstract

Aim: The present study aimed both to gain knowledge on the distinctive clinical characteristics of older adults with coronavirus disease 2019 (COVID-19), in comparison with those of younger patients, and to identify risk factors for mortality.

Methods: A retrospective observational study was carried out of patients consecutively admitted to Doctor Peset University Hospital, Valencia (Spain) for COVID-19 from 11 March to 28 April 2020. Every case was diagnosed by reverse transcription polymerase chain reaction or by serology test to detect antibodies. Demographic details, clinical characteristics, laboratory findings on admission and complications of each case were collected from electronic medical records.

Results: The dataset comprised 340 patients. Of them, 152 (44.6%) were aged >70 years. Comorbidities were more common in the older groups. Confusion was more common in older adults, whereas typical symptoms of COVID-19, such as fever, cough and myalgia, were less common. Oxygen saturation ≤93% on room air, neutrophilia, D-dimer >0.5 μg/mL, creatinine >1.5 mg/dL, lactate dehydrogenase ≥250 U/L and elevation of creatine kinase were higher in the older adult groups. Complications during hospitalization, such as acute respiratory distress syndrome (53.3% vs 33.2%, $P < 0.001$), acute kidney injury (11.8% vs 5.3%; $P = 0.030$) and mortality (28.9% vs 6.5%; $P < 0.001$) were more common in patients aged >70 years. Oxygen saturation ≤93% on room air on admission was a predictor of mortality (odds ratio 11.65, 95% confidence interval 3.26-41.66, $P < 0.001$) in patients aged >70 years.

Conclusions: Older adults with COVID-19 have more atypical presentation, more complications and higher mortality. Oxygen saturation ≤93% on room air on admission is a predictive factor of death. Geriatr Gerontol Int 2021; 21: 60-65.

Keywords: COVID-19; clinical characteristics; coronavirus; mortality; older adults.

© 2020 Japan Geriatrics Society.

- 30 references
- 1 figure

Supplementary info

Publication types, MeSH terms Expand

Publication types

- Observational Study

MeSH terms

- Adolescent
- Adult

- Age Factors
- Aged
- Aged, 80 and over
- COVID-19 / epidemiology*
- Comorbidity
- Cough
- Female
- Fever
- Hospital Mortality
- Hospitalization / statistics & numerical data
- Humans
- Male
- Middle Aged
- Retrospective Studies
- Risk Factors
- SARS-CoV-2
- Spain / epidemiology
- Young Adult

Full text links

WILEY Full Text Article Wiley Free PMC article

Proceed to details

Cite

Share

☐ 153

Observational Study

Herz

. 2020 Nov;45(7):663-667.

doi: 10.1007/s00059-020-04991-3. Epub 2020 Oct 7.

Reduced rate of admissions for acute coronary syndromes during the COVID-19 pandemic: an observational analysis from a tertiary hospital in Germany

Gaetano Vacanti [1], Peter Bramlage [2], Gerhard Schymik [1], Claus Schmitt [1], Armin Luik [1], Patrick Swojanowsky [1], Panagiotis Tzamalis [3]

Affiliations Expand

Affiliations

- [1] Medical Clinic IV-Department of Cardiology, Municipal Hospital Karlsruhe, Academic Teaching Hospital of the University of Freiburg, Moltkestr. 90, 76133, Karlsruhe, Germany.
- [2] Institute for Pharmacology and Preventive Medicine, Cloppenburg, Germany.
- [3] Medical Clinic IV-Department of Cardiology, Municipal Hospital Karlsruhe, Academic Teaching Hospital of the University of Freiburg, Moltkestr. 90, 76133, Karlsruhe, Germany. tzamalisp@gmail.com.

- PMID: **33026483**
- PMCID: PMC7539285
- DOI: 10.1007/s00059-020-04991-3

Free PMC article
Observational Study

Reduced rate of admissions for acute coronary syndromes during the COVID-19 pandemic: an observational analysis from a tertiary hospital in Germany

Gaetano Vacanti et al. Herz. 2020 Nov.
Free PMC article
Show details
Herz

. 2020 Nov;45(7):663-667.
doi: 10.1007/s00059-020-04991-3. Epub 2020 Oct 7.

Authors

Gaetano Vacanti [1], Peter Bramlage [2], Gerhard Schymik [1], Claus Schmitt [1], Armin Luik [1], Patrick Swojanowsky [1], Panagiotis Tzamalis [3]

Affiliations

- [1] Medical Clinic IV-Department of Cardiology, Municipal Hospital Karlsruhe, Academic Teaching Hospital of the University of Freiburg, Moltkestr. 90, 76133, Karlsruhe, Germany.
- [2] Institute for Pharmacology and Preventive Medicine, Cloppenburg, Germany.
- [3] Medical Clinic IV-Department of Cardiology, Municipal Hospital Karlsruhe, Academic Teaching Hospital of the University of Freiburg, Moltkestr. 90, 76133, Karlsruhe, Germany. tzamalisp@gmail.com.

- PMID: **33026483**
- PMCID: PMC7539285
- DOI: 10.1007/s00059-020-04991-3

Abstract
in English, German

Background: Several observational studies have suggested a worrying reduction in hospitalisations for acute coronary syndromes in the emergency cardiology department in the last few months all over the world. The aim of the present study is to assess the impact of the current COVID-19 health crisis on admission for acute coronary syndrome (ACS) in the cardiology department of a tertiary general hospital in Germany with a COVID-19 ward.

Methods and results: The authors retrieved clinical data evaluating consecutive patients with ACS admitted to their emergency cardiology department. Data from January to June 2020, as well as for a 5-week period corresponding to this year's COVID-19 outbreak in south-west Germany (23rd March-26th April), were analysed and compared to data from equivalent weeks in the previous 2 years. A trend of reduction in admissions for ACS was observed from the beginning of the outbreak in the region at the end of March 2020. This trend continued and even intensified after a fall in COVID-19 cases in the area; the number of ACS patients in April 2020 was 25% and in June 29% lower than in January 2020 (p-value for linear trend <0.001). An even more consistent reduction was observed as compared with the equivalent weeks in the previous 2 years (38% and 30% lower than in 2019 and 2018, respectively; p = 0.009).

Conclusions: The COVID-19 health and social crisis has caused a worrying trend of reduced cardiological admissions for ACS, without evidence of a decrease in its incidence. Understanding and counteracting the causes appears to be crucial to avoiding major long-term consequences for healthcare systems worldwide.

Hintergrund: Mehrere Beobachtungsstudien weisen auf einen besorgniserregenden Rückgang der Hospitalisierungen wegen eines akuten Koronarsyndroms (ACS) in kardiologischen Notaufnahmen über die vergangenen Monate hin – und das weltweit. Ziel der vorliegenden Studie war es, die Auswirkungen der gegenwärtigen COVID-19-Gesundheitskrise auf die Aufnahmen wegen ACS in der kardiologischen Abteilung eines deutschen Krankenhauses der Tertiärversorgung mit COVID-19-Station zu untersuchen.

Methoden und Ergebnisse: Ausgewertet wurden klinische Daten konsekutiver Patienten mit ACS, die in der kardiologischen Notaufnahme der Autoren behandelt worden waren. Daten von Januar bis Juni 2020 sowie aus einer 5-wöchigen Phase, die den diesjährigen COVID-19-Ausbruch in Südwestdeutschland abdeckt (23. März bis 26. April), wurden analysiert und mit Daten aus den entsprechenden Wochen der beiden Vorjahre verglichen. Ein Trend zu reduzierten Aufnahmen wegen ACS zeigte sich ab Beginn des Ausbruchs in der Region Ende März 2020. Dieser Trend hielt an und verstärkte sich nach einem Abfall der COVID-19-Fälle in der Region sogar; die Zahl der Patienten mit ACS war im April 2020 25% und im Juni 29 % niedriger als im Januar 2020 (p-Wert für linearen Trend <0,001). Eine noch beständigere Reduktion zeigte sich im Vergleich mit den entsprechenden Wochen in den beiden Vorjahren (38 % und 30 % niedriger als 2019 bzw. 2018; $p = 0{,}009$).

Schlussfolgerungen: Die gesundheitliche und gesellschaftliche Krise durch COVID-19 hat zu einem besorgniserregenden Rückgang kardiologischer Aufnahmen wegen ACS geführt, ohne dass es Hinweise auf eine reduzierte Inzidenz geben würde. Es erscheint von wesentlicher Bedeutung, die Ursachen zu verstehen und ihnen entgegenzuwirken, um schwerwiegende Langzeitfolgen für Gesundheitssysteme weltweit zu vermeiden.

Keywords: Acute coronary events; Coronary angiography; Myocardial infarction; Percutaneous coronary intervention; SARS-CoV-2.

Conflict of interest statement

G. Vacanti, P. Bramlage, G. Schymik,C. Schmitt, A. Luik, P. Swojanowsky and P. Tzamalis declare that they have no competing interests.

- 23 references
- 3 figures

Supplementary info

Publication types, MeSH terms Expand

Publication types

- Observational Study

MeSH terms

- Acute Coronary Syndrome / epidemiology*
- Betacoronavirus
- COVID-19
- Coronavirus Infections / epidemiology*
- Germany / epidemiology
- Hospitalization
- Humans
- Pandemics
- Pneumonia, Viral / epidemiology*
- Retrospective Studies
- SARS-CoV-2
- Tertiary Care Centers

Full text links

SpringerLink FULL-TEXT ARTICLE Springer Free PMC article
Proceed to details
Cite
Share
☐ 154
Observational Study
BMC Med Res Methodol
. 2022 Jan 20;22(1):23.
doi: 10.1186/s12874-021-01501-9.

Poor reporting quality of observational clinical studies comparing treatments of COVID-19 - a retrospective cross-sectional study

Sebastian Ziemann [1], Irina Paetzolt [2], Linda Grüßer [2], Mark Coburn [3], Rolf Rossaint [2], Ana Kowark [2]
Affiliations Expand

Affiliations

- [1] Department of Anaesthesiology, Medical Faculty, RWTH Aachen University, Aachen, Germany. sziemann@ukaachen.de.
- [2] Department of Anaesthesiology, Medical Faculty, RWTH Aachen University, Aachen, Germany.
- [3] Department of Anaesthesiology and Intensive Care Medicine, University Hospital Bonn, Bonn, Germany.

- PMID: **35057739**
- PMCID: PMC8771183
- DOI: 10.1186/s12874-021-01501-9

Free PMC article
Observational Study

Poor reporting quality of observational clinical studies comparing treatments of COVID-19 - a retrospective cross-sectional study

Sebastian Ziemann et al. BMC Med Res Methodol. 2022.
Free PMC article
Show details
BMC Med Res Methodol
. 2022 Jan 20;22(1):23.
doi: 10.1186/s12874-021-01501-9.

Authors

Sebastian Ziemann [1], Irina Paetzolt [2], Linda Grüßer [2], Mark Coburn [3], Rolf Rossaint [2], Ana Kowark [2]

Affiliations

- [1] Department of Anaesthesiology, Medical Faculty, RWTH Aachen University, Aachen, Germany. sziemann@ukaachen.de.
- [2] Department of Anaesthesiology, Medical Faculty, RWTH Aachen University, Aachen, Germany.
- [3] Department of Anaesthesiology and Intensive Care Medicine, University Hospital Bonn, Bonn, Germany.

- PMID: **35057739**
- PMCID: PMC8771183
- DOI: 10.1186/s12874-021-01501-9

Abstract

Background: During the COVID-19 pandemic, the scientific world is in urgent need for new evidence on the treatment of COVID patients. The reporting quality is crucial for transparent scientific publication. Concerns of data integrity, methodology and transparency were raised. Here, we assessed the adherence of observational studies comparing treatments of COVID 19 to the STROBE checklist in 2020.

Methods: Design: We performed a retrospective, cross-sectional study.

Setting: We conducted a systematic literature search in the Medline database. This study was performed at the RWTH Aachen University Hospital, Department of Anaesthesiology Participants: We extracted all observational studies on the treatment of COVID-19 patients from the year 2020.

Main outcome measures: The adherence of each publication to the STROBE checklist items was analysed. The journals' impact factor (IF), the country of origin, the kind of investigated treatment and the month of publication were assessed.

Results: We analysed 147 observational studies and found a mean adherence of 45.6% to the STROBE checklist items. The percentage adherence per publication correlated significantly with the journals' IF (point estimate for the difference between 1st and 4th quartile 11.07%, 95% CI 5.12 to 17.02, $p < 0.001$). U.S. American authors gained significantly higher adherence to the checklist than Chinese authors, mean difference 9.10% (SD 2.85%, $p = 0.023$).

Conclusions: We conclude a poor reporting quality of observational studies on the treatment of COVID-19 throughout the year 2020. A considerable improvement is mandatory.

Keywords: COVID-19; Observational studies; Reporting quality; STROBE statement.

© 2022. The Author(s).

Conflict of interest statement

The authors declare that they have no competing interests.

- 18 references
- 3 figures

Supplementary info

Publication types, MeSH terms Expand

Publication types

- Observational Study
- Research Support, Non-U.S. Gov't

MeSH terms

- COVID-19*
- Cross-Sectional Studies
- Humans
- Pandemics
- Retrospective Studies
- SARS-CoV-2
- United States

Full text links

Read free full text at BMC BioMed Central Free PMC article
Proceed to details
Cite
Share
155
Observational Study
Acta Orthop
. 2020 Dec;91(6):633-638.
doi: 10.1080/17453674.2020.1807092. Epub 2020 Aug 24.

Impact of the COVID-19 pandemic on paediatric orthopaedic trauma workload in central London: a multi-centre longitudinal observational study over the "golden weeks"

Kapil Sugand [1], Chang Park [1], Catrin Morgan [2], Rory Dyke [1], Arash Aframian [2], Alison Hulme [2], Stuart Evans [2], Khaled M Sarraf [1], Camilla Baker [2], Katharine Bennett-Brown [2], Henry Simon [2], Edward Bray [2], Lily Li [2], Noel Lee [2], Nadia Pakroo [2], Kashed Rahman [2], Andrew Harrison [1]
Affiliations Expand

Affiliations

- [1] Imperial College Healthcare NHS Trust, London.
- [2] Chelsea and Westminster Hospital, London, UK.

- PMID: **32835573**
- PMCID: PMC8023947
- DOI: 10.1080/17453674.2020.1807092

Free PMC article
Observational Study

Impact of the COVID-19 pandemic on paediatric orthopaedic trauma workload in central London: a multi-centre longitudinal observational study over the "golden weeks"

Kapil Sugand et al. Acta Orthop. 2020 Dec.
Free PMC article
Show details
Acta Orthop
. 2020 Dec;91(6):633-638.
doi: 10.1080/17453674.2020.1807092. Epub 2020 Aug 24.

Authors

Kapil Sugand [1], Chang Park [1], Catrin Morgan [2], Rory Dyke [1], Arash Aframian [2], Alison Hulme [2], Stuart Evans [2], Khaled M Sarraf [1], Camilla Baker [2], Katharine Bennett-Brown [2], Henry Simon [2], Edward Bray [2], Lily Li [2], Noel Lee [2], Nadia Pakroo [2], Kashed Rahman [2], Andrew Harrison [1]

Affiliations

- [1] Imperial College Healthcare NHS Trust, London.
- [2] Chelsea and Westminster Hospital, London, UK.

- PMID: **32835573**
- PMCID: PMC8023947
- DOI: 10.1080/17453674.2020.1807092

Abstract

Background and purpose - The COVID-19 pandemic has been recognised as an unprecedented global health crisis. This study assesses the impact on a large acute paediatric hospital service in London, evaluating the trends in the acute paediatric orthopaedic trauma referral caseload and operative casemix before (2019) and during (2020) COVID-19 lockdown. Patients and methods -

A longitudinal retrospective observational prevalence study of both acute paediatric orthopaedic trauma referrals and operative caseload was performed for the first 6 "golden weeks" of lockdown. These data were compared with the same period in 2019. Statistical analyses included median (± median absolute deviation), risk and odds ratios as well as Fisher's exact test to calculate the statistical significance, set at $p \leq 0.05$. Results - Acute paediatric trauma referrals in 2020 were reduced by two-thirds compared with 2019 (n = 302 vs. 97) with a halving risk (RR 0.55) and odds ratios (OR 0.43) of sporting-related mechanism of injuries ($p = 0.002$). There was a greater use of outpatient telemedicine in the COVID-19 period with more Virtual Fracture Clinic use (OR 97, RR 84, $p < 0.001$), and fewer patients being seen for consultation and followed up face to face (OR 0.55, RR 0.05, $p < 0.001$). Interpretation - The impact of the COVID-19 pandemic has led to a decline in the number of acute paediatric trauma referrals, admissions, and operations during the COVID period. There has also been a significant change in the patient pathway with more being reviewed via the means of telemedicine to reduce the risk of COVID-19 transmission and exposure. More work is required to observe for similar trends nationwide and globally as the pandemic has permanently affected the entire healthcare infrastructure.

- 26 references

Supplementary info

Publication types, MeSH terms Expand

Publication types

- Multicenter Study
- Observational Study

MeSH terms

- Athletic Injuries* / epidemiology
- Athletic Injuries* / therapy
- COVID-19* / epidemiology
- COVID-19* / prevention & control
- Child
- Communicable Disease Control / methods*
- Delivery of Health Care / trends
- Female
- Hospitalization / statistics & numerical data
- Hospitals, Pediatric* / organization & administration
- Hospitals, Pediatric* / statistics & numerical data
- Humans
- London / epidemiology
- Male
- Risk Management / organization & administration
- SARS-CoV-2
- Telemedicine* / methods

- Telemedicine* / organization & administration
- Telemedicine* / statistics & numerical data
- Workload / statistics & numerical data
- Wounds and Injuries* / epidemiology
- Wounds and Injuries* / therapy

Full text links

Free PMC article
Proceed to details
Cite
Share
156
Clinical Trial
PLoS One
. 2020 Dec 31;15(12):e0244857.
doi: 10.1371/journal.pone.0244857. eCollection 2020.

The role of CPAP as a potential bridge to invasive ventilation and as a ceiling-of-care for patients hospitalized with Covid-19-An observational study

Jonathan Walker [1], Shaman Dolly [1], Liji Ng [1], Melissa Prior-Ong [1], Kalpana Sabapathy [2]
Affiliations Expand

Affiliations

- [1] Calderdale Royal Hospital, Calderdale and Huddersfield NHS Foundation Trust, Halifax, United Kingdom.
- [2] Department of Infectious Disease Epidemiology, The London School of Hygiene and Tropical Medicine, London, United Kingdom.

- PMID: **33382796**
- PMCID: PMC7774971
- DOI: 10.1371/journal.pone.0244857

Free PMC article
Clinical Trial

The role of CPAP as a potential bridge to invasive ventilation and as a ceiling-of-care

for patients hospitalized with Covid-19-An observational study

Jonathan Walker et al. PLoS One. 2020.
Free PMC article
Show details
PLoS One
. 2020 Dec 31;15(12):e0244857.
doi: 10.1371/journal.pone.0244857. eCollection 2020.

Authors

Jonathan Walker [1], Shaman Dolly [1], Liji Ng [1], Melissa Prior-Ong [1], Kalpana Sabapathy [2]

Affiliations

- [1] Calderdale Royal Hospital, Calderdale and Huddersfield NHS Foundation Trust, Halifax, United Kingdom.
- [2] Department of Infectious Disease Epidemiology, The London School of Hygiene and Tropical Medicine, London, United Kingdom.

- PMID: **33382796**
- PMCID: PMC7774971
- DOI: 10.1371/journal.pone.0244857

Abstract

Background: Continuous positive airway pressure (CPAP) ventilation may be used as a potential bridge to invasive mechanical ventilation (IMV), or as a ceiling-of-care for persistent hypoxaemia despite standard oxygen therapy, according to UK guidelines. We examined the association of mode of respiratory support and ceiling-of-care on mortality.

Methods: We conducted a retrospective cohort analysis of routinely collected de-identified data of adults with nasal/throat SARs-CoV-2 swab-positive results, at the Calderdale and Huddersfield NHS Foundation Trust between 10th March-19th April 2020 (outcomes determined on 22nd May).

Findings: Of 347 patients with SARs-CoV-2 swab-positive results, 294 (84.7%) patients admitted for Covid-19 were included in the study. Sixty-nine patients were trialled on CPAP, mostly delivered by face mask, either as an early ceiling of care instituted within 24 hours of admission (N = 19), or as a potential bridge to IMV (N = 44). Patients receiving a ceiling of care more than 24 hours after admission (N = 6) were excluded from the analysis. Two hundred and fifteen patients (73.1%) maximally received air/standard oxygen therapy, and 45 (15.3%) patients maximally received CPAP. Thirty-four patients (11.6%) required IMV, of which 24 had received prior CPAP. There were 138 patients with an early ceiling-of-care plan (pre-admission/within 24h). Overall, 103(35.0%) patients died and 191(65.0%) were alive at study end. Among all patients trialled on CPAP either as a potential bridge to IMV (N = 44) or as a ceiling-of-care (N = 19) mortality was 25% and 84%, respectively. Overall, there was strong evidence for higher mortality among patients who required CPAP or IMV, compared to those who required only

air/oxygen (aOR 5.24 95%CI: 1.38, 19.81 and aOR 46.47 95%CI: 7.52, 287.08, respectively; p<0.001), and among patients with early ceiling-of-care compared to those without a ceiling (aOR 41.81 95%CI: 8.28, 211.17; p<0.001). Among patients without a ceiling of care (N = 137), 10 patients required prompt intubation following failed oxygen therapy, but 44 patients received CPAP. CPAP failure, defined as death (N = 1) or intubation (N = 24), occurred in 57% (N = 25) of patients. But in total, 75% (N = 33) of those started on CPAP with no ceiling of care recovered to discharge-19 without the need for IMV, and 14 following IMV.

Conclusion: Our data suggest that among patients with no ceiling-of-care, an initial trial of CPAP as a potential bridge to IMV offers a favourable therapeutic alternative to early intubation. In contrast, among patients with a ceiling-of care, CPAP seems to offer little additional survival benefit beyond oxygen therapy alone. Information on ceilings of respiratory support is vital to interpreting mortality from Covid-19.

Strengths and limitations of this study: Sample size relatively small.Study sample representative of hospitalised Covid-19 patients in UK.Previously unreported data on role of ceilings-of-care in hospitalised Covid-19 patients.Novel data on use of CPAP separated by indication.

Conflict of interest statement

The authors have declared that no competing interests exist.

- 33 references
- 2 figures

Supplementary info

Publication types, MeSH terms, Substances, Grant support Expand

Publication types

- Clinical Trial
- Multicenter Study
- Observational Study
- Research Support, Non-U.S. Gov't

MeSH terms

- Adult
- COVID-19* / economics
- COVID-19* / epidemiology
- COVID-19* / therapy
- Continuous Positive Airway Pressure / economics*
- Female
- Hospitalization / economics*
- Humans
- Japan / epidemiology

- Male
- Middle Aged
- Oxygen / administration & dosage*
- Retrospective Studies
- SARS-CoV-2*

Substances

- Oxygen

Grant support

- MR/R010161/1/MRC_/Medical Research Council/United Kingdom

Full text links

OPEN ACCESS TO FULL TEXT PLOS ONE Public Library of Science Free PMC article
Proceed to details
Cite
Share
157
Observational Study
Intern Med
. 2020 Dec 15;59(24):3131-3133.
doi: 10.2169/internalmedicine.5614-20. Epub 2020 Nov 2.

Non-COVID-19 Patients with Life-threatening Diseases Who Visited a Fever Clinic: A Single-center, Observational Study in Tokyo, Japan

Yutaro Akiyama [1], Shinichiro Morioka [1,2], Yuji Wakimoto [1], Akira Kawashima [1], Kohei Kanda [1], Ayako Okuhama [1], Tetsuya Suzuki [1,2], Yusuke Miyazato [1], Hidetoshi Nomoto [1,2], Satoshi Ide [1,2], Takato Nakamoto [1], Keiji Nakamura [1], Masayuki Ota [1], Yuki Moriyama [1,2], Saho Takaya [1], Kota Yamada [3], Maho Taguchi [3], Erika Sugito [3], Shinji Izuka [4], Kenji Ishiguro [4], Toshiaki Kobayashi [4], Wataru Miyake [5], Shuji Kubota [5], Masahiro Ishikane [1], Noriko Kinoshita [1,2], Kei Yamamoto [1], Mugen Ujiie [1], Satoshi Kutsuna [1], Kayoko Hayakawa [1], Sho Saito [1,2], Norio Ohmagari [1]
Affiliations Expand

Affiliations

- [1] Department of Infectious Diseases, Disease Control and Prevention Center, National Center for Global Health and Medicine, Japan.
- [2] Emerging and Reemerging Infectious Diseases, Graduate School of Medicine, Tohoku University, Japan.
- [3] Department of Diabetes, Endocrinology, and Metabolism, National Center for Global Health and Medicine, Japan.
- [4] Division of Rheumatic Diseases, National Center for Global Health and Medicine, Japan.
- [5] Department of Cardiology, National Center for Global Health and Medicine, Japan.

- PMID: **33132334**
- PMCID: PMC7807116
- DOI: 10.2169/internalmedicine.5614-20

Free PMC article
Observational Study

Non-COVID-19 Patients with Life-threatening Diseases Who Visited a Fever Clinic: A Single-center, Observational Study in Tokyo, Japan

Yutaro Akiyama et al. Intern Med. 2020.
Free PMC article
Show details
Intern Med

. 2020 Dec 15;59(24):3131-3133.
doi: 10.2169/internalmedicine.5614-20. Epub 2020 Nov 2.

Authors

Yutaro Akiyama [1], Shinichiro Morioka [1, 2], Yuji Wakimoto [1], Akira Kawashima [1], Kohei Kanda [1], Ayako Okuhama [1], Tetsuya Suzuki [1, 2], Yusuke Miyazato [1], Hidetoshi Nomoto [1, 2], Satoshi Ide [1, 2], Takato Nakamoto [1], Keiji Nakamura [1], Masayuki Ota [1], Yuki Moriyama [1, 2], Saho Takaya [1], Kota Yamada [3], Maho Taguchi [3], Erika Sugito [3], Shinji Izuka [4], Kenji Ishiguro [4], Toshiaki Kobayashi [4], Wataru Miyake [5], Shuji Kubota [5], Masahiro Ishikane [1], Noriko Kinoshita [1, 2], Kei Yamamoto [1], Mugen Ujiie [1], Satoshi Kutsuna [1], Kayoko Hayakawa [1], Sho Saito [1, 2], Norio Ohmagari [1]

Affiliations

- [1] Department of Infectious Diseases, Disease Control and Prevention Center, National Center for Global Health and Medicine, Japan.
- [2] Emerging and Reemerging Infectious Diseases, Graduate School of Medicine, Tohoku University, Japan.

- [3] Department of Diabetes, Endocrinology, and Metabolism, National Center for Global Health and Medicine, Japan.
- [4] Division of Rheumatic Diseases, National Center for Global Health and Medicine, Japan.
- [5] Department of Cardiology, National Center for Global Health and Medicine, Japan.

- PMID: **33132334**
- PMCID: PMC7807116
- DOI: 10.2169/internalmedicine.5614-20

Abstract

Objective In fever clinics screening coronavirus disease (COVID-19), there could be patients with life-threatening diseases that physicians should not overlook. We exploratorily investigated the final diagnosis among non-COVID-19 hospitalized patients who visited the fever clinic. Methods This was a retrospective, observational, and single-centered study conducted in the National Center for Global Health and Medicine (NCGM), Tokyo, Japan. We conducted a retrospective chart review of patients who visited the fever clinic in the NCGM from 11 March 2020 to 24 April 2020. Patients Patients who met the following clinical criteria visited the fever clinic in the NCGM: (1) body temperature >37.5°C, (2) any symptoms consistent with COVID-19 or (3) referral from local healthcare facilities. In the fever clinic, all patients who met the above criteria had severe acute respiratory syndrome coronavirus 2 polymerase chain reaction test with nasopharyngeal swab specimens. Patients with severe symptoms or an unstable condition were sent to an outpatient clinic for infectious diseases for further evaluation and treatment. Results Among 1,470 patients who visited the fever clinic, 84 patients were hospitalized, and 45 of them were diagnosed as having COVID-19. Among the remaining 39 non-COVID-19 patients, there were nine patients with life-threatening diseases. The life-threatening diseases included acute heart failure, septic shock, pneumocystis pneumonia, peritonsillar abscess, and necrotizing fasciitis. Conclusion Physicians should evaluate each patient carefully while considering other life-threatening conditions even in such a COVID-19 pandemic era.

Keywords: COVID-19; coronavirus disease; differential diagnosis; fever clinic; life-threatening diseases.

Conflict of interest statement

The authors state that they have no Conflict of Interest (COI).

- 7 references

Supplementary info

Publication types, MeSH terms, Substances Expand

Publication types

- Observational Study

MeSH terms

- COVID-19 / epidemiology*
- COVID-19 / virology
- Comorbidity
- Fever / diagnosis
- Fever / epidemiology*
- Humans
- Japan / epidemiology
- Pandemics*
- RNA, Viral / analysis*
- Retrospective Studies
- SARS-CoV-2 / genetics*
- Tokyo / epidemiology

Substances

- RNA, Viral

Full text links

J-STAGE, Japan Science and Technology Information Aggregator, Electronic
Free PMC article
Proceed to details
Cite
Share
158
Observational Study
Cardiovasc Diabetol
. 2021 Jul 10;20(1):140.
doi: 10.1186/s12933-021-01336-0.

Statins and clinical outcomes in hospitalized COVID-19 patients with and without Diabetes Mellitus: a retrospective cohort study with propensity score matching

Prateek Lohia[#][1], Shweta Kapur[#][2], Sindhuri Benjaram[2], Zachary Cantor[2], Navid Mahabadi[2], Tanveer Mir[2], M Safwan Badr[2]
Affiliations Expand

Affiliations

- [1] Department of Internal Medicine, Wayne State University, Detroit, MI, 48201, USA. plohia@med.wayne.edu.
- [2] Department of Internal Medicine, Wayne State University, Detroit, MI, 48201, USA.

[#] Contributed equally.

- PMID: **34246277**
- PMCID: PMC8272452
- DOI: 10.1186/s12933-021-01336-0

Free PMC article
Observational Study

Statins and clinical outcomes in hospitalized COVID-19 patients with and without Diabetes Mellitus: a retrospective cohort study with propensity score matching

Prateek Lohia et al. Cardiovasc Diabetol. 2021.
Free PMC article
Show details
Cardiovasc Diabetol
. 2021 Jul 10;20(1):140.
doi: 10.1186/s12933-021-01336-0.

Authors

Prateek Lohia [#] [1], Shweta Kapur [#] [2], Sindhuri Benjaram [2], Zachary Cantor [2], Navid Mahabadi [2], Tanveer Mir [2], M Safwan Badr [2]

Affiliations

- [1] Department of Internal Medicine, Wayne State University, Detroit, MI, 48201, USA. plohia@med.wayne.edu.
- [2] Department of Internal Medicine, Wayne State University, Detroit, MI, 48201, USA.

[#] Contributed equally.

- PMID: **34246277**
- PMCID: PMC8272452
- DOI: 10.1186/s12933-021-01336-0

Abstract

Background: The pleiotropic effects of statins may reduce the severity of COVID-19 disease. This study aims to determine the association between inpatient statin use and severe disease outcomes among hospitalized COVID-19 patients, especially those with Diabetes Mellitus (DM).

Research design and methods: A retrospective cohort study on hospitalized patients with confirmed COVID-19 diagnosis. The primary outcome was mortality during hospitalization. Patients were classified into statin and non-statin groups based on the administration of statins during hospitalization. Analysis included multivariable regression analysis adjusting for confounders and propensity score matching to achieve a 1:1 balanced cohort. Subgroup analyses based on presence of DM were conducted.

Results: In the cohort of 922 patients, 413 had a history of DM. About 27.1% patients (n = 250) in the total cohort (TC) and 32.9% patients (n = 136) in DM cohort received inpatient statins. Atorvastatin (n = 205, 82%) was the most commonly prescribed statin medication in TC. On multivariable analysis in TC, inpatient statin group had reduced mortality compared to the non-statin group (OR, 0.61; 95% CI, 0.42-0.90; p = 0.01). DM modified this association between inpatient statins and mortality. Patients with DM who received inpatient statins had reduced mortality (OR, 0.35; 95% CI, 0.21-0.61; p < 0.001). However, no such association was noted among patients without DM (OR, 1.21; 95% CI, 0.67-2.17; p = 0.52). These results were further validated using propensity score matching.

Conclusions: Inpatient statin use was associated with significant reduction in mortality among COVID-19 patients especially those with DM. These findings support the pursuit of randomized clinical trials and inpatient statin use appears safe among COVID-19 patients.

Keywords: COVID-19; Diabetes Mellitus; Inpatient; Intensive care; Mechanical ventilation; Mortality; Race; Statins.

© 2021. The Author(s).

Conflict of interest statement

All authors declare that they have no competing interests.

- 45 references
- 2 figures

Supplementary info

Publication types, MeSH terms, Substances Expand

Publication types

- Comparative Study
- Observational Study

MeSH terms

- Aged
- COVID-19 / diagnosis

- COVID-19 / drug therapy*
- COVID-19 / mortality*
- Cohort Studies
- Diabetes Mellitus / diagnosis
- Diabetes Mellitus / drug therapy*
- Diabetes Mellitus / mortality*
- Female
- Hospitalization / trends*
- Humans
- Hydroxymethylglutaryl-CoA Reductase Inhibitors / therapeutic use*
- Male
- Middle Aged
- Propensity Score
- Retrospective Studies
- Treatment Outcome

Substances

- Hydroxymethylglutaryl-CoA Reductase Inhibitors

Full text links

Read free full text at BMC BioMed Central Free PMC article

Proceed to details

Cite

Share

☐ 159

Observational Study

Medicina (Kaunas)

. 2021 Jun 29;57(7):674.

doi: 10.3390/medicina57070674.

Obesity May Not Be Associated with 28-Day Mortality, Duration of Invasive Mechanical Ventilation and Length of Intensive Care Unit and Hospital Stay in Critically Ill Patients with Severe Acute Respiratory Syndrome Coronavirus-2: A Retrospective Cohort Study

Sjaak Pouwels [1], Dharmanand Ramnarain [1], Emily Aupers [1], Laura Rutjes-Weurding [1], Jos van Oers [1]
Affiliations Expand

Affiliation

- [1] Department of Intensive Care Medicine, Elisabeth-Tweesteden Hospital, Hilvarenbeekseweg 60, P.O. Box 90151, 5000 LC Tilburg, The Netherlands.

- PMID: **34210077**
- PMCID: PMC8306227
- DOI: 10.3390/medicina57070674

Free PMC article
Observational Study

Obesity May Not Be Associated with 28-Day Mortality, Duration of Invasive Mechanical Ventilation and Length of Intensive Care Unit and Hospital Stay in Critically Ill Patients with Severe Acute Respiratory Syndrome Coronavirus-2: A Retrospective Cohort Study

Sjaak Pouwels et al. Medicina (Kaunas). 2021.
Free PMC article
Show details
Medicina (Kaunas)
. 2021 Jun 29;57(7):674.
doi: 10.3390/medicina57070674.

Authors

Sjaak Pouwels [1], Dharmanand Ramnarain [1], Emily Aupers [1], Laura Rutjes-Weurding [1], Jos van Oers [1]

Affiliation

- [1] Department of Intensive Care Medicine, Elisabeth-Tweesteden Hospital, Hilvarenbeekseweg 60, P.O. Box 90151, 5000 LC Tilburg, The Netherlands.

- PMID: **34210077**
- PMCID: PMC8306227
- DOI: 10.3390/medicina57070674

Abstract

Background and Objectives: The aim of this study was to investigate the association between obesity and 28-day mortality, duration of invasive mechanical ventilation and length of stay at the Intensive Care Unit (ICU) and hospital in patients admitted to the ICU for SARS-CoV-2 pneumonia. *Materials and Methods:* This was a retrospective observational cohort study in patients admitted to the ICU for SARS-CoV-2 pneumonia, in a single Dutch center. The association between obesity (body mass index > 30 kg/m^2) and 28-day mortality, duration of invasive mechanical ventilation and length of ICU and hospital stay was investigated. *Results:* In 121 critically ill patients, pneumonia due to SARS-CoV-2 was confirmed by RT-PCR. Forty-eight patients had obesity (33.5%). The 28-day all-cause mortality was 28.1%. Patients with obesity had no significant difference in 28-day survival in Kaplan-Meier curves (log rank p 0.545) compared with patients without obesity. Obesity made no significant contribution in a multivariate Cox regression model for prediction of 28-day mortality ($p = 0.124$), but age and the Sequential Organ Failure Assessment (SOFA) score were significant independent factors ($p < 0.001$ and 0.002, respectively). No statistically significant correlation was observed between obesity and duration of invasive mechanical ventilation and length of ICU and hospital stay. *Conclusion:* One-third of the patients admitted to the ICU for SARS-CoV-2 pneumonia had obesity. The present study showed no relationship between obesity and 28-day mortality, duration of invasive mechanical ventilation, ICU and hospital length of stay. Further studies are needed to substantiate these findings.

Keywords: SARS-CoV-2; intensive care unit; invasive mechanical ventilation; length of stay; obesity; severe acute respiratory syndrome coronavirus-2.

Conflict of interest statement

The authors declare no conflict of interest.

- 26 references
- 2 figures

Supplementary info

Publication types, MeSH terms Expand

Publication types

- Observational Study

MeSH terms

- COVID-19*
- Cohort Studies
- Critical Illness*
- Hospital Mortality
- Humans
- Intensive Care Units
- Length of Stay

- Obesity / complications
- Respiration, Artificial
- Retrospective Studies
- SARS-CoV-2

Full text links

FULL TEXT OPEN ACCESS MDPI Multidisciplinary Digital Publishing Institute (MDPI) Free PMC article
Proceed to details
Cite
Share
160
Observational Study
West J Emerg Med
. 2021 Apr 2;22(3):580-586.
doi: 10.5811/westjem.2020.12.49206.

Rate of Decompensation of Normoxic Emergency Department Patients with SARS-CoV-2

Kraftin E Schreyer [1], Derek L Isenberg [1], Wayne A Satz [1], Nicole V Lucas [1], Jennifer Rosenbaum [1], Gregory Zandrow [1], Nina T Gentile [1]
Affiliations Expand

Affiliation

- [1] Lewis Katz School of Medicine at Temple University, Department of Emergency Medicine, Philadelphia, Pennsylvania.

- PMID: **34125030**
- PMCID: PMC8203021
- DOI: 10.5811/westjem.2020.12.49206

Free PMC article
Observational Study

Rate of Decompensation of Normoxic Emergency Department Patients with SARS-CoV-2

Kraftin E Schreyer et al. West J Emerg Med. 2021.

Free PMC article
Show details
West J Emerg Med
. 2021 Apr 2;22(3):580-586.
doi: 10.5811/westjem.2020.12.49206.

Authors

Kraftin E Schreyer [1], Derek L Isenberg [1], Wayne A Satz [1], Nicole V Lucas [1], Jennifer Rosenbaum [1], Gregory Zandrow [1], Nina T Gentile [1]

Affiliation

- [1] Lewis Katz School of Medicine at Temple University, Department of Emergency Medicine, Philadelphia, Pennsylvania.

- PMID: **34125030**
- PMCID: PMC8203021
- DOI: 10.5811/westjem.2020.12.49206

Abstract

Introduction: As of October 30, 2020, severe acute respiratory syndrome coronavirus 2 (SARS-CoV-2) has infected over 44 million people worldwide and killed over 1.1 million people. In the emergency department (ED), patients who need supplemental oxygen or respiratory support are admitted to the hospital, but the course of normoxic patients with SARS-CoV-2 infection is unknown. In our health system, the policy during the coronavirus 2019 (COVID-19) pandemic was to admit all patients with abnormal chest imaging (CXR) regardless of their oxygen level. We also admitted febrile patients with respiratory complaints who resided in congregate living. We describe the rate of decompensation among patients admitted with suspected SARS-CoV-2 infection but who were not hypoxemic in the ED.

Methods: This is a retrospective observational study of patients admitted to our health system between March 1-May 5, 2020 with suspected SARS-CoV-2 infection. We queried our registry to find patients who were admitted to the hospital but had no recorded oxygen saturation of <92% in the ED and received no supplemental oxygen prior to admission. Our primary outcome was decompensation at 72 hours, defined by the need for respiratory support (oxygen, high-flow nasal cannula, non-invasive ventilation, or intubation).

Results: A total of 840 patients met our inclusion criteria. Of those patients, 376 (45%) tested positive for SARS-CoV-2. Sixty patients (7.1%) with suspected COVID-19 required respiratory support at 72 hours including 27 (3%) of confirmed SARS-CoV-2 positive patients. Among the 376 patients who tested positive for SARS-CoV-2, 54 patients (14%) had normal CXR in the ED. One-third of patients with normal CXRs decompensated at 72 hours. Seven SARS-CoV-2 positive patients in our cohort died during their hospitalization, of whom five had normal CXRs on admission.

Conclusion: Sixty (7.1%) of suspected COVID-19 patients hospitalized at 72 hours required respiratory support despite being normoxic in the ED. Further research should look to identify the normoxic SARS-CoV-2 patients at risk for decompensation.

Conflict of interest statement

Conflicts of Interest: By the WestJEM article submission agreement, all authors are required to disclose all affiliations, funding sources and financial or management relationships that could be perceived as potential sources of bias. No author has professional or financial relationships with any companies that are relevant to this study. There are no conflicts of interest or sources of funding to declare.

- 19 references
- 2 figures

Supplementary info

Publication types, MeSH terms, Substances Expand

Publication types

- Observational Study

MeSH terms

- COVID-19 / diagnosis*
- COVID-19 / epidemiology
- COVID-19 / therapy
- Disease Progression
- Emergency Service, Hospital / statistics & numerical data*
- Female
- Hospitalization / statistics & numerical data
- Humans
- Male
- Middle Aged
- Oxygen / blood*
- Pandemics
- Registries
- Respiration, Artificial / statistics & numerical data*
- Retrospective Studies
- SARS-CoV-2

Substances

- Oxygen

Full text links

Free PMC article

Proceed to details
Cite
Share
☐ 161
Observational Study
Epidemiol Prev
. Sep-Dec 2020;44(5-6 Suppl 2):315-322.
doi: 10.19191/EP20.5-6.S2.132.

[Can diabetes and its related hypoglycemic drug treatment be considered risk factors for health outcomes in COVID-19 patients? The results of a study in the population residing in Sicily Region (Southern Italy)]

[Article in Italian]
Achille Cernigliaro [1], Alessandra Vincenza Allotta [2], Salvatore Scondotto [3]
Affiliations Expand

Affiliations

- [1] Dipartimento per le attività sanitarie e Osservatorio epidemiologico, Assessorato della salute, Regione Siciliana, Palermo; achille.cernigliaro@regione.sicilia.it.
- [2] Dipartimento per le attività sanitarie e osservatorio epidemiologico, Assessorato della salute, Regione Siciliana, Palermo.
- [3] Dipartimento per le attività sanitarie e Osservatorio epidemiologico, Assessorato della salute, Regione Siciliana, Palermo.

- PMID: **33412824**
- DOI: 10.19191/EP20.5-6.S2.132

Free article
Observational Study

[Can diabetes and its related hypoglycemic drug treatment be considered risk factors for health outcomes in COVID-19 patients? The results of a study in the population residing in Sicily Region (Southern Italy)]

[Article in Italian]
Achille Cernigliaro et al. Epidemiol Prev. Sep-Dec 2020.
Free article
Show details
Epidemiol Prev
. Sep-Dec 2020;44(5-6 Suppl 2):315-322.
doi: 10.19191/EP20.5-6.S2.132.

Authors

Achille Cernigliaro [1], Alessandra Vincenza Allotta [2], Salvatore Scondotto [3]

Affiliations

- [1] Dipartimento per le attività sanitarie e Osservatorio epidemiologico, Assessorato della salute, Regione Siciliana, Palermo; achille.cernigliaro@regione.sicilia.it.
- [2] Dipartimento per le attività sanitarie e osservatorio epidemiologico, Assessorato della salute, Regione Siciliana, Palermo.
- [3] Dipartimento per le attività sanitarie e Osservatorio epidemiologico, Assessorato della salute, Regione Siciliana, Palermo.

- PMID: **33412824**
- DOI: 10.19191/EP20.5-6.S2.132

Abstract

Objectives: to evaluate the effects of a pre-existing condition of diabetes and of the use of antidiabetic drugs in the Sicilian population on different outcomes of the COVID-19 disease.

Design: a retrospective observational study based was used. Data deriving from the COVID-19 epidemic surveillance and from the collection of information on drugs consume by Sicilian residents.

Setting and participants: due to the data availability, the study was calibrated on the Region and included all population distinguishing by gender and age groups.

Main outcome measures: the risks of cumulative incidence for COVID-19 were investigated in people who had diabetes comorbidities to incur a hospitalization for COVID-19, to be treated within an intensive care unit, and lethality. The role of previous antidiabetic drug treatments with respect to each study outcome was also investigated.

Results: in Sicily, from 01.03.2020 to 26.06.2020, a number of 172 cases of COVID-19 disease with diabetes comorbidity were diagnosed. The data did not show any difference in the cumulative incidence for COVID-19 between diabetics (64.2/100,000 inhabitants) and non-diabetics (56.9/100,000 inhabitants) patients. Diabetes increases the risk of hospitalization in the under 80 in both men and women (men: OR 2.62; women OR 4.31), for treatment in intensive care (men: OR 4,41; women: OR 7.74), and for death (men: OR 5.21; women OR 5.92). The analysis of drug using showed risks effect of insulin (OR 2.13) on hospitalization, sulfonylureas/glinides (OR 2.58) on intensive care and protective of metformin on death both in single component (OR 0.44) and in multicomponent (OR 0.43).

Conclusions: data availability made it possible to monitor the occurrence and explore some of the characteristics of the cases with COVID-19 in Sicily. Diabetes does not seem to represent a risk factor for SARS-CoV-2 infection in Sicily, while previous diabetes condition seems to determine greater risk of hospitalization, treatment in intensive care, and lethality among over 80. There are also gender differences with almost double risks in women for hospitalization and intensive care only. Among the antidiabetic drugs investigated, there was a risk for hospitalization and intensive care while protective for deaths. This study represents an important tool for the activation of intervention programmes in the area aimed at populations with greater health risk deriving from the effects of this new pandemic.

Keywords: COVID-19; SARS-CoV-2; antidiabetic drugs; diabetes; hospitalization; intensive care; lethality.

Supplementary info

Publication types, MeSH terms, Substances, Supplementary concepts Expand

Publication types

- Observational Study

MeSH terms

- Adolescent
- Adult
- Aged
- Aged, 80 and over
- COVID-19 / drug therapy
- COVID-19 / epidemiology*
- COVID-19 / therapy
- Child
- Child, Preschool
- Comorbidity
- Diabetes Mellitus / drug therapy
- Diabetes Mellitus / epidemiology*
- Female
- Humans
- Hypoglycemic Agents / adverse effects*
- Hypoglycemic Agents / therapeutic use
- Incidence
- Infant
- Infant, Newborn
- Intensive Care Units / statistics & numerical data
- Male
- Middle Aged

- Pandemics*
- Retrospective Studies
- Risk Factors
- SARS-CoV-2*
- Sicily / epidemiology
- Survival Analysis
- Treatment Outcome
- Young Adult

Substances

- Hypoglycemic Agents

Supplementary concepts

- COVID-19 drug treatment

Full text links

Free Full Text Inferenze scarl
Proceed to details
Cite
Share
162
Observational Study
Aging (Albany NY)
. 2020 Dec 9;13(2):1591-1607.
doi: 10.18632/aging.202223. Epub 2020 Dec 9.

Coagulation dysfunction in ICU patients with coronavirus disease 2019 in Wuhan, China: a retrospective observational study of 75 fatal cases

Jiaran Shi [1], Wang Zhang [2], Ling Sang [3], Zhaohui Qu [4], Ming Zhong [5], Li Jiang [6], Bin Song [7], Liang Kang [4], Yun Zhang [8], Xingxiang Wang [1], Dingyu Zhang [9 10], Xia Zheng [8]
Affiliations Expand

Affiliations

- [1] Department of Cardiology, The First Affiliated Hospital, Zhejiang University School of Medicine, Hangzhou, Zhejiang, PR China.
- [2] Department of Infectious Diseases, Sir Run Run Shaw Hospital, Zhejiang University School of Medicine, Hangzhou, Zhejiang, PR China.
- [3] Department of Critical Care Medicine, The First Affiliated Hospital of GuangZhou Medical University, GuangZhou Institute of Respiratory Health, Guangzhou, Guangdong, PR China.
- [4] Department of Critical Care Medicine, Jinyintan Hospital, Wuhan, Hubei, PR China.
- [5] Department of Critical Care Medicine, Zhongshan Hospital, Fudan University, Shanghai, PR China.
- [6] Department of Critical Care Medicine, Xuanwu Hospital, Capital Medical University, Beijing, PR China.
- [7] Department of Tuberculosis and Respiratory Disease, Jinyintan Hospital, Wuhan, Hubei, PR China.
- [8] Department of Critical Care Medicine, The First Affiliated Hospital, Zhejiang University School of Medicine, Hangzhou, Zhejiang, PR China.
- [9] Research Center for Translational Medicine, Wuhan Jinyintan Hospital, Hubei, PR China.
- [10] Joint Laboratory of Infectious Diseases and Health, Wuhan Institute of Virology and Wuhan Jinyintan Hospital, Chinese Academy of Sciences, Hubei, PR China.

- PMID: **33318314**
- PMCID: PMC7880373
- DOI: 10.18632/aging.202223

Free PMC article
Observational Study

Coagulation dysfunction in ICU patients with coronavirus disease 2019 in Wuhan, China: a retrospective observational study of 75 fatal cases

Jiaran Shi et al. Aging (Albany NY). 2020.
Free PMC article
Show details
Aging (Albany NY)
. 2020 Dec 9;13(2):1591-1607.
doi: 10.18632/aging.202223. Epub 2020 Dec 9.

Authors

Jiaran Shi [1], Wang Zhang [2], Ling Sang [3], Zhaohui Qu [4], Ming Zhong [5], Li Jiang [6], Bin Song [7], Liang Kang [4], Yun Zhang [8], Xingxiang Wang [1], Dingyu Zhang [9] [10], Xia Zheng [8]

Affiliations

- [1] Department of Cardiology, The First Affiliated Hospital, Zhejiang University School of Medicine, Hangzhou, Zhejiang, PR China.
- [2] Department of Infectious Diseases, Sir Run Run Shaw Hospital, Zhejiang University School of Medicine, Hangzhou, Zhejiang, PR China.
- [3] Department of Critical Care Medicine, The First Affiliated Hospital of GuangZhou Medical University, GuangZhou Institute of Respiratory Health, Guangzhou, Guangdong, PR China.
- [4] Department of Critical Care Medicine, Jinyintan Hospital, Wuhan, Hubei, PR China.
- [5] Department of Critical Care Medicine, Zhongshan Hospital, Fudan University, Shanghai, PR China.
- [6] Department of Critical Care Medicine, Xuanwu Hospital, Capital Medical University, Beijing, PR China.
- [7] Department of Tuberculosis and Respiratory Disease, Jinyintan Hospital, Wuhan, Hubei, PR China.
- [8] Department of Critical Care Medicine, The First Affiliated Hospital, Zhejiang University School of Medicine, Hangzhou, Zhejiang, PR China.
- [9] Research Center for Translational Medicine, Wuhan Jinyintan Hospital, Hubei, PR China.
- [10] Joint Laboratory of Infectious Diseases and Health, Wuhan Institute of Virology and Wuhan Jinyintan Hospital, Chinese Academy of Sciences, Hubei, PR China.

- PMID: **33318314**
- PMCID: PMC7880373
- DOI: 10.18632/aging.202223

Abstract

Coagulation dysfunction in critically ill patients with coronavirus disease 2019 (COVID-19) has not been well described, and the efficacy of anticoagulant therapy is unclear. In this study, we retrospectively reviewed 75 fatal COVID-19 cases who were admitted to the intensive care unit at Jinyintan Hospital (Wuhan, China). The median age of the cases was 67 (62-74) years, and 47 (62.7%) were male. Fifty patients (66.7%) were diagnosed with disseminated intra-vascular coagulation. Approximately 90% of patients had elevated D-dimer and fibrinogen degradation products, which decreased continuously after anticoagulant treatment and was accompanied by elevated albumin (all $P<0.05$). The median survival time of patients treated with anticoagulant was 9.0 (6.0-14.0) days compared with 7.0 (3.0-10.0) days in patients without anticoagulant therapy ($P=0.008$). After anticoagulation treatment, C-reactive protein levels decreased ($P=0.004$), as did high-sensitivity troponin ($P=0.018$), lactate dehydrogenase ($P<0.001$), and hydroxybutyrate dehydrogenase ($P<0.001$). In conclusion, coagulation disorders were widespread among fatal COVID-19 cases. Anticoagulant treatment partially improved hypercoagulability, prolonged median survival time, and may have postponed inflammatory processes and cardiac injury.

Keywords: COVID-19; cardiac injury; coagulation dysfunction; coronavirus disease 2019; death; inflammation.

Conflict of interest statement

CONFLICTS OF INTEREST: All authors certify that they have no affiliation with or involvement in any organization or entity with any financial interest, or non-financial interest in the subject matter or materials discussed in this manuscript.

- 37 references

- 5 figures

Supplementary info

Publication types, MeSH terms, Substances Expand

Publication types

- Observational Study
- Research Support, Non-U.S. Gov't

MeSH terms

- Aged
- Anticoagulants / therapeutic use
- Blood Coagulation Disorders / drug therapy
- Blood Coagulation Disorders / virology*
- COVID-19 / complications*
- China
- Female
- Humans
- Intensive Care Units
- Male
- Middle Aged
- Retrospective Studies
- SARS-CoV-2

Substances

- Anticoagulants

Full text links

Impact Journals, LLC Free PMC article
Proceed to details
Cite
Share
163
Meta-Analysis
Diabetes Metab
. 2021 Nov;47(6):101220.
doi: 10.1016/j.diabet.2020.101220. Epub 2020 Dec 23.

Statins and clinical outcomes with COVID-19: Meta-analyses of observational studies

André J Scheen [1]
Affiliations Expand

Affiliation

- [1] Division of Diabetes, Nutrition and Metabolic Disorders, Department of Medicine, CHU Liège, Liège University, Liège, Belgium; Clinical Pharmacology Unit, CHU Liège, Center for Interdisciplinary Research on Medicines (CIRM), Liège University, Liège, Belgium. Electronic address: andre.scheen@chuliege.be.

- PMID: **33359486**
- PMCID: PMC7757378
- DOI: 10.1016/j.diabet.2020.101220

Free PMC article
Meta-Analysis

Statins and clinical outcomes with COVID-19: Meta-analyses of observational studies

André J Scheen. Diabetes Metab. 2021 Nov.
Free PMC article
Show details
Diabetes Metab
. 2021 Nov;47(6):101220.
doi: 10.1016/j.diabet.2020.101220. Epub 2020 Dec 23.

Author

André J Scheen [1]

Affiliation

- [1] Division of Diabetes, Nutrition and Metabolic Disorders, Department of Medicine, CHU Liège, Liège University, Liège, Belgium; Clinical Pharmacology Unit, CHU Liège, Center for Interdisciplinary Research on Medicines (CIRM), Liège University, Liège, Belgium. Electronic address: andre.scheen@chuliege.be.

- PMID: **33359486**
- PMCID: PMC7757378
- DOI: 10.1016/j.diabet.2020.101220

Abstract

Aims: People with cardiovascular disease or risk factors are at increased risk when exposed to SARS-CoV-2. Most are treated with statins, but the impact of these drugs on clinical outcomes of COVID-19 remains unclear. This report is therefore based on meta-analyses of retrospective observational studies aimed at investigating the impact of previous statin therapy in patients hospitalized for COVID-19.

Methods: In studies reporting on the clinical outcomes of COVID-19 in statin users vs non-users, two endpoints have been used-in-hospital death rates, and disease severity as assessed by admission to intensive care units (ICUs)-with a special focus on patients with diabetes.

Results: Regarding mortality, 13 studies were included in the meta-analysis for a total of 10,829 statin users (2517 deaths) and 31,893 non-users (7516 deaths): univariate analysis showed no statistically significant reduction in deaths (OR: 0.97, 95% CI: 0.92-1.03), although between-study heterogeneity was high ($I^2 = 97\%$). As for disease severity, 11 studies were selected for a total of 3462 statin users (724 endpoints) and 10,560 non-users (1763 endpoints): here again, univariate analysis showed no reduction in severity (OR: 1.09, 95% CI: 0.99-1.22; $I^2 = 93\%$). Collectively, in 10 studies using multivariable analysis adjusted for the more prevalent baseline risk factors among statin users, lower OR values were reported than with univariate analyses (0.73 ± 0.31 vs 1.44 ± 0.84, respectively; $P = 0.0028$; adjusted OR: $P = 0.0237$ vs non-users). Limited but conflicting findings were observed for diabetes patients.

Conclusion: Although no significant reductions in either in-hospital mortality or COVID-19 severity were reported among statin users compared with non-users after univariate comparisons, such reductions were observed after adjusting for confounding factors. These highly heterogeneous observational findings now require confirmation by ongoing randomized clinical trials.

Keywords: COVID-19; Intensive care unit; Mortality; SARS-CoV-2; Statin; Type 2 diabetes.

Copyright © 2020 Elsevier Masson SAS. All rights reserved.

- 82 references
- 4 figures

Supplementary info

Publication types, MeSH terms, Substances Expand

Publication types

- Meta-Analysis

MeSH terms

- COVID-19* / drug therapy
- COVID-19* / mortality
- Diabetes Mellitus* / drug therapy
- Hospital Mortality

- Humans
- Hydroxymethylglutaryl-CoA Reductase Inhibitors* / therapeutic use
- Observational Studies as Topic
- Retrospective Studies
- Severity of Illness Index
- Treatment Outcome

Substances

- Hydroxymethylglutaryl-CoA Reductase Inhibitors

Full text links

ELSEVIER FULL-TEXT ARTICLE Elsevier Science Free PMC article
Proceed to details
Cite
Share
164
Observational Study
J Med Virol
. 2021 Sep;93(9):5367-5375.
doi: 10.1002/jmv.27049. Epub 2021 May 31.

Treatment patterns in US patients hospitalized with COVID-19 and pulmonary involvement

Jennie H Best [1], Amanda M Kong [2], Emma Kaplan-Lewis [3], Otis W Brawley [4], Rachel Baden [5], James L Zazzali [1], Karen S Miller [6], James Loveless [6], Krutika Jariwala-Parikh [2], Shalini V Mohan [1]
Affiliations Expand

Affiliations

- [1] US Medical Affairs, Genentech, Inc, South San Francisco, California, USA.
- [2] Life Sciences, IBM Watson Health, Cambridge, Massachusetts, USA.
- [3] Department of Medicine, NYC Health and Hospitals, Elmhurst Hospital Center, Queens, New York, USA.
- [4] Department of Oncology, Johns Hopkins Medicine, Baltimore, Maryland, USA.
- [5] Department of Medicine, Alameda Health System-Highland Hospital, Oakland, California, USA.
- [6] Idaho Pulmonary Associates, St. Luke's Health System, Boise, Idaho, USA.

- PMID: **33913536**
- PMCID: PMC8242555
- DOI: 10.1002/jmv.27049

Free PMC article
Observational Study

Treatment patterns in US patients hospitalized with COVID-19 and pulmonary involvement

Jennie H Best et al. J Med Virol. 2021 Sep.
Free PMC article
Show details
J Med Virol
. 2021 Sep;93(9):5367-5375.
doi: 10.1002/jmv.27049. Epub 2021 May 31.

Authors

Jennie H Best [1], Amanda M Kong [2], Emma Kaplan-Lewis [3], Otis W Brawley [4], Rachel Baden [5], James L Zazzali [1], Karen S Miller [6], James Loveless [6], Krutika Jariwala-Parikh [2], Shalini V Mohan [1]

Affiliations

- [1] US Medical Affairs, Genentech, Inc, South San Francisco, California, USA.
- [2] Life Sciences, IBM Watson Health, Cambridge, Massachusetts, USA.
- [3] Department of Medicine, NYC Health and Hospitals, Elmhurst Hospital Center, Queens, New York, USA.
- [4] Department of Oncology, Johns Hopkins Medicine, Baltimore, Maryland, USA.
- [5] Department of Medicine, Alameda Health System-Highland Hospital, Oakland, California, USA.
- [6] Idaho Pulmonary Associates, St. Luke's Health System, Boise, Idaho, USA.

- PMID: **33913536**
- PMCID: PMC8242555
- DOI: 10.1002/jmv.27049

Abstract

This study describes the baseline characteristics and treatment patterns of US patients hospitalized with a diagnosis of coronavirus disease 2019 (COVID-19) and pulmonary involvement. Patients hospitalized with pulmonary involvement due to COVID-19 (first hospitalization) were identified in the IBM Explorys® electronic health records database. Demographics, baseline clinical characteristics, and in-hospital medications were assessed. For evaluation of in-hospital medications, results were stratified by race, geographic region, age, and month of admission. Of

6564 hospitalized patients with COVID-19-related pulmonary involvement, 50.4% were male, and mean (SD) age was 62.6 (16.4) years; 75.2% and 23.6% of patients were from the South and Midwest, respectively, and 50.2% of patients were African American. Compared with African American patients, a numerically higher proportion of White patients received dexamethasone (19.7% vs. 31.8%, respectively), nonsteroidal anti-inflammatory drugs (NSAIDs; 27.1% vs. 34.9%), bronchodilators (19.8% vs. 29.5%), and remdesivir (9.3% vs. 21.0%). Numerically higher proportions of White patients than African American patients received select medications in the South but not in the Midwest. Compared with patients in the South, a numerically higher proportion of patients in the Midwest received dexamethasone (20.1% vs. 34.5%, respectively), NSAIDs (19.6% vs. 55.7%), bronchodilators (15.9% vs. 41.3%), and remdesivir (10.6% vs. 23.1%). Inpatient use of hydroxychloroquine decreased over time, whereas the use of dexamethasone and remdesivir increased over time. Among US patients predominantly from the South and Midwest hospitalized with COVID-19 and pulmonary involvement, differences were seen in medication use between different races, geographic regions, and months of hospitalization.

Keywords: SARS coronavirus; antiviral agents; cytokine/chemokine; disease control; immune responses; immunodulators; inflammation; respiratory tract.

© 2021 The Authors. Journal of Medical Virology Published by Wiley Periodicals LLC.

Conflict of interest statement

Jennie H. Best, Shalini V. Mohan, and James L. Zazzali are employees and shareholders of Genentech, Inc. Amanda M. Kong was an employee of IBM Watson Health at the time of this study and manuscript preparation. Krutika Jariwala-Parikh is an employee of IBM Watson Health. Emma Kaplan-Lewis is a principal investigator for a Genentech-sponsored clinical trial at Elmhurst Hospital Center, with no direct financial support received, and has served on medical advisory boards for ViiV Pharmaceuticals. Otis W. Brawley reports no conflicts of interest. Rachel Baden is a principal investigator for a Genentech-sponsored clinical trial at Highland Hospital, with no direct financial support received. Karen S. Miller is a principal investigator for a Genentech-sponsored clinical trial at St Luke′s Health System, with no direct financial support received. James Loveless has served as an investigator for Genentech-sponsored clinical trials, and as an advisor and on a speaker′s bureau for Genentech, all outside of the submitted work.

- 21 references
- 1 figure

Supplementary info

Publication types, MeSH terms, Substances, Grant support Expand

Publication types

- Multicenter Study
- Observational Study
- Research Support, Non-U.S. Gov't

MeSH terms

- Adenosine Monophosphate / analogs & derivatives*

- Adenosine Monophosphate / therapeutic use
- Adolescent
- Adult
- Aged
- Aged, 80 and over
- Alanine / analogs & derivatives*
- Alanine / therapeutic use
- Anti-Inflammatory Agents / therapeutic use
- Antiviral Agents / therapeutic use
- Blacks
- Bronchodilator Agents / therapeutic use*
- COVID-19 / drug therapy*
- COVID-19 / ethnology
- COVID-19 / pathology
- COVID-19 / virology
- Dexamethasone / therapeutic use*
- Female
- Hospitalization
- Humans
- Hydroxychloroquine / therapeutic use*
- Lung / drug effects
- Lung / pathology
- Lung / virology
- Male
- Middle Aged
- Pneumonia / drug therapy*
- Pneumonia / ethnology
- Pneumonia / pathology
- Pneumonia / virology
- Retrospective Studies
- SARS-CoV-2 / drug effects*
- SARS-CoV-2 / pathogenicity
- SARS-CoV-2 / physiology
- United States
- Whites

Substances

- Anti-Inflammatory Agents
- Antiviral Agents
- Bronchodilator Agents
- remdesivir

- Adenosine Monophosphate
- Hydroxychloroquine
- Dexamethasone
- Alanine

Grant support

- Genentech, Inc.

Full text links

WILEY Full Text Article Wiley Free PMC article
Proceed to details
Cite
Share
165
Observational Study
Intern Emerg Med
. 2021 Jun;16(4):1051-1060.
doi: 10.1007/s11739-020-02548-0. Epub 2020 Nov 11.

Clinical characteristics and respiratory support of 310 COVID-19 patients, diagnosed at the emergency room: a single-center retrospective study

Sandro Luigi Di Domenico [1], Daniele Coen [2], Marta Bergamaschi [3], Valentina Albertini [3], Leonardo Ghezzi [2], Michela Maria Cazzaniga [2], Valeria Tombini [2], Riccardo Colombo [2], Nicolò Capsoni [3], Tommaso Coen [4], Katia Barbara Cazzola [2], Marina Di Fiore [2], Laura Angaroni [2], Marco Alberto Strozzi [2]
Affiliations Expand

Affiliations

- [1] Department of Emergency Medicine, ASST Grande Ospedale Metropolitano Niguarda, Piazza Ospedale Maggiore 3, 20162, Milan, Italy. didomenico.sandro@gmail.com.
- [2] Department of Emergency Medicine, ASST Grande Ospedale Metropolitano Niguarda, Piazza Ospedale Maggiore 3, 20162, Milan, Italy.
- [3] Università Degli Studi Milano Bicocca, Piazza dell'Ateneo Nuovo 1; 20126, Milan, Italy.
- [4] Department of Economics, Brown University, Providence, RI, USA.

- PMID: **33175297**
- PMCID: PMC7656099
- DOI: 10.1007/s11739-020-02548-0

Free PMC article
Observational Study

Clinical characteristics and respiratory support of 310 COVID-19 patients, diagnosed at the emergency room: a single-center retrospective study

Sandro Luigi Di Domenico et al. Intern Emerg Med. 2021 Jun.
Free PMC article
Show details
Intern Emerg Med
. 2021 Jun;16(4):1051-1060.
doi: 10.1007/s11739-020-02548-0. Epub 2020 Nov 11.

Authors

Sandro Luigi Di Domenico [1], Daniele Coen [2], Marta Bergamaschi [3], Valentina Albertini [3], Leonardo Ghezzi [2], Michela Maria Cazzaniga [2], Valeria Tombini [2], Riccardo Colombo [2], Nicolò Capsoni [3], Tommaso Coen [4], Katia Barbara Cazzola [2], Marina Di Fiore [2], Laura Angaroni [2], Marco Alberto Strozzi [2]

Affiliations

- [1] Department of Emergency Medicine, ASST Grande Ospedale Metropolitano Niguarda, Piazza Ospedale Maggiore 3, 20162, Milan, Italy. didomenico.sandro@gmail.com.
- [2] Department of Emergency Medicine, ASST Grande Ospedale Metropolitano Niguarda, Piazza Ospedale Maggiore 3, 20162, Milan, Italy.
- [3] Università Degli Studi Milano Bicocca, Piazza dell'Ateneo Nuovo 1; 20126, Milan, Italy.
- [4] Department of Economics, Brown University, Providence, RI, USA.

- PMID: **33175297**
- PMCID: PMC7656099
- DOI: 10.1007/s11739-020-02548-0

Abstract

An ongoing outbreak of pneumonia associated with severe acute respiratory coronavirus 2 (SARS-CoV-2) occurred at the end of February 2020 in Lombardy, Italy. We analyzed data from a retrospective, single-center case series of 310 consecutive patients, with confirmed SARS-CoV-2 infection, admitted to the emergency room. We aimed to describe the clinical course, treatment and outcome of a cohort of patients with COVID-19 pneumonia, with special attention to oxygen delivery and ventilator support. Throughout the study period, 310 consecutive patients, with confirmed SARS-CoV-2 infection, attended the Emergency Room (ER), of these, 34 were discharged home directly from the ER. Of the remaining 276 patients, the overall mortality was 30.4%: 7 patients died in the ER and 77 during hospitalization. With respect to oxygen delivery:

22 patients did not need any oxygen support (8.0%), 151 patients were treated with oxygen only (54.7%), and 49 (17.8%) were intubated. 90 patients (32.6%) were treated with CPAP (Continuous Positive Airway Pressure) or NIV (Non Invasive Ventilation); in this group, 27 patients had a Do Not Intubate (DNI) order and were treated with CPAP/NIV as an upper threshold therapy, showing high mortality rate (88.9%). Among the 63 patients treated with CPAP/NIV without DNI, NIV failure occurred in 36 patients (57.1%), with mortality rate of 47.2%. Twenty-seven (27) patients were treated with CPAP/NIV without needing mechanical ventilation and 26 were discharged alive (96.3%). The study documents the poor prognosis of patients with severe respiratory failure, although a considerable minority of patients treated with CPAP/NIV had a positive outcome.

Keywords: COVID-19; CPAP (continuous positive airway pressure); Epidemiology; NIV (non-invasive ventilation); SARS-CoV-2 infection.

Conflict of interest statement

The authors declare no conflict of interest.

- 31 references
- 2 figures

Supplementary info

Publication types, MeSH terms Expand

Publication types

- Observational Study

MeSH terms

- Adult
- Aged
- COVID-19 / complications*
- COVID-19 / mortality
- COVID-19 / therapy*
- Emergency Service, Hospital*
- Female
- Hospitalization
- Humans
- Italy
- Male
- Middle Aged
- Outcome Assessment, Health Care
- Oxygen Inhalation Therapy
- Respiration, Artificial

- Retrospective Studies
- Survival Rate

Full text links

SpringerLink FULL-TEXT ARTICLE Springer Free PMC article
Proceed to details
Cite
Share
☐ 166
Observational Study
Neurol Neurochir Pol
. 2021;55(3):295-299.
doi: 10.5603/PJNNS.a2021.0037. Epub 2021 May 5.

4C Mortality Score correlates with in-hospital functional outcome after COVID-19-associated ischaemic stroke

Katarzyna Sawczyńska [1, 2], Marcin Wnuk [3, 4], Jeremiasz Jagiełła [3, 4], Tomasz Kęsek [4], Magdalena Wolska-Sikora [5], Magdalena Szara-Cichoń [5], Kinga Zagata-Szewczyk [6], Adela Uchacz [6], Katarzyna Filipowicz [6], Marcin Plaszczak [6], Katarzyna Spisak-Borowska [7], Anna Baranowska [8], Magdalena Wójcik-Pędziwiatr [9], Marta Swarowska-Skuza [9], Elżbieta Szczygieł-Pilut [9], Mariusz Kłos [10], Piotr Grzyb [10], Michał Biela [10], Joanna Mierzwińska [5], Iwona Sinkiewicz [6], Jerzy Machowski [7], Anna Węgrzyn [8], Michał Michalski [9], Ryszard Nowak [10], Agnieszka Słowik [3, 4]
Affiliations Expand

Affiliations

- [1] Department of Neurology, Jagiellonian University Medical College, Krakow, Poland. katarzyna.sawczynska@gmail.com.
- [2] Department of Neurology, University Hospital in Krakow, Poland. katarzyna.sawczynska@gmail.com.
- [3] Department of Neurology, Jagiellonian University Medical College, Krakow, Poland.
- [4] Department of Neurology, University Hospital in Krakow, Poland.
- [5] Department of Neurology, Henryk Klimontowicz Specialist Hospital, Gorlice, Poland.
- [6] Department of Neurology, John Paul II Podhale Specialist Hospital, Nowy Targ, Poland.
- [7] Department of Neurology, Saint Maximillian County Hospital, Oswiecim, Poland.
- [8] Department of Neurology, Jedrzej Sniadecki Specialist Hospital, Nowy Sacz, Poland.
- [9] Department of Neurology, John Paul II Specialist Hospital, Krakow, Poland.
- [10] Department of Neurology, Ludwik Rydygier Specialist Hospital, Krakow, Poland.

- PMID: **33949676**

- DOI: 10.5603/PJNNS.a2021.0037

Free article
Observational Study

4C Mortality Score correlates with in-hospital functional outcome after COVID-19-associated ischaemic stroke

Katarzyna Sawczyńska et al. Neurol Neurochir Pol. 2021.
Free article

Show details

Neurol Neurochir Pol
. 2021;55(3):295-299.
doi: 10.5603/PJNNS.a2021.0037. Epub 2021 May 5.

Authors

Katarzyna Sawczyńska [1,2], Marcin Wnuk [3,4], Jeremiasz Jagiełła [3,4], Tomasz Kęsek [4], Magdalena Wolska-Sikora [5], Magdalena Szara-Cichoń [5], Kinga Zagata-Szewczyk [6], Adela Uchacz [6], Katarzyna Filipowicz [6], Marcin Plaszczak [6], Katarzyna Spisak-Borowska [7], Anna Baranowska [8], Magdalena Wójcik-Pędziwiatr [9], Marta Swarowska-Skuza [9], Elżbieta Szczygieł-Pilut [9], Mariusz Kłos [10], Piotr Grzyb [10], Michał Biela [10], Joanna Mierzwińska [5], Iwona Sinkiewicz [6], Jerzy Machowski [7], Anna Węgrzyn [8], Michał Michalski [9], Ryszard Nowak [10], Agnieszka Słowik [3,4]

Affiliations

- [1] Department of Neurology, Jagiellonian University Medical College, Krakow, Poland. katarzyna.sawczynska@gmail.com.
- [2] Department of Neurology, University Hospital in Krakow, Poland. katarzyna.sawczynska@gmail.com.
- [3] Department of Neurology, Jagiellonian University Medical College, Krakow, Poland.
- [4] Department of Neurology, University Hospital in Krakow, Poland.
- [5] Department of Neurology, Henryk Klimontowicz Specialist Hospital, Gorlice, Poland.
- [6] Department of Neurology, John Paul II Podhale Specialist Hospital, Nowy Targ, Poland.
- [7] Department of Neurology, Saint Maximillian County Hospital, Oswiecim, Poland.
- [8] Department of Neurology, Jedrzej Sniadecki Specialist Hospital, Nowy Sacz, Poland.
- [9] Department of Neurology, John Paul II Specialist Hospital, Krakow, Poland.
- [10] Department of Neurology, Ludwik Rydygier Specialist Hospital, Krakow, Poland.

- PMID: **33949676**
- DOI: 10.5603/PJNNS.a2021.0037

Abstract

Aim of the study: The 4C Mortality Score was created to predict mortality in hospitalised patients with COVID-19 and has to date been evaluated only in respiratory system disorders. The aim of this study was to investigate its application in patients with COVID-19-associated acute ischaemic stroke (AIS).

Clinical rationale for study: COVID-19 is a risk factor for AIS. COVID-19-associated AIS results in higher mortality and worse functional outcome. Predictors of functional outcome in COVID-19-associated AIS are required.

Materials and methods: This was a retrospective observational study of patients with AIS hospitalised in seven neurological wards in Małopolska Voivodship (Poland) between August and December 2020. We gathered data concerning the patients' age, sex, presence of cardiovascular risk factors, type of treatment received, and the presence of stroke-associated infections (including pneumonia, urinary tract infection and infection of unknown source). We calculated 4C Mortality Score at stroke onset, and investigated whether there was a correlation with neurological deficit measured using the National Health Institute Stroke Scale (NIHSS) and functional outcome assessed using the modified Rankin Scale (mRS) at discharge.

Results: The study included 52 patients with COVID-19-associated AIS. The 4C Mortality Score at stroke onset correlated with mRS ($r_s = 0.565$, $p < 0.01$) at discharge. There was also a statistically significant difference in the mean 4C Mortality Score between patients who died and patients who survived the stroke (13.08 ± 2.71 vs. 9.85 ± 3.47, $p = 0.04$).

Conclusions and clinical implications: 4C Mortality Score predicts functional outcome at discharge in COVID-19-associated AIS patients.

Keywords: 4C Mortality Score; COVID-19; acute ischaemic stroke; modified Rankin Scale.

Supplementary info

Publication types, MeSH terms Expand

Publication types

- Observational Study

MeSH terms

- Brain Ischemia*
- COVID-19*
- Hospitals
- Humans
- Ischemic Stroke*
- Poland
- SARS-CoV-2
- Stroke*

- Treatment Outcome

Full text links

Full-text Via Medica Medical Publishers

Proceed to details

Cite

Share

167

Observational Study

Int J Infect Dis
. 2020 Nov;100:258-263.
doi: 10.1016/j.ijid.2020.09.012. Epub 2020 Sep 11.

Risk factors for non-invasive/invasive ventilatory support in patients with COVID-19 pneumonia: A retrospective study within a multidisciplinary approach

Lorenzo Roberto Suardi [1], Carlo Pallotto [2], Sara Esperti [2], Elisa Tazzioli [2], Filippo Baragli [2], Elena Salomoni [2], Annarita Botta [3], Francesca Covani Frigieri [4], Maddalena Pazzi [4], Caterina Stera [5], Martina Carlucci [4], Raffaella Papa [4], Tommaso Meconi [4], Vittorio Pavoni [4], Pierluigi Blanc [2]

Affiliations Expand

Affiliations

- [1] Infectious Diseases Unit, Santa Maria Annunziata Hospital, Azienda USL Toscana Centro, Bagno a Ripoli, Florence, Italy. Electronic address: lorenzoroberto.suardi@gmail.com.
- [2] Infectious Diseases Unit, Santa Maria Annunziata Hospital, Azienda USL Toscana Centro, Bagno a Ripoli, Florence, Italy.
- [3] Infectious Diseases Unit, Santa Maria Annunziata Hospital, Azienda USL Toscana Centro, Bagno a Ripoli, Florence, Italy; Department of Experimental and Clinical Medicine, University of Florence, Florence, Italy.
- [4] Intensive Care Unit, Santa Maria Annunziata Hospital, Azienda USL Toscana Centro, Bagno a Ripoli, Florence, Italy.
- [5] Intensive Care Unit, Santa Maria Annunziata Hospital, Azienda USL Toscana Centro, Bagno a Ripoli, Florence, Italy; Department of Anaesthesia, University of Pisa, Pisa, Italy.

- PMID: **32920232**
- PMCID: PMC7484622
- DOI: 10.1016/j.ijid.2020.09.012

Free PMC article
Observational Study

Risk factors for non-invasive/invasive ventilatory support in patients with COVID-19 pneumonia: A retrospective study within a multidisciplinary approach

Lorenzo Roberto Suardi et al. Int J Infect Dis. 2020 Nov.
Free PMC article
Show details
Int J Infect Dis
. 2020 Nov;100:258-263.
doi: 10.1016/j.ijid.2020.09.012. Epub 2020 Sep 11.

Authors

Lorenzo Roberto Suardi [1], Carlo Pallotto [2], Sara Esperti [2], Elisa Tazzioli [2], Filippo Baragli [2], Elena Salomoni [2], Annarita Botta [3], Francesca Covani Frigieri [4], Maddalena Pazzi [4], Caterina Stera [5], Martina Carlucci [4], Raffaella Papa [4], Tommaso Meconi [4], Vittorio Pavoni [4], Pierluigi Blanc [2]

Affiliations

- [1] Infectious Diseases Unit, Santa Maria Annunziata Hospital, Azienda USL Toscana Centro, Bagno a Ripoli, Florence, Italy. Electronic address: lorenzoroberto.suardi@gmail.com.
- [2] Infectious Diseases Unit, Santa Maria Annunziata Hospital, Azienda USL Toscana Centro, Bagno a Ripoli, Florence, Italy.
- [3] Infectious Diseases Unit, Santa Maria Annunziata Hospital, Azienda USL Toscana Centro, Bagno a Ripoli, Florence, Italy; Department of Experimental and Clinical Medicine, University of Florence, Florence, Italy.
- [4] Intensive Care Unit, Santa Maria Annunziata Hospital, Azienda USL Toscana Centro, Bagno a Ripoli, Florence, Italy.
- [5] Intensive Care Unit, Santa Maria Annunziata Hospital, Azienda USL Toscana Centro, Bagno a Ripoli, Florence, Italy; Department of Anaesthesia, University of Pisa, Pisa, Italy.

- PMID: **32920232**
- PMCID: PMC7484622
- DOI: 10.1016/j.ijid.2020.09.012

Abstract

Objectives: To investigate risk factors for non-invasive/invasive ventilatory support (NI/I-VS) in patients with coronavirus disease 2019 (COVID-19).

Methods: All consecutive patients admitted to the Infectious Diseases Unit and Intensive Care Unit (ICU) of Santa Maria Annunziata Hospital (Florence, Italy), from February 25 to April 25, 2020, with a confirmed COVID-19 diagnosis were enrolled in this retrospective cohort study. NI/I-VS was defined as the need for continuous positive airway pressure (CPAP) or bilevel

positive airway pressure (BPAP) (non-invasive ventilation) or mechanical ventilation, not including low-flow systems of oxygen therapy such as the Venturi mask or nasal cannula.

Results: Ninety-seven patients were enrolled; 61.9% (60/97) were male and the median patient age was 64 years. The in-hospital mortality was 9.3%. Thirty-five of the 97 patients (36%) required ICU admission and 94.8% (92/97) were prescribed oxygen therapy: 10.8% (10/92) by nasal cannula, 44.5% (41/92) by Venturi mask, 31.5% (29/92) by CPAP, 2.2% (2/92) by BPAP, and 10.8% (10/92) by mechanical ventilation following intubation. On univariate analysis, patients with a body mass index >30, type II diabetes mellitus, and those presenting with dyspnoea, asthenia, SOFA score ≥2 points, PaO_2/FiO_2 <300, temperature >38 °C, increased levels of lactate dehydrogenase (LDH), alanine aminotransferase, and C-reactive protein, and a d-dimer >1000 ng/mL at admission more frequently underwent NI/I-VS. Multivariate logistic regression analysis confirmed temperature >38 °C (odds ratio (OR) 21.2, 95% confidential interval (95% CI) 3.5-124.5, p = 0.001), LDH >250 U/l (OR 15.2, 95% CI 1.8-128.8, p = 0.012), and d-dimer >1000 ng/mL (OR 4.5, 95% CI 1.2-17.3, p = 0.027) as significantly associated with the requirement for NI/I-VS. A non-significant trend (p = 0.051) was described for PaO_2/FiO_2 <300.

Conclusions: Temperature >38 °C, LDH > 250 U/l, and d-dimer >1000 ng/mL were found to be independent risk factors for NI/I-VS in COVID-19 patients. In order to quickly identify patients likely at risk of developing a critical illness, inflammatory markers should be assessed upon hospital admission.

Keywords: COVID-19; Invasive ventilation; Italy; Multidisciplinary; Non-invasive ventilation; Risk factors.

Copyright © 2020 The Authors. Published by Elsevier Ltd.. All rights reserved.

- 18 references

Supplementary info

Publication types, MeSH terms Expand

Publication types

- Observational Study

MeSH terms

- Adult
- Aged
- Aged, 80 and over
- COVID-19 / therapy*
- Cohort Studies
- Critical Illness
- Diabetes Mellitus, Type 2 / complications
- Female
- Hospital Mortality

- Hospitalization
- Humans
- Intensive Care Units
- Male
- Middle Aged
- Pandemics
- Respiration, Artificial*
- Retrospective Studies
- Risk Factors
- SARS-CoV-2

Full text links

ELSEVIER OPEN ACCESS Elsevier Science Free PMC article
Proceed to details
Cite
Share
168
Observational Study
Rev Assoc Med Bras (1992)
. 2021 Mar;67(3):437-442.
doi: 10.1590/1806-9282.20200896.

Mean serum D-dimer level to predict in-hospital mortality in COVID-19

Mert İlker Hayıroğlu [1], Vedat Çiçek [2], Şahhan Kılıç [2], Tufan Çınar [2]
Affiliations Expand

Affiliations

- [1] Department of Cardiology, Dr. Siyami Ersek Thoracic and Cardiovascular Surgery Training and Research Hospital, Istanbul, Turkey.
- [2] Department of Cardiology, Haydarpasa Sultan Abdulhamid Han Training and Research Hospital, Istanbul, Turkey.

- PMID: **34468611**
- DOI: 10.1590/1806-9282.20200896

Free article
Observational Study

Mean serum D-dimer level to predict in-hospital mortality in COVID-19

Mert İlker Hayıroğlu et al. Rev Assoc Med Bras (1992). 2021 Mar.
Free article
Show details
Rev Assoc Med Bras (1992)
. 2021 Mar;67(3):437-442.
doi: 10.1590/1806-9282.20200896.

Authors

Mert İlker Hayıroğlu [1], Vedat Çiçek [2], Şahhan Kılıç [2], Tufan Çınar [2]

Affiliations

- [1] Department of Cardiology, Dr. Siyami Ersek Thoracic and Cardiovascular Surgery Training and Research Hospital, Istanbul, Turkey.
- [2] Department of Cardiology, Haydarpasa Sultan Abdulhamid Han Training and Research Hospital, Istanbul, Turkey.

- PMID: **34468611**
- DOI: 10.1590/1806-9282.20200896

Abstract

Objective: The prognostic effect of the mean serum D-dimer levels, which was calculated from the first five days of hospitalization of the patients, has not been elucidated. This study aimed to evaluate the effect of mean D-dimer level about in-hospital mortality in patients hospitalized due to coronavirus disease-2019 (COVID-19) infection.

Methods: In this observational retrospective study, we examined the in-hospital prognostic value of mean D-dimer [D-dimerfirst day+D-dimerthird day+D-dimerfifth day)/3 on 240 consecutive adult patients with COVID-19. Patients were stratified into tertiles according to their mean D-dimer starting from the lowest one. In-hospital mortality rates were compared between tertiles and the power of the mean D-dimer level was also presented by a receiver operating curve analysis.

Results: After adjustment for confounding baseline variables, mean D-dimer in tertile 3 was associated with 4.2-fold hazard ratio of in-hospital mortality (odds ratio [OR] 4.2; 95% confidence interval [CI] 1.8-20.1, $p<0.001$). A receiver-operating curve analysis revealed that the optimal cutoff value of the mean D-dimer to predict in-hospital mortality was 779 µg/L with 77% sensitivity and 83% specificity (area under the curve [AUC] 0.87; 95%CI 0.81-0.94; $p<0.001$).

Conclusion: Patients with a higher mean D-dimer level should be followed-up more closely as they may be a candidate for a more aggressive treatment modality, such as biologic agents or convalescent plasma.

Supplementary info

Publication types, MeSH terms, Substances, Supplementary concepts Expand

Publication types

- Observational Study

MeSH terms

- Adult
- Biomarkers
- COVID-19* / therapy
- Fibrin Fibrinogen Degradation Products
- Hospital Mortality
- Humans
- Immunization, Passive
- Prognosis
- Retrospective Studies
- SARS-CoV-2

Substances

- Biomarkers
- Fibrin Fibrinogen Degradation Products
- fibrin fragment D

Supplementary concepts

- COVID-19 serotherapy

Full text links

free full text available at SciELO.org Scientific Electronic Library Online

Proceed to details

Cite

Share

169

Observational Study

Pulm Med

. 2021 Jun 23;2021:5533123.
doi: 10.1155/2021/5533123. eCollection 2021.

The Impact of COVID-19 on Hospitalised COPD Exacerbations in Malta

Yvette Farrugia [1], Bernard Paul Spiteri Meilak [1], Neil Grech [1], Rachelle Asciak [1], Liberato Camilleri [1], Stephen Montefort [1], Christopher Zammit [1]
Affiliations Expand

Affiliation

- [1] Mater Dei Hospital, Triq id-Donaturi tad-Demm, Msida MSD2090, Malta.

- PMID: **34258061**
- PMCID: PMC8241528
- DOI: 10.1155/2021/5533123

Free PMC article
Observational Study

The Impact of COVID-19 on Hospitalised COPD Exacerbations in Malta

Yvette Farrugia et al. Pulm Med. 2021.
Free PMC article
Show details
Pulm Med
. 2021 Jun 23;2021:5533123.
doi: 10.1155/2021/5533123. eCollection 2021.

Authors

Yvette Farrugia [1], Bernard Paul Spiteri Meilak [1], Neil Grech [1], Rachelle Asciak [1], Liberato Camilleri [1], Stephen Montefort [1], Christopher Zammit [1]

Affiliation

- [1] Mater Dei Hospital, Triq id-Donaturi tad-Demm, Msida MSD2090, Malta.

- PMID: **34258061**
- PMCID: PMC8241528
- DOI: 10.1155/2021/5533123

Abstract

Method: Data was collected retrospectively from electronic hospital records during the periods 1st March until 10th May in 2019 and 2020.

Results: There was a marked decrease in AECOPD admissions in 2020, with a 54.2% drop in admissions (n = 119 in 2020 vs. n = 259 in 2019). There was no significant difference in patient demographics or medical comorbidities. In 2020, there was a significantly lower number of patients with AECOPD who received nebulised medications during admission (60.4% in 2020 vs. 84.9% in 2019; $p \leq 0.001$). There were also significantly lower numbers of AECOPD patients admitted in 2020 who received controlled oxygen *via* venturi masks (69.0% in 2020 vs. 84.5% in 2019; $p = 0.006$). There was a significant increase in inpatient mortality in 2020 (19.3% [n = 23] and 8.4% [n = 22] for 2020 and 2019, respectively, $p = 0.003$). Year was found to be the best predictor of mortality outcome ($p = 0.001$). The lack of use of SABA pre-admission treatment (p = 0.002), active malignancy ($p = 0.003$), and increased length of hospital stay ($p = 0.046$) were also found to be predictors of mortality for AECOPD patients; however, these parameters were unchanged between 2019 and 2020 and therefore could not account for the increase in mortality.

Conclusions: There was a decrease in the number of admissions with AECOPD in 2020 during the COVID-19 pandemic, when compared to 2019. The year 2020 proved to be a significant predictor for inpatient mortality, with a significant increase in mortality in 2020. The decrease in nebuliser and controlled oxygen treatment noted in the study period did not prove to be a significant predictor of mortality when corrected for other variables. Therefore, the difference in mortality cannot be explained with certainty in this retrospective cohort study.

Copyright © 2021 Yvette Farrugia et al.

Conflict of interest statement

We have no conflict of interest to declare.

- 29 references
- 3 figures

Supplementary info

Publication types, MeSH terms Expand

Publication types

- Observational Study

MeSH terms

- Aged
- COVID-19*
- Cross-Sectional Studies
- Disease Progression
- Female
- Hospitalization / statistics & numerical data*
- Humans
- Male
- Malta

- Pulmonary Disease, Chronic Obstructive / therapy*
- Retrospective Studies

Full text links

Hindawi Limited Free PMC article
Proceed to details
Cite
Share
170
Observational Study
PLoS One
. 2021 Jun 11;16(6):e0252591.
doi: 10.1371/journal.pone.0252591. eCollection 2021.

COVID-19 treatment combinations and associations with mortality in a large multi-site healthcare system

Dagan Coppock [1], Michael Baram [2], Anna Marie Chang [3], Patricia Henwood [3], Alan Kubey [4] [5], Ross Summer [2], John Zurlo [1], Michael Li [6], Bryan Hess [1]
Affiliations Expand

Affiliations

- [1] Division of Infectious Diseases, Department of Medicine, Thomas Jefferson University, Sidney Kimmel Medical College, Philadelphia, PA, United States of America.
- [2] Division of Pulmonary, Department of Medicine, Allergy, and Critical Care Medicine, Thomas Jefferson University, Sidney Kimmel Medical College, Philadelphia, PA, United States of America.
- [3] Department of Emergency Medicine, Thomas Jefferson University, Sidney Kimmel Medical College, Philadelphia, PA, United States of America.
- [4] Division of Hospital Medicine, Department of Medicine, Thomas Jefferson University, Sidney Kimmel Medical College, Philadelphia, PA, United States of America.
- [5] Division of Hospital Internal Medicine, Department of Internal Medicine, Mayo Clinic, Rochester, MN, United States of America.
- [6] Institute of Emerging Health Professions, Center for Digital Health and Data Science, Thomas Jefferson University, Philadelphia, PA, United States of America.

- PMID: **34115801**
- PMCID: PMC8195431
- DOI: 10.1371/journal.pone.0252591

Free PMC article
Observational Study

COVID-19 treatment combinations and associations with mortality in a large multi-site healthcare system

Dagan Coppock et al. PLoS One. 2021.
Free PMC article
Show details
PLoS One
. 2021 Jun 11;16(6):e0252591.
doi: 10.1371/journal.pone.0252591. eCollection 2021.

Authors

Dagan Coppock [1], Michael Baram [2], Anna Marie Chang [3], Patricia Henwood [3], Alan Kubey [4] [5], Ross Summer [2], John Zurlo [1], Michael Li [6], Bryan Hess [1]

Affiliations

- [1] Division of Infectious Diseases, Department of Medicine, Thomas Jefferson University, Sidney Kimmel Medical College, Philadelphia, PA, United States of America.
- [2] Division of Pulmonary, Department of Medicine, Allergy, and Critical Care Medicine, Thomas Jefferson University, Sidney Kimmel Medical College, Philadelphia, PA, United States of America.
- [3] Department of Emergency Medicine, Thomas Jefferson University, Sidney Kimmel Medical College, Philadelphia, PA, United States of America.
- [4] Division of Hospital Medicine, Department of Medicine, Thomas Jefferson University, Sidney Kimmel Medical College, Philadelphia, PA, United States of America.
- [5] Division of Hospital Internal Medicine, Department of Internal Medicine, Mayo Clinic, Rochester, MN, United States of America.
- [6] Institute of Emerging Health Professions, Center for Digital Health and Data Science, Thomas Jefferson University, Philadelphia, PA, United States of America.

- PMID: **34115801**
- PMCID: PMC8195431
- DOI: 10.1371/journal.pone.0252591

Abstract

Introduction: During the early months of the COVID-19 pandemic, mortality associated with the disease declined in the United States. The standard of care for pharmacological interventions evolved during this period as new and repurposed treatments were used alone and in combination. Though these medications have been studied individually, data are limited regarding the relative impact of different medication combinations. The objectives of this study were to evaluate the association of COVID-19-related mortality and observed medication combinations and to determine whether changes in medication-related practice patterns and measured patient characteristics, alone, explain the decline in mortality seen early in the COVID-19 pandemic.

Methods: A retrospective cohort study was conducted at a multi-hospital healthcare system exploring the association of mortality and combinations of remdesivir, corticosteroids, anticoagulants, tocilizumab, and hydroxychloroquine. Multivariable logistic regression was used to identify predictors of mortality for both the overall population and the population stratified by intensive care and non-intensive care unit admissions. A separate model was created to control for the change in unmeasured variables over time.

Results: For all patients, four treatment combinations were associated with lower mortality: Anticoagulation Only (OR 0.24, $p < 0.0001$), Anticoagulation and Remdesivir (OR 0.25, $p = 0.0031$), Anticoagulation and Corticosteroids (OR 0.53, $p = 0.0263$), and Anticoagulation, Corticosteroids and Remdesivir (OR 0.42, $p = 0.026$). For non-intensive care unit patients, the same combinations were significantly associated with lower mortality. For patients admitted to the intensive care unit, Anticoagulation Only was the sole treatment category associated with decreased mortality. When adjusted for demographics, clinical characteristics, and all treatment combinations there was an absolute decrease in the mortality rate by 2.5% between early and late periods of the study. However, when including an additional control for changes in unmeasured variables overtime, the absolute mortality rate decreased by 5.4%.

Conclusions: This study found that anticoagulation was the most significant treatment for the reduction of COVID-related mortality. Anticoagulation Only was the sole treatment category associated with a significant decrease in mortality for both intensive care and non-intensive care patients. Treatment combinations that additionally included corticosteroids and/or remdesivir were also associated with decreased mortality, though only in the non-intensive care stratum. Further, we found that factors other than measured changes in demographics, clinical characteristics or pharmacological interventions accounted for an additional decrease in the COVID-19-related mortality rate over time.

Conflict of interest statement

The authors have declared that no competing interests exist.

- 37 references
- 1 figure

Supplementary info

Publication types, MeSH terms, Substances, Supplementary concepts, Grant support Expand

Publication types

- Multicenter Study
- Observational Study

MeSH terms

- Adenosine Monophosphate / analogs & derivatives*
- Adenosine Monophosphate / therapeutic use
- Adrenal Cortex Hormones / therapeutic use*
- Adult

- Aged
- Aged, 80 and over
- Alanine / analogs & derivatives*
- Alanine / therapeutic use
- Antibodies, Monoclonal, Humanized / therapeutic use*
- Anticoagulants / therapeutic use*
- Antiviral Agents / therapeutic use*
- COVID-19 / drug therapy*
- COVID-19 / epidemiology
- COVID-19 / mortality
- COVID-19 / virology
- Delivery of Health Care / methods
- Drug Therapy, Combination
- Humans
- Hydroxychloroquine / therapeutic use*
- Intensive Care Units
- Length of Stay
- Male
- Middle Aged
- Pandemics*
- Retrospective Studies
- SARS-CoV-2 / isolation & purification*
- Treatment Outcome
- United States / epidemiology

Substances

- Adrenal Cortex Hormones
- Antibodies, Monoclonal, Humanized
- Anticoagulants
- Antiviral Agents
- remdesivir
- Adenosine Monophosphate
- Hydroxychloroquine
- tocilizumab
- Alanine

Supplementary concepts

- COVID-19 drug treatment

Grant support

The authors received no specific funding for this work.

Full text links

OPEN ACCESS TO FULL TEXT PLOS ONE Public Library of Science Free PMC article
Proceed to details
Cite
Share
☐ 171
Observational Study
Public Health
. 2021 Jan;190:93-98.
doi: 10.1016/j.puhe.2020.11.021. Epub 2020 Nov 28.

Racial disparities in COVID-19 hospitalizations do not lead to disparities in outcomes

G Krishnamoorthy[1], C Arsene[2], N Jena[1], S M Mogulla[1], R Coakley[3], J Khine[1], N Khosrodad[1], A Klein[3], A A Sule[4]
Affiliations Expand

Affiliations

- [1] St Joseph Mercy Oakland Hospital, Pontiac, MI, United States.
- [2] ProMedica Health System, Toledo, OH, United States.
- [3] Ross University School of Medicine, United States.
- [4] St Joseph Mercy Oakland Hospital, Pontiac, MI, United States. Electronic address: Anupam.A.Sule@stjoeshealth.org.

- PMID: **33385640**
- PMCID: PMC7698674
- DOI: 10.1016/j.puhe.2020.11.021

Free PMC article
Observational Study

Racial disparities in COVID-19 hospitalizations do not lead to disparities in outcomes

G Krishnamoorthy et al. Public Health. 2021 Jan.
Free PMC article
Show details
Public Health
. 2021 Jan;190:93-98.
doi: 10.1016/j.puhe.2020.11.021. Epub 2020 Nov 28.

Authors

G Krishnamoorthy [1], C Arsene [2], N Jena [1], S M Mogulla [1], R Coakley [3], J Khine [1], N Khosrodad [1], A Klein [3], A A Sule [4]

Affiliations

- [1] St Joseph Mercy Oakland Hospital, Pontiac, MI, United States.
- [2] ProMedica Health System, Toledo, OH, United States.
- [3] Ross University School of Medicine, United States.
- [4] St Joseph Mercy Oakland Hospital, Pontiac, MI, United States. Electronic address: Anupam.A.Sule@stjoeshealth.org.

- PMID: **33385640**
- PMCID: PMC7698674
- DOI: 10.1016/j.puhe.2020.11.021

Abstract

Objectives: The objective of the study is the identification of racial differences in characteristics and comorbidities in patients hospitalized for COVID-19 and the impact on outcomes.

Study design: The study design is a retrospective observational study.

Methods: Data for all patients admitted to seven community hospitals in Michigan, United States, with polymerase chain reaction confirmed diagnosis of COVID-19 from March 10 to April 15, 2020 were analyzed. The primary outcomes of racial disparity in inpatient mortality and intubation were analyzed using descriptive statistics and multivariate regression models.

Results: The study included 336 Black and 408 White patients. Black patients were younger (62.9 ± 15.0 years vs 71.8 ± 16.4, P < .001), had a higher mean body mass index (32.4 ± 8.6 kg/m^2 vs 28.8 ± 7.5, P < .001), had higher prevalence of diabetes (136/336 vs 130/408, P = .02), and presented later (6.6 ± 5.3 days after symptom onset vs. 5.4 ± 5.4, P = .006) compared with White patients. Younger Black patients had a higher prevalence of obesity (age <65 years, 69.9%) than older Black patients (age >65 years, 39.2%) and younger White patients (age < 65, 55.1%). Intubation did not reach statistical significance for racial difference (Black patients 61/335 vs. 54/406, P = .08). Mortality was not higher in Black patients (65/335 vs. 142/406 in White patients, odds ratio 0.61, 95% confidence interval: 0.37 to 0.99, 2-sided P = .05) in multivariate analysis, accounting for other risk factors associated with mortality.

Conclusions: Higher prevalence of obesity and diabetes in young Black populations may be the critical factor driving disproportionate COVID-19 hospitalizations in Black populations. Hospitalized Black patients do not have worse outcomes compared with White patients.

Keywords: COVID-19; Diabetes; Hospitalization; Obesity; Outcomes; Racial disparities.

Copyright © 2020 The Royal Society for Public Health. Published by Elsevier Ltd. All rights reserved.

- 38 references
- 2 figures

Supplementary info

Publication types, MeSH terms Expand

Publication types

- Observational Study

MeSH terms

- African Americans / statistics & numerical data
- Aged
- Body Mass Index
- COVID-19 / ethnology*
- COVID-19 / mortality
- COVID-19 / therapy*
- COVID-19 / virology
- Comorbidity
- Diabetes Mellitus / epidemiology*
- Female
- Health Status Disparities
- Healthcare Disparities / ethnology
- Healthcare Disparities / statistics & numerical data
- Hospitalization / statistics & numerical data*
- Hospitals, Community
- Humans
- Intensive Care Units
- Male
- Michigan / epidemiology
- Middle Aged
- Obesity / epidemiology*
- Pandemics
- Prevalence
- Pulmonary Disease, Chronic Obstructive / epidemiology
- Racial Groups / ethnology
- Racial Groups / statistics & numerical data*

- Retrospective Studies
- Risk Factors
- SARS-CoV-2*
- Whites / statistics & numerical data

Full text links

ELSEVIER FULL-TEXT ARTICLE Elsevier Science Free PMC article
Proceed to details
Cite
Share
☐ 172
Observational Study
BMC Infect Dis
. 2020 Dec 22;20(1):964.
doi: 10.1186/s12879-020-05701-4.

Effects of Tocilizumab in COVID-19 patients: a cohort study

Christine A Vu [1] [2], Kailynn J DeRonde [3], Ana D Vega [3], Meshell Maxam [3], Gregory Holt [4], Yoichiro Natori [5], Jose Gonzales Zamora [5], Veronica Salazar [3], Renata Boatwright [3], Stephen R Morris [5], Daniela de Lima Corvino [5], Anmary Fernandez Betances [5], Leah Colucci [6], James Keegan [6], Andy Lopez [6], Andrew Hany Rezk [6], Yvette Rodriguez [6], Gabriela M Moraru [7], Susanne Doblecki [5], David J De La Zerda [4], Lilian M Abbo [5]
Affiliations Expand

Affiliations

- [1] Department of Pharmacy, Jackson Health System, Miami, FL, USA. christine.vu@jhsmiami.org.
- [2] Jackson Memorial Hospital, Pharmacy Services, B069, 1611 NW 12th Ave, Miami, FL, 33136, USA. christine.vu@jhsmiami.org.
- [3] Department of Pharmacy, Jackson Health System, Miami, FL, USA.
- [4] Division of Pulmonary Critical Care, Department of Medicine, University of Miami Miller School of Medicine, Miami, FL, USA.
- [5] Division of Infectious Diseases, Department of Medicine, University of Miami Miller School of Medicine, Miami, FL, USA.
- [6] University of Miami Miller School of Medicine, Miami, FL, USA.
- [7] Department of Pediatrics, Jackson Health System/Holtz Children's Hospital, Miami, FL, USA.

- PMID: **33353546**
- PMCID: PMC7755065
- DOI: 10.1186/s12879-020-05701-4

Free PMC article
Observational Study

Effects of Tocilizumab in COVID-19 patients: a cohort study

Christine A Vu et al. BMC Infect Dis. 2020.
Free PMC article
Show details
BMC Infect Dis
. 2020 Dec 22;20(1):964.
doi: 10.1186/s12879-020-05701-4.

Authors

Christine A Vu [1, 2], Kailynn J DeRonde [3], Ana D Vega [3], Meshell Maxam [3], Gregory Holt [4], Yoichiro Natori [5], Jose Gonzales Zamora [5], Veronica Salazar [3], Renata Boatwright [3], Stephen R Morris [5], Daniela de Lima Corvino [5], Anmary Fernandez Betances [5], Leah Colucci [6], James Keegan [6], Andy Lopez [6], Andrew Hany Rezk [6], Yvette Rodriguez [6], Gabriela M Moraru [7], Susanne Doblecki [5], David J De La Zerda [4], Lilian M Abbo [5]

Affiliations

- [1] Department of Pharmacy, Jackson Health System, Miami, FL, USA. christine.vu@jhsmiami.org.
- [2] Jackson Memorial Hospital, Pharmacy Services, B069, 1611 NW 12th Ave, Miami, FL, 33136, USA. christine.vu@jhsmiami.org.
- [3] Department of Pharmacy, Jackson Health System, Miami, FL, USA.
- [4] Division of Pulmonary Critical Care, Department of Medicine, University of Miami Miller School of Medicine, Miami, FL, USA.
- [5] Division of Infectious Diseases, Department of Medicine, University of Miami Miller School of Medicine, Miami, FL, USA.
- [6] University of Miami Miller School of Medicine, Miami, FL, USA.
- [7] Department of Pediatrics, Jackson Health System/Holtz Children's Hospital, Miami, FL, USA.

- PMID: **33353546**
- PMCID: PMC7755065
- DOI: 10.1186/s12879-020-05701-4

Abstract

Background: Due to the lack of proven therapies, we evaluated the effects of early administration of tocilizumab for COVID-19. By inhibition of the IL-6 receptor, tocilizumab may help to mitigate the hyperinflammatory response associated with progressive respiratory failure from SARS-CoV-2.

Methods: A retrospective, observational study was conducted on hospitalized adults who received intravenous tocilizumab for COVID-19 between March 23, 2020 and April 10, 2020.

Results: Most patients were male (66.7%), Hispanic (63.3%) or Black (23.3%), with a median age of 54 years. Tocilizumab was administered at a median of 8 days (range 1-21) after initial symptoms and 2 days (range 0-12) after hospital admission. Within 30 days from receiving tocilizumab, 36 patients (60.0%) demonstrated clinical improvement, 9 (15.0%) died, 33 (55.0%) were discharged alive, and 18 (30.0%) remained hospitalized. Successful extubation occurred in 13 out of 29 patients (44.8%). Infectious complications occurred in 16 patients (26.7%) at a median of 10.5 days. After tocilizumab was administered, there was a slight increase in PaO_2/FiO_2 and an initial reduction in CRP, but this effect was not sustained beyond day 10.

Conclusions: Majority of patients demonstrated clinical improvement and were successfully discharged alive from the hospital after receiving tocilizumab. We observed a rebound effect with CRP, which may suggest the need for higher or subsequent doses to adequately manage cytokine storm. Based on our findings, we believe that tocilizumab may have a role in the early treatment of COVID-19, however larger randomized controlled studies are needed to confirm this.

Keywords: COVID-19; Coronavirus; Cytokine release syndrome; Tocilizumab.

Conflict of interest statement

The authors declare that they have no competing interests.

- 35 references
- 2 figures

Supplementary info

Publication types, MeSH terms, Substances Expand

Publication types

- Observational Study

MeSH terms

- Adult
- Aged
- Aged, 80 and over
- Antibodies, Monoclonal, Humanized / pharmacology
- Antibodies, Monoclonal, Humanized / therapeutic use*
- COVID-19 / complications
- COVID-19 / drug therapy*
- Cohort Studies
- Female
- Hospitalization
- Humans

- Male
- Middle Aged
- Receptors, Interleukin-6 / antagonists & inhibitors*
- Respiratory Insufficiency / drug therapy*
- Respiratory Insufficiency / virology
- Retrospective Studies
- SARS-CoV-2
- Treatment Outcome

Substances

- Antibodies, Monoclonal, Humanized
- Receptors, Interleukin-6
- tocilizumab

Full text links

Read free full text at BMC BioMed Central Free PMC article
Proceed to details
Cite
Share
☐ 173
Observational Study
Future Oncol
. 2022 Feb;18(6):719-725.
doi: 10.2217/fon-2021-1116. Epub 2022 Feb 2.

An observational study of hospitalized COVID-19 patients with cancer in San Diego county

David J Hermel[1], Jason Cham[1], Samantha R Spierling Bagsic[1], Lee K Hong[1], Carrie L Costantini[1], James R Mason[1], Alan Saven[1], Darren S Sigal[1]
Affiliations Expand

Affiliation

- [1] Division of Hematology & Oncology, Scripps Clinic, La Jolla, CA 92037, USA.

- PMID: **35105156**
- PMCID: PMC8809375
- DOI: 10.2217/fon-2021-1116

Free PMC article
Observational Study

An observational study of hospitalized COVID-19 patients with cancer in San Diego county

David J Hermel et al. Future Oncol. 2022 Feb.
Free PMC article
Show details
Future Oncol
. 2022 Feb;18(6):719-725.
doi: 10.2217/fon-2021-1116. Epub 2022 Feb 2.

Authors

David J Hermel [1], Jason Cham [1], Samantha R Spierling Bagsic [1], Lee K Hong [1], Carrie L Costantini [1], James R Mason [1], Alan Saven [1], Darren S Sigal [1]

Affiliation

- [1] Division of Hematology & Oncology, Scripps Clinic, La Jolla, CA 92037, USA.

- PMID: **35105156**
- PMCID: PMC8809375
- DOI: 10.2217/fon-2021-1116

Abstract

Aim: To delineate clinical correlates of COVID-19 infection severity in hospitalized patients with malignancy. **Methods:** The authors conducted a retrospective review of all hospitalized patients with a hematologic and/or solid tumor malignancy presenting to the authors' institution between 1 March 2020 and 5 January 2021, with a laboratory confirmed diagnosis of COVID-19. Univariate and multivariate logistic regression analyses were used to determine associations between specific severity outcomes and clinical characteristics. **Results:** Among 2771 hospitalized patients with COVID-19, 246 (8.88%) met inclusion criteria. Patients who were actively receiving treatment had an increased rate of death following admission (odds ratio [OR]: 2.7). After adjusting for significant covariates, the odds ratio increased to 4.4. Patients with cancer involvement of the lungs had a trend toward increased odds of death after adjusting for covariates (OR: 2.3). **Conclusions:** Among COVID-19 positive hospitalized cancer patients, systemic anti-cancer therapy was associated with significantly increased odds of mortality.

Keywords: COVID-19; SARS-CoV-2; cancer; hematologic malignancy; lung cancer; lung metastasis; systemic anti-cancer therapy.

Plain Language Summary

Plain language summary Though cancer is a biologically heterogenous disease with a wide spectrum of clinical features and behavior, accumulating evidence suggests that cancer patients are at greater susceptibility to COVID-19 infection and more likely to experience morbidity and mortality from COVID-19 infection than non-cancer patients. In this study, the authors reviewed the clinical characteristics of patients with a diagnosis of cancer hospitalized with COVID-19 to assess potential correlates of COVID-19 severity in this population. Notably, analysis of the hospital data revealed a statistically significant increased incidence of mortality in cancer patients who were receiving systemic anti-cancer treatment, including chemotherapy, immunotherapy or targeted therapy, than in those not on therapy. Likewise, there was a trend toward increased mortality in those with either primary or metastatic tumor involvement of the lung compared with those without lung involvement.

- 17 references
- 1 figure

Supplementary info

Publication types, MeSH terms, Substances Expand

Publication types

- Observational Study

MeSH terms

- Aged
- Aged, 80 and over
- Antineoplastic Agents / therapeutic use
- COVID-19 / complications*
- COVID-19 / mortality*
- California / epidemiology
- Female
- Hospitalization
- Humans
- Immune Checkpoint Inhibitors / therapeutic use
- Immunologic Factors / therapeutic use
- Lung Neoplasms / complications
- Male
- Middle Aged
- Molecular Targeted Therapy
- Neoplasms / complications*
- Neoplasms / drug therapy*
- Patient Acuity
- Retrospective Studies
- SARS-CoV-2

Substances

- Antineoplastic Agents
- Immune Checkpoint Inhibitors
- Immunologic Factors

Full text links

FREE ARTICLE Future Medicine Atypon Free PMC article
Proceed to details
Cite
Share
174
Observational Study
HNO
. 2021 Apr;69(4):303-311.
doi: 10.1007/s00106-021-01021-4. Epub 2021 Mar 5.

Surgical tracheostomy in a cohort of COVID-19 patients

Patrick J Schuler [1], Jens Greve [2], Thomas K Hoffmann [2], Janina Hahn [2], Felix Boehm [2], Bastian Bock [3], Johannes Reins [3], Ulrich Ehrmann [4], Eberhard Barth [3], Karl Traeger [3], Bettina Jungwirth [3], Martin Wepler [3]
Affiliations Expand

Affiliations

- [1] Department of Oto-Rhino-Laryngology, Head and Neck Surgery, Ulm University Medical Center, Frauensteige 12, 89075, Ulm, Germany. patrick.schuler@uniklinik-ulm.de.
- [2] Department of Oto-Rhino-Laryngology, Head and Neck Surgery, Ulm University Medical Center, Frauensteige 12, 89075, Ulm, Germany.
- [3] Department of Anesthesiology and Intensive Care, Ulm University Medical Center, Ulm, Germany.
- [4] Department of Anesthesiology and Intensive Care, Ehingen Hospital, Ehingen, Germany.

- PMID: **33666682**
- PMCID: PMC7934348
- DOI: 10.1007/s00106-021-01021-4

Free PMC article
Observational Study

Surgical tracheostomy in a cohort of COVID-19 patients

Patrick J Schuler et al. HNO. 2021 Apr.
Free PMC article
Show details
HNO
. 2021 Apr;69(4):303-311.
doi: 10.1007/s00106-021-01021-4. Epub 2021 Mar 5.

Authors

Patrick J Schuler [1], Jens Greve [2], Thomas K Hoffmann [2], Janina Hahn [2], Felix Boehm [2], Bastian Bock [3], Johannes Reins [3], Ulrich Ehrmann [4], Eberhard Barth [3], Karl Traeger [3], Bettina Jungwirth [3], Martin Wepler [3]

Affiliations

- [1] Department of Oto-Rhino-Laryngology, Head and Neck Surgery, Ulm University Medical Center, Frauensteige 12, 89075, Ulm, Germany. patrick.schuler@uniklinik-ulm.de.
- [2] Department of Oto-Rhino-Laryngology, Head and Neck Surgery, Ulm University Medical Center, Frauensteige 12, 89075, Ulm, Germany.
- [3] Department of Anesthesiology and Intensive Care, Ulm University Medical Center, Ulm, Germany.
- [4] Department of Anesthesiology and Intensive Care, Ehingen Hospital, Ehingen, Germany.

- PMID: **33666682**
- PMCID: PMC7934348
- DOI: 10.1007/s00106-021-01021-4

Abstract
in English, German

Background: One of the main symptoms of severe infection with the new coronavirus-2 (SARS-CoV-2) is hypoxemic respiratory failure because of viral pneumonia with the need for mechanical ventilation. Prolonged mechanical ventilation may require a tracheostomy, but the increased risk for contamination is a matter of considerable debate.

Objective: Evaluation of safety and effects of surgical tracheostomy on ventilation parameters and outcome in patients with COVID-19.

Study design: Retrospective observational study between March 27 and May 18, 2020, in a single-center coronavirus disease-designated ICU at a tertiary care German hospital.

Patients: Patients with COVID-19 were treated with open surgical tracheostomy due to severe hypoxemic respiratory failure requiring mechanical ventilation.

Measurements: Clinical and ventilation data were obtained from medical records in a retrospective manner.

Results: A total of 18 patients with confirmed SARS-CoV-2 infection and surgical tracheostomy were analyzed. The age range was 42-87 years. All patients received open tracheostomy between 2-16 days after admission. Ventilation after tracheostomy was less invasive (reduction in PEAK and positive end-expiratory pressure [PEEP]) and lung compliance increased over time after tracheostomy. Also, sedative drugs could be reduced, and patients had a reduced need of norepinephrine to maintain hemodynamic stability. Six of 18 patients died. All surgical staff were equipped with N99-masks and facial shields or with powered air-purifying respirators (PAPR).

Conclusion: Our data suggest that open surgical tracheostomy can be performed without severe complications in patients with COVID-19. Tracheostomy may reduce invasiveness of mechanical ventilation and the need for sedative drugs and norepinehprine. Recommendations for personal protective equipment (PPE) for surgical staff should be followed when PPE is available to avoid contamination of the personnel.

Zusammenfassung: HINTERGRUND: Ein Hauptsymptom der schweren Infektion mit dem neuen Coronavirus-2 (SARS-CoV-2) ist das hypoxämische Atemversagen aufgrund einer Viruspneumonie, welches eine mechanische Beatmung erfordert. Eine längere mechanische Beatmung erfordert ggf. eine Tracheostomie, welche aufgrund des erhöhten Kontaminationsrisikos jedoch umstritten ist. ZIEL: Bewertung von Sicherheit und Auswirkungen der chirurgischen Tracheostomie auf Beatmungsparameter und Überleben bei Patienten mit COVID-19.

Studiendesign: Retrospektive Beobachtungsstudie zwischen 27. März und 18. Mai 2020 auf einer COVID-Intensivstation in einem Krankenhaus der Tertiärversorgung.

Patienten: Patienten mit COVID-19 wurden wegen schweren hypoxämischen Atemversagens mit offener chirurgischer Tracheostomie behandelt.

Messungen: Klinische Daten und Beatmungsparameter wurden retrospektiv aus medizinischen Unterlagen entnommen.

Ergebnisse: Analysiert wurden die Daten von 18 Patienten mit chirurgischer Tracheostomie bei bestätigter SARS-CoV-2-Infektion. Die Altersspanne betrug 42–87 Jahre. Bei allen Patienten erfolgte zwischen 2 und 16 Tage nach der Aufnahme eine offene Tracheostomie. Anschließend war die Beatmung weniger invasiv, die Lungen-Compliance nahm zu und die Sedierung konnte reduziert werden. Es starben 6 von 18 Patienten. Alle chirurgischen Mitarbeiter waren mit FFP3-Masken oder mit motorbetriebenen luftreinigenden Atemschutzmasken ausgestattet.

Schlussfolgerung: Die vorliegenden Daten legen nahe, dass eine offene chirurgische Tracheostomie ohne schwerwiegende Komplikationen durchgeführt werden kann. Diese kann die Invasivität der mechanischen Beatmung sowie den Bedarf an Beruhigungsmitteln und Katecholaminen verringern. Falls verfügbar, sollten Empfehlungen für die persönliche Schutzausrüstung befolgt werden.

Keywords: Coronavirus; Mortality; Surgery; Tracheostomy; Ventilation.

Conflict of interest statement

P.J. Schuler, J. Greve, T.K. Hoffmann, J. Hahn, F. Boehm, B. Bock, J. Reins, U. Ehrmann, E. Barth, K. Traeger, B. Jungwirth and M. Wepler declare that they have no competing interests.

- 38 references
- 4 figures

Supplementary info

Publication types, MeSH terms Expand

Publication types

- Observational Study

MeSH terms

- Adult
- Aged
- Aged, 80 and over
- COVID-19*
- Humans
- Middle Aged
- Pneumonia, Viral*
- Retrospective Studies
- SARS-CoV-2
- Tracheostomy / adverse effects

Full text links

SpringerLink FULL-TEXT ARTICLE Springer Free PMC article
Proceed to details
Cite
Share

175

Clinical Trial

Proc Natl Acad Sci U S A
. 2021 Jun 22;118(25):e2101708118.
doi: 10.1073/pnas.2101708118.

Altered amino acid profile in patients with SARS-CoV-2 infection

Chris A Rees [1], Christina A Rostad [2 3], Grace Mantus [2], Evan J Anderson [2 3], Ann Chahroudi [2 3], Preeti Jaggi [2 3], Jens Wrammert [2], Juan B Ochoa [4], Augusto Ochoa [5], Rajit K Basu [2 3], Stacy Heilman [2], Frank Harris [2], Stacey A Lapp [2 3], Laila Hussaini [2 3], Miriam B Vos [2 3], Lou Ann Brown [2], Claudia R Morris [6 3]

Affiliations Expand

Affiliations

- [1] Division of Pediatric Emergency Medicine, Boston Children's Hospital, Harvard Medical School, Boston, MA 02115.
- [2] Department of Pediatrics, Emory University School of Medicine, Atlanta, GA 30322.
- [3] Children's Healthcare of Atlanta, Atlanta, GA 30329.
- [4] Department of Pulmonary/Critical Care, Hunterdon Medical Center, Milford, NJ 08848.
- [5] Stanley S. Scott Cancer Center, Louisiana State University Health, New Orleans, LA 70112.
- [6] Department of Pediatrics, Emory University School of Medicine, Atlanta, GA 30322; claudia.r.morris@emory.edu.

- PMID: **34088793**
- PMCID: PMC8237604
- DOI: 10.1073/pnas.2101708118

Free PMC article
Clinical Trial

Altered amino acid profile in patients with SARS-CoV-2 infection

Chris A Rees et al. Proc Natl Acad Sci U S A. 2021.
Free PMC article
Show details
Proc Natl Acad Sci U S A

. 2021 Jun 22;118(25):e2101708118.
doi: 10.1073/pnas.2101708118.

Authors

Chris A Rees [1], Christina A Rostad [2] [3], Grace Mantus [2], Evan J Anderson [2] [3], Ann Chahroudi [2] [3], Preeti Jaggi [2] [3], Jens Wrammert [2], Juan B Ochoa [4], Augusto Ochoa [5], Rajit K Basu [2] [3], Stacy Heilman [2], Frank Harris [2], Stacey A Lapp [2] [3], Laila Hussaini [2] [3], Miriam B Vos [2] [3], Lou Ann Brown [2], Claudia R Morris [6] [3]

Affiliations

- [1] Division of Pediatric Emergency Medicine, Boston Children's Hospital, Harvard Medical School, Boston, MA 02115.
- [2] Department of Pediatrics, Emory University School of Medicine, Atlanta, GA 30322.
- [3] Children's Healthcare of Atlanta, Atlanta, GA 30329.
- [4] Department of Pulmonary/Critical Care, Hunterdon Medical Center, Milford, NJ 08848.
- [5] Stanley S. Scott Cancer Center, Louisiana State University Health, New Orleans, LA 70112.

- [6] Department of Pediatrics, Emory University School of Medicine, Atlanta, GA 30322; claudia.r.morris@emory.edu.

- PMID: **34088793**
- PMCID: PMC8237604
- DOI: 10.1073/pnas.2101708118

Abstract

Low plasma arginine bioavailability has been implicated in endothelial dysfunction and immune dysregulation. The role of arginine in COVID-19 is unknown, but could contribute to cellular damage if low. Our objective was to determine arginine bioavailability in adults and children with COVID-19 vs. healthy controls. We hypothesized that arginine bioavailability would be low in patients with COVID-19 and multisystem inflammatory syndrome in children (MIS-C). We conducted a prospective observational study of three patient cohorts; arginine bioavailability was determined in asymptomatic healthy controls, adults hospitalized with COVID-19, and hospitalized children/adolescents <21 y old with COVID-19, MIS-C, or asymptomatic severe acute respiratory syndrome coronavirus 2 (SARS-CoV-2) infection identified on admission screen. Mean patient plasma amino acids were compared to controls using the Student's *t* test. Arginine-to-ornithine ratio, a biomarker of arginase activity, and global arginine bioavailability ratio (GABR, arginine/[ornithine+citrulline]) were assessed in all three groups. A total of 80 patients were included (28 controls, 32 adults with COVID-19, and 20 pediatric patients with COVID-19/MIS-C). Mean plasma arginine and arginine bioavailability ratios were lower among adult and pediatric patients with COVID-19/MIS-C compared to controls. There was no difference between arginine bioavailability in children with COVID-19 vs. MIS-C. Adults and children with COVID-19 and MIS-C in our cohort had low arginine bioavailability compared to healthy adult controls. This may contribute to immune dysregulation and endothelial dysfunction in COVID-19. Low arginine-to-ornithine ratio in patients with COVID-19 or MIS-C suggests an elevation of arginase activity. Further study is merited to explore the role of arginine dysregulation in COVID-19.

Keywords: COVID-19; arginine; multisystem inflammatory syndrome in children; nitric oxide; tetrahydrobiopterin.

Copyright © 2021 the Author(s). Published by PNAS.

Conflict of interest statement

Competing interest statement: C.R.M. is the inventor or coinventor of several UCSF Benioff Children's Hospital Oakland patents/patent-pending applications that include nutritional supplements, and biomarkers of cardiovascular disease related to arginine bioavailability; is an inventor of several Emory University School of Medicine patents/patent applications, including a patent filing for nutritional therapies that target coronaviruses; and is a consultant for Pfizer, Hoffmann-La Roche Ltd., and CSL Behring. M.B.V. is a consultant for Boehringer Ingelheim, Bristol Myers Squibb, Intercept, Eli Lilly, Novo Nordisk, and Target Pharmasolutions and has research funding from Bristol Myers Squibb and Target Pharmasolutions. J.B.O. has worked for Nestle Healthcare Nutrition, Inc.

- 15 references
- 1 figure

Supplementary info

Publication types, MeSH terms, Substances, Grant support Expand

Publication types

- Clinical Trial
- Observational Study
- Research Support, N.I.H., Extramural
- Research Support, Non-U.S. Gov't

MeSH terms

- Adult
- Amino Acids / blood*
- COVID-19 / blood*
- COVID-19 / therapy
- Female
- Hospitalization*
- Humans
- Male
- Middle Aged
- Retrospective Studies
- SARS-CoV-2 / metabolism*

Substances

- Amino Acids

Grant support

- K24 AT009893/AT/NCCIH NIH HHS/United States

Full text links

Open Access PNAS Atypon Free PMC article
Proceed to details
Cite
Share
☐ 176
Observational Study
Turk J Med Sci
. 2020 Dec 17;50(8):1771-1780.
doi: 10.3906/sag-2009-140.

Influence of aluminum salts on COVID-19 infected patients

Ali Demir [1], İbrahim Erayman [2], Oğuz Doğan [3], Murat Kekilli̇ [4]
Affiliations Expand

Affiliations

- [1] Department of Gastroenterology, Meram School of Medicine, Necmettin Erbakan University, Konya, Turkey
- [2] Department of Infectious Diseases, Meram School of Medicine, Necmettin Erbakan University, Konya, Turkey
- [3] Department of Mathematics and Science Education, Department of Physics Education, Ahmet Keleşoğlu Faculty of Education, Necmettin Erbakan University, Konya, Turkey
- [4] Department of Gastroenterology, Faculty of Medicine, Gazi University, Ankara, Turkey

- PMID: **33315350**
- PMCID: PMC7775704
- DOI: 10.3906/sag-2009-140

Free PMC article
Observational Study

Influence of aluminum salts on COVID-19 infected patients

Ali Demir et al. Turk J Med Sci. 2020.
Free PMC article
Show details
Turk J Med Sci
. 2020 Dec 17;50(8):1771-1780.
doi: 10.3906/sag-2009-140.

Authors

Ali Demir [1], İbrahim Erayman [2], Oğuz Doğan [3], Murat Kekilli̇ [4]

Affiliations

- [1] Department of Gastroenterology, Meram School of Medicine, Necmettin Erbakan University, Konya, Turkey
- [2] Department of Infectious Diseases, Meram School of Medicine, Necmettin Erbakan University, Konya, Turkey
- [3] Department of Mathematics and Science Education, Department of Physics Education, Ahmet Keleşoğlu Faculty of Education, Necmettin Erbakan University, Konya, Turkey
- [4] Department of Gastroenterology, Faculty of Medicine, Gazi University, Ankara, Turkey

- PMID: **33315350**
- PMCID: PMC7775704
- DOI: 10.3906/sag-2009-140

Abstract

Background/aim: Based on the antiviral and antibacterial properties of aluminum salts, we aimed to find out the influence of aluminum salts on COVID-19 infected patients.

Materials and methods: We performed an observational retrospective cohort study which includes the patients diagnosed as COVID-19 and received aluminum salts in addition to actual treatments during hospitalization as the treatment group (Alum Group). Patients who received standard COVID-19 treatment protocols in the Infectious Diseases Clinics were included as the Control Group. Clinical findings, laboratory parameters, length of stay, survival, radiological follow-up, intensive care and mechanical ventilation needs, the presence of comorbidity, polymerase chain reaction (PCR) tests, symptoms, symptom recovery times, hospital stay times, treatment protocols, and clinical presence of pneumonia were examined in all patients. Advanced chemical composition analyzes of existing aluminum salts were also performed.

Results: A total of 109 patients, 54 in the alum group and 55 in the control group, were included in the study. None of the patients in the aluminum group developed side effects due to the intake of aluminum salt. Survival status was significantly different between the two groups as there were 5 loss in the Control Group and none in the Alum Group ($P = 0.023$). The symptom recovery time was significantly shorter in the Alum Group; 2 (1–3) vs. 1 (1–2) days, $P = 0.003$. According to the paired samples analyses of the comparison between hospitalization and discharge, CRP levels significantly drops in the Alum Group (from 54.09 to 27, $P = 0.001$) but not in the Control Group. The drop was significantly same for the lactate dehydrogenase (LDH) and procalcitonin levels with $P = 0.001$.

Conclusion: It has been observed that aluminum salts have beneficial effects in COVID-19 infected cases. Considering the low systemic toxicity of intermittent oral intake of aluminum salts as food supplements and the fact that pandemic control is still not achieved, the use of aluminum salts is promising.

Keywords: Coronavirus (COVID-19); aluminum salts.

This work is licensed under a Creative Commons Attribution 4.0 International License.

Conflict of interest statement

none declared

- 17 references
- 6 figures

Supplementary info

Publication types, MeSH terms, Substances Expand

Publication types

- Observational Study

MeSH terms

- Alum Compounds* / administration & dosage
- Alum Compounds* / adverse effects
- Antiviral Agents / administration & dosage
- Antiviral Agents / adverse effects
- COVID-19 Testing / methods
- COVID-19* / diagnosis
- COVID-19* / drug therapy
- COVID-19* / epidemiology
- COVID-19* / physiopathology
- Critical Care / methods
- Critical Care / statistics & numerical data
- Female
- Hospitalization / statistics & numerical data*
- Humans
- Male
- Middle Aged
- Recovery of Function / drug effects*
- Respiration, Artificial / statistics & numerical data
- Retrospective Studies
- SARS-CoV-2 / drug effects
- Survival Analysis
- Treatment Outcome
- Turkey / epidemiology

Substances

- Alum Compounds
- Antiviral Agents

Full text links

TUBITAK Free PMC article

Proceed to details

Cite

Share

☐ 177

Observational Study

Sci Rep

. 2021 Jul 2;11(1):13733.
doi: 10.1038/s41598-021-93076-0.

Frequency, risk factors, and outcomes of hospital readmissions of COVID-19 patients

Antonio Ramos-Martínez [1] [2], Lina Marcela Parra-Ramírez [3], Ignacio Morrás [4], María Carnevali [5], Lorena Jiménez-Ibañez [6], Manuel Rubio-Rivas [7], Francisco Arnalich [8], José Luis Beato [9], Daniel Monge [10], Uxua Asín [11], Carmen Suárez [12], Santiago Jesús Freire [13], Manuel Méndez-Bailón [14], Isabel Perales [15], José Loureiro-Amigo [16], Ana Belén Gómez-Belda [17], Paula María Pesqueira [18], Ricardo Gómez-Huelgas [19], Carmen Mella [20], Luis Felipe Díez-García [21], Joaquim Fernández-Sola [22], Ruth González-Ferrer [23], Marina Aroza [24], Juan Miguel Antón-Santos [25], Carlos Lumbreras Bermejo [5]

Affiliations Expand

Affiliations

- [1] Infectious Diseases Unit, Internal Medicine Department, Hospital Universitario Puerta de Hierro-Majadahonda UAM, IDIPHSA, Maestro Rodrigo 2, 28222, Majadahonda, Madrid, Spain. aramos220@gmail.com.
- [2] Infectious Diseases Unit, Hospital Universitario Puerte de Hierro-Majadahonda Majadahonda, Calle Maestro Rodrigo 2, 28222, Majadahonda, Madrid, Spain. aramos220@gmail.com.
- [3] Preventive Medicine Department, Hospital Universitario Puerta de Hierro-Majadahonda UAM, IDIPHSA, Maestro Rodrigo 2, 28222, Majadahonda, Madrid, Spain.
- [4] Internal Medicine Department, Hospital Universitario Puerta de Hierro-Majadahonda UAM, IDIPHSA, Maestro Rodrigo 2, 28222, Majadahonda, Madrid, Spain.
- [5] Internal Medicine Department, 12 de Octubre University Hospital, Av. de Córdoba, s/n, 28041, Madrid, Spain.
- [6] Internal Medicine Department, Gregorio Marañon University Hospital, Dr. Esquerdo, 46, 28007, Madrid, Spain.
- [7] Internal Medicine Department, Bellvitge University Hospital, Carrer de La Feixa Llarga, s/n, 08907, L'Hospitalet de Llobregat, Barcelona, Spain.
- [8] Internal Medicine Department, La Paz University Hospital, Paseo de La Castellana, 261, 28046, Madrid, Spain.
- [9] Internal Medicine Department, Albacete University Hospital Complex, Hermanos Falco, 37, 02006, Albacete, Spain.
- [10] Internal Medicine Department, Segovia Hospital Complex, Luis Erik Clavería Neurólogo s/n, 40002, Segovia, Spain.
- [11] Internal Medicine Department, Miguel Servet Hospital, Paseo Isabel la Católica, 1-3, 50009, Zaragoza, Spain.
- [12] Internal Medicine Department, La Princesa University Hospital, Diego de León, 62, 28006, Madrid, Spain.
- [13] Internal Medicine Department, A Coruña University Hospital, Xubias de Arriba, 84, 15006 A, Coruña, Spain.
- [14] Internal Medicine Department, San Carlos Clinical Hospital, Prof Martín Lagos, s/n, 28040, Madrid, Spain.

- [15] Internal Medicine Department, Infanta Sofía Hospital, Paseo de Europa, 34, 28703, San Sebastián de los Reyes, Madrid, Spain.
- [16] Internal Medicine Department, Moisès Broggi Hospital, Carrer de Jacint Verdaguer, 90, 08970, Sant Joan Despí, Barcelona, Spain.
- [17] Internal Medicine Department, Dr. Peset University Hospital, Av. de Gaspar Aguilar, 90, 46017, Valencia, Spain.
- [18] Internal Medicine Department, Santiago Clinical Hospital, Rúa da Choupana, s/n, 15706, Santiago de Compostela, A Coruña, Spain.
- [19] Internal Medicine Department, Regional University Hospital of Málaga, Biomedical Research Institute of Málaga (IBIMA), University of Málaga (UMA), Av. de Carlos Haya, 84, 29010, Málaga, Spain.
- [20] Internal Medicine Department, Hospital Architect Marcide-Novoa Santos, Rúa Pardo Bazán, s/n, 15404, Ferrol, A Coruña, Spain.
- [21] Internal Medicine Department, Torrecárdenas Hospital, Hermandad de Donantes de Sangre, s/n, 04009, Almería, Spain.
- [22] Internal Medicine Department, Clinic Barcelona Hospital, Villarroel, 170, 08036, Barcelona, Spain.
- [23] Internal Medicine Department, Tajo Hospital, Av. Amazonas Central, s/n, 28300, Aranjuez, Madrid, Spain.
- [24] Internal Medicine Department, Insular de Gran Canaria Hospital, Av. Marítima del Sur, s/n, 35016, Las Palmas de Gran Canaria, Las Palmas, Spain.
- [25] Internal Medicine Department, Infanta Cristina University Hospital, Av. 9 de Junio, 2, 28981, Parla, Madrid, Spain.

- PMID: **34215803**
- PMCID: PMC8253752
- DOI: 10.1038/s41598-021-93076-0

Free PMC article
Observational Study

Frequency, risk factors, and outcomes of hospital readmissions of COVID-19 patients

Antonio Ramos-Martínez et al. Sci Rep. 2021.
Free PMC article
Show details
Sci Rep
. 2021 Jul 2;11(1):13733.
doi: 10.1038/s41598-021-93076-0.

Authors

Antonio Ramos-Martínez [1, 2], Lina Marcela Parra-Ramírez [3], Ignacio Morrás [4], María Carnevali [5], Lorena Jiménez-Ibañez [6], Manuel Rubio-Rivas [7], Francisco Arnalich [8], José Luis Beato [9], Daniel Monge [10], Uxua Asín [11], Carmen Suárez [12], Santiago Jesús Freire [13], Manuel Méndez-Bailón [14], Isabel Perales [15], José Loureiro-Amigo [16], Ana Belén Gómez-Belda [17], Paula María Pesqueira [18], Ricardo Gómez-Huelgas [19], Carmen Mella [20], Luis Felipe Díez-García [21], Joaquim

Fernández-Sola [22], Ruth González-Ferrer [23], Marina Aroza [24], Juan Miguel Antón-Santos [25], Carlos Lumbreras Bermejo [5]

Affiliations

- [1] Infectious Diseases Unit, Internal Medicine Department, Hospital Universitario Puerta de Hierro-Majadahonda UAM, IDIPHSA, Maestro Rodrigo 2, 28222, Majadahonda, Madrid, Spain. aramos220@gmail.com.
- [2] Infectious Diseases Unit, Hospital Universitario Puerte de Hierro-Majadahonda Majadahonda, Calle Maestro Rodrigo 2, 28222, Majadahonda, Madrid, Spain. aramos220@gmail.com.
- [3] Preventive Medicine Department, Hospital Universitario Puerta de Hierro-Majadahonda UAM, IDIPHSA, Maestro Rodrigo 2, 28222, Majadahonda, Madrid, Spain.
- [4] Internal Medicine Department, Hospital Universitario Puerta de Hierro-Majadahonda UAM, IDIPHSA, Maestro Rodrigo 2, 28222, Majadahonda, Madrid, Spain.
- [5] Internal Medicine Department, 12 de Octubre University Hospital, Av. de Córdoba, s/n, 28041, Madrid, Spain.
- [6] Internal Medicine Department, Gregorio Marañon University Hospital, Dr. Esquerdo, 46, 28007, Madrid, Spain.
- [7] Internal Medicine Department, Bellvitge University Hospital, Carrer de La Feixa Llarga, s/n, 08907, L'Hospitalet de Llobregat, Barcelona, Spain.
- [8] Internal Medicine Department, La Paz University Hospital, Paseo de La Castellana, 261, 28046, Madrid, Spain.
- [9] Internal Medicine Department, Albacete University Hospital Complex, Hermanos Falco, 37, 02006, Albacete, Spain.
- [10] Internal Medicine Department, Segovia Hospital Complex, Luis Erik Clavería Neurólogo s/n, 40002, Segovia, Spain.
- [11] Internal Medicine Department, Miguel Servet Hospital, Paseo Isabel la Católica, 1-3, 50009, Zaragoza, Spain.
- [12] Internal Medicine Department, La Princesa University Hospital, Diego de León, 62, 28006, Madrid, Spain.
- [13] Internal Medicine Department, A Coruña University Hospital, Xubias de Arriba, 84, 15006 A, Coruña, Spain.
- [14] Internal Medicine Department, San Carlos Clinical Hospital, Prof Martín Lagos, s/n, 28040, Madrid, Spain.
- [15] Internal Medicine Department, Infanta Sofía Hospital, Paseo de Europa, 34, 28703, San Sebastián de los Reyes, Madrid, Spain.
- [16] Internal Medicine Department, Moisès Broggi Hospital, Carrer de Jacint Verdaguer, 90, 08970, Sant Joan Despí, Barcelona, Spain.
- [17] Internal Medicine Department, Dr. Peset University Hospital, Av. de Gaspar Aguilar, 90, 46017, Valencia, Spain.
- [18] Internal Medicine Department, Santiago Clinical Hospital, Rúa da Choupana, s/n, 15706, Santiago de Compostela, A Coruña, Spain.
- [19] Internal Medicine Department, Regional University Hospital of Málaga, Biomedical Research Institute of Málaga (IBIMA), University of Málaga (UMA), Av. de Carlos Haya, 84, 29010, Málaga, Spain.
- [20] Internal Medicine Department, Hospital Architect Marcide-Novoa Santos, Rúa Pardo Bazán, s/n, 15404, Ferrol, A Coruña, Spain.

- [21] Internal Medicine Department, Torrecárdenas Hospital, Hermandad de Donantes de Sangre, s/n, 04009, Almería, Spain.
- [22] Internal Medicine Department, Clinic Barcelona Hospital, Villarroel, 170, 08036, Barcelona, Spain.
- [23] Internal Medicine Department, Tajo Hospital, Av. Amazonas Central, s/n, 28300, Aranjuez, Madrid, Spain.
- [24] Internal Medicine Department, Insular de Gran Canaria Hospital, Av. Marítima del Sur, s/n, 35016, Las Palmas de Gran Canaria, Las Palmas, Spain.
- [25] Internal Medicine Department, Infanta Cristina University Hospital, Av. 9 de Junio, 2, 28981, Parla, Madrid, Spain.

- PMID: **34215803**
- PMCID: PMC8253752
- DOI: 10.1038/s41598-021-93076-0

Abstract

To determine the proportion of patients with COVID-19 who were readmitted to the hospital and the most common causes and the factors associated with readmission. Multicenter nationwide cohort study in Spain. Patients included in the study were admitted to 147 hospitals from March 1 to April 30, 2020. Readmission was defined as a new hospital admission during the 30 days after discharge. Emergency department visits after discharge were not considered readmission. During the study period 8392 patients were admitted to hospitals participating in the SEMI-COVID-19 network. 298 patients (4.2%) out of 7137 patients were readmitted after being discharged. 1541 (17.7%) died during the index admission and 35 died during hospital readmission (11.7%, p = 0.007). The median time from discharge to readmission was 7 days (IQR 3-15 days). The most frequent causes of hospital readmission were worsening of previous pneumonia (54%), bacterial infection (13%), venous thromboembolism (5%), and heart failure (5%). Age [odds ratio (OR): 1.02; 95% confident interval (95% CI): 1.01-1.03], age-adjusted Charlson comorbidity index score (OR: 1.13; 95% CI: 1.06-1.21), chronic obstructive pulmonary disease (OR: 1.84; 95% CI: 1.26-2.69), asthma (OR: 1.52; 95% CI: 1.04-2.22), hemoglobin level at admission (OR: 0.92; 95% CI: 0.86-0.99), ground-glass opacification at admission (OR: 0.86; 95% CI:0.76-0.98) and glucocorticoid treatment (OR: 1.29; 95% CI: 1.00-1.66) were independently associated with hospital readmission. The rate of readmission after hospital discharge for COVID-19 was low. Advanced age and comorbidity were associated with increased risk of readmission.

Conflict of interest statement

The authors declare no competing interests.

- 34 references
- 1 figure

Supplementary info

Publication types, MeSH terms Expand

Publication types

- Observational Study

MeSH terms

- Age Factors
- Aged
- Aged, 80 and over
- COVID-19 / complications
- COVID-19 / epidemiology
- COVID-19 / therapy*
- Female
- Humans
- Male
- Middle Aged
- Patient Discharge
- Patient Readmission*
- Retrospective Studies
- Risk Factors
- SARS-CoV-2 / isolation & purification

Full text links

Nature Publishing Group Free PMC article
Proceed to details
Cite
Share
178

J Gen Intern Med
. 2020 Sep;35(9):2698-2706.
doi: 10.1007/s11606-020-05906-y. Epub 2020 Jun 17.

Antithrombotic Therapies in COVID-19 Disease: a Systematic Review

Edward Maldonado [1], Derrick Tao [1], Katherine Mackey [2]
Affiliations Expand

Affiliations

- [1] Department of Medicine, Oregon Health & Science University, Portland, OR, USA.
- [2] Department of Veterans Affairs Evidence Synthesis Program (ESP), VA Portland Healthcare System, Portland, OR, USA. katherine.mackey@va.gov.

- PMID: **32556875**
- PMCID: PMC7299557

- DOI: 10.1007/s11606-020-05906-y

Free PMC article

Antithrombotic Therapies in COVID-19 Disease: a Systematic Review

Edward Maldonado et al. J Gen Intern Med. 2020 Sep.
Free PMC article

Show details

J Gen Intern Med

. 2020 Sep;35(9):2698-2706.
doi: 10.1007/s11606-020-05906-y. Epub 2020 Jun 17.

Authors

Edward Maldonado [1], Derrick Tao [1], Katherine Mackey [2]

Affiliations

- [1] Department of Medicine, Oregon Health & Science University, Portland, OR, USA.
- [2] Department of Veterans Affairs Evidence Synthesis Program (ESP), VA Portland Healthcare System, Portland, OR, USA. katherine.mackey@va.gov.

- PMID: **32556875**
- PMCID: PMC7299557
- DOI: 10.1007/s11606-020-05906-y

Abstract

Background: Infection with coronavirus SARS-CoV-2, causing COVID-19 disease, leads to inflammation and a prothrombotic state.

Objective: This rapid systematic review aims to synthesize evidence on thromboembolism incidence and outcomes with antithrombotic therapies in COVID-19.

Data sources: We searched MEDLINE (Ovid), Cochrane Rapid Reviews, PROSPERO, and the WHO COVID-19 Database from January 1, 2003, to April 22, 2020, for studies meeting pre-specified inclusion criteria.

Study selection, data extraction, and synthesis: One investigator identified articles for inclusion, abstracted data, and performed quality assessment, with second reviewer checking.

Results: Incidence of thromboembolism among hospitalized patients with COVID-19 ranged from 25 to 53% in 4 retrospective series. We identified 3 studies (1 retrospective cohort study, 1 prospective uncontrolled observational study, and 1 case series) examining outcomes among COVID-19 patients who received antithrombotic therapies. These studies all included different interventions (thromboprophylaxis with unfractionated heparin (UFH) or low molecular-weight heparin (LMWH); an intensive thromboprophylaxis protocol with LMWH, antithrombin, and

clopidogrel; and salvage therapy with tissue plasminogen activator and heparin). These studies are overall poor quality due to methodological limitations including unclear patient selection protocols, lack of reporting or adjustment for patient baseline characteristics, inadequate duration of follow-up, and partial reporting of outcomes.

Conclusions: New evidence on thromboembolism in COVID-19 does not warrant a change in current guidance on thromboprophylaxis among hospitalized patients. Prospective trials of antithrombotic treatment strategies among patients with COVID-19 are urgently needed.

Keywords: COVID-19; anticoagulants; sepsis; thromboembolism.

Conflict of interest statement

The authors declare that they do not have a conflict of interest.

- 44 references
- 1 figure

Supplementary info

Publication types, MeSH terms, Substances Expand

Publication types

- Systematic Review

MeSH terms

- Anticoagulants / therapeutic use
- Betacoronavirus*
- COVID-19
- Coronavirus Infections / drug therapy*
- Coronavirus Infections / epidemiology*
- Fibrinolytic Agents / therapeutic use*
- Humans
- Observational Studies as Topic / methods
- Pandemics
- Pneumonia, Viral / drug therapy*
- Pneumonia, Viral / epidemiology*
- Prospective Studies
- Retrospective Studies
- SARS-CoV-2
- Venous Thromboembolism / drug therapy*
- Venous Thromboembolism / epidemiology*

Substances

- Anticoagulants
- Fibrinolytic Agents

Full text links

SpringerLink FULL-TEXT ARTICLE Springer Free PMC article
Proceed to details
Cite
Share
179
Observational Study

Endocrine
. 2021 Dec;74(3):443-454.
doi: 10.1007/s12020-021-02881-0. Epub 2021 Oct 19.

Finding answers in lipid profile in COVID-19 patients

M Sampedro-Nuñez[1 2 3], N Aguirre-Moreno[4], L García-Fraile Fraile[5], S Jiménez-Blanco[1 2 3], C Knott-Torcal[1 2 3], P Sanz-Martin[6], G Fernández-Jiménez[7], M Marazuela[8 9 10]
Affiliations Expand

Affiliations

- [1] Department of Endocrinology and Nutrition, Hospital Universitario de la Princesa, 28006, Madrid, Spain.
- [2] Department of Medicine, Universidad Autónoma de Madrid, 28049, Madrid, Spain.
- [3] Endocrinology Unit, Instituto de Investigación Sanitaria Princesa, 28006, Madrid, Spain.
- [4] Department of Endocrinology and Nutrition, Hospital Universitario Rey Juan Carlos, 28933, Madrid, Spain.
- [5] Department of Internal Medicine, Hospital Universitario de la Princesa, Universidad Autónoma de Madrid, 28006, Madrid, Spain.
- [6] Department of Clinical Chemistry, Hospital Universitario de la Princesa, Universidad Autónoma de Madrid, Instituto Princesa, 28006, Madrid, Spain.
- [7] Clinical Information Unit, Hospital Universitario de la Princesa, Universidad Autónoma de Madrid, Instituto Princesa, 28006, Madrid, Spain.
- [8] Department of Endocrinology and Nutrition, Hospital Universitario de la Princesa, 28006, Madrid, Spain. monica.marazuela@uam.es.
- [9] Department of Medicine, Universidad Autónoma de Madrid, 28049, Madrid, Spain. monica.marazuela@uam.es.
- [10] Endocrinology Unit, Instituto de Investigación Sanitaria Princesa, 28006, Madrid, Spain. monica.marazuela@uam.es.

- PMID: **34668172**
- PMCID: PMC8525620
- DOI: 10.1007/s12020-021-02881-0

Free PMC article
Observational Study

Finding answers in lipid profile in COVID-19 patients

M Sampedro-Nuñez et al. Endocrine. 2021 Dec.
Free PMC article

Show details

Endocrine
. 2021 Dec;74(3):443-454.
doi: 10.1007/s12020-021-02881-0. Epub 2021 Oct 19.

Authors

M Sampedro-Nuñez [1 2 3], N Aguirre-Moreno [4], L García-Fraile Fraile [5], S Jiménez-Blanco [1 2 3], C Knott-Torcal [1 2 3], P Sanz-Martin [6], G Fernández-Jiménez [7], M Marazuela [8 9 10]

Affiliations

- [1] Department of Endocrinology and Nutrition, Hospital Universitario de la Princesa, 28006, Madrid, Spain.
- [2] Department of Medicine, Universidad Autónoma de Madrid, 28049, Madrid, Spain.
- [3] Endocrinology Unit, Instituto de Investigación Sanitaria Princesa, 28006, Madrid, Spain.
- [4] Department of Endocrinology and Nutrition, Hospital Universitario Rey Juan Carlos, 28933, Madrid, Spain.
- [5] Department of Internal Medicine, Hospital Universitario de la Princesa, Universidad Autónoma de Madrid, 28006, Madrid, Spain.
- [6] Department of Clinical Chemistry, Hospital Universitario de la Princesa, Universidad Autónoma de Madrid, Instituto Princesa, 28006, Madrid, Spain.
- [7] Clinical Information Unit, Hospital Universitario de la Princesa, Universidad Autónoma de Madrid, Instituto Princesa, 28006, Madrid, Spain.
- [8] Department of Endocrinology and Nutrition, Hospital Universitario de la Princesa, 28006, Madrid, Spain. monica.marazuela@uam.es.
- [9] Department of Medicine, Universidad Autónoma de Madrid, 28049, Madrid, Spain. monica.marazuela@uam.es.
- [10] Endocrinology Unit, Instituto de Investigación Sanitaria Princesa, 28006, Madrid, Spain. monica.marazuela@uam.es.

- PMID: **34668172**
- PMCID: PMC8525620
- DOI: 10.1007/s12020-021-02881-0

Abstract

Introduction: A small percentage of patients will develop a severe form of COVID-19 caused by SARS-CoV-2 infection. Thus, it is important to predict the potential outcomes identifying early markers of poor prognosis. In this context, we evaluated the association of SARS-CoV-2 infection with lipid abnormalities and their role in prognosis.

Methods: Single-center, retrospective, observational study of COVID-19 patients admitted from March to October 2020. Clinical and laboratory data, comorbidities, and treatments for COVID-19 were evaluated. Main outcomes including intensive care unit (ICU) admission and mortality were analyzed with a multivariable Cox proportional hazards regression model.

Results: We selected 1489 from a total of 2038 consecutive patients with confirmed COVID-19, who had a complete lipid profile before ICU admission. During the follow-up performed in 1109 patients, we observed a decrease in T-c, HDL-c, and LDL-c in 28.6%, 42.9%, and 30.4% of patients, respectively, and an increase in TG in 76.8%. The decrease of both T-c and HDL- c was correlated with a decrease in albumin levels ($r = 0.39$ and $r = 0.37$, respectively). Kaplan-Meier survival curves found an increased ICU admission in patients with lower T-c (HR 0.55, CI 0.36-0.86), HDL-c (HR 0.61, CI 0.45-0.84), and LDL-c (HR 0.85, CI 0.74-0.97). Higher values of T-c (HR 0.45, CI 0.36-0.57), HDL-c (HR 0.66, CI 0.54-0.81), and LDL-c (HR 0.86, CI 0.78-0.94) showed a protective effect on mortality.

Conclusions: Abnormalities in lipid profile are a frequent complication of SARS-CoV-2 infection and might be related to morbidity and mortality.

Funding: Proyectos de Investigación en Salud (FIS) and cofinanced by FEDER.

Keywords: COVID-19; Lipid profile; biomarker; prognosis.

© 2021. The Author(s).

Conflict of interest statement

The authors declare no competing interests.

- 30 references
- 3 figures

Supplementary info

Publication types, MeSH terms, Substances Expand

Publication types

- Observational Study
- Research Support, Non-U.S. Gov't

MeSH terms

- COVID-19*

- Humans
- Intensive Care Units
- Lipids
- Retrospective Studies
- Risk Factors
- SARS-CoV-2

Substances

- Lipids

Full text links

Springer Free PMC article
Proceed to details
Cite
Share
180
Observational Study
Medicina (B Aires)
. 2021;81(4):527-535.

Clinical characteristics of critically ill patients with COVID-19

Indalecio Carboni Bisso [1] [2] [3], Iván Huespe [4] [5] [6], Carolina Lockhart [4], Agustín Massó [4], Julieta Gonzalez Anaya [4], Micaela Hornos [4], Romina Famiglietti [7], Marcelo Di Grazia [7], Pablo Coria [7], Eduardo San Román [4], Marcos Las Heras [4]
Affiliations Expand

Affiliations

- [1] Unidad de Cuidados Intensivos, Hospital Italiano de Buenos Aires, Argentina. E-mail: indalecio.carbonibisso@hospitalitaliano.org.ar.
- [2] Unidad de Cuidados Intensivos, Hospital San Antonio, Gualeguay, Entre Ríos, Argentina.
- [3] Unidad de Cuidados Intensivos, Sanatorio Franchin, Buenos Aires, Argentina.
- [4] Unidad de Cuidados Intensivos, Hospital Italiano de Buenos Aires, Argentina.
- [5] Instituto de Medicina Traslacional e Ingeniería Biomédica, Hospital Italiano de Buenos Aires, Argentina.
- [6] Instituto de Ciencias Aplicadas Provenzano (ICAP), Facultad de Medicina, Universidad de Buenos Aires, Argentina.
- [7] Rehabilitación y Cuidados Respiratorios, Servicio de Kinesiología, Hospital Italiano de Buenos Aires, Argentina.

- PMID: **34453793**

Free article
Observational Study

Clinical characteristics of critically ill patients with COVID-19

Indalecio Carboni Bisso et al. Medicina (B Aires). 2021.
Free article
Show details
Medicina (B Aires)
. 2021;81(4):527-535.

Authors

Indalecio Carboni Bisso [1] [2] [3], Iván Huespe [4] [5] [6], Carolina Lockhart [4], Agustín Massó [4], Julieta Gonzalez Anaya [4], Micaela Hornos [4], Romina Famiglietti [7], Marcelo Di Grazia [7], Pablo Coria [7], Eduardo San Román [4], Marcos Las Heras [4]

Affiliations

- [1] Unidad de Cuidados Intensivos, Hospital Italiano de Buenos Aires, Argentina. E-mail: indalecio.carbonibisso@hospitalitaliano.org.ar.
- [2] Unidad de Cuidados Intensivos, Hospital San Antonio, Gualeguay, Entre Ríos, Argentina.
- [3] Unidad de Cuidados Intensivos, Sanatorio Franchin, Buenos Aires, Argentina.
- [4] Unidad de Cuidados Intensivos, Hospital Italiano de Buenos Aires, Argentina.
- [5] Instituto de Medicina Traslacional e Ingeniería Biomédica, Hospital Italiano de Buenos Aires, Argentina.
- [6] Instituto de Ciencias Aplicadas Provenzano (ICAP), Facultad de Medicina, Universidad de Buenos Aires, Argentina.
- [7] Rehabilitación y Cuidados Respiratorios, Servicio de Kinesiología, Hospital Italiano de Buenos Aires, Argentina.

- PMID: **34453793**

Abstract
in English, Spanish

Coronavirus disease 2019 (COVID-19) pandemic poses a major challenge for healthcare systems. In South America, local information about the incidence and clinical characteristics of critically ill patients diagnosed with COVID-19 is still limited. In this observational and retrospective study, we aimed to describe critically ill patients' clinical and respiratory characteristics with COVID-19. The study was performed over 6 months in an intensive care unit (ICU) of a high complexity hospital in Buenos Aires, Argentina. Patients older than 18 years with laboratory-confirmed COVID-19 by reverse tran scriptase-polymerase chain reaction (RT-PCR) for SARS-CoV-2 were included in the study. Demographic characteristics such as sex and age, comorbidities, laboratory

results, imaging results, ventilatory mechanics data, complications, and mortality were recorded. A total of 168 critically ill patients with COVID-19 were included. Sixty-six percent were men with a median age of 65 years (58-75); 79.7% had at least one comorbidity. The most frequent comorbidity was arterial hypertension, affecting 52.4%. A 67.9% required invasive mechanical ventilation (MV), and no one was treated with non-invasive ventilation. Most of the patients in MV (73.7%) required neuromuscular blockade due to severe hypoxemia. A 36% was ventilated in the prone position. The length of stay in the ICU was 13 days (6-24) and ICU's mortality was 25%.

La pandemia de la enfermedad por coronavirus 2019 (COVID-19) plantea un gran desafío para los sistemas de salud. En América del Sur, la información local sobre la incidencia y las características clínicas de los pacientes críticamente enfermos diagnosticados con COVID-19 aún es limitada. En este estudio observacional y retrospectivo, nuestro objetivo fue describir las características clínicas y respiratorias de los pacientes críticamente enfermos con COVID-19. El estudio se realizó durante 6 meses en una unidad de cuidados intensivos (UCI) de un hospital de alta complejidad en Buenos Aires, Argentina. Se incluyeron en el estudio pacientes mayores de 18 años con COVID-19 confirmado por laboratorio mediante la reacción en cadena de la polimerasa con transcriptasa inversa (RT-PCR) para SARS-CoV-2. Se registraron características demográficas como sexo y edad, comorbilidades, resultados de laboratorio, resultados de imagen, datos de mecánica ventilatoria, complicaciones y mortalidad. Se incluyeron un total de 168 pacientes críticamente enfermos con COVID-19. El 66% eran hombres con una mediana de edad de 65 años (58-75). El 79.7% presentaba al menos una comorbilidad. La comorbilidad más frecuente fue la hipertensión arterial, afectando al 52.4%. El 67.9% requirió ventilación mecánica invasiva (VM) y ninguno fue tratado con ventilación no invasiva. La mayoría de los pacientes en VM (73.7%) requirieron bloqueo neuromuscular por hipoxemia grave. Un 36% de ellos fueron ventilados en decúbito prono. La estancia en UCI fue de 13 días (6-24) y la mortalidad en UCI fue del 25%.

Keywords: Argentina; COVID-19; SARS-CoV-2; South America; intensive care; pandemic.

Supplementary info

Publication types, MeSH terms Expand

Publication types

- Observational Study

MeSH terms

- Aged
- COVID-19*
- Critical Illness*
- Humans
- Intensive Care Units
- Male
- Middle Aged
- Pandemics

- Respiration, Artificial
- Retrospective Studies
- SARS-CoV-2

Full text links

Free Full Text medicina Fundacion Revista Medicina (Buenos Aires)
Proceed to details
Cite
Share
☐ 181
Observational Study
Eur Radiol
. 2020 Dec;30(12):6940-6949.
doi: 10.1007/s00330-020-07041-y. Epub 2020 Jun 30.

Longitudinal study of interventional radiology activity in a large metropolitan Italian tertiary care hospital: how the COVID-19 pandemic emergency has changed our activity

Roberto Iezzi [1] [2], Iacopo Valente [3], Alessandro Cina [3], Alessandro Posa [3], Andrea Contegiacomo [3], Andrea Alexandre [3], Francesco D'Argento [3], Emilio Lozupone [3], Michele Barone [3], Francesca Giubbolini [3], Luca Milonia [3], Andrea Romi [3], Anna Rita Scrofani [3], Alessandro Pedicelli [3], Riccardo Manfredi [3] [4], Cesare Colosimo [3] [4]
Affiliations Expand

Affiliations

- [1] Fondazione Policlinico Universitario "A. Gemelli" - IRCCS, Dipartimento di Diagnostica per Immagini, Radioterapia, Oncologia ed Ematologia - Area di Diagnostica per Immagini, UOC Radiologia Diagnostica e Interventistica Generale, L.go A. Gemelli 8, 00168, Rome, Italy. roberto.iezzi.md@gmail.com.
- [2] Università Cattolica del Sacro Cuore, L.go F. Vito 1, 00168, Rome, Italy. roberto.iezzi.md@gmail.com.
- [3] Fondazione Policlinico Universitario "A. Gemelli" - IRCCS, Dipartimento di Diagnostica per Immagini, Radioterapia, Oncologia ed Ematologia - Area di Diagnostica per Immagini, UOC Radiologia Diagnostica e Interventistica Generale, L.go A. Gemelli 8, 00168, Rome, Italy.
- [4] Università Cattolica del Sacro Cuore, L.go F. Vito 1, 00168, Rome, Italy.

- PMID: **32607633**

- PMCID: PMC7326392
- DOI: 10.1007/s00330-020-07041-y

Free PMC article
Observational Study

Longitudinal study of interventional radiology activity in a large metropolitan Italian tertiary care hospital: how the COVID-19 pandemic emergency has changed our activity

Roberto Iezzi et al. Eur Radiol. 2020 Dec.
Free PMC article
Show details
Eur Radiol
. 2020 Dec;30(12):6940-6949.
doi: 10.1007/s00330-020-07041-y. Epub 2020 Jun 30.

Authors

Roberto Iezzi [1, 2], Iacopo Valente [3], Alessandro Cina [3], Alessandro Posa [3], Andrea Contegiacomo [3], Andrea Alexandre [3], Francesco D'Argento [3], Emilio Lozupone [3], Michele Barone [3], Francesca Giubbolini [3], Luca Milonia [3], Andrea Romi [3], Anna Rita Scrofani [3], Alessandro Pedicelli [3], Riccardo Manfredi [3, 4], Cesare Colosimo [3, 4]

Affiliations

- [1] Fondazione Policlinico Universitario "A. Gemelli" - IRCCS, Dipartimento di Diagnostica per Immagini, Radioterapia, Oncologia ed Ematologia - Area di Diagnostica per Immagini, UOC Radiologia Diagnostica e Interventistica Generale, L.go A. Gemelli 8, 00168, Rome, Italy. roberto.iezzi.md@gmail.com.
- [2] Università Cattolica del Sacro Cuore, L.go F. Vito 1, 00168, Rome, Italy. roberto.iezzi.md@gmail.com.
- [3] Fondazione Policlinico Universitario "A. Gemelli" - IRCCS, Dipartimento di Diagnostica per Immagini, Radioterapia, Oncologia ed Ematologia - Area di Diagnostica per Immagini, UOC Radiologia Diagnostica e Interventistica Generale, L.go A. Gemelli 8, 00168, Rome, Italy.
- [4] Università Cattolica del Sacro Cuore, L.go F. Vito 1, 00168, Rome, Italy.

- PMID: **32607633**
- PMCID: PMC7326392
- DOI: 10.1007/s00330-020-07041-y

Abstract

Objectives: To retrospectively analyze interventional radiology (IR) activity changes in the COVID-19 era and to describe how to safely and effectively reorganize IR activity.

Methods: All IR procedures performed between January 30 and April 8, 2020 (COVID-era group) and the same 2019 period (non-COVID-era group) were retrospectively included and compared. A sub-analysis for the lockdown period (LDP: 11 March-8 April) was also conducted. Demographic, hospitalization, clinical, and procedural data were obtained for both groups and statistically compared with univariable analysis.

Results: A total of 1496 procedures (non-COVID era, 825; COVID era, 671) performed in 1226 patients (64.9 ± 15.1 years, 618 women) were included. The number of procedures decreased by 18.6% between 2019 and 2020 (825 vs 671, $p < .001$), with a reduction by 48.2% in LDP (188 vs 363, $p < .0001$). In the LDP COVID era, bedside procedures were preferred ($p = .013$), with an increase in procedures from the intensive care unit compared with the emergency department and outpatients ($p = .048$), and an increased activity for oncological patients ($p = .003$). No incidents of cross-infection of non-infected from infected patients and no evidence of COVID-19 infection of healthcare workers in the IR service was registered.

Conclusions: Coronavirus disease outbreak changed the interventional radiology activity with an overall reduction in the number of procedures. However, this study confirms that interventional radiology continuum of care can be safely performed also during the pandemic, following defined measures and protocols, taking care of all patients.

Key points: • Coronavirus disease pandemic determined a reduction of interventional radiology activity as compared to the same period of the previous year. • Interventional radiology procedures for life-threatening conditions and non-deferrable oncologic treatments were prioritized as opposed to elective procedures. • Strict adoption of safe procedures allowed us to have until now no incidents of cross-infection of non-infected from infected patients and no evidence of COVID-19 infection of HCWs in the IR service.

Keywords: Infections; Interventional radiology; Neoplasms; Safety; Virus diseases.

Conflict of interest statement

The authors of this manuscript declare no relationships with any companies, whose products or services may be related to the subject matter of the article.

- 28 references

Supplementary info

Publication types, MeSH terms Expand

Publication types

- Observational Study

MeSH terms

- Aged
- Betacoronavirus*
- COVID-19
- Coronavirus Infections / diagnosis*
- Coronavirus Infections / epidemiology
- Emergency Service, Hospital / statistics & numerical data
- Female
- Hospitalization / trends
- Humans
- Italy / epidemiology
- Longitudinal Studies
- Male
- Middle Aged
- Pandemics*
- Pneumonia, Viral / diagnosis*
- Pneumonia, Viral / epidemiology
- Radiography / methods*
- Radiology, Interventional / methods
- Retrospective Studies
- SARS-CoV-2
- Tertiary Care Centers / statistics & numerical data*

Full text links

SpringerLink FULL-TEXT ARTICLE Springer Free PMC article
Proceed to details
Cite
Share
☐ 182
Observational Study
Am J Emerg Med
. 2021 Sep;47:244-247.
doi: 10.1016/j.ajem.2021.04.070. Epub 2021 Apr 27.

Outcomes for in-hospital cardiac arrest for COVID-19 patients at a rural hospital in Southern California

Rahul V Nene [1], Nicole Amidon [2], Christian A Tomaszewski [3], Gabriel Wardi [4], Andrew Lafree [3]
Affiliations Expand

Affiliations

- [1] Department of Emergency Medicine, University of California, San Diego, CA, United States of America; Department of Emergency Medicine, El Centro Regional Medical Center, El Centro, CA, United States of America. Electronic address: rnene@ucsd.edu.
- [2] Department of Emergency Medicine, El Centro Regional Medical Center, El Centro, CA, United States of America.
- [3] Department of Emergency Medicine, University of California, San Diego, CA, United States of America; Department of Emergency Medicine, El Centro Regional Medical Center, El Centro, CA, United States of America.
- [4] Department of Emergency Medicine, University of California, San Diego, CA, United States of America; Division of Pulmonary, Critical Care, and Sleep Medicine, University of California, San Diego, CA, United States of America.

- PMID: **33957412**
- PMCID: PMC8076731
- DOI: 10.1016/j.ajem.2021.04.070

Free PMC article
Observational Study

Outcomes for in-hospital cardiac arrest for COVID-19 patients at a rural hospital in Southern California

Rahul V Nene et al. Am J Emerg Med. 2021 Sep.
Free PMC article
Show details
Am J Emerg Med
. 2021 Sep;47:244-247.
doi: 10.1016/j.ajem.2021.04.070. Epub 2021 Apr 27.

Authors

Rahul V Nene [1], Nicole Amidon [2], Christian A Tomaszewski [3], Gabriel Wardi [4], Andrew Lafree [3]

Affiliations

- [1] Department of Emergency Medicine, University of California, San Diego, CA, United States of America; Department of Emergency Medicine, El Centro Regional Medical Center, El Centro, CA, United States of America. Electronic address: rnene@ucsd.edu.
- [2] Department of Emergency Medicine, El Centro Regional Medical Center, El Centro, CA, United States of America.
- [3] Department of Emergency Medicine, University of California, San Diego, CA, United States of America; Department of Emergency Medicine, El Centro Regional Medical Center, El Centro, CA, United States of America.

- [4] Department of Emergency Medicine, University of California, San Diego, CA, United States of America; Division of Pulmonary, Critical Care, and Sleep Medicine, University of California, San Diego, CA, United States of America.

- PMID: **33957412**
- PMCID: PMC8076731
- DOI: 10.1016/j.ajem.2021.04.070

Abstract

Background: In-hospital cardiac arrest (IHCA) carries a high mortality and providing resuscitation to COVID-19 patients presents additional challenges for emergency physicians. Our objective was to describe outcomes of COVID-19 patients suffering IHCA at a rural hospital in Southern California.

Methods: Single-center retrospective observational study. A hospital registry of COVID-19 patients was queried for all patients who suffered IHCA and received cardiopulmonary resuscitation (CPR) between May 1st and July 31st, 2020. A manual chart review was performed to obtain patient demographics, oxygen requirement prior to cardiac arrest (CA), details of the resuscitation including presence of an emergency physician, and final disposition.

Results: Twenty-one patients were identified, most of whom were Hispanic, male, and aged 50-70. The most common medical comorbidities were diabetes and hypertension. Most patients suffered respiratory arrest, with an initial rhythm of pulseless electrical activity or asystole. Return of spontaneous circulation (ROSC) was achieved in 3/9 patients already receiving mechanical ventilation, but all 3 expired within the following 24 h. ROSC was achieved in 10/12 patients not already intubated, though most also expired within a few days. The only 2 patients who survived to discharge suffered respiratory arrest after their oxygen delivery device dislodged.

Conclusion: At a small rural hospital with limited resources and a predominantly Hispanic population, cardiac arrest in a COVID-19 patient portends an extremely poor prognosis. A better appreciation of these outcomes should help inform emergency providers and patients when discussing code status and attempts at resuscitation, particularly in resource limited settings.

Keywords: COVID-19; Cardiac arrest; Rural hospitals.

Copyright © 2021 The Authors. Published by Elsevier Inc. All rights reserved.

Conflict of interest statement

Declaration of Competing Interest Dr. Wardi is supported by the National Foundation of Emergency Medicine and funding from the Gordon and Betty Moore Foundation (#GBMF9052). He has received speaker's fees from Thermo-Fisher and consulting fees from General Electric. The remaining authors have no disclosures to report.

- 25 references

Supplementary info

Publication types, MeSH terms Expand

Publication types

- Observational Study

MeSH terms

- Aged
- COVID-19 / complications*
- California
- Cardiopulmonary Resuscitation / methods*
- Comorbidity
- Female
- Heart Arrest / etiology
- Heart Arrest / mortality*
- Heart Arrest / therapy
- Hospital Mortality*
- Hospitals, Rural
- Humans
- Male
- Middle Aged
- Prognosis
- Retrospective Studies
- Return of Spontaneous Circulation
- SARS-CoV-2*

Full text links

ELSEVIER OPEN ACCESS Elsevier Science Free PMC article
Proceed to details
Cite
Share
☐ 183
Observational Study
Rev Esp Quimioter
. 2021 Apr;34(2):145-150.
doi: 10.37201/req/130.2020. Epub 2021 Feb 1.

[Clinical and pharmacological data in COVID-19 hospitalized nonagenarian patients]

[Article in Spanish]

S Ortonobes Roig [1], N Soler-Blanco, I Torrente Jiménez, E Van den Eynde Otero, M Moreno-Ariño, M Gómez-Valent
Affiliations Expand

Affiliation

- [1] Sara Ortonobes Roig, Servicio de Farmacia Hospitalaria. Corporació Sanitària Parc Taulí. Parc del Taulí, 1. 08028 Sabadell (Barcelona), Spain. sortonobes@tauli.cat.

- PMID: **33522213**
- PMCID: PMC8019469
- DOI: 10.37201/req/130.2020

Free PMC article
Observational Study

[Clinical and pharmacological data in COVID-19 hospitalized nonagenarian patients]

[Article in Spanish]
S Ortonobes Roig et al. Rev Esp Quimioter. 2021 Apr.
Free PMC article
Show details
Rev Esp Quimioter

. 2021 Apr;34(2):145-150.
doi: 10.37201/req/130.2020. Epub 2021 Feb 1.

Authors

S Ortonobes Roig [1], N Soler-Blanco, I Torrente Jiménez, E Van den Eynde Otero, M Moreno-Ariño, M Gómez-Valent

Affiliation

- [1] Sara Ortonobes Roig, Servicio de Farmacia Hospitalaria. Corporació Sanitària Parc Taulí. Parc del Taulí, 1. 08028 Sabadell (Barcelona), Spain. sortonobes@tauli.cat.

- PMID: **33522213**
- PMCID: PMC8019469
- DOI: 10.37201/req/130.2020

Abstract
in English, Spanish

Objective: Despite the impact of SARS-CoV-2 infection in geriatrics, data on nonagenarian patients is scarce. The aim of this study is to describe the clinical features of COVID19-diagnosed nonagenarians, as well as its clinical evolution and therapeutic response.

Methods: Retrospective observational study of nonagenarians, admitted for COVID-19. Sociodemographic and clinical variables were registered, including previous polypharmacy. Blood analysis data and COVID-19-specific treatment were registered.

Results: A total of 79 patients were included, with 50.6% (40 patients) of mortality. None of the comorbidities registered correlated with mortality, which was significantly higher among patients with moderate/complete functional dependence, compared to those mild-dependents/independents (59.5% vs 40.5%; p=0.015). Most prescribed drugs were hydroxychloroquine/chloroquine and azithromycin. Non-survivors presented higher counts of leukocytes and neutrophils, and higher lymphopenia.

Conclusions: Nonagenarians with functional dependence presented higher mortality, irrespective of comorbidities or treatment received. Implementing an integral geriatric evaluation would enhance the implementation of personalized therapeutic strategies for nonagenarians.

Introducción: A pesar del impacto del SARS-CoV-2 en geriatría, disponemos de escasa información en pacientes nonagenarios. Nuestro objetivo es describir características clínicas, respuesta al tratamiento y factores de riesgo de mortalidad en nonagenarios con COVID-19.

Material y métodos: Estudio retrospectivo observacional de pacientes nonagenarios hospitalizados por COVID-19. Se registraron variables sociodemográficas, clínicas y polifarmacia previa, parámetros analíticos y tratamiento específico.

Resultados: Se incluyeron 79 pacientes. No se relacionó con mortalidad ninguna de las comorbilidades. La mortalidad fue del 50,6%, siendo mayor en pacientes con dependencia funcional moderada/grave respecto aquéllos independientes/ dependientes leves (59,5% *vs* 40,5%; p=0,015). Los fármacos específicos más prescritos fueron hidroxicloroquina/cloroquina y azitromicina. Los pacientes fallecidos presentaron más leucocitos y neutrófilos, y mayor linfopenia.

Conclusión: En nuestra cohorte, el estado funcional es el principal factor de riesgo de mortalidad, independientemente de las comorbilidades y el tratamiento recibido. Implementar la valoración geriátrica integral permitiría individualizar las estrategias terapéuticas en nonagenarios.

Keywords: COVID-19; functional dependence; nonagenarians.

©The Author 2021. Published by Sociedad Española de Quimioterapia. This article is distributed under the terms of the Creative Commons Attribution-NonCommercial 4.0 International (CC BY-NC 4.0)(https://creativecommons.org/licenses/by-nc/4.0/).

Conflict of interest statement

Los autores no tienen ningún conflicto de intereses a declarar.

- 19 references

Supplementary info

Publication types, MeSH terms, Substances Expand

Publication types

- Observational Study

MeSH terms

- Aged, 80 and over*
- Antiviral Agents / therapeutic use
- Azithromycin / therapeutic use
- COVID-19 / blood
- COVID-19 / drug therapy*
- COVID-19 / mortality*
- Chloroquine / therapeutic use
- Female
- Hospitalization*
- Humans
- Hydroxychloroquine / therapeutic use
- Male
- Physical Functional Performance
- Polypharmacy
- Retrospective Studies
- Risk Factors
- Treatment Outcome

Substances

- Antiviral Agents
- Hydroxychloroquine
- Azithromycin
- Chloroquine

Full text links

FREE Full Text www.seq.es Sociedad Espanola de Quimioterapia Free PMC article
Proceed to details
Cite
Share
☐ 184
Observational Study
J Med Virol
. 2021 Feb;93(2):1023-1028.
doi: 10.1002/jmv.26471. Epub 2020 Oct 5.

Tocilizumab use in COVID-19-associated pneumonia

Alexis K Okoh [1], Eliahu Bishburg [1], Sagy Grinberg [1], Sandhya Nagarakanti [1]
Affiliations Expand

Affiliation

- [1] Department of Medicine, Division of Infectious Diseases, Newark Beth Israel Medical Center, Newark, New Jersey, USA.

- PMID: **32860432**
- DOI: 10.1002/jmv.26471

Observational Study

Tocilizumab use in COVID-19-associated pneumonia

Alexis K Okoh et al. J Med Virol. 2021 Feb.
Show details
J Med Virol
. 2021 Feb;93(2):1023-1028.
doi: 10.1002/jmv.26471. Epub 2020 Oct 5.

Authors

Alexis K Okoh [1], Eliahu Bishburg [1], Sagy Grinberg [1], Sandhya Nagarakanti [1]

Affiliation

- [1] Department of Medicine, Division of Infectious Diseases, Newark Beth Israel Medical Center, Newark, New Jersey, USA.

- PMID: **32860432**
- DOI: 10.1002/jmv.26471

Abstract

Background: We sought to evaluate the effect of tocilizumab (TCB), a recombinant humanized monoclonal antibody against soluble interleukin-6 receptors, in patients hospitalized for coronavirus disease 2019 (COVID-19).

Methods: We included all patients with laboratory-confirmed COVID-19 who had completed hospitalization between March 10, 2020 and April 10, 2020 with follow-up through April 20, 2020. Patients who received TCB in addition to standard of care within 48 h of admission were

matched in a 1:2 fashion to a similar cohort who received standard of care alone. Clinical outcomes were compared between matched groups. The primary outcome was de-escalation in oxygen therapy. Secondary outcomes were in-hospital death, septic shock, and acute kidney injury (AKI) requiring hemodialysis.

Results: Out of 77 patients who received TCB in addition to standard of care, 34% (n = 26) received TCB within 48 h of admission. One-to-two propensity matching identified 20 versus 40 patients in the TCB and no-TCB treatment arms. In the TCB group, an improvement in oxygenation was observed in 80% (n = 16) of the patients by 7 days post TCB administration. After matching, there was no difference in clinical outcomes between TCB and no-TCB patients. In-hospital death: 10% versus 8%; p = .823, septic shock: 10% versus 11%, p = .912, AKI requiring hemodialysis (10% vs. 13%; p = .734).

Conclusions: Early treatment with TCB in patients admitted for COVID-19 led to an improvement in their oxygen status during hospitalization. This change however did not translate into improved survival when compared to a matched cohort with a similar clinical profile.

Keywords: coronavirus; disease control; epidemiology; pandemics; virus classification.

© 2020 Wiley Periodicals LLC.

- 14 references

Supplementary info

Publication types, MeSH terms, Substances Expand

Publication types

- Observational Study

MeSH terms

- Acute Kidney Injury / virology
- Adult
- Aged
- Antibodies, Monoclonal, Humanized / therapeutic use*
- COVID-19 / complications*
- COVID-19 / therapy*
- Female
- Hospital Mortality
- Hospitalization / statistics & numerical data*
- Humans
- Male
- Middle Aged
- Oxygen / metabolism
- Receptors, Interleukin-6 / antagonists & inhibitors

- Renal Dialysis
- Retrospective Studies
- Shock, Septic / virology
- Time Factors
- Treatment Outcome
- United States

Substances

- Antibodies, Monoclonal, Humanized
- Receptors, Interleukin-6
- tocilizumab
- Oxygen

Full text links

WILEY Full Text Article Wiley

Proceed to details

Cite

Share

☐ 185

Observational Study

Gac Med Mex
. 2021;157(1):76-83.
doi: 10.24875/GMM.M21000525.

Descriptive study of a cohort of COVID-19 hospitalized patients in Spain

Laura Álvarez-Arroyo [1], Francisco J Carrera-Hueso [1], David El-Qutob [2], Montserrat Robustillo-Villarino [3], Ana M Girona-Sanz [4], María T Pin-Godos [5], Paula Sánchez-Monzó [6], Rafael Martínez-Gonzálbez [7], Sonia Cepeda-Madrigal [8], Fernando Martínez-Martínez [9]

Affiliations Expand

Affiliations

- [1] Department of Pharmacy. Hospital Universitario de La Plana, Castellón, Spain.
- [2] Internal Medicine Department, Allergology Unit. Hospital Universitario de La Plana, Castellón, Spain.
- [3] Internal Medicine Department, Rheumatology Department. Hospital Universitario de La Plana, Castellón, Spain.
- [4] Internal Medicine Department, Digestive Medicine. Hospital Universitario de La Plana, Castellón, Spain.

- [5] Internal Medicine Department, Nephrology Department. Hospital Universitario de La Plana, Castellón, Spain.
- [6] Neurophysiology Unit. Hospital Universitario de La Plana, Castellón, Spain.
- [7] Informatics Unit. Hospital Universitario de La Plana, Castellón, Spain.
- [8] Internal Medicine Department, Pulmonology Department. Hospital Universitario de La Plana, Castellón, Spain.
- [9] Department of Pharmacy and Pharmaceutical Technology, Universidad de Granada, Granada, Spain.

- PMID: **34125825**
- DOI: 10.24875/GMM.M21000525

Free article
Observational Study

Descriptive study of a cohort of COVID-19 hospitalized patients in Spain

Laura Álvarez-Arroyo et al. Gac Med Mex. 2021.
Free article
Show details
Gac Med Mex
. 2021;157(1):76-83.
doi: 10.24875/GMM.M21000525.

Authors

Laura Álvarez-Arroyo [1], Francisco J Carrera-Hueso [1], David El-Qutob [2], Montserrat Robustillo-Villarino [3], Ana M Girona-Sanz [4], María T Pin-Godos [5], Paula Sánchez-Monzó [6], Rafael Martínez-Gonzálbez [7], Sonia Cepeda-Madrigal [8], Fernando Martínez-Martínez [9]

Affiliations

- [1] Department of Pharmacy. Hospital Universitario de La Plana, Castellón, Spain.
- [2] Internal Medicine Department, Allergology Unit. Hospital Universitario de La Plana, Castellón, Spain.
- [3] Internal Medicine Department, Rheumatology Department. Hospital Universitario de La Plana, Castellón, Spain.
- [4] Internal Medicine Department, Digestive Medicine. Hospital Universitario de La Plana, Castellón, Spain.
- [5] Internal Medicine Department, Nephrology Department. Hospital Universitario de La Plana, Castellón, Spain.
- [6] Neurophysiology Unit. Hospital Universitario de La Plana, Castellón, Spain.
- [7] Informatics Unit. Hospital Universitario de La Plana, Castellón, Spain.
- [8] Internal Medicine Department, Pulmonology Department. Hospital Universitario de La Plana, Castellón, Spain.

- [9] Department of Pharmacy and Pharmaceutical Technology, Universidad de Granada, Granada, Spain.

- PMID: **34125825**
- DOI: 10.24875/GMM.M21000525

Abstract
in English, Spanish

Background: Several descriptive cohort studies of patients affected by COVID-19 have been published.

Objective: To describe the characteristics of patients with SARS-CoV-2 infection who were admitted to Hospital Universitario la Plana, Castellón, Spain.

Methods: Retrospective, observational cohort study that included 18-year-old or older patients who were consecutively admitted with SARS-CoV2 confirmed infection. Demographic characteristics, comorbidities, clinical symptoms, laboratory results and radiological tests are described.

Results: The study included 255 patients with a mean age of 70 years; 54.9 % were males. Most common comorbidities were high blood pressure (58 %), dyslipidemia (42.4 %), diabetes (25.5 %) and obesity (24.3 %). Median number of days from the onset of clinical symptoms prior to hospital admission was seven. Most common manifestations prior to admission were fever (74.5 %), dry cough (61.2 %), malaise (51.8 %) and dyspnea (51.0 %); 19 patients (7.4 %) were admitted to the intensive care unit, where mortality was 50 %; overall mortality was 16.9 %.

Conclusions: Our cohort reflects similar characteristics to those of other European series. Mortality was lower than that in similar studies.

Antecedentes: Se han publicado varios estudios descriptivos de cohortes de pacientes afectados por COVID-19.

Objetivo: Describir las características de pacientes con infección por SARS-CoV-2 que ingresaron al Hospital Universitario de La Plana, Castellón, España.

Métodos: Estudio observacional de cohortes retrospectivo, que incluyó pacientes de 18 años o mayores que ingresaron en forma consecutiva con infección confirmada por SARS-CoV2; se describen características demográficas, comorbilidades, síntomas clínicos, resultados de laboratorio y pruebas radiológicas.

Resultados: El estudio incluyó 255 pacientes con edad promedio de 70 años; 54.9 % fue del sexo masculino. Las comorbilidades más frecuentes fueron hipertensión arterial (58 %), dislipemia (42.4 %), diabetes (25.5 %) y obesidad (24.3 %). La mediana de días del inicio de síntomas clínicos antes del ingreso fue de siete. Las manifestaciones más frecuentes previas al ingreso fueron fiebre (74.5 %), tos seca (61.2 %), malestar general (51.8 %) y disnea (51.0 %); 19 pacientes (7.4 %) ingresaron a la unidad de cuidados intensivos, donde la mortalidad fue de 50 %; la mortalidad total fue de 16.9 %.

Conclusiones: Nuestra cohorte refleja características similares a las de otras series europeas. La mortalidad fue inferior a la de estudios similares.

Keywords: COVID-19; Cohort study; Coronavirus; Estudio de cohortes; Neumonía viral; SARS-CoV-2; Viral pneumonia.

Copyright: © 2020 Permanyer.

Supplementary info

Publication types, MeSH terms Expand

Publication types

- Observational Study

MeSH terms

- Adult
- Aged
- Aged, 80 and over
- COVID-19* / complications
- COVID-19* / diagnosis
- COVID-19* / mortality
- COVID-19* / therapy
- Cohort Studies
- Female
- Hospitalization
- Humans
- Male
- Middle Aged
- Retrospective Studies
- Spain

Full text links

Gaceta Médica de México Permanyer Publications

Proceed to details

Cite

Share

☐ 186

Observational Study

Clin Transplant

. 2021 Oct;35(10):e14423.

doi: 10.1111/ctr.14423. Epub 2021 Jul 19.

A retrospective multi-center experience of renal transplants from India during COVID-19 pandemic

Pranaw Kumar Jha [1], Dinesh Kumar Yadav [1], Vishwanath Siddini [2], Shyam Bihari Bansal [1], Reetesh Sharma [3], Urmila Anandh [4], Tarun Jeloka [5], Sreedhar Reddy [6], Saurabh Pokhariyal [7], Ashish Nandwani [7], Salil Jain [8], Vishal Saxena [8], Sidharth Kumar Sethi [1], Dinesh Bansal [1], Manish Jain [1], Puneet Sodhi [1], Ashwini Gadde [1], Rohan Augustine [2], Feroz Amir Zafar [9], Prasun Ghosh [9], Aseem Kumar Tiwari [10], Rajesh Ahlawat [9], Vijay Kher [1]
Affiliations Expand

Affiliations

- [1] Department of Nephrology, Medanta Institute of Kidney and Urology, Medanta - The Medicity, Gurugram, Haryana, India.
- [2] Department of Nephrology, Manipal Hospitals, Old Airport Road, Bengaluru, Karnataka, India.
- [3] Nephrology and Kidney Transplant Medicine, Asian Institute of Medical Sciences, Faridabad, Haryana, India.
- [4] Department of Nephrology, Yashoda Hospitals, Secunderabad, Telangana, India.
- [5] Department of Nephrology and Renal Transplant, Aditya Birla Memorial Hospital, Pune, Maharashtra, India.
- [6] Department of Nephrology, Krishna Institute of Medical Sciences, Secunderabad, Telangana, India.
- [7] Department of Nephrology, Manipal Hospital Delhi, Delhi, India.
- [8] Department of Nephrology, Fortis Memorial Research Institute, Gurugram, Haryana, India.
- [9] Department of Urology, Medanta Institute of Kidney and Urology, Medanta - The Medicity, Gurugram, Haryana, India.
- [10] Department of Transfusion Medicine, Medanta - The Medicity, Gurugram, Haryana, India.

- PMID: **34255903**
- PMCID: PMC8420412
- DOI: 10.1111/ctr.14423

Free PMC article
Observational Study

A retrospective multi-center experience of renal transplants from India during COVID-19 pandemic

Pranaw Kumar Jha et al. Clin Transplant. 2021 Oct.

Free PMC article
Show details
Clin Transplant
. 2021 Oct;35(10):e14423.
doi: 10.1111/ctr.14423. Epub 2021 Jul 19.

Authors

Pranaw Kumar Jha [1], Dinesh Kumar Yadav [1], Vishwanath Siddini [2], Shyam Bihari Bansal [1], Reetesh Sharma [3], Urmila Anandh [4], Tarun Jeloka [5], Sreedhar Reddy [6], Saurabh Pokhariyal [7], Ashish Nandwani [7], Salil Jain [8], Vishal Saxena [8], Sidharth Kumar Sethi [1], Dinesh Bansal [1], Manish Jain [1], Puneet Sodhi [1], Ashwini Gadde [1], Rohan Augustine [2], Feroz Amir Zafar [9], Prasun Ghosh [9], Aseem Kumar Tiwari [10], Rajesh Ahlawat [9], Vijay Kher [1]

Affiliations

- [1] Department of Nephrology, Medanta Institute of Kidney and Urology, Medanta - The Medicity, Gurugram, Haryana, India.
- [2] Department of Nephrology, Manipal Hospitals, Old Airport Road, Bengaluru, Karnataka, India.
- [3] Nephrology and Kidney Transplant Medicine, Asian Institute of Medical Sciences, Faridabad, Haryana, India.
- [4] Department of Nephrology, Yashoda Hospitals, Secunderabad, Telangana, India.
- [5] Department of Nephrology and Renal Transplant, Aditya Birla Memorial Hospital, Pune, Maharashtra, India.
- [6] Department of Nephrology, Krishna Institute of Medical Sciences, Secunderabad, Telangana, India.
- [7] Department of Nephrology, Manipal Hospital Delhi, Delhi, India.
- [8] Department of Nephrology, Fortis Memorial Research Institute, Gurugram, Haryana, India.
- [9] Department of Urology, Medanta Institute of Kidney and Urology, Medanta - The Medicity, Gurugram, Haryana, India.
- [10] Department of Transfusion Medicine, Medanta - The Medicity, Gurugram, Haryana, India.

- PMID: **34255903**
- PMCID: PMC8420412
- DOI: 10.1111/ctr.14423

Abstract

Introduction: Coronavirus disease 2019 (COVID-19) pandemic led to a sudden drop in renal transplant numbers across India in the initial months of 2020. Although the transplant numbers increased with easing of lockdown, the outcome of these transplants remains unknown.

Methods: This was a retrospective, observational, multi-center study done across eight different transplant centers in India. All the transplants done from January 30, 2020 to December 31, 2020 were included. The primary outcomes studied were patient and death censored graft survival as well as incidence of COVID-19 infection and its outcomes.

Results: During the study period a total of 297 kidney transplants were done. After a median follow up of 265 days the patient and death censored graft survival was 95.3% and 97.6%, respectively. Forty-one patients (13.8%) developed COVID-19 post-transplant. Majority (58.5%) were asymptomatic to mildly symptomatic and the case fatality ratio was 14.6%. On multivariable logistic regression analysis older age was associated with higher likelihood of COVID-19 infection (odds ratio 1.038; CI 1.002-1.077).

Conclusions: Patient and graft outcome of kidney transplants done during the COVID-19 pandemic in India was acceptable. The incidence of COVID-19 was 13.8% with a high case fatality ratio.

Keywords: COVID-19; India; SARS-CoV-2; coronavirus; kidney transplantation; pandemic.

© 2021 John Wiley & Sons A/S. Published by John Wiley & Sons Ltd.

Conflict of interest statement

Vijay Kher has received research funding from Novartis India, Sanofi Aventis India, Astellas India. He has been scientific advisor for Roche India, Novartis India, Astellas India, Torrent India, Reddy's India, Biocon India, Medtronics, Wockhardt India and declared having received honoraria and speaker fees from them. All other authors have declared no competing interests.

- 28 references
- 5 figures

Supplementary info

Publication types, MeSH terms Expand

Publication types

- Multicenter Study
- Observational Study

MeSH terms

- Aged
- COVID-19*
- Communicable Disease Control
- Humans
- India / epidemiology
- Kidney Transplantation*
- Pandemics
- Retrospective Studies
- SARS-CoV-2

Full text links

WILEY Full Text Article Wiley Free PMC article
Proceed to details
Cite
Share
☐ 187

Observational Study

Med Clin (Barc)
. 2021 Mar 12;156(5):221-228.
doi: 10.1016/j.medcli.2020.11.004. Epub 2020 Dec 5.

Effectiveness of glucocorticoids in patients hospitalized for severe SARS-CoV-2 pneumonia

[Article in English, Spanish]

José Francisco Pascual Pareja [1], Rebeca García-Caballero [2], Llanos Soler Rangel [3], Miguel Angel Vázquez-Ronda [3], Silvia Roa Franco [2], Gema Navarro Jiménez [2], Miguel Angel Moreno Palanco [3], Patricia González-Ruano [3], Ramiro López-Menchaca [2], Pilar Ruíz-Seco [3], Bárbara Pagán Muñoz [2], Alejandro Gómez Gómez [2], Beatriz Pérez-Monte [2], Rebeca Fuerte Martínez [2], Jose Luis Valle López [2], Arturo Muñoz Blanco [2], Isabel Rábago Lorite [2], Patricia Martínez Martín [2], Gonzalo Serralta San Martín [3], Jorge Francisco Gómez-Cerezo [3], en nombre del grupo de trabajo HUIS-COVID-19
Affiliations Expand

Affiliations

- [1] Hospital Universitario Infanta Sofía, San Sebastián de los Reyes, Madrid, España; Universidad Europea de Madrid Villaviciosa de Odón, Madrid, España. Electronic address: josefrancisco.pascual@salud.madrid.org.
- [2] Hospital Universitario Infanta Sofía, San Sebastián de los Reyes, Madrid, España.
- [3] Hospital Universitario Infanta Sofía, San Sebastián de los Reyes, Madrid, España; Universidad Europea de Madrid Villaviciosa de Odón, Madrid, España.

- PMID: **33380371**
- PMCID: PMC7719003
- DOI: 10.1016/j.medcli.2020.11.004

Free PMC article
Observational Study

Effectiveness of glucocorticoids in patients hospitalized for severe SARS-CoV-2 pneumonia

[Article in English, Spanish]
José Francisco Pascual Pareja et al. Med Clin (Barc). 2021.
Free PMC article
Show details
Med Clin (Barc)
. 2021 Mar 12;156(5):221-228.
doi: 10.1016/j.medcli.2020.11.004. Epub 2020 Dec 5.

Authors

José Francisco Pascual Pareja [1], Rebeca García-Caballero [2], Llanos Soler Rangel [3], Miguel Angel Vázquez-Ronda [3], Silvia Roa Franco [2], Gema Navarro Jiménez [2], Miguel Angel Moreno Palanco [3], Patricia González-Ruano [3], Ramiro López-Menchaca [2], Pilar Ruíz-Seco [3], Bárbara Pagán Muñoz [2], Alejandro Gómez Gómez [2], Beatriz Pérez-Monte [2], Rebeca Fuerte Martínez [2], Jose Luis Valle López [2], Arturo Muñoz Blanco [2], Isabel Rábago Lorite [2], Patricia Martínez Martín [2], Gonzalo Serralta San Martín [3], Jorge Francisco Gómez-Cerezo [3], en nombre del grupo de trabajo HUIS-COVID-19

Affiliations

- [1] Hospital Universitario Infanta Sofía, San Sebastián de los Reyes, Madrid, España; Universidad Europea de Madrid Villaviciosa de Odón, Madrid, España. Electronic address: josefrancisco.pascual@salud.madrid.org.
- [2] Hospital Universitario Infanta Sofía, San Sebastián de los Reyes, Madrid, España.
- [3] Hospital Universitario Infanta Sofía, San Sebastián de los Reyes, Madrid, España; Universidad Europea de Madrid Villaviciosa de Odón, Madrid, España.

- PMID: **33380371**
- PMCID: PMC7719003
- DOI: 10.1016/j.medcli.2020.11.004

Abstract

Introduction: Several studies have reported the beneficial effect of glucocorticoids in the treatment of cytokine storm that occurs in patients with severe COVID-19. Various glucocorticoids regimens have been proposed.

Methods: Retrospective observational study that includes patients with severe SARS-CoV-2 pneumonia and compares admission to an Intensive Care Unit (ICU) or death during hospitalization in three groups of patients: no glucocorticoids treatment, use of glucocorticoids doses equivalent to less than 250mg of prednisone daily and use of equivalent doses greater than or equal to 250mg of prednisone daily. Multivariate analysis was performed using logistic regression, using the propensity index as a covariant.

Results: Of the 259 patients enrolled in the study, 67 (25.9%) had an unfavorable evolution, dying or requiring ICU admission. Comparative analyzes between different glucocorticoids treatments and the association with ICU admission or death were: glucocorticoids treatment (any dose) versus no glucocorticoids treatment (OR: 0.71 [0.30-1.66]), treatment with glucocorticoids (≥250mg prednisone daily) versus no glucocorticoids treatment (OR: 0.35 [0.11-1.08]) and glucocorticoids treatment (≥250mg prednisone daily) versus patients with glucocorticoids doses <250mg prednisone daily or without glucocorticoids treatment (OR: 0.30 [0.10-0.88]).

Conclusion: The results of this study show that patients with severe SARS-CoV-2 pneumonia treated with glucocorticoids pulses with equivalent doses of prednisone greater than or equal to 250mg have a more favorable evolution (less mortality and less admission to ICU).

Keywords: Coronavirus infection disease 2019 (COVID-19); Corticoides; Corticosteroids; Enfermedad por coronavirus 2019 (COVID-19); Propensity index; Severe acute respiratory syndrome coronavirus 2 (SARS-CoV-2); Síndrome agudo respiratorio severo por coronavirus 2 (SARS-CoV-2); Índice de propensión.

Copyright © 2020 Elsevier España, S.L.U. All rights reserved.

- 23 references
- 1 figure

Supplementary info

Publication types, MeSH terms, Substances Expand

Publication types

- Observational Study

MeSH terms

- Adolescent
- Adult
- Aged
- Anti-Inflammatory Agents / therapeutic use*
- COVID-19 / complications
- COVID-19 / drug therapy*
- COVID-19 / mortality
- Dose-Response Relationship, Drug
- Drug Administration Schedule
- Female
- Glucocorticoids / therapeutic use*
- Hospitalization
- Humans
- Logistic Models
- Male

- Middle Aged
- Retrospective Studies
- Severity of Illness Index
- Treatment Outcome
- Young Adult

Substances

- Anti-Inflammatory Agents
- Glucocorticoids

Full text links

Full text at MEDICINA CLINICA Ediciones Doyma, S.L. Free PMC article
Proceed to details
Cite
Share
188
Observational Study
J Diabetes Complications
. 2021 Aug;35(8):107967.
doi: 10.1016/j.jdiacomp.2021.107967. Epub 2021 May 28.

Preadmission predictors of severe COVID-19 in patients with diabetes mellitus

Alpana P Shukla [1], Beverly G Tchang [2], Tiffany Lam [3], Ian Steller [4], Samir Touhamy 2nd [5], Gulce Askin [6], Felicia A Mendelsohn Curanaj [7], Jane J Seley [8], Daniel Lorber [9], Monika M Safford [10], Louis J Aronne [11], Laura C Alonso [12]
Affiliations Expand

Affiliations

- [1] Weill Cornell Medicine, Department of Medicine, Division of Endocrinology, Diabetes and Metabolism, Comprehensive Weight Control Center, New York, NY 10065, USA. Electronic address: aps2004@med.cornell.edu.
- [2] Weill Cornell Medicine, Department of Medicine, Division of Endocrinology, Diabetes and Metabolism, Comprehensive Weight Control Center, New York, NY 10065, USA. Electronic address: bgt9001@med.cornell.edu.
- [3] Weill Cornell Medical College, New York, NY 10065, USA. Electronic address: til4007@med.cornell.edu.
- [4] Western University of Health Sciences, Lebanon, OR 97355, USA. Electronic address: ian.steller@westernu.edu.

- [5] Weill Cornell Medical College, New York, NY 10065, USA. Electronic address: sat2032@med.cornell.edu.
- [6] Weill Cornell Medicine, Department of Population Health Sciences, Division of Biostatistics, New York, NY 10065, USA.
- [7] Weill Cornell Medicine, Department of Medicine, Division of Endocrinology, Diabetes and Metabolism, New York, NY 10065, USA. Electronic address: fam9025@med.cornell.edu.
- [8] Weill Cornell Medicine, Department of Medicine, Division of Endocrinology, Diabetes and Metabolism, New York, NY 10065, USA. Electronic address: jas9067@med.cornell.edu.
- [9] Weill Cornell Medicine and New-York Presbyterian Hospital Queens, Department of Medicine, Division of Endocrinology, New York, NY 10065, USA. Electronic address: dll9004@nyp.org.
- [10] Weill Cornell Medicine, Department of Medicine, New York, NY 10065, USA. Electronic address: mms9024@med.cornell.edu.
- [11] Weill Cornell Medicine, Department of Medicine, Division of Endocrinology, Diabetes and Metabolism, Comprehensive Weight Control Center, New York, NY 10065, USA. Electronic address: ljaronne@med.cornell.edu.
- [12] Weill Cornell Medicine, Department of Medicine, Division of Endocrinology, Diabetes and Metabolism, New York, NY 10065, USA. Electronic address: lca4001@med.cornell.edu.

- PMID: **34099384**
- PMCID: PMC8162023
- DOI: 10.1016/j.jdiacomp.2021.107967

Free PMC article
Observational Study

Preadmission predictors of severe COVID-19 in patients with diabetes mellitus

Alpana P Shukla et al. J Diabetes Complications. 2021 Aug.
Free PMC article
Show details
J Diabetes Complications
. 2021 Aug;35(8):107967.
doi: 10.1016/j.jdiacomp.2021.107967. Epub 2021 May 28.

Authors

Alpana P Shukla [1], Beverly G Tchang [2], Tiffany Lam [3], Ian Steller [4], Samir Touhamy 2nd [5], Gulce Askin [6], Felicia A Mendelsohn Curanaj [7], Jane J Seley [8], Daniel Lorber [9], Monika M Safford [10], Louis J Aronne [11], Laura C Alonso [12]

Affiliations

- [1] Weill Cornell Medicine, Department of Medicine, Division of Endocrinology, Diabetes and Metabolism, Comprehensive Weight Control Center, New York, NY 10065, USA. Electronic address: aps2004@med.cornell.edu.
- [2] Weill Cornell Medicine, Department of Medicine, Division of Endocrinology, Diabetes and Metabolism, Comprehensive Weight Control Center, New York, NY 10065, USA. Electronic address: bgt9001@med.cornell.edu.
- [3] Weill Cornell Medical College, New York, NY 10065, USA. Electronic address: til4007@med.cornell.edu.
- [4] Western University of Health Sciences, Lebanon, OR 97355, USA. Electronic address: ian.steller@westernu.edu.
- [5] Weill Cornell Medical College, New York, NY 10065, USA. Electronic address: sat2032@med.cornell.edu.
- [6] Weill Cornell Medicine, Department of Population Health Sciences, Division of Biostatistics, New York, NY 10065, USA.
- [7] Weill Cornell Medicine, Department of Medicine, Division of Endocrinology, Diabetes and Metabolism, New York, NY 10065, USA. Electronic address: fam9025@med.cornell.edu.
- [8] Weill Cornell Medicine, Department of Medicine, Division of Endocrinology, Diabetes and Metabolism, New York, NY 10065, USA. Electronic address: jas9067@med.cornell.edu.
- [9] Weill Cornell Medicine and New-York Presbyterian Hospital Queens, Department of Medicine, Division of Endocrinology, New York, NY 10065, USA. Electronic address: dll9004@nyp.org.
- [10] Weill Cornell Medicine, Department of Medicine, New York, NY 10065, USA. Electronic address: mms9024@med.cornell.edu.
- [11] Weill Cornell Medicine, Department of Medicine, Division of Endocrinology, Diabetes and Metabolism, Comprehensive Weight Control Center, New York, NY 10065, USA. Electronic address: ljaronne@med.cornell.edu.
- [12] Weill Cornell Medicine, Department of Medicine, Division of Endocrinology, Diabetes and Metabolism, New York, NY 10065, USA. Electronic address: lca4001@med.cornell.edu.

- PMID: **34099384**
- PMCID: PMC8162023
- DOI: 10.1016/j.jdiacomp.2021.107967

Abstract

Objective: To explore predictors of severe COVID-19 disease in patients with diabetes hospitalized for COVID-19.

Methods: This is a retrospective observational study of adults with diabetes admitted for COVID-19. Bivariate tests and multivariable Cox regression were used to identify risk factors for severe COVID-19, defined as a composite endpoint of intensive care unit admission/intubation or in-hospital death.

Results: In 1134 patients with diabetes admitted for COVID-19, more severe disease was associated with older age (HR 1.02, p<0.001), male sex (HR 1.28, p=0.017), Asian race (HR 1.34, p=0.029 [reference: white]), and greater obesity (moderate obesity HR 1.59, p=0.015; severe obesity HR 2.07, p=0.002 [reference: normal body mass index]). Outpatient diabetes medications were not associated with outcomes.

Conclusions: Age, male sex, Asian race, and obesity were associated with increased risk of severe COVID-19 disease in adults with type 2 diabetes hospitalized for COVID-19.

Summary: In patients with type 2 diabetes hospitalized for COVID-19 disease, we observed that age, male sex, Asian race, and obesity predicted severe COVID-19 outcomes of intensive care unit admission, intubation, or in-hospital death. The risk conferred by obesity increased with worsening obesity. Outpatient diabetes medications were not observed to be significant predictors of study outcomes.

Keywords: Asian; COVID-19; Mortality; Obesity; Race; Type 2 diabetes mellitus.

Copyright © 2021 Elsevier Inc. All rights reserved.

Conflict of interest statement

LJA reports receiving consulting fees from and serving on advisory boards for Jamieson Laboratories, Pfizer, Novo Nordisk, Eisai, Erx Pharmaceuticals, Real Appeal, Janssen Pharmaceuticals, and Gelesis; receiving research funding from Aspire Bariatrics, Allurion, Eisai, AstraZeneca, Gelesis, Janssen Pharmaceuticals and Novo Nordisk; having equity interests in Intellihealth Corp, Allurion, Erx Pharmaceuticals, Zafgen, Gelesis, Myos Corp., and Jamieson Laboratories; and serving on a board of directors for Intellihealth Corp., Myos Corp. and Jamieson Laboratories. MMS reports receiving salary support for investigator-initiated research unrelated to the topic from Amgen, Inc. BGT serves as a consultant for Novo Nordisk. All other authors have nothing to disclose.

- 14 references

Supplementary info

Publication types, MeSH terms, Grant support Expand

Publication types

- Observational Study
- Research Support, N.I.H., Extramural
- Research Support, Non-U.S. Gov't

MeSH terms

- Adult
- Aged
- Aged, 80 and over
- Body Mass Index
- COVID-19 / diagnosis*
- COVID-19 / epidemiology*
- COVID-19 / pathology
- COVID-19 / therapy
- Comorbidity

- Diabetes Mellitus, Type 2 / complications
- Diabetes Mellitus, Type 2 / diagnosis*
- Diabetes Mellitus, Type 2 / epidemiology*
- Diabetes Mellitus, Type 2 / therapy
- Female
- Humans
- Intensive Care Units / statistics & numerical data
- Male
- Middle Aged
- New York / epidemiology
- Obesity / complications
- Obesity / diagnosis
- Obesity / epidemiology
- Obesity / therapy
- Patient Admission / statistics & numerical data*
- Prognosis
- Racial Groups / statistics & numerical data
- Retrospective Studies
- Risk Factors
- SARS-CoV-2 / physiology
- Severity of Illness Index

Grant support

- P30 DK020541/DK/NIDDK NIH HHS/United States
- P30 DK111022/DK/NIDDK NIH HHS/United States
- P60 DK020541/DK/NIDDK NIH HHS/United States
- UL1 TR002384/TR/NCATS NIH HHS/United States

Full text links

ELSEVIER FULL-TEXT ARTICLE Elsevier Science Free PMC article

Proceed to details

Cite

Share

☐ 189

Observational Study

Clin Neurol Neurosurg

. 2021 Nov;210:106985.

doi: 10.1016/j.clineuro.2021.106985. Epub 2021 Oct 11.

Neurological symptoms of COVID-19 infection; a cross-sectional study on hospitalized COVID-19 patients in Iran

Javad Hosseini Nejad [1], Fakhri Allahyari [1], Ramin Hosseinzadeh [2], Mohammad Heiat [3], Reza Ranjbar [4]
Affiliations Expand

Affiliations

- [1] Neuroscience research center, Baqiyatallah University of Medical Sciences, Tehran, Iran.
- [2] Faculty of Medicine, Mashhad University of Medical Sciences, Mashhad, Iran.
- [3] Baqiyatallah Research Center for Gastroenterology and Liver Diseases, Baqiyatallah University of Medical Sciences, Tehran, Iran.
- [4] Molecular Biology Research Center, Systems Biology and Poisonings Institute, Baqiyatallah University of Medical Sciences, Tehran. Electronic address: ranjbarre@gmail.com.

- PMID: **34700274**
- PMCID: PMC8502683
- DOI: 10.1016/j.clineuro.2021.106985

Free PMC article
Observational Study

Neurological symptoms of COVID-19 infection; a cross-sectional study on hospitalized COVID-19 patients in Iran

Javad Hosseini Nejad et al. Clin Neurol Neurosurg. 2021 Nov.
Free PMC article
Show details
Clin Neurol Neurosurg
. 2021 Nov;210:106985.
doi: 10.1016/j.clineuro.2021.106985. Epub 2021 Oct 11.

Authors

Javad Hosseini Nejad [1], Fakhri Allahyari [1], Ramin Hosseinzadeh [2], Mohammad Heiat [3], Reza Ranjbar [4]

Affiliations

- [1] Neuroscience research center, Baqiyatallah University of Medical Sciences, Tehran, Iran.

- [2] Faculty of Medicine, Mashhad University of Medical Sciences, Mashhad, Iran.
- [3] Baqiyatallah Research Center for Gastroenterology and Liver Diseases, Baqiyatallah University of Medical Sciences, Tehran, Iran.
- [4] Molecular Biology Research Center, Systems Biology and Poisonings Institute, Baqiyatallah University of Medical Sciences, Tehran. Electronic address: ranjbarre@gmail.com.

- PMID: **34700274**
- PMCID: PMC8502683
- DOI: 10.1016/j.clineuro.2021.106985

Abstract

Background: Coronavirus disease 2019 (COVID-19), a global issue now, can have a variety of clinical manifestations. Hundreds of articles have discussed different aspects of this infectious disease, such as physiopathology, epidemiology, clinical manifestations and treatment protocols. Recently, neurological manifestations of the disease have been found to be pretty common among COVID-19 patients. Here, neurological symptoms of COVID-19 infection with a focus on non-cerebrovascular complications are discussed in a large study population.

Methods: Neurological symptoms of 891hospitalized COVID-19 patients from March to June 2020 in a major Hospital, Tehran, Iran, were reviewed. Demographic characteristics and neurological manifestations were analyzed.

Results: Among 891 hospitalized COVID-19 patients, the following symptoms were observed: headache (63.9%), sleeping problems (51.3%), hyposmia/anosmia (46%), dizziness (45.4%), hypogeusia (42.1%), memory issues (31.5%), auditory disturbances (17.5%), paralysis (3.7%) and seizures (1.7%). In 29.7% of the patients, a neurological symptom was the initiating symptoms of the infection. Females were more likely to show headache and dizziness compared to males (p value<0.05). Headache intensity was also higher in females compared to males (p value<0.05). Headache prevalence was lower in older patients (p value<0.05), while memory loss and impaired consciousness were higher by increasing age (p values=0.002 and 0.001, respectively).

Conclusion: Neurological manifestations were common among COVID-19 patients under study. Headache, as the most common neurological symptom among COVID-19 patients, was the most prevalent and intense among the female population. Headache, dizziness, sleeping problems, hyposmia/anosmia and hypogeusia were common COVID-19 neurological manifestations, while memory issues, auditory disturbances, paralysis, and seizures were less common.

Keywords: COVID-19; Headache; Neurological; Seizures; Symptom.

Copyright © 2021 Elsevier B.V. All rights reserved.

- 44 references

Supplementary info

Publication types, MeSH terms Expand

Publication types

- Observational Study

MeSH terms

- Adolescent
- Adult
- Aged
- Aged, 80 and over
- COVID-19 / diagnosis
- COVID-19 / epidemiology*
- COVID-19 / therapy*
- Child
- Cross-Sectional Studies
- Dizziness / diagnosis
- Dizziness / epidemiology
- Dizziness / therapy
- Female
- Headache / diagnosis
- Headache / epidemiology
- Headache / therapy
- Hospitalization / trends*
- Humans
- Iran / epidemiology
- Male
- Middle Aged
- Nervous System Diseases / diagnosis
- Nervous System Diseases / epidemiology*
- Nervous System Diseases / therapy*
- Retrospective Studies
- Seizures / diagnosis
- Seizures / epidemiology
- Seizures / therapy
- Young Adult

Full text links

ELSEVIER FULL-TEXT ARTICLE Elsevier Science Free PMC article

Proceed to details

Cite

Share

☐ 190

Observational Study

J Trop Pediatr
. 2021 Jul 2;67(3):fmab052.
doi: 10.1093/tropej/fmab052.

Severe Coronavirus Disease Pneumonia in Pediatric Patients in a Referral Hospital

Serhan Ozcan [1], Serhat Emeksiz [1], Oktay Perk [1], Emel Uyar [1], Saliha Kanik Yüksek [2]
Affiliations Expand

Affiliations

- [1] Department of Pediatric Intensive Care, Ankara City Hospital, Ankara, Turkey.
- [2] Department of Pediatric Infectious Disease, Ankara City Hospital, Ankara, Turkey.

- PMID: **34081145**
- PMCID: PMC8194880
- DOI: 10.1093/tropej/fmab052

Free PMC article
Observational Study

Severe Coronavirus Disease Pneumonia in Pediatric Patients in a Referral Hospital

Serhan Ozcan et al. J Trop Pediatr. 2021.
Free PMC article
Show details
J Trop Pediatr
. 2021 Jul 2;67(3):fmab052.
doi: 10.1093/tropej/fmab052.

Authors

Serhan Ozcan [1], Serhat Emeksiz [1], Oktay Perk [1], Emel Uyar [1], Saliha Kanik Yüksek [2]

Affiliations

- [1] Department of Pediatric Intensive Care, Ankara City Hospital, Ankara, Turkey.
- [2] Department of Pediatric Infectious Disease, Ankara City Hospital, Ankara, Turkey.

- PMID: **34081145**
- PMCID: PMC8194880
- DOI: 10.1093/tropej/fmab052

Abstract

Objective: We aimed to evaluate the characteristics and outcomes of critically ill children managed in an intensive care unit because of coronavirus disease (COVID-19) pneumonia with respiratory support requirements.

Methods: We performed a single-center retrospective observational study in a pediatric intensive care unit (PICU) with 32 beds in Ankara City Hospital, Ankara, Turkey, from 13 March 2020 to 31 December 2020. Patients who needed positive-pressure ventilation (PPV) therapy for COVID-19 pneumonia were included in the study. Demographic, clinical and laboratory data were extracted from the patients' electronic medical records. As outcomes, the hospitalization rate of all pediatric patients diagnosed as having with COVID-19 by Polymerase Chaın Reactıon(PCR), PICU admission rate for COVID-19 pneumonia among all hospitalized patients, PPV support rate, intensive care hospitalization duration (days), total hospitalization duration (days), survival rate and tracheotomy requirement were evaluated.

Results: During the study period, 7033 children tested positive for COVID-19 in PCR tests. Of these patients, 1219 were hospitalized for COVID-19. Seventeen patients needed PPV support because of COVID-19 pneumonia. High proportion (65%) of patients admitted to the PICU had comorbid diseases. Noninvasive ventilation was applied in 15 patients (88%). The hospitalization rate among the children with COVID-19 was 17%, of whom 1.6% were admitted to the PICU. Mortality rates were 0.056% of all the cases and 0.32% of the hospitalized patients in our hospital.

Conclusion: The presence of a comorbid disease could be a sign of severe disease in children with higher lethality. Very few children required PPV support because of severe COVID-19 pneumonia.

Keywords: COVID-19; children; intensive care; respiratory support.

Plain Language Summary

Coronavirus disease (COVID-19) spread from Wuhan, China, and caused an outbreak that threatened human health globally. Reports worldwide have shown that the outbreak mainly affected the adult population. Data about severe COVID-19 pneumonia in children are limited. Treatment interventions for the adult population have been adapted for children. Our article was aimed at building an opinion about this patient group. We found that severe COVID-19 pneumonia occurred in only a small population. Cardiac and neurological comorbidities are associated with higher mortality rates. Only a few patients with COVID-19 required mechanical ventilation support.

© The Author(s) [2021]. Published by Oxford University Press. All rights reserved. For permissions, please email: journals.permissions@oup.com.

Supplementary info

Publication types, MeSH terms Expand

Publication types

- Observational Study

MeSH terms

- COVID-19*
- Child
- Hospitalization
- Hospitals
- Humans
- Referral and Consultation
- Retrospective Studies
- SARS-CoV-2
- Turkey / epidemiology

Full text links

OXFORD ACADEMIC Silverchair Information Systems Free PMC article
Proceed to details
Cite
Share
191
Observational Study
Influenza Other Respir Viruses
. 2022 Mar;16(2):193-203.
doi: 10.1111/irv.12919. Epub 2021 Oct 13.

Impact of varying wave periods of COVID-19 on in-hospital mortality and length of stay for admission through emergency department: A territory-wide observational cohort study

Xi Xiong [1], Abraham K C Wai [2, 3], Janet Y H Wong [4], Eric H M Tang [5], Owen C K Chu [2], Carlos K H Wong [1, 5, 6], Timothy H Rainer [2, 3]
Affiliations Expand

Affiliations

- [1] Centre for Safe Medication Practice and Research, Department of Pharmacology and Pharmacy, Li Ka Shing Faculty of Medicine, The University of Hong Kong, Hong Kong SAR, China.
- [2] Emergency Medicine Unit, Li Ka Shing Faculty of Medicine, The University of Hong Kong, Hong Kong, SAR, China.
- [3] Accident and Emergency Department, Queen Mary Hospital, Hong Kong SAR, China.

- [4] School of Nursing, Li Ka Shing Faculty of Medicine, The University of Hong Kong, Hong Kong SAR, China.
- [5] Department of Family Medicine and Primary Care, Li Ka Shing Faculty of Medicine, The University of Hong Kong, Hong Kong SAR, China.
- [6] Laboratory of Data Discovery for Health (D24H), Hong Kong Science and Technology Park, Hong Kong SAR, China.

- PMID: **34643047**
- PMCID: PMC8653231
- DOI: 10.1111/irv.12919

Free PMC article
Observational Study

Impact of varying wave periods of COVID-19 on in-hospital mortality and length of stay for admission through emergency department: A territory-wide observational cohort study

Xi Xiong et al. Influenza Other Respir Viruses. 2022 Mar.
Free PMC article
Show details
Influenza Other Respir Viruses
. 2022 Mar;16(2):193-203.
doi: 10.1111/irv.12919. Epub 2021 Oct 13.

Authors

Xi Xiong [1], Abraham K C Wai [2, 3], Janet Y H Wong [4], Eric H M Tang [5], Owen C K Chu [2], Carlos K H Wong [1, 5, 6], Timothy H Rainer [2, 3]

Affiliations

- [1] Centre for Safe Medication Practice and Research, Department of Pharmacology and Pharmacy, Li Ka Shing Faculty of Medicine, The University of Hong Kong, Hong Kong SAR, China.
- [2] Emergency Medicine Unit, Li Ka Shing Faculty of Medicine, The University of Hong Kong, Hong Kong, SAR, China.
- [3] Accident and Emergency Department, Queen Mary Hospital, Hong Kong SAR, China.
- [4] School of Nursing, Li Ka Shing Faculty of Medicine, The University of Hong Kong, Hong Kong SAR, China.
- [5] Department of Family Medicine and Primary Care, Li Ka Shing Faculty of Medicine, The University of Hong Kong, Hong Kong SAR, China.
- [6] Laboratory of Data Discovery for Health (D24H), Hong Kong Science and Technology Park, Hong Kong SAR, China.

- PMID: **34643047**
- PMCID: PMC8653231
- DOI: 10.1111/irv.12919

Abstract

Background: The COVID-19 pandemic has been associated with excess mortality and reduced emergency department attendance. However, the effect of varying wave periods of COVID-19 on in-hospital mortality and length of stay (LOS) for non-COVID disease for non-COVID diseases remains unexplored.

Methods: We examined a territory-wide observational cohort of 563,680 emergency admissions between January 1 and November 30, 2020, and 709,583 emergency admissions during the same 2019 period in Hong Kong, China. Differences in 28-day in-hospital mortality risk and LOS due to COVID-19 were evaluated.

Results: The cumulative incidence of 28-day in-hospital mortality increased overall from 2.9% in 2019 to 3.6% in 2020 (adjusted hazard ratio [aHR] = 1.22, 95% CI 1.20 to 1.25). The aHR was higher among patients with lower respiratory tract infection (aHR: 1.30 95% CI 1.26 to 1.34), airway disease (aHR: 1.35 95% CI 1.22 to 1.49), and mental disorders (aHR: 1.26 95% CI 1.15 to 1.37). Mortality risk in the first- and third-wave periods was significantly greater than that in the inter-wave period (p-interaction < 0.001). The overall average LOS in the pandemic year was significantly shorter than that in 2019 (Mean difference = -0.40 days; 95% CI -0.43 to -0.36). Patients with mental disorders and cerebrovascular disease in 2020 had a 3.91-day and 2.78-day shorter LOS than those in 2019, respectively.

Conclusions: Increased risk of in-hospital deaths was observed overall and by all major subgroups of disease during the pandemic period. Together with significantly reduced LOS for patients with mental disorders and cerebrovascular disease, this study shows the spillover effect of the COVID-19 pandemic.

Keywords: COVID-19; cerebrovascular disease; emergency admission; hospital mortality; length of stay; mental health.

© 2021 The Authors. Influenza and Other Respiratory Viruses published by John Wiley & Sons Ltd.

- 42 references
- 5 figures

Supplementary info

Publication types, MeSH terms Expand

Publication types

- Observational Study

MeSH terms

- COVID-19*
- Cohort Studies
- Emergency Service, Hospital
- Hospital Mortality
- Humans
- Length of Stay
- Pandemics
- Retrospective Studies
- SARS-CoV-2

Full text links

Wiley Free PMC article
Proceed to details
Cite
Share
☐ 192
Observational Study
J Clin Endocrinol Metab
. 2021 Aug 18;106(9):e3364-e3368.
doi: 10.1210/clinem/dgab393. Epub 2021 Jun 1.

Diabetes Increases Severe COVID-19 Outcomes Primarily in Younger Adults

Marc Diedisheim [1, 2], Etienne Dancoisne [3, 4], Jean-François Gautier [1, 5], Etienne Larger [2], Emmanuel Cosson [6, 7], Bruno Fève [8, 9, 10], Philippe Chanson [11, 12], Sébastien Czernichow [13, 14], Sopio Tatulashvili [6, 7], Marie-Laure Raffin-Sanson [15, 16], Kankoé Sallah [4], Muriel Bourgeon [17], Christiane Ajzenberg [18], Agnès Hartemann [19], Christel Daniel [3, 20], Thomas Moreau [21], Ronan Roussel [1, 22], Louis Potier [1, 22]
Affiliations Expand

Affiliations

- [1] Cordeliers Research Centre, ImMeDiab team, INSERM, Université de Paris, 75006 Paris, France.
- [2] Hôpital Cochin, APHP, Diabetology Department, 75014 Paris, France.
- [3] Assistance Publique-Hôpitaux de Paris, DSI WIND, Web Innovation Données, 75012 Paris, France.
- [4] Hôpital Bichat - Claude-Bernard, APHP, URC PNVS, CIC-EC 1425, INSERM, 75018 Paris, France.
- [5] GH Lariboisiere Fernand-Widal, APHP, Department of Diabetes and Endocrinology, 75010 Paris, France.

- [6] Hospital Avicenne, APHP, Department of Endocrinology-Diabetology-Nutrition, CRNH-IdF, CINFO, 93000 Bobigny, France.
- [7] Université Sorbonne Paris Cité, UMR U557 INSERM/U11125 INRAE, Unité de Recherche Epidémiologique Nutritionnelle, 93000 Bobigny, France.
- [8] Hôpital Saint-Antoine, APHP, Department of Endocrinology-Diabetology, 75012 Paris, France.
- [9] Institut Hospitalo-Universitaire ICAN, 75013 Paris, France.
- [10] Sorbonne Université, INSERM, UMR_S938, CRMR PRISIS, 75012 Paris, France.
- [11] Hôpital Bicêtre, APHP, Service d'Endocrinologie et des Maladies de la Reproduction, 94270 Le Kremlin-Bicetre, France.
- [12] Université Paris-Saclay, INSERM, Physiologie et Physiopathologie Endocriniennes, 94270 Le Kremlin-Bicetre, France.
- [13] Hôpital Européen Georges Pompidou, APHP, Service de Nutrition, Centre Spécialisé Obésité, 75015 Paris, France.
- [14] Université de Paris, INSERM, UMR1153, Epidemiology and Biostatistics Sorbonne Paris Cité Centre (CRESS), 75015 Paris, France.
- [15] Hospital Ambroise Paré, APHP, Service d'Endocrinologie Diabétologie et Nutrition, 92100 Boulogne-Billancourt, France.
- [16] Université de Versailles Saint-Quentin-en-Yvelines, 78000 Versailles, France.
- [17] Hôpital Antoine-Béclère, APHP, Service de Médecine Interne, 92140 Clamart, France.
- [18] Hôpital Henri Mondor, APHP, Service de Médecine Interne, 94000 Creteil, France.
- [19] Hôpital Pitié Salpêtrière, APHP, Diabetology Department, 75013 Paris, France.
- [20] Sorbonne Université, University Paris 13, Sorbonne Paris Cité, INSERM UMR_S 1142, 75006 Paris, France.
- [21] Université Paris-Saclay, INRIA, CEA, 91120 Palaiseau, France.
- [22] Hôpital Bichat - Claude-Bernard, APHP, Department of Diabetology, 75018 Paris, France.

- PMID: **34406396**
- PMCID: PMC8195170
- DOI: 10.1210/clinem/dgab393

Free PMC article
Observational Study

Diabetes Increases Severe COVID-19 Outcomes Primarily in Younger Adults

Marc Diedisheim et al. J Clin Endocrinol Metab. 2021.
Free PMC article

Show details

J Clin Endocrinol Metab
. 2021 Aug 18;106(9):e3364-e3368.
doi: 10.1210/clinem/dgab393. Epub 2021 Jun 1.

Authors

Marc Diedisheim [1 2], Etienne Dancoisne [3 4], Jean-François Gautier [1 5], Etienne Larger [2], Emmanuel Cosson [6 7], Bruno Fève [8 9 10], Philippe Chanson [11 12], Sébastien Czernichow [13 14], Sopio Tatulashvili [6 7], Marie-Laure Raffin-Sanson [15 16], Kankoé Sallah [4], Muriel Bourgeon [17], Christiane Ajzenberg [18], Agnès Hartemann [19], Christel Daniel [3 20], Thomas Moreau [21], Ronan Roussel [1 22], Louis Potier [1 22]

Affiliations

- [1] Cordeliers Research Centre, ImMeDiab team, INSERM, Université de Paris, 75006 Paris, France.
- [2] Hôpital Cochin, APHP, Diabetology Department, 75014 Paris, France.
- [3] Assistance Publique-Hôpitaux de Paris, DSI WIND, Web Innovation Données, 75012 Paris, France.
- [4] Hôpital Bichat - Claude-Bernard, APHP, URC PNVS, CIC-EC 1425, INSERM, 75018 Paris, France.
- [5] GH Lariboisiere Fernand-Widal, APHP, Department of Diabetes and Endocrinology, 75010 Paris, France.
- [6] Hospital Avicenne, APHP, Department of Endocrinology-Diabetology-Nutrition, CRNH-IdF, CINFO, 93000 Bobigny, France.
- [7] Université Sorbonne Paris Cité, UMR U557 INSERM/U11125 INRAE, Unité de Recherche Epidémiologique Nutritionnelle, 93000 Bobigny, France.
- [8] Hôpital Saint-Antoine, APHP, Department of Endocrinology-Diabetology, 75012 Paris, France.
- [9] Institut Hospitalo-Universitaire ICAN, 75013 Paris, France.
- [10] Sorbonne Université, INSERM, UMR_S938, CRMR PRISIS, 75012 Paris, France.
- [11] Hôpital Bicêtre, APHP, Service d'Endocrinologie et des Maladies de la Reproduction, 94270 Le Kremlin-Bicetre, France.
- [12] Université Paris-Saclay, INSERM, Physiologie et Physiopathologie Endocriniennes, 94270 Le Kremlin-Bicetre, France.
- [13] Hôpital Européen Georges Pompidou, APHP, Service de Nutrition, Centre Spécialisé Obésité, 75015 Paris, France.
- [14] Université de Paris, INSERM, UMR1153, Epidemiology and Biostatistics Sorbonne Paris Cité Centre (CRESS), 75015 Paris, France.
- [15] Hospital Ambroise Paré, APHP, Service d'Endocrinologie Diabétologie et Nutrition, 92100 Boulogne-Billancourt, France.
- [16] Université de Versailles Saint-Quentin-en-Yvelines, 78000 Versailles, France.
- [17] Hôpital Antoine-Béclère, APHP, Service de Médecine Interne, 92140 Clamart, France.
- [18] Hôpital Henri Mondor, APHP, Service de Médecine Interne, 94000 Creteil, France.
- [19] Hôpital Pitié Salpêtrière, APHP, Diabetology Department, 75013 Paris, France.
- [20] Sorbonne Université, University Paris 13, Sorbonne Paris Cité, INSERM UMR_S 1142, 75006 Paris, France.
- [21] Université Paris-Saclay, INRIA, CEA, 91120 Palaiseau, France.
- [22] Hôpital Bichat - Claude-Bernard, APHP, Department of Diabetology, 75018 Paris, France.

- PMID: **34406396**
- PMCID: PMC8195170
- DOI: 10.1210/clinem/dgab393

Abstract

Context: Diabetes is reported as a risk factor for severe coronavirus disease 2019 (COVID-19), but whether this risk is similar in all categories of age remains unclear.

Objective: To investigate the risk of severe COVID-19 outcomes in hospitalized patients with and without diabetes according to age categories.

Design setting and participants: We conducted a retrospective observational cohort study of 6314 consecutive patients hospitalized for COVID-19 between February and 30 June 2020 in the Paris metropolitan area, France; follow-up was recorded until 30 September 2020.

Main outcome measure(s): The main outcome was a composite outcome of mortality and orotracheal intubation in subjects with diabetes compared with subjects without diabetes, after adjustment for confounding variables and according to age categories.

Results: Diabetes was recorded in 39% of subjects. Main outcome was higher in patients with diabetes, independently of confounding variables (hazard ratio [HR] 1.13 [1.03-1.24]) and increased with age in individuals without diabetes, from 23% for those <50 to 35% for those >80 years but reached a plateau after 70 years in those with diabetes. In direct comparison between patients with and without diabetes, diabetes-associated risk was inversely proportional to age, highest in <50 years and similar after 70 years. Similarly, mortality was higher in patients with diabetes (26%) than in those without diabetes (22%, $P < 0.001$), but adjusted HR for diabetes was significant only in patients younger than age 50 years (HR 1.81 [1.14-2.87]).

Conclusions: Diabetes should be considered as an independent risk factor for the severity of COVID-19 in young adults more so than in older adults, especially for individuals younger than 70 years.

Keywords: age; covid-19; diabetes; mortality.

© The Author(s) 2021. Published by Oxford University Press on behalf of the Endocrine Society. All rights reserved. For permissions, please e-mail: journals.permissions@oup.com.

Comment in

- Letter to the Editor From Woolcott and Castilla-Bancayán: "Diabetes Increases Severe COVID-19 Outcomes Primarily in Younger Adults: Age and Diabetes in COVID-19 Severity".
  Woolcott OO, Castilla-Bancayán JP. Woolcott OO, et al. J Clin Endocrinol Metab. 2021 Nov 19;106(12):e5273-e5274. doi: 10.1210/clinem/dgab582. J Clin Endocrinol Metab. 2021. PMID: 34363481 Free PMC article. No abstract available.
- Letter to the Editor From Singhania et al: "Diabetes Increases Severe COVID-19 Outcomes Primarily in Younger Adults".
  Singhania P, Bhattacharjee R. Singhania P, et al. J Clin Endocrinol Metab. 2022 Jan 8:dgab918. doi: 10.1210/clinem/dgab918. Online ahead of print. J Clin Endocrinol Metab. 2022. PMID: 35018443 Free PMC article. No abstract available.

Supplementary info

Publication types, MeSH terms Expand

Publication types

- Observational Study

MeSH terms

- Aged
- Aged, 80 and over
- COVID-19 / epidemiology*
- COVID-19 / virology
- Diabetes Mellitus / physiopathology*
- Female
- France / epidemiology
- Hospital Mortality / trends*
- Hospitalization / statistics & numerical data*
- Humans
- Male
- Middle Aged
- Prognosis
- Retrospective Studies
- Risk Factors
- SARS-CoV-2 / isolation & purification*
- Severity of Illness Index*

Full text links

ENDOCRINE SOCIETY Silverchair Information Systems Free PMC article
Proceed to details
Cite
Share
193
Observational Study
Med Clin (Barc)
. 2021 Aug 13;157(3):99-105.
doi: 10.1016/j.medcli.2021.01.002. Epub 2021 Jan 28.

Application of validated severity scores for pneumonia caused by SARS-CoV-2

[Article in English, Spanish]

Violeta Esteban Ronda [1], Sandra Ruiz Alcaraz [2], Paloma Ruiz Torregrosa [2], Mario Giménez Suau [2], Ester Nofuentes Pérez [2], José Manuel León Ramírez [3], Mariano Andrés [4], Óscar Moreno-Pérez [5], Alfredo Candela Blanes [3], Joan Gil Carbonell [3], Esperanza Merino de Lucas [6]
Affiliations Expand

Affiliations

- [1] Servicio de Neumología, Hospital General Universitario de Alicante, Alicante, España. Electronic address: esteban_vio@gva.es.
- [2] Servicio de Neumología, Hospital General Universitario de Alicante, Alicante, España.
- [3] Servicio de Neumología, Hospital General Universitario de Alicante, Alicante, España; Instituto de Investigación Sanitaria y Biomédica de Alicante (ISABIAL), Hospital General Universitario de Alicante, Alicante, España.
- [4] Servicio de Reumatología, Hospital General Universitario de Alicante, Alicante, España; Instituto de Investigación Sanitaria y Biomédica de Alicante (ISABIAL), Hospital General Universitario de Alicante, Alicante, España; Departamento de Medicina Clínica, Universidad Miguel Hernández de Elche; Hospital General Universitario de Alicante, Elche (Alicante), España.
- [5] Servicio de Endocrinología y Nutrición, Hospital General Universitario de Alicante, Alicante, España; Instituto de Investigación Sanitaria y Biomédica de Alicante (ISABIAL), Hospital General Universitario de Alicante, Alicante, España; Departamento de Medicina Clínica, Universidad Miguel Hernández de Elche; Hospital General Universitario de Alicante, Elche (Alicante), España.
- [6] Unidad de Enfermedades Infecciosas, Hospital General Universitario de Alicante, Alicante, España; Instituto de Investigación Sanitaria y Biomédica de Alicante (ISABIAL), Hospital General Universitario de Alicante, Alicante, España.

- PMID: **33637335**
- PMCID: PMC7843026
- DOI: 10.1016/j.medcli.2021.01.002

Free PMC article
Observational Study

Application of validated severity scores for pneumonia caused by SARS-CoV-2

[Article in English, Spanish]
Violeta Esteban Ronda et al. Med Clin (Barc). 2021.
Free PMC article
Show details
Med Clin (Barc)
. 2021 Aug 13;157(3):99-105.
doi: 10.1016/j.medcli.2021.01.002. Epub 2021 Jan 28.

Authors

Violeta Esteban Ronda [1], Sandra Ruiz Alcaraz [2], Paloma Ruiz Torregrosa [2], Mario Giménez Suau [2], Ester Nofuentes Pérez [2], José Manuel León Ramírez [3], Mariano Andrés [4], Óscar Moreno-Pérez [5], Alfredo Candela Blanes [3], Joan Gil Carbonell [3], Esperanza Merino de Lucas [6]

Affiliations

- [1] Servicio de Neumología, Hospital General Universitario de Alicante, Alicante, España. Electronic address: esteban_vio@gva.es.
- [2] Servicio de Neumología, Hospital General Universitario de Alicante, Alicante, España.
- [3] Servicio de Neumología, Hospital General Universitario de Alicante, Alicante, España; Instituto de Investigación Sanitaria y Biomédica de Alicante (ISABIAL), Hospital General Universitario de Alicante, Alicante, España.
- [4] Servicio de Reumatología, Hospital General Universitario de Alicante, Alicante, España; Instituto de Investigación Sanitaria y Biomédica de Alicante (ISABIAL), Hospital General Universitario de Alicante, Alicante, España; Departamento de Medicina Clínica, Universidad Miguel Hernández de Elche; Hospital General Universitario de Alicante, Elche (Alicante), España.
- [5] Servicio de Endocrinología y Nutrición, Hospital General Universitario de Alicante, Alicante, España; Instituto de Investigación Sanitaria y Biomédica de Alicante (ISABIAL), Hospital General Universitario de Alicante, Alicante, España; Departamento de Medicina Clínica, Universidad Miguel Hernández de Elche; Hospital General Universitario de Alicante, Elche (Alicante), España.
- [6] Unidad de Enfermedades Infecciosas, Hospital General Universitario de Alicante, Alicante, España; Instituto de Investigación Sanitaria y Biomédica de Alicante (ISABIAL), Hospital General Universitario de Alicante, Alicante, España.

- PMID: **33637335**
- PMCID: PMC7843026
- DOI: 10.1016/j.medcli.2021.01.002

Abstract

Objectives: Compare the accuracy of PSI, CURB-65, MuLBSTA and COVID-GRAM prognostic scores to predict mortality, the need for invasive mechanical ventilation in patients with pneumonia caused by SARS-CoV-2 and assess the coexistence of bacterial respiratory tract infection during admission.

Methods: Retrospective observational study that included hospitalized adults with pneumonia caused by SARS-CoV-2 from 15/03 to 15/05/2020. We excluded immunocompromised patients, nursing home residents and those admitted in the previous 14 days for another reasons. Analysis of ROC curves was performed, calculating the area under the curve for the different scales, as well as sensitivity, specificity and predictive values.

Results: A total of 208 patients were enrolled, aged 63±17 years, 57,7% were men; 38 patients were admitted to ICU (23,5%), of these patients 33 required invasive mechanical ventilation (86,8%), with an overall mortality of 12,5%. Area under the ROC curves for mortality of the scores were: PSI 0,82 (95% CI: 0,73-0,91), CURB-65 0,82 (0,73-0,91), MuLBSTA 0,72 (0,62-0,81) and COVID-GRAM 0,86 (0,70-1). Area under the curve for needing invasive mechanical ventilation was: PSI 0,73 (95% CI: 0,64-0,82), CURB-65 0,66 (0,55-0,77), MuLBSTA 0,78 (0,69-0,86) and COVID-GRAM 0,76 (0,67-0,85), respectively. Patients with

bacterial co-infections of the respiratory tract were 20 (9,6%), the most frequent strains being Pseudomonas aeruginosa and Klebsiella pneumoniae.

Conclusions: In our study, the COVID-GRAM score was the most accurate to identify patients with higher mortality with pneumonia caused by SARS-CoV-2; however, none of these scores accurately predicts the need for invasive mechanical ventilation with ICU admission. The 10% of patients admitted presented bacterial respiratory co-infection.

Keywords: COVID-19; Coronavirus; Escalas pronósticas; Neumonía; Penumonia; Severity scores.

Copyright © 2021 Elsevier España, S.L.U. All rights reserved.

- 30 references
- 2 figures

Supplementary info

Publication types, MeSH terms Expand

Publication types

- Observational Study

MeSH terms

- Aged
- COVID-19* / pathology
- Female
- Hospitalization
- Humans
- Male
- Middle Aged
- Pneumonia* / pathology
- Respiration, Artificial
- Retrospective Studies
- Severity of Illness Index

Full text links

Full text at MEDICINA CLINICA Ediciones Doyma, S.L. Free PMC article
Proceed to details
Cite
Share
☐ 194
Observational Study

J Korean Med Sci
. 2021 Nov 15;36(44):e309.
doi: 10.3346/jkms.2021.36.e309.

Maternal and Neonatal Outcomes of Critically Ill Pregnant and Puerperal Patients Diagnosed with COVID-19 Disease: Retrospective Comparative Study

Ali Eman [1], Onur Balaban [2], Havva Kocayiğit [3], Kezban Özmen Süner [4], Y Cırdı [4], Ali Fuat Erdem [2]
Affiliations Expand

Affiliations

- [1] Department of Anesthesiology and Reanimation, Sakarya Training and Research Hospital, Sakarya, Turkey. dralieman02@gmail.com.
- [2] Department of Anesthesiology and Reanimation, Sakarya University Faculty of Medicine, Training and Research Hospital, Sakarya, Turkey.
- [3] Department of Anesthesiology and Reanimation, Sakarya Training and Research Hospital, Sakarya, Turkey.
- [4] Department of Intensive Care, Sakarya Training and Research Hospital, Sakarya, Turkey.

- PMID: **34783218**
- PMCID: PMC8593409
- DOI: 10.3346/jkms.2021.36.e309

Free PMC article
Observational Study

Maternal and Neonatal Outcomes of Critically Ill Pregnant and Puerperal Patients Diagnosed with COVID-19 Disease: Retrospective Comparative Study

Ali Eman et al. J Korean Med Sci. 2021.
Free PMC article
Show details
J Korean Med Sci
. 2021 Nov 15;36(44):e309.
doi: 10.3346/jkms.2021.36.e309.

Authors

Ali Eman [1], Onur Balaban [2], Havva Kocayiğit [3], Kezban Özmen Süner [4], Y Cırdı [4], Ali Fuat Erdem [2]

Affiliations

- [1] Department of Anesthesiology and Reanimation, Sakarya Training and Research Hospital, Sakarya, Turkey. dralieman02@gmail.com.
- [2] Department of Anesthesiology and Reanimation, Sakarya University Faculty of Medicine, Training and Research Hospital, Sakarya, Turkey.
- [3] Department of Anesthesiology and Reanimation, Sakarya Training and Research Hospital, Sakarya, Turkey.
- [4] Department of Intensive Care, Sakarya Training and Research Hospital, Sakarya, Turkey.

- PMID: **34783218**
- PMCID: PMC8593409
- DOI: 10.3346/jkms.2021.36.e309

Abstract

Background: We assessed maternal and neonatal outcomes of critically ill pregnant and puerperal patients in the clinical course of coronavirus disease 2019 (COVID-19).

Methods: Records of pregnant and puerperal women with polymerase chain reaction positive COVID-19 virus who were admitted to our intensive care unit (ICU) from March 2020 to August 2021 were investigated. Demographic, clinical and laboratory data, pharmacotherapy, and neonatal outcomes were analyzed. These outcomes were compared between patients that were discharged from ICU and patients who died in ICU.

Results: Nineteen women were included in this study. Additional oxygen was required in all cases (100%). Eight patients (42%) were intubated and mechanically ventilated. All patients that were mechanically ventilated have died. Increased levels of C-reactive protein (CRP) was seen in all patients (100%). D-dimer values increased in 15 patients (78.9%); interleukin-6 (IL-6) increased in 16 cases (84.2%). Sixteen patients used antiviral drugs. Eleven patients were discharged from the ICU and eight patients have died due to complications of COVID-19 showing an ICU mortality rate of 42.1%. Mean number of hospitalized days in ICU was significantly lower in patients that were discharged ($P = 0.037$). Seventeen patients underwent cesarean-section (C/S) (89.4%). Mean birth week was significantly lower in patients who died in ICU ($P = 0.024$). Eleven preterm (57.8%) and eight term deliveries (42.1%) occurred.

Conclusion: High mortality rate was detected among critically ill pregnant/parturient patients followed in the ICU. Main predictors of mortality were the need of invasive mechanical ventilation and higher number of days hospitalized in ICU. Rate of C/S operations and preterm delivery were high. Pleasingly, the rate of neonatal death was low and no neonatal COVID-19 occurred.

Keywords: COVID-19; Clinical Outcome; Critical Ill; Pregnancy; Puerperium; SARS-CoV-2.

© 2021 The Korean Academy of Medical Sciences.

Conflict of interest statement

The authors have no potential conflicts of interest to disclose.

- 26 references
- 2 figures

Supplementary info

Publication types, MeSH terms, Substances Expand

Publication types

- Comparative Study
- Observational Study

MeSH terms

- Adult
- Antiviral Agents / therapeutic use
- COVID-19 / blood
- COVID-19 / diagnostic imaging
- COVID-19 / mortality*
- COVID-19 / therapy
- Cesarean Section
- Combined Modality Therapy
- Critical Illness / mortality
- Delivery, Obstetric / statistics & numerical data
- Female
- Hospital Mortality
- Humans
- Infant, Newborn
- Intensive Care Units / statistics & numerical data
- Length of Stay / statistics & numerical data
- Lung / diagnostic imaging
- Oxygen Inhalation Therapy
- Pregnancy
- Pregnancy Complications, Infectious / mortality*
- Pregnancy Outcome
- Puerperal Disorders / mortality*
- Respiration, Artificial
- Retrospective Studies
- SARS-CoV-2*

- Treatment Outcome
- Young Adult

Substances

- Antiviral Agents

Full text links

JKMS FULL TEXT FREE Journal of Korean Medical Science Korean Academy of Medical Sciences Free PMC article

Proceed to details

Cite

Share

☐ 195

Observational Study

Clin Microbiol Infect

. 2020 Dec;26(12):1663-1669.

doi: 10.1016/j.cmi.2020.08.011. Epub 2020 Aug 18.

Clinical course and factors associated with outcomes among 1904 patients hospitalized with COVID-19 in Germany: an observational study

Irit Nachtigall [1], Pavlina Lenga [2], Katarzyna Jóźwiak [3], Petra Thürmann [4], Andreas Meier-Hellmann [5], Ralf Kuhlen [6], Joerg Brederlau [7], Torsten Bauer [8], Juergen Tebbenjohanns [9], Karin Schwegmann [10], Michael Hauptmann [3], Julius Dengler [11]

Affiliations Expand

Affiliations

- [1] Department of Preventive Medicine and Hygiene, HELIOS Hospital Bad Saarow, Bad Saarow, Germany; Department of Anaesthesiology and Operative Intensive Care Medicine (CCM, CVK), Charité-Universitätsmedizin Berlin, Berlin, Germany.
- [2] Brandenburg Medical School Theodor Fontane, Campus Bad Saarow, Bad Saarow, Germany; Department of Neurosurgery, HELIOS Hospital Bad Saarow, Bad Saarow, Germany.
- [3] Institute of Biostatistics and Registry Research, Brandenburg Medical School Theodor Fontane, Neuruppin, Germany.
- [4] Philipp Klee-Institute for Clinical Pharmacology, HELIOS University Hospital Wuppertal, Wuppertal, Germany; Department of Clinical Pharmacology, University Witten Herdecke Faculty of Health Witten, Witten, Germany.
- [5] HELIOS Kliniken GmbH, Berlin, Germany.

- [6] HELIOS Health GmbH, Berlin, Germany.
- [7] Department of Intensive Care Medicine, HELIOS Hospital Berlin-Buch, Berlin, Germany.
- [8] Respiratory Diseases Clinic Heckeshorn, Department of Pneumology, HELIOS Hospital Emil von Behring Berlin-Zehlendorf, Berlin, Germany.
- [9] Department of Cardiology, HELIOS Hospital Hildesheim, Hildesheim, Germany.
- [10] Central Department of Hygiene, HELIOS Hospital Hildesheim, Hildesheim, Germany.
- [11] Brandenburg Medical School Theodor Fontane, Campus Bad Saarow, Bad Saarow, Germany; Department of Neurosurgery, HELIOS Hospital Bad Saarow, Bad Saarow, Germany. Electronic address: julius.dengler@helios-gesundheit.de.

- PMID: **32822883**
- PMCID: PMC7434317
- DOI: 10.1016/j.cmi.2020.08.011

Free PMC article
Observational Study

Clinical course and factors associated with outcomes among 1904 patients hospitalized with COVID-19 in Germany: an observational study

Irit Nachtigall et al. Clin Microbiol Infect. 2020 Dec.
Free PMC article
Show details
Clin Microbiol Infect
. 2020 Dec;26(12):1663-1669.
doi: 10.1016/j.cmi.2020.08.011. Epub 2020 Aug 18.

Authors

Irit Nachtigall [1], Pavlina Lenga [2], Katarzyna Jóźwiak [3], Petra Thürmann [4], Andreas Meier-Hellmann [5], Ralf Kuhlen [6], Joerg Brederlau [7], Torsten Bauer [8], Juergen Tebbenjohanns [9], Karin Schwegmann [10], Michael Hauptmann [3], Julius Dengler [11]

Affiliations

- [1] Department of Preventive Medicine and Hygiene, HELIOS Hospital Bad Saarow, Bad Saarow, Germany; Department of Anaesthesiology and Operative Intensive Care Medicine (CCM, CVK), Charité-Universitätsmedizin Berlin, Berlin, Germany.
- [2] Brandenburg Medical School Theodor Fontane, Campus Bad Saarow, Bad Saarow, Germany; Department of Neurosurgery, HELIOS Hospital Bad Saarow, Bad Saarow, Germany.
- [3] Institute of Biostatistics and Registry Research, Brandenburg Medical School Theodor Fontane, Neuruppin, Germany.

- [4] Philipp Klee-Institute for Clinical Pharmacology, HELIOS University Hospital Wuppertal, Wuppertal, Germany; Department of Clinical Pharmacology, University Witten Herdecke Faculty of Health Witten, Witten, Germany.
- [5] HELIOS Kliniken GmbH, Berlin, Germany.
- [6] HELIOS Health GmbH, Berlin, Germany.
- [7] Department of Intensive Care Medicine, HELIOS Hospital Berlin-Buch, Berlin, Germany.
- [8] Respiratory Diseases Clinic Heckeshorn, Department of Pneumology, HELIOS Hospital Emil von Behring Berlin-Zehlendorf, Berlin, Germany.
- [9] Department of Cardiology, HELIOS Hospital Hildesheim, Hildesheim, Germany.
- [10] Central Department of Hygiene, HELIOS Hospital Hildesheim, Hildesheim, Germany.
- [11] Brandenburg Medical School Theodor Fontane, Campus Bad Saarow, Bad Saarow, Germany; Department of Neurosurgery, HELIOS Hospital Bad Saarow, Bad Saarow, Germany. Electronic address: julius.dengler@helios-gesundheit.de.

- PMID: **32822883**
- PMCID: PMC7434317
- DOI: 10.1016/j.cmi.2020.08.011

Abstract

Objectives: In Germany the coronavirus disease 2019 (COVID-19) pandemic situation is unique among large European countries in that incidence and case fatality rate are distinctly lower. We describe the clinical course and examine factors associated with outcomes among patients hospitalized with COVID-19 in Germany.

Methods: In this retrospective cohort study we included patients with COVID-19 admitted to a national network of German hospitals between February 12 and June 12, 2020. We examined demographic characteristics, comorbidities and clinical outcomes.

Results: We included 1904 patients with a median age of 73 years, 48.5% (924/1904) of whom were female. The mortality rate was 17% (317/1835; 95% confidence interval (95%CI) 16-19), the rate of admission to the intensive care unit (ICU) was 21% (399/1860; 95%CI 20-23), and the rate of invasive mechanical ventilation was 14% (250/1850: 95%CI 12-15). The most prominent risk factors for death were male sex (hazard ratio (HR) 1.45; 95%CI 1.15-1.83), pre-existing lung disease (HR 1.61; 95%CI 1.20-2.16), and increased patient age (HR 4.11 (95%CI 2.57-6.58) for age >79 years versus <60 years). Among patients admitted to the ICU, the mortality rate was 29% (109/374; 95%CI 25-34) and higher in ventilated (33% [77/235; 95%CI 27-39]) than in non-ventilated ICU patients (23%, 32/139; 95%CI 16-30; $p < 0.05$).

Conclusions: In this nationwide series of patients hospitalized with COVID-19 in Germany, in-hospital and ICU mortality rates were substantial. The most prominent risk factors for death were male sex, pre-existing lung disease, and greater patient age.

Keywords: COVID-19; Coronavirus; Germany; Observational cohort study; Pandemic.

Copyright © 2020 The Author(s). Published by Elsevier Ltd.. All rights reserved.

- 22 references
- 2 figures

Supplementary info

Publication types, MeSH terms Expand

Publication types

- Multicenter Study
- Observational Study

MeSH terms

- Age Factors
- Aged
- Aged, 80 and over
- COVID-19 / mortality*
- COVID-19 / physiopathology
- Comorbidity
- Critical Care
- Female
- Germany / epidemiology
- Hospital Mortality
- Hospitalization / statistics & numerical data*
- Humans
- Incidence
- Intensive Care Units / statistics & numerical data
- Male
- Middle Aged
- Pandemics / statistics & numerical data*
- Respiration, Artificial / statistics & numerical data
- Retrospective Studies
- Risk Factors
- Sex Factors

Full text links

ELSEVIER OPEN ACCESS Elsevier Science Free PMC article
Proceed to details
Cite
Share
196
Observational Study
Nord J Psychiatry
. 2021 Aug;75(6):465-471.
doi: 10.1080/08039488.2021.1885061. Epub 2021 Feb 25.

Emerging cycloid psychosis episodes during COVID-19 pandemic: a case series

Eloi Giné Servén [1], Maria Martinez Ramirez [1], Ester Boix Quintana [1], Araitz Petrizan Aleman [1], Francisco-Javier Barón Fernández [1], Paloma Fernández Corcuera [1], Maria Serra Buil [1], Josep Cañete Crespillo [1]
Affiliations Expand

Affiliation

- [1] Psychiatry Department, Hospital de Mataró, Consorci Sanitari del Maresme, Mataró, Spain.

- PMID: **33630694**
- DOI: 10.1080/08039488.2021.1885061

Observational Study

Emerging cycloid psychosis episodes during COVID-19 pandemic: a case series

Eloi Giné Servén et al. Nord J Psychiatry. 2021 Aug.
Show details
Nord J Psychiatry
. 2021 Aug;75(6):465-471.
doi: 10.1080/08039488.2021.1885061. Epub 2021 Feb 25.

Authors

Eloi Giné Servén [1], Maria Martinez Ramirez [1], Ester Boix Quintana [1], Araitz Petrizan Aleman [1], Francisco-Javier Barón Fernández [1], Paloma Fernández Corcuera [1], Maria Serra Buil [1], Josep Cañete Crespillo [1]

Affiliation

- [1] Psychiatry Department, Hospital de Mataró, Consorci Sanitari del Maresme, Mataró, Spain.

- PMID: **33630694**
- DOI: 10.1080/08039488.2021.1885061

Abstract

Aims: Cycloid psychosis (CP) is a clinical entity characterized by sudden onset of psychotic polymorphic symptomatology and fluctuant course. It has a reported rate of psychosocial

precipitating factors ranging 30-65%. The aim of the study was to describe all cases of CP, admitted in our Psychiatry ward, during the first two months of the COVID-19 pandemic.

Method: In this retrospective and observational study, we reported a sample of eight patients who were treated as inpatients in the psychiatric ward of our hospital during the first two months of COVID-19 pandemic (mid-March to mid-May 2020) and compared it with previous years. All our patients fulfilled all four Perris & Brockington criteria for CP. We reported the sociodemographic, clinical and biological parameters.

Results: In our sample, all of the patients had maladaptive personality traits; the major external stressing factor was COVID-19; all our patients had short prodromal symptomatology, short Duration of Untreated Psychosis (DUP) and high score at the Positive Scale at Positive and Negative Syndrome Scale (PANSS-P) at hospital admission with the majority showing psychotic symptoms related to the actual COVID-19 pandemic. The predominant treatment during admission was olanzapine and a short time to full remission of psychotic symptoms was observed in all patients.

Conclusion: We found an increase in the admission of patients with CP during the first two months of the actual pandemic. Stress caused by the COVID-19 situation has possibly incremented the frequency of stress-related disorders and it has also influenced its clinical presentation.

Keywords: Atypical psychosis; SARS-CoV-2; epidemic; first psychotic episode; reactive psychosis.

Supplementary info

Publication types, MeSH terms Expand

Publication types

- Observational Study

MeSH terms

- COVID-19*
- Humans
- Pandemics
- Psychotic Disorders* / epidemiology
- Retrospective Studies
- SARS-CoV-2

Full text links

View full text Taylor & Francis

Proceed to details

Cite

Share

☐ 197
Observational Study
Medicina (B Aires)
. 2021;81(3):401-407.

[Clinical characteristics of SARS-CoV-2 disease (COVID-19) in middle-aged patients]

[Article in Spanish]
Ariel A Baletto [1], S Gonzalo Acosta [1], Franco Piasterlini [1], Cecilia Barrios [2] [3] [4], Indalecio Carboni Bisso [2] [5], Eleno Martinez Aquino [1]
Affiliations Expand

Affiliations

- [1] Servicio de Clínica Médica, Sanatorio Franchin, Buenos Aires, Argentina.
- [2] Terapia Intensiva de Adultos, Sanatorio Franchin, Buenos Aires, Argentina.
- [3] Terapia Intensiva de Adultos, Hospital Italiano de Buenos Aires, Buenos Aires, Argentina.
- [4] Terapia Intensiva de Adultos, Hospital Ignacio Pirovano, Buenos Aires, Argentina.
- [5] Terapia Intensiva de Adultos, Hospital Italiano de Buenos Aires, Buenos Aires, Argentina. E-mail: indalecio.carbonibisso@hospitalitaliano.org.ar.

- PMID: **34137700**

Free article
Observational Study

[Clinical characteristics of SARS-CoV-2 disease (COVID-19) in middle-aged patients]

[Article in Spanish]
Ariel A Baletto et al. Medicina (B Aires). 2021.
Free article
Show details
Medicina (B Aires)
. 2021;81(3):401-407.

Authors

Ariel A Baletto [1], S Gonzalo Acosta [1], Franco Piasterlini [1], Cecilia Barrios [2] [3] [4], Indalecio Carboni Bisso [2] [5], Eleno Martinez Aquino [1]

Affiliations

- [1] Servicio de Clínica Médica, Sanatorio Franchin, Buenos Aires, Argentina.

- [2] Terapia Intensiva de Adultos, Sanatorio Franchin, Buenos Aires, Argentina.
- [3] Terapia Intensiva de Adultos, Hospital Italiano de Buenos Aires, Buenos Aires, Argentina.
- [4] Terapia Intensiva de Adultos, Hospital Ignacio Pirovano, Buenos Aires, Argentina.
- [5] Terapia Intensiva de Adultos, Hospital Italiano de Buenos Aires, Buenos Aires, Argentina. E-mail: indalecio.carbonibisso@hospitalitaliano.org.ar.

- PMID: **34137700**

Abstract
in English, Spanish

Coronavirus disease 2019 (COVID-19) meant an unprecedented global crisis, which involved the reorganization of health systems and the rationalization of available diagnostic and therapeutic resources. The objective of this observational and retrospective study was to analyze the clinical characteristics and evolution of patients admitted to general ward, intensive care unit and emergency department of a high complexity hospital in Buenos Aires city, during the first seven months of viral circulation. A total of 1005 patients with laboratory-confirmed COVID-19 were included. The median age was 45 years, and 73.7% were men. Half of the patients had at least one comorbidity. Among the laboratory findings, the median of total leukocytes was 6300 cells/mm3 and that of lymphocytes 818 cells/mm3; 82.3% of the patients presented alterations in the chest tomography, and the most frequently observed radiological pattern was ground-glass opacity (33%); 82.4% of them received empirical antibiotic therapy directed to the respiratory focus and, in addition, 18.7% were treated with dexamethasone. Regarding severity, 14.7% of the patients presented uncomplicated disease, 55.2% mild pneumonia, 20.8% moderate pneumonia, and 9.2% severe pneumonia. Likewise, 8.7% of them were transferred to intensive care. In-hospital mortality was 2.3%, and 20.5% among critically ill patients. A statistically significant association was found between mortality and age, with an age difference of 9.6 years, being greater among the deceased ($p = 0.0004$; 95% CI 4-14). However, there was no association between the presence of comorbidities and sex vs. mortality and severity of the disease.

La pandemia por COVID-19 significó una crisis mundial sin precedentes, que implicó la reorganización de los sistemas de salud y la racionalización de los recursos diagnósticos y terapéuticos disponibles. El objetivo de este estudio observacional y retrospectivo fue analizar características clínicas y evolución de los pacientes internados en guardia, sala general y terapia intensiva en un hospital privado de alta complejidad de la Ciudad de Buenos Aires, durante los primeros siete meses de circulación viral. Se incluyeron 1005 pacientes con COVID-19 confirmado por laboratorio. La mediana de edad fue de 45 años; 73.7% eran varones. La mitad de los pacientes presentaba al menos una comorbilidad. La mediana de leucocitos totales fue 6300 células/mm3 y de linfocitos 818 células/mm3. El 82.3% presentó alteraciones en la tomografía de tórax; y el patrón radiológico observado con mayor frecuencia fue opacidad tipo vidrio esmerilado (33%). El 82.4% recibió antibioticoterapia empírica dirigida a foco respiratorio y, además, el 18.7% fu e tratado con dexametasona. Respecto de la gravedad, el 14.7% presentó enfermedad no complicada, el 55.2% neumonía leve, el 20.8% neumonía moderada y el 9.2% neumonía grave. Asimismo, el 8.7% fue transferido a terapia intensiva. Se registró una mortalidad hospitalaria del 2.3% y del 20.5% en terapia intensiva. Se encontró asociación estadísticamente significativa entre mortalidad y edad, con una diferencia de edad de 9.6 años, siendo mayor entre los fallecidos ($p = 0.0004$; IC 95% 4-14). Sin embargo, no hubo asociación entre presencia de comorbilidades y sexo vs. mortalidad y gravedad de la enfermedad.

Keywords: Argentina; COVID-19; comorbidity; pneumonia; severity of illness index; signs and symptoms.

Supplementary info

Publication types, MeSH terms Expand

Publication types

- Observational Study

MeSH terms

- COVID-19*
- Child
- Comorbidity
- Critical Illness
- Female
- Humans
- Intensive Care Units
- Male
- Middle Aged
- Retrospective Studies
- SARS-CoV-2*

Full text links

Free Full Text medicina Fundacion Revista Medicina (Buenos Aires)
Proceed to details
Cite
Share
198
Observational Study
QJM
. 2022 Feb 21;115(2):77-83.
doi: 10.1093/qjmed/hcab184.

Efficacy of diammonium glycyrrhizinate combined with vitamin C for treating hospitalized COVID-19 patients: a retrospective, observational study

R Tan [1], X Xiang [2], W Chen [3], Z Yang [4], W Hu [5], H Qu [1], J Liu [1]
Affiliations Expand

Affiliations

- [1] From the Department of Critical Care Medicine, Ruijin Hospital, Shanghai Jiao Tong University School of Medicine, Shanghai 200025, China.
- [2] Department of Infectious Diseases, Ruijin Hospital, Shanghai Jiao Tong University School of Medicine, Shanghai 200025, China.
- [3] Department of Pulmonary and Critical Care Medicine, Ruijin Hospital, Shanghai Jiao Tong University School of Medicine, Shanghai 200025, China.
- [4] Department of Emergency, Ruijin Hospital, Shanghai Jiao Tong University School of Medicine, Shanghai 200025, China.
- [5] Department of Surgery, Ruijin Hospital, Shanghai Jiao Tong University School of Medicine, Shanghai 200025, China.

- PMID: **34314507**
- PMCID: PMC8420637
- DOI: 10.1093/qjmed/hcab184

Free PMC article
Observational Study

Efficacy of diammonium glycyrrhizinate combined with vitamin C for treating hospitalized COVID-19 patients: a retrospective, observational study

R Tan et al. QJM. 2022.
Free PMC article
Show details
QJM
. 2022 Feb 21;115(2):77-83.
doi: 10.1093/qjmed/hcab184.

Authors

R Tan [1], X Xiang [2], W Chen [3], Z Yang [4], W Hu [5], H Qu [1], J Liu [1]

Affiliations

- [1] From the Department of Critical Care Medicine, Ruijin Hospital, Shanghai Jiao Tong University School of Medicine, Shanghai 200025, China.
- [2] Department of Infectious Diseases, Ruijin Hospital, Shanghai Jiao Tong University School of Medicine, Shanghai 200025, China.
- [3] Department of Pulmonary and Critical Care Medicine, Ruijin Hospital, Shanghai Jiao Tong University School of Medicine, Shanghai 200025, China.
- [4] Department of Emergency, Ruijin Hospital, Shanghai Jiao Tong University School of Medicine, Shanghai 200025, China.

- [5] Department of Surgery, Ruijin Hospital, Shanghai Jiao Tong University School of Medicine, Shanghai 200025, China.

- PMID: **34314507**
- PMCID: PMC8420637
- DOI: 10.1093/qjmed/hcab184

Abstract

Background: The current global coronavirus disease 2019 (COVID-19) pandemic caused by severe acute respiratory syndrome coronavirus 2 (SARS-CoV-2) has shown limited responses to medical treatments.

Aims: To observe the effect of combination treatment of giammonium glycyrrhizinate and vitamin C (DV) on the prognoses of patients with COVID-19.

Methods: This retrospective observational study recruited 207 COVID-19 patients from Tongji Hospital, patients were assigned to DV and non-DV groups on the basis of the DV treatment. To make the results more credible, a propensity score matching (PSM) approach was adopted at a 1:3 ratio to determine the participants. Logistic analysis was used to assess the effect of DV therapy in the progress of COVID-19.

Results: In the DV group, the new-onset incidence rate of acute respiratory distress syndrome (ARDS) after admission was clearly lower than that in the non-DV group (DV vs. non-DV groups, 15.2% vs. 35.7%; P = 0.002). Compared with the non-DV group, the DV group showed fewer new onset of complications (such as ARDS, acute liver injury and acute myocardial injury) (DV vs. non-DV groups, 19.6% vs. 46.1%; P = 0.000). Moreover, DG+VC may help to recover the count of NK cells and decrease the level of sIL-2R.

Conclusions: DG+VC might be a promising candidate for preventing the deterioration of COVID-19 patients, which is worthy to be studied in large and perspective cohort.

© The Author(s) 2021. Published by Oxford University Press on behalf of the Association of Physicians. All rights reserved. For permissions, please email: journals.permissions@oup.com.

Supplementary info

Publication types, MeSH terms, Substances, Grant support Expand

Publication types

- Observational Study

MeSH terms

- Ascorbic Acid
- COVID-19*
- Glycyrrhizic Acid
- Humans

- Retrospective Studies
- SARS-CoV-2

Substances

- Glycyrrhizic Acid
- Ascorbic Acid

Grant support

- YG2020YQ30/Medical-engineering Cross Foundation of Shanghai Jiao Tong University
- GWV-10.2-XD03/Three-year Plan For Developing a Public Health System of Shanghai, Talent Training
- 81770005/National Natural Science Foundation of China

Full text links

Free PMC article
Proceed to details
Cite
Share
☐ 199
Observational Study
S Afr Med J
. 2020 Aug 31;110(9):910-915.
doi: 10.7196/SAMJ.2020.v110i9.15025.

The effect of COVID-19 on essential surgical admissions in South Africa: A retrospective observational analysis of admissions before and during lockdown at a tertiary healthcare complex

J Moustakis [1], A A Piperidis, A B Ogunrombi
Affiliations Expand

Affiliation

- [1] Johannesburg District Health, Gauteng Department of Health, Johannesburg, South Africa. moustakisy@gmail.com.

- PMID: **32880277**
- DOI: 10.7196/SAMJ.2020.v110i9.15025

Observational Study

The effect of COVID-19 on essential surgical admissions in South Africa: A retrospective observational analysis of admissions before and during lockdown at a tertiary healthcare complex

J Moustakis et al. S Afr Med J. 2020.
Show details
S Afr Med J
. 2020 Aug 31;110(9):910-915.
doi: 10.7196/SAMJ.2020.v110i9.15025.

Authors

J Moustakis[1], A A Piperidis, A B Ogunrombi

Affiliation

- [1] Johannesburg District Health, Gauteng Department of Health, Johannesburg, South Africa. moustakisy@gmail.com.

- PMID: **32880277**
- DOI: 10.7196/SAMJ.2020.v110i9.15025

Abstract

Background: With COVID-19 having spread across the globe, it has become standard to implement infection control strategies (colloquially known as lockdown) with the intention of reducing the magnitude and delaying the peak of the epidemiological curve. Personal infection mitigation strategies coupled with lockdown have caused a change in healthcare-seeking behaviour, with individuals not attending to their ill health as they previously did.

Objectives: To determine whether admissions for urgent and emergency surgical pathologies have declined during the COVID-19 lockdown period, and the magnitude of the decline.

Methods: A retrospective analysis was conducted, comparing pre-lockdown (3 February - 26 March 2020) and lockdown (27 March - 30 April 2020) admission incidences for surgical pathologies at a tertiary healthcare complex in North West Province, South Africa. Poisson regression models were created to determine admission incidence rate ratios (IRRs).

Results: Of 769 surgical admissions included in the analysis, 49.7% were male and 67.2% were unemployed. There was a 44% reduction in the incidence of non-trauma admissions during lockdown (IRR 0.56; 95% confidence interval (CI) 0.47 - 0.68; p<0.001) and a 53% reduction in the incidence of trauma-related admissions (IRR 0.47; 95% CI 0.34 - 0.66; p<0.001).

Conclusions: Even when the prevalence of SARS-CoV-2 infection was minimal, COVID-19 lockdown in North West was associated with a significant reduction in surgical admissions. In order to ensure an overall benefit to public health, a balance between maintaining the integrity of COVID-19 control mechanisms and access to healthcare services is essential.

Supplementary info

Publication types, MeSH terms Expand

Publication types

- Observational Study

MeSH terms

- Adult
- Aged
- Appendicitis / epidemiology*
- Betacoronavirus
- COVID-19
- Coronavirus Infections / epidemiology*
- Emergencies
- Employment / statistics & numerical data
- Female
- Gastrointestinal Hemorrhage / epidemiology
- Hospitalization / statistics & numerical data*
- Humans
- Intestinal Obstruction / epidemiology
- Male
- Middle Aged
- Neoplasms / epidemiology*
- Pandemics
- Pneumonia, Viral / epidemiology*
- Retrospective Studies
- SARS-CoV-2
- Soft Tissue Infections / epidemiology*
- South Africa / epidemiology
- Surgery Department, Hospital*
- Tertiary Care Centers
- Wounds and Injuries / epidemiology*
- Young Adult

Proceed to details

Cite

Share

☐ 200

Observational Study

Neurocrit Care
. 2021 Dec;35(3):693-706.
doi: 10.1007/s12028-021-01220-5. Epub 2021 Mar 16.

Toxic Metabolic Encephalopathy in Hospitalized Patients with COVID-19

Jennifer A Frontera [1], Kara Melmed [2], Taolin Fang [2], Andre Granger [2], Jessica Lin [2], Shadi Yaghi [3], Ting Zhou [2], Ariane Lewis [2], Sebastian Kurz [4], D Ethan Kahn [2], Adam de Havenon [5], Joshua Huang [6], Barry M Czeisler [2], Aaron Lord [2], Sharon B Meropol [7], Andrea B Troxel [7], Thomas Wisniewski [2], Laura Balcer [2], Steven Galetta [2]

Affiliations Expand

Affiliations

- [1] Department of Neurology, New York University Grossman School of Medicine, New York, NY, USA. jennifer.frontera@nyulangone.org.
- [2] Department of Neurology, New York University Grossman School of Medicine, New York, NY, USA.
- [3] Brown University School of Medicine, Providence, RI, USA.
- [4] Department of Medicine, Icahn School of Medicine at Mount Sinai, New York, NY, USA.
- [5] Department of Neurology, University of Utah School of Medicine, Salt Lake City, UT, USA.
- [6] New York University Langone Hospitals, New York, NY, USA.
- [7] Department of Population Health, New York University Grossman School of Medicine, New York, NY, USA.

- PMID: **33725290**
- PMCID: PMC7962078
- DOI: 10.1007/s12028-021-01220-5

Free PMC article

Observational Study

Toxic Metabolic Encephalopathy in Hospitalized Patients with COVID-19

Jennifer A Frontera et al. Neurocrit Care. 2021 Dec.

Free PMC article

Show details

Neurocrit Care
. 2021 Dec;35(3):693-706.

doi: 10.1007/s12028-021-01220-5. Epub 2021 Mar 16.

Authors

Jennifer A Frontera [1], Kara Melmed [2], Taolin Fang [2], Andre Granger [2], Jessica Lin [2], Shadi Yaghi [3], Ting Zhou [2], Ariane Lewis [2], Sebastian Kurz [4], D Ethan Kahn [2], Adam de Havenon [5], Joshua Huang [6], Barry M Czeisler [2], Aaron Lord [2], Sharon B Meropol [7], Andrea B Troxel [7], Thomas Wisniewski [2], Laura Balcer [2], Steven Galetta [2]

Affiliations

- [1] Department of Neurology, New York University Grossman School of Medicine, New York, NY, USA. jennifer.frontera@nyulangone.org.
- [2] Department of Neurology, New York University Grossman School of Medicine, New York, NY, USA.
- [3] Brown University School of Medicine, Providence, RI, USA.
- [4] Department of Medicine, Icahn School of Medicine at Mount Sinai, New York, NY, USA.
- [5] Department of Neurology, University of Utah School of Medicine, Salt Lake City, UT, USA.
- [6] New York University Langone Hospitals, New York, NY, USA.
- [7] Department of Population Health, New York University Grossman School of Medicine, New York, NY, USA.

- PMID: **33725290**
- PMCID: PMC7962078
- DOI: 10.1007/s12028-021-01220-5

Abstract

Background: Toxic metabolic encephalopathy (TME) has been reported in 7-31% of hospitalized patients with coronavirus disease 2019 (COVID-19); however, some reports include sedation-related delirium and few data exist on the etiology of TME. We aimed to identify the prevalence, etiologies, and mortality rates associated with TME in severe acute respiratory syndrome coronavirus 2 (SARS-CoV-2)-positive patients.

Methods: We conducted a retrospective, multicenter, observational cohort study among patients with reverse transcriptase-polymerase chain reaction-confirmed SARS-CoV-2 infection hospitalized at four New York City hospitals in the same health network between March 1, 2020, and May 20, 2020. TME was diagnosed in patients with altered mental status off sedation or after an adequate sedation washout. Patients with structural brain disease, seizures, or primary neurological diagnoses were excluded. The coprimary outcomes were the prevalence of TME stratified by etiology and in-hospital mortality (excluding comfort care only patients) assessed by using a multivariable time-dependent Cox proportional hazards models with adjustment for age, race, sex, intubation, intensive care unit requirement, Sequential Organ Failure Assessment scores, hospital location, and date of admission.

Results: Among 4491 patients with COVID-19, 559 (12%) were diagnosed with TME, of whom 435 of 559 (78%) developed encephalopathy immediately prior to hospital admission. The most common etiologies were septic encephalopathy (n = 247 of 559 [62%]), hypoxic-ischemic encephalopathy (HIE) (n = 331 of 559 [59%]), and uremia (n = 156 of 559 [28%]). Multiple

etiologies were present in 435 (78%) patients. Compared with those without TME (n = 3932), patients with TME were older (76 vs. 62 years), had dementia (27% vs. 3%) or psychiatric history (20% vs. 10%), were more often intubated (37% vs. 20%), had a longer hospital length of stay (7.9 vs. 6.0 days), and were less often discharged home (25% vs. 66% [all P < 0.001]). Excluding comfort care patients (n = 267 of 4491 [6%]) and after adjustment for confounders, TME remained associated with increased risk of in-hospital death (n = 128 of 425 [30%] patients with TME died, compared with n = 600 of 3799 [16%] patients without TME; adjusted hazard ratio [aHR] 1.24, 95% confidence interval [CI] 1.02-1.52, P = 0.031), and TME due to hypoxemia conferred the highest risk (n = 97 of 233 [42%] patients with HIE died, compared with n = 631 of 3991 [16%] patients without HIE; aHR 1.56, 95% CI 1.21-2.00, P = 0.001).

Conclusions: TME occurred in one in eight hospitalized patients with COVID-19, was typically multifactorial, and was most often due to hypoxemia, sepsis, and uremia. After we adjustment for confounding factors, TME was associated with a 24% increased risk of in-hospital mortality.

Keywords: COVID-19; Confusion; Delirium; Encephalopathy; Mental status; SARS-CoV-2.

© 2021. Springer Science+Business Media, LLC, part of Springer Nature and Neurocritical Care Society.

Conflict of interest statement

LB reports grant support from NIH/NIA grant 3P30AG066512-01S1, outside the submitted work; JAF, ABT, SBM and SY report grant support from NIH/NIA grant 3P30AG066512-01S1 and NIH/NINDS grant 3U24NS11384401S1, outside the submitted work; TW reports grant support from NIH/NIA grant 3P30AG066512-01S1, outside the submitted work; the other authors have nothing to disclose.

- 49 references
- 3 figures

Supplementary info

Publication types, MeSH terms, Grant support Expand

Publication types

- Multicenter Study
- Observational Study
- Research Support, N.I.H., Extramural

MeSH terms

- Brain Diseases*
- Brain Diseases, Metabolic*
- COVID-19*
- Hospital Mortality
- Hospitalization
- Humans

- Retrospective Studies
- SARS-CoV-2

Grant support

- P30 AG066512/AG/NIA NIH HHS/United States
- 3U24NS11384401S1/NS/NINDS NIH HHS/United States
- 3P30AG066512-01S1/AG/NIA NIH HHS/United States

Full text links

SpringerLink FULL-TEXT ARTICLE Springer Free PMC article
Proceed to details
Cite
Share
1,388 results
Show more results
[x]
Cite
Copy
Download .nbib
Format: NLM
[x]
Share

-
-

Permalink
Copy
first first First previous previous Prev
Page
1
of 7
Next next next Last last last

Send To

- Clipboard
- Email
- Save
- My Bibliography
- Collections
- Citation Manager

[x]

- Article type

- Species
- Language
- Sex
- Journal
- Age

- ☐ Address
- ☐ Autobiography
- ☐ Bibliography
- ☐ Biography
- ☐ Case Reports
- ☐ Classical Article
- ☐ Clinical Conference
- ☐ Clinical Study
- ☐ Clinical Trial Protocol
- ☐ Clinical Trial, Phase I
- ☐ Clinical Trial, Phase II
- ☐ Clinical Trial, Phase III
- ☐ Clinical Trial, Phase IV
- ☐ Clinical Trial, Veterinary
- ☐ Comment
- ☐ Comparative Study
- ☐ Congress
- ☐ Consensus Development Conference
- ☐ Consensus Development Conference, NIH
- ☐ Controlled Clinical Trial
- ☐ Corrected and Republished Article
- ☐ Dataset
- ☐ Dictionary
- ☐ Directory
- ☐ Duplicate Publication
- ☐ Editorial
- ☐ Electronic Supplementary Materials
- ☐ English Abstract
- ☐ Evaluation Study
- ☐ Festschrift
- ☐ Government Publication
- ☐ Guideline
- ☐ Historical Article
- ☐ Interactive Tutorial
- ☐ Interview
- ☐ Introductory Journal Article
- ☐ Lecture
- ☐ Legal Case
- ☐ Legislation
- ☐ Letter
- ☐ Multicenter Study
- ☐ News
- ☐ Newspaper Article

- ☐ Observational Study
- ☐ Observational Study, Veterinary
- ☐ Overall
- ☐ Patient Education Handout
- ☐ Periodical Index
- ☐ Personal Narrative
- ☐ Portrait
- ☐ Practice Guideline
- ☐ Pragmatic Clinical Trial
- ☐ Preprint
- ☐ Published Erratum
- ☐ Research Support, American Recovery and Reinvestment Act
- ☐ Research Support, N.I.H., Extramural
- ☐ Research Support, N.I.H., Intramural
- ☐ Research Support, Non-U.S. Gov't
- ☐ Research Support, U.S. Gov't, Non-P.H.S.
- ☐ Research Support, U.S. Gov't, P.H.S.
- ☐ Research Support, U.S. Gov't
- ☐ Retracted Publication
- ☐ Retraction of Publication
- ☐ Scientific Integrity Review
- ☐ Technical Report
- ☐ Twin Study
- ☐ Validation Study
- ☐ Video-Audio Media
- ☐ Webcast

- ☐ Humans
- ☐ Other Animals

- ☐ Afrikaans
- ☐ Albanian
- ☐ Arabic
- ☐ Armenian
- ☐ Azerbaijani
- ☐ Bosnian
- ☐ Bulgarian
- ☐ Catalan
- ☐ Chinese
- ☐ Croatian
- ☐ Czech
- ☐ Danish
- ☐ Dutch
- ☐ English
- ☐ Esperanto
- ☐ Estonian
- ☐ Finnish
- ☐ French
- ☐ Georgian

- ☐ German
- ☐ Greek, Modern
- ☐ Hebrew
- ☐ Hindi
- ☐ Hungarian
- ☐ Icelandic
- ☐ Indonesian
- ☐ Italian
- ☐ Japanese
- ☐ Kinyarwanda
- ☐ Korean
- ☐ Latin
- ☐ Latvian
- ☐ Lithuanian
- ☐ Macedonian
- ☐ Malay
- ☐ Malayalam
- ☐ Maori
- ☐ Multiple Languages
- ☐ Norwegian
- ☐ Persian
- ☐ Polish
- ☐ Portuguese
- ☐ Pushto
- ☐ Romanian
- ☐ Russian
- ☐ Sanskrit
- ☐ Scottish gaelic
- ☐ Serbian
- ☐ Slovak
- ☐ Slovenian
- ☐ Spanish
- ☐ Swedish
- ☐ Thai
- ☐ Turkish
- ☐ Ukrainian
- ☐ Undetermined
- ☐ Vietnamese
- ☐ Welsh

- ☐ Female
- ☐ Male

- ☐ MEDLINE

- ☐ Child: birth-18 years
- ☐ Newborn: birth-1 month
- ☐ Infant: birth-23 months
- ☐ Infant: 1-23 months

- ☐ Preschool Child: 2-5 years
- ☐ Child: 6-12 years
- ☐ Adolescent: 13-18 years
- ☐ Adult: 19+ years
- ☐ Young Adult: 19-24 years
- ☐ Adult: 19-44 years
- ☐ Middle Aged + Aged: 45+ years
- ☐ Middle Aged: 45-64 years
- ☐ Aged: 65+ years
- ☐ 80 and over: 80+ years

Cancel Show
Close dialog
Back to Top
Jump to page Close dialog
2 of 7
Jump

NCBI Literature Resources

MeSH PMC Bookshelf Disclaimer

Follow NCBI

[Connect with NLM]

-
-
-

National Library of Medicine
8600 Rockville Pike
Bethesda, MD 20894

Web Policies
FOIA
HHS Vulnerability Disclosure

[Help](#)
[Accessibility](#)
[Careers](#)

- NLM
- NIH
- HHS
- USA.gov

ERREUR [illegible]
du site :
Domaine
[illegible]
