# Supplementary material for: COVID-19 treatment of hospital patients worldwide at the onset of the pandemic in 2020: a systematic review
Source: BMC Infect Dis. 2025 Dec 17;26:107. doi: 10.1186/s12879-025-12368-2 (PMC12822144; doi:10.1186/s12879-025-12368-2)
Supplement: Supplementary file 4 — Supplementary Material 4 [file 12879_2025_12368_MOESM4_ESM.zip › 12879_2025_12368_MOESM4_ESM/Search Pubmed 2022 03 28 retrospective observational study hospital treatment covid 401-600.pdf]

Skip to main page content

COVID-19 Information

Public health information (CDC)

Research information (NIH)

SARS-CoV-2 data (NCBI)

Prevention and treatment information (HHS)

Español

Close

Account

Logged in as:
username

- Dashboard
- Publications
- Account settings
- Log out

Access keys NCBI Homepage MyNCBI Homepage Main Content Main Navigation

Search Page

pubmed

Search: retrospective observation Search
Advanced Create alert Create RSS Clipboard
User Guide
Filters 0 Timeline
Sorted by: Best match
Sorted by: Best match Display options
Save Email
Send to

Save citations to file

Selection: All results on this page

Format: Summary (text)
Create file Cancel

# Email citations

Subject: retrospective observational study hospital treatm - PubMed
To: antoine.bosquet@lmr.apl
Selection: All results on this page
Format: Summary
MeSH and other data
Send email Cancel

Send citations to clipboard

Selection: All results on this page
Send Cancel

Add to Collections

Selection: All results on this page

- Create a new collection
- Add to an existing collection

Name your collection:
Name must be less than 100 characters
Choose a collection:
Unable to load your collection due to an error
Please try again
Add Cancel

Add to My Bibliography

Selection: All results on this page

- My Bibliography

Unable to load your delegates due to an error
Please try again
Add Cancel

Create a file for external citation management software

Selection: All results on this page
Create file Cancel

Your saved search

Name of saved search: retrospective observation

Search terms: retrospective observational study

Test search terms

Would you like email updates of new search results?

Saved Search Alert Radio Buttons

- Yes
- No

Email: antoine.bosquet@lmr.aphp.fr (change)

Frequency: Monthly

Which day? The first Sunday

Which day? Sunday

Report format: Summary

Send at most: 5 items

Send even when there aren't any new results

Optional text in email:

Save Cancel

Your RSS Feed

Name of RSS Feed: retrospective observation

Number of items displayed: 15

Create RSS Cancel

RSS Link Your RSS Feed Link Copy

My NCBI Filters

- All (1,388)
- Assistance Publique Hopitaux de Paris (0)
- clinical trial (17)
- Review (1)

Show Fewer

Results by year Expand/collapse timeline

Reset

Table representation of search results timeline featuring number of search results per year.

Year	Number of Results
2020	548
2021	893
2022	147

Text availability

- Abstract
- Free full text
- Full text

Article attribute

- Associated data

Article type

- Books and Documents
- Clinical Trial
- Meta-Analysis
- Randomized Controlled Trial
- Review
- Systematic Review

Publication date

- 1 year
- 5 years
- 10 years
- Custom Range

Additional filters Reset all filters

Search Results

clear all

1,388 results

first first previous previous

Page

3

of 7

next next last last

Use COVID-19 filters from PubMed Clinical Queries to refine your search

- Treatment
- Mechanism
- Transmission
- More filters

See more SARS-CoV-2 literature, sequence, and clinical content from NCBI

Results by year Expand/collapse timeline

Reset

Filters applied: . *Clear all* Select search result to email or save

Page 3

☐ 401

Observational Study

Med Sci Monit

. 2020 Sep 26;26:e926974.

doi: 10.12659/MSM.926974.

Intensive Care Unit Admissions During the First 3 Months of the COVID-19 Pandemic in Poland: A Single-Center, Cross-Sectional Study

Izabela Kokoszka-Bargieł [1], Paweł Cyprys [2], Katarzyna Rutkowska [3], Jarosław Madowicz [4] [5], Piotr Knapik [3]

Affiliations Expand

Affiliations

- [1] Department of Anesthesiology and Intensive Therapy, Provincial Specialist Hospital, Tychy, Poland.
- [2] Students' Scientific Society, Department of Cardiac Anesthesia and Intensive Care, Medical University of Silesia, Katowice, Poland.
- [3] Department of Anesthesiology, Intensive Therapy and Emergency Medicine, Silesian Centre for Heart Diseases in Zabrze, Medical University of Silesia, Zabrze, Poland.
- [4] Provincial Specialist Hospital, Tychy, Poland.
- [5] Department of Health Sciences, Higher School of Strategic Planning, Dąbrowa Górnicza, Poland.

- PMID: **32979262**
- PMCID: PMC7526339
- DOI: 10.12659/MSM.926974

Free PMC article
Observational Study

Intensive Care Unit Admissions During the First 3 Months of the COVID-19 Pandemic in Poland: A Single-Center, Cross-Sectional Study

Izabela Kokoszka-Bargieł et al. Med Sci Monit. 2020.
Free PMC article
Show details
Med Sci Monit
. 2020 Sep 26;26:e926974.
doi: 10.12659/MSM.926974.

Authors

Izabela Kokoszka-Bargieł [1], Paweł Cyprys [2], Katarzyna Rutkowska [3], Jarosław Madowicz [4] [5], Piotr Knapik [3]

Affiliations

- [1] Department of Anesthesiology and Intensive Therapy, Provincial Specialist Hospital, Tychy, Poland.
- [2] Students' Scientific Society, Department of Cardiac Anesthesia and Intensive Care, Medical University of Silesia, Katowice, Poland.
- [3] Department of Anesthesiology, Intensive Therapy and Emergency Medicine, Silesian Centre for Heart Diseases in Zabrze, Medical University of Silesia, Zabrze, Poland.
- [4] Provincial Specialist Hospital, Tychy, Poland.
- [5] Department of Health Sciences, Higher School of Strategic Planning, Dąbrowa Górnicza, Poland.

- PMID: **32979262**
- PMCID: PMC7526339
- DOI: 10.12659/MSM.926974

Abstract

BACKGROUND Data on the outcomes of patients with coronavirus disease 2019 (COVID-19) requiring Intensive Care Unit (ICU) care in Poland are limited. There are no data on critically ill patients with COVID-19 who did not meet criteria for ICU admission. MATERIAL AND METHODS We analyzed patients admitted to the ICU and those ineligible for ICU admission in a large COVID-19-dedicated hospital, during the first 3 months of the pandemic in Poland. Data from 67 patients considered for ICU admissions due to COVID-19 infection, treated between 10 March and 10 June 2020, were reviewed. Following exclusions, data on 32 patients admitted to the ICU and 21 patients ineligible for ICU admission were analyzed. RESULTS In 38% of

analyzed patients, symptoms of COVID-19 infection occurred during a hospital stay for an unrelated medical issue. The mean age of ICU patients was 62.4 (10.4) years, and the majority of patients were male (69%), with at least one comorbidity (88%). The mean admission APACHE II and SAPS II scores were 20.1 (8.1) points and 51.2 (15.3) points, respectively. The Charlson Comorbidity Index and Clinical Frailty Scale were lower in ICU patients compared with those disqualified: 5.9 (4.3) vs. 9.1 (3.5) points, P=0.01, and 4.7 (1.7) vs. 6.9 (1.2) points, P<0.01, respectively. All ICU patients required intubation and mechanical ventilation. ICU mortality was 67%. Hospital mortality among patients admitted to the ICU and those who were disqualified was 70% and 79%, respectively. CONCLUSIONS Patients with COVID-19 requiring ICU admission in our studied population were frail and had significant comorbidities. The outcomes in this group were poor and did not seem to be influenced by ICU admission.

Conflict of interest statement

Conflict of interest

None.

- Cited by 12 articles
- 39 references
- 1 figure

Supplementary info

Publication types, MeSH terms Expand

Publication types

- Observational Study

MeSH terms

- Aged
- Betacoronavirus
- COVID-19
- Comorbidity
- Coronavirus Infections / epidemiology*
- Coronavirus Infections / therapy
- Cross-Sectional Studies
- Female
- Health Status Indicators
- Hospital Mortality
- Hospitals, State / statistics & numerical data
- Humans
- Intensive Care Units / statistics & numerical data*
- Intubation, Intratracheal
- Male

- Middle Aged
- Pandemics*
- Patient Admission / statistics & numerical data*
- Pneumonia, Viral / epidemiology*
- Pneumonia, Viral / therapy
- Poland / epidemiology
- Respiration, Artificial
- Retrospective Studies
- SARS-CoV-2
- Survivors
- Treatment Outcome

Full text links

International Scientific Literature, Ltd. Free PMC article
Proceed to details
Cite
Share
402
Comment
Intern Med J
. 2021 Nov;51(11):1810-1815.
doi: 10.1111/imj.15345.

Six-month respiratory outcomes and exercise capacity of COVID-19 acute respiratory failure patients treated with continuous positive airway pressure

Sarah Damanti [1], Giuseppe Alvise Ramirez [2], Enrica Paola Bozzolo [1], Patrizia Rovere-Querini [3] [4], Rebecca De Lorenzo [3], Cristiano Magnaghi [4], Raffaella Scotti [1], Giuseppe Di Lucca [1], Alessandro Marinosci [1], Silvia Strada [1], Gaetano Di Terlizzi [1], Giordano Vitali [5], Sabina Martinenghi [5] [6], Nicola Compagnone [3], Giovanni Landoni [7], Moreno Tresoldi [1]
Affiliations Expand

Affiliations

- [1] Unit of General Medicine and Advanced Care, IRCCS San Raffaele Scientific Institute, Milan, Italy.
- [2] Unit of Immunology, Rheumatology, Allergy and Rare Diseases, IRCCS San Raffaele Scientific Institute, Milan, Italy.
- [3] Division of Medicine, Vita-Salute San Raffaele University, Milan, Italy.

- [4] Division of Immunology, Transplantation and Infectious Diseases, IRCCS San Raffaele Scientific Institute, Milan, Italy.
- [5] Internal Medicine, Diabetes and Endocrinology Unit, San Raffaele Hospital and Scientific Institute, Milan, Italy.
- [6] Diabetes Research Institute, San Raffaele Hospital and Scientific Institute, San Raffaele Vita Salute University, Milan, Italy.
- [7] Department of Anesthesia and Intensive Care, IRCCS San Raffaele Scientific Institute, Milan, Italy.

- PMID: **33961728**
- PMCID: PMC8242499
- DOI: 10.1111/imj.15345

Free PMC article
Comment

Six-month respiratory outcomes and exercise capacity of COVID-19 acute respiratory failure patients treated with continuous positive airway pressure

Sarah Damanti et al. Intern Med J. 2021 Nov.
Free PMC article
Show details
Intern Med J
. 2021 Nov;51(11):1810-1815.
doi: 10.1111/imj.15345.

Authors

Sarah Damanti [1], Giuseppe Alvise Ramirez [2], Enrica Paola Bozzolo [1], Patrizia Rovere-Querini [3] [4], Rebecca De Lorenzo [3], Cristiano Magnaghi [4], Raffaella Scotti [1], Giuseppe Di Lucca [1], Alessandro Marinosci [1], Silvia Strada [1], Gaetano Di Terlizzi [1], Giordano Vitali [5], Sabina Martinenghi [5] [6], Nicola Compagnone [3], Giovanni Landoni [7], Moreno Tresoldi [1]

Affiliations

- [1] Unit of General Medicine and Advanced Care, IRCCS San Raffaele Scientific Institute, Milan, Italy.
- [2] Unit of Immunology, Rheumatology, Allergy and Rare Diseases, IRCCS San Raffaele Scientific Institute, Milan, Italy.
- [3] Division of Medicine, Vita-Salute San Raffaele University, Milan, Italy.
- [4] Division of Immunology, Transplantation and Infectious Diseases, IRCCS San Raffaele Scientific Institute, Milan, Italy.
- [5] Internal Medicine, Diabetes and Endocrinology Unit, San Raffaele Hospital and Scientific Institute, Milan, Italy.

- [6] Diabetes Research Institute, San Raffaele Hospital and Scientific Institute, San Raffaele Vita Salute University, Milan, Italy.
- [7] Department of Anesthesia and Intensive Care, IRCCS San Raffaele Scientific Institute, Milan, Italy.

- PMID: **33961728**
- PMCID: PMC8242499
- DOI: 10.1111/imj.15345

Abstract

Background: COVID-19 long-term sequelae are ill-defined since only a few studies have explored the long-term consequences of this disease so far.

Aims: To evaluate the 6-month respiratory outcome and exercise capacity of COVID-19 acute respiratory failure (ARF) patients treated with continuous positive airway pressure (CPAP) during the first wave of the ongoing COVID-19 pandemic.

Methods: A retrospective observational study included COVID-19 patients with ARF. Interventions included CPAP during hospitalisation and 6-month follow up. Frailty assessment was carried out through frailty index (FI), pO_2 /FiO_2 during hospitalisation and at follow up, respiratory parameters, 6-min walking test (6MWT) and the modified British Medical Research Council (mMRC) and Borg scale at follow up.

Results: More than half of the patients had no dyspnoea according to the mMRC scale. Lower in-hospital pO_2 /FiO_2 correlated with higher Borg scale levels after 6MWT (ρ 0.27; P 0.04) at the follow-up visit. FI was positively correlated with length of hospitalisation (ρ 0.3; P 0.03) and negatively with the 6MWT distance walked (ρ -0.36; P 0.004).

Conclusions: Robust and frail patients with COVID-19 ARF treated with CPAP outside the intensive care unit setting had good respiratory parameters and exercise capacity at 6-month follow up, although more severe patients had slightly poorer respiratory performance compared with patients with higher PaO_2 /FiO_2 and lower FI.

Keywords: COVID-19; acute respiratory failure; exercise capacity; follow up.

© 2021 Royal Australasian College of Physicians.

Comment on

- 6-month consequences of COVID-19 in patients discharged from hospital: a cohort study.
  Huang C, Huang L, Wang Y, Li X, Ren L, Gu X, Kang L, Guo L, Liu M, Zhou X, Luo J, Huang Z, Tu S, Zhao Y, Chen L, Xu D, Li Y, Li C, Peng L, Li Y, Xie W, Cui D, Shang L, Fan G, Xu J, Wang G, Wang Y, Zhong J, Wang C, Wang J, Zhang D, Cao B. Huang C, et al. Lancet. 2021 Jan 16;397(10270):220-232. doi: 10.1016/S0140-6736(20)32656-8. Epub 2021 Jan 8. Lancet. 2021. PMID: 33428867 Free PMC article.

- Cited by 4 articles
- 37 references

Supplementary info

Publication types, MeSH terms Expand

Publication types

- Comment

MeSH terms

- COVID-19*
- Continuous Positive Airway Pressure
- Exercise Tolerance
- Humans
- Pandemics
- Respiratory Insufficiency* / epidemiology
- Respiratory Insufficiency* / therapy
- Retrospective Studies
- SARS-CoV-2

Full text links

WILEY Full Text Article Wiley Free PMC article
Proceed to details
Cite
Share
☐ 403
Observational Study
Ethiop J Health Sci
. 2021 Jul;31(4):699-708.
doi: 10.4314/ejhs.v31i4.3.

Duration of Supplemental Oxygen Requirement and Predictors in Severe COVID-19 Patients in Ethiopia: A Survival Analysis

Tigist W Leulseged [1], Ishmael S Hassen [1], Mesay G Edo [1], Daniel S Abebe [1], Endalkachew H Maru [1], Wuletaw C Zewde [1], Negat W Chamiso [1], Tariku B Jagema [1]
Affiliations Expand

Affiliation

- [1] Millennium COVID-19 Care Center, St. Paul's Hospital Millennium Medical College, Addis Ababa, Ethiopia.

- PMID: **34703168**
- PMCID: PMC8512943
- DOI: 10.4314/ejhs.v31i4.3

Free PMC article
Observational Study

Duration of Supplemental Oxygen Requirement and Predictors in Severe COVID-19 Patients in Ethiopia: A Survival Analysis

Tigist W Leulseged et al. Ethiop J Health Sci. 2021 Jul.
Free PMC article
Show details
Ethiop J Health Sci
. 2021 Jul;31(4):699-708.
doi: 10.4314/ejhs.v31i4.3.

Authors

Tigist W Leulseged [1], Ishmael S Hassen [1], Mesay G Edo [1], Daniel S Abebe [1], Endalkachew H Maru [1], Wuletaw C Zewde [1], Negat W Chamiso [1], Tariku B Jagema [1]

Affiliation

- [1] Millennium COVID-19 Care Center, St. Paul's Hospital Millennium Medical College, Addis Ababa, Ethiopia.

- PMID: **34703168**
- PMCID: PMC8512943
- DOI: 10.4314/ejhs.v31i4.3

Abstract

Background: With the rising number of new cases of COVID-19, understanding the oxygen requirement of severe patients assists in identifying at risk groups and in making an informed decision on building hospitals capacity in terms of oxygen facility arrangement. Therefore, the study aimed to estimate time to getting off supplemental oxygen therapy and identify predictors among COVID-19 patients admitted to Millennium COVID-19 Care Center in Ethiopia.

Methods: A prospective observational study was conducted among 244 consecutively admitted COVID-19 patients from July to September, 2020. Kaplan Meier plots, median survival times and Log-rank test were used to describe the data and compare survival distribution between groups.

Cox proportional hazard survival model was used to identify determinants of time to getting off supplemental oxygen therapy, where hazard ratio (HR), P-value and 95%CI for HR were used for testing significance and interpretation of results.

Results: Median time to getting off supplemental oxygen therapy among the studied population was 6 days (IQR,4.3-20.0). Factors that affect time to getting off supplemental oxygen therapy were age group (AHR=0.52,95%CI=0.32,0.84, p-value=0.008 for ≥70 years) and shortness of breath (AHR=0.71,95%CI=0.52,0.96, p-value=0.026).

Conclusion: Average duration of supplemental oxygen therapy requirement among COVID-19 patients was 6 days and being 70 years and older and having shortness of breath were found to be associated with prolonged duration of supplemental oxygen therapy requirement. This result can be used as a guide in planning institutional resource allocation and patient management to provide a well-equipped care to prevent complications and death from the disease.

Keywords: COVID-19; Ethiopia; predictors; prospective observational study; supplemental oxygen; survival analysis.

© 2021 Tigist W. Leulseged.

- Cited by 5 articles
- 22 references
- 2 figures

Supplementary info

Publication types, MeSH terms, Substances Expand

Publication types

- Observational Study

MeSH terms

- Aged
- COVID-19*
- Ethiopia
- Humans
- Oxygen
- Retrospective Studies
- SARS-CoV-2
- Survival Analysis

Substances

- Oxygen

Full text links

Free PMC article
Proceed to details
Cite
Share
☐ 404
Observational Study
Respir Res
. 2021 Jan 15;22(1):16.
doi: 10.1186/s12931-021-01613-2.

More skilled clinical management of COVID-19 patients modified mortality in an intermediate respiratory intensive care unit in Italy

Giovanna E Carpagnano [1], Giovanni Migliore [2], Salvatore Grasso [3], Vito Procacci [4], Emanuela Resta [5], Francesco Panza [6], Onofrio Resta [7]
Affiliations Expand

Affiliations

- [1] Department of Basic Medical Science, Institute of Respiratory Disease, Neuroscience, and Sense Organs, University of Bari "Aldo Moro", Bari, Italy.
- [2] General Direction, Policlinico Hospital, Bari, Italy.
- [3] Department of Emergency and Organ Transplantation, Section of Anesthesia and Intensive Care, University of Bari "Aldo Moro", Bari, Italy.
- [4] Emergency Department, Policlinico Hospital, Bari, Italy.
- [5] Translational Medicine and Health System Management, University of Foggia, Foggia, Italy.
- [6] Population Health Unit, Healthy Aging Phenotypes Research Unit, "Salus in Apulia Study", National Institute of Gastroenterology "Saverio de Bellis", Research Hospital, Castellana Grotte, Bari, Italy. f_panza@hotmail.com.
- [7] Department of Basic Medical Science, Institute of Respiratory Disease, Neuroscience, and Sense Organs, University of Bari "Aldo Moro", Bari, Italy. onofrio.resta@uniba.it.

- PMID: **33451327**
- PMCID: PMC7809547
- DOI: 10.1186/s12931-021-01613-2

Free PMC article
Observational Study

More skilled clinical management of COVID-19 patients modified mortality in an intermediate respiratory intensive care unit in Italy

Giovanna E Carpagnano et al. Respir Res. 2021.
Free PMC article
Show details
Respir Res
. 2021 Jan 15;22(1):16.
doi: 10.1186/s12931-021-01613-2.

Authors

Giovanna E Carpagnano [1], Giovanni Migliore [2], Salvatore Grasso [3], Vito Procacci [4], Emanuela Resta [5], Francesco Panza [6], Onofrio Resta [7]

Affiliations

- [1] Department of Basic Medical Science, Institute of Respiratory Disease, Neuroscience, and Sense Organs, University of Bari "Aldo Moro", Bari, Italy.
- [2] General Direction, Policlinico Hospital, Bari, Italy.
- [3] Department of Emergency and Organ Transplantation, Section of Anesthesia and Intensive Care, University of Bari "Aldo Moro", Bari, Italy.
- [4] Emergency Department, Policlinico Hospital, Bari, Italy.
- [5] Translational Medicine and Health System Management, University of Foggia, Foggia, Italy.
- [6] Population Health Unit, Healthy Aging Phenotypes Research Unit, "Salus in Apulia Study", National Institute of Gastroenterology "Saverio de Bellis", Research Hospital, Castellana Grotte, Bari, Italy. f_panza@hotmail.com.
- [7] Department of Basic Medical Science, Institute of Respiratory Disease, Neuroscience, and Sense Organs, University of Bari "Aldo Moro", Bari, Italy. onofrio.resta@uniba.it.

- PMID: **33451327**
- PMCID: PMC7809547
- DOI: 10.1186/s12931-021-01613-2

Abstract

Background: Some studies investigated epidemiological and clinical features of laboratory-confirmed patients with severe acute respiratory syndrome coronavirus 2 (SARS-CoV-2) the virus causing coronavirus disease 2019 (COVID-19), but limited attention has been paid to the follow-up of hospitalized patients on the basis of clinical setting and the expertise of clinical management.

Methods: In the present single-centered, retrospective, observational study, we reported findings from 87 consecutive laboratory-confirmed COVID-19 patients with moderate-to-severe acute respiratory syndrome hospitalized in an intermediate Respiratory Intensive Care Unit (RICU), subdividing the patients in two groups according to the admission date (before and after March 29, 2020).

Results: With improved skills in the clinical management of COVID-19, we observed a significant lower mortality in the T2 group compared with the T1 group and a significantly difference in terms of mortality among the patients transferred in Intensive Care Unit (ICU) from our intermediate RICU (100% in T1 group vs. 33.3% in T2 group). The average length of stay in intermediate RICU of ICU-transferred patients who survived in T1 and T2 was significantly longer than those who died (who died 3.3 ± 2.8 days vs. who survived 6.4 ± 3.3 days).

T conclusions: The present findings suggested that an intermediate level of hospital care may have the potential to modify survival in COVID-19 patients, particularly in the present phase of a more skilled clinical management of the pandemic.

Keywords: Assisted ventilation; Critical care; Intermediate RICU; Italy; Pandemic; Survival.

Conflict of interest statement

None reported.

- Cited by 5 articles
- 26 references

Supplementary info

Publication types, MeSH terms Expand

Publication types

- Comparative Study
- Observational Study

MeSH terms

- Aged
- Aged, 80 and over
- COVID-19 / diagnosis
- COVID-19 / mortality
- COVID-19 / therapy*
- Clinical Competence*
- Critical Care*
- Female
- Hospital Mortality
- Humans
- Intensive Care Units*

- Italy
- Length of Stay
- Male
- Middle Aged
- Patient Admission
- Retrospective Studies
- Severity of Illness Index
- Time Factors
- Treatment Outcome

Full text links

Read free full text at BMC BioMed Central Free PMC article
Proceed to details
Cite
Share
405
Observational Study

J Neurol Sci
. 2020 Dec 15;419:117183.
doi: 10.1016/j.jns.2020.117183. Epub 2020 Oct 14.

Cerebral venous sinus thrombosis associated with SARS-CoV-2; a multinational case series

Ashkan Mowla [1], Banafsheh Shakibajahromi [2], Shima Shahjouei [3], Afshin Borhani-Haghighi [4], Nasrin Rahimian [5], Humain Baharvahdat [6], Soheil Naderi [7], Fariborz Khorvash [8], Davar Altafi [9], Seyed Amir Ebrahimzadeh [10], Ghasem Farahmand [11], Alaleh Vaghefi Far [11], Vijay K Sharma [12], Saeideh Aghayari Sheikh Neshin [13], Georgios Tsivgoulis [14], Ramin Zand [15]
Affiliations Expand

Affiliations

- [1] Division of Stroke and Endovascular Neurosurgery, Department of Neurological Surgery, Keck School of Medicine, University of Southern California, California, USA.
- [2] Clinical Neurology Research Center, Shiraz University of Medical Sciences, Shiraz, Iran.
- [3] Neurology Department, Neuroscience Institute, Geisinger Health System, PA, USA.
- [4] Clinical Neurology Research Center, Shiraz University of Medical Sciences, Shiraz, Iran. Electronic address: neuro.ab@gmail.com.
- [5] Neurology Department, Yasrebi Hospital, Kashan, Iran.
- [6] Division of Neuroendovascular Surgery, Department of Neurosurgery, Ghaem Hospital, Mashhad University of Medical Sciences, Mashhad, Iran.
- [7] Neurosurgery Department, Tehran University of Medical Sciences, Tehran, Iran.
- [8] Department of Neurology, Isfahan University of Medical Sciences, Isfahan, Iran.

- [9] Department of Neurology, Alavi Hospital, Ardabil University of Medical Sciences, Ardabil, Iran.
- [10] Department of Radiology, Yasrebi Hospital, Kashan, Iran.
- [11] Iranian Center of Neurological Research, Neuroscience Institute, Tehran University of Medical Sciences, Tehran, Iran.
- [12] Division of Neurology, National University Health System, School of Medicine, National University of Singapore, Singapore.
- [13] Neuroscience Research Center, Guilan University of Medical Sciences, Guilan, Iran.
- [14] Second Department of Neurology, National and Kapodistrian University of Athens, School of Medicine, "Attikon" University Hospital, Athens, Greece; Neurology Department, University of Tennessee Health Science Center, TN, USA.
- [15] Neurology Department, Neuroscience Institute, Geisinger Health System, PA, USA; Neurology Department, University of Tennessee Health Science Center, TN, USA. Electronic address: rzand@geisinger.edu.

- PMID: **33075595**
- PMCID: PMC7556283
- DOI: 10.1016/j.jns.2020.117183

Free PMC article
Observational Study

Cerebral venous sinus thrombosis associated with SARS-CoV-2; a multinational case series

Ashkan Mowla et al. J Neurol Sci. 2020.
Free PMC article
Show details
J Neurol Sci
. 2020 Dec 15;419:117183.
doi: 10.1016/j.jns.2020.117183. Epub 2020 Oct 14.

Authors

Ashkan Mowla [1], Banafsheh Shakibajahromi [2], Shima Shahjouei [3], Afshin Borhani-Haghighi [4], Nasrin Rahimian [5], Humain Baharvahdat [6], Soheil Naderi [7], Fariborz Khorvash [8], Davar Altafi [9], Seyed Amir Ebrahimzadeh [10], Ghasem Farahmand [11], Alaleh Vaghefi Far [11], Vijay K Sharma [12], Saeideh Aghayari Sheikh Neshin [13], Georgios Tsivgoulis [14], Ramin Zand [15]

Affiliations

- [1] Division of Stroke and Endovascular Neurosurgery, Department of Neurological Surgery, Keck School of Medicine, University of Southern California, California, USA.
- [2] Clinical Neurology Research Center, Shiraz University of Medical Sciences, Shiraz, Iran.
- [3] Neurology Department, Neuroscience Institute, Geisinger Health System, PA, USA.
- [4] Clinical Neurology Research Center, Shiraz University of Medical Sciences, Shiraz, Iran. Electronic address: neuro.ab@gmail.com.

- [5] Neurology Department, Yasrebi Hospital, Kashan, Iran.
- [6] Division of Neuroendovascular Surgery, Department of Neurosurgery, Ghaem Hospital, Mashhad University of Medical Sciences, Mashhad, Iran.
- [7] Neurosurgery Department, Tehran University of Medical Sciences, Tehran, Iran.
- [8] Department of Neurology, Isfahan University of Medical Sciences, Isfahan, Iran.
- [9] Department of Neurology, Alavi Hospital, Ardabil University of Medical Sciences, Ardabil, Iran.
- [10] Department of Radiology, Yasrebi Hospital, Kashan, Iran.
- [11] Iranian Center of Neurological Research, Neuroscience Institute, Tehran University of Medical Sciences, Tehran, Iran.
- [12] Division of Neurology, National University Health System, School of Medicine, National University of Singapore, Singapore.
- [13] Neuroscience Research Center, Guilan University of Medical Sciences, Guilan, Iran.
- [14] Second Department of Neurology, National and Kapodistrian University of Athens, School of Medicine, "Attikon" University Hospital, Athens, Greece; Neurology Department, University of Tennessee Health Science Center, TN, USA.
- [15] Neurology Department, Neuroscience Institute, Geisinger Health System, PA, USA; Neurology Department, University of Tennessee Health Science Center, TN, USA. Electronic address: rzand@geisinger.edu.

- PMID: **33075595**
- PMCID: PMC7556283
- DOI: 10.1016/j.jns.2020.117183

Abstract

Background: SARS-CoV-2 induced coagulopathy can lead to thrombotic complications such as stroke. Cerebral venous sinus thrombosis (CVST) is a less common type of stroke which might be triggered by COVID-19. We present a series of CVST cases with SARS-CoV-2 infection.

Methods: In a multinational retrospective study, we collected all cases of CVST in SARS-CoV-2 infected patients admitted to nine tertiary stroke centers from the beginning of the pandemic to June 30th, 2020. We compared the demographics, clinical and radiological characteristics, risk factors, and outcome of these patients with a control group of non-SARS-CoV-2 infected CVST patients in the same seasonal period of the years 2012-2016 from the country where the majority of cases were recruited.

Results: A total of 13 patients fulfilled the inclusion criteria (62% women, mean age 50.9 ± 11.2 years). Six patients were discharged with good outcomes (mRS ≤ 2) and three patients died in hospital. Compared to the control group, the SARS-CoV-2 infected patients were significantly older (50.9 versus 36.7 years, $p < 0.001$), had a lower rate of identified CVST risk factors (23.1% versus 84.2%, $p < 0.001$), had more frequent cortical vein involvement (38.5% versus 10.5%, p: 0.025), and a non-significant higher rate of in-hospital mortality (23.1% versus 5.3%, p: 0.073).

Conclusion: CVST should be considered as potential comorbidity in SARS-CoV-2 infected patients presenting with neurological symptoms. Our data suggest that compared to non-SARS-CoV-2 infected patients, CVST occurs in older patients, with lower rates of known CVST risk factors and might lead to a poorer outcome in the SARS-CoV-2 infected group.

Keywords: COVID-19; Cerebral venous sinus thrombosis; Cerebral venous thrombosis; SARS-CoV-2; Stroke; Thrombosis.

Copyright © 2020 Elsevier B.V. All rights reserved.

- Cited by 25 articles
- 30 references
- 1 figure

Supplementary info

Publication types, MeSH terms Expand

Publication types

- Multicenter Study
- Observational Study

MeSH terms

- Adult
- Aged
- COVID-19 / blood
- COVID-19 / complications*
- Female
- Humans
- Length of Stay / statistics & numerical data
- Male
- Middle Aged
- Pandemics
- Retrospective Studies
- SARS-CoV-2*
- Sinus Thrombosis, Intracranial / diagnostic imaging
- Sinus Thrombosis, Intracranial / etiology*
- Tertiary Care Centers / statistics & numerical data
- Thrombophilia / etiology

Full text links

ELSEVIER FULL-TEXT ARTICLE Elsevier Science Free PMC article
Proceed to details
Cite
Share
☐ 406
Observational Study
HNO
. 2022 Feb;70(2):133-139.

doi: 10.1007/s00106-021-01121-1. Epub 2021 Nov 17.

[Analysis of ENT emergency patients during the COVID-19 pandemic in Germany]

[Article in German]

Olcay Cem Bulut [1], Maximilian Pilz [2], Sina M Beisel [3], Burkard M Lippert [3], Katrin Knopf [3]
Affiliations Expand

Affiliations

- [1] Klinik für Hals-Nasen-Ohrenheilkunde, Kopf- und Halschirurgie, plastische Operationen, SLK-Kliniken am Gesundbrunnen, Am Gesundbrunnen 20-26, 74078, Heilbronn, Deutschland. ocbulut@hotmail.com.
- [2] Institut für Medizinische Biometrie und Informatik, Universitätsklinikum Heidelberg, Im Neuenheimer Feld 130.3, 69120, Heidelberg, Deutschland.
- [3] Klinik für Hals-Nasen-Ohrenheilkunde, Kopf- und Halschirurgie, plastische Operationen, SLK-Kliniken am Gesundbrunnen, Am Gesundbrunnen 20-26, 74078, Heilbronn, Deutschland.

- PMID: **34791514**
- PMCID: PMC8597875
- DOI: 10.1007/s00106-021-01121-1

Free PMC article
Observational Study

[Analysis of ENT emergency patients during the COVID-19 pandemic in Germany]

[Article in German]
Olcay Cem Bulut et al. HNO. 2022 Feb.
Free PMC article
Show details
HNO
. 2022 Feb;70(2):133-139.
doi: 10.1007/s00106-021-01121-1. Epub 2021 Nov 17.

Authors

Olcay Cem Bulut [1], Maximilian Pilz [2], Sina M Beisel [3], Burkard M Lippert [3], Katrin Knopf [3]

Affiliations

- [1] Klinik für Hals-Nasen-Ohrenheilkunde, Kopf- und Halschirurgie, plastische Operationen, SLK-Kliniken am Gesundbrunnen, Am Gesundbrunnen 20-26, 74078, Heilbronn, Deutschland. ocbulut@hotmail.com.
- [2] Institut für Medizinische Biometrie und Informatik, Universitätsklinikum Heidelberg, Im Neuenheimer Feld 130.3, 69120, Heidelberg, Deutschland.
- [3] Klinik für Hals-Nasen-Ohrenheilkunde, Kopf- und Halschirurgie, plastische Operationen, SLK-Kliniken am Gesundbrunnen, Am Gesundbrunnen 20-26, 74078, Heilbronn, Deutschland.

- PMID: **34791514**
- PMCID: PMC8597875
- DOI: 10.1007/s00106-021-01121-1

Abstract
in English, German

Background: This retrospective observational study was undertaken to assess the ENT emergency workload during the COVID-19 pandemic caused by the severe acute respiratory coronavirus-2 (SARS-CoV-2).

Materials and methods: All 3230 patients who were treated as an emergency from 23.01.2020 to 06.08.2020 in the Department of Otolaryngology at the SLK-Kliniken Heilbronn were included in this study. Demographic data, diagnostics, diagnosis, and treatment (in-/outpatient) were retrospectively retrieved. Not only did the physicians on call triage the emergency department (ED) ENT patients, but the patients also self-assessed their urgency of treatment.

Results: The number of patients consulting our ED decreased significantly during the pandemic, by 42.2%. However, the top diagnoses remained almost constant, with epistaxis being the most frequent diagnosis before, during, and after COVID-19. Facial trauma remained the second most frequent consultation reason. The hospitalization rate decreased from 21.9% before COVID-19 to 16.2% during the pandemic. Surgical therapy was necessary in 17.6% of patients before COVID-19 and this increased to 23.5% during COVID-19. The self-referral rate increased from 61 to 66% during the pandemic. More men than women consulted the ED during COVID-19. Regarding the triage assessment by the physician on call and the patient's self-assessment, a significant discrepancy was noted before, during, and after COVID-19.

Conclusion: The reasons for reduction in ENT ED visits are multifactorial. The clinical consequences of decreased hospitalizations remain uncertain. However, health authorities need to advocate the safety of the hospital environment to limit potential damage.

Zusammenfassung: HINTERGRUND: Diese retrospektive Beobachtungsstudie wurde durchgeführt, um Veränderungen der HNO-Notfälle während der durch „severe acute respiratory coronavirus-2" (SARS-CoV-2) verursachten (COVID-19-)Pandemie zu analysieren.

Methodik: In diese Studie wurden insgesamt 3230 Patienten eingeschlossen, die sich vom 23.01.2020 bis zum 06.08.2020 in der Klinik für Hals-Nasen-Ohren-Heilkunde, Kopf- und Halschirurgie, plastische Operationen des SLK-Klinikums Heilbronn als Notfall vorstellten. Demografische Daten, Diagnostik, Diagnose, Therapie und Behandlung (ambulant/stationär) wurden retrospektiv erhoben. Die Triage der Notfallpatienten erfolgte nicht nur durch die diensthabenden Ärzte, sondern auch durch den Patienten selbst, der die Dringlichkeit seiner Behandlung einschätzte.

Ergebnisse: Die Anzahl der Patienten, die unsere Notaufnahme aufsuchten, ging während der Pandemie um 42,2 % zurück. Die häufigsten Diagnosen blieben jedoch fast gleich, wobei Epistaxis die häufigste Diagnose vor, während und nach COVID-19 war. Das Gesichtstrauma blieb der zweithäufigste Vorstellungsgrund. Die Hospitalisierungsrate sank von 21,9 % vor COVID-19 auf 16,2 % während der Pandemie. Eine chirurgische Therapie war bei 17,6 % der Patienten vor COVID-19 notwendig und stieg während COVID-19 auf 23,5 %. Die Selbsteinweisungsrate stieg während der Pandemie von 61 auf 66 %. Während COVID-19 suchten mehr Männer als Frauen die Notaufnahme auf. Hinsichtlich der Dringlichkeitseinschätzung der Behandlung durch den diensthabenden Arzt und durch den Patienten selbst wurde eine signifikante Diskrepanz vor, während und nach COVID-19 festgestellt.

Schlussfolgerung: Verschiedene Faktoren könnten für den Rückgang der notfallmäßigen Vorstellungen in der HNO-Klinik verantwortlich sein. Die Folgen der verringerten Rate an stationären Behandlungen von Patienten bleiben ungewiss. Sicher ist, dass sich die Gesundheitsbehörden vermehrt für die Sicherheit der Krankenhausumgebung, in diesem Fall besonders bezüglich des Infektionsschutzes einsetzen müssen, um potenzielle kurz- und langfristige gesundheitliche Folgen für die Patienten zu vermeiden.

Keywords: Ambulatory care; COVID-19; Emergency medicine; Hospitalisation; Triage.

© 2021. The Author(s), under exclusive licence to Springer Medizin Verlag GmbH, ein Teil von Springer Nature.

- 18 references
- 3 figures

Supplementary info

Publication types, MeSH terms Expand

Publication types

- Observational Study

MeSH terms

- COVID-19*
- Emergency Service, Hospital
- Female
- Humans
- Male
- Pandemics*
- Retrospective Studies
- SARS-CoV-2

Full text links

SpringerLink FULL-TEXT ARTICLE Springer Free PMC article

Proceed to details

Cite

Share

☐ 407

Observational Study

J Public Health (Oxf)

. 2021 Apr 12;43(1):26-34.

doi: 10.1093/pubmed/fdaa195.

The experience of the health care workers of a severely hit SARS-CoV-2 referral Hospital in Italy: incidence, clinical course and modifiable risk factors for COVID-19 infection

Marta Colaneri [1], Viola Novelli [2], Sara Cutti [2], Alba Muzzi [2], Guido Resani [2] [3], Maria Cristina Monti [4], Claudia Rona [2], Anna Maria Grugnetti [5], Marco Rettani [6], Francesca Rovida [7], Valentina Zuccaro [1], Antonio Triarico [8], Carlo Marena [2]

Affiliations Expand

Affiliations

- [1] Division of Infectious Diseases I, Fondazione IRCCS Policlinico San Matteo, Pavia, Italy.
- [2] Medical Direction, Fondazione IRCCS Policlinico San Matteo, Pavia, Italy.
- [3] Department of Public Health, Experimental and Forensic Medicine, Section of Hygiene, University of Pavia, Pavia, Italy.
- [4] Department of Public Health, Experimental and Forensic Medicine, Unit of Biostatistics and Clinical Epidemiology, University of Pavia, Pavia, Italy.
- [5] Department of Health Professions, Fondazione IRCCS Policlinico San Matteo, Pavia, Italy.
- [6] Information Tecnology, Fondazione IRCCS Policlinico San Matteo, Pavia, Italy.
- [7] Department of Microbiology and Virology Unit, Fondazione IRCCS Policlinico San Matteo, Pavia, Italy.
- [8] Department of Hospital Leadership, Fondazione IRCCS Policlinico San Matteo, Pavia, Italy.

- PMID: **33140084**
- PMCID: PMC7665642
- DOI: 10.1093/pubmed/fdaa195

Free PMC article

Observational Study

The experience of the health care workers of a severely hit SARS-CoV-2 referral Hospital in Italy: incidence, clinical course and modifiable risk factors for COVID-19 infection

Marta Colaneri et al. J Public Health (Oxf). 2021.
Free PMC article
Show details
J Public Health (Oxf)
. 2021 Apr 12;43(1):26-34.
doi: 10.1093/pubmed/fdaa195.

Authors

Marta Colaneri [1], Viola Novelli [2], Sara Cutti [2], Alba Muzzi [2], Guido Resani [2] [3], Maria Cristina Monti [4], Claudia Rona [2], Anna Maria Grugnetti [5], Marco Rettani [6], Francesca Rovida [7], Valentina Zuccaro [1], Antonio Triarico [8], Carlo Marena [2]

Affiliations

- [1] Division of Infectious Diseases I, Fondazione IRCCS Policlinico San Matteo, Pavia, Italy.
- [2] Medical Direction, Fondazione IRCCS Policlinico San Matteo, Pavia, Italy.
- [3] Department of Public Health, Experimental and Forensic Medicine, Section of Hygiene, University of Pavia, Pavia, Italy.
- [4] Department of Public Health, Experimental and Forensic Medicine, Unit of Biostatistics and Clinical Epidemiology, University of Pavia, Pavia, Italy.
- [5] Department of Health Professions, Fondazione IRCCS Policlinico San Matteo, Pavia, Italy.
- [6] Information Tecnology, Fondazione IRCCS Policlinico San Matteo, Pavia, Italy.
- [7] Department of Microbiology and Virology Unit, Fondazione IRCCS Policlinico San Matteo, Pavia, Italy.
- [8] Department of Hospital Leadership, Fondazione IRCCS Policlinico San Matteo, Pavia, Italy.

- PMID: **33140084**
- PMCID: PMC7665642
- DOI: 10.1093/pubmed/fdaa195

Abstract

Background: During the COVID-19 pandemic, the health care workers (HCWs) at the frontline have been largely exposed to infected patients, running a high risk of being infected by the SARS-CoV-2 virus.Since limiting transmission of severe acute respiratory syndrome coronavirus 2

(SARS-CoV-2) in health care setting is crucial to avoid the community spread of SARS-CoV-2, we want to share our experience as an early hit hospital where standard infection control practices have been conscientiously applied and effective. We believe that our example, as first and hardest hit country, might be a warning and aid not only for those who have been hit later, but also for a second fearful wave of contagion. In addition, we want to offer an insight on modifiable risk factors for HWs-related infection.

Methods: Demographic, lifestyle, work-related and comorbidities data of 1447 HCWs, which underwent a nasopharyngeal swab for SARS-CoV-2, were retrospectively collected. For the 164 HCWs positive for SARS-CoV-2, data about safety in the workplace, symptoms and clinical course of COVID-19 were also collected. Cumulative incidence of SARS-CoV-2 infection was estimated. Risk factors for SARS-CoV-2 infection were assessed using a multivariable Poisson regression.

Results: The cumulative incidence of SARS-CoV-2 infection among the screened HCWs was 11.33% (9.72-13.21). Working in a COVID-19 ward, being a former smoker (versus being a person who never smoked) and BMI was positively associated with SARS-CoV-2 infection, whereas being a current smoker was negatively associated with this variable.

Conclusions: Assuming an equal accessibility and proper use of personal protective equipment of all the HCWs of our Hospital, the great and more prolonged contact with COVID-19 patients remains the crucial risk factor for SARS-CoV-2. Therefore, increased and particular care needs to be focused specifically on the most exposed HCWs groups, which should be safeguarded. Furthermore, in order to limit the risk of asymptomatic spread of SARS-CoV-2 infection, the HCWs mild symptoms of COVID-19 should be considered when evaluating the potential benefits of universal staff testing.

Keywords: COVID-19; SARS-CoV-2; health care workers; retrospective cohort study; risk of infection.

© The Author(s) 2020. Published by Oxford University Press on behalf of Faculty of Public Health. All rights reserved. For permissions, please e-mail: journals.permissions@oup.com.

- Cited by 14 articles
- 46 references

Supplementary info

Publication types, MeSH terms Expand

Publication types

- Observational Study

MeSH terms

- Adult
- Body Mass Index
- COVID-19 / diagnosis
- COVID-19 / epidemiology*

- COVID-19 / prevention & control
- COVID-19 Nucleic Acid Testing
- Female
- Humans
- Incidence
- Infection Control
- Infectious Disease Transmission, Patient-to-Professional / prevention & control
- Italy
- Male
- Middle Aged
- Multivariate Analysis
- Personnel, Hospital*
- Referral and Consultation
- Retrospective Studies
- Risk Factors
- SARS-CoV-2 / isolation & purification
- Smoking

Full text links

OXFORD ACADEMIC Silverchair Information Systems Free PMC article
Proceed to details
Cite
Share

408

Review

Int J Infect Dis
. 2021 Dec;113:282-287.
doi: 10.1016/j.ijid.2021.10.032. Epub 2021 Oct 21.

Measuring the impact of a single dose of ChAdOx1 nCoV-19 (recombinant) coronavirus vaccine on hospital stay, ICU requirement, and mortality outcome in a tertiary care centre

Anuja Desai [1], Parth Desai [2], Jigar Mehta [3], Wasimahmed Sachora [4], Neeraj Bharti [5], Tushar Patel [6], Kalpesh Sukhwani [7], Ankita Jain [8], Dipesh Sorathiya [9], Vivek Nanda [10], Parin Mehta [11], Adit Desai [12]
Affiliations Expand

Affiliations

- [1] Department of Ophthalmology, Kusum Dhirajlal Hospital, Vaishnodevi Circle, SG Road, Ahmedabad, 382421, Gujarat, India. Electronic address: dranuja@kdhospital.co.in.
- [2] Kusum Dhirajlal Hospital, Vaishnodevi Circle, SG Road, Ahmedabad, 382421, Gujarat, India. Electronic address: parth@kdhospital.co.in.
- [3] Department of Critical Care Medicine, Kusum Dhirajlal Hospital, Vaishnodevi Circle, SG Road, Ahmedabad, 382421, Gujarat, India. Electronic address: drjigar74@yahoo.com.
- [4] Department of General Medicine, Kusum Dhirajlal Hospital, Vaishnodevi Circle, SG Road, Ahmedabad, 382421, Gujarat, India. Electronic address: wasimsachora@yahoo.com.
- [5] Department of General Medicine, Kusum Dhirajlal Hospital, Vaishnodevi Circle, SG Road, Ahmedabad, 382421, Gujarat, India. Electronic address: neerajbharti2001@gmail.com.
- [6] Department of Pulmonary Medicine, Kusum Dhirajlal Hospital, Vaishnodevi Circle, SG Road, Ahmedabad, 382421, Gujarat, India. Electronic address: drtusharpatel@yahoo.com.
- [7] Department of Infectious Disease, Kusum Dhirajlal Hospital, Vaishnodevi Circle, SG Road, Ahmedabad, 382421, Gujarat, India. Electronic address: kalpesh.sukhwani@gmail.com.
- [8] Department of Obstetrics and Gynecology, Kusum Dhirajlal Hospital, Vaishnodevi Circle, SG Road, Ahmedabad, 382421, Gujarat, India. Electronic address: drankitajain@yahoo.com.
- [9] Department of In-vitro Fertilization, Kusum Dhirajlal Hospital, Vaishnodevi Circle, SG Road, Ahmedabad, 382421, Gujarat, India. Electronic address: drdipeshsorathiya@gmail.com.
- [10] Department of Emergency Medicine, Kusum Dhirajlal Hospital, Vaishnodevi Circle, SG Road, Ahmedabad,382421, Gujarat, India. Electronic address: dr.viveknanda@gmail.com.
- [11] Department of Ophthalmology, Kusum Dhirajlal Hospital, Vaishnodevi Circle, SG Road, Ahmedabad, 382421, Gujarat, India. Electronic address: parinmehta23@gmail.com.
- [12] Kusum Dhirajlal Hospital, Vaishnodevi Circle, SG Road, Ahmedabad, 382421, Gujarat, India. Electronic address: adit@kdhospital.co.in.

- PMID: **34688949**
- PMCID: PMC8529545
- DOI: 10.1016/j.ijid.2021.10.032

Free PMC article
Review

Measuring the impact of a single dose of ChAdOx1 nCoV-19 (recombinant) coronavirus vaccine on hospital stay, ICU requirement, and mortality outcome in a tertiary care centre

Anuja Desai et al. Int J Infect Dis. 2021 Dec.
Free PMC article

Show details
Int J Infect Dis
. 2021 Dec;113:282-287.
doi: 10.1016/j.ijid.2021.10.032. Epub 2021 Oct 21.

Authors

Anuja Desai [1], Parth Desai [2], Jigar Mehta [3], Wasimahmed Sachora [4], Neeraj Bharti [5], Tushar Patel [6], Kalpesh Sukhwani [7], Ankita Jain [8], Dipesh Sorathiya [9], Vivek Nanda [10], Parin Mehta [11], Adit Desai [12]

Affiliations

- [1] Department of Ophthalmology, Kusum Dhirajlal Hospital, Vaishnodevi Circle, SG Road, Ahmedabad, 382421, Gujarat, India. Electronic address: dranuja@kdhospital.co.in.
- [2] Kusum Dhirajlal Hospital, Vaishnodevi Circle, SG Road, Ahmedabad, 382421, Gujarat, India. Electronic address: parth@kdhospital.co.in.
- [3] Department of Critical Care Medicine, Kusum Dhirajlal Hospital, Vaishnodevi Circle, SG Road, Ahmedabad, 382421, Gujarat, India. Electronic address: drjigar74@yahoo.com.
- [4] Department of General Medicine, Kusum Dhirajlal Hospital, Vaishnodevi Circle, SG Road, Ahmedabad, 382421, Gujarat, India. Electronic address: wasimsachora@yahoo.com.
- [5] Department of General Medicine, Kusum Dhirajlal Hospital, Vaishnodevi Circle, SG Road, Ahmedabad, 382421, Gujarat, India. Electronic address: neerajbharti2001@gmail.com.
- [6] Department of Pulmonary Medicine, Kusum Dhirajlal Hospital, Vaishnodevi Circle, SG Road, Ahmedabad, 382421, Gujarat, India. Electronic address: drtusharpatel@yahoo.com.
- [7] Department of Infectious Disease, Kusum Dhirajlal Hospital, Vaishnodevi Circle, SG Road, Ahmedabad, 382421, Gujarat, India. Electronic address: kalpesh.sukhwani@gmail.com.
- [8] Department of Obstetrics and Gynecology, Kusum Dhirajlal Hospital, Vaishnodevi Circle, SG Road, Ahmedabad, 382421, Gujarat, India. Electronic address: drankitajain@yahoo.com.
- [9] Department of In-vitro Fertilization, Kusum Dhirajlal Hospital, Vaishnodevi Circle, SG Road, Ahmedabad, 382421, Gujarat, India. Electronic address: drdipeshsorathiya@gmail.com.
- [10] Department of Emergency Medicine, Kusum Dhirajlal Hospital, Vaishnodevi Circle, SG Road, Ahmedabad,382421, Gujarat, India. Electronic address: dr.viveknanda@gmail.com.
- [11] Department of Ophthalmology, Kusum Dhirajlal Hospital, Vaishnodevi Circle, SG Road, Ahmedabad, 382421, Gujarat, India. Electronic address: parinmehta23@gmail.com.
- [12] Kusum Dhirajlal Hospital, Vaishnodevi Circle, SG Road, Ahmedabad, 382421, Gujarat, India. Electronic address: adit@kdhospital.co.in.

- PMID: **34688949**
- PMCID: PMC8529545
- DOI: 10.1016/j.ijid.2021.10.032

Abstract

Objective: To comparatively evaluate ICU requirement, length of stay, and mortality between single-dose vaccinated and non-vaccinated hospitalized COVID-19 patients.

Design: A retrospective observational study was carried out in a tertiary care hospital in western Indian, from April 1 to June 30, 2021.

Results: Of the 569 patients who fulfilled the eligibility criteria and were enrolled in the study, 137 (24.08%) patients had received a single dose of ChAdOx1 nCoV-19 vaccine, while 432 (75.92%) patients had not received any form of vaccination. The overall length of stay in hospital was similar for both groups; however, a significant difference was seen in length of stay in the ward and in the ICU. Vaccinated patients were admitted to the ward for 6.21 ± 3.204 days, while non-vaccinated patients were admitted for 5.56 ± 4.55 days ($p < 0.001$). The mean length of ICU stay for the 21 vaccinated patients requiring intensive care was 4.47 ± 2.3 days, while that for the 145 non-vaccinated patients was 6.29 ± 2.19 days ($p < 0.001$). Mortality was observed in four patients in the vaccinated group and in 95 patients in the non-vaccinated group.

Conclusion: A single dose of ChAdOx1 nCoV-19 vaccine was associated with a significantly lower severity of SARS-CoV-2 infection compared with no vaccination.

Keywords: COVID-19; ChAdOx1 nCoV-19 vaccine; SARS-CoV-2 infection; vaccination; vaccine hesitancy.

Copyright © 2021 The Author(s). Published by Elsevier Ltd.. All rights reserved.

- 31 references
- 3 figures

Supplementary info

Publication types, MeSH terms, Substances Expand

Publication types

- Review

MeSH terms

- COVID-19 Vaccines
- COVID-19*
- ChAdOx1 nCoV-19*
- Humans
- Intensive Care Units
- Length of Stay
- Observational Studies as Topic
- SARS-CoV-2
- Tertiary Care Centers
- Vaccination

Substances

- COVID-19 Vaccines
- ChAdOx1 nCoV-19

Full text links

Elsevier Science Free PMC article
Proceed to details
Cite
Share
409
Observational Study
Int Orthop
. 2020 Aug;44(8):1473-1480.
doi: 10.1007/s00264-020-04619-5. Epub 2020 May 25.

Staying home during "COVID-19" decreased fractures, but trauma did not quarantine in one hundred and twelve adults and twenty eight children and the "tsunami of recommendations" could not lockdown twelve elective operations

Jacques Hernigou [1], Xavier Morel [1], Antoine Callewier [1], Olivier Bath [1], Philippe Hernigou [2]
Affiliations Expand

Affiliations

- [1] EpiCURA Hospital, Orthopedic Department, Baudour, Hornu, Belgium.
- [2] Hospital Henri Mondor, University of Paris-Est, Creteil, France. philippe.hernigou@wanadoo.fr.

- PMID: **32451655**
- PMCID: PMC7247744
- DOI: 10.1007/s00264-020-04619-5

Free PMC article
Observational Study

Staying home during "COVID-19" decreased fractures, but trauma did not quarantine in one hundred and twelve adults and twenty eight children and the "tsunami of recommendations" could not lockdown twelve elective operations

Jacques Hernigou et al. Int Orthop. 2020 Aug.
Free PMC article
Show details
Int Orthop
. 2020 Aug;44(8):1473-1480.
doi: 10.1007/s00264-020-04619-5. Epub 2020 May 25.

Authors

Jacques Hernigou [1], Xavier Morel [1], Antoine Callewier [1], Olivier Bath [1], Philippe Hernigou [2]

Affiliations

- [1] EpiCURA Hospital, Orthopedic Department, Baudour, Hornu, Belgium.
- [2] Hospital Henri Mondor, University of Paris-Est, Creteil, France. philippe.hernigou@wanadoo.fr.

- PMID: **32451655**
- PMCID: PMC7247744
- DOI: 10.1007/s00264-020-04619-5

Abstract

Purpose: The current pandemic caused by COVID-19 is the biggest challenge for national health systems for a century. While most medical resources are allocated to treat COVID-19 patients, fractures still need to be treated, as some patients with non-deferrable pathologies. The aim of this paper is to report the early experience of an integrated team of orthopaedic surgeons during this period.

Material and methods: This is a mono-geographic, observational, retrospective, descriptive study. We collected data from the beginning of the epidemic (1 March 2020), during the pandemic lockdown period (declared in the country on March 16, 2020) until the end of our study period on April 15, 2020. All the 140 patients presented to the Emergency Department of the hospital during this period with a diagnosis of fracture, or trauma (sprains, dislocations, wounds) were included in the cohort. In addition, 12 patients needing hospitalization for planning a non-deferrable elective surgical treatment were included. A group of patients from the two same hospitals and treated during the same period (1st March 2018 to April 15, 2018) but previously was used as control.

Results: Of these 152 patients (mean age 45.5 years; range 1 to 103), 100 underwent a surgical procedure and 52 were managed non-operatively. Twenty-eight were children and 124 were adults. The COVID-19 diagnosis was confirmed for four patients. The frequency of patients with confirmed COVID-19 diagnosis among this population treated in emergency was ten fold higher (2.6%; 4 among 152) than in the general population (0.30%) of the country. The mortality rate for patients with surgery was 2% (2 of 100 patients) and 50% (2 of 4) for those older than 60 years with COVID-19; it was null for patients who were managed non-operatively. As compared to the year 2018, the number of patients seen with trauma had decreased of 32% during the epidemic.

Conclusion: Staying home during the COVID-19 pandemic decreased trauma frequency of 32%. The structural organization in our hospital allowed us to reduce the time to surgery and ultimately hospital stay, thereby maximizing the already stretched medical resources available to treat all the patients who needed orthopedic care during this period.

Keywords: Adult trauma; COVID-19; Children fractures; Dislocation; Fracture; Lockdown; Paediatrics; Quarantine; Recommendation; Sprain.

Conflict of interest statement

The authors declare that they have no conflict of interest.

- Cited by 41 articles
- 11 references
- 1 figure

Supplementary info

Publication types, MeSH terms Expand

Publication types

- Observational Study

MeSH terms

- Adolescent
- Adult
- Aged
- Aged, 80 and over
- Betacoronavirus*
- COVID-19
- Child
- Child, Preschool
- Coronavirus Infections*
- Elective Surgical Procedures
- Emergency Service, Hospital
- Female

- Fractures, Bone / epidemiology*
- Humans
- Infant
- Length of Stay
- Male
- Middle Aged
- Pandemics*
- Pneumonia, Viral*
- Quarantine
- Retrospective Studies
- SARS-CoV-2
- Wounds and Injuries / epidemiology*
- Young Adult

Full text links

Springer Free PMC article
Proceed to details
Cite
Share
☐ 410
Observational Study
Rev Paul Pediatr
. 2020 Nov 27;39:e2020305.
doi: 10.1590/1984-0462/2021/39/2020305. eCollection 2020.

CLINICAL MANIFESTATIONS OF CHILDREN AND ADOLESCENTS WITH COVID-19: REPORT OF THE FIRST 115 CASES FROM SABARÁ HOSPITAL INFANTIL

[Article in English, Portuguese]
Anna Clara Rabha [1], Francisco Ivanildo de Oliveira Junior [1], Thales Araújo de Oliveira [1], Regina Grigolli Cesar [1], Giuliana Fongaro [1], Roberta Ferreira Mariano [1], Clarice Neves Camargo [1], Fátima Rodrigues Fernandes [1], Gustavo Falbo Wandalsen [2]
Affiliations Expand

Affiliations

- [1] Instituto Pensi, Sabará Hospital Infantil, Fundação José Luiz Egydio Setúbal, São Paulo, SP, Brazil.
- [2] Escola Paulista de Medicina, Universidade Federal de São Paulo, São Paulo, SP, Brazil.

- PMID: **33263697**
- PMCID: PMC7695045
- DOI: 10.1590/1984-0462/2021/39/2020305

Free PMC article
Observational Study

CLINICAL MANIFESTATIONS OF CHILDREN AND ADOLESCENTS WITH COVID-19: REPORT OF THE FIRST 115 CASES FROM SABARÁ HOSPITAL INFANTIL

[Article in English, Portuguese]
Anna Clara Rabha et al. Rev Paul Pediatr. 2020.
Free PMC article
Show details
Rev Paul Pediatr
. 2020 Nov 27;39:e2020305.
doi: 10.1590/1984-0462/2021/39/2020305. eCollection 2020.

Authors

Anna Clara Rabha [1], Francisco Ivanildo de Oliveira Junior [1], Thales Araújo de Oliveira [1], Regina Grigolli Cesar [1], Giuliana Fongaro [1], Roberta Ferreira Mariano [1], Clarice Neves Camargo [1], Fátima Rodrigues Fernandes [1], Gustavo Falbo Wandalsen [2]

Affiliations

- [1] Instituto Pensi, Sabará Hospital Infantil, Fundação José Luiz Egydio Setúbal, São Paulo, SP, Brazil.
- [2] Escola Paulista de Medicina, Universidade Federal de São Paulo, São Paulo, SP, Brazil.

- PMID: **33263697**
- PMCID: PMC7695045
- DOI: 10.1590/1984-0462/2021/39/2020305

Abstract
in English, Portuguese

Objective: To describe the clinical manifestations and severity of children and adolescents affected by COVID-19 treated at Sabará Hospital Infantil.

Methods: This is a cross-sectional, retrospective, and observational study. All cases of COVID-19 confirmed by RT-qPCR of patients seen at the hospital (emergency room, first-aid room, and ICU) were analyzed. The severity of the cases was classified according to the Chinese Consensus.

Results: Among the 115 children included, a predominance of boys (57%) was verified, and the median age was two years. A total of 22 children were hospitalized, 12 in the ICU. Of the total, 26% had comorbidities with a predominance of asthma (13%). Fever, cough, and nasal discharge were the most frequent symptoms. Respiratory symptoms were reported by 58% of children and gastrointestinal symptoms, by 34%. Three children were asymptomatic, 81 (70%) had upper airway symptoms, 15 (13%) had mild pneumonia, and 16 (14%) had severe pneumonia. Hospitalized children were younger than non-hospitalized children (7 months vs. 36 months). In hospitalized patients, a higher frequency of irritability, dyspnea, drowsiness, respiratory distress, low oxygen saturation, and hepatomegaly was observed. Chest radiography was performed in 69 children with 45% of abnormal exams. No child required mechanical ventilation and there were no deaths.

Conclusions: Most of children and adolescents affected by COVID-19 had mild upper airway symptoms. Clinical manifestations of COVID-19 were more severe among younger children who exhibited gastrointestinal and respiratory symptoms more frequently.

Objetivo:: Descrever as manifestações clínicas e a gravidade de crianças e adolescentes acometidos pela COVID-19 atendidos no Sabará Hospital Infantil.

Métodos:: Trata-se de estudo transversal, retrospectivo e observacional. Foram analisados os atendimentos (pronto-socorro, enfermaria e Unidade de Terapia Intensiva - UTI) que apresentavam diagnóstico de COVID-19 confirmado por RT-qPCR. A gravidade dos casos foi classificada de acordo com o Consenso Chinês.

Resultados:: Entre as 115 crianças incluídas, houve predominância do sexo masculino (57%) e a mediana de idade foi de 2 anos. Vinte e duas crianças foram hospitalizadas, sendo 12 em UTI. Do total, 26% apresentava comorbidades com predomínio de asma (13%). Febre, tosse e coriza foram os sintomas mais frequentes. Sintomas respiratórios foram relatados por 58% das crianças e gastrintestinais por 34%. Três crianças apresentavam-se assintomáticas, 81 (70%) com sintomas de vias aéreas superiores, 15 (13%) com quadro de pneumonia leve e 16 (14%) com pneumonia grave. As crianças hospitalizadas eram mais jovens do que as não hospitalizadas (7 meses *vs*. 36 meses). Nas hospitalizadas, observamos maior frequência de irritabilidade, dispneia, sonolência, desconforto respiratório, baixa saturação de oxigênio e hepatomegalia. Radiografia de tórax foi realizada por 69 crianças com 45% de exames alterados. Nenhuma criança necessitou de ventilação mecânica e não houve óbitos.

Conclusões:: Observamos que crianças e adolescentes acometidos pela Covid-19 apresentaram, em sua maioria, quadros leves e limitados a sintomas de via aérea superior. A gravidade do quadro clínico da Covid-19 foi maior entre as crianças de menor idade que tinham com maior frequência sintomas gastrintestinais e pulmonares.

Conflict of interest statement

The authors declare there is no conflict of interests.

- Cited by 6 articles
- 14 references

Supplementary info

Publication types, MeSH terms Expand

Publication types

- Observational Study

MeSH terms

- Adolescent
- Brazil
- COVID-19 / complications
- COVID-19 / diagnosis*
- COVID-19 / therapy
- COVID-19 Testing / statistics & numerical data*
- Child
- Child, Preschool
- Cough / etiology
- Cross-Sectional Studies
- Female
- Fever / etiology
- Humans
- Infant
- Length of Stay / statistics & numerical data
- Male
- Pneumonia / etiology
- Retrospective Studies
- SARS-CoV-2
- Severity of Illness Index*

Full text links

free full text available at SciELO.org Scientific Electronic Library Online Free PMC article

Proceed to details

Cite

Share

☐ 411

Observational Study

Am J Trop Med Hyg

. 2020 Dec 23;104(2):540-545.

doi: 10.4269/ajtmh.20-1427.

Lack of Association of Initial Viral Load in SARS-CoV-2 Patients with In-Hospital Mortality

Anna Carrasquer [1 2 3], Óscar M Peiró [1 2 3], Raul Sanchez-Gimenez [1 2 3], Nisha Lal-Trehan [1 2 3], Victor Del-Moral-Ronda [1 2 3], Gil Bonet [1 2 3], Cristina Gutierrez [4], Isabel Fort-Gallifa [4], Carla Martin-Grau [4], Clara Benavent [4], Francesc Vidal [2 3 5], Alfredo Bardají [1 2 3]
Affiliations Expand

Affiliations

- [1] 1Department of Cardiology, Joan XXIII University Hospital, Tarragona, Spain.
- [2] 2Pere Virgili Health Research Institute (IISPV), Tarragona, Spain.
- [3] 3Rovira i Virgili University, Tarragona, Spain.
- [4] 4Clinical Laboratory, Catalan Institute of HealthTarragona, Spain.
- [5] 5Infectious Disease Unit, Department of Internal Medicine, Joan XXIII University Hospital, Tarragona, Spain.

- PMID: **33357280**
- PMCID: PMC7866314
- DOI: 10.4269/ajtmh.20-1427

Free PMC article
Observational Study

Lack of Association of Initial Viral Load in SARS-CoV-2 Patients with In-Hospital Mortality

Anna Carrasquer et al. Am J Trop Med Hyg. 2020.
Free PMC article
Show details
Am J Trop Med Hyg
. 2020 Dec 23;104(2):540-545.
doi: 10.4269/ajtmh.20-1427.

Authors

Anna Carrasquer [1 2 3], Óscar M Peiró [1 2 3], Raul Sanchez-Gimenez [1 2 3], Nisha Lal-Trehan [1 2 3], Victor Del-Moral-Ronda [1 2 3], Gil Bonet [1 2 3], Cristina Gutierrez [4], Isabel Fort-Gallifa [4], Carla Martin-Grau [4], Clara Benavent [4], Francesc Vidal [2 3 5], Alfredo Bardají [1 2 3]

Affiliations

- [1] 1Department of Cardiology, Joan XXIII University Hospital, Tarragona, Spain.
- [2] 2Pere Virgili Health Research Institute (IISPV), Tarragona, Spain.
- [3] 3Rovira i Virgili University, Tarragona, Spain.
- [4] 4Clinical Laboratory, Catalan Institute of HealthTarragona, Spain.
- [5] 5Infectious Disease Unit, Department of Internal Medicine, Joan XXIII University Hospital, Tarragona, Spain.

- PMID: **33357280**
- PMCID: PMC7866314
- DOI: 10.4269/ajtmh.20-1427

Abstract

Controversy exists in the literature regarding the possible prognostic implications of the nasopharyngeal SARS-CoV-2 viral load. We carried out a retrospective observational study of 169 patients, 96 (58.9%) of whom had a high viral load and the remaining had a low viral load. Compared with patients with a low viral load, patients with a high viral load did not exhibit differences regarding preexisting cardiovascular risk factors or comorbidities. There were no differences in symptoms, vital signs, or laboratory tests in either group, except for the maximum cardiac troponin I (cTnI), which was higher in the group with a higher viral load (24 [interquartile range 9.5-58.5] versus 8.5 [interquartile range 3-22.5] ng/L, P = 0.007). There were no differences in the need for hospital admission, admission to the intensive care unit, or the need for mechanical ventilation in clinical management. In-hospital mortality was greater in patients who had a higher viral load than in those with low viral load (24% versus 10.4%, P = 0.029). High viral loads were associated with in-hospital mortality in the binary logistic regression analysis (odds ratio: 2.701, 95% Charlson Index (CI): 1.084-6.725, P = 0.033). However, in an analysis adjusted for age, gender, CI, and cTnI, viral load was no longer a predictor of mortality. In conclusion, an elevated nasopharyngeal viral load was not a determinant of in-hospital mortality in patients with COVID-19, as much as age, comorbidity, and myocardial damage determined by elevated cTnI are.

- Cited by 6 articles
- 31 references
- 2 figures

Supplementary info

Publication types, MeSH terms Expand

Publication types

- Observational Study

MeSH terms

- Aged
- COVID-19 / mortality*

- COVID-19 / virology*
- Comorbidity
- Female
- Hospital Mortality*
- Hospitalization / statistics & numerical data
- Hospitals, University / statistics & numerical data*
- Humans
- Male
- Middle Aged
- Nasopharynx / virology
- Prognosis
- Retrospective Studies
- Viral Load / statistics & numerical data*

Full text links

Full Text THE AMERICAN JOURNAL OF TROPICAL MEDICINE AND HYGIENE Sheridan PubFactory Free PMC article
Proceed to details
Cite
Share
☐ 412
Clinical Trial
PLoS One
. 2021 Oct 19;16(10):e0258754.
doi: 10.1371/journal.pone.0258754. eCollection 2021.

Feasibility of CPAP application and variables related to worsening of respiratory failure in pregnant women with SARS-CoV-2 pneumonia: Experience of a tertiary care centre

Paola Faverio [1], Sara Ornaghi [2], Anna Stainer [1], Francesca Invernizzi [2], Mara Borelli [1], Federica Brunetti [3], Laura La Milia [2], Valentina Paolini [1], Roberto Rona [1] [4], Giuseppe Foti [1] [4], Fabrizio Luppi [1], Patrizia Vergani [2], Alberto Pesci [1]
Affiliations Expand

Affiliations

- [1] Respiratory Unit, School of Medicine and Surgery, University of Milano Bicocca, San Gerardo Hospital, ASST Monza, Monza, Italy.

- [2] Obstetric Unit, School of Medicine and Surgery, University of Milano Bicocca, MBBM Foundation Onlus at San Gerardo Hospital, Monza, Italy.
- [3] Department of Obstetrics and Gynaecology, Desio Hospital, ASST Monza, Desio, Italy.
- [4] Department of Anesthesia and Intensive Care Medicine, ASST Monza, Monza, Italy.

- PMID: **34665818**
- PMCID: PMC8525751
- DOI: 10.1371/journal.pone.0258754

Free PMC article
Clinical Trial

Feasibility of CPAP application and variables related to worsening of respiratory failure in pregnant women with SARS-CoV-2 pneumonia: Experience of a tertiary care centre

Paola Faverio et al. PLoS One. 2021.
Free PMC article
Show details
PLoS One

. 2021 Oct 19;16(10):e0258754.
doi: 10.1371/journal.pone.0258754. eCollection 2021.

Authors

Paola Faverio [1], Sara Ornaghi [2], Anna Stainer [1], Francesca Invernizzi [2], Mara Borelli [1], Federica Brunetti [3], Laura La Milia [2], Valentina Paolini [1], Roberto Rona [1] [4], Giuseppe Foti [1] [4], Fabrizio Luppi [1], Patrizia Vergani [2], Alberto Pesci [1]

Affiliations

- [1] Respiratory Unit, School of Medicine and Surgery, University of Milano Bicocca, San Gerardo Hospital, ASST Monza, Monza, Italy.
- [2] Obstetric Unit, School of Medicine and Surgery, University of Milano Bicocca, MBBM Foundation Onlus at San Gerardo Hospital, Monza, Italy.
- [3] Department of Obstetrics and Gynaecology, Desio Hospital, ASST Monza, Desio, Italy.
- [4] Department of Anesthesia and Intensive Care Medicine, ASST Monza, Monza, Italy.

- PMID: **34665818**
- PMCID: PMC8525751
- DOI: 10.1371/journal.pone.0258754

Abstract

Continuous positive airway pressure (CPAP) has been successfully applied to patients with COVID-19 to prevent endotracheal intubation. However, experience of CPAP application in pregnant women with acute respiratory failure (ARF) due to SARS-CoV-2 pneumonia is scarce. This study aimed to describe the natural history and outcome of ARF in a cohort of pregnant women with SARS-CoV-2 pneumonia, focusing on the feasibility of helmet CPAP (h-CPAP) application and the variables related to ARF worsening. A retrospective, observational study enrolling 41 consecutive pregnant women hospitalised for SARS-CoV-2 pneumonia in a tertiary care center between March 2020 and March 2021. h-CPAP was applied if arterial partial pressure of oxygen to fraction of inspired oxygen ratio (PaO2/FiO2) was inferior to 200 and/or patients had respiratory distress despite adequate oxygen supplementation. Characteristics of patients requiring h-CPAP vs those in room air or oxygen only were compared. Twenty-seven (66%) patients showed hypoxemic ARF requiring oxygen supplementation and h-CPAP was needed in 10 cases (24%). PaO2/FiO2 was significantly improved during h-CPAP application. The device was well-tolerated in all cases with no adverse events. Higher serum C reactive protein and more extensive (≥3 lobes) involvement at chest X-ray upon admission were observed in the h-CPAP group. Assessment of temporal distribution of cases showed a substantially increased rate of CPAP requirement during the third pandemic wave (January-March 2021). In conclusion, h-CPAP was feasible, safe, well-tolerated and improved oxygenation in pregnant women with moderate-to-severe ARF due to SARS-CoV-2 pneumonia. Moderate-to-severe ARF was more frequently observed during the third pandemic wave.

Conflict of interest statement

The authors have declared that no competing interests exist.

- 22 references
- 3 figures

Supplementary info

Publication types, MeSH terms, Substances, Grant support Expand

Publication types

- Clinical Trial

MeSH terms

- Acute Disease
- Adult
- COVID-19* / blood
- COVID-19* / therapy
- Continuous Positive Airway Pressure*
- Female
- Humans
- Oxygen / administration & dosage*

- Oxygen / blood
- Pregnancy
- Pregnancy Complications, Infectious* / blood
- Pregnancy Complications, Infectious* / therapy
- Protein C / metabolism
- Respiratory Insufficiency* / blood
- Respiratory Insufficiency* / therapy
- Retrospective Studies
- SARS-CoV-2 / metabolism*
- Tertiary Care Centers*

Substances

- Protein C
- Oxygen

Grant support

The authors received no specific funding for this work.

Full text links

OPEN ACCESS TO FULL TEXT PLOS ONE Public Library of Science Free PMC article
Proceed to details
Cite
Share
☐ 413
Observational Study
Clin Res Cardiol
. 2020 Dec;109(12):1483-1489.
doi: 10.1007/s00392-020-01682-1. Epub 2020 Jun 6.

The mystery of "missing" visits in an emergency cardiology department, in the era of COVID-19.; a time-series analysis in a tertiary Greek General Hospital

Konstantinos Tsioufis [1], Christina Chrysohoou [2], Maria Kariori [2], Ioannis Leontsinis [2], Ioannis Dalakouras [2], Angelos Papanikolaou [2], Georgios Charalambus [3], Helen Sambatakou [4], Gerasimos Siasos [2], Demosthenes Panagiotakos [5], Dimitrios Tousoulis [2]
Affiliations Expand

Affiliations

- [1] First Cardiology Clinic, Medical School, National and Kapodistrian University of Athens, Hippokration Hospital, 114 Vass Sofias Ave, 11527, Athens, Greece. ktsioufis@hippocratio.gr.
- [2] First Cardiology Clinic, Medical School, National and Kapodistrian University of Athens, Hippokration Hospital, 114 Vass Sofias Ave, 11527, Athens, Greece.
- [3] Emergency Department, Hippocratio Hospital, Athens, Greece.
- [4] 2nd Department of Internal Medicine, HIV Unit, Medical School, Hippokration General Hospital, National and Kapodistrian University of Athens, Athens, Greece.
- [5] School of Health Science and Education, Harokopio University, Athens, Greece.

- PMID: **32506198**
- PMCID: PMC7275652
- DOI: 10.1007/s00392-020-01682-1

Free PMC article
Observational Study

The mystery of "missing" visits in an emergency cardiology department, in the era of COVID-19.; a time-series analysis in a tertiary Greek General Hospital

Konstantinos Tsioufis et al. Clin Res Cardiol. 2020 Dec.
Free PMC article

Show details

Clin Res Cardiol

. 2020 Dec;109(12):1483-1489.
doi: 10.1007/s00392-020-01682-1. Epub 2020 Jun 6.

Authors

Konstantinos Tsioufis [1], Christina Chrysohoou [2], Maria Kariori [2], Ioannis Leontsinis [2], Ioannis Dalakouras [2], Angelos Papanikolaou [2], Georgios Charalambus [3], Helen Sambatakou [4], Gerasimos Siasos [2], Demosthenes Panagiotakos [5], Dimitrios Tousoulis [2]

Affiliations

- [1] First Cardiology Clinic, Medical School, National and Kapodistrian University of Athens, Hippokration Hospital, 114 Vass Sofias Ave, 11527, Athens, Greece. ktsioufis@hippocratio.gr.
- [2] First Cardiology Clinic, Medical School, National and Kapodistrian University of Athens, Hippokration Hospital, 114 Vass Sofias Ave, 11527, Athens, Greece.
- [3] Emergency Department, Hippocratio Hospital, Athens, Greece.

- [4] 2nd Department of Internal Medicine, HIV Unit, Medical School, Hippokration General Hospital, National and Kapodistrian University of Athens, Athens, Greece.
- [5] School of Health Science and Education, Harokopio University, Athens, Greece.

- PMID: **32506198**
- PMCID: PMC7275652
- DOI: 10.1007/s00392-020-01682-1

Erratum in

- Correction to: The mystery of "missing" visits in an emergency cardiology department, in the era of COVID-19.; a time-series analysis in a tertiary Greek General Hospital.
  Tsioufis K, Chrysohoou C, Kariori M, Leontsinis I, Dalakouras I, Papanikolaou A, Charalambus G, Sambatakou H, Siasos G, Panagiotakos D, Tousoulis D. Tsioufis K, et al. Clin Res Cardiol. 2020 Dec;109(12):1490. doi: 10.1007/s00392-020-01716-8. Clin Res Cardiol. 2020. PMID: 32770261 Free PMC article.

Abstract

Background: In the era of the current COVID-19 health crisis, the aim of the present study was to explore population behavior as regards the visits in the Emergency Cardiology department (ECD) of a tertiary General Hospital that does not hospitalize SARS-CoV-2 infected patients METHODS AND RESULTS: Daily number of visits at the EDC and admissions to Cardiology Wards and Intensive Care Unit of a tertiary General Hospital, in Athens, Greece, were retrieved from hospital's database (January 1st-April 30th 2018, 2019 and 2020). A highly significant reduction in the visits at ECD of the hospital during March and April 2020 was observed as compared with January and February of the same year (p for linear trend < ·001); in particular the number of visits was 41.1% lower in March 2020 and 32.7% lower in April 2020, as compared to January 2020. As the number of confirmed COVID-19 cases throughout the country increased (i.e., from February 26th to April 2nd) the number of visits at ECD decreased (p = 0.01), whereas, the opposite was observed in the period afterwards (p = 0.01).The number of acute Myocardial infarctions (MI) cases in March 2020 was the lowest compared to the entire three year period (p < 0·001); however, the number of acute MI cases in April 2020 was doubled as compared to March 2020, but still was lower than the preceding years (p < 0·001).

Conclusions: It is hard to explain the mystery of the "missing" emergency hospital visits. However, if this decline in cardiovascular disease related hospital visits is "true", it is something that needs to be rigorously studied, to learn how to keep these rates down.

Keywords: Covid19; Emergency cardiological department; Missing visits.

Conflict of interest statement

The author declare that there is no conflict of interest.

Comment in

- Response to paper by Tsioufis et al.
  Liebert C, Patel R, Kirresh A, Ahmad M. Liebert C, et al. Clin Res Cardiol. 2020 Dec;109(12):1579. doi: 10.1007/s00392-020-01699-6. Epub 2020 Jul 10. Clin Res Cardiol. 2020. PMID: 32651655 Free PMC article. No abstract available.

- Cited by 23 articles
- 25 references
- 3 figures

Supplementary info

Publication types, MeSH terms Expand

Publication types

- Observational Study

MeSH terms

- COVID-19 / epidemiology
- COVID-19 / prevention & control*
- COVID-19 / transmission
- Cardiology Service, Hospital / trends*
- Cardiovascular Diseases / diagnosis
- Cardiovascular Diseases / epidemiology
- Cardiovascular Diseases / therapy*
- Emergency Service, Hospital / trends*
- Greece / epidemiology
- Health Knowledge, Attitudes, Practice
- Health Services Accessibility / trends
- Health Services Needs and Demand / trends
- Hospitals, General / trends*
- Humans
- Patient Acceptance of Health Care*
- Patient Admission / trends*
- Retrospective Studies
- Tertiary Care Centers / trends*
- Time Factors

Full text links

SpringerLink FULL-TEXT ARTICLE Springer Free PMC article
Proceed to details
Cite
Share
☐ 414
Observational Study
Am Heart J
. 2020 Aug;226:45-48.

doi: 10.1016/j.ahj.2020.04.022. Epub 2020 May 11.

Impact of COVID-19 pandemic on patients with ST-segment elevation myocardial infarction: Insights from a British cardiac center

Hesham K Abdelaziz [1], Amr Abdelrahman [2], Amjad Nabi [2], Maciej Debski [2], Amgad Mentias [3], Tawfiq Choudhury [2], Billal Patel [2], Marwan Saad [4]
Affiliations Expand

Affiliations

- [1] Lancashire Cardiac Centre, Blackpool Victoria Hospital, Blackpool, UK; Division of Cardiology, Ain Shams University, Cairo, Egypt.
- [2] Lancashire Cardiac Centre, Blackpool Victoria Hospital, Blackpool, UK.
- [3] Division of Cardiovascular Medicine, University of Iowa Carver College of Medicine, Iowa City, IA, USA.
- [4] Division of Cardiology, Ain Shams University, Cairo, Egypt; Cardiovascular Institute, The Warren Alpert Medical School of Brown University and Lifespan Cardiovascular Institute, Providence, RI, USA. Electronic address: marwanssaad@gmail.com.

- PMID: **32497914**
- PMCID: PMC7211651
- DOI: 10.1016/j.ahj.2020.04.022

Free PMC article
Observational Study

Impact of COVID-19 pandemic on patients with ST-segment elevation myocardial infarction: Insights from a British cardiac center

Hesham K Abdelaziz et al. Am Heart J. 2020 Aug.
Free PMC article
Show details
Am Heart J
. 2020 Aug;226:45-48.
doi: 10.1016/j.ahj.2020.04.022. Epub 2020 May 11.

Authors

Hesham K Abdelaziz [1], Amr Abdelrahman [2], Amjad Nabi [2], Maciej Debski [2], Amgad Mentias [3], Tawfiq Choudhury [2], Billal Patel [2], Marwan Saad [4]

Affiliations

- [1] Lancashire Cardiac Centre, Blackpool Victoria Hospital, Blackpool, UK; Division of Cardiology, Ain Shams University, Cairo, Egypt.
- [2] Lancashire Cardiac Centre, Blackpool Victoria Hospital, Blackpool, UK.
- [3] Division of Cardiovascular Medicine, University of Iowa Carver College of Medicine, Iowa City, IA, USA.
- [4] Division of Cardiology, Ain Shams University, Cairo, Egypt; Cardiovascular Institute, The Warren Alpert Medical School of Brown University and Lifespan Cardiovascular Institute, Providence, RI, USA. Electronic address: marwanssaad@gmail.com.

- PMID: **32497914**
- PMCID: PMC7211651
- DOI: 10.1016/j.ahj.2020.04.022

Abstract

The current study aimed to examine the impact of COVID-19 pandemic on patient-related delay with ST-segment elevation myocardial infarction (STEMI) at a tertiary center in the United Kingdom. The study demonstrated a significant delay in symptom-to-first medical contact and a higher cardiac troponin-I level on admission in patients with STEMI during the COVID-19 pandemic versus the pre-COVID era.

Copyright © 2020 Elsevier Inc. All rights reserved.

- Cited by 27 articles
- 14 references
- 1 figure

Supplementary info

Publication types, MeSH terms Expand

Publication types

- Letter
- Observational Study

MeSH terms

- Betacoronavirus*
- COVID-19
- Cardiac Care Facilities

- Coronavirus Infections / epidemiology*
- Emergency Medical Services / statistics & numerical data
- Female
- Humans
- Male
- Middle Aged
- Myocardial Reperfusion / statistics & numerical data
- Pandemics
- Pneumonia, Viral / epidemiology*
- Retrospective Studies
- SARS-CoV-2
- ST Elevation Myocardial Infarction / complications
- ST Elevation Myocardial Infarction / therapy*
- Tertiary Care Centers
- Time-to-Treatment / statistics & numerical data*
- United Kingdom / epidemiology

Full text links

ELSEVIER FULL-TEXT ARTICLE Elsevier Science Free PMC article
Proceed to details
Cite
Share
415
Observational Study
Sci Rep
. 2021 Jun 23;11(1):13134.
doi: 10.1038/s41598-021-92497-1.

Detection of SARS-CoV-2 RNA in serum is associated with increased mortality risk in hospitalized COVID-19 patients

Diego A Rodríguez-Serrano [#] [1], Emilia Roy-Vallejo [#] [2], Nelly D Zurita Cruz [3], Alexandra Martín Ramírez [3], Sebastián C Rodríguez-García [4], Nuria Arevalillo-Fernández [5], José María Galván-Román [2], Leticia Fontán García-Rodrigo [3], Lorena Vega-Piris [6], Marta Chicot Llano [5], David Arribas Méndez [7], Begoña González de Marcos [5], Julia Hernando Santos [7], Ana Sánchez Azofra [8], Elena Ávalos Pérez-Urria [8], Pablo Rodriguez-Cortes [2], Laura Esparcia [9], Ana Marcos-Jimenez [9], Santiago Sánchez-Alonso [9], Irene Llorente [4], Joan Soriano [8] [10], Carmen Suárez Fernández [2] [10], Rosario García-Vicuña [4], Julio Ancochea [8] [10], Jesús Sanz [2], Cecilia Muñoz-Calleja [9] [10], Rafael de la Cámara [11], Alfonso Canabal Berlanga [5], Isidoro González-Álvaro [#] [4], Laura Cardeñoso [#] [3], REINMUN-COVID Group

Collaborators, Affiliations Expand

Collaborators

- **REINMUN-COVID Group**:
  Rosa Méndez Hernández, Mar Orts, Carlos Figueroa, Carlos Román, Antonio Planas, Lourdes Domínguez Arganda, Pablo Martínez Vives, Guillermo Diego Nieto, Alberto Cecconi, Amparo Benedicto, Antonio Rojas González, Jesús Jiménez-Borreguero, Carmen Del Arco, Juan Mariano Aguilar, Natalia Villalba, Mónica Negro, Elvira Contreras, Ana Del Rey, Cristina Santiago, Manuel Junquera, Raquel Caminero, Francisco Javier Val, Sonia González, Marta Caño, Isabel López, Andrés von Wernitz, Iñigo Guerra, Jorge Sorando, Lydia Chao, María José Cárdenas, Verónica Espiga, Alberto Pizarro, Ángela Figuera Álvarez, Beatriz Aguado, Jimena Cannata, Javier Ortiz, Alberto Morell, Esther Ramírez, Amparo Ibáñez Zurriaga, María Pérez Abanades, Silvia Ruiz García, Tomás Gallego Aranda, María Ruiz, Concepción Martínez Nieto, José María Serra, Francisco Sánchez-Madrid, Ildefonso Sánchez-Cerrillo, Pedro Martínez-Fleta, Celia López-Sanz, Ligia Gabrie, Luciana Del Campo Guerola, Elena Fernández, Reyes Tejedor, Pablo A Patiño Haro, Marina Trigueros Genao, Begoña Quicios Dorado, David Jiménez Jiménez, Macarena Alonso González, Pablo Villamayor, Ignacio de Los Santos, Eduardo Sánchez, Fernando Moldenhauer, Pedro Casado, Jose Curbelo, Angela Gutiérrez, Azucena Bautista, Nuria Ruiz Giménez, Angelica Fernández, Lucio García Fraile, Pedro Parra, Berta Moyano, Ana Barrios, Paloma Gil, Iluminada García Polo, Diego Real de Asúa, Beatriz Sánchez, Carmen Sáez, Marianela Ciudad, Marta Fernández Rico, Cristina Arévalo Román, Esperanza Morillo Rodríguez, Desiré Navas, María Del Carmen Cuevas Torresano, Diego Domingo García, Teresa Alarcón Cavero, Alicia García Blanco, María Auxiliadora Semiglia Chong, Ainhoa Gutiérrez Cobos, Arturo Manuel Fraile Torres, Tamara Alonso, Pedro Landete, Joan Soriano, Carolina Cisneros, Elena García Castillo, Claudia Valenzuela, Francisco Javier García Pérez, Rosa María Girón, Javier Aspa, Celeste Marcos, Enrique Zamora, Gorane Iturricastillo, Mar Barrio Mayo, Encarna Rubia Garrido, Santos Castañeda, Carlos Fernández-Díaz, Eva G Tomero, Noelia García Castañeda, Ana Ma Ortiz, Cristina Valero, Miren Uriarte, Nuria Montes, Iñigo García Sanz, Francisco Eduardo Viamontes, Jesús Delgado Valdueza

Affiliations

- [1] Intensive Care Unit, Hospital Universitario La Princesa, Madrid, Spain. cancabrilla@hotmail.com.
- [2] Internal Medicine Department, Hospital Universitario La Princesa, IIS-IP, Madrid, Spain.
- [3] Microbiology Department, Hospital Universitario La Princesa, IIS-IP, Madrid, Spain.
- [4] Rheumatology Department, Hospital Universitario La Princesa, IIS-IP, Madrid, Spain.
- [5] Intensive Care Unit, Hospital Universitario La Princesa, Madrid, Spain.
- [6] Methodology Unit, Biomedical Research Institute, Hospital Universitario La Princesa, IIS-IP, Madrid, Spain.
- [7] Anaesthesiology Department, Hospital Universitario La Princesa, IIS-IP, Madrid, Spain.
- [8] Pneumology Department, Hospital Universitario La Princesa, IIS-IP, Madrid, Spain.
- [9] Immunology Department, Hospital Universitario La Princesa, IIS-IP, Madrid, Spain.
- [10] Universidad Autónoma de Madrid, Madrid, Spain.
- [11] Hematology Department, Hospital Universitario La Princesa, IIS-IP, Madrid, Spain.

[#] Contributed equally.

- PMID: **34162948**
- PMCID: PMC8222315
- DOI: 10.1038/s41598-021-92497-1

Free PMC article
Observational Study

Detection of SARS-CoV-2 RNA in serum is associated with increased mortality risk in hospitalized COVID-19 patients

Diego A Rodríguez-Serrano et al. Sci Rep. 2021.
Free PMC article
Show details
Sci Rep
. 2021 Jun 23;11(1):13134.
doi: 10.1038/s41598-021-92497-1.

Authors

Diego A Rodríguez-Serrano [#, 1], Emilia Roy-Vallejo [#, 2], Nelly D Zurita Cruz [3], Alexandra Martín Ramírez [3], Sebastián C Rodríguez-García [4], Nuria Arevalillo-Fernández [5], José María Galván-Román [2], Leticia Fontán García-Rodrigo [3], Lorena Vega-Piris [6], Marta Chicot Llano [5], David Arribas Méndez [7], Begoña González de Marcos [5], Julia Hernando Santos [7], Ana Sánchez Azofra [8], Elena Ávalos Pérez-Urria [8], Pablo Rodriguez-Cortes [2], Laura Esparcia [9], Ana Marcos-Jimenez [9], Santiago Sánchez-Alonso [9], Irene Llorente [4], Joan Soriano [8, 10], Carmen Suárez Fernández [2, 10], Rosario García-Vicuña [4], Julio Ancochea [8, 10], Jesús Sanz [2], Cecilia Muñoz-Calleja [9, 10], Rafael de la Cámara [11], Alfonso Canabal Berlanga [5], Isidoro González-Álvaro [#, 4], Laura Cardeñoso [#, 3], REINMUN-COVID Group

Collaborators

- **REINMUN-COVID Group**: Rosa Méndez Hernández, Mar Orts, Carlos Figueroa, Carlos Román, Antonio Planas, Lourdes Domínguez Arganda, Pablo Martínez Vives, Guillermo Diego Nieto, Alberto Cecconi, Amparo Benedicto, Antonio Rojas González, Jesús Jiménez-Borreguero, Carmen Del Arco, Juan Mariano Aguilar, Natalia Villalba, Mónica Negro, Elvira Contreras, Ana Del Rey, Cristina Santiago, Manuel Junquera, Raquel Caminero, Francisco Javier Val, Sonia González, Marta Caño, Isabel López, Andrés von Wernitz, Iñigo Guerra, Jorge Sorando, Lydia Chao, María José Cárdenas, Verónica Espiga, Alberto Pizarro, Ángela Figuera Álvarez, Beatriz Aguado, Jimena Cannata, Javier Ortiz, Alberto Morell, Esther Ramírez, Amparo Ibáñez Zurriaga, María Pérez Abanades, Silvia Ruiz García, Tomás Gallego Aranda, María Ruiz, Concepción Martínez Nieto, José María Serra, Francisco Sánchez-Madrid, Ildefonso Sánchez-Cerrillo, Pedro Martínez-Fleta, Celia López-Sanz, Ligia Gabrie, Luciana Del Campo Guerola, Elena Fernández, Reyes Tejedor, Pablo A Patiño Haro, Marina Trigueros Genao, Begoña Quicios Dorado, David Jiménez Jiménez, Macarena Alonso González, Pablo Villamayor, Ignacio de

Los Santos, Eduardo Sánchez, Fernando Moldenhauer, Pedro Casado, Jose Curbelo, Angela Gutiérrez, Azucena Bautista, Nuria Ruiz Giménez, Angelica Fernández, Lucio García Fraile, Pedro Parra, Berta Moyano, Ana Barrios, Paloma Gil, Iluminada García Polo, Diego Real de Asúa, Beatriz Sánchez, Carmen Sáez, Marianela Ciudad, Marta Fernández Rico, Cristina Arévalo Román, Esperanza Morillo Rodríguez, Desiré Navas, María Del Carmen Cuevas Torresano, Diego Domingo García, Teresa Alarcón Cavero, Alicia García Blanco, María Auxiliadora Semiglia Chong, Ainhoa Gutiérrez Cobos, Arturo Manuel Fraile Torres, Tamara Alonso, Pedro Landete, Joan Soriano, Carolina Cisneros, Elena García Castillo, Claudia Valenzuela, Francisco Javier García Pérez, Rosa María Girón, Javier Aspa, Celeste Marcos, Enrique Zamora, Gorane Iturricastillo, Mar Barrio Mayo, Encarna Rubia Garrido, Santos Castañeda, Carlos Fernández-Díaz, Eva G Tomero, Noelia García Castañeda, Ana Ma Ortiz, Cristina Valero, Miren Uriarte, Nuria Montes, Iñigo García Sanz, Francisco Eduardo Viamontes, Jesús Delgado Valdueza

Affiliations

- [1] Intensive Care Unit, Hospital Universitario La Princesa, Madrid, Spain. cancabrilla@hotmail.com.
- [2] Internal Medicine Department, Hospital Universitario La Princesa, IIS-IP, Madrid, Spain.
- [3] Microbiology Department, Hospital Universitario La Princesa, IIS-IP, Madrid, Spain.
- [4] Rheumatology Department, Hospital Universitario La Princesa, IIS-IP, Madrid, Spain.
- [5] Intensive Care Unit, Hospital Universitario La Princesa, Madrid, Spain.
- [6] Methodology Unit, Biomedical Research Institute, Hospital Universitario La Princesa, IIS-IP, Madrid, Spain.
- [7] Anaesthesiology Department, Hospital Universitario La Princesa, IIS-IP, Madrid, Spain.
- [8] Pneumology Department, Hospital Universitario La Princesa, IIS-IP, Madrid, Spain.
- [9] Immunology Department, Hospital Universitario La Princesa, IIS-IP, Madrid, Spain.
- [10] Universidad Autónoma de Madrid, Madrid, Spain.
- [11] Hematology Department, Hospital Universitario La Princesa, IIS-IP, Madrid, Spain.

[#] Contributed equally.

- PMID: **34162948**
- PMCID: PMC8222315
- DOI: 10.1038/s41598-021-92497-1

Abstract

COVID-19 has overloaded national health services worldwide. Thus, early identification of patients at risk of poor outcomes is critical. Our objective was to analyse SARS-CoV-2 RNA detection in serum as a severity biomarker in COVID-19. Retrospective observational study including 193 patients admitted for COVID-19. Detection of SARS-CoV-2 RNA in serum (viremia) was performed with samples collected at 48-72 h of admission by two techniques from Roche and Thermo Fischer Scientific (TFS). Main outcome variables were mortality and need for ICU admission during hospitalization for COVID-19. Viremia was detected in 50-60% of patients depending on technique. The correlation of Ct in serum between both techniques was good (intraclass correlation coefficient: 0.612; $p < 0.001$). Patients with viremia were older ($p = 0.006$), had poorer baseline oxygenation (PaO_2/FiO_2; $p < 0.001$), more severe lymphopenia ($p < 0.001$) and higher LDH ($p < 0.001$), IL-6 ($p = 0.021$), C-reactive protein (CRP; $p = 0.022$) and procalcitonin ($p = 0.002$) serum levels. We defined "relevant viremia" when detection Ct was < 34

with Roche and < 31 for TFS. These thresholds had 95% sensitivity and 35% specificity. Relevant viremia predicted death during hospitalization (OR 9.2 [3.8-22.6] for Roche, OR 10.3 [3.6-29.3] for TFS; p < 0.001). Cox regression models, adjusted by age, sex and Charlson index, identified increased LDH serum levels and relevant viremia (HR = 9.87 [4.13-23.57] for TFS viremia and HR = 7.09 [3.3-14.82] for Roche viremia) as the best markers to predict mortality. Viremia assessment at admission is the most useful biomarker for predicting mortality in COVID-19 patients. Viremia is highly reproducible with two different techniques (TFS and Roche), has a good consistency with other severity biomarkers for COVID-19 and better predictive accuracy.

Conflict of interest statement

The authors of this manuscript have the following competing interests: SCR-G reports grants from Spanish Rheumatology Foundation, during the conduct of the study; nonfinancial support from Roche, Lilly, Pfizer, and Abbvie; personal fees and nonfinancial support from Novartis, Sanofi, and MSD and from UCB-Pharma, outside the submitted work. JA reports grants and personal fees from GlaxoSmithKline and Boehringer Ingelheim; grants from Linde Healthcare; and grants, personal fees, and nonfinancial support from Roche and from Chiesi, outside the submitted work. DAR-S reports personal fees from MSD, outside the submitted work. RdC reports personal fees from MSD, ASTELLAS, Clinigen, Janssen, Roche, and IQONE Health Care outside the submitted work. RG-V reports grants, personal fees, and nonfinancial support from Abbvie, BMS, Lilly, Novartis, Sanofi, Sandoz, and MSD; personal fees from Biogen and Celltrion and from Mylan, outside the submitted work; personal fees and nonfinancial support from Pfizer; grants from Roche; and grants and personal fees from Janssen. CSF reports personal fees from Bayer, BMS, Daichi Sankyo, MSD, and Pfizer, outside the submitted work. CM-C reports competitive grants from ISCIII during the conduct of the study. IG-A reports grants from Instituto de Salud Carlos III, during the course of the study; Personal fees from Lilly and Sanofi; personal fees and nonfinancial support from BMS and Abbvie; research support, personal fees, and nonfinancial support from Roche Laboratories; and nonfinancial support from MSD, Pfizer, and Novartis, not related to the submitted work. The rest of the authors declare that they have no relevant competing interests.

- Cited by 4 articles
- 26 references
- 4 figures

Supplementary info

Publication types, MeSH terms, Substances, Grant support Expand

Publication types

- Observational Study
- Research Support, Non-U.S. Gov't

MeSH terms

- Aged
- Biomarkers / blood
- COVID-19 / blood*

- COVID-19 / mortality
- COVID-19 / virology
- Critical Care
- Female
- Hospitalization
- Humans
- Interleukin-6 / blood
- Male
- Middle Aged
- Patient Acuity
- RNA, Viral / blood*
- Real-Time Polymerase Chain Reaction
- Retrospective Studies
- Risk Factors
- SARS-CoV-2 / genetics*
- Spain
- Viremia / blood*
- Viremia / virology

Substances

- Biomarkers
- Interleukin-6
- RNA, Viral

Grant support

- CM19/00149/Instituto de Salud Carlos III
- RD16/0011/0012/Instituto de Salud Carlos III
- Fondos Supera COVID-19/Fundación Banco Santander

Full text links

Nature Publishing Group Free PMC article

Proceed to details

Cite

Share

☐ 416

Observational Study

Cardiol J

. 2021;28(3):360-368.

doi: 10.5603/CJ.a2021.0034. Epub 2021 Apr 12.

Impact of the presence of heart disease, cardiovascular medications and cardiac events on outcome in COVID-19

Gonzalo Cabezón Villalba [1], Ignacio J Amat-Santos [1], Carlos Dueñas [2], Diego Lopez Otero [3], Pablo Catala [1], Alvaro Aparisi [1], Javier López-Pais [3], Carla Eugenia Cacho Antonio [3], Jordi Candela [1], Pablo Antúnez Muiños [3], Jose Francisco Gil [1], Teba Gonzalez Ferrero [3], Marta Marcos [1], Marta Pérez-Poza [3], Gino Rojas [1], Oscar Otero Garcia [3], Carlos Veras [1], Victor Jiménez Ramos [3], Aitor Uribarri [1], Ana Revilla [1], Pablo Elpidio Garcia-Granja [1], Itziar Gómez [1], José Ramón González-Juanatey [3], J Alberto San Román [4]

Affiliations Expand

Affiliations

- [1] Department of Cardiology, Institute of Heart Sciences (ICICOR), Hospital Clínico Universitario, Valladolid, Spain.
- [2] Hospital Clínico Universitario de Valladolid, Valladolid, Spain.
- [3] Complejo Hospitalario Universitario de Santiago de Compostela, Rua da Choupana s/n, 15702 Santiago de Compostela, Spain.
- [4] Department of Cardiology, Institute of Heart Sciences (ICICOR), Hospital Clínico Universitario, Valladolid, Spain. asanroman@secardiologia.es.

- PMID: **33843043**
- PMCID: PMC8169179
- DOI: 10.5603/CJ.a2021.0034

Free PMC article
Observational Study

Impact of the presence of heart disease, cardiovascular medications and cardiac events on outcome in COVID-19

Gonzalo Cabezón Villalba et al. Cardiol J. 2021.
Free PMC article
Show details
Cardiol J
. 2021;28(3):360-368.
doi: 10.5603/CJ.a2021.0034. Epub 2021 Apr 12.

Authors

Gonzalo Cabezón Villalba [1], Ignacio J Amat-Santos [1], Carlos Dueñas [2], Diego Lopez Otero [3], Pablo Catala [1], Alvaro Aparisi [1], Javier López-Pais [3], Carla Eugenia Cacho Antonio [3], Jordi Candela [1], Pablo Antúnez Muiños [3], Jose Francisco Gil [1], Teba Gonzalez Ferrero [3], Marta Marcos [1], Marta Pérez-Poza [3], Gino Rojas [1], Oscar Otero Garcia [3], Carlos Veras [1], Victor Jiménez Ramos [3], Aitor Uribarri [1], Ana Revilla [1], Pablo Elpidio Garcia-Granja [1], Itziar Gómez [1], José Ramón González-Juanatey [3], J Alberto San Román [4]

Affiliations

- [1] Department of Cardiology, Institute of Heart Sciences (ICICOR), Hospital Clínico Universitario, Valladolid, Spain.
- [2] Hospital Clínico Universitario de Valladolid, Valladolid, Spain.
- [3] Complejo Hospitalario Universitario de Santiago de Compostela, Rua da Choupana s/n, 15702 Santiago de Compostela, Spain.
- [4] Department of Cardiology, Institute of Heart Sciences (ICICOR), Hospital Clínico Universitario, Valladolid, Spain. asanroman@secardiologia.es.

- PMID: **33843043**
- PMCID: PMC8169179
- DOI: 10.5603/CJ.a2021.0034

Abstract

Background: Cardiovascular risk factors and usage of cardiovascular medication are prevalent among coronavirus disease 2019 (COVID-19) patients. Little is known about the cardiovascular implications of COVID-19. The goal herein, was to evaluate the prognostic impact of having heart disease (HD) and taking cardiovascular medications in a population diagnosed of COVID-19 who required hospitalization. Also, we studied the development of cardiovascular events during hospitalization.

Methods: Consecutive patients with definitive diagnosis of COVID-19 made by a positive real time- -polymerase chain reaction of nasopharyngeal swabs who were admitted to the hospital from March 15 to April 14 were included in a retrospective registry. The association of HD with mortality and with mortality or respiratory failure were the primary and secondary objectives, respectively.

Results: A total of 859 patients were included in the present analysis. Cardiovascular risk factors were related to death, particularly diabetes mellitus (hazard ratio in the multivariate analysis: 1.810 [1.159- -2.827], $p = 0.009$). A total of 113 (13.1%) patients had HD. The presence of HD identified a group of patients with higher mortality (35.4% vs. 18.2%, $p < 0.001$) but HD was not independently related to prognosis; renin-angiotensin-aldosterone system inhibitors, calcium channel blockers, diuretics and beta-blockers did not worsen prognosis. Statins were independently associated with decreased mortality (0.551 [0.329-0.921], $p = 0.023$). Cardiovascular events during hospitalization identified a group of patients with poor outcome (mortality 31.8% vs. 19.3% without cardiovascular events, $p = 0.007$).

Conclusions: The presence of HD is related to higher mortality. Cardiovascular medications taken before admission are not harmful, statins being protective. The development of cardiovascular events during the course of the disease is related to poor outcome.

Keywords: COVID-19; cardiovascular diseases; diabetes mellitus; heart failure; statins.

Conflict of interest statement

Conflict of interest: None declared

- Cited by 1 article

Supplementary info

Publication types, MeSH terms, Substances Expand

Publication types

- Multicenter Study
- Observational Study

MeSH terms

- Aged
- COVID-19 / epidemiology*
- Cardiovascular Agents / therapeutic use*
- Comorbidity
- Female
- Heart Diseases / drug therapy
- Heart Diseases / epidemiology*
- Humans
- Male
- Pandemics*
- Prognosis
- Retrospective Studies
- SARS-CoV-2

Substances

- Cardiovascular Agents

Full text links

full text provider Via Medica Medical Publishers Free PMC article

Proceed to details

Cite

Share

☐ 417

Observational Study

Int J Artif Organs

. 2022 Feb;45(2):216-220.
doi: 10.1177/03913988211052572. Epub 2021 Oct 26.

CytoSorb purification in critically ill SARS-CoV-2 patients

Marina Pieri [1], Evgeny Fominskiy [1], Pasquale Nardelli [1], Matteo A Bonizzoni [1], Anna M Scandroglio [1]
Affiliations Expand

Affiliation

- [1] Department of Anesthesia and Intensive Care, IRCCS San Raffaele Scientific Institute, Milan, Italy.

- PMID: **34702109**
- DOI: 10.1177/03913988211052572

Observational Study

CytoSorb purification in critically ill SARS-CoV-2 patients

Marina Pieri et al. Int J Artif Organs. 2022 Feb.
Show details
Int J Artif Organs
. 2022 Feb;45(2):216-220.
doi: 10.1177/03913988211052572. Epub 2021 Oct 26.

Authors

Marina Pieri [1], Evgeny Fominskiy [1], Pasquale Nardelli [1], Matteo A Bonizzoni [1], Anna M Scandroglio [1]

Affiliation

- [1] Department of Anesthesia and Intensive Care, IRCCS San Raffaele Scientific Institute, Milan, Italy.

- PMID: **34702109**
- DOI: 10.1177/03913988211052572

Abstract

Objective: To describe the experience with CytoSorb treatment in patients with refractory acute respiratory distress syndrome (ARDS) following SARS-CoV-2 infection.

Methods: Retrospective observational study on 15 patients treated in a University Hospital.

Results: All patients were male, with a mean age of 55 ± 14 years; eight patients (53%) were on venovenous extracorporeal membrane oxygenation (VV ECMO) due to refractory ARDS and all (100%) under mechanical ventilation at the time of CytoSorb use. We observed reduction in the level of C reactive protein (-52%, $p = 0.002$), total bilirubin (-46%, $p = 0.03$), direct bilirubin (-50%, $p = 0.02$), and D-dimers (-39%, $p = 0.04$) during CytoSorb treatment and a trend toward reduction in lactate dehydrogenase (-20%, $p = 0.2$), creatine phosphokinase (-38%, $p = 0.1$), and fibrinogen (-15%, $p = 0.07$). Eight patients died (53%) and seven (47%) were discharged from the ICU, of which five had recovery of the native lung function and two were successfully bridged to lung transplantation on VV ECMO support. No difference between survivors and non-survivors was present at baseline. Patients received three CytoSorb cycles on average: mean duration of CytoSorb cycle was 17 h 21 min, but premature circuit clotting despite appropriate level of systemic anticoagulation was frequently observed.

Conclusions: CytoSorb treatment was effective in improving several laboratory parameters and inflammation in our experience and no treatment-related adverse effects were recorded. In the light of the unique pathophysiology of SARS-CoV-2 infection, CytoSorb treatment is extremely promising, since it might both reduce inflammation and activation of coagulation.

Keywords: Extracorporeal circuit; SARS-CoV-2; intensive care unit; mortality; sepsis.

Supplementary info

Publication types, MeSH terms Expand

Publication types

- Observational Study

MeSH terms

- Adult
- Aged
- COVID-19*
- Critical Illness
- Extracorporeal Membrane Oxygenation*
- Humans
- Male
- Middle Aged
- Respiratory Distress Syndrome* / therapy
- Retrospective Studies
- SARS-CoV-2

Full text links

SAGE journals Atypon
Proceed to details
Cite
Share
418

Observational Study

Infection
. 2021 Aug;49(4):757-762.
doi: 10.1007/s15010-021-01606-9. Epub 2021 Apr 6.

CD169/SIGLEC1 is expressed on circulating monocytes in COVID-19 and expression levels are associated with disease severity

Jan-Moritz Doehn [#, 1], Christoph Tabeling [#, 1, 2, 3], Robert Biesen [4], Jacopo Saccomanno [1], Elena Madlung [1], Eva Pappe [1], Frieder Gabriel [1], Florian Kurth [1, 5], Christian Meisel [6, 7], Victor M Corman [8, 9], Leif G Hanitsch [6], Sascha Treskatsch [10], Kathrin Heim [1], Miriam S Stegemann [1], Christoph Ruwwe-Glösenkamp [1], Holger C Müller-Redetzky [1], Alexander Uhrig [1], Rajan Somasundaram [11], Claudia Spies [12], Horst von Bernuth [13], Jörg Hofmann [7, 8, 9], Christian Drosten [8, 9], Norbert Suttorp [1, 14], Martin Witzenrath [1, 2, 14], Leif E Sander [1, 14], Ralf-Harto Hübner [15]

Affiliations Expand

Affiliations

- [1] Department of Infectious Diseases and Respiratory Medicine, Charité-Universitätsmedizin Berlin, corporate member of Freie Universität Berlin, Humboldt-Universität zu Berlin, and Berlin Institute of Health, 10117, Berlin, Germany.
- [2] Division of Pulmonary Inflammation, Charité-Universitätsmedizin Berlin, corporate member of Freie Universität Berlin, Humboldt-Universität zu Berlin, and Berlin Institute of Health, Berlin, Germany.
- [3] Berlin Institute of Health at Charité - Universitätsmedizin Berlin, Berlin, Germany.
- [4] Department of Rheumatology and Clinical Immunology, Charité-Universitätsmedizin Berlin, corporate member of Freie Universität Berlin, Humboldt-Universität zu Berlin, and Berlin Institute of Health, Berlin, Germany.
- [5] Department of Tropical Medicine, Bernhard Nocht Institute for Tropical Medicine, Hamburg, Germany.
- [6] Institute of Medical Immunology, Charité-Universitätsmedizin Berlin, corporate member of Freie Universität Berlin, Humboldt-Universität zu Berlin, and Berlin Institute of Health, Berlin, Germany.
- [7] Labor Berlin GmbH, Berlin, Germany.

- [8] Institute of Virology, Charité-Universitätsmedizin Berlin, corporate member of Freie Universität Berlin, Humboldt-Universität zu Berlin, and Berlin Institute of Health, Berlin, Germany.
- [9] German Centre for Infection Research (DZIF), Berlin, Germany.
- [10] Department of Anesthesiology and Intensive Care Medicine, Charité Campus Benjamin Franklin, Charité-Universitätsmedizin Berlin, corporate member of Freie Universität Berlin, Humboldt-Universität zu Berlin, and Berlin Institute of Health, Berlin, Germany.
- [11] Emergency Department, Charité-Universitätsmedizin Berlin, corporate member of Freie Universität Berlin, Humboldt-Universität zu Berlin, and Berlin Institute of Health, Berlin, Germany.
- [12] Department of Anesthesiology and Intensive Care Medicine, Charité Campus Mitte and Campus-Virchow-Klinikum, Charité-Universitätsmedizin Berlin, corporate member of Freie Universität Berlin, Humboldt-Universität zu Berlin, and Berlin Institute of Health, Berlin, Germany.
- [13] Department of Pediatric Pneumology, Immunology and Intensive Care Medicine, Charité-Universitätsmedizin Berlin, Berlin, Germany.
- [14] Associate member of the German Center for Lung Research (DZL), Marburg, Germany.
- [15] Department of Infectious Diseases and Respiratory Medicine, Charité-Universitätsmedizin Berlin, corporate member of Freie Universität Berlin, Humboldt-Universität zu Berlin, and Berlin Institute of Health, 10117, Berlin, Germany. ralf-harto.huebner@charite.de.

[#] Contributed equally.

- PMID: **33825125**
- PMCID: PMC8023546
- DOI: 10.1007/s15010-021-01606-9

Free PMC article
Observational Study

CD169/SIGLEC1 is expressed on circulating monocytes in COVID-19 and expression levels are associated with disease severity

Jan-Moritz Doehn et al. Infection. 2021 Aug.
Free PMC article
Show details
Infection
. 2021 Aug;49(4):757-762.
doi: 10.1007/s15010-021-01606-9. Epub 2021 Apr 6.

Authors

Jan-Moritz Doehn [#] [1], Christoph Tabeling [#] [1] [2] [3], Robert Biesen [4], Jacopo Saccomanno [1], Elena Madlung [1], Eva Pappe [1], Frieder Gabriel [1], Florian Kurth [1] [5], Christian Meisel [6] [7], Victor M Corman [8] [9], Leif G Hanitsch [6], Sascha Treskatsch [10], Kathrin Heim [1], Miriam S Stegemann

[1], Christoph Ruwwe-Glösenkamp [1], Holger C Müller-Redetzky [1], Alexander Uhrig [1], Rajan Somasundaram [11], Claudia Spies [12], Horst von Bernuth [13], Jörg Hofmann [7] [8] [9], Christian Drosten [8] [9], Norbert Suttorp [1] [14], Martin Witzenrath [1] [2] [14], Leif E Sander [1] [14], Ralf-Harto Hübner [15]

Affiliations

- [1] Department of Infectious Diseases and Respiratory Medicine, Charité-Universitätsmedizin Berlin, corporate member of Freie Universität Berlin, Humboldt-Universität zu Berlin, and Berlin Institute of Health, 10117, Berlin, Germany.
- [2] Division of Pulmonary Inflammation, Charité-Universitätsmedizin Berlin, corporate member of Freie Universität Berlin, Humboldt-Universität zu Berlin, and Berlin Institute of Health, Berlin, Germany.
- [3] Berlin Institute of Health at Charité - Universitätsmedizin Berlin, Berlin, Germany.
- [4] Department of Rheumatology and Clinical Immunology, Charité-Universitätsmedizin Berlin, corporate member of Freie Universität Berlin, Humboldt-Universität zu Berlin, and Berlin Institute of Health, Berlin, Germany.
- [5] Department of Tropical Medicine, Bernhard Nocht Institute for Tropical Medicine, Hamburg, Germany.
- [6] Institute of Medical Immunology, Charité-Universitätsmedizin Berlin, corporate member of Freie Universität Berlin, Humboldt-Universität zu Berlin, and Berlin Institute of Health, Berlin, Germany.
- [7] Labor Berlin GmbH, Berlin, Germany.
- [8] Institute of Virology, Charité-Universitätsmedizin Berlin, corporate member of Freie Universität Berlin, Humboldt-Universität zu Berlin, and Berlin Institute of Health, Berlin, Germany.
- [9] German Centre for Infection Research (DZIF), Berlin, Germany.
- [10] Department of Anesthesiology and Intensive Care Medicine, Charité Campus Benjamin Franklin, Charité-Universitätsmedizin Berlin, corporate member of Freie Universität Berlin, Humboldt-Universität zu Berlin, and Berlin Institute of Health, Berlin, Germany.
- [11] Emergency Department, Charité-Universitätsmedizin Berlin, corporate member of Freie Universität Berlin, Humboldt-Universität zu Berlin, and Berlin Institute of Health, Berlin, Germany.
- [12] Department of Anesthesiology and Intensive Care Medicine, Charité Campus Mitte and Campus-Virchow-Klinikum, Charité-Universitätsmedizin Berlin, corporate member of Freie Universität Berlin, Humboldt-Universität zu Berlin, and Berlin Institute of Health, Berlin, Germany.
- [13] Department of Pediatric Pneumology, Immunology and Intensive Care Medicine, Charité-Universitätsmedizin Berlin, Berlin, Germany.
- [14] Associate member of the German Center for Lung Research (DZL), Marburg, Germany.
- [15] Department of Infectious Diseases and Respiratory Medicine, Charité-Universitätsmedizin Berlin, corporate member of Freie Universität Berlin, Humboldt-Universität zu Berlin, and Berlin Institute of Health, 10117, Berlin, Germany. ralf-harto.huebner@charite.de.

[#] Contributed equally.

- PMID: **33825125**
- PMCID: PMC8023546
- DOI: 10.1007/s15010-021-01606-9

Abstract

Coronavirus disease 2019 (COVID-19) is caused by infection with severe acute respiratory syndrome coronavirus 2 (SARS-CoV-2). Type I interferons are important in the defense of viral infections. Recently, neutralizing IgG auto-antibodies against type I interferons were found in patients with severe COVID-19 infection. Here, we analyzed expression of CD169/SIGLEC1, a well described downstream molecule in interferon signaling, and found increased monocytic CD169/SIGLEC1 expression levels in patients with mild, acute COVID-19, compared to patients with severe disease. We recommend further clinical studies to evaluate the value of CD169/SIGLEC1 expression in patients with COVID-19 with or without auto-antibodies against type I interferons.

Keywords: CD169; COVID-19; SARS-CoV-2; SIGLEC1; Type I interferons.

© 2021. The Author(s).

Conflict of interest statement

Dr. Victor M Corman is named together with Euroimmun on a patent application filed recently regarding the diagnostic of SARS-CoV-2 by antibody testing. Dr. Christoph Tabeling is participant in the BIH-Charité Clinician Scientist Program funded by the Charité-Universitätsmedizin Berlin and the Berlin Institute of Health. The other authors declare no conflicts of interest.

- Cited by 10 articles
- 10 references
- 3 figures

Supplementary info

Publication types, MeSH terms, Substances, Grant support Expand

Publication types

- Observational Study

MeSH terms

- Aged
- COVID-19 / immunology*
- Female
- Hospitalization
- Humans
- Longitudinal Studies
- Male
- Middle Aged
- Monocytes / immunology*
- Retrospective Studies

- SARS-CoV-2 / physiology*
- Severity of Illness Index
- Sialic Acid Binding Ig-like Lectin 1 / biosynthesis
- Sialic Acid Binding Ig-like Lectin 1 / blood*
- Up-Regulation

Substances

- Sialic Acid Binding Ig-like Lectin 1

Grant support

- C8/SFB-TR84
- C6/SFB-TR84
- C9/SFB-TR84
- 01KI07114/German Ministry of Education and Research in the framework of the PROGRESS
- 01ZX1304B/CAPSyS

- 01ZX1906A/SYMPATH
- 01KI20160A/PROVID

Show all 7 grants

Full text links

SpringerLink FULL-TEXT ARTICLE Springer Free PMC article

Proceed to details

Cite

Share

419

Case Reports

World Neurosurg
. 2020 Nov;143:502-506.e1.
doi: 10.1016/j.wneu.2020.08.007. Epub 2020 Aug 7.

The Perioperative Management of Subarachnoid Hemorrhage During the Coronavirus Disease 2019 Pandemic in China

Min Zeng [1], Shu Li [1], Muhan Li [1], Xiang Yan [1], Ruowen Li [1], Jia Dong [1], Yuewei Zhang [2], Zhongrong Miao [3], Shuo Wang [4], Yuming Peng [5], Ruquan Han [1]
Affiliations Expand

Affiliations

- [1] Department of Anesthesiology, Beijing Tiantan Hospital, Capital Medical University, Beijing, China.
- [2] Department of Infection Prevention and Control, Beijing Tiantan Hospital, Capital Medical University, Beijing, China.
- [3] Department of Neuro-intervention, Beijing Tiantan Hospital, Capital Medical University, Beijing, China.
- [4] Department of Neurosurgery, Beijing Tiantan Hospital, Capital Medical University, Beijing, China.
- [5] Department of Anesthesiology, Beijing Tiantan Hospital, Capital Medical University, Beijing, China. Electronic address: florapym766@163.com.

- PMID: **32777392**
- PMCID: PMC7413212
- DOI: 10.1016/j.wneu.2020.08.007

Free PMC article
Case Reports

The Perioperative Management of Subarachnoid Hemorrhage During the Coronavirus Disease 2019 Pandemic in China

Min Zeng et al. World Neurosurg. 2020 Nov.
Free PMC article
Show details
World Neurosurg
. 2020 Nov;143:502-506.e1.
doi: 10.1016/j.wneu.2020.08.007. Epub 2020 Aug 7.

Authors

Min Zeng [1], Shu Li [1], Muhan Li [1], Xiang Yan [1], Ruowen Li [1], Jia Dong [1], Yuewei Zhang [2], Zhongrong Miao [3], Shuo Wang [4], Yuming Peng [5], Ruquan Han [1]

Affiliations

- [1] Department of Anesthesiology, Beijing Tiantan Hospital, Capital Medical University, Beijing, China.
- [2] Department of Infection Prevention and Control, Beijing Tiantan Hospital, Capital Medical University, Beijing, China.
- [3] Department of Neuro-intervention, Beijing Tiantan Hospital, Capital Medical University, Beijing, China.
- [4] Department of Neurosurgery, Beijing Tiantan Hospital, Capital Medical University, Beijing, China.

- [5] Department of Anesthesiology, Beijing Tiantan Hospital, Capital Medical University, Beijing, China. Electronic address: florapym766@163.com.

- PMID: **32777392**
- PMCID: PMC7413212
- DOI: 10.1016/j.wneu.2020.08.007

Erratum in

- Erratum to "The Perioperative Management of Subarachnoid Hemorrhage During the Coronavirus Disease 2019 Pandemic in China. [World Neurosurg. 143 (2020), 502-506]". Zeng M, Li S, Li M, Yan X, Li R, Dong J, Zhang Y, Miao Z, Wang S, Peng Y, Han R. Zeng M, et al. World Neurosurg. 2021 Oct;154:232. doi: 10.1016/j.wneu.2021.07.111. Epub 2021 Aug 5. World Neurosurg. 2021. PMID: 34417102 Free PMC article. No abstract available.

Abstract

Background: For most of the international community outside the epicenter, coronavirus disease 2019 (COVID-19) containment is normalizing, and daily medical practice runs parallel to preventing and treating COVID-19. This experience of simultaneously conducting emergent surgery and infection control for COVID-19 disease is useful outside the epicenter during the pandemic.

Case description: In this single-center retrospective observational study, we enrolled patients with subarachnoid hemorrhage (SAH) who were emergently admitted from January 23 to April 8, 2020. Based on the COVID-19 triage, patients with SAH were divided into 3 categories: positive, negative, and under investigation. During 77 days, 90 patients with SAH were admitted at the center. The median age was 55 years (range, 18-80 years) and 40 patients (44.4%) were male. None was positive, 42 patients were negative, and 48 patients were under investigation for COVID-19 before surgery. During the same period, 9 patients were diagnosed with COVID-19 without nosocomial infection.

Conclusions: Rescuing patients with SAH and containment of COVID-19 benefit from joint prevention and control, a centralized system of equipment distribution and personnel assignment, and quick workflow establishment.

Keywords: Coronavirus disease 2019; Perioperative management; Subarachnoid hemorrhage.

Copyright © 2020 Elsevier Inc. All rights reserved.

- 20 references
- 1 figure

Supplementary info

Publication types, MeSH terms Expand

Publication types

- Case Reports

MeSH terms

- Adolescent
- Adult
- Aged
- Aged, 80 and over
- COVID-19 / surgery*
- COVID-19 / virology
- China
- Female
- Hospitalization / statistics & numerical data
- Humans
- Infection Control / methods
- Male
- Middle Aged
- Retrospective Studies
- SARS-CoV-2 / pathogenicity*
- Subarachnoid Hemorrhage / etiology*
- Subarachnoid Hemorrhage / virology
- Young Adult

Full text links

ELSEVIER FULL-TEXT ARTICLE Elsevier Science Free PMC article

Proceed to details

Cite

Share

☐ 420

Observational Study

Respir Investig

. 2021 Sep;59(5):602-607.

doi: 10.1016/j.resinv.2021.05.002. Epub 2021 Jun 1.

Risk factors for transfer from Respiratory Intermediate Care Unit to Intensive Care Unit in COVID-19

Enrico Buonamico [1], Vitaliano Nicola Quaranta [2], Esterina Boniello [1], Michela Dimitri [1], Valentina Di Lecce [3], Luciana Labate [1], Paola Pierucci [1], Elena Capozza [1], Giovanna Elisiana Carpagnano [1], Onofrio Resta [1]

Affiliations Expand

Affiliations

- [1] Institute of Respiratory Disease, Department of Basic Medical Science, Neuroscience, and Sense Organs, University of Bari "Aldo Moro", Piazza Giulio Cesare 11, 70125, Bari, Italy.
- [2] Pneumology Department, "Di Venere" Hospital, Via Ospedale Di Venere 1, 70131, Bari, Italy.
- [3] Institute of Respiratory Disease, Department of Basic Medical Science, Neuroscience, and Sense Organs, University of Bari "Aldo Moro", Piazza Giulio Cesare 11, 70125, Bari, Italy. Electronic address: valentina.dilecce@policlinico.ba.it.

- PMID: **34130948**
- PMCID: PMC8166522
- DOI: 10.1016/j.resinv.2021.05.002

Free PMC article
Observational Study

Risk factors for transfer from Respiratory Intermediate Care Unit to Intensive Care Unit in COVID-19

Enrico Buonamico et al. Respir Investig. 2021 Sep.
Free PMC article
Show details
Respir Investig
. 2021 Sep;59(5):602-607.
doi: 10.1016/j.resinv.2021.05.002. Epub 2021 Jun 1.

Authors

Enrico Buonamico [1], Vitaliano Nicola Quaranta [2], Esterina Boniello [1], Michela Dimitri [1], Valentina Di Lecce [3], Luciana Labate [1], Paola Pierucci [1], Elena Capozza [1], Giovanna Elisiana Carpagnano [1], Onofrio Resta [1]

Affiliations

- [1] Institute of Respiratory Disease, Department of Basic Medical Science, Neuroscience, and Sense Organs, University of Bari "Aldo Moro", Piazza Giulio Cesare 11, 70125, Bari, Italy.
- [2] Pneumology Department, "Di Venere" Hospital, Via Ospedale Di Venere 1, 70131, Bari, Italy.
- [3] Institute of Respiratory Disease, Department of Basic Medical Science, Neuroscience, and Sense Organs, University of Bari "Aldo Moro", Piazza Giulio Cesare 11, 70125, Bari, Italy. Electronic address: valentina.dilecce@policlinico.ba.it.

- PMID: **34130948**
- PMCID: PMC8166522
- DOI: 10.1016/j.resinv.2021.05.002

Abstract

Background: Patients hospitalized for COVID-19-related pneumonia often need several degrees of ventilatory support, which are performed between Respiratory Intermediate Care Units (RICUs) and Intensive Care Units (ICUs), and which depend on the severity of acute respiratory distress syndrome. There is no firm consensus on transfer predictors from the RICU to the ICU.

Methods: In this retrospective observational single center study, we evaluated 96 COVID-19 patients referred to the RICU for acute respiratory failure (ARF) according to their transferal to the ICU or their stay at the RICU. We compared demographic data, baseline laboratory profile, and final clinical outcomes to identify early risk factors for transfer.

Results: The best predictors for transfer to the ICU were elevated C-reactive protein and lymphopenia. The mortality rate was lower in the RICU than in the ICU, where transferred patients who died were mostly younger men and with less comorbidities than those in the RICU.

Conclusions: Few inflammatory markers can predict the need for transfer from the RICU to the ICU. Due to the ongoing COVID-19 pandemic, we urge better clinical stratification by early and meaningful profiles in patients admitted to the RICU who are at risk of transferal to the ICU.

Keywords: COVID-19; COVID-19 ICU transfer risk factors; COVID-19 management Italy; Respiratory intermediate care unit.

Copyright © 2021 The Japanese Respiratory Society. Published by Elsevier B.V. All rights reserved.

Conflict of interest statement

Conflict of Interest The authors have no conflicts of interest.

- Cited by 1 article
- 20 references

Supplementary info

Publication types, MeSH terms Expand

Publication types

- Observational Study

MeSH terms

- COVID-19*
- Humans
- Intensive Care Units
- Male
- Pandemics
- Respiratory Insufficiency* / epidemiology

- Respiratory Insufficiency* / etiology
- Respiratory Insufficiency* / therapy
- Retrospective Studies
- Risk Factors
- SARS-CoV-2

Full text links

ELSEVIER FULL-TEXT ARTICLE Elsevier Science Free PMC article
Proceed to details
Cite
Share
421
Observational Study
BMC Infect Dis
. 2020 Dec 9;20(1):934.
doi: 10.1186/s12879-020-05647-7.

Clinical characteristics and laboratory biomarkers changes in COVID-19 patients requiring or not intensive or sub-intensive care: a comparative study

Anna Maria Cattelan [1], Eugenia Di Meco [2], Marco Trevenzoli [2], Alessia Frater [2], Anna Ferrari [2], Marco Villano [3], Federica Gomiero [3], Giovanni Carretta [4], Lolita Sasset [2]
Affiliations Expand

Affiliations

- [1] Infectious Diseases Unit, Azienda Ospedale Università di Padova, Via Nicolò Giustiniani 2, 35128, Padova, Italy. annamaria.cattelan@aopd.veneto.it.
- [2] Infectious Diseases Unit, Azienda Ospedale Università di Padova, Via Nicolò Giustiniani 2, 35128, Padova, Italy.
- [3] Information Technology System Unit, Azienda Ospedale Università di Padova, Padova, Italy.
- [4] Department of Directional Hospital Management, Azienda Ospedale Università di Padova, Padova, Italy.

- PMID: **33297986**
- PMCID: PMC7724444
- DOI: 10.1186/s12879-020-05647-7

Free PMC article
Observational Study

Clinical characteristics and laboratory biomarkers changes in COVID-19 patients requiring or not intensive or sub-intensive care: a comparative study

Anna Maria Cattelan et al. BMC Infect Dis. 2020.
Free PMC article
Show details
BMC Infect Dis
. 2020 Dec 9;20(1):934.
doi: 10.1186/s12879-020-05647-7.

Authors

Anna Maria Cattelan [1], Eugenia Di Meco [2], Marco Trevenzoli [2], Alessia Frater [2], Anna Ferrari [2], Marco Villano [3], Federica Gomiero [3], Giovanni Carretta [4], Lolita Sasset [2]

Affiliations

- [1] Infectious Diseases Unit, Azienda Ospedale Università di Padova, Via Nicolò Giustiniani 2, 35128, Padova, Italy. annamaria.cattelan@aopd.veneto.it.
- [2] Infectious Diseases Unit, Azienda Ospedale Università di Padova, Via Nicolò Giustiniani 2, 35128, Padova, Italy.
- [3] Information Technology System Unit, Azienda Ospedale Università di Padova, Padova, Italy.
- [4] Department of Directional Hospital Management, Azienda Ospedale Università di Padova, Padova, Italy.

- PMID: **33297986**
- PMCID: PMC7724444
- DOI: 10.1186/s12879-020-05647-7

Abstract

Background: Identifying risk factors for severe novel-coronavirus disease (COVID-19) is useful to ascertain which patients may benefit from advanced supportive care. The study offers a description of COVID-19 patients, admitted to a general ward for a non-critical clinical picture, with the aim to analyse the differences between those transferred to the intensive (ICU) and/or sub-intensive care (SICU) units and those who were not.

Methods: This observational retrospective study includes all COVID-19 patients admitted to the Infectious Diseases Unit. Clinical, laboratory, radiological and treatment data were collected. The primary outcome was a composite of need of transfer to the ICU and/or SICU during the hospitalization. Patients who did not require to be transferred are defined as Group 1; patients who were transferred to the ICU and/or SICU are defined as Group 2. Demographic, clinical

characteristics and laboratory findings at the 1st, 3rd and last measurements were compared between the two groups.

Results: 303 were included. The median age was 62 years. 69 patients (22.8%) met the primary outcome and were defined as Group 2. The overall fatality rate was 6.8%. Group 2 patients were predominantly male (76.8% vs. 55.1%, p < 0.01), had a higher fatality rate (14.5% vs. 3.8%, p < 0,01), had more hypertension (72.4% vs. 44%, p < 0,01) and diabetes (31.9% vs. 21%, p = 0.04) and were more likely to present dry cough (49.3% vs. 25.2%, p < 0.01). Overall, chest X-ray at admission showed findings suggestive of pneumonia in 63.2%, and Group 2 were more likely to develop pathological findings during the hospitalization (72.7% vs. 17.2%, p = 0.01). At admission, Group 2 presented significantly higher neutrophil count, aspartate-transaminase and C-Reactive-Protein. At the 3rd measurement, Group 2 presented persistently higher neutrophil count, hepatic inflammation markers and C-Reactive-Protein. Group 1 presented a shorter duration from admission to negativization of follow-up swabs (20 vs. 35 days, p < 0.01).

Conclusions: The presence of comorbidities and the persistent observation of abnormal laboratory findings should be regarded as predisposing factors for clinical worsening.

Keywords: COVID-19; Disease severity; Intensive care; Novel coronavirus; Outcome; SARS-CoV-2; Sub-intensive care.

Conflict of interest statement

The authors declare that they have no competing interests.

- Cited by 9 articles
- 28 references

Supplementary info

Publication types, MeSH terms, Substances Expand

Publication types

- Comparative Study
- Observational Study

MeSH terms

- Aged
- Aspartate Aminotransferases / blood
- Biomarkers / blood
- C-Reactive Protein / analysis
- COVID-19 / blood*
- COVID-19 / epidemiology
- COVID-19 / therapy*
- COVID-19 / virology
- Comorbidity

- Critical Care / methods*
- Female
- Follow-Up Studies
- Humans
- Intensive Care Units
- Italy / epidemiology
- Leukocyte Count
- Male
- Middle Aged
- Neutrophils / immunology
- Patient Transfer*
- Retrospective Studies
- Risk Factors
- SARS-CoV-2 / genetics*
- Time Factors

Substances

- Biomarkers
- C-Reactive Protein
- Aspartate Aminotransferases

Full text links

Read free full text at BMC BioMed Central Free PMC article

Proceed to details

Cite

Share

422

Observational Study

J Med Virol

. 2021 Jul;93(7):4446-4453.

doi: 10.1002/jmv.26798. Epub 2021 Apr 23.

Clinical efficacy of methylprednisolone and the combined use of lopinavir/ritonavir with arbidol in treatment of coronavirus disease 2019

Qi Xia [1 2], Wanrong Dai [3], Kaijin Xu [1], Qin Ni [1], Yongtao Li [1], Jun Liu [1], Hong Zhao [1], Yongzheng Guo [1], Liang Yu [1], Ping Yi [1], Junwei Su [1], Guanjing Lang [1], Jingjing Tao

[1], Ding Shi [1], Wenrui Wu [1], Xiaoxin Wu [1], Yan Xu [1], Min Xu [1], Ling Yu [1], Xiaoyan Wang [1], Hongliu Cai [1], Qiang Fang [1], Jianying Zhou [1], Yunqing Qiu [1], Lanjuan Li [1] [2]
Affiliations Expand

Affiliations

- [1] Department of Infectious Disease, State Key Laboratory for Diagnosis and Treatment of Infectious Diseases, National Clinical Research Center for Infectious Diseases, Collaborative Innovation Center for Diagnosis and Treatment of Infectious Diseases, The First Affiliated Hospital, College of Medicine, Zhejiang University, Hangzhou, Zhejiang, China.
- [2] Key Laboratory for Biomedical Engineering of Ministry of Education, Zhejiang University, Hangzhou, Zhejiang, China.
- [3] The First Affiliated Hospital, College of Medicine, Zhejiang University, Hangzhou, Zhejiang Province, China.

- PMID: **33448426**
- PMCID: PMC8013375
- DOI: 10.1002/jmv.26798

Free PMC article
Observational Study

Clinical efficacy of methylprednisolone and the combined use of lopinavir/ritonavir with arbidol in treatment of coronavirus disease 2019

Qi Xia et al. J Med Virol. 2021 Jul.
Free PMC article
Show details
J Med Virol
. 2021 Jul;93(7):4446-4453.
doi: 10.1002/jmv.26798. Epub 2021 Apr 23.

Authors

Qi Xia [1] [2], Wanrong Dai [3], Kaijin Xu [1], Qin Ni [1], Yongtao Li [1], Jun Liu [1], Hong Zhao [1], Yongzheng Guo [1], Liang Yu [1], Ping Yi [1], Junwei Su [1], Guanjing Lang [1], Jingjing Tao [1], Ding Shi [1], Wenrui Wu [1], Xiaoxin Wu [1], Yan Xu [1], Min Xu [1], Ling Yu [1], Xiaoyan Wang [1], Hongliu Cai [1], Qiang Fang [1], Jianying Zhou [1], Yunqing Qiu [1], Lanjuan Li [1] [2]

Affiliations

- [1] Department of Infectious Disease, State Key Laboratory for Diagnosis and Treatment of Infectious Diseases, National Clinical Research Center for Infectious Diseases,

Collaborative Innovation Center for Diagnosis and Treatment of Infectious Diseases, The First Affiliated Hospital, College of Medicine, Zhejiang University, Hangzhou, Zhejiang, China.

- [2] Key Laboratory for Biomedical Engineering of Ministry of Education, Zhejiang University, Hangzhou, Zhejiang, China.
- [3] The First Affiliated Hospital, College of Medicine, Zhejiang University, Hangzhou, Zhejiang Province, China.

- PMID: **33448426**
- PMCID: PMC8013375
- DOI: 10.1002/jmv.26798

Abstract

This study aims to comparatively analyze the therapeutic efficacy upon multiple medication plans over lopinavir/ritonavir (LPV/r), arbidol (ARB), and methylprednisolone on patients with coronavirus disease 2019 (COVID-19). Totally, 75 COVID-19 patients admitted to The First Affiliated Hospital, Zhejiang University School of Medicine from January 22, 2020 to February 29, 2020 were recruited and grouped based on whether or not LPV/r and ARB were jointly used and whether or not methylprednisolone was used. Indexes including body temperature, time for nucleic acid negative conversion, hospital stays, and laboratory indexes were examined and compared. For all patients, there were no significant differences in the change of body temperature, the time for negative conversion, and hospital stays whether LPV/r and ARB were jointly used or not. While for severe and critically severe patients, methylprednisolone noticeably reduced the time for negative conversion. Meanwhile, the clinical efficacy was superior on patients receiving methylprednisolone within 3 days upon admission, and the duration of hospital stays was much shorter when methylprednisolone was given at a total dose of 0-400 mg than a higher dose of >400 mg if all patients received a similar dose per day. Nonetheless, no significant changes across hepatic, renal, and myocardial function indexes were observed. LPV/r combined with ARB produced no noticeably better effect on COVID-19 patients relative to the single-agent treatment. Additionally, methylprednisolone was efficient in severe and critically severe cases, and superior efficacy could be realized upon its early, appropriate, and short-term application.

Keywords: COVID-19; arbidol; coronavirus; lopinavir/ritonavir; methylprednisolone.

© 2021 Wiley Periodicals LLC.

- Cited by 1 article
- 19 references

Supplementary info

Publication types, MeSH terms, Substances, Supplementary concepts, Grant support Expand

Publication types

- Observational Study
- Research Support, Non-U.S. Gov't

MeSH terms

- Antiviral Agents / therapeutic use*
- COVID-19 / drug therapy*
- China
- Drug Combinations
- Female
- Fever / drug therapy
- Humans
- Indoles / therapeutic use*
- Length of Stay
- Lopinavir / therapeutic use*
- Male
- Methylprednisolone / therapeutic use*
- Middle Aged
- Retrospective Studies
- Ritonavir / therapeutic use*
- SARS-CoV-2 / drug effects

Substances

- Antiviral Agents
- Drug Combinations
- Indoles
- lopinavir-ritonavir drug combination
- Lopinavir
- umifenovir
- Ritonavir
- Methylprednisolone

Supplementary concepts

- COVID-19 drug treatment

Grant support

- 2019YFC0840600 and 2019YFC0840609/Ministry of Science and Technology of the People's Republic of China, 13th Five-Year National Key Research and Development Program of China
- 2018ZX10715-013-003/Ministry of Science and Technology of the People's Republic of China, 13th Five-Year National Science and Technology Major Project
- Fundamental Research Funds for the Central Universities

Full text links

Wiley Full Text Article Wiley Free PMC article
Proceed to details
Cite
Share
423
Observational Study
J Autoimmun
. 2021 Feb;117:102580.
doi: 10.1016/j.jaut.2020.102580. Epub 2020 Nov 30.

A multidisciplinary registry of patients with autoimmune and immune-mediated diseases with symptomatic COVID-19 from a single center

Juan C Sarmiento-Monroy [1], Gerard Espinosa [2], Maria-Carlota Londoño [3], Fernanda Meira [4], Berta Caballol [5], Sara Llufriu [6], Josep Lluis Carrasco [7], Aina Moll-Udina [8], Luis F Quintana [9], Priscila Giavedoni [10], Julio Ramírez [11], Jose Inciarte-Mundo [1], Elisabeth Solana [6], Yolanda Blanco [6], Eugenia Martinez-Hernandez [6], Maria Sepúlveda [6], Victor Llorenç [8], Sergio Prieto-González [2], Georgina Espígol-Frigolé [2], Jose C Milisenda [11], Maria C Cid [2], Jose M Mascaró Jr [10], Isabel Blanco [12], Joan Albert Barberá [12], Oriol Sibila [12], Jordi Gratacos-Ginès [5], Alfredo Adán [8], Alvaro Agustí [12], Raimon Sanmartí [1], Julian Panés [5], Ricard Cervera [2], Jordi Vila [13], Alex Soriano [4], José A Gómez-Puerta [14], Immunocovid Clinic
Affiliations Expand

Affiliations

- [1] Rheumatology Department, Hospital Clínic, Barcelona, Catalonia, Spain.
- [2] Department of Autoimmune Diseases, IDIBAPS, University of Barcelona, Hospital Clínic, Barcelona, Catalonia, Spain.
- [3] Liver Unit, Hospital Clínic, Institut d'Investigacions Biomèdiques August Pi I Sunyer (IDIBAPS) and Centro de Investigación en Red de Enfermedades Hepáticas y Digestivas (CIBERehd), University of Barcelona, Barcelona, Catalonia, Spain.
- [4] Department of Infectious Diseases, Hospital Clinic, IDIBAPS, Barcelona, Spain.
- [5] Department of Gastroenterology, Hospital Clínic, Barcelona, Catalonia, Spain.
- [6] Department of Neurology, Hospital Clínic, Barcelona, Catalonia, Spain.
- [7] Biostatistics, Department of Basic Clinical Practice, University of Barcelona, Barcelona, Catalonia, Spain.
- [8] Group of Ocular Inflammation, Clinical and Experimental Studies, Institut d'Investigacions Biomèdiques Agustí Pi i Sunyer (IDIBAPS), Hospital Clínic, Barcelona, Catalonia, Spain.

- [9] Department of Nephrology and Renal Transplantation, Hospital Clínic, Centro de Referencia en Enfermedad Glomerular Compleja del Sistema Nacional de Salud (CSUR), Department of Medicine, University of Barcelona, IDIBAPS, Barcelona, Catalonia, Spain.
- [10] Department of Dermatology, Hospital Clínic, Universitat de Barcelona, Barcelona, Catalonia, Spain.
- [11] Muscle Research Unit, Department of Internal Medicine, Hospital Clínic, University of Barcelona, CIBERER, Barcelona, Catalonia, Spain.
- [12] Department of Pulmonary Medicine, Hospital Clínic-Institut d'Investigacions Biomèdiques August Pi i Sunyer (IDIBAPS), University of Barcelona, Barcelona, Catalonia, Spain.
- [13] Department of Clinical Microbiology, Biomedical Diagnostic Center, Hospital Clinic, Barcelona Institute for Global Health, University of Barcelona, Barcelona, Catalonia, Spain.
- [14] Rheumatology Department, Hospital Clínic, Barcelona, Catalonia, Spain. Electronic address: jagomez@clinic.cat.

- PMID: **33338707**
- PMCID: PMC7836738
- DOI: 10.1016/j.jaut.2020.102580

Free PMC article
Observational Study

A multidisciplinary registry of patients with autoimmune and immune-mediated diseases with symptomatic COVID-19 from a single center

Juan C Sarmiento-Monroy et al. J Autoimmun. 2021 Feb.
Free PMC article

Show details

J Autoimmun

. 2021 Feb;117:102580.
doi: 10.1016/j.jaut.2020.102580. Epub 2020 Nov 30.

Authors

Juan C Sarmiento-Monroy [1], Gerard Espinosa [2], Maria-Carlota Londoño [3], Fernanda Meira [4], Berta Caballol [5], Sara Llufriu [6], Josep Lluis Carrasco [7], Aina Moll-Udina [8], Luis F Quintana [9], Priscila Giavedoni [10], Julio Ramírez [11], Jose Inciarte-Mundo [1], Elisabeth Solana [6], Yolanda Blanco [6], Eugenia Martinez-Hernandez [6], Maria Sepúlveda [6], Victor Llorenç [8], Sergio Prieto-González [2], Georgina Espígol-Frigolé [2], Jose C Milisenda [11], Maria C Cid [2], Jose M Mascaró Jr [10], Isabel Blanco [12], Joan Albert Barberá [12], Oriol Sibila [12], Jordi Gratacos-Ginès [5], Alfredo Adán [8], Alvaro Agustí [12], Raimon Sanmartí [1], Julian Panés [5], Ricard Cervera [2], Jordi Vila [13], Alex Soriano [4], José A Gómez-Puerta [14], Immunocovid Clinic

Affiliations

- [1] Rheumatology Department, Hospital Clínic, Barcelona, Catalonia, Spain.
- [2] Department of Autoimmune Diseases, IDIBAPS, University of Barcelona, Hospital Clínic, Barcelona, Catalonia, Spain.
- [3] Liver Unit, Hospital Clínic, Institut d'Investigacions Biomèdiques August Pi I Sunyer (IDIBAPS) and Centro de Investigación en Red de Enfermedades Hepáticas y Digestivas (CIBERehd), University of Barcelona, Barcelona, Catalonia, Spain.
- [4] Department of Infectious Diseases, Hospital Clinic, IDIBAPS, Barcelona, Spain.
- [5] Department of Gastroenterology, Hospital Clínic, Barcelona, Catalonia, Spain.
- [6] Department of Neurology, Hospital Clínic, Barcelona, Catalonia, Spain.
- [7] Biostatistics, Department of Basic Clinical Practice, University of Barcelona, Barcelona, Catalonia, Spain.
- [8] Group of Ocular Inflammation, Clinical and Experimental Studies, Institut d'Investigacions Biomèdiques Agustí Pi i Sunyer (IDIBAPS), Hospital Clínic, Barcelona, Catalonia, Spain.
- [9] Department of Nephrology and Renal Transplantation, Hospital Clínic, Centro de Referencia en Enfermedad Glomerular Compleja del Sistema Nacional de Salud (CSUR), Department of Medicine, University of Barcelona, IDIBAPS, Barcelona, Catalonia, Spain.
- [10] Department of Dermatology, Hospital Clínic, Universitat de Barcelona, Barcelona, Catalonia, Spain.
- [11] Muscle Research Unit, Department of Internal Medicine, Hospital Clínic, University of Barcelona, CIBERER, Barcelona, Catalonia, Spain.
- [12] Department of Pulmonary Medicine, Hospital Clínic-Institut d'Investigacions Biomèdiques August Pi i Sunyer (IDIBAPS), University of Barcelona, Barcelona, Catalonia, Spain.
- [13] Department of Clinical Microbiology, Biomedical Diagnostic Center, Hospital Clinic, Barcelona Institute for Global Health, University of Barcelona, Barcelona, Catalonia, Spain.
- [14] Rheumatology Department, Hospital Clínic, Barcelona, Catalonia, Spain. Electronic address: jagomez@clinic.cat.

- PMID: **33338707**
- PMCID: PMC7836738
- DOI: 10.1016/j.jaut.2020.102580

Abstract

Background and aim: There is increasing interest regarding SARS-CoV-2 infection in patients with autoimmune and immune-mediated inflammatory diseases (AI/IMID) with some discrepancies in different cohorts about their risk and outcomes. The aim was to describe a multidisciplinary cohort of patients with AI/IMID and symptomatic SARS-CoV-2 infection in a single tertiary center and analyze sociodemographic, clinical, and therapeutic factors associated with poor outcomes.

Methods: A retrospective observational study was conducted from the 1st of March until May 29th, 2020 in a University tertiary hospital in Barcelona, Spain. Patients with an underlying AI/IMID and symptomatic SARS-CoV-2 infection were identified in our local SARS-CoV-2 infection database. Controls (2:1) were selected from the same database and matched by age and gender. The primary outcome was severe SARS-CoV-2 infection, which was a composite endpoint including admission to the intensive care unit (ICU), need for mechanical ventilation

(MV), and/or death. Several covariates including age, sex, and comorbidities among others were combined into a multivariate model having severe SARS-CoV-2 as the dependent variable. Also, a sensitivity analysis was performed evaluating AID and IMID separately.

Results: The prevalence of symptomatic SARS-CoV-2 infection in a cohort of AI/IMID patients was 1.3%. Eighty-five patients with AI/IMID and symptomatic SARS-CoV-2 were identified, requiring hospitalization in 58 (68%) cases. A total of 175 patients admitted for SARS-CoV-2 (58 with AI/IMID and 117 matched-controls) were analyzed. In logistic regression analysis, a significant inverse association between AI/IMID group and severe SARS-CoV-2 (OR 0.28; 95% CI 0.12-0.61; p = 0.001), need of MV (OR 0.20; IC 95% 0.05-0.71; p = 0.014), and ICU admission (OR 0.25; IC 95% 0.10-0.62; p = 0.003) was found.

Conclusions: Patients with AI/IMID who require admission for SARS-CoV-2 infection have a lower risk of developing severe disease, including the need to stay in the ICU and MV.

Keywords: Adverse outcome; Autoimmune diseases; COVID-19; Immunosuppression; Severe acute respiratory syndrome coronavirus 2.

Copyright © 2020 Elsevier Ltd. All rights reserved.

- Cited by 7 articles
- 49 references
- 2 figures

Supplementary info

Publication types, MeSH terms Expand

Publication types

- Observational Study

MeSH terms

- Aged
- Autoimmune Diseases / epidemiology*
- Autoimmune Diseases / mortality
- COVID-19 / epidemiology*
- COVID-19 / mortality
- Cohort Studies
- Female
- Hospitalization / statistics & numerical data
- Humans
- Intensive Care Units / statistics & numerical data
- Interdisciplinary Communication
- Male
- Middle Aged
- Prevalence

- Registries*
- Respiration, Artificial / statistics & numerical data
- Retrospective Studies
- Risk Factors
- SARS-CoV-2 / physiology*
- Spain / epidemiology
- Survival Analysis
- Treatment Outcome

Full text links

ELSEVIER FULL-TEXT ARTICLE Elsevier Science Free PMC article
Proceed to details
Cite
Share
☐ 424
Observational Study
Jpn J Infect Dis
. 2021 Jul 21;74(4):273-279.
doi: 10.7883/yoken.JJID.2020.781. Epub 2020 Dec 1.

Comparison of Clinical and Laboratory Features and Treatment Options of 237 Symptomatic and Asymptomatic Children Infected with SARS-CoV-2 in the Early Phase of the COVID-19 Pandemic in Turkey

Ahmet Soysal [1], Erdem Gönüllü [1], Hüseyin Arslan [2], Büşra Sultan Kibar [3], Serdar Pop [4], Gözde Nur Yurttaş [5], Hacer Demirbacak [3], Füsun Ünal [2], Sedat Öktem [2], Serkan Atıcı [6], Arife Derda Yücel Şen [3], Nalan Karabayır [2], Metin Karaböcüoğlu [1], PEDCOVID19 study group
Affiliations Expand

Affiliations

- [1] Memorial Ataşehir Hospital, Division of Pediatric Infectious Diseases, Turkey.
- [2] Medipol University, Department of Pediatrics, Turkey.
- [3] Yalova State Hospital, Turkey.
- [4] Erbaa State Hospital, Turkey.
- [5] Tokat State Hospital, Turkey.
- [6] Okan University Hospital, Division of Pediatric Infectious Diseases, Turkey.

- PMID: **33250495**
- DOI: 10.7883/yoken.JJID.2020.781

Free article
Observational Study

Comparison of Clinical and Laboratory Features and Treatment Options of 237 Symptomatic and Asymptomatic Children Infected with SARS-CoV-2 in the Early Phase of the COVID-19 Pandemic in Turkey

Ahmet Soysal et al. Jpn J Infect Dis. 2021.
Free article
Show details
Jpn J Infect Dis
. 2021 Jul 21;74(4):273-279.
doi: 10.7883/yoken.JJID.2020.781. Epub 2020 Dec 1.

Authors

Ahmet Soysal[1], Erdem Gönüllü[1], Hüseyin Arslan[2], Büşra Sultan Kibar[3], Serdar Pop[4], Gözde Nur Yurttaş[5], Hacer Demirbacak[3], Füsun Ünal[2], Sedat Öktem[2], Serkan Atıcı[6], Arife Derda Yücel Şen[3], Nalan Karabayır[2], Metin Karaböcüoğlu[1], PEDCOVID19 study group

Affiliations

- [1] Memorial Ataşehir Hospital, Division of Pediatric Infectious Diseases, Turkey.
- [2] Medipol University, Department of Pediatrics, Turkey.
- [3] Yalova State Hospital, Turkey.
- [4] Erbaa State Hospital, Turkey.
- [5] Tokat State Hospital, Turkey.
- [6] Okan University Hospital, Division of Pediatric Infectious Diseases, Turkey.

- PMID: **33250495**
- DOI: 10.7883/yoken.JJID.2020.781

Abstract

Little is known about the therapeutic use of hydroxychloroquine in pediatric patients with coronavirus disease 2019 (COVID-19). Here, we retrospectively retrieved data of severe acute respiratory syndrome coronavirus 2 (SARS-CoV-2) PCR-positive pediatric patients from 20 hospitals in 8 Turkish cities. We obtained epidemiological, clinical, and laboratory features of the patients, as well as the drugs used for treating COVID-19. A total of 237 nasopharyngeal swab

SARS-CoV-2 PCR-positive children were included in the study from March 26, 2020 to June 20, 2020. The mean age of asymptomatic children (118 ± 62 months) was higher than that of symptomatic children (89 ± 69 months). Symptomatic children had significantly lower mean lymphocyte counts and higher mean CRP, D-dimer, procalcitonin, and LDH levels than asymptomatic children in the univariate analysis. Among 156 children, 78 (50%), 15, 44, and 21 were treated with a hydroxychloroquine-containing regimen, hydroxychloroquine + azithromycin + oseltamivir, hydroxychloroquine + azithromycin, and hydroxychloroquine alone, respectively. Among 156 patients who received medical treatment, 90 (58%) underwent pre- and/or post-treatment electrocardiogram (ECG). However, none of them had ECG abnormalities or required hydroxychloroquine discontinuation due to adverse drug reactions.

Keywords: SARS-CoV-2; Turkey; hydroxychloroquine.

- Cited by 2 articles

Supplementary info

Publication types, MeSH terms, Substances Expand

Publication types

- Observational Study

MeSH terms

- Adolescent
- Antiviral Agents / therapeutic use*
- Asymptomatic Infections
- COVID-19 / drug therapy*
- Child
- Child, Preschool
- Electrocardiography
- Female
- Humans
- Hydroxychloroquine / therapeutic use
- Infant
- Infant, Newborn
- Laboratories
- Lymphocyte Count / methods
- Male
- Pandemics / prevention & control*
- Retrospective Studies
- SARS-CoV-2 / drug effects*
- Turkey

Substances

- Antiviral Agents
- Hydroxychloroquine

Full text links

J-STAGE, Japan Science and Technology Information Aggregator, Electronic
Proceed to details
Cite
Share
425
Observational Study
J Laparoendosc Adv Surg Tech A
. 2020 Sep;30(9):1001-1007.
doi: 10.1089/lap.2020.0465. Epub 2020 Jun 23.

The Impact of the Coronavirus Disease 2019 Outbreak on the Attendance of Patients with Surgical Complaints at a Tertiary Hospital Emergency Department

Roi Anteby [1, 2], Yaniv Zager [1, 2], Yiftach Barash [1, 3, 4], Roy Nadler [1, 2], Mordehay Cordoba [1, 2], Eyal Klang [1, 3, 4], Yoram Klein [1, 2], Edward Ram [1, 2], Mordechai Gutman [1, 2], Nir Horesh [1, 2]

Affiliations Expand

Affiliations

- [1] Faculty of Medicine, Tel Aviv University, Tel Aviv, Israel.
- [2] Department of Surgery and Transplantation B, Chaim Sheba Medical Center, Tel Hashomer, Israel.
- [3] Department of Diagnostic Imaging, The Chaim Sheba Medical Center, Tel Hashomer, Israel.
- [4] Deep Vision Lab, Sheba Medical Center, Tel Hashomer, Israel.

- PMID: **32589496**
- DOI: 10.1089/lap.2020.0465

Observational Study

The Impact of the Coronavirus Disease 2019 Outbreak on the Attendance of Patients with Surgical Complaints at a Tertiary Hospital Emergency Department

Roi Anteby et al. J Laparoendosc Adv Surg Tech A. 2020 Sep.
Show details
J Laparoendosc Adv Surg Tech A
. 2020 Sep;30(9):1001-1007.
doi: 10.1089/lap.2020.0465. Epub 2020 Jun 23.

Authors

Roi Anteby [1, 2], Yaniv Zager [1, 2], Yiftach Barash [1, 3, 4], Roy Nadler [1, 2], Mordehay Cordoba [1, 2], Eyal Klang [1, 3, 4], Yoram Klein [1, 2], Edward Ram [1, 2], Mordechai Gutman [1, 2], Nir Horesh [1, 2]

Affiliations

- [1] Faculty of Medicine, Tel Aviv University, Tel Aviv, Israel.
- [2] Department of Surgery and Transplantation B, Chaim Sheba Medical Center, Tel Hashomer, Israel.
- [3] Department of Diagnostic Imaging, The Chaim Sheba Medical Center, Tel Hashomer, Israel.
- [4] Deep Vision Lab, Sheba Medical Center, Tel Hashomer, Israel.

- PMID: **32589496**
- DOI: 10.1089/lap.2020.0465

Abstract

Introduction: Emergency departments (EDs) during the novel coronavirus disease 2019 (COVID-19) pandemic are perceived as possible sources of infection. The effects of COVID-19 on patients presenting to the hospital with surgical complaints remain uncertain. ***Methods:*** A single tertiary center retrospective study analysis compared the ED attendance rate and severity of patients with surgical complaints between March 2020 (COVID-19 outbreak) and pre-COVID-19 periods: February 2020 and the same 2 months in 2019 and 2018. ***Results:*** Overall, 6,017 patients were included. The mean daily ED visits of patients with nontrauma surgical complaints in the COVID-19 outbreak period declined by 27%-32% (P value <.01) compared with pre-COVID-19 periods. The log number of confirmed severe acute respiratory syndrome coronavirus 2 (SARS-CoV-2) cases in Israel in March 2020 was negatively correlated with the number of ED visits (Pearson's r = -0.59, $P < .01$). The proportion of patients requiring hospitalization increased by up to 8% during the outbreak period ($P < .01$), and there was a higher proportion of tachycardic patients (20% versus 15.5%, $P = .01$). The percentage of visits to the ED by men declined by 5% ($P < .01$). The ED diagnosis distribution significantly changed during COVID-19 ($P = .013$), with an 84% decrease in the number of patients hospitalized for diverticular disease ($P < .05$). ***Conclusion:***

During the COVID-19 outbreak, the overall number of patients presenting at the ED with surgical complaints decreased significantly, and there was a higher admissions ratio. The extent to which the pandemic affects hospital ED attendance can help health care professionals prepare for future such events. ClinicalTrials.gov ID: NCT04338672.

Keywords: COVID-19; SARS-COV-19; acute care surgery; hospital visit.

- Cited by 10 articles

Supplementary info

Publication types, MeSH terms, Associated data Expand

Publication types

- Observational Study

MeSH terms

- Adolescent
- Adult
- Aged
- Betacoronavirus
- COVID-19
- Coronavirus Infections / epidemiology*
- Emergency Service, Hospital / statistics & numerical data*
- Female
- Health Personnel
- Hospitalization
- Humans
- Intestinal Diseases / epidemiology
- Israel / epidemiology
- Male
- Middle Aged
- Pandemics
- Pneumonia, Viral / epidemiology*
- Retrospective Studies
- SARS-CoV-2
- Sex Factors
- Surgery Department, Hospital / statistics & numerical data*
- Tachycardia / epidemiology
- Tertiary Care Centers / statistics & numerical data*
- Young Adult

Associated data

- ClinicalTrials.gov/NCT04338672

Full text links

Atypon
Proceed to details
Cite
Share
426
Observational Study
South Med J
. 2021 Mar;114(3):144-149.
doi: 10.14423/SMJ.0000000000001222.

Epidemiology, Clinical Features, and Outcomes of Hospitalized Adults with COVID-19: Early Experience from an Academic Medical Center in Mississippi

Jose Lucar [1], Mary Joyce B Wingler [1], David A Cretella [1], Lori M Ward [1], Courtney E Sims Gomillia [1], Nicholas Chamberlain [1], Luis A Shimose [1], James B Brock [1], Jessie Harvey [1], Andrew Wilhelm [1], Lance T Majors [1], Joshua B Jeter [1], Maria X Bueno [1], Svenja Albrecht [1], Bhagyashri Navalkele [1], Leandro A Mena [1], Jason Parham [1]
Affiliations Expand

Affiliation

- [1] From the Department of Medicine, Divisions of Infectious Diseases, Pulmonary, and Critical Care, and Hospital Medicine, and the Department of Population Health Science, John D. Bower School of Population Health, University of Mississippi Medical Center, Jackson.

- PMID: **33655307**
- PMCID: PMC7904045
- DOI: 10.14423/SMJ.0000000000001222

Free PMC article
Observational Study

Epidemiology, Clinical Features, and Outcomes of Hospitalized Adults with

COVID-19: Early Experience from an Academic Medical Center in Mississippi

Jose Lucar et al. South Med J. 2021 Mar.
Free PMC article
Show details
South Med J
. 2021 Mar;114(3):144-149.
doi: 10.14423/SMJ.0000000000001222.

Authors

Jose Lucar [1], Mary Joyce B Wingler [1], David A Cretella [1], Lori M Ward [1], Courtney E Sims Gomillia [1], Nicholas Chamberlain [1], Luis A Shimose [1], James B Brock [1], Jessie Harvey [1], Andrew Wilhelm [1], Lance T Majors [1], Joshua B Jeter [1], Maria X Bueno [1], Svenja Albrecht [1], Bhagyashri Navalkele [1], Leandro A Mena [1], Jason Parham [1]

Affiliation

- [1] From the Department of Medicine, Divisions of Infectious Diseases, Pulmonary, and Critical Care, and Hospital Medicine, and the Department of Population Health Science, John D. Bower School of Population Health, University of Mississippi Medical Center, Jackson.

- PMID: **33655307**
- PMCID: PMC7904045
- DOI: 10.14423/SMJ.0000000000001222

Abstract

Objectives: To describe the demographics, clinical characteristics, and outcomes of hospitalized adults with coronavirus disease 2019 (COVID-19) in an academic medical center in the southern United States.

Methods: Retrospective, observational cohort study of all adult patients (18 years and older) consecutively admitted with laboratory-confirmed severe acute respiratory syndrome-coronavirus-2 infection between March 13 and April 25, 2020 at the University of Mississippi Medical Center. All of the patients either survived to hospital discharge or died during hospitalization. Demographics, body mass index, comorbidities, clinical manifestations, and laboratory findings were collected. Patient outcomes (need for invasive mechanical ventilation and in-hospital death) were analyzed.

Results: One hundred patients were included, 53% of whom were women. Median age was 59 years (interquartile range 44-70) and 66% were younger than 65. Seventy-five percent identified themselves as Black, despite representing 58% of hospitalized patients at our institution in 2019. Common comorbid conditions included hypertension (68%), obesity (65%), and diabetes mellitus (31%). Frequent clinical manifestations included shortness of breath (76%), cough (75%), and fever (64%). Symptoms were present for a median of 7 days (interquartile range 4-7) on presentation. Twenty-four percent of patients required mechanical ventilation and, overall, 19%

died (67% of those requiring mechanical ventilation). Eighty-four percent of those who died were Black. On multivariate analysis, ever smoking (odds ratio [OR] 5.9, 95% confidence interval [CI] 1.2-28.6) and history of diabetes mellitus (OR 5.9, 95% CI 1.5-24.3) were associated with mortality, and those admitted from home were less likely to die (vs outside facility, OR 0.2, 95% CI 0.0-0.7). Neither age, sex, race, body mass index, insurance status, nor rural residence was independently associated with mortality.

Conclusions: Our study adds evidence that Black patients appear to be overrepresented in those hospitalized with and those who die from COVID-19, likely a manifestation of adverse social determinants of health. These findings should help guide preventive interventions targeting groups at higher risk of acquiring and developing severe COVID-19 disease.

Conflict of interest statement

L.A.M. has received compensation from Evofem, Gilead Science, Melinta, Merck, Rheonix, Roche Molecular, ViiV Heathcare|GSK, and Visby. The remaining authors did not report any financial relationships or conflicts of interest.

Supplementary info

Publication types, MeSH terms Expand

Publication types

- Observational Study

MeSH terms

- Academic Medical Centers
- Adult
- African Americans / statistics & numerical data
- Aged
- Body Mass Index
- COVID-19 / diagnosis
- COVID-19 / epidemiology*
- COVID-19 / therapy
- Female
- Hispanic or Latino / statistics & numerical data
- Hospital Mortality
- Hospitalization*
- Humans
- Male
- Middle Aged
- Mississippi
- Respiration, Artificial
- Retrospective Studies

- Risk Factors
- Whites / statistics & numerical data

Full text links

SMJ Full Text Southern Medical Association Free PMC article

Proceed to details

Cite

Share

427

Observational Study

Emergencias

. 2021 Jun;33(3):236-238.

Collateral damage in pediatric scenarios requiring urgent care during the COVID-19 pandemic

[Article in English, Spanish]

José Antonio Ruiz Domínguez [1], Miguel Ángel Molina Gutiérrez [1], Cristina de Miguel Cáceres [1], Irene Martín Espín [1], Marta Plata Gallardo [1], Julia Martín Sánchez [1]

Affiliations Expand

Affiliation

- [1] Servicio de Urgencias Pediátricas, Hospital Universitario La Paz, Madrid, España.

- PMID: **33978342**

Free article

Observational Study

Collateral damage in pediatric scenarios requiring urgent care during the COVID-19 pandemic

[Article in English, Spanish]

José Antonio Ruiz Domínguez et al. Emergencias. 2021 Jun.

Free article

Show details

Emergencias

. 2021 Jun;33(3):236-238.

Authors

José Antonio Ruiz Domínguez [1], Miguel Ángel Molina Gutiérrez [1], Cristina de Miguel Cáceres [1], Irene Martín Espín [1], Marta Plata Gallardo [1], Julia Martín Sánchez [1]

Affiliation

- [1] Servicio de Urgencias Pediátricas, Hospital Universitario La Paz, Madrid, España.

- PMID: **33978342**

No abstract available

- Cited by 1 article

Supplementary info

Publication types, MeSH terms Expand

Publication types

- Observational Study

MeSH terms

- Adolescent
- Ambulatory Care / organization & administration
- Ambulatory Care / statistics & numerical data*
- Ambulatory Care Facilities / statistics & numerical data
- Appendicitis / epidemiology
- COVID-19*
- Child
- Child, Preschool
- Diabetes Mellitus, Type 1 / epidemiology
- Diagnosis-Related Groups
- Emergencies*
- Fractures, Bone / epidemiology
- Hospitals, University / organization & administration
- Hospitals, University / statistics & numerical data
- Humans
- Hyperbilirubinemia, Neonatal / epidemiology
- Infant
- Infant, Newborn
- Pandemics*
- Retrospective Studies

- SARS-CoV-2*
- Spain / epidemiology
- Time-to-Treatment* / statistics & numerical data
- Triage
- Urinary Tract Infections / epidemiology

Full text links

EMERGENCIAS Grupo Saned
Proceed to details
Cite
Share
428
Observational Study
Ren Fail
. 2021 Dec;43(1):49-57.
doi: 10.1080/0886022X.2020.1853571.

Clinical features and outcome of maintenance hemodialysis patients with COVID-19 from a tertiary nephrology care center in Romania

Gabriel Stefan [1], Ana Maria Mehedinti [1 2], Iuliana Andreiana [1 2], Adrian Dorin Zugravu [1 2], Simona Cinca [1], Ruxandra Busuioc [1], Ioana Miler [1], Simona Stancu [1 2], Ligia Petrescu [1 2], Ioana Dimitriu [1], Elena Moldovanu [1], Diana Elena Crasnaru [1], Georgeta Gugonea [1], Valentin Georgescu [1], Victor Dan Strambu [1 3], Cristina Capusa [1 2]
Affiliations Expand

Affiliations

- [1] "Dr. Carol Davila" Teaching Hospital of Nephrology, Bucharest, Romania.
- [2] Department of Nephrology, "Carol Davila" University of Medicine and Pharmacy, Bucharest, Romania.
- [3] Department of Surgery,"Carol Davila" University of Medicine and Pharmacy, Bucharest, Romania.

- PMID: **33307933**
- PMCID: PMC7745841
- DOI: 10.1080/0886022X.2020.1853571

Free PMC article
Observational Study

Clinical features and outcome of maintenance hemodialysis patients with COVID-19 from a tertiary nephrology care center in Romania

Gabriel Stefan et al. Ren Fail. 2021 Dec.
Free PMC article
Show details
Ren Fail
. 2021 Dec;43(1):49-57.
doi: 10.1080/0886022X.2020.1853571.

Authors

Gabriel Stefan [1], Ana Maria Mehedinti [1 2], Iuliana Andreiana [1 2], Adrian Dorin Zugravu [1 2], Simona Cinca [1], Ruxandra Busuioc [1], Ioana Miler [1], Simona Stancu [1 2], Ligia Petrescu [1 2], Ioana Dimitriu [1], Elena Moldovanu [1], Diana Elena Crasnaru [1], Georgeta Gugonea [1], Valentin Georgescu [1], Victor Dan Strambu [1 3], Cristina Capusa [1 2]

Affiliations

- [1] "Dr. Carol Davila" Teaching Hospital of Nephrology, Bucharest, Romania.
- [2] Department of Nephrology, "Carol Davila" University of Medicine and Pharmacy, Bucharest, Romania.
- [3] Department of Surgery,"Carol Davila" University of Medicine and Pharmacy, Bucharest, Romania.

- PMID: **33307933**
- PMCID: PMC7745841
- DOI: 10.1080/0886022X.2020.1853571

Abstract

Background: There is limited information about the clinical characteristics, treatment and outcome of maintenance hemodialysis patients with COVID-19. Moreover, regional differences are also conceivable since the extend and severity of outbreaks varied among countries.

Methods: In this retrospective, observational, single-center study, we analyzed the clinical course and outcomes of 37 maintenance hemodialysis patients (median age 64 years, 51% men) hospitalized with COVID-19 from 24 March to 22 May 2020 as confirmed by real-time PCR.

Results: The most common symptoms at admission were fatigue (51%), fever (43%), dyspnea (38%) and cough (35%). There were 59% mild/moderate patients and 41% severe/critical patients. Patients in the severe/critical group had a significantly higher atherosclerotic burden since diabetic kidney disease and vascular nephropathies were the most common primary kidney diseases and eighty percent of them had coronary heart disease. Also, Charlson comorbidity score was higher in this group. At admission chest X-ray, 46% had ground-glass abnormalities. Overall, 60% patients received hydroxychloroquine, 22% lopinavir-ritonavir, 11% tocilizumab, 24% systemic

glucocorticoids, and 54% received prophylactic anticoagulation. Seven (19%) patients died during hospitalization and 30 were discharged. The main causes of death were cardiovascular (5 patients) and respiratory distress syndrome (2 patients). In Cox regression analysis, lower oxygen saturation, anemia and hypoalbuminemia at admission were associated with increased mortality.

Conclusions: In conclusion, we observed a high mortality rate among maintenance hemodialysis patients hospitalized for COVID-19. Anemia, lower serum albumin and lower basal oxygen saturation at admission were factors associated with poor prognosis.

Keywords: COVID-19; chronic hemodialysis; clinical features; mortality.

Conflict of interest statement

No potential conflict of interest was reported by the author(s).

- Cited by 11 articles
- 39 references

Supplementary info

Publication types, MeSH terms, Substances Expand

Publication types

- Observational Study

MeSH terms

- Aged
- COVID-19 / diagnosis
- COVID-19 / mortality*
- COVID-19 / therapy
- COVID-19 / virology
- Cause of Death
- Comorbidity
- Female
- Hospital Mortality
- Humans
- Kidney Failure, Chronic / blood
- Kidney Failure, Chronic / etiology
- Kidney Failure, Chronic / mortality
- Kidney Failure, Chronic / therapy*
- Male
- Middle Aged
- Oxygen / blood
- Patient Admission

- Prognosis
- Renal Dialysis*
- Retrospective Studies
- Risk Factors
- Romania / epidemiology
- SARS-CoV-2 / isolation & purification
- Serum Albumin, Human / analysis
- Severity of Illness Index

Substances

- Oxygen
- Serum Albumin, Human

Full text links

View full text Taylor & Francis Free PMC article
Proceed to details
Cite
Share
429
Observational Study
G Ital Nefrol
. 2020 Dec 7;37(6):2020-vol6.

[SARS CoV-2 related disease features in a population of chronic hemodialysis patients]

[Article in Italian]
Cristina Silvestri[1], Silvio Di Stante[1], Veronica Bertuzzi[1], Mauro Martello[1], Marco Palladino[1], Xhensila Grabocka[1], Hrissanthi Kulurianu[1], Flavia Manenti[1], Francesca Pizzolante[1], Assunta Cardillo[1], Angelo Francioso[1], Osmy Paci Della Costanza[1], Chiara Valentini[1], Marina Di Luca[1]
Affiliations Expand

Affiliation

- [1] U.O.C. Nefrologia e Dialisi Azienda Ospedaliera Ospedali Riuniti Marche Nord Pesaro - Fano, Italy.

- PMID: **33295704**

Observational Study

[SARS CoV-2 related disease features in a population of chronic hemodialysis patients]

[Article in Italian]
Cristina Silvestri et al. G Ital Nefrol. 2020.
Show details
G Ital Nefrol
. 2020 Dec 7;37(6):2020-vol6.

Authors

Cristina Silvestri [1], Silvio Di Stante [1], Veronica Bertuzzi [1], Mauro Martello [1], Marco Palladino [1], Xhensila Grabocka [1], Hrissanthi Kulurianu [1], Flavia Manenti [1], Francesca Pizzolante [1], Assunta Cardillo [1], Angelo Francioso [1], Osmy Paci Della Costanza [1], Chiara Valentini [1], Marina Di Luca [1]

Affiliation

- [1] U.O.C. Nefrologia e Dialisi Azienda Ospedaliera Ospedali Riuniti Marche Nord Pesaro - Fano, Italy.

- PMID: **33295704**

Abstract

Patients on chronic dialysis have an increased risk for SARS CoV-2 virus disease and its complications because of multiple comorbidities and alterations in the immune response caused by renal disease. In this retrospective observational study we describe the clinical features and the evolution of SARS CoV-2-related disease in 19 patients of our Pesaro and Fano facilities, where incidence and mortality of the epidemic were among the highest in Italy. A total of 176 patients were undergoing chronic treatment, 153 hemodialysis and 23 peritoneal dialysis. The incidence of infection was 10,8%, with 84% needing hospitalization and mortality amounting to 53%. The most frequent onset symptom was fever (84,2%) and the most used therapy was an association of low molecular weight heparin and hydroxychloroquine (57,9%). Comparing the deceased and survivor populations we noticed significant differences in age and presence of cardiopathy for what concerns anamnestic data and in fatigue and dyspnea in terms of clinical presentation. LDH and CPK resulted highest among deceased patients, while the use of enoxaparin was more frequent in survivors. By observing contagions over time, we also noticed that most of the cases, and the ones with worse clinical condition and outcome, all occurred in the early stage of the epidemic and in particular within the first 20 days from the implementation and codification of the measures to prevent its spread, the only modifiable factor that had an unmistakable effect on the evolution of events.

Keywords: COVID-19; SARS Cov-2; dialysis; nephrology.

- Cited by 1 article

Supplementary info

Publication types, MeSH terms, Supplementary concepts Expand

Publication types

- Multicenter Study
- Observational Study

MeSH terms

- Aged
- Aged, 80 and over
- COVID-19 / diagnostic imaging
- COVID-19 / drug therapy
- COVID-19 / epidemiology*
- COVID-19 / prevention & control
- COVID-19 / therapy
- COVID-19 Testing
- Combined Modality Therapy
- Comorbidity
- Humans
- Infection Control
- Italy / epidemiology
- Kaplan-Meier Estimate
- Kidney Failure, Chronic / epidemiology*
- Kidney Failure, Chronic / therapy
- Middle Aged
- Pandemics*
- Proportional Hazards Models
- Renal Dialysis*
- Retrospective Studies
- SARS-CoV-2*
- Symptom Assessment
- Tomography, X-Ray Computed

Supplementary concepts

- COVID-19 drug treatment

Proceed to details

Cite

Share

☐ 430
Observational Study
Int Heart J
. 2021 May 29;62(3):540-545.
doi: 10.1536/ihj.20-522. Epub 2021 May 1.

Reduction of Inhospital Mortality of Patient Admissions to Cardiac Intensive Care Units During the COVID-19 Pandemic in Hunan, China

Zhijian Wu [1], Mingxian Chen [1], Qingdan Hu [1], Yaqin Chen [1], Jianjun Tang [1]
Affiliations Expand

Affiliation

- [1] Department of Cardiology, Cardiac Intensive Care Units, The Second Xiangya Hospital of Central South University.

- PMID: **33952805**
- DOI: 10.1536/ihj.20-522

Free article
Observational Study

Reduction of Inhospital Mortality of Patient Admissions to Cardiac Intensive Care Units During the COVID-19 Pandemic in Hunan, China

Zhijian Wu et al. Int Heart J. 2021.
Free article
Show details
Int Heart J
. 2021 May 29;62(3):540-545.
doi: 10.1536/ihj.20-522. Epub 2021 May 1.

Authors

Zhijian Wu [1], Mingxian Chen [1], Qingdan Hu [1], Yaqin Chen [1], Jianjun Tang [1]

Affiliation

- [1] Department of Cardiology, Cardiac Intensive Care Units, The Second Xiangya Hospital of Central South University.

- PMID: **33952805**
- DOI: 10.1536/ihj.20-522

Abstract

This study aims to evaluate the impact of the coronavirus disease 2019 (COVID-19) pandemic on patient admissions to Hunan's cardiac intensive care units (CCUs).We conducted a retrospective, single-center study. Data were collected from patients who were confirmed to have critical cardiovascular disease and admitted to the CCU of the Second Xiangya Hospital of Central South University, Hunan, from January 23 to April 23, 2020. Compared with the same period in 2019, the results show that the number of hospitalization decreased by 19.6%; the inhospital mortality rate of CCU was decreased (28.57% versus 16.67%; odds ratio (OR), 0.50; 95% confidence interval (CI), 0.251-0.996; P = 0.047); hospital stay was decreased (7.97 versus 12.36, P < 0.001); hospital emergency percutaneous coronary intervention (PCI) rate in patients with acute coronary syndromes (ACS) significantly decreased (76.00% versus 39.00%, P < 0.001); among this, the PCI rate of patients with ST-segment elevation myocardial infarction (STEMI) decreased (76.32% versus 55.17%, P = 0.028) as well. In addition, the number of patients transferred from other hospitals significantly decreased (76.79% versus 56.67%, P = 0.002), and the number of patients transferred from other cities also decreased by 10.75%.During the outbreak of the COVID-19 epidemic in Hunan Province, the number of patients admitted to CCU decreased, as well as the mortality rate; fewer patients with severe cardiovascular disease can be transported to better hospitals from remote rural areas. In addition to epidemic prevention and control, experts in China should focus on improved emergency transport medical services to reduce this impact.

Keywords: Acute coronary syndrome; CCU; Heart failure; PCI; SARS-CoV-2.

Supplementary info

Publication types, MeSH terms Expand

Publication types

- Observational Study

MeSH terms

- Adult
- Aged
- Aged, 80 and over
- COVID-19* / epidemiology
- COVID-19* / prevention & control
- Cardiovascular Diseases / diagnosis
- Cardiovascular Diseases / mortality*

- Cardiovascular Diseases / therapy
- China / epidemiology
- Coronary Care Units / trends*
- Female
- Hospital Mortality / trends*
- Humans
- Male
- Middle Aged
- Pandemics
- Patient Admission / trends*
- Patient Transfer / trends*
- Retrospective Studies

Full text links

J-STAGE, Japan Science and Technology Information Aggregator, Electronic

Proceed to details

Cite

Share

431

Observational Study

Clin Infect Dis

. 2021 Dec 6;73(11):e4141-e4151.

doi: 10.1093/cid/ciaa1459.

Predictors at Admission of Mechanical Ventilation and Death in an Observational Cohort of Adults Hospitalized With Coronavirus Disease 2019

Brendan R Jackson [1, 2], Jeremy A W Gold [1, 3], Pavithra Natarajan [1], John Rossow [1, 2, 3], Robyn Neblett Fanfair [1, 2], Juliana da Silva [1], Karen K Wong [1, 2], Sean D Browning [1, 4], Sapna Bamrah Morris [1, 2], Jessica Rogers-Brown [1, 4], Alfonso C Hernandez-Romieu [1, 2, 3, 5], Christine M Szablewski [1, 2, 3, 6], Nadine Oosmanally [6], Melissa Tobin-D'Angelo [6], Cherie Drenzek [6], David J Murphy [5], Julie Hollberg [5], James M Blum [5, 7], Robert Jansen [8], David W Wright [5, 8], William M Sewell [9], Jack D Owens [9], Benjamin Lefkove [10], Frank W Brown [5, 10], Deron C Burton [1, 2], Timothy M Uyeki [1, 2], Stephanie R Bialek [1, 2], Priti R Patel [1, 2], Beau B Bruce [1]

Affiliations Expand

Affiliations

- [1] COVID-19 Emergency Response, Centers for Disease Control and Prevention, Atlanta, Georgia, USA.
- [2] United States Public Health Service, Atlanta, GA, USA.
- [3] Epidemic Intelligence Service, Centers for Disease Control and Prevention, Atlanta, Georgia, USA.
- [4] Oak Ridge Institute for Science and Education, Oak Ridge, Tennessee, USA.
- [5] Emory University School of Medicine, Atlanta, Georgia, USA.
- [6] Georgia Department of Public Health, Atlanta, Georgia, USA.
- [7] Georgia Clinical and Translational Science Alliance, Atlanta, Georgia, USA.
- [8] Grady Health System, Atlanta, Georgia, USA.
- [9] Phoebe Putney Memorial Hospital, Albany, Georgia, USA.
- [10] Emory Decatur Hospital, Decatur, Georgia, USA.

- PMID: **32971532**
- PMCID: PMC7543323
- DOI: 10.1093/cid/ciaa1459

Free PMC article
Observational Study

Predictors at Admission of Mechanical Ventilation and Death in an Observational Cohort of Adults Hospitalized With Coronavirus Disease 2019

Brendan R Jackson et al. Clin Infect Dis. 2021.
Free PMC article
Show details
Clin Infect Dis
. 2021 Dec 6;73(11):e4141-e4151.
doi: 10.1093/cid/ciaa1459.

Authors

Brendan R Jackson [1,2], Jeremy A W Gold [1,3], Pavithra Natarajan [1], John Rossow [1,2,3], Robyn Neblett Fanfair [1,2], Juliana da Silva [1], Karen K Wong [1,2], Sean D Browning [1,4], Sapna Bamrah Morris [1,2], Jessica Rogers-Brown [1,4], Alfonso C Hernandez-Romieu [1,2,3,5], Christine M Szablewski [1,2,3,6], Nadine Oosmanally [6], Melissa Tobin-D'Angelo [6], Cherie Drenzek [6], David J Murphy [5], Julie Hollberg [5], James M Blum [5,7], Robert Jansen [8], David W Wright [5,8], William M Sewell [9], Jack D Owens [9], Benjamin Lefkove [10], Frank W Brown [5,10], Deron C Burton [1,2], Timothy M Uyeki [1,2], Stephanie R Bialek [1,2], Priti R Patel [1,2], Beau B Bruce [1]

Affiliations

- [1] COVID-19 Emergency Response, Centers for Disease Control and Prevention, Atlanta, Georgia, USA.
- [2] United States Public Health Service, Atlanta, GA, USA.
- [3] Epidemic Intelligence Service, Centers for Disease Control and Prevention, Atlanta, Georgia, USA.
- [4] Oak Ridge Institute for Science and Education, Oak Ridge, Tennessee, USA.
- [5] Emory University School of Medicine, Atlanta, Georgia, USA.
- [6] Georgia Department of Public Health, Atlanta, Georgia, USA.
- [7] Georgia Clinical and Translational Science Alliance, Atlanta, Georgia, USA.
- [8] Grady Health System, Atlanta, Georgia, USA.
- [9] Phoebe Putney Memorial Hospital, Albany, Georgia, USA.
- [10] Emory Decatur Hospital, Decatur, Georgia, USA.

- PMID: **32971532**
- PMCID: PMC7543323
- DOI: 10.1093/cid/ciaa1459

Abstract

Background: Coronavirus disease (COVID-19) can cause severe illness and death. Predictors of poor outcome collected on hospital admission may inform clinical and public health decisions.

Methods: We conducted a retrospective observational cohort investigation of 297 adults admitted to 8 academic and community hospitals in Georgia, United States, during March 2020. Using standardized medical record abstraction, we collected data on predictors including admission demographics, underlying medical conditions, outpatient antihypertensive medications, recorded symptoms, vital signs, radiographic findings, and laboratory values. We used random forest models to calculate adjusted odds ratios (aORs) and 95% confidence intervals (CIs) for predictors of invasive mechanical ventilation (IMV) and death.

Results: Compared with age <45 years, ages 65-74 years and ≥75 years were predictors of IMV (aORs, 3.12 [95% CI, 1.47-6.60] and 2.79 [95% CI, 1.23-6.33], respectively) and the strongest predictors for death (aORs, 12.92 [95% CI, 3.26-51.25] and 18.06 [95% CI, 4.43-73.63], respectively). Comorbidities associated with death (aORs, 2.4-3.8; P < .05) included end-stage renal disease, coronary artery disease, and neurologic disorders, but not pulmonary disease, immunocompromise, or hypertension. Prehospital use vs nonuse of angiotensin receptor blockers (aOR, 2.02 [95% CI, 1.03-3.96]) and dihydropyridine calcium channel blockers (aOR, 1.91 [95% CI, 1.03-3.55]) were associated with death.

Conclusions: After adjustment for patient and clinical characteristics, older age was the strongest predictor of death, exceeding comorbidities, abnormal vital signs, and laboratory test abnormalities. That coronary artery disease, but not chronic lung disease, was associated with death among hospitalized patients warrants further investigation, as do associations between certain antihypertensive medications and death.

Keywords: COVID-19; SARS-CoV-2; angiotensin receptor antagonists; hospitalization; mortality.

Published by Oxford University Press for the Infectious Diseases Society of America 2020.

- Cited by 19 articles

Supplementary info

Publication types, MeSH terms, Grant support Expand

Publication types

- Observational Study
- Research Support, U.S. Gov't, P.H.S.

MeSH terms

- Aged
- COVID-19*
- Hospitalization
- Humans
- Middle Aged
- Respiration, Artificial
- Retrospective Studies
- Risk Factors
- SARS-CoV-2
- United States

Grant support

- UL1 TR002378/TR/NCATS NIH HHS/United States
- CC/CDC HHS/United States

Full text links

OXFORD ACADEMIC Silverchair Information Systems Free PMC article
Proceed to details
Cite
Share
☐ 432
Observational Study
Rev Esp Anestesiol Reanim (Engl Ed)
. 2021 Nov;68(9):513-522.
doi: 10.1016/j.redare.2020.11.008. Epub 2021 Nov 4.

C-Reactive protein and SOFA scale: A simple score as early predictor of critical care

requirement in patients with COVID-19 pneumonia in Spain

L M Vaquero-Roncero [1], E Sánchez-Barrado [2], D Escobar-Macias [1], P Arribas-Pérez [1], R González de Castro [3], J R González-Porras [4], M V Sánchez-Hernandez [1], COVID Working Group
Affiliations Expand

Affiliations

- [1] Departamento de Anestesiología y Reanimación, Hospital Universitario de Salamanca-IBSAL, Departamento de Medicina, Universidad de Salamanca, Salamanca, Spain.
- [2] Departamento de Anestesiología y Reanimación, Hospital Universitario de Salamanca-IBSAL, Departamento de Medicina, Universidad de Salamanca, Salamanca, Spain. Electronic address: mesanchezba@saludcastillayleon.es.
- [3] Departamento de Anestesiología y Reanimación, Hospital Universitario de León, Universidad de León, León, Spain.
- [4] Departamento de Hematología, Hospital Universitario de Salamanca-IBSAL, Departamento de Medicina, Universidad de Salamanca, Salamanca, Spain.

- PMID: **34743905**
- PMCID: PMC8568297
- DOI: 10.1016/j.redare.2020.11.008

Free PMC article
Observational Study

C-Reactive protein and SOFA scale: A simple score as early predictor of critical care requirement in patients with COVID-19 pneumonia in Spain

L M Vaquero-Roncero et al. Rev Esp Anestesiol Reanim (Engl Ed). 2021 Nov.
Free PMC article
Show details
Rev Esp Anestesiol Reanim (Engl Ed)
. 2021 Nov;68(9):513-522.
doi: 10.1016/j.redare.2020.11.008. Epub 2021 Nov 4.

Authors

L M Vaquero-Roncero [1], E Sánchez-Barrado [2], D Escobar-Macias [1], P Arribas-Pérez [1], R González de Castro [3], J R González-Porras [4], M V Sánchez-Hernandez [1], COVID Working Group

Affiliations

- [1] Departamento de Anestesiología y Reanimación, Hospital Universitario de Salamanca-IBSAL, Departamento de Medicina, Universidad de Salamanca, Salamanca, Spain.
- [2] Departamento de Anestesiología y Reanimación, Hospital Universitario de Salamanca-IBSAL, Departamento de Medicina, Universidad de Salamanca, Salamanca, Spain. Electronic address: mesanchezba@saludcastillayleon.es.
- [3] Departamento de Anestesiología y Reanimación, Hospital Universitario de León, Universidad de León, León, Spain.
- [4] Departamento de Hematología, Hospital Universitario de Salamanca-IBSAL, Departamento de Medicina, Universidad de Salamanca, Salamanca, Spain.

- PMID: **34743905**
- PMCID: PMC8568297
- DOI: 10.1016/j.redare.2020.11.008

Abstract
in English, Spanish

Objective: To identify potential markers at admission predicting the need for critical care in patients with COVID-19 pneumonia.

Material and methods: An approved, observational, retrospective study was conducted between March 15 to April 15, 2020. 150 adult patients aged less than 75 with Charlson comorbidity index ≤6 diagnosed with COVID-19 pneumonia were included. Seventy-five patients were randomly selected from those admitted to the critical care units (critical care group [CG]) and seventy-five hospitalized patients who did not require critical care (non-critical care group [nCG]) represent the control group. One additional cohort of hospitalized patients with COVID-19 were used to validate the score.

Measurements and main results: Multivariable regression showed increasing odds of in-hospital critical care associated with increased C-reactive protein (CRP) (odds ratio 1.052 [1.009-1.101]; $P = 0.0043$) and higher Sequential Organ Failure Assessment (SOFA) score (1.968 [1.389-2.590]; $P < 0.0001$), both at the time of hospital admission. The AUC-ROC for the combined model was 0.83 (0.76-0.90) (vs AUC-ROC SOFA $P < 0.05$). The AUC-ROC for the validation cohort was 0.89 (0.82-0.95) ($P > 0.05$ vs AUC-ROC development).

Conclusion: Patients COVID-19 presenting at admission SOFA score ≥ 2 combined with CRP ≥ 9.1 mg/mL could be at high risk to require critical care.

Objetivo: Identificar marcadores potenciales durante el ingreso que predigan la necesidad de cuidados críticos en pacientes con neumonía causada por COVID-19.

Material y métodos: Estudio autorizado, observacional y retrospectivo realizado entre el 15 de marzo y el 15 de abril de 2020; incluyó a 150 pacientes adultos menores de 75 años con índice de comorbilidad de Charlson ≤ 6 diagnosticados de neumonía por COVID-19. Se seleccionaron aleatoriamente 75 pacientes de entre los ingresados en las unidades de cuidados críticos (grupo de cuidados críticos [GC]) y 75 pacientes hospitalizados que no requirieron cuidados críticos (grupo de cuidados no críticos [GnC]) que representaron el grupo control. Se utilizó una cohorte adicional de pacientes hospitalizados con COVID-19 para validar la escala.

Medidas y resultados principales: La regresión multivariante reflejó unos incrementos de los odds ratio de cuidados críticos hospitalarios asociados al incremento de proteína C reactiva (PCR) (odds ratio: 1,052 [1,009–1,101]; p = 0,0043) y puntuaciones en Sequential Organ Failure Assessment (SOFA) más altas (1,968 [1,389–2,590]; p < 0,0001) en el momento del ingreso hospitalario. El valor de la curva AUC-ROC para el modelo combinado fue de 0,83 (0,76–0,90) (frente a AUC-ROC SOFA p < 0,05). El valor de AUC-ROC para la cohorte de validación fue de 0,89 (0,82–0,95) (p > 0,05 frente a AUC-ROC de la cohorte desarrollo).

Conclusión: Los pacientes de COVID-19 que presentan al ingreso una puntuación SOFA ≥ 2 combinada con PCR ≥ 9,1 mg/mL podrían ser de alto riesgo a la hora de requerir cuidados críticos.

Keywords: C-reactive protein; COVID-19 pneumonia; Critical care; Cuidados críticos; Neumonía por COVID-19; Proteína C reactiva; SOFA - evaluación de fallo orgánico secuencial; Sequential organ failure assessment - SOFA.

Copyright © 2021 Sociedad Española de Anestesiología, Reanimación y Terapéutica del Dolor. Publicado por Elsevier España, S.L.U. All rights reserved.

- 30 references

Supplementary info

Publication types, MeSH terms, Substances Expand

Publication types

- Observational Study

MeSH terms

- Adult
- C-Reactive Protein
- COVID-19*
- Critical Care
- Humans
- Prognosis
- ROC Curve
- Retrospective Studies
- SARS-CoV-2
- Sepsis*
- Spain

Substances

- C-Reactive Protein

Full text links

ELSEVIER FULL-TEXT ARTICLE Elsevier Science Free PMC article
Proceed to details
Cite
Share
433
Observational Study
Rev Esp Geriatr Gerontol
. Sep-Oct 2021;56(5):259-267.
doi: 10.1016/j.regg.2020.09.006. Epub 2020 Nov 11.

[Mortality and associated prognostic factors in elderly and very elderly hospitalized patients with respiratory disease COVID-19]

[Article in Spanish]
Daniel Águila-Gordo [1], Jorge Martínez-Del Río [2], Virginia Mazoteras-Muñoz [3], Martín Negreira-Caamaño [2], Patricia Nieto-Sandoval Martín de la Sierra [4], Jesús Piqueras-Flores [2]
Affiliations Expand

Affiliations

- [1] Servicio de Cardiología, Hospital General Universitario de Ciudad Real, Ciudad Real, España. Electronic address: danielaguilagordo@gmail.com.
- [2] Servicio de Cardiología, Hospital General Universitario de Ciudad Real, Ciudad Real, España.
- [3] Servicio de Geriatría, Hospital General Universitario de Ciudad Real, Ciudad Real, España.
- [4] Servicio de Farmacia, Hospital General Universitario de Ciudad Real, Ciudad Real, España.

- PMID: **33610380**
- PMCID: PMC7656995
- DOI: 10.1016/j.regg.2020.09.006

Free PMC article
Observational Study

[Mortality and associated prognostic factors in elderly and very elderly hospitalized patients with respiratory disease COVID-19]

[Article in Spanish]
Daniel Águila-Gordo et al. Rev Esp Geriatr Gerontol. Sep-Oct 2021.
Free PMC article
Show details
Rev Esp Geriatr Gerontol
. Sep-Oct 2021;56(5):259-267.
doi: 10.1016/j.regg.2020.09.006. Epub 2020 Nov 11.

Authors

Daniel Águila-Gordo [1], Jorge Martínez-Del Río [2], Virginia Mazoteras-Muñoz [3], Martín Negreira-Caamaño [2], Patricia Nieto-Sandoval Martín de la Sierra [4], Jesús Piqueras-Flores [2]

Affiliations

- [1] Servicio de Cardiología, Hospital General Universitario de Ciudad Real, Ciudad Real, España. Electronic address: danielaguilagordo@gmail.com.
- [2] Servicio de Cardiología, Hospital General Universitario de Ciudad Real, Ciudad Real, España.
- [3] Servicio de Geriatría, Hospital General Universitario de Ciudad Real, Ciudad Real, España.
- [4] Servicio de Farmacia, Hospital General Universitario de Ciudad Real, Ciudad Real, España.

- PMID: **33610380**
- PMCID: PMC7656995
- DOI: 10.1016/j.regg.2020.09.006

Abstract
in English, Spanish

Introduction: Elderly patients with COVID-19 has a worse clinical evolution, being more susceptible to develop serious manifestations. The differences between the elderly and very elderly population, mortality and associated prognostic factors of SARS-CoV-2 infection have not been enough studied yet.

Methods: An observational study of 416 elderly patients admitted consecutively to Hospital General Universitario de Ciudad Real for COVID-19 respiratory infection from March 1st to April 30th, 2020. Data were collected including patient demographic information, medical history, clinical characteristics, laboratory data, therapeutic interventions and clinical outcomes during the hospitalization and after discharge, until June 15, 2020 with the aim of analyzing mortality, and associated prognostic factors.

Results: The mean age was 84.43±5.74 years old; elderly patients (75-84 years) were 50.2% of the sample and very elderly (≥85 years) the remaining 49.8%. In Cox regression model, mortality rate was higher in very elderly group (HR = 2.58; 95% CI: 1.23-5.38; P = .01), hypertensive (HR = 3, 45; 95% CI: 1.13-10.5; P = .03) and chronic kidney disease patients (HR = 3.86; 95% CI: 1.3-11.43; P = .02). In contrast, calcium antagonists (HR = 0.27; 95% CI: 0.12-0.62; P = .002) and anticoagulant therapy during hospitalization (HR = 0.26; 95% CI: 0.08 0, 83; P = .02) were associated with a longer time free of mortality.

Conclusions: Mortality rate was higher in very eldery patients compared with eldery; and in hypertensive and chronic kidney disease patients. Anticoagulation therapy and calcium chanel bloquers treatment during hospitalization were associated with a higher survival in the short-term follow-up in patients hospitalized with COVID-19.

Introducción: La población de mayor edad infectada por COVID-19 presenta una peor evolución clínica, siendo más susceptible a desarrollar manifestaciones graves. Las diferencias entre la población anciana y muy anciana, la mortalidad y los factores pronósticos asociados a la infección por SARS-CoV-2 no han sido suficientemente estudiados.

Métodos: Registro observacional de 416 pacientes ancianos ingresados de forma consecutiva por infección respiratoria COVID-19 entre el 1 de marzo y el 30 abril de 2020 en el Hospital General Universitario de Ciudad Real. Se registraron variables clínicas y analíticas, intervenciones terapéuticas y desarrollo de eventos durante el ingreso y tras el alta hasta el 15 de junio de 2020, con el objetivo de analizar la mortalidad y factores pronósticos asociados.

Resultados: La edad media fue de 84,43 ± 5,74 años. Los pacientes ancianos (entre 75 y 84 años) constituyeron un 50,2% de la muestra y los muy ancianos (≥ 85 años) el 49,8% restante. En el modelo de regresión de Cox multivariante, el riesgo de mortalidad fue mayor en pacientes muy ancianos (HR = 2,58; IC95%: 1,23-5,38; p = 0,01), hipertensos (HR = 3,45; IC95%: 1,13-10,5; p = 0,03) y con enfermedad renal crónica (HR = 3,86; IC95%: 1,3-11,43; p = 0,02). En cambio, la prescripción de calcioantagonistas (HR = 0,27; IC95%: 0,12-0,62; p = 0,002) y la terapia anticoagulante durante la hospitalización (HR = 0,26; IC95%: 0,08-0,83; p = 0,02) se asociaron con mayor tiempo libre de mortalidad.

Conclusiones: Los pacientes muy ancianos, hipertensos y con enfermedad renal crónica presentaron mayor riesgo de mortalidad por infección respiratoria COVID-19. En cambio, el empleo de anticoagulación terapéutica y calcioantagonistas durante el ingreso se asociaron con mayor probabilidad de supervivencia en el seguimiento a corto plazo.

Keywords: Aged; Anciano; COVID-19; Muy anciano; Oldest old.

Copyright © 2020 SEGG. Publicado por Elsevier España, S.L.U. All rights reserved.

- Cited by 1 article
- 39 references
- 2 figures

Supplementary info

Publication types, MeSH terms Expand

Publication types

- Observational Study

MeSH terms

- Aged
- Aged, 80 and over
- COVID-19 / mortality*

- Hospital Mortality*
- Hospitalization / statistics & numerical data*
- Humans
- Pandemics
- Prognosis
- Retrospective Studies
- Risk Factors
- SARS-CoV-2

Full text links

FULL TEXT AT: Geriatría y Gerontología Ediciones Doyma, S.L. Free PMC article
Proceed to details
Cite
Share
434
Observational Study
Med J Malaysia
. 2020 Sep;75(5):479-484.

Clinical characteristics of severe acute respiratory syndrome Coronavirus 2 (SARS-CoV2) patients in Hospital Tengku Ampuan Afzan

T V Soh [1], M Dzawani [2], N Noorlina [2], F Nik [2], A Norazmi [2]
Affiliations Expand

Affiliations

- [1] Hospital Tengku Ampuan Afzan, 25100 Kuantan, Pahang, Malaysia. sohtzevee@hotmail.com.
- [2] Hospital Tengku Ampuan Afzan, 25100 Kuantan, Pahang, Malaysia.

- PMID: **32918413**

Free article
Observational Study

Clinical characteristics of severe acute respiratory syndrome Coronavirus 2 (SARS-

CoV2) patients in Hospital Tengku Ampuan Afzan

T V Soh et al. Med J Malaysia. 2020 Sep.
Free article
Show details
Med J Malaysia
. 2020 Sep;75(5):479-484.

Authors

T V Soh [1], M Dzawani [2], N Noorlina [2], F Nik [2], A Norazmi [2]

Affiliations

- [1] Hospital Tengku Ampuan Afzan, 25100 Kuantan, Pahang, Malaysia. sohtzevee@hotmail.com.
- [2] Hospital Tengku Ampuan Afzan, 25100 Kuantan, Pahang, Malaysia.

- PMID: **32918413**

Abstract

Background: The COVID-19 is a disease caused by severe acute respiratory syndrome coronavirus 2 (SARS-CoV-2). This study aims to describe the clinical characteristics of COVID-19 patients admitted to Hospital Tengku Ampuan Afzan (HTAA), Pahang, Malaysia and to identify the clinical and laboratory markers for severe disease, complications and virologic clearance according to clinical staging.

Methods: This was a single-centre, retrospective, descriptive study. All COVID-19 patients admitted to HTAA from March 9 to April 15, 2020, were included in the study. Patients were categorised according to clinical staging. Data obtained from the medical report includes baseline characteristics of patients, comorbidities, presenting symptoms, laboratory findings, treatments, complications, and outcomes.

Results: Of the total of 247 patients hospitalised, the majority consisted at clinical-stage 1 (43%) and stage 2 (39%) disease. Older patients, diabetes mellitus, hypertension, cardiovascular diseases, and chronic kidney disease were found more common among patients with severe disease. Fever was uncommon and the majority had normal haemoglobin levels, white cell counts, and platelet counts. C-reactive protein (CRP) was found statistically significant to predict pneumonia or hypoxia at a cut-off value of 14mg/L (sensitivity 73.8%, specificity 91.3%) and 50mg/L (sensitivity 100%, specificity 96.4%) respectively. Pneumonia was mostly diagnosed radiologically using chest radiography, especially among clinical stage 3. Acute kidney injury (AKI) was a significant complication, with 31% of clinical stage 3 and above developed AKI and 44% of them requiring haemodialysis. Median virologic clearance time was 15 days from onset of illness, and asymptomatic patients had longer clearance time.

Conclusion: COVID-19 presented with a wide spectrum of clinical patterns. CRP was a valuable predictor of severe disease. In this study risk and severity of acute kidney injury were found to be higher. A longer duration of virologic clearance was observed among the asymptomatic patients.

- Cited by 2 articles

Supplementary info

Publication types, MeSH terms Expand

Publication types

- Observational Study

MeSH terms

- Adolescent
- Adult
- Aged
- Betacoronavirus*
- COVID-19
- Coronavirus Infections / complications*
- Coronavirus Infections / diagnosis*
- Coronavirus Infections / therapy
- Female
- Hospitalization
- Humans
- Malaysia
- Male
- Middle Aged
- Pandemics
- Pneumonia, Viral / complications*
- Pneumonia, Viral / diagnosis*
- Pneumonia, Viral / therapy
- Retrospective Studies
- SARS-CoV-2
- Severity of Illness Index
- Symptom Assessment
- Treatment Outcome
- Young Adult

Full text links

Malaysian Medical Association
Proceed to details
Cite
Share
435
Comparative Study
J Nutr Health Aging
. 2020;24(9):928-937.
doi: 10.1007/s12603-020-1477-2.

Older Adults Hospitalized with Covid-19: Clinical Characteristics and Early Outcomes from a Single Center in Istanbul, Turkey

A Medetalibeyoglu [1], N Senkal, M Kose, Y Catma, E Bilge Caparali, M Erelel, M Oral Oncul, G Bahat, T Tukek
Affiliations Expand

Affiliation

- [1] Gulistan Bahat, Istanbul Universitesi Istanbul Tip Fakultesi, Istanbul, Turkey, gbahatozturk@yahoo.com.

- PMID: **33155617**
- PMCID: PMC7597420
- DOI: 10.1007/s12603-020-1477-2

Free PMC article
Comparative Study

Older Adults Hospitalized with Covid-19: Clinical Characteristics and Early Outcomes from a Single Center in Istanbul, Turkey

A Medetalibeyoglu et al. J Nutr Health Aging. 2020.
Free PMC article
Show details
J Nutr Health Aging
. 2020;24(9):928-937.
doi: 10.1007/s12603-020-1477-2.

Authors

A Medetalibeyoglu[1], N Senkal, M Kose, Y Catma, E Bilge Caparali, M Erelel, M Oral Oncul, G Bahat, T Tukek

Affiliation

- [1] Gulistan Bahat, Istanbul Universitesi Istanbul Tip Fakultesi, Istanbul, Turkey, gbahatozturk@yahoo.com.

- PMID: **33155617**
- PMCID: PMC7597420
- DOI: 10.1007/s12603-020-1477-2

Abstract

Objective: Older adults have been continuously reported to be at higher risk for adverse outcomes of Covid-19. We aimed to describe clinical characteristics and early outcomes of the older Covid-19 patients hospitalized in our center comparatively with the younger patients, and also to analyze the triage factors that were related to the in-hospital mortality of older adults.

Design: Retrospective; observational study.

Setting: Istanbul Faculty of Medicine hospital, Turkey.

Participants: 362 hospitalized patients with laboratory-confirmed Covid-19 from March 11 to May 11, 2020.

Measurements: The demographic information; associated comorbidities; presenting clinical, laboratory, radiological characteristics on admission and outcomes from the electronic medical records were analyzed comparatively between the younger (<65 years) and older (≥65 years) adults. Factors associated with in-hospital mortality of the older adults were analyzed by multivariate regression analyses.

Results: The median age was 56 years (interquartile range [IQR], 46-67), and 224 (61.9%) were male. There were 104 (28.7%) patients ≥65 years of age. More than half of the patients (58%) had one or more chronic comorbidity. The three most common presenting symptoms in the older patients were fatigue/myalgia (89.4%), dry cough (72.1%), and fever (63.5%). Cough and fever were significantly less prevalent in older adults compared to younger patients (p=0.001 and 0.008, respectively). Clinically severe pneumonia was present in 31.5% of the study population being more common in older adults (49% vs. 24.4%) (p<0.001). The laboratory parameters that were significantly different between the older and younger adults were as follows: the older patients had significantly higher CRP, D-dimer, TnT, pro-BNP, procalcitonin levels, higher prevalence of lymphopenia, neutrophilia, increased creatinine, and lower hemoglobin, ALT, albumin level (p<0.05). In the radiological evaluation, more than half of the patients (54.6%) had moderate-severe pneumonia, which was more prevalent in older patients (66% vs. 50%) (p=0.006). The adverse outcomes were significantly more prevalent in older adults compared to the younger patients (ICU admission, 28.8% vs. 8.9%; mortality, 23.1% vs. 4.3%, p<0.001). Among the triage evaluation parameters, the only factor associated with higher mortality was the presence of clinically severe pneumonia on admission (Odds Ratio=12.3, 95% confidence interval=2.7-55.5, p=0.001).

Conclusion: Older patients presented with more prevalent chronic comorbidities, less prevalent symptomatology but more severe respiratory signs and laboratory abnormalities than the younger

patients. Among the triage assessment factors, the clinical evaluation of pulmonary involvement came in front to help clinicians to stratify the patients for mortality risk.

Keywords: Covid-19; characteristics; clinical; elderly; mortality.

Conflict of interest statement

Alpay Medetalibeyoğlu declares that there is no conflict of interest. Naci Şenkal declares that there is no conflict of interest. Murat Köse declares that there is no conflict of interest. Yunus Çatma declares that there is no conflict of interest. Emine Bilge Çaparali declares that there is no conflict of interest. Mustafa Erelel declares that there is no conflict of interest. Mustafa Oral Öncül declares that there is no conflict of interest. Gülistan Bahat Öztürk declares that there is no conflict of interest. Tufan Tükek declares that there is no conflict of interest.

- Cited by 9 articles
- 20 references

Supplementary info

Publication types, MeSH terms, Substances Expand

Publication types

- Comparative Study

MeSH terms

- Age Factors
- Aged
- COVID-19* / complications
- COVID-19* / mortality
- Comorbidity
- Female
- Fibrin Fibrinogen Degradation Products / metabolism
- Hospital Mortality*
- Hospitalization*
- Humans
- Male
- Middle Aged
- Pandemics*
- Pneumonia / etiology
- Pneumonia / mortality
- Retrospective Studies
- Risk Factors
- SARS-CoV-2
- Triage

- Turkey / epidemiology

Substances

- Fibrin Fibrinogen Degradation Products
- fibrin fragment D

Full text links

Springer Free PMC article
Proceed to details
Cite
Share
436
Observational Study
Nutr Hosp
. 2022 Feb 9;39(1):93-100.
doi: 10.20960/nh.03738.

[Nutritional risk and clinical outcomes in patients diagnosed with COVID-19 in a high-complexity hospital network]

[Article in Spanish]
Olga Lucía Pinzón-Espitia [1], Juan Mauricio Pardo-Oviedo [2], Luisa Fernanda Murcia Soriano [3]
Affiliations Expand

Affiliations

- [1] Departamento de Nutrición Humana. Facultad de Medicina. Universidad Nacional de Colombia.
- [2] Universidad del Rosario.
- [3] Hospital Universitario Mayor Méderi. Universidad del Rosario.

- PMID: **34756055**
- DOI: 10.20960/nh.03738

Free article
Observational Study

[Nutritional risk and clinical outcomes in patients diagnosed with COVID-19 in a high-complexity hospital network]

[Article in Spanish]
Olga Lucía Pinzón-Espitia et al. Nutr Hosp. 2022.
Free article
Show details
Nutr Hosp
. 2022 Feb 9;39(1):93-100.
doi: 10.20960/nh.03738.

Authors

Olga Lucía Pinzón-Espitia [1], Juan Mauricio Pardo-Oviedo [2], Luisa Fernanda Murcia Soriano [3]

Affiliations

- [1] Departamento de Nutrición Humana. Facultad de Medicina. Universidad Nacional de Colombia.
- [2] Universidad del Rosario.
- [3] Hospital Universitario Mayor Méderi. Universidad del Rosario.

- PMID: **34756055**
- DOI: 10.20960/nh.03738

Abstract
in English, Spanish

Introduction: the identification of nutritional risk at hospital admission is important to establish timely interventions in the COVID-19 patient care cycle, due to a high risk of it being associated with complications. Objective: to determine the association between the level of nutritional risk upon admission and in-hospital mortality at 28 days in patients diagnosed with COVID-19 treated between March and October 2020 in two hospital institutions in Colombia. Methods: a retrospective, observational study. Hospitalized patients with a diagnosis of COVID-19 were included and assessed by the Nutrition Service using the nutritional risk identification in emergencies scale, adapted from the NRS 2002 scale. In-hospital mortality at 28 days was analyzed as the primary endpoint, and hospital stay, admission to Intensive Care Unit (ICU), and requirement for mechanical ventilation as secondary endpoints. Results: a total of 1230 patients were included, with a mean age of 65.43 ± 15.90 years, mainly men (57.1 %, n = 702). A high nutritional risk (≥ 2 points) was identified in 74.3 % (n = 914). Patients with a high nutritional risk had a greater probability of in-hospital death at 28 days (HRadj: 1.64; 95 % CI: 1.11-2.44), and a greater risk of requiring mechanical ventilation (OR = 1.78; 95 % CI: 1.11-2.86) or ICU admission (OR = 1.478; 95 % CI: 1.05-2.09), as well as hospital stay longer than 7 days (OR = 1.91; 95 % CI: 1.47-2.48). Conclusions: patients with a diagnosis of COVID-19 at high nutritional risk had a significantly higher in-hospital mortality at 28 days and a higher probability of requiring mechanical ventilation, ICU admission, and prolonged hospital stay.

Introducción: la identificación del riesgo nutricional al ingreso hospitalario es importante para establecer intervenciones oportunas en el ciclo de atención del paciente con COVID-19, debido al alto riesgo de asociarse a complicaciones. Objetivo: determinar la asociación entre el nivel de riesgo nutricional al ingreso y la mortalidad intrahospitalaria a 28 días en pacientes con diagnóstico de COVID-19 atendidos entre marzo y octubre de 2020 en una red de dos instituciones hospitalarias de Colombia. Metodología: estudio observacional retrospectivo. Se incluyeron pacientes hospitalizados con diagnóstico de COVID-19 y valorados por el Servicio de Nutrición con la escala de identificación de riesgo nutricional en emergencias, adaptada de la escala NRS 2002. Se analizó como resultado principal la mortalidad intrahospitalaria a 28 días y como secundarios, la estancia hospitalaria, el ingreso a la Unidad de Cuidados Intensivos (UCI) y el requerimiento de ventilación mecánica. Resultados: se incluyeron 1230 pacientes, con edad promedio de 65,43 ± 15,90 años, principalmente hombres (57,1 %, n = 702). Se identificó un alto riesgo nutricional (≥ 2 puntos) en el 74,3 % (n = 914). Se evidenció que los pacientes con alto riesgo nutricional tenían una mayor probabilidad de muerte intrahospitalaria a 28 días (HR: 1,64; IC 95 %: 1,11-2,44) y un mayor riesgo de requerir ventilación mecánica (OR = 1,78; IC 95 %: 1,11-2,86), de ingreso en la UCI (OR = 1,478; IC 95 %: 1,05-2,09) y de estancia hospitalaria superior a 7 días (OR = 1,91; IC 95 %: 1,47-2,48). Conclusiones: los pacientes con diagnóstico de COVID-19 y riesgo nutricional alto presentaron una mortalidad intrahospitalaria a 28 días significativamente mayor y una mayor probabilidad de requerir ventilación mecánica y atención en la UCI, así como estancias hospitalarias prolongadas.

Keywords: Desnutrición. Evaluación nutricional. Servicios de salud para ancianos. Infecciones por coronavirus..

Supplementary info

Publication types, MeSH terms Expand

Publication types

- Observational Study

MeSH terms

- Aged
- Aged, 80 and over
- COVID-19*
- Hospital Mortality
- Hospitals
- Humans
- Intensive Care Units
- Male
- Middle Aged
- Respiration, Artificial
- Retrospective Studies
- SARS-CoV-2

Full text links

Free full text Nutrición Hospitalaria Aran ediciones, S.L.

Proceed to details

Cite

Share

437

Observational Study

Monaldi Arch Chest Dis

. 2020 Nov 27;90(4).

doi: 10.4081/monaldi.2020.1357.

Clinical and epidemiological profile of patients infected by COVID-19 at a tertiary care centre in North India

Sadanand Prakash [1], Manas Mani Agrawal [2], Rajendra Kumar [3], Shubhangi Yadav [4]

Affiliations Expand

Affiliations

- [1] Department of Radiation Oncology, King George's Medical University, Lucknow. sadanand3030@gmail.com.
- [2] Department of Radiation Oncology, King George's Medical University, Lucknow. dr_sadanand30@rediffmail.com.
- [3] Department of Radiation Oncology, King George's Medical University, Lucknow. sadanandprakash30@gmail.com.
- [4] Department of Anatomy, All India Institute of Medical Sciences, Raebareli. dr_shubhangi4@rediffmail.com.

- PMID: **33305553**
- DOI: 10.4081/monaldi.2020.1357

Free article

Observational Study

Clinical and epidemiological profile of patients infected by COVID-19 at a tertiary care centre in North India

Sadanand Prakash et al. Monaldi Arch Chest Dis. 2020.

Free article

Show details

Monaldi Arch Chest Dis
. 2020 Nov 27;90(4).
doi: 10.4081/monaldi.2020.1357.

Authors

Sadanand Prakash [1], Manas Mani Agrawal [2], Rajendra Kumar [3], Shubhangi Yadav [4]

Affiliations

- [1] Department of Radiation Oncology, King George's Medical University, Lucknow. sadanand3030@gmail.com.
- [2] Department of Radiation Oncology, King George's Medical University, Lucknow. dr_sadanand30@rediffmail.com.
- [3] Department of Radiation Oncology, King George's Medical University, Lucknow. sadanandprakash30@gmail.com.
- [4] Department of Anatomy, All India Institute of Medical Sciences, Raebareli. dr_shubhangi4@rediffmail.com.

- PMID: **33305553**
- DOI: 10.4081/monaldi.2020.1357

Abstract

A worldwide outbreak of a respiratory illness, first detected in December 2019 in Wuhan city, Hubei province, China is ongoing. The disease is caused by a novel coronavirus, SARS-CoV-2 and on February 11, 2020, was officially named Coronavirus Disease 2019 (COVID-19) by the World Health Organization. Within few weeks, it has spread globally to the extent that World Health Organization declared it as a global pandemic on March 11, 2020. India's first positive case was reported on January 30th in Kerala. Before March 3rd, India had 3 cases of coronavirus in Kerala all of which were treated and discharged. On March 3rd, India's 4th case was diagnosed in the state of Rajasthan. Indian government had announced a number of preventive measures to minimize the entry and spread of coronavirus. On March 3rd, India announced the suspension of all visas issued to Italy, Iran, South Korea and Japan. India banned international flights from March 22nd. A 21-day lockdown across the country was imposed from March 26th, which later got further extended. Rigorous contact tracing and tracking of COVID patients and monitoring home quarantine helped in preventing community transmission. The aim of this work is to describe the experience with clinical and epidemiologic features, as well as with the management of COVID-19 patients in north India. This is a descriptive study of the 17 COVID-19 infected patients confirmed with polymerase chain reaction (PCR) and admitted to a tertiary care centre in India from March 11th 2020 to April 16th 2020. The present work also provides insight in to treatment provided and final outcome of the patients infected with COVID-19 in India. Laboratory investigations in COVID-19 patients in the Indian subcontinent reveal lymphopenia as predominant finding in hemogram. Patients with older age and associated comorbidities (COPD, hypertension and diabetes) seem to have greater risk for lung injury, thereby requiring oxygen support during the course of disease.

- Cited by 1 article

Supplementary info

Publication types, MeSH terms Expand

Publication types

- Observational Study

MeSH terms

- Adult
- COVID-19 / diagnosis*
- COVID-19 / epidemiology*
- COVID-19 / therapy
- COVID-19 / virology
- Comorbidity
- Contact Tracing / methods
- Disease Management
- Female
- Humans
- India / epidemiology
- Length of Stay
- Male
- Middle Aged
- Pandemics / prevention & control
- Quarantine / methods
- Retrospective Studies
- Reverse Transcriptase Polymerase Chain Reaction / methods
- SARS-CoV-2 / genetics*
- Tertiary Care Centers

Full text links

Monaldi Arch Chest Dis Pagepress Publications

Proceed to details

Cite

Share

☐ 438

Observational Study

Allergol Int

. 2021 Oct;70(4):489-491.
doi: 10.1016/j.alit.2021.06.001. Epub 2021 Jul 16.

Impact of the COVID-19 pandemic on asthma exacerbations in children: A multi-center survey using an administrative database in Japan

Seiko Bun [1], Kenji Kishimoto [2], Jung-Ho Shin [2], Takanobu Maekawa [3], Daisuke Takada [2], Tetsuji Morishita [2], Susumu Kunisawa [2], Yuichi Imanaka [4]
Affiliations Expand

Affiliations

- [1] Department of Healthcare Economics and Quality Management, Graduate School of Medicine, Kyoto University, Kyoto, Japan; Department of Pharmacy, National Center for Child Health and Development Hospital, Tokyo, Japan.
- [2] Department of Healthcare Economics and Quality Management, Graduate School of Medicine, Kyoto University, Kyoto, Japan.
- [3] Department of General Pediatrics & Interdisciplinary Medicine, National Center for Child Health and Development Hospital, Tokyo, Japan.
- [4] Department of Healthcare Economics and Quality Management, Graduate School of Medicine, Kyoto University, Kyoto, Japan. Electronic address: imanaka-y@umin.net.

- PMID: **34281768**
- PMCID: PMC8282991
- DOI: 10.1016/j.alit.2021.06.001

Free PMC article
Observational Study

Impact of the COVID-19 pandemic on asthma exacerbations in children: A multi-center survey using an administrative database in Japan

Seiko Bun et al. Allergol Int. 2021 Oct.
Free PMC article
Show details
Allergol Int
. 2021 Oct;70(4):489-491.
doi: 10.1016/j.alit.2021.06.001. Epub 2021 Jul 16.

Authors

Seiko Bun [1], Kenji Kishimoto [2], Jung-Ho Shin [2], Takanobu Maekawa [3], Daisuke Takada [2], Tetsuji Morishita [2], Susumu Kunisawa [2], Yuichi Imanaka [4]

Affiliations

- [1] Department of Healthcare Economics and Quality Management, Graduate School of Medicine, Kyoto University, Kyoto, Japan; Department of Pharmacy, National Center for Child Health and Development Hospital, Tokyo, Japan.
- [2] Department of Healthcare Economics and Quality Management, Graduate School of Medicine, Kyoto University, Kyoto, Japan.
- [3] Department of General Pediatrics & Interdisciplinary Medicine, National Center for Child Health and Development Hospital, Tokyo, Japan.
- [4] Department of Healthcare Economics and Quality Management, Graduate School of Medicine, Kyoto University, Kyoto, Japan. Electronic address: imanaka-y@umin.net.

- PMID: **34281768**
- PMCID: PMC8282991
- DOI: 10.1016/j.alit.2021.06.001

No abstract available

- Cited by 1 article
- 10 references
- 2 figures

Supplementary info

Publication types, MeSH terms Expand

Publication types

- Letter
- Multicenter Study
- Observational Study

MeSH terms

- Adolescent
- Asthma / epidemiology*
- COVID-19*
- Child
- Child, Preschool
- Disease Progression
- Hospitalization / statistics & numerical data*
- Humans
- Infant
- Japan

- Retrospective Studies
- SARS-CoV-2
- Surveys and Questionnaires

Full text links

ELSEVIER OPEN ACCESS Elsevier Science Free PMC article
Proceed to details
Cite
Share
439

Expert Rev Respir Med
. 2021 Jun;15(6):853-857.
doi: 10.1080/17476348.2021.1866546. Epub 2020 Dec 23.

Bilevel and continuous positive airway pressure and factors linked to all-cause mortality in COVID-19 patients in an intermediate respiratory intensive care unit in Italy

Giovanna E Carpagnano [1], Enrico Buonamico [1], Giovanni Migliore [2], Emanuela Resta [3], Valentina Di Lecce [1], Maria Luisa de Candia [1], Vincenzo Solfrizzi [4], Francesco Panza [5], Onofrio Resta [1]
Affiliations Expand

Affiliations

- [1] Institute of Respiratory Disease, Department of Basic Medical Science, Neuroscience, and Sense Organs, University of Bari "Aldo Moro", Bari, Italy.
- [2] Azienda Universitaria Ospedaliera Consorziale, General Director Office, Policlinico Bari, Italy.
- [3] Translational Medicine and Health System Management, University of Foggia, Foggia, Italy.
- [4] "Cesare Frugoni" Internal and Geriatric Medicine and Memory Unit, University of Bari "Aldo Moro", Bari, Italy.
- [5] Population Health Unit, "Salus in Apulia Study", National Institute of Gastroenterology "Saverio De Bellis", Research Hospital, Bari, Italy.

- PMID: **33334197**
- PMCID: PMC7784777
- DOI: 10.1080/17476348.2021.1866546

Free PMC article

Bilevel and continuous positive airway pressure and factors linked to all-cause mortality in COVID-19 patients in an intermediate respiratory intensive care unit in Italy

Giovanna E Carpagnano et al. Expert Rev Respir Med. 2021 Jun.
Free PMC article
Show details
Expert Rev Respir Med
. 2021 Jun;15(6):853-857.
doi: 10.1080/17476348.2021.1866546. Epub 2020 Dec 23.

Authors

Giovanna E Carpagnano [1], Enrico Buonamico [1], Giovanni Migliore [2], Emanuela Resta [3], Valentina Di Lecce [1], Maria Luisa de Candia [1], Vincenzo Solfrizzi [4], Francesco Panza [5], Onofrio Resta [1]

Affiliations

- [1] Institute of Respiratory Disease, Department of Basic Medical Science, Neuroscience, and Sense Organs, University of Bari "Aldo Moro", Bari, Italy.
- [2] Azienda Universitaria Ospedaliera Consorziale, General Director Office, Policlinico Bari, Italy.
- [3] Translational Medicine and Health System Management, University of Foggia, Foggia, Italy.
- [4] "Cesare Frugoni" Internal and Geriatric Medicine and Memory Unit, University of Bari "Aldo Moro", Bari, Italy.
- [5] Population Health Unit, "Salus in Apulia Study", National Institute of Gastroenterology "Saverio De Bellis", Research Hospital, Bari, Italy.

- PMID: **33334197**
- PMCID: PMC7784777
- DOI: 10.1080/17476348.2021.1866546

Abstract

Objectives: In the present single-centered, retrospective, observational study, we reported findings from 78 consecutive laboratory-confirmed COVID-19 patients with moderate-to-severe acute respiratory distress syndrome (ARDS) hospitalized in an intermediate Respiratory Intensive Care Unit, subdividing the patients into two groups according to their clinical outcome, dead patients and discharged patients.**Methods**: We further subdivided patients depending on the

noninvasive respiratory support used during hospitalization.**Results**: In those patients who died, we found significant older age and higher multimorbidity and higher values of serum lactate dehydrogenase, C-reactive protein, and D-dimer. Among patients who were submitted to bilevel positive airway pressure (BPAP), those who died had a significant shorter number of days in overall length of stay and lower values of arterial oxygen partial pressure to fractional inspired oxygen ratio (PaO2/FiO2 ratio) compared to those who survived. No difference in all-cause mortality was observed between the two different noninvasive respiratory support groups [48% for continuous positive airway pressure (CPAP) and 52% for BPAP].**Conclusion**: In COVID-19 patients with moderate-to-severe ARDS using BPAP in an intermediate level of hospital care had more factors associated to all-cause mortality (shorter length of stay and lower baseline PaO2/FiO2 ratio) compared to those who underwent CPAP.

Keywords: Critical care; SARS-CoV-2; assisted ventilation; intermediate RICU; respiratory infection; viral infection.

- Cited by 6 articles
- 16 references

Supplementary info

MeSH terms, Substances, Grant support Expand

MeSH terms

- Adult
- Aged
- Aged, 80 and over
- COVID-19 / etiology
- COVID-19 / mortality*
- COVID-19 / pathology
- COVID-19 / therapy*
- Cause of Death
- Comorbidity
- Continuous Positive Airway Pressure / methods*
- Critical Care / statistics & numerical data
- Female
- Hospital Mortality
- Hospitalization / statistics & numerical data
- Humans
- Intensive Care Units / statistics & numerical data*
- Italy / epidemiology
- Male
- Middle Aged
- Oxygen / therapeutic use
- Respiratory Distress Syndrome / mortality
- Respiratory Distress Syndrome / therapy

- Respiratory Distress Syndrome / virology
- Retrospective Studies
- Risk Factors
- SARS-CoV-2 / physiology

Substances

- Oxygen

Grant support

This paper was not funded.

Full text links

View full text Taylor & Francis Free PMC article
Proceed to details
Cite
Share
☐ 440
Observational Study
Am J Cardiol
. 2021 Jan 1;138:100-106.
doi: 10.1016/j.amjcard.2020.09.060. Epub 2020 Oct 13.

Usefulness of Elevated Troponin to Predict Death in Patients With COVID-19 and Myocardial Injury

David T Majure [1], Luis Gruberg [2], Shahryar G Saba [3], Charlotte Kvasnovsky [4], Jamie S Hirsch [5], Rajiv Jauhar [3], Northwell Health COVID-19 Research Consortium
Affiliations Expand

Affiliations

- [1] Donald and Barbara Zucker School of Medicine at Hofstra/Northwell, Northwell Health, Hempstead, New York; Department of Cardiology, North Shore University Hospital, Manhasset, New York. Electronic address: dmajure@northwell.edu.
- [2] Department of Cardiology, Southside Hospital, Bay Shore, New York.
- [3] Donald and Barbara Zucker School of Medicine at Hofstra/Northwell, Northwell Health, Hempstead, New York; Department of Cardiology, North Shore University Hospital, Manhasset, New York.
- [4] Donald and Barbara Zucker School of Medicine at Hofstra/Northwell, Northwell Health, Hempstead, New York.

- [5] Division of Kidney Diseases and Hypertension, Department of Medicine, Donald and Barbara Zucker School of Medicine at Hofstra/Northwell, Great Neck, New York; Institute of Health Innovations and Outcomes Research, Feinstein Institutes for Medical Research, Manhasset, New York; Department of Information Services, Northwell Health, New Hyde Park, New York.

- PMID: **33058800**
- PMCID: PMC7550867
- DOI: 10.1016/j.amjcard.2020.09.060

Free PMC article
Observational Study

Usefulness of Elevated Troponin to Predict Death in Patients With COVID-19 and Myocardial Injury

David T Majure et al. Am J Cardiol. 2021.
Free PMC article
Show details
Am J Cardiol
. 2021 Jan 1;138:100-106.
doi: 10.1016/j.amjcard.2020.09.060. Epub 2020 Oct 13.

Authors

David T Majure [1], Luis Gruberg [2], Shahryar G Saba [3], Charlotte Kvasnovsky [4], Jamie S Hirsch [5], Rajiv Jauhar [3], Northwell Health COVID-19 Research Consortium

Affiliations

- [1] Donald and Barbara Zucker School of Medicine at Hofstra/Northwell, Northwell Health, Hempstead, New York; Department of Cardiology, North Shore University Hospital, Manhasset, New York. Electronic address: dmajure@northwell.edu.
- [2] Department of Cardiology, Southside Hospital, Bay Shore, New York.
- [3] Donald and Barbara Zucker School of Medicine at Hofstra/Northwell, Northwell Health, Hempstead, New York; Department of Cardiology, North Shore University Hospital, Manhasset, New York.
- [4] Donald and Barbara Zucker School of Medicine at Hofstra/Northwell, Northwell Health, Hempstead, New York.
- [5] Division of Kidney Diseases and Hypertension, Department of Medicine, Donald and Barbara Zucker School of Medicine at Hofstra/Northwell, Great Neck, New York; Institute of Health Innovations and Outcomes Research, Feinstein Institutes for Medical Research, Manhasset, New York; Department of Information Services, Northwell Health, New Hyde Park, New York.

- PMID: **33058800**
- PMCID: PMC7550867

- DOI: 10.1016/j.amjcard.2020.09.060

Abstract

Elevations in troponin levels have been shown to predict mortality in patients with coronavirus disease 2019 (COVID-19). The role of inflammation in myocardial injury remains unclear. We sought to determine the association of elevated troponin with mortality in a large, ethnically diverse population of patients hospitalized with COVID-19, and to determine the association of elevated inflammatory markers with increased troponin levels. We reviewed all patients admitted at our health system with COVID-19 from March 1 to April 27, 2020, who had a troponin assessment within 48 hours of admission. We used logistic regression to calculate odds ratios (ORs) for mortality during hospitalization, controlling for demographics, co-morbidities, and markers of inflammation. Of 11,159 patients hospitalized with COVID-19, 6,247 had a troponin assessment within 48 hours. Of these, 4,426 (71%) patients had normal, 919 (15%) had mildly elevated, and 902 (14%) had severely elevated troponin. Acute phase and inflammatory markers were significantly elevated in patients with mildly and severely elevated troponin compared with normal troponin. Patients with elevated troponin had significantly increased odds of death for mildly elevated compared with normal troponin (adjusted OR, 2.06; 95% confidence interval, 1.68 to 2.53; $p < 0.001$) and for severely elevated compared with normal troponin (OR, 4.51; 95% confidence interval, 3.66 to 5.54; $p < 0.001$) independently of elevation in inflammatory markers. In conclusion, patients hospitalized with COVID-19 and elevated troponin had markedly increased mortality compared with patients with normal troponin levels. This risk was independent of cardiovascular co-morbidities and elevated markers of inflammation.

Copyright © 2020 Elsevier Inc. All rights reserved.

- Cited by 20 articles
- 17 references
- 3 figures

Supplementary info

Publication types, MeSH terms, Substances, Grant support Expand

Publication types

- Observational Study

MeSH terms

- Aged
- Biomarkers / blood
- COVID-19 / blood*
- COVID-19 / diagnosis
- COVID-19 / mortality*
- Female
- Hospital Mortality
- Hospitalization

- Humans
- Logistic Models
- Male
- Middle Aged
- Odds Ratio
- Predictive Value of Tests
- Retrospective Studies
- Risk Factors
- SARS-CoV-2*
- Survival Rate
- Troponin / blood*

Substances

- Biomarkers
- Troponin

Grant support

- R01 LM012836/LM/NLM NIH HHS/United States
- R24 AG064191/AG/NIA NIH HHS/United States

Full text links

ELSEVIER FULL-TEXT ARTICLE Elsevier Science Free PMC article
Proceed to details
Cite
Share
441
Observational Study
Medicine (Baltimore)
. 2020 Dec 18;99(51):e23797.
doi: 10.1097/MD.0000000000023797.

A retrospective view of pediatric cases infected with SARS-CoV-2 of a middle-sized city in mainland China

Yanjun Kang [1], Zhong You [2], Kang Wang [1], Zijuan Dong [3], Jiajia Zhang [3], Yuanwang Qiu [2], Guizhi Ge [3]
Affiliations Expand

Affiliations

- [1] Department of Pediatric Laboratory, The Affiliated Wuxi Children's Hospital of Nanjing Medical University.
- [2] Infectious Disease Department, The Fifth People's Hospital of Wuxi, Jiangsu Province.
- [3] Department of Infectious Disease, The Affiliated Wuxi Children's Hospital of Nanjing Medical University, Wuxi, China.

- PMID: **33371153**
- PMCID: PMC7748305
- DOI: 10.1097/MD.0000000000023797

Free PMC article
Observational Study

A retrospective view of pediatric cases infected with SARS-CoV-2 of a middle-sized city in mainland China

Yanjun Kang et al. Medicine (Baltimore). 2020.
Free PMC article
Show details
Medicine (Baltimore)

. 2020 Dec 18;99(51):e23797.
doi: 10.1097/MD.0000000000023797.

Authors

Yanjun Kang [1], Zhong You [2], Kang Wang [1], Zijuan Dong [3], Jiajia Zhang [3], Yuanwang Qiu [2], Guizhi Ge [3]

Affiliations

- [1] Department of Pediatric Laboratory, The Affiliated Wuxi Children's Hospital of Nanjing Medical University.
- [2] Infectious Disease Department, The Fifth People's Hospital of Wuxi, Jiangsu Province.
- [3] Department of Infectious Disease, The Affiliated Wuxi Children's Hospital of Nanjing Medical University, Wuxi, China.

- PMID: **33371153**
- PMCID: PMC7748305
- DOI: 10.1097/MD.0000000000023797

Abstract

The coronavirus disease 2019 (COVID-19) caused by SARS-CoV-2 had resulted in a global pandemic. A comprehensive analysis of pediatric COVID-19 cases is essential to decipher the

natural features of children under the risk of this disease.In the epidemic period, all the children infected with SARS-CoV-2 in Wuxi, a city with a stable medical system during the COVID-19 outbreak in China, were enrolled for comprehensive data documenting their clinical, prognosis, follow-up, treatment and various tests results. Combing their family cluster characteristics, the epidemiological, hospitalization, and transmission features of children with SARS-CoV-2 were analyzed and discussed.A total of 7 children were enrolled, including 4 mild cases, 1 moderate case, and 2 asymptomatic cases. The common symptoms were fever and dry cough. The length of viral nucleic acid duration in nasopharynx varied and was irrelevant to the severity of the symptom, whether symptomatic or asymptomatic. Two cases showed viral nucleic acid positive recurrence after discharge from the hospital. A child with type 1 diabetes was also focused, for the elevated blood sugar during hospitalization. All these children had close contacts with their family members, some of those were confirmed COVID-19 cases.We provided a holistic and detailed portrayal of the pediatric COVID-19 cases in a typical city of timely response to the epidemic. While the family cluster exhibits the major transmission mode, attention should be paid for the potential risk since the expanded social space of children in future.

Copyright © 2020 the Author(s). Published by Wolters Kluwer Health, Inc.

Conflict of interest statement

The authors have no conflicts of interests to disclose.

- Cited by 1 article
- 20 references
- 2 figures

Supplementary info

Publication types, MeSH terms, Substances, Supplementary concepts, Grant support Expand

Publication types

- Observational Study

MeSH terms

- Adolescent
- Antiviral Agents / therapeutic use
- Asymptomatic Infections
- COVID-19 / blood*
- COVID-19 / diagnostic imaging
- COVID-19 / drug therapy
- COVID-19 / virology
- Child
- Child, Preschool
- Female
- Humans

- Male
- Radiography, Thoracic
- Retrospective Studies
- SARS-CoV-2 / isolation & purification*
- Tomography, X-Ray Computed

Substances

- Antiviral Agents

Supplementary concepts

- COVID-19 drug treatment

Grant support

- FZXK006/Wuxi Medical Development Discipline

Full text links

Wolters Kluwer Wolters Kluwer Free PMC article
Proceed to details
Cite
Share
☐ 442
Observational Study
Emergencias
. 2022 Feb;34(1):38-46.

Predictors of revisits within 1 year by patients after acute COVID-19: the HUBCOVID365 cohort study

[Article in English, Spanish]
Arantxa Albert [1], Javier Jacob [1], Pierre Malchair [1], Ferrán Llopis [1], Lidia Fuentes [1], Cristina Martín [1], Cristina García [1], Orlando Rodríguez [1], José Carlos Ruibal [1], Concepción Martínez [1], Elena Fuentes [1], Marco Cordero [1], Lara Guillén [1], Francisco Chamorro [1], Sebastián Quetglas [1], Carles Ferre [1]
Affiliations Expand

Affiliation

- [1] Servei d'Urgències Hospital Universitari de Bellvitge, Barcelona, España.

- PMID: **35103442**

Free article
Observational Study

Predictors of revisits within 1 year by patients after acute COVID-19: the HUBCOVID365 cohort study

[Article in English, Spanish]
Arantxa Albert et al. Emergencias. 2022 Feb.
Free article
Show details
Emergencias
. 2022 Feb;34(1):38-46.

Authors

Arantxa Albert [1], Javier Jacob [1], Pierre Malchair [1], Ferrán Llopis [1], Lidia Fuentes [1], Cristina Martín [1], Cristina García [1], Orlando Rodríguez [1], José Carlos Ruibal [1], Concepción Martínez [1], Elena Fuentes [1], Marco Cordero [1], Lara Guillén [1], Francisco Chamorro [1], Sebastián Quetglas [1], Carles Ferre [1]

Affiliation

- [1] Servei d'Urgències Hospital Universitari de Bellvitge, Barcelona, España.

- PMID: **35103442**

Abstract
in English, Spanish

Objectives: To analyze the frequencies of 3 types of hospital revisits by patients after treatment for COVID-19 in the emergency department.

Material and methods: Retrospective observational study of consecutive patients who came to the emergency department in March and April 2020 and were discharged alive with a diagnosis of COVID-19. Baseline and acute episode data were collected and the patients were followed for 1 year. We analyzed variables associated with revisits for any reason, revisits related to COVID-19, and early COVID-19-related revisits (within 30 days).

Results: A total of 1352 patients with a mean age of 62.1 years (52.9% male) were studied. A total of 553 revisits were made by 342 patients (25.3%) for any reason; 132 (9.8%) revisited in relation to COVID-19 at least once. Of those, 103 (7.6%) revisited within 30 days (early) and 29 (2.2%) came later. COVID-19-related revisits were associated with thrombotic events (odds ratio [OR], 7.58; 95% CI, 1.75-32.81) and pulmonary fibrosis (OR, 4.95; 95% CI, 1.27-19.24); early revisits were inversely associated with follow-up management by a contracted health care support

service (OR, 0.18; 95% CI, 0.03-0.92). Hospital admission during the initial visit was significantly associated with fewer revisits for any reason or related to COVID-19 at any time.

Conclusion: Fewer than half the total number of emergency department revisits after initial care for COVID-19 were related to the novel coronavirus infection. Revisits occurred more often in the first 30 days after discharge. Later COVID-19-related revisits were uncommon, but given the large number of patients with this infection, such visits can be expected.

Objetivo: Analizar diferentes categorías de revisita (RV) al año en pacientes con infección COVID-19 que consultan en un servicio de urgencias hospitalario (SUH).

Metodo: Estudio observacional, retrospectivo, que incluyó pacientes consecutivos que consultaron al SUH en los meses de marzo y abril de 2020 con diagnóstico de COVID-19 y fueron dados de alta vivos del hospital. Se recogieron variables basales y del episodio agudo y se realizó un seguimiento al año. Se hicieron tres comparaciones identificando variables asociadas a la RV total, RV relacionada con COVID-19 (RCovid) y RCovid precoz (# 30 días).

Resultados: Se analizaron 1.352 pacientes con edad media de 62,1 años y 52,9% varones. En el seguimiento al año hubo 553 RV en 342 (25,3%) pacientes, 132 (9,8%) con al menos una RCovid, 103 (7,6%) precoz y 29 (2,2%) tardía. La RCovid se relacionó con la presencia de fenómenos trombóticos [OR 7,58 (IC 95%: 1,75-32,81)] y la fibrosis pulmonar [OR 4,95 (IC 95%: 1,27-19,24)]; y la RCovid precoz se relacionó inversamente con alta a dispositivo de soporte sanitario [OR 0,18 (IC 95%: 0,03-0,92)]. El ingreso hospitalario en el evento índice disminuyó la RV total y RCovid y las hospitalizaciones derivadas de esta RV de manera significativa a largo plazo.

Conclusiones: Menos de la mitad de la RV total tras una infección COVID-19 está relacionada con la infección, y es más frecuente en los primeros 30 días. La RCovid tardía no es frecuente, pero dado el elevado número de pacientes que han sido infectados por COVID-19 se debe tener en cuenta.

Keywords: COVID-19.; Early revisits.; Emergency department.; Late revisits.; Revisita precoz.; Revisita tardía.; Revisita total.; Revisits.; Urgencias..

Supplementary info

Publication types, MeSH terms Expand

Publication types

- Observational Study

MeSH terms

- COVID-19*
- Cohort Studies
- Female
- Humans
- Male
- Middle Aged
- Patient Readmission

- Retrospective Studies
- SARS-CoV-2

Full text links

EMERGENCIAS Grupo Saned
Proceed to details
Cite
Share
443
Observational Study
BJU Int
. 2020 Aug;126(2):256-258.
doi: 10.1111/bju.15109. Epub 2020 Jun 14.

The impact of the coronavirus disease 2019 pandemic on the utilisation of emergency urological services

Mariana Madanelo [1], Carlos Ferreira [1], Diogo Nunes-Carneiro [1], André Pinto [1], Maria Alexandra Rocha [1], Jorge Correia [1], Bernardo Teixeira [1], Gonçalo Mendes [1], Catarina Tavares [1], Sofia Mesquita [1], Avelino Fraga [1]
Affiliations Expand

Affiliation

- [1] Department of Urology, Centro Hospitalar Universitário do Porto, Porto, Portugal.

- PMID: **32406551**
- PMCID: PMC7272803
- DOI: 10.1111/bju.15109

Free PMC article
Observational Study

The impact of the coronavirus disease 2019 pandemic on the utilisation of emergency urological services

Mariana Madanelo et al. BJU Int. 2020 Aug.
Free PMC article
Show details

BJU Int
. 2020 Aug;126(2):256-258.
doi: 10.1111/bju.15109. Epub 2020 Jun 14.

Authors

Mariana Madanelo [1], Carlos Ferreira [1], Diogo Nunes-Carneiro [1], André Pinto [1], Maria Alexandra Rocha [1], Jorge Correia [1], Bernardo Teixeira [1], Gonçalo Mendes [1], Catarina Tavares [1], Sofia Mesquita [1], Avelino Fraga [1]

Affiliation

- [1] Department of Urology, Centro Hospitalar Universitário do Porto, Porto, Portugal.

- PMID: **32406551**
- PMCID: PMC7272803
- DOI: 10.1111/bju.15109

Abstract

Objectives: To compare the number of patients attending the Urology Emergency Department (ED) of the Centro Hospitalar Universitário do Porto (CHUP), as well as their demographic characteristics, the reasons for admission, the clinical severity under the Manchester triage system (MTS), and the need for emergency surgery or hospitalisation, during the coronavirus disease 2019 (COVID-19) pandemic and the equivalent period in 2019.

Patients and methods: Data were collected from patients attending the Urology ED of the CHUP over 3 weeks, from 11 March to 1 April 2020, and from the same period in the previous year (from 11 March to 1 April 2019).

Results: During the pandemic, 46.4% fewer patients visited our urological ED (122 vs 263). There was no significant difference in the mean age or the number of old patients (aged ≥65 years) between the two periods. However, significantly fewer female patients sought emergency urological services during the COVID-19 pandemic period (32.7% vs 14.8%, $P < 0.05$). No significant differences were noted between different clinical severity groups under the MTS. In 2019, significantly less patients required hospitalisation. The most common reasons for admission, during both periods, were haematuria, renal colic and urinary tract infections. The authors recognise that the study has several limitations, namely, those inherent to its retrospective nature.

Conclusion: COVID-19 significantly influenced people's urological care-seeking behaviour. Understanding the present situation is helpful for predicting future urological needs. Based on the results of this study, we have reason to speculate that people's requirements for urological services might grow explosively in the post-COVID-19 period. There should be further studies about the real state of long-term urological services and the consequences that this pandemic may have in terms of morbimortality not directly related to the severe acute respiratory syndrome coronavirus 2.

Keywords: emergency department; pandemics; severe acute respiratory syndrome coronavirus 2 (SARS-Cov-2); state of emergency; urology.

© 2020 The Authors BJU International © 2020 BJU International Published by John Wiley & Sons Ltd.

- Cited by 15 articles
- 7 references

Supplementary info

Publication types, MeSH terms Expand

Publication types

- Observational Study

MeSH terms

- Adult
- Aged
- Betacoronavirus*
- COVID-19
- Coronavirus Infections / complications*
- Coronavirus Infections / epidemiology
- Emergency Medical Services / statistics & numerical data*
- Female
- Hospitals / statistics & numerical data*
- Humans
- Incidence
- Male
- Middle Aged
- Pandemics*
- Pneumonia, Viral / complications*
- Pneumonia, Viral / epidemiology
- Portugal / epidemiology
- Retrospective Studies
- SARS-CoV-2
- Urologic Diseases / complications
- Urologic Diseases / epidemiology
- Urologic Diseases / therapy*

Full text links

WILEY Full Text Article Wiley Free PMC article

Proceed to details

Cite

Share

☐ 444

Tuberk Toraks
. 2020 Sep;68(3):218-226.
doi: 10.5578/tt.70128.

Clinical presentation of health care workers with symptoms of coronavirus disease 2019 at the İzmir tertiary education hospital, during an early phase of the pandemic

Filiz Güldaval [1], Ceyda Anar [2], Mine Gayaf [1], Melih Büyükşirin [1], Gülru Polat [1], Gülistan Karadeniz [1], Aylin Alpözen [1], Aysu Ayrancı [1], Fatma Üçsular [1], Zeynep Seymenoğlu [1], Özgür Batum [1]
Affiliations Expand

Affiliations

- [1] Clinic of Chest Diseases, Izmir Dr. Suat Seren Chest Diseases and Thoracic Surgery Training and Research Hospital, Izmir, Turkey.
- [2] Department of Chest Diseases, Faculty of Medicine, Izmir Katip Celebi University, Izmir, Turkey.

- PMID: **33295719**
- DOI: 10.5578/tt.70128

Free article

Clinical presentation of health care workers with symptoms of coronavirus disease 2019 at the İzmir tertiary education hospital, during an early phase of the pandemic

Filiz Güldaval et al. Tuberk Toraks. 2020 Sep.
Free article
Show details
Tuberk Toraks
. 2020 Sep;68(3):218-226.
doi: 10.5578/tt.70128.

Authors

Filiz Güldaval [1], Ceyda Anar [2], Mine Gayaf [1], Melih Büyükşirin [1], Gülru Polat [1], Gülistan Karadeniz [1], Aylin Alpözen [1], Aysu Ayrancı [1], Fatma Üçsular [1], Zeynep Seymenoğlu [1], Özgür Batum [1]

Affiliations

- [1] Clinic of Chest Diseases, Izmir Dr. Suat Seren Chest Diseases and Thoracic Surgery Training and Research Hospital, Izmir, Turkey.
- [2] Department of Chest Diseases, Faculty of Medicine, Izmir Katip Celebi University, Izmir, Turkey.

- PMID: **33295719**
- DOI: 10.5578/tt.70128

Abstract

Introduction: The aim of this study is to investigate and report on the data regarding the clinical characteristics and outcomes of healthcare workers with COVID-19 at tertiary education hospitals from Turkey.

Materials and methods: This was a single center, retrospective, descriptive and observational study using cross-sectional data, which were collected from confirmed COVID-19 patients at a tertiary education hospital. Patients' demographic and clinical characteristics, mortality rates, and the factors associated with hospitalization were analyzed.

Result: By May 15, 2020, 480 patients were diagnosed with COVID-19 in our hospital where 49 (10.2%) of whom were HCWs. The mean age was 40.0 ± 8.45 (75.5% female). The most common symptoms were cough (32.7%), fever (30.6%), and myalgia (14.3%). Comorbidities were present in 32.7% of the patients. Most of the HCWs were nurses (53.1%) and physicians (18.4%), and the remaining 14 (28.6%) were cleaning and administrative staff. The severity of the disease was mild in 65.3% and severe in 34.7% HCWs. Leukocyte, neutrophil, lymphocyte and platelet values were statistically lower in hospitalized patients. There was a statistically significant relationship between the presence of infiltration on the chest X-ray, and the patient's symptoms with the severity of the disease (respectively p= 0.002 and 0.009).

Conclusions: In conclusion, the frequency of COVID-19 in healthcare workers is high. The study presents the characteristics of HCWs infected with coronavirus from a single center in Turkey.

- Cited by 1 article

Supplementary info

MeSH terms Expand

MeSH terms

- Adult
- COVID-19 / diagnosis*
- COVID-19 / epidemiology
- Comorbidity

- Cross-Sectional Studies
- Female
- Health Personnel / statistics & numerical data*
- Hospitalization
- Humans
- Male
- Middle Aged
- Pneumonia, Viral / diagnosis
- Retrospective Studies
- SARS-CoV-2 / isolation & purification*
- Tertiary Care Centers*
- Turkey

Full text links

Bilimsel Tip Publishing House
Proceed to details
Cite
Share
☐ 445
Observational Study
Sci Rep
. 2021 Jun 17;11(1):12801.
doi: 10.1038/s41598-021-92146-7.

Multivariable mortality risk prediction using machine learning for COVID-19 patients at admission (AICOVID)

Sujoy Kar [1], Rajesh Chawla [2], Sai Praveen Haranath [3], Suresh Ramasubban [4], Nagarajan Ramakrishnan [5], Raju Vaishya [2], Anupam Sibal [2], Sangita Reddy [3]
Affiliations Expand

Affiliations

- [1] Apollo Hospitals, Jubilee Hills, Hyderabad, 500033, India. drsujoy_k@apollohospitals.com.
- [2] Indraprastha Apollo Hospitals, Sarita Vihar, New Delhi, India.
- [3] Apollo Hospitals, Jubilee Hills, Hyderabad, 500033, India.
- [4] Apollo Multispecialty Hospitals, Kolkata, 700054, India.
- [5] Apollo Hospitals, Greams Road, Chennai, India.

- PMID: **34140592**

- PMCID: PMC8211710
- DOI: 10.1038/s41598-021-92146-7

Free PMC article
Observational Study

Multivariable mortality risk prediction using machine learning for COVID-19 patients at admission (AICOVID)

Sujoy Kar et al. Sci Rep. 2021.
Free PMC article
Show details
Sci Rep
. 2021 Jun 17;11(1):12801.
doi: 10.1038/s41598-021-92146-7.

Authors

Sujoy Kar [1], Rajesh Chawla [2], Sai Praveen Haranath [3], Suresh Ramasubban [4], Nagarajan Ramakrishnan [5], Raju Vaishya [2], Anupam Sibal [2], Sangita Reddy [3]

Affiliations

- [1] Apollo Hospitals, Jubilee Hills, Hyderabad, 500033, India. drsujoy_k@apollohospitals.com.
- [2] Indraprastha Apollo Hospitals, Sarita Vihar, New Delhi, India.
- [3] Apollo Hospitals, Jubilee Hills, Hyderabad, 500033, India.
- [4] Apollo Multispecialty Hospitals, Kolkata, 700054, India.
- [5] Apollo Hospitals, Greams Road, Chennai, India.

- PMID: **34140592**
- PMCID: PMC8211710
- DOI: 10.1038/s41598-021-92146-7

Abstract

In Coronavirus disease 2019 (COVID-19), early identification of patients with a high risk of mortality can significantly improve triage, bed allocation, timely management, and possibly, outcome. The study objective is to develop and validate individualized mortality risk scores based on the anonymized clinical and laboratory data at admission and determine the probability of Deaths at 7 and 28 days. Data of 1393 admitted patients (Expired-8.54%) was collected from six Apollo Hospital centers (from April to July 2020) using a standardized template and electronic medical records. 63 Clinical and Laboratory parameters were studied based on the patient's initial clinical state at admission and laboratory parameters within the first 24 h. The Machine Learning (ML) modelling was performed using eXtreme Gradient Boosting (XGB) Algorithm. 'Time to event' using Cox Proportional Hazard Model was used and combined with XGB Algorithm. The

prospective validation cohort was selected of 977 patients (Expired-8.3%) from six centers from July to October 2020. The Clinical API for the Algorithm is http://20.44.39.47/covid19v2/page1.php being used prospectively. Out of the 63 clinical and laboratory parameters, Age [adjusted hazard ratio (HR) 2.31; 95% CI 1.52-3.53], Male Gender (HR 1.72, 95% CI 1.06-2.85), Respiratory Distress (HR 1.79, 95% CI 1.32-2.53), Diabetes Mellitus (HR 1.21, 95% CI 0.83-1.77), Chronic Kidney Disease (HR 3.04, 95% CI 1.72-5.38), Coronary Artery Disease (HR 1.56, 95% CI - 0.91 to 2.69), respiratory rate > 24/min (HR 1.54, 95% CI 1.03-2.3), oxygen saturation below 90% (HR 2.84, 95% CI 1.87-4.3), Lymphocyte% in DLC (HR 1.99, 95% CI 1.23-2.32), INR (HR 1.71, 95% CI 1.31-2.13), LDH (HR 4.02, 95% CI 2.66-6.07) and Ferritin (HR 2.48, 95% CI 1.32-4.74) were found to be significant. The performance parameters of the current model is at AUC ROC Score of 0.8685 and Accuracy Score of 96.89. The validation cohort had the AUC of 0.782 and Accuracy of 0.93. The model for Mortality Risk Prediction provides insight into the COVID Clinical and Laboratory Parameters at admission. It is one of the early studies, reflecting on 'time to event' at the admission, accurately predicting patient outcomes.

Conflict of interest statement

The authors declare no competing interests.

- Cited by 8 articles
- 28 references
- 6 figures

Supplementary info

Publication types, MeSH terms Expand

Publication types

- Multicenter Study
- Observational Study

MeSH terms

- Aged
- COVID-19 / epidemiology*
- COVID-19 / mortality*
- COVID-19 / virology
- Electronic Health Records
- Female
- Humans
- India / epidemiology
- Machine Learning*
- Male
- Middle Aged
- Patient Admission*

- Prognosis
- Propensity Score
- Proportional Hazards Models
- Prospective Studies
- Retrospective Studies
- Risk Assessment
- Risk Factors
- SARS-CoV-2*
- Triage

Full text links

Nature Publishing Group Free PMC article
Proceed to details
Cite
Share
446
Observational Study
Obstet Gynecol
. 2021 Oct 1;138(4):660-662.
doi: 10.1097/AOG.0000000000004529.

Pregnancy Outcomes and Maternal Complications During the Second Wave of Coronavirus Disease 2019 (COVID-19) in India

Niraj N Mahajan [1], Madhura Pophalkar, Sarika Patil, Bhagyashree Yewale, Itta Krishna Chaaithanya, Smita D Mahale, Rahul K Gajbhiye
Affiliations Expand

Affiliation

- [1] Department of Obstetrics and Gynecology, Topiwala National Medical College & BYL Nair Charitable Hospital, and the ICMR-National Institute for Research in Reproductive Health, Mumbai, India.

- PMID: **34233345**
- PMCID: PMC8454281
- DOI: 10.1097/AOG.0000000000004529

Free PMC article
Observational Study

Pregnancy Outcomes and Maternal Complications During the Second Wave of Coronavirus Disease 2019 (COVID-19) in India

Niraj N Mahajan et al. Obstet Gynecol. 2021.
Free PMC article
Show details
Obstet Gynecol
. 2021 Oct 1;138(4):660-662.
doi: 10.1097/AOG.0000000000004529.

Authors

Niraj N Mahajan [1], Madhura Pophalkar, Sarika Patil, Bhagyashree Yewale, Itta Krishna Chaaithanya, Smita D Mahale, Rahul K Gajbhiye

Affiliation

- [1] Department of Obstetrics and Gynecology, Topiwala National Medical College & BYL Nair Charitable Hospital, and the ICMR-National Institute for Research in Reproductive Health, Mumbai, India.

- PMID: **34233345**
- PMCID: PMC8454281
- DOI: 10.1097/AOG.0000000000004529

Abstract

Rates of severe coronavirus disease 2019 (COVID-19), intensive care unit admission, and maternal mortality increased among pregnant and postpartum women admitted for COVID-19 in the second wave compared with the first wave in India.

Conflict of interest statement

Financial Disclosure The authors did not report any potential conflicts of interest.

- Cited by 5 articles
- 10 references
- 1 figure

Supplementary info

Publication types, MeSH terms, Grant support Expand

Publication types

- Observational Study
- Research Support, Non-U.S. Gov't

MeSH terms

- Adult
- COVID-19 / complications
- COVID-19 / mortality*
- Female
- Hospitalization / statistics & numerical data
- Humans
- India / epidemiology
- Infant, Newborn
- Intensive Care Units
- Maternal Mortality
- Pregnancy
- Pregnancy Complications, Infectious / mortality*
- Pregnancy Complications, Infectious / virology
- Pregnancy Outcome / epidemiology*
- Premature Birth / epidemiology
- Premature Birth / virology
- Prenatal Care / statistics & numerical data
- Retrospective Studies
- SARS-CoV-2*
- Stillbirth / epidemiology

Grant support

- WT_/Wellcome Trust/United Kingdom
- IA/CPHI/18/1/503933/WTDBT_/DBT-Wellcome Trust India Alliance/India

Full text links

Wolters Kluwer Wolters Kluwer Free PMC article
Proceed to details
Cite
Share
☐ 447
Observational Study
Ultrasound Med Biol
. 2021 Dec;47(12):3323-3332.

doi: 10.1016/j.ultrasmedbio.2021.07.014. Epub 2021 Jul 24.

Coronavirus Disease 2019 Phenotypes, Lung Ultrasound, Chest Computed Tomography and Clinical Features in Critically Ill Mechanically Ventilated Patients

Davide Orlandi [1], Denise Battaglini [2], Chiara Robba [3], Marco Viganò [4], Giulio Bergamaschi [5], Tiziana Mignatti [5], Maria Luisa Radice [6], Antonio Lapolla [6], Giovanni Turtulici [5], Paolo Pelosi [3]
Affiliations Expand

Affiliations

- [1] Department of Radiology, Ospedale Evangelico Internazionale, Genoa, Italy. Electronic address: my.davideorlandi@gmail.com.
- [2] Anesthesia and Intensive Care, San Martino Policlinico Hospital, Scientific Institute for Research, Hospitalization and Healthcare (IRCCS) for Oncology and Neurosciences, Genoa, Italy; Department of Medicine, University of Barcelona, Barcelona, Spain.
- [3] Anesthesia and Intensive Care, San Martino Policlinico Hospital, Scientific Institute for Research, Hospitalization and Healthcare (IRCCS) for Oncology and Neurosciences, Genoa, Italy; Department of Surgical Sciences and Integrated Diagnostic (DISC), University of Genoa, Genoa, Italy.
- [4] Orthopedics Biotechnology Laboratory, Scientific Institute for Research, Hospitalization and Healthcare (IRCCS) Istituto Ortopedico Galeazzi, Milan, Italy.
- [5] Department of Radiology, Ospedale Evangelico Internazionale, Genoa, Italy.
- [6] Anesthesia and Intensive Care, Ospedale Evangelico Internazionale, Genoa, Italy.

- PMID: **34551862**
- PMCID: PMC8302846
- DOI: 10.1016/j.ultrasmedbio.2021.07.014

Free PMC article
Observational Study

Coronavirus Disease 2019 Phenotypes, Lung Ultrasound, Chest Computed Tomography and Clinical Features in Critically Ill Mechanically Ventilated Patients

Davide Orlandi et al. Ultrasound Med Biol. 2021 Dec.
Free PMC article

Show details

Ultrasound Med Biol

. 2021 Dec;47(12):3323-3332.
doi: 10.1016/j.ultrasmedbio.2021.07.014. Epub 2021 Jul 24.

Authors

Davide Orlandi [1], Denise Battaglini [2], Chiara Robba [3], Marco Viganò [4], Giulio Bergamaschi [5], Tiziana Mignatti [5], Maria Luisa Radice [6], Antonio Lapolla [6], Giovanni Turtulici [5], Paolo Pelosi [3]

Affiliations

- [1] Department of Radiology, Ospedale Evangelico Internazionale, Genoa, Italy. Electronic address: my.davideorlandi@gmail.com.
- [2] Anesthesia and Intensive Care, San Martino Policlinico Hospital, Scientific Institute for Research, Hospitalization and Healthcare (IRCCS) for Oncology and Neurosciences, Genoa, Italy; Department of Medicine, University of Barcelona, Barcelona, Spain.
- [3] Anesthesia and Intensive Care, San Martino Policlinico Hospital, Scientific Institute for Research, Hospitalization and Healthcare (IRCCS) for Oncology and Neurosciences, Genoa, Italy; Department of Surgical Sciences and Integrated Diagnostic (DISC), University of Genoa, Genoa, Italy.
- [4] Orthopedics Biotechnology Laboratory, Scientific Institute for Research, Hospitalization and Healthcare (IRCCS) Istituto Ortopedico Galeazzi, Milan, Italy.
- [5] Department of Radiology, Ospedale Evangelico Internazionale, Genoa, Italy.
- [6] Anesthesia and Intensive Care, Ospedale Evangelico Internazionale, Genoa, Italy.

- PMID: **34551862**
- PMCID: PMC8302846
- DOI: 10.1016/j.ultrasmedbio.2021.07.014

Abstract

Chest computed tomography (CT) may provide insights into the pathophysiology of coronavirus disease 2019 (COVID-19), although it is not suitable for a timely bedside dynamic assessment of patients admitted to intensive care unit (ICU); therefore, lung ultrasound (LUS) has been proposed as a complementary diagnostic tool. The aims of this study were to investigate different lungs phenotypes in patients with COVID-19 and to assess the differences in CT and LUS scores between ICU survivors and non-survivors. We also explored the association between CT and LUS, and oxygenation (arterial partial pressure of oxygen [PaO_2]/fraction of inspired oxygen [FiO_2]) and clinical parameters. The study included 39 patients with COVID-19. CT scans revealed types 1, 2 and 3 phenotypes in 62%, 28% and 10% of patients, respectively. Among survivors, pattern 1 was prevalent ($p < 0.005$). Chest CT and LUS scores differed between survivors and non-survivors both at ICU admission and 10 days after and were associated with ICU mortality. Chest CT score was positively correlated with LUS findings at ICU admission ($r = 0.953$, $p < 0.0001$) and was inversely correlated with PaO_2/FiO_2 ($r = -0.375$, $p = 0.019$) and C-reactive protein ($r = 0.329$, $p = 0.041$). LUS score was inversely correlated with PaO_2/FiO_2 ($r = -0.345$, $p = 0.031$). COVID-19 presents distinct phenotypes with differences between survivors and non-survivors. LUS is a valuable monitoring tool in an ICU setting because it may correlate with CT findings and mortality, although it cannot predict oxygenation changes.

Keywords: COVID-19; CT scan; ICU; LUS; Lung ultrasound; Phenotypes; SARS-CoV-2.

Copyright © 2021 World Federation for Ultrasound in Medicine & Biology. Published by Elsevier Inc. All rights reserved.

Conflict of interest statement

Declaration of Competing Interest All authors have no conflict of interest to disclose.

- Cited by 1 article
- 51 references
- 3 figures

Supplementary info

Publication types, MeSH terms, Substances Expand

Publication types

- Observational Study

MeSH terms

- Aged
- COVID-19 / blood
- COVID-19 / diagnostic imaging*
- COVID-19 / therapy*
- Critical Care
- Critical Illness
- Female
- Humans
- Lung / diagnostic imaging*
- Male
- Middle Aged
- Oxygen / blood
- Phenotype
- Prognosis
- Respiration, Artificial*
- Retrospective Studies
- SARS-CoV-2
- Tomography, X-Ray Computed
- Ultrasonography

Substances

- Oxygen

Full text links

ELSEVIER FULL-TEXT ARTICLE Elsevier Science Free PMC article

Proceed to details

Cite

Share

448

Case Reports

Ital J Pediatr

. 2020 Dec 7;46(1):180.

doi: 10.1186/s13052-020-00947-9.

A neonatal cluster of novel coronavirus disease 2019: clinical management and considerations

Nicole Olivini [1], Francesca Ippolita Calò Carducci [2], Veronica Santilli [2], Maria Antonietta De Ioris [3], Alessia Scarselli [3], Dario Alario [4], Caterina Geremia [3], Mary Haywood Lombardi [3], Caterina Marabotto [3], Rosanna Mariani [3], Raffaele Edo Papa [3], Emanuela Peschiaroli [3], Raffaella Scrocca [3], Serena Sinibaldi [3], Andrea Smarrazzo [3], Pietro Stella [3], Stefania Bernardi [2], Sara Chiurchiù [5], Paola Pansa [5], Lorenza Romani [5], Carletti Michaela [6], Carlo Concato [7], Domenico Umberto De Rose [8], Gugliemo Salvatori [8], Paolo Rossi [2] [9], Alberto Villani [5], Andrea Dotta [8], Patrizia D'Argenio [2], Andrea Campana [3]

Affiliations Expand

Affiliations

- [1] Pediatrics Unit, University Department of Pediatrics (DPUO), Bambino Gesù Children's Hospital - IRCCS, Via della Torre di Palidoro, 00050, Fiumicino, Rome, Italy. nicole.olivini@opbg.net.
- [2] Research Unit in Congenital and Perinatal Infection, Immune and Infectious Diseases Division, University Department of Pediatrics (DPUO), Bambino Gesù Children's Hospital - IRCCS, Rome, Italy.
- [3] Pediatrics Unit, University Department of Pediatrics (DPUO), Bambino Gesù Children's Hospital - IRCCS, Via della Torre di Palidoro, 00050, Fiumicino, Rome, Italy.
- [4] Pediatrics and Neonatology Unit, San Paolo Hospital, Civitavecchia, Italy.
- [5] Pediatrics and Infectious Diseases Unit, Bambino Gesù Children's Hospital - IRCCS, Rome, Italy.
- [6] Laboratory Unit, Bambino Gesù Children's Hospital - IRCCS, Rome, Italy.
- [7] Virology Unit, Bambino Gesù Children's Hospital - IRCCS, Rome, Italy.
- [8] Department of Neonatology, Bambino Gesù Children's Hospital - IRCCS, Rome, Italy.
- [9] Department of Systems Medicine, University of Rome Tor Vergata, Rome, Italy.

- PMID: **33287880**
- PMCID: PMC7720265
- DOI: 10.1186/s13052-020-00947-9

Free PMC article
Case Reports

A neonatal cluster of novel coronavirus disease 2019: clinical management and considerations

Nicole Olivini et al. Ital J Pediatr. 2020.
Free PMC article
Show details
Ital J Pediatr
. 2020 Dec 7;46(1):180.
doi: 10.1186/s13052-020-00947-9.

Authors

Nicole Olivini [1], Francesca Ippolita Calò Carducci [2], Veronica Santilli [2], Maria Antonietta De Ioris [3], Alessia Scarselli [3], Dario Alario [4], Caterina Geremia [3], Mary Haywood Lombardi [3], Caterina Marabotto [3], Rosanna Mariani [3], Raffaele Edo Papa [3], Emanuela Peschiaroli [3], Raffaella Scrocca [3], Serena Sinibaldi [3], Andrea Smarrazzo [3], Pietro Stella [3], Stefania Bernardi [2], Sara Chiurchiù [5], Paola Pansa [5], Lorenza Romani [5], Carletti Michaela [6], Carlo Concato [7], Domenico Umberto De Rose [8], Gugliemo Salvatori [8], Paolo Rossi [2] [9], Alberto Villani [5], Andrea Dotta [8], Patrizia D'Argenio [2], Andrea Campana [3]

Affiliations

- [1] Pediatrics Unit, University Department of Pediatrics (DPUO), Bambino Gesù Children's Hospital - IRCCS, Via della Torre di Palidoro, 00050, Fiumicino, Rome, Italy. nicole.olivini@opbg.net.
- [2] Research Unit in Congenital and Perinatal Infection, Immune and Infectious Diseases Division, University Department of Pediatrics (DPUO), Bambino Gesù Children's Hospital - IRCCS, Rome, Italy.
- [3] Pediatrics Unit, University Department of Pediatrics (DPUO), Bambino Gesù Children's Hospital - IRCCS, Via della Torre di Palidoro, 00050, Fiumicino, Rome, Italy.
- [4] Pediatrics and Neonatology Unit, San Paolo Hospital, Civitavecchia, Italy.
- [5] Pediatrics and Infectious Diseases Unit, Bambino Gesù Children's Hospital - IRCCS, Rome, Italy.
- [6] Laboratory Unit, Bambino Gesù Children's Hospital - IRCCS, Rome, Italy.
- [7] Virology Unit, Bambino Gesù Children's Hospital - IRCCS, Rome, Italy.
- [8] Department of Neonatology, Bambino Gesù Children's Hospital - IRCCS, Rome, Italy.
- [9] Department of Systems Medicine, University of Rome Tor Vergata, Rome, Italy.

- PMID: **33287880**
- PMCID: PMC7720265
- DOI: 10.1186/s13052-020-00947-9

Abstract

Background: Lately, one of the major clinical and public health issues has been represented by Coronavirus disease of 2019 (COVID-19) during pregnancy and the risk of transmission of the infection from mother to child. Debate on perinatal management and postnatal care is still ongoing, principally questioning the option of the joint management of mother and child after birth and the safety of breastfeeding. According to the available reports, neonatal COVID-19 appears to have a horizontal transmission and seems to be paucisymptomatic or asymptomatic, compared to older age groups. The aim of this work is to describe a cluster of neonatal COVID-19 and discuss our experience, with reference to current evidence on postnatal care and perinatal management.

Methods: This is a retrospective observational case series of five mother-child dyads, who attended the Labor and Delivery Unit of a first-level hospital in Italy, in March 2020. Descriptive statistics for continuous variables consisted of number of observations, mean and the range of the minimum and maximum values.

Results: Five women and four neonates tested positive for Severe Acute Respiratory Syndrome Coronavirus 2 (SARS-CoV-2). In one case, the mother-child dyad was separated and the neonate remained negative on two consecutive tests. Two positive neonates developed symptoms, with a predominant involvement of the gastrointestinal tract. Blood tests were unremarkable, except for a single patient who developed mild neutropenia. No complications occurred.

Conclusions: We agree that the decision on whether or not to separate a positive/suspected mother from her child should be made on an individual basis, taking into account the parent's will, clinical condition, hospital logistics and the local epidemiological situation. In conformity with literature, in our study, affected neonates were asymptomatic or paucisymptomatic. Despite these reassuring findings, a few cases of severe presentation in the neonatal population have been reported. Therefore, we agree on encouraging clinicians to monitor the neonates with a suspected or confirmed infection.

Keywords: COVID-19; Infection; Newborns; Pandemic; SARS-CoV-2.

Conflict of interest statement

The authors declare that they have no competing interests.

- Cited by 9 articles
- 60 references

Supplementary info

Publication types, MeSH terms Expand

Publication types

- Case Reports

- Observational Study

MeSH terms

- Adult
- COVID-19 / epidemiology
- COVID-19 / therapy*
- COVID-19 / transmission*
- COVID-19 Testing
- Disease Transmission, Infectious*
- Female
- Humans
- Infant, Newborn
- Italy / epidemiology
- Male
- Mothers*
- Pandemics
- Postnatal Care*
- Retrospective Studies
- SARS-CoV-2

Full text links

Read free full text at BMC BioMed Central Free PMC article

Proceed to details

Cite

Share

☐ 449

Observational Study

Clin Cardiol

. 2020 Oct;43(10):1142-1149.

doi: 10.1002/clc.23424. Epub 2020 Jul 21.

"Missing" acute coronary syndrome hospitalizations during the COVID-19 era in Greece: Medical care avoidance combined with a true reduction in incidence?

Michail I Papafaklis [1], Christos S Katsouras [1], Grigorios Tsigkas [2], Konstantinos Toutouzas [3], Periklis Davlouros [2], George N Hahalis [2], Maria S Kousta [4], Ioannis G Styliadis [5], Konstantinos Triantafyllou [6], Loukas Pappas [7], Fotini Tsiourantani [8], Efthymia Varytimiadi

[9], Zacharias-Alexandros Anyfantakis [10], Nikolaos Iakovis [10], Paraskevi Grammata [11], Haralambos Karvounis [12], Antonios Ziakas [12], George Sianos [12], Dimitrios Tziakas [13], Evgenia Pappa [14], Anna Dagre [15], Sotirios Patsilinakos [16], Athanasios Trikas [17], Thomais Lamprou [18], Ioannis Mamarelis [19], Georgios Katsimagklis [20], Dimitri Karmpaliotis [21], Katerina Naka [1], Lampros K Michalis [1]

Affiliations Expand

Affiliations

- [1] 2nd Department of Cardiology, University Hospital of Ioannina, Ioannina, Greece.
- [2] Department of Cardiology, Patras University Hospital, Patras, Greece.
- [3] 1st Department of Cardiology, "Hippokration" University Hospital, Athens, Greece.
- [4] Cardiology Department, General Hospital "G. Gennimatas", Athens, Greece.
- [5] 2nd Department of Cardiology, "Papageorgiou" General Hospital, Thessaloniki, Greece.
- [6] 1st Department of Cardiology, "Evaggelismos" General Hospital, Athens, Greece.
- [7] 2nd Department of Cardiology, "Evaggelismos" General Hospital, Athens, Greece.
- [8] 2nd Department of Cardiology, Hellenic Red Cross Hospital, Athens, Greece.
- [9] Department of Cardiology, "Attikon" University Hospital, Athens, Greece.
- [10] Department of Cardiology, University Hospital of Larissa, Larissa, Greece.
- [11] Department of Cardiology, "Sismanogleio" General Hospital, Athens, Greece.
- [12] Department of Cardiology, "AHEPA" University Hospital, Thessaloniki, Greece.
- [13] Department of Cardiology, University Hospital of Alexandroupolis, Thrace, Greece.
- [14] Department of Cardiology, General Hospital "G. Hatzikosta", Ioannina, Greece.
- [15] Department of Cardiology, "Thriasion" General Hospital of Elefsina, Attiki, Greece.
- [16] Department of Cardiology, "Konstandopoulio" General Hospital, Athens, Greece.
- [17] Department of Cardiology, "Elpis" General Hospital, Athens, Greece.
- [18] 2nd Department of Cardiology, General Hospital of Nikea-Piraeus "Agios Panteleimon", Piraeus, Greece.
- [19] Department of Cardiology, 401 Army General Hospital, Athens, Greece.
- [20] Department of Cardiology, Athens Naval Hospital, Athens, Greece.
- [21] Department of Cardiology, New York Presbyterian Hospital/Columbia University Irving Medical Center, New York, New York, USA.

- PMID: **32691901**
- PMCID: PMC7404667
- DOI: 10.1002/clc.23424

Free PMC article
Observational Study

"Missing" acute coronary syndrome hospitalizations during the COVID-19 era in Greece: Medical care avoidance combined with a true reduction in incidence?

Michail I Papafaklis et al. Clin Cardiol. 2020 Oct.
Free PMC article
Show details
Clin Cardiol
. 2020 Oct;43(10):1142-1149.
doi: 10.1002/clc.23424. Epub 2020 Jul 21.

Authors

Michail I Papafaklis [1], Christos S Katsouras [1], Grigorios Tsigkas [2], Konstantinos Toutouzas [3], Periklis Davlouros [2], George N Hahalis [2], Maria S Kousta [4], Ioannis G Styliadis [5], Konstantinos Triantafyllou [6], Loukas Pappas [7], Fotini Tsiourantani [8], Efthymia Varytimiadi [9], Zacharias-Alexandros Anyfantakis [10], Nikolaos Iakovis [10], Paraskevi Grammata [11], Haralambos Karvounis [12], Antonios Ziakas [12], George Sianos [12], Dimitrios Tziakas [13], Evgenia Pappa [14], Anna Dagre [15], Sotirios Patsilinakos [16], Athanasios Trikas [17], Thomais Lamprou [18], Ioannis Mamarelis [19], Georgios Katsimagklis [20], Dimitri Karmpaliotis [21], Katerina Naka [1], Lampros K Michalis [1]

Affiliations

- [1] 2nd Department of Cardiology, University Hospital of Ioannina, Ioannina, Greece.
- [2] Department of Cardiology, Patras University Hospital, Patras, Greece.
- [3] 1st Department of Cardiology, "Hippokration" University Hospital, Athens, Greece.
- [4] Cardiology Department, General Hospital "G. Gennimatas", Athens, Greece.
- [5] 2nd Department of Cardiology, "Papageorgiou" General Hospital, Thessaloniki, Greece.
- [6] 1st Department of Cardiology, "Evaggelismos" General Hospital, Athens, Greece.
- [7] 2nd Department of Cardiology, "Evaggelismos" General Hospital, Athens, Greece.
- [8] 2nd Department of Cardiology, Hellenic Red Cross Hospital, Athens, Greece.
- [9] Department of Cardiology, "Attikon" University Hospital, Athens, Greece.
- [10] Department of Cardiology, University Hospital of Larissa, Larissa, Greece.
- [11] Department of Cardiology, "Sismanogleio" General Hospital, Athens, Greece.
- [12] Department of Cardiology, "AHEPA" University Hospital, Thessaloniki, Greece.
- [13] Department of Cardiology, University Hospital of Alexandroupolis, Thrace, Greece.
- [14] Department of Cardiology, General Hospital "G. Hatzikosta", Ioannina, Greece.
- [15] Department of Cardiology, "Thriasion" General Hospital of Elefsina, Attiki, Greece.
- [16] Department of Cardiology, "Konstandopoulio" General Hospital, Athens, Greece.
- [17] Department of Cardiology, "Elpis" General Hospital, Athens, Greece.
- [18] 2nd Department of Cardiology, General Hospital of Nikea-Piraeus "Agios Panteleimon", Piraeus, Greece.

- [19] Department of Cardiology, 401 Army General Hospital, Athens, Greece.
- [20] Department of Cardiology, Athens Naval Hospital, Athens, Greece.
- [21] Department of Cardiology, New York Presbyterian Hospital/Columbia University Irving Medical Center, New York, New York, USA.

- PMID: **32691901**
- PMCID: PMC7404667
- DOI: 10.1002/clc.23424

Abstract

Background: Reports from countries severely hit by the COVID-19 pandemic suggest a decline in acute coronary syndrome (ACS)-related hospitalizations. The generalizability of this observation on ACS admissions and possible related causes in countries with low COVID-19 incidence are not known.

Hypothesis: ACS admissions were reduced in a country spared by COVID-19.

Methods: We conducted a nationwide study on the incidence rates of ACS-related admissions during a 6-week period of the COVID-19 outbreak and the corresponding control period in 2019 in Greece, a country with strict social measures, low COVID-19 incidence, and no excess in mortality.

Results: ACS admissions in the COVID-19 (n = 771) compared with the control (n = 1077) period were reduced overall (incidence rate ratio [IRR]: 0.72, $P < .001$) and for each ACS type (ST-segment elevation myocardial infarction [STEMI]: IRR: 0.76, P = .001; non-STEMI: IRR: 0.74, $P < .001$; and unstable angina [UA]: IRR: 0.63, P = .002). The decrease in STEMI admissions was stable throughout the COVID-19 period (temporal correlation; $R^2 = 0.11$, P = .53), whereas there was a gradual decline in non-STEMI/UA admissions ($R^2 = 0.75$, P = .026) following the progressively stricter social measures. During the COVID-19 period, patients admitted with ACS presented more frequently with left ventricular systolic impairment (22.2 vs 15.5% control period; $P < .001$).

Conclusions: We observed a reduction in ACS hospitalizations during the COVID-19 outbreak in a country with strict social measures, low community transmission, and no excess in mortality. Medical care avoidance behavior is an important factor for these observations, while a true reduction of the ACS incidence due to self-isolation/quarantining may have also played a role.

Keywords: COVID-19; acute cardiac care; acute coronary syndrome; myocardial infarction; public health.

© 2020 The Authors. Clinical Cardiology published by Wiley Periodicals LLC.

Conflict of interest statement

The authors declare no potential conflict of interest.

- Cited by 14 articles
- 15 references
- 3 figures

Supplementary info

Publication types, MeSH terms Expand

Publication types

- Multicenter Study
- Observational Study

MeSH terms

- Acute Coronary Syndrome / epidemiology*
- Aged
- COVID-19 / epidemiology*
- Coronary Angiography
- Female
- Greece / epidemiology
- Hospitalization / statistics & numerical data*
- Humans
- Incidence
- Male
- Middle Aged
- Pandemics
- Retrospective Studies
- SARS-CoV-2

Full text links

WILEY Free Full Text Wiley Free PMC article

Proceed to details

Cite

Share

☐ 450

Observational Study

Actas Urol Esp (Engl Ed)

. 2020 Dec;44(10):665-673.

doi: 10.1016/j.acuro.2020.09.007. Epub 2020 Sep 29.

Urological surgery during SARS-CoV-2 pandemic. Descriptive analysis of the

experience in a Urology Department across the pandemic phases

[Article in English, Spanish]

A González-Díaz [1], P Abad-López [2], E Peña-Vallejo [2], M P Caro-González [2], C Calzas-Montalvo [2], J Gil-Moradillo [2], N Miranda-Utrera [2], J Díez-Sebastián [3], C Varela-Rodríguez [4], A Rodríguez-Antolín [2], A Tejido-Sánchez [2]
Affiliations Expand

Affiliations

- [1] Servicio de Urología, Hospital Universitario 12 de Octubre, Instituto de Investigación Sanitaria 12 de Octubre i+12 (imas12), Madrid, España. Electronic address: alejandroglezdiaz@gmail.com.
- [2] Servicio de Urología, Hospital Universitario 12 de Octubre, Instituto de Investigación Sanitaria 12 de Octubre i+12 (imas12), Madrid, España.
- [3] Servicio de Medicina Preventiva y Salud Pública, Hospital Universitario La Paz, Madrid, España.
- [4] Unidad de Calidad Asistencial, Hospital Universitario 12 de Octubre, Instituto de Investigación Sanitaria 12 de Octubre i+12 (imas12), Madrid, España.

- PMID: **33069489**
- PMCID: PMC7522646
- DOI: 10.1016/j.acuro.2020.09.007

Free PMC article
Observational Study

Urological surgery during SARS-CoV-2 pandemic. Descriptive analysis of the experience in a Urology Department across the pandemic phases

[Article in English, Spanish]
A González-Díaz et al. Actas Urol Esp (Engl Ed). 2020 Dec.
Free PMC article
Show details
Actas Urol Esp (Engl Ed)

. 2020 Dec;44(10):665-673.
doi: 10.1016/j.acuro.2020.09.007. Epub 2020 Sep 29.

Authors

A González-Díaz [1], P Abad-López [2], E Peña-Vallejo [2], M P Caro-González [2], C Calzas-Montalvo [2], J Gil-Moradillo [2], N Miranda-Utrera [2], J Díez-Sebastián [3], C Varela-Rodríguez [4], A Rodríguez-Antolín [2], A Tejido-Sánchez [2]

Affiliations

- [1] Servicio de Urología, Hospital Universitario 12 de Octubre, Instituto de Investigación Sanitaria 12 de Octubre i+12 (imas12), Madrid, España. Electronic address: alejandroglezdiaz@gmail.com.
- [2] Servicio de Urología, Hospital Universitario 12 de Octubre, Instituto de Investigación Sanitaria 12 de Octubre i+12 (imas12), Madrid, España.
- [3] Servicio de Medicina Preventiva y Salud Pública, Hospital Universitario La Paz, Madrid, España.
- [4] Unidad de Calidad Asistencial, Hospital Universitario 12 de Octubre, Instituto de Investigación Sanitaria 12 de Octubre i+12 (imas12), Madrid, España.

- PMID: **33069489**
- PMCID: PMC7522646
- DOI: 10.1016/j.acuro.2020.09.007

Abstract

Introduction: The SARS-CoV-2 pandemic has changed the urological practice around the world. Our objective is to describe the outcomes presented by patients undergoing surgery in the urology department of a tertiary hospital, across the pandemic phases.

Methods: Observational, cohort study including all patients undergoing surgery from March 1 to May 14. According to the hospital organization, we identified three periods: there were no changes during the first two weeks (1st. period), the following seven weeks, when only urgent interventions were carried out after performance of nasopharyngeal swab test (2nd. period), and finally, elective surgery was resumed on May 4, after the implementation of a multidisciplinary screening protocol (3rd. period). Demographic, baseline, surgical and perioperative variables, as well as postoperative outcomes, were obtained in a retrospective (periods 1 and 2) and prospective (period 3) manner. Telephone follow-up was initiated at least 3 weeks after hospital discharge.

Results: 103 urological surgeries were performed, and 11 patients were diagnosed with COVID-19, 8 of them within the 1st.

Period: The diagnosis was already known in 1 patient, while the other 10 developed the disease in an average of 25 days after the intervention and 16,6 days after discharge. Of seven transplant patients, four got the infection. Three deaths were recorded due to the disease: a 69-year-old woman transplanted and two men over 80 with comorbidities and high anesthetic risk who underwent drainage of retroperitoneal abscess and retrograde intrarenal surgery, respectively.

Conclusions: SARS-CoV-2 infection mainly affected renal transplant recipients or elderly patients with high anesthetic risk, during the first 2 weeks of the pandemic. After implementing preoperative PCR tests and a comprehensive screening protocol, cases were substantially reduced, and safe surgical procedures were achieved.

Keywords: COVID-19; Cirugía; Infección postoperatoria; Kidney transplantation; Postoperative infection; Protocolo de cribado; SARS-CoV-2; Screening protocol; Surgery; Trasplante renal.

Copyright © 2020 AEU. Publicado por Elsevier España, S.L.U. All rights reserved.

- 24 references

Supplementary info

Publication types, MeSH terms Expand

Publication types

- Observational Study

MeSH terms

- Aged
- Aged, 80 and over
- COVID-19 / epidemiology*
- COVID-19 / mortality
- Cohort Studies
- Elective Surgical Procedures / statistics & numerical data*
- Female
- Humans
- Kidney Transplantation / statistics & numerical data
- Male
- Middle Aged
- Pandemics*
- Prospective Studies
- Retrospective Studies
- SARS-CoV-2*
- Spain / epidemiology
- Symptom Assessment
- Tertiary Care Centers
- Urologic Surgical Procedures / statistics & numerical data*
- Urology Department, Hospital / statistics & numerical data

Full text links

ELSEVIER FULL-TEXT ARTICLE Elsevier Science Free PMC article

Proceed to details

Cite

Share

☐ 451

Observational Study

Eur J Clin Microbiol Infect Dis

. 2021 Oct;40(10):2227-2234.
doi: 10.1007/s10096-021-04213-6. Epub 2021 Mar 17.

Impact of rapid multiplex PCR on management of antibiotic therapy in COVID-19-positive patients hospitalized in intensive care unit

Naouale Maataoui [1, 2], Lotfi Chemali [3], Juliette Patrier [4], Alexy Tran Dinh [5, 6], Lucie Le Fèvre [4], Brice Lortat-Jacob [5], Mehdi Marzouk [4], Camille d'Humières [7, 3], Emilie Rondinaud [7, 3], Etienne Ruppé [7, 3], Philippe Montravers [5, 6], Jean-François Timsit [7, 4], Laurence Armand-Lefèvre [7, 3]

Affiliations Expand

Affiliations

- [1] Université de Paris, INSERM, IAME, F-75006, Paris, France. naouale.maataoui@aphp.fr.
- [2] Service de Bactériologie, Hôpital Bichat Claude Bernard, AP-HP Nord, Université de Paris, 46 rue Henri Huchard, 75877, Paris Cedex 18, France. naouale.maataoui@aphp.fr.
- [3] Service de Bactériologie, Hôpital Bichat Claude Bernard, AP-HP Nord, Université de Paris, 46 rue Henri Huchard, 75877, Paris Cedex 18, France.
- [4] Medical and Infectious Diseases ICU (MI2), Hôpital Bichat, AP-HP, F-75018, Paris, France.
- [5] Department of Anesthesiology and Surgical Critical Care, Hôpital Bichat, AP-HP, F-75018, Paris, France.
- [6] Université de Paris, INSERM U 1148, F-75006, Paris, France.
- [7] Université de Paris, INSERM, IAME, F-75006, Paris, France.

- PMID: **33733394**
- PMCID: PMC7968559
- DOI: 10.1007/s10096-021-04213-6

Free PMC article
Observational Study

Impact of rapid multiplex PCR on management of antibiotic therapy in COVID-19-positive patients hospitalized in intensive care unit

Naouale Maataoui et al. Eur J Clin Microbiol Infect Dis. 2021 Oct.
Free PMC article

Show details

Eur J Clin Microbiol Infect Dis

. 2021 Oct;40(10):2227-2234.
doi: 10.1007/s10096-021-04213-6. Epub 2021 Mar 17.

Authors

Naouale Maataoui [1] [2], Lotfi Chemali [3], Juliette Patrier [4], Alexy Tran Dinh [5] [6], Lucie Le Fèvre [4], Brice Lortat-Jacob [5], Mehdi Marzouk [4], Camille d'Humières [7] [3], Emilie Rondinaud [7] [3], Etienne Ruppé [7] [3], Philippe Montravers [5] [6], Jean-François Timsit [7] [4], Laurence Armand-Lefèvre [7] [3]

Affiliations

- [1] Université de Paris, INSERM, IAME, F-75006, Paris, France. naouale.maataoui@aphp.fr.
- [2] Service de Bactériologie, Hôpital Bichat Claude Bernard, AP-HP Nord, Université de Paris, 46 rue Henri Huchard, 75877, Paris Cedex 18, France. naouale.maataoui@aphp.fr.
- [3] Service de Bactériologie, Hôpital Bichat Claude Bernard, AP-HP Nord, Université de Paris, 46 rue Henri Huchard, 75877, Paris Cedex 18, France.
- [4] Medical and Infectious Diseases ICU (MI2), Hôpital Bichat, AP-HP, F-75018, Paris, France.
- [5] Department of Anesthesiology and Surgical Critical Care, Hôpital Bichat, AP-HP, F-75018, Paris, France.
- [6] Université de Paris, INSERM U 1148, F-75006, Paris, France.
- [7] Université de Paris, INSERM, IAME, F-75006, Paris, France.

- PMID: **33733394**
- PMCID: PMC7968559
- DOI: 10.1007/s10096-021-04213-6

Abstract

Because the diagnosis of co/superinfection in COVID-19 patients is challenging, empirical antibiotic therapy is frequently initiated until microbiological analysis results. We evaluated the performance and the impact of the BioFire® FilmArray® Pneumonia plus Panel on 112 respiratory samples from 67 COVID-19 ICU patients suspected of co/superinfections. Globally, the sensitivity and specificity of the test were 89.3% and 99.1%, respectively. Positive tests led to antibiotic initiation or adaptation in 15% of episodes and de-escalation in 4%. When negative, 28% of episodes remained antibiotic-free (14% no initiation, 14% withdrawal). Rapid multiplex PCRs can help to improve antibiotic stewardship by administering appropriate antibiotics earlier and avoiding unnecessary prescriptions.

Keywords: Antibiotic stewardship; COVID-19; Coinfection; Multiplex PCR; Superinfection.

© 2021. The Author(s), under exclusive licence to Springer-Verlag GmbH Germany, part of Springer Nature.

Conflict of interest statement

ERu received funds from bioMérieux and speaking fees from Mobidiag. JFT received lecture fees from bioMérieux and participates, outside of the submitted work, on the advisory boards of MSD, Pfizer, Bayer, Nabriva, Gilead, BD, 3M, Paratek. LA received speaking fees from bioMérieux.

- Cited by 12 articles
- 19 references

Supplementary info

Publication types, MeSH terms, Substances Expand

Publication types

- Observational Study

MeSH terms

- Aged
- Anti-Bacterial Agents / therapeutic use*
- Antimicrobial Stewardship
- Bacteria / classification
- Bacteria / drug effects
- Bacteria / genetics
- Bacteria / isolation & purification*
- Bacterial Infections / diagnosis
- Bacterial Infections / drug therapy*
- Bacterial Infections / microbiology
- COVID-19 / complications*
- COVID-19 / virology
- Female
- Hospitalization
- Humans
- Intensive Care Units / statistics & numerical data
- Male
- Middle Aged
- Multiplex Polymerase Chain Reaction / methods*
- Retrospective Studies
- SARS-CoV-2 / genetics
- SARS-CoV-2 / physiology

Substances

- Anti-Bacterial Agents

Full text links

SpringerLink FULL-TEXT ARTICLE Springer Free PMC article

Proceed to details

Cite

Share

452

Observational Study

Eur J Hosp Pharm

. 2022 Mar;29(e1):e41-e45.

doi: 10.1136/ejhpharm-2021-002741. Epub 2021 Jul 28.

Baricitinib against severe COVID-19: effectiveness and safety in hospitalised pretreated patients

Rubén Iglesias Gómez [1], Raúl Méndez [2], Tomás Palanques-Pastor [3], Octavio Ballesta-López [3], Conxa Borrás Almenar [3], Juan Eduardo Megías Vericat [3], Eduardo López-Briz [3], Isabel Font-Noguera [3], Rosario Menéndez Villanueva [2], José Andrés Román Iborra [4], José Luis Poveda Andrés [3]

Affiliations Expand

Affiliations

- [1] Pharmacy Department, Hospital Universitari i Politècnic La Fe, Valencia, Spain iglesis_rub@gva.es.
- [2] Pneumology Department, Hospital Universitari i Politècnic La Fe, Valencia, Spain.
- [3] Pharmacy Department, Hospital Universitari i Politècnic La Fe, Valencia, Spain.
- [4] Rheumatology Department, Hospital Universitari i Politècnic La Fe, Valencia, Spain.

- PMID: **34321249**
- PMCID: PMC8326024
- DOI: 10.1136/ejhpharm-2021-002741

Free PMC article

Observational Study

Baricitinib against severe COVID-19: effectiveness and safety in hospitalised pretreated patients

Rubén Iglesias Gómez et al. Eur J Hosp Pharm. 2022 Mar.

Free PMC article

Show details

Eur J Hosp Pharm
. 2022 Mar;29(e1):e41-e45.
doi: 10.1136/ejhpharm-2021-002741. Epub 2021 Jul 28.

Authors

Rubén Iglesias Gómez [1], Raúl Méndez [2], Tomás Palanques-Pastor [3], Octavio Ballesta-López [3], Conxa Borrás Almenar [3], Juan Eduardo Megías Vericat [3], Eduardo López-Briz [3], Isabel Font-Noguera [3], Rosario Menéndez Villanueva [2], José Andrés Román Iborra [4], José Luis Poveda Andrés [3]

Affiliations

- [1] Pharmacy Department, Hospital Universitari i Politècnic La Fe, Valencia, Spain iglesis_rub@gva.es.
- [2] Pneumology Department, Hospital Universitari i Politècnic La Fe, Valencia, Spain.
- [3] Pharmacy Department, Hospital Universitari i Politècnic La Fe, Valencia, Spain.
- [4] Rheumatology Department, Hospital Universitari i Politècnic La Fe, Valencia, Spain.

- PMID: **34321249**
- PMCID: PMC8326024
- DOI: 10.1136/ejhpharm-2021-002741

Abstract

Objectives: To analyse the effectiveness and safety of baricitinib for severe COVID-19 in cytokine storm syndrome based on its potential role as an anti-inflammatory immunomodulator and inhibitor of viral endocytosis.

Methods: This was an observational retrospective study of hospitalised patients treated with baricitinib for severe COVID-19. Outcomes were clinical improvement on an ordinal scale of 1-8 on day 1 of baricitinib compared with day 14 (where 8=death and 1=not hospitalised with no limitations of activities), overall survival, time to recovery since baricitinib treatment started (days until hospital discharge) and laboratory parameters related to COVID-19 poor prognosis. Adverse events related to baricitinib during the admission period were also reported.

Results: Forty-three patients (70% men, mean age 70 years (IQR 54-79)) treated with baricitinib daily for 6 days (IQR 5-7) were included. Thirty-six patients were treated with corticosteroids (84%). Clinical improvement was 3 points (IQR 1-4) in patients on an ordinal scale of 4-6, overall survival was 100% at day 30 and day 60 with a mean time to recovery of 12 days (IQR 9-25) from start of baricitinib treatment. No adverse events of interest were found and all poor prognosis risk factors improved at day 14: interleukin-6, C-reactive protein, ferritin, lymphocytes, platelets and D-dimers.

Conclusions: Patients treated with baricitinib for severe COVID-19 showed improvements in clinical and analytical values without relevant adverse events and 100% overall survival. Clinical randomised trials are needed to confirm the clinical benefit of baricitinib.

Keywords: COVID-19; critical care; education; evidence-based medicine; pharmacy; pulmonary medicine; virology.

© European Association of Hospital Pharmacists 2022. No commercial re-use. See rights and permissions. Published by BMJ.

Conflict of interest statement

Competing interests: None declared.

- Cited by 2 articles
- 23 references
- 1 figure

Supplementary info

Publication types, MeSH terms, Substances, Supplementary concepts Expand

Publication types

- Observational Study

MeSH terms

- Aged
- Azetidines
- COVID-19* / drug therapy
- Female
- Humans
- Male
- Purines
- Pyrazoles
- Retrospective Studies
- SARS-CoV-2
- Sulfonamides

Substances

- Azetidines
- Purines
- Pyrazoles
- Sulfonamides
- baricitinib

Supplementary concepts

- COVID-19 drug treatment

Full text links

BMJ Full Text HighWire Free PMC article
Proceed to details
Cite
Share
453
Observational Study
J Cardiol
. 2022 Apr;79(4):494-500.
doi: 10.1016/j.jjcc.2021.12.012. Epub 2021 Dec 22.

The association of statins use with survival of patients with COVID-19

Toshiki Kuno [1], Matsuo So [2], Masao Iwagami [3], Mai Takahashi [2], Natalia N Egorova [4]
Affiliations Expand

Affiliations

- [1] Department of Medicine, Icahn School of Medicine at Mount Sinai, Mount Sinai Beth Israel, NY, USA; Department of Cardiology, Montefiore Medical Center, Albert Einstein Medical College, New York, NY, USA. Electronic address: tkuno@montefiore.org.
- [2] Department of Medicine, Icahn School of Medicine at Mount Sinai, Mount Sinai Beth Israel, NY, USA.
- [3] Department of Health Services Research, University of Tsukuba, Ibaraki, Japan.
- [4] Department of Population Health Science and Policy, Icahn School of Medicine at Mount Sinai, Mount Sinai Beth Israel, NY, USA.

- PMID: **34974938**
- PMCID: PMC8692086
- DOI: 10.1016/j.jjcc.2021.12.012

Free PMC article
Observational Study

The association of statins use with survival of patients with COVID-19

Toshiki Kuno et al. J Cardiol. 2022 Apr.
Free PMC article
Show details
J Cardiol
. 2022 Apr;79(4):494-500.
doi: 10.1016/j.jjcc.2021.12.012. Epub 2021 Dec 22.

Authors

Toshiki Kuno [1], Matsuo So [2], Masao Iwagami [3], Mai Takahashi [2], Natalia N Egorova [4]

Affiliations

- [1] Department of Medicine, Icahn School of Medicine at Mount Sinai, Mount Sinai Beth Israel, NY, USA; Department of Cardiology, Montefiore Medical Center, Albert Einstein Medical College, New York, NY, USA. Electronic address: tkuno@montefiore.org.
- [2] Department of Medicine, Icahn School of Medicine at Mount Sinai, Mount Sinai Beth Israel, NY, USA.
- [3] Department of Health Services Research, University of Tsukuba, Ibaraki, Japan.
- [4] Department of Population Health Science and Policy, Icahn School of Medicine at Mount Sinai, Mount Sinai Beth Israel, NY, USA.

- PMID: **34974938**
- PMCID: PMC8692086
- DOI: 10.1016/j.jjcc.2021.12.012

Abstract

Background: Statins are frequently prescribed for patients with dyslipidemia and diabetes mellitus. These comorbidities are highly prevalent in coronavirus disease 2019 (COVID-19) patients. Statin's beneficial effect on mortality in COVID-19 infection has been reported in several studies. However, these findings are still inconclusive.

Methods: We conducted a retrospective observational study among 6,095 patients with laboratory confirmed COVID-19 hospitalized in Mount Sinai Health System between March 1st 2020 and May 7th 2020. Patients were stratified into two groups: statin use prior to or during hospitalization (N = 2,423) versus no statins (N = 3,672). We evaluated in-hospital mortality as a primary outcome using propensity score matching and inverse probability treatment weighted (IPTW) analysis. In additional analysis, we compared continuous use of statins (N = 1,108) with no statins, continuous use of statins with discontinuation of statins (N = 644), and discontinuation of statins with no statins.

Results: Among 6,095 COVID-19 patients, statin use prior to or during hospitalization group were older (70.8 ± 12.7 years versus 59.2 ± 18.2 years, $p<0.001$) and had more comorbidities compared to no statins group. After matching by propensity score (1,790 pairs), there were no significant differences in-hospital mortality between patients with statins and those without [28.9% versus 31.0%, p = 0.19, odds ratio (OR) 95% confidence interval (CI): 0.91 (0.79-1.05)]. This result was confirmed by IPTW analysis [OR (95% CI): 0.96 (0.81-1.12), p = 0.53]. In the additional analysis comparing continuous use of statins with no statins group, in-hospital mortality was significantly lower in continuous use of statins compared to no statins group [26.3% versus 34.5%, $p<0.001$, OR (95% CI): 0.68 (0.55-0.82)] after matching by propensity score (944 pairs), as well as IPTW analysis [OR (95% CI): 0.77 (0.64-0.94), p = 0.009]. Finally, comparison of continuous use of statins with discontinuation of statins showed lower in-hospital mortality in continuous use of statins group [27.9% versus 42.1%, $p<0.001$, OR (95% CI): 0.53 (0.41-0.68)].

Conclusions: Use of statins prior to or during hospitalization was not associated with a decreased risk of in-hospital mortality, however, continuous use of statins was associated with lower in-hospital mortality compared to no statin use and discontinuation of statins.

Keywords: COVID-19; Statin.

Copyright © 2021. Published by Elsevier Ltd.

- Cited by 1 article
- 36 references
- 2 figures

Supplementary info

Publication types, MeSH terms, Substances Expand

Publication types

- Observational Study

MeSH terms

- COVID-19*
- Dyslipidemias*
- Hospital Mortality
- Humans
- Hydroxymethylglutaryl-CoA Reductase Inhibitors* / therapeutic use
- Retrospective Studies
- SARS-CoV-2

Substances

- Hydroxymethylglutaryl-CoA Reductase Inhibitors

Full text links

ELSEVIER FULL-TEXT ARTICLE Elsevier Science Free PMC article
Proceed to details
Cite
Share
☐ 454
Observational Study
Emergencias
. 2020 Nov;32(6):386-394.

Factors associated with revisits by patients with SARS-CoV-2 infection discharged from a hospital emergency department

[Article in Spanish, English]

Beatriz López-Barbeito [1], Ana García-Martínez [1], Blanca Coll-Vinent [1], Arrate Placer [1], Carme Font [1], Carmen Rosa Vargas [1], Carolina Sánchez [1], Daniela Piñango [1], Elisenda Gómez-Angelats [1], David Curtelin [1], Emilio Salgado [1], Francisco Aya [1], Gemma Martínez-Nada [1], José Ramón Alonso [1], Julia García-Gozalbes [1], Leticia Fresco [1], Miguel Galicia [1], Milagrosa Perea [1], Miriam Carbó [1], Nerea Iniesta [1], Ona Escoda [1], Rafael Perelló [1], Sandra Cuerpo [1], Vanesa Flores [1], Xavier Alemany [1], Óscar Miró [1], Mª Del Mar Ortega [1], Grupo de Trabajo sobre la atención de la COVID-19 en Urgencias (COVID19-URG)
Affiliations Expand

Affiliation

- [1] Área de Urgencias, Hospital Clinic, Universitat de Barcelona, España.

- PMID: **33275358**

Free article
Observational Study

Factors associated with revisits by patients with SARS-CoV-2 infection discharged from a hospital emergency department

[Article in Spanish, English]

Beatriz López-Barbeito et al. Emergencias. 2020 Nov.
Free article
Show details
Emergencias
. 2020 Nov;32(6):386-394.

Authors

Beatriz López-Barbeito [1], Ana García-Martínez [1], Blanca Coll-Vinent [1], Arrate Placer [1], Carme Font [1], Carmen Rosa Vargas [1], Carolina Sánchez [1], Daniela Piñango [1], Elisenda Gómez-Angelats [1], David Curtelin [1], Emilio Salgado [1], Francisco Aya [1], Gemma Martínez-Nada [1], José Ramón Alonso [1], Julia García-Gozalbes [1], Leticia Fresco [1], Miguel Galicia [1], Milagrosa Perea [1], Miriam Carbó [1], Nerea Iniesta [1], Ona Escoda [1], Rafael Perelló [1], Sandra Cuerpo [1], Vanesa Flores [1], Xavier Alemany [1], Óscar Miró [1], Mª Del Mar Ortega [1], Grupo de Trabajo sobre la atención de la COVID-19 en Urgencias (COVID19-URG)

Affiliation

- [1] Área de Urgencias, Hospital Clinic, Universitat de Barcelona, España.

- PMID: **33275358**

Abstract
in English, Spanish

Objectives: To analyze emergency department (ED) revisits from patients discharged with possible coronavirus disease 2019 (COVID-19).

Material and methods: Retrospective observational study of consecutive patients who came to the ED over a period of 2 months and were diagnosed with possible COVID-19. We analyzed clinical and epidemiologic variables, treatments given in the ED, discharge destination, need to revisit, and reasons for revisits. Patients who did or did not revisit were compared, and factors associated with revisits were explored.

Results: The 2378 patients included had a mean age of 57 years; 49% were women. Of the 925 patients (39%) discharged, 170 (20.5%) revisited the ED, mainly for persistence or progression of symptoms. Sixty-six (38.8%) were hospitalized. Odds ratios (ORs) for the following factors showed an association with revisits: history of rheumatologic disease (OR, 2.97; 95% CI, 1.10-7.99; P = .03), digestive symptoms (OR, 1.73; 95% CI, 1.14-2.63; P = .01), respiratory rate over 20 breaths per minute (OR, 1.03; 95% CI, 1.0-1.06; P = .05), and corticosteroid therapy given in the ED (OR, 7.78; 95% CI, 1.77-14.21, P = .01). Factors associated with hospitalization after revisits were age over 48 years (OR, 2.57; 95% CI, 1 42-4.67; P = .002) and fever (OR, 4.73; 95% CI, 1.99-11.27; P = .001).

Conclusion: Patients under the age of 48 years without comorbidity and with normal vitals can be discharged from the ED without fear of complications. A history of rheumatologic disease, fever, digestive symptoms, and a respiratory rate over 20 breaths per minute, or a need for corticosteroid therapy were independently associated with revisits. Fever and age over 48 years were associated with a need for hospitalization.

Objetivo: Analizar las revisitas y los factores asociados a la misma en pacientes con diagnóstico de posible COVID-19 dados de alta de un servicio de urgencias hospitalario (SUH).

Metodo: Estudio observacional, retrospectivo que incluyó pacientes consecutivos que consultaron al SUH en un periodo de 2 meses y fueron diagnosticados de posible de COVID-19. Se analizaron variables clínico-epidemiológicas, tratamiento administrado en urgencias, destino final, revisita al SUH y motivo de esta. Se hizo un análisis comparativo entre ambos grupos (revisita sí/no) y se identificaron factores asociados a la revisita.

Resultados: Se incluyeron 2.378 pacientes (edad media 57 años; 49% mujeres). De los pacientes dados de alta (39% del total; n = 925), 170 (20,5%) reconsultaron al SUH, principalmente por persistencia o progresión de síntomas, y 66 (38,8%) precisaron ingreso. Los factores relacionados con la revisita fueron: antecedentes de enfermedad reumatológica [OR: 2,97 (IC 95%: 1,10-7,99, p = 0,03)], síntomas digestivos [OR: 1,73 (IC 95%: 1,14-2,63, p = 0,01)], frecuencia respiratoria $ 20 [OR: 1,03 (IC 95%: 1,0-1,06, p = 0,05)] y haber recibido tratamiento con esteroides en urgencias [OR: 7,78 (IC 95%: 1,77-14,21, p = 0,01)]. Los factores asociados al ingreso en la revisita fueron la edad $ 48 años [OR: 2,57 (IC 95%: 1,42-4,67, p = 0,002)] y presentar fiebre [OR: 4,73 (IC 95%: 1,99-11,27, p = 0,001)].

Conclusiones: Los pacientes con posible COVID-19 menores de 48 años, sin comorbilidad y con signos vitales normales podrían ser dados de alta desde urgencias sin temor a sufrir complicaciones. Los antecedentes de enfermedad reumatológica, fiebre, sintomas digestivos, frecuencia respiratoria $ 20/min o necesidad de tratamiento con esteroides fueron factores independientes de revisita, y la fiebre y edad $ 48 años de necesidad de ingreso.

Keywords: COVID-19; Emergency health services; Infección; Revisita; Revisits; SARS-CoV-2; Urgencias.

- Cited by 3 articles

Supplementary info

Publication types, MeSH terms Expand

Publication types

- Observational Study

MeSH terms

- Adult
- Aged
- COVID-19 / complications
- COVID-19 / diagnosis
- COVID-19 / therapy*
- Emergency Service, Hospital*
- Female
- Humans
- Male
- Middle Aged
- Odds Ratio
- Patient Discharge / standards*
- Patient Readmission / statistics & numerical data*
- Retrospective Studies
- Risk Assessment
- Risk Factors

Full text links

EMERGENCIAS Grupo Saned
Proceed to details
Cite
Share
☐ 455

Observational Study
Rev Esp Quimioter
. 2020 Dec;33(6):444-447.
doi: 10.37201/req/077.2020. Epub 2020 Sep 22.

[Description of Influenza B in seasonal epidemic in Cantabria during the beginning of the pandemia due to SARS-CoV-2]

[Article in Spanish]

D Pablo-Marcos [1], A Rodríguez-Fernández, M Gozalo, J Agüero, F Arnaiz de Las Revillas, J Calvo
Affiliations Expand

Affiliation

- [1] Daniel Pablo Marcos, Servicio de Microbiología, Hospital Universitario Marqués de Valdecilla-IDIVAL. Santander, Spain. daniel.pablo@scsalud.es.

- PMID: **32957746**
- PMCID: PMC7712335
- DOI: 10.37201/req/077.2020

Free PMC article
Observational Study

[Description of Influenza B in seasonal epidemic in Cantabria during the beginning of the pandemia due to SARS-CoV-2]

[Article in Spanish]
D Pablo-Marcos et al. Rev Esp Quimioter. 2020 Dec.
Free PMC article
Show details
Rev Esp Quimioter
. 2020 Dec;33(6):444-447.
doi: 10.37201/req/077.2020. Epub 2020 Sep 22.

Authors

D Pablo-Marcos [1], A Rodríguez-Fernández, M Gozalo, J Agüero, F Arnaiz de Las Revillas, J Calvo

Affiliation

- [1] Daniel Pablo Marcos, Servicio de Microbiología, Hospital Universitario Marqués de Valdecilla-IDIVAL. Santander, Spain. daniel.pablo@scsalud.es.

- PMID: **32957746**
- PMCID: PMC7712335
- DOI: 10.37201/req/077.2020

Abstract
in English, Spanish

Objective: Co-circulation of the two Influenza B lineages hinders forecast of strain to include in trivalent vaccine. Autonomous Communities such as Cantabria continue without supplying tetravalent vaccine. The aim of this study was to analyse epidemiological characteristics of influenza type B in Cantabria (2019-2020 season) as well as to establish the predominant lineage and its relation to the recommended vaccine.

Methods: Retrospective study whereby flu diagnosis and lineage analysis were determined by RT-PCR.

Results: All samples belonged to the Victoria lineage. Most prevalent viral co-infection was due to SARS-CoV-2. The population affected by influenza B was mainly paediatric and non-vaccinated patients more frequently required hospital admittance.

Conclusions: Influenza type B has a higher incidence in the paediatric population and type A affects more the adult population. Only 28.8% of patients with Influenza B that presented with some underlying condition or risk factor were vaccinated. This shows the need to increase coverage with tetravalent vaccines in order to reduce the burden of disease associated with the Influenza B virus.

Introducción: La cocirculación de los dos linajes de gripe B dificulta la predicción de la cepa a incluir en la vacuna trivalente. Comunidades autónomas (CCAA) como Cantabria continúan sin suministrar la vacuna tetravalente. El objetivo de este estudio fue analizar las características epidemiológicas de la gripe B en Cantabria (temporada 2019-2020), y determinar el linaje predominante y su relación con la vacuna recomendada.

Métodos: Estudio retrospectivo en el que el diagnóstico de gripe y los linajes de gripe B se determinaron mediante RTPCR.

Resultados: Todas las muestras pertenecieron al linaje Victoria. La coinfección vírica más frecuente fue por SARSCoV-2. La población afectada por gripe B fue fundamental-mente pediátrica y los pacientes no vacunados requirieron más frecuentemente ingreso hospitalario.

Conclusión: La gripe B presenta una mayor incidencia sobre población pediátrica, y la gripe A afecta más a población adulta. Sólo el 28,8% de los pacientes con gripe B que presentaban algún factor de riesgo estaban vacunados, existiendo la necesidad de aumentar la cobertura con vacunas tetravalentes para reducir la carga de enfermedad asociada al virus gripal B.

Keywords: Influenza; epidemiology; vaccination.

©The Author 2020. Published by Sociedad Española de Quimioterapia. This article is distributed under the terms of the Creative Commons Attribution-NonCommercial 4.0 International (CC BY-NC 4.0)(https://creativecommons.org/licenses/by-nc/4.0/).

Conflict of interest statement

Los autores declaran no tener conflictos de intereses.

- 14 references

Supplementary info

Publication types, MeSH terms, Substances Expand

Publication types

- Observational Study

MeSH terms

- Adult
- COVID-19 / epidemiology*
- COVID-19 / virology
- Chi-Square Distribution
- Child
- Coinfection / epidemiology
- Coinfection / virology
- Epidemics
- Female
- Hospitalization / statistics & numerical data
- Humans
- Influenza B virus*
- Influenza Vaccines / administration & dosage
- Influenza, Human / epidemiology*
- Influenza, Human / prevention & control
- Influenza, Human / virology
- Male
- Pandemics*
- Retrospective Studies
- SARS-CoV-2*
- Seasons
- Spain / epidemiology
- Statistics, Nonparametric

Substances

- Influenza Vaccines

Full text links

FREE Full Text www.seq.es Sociedad Espanola de Quimioterapia Free PMC article
Proceed to details
Cite
Share
456
Observational Study
CMAJ Open
. 2020 Nov 24;8(4):E788-E795.
doi: 10.9778/cmajo.20200159. Print Oct-Dec 2020.

Outcomes and clinical practice in patients with COVID-19 admitted to the intensive care unit in Montréal, Canada: a descriptive analysis

Stephen Su Yang [1], Jed Lipes [2], Sandra Dial [2], Blair Schwartz [2], Denny Laporta [2], Evan Wong [2], Craig Baldry [2], Paul Warshawsky [2], Patricia McMillan [2], David Hornstein [2], Michel de Marchie [2], Dev Jayaraman [2]
Affiliations Expand

Affiliations

- [1] Departments of Anesthesia (Yang, Baldry, McMillan), Medicine (Lipes, Dial, Schwartz, Laporta, Warshawsky, Hornstein, de Marchie, Jayaraman) and Surgery (Wong), and Division of Critical Care (Yang, Lipes, Dial, Schwartz, Laporta, Wong, Baldry, Warshawsky, McMillan, Hornstein, de Marchie, Jayaraman), Jewish General Hospital, McGill University, Montréal, Que. Stephen.yang@mail.mcgill.ca.
- [2] Departments of Anesthesia (Yang, Baldry, McMillan), Medicine (Lipes, Dial, Schwartz, Laporta, Warshawsky, Hornstein, de Marchie, Jayaraman) and Surgery (Wong), and Division of Critical Care (Yang, Lipes, Dial, Schwartz, Laporta, Wong, Baldry, Warshawsky, McMillan, Hornstein, de Marchie, Jayaraman), Jewish General Hospital, McGill University, Montréal, Que.

- PMID: **33234586**
- PMCID: PMC7721255
- DOI: 10.9778/cmajo.20200159

Free PMC article
Observational Study

Outcomes and clinical practice in patients with COVID-19 admitted to the intensive care unit in Montréal, Canada: a descriptive analysis

Stephen Su Yang et al. CMAJ Open. 2020.
Free PMC article
Show details
CMAJ Open
. 2020 Nov 24;8(4):E788-E795.
doi: 10.9778/cmajo.20200159. Print Oct-Dec 2020.

Authors

Stephen Su Yang [1], Jed Lipes [2], Sandra Dial [2], Blair Schwartz [2], Denny Laporta [2], Evan Wong [2], Craig Baldry [2], Paul Warshawsky [2], Patricia McMillan [2], David Hornstein [2], Michel de Marchie [2], Dev Jayaraman [2]

Affiliations

- [1] Departments of Anesthesia (Yang, Baldry, McMillan), Medicine (Lipes, Dial, Schwartz, Laporta, Warshawsky, Hornstein, de Marchie, Jayaraman) and Surgery (Wong), and Division of Critical Care (Yang, Lipes, Dial, Schwartz, Laporta, Wong, Baldry, Warshawsky, McMillan, Hornstein, de Marchie, Jayaraman), Jewish General Hospital, McGill University, Montréal, Que. Stephen.yang@mail.mcgill.ca.
- [2] Departments of Anesthesia (Yang, Baldry, McMillan), Medicine (Lipes, Dial, Schwartz, Laporta, Warshawsky, Hornstein, de Marchie, Jayaraman) and Surgery (Wong), and Division of Critical Care (Yang, Lipes, Dial, Schwartz, Laporta, Wong, Baldry, Warshawsky, McMillan, Hornstein, de Marchie, Jayaraman), Jewish General Hospital, McGill University, Montréal, Que.

- PMID: **33234586**
- PMCID: PMC7721255
- DOI: 10.9778/cmajo.20200159

Abstract

Background: The coronavirus disease 2019 (COVID-19) pandemic is responsible for millions of infections worldwide, and a substantial number of these patients will be admitted to the intensive care unit (ICU). Our objective was to describe the characteristics, outcomes and management of critically ill patients with COVID-19 pneumonia at a single designated pandemic centre in Montréal, Canada.

Methods: A descriptive analysis was performed on consecutive critically ill patients with COVID-19 pneumonia admitted to the ICU at the Jewish General Hospital, a designated pandemic centre in Montréal, between Mar. 5 and May 21, 2020. Complete follow-up data corresponding to

death or discharge from hospital health records were included to Aug. 4, 2020. We summarized baseline characteristics, management and outcomes, including mortality.

Results: A total of 106 patients were included in this study. Twenty-one patients (19.8%) died during their hospital stay, and the ICU mortality was 17.0% (18/106); all patients were discharged home or died, except for 4 patients (2 awaiting a rehabilitation bed and 2 awaiting long-term care). Twelve of 65 patients (18.5%) requiring mechanical ventilation died. Prone positioning was used in 29 patients (27.4%), including in 10 patients who were spontaneously breathing; no patient was placed on extracorporeal membrane oxygenation. High-flow nasal cannula was used in 51 patients (48.1%). Acute kidney injury was the most common complication, seen in 20 patients (18.9%), and 12 patients (11.3%) required renal replacement therapy. A total of 53 patients (50.0%) received corticosteroids.

Interpretation: Our cohort of critically ill patients with COVID-19 had lower mortality than that previously described in other jurisdictions. These findings may help guide critical care decision-making in similar health care systems in further COVID-19 surges.

Copyright 2020, Joule Inc. or its licensors.

Conflict of interest statement

Competing interests: Paul Warshawsky reports personal fees from Gilead Sciences Canada, outside the submitted work. No other competing interests were declared.

- Cited by 6 articles
- 3 figures

Supplementary info

Publication types, MeSH terms, Substances Expand

Publication types

- Observational Study

MeSH terms

- Acute Kidney Injury / epidemiology
- Acute Kidney Injury / therapy
- Adrenal Cortex Hormones / therapeutic use
- Aged
- COVID-19 / diagnosis*
- COVID-19 / epidemiology
- COVID-19 / mortality
- COVID-19 / virology
- Canada / epidemiology
- Cannula / statistics & numerical data
- Cohort Studies

- Critical Illness / mortality*
- Critical Illness / nursing
- Female
- Hospitalization / statistics & numerical data
- Humans
- Intensive Care Units / statistics & numerical data*
- Length of Stay / statistics & numerical data
- Male
- Middle Aged
- Practice Patterns, Physicians' / trends
- Prone Position
- Renal Replacement Therapy / methods
- Respiration, Artificial / mortality
- Respiration, Artificial / statistics & numerical data
- Retrospective Studies
- SARS-CoV-2 / genetics*
- Treatment Outcome

Substances

- Adrenal Cortex Hormones

Full text links

Free full text on cmajopen.ca HighWire Free PMC article
Proceed to details
Cite
Share
457
Observational Study
Medicine (Baltimore)
. 2021 Jun 18;100(24):e26371.
doi: 10.1097/MD.0000000000026371.

Mild to moderate COVID-19 illness in adult outpatients: Characteristics, symptoms, and outcomes in the first 4 weeks of illness

Janis E Blair[1], Ashwini Gotimukul[1,2], Fangfang Wang[3], Syeda A Mina[3], Helen C Bartels[3], Mark W Burns[1], Amy E Kole[1], Holenarasipur R Vikram[1], Juan C Gea-Banacloche[1,4], M Teresa Seville[1], Skye A Buckner Petty[5], Avinash Vikram[1,2], Robert Orenstein[1]

Affiliations Expand

Affiliations

- [1] Division of Infectious Diseases.
- [2] Division of Infectious Diseases, University of Louisville, Louisville, Kentucky.
- [3] Department of Internal Medicine, Mayo Clinic Hospital, Phoenix.
- [4] Division of Clinical Research, National Institutes of Allergy and Infectious Diseases, Bethesda, Maryland, U.S.A.
- [5] Department of Biostatistics (Mr Buckner Petty), Mayo Clinic, Scottsdale.

- PMID: **34128896**
- PMCID: PMC8213280
- DOI: 10.1097/MD.0000000000026371

Free PMC article
Observational Study

Mild to moderate COVID-19 illness in adult outpatients: Characteristics, symptoms, and outcomes in the first 4 weeks of illness

Janis E Blair et al. Medicine (Baltimore). 2021.
Free PMC article
Show details
Medicine (Baltimore)
. 2021 Jun 18;100(24):e26371.
doi: 10.1097/MD.0000000000026371.

Authors

Janis E Blair [1], Ashwini Gotimukul [1] [2], Fangfang Wang [3], Syeda A Mina [3], Helen C Bartels [3], Mark W Burns [1], Amy E Kole [1], Holenarasipur R Vikram [1], Juan C Gea-Banacloche [1] [4], M Teresa Seville [1], Skye A Buckner Petty [5], Avinash Vikram [1] [2], Robert Orenstein [1]

Affiliations

- [1] Division of Infectious Diseases.
- [2] Division of Infectious Diseases, University of Louisville, Louisville, Kentucky.
- [3] Department of Internal Medicine, Mayo Clinic Hospital, Phoenix.
- [4] Division of Clinical Research, National Institutes of Allergy and Infectious Diseases, Bethesda, Maryland, U.S.A.
- [5] Department of Biostatistics (Mr Buckner Petty), Mayo Clinic, Scottsdale.

- PMID: **34128896**
- PMCID: PMC8213280

- DOI: 10.1097/MD.0000000000026371

Abstract

Most patients with coronavirus disease 2019 (COVID-19) have mild to moderate illness not requiring hospitalization. However, no study has detailed the evolution of symptoms in the first month of illness.At our institution, we conducted remote (telephone and video) visits for all adult outpatients diagnosed with COVID-19 within 24 h of a positive nasopharyngeal polymerase chain test for SARS-CoV-2. We repeated regular video visits at 7, 14, and 28 days after the positive test, retrospectively reviewed the prospective data collected in the remote visits, and constructed a week by week profile of clinical illness, through week 4 of illness.We reviewed the courses of 458 symptomatic patients diagnosed between March 12, 2020, and June 22, 2020, and characterized their weekly courses. Common initial symptoms included fever, headache, cough, and chest pain, which frequently persisted through week 3 or longer. Upper respiratory or gastrointestinal symptoms were much shorter lived, present primarily in week 1. Anosmia/ageusia peaked in weeks 2 to 3. Emergency department visits were frequent, with 128 visits in the 423 patients who were not hospitalized and 48 visits among the 35 outpatients (7.6%) who were eventually hospitalized (2 subsequently died). By the fourth week, 28.9% said their illness had completely resolved. After the 4-week follow up, 20 (4.7%) of the 423 nonhospitalized patients had further medical evaluation and management for subacute or chronic COVID-19 symptoms.Mild to moderate outpatient COVID-19 is a prolonged illness, with evolving symptoms commonly lasting into the fourth week of illness.

Copyright © 2021 the Author(s). Published by Wolters Kluwer Health, Inc.

Conflict of interest statement

The authors have no conflicts of interest to disclose.

- Cited by 2 articles
- 13 references
- 1 figure

Supplementary info

Publication types, MeSH terms Expand

Publication types

- Observational Study

MeSH terms

- Adolescent
- Adult
- Aged
- Aged, 80 and over
- Ambulatory Care*

- Anosmia / etiology
- COVID-19 / complications*
- COVID-19 / diagnosis
- COVID-19 / therapy*
- Chest Pain / etiology
- Cough / etiology
- Dyspnea / etiology
- Emergency Service, Hospital
- Fatigue / etiology
- Female
- Fever / etiology
- Humans
- Male
- Middle Aged
- Myalgia / etiology
- Retrospective Studies
- SARS-CoV-2
- Severity of Illness Index
- Treatment Outcome
- Young Adult

Full text links

Wolters Kluwer Wolters Kluwer Free PMC article

Proceed to details

Cite

Share

☐ 458

Observational Study

Nat Commun

. 2021 Jan 19;12(1):434.

doi: 10.1038/s41467-020-20688-x.

Genomic epidemiology of SARS-CoV-2 reveals multiple lineages and early spread of SARS-CoV-2 infections in Lombardy, Italy

Claudia Alteri [#, 1], Valeria Cento [#, 1], Antonio Piralla [#, 2], Valentino Costabile [3], Monica Tallarita [2], Luna Colagrossi [4], Silvia Renica [1], Federica Giardina [2], Federica Novazzi [2], Stefano Gaiarsa [2], Elisa Matarazzo [5], Maria Antonello [1], Chiara Vismara [6], Roberto Fumagalli [7], Oscar Massimiliano Epis [8], Massimo Puoti [9], Carlo Federico Perno [10], Fausto Baldanti [2, 11]

Affiliations Expand

Affiliations

- [1] Department of Oncology and Hemato-oncology, University of Milan, Milan, Italy.
- [2] Molecular Virology Unit, Microbiology and Virology Department, Fondazione IRCCS Policlinico San Matteo, Pavia, Italy.
- [3] Department of Pathophysiology and Transplantation, University of Milan, Milan, Italy.
- [4] Microbiology and Diagnostic Immunology Unit, Bambino Gesù Children's Hospital, IRCCS, Rome, Italy.
- [5] Residency in Microbiology and Virology, University of Milan, Milan, Italy.
- [6] Chemico-clinical and Microbiological Analyses, ASST Grande Ospedale Metropolitano Niguarda, Milan, Italy.
- [7] Department of Anesthesiology, Critical Care and Pain Medicine, ASST Grande Ospedale Metropolitano Niguarda, Milan, Italy.
- [8] Rheumatology Unit, ASST Grande Ospedale Metropolitano Niguarda, Milan, Italy.
- [9] Infectious Diseases Unit, ASST Grande Ospedale Metropolitano Niguarda, Milan, Italy.
- [10] Microbiology and Diagnostic Immunology Unit, Bambino Gesù Children's Hospital, IRCCS, Rome, Italy. cf.perno@uniroma2.it.
- [11] Department of Clinical, Surgical, Diagnostic and Paediatric Sciences, University of Pavia, Pavia, Italy.

[#] Contributed equally.

- PMID: **33469026**
- PMCID: PMC7815831
- DOI: 10.1038/s41467-020-20688-x

Free PMC article
Observational Study

Genomic epidemiology of SARS-CoV-2 reveals multiple lineages and early spread of SARS-CoV-2 infections in Lombardy, Italy

Claudia Alteri et al. Nat Commun. 2021.
Free PMC article
Show details
Nat Commun

. 2021 Jan 19;12(1):434.
doi: 10.1038/s41467-020-20688-x.

Authors

Claudia Alteri [#1], Valeria Cento [#1], Antonio Piralla [#2], Valentino Costabile [3], Monica Tallarita [2], Luna Colagrossi [4], Silvia Renica [1], Federica Giardina [2], Federica Novazzi [2], Stefano Gaiarsa

[2], Elisa Matarazzo [5], Maria Antonello [1], Chiara Vismara [6], Roberto Fumagalli [7], Oscar Massimiliano Epis [8], Massimo Puoti [9], Carlo Federico Perno [10], Fausto Baldanti [2] [11]

Affiliations

- [1] Department of Oncology and Hemato-oncology, University of Milan, Milan, Italy.
- [2] Molecular Virology Unit, Microbiology and Virology Department, Fondazione IRCCS Policlinico San Matteo, Pavia, Italy.
- [3] Department of Pathophysiology and Transplantation, University of Milan, Milan, Italy.
- [4] Microbiology and Diagnostic Immunology Unit, Bambino Gesù Children's Hospital, IRCCS, Rome, Italy.
- [5] Residency in Microbiology and Virology, University of Milan, Milan, Italy.
- [6] Chemico-clinical and Microbiological Analyses, ASST Grande Ospedale Metropolitano Niguarda, Milan, Italy.
- [7] Department of Anesthesiology, Critical Care and Pain Medicine, ASST Grande Ospedale Metropolitano Niguarda, Milan, Italy.
- [8] Rheumatology Unit, ASST Grande Ospedale Metropolitano Niguarda, Milan, Italy.
- [9] Infectious Diseases Unit, ASST Grande Ospedale Metropolitano Niguarda, Milan, Italy.
- [10] Microbiology and Diagnostic Immunology Unit, Bambino Gesù Children's Hospital, IRCCS, Rome, Italy. cf.perno@uniroma2.it.
- [11] Department of Clinical, Surgical, Diagnostic and Paediatric Sciences, University of Pavia, Pavia, Italy.

[#] Contributed equally.

- PMID: **33469026**
- PMCID: PMC7815831
- DOI: 10.1038/s41467-020-20688-x

Abstract

From February to April 2020, Lombardy (Italy) reported the highest numbers of SARS-CoV-2 cases worldwide. By analyzing 346 whole SARS-CoV-2 genomes, we demonstrate the presence of seven viral lineages in Lombardy, frequently sustained by local transmission chains and at least two likely to have originated in Italy. Six single nucleotide polymorphisms (five of them non-synonymous) characterized the SARS-CoV-2 sequences, none of them affecting N-glycosylation sites. The seven lineages, and the presence of local transmission clusters within three of them, revealed that sustained community transmission was underway before the first COVID-19 case had been detected in Lombardy.

Conflict of interest statement

The authors declare no competing interests.

- Cited by 35 articles
- 46 references
- 5 figures

Supplementary info

Publication types, MeSH terms Expand

Publication types

- Observational Study
- Research Support, Non-U.S. Gov't

MeSH terms

- Adult
- Aged
- Aged, 80 and over
- COVID-19 / epidemiology
- COVID-19 / prevention & control*
- COVID-19 / virology
- Epidemics
- Female
- Genome, Viral / genetics*
- Genomics / methods*
- Geography
- Humans
- Italy / epidemiology
- Male
- Middle Aged
- Phylogeny
- Polymorphism, Single Nucleotide*
- Prevalence
- Retrospective Studies
- SARS-CoV-2 / classification
- SARS-CoV-2 / genetics*
- SARS-CoV-2 / physiology

Full text links

nature publishing group Nature Publishing Group Free PMC article

Proceed to details

Cite

Share

☐ 459

BMC Infect Dis
. 2022 Jan 20;22(1):70.
doi: 10.1186/s12879-021-07019-1.

[Protocol for SARS-CoV-2 post-vaccine surveillance study in Australian adults and children with cancer: an observational study of safety and serological and immunological response to SARS-CoV-2 vaccination (SerOzNET)]

Amy Body [1, 2], Elizabeth Ahern [3, 4], Luxi Lal [3, 4], Karen Gillett [3], Hesham Abdulla [3], Stephen Opat [3, 4], Tracey O'Brien [5, 6], Peter Downie [7], Stuart Turville [8], C Mee Ling Munier [8], Corey Smith [9], C Raina MacIntyre [10], Eva Segelov [3, 4]
Affiliations Expand

Affiliations

- [1] Level 7, Monash Health Translational Precinct, 246 Clayton Rd, Clayton, Melbourne, VIC, 3168, Australia. amy.body@monash.edu.
- [2] Monash University, Clayton, Melbourne, VIC, 3168, Australia. amy.body@monash.edu.
- [3] Level 7, Monash Health Translational Precinct, 246 Clayton Rd, Clayton, Melbourne, VIC, 3168, Australia.
- [4] Monash University, Clayton, Melbourne, VIC, 3168, Australia.
- [5] Kids Cancer Centre, Sydney Children's Hospital', Randwick, NSW, 2031, Australia.
- [6] School of Women's & Children's Health, Faculty of Medicine, University of New South Wales, Sydney, 2052, Australia.
- [7] Children's Cancer Centre, Monash Children's Hospital, 246 Clayton Rd, Clayton, Melbourne, VIC, 3168, Australia.
- [8] Immunovirology and Pathogenesis Program, The Kirby Institute, University of New South Wales, Kensington, Sydney NSW, 2052, Australia.
- [9] QIMR Berghofer Centre for Immunotherapy and Vaccine Development and Translational and Human Immunology Laboratory, QIMR Berghofer Medical Research Institute, Brisbane, QLD, Australia.
- [10] The Kirby Institute, University of New South Wales, Kensington, Sydney NSW, 2052, Australia.

- PMID: **35057745**
- PMCID: PMC8771167
- DOI: 10.1186/s12879-021-07019-1

Free PMC article

Protocol for SARS-CoV-2 post-vaccine surveillance study in Australian adults and

children with cancer: an observational study of safety and serological and immunological response to SARS-CoV-2 vaccination (SerOzNET)

Amy Body et al. BMC Infect Dis. 2022.
Free PMC article
Show details
BMC Infect Dis
. 2022 Jan 20;22(1):70.
doi: 10.1186/s12879-021-07019-1.

Authors

Amy Body [1 2], Elizabeth Ahern [3 4], Luxi Lal [3 4], Karen Gillett [3], Hesham Abdulla [3], Stephen Opat [3 4], Tracey O'Brien [5 6], Peter Downie [7], Stuart Turville [8], C Mee Ling Munier [8], Corey Smith [9], C Raina MacIntyre [10], Eva Segelov [3 4]

Affiliations

- [1] Level 7, Monash Health Translational Precinct, 246 Clayton Rd, Clayton, Melbourne, VIC, 3168, Australia. amy.body@monash.edu.
- [2] Monash University, Clayton, Melbourne, VIC, 3168, Australia. amy.body@monash.edu.
- [3] Level 7, Monash Health Translational Precinct, 246 Clayton Rd, Clayton, Melbourne, VIC, 3168, Australia.
- [4] Monash University, Clayton, Melbourne, VIC, 3168, Australia.
- [5] Kids Cancer Centre, Sydney Children's Hospital', Randwick, NSW, 2031, Australia.
- [6] School of Women's & Children's Health, Faculty of Medicine, University of New South Wales, Sydney, 2052, Australia.
- [7] Children's Cancer Centre, Monash Children's Hospital, 246 Clayton Rd, Clayton, Melbourne, VIC, 3168, Australia.
- [8] Immunovirology and Pathogenesis Program, The Kirby Institute, University of New South Wales, Kensington, Sydney NSW, 2052, Australia.
- [9] QIMR Berghofer Centre for Immunotherapy and Vaccine Development and Translational and Human Immunology Laboratory, QIMR Berghofer Medical Research Institute, Brisbane, QLD, Australia.
- [10] The Kirby Institute, University of New South Wales, Kensington, Sydney NSW, 2052, Australia.

- PMID: **35057745**
- PMCID: PMC8771167
- DOI: 10.1186/s12879-021-07019-1

Abstract

Background: Cancer is associated with excess morbidity and mortality from coronavirus disease 2019 (COVID-19) following infection by the novel pandemic coronavirus SARS-CoV-2. Vaccinations against SARS-CoV-2 have been rapidly developed and proved highly effective in reducing the incidence of severe COVID-19 in clinical trials of healthy populations. However, patients with cancer were excluded from pivotal clinical trials. Early data suggest that vaccine response is less robust in patients with immunosuppressive conditions or treatments, while toxicity and acceptability of COVID-19 vaccines in the cancer population is unknown. Unanswered questions remain about the impact of various cancer characteristics (such as treatment modality and degree of immunosuppression) on serological response to and safety of COVID-19 vaccinations. Furthermore, as the virus and disease manifestations evolve, ongoing data is required to address the impact of new variants.

Methods: SerOzNET is a prospective observational study of adults and children with cancer undergoing routine SARS-CoV-2 vaccination in Australia. Peripheral blood will be collected and processed at five timepoints (one pre-vaccination and four post-vaccination) for analysis of serologic responses to vaccine and exploration of T-cell immune correlates. Cohorts include: solid organ cancer (SOC) or haematological malignancy (HM) patients currently receiving (1) chemotherapy, (2) immune checkpoint inhibitors (3) hormonal or targeted therapy; (4) patients who completed chemotherapy within 6-12 months of vaccination; (5) HM patients with conditions associated with hypogammaglobulinaemia or immunocompromise; (6) SOC or HM patients with allergy to PEG or polysorbate 80. Data from healthy controls already enrolled on several parallel studies with comparable time points will be used for comparison. For children, patients with current or prior cancer who have not received recent systemic therapy will act as controls. Standardised scales for quality-of-life assessment, patient-reported toxicity and vaccine hesitancy will be obtained.

Discussion: The SerOzNET study was commenced in June 2021 to prospectively study immune correlates of vaccination in specific cancer cohorts. The high proportion of the Australian population naïve to COVID-19 infection and vaccination at study commencement has allowed a unique window of opportunity to study vaccine-related immunity. Quality of life and patient-reported adverse events have not yet been reported in detail post-vaccination for cancer patients. Trial registration This trial is registered on the Australia New Zealand Clinical Trials Registry (ANZCTR) ACTRN12621001004853. Submitted for registration 25 June 2021. Registered 30 July 2021 (Retrospectively registered). https://www.anzctr.org.au/Trial/Registration/TrialReview.aspx?id=382281&isReview=true.

Keywords: Cancer; Covid-19 vaccine; Immune response; SARS-CoV-2 vaccine; Vaccine response.

© 2022. The Author(s).

Conflict of interest statement

RM has consulted for or been on advisory boards for Seqirus, AstraZeneca and Janssen on COVID-19 vaccines. She has been a panellist on a WHO research and development consultation on COVID-19 vaccines.

- 52 references

Supplementary info

Publication types, MeSH terms, Substances, Associated data, Grant support Expand

Publication types

- Clinical Trial Protocol

MeSH terms

- Australia / epidemiology
- COVID-19 Vaccines
- COVID-19*
- Humans
- Neoplasms* / complications
- Observational Studies as Topic
- Quality of Life
- SARS-CoV-2
- Vaccination
- Vaccination Hesitancy
- Viral Vaccines*

Substances

- COVID-19 Vaccines
- Viral Vaccines

Associated data

- ANZCTR/ACTRN12621001004853

Grant support

- COMMONWEALTH APPROACH TO MARKET (ATM) REFERENCE NO. F21/113/Cancer Australia

Full text links

Read free full text at BMC BioMed Central Free PMC article
Proceed to details
Cite
Share
460
Case Reports
Rev Esp Anestesiol Reanim (Engl Ed)
. Jun-Jul 2021;68(6):346-352.
doi: 10.1016/j.redare.2020.10.002. Epub 2021 Jun 9.

Utility of preoperative polymerase chain reaction testing during SARS-CoV-2 pandemic: The challenge of evolving incidence

M de la Matta [1], J M Delgado-Sánchez [2], G M Gutiérrez [3], J L López Romero [4], M M Martínez Gómez [4], A Domínguez Blanco [4]
Affiliations Expand

Affiliations

- [1] Servicio de Anestesiología y Reanimación, Hospital Universitario Virgen del Rocío, Sevilla, Spain. Electronic address: mdlmattam@hotmail.com.
- [2] Departamento de Matemática Aplicada I, Escuela Técnica Superior de Arquitectura, Universidad de Sevilla, Sevilla, Spain.
- [3] Unidad de Enfermedades Infecciosas, Microbiología y Medicina Preventiva, Hospital Universitario Virgen del Rocío, Sevilla, Spain.
- [4] Servicio de Anestesiología y Reanimación, Hospital Universitario Virgen del Rocío, Sevilla, Spain.

- PMID: **34147408**
- PMCID: PMC8188388
- DOI: 10.1016/j.redare.2020.10.002

Free PMC article
Case Reports

Utility of preoperative polymerase chain reaction testing during SARS-CoV-2 pandemic: The challenge of evolving incidence

M de la Matta et al. Rev Esp Anestesiol Reanim (Engl Ed). Jun-Jul 2021.
Free PMC article
Show details
Rev Esp Anestesiol Reanim (Engl Ed)
. Jun-Jul 2021;68(6):346-352.
doi: 10.1016/j.redare.2020.10.002. Epub 2021 Jun 9.

Authors

M de la Matta [1], J M Delgado-Sánchez [2], G M Gutiérrez [3], J L López Romero [4], M M Martínez Gómez [4], A Domínguez Blanco [4]

Affiliations

- [1] Servicio de Anestesiología y Reanimación, Hospital Universitario Virgen del Rocío, Sevilla, Spain. Electronic address: mdlmattam@hotmail.com.
- [2] Departamento de Matemática Aplicada I, Escuela Técnica Superior de Arquitectura, Universidad de Sevilla, Sevilla, Spain.
- [3] Unidad de Enfermedades Infecciosas, Microbiología y Medicina Preventiva, Hospital Universitario Virgen del Rocío, Sevilla, Spain.
- [4] Servicio de Anestesiología y Reanimación, Hospital Universitario Virgen del Rocío, Sevilla, Spain.

- PMID: **34147408**
- PMCID: PMC8188388
- DOI: 10.1016/j.redare.2020.10.002

Abstract
in English, Spanish

Introduction: Due to its high transmissibility, measures aimed at reducing the spread of SARS CoV2 have become mandatory. Different organizations have recommended performing polymerase chain reaction tests (PCR) as part of the preoperative screening of surgical patients. We aimed to determine the performance of PCR testing to detect asymptomatic carriers.

Methods: Observational study carried out at a tertiary care center. We compared the results of preoperative real-time reverse-transcription-PCR test (RT-PCR) performed on a cohort of patients pending surgery with the results we would have expected assuming the epidemiological data released by government offices.

Results: We registered no positives in the 2,722 preoperative RT-PCR tests performed in our health care area between epidemiological Weeks 18 to 21, meaning a cumulative incidence trending to zero. Assuming public epidemiological data, the probabilistic projection of potential asymptomatic individuals ranged from $0.27 \times 10e^{-4}$ (according to official data of new cases diagnosed by PCR) to $4.69 \times 10e^{-4}$ if we assumed cases confirmed by IgG test in our province. Assuming a RT-PCR sensitivity of 95%, to obtain a positive result we should perform 38,461 and 2,028 tests respectively.

Conclusions: In scenarios of very low prevalence and despite high sensitivity scores, indiscriminate preoperative RT-PCR screening is of a questionable effectiveness for detecting asymptomatic carriers. Our findings evidence the difficulty of establishing reliable predictive models for the episodic and rapidly evolving incidence of infections such as has characterized the SARS CoV2 pandemic.

Antecedentes y objetivo: La alta transmisibilidad de la infección por SARS CoV2 ha obligado a los sistemas de salud mundiales a arbitrar medidas para evitar su expansión. En España, el consenso alcanzado entre diferentes sociedades científicas recomienda la realización de la prueba de reacción en cadena de la polimerasa (PCR) como cribado preoperatorio de portadores asintomáticos. Nos propusimos evaluar el rendimiento de la PCR preoperatoria para detectar portadores asintomáticos.

Material y métodos: Estudio observacional realizado en un hospital de tercer nivel. Comparamos los resultados de la prueba de PCR en tiempo real (RT-PCR) realizada en una cohorte de pacientes quirúrgicos de nuestra área asistencial con los resultados que hubiéramos esperado asumiendo los datos epidemiológicos publicados por las oficinas gubernamentales.

Resultados: No registramos resultados positivos en las 2722 RT-PCR realizadas en nuestra área entre las semanas epidemiológicas 18 a 21, lo que implica una incidencia acumulada de nuevos casos tendente a cero. Asumiendo los datos epidemiológicos publicados, la proyección probabilística de individuos asintomáticos varió de $0.27 \times 10e^{-4}$ (datos oficiales de nuevos casos diagnosticados por PCR) a $4.69 \times 10e^{-4}$ si asumimos casos confirmados por IgG en nuestra provincia. Suponiendo una sensibilidad de RT-PCR del 95%, para obtener un resultado positivo, deberíamos realizar 38,461 y 2,028 pruebas respectivamente.

Conclusiones: En escenarios de muy baja prevalencia y a pesar de su alta sensibilidad, la detección preoperatoria de portadores asintomáticos mediante de RT-PCR es de una efectividad cuestionable. Nuestros hallazgos evidencian la dificultad de establecer modelos predictivos fiables en el contexto de epidemias de evolución rápida, como la pandemia de SARS CoV2.

Keywords: Asymptomatic patient; COVID-19; Coronavirus; Paciente asintomático; Periodo preoperatorio; Preoperative period; Prevalence; Prevalencia; SARS; Sensibilidad y especificidad; Sensitivity and specificity.

Copyright © 2020 Sociedad Española de Anestesiología, Reanimación y Terapéutica del Dolor. Publicado por Elsevier España, S.L.U. All rights reserved.

- 18 references
- 3 figures

Supplementary info

Publication types, MeSH terms Expand

Publication types

- Case Reports
- Observational Study

MeSH terms

- COVID-19 / diagnosis*
- COVID-19 / epidemiology
- COVID-19 Nucleic Acid Testing* / statistics & numerical data
- Carrier State / diagnosis*
- Carrier State / epidemiology
- Humans
- Incidence
- Pandemics*
- Preoperative Care*

- Prevalence
- Retrospective Studies
- SARS-CoV-2*
- Spain / epidemiology

Full text links

ELSEVIER FULL-TEXT ARTICLE Elsevier Science Free PMC article
Proceed to details
Cite
Share
☐ 461
Observational Study
Diabetes Metab Syndr
. 2022 Feb;16(2):102407.
doi: 10.1016/j.dsx.2022.102407. Epub 2022 Jan 19.

Effect of COVID 19 pneumonia on hyperglycemia: Is it different from non COVID pneumonia?

Daniel B Knox [1], Eliotte L Hirshberg [2], James Orme [2], Ithan Peltan [2], Michael J Lanspa [2]
Affiliations Expand

Affiliations

- [1] Division of Pulmonary and Critical Care Medicine, Intermountain Medical Center, Murray, UT, USA; Division of Pulmonary and Critical Care Medicine, University of Utah, Salt Lake City, UT, USA. Electronic address: dan.knox@imail.org.
- [2] Division of Pulmonary and Critical Care Medicine, Intermountain Medical Center, Murray, UT, USA; Division of Pulmonary and Critical Care Medicine, University of Utah, Salt Lake City, UT, USA.

- PMID: **35074624**
- PMCID: PMC8767985
- DOI: 10.1016/j.dsx.2022.102407

Free PMC article
Observational Study

Effect of COVID 19 pneumonia on hyperglycemia: Is it different from non COVID pneumonia?

Daniel B Knox et al. Diabetes Metab Syndr. 2022 Feb.
Free PMC article
Show details
Diabetes Metab Syndr
. 2022 Feb;16(2):102407.
doi: 10.1016/j.dsx.2022.102407. Epub 2022 Jan 19.

Authors

Daniel B Knox [1], Eliotte L Hirshberg [2], James Orme [2], Ithan Peltan [2], Michael J Lanspa [2]

Affiliations

- [1] Division of Pulmonary and Critical Care Medicine, Intermountain Medical Center, Murray, UT, USA; Division of Pulmonary and Critical Care Medicine, University of Utah, Salt Lake City, UT, USA. Electronic address: dan.knox@imail.org.
- [2] Division of Pulmonary and Critical Care Medicine, Intermountain Medical Center, Murray, UT, USA; Division of Pulmonary and Critical Care Medicine, University of Utah, Salt Lake City, UT, USA.

- PMID: **35074624**
- PMCID: PMC8767985
- DOI: 10.1016/j.dsx.2022.102407

Abstract

Background and aims: Glycemic control in critical illness has been linked to outcomes. We sought to investigate if COVID pneumonia was causing disrupted glycemic control compared to historically similar diseases.

Methods: At Intermountain Healthcare, a 23-hospital healthcare system in the intermountain west, we performed a multicenter, retrospective cohort observational study. We compared 13,268 hospitalized patients with COVID pneumonia to 6673 patients with non -COVID-pneumonia.

Results: Patients with COVID-19 were younger had fewer comorbidities, had lower mortality and greater length of hospital stay. Our regression models demonstrated that daily insulin dose, indexed for weight, was associated with COVID-19, age, diabetic status, HgbA1c, admission SOFA, ICU length of stay and receipt of corticosteroids. There was significant interaction between a diagnosis of diabetes and having COVID-19. Time in range for our IV insulin protocol was not correlated with having COVID after adjustment. It was correlated with ICU length of stay, diabetic control (HgbA1C) and prior history of diabetes. Among patients with subcutaneous (SQ) insulin only percent of glucose checks in range was correlated with diabetic status, having Covid-19, HgbA1c, total steroids given and Elixhauser comorbidity score even when controlled for other factors.

Conclusions: Hospitalized patients with COVID-19 pneumonia who receive insulin for glycemic control require both more SQ and IV insulin than the non-COVID-19 pneumonia counterparts. Patients with COVID-19 who received SQ insulin only had a lower percent of glucose checks in range.

Keywords: COVID-19; Critical illness; Glucose control; Pneumonia.

Copyright © 2022 Diabetes India. Published by Elsevier Ltd. All rights reserved.

Conflict of interest statement

Declaration of competing interest We have no conflicts of interest to disclose. All authors have a) contributed substantively to the conception, design, or analysis and interpretation of the data, b) contributed substantively to the drafting of the manuscript or critical revision for important intellectual content, c) given final approval of the version to be published, and d) agree to be accountable for all aspects of the work in ensuring that questions related to the accuracy or integrity of any part of the work are appropriately investigated and resolved. This study was approved by the Intermountain Healthcare IRB #1051342.

- 13 references

Supplementary info

Publication types, MeSH terms, Substances Expand

Publication types

- Comparative Study
- Multicenter Study
- Observational Study

MeSH terms

- Aged
- COVID-19 / blood
- COVID-19 / epidemiology*
- Cohort Studies
- Comorbidity
- Diabetes Mellitus / blood
- Diabetes Mellitus / drug therapy
- Diabetes Mellitus / epidemiology*
- Female
- Glycated Hemoglobin A / analysis
- Glycemic Control / methods
- Glycemic Control / statistics & numerical data*
- Hospitalization
- Humans

- Hyperglycemia / drug therapy
- Hyperglycemia / epidemiology*
- Insulin / administration & dosage
- Length of Stay
- Male
- Middle Aged
- Pneumonia / blood
- Pneumonia / epidemiology*
- Retrospective Studies
- SARS-CoV-2*

Substances

- Glycated Hemoglobin A
- Insulin

Full text links

ELSEVIER FULL-TEXT ARTICLE Elsevier Science Free PMC article

Proceed to details

Cite

Share

☐ 462

Observational Study

J Clin Virol
. 2020 Dec;133:104661.
doi: 10.1016/j.jcv.2020.104661. Epub 2020 Oct 14.

Factors associated with clinical outcomes in patients with Coronavirus Disease 2019 in Guangzhou, China

Chunliang Lei [1], Weiyin Lin [1], Xilong Deng [1], Fengyu Hu [1], Fengjuan Chen [1], Weiping Cai [1], Yueping Li [1], Chunyan Wen [1], Yujuan Guan [1], Jian Wang [1], Xiaoting Chen [1], Yi Cao [1], Feng Li [1], Xiaoping Tang [2], Linghua Li [3]

Affiliations Expand

Affiliations

- [1] Guangzhou Eighth People's Hospital, Guangzhou Medical University, Guangzhou, Guangdong, 510060, China.
- [2] Guangzhou Eighth People's Hospital, Guangzhou Medical University, Guangzhou, Guangdong, 510060, China. Electronic address: tangxiaopinggz@163.com.

- [3] Guangzhou Eighth People's Hospital, Guangzhou Medical University, Guangzhou, Guangdong, 510060, China. Electronic address: llheliza@126.com.

- PMID: **33096290**
- PMCID: PMC7554493
- DOI: 10.1016/j.jcv.2020.104661

Free PMC article
Observational Study

Factors associated with clinical outcomes in patients with Coronavirus Disease 2019 in Guangzhou, China

Chunliang Lei et al. J Clin Virol. 2020 Dec.
Free PMC article
Show details
J Clin Virol
. 2020 Dec;133:104661.
doi: 10.1016/j.jcv.2020.104661. Epub 2020 Oct 14.

Authors

Chunliang Lei [1], Weiyin Lin [1], Xilong Deng [1], Fengyu Hu [1], Fengjuan Chen [1], Weiping Cai [1], Yueping Li [1], Chunyan Wen [1], Yujuan Guan [1], Jian Wang [1], Xiaoting Chen [1], Yi Cao [1], Feng Li [1], Xiaoping Tang [2], Linghua Li [3]

Affiliations

- [1] Guangzhou Eighth People's Hospital, Guangzhou Medical University, Guangzhou, Guangdong, 510060, China.
- [2] Guangzhou Eighth People's Hospital, Guangzhou Medical University, Guangzhou, Guangdong, 510060, China. Electronic address: tangxiaopinggz@163.com.
- [3] Guangzhou Eighth People's Hospital, Guangzhou Medical University, Guangzhou, Guangdong, 510060, China. Electronic address: llheliza@126.com.

- PMID: **33096290**
- PMCID: PMC7554493
- DOI: 10.1016/j.jcv.2020.104661

Abstract

Background: Coronavirus Disease 2019 (COVID-19) is threatening billions of people. We described the clinical characteristics and explore virological and immunological factors associated with clinical outcomes.

Methods: 297 COVID-19 patients hospitalized in Guangzhou Eighth People's Hospital between January 20 and February 20, 2020 were included. Epidemiological, clinical and laboratory data were collected and analyzed. Severe Acute Respiratory Syndrome Coronavirus 2 (SARS-CoV-2) RNA in respiratory tract, blood samples and digestive tract was detected and lymphocyte subsets were tested periodically.

Result: Among the 297 patients (median age of 48 years), 154 (51.9 %) were female, 245 (82.5 %) mild/moderate cases, and 52 (17.5 %) severe/critical cases. 270 patients were detected for SARS-CoV-2 RNA in anal swabs and/or blood samples, and the overall positive rate was 23.0 % (62/270), higher in severe/critical cases than in mild/moderate cases (52.0 % vs. 16.4 %, P < 0.001). The CD4/CD8 ratio on admission was significantly higher in severe/critical cases than in mild/moderate cases (1.84 vs. 1.50, P = 0.022). During a median follow-up period of 17 days, 36 (12.1 %) patients were admitted to intensive care unit (ICU), 16 (5.4 %) patients developed respiratory failure and underwent mechanical ventilation, four (1.3 %) patients needed extracorporeal membrane oxygenation (ECMO), only one (0.34 %) patients died of multiple organ failure. Detectable SARS-CoV-2 RNA in anal swabs and/or blood samples, as well as higher CD4/CD8 ratio were independent risk factors of respiratory failure and ICU admission.

Conclusions: Most of COVID-19 patients in Guangzhou are mild/moderate, and presence of extrapulmonary virus and higher CD4/CD8 ratio are associated with higher risk of worse outcomes.

Keywords: CD4/CD8 ratio; COVID-19; Extrapulmonary; ICU admission; Respiratory failure; SARS-CoV-2; Virus.

Copyright © 2020 The Authors. Published by Elsevier B.V. All rights reserved.

Conflict of interest statement

The authors report no declarations of interest.

- Cited by 3 articles
- 23 references
- 2 figures

Supplementary info

Publication types, MeSH terms Expand

Publication types

- Observational Study
- Research Support, Non-U.S. Gov't

MeSH terms

- Adult
- CD4-CD8 Ratio
- COVID-19 / epidemiology*
- COVID-19 / mortality

- COVID-19 / therapy
- China
- Female
- Hospitalization / statistics & numerical data*
- Humans
- Intensive Care Units
- Male
- Middle Aged
- Respiration, Artificial / statistics & numerical data
- Retrospective Studies
- Risk Factors

Full text links

ELSEVIER OPEN ACCESS Elsevier Science Free PMC article
Proceed to details
Cite
Share
463
Observational Study
Crit Care Med
. 2021 May 1;49(5):804-815.
doi: 10.1097/CCM.0000000000004890.

Prevalence of Thrombotic Complications in ICU-Treated Patients With Coronavirus Disease 2019 Detected With Systematic CT Scanning

Saeed Mirsadraee [1, 2], Diana A Gorog [2, 3], Ciara F Mahon [1], Bhavin Rawal [1], Thomas R Semple [1, 2], Edward D Nicol [1, 2], Deepa R J Arachchillage [4], Anand Devaraj [1, 2], Susanna Price [1, 2], Sujal R Desai [1, 2], Carole A Ridge [1, 2], Suveer Singh [1, 2], Simon P G Padley [1, 2]
Affiliations Expand

Affiliations

- [1] Royal Brompton Hospital, London, United Kingdom.
- [2] National Heart and Lung Institute, Imperial College London, London, United Kingdom.
- [3] Lister Hospital, Coreys Mill Lane, Stevenage, United Kingdom.
- [4] Centre for Haematology, Imperial College London, London, United Kingdom.

- PMID: **33470780**

- DOI: 10.1097/CCM.0000000000004890

Observational Study

Prevalence of Thrombotic Complications in ICU-Treated Patients With Coronavirus Disease 2019 Detected With Systematic CT Scanning

Saeed Mirsadraee et al. Crit Care Med. 2021.

Show details

Crit Care Med

. 2021 May 1;49(5):804-815.
doi: 10.1097/CCM.0000000000004890.

Authors

Saeed Mirsadraee [1, 2], Diana A Gorog [2, 3], Ciara F Mahon [1], Bhavin Rawal [1], Thomas R Semple [1, 2], Edward D Nicol [1, 2], Deepa R J Arachchillage [4], Anand Devaraj [1, 2], Susanna Price [1, 2], Sujal R Desai [1, 2], Carole A Ridge [1, 2], Suveer Singh [1, 2], Simon P G Padley [1, 2]

Affiliations

- [1] Royal Brompton Hospital, London, United Kingdom.
- [2] National Heart and Lung Institute, Imperial College London, London, United Kingdom.
- [3] Lister Hospital, Coreys Mill Lane, Stevenage, United Kingdom.
- [4] Centre for Haematology, Imperial College London, London, United Kingdom.

- PMID: **33470780**
- DOI: 10.1097/CCM.0000000000004890

Abstract

Objectives: Severe coronavirus disease 2019 is associated with an extensive pneumonitis and frequent coagulopathy. We sought the true prevalence of thrombotic complications in critically ill patients with severe coronavirus disease 2019 on the ICU, with or without extracorporeal membrane oxygenation.

Design: We undertook a single-center, retrospective analysis of 72 critically ill patients with coronavirus disease 2019-associated acute respiratory distress syndrome admitted to ICU. CT angiography of the thorax, abdomen, and pelvis were performed at admission as per routine institution protocols, with further imaging as clinically indicated. The prevalence of thrombotic complications and the relationship with coagulation parameters, other biomarkers, and survival were evaluated.

Setting: Coronavirus disease 2019 ICUs at a specialist cardiorespiratory center.

Patients: Seventy-two consecutive patients with coronavirus disease 2019 admitted to ICU during the study period (March 19, 2020, to June 23, 2020).

Interventions: None.

Measurements and main results: All but one patient received thromboprophylaxis or therapeutic anticoagulation. Among 72 patients (male:female = 74%; mean age: 52 ± 10; 35 on extracorporeal membrane oxygenation), there were 54 thrombotic complications in 42 patients (58%), comprising 34 pulmonary arterial (47%), 15 peripheral venous (21%), and five (7%) systemic arterial thromboses/end-organ embolic complications. In those with pulmonary arterial thromboses, 93% were identified incidentally on first screening CT with only 7% suspected clinically. Biomarkers of coagulation (e.g., d-dimer, fibrinogen level, and activated partial thromboplastin time) or inflammation (WBC count, C-reactive protein) did not discriminate between patients with or without thrombotic complications. Fifty-one patients (76%) survived to discharge; 17 (24%) patients died. Mortality was significantly greater in patients with detectable thrombus (33% vs 10%; p = 0.022).

Conclusions: There is a high prevalence of thrombotic complications, mainly pulmonary, among coronavirus disease 2019 patients admitted to ICU, despite anticoagulation. Detection of thrombus was usually incidental, not predicted by coagulation or inflammatory biomarkers, and associated with increased risk of death. Systematic CT imaging at admission should be considered in all coronavirus disease 2019 patients requiring ICU.

Copyright © 2021 by the Society of Critical Care Medicine and Wolters Kluwer Health, Inc. All Rights Reserved.

Conflict of interest statement

Dr. Devaraj received funding from Boehringer-Ingelheim, Galapagos, Galecto Biotech, and GlaxoSmithKline. Dr. Desai received funding from Boehringer-Ingelheim. The remaining authors have disclosed that they do not have any potential conflicts of interest.

Comment in

- Optimal Management of Thrombotic Complications in Patients With Coronavirus Disease 2019.
  Imamura T. Imamura T. Crit Care Med. 2021 Nov 1;49(11):e1189. doi: 10.1097/CCM.0000000000005178. Crit Care Med. 2021. PMID: 34074855 Free PMC article. No abstract available.
- The authors reply.
  Singh S, Gorog DA, Mahon CF, Rawal B, Semple TR, Nicol ED, Arachchillage DRJ, Price S, Desai S, Ridge CA, Padley SPG, Mirsadraee S. Singh S, et al. Crit Care Med. 2021 Nov 1;49(11):e1190-e1191. doi: 10.1097/CCM.0000000000005259. Crit Care Med. 2021. PMID: 34369426 Free PMC article. No abstract available.

- Cited by 11 articles
- 27 references

Supplementary info

Publication types, MeSH terms Expand

Publication types

- Observational Study

MeSH terms

- Adult
- Aged
- COVID-19 / complications*
- COVID-19 / diagnostic imaging*
- Computed Tomography Angiography*
- Critical Illness*
- Female
- Humans
- Intensive Care Units
- Length of Stay / statistics & numerical data
- Male
- Middle Aged
- Mortality
- Patient Discharge / statistics & numerical data
- Prevalence
- Retrospective Studies
- SARS-CoV-2
- Thrombosis / diagnostic imaging*
- Thrombosis / etiology*

Full text links

Wolters Kluwer Wolters Kluwer
Proceed to details
Cite
Share
☐ 464
Observational Study
Sci Prog
. Jan-Mar 2022;105(1):368504221074574.
doi: 10.1177/00368504221074574.

Use of N-Acetylcysteine at high doses as an oral treatment for patients hospitalized with COVID-19

José Luis Izquierdo [1] [2], Joan B Soriano [3] [4] [5], Yolanda González [6], Sara Lumbreras [6] [7], Julio Ancochea [3] [4] [5], Christian Echeverry [6], José Miguel Rodríguez [1] [8]
Affiliations Expand

Affiliations

- [1] Department of Medicine and Medical Specialties, Universidad de Alcalá, Madrid, Spain.
- [2] Respiratory Medicine, Hospital Universitario de Guadalajara, Guadalajara, Spain.
- [3] Respiratory Medicine, 16517Hospital Universitario de La Princesa, Madrid, Spain.
- [4] Universidad Autónoma de Madrid, Madrid, Spain.
- [5] Respiratory Diseases Networking Biomedical Research Centre (CIBERES), Institute of Health Carlos III (ISCIII), Madrid, Spain.
- [6] SAVANA.
- [7] 16768Universidad Pontificia Comillas-IIT, Madrid, Spain.
- [8] Respiratory Medicine, 16269Hospital Universitario Príncipe de Asturias, Alcalá de Henares, Madrid, Spain.

- PMID: **35084258**
- PMCID: PMC8795755
- DOI: 10.1177/00368504221074574

Free PMC article
Observational Study

Use of N-Acetylcysteine at high doses as an oral treatment for patients hospitalized with COVID-19

José Luis Izquierdo et al. Sci Prog. Jan-Mar 2022.
Free PMC article
Show details
Sci Prog
. Jan-Mar 2022;105(1):368504221074574.
doi: 10.1177/00368504221074574.

Authors

José Luis Izquierdo [1] [2], Joan B Soriano [3] [4] [5], Yolanda González [6], Sara Lumbreras [6] [7], Julio Ancochea [3] [4] [5], Christian Echeverry [6], José Miguel Rodríguez [1] [8]

Affiliations

- [1] Department of Medicine and Medical Specialties, Universidad de Alcalá, Madrid, Spain.
- [2] Respiratory Medicine, Hospital Universitario de Guadalajara, Guadalajara, Spain.
- [3] Respiratory Medicine, 16517Hospital Universitario de La Princesa, Madrid, Spain.
- [4] Universidad Autónoma de Madrid, Madrid, Spain.
- [5] Respiratory Diseases Networking Biomedical Research Centre (CIBERES), Institute of Health Carlos III (ISCIII), Madrid, Spain.
- [6] SAVANA.
- [7] 16768Universidad Pontificia Comillas-IIT, Madrid, Spain.
- [8] Respiratory Medicine, 16269Hospital Universitario Príncipe de Asturias, Alcalá de Henares, Madrid, Spain.

- PMID: **35084258**
- PMCID: PMC8795755
- DOI: 10.1177/00368504221074574

Abstract

Infection by SARS-CoV-2 causing coronavirus disease 2019 (COVID-19) can be associated with serious and life-threatening conditions, including acute respiratory distress syndrome (ARDS). Severity and mortality have been related to a cytokine storm, an imbalance of oxidative stress, and a pro-thrombotic state.We conducted an observational retrospective cohort study from a community-based large population of hospitalized COVID-19 PCR + patients admitted from March 01, 2020, to January 24, 2021, with integrated primary to tertiary care information in Castilla la Mancha, Spain. We explored the potential benefits of the antioxidant, anti-inflammatory and anti-thrombotic drug N-acetylcysteine (NAC) administered orally in high doses (600 mg every 8 h), added to standard of care in COVID-19 patients by using the free text information contained in their electronic health records (EHRs).Out of 19,208 patients with a diagnosis of COVID-19 hospitalized, we studied 2071 (10.8%) users of oral NAC at high doses. COVID-19 patients treated with NAC were older, predominantly male, and with more comorbidities such as hypertension, dyslipidemia, diabetes, and COPD when compared with those not on NAC (all $p < 0.05$). Despite greater baseline risk, use of NAC in COVID-19 patients was associated with significantly lower mortality (OR 0.56; 95%CI 0.47-0.67), a finding that remained significant in a multivariate analysis adjusting by baseline characteristics and concomitant use of corticosteroids. There were no significant differences with the use of NAC on the mean duration of hospitalization, admission to the intensive care unit or use of invasive mechanical ventilation. The observed association signaling to better relevant outcomes in COVID-19 patients treated with NAC at high doses should be further explored in other settings and populations and in randomized controlled trials.

Keywords: COVID-19; N-acetylcysteine; mortality; treatment; use of health services.

Conflict of interest statement

Declaration of conflicting interests: The author(s) declared no potential conflicts of interest with respect to the research, authorship, and/or publication of this article.

- 33 references
- 1 figure

Supplementary info

Publication types, MeSH terms, Substances Expand

Publication types

- Observational Study

MeSH terms

- Acetylcysteine / therapeutic use
- COVID-19*
- Hospitalization
- Humans
- Male
- Retrospective Studies
- SARS-CoV-2
- Treatment Outcome

Substances

- Acetylcysteine

Full text links

SAGE journals Open access full text Atypon Free PMC article

Proceed to details

Cite

Share

465

Observational Study

Medicine (Baltimore)

. 2020 Dec 4;99(49):e22899.

doi: 10.1097/MD.0000000000022899.

Corona Virus Disease 2019 patients with different disease severity or age range: A single-center study of clinical features and prognosis

Lingyun Ren [1], Dan Yao, Zuowei Cui, Shanshan Chen, Hong Yan

Affiliations Expand

Affiliation

- [1] Department of Anesthesiology, The Central Hospital of Wuhan, Tongji Medical College, Huazhong University of Science and Technology, Wuhan, P.R. China.

- PMID: **33285678**
- PMCID: PMC7717834
- DOI: 10.1097/MD.0000000000022899

Free PMC article
Observational Study

Corona Virus Disease 2019 patients with different disease severity or age range: A single-center study of clinical features and prognosis

Lingyun Ren et al. Medicine (Baltimore). 2020.
Free PMC article
Show details
Medicine (Baltimore)
. 2020 Dec 4;99(49):e22899.
doi: 10.1097/MD.0000000000022899.

Authors

Lingyun Ren [1], Dan Yao, Zuowei Cui, Shanshan Chen, Hong Yan

Affiliation

- [1] Department of Anesthesiology, The Central Hospital of Wuhan, Tongji Medical College, Huazhong University of Science and Technology, Wuhan, P.R. China.

- PMID: **33285678**
- PMCID: PMC7717834
- DOI: 10.1097/MD.0000000000022899

Abstract

This study aimed to describe clinical characteristics and prognosis of Corona Virus Disease 2019 (COVID-19) patients, and to compare these features among COVID-19 patients with different disease severity or age range.Totally, 129 COVID-19 patients were retrospectively enrolled, and the information about demographics, comorbidities, medical histories, clinical symptoms, and laboratory findings at the time of hospital admission were collected. Meanwhile, their clinical

outcomes were recorded. According to the fourth version of the guidelines on the Diagnosis and Treatment of COVID-19 by the National Health Commission of China, patients were divided into subgroups according to disease severity (moderate and severe/critical) or age (<40 years, 40-64 years and ≥65 years).In total patients, the most common clinical symptoms were fever and cough (all incidences over 50%). Other common clinical symptoms included tiredness/anorexia, shortness of breath, dyspnea, aching pain, expectoration, diarrhea, shivering, and nausea/vomiting. The mortality rate was 5.4%, and the median value of hospital stay was 16.0 (11.0-23.0) days. Subgroup analyses disclosed that severe/critical patients exhibited increased neutrophil count, neutrophils, C-reactive protein, calcitonin, alpha-hydroxybutyric dehydrogenase, lactate dehydrogenase, aspartate aminotransferase, gamma-glutamyl transferase, creatinine, and D-dimer levels, and more deaths compared with that in moderate patients. Regarding age, it correlated with more common fever, higher levels of red blood cell, neutrophil count, lymphocyte count, neutrophils, red cell volume distribution width standard deviation-coefficient of variation, calcitonin, alpha-hydroxybutyric dehydrogenase, Creatine Kinase, aspartate aminotransferase, gamma-glutamyl transferase, and D-dimer, raised death rate and prolonged hospital stay.Our findings provide valuable evidence regarding clinical characteristics and prognosis of COVID-19 patients to help with the understanding of the disease and prognosis improvement.

Conflict of interest statement

The authors have no conflicts of interest to disclose.

- Cited by 2 articles
- 19 references

Supplementary info

Publication types, MeSH terms Expand

Publication types

- Observational Study

MeSH terms

- Age Distribution
- COVID-19 / epidemiology*
- COVID-19 / immunology
- COVID-19 / mortality
- COVID-19 / physiopathology*
- Comorbidity
- Female
- Humans
- Length of Stay
- Male
- Prognosis
- Retrospective Studies

- SARS-CoV-2
- Severity of Illness Index
- Sex Distribution
- Socioeconomic Factors

Full text links

Wolters Kluwer Wolters Kluwer Free PMC article
Proceed to details
Cite
Share
466
Observational Study

Healthc (Amst)
. 2022 Mar;10(1):100611.
doi: 10.1016/j.hjdsi.2021.100611. Epub 2021 Dec 22.

Association of community-level social vulnerability with US acute care hospital intensive care unit capacity during COVID-19

Thomas C Tsai [1], Benjamin H Jacobson [2], E John Orav [3], Ashish K Jha [4]
Affiliations Expand

Affiliations

- [1] Department of Health Policy and Management, Harvard T.H. Chan School of Public Health, Boston, MA, USA; Department of Surgery, Brigham and Women's Hospital, Boston, MA, USA. Electronic address: ttsai@bwh.harvard.edu.
- [2] Department of Health Policy and Management, Harvard T.H. Chan School of Public Health, Boston, MA, USA.
- [3] Division of General Internal Medicine, Brigham and Women's Hospital, Boston, MA, USA.
- [4] Brown University School of Public Health, Providence, RI, USA.

- PMID: **34979442**
- PMCID: PMC8692088
- DOI: 10.1016/j.hjdsi.2021.100611

Free PMC article
Observational Study

Association of community-level social vulnerability with US acute care hospital intensive care unit capacity during COVID-19

Thomas C Tsai et al. Healthc (Amst). 2022 Mar.
Free PMC article
Show details
Healthc (Amst)
. 2022 Mar;10(1):100611.
doi: 10.1016/j.hjdsi.2021.100611. Epub 2021 Dec 22.

Authors

Thomas C Tsai [1], Benjamin H Jacobson [2], E John Orav [3], Ashish K Jha [4]

Affiliations

- [1] Department of Health Policy and Management, Harvard T.H. Chan School of Public Health, Boston, MA, USA; Department of Surgery, Brigham and Women's Hospital, Boston, MA, USA. Electronic address: ttsai@bwh.harvard.edu.
- [2] Department of Health Policy and Management, Harvard T.H. Chan School of Public Health, Boston, MA, USA.
- [3] Division of General Internal Medicine, Brigham and Women's Hospital, Boston, MA, USA.
- [4] Brown University School of Public Health, Providence, RI, USA.

- PMID: **34979442**
- PMCID: PMC8692088
- DOI: 10.1016/j.hjdsi.2021.100611

Abstract

The COVID-19 pandemic has placed unprecedented stress on US acute care hospitals, leading to overburdened ICUs. It remains unknown if increased COVID-19 ICU occupancy is crowding out non-COVID-related care and whether hospitals in vulnerable communities may be more susceptible to ICUs reaching capacity. Using facility-level hospitalization data, we conducted a retrospective observational cohort study of 1753 US acute care hospitals reporting to the US Department of Health and Human Services Protect database from September 4, 2020 to February 25, 2021. 63% of hospitals reached critical ICU capacity for at least two weeks during the study period, and the surge of COVID-19 cases appeared to be crowding out non-COVID-19-related intensive care needs. Hospitals in the South (OR = 3.31, 95% CI OR 2.31-4.78) and West (OR = 2.28, 95% CI OR 1.51-3.46) were more likely to reach critical capacity than those in the Northeast, and hospitals in areas with the highest social vulnerability were more than twice as likely to reach capacity as those in the least vulnerable areas (OR = 2.15, 95% CI OR 1.41-3.29). The association between social vulnerability and critical ICU capacity highlights underlying structural inequities in health care access and provides an opportunity for policymakers to take action to prevent strained ICU capacity from compounding COVID-19 inequities.

Keywords: COVID-19; Critical care; Healthcare equity; Healthcare supply.

Copyright © 2021 Elsevier Inc. All rights reserved.

Conflict of interest statement

The authors declare that they have no known competing financial interests or personal relationships that could have appeared to influence the work reported in this paper.

- 20 references
- 2 figures

Supplementary info

Publication types, MeSH terms Expand

Publication types

- Observational Study

MeSH terms

- COVID-19*
- Hospitals
- Humans
- Intensive Care Units
- Pandemics / prevention & control
- Retrospective Studies
- SARS-CoV-2
- Social Vulnerability

Full text links

ELSEVIER FULL-TEXT ARTICLE Elsevier Science Free PMC article

Proceed to details

Cite

Share

☐ 467

Observational Study

Acta Med Okayama
. 2020 Dec;74(6):513-520.
doi: 10.18926/AMO/61210.

Delay in Emergency Medical Service Transportation Responsiveness during the

COVID-19 Pandemic in a Minimally Affected Region

Kohei Ageta [1], Hiromichi Naito [1], Takashi Yorifuji [2], Takafumi Obara [1], Tsuyoshi Nojima [1], Taihei Yamada [1], Kohei Tsukahara [1], Hiromasa Yakushiji [1], Atsunori Nakao [1]
Affiliations Expand

Affiliations

- [1] Department of Emergency, Critical Care and Disaster Medicine, Okayama University Graduate School of Medicine, Dentistry and Pharmaceutical Sciences.
- [2] Department of Epidemiology, Okayama University Graduate School of Medicine, Dentistry and Pharmaceutical Sciences.

- PMID: **33361871**
- DOI: 10.18926/AMO/61210

Free article
Observational Study

Delay in Emergency Medical Service Transportation Responsiveness during the COVID-19 Pandemic in a Minimally Affected Region

Kohei Ageta et al. Acta Med Okayama. 2020 Dec.
Free article
Show details
Acta Med Okayama
. 2020 Dec;74(6):513-520.
doi: 10.18926/AMO/61210.

Authors

Kohei Ageta [1], Hiromichi Naito [1], Takashi Yorifuji [2], Takafumi Obara [1], Tsuyoshi Nojima [1], Taihei Yamada [1], Kohei Tsukahara [1], Hiromasa Yakushiji [1], Atsunori Nakao [1]

Affiliations

- [1] Department of Emergency, Critical Care and Disaster Medicine, Okayama University Graduate School of Medicine, Dentistry and Pharmaceutical Sciences.
- [2] Department of Epidemiology, Okayama University Graduate School of Medicine, Dentistry and Pharmaceutical Sciences.

- PMID: **33361871**
- DOI: 10.18926/AMO/61210

Abstract

Few studies have investigated the influence of the Coronavirus Disease 2019 (COVID-19) pandemic on emer-gency medical service (EMS) systems, especially in areas less affected or unaffected by COVID-19. In this study, we investigated changes in prehospital EMS activity and transport times during the COVID-19 pandemic. All patients transported by EMS in the city of Okayama from March-May 2019 or March-May 2020 were included. Interfacility transports were excluded. The primary outcome was the time from a patient's first emergency call until hospital arrival (total prehospital time). Secondary outcomes included three segments of total prehospital time: the response time, on-scene time, and transportation time. Total prehospital time and the durations of each segment were compared between corresponding months in 2020 (COVID19-affected) and 2019 (control). The results showed that total prehospital times in April 2020 were significantly higher than those in 2019 (33.8 ± 11.6 vs. 32.2 ± 10.8 min, $p < 0.001$). Increases in total prehospital time were caused by longer response time (9.3 ± 3.8 vs. 8.7 ± 3.7 min, $p < 0.001$) and on-scene time (14.4 ± 7.9 vs. 13.5 ± 6.2min, $p < 0.001$). The COVID-19 pandemic was thus shown to affect EMS and delayed arrival/response even in a minimally affected region. A system to minimize transportation delays should be developed for emerging pandemics.

Keywords: coronavirus; emergency medical services; emergency transport; health care system; infection.

Conflict of interest statement

No potential conflict of interest relevant to this article was reported.

- Cited by 5 articles
- 14 references

Supplementary info

Publication types, MeSH terms Expand

Publication types

- Observational Study

MeSH terms

- Adult
- Aged
- Aged, 80 and over
- COVID-19 / epidemiology*
- Emergency Medical Services*
- Female
- Humans

- Male
- Middle Aged
- Retrospective Studies
- SARS-CoV-2*
- Time Factors
- Transportation of Patients*

Full text links

ACTA MED OKAYAMA FULL-TEXT ARTICLE Okayama University Medical School

Proceed to details

Cite

Share

☐ 468

Observational Study

Indian J Ophthalmol
. 2022 Mar;70(3):1000-1006.
doi: 10.4103/ijo.IJO_2735_21.

An analysis of the clinical profile of patients with uveitis following COVID-19 infection

Sudha K Ganesh [1], Amanda Mohanan-Earatt [1]
Affiliations Expand

Affiliation

- [1] Department of Uveitis, Medical Research Foundation, Sankara Nethralaya, Chennai, Tamil Nadu, India.

- PMID: **35225561**
- DOI: 10.4103/ijo.IJO_2735_21

Observational Study

An analysis of the clinical profile of patients with uveitis following COVID-19 infection

Sudha K Ganesh et al. Indian J Ophthalmol. 2022 Mar.

Show details

Indian J Ophthalmol
. 2022 Mar;70(3):1000-1006.
doi: 10.4103/ijo.IJO_2735_21.

Authors

Sudha K Ganesh [1], Amanda Mohanan-Earatt [1]

Affiliation

- [1] Department of Uveitis, Medical Research Foundation, Sankara Nethralaya, Chennai, Tamil Nadu, India.

- PMID: **35225561**
- DOI: 10.4103/ijo.IJO_2735_21

Abstract

Purpose: To describe the clinical profile of patients presenting with uveitis following COVID-19 infection at a tertiary care eye hospital in South India.

Methods: In this retrospective chart review, all consecutive cases presenting with an acute episode of intraocular inflammation and a history of COVID-19 infection diagnosed within the preceding 6 weeks, between March 2020 and September 2021, were included. Data retrieved and analyzed included age, sex, laterality of uveitis, and site of inflammation. The diagnosis was categorized based on the SUN working group classification criteria for uveitis. Details regarding clinical features, investigations, ophthalmic treatment given, response to treatment, ocular complications, and status at last visit were also accessed. Statistical analysis of demographical data was done using Microsoft Excel 2019.

Results: Twenty-one eyes of 13 patients were included in this hospital-based retrospective observational study. The study included six male and seven female patients. The mean age was 38 ± 16.8 years. Eight patients had bilateral involvement. Seven patients were diagnosed with anterior uveitis, three with intermediate uveitis, one with posterior uveitis, and two with panuveitis. All patients responded well to treatment and were doing well at their last visit. Two patients had complications that necessitated surgical treatment, following which they recovered good visual outcomes.

Conclusion: With prompt diagnosis and appropriate management, all the patients with uveitis post-COVID-19 infection recovered with good visual outcomes. Thus, ophthalmologists must be aware of the possible uveitic manifestations following even uneventful COVID-19 infection.

Keywords: Anterior uveitis; COVID-19; intermediate uveitis; neuroretinitis; panuveitis; uveitis.

Conflict of interest statement

None

Supplementary info

Publication types, MeSH terms Expand

Publication types

- Observational Study

MeSH terms

- Adult
- COVID-19* / complications
- Female
- Humans
- Male
- Middle Aged
- Panuveitis*
- Retrospective Studies
- SARS-CoV-2
- Uveitis* / diagnosis
- Uveitis* / drug therapy
- Uveitis* / etiology
- Young Adult

Proceed to details
Cite
Share
☐ 469
Observational Study

Ital J Pediatr
. 2022 Feb 5;48(1):23.
doi: 10.1186/s13052-022-01213-w.

Sociodemographic and clinical characteristics of paediatric patients admitted to a neuropsychiatric care hospital in the COVID-19 era

Michela Gatta [1], Alessia Raffagnato [2], Federica Mason [2], Rachele Fasolato [2], Annalisa Traverso [2], Silvia Zanato [2], Marina Miscioscia [2] [3]
Affiliations Expand

Affiliations

- [1] Child and Adolescent Neuropsychiatric Unit, Department of Women's and Children's Health, University Hospital of Padua, 35128, Padua, Italy. michela.gatta@unipd.it.
- [2] Child and Adolescent Neuropsychiatric Unit, Department of Women's and Children's Health, University Hospital of Padua, 35128, Padua, Italy.

- [3] Department of Developmental Psychology and Socialisation, University of Padua, 35131, Padua, Italy.

- PMID: **35123540**
- PMCID: PMC8817472
- DOI: 10.1186/s13052-022-01213-w

Free PMC article
Observational Study

Sociodemographic and clinical characteristics of paediatric patients admitted to a neuropsychiatric care hospital in the COVID-19 era

Michela Gatta et al. Ital J Pediatr. 2022.
Free PMC article
Show details
Ital J Pediatr
. 2022 Feb 5;48(1):23.
doi: 10.1186/s13052-022-01213-w.

Authors

Michela Gatta [1], Alessia Raffagnato [2], Federica Mason [2], Rachele Fasolato [2], Annalisa Traverso [2], Silvia Zanato [2], Marina Miscioscia [2] [3]

Affiliations

- [1] Child and Adolescent Neuropsychiatric Unit, Department of Women's and Children's Health, University Hospital of Padua, 35128, Padua, Italy. michela.gatta@unipd.it.
- [2] Child and Adolescent Neuropsychiatric Unit, Department of Women's and Children's Health, University Hospital of Padua, 35128, Padua, Italy.
- [3] Department of Developmental Psychology and Socialisation, University of Padua, 35131, Padua, Italy.

- PMID: **35123540**
- PMCID: PMC8817472
- DOI: 10.1186/s13052-022-01213-w

Abstract

Background: Since the first months of 2020, Italy and the world have been facing the COVID-19 pandemic. In addition to the dangerous and potentially deadly effects on physical health, it has caused a radical change in the lifestyle of the population and a potential danger for mental health

too. These events were inserted into the context of a growing epidemiological trend regarding children's psychiatric disorders in the past decade.

Aim: To study the population of patients admitted to a Neuropsychiatric Hospital Unit of North Italy in the first COVID-19 year, comparing them with the population of patients hospitalised during the year immediately before, according to sociodemographic and clinical variables.

Methodology: The study is an observational retrospective cohort. In total, 198 patients hospitalised due to neuropsychiatric problems from February 2019 to March 2021 were recruited. Data were analysed through mean and standard deviation, t-test, percentages, chi square test, and the Fischer exact test.

Results: Risk factors associated with mental health disorders were similar between the two years. The hospitalisation modality showed a decrease in scheduled hospitalisations compared to urgent ones, and among the reasons that led patients to hospitalisation there was a conspicuous increase in eating disorders. More suicidal and self-harming behaviours occurred in the COVID-19 group too, compared to the previous year. The methods used to attempt suicide were changed considerably, with a prevalence of that attempted within the home. Changes in pharmacological therapies also occurred, necessary for more than 80% of inpatients during the COVID year, with a greater use of neuroleptics. There were alarming data about hospitalisation relapses, which increased from 12.2% in the pre-COVID year to 35.0% in the COVID year.

Conclusion: Data shed light on clinical and policy issues in mental health care during the developmental age. Since the COVID-19 health emergency is not yet over, and its effects, especially on mental health, will be long-term, it is necessary to implement services and activities dedicated to both primary and secondary prevention of neuropsychiatric diseases especially during adolescent ages.

Keywords: Adolescents; COVID-19; Children; Hospitalisation; Mental health; Neuropsychiatry; Paediatric age.

© 2022. The Author(s).

Conflict of interest statement

The authors declare that they have no competing interests.

- 57 references
- 1 figure

Supplementary info

Publication types, MeSH terms Expand

Publication types

- Observational Study

MeSH terms

- Adolescent

- COVID-19*
- Child
- Hospitalization
- Hospitals
- Humans
- Pandemics
- Retrospective Studies
- SARS-CoV-2

Full text links

Read free full text at BMC BioMed Central Free PMC article
Proceed to details
Cite
Share
470
Observational Study
Rom J Intern Med
. 2022 Mar 17;60(1):49-55.
doi: 10.2478/rjim-2021-0027. Print 2022 Mar 1.

Characteristics and outcomes of patients with COVID-19 and liver injury: a retrospective analysis and a multicenter experience

Andrei Voiosu [1, 2], Adina Roman [3, 4], Ruxandra Pop [1], Alina Boeriu [3, 4], Cristiana Popp [5, 2], Sabina Zurac [5, 2], Theodor Voiosu [1, 2], Daniela Dobru [3, 4], Bogdan Mateescu [1, 2]
Affiliations Expand

Affiliations

- [1] Gastroenterology Department, Colentina Clinical Hospital, Bucharest, Romania.
- [2] Carol Davila University of Medicine and Pharmacy, Bucharest, Romania.
- [3] Gastroenterology Department, Mureș County Clinical Hospital, Romania.
- [4] University of Medicine, Pharmacy Sciences and Technology "George Emil Palade" Târgu Mureș, Romania.
- [5] Pathology Department, Colentina Clinical Hospital, Bucharest, Romania.

- PMID: **34253002**
- DOI: 10.2478/rjim-2021-0027

Free article
Observational Study

Characteristics and outcomes of patients with COVID-19 and liver injury: a retrospective analysis and a multicenter experience

Andrei Voiosu et al. Rom J Intern Med. 2022.
Free article
Show details
Rom J Intern Med
. 2022 Mar 17;60(1):49-55.
doi: 10.2478/rjim-2021-0027. Print 2022 Mar 1.

Authors

Andrei Voiosu [1, 2], Adina Roman [3, 4], Ruxandra Pop [1], Alina Boeriu [3, 4], Cristiana Popp [5, 2], Sabina Zurac [5, 2], Theodor Voiosu [1, 2], Daniela Dobru [3, 4], Bogdan Mateescu [1, 2]

Affiliations

- [1] Gastroenterology Department, Colentina Clinical Hospital, Bucharest, Romania.
- [2] Carol Davila University of Medicine and Pharmacy, Bucharest, Romania.
- [3] Gastroenterology Department, Mureș County Clinical Hospital, Romania.
- [4] University of Medicine, Pharmacy Sciences and Technology "George Emil Palade" Târgu Mureș, Romania.
- [5] Pathology Department, Colentina Clinical Hospital, Bucharest, Romania.

- PMID: **34253002**
- DOI: 10.2478/rjim-2021-0027

Abstract

Background and aims. Patients with COVID-19 frequently present abnormal elevated liver function tests of unknown clinical significance. We aimed to investigate the characteristics and factors influencing outcome in patients with confirmed SARS-CoV-2 infection and liver injury on admission.**Methods.** This is a retrospective observational study of patients hospitalized in two COVID units in Romania. Relevant data on clinical and laboratory parameters and medication administered during the admission were analyzed to identify predictors of a negative outcome. Patients with confirmed COVID-19 and liver function tests (LFTs) above the upper limit of normal were included in the analysis.**Results.** From 1,207 patients, we identified 134 patients (11%) with abnormal LFTs during hospitalization. The majority of patients had mildly elevated levels and a predominantly cholestatic pattern of liver injury. Patients who received lopinavir/ritonavir were more likely to have increased ALAT levels ($p<0.0001$). Sixteen patients had pre-existing chronic liver disease, and they were more likely to suffer from severe COVID-19 (p=0.009) and have a negative outcome (p<0.001), but on multivariate analysis, only the severity of COVID-19 was predictive of death (OR 69.9; 95% CI 6.4-761.4).**Conclusions.** Mild liver injury is relatively common in COVID-19 and possibly influenced by medication. Patients with chronic liver disease are at high risk for negative outcome, but the severity of the infection is the only predictor of death.

Keywords: COVID-19; SARS-CoV-2; antiviral therapy; drug-induced liver injury; hepatitis; hydroxychloroquine; liver injury; lopinavir/ritonavir.

© 2022 Andrei Voiosu et al., published by Sciendo.

- Cited by 1 article
- 18 references

Supplementary info

Publication types, MeSH terms, Substances Expand

Publication types

- Multicenter Study
- Observational Study

MeSH terms

- Antiviral Agents / therapeutic use
- COVID-19* / complications
- Humans
- Liver
- Retrospective Studies
- SARS-CoV-2

Substances

- Antiviral Agents

Full text links

sciendo De Gruyter
Proceed to details
Cite
Share
☐ 471
Observational Study
J Clin Virol
. 2022 Jan;146:105031.
doi: 10.1016/j.jcv.2021.105031. Epub 2021 Nov 23.

Wave comparisons of clinical characteristics and outcomes of COVID-19 admissions -

Exploring the impact of treatment and strain dynamics

Anna Freeman [1], Alastair Watson [2], Paul O'Regan [3], Oskar Wysocki [4], Hannah Burke [2], Andre Freitas [5], Robert Livingstone [2], Ahilanadan Dushianthan [6], Michael Celinski [2], James Batchelor [7], Hang Phan [8], Florina Borca [9], Paul Fitzpatrick [3], Donal Landers [3], Tom Ma Wilkinson [2]
Affiliations Expand

Affiliations

- [1] Faculty of Medicine, University of Southampton, United Kingdom; University Hospitals Southampton NHS Foundation Trust, United Kingdom. Electronic address: a.freeman@soton.ac.uk.
- [2] Faculty of Medicine, University of Southampton, United Kingdom; University Hospitals Southampton NHS Foundation Trust, United Kingdom.
- [3] Digital Experimental Cancer Medicine Team, Cancer Biomarker Centre, Cancer Research UK Manchester Institute, The University of Manchester, United Kingdom.
- [4] Digital Experimental Cancer Medicine Team, Cancer Biomarker Centre, Cancer Research UK Manchester Institute, The University of Manchester, United Kingdom; Department of Computer Science, The University of Manchester, United Kingdom.
- [5] Digital Experimental Cancer Medicine Team, Cancer Biomarker Centre, Cancer Research UK Manchester Institute, The University of Manchester, United Kingdom; Department of Computer Science, The University of Manchester, United Kingdom; Idiap Research Institute, Switzerland.
- [6] Faculty of Medicine, University of Southampton, United Kingdom; University Hospitals Southampton NHS Foundation Trust, United Kingdom; NIHR Southampton Biomedical Research Centre, University Hospital Southampton NHS Foundation Trust, United Kingdom.
- [7] Faculty of Medicine, University of Southampton, United Kingdom; University Hospitals Southampton NHS Foundation Trust, United Kingdom; Institute for Life Sciences, University of Southampton, United Kingdom; Clinical Informatics Research Unit Faculty of Medicine, University of Southampton,United Kingdom.
- [8] Faculty of Medicine, University of Southampton, United Kingdom; Clinical Informatics Research Unit Faculty of Medicine, University of Southampton,United Kingdom; NIHR Southampton Biomedical Research Centre, University Hospital Southampton NHS Foundation Trust, United Kingdom.
- [9] Faculty of Medicine, University of Southampton, United Kingdom; University Hospitals Southampton NHS Foundation Trust, United Kingdom; Clinical Informatics Research Unit Faculty of Medicine, University of Southampton,United Kingdom; NIHR Southampton Biomedical Research Centre, University Hospital Southampton NHS Foundation Trust, United Kingdom.

- PMID: **34844145**
- PMCID: PMC8608665
- DOI: 10.1016/j.jcv.2021.105031

Free PMC article
Observational Study

Wave comparisons of clinical characteristics and outcomes of COVID-19 admissions - Exploring the impact of treatment and strain dynamics

Anna Freeman et al. J Clin Virol. 2022 Jan.
Free PMC article
Show details
J Clin Virol
. 2022 Jan;146:105031.
doi: 10.1016/j.jcv.2021.105031. Epub 2021 Nov 23.

Authors

Anna Freeman [1], Alastair Watson [2], Paul O'Regan [3], Oskar Wysocki [4], Hannah Burke [2], Andre Freitas [5], Robert Livingstone [2], Ahilanadan Dushianthan [6], Michael Celinski [2], James Batchelor [7], Hang Phan [8], Florina Borca [9], Paul Fitzpatrick [3], Donal Landers [3], Tom Ma Wilkinson [2]

Affiliations

- [1] Faculty of Medicine, University of Southampton, United Kingdom; University Hospitals Southampton NHS Foundation Trust, United Kingdom. Electronic address: a.freeman@soton.ac.uk.
- [2] Faculty of Medicine, University of Southampton, United Kingdom; University Hospitals Southampton NHS Foundation Trust, United Kingdom.
- [3] Digital Experimental Cancer Medicine Team, Cancer Biomarker Centre, Cancer Research UK Manchester Institute, The University of Manchester, United Kingdom.
- [4] Digital Experimental Cancer Medicine Team, Cancer Biomarker Centre, Cancer Research UK Manchester Institute, The University of Manchester, United Kingdom; Department of Computer Science, The University of Manchester, United Kingdom.
- [5] Digital Experimental Cancer Medicine Team, Cancer Biomarker Centre, Cancer Research UK Manchester Institute, The University of Manchester, United Kingdom; Department of Computer Science, The University of Manchester, United Kingdom; Idiap Research Institute, Switzerland.
- [6] Faculty of Medicine, University of Southampton, United Kingdom; University Hospitals Southampton NHS Foundation Trust, United Kingdom; NIHR Southampton Biomedical Research Centre, University Hospital Southampton NHS Foundation Trust, United Kingdom.
- [7] Faculty of Medicine, University of Southampton, United Kingdom; University Hospitals Southampton NHS Foundation Trust, United Kingdom; Institute for Life Sciences, University of Southampton, United Kingdom; Clinical Informatics Research Unit Faculty of Medicine, University of Southampton,United Kingdom.
- [8] Faculty of Medicine, University of Southampton, United Kingdom; Clinical Informatics Research Unit Faculty of Medicine, University of Southampton,United Kingdom; NIHR Southampton Biomedical Research Centre, University Hospital Southampton NHS Foundation Trust, United Kingdom.

- [9] Faculty of Medicine, University of Southampton, United Kingdom; University Hospitals Southampton NHS Foundation Trust, United Kingdom; Clinical Informatics Research Unit Faculty of Medicine, University of Southampton,United Kingdom; NIHR Southampton Biomedical Research Centre, University Hospital Southampton NHS Foundation Trust, United Kingdom.

- PMID: **34844145**
- PMCID: PMC8608665
- DOI: 10.1016/j.jcv.2021.105031

Abstract

Objectives: Dexamethasone has now been incorporated into the standard of care for COVID-19 hospital patients. However, larger intensive care unit studies have failed to show discernible improvements in mortality in the recent wave. We aimed to investigate the impacts of these factors on disease outcomes in a UK hospital study.

Methods: This retrospective observational study reports patient characteristics, interventions and outcomes in COVID-19 patients from a UK teaching hospital; cohort 1, pre 16th June-2020 (pre-dexamethasone); cohort 2, 17th June to 30th November-2020 (post-dexamethasone, pre-VOC 202,012/01 as dominant strain); cohort 3, 1st December-2020 to 3rd March-2021 (during establishment of VOC202012/01 as the dominant strain).

Results: Dexamethasone treatment was more common in cohorts 2 and 3 (42.7% and 51.6%) compared with cohort 1 (2.5%). After adjusting for risk, odds of death within 28 days were 2-fold lower in cohort 2 vs 1 (OR:0.47,[0.27,0.79],p = 0.006). Mortality was higher cohort 3 vs 2 (20% vs 14%); but not significantly different to cohort 1 (OR: 0.86,[0.64, 1.15],p = 0.308).

Conclusions: The real world finding of lower mortality following dexamethasone supports the published trial evidence and highlights ongoing need for research with introduction of new treatments and ongoing concern over new COVID-19 variants.

Keywords: COVID-19 variants; COVID-19 waves; Dexamethasone.

Copyright © 2021. Published by Elsevier B.V.

- Cited by 2 articles
- 29 references
- 2 figures

Supplementary info

Publication types, MeSH terms, Substances, Supplementary concepts Expand

Publication types

- Observational Study

MeSH terms

- COVID-19* / drug therapy
- COVID-19* / epidemiology
- Dexamethasone / therapeutic use
- Hospitalization / statistics & numerical data
- Hospitals, Teaching
- Humans
- Intensive Care Units
- SARS-CoV-2
- United Kingdom / epidemiology

Substances

- Dexamethasone

Supplementary concepts

- SARS-CoV-2 variants

Full text links

ELSEVIER FULL-TEXT ARTICLE Elsevier Science Free PMC article

Proceed to details

Cite

Share

☐ 472

Observational Study

Gastroenterology

. 2020 Dec;159(6):2226-2228.e2.

doi: 10.1053/j.gastro.2020.08.044. Epub 2020 Aug 26.

Prevalence, Risk Factors, and Outcomes of Hospitalized Patients With Coronavirus Disease 2019 Presenting as Acute Pancreatitis

Sumant Inamdar [1], Petros C Benias [2], Yan Liu [3], Divyesh V Sejpal [4], Sanjaya K Satapathy [5], Arvind J Trindade [6], Northwell COVID-19 Research Consortium

Affiliations Expand

Affiliations

- [1] Division of Gastroenterology, Department of Medicine, University of Arkansas for Medical Sciences, Little Rock, Arkansas.

- [2] Division of Gastroenterology, Lenox Hill Hospital, Zucker School of Medicine at Hofstra/Northwell, Northwell Health System, New York, New York.
- [3] Feinstein Institutes for Medical Research, Northwell Health, Manhasset, New York.
- [4] Division of Gastroenterology, North Shore University Hospital, Zucker School of Medicine at Hofstra/Northwell, Northwell Health System, Manhasset, New York.
- [5] Division of Hepatology, North Shore University Hospital, Zucker School of Medicine at Hofstra/Northwell, Northwell Health System, New York, New York.
- [6] Institute of Health Innovations and Outcomes Research, Feinstein Institutes for Medical Research, Northwell Health, Manhasset, New York; Division of Gastroenterology, Long Island Jewish Medical Center Zucker School of Medicine at Hofstra/Northwell, Northwell Health System, New Hyde Park, New York. Electronic address: arvind.trindade@gmail.com.

- PMID: **32860787**
- PMCID: PMC7448741
- DOI: 10.1053/j.gastro.2020.08.044

Free PMC article
Observational Study

Prevalence, Risk Factors, and Outcomes of Hospitalized Patients With Coronavirus Disease 2019 Presenting as Acute Pancreatitis

Sumant Inamdar et al. Gastroenterology. 2020 Dec.
Free PMC article
Show details
Gastroenterology
. 2020 Dec;159(6):2226-2228.e2.
doi: 10.1053/j.gastro.2020.08.044. Epub 2020 Aug 26.

Authors

Sumant Inamdar [1], Petros C Benias [2], Yan Liu [3], Divyesh V Sejpal [4], Sanjaya K Satapathy [5], Arvind J Trindade [6], Northwell COVID-19 Research Consortium

Affiliations

- [1] Division of Gastroenterology, Department of Medicine, University of Arkansas for Medical Sciences, Little Rock, Arkansas.
- [2] Division of Gastroenterology, Lenox Hill Hospital, Zucker School of Medicine at Hofstra/Northwell, Northwell Health System, New York, New York.
- [3] Feinstein Institutes for Medical Research, Northwell Health, Manhasset, New York.
- [4] Division of Gastroenterology, North Shore University Hospital, Zucker School of Medicine at Hofstra/Northwell, Northwell Health System, Manhasset, New York.
- [5] Division of Hepatology, North Shore University Hospital, Zucker School of Medicine at Hofstra/Northwell, Northwell Health System, New York, New York.

- [6] Institute of Health Innovations and Outcomes Research, Feinstein Institutes for Medical Research, Northwell Health, Manhasset, New York; Division of Gastroenterology, Long Island Jewish Medical Center Zucker School of Medicine at Hofstra/Northwell, Northwell Health System, New Hyde Park, New York. Electronic address: arvind.trindade@gmail.com.

- PMID: **32860787**
- PMCID: PMC7448741
- DOI: 10.1053/j.gastro.2020.08.044

No abstract available

Keywords: COVID; Gastrointestinal; Pancreas; Pandemic; SARS-CoV-2.

- Cited by 47 articles
- 15 references

Supplementary info

Publication types, MeSH terms, Substances Expand

Publication types

- Comparative Study
- Observational Study

MeSH terms

- Adult
- African Americans / statistics & numerical data
- Aged
- Asian Americans / statistics & numerical data
- COVID-19 / complications
- COVID-19 / diagnosis
- COVID-19 / epidemiology*
- COVID-19 / virology
- COVID-19 Nucleic Acid Testing / statistics & numerical data
- Female
- Hispanic or Latino / statistics & numerical data
- Hospital Mortality
- Humans
- Length of Stay / statistics & numerical data
- Male
- Middle Aged
- New York / epidemiology
- Pancreatitis / diagnosis

- Pancreatitis / epidemiology*
- Pancreatitis / virology
- Prevalence
- RNA, Viral / isolation & purification
- Retrospective Studies
- Risk Factors
- SARS-CoV-2 / genetics
- SARS-CoV-2 / isolation & purification*
- SARS-CoV-2 / pathogenicity
- Severity of Illness Index
- Whites / statistics & numerical data

Substances

- RNA, Viral

Full text links

ELSEVIER FULL-TEXT ARTICLE Elsevier Science Free PMC article
Proceed to details
Cite
Share
473
Observational Study
J Med Virol
. 2022 Jan;94(1):384-387.
doi: 10.1002/jmv.27280. Epub 2021 Aug 23.

Elevated inflammatory markers are associated with poor outcomes in COVID-19 patients treated with remdesivir

Kate Stoeckle [1, 2], Britta Witting [3], Shashi Kapadia [2, 3], Anjile An [4], Kristen Marks [2, 3]
Affiliations Expand

Affiliations

- [1] Department of Medicine, Weill Cornell Medicine, New York, New York, USA.
- [2] NewYork-Presbyterian Hospital, New York, New York, USA.
- [3] Division of Infectious Diseases, Weill Cornell Medicine, New York, New York, USA.
- [4] Division of Biostatistics, Weill Cornell Medicine, New York, New York, USA.

- PMID: **34406670**

- PMCID: PMC8426873
- DOI: 10.1002/jmv.27280

Free PMC article
Observational Study

Elevated inflammatory markers are associated with poor outcomes in COVID-19 patients treated with remdesivir

Kate Stoeckle et al. J Med Virol. 2022 Jan.
Free PMC article
Show details
J Med Virol
. 2022 Jan;94(1):384-387.
doi: 10.1002/jmv.27280. Epub 2021 Aug 23.

Authors

Kate Stoeckle [1, 2], Britta Witting [3], Shashi Kapadia [2, 3], Anjile An [4], Kristen Marks [2, 3]

Affiliations

- [1] Department of Medicine, Weill Cornell Medicine, New York, New York, USA.
- [2] NewYork-Presbyterian Hospital, New York, New York, USA.
- [3] Division of Infectious Diseases, Weill Cornell Medicine, New York, New York, USA.
- [4] Division of Biostatistics, Weill Cornell Medicine, New York, New York, USA.

- PMID: **34406670**
- PMCID: PMC8426873
- DOI: 10.1002/jmv.27280

Abstract

The antiviral remdesivir has been shown to decrease the length of hospital stay in coronavirus disease 2019 (COVID-19) patients requiring supplemental oxygen. However many patients decompensate despite being treated with remdesivir. To identify potential prognostic factors in remdesivir-treated patients, we performed a retrospective cohort study of patients hospitalized at NewYork-Presbyterian Hospital/Weill Cornell Medical Center between March 23, 2020 and May 27, 2020. We identified 55 patients who were treated with remdesivir for COVID-19 and analyzed inflammatory markers and clinical outcomes. C-reactive protein (CRP), d-dimer, and lactate dehydrogenase levels were significantly higher in patients who progressed to intubation or death by 14 days compared to those who remained stable. CRP levels decreased significantly after remdesivir administration in patients who remained nonintubated over the study period. To our knowledge, this is the largest study to date examining inflammatory markers before and after remdesivir administration. Our findings support further investigation into COVID-19 treatment strategies that modify the inflammatory response.

Keywords: coronavirus disease 2019; inflammatory markers; remdesivir.

© 2021 Wiley Periodicals LLC.

Conflict of interest statement

Kristen Marks and Shashi Kapadia are investigators on research grants paid to the institution from Gilead Sciences Inc., for the study of hepatitis C unrelated to the current work. Shashi Kapadia receives research funding paid to the institution from Verily Life Sciences for research related to coronavirus disease 2019.

- 17 references
- 1 figure

Supplementary info

Publication types, MeSH terms, Substances, Supplementary concepts, Grant support Expand

Publication types

- Observational Study
- Research Support, N.I.H., Extramural
- Research Support, Non-U.S. Gov't

MeSH terms

- Adenosine Monophosphate / analogs & derivatives*
- Adenosine Monophosphate / therapeutic use
- Aged
- Alanine / analogs & derivatives*
- Alanine / therapeutic use
- Antiviral Agents / therapeutic use*
- Biomarkers / blood
- C-Reactive Protein / analysis
- COVID-19 / drug therapy*
- COVID-19 / mortality*
- COVID-19 / pathology
- Female
- Fibrin Fibrinogen Degradation Products / analysis
- Humans
- Inflammation / drug therapy
- L-Lactate Dehydrogenase / blood
- Length of Stay / statistics & numerical data
- Male
- Middle Aged

- Prognosis
- Retrospective Studies
- SARS-CoV-2 / drug effects*
- SARS-CoV-2 / immunology

Substances

- Antiviral Agents
- Biomarkers
- Fibrin Fibrinogen Degradation Products
- fibrin fragment D
- remdesivir
- Adenosine Monophosphate
- C-Reactive Protein
- L-Lactate Dehydrogenase
- Alanine

Supplementary concepts

- COVID-19 drug treatment

Grant support

- UL1 TR002384/TR/NCATS NIH HHS/United States

Full text links

WILEY Full Text Article Wiley Free PMC article
Proceed to details
Cite
Share
☐ 474
Observational Study
Eur Rev Med Pharmacol Sci
. 2022 Feb;26(4):1414-1429.
doi: 10.26355/eurrev_202202_28135.

Acute intestinal ischemia in patients with COVID-19: single-centre experience and literature review

P Fransvea [1], G Costa, G Pepe, A La Greca, S Magalini, C Puccioni, L d'Agostino, G Altieri, A Borello, V Cozza, G Sganga
Affiliations Expand

Affiliation

- [1] Emergency Surgery and Trauma - Fondazione Policlinico Universitario "A. Gemelli" IRCCS, Rome, Italy. gabriele.sganga@policlinicogemelli.it.

- PMID: **35253199**
- DOI: 10.26355/eurrev_202202_28135

Free article
Observational Study

Acute intestinal ischemia in patients with COVID-19: single-centre experience and literature review

P Fransvea et al. Eur Rev Med Pharmacol Sci. 2022 Feb.
Free article
Show details
Eur Rev Med Pharmacol Sci
. 2022 Feb;26(4):1414-1429.
doi: 10.26355/eurrev_202202_28135.

Authors

P Fransvea [1], G Costa, G Pepe, A La Greca, S Magalini, C Puccioni, L d'Agostino, G Altieri, A Borello, V Cozza, G Sganga

Affiliation

- [1] Emergency Surgery and Trauma - Fondazione Policlinico Universitario "A. Gemelli" IRCCS, Rome, Italy. gabriele.sganga@policlinicogemelli.it.

- PMID: **35253199**
- DOI: 10.26355/eurrev_202202_28135

Abstract

Objective: Acute Intestinal ischemia (AII) may involve the small and/or large bowel after any process affecting intestinal blood flow. COVID-19-related gastrointestinal manifestations, including AII, have been attributed to pharmacologic effects, metabolic disorders in ICU patients and other opportunistic colonic pathogens. AII in COVID-19 patients may be due also to "viral enteropathy" and SARS-CoV-2-induced small vessel thrombosis. A critical appraisal of personal

experience regarding COVID-19 and AII was carried out comparing this with a systematic literature review of published series.

Patients and methods: A retrospective observational clinical cohort study and a systematic literature review including only COVID-19 positive patients with acute arterial or venous intestinal ischemia were performed. The primary endpoint of the study was the mortality rate. Secondary endpoints were occurrence of major complications and length of hospital stay.

Results: Patient mean age was 62.9±14.9, with a prevalence of male gender (23 male, 72% vs. 9 female, 28%). The mean Charlson Comorbidity Index was 3.1±2.7. Surgery was performed in 24/32 patients (75.0%), with a mean delay time from admission to surgery of 6.0 ±5.6 days. Small bowel ischemia was confirmed to be the most common finding at surgical exploration (22/24, 91.7%). Acute abdomen at admission to the ED (Group 1) was observed in 10 (31.2%) cases, while 16 (50%) patients developed an acute abdomen condition during hospitalization (Group 2) for SARS-CoV-2 infection.

Conclusions: Our literature review showed how intestinal ischemia in patients with SARS-CoV-2 has been reported all over the world. The majority of the patients have a high CCI with multiple comorbidities, above all hypertension and cardiovascular disease. GI symptoms were not always present at the admission. A high level of suspicion for intestinal ischemia should be maintained in COVID-19 patients presenting with GI symptoms or with incremental abdominal pain. Nevertheless, a prompt thromboelastogram and laboratory test may confirm the need of improving and fastening the use of anticoagulants and trigger an extended indication for early abdominal CECT in patients with suggestive symptoms or biochemical markers of intestinal ischemia.

Supplementary info

Publication types, MeSH terms Expand

Publication types

- Comparative Study
- Observational Study

MeSH terms

- Aged
- COVID-19 / complications
- COVID-19 / epidemiology*
- Emergency Service, Hospital
- Female
- Hospitalization
- Humans
- Length of Stay
- Male
- Mesenteric Ischemia / diagnostic imaging
- Mesenteric Ischemia / epidemiology*
- Mesenteric Ischemia / etiology

- Mesenteric Ischemia / surgery
- Meta-Analysis as Topic
- Middle Aged
- Retrospective Studies
- Systematic Reviews as Topic
- Tomography, X-Ray Computed
- Treatment Outcome

Full text links

FREE FULL TEXT European Review for Medical and Pharmacological Sciences

Proceed to details

Cite

Share

☐ 475

Observational Study

Clin Pediatr (Phila)

. 2022 Feb;61(2):206-211.

doi: 10.1177/00099228211065898. Epub 2021 Dec 13.

Impact of Prenatal SARS-CoV-2 Infection on Infant Emergency Department Visits and Hospitalization

Stephanie P Ungar [1], Sadie Solomon [1], Anna Stachel [1], Kathleen Demarco [1], Ashley S Roman [1], Jennifer Lighter [1]

Affiliations Expand

Affiliation

- [1] New York University, New York, NY, USA.

- PMID: **34903074**
- DOI: 10.1177/00099228211065898

Observational Study

Impact of Prenatal SARS-CoV-2 Infection on Infant Emergency Department Visits and Hospitalization

Stephanie P Ungar et al. Clin Pediatr (Phila). 2022 Feb.

Show details

Clin Pediatr (Phila)
. 2022 Feb;61(2):206-211.
doi: 10.1177/00099228211065898. Epub 2021 Dec 13.

Authors

Stephanie P Ungar [1], Sadie Solomon [1], Anna Stachel [1], Kathleen Demarco [1], Ashley S Roman [1], Jennifer Lighter [1]

Affiliation

- [1] New York University, New York, NY, USA.

- PMID: **34903074**
- DOI: 10.1177/00099228211065898

Abstract

To better understand the impact of prenatal severe acute respiratory syndrome coronavirus 2 (SARS-CoV-2) infection on infants, this study sought to compare the risk of hospital visits and of postnatal SARS-CoV-2 infection between infants born to mothers with and without prenatal SARS-CoV-2 infection. In this retrospective observational cohort study of 6871 mothers and their infants, overall rates of emergency department (ED) visits and hospital admissions in the first 90 days of life were similar for infants born to mothers with and without prenatal SARS-CoV-2 infection. Infants born to negative mothers were more likely than infants of positive mothers to be hospitalized after ED visit (relative risk: 3.76; 95% confidence interval: 1.27-11.13, P = .003). Five infants tested positive; all were born to negative mothers, suggesting that maternal prenatal SARS-CoV-2 infection may protect infants from postnatal infection. The lower acuity ED visits for infants born to mothers with prenatal SARS-CoV-2 infection may reflect a heightened level of concern among these mothers.

Keywords: COVID-19; SARS-CoV-2; neonates; prenatal infection.

Supplementary info

Publication types, MeSH terms Expand

Publication types

- Multicenter Study
- Observational Study

MeSH terms

- Adult
- COVID-19 / complications*
- COVID-19 / epidemiology

- Cohort Studies
- Emergency Service, Hospital / organization & administration
- Emergency Service, Hospital / statistics & numerical data*
- Female
- Hospitalization / statistics & numerical data*
- Humans
- Infant
- Infant, Newborn
- Male
- New York City / epidemiology
- Pregnancy
- Pregnancy Complications, Infectious / diagnosis*
- Pregnancy Complications, Infectious / epidemiology
- Retrospective Studies

Full text links

SAGE journals Atypon
Proceed to details
Cite
Share
476
Observational Study

J Natl Med Assoc
. 2022 Jan;113(6):701-705.
doi: 10.1016/j.jnma.2021.08.036. Epub 2021 Sep 11.

Implementation and outcomes of monoclonal antibody infusion for COVID-19 in an inner-city safety net hospital: A South-Bronx experience

Sridhar Chilimuri [1], Nikhitha Mantri [2], Hitesh Gurjar [3], Karnokjun Annie Youthjug [4], Haozhe Sun [3], Sudharsan Gongati [3], Maleeha Zahid [3], Diana Maria Ronderos [3], Angel De La Cruz [3], Paavana Varanasi [3], Dongmin Shin [3], Suresh Kumar Nayudu [1]
Affiliations Expand

Affiliations

- [1] Department of Medicine, Bronx Care Health System, Affiliated with Icahn School of Medicine at Mount Sinai, Bronx, NY, USA; Division of Gastroenterology, Bronx Care Health System, Affiliated with Icahn School of Medicine at Mount Sinai, Bronx, NY, USA.

- [2] Department of Medicine, Bronx Care Health System, Affiliated with Icahn School of Medicine at Mount Sinai, Bronx, NY, USA. Electronic address: nmantri@bronxcare.org.
- [3] Department of Medicine, Bronx Care Health System, Affiliated with Icahn School of Medicine at Mount Sinai, Bronx, NY, USA.
- [4] Department of Nursing, Bronx Care Health System, Affiliated with Icahn School of Medicine at Mount Sinai, Bronx, NY, USA.

- PMID: **34521513**
- PMCID: PMC8433571
- DOI: 10.1016/j.jnma.2021.08.036

Free PMC article
Observational Study

Implementation and outcomes of monoclonal antibody infusion for COVID-19 in an inner-city safety net hospital: A South-Bronx experience

Sridhar Chilimuri et al. J Natl Med Assoc. 2022 Jan.
Free PMC article

Show details

J Natl Med Assoc
. 2022 Jan;113(6):701-705.
doi: 10.1016/j.jnma.2021.08.036. Epub 2021 Sep 11.

Authors

Sridhar Chilimuri [1], Nikhitha Mantri [2], Hitesh Gurjar [3], Karnokjun Annie Youthjug [4], Haozhe Sun [3], Sudharsan Gongati [3], Maleeha Zahid [3], Diana Maria Ronderos [3], Angel De La Cruz [3], Paavana Varanasi [3], Dongmin Shin [3], Suresh Kumar Nayudu [1]

Affiliations

- [1] Department of Medicine, Bronx Care Health System, Affiliated with Icahn School of Medicine at Mount Sinai, Bronx, NY, USA; Division of Gastroenterology, Bronx Care Health System, Affiliated with Icahn School of Medicine at Mount Sinai, Bronx, NY, USA.
- [2] Department of Medicine, Bronx Care Health System, Affiliated with Icahn School of Medicine at Mount Sinai, Bronx, NY, USA. Electronic address: nmantri@bronxcare.org.
- [3] Department of Medicine, Bronx Care Health System, Affiliated with Icahn School of Medicine at Mount Sinai, Bronx, NY, USA.
- [4] Department of Nursing, Bronx Care Health System, Affiliated with Icahn School of Medicine at Mount Sinai, Bronx, NY, USA.

- PMID: **34521513**
- PMCID: PMC8433571

- DOI: 10.1016/j.jnma.2021.08.036

Abstract

Background: Monoclonal antibody therapy (MAT) is recommended in mild to moderate Coronavirus disease 2019 (COVID-19) patients who are at risk of progressing to severe disease. Due to limited data on its outcomes and the logistic challenges in administering the drug, MAT has not been widely used in the United States (US) despite of emergency use authorization (EUA) approval by the Food and Drug Administration (FDA).

Aim: We aim to study the outcomes of MAT in patients predominantly from ethnic minority groups and the challenges we experienced in implementing the infusion therapy protocol in an inner-city safety-net-hospital in the South Bronx.

Methods and results: We conducted a retrospective observational study of 49 patients who were offered MAT as per EUA protocol of FDA. Patient who met the criteria for MAT and received therapy were included in treatment group (n = 38) and the remaining (n = 11) who declined treatment were included in the control group. A majority of patients (76%) in the study group reported symptomatic improvement, the day after infusion. There was statistically significant reduction in COVID-19 related hospitalizations (7.8 vs 54.5%, P = < 0.001) mortality (0 vs 18.1%, P value = 0.008) in the treatment group.

Conclusion: MAT reduced both hospitalization and mortality in this predominantly Hispanic patient population with mild to moderate COVID-19 with high risk factors for disease progression.

Keywords: Bamlanivimab; Bamlanivimab-etesevimab; Casirivimab-imdevimab; Mild-moderate COVID-19; Mortality.

Copyright © 2021 The Authors. Published by Elsevier Inc. All rights reserved.

- 13 references

Supplementary info

Publication types, MeSH terms, Substances Expand

Publication types

- Observational Study

MeSH terms

- Antibodies, Monoclonal / therapeutic use*
- COVID-19* / therapy
- Hispanic or Latino
- Hospital Mortality
- Hospitalization
- Humans

- Minority Groups
- New York City
- Retrospective Studies
- Safety-net Providers

Substances

- Antibodies, Monoclonal

Full text links

ELSEVIER OPEN ACCESS Elsevier Science Free PMC article

Proceed to details

Cite

Share

477

Observational Study

N Z Med J

. 2020 Aug 21;133(1520):153-156.

Pandemic control: getting to the heart of unintended consequences

Bernard Wong [1], Seif El-Jack [2], Guy Armstrong [2]

Affiliations Expand

Affiliations

- [1] Cardiology Trainee, Department of Cardiology, Waitemata District Health Board, Auckland.
- [2] Cardiologist, Department of Cardiology, Waitemata District Health Board, Auckland.

- PMID: **32994610**

Observational Study

Pandemic control: getting to the heart of unintended consequences

Bernard Wong et al. N Z Med J. 2020.

Show details

N Z Med J

. 2020 Aug 21;133(1520):153-156.

Authors

Bernard Wong [1], Seif El-Jack [2], Guy Armstrong [2]

Affiliations

- [1] Cardiology Trainee, Department of Cardiology, Waitemata District Health Board, Auckland.
- [2] Cardiologist, Department of Cardiology, Waitemata District Health Board, Auckland.

- PMID: **32994610**

No abstract available

Conflict of interest statement

Nil.

Supplementary info

Publication types, MeSH terms Expand

Publication types

- Letter
- Observational Study

MeSH terms

- Acute Coronary Syndrome / epidemiology
- Aged
- Aged, 80 and over
- Betacoronavirus / isolation & purification
- COVID-19 / epidemiology
- COVID-19 / prevention & control*
- COVID-19 / virology
- Cardiovascular Diseases / epidemiology*
- Case-Control Studies
- Coronavirus Infections / epidemiology
- Coronavirus Infections / prevention & control*
- Coronavirus Infections / virology
- Female
- Heart Failure / epidemiology
- Hospitalization / statistics & numerical data
- Hospitalization / trends*

- Humans
- Male
- Middle Aged
- New Zealand / epidemiology
- Pandemics / prevention & control*
- Physical Distancing
- Pneumonia, Viral / epidemiology
- Pneumonia, Viral / prevention & control*
- Pneumonia, Viral / virology
- Retrospective Studies
- SARS-CoV-2

Proceed to details
Cite
Share
☐ 478
Observational Study
Air Med J
. Jan-Feb 2022;41(1):68-72.
doi: 10.1016/j.amj.2021.10.012. Epub 2021 Oct 25.

Helicopter Emergency Medical Services Out-of-Hospital Cardiac Arrests During the Initial COVID-19 Lockdown Versus Nonpandemic: A Comparison

Sarah Morton [1], Jonathan Dawson [2], Sarah McLachlan [3], William McGuinness [4]
Affiliations Expand

Affiliations

- [1] Essex and Herts Air Ambulance, Earls Colne, Colchester, Essex, United Kingdom. Electronic address: sarah.morton@doctors.org.uk.
- [2] Essex and Herts Air Ambulance, Earls Colne, Colchester, Essex, United Kingdom.
- [3] Essex and Herts Air Ambulance, Earls Colne, Colchester, Essex, United Kingdom; Anglia Ruskin University, Chelmsford and Cambridge, United Kingdom.
- [4] Essex and Herts Air Ambulance, Earls Colne, Colchester, Essex, United Kingdom; Emergency Department, St George's Hospital, London, United Kingdom.

- PMID: **35248347**
- PMCID: PMC8570121
- DOI: 10.1016/j.amj.2021.10.012

Free PMC article

Observational Study

Helicopter Emergency Medical Services Out-of-Hospital Cardiac Arrests During the Initial COVID-19 Lockdown Versus Nonpandemic: A Comparison

Sarah Morton et al. Air Med J. Jan-Feb 2022.
Free PMC article
Show details
Air Med J
. Jan-Feb 2022;41(1):68-72.
doi: 10.1016/j.amj.2021.10.012. Epub 2021 Oct 25.

Authors

Sarah Morton [1], Jonathan Dawson [2], Sarah McLachlan [3], William McGuinness [4]

Affiliations

- [1] Essex and Herts Air Ambulance, Earls Colne, Colchester, Essex, United Kingdom. Electronic address: sarah.morton@doctors.org.uk.
- [2] Essex and Herts Air Ambulance, Earls Colne, Colchester, Essex, United Kingdom.
- [3] Essex and Herts Air Ambulance, Earls Colne, Colchester, Essex, United Kingdom; Anglia Ruskin University, Chelmsford and Cambridge, United Kingdom.
- [4] Essex and Herts Air Ambulance, Earls Colne, Colchester, Essex, United Kingdom; Emergency Department, St George's Hospital, London, United Kingdom.

- PMID: **35248347**
- PMCID: PMC8570121
- DOI: 10.1016/j.amj.2021.10.012

Abstract

Objective: COVID-19 may have contributed to an excess of out-of-hospital cardiac arrests (OOHCAs). This observational study identified changes in OOHCA epidemiology pre- and post-COVID-19 lockdown in a single UK helicopter emergency medical service (HEMS).

Methods: A retrospective, single-center (Essex & Herts Air Ambulance), observational study was undertaken with anonymized OOHCA data (demographics, etiology, and outcomes) from March 23, 2020, to June 23, 2020, and comparative data from March 23, 2019, to June 23, 2019. Supplementary data (total OOHCAs and patient outcomes) were provided by the East of England Ambulance Service National Health Service Trust. Data were analyzed using the Mann-Whitney U test and chi-square test; P < .05 was statistically significant.

Results: Of the HEMS activations during national lockdown, 33.6% were for OOHCAs compared with 25.8% during the reference time frame. The frequency of young and female OOHCAs demonstrated a statistically significant increase. Statistically significant variations in medical etiology and initial cardiac rhythm were identified.

Conclusion: During the initial UK-wide lockdown, the OOHCA characteristics attended by 1 HEMS team were altered. The changes seen may be due to the pathophysiology of COVID-19 or an alteration in dispatch due to the demand placed on the wider ambulance service; this may require further consideration for any future lockdowns or pandemics.

Copyright © 2021 Air Medical Journal Associates. Published by Elsevier Inc. All rights reserved.

- 24 references
- 2 figures

Supplementary info

Publication types, MeSH terms Expand

Publication types

- Observational Study

MeSH terms

- Air Ambulances*
- Aircraft
- COVID-19* / epidemiology
- Communicable Disease Control
- Emergency Medical Services*
- Female
- Humans
- Out-of-Hospital Cardiac Arrest* / epidemiology
- Out-of-Hospital Cardiac Arrest* / therapy
- Retrospective Studies
- SARS-CoV-2
- State Medicine

Full text links

ELSEVIER FULL-TEXT ARTICLE Elsevier Science Free PMC article

Proceed to details

Cite

Share

☐ 479

Observational Study

J Thromb Thrombolysis
. 2022 Jan;53(1):96-102.
doi: 10.1007/s11239-021-02507-2. Epub 2021 Jun 17.

Impact of pre-admission antithrombotic therapy on disease severity and mortality in patients hospitalized for COVID-19

Mariana Corrochano [1], René Acosta-Isaac [2], Sergi Mojal [2], Sara Miqueleiz [3], Diana Rodriguez [3], María Ángeles Quijada-Manuitt [4], Edmundo Fraga [5], Marta Castillo-Ocaña [4], Kristopher Amaro-Hosey [4], Nil Albiol [2], José Manuel Soria [6], Rosa Maria Antonijoan [4] [7], Joan Carles Souto [2]
Affiliations Expand

Affiliations

- [1] Haemostasis and Thrombosis Unit, Hospital de La Santa Creu I Sant Pau. Carrer de Sant Quintí 89, 08041, Barcelona, Spain. mcorrochano@santpau.cat.
- [2] Haemostasis and Thrombosis Unit, Hospital de La Santa Creu I Sant Pau. Carrer de Sant Quintí 89, 08041, Barcelona, Spain.
- [3] Clinical Trials Unit (AGDAC), Hospital de la Santa Creu i Sant Pau, Barcelona, Spain.
- [4] Clinical Pharmacology Service, Hospital de la Santa Creu i Sant Pau, Barcelona, Spain.
- [5] ALBA Synchrotron, Barcelona, Spain.
- [6] Unit of Genomics of Complex Diseases, Institut d'Investigació Biomèdica Sant Pau, IIB-Sant Pau, Barcelona, Spain.
- [7] Drug Research Center, Institut d'Investigació Biomèdica Sant Pau, IIB-Sant Pau, Barcelona, Spain.

- PMID: **34138399**
- PMCID: PMC8210515
- DOI: 10.1007/s11239-021-02507-2

Free PMC article
Observational Study

Impact of pre-admission antithrombotic therapy on disease severity and mortality in patients hospitalized for COVID-19

Mariana Corrochano et al. J Thromb Thrombolysis. 2022 Jan.
Free PMC article
Show details
J Thromb Thrombolysis

. 2022 Jan;53(1):96-102.
doi: 10.1007/s11239-021-02507-2. Epub 2021 Jun 17.

Authors

Mariana Corrochano [1], René Acosta-Isaac [2], Sergi Mojal [2], Sara Miqueleiz [3], Diana Rodriguez [3], María Ángeles Quijada-Manuitt [4], Edmundo Fraga [5], Marta Castillo-Ocaña [4], Kristopher Amaro-Hosey [4], Nil Albiol [2], José Manuel Soria [6], Rosa Maria Antonijoan [4] [7], Joan Carles Souto [2]

Affiliations

- [1] Haemostasis and Thrombosis Unit, Hospital de La Santa Creu I Sant Pau. Carrer de Sant Quintí 89, 08041, Barcelona, Spain. mcorrochano@santpau.cat.
- [2] Haemostasis and Thrombosis Unit, Hospital de La Santa Creu I Sant Pau. Carrer de Sant Quintí 89, 08041, Barcelona, Spain.
- [3] Clinical Trials Unit (AGDAC), Hospital de la Santa Creu i Sant Pau, Barcelona, Spain.
- [4] Clinical Pharmacology Service, Hospital de la Santa Creu i Sant Pau, Barcelona, Spain.
- [5] ALBA Synchrotron, Barcelona, Spain.
- [6] Unit of Genomics of Complex Diseases, Institut d'Investigació Biomèdica Sant Pau, IIB-Sant Pau, Barcelona, Spain.
- [7] Drug Research Center, Institut d'Investigació Biomèdica Sant Pau, IIB-Sant Pau, Barcelona, Spain.

- PMID: **34138399**
- PMCID: PMC8210515
- DOI: 10.1007/s11239-021-02507-2

Abstract

Anticoagulant therapy is a cornerstone treatment for coronavirus disease 2019 (COVID-19) due to the high rates of thromboembolic complications associated with this disease. We hypothesized that chronic antithrombotic therapy could play a protective role in patients hospitalized for COVID-19. Retrospective, observational study of all patients admitted to our hospital for ≥ 24 h from March 1 to May 31, 2020 with SARS-CoV-2. The objective was to evaluate clinical outcomes and mortality in COVID-19 patients receiving chronic anticoagulation (AC) or antiplatelet therapy (AP) prior to hospital admission. A total of 1612 patients were evaluated. The mean (standard deviation; SD) age was 66.5 (17.1) years. Patients were divided into three groups according to the use of antithrombotic therapy prior to admission (AP, AC, or no-antithrombotic treatment). At admission, 9.6% of the patients were taking anticoagulants and 19.1% antiplatelet therapy. The overall mortality rate was 19.3%. On the multivariate analysis there were no significant differences in mortality between the antithrombotic groups (AC or AP) and the no-antithrombotic group (control group). Patients on AC had lower ICU admission rates than the control group (OR: 0.41, 95% CI, 0.18-0.93). Anticoagulation therapy prior to hospitalization for COVID-19 was associated with lower ICU admission rates. However, there were no significant differences in mortality between the patients receiving chronic antithrombotic therapy and patients not taking antithrombotic medications. These findings suggest that chronic anticoagulation therapy at the time of COVID-19 infection may reduce disease severity and thus the need for ICU admission.

Keywords: Anticoagulants; Antiplatelet drugs; Covid-19; Intensive care; Mortality.

© 2021. The Author(s), under exclusive licence to Springer Science+Business Media, LLC, part of Springer Nature.

Conflict of interest statement

No authors declare competing financial interests.

- Cited by 2 articles
- 37 references

Supplementary info

Publication types, MeSH terms, Substances Expand

Publication types

- Observational Study

MeSH terms

- Aged
- Aged, 80 and over
- Anticoagulants / therapeutic use
- COVID-19*
- Fibrinolytic Agents* / therapeutic use
- Hospital Mortality
- Hospitalization
- Humans
- Intensive Care Units
- Middle Aged
- Platelet Aggregation Inhibitors / therapeutic use
- Retrospective Studies
- Severity of Illness Index

Substances

- Anticoagulants
- Fibrinolytic Agents
- Platelet Aggregation Inhibitors

Full text links

SpringerLink FULL-TEXT ARTICLE Springer Free PMC article

Proceed to details

Cite

Share

480

Observational Study

J Med Internet Res

. 2020 Sep 10;22(9):e21562.

doi: 10.2196/21562.

Excess Patient Visits for Cough and Pulmonary Disease at a Large US Health System in the Months Prior to the COVID-19 Pandemic: Time-Series Analysis

Joann G Elmore [1], Pin-Chieh Wang [1], Kathleen F Kerr [2], David L Schriger [3], Douglas E Morrison [4], Ron Brookmeyer [4], Michael A Pfeffer [1], Thomas H Payne [5], Judith S Currier [1]

Affiliations Expand

Affiliations

- [1] Department of Medicine, David Geffen School of Medicine, UCLA, Los Angeles, CA, United States.
- [2] Department of Biostatistics, UW School of Public Health, Seattle, WA, United States.
- [3] Department of Emergency Medicine, David Geffen School of Medicine, UCLA, Los Angeles, CA, United States.
- [4] Department of Biostatistics, Fielding School of Public Health, UCLA, Los Angeles, CA, United States.
- [5] Department of Medicine, UW School of Medicine, Seattle, WA, United States.

- PMID: **32791492**
- PMCID: PMC7485935
- DOI: 10.2196/21562

Free PMC article

Observational Study

Excess Patient Visits for Cough and Pulmonary Disease at a Large US Health System in the Months Prior to the COVID-19 Pandemic: Time-Series Analysis

Joann G Elmore et al. J Med Internet Res. 2020.

Free PMC article

Show details

J Med Internet Res
. 2020 Sep 10;22(9):e21562.
doi: 10.2196/21562.

Authors

Joann G Elmore[1], Pin-Chieh Wang[1], Kathleen F Kerr[2], David L Schriger[3], Douglas E Morrison[4], Ron Brookmeyer[4], Michael A Pfeffer[1], Thomas H Payne[5], Judith S Currier[1]

Affiliations

- [1] Department of Medicine, David Geffen School of Medicine, UCLA, Los Angeles, CA, United States.
- [2] Department of Biostatistics, UW School of Public Health, Seattle, WA, United States.
- [3] Department of Emergency Medicine, David Geffen School of Medicine, UCLA, Los Angeles, CA, United States.
- [4] Department of Biostatistics, Fielding School of Public Health, UCLA, Los Angeles, CA, United States.
- [5] Department of Medicine, UW School of Medicine, Seattle, WA, United States.

- PMID: **32791492**
- PMCID: PMC7485935
- DOI: 10.2196/21562

Abstract

Background: Accurately assessing the regional activity of diseases such as COVID-19 is important in guiding public health interventions. Leveraging electronic health records (EHRs) to monitor outpatient clinical encounters may lead to the identification of emerging outbreaks.

Objective: The aim of this study is to investigate whether excess visits where the word "cough" was present in the EHR reason for visit, and hospitalizations with acute respiratory failure were more frequent from December 2019 to February 2020 compared with the preceding 5 years.

Methods: A retrospective observational cohort was identified from a large US health system with 3 hospitals, over 180 clinics, and 2.5 million patient encounters annually. Data from patient encounters from July 1, 2014, to February 29, 2020, were included. Seasonal autoregressive integrated moving average (SARIMA) time-series models were used to evaluate if the observed winter 2019/2020 rates were higher than the forecast 95% prediction intervals. The estimated excess number of visits and hospitalizations in winter 2019/2020 were calculated compared to previous seasons.

Results: The percentage of patients presenting with an EHR reason for visit containing the word "cough" to clinics exceeded the 95% prediction interval the week of December 22, 2019, and was consistently above the 95% prediction interval all 10 weeks through the end of February 2020. Similar trends were noted for emergency department visits and hospitalizations starting December 22, 2019, where observed data exceeded the 95% prediction interval in 6 and 7 of the 10 weeks, respectively. The estimated excess over the 3-month 2019/2020 winter season, obtained by either subtracting the maximum or subtracting the average of the five previous seasons from the current

season, was 1.6 or 2.0 excess visits for cough per 1000 outpatient visits, 11.0 or 19.2 excess visits for cough per 1000 emergency department visits, and 21.4 or 39.1 excess visits per 1000 hospitalizations with acute respiratory failure, respectively. The total numbers of excess cases above the 95% predicted forecast interval were 168 cases in the outpatient clinics, 56 cases for the emergency department, and 18 hospitalized with acute respiratory failure.

Conclusions: A significantly higher number of patients with respiratory complaints and diseases starting in late December 2019 and continuing through February 2020 suggests community spread of SARS-CoV-2 prior to established clinical awareness and testing capabilities. This provides a case example of how health system analytics combined with EHR data can provide powerful and agile tools for identifying when future trends in patient populations are outside of the expected ranges.

Keywords: COVID-19; electronic health record; forecast; pandemic; prediction; time-series analysis.

©Joann G Elmore, Pin-Chieh Wang, Kathleen F Kerr, David L Schriger, Douglas E Morrison, Ron Brookmeyer, Michael A Pfeffer, Thomas H Payne, Judith S Currier. Originally published in the Journal of Medical Internet Research (http://www.jmir.org), 10.09.2020.

Conflict of interest statement

Conflicts of Interest: JGE serves as Editor-in-Chief for Adult Primary Care topics at UpToDate. All other authors declare no conflicts of interest.

- Cited by 6 articles
- 36 references
- 4 figures

Supplementary info

Publication types, MeSH terms, Grant support Expand

Publication types

- Observational Study

MeSH terms

- Acute Disease
- Adult
- Ambulatory Care Facilities
- Betacoronavirus
- COVID-19
- California / epidemiology
- Coronavirus Infections
- Cough / epidemiology*
- Electronic Health Records

- Emergency Service, Hospital
- Female
- Hospitalization / statistics & numerical data
- Humans
- Male
- Middle Aged
- Pandemics
- Pneumonia, Viral
- Respiratory Insufficiency / epidemiology*
- Retrospective Studies
- SARS-CoV-2
- Seasons

Grant support

- P2C HD041022/HD/NICHD NIH HHS/United States

Full text links

JMIR Publications Free PMC article
Proceed to details
Cite
Share
481
Observational Study
South Med J
. 2022 Mar;115(3):175-180.
doi: 10.14423/SMJ.0000000000001368.

Analysis of the Effects of a Texas State-Wide Mask Mandate (Executive Order GA-29) on Case Load, Hospitalizations, and Mortality

Michael D April[1], Jason F Naylor[1], Brit Long[1]
Affiliations Expand

Affiliation

- [1] From the Department of Military and Emergency Medicine, Uniformed Services University of the Health Sciences, Bethesda, Maryland, the Department of Emergency Medicine, Madigan Army Medical Center, Joint Base Lewis McChord, Washington, and the Department of Emergency Medicine, Brooke Army Medical Center, JBSA Fort Sam Houston, Texas.

- PMID: **35237834**
- PMCID: PMC8865024
- DOI: 10.14423/SMJ.0000000000001368

Free PMC article
Observational Study

Analysis of the Effects of a Texas State-Wide Mask Mandate (Executive Order GA-29) on Case Load, Hospitalizations, and Mortality

Michael D April et al. South Med J. 2022 Mar.
Free PMC article
Show details
South Med J
. 2022 Mar;115(3):175-180.
doi: 10.14423/SMJ.0000000000001368.

Authors

Michael D April[1], Jason F Naylor[1], Brit Long[1]

Affiliation

- [1] From the Department of Military and Emergency Medicine, Uniformed Services University of the Health Sciences, Bethesda, Maryland, the Department of Emergency Medicine, Madigan Army Medical Center, Joint Base Lewis McChord, Washington, and the Department of Emergency Medicine, Brooke Army Medical Center, JBSA Fort Sam Houston, Texas.

- PMID: **35237834**
- PMCID: PMC8865024
- DOI: 10.14423/SMJ.0000000000001368

Abstract

Objectives: The coronavirus disease 2019 (COVID-19) pandemic has resulted in unprecedented hospitalizations, ventilator use, and deaths. Because of concerns for resource utilization and surges in hospital capacity use, Texas Executive Order GA-29 required statewide mask wear beginning July 3, 2020. Our objective was to compare COVID-19 case load, hospital bed use, and deaths before and after implementation of this mask order.

Methods: This was a retrospective observational study using publicly reported statewide data to perform a mixed-methods interrupted time series analysis. We compared outcomes before and after the statewide mask wear mandate per Executive Order GA-29. The preorder period was from June 19 to July 2, 2020. The postorder period was July 17 to September 17, 2020. Outcomes included daily COVID-19 case load, hospitalizations, and mortality.

Results: The daily case load before the mask order per 100,000 individuals was 187.5 (95% confidence interval [CI] 157.0-217.0) versus 200.7 (95% CI 179.8-221.6) after GA-29. The number of daily hospitalized patients with COVID-19 was 171.4 (95% CI 143.8-199.0) before GA-29 versus 225.1 (95% CI 202.9-247.3) after. Daily mortality was 2.4 (95% CI 1.9-2.9) before GA-29 versus 5.2 (95% CI 4.6-5.8). There was no material impact on our results after controlling for economic activity.

Conclusions: In both adjusted and unadjusted analyses, we were unable to detect a reduction in case load, hospitalization rates, or mortality associated with the implementation of an executive order requiring a statewide mask order. These results suggest that during a period of rapid virus spread, additional public health measures may be necessary to mitigate transmission at the population level.

Conflict of interest statement

The authors did not report any financial relationships or conflicts of interest.

- 31 references
- 3 figures

Supplementary info

Publication types, MeSH terms Expand

Publication types

- Observational Study

MeSH terms

- COVID-19 / diagnosis
- COVID-19 / epidemiology*
- COVID-19 / prevention & control
- Communicable Disease Control*
- Facilities and Services Utilization
- Hospital Mortality
- Hospitalization / statistics & numerical data*
- Humans
- Interrupted Time Series Analysis
- Mandatory Programs*
- Masks*
- Retrospective Studies
- Survival Rate
- Texas
- Workload / statistics & numerical data*

Full text links

Southern Medical Association Free PMC article
Proceed to details
Cite
Share
482

Observational Study

Anaesth Crit Care Pain Med
. 2020 Dec;39(6):709-715.
doi: 10.1016/j.accpm.2020.09.007. Epub 2020 Oct 5.

A national healthcare response to intensive care bed requirements during the COVID-19 outbreak in France

Jean-Yves Lefrant[1], Marc-Olivier Fischer[2], Hugo Potier[3], Cécile Degryse[4], Samir Jaber[5], Laurent Muller[6], Julien Pottecher[7], Hélène Charboneau[8], Eric Meaudre[9], Pierre Lanot[10], Vincent Bruckert[11], Benoît Plaud[12], Bertrand Dureuil[13], Emmanuel Samain[14], Hervé Bouaziz[15], Claude Ecoffey[16], Xavier Capdevila[17], French ICU study investigators group

Affiliations Expand

Affiliations

- [1] EA 2992 IMAGINE, Univ Montpellier, Pôle Anesthésie Réanimation Douleur Urgence, CHU Nîmes, Nîmes, France. Electronic address: jean.yves.lefrant@chu-nimes.fr.
- [2] Normandie Univ, UNICAEN, CHU de Caen Normandie, Service d'Anesthésie Réanimation, 14000 Caen, France.
- [3] Laboratoire de Biostatistique, Epidémiologie clinique, Santé Publique Innovation et Méthodologie (BESPIM), Pôle Pharmacie, Santé publique, CHU Nîmes, Nîmes, University of Montpellier, France.
- [4] Service d'Anesthésie Réanimation Pellegrin Tripode, CHU Bordeaux, Bordeaux, France.
- [5] Department of Anaesthesia & Critical Care Medicine, University of Montpellier Saint Eloi Hospital, and PhyMedExp, University of Montpellier, INSERM, CNRS, Montpellier, France.
- [6] EA 2992 IMAGINE, Univ Montpellier, Pôle Anesthésie Réanimation Douleur Urgence, CHU Nîmes, Nîmes, France.
- [7] Hôpitaux Universitaires de Strasbourg, Pôle d'Anesthésie-Réanimation & Médecine Péri-Opératoire, Service d'Anesthésie-Réanimation & Médecine Péri-Opératoire Hôpital de Hautepierre - Université de Strasbourg, Faculté de Médecine, Fédération de Médecine Translationnelle de Strasbourg (FMTS), UR3072, Strasbourg, France.
- [8] Service d'Anesthésie, Clinique Pasteur, 31300 Toulouse, France.
- [9] Fédération d'Anesthésie-Réanimation, Hôpital d'Instruction des Armées Sainte-Anne, Toulon; Chaire d'Anesthésie-réanimation, Médecine d'Urgence, École du Val-de-Grâce, Paris, France.

- [10] GARHPA, groupe de anesthésistes réanimateurs de l'Hôpital Privé d'Antony, 92160 Antony, France.
- [11] Pôle d'Anesthésie-Réanimation Médecine péri-opératoire et Urgences, Hôpital l'Archet 2, Centre Hospitalier Universitaire de Nice, Université de Nice, 06000 Nice, France.
- [12] Paris University & APHP. Nord. DMU PARABOL, Department of Anaesthesiology, Critical Care & Burn Unit, Saint-Louis hospital, 1, Avenue Claude Vellefaux, 75010 Paris, France.
- [13] Departement of Anaesthesia and Critical Care, Rouen University Hospital, Rouen, France.
- [14] Département d'Anesthésie Réanimation, Hôpital Jean Minjoz - C.H.U. de Besançon, Besançon, France.
- [15] Département d'Anesthésie Réanimation, Hôpital Central - CHRU Nancy, Nancy, France.
- [16] Département d'Anesthésie-Réanimation et Médecine Péri-Opératoire, Hôpital Pontchaillou, Université Rennes 1, Rennes, France.
- [17] Department of Anaesthesiology and Critical Care Medicine, Lapeyronie University Hospital and Montpellier University. INSERM unit 1051, Montpellier Neurosciences Institute, Montpellier, France.

- PMID: **33031979**
- PMCID: PMC7534597
- DOI: 10.1016/j.accpm.2020.09.007

Free PMC article
Observational Study

A national healthcare response to intensive care bed requirements during the COVID-19 outbreak in France

Jean-Yves Lefrant et al. Anaesth Crit Care Pain Med. 2020 Dec.
Free PMC article
Show details
Anaesth Crit Care Pain Med
. 2020 Dec;39(6):709-715.
doi: 10.1016/j.accpm.2020.09.007. Epub 2020 Oct 5.

Authors

Jean-Yves Lefrant [1], Marc-Olivier Fischer [2], Hugo Potier [3], Cécile Degryse [4], Samir Jaber [5], Laurent Muller [6], Julien Pottecher [7], Hélène Charbonneau [8], Eric Meaudre [9], Pierre Lanot [10], Vincent Bruckert [11], Benoît Plaud [12], Bertrand Dureuil [13], Emmanuel Samain [14], Hervé Bouaziz [15], Claude Ecoffey [16], Xavier Capdevila [17], French ICU study investigators group

Affiliations

- [1] EA 2992 IMAGINE, Univ Montpellier, Pôle Anesthésie Réanimation Douleur Urgence, CHU Nîmes, Nîmes, France. Electronic address: jean.yves.lefrant@chu-nimes.fr.

- [2] Normandie Univ, UNICAEN, CHU de Caen Normandie, Service d'Anesthésie Réanimation, 14000 Caen, France.
- [3] Laboratoire de Biostatistique, Epidémiologie clinique, Santé Publique Innovation et Méthodologie (BESPIM), Pôle Pharmacie, Santé publique, CHU Nîmes, Nîmes, University of Montpellier, France.
- [4] Service d'Anesthésie Réanimation Pellegrin Tripode, CHU Bordeaux, Bordeaux, France.
- [5] Department of Anaesthesia & Critical Care Medicine, University of Montpellier Saint Eloi Hospital, and PhyMedExp, University of Montpellier, INSERM, CNRS, Montpellier, France.
- [6] EA 2992 IMAGINE, Univ Montpellier, Pôle Anesthésie Réanimation Douleur Urgence, CHU Nîmes, Nîmes, France.
- [7] Hôpitaux Universitaires de Strasbourg, Pôle d'Anesthésie-Réanimation & Médecine Péri-Opératoire, Service d'Anesthésie-Réanimation & Médecine Péri-Opératoire Hôpital de Hautepierre - Université de Strasbourg, Faculté de Médecine, Fédération de Médecine Translationnelle de Strasbourg (FMTS), UR3072, Strasbourg, France.
- [8] Service d'Anesthésie, Clinique Pasteur, 31300 Toulouse, France.
- [9] Fédération d'Anesthésie-Réanimation, Hôpital d'Instruction des Armées Sainte-Anne, Toulon; Chaire d'Anesthésie-réanimation, Médecine d'Urgence, École du Val-de-Grâce, Paris, France.
- [10] GARHPA, groupe de anesthésistes réanimateurs de l'Hôpital Privé d'Antony, 92160 Antony, France.
- [11] Pôle d'Anesthésie-Réanimation Médecine péri-opératoire et Urgences, Hôpital l'Archet 2, Centre Hospitalier Universitaire de Nice, Université de Nice, 06000 Nice, France.
- [12] Paris University & APHP. Nord. DMU PARABOL, Department of Anaesthesiology, Critical Care & Burn Unit, Saint-Louis hospital, 1, Avenue Claude Vellefaux, 75010 Paris, France.
- [13] Departement of Anaesthesia and Critical Care, Rouen University Hospital, Rouen, France.
- [14] Département d'Anesthésie Réanimation, Hôpital Jean Minjoz - C.H.U. de Besançon, Besançon, France.
- [15] Département d'Anesthésie Réanimation, Hôpital Central - CHRU Nancy, Nancy, France.
- [16] Département d'Anesthésie-Réanimation et Médecine Péri-Opératoire, Hôpital Pontchaillou, Université Rennes 1, Rennes, France.
- [17] Department of Anaesthesiology and Critical Care Medicine, Lapeyronie University Hospital and Montpellier University. INSERM unit 1051, Montpellier Neurosciences Institute, Montpellier, France.

- PMID: **33031979**
- PMCID: PMC7534597
- DOI: 10.1016/j.accpm.2020.09.007

Abstract

Background: Whereas 5415 Intensive Care Unit (ICU) beds were initially available, 7148 COVID-19 patients were hospitalised in the ICU at the peak of the outbreak. The present study reports how the French Health Care system created temporary ICU beds to avoid being overwhelmed.

Methods: All French ICUs were contacted for answering a questionnaire focusing on the available beds and health care providers before and during the outbreak.

Results: Among 336 institutions with ICUs before the outbreak, 315 (94%) participated, covering 5054/5531 (91%) ICU beds. During the outbreak, 4806 new ICU beds (+95% increase) were created from Acute Care Unit (ACU, 2283), Post Anaesthetic Care Unit and Operating Theatre (PACU & OT, 1522), other units (374) or real build-up of new ICU beds (627), respectively. At the peak of the outbreak, 9860, 1982 and 3089 ICU, ACU and PACU beds were made available. Before the outbreak, 3548 physicians (2224 critical care anaesthesiologists, 898 intensivists and 275 from other specialties, 151 paediatrics), 1785 residents, 11,023 nurses and 6763 nursing auxiliaries worked in established ICUs. During the outbreak, 2524 physicians, 715 residents, 7722 nurses and 3043 nursing auxiliaries supplemented the usual staff in all ICUs. A total number of 3212 new ventilators were added to the 5997 initially available in ICU.

Conclusion: During the COVID-19 outbreak, the French Health Care system created 4806 ICU beds (+95% increase from baseline), essentially by transforming beds from ACUs and PACUs. Collaboration between intensivists, critical care anaesthesiologists, emergency physicians as well as the mobilisation of nursing staff were primordial in this context.

Keywords: Bed availability; COVID-19; France; ICU.

Copyright © 2020 Société française d'anesthésie et de réanimation (Sfar). Published by Elsevier Masson SAS. All rights reserved.

- Cited by 17 articles
- 32 references
- 3 figures

Supplementary info

Publication types, MeSH terms Expand

Publication types

- Multicenter Study
- Observational Study

MeSH terms

- Bed Conversion / statistics & numerical data
- COVID-19 / epidemiology*
- France / epidemiology
- Health Care Surveys / statistics & numerical data
- Hospital Bed Capacity / statistics & numerical data*
- Hospitalization / statistics & numerical data
- Humans
- Intensive Care Units / statistics & numerical data*
- National Health Programs*
- Pandemics*
- Personnel Staffing and Scheduling / statistics & numerical data
- Personnel, Hospital / supply & distribution

- Retrospective Studies
- SARS-CoV-2*
- Ventilators, Mechanical / supply & distribution

Full text links

ELSEVIER FULL-TEXT ARTICLE Elsevier Science Free PMC article
Proceed to details
Cite
Share
483
Observational Study
Clin Lab
. 2022 Mar 1;68(3).
doi: 10.7754/Clin.Lab.2021.210745.

A Nomogram Based on Myocardial Damage and Novel Inflammatory Indexes for Post-Discharge Survival Rates of COVID-19

Seyda Gunay, Serhat Caliskan, Deniz Sigirli

- PMID: **35254020**
- DOI: 10.7754/Clin.Lab.2021.210745

Observational Study

A Nomogram Based on Myocardial Damage and Novel Inflammatory Indexes for Post-Discharge Survival Rates of COVID-19

Seyda Gunay et al. Clin Lab. 2022.
Show details
Clin Lab
. 2022 Mar 1;68(3).
doi: 10.7754/Clin.Lab.2021.210745.

Authors

Seyda Gunay, Serhat Caliskan, Deniz Sigirli

- PMID: **35254020**
- DOI: 10.7754/Clin.Lab.2021.210745

Abstract

Background: In the course of SARS-CoV-2 infection, early prognostic evaluation is important since clinical symptoms may worsen rapidly and may be fatal. Inflammation plays an important role in the pathogenesis of COVID-19 and can cause myocardial damage which is common in severe COVID-19 patients. Therefore, novel inflammatory indexes and myocardial damage may be predictive of prognosis in patients with COVID-19. The aim of the study was to evaluate the role of cardiac troponin I (cTnI), modified Glasgow prognostic score (mGPS), systemic immune inflammation index (SII), prognostic nutritional index (PNI), and CRP to albumin ratio (CAR) in the outcome estimation of COVID-19 and to develop a risk model predicting the survival probability of COVID-19 survivors during early post-discharge.

Methods: This was a single-center, observational, retrospective cohort study. Laboratory confirmed COVID-19 patients (n = 265) were included and grouped according to in-hospital mortality. ROC curve analysis was performed and Youden's J index was used to obtain optimal cutoff values for inflammatory indexes in discriminating survivors and non-survivors. Cox regression analysis was performed to assess the possible predictors of in-hospital mortality. A nomogram was constructed based on the Cox regression model, to calculate 7- and 14-day survival.

Results: The area under the ROC curve (AUC) of the variables ranged between 0.79 and 0.92 with the three highest AUC values for albumin, PNI, and cTnI (0.919, 0.918, and 0.911, respectively). Optimal threshold value for cTnI was 9.7 pg/mL. Univariate analysis showed that gender, albumin, CRP, CAR, PNI, SII, cTnI, and mGPS were significantly related to in-hospital mortality. The Cox regression analysis indicated that mGPS ($p = 0.001$), CRP ($p = 0.026$), and cTnI ($p = 0.001$) were significant prognostic factors.

Conclusions: cTnI should not be considered merely as an indicator of myocardial damage. It also reflects the inflammatory phase and, along with other inflammatory markers, it should be included in risk models as a prognostic factor for COVID-19.

Supplementary info

Publication types, MeSH terms Expand

Publication types

- Observational Study

MeSH terms

- Aftercare
- COVID-19*
- Humans
- Nomograms
- Patient Discharge
- Prognosis
- Retrospective Studies

- SARS-CoV-2
- Survival Rate

Full text links

Clin Lab FULL TEXT Clinical Laboratory
Proceed to details
Cite
Share
484
Observational Study
Acta Anaesthesiol Scand
. 2022 Jan;66(1):48-55.
doi: 10.1111/aas.13982. Epub 2021 Oct 11.

Chronic dysglycemia and risk of SARS-CoV-2 associated respiratory failure in hospitalized patients

Susanne Rysz [1, 2], Malin Jonsson Fagerlund [1, 3], Claire Rimes-Stigare [1], Emma Larsson [1, 3], Francesca Campoccia Jalde [1, 4], Johan Mårtensson [1, 3]
Affiliations Expand

Affiliations

- [1] Department of Perioperative Medicine and Intensive Care, Karolinska University Hospital, Stockholm, Sweden.
- [2] Department of Medicine Solna, Karolinska Institutet, Stockholm, Sweden.
- [3] Department of Physiology and Pharmacology, Karolinska Institutet, Stockholm, Sweden.
- [4] Department of Molecular Medicine and Surgery, Karolinska Institutet, Stockholm, Sweden.

- PMID: **34582033**
- PMCID: PMC8653023
- DOI: 10.1111/aas.13982

Free PMC article
Observational Study

Chronic dysglycemia and risk of SARS-CoV-2 associated respiratory failure in hospitalized patients

Susanne Rysz et al. Acta Anaesthesiol Scand. 2022 Jan.
Free PMC article
Show details
Acta Anaesthesiol Scand
. 2022 Jan;66(1):48-55.
doi: 10.1111/aas.13982. Epub 2021 Oct 11.

Authors

Susanne Rysz [1 2], Malin Jonsson Fagerlund [1 3], Claire Rimes-Stigare [1], Emma Larsson [1 3], Francesca Campoccia Jalde [1 4], Johan Mårtensson [1 3]

Affiliations

- [1] Department of Perioperative Medicine and Intensive Care, Karolinska University Hospital, Stockholm, Sweden.
- [2] Department of Medicine Solna, Karolinska Institutet, Stockholm, Sweden.
- [3] Department of Physiology and Pharmacology, Karolinska Institutet, Stockholm, Sweden.
- [4] Department of Molecular Medicine and Surgery, Karolinska Institutet, Stockholm, Sweden.

- PMID: **34582033**
- PMCID: PMC8653023
- DOI: 10.1111/aas.13982

Abstract

Background: Diabetes is common among patients with severe acute respiratory syndrome coronavirus 2 (SARS-CoV-2)-induced respiratory failure. We aimed to investigate the relationship between different stages of chronic dysglycemia and development of respiratory failure in hospitalized SARS-CoV-2 positive patients.

Methods: In this retrospective observational study, we included 385 hospitalized SARS-CoV-2 positive patients at Karolinska University Hospital, Sweden with an HbA1c test obtained within 3 months before admission. Based on HbA1c level and previous diabetes history, we classified patients into the following dysglycemia categories: prediabetes, unknown diabetes, controlled diabetes, or uncontrolled diabetes. We used multivariable logistic regression analysis adjusted for age, sex, and body mass index, to assess the association between dysglycemia categories and development of SARS-CoV-2-induced respiratory failure.

Results: Of the 385 study patients, 88 (22.9%) had prediabetes, 68 (17.7%) had unknown diabetes, 36 (9.4%) had controlled diabetes, and 83 (21.6%) had uncontrolled diabetes. Overall, 299 (77.7%) patients were admitted with or developed SARS-CoV-2-induced respiratory failure

during hospitalization. In multivariable logistic regression analysis compared with no chronic dysglycemia, prediabetes (OR 14.41, 95% CI 5.27-39.43), unknown diabetes (OR 15.86, 95% CI 4.55-55.36), and uncontrolled diabetes (OR 17.61, 95% CI 5.77-53.74) was independently associated with increased risk of SARS-CoV-2-induced respiratory failure.

Conclusion: In our cohort of hospitalized SARS-CoV-2 positive patients with available HbA1c data, prediabetes, undiagnosed diabetes, and poorly controlled diabetes were associated with a markedly increased risk of SARS-CoV-2-associated respiratory failure.

Keywords: dysglycemia; respiratory failure; severe Covid-19.

© 2021 The Authors. Acta Anaesthesiologica Scandinavica published by John Wiley & Sons Ltd on behalf of Acta Anaesthesiologica Scandinavica Foundation.

Conflict of interest statement

None of the authors have any conflict of interests.

- 22 references
- 2 figures

Supplementary info

Publication types, MeSH terms Expand

Publication types

- Observational Study

MeSH terms

- COVID-19*
- Diabetes Mellitus* / epidemiology
- Hospitalization
- Humans
- Respiratory Insufficiency* / epidemiology
- Respiratory Insufficiency* / etiology
- Retrospective Studies
- Risk Factors
- SARS-CoV-2

Full text links

WILEY Full Text Article Wiley Free PMC article

Proceed to details

Cite

Share

☐ 485
Observational Study
Pediatr Pulmonol
. 2022 Feb;57(2):361-366.
doi: 10.1002/ppul.25752. Epub 2021 Nov 15.

Severe respiratory viral infections in children with history of asymptomatic or mild COVID-19

Nooralam Rai [1], Joseph A Cornett [2], Philip Zachariah [3], Lynne Quittell [1], Stephanie Lovinsky-Desir [1]
Affiliations Expand

Affiliations

- [1] Division of Pediatric Pulmonary, Department of Pediatrics, Vagelos College of Physicians and Surgeons, Columbia University, New York, New York, USA.
- [2] Vagelos College of Physicians and Surgeons, Columbia University, 630 W 168th street, New York, New York, USA.
- [3] Division of Pediatric Infectious Diseases, Department of Pediatrics, Vagelos College of Physicians and Surgeons, Columbia University, New York, New York, USA.

- PMID: **34741579**
- PMCID: PMC8661820
- DOI: 10.1002/ppul.25752

Free PMC article
Observational Study

Severe respiratory viral infections in children with history of asymptomatic or mild COVID-19

Nooralam Rai et al. Pediatr Pulmonol. 2022 Feb.
Free PMC article
Show details
Pediatr Pulmonol
. 2022 Feb;57(2):361-366.
doi: 10.1002/ppul.25752. Epub 2021 Nov 15.

Authors

Nooralam Rai [1], Joseph A Cornett [2], Philip Zachariah [3], Lynne Quittell [1], Stephanie Lovinsky-Desir [1]

Affiliations

- [1] Division of Pediatric Pulmonary, Department of Pediatrics, Vagelos College of Physicians and Surgeons, Columbia University, New York, New York, USA.
- [2] Vagelos College of Physicians and Surgeons, Columbia University, 630 W 168th street, New York, New York, USA.
- [3] Division of Pediatric Infectious Diseases, Department of Pediatrics, Vagelos College of Physicians and Surgeons, Columbia University, New York, New York, USA.

- PMID: **34741579**
- PMCID: PMC8661820
- DOI: 10.1002/ppul.25752

Abstract

Importance: The spectrum of complications of COVID-19 in children, including the effect of COVID-19 on later viral infection, is not known.

Objective: To examine the features of children hospitalized for respiratory illness with history of prior COVID-19.

Design: Retrospective observational case series at a single pediatric quaternary medical center in New York City. Data were obtained from review of medical records.

Participants: Children with prior mild or asymptomatic COVID-19 and no known risk factors for severe respiratory disease, who were hospitalized at our center for acute respiratory illness from October 2020 to May 2021, were reviewed.

Main outcomes and measures: Co-morbidities, history of prior COVID-19 symptoms, respiratory viral panel findings, acuity of illness, degree of respiratory decompensation based on support and interventions required, duration of hospitalization, and overall clinical course were assessed from the medical record.

Results: This study included 5 patients (median age, 4 years; age range: 0.8-9 years; 4 [80%] male). All had positive COVID-19 serology, 1 (20%) had mild symptoms, while the others had no symptoms of prior Sars-CoV-2 infection, 3 (60%) had asthma, and the remaining had no co-morbidities. All were admitted between April and May 2021. Two were re-admitted for respiratory symptoms in the subsequent 3 months.

Conclusions and relevance: This case series describes a possible association between severe lower respiratory tract infection and prior mild COVID-19 in children. Larger cohort studies describing the respiratory effects of prior COVID-19 in children are needed.

Keywords: COVID-19; asymptomatic; lower respiratory tract infection; pediatric.

© 2021 Wiley Periodicals LLC.

- 28 references

Supplementary info

Publication types, MeSH terms Expand

Publication types

- Observational Study

MeSH terms

- COVID-19*
- Child
- Child, Preschool
- Female
- Hospitalization
- Humans
- Infant
- Male
- Retrospective Studies
- SARS-CoV-2
- Virus Diseases* / complications
- Virus Diseases* / epidemiology

Full text links

WILEY Full Text Article Wiley Free PMC article
Proceed to details
Cite
Share
486
Observational Study
Eur J Clin Invest
. 2022 Jan;52(1):e13703.
doi: 10.1111/eci.13703. Epub 2021 Nov 11.

Myocardial injury in patients with SARS-CoV-2 pneumonia: Pivotal role of inflammation in COVID-19

Francesco Melillo [1], Antonio Napolano [1], Marco Loffi [2], Valentina Regazzoni [2], Antonio Boccellino [1], Gian Battista Danzi [2], Alberto Maria Cappelletti [3], Patrizia Rovere-Querini [4] [5], Giovanni Landoni [5] [6], Giacomo Ingallina [1], Stefano Stella [1], Francesco Ancona [1], Lorenzo

Dagna[5 7], Paolo Scarpellini[8], Marco Ripa[8], Antonella Castagna[5 8], Moreno Tresoldi[9], Alberto Zangrillo[5 6], Fabio Ciceri[5 10], Eustachio Agricola[1 5]
Affiliations Expand

Affiliations

- [1] Cardiovascular Imaging Unit, Cardiothoracic Department, San Raffaele Scientific Institute, Milan, Italy.
- [2] Division of Cardiology, Cremona Hospital, Cremona, Italy.
- [3] Cardiac Intensive Care Unit, San Raffaele Scientific Institute, Milan, Italy.
- [4] Internal Medicine, Diabetes and Endocrinology Unit, San Raffaele Scientific Institute, Milan, Italy.
- [5] Vita-Salute San Raffaele University, Milan, Italy.
- [6] Department of Anesthesia and Intensive Care, San Raffaele Scientific Institute, Milan, Italy.
- [7] Unit of Immunology, Rheumatology, Allergy and Rare Diseases, San Raffaele Scientific Institute, Milan, Italy.
- [8] Department of Infectious Diseases, San Raffaele Scientific Institute, Milan, Italy.
- [9] Department of Internal Medicine and Advanced Therapies, San Raffaele Scientific Institute, Milan, Italy.
- [10] Hematology and Bone Marrow Transplantation Unit, San Raffaele Scientific Institute, Milan, Italy.

- PMID: **34706062**
- PMCID: PMC8646244
- DOI: 10.1111/eci.13703

Free PMC article
Observational Study

Myocardial injury in patients with SARS-CoV-2 pneumonia: Pivotal role of inflammation in COVID-19

Francesco Melillo et al. Eur J Clin Invest. 2022 Jan.
Free PMC article
Show details
Eur J Clin Invest
. 2022 Jan;52(1):e13703.
doi: 10.1111/eci.13703. Epub 2021 Nov 11.

Authors

Francesco Melillo[1], Antonio Napolano[1], Marco Loffi[2], Valentina Regazzoni[2], Antonio Boccellino[1], Gian Battista Danzi[2], Alberto Maria Cappelletti[3], Patrizia Rovere-Querini[4 5], Giovanni Landoni[5 6], Giacomo Ingallina[1], Stefano Stella[1], Francesco Ancona[1], Lorenzo

Dagna [5, 7], Paolo Scarpellini [8], Marco Ripa [8], Antonella Castagna [5, 8], Moreno Tresoldi [9], Alberto Zangrillo [5, 6], Fabio Ciceri [5, 10], Eustachio Agricola [1, 5]

Affiliations

- [1] Cardiovascular Imaging Unit, Cardiothoracic Department, San Raffaele Scientific Institute, Milan, Italy.
- [2] Division of Cardiology, Cremona Hospital, Cremona, Italy.
- [3] Cardiac Intensive Care Unit, San Raffaele Scientific Institute, Milan, Italy.
- [4] Internal Medicine, Diabetes and Endocrinology Unit, San Raffaele Scientific Institute, Milan, Italy.
- [5] Vita-Salute San Raffaele University, Milan, Italy.
- [6] Department of Anesthesia and Intensive Care, San Raffaele Scientific Institute, Milan, Italy.
- [7] Unit of Immunology, Rheumatology, Allergy and Rare Diseases, San Raffaele Scientific Institute, Milan, Italy.
- [8] Department of Infectious Diseases, San Raffaele Scientific Institute, Milan, Italy.
- [9] Department of Internal Medicine and Advanced Therapies, San Raffaele Scientific Institute, Milan, Italy.
- [10] Hematology and Bone Marrow Transplantation Unit, San Raffaele Scientific Institute, Milan, Italy.

- PMID: **34706062**
- PMCID: PMC8646244
- DOI: 10.1111/eci.13703

Abstract

Aims: Infection by SARS-CoV-2 may result in a systemic disease and a proportion of patients ranging 15%-44% experienced cardiac injury (CI) diagnosed by abnormal troponin levels. The aim of the present study was to analyse the clinical characteristics of a large series of hospitalized patients for COVID-19 in order to identify predisposing and/or protective factors of CI and the outcome.

Methods and results: This is an observational, retrospective study on patients hospitalized in two Italian centres (San Raffaele Hospital and Cremona Hospital) for COVID-19 and at least one high-sensitivity cardiac troponin (hs-cTnt) measurement during hospitalization. CI was defined if at least one hs-cTnt value was above the 99th percentile. The primary end-point was the occurrence of CI during hospitalization. We included 750 patients (median age 67, IQR 56-77 years; 69% males), of whom 46.9% had history of hypertension, 14.7% of chronic coronary disease and 22.3% of chronic kidney disease (CKD). Abnormal troponin levels (median troponin 74, IQR 34-147 ng/l) were detected in 390 patients (52%) during the hospitalization. At multivariable analysis age, CKD, cancer, C-reactive protein (CRP) levels were independently associated with CI. Independent predictors of very high troponin levels were chronic kidney disease and CRP levels. Patients with CI showed higher rate of all-cause mortality (40.0% vs. 9.1%, p = 0.001) compared to those without CI.

Conclusion: This large, multicentre Italian study confirmed the high prevalence of CI and its prognostic role in hospitalized patients with COVID-19, highlighting the leading role of systemic inflammation for the occurrence of CI.

Keywords: COVID-19; SARS-coronavirus-2; cardiac complications; myocardial injury; troponin.

© 2021 Stichting European Society for Clinical Investigation Journal Foundation. Published by John Wiley & Sons Ltd.

Conflict of interest statement

All authors declare that they have no conflict of interest.

- Cited by 2 articles
- 27 references
- 2 figures

Supplementary info

Publication types, MeSH terms, Substances Expand

Publication types

- Multicenter Study
- Observational Study

MeSH terms

- Aged
- COVID-19 / diagnosis*
- COVID-19 / mortality
- Female
- Heart Diseases / virology*
- Hospitalization
- Humans
- Inflammation / virology*
- Italy / epidemiology
- Male
- Middle Aged
- Prognosis
- Retrospective Studies
- Troponin / blood

Substances

- Troponin

Full text links

WILEY Full Text Article Wiley Free PMC article
Proceed to details
Cite
Share
487

Observational Study

Obesity (Silver Spring)
. 2022 Mar;30(3):599-605.
doi: 10.1002/oby.23314. Epub 2022 Feb 9.

History of bariatric surgery and COVID-19 outcomes in patients with type 2 diabetes: Results from the CORONADO study

Claire Blanchard[1, 2], Tanguy Perennec[3], Sarra Smati[1], Blandine Tramunt[4], Béatrice Guyomarch[1], Edith Bigot-Corbel[5], Lyse Bordier[6], Sophie Borot[7], Olivier Bourron[8], Cyrielle Caussy[9, 10], Christine Coffin-Boutreux[11], Anne Dutour[12], Natacha Germain[13, 14], Céline Gonfroy-Leymarie[15], Laurent Meyer[16], Gaëtan Prevost[17], Ronan Roussel[18], Dominique Seret-Bégué[19], Charles Thivolet[20], Bruno Vergès[21], Matthieu Pichelin[1], Pierre Gourdy[4], Samy Hadjadj[1], Matthieu Wargny[1, 3], François Pattou[22], Bertrand Cariou[1], CORONADO investigators
Affiliations Expand

Affiliations

- [1] Université de Nantes, CHU Nantes, CNRS, INSERM, l'institut Du Thorax, Nantes, France.
- [2] Chirurgie Cancérologique Digestive et Endocrinienne (CCDE), Institut des Maladies de l'Appareil Digestif (IMAD), Centre Hospitalo-universitaire de Nantes (CHU) Hôtel-Dieu, Nantes, France.
- [3] CHU de Nantes, INSERM CIC 1413, Pôle Hospitalo-Universitaire 11 : Santé Publique, Clinique des données, Nantes, France.
- [4] Département d'Endocrinologie, Diabétologie et Nutrition, CHU Toulouse, Institut des Maladies Métaboliques et Cardiovasculaires, UMR1297 INSERM/UPS, Université de Toulouse, Toulouse, France.
- [5] Laboratoire de Biochimie, CHU de Nantes, Hôpital G et R Laënnec, Nantes, France.
- [6] Hôpital d'instruction des Armées Bégin, Saint Mandé, France.
- [7] Département d'Endocrinologie, Diabétologie et Nutrition, CHU de Besançon, Besançon, France.
- [8] Département de Diabétologie, CHU La Pitié Salpêtrière-Charles Foix, Inserm, UMR_S 1138, Centre de Recherche des Cordeliers, Paris 06, Institute of Cardiometabolism and Nutrition ICAN, Sorbonne Université, Assistance Publique-Hôpitaux de Paris, Paris, France.
- [9] Univ-Lyon, laboratoire CarMeN, Inserm U1060, INRA U1397, Université Claude Bernard Lyon 1, INSA Lyon, Villeurbanne, France.

- [10] Département Endocrinologie, Diabète et Nutrition, Hospices Civils de Lyon, Hôpital Lyon Sud, Pierre-Bénite, France.
- [11] Département d'Endocrinologie, Diabétologie, Maladies Métaboliques, CH de Périgueux, Périgueux, France.
- [12] Aix Marseille Univ, APHM, INSERM, INRAE, C2VN, Hôpital Nord Département d'Endocrinologie et de Diabétologie, Marseille, France.
- [13] Département d'Endocrinologie, CHU de Saint-Etienne, Saint-Etienne, France.
- [14] Laboratoire TAPE, Eating desorders, Addiction and Extreme bodyweight, Université Jean Monnet, Saint-Etienne, France.
- [15] Service d'Endocrinologie, Diabétologie, CH de Pontoise, Pontoise, France.
- [16] Département d'Endocrinologie, Diabétologie et Nutrition, Hôpitaux Universitaires de Strasbourg, Strasbourg, France.
- [17] Département d'Endocrinologie, Diabète et Maladies Métaboliques, Normandie Univ, UNIROUEN, CHU de Rouen, Rouen, France.
- [18] Département d'Endocrinologie, Diabétologie et Nutrition, Hôpital Bichat, Assistance Publique-Hôpitaux de Paris, Centre de Recherche des Cordeliers, INSERM, U-1138, Université de Paris, Paris, France.
- [19] Service de Diabétologie, CH Gonesse, Gonesse, France.
- [20] Centre du Diabète DIAB-eCARE, Hospices Civils de Lyon, Lyon, France.
- [21] Service Endocrinologie, Diabétologie et Maladies Métaboliques, Hôpital du Bocage, Dijon, France.
- [22] Univ Lille, Inserm, CHU Lille, Institut Pasteur de Lille, European Genomic Institute of Diabetes, Chirurge Endocrinienne et Métabolique, Cente Intégré de l'Obésité, Lille, France.

- PMID: **34586754**
- PMCID: PMC8661775
- DOI: 10.1002/oby.23314

Free PMC article
Observational Study

History of bariatric surgery and COVID-19 outcomes in patients with type 2 diabetes: Results from the CORONADO study

Claire Blanchard et al. Obesity (Silver Spring). 2022 Mar.
Free PMC article
Show details
Obesity (Silver Spring)
. 2022 Mar;30(3):599-605.
doi: 10.1002/oby.23314. Epub 2022 Feb 9.

Authors

Claire Blanchard [1, 2], Tanguy Perennec [3], Sarra Smati [1], Blandine Tramunt [4], Béatrice Guyomarch [1], Edith Bigot-Corbel [5], Lyse Bordier [6], Sophie Borot [7], Olivier Bourron [8], Cyrielle

Caussy[9, 10], Christine Coffin-Boutreux[11], Anne Dutour[12], Natacha Germain[13, 14], Céline Gonfroy-Leymarie[15], Laurent Meyer[16], Gaëtan Prevost[17], Ronan Roussel[18], Dominique Seret-Bégué[19], Charles Thivolet[20], Bruno Vergès[21], Matthieu Pichelin[1], Pierre Gourdy[4], Samy Hadjadj[1], Matthieu Wargny[1, 3], François Pattou[22], Bertrand Cariou[1], CORONADO investigators

Affiliations

- [1] Université de Nantes, CHU Nantes, CNRS, INSERM, l'institut Du Thorax, Nantes, France.
- [2] Chirurgie Cancérologique Digestive et Endocrinienne (CCDE), Institut des Maladies de l'Appareil Digestif (IMAD), Centre Hospitalo-universitaire de Nantes (CHU) Hôtel-Dieu, Nantes, France.
- [3] CHU de Nantes, INSERM CIC 1413, Pôle Hospitalo-Universitaire 11 : Santé Publique, Clinique des données, Nantes, France.
- [4] Département d'Endocrinologie, Diabétologie et Nutrition, CHU Toulouse, Institut des Maladies Métaboliques et Cardiovasculaires, UMR1297 INSERM/UPS, Université de Toulouse, Toulouse, France.
- [5] Laboratoire de Biochimie, CHU de Nantes, Hôpital G et R Laënnec, Nantes, France.
- [6] Hôpital d'instruction des Armées Bégin, Saint Mandé, France.
- [7] Département d'Endocrinologie, Diabétologie et Nutrition, CHU de Besançon, Besançon, France.
- [8] Département de Diabétologie, CHU La Pitié Salpêtrière-Charles Foix, Inserm, UMR_S 1138, Centre de Recherche des Cordeliers, Paris 06, Institute of Cardiometabolism and Nutrition ICAN, Sorbonne Université, Assistance Publique-Hôpitaux de Paris, Paris, France.
- [9] Univ-Lyon, laboratoire CarMeN, Inserm U1060, INRA U1397, Université Claude Bernard Lyon 1, INSA Lyon, Villeurbanne, France.
- [10] Département Endocrinologie, Diabète et Nutrition, Hospices Civils de Lyon, Hôpital Lyon Sud, Pierre-Bénite, France.
- [11] Département d'Endocrinologie, Diabétologie, Maladies Métaboliques, CH de Périgueux, Périgueux, France.
- [12] Aix Marseille Univ, APHM, INSERM, INRAE, C2VN, Hôpital Nord Département d'Endocrinologie et de Diabétologie, Marseille, France.
- [13] Département d'Endocrinologie, CHU de Saint-Etienne, Saint-Etienne, France.
- [14] Laboratoire TAPE, Eating desorders, Addiction and Extreme bodyweight, Université Jean Monnet, Saint-Etienne, France.
- [15] Service d'Endocrinologie, Diabétologie, CH de Pontoise, Pontoise, France.
- [16] Département d'Endocrinologie, Diabétologie et Nutrition, Hôpitaux Universitaires de Strasbourg, Strasbourg, France.
- [17] Département d'Endocrinologie, Diabète et Maladies Métaboliques, Normandie Univ, UNIROUEN, CHU de Rouen, Rouen, France.
- [18] Département d'Endocrinologie, Diabétologie et Nutrition, Hôpital Bichat, Assistance Publique-Hôpitaux de Paris, Centre de Recherche des Cordeliers, INSERM, U-1138, Université de Paris, Paris, France.
- [19] Service de Diabétologie, CH Gonesse, Gonesse, France.
- [20] Centre du Diabète DIAB-eCARE, Hospices Civils de Lyon, Lyon, France.
- [21] Service Endocrinologie, Diabétologie et Maladies Métaboliques, Hôpital du Bocage, Dijon, France.

- [22] Univ Lille, Inserm, CHU Lille, Institut Pasteur de Lille, European Genomic Institute of Diabetes, Chirurge Endocrinienne et Métabolique, Cente Intégré de l'Obésité, Lille, France.

- PMID: **34586754**
- PMCID: PMC8661775
- DOI: 10.1002/oby.23314

Abstract

Objective: This study assessed the impact of a history of metabolic and bariatric surgery (MBS) on the clinical outcomes in patients with type 2 diabetes (T2D) and severe obesity hospitalized for COVID-19.

Methods: In this post hoc analysis from the nationwide observational CORONADO (Coronavirus SARS-CoV2 and Diabetes Outcomes) study, patients with T2D and a history of MBS were matched with patients without MBS for age, sex, and BMI either at the time of MBS or on admission for COVID-19. The composite primary outcome (CPO) combined invasive mechanical ventilation and/or death within 7 and 28 days following admission.

Results: Out of 2,398 CORONADO participants, 20 had a history of MBS. When matching for BMI at the time of MBS and after adjustment for diabetes duration, the CPO occurred less frequently within 7 days (3 vs. 17 events, OR: 0.15 [0.01 to 0.94], p = 0.03) and 28 days (3 vs. 19 events, OR: 0.11 [0.01 to 0.71], p = 0.02) in patients with MBS (n = 16) vs. controls (n = 44). There was no difference in CPO rate between patients with MBS and controls when matching for BMI on admission.

Conclusions: These data are reassuring regarding COVID-19 prognosis in patients with diabetes and a history of MBS compared with those without MBS.

© 2021 The Obesity Society.

- Cited by 1 article
- 18 references

Supplementary info

Publication types, MeSH terms, Substances, Grant support Expand

Publication types

- Observational Study
- Research Support, Non-U.S. Gov't

MeSH terms

- Bariatric Surgery*
- COVID-19*
- Diabetes Mellitus, Type 2*
- Humans

- RNA, Viral
- Retrospective Studies
- SARS-CoV-2

Substances

- RNA, Viral

Grant support

- Fondation Francophone de Recherche sur le Diabète

Full text links

WILEY Full Text Article Wiley Free PMC article

Proceed to details

Cite

Share

☐ 488

Observational Study

Clin Exp Nephrol

. 2022 Jan;26(1):36-44.

doi: 10.1007/s10157-021-02123-7. Epub 2021 Aug 16.

Clinical profile and outcomes of COVID-19 patients with acute kidney injury: a tertiary centre experience from South India

Chaganti Sindhu [1], Pallavi Prasad [2] [3], Ramprasad Elumalai [1], Jayakumar Matcha [1]

Affiliations Expand

Affiliations

- [1] Department of Nephrology, Sri Ramachandra Institute of Higher Education and Research, Chennai, India.
- [2] Department of Nephrology, Sri Ramachandra Institute of Higher Education and Research, Chennai, India. pallaviprasad1986@gmail.com.
- [3] , 37&38, Sri Lakshmi Nagar, 10th Cross Street, Valasarvakkam, Chennai, 600087, India. pallaviprasad1986@gmail.com.

- PMID: **34401969**
- PMCID: PMC8366740
- DOI: 10.1007/s10157-021-02123-7

Free PMC article

Observational Study

Clinical profile and outcomes of COVID-19 patients with acute kidney injury: a tertiary centre experience from South India

Chaganti Sindhu et al. Clin Exp Nephrol. 2022 Jan.
Free PMC article
Show details
Clin Exp Nephrol
. 2022 Jan;26(1):36-44.
doi: 10.1007/s10157-021-02123-7. Epub 2021 Aug 16.

Authors

Chaganti Sindhu [1], Pallavi Prasad [2] [3], Ramprasad Elumalai [1], Jayakumar Matcha [1]

Affiliations

- [1] Department of Nephrology, Sri Ramachandra Institute of Higher Education and Research, Chennai, India.
- [2] Department of Nephrology, Sri Ramachandra Institute of Higher Education and Research, Chennai, India. pallaviprasad1986@gmail.com.
- [3] , 37&38, Sri Lakshmi Nagar, 10th Cross Street, Valasarvakkam, Chennai, 600087, India. pallaviprasad1986@gmail.com.

- PMID: **34401969**
- PMCID: PMC8366740
- DOI: 10.1007/s10157-021-02123-7

Abstract

Aim: The rates of development of acute kidney injury (AKI) in COVID-19 have been variably reported from across the world. Prevalence and outcomes of AKI in hospitalised COVID-19 patients in India has not been studied well.

Methods: This was a retrospective observational study amongst adult hospitalised COVID-19 patients admitted at a tertiary care centre between May 1 and October 31, 2020. We estimated the prevalence of AKI and outcomes including mortality and acute kidney disease (AKD) at the time of discharge. Regression analysis was done to study the factors associated with mortality and AKD.

Results: Out of 2650 hospitalised patients with COVID-19, 190 (7.2%) patients developed AKI. Mean age of patients with AKI was 62.6 years, 81.6% were male. Comorbidities included diabetes mellitus in 72.1%, hypertension in 66.8%, heart disease in 30% and chronic kidney disease (CKD) in 22.6%. Most patients had stage 1 AKI (71.1%). Overall mortality in patients with AKI was 22.1%, 75% in those requiring dialysis and 74.5% in those requiring ICU. Amongst survivors without pre-existing CKD, 40.9% patients had acute kidney disease at the time of discharge.

Higher age, stage 3 AKI and need for mechanical ventilation were associated with higher mortality. On multivariable regression, factors associated with AKD at discharge included pre-existing heart disease and severe albuminuria during hospitalisation.

Conclusion: In our study population, we found a low prevalence of AKI. Mortality was high in AKI patients requiring ICU care and dialysis. Amongst survivors, a significant percentage had AKD at the time of discharge.

Keywords: AKI; CKD; COVID-19; Kidney; Mortality.

© 2021. Japanese Society of Nephrology.

Conflict of interest statement

The authors have declared that no conflict of interest exists.

- Cited by 1 article
- 23 references
- 3 figures

Supplementary info

Publication types, MeSH terms Expand

Publication types

- Observational Study

MeSH terms

- Acute Kidney Injury / epidemiology*
- Acute Kidney Injury / mortality
- Acute Kidney Injury / therapy
- Acute Kidney Injury / virology
- Aged
- Aged, 80 and over
- COVID-19 / epidemiology*
- COVID-19 / mortality
- COVID-19 / therapy
- COVID-19 / virology
- Female
- Hospital Mortality
- Hospitalization
- Humans
- India / epidemiology
- Intensive Care Units

- Male
- Middle Aged
- Prevalence
- Renal Dialysis
- Retrospective Studies
- Risk Assessment
- Risk Factors
- Tertiary Care Centers
- Time Factors
- Treatment Outcome

Full text links

SpringerLink FULL-TEXT ARTICLE Springer Free PMC article
Proceed to details
Cite
Share
489
Observational Study
Sr Care Pharm
. 2022 Feb 1;37(2):62-72.
doi: 10.4140/TCP.n.2022.62.

The Impact of a Pharmacist-Led Hypertension Medication Management Program on Older People in a Skilled Nursing Facility

Rachel Stone [1], Aida Oganesyan [2], Noachim Marco [2], Rick Smith [2], Janice Hoffman [1]
Affiliations Expand

Affiliations

- [1] 1 Western University of Health Sciences, Pomona, California.
- [2] 2 Los Angeles Jewish Home, Reseda, California.

- PMID: **35082011**
- DOI: 10.4140/TCP.n.2022.62

Observational Study

The Impact of a Pharmacist-Led Hypertension Medication Management Program on Older People in a Skilled Nursing Facility

Rachel Stone et al. Sr Care Pharm. 2022.
Show details
Sr Care Pharm
. 2022 Feb 1;37(2):62-72.
doi: 10.4140/TCP.n.2022.62.

Authors

Rachel Stone [1], Aida Oganesyan [2], Noachim Marco [2], Rick Smith [2], Janice Hoffman [1]

Affiliations

- [1] 1 Western University of Health Sciences, Pomona, California.
- [2] 2 Los Angeles Jewish Home, Reseda, California.

- PMID: **35082011**
- DOI: 10.4140/TCP.n.2022.62

Abstract

Objective To illustrate the impact of a pharmacist-led hypertension medication management program on skilled nursing facility residents. **Design** Sixteen-week retrospective, observational study. **Setting** Long-term care, local skilled nursing facility. **Patients, Participants** Subjects with a diagnosis of hypertension and treated with at least one antihypertensive medication were identified using electronic health records. The subjects also needed to be enrolled in the Hypertension Medication Management Program, and were excluded if receiving hospice or psychiatric treatment or had active infection with COVID-19. Initially, 120 residents were eligible with 54 in an intervention and 66 in a control group. At 16-weeks, a total of 67 residents remained after some were lost to follow-up. **Interventions** Under a collaborative practice agreement, a pharmacist optimized medications, ordered monitoring of vital signs, and relevant labs in conjunction with standard physician care (intervention): these subjects were compared to those who received standard physician care alone (control). **Results** There was a significant difference in the proportion of subjects who attained treatment goals for diastolic BP, but not for systolic BP or mean arterial pressure. The proportion of subjects in the intervention group who had falls, hospitalization or death was not significantly different between groups. diastolic pressure (90.9% and 38.0%; $P < 0.0001$; 86.7% and 32.4%; $P < 0.0001$) but not for systolic and mean arterial pressure at 8 and 16 weeks. For secondary endpoints, there was clinical significance in de-prescribing incidence ($P < 0.0001$) but not for fall events, hospitalizations, and death. However, control group had 11% more falls and 1.2% more hospitalizations. **Conclusion** A pharmacist-led hypertension program appeared to impact skilled nursing facility residents by allowing attainment of maintaining diastolic blood pressure, goals de-prescribing events, and reducing fall incidence

and hospitalization. The intervention has the potential to promote de-prescribing but does not appear to have increased the prevalence of serious adverse outcomes relative to standard practice.

Supplementary info

Publication types, MeSH terms Expand

Publication types

- Observational Study

MeSH terms

- Aged
- COVID-19*
- Humans
- Hypertension* / drug therapy
- Hypertension* / epidemiology
- Medication Therapy Management
- Pharmacists
- Retrospective Studies
- SARS-CoV-2
- Skilled Nursing Facilities

Full text links

Ingenta plc
Proceed to details
Cite
Share
☐ 490
Observational Study
J Prim Care Community Health
. Jan-Dec 2022;13:21501319211069748.
doi: 10.1177/21501319211069748.

Utilization of an Electronic Health Record Integrated Risk Score to Predict Hospitalization Among COVID-19 Patients

Mark A Nyman [1], Thulasee Jose [1 2], Ivana T Croghan [1], Mark A Parkulo [3], Charles D Burger [3], Darrell R Schroeder [1], Ryan T Hurt [1], John C O'Horo [1]

Affiliations Expand

Affiliations

- [1] Mayo Clinic, Rochester, MN, USA.
- [2] Baptist Hospitals of Southeast Texas, Beaumont, TX, USA.
- [3] Mayo Clinic, Jacksonville, FL, USA.

- PMID: **35068257**
- PMCID: PMC8796071
- DOI: 10.1177/21501319211069748

Free PMC article
Observational Study

Utilization of an Electronic Health Record Integrated Risk Score to Predict Hospitalization Among COVID-19 Patients

Mark A Nyman et al. J Prim Care Community Health. Jan-Dec 2022.
Free PMC article
Show details
J Prim Care Community Health
. Jan-Dec 2022;13:21501319211069748.
doi: 10.1177/21501319211069748.

Authors

Mark A Nyman [1], Thulasee Jose [1, 2], Ivana T Croghan [1], Mark A Parkulo [3], Charles D Burger [3], Darrell R Schroeder [1], Ryan T Hurt [1], John C O'Horo [1]

Affiliations

- [1] Mayo Clinic, Rochester, MN, USA.
- [2] Baptist Hospitals of Southeast Texas, Beaumont, TX, USA.
- [3] Mayo Clinic, Jacksonville, FL, USA.

- PMID: **35068257**
- PMCID: PMC8796071
- DOI: 10.1177/21501319211069748

Abstract

Objective: To evaluate the performance of an Electronic Health Record (EHR) integrated risk score for COVID-19 positive outpatients to predict 30-day risk of hospitalization.

Patients and methods: A retrospective observational study of 67 470 patients with COVID-19 confirmed by polymerase chain reaction (PCR) test between March 12, 2020 and February 8, 2021. Risk scores were calculated based on data in the chart at the time of the incident infection.

Results: The Mayo Clinic COVID-19 risk score consisted of 13 components included age, sex, chronic lung disease, congenital heart disease, congestive heart failure, coronary artery disease, diabetes mellitus, end stage liver disease, end stage renal disease, hypertension, immune compromised, nursing home resident, and pregnant. Univariate analysis showed all components, except pregnancy, have significant ($P < .001$) association with admission. The Mayo Clinic COVID-19 risk score showed a Receiver Operating Characteristic Area Under Curve (AUC) of 0.837 for the prediction of admission for this large cohort of COVID-19 positive patients.

Conclusion: The Mayo Clinic COVID-19 risk score is a simple score that is easily integrated into the EHR with excellent predictive performance for severe COVID-19. It can be leveraged to stratify risk for severe COVID-19 at initial contact, when considering therapeutics or in the allocation of vaccine supply.

Keywords: COVID-19; EHR; SARS-CoV2; pandemic; risk score.

Conflict of interest statement

Declaration of Conflicting Interests: The author(s) declared no potential conflicts of interest with respect to the research, authorship, and/or publication of this article.

- 21 references
- 3 figures

Supplementary info

Publication types, MeSH terms Expand

Publication types

- Observational Study
- Research Support, Non-U.S. Gov't

MeSH terms

- COVID-19*
- Electronic Health Records
- Female
- Hospitalization
- Humans
- Pregnancy
- Retrospective Studies
- Risk Factors
- SARS-CoV-2

Full text links

SAGE journals Open access full text Atypon Free PMC article
Proceed to details
Cite
Share
491
Multicenter Study
Front Endocrinol (Lausanne)
. 2022 Jan 13;12:777130.
doi: 10.3389/fendo.2021.777130. eCollection 2021.

Clinical Characteristics of COVID-19 Patients in a Regional Population With Diabetes Mellitus: The ACCREDIT Study

Daniel Kevin Llanera [1], Rebekah Wilmington [1], Haika Shoo [1], Paulo Lisboa [2], Ian Jarman [2], Stephanie Wong [3], Jael Nizza [3], Dushyant Sharma [4], Dhanya Kalathil [4], Surya Rajeev [4], Scott Williams [5], Rahul Yadav [6], Zubair Qureshi [7], Ram Prakash Narayanan [8], Niall Furlong [8], Sam Westall [8], Sunil Nair [1]
Affiliations Expand

Affiliations

- [1] Department of Diabetes and Endocrinology, Countess of Chester Hospital NHS Foundation Trust, Chester, United Kingdom.
- [2] School of Computer Science and Mathematics, Liverpool John Moores University, Liverpool, United Kingdom.
- [3] Department of Diabetes and Endocrinology, Arrowe Park Hospital, Birkenhead, United Kingdom.
- [4] Department of Diabetes and Endocrinology, The Royal Liverpool University Hospital, Liverpool, United Kingdom.
- [5] Department of Diabetes and Endocrinology, Aintree University Hospital, Liverpool, United Kingdom.
- [6] Department of Diabetes and Endocrinology, Warrington Hospital, Warrington, United Kingdom.
- [7] Department of Diabetes and Endocrinology, Leighton Hospital, Crewe, United Kingdom.
- [8] Department of Diabetes and Endocrinology, Whiston Hospital, Prescot, United Kingdom.

- PMID: **35095757**
- PMCID: PMC8793829
- DOI: 10.3389/fendo.2021.777130

Free PMC article
Multicenter Study

Clinical Characteristics of COVID-19 Patients in a Regional Population With Diabetes Mellitus: The ACCREDIT Study

Daniel Kevin Llanera et al. Front Endocrinol (Lausanne). 2022.
Free PMC article
Show details
Front Endocrinol (Lausanne)
. 2022 Jan 13;12:777130.
doi: 10.3389/fendo.2021.777130. eCollection 2021.

Authors

Daniel Kevin Llanera [1], Rebekah Wilmington [1], Haika Shoo [1], Paulo Lisboa [2], Ian Jarman [2], Stephanie Wong [3], Jael Nizza [3], Dushyant Sharma [4], Dhanya Kalathil [4], Surya Rajeev [4], Scott Williams [5], Rahul Yadav [6], Zubair Qureshi [7], Ram Prakash Narayanan [8], Niall Furlong [8], Sam Westall [8], Sunil Nair [1]

Affiliations

- [1] Department of Diabetes and Endocrinology, Countess of Chester Hospital NHS Foundation Trust, Chester, United Kingdom.
- [2] School of Computer Science and Mathematics, Liverpool John Moores University, Liverpool, United Kingdom.
- [3] Department of Diabetes and Endocrinology, Arrowe Park Hospital, Birkenhead, United Kingdom.
- [4] Department of Diabetes and Endocrinology, The Royal Liverpool University Hospital, Liverpool, United Kingdom.
- [5] Department of Diabetes and Endocrinology, Aintree University Hospital, Liverpool, United Kingdom.
- [6] Department of Diabetes and Endocrinology, Warrington Hospital, Warrington, United Kingdom.
- [7] Department of Diabetes and Endocrinology, Leighton Hospital, Crewe, United Kingdom.
- [8] Department of Diabetes and Endocrinology, Whiston Hospital, Prescot, United Kingdom.

- PMID: **35095757**
- PMCID: PMC8793829
- DOI: 10.3389/fendo.2021.777130

Abstract

Objective: To identify clinical and biochemical characteristics associated with 7- & 30-day mortality and intensive care admission amongst diabetes patients admitted with COVID-19.

Research design and methods: We conducted a cohort study collecting data from medical notes of hospitalised people with diabetes and COVID-19 in 7 hospitals within the Mersey-Cheshire

region from 1 January to 30 June 2020. We also explored the impact on inpatient diabetes team resources. Univariate and multivariate logistic regression analyses were performed and optimised by splitting the dataset into a training, test, and validation sets, developing a robust predictive model for the primary outcome.

Results: We analyzed data from 1004 diabetes patients (mean age 74.1 (± 12.6) years, predominantly men 60.7%). 45% belonged to the most deprived population quintile in the UK. Median BMI was 27.6 (IQR 23.9-32.4) kg/m^2. The primary outcome (7-day mortality) occurred in 24%, increasing to 33% by day 30. Approximately one in ten patients required insulin infusion (9.8%). In univariate analyses, patients with type 2 diabetes had a higher risk of 7-day mortality [p < 0.05, OR 2.52 (1.06, 5.98)]. Patients requiring insulin infusion had a lower risk of death [p = 0.02, OR 0.5 (0.28, 0.9)]. CKD in younger patients (<70 years) had a greater risk of death [OR 2.74 (1.31-5.76)]. BMI, microvascular and macrovascular complications, HbA1c, and random non-fasting blood glucose on admission were not associated with mortality. On multivariate analysis, CRP and age remained associated with the primary outcome [OR 3.44 (2.17, 5.44)] allowing for a validated predictive model for death by day 7.

Conclusions: Higher CRP and advanced age were associated with and predictive of death by day 7. However, BMI, presence of diabetes complications, and glycaemic control were not. A high proportion of these patients required insulin infusion warranting increased input from the inpatient diabetes teams.

Keywords: COVID-19; CRP; diabetes; mortality; observational study; risk factors.

Copyright © 2022 Llanera, Wilmington, Shoo, Lisboa, Jarman, Wong, Nizza, Sharma, Kalathil, Rajeev, Williams, Yadav, Qureshi, Narayanan, Furlong, Westall and Nair.

Conflict of interest statement

The authors declare that the research was conducted in the absence of any commercial or financial relationships that could be construed as a potential conflict of interest.

- 32 references
- 2 figures

Supplementary info

Publication types, MeSH terms, Substances Expand

Publication types

- Multicenter Study

MeSH terms

- Age Factors
- Aged
- Aged, 80 and over
- Biomarkers / blood*
- Blood Glucose / analysis

- COVID-19 / complications*
- COVID-19 / transmission
- COVID-19 / virology
- Diabetes Mellitus, Type 2 / blood
- Diabetes Mellitus, Type 2 / epidemiology
- Diabetes Mellitus, Type 2 / mortality*
- Diabetes Mellitus, Type 2 / virology
- Female
- Follow-Up Studies
- Glycated Hemoglobin A / analysis
- Hospitalization
- Humans
- Male
- Middle Aged
- Prognosis
- Receptors, Immunologic / blood*
- Retrospective Studies
- SARS-CoV-2 / isolation & purification*
- Survival Rate
- United Kingdom / epidemiology

Substances

- Biomarkers
- Blood Glucose
- CRP protein, human
- Glycated Hemoglobin A
- Receptors, Immunologic
- hemoglobin A1c protein, human

Full text links

OPEN ACCESS frontiers Frontiers Media SA Free PMC article

Proceed to details

Cite

Share

☐ 492

Observational Study

BMC Infect Dis

. 2022 Feb 23;22(1):185.
doi: 10.1186/s12879-022-07176-x.

The changing pattern of bacterial and fungal respiratory isolates in patients with and without COVID-19 admitted to intensive care unit

Gianluca Zuglian [1], Diego Ripamonti [2], Alessandra Tebaldi [2], Marina Cuntrò [3], Ivano Riva [4], Claudio Farina [3], Marco Rizzi [2]
Affiliations Expand

Affiliations

- [1] Infectious Diseases Unit, ASST "Papa Giovanni XXIII", Piazza OMS, 1, 24127, Bergamo, Italy. gianluca.zuglian@gmail.com.
- [2] Infectious Diseases Unit, ASST "Papa Giovanni XXIII", Piazza OMS, 1, 24127, Bergamo, Italy.
- [3] Microbiology and Virology Laboratory, ASST "Papa Giovanni XXIII", Bergamo, Italy.
- [4] Intensive Care Unit, ASST "Papa Giovanni XXIII", Bergamo, Italy.

- PMID: **35196993**
- PMCID: PMC8865172
- DOI: 10.1186/s12879-022-07176-x

Free PMC article
Observational Study

The changing pattern of bacterial and fungal respiratory isolates in patients with and without COVID-19 admitted to intensive care unit

Gianluca Zuglian et al. BMC Infect Dis. 2022.
Free PMC article
Show details
BMC Infect Dis
. 2022 Feb 23;22(1):185.
doi: 10.1186/s12879-022-07176-x.

Authors

Gianluca Zuglian [1], Diego Ripamonti [2], Alessandra Tebaldi [2], Marina Cuntrò [3], Ivano Riva [4], Claudio Farina [3], Marco Rizzi [2]

Affiliations

- [1] Infectious Diseases Unit, ASST "Papa Giovanni XXIII", Piazza OMS, 1, 24127, Bergamo, Italy. gianluca.zuglian@gmail.com.
- [2] Infectious Diseases Unit, ASST "Papa Giovanni XXIII", Piazza OMS, 1, 24127, Bergamo, Italy.
- [3] Microbiology and Virology Laboratory, ASST "Papa Giovanni XXIII", Bergamo, Italy.
- [4] Intensive Care Unit, ASST "Papa Giovanni XXIII", Bergamo, Italy.

- PMID: **35196993**
- PMCID: PMC8865172
- DOI: 10.1186/s12879-022-07176-x

Abstract

Objectives: Severe acute respiratory syndrome 2 (SARS-CoV-2) pandemic has had a heavy impact on national health system, especially in the first wave. That impact hit principally the intensive care units (ICUs). The large number of patients requiring hospitalization in ICUs lead to a complete upheaval of intensive wards. The increase in bed, the fewer number of nurses per patient, the constant use of personal protective equipment, the new antimicrobial surveillance protocols could have had deeply effects on microbiological flora of these wards. Moreover, the overconsumption of antimicrobial therapy in COVID-19 patients, like several studies report, could have impact of this aspect. Aim of this study is to evaluate the changing pattern of microbiological respiratory isolates during and before COVID-19 pandemic in a tertiary hospital ICUs.

Methods: A retrospective, observational study was conducted in ICUs of "ASST Papa Giovanni XXIII", a large tertiary referral hospital in Northern Italy. We have retrospectively collected the microbiological data from bronchoalveolar lavage (BAL) and tracheal aspirate (TA) of patients with COVID-19, hospitalized in ICUs from 22nd February 2020 to 31st May 2020 (Period 1), and without COVID-19, from 22nd February 2019 to 31st May 2019 (Period 2). We compared the prevalence and the antibiotic profile of bacterial and fungal species in the two time periods.

Results: The prevalence of Pseudomonas spp. shows a statistically significant increase from patients without COVID-19 compared to COVID-19 positive as well as the prevalence of Enterococcus spp. On the contrary, the prevalence of Gram negative non fermenting bacteria (GN-NFB), Haemophilus influenzae and Streptococcus pneumoniae showed a significant reduction between two periods. There was a statistically significant increase in resistance of Pseudomonas spp. to carbapenems and piperacillin/tazobactam and Enterobacterales spp. for piperacillin/tazobactam, in COVID-19 positive patients compared to patients without COVID-19. We did not observe significant changing in fungal respiratory isolates.

Conclusions: A changing pattern in prevalence and resistance profiles of bacterial and fungal species was observed during COVID-19 pandemic.

© 2022. The Author(s).

Conflict of interest statement

The authors declare that they have no competing interests.

- 19 references

Supplementary info

Publication types, MeSH terms, Substances Expand

Publication types

- Observational Study

MeSH terms

- Anti-Bacterial Agents / pharmacology
- Anti-Bacterial Agents / therapeutic use
- Bacteria
- COVID-19*
- Drug Resistance, Bacterial
- Hospitalization
- Humans
- Intensive Care Units
- Microbial Sensitivity Tests
- Pandemics
- Retrospective Studies
- SARS-CoV-2

Substances

- Anti-Bacterial Agents

Full text links

Read free full text at BMC BioMed Central Free PMC article
Proceed to details
Cite
Share
493
Observational Study
Clin Microbiol Infect
. 2022 Jan;28(1):107-113.
doi: 10.1016/j.cmi.2021.08.022. Epub 2021 Sep 30.

Association between first language and SARS-CoV-2 infection rates, hospitalization,

intensive care admissions and death in Finland: a population-based observational cohort study

Ville Holmberg [1], Heli Salmi [2], Salla Kattainen [2], Jukka Ollgren [3], Anu Kantele [4], Juulia Pynnönen [4], Asko Järvinen [4], Erik Forsblom [4], Suvi Silén [5], Sanna-Maria Kivivuori [6], Atte Meretoja [7], Johanna Hästbacka [2]
Affiliations Expand

Affiliations

- [1] Department of Infectious Diseases, University of Helsinki and Helsinki University Hospital, Helsinki, Finland. Electronic address: ville.holmberg@hus.fi.
- [2] Department of Anaesthesiology, Intensive Care and Pain Medicine, University of Helsinki and Helsinki University Hospital, Helsinki, Finland.
- [3] Department of Health Security, Finnish Institute for Health and Welfare (THL), Finland.
- [4] Department of Infectious Diseases, University of Helsinki and Helsinki University Hospital, Helsinki, Finland.
- [5] Department of Otorhinolaryngology, Head and Neck Surgery, University of Helsinki and Helsinki University Hospital, Helsinki, Finland; Department of Biosciences and Nutrition, Karolinska Institutet, Stockholm, Sweden.
- [6] Helsinki University Hospital, Helsinki, Finland.
- [7] General Administration, Helsinki University Hospital, Helsinki, Finland.

- PMID: **34949510**
- PMCID: PMC8482016
- DOI: 10.1016/j.cmi.2021.08.022

Free PMC article
Observational Study

Association between first language and SARS-CoV-2 infection rates, hospitalization, intensive care admissions and death in Finland: a population-based observational cohort study

Ville Holmberg et al. Clin Microbiol Infect. 2022 Jan.
Free PMC article
Show details
Clin Microbiol Infect
. 2022 Jan;28(1):107-113.

doi: 10.1016/j.cmi.2021.08.022. Epub 2021 Sep 30.

Authors

Ville Holmberg [1], Heli Salmi [2], Salla Kattainen [2], Jukka Ollgren [3], Anu Kantele [4], Juulia Pynnönen [4], Asko Järvinen [4], Erik Forsblom [4], Suvi Silén [5], Sanna-Maria Kivivuori [6], Atte Meretoja [7], Johanna Hästbacka [2]

Affiliations

- [1] Department of Infectious Diseases, University of Helsinki and Helsinki University Hospital, Helsinki, Finland. Electronic address: ville.holmberg@hus.fi.
- [2] Department of Anaesthesiology, Intensive Care and Pain Medicine, University of Helsinki and Helsinki University Hospital, Helsinki, Finland.
- [3] Department of Health Security, Finnish Institute for Health and Welfare (THL), Finland.
- [4] Department of Infectious Diseases, University of Helsinki and Helsinki University Hospital, Helsinki, Finland.
- [5] Department of Otorhinolaryngology, Head and Neck Surgery, University of Helsinki and Helsinki University Hospital, Helsinki, Finland; Department of Biosciences and Nutrition, Karolinska Institutet, Stockholm, Sweden.
- [6] Helsinki University Hospital, Helsinki, Finland.
- [7] General Administration, Helsinki University Hospital, Helsinki, Finland.

- PMID: **34949510**
- PMCID: PMC8482016
- DOI: 10.1016/j.cmi.2021.08.022

Abstract

Objectives: Motivated by reports of increased risk of coronavirus disease 2019 (COVID-19) in ethnic minorities of high-income countries, we explored whether patients with a foreign first language are at an increased risk of COVID-19 infections, more serious presentations, or worse outcomes.

Methods: In a retrospective observational population-based quality registry study covering a population of 1.7 million, we studied the incidence of severe acute respiratory syndrome coronavirus 2 (SARS-CoV-2), admissions to specialist healthcare and the intensive care unit (ICU), and all-cause case fatality in different language groups between 27th February and 3rd August 2020 in Southern Finland. A first language other than Finnish, Swedish or Sámi served as a surrogate marker for a foreign ethnic background.

Results: In total, 124 240 individuals were tested, and among the 118 300 (95%) whose first language could be determined, 4005 (3.4%) were COVID-19-positive, 623 (0.5%) were admitted to specialized hospitals, and 147 (0.1%) were admitted to the ICU; 254 (0.2%) died. Those with a foreign first language had lower testing rates (348, 95%CI 340-355 versus 758, 95%CI 753-762 per 10 000, $p < 0.0001$), higher incidence (36, 95%CI 33-38 versus 22, 95%CI 21-23 per 10 000, $p < 0.0001$), and higher positivity rates (103, 95%CI 96-109 versus 29, 95%CI 28-30 per 1000, $p < 0.0001$). There was no significant difference in ICU admissions, disease severity at ICU admission, or ICU outcomes. Case fatality by 90 days was 7.7% in domestic cases and 1.2% in those with a foreign first language, explained by demographics (age- and sex-adjusted HR 0.49, 95%CI 0.21-1.15).

Conclusions: The population with a foreign first language was at an increased risk for testing positive for SARS-CoV-2, but when hospitalized they had outcomes similar to those in the native, domestic language population. This suggests that special attention should be paid to the prevention and control of infectious diseases among language minorities.

Keywords: COVID-19; Ethnic; Language; Migrants; Minority; SARS-CoV-2; Vulnerable.

Copyright © 2021 The Authors. Published by Elsevier Ltd.. All rights reserved.

- 17 references
- 2 figures

Supplementary info

Publication types, MeSH terms Expand

Publication types

- Observational Study

MeSH terms

- COVID-19* / epidemiology
- COVID-19* / ethnology
- Cohort Studies
- Critical Care
- Ethnic and Racial Minorities / statistics & numerical data*
- Finland / epidemiology
- Hospitalization
- Humans
- Intensive Care Units
- Language
- Retrospective Studies

Full text links

ELSEVIER OPEN ACCESS Elsevier Science Free PMC article

Proceed to details

Cite

Share

☐ 494

Observational Study

Anaesthesia

. 2022 Feb;77(2):143-152.
doi: 10.1111/anae.15581. Epub 2021 Sep 20.

The effect of patient ethnicity on the accuracy of peripheral pulse oximetry in patients with COVID-19 pneumonitis: a single-centre, retrospective analysis

M D Wiles [1,2], A El-Nayal [1], G Elton [1], M Malaj [1], J Winterbottom [1], C Gillies [1], I K Moppett [3], K Bauchmuller [1]
Affiliations Expand

Affiliations

- [1] Department of Critical Care, Sheffield Teaching Hospitals NHS Foundation Trust, Sheffield, UK.
- [2] University of Sheffield Medical School, Sheffield, UK.
- [3] Department of Anaesthesia and Peri-operative Medicine, University of Nottingham, Nottingham, UK.

- PMID: **34542168**
- PMCID: PMC8653100
- DOI: 10.1111/anae.15581

Free PMC article
Observational Study

The effect of patient ethnicity on the accuracy of peripheral pulse oximetry in patients with COVID-19 pneumonitis: a single-centre, retrospective analysis

M D Wiles et al. Anaesthesia. 2022 Feb.
Free PMC article
Show details
Anaesthesia
. 2022 Feb;77(2):143-152.
doi: 10.1111/anae.15581. Epub 2021 Sep 20.

Authors

M D Wiles [1,2], A El-Nayal [1], G Elton [1], M Malaj [1], J Winterbottom [1], C Gillies [1], I K Moppett [3], K Bauchmuller [1]

Affiliations

- [1] Department of Critical Care, Sheffield Teaching Hospitals NHS Foundation Trust, Sheffield, UK.
- [2] University of Sheffield Medical School, Sheffield, UK.
- [3] Department of Anaesthesia and Peri-operative Medicine, University of Nottingham, Nottingham, UK.

- PMID: **34542168**
- PMCID: PMC8653100
- DOI: 10.1111/anae.15581

Abstract

Pulse oximetry is used widely to titrate oxygen therapy and for triage in patients who are critically ill. However, there are concerns regarding the accuracy of pulse oximetry in patients with COVID-19 pneumonitis and in patients who have a greater degree of skin pigmentation. We aimed to determine the impact of patient ethnicity on the accuracy of peripheral pulse oximetry in patients who were critically ill with COVID-19 pneumonitis by conducting a retrospective observational study comparing paired measurements of arterial oxygen saturation measured by co-oximetry on arterial blood gas analysis (SaO_2) and the corresponding peripheral oxygenation saturation measured by pulse oximetry (S_p O_2). Bias was calculated as the mean difference between SaO_2 and S_p O_2 measurements and limits of agreement were calculated as bias ±1.96 SD. Data from 194 patients (135 White ethnic origin, 34 Asian ethnic origin, 19 Black ethnic origin and 6 other ethnic origin) were analysed consisting of 6216 paired SaO_2 and S_p O_2 measurements. Bias (limits of agreement) between SaO_2 and S_p O_2 measurements was 0.05% (-2.21-2.30). Patient ethnicity did not alter this to a clinically significant degree: 0.28% (1.79-2.35), -0.33% (-2.47-2.35) and -0.75% (-3.47-1.97) for patients of White, Asian and Black ethnic origin, respectively. In patients with COVID-19 pneumonitis, S_p O_2 measurements showed a level of agreement with SaO_2 values that was in line with previous work, and this was not affected by patient ethnicity.

Keywords: COVID-19 pneumonitis; critical care; ethnicity; pulse oximetry; skin pigmentation.

© 2021 Association of Anaesthetists.

Comment in

- Variation in pulse oximetry readings: melanin, not ethnicity, is the appropriate variable to use when investigating bias.
  Norton HL. Norton HL. Anaesthesia. 2022 Mar;77(3):354-355. doi: 10.1111/anae.15620. Epub 2021 Nov 11. Anaesthesia. 2022. PMID: 34766336 No abstract available.
- Racial discrepancies in oximetry: where do we stand?
  Knight MJ, Subbe CP, Inada-Kim M. Knight MJ, et al. Anaesthesia. 2022 Feb;77(2):129-131. doi: 10.1111/anae.15635. Epub 2021 Nov 29. Anaesthesia. 2022. PMID: 34844284 No abstract available.

- Cited by 2 articles
- 15 references
- 4 figures

Supplementary info

Publication types, MeSH terms Expand

Publication types

- Observational Study

MeSH terms

- COVID-19 / physiopathology*
- COVID-19 / therapy
- Critical Care / methods
- Ethnicity / statistics & numerical data*
- Female
- Humans
- Male
- Middle Aged
- Oximetry / methods*
- Oximetry / standards*
- Oxygen Inhalation Therapy / methods
- Oxygen Saturation / physiology*
- Reproducibility of Results
- Retrospective Studies
- SARS-CoV-2

Full text links

WILEY Full Text Article Wiley Free PMC article

Proceed to details

Cite

Share

☐ 495

Acta Anaesthesiol Scand
. 2022 Jan;66(1):65-75.
doi: 10.1111/aas.13991. Epub 2021 Oct 15.

Rapid Evaluation of Coronavirus Illness Severity (RECOILS) in intensive care:

Development and validation of a prognostic tool for in-hospital mortality

Drago Plečko [1, 2], Nicolas Bennett [2], Johan Mårtensson [3, 4], Tariq A Dam [1], Robert Entjes [5], Thijs C D Rettig [6], Dave A Dongelmans [1], Age D Boelens [7], Sander Rigter [8], Stefaan H A Hendriks [9], Remko de Jong [10], Marlijn J A Kamps [11], Marco Peters [12], Attila Karakus [13], Diederik Gommers [14], Dharmanand Ramnarain [15], Evert-Jan Wils [16], Sefanja Achterberg [17], Ralph Nowitzky [18], Walter van den Tempel [19], Cornelis P C de Jager [20], Fleur G C A Nooteboom [21], Evelien Oostdijk [22], Peter Koetsier [23], Alexander D Cornet [24], Auke C Reidinga [25], Wouter de Ruijter [26], Rob J Bosman [27], Tim Frenzel [28], Louise C Urlings-Strop [29], Paul de Jong [30], Ellen G M Smit [31], Olaf L Cremer [32], D Jannet Mehagnoul-Schipper [33], Harald J Faber [34], Judith Lens [35], Gert B Brunnekreef [36], Barbara Festen-Spanjer [37], Tom Dormans [38], Daan P de Bruin [39], Robbert C A Lalisang [39], Sebastiaan J J Vonk [39], Martin E Haan [1], Lucas M Fleuren [1], Patrick J Thoral [1], Paul W G Elbers [1], Rinaldo Bellomo [40, 41, 42, 43]

Affiliations Expand

Affiliations

- [1] Department of Intensive Care Medicine, Laboratory for Critical Care Computational Intelligence, Amsterdam Medical Data Science, Amsterdam UMC, Amsterdam, The Netherlands.
- [2] Department of Mathematics, Seminar for Statistics, ETH Zürich, Zurich, Switzerland.
- [3] Department of Physiology and Pharmacology, Section of Anaesthesia and Intensive Care, Karolinska Institutet, Stockholm, Sweden.
- [4] Department of Perioperative Medicine and Intensive Care, Karolinska University Hospital, Stockholm, Sweden.
- [5] Department of Intensive Care, Admiraal De Ruyter Ziekenhuis, Goes, The Netherlands.
- [6] Department of Intensive Care, Amphia Ziekenhuis, Breda, The Netherlands.
- [7] Antonius Ziekenhuis Sneek, Sneek, The Netherlands.
- [8] Department of Anesthesiology and Intensive Care, St. Antonius Hospital, Nieuwegein, The Netherlands.
- [9] Intensive Care, Albert Schweitzerziekenhuis, Dordrecht, The Netherlands.
- [10] Intensive Care, Bovenij Ziekenhuis, Amsterdam, The Netherlands.
- [11] Intensive Care, Catharina Ziekenhuis Eindhoven, Eindhoven, The Netherlands.
- [12] Intensive Care, Canisius Wilhelmina Ziekenhuis, Nijmegen, The Netherlands.
- [13] Department of Intensive Care, Diakonessenhuis Hospital, Utrecht, The Netherlands.
- [14] Department of Intensive Care, Erasmus Medical Center, Rotterdam, The Netherlands.
- [15] Intensive Care, ETZ Tilburg, Tilburg, The Netherlands.
- [16] Department of Intensive Care, Franciscus Gasthuis & Vlietland, Rotterdam, The Netherlands.
- [17] ICU, Haaglanden Medisch Centrum, Den Haag, The Netherlands.
- [18] Intensive Care, HagaZiekenhuis, Den Haag, The Netherlands.
- [19] Department of Intensive Care, Ikazia Ziekenhuis Rotterdam, Rotterdam, The Netherlands.
- [20] Department of Intensive Care, Jeroen Bosch Ziekenhuis, Den Bosch, The Netherlands.
- [21] Intensive Care, Laurentius Ziekenhuis, Roermond, The Netherlands.

- [22] ICU, Maasstad Ziekenhuis Rotterdam, Rotterdam, The Netherlands.
- [23] Intensive Care, Medisch Centrum Leeuwarden, Leeuwarden, The Netherlands.
- [24] Department of Intensive Care, Medisch Spectrum Twente, Enschede, The Netherlands.
- [25] ICU, SEH, BWC, Martiniziekenhuis, Groningen, The Netherlands.
- [26] Department of Intensive Care Medicine, Northwest Clinics, Alkmaar, The Netherlands.
- [27] ICU, OLVG, Amsterdam, The Netherlands.
- [28] Department of Intensive Care Medicine, Radboud University Medical Center, Nijmegen, The Netherlands.
- [29] Intensive Care, Reinier de Graaf Gasthuis, Delft, The Netherlands.
- [30] Department of Anesthesia and Intensive Care, Slingeland Ziekenhuis, Doetinchem, The Netherlands.
- [31] Intensive Care, Spaarne Gasthuis, Haarlem en Hoofddorp, The Netherlands.
- [32] Intensive Care, UMC Utrecht, Utrecht, The Netherlands.
- [33] Intensive Care, VieCuri Medisch Centrum, Venlo, The Netherlands.
- [34] ICU, WZA, Assen, The Netherlands.
- [35] ICU, ICU, IJsselland Ziekenhuis, Capelle aan den IJssel, The Netherlands.
- [36] Department of Intensive Care, Ziekenhuisgroep Twente, Almelo, The Netherlands.
- [37] Intensive Care, Ziekenhuis Gelderse Vallei, Ede, The Netherlands.
- [38] Intensive care, Zuyderland MC, Heerlen, The Netherlands.
- [39] Pacmed, Amsterdam, Amsterdam, The Netherlands.
- [40] Australian and New Zealand Intensive Care Research Centre, School of Public Health and Preventative Medicine, Monash University, Melbourne, Australia.
- [41] Department of Critical Care, The University of Melbourne, Melbourne, Australia.
- [42] Data Analytics Research and Evaluation Centre, Department of Medicine and Radiology, The University of Melbourne.
- [43] Austin Hospital, Melbourne, Australia.

- PMID: **34622441**
- PMCID: PMC8652966
- DOI: 10.1111/aas.13991

Free PMC article

Rapid Evaluation of Coronavirus Illness Severity (RECOILS) in intensive care: Development and validation of a prognostic tool for in-hospital mortality

Drago Plečko et al. Acta Anaesthesiol Scand. 2022 Jan.
Free PMC article

Show details

Acta Anaesthesiol Scand
. 2022 Jan;66(1):65-75.
doi: 10.1111/aas.13991. Epub 2021 Oct 15.

Authors

Drago Plečko [1, 2], Nicolas Bennett [2], Johan Mårtensson [3, 4], Tariq A Dam [1], Robert Entjes [5], Thijs C D Rettig [6], Dave A Dongelmans [1], Age D Boelens [7], Sander Rigter [8], Stefaan H A Hendriks [9], Remko de Jong [10], Marlijn J A Kamps [11], Marco Peters [12], Attila Karakus [13], Diederik Gommers [14], Dharmanand Ramnarain [15], Evert-Jan Wils [16], Sefanja Achterberg [17], Ralph Nowitzky [18], Walter van den Tempel [19], Cornelis P C de Jager [20], Fleur G C A Nooteboom [21], Evelien Oostdijk [22], Peter Koetsier [23], Alexander D Cornet [24], Auke C Reidinga [25], Wouter de Ruijter [26], Rob J Bosman [27], Tim Frenzel [28], Louise C Urlings-Strop [29], Paul de Jong [30], Ellen G M Smit [31], Olaf L Cremer [32], D Jannet Mehagnoul-Schipper [33], Harald J Faber [34], Judith Lens [35], Gert B Brunnekreef [36], Barbara Festen-Spanjer [37], Tom Dormans [38], Daan P de Bruin [39], Robbert C A Lalisang [39], Sebastiaan J J Vonk [39], Martin E Haan [1], Lucas M Fleuren [1], Patrick J Thoral [1], Paul W G Elbers [1], Rinaldo Bellomo [40, 41, 42, 43]

Affiliations

- [1] Department of Intensive Care Medicine, Laboratory for Critical Care Computational Intelligence, Amsterdam Medical Data Science, Amsterdam UMC, Amsterdam, The Netherlands.
- [2] Department of Mathematics, Seminar for Statistics, ETH Zürich, Zurich, Switzerland.
- [3] Department of Physiology and Pharmacology, Section of Anaesthesia and Intensive Care, Karolinska Institutet, Stockholm, Sweden.
- [4] Department of Perioperative Medicine and Intensive Care, Karolinska University Hospital, Stockholm, Sweden.
- [5] Department of Intensive Care, Admiraal De Ruyter Ziekenhuis, Goes, The Netherlands.
- [6] Department of Intensive Care, Amphia Ziekenhuis, Breda, The Netherlands.
- [7] Antonius Ziekenhuis Sneek, Sneek, The Netherlands.
- [8] Department of Anesthesiology and Intensive Care, St. Antonius Hospital, Nieuwegein, The Netherlands.
- [9] Intensive Care, Albert Schweitzerziekenhuis, Dordrecht, The Netherlands.
- [10] Intensive Care, Bovenij Ziekenhuis, Amsterdam, The Netherlands.
- [11] Intensive Care, Catharina Ziekenhuis Eindhoven, Eindhoven, The Netherlands.
- [12] Intensive Care, Canisius Wilhelmina Ziekenhuis, Nijmegen, The Netherlands.
- [13] Department of Intensive Care, Diakonessenhuis Hospital, Utrecht, The Netherlands.
- [14] Department of Intensive Care, Erasmus Medical Center, Rotterdam, The Netherlands.
- [15] Intensive Care, ETZ Tilburg, Tilburg, The Netherlands.
- [16] Department of Intensive Care, Franciscus Gasthuis & Vlietland, Rotterdam, The Netherlands.
- [17] ICU, Haaglanden Medisch Centrum, Den Haag, The Netherlands.
- [18] Intensive Care, HagaZiekenhuis, Den Haag, The Netherlands.
- [19] Department of Intensive Care, Ikazia Ziekenhuis Rotterdam, Rotterdam, The Netherlands.
- [20] Department of Intensive Care, Jeroen Bosch Ziekenhuis, Den Bosch, The Netherlands.
- [21] Intensive Care, Laurentius Ziekenhuis, Roermond, The Netherlands.
- [22] ICU, Maasstad Ziekenhuis Rotterdam, Rotterdam, The Netherlands.
- [23] Intensive Care, Medisch Centrum Leeuwarden, Leeuwarden, The Netherlands.
- [24] Department of Intensive Care, Medisch Spectrum Twente, Enschede, The Netherlands.
- [25] ICU, SEH, BWC, Martiniziekenhuis, Groningen, The Netherlands.

- [26] Department of Intensive Care Medicine, Northwest Clinics, Alkmaar, The Netherlands.
- [27] ICU, OLVG, Amsterdam, The Netherlands.
- [28] Department of Intensive Care Medicine, Radboud University Medical Center, Nijmegen, The Netherlands.
- [29] Intensive Care, Reinier de Graaf Gasthuis, Delft, The Netherlands.
- [30] Department of Anesthesia and Intensive Care, Slingeland Ziekenhuis, Doetinchem, The Netherlands.
- [31] Intensive Care, Spaarne Gasthuis, Haarlem en Hoofddorp, The Netherlands.
- [32] Intensive Care, UMC Utrecht, Utrecht, The Netherlands.
- [33] Intensive Care, VieCuri Medisch Centrum, Venlo, The Netherlands.
- [34] ICU, WZA, Assen, The Netherlands.
- [35] ICU, ICU, IJsselland Ziekenhuis, Capelle aan den IJssel, The Netherlands.
- [36] Department of Intensive Care, Ziekenhuisgroep Twente, Almelo, The Netherlands.
- [37] Intensive Care, Ziekenhuis Gelderse Vallei, Ede, The Netherlands.
- [38] Intensive care, Zuyderland MC, Heerlen, The Netherlands.
- [39] Pacmed, Amsterdam, Amsterdam, The Netherlands.
- [40] Australian and New Zealand Intensive Care Research Centre, School of Public Health and Preventative Medicine, Monash University, Melbourne, Australia.
- [41] Department of Critical Care, The University of Melbourne, Melbourne, Australia.
- [42] Data Analytics Research and Evaluation Centre, Department of Medicine and Radiology, The University of Melbourne.
- [43] Austin Hospital, Melbourne, Australia.

- PMID: **34622441**
- PMCID: PMC8652966
- DOI: 10.1111/aas.13991

Abstract

Background: The prediction of in-hospital mortality for ICU patients with COVID-19 is fundamental to treatment and resource allocation. The main purpose was to develop an easily implemented score for such prediction.

Methods: This was an observational, multicenter, development, and validation study on a national critical care dataset of COVID-19 patients. A systematic literature review was performed to determine variables possibly important for COVID-19 mortality prediction. Using a logistic multivariable model with a LASSO penalty, we developed the Rapid Evaluation of Coronavirus Illness Severity (RECOILS) score and compared its performance against published scores.

Results: Our development (validation) cohort consisted of 1480 (937) adult patients from 14 (11) Dutch ICUs admitted between March 2020 and April 2021. Median age was 65 (65) years, 31% (26%) died in hospital, 74% (72%) were males, average length of ICU stay was 7.83 (10.25) days and average length of hospital stay was 15.90 (19.92) days. Age, platelets, PaO2/FiO2 ratio, pH, blood urea nitrogen, temperature, PaCO2, Glasgow Coma Scale (GCS) score measured within +/- 24 h of ICU admission were used to develop the score. The AUROC of RECOILS score was 0.75 (CI 0.71-0.78) which was higher than that of any previously reported predictive scores (0.68 [CI 0.64-0.71], 0.61 [CI 0.58-0.66], 0.67 [CI 0.63-0.70], 0.70 [CI 0.67-0.74] for ISARIC 4C Mortality Score, SOFA, SAPS-III, and age, respectively).

Conclusions: Using a large dataset from multiple Dutch ICUs, we developed a predictive score for mortality of COVID-19 patients admitted to ICU, which outperformed other predictive scores reported so far.

Keywords: COVID-19; corona virus; intensive care; mechanical ventilation; respiratory failure.

© 2021 The Authors. Acta Anaesthesiologica Scandinavica published by John Wiley & Sons Ltd on behalf of Acta Anaesthesiologica Scandinavica Foundation.

Conflict of interest statement

The authors report no conflicts of interest.

- Cited by 1 article
- 40 references
- 2 figures

Supplementary info

Publication types, MeSH terms, Grant support Expand

Publication types

- Systematic Review

MeSH terms

- Adult
- Aged
- COVID-19*
- Critical Care
- Hospital Mortality
- Humans
- Intensive Care Units
- Male
- Multicenter Studies as Topic
- Observational Studies as Topic
- Patient Acuity
- Prognosis
- Retrospective Studies
- SARS-CoV-2

Grant support

- #2017-110/Personalized Health and Related Technologies (PHRT)

Full text links

Wiley Full Text Article Wiley Free PMC article
Proceed to details
Cite
Share
496
Observational Study
Nat Commun
. 2022 Feb 1;13(1):612.
doi: 10.1038/s41467-022-28233-8.

Impacts of rapid mass vaccination against SARS-CoV2 in an early variant of concern hotspot

Jörg Paetzold[#,1], Janine Kimpel[2], Katie Bates[3], Michael Hummer[4], Florian Krammer[5], Dorothee von Laer[2], Hannes Winner[#,6]
Affiliations Expand

Affiliations

- [1] University of Salzburg, Department of Economics, Residenzplatz 9, A-5010, Salzburg, Austria. Joerg.Paetzold@sbg.ac.at.
- [2] Institute of Virology, Department of Hygiene, Microbiology and Public Health, Medical University of Innsbruck, Peter-Mayr-Str. 4b, 6020, Innsbruck, Austria.
- [3] Department of Medical Statistics, Informatics and Health Economics, Medical University of Innsbruck, Innsbruck, Austria.
- [4] The Austrian National Public Health Institute (Gesundheit Österreich GmbH, GÖG), Stubenring 6, 1010, Vienna, Austria.
- [5] Department of Microbiology, Icahn School of Medicine at Mount Sinai, One Gustave L. Levy Place, Box 1124, New York, NY, 10029, USA.
- [6] University of Salzburg, Department of Economics, Residenzplatz 9, A-5010, Salzburg, Austria.

[#] Contributed equally.

- PMID: **35105889**
- PMCID: PMC8807735
- DOI: 10.1038/s41467-022-28233-8

Free PMC article
Observational Study

Impacts of rapid mass vaccination against SARS-CoV2 in an early variant of concern hotspot

Jörg Paetzold et al. Nat Commun. 2022.
Free PMC article
Show details
Nat Commun
. 2022 Feb 1;13(1):612.
doi: 10.1038/s41467-022-28233-8.

Authors

Jörg Paetzold[# 1], Janine Kimpel[2], Katie Bates[3], Michael Hummer[4], Florian Krammer[5], Dorothee von Laer[2], Hannes Winner[# 6]

Affiliations

- [1] University of Salzburg, Department of Economics, Residenzplatz 9, A-5010, Salzburg, Austria. Joerg.Paetzold@sbg.ac.at.
- [2] Institute of Virology, Department of Hygiene, Microbiology and Public Health, Medical University of Innsbruck, Peter-Mayr-Str. 4b, 6020, Innsbruck, Austria.
- [3] Department of Medical Statistics, Informatics and Health Economics, Medical University of Innsbruck, Innsbruck, Austria.
- [4] The Austrian National Public Health Institute (Gesundheit Österreich GmbH, GÖG), Stubenring 6, 1010, Vienna, Austria.
- [5] Department of Microbiology, Icahn School of Medicine at Mount Sinai, One Gustave L. Levy Place, Box 1124, New York, NY, 10029, USA.
- [6] University of Salzburg, Department of Economics, Residenzplatz 9, A-5010, Salzburg, Austria.

[#] Contributed equally.

- PMID: **35105889**
- PMCID: PMC8807735
- DOI: 10.1038/s41467-022-28233-8

Abstract

We study the real-life effect of an unprecedented rapid mass vaccination campaign. Following a large outbreak of the Beta variant in the district of Schwaz/Austria, 100,000 doses of BNT162b2 (Pfizer/BioNTech) were procured to mass vaccinate the entire adult population of the district between the 11th and 16th of March 2021. This made the district the first widely inoculated region in Europe. We examine the effect of this campaign on the number of infections, cases of variants of concern, hospital and ICU admissions. We compare Schwaz with (i) a control group of highly similar districts, and (ii) with populations residing in municipalities along the border of Schwaz which were just excluded from the campaign. We find large and significant decreases for all

outcomes after the campaign. Our results suggest that rapid mass vaccination is an effective tool to curb the spread of SARS-CoV-2.

© 2022. The Author(s).

Conflict of interest statement

The Icahn School of Medicine at Mount Sinai has filed patent applications relating to SARS-CoV-2 serological assays and NDV-based SARS-CoV-2 vaccines which list Florian Krammer as co-inventor. Mount Sinai has spun out a company, Kantaro, to market serological tests for SARS-CoV-2. Florian Krammer has consulted for Merck and Pfizer (before 2020), and is currently consulting for Pfizer, Seqirus, and Avimex. The Krammer laboratory is also collaborating with Pfizer on animal models of SARS-CoV-2. The funders had no role in the design of the study; in the collection or analyses of data, in the writing of the manuscript, or influence on the judgments and actions with regard to objective data presentation and interpretation. Thus, there was no threat to the objectivity, integrity, and value of a publication. For all other authors, no competing interests exist.

- 20 references
- 4 figures

Supplementary info

Publication types, MeSH terms, Substances, Supplementary concepts Expand

Publication types

- Observational Study
- Research Support, Non-U.S. Gov't

MeSH terms

- Adult
- Austria / epidemiology
- BNT162 Vaccine / administration & dosage*
- COVID-19 / epidemiology
- COVID-19 / prevention & control*
- COVID-19 / therapy
- COVID-19 / virology
- Hospitalization / statistics & numerical data
- Humans
- Intensive Care Units / statistics & numerical data
- Mass Vaccination
- Middle Aged
- Retrospective Studies
- SARS-CoV-2 / genetics

- SARS-CoV-2 / immunology*

Substances

- BNT162 Vaccine

Supplementary concepts

- SARS-CoV-2 variants

Full text links

Nature Publishing Group Free PMC article

Proceed to details

Cite

Share

497

Observational Study

Transpl Infect Dis

. 2020 Oct;22(5):e13372.

doi: 10.1111/tid.13372. Epub 2020 Jul 1.

Varied clinical presentation and outcome of SARS-CoV-2 infection in liver transplant recipients: Initial experience at a single center in Madrid, Spain

Carmelo Loinaz [1], Alberto Marcacuzco [1], Mario Fernández-Ruiz [2], Oscar Caso [1], Félix Cambra [1], Rafael San Juan [2], Iago Justo [1], Jorge Calvo [1], Alvaro García-Sesma [1], Alejandro Manrique [1], María Asunción Pérez-Jacoiste Asín [3], María Dolores Folgueira [4], José María Aguado [2], Carlos Lumbreras [3]

Affiliations Expand

Affiliations

- [1] HBP and Transplant Surgery Unit, Department of General Surgery, Digestive Tract and Abdominal Organ Transplantation, Hospital Universitario "12 de Octubre", Instituto de Investigación Sanitaria Hospital, Universidad Complutense de Madrid, Madrid, Spain.
- [2] Unit of infectious Diseases, Hospital Universitario "12 de Octubre", Instituto de Investigación Sanitaria Hospital, Universidad Complutense de Madrid, Madrid, Spain.
- [3] Department of Internal Medicine, Hospital Universitario "12 de Octubre", Instituto de Investigación Sanitaria Hospital ", Universidad Complutense de Madrid, Madrid, Spain.

- [4] Department of Microbiology, Hospital Universitario "12 de Octubre", Instituto de Investigación Sanitaria Hospital ", Universidad Complutense de Madrid Madrid, Madrid, Spain.

- PMID: **32562561**
- PMCID: PMC7323090
- DOI: 10.1111/tid.13372

Free PMC article
Observational Study

Varied clinical presentation and outcome of SARS-CoV-2 infection in liver transplant recipients: Initial experience at a single center in Madrid, Spain

Carmelo Loinaz et al. Transpl Infect Dis. 2020 Oct.
Free PMC article
Show details
Transpl Infect Dis
. 2020 Oct;22(5):e13372.
doi: 10.1111/tid.13372. Epub 2020 Jul 1.

Authors

Carmelo Loinaz [1], Alberto Marcacuzco [1], Mario Fernández-Ruiz [2], Oscar Caso [1], Félix Cambra [1], Rafael San Juan [2], Iago Justo [1], Jorge Calvo [1], Alvaro García-Sesma [1], Alejandro Manrique [1], María Asunción Pérez-Jacoiste Asín [3], María Dolores Folgueira [4], José María Aguado [2], Carlos Lumbreras [3]

Affiliations

- [1] HBP and Transplant Surgery Unit, Department of General Surgery, Digestive Tract and Abdominal Organ Transplantation, Hospital Universitario "12 de Octubre", Instituto de Investigación Sanitaria Hospital, Universidad Complutense de Madrid, Madrid, Spain.
- [2] Unit of infectious Diseases, Hospital Universitario "12 de Octubre", Instituto de Investigación Sanitaria Hospital, Universidad Complutense de Madrid, Madrid, Spain.
- [3] Department of Internal Medicine, Hospital Universitario "12 de Octubre", Instituto de Investigación Sanitaria Hospital ", Universidad Complutense de Madrid, Madrid, Spain.
- [4] Department of Microbiology, Hospital Universitario "12 de Octubre", Instituto de Investigación Sanitaria Hospital ", Universidad Complutense de Madrid Madrid, Madrid, Spain.

- PMID: **32562561**
- PMCID: PMC7323090
- DOI: 10.1111/tid.13372

Abstract

Background: Which are the consequences of severe acute respiratory syndrome coronavirus 2 (SARS-CoV-2) infection in liver transplant (LT) recipients?

Methods: We attempted to address this question by reviewing our single-center experience during the first 2 months of the pandemics at a high incidence area.

Results: Nineteen adult patients (5 females) were diagnosed by May 5, 2020. Median age was 58 (range 55-72), and median follow-up since transplantation was 83 (range 20-183) months. Cough (84.2%), fever (57.9%), and dyspnea (47.4%) were the most common symptoms. Thirteen patients (68.4%) had pneumonia in x-ray/CT scan. Hydroxychloroquine was administered in 11 patients, associated with lopinavir/ritonavir and interferon β in 2 cases each. Immunomodulatory therapy with tocilizumab was used in 2 patients. Immunosuppression (IS) was halted in one patient and modified in only other two due to potential drug interactions. Five (26.3%) patients were managed as outpatient. Two patients (10.5%) died, 10 (52.6%) were discharged home, and 2 (10.5%) were still hospitalized after a median follow-up of 41 days from the onset of symptoms. Baseline IS regimen remained unchanged in all surviving recipients, with good liver function.

Conclusions: Our preliminary experience shows a broad spectrum of disease severity in LT patients with COVID-19, with a favorable outcome in most of them without needing to modify baseline IS.

Keywords: COVID-19; SARS-CoV2; immunosuppression; liver transplant; prognosis.

© 2020 Wiley Periodicals LLC.

- Cited by 11 articles
- 25 references

Supplementary info

Publication types, MeSH terms, Substances, Grant support Expand

Publication types

- Observational Study

MeSH terms

- Aged
- Antibodies, Monoclonal, Humanized / therapeutic use
- Antiviral Agents / therapeutic use
- COVID-19 / diagnosis*
- COVID-19 / drug therapy
- COVID-19 / epidemiology
- COVID-19 / immunology
- Female

- Follow-Up Studies
- Graft Rejection / immunology
- Graft Rejection / prevention & control
- Humans
- Hydroxychloroquine / therapeutic use
- Immunocompromised Host
- Immunosuppressive Agents / adverse effects*
- Liver Transplantation / adverse effects*
- Male
- Middle Aged
- Pandemics
- Prospective Studies
- Retrospective Studies
- SARS-CoV-2 / immunology*
- SARS-CoV-2 / isolation & purification
- Severity of Illness Index
- Spain / epidemiology
- Transplant Recipients
- Treatment Outcome

Substances

- Antibodies, Monoclonal, Humanized
- Antiviral Agents
- Immunosuppressive Agents
- Hydroxychloroquine
- tocilizumab

Grant support

- 18/00073/Spanish Ministry of Science and Innovation, Instituto de Salud Carlos III

Full text links

WILEY Full Text Article Wiley Free PMC article

Proceed to details

Cite

Share

☐ 498

Observational Study

J Med Virol

. 2022 Apr;94(4):1540-1549.

doi: 10.1002/jmv.27488. Epub 2021 Dec 7.

Prognostic factors and combined use of tocilizumab and corticosteroids in a Spanish cohort of elderly COVID-19 patients

Miguel A Duarte-Millán [1], Nieves Mesa-Plaza [1], Marta Guerrero-Santillán [1], Alejandro Morales-Ortega [1], David Bernal-Bello [1], Ana I Farfán-Sedano [1], Vanessa García de Viedma-García [1], Laura Velázquez-Ríos [1], Begoña Frutos-Pérez [1], Cristina L De Ancos-Aracil [1], Guillermo Soria Fernández-Llamazares [1], María Toledano-Macías [1], Rafael Cristóbal-Bilbao [1], Nuria Luquín-Ciuro [1], Jorge Marrero-Francés [1], Sara I Piedrabuena-García [1], José A Satué-Bartolomé [1], Sonia Gonzalo-Pascua [1], Marta Rivilla-Jiménez [1], Lorena Carpintero-García [1], Ibone Ayala-Larrañaga [1], Virginia García-Bermúdez [1], Celia Lara-Montes [1], Álvaro R Llerena-Riofrío [1], Luis RIvas-Prado [1], Stefan Walter [2], Almudena Escriba-Barcena [3], Juan V San Martín López [1], José M Ruíz-Giardín [1]
Affiliations Expand

Affiliations

- [1] Department of Internal Medicine, Hospital Universitario de Fuenlabrada, Madrid, Spain.
- [2] Methodological Support Unit, Hospital Universitario de Fuenlabrada, Madrid, Spain.
- [3] Intensive Care Unit, Hospital Universitario de Fuenlabrada, Madrid, Spain.

- PMID: **34845754**
- DOI: 10.1002/jmv.27488

Observational Study

Prognostic factors and combined use of tocilizumab and corticosteroids in a Spanish cohort of elderly COVID-19 patients

Miguel A Duarte-Millán et al. J Med Virol. 2022 Apr.
Show details
J Med Virol
. 2022 Apr;94(4):1540-1549.
doi: 10.1002/jmv.27488. Epub 2021 Dec 7.

Authors

Miguel A Duarte-Millán [1], Nieves Mesa-Plaza [1], Marta Guerrero-Santillán [1], Alejandro Morales-Ortega [1], David Bernal-Bello [1], Ana I Farfán-Sedano [1], Vanessa García de Viedma-García [1], Laura Velázquez-Ríos [1], Begoña Frutos-Pérez [1], Cristina L De Ancos-Aracil [1], Guillermo Soria Fernández-Llamazares [1], María Toledano-Macías [1], Rafael Cristóbal-Bilbao [1], Nuria Luquín-Ciuro [1], Jorge Marrero-Francés [1], Sara I Piedrabuena-García [1], José A Satué-

Bartolomé [1], Sonia Gonzalo-Pascua [1], Marta Rivilla-Jiménez [1], Lorena Carpintero-García [1], Ibone Ayala-Larrañaga [1], Virginia García-Bermúdez [1], Celia Lara-Montes [1], Álvaro R Llerena-Riofrío [1], Luis RIvas-Prado [1], Stefan Walter [2], Almudena Escriba-Barcena [3], Juan V San Martín López [1], José M Ruíz-Giardín [1]

Affiliations

- [1] Department of Internal Medicine, Hospital Universitario de Fuenlabrada, Madrid, Spain.
- [2] Methodological Support Unit, Hospital Universitario de Fuenlabrada, Madrid, Spain.
- [3] Intensive Care Unit, Hospital Universitario de Fuenlabrada, Madrid, Spain.

- PMID: **34845754**
- DOI: 10.1002/jmv.27488

Abstract

Coronavirus disease 2019 (COVID-19) infection in elderly patients is more aggressive and treatments have shown limited efficacy. Our objective is to describe the clinical course and to analyze the prognostic factors associated with a higher risk of mortality of a cohort of patients older than 80 years. In addition, we assess the efficacy of immunosuppressive treatments in this population. We analyzed the data from 163 patients older than 80 years admitted to our institution for COVID-19, during March and April 2020. A Lasso regression model and subsequent multivariate Cox regression were performed to select variables predictive of death. We evaluated the efficacy of immunomodulatory therapy in three cohorts using adjusted survival analysis. The mortality rate was 43%. The mean age was 85.2 years. The disease was considered severe in 76.1% of the cases. Lasso regression and multivariate Cox regression indicated that factors correlated with hospital mortality were: age (hazard ratio [HR] 1.12, 95% confidence interval [CI]: 1.03-1.22), alcohol consumption (HR 3.15, 95% CI: 1.27-7.84), CRP > 10 mg/dL (HR 2.67, 95% CI: 1.36-5.24), and oxygen support with Venturi Mask (HR 6.37, 95% CI: 2.18-18.62) or reservoir (HR 7.87, 95% CI: 3.37-18.38). Previous treatment with antiplatelets was the only protective factor (HR 0.47, 95% CI: 0.23-0.96). In the adjusted treatment efficacy analysis, we found benefit in the combined use of tocilizumab (TCZ) and corticosteroids (CS) (HR 0.09, 95% CI: 0.01-0.74) compared to standard treatment, with no benefit of CS alone (HR 0.95, 95% CI: 0.53-1.71). Hospitalized elderly patients suffer from a severe and often fatal form of COVID-19 disease. In this regard, several parameters might identify high-risk patients upon admission. Combined use of TCZ and CS could improve survival.

Keywords: COVID-19; corticosteroids; elderly; prognosis; tocilizumab.

© 2021 Wiley Periodicals LLC.

- Cited by 2 articles
- 29 references

Supplementary info

Publication types, MeSH terms, Substances Expand

Publication types

- Observational Study

MeSH terms

- Adrenal Cortex Hormones / administration & dosage*
- Aged, 80 and over
- Antibodies, Monoclonal, Humanized / administration & dosage*
- COVID-19 / drug therapy*
- COVID-19 / mortality*
- COVID-19 / virology
- Comorbidity
- Drug Therapy, Combination
- Female
- Hospital Mortality
- Hospitalization
- Humans
- Male
- Prognosis
- Retrospective Studies
- SARS-CoV-2 / drug effects
- SARS-CoV-2 / physiology
- Spain / epidemiology
- Survival Analysis

Substances

- Adrenal Cortex Hormones
- Antibodies, Monoclonal, Humanized
- tocilizumab

Full text links

WILEY Full Text Article Wiley

Proceed to details

Cite

Share

☐ 499

Observational Study

Mol Genet Metab

. 2022 Feb;135(2):115-121.

doi: 10.1016/j.ymgme.2021.08.004. Epub 2021 Aug 13.

The clinical spectrum of SARS-CoV-2 infection in Gaucher disease: Effect of both a pandemic and a rare disease that disrupts the immune system

Praveena Narayanan [1], Shiny Nair [2], Manisha Balwani [3], Maricar Malinis [4], Pramod Mistry [5]
Affiliations Expand

Affiliations

- [1] Section of Digestive Diseases, Department of Internal Medicine, Yale University School of Medicine, New Haven, CT, United States of America. Electronic address: praveena.narayanan@yale.edu.
- [2] Section of Digestive Diseases, Department of Internal Medicine, Yale University School of Medicine, New Haven, CT, United States of America.
- [3] Department of Genetics and Genomic Sciences, Icahn School of Medicine at Mount Sinai, New York, NY, United States of America. Electronic address: manisha.balwani@mssm.edu.
- [4] Section of Infectious Diseases, Department of Internal Medicine, Yale University School of Medicine, New Haven, CT, United States of America. Electronic address: maricar.malinis@yale.edu.
- [5] Section of Digestive Diseases, Department of Internal Medicine, Yale University School of Medicine, New Haven, CT, United States of America. Electronic address: pramod.mistry@yale.edu.

- PMID: **34412940**
- PMCID: PMC8361210
- DOI: 10.1016/j.ymgme.2021.08.004

Free PMC article
Observational Study

The clinical spectrum of SARS-CoV-2 infection in Gaucher disease: Effect of both a pandemic and a rare disease that disrupts the immune system

Praveena Narayanan et al. Mol Genet Metab. 2022 Feb.
Free PMC article
Show details
Mol Genet Metab
. 2022 Feb;135(2):115-121.
doi: 10.1016/j.ymgme.2021.08.004. Epub 2021 Aug 13.

Authors

Praveena Narayanan [1], Shiny Nair [2], Manisha Balwani [3], Maricar Malinis [4], Pramod Mistry [5]

Affiliations

- [1] Section of Digestive Diseases, Department of Internal Medicine, Yale University School of Medicine, New Haven, CT, United States of America. Electronic address: praveena.narayanan@yale.edu.
- [2] Section of Digestive Diseases, Department of Internal Medicine, Yale University School of Medicine, New Haven, CT, United States of America.
- [3] Department of Genetics and Genomic Sciences, Icahn School of Medicine at Mount Sinai, New York, NY, United States of America. Electronic address: manisha.balwani@mssm.edu.
- [4] Section of Infectious Diseases, Department of Internal Medicine, Yale University School of Medicine, New Haven, CT, United States of America. Electronic address: maricar.malinis@yale.edu.
- [5] Section of Digestive Diseases, Department of Internal Medicine, Yale University School of Medicine, New Haven, CT, United States of America. Electronic address: pramod.mistry@yale.edu.

- PMID: **34412940**
- PMCID: PMC8361210
- DOI: 10.1016/j.ymgme.2021.08.004

Abstract

Introduction: The impact of SARS-CoV-2 in rare disease populations has been underreported. Gaucher disease (GD) is a prototype rare disease that shares with SARS-CoV-2 a disruption of the lysosomal pathway.

Materials-methods: Retrospective analysis of 11 patients with Type 1 GD who developed COVID-19 between March 2020 and March 2021.

Results: Seven male and 4 female patients with Type 1 GD developed COVID-19. One was a pediatric patient (8 years old) while the remainder were adults, median age of 44 years old (range 21 to 64 years old). Two patients required hospitalization though none required intensive care or intubation. All 11 patients recovered from COVID-19 and there were no reported deaths.

Conclusions: Our case series suggests that GD patients acquired COVID-19 at a similar frequency as the general population, though experienced a milder overall course despite harboring underlying immune system dysfunction and other known co-morbidities that confer high risk of adverse outcomes from SARS-CoV-2 infection.

Keywords: Gaucher disease; SARS-CoV-2.

Copyright © 2021 Elsevier Inc. All rights reserved.

- 35 references
- 6 figures

Supplementary info

Publication types, MeSH terms, Grant support Expand

Publication types

- Observational Study
- Research Support, Non-U.S. Gov't

MeSH terms

- Adult
- COVID-19 / immunology*
- COVID-19 / virology
- Child
- Comorbidity
- Female
- Gaucher Disease / immunology*
- Gaucher Disease / virology*
- Hospitalization
- Humans
- Immune System / immunology*
- Male
- Middle Aged
- Pandemics / prevention & control
- Rare Diseases / immunology*
- Retrospective Studies
- SARS-CoV-2 / immunology*
- Young Adult

Grant support

- T32 DK007356/DK/NIDDK NIH HHS/United States
- UL1 TR001863/TR/NCATS NIH HHS/United States

Full text links

ELSEVIER FULL-TEXT ARTICLE Elsevier Science Free PMC article

Proceed to details

Cite

Share

☐ 500

Observational Study

J Med Virol

. 2022 Jan;94(1):291-297.
doi: 10.1002/jmv.27319. Epub 2021 Sep 15.

Comparison of demographic and clinical characteristics of hospitalized COVID-19 patients with severe/critical illness in the first wave versus the second wave

Elif Sargin Altunok [1], Celal Satici [2], Veysel Dinc [3], Sadettin Kamat [4], Mustafa Alkan [1], Mustafa Asim Demirkol [4], Ilkim Deniz Toprak [5], Muhammed Emin Kostek [5], Semih Yazla [6], Sinem Nihal Esatoglu [7]

Affiliations Expand

Affiliations

- [1] Department of Infectious Diseases and Clinical Microbiology, Istanbul Gaziosmanpasa Training and Research Hospital, University of Health Sciences, Istanbul, Turkey.
- [2] Department of Chest Diseases, Istanbul Yedikule Chest Diseases and Chest Surgery Training and Research Hospital, Istanbul, Turkey.
- [3] Department of Anesthesia and Reanimation, Istanbul Gaziosmanpasa Training and Research Hospital, University of Health Sciences, Istanbul, Turkey.
- [4] Department of Chest Diseases, Istanbul Gaziosmanpasa Training and Research Hospital, University of Health Sciences, Istanbul, Turkey.
- [5] Department of Internal Medicine, Istanbul Gaziosmanpasa Training and Research Hospital, University of Health Sciences, Istanbul, Turkey.
- [6] Department of Otolaryngology, Head and Neck Surgery, Istanbul Gaziosmanpasa Training and Research Hospital, University of Health Sciences, Istanbul, Turkey.
- [7] Department of Rheumatology, Istanbul Gaziosmanpasa Training and Research Hospital, University of Health Sciences, Istanbul, Turkey.

- PMID: **34491575**
- PMCID: PMC8661950
- DOI: 10.1002/jmv.27319

Free PMC article
Observational Study

Comparison of demographic and clinical characteristics of hospitalized COVID-19 patients with severe/critical illness in the first wave versus the second wave

Elif Sargin Altunok et al. J Med Virol. 2022 Jan.
Free PMC article
Show details
J Med Virol
. 2022 Jan;94(1):291-297.
doi: 10.1002/jmv.27319. Epub 2021 Sep 15.

Authors

Elif Sargin Altunok [1], Celal Satici [2], Veysel Dinc [3], Sadettin Kamat [4], Mustafa Alkan [1], Mustafa Asim Demirkol [4], Ilkim Deniz Toprak [5], Muhammed Emin Kostek [5], Semih Yazla [6], Sinem Nihal Esatoglu [7]

Affiliations

- [1] Department of Infectious Diseases and Clinical Microbiology, Istanbul Gaziosmanpasa Training and Research Hospital, University of Health Sciences, Istanbul, Turkey.
- [2] Department of Chest Diseases, Istanbul Yedikule Chest Diseases and Chest Surgery Training and Research Hospital, Istanbul, Turkey.
- [3] Department of Anesthesia and Reanimation, Istanbul Gaziosmanpasa Training and Research Hospital, University of Health Sciences, Istanbul, Turkey.
- [4] Department of Chest Diseases, Istanbul Gaziosmanpasa Training and Research Hospital, University of Health Sciences, Istanbul, Turkey.
- [5] Department of Internal Medicine, Istanbul Gaziosmanpasa Training and Research Hospital, University of Health Sciences, Istanbul, Turkey.
- [6] Department of Otolaryngology, Head and Neck Surgery, Istanbul Gaziosmanpasa Training and Research Hospital, University of Health Sciences, Istanbul, Turkey.
- [7] Department of Rheumatology, Istanbul Gaziosmanpasa Training and Research Hospital, University of Health Sciences, Istanbul, Turkey.

- PMID: **34491575**
- PMCID: PMC8661950
- DOI: 10.1002/jmv.27319

Abstract

Due to current advances and growing experience in the management of coronavirus Disease 2019 (COVID-19), the outcome of COVID-19 patients with severe/critical illness would be expected to be better in the second wave compared with the first wave. As our hospitalization criteria changed in the second wave, we aimed to investigate whether a favorable outcome occurred in hospitalized COVID-19 patients with only severe/critical illness. Among 642 laboratory-confirmed hospitalized COVID-19 patients in the first wave and 1121 in the second wave, those who met World Health Organization (WHO) definitions for severe or critical illness on admission or during follow-up were surveyed. Data on demographics, comorbidities, C-reactive protein (CRP) levels on admission, and outcomes were obtained from an electronic hospital database. Univariate analysis was performed to compare the characteristics of patients in the first and second waves. There were 228 (35.5%) patients with severe/critical illness in the first wave and 681 (60.7%) in the second wave. Both groups were similar in terms of age, gender, and comorbidities, other than chronic kidney disease. Median serum CRP levels were significantly higher in patients in the second wave compared with those in the first wave [109 mg/L (interquartile range [IQR]: 65-157)

vs. 87 mg/L (IQR: 39-140); p < 0.001]. However, intensive care unit admission and mortality rates were similar among the waves. Even though a lower mortality rate in the second wave has been reported in previous studies, including all hospitalized COVID-19 patients, we found similar demographics and outcomes among hospitalized COVID-19 patients with severe/critical illness in the first and second wave.

Keywords: COVID-19; Turkey; demographic characteristics; mortality; the first versus second wave.

© 2021 Wiley Periodicals LLC.

Conflict of interest statement

The authors declare that there are no conflict of interests.

- Cited by 1 article
- 20 references
- 1 figure

Supplementary info

Publication types, MeSH terms, Substances Expand

Publication types

- Comparative Study
- Observational Study

MeSH terms

- Aged
- Amides / therapeutic use
- Antibodies, Monoclonal, Humanized / therapeutic use
- Azithromycin / therapeutic use
- C-Reactive Protein / analysis
- COVID-19 / drug therapy*
- COVID-19 / epidemiology
- COVID-19 / mortality*
- COVID-19 / pathology
- Comorbidity
- Critical Care / statistics & numerical data*
- Drug Combinations
- Enoxaparin / therapeutic use
- Female
- Hospital Mortality
- Hospitalization / statistics & numerical data

- Humans
- Hydroxychloroquine / therapeutic use
- Interleukin 1 Receptor Antagonist Protein / therapeutic use
- Lopinavir / therapeutic use
- Male
- Methylprednisolone / therapeutic use
- Middle Aged
- Pyrazines / therapeutic use
- Retrospective Studies
- Ritonavir / therapeutic use
- SARS-CoV-2
- Severity of Illness Index*
- Treatment Outcome
- Turkey / epidemiology

Substances

- Amides
- Antibodies, Monoclonal, Humanized
- Drug Combinations
- Enoxaparin
- Interleukin 1 Receptor Antagonist Protein
- Pyrazines
- lopinavir-ritonavir drug combination
- Lopinavir
- Hydroxychloroquine
- Azithromycin
- C-Reactive Protein
- favipiravir
- tocilizumab
- Ritonavir
- Methylprednisolone

Full text links

WILEY Full Text Article Wiley Free PMC article
Proceed to details
Cite
Share
☐ 501
Observational Study
J Med Virol

. 2022 Jan;94(1):272-278.
doi: 10.1002/jmv.27315. Epub 2021 Sep 7.

COVID-19 disease in hospitalized young adults in India and China: Evaluation of risk factors predicting progression across two major ethnic groups

Smriti Panda [1], Sankanika Roy [2] [3], Rohit K Garg [4], Gan Hui [5], Jack Gorard [6], Mayank Bhutada [1], Yuanli Sun [5], Sushma Bhatnagar [7], Anant Mohan [8], Lalit Dar [9], Mao Liu [10]
Affiliations Expand

Affiliations

- [1] Department of Otorhinolaryngology, All India Institute of Medical Sciences, New Delhi, Delhi, India.
- [2] Department of Neurology, Nottingham University Hospitals, Nottingham, UK.
- [3] Cardiovascular sciences, Leicester Royal Infirmary, University of Leicester, Leicester, UK.
- [4] Department of Medicine, All India Institute of Medical Sciences, New Delhi, Delhi, India.
- [5] Department of Allergy, Zhongnan Hospital of Wuhan University, Wuhan University, Wuhan, Hubei, China.
- [6] Department of Internal Medicine, Lincoln County Hospital, Lincoln, UK.
- [7] Department of Onco-Anaesthesia, All India Institute of Medical Sciences, New Delhi, Delhi, India.
- [8] Department of Pulmonary Medicine, All India Institute of Medical Sciences, New Delhi, Delhi, India.
- [9] Department of Microbiology, All India Institute of Medical Sciences, New Delhi, Delhi, India.
- [10] Department of Neurology, Tongji Hospital, Tongji Medical College of Huazhong University of Science and Technology, Wuhan, Hubei, China.

- PMID: **34468994**
- PMCID: PMC8662198
- DOI: 10.1002/jmv.27315

Free PMC article
Observational Study

COVID-19 disease in hospitalized young adults in India and China: Evaluation of risk

factors predicting progression across two major ethnic groups

Smriti Panda et al. J Med Virol. 2022 Jan.
Free PMC article
Show details
J Med Virol
. 2022 Jan;94(1):272-278.
doi: 10.1002/jmv.27315. Epub 2021 Sep 7.

Authors

Smriti Panda [1], Sankanika Roy [2] [3], Rohit K Garg [4], Gan Hui [5], Jack Gorard [6], Mayank Bhutada [1], Yuanli Sun [5], Sushma Bhatnagar [7], Anant Mohan [8], Lalit Dar [9], Mao Liu [10]

Affiliations

- [1] Department of Otorhinolaryngology, All India Institute of Medical Sciences, New Delhi, Delhi, India.
- [2] Department of Neurology, Nottingham University Hospitals, Nottingham, UK.
- [3] Cardiovascular sciences, Leicester Royal Infirmary, University of Leicester, Leicester, UK.
- [4] Department of Medicine, All India Institute of Medical Sciences, New Delhi, Delhi, India.
- [5] Department of Allergy, Zhongnan Hospital of Wuhan University, Wuhan University, Wuhan, Hubei, China.
- [6] Department of Internal Medicine, Lincoln County Hospital, Lincoln, UK.
- [7] Department of Onco-Anaesthesia, All India Institute of Medical Sciences, New Delhi, Delhi, India.
- [8] Department of Pulmonary Medicine, All India Institute of Medical Sciences, New Delhi, Delhi, India.
- [9] Department of Microbiology, All India Institute of Medical Sciences, New Delhi, Delhi, India.
- [10] Department of Neurology, Tongji Hospital, Tongji Medical College of Huazhong University of Science and Technology, Wuhan, Hubei, China.

- PMID: **34468994**
- PMCID: PMC8662198
- DOI: 10.1002/jmv.27315

Abstract

Data pertaining to risk factor analysis in coronavirus disease 2019 (COVID-19) is confounded by the lack of data from an ethnically diverse population. In addition, there is a lack of data for young adults. This study was conducted to assess risk factors predicting COVID-19 severity and mortality in hospitalized young adults. A retrospective observational study was conducted at two centers from China and India on COVID-19 patients aged 20-50 years. Regression analysis to predict adverse outcomes was performed using parameters including age, sex, country of origin,

hospitalization duration, comorbidities, lymphocyte count, and National Early Warning Score 2 (NEWS2) score at admission. A total of 420 patients (172 East Asians and 248 South Asians) were included. The predictive model for intensive care unit (ICU) admission with variables NEWS2 Category II and higher, diabetes mellitus, liver dysfunction, and low lymphocyte counts had an area under the curve (AUC) value of 0.930 with a sensitivity of 0.931 and a specificity of 0.784. The predictive model for mortality with NEWS2 Category III, cancer, and decreasing lymphocyte count had an AUC value of 0.883 with a sensitivity of 0.903 and a specificity of 0.701. A combined predictive model with bronchial asthma and low lymphocyte count, in contrast, had an AUC value of 0.768 with a sensitivity of 0.828 and a specificity of 0.719 for NEWS2 score (5 or above) at presentation. NEWS2 supplemented with comorbidity profile and lymphocyte count could help identify hospitalized young adults at risk of adverse COVID-19 outcomes.

Keywords: COVID-19; NEWS; NEWS2; SARS-CoV-2; lymphocyte; lymphopenia; prognostic factors.

© 2021 Wiley Periodicals LLC.

- 18 references

Supplementary info

Publication types, MeSH terms Expand

Publication types

- Observational Study

MeSH terms

- Adult
- Asians
- COVID-19 / diagnosis*
- COVID-19 / ethnology*
- COVID-19 / mortality
- COVID-19 / physiopathology
- China
- Comorbidity
- Disease Progression
- Early Warning Score
- Female
- Hospitalization
- Humans
- India
- Intensive Care Units
- Lymphocyte Count

- Male
- Middle Aged
- Prognosis
- Retrospective Studies
- Risk Factors
- Severity of Illness Index
- Young Adult

Full text links

WILEY Full Text Article Wiley Free PMC article
Proceed to details
Cite
Share
502
Observational Study
Public Health
. 2021 Sep;198:85-88.
doi: 10.1016/j.puhe.2021.06.025. Epub 2021 Jul 10.

Essential public healthcare services utilization and excess non-COVID-19 mortality in Greece

E Kondilis [1], F Tarantilis [2], A Benos [2]
Affiliations Expand

Affiliations

- [1] School of Medicine, Aristotle University of Thessaloniki, Thessaloniki, Greece. Electronic address: ekondilis@auth.gr.
- [2] School of Medicine, Aristotle University of Thessaloniki, Thessaloniki, Greece.

- PMID: **34365111**
- DOI: 10.1016/j.puhe.2021.06.025

Observational Study

Essential public healthcare services utilization and excess non-COVID-19 mortality in Greece

E Kondilis et al. Public Health. 2021 Sep.
Show details
Public Health
. 2021 Sep;198:85-88.
doi: 10.1016/j.puhe.2021.06.025. Epub 2021 Jul 10.

Authors

E Kondilis [1], F Tarantilis [2], A Benos [2]

Affiliations

- [1] School of Medicine, Aristotle University of Thessaloniki, Thessaloniki, Greece. Electronic address: ekondilis@auth.gr.
- [2] School of Medicine, Aristotle University of Thessaloniki, Thessaloniki, Greece.

- PMID: **34365111**
- DOI: 10.1016/j.puhe.2021.06.025

Abstract

Objectives: Ensuring access to care for all patients-especially those with life-threatening and chronic conditions-during a pandemic is a challenge for all healthcare systems. During the COVID-19 pandemic, many countries faced excess mortality partly attributed to disruptions in essential healthcare services provision. This study aims to estimate the utilization of public primary care and hospital services during the COVID-19 epidemic in Greece and its potential association with excess non-COVID-19 mortality in the country.

Study design: This is an observational study.

Methods: A retrospective analysis of national secondary utilization and mortality data from multiple official sources, covering the first nine months of the COVID-19 epidemic in Greece (February 26th to November 30th, 2020), was carried out.

Results: Utilization rates of all public healthcare services during the first nine months of the epidemic dropped significantly compared to the average utilization rates of the 2017-19 control period; hospital admissions, hospital surgical procedures, and primary care visits dropped by 17.3% (95% CI: 6.6%-28.0%), 23.1% (95% CI: 7.3%-38.9%), and 24.8% (95% CI: 13.3%-36.3%) respectively. This underutilization of essential public services-mainly due to supply restrictions such as suspension of outpatient care and cancelation of elective surgeries-is most probably related to the 3778 excess non-COVID-19 deaths (representing 62% of all-cause excess deaths) that have been reported during the first 9 months of the epidemic in the country.

Conclusions: Greece's healthcare system, deeply wounded by the 2008-18 recession and austerity, was ill-resourced to cope with the challenges of the COVID-19 epidemic. Early and prolonged lockdowns have kept COVID-19 infections and deaths at relatively low levels. However, this "success" seems to have been accomplished at the expense of non-COVID-19 patients. It is important to acknowledge the "hidden epidemic" of unmet non-COVID-19 needs and increased non-COVID-19 deaths in the country and urgently strengthen public healthcare services to address it.

Keywords: COVID-19; Essential health services; Excess mortality; Excess non-COVID-19 deaths; Health services utilization.

Copyright © 2021 The Royal Society for Public Health. Published by Elsevier Ltd. All rights reserved.

- Cited by 2 articles

Supplementary info

Publication types, MeSH terms Expand

Publication types

- Observational Study

MeSH terms

- Ambulatory Care
- COVID-19*
- Communicable Disease Control
- Delivery of Health Care
- Facilities and Services Utilization
- Greece / epidemiology
- Humans
- Mortality
- Pandemics*
- Retrospective Studies
- SARS-CoV-2

Full text links

ELSEVIER FULL-TEXT ARTICLE Elsevier Science
Proceed to details
Cite
Share
503
Observational Study
Intern Emerg Med
. 2021 Sep;16(6):1593-1603.
doi: 10.1007/s11739-021-02655-6. Epub 2021 Feb 5.

Timing of corticosteroids impacts mortality in hospitalized COVID-19 patients

Amit Bahl [1], Steven Johnson [2], Nai-Wei Chen [3]
Affiliations Expand

Affiliations

- [1] Department of Emergency Medicine, Beaumont Hospital, Royal Oak, MI, USA. Amit.bahl@beaumont.edu.
- [2] Department of Emergency Medicine, Beaumont Hospital, Royal Oak, MI, USA.
- [3] Beaumont Health Research Institute, Royal Oak, MI, USA.

- PMID: **33547620**
- PMCID: PMC7864133
- DOI: 10.1007/s11739-021-02655-6

Free PMC article
Observational Study

Timing of corticosteroids impacts mortality in hospitalized COVID-19 patients

Amit Bahl et al. Intern Emerg Med. 2021 Sep.
Free PMC article
Show details
Intern Emerg Med
. 2021 Sep;16(6):1593-1603.
doi: 10.1007/s11739-021-02655-6. Epub 2021 Feb 5.

Authors

Amit Bahl [1], Steven Johnson [2], Nai-Wei Chen [3]

Affiliations

- [1] Department of Emergency Medicine, Beaumont Hospital, Royal Oak, MI, USA. Amit.bahl@beaumont.edu.
- [2] Department of Emergency Medicine, Beaumont Hospital, Royal Oak, MI, USA.
- [3] Beaumont Health Research Institute, Royal Oak, MI, USA.

- PMID: **33547620**
- PMCID: PMC7864133
- DOI: 10.1007/s11739-021-02655-6

Abstract

The optimal timing of initiating corticosteroid treatment in hospitalized patients is unknown. We aimed to assess the relationship between timing of initial corticosteroid treatment and in-hospital mortality in COVID-19 patients. In this observational study through medical record analysis, we quantified the mortality benefit of corticosteroids in two equally matched groups of hospitalized COVID-19 patients. We subsequently evaluated the timing of initiating corticosteroids and its effect on mortality in all patients receiving corticosteroids. Demographic, clinical, and laboratory variables were collected and employed for multivariable regression analyses. 1461 hospitalized patients with confirmed COVID-19 were analyzed. Of these, 760 were also matched into two equal groups based on having received corticosteroid therapy. Patients receiving corticosteroids had a lower risk of death than those who did not (HR 0.67, 95% CI 0.67-0.90; p = 0.01). Timing of corticosteroids was assessed for all 615 patients receiving corticosteroids during admission. Patients receiving first dose of corticosteroids > 72 h into hospitalization had a lower risk of death compared to patients with first dose at earlier time intervals (HR 0.56, 95% CI 0.38-0.82; p = 0.003). There was a mortality benefit in patients with > 7 days of symptom onset to initiation of corticosteroids (HR 0.56, 95% CI 0.33-0.95; p = 0.03). In patients receiving oxygen therapy, corticosteroids reduced risk of death in mechanically ventilated patients (HR 0.38, 95% CI 0.24-0.60; p < 0.001) but not in patients on high-flow or other oxygen therapy (HR 0.46, 95% CI 0.20-1.07; p = 0.07) and (HR 0.84, 95% CI 0.35-2.00; p = 0.69), respectively. Timing of corticosteroids initiation was related to in-hospital mortality for COVID-19 patients. Time from symptom onset > 7 days should trigger initiation of corticosteroids. In the absence of invasive mechanical ventilation, corticosteroids should be initiated if the patient remains hospitalized at 72 h. Hypoxia requiring supplemental oxygen therapy should not be a trigger for initiation of corticosteroids unless the timing is appropriate.

Keywords: COVID-19; Coronavirus; Corticosteroids; Mortality; Timing; Treatment.

© 2021. Società Italiana di Medicina Interna (SIMI).

Conflict of interest statement

This manuscript in part or in full has not been submitted or published anywhere. No authors have any relevant conflict of interest disclosures.

- Cited by 10 articles
- 15 references
- 3 figures

Supplementary info

Publication types, MeSH terms, Substances Expand

Publication types

- Observational Study

MeSH terms

- Adrenal Cortex Hormones / therapeutic use*

- Adult
- Aged
- COVID-19 / drug therapy*
- COVID-19 / mortality*
- Critical Illness / mortality
- Hospital Mortality*
- Humans
- Hydroxychloroquine / therapeutic use
- Length of Stay / statistics & numerical data
- Middle Aged
- Retrospective Studies
- Survival Rate
- Treatment Outcome

Substances

- Adrenal Cortex Hormones
- Hydroxychloroquine

Full text links

SpringerLink FULL-TEXT ARTICLE Springer Free PMC article
Proceed to details
Cite
Share
504
Observational Study
Ann Intern Med
. 2021 Jun;174(6):777-785.
doi: 10.7326/M20-6754. Epub 2021 Mar 2.

Development of Severe COVID-19 Adaptive Risk Predictor (SCARP), a Calculator to Predict Severe Disease or Death in Hospitalized Patients With COVID-19

Shannon Wongvibulsin [1], Brian T Garibaldi [1], Annukka A R Antar [1], Jiyang Wen [2], Mei-Cheng Wang [2], Amita Gupta [1], Robert Bollinger [1], Yanxun Xu [3], Kunbo Wang [3], Joshua F Betz [2], John Muschelli [2], Karen Bandeen-Roche [2], Scott L Zeger [2], Matthew L Robinson [1]
Affiliations Expand

Affiliations

- [1] Johns Hopkins University School of Medicine, Baltimore, Maryland (S.W., B.T.G., A.A.A., A.G., R.B., M.L.R.).
- [2] Johns Hopkins Bloomberg School of Public Health, Baltimore, Maryland (J.W., M.W., J.F.B., J.M., K.B., S.L.Z.).
- [3] Johns Hopkins University, Baltimore, Maryland (Y.X., K.W.).

- PMID: **33646849**
- PMCID: PMC7934337
- DOI: 10.7326/M20-6754

Free PMC article
Observational Study

Development of Severe COVID-19 Adaptive Risk Predictor (SCARP), a Calculator to Predict Severe Disease or Death in Hospitalized Patients With COVID-19

Shannon Wongvibulsin et al. Ann Intern Med. 2021 Jun.
Free PMC article
Show details
Ann Intern Med

. 2021 Jun;174(6):777-785.
doi: 10.7326/M20-6754. Epub 2021 Mar 2.

Authors

Shannon Wongvibulsin [1], Brian T Garibaldi [1], Annukka A R Antar [1], Jiyang Wen [2], Mei-Cheng Wang [2], Amita Gupta [1], Robert Bollinger [1], Yanxun Xu [3], Kunbo Wang [3], Joshua F Betz [2], John Muschelli [2], Karen Bandeen-Roche [2], Scott L Zeger [2], Matthew L Robinson [1]

Affiliations

- [1] Johns Hopkins University School of Medicine, Baltimore, Maryland (S.W., B.T.G., A.A.A., A.G., R.B., M.L.R.).
- [2] Johns Hopkins Bloomberg School of Public Health, Baltimore, Maryland (J.W., M.W., J.F.B., J.M., K.B., S.L.Z.).
- [3] Johns Hopkins University, Baltimore, Maryland (Y.X., K.W.).

- PMID: **33646849**
- PMCID: PMC7934337
- DOI: 10.7326/M20-6754

Abstract

Background: Predicting the clinical trajectory of individual patients hospitalized with coronavirus disease 2019 (COVID-19) is challenging but necessary to inform clinical care. The majority of COVID-19 prognostic tools use only data present upon admission and do not incorporate changes occurring after admission.

Objective: To develop the Severe COVID-19 Adaptive Risk Predictor (SCARP) (https://rsconnect.biostat.jhsph.edu/covid_trajectory/), a novel tool that can provide dynamic risk predictions for progression from moderate disease to severe illness or death in patients with COVID-19 at any time within the first 14 days of their hospitalization.

Design: Retrospective observational cohort study.

Settings: Five hospitals in Maryland and Washington, D.C.

Patients: Patients who were hospitalized between 5 March and 4 December 2020 with severe acute respiratory syndrome coronavirus 2 (SARS-CoV-2) confirmed by nucleic acid test and symptomatic disease.

Measurements: A clinical registry for patients hospitalized with COVID-19 was the primary data source; data included demographic characteristics, admission source, comorbid conditions, time-varying vital signs, laboratory measurements, and clinical severity. Random forest for survival, longitudinal, and multivariate (RF-SLAM) data analysis was applied to predict the 1-day and 7-day risks for progression to severe disease or death for any given day during the first 14 days of hospitalization.

Results: Among 3163 patients admitted with moderate COVID-19, 228 (7%) became severely ill or died in the next 24 hours; an additional 355 (11%) became severely ill or died in the next 7 days. The area under the receiver-operating characteristic curve (AUC) for 1-day risk predictions for progression to severe disease or death was 0.89 (95% CI, 0.88 to 0.90) and 0.89 (CI, 0.87 to 0.91) during the first and second weeks of hospitalization, respectively. The AUC for 7-day risk predictions for progression to severe disease or death was 0.83 (CI, 0.83 to 0.84) and 0.87 (CI, 0.86 to 0.89) during the first and second weeks of hospitalization, respectively.

Limitation: The SCARP tool was developed by using data from a single health system.

Conclusion: Using the predictive power of RF-SLAM and longitudinal data from more than 3000 patients hospitalized with COVID-19, an interactive tool was developed that rapidly and accurately provides the probability of an individual patient's progression to severe illness or death on the basis of readily available clinical information.

Primary funding source: Hopkins inHealth and COVID-19 Administrative Supplement for the HHS Region 3 Treatment Center from the Office of the Assistant Secretary for Preparedness and Response.

Conflict of interest statement

Disclosures: Disclosures can be viewed at www.acponline.org/authors/icmje/ConflictOfInterestForms.do?msNum=M20-6754.

- Cited by 9 articles
- 29 references

- 6 figures

Supplementary info

Publication types, MeSH terms, Grant support Expand

Publication types

- Observational Study

MeSH terms

- Aged
- Aged, 80 and over
- COVID-19 / mortality*
- COVID-19 / pathology*
- Disease Progression
- District of Columbia / epidemiology
- Female
- Hospital Mortality*
- Hospitalization
- Humans
- Male
- Maryland / epidemiology
- Middle Aged
- Pandemics
- Patient Acuity*
- Pneumonia, Viral / mortality*
- Pneumonia, Viral / virology
- Predictive Value of Tests
- Prognosis
- Registries
- Retrospective Studies
- Risk Assessment / methods*
- Risk Factors
- SARS-CoV-2

Grant support

- K08 AI143391/AI/NIAID NIH HHS/United States

Full text links

Annals FREE FULL TEXT Atypon Free PMC article
Proceed to details
Cite
Share
☐ 505
Observational Study
J Bras Pneumol
. 2021 Apr 30;47(2):e20200545.
doi: 10.36416/1806-3756/e20200545. eCollection 2021.

Implementation of Tele-ICU during the COVID-19 pandemic

[Article in English, Portuguese]
Bruno Rocha de Macedo [1], Marcos Vinicius Fernandes Garcia [1], Michelle Louvaes Garcia [1], Marcia Volpe [1] [2], Mayson Laércio de Araújo Sousa [1], Talita Freitas Amaral [1], Marco Antônio Gutierrez [1], Antonio Pires Barbosa [1], Paula Gobi Scudeller [1], Pedro Caruso [1], Carlos Roberto Ribeiro Carvalho [1]
Affiliations Expand

Affiliations

- [1] . Divisão de Pneumologia, Instituto do Coração - InCor - Hospital das Clínicas, Faculdade de Medicina, Universidade de São Paulo, São Paulo (SP) Brasil.
- [2] . Departamento de Ciências do Movimento Humano, Universidade Federal de São Paulo - UNIFESP - Santos (SP) Brasil.

- PMID: **33950091**
- PMCID: PMC8332846
- DOI: 10.36416/1806-3756/e20200545

Free PMC article
Observational Study

Implementation of Tele-ICU during the COVID-19 pandemic

[Article in English, Portuguese]
Bruno Rocha de Macedo et al. J Bras Pneumol. 2021.
Free PMC article
Show details
J Bras Pneumol
. 2021 Apr 30;47(2):e20200545.
doi: 10.36416/1806-3756/e20200545. eCollection 2021.

Authors

Bruno Rocha de Macedo [1], Marcos Vinicius Fernandes Garcia [1], Michelle Louvaes Garcia [1], Marcia Volpe [1] [2], Mayson Laércio de Araújo Sousa [1], Talita Freitas Amaral [1], Marco Antônio Gutierrez [1], Antonio Pires Barbosa [1], Paula Gobi Scudeller [1], Pedro Caruso [1], Carlos Roberto Ribeiro Carvalho [1]

Affiliations

- [1] . Divisão de Pneumologia, Instituto do Coração - InCor - Hospital das Clínicas, Faculdade de Medicina, Universidade de São Paulo, São Paulo (SP) Brasil.
- [2] . Departamento de Ciências do Movimento Humano, Universidade Federal de São Paulo - UNIFESP - Santos (SP) Brasil.

- PMID: **33950091**
- PMCID: PMC8332846
- DOI: 10.36416/1806-3756/e20200545

Abstract
in English, Portuguese

Objective: To describe the implementation of a Tele-ICU program during the COVID-19 pandemic, as well as to describe and analyze the results of the first four months of operation of the program.

Methods: This was a descriptive observational study of the implementation of a Tele-ICU program, followed by a retrospective analysis of clinical data of patients with COVID-19 admitted to ICUs between April and July of 2020.

Results: The Tele-ICU program was implemented over a four-week period and proved to be feasible during the pandemic. Participants were trained remotely, and the program had an evidence-based design, the objective being to standardize care for patients with COVID-19. More than 100,000 views were recorded on the free online platforms and the mobile application. During the study period, the cases of 326 patients with COVID-19 were evaluated through the program. The median age was 60 years (IQR, 49-68 years). There was a predominance of males (56%). There was also a high prevalence of hypertension (49.1%) and diabetes mellitus (38.4%). At ICU admission, 83.7% of patients were on invasive mechanical ventilation, with a median PaO2/FiO2 ratio < 150. It was possible to use lung-protective ventilation in 75% of the patients. Overall, in-hospital mortality was 68%, and ICU mortality was 65%.

Conclusions: Our Tele-ICU program provided multidisciplinary training to health care professionals and clinical follow-up for hundreds of critically ill patients. This public health care network initiative was unprecedented and proved to be feasible during the COVID-19 pandemic, encouraging the creation of similar projects that combine evidence-based practices, training, and Tele-ICU.

Objetivo:: Descrever a implantação de um serviço de telemedicina de UTI durante a pandemia de COVID-19, assim como descrever e analisar os resultados dos primeiros quatro meses de funcionamento do programa.

Métodos:: Estudo observacional descritivo da implantação de um serviço de telemedicina de UTI seguido de análise retrospectiva dos dados clínicos de pacientes com COVID-19 internados em UTI entre abril e julho de 2020.

Resultados:: O serviço foi implantado em quatro semanas e mostrou-se viável em meio à pandemia. O treinamento foi desenhado para ser remoto e baseado em evidências, promovendo a padronização do atendimento aos pacientes com COVID-19. Mais de 100.000 visualizações foram registradas nas plataformas on-line de acesso livre e no aplicativo móvel. Durante o período do estudo, os casos de 326 pacientes com COVID-19 foram avaliados no programa. A mediana de idade foi de 60 anos (variação: 49-68 anos). Houve predomínio do sexo masculino (56%) e alta prevalência de hipertensão arterial (49,1%) e diabetes mellitus (38,4%). Na admissão na UTI, 83,7% dos pacientes estavam em ventilação mecânica invasiva, com uma mediana da relação $PaO_2/FiO_2 < 150$. Ventilação pulmonar protetora foi possível em 75% dos casos. A mortalidade na UTI foi de 65%, e a mortalidade hospitalar foi de 68%.

Conclusões:: A telemedicina de UTI forneceu treinamento multidisciplinar aos profissionais de saúde e acompanhamento clínico de centenas de pacientes críticos. A iniciativa na rede pública foi pioneira e mostrou-se viável em meio à pandemia de COVID-19, incentivando a criação de projetos semelhantes que combinem práticas baseadas em evidências, treinamento e telemedicina.

- Cited by 1 article
- 40 references
- 3 figures

Supplementary info

Publication types, MeSH terms Expand

Publication types

- Observational Study

MeSH terms

- COVID-19*
- Critical Illness
- Humans
- Intensive Care Units
- Male
- Middle Aged
- Pandemics*
- Respiration, Artificial
- Retrospective Studies
- SARS-CoV-2

Full text links

free full text available at SciELO.org Scientific Electronic Library Online Free PMC article
Proceed to details
Cite
Share
506

PLoS One
. 2021 Dec 9;16(12):e0260743.
doi: 10.1371/journal.pone.0260743. eCollection 2021.

Changes in motor paralysis involving upper extremities of outpatient chronic stroke patients from temporary rehabilitation interruption due to spread of COVID-19 infection: An observational study on pre- and post-survey data without a control group

Daigo Sakamoto [1] [2], Toyohiro Hamaguchi [2], Yasuhide Nakayama [1] [3], Takuya Hada [3], Masahiro Abo [3]
Affiliations Expand

Affiliations

- [1] Department of Rehabilitation Medicine, The Jikei University School of Medicine Hospital, Tokyo, Japan.
- [2] Department of Rehabilitation, Graduate School of Health Science, Saitama Prefectural University, Saitama, Japan.
- [3] Department of Rehabilitation Medicine, The Jikei University School of Medicine, Tokyo, Japan.

- PMID: **34882736**
- PMCID: PMC8659304
- DOI: 10.1371/journal.pone.0260743

Free PMC article

Changes in motor paralysis involving upper extremities of outpatient chronic stroke patients from temporary rehabilitation interruption due to spread of COVID-19

infection: An observational study on pre- and post-survey data without a control group

Daigo Sakamoto et al. PLoS One. 2021.
Free PMC article
Show details
PLoS One
. 2021 Dec 9;16(12):e0260743.
doi: 10.1371/journal.pone.0260743. eCollection 2021.

Authors

Daigo Sakamoto [1] [2], Toyohiro Hamaguchi [2], Yasuhide Nakayama [1] [3], Takuya Hada [3], Masahiro Abo [3]

Affiliations

- [1] Department of Rehabilitation Medicine, The Jikei University School of Medicine Hospital, Tokyo, Japan.
- [2] Department of Rehabilitation, Graduate School of Health Science, Saitama Prefectural University, Saitama, Japan.
- [3] Department of Rehabilitation Medicine, The Jikei University School of Medicine, Tokyo, Japan.

- PMID: **34882736**
- PMCID: PMC8659304
- DOI: 10.1371/journal.pone.0260743

Abstract

Background: Outpatient rehabilitation was temporarily suspended because of coronavirus disease (COVID-19), and there was a risk that patients' activities of daily living (ADLs) would decrease and physical functions unmaintained. Therefore, we investigated the ADLs and motor functions of chronic stroke patients whose outpatient rehabilitation was temporarily interrupted.

Methods: In this observational study, the Fugl-Meyer Assessment of the Upper Extremity (FMA-UE), Action Research Arm Test (ARAT), and Barthel Index (BI) scores of 49 stroke hemiplegic patients at 6 and 3 months before rehabilitation interruptions were retrospectively determined and were prospectively investigated on resumption of outpatient rehabilitation. Presence or absence of symptoms and difficulties caused by the interruption period (IP) was investigated using a binomial method. Deltas were analyzed using a generalized linear model (GLM) according to the survey period. Age, sex, severity of FMA-UE immediately post-resumption and post-onset period were used as covariates. For survey items showing significant model fit, the 95% confidence interval of minimum detectable change (MDC95) was calculated, and the amount of change was compared. Questionnaire responses were tested via proportion ratio. Statistical significance was set at 5%.

Results: The FMA-UE part A and total scores were significantly model fit depending on periods. The estimated FMA-UE total score decreased by 1.64 (z = -2.38, p = 0.02) during the 3-month IP. No fits were observed by GLM in other parts of the FMA-UE, ARAT, or BI. The calculated

MDC95 was 3.58 for FMA-UE part A and 4.50 for FMA-UE overall. Answers to questions regarding sleep disturbance and physical pain were significantly biased toward "no" in the psychosomatic function items ($p<0.05$). There was no bias in the distribution of answers to questions regarding joint stiffness, muscle weakness, muscle stiffness, and difficulty in moving arms and hands. All 16 questions regarding activities and participation items were significantly biased toward answers "no" ($p<0.05$).

Conclusions: The FMA-UE part A and total scores were affected. Patients complained of subjective symptoms related to upper limb paralysis after the IP. Since ADLs of patients were maintained, the therapist can recommend that patients not receiving outpatient treatments be evaluated in relation to the shoulder, elbow, and forearm and instructed on self-training to maintain motor function.

Conflict of interest statement

The authors have declared that no competing interests exist.

- 32 references
- 3 figures

Supplementary info

Publication types, MeSH terms, Grant support Expand

Publication types

- Research Support, Non-U.S. Gov't

MeSH terms

- Adult
- COVID-19 / epidemiology*
- COVID-19 / virology
- Chronic Disease
- Female
- Humans
- Interrupted Time Series Analysis
- Male
- Middle Aged
- Muscle Strength
- Muscle Weakness / physiopathology
- Observational Studies as Topic
- Outpatients / psychology*
- SARS-CoV-2 / isolation & purification
- Stroke / physiopathology*
- Stroke Rehabilitation

- Surveys and Questionnaires
- Upper Extremity / physiopathology*

Grant support

This work was supported by JSPS KAKENHI Grant Number 18K10691.

Full text links

OPEN ACCESS TO FULL TEXT PLOS ONE Public Library of Science Free PMC article
Proceed to details
Cite
Share
507
Observational Study
Hypertension
. 2021 Jul;78(1):165-173.
doi: 10.1161/HYPERTENSIONAHA.121.17328. Epub 2021 Jun 9.

Discontinuation of Antihypertensive Medications on the Outcome of Hospitalized Patients With Severe Acute Respiratory Syndrome-Coronavirus 2

Sandeep Singh [1, 2], Annette K Offringa-Hup [3], Susan J J Logtenberg [4], Paul D Van der Linden [5], Wilbert M T Janssen [6], Hubertina Klein [7], Femke Waanders [8], Suat Simsek [9, 10], Cornelis P C de Jager [11], Paul Smits [12], Machteld van der Feltz [13], Gerrit Jan Beumer [14], Christine Widrich [15], Martijn Nap [15], Sara-Joan Pinto-Sietsma [1, 2]
Affiliations Expand

Affiliations

- [1] From the Department of Clinical Epidemiology, Biostatistics and Bioinformatics (S.S., S.-J.P.-S.), Amsterdam UMC, Academic Medical Center Amsterdam, the Netherlands.
- [2] Department of Vascular Medicine (S.S., S.-J.P.-S.), Amsterdam UMC, Academic Medical Center Amsterdam, the Netherlands.
- [3] Microbiology and System Biology, Netherlands Organization for Applied Scientific Research, the Hague (A.K.O.-H.).
- [4] Department of Internal Medicine, Diakonessenhuis, Utrecht, the Netherlands (S.J.J.L.).
- [5] Department of Clinical Pharmacy, Tergooi, the Netherlands (P.D.V.d.L.).
- [6] Department of Internal Medicine, Martini Hospital, the Netherlands (W.M.T.J.).
- [7] Department of Internal Medicine, Slingeland Hospital, Doetinchem, the Netherlands (H.K.).

- [8] Department of Internal Medicine, Isala, Zwolle, the Netherlands (F.W.).
- [9] Department of Internal Medicine/Endocrinology, Northwest Clinics, Alkmaar, the Netherlands (S.S.).
- [10] Department of Internal Medicine/Endocrinology, Amsterdam UMC, VU University Medical Center, the Netherlands (S.S.).
- [11] Department of Intensive Care Medicine, Jeroen Bosch Ziekenhuis, the Netherlands (C.P.C.d.J.).
- [12] Department of Pharmacology and Toxicology, Radboud university medical center, Radboud Institute for Health Sciences, the Netherlands (P.S.).
- [13] Department of Internal Medicine, Alrijne Hospital, Leiderdorp, the Netherlands (M.v.d.F.).
- [14] Life Sciences TNO, Leiden, the Netherlands (G.J.B.).
- [15] IQVIA, Amsterdam, the Netherlands (C.W., M.N.).

- PMID: **34106731**
- PMCID: PMC8189257
- DOI: 10.1161/HYPERTENSIONAHA.121.17328

Free PMC article
Observational Study

Discontinuation of Antihypertensive Medications on the Outcome of Hospitalized Patients With Severe Acute Respiratory Syndrome-Coronavirus 2

Sandeep Singh et al. Hypertension. 2021 Jul.
Free PMC article
Show details
Hypertension
. 2021 Jul;78(1):165-173.
doi: 10.1161/HYPERTENSIONAHA.121.17328. Epub 2021 Jun 9.

Authors

Sandeep Singh [1] [2], Annette K Offringa-Hup [3], Susan J J Logtenberg [4], Paul D Van der Linden [5], Wilbert M T Janssen [6], Hubertina Klein [7], Femke Waanders [8], Suat Simsek [9] [10], Cornelis P C de Jager [11], Paul Smits [12], Machteld van der Feltz [13], Gerrit Jan Beumer [14], Christine Widrich [15], Martijn Nap [15], Sara-Joan Pinto-Sietsma [1] [2]

Affiliations

- [1] From the Department of Clinical Epidemiology, Biostatistics and Bioinformatics (S.S., S.-J.P.-S.), Amsterdam UMC, Academic Medical Center Amsterdam, the Netherlands.
- [2] Department of Vascular Medicine (S.S., S.-J.P.-S.), Amsterdam UMC, Academic Medical Center Amsterdam, the Netherlands.

- [3] Microbiology and System Biology, Netherlands Organization for Applied Scientific Research, the Hague (A.K.O.-H.).
- [4] Department of Internal Medicine, Diakonessenhuis, Utrecht, the Netherlands (S.J.J.L.).
- [5] Department of Clinical Pharmacy, Tergooi, the Netherlands (P.D.V.d.L.).
- [6] Department of Internal Medicine, Martini Hospital, the Netherlands (W.M.T.J.).
- [7] Department of Internal Medicine, Slingeland Hospital, Doetinchem, the Netherlands (H.K.).
- [8] Department of Internal Medicine, Isala, Zwolle, the Netherlands (F.W.).
- [9] Department of Internal Medicine/Endocrinology, Northwest Clinics, Alkmaar, the Netherlands (S.S.).
- [10] Department of Internal Medicine/Endocrinology, Amsterdam UMC, VU University Medical Center, the Netherlands (S.S.).
- [11] Department of Intensive Care Medicine, Jeroen Bosch Ziekenhuis, the Netherlands (C.P.C.d.J.).
- [12] Department of Pharmacology and Toxicology, Radboud university medical center, Radboud Institute for Health Sciences, the Netherlands (P.S.).
- [13] Department of Internal Medicine, Alrijne Hospital, Leiderdorp, the Netherlands (M.v.d.F.).
- [14] Life Sciences TNO, Leiden, the Netherlands (G.J.B.).
- [15] IQVIA, Amsterdam, the Netherlands (C.W., M.N.).

- PMID: **34106731**
- PMCID: PMC8189257
- DOI: 10.1161/HYPERTENSIONAHA.121.17328

Abstract

[Figure: see text].

Keywords: angiotensins; coronary artery disease; diuretics; obesity; pandemic.

- Cited by 5 articles
- 27 references
- 1 figure

Supplementary info

Publication types, MeSH terms, Substances Expand

Publication types

- Observational Study

MeSH terms

- Adrenergic beta-Antagonists / pharmacology
- Adrenergic beta-Antagonists / therapeutic use
- Adult

- Aged
- Aged, 80 and over
- Angiotensin Receptor Antagonists / pharmacology
- Angiotensin Receptor Antagonists / therapeutic use
- Angiotensin-Converting Enzyme Inhibitors / pharmacology
- Angiotensin-Converting Enzyme Inhibitors / therapeutic use
- Antihypertensive Agents / pharmacology*
- Antihypertensive Agents / therapeutic use
- COVID-19 / complications
- COVID-19 / mortality*
- COVID-19 / physiopathology
- Female
- Hospitalization
- Humans
- Hypertension / complications
- Hypertension / drug therapy*
- Male
- Middle Aged
- Renin-Angiotensin System / drug effects*
- Retrospective Studies
- SARS-CoV-2
- Treatment Outcome
- Withholding Treatment

Substances

- Adrenergic beta-Antagonists
- Angiotensin Receptor Antagonists
- Angiotensin-Converting Enzyme Inhibitors
- Antihypertensive Agents

Full text links

OPEN ACCESS Atypon Free PMC article
Proceed to details
Cite
Share
☐ 508
Observational Study
PLoS One
. 2021 Oct 28;16(10):e0259061.
doi: 10.1371/journal.pone.0259061. eCollection 2021.

Disulfiram use is associated with lower risk of COVID-19: A retrospective cohort study

Nathanael Fillmore [1, 2], Steven Bell [3], Ciyue Shen [4, 5, 6], Vinh Nguyen [1], Jennifer La [1], Maureen Dubreuil [7, 8], Judith Strymish [2, 9], Mary Brophy [1, 10], Gautam Mehta [11, 12], Hao Wu [13, 14], Judy Lieberman [14, 15], Nhan Do [1, 16], Chris Sander [4, 5, 6]

Affiliations Expand

Affiliations

- [1] Boston VA Cooperative Studies Program (CSP) Center, VA Boston Healthcare System, Boston, Massachusetts, United States of America.
- [2] Department of Medicine, Harvard Medical School, Boston, Massachusetts, United States of America.
- [3] Department of Clinical Neurosciences, University of Cambridge, Cambridge, United Kingdom.
- [4] Department of Cell Biology, Harvard Medical School, Boston, Massachusetts, United States of America.
- [5] Department of Data Science, Dana-Farber Cancer Institute, Boston, Massachusetts, United States of America.
- [6] Broad Institute of Harvard and MIT, Boston, Massachusetts, United States of America.
- [7] Section of Rheumatology, Boston University School of Medicine, Boston, Massachusetts, United States of America.
- [8] Rheumatology, VA Boston Healthcare System, Boston, Massachusetts, United States of America.
- [9] Infection Disease, VA Boston Healthcare System, Boston, Massachusetts, United States of America.
- [10] Section of Hematology and Medical Oncology, Boston University School of Medicine, Boston, Massachusetts, United States of America.
- [11] Institute for Liver and Digestive Health, University College London, London, United Kingdom.
- [12] Institute of Hepatology, Foundation for Liver Research, London, United Kingdom.
- [13] Department of Biological Chemistry and Molecular Pharmacology, Harvard Medical School, Boston, Massachusetts, United States of America.
- [14] Program in Cellular and Molecular Medicine, Boston Children's Hospital, Boston, Massachusetts, United States of America.
- [15] Department of Pediatrics, Harvard Medical School, Boston, Massachusetts, United States of America.
- [16] Section of General Internal Medicine, Boston University School of Medicine, Boston, Massachusetts, United States of America.

- PMID: **34710137**
- PMCID: PMC8553043
- DOI: 10.1371/journal.pone.0259061

Free PMC article
Observational Study

Disulfiram use is associated with lower risk of COVID-19: A retrospective cohort study

Nathanael Fillmore et al. PLoS One. 2021.
Free PMC article
Show details
PLoS One
. 2021 Oct 28;16(10):e0259061.
doi: 10.1371/journal.pone.0259061. eCollection 2021.

Authors

Nathanael Fillmore [1] [2], Steven Bell [3], Ciyue Shen [4] [5] [6], Vinh Nguyen [1], Jennifer La [1], Maureen Dubreuil [7] [8], Judith Strymish [2] [9], Mary Brophy [1] [10], Gautam Mehta [11] [12], Hao Wu [13] [14], Judy Lieberman [14] [15], Nhan Do [1] [16], Chris Sander [4] [5] [6]

Affiliations

- [1] Boston VA Cooperative Studies Program (CSP) Center, VA Boston Healthcare System, Boston, Massachusetts, United States of America.
- [2] Department of Medicine, Harvard Medical School, Boston, Massachusetts, United States of America.
- [3] Department of Clinical Neurosciences, University of Cambridge, Cambridge, United Kingdom.
- [4] Department of Cell Biology, Harvard Medical School, Boston, Massachusetts, United States of America.
- [5] Department of Data Science, Dana-Farber Cancer Institute, Boston, Massachusetts, United States of America.
- [6] Broad Institute of Harvard and MIT, Boston, Massachusetts, United States of America.
- [7] Section of Rheumatology, Boston University School of Medicine, Boston, Massachusetts, United States of America.
- [8] Rheumatology, VA Boston Healthcare System, Boston, Massachusetts, United States of America.
- [9] Infection Disease, VA Boston Healthcare System, Boston, Massachusetts, United States of America.
- [10] Section of Hematology and Medical Oncology, Boston University School of Medicine, Boston, Massachusetts, United States of America.
- [11] Institute for Liver and Digestive Health, University College London, London, United Kingdom.
- [12] Institute of Hepatology, Foundation for Liver Research, London, United Kingdom.
- [13] Department of Biological Chemistry and Molecular Pharmacology, Harvard Medical School, Boston, Massachusetts, United States of America.
- [14] Program in Cellular and Molecular Medicine, Boston Children's Hospital, Boston, Massachusetts, United States of America.
- [15] Department of Pediatrics, Harvard Medical School, Boston, Massachusetts, United States of America.

- [16] Section of General Internal Medicine, Boston University School of Medicine, Boston, Massachusetts, United States of America.

- PMID: **34710137**
- PMCID: PMC8553043
- DOI: 10.1371/journal.pone.0259061

Abstract

Effective, low-cost therapeutics are needed to prevent and treat COVID-19. Severe COVID-19 disease is linked to excessive inflammation. Disulfiram is an approved oral drug used to treat alcohol use disorder that is a potent anti-inflammatory agent and an inhibitor of the viral proteases. We investigated the potential effects of disulfiram on SARS-CoV-2 infection and disease severity in an observational study using a large database of clinical records from the national US Veterans Affairs healthcare system. A multivariable Cox regression adjusted for demographic information and diagnosis of alcohol use disorder revealed a reduced risk of SARS-CoV-2 infection with disulfiram use at a hazard ratio of 0.66 (34% lower risk, 95% confidence interval 24-43%). There were no COVID-19 related deaths among the 188 SARS-CoV-2 positive patients treated with disulfiram, in contrast to 5-6 statistically expected deaths based on the untreated population (P = 0.03). Our epidemiological results suggest that disulfiram may contribute to the reduced incidence and severity of COVID-19. These results support carefully planned clinical trials to assess the potential therapeutic effects of disulfiram in COVID-19.

Conflict of interest statement

The authors have declared that no competing interests exist.

- Cited by 3 articles
- 19 references
- 1 figure

Supplementary info

Publication types, MeSH terms, Substances, Grant support Expand

Publication types

- Observational Study
- Research Support, N.I.H., Extramural
- Research Support, Non-U.S. Gov't
- Research Support, U.S. Gov't, Non-P.H.S.

MeSH terms

- Adult
- Alcoholism / complications
- COVID-19 / drug therapy*
- COVID-19 / epidemiology

- COVID-19 / metabolism
- Cohort Studies
- Disulfiram / metabolism
- Disulfiram / therapeutic use*
- Female
- Humans
- Male
- Middle Aged
- Proportional Hazards Models
- Retrospective Studies
- Risk Factors
- SARS-CoV-2 / drug effects
- SARS-CoV-2 / pathogenicity
- Severity of Illness Index
- Veterans

Substances

- Disulfiram

Grant support

- DP1 HD087988/HD/NICHD NIH HHS/United States
- BHF_/British Heart Foundation/United Kingdom
- K23 AR069127/AR/NIAMS NIH HHS/United States

Full text links

OPEN ACCESS TO FULL TEXT PLOS ONE Public Library of Science Free PMC article

Proceed to details

Cite

Share

☐ 509

Observational Study

Cancer

. 2020 Oct 1;126(19):4294-4303.

doi: 10.1002/cncr.33084. Epub 2020 Jul 30.

COVID-19 outcomes of patients with gynecologic cancer in New York City

Olivia D Lara [1], Roisin E O'Cearbhaill [2], Maria J Smith [1], Megan E Sutter [1 3], Anne Knisely [4], Jennifer McEachron [5], Lisa R Gabor [6], Justin Jee [2], Julia E Fehniger [1], Yi-Chun Lee [5], Sara S Isani [6], Jason D Wright [4], Bhavana Pothuri [1]
Affiliations Expand

Affiliations

- [1] Department of Obstetrics and Gynecology, Perlmutter Cancer Center, NYU Langone Health, New York, New York.
- [2] Department of Medical Oncology, Memorial Sloan Kettering Cancer Center, New York, New York.
- [3] Department of Population Health, NYU Langone Health, New York, New York.
- [4] Department of Obstetrics and Gynecology, College of Physicians and Surgeons, Columbia University, New York, New York.
- [5] Department of Obstetrics and Gynecology, State University of New York Downstate Medical Center, Brooklyn, New York.
- [6] Department of Obstetrics & Gynecology and Women's Health, Montefiore Medical Center and Albert Einstein College of Medicine, Bronx, New York.

- PMID: **32729142**
- PMCID: PMC8654115
- DOI: 10.1002/cncr.33084

Free PMC article
Observational Study

COVID-19 outcomes of patients with gynecologic cancer in New York City

Olivia D Lara et al. Cancer. 2020.
Free PMC article
Show details
Cancer
. 2020 Oct 1;126(19):4294-4303.
doi: 10.1002/cncr.33084. Epub 2020 Jul 30.

Authors

Olivia D Lara [1], Roisin E O'Cearbhaill [2], Maria J Smith [1], Megan E Sutter [1 3], Anne Knisely [4], Jennifer McEachron [5], Lisa R Gabor [6], Justin Jee [2], Julia E Fehniger [1], Yi-Chun Lee [5], Sara S Isani [6], Jason D Wright [4], Bhavana Pothuri [1]

Affiliations

- [1] Department of Obstetrics and Gynecology, Perlmutter Cancer Center, NYU Langone Health, New York, New York.

- [2] Department of Medical Oncology, Memorial Sloan Kettering Cancer Center, New York, New York.
- [3] Department of Population Health, NYU Langone Health, New York, New York.
- [4] Department of Obstetrics and Gynecology, College of Physicians and Surgeons, Columbia University, New York, New York.
- [5] Department of Obstetrics and Gynecology, State University of New York Downstate Medical Center, Brooklyn, New York.
- [6] Department of Obstetrics & Gynecology and Women's Health, Montefiore Medical Center and Albert Einstein College of Medicine, Bronx, New York.

- PMID: **32729142**
- PMCID: PMC8654115
- DOI: 10.1002/cncr.33084

Abstract

Background: New York City (NYC) is the epicenter of severe acute respiratory syndrome coronavirus 2 (coronavirus disease 2019 [COVID-19]) in the United States. Clinical characteristics and outcomes of vulnerable populations, such as those with gynecologic cancer who develop COVID-19 infections, is limited.

Methods: Patients from 6 NYC-area hospital systems with known gynecologic cancer and a COVID-19 diagnosis were identified. Demographic and clinical outcome data were abstracted through a review of electronic medical records.

Results: Records for 121 patients with gynecologic cancer and COVID-19 were abstracted; the median age at the COVID-19 diagnosis was 64.0 years (interquartile range, 51.0-73.0 years). Sixty-six of the 121 patients (54.5%) required hospitalization; among the hospitalized patients, 45 (68.2%) required respiratory intervention, 20 (30.3%) were admitted to the intensive care unit, and 9 (13.6%) underwent invasive mechanical ventilation. Seventeen patients (14.0%) died of COVID-19 complications. No patient requiring mechanical ventilation survived. On multivariable analysis, hospitalization was associated with an age ≥64 years (risk ratio [RR], 1.73; 95% confidence interval [CI], 1.18-2.51), African American race (RR, 1.56; 95% CI, 1.13-2.15), and 3 or more comorbidities (RR, 1.43; 95% CI, 1.03-1.98). Only recent immunotherapy use (RR, 3.49; 95% CI, 1.08-11.27) was associated with death due to COVID-19 on multivariable analysis; chemotherapy treatment and recent major surgery were not predictive of COVID-19 severity or mortality.

Conclusions: The case fatality rate among gynecologic oncology patients with a COVID-19 infection is 14.0%. Recent immunotherapy use is associated with an increased risk of mortality related to COVID-19 infection.

Lay summary: The case fatality rate among gynecologic oncology patients with a coronavirus disease 2019 (COVID-19) infection is 14.0%; there is no association between cytotoxic chemotherapy and cancer-directed surgery and COVID-19 severity or death. As such, patients can be counseled regarding the safety of continued anticancer treatments during the pandemic. This is important because the ability to continue cancer therapies for cancer control and cure is critical.

Keywords: coronavirus disease 2019 (COVID-19); gynecologic cancer; outcomes; severe acute respiratory syndrome coronavirus 2 (SARS-CoV-2).

© 2020 American Cancer Society.

- Cited by 27 articles
- 1 figure

Supplementary info

Publication types, MeSH terms, Grant support Expand

Publication types

- Multicenter Study
- Observational Study
- Research Support, N.I.H., Extramural

MeSH terms

- Aged
- COVID-19 / epidemiology
- COVID-19 / etiology
- COVID-19 / mortality*
- COVID-19 / therapy*
- Comorbidity
- Female
- Genital Neoplasms, Female / epidemiology*
- Genital Neoplasms, Female / therapy
- Hospitalization
- Humans
- Immunotherapy
- Intensive Care Units
- Middle Aged
- New York City
- Respiration, Artificial
- Retrospective Studies
- Risk Factors
- Treatment Outcome

Grant support

- L60 MD014442/MD/NIMHD NIH HHS/United States
- P30 CA008748/CA/NCI NIH HHS/United States
- T32 HS026120/HS/AHRQ HHS/United States
- T32HS026120/Agency for Healthcare Research and Quality/International

Full text links

WILEY Free Full Text Wiley Free PMC article
Proceed to details
Cite
Share
510

Observational Study

BMC Pulm Med
. 2021 Apr 14;21(1):120.
doi: 10.1186/s12890-021-01487-6.

Risk stratification scores for hospitalization duration and disease progression in moderate and severe patients with COVID-19

Jiaqi Huang[#1], Yu Xu[#2], Bin Wang[#2], Ying Xiang[1], Na Wu[1], Wenjing Zhang[2], Tingting Xia[1], Zhiquan Yuan[1], Chengying Li[1], Xiaoyue Jia[1], Yifan Shan[1], Menglei Chen[1], Qi Li[#2], Li Bai[#2], Yafei Li[#3]
Affiliations Expand

Affiliations

- [1] Department of Epidemiology, College of Preventive Medicine, Army Medical University (Third Military Medical University), No. 30 Gaotanyan Street, Chongqing, 400038, People's Republic of China.
- [2] Department of Respiratory and Critical Care Medicine, The Second Affiliated Hospital of The Army Medical University, Chongqing, 400037, People's Republic of China.
- [3] Department of Epidemiology, College of Preventive Medicine, Army Medical University (Third Military Medical University), No. 30 Gaotanyan Street, Chongqing, 400038, People's Republic of China. liyafei2008@tmmu.edu.cn.

[#] Contributed equally.

- PMID: **33853568**
- PMCID: PMC8045569
- DOI: 10.1186/s12890-021-01487-6

Free PMC article
Observational Study

Risk stratification scores for hospitalization duration and disease progression in moderate and severe patients with COVID-19

Jiaqi Huang et al. BMC Pulm Med. 2021.
Free PMC article
Show details
BMC Pulm Med
. 2021 Apr 14;21(1):120.
doi: 10.1186/s12890-021-01487-6.

Authors

Jiaqi Huang [# 1], Yu Xu [# 2], Bin Wang [# 2], Ying Xiang [1], Na Wu [1], Wenjing Zhang [2], Tingting Xia [1], Zhiquan Yuan [1], Chengying Li [1], Xiaoyue Jia [1], Yifan Shan [1], Menglei Chen [1], Qi Li [# 2], Li Bai [# 2], Yafei Li [# 3]

Affiliations

- [1] Department of Epidemiology, College of Preventive Medicine, Army Medical University (Third Military Medical University), No. 30 Gaotanyan Street, Chongqing, 400038, People's Republic of China.
- [2] Department of Respiratory and Critical Care Medicine, The Second Affiliated Hospital of The Army Medical University, Chongqing, 400037, People's Republic of China.
- [3] Department of Epidemiology, College of Preventive Medicine, Army Medical University (Third Military Medical University), No. 30 Gaotanyan Street, Chongqing, 400038, People's Republic of China. liyafei2008@tmmu.edu.cn.

[#] Contributed equally.

- PMID: **33853568**
- PMCID: PMC8045569
- DOI: 10.1186/s12890-021-01487-6

Abstract

Background: During outbreak of Coronavirus Disease 2019 (COVID-19), healthcare providers are facing critical clinical decisions based on the prognosis of patients. Decision support tools of risk stratification are needed to predict outcomes in patients with different clinical types of COVID-19.

Methods: This retrospective cohort study recruited 2425 patients with moderate or severe COVID-19. A logistic regression model was used to select and estimate the factors independently associated with outcomes. Simplified risk stratification score systems were constructed to predict outcomes in moderate and severe patients with COVID-19, and their performances were evaluated by discrimination and calibration.

Results: We constructed two risk stratification score systems, named as STPCAL (including significant factors in the prediction model: number of clinical symptoms, the maximum body temperature during hospitalization, platelet count, C-reactive protein, albumin and lactate dehydrogenase) and TRPNCLP (including maximum body temperature during hospitalization, history of respiratory diseases, platelet count, neutrophil-to-lymphocyte ratio, creatinine, lactate dehydrogenase, and prothrombin time), to predict hospitalization duration for moderate patients and disease progression for severe patients, respectively. According to STPCAL score, moderate patients were classified into three risk categories for a longer hospital duration: low (Score 0-1,

median = 8 days, with less than 20.0% probabilities), intermediate (Score 2-6, median = 13 days, with 30.0-78.9% probabilities), high (Score 7-9, median = 19 days, with more than 86.5% probabilities). Severe patients were stratified into three risk categories for disease progression: low risk (Score 0-5, with less than 12.7% probabilities), intermediate risk (Score 6-11, with 18.6-69.1% probabilities), and high risk (Score 12-16, with more than 77.9% probabilities) by TRPNCLP score. The two risk scores performed well with good discrimination and calibration.

Conclusions: Two easy-to-use risk stratification score systems were built to predict the outcomes in COVID-19 patients with different clinical types. Identifying high risk patients with longer stay or poor prognosis could assist healthcare providers in triaging patients when allocating limited healthcare during COVID-19 outbreak.

Keywords: COVID-19; Disease progression; Length of hospital stay; Risk stratification score.

Conflict of interest statement

We declare no competing interests.

- Cited by 2 articles
- 44 references
- 3 figures

Supplementary info

Publication types, MeSH terms Expand

Publication types

- Observational Study

MeSH terms

- Adolescent
- Adult
- Aged
- Aged, 80 and over
- COVID-19 / diagnosis*
- COVID-19 / therapy*
- COVID-19 Testing / methods*
- Clinical Decision Rules*
- Clinical Decision-Making / methods
- Disease Progression*
- Female
- Hospitalization / statistics & numerical data*
- Humans
- Logistic Models
- Male

- Middle Aged
- Prognosis
- Retrospective Studies
- Risk Assessment
- Risk Factors
- Sensitivity and Specificity
- Severity of Illness Index*
- Triage / methods
- Young Adult

Full text links

Read free full text at BMC BioMed Central Free PMC article
Proceed to details
Cite
Share
511
Observational Study
Chest
. 2021 Jul;160(1):89-93.
doi: 10.1016/j.chest.2021.01.073. Epub 2021 Feb 3.

Dihydropyridine Calcium Channel Blockers and the Risk of Severe COVID-19

Sean R Mendez [1], Rachel C Frank [2], Elizabeth K Stevenson [3], Mabel Chung [4], Michael G Silverman [5]
Affiliations Expand

Affiliations

- [1] Department of Medicine, Massachusetts General Hospital, Boston, MA.
- [2] Cardiology Division, Department of Medicine, Massachusetts General Hospital, Boston, MA.
- [3] Division of Pulmonary, Critical Care, and Sleep Medicine, North Shore Medical Center, Salem, MA.
- [4] Department of Anesthesia, Critical Care and Pain Medicine, Massachusetts General Hospital, Boston, MA; Heart Center Intensive Care Unit, Massachusetts General Hospital, Boston, MA; Smith Center for Outcomes Research in Cardiology, Division of Cardiovascular Medicine, Department of Medicine, Beth Israel Deaconess Medical Center, Boston, MA.
- [5] Cardiology Division, Department of Medicine, Massachusetts General Hospital, Boston, MA; Heart Center Intensive Care Unit, Massachusetts General Hospital, Boston, MA. Electronic address: mgsilverman@mgh.harvard.edu.

- PMID: **33548220**
- PMCID: PMC7857077
- DOI: 10.1016/j.chest.2021.01.073

Free PMC article
Observational Study

Dihydropyridine Calcium Channel Blockers and the Risk of Severe COVID-19

Sean R Mendez et al. Chest. 2021 Jul.
Free PMC article
Show details
Chest

. 2021 Jul;160(1):89-93.
doi: 10.1016/j.chest.2021.01.073. Epub 2021 Feb 3.

Authors

Sean R Mendez [1], Rachel C Frank [2], Elizabeth K Stevenson [3], Mabel Chung [4], Michael G Silverman [5]

Affiliations

- [1] Department of Medicine, Massachusetts General Hospital, Boston, MA.
- [2] Cardiology Division, Department of Medicine, Massachusetts General Hospital, Boston, MA.
- [3] Division of Pulmonary, Critical Care, and Sleep Medicine, North Shore Medical Center, Salem, MA.
- [4] Department of Anesthesia, Critical Care and Pain Medicine, Massachusetts General Hospital, Boston, MA; Heart Center Intensive Care Unit, Massachusetts General Hospital, Boston, MA; Smith Center for Outcomes Research in Cardiology, Division of Cardiovascular Medicine, Department of Medicine, Beth Israel Deaconess Medical Center, Boston, MA.
- [5] Cardiology Division, Department of Medicine, Massachusetts General Hospital, Boston, MA; Heart Center Intensive Care Unit, Massachusetts General Hospital, Boston, MA. Electronic address: mgsilverman@mgh.harvard.edu.

- PMID: **33548220**
- PMCID: PMC7857077
- DOI: 10.1016/j.chest.2021.01.073

No abstract available

- Cited by 3 articles
- 8 references
- 1 figure

Supplementary info

Publication types, MeSH terms, Substances, Grant support Expand

Publication types

- Observational Study

MeSH terms

- Antihypertensive Agents / therapeutic use
- COVID-19* / diagnosis
- COVID-19* / epidemiology
- COVID-19* / physiopathology
- COVID-19* / therapy
- Calcium Channel Blockers / therapeutic use*
- Comorbidity
- Correlation of Data
- Dihydropyridines / therapeutic use*
- Female
- Humans
- Hypertension / drug therapy*
- Hypertension / epidemiology
- Hypoxia* / etiology
- Hypoxia* / physiopathology
- Hypoxia* / prevention & control
- Male
- Middle Aged
- Proportional Hazards Models
- Retrospective Studies
- SARS-CoV-2 / isolation & purification
- United States / epidemiology
- Vasoconstriction / drug effects*
- Withholding Treatment / statistics & numerical data

Substances

- Antihypertensive Agents
- Calcium Channel Blockers
- Dihydropyridines

Grant support

- T32 GM007592/GM/NIGMS NIH HHS/United States

Full text links

ELSEVIER FULL-TEXT ARTICLE Elsevier Science Free PMC article
Proceed to details
Cite
Share
512
Observational Study
Int J Environ Res Public Health
. 2021 Nov 10;18(22):11786.
doi: 10.3390/ijerph182211786.

Baseline Drug Treatments as Indicators of Increased Risk of COVID-19 Mortality in Spain and Italy

Kevin Bliek-Bueno [1] [2], Sara Mucherino [3], Beatriz Poblador-Plou [1] [4], Francisca González-Rubio [1] [4] [5] [6], Mercedes Aza-Pascual-Salcedo [1] [4] [7], Valentina Orlando [3], Mercedes Clerencia-Sierra [1] [4] [8], Ignatios Ioakeim-Skoufa [1] [6] [9] [10], Enrico Coscioni [11], Jonás Carmona-Pírez [1] [4] [5], Alessandro Perrella [12], Ugo Trama [13], Alexandra Prados-Torres [1] [4], Enrica Menditto [3], Antonio Gimeno-Miguel [1] [4]
Affiliations Expand

Affiliations

- [1] EpiChron Research Group, Aragon Health Sciences Institute (IACS), IIS Aragón, Miguel Servet University Hospital, 50009 Zaragoza, Spain.
- [2] Teaching Unit of Preventive Medicine and Public Health, Miguel Servet University Hospital, 50009 Zaragoza, Spain.
- [3] Centro Interdipartimentale di Ricerca in Farmacoeconomia e Farmacoutilizzazione (CIRFF), Center of Drug Utilization and Pharmacoeconomics, Department of Pharmacy, University of Naples Federico II, 80131 Naples, Italy.
- [4] Health Services Research on Chronic Patients Network (REDISSEC), Institute of Health Carlos III (ISCIII), 28222 Madrid, Spain.
- [5] Delicias-Sur Primary Care Health Centre, Aragon Health Service (SALUD), 50009 Zaragoza, Spain.
- [6] Drug Utilization Work Group, Spanish Society of Family and Community Medicine (semFYC), 08009 Barcelona, Spain.
- [7] Primary Care Pharmacy Service Zaragoza III, Aragon Health Service (SALUD), 50017 Zaragoza, Spain.
- [8] Aragon Health Service (SALUD), Miguel Servet University Hospital, 50009 Zaragoza, Spain.

- [9] WHO Collaborating Centre for Drug Statistics Methodology, Norwegian Institute of Public Health, 0213 Oslo, Norway.
- [10] Department of Drug Statistics, Division of Health Data and Digitalisation, Norwegian Institute of Public Health, 0213 Oslo, Norway.
- [11] Division of Cardiac Surgery, AOU San Giovanni di Dio e Ruggi d'Aragona, 84131 Salerno, Italy.
- [12] Infectious Disease of Healthcare Direction, AORN Antonio Cardarelli, 80131 Naples, Italy.
- [13] Regional Pharmaceutical Unit, Campania Region, 80143 Naples, Italy.

- PMID: **34831541**
- PMCID: PMC8623536
- DOI: 10.3390/ijerph182211786

Free PMC article
Observational Study

Baseline Drug Treatments as Indicators of Increased Risk of COVID-19 Mortality in Spain and Italy

Kevin Bliek-Bueno et al. Int J Environ Res Public Health. 2021.
Free PMC article
Show details
Int J Environ Res Public Health
. 2021 Nov 10;18(22):11786.
doi: 10.3390/ijerph182211786.

Authors

Kevin Bliek-Bueno [1, 2], Sara Mucherino [3], Beatriz Poblador-Plou [1, 4], Francisca González-Rubio [1, 4, 5, 6], Mercedes Aza-Pascual-Salcedo [1, 4, 7], Valentina Orlando [3], Mercedes Clerencia-Sierra [1, 4, 8], Ignatios Ioakeim-Skoufa [1, 6, 9, 10], Enrico Coscioni [11], Jonás Carmona-Pírez [1, 4, 5], Alessandro Perrella [12], Ugo Trama [13], Alexandra Prados-Torres [1, 4], Enrica Menditto [3], Antonio Gimeno-Miguel [1, 4]

Affiliations

- [1] EpiChron Research Group, Aragon Health Sciences Institute (IACS), IIS Aragón, Miguel Servet University Hospital, 50009 Zaragoza, Spain.
- [2] Teaching Unit of Preventive Medicine and Public Health, Miguel Servet University Hospital, 50009 Zaragoza, Spain.
- [3] Centro Interdipartimentale di Ricerca in Farmacoeconomia e Farmacoutilizzazione (CIRFF), Center of Drug Utilization and Pharmacoeconomics, Department of Pharmacy, University of Naples Federico II, 80131 Naples, Italy.
- [4] Health Services Research on Chronic Patients Network (REDISSEC), Institute of Health Carlos III (ISCIII), 28222 Madrid, Spain.

- [5] Delicias-Sur Primary Care Health Centre, Aragon Health Service (SALUD), 50009 Zaragoza, Spain.
- [6] Drug Utilization Work Group, Spanish Society of Family and Community Medicine (semFYC), 08009 Barcelona, Spain.
- [7] Primary Care Pharmacy Service Zaragoza III, Aragon Health Service (SALUD), 50017 Zaragoza, Spain.
- [8] Aragon Health Service (SALUD), Miguel Servet University Hospital, 50009 Zaragoza, Spain.
- [9] WHO Collaborating Centre for Drug Statistics Methodology, Norwegian Institute of Public Health, 0213 Oslo, Norway.
- [10] Department of Drug Statistics, Division of Health Data and Digitalisation, Norwegian Institute of Public Health, 0213 Oslo, Norway.
- [11] Division of Cardiac Surgery, AOU San Giovanni di Dio e Ruggi d'Aragona, 84131 Salerno, Italy.
- [12] Infectious Disease of Healthcare Direction, AORN Antonio Cardarelli, 80131 Naples, Italy.
- [13] Regional Pharmaceutical Unit, Campania Region, 80143 Naples, Italy.

- PMID: **34831541**
- PMCID: PMC8623536
- DOI: 10.3390/ijerph182211786

Abstract

This study aims to identify baseline medications that, as a proxy for the diseases they are dispensed for, are associated with increased risk of mortality in COVID-19 patients from two regions in Spain and Italy using real-world data. We conducted a cross-country, retrospective, observational study including 8570 individuals from both regions with confirmed SARS-CoV-2 infection between 4 March and 17 April 2020, and followed them for a minimum of 30 days to allow sufficient time for the studied event, in this case death, to occur. Baseline demographic variables and all drugs dispensed in community pharmacies three months prior to infection were extracted from the PRECOVID Study cohort (Aragon, Spain) and the Campania Region Database (Campania, Italy) and analyzed using logistic regression models. Results show that the presence at baseline of potassium-sparing agents, antipsychotics, vasodilators, high-ceiling diuretics, antithrombotic agents, vitamin B12, folic acid, and antiepileptics were systematically associated with mortality in COVID-19 patients from both countries. Treatments for chronic cardiovascular and metabolic diseases, systemic inflammation, and processes with increased risk of thrombosis as proxies for the conditions they are intended for can serve as timely indicators of an increased likelihood of mortality after the infection, and the assessment of pharmacological profiles can be an additional approach to the identification of at-risk individuals in clinical practice.

Keywords: COVID-19; drugs; medications; mortality; real-world data.

Conflict of interest statement

The authors declare no conflict of interest. The funders had no role in the design of the study; in the collection, analyses, or interpretation of data, in the writing of the manuscript or in the decision to publish the results.

- 23 references

Supplementary info

Publication types, MeSH terms, Substances, Supplementary concepts, Grant support Expand

Publication types

- Observational Study
- Research Support, Non-U.S. Gov't

MeSH terms

- COVID-19* / drug therapy
- Humans
- Pharmaceutical Preparations*
- Retrospective Studies
- SARS-CoV-2
- Spain / epidemiology

Substances

- Pharmaceutical Preparations

Supplementary concepts

- COVID-19 drug treatment

Grant support

- Recognized Groups Grant reference B01_20R and Decree-Law 3/2020, published on 3 June/Government of Aragon

Full text links

FULL TEXT OPEN ACCESS MDPI Multidisciplinary Digital Publishing Institute (MDPI) Free PMC article

Proceed to details

Cite

Share

513

Observational Study

Updates Surg
. 2021 Dec;73(6):2205-2213.
doi: 10.1007/s13304-021-01126-z. Epub 2021 Jul 4.

Appendectomy during the COVID-19 pandemic in Italy: a multicenter ambispective cohort study by the Italian Society of Endoscopic Surgery and new technologies (the CRAC study)

Alberto Sartori [1], Mauro Podda [2], Emanuele Botteri [3], Roberto Passera [4], Ferdinando Agresta [5], Alberto Arezzo [6], CRAC Study Collaboration Group

Collaborators, Affiliations Expand

Collaborators

- **CRAC Study Collaboration Group**: M Guerrieri, M Ortenzi, F Cavallo, M Zese, D Prando, E Restini, P Cianci, P Millo, R Brachet Contul, A Serrao, F Abatini, D F Altomare, A Picciariello, G Chetta, F Lattanzio, V Tonini, A Gori, E Jovine, L Mastrangelo, L Sartarelli, A Frena, A Malpaga, F Bertelli, G Pignata, J Andreuccetti, S Sanna, B Lares, R Sechi, N Cillara, A Pisanu, D Delogu, G Ciaccio, M Farulla, M Casati, L Laface, M De Luca, D Russello, S Latteri, M Longoni, E Masci, S Vigna, F C Campanile, N Foti, P Lepiane, A Balla, F Cantore, V Raveglia, F Borghi, G Giraudo, A Verzelli, A Budassi, A Patriti, D Foghetti, U Montin, L Amadio, G Anania, C Bombardini, Niccolò Fabbri, Carlo Feo, F Cianchi, A Manetti, M Lucchese, E Soricelli, G Ceccarelli, M Patiti, M Frascio, C Stabilini, M Filauro, A Barberis, M Troian, C Nagliati, R Campagnacci, A Maurizi, S Berti, A Gennai, A Marvaso, D D'Antonio, C V Feo, N Fabbri, L Mazzola, F Selvaggi, S Carini, F Costanzo, L Boccia, A Pascariello, N Perrotta, M Celiento, E Opocher, M Giovenzana, M Stella, F Ferrara, L Boni, E Abate, C Da Lio, V Valli, R Gelmini, F Serra, M Piccoli, D Gozzo, A Gattolin, D Sasia, A Balani, B Petronio, P G Calò, G L Canu, E Contarini, G Piatto, N Vettoretto, M Caprioli, M Braga, M F Chiappetta, P Maida, P Tammaro, G De Palma, M Milone, V Bottino, A Canfora, F Selvaggi, G Bagaglini, A Agrusa, M Barone, A Mirabella, M V Marino, G Gulotta, G Romano, M Sorrentino, S Ferfoglia, V Papagni, S Eramo, C Boselli, M Basti, V Caracino, G Moretto, M Inama, P Capelli, L Conti, A Muratore, M M Cuoghi, A Zerbinati, S Corso, M C Vasino, M Montuori, F Fidanza, A Lucchetta, A Giuliani, G Dinatale, F Zanzi, A Guariniello, S Bonilauri, G Frazzetta, M Garino, C Marafante, A Gioffrè, S R Del Monte, G Sganga, P Fransvea, M Grande, L Siragusa, G Sica, M Paola, D G Passantino, Marco Catani, F Ricci, E Lauro, E Facci, D Parini, M F Armellino, G Argenio, A Porcu, T Perra, P Bordoni, F Fleres, A Parisi, S Rossi, R Saracco, D Bono, T Viora, F Orlando, A Ferrero, A P Fontana, P De Paolis, D Visconti, F Quaglino, F Festa, S Palagi, G Lo Secco, M Morino, M E Allaix, A Salzano, G Tirone, M Motter, G Zanus, N Passuello, M Massani, R Tutino, N Manzini, S Terranova, R Merenda, S Nordio, S Zonta, F Lovisetto, A Guglielmi, T Campagnaro, E Amedeo, M Scollica, P Amodio, D Giannotti, S Olmi, A Oldani

Affiliations

- [1] Department of General Surgery, Ospedale Di Montebelluna, Montebelluna, Italy.

- [2] Department of Emergency Surgery, Azienda Ospedaliero-Universitaria Di Cagliari, Policlinico Universitario Di Monserrato "Duilio Casula" University of Cagliari, Cagliari, Italy.
- [3] General Surgery, ASST Spedali Civili Di Brescia, Montichiari, Italy.
- [4] Division of Nuclear Medicine, University of Torino, Torino, Italy.
- [5] Department of General Surgery, Ospedale Civile, Adria, Italy.
- [6] Department of Surgical Sciences, University of Torino, corso AM Dogliotti 14, 10126, Torino, Italy. alberto.arezzo@unito.it.

- PMID: **34219197**
- PMCID: PMC8255092
- DOI: 10.1007/s13304-021-01126-z

Free PMC article
Observational Study

Appendectomy during the COVID-19 pandemic in Italy: a multicenter ambispective cohort study by the Italian Society of Endoscopic Surgery and new technologies (the CRAC study)

Alberto Sartori et al. Updates Surg. 2021 Dec.
Free PMC article
Show details
Updates Surg
. 2021 Dec;73(6):2205-2213.
doi: 10.1007/s13304-021-01126-z. Epub 2021 Jul 4.

Authors

Alberto Sartori [1], Mauro Podda [2], Emanuele Botteri [3], Roberto Passera [4], Ferdinando Agresta [5], Alberto Arezzo [6], CRAC Study Collaboration Group

Collaborators

- **CRAC Study Collaboration Group**:
M Guerrieri, M Ortenzi, F Cavallo, M Zese, D Prando, E Restini, P Cianci, P Millo, R Brachet Contul, A Serrao, F Abatini, D F Altomare, A Picciariello, G Chetta, F Lattanzio, V Tonini, A Gori, E Jovine, L Mastrangelo, L Sartarelli, A Frena, A Malpaga, F Bertelli, G Pignata, J Andreuccetti, S Sanna, B Lares, R Sechi, N Cillara, A Pisanu, D Delogu, G Ciaccio, M Farulla, M Casati, L Laface, M De Luca, D Russello, S Latteri, M Longoni, E Masci, S Vigna, F C Campanile, N Foti, P Lepiane, A Balla, F Cantore, V Raveglia, F Borghi, G Giraudo, A Verzelli, A Budassi, A Patriti, D Foghetti, U Montin, L Amadio, G Anania, C Bombardini, Niccolò Fabbri, Carlo Feo, F Cianchi, A Manetti, M Lucchese, E Soricelli, G Ceccarelli, M Patiti, M Frascio, C Stabilini, M Filauro, A Barberis, M Troian, C

Nagliati, R Campagnacci, A Maurizi, S Berti, A Gennai, A Marvaso, D D'Antonio, C V Feo, N Fabbri, L Mazzola, F Selvaggi, S Carini, F Costanzo, L Boccia, A Pascariello, N Perrotta, M Celiento, E Opocher, M Giovenzana, M Stella, F Ferrara, L Boni, E Abate, C Da Lio, V Valli, R Gelmini, F Serra, M Piccoli, D Gozzo, A Gattolin, D Sasia, A Balani, B Petronio, P G Calò, G L Canu, E Contarini, G Piatto, N Vettoretto, M Caprioli, M Braga, M F Chiappetta, P Maida, P Tammaro, G De Palma, M Milone, V Bottino, A Canfora, F Selvaggi, G Bagaglini, A Agrusa, M Barone, A Mirabella, M V Marino, G Gulotta, G Romano, M Sorrentino, S Ferfoglia, V Papagni, S Eramo, C Boselli, M Basti, V Caracino, G Moretto, M Inama, P Capelli, L Conti, A Muratore, M M Cuoghi, A Zerbinati, S Corso, M C Vasino, M Montuori, F Fidanza, A Lucchetta, A Giuliani, G Dinatale, F Zanzi, A Guariniello, S Bonilauri, G Frazzetta, M Garino, C Marafante, A Gioffrè, S R Del Monte, G Sganga, P Fransvea, M Grande, L Siragusa, G Sica, M Paola, D G Passantino, Marco Catani, F Ricci, E Lauro, E Facci, D Parini, M F Armellino, G Argenio, A Porcu, T Perra, P Bordoni, F Fleres, A Parisi, S Rossi, R Saracco, D Bono, T Viora, F Orlando, A Ferrero, A P Fontana, P De Paolis, D Visconti, F Quaglino, F Festa, S Palagi, G Lo Secco, M Morino, M E Allaix, A Salzano, G Tirone, M Motter, G Zanus, N Passuello, M Massani, R Tutino, N Manzini, S Terranova, R Merenda, S Nordio, S Zonta, F Lovisetto, A Guglielmi, T Campagnaro, E Amedeo, M Scollica, P Amodio, D Giannotti, S Olmi, A Oldani

Affiliations

- [1] Department of General Surgery, Ospedale Di Montebelluna, Montebelluna, Italy.
- [2] Department of Emergency Surgery, Azienda Ospedaliero-Universitaria Di Cagliari, Policlinico Universitario Di Monserrato "Duilio Casula" University of Cagliari, Cagliari, Italy.
- [3] General Surgery, ASST Spedali Civili Di Brescia, Montichiari, Italy.
- [4] Division of Nuclear Medicine, University of Torino, Torino, Italy.
- [5] Department of General Surgery, Ospedale Civile, Adria, Italy.
- [6] Department of Surgical Sciences, University of Torino, corso AM Dogliotti 14, 10126, Torino, Italy. alberto.arezzo@unito.it.

- PMID: **34219197**
- PMCID: PMC8255092
- DOI: 10.1007/s13304-021-01126-z

Abstract

Major surgical societies advised using non-operative management of appendicitis and suggested against laparoscopy during the COVID-19 pandemic. The hypothesis is that a significant reduction in the number of emergent appendectomies was observed during the pandemic, restricted to complex cases. The study aimed to analyse emergent surgical appendectomies during pandemic on a national basis and compare it to the same period of the previous year. This is a multicentre, retrospective, observational study investigating the outcomes of patients undergoing emergent appendectomy in March-April 2019 vs March-April 2020. The primary outcome was the number of appendectomies performed, classified according to the American Association for the Surgery of Trauma (AAST) score. Secondary outcomes were the type of surgical technique employed (laparoscopic vs open) and the complication rates. One thousand five hundred forty one patients with acute appendicitis underwent surgery during the two study periods. 1337 (86.8%) patients met the inclusion criteria: 546 (40.8%) patients underwent surgery for acute appendicitis in 2020 and 791 (59.2%) in 2019. According to AAST, patients with complicated appendicitis

operated in 2019 were 30.3% vs 39.9% in 2020 ($p = 0.001$). We observed an increase in the number of post-operative complications in 2020 (15.9%) compared to 2019 (9.6%) ($p < 0.001$). The following determinants increased the likelihood of complication occurrence: undergoing surgery during 2020 (+ 67%), the increase of a unit in the AAST score (+ 26%), surgery performed > 24 h after admission (+ 58%), open surgery (+ 112%) and conversion to open surgery (+ 166%). In Italian hospitals, in March and April 2020, the number of appendectomies has drastically dropped. During the first pandemic wave, patients undergoing surgery were more frequently affected by more severe appendicitis than the previous year's timeframe and experienced a higher number of complications. Trial registration number and date: Research Registry ID 5789, May 7th, 2020.

Keywords: Appendectomy; Appendicitis; COVID-19 Pandemic; Machine learning.

© 2021. The Author(s).

Conflict of interest statement

Alberto Sartori, Mauro Podda, Emanuele Botteri, Roberto Passera, Alberto Arezzo, Ferdinando Agresta have no conflict of interest or financial ties to disclose.

- Cited by 2 articles
- 38 references
- 3 figures

Supplementary info

Publication types, MeSH terms Expand

Publication types

- Multicenter Study
- Observational Study

MeSH terms

- Appendectomy
- Appendicitis* / epidemiology
- Appendicitis* / surgery
- COVID-19*
- Cohort Studies
- Humans
- Italy / epidemiology
- Laparoscopy*
- Length of Stay
- Pandemics
- Postoperative Complications / epidemiology
- Retrospective Studies

- SARS-CoV-2

Full text links

SpringerLink FULL-TEXT ARTICLE Springer Free PMC article
Proceed to details
Cite
Share
514
Observational Study
Rev Esp Quimioter
. 2021 Aug;34(4):342-352.
doi: 10.37201/req/050.2021. Epub 2021 May 19.

Clinical characteristics and outcomes of 1,331 patients with COVID-19: HM Spanish Cohort

P Cardinal-Fernández[#, 1], E Garcia Cuesta[#], J Barberán[#], J F Varona, A Estirado, A Moreno, J Villanueva, M Villareal, O Baez-Pravia, J Menéndez, P Villares, A López Escobar, J Rodríguez-Pascual, C Almirall, E Domínguez, C Pey, A Ferreiro, M Revilla Amores, N Sánchez, S Ruiz de Aguiar, J M Castellano
Affiliations Expand

Affiliation

- [1] Pablo Cardinal-Fernández, Intensive care unit coordinator, HM Torrelodones University Hospital, Av. Castillo Olivares, s/n, CP 28250, Torrelodones, Madrid, Spain. pablocardinal@hotmail.com.

[#] Contributed equally.

- PMID: **34008930**
- PMCID: PMC8329575
- DOI: 10.37201/req/050.2021

Free PMC article
Observational Study

Clinical characteristics and outcomes of 1,331 patients with COVID-19: HM Spanish Cohort

P Cardinal-Fernández et al. Rev Esp Quimioter. 2021 Aug.
Free PMC article
Show details
Rev Esp Quimioter

. 2021 Aug;34(4):342-352.
doi: 10.37201/req/050.2021. Epub 2021 May 19.

Authors

P Cardinal-Fernández [#, 1], E Garcia Cuesta [#], J Barberán [#], J F Varona, A Estirado, A Moreno, J Villanueva, M Villareal, O Baez-Pravia, J Menéndez, P Villares, A López Escobar, J Rodríguez-Pascual, C Almirall, E Domínguez, C Pey, A Ferreiro, M Revilla Amores, N Sánchez, S Ruiz de Aguiar, J M Castellano

Affiliation

- [1] Pablo Cardinal-Fernández, Intensive care unit coordinator, HM Torrelodones University Hospital, Av. Castillo Olivares, s/n, CP 28250, Torrelodones, Madrid, Spain. pablocardinal@hotmail.com.

[#] Contributed equally.

- PMID: **34008930**
- PMCID: PMC8329575
- DOI: 10.37201/req/050.2021

Abstract
in English, Spanish

Objective: Spain is one of the European countries most affected by the COVID-19 pandemic. Epidemiologic studies are warranted to improve the disease understanding, evaluate the care procedure and prepare for futures waves. The aim of the study was to describe epidemiologic characteristics associated with hospitalized patients with COVID-19.

Methods: This real-world, observational, multicenter and retrospective study screened all consecutive patients admitted to 8 Spanish private hospitals. Inclusion criteria: hospitalized adults (age≥18 years old) with clinically and radiologically findings compatible with COVID-19 disease from March 1st to April 5th, 2020. Exclusion criteria: patients presenting negative PCR for SARS-CoV-2 during the first 7 days from hospital admission, transfer to a hospital not belonging to the HM consortium, lack of data and discharge against medical advice in emergency departments.

Results: One thousand and three hundred thirty-one COVID-19 patients (medium age 66.9 years old; males n= 841, medium length of hospital stayed 8 days, non-survivors n=233) were analyzed. One hundred and fifteen were admitted to intensive care unit (medium length of stay 16 days, invasive mechanical ventilation n= 95, septic shock n= 37 and renal replacement therapy n= 17). Age, male gender, leukocytes, platelets, oxygen saturation, chronic therapy with steroids and treatment with hydroxychloroquine/azithromycin were independent factors associated with mortality. The proportion of patients that survive and received tocilizumab and steroids were lesser and higher respectively than those that die, but their association was not significant.

Conclusions: Overall crude mortality rate was 17.5%, rising up to 36.5% in the subgroup of patients that were admitted to the intensive care unit. Seven factors impact in hospital mortality. No immunomodulatory intervention were associated with in-hospital mortality.

Introducción: España es uno de los países europeos más afectados por la pandemia de COVID-19. Conocer las características epidemiológicas y evolutivas permitirá mejorar la comprensión de la enfermedad, evaluar el procedimiento de atención y prepararse para las olas futuras. El objetivo del estudio fue describir las características epidemiológicas asociadas a los pacientes hospitalizados por COVID-19.

Material y métodos: Diseño observacional, multicéntrico y retrospectivo del mundo real realizado en 8 hospitales privados de España. Criterios de inclusión: adultos hospitalizados (edad≥18 años) con hallazgos clínicos y radiológicos compatibles con enfermedad COVID-19 entre el 1 de marzo al 5 de abril de 2020. Criterios de exclusión: PCR negativa para SARSCoV-2 durante los primeros 7 días de ingreso hospitalario, traslado a un hospital no perteneciente al consorcio HM, falta de datos y alta contra consejo médico en urgencias.

Resultados: Se analizaron 1.331 pacientes con COVID-19 (edad media 66,9 años; varones n = 841, estancia media hospitalaria 8 días, no supervivientes n = 233). Ciento quince ingresaron en la unidad de cuidados intensivos (estancia media 16 días, ventilación mecánica invasiva n = 95, choque séptico n = 37 y terapia renal sustitutiva n = 17). La edad, el sexo masculino, los leucocitos, las plaquetas, la saturación de oxígeno, la terapia crónica con esteroides y el tratamiento con hidroxicloroquina / azitromicina fueron factores independientes asociados con la mortalidad.

Conclusiones: La tasa de mortalidad bruta global fue del 17,5%, elevándose hasta el 36,5% en el subgrupo de pacientes que ingresaron en la unidad de cuidados intensivos. Siete factores impactan en la mortalidad hospitalaria.

Keywords: COVID-19; SARS-CoV-2; epidemiology; pandemic.

©The Author 2021. Published by Sociedad Española de Quimioterapia. This article is distributed under the terms of the Creative Commons Attribution-NonCommercial 4.0 International (CC BY-NC 4.0)(https://creativecommons.org/licenses/by-nc/4.0/).

Conflict of interest statement

all authors declare no conflict of interest

- 30 references
- 2 figures

Supplementary info

Publication types, MeSH terms Expand

Publication types

- Multicenter Study
- Observational Study

MeSH terms

- Aged

- Aged, 80 and over
- COVID-19 / drug therapy
- COVID-19 / mortality*
- COVID-19 / therapy*
- Cohort Studies
- Comorbidity
- Critical Care
- Female
- Hospital Mortality
- Humans
- Length of Stay
- Male
- Middle Aged
- Respiration, Artificial / statistics & numerical data
- Spain
- Survival Analysis
- Treatment Outcome

Full text links

FREE Full Text www.seq.es Sociedad Espanola de Quimioterapia Free PMC article

Proceed to details

Cite

Share

515

Observational Study

Ann Vasc Surg

. 2020 Nov;69:100-104.

doi: 10.1016/j.avsg.2020.08.002. Epub 2020 Aug 11.

Is There an Impact of COVID-19 on Admission of Patients to the Emergency Department for Vascular Surgery?

Mafalda Correia [1], Vânia Constâncio [2], Joana Cruz Silva [2], Pedro Lima [2], Mário Moreira [2], Luís F Antunes [3], Manuel Fonseca [2]

Affiliations Expand

Affiliations

- [1] Cirurgia Vascular, Centro Hospitalar e Universitário de Coimbra, Coimbra, Portugal. Electronic address: mafaldabmcorreia@gmail.com.

- [2] Cirurgia Vascular, Centro Hospitalar e Universitário de Coimbra, Coimbra, Portugal.
- [3] Cirurgia Vascular, Centro Hospitalar e Universitário de Coimbra, Coimbra, Portugal; Faculdade de Medicina da Universidade de Coimbra, Coimbra, Portugal.

- PMID: **32791192**
- PMCID: PMC7417287
- DOI: 10.1016/j.avsg.2020.08.002

Free PMC article
Observational Study

Is There an Impact of COVID-19 on Admission of Patients to the Emergency Department for Vascular Surgery?

Mafalda Correia et al. Ann Vasc Surg. 2020 Nov.
Free PMC article
Show details
Ann Vasc Surg
. 2020 Nov;69:100-104.
doi: 10.1016/j.avsg.2020.08.002. Epub 2020 Aug 11.

Authors

Mafalda Correia [1], Vânia Constâncio [2], Joana Cruz Silva [2], Pedro Lima [2], Mário Moreira [2], Luís F Antunes [3], Manuel Fonseca [2]

Affiliations

- [1] Cirurgia Vascular, Centro Hospitalar e Universitário de Coimbra, Coimbra, Portugal. Electronic address: mafaldabmcorreia@gmail.com.
- [2] Cirurgia Vascular, Centro Hospitalar e Universitário de Coimbra, Coimbra, Portugal.
- [3] Cirurgia Vascular, Centro Hospitalar e Universitário de Coimbra, Coimbra, Portugal; Faculdade de Medicina da Universidade de Coimbra, Coimbra, Portugal.

- PMID: **32791192**
- PMCID: PMC7417287
- DOI: 10.1016/j.avsg.2020.08.002

Abstract

Background: On March 2020, the World Health Organization declared the coronavirus disease 2019 outbreak a pandemic. During this period, surgical activity and admission to the Emergency Department (ED) decreased globally. The aim of this article is to understand how the admission of a patient to the ED for vascular surgery changed in our center in Portugal and if this situation prevented urgent surgical procedures.

Methods: Through a retrospective study, we compared the volume of patients admitted to the ED during the emergency state (ES) in Portugal with the same period in 2019. In addition, we analyzed the urgent surgical activity during the ES and in the correspondent period of the previous 10 years, regarding limb acute ischemia, acute aortic pathology, and vascular trauma. Two groups of patients were formed-patients operated during the ES and during the non-ES, for control. Statistical analysis was performed using IBM SPSS® Statistics, version 25.

Results: In the ES, 115 patients were observed at the ED and 179 in the 2019 corresponding period. During the ES, patients significantly recurred less to the ED directly from home ($P < 0.001$) and were less referred to the ED by primary care doctors ($P < 0.001$). Patients observed at the ED were significantly more urgent-required urgent surgery or were admitted to the department-than those in 2019 (40% vs. 24%). However, there were no differences when only considering urgent surgery (14% in ES vs. 10% in 2019). In the ES, 38% of patients observed at the ED were discharged with no follow-up related to vascular surgery against 60% in 2019, although this difference was not significant. Compared with the preceding 10 years, there are not significant differences in the number of patients who underwent urgent surgery in both ES and non-ES periods. In patients with acute limb ischemia, we did not find an increase in the time between onset of symptoms and ED admission, during the ES.

Conclusions: Fewer patients were admitted at the ED during the ES, and those admitted were significantly more urgent. We did not find a decrease in the number of urgent surgeries when compared with the preceding 10 years. Therefore, we cannot assume that coronavirus pandemic precluded urgent surgical procedures.

Copyright © 2020 Elsevier Inc. All rights reserved.

- Cited by 6 articles
- 6 references
- 3 figures

Supplementary info

Publication types, MeSH terms Expand

Publication types

- Observational Study

MeSH terms

- Aged
- Betacoronavirus
- COVID-19
- Coronavirus Infections / epidemiology*
- Emergency Service, Hospital / statistics & numerical data*
- Female
- Health Services Accessibility
- Humans
- Male

- Pandemics
- Patient Admission / statistics & numerical data*
- Pneumonia, Viral / epidemiology*
- Portugal / epidemiology
- Retrospective Studies
- SARS-CoV-2
- Vascular Surgical Procedures*

Full text links

ELSEVIER FULL-TEXT ARTICLE Elsevier Science Free PMC article
Proceed to details
Cite
Share
516
Observational Study
Ann Saudi Med
. Nov-Dec 2021;41(6):327-335.
doi: 10.5144/0256-4947.2021.327. Epub 2021 Dec 2.

Prognosis of patients hospitalized with a diagnosis of COVID-19 pneumonia in a tertiary hospital in Turkey

Tayfun Birtay [1], Suzan Bahadir [1], Ebru Kabacaoglu [2], Ozgur Yetiz [1], Mehmet Fatih Demirci [1], Gultekin Genctoy [3]
Affiliations Expand

Affiliations

- [1] From the Department of Anesthesia, Baskent University, Antalya, Turkey.
- [2] From the Department of Chest Diseases, Baskent University, Antalya, Turkey.
- [3] From the Department of Nephrology, Baskent University, Antalya, Turkey.

- PMID: **34873938**
- PMCID: PMC8650597
- DOI: 10.5144/0256-4947.2021.327

Free PMC article
Observational Study

Prognosis of patients hospitalized with a diagnosis of COVID-19 pneumonia in a tertiary hospital in Turkey

Tayfun Birtay et al. Ann Saudi Med. Nov-Dec 2021.
Free PMC article
Show details
Ann Saudi Med
. Nov-Dec 2021;41(6):327-335.
doi: 10.5144/0256-4947.2021.327. Epub 2021 Dec 2.

Authors

Tayfun Birtay [1], Suzan Bahadir [1], Ebru Kabacaoglu [2], Ozgur Yetiz [1], Mehmet Fatih Demirci [1], Gultekin Genctoy [3]

Affiliations

- [1] From the Department of Anesthesia, Baskent University, Antalya, Turkey.
- [2] From the Department of Chest Diseases, Baskent University, Antalya, Turkey.
- [3] From the Department of Nephrology, Baskent University, Antalya, Turkey.

- PMID: **34873938**
- PMCID: PMC8650597
- DOI: 10.5144/0256-4947.2021.327

Abstract

Background: SARS-CoV2/COVID-19 emerged in China and caused a global pandemic in 2020. The mortality rate has been reported to be between 0% and 14.6% in all patients. In this study, we determined the clinical and laboratory parameters of COVID-19 related morbidity and mortality in our hospital.

Objectives: Investigate the relationship between demographic, clinical, and laboratory parameters on COVID-19-related morbidity and mortality.

Design: Retrospective observational study.

Settings: Tertiary care hospital.

Patients and methods: Patients diagnosed with COVID-19 pneumonia from March until the end of December were included in the study.

Main outcome measures: The relationship between demographic, clinical, and laboratory parameters and the morbidity and mortality rates of patients diagnosed with COVID-19.

Sample size: 124 patients RESULTS: The mortality rate was 9.6% (12/124). Coronary artery disease (*P*<.0001) diabetes mellitus (*P*=.04) fever (>38.3°C) at presentation (*P*=.04) hypertension

(*P*<.0001), and positive smoking history (*P*<.0001) were significantly associated with mortality. Patients who died were older, had a higher comorbid disease index, pneumonia severity index, fasting blood glucose, baseline serum creatinine, D-dimer, and had lower baseline haemoglobin, SaO_2, percentage of lymphocyte counts and diastolic blood pressure. Patients admitted to the ICU were older, had a higher comorbidity disease index, pneumonia severity index, C-reactive protein, WBC, D-dimer, creatinine, number of antibiotics used, longer O_2 support duration, lower hemoglobin, lymphocyte (%), and baseline SaO_2 (%).

Conclusions: Our results were consistent with much of the reported data. We suggest that the frequency, dosage, and duration of steroid treatment should be limited.

Limitations: Low patient number, uncertain reason of mortality, no standard treatment regimen, limited treatment options, like ECMO.

Conflict of interest: None.

- 40 references
- 1 figure

Supplementary info

Publication types, MeSH terms, Substances, Grant support Expand

Publication types

- Observational Study

MeSH terms

- COVID-19*
- Humans
- Pneumonia* / diagnosis
- Pneumonia* / epidemiology
- Prognosis
- RNA, Viral
- SARS-CoV-2
- Tertiary Care Centers
- Turkey / epidemiology

Substances

- RNA, Viral

Grant support

None

Full text links

OPEN ACCESS Atypon Free PMC article

Proceed to details

Cite

Share

517

Observational Study

Blood Purif

. 2022;51(1):47-54.

doi: 10.1159/000515628. Epub 2021 Apr 15.

Endotoxin Adsorbent Therapy in Severe COVID-19 Pneumonia

Sadudee Peerapornratana [1 2 3 4 5], Phatadon Sirivongrangson [1 2 3], Somkanya Tungsanga [1], Kanitha Tiankanon [1], Win Kulvichit [1 2 3], Opass Putcharoen [6], John A Kellum [5], Nattachai Srisawat [1 2 3 5 7 8]

Affiliations Expand

Affiliations

- [1] Division of Nephrology, Department of Medicine, Faculty of Medicine, Chulalongkorn University, Bangkok, Thailand.
- [2] Excellence Center for Critical Care Nephrology, King Chulalongkorn Memorial Hospital, Bangkok, Thailand.
- [3] Critical Care Nephrology Research Unit, Chulalongkorn University, Bangkok, Thailand.
- [4] Department of Laboratory Medicine, Faculty of Medicine, Chulalongkorn University, Bangkok, Thailand.
- [5] Center for Critical Care Nephrology, The CRISMA Center, Department of Critical Care Medicine, University of Pittsburgh, School of Medicine, Pittsburgh, Pennsylvania, USA.
- [6] Division of Infectious Diseases, Department of Medicine, Faculty of Medicine, Chulalongkorn University, Bangkok, Thailand.
- [7] Tropical Medicine Cluster, Chulalongkorn University, Bangkok, Thailand.
- [8] Academy of Science, Royal Society of Thailand, Bangkok, Thailand.

- PMID: **33857940**
- PMCID: PMC8089445
- DOI: 10.1159/000515628

Free PMC article

Observational Study

Endotoxin Adsorbent Therapy in Severe COVID-19 Pneumonia

Sadudee Peerapornratana et al. Blood Purif. 2022.
Free PMC article
Show details
Blood Purif
. 2022;51(1):47-54.
doi: 10.1159/000515628. Epub 2021 Apr 15.

Authors

Sadudee Peerapornratana [1 2 3 4 5], Phatadon Sirivongrangson [1 2 3], Somkanya Tungsanga [1], Kanitha Tiankanon [1], Win Kulvichit [1 2 3], Opass Putcharoen [6], John A Kellum [5], Nattachai Srisawat [1 2 3 5 7 8]

Affiliations

- [1] Division of Nephrology, Department of Medicine, Faculty of Medicine, Chulalongkorn University, Bangkok, Thailand.
- [2] Excellence Center for Critical Care Nephrology, King Chulalongkorn Memorial Hospital, Bangkok, Thailand.
- [3] Critical Care Nephrology Research Unit, Chulalongkorn University, Bangkok, Thailand.
- [4] Department of Laboratory Medicine, Faculty of Medicine, Chulalongkorn University, Bangkok, Thailand.
- [5] Center for Critical Care Nephrology, The CRISMA Center, Department of Critical Care Medicine, University of Pittsburgh, School of Medicine, Pittsburgh, Pennsylvania, USA.
- [6] Division of Infectious Diseases, Department of Medicine, Faculty of Medicine, Chulalongkorn University, Bangkok, Thailand.
- [7] Tropical Medicine Cluster, Chulalongkorn University, Bangkok, Thailand.
- [8] Academy of Science, Royal Society of Thailand, Bangkok, Thailand.

- PMID: **33857940**
- PMCID: PMC8089445
- DOI: 10.1159/000515628

Abstract

Introduction: Uncontrolled systemic inflammation may occur in severe coronavirus disease 19 (COVID-19). We have previously shown that endotoxemia, presumably from the gut, may complicate COVID-19. However, the role of endotoxin adsorbent (EA) therapy to mitigate organ dysfunction in COVID-19 has not been explored.

Methods: We conducted a retrospective observational study in COVID-19 patients who received EA therapy at the King Chulalongkorn Memorial Hospital, Bangkok, Thailand, between March 13 and April 17, 2020. Relevant clinical and laboratory data were collected by inpatient chart review.

Results: Among 147 hospitalized COVID-19 patients, 6 patients received EA therapy. All of the 6 patients had severe COVID-19 infection with acute respiratory distress syndrome (ARDS). Among these, 5 of them were mechanically ventilated and 4 had complications of secondary bacterial infection. The endotoxin activity assay (EAA) results of pre-EA therapy ranged from 0.47 to 2.79. The choices of EA therapy were at the discretion of attending physicians. One patient was treated with oXiris® along with continuous renal replacement therapy, and the others received polymyxin B hemoperfusion sessions. All patients have survived and were finally free from the mechanical ventilation as well as had improvement in PaO2/FiO2 ratio and decreased EAA level after EA therapy.

Conclusions: We demonstrated the clinical improvement of severe COVID-19 patients with elevated EAA level upon receiving EA therapy. However, the benefit of EA therapy in COVID-19 ARDS is still unclear and needs to be elucidated with randomized controlled study.

Keywords: Acute respiratory distress syndrome; Adsorption therapy; Coronavirus disease 19; Critical care; Endotoxemia; Endotoxin adsorbent.

© 2021 S. Karger AG, Basel.

Conflict of interest statement

Toray Industries provided the polymyxin B cartridge and endotoxin activity assay kits for use in this study and Baxter company provided oXiris® set. Both companies had no influence on the study design or analysis or on the comment of this article. None of the authors have any conflicts to disclosure.

- Cited by 2 articles
- 40 references
- 1 figure

Supplementary info

Publication types, MeSH terms, Substances Expand

Publication types

- Observational Study
- Research Support, Non-U.S. Gov't

MeSH terms

- Acute Kidney Injury / etiology
- Acute Kidney Injury / therapy
- Adsorption
- COVID-19 / complications
- COVID-19 / therapy*
- Critical Care / methods
- Endotoxemia / etiology
- Endotoxemia / therapy*

- Female
- Hemoperfusion / methods*
- Heparin / administration & dosage
- Humans
- Male
- Membranes, Artificial
- Middle Aged
- Polymyxin B / administration & dosage
- Renal Replacement Therapy
- Respiratory Distress Syndrome / etiology
- Retrospective Studies
- SARS-CoV-2*
- Treatment Outcome

Substances

- Membranes, Artificial
- Heparin
- Polymyxin B

Full text links

S. Karger AG, Basel, Switzerland Free PMC article

Proceed to details

Cite

Share

518

Observational Study

BMJ Open

. 2022 Mar 11;12(3):e053722.

doi: 10.1136/bmjopen-2021-053722.

Clinical profile, risk factors and outcomes of ric COVID-19: a retrospective cohort multicentre study in Saudi Arabia

Waleed H Albuali [1,2], Amal A AlGhamdi [3], Shaikha J Aldossary [1,2], Saleh A AlHarbi [4,5], Sami I Al Majed [6], Ahmed Alenizi [7], Mohammad H Al-Qahtani [1,8], Amer A Lardhi [1,2], Shams A Al-Turki [1,2], Abdulaziz S AlSanea [1,2], Dalal K Bubshait [1,2], Sumayyah A Kobeisy [5], Noor H Herzallah [6], Wejdan A Alqarni [6], Abeer H AlHarbi [7], Hamad W Albuali [2], Bader J Aldossary [2], Faisal O AlQurashi [9,2], Abdullah A Yousef [1,2]

Affiliations Expand

Affiliations

- [1] Department of Pediatrics, Imam Abdulrahman Bin Faisal University, King Fahd Hospital of the University, AlKhobar, Saudi Arabia.
- [2] College of Medicine, Imam Abdulrahman Bin Faisal University, Dammam, Saudi Arabia.
- [3] Department of Family and Community Medicine, College of Medicine, Imam Abdulrahman Bin Faisal University, Dammam, Saudi Arabia.
- [4] Department of Pediatrics, Umm Al-Qura University, Makkah, Saudi Arabia.
- [5] Department of Pediatrics, Dr Soliman Fakeeh Hospital, Jeddah, Saudi Arabia.
- [6] Department of Pediatrics, John Hopkins Aramco Healthcare, Dhahran, Saudi Arabia.
- [7] Pediatric Pulmonology and Sleep Medicine Department, King Saud Medical City, Riyadh, Saudi Arabia.
- [8] Department of Pediatrics, Imam Abdulrahman Bin Faisal University College of Medicine, Dammam, Saudi Arabia.
- [9] Department of Pediatrics, Imam Abdulrahman Bin Faisal University, King Fahd Hospital of the University, AlKhobar, Saudi Arabia faisal.alqurashi@yahoo.com.

- PMID: **35277403**
- PMCID: PMC8919130
- DOI: 10.1136/bmjopen-2021-053722

Free PMC article
Observational Study

Clinical profile, risk factors and outcomes of ric COVID-19: a retrospective cohort multicentre study in Saudi Arabia

Waleed H Albuali et al. BMJ Open. 2022.
Free PMC article
Show details
BMJ Open
. 2022 Mar 11;12(3):e053722.
doi: 10.1136/bmjopen-2021-053722.

Authors

Waleed H Albuali [1, 2], Amal A AlGhamdi [3], Shaikha J Aldossary [1, 2], Saleh A AlHarbi [4, 5], Sami I Al Majed [6], Ahmed Alenizi [7], Mohammad H Al-Qahtani [1, 8], Amer A Lardhi [1, 2], Shams A Al-Turki [1, 2], Abdulaziz S AlSanea [1, 2], Dalal K Bubshait [1, 2], Sumayyah A Kobeisy [5], Noor H Herzallah [6], Wejdan A Alqarni [6], Abeer H AlHarbi [7], Hamad W Albuali [2], Bader J Aldossary [2], Faisal O AlQurashi [9, 2], Abdullah A Yousef [1, 2]

Affiliations

- [1] Department of Pediatrics, Imam Abdulrahman Bin Faisal University, King Fahd Hospital of the University, AlKhobar, Saudi Arabia.
- [2] College of Medicine, Imam Abdulrahman Bin Faisal University, Dammam, Saudi Arabia.
- [3] Department of Family and Community Medicine, College of Medicine, Imam Abdulrahman Bin Faisal University, Dammam, Saudi Arabia.
- [4] Department of Pediatrics, Umm Al-Qura University, Makkah, Saudi Arabia.
- [5] Department of Pediatrics, Dr Soliman Fakeeh Hospital, Jeddah, Saudi Arabia.
- [6] Department of Pediatrics, John Hopkins Aramco Healthcare, Dhahran, Saudi Arabia.
- [7] Pediatric Pulmonology and Sleep Medicine Department, King Saud Medical City, Riyadh, Saudi Arabia.
- [8] Department of Pediatrics, Imam Abdulrahman Bin Faisal University College of Medicine, Dammam, Saudi Arabia.
- [9] Department of Pediatrics, Imam Abdulrahman Bin Faisal University, King Fahd Hospital of the University, AlKhobar, Saudi Arabia faisal.alqurashi@yahoo.com.

- PMID: **35277403**
- PMCID: PMC8919130
- DOI: 10.1136/bmjopen-2021-053722

Abstract

Objective: To describe the risk factors, clinical profile and outcomes of COVID-19 in the paediatric population.

Design: Multicentre, retrospective observational study.

Setting: Four tertiary hospitals in Saudi Arabia.

Patients: We recruited 390 paediatric patients aged 0-18 years who presented from March to December 2020 and tested positive for COVID-19 on PCR.

Main outcome measures: We retrospectively analysed medical records for sociodemographics, health indicators, clinical presentations, laboratory findings, clinical complications, and outcomes.

Results: The mean participant age was 5.66±4.90 years, and the mean hospital stay was 2.17±3.48 days. Forty patients, mostly school-aged children (16, 40.00%; p=0.005) and children with comorbidities (25, 62.50%; p<0.001), received more than just supportive care. Complications were seen in 15 (3.9%) patients, bacterial infection being the most common (6, 40.00%). Patients presented with dyspnoea (OR 6.89; 95% CI 2.89 to 20.72), abnormal chest radiographs (OR 6.11; 95% CI 1.26 to 29.38), lethargy (OR 9.04; 95% CI 2.91 to 28.06) and elevated ferritin (OR 14.21; 95% CI 4.18 to 48.37) and D-dimer (OR 48.40; 95% CI 14.32 to 163.62), with higher odds of developing complications. The odds of paediatric intensive care unit (ICU) admission were higher for patients with dyspnoea (adjusted OR 4.66; 95% CI 1.24 to 17.50) and elevated white blood cell count (adjusted OR 3.54; 95% CI 1.02 to 12.30).

Conclusions: COVID-19 complications were limited among our patients. However, dyspnoea, abnormal chest radiographs, lethargy and elevated ferritin and D-dimer were associated with an increased risk of complications. Dyspnoea, leucocytosis, comorbidities and abnormal chest radiographs at presentation increased the risk of ICU admission.

Keywords: COVID-19; paediatric intensive & critical care; paediatrics.

© Author(s) (or their employer(s)) 2022. Re-use permitted under CC BY-NC. No commercial re-use. See rights and permissions. Published by BMJ.

Conflict of interest statement

Competing interests: None declared.

- 48 references

Supplementary info

Publication types, MeSH terms Expand

Publication types

- Multicenter Study
- Observational Study

MeSH terms

- Adolescent
- COVID-19* / epidemiology
- Child
- Child, Preschool
- Hospitalization
- Humans
- Infant
- Infant, Newborn
- Retrospective Studies
- Risk Factors
- Saudi Arabia / epidemiology

Full text links

BMJ Free Full Text HighWire Free PMC article

Proceed to details

Cite

Share

519

Observational Study

BMJ Open

. 2021 Dec 7;11(12):e053810.
doi: 10.1136/bmjopen-2021-053810.

Retrospective case-control study to evaluate hypocalcaemia as a distinguishing feature of COVID-19 compared with other infective pneumonias and its association with disease severity

Meera Mehta [1], Hakim Ghani [2], Felix Chua [3] [4], Adrian Draper [5], Sam Calmonson [2], Meghna Prabhakar [2], Rijul Shah [2], Alessio Navarra [2], Tejal Vaghela [2], Andrew Barlow [2], Rama Vancheeswaran [2]

Affiliations Expand

Affiliations

- [1] Respiratory Medicine, West Hertfordshire Hospitals NHS Trust, Watford, UK meera.mehta2@nhs.net.
- [2] Respiratory Medicine, West Hertfordshire Hospitals NHS Trust, Watford, UK.
- [3] Interstitial Lung Disease Unit, Royal Brompton and Harefield NHS Foundation Trust, London, UK.
- [4] National Heart and Lung Institute, Imperial College London, London, UK.
- [5] Respiratory Medicine, St George's Hospital, London, UK.

- PMID: **34876435**
- PMCID: PMC8655344
- DOI: 10.1136/bmjopen-2021-053810

Free PMC article
Observational Study

Retrospective case-control study to evaluate hypocalcaemia as a distinguishing feature of COVID-19 compared with other infective pneumonias and its association with disease severity

Meera Mehta et al. BMJ Open. 2021.
Free PMC article
Show details
BMJ Open
. 2021 Dec 7;11(12):e053810.
doi: 10.1136/bmjopen-2021-053810.

Authors

Meera Mehta [1], Hakim Ghani [2], Felix Chua [3] [4], Adrian Draper [5], Sam Calmonson [2], Meghna Prabhakar [2], Rijul Shah [2], Alessio Navarra [2], Tejal Vaghela [2], Andrew Barlow [2], Rama Vancheeswaran [2]

Affiliations

- [1] Respiratory Medicine, West Hertfordshire Hospitals NHS Trust, Watford, UK meera.mehta2@nhs.net.
- [2] Respiratory Medicine, West Hertfordshire Hospitals NHS Trust, Watford, UK.
- [3] Interstitial Lung Disease Unit, Royal Brompton and Harefield NHS Foundation Trust, London, UK.
- [4] National Heart and Lung Institute, Imperial College London, London, UK.
- [5] Respiratory Medicine, St George's Hospital, London, UK.

- PMID: **34876435**
- PMCID: PMC8655344
- DOI: 10.1136/bmjopen-2021-053810

Abstract

Objectives: To investigate whether calcium derangement was a specific feature of COVID-19 that distinguishes it from other infective pneumonias, and its association with disease severity.

Design: A retrospective observational case-control study looking at serum calcium on adult patients with COVID-19, and community-acquired pneumonia (CAP) or viral pneumonia (VP).

Setting: A district general hospital on the outskirts of London, UK.

Participants: 506 patients with COVID-19, 95 patients with CAP and 152 patients with VP.

Outcome measures: Baseline characteristics including hypocalcaemia in patients with COVID-19, CAP and VP were detailed. For patients with COVID-19, the impact of an abnormally low calcium level on the maximum level of hospital care, as a surrogate of COVID-19 severity, was evaluated. The primary outcome of maximal level of care was based on the WHO Clinical Progression Scale for COVID-19.

Results: Hypocalcaemia was a specific and common clinical finding in patients with COVID-19 that distinguished it from other respiratory infections. Calcium levels were significantly lower in those with severe disease. Ordinal regression of risk estimates for categorised care levels showed that baseline hypocalcaemia was incrementally associated with OR of 2.33 (95% CI 1.5 to 3.61) for higher level of care, superior to other variables that have previously been shown to predict worse COVID-19 outcome. Serial calcium levels showed improvement by days 7-9 of admission, only in survivors of COVID-19.

Conclusion: Hypocalcaemia is specific to COVID-19 and may help distinguish it from other infective pneumonias. Hypocalcaemia may independently predict severe disease and warrants detailed prognostic investigation. The fact that decreased serum calcium is observed at the time of clinical presentation in COVID-19, but not other infective pneumonias, suggests that its early derangement is pathophysiological and may influence the deleterious evolution of this disease.

Trial registration number: 20/HRA/2344.

Keywords: COVID-19; clinical chemistry; respiratory infections.

© Author(s) (or their employer(s)) 2021. Re-use permitted under CC BY-NC. No commercial re-use. See rights and permissions. Published by BMJ.

Conflict of interest statement

Competing interests: None declared.

- 35 references
- 4 figures

Supplementary info

Publication types, MeSH terms Expand

Publication types

- Observational Study
- Research Support, Non-U.S. Gov't

MeSH terms

- Adult
- COVID-19*
- Case-Control Studies
- Humans
- Hypocalcemia* / diagnosis
- Retrospective Studies
- SARS-CoV-2
- Severity of Illness Index
- Treatment Outcome

Full text links

BMJ Free Full Text HighWire Free PMC article
Proceed to details
Cite
Share
520
Observational Study
BMC Infect Dis
. 2021 Aug 16;21(1):820.
doi: 10.1186/s12879-021-06502-z.

Evaluation of China's Hubei control strategy for COVID-19 epidemic: an observational study

Yu Liu [1], Fangfang Zheng [2], Zhicheng Du [1], Jinghua Li [1], Jing Gu [1], Mei Jiang [3], Daisuke Yoneoka [4], Stuart Gilmour [4], Yuantao Hao [5]
Affiliations Expand

Affiliations

- [1] Department of Medical Statistics and Epidemiology, School of Public Health, Sun Yat-Sen University, Guangzhou, 510080, China.
- [2] School of Traditional Chinese Medicine Healthcare, Guangdong Food and Drug Vocational College, Guangzhou, 510520, China.
- [3] National Clinical Research Center for Respiratory Disease, State Key Laboratory of Respiratory Disease, Guangzhou Institute of Respiratory Health, The First Affiliated Hospital of Guangzhou Medical University, Guangzhou, 510120, China.
- [4] Graduate School of Public Health, St. Luke's International University, Tokyo, 104-0045, Japan.
- [5] Department of Medical Statistics and Epidemiology, School of Public Health, Sun Yat-Sen University, Guangzhou, 510080, China. haoyt@mail.sysu.edu.cn.

- PMID: **34399697**
- PMCID: PMC8366153
- DOI: 10.1186/s12879-021-06502-z

Free PMC article
Observational Study

Evaluation of China's Hubei control strategy for COVID-19 epidemic: an observational study

Yu Liu et al. BMC Infect Dis. 2021.
Free PMC article
Show details
BMC Infect Dis
. 2021 Aug 16;21(1):820.
doi: 10.1186/s12879-021-06502-z.

Authors

Yu Liu [1], Fangfang Zheng [2], Zhicheng Du [1], Jinghua Li [1], Jing Gu [1], Mei Jiang [3], Daisuke Yoneoka [4], Stuart Gilmour [4], Yuantao Hao [5]

Affiliations

- [1] Department of Medical Statistics and Epidemiology, School of Public Health, Sun Yat-Sen University, Guangzhou, 510080, China.
- [2] School of Traditional Chinese Medicine Healthcare, Guangdong Food and Drug Vocational College, Guangzhou, 510520, China.
- [3] National Clinical Research Center for Respiratory Disease, State Key Laboratory of Respiratory Disease, Guangzhou Institute of Respiratory Health, The First Affiliated Hospital of Guangzhou Medical University, Guangzhou, 510120, China.
- [4] Graduate School of Public Health, St. Luke's International University, Tokyo, 104-0045, Japan.
- [5] Department of Medical Statistics and Epidemiology, School of Public Health, Sun Yat-Sen University, Guangzhou, 510080, China. haoyt@mail.sysu.edu.cn.

- PMID: **34399697**
- PMCID: PMC8366153
- DOI: 10.1186/s12879-021-06502-z

Abstract

Background: To fight against COVID-19, many policymakers are wavering on stricter public health interventions. Examining the different strategies both in and out of China's Hubei province, which contained the epidemic in late February 2020, could yield valuable guidance for the management of future pandemics. This study assessed the response process and estimated the time-varying effects of the Hubei control strategy. Analysis of these strategies provides insights for the design and implementation of future policy interventions.

Methods: We retrospectively compared the spread and control of COVID-19 between China's Hubei (excluding Wuhan) and non-Hubei areas using data that includes case reports, human mobility, and public health interventions from 1 January to 29 February 2020. Static and dynamic risk assessment models were developed to statistically investigate the effects of the Hubei control strategy on the virus case growth after adjusting importation risk and policy response timing with the non-Hubei strategy as a control.

Results: The analysis detected much higher but differential importation risk in Hubei. The response timing largely coincided with the importation risk in non-Hubei areas, but Hubei areas showed an opposite pattern. Rather than a specific intervention assessment, a comprehensive comparison showed that the Hubei control strategy implemented severe interventions characterized by unprecedentedly strict and 'monitored' self-quarantine at home, while the non-Hubei strategy included physical distancing measures to reduce contact among individuals within or between populations. In contrast with the non-Hubei control strategy, the Hubei strategy showed a much higher, non-linear and gradually diminishing protective effect with at least 3 times fewer cases.

Conclusions: A risk-based control strategy was crucial to the design of an effective response to the COVID-19 outbreak. Our study demonstrates that the stricter Hubei strategy achieves a stronger controlling effect compared to other strategies. These findings highlight the health benefits and policy impacts of precise and differentiated strategies informed by constant monitoring of outbreak risk.

Keywords: COVID-19; Control strategy; Public health interventions; Time-varying effect.

© 2021. The Author(s).

Conflict of interest statement

The authors declare that they have no competing interests.

- 27 references
- 3 figures

Supplementary info

Publication types, MeSH terms, Grant support Expand

Publication types

- Observational Study

MeSH terms

- COVID-19 / epidemiology
- COVID-19 / prevention & control*
- China / epidemiology
- Humans
- Pandemics / prevention & control*
- Retrospective Studies
- SARS-CoV-2

Grant support

- 2021A1515011591/Guangdong Basic and Applied Basic Research Foundation
- A2021104/Medical Science and Technology Foundation of Guangdong Province

Full text links

Read free full text at BMC BioMed Central Free PMC article

Proceed to details

Cite

Share

☐ 521

Observational Study

Acta Biomed

. 2020 Nov 10;91(4):e2020145.
doi: 10.23750/abm.v91i4.10175.

The characteristics of cancerous patients infected with COVID-19 in hospital setting

Amir Sadeghi [1], Arash Dooghaie Moghadam [2], Pegah Eslami [3], Ali Pirsalehi [4], Sajad Shojaee [5], Ghazal Sanadgol [6], Laya Jalilian Khave [7], Mohammad Vahidi [8], Hamid Asadzadeh Aghdaei [9], Ehsan Nazemalhosseini Mojarad [1]
Affiliations Expand

Affiliations

- [1] Gastroenterology and Liver Diseases Research Center, Research Institute for Gastroenterology and Liver Diseases, Shahid Beheshti University of Medical Sciences, Tehran, Iran.. ehsanmojarad@gmail.com.
- [2] Gastroenterology and Liver Diseases Research Center, Research Institute for Gastroenterology and Liver Diseases, Shahid Beheshti University of Medical Sciences, Tehran, Iran.. Arashddm@gmail.com.
- [3] Gastroenterology and Liver Diseases Research Center, Research Institute for Gastroenterology and Liver Diseases, Shahid Beheshti University of Medical Sciences, Tehran, Iran.. Pegahslm@gmail.com.
- [4] Research Institute for Gastroenterology and Liver Diseases, Shahid Beheshti University of Medical Sciences, Tehran, Iran. Pirsalehi@sbmu.ac.ir.
- [5] Gastroenterology and Liver Diseases Research Center, Research Institute for Gastroenterology and Liver Diseases, Shahid Beheshti University of Medical Sciences, Tehran, Iran.. sajad.shojaei.stat@gmail.com.
- [6] Gastroenterology and Liver Diseases Research Center, Research Institute for Gastroenterology and Liver Diseases, Shahid Beheshti University of Medical Sciences, Tehran, Iran.. ghsanadgol@gmail.com.
- [7] Gastroenterology and Liver Diseases Research Center, Research Institute for Gastroenterology and Liver Diseases, Shahid Beheshti University of Medical Sciences, Tehran, Iran.. layajalilian@gmail.com.
- [8] Gastroenterology and Liver Diseases Research Center, Research Institute for Gastroenterology and Liver Diseases, Shahid Beheshti University of Medical Sciences, Tehran, Iran.. mammad.vahidi@gmail.com.
- [9] Basic and Molecular Epidemiology of Gastrointestinal Disorders Research Center, Research Institute for Gastroenterology and Liver Diseases, Shahid Beheshti University of Medical Sciences, Tehran, Iran.. hamid.asadzadeh@sbmu.ac.ir.

- PMID: **33525203**
- PMCID: PMC7927459
- DOI: 10.23750/abm.v91i4.10175

Free PMC article
Observational Study

The characteristics of cancerous patients infected with COVID-19 in hospital setting

Amir Sadeghi et al. Acta Biomed. 2020.
Free PMC article
Show details
Acta Biomed
. 2020 Nov 10;91(4):e2020145.
doi: 10.23750/abm.v91i4.10175.

Authors

Amir Sadeghi [1], Arash Dooghaie Moghadam [2], Pegah Eslami [3], Ali Pirsalehi [4], Sajad Shojaee [5], Ghazal Sanadgol [6], Laya Jalilian Khave [7], Mohammad Vahidi [8], Hamid Asadzadeh Aghdaei [9], Ehsan Nazemalhosseini Mojarad [1]

Affiliations

- [1] Gastroenterology and Liver Diseases Research Center, Research Institute for Gastroenterology and Liver Diseases, Shahid Beheshti University of Medical Sciences, Tehran, Iran.. ehsanmojarad@gmail.com.
- [2] Gastroenterology and Liver Diseases Research Center, Research Institute for Gastroenterology and Liver Diseases, Shahid Beheshti University of Medical Sciences, Tehran, Iran.. Arashddm@gmail.com.
- [3] Gastroenterology and Liver Diseases Research Center, Research Institute for Gastroenterology and Liver Diseases, Shahid Beheshti University of Medical Sciences, Tehran, Iran.. Pegahslm@gmail.com.
- [4] Research Institute for Gastroenterology and Liver Diseases, Shahid Beheshti University of Medical Sciences, Tehran, Iran. Pirsalehi@sbmu.ac.ir.
- [5] Gastroenterology and Liver Diseases Research Center, Research Institute for Gastroenterology and Liver Diseases, Shahid Beheshti University of Medical Sciences, Tehran, Iran.. sajad.shojaei.stat@gmail.com.
- [6] Gastroenterology and Liver Diseases Research Center, Research Institute for Gastroenterology and Liver Diseases, Shahid Beheshti University of Medical Sciences, Tehran, Iran.. ghsanadgol@gmail.com.
- [7] Gastroenterology and Liver Diseases Research Center, Research Institute for Gastroenterology and Liver Diseases, Shahid Beheshti University of Medical Sciences, Tehran, Iran.. layajalilian@gmail.com.
- [8] Gastroenterology and Liver Diseases Research Center, Research Institute for Gastroenterology and Liver Diseases, Shahid Beheshti University of Medical Sciences, Tehran, Iran.. mammad.vahidi@gmail.com.
- [9] Basic and Molecular Epidemiology of Gastrointestinal Disorders Research Center, Research Institute for Gastroenterology and Liver Diseases, Shahid Beheshti University of Medical Sciences, Tehran, Iran.. hamid.asadzadeh@sbmu.ac.ir.

- PMID: **33525203**
- PMCID: PMC7927459
- DOI: 10.23750/abm.v91i4.10175

Abstract

Introduction: Recently, Covid 19 as a fatal virus has been known as the cause of the pandemic. Different number of the mortality rate in various societies have been reported. However, it seems the underlying comorbidities increase the risk of mortality and the severity of presentation. In this study we evaluated the pattern of presentation of COVID-19 among cancerous patients in terms of severity.

Method: between 20th February to 22nd April of 2020, among 214 hospitalized patients because of COVID-19. 41 patients revealed the cancer as a synchronous comorbidity. These patients based on the severity of COVID-19 infection presentation were divided to mild and severe groups. Then, the demographic characteristics, manifestation and laboratory data between these groups were compared.

Result: about 19 (46.34%) of 41 cases were categorized as severe forms of COVID-19 with malignancy. The mean age of severe groups was significantly higher (P=0.00). Dyspnea (48.78%), cough (46.34%) and myalgia (24.39%) were the most common clinical features among cancerous patients with COVID-19. diarrhea and nearly cough caused significant effects on severe form of presentation of COVID-19 infection (P=0.05, P=0.06, respectively). Hematological cancers were the most frequent types of cancer among these patients (46.34%). White Blood Cell counts were significantly lower in severe groups (P=0.03, P=.0.06, respectively). C-reactive protein is another item that nearly significantly was higher in severe groups of cancerous patients (P=0.06).

Conclusion: The elderly age, the positive chemotherapy history, diarrhea, cough, declined WBC, PLT and elevated CRP correlated with a severe form of this infection in malignant cases.

Conflict of interest statement

Each author declares that he or she has no commercial associations (e.g. consultancies, stock ownership, equity interest, patent/licensing arrangement etc.) that might pose a conflict of interest in connection with the submitted article

- Cited by 2 articles
- 22 references

Supplementary info

Publication types, MeSH terms Expand

Publication types

- Observational Study

MeSH terms

- Adult
- Aged
- Aged, 80 and over
- COVID-19 / complications*

- COVID-19 / diagnosis*
- Female
- Hospitalization
- Humans
- Male
- Middle Aged
- Neoplasms / complications*
- Retrospective Studies
- Severity of Illness Index

Full text links

Free PMC article
Proceed to details
Cite
Share
☐ 522
Observational Study
Eur J Anaesthesiol
. 2021 Dec 1;38(12):1274-1283.
doi: 10.1097/EJA.0000000000001565.

Association of early positive end-expiratory pressure settings with ventilator-free days in patients with coronavirus disease 2019 acute respiratory distress syndrome: A secondary analysis of the Practice of VENTilation in COVID-19 study

Christel M A Valk [1], Anissa M Tsonas, Michela Botta, Lieuwe D J Bos, Janesh Pillay, Ary Serpa Neto, Marcus J Schultz, Frederique Paulus, Writing Committee for the PRoVENT-COVID∗ Collaborative Group
Affiliations Expand

Affiliation

- [1] From the Department of Intensive Care & Laboratory of Experimental Intensive Care and Anaesthesiology (LEICA), Amsterdam UMC, Location AMC, Amsterdam (CMAV, AMT, MB, LDJB, ASN, MJS, FP), Department of Critical Care, University Medical Center Groningen, University of Groningen, Groningen, The Netherlands (JP), Department of Critical Care Medicine, Hospital Israelita Albert Einstein, São Paulo, Brazil (ASN), Australian and New Zealand Intensive Care Research Centre (ANZIC-RC), Monash

University (ASN), Data Analytics Research & Evaluation (DARE) Centre, Austin Hospital and University of Melbourne, Melbourne, Victoria, Australia (ASN), Nuffield Department of Medicine, Oxford University, Oxford, UK (MJS), Mahidol-Oxford Tropical Medicine Research Unit (MORU), Mahidol University, Bangkok, Thailand (MJS) and ACHIEVE Centre of Expertise, Faculty of Health, Amsterdam University of Applied Sciences, Amsterdam, The Netherlands (FP).

- PMID: **34238782**
- PMCID: PMC8630930
- DOI: 10.1097/EJA.0000000000001565

Free PMC article
Observational Study

Association of early positive end-expiratory pressure settings with ventilator-free days in patients with coronavirus disease 2019 acute respiratory distress syndrome: A secondary analysis of the Practice of VENTilation in COVID-19 study

Christel M A Valk et al. Eur J Anaesthesiol. 2021.
Free PMC article
Show details
Eur J Anaesthesiol
. 2021 Dec 1;38(12):1274-1283.
doi: 10.1097/EJA.0000000000001565.

Authors

Christel M A Valk [1], Anissa M Tsonas, Michela Botta, Lieuwe D J Bos, Janesh Pillay, Ary Serpa Neto, Marcus J Schultz, Frederique Paulus, Writing Committee for the PRoVENT-COVID* Collaborative Group

Affiliation

- [1] From the Department of Intensive Care & Laboratory of Experimental Intensive Care and Anaesthesiology (LEICA), Amsterdam UMC, Location AMC, Amsterdam (CMAV, AMT, MB, LDJB, ASN, MJS, FP), Department of Critical Care, University Medical Center Groningen, University of Groningen, Groningen, The Netherlands (JP), Department of Critical Care Medicine, Hospital Israelita Albert Einstein, São Paulo, Brazil (ASN), Australian and New Zealand Intensive Care Research Centre (ANZIC-RC), Monash University (ASN), Data Analytics Research & Evaluation (DARE) Centre, Austin Hospital and University of Melbourne, Melbourne, Victoria, Australia (ASN), Nuffield Department of Medicine, Oxford University, Oxford, UK (MJS), Mahidol-Oxford Tropical Medicine

Research Unit (MORU), Mahidol University, Bangkok, Thailand (MJS) and ACHIEVE Centre of Expertise, Faculty of Health, Amsterdam University of Applied Sciences, Amsterdam, The Netherlands (FP).

- PMID: **34238782**
- PMCID: PMC8630930
- DOI: 10.1097/EJA.0000000000001565

Abstract

Background: There is uncertainty about how much positive end-expiratory pressure (PEEP) should be used in patients with acute respiratory distress syndrome (ARDS) due to coronavirus disease 2019 (COVID-19).

Objective: To investigate whether a higher PEEP strategy is superior to a lower PEEP strategy regarding the number of ventilator-free days (VFDs).

Design: Multicentre observational study conducted from 1 March to 1 June 2020.

Setting and patients: Twenty-two ICUs in The Netherlands and 933 invasively ventilated COVID-19 ARDS patients.

Interventions: Patients were categorised retrospectively as having received invasive ventilation with higher (n=259) or lower PEEP (n=674), based on the high and low PEEP/FiO2 tables of the ARDS Network, and using ventilator settings and parameters in the first hour of invasive ventilation, and every 8 h thereafter at fixed time points during the first four calendar days. We also used propensity score matching to control for observed confounding factors that might influence outcomes.

Main outcomes and measures: The primary outcome was the number of VFDs. Secondary outcomes included distant organ failures including acute kidney injury (AKI) and use of renal replacement therapy (RRT), and mortality.

Results: In the unmatched cohort, the higher PEEP strategy had no association with the median [IQR] number of VFDs (2.0 [0.0 to 15.0] vs. 0.0 [0.0 to 16.0] days). The median (95% confidence interval) difference was 0.21 (-3.34 to 3.78) days, P = 0.905. In the matched cohort, the higher PEEP group had an association with a lower median number of VFDs (0.0 [0.0 to 14.0] vs. 6.0 [0.0 to 17.0] days) a median difference of -4.65 (-8.92 to -0.39) days, P = 0.032. The higher PEEP strategy had associations with higher incidence of AKI (in the matched cohort) and more use of RRT (in the unmatched and matched cohorts). The higher PEEP strategy had no association with mortality.

Conclusion: In COVID-19 ARDS, use of higher PEEP may be associated with a lower number of VFDs, and may increase the incidence of AKI and need for RRT.

Trial registration: Practice of VENTilation in COVID-19 is registered at ClinicalTrials.gov, NCT04346342.

Copyright © 2021 European Society of Anaesthesiology and Intensive Care. Unauthorized reproduction of this article is prohibited.

Conflict of interest statement

Subdistribution hazard ratio from a clustered Fine–Gray competing risk model, with death before extubation treated as competing risk.

Hazard ratio from a (shared-frailty) Cox proportional hazard model. For the ICU and hospital length of stay analyses, all patients who died prior to discharge were assigned the maximum length of stay to account for death as a competing risk in this model. P value for Schoenfeld residuals: ICU length of stay (P = 0.420 in the unmatched cohort and P = 0.330 for the matched cohort); hospital length of stay (P = 0.830 in the unmatched cohort and P = 0.770 for the matched cohort); 7-day mortality (P = 0.380 in the unmatched cohort and P = 0.780 in the matched cohort); 28-day mortality (P = 0.260 in the unmatched cohort and P = 0.110 in the matched cohort); 90-day mortality (P = 0.100 in the unmatched cohort and P = 0.055 in the matched cohort).

- Cited by 2 articles
- 36 references
- 3 figures

Supplementary info

Publication types, MeSH terms, Associated data Expand

Publication types

- Observational Study
- Research Support, Non-U.S. Gov't

MeSH terms

- COVID-19*
- Humans
- Positive-Pressure Respiration
- Respiratory Distress Syndrome* / diagnosis
- Respiratory Distress Syndrome* / epidemiology
- Respiratory Distress Syndrome* / therapy
- Retrospective Studies
- SARS-CoV-2
- Ventilators, Mechanical

Associated data

- ClinicalTrials.gov/NCT04346342

Full text links

Wolters Kluwer Wolters Kluwer Free PMC article

Proceed to details

Cite

Share

523

Observational Study

Clin Transplant

. 2020 Nov;34(11):e14072.
doi: 10.1111/ctr.14072. Epub 2020 Sep 27.

Tocilizumab use in Kidney Transplant Patients with COVID-19

Hernando Trujillo [1], Fernando Caravaca-Fontán [1 2], Ángel Sevillano [1], Eduardo Gutiérrez [1], Mario Fernández-Ruiz [2 3], Francisco López-Medrano [2 3 4], Ana Hernández [1], José María Aguado [2 3 4], Manuel Praga [1 2 4], Amado Andrés [1 2 4]

Affiliations Expand

Affiliations

- [1] Department of Nephrology, Hospital Universitario, Madrid, Spain.
- [2] Research Institute Hospital, Madrid, Spain.
- [3] Unit of Infectious Diseases, Hospital Universitario, Madrid, Spain.
- [4] Department of Medicine, Universidad Complutense de Madrid, Madrid, Spain.

- PMID: **32862472**
- DOI: 10.1111/ctr.14072

Observational Study

Tocilizumab use in Kidney Transplant Patients with COVID-19

Hernando Trujillo et al. Clin Transplant. 2020 Nov.

Show details

Clin Transplant

. 2020 Nov;34(11):e14072.
doi: 10.1111/ctr.14072. Epub 2020 Sep 27.

Authors

Hernando Trujillo [1], Fernando Caravaca-Fontán [1 2], Ángel Sevillano [1], Eduardo Gutiérrez [1], Mario Fernández-Ruiz [2 3], Francisco López-Medrano [2 3 4], Ana Hernández [1], José María Aguado [2 3 4], Manuel Praga [1 2 4], Amado Andrés [1 2 4]

Affiliations

- [1] Department of Nephrology, Hospital Universitario, Madrid, Spain.

- [2] Research Institute Hospital, Madrid, Spain.
- [3] Unit of Infectious Diseases, Hospital Universitario, Madrid, Spain.
- [4] Department of Medicine, Universidad Complutense de Madrid, Madrid, Spain.

- PMID: **32862472**
- DOI: 10.1111/ctr.14072

Abstract

A potential benefit of immunomodulatory agents such as tocilizumab (TCZ) has been reported in patients with coronavirus disease 2019 (COVID-19) and severe pulmonary involvement. However, this therapy has been scarcely studied in kidney transplant (KT) recipients. Herein, we describe the clinical course and outcome of 10 KT patients with severe COVID-19 that were treated with TCZ. Mean age of the study group was 54 ± 10 years (70% females), and 30% of the cases were within 6 months from transplant. Mycophenolate mofetil was discontinued in all cases upon admission, whereas baseline steroids were maintained and tacrolimus dose was reduced. Initial treatment included hydroxychloroquine, antibiotics, and prophylactic anticoagulation. Before treatment with TCZ, 3 patients were receiving high-flow oxygen, 4 patients low-flow oxygen and 1 case non-invasive ventilation. All patients received a single dose of intravenous TCZ within a mean time of 7 ± 4 days since admission. During a median follow-up of 16 days (IQR: 10-29), 7 patients (70%) gradually improved and were finally discharged while three cases (30%) did not exhibited clinical improvement and ultimately died. In conclusion, although treatment with TCZ could be associated with improved clinical outcomes in a subset of KT recipients with COVID-19, further studies are warranted before drawing firm conclusions.

Keywords: coronavirus disease 2019; kidney transplantation; outcomes; severe acute respiratory syndrome coronavirus 2; tocilizumab.

© 2020 John Wiley & Sons A/S. Published by John Wiley & Sons Ltd.

- Cited by 11 articles
- 17 references

Supplementary info

Publication types, MeSH terms, Substances, Grant support Expand

Publication types

- Observational Study
- Research Support, Non-U.S. Gov't

MeSH terms

- Adult
- Aged
- Antibodies, Monoclonal, Humanized / therapeutic use*
- COVID-19 / drug therapy*

- COVID-19 / etiology
- Female
- Follow-Up Studies
- Hospitalization
- Humans
- Immunologic Factors / therapeutic use*
- Kidney Transplantation*
- Male
- Middle Aged
- Postoperative Complications / drug therapy*
- Retrospective Studies
- Severity of Illness Index
- Treatment Outcome

Substances

- Antibodies, Monoclonal, Humanized
- Immunologic Factors
- tocilizumab

Grant support

- CP 18/00073/Instituto de Salud Carlos III/International

Full text links

WILEY Full Text Article Wiley
Proceed to details
Cite
Share
524
Observational Study
Diabetes Obes Metab
. 2021 Jul;23(7):1624-1630.
doi: 10.1111/dom.14380. Epub 2021 Apr 6.

Effects of the COVID-19 lockdown on glycaemic control in subjects with type 2 diabetes: the glycalock study

Luca D'Onofrio [1], Silvia Pieralice [2], Ernesto Maddaloni [1], Carmen Mignogna [1], Sara Sterpetti [1], Lucia Coraggio [1], Cecilia Luordi [1], Gloria Guarisco [3], Gaetano Leto [3], Frida Leonetti [3], Silvia Manfrini [2], Raffaella Buzzetti [1]
Affiliations Expand

Affiliations

- [1] Experimental Medicine Department, Sapienza University of Rome, Latina, Italy.
- [2] Endocrinology and Diabetes Unit, Campus Bio-Medico University of Rome, Rome, Italy.
- [3] Diabetes Unit, Department of Medical-Surgical Sciences and Biotechnologies, Santa Maria Goretti Hospital, Sapienza University of Rome, Latina, Italy.

- PMID: **33764666**
- PMCID: PMC8251001
- DOI: 10.1111/dom.14380

Free PMC article
Observational Study

Effects of the COVID-19 lockdown on glycaemic control in subjects with type 2 diabetes: the glycalock study

Luca D'Onofrio et al. Diabetes Obes Metab. 2021 Jul.
Free PMC article
Show details
Diabetes Obes Metab
. 2021 Jul;23(7):1624-1630.
doi: 10.1111/dom.14380. Epub 2021 Apr 6.

Authors

Luca D'Onofrio [1], Silvia Pieralice [2], Ernesto Maddaloni [1], Carmen Mignogna [1], Sara Sterpetti [1], Lucia Coraggio [1], Cecilia Luordi [1], Gloria Guarisco [3], Gaetano Leto [3], Frida Leonetti [3], Silvia Manfrini [2], Raffaella Buzzetti [1]

Affiliations

- [1] Experimental Medicine Department, Sapienza University of Rome, Latina, Italy.
- [2] Endocrinology and Diabetes Unit, Campus Bio-Medico University of Rome, Rome, Italy.
- [3] Diabetes Unit, Department of Medical-Surgical Sciences and Biotechnologies, Santa Maria Goretti Hospital, Sapienza University of Rome, Latina, Italy.

- PMID: **33764666**
- PMCID: PMC8251001
- DOI: 10.1111/dom.14380

Abstract

Aim: To assess the effect of the coronavirus disease 2019 (COVID-19) lockdown on glycaemic control in subjects with type 2 diabetes (T2D).

Materials and methods: In this observational, multicentre, retrospective study conducted in the Lazio region, Italy, we compared the differences in the HbA1c levels of 141 subjects with T2D exposed to lockdown with 123 matched controls with T2D who attended the study centres 1 year before. Basal data were collected from 9 December to 9 March and follow-up data from 3 June to 10 July in 2020 for the lockdown group, and during the same timeframes in 2019 for the control groups. Changes in HbA1c (ΔHbA1c) and body mass index (ΔBMI) during lockdown were compared among patients with different psychological well-being, as evaluated by tertiles of the Psychological General Well-Being Index (PGWBS).

Results: No difference in ΔHbA1c was found between the lockdown and control groups (lockdown group -0.1% [-0.5%-0.3%] vs. control group -0.1% [-0.4%-0.2%]; p = .482). Also, no difference was found in ΔBMI (p = .316) or ΔGlucose (p = .538). In the lockdown group, subjects with worse PGWBS showed a worsening of HbA1c (p = .041 for the trend among PGWBS tertiles) and BMI (p = .022).

Conclusions: The COVID-19 lockdown did not significantly impact glycaemic control in people with T2D. People with poor psychological well-being may experience a worsening a glycaemic control because of restrictions resulting from lockdown. These findings may aid healthcare providers in diabetes management once the second wave of COVID-19 has ended.

Keywords: covid-19 lockdown; glycaemic control; psychological health; type 2 diabetes.

© 2021 The Authors. Diabetes, Obesity and Metabolism published by John Wiley & Sons Ltd.

Conflict of interest statement

All the authors declare no conflicts of interests related to this manuscript.

- Cited by 9 articles
- 18 references
- 1 figure

Supplementary info

Publication types, MeSH terms, Substances, Grant support Expand

Publication types

- Multicenter Study
- Observational Study

MeSH terms

- Blood Glucose
- COVID-19*

- Communicable Disease Control
- Diabetes Mellitus, Type 2* / epidemiology
- Glycemic Control
- Humans
- Italy / epidemiology
- Retrospective Studies
- SARS-CoV-2

Substances

- Blood Glucose

Grant support

- No funding supported this study.

Full text links

WILEY Full Text Article Wiley Free PMC article

Proceed to details

Cite

Share

☐ 525

Observational Study

Br J Radiol

. 2020 Sep 1;93(1113):20200407.

doi: 10.1259/bjr.20200407. Epub 2020 Jul 31.

Pulmonary thromboembolism in hospitalised COVID-19 patients at moderate to high risk by Wells score: a report from Lombardy, Italy

Lorenzo Monfardini [1], Mauro Morassi [1], Paolo Botti [1], Roberto Stellini [2], Luca Bettari [3], Stefania Pezzotti [1], Marco Alì [4] [5], Cristian Giuseppe Monaco [4], Veronica Magni [6], Andrea Cozzi [7], Simone Schiaffino [4], Claudio Bnà [1]

Affiliations Expand

Affiliations

- [1] Department of Radiology, Fondazione Poliambulanza Istituto Ospedaliero, Via Leonida Bissolati 57, Brescia, Italy.

- [2] Infectious Diseases Service, Fondazione Poliambulanza Istituto Ospedaliero, Via Leonida Bissolati 57, Brescia, Italy.
- [3] Cardiology Unit, Cardiovascular Department, Fondazione Poliambulanza Istituto Ospedaliero, Via Leonida Bissolati 57, Brescia, Italy.
- [4] Unit of Radiology, IRCCS Policlinico San Donato, Via Rodolfo Morandi 30, San Donato Milanese, Italy.
- [5] Unit of Diagnostic Imaging and Stereotactic Radiosurgery, C.D.I. Centro Diagnostico Italiano S.p.A., Via Simone Saint Bon 20, Milano, Italy.
- [6] Medical School, Università degli Studi di Milano, Via Festa del Perdono 7, Milano, Italy.
- [7] Department of Biomedical Sciences for Health, Università degli Studi di Milano, Via Luigi Mangiagalli 31, Milano, Italy.

- PMID: **32735448**
- PMCID: PMC7465860
- DOI: 10.1259/bjr.20200407

Free PMC article
Observational Study

Pulmonary thromboembolism in hospitalised COVID-19 patients at moderate to high risk by Wells score: a report from Lombardy, Italy

Lorenzo Monfardini et al. Br J Radiol. 2020.
Free PMC article
Show details
Br J Radiol
. 2020 Sep 1;93(1113):20200407.
doi: 10.1259/bjr.20200407. Epub 2020 Jul 31.

Authors

Lorenzo Monfardini [1], Mauro Morassi [1], Paolo Botti [1], Roberto Stellini [2], Luca Bettari [3], Stefania Pezzotti [1], Marco Alì [4] [5], Cristian Giuseppe Monaco [4], Veronica Magni [6], Andrea Cozzi [7], Simone Schiaffino [4], Claudio Bnà [1]

Affiliations

- [1] Department of Radiology, Fondazione Poliambulanza Istituto Ospedaliero, Via Leonida Bissolati 57, Brescia, Italy.
- [2] Infectious Diseases Service, Fondazione Poliambulanza Istituto Ospedaliero, Via Leonida Bissolati 57, Brescia, Italy.
- [3] Cardiology Unit, Cardiovascular Department, Fondazione Poliambulanza Istituto Ospedaliero, Via Leonida Bissolati 57, Brescia, Italy.

- [4] Unit of Radiology, IRCCS Policlinico San Donato, Via Rodolfo Morandi 30, San Donato Milanese, Italy.
- [5] Unit of Diagnostic Imaging and Stereotactic Radiosurgery, C.D.I. Centro Diagnostico Italiano S.p.A., Via Simone Saint Bon 20, Milano, Italy.
- [6] Medical School, Università degli Studi di Milano, Via Festa del Perdono 7, Milano, Italy.
- [7] Department of Biomedical Sciences for Health, Università degli Studi di Milano, Via Luigi Mangiagalli 31, Milano, Italy.

- PMID: **32735448**
- PMCID: PMC7465860
- DOI: 10.1259/bjr.20200407

Abstract

Objectives: To present a single-centre experience on CT pulmonary angiography (CTPA) for the assessment of hospitalised COVID-19 patients with moderate-to-high risk of pulmonary thromboembolism (PTE).

Methods: We analysed consecutive COVID-19 patients (RT-PCR confirmed) undergoing CTPA in March 2020 for PTE clinical suspicion. Clinical data were retrieved. Two experienced radiologists reviewed CTPAs to assess pulmonary parenchyma and vascular findings.

Results: Among 34 patients who underwent CTPA, 26 had PTE (76%, 20 males, median age 61 years, interquartile range 54-70), 20/26 (77%) with comorbidities (mainly hypertension, 44%), and 8 (31%) subsequently dying. Eight PTE patients were under thromboprophylaxis with low-molecular-weight heparin, four PTE patients had lower-limbs deep vein thrombosis at ultrasound examination (performed in 33/34 patients). Bilateral PTE characterised 19/26 cases, with main branches involved in 10/26 cases. Twelve patients had a parenchymal involvement >75%, the predominant pneumonia pattern being consolidation in 10/26 patients, ground glass opacities in 9/26, crazy paving in 5/26, and both ground glass opacities and consolidation in 2/26.

Conclusion: COVID-19 patients are prone to PTE.

Advances in knowledge: PTE, potentially attributable to an underlying thrombophilic status, may be more frequent than expected in COVID-19 patients. Extension of prophylaxis and adaptation of diagnostic criteria should be considered.

Conflict of interest statement

Conflicts of interest: L. Monfardini, M. Morassi, P. Botti, R. Stellini, L. Bettari, S. Pezzotti, M. Alì, C. G. Monaco, V. Magni, A. Cozzi, and C. Bnà all declare that they have no conflict of interest and that they have nothing to disclose.

S. Schiaffino declares to be member of speakers' bureau for General Electric and to have received travel support from Bracco Imaging.

- Cited by 11 articles
- 15 references
- 1 figure

Supplementary info

Publication types, MeSH terms Expand

Publication types

- Observational Study

MeSH terms

- Aged
- Betacoronavirus*
- COVID-19
- Comorbidity
- Computed Tomography Angiography / methods
- Coronavirus Infections / epidemiology*
- Female
- Hospitalization
- Humans
- Inpatients / statistics & numerical data*
- Italy / epidemiology
- Lung / diagnostic imaging
- Male
- Middle Aged
- Pandemics
- Pneumonia, Viral / epidemiology*
- Pulmonary Embolism / epidemiology*
- Retrospective Studies
- Risk
- SARS-CoV-2

Full text links

BIR Full text Atypon Free PMC article
Proceed to details
Cite
Share
☐ 526
Observational Study
Eur J Med Res
. 2020 Nov 25;25(1):61.
doi: 10.1186/s40001-020-00462-x.

Clinical, epidemiological, and laboratory characteristics of mild-to-moderate COVID-19 patients in Saudi Arabia: an observational cohort study

Abbas Al Mutair [1, 2], Saad Alhumaid [3], Waad N Alhuqbani [1, 4], Abdul Rehman Z Zaidi [1, 5], Safug Alkoraisi [6], Maha F Al-Subaie [1, 5], Alanoud M AlHindi [4], Ahmed K Abogosh [4], Aljwhara K Alrasheed [4], Aya A Alsharafi [4], Mohammed N Alhuqbani [4], Njoud A Alhowar [5], Samer Salih [1], Mogbil A Alhedaithy [1], Jaffar A Al-Tawfiq [7, 8, 9], Haifa Al-Shammari [10], Rayid Abdulqawi [1, 2], Alaa F Ismail [1], Noura Hamdan [1], Fares Saad [1], Fahad A Olhaye [11], Tarig A Eltahir [11], Ali A Rabaan [12], Awad Al-Omari [1, 5]

Affiliations Expand

Affiliations

- [1] Research Center, Dr. Sulaiman Al Habib Medical Group, Riyadh, Saudi Arabia.
- [2] University of Wollongong, Wollongong, Australia.
- [3] Administration of Pharmaceutical Care, Alahsa, Ministry of Health, Rashdiah Street, P. O. Box 12944, Alahsa, 31982, Saudi Arabia. saalhumaid@moh.gov.sa.
- [4] College of Pharmacy, King Saud University, Riyadh, Saudi Arabia.
- [5] College of Medicine, Alfaisal University, Riyadh, Saudi Arabia.
- [6] Department of Critical Care, Al Hammadi Hospital, Riyadh, Saudi Arabia.
- [7] Infectious Disease Unit, Specialty Internal Medicine, Johns Hopkins Aramco Healthcare, Dhahran, Saudi Arabia.
- [8] Department of Medicine, Indiana University School of Medicine, Indianapolis, IN, USA.
- [9] Department of Medicine, Johns Hopkins University School of Medicine, Baltimore, MD, USA.
- [10] Department of Histopathology, King Saud Medical City, Riyadh, Saudi Arabia.
- [11] Department of Internal Medicine, Al Hammadi Hospital, Riyadh, Saudi Arabia.
- [12] Molecular Diagnostics Laboratory, Johns Hopkins Aramco Healthcare, Dhahran, Saudi Arabia.

- PMID: **33239068**
- PMCID: PMC7686832
- DOI: 10.1186/s40001-020-00462-x

Free PMC article
Observational Study

Clinical, epidemiological, and laboratory characteristics of mild-to-moderate COVID-

[19 patients in Saudi Arabia: an observational cohort study]

Abbas Al Mutair et al. Eur J Med Res. 2020.
Free PMC article
Show details
Eur J Med Res
. 2020 Nov 25;25(1):61.
doi: 10.1186/s40001-020-00462-x.

Authors

Abbas Al Mutair [1, 2], Saad Alhumaid [3], Waad N Alhuqbani [1, 4], Abdul Rehman Z Zaidi [1, 5], Safug Alkoraisi [6], Maha F Al-Subaie [1, 5], Alanoud M AlHindi [4], Ahmed K Abogosh [4], Aljwhara K Alrasheed [4], Aya A Alsharafi [4], Mohammed N Alhuqbani [4], Njoud A Alhowar [5], Samer Salih [1], Mogbil A Alhedaithy [1], Jaffar A Al-Tawfiq [7, 8, 9], Haifa Al-Shammari [10], Rayid Abdulqawi [1, 2], Alaa F Ismail [1], Noura Hamdan [1], Fares Saad [1], Fahad A Olhaye [11], Tarig A Eltahir [11], Ali A Rabaan [12], Awad Al-Omari [1, 5]

Affiliations

- [1] Research Center, Dr. Sulaiman Al Habib Medical Group, Riyadh, Saudi Arabia.
- [2] University of Wollongong, Wollongong, Australia.
- [3] Administration of Pharmaceutical Care, Alahsa, Ministry of Health, Rashdiah Street, P. O. Box 12944, Alahsa, 31982, Saudi Arabia. saalhumaid@moh.gov.sa.
- [4] College of Pharmacy, King Saud University, Riyadh, Saudi Arabia.
- [5] College of Medicine, Alfaisal University, Riyadh, Saudi Arabia.
- [6] Department of Critical Care, Al Hammadi Hospital, Riyadh, Saudi Arabia.
- [7] Infectious Disease Unit, Specialty Internal Medicine, Johns Hopkins Aramco Healthcare, Dhahran, Saudi Arabia.
- [8] Department of Medicine, Indiana University School of Medicine, Indianapolis, IN, USA.
- [9] Department of Medicine, Johns Hopkins University School of Medicine, Baltimore, MD, USA.
- [10] Department of Histopathology, King Saud Medical City, Riyadh, Saudi Arabia.
- [11] Department of Internal Medicine, Al Hammadi Hospital, Riyadh, Saudi Arabia.
- [12] Molecular Diagnostics Laboratory, Johns Hopkins Aramco Healthcare, Dhahran, Saudi Arabia.

- PMID: **33239068**
- PMCID: PMC7686832
- DOI: 10.1186/s40001-020-00462-x

Abstract

Background: Severe acute respiratory syndrome coronavirus 2 (SARS-CoV-2) emerged from China in December 2019 and has presented as a substantial and serious threat to global health. We

aimed to describe the clinical, epidemiological, and laboratory findings of patients in Saudi Arabia infected with SARS-CoV-2 to direct us in helping prevent and treat coronavirus disease 2019 (COVID-19) across Saudi Arabia and around the world.

Materials and methods: Clinical, epidemiological, laboratory, and radiological characteristics, treatment, and outcomes of pediatric and adult patients in five hospitals in Riyadh, Saudi Arabia, were surveyed in this study.

Results: 401 patients (mean age 38.16 ± 13.43 years) were identified to be SARS-CoV-2 positive and 80% of cases were male. 160 patients had moderate severity and 241 were mild in severity. The most common signs and symptoms at presentation were cough, fever, fatigue, and shortness of breath. Neutrophil and lymphocyte counts, aspartate aminotransferase, C-reactive protein, and ferritin were higher in the COVID-19 moderate severity patient group. Mild severity patients spent a shorter duration hospitalized and had slightly higher percentages of abnormal CT scans and X-ray imaging.

Conclusions: This study provides an understanding of the features of non-ICU COVID-19 patients in Saudi Arabia. Further national collaborative studies are needed to streamline screening and treatment procedures for COVID-19.

Keywords: COVID-19; Comorbidities; Epidemiology; SARS-CoV-2; Saudi Arabia; Symptoms.

Conflict of interest statement

The authors have no conflicts of interest to declare.

- Cited by 30 articles
- 28 references

Supplementary info

Publication types, MeSH terms, Substances Expand

Publication types

- Observational Study

MeSH terms

- Adolescent
- Adult
- Aged
- Aged, 80 and over
- Biomarkers / analysis*
- COVID-19 / diagnostic imaging
- COVID-19 / epidemiology*
- COVID-19 / pathology
- COVID-19 / virology

- Child
- Child, Preschool
- Female
- Follow-Up Studies
- Humans
- Infant
- Male
- Middle Aged
- Prognosis
- Retrospective Studies
- Risk Factors
- SARS-CoV-2 / isolation & purification*
- Saudi Arabia / epidemiology
- Tomography, X-Ray Computed / methods*
- Young Adult

Substances

- Biomarkers

Full text links

Read free full text at BMC BioMed Central Free PMC article

Proceed to details

Cite

Share

527

Observational Study

Medicine (Baltimore)
. 2021 Feb 26;100(8):e24750.
doi: 10.1097/MD.0000000000024750.

Clinical characteristics and outcome of patients aged over 80 years with covid-19

Aina Capdevila-Reniu [1], Martina Pellice [1], Sergio Prieto-González [1], Helena Ventosa [1], Andrea Ladino [1], Jose Naval [1], Olga Rodriguez-Nuñez [1], Jose César Milisenda [1], Pedro Juan Moreno-Lozano [1], Alex Soriano [2], Xavier Bosch [1], Alfonso López-Soto [1], Hospital Clínic COVID-19 Research Group
Affiliations Expand

Affiliations

- [1] Department of Internal Medicine.
- [2] Department of Infectious Diseases, Hospital Clínic of Barcelona, Institut d'Investigacions Biomèdiques August Pi i Sunyer (IDIBAPS), University of Barcelona, Spain.

- PMID: **33663089**
- PMCID: PMC7909132
- DOI: 10.1097/MD.0000000000024750

Free PMC article
Observational Study

Clinical characteristics and outcome of patients aged over 80 years with covid-19

Aina Capdevila-Reniu et al. Medicine (Baltimore). 2021.
Free PMC article
Show details
Medicine (Baltimore)

. 2021 Feb 26;100(8):e24750.
doi: 10.1097/MD.0000000000024750.

Authors

Aina Capdevila-Reniu [1], Martina Pellice [1], Sergio Prieto-González [1], Helena Ventosa [1], Andrea Ladino [1], Jose Naval [1], Olga Rodriguez-Nuñez [1], Jose César Milisenda [1], Pedro Juan Moreno-Lozano [1], Alex Soriano [2], Xavier Bosch [1], Alfonso López-Soto [1], Hospital Clínic COVID-19 Research Group

Affiliations

- [1] Department of Internal Medicine.
- [2] Department of Infectious Diseases, Hospital Clínic of Barcelona, Institut d'Investigacions Biomèdiques August Pi i Sunyer (IDIBAPS), University of Barcelona, Spain.

- PMID: **33663089**
- PMCID: PMC7909132
- DOI: 10.1097/MD.0000000000024750

Abstract

To investigate the clinical characteristics and outcome of octogenarians with covid-19.This is a observational, retrospective, descriptive study.Consecutive patients aged >80 years who were admitted for covid-19 pneumonia during a 6 weeks period (March 20-April 30, 2020).Illness severity on admission was classified according to World Health Organization (WHO) criteria: mild, moderate, severe, and critical. Data collected included demographics, presenting symptoms, radiological and laboratory findings, comorbidities, functional status, treatment, and clinical outcome.There were 159 patients (52.2% women) with a median age of 85.99 (IQR: 80-98). The median Barthel index was 90 (40-100) and Charlson index was 5 (5-6). Most common presenting

symptoms were fever, dyspnea, and cough. Patients had mild (8.2%), moderate (52.2%), or severe (39.6%) illness according to WHO criteria. A bilateral pulmonary involvement was seen in 86% of patients. Laboratory analysis revealed increased serum concentrations of inflammatory parameters (C-reactive protein, ferritin, lactate dehydrogenase, and D-dimer) with an abnormal lymphocyte count [0.88 × 109/L (0.5)]. Treatments included corticosteroids in 37%, and biological therapies in 17.6%. Fifty three (33.3%) patients died during hospitalization, with a median time from admission to death of 3 (IQR 1-6) days. Mortality was higher in men (55%). Deceased patients had a significantly higher frequency of dyspnea, increased inflammatory parameters, and illness severity compared to survivors.One-third of octogenarians with covid-19 died during hospitalization and most had bilateral lung involvement. A further knowledge of the characteristics and outcome of this population may assist clinicians in the decision-making process in these patients.

Copyright © 2021 the Author(s). Published by Wolters Kluwer Health, Inc.

Conflict of interest statement

The authors have no funding and conflicts of interests to disclose.

- Cited by 2 articles
- 16 references
- 2 figures

Supplementary info

Publication types, MeSH terms, Substances Expand

Publication types

- Observational Study

MeSH terms

- Adrenal Cortex Hormones
- Aged, 80 and over
- Biological Products / therapeutic use
- COVID-19 / mortality
- COVID-19 / physiopathology*
- Female
- Hospital Mortality
- Humans
- Inflammation Mediators / metabolism
- Lymphocyte Count
- Male
- Retrospective Studies
- SARS-CoV-2
- Severity of Illness Index

- Sex Factors

Substances

- Adrenal Cortex Hormones
- Biological Products
- Inflammation Mediators

Full text links

Wolters Kluwer Wolters Kluwer Free PMC article
Proceed to details
Cite
Share
528
Observational Study
Ann R Coll Surg Engl
. 2021 Feb;103(2):114-119.
doi: 10.1308/rcsann.2020.7026.

The effect of the COVID-19 pandemic on mental health associated trauma, admissions and fractures at a London major trauma centre

D Hay [1], M S Jamal [1], K Al-Tawil [1], A Petohazi [1], V Gulli [1], N F Bednarczuk [1], R Baldwin-Smith [1], J Gibbons [2], J Sinha [1]
Affiliations Expand

Affiliations

- [1] Department of Trauma and Orthopaedic Surgery, Kings College Hospital NHS Foundation Trust, London, UK.
- [2] GKT School of Medical Education, Kings College London, London, UK.

- PMID: **33559558**
- DOI: 10.1308/rcsann.2020.7026

Observational Study

The effect of the COVID-19 pandemic on mental health associated trauma, admissions

and fractures at a London major trauma centre

D Hay et al. Ann R Coll Surg Engl. 2021 Feb.
Show details
Ann R Coll Surg Engl
. 2021 Feb;103(2):114-119.
doi: 10.1308/rcsann.2020.7026.

Authors

D Hay [1], M S Jamal [1], K Al-Tawil [1], A Petohazi [1], V Gulli [1], N F Bednarczuk [1], R Baldwin-Smith [1], J Gibbons [2], J Sinha [1]

Affiliations

- [1] Department of Trauma and Orthopaedic Surgery, Kings College Hospital NHS Foundation Trust, London, UK.
- [2] GKT School of Medical Education, Kings College London, London, UK.

- PMID: **33559558**
- DOI: 10.1308/rcsann.2020.7026

Abstract

Introduction: Non-injury-related factors have been extensively studied in major trauma and have been shown to have a significant impact on patient outcomes. Mental illness and associated medication use has been proven to have a negative effect on bone health and fracture healing.

Materials and methods: We collated data retrospectively from the records of orthopaedic inpatients in a non-COVID and COVID period. We analysed demographic data, referral and admission numbers, orthopaedic injuries, surgery performed and patient comorbidities, including psychiatric history.

Results: There were 824 orthopaedic referrals and 358 admissions (six/day) in the non-COVID period, with 38/358 (10.6%) admissions having a psychiatric diagnosis and 30/358 (8.4%) also having a fracture. This was compared with 473 referrals and 195 admissions (three/day) in the COVID period, with 73/195 (37.4%) admissions having a documented psychiatric diagnosis and 47/195 (24.1%) having a fracture.

Discussion: There was a reduction in the number of admissions and referrals during the pandemic, but a simultaneous three-fold rise in admissions with a psychiatric diagnosis. The proportion of patients with both a fracture and a psychiatric diagnosis more than doubled and the number of patients presenting due to a traumatic suicide attempt almost tripled.

Conclusion: While total numbers using the orthopaedic service decreased, the impact of the pandemic and lockdown disproportionately affects those with mental health problems, a group already at higher risk of poorer functional outcomes and non-union. It is imperative that adequate

support is in place for patients with vulnerable mental health during these periods, particularly as we look towards a potential 'second wave' of COVID-19.

Keywords: Bone fractures; COVID-19; Depression; Mental health; Pandemic; Trauma.

Supplementary info

Publication types, MeSH terms, Substances Expand

Publication types

- Observational Study

MeSH terms

- Adult
- Alcoholism / epidemiology
- Anxiety Disorders / epidemiology
- COVID-19*
- Comorbidity
- Depressive Disorder / epidemiology
- Female
- Foreign Bodies / epidemiology
- Foreign Bodies / surgery
- Fractures, Bone / epidemiology*
- Fractures, Bone / surgery
- Hospitalization / trends*
- Humans
- Joint Dislocations / epidemiology
- Joint Dislocations / surgery
- London / epidemiology
- Male
- Mental Disorders / drug therapy
- Mental Disorders / epidemiology*
- Orthopedic Procedures
- Psychotropic Drugs / therapeutic use
- Referral and Consultation / trends*
- Retrospective Studies
- SARS-CoV-2
- Soft Tissue Injuries / epidemiology
- Soft Tissue Injuries / surgery
- Substance-Related Disorders / epidemiology
- Suicide, Attempted / trends*

- Wounds and Injuries / epidemiology
- Wounds and Injuries / surgery

Substances

- Psychotropic Drugs

Full text links

annals FULL TEXT Atypon
Proceed to details
Cite
Share
☐ 529
Observational Study
Eur Respir J
. 2022 Feb 24;59(2):2100265.
doi: 10.1183/13993003.00265-2021. Print 2022 Feb.

Awake prone positioning and oxygen therapy in patients with COVID-19: the APRONOX study

Orlando R Perez-Nieto [1], Diego Escarraman-Martinez [2], Manuel A Guerrero-Gutierrez [3], Eder I Zamarron-Lopez [4], Javier Mancilla-Galindo [5] [6], Ashuin Kammar-García [7], Miguel A Martinez-Camacho [8], Ernesto Deloya-Tomás [9], Jesús S Sanchez-Díaz [10], Luis A Macías-García [11], Raúl Soriano-Orozco [12], Gabriel Cruz-Sánchez [13], José D Salmeron-Gonzalez [14], Marco A Toledo-Rivera [15], Ivette Mata-Maqueda [16], Luis A Morgado-Villaseñor [17], Jenner J Martinez-Mazariegos [18], Raymundo Flores Ramirez [19], Josue L Medina-Estrada [20], Silvio A Ñamendys-Silva, APRONOX Group
Affiliations Expand

Affiliations

- [1] Intensive Care Unit, Hospital General San Juan del Rio, Querétaro, Mexico orlando_rpn@hotmail.com.
- [2] Dept of Anaesthesia, Hospital de Especialidades Centro Médico Nacional "LaRaza", Mexico City, Mexico.
- [3] Dept of Critical Care Medicine, Instituto Nacional de Cancerología, Mexico City, Mexico.
- [4] Intensive Care Unit, Hospital CEMAIN Tampico, Tamaulipas, Mexico.
- [5] Unidad de Investigación UNAM-INC, Instituto Nacional de Cardiología Ignacio Chávez, Mexico City, Mexico.
- [6] Respiratory Medicine, Instituto Nacional de Enfermedades Respiratorias, Mexico City, Mexico.

- [7] Emergency Dept, Instituto Nacional de Ciencias Médicas y Nutrición "Salvador Zubirán", Mexico City, Mexico.
- [8] Intensive Care Unit, Hospital General de México, Mexico City, Mexico.
- [9] Intensive Care Unit, Hospital General San Juan del Rio, Querétaro, Mexico.
- [10] Intensive Care Unit, Hospital de Alta Especialidad IMSS "Adolfo Ruiz Cortines" Veracruz, Veracruz, Mexico.
- [11] Intensive Care Unit, Hospital Regional ISSSTE "Fernando Quiroz Gutiérrez", Mexico City, Mexico.
- [12] Intensive Care Unit, Hospital de Alta Especialidad T1 IMSS, León, Mexico.
- [13] Intensive Care Unit, Clínica Hospital Mérida ISSSTE, Yucatán, Mexico.
- [14] Intensive Care Unit, Hospital General "Miguel Silva", Morelia, Mexico.
- [15] Intensive Care Unit, Hospital SEDNA, Mexico City, Mexico.
- [16] Secretaría de Salud del Estado de Querétaro, Ethics and Research Committee, Mexico.
- [17] Intensive Care Unit, Hospital General de Zona IMSS No. 15 Reynosa, Tamaulipas, Mexico.
- [18] Intensive Care Unit, Hospital Vida Mejor ISSSTECH Tuxtla Gutiérrez, Chiapas, Mexico.
- [19] Intensive Care Unit, Hospital de Especialidades "5 de Mayo" ISSSTEP, Puebla, Mexico.
- [20] Intensive Care Unit, Hospital Regional No. 1 IMSS "Vicente Guerrero", Acapulco, Mexico.

- PMID: **34266942**
- PMCID: PMC8576803
- DOI: 10.1183/13993003.00265-2021

Free PMC article
Observational Study

Awake prone positioning and oxygen therapy in patients with COVID-19: the APRONOX study

Orlando R Perez-Nieto et al. Eur Respir J. 2022.
Free PMC article

Show details

Eur Respir J
. 2022 Feb 24;59(2):2100265.
doi: 10.1183/13993003.00265-2021. Print 2022 Feb.

Authors

Orlando R Perez-Nieto [1], Diego Escarraman-Martinez [2], Manuel A Guerrero-Gutierrez [3], Eder I Zamarron-Lopez [4], Javier Mancilla-Galindo [5] [6], Ashuin Kammar-García [7], Miguel A Martinez-Camacho [8], Ernesto Deloya-Tomás [9], Jesús S Sanchez-Díaz [10], Luis A Macías-García [11], Raúl Soriano-Orozco [12], Gabriel Cruz-Sánchez [13], José D Salmeron-Gonzalez [14], Marco A Toledo-Rivera [15], Ivette Mata-Maqueda [16], Luis A Morgado-Villaseñor [17], Jenner J Martinez-

Mazariegos [18], Raymundo Flores Ramirez [19], Josue L Medina-Estrada [20], Silvio A Ñamendys-Silva, APRONOX Group

Affiliations

- [1] Intensive Care Unit, Hospital General San Juan del Rio, Querétaro, Mexico orlando_rpn@hotmail.com.
- [2] Dept of Anaesthesia, Hospital de Especialidades Centro Médico Nacional "LaRaza", Mexico City, Mexico.
- [3] Dept of Critical Care Medicine, Instituto Nacional de Cancerología, Mexico City, Mexico.
- [4] Intensive Care Unit, Hospital CEMAIN Tampico, Tamaulipas, Mexico.
- [5] Unidad de Investigación UNAM-INC, Instituto Nacional de Cardiología Ignacio Chávez, Mexico City, Mexico.
- [6] Respiratory Medicine, Instituto Nacional de Enfermedades Respiratorias, Mexico City, Mexico.
- [7] Emergency Dept, Instituto Nacional de Ciencias Médicas y Nutrición "Salvador Zubirán", Mexico City, Mexico.
- [8] Intensive Care Unit, Hospital General de México, Mexico City, Mexico.
- [9] Intensive Care Unit, Hospital General San Juan del Rio, Querétaro, Mexico.
- [10] Intensive Care Unit, Hospital de Alta Especialidad IMSS "Adolfo Ruiz Cortines" Veracruz, Veracruz, Mexico.
- [11] Intensive Care Unit, Hospital Regional ISSSTE "Fernando Quiroz Gutiérrez", Mexico City, Mexico.
- [12] Intensive Care Unit, Hospital de Alta Especialidad T1 IMSS, León, Mexico.
- [13] Intensive Care Unit, Clínica Hospital Mérida ISSSTE, Yucatán, Mexico.
- [14] Intensive Care Unit, Hospital General "Miguel Silva", Morelia, Mexico.
- [15] Intensive Care Unit, Hospital SEDNA, Mexico City, Mexico.
- [16] Secretaría de Salud del Estado de Querétaro, Ethics and Research Committee, Mexico.
- [17] Intensive Care Unit, Hospital General de Zona IMSS No. 15 Reynosa, Tamaulipas, Mexico.
- [18] Intensive Care Unit, Hospital Vida Mejor ISSSTECH Tuxtla Gutiérrez, Chiapas, Mexico.
- [19] Intensive Care Unit, Hospital de Especialidades "5 de Mayo" ISSSTEP, Puebla, Mexico.
- [20] Intensive Care Unit, Hospital Regional No. 1 IMSS "Vicente Guerrero", Acapulco, Mexico.

- PMID: **34266942**
- PMCID: PMC8576803
- DOI: 10.1183/13993003.00265-2021

Abstract

Background: The awake prone positioning strategy for patients with acute respiratory distress syndrome is a safe, simple and cost-effective technique used to improve hypoxaemia. We aimed to evaluate intubation and mortality risk in patients with coronavirus disease 2019 (COVID-19) who underwent awake prone positioning during hospitalisation.

Methods: In this retrospective, multicentre observational study conducted between 1 May 2020 and 12 June 2020 in 27 hospitals in Mexico and Ecuador, nonintubated patients with COVID-19 managed with awake prone or awake supine positioning were included to evaluate intubation and

mortality risk through logistic regression models; multivariable and centre adjustment, propensity score analyses, and E-values were calculated to limit confounding.

Results: 827 nonintubated patients with COVID-19 in the awake prone (n=505) and awake supine (n=322) groups were included for analysis. Fewer patients in the awake prone group required endotracheal intubation (23.6% *versus* 40.4%) or died (19.8% *versus* 37.3%). Awake prone positioning was a protective factor for intubation even after multivariable adjustment (OR 0.35, 95% CI 0.24-0.52; p<0.0001, E=2.12), which prevailed after propensity score analysis (OR 0.41, 95% CI 0.27-0.62; p<0.0001, E=1.86) and mortality (adjusted OR 0.38, 95% CI 0.26-0.55; p<0.0001, E=2.03). The main variables associated with intubation among awake prone patients were increasing age, lower baseline peripheral arterial oxygen saturation/inspiratory oxygen fraction ratio (P_{aO_2}/F_{IO_2}) and management with a nonrebreather mask.

Conclusions: Awake prone positioning in hospitalised nonintubated patients with COVID-19 is associated with a lower risk of intubation and mortality.

Trial registration: ClinicalTrials.gov NCT04407468.

Copyright ©The authors 2022.

Conflict of interest statement

Conflict of interest: O.R. Perez-Nieto has nothing to disclose. Conflict of interest: D. Escarraman-Martínez has nothing to disclose. Conflict of interest: M.A. Guerrero-Gutierrez has nothing to disclose. Conflict of interest: E.I. Zamarron-Lopez has nothing to disclose. Conflict of interest: J. Mancilla-Galindo has nothing to disclose. Conflict of interest: A. Kammar-García has nothing to disclose. Conflict of interest: M.A. Martinez-Camacho has nothing to disclose. Conflict of interest: E. Deloya-Tomás has nothing to disclose. Conflict of interest: J.S. Sanchez-Díaz has nothing to disclose. Conflict of interest: L.A. Macías-García has nothing to disclose. Conflict of interest: R. Soriano-Orozco has nothing to disclose. Conflict of interest: G. Cruz-Sánchez has nothing to disclose. Conflict of interest: J.D. Salmeron-Gonzalez has nothing to disclose. Conflict of interest: M.A. Toledo-Rivera has nothing to disclose. Conflict of interest: I. Mata-Maqueda has nothing to disclose. Conflict of interest: L.A. Morgado-Villaseñor has nothing to disclose. Conflict of interest: J.J. Martinez-Mazariegos has nothing to disclose. Conflict of interest: R. Flores Ramirez has nothing to disclose. Conflict of interest: J.L. Medina-Estrada has nothing to disclose. Conflict of interest: S.A. Ñamendys-Silva has nothing to disclose.

Comment in

- Prone positioning for non-intubated hypoxaemic patients with COVID-19: cheap, easy and makes sense, but does it work?
  Morrell ED, Wurfel MM. Morrell ED, et al. Eur Respir J. 2022 Feb 24;59(2):2102416. doi: 10.1183/13993003.02416-2021. Print 2022 Feb. Eur Respir J. 2022. PMID: 34649977 Free PMC article.

- Cited by 6 articles
- 37 references
- 3 figures

Supplementary info

Publication types, MeSH terms, Substances, Associated data Expand

Publication types

- Multicenter Study
- Observational Study

MeSH terms

- COVID-19* / therapy
- Humans
- Oxygen / therapeutic use
- Prone Position
- Respiratory Insufficiency* / therapy
- Retrospective Studies
- SARS-CoV-2
- Wakefulness

Substances

- Oxygen

Associated data

- ClinicalTrials.gov/NCT04407468

Full text links

Free full text at ersjournals.com HighWire Free PMC article
Proceed to details
Cite
Share
☐ 530
Observational Study
Hosp Pediatr
. 2021 Jun;11(6):e90-e94.
doi: 10.1542/hpeds.2021-005866. Epub 2021 Mar 30.

COVID-19-Associated Pulmonary Embolism in Pediatric Patients

Melissa Chima [1], Duane Williams [2], Neal J Thomas [2] [3], Conrad Krawiec [4]
Affiliations Expand

Affiliations

- [1] College of Medicine and.
- [2] Pediatric Critical Care, Department of Pediatrics, College of Medicine, Penn State Health Children's Hospital, Hershey, Pennsylvania.
- [3] Department of Public Health Sciences Pennsylvania State University, Hershey, Pennsylvania; and.
- [4] Pediatric Critical Care, Department of Pediatrics, College of Medicine, Penn State Health Children's Hospital, Hershey, Pennsylvania ckrawiec@pennstatehealth.psu.edu.

- PMID: **33785517**
- DOI: 10.1542/hpeds.2021-005866

Observational Study

COVID-19-Associated Pulmonary Embolism in Pediatric Patients

Melissa Chima et al. Hosp Pediatr. 2021 Jun.

Show details

Hosp Pediatr

. 2021 Jun;11(6):e90-e94.

doi: 10.1542/hpeds.2021-005866. Epub 2021 Mar 30.

Authors

Melissa Chima [1], Duane Williams [2], Neal J Thomas [2] [3], Conrad Krawiec [4]

Affiliations

- [1] College of Medicine and.
- [2] Pediatric Critical Care, Department of Pediatrics, College of Medicine, Penn State Health Children's Hospital, Hershey, Pennsylvania.
- [3] Department of Public Health Sciences Pennsylvania State University, Hershey, Pennsylvania; and.
- [4] Pediatric Critical Care, Department of Pediatrics, College of Medicine, Penn State Health Children's Hospital, Hershey, Pennsylvania ckrawiec@pennstatehealth.psu.edu.

- PMID: **33785517**
- DOI: 10.1542/hpeds.2021-005866

Abstract

Background and objectives: Coronavirus disease 2019 (COVID-19) is associated with pulmonary embolism in adults, but the clinical circumstances surrounding its presence are unknown in children. The objectives of this study are to determine the prevalence of pulmonary embolism in pediatric subjects with COVID-19, evaluate patient characteristics, and describe treatments applied.

Methods: We performed a retrospective cohort study using TriNetX electronic health record data of subjects aged <18 years who were diagnosed with COVID-19 infection (*International Classification of Diseases, 10th Revision*, code U07.1). Pulmonary embolism was identified by using *International Classification of Diseases, 10th Revision*, code I26. We additionally collected data on age, sex, race, ethnicity, all diagnostic codes, medications, procedures, laboratory results, comorbidities, and outcomes.

Results: During the study period, 24 723 pediatric subjects were reported to have a COVID-19 infection diagnosis among 41 health care organizations, of which 693 (2.8%) were hospitalized. Eight subjects (0.03% overall and 1.2% of hospitalized patients) were diagnosed with pulmonary embolism. The median age (25th to 75th percentile) of patients diagnosed with pulmonary embolism was 16.5 years, and median (25th to 75th percentile) BMI was 22.1 (19.6-47.9). Three (37.5%) received critical care services, and 1 (12.5%) underwent mechanical ventilation. Five (62.5%) subjects had potentially significant risk factors (obesity, malignancy, recent surgery, and oral contraceptive use). All patients received anticoagulation, but none underwent thrombolysis. There were no reported deaths.

Conclusions: Although pulmonary embolism is diagnosed less commonly in children than in adults, its occurrence appears to be more frequent in children hospitalized with COVID-19, as compared with previous reports in hospitalized children in general. All patients survived, with only 1 requiring mechanical ventilation.

Copyright © 2021 by the American Academy of Pediatrics.

Conflict of interest statement

POTENTIAL CONFLICT OF INTEREST: The authors have indicated they have no potential conflicts of interest to disclose.

- Cited by 1 article

Supplementary info

Publication types, MeSH terms, Grant support Expand

Publication types

- Observational Study
- Research Support, N.I.H., Extramural

MeSH terms

- Adolescent
- Age Factors
- Body Mass Index
- COVID-19 / epidemiology*
- Causality
- Cohort Studies
- Comorbidity

- Critical Care / statistics & numerical data
- Female
- Humans
- Male
- Prevalence
- Pulmonary Embolism / epidemiology*
- Respiration, Artificial / statistics & numerical data
- Retrospective Studies
- Risk Factors
- SARS-CoV-2
- United States / epidemiology

Grant support

- UL1 TR002014/TR/NCATS NIH HHS/United States

Full text links

AAP Publications Silverchair Information Systems
Proceed to details
Cite
Share
531
Observational Study
Med Clin (Barc)
. 2021 Mar 26;156(6):277-280.
doi: 10.1016/j.medcli.2020.11.002. Epub 2020 Dec 5.

Patient characterization and adverse health care-related events in SARS-CoV-2 infected patients who died in a tertiary hospital

[Article in English, Spanish]
Guillermo Mena [1], Eva Montané [2], Mónica Rodríguez [3], Patricia Beroiz [4], Juan J López-Núñez [5], Mónica Ballester [6]
Affiliations Expand

Affiliations

- [1] Servicio de Medicina Preventiva, Hospital Universitari Germans Trias i Pujol, Badalona, España; Universitat Autònoma de Barcelona, Bellaterra, Barcelona, España. Electronic address: guillemena.germanstrias@gencat.cat.
- [2] Universitat Autònoma de Barcelona, Bellaterra, Barcelona, España; Servicio de Farmacología Clínica, Hospital Universitari Germans Trias i Pujol, Badalona, España.

- [3] Universitat Autònoma de Barcelona, Bellaterra, Barcelona, España; Servicio de Anestesiología y Reanimación, Hospital Universitari Germans Trias i Pujol, Badalona, España.
- [4] Servicio de Geriatría, Hospital Universitari Germans Trias i Pujol, Badalona, España.
- [5] Servicio de Medicina Interna, Hospital Universitari Germans Trias i Pujol, Badalona, España.
- [6] Dirección de Calidad, Hospital Universitari Germans Trias i Pujol, Badalona, España.

- PMID: **33358536**
- PMCID: PMC7832921
- DOI: 10.1016/j.medcli.2020.11.002

Free PMC article
Observational Study

Patient characterization and adverse health care-related events in SARS-CoV-2 infected patients who died in a tertiary hospital

[Article in English, Spanish]
Guillermo Mena et al. Med Clin (Barc). 2021.
Free PMC article
Show details
Med Clin (Barc)
. 2021 Mar 26;156(6):277-280.
doi: 10.1016/j.medcli.2020.11.002. Epub 2020 Dec 5.

Authors

Guillermo Mena [1], Eva Montané [2], Mónica Rodríguez [3], Patricia Beroiz [4], Juan J López-Núñez [5], Mónica Ballester [6]

Affiliations

- [1] Servicio de Medicina Preventiva, Hospital Universitari Germans Trias i Pujol, Badalona, España; Universitat Autònoma de Barcelona, Bellaterra, Barcelona, España. Electronic address: guillemena.germanstrias@gencat.cat.
- [2] Universitat Autònoma de Barcelona, Bellaterra, Barcelona, España; Servicio de Farmacología Clínica, Hospital Universitari Germans Trias i Pujol, Badalona, España.
- [3] Universitat Autònoma de Barcelona, Bellaterra, Barcelona, España; Servicio de Anestesiología y Reanimación, Hospital Universitari Germans Trias i Pujol, Badalona, España.
- [4] Servicio de Geriatría, Hospital Universitari Germans Trias i Pujol, Badalona, España.
- [5] Servicio de Medicina Interna, Hospital Universitari Germans Trias i Pujol, Badalona, España.
- [6] Dirección de Calidad, Hospital Universitari Germans Trias i Pujol, Badalona, España.

- PMID: **33358536**
- PMCID: PMC7832921
- DOI: 10.1016/j.medcli.2020.11.002

Abstract

Objective: To characterize health care-related adverse events in patients with SARS-CoV-2 infection who died in a tertiary hospital.

Methods: This is a retrospective, observational study, that included patients who died at HUGTiP hospital between 16 March and 10 April 2020. Data was extracted from the electronic medical record.

Results: The median age of the 164 SARS-CoV-2 infected patients who died in the center in the study period was 77.5 years and> 90% of patients had ≥ 1 comorbidity. Forty point two percent of patients had at least ≥ 1 health care-related adverse event. Twenty three point eight of patients had an adverse drug reaction, the leading cause of adverse events in patients who died. Of patients who died in intensive care units, the frequency of problems related to mechanical ventilation was 8.8%.

Conclusions: Although the case fatality rate associated with the adverse events detected was very low, close monitoring of potential health care-related adverse events, especially drug reactions, as the therapeutic management of the disease remains unclear.

Keywords: Adverse drug reactions; Adverse events; COVID-19; Episodios adversos; Mortalidad; Mortality; Pandemia; Pandemic; RAM; SARS-CoV-2.

Copyright © 2020 Elsevier España, S.L.U. All rights reserved.

- Cited by 2 articles
- 13 references

Supplementary info

Publication types, MeSH terms, Substances Expand

Publication types

- Observational Study

MeSH terms

- Adult
- Aged
- Aged, 80 and over
- Antiviral Agents / adverse effects*
- Antiviral Agents / therapeutic use
- COVID-19 / diagnosis
- COVID-19 / mortality*

- COVID-19 / therapy*
- Combined Modality Therapy
- Drug-Related Side Effects and Adverse Reactions / epidemiology*
- Drug-Related Side Effects and Adverse Reactions / etiology
- Female
- Humans
- Male
- Middle Aged
- Respiration, Artificial / adverse effects*
- Respiration, Artificial / mortality
- Retrospective Studies
- Spain / epidemiology
- Tertiary Care Centers*

Substances

- Antiviral Agents

Full text links

Full text at MEDICINA CLINICA Ediciones Doyma, S.L. Free PMC article
Proceed to details
Cite
Share
532
Observational Study
J Intensive Care Med
. 2021 Jun;36(6):646-654.
doi: 10.1177/0885066621989959. Epub 2021 Mar 15.

High Incidence of Barotrauma in Patients With Severe Coronavirus Disease 2019

Michael R Kahn [1], Richard L Watson [2], Jay T Thetford [1], Joseph Isaac Wong [1], Nader Kamangar [3]
Affiliations Expand

Affiliations

- [1] Department of Medicine, 12222UCLA-Olive View Medical Center, David Geffen School of Medicine at UCLA, Los Angeles, CA, USA.
- [2] Division of Pulmonary and Critical Care Medicine, Ronald Reagan 12222UCLA Medical Center, David Geffen School of Medicine at UCLA, Los Angeles, CA, USA.

- [3] Division of Pulmonary and Critical Care Medicine, 12222UCLA-Olive View Medical Center, David Geffen School of Medicine at UCLA, Los Angeles, CA, USA.

- PMID: **33722090**
- PMCID: PMC7967021
- DOI: 10.1177/0885066621989959

Free PMC article
Observational Study

High Incidence of Barotrauma in Patients With Severe Coronavirus Disease 2019

Michael R Kahn et al. J Intensive Care Med. 2021 Jun.
Free PMC article
Show details
J Intensive Care Med
. 2021 Jun;36(6):646-654.
doi: 10.1177/0885066621989959. Epub 2021 Mar 15.

Authors

Michael R Kahn [1], Richard L Watson [2], Jay T Thetford [1], Joseph Isaac Wong [1], Nader Kamangar [3]

Affiliations

- [1] Department of Medicine, 12222UCLA-Olive View Medical Center, David Geffen School of Medicine at UCLA, Los Angeles, CA, USA.
- [2] Division of Pulmonary and Critical Care Medicine, Ronald Reagan 12222UCLA Medical Center, David Geffen School of Medicine at UCLA, Los Angeles, CA, USA.
- [3] Division of Pulmonary and Critical Care Medicine, 12222UCLA-Olive View Medical Center, David Geffen School of Medicine at UCLA, Los Angeles, CA, USA.

- PMID: **33722090**
- PMCID: PMC7967021
- DOI: 10.1177/0885066621989959

Abstract

Objective.: To report the high incidence of barotrauma in critically ill patients admitted to the intensive care unit (ICU) with coronavirus disease 2019 (COVID-19) and to discuss its implications.

Design.: Retrospective cohort study.

Setting.: ICU of an academic county hospital in Los Angeles, CA admitted from March 15-June 20, 2020.

Patients.: 77 patients with COVID-19 pneumonia. 75 patients met inclusion criteria.

Results.: 21% of patients with severe COVID-19 sustained barotrauma (33% of patients receiving IMV, 8% of patients receiving (NIV). There were no differences between the barotrauma and non-barotrauma groups regarding demographics, illness severity, or medications received, nor tidal volume or average/peak airway pressures in those receiving IMV. In the barotrauma group there was a greater proportion of patients receiving therapeutic anticoagulation (81% vs. 47%, p = 0.023) and ventilated using airway pressure release ventilation mode (13% vs. 0%, p = 0.043). Barotrauma was associated with increased likelihood of receiving a tracheostomy (OR 2.58 [0.23-4.9], p = 0.018]), longer median ICU length of stay (17 days vs. 7 days, p = 0.03), and longer median length of hospitalization (26 days vs. 14 days, p < 0.001). There was also a trend toward prolonged median duration of IMV (12.5 days vs 7 days, p = 0.13) and higher average mortality (56% vs 37%, p = 0.25).

Conclusions.: Barotrauma is seen in 5-12% of patients with ARDS receiving IMV and is exceedingly rare in patients receiving NIV. We report a high incidence of barotrauma observed in critically ill patients with COVID-19 requiring either NIV or IMV. While there was a trend toward increased mortality in patients with barotrauma, this did not reach statistical significance. The increased incidence of barotrauma with COVID-19 may be a product of the pathophysiology of this disease state and a heightened inflammatory response causing rampant acute lung injury. Evidence-based medicine and lung-protective ventilation should remain the mainstay of treatment.

Keywords: COVID-19; adult; artificial; barotrauma; inflammation; physiology; respiration; respiratory distress syndrome.

Conflict of interest statement

Declaration of Conflicting Interests: The author(s) declared no potential conflicts of interest with respect to the research, authorship, and/or publication of this article.

- Cited by 6 articles
- 43 references
- 2 figures

Supplementary info

Publication types, MeSH terms Expand

Publication types

- Observational Study

MeSH terms

- Adult
- Aged
- Barotrauma / diagnosis
- Barotrauma / epidemiology*
- Barotrauma / therapy

- COVID-19 / complications*
- COVID-19 / mortality
- COVID-19 / therapy*
- California
- Critical Care*
- Critical Illness
- Female
- Hospitalization
- Humans
- Incidence
- Male
- Middle Aged
- Respiration, Artificial*
- Retrospective Studies
- Risk Factors
- Survival Rate

Full text links

SAGE journals Open access full text Atypon Free PMC article
Proceed to details
Cite
Share
533
Observational Study
N Engl J Med
. 2020 Jun 25;382(26):2534-2543.
doi: 10.1056/NEJMsa2011686. Epub 2020 May 27.

Hospitalization and Mortality among Black Patients and White Patients with Covid-19

Eboni G Price-Haywood [1], Jeffrey Burton [1], Daniel Fort [1], Leonardo Seoane [1]
Affiliations Expand

Affiliation

- [1] From the Ochsner Health Center for Outcomes and Health Services Research (E.G.P.-H., J.B., D.F.) and the University of Queensland Ochsner Clinical School (E.G.P.-H., L.S.) - both in New Orleans.

- PMID: **32459916**
- PMCID: PMC7269015

- DOI: 10.1056/NEJMsa2011686

Free PMC article
Observational Study

Hospitalization and Mortality among Black Patients and White Patients with Covid-19

Eboni G Price-Haywood et al. N Engl J Med. 2020.
Free PMC article
Show details
N Engl J Med
. 2020 Jun 25;382(26):2534-2543.
doi: 10.1056/NEJMsa2011686. Epub 2020 May 27.

Authors

Eboni G Price-Haywood [1], Jeffrey Burton [1], Daniel Fort [1], Leonardo Seoane [1]

Affiliation

- [1] From the Ochsner Health Center for Outcomes and Health Services Research (E.G.P.-H., J.B., D.F.) and the University of Queensland Ochsner Clinical School (E.G.P.-H., L.S.) - both in New Orleans.

- PMID: **32459916**
- PMCID: PMC7269015
- DOI: 10.1056/NEJMsa2011686

Abstract

Background: Many reports on coronavirus disease 2019 (Covid-19) have highlighted age- and sex-related differences in health outcomes. More information is needed about racial and ethnic differences in outcomes from Covid-19.

Methods: In this retrospective cohort study, we analyzed data from patients seen within an integrated-delivery health system (Ochsner Health) in Louisiana between March 1 and April 11, 2020, who tested positive for severe acute respiratory syndrome coronavirus 2 (SARS-CoV-2, the virus that causes Covid-19) on qualitative polymerase-chain-reaction assay. The Ochsner Health population is 31% black non-Hispanic and 65% white non-Hispanic. The primary outcomes were hospitalization and in-hospital death.

Results: A total of 3626 patients tested positive, of whom 145 were excluded (84 had missing data on race or ethnic group, 9 were Hispanic, and 52 were Asian or of another race or ethnic group). Of the 3481 Covid-19-positive patients included in our analyses, 60.0% were female, 70.4% were black non-Hispanic, and 29.6% were white non-Hispanic. Black patients had higher prevalences of obesity, diabetes, hypertension, and chronic kidney disease than white patients. A total of 39.7% of Covid-19-positive patients (1382 patients) were hospitalized, 76.9% of whom were black. In multivariable analyses, black race, increasing age, a higher score on the Charlson

Comorbidity Index (indicating a greater burden of illness), public insurance (Medicare or Medicaid), residence in a low-income area, and obesity were associated with increased odds of hospital admission. Among the 326 patients who died from Covid-19, 70.6% were black. In adjusted time-to-event analyses, variables that were associated with higher in-hospital mortality were increasing age and presentation with an elevated respiratory rate; elevated levels of venous lactate, creatinine, or procalcitonin; or low platelet or lymphocyte counts. However, black race was not independently associated with higher mortality (hazard ratio for death vs. white race, 0.89; 95% confidence interval, 0.68 to 1.17).

Conclusions: In a large cohort in Louisiana, 76.9% of the patients who were hospitalized with Covid-19 and 70.6% of those who died were black, whereas blacks comprise only 31% of the Ochsner Health population. Black race was not associated with higher in-hospital mortality than white race, after adjustment for differences in sociodemographic and clinical characteristics on admission.

Copyright © 2020 Massachusetts Medical Society.

- Cited by 709 articles
- 25 references

Supplementary info

Publication types, MeSH terms, Grant support Expand

Publication types

- Observational Study

MeSH terms

- Adult
- African Americans / statistics & numerical data*
- Aged
- Betacoronavirus
- COVID-19
- Comorbidity
- Coronavirus Infections / ethnology*
- Coronavirus Infections / mortality*
- Female
- Hospital Mortality
- Hospitalization
- Humans
- Louisiana
- Male
- Middle Aged
- Pandemics

- Pneumonia, Viral / ethnology*
- Pneumonia, Viral / mortality*
- Retrospective Studies
- SARS-CoV-2
- Socioeconomic Factors
- Whites / statistics & numerical data*

Grant support

- U54 GM104940/GM/NIGMS NIH HHS/United States

Full text links

NEJM FULL TEXT Atypon Free PMC article
Proceed to details
Cite
Share
534
Observational Study
Clin Nutr
. 2021 Feb;40(2):534-541.
doi: 10.1016/j.clnu.2020.05.051. Epub 2020 Jun 5.

The modified NUTRIC score can be used for nutritional risk assessment as well as prognosis prediction in critically ill COVID-19 patients

Ping Zhang [1], Zhigang He [2], Gang Yu [2], Dan Peng [2], Yikuan Feng [2], Jianmin Ling [2], Ye Wang [2], Shusheng Li [2], Yi Bian [3]
Affiliations Expand

Affiliations

- [1] Department of Neurology, Tongji Hospital, Tongji Medical College, Huazhong University of Science and Technology, Wuhan, China.
- [2] Department of Emergency, Intensive Care Unit, Tongji Hospital, Tongji Medical College, Huazhong University of Science and Technology, Wuhan, China.
- [3] Department of Emergency, Intensive Care Unit, Tongji Hospital, Tongji Medical College, Huazhong University of Science and Technology, Wuhan, China. Electronic address: bianyi2526@163.com.

- PMID: **32527576**
- PMCID: PMC7273137

- DOI: 10.1016/j.clnu.2020.05.051

Free PMC article
Observational Study

The modified NUTRIC score can be used for nutritional risk assessment as well as prognosis prediction in critically ill COVID-19 patients

Ping Zhang et al. Clin Nutr. 2021 Feb.
Free PMC article
Show details
Clin Nutr

. 2021 Feb;40(2):534-541.
doi: 10.1016/j.clnu.2020.05.051. Epub 2020 Jun 5.

Authors

Ping Zhang [1], Zhigang He [2], Gang Yu [2], Dan Peng [2], Yikuan Feng [2], Jianmin Ling [2], Ye Wang [2], Shusheng Li [2], Yi Bian [3]

Affiliations

- [1] Department of Neurology, Tongji Hospital, Tongji Medical College, Huazhong University of Science and Technology, Wuhan, China.
- [2] Department of Emergency, Intensive Care Unit, Tongji Hospital, Tongji Medical College, Huazhong University of Science and Technology, Wuhan, China.
- [3] Department of Emergency, Intensive Care Unit, Tongji Hospital, Tongji Medical College, Huazhong University of Science and Technology, Wuhan, China. Electronic address: bianyi2526@163.com.

- PMID: **32527576**
- PMCID: PMC7273137
- DOI: 10.1016/j.clnu.2020.05.051

Abstract

Background & aims: In the newly emerged Coronavirus Disease 2019 (COVID-19) disaster, little is known about the nutritional risks for critically ill patients. It is also unknown whether the modified Nutrition Risk in the Critically ill (mNUTRIC) score is applicable for nutritional risk assessment in intensive care unit (ICU) COVID-19 patients. We set out to investigate the applicability of the mNUTRIC score for assessing nutritional risks and predicting outcomes for these critically ill COVID-19 patients.

Methods: This retrospective observational study was conducted in three ICUs which had been specially established and equipped for COVID-19 in Wuhan, China. The study population was critically ill COVID-19 patients who had been admitted to these ICUs between January 28 and February 21, 2020. Exclusion criteria were as follows: 1) patients of <18 years; 2) patients who were pregnant; 3) length of ICU stay of <24 h; 4) insufficient medical information available. Patients' characteristics and clinical information were obtained from electronic medical and nursing records. The nutritional risk for each patient was assessed at their ICU admission using the mNUTRIC score. A score of ≥ 5 indicated high nutritional risk. Mortality was calculated according to patients' outcomes following 28 days of hospitalization in ICU.

Results: A total of 136 critically ill COVID-19 patients with a median age of 69 years (IQR: 57-77), 86 (63%) males and 50 (37%) females, were included in the study. Based on the mNUTRIC score at ICU admission, a high nutritional risk (≥5 points) was observed in 61% of the critically ill COVID-19 patients, while a low nutritional risk (<5 points) was observed in 39%. The mortality of ICU 28-day was significantly higher in the high nutritional risk group than in the low nutritional risk group (87% vs 49%, $P < 0.001$). Patients in the high nutritional risk group exhibited significantly higher incidences of acute respiratory distress syndrome, acute myocardial injury, secondary infection, shock and use of vasopressors. Additionally, use of a multivariate Cox analysis showed that patients with high nutritional risk had a higher probability of death at ICU 28-day than those with low nutritional risk (adjusted HR = 2.01, 95% CI: 1.22-3.32, P = 0.006).

Conclusions: A large proportion of critically ill COVID-19 patients had a high nutritional risk, as revealed by their mNUTRIC score. Patients with high nutritional risk at ICU admission exhibited significantly higher mortality of ICU 28-day, as well as twice the probability of death at ICU 28-day than those with low nutritional risk. Therefore, the mNUTRIC score may be an appropriate tool for nutritional risk assessment and prognosis prediction for critically ill COVID-19 patients.

Keywords: 28-Day mortality; Coronavirus disease 2019; Intensive care unit; Modified NUTRIC score; Nutritional risk.

Copyright © 2020 Elsevier Ltd and European Society for Clinical Nutrition and Metabolism. All rights reserved.

Conflict of interest statement

Conflict of interest The authors declare no conflicts of interest.

- Cited by 36 articles
- 32 references
- 2 figures

Supplementary info

Publication types, MeSH terms Expand

Publication types

- Observational Study
- Research Support, Non-U.S. Gov't

MeSH terms

- Aged
- COVID-19 / diagnosis*
- COVID-19 / mortality
- China
- Critical Illness
- Female
- Hospitalization
- Humans
- Intensive Care Units
- Male
- Middle Aged
- Nutrition Assessment*
- Nutritional Status*
- Nutritional Support
- Prognosis
- Retrospective Studies
- Risk Assessment
- Risk Factors

Full text links

ELSEVIER FULL-TEXT ARTICLE Elsevier Science Free PMC article
Proceed to details
Cite
Share
535
Observational Study
World Neurosurg
. 2021 Aug;152:e603-e609.
doi: 10.1016/j.wneu.2021.06.046. Epub 2021 Jun 16.

Does Coronavirus Disease 2019 (COVID-19) Affect Perioperative Morbidity and Mortality for Patients Requiring Emergency Instrumented Spinal Surgery? A Single-Center Cohort Study

Mathew Sewell [1], Fahid Rasul [2], Kathak Vachhani [3], Fady Sedra [2], Syed Aftab [2], Suresh Pushpananthan [2], Jonathan Bull [2], Arun Ranganathan [2], Alex Montgomery [2]
Affiliations Expand

Affiliations

- [1] Department of Spinal Surgery, Royal London Hospital, London, United Kingdom. Electronic address: mathew.sewell2@nhs.net.
- [2] Department of Spinal Surgery, Royal London Hospital, London, United Kingdom.
- [3] Faculty of Medicine, University of Toronto, Toronto, Canada.

- PMID: **34144165**
- PMCID: PMC8205544
- DOI: 10.1016/j.wneu.2021.06.046

Free PMC article
Observational Study

Does Coronavirus Disease 2019 (COVID-19) Affect Perioperative Morbidity and Mortality for Patients Requiring Emergency Instrumented Spinal Surgery? A Single-Center Cohort Study

Mathew Sewell et al. World Neurosurg. 2021 Aug.
Free PMC article
Show details
World Neurosurg
. 2021 Aug;152:e603-e609.
doi: 10.1016/j.wneu.2021.06.046. Epub 2021 Jun 16.

Authors

Mathew Sewell [1], Fahid Rasul [2], Kathak Vachhani [3], Fady Sedra [2], Syed Aftab [2], Suresh Pushpananthan [2], Jonathan Bull [2], Arun Ranganathan [2], Alex Montgomery [2]

Affiliations

- [1] Department of Spinal Surgery, Royal London Hospital, London, United Kingdom. Electronic address: mathew.sewell2@nhs.net.
- [2] Department of Spinal Surgery, Royal London Hospital, London, United Kingdom.
- [3] Faculty of Medicine, University of Toronto, Toronto, Canada.

- PMID: **34144165**
- PMCID: PMC8205544

- DOI: 10.1016/j.wneu.2021.06.046

Abstract

Background: The coronavirus disease 2019 (COVID-19) pandemic sent shockwaves through health services worldwide. Resources were reallocated. Patients with COVID-19 still required instrumented spinal surgery for emergencies. Clinical outcomes for these patients are not known. The objective of this study was to evaluate the effects of COVID-19 on perioperative morbidity and mortality for patients undergoing emergency instrumented spinal surgery and to determine risk factors for increased morbidity/mortality.

Methods: This retrospective cohort study included 11 patients who were negative for COVID-19 and 8 patients who were positive for COVID-19 who underwent emergency instrumented spinal surgery in 1 hospital in the United Kingdom during the pandemic peak. Data collection was performed through case note review. Patients in both treatment groups were comparable for age, sex, body mass index (BMI), comorbidities, surgical indication, and preoperative neurologic status. Predefined perioperative outcomes were recorded within a 30-day postoperative period. Univariable analysis was used to identify risk factors for increased morbidity.

Results: There were no mortalities in either treatment group. Four patients positive for COVID-19 (50%) developed a complication compared with 6 (55%) in the COVID-19-negative group ($P > 0.05$). The commonest complication in both groups was respiratory infection. Three patients positive for COVID-19 (37.5%) required intensive care unit admission, compared with 4 (36%) in the COVID-19-negative group ($P > 0.05$). The average time between surgery and discharge was 19 and 10 days in COVID-19-positive and -negative groups, respectively ($P = 0.02$). In the COVID-19 positive group, smoking, abnormal BMI, preoperative oxygen requirement, presence of fever, and oxygen saturations <95% correlated with increased risk of complications.

Conclusions: Emergency instrumented spinal surgery in patients positive for COVID-19 was associated with increased length of hospital stay. There was no difference in occurrence of complications or intensive care unit admission. Risk factors for increased morbidity in patients with COVID-19 included smoking, abnormal BMI, preoperative oxygen requirement, fever and saturations <95%.

Keywords: COVID-19; Clinical outcome; Cohort study; Emergency; Instrumented spine surgery (ISS); Major trauma center (MTC).

Copyright © 2021 Elsevier Inc. All rights reserved.

- 18 references

Supplementary info

Publication types, MeSH terms Expand

Publication types

- Observational Study

MeSH terms

- Adult
- Aged
- COVID-19 / complications*
- COVID-19 / mortality
- Cohort Studies
- Emergency Treatment / adverse effects
- Emergency Treatment / methods
- Female
- Humans
- Length of Stay
- Male
- Middle Aged
- Postoperative Complications / epidemiology
- Risk Factors
- SARS-CoV-2
- Spinal Fusion* / adverse effects
- Spinal Fusion* / mortality
- Spinal Injuries / surgery*
- Spinal Injuries / virology*
- Treatment Outcome
- United Kingdom

Full text links

ELSEVIER FULL-TEXT ARTICLE Elsevier Science Free PMC article

Proceed to details

Cite

Share

536

Observational Study

Ther Adv Respir Dis

. Jan-Dec 2022;16:17534666221081035.

doi: 10.1177/17534666221081035.

Lung transplantation for severe COVID-19-related ARDS

Ryoung-Eun Ko [1], Dong Kyu Oh [2], Sun Mi Choi [3], Sunghoon Park [4], Ji Eun Park [5], Jin Gu Lee [6], Young Tae Kim [7], Kyeongman Jeon [8]

Affiliations Expand

Affiliations

- [1] Department of Critical Care Medicine, Samsung Medical Center, Sungkyunkwan University School of Medicine, Seoul, South Korea.
- [2] Department of Pulmonary and Critical Care Medicine, Asan Medical Center, University of Ulsan College of Medicine, Seoul, South Korea.
- [3] Division of Pulmonary and Critical Care Medicine, Department of Internal Medicine, Seoul National University Hospital, Seoul National University College of Medicine, Seoul, South Korea.
- [4] Department of Pulmonary, Allergy and Critical Care Medicine, Hallym University Sacred Heart Hospital, Anyang, South Korea.
- [5] Department of Pulmonary and Critical Care Medicine, Ajou University School of Medicine, Suwon, South Korea.
- [6] Department of Thoracic and Cardiovascular Surgery, Severance Hospital, Yonsei University College of Medicine, Seoul, South Korea.
- [7] Department of Thoracic and Cardiovascular Surgery, Seoul National University Hospital, Seoul National University College of Medicine, Seoul, South Korea.
- [8] Division of Pulmonary and Critical Care Medicine, Department of Medicine, Samsung Medical Center, Sungkyunkwan University School of Medicine, 81 Irwon-ro, Gangnam-gu, Seoul 06351, South Korea.

- PMID: **35253546**
- PMCID: PMC8902188
- DOI: 10.1177/17534666221081035

Free PMC article
Observational Study

Lung transplantation for severe COVID-19-related ARDS

Ryoung-Eun Ko et al. Ther Adv Respir Dis. Jan-Dec 2022.
Free PMC article

Show details

Ther Adv Respir Dis

. Jan-Dec 2022;16:17534666221081035.
doi: 10.1177/17534666221081035.

Authors

Ryoung-Eun Ko [1], Dong Kyu Oh [2], Sun Mi Choi [3], Sunghoon Park [4], Ji Eun Park [5], Jin Gu Lee [6], Young Tae Kim [7], Kyeongman Jeon [8]

Affiliations

- [1] Department of Critical Care Medicine, Samsung Medical Center, Sungkyunkwan University School of Medicine, Seoul, South Korea.

- [2] Department of Pulmonary and Critical Care Medicine, Asan Medical Center, University of Ulsan College of Medicine, Seoul, South Korea.
- [3] Division of Pulmonary and Critical Care Medicine, Department of Internal Medicine, Seoul National University Hospital, Seoul National University College of Medicine, Seoul, South Korea.
- [4] Department of Pulmonary, Allergy and Critical Care Medicine, Hallym University Sacred Heart Hospital, Anyang, South Korea.
- [5] Department of Pulmonary and Critical Care Medicine, Ajou University School of Medicine, Suwon, South Korea.
- [6] Department of Thoracic and Cardiovascular Surgery, Severance Hospital, Yonsei University College of Medicine, Seoul, South Korea.
- [7] Department of Thoracic and Cardiovascular Surgery, Seoul National University Hospital, Seoul National University College of Medicine, Seoul, South Korea.
- [8] Division of Pulmonary and Critical Care Medicine, Department of Medicine, Samsung Medical Center, Sungkyunkwan University School of Medicine, 81 Irwon-ro, Gangnam-gu, Seoul 06351, South Korea.

- PMID: **35253546**
- PMCID: PMC8902188
- DOI: 10.1177/17534666221081035

Abstract

Background: Lung transplantation (LT) is the gold standard for various end-stage chronic lung diseases and could be a salvage therapeutic option in acute respiratory distress syndrome (ARDS). However, LT is uncertain in patients with coronavirus disease 2019 (COVID-19)-related ARDS who failed to recover despite optimal management including extracorporeal membrane oxygenation (ECMO). This study aims to describe the pooled experience of LT for patients with severe COVID-19-related ARDS in Korea.

Methods: A nationwide multicenter retrospective observational study was performed with consecutive LT for severe COVID-19-related ARDS in South Korea (June 2020-June 2021). Data were collected and compared with other LTs after bridging with ECMO from the Korean Organ Transplantation Registry.

Results: Eleven patients with COVID-19-related ARDS underwent LT. The median age was 60.0 years [interquartile range (IQR), 57.5-62.5; six males]. All patients were supported with venovenous ECMO at LT listing and received rehabilitation before LT. Patients were transplanted at a median of 49 (IQR, 32-66) days after ECMO cannulation. Primary graft dysfunction within 72 h of LT developed in two (18.2%). One patient expired 4 days after LT due to sepsis and one patient underwent retransplantation for graft failure. After a median follow-up of 322 (IQR, 299-397) days, 10 patients are alive and recovering well. Compared with other LTs after bridging with ECMO ($n = 27$), post-transplant outcomes were similar between the two groups.

Conclusions: LT in patients with unresolving COVID-19-related ARDS were effective with reasonable short-term outcome.

Keywords: COVID-19; extracorporeal membrane oxygenations; frailty; lung transplantation; treatment outcome.

Conflict of interest statement

Conflict of interest statement: The authors declared no potential conflicts of interest with respect to the research, authorship, and/or publication of this article.

- 31 references
- 1 figure

Supplementary info

Publication types, MeSH terms Expand

Publication types

- Multicenter Study
- Observational Study
- Research Support, Non-U.S. Gov't

MeSH terms

- COVID-19*
- Extracorporeal Membrane Oxygenation*
- Humans
- Lung Transplantation* / adverse effects
- Male
- Middle Aged
- Respiratory Distress Syndrome* / etiology
- Respiratory Distress Syndrome* / therapy
- Retrospective Studies
- SARS-CoV-2

Full text links

SAGE journals Open access full text Atypon Free PMC article

Proceed to details

Cite

Share

☐ 537

Observational Study

Am J Cardiol

. 2020 Dec 1;136:149-155.
doi: 10.1016/j.amjcard.2020.09.012. Epub 2020 Sep 16.

Relation of Statin Use Prior to Admission to Severity and Recovery Among COVID-19 Inpatients

Lori B Daniels [1], Amy M Sitapati [2], Jing Zhang [3], Jingjing Zou [4], Quan M Bui [5], Junting Ren [4], Christopher A Longhurst [2], Michael H Criqui [6], Karen Messer [7]
Affiliations Expand

Affiliations

- [1] Division of Cardiovascular Medicine, Department of Medicine, University of California San Diego, La Jolla, California; Division of Epidemiology, Department of Family Medicine and Public Health, University of California San Diego, La Jolla, California. Electronic address: lbdaniels@health.ucsd.edu.
- [2] Department of Medicine, University of California San Diego, La Jolla, California.
- [3] University of California San Diego Health Moores Cancer Center, University of California San Diego, La Jolla, California.
- [4] Division of Biostatistics and Bioinformatics, Department of Family Medicine and Public Health, University of California San Diego, La Jolla, California.
- [5] Division of Cardiovascular Medicine, Department of Medicine, University of California San Diego, La Jolla, California.
- [6] Division of Cardiovascular Medicine, Department of Medicine, University of California San Diego, La Jolla, California; Division of Epidemiology, Department of Family Medicine and Public Health, University of California San Diego, La Jolla, California.
- [7] University of California San Diego Health Moores Cancer Center, University of California San Diego, La Jolla, California; Division of Biostatistics and Bioinformatics, Department of Family Medicine and Public Health, University of California San Diego, La Jolla, California.

- PMID: **32946859**
- PMCID: PMC7492151
- DOI: 10.1016/j.amjcard.2020.09.012

Free PMC article
Observational Study

Relation of Statin Use Prior to Admission to Severity and Recovery Among COVID-19 Inpatients

Lori B Daniels et al. Am J Cardiol. 2020.
Free PMC article
Show details
Am J Cardiol

. 2020 Dec 1;136:149-155.
doi: 10.1016/j.amjcard.2020.09.012. Epub 2020 Sep 16.

Authors

Lori B Daniels [1], Amy M Sitapati [2], Jing Zhang [3], Jingjing Zou [4], Quan M Bui [5], Junting Ren [4], Christopher A Longhurst [2], Michael H Criqui [6], Karen Messer [7]

Affiliations

- [1] Division of Cardiovascular Medicine, Department of Medicine, University of California San Diego, La Jolla, California; Division of Epidemiology, Department of Family Medicine and Public Health, University of California San Diego, La Jolla, California. Electronic address: lbdaniels@health.ucsd.edu.
- [2] Department of Medicine, University of California San Diego, La Jolla, California.
- [3] University of California San Diego Health Moores Cancer Center, University of California San Diego, La Jolla, California.
- [4] Division of Biostatistics and Bioinformatics, Department of Family Medicine and Public Health, University of California San Diego, La Jolla, California.
- [5] Division of Cardiovascular Medicine, Department of Medicine, University of California San Diego, La Jolla, California.
- [6] Division of Cardiovascular Medicine, Department of Medicine, University of California San Diego, La Jolla, California; Division of Epidemiology, Department of Family Medicine and Public Health, University of California San Diego, La Jolla, California.
- [7] University of California San Diego Health Moores Cancer Center, University of California San Diego, La Jolla, California; Division of Biostatistics and Bioinformatics, Department of Family Medicine and Public Health, University of California San Diego, La Jolla, California.

- PMID: **32946859**
- PMCID: PMC7492151
- DOI: 10.1016/j.amjcard.2020.09.012

Abstract

The impact of statins, angiotensin-converting enzyme inhibitors and angiotensin II receptor blockers (ARBs) on coronavirus disease 2019 (COVID-19) severity and recovery is important given their high prevalence of use among individuals at risk for severe COVID-19. We studied the association between use of statin/angiotensin-converting enzyme inhibitors/ARB in the month before hospital admission, with risk of severe outcome, and with time to severe outcome or disease recovery, among patients hospitalized for COVID-19. We performed a retrospective single-center study of all patients hospitalized at University of California San Diego Health between February 10, 2020 and June 17, 2020 (n = 170 hospitalized for COVID-19, n = 5,281 COVID-negative controls). Logistic regression and competing risks analyses were used to investigate progression to severe disease (death or intensive care unit admission), and time to discharge without severe disease. Severe disease occurred in 53% of COVID-positive inpatients. Median time from hospitalization to severe disease was 2 days; median time to recovery was 7 days. Statin use prior to admission was associated with reduced risk of severe COVID-19 (adjusted OR 0.29, 95%CI 0.11 to 0.71, $p < 0.01$) and faster time to recovery among those without severe disease (adjusted HR for recovery 2.69, 95%CI 1.36 to 5.33, $p < 0.01$). The association

between statin use and severe disease was smaller in the COVID-negative cohort (p for interaction = 0.07). There was potential evidence of faster time to recovery with ARB use (adjusted HR 1.92, 95%CI 0.81 to 4.56). In conclusion, statin use during the 30 days prior to admission for COVID-19 was associated with a lower risk of developing severe COVID-19, and a faster time to recovery among patients without severe disease.

Copyright © 2020 The Author(s). Published by Elsevier Inc. All rights reserved.

- Cited by 71 articles
- 30 references
- 2 figures

Supplementary info

Publication types, MeSH terms, Substances Expand

Publication types

- Observational Study
- Research Support, Non-U.S. Gov't

MeSH terms

- Adult
- Aged
- Angiotensin Receptor Antagonists / therapeutic use*
- Angiotensin-Converting Enzyme Inhibitors / therapeutic use*
- Betacoronavirus*
- COVID-19
- Coronavirus Infections / diagnosis
- Coronavirus Infections / epidemiology*
- Coronavirus Infections / therapy
- Critical Care
- Female
- Hospitalization
- Humans
- Hydroxymethylglutaryl-CoA Reductase Inhibitors / therapeutic use*
- Male
- Middle Aged
- Pandemics
- Pneumonia, Viral / diagnosis
- Pneumonia, Viral / epidemiology*
- Pneumonia, Viral / therapy
- Recovery of Function
- Retrospective Studies

- Risk Factors
- SARS-CoV-2
- Severity of Illness Index

Substances

- Angiotensin Receptor Antagonists
- Angiotensin-Converting Enzyme Inhibitors
- Hydroxymethylglutaryl-CoA Reductase Inhibitors

Full text links

ELSEVIER OPEN ACCESS Elsevier Science Free PMC article

Proceed to details

Cite

Share

☐ 538

Observational Study

J Infect Dev Ctries
. 2021 Jun 30;15(6):766-772.
doi: 10.3855/jidc.14072.

Diagnostic and early prognostic value of serum CRP and LDH levels in patients with possible COVID-19 at the first admission

Dogan Akdogan [1], Mustafa Guzel [2], Dervis Tosun [3], Orhan Akpinar [4]

Affiliations Expand

Affiliations

- [1] Pursaklar State Hospital Department of Medical Microbiology, Ankara, Turkey.
- [2] Maltepe Medical Center Department of Medical Microbiology, Istanbul, Turkey.
- [3] Pursaklar State Hospital Department of Infectious Diseases, Ankara, Turkey.
- [4] Department of Medical Microbiology, Health Sciences Institute, University of Süleyman Demirel, Isparta, Turkey. orhanakpnr@hotmail.com.

- PMID: **34242184**
- DOI: 10.3855/jidc.14072

Free article
Observational Study

Diagnostic and early prognostic value of serum CRP and LDH levels in patients with possible COVID-19 at the first admission

Dogan Akdogan et al. J Infect Dev Ctries. 2021.
Free article
Show details
J Infect Dev Ctries
. 2021 Jun 30;15(6):766-772.
doi: 10.3855/jidc.14072.

Authors

Dogan Akdogan [1], Mustafa Guzel [2], Dervis Tosun [3], Orhan Akpinar [4]

Affiliations

- [1] Pursaklar State Hospital Department of Medical Microbiology, Ankara, Turkey.
- [2] Maltepe Medical Center Department of Medical Microbiology, Istanbul, Turkey.
- [3] Pursaklar State Hospital Department of Infectious Diseases, Ankara, Turkey.
- [4] Department of Medical Microbiology, Health Sciences Institute, University of Süleyman Demirel, Isparta, Turkey. orhanakpnr@hotmail.com.

- PMID: **34242184**
- DOI: 10.3855/jidc.14072

Abstract

Introduction: COVID-19 is the infection caused by the new coronavirus. Specific treatment for COVID-19 has not been established, yet. It is important to determine the disease severity of the patients at the first admission. Therefore, the exploration of biomarkers is deemed necessary. We aimed to assess the diagnostic and early prognostic value of CRP and LDH levels in possible COVID-19 patients presenting with a severe clinical picture.

Methodology: We evaluated the correlations of relevant routine laboratory test results with disease severity in COVID-19 patients admitted to our infectious diseases clinic. Patients were divided into severe and non-severe disease groups based on clinical findings, oxygen saturation levels in the arterial blood, biochemical test results, and radiological findings. Differences in the findings between the two disease severity groups were examined to determine potential biomarkers.

Results: Median age and the CRP and LDH levels in the severe disease group were statistically significantly higher compared to the nonsevere group ($p < 0.0001$). No other parameters statistically significant differences have been observed between the two groups ($P > 0.05$).

Conclusions: CRP and LDH levels were positively correlated with lung lesions in early-stage COVID-19, potentially reflecting disease severity. Because LDH and CRP levels can potentially reflect the pulmonary function, they can be potential predictors of COVID-19- related respiratory

failure. For avoiding poor prognosis; LDH and CRP should be considered as potential predictors for identifying the need for thoracic CT scans, close monitoring of pulmonary function, and aggressive supportive therapy early in the course of COVID-19.

Keywords: C-reactive protein; COVID-19; SARS-CoV-2; laboratory findings; lactate dehydrogenase.

Copyright (c) 2021 Dogan Akdogan, Mustafa Guzel, Dervis Tosun, Orhan Akpinar.

Conflict of interest statement

No Conflict of Interest is declared

- Cited by 2 articles

Supplementary info

Publication types, MeSH terms, Substances Expand

Publication types

- Observational Study

MeSH terms

- Adult
- Biomarkers / blood
- C-Reactive Protein / analysis*
- COVID-19 / blood*
- COVID-19 / classification
- COVID-19 / diagnosis*
- Female
- Hospitalization
- Humans
- L-Lactate Dehydrogenase / blood*
- Lung / pathology
- Lung / virology
- Male
- Middle Aged
- Prognosis
- Retrospective Studies
- Risk Factors
- Severity of Illness Index*
- Turkey

Substances

- Biomarkers
- C-Reactive Protein
- L-Lactate Dehydrogenase

Full text links

The Journal of Infection in Developing Countries
Proceed to details
Cite
Share
539
Observational Study
PLoS One
. 2021 Jun 23;16(6):e0253465.
doi: 10.1371/journal.pone.0253465. eCollection 2021.

COVID-19 pneumonia in Galicia (Spain): Impact of prognostic factors and therapies on mortality and need for mechanical ventilation

Luis Pérez-de-Llano [1], Eva María Romay-Lema [2], Adolfo Baloira-Villar [3], Christian Anchorena [3], María Luisa Torres-Durán [4], Adrián Sousa [5], Dolores Corbacho-Abelaira [6], José Paz-Ferrin [7], Carmen Diego-Roza [8], Laura Vilariño-Maneiro [9], Pedro J Marcos [10], Carmen Montero-Martínez [10], Fernando de la Iglesia-Martínez [11], Vanessa Riveiro-Blanco [12], Nuria Rodríguez-Núñez [12], José Abal-Arca [13], María Bustillo-Casado [14], Rafael Golpe [1]
Affiliations Expand

Affiliations

- [1] Pneumology Service, Lucus Augusti University Hospital, EOXI Lugo, Monforte, Cervo, Lugo, Spain.
- [2] Infectious Diseases Unit, Lucus Augusti University Hospital, EOXI Lugo, Monforte, Cervo, Lugo, Spain.
- [3] Pneumology Service, Complexo Hospitalario Universitario, Pontevedra, Spain.
- [4] Pneumology Service, Complexo Hospitalario Universitario de Vigo, Vigo, Spain.
- [5] Internal Medicine Service, Complexo Hospitalario Universitario de Vigo, Vigo, Spain.
- [6] Pneumology Service, Hospital POVISA, Vigo, Spain.
- [7] Internal Medicine Service, Hospital POVISA, Vigo, Spain.
- [8] Pneumology Service, Hospital Arquitecto Marcide, Ferrol, Spain.
- [9] Internal Medicine Service, Hospital Arquitecto Marcide, Ferrol, Spain.
- [10] Pneumology Service, Complexo Universitario de A Coruña, A Coruña, Spain.

- [11] Internal Medicine Service, Complexo Universitario de A Coruña, A Coruña, Spain.
- [12] Pneumology Service, Complexo Hospitalario Universitario de Santiago, Santiago de Compostela, Spain.
- [13] Pneumology Service, Complexo Hospitalario Universitario de Ourense, Ourense, Spain.
- [14] Infectious Diseases Unit, Complexo Hospitalario Universitario de Ourense, Ourense, Spain.

- PMID: **34161387**
- PMCID: PMC8221482
- DOI: 10.1371/journal.pone.0253465

Free PMC article
Observational Study

COVID-19 pneumonia in Galicia (Spain): Impact of prognostic factors and therapies on mortality and need for mechanical ventilation

Luis Pérez-de-Llano et al. PLoS One. 2021.
Free PMC article

Show details

PLoS One
. 2021 Jun 23;16(6):e0253465.
doi: 10.1371/journal.pone.0253465. eCollection 2021.

Authors

Luis Pérez-de-Llano [1], Eva María Romay-Lema [2], Adolfo Baloira-Villar [3], Christian Anchorena [3], María Luisa Torres-Durán [4], Adrián Sousa [5], Dolores Corbacho-Abelaira [6], José Paz-Ferrin [7], Carmen Diego-Roza [8], Laura Vilariño-Maneiro [9], Pedro J Marcos [10], Carmen Montero-Martínez [10], Fernando de la Iglesia-Martínez [11], Vanessa Riveiro-Blanco [12], Nuria Rodríguez-Núñez [12], José Abal-Arca [13], María Bustillo-Casado [14], Rafael Golpe [1]

Affiliations

- [1] Pneumology Service, Lucus Augusti University Hospital, EOXI Lugo, Monforte, Cervo, Lugo, Spain.
- [2] Infectious Diseases Unit, Lucus Augusti University Hospital, EOXI Lugo, Monforte, Cervo, Lugo, Spain.
- [3] Pneumology Service, Complexo Hospitalario Universitario, Pontevedra, Spain.
- [4] Pneumology Service, Complexo Hospitalario Universitario de Vigo, Vigo, Spain.
- [5] Internal Medicine Service, Complexo Hospitalario Universitario de Vigo, Vigo, Spain.
- [6] Pneumology Service, Hospital POVISA, Vigo, Spain.
- [7] Internal Medicine Service, Hospital POVISA, Vigo, Spain.
- [8] Pneumology Service, Hospital Arquitecto Marcide, Ferrol, Spain.
- [9] Internal Medicine Service, Hospital Arquitecto Marcide, Ferrol, Spain.

- [10] Pneumology Service, Complexo Universitario de A Coruña, A Coruña, Spain.
- [11] Internal Medicine Service, Complexo Universitario de A Coruña, A Coruña, Spain.
- [12] Pneumology Service, Complexo Hospitalario Universitario de Santiago, Santiago de Compostela, Spain.
- [13] Pneumology Service, Complexo Hospitalario Universitario de Ourense, Ourense, Spain.
- [14] Infectious Diseases Unit, Complexo Hospitalario Universitario de Ourense, Ourense, Spain.

- PMID: **34161387**
- PMCID: PMC8221482
- DOI: 10.1371/journal.pone.0253465

Abstract

Introduction: This study was aimed to identify risk factors associated with unfavorable outcomes (composite outcome variable: mortality and need for mechanical ventilation) in patients hospitalized in Galicia with COVID-19 pneumonia.

Methods: Retrospective, multicenter, observational study carried out in the 8 Galician tertiary hospitals. All Patients admitted with confirmed COVID-19 pneumonia from 1st of March to April 24th, 2020 were included. A multivariable logistic regression analysis was performed in order to identify the relationship between risk factors, therapeutic interventions and the composite outcome variable.

Results: A total of 1292 patients (56.1% male) were included. Two hundred and twenty-five (17.4%) died and 327 (25.3%) reached the main outcome variable. Age [odds ratio (OR) = 1.03 (95% confidence interval (CI): 1.01-1.04)], CRP quartiles 3 and 4 [OR = 2.24 (95% CI: 1.39-3.63)] and [OR = 3.04 (95% CI: 1.88-4.92)], respectively, Charlson index [OR = 1.16 (95% CI: 1.06-1.26)], SaO2 upon admission [OR = 0.93 (95% CI: 0.91-0.95)], hydroxychloroquine prescription [OR = 0.22 (95%CI: 0.12-0.37)], systemic corticosteroids prescription [OR = 1.99 (95%CI: 1.45-2.75)], and tocilizumab prescription [OR = 3.39 (95%CI: 2.15-5.36)], significantly impacted the outcome. Sensitivity analysis using different alternative logistic regression models identified consistently the ratio admissions/hospital beds as a predictor of the outcome [OR = 1.06 (95% CI: 1.02-1.11)].

Conclusion: These findings may help to identify patients at hospital admission with a higher risk of death and may urge healthcare authorities to implement policies aimed at reducing deaths by increasing the availability of hospital beds.

Conflict of interest statement

The authors have declared that no competing interests exist.

- Cited by 1 article
- 61 references

Supplementary info

Publication types, MeSH terms, Substances, Grant support Expand

Publication types

- Multicenter Study
- Observational Study
- Research Support, Non-U.S. Gov't

MeSH terms

- Adrenal Cortex Hormones / therapeutic use
- Aged
- Aged, 80 and over
- Antiviral Agents / therapeutic use*
- COVID-19 / epidemiology
- COVID-19 / mortality*
- COVID-19 / therapy*
- Comorbidity
- Female
- Hospitals / statistics & numerical data
- Humans
- Hydroxychloroquine / therapeutic use
- Male
- Middle Aged
- Respiration, Artificial
- Retrospective Studies
- Risk Factors
- Spain / epidemiology
- Treatment Outcome

Substances

- Adrenal Cortex Hormones
- Antiviral Agents
- Hydroxychloroquine

Grant support

This project was supported by an unconditional grant from AstraZeneca, with no role in the analysis, decision to publish or preparation of the manuscript. The design, analysis, and writing of this report are entirely the work and responsibility of the authors, and Dr. Pérez de Llano had full access to all data and final responsibility for the decision to submit this work for publication.

Full text links

OPEN ACCESS TO FULL TEXT PLOS ONE Public Library of Science Free PMC article
Proceed to details
Cite
Share
540

Observational Study

Anaesth Crit Care Pain Med
. 2021 Oct;40(5):100937.
doi: 10.1016/j.accpm.2021.100937. Epub 2021 Aug 12.

Clinical, obstetrical and anaesthesia outcomes in pregnant women during the first COVID-19 surge in France: A prospective multicentre observational cohort study

Hawa Keita [1], Arthur James [2], Lionel Bouvet [3], Emilie Herrmann [4], Agnès Le Gouez [5], Jean-Xavier Mazoit [6], Frédéric-Jean Mercier [5], Dan Benhamou [6], Obstetric Anaesthesia COVID-19 Collaboration Network
Affiliations Expand

Affiliations

- [1] Assistance Publique des Hôpitaux Paris, Hôpital Necker-Enfants-Malades, Service d'Anesthésie-Réanimation, AP-HP, Centre - Université de Paris, Paris, France; Unité de Recherche EA 7323 Pharmacologie et Évaluation des Thérapeutiques Chez l'Enfant et la Femme Enceinte, Université de Paris, Paris, France. Electronic address: hawa.keita@aphp.fr.
- [2] Sorbonne Université, GRC 29, AP-HP, DMU DREAM, Département d'Anesthésie-Réanimation, Hôpital Pitié-Salpêtrière, Paris, France.
- [3] Service d'Anesthésie-Réanimation, Hospices Civils de Lyon, Groupement Hospitalier Est, Hôpital Femme-Mère-Enfant, 59, Boulevard Pinel, 69500 Bron, France.
- [4] Service d'Anesthésie Réanimation, Hôpital de Hautepierre, 1 Avenue Molière, 67200 Strasbourg, France.
- [5] Département d'Anesthésie-Réanimation, Hôpital Antoine-Béclère - APHP, Université Paris-Saclay, 157, rue de la Porte de Trivaux, 92140 Clamart, France.
- [6] Département d'Anesthésie-Réanimation, Hôpital Bicêtre, Université Paris-Saclay, 48 rue du Général Leclerc, 94275, Le Kremlin-Bicêtre, France.

- PMID: **34391984**
- PMCID: PMC8359490
- DOI: 10.1016/j.accpm.2021.100937

Free PMC article
Observational Study

Clinical, obstetrical and anaesthesia outcomes in pregnant women during the first COVID-19 surge in France: A prospective multicentre observational cohort study

Hawa Keita et al. Anaesth Crit Care Pain Med. 2021 Oct.
Free PMC article
Show details
Anaesth Crit Care Pain Med
. 2021 Oct;40(5):100937.
doi: 10.1016/j.accpm.2021.100937. Epub 2021 Aug 12.

Authors

Hawa Keita [1], Arthur James [2], Lionel Bouvet [3], Emilie Herrmann [4], Agnès Le Gouez [5], Jean-Xavier Mazoit [6], Frédéric-Jean Mercier [5], Dan Benhamou [6], Obstetric Anaesthesia COVID-19 Collaboration Network

Affiliations

- [1] Assistance Publique des Hôpitaux Paris, Hôpital Necker-Enfants-Malades, Service d'Anesthésie-Réanimation, AP-HP, Centre - Université de Paris, Paris, France; Unité de Recherche EA 7323 Pharmacologie et Évaluation des Thérapeutiques Chez l'Enfant et la Femme Enceinte, Université de Paris, Paris, France. Electronic address: hawa.keita@aphp.fr.
- [2] Sorbonne Université, GRC 29, AP-HP, DMU DREAM, Département d'Anesthésie-Réanimation, Hôpital Pitié-Salpêtrière, Paris, France.
- [3] Service d'Anesthésie-Réanimation, Hospices Civils de Lyon, Groupement Hospitalier Est, Hôpital Femme-Mère-Enfant, 59, Boulevard Pinel, 69500 Bron, France.
- [4] Service d'Anesthésie Réanimation, Hôpital de Hautepierre, 1 Avenue Molière, 67200 Strasbourg, France.
- [5] Département d'Anesthésie-Réanimation, Hôpital Antoine-Béclère - APHP, Université Paris-Saclay, 157, rue de la Porte de Trivaux, 92140 Clamart, France.
- [6] Département d'Anesthésie-Réanimation, Hôpital Bicêtre, Université Paris-Saclay, 48 rue du Général Leclerc, 94275, Le Kremlin-Bicêtre, France.

- PMID: **34391984**
- PMCID: PMC8359490
- DOI: 10.1016/j.accpm.2021.100937

Abstract

Introduction: Clinical outcomes and critical care utilisation associated with Coronavirus Disease 2019 (COVID-19) in obstetric patients remain limited particularly in relation to severe cases.

Methods: A retrospective multicentre cohort study was conducted during the first wave of COVID-19 in France in 18 tertiary referral maternity units. Consecutive women with confirmed or suspected COVID-19 during pregnancy or the delivery hospitalisation were included between March and July 2020 (17-week period). We report clinical, obstetrical and anaesthetic outcomes of pregnant women with COVID-19 and report the prevalence of severe forms and risk factors for respiratory support in this cohort.

Results: There were 126 included cases; RT-PCR testing occurred in 82 cases, of which 64 (78%) had a positive test. The caesarean section rate was 52%, and preterm delivery (< 37 weeks) rate was 40%. Neuraxial anaesthesia was performed in 108 (86%) cases with an increasing proportion compared to general anaesthesia over time ($p < 0.0002$). Twenty-eight cases received oxygen supplementation (nasal oxygen therapy or mechanical ventilation); the SOFAresp score was associated with gestational age at the time of COVID-19 presentation ($p = 0.0036$) and at delivery ($p < 0.0001$). Postpartum intensive care unit (ICU) admission occurred in 21 cases (17%) with 17 (13%) receiving invasive or non-invasive ventilation. Pre-delivery factors associated with postpartum ventilation were oxygen support, oxygen saturation and haemoglobin levels.

Conclusion: In our cohort, COVID-19 was associated with significant maternal morbidity resulting in high ICU admission rates (17%) and invasive or non-invasive ventilation utilisation (10%).

Keywords: COVID-19; Caesarean section; Critical care; Labour; Pregnancy; SARS-CoV-2; SOFAresp; Ventilation.

Copyright © 2021 Société française d'anesthésie et de réanimation (Sfar). Published by Elsevier Masson SAS. All rights reserved.

- Cited by 2 articles
- 31 references
- 3 figures

Supplementary info

Publication types, MeSH terms Expand

Publication types

- Multicenter Study
- Observational Study

MeSH terms

- Anesthesia*
- COVID-19*
- Cesarean Section
- Cohort Studies
- Female
- Humans
- Infant, Newborn

- Pregnancy
- Pregnancy Complications, Infectious*
- Pregnant Women
- Prospective Studies
- Retrospective Studies
- SARS-CoV-2

Full text links

ELSEVIER FULL-TEXT ARTICLE Elsevier Science Free PMC article
Proceed to details
Cite
Share
541
Observational Study
Medicine (Baltimore)
. 2021 Jul 16;100(28):e26538.
doi: 10.1097/MD.0000000000026538.

Corrected QT interval in hospitalized patients with coronavirus disease 2019: Focus on drugs therapy

Jiaxing Ding [1], Wei Liu, Hongquan Guan, Yu Feng, Yintu Bao, Huili Li, Xuehua Wang, Zihua Zhou, Zhijian Chen
Affiliations Expand

Affiliation

- [1] Department of Cardiology, Union Hospital, Tongji Medical College, Huazhong University of Science and Technology, Wuhan, China.

- PMID: **34260531**
- PMCID: PMC8284736
- DOI: 10.1097/MD.0000000000026538

Free PMC article
Observational Study

Corrected QT interval in hospitalized patients with coronavirus disease 2019: Focus on drugs therapy

Jiaxing Ding et al. Medicine (Baltimore). 2021.
Free PMC article
Show details
Medicine (Baltimore)
. 2021 Jul 16;100(28):e26538.
doi: 10.1097/MD.0000000000026538.

Authors

Jiaxing Ding [1], Wei Liu, Hongquan Guan, Yu Feng, Yintu Bao, Huili Li, Xuehua Wang, Zihua Zhou, Zhijian Chen

Affiliation

- [1] Department of Cardiology, Union Hospital, Tongji Medical College, Huazhong University of Science and Technology, Wuhan, China.

- PMID: **34260531**
- PMCID: PMC8284736
- DOI: 10.1097/MD.0000000000026538

Abstract

Corrected QT (QTc) interval prolongation has been associated with poor patient prognosis. In this study, we assessed the effects of different drugs and cardiac injury on QTc interval prolongation in patients with coronavirus disease 2019 (COVID-19).The study cohort consisted of 395 confirmed COVID-19 cases from the Wuhan Union Hospital West Campus. All hospitalized patients were treated with chloroquine/hydroxychloroquine (CQ/HCQ), lopinavir/ritonavir (LPV/r), quinolones, interferon, Arbidol, or Qingfei Paidu decoction (QPD) and received at least 1 electrocardiogram after drug administration.Fifty one (12.9%) patients exhibited QTc prolongation (QTc ≥ 470 ms). QTc interval prolongation was associated with COVID-19 severity and mortality (both P < .001). Administration of CQ/HCQ (odds ratio [OR], 2.759; 95% confidence interval [CI], 1.318-5.775; P = .007), LPV/r (OR, 2.342; 95% CI, 1.152-4.760; P = .019), and quinolones (OR, 2.268; 95% CI, 1.171-4.392; P = .015) increased the risk of QTc prolongation. In contrast, the administration of Arbidol, interferon, or QPD did not increase the risk of QTc prolongation. Notably, patients treated with QPD had a shorter QTc duration than those without QPD treatment (412.10 [384.39-433.77] vs 420.86 [388.19-459.58]; P = .042). The QTc interval was positively correlated with the levels of cardiac biomarkers (creatine kinase-MB fraction [rho = 0.14, P = .016], high-sensitivity troponin I [rho = .22, P < .001], and B-type natriuretic peptide [rho = 0.27, P < .001]).In conclusion, QTc prolongation was associated with COVID-19 severity and mortality. The risk of QTc prolongation was higher in patients receiving CQ/HCQ, LPV/r, and quinolones. QPD had less significant effects on QTc prolongation than other antiviral agents.

Copyright © 2021 the Author(s). Published by Wolters Kluwer Health, Inc.

Conflict of interest statement

The authors have no conflicts of interest to disclose.

- Cited by 1 article
- 28 references
- 4 figures

Supplementary info

Publication types, MeSH terms, Substances, Grant support Expand

Publication types

- Observational Study

MeSH terms

- Aged
- Antiviral Agents / adverse effects*
- COVID-19 / drug therapy*
- COVID-19 / mortality*
- COVID-19 / virology
- Chloroquine / adverse effects
- Drug Therapy, Combination
- Drugs, Chinese Herbal / adverse effects
- Electrocardiography
- Female
- Hospital Mortality
- Hospitalization / statistics & numerical data
- Humans
- Hydroxychloroquine / adverse effects
- Indoles / adverse effects
- Interferons / adverse effects
- Long QT Syndrome / chemically induced
- Long QT Syndrome / mortality*
- Lopinavir / adverse effects
- Male
- Middle Aged
- Odds Ratio
- Quinolones / adverse effects
- Retrospective Studies
- Ritonavir / adverse effects
- SARS-CoV-2*

- Severity of Illness Index

Substances

- Antiviral Agents
- Drugs, Chinese Herbal
- Indoles
- Quinolones
- qingfei paidu decoction
- Lopinavir
- Hydroxychloroquine
- Chloroquine
- Interferons
- umifenovir
- Ritonavir

Grant support

- 81770330/National Natural Science Foundation of China

Full text links

Wolters Kluwer Wolters Kluwer Free PMC article
Proceed to details
Cite
Share
542
Observational Study
Clin Exp Med
. 2021 May;21(2):249-268.
doi: 10.1007/s10238-021-00684-1. Epub 2021 Feb 8.

Clinical presentation, therapeutic approach, and outcome of young patients admitted for COVID-19, with respect to the elderly counterpart

Martino Pepe [1], Charbel Maroun-Eid [2], Rodolfo Romero [3], Ramón Arroyo-Espliguero [4], Inmaculada Fernàndez-Rozas [5], Alvaro Aparisi [6], Víctor Manuel Becerra-Muñoz [7], Marcos Garcìa Aguado [8], Gaetano Brindicci [9], Jia Huang [10], Emilio Alfonso-Rodríguez [11], Alex Fernando Castro-Mejía [12], Serena Favretto [13], Enrico Cerrato [14], Paloma Albiol [15], Sergio Raposeiras-Roubin [16], Oscar Vedia [17], Gisela Feltes Guzmãn [18], Ana Carrero-Fernández

[19], Clara Perez Cimarra [20], Luis Buzón [21], Jorge Luis Jativa Mendez [22], Mohammad Abumayyaleh [23], Miguel Corbi-Pascual [24], Carlos Macaya [17], Vicente Estrada [17], Palma Luisa Nestola [9], Giuseppe Biondi-Zoccai [25] [26], Iván J Núñez-Gil [17]
Affiliations Expand

Affiliations

- [1] Azienda Ospedaliero-Universitaria Consorziale Policlinico di Bari, Piazza G. Cesare 11, Bari, Italy. drmartinopepe@libero.it.
- [2] Hospital Universitario La Paz. Instituto de Investigación Hospital Universitario La Paz (IdiPAZ), Madrid, Spain.
- [3] Hospital Universitario Getafe, Getafe, Madrid, Spain.
- [4] Hospital Universitario Guadalajara, Guadalajara, Spain.
- [5] Hospital Universitario Severo Ochoa, Leganés, Spain.
- [6] Hospital Clinico Universitario de Valladolid, Valladolid, Spain.
- [7] Unidad de Gestión Clínica Área del Corazón, Instituto de Investigación Biomédica de Málaga (IBIMA), Centro de Investigación Biomédica en Red de Enfermedades Cardiovasculares (CIBERCV), Hospital Universitario Virgen de la Victoria, Universidad de Málaga (UMA), Málaga, Spain.
- [8] Hospital Puerta de Hierro de Majadahonda, Majadahonda, Madrid, Spain.
- [9] Azienda Ospedaliero-Universitaria Consorziale Policlinico di Bari, Piazza G. Cesare 11, Bari, Italy.
- [10] The Second Affiliated Hospital of Southern University of Science and Technology, Shenzhen, China.
- [11] Institute of Cardiology and Cardiovascular Surgery, Havana, Cuba.
- [12] Hospital General del norte de Guayaquil IESS Los Ceibos, Guayaquil, Ecuador.
- [13] Sant'Andrea Hospital, Vercelli, Italy.
- [14] Orbassano and Rivoli Infermi Hospital, San Luigi Gonzaga University Hospital, Rivoli (Turin), Italy.
- [15] Hospital Clinico, INCLIVA, Valencia, Spain.
- [16] University Hospital Alvaro Cunqueiro, Vigo, Spain.
- [17] Hospital Clinico San Carlos, Madrid, Spain.
- [18] Nuestra Señora De America, Madrid, Spain.
- [19] Hospital Universitario Príncipe de Asturias, Madrid, Spain.
- [20] Hospital Universitario Infanta Sofia. San Sebastian de los Reyes, Madrid, Spain.
- [21] Hospital Universitario de Burgos, Burgos, Spain.
- [22] Hospital De Especialidades De Las Fuerzas Armadas N1, Quito, Ecuador.
- [23] First Department of Medicine, Medical Faculty Mannheim, University Heidelberg, Mannheim, Germany.
- [24] Hospital General de Albacete, Albacete, Spain.
- [25] Department of Medico-Surgical Sciences and Biotechnologies, Sapienza University of Rome, Latina, Italy.
- [26] Mediterranea Cardiocentro, Napoli, Italy.

- PMID: **33555436**
- PMCID: PMC7868661
- DOI: 10.1007/s10238-021-00684-1

Free PMC article
Observational Study

Clinical presentation, therapeutic approach, and outcome of young patients admitted for COVID-19, with respect to the elderly counterpart

Martino Pepe et al. Clin Exp Med. 2021 May.
Free PMC article
Show details
Clin Exp Med
. 2021 May;21(2):249-268.
doi: 10.1007/s10238-021-00684-1. Epub 2021 Feb 8.

Authors

Martino Pepe [1], Charbel Maroun-Eid [2], Rodolfo Romero [3], Ramón Arroyo-Espliguero [4], Inmaculada Fernàndez-Rozas [5], Alvaro Aparisi [6], Víctor Manuel Becerra-Muñoz [7], Marcos Garcìa Aguado [8], Gaetano Brindicci [9], Jia Huang [10], Emilio Alfonso-Rodríguez [11], Alex Fernando Castro-Mejía [12], Serena Favretto [13], Enrico Cerrato [14], Paloma Albiol [15], Sergio Raposeiras-Roubin [16], Oscar Vedia [17], Gisela Feltes Guzmãn [18], Ana Carrero-Fernández [19], Clara Perez Cimarra [20], Luis Buzón [21], Jorge Luis Jativa Mendez [22], Mohammad Abumayyaleh [23], Miguel Corbi-Pascual [24], Carlos Macaya [17], Vicente Estrada [17], Palma Luisa Nestola [9], Giuseppe Biondi-Zoccai [25] [26], Iván J Núñez-Gil [17]

Affiliations

- [1] Azienda Ospedaliero-Universitaria Consorziale Policlinico di Bari, Piazza G. Cesare 11, Bari, Italy. drmartinopepe@libero.it.
- [2] Hospital Universitario La Paz. Instituto de Investigación Hospital Universitario La Paz (IdiPAZ), Madrid, Spain.
- [3] Hospital Universitario Getafe, Getafe, Madrid, Spain.
- [4] Hospital Universitario Guadalajara, Guadalajara, Spain.
- [5] Hospital Universitario Severo Ochoa, Leganés, Spain.
- [6] Hospital Clinico Universitario de Valladolid, Valladolid, Spain.
- [7] Unidad de Gestión Clínica Área del Corazón, Instituto de Investigación Biomédica de Málaga (IBIMA), Centro de Investigación Biomédica en Red de Enfermedades Cardiovasculares (CIBERCV), Hospital Universitario Virgen de la Victoria, Universidad de Málaga (UMA), Málaga, Spain.
- [8] Hospital Puerta de Hierro de Majadahonda, Majadahonda, Madrid, Spain.
- [9] Azienda Ospedaliero-Universitaria Consorziale Policlinico di Bari, Piazza G. Cesare 11, Bari, Italy.
- [10] The Second Affiliated Hospital of Southern University of Science and Technology, Shenzhen, China.

- [11] Institute of Cardiology and Cardiovascular Surgery, Havana, Cuba.
- [12] Hospital General del norte de Guayaquil IESS Los Ceibos, Guayaquil, Ecuador.
- [13] Sant'Andrea Hospital, Vercelli, Italy.
- [14] Orbassano and Rivoli Infermi Hospital, San Luigi Gonzaga University Hospital, Rivoli (Turin), Italy.
- [15] Hospital Clinico, INCLIVA, Valencia, Spain.
- [16] University Hospital Alvaro Cunqueiro, Vigo, Spain.
- [17] Hospital Clinico San Carlos, Madrid, Spain.
- [18] Nuestra Señora De America, Madrid, Spain.
- [19] Hospital Universitario Príncipe de Asturias, Madrid, Spain.
- [20] Hospital Universitario Infanta Sofia. San Sebastian de los Reyes, Madrid, Spain.
- [21] Hospital Universitario de Burgos, Burgos, Spain.
- [22] Hospital De Especialidades De Las Fuerzas Armadas N1, Quito, Ecuador.
- [23] First Department of Medicine, Medical Faculty Mannheim, University Heidelberg, Mannheim, Germany.
- [24] Hospital General de Albacete, Albacete, Spain.
- [25] Department of Medico-Surgical Sciences and Biotechnologies, Sapienza University of Rome, Latina, Italy.
- [26] Mediterranea Cardiocentro, Napoli, Italy.

- PMID: **33555436**
- PMCID: PMC7868661
- DOI: 10.1007/s10238-021-00684-1

Abstract

There is limited information on the presenting characteristics, prognosis, and therapeutic approaches of young patients hospitalized for coronavirus disease 2019 (COVID-19). We sought to investigate the baseline characteristics, in-hospital treatment, and outcomes of a wide cohort < 65 years admitted for COVID-19. Using the international multicenter HOPE-COVID-19 registry, we evaluated the baseline characteristics, clinical presentation, therapeutic approach, and prognosis of patients < 65 years discharged (deceased or alive) after hospital admission for COVID-19, also compared with the elderly counterpart. Of the included 5746 patients, 2676 were < 65 and 3070 ≥ 65 years. All risk factors and several parameters suggestive of worse clinical presentation augmented through increasing age classes. In-hospital mortality rates were 6.8% and 32.1% in the younger and older cohort, respectively ($p < 0.001$). Among young patients, mortality, access to ICU and treatment with IMVwere positively correlated with age. Contrariwise, over 65 years of age this trend was broken so that only the association between age and mortality was persistent, while the rates of access to ICU and IMV started to decline. Younger patients also recognized specific predictors of case fatality, such as obesity and gender. Age negatively impacts on mortality, access to ICU and treatment with IMV in patients < 65 years. In elderly patients only case fatality rate keeps augmenting in a stepwise manner through increasing age categories, while therapeutic approaches become more conservative. Besides age, obesity, gender, history of cancer, and severe dyspnea, tachypnea, chest X-ray bilateral abnormalities, abnormal level of creatinine and leucocyte among admission parameters seem to play a central role in the outcome of patients younger than 65 years.

Trial registration: ClinicalTrials.gov NCT04334291.

Keywords: Coronavirus disease 2019; Intensive care unit; Invasive mechanical ventilation; SARS-CoV-2 infection.

Conflict of interest statement

The authors declare that they have no conflict of interest.

- Cited by 8 articles
- 18 references
- 4 figures

Supplementary info

Publication types, MeSH terms, Substances, Associated data Expand

Publication types

- Comparative Study
- Multicenter Study
- Observational Study

MeSH terms

- Adult
- Age Factors
- Aged
- Aged, 80 and over
- Aging*
- Antiviral Agents / therapeutic use
- COVID-19 / diagnosis*
- COVID-19 / drug therapy
- COVID-19 / mortality*
- Comorbidity
- Female
- Humans
- Male
- Middle Aged
- Registries
- Retrospective Studies
- SARS-CoV-2 / drug effects
- Treatment Outcome
- Young Adult

Substances

- Antiviral Agents

Associated data

- ClinicalTrials.gov/NCT04334291

Full text links

Springer Free PMC article
Proceed to details
Cite
Share
543
PLoS One
. 2022 Jan 26;17(1):e0262908.
doi: 10.1371/journal.pone.0262908. eCollection 2022.

Predictive factors of clinical outcomes in patients with COVID-19 treated with tocilizumab: A monocentric retrospective analysis

Giulia Cassone [1] [2], Giovanni Dolci [3], Giulia Besutti [2] [4], Luca Braglia [5], Paolo Pavone [6], Romina Corsini [6], Fabio Sampaolesi [6], Valentina Iotti [4], Elisabetta Teopompi [7], Marco Massari [6], Matteo Fontana [8], Giulia Ghidoni [8], Anaflorina Matei [9], Stefania Croci [10], Emanuele Alberto Negri [11], Massimo Costantini [5], Nicola Facciolongo [8], Carlo Salvarani [1] [12]
Affiliations Expand

Affiliations

- [1] Rheumatology Unit, IRCCS Arcispedale Santa Maria Nuova, Azienda Unità Sanitaria Locale-IRCCS di Reggio Emilia, Reggio Emilia, Italy.
- [2] Clinical and Experimental Medicine PhD Program, University of Modena and Reggio Emilia, Modena, Italy.
- [3] Infectious Disease Unit, University of Modena and Reggio Emilia, Modena, Italy.
- [4] Radiology Unit, Department of Imaging and Laboratory Medicine, Azienda USL-IRCCS di Reggio Emilia, Reggio Emilia, Italy.
- [5] Azienda USL-IRCCS di Reggio Emilia, Reggio Emilia, Italy.
- [6] Infectious Disease Unit, Azienda USL-IRCCS di Reggio Emilia, Reggio Emilia, Italy.
- [7] SOC Internistica Multidisciplinare, Ospedale Civile Guastalla, Azienda USL-IRCCS di Reggio Emilia, Reggio Emilia, Italy.

- [8] Pneumology Unit, Azienda USL-IRCCS di Reggio Emilia, Reggio Emilia, Italy.
- [9] Department of Anesthesia and Intensive Care, Azienda USL-IRCCS di Reggio Emilia, Reggio Emilia, Italy.
- [10] Clinical Immunology, Allergy and Advanced Biotechnologies Unit, Azienda USL-IRCCS di Reggio Emilia, Reggio Emilia, Italy.
- [11] High Intensity Unit, Azienda USL-IRCCS di Reggio Emilia, Reggio Emilia, Italy.
- [12] Rheumatology Unit, University of Modena and Reggio Emilia, Modena, Italy.

- PMID: **35081151**
- PMCID: PMC8791493
- DOI: 10.1371/journal.pone.0262908

Free PMC article

Predictive factors of clinical outcomes in patients with COVID-19 treated with tocilizumab: A monocentric retrospective analysis

Giulia Cassone et al. PLoS One. 2022.
Free PMC article
Show details
PLoS One
. 2022 Jan 26;17(1):e0262908.
doi: 10.1371/journal.pone.0262908. eCollection 2022.

Authors

Giulia Cassone [1] [2], Giovanni Dolci [3], Giulia Besutti [2] [4], Luca Braglia [5], Paolo Pavone [6], Romina Corsini [6], Fabio Sampaolesi [6], Valentina Iotti [4], Elisabetta Teopompi [7], Marco Massari [6], Matteo Fontana [8], Giulia Ghidoni [8], Anaflorina Matei [9], Stefania Croci [10], Emanuele Alberto Negri [11], Massimo Costantini [5], Nicola Facciolongo [8], Carlo Salvarani [1] [12]

Affiliations

- [1] Rheumatology Unit, IRCCS Arcispedale Santa Maria Nuova, Azienda Unità Sanitaria Locale-IRCCS di Reggio Emilia, Reggio Emilia, Italy.
- [2] Clinical and Experimental Medicine PhD Program, University of Modena and Reggio Emilia, Modena, Italy.
- [3] Infectious Disease Unit, University of Modena and Reggio Emilia, Modena, Italy.
- [4] Radiology Unit, Department of Imaging and Laboratory Medicine, Azienda USL-IRCCS di Reggio Emilia, Reggio Emilia, Italy.
- [5] Azienda USL-IRCCS di Reggio Emilia, Reggio Emilia, Italy.
- [6] Infectious Disease Unit, Azienda USL-IRCCS di Reggio Emilia, Reggio Emilia, Italy.

- [7] SOC Internistica Multidisciplinare, Ospedale Civile Guastalla, Azienda USL-IRCCS di Reggio Emilia, Reggio Emilia, Italy.
- [8] Pneumology Unit, Azienda USL-IRCCS di Reggio Emilia, Reggio Emilia, Italy.
- [9] Department of Anesthesia and Intensive Care, Azienda USL-IRCCS di Reggio Emilia, Reggio Emilia, Italy.
- [10] Clinical Immunology, Allergy and Advanced Biotechnologies Unit, Azienda USL-IRCCS di Reggio Emilia, Reggio Emilia, Italy.
- [11] High Intensity Unit, Azienda USL-IRCCS di Reggio Emilia, Reggio Emilia, Italy.
- [12] Rheumatology Unit, University of Modena and Reggio Emilia, Modena, Italy.

- PMID: **35081151**
- PMCID: PMC8791493
- DOI: 10.1371/journal.pone.0262908

Abstract

Objective: The aim of this retrospective observational study is to analyse clinical, serological and radiological predictors of outcome in patients with COVID-19 pneumonia treated with tocilizumab, providing clinical guidance to its use in real-life.

Method: This is a retrospective, monocentric observational cohort study. All consecutive patients hospitalized between February the 11th and April 14th 2020 for severe COVID-19 pneumonia at Reggio Emilia AUSL and treated with tocilizumab were enrolled. The patient's clinical status was recorded every day using the WHO ordinal scale for clinical improvement. Response to treatment was defined as an improvement of one point (from the status at the beginning of tocilizumab treatment) during the follow-up on this scale. Bivariate association of main patients' characteristics with outcomes was explored by descriptive statistics and Fisher or Kruskal Wallis tests (respectively for qualitative or quantitative variables). Each clinically significant predictor was checked by a loglikelihood ratio test (in univariate logistic models for each of the considered outcomes) against the null model.

Results: A total of 173 patients were included. Only hypertension, the use of angiotensin-converting enzyme inhibitors, PaO2/FiO2, respiratory rate and C-reactive protein were selected for the multivariate analysis. In the multivariable model, none of them was significantly associated with response.

Conclusions: Evaluating a large number of clinical variables, our study did not find new predictors of outcome in COVID19 patients treated with tocilizumab. Further studies are needed to investigate the use of tocilizumab in COVID-19 and to better identify clinical phenotypes which could benefit from this treatment.

Conflict of interest statement

The authors have declared that no competing interests exist.

- 18 references

Supplementary info

MeSH terms, Substances, Grant support Expand

MeSH terms

- Aged
- Antibodies, Monoclonal, Humanized / therapeutic use*
- C-Reactive Protein / analysis
- COVID-19 / drug therapy*
- COVID-19 / virology
- Female
- Humans
- Male
- Middle Aged
- Oxygen Consumption
- Respiratory Rate
- Retrospective Studies
- SARS-CoV-2 / isolation & purification
- Treatment Outcome

Substances

- Antibodies, Monoclonal, Humanized
- C-Reactive Protein
- tocilizumab

Grant support

The authors received no specific funding for this work.

Full text links

OPEN ACCESS TO FULL TEXT PLOS ONE Public Library of Science Free PMC article
Proceed to details
Cite
Share
☐ 544
Observational Study
Vector Borne Zoonotic Dis
. 2021 Oct;21(10):777-784.
doi: 10.1089/vbz.2021.0023. Epub 2021 Aug 10.

Soaring Asymptomatic Infected Individuals Bring About Barriers and Difficulties for

Interruption of COVID-19 Prevalence in China

Muxin Chen [1, 2], Lin Ai [2, 3], Dana Huang [1], Jiaxu Chen [2], Tiejian Feng [1], Shujiang Mei [1], Yalan Huang [1], Bo Peng [1], Shunxian Zhang [4], Renli Zhang [1], Xiaonong Zhou [2, 3]
Affiliations Expand

Affiliations

- [1] Institute of Pathogenic Biology, Shenzhen Center for Disease Control and Prevention, Shenzhen, China.
- [2] Health Education and Detection Center, National Institute of Parasitic Diseases, Chinese Center for Disease Control and Prevention (Chinese Center for Tropical Diseases Research); NHC Key Laboratory for Parasitology and Vector Biology; WHO Collaborating Center for Tropical Diseases; National Center for International Research on Tropical Diseases, Shanghai, China.
- [3] Department of One Health, School of Global Health, Chinese Center for Tropical Diseases Research, Shanghai Jiao Tong University School of Medicine, Shanghai, China.
- [4] Clinical Research Center, Longhua Hospital Shanghai University of Traditional Chinese Medicine, Shanghai, China.

- PMID: **34375121**
- DOI: 10.1089/vbz.2021.0023

Observational Study

Soaring Asymptomatic Infected Individuals Bring About Barriers and Difficulties for Interruption of COVID-19 Prevalence in China

Muxin Chen et al. Vector Borne Zoonotic Dis. 2021 Oct.
Show details
Vector Borne Zoonotic Dis
. 2021 Oct;21(10):777-784.
doi: 10.1089/vbz.2021.0023. Epub 2021 Aug 10.

Authors

Muxin Chen [1, 2], Lin Ai [2, 3], Dana Huang [1], Jiaxu Chen [2], Tiejian Feng [1], Shujiang Mei [1], Yalan Huang [1], Bo Peng [1], Shunxian Zhang [4], Renli Zhang [1], Xiaonong Zhou [2, 3]

Affiliations

- [1] Institute of Pathogenic Biology, Shenzhen Center for Disease Control and Prevention, Shenzhen, China.
- [2] Health Education and Detection Center, National Institute of Parasitic Diseases, Chinese Center for Disease Control and Prevention (Chinese Center for Tropical Diseases Research); NHC Key Laboratory for Parasitology and Vector Biology; WHO Collaborating Center for Tropical Diseases; National Center for International Research on Tropical Diseases, Shanghai, China.
- [3] Department of One Health, School of Global Health, Chinese Center for Tropical Diseases Research, Shanghai Jiao Tong University School of Medicine, Shanghai, China.
- [4] Clinical Research Center, Longhua Hospital Shanghai University of Traditional Chinese Medicine, Shanghai, China.

- PMID: **34375121**
- DOI: 10.1089/vbz.2021.0023

Abstract

Background: Coronavirus disease 2019 (COVID-19) caused by severe acute respiratory syndrome coronavirus 2 (SARS-CoV-2) has become a global pandemic, which has caused unprecedented damage to human health and life. The present study aimed to carry out and discover asymptomatic infected individuals in Shenzhen, China. The data will provide the control measures to stop COVID-19 prevalence. ***Methods:*** The study was a retrospective review of medical records from 462 confirmed patients with COVID-19 and 45 asymptomatic infected individuals in Shenzhen from January 19 to April 30, 2020; this is a retrospective, observational multicenter study. ***Results:*** A total of 462 confirmed cases were diagnosed in Shenzhen from January 19 to April 30, 2020. The cohort included 423 domestic cases (91.56%, 95% confidence interval [CI]: 88.67-93.76) and 39 (8.44%, 95% CI: 6.24-11.33) imported cases from other countries. Moreover, a total of 45 asymptomatic infections were found, encompassing 31 (68.89%, 95% CI: 54.34-80.47) local infections and 14 (31.11%, 95% CI: 19.53-45.66) individuals imported from other countries. The proportion of asymptomatic infected persons in Shenzhen is continuously increasing ($Z = 13.19$, $p < 0.0001$). The total number of local asymptomatic infections was more than that in other provinces ($\chi^2 = 118.83$, $p < 0.0001$). The proportion of asymptomatic infected individuals among cases imported from other countries was higher than the domestic cases ($\chi^2 = 22.51$, $p < 0.0001$, odds ratio = 4.90, 95% CI: 2.40-9.98). ***Conclusions:*** The proportion of asymptomatic infection is increasing. Hence, development and application of the diagnosis method with high sensitivity and specificity play a critical role in reducing COVID-19 global epidemics.

Keywords: COVID-19; China; SARS-CoV-2; asymptomatic infection; pathogen carrier; transmission in incubation period.

- Cited by 1 article

Supplementary info

Publication types, MeSH terms Expand

Publication types

- Multicenter Study

- Observational Study
- Research Support, Non-U.S. Gov't

MeSH terms

- Asymptomatic Infections / epidemiology*
- COVID-19 / epidemiology*
- COVID-19 / prevention & control*
- China / epidemiology
- Cohort Studies
- Humans
- Prevalence
- Retrospective Studies
- SARS-CoV-2*
- Time Factors

Full text links

Mary Ann Liebert Atypon
Proceed to details
Cite
Share
545
Observational Study
Pathog Dis
. 2021 Jan 9;79(1):ftaa064.
doi: 10.1093/femspd/ftaa064.

SARS-CoV-2 RT-PCR profile in 298 Indian COVID-19 patients: a retrospective observational study

Bisakh Bhattacharya [1], Rohit Kumar [1], Ved Prakash Meena [1], Manish Soneja [1], Amit Singh [2], Rojaleen Das [2], Ashit Xess [2], Nazneen Arif [2], Saurabh Vig [3], Vandana Rastogi [4], Pavan Tiwari [5], Sushma Bhatnagar [3], Anant Mohan [5], Naveet Wig [1], Lalit Dar [2]
Affiliations Expand

Affiliations

- [1] Department of Medicine, All India Institute of Medical Sciences, Medicine Office, 3rd floor, Teaching block, AIIMS, New Delhi, India.

- [2] Department of Microbiology, Teaching block, 2nd floor, All India Institute of Medical Sciences, New Delhi, India.
- [3] Department of Onco-anesthesia and Palliative Medicine, Dr. B.R.A.I.R.C.H, All India Institute of Medical Sciences, New Delhi, India.
- [4] Department of Biostatistics, All India Institute of Medical Sciences, New Delhi, India.
- [5] Department of Pulmonary Medicine and Sleep Disorders, All India Institute of Medical Sciences, New Delhi, India.

- PMID: **33053181**
- PMCID: PMC7665504
- DOI: 10.1093/femspd/ftaa064

Free PMC article
Observational Study

SARS-CoV-2 RT-PCR profile in 298 Indian COVID-19 patients: a retrospective observational study

Bisakh Bhattacharya et al. Pathog Dis. 2021.
Free PMC article
Show details
Pathog Dis
. 2021 Jan 9;79(1):ftaa064.
doi: 10.1093/femspd/ftaa064.

Authors

Bisakh Bhattacharya [1], Rohit Kumar [1], Ved Prakash Meena [1], Manish Soneja [1], Amit Singh [2], Rojaleen Das [2], Ashit Xess [2], Nazneen Arif [2], Saurabh Vig [3], Vandana Rastogi [4], Pavan Tiwari [5], Sushma Bhatnagar [3], Anant Mohan [5], Naveet Wig [1], Lalit Dar [2]

Affiliations

- [1] Department of Medicine, All India Institute of Medical Sciences, Medicine Office, 3rd floor, Teaching block, AIIMS, New Delhi, India.
- [2] Department of Microbiology, Teaching block, 2nd floor, All India Institute of Medical Sciences, New Delhi, India.
- [3] Department of Onco-anesthesia and Palliative Medicine, Dr. B.R.A.I.R.C.H, All India Institute of Medical Sciences, New Delhi, India.
- [4] Department of Biostatistics, All India Institute of Medical Sciences, New Delhi, India.
- [5] Department of Pulmonary Medicine and Sleep Disorders, All India Institute of Medical Sciences, New Delhi, India.

- PMID: **33053181**
- PMCID: PMC7665504
- DOI: 10.1093/femspd/ftaa064

Abstract

Background: despite being in the 5th month of pandemic, knowledge with respect to viral dynamics, infectivity and RT-PCR positivity continues to evolve.

Aim: to analyse the SARS CoV-2 nucleic acid RT-PCR profiles in COVID-19 patients.

Design: it was a retrospective, observational study conducted at COVID facilities under AIIMS, New Delhi.

Methods: patients admitted with laboratory confirmed COVID-19 were eligible for enrolment. Patients with incomplete details, or only single PCR tests were excluded. Data regarding demographic details, comorbidities, treatment received and results of SARS-CoV-2 RT-PCR performed on nasopharyngeal and oropharyngeal swabs, collected at different time points, was retrieved from the hospital records.

Results: a total of 298 patients were included, majority were males (75·8%) with mean age of 39·07 years (0·6-88 years). The mean duration from symptom onset to first positive RT-PCR was 4·7 days (SD 3·67), while that of symptom onset to last positive test was 17·83 days (SD 6·22). Proportions of positive RT-PCR tests were 100%, 49%, 24%, 8·7% and 20·6% in the 1st, 2nd, 3rd, 4th and >4 weeks of illness. A total of 12 symptomatic patients had prolonged positive test results even after 3 weeks of symptom onset. Age > = 60 years was associated with prolonged RT-PCR positivity (statistically significant).

Conclusion: this study showed that the average period of PCR positivity is more than 2 weeks in COVID-19 patients; elderly patients have prolonged duration of RT-PCR positivity and requires further follow up.

Keywords: COVID-19; RT-PCR; SARS-CoV-2; profile.

© The Author(s) 2020. Published by Oxford University Press on behalf of FEMS.

- Cited by 1 article

Supplementary info

Publication types, MeSH terms, Substances Expand

Publication types

- Observational Study
- Research Support, Non-U.S. Gov't

MeSH terms

- Adolescent
- Adult
- Aged
- Aged, 80 and over

- Asymptomatic Diseases
- COVID-19 / diagnosis*
- COVID-19 / epidemiology
- COVID-19 / pathology
- COVID-19 / virology
- COVID-19 Nucleic Acid Testing / methods
- Child
- Child, Preschool
- Comorbidity
- Diabetes Mellitus / diagnosis*
- Diabetes Mellitus / epidemiology
- Diabetes Mellitus / pathology
- Diabetes Mellitus / virology
- Humans
- Hypertension / diagnosis*
- Hypertension / epidemiology
- Hypertension / pathology
- Hypertension / virology
- India / epidemiology
- Infant
- Infectious Disease Incubation Period
- Male
- Middle Aged
- Nasopharynx / virology
- Oropharynx / virology
- RNA, Viral / genetics*
- Retrospective Studies
- Reverse Transcriptase Polymerase Chain Reaction
- SARS-CoV-2 / genetics
- SARS-CoV-2 / pathogenicity*
- Severity of Illness Index

Substances

- RNA, Viral

Full text links

OXFORD ACADEMIC Silverchair Information Systems Free PMC article
Proceed to details
Cite
Share

☐ 546

Observational Study

Indian J Pediatr
. 2021 Oct;88(10):974-978.
doi: 10.1007/s12098-020-03590-8. Epub 2021 Jan 4.

Role of Telemedicine in Follow-up Care of Children with Respiratory Illnesses at a Tertiary Care Hospital - An Ambispective Observational Study

Jyoti Kumari [1], Kana Ram Jat [2], S K Kabra [1]

Affiliations Expand

Affiliations

- [1] Department of Pediatrics, All India Institute of Medical Sciences, New Delhi, 110029, India.
- [2] Department of Pediatrics, All India Institute of Medical Sciences, New Delhi, 110029, India. drkanaram@gmail.com.

- PMID: **33394296**
- PMCID: PMC7780214
- DOI: 10.1007/s12098-020-03590-8

Free PMC article

Observational Study

Role of Telemedicine in Follow-up Care of Children with Respiratory Illnesses at a Tertiary Care Hospital - An Ambispective Observational Study

Jyoti Kumari et al. Indian J Pediatr. 2021 Oct.

Free PMC article

Show details

Indian J Pediatr
. 2021 Oct;88(10):974-978.
doi: 10.1007/s12098-020-03590-8. Epub 2021 Jan 4.

Authors

Jyoti Kumari [1], Kana Ram Jat [2], S K Kabra [1]

Affiliations

- [1] Department of Pediatrics, All India Institute of Medical Sciences, New Delhi, 110029, India.
- [2] Department of Pediatrics, All India Institute of Medical Sciences, New Delhi, 110029, India. drkanaram@gmail.com.

- PMID: **33394296**
- PMCID: PMC7780214
- DOI: 10.1007/s12098-020-03590-8

Abstract

Objective: To assess if telemedicine can be used successfully for follow-up care of children with respiratory illnesses. The authors also assessed problems faced by the doctors and satisfaction of caregivers of these patients with telemedicine.

Methods: The authors conducted an ambispective observational study. Data related to demographic details and diagnoses of patients who had telemedicine consultation (teleconsultation) appointments between 2nd April 2020 to 15th May 2020 were reviewed retrospectively. They noted proportion of patients having successful prescription. To assess problems faced by doctors and satisfaction of caregiver of patients with teleconsultation, a prospective questionnaire was sent via Google Forms 6-10 wk after the initial appointment date. Those who did not respond to Google Forms were called by phone to assess the same.

Results: A total of 188 patients received teleconsultation during the study period. Team was able to prescribe treatment in 181 (96.3%) patients via teleconsultation and other seven (3.7%) required physical evaluation. Mean (SD) age of patients was 9.7 (4.9) y, range 3 mo to 18 y. There were 117 (62.2%) boys and 71 (37.8%) girls. Majority (58%) of the patients were asthmatics. The team advised refill prescription in 83% patients as symptoms were controlled. Three out of five residents faced minor problems while providing teleconsultation. In satisfaction assessment, 78% of caregivers rated teleconsultation 8 or more, out of 10 points, suggesting that most of them were satisfied with telemedicine.

Conclusion: In majority of children with respiratory illnesses, successful follow-up care can be provided by telemedicine.

Keywords: COVID-19; Children; Respiratory; Telemedicine.

© 2021. Dr. K C Chaudhuri Foundation.

Conflict of interest statement

None.

Comment in

- Telemedicine During the COVID-19 Pandemic: Moving from Physical to Virtual Outpatient Care.

Goyal JP, Kumar P. Goyal JP, et al. Indian J Pediatr. 2021 Oct;88(10):959-960. doi: 10.1007/s12098-021-03924-0. Epub 2021 Aug 27. Indian J Pediatr. 2021. PMID: 34449026
Free PMC article. No abstract available.

- Cited by 5 articles
- 15 references
- 2 figures

Supplementary info

Publication types, MeSH terms Expand

Publication types

- Observational Study

MeSH terms

- Aftercare
- COVID-19*
- Child
- Female
- Humans
- Male
- Prospective Studies
- Remote Consultation*
- Retrospective Studies
- Telemedicine*
- Tertiary Care Centers

Full text links

SpringerLink FULL-TEXT ARTICLE Springer Free PMC article
Proceed to details
Cite
Share
☐ 547
Observational Study
Echocardiography
. 2020 Sep;37(9):1362-1365.
doi: 10.1111/echo.14825. Epub 2020 Aug 13.

The utility of bedside echocardiography in critically ill COVID-19 patients: Early

observational findings from three Northern New Jersey hospitals

Rahul Vasudev [1], Nirmal Guragai [1], Habib Habib [1], Kevin Hosein [1], Hartaj Virk [1], Irvin Goldfarb [2], Mahesh Bikkina [1], Fayez Shamoon [1], Raja Pullatt [3]
Affiliations Expand

Affiliations

- [1] Department of Cardiology, St Joseph's University Medical Center, Paterson, NJ, USA.
- [2] Department of Cardiology, St Michael's Medical Center, Newark, NJ, USA.
- [3] Department of Cardiology, Trinitas Regional Medical Center, Elizabeth, NJ, USA.

- PMID: **32789869**
- PMCID: PMC7436602
- DOI: 10.1111/echo.14825

Free PMC article
Observational Study

The utility of bedside echocardiography in critically ill COVID-19 patients: Early observational findings from three Northern New Jersey hospitals

Rahul Vasudev et al. Echocardiography. 2020 Sep.
Free PMC article
Show details
Echocardiography
. 2020 Sep;37(9):1362-1365.
doi: 10.1111/echo.14825. Epub 2020 Aug 13.

Authors

Rahul Vasudev [1], Nirmal Guragai [1], Habib Habib [1], Kevin Hosein [1], Hartaj Virk [1], Irvin Goldfarb [2], Mahesh Bikkina [1], Fayez Shamoon [1], Raja Pullatt [3]

Affiliations

- [1] Department of Cardiology, St Joseph's University Medical Center, Paterson, NJ, USA.
- [2] Department of Cardiology, St Michael's Medical Center, Newark, NJ, USA.
- [3] Department of Cardiology, Trinitas Regional Medical Center, Elizabeth, NJ, USA.

- PMID: **32789869**

- PMCID: PMC7436602
- DOI: 10.1111/echo.14825

Abstract

Introduction: Cardiovascular complications related to coronavirus disease 2019 (COVID-19) have led to the need for echocardiographic services during the pandemic. The present study aimed to identify the echocardiographic findings in hospitalized COVID-19 patients and their utility in disease management.

Methods: We included patients who were diagnosed with COVID-19 using polymerase chain reaction and those who underwent echocardiographic examination during their hospitalization.

Results: Altogether, 45 patients were evaluated. The mean age was 61.4 ± 12.2 years. Hypertension (n = 29, 64%) and diabetes mellitus (n = 25, 55%) were the most common comorbidities followed by congestive heart failure (n = 11, 24%), coronary artery disease (n = 9, 20%), and valvular heart disease (n = 3, 7%). Eight patients (18%) showed evidence of myocardial injury, as suggested by elevated troponin levels. Brain natriuretic peptide was elevated in 14 patients (36%), and 14 patients had left ventricular dysfunction in the form of reduced ejection fraction (31%). Right ventricular (RV) dilatation was observed in six patients, and five patients had reduced RV ejection fraction. RV pressure and volume overload were observed in three patients. RV thrombus was observed in one patient. Pulmonary pressure was elevated in 10 patients (24%).

Conclusion: Two-dimensional echocardiography can be an important bedside tool for the assessment of cardiovascular abnormalities and hemodynamic status of COVID-19 patients.

© 2020 Wiley Periodicals LLC.

Conflict of interest statement

None.

- Cited by 10 articles
- 15 references

Supplementary info

Publication types, MeSH terms Expand

Publication types

- Multicenter Study
- Observational Study

MeSH terms

- Adult
- Aged

- Aged, 80 and over
- COVID-19 / complications*
- COVID-19 / physiopathology
- Critical Care / methods*
- Critical Illness
- Echocardiography / methods*
- Female
- Heart Diseases / diagnostic imaging*
- Heart Diseases / etiology*
- Heart Diseases / physiopathology
- Hospitals
- Humans
- Male
- Middle Aged
- New Jersey
- Point-of-Care Systems*
- Retrospective Studies

Full text links

Wiley Full Text Article Wiley Free PMC article

Proceed to details

Cite

Share

548

Observational Study

Clin Pharmacol Ther

. 2021 Jun;109(6):1660-1667.

doi: 10.1002/cpt.2245. Epub 2021 Apr 19.

Effect of Corticosteroids on Mortality in Hospitalized COVID-19 Patients Not Receiving Invasive Mechanical Ventilation

Federica Fusina [1], Filippo Albani [1], Enza Granato [1], Angelo Meloni [2], Renzo Rozzini [3], Tony Sabatini [4], Roberto Stellini [5], Paolo Terragnoli [6], Antonio Rosano [1], Mohammed Abu Hilal [7], Giuseppe Natalini [1]

Affiliations Expand

Affiliations

- [1] Department of Anesthesia, Intensive Care and Pain medicine, Fondazione Poliambulanza Istituto Ospedaliero, Brescia, Italy.
- [2] Department of Laboratory Medicine, Fondazione Poliambulanza Istituto Ospedaliero, Brescia, Italy.
- [3] Department of Geriatrics, Fondazione Poliambulanza Istituto Ospedaliero, Brescia, Italy.
- [4] Department of Internal Medicine, Gastroenterology and Endoscopy, Fondazione Poliambulanza Istituto Ospedaliero, Brescia, Italy.
- [5] Infectious Diseases, Fondazione Poliambulanza Istituto Ospedaliero, Brescia, Italy.
- [6] Emergency Department, Fondazione Poliambulanza Istituto Ospedaliero, Brescia, Italy.
- [7] Department of Surgery, Fondazione Poliambulanza Istituto Ospedaliero, Brescia, Italy.

- PMID: **33792037**
- PMCID: PMC8251340
- DOI: 10.1002/cpt.2245

Free PMC article
Observational Study

Effect of Corticosteroids on Mortality in Hospitalized COVID-19 Patients Not Receiving Invasive Mechanical Ventilation

Federica Fusina et al. Clin Pharmacol Ther. 2021 Jun.
Free PMC article
Show details
Clin Pharmacol Ther
. 2021 Jun;109(6):1660-1667.
doi: 10.1002/cpt.2245. Epub 2021 Apr 19.

Authors

Federica Fusina [1], Filippo Albani [1], Enza Granato [1], Angelo Meloni [2], Renzo Rozzini [3], Tony Sabatini [4], Roberto Stellini [5], Paolo Terragnoli [6], Antonio Rosano [1], Mohammed Abu Hilal [7], Giuseppe Natalini [1]

Affiliations

- [1] Department of Anesthesia, Intensive Care and Pain medicine, Fondazione Poliambulanza Istituto Ospedaliero, Brescia, Italy.
- [2] Department of Laboratory Medicine, Fondazione Poliambulanza Istituto Ospedaliero, Brescia, Italy.
- [3] Department of Geriatrics, Fondazione Poliambulanza Istituto Ospedaliero, Brescia, Italy.
- [4] Department of Internal Medicine, Gastroenterology and Endoscopy, Fondazione Poliambulanza Istituto Ospedaliero, Brescia, Italy.
- [5] Infectious Diseases, Fondazione Poliambulanza Istituto Ospedaliero, Brescia, Italy.
- [6] Emergency Department, Fondazione Poliambulanza Istituto Ospedaliero, Brescia, Italy.

- [7] Department of Surgery, Fondazione Poliambulanza Istituto Ospedaliero, Brescia, Italy.

- PMID: **33792037**
- PMCID: PMC8251340
- DOI: 10.1002/cpt.2245

Abstract

The most beneficial effect of corticosteroid therapy in COVID-19 patients has been shown in subjects receiving invasive mechanical ventilation (IMV), corresponding to a score of 6 on the World Health Organization (WHO) COVID-19 Ordinal Scale for Clinical Improvement (OSCI). The aim of this observational, single-center, prospective study was to assess the association between corticosteroids and hospital mortality in coronavirus disease 2019 (COVID-19) patients who did not receive IMV (OSCI 3-5). Included were 1,311 COVID-19 patients admitted to nonintensive care wards, and they were divided in two cohorts: (i) 480 patients who received corticosteroid therapy and (ii) 831 patients who did not. The median daily dose was of 8 mg of dexamethasone or equivalent, with a mean therapy duration of 5 (3-9) days. The indication to administer or withhold corticosteroids was given by the treating physician. In-hospital mortality was similar between the two cohorts after adjusting for possible confounders (adjusted odds ratio (ORadj) 1.04, 95% confidence interval (CI), 0.81-1.34, P = 0.74). There was also no difference in Intensive Care Unit (ICU) admission (ORadj 0.81, 95% CI, 0.56-1.17, P = 0.26). COVID-19 patients with noninvasive mechanical ventilation (NIMV) had a lower risk for ICU admission if they received steroid therapy (ORadj 0.58, 95% CI, 0.35-0.94, P = 0.03). In conclusion, corticosteroids were overall not associated with a difference in hospital mortality for patients with COVID-19 with OSCI 3-5. In the subgroup of patients with NIMV (OSCI 5), corticosteroids reduced ICU admission, whereas the effect on mortality requires further studies.

© 2021 The Authors. Clinical Pharmacology & Therapeutics © 2021 American Society for Clinical Pharmacology and Therapeutics.

Conflict of interest statement

The authors declared no competing interests for this work.

- Cited by 4 articles
- 30 references
- 4 figures

Supplementary info

Publication types, MeSH terms, Substances Expand

Publication types

- Observational Study

MeSH terms

- Adrenal Cortex Hormones / administration & dosage

- Adrenal Cortex Hormones / therapeutic use*
- Aged
- Aged, 80 and over
- COVID-19 / drug therapy*
- COVID-19 / mortality*
- Female
- Hospital Mortality / trends*
- Humans
- Intensive Care Units / statistics & numerical data*
- Male
- Middle Aged
- Prospective Studies
- Respiration, Artificial / statistics & numerical data*
- Retrospective Studies
- SARS-CoV-2
- Sex Factors

Substances

- Adrenal Cortex Hormones

Full text links

WILEY Full Text Article Wiley Free PMC article

Proceed to details

Cite

Share

549

Observational Study

QJM

. 2021 Nov 5;114(7):464-470.

doi: 10.1093/qjmed/hcab190.

COVID-19-associated mucormycosis presenting to the Emergency Department-an observational study of 70 patients

A Ramaswami [1], A K Sahu [1], A Kumar [1], S Suresh [1], A Nair [1], D Gupta [1], R Chouhan [1], R Bhat [1], R Mathew [1], J A Majeed [1], P Aggarwal [1], J Nayer [1], M Ekka [1], A Thakar [2], G Singh [3], I Xess [3], N Wig [4]

Affiliations Expand

Affiliations

- [1] Department of Emergency Medicine, All India Institute of Medical Sciences, Ansari Nagar, New Delhi 110029, India.
- [2] Department of Otorhinolaryngology, All India Institute of Medical Sciences, Ansari Nagar, New Delhi 110029, India.
- [3] Department of Microbiology, All India Institute of Medical Sciences, Ansari Nagar, New Delhi 110029, India.
- [4] Department of Medicine, All India Institute of Medical Sciences, Ansari Nagar, New Delhi 110029, India.

- PMID: **34254132**
- PMCID: PMC8420631
- DOI: 10.1093/qjmed/hcab190

Free PMC article
Observational Study

COVID-19-associated mucormycosis presenting to the Emergency Department-an observational study of 70 patients

A Ramaswami et al. QJM. 2021.
Free PMC article
Show details
QJM
. 2021 Nov 5;114(7):464-470.
doi: 10.1093/qjmed/hcab190.

Authors

A Ramaswami [1], A K Sahu [1], A Kumar [1], S Suresh [1], A Nair [1], D Gupta [1], R Chouhan [1], R Bhat [1], R Mathew [1], J A Majeed [1], P Aggarwal [1], J Nayer [1], M Ekka [1], A Thakar [2], G Singh [3], I Xess [3], N Wig [4]

Affiliations

- [1] Department of Emergency Medicine, All India Institute of Medical Sciences, Ansari Nagar, New Delhi 110029, India.
- [2] Department of Otorhinolaryngology, All India Institute of Medical Sciences, Ansari Nagar, New Delhi 110029, India.
- [3] Department of Microbiology, All India Institute of Medical Sciences, Ansari Nagar, New Delhi 110029, India.
- [4] Department of Medicine, All India Institute of Medical Sciences, Ansari Nagar, New Delhi 110029, India.

- PMID: **34254132**

- PMCID: PMC8420631
- DOI: 10.1093/qjmed/hcab190

Abstract

Background: Mucormycosis (MM) is a deadly opportunistic fungal infection and a large surge in COVID-19-associated mucormycosis (CAM) is occurring in India.

Aim: Our aim was to delineate the clinico-epidemiological profile and identify risk factors of CAM patients presenting to the Emergency Department (ED).

Design: This was a retrospective, single-centre, observational study.

Methods: We included patients who presented with clinical features or diagnosed MM and who were previously treated for COVID-19 in last 3 months of presentation (recent COVID-19) or currently being treated for COVID-19 (active COVID-19). Information regarding clinical features of CAM, possible risk factors, examination findings, diagnostic workup including imaging and treatment details were collected.

Results: Seventy CAM patients (median age: 44.5 years, 60% males) with active (75.7%) or recent COVID-19 (24.3%) who presented to the ED in between 6 May 2021 and 1 June 2021, were included. A median duration of 20 days (interquartile range: 13.5-25) was present between the onset of COVID-19 symptoms and the onset of CAM symptoms. Ninety-three percent patients had at least one risk factor. Most common risk factors were diabetes mellitus (70%) and steroid use for COVID-19 disease (70%). After clinical, microbiological and radiological workup, final diagnosis of rhino-orbital CAM was made in most patients (68.6%). Systemic antifungals were started in the ED and urgent surgical debridement was planned.

Conclusion: COVID-19 infection along with its medical management have increased patient susceptibility to MM.

© The Author(s) 2021. Published by Oxford University Press on behalf of the Association of Physicians. All rights reserved. For permissions, please email: journals.permissions@oup.com.

- Cited by 4 articles
- 35 references
- 2 figures

Supplementary info

Publication types, MeSH terms Expand

Publication types

- Observational Study

MeSH terms

- Adult
- COVID-19*

- Emergency Service, Hospital
- Female
- Humans
- Male
- Mucormycosis* / diagnosis
- Mucormycosis* / epidemiology
- Retrospective Studies
- SARS-CoV-2

Full text links

Free PMC article
Proceed to details
Cite
Share
☐ 550
Observational Study
BMC Neurol
. 2021 Feb 19;21(1):83.
doi: 10.1186/s12883-021-02109-8.

Coronavirus disease 2019 (COVID-19) can predispose young to Intracerebral hemorrhage: a retrospective observational study

Michael T Lawton [1], Ehsan Alimohammadi [2], Seyed Reza Bagheri [3], Arash Bostani [4], Siavash Vaziri [5], Ali Karbasforoushan [6], Kossar Mozaffari [7], Mehran Bahrami Bukani [3], Alireza Abdi [8]
Affiliations Expand

Affiliations

- [1] Department of Neurological Surgery, Barrow Neurological Institute, St. Joseph's Hospital and Medical Center, Phoenix, AZ, USA.
- [2] Department of neurosurgery, Kermanshah University of Medical Sciences, Kermanshah, Iran. hafez125@gmail.com.
- [3] Department of neurosurgery, Kermanshah University of Medical Sciences, Kermanshah, Iran.
- [4] Department of neurology, Kermanshah University of Medical Sciences, Kermanshah, Iran.
- [5] Infectious Disease Research Center, Kermanshah University of Medical Sciences, Kermanshah, Iran.
- [6] Department of anesthesiology, Kermanshah University of Medical Sciences, Kermanshah, Iran.

- [7] Clinical Research Development Center, Imam Reza hospital Kermanshah University of Medical Sciences, Imam Reza hospital, Kermanshah, Iran.
- [8] Nursing and midwifery school, Kermanshah University of Medical Sciences, Kermanshah, Iran.

- PMID: **33607952**
- PMCID: PMC7892324
- DOI: 10.1186/s12883-021-02109-8

Free PMC article
Observational Study

Coronavirus disease 2019 (COVID-19) can predispose young to Intracerebral hemorrhage: a retrospective observational study

Michael T Lawton et al. BMC Neurol. 2021.
Free PMC article
Show details
BMC Neurol

. 2021 Feb 19;21(1):83.
doi: 10.1186/s12883-021-02109-8.

Authors

Michael T Lawton [1], Ehsan Alimohammadi [2], Seyed Reza Bagheri [3], Arash Bostani [4], Siavash Vaziri [5], Ali Karbasforoushan [6], Kossar Mozaffari [7], Mehran Bahrami Bukani [3], Alireza Abdi [8]

Affiliations

- [1] Department of Neurological Surgery, Barrow Neurological Institute, St. Joseph's Hospital and Medical Center, Phoenix, AZ, USA.
- [2] Department of neurosurgery, Kermanshah University of Medical Sciences, Kermanshah, Iran. hafez125@gmail.com.
- [3] Department of neurosurgery, Kermanshah University of Medical Sciences, Kermanshah, Iran.
- [4] Department of neurology, Kermanshah University of Medical Sciences, Kermanshah, Iran.
- [5] Infectious Disease Research Center, Kermanshah University of Medical Sciences, Kermanshah, Iran.
- [6] Department of anesthesiology, Kermanshah University of Medical Sciences, Kermanshah, Iran.
- [7] Clinical Research Development Center, Imam Reza hospital Kermanshah University of Medical Sciences, Imam Reza hospital, Kermanshah, Iran.
- [8] Nursing and midwifery school, Kermanshah University of Medical Sciences, Kermanshah, Iran.

- PMID: **33607952**
- PMCID: PMC7892324
- DOI: 10.1186/s12883-021-02109-8

Abstract

Background: The respiratory system involvement is the most common presentation of Coronavirus disease 2019 (COVID-19). However, other organs including the central nervous system (CNS) could be affected by the virus. Strokes, seizures, change in mental status, and encephalitis have been reported as the neurological manifestation of the disease. We hypothesized that COVID-19 could predispose younger patients to spontaneous intracerebral hemorrhage (ICH). The present study aimed to investigate whether COVID-19 has any relationship with the occurrence of spontaneous ICH in young or not.

Methods: We retrospectively evaluated all the patients with spontaneous ICH who were referred to our center between 20 Feb and 1 Sep 2020. The demographic, clinical, radiological, and laboratory test data were evaluated. Patients were divided into two groups. The COVID-19 positive patients and COVID-19 negative ones. All the variables including age, sex, history of hypertension, diabetes mellitus, smoking, Glasgow Coma Scale (GCS), hematoma volume and location, the presence of intraventricular hemorrhage and hydrocephalus on admission, the length of hospital stay, the lab test results and the clinical outcome at last visit or discharge as Glasgow Outcome Scale (GOS) were compared between the two groups.

Results: There were 22 COVID-19 positive patients (20.8%) and 84 COVID-19 negative ones (79.2%). The mean age of the patients in the case group (54.27 ± 4.67) was significantly lower than that in the control group (69.88 ± 4.47) ($p < 0.05$). Meanwhile, our results showed a significant difference between the two groups based on the presence of chronic arterial hypertension ($p < 0.05$). There were no significant differences between the two groups based on gender, diabetes mellitus, smoking, Glasgow Coma Scale (GCS), hematoma volume, need for surgery, the presence of intraventricular hemorrhage and hydrocephalus on admission, White Blood Cell (WBC) count, platelet count, Prothrombin Time (PT), and Partial Thromboplastin Time (PTT) ($p > 0.05$).

Conclusions: Our results show that COVID positive patients with ICH are younger and with less predisposing factors than COVID negative subjects with ICH.

Keywords: Central nervous system; Coronavirus disease 2019; Intracerebral hemorrhage; chronic arterial hypertension, hematoma..

Conflict of interest statement

All authors declare that they have no conflict of interest.

- Cited by 3 articles
- 24 references
- 1 figure

Supplementary info

Publication types, MeSH terms Expand

Publication types

- Observational Study

MeSH terms

- Aged
- COVID-19 / complications*
- Case-Control Studies
- Cerebral Hemorrhage / epidemiology*
- Female
- Glasgow Coma Scale
- Glasgow Outcome Scale
- Hematoma / epidemiology*
- Hematoma / surgery
- Hospitalization
- Humans
- Hydrocephalus / epidemiology
- Length of Stay
- Leukocyte Count
- Male
- Middle Aged
- Retrospective Studies

Full text links

Read free full text at BMC BioMed Central Free PMC article

Proceed to details

Cite

Share

551

Observational Study

J Am Soc Nephrol

. 2021 Jan;32(1):151-160.

doi: 10.1681/ASN.2020050615. Epub 2020 Sep 3.

AKI in Hospitalized Patients with COVID-19

Lili Chan [1 2 3 4], Kumardeep Chaudhary [3 4 5], Aparna Saha [3 4], Kinsuk Chauhan [1], Akhil Vaid [6], Shan Zhao [6 7], Ishan Paranjpe [6], Sulaiman Somani [6], Felix Richter [5 6], Riccardo Miotto [5 6], Anuradha Lala [7 8], Arash Kia [9 10], Prem Timsina [9 10], Li Li [5 11], Robert Freeman [9 10], Rong Chen [5 11], Jagat Narula [12 13], Allan C Just [14], Carol Horowitz [2 9], Zahi Fayad [15 16], Carlos Cordon-Cardo [17], Eric Schadt [5 11], Matthew A Levin [7], David L Reich [7], Valentin

Fuster [8], Barbara Murphy [1 2], John C He [1 2], Alexander W Charney [5 18 19], Erwin P Böttinger [6 20], Benjamin S Glicksberg [5 6], Steven G Coca [21 2], Girish N Nadkarni [21 2 3 4 6], Mount Sinai COVID Informatics Center (MSCIC)
Collaborators, Affiliations Expand

Collaborators

- **Mount Sinai COVID Informatics Center (MSCIC)**:
Lili Chan, Kumardeep Chaudhary, Aparna Saha, Kinsuk Chauhan, Akhil Vaid, Shan Zhao, Ishan Paranjpe, Sulaiman Somani, Felix Richter, Riccardo Miotto, Anuradha Lala, Arash Kia, Prem Timsina, Li Li, Robert Freeman, Rong Chen, Jagat Narula, Allan C Just, Carol Horowitz, Zahi Fayad, Carlos Cordon-Cardo, Eric Schadt, Matthew A Levin, David L Reich, Valentin Fuster, Barbara Murphy, John C He, Alexander W Charney, Erwin P Böttinger, Benjamin S Glicksberg, Steven G Coca, Girish N Nadkarni

Affiliations

- [1] Division of Nephrology, Department of Medicine, Icahn School of Medicine at Mount Sinai, New York, New York.
- [2] Department of Medicine, Icahn School of Medicine at Mount Sinai, New York, New York.
- [3] The Charles Bronfman Institute for Personalized Medicine, Icahn School of Medicine at Mount Sinai, New York, New York.
- [4] BioMe Phenomics Center, Icahn School of Medicine at Mount Sinai, New York, New York.
- [5] Department of Genetics and Genomic Sciences, Icahn School of Medicine at Mount Sinai, New York, New York.
- [6] The Hasso Plattner Institute for Digital Health at Mount Sinai, New York, New York.
- [7] Department of Anesthesiology, Perioperative and Pain Medicine, Icahn School of Medicine at Mount Sinai, New York, New York.
- [8] The Zena and Michael A. Wiener Cardiovascular Institute, Icahn School of Medicine at Mount Sinai, New York, New York.
- [9] Department of Population Health Science and Policy, Icahn School of Medicine at Mount Sinai, New York, New York.
- [10] Institute for Healthcare Delivery Science, Icahn School of Medicine at Mount Sinai, New York, New York.
- [11] Icahn Institute for Data Science and Genomic Technology, Icahn School of Medicine at Mount Sinai, New York, New York.
- [12] Mount Sinai Heart, Icahn School of Medicine at Mount Sinai, New York, New York.
- [13] Department of Cardiology, Icahn School of Medicine at Mount Sinai, New York, New York.
- [14] Department of Environmental Medicine and Public Health, Icahn School of Medicine at Mount Sinai, New York, New York.
- [15] BioMedical Engineering and Imaging Institute, Icahn School of Medicine at Mount Sinai, New York, New York.
- [16] Department of Radiology, Icahn School of Medicine at Mount Sinai, New York, New York.
- [17] Department of Pathology, Icahn School of Medicine at Mount Sinai, New York, New York.

- [18] The Pamela Sklar Division of Psychiatric Genomics, Icahn School of Medicine at Mount Sinai, New York, New York.
- [19] Department of Psychiatry, Icahn School of Medicine at Mount Sinai, New York, New York.
- [20] Digital Health Center, Hasso Plattner Institute, University of Potsdam, Potsdam, Germany.
- [21] Division of Nephrology, Department of Medicine, Icahn School of Medicine at Mount Sinai, New York, New York steven.coca@mssm.edu girish.nadkarni@mountsinai.org.

- PMID: **32883700**
- PMCID: PMC7894657
- DOI: 10.1681/ASN.2020050615

Free PMC article
Observational Study

AKI in Hospitalized Patients with COVID-19

Lili Chan et al. J Am Soc Nephrol. 2021 Jan.
Free PMC article

Show details

J Am Soc Nephrol
. 2021 Jan;32(1):151-160.
doi: 10.1681/ASN.2020050615. Epub 2020 Sep 3.

Authors

Lili Chan [1 2 3 4], Kumardeep Chaudhary [3 4 5], Aparna Saha [3 4], Kinsuk Chauhan [1], Akhil Vaid [6], Shan Zhao [6 7], Ishan Paranjpe [6], Sulaiman Somani [6], Felix Richter [5 6], Riccardo Miotto [5 6], Anuradha Lala [7 8], Arash Kia [9 10], Prem Timsina [9 10], Li Li [5 11], Robert Freeman [9 10], Rong Chen [5 11], Jagat Narula [12 13], Allan C Just [14], Carol Horowitz [2 9], Zahi Fayad [15 16], Carlos Cordon-Cardo [17], Eric Schadt [5 11], Matthew A Levin [7], David L Reich [7], Valentin Fuster [8], Barbara Murphy [1 2], John C He [1 2], Alexander W Charney [5 18 19], Erwin P Böttinger [6 20], Benjamin S Glicksberg [5 6], Steven G Coca [21 2], Girish N Nadkarni [21 2 3 4 6], Mount Sinai COVID Informatics Center (MSCIC)

Collaborators

- **Mount Sinai COVID Informatics Center (MSCIC)**: Lili Chan, Kumardeep Chaudhary, Aparna Saha, Kinsuk Chauhan, Akhil Vaid, Shan Zhao, Ishan Paranjpe, Sulaiman Somani, Felix Richter, Riccardo Miotto, Anuradha Lala, Arash Kia, Prem Timsina, Li Li, Robert Freeman, Rong Chen, Jagat Narula, Allan C Just, Carol Horowitz, Zahi Fayad, Carlos Cordon-Cardo, Eric Schadt, Matthew A Levin, David L Reich, Valentin Fuster, Barbara Murphy, John C He, Alexander W Charney, Erwin P Böttinger, Benjamin S Glicksberg, Steven G Coca, Girish N Nadkarni

Affiliations

- [1] Division of Nephrology, Department of Medicine, Icahn School of Medicine at Mount Sinai, New York, New York.
- [2] Department of Medicine, Icahn School of Medicine at Mount Sinai, New York, New York.
- [3] The Charles Bronfman Institute for Personalized Medicine, Icahn School of Medicine at Mount Sinai, New York, New York.
- [4] BioMe Phenomics Center, Icahn School of Medicine at Mount Sinai, New York, New York.
- [5] Department of Genetics and Genomic Sciences, Icahn School of Medicine at Mount Sinai, New York, New York.
- [6] The Hasso Plattner Institute for Digital Health at Mount Sinai, New York, New York.
- [7] Department of Anesthesiology, Perioperative and Pain Medicine, Icahn School of Medicine at Mount Sinai, New York, New York.
- [8] The Zena and Michael A. Wiener Cardiovascular Institute, Icahn School of Medicine at Mount Sinai, New York, New York.
- [9] Department of Population Health Science and Policy, Icahn School of Medicine at Mount Sinai, New York, New York.
- [10] Institute for Healthcare Delivery Science, Icahn School of Medicine at Mount Sinai, New York, New York.
- [11] Icahn Institute for Data Science and Genomic Technology, Icahn School of Medicine at Mount Sinai, New York, New York.
- [12] Mount Sinai Heart, Icahn School of Medicine at Mount Sinai, New York, New York.
- [13] Department of Cardiology, Icahn School of Medicine at Mount Sinai, New York, New York.
- [14] Department of Environmental Medicine and Public Health, Icahn School of Medicine at Mount Sinai, New York, New York.
- [15] BioMedical Engineering and Imaging Institute, Icahn School of Medicine at Mount Sinai, New York, New York.
- [16] Department of Radiology, Icahn School of Medicine at Mount Sinai, New York, New York.
- [17] Department of Pathology, Icahn School of Medicine at Mount Sinai, New York, New York.
- [18] The Pamela Sklar Division of Psychiatric Genomics, Icahn School of Medicine at Mount Sinai, New York, New York.
- [19] Department of Psychiatry, Icahn School of Medicine at Mount Sinai, New York, New York.
- [20] Digital Health Center, Hasso Plattner Institute, University of Potsdam, Potsdam, Germany.
- [21] Division of Nephrology, Department of Medicine, Icahn School of Medicine at Mount Sinai, New York, New York steven.coca@mssm.edu girish.nadkarni@mountsinai.org.

- PMID: **32883700**
- PMCID: PMC7894657
- DOI: 10.1681/ASN.2020050615

Abstract

Background: Early reports indicate that AKI is common among patients with coronavirus disease 2019 (COVID-19) and associated with worse outcomes. However, AKI among hospitalized patients with COVID-19 in the United States is not well described.

Methods: This retrospective, observational study involved a review of data from electronic health records of patients aged ≥18 years with laboratory-confirmed COVID-19 admitted to the Mount Sinai Health System from February 27 to May 30, 2020. We describe the frequency of AKI and dialysis requirement, AKI recovery, and adjusted odds ratios (aORs) with mortality.

Results: Of 3993 hospitalized patients with COVID-19, AKI occurred in 1835 (46%) patients; 347 (19%) of the patients with AKI required dialysis. The proportions with stages 1, 2, or 3 AKI were 39%, 19%, and 42%, respectively. A total of 976 (24%) patients were admitted to intensive care, and 745 (76%) experienced AKI. Of the 435 patients with AKI and urine studies, 84% had proteinuria, 81% had hematuria, and 60% had leukocyturia. Independent predictors of severe AKI were CKD, men, and higher serum potassium at admission. In-hospital mortality was 50% among patients with AKI versus 8% among those without AKI (aOR, 9.2; 95% confidence interval, 7.5 to 11.3). Of survivors with AKI who were discharged, 35% had not recovered to baseline kidney function by the time of discharge. An additional 28 of 77 (36%) patients who had not recovered kidney function at discharge did so on posthospital follow-up.

Conclusions: AKI is common among patients hospitalized with COVID-19 and is associated with high mortality. Of all patients with AKI, only 30% survived with recovery of kidney function by the time of discharge.

Keywords: COVID-19; acute renal failure; clinical nephrology; dialysis.

Copyright © 2021 by the American Society of Nephrology.

Update of

- Acute Kidney Injury in Hospitalized Patients with COVID-19.
  Chan L, Chaudhary K, Saha A, Chauhan K, Vaid A, Baweja M, Campbell K, Chun N, Chung M, Deshpande P, Farouk SS, Kaufman L, Kim T, Koncicki H, Lapsia V, Leisman S, Lu E, Meliambro K, Menon MC, Rein JL, Sharma S, Tokita J, Uribarri J, Vassalotti JA, Winston J, Mathews KS, Zhao S, Paranjpe I, Somani S, Richter F, Do R, Miotto R, Lala A, Kia A, Timsina P, Li L, Danieletto M, Golden E, Glowe P, Zweig M, Singh M, Freeman R, Chen R, Nestler E, Narula J, Just AC, Horowitz C, Aberg J, Loos RJF, Cho J, Fayad Z, Cordon-Cardo C, Schadt E, Levin MA, Reich DL, Fuster V, Murphy B, He JC, Charney AW, Bottinger EP, Glicksberg BS, Coca SG, Nadkarni GN. Chan L, et al. medRxiv. 2020 May 8:2020.05.04.20090944. doi: 10.1101/2020.05.04.20090944. Preprint. medRxiv. 2020. PMID: 32511564 Free PMC article. Updated.

Comment in

- COVID-19-Associated Acute Kidney Injury: Learning from the First Wave.
  Wald R, Bagshaw SM. Wald R, et al. J Am Soc Nephrol. 2021 Jan;32(1):4-6. doi: 10.1681/ASN.2020101401. Epub 2020 Oct 28. J Am Soc Nephrol. 2021. PMID: 33115918 Free PMC article. No abstract available.

- Cited by 167 articles
- 5 figures

Supplementary info

Publication types, MeSH terms, Grant support Expand

Publication types

- Observational Study

MeSH terms

- Acute Kidney Injury / epidemiology
- Acute Kidney Injury / etiology*
- Acute Kidney Injury / therapy
- Acute Kidney Injury / urine
- Aged
- Aged, 80 and over
- COVID-19 / complications*
- COVID-19 / mortality
- Female
- Hematuria / etiology
- Hospital Mortality
- Hospitals, Private / statistics & numerical data
- Hospitals, Urban / statistics & numerical data
- Humans
- Incidence
- Inpatients
- Leukocytes
- Male
- Middle Aged
- New York City / epidemiology
- Proteinuria / etiology
- Renal Dialysis
- Retrospective Studies
- SARS-CoV-2*
- Treatment Outcome
- Urine / cytology

Grant support

- R01 HL085757/HL/NHLBI NIH HHS/United States
- U01 DK106962/DK/NIDDK NIH HHS/United States
- R01 DK108803/DK/NIDDK NIH HHS/United States
- U01OH011326/ACL/ACL HHS/United States

- U01 DK116100/DK/NIDDK NIH HHS/United States
- R01 DK115562/DK/NIDDK NIH HHS/United States
- U01 HG007278/HG/NHGRI NIH HHS/United States
- R01 DK112258/DK/NIDDK NIH HHS/United States
- K23 DK107908/DK/NIDDK NIH HHS/United States

Show all 9 grants

Full text links

Final Version FREE J Am Soc Nephrol HighWire Free PMC article

Proceed to details

Cite

Share

552

Observational Study

Aust Crit Care

. 2021 Mar;34(2):167-175.

doi: 10.1016/j.aucc.2020.10.009. Epub 2020 Oct 27.

Mechanical ventilation and mortality among 223 critically ill patients with coronavirus disease 2019: A multicentric study in Germany

Kevin Roedl [1], Dominik Jarczak [2], Liina Thasler [2], Martin Bachmann [3], Frank Schulte [4], Berthold Bein [5], Christian Friedrich Weber [6], Ulrich Schäfer [7], Carsten Veit [8], Hans-Peter Hauber [9], Sebastian Kopp [10], Karsten Sydow [11], Andreas de Weerth [12], Marc Bota [13], Rüdiger Schreiber [14], Oliver Detsch [15], Jan-Peer Rogmann [16], Daniel Frings [2], Barbara Sensen [2], Christoph Burdelski [2], Olaf Boenisch [2], Axel Nierhaus [2], Geraldine de Heer [2], Stefan Kluge [2]

Affiliations Expand

Affiliations

- [1] Department of Intensive Care Medicine, University Medical Center Hamburg-Eppendorf, Hamburg, Germany. Electronic address: k.roedl@uke.de.
- [2] Department of Intensive Care Medicine, University Medical Center Hamburg-Eppendorf, Hamburg, Germany.
- [3] Department of Intensive Care and Respiratory Medicine, Clinic for Airway-, Thorax and Respiratory Medicine, Asklepios Hospital Harburg, Hamburg, Germany.
- [4] Department of Pneumonology and Intensive Care Medicine, Weaningcenter, Asklepios Hospital Barmbek, Hamburg, Germany.

- [5] Department of Anaesthesiology, Intensive Care, Emergency Medicine and Pain Medicine, Asklepios Hospital St. Georg, Hamburg, Germany.
- [6] Department of Anaesthesiology, Intensive Care and Emergency Medicine, Asklepios Hospital Wandsbek, Hamburg, Germany.
- [7] Department of Cardiology, Angiology and Intensive Care Medicine, Marien Hospital, Hamburg, Germany.
- [8] Department of Interdisciplinary Intensive Care Medicine, Bundeswehr Hospital, Hamburg, Germany.
- [9] Department of Cardiology, Pneumology and Intensive Care Medicine, Asklepios Hospital Altona, Hamburg, Germany.
- [10] Departement for Anaesthesiology and Intensive Care Medicine, Amalie Sieveking Hospital, Hamburg, Germany.
- [11] Department of Cardiology, Albertinen Hospital, Hamburg, Germany.
- [12] Department of Internal Medicine, Agaplesion Diakonie Hospital Hamburg, Hamburg, Germany.
- [13] Department of Internal Medicine, Bethesda Hospital Bergedorf, Hamburg, Germany.
- [14] Department of Anaesthesiology, Intensive Care and Emergency Medicine, Asklepios West Hospital Hamburg, Hamburg, Germany.
- [15] Department of Anaesthesiology, Intensive Care, Emergency Medicine and Pain Medicine, Asklepios Hospital Nord, Hamburg, Germany.
- [16] Department of Anaesthesiology and Intensive Care Medicine, Israelitic Hospital, Hamburg, Germany.

- PMID: **33250401**
- PMCID: PMC7590821
- DOI: 10.1016/j.aucc.2020.10.009

Free PMC article
Observational Study

Mechanical ventilation and mortality among 223 critically ill patients with coronavirus disease 2019: A multicentric study in Germany

Kevin Roedl et al. Aust Crit Care. 2021 Mar.
Free PMC article
Show details
Aust Crit Care
. 2021 Mar;34(2):167-175.
doi: 10.1016/j.aucc.2020.10.009. Epub 2020 Oct 27.

Authors

Kevin Roedl [1], Dominik Jarczak [2], Liina Thasler [2], Martin Bachmann [3], Frank Schulte [4], Berthold Bein [5], Christian Friedrich Weber [6], Ulrich Schäfer [7], Carsten Veit [8], Hans-Peter

Hauber [9], Sebastian Kopp [10], Karsten Sydow [11], Andreas de Weerth [12], Marc Bota [13], Rüdiger Schreiber [14], Oliver Detsch [15], Jan-Peer Rogmann [16], Daniel Frings [2], Barbara Sensen [2], Christoph Burdelski [2], Olaf Boenisch [2], Axel Nierhaus [2], Geraldine de Heer [2], Stefan Kluge [2]

Affiliations

- [1] Department of Intensive Care Medicine, University Medical Center Hamburg-Eppendorf, Hamburg, Germany. Electronic address: k.roedl@uke.de.
- [2] Department of Intensive Care Medicine, University Medical Center Hamburg-Eppendorf, Hamburg, Germany.
- [3] Department of Intensive Care and Respiratory Medicine, Clinic for Airway-, Thorax and Respiratory Medicine, Asklepios Hospital Harburg, Hamburg, Germany.
- [4] Department of Pneumonology and Intensive Care Medicine, Weaningcenter, Asklepios Hospital Barmbek, Hamburg, Germany.
- [5] Department of Anaesthesiology, Intensive Care, Emergency Medicine and Pain Medicine, Asklepios Hospital St. Georg, Hamburg, Germany.
- [6] Department of Anaesthesiology, Intensive Care and Emergency Medicine, Asklepios Hospital Wandsbek, Hamburg, Germany.
- [7] Department of Cardiology, Angiology and Intensive Care Medicine, Marien Hospital, Hamburg, Germany.
- [8] Department of Interdisciplinary Intensive Care Medicine, Bundeswehr Hospital, Hamburg, Germany.
- [9] Department of Cardiology, Pneumology and Intensive Care Medicine, Asklepios Hospital Altona, Hamburg, Germany.
- [10] Departement for Anaesthesiology and Intensive Care Medicine, Amalie Sieveking Hospital, Hamburg, Germany.
- [11] Department of Cardiology, Albertinen Hospital, Hamburg, Germany.
- [12] Department of Internal Medicine, Agaplesion Diakonie Hospital Hamburg, Hamburg, Germany.
- [13] Department of Internal Medicine, Bethesda Hospital Bergedorf, Hamburg, Germany.
- [14] Department of Anaesthesiology, Intensive Care and Emergency Medicine, Asklepios West Hospital Hamburg, Hamburg, Germany.
- [15] Department of Anaesthesiology, Intensive Care, Emergency Medicine and Pain Medicine, Asklepios Hospital Nord, Hamburg, Germany.
- [16] Department of Anaesthesiology and Intensive Care Medicine, Israelitic Hospital, Hamburg, Germany.

- PMID: **33250401**
- PMCID: PMC7590821
- DOI: 10.1016/j.aucc.2020.10.009

Abstract

Background: There are large uncertainties with regard to the outcome of patients with coronavirus disease 2019 (COVID-19) and mechanical ventilation (MV). High mortality (50-97%) was proposed by some groups, leading to considerable uncertainties with regard to outcomes of critically ill patients with COVID-19.

Objectives: The aim was to investigate the characteristics and outcomes of critically ill patients with COVID-19 requiring intensive care unit (ICU) admission and MV.

Methods: A multicentre retrospective observational cohort study at 15 hospitals in Hamburg, Germany, was performed. Critically ill adult patients with COVID-19 who completed their ICU stay between February and June 2020 were included. Patient demographics, severity of illness, and ICU course were retrospectively evaluated.

Results: A total of 223 critically ill patients with COVID-19 were included. The majority, 73% (n = 163), were men; the median age was 69 (interquartile range = 58-77.5) years, with 68% (n = 151) patients having at least one chronic medical condition. Their Sequential Organ Failure Assessment score was a median of 5 (3-9) points on admission. Overall, 167 (75%) patients needed MV. Noninvasive ventilation and high-flow nasal cannula were used in 31 (14%) and 26 (12%) patients, respectively. Subsequent MV, due to noninvasive ventilation/high-flow nasal cannula therapy failure, was necessary in 46 (81%) patients. Renal replacement therapy was initiated in 33% (n = 72) of patients, and owing to severe respiratory failure, extracorporeal membrane oxygenation was necessary in 9% (n = 20) of patients. Experimental antiviral therapy was used in 9% (n = 21) of patients. Complications during the ICU stay were as follows: septic shock (40%, n = 90), heart failure (8%, n = 17), and pulmonary embolism (6%, n = 14). The length of ICU stay was a median of 13 days (5-24), and the duration of MV was 15 days (8-25). The ICU mortality was 35% (n = 78) and 44% (n = 74) among mechanically ventilated patients.

Conclusion: In this multicentre observational study of 223 critically ill patients with COVID-19, the survival to ICU discharge was 65%, and it was 56% among patients requiring MV. Patients showed high rate of septic complications during their ICU stay.

Keywords: ARDS; COVID-19; Mechanical ventilation; Mortality; Multiple organ failure; SARS-CoV-2.

Copyright © 2020 Australian College of Critical Care Nurses Ltd. Published by Elsevier Ltd. All rights reserved.

Conflict of interest statement

Conflict of Interest K.R., D.J., L.T., M.B., F.S., C.F.W., U.S., C.V., H.-P.H., S.Ko., K.S., A.d.W., M.B., R.S., O.D., J.-P.R., B.S., C.B., O.B., B.B., and G.d.H. do not report any conflicts of interest. S.K. received research support from Ambu, E.T.View Ltd., Fisher & Paykel, Pfizer, and Xenios; lecture honoraria from ArjoHuntleigh, Astellas, Astra, Basilea, Bard, Baxter, Biotest, CSL Behring, CytoSorbents, Fresenius, Gilead, MSD, Orion, Pfizer, Philips, Sedana, Sorin, Xenios, and Zoll; and consultant honorarium from AMOMED, Astellas, Baxter, Bayer, Fresenius, Gilead, MSD, Pfizer, and Xenios. A.N. received research funds, lecture honoraria, and travel reimbursement within the last 5 years from CytoSorbents Europe, Biotest AG, and Thermo Fisher Scientific. D.F. reports lecture honoraria within the last 5 years from Xenios AG.

Comment in

- Renal failure in COVID-19 ARDS: Could it be partially avoided?
  Zakynthinos GE, Tsolaki V. Zakynthinos GE, et al. Aust Crit Care. 2021 Nov;34(6):523. doi: 10.1016/j.aucc.2021.01.003. Epub 2021 Feb 11. Aust Crit Care. 2021. PMID: 33707068 Free PMC article. No abstract available.

- Cited by 28 articles
- 37 references

- 1 figure

Supplementary info

Publication types, MeSH terms Expand

Publication types

- Multicenter Study
- Observational Study

MeSH terms

- Aged
- COVID-19 / mortality*
- COVID-19 / therapy*
- Critical Illness*
- Female
- Germany / epidemiology
- Humans
- Male
- Middle Aged
- Pneumonia, Viral / mortality*
- Pneumonia, Viral / therapy*
- Pneumonia, Viral / virology
- Respiration, Artificial*
- Retrospective Studies
- SARS-CoV-2

Full text links

ELSEVIER FULL-TEXT ARTICLE Elsevier Science Free PMC article
Proceed to details
Cite
Share
☐ 553
Observational Study
Eur Arch Otorhinolaryngol
. 2021 May;278(5):1605-1612.
doi: 10.1007/s00405-020-06220-3. Epub 2020 Aug 4.

Outcome of 1890 tracheostomies for critical COVID-19 patients: a national cohort study in Spain

Cristina Martin-Villares [1, 2], Carmen Perez Molina-Ramirez [3], Margarita Bartolome-Benito [4], Manuel Bernal-Sprekelsen [5], COVID ORL ESP Collaborative Group (*)

Collaborators, Affiliations Expand

Collaborators

- **COVID ORL ESP Collaborative Group (*)**: Aranzazu Perez-Fernandez, Sara Alcantara-Armenteros, Irene Monjas-Cánovas, Manuela Sancho-Mestre, Oscar Alemán-Lopez, M Dolors Deola-Trasserra, Vanessa Villarraga-Cova, Azor Carreras-Alcaraz, Esther Montaner-Sala, Esther Sota-Eguizabal, Aihnoa Tolosa, Belen De la Iglesia, Rafael Garcia-Sardon, Laura Diez, Eduardo Lehrer, Francisco Xavier Aviles-Jurado, Kiara Tudela-Cabello, Gabriel Huguet-Llull, Marta Mesalles-Ruíz, Ramon Jimenez-Montoya, Ana Navazo-Eguïa, Blanca Galindo-Torres, Marta Fernandez-Pello, Nuria Rodriguez-Prado, Carmen Salazar, Francisco Ramos, Juan Carlos Amor-Dorado, Marta Faubel-Serra, Valery Nuñez-Carrasco, Adriana Agüero-Medina, Juan Carlos-Villatoro, Roser Lopez-Diu, Selvyn Gonzalez-Melgan, María Uzcanga-Lacabe, Marisela Cardier-Suárez, Esther Úbeda-Fernández, Miguel Ángel Alañon-Fernández, Carlos Sanchez-Herrero, Jose Antonio Municio-Martin, Luis Pascua-Gomez, Sergio Andrino-Martin, Sandra Ayala-Mejias, Bárbara Molina-Gil, Jorge De Abajo-Larriba, Zenaida Piñeiro-Aguín, Elisa Gil-Carcedo, Javier Herrero-Agustín, Jorge Freijanes-Otero, Mario Cuetos-Azcona, Alfredo Garcia-Fernandez, Ignacio Jimenez-Huerta, Rosa Babarro-Fernández, Felipe Junjgohann-Jofre, Antonio Martel-Lopez, Maria Dolores Martin-Sanchez, Jesus Benitez-Del Rosario, Diana Luorido, Monica Granda, Jose Miguel Tejeda, Antonio Martinez Ruiz-Coello, Antonio Almodovar-Iniesta, Jesus Bonnin, Jacobo Chao-Vietes, Estefanía Hernández-Garcia, Guillermo Plaza, Jose Miguel Villacampa-Auba, Alfonso Campos-Gonzalez, Jose Carlos Casqueiro, Lucia Baguena-Campos, Alberto Encinas-Vicente, Raul Rubio-Yanguas, Alejandro Lowy-Benoliel, Daniel Poletti-Serafini, Juan Antonio Pasamontes-Pingarron, Miguel Aristegui-Ruiz, Monica Hernando-Cuñado, Rosalia Souviron-Encabo, Tomas Martinez-Guirado, Mario Fernandez-Fernandez, Ricardo Gonzalez-Orus, Beatriz Molina-Montes, Raquel De la Fuente-Hernandez, Jesús Crovato-Rojas, Cristian Ruminot, Daniella Laguado, Hander Acosta, María José Hernández-Garcia, Alejandro Zuaza-Gonzalez, Carlos Domingo-Carrasco, Cristina Valor-García, Javier Lopez-Martin, Tomas Mogollon-Cano, Carla Meler-Claramonte, Juan Carlos Flores-Martín, Juanjo Arzok Del Toro, Alfonso Garcia-Piñero, Noelia Muñoz-Fernandez, Antonio Del Palacio, Blanca Mateos-Serrano, Isabel Garcia-Lopez, Laura Rodrigañez-Riesco, Paula Aragon, Teresa Rivera, Gustavo Eisenberg-Plaza, Inmaculada Fernandez-Robledo, Jorge Prada-Pendolero, Ignacio Alvarez-Alvarez, Maria Puente-Verez, Ana Quintana-Sanjuas, Pablo Parente-Arias, Francisco García-Cordoba, Francisco Jose Garcia-Purriños, Carmelo Morales-Angulo, Simara Rodriguez-Rondon, Diana Lopez-Lopez, Rafael Fernandez-Liesa, Amaya Lázaro-Sánchez, I Alonso-Alonso, Michalina Rusiecka, Rosa Delia Ramirez, Javier Vila, Angela Bellmunt-Fontanet, Victoria Montoro-Martinez, Francisco Piqueras, Vania Novoa-Morales, Gabriela Simonetti, Enrique Guillen-Lozada, Maria Jesus Velasco, Ignacio Alonso-Castiñeira, Maria Hernandez, Carlota Rovira-Ramos, Juan Jose Diaz-Argüello, Yolanda Escamilla-Carpintero, Pablo Torrico-Roman, Carlos Calvo-

Navarro, Ignacio Viza-Puiggros, Cristina Vaduva, Juan Higuera-Lucas, Jose Ignacio Tato, Teresa Rivera-Rodriguez, Diego Rodriguez-Contreras, Antonio Caravaca-Garcia, Alejandra Ayala, José Manuel Morales-Puebla, Carlos Ruiz-Escudero, E Lozano-Reina, Juan Manuel Maza-Solano, Jorge Alfaro-Garcia, Fatima Sanchez, Luordes Montes-Jovellar, Mar Medina, Rafael Barbera, Alfonso Marco-Garrido, Isabel Cremades-Navalon, Javier García-Callejo, C Garcia Bastida, Raimundo Gutierrez-Fonseca, Jorge Prada-Pendolero, Angel Muñoz-Herrera, Marta Calvo, Carmen Rosal-Fraga, Mari Cruz Iglesias-Moreno, Jesus Gimeno-Hernández, Cristina A Vázquez-Romero, Fabian Alzate-Amaya, Estefanía Hernández-Garcia, Laura Palomino, Marta Alcaraz, Coia Romeu-Figuerola, Maria Foglia-Fernandez, Cristina Dios-Loureiro, Isabel Gonzalez-Guijarro, Rafael Vera-Llao, Rosa Sancho-Calvo, Jesus Jose Ramos-Fernandez, Mar Lasso-delaVega, Paula Martinez-Pascual, Silvia Dominguez-Ovejas, Rafael Vera-Llao, Eduardo Morera-Senra, Jacoba Alba-Mesquida, Alfonso Bonilla, Javier Martinez-Subias, Jesus M Martinez-Salazar, Antonio Martinez-Lapeña, Rocio Corrales-Millan, Rosario Ruiz, Nieves Mata, Alfredo Espinosa, Laura Gerarda Cianci-Jaimes, Enrique Zapater-Latorre, Jose Ramon Alba-Garcia, Miriam Natsuki Oishi-Konari, Beatriz Pallares-Marti, Andrea Rubio-Fernandez, Jaime Santos-Pérez, Albert Idigora-Planas, Alvaro Sanchez-Barrueco, Ignacio Alcala-Rueda, Ruben Jara-Rubio, Estefanía Lozano, Isabel Tirado-Zamora, Enrique Coscaron-Blanco, Soledad Suarez-Ortega, Guillermo Gil-Grasa, Maria Jose Lavilla-Martin de Valmaseda, Jose Miguel Sebastian, Hazem Nijim, Jesus Dominguez-Calvo, Maria Jose Gonzalez-Gimeno, Ana Carvajal-Urueña, Luis Anel, Manuel Gonzalo-Orden

Affiliations

- [1] Department of Otorhinolaryngology, Hospital Universitario El Bierzo, Médicos sin Fronteras s/n, 24401, Ponferrada, León, Spain. crismvillares@gmail.com.
- [2] Department of Medicine, Surgery and Anatomy, University of León, León, Spain. crismvillares@gmail.com.
- [3] Department of Otorhinolaryngology, Complejo Universitario Hospitalario de Segovia, University of Valladolid, Segovia, Spain.
- [4] Department of Otorhinolaryngology, Hospital Infantil Universitario Niño Jesús, Autonomous University of Madrid, Madrid, Spain.
- [5] Department of Surgery, Otorhinolaryngology, University of Valencia, Valencia, Spain.

- PMID: **32749607**
- PMCID: PMC7399582
- DOI: 10.1007/s00405-020-06220-3

Free PMC article
Observational Study

Outcome of 1890 tracheostomies for critical COVID-19 patients: a national cohort study in Spain

Cristina Martin-Villares et al. Eur Arch Otorhinolaryngol. 2021 May.
Free PMC article
Show details

Eur Arch Otorhinolaryngol
. 2021 May;278(5):1605-1612.
doi: 10.1007/s00405-020-06220-3. Epub 2020 Aug 4.

Authors

Cristina Martin-Villares [1] [2], Carmen Perez Molina-Ramirez [3], Margarita Bartolome-Benito [4], Manuel Bernal-Sprekelsen [5], COVID ORL ESP Collaborative Group (*)

Collaborators

- **COVID ORL ESP Collaborative Group (*)**: Aranzazu Perez-Fernandez, Sara Alcantara-Armenteros, Irene Monjas-Cánovas, Manuela Sancho-Mestre, Oscar Alemán-Lopez, M Dolors Deola-Trasserra, Vanessa Villarraga-Cova, Azor Carreras-Alcaraz, Esther Montaner-Sala, Esther Sota-Eguizabal, Aihnoa Tolosa, Belen De la Iglesia, Rafael Garcia-Sardon, Laura Diez, Eduardo Lehrer, Francisco Xavier Aviles-Jurado, Kiara Tudela-Cabello, Gabriel Huguet-Llull, Marta Mesalles-Ruíz, Ramon Jimenez-Montoya, Ana Navazo-Eguïa, Blanca Galindo-Torres, Marta Fernandez-Pello, Nuria Rodriguez-Prado, Carmen Salazar, Francisco Ramos, Juan Carlos Amor-Dorado, Marta Faubel-Serra, Valery Nuñez-Carrasco, Adriana Agüero-Medina, Juan Carlos-Villatoro, Roser Lopez-Diu, Selvyn Gonzalez-Melgan, María Uzcanga-Lacabe, Marisela Cardier-Suárez, Esther Úbeda-Fernández, Miguel Ángel Alañon-Fernández, Carlos Sanchez-Herrero, Jose Antonio Municio-Martin, Luis Pascua-Gomez, Sergio Andrino-Martin, Sandra Ayala-Mejias, Bárbara Molina-Gil, Jorge De Abajo-Larriba, Zenaida Piñeiro-Aguín, Elisa Gil-Carcedo, Javier Herrero-Agustín, Jorge Freijanes-Otero, Mario Cuetos-Azcona, Alfredo Garcia-Fernandez, Ignacio Jimenez-Huerta, Rosa Babarro-Fernández, Felipe Junjgohann-Jofre, Antonio Martel-Lopez, Maria Dolores Martin-Sanchez, Jesus Benitez-Del Rosario, Diana Luorido, Monica Granda, Jose Miguel Tejeda, Antonio Martinez Ruiz-Coello, Antonio Almodovar-Iniesta, Jesus Bonnin, Jacobo Chao-Vietes, Estefanía Hernández-Garcia, Guillermo Plaza, Jose Miguel Villacampa-Auba, Alfonso Campos-Gonzalez, Jose Carlos Casqueiro, Lucia Baguena-Campos, Alberto Encinas-Vicente, Raul Rubio-Yanguas, Alejandro Lowy-Benoliel, Daniel Poletti-Serafini, Juan Antonio Pasamontes-Pingarron, Miguel Aristegui-Ruiz, Monica Hernando-Cuñado, Rosalia Souviron-Encabo, Tomas Martinez-Guirado, Mario Fernandez-Fernandez, Ricardo Gonzalez-Orus, Beatriz Molina-Montes, Raquel De la Fuente-Hernandez, Jesús Crovato-Rojas, Cristian Ruminot, Daniella Laguado, Hander Acosta, María José Hernández-Garcia, Alejandro Zuaza-Gonzalez, Carlos Domingo-Carrasco, Cristina Valor-García, Javier Lopez-Martin, Tomas Mogollon-Cano, Carla Meler-Claramonte, Juan Carlos Flores-Martín, Juanjo Arzok Del Toro, Alfonso Garcia-Piñero, Noelia Muñoz-Fernandez, Antonio Del Palacio, Blanca Mateos-Serrano, Isabel Garcia-Lopez, Laura Rodrigañez-Riesco, Paula Aragon, Teresa Rivera, Gustavo Eisenberg-Plaza, Inmaculada Fernandez-Robledo, Jorge Prada-Pendolero, Ignacio Alvarez-Alvarez, Maria Puente-Verez, Ana Quintana-Sanjuas, Pablo Parente-Arias, Francisco García-Cordoba, Francisco Jose Garcia-Purriños, Carmelo Morales-Angulo, Simara Rodriguez-Rondon, Diana Lopez-Lopez, Rafael Fernandez-Liesa, Amaya Lázaro-Sánchez, I Alonso-Alonso, Michalina Rusiecka, Rosa Delia Ramirez, Javier Vila, Angela Bellmunt-Fontanet, Victoria Montoro-Martinez, Francisco Piqueras, Vania Novoa-Morales, Gabriela Simonetti, Enrique Guillen-Lozada, Maria Jesus Velasco, Ignacio Alonso-Castiñeira, Maria Hernandez, Carlota Rovira-Ramos, Juan Jose Diaz-Argüello, Yolanda Escamilla-Carpintero, Pablo Torrico-Roman, Carlos Calvo-Navarro, Ignacio Viza-Puiggros, Cristina Vaduva, Juan Higuera-Lucas, Jose Ignacio Tato, Teresa Rivera-Rodriguez, Diego Rodriguez-Contreras, Antonio Caravaca-

Garcia, Alejandra Ayala, José Manuel Morales-Puebla, Carlos Ruiz-Escudero, E Lozano-Reina, Juan Manuel Maza-Solano, Jorge Alfaro-Garcia, Fatima Sanchez, Luordes Montes-Jovellar, Mar Medina, Rafael Barbera, Alfonso Marco-Garrido, Isabel Cremades-Navalon, Javier García-Callejo, C Garcia Bastida, Raimundo Gutierrez-Fonseca, Jorge Prada-Pendolero, Angel Muñoz-Herrera, Marta Calvo, Carmen Rosal-Fraga, Mari Cruz Iglesias-Moreno, Jesus Gimeno-Hernández, Cristina A Vázquez-Romero, Fabian Alzate-Amaya, Estefanía Hernández-Garcia, Laura Palomino, Marta Alcaraz, Coia Romeu-Figuerola, Maria Foglia-Fernandez, Cristina Dios-Loureiro, Isabel Gonzalez-Guijarro, Rafael Vera-Llao, Rosa Sancho-Calvo, Jesus Jose Ramos-Fernandez, Mar Lasso-delaVega, Paula Martinez-Pascual, Silvia Dominguez-Ovejas, Rafael Vera-Llao, Eduardo Morera-Senra, Jacoba Alba-Mesquida, Alfonso Bonilla, Javier Martinez-Subias, Jesus M Martinez-Salazar, Antonio Martinez-Lapeña, Rocio Corrales-Millan, Rosario Ruiz, Nieves Mata, Alfredo Espinosa, Laura Gerarda Cianci-Jaimes, Enrique Zapater-Latorre, Jose Ramon Alba-Garcia, Miriam Natsuki Oishi-Konari, Beatriz Pallares-Marti, Andrea Rubio-Fernandez, Jaime Santos-Pérez, Albert Idigora-Planas, Alvaro Sanchez-Barrueco, Ignacio Alcala-Rueda, Ruben Jara-Rubio, Estefanía Lozano, Isabel Tirado-Zamora, Enrique Coscaron-Blanco, Soledad Suarez-Ortega, Guillermo Gil-Grasa, Maria Jose Lavilla-Martin de Valmaseda, Jose Miguel Sebastian, Hazem Nijim, Jesus Dominguez-Calvo, Maria Jose Gonzalez-Gimeno, Ana Carvajal-Urueña, Luis Anel, Manuel Gonzalo-Orden

Affiliations

- [1] Department of Otorhinolaryngology, Hospital Universitario El Bierzo, Médicos sin Fronteras s/n, 24401, Ponferrada, León, Spain. crismvillares@gmail.com.
- [2] Department of Medicine, Surgery and Anatomy, University of León, León, Spain. crismvillares@gmail.com.
- [3] Department of Otorhinolaryngology, Complejo Universitario Hospitalario de Segovia, University of Valladolid, Segovia, Spain.
- [4] Department of Otorhinolaryngology, Hospital Infantil Universitario Niño Jesús, Autonomous University of Madrid, Madrid, Spain.
- [5] Department of Surgery, Otorhinolaryngology, University of Valencia, Valencia, Spain.

- PMID: **32749607**
- PMCID: PMC7399582
- DOI: 10.1007/s00405-020-06220-3

Abstract

Background: The question of an optimal strategy and outcomes in COVID-19 tracheostomy has not been answered yet. The critical focus in our case study is to evaluate the outcomes of tracheostomy on intubated COVID-19 patients.

Methods: A multicentric prospective observational study of 1890 COVID-19 patients undergoing tracheostomy across 120 hospitals was conducted over 7 weeks in Spain (March 28 to May 15, 2020). Data were collected with an innovative approach: instant messaging via WhatsApp.

Outcome measurements: complications, achieved weaning and decannulation and survival.

Results: We performed 1,461 surgical (81.3%) and 429 percutaneous tracheostomies. Median timing of tracheostomy was 12 days (4-42 days) since orotracheal intubation. A close follow-up of 1616/1890 (85.5%) patients at the cut-off time of 1-month follow-up showed that in 842 (52.1%) patients, weaning was achieved, while 391 (24.2%) were still under mechanical ventilation and

383 (23.7%) patients had died from COVID-19. Decannulation among those in whom weaning was successful (n = 842) was achieved in 683 (81%) patients.

Conclusion: To the best of our knowledge, this is the largest cohort of COVID-19 patients undergoing tracheostomy. The critical focus is the unprecedented amount of tracheostomies: 1890 in 7 weeks. Weaning could be achieved in over half of the patients with follow-up. Almost one out of four tracheotomized patients died from COVID-19.

Keywords: COVID-19; Intensive care unit; Mechanical ventilation; Tracheostomy.

Conflict of interest statement

We have no conflicts of interest to disclose.

- Cited by 34 articles
- 30 references
- 4 figures

Supplementary info

Publication types, MeSH terms Expand

Publication types

- Observational Study

MeSH terms

- COVID-19*
- Cohort Studies
- Humans
- Respiration, Artificial
- Retrospective Studies
- SARS-CoV-2
- Spain
- Tracheostomy*

Full text links

SpringerLink FULL-TEXT ARTICLE Springer Free PMC article
Proceed to details
Cite
Share
☐ 554
Observational Study
JMIR Public Health Surveill
. 2021 May 27;7(5):e28594.

doi: 10.2196/28594.

An Overview of the Treatment Options Used for the Management of COVID-19 in Pakistan: Retrospective Observational Study

Hashaam Akhtar [1], Samar Akhtar [1], Fazal-Ul Rahman [2], Maham Afridi [3], Sundas Khalid [4], Sabahat Ali [5], Nasim Akhtar [6], Yousef S Khader [7], Hamaad Ahmad [1], Muhammad Mujeeb Khan [8]
Affiliations Expand

Affiliations

- [1] Yusra Institute of Pharmaceutical Sciences, Yusra Medical and Dental College, Islamabad, Pakistan.
- [2] Department of Medicine, Benazir Bhutto Hospital, Rawalpindi, Pakistan.
- [3] Department of Biotechnology, Quaid-i-Azam University, Islamabad, Pakistan.
- [4] School of Chemical and Materials Engineering, National University of Science and Technology, Islamabad, Pakistan.
- [5] Department of Gynecology and Obstetrics, Pakistan Air Force Hospital, Islamabad, Pakistan.
- [6] Department of Infectious Diseases, Pakistan Institute of Medical Sciences, Islamabad, Pakistan.
- [7] Medical Education and Biostatistics, Department of Community Medicine, Public Health and Family Medicine, Faculty of Medicine, Jordan University of Science and Technology, Irbid, Jordan.
- [8] Department of Infectious Diseases, Rawalpindi Medical University, Rawalpindi, Pakistan.

- PMID: **33945498**
- PMCID: PMC8163494
- DOI: 10.2196/28594

Free PMC article
Observational Study

An Overview of the Treatment Options Used for the Management of COVID-19 in Pakistan: Retrospective Observational Study

Hashaam Akhtar et al. JMIR Public Health Surveill. 2021.
Free PMC article
Show details
JMIR Public Health Surveill
. 2021 May 27;7(5):e28594.

doi: 10.2196/28594.

Authors

Hashaam Akhtar [1], Samar Akhtar [1], Fazal-Ul Rahman [2], Maham Afridi [3], Sundas Khalid [4], Sabahat Ali [5], Nasim Akhtar [6], Yousef S Khader [7], Hamaad Ahmad [1], Muhammad Mujeeb Khan [8]

Affiliations

- [1] Yusra Institute of Pharmaceutical Sciences, Yusra Medical and Dental College, Islamabad, Pakistan.
- [2] Department of Medicine, Benazir Bhutto Hospital, Rawalpindi, Pakistan.
- [3] Department of Biotechnology, Quaid-i-Azam University, Islamabad, Pakistan.
- [4] School of Chemical and Materials Engineering, National University of Science and Technology, Islamabad, Pakistan.
- [5] Department of Gynecology and Obstetrics, Pakistan Air Force Hospital, Islamabad, Pakistan.
- [6] Department of Infectious Diseases, Pakistan Institute of Medical Sciences, Islamabad, Pakistan.
- [7] Medical Education and Biostatistics, Department of Community Medicine, Public Health and Family Medicine, Faculty of Medicine, Jordan University of Science and Technology, Irbid, Jordan.
- [8] Department of Infectious Diseases, Rawalpindi Medical University, Rawalpindi, Pakistan.

- PMID: **33945498**
- PMCID: PMC8163494
- DOI: 10.2196/28594

Abstract

Background: Since the first reports of COVID-19 infection, the foremost requirement has been to identify a treatment regimen that not only fights the causative agent but also controls the associated complications of the infection. Due to the time-consuming process of drug discovery, physicians have used readily available drugs and therapies for treatment of infections to minimize the death toll.

Objective: The aim of this study is to provide a snapshot analysis of the major drugs used in a cohort of 1562 Pakistani patients during the period from May to July 2020, when the first wave of COVID-19 peaked in Pakistan.

Methods: A retrospective observational study was performed to provide an overview of the major drugs used in a cohort of 1562 patients with COVID-19 admitted to the four major tertiary-care hospitals in the Rawalpindi-Islamabad region of Pakistan during the peak of the first wave of COVID-19 in the country (May-July 2020).

Results: Antibiotics were the most common choice out of all the therapies employed, and they were used as first line of treatment for COVID-19. Azithromycin was the most prescribed drug for treatment. No monthly trend was observed in the choice of antibiotics, and these drugs appeared to be a random but favored choice throughout the months of the study. It was also noted that even

antibiotics used for multidrug resistant infections were prescribed irrespective of the severity or progression of the infection. The results of the analysis are alarming, as this approach may lead to antibiotic resistance and complications in immunocompromised patients with COVID-19. A total of 1562 patients (1064 male, 68.1%, and 498 female, 31.9%) with a mean age of 47.35 years (SD 17.03) were included in the study. The highest frequency of patient hospitalizations occurred in June (846/1562, 54.2%).

Conclusions: Guidelines for a targeted treatment regime are needed to control related complications and to limit the misuse of antibiotics in the management of COVID-19.

Keywords: COVID-19; Pakistan; antibiotic resistance; antibiotics; first wave; multidrug resistant infections.

©Hashaam Akhtar, Samar Akhtar, Fazal-Ul Rahman, Maham Afridi, Sundas Khalid, Sabahat Ali, Nasim Akhtar, Yousef S Khader, Hamaad Ahmad, Muhammad Mujeeb Khan. Originally published in JMIR Public Health and Surveillance (https://publichealth.jmir.org), 27.05.2021.

Conflict of interest statement

Conflicts of Interest: None declared.

- Cited by 2 articles
- 40 references
- 2 figures

Supplementary info

Publication types, MeSH terms, Substances Expand

Publication types

- Observational Study
- Research Support, N.I.H., Extramural
- Research Support, Non-U.S. Gov't

MeSH terms

- Adult
- Anti-Bacterial Agents / therapeutic use
- COVID-19 / drug therapy*
- COVID-19 / epidemiology
- Female
- Humans
- Male
- Middle Aged
- Pakistan / epidemiology
- Retrospective Studies

- Tertiary Care Centers

Substances

- Anti-Bacterial Agents

Full text links

JMIR Publications Free PMC article
Proceed to details
Cite
Share
555
Observational Study
Medicine (Baltimore)
. 2020 Dec 18;99(51):e23547.
doi: 10.1097/MD.0000000000023547.

Clinical characteristics and viral shedding kinetics of 38 asymptomatic patients with coronavirus disease 2019: A retrospective observational study

Yanyan Li [1], Kaishu Li [1], Wei Xiong [2], Xinan Wang [3], Chaowu Liu [2], Chun Liu [4], Weiping Tan [5], Baowei Luo [6], Yongfeng Zhu [7], Yanbin Wu [8], Huiming Yin [9], Xueqin Li [10], Zunchang Li [11]
Affiliations Expand

Affiliations

- [1] Department of Respiratory Medicine, The People's Hospital of Binzhou City, Binzhou, China.
- [2] Department of Respiration, First Teaching Hospital of Tianjin University of Traditional Chinese Medicine, Tianjin.
- [3] Medical Department,The People's Hospital of Binzhou City, Binzhou, China.
- [4] Department of Respirology & Critical Care Medicine, Third Xiangya Hospital, Central South University, Changsha.
- [5] Pulmonary and Critical Care Medicine,First Affiliated Hospital, Sun Yat-Sen University.
- [6] Pulmonary and Critical Care Medicine, ShuangFeng People's Hospital, Hunan Province.
- [7] Department of Respiration, Changsha Central Hospital Affiliated to Nanhua University.
- [8] Pulmonary and Critical Care Medicine, First Affiliated Hospital, Guangxi Medical University.
- [9] Pulmonary and Critical Care Medicine, First Affiliated Hospital, Hunan Medical College.

- [10] Nursing Department.
- [11] Hematology Department, The People's Hospital of Binzhou City, Binzhou, China.

- PMID: **33371079**
- PMCID: PMC7748189
- DOI: 10.1097/MD.0000000000023547

Free PMC article
Observational Study

Clinical characteristics and viral shedding kinetics of 38 asymptomatic patients with coronavirus disease 2019: A retrospective observational study

Yanyan Li et al. Medicine (Baltimore). 2020.
Free PMC article
Show details
Medicine (Baltimore)
. 2020 Dec 18;99(51):e23547.
doi: 10.1097/MD.0000000000023547.

Authors

Yanyan Li [1], Kaishu Li [1], Wei Xiong [2], Xinan Wang [3], Chaowu Liu [2], Chun Liu [4], Weiping Tan [5], Baowei Luo [6], Yongfeng Zhu [7], Yanbin Wu [8], Huiming Yin [9], Xueqin Li [10], Zunchang Li [11]

Affiliations

- [1] Department of Respiratory Medicine, The People's Hospital of Binzhou City, Binzhou, China.
- [2] Department of Respiration, First Teaching Hospital of Tianjin University of Traditional Chinese Medicine, Tianjin.
- [3] Medical Department,The People's Hospital of Binzhou City, Binzhou, China.
- [4] Department of Respirology & Critical Care Medicine, Third Xiangya Hospital, Central South University, Changsha.
- [5] Pulmonary and Critical Care Medicine,First Affiliated Hospital, Sun Yat-Sen University.
- [6] Pulmonary and Critical Care Medicine, ShuangFeng People's Hospital, Hunan Province.
- [7] Department of Respiration, Changsha Central Hospital Affiliated to Nanhua University.
- [8] Pulmonary and Critical Care Medicine, First Affiliated Hospital, Guangxi Medical University.
- [9] Pulmonary and Critical Care Medicine, First Affiliated Hospital, Hunan Medical College.
- [10] Nursing Department.
- [11] Hematology Department, The People's Hospital of Binzhou City, Binzhou, China.

- PMID: **33371079**
- PMCID: PMC7748189
- DOI: 10.1097/MD.0000000000023547

Abstract

This study aims to investigate the clinical characteristics and viral shedding kinetics of asymptomatic patients with coronavirus disease 2019 (COVID-19).The data of 38 asymptomatic patients positive for SARS-CoV-2 nucleic acid were collected from February to March 2020 in Tuanfeng County, Huanggang, Hubei, China. The epidemiology, laboratory examination, chest imaging, viral nucleic acid test results, clinical characteristics, and viral shedding time were summarized in this retrospective study.The study included 20 family members of patients with COVID-19, 10 medical personnel participating in COVID-19 treatment or working in a fever clinic, 6 personnel from quarantine places, 1 individual with a close contact history with confirmed patients, and 1 local epidemic prevention personnel. All were positive for SARS-CoV-2 nucleic acid. The white blood cell (WBC) count, the absolute value of lymphocytes, C-reactive protein (CRP), and D-dimer were normal. Pneumonia manifestations were not found in the chest computed tomography (CT) scan of 36 patients; the remaining 2 cases included a 1-year-old child and a pregnant woman, and they did not undergo chest CT. The viral shedding time was 6 days.All asymptomatic patients with COVID-19 had a history of close contact or exposure. Laboratory tests were normal. Chest imaging did not show any pneumonia manifestation. The viral shedding time was <10 days, which is shorter than that of patients with COVID-19. A timely discovery of such asymptomatic infections is crucial for blocking the spread of the virus and strengthening the prevention and control measures.

Copyright © 2020 the Author(s). Published by Wolters Kluwer Health, Inc.

Conflict of interest statement

The authors have no conflicts of interest to disclose.

- Cited by 1 article
- 17 references
- 1 figure

Supplementary info

Publication types, MeSH terms, Substances Expand

Publication types

- Observational Study

MeSH terms

- Adolescent
- Adult
- Asymptomatic Infections / epidemiology*
- Asymptomatic Infections / therapy

- COVID-19 / blood
- COVID-19 / diagnostic imaging
- COVID-19 / epidemiology
- COVID-19 / virology*
- Child
- China / epidemiology
- Female
- Humans
- Indoles / therapeutic use
- Infant
- Male
- Medicine, Chinese Traditional
- Middle Aged
- Radiography, Thoracic
- Retrospective Studies
- SARS-CoV-2*
- Virus Shedding*
- Young Adult

Substances

- Indoles
- umifenovir

Full text links

Wolters Kluwer Wolters Kluwer Free PMC article
Proceed to details
Cite
Share
☐ 556
Observational Study
Am J Otolaryngol
. Sep-Oct 2021;42(5):103123.
doi: 10.1016/j.amjoto.2021.103123. Epub 2021 Jun 19.

The impact of the COVID-19 pandemic on otolaryngologic emergency department visits at two major NYC hospital systems

Viraj M Patel [1], Evan Kominsky [2], Tristan Tham [3], Danielle Bottalico [1], Michael Setzen [4], Denisa Ferastraoaru [5], Nadeem Akbar [1], Judd H Fastenberg [6]
Affiliations Expand

Affiliations

- [1] Department of Otolaryngology - Head and Neck Surgery, Montefiore Medical Center, Bronx, NY, USA.
- [2] Albert Einstein College of Medicine, Bronx, NY, USA.
- [3] Department of Otolaryngology - Head and Neck Surgery, Long Island Jewish Medical Center and North Shore University Hospital, Northwell Health, New Hyde Park, NY, USA.
- [4] Weill Cornell Medical College, Michael Setzen Otolaryngology, PC, Great Neck, NY, USA.
- [5] Department of Allergy and Immunology, Montefiore Medical Center, Bronx, NY, USA.
- [6] Department of Otolaryngology - Head and Neck Surgery, Long Island Jewish Medical Center and North Shore University Hospital, Northwell Health, New Hyde Park, NY, USA.. Electronic address: jfastenberg@northwell.edu.

- PMID: **34186437**
- PMCID: PMC8214322
- DOI: 10.1016/j.amjoto.2021.103123

Free PMC article
Observational Study

The impact of the COVID-19 pandemic on otolaryngologic emergency department visits at two major NYC hospital systems

Viraj M Patel et al. Am J Otolaryngol. Sep-Oct 2021.
Free PMC article
Show details
Am J Otolaryngol
. Sep-Oct 2021;42(5):103123.
doi: 10.1016/j.amjoto.2021.103123. Epub 2021 Jun 19.

Authors

Viraj M Patel [1], Evan Kominsky [2], Tristan Tham [3], Danielle Bottalico [1], Michael Setzen [4], Denisa Ferastraoaru [5], Nadeem Akbar [1], Judd H Fastenberg [6]

Affiliations

- [1] Department of Otolaryngology - Head and Neck Surgery, Montefiore Medical Center, Bronx, NY, USA.
- [2] Albert Einstein College of Medicine, Bronx, NY, USA.

- [3] Department of Otolaryngology - Head and Neck Surgery, Long Island Jewish Medical Center and North Shore University Hospital, Northwell Health, New Hyde Park, NY, USA.
- [4] Weill Cornell Medical College, Michael Setzen Otolaryngology, PC, Great Neck, NY, USA.
- [5] Department of Allergy and Immunology, Montefiore Medical Center, Bronx, NY, USA.
- [6] Department of Otolaryngology - Head and Neck Surgery, Long Island Jewish Medical Center and North Shore University Hospital, Northwell Health, New Hyde Park, NY, USA.. Electronic address: jfastenberg@northwell.edu.

- PMID: **34186437**
- PMCID: PMC8214322
- DOI: 10.1016/j.amjoto.2021.103123

Abstract

Purpose: Since the COVID-19 pandemic began, emergency departments (ED) across the country have seen a significant decrease in patient visits. We aim to evaluate the impact of COVID-19 on ED visits for acute otolaryngologic complaints in New York City, one of the first epicenters of the pandemic in the US.

Materials and methods: We conducted a retrospective study of patients who presented to the ED with a primary diagnosis of an acute otolaryngologic complaint between March 1 and May 31 in 2019 and 2020. This was a multicenter study, including two tertiary care hospital systems encompassing Manhattan, Bronx, Queens, and Long Island.

Results: A total of 10,162 patients were identified. Significantly fewer patients presented to the ED for acute otolaryngologic complaints in 2020 (7332 vs 2830, $p < 0.001$). The rate of total otolaryngology-related ED visits was decreased by a factor of 0.635 (95% CI 0.6079 to 0.6634). In a subgroup analysis of each individual diagnosis, there was a significant decrease in rate of ED visits for 13 out of 18 diagnoses, including for life-threatening conditions, such as anaphylaxis. There was no significant difference based on which borough in New York City. Pediatric patients (age 0-17) were more significantly impacted by the pandemic compared to other age groups.

Conclusion: The COVID-19 pandemic has led to a reduction in the utilization of ED for acute otolaryngologic complaints, including those requiring emergent management, and an even more significant reduction in the pediatric population. Healthcare providers should encourage patients to seek appropriate care, particularly for those illnesses with significant associated morbidity and mortality.

Keywords: COVID-19; ENT; Emergency; Otolaryngology; Pandemic.

Copyright © 2021 Elsevier Inc. All rights reserved.

Conflict of interest statement

The authors of this manuscript have no conflicts of interest to disclose.

- 31 references
- 2 figures

Supplementary info

Publication types, MeSH terms Expand

Publication types

- Multicenter Study
- Observational Study

MeSH terms

- Adolescent
- Adult
- Aged
- COVID-19 / complications*
- COVID-19 / diagnosis
- COVID-19 / therapy
- Child
- Child, Preschool
- Emergency Service, Hospital*
- Female
- Hospitalization
- Humans
- Infant
- Infant, Newborn
- Male
- Middle Aged
- New York City
- Otorhinolaryngologic Diseases / diagnosis
- Otorhinolaryngologic Diseases / epidemiology*
- Otorhinolaryngologic Diseases / virology*
- Retrospective Studies
- Symptom Assessment
- Young Adult

Full text links

ELSEVIER FULL-TEXT ARTICLE Elsevier Science Free PMC article

Proceed to details

Cite

Share

557

Observational Study

Rev Neurol

. 2021 Aug 1;73(3):89-95.

doi: 10.33588/rn.7303.2020445.

The impact of COVID-19 pandemic in stroke code activation and time from symptom onset to hospital arrival in a Portuguese comprehensive stroke centre

[Article in English, Spanish]

M Rodrigues [1], M Grunho [1], A Rachão [1], E Silva [1], A Cordeiro [1], M Guilherme [1], L Pereira [1]
Affiliations Expand

Affiliation

- [1] Hospital Garcia de Orta, Almada, Portugal.

- PMID: **34291445**
- DOI: 10.33588/rn.7303.2020445

Free article
Observational Study

The impact of COVID-19 pandemic in stroke code activation and time from symptom onset to hospital arrival in a Portuguese comprehensive stroke centre

[Article in English, Spanish]
M Rodrigues et al. Rev Neurol. 2021.
Free article

Show details

Rev Neurol
. 2021 Aug 1;73(3):89-95.
doi: 10.33588/rn.7303.2020445.

Authors

M Rodrigues [1], M Grunho [1], A Rachão [1], E Silva [1], A Cordeiro [1], M Guilherme [1], L Pereira [1]

Affiliation

- [1] Hospital Garcia de Orta, Almada, Portugal.

- PMID: **34291445**
- DOI: 10.33588/rn.7303.2020445

Abstract
in English, Spanish

Introduction: Coronavirus disease 2019 (COVID-19) impacted emergency services worldwide.

Aim: We aimed to evaluate COVID-19 effect on the number of stroke code activations and timings during the first two months of the pandemic.

Material and methods: We reviewed the stroke code database of a single comprehensive stroke centre in Portugal for the number of activations through 2019-2020. We compared the pathway timings between March and April 2020 (COVID-19 period) and the homologous months of the previous four years (pre-COVID-19 period), whilst using February as a control.

Results: Monthly stroke code activation rates decreased up to 34.2% during COVID-19 pandemic. Compared to the pre-COVID-19 period, we observed an increase in the time from symptom onset to emergency call, with a significant number of patients waiting more than four hours (March 20.8% vs. 6.8%, p = 0.034; April 23.8% vs. 6%, p = 0.01); as well as an increase in the time from symptom onset to hospital arrival (March: median 136 minutes [IQR 106-410] vs. 100 [IQR 64-175], p = 0.001; April: median 188 [IQR 96-394] vs. 98 [IQR 66-168], p = 0.007). No difference between both periods was found concerning in-hospital times, patient characteristics, stroke/mimic diagnosis, stroke severity, and mortality.

Conclusion: COVID-19 related factors probably reduced healthcare services utilization, and delayed emergency calls and hospital arrival after stroke onset. These highlight the importance of health education to improve the effectiveness of medical assistance. The preservation of in-hospital times validates the feasibility of the protected stroke code protocol.

Title: El impacto de la pandemia de COVID-19 en la activación del Código Ictus y en el tiempo desde el inicio de los síntomas hasta la llegada al hospital en un centro de ictus portugués.

Introducción. La enfermedad por coronavirus 2019 (COVID-19) provocó un considerable impacto mundial en los servicios de emergencia. Objetivo. Se pretende evaluar el efecto de la COVID-19 sobre el número y los tiempos de activaciones del Código Ictus en el comienzo de la pandemia. Material y métodos. Se revisó la base de datos del Código Ictus de un centro de ictus de Portugal entre 2016 y 2020. Se compararon los tiempos de activación entre marzo y abril de 2020 (período COVID-19) y los meses homólogos de los cuatro años anteriores, mientras que se utilizó febrero como control. Resultados. Las tasas mensuales de activación disminuyeron hasta el 34,2% durante la pandemia. En comparación con el período previo, se observó un aumento del tiempo desde los síntomas hasta la llamada de emergencia, con un aumento de pacientes que esperaron más de cuatro horas (marzo: 20,8 frente a 6,8%, p = 0,034; abril: 23,8 frente a 6%, p = 0,01) y del tiempo desde los síntomas hasta la llegada al hospital –marzo: mediana de 136 minutos (rango intercuartílico [RIC]: 106-410) frente a 100 (RIC: 64-175), p = 0,001; abril: mediana de 188 (RIC: 96-394) frente a 98 (RIC: 66-168), p = 0,007–. No hubo diferencias en los tiempos de internamiento, las características de los pacientes, el diagnóstico de ictus/stroke mimics, la gravedad del ictus o la mortalidad. Conclusión. Los factores relacionados con la COVID-19 redujeron la utilización de los servicios sanitarios y retrasaron las llamadas de emergencia y el tiempo de llegada al hospital. Esto demuestra la importancia de la educación sanitaria para mejorar la eficacia de la asistencia médica.

Supplementary info

Publication types, MeSH terms Expand

Publication types

- Observational Study

MeSH terms

- Aged
- Aged, 80 and over
- COVID-19*
- Emergencies / epidemiology*
- Emergency Service, Hospital / statistics & numerical data
- Emergency Treatment / statistics & numerical data*
- Endovascular Procedures / statistics & numerical data
- Female
- Humans
- Incidence
- Length of Stay
- Male
- Middle Aged
- Pandemics*
- Patient Acceptance of Health Care / statistics & numerical data*
- Portugal / epidemiology
- Retrospective Studies
- SARS-CoV-2*
- Stroke / diagnosis
- Stroke / epidemiology*
- Stroke / therapy
- Thrombectomy / statistics & numerical data
- Thrombolytic Therapy / statistics & numerical data
- Time-to-Treatment / statistics & numerical data*

Full text links

Revista de Neurologia Free full text article Viguera Editores, S. L.

Proceed to details

Cite

Share

☐ 558

Observational Study

Sci Rep
. 2022 Feb 10;12(1):2258.
doi: 10.1038/s41598-022-06276-7.

Mortality and readmission rates among hospitalized COVID-19 patients with varying stages of chronic kidney disease: a multicenter retrospective cohort

Brent Appelman [1 2], Jetta J Oppelaar [3], Lani Broeders [3], Willem Joost Wiersinga [1 2 4], Hessel Peters-Sengers [1 2], Liffert Vogt [5], CovidPredict Study Group
Collaborators, Affiliations Expand

Collaborators

- **CovidPredict Study Group**: Brent Appelman, Michiel Schinkel, David Buis, Kim C E Sigaloff, Paul W G Elbers, Daisy Rusch, Auke Reidinga, Hazra Moeniralam, Caroline Wyers, Joop van den Bergh, Suat Simsek, Bastiaan van Dam, Niels C van den Gritters, Nejma Bokhizzou, Kees Brinkman, Martijn de Kruif, Tom Dormans, Renée Douma, Lianne R de Haan, Tsz Yeung Fung, Martijn Beudel

Affiliations

- [1] Center for Experimental and Molecular Medicine, Amsterdam UMC, Amsterdam, The Netherlands.
- [2] The Amsterdam Institute for Infection and Immunity, Amsterdam UMC, Amsterdam, The Netherlands.
- [3] Department of Internal Medicine, Section of Nephrology, Amsterdam UMC, Amsterdam, The Netherlands.
- [4] Division of Infectious Diseases, Location Academic Medical Center, University of Amsterdam, Amsterdam UMC, Amsterdam, The Netherlands.
- [5] Department of Internal Medicine, Section of Nephrology, Amsterdam UMC, Amsterdam, The Netherlands. l.vogt@amsterdamumc.nl.

- PMID: **35145189**
- PMCID: PMC8831646
- DOI: 10.1038/s41598-022-06276-7

Free PMC article
Observational Study

Mortality and readmission rates among hospitalized COVID-19 patients with varying

stages of chronic kidney disease: a multicenter retrospective cohort

Brent Appelman et al. Sci Rep. 2022.
Free PMC article
Show details
Sci Rep
. 2022 Feb 10;12(1):2258.
doi: 10.1038/s41598-022-06276-7.

Authors

Brent Appelman [1] [2], Jetta J Oppelaar [3], Lani Broeders [3], Willem Joost Wiersinga [1] [2] [4], Hessel Peters-Sengers [1] [2], Liffert Vogt [5], CovidPredict Study Group

Collaborators

- **CovidPredict Study Group**:
Brent Appelman, Michiel Schinkel, David Buis, Kim C E Sigaloff, Paul W G Elbers, Daisy Rusch, Auke Reidinga, Hazra Moeniralam, Caroline Wyers, Joop van den Bergh, Suat Simsek, Bastiaan van Dam, Niels C van den Gritters, Nejma Bokhizzou, Kees Brinkman, Martijn de Kruif, Tom Dormans, Renée Douma, Lianne R de Haan, Tsz Yeung Fung, Martijn Beudel

Affiliations

- [1] Center for Experimental and Molecular Medicine, Amsterdam UMC, Amsterdam, The Netherlands.
- [2] The Amsterdam Institute for Infection and Immunity, Amsterdam UMC, Amsterdam, The Netherlands.
- [3] Department of Internal Medicine, Section of Nephrology, Amsterdam UMC, Amsterdam, The Netherlands.
- [4] Division of Infectious Diseases, Location Academic Medical Center, University of Amsterdam, Amsterdam UMC, Amsterdam, The Netherlands.
- [5] Department of Internal Medicine, Section of Nephrology, Amsterdam UMC, Amsterdam, The Netherlands. l.vogt@amsterdamumc.nl.

- PMID: **35145189**
- PMCID: PMC8831646
- DOI: 10.1038/s41598-022-06276-7

Abstract

Chronic kidney disease (CKD) has been recognized as a highly prevalent risk factor for both the severity of coronavirus disease 2019 (COVID-19) and COVID-19 associated adverse outcomes. In this multicenter observational cohort study, we aim to determine mortality and readmission rates of patients hospitalized for COVID-19 across varying CKD stages. We performed a multicenter cohort study among COVID-19 patients included in the Dutch COVIDPredict cohort. The cohort

consists of hospitalized patients from March 2020 until July 2021 with PCR-confirmed SARS-CoV-2 infection or a highly suspected CT scan-based infection with a CORADS score ≥ 4. A total of 4151 hospitalized COVID-19 patients were included of who 389 had a history of CKD before admission. After adjusting for all confounding covariables, in patients with CKD stage 3a, stage 3b, stage 4 and patients with KTX (kidney transplantation), odds ratios of death and readmission compared to patients without CKD ranged from 1.96 to 8.94. We demonstrate an evident increased 12-week mortality and readmission rate in patients with chronic kidney disease. Besides justified concerns for kidney transplant patients, clinicians should also be aware of more severe COVID-19 outcomes and increased vulnerability in CKD patients.

© 2022. The Author(s).

Conflict of interest statement

The authors declare no competing interests.

- 18 references
- 1 figure

Supplementary info

Publication types, MeSH terms Expand

Publication types

- Multicenter Study
- Observational Study
- Research Support, Non-U.S. Gov't

MeSH terms

- Aged
- COVID-19 / complications
- COVID-19 / mortality*
- Female
- Humans
- Male
- Middle Aged
- Netherlands / epidemiology
- Patient Readmission / statistics & numerical data*
- Renal Insufficiency, Chronic / complications*
- Retrospective Studies
- Risk Factors

Full text links

nature publishing group Nature Publishing Group Free PMC article
Proceed to details
Cite
Share
559
Observational Study
BMC Palliat Care
. 2021 Jan 11;20(1):10.
doi: 10.1186/s12904-021-00711-8.

Advanced care planning during the COVID-19 pandemic: ceiling of care decisions and their implications for observational data

Sam Straw [1], Melanie McGinlay [2], Michael Drozd [1], Thomas A Slater [1], Alice Cowley [2], Stephe Kamalathasan [2], Nicholas Maxwell [3], Rory A Bird [3], Aaron O Koshy [1], Milos Prica [2], Peysh A Patel [2], Samuel D Relton [4], John Gierula [1], Richard M Cubbon [1], Mark T Kearney [1], Klaus K Witte [5]
Affiliations Expand

Affiliations

- [1] Leeds Institute of Cardiovascular and Metabolic Medicine, University of Leeds, Leeds, UK.
- [2] Leeds Teaching Hospitals NHS Trust, Leeds, UK.
- [3] School of Medicine, University of Leeds, Leeds, UK.
- [4] Leeds Institute of Health Sciences, University of Leeds, Leeds, UK.
- [5] Leeds Institute of Cardiovascular and Metabolic Medicine, University of Leeds, Leeds, UK. k.k.witte@leeds.ac.uk.

- PMID: **33430850**
- PMCID: PMC7797882
- DOI: 10.1186/s12904-021-00711-8

Free PMC article
Observational Study

Advanced care planning during the COVID-19 pandemic: ceiling of care decisions and their implications for observational data

Sam Straw et al. BMC Palliat Care. 2021.
Free PMC article

Show details

BMC Palliat Care
. 2021 Jan 11;20(1):10.
doi: 10.1186/s12904-021-00711-8.

Authors

Sam Straw [1], Melanie McGinlay [2], Michael Drozd [1], Thomas A Slater [1], Alice Cowley [2], Stephe Kamalathasan [2], Nicholas Maxwell [3], Rory A Bird [3], Aaron O Koshy [1], Milos Prica [2], Peysh A Patel [2], Samuel D Relton [4], John Gierula [1], Richard M Cubbon [1], Mark T Kearney [1], Klaus K Witte [5]

Affiliations

- [1] Leeds Institute of Cardiovascular and Metabolic Medicine, University of Leeds, Leeds, UK.
- [2] Leeds Teaching Hospitals NHS Trust, Leeds, UK.
- [3] School of Medicine, University of Leeds, Leeds, UK.
- [4] Leeds Institute of Health Sciences, University of Leeds, Leeds, UK.
- [5] Leeds Institute of Cardiovascular and Metabolic Medicine, University of Leeds, Leeds, UK. k.k.witte@leeds.ac.uk.

- PMID: **33430850**
- PMCID: PMC7797882
- DOI: 10.1186/s12904-021-00711-8

Abstract

Background: Observational studies investigating risk factors in coronavirus disease 2019 (COVID-19) have not considered the confounding effects of advanced care planning, such that a valid picture of risk for elderly, frail and multi-morbid patients is unknown. We aimed to report ceiling of care and cardiopulmonary resuscitation (CPR) decisions and their association with demographic and clinical characteristics as well as outcomes during the COVID-19 pandemic.

Methods: Retrospective, observational study conducted between 5th March and 7th May 2020 of all hospitalised patients with COVID-19. Ceiling of care and CPR decisions were documented using the Recommended Summary Plan for Emergency Care and Treatment (ReSPECT) process. Unadjusted and multivariable regression analyses were used to determine factors associated with ceiling of care decisions and death during hospitalisation.

Results: A total of 485 patients were included, of whom 409 (84·3%) had a documented ceiling of care; level one for 208 (50·9%), level two for 75 (18·3%) and level three for 126 (30·8%). CPR decisions were documented for 451 (93·0%) of whom 336 (74·5%) were 'not for resuscitation'. Advanced age, frailty, White-European ethnicity, a diagnosis of any co-morbidity and receipt of cardiovascular medications were associated with ceiling of care decisions. In a multivariable model only advanced age (odds 0·89, 0·86-0·93 p < 0·001), frailty (odds 0·48, 0·38-0·60, p < 0·001) and the cumulative number of co-morbidities (odds 0·72, 0·52-1·0, p = 0·048) were independently associated. Death during hospitalisation was independently associated with age, frailty and requirement for level two or three care.

Conclusion: Ceiling of care decisions were made for the majority of patients during the COVID-19 pandemic, broadly in line with known predictors of poor outcomes in COVID-19, but with a focus on co-morbidities suggesting ICU admission might not be a reliable end-point for observational studies where advanced care planning is routine.

Keywords: Advanced care planning; COVID-19; Comorbidity; Elderly; Geriatrics; Resuscitation.

Conflict of interest statement

There are no competing interests for any of the authors.

- Cited by 2 articles
- 32 references
- 3 figures

Supplementary info

Publication types, MeSH terms, Grant support Expand

Publication types

- Observational Study

MeSH terms

- Adult
- Advance Care Planning*
- Aged
- Aged, 80 and over
- COVID-19 / therapy*
- Cardiopulmonary Resuscitation
- Clinical Decision-Making*
- Female
- Humans
- Life Support Care
- Male
- Middle Aged
- Retrospective Studies

Grant support

- FS/12/80/29821/BHF_/British Heart Foundation/United Kingdom
- FS/18/44/33792/BHF_/British Heart Foundation/United Kingdom

Full text links

Read free full text at BMC BioMed Central Free PMC article
Proceed to details
Cite
Share

560
Observational Study
Clin Transl Sci
. 2021 Jan;14(1):163-169.
doi: 10.1111/cts.12883. Epub 2020 Oct 23.

Insights on the Evidence of Cardiotoxicity of Hydroxychloroquine Prior and During COVID-19 Epidemic

Serena Romani [1], Alexandre Gérard [1], Audrey Fresse [1], Delphine Viard [1], Élise Van-Obberghen [1], Joëlle Micallef [2], Fanny Rocher [1], Milou-Daniel Drici [1], French Pharmacovigilance Network
Affiliations Expand

Affiliations

- [1] Pharmacovigilance, Department of Clinical Pharmacology, Université Côte d'Azur Medical Center, Pasteur Hospital, Nice, France.
- [2] Pharmacovigilance, Department of Clinical Pharmacology and Pharmacovigilance, Aix Marseille University, APHM, INSERM, Institute for Neuroscience Systems, UMR 1106, Marseille, France.

- PMID: **32964653**
- PMCID: PMC7877831
- DOI: 10.1111/cts.12883

Free PMC article
Observational Study

Insights on the Evidence of Cardiotoxicity of Hydroxychloroquine Prior and During COVID-19 Epidemic

Serena Romani et al. Clin Transl Sci. 2021 Jan.
Free PMC article
Show details
Clin Transl Sci
. 2021 Jan;14(1):163-169.

doi: 10.1111/cts.12883. Epub 2020 Oct 23.

Authors

Serena Romani [1], Alexandre Gérard [1], Audrey Fresse [1], Delphine Viard [1], Élise Van-Obberghen [1], Joëlle Micallef [2], Fanny Rocher [1], Milou-Daniel Drici [1], French Pharmacovigilance Network

Affiliations

- [1] Pharmacovigilance, Department of Clinical Pharmacology, Université Côte d'Azur Medical Center, Pasteur Hospital, Nice, France.
- [2] Pharmacovigilance, Department of Clinical Pharmacology and Pharmacovigilance, Aix Marseille University, APHM, INSERM, Institute for Neuroscience Systems, UMR 1106, Marseille, France.

- PMID: **32964653**
- PMCID: PMC7877831
- DOI: 10.1111/cts.12883

Abstract

The recent empirical use of hydroxychloroquine (HCQ) in coronavirus disease 2019 (COVID-19) revived the interest in its cardiac toxicity, increasingly sidelined over time. We aimed to assess and compare the profile of cardiac adverse drug reactions (CADRs) associated with HCQ before and during COVID-19. We performed a retrospective comparative observational study using the French Pharmacovigilance network database between 1985 and May 2020 to assess all postmarketing CADRs associated with HCQ notified before COVID-19 in its approved indications for lupus and rheumatoid arthritis (preCOV), and those concerning its empirical use in COVID-19 (COV). Eighty-five CADR in preCOV were compared with 141 CADRs in COV. The most common CADR of preCOV were cardiomyopathies (42.4%) and conduction disorders (28.2%), both statistically more frequent than in COV ($P < 0.001$). COV notifications significantly highlighted repolarization and ventricular rhythm disorders (78.0%, $P < 0.001$) as well as sinus bradycardias (14.9%, $P = 0.01$) as compared with preCOV. Estimated incidence of CADR was significantly higher among patients exposed to off-label use of HCQ in COVID-19 (2.9%) than before COVID-19 in its approved indications (0.01%, $P < 0.001$). The use of HCQ in COVID-19 sheds a new light on the spectrum of its cardiac toxicity. This fosters the value of a closer monitoring of all patients treated with HCQ, regardless of its indication, and the importance of an update of its summary of product characteristics.

© 2020 The Authors. Clinical and Translational Science published by Wiley Periodicals LLC on behalf of the American Society for Clinical Pharmacology and Therapeutics.

Conflict of interest statement

All authors declared no competing interests for this work.

- Cited by 2 articles
- 50 references
- 4 figures

Supplementary info

Publication types, MeSH terms, Substances, Supplementary concepts Expand

Publication types

- Observational Study

MeSH terms

- Adult
- Aged
- COVID-19 / drug therapy*
- Cardiomyopathies / chemically induced
- Cardiotoxicity / etiology*
- Female
- Heart Conduction System / drug effects
- Humans
- Hydroxychloroquine / adverse effects*
- Male
- Middle Aged
- Retrospective Studies
- SARS-CoV-2*

Substances

- Hydroxychloroquine

Supplementary concepts

- COVID-19 drug treatment

Full text links

WILEY Full Text Article Wiley Free PMC article

Proceed to details

Cite

Share

☐ 561

Observational Study

Epidemiol Infect

. 2021 Oct 22;149:e230.
doi: 10.1017/S095026882100234X.

COVID-19 patients with increasing age experience differential time to initial medical care and severity of symptoms

J Mancilla-Galindo [1], A Kammar-García [2, 3], A Martínez-Esteban [4], H D Meza-Comparán [4], J Mancilla-Ramírez [2, 5], N Galindo-Sevilla [2, 6]
Affiliations Expand

Affiliations

- [1] Unidad de Investigación UNAM-INC, Instituto Nacional de Cardiología Ignacio Chávez, Mexico City, Mexico.
- [2] Sección de Estudios de Posgrado e Investigación, Escuela Superior de Medicina, Instituto Politécnico Nacional, Mexico City, Mexico.
- [3] Dirección de Investigación, Instituto Nacional de Geriatría, Mexico City, Mexico.
- [4] Facultad de Medicina, Universidad Nacional Autónoma de México, Mexico City, Mexico.
- [5] Hospital de la Mujer, Secretaría de Salud, Mexico City, Mexico.
- [6] Departamento de Infectología e Inmunología, Instituto Nacional de Perinatología, Secretaría de Salud, Mexico City, Mexico.

- PMID: **34674789**
- PMCID: PMC8576123
- DOI: 10.1017/S095026882100234X

Free PMC article
Observational Study

COVID-19 patients with increasing age experience differential time to initial medical care and severity of symptoms

J Mancilla-Galindo et al. Epidemiol Infect. 2021.
Free PMC article
Show details
Epidemiol Infect
. 2021 Oct 22;149:e230.
doi: 10.1017/S095026882100234X.

Authors

J Mancilla-Galindo [1], A Kammar-García [2, 3], A Martínez-Esteban [4], H D Meza-Comparán [4], J Mancilla-Ramírez [2, 5], N Galindo-Sevilla [2, 6]

Affiliations

- [1] Unidad de Investigación UNAM-INC, Instituto Nacional de Cardiología Ignacio Chávez, Mexico City, Mexico.
- [2] Sección de Estudios de Posgrado e Investigación, Escuela Superior de Medicina, Instituto Politécnico Nacional, Mexico City, Mexico.
- [3] Dirección de Investigación, Instituto Nacional de Geriatría, Mexico City, Mexico.
- [4] Facultad de Medicina, Universidad Nacional Autónoma de México, Mexico City, Mexico.
- [5] Hospital de la Mujer, Secretaría de Salud, Mexico City, Mexico.
- [6] Departamento de Infectología e Inmunología, Instituto Nacional de Perinatología, Secretaría de Salud, Mexico City, Mexico.

- PMID: **34674789**
- PMCID: PMC8576123
- DOI: 10.1017/S095026882100234X

Abstract

We conducted a retrospective observational study in patients with laboratory-confirmed coronavirus disease (COVID-19) who received medical care in 688 COVID-19 ambulatory units and hospitals in Mexico City between 24 February 2020 and 24 December 2020, to study if the elderly seek medical care later than younger patients and their severity of symptoms at initial medical evaluation. Patients were categorised into eight groups (<20, 20-29, 30-39, 40-49, 50-59, 60-69, 70-79 and ≥80 years). Symptoms at initial evaluation were classified according to a previously validated classification into respiratory and non-respiratory symptoms. Comparisons between time from symptom onset to medical care for every age category were performed through variance analyses. Logistic regression models were applied to determine the risk of presenting symptoms of severity according to age, and mortality risk according to delays in medical care. In total, 286 020 patients were included (mean age: 42.8, s.d.: 16.8 years; 50.4% were women). Mean time from symptom onset to medical care was 4.04 (s.d.: 3.6) days and increased with older age categories ($P < 0.0001$). Mortality risk increased by 6.4% for each day of delay in medical care from symptom onset. The risk of presenting with the symptoms of severity was greater with increasing age categories. In conclusion, COVID-19 patients with increasing ages tend to seek medical care later, with higher rates of symptoms of severity at initial presentation in both ambulatory units and hospitals.

Keywords: Ageing; COVID-19; coronavirus; epidemiology; pandemic.

Conflict of interest statement

None.

- Cited by 1 article
- 21 references
- 2 figures

Supplementary info

Publication types, MeSH terms Expand

Publication types

- Observational Study

MeSH terms

- Adolescent
- Adult
- Aged
- Aged, 80 and over
- Aging*
- COVID-19 / diagnosis
- COVID-19 / epidemiology*
- Child
- Child, Preschool
- Female
- Humans
- Infant
- Infant, Newborn
- Male
- Mexico / epidemiology
- Middle Aged
- Patient Acceptance of Health Care / statistics & numerical data
- Retrospective Studies
- SARS-CoV-2
- Severity of Illness Index
- Time-to-Treatment / statistics & numerical data*
- Young Adult

Full text links

Cambridge University Press Free PMC article

Proceed to details

Cite

Share

562

Observational Study

Obes Res Clin Pract

. May-Jun 2020;14(3):205-209.

doi: 10.1016/j.orcp.2020.05.009. Epub 2020 Jun 4.

Obesity prolongs the hospital stay in patients affected by COVID-19, and may impact on SARS-COV-2 shedding

Diego Moriconi [1], Stefano Masi [2], Eleni Rebelos [3], Agostino Virdis [4], Maria Laura Manca [5], Salvatore De Marco [6], Stefano Taddei [7], Monica Nannipieri [8]
Affiliations Expand

Affiliations

- [1] Department of Clinical and Experimental Medicine, University of Pisa, Italy. Electronic address: d.moriconi@ao-pisa.toscana.it.
- [2] Department of Clinical and Experimental Medicine, University of Pisa, Italy. Electronic address: stefano.masi@unipi.it.
- [3] Department of Clinical and Experimental Medicine, University of Pisa, Italy. Electronic address: elenirebelos@gmail.com.
- [4] Department of Clinical and Experimental Medicine, University of Pisa, Italy. Electronic address: agostino.virdis@unipi.it.
- [5] Department of Clinical and Experimental Medicine, University of Pisa, Italy. Electronic address: l.manca@med.unipi.it.
- [6] Azienda Ospedaliero Universitaria Pisa, AOUP, Pisa, Italy. Electronic address: s.demarco@ao-pisa.toscana.it.
- [7] Department of Clinical and Experimental Medicine, University of Pisa, Italy. Electronic address: stefano.taddei@unipi.it.
- [8] Department of Clinical and Experimental Medicine, University of Pisa, Italy. Electronic address: monica.nannipieri@dmi.unipi.it.

- PMID: **32534848**
- PMCID: PMC7269944
- DOI: 10.1016/j.orcp.2020.05.009

Free PMC article
Observational Study

Obesity prolongs the hospital stay in patients affected by COVID-19, and may impact on SARS-COV-2 shedding

Diego Moriconi et al. Obes Res Clin Pract. May-Jun 2020.
Free PMC article
Show details
Obes Res Clin Pract
. May-Jun 2020;14(3):205-209.
doi: 10.1016/j.orcp.2020.05.009. Epub 2020 Jun 4.

Authors

Diego Moriconi [1], Stefano Masi [2], Eleni Rebelos [3], Agostino Virdis [4], Maria Laura Manca [5], Salvatore De Marco [6], Stefano Taddei [7], Monica Nannipieri [8]

Affiliations

- [1] Department of Clinical and Experimental Medicine, University of Pisa, Italy. Electronic address: d.moriconi@ao-pisa.toscana.it.
- [2] Department of Clinical and Experimental Medicine, University of Pisa, Italy. Electronic address: stefano.masi@unipi.it.
- [3] Department of Clinical and Experimental Medicine, University of Pisa, Italy. Electronic address: elenirebelos@gmail.com.
- [4] Department of Clinical and Experimental Medicine, University of Pisa, Italy. Electronic address: agostino.virdis@unipi.it.
- [5] Department of Clinical and Experimental Medicine, University of Pisa, Italy. Electronic address: l.manca@med.unipi.it.
- [6] Azienda Ospedaliero Universitaria Pisa, AOUP, Pisa, Italy. Electronic address: s.demarco@ao-pisa.toscana.it.
- [7] Department of Clinical and Experimental Medicine, University of Pisa, Italy. Electronic address: stefano.taddei@unipi.it.
- [8] Department of Clinical and Experimental Medicine, University of Pisa, Italy. Electronic address: monica.nannipieri@dmi.unipi.it.

- PMID: **32534848**
- PMCID: PMC7269944
- DOI: 10.1016/j.orcp.2020.05.009

Abstract

Introduction: On the last three months the new SARS-COV-2 coronavirus has created a pandemic, rapidly spreading all around the world. The aim of the study is to investigate whether obesity impacts on COVID-19 morbidity.

Methods: One hundred consecutive patients with COVID-19 pneumonia admitted in our Medical Unit were evaluated. Anthropometric parameters and past medical history were registered. Nasopharyngeal swab samples and biochemical analysis were obtained at admission and during hospital stay.

Results: Patients with (OB, 29) and without obesity (N-OB, 71) were similar in age, gender and comorbidities, with the exception of hypertension that was more frequent in OB group. At admission, inflammatory markers were higher in OB than N-OB group. OB group showed a worse pulmonary clinical picture, with lower PaO2 (57 ± 15 vs. 68 ± 14 mmHg, $p = 0.042$), and SaO2 (88 ± 6 vs. 92 ± 5%, $p = 0.049$) at admission consequently requiring higher volumes of oxygen (Fi02: 38 ± 15 vs. 29 ± 19%, $p = 0.047$) and a longer period to achieve oxygen weaning (10 ± 6 vs. 15 ± 7 days, $p = 0.03$). OB group also had positive swabs for longer time (19 ± 8 vs. 13 ± 7, days, $p = 0.002$), and required longer hospital stay (21 ± 8 vs. 13 ± 8, days, $p = 0.0008$). Partial least square regression analysis showed that BMI, age and CRP at admission were related to longer length of hospital stay, and time for negative swab. On the contrary, in this cohort, obesity did not predict higher mortality.

Conclusions: Subjects with obesity affected by COVID-19 require longer hospitalization, more intensive and longer oxygen treatment, and they may have longer SARS-COV-2 shedding.

Keywords: COVID-19; CRP; Citokynes; Obesity; Viral shedding.

Copyright © 2020 Asia Oceania Association for the Study of Obesity. Published by Elsevier Ltd. All rights reserved.

- Cited by 37 articles
- 30 references
- 2 figures

Supplementary info

Publication types, MeSH terms Expand

Publication types

- Observational Study

MeSH terms

- Aged
- Betacoronavirus*
- COVID-19
- Cohort Studies
- Coronavirus Infections / complications*
- Coronavirus Infections / physiopathology*
- Female
- Humans
- Italy
- Length of Stay / statistics & numerical data*
- Male
- Obesity / complications*
- Obesity / physiopathology*
- Pandemics
- Pneumonia, Viral / complications*
- Pneumonia, Viral / physiopathology*
- Retrospective Studies
- SARS-CoV-2
- Virus Shedding / physiology*

Full text links

ELSEVIER FULL-TEXT ARTICLE Elsevier Science Free PMC article

Proceed to details

Cite

Share

563

Observational Study

J Intensive Care Med

. 2021 Mar;36(3):319-326.

doi: 10.1177/0885066620970858. Epub 2020 Dec 3.

Clinical Characteristics and Outcomes of Patients With Severe COVID-19 Induced Acute Kidney Injury

Jingyuan Xu [1], Jianfeng Xie [1], Bin Du [2], Zhaohui Tong [3], Haibo Qiu [1], Sean M Bagshaw [4]

Affiliations Expand

Affiliations

- [1] Department of Critical Care Medicine, Zhongda Hospital, Jiangsu Provincial Key Laboratory of Critical Care Medicine, School of Medicine, 12579Southeast University, Nanjing, China.
- [2] Peking Union Medical College Hospital, Peking Union Medical College & Chinese Academy of Medical Sciences, Beijing, China.
- [3] Department of Respiratory and Critical Care Medicine, Beijing Institute of Respiratory Medicine, 74639Beijing Chao-yang Hospital, Capital Medical University, Beijing, China.
- [4] Department of Critical Care Medicine, Faculty of Medicine and Dentistry, 3158University of Alberta, Edmonton, Alberta, Canada.

- PMID: **33267722**
- DOI: 10.1177/0885066620970858

Observational Study

Clinical Characteristics and Outcomes of Patients With Severe COVID-19 Induced Acute Kidney Injury

Jingyuan Xu et al. J Intensive Care Med. 2021 Mar.

Show details

J Intensive Care Med

. 2021 Mar;36(3):319-326.

doi: 10.1177/0885066620970858. Epub 2020 Dec 3.

Authors

Jingyuan Xu [1], Jianfeng Xie [1], Bin Du [2], Zhaohui Tong [3], Haibo Qiu [1], Sean M Bagshaw [4]

Affiliations

- [1] Department of Critical Care Medicine, Zhongda Hospital, Jiangsu Provincial Key Laboratory of Critical Care Medicine, School of Medicine, 12579Southeast University, Nanjing, China.
- [2] Peking Union Medical College Hospital, Peking Union Medical College & Chinese Academy of Medical Sciences, Beijing, China.
- [3] Department of Respiratory and Critical Care Medicine, Beijing Institute of Respiratory Medicine, 74639Beijing Chao-yang Hospital, Capital Medical University, Beijing, China.
- [4] Department of Critical Care Medicine, Faculty of Medicine and Dentistry, 3158University of Alberta, Edmonton, Alberta, Canada.

- PMID: **33267722**
- DOI: 10.1177/0885066620970858

Abstract

Background: The incidence and outcome of Coronavirus disease 2019 (COVID-19)-induced kidney injury have been variably described. We aimed to describe the clinical characteristics, correlates and outcomes of critically ill patients with severe COVID-19 complicated by acute kidney injury (AKI).

Methods: We performed a multicenter retrospective cohort study of 671 critically ill adults with laboratory-confirmed COVID-19 from 19 hospitals in China between January 1 to February 29, 2020. Data were captured on demographics, comorbidities, symptoms, acute physiology, laboratory parameters, interventions, and outcomes. The primary exposure was ICU admission for confirmed COVID-19 related critically illness. The primary outcome was 28-day mortality. Secondary outcomes included factors associated with AKI, organ dysfunction, treatment intensity, and health services use.

Measurements and main results: Of 671 severe COVID-19 patients (median [IQR] 65 [56-73] years; male sex 65% (n = 434); hypertension 43% (n = 287) and APACHE II score 10 [7-14]), 39% developed AKI. Patients with AKI were older, had greater markers of inflammation and coagulation activation, and had greater acuity and organ dysfunction as presentation. Despite similar treatment with antivirals, patients with AKI had lower viral conversion negative rates than those without AKI. The 28-day mortality was much higher in AKI patients than patients without AKI (72% vs. 42%), and there was an increase in 28-day mortality according to the severity of AKI. Non-survivors were less likely to receive antiviral therapy [132 (70%) vs. 65 (88%)] compared with survivors and have lower viral negative conversion rate [17 (9%) vs. 47 (64%)].

Conclusions: Acute kidney injury was quite common in severe COVID-19 pneumonia, which associated with higher mortality.

Keywords: AKI; COVID-19; Mortality; critically ill.

- Cited by 8 articles

Supplementary info

Publication types, MeSH terms, Substances, Supplementary concepts Expand

Publication types

- Observational Study

MeSH terms

- APACHE
- Acute Kidney Injury / epidemiology*
- Acute Kidney Injury / physiopathology
- Acute Kidney Injury / therapy
- Aged
- Antiviral Agents / therapeutic use
- COVID-19 / drug therapy
- COVID-19 / epidemiology
- COVID-19 / physiopathology*
- COVID-19 / therapy
- Case-Control Studies
- China
- Cohort Studies
- Extracorporeal Membrane Oxygenation
- Female
- Humans
- Incidence
- Inflammation
- Intensive Care Units
- Male
- Middle Aged
- Mortality*
- Organ Dysfunction Scores
- Proportional Hazards Models
- Renal Replacement Therapy
- Respiration, Artificial
- Retrospective Studies
- SARS-CoV-2
- Severity of Illness Index
- Vasoconstrictor Agents / therapeutic use

Substances

- Antiviral Agents
- Vasoconstrictor Agents

Supplementary concepts

- COVID-19 drug treatment

Full text links

SAGE journals Atypon
Proceed to details
Cite
Share
564
PLoS One
. 2021 Nov 12;16(11):e0259822.
doi: 10.1371/journal.pone.0259822. eCollection 2021.

Chronic diseases associated with increased likelihood of hospitalization and mortality in 68,913 COVID-19 confirmed cases in Spain: A population-based cohort study

Antonio Gimeno-Miguel [1, 2], Kevin Bliek-Bueno [1, 3], Beatriz Poblador-Plou [1, 2], Jonás Carmona-Pírez [1, 2, 4], Antonio Poncel-Falcó [1, 2, 5], Francisca González-Rubio [1, 2, 4, 6], Ignatios Ioakeim-Skoufa [1, 6, 7], Victoria Pico-Soler [1, 2, 8], Mercedes Aza-Pascual-Salcedo [1, 2, 9], Alexandra Prados-Torres [1, 2], Luis Andrés Gimeno-Feliu [1, 2, 10], PRECOVID Group
Affiliations Expand

Affiliations

- [1] EpiChron Research Group, Aragon Health Sciences Institute (IACS), IIS Aragón, Miguel Servet University Hospital, Zaragoza, Spain.
- [2] Health Services Research on Chronic Patients Network (REDISSEC), ISCIII, Madrid, Spain.
- [3] Preventive Medicine and Public Health Teaching Unit, Miguel Servet University Hospital, Zaragoza, Spain.
- [4] Delicias-Sur Primary Care Health Centre, Aragon Health Service (SALUD), Zaragoza, Spain.
- [5] Aragon Health Service (SALUD), Zaragoza, Spain.

- [6] Drug Utilization Work Group, Spanish Society of Family and Community Medicine (semFYC), Barcelona, Spain.
- [7] WHO Collaborating Centre for Drug Statistics Methodology, Department of Drug Statistics, Division of Health Data and Digitalisation, Norwegian Institute of Public Health, Oslo, Norway.
- [8] Torrero-La Paz Primary Care Health Centre, Aragon Health Service (SALUD), Zaragoza, Spain.
- [9] Primary Care Pharmacy Service Zaragoza III, Aragon Health Service (SALUD), Zaragoza, Spain.
- [10] San Pablo Primary Care Health Centre, Aragon Health Service (SALUD), University of Zaragoza, Zaragoza, Spain.

- PMID: **34767594**
- PMCID: PMC8589220
- DOI: 10.1371/journal.pone.0259822

Free PMC article

Chronic diseases associated with increased likelihood of hospitalization and mortality in 68,913 COVID-19 confirmed cases in Spain: A population-based cohort study

Antonio Gimeno-Miguel et al. PLoS One. 2021.
Free PMC article

Show details

PLoS One

. 2021 Nov 12;16(11):e0259822.
doi: 10.1371/journal.pone.0259822. eCollection 2021.

Authors

Antonio Gimeno-Miguel [1, 2], Kevin Bliek-Bueno [1, 3], Beatriz Poblador-Plou [1, 2], Jonás Carmona-Pírez [1, 2, 4], Antonio Poncel-Falcó [1, 2, 5], Francisca González-Rubio [1, 2, 4, 6], Ignatios Ioakeim-Skoufa [1, 6, 7], Victoria Pico-Soler [1, 2, 8], Mercedes Aza-Pascual-Salcedo [1, 2, 9], Alexandra Prados-Torres [1, 2], Luis Andrés Gimeno-Feliu [1, 2, 10], PRECOVID Group

Affiliations

- [1] EpiChron Research Group, Aragon Health Sciences Institute (IACS), IIS Aragón, Miguel Servet University Hospital, Zaragoza, Spain.
- [2] Health Services Research on Chronic Patients Network (REDISSEC), ISCIII, Madrid, Spain.
- [3] Preventive Medicine and Public Health Teaching Unit, Miguel Servet University Hospital, Zaragoza, Spain.

- [4] Delicias-Sur Primary Care Health Centre, Aragon Health Service (SALUD), Zaragoza, Spain.
- [5] Aragon Health Service (SALUD), Zaragoza, Spain.
- [6] Drug Utilization Work Group, Spanish Society of Family and Community Medicine (semFYC), Barcelona, Spain.
- [7] WHO Collaborating Centre for Drug Statistics Methodology, Department of Drug Statistics, Division of Health Data and Digitalisation, Norwegian Institute of Public Health, Oslo, Norway.
- [8] Torrero-La Paz Primary Care Health Centre, Aragon Health Service (SALUD), Zaragoza, Spain.
- [9] Primary Care Pharmacy Service Zaragoza III, Aragon Health Service (SALUD), Zaragoza, Spain.
- [10] San Pablo Primary Care Health Centre, Aragon Health Service (SALUD), University of Zaragoza, Zaragoza, Spain.

- PMID: **34767594**
- PMCID: PMC8589220
- DOI: 10.1371/journal.pone.0259822

Abstract

Background: Clinical outcomes among COVID-19 patients vary greatly with age and underlying comorbidities. We aimed to determine the demographic and clinical factors, particularly baseline chronic conditions, associated with an increased risk of severity in COVID-19 patients from a population-based perspective and using data from electronic health records (EHR).

Methods: Retrospective, observational study in an open cohort analyzing all 68,913 individuals (mean age 44.4 years, 53.2% women) with SARS-CoV-2 infection between 15 June and 19 December 2020 using exhaustive electronic health registries. Patients were followed for 30 days from inclusion or until the date of death within that period. We performed multivariate logistic regression to analyze the association between each chronic disease and severe infection, based on hospitalization and all-cause mortality.

Results: 5885 (8.5%) individuals showed severe infection and old age was the most influencing factor. Congestive heart failure (odds ratio -OR- men: 1.28, OR women: 1.39), diabetes (1.37, 1.24), chronic renal failure (1.31, 1.22) and obesity (1.21, 1.26) increased the likelihood of severe infection in both sexes. Chronic skin ulcers (1.32), acute cerebrovascular disease (1.34), chronic obstructive pulmonary disease (1.21), urinary incontinence (1.17) and neoplasms (1.26) in men, and infertility (1.87), obstructive sleep apnea (1.43), hepatic steatosis (1.43), rheumatoid arthritis (1.39) and menstrual disorders (1.18) in women were also associated with more severe outcomes.

Conclusions: Age and specific cardiovascular and metabolic diseases increased the risk of severe SARS-CoV-2 infections in men and women, whereas the effects of certain comorbidities are sex specific. Future studies in different settings are encouraged to analyze which profiles of chronic patients are at higher risk of poor prognosis and should therefore be the targets of prevention and shielding strategies.

Conflict of interest statement

The authors have declared that no competing interests exist.

- Cited by 2 articles
- 53 references
- 1 figure

Supplementary info

Publication types, MeSH terms, Grant support Expand

Publication types

- Research Support, Non-U.S. Gov't

MeSH terms

- Adult
- Aged
- COVID-19 / complications
- COVID-19 / epidemiology*
- COVID-19 / pathology
- COVID-19 / virology
- Chronic Disease / mortality*
- Cohort Studies
- Comorbidity
- Female
- Hospitalization / statistics & numerical data
- Humans
- Logistic Models
- Male
- Middle Aged
- Pulmonary Disease, Chronic Obstructive / complications
- Pulmonary Disease, Chronic Obstructive / epidemiology*
- Pulmonary Disease, Chronic Obstructive / pathology
- Risk Factors
- SARS-CoV-2 / pathogenicity*
- Spain / epidemiology

Grant support

This research was funded by Gobierno de Aragón (Grant numbers B01_20R and DECRETO-LEY 3/2020, de 3 de junio, del Gobierno de Aragón), which provided financial support to the EpiChron Research Group and financed the article processing charge. The funders had no role in study design, data collection and analysis, decision to publish, or preparation of the manuscript.

Full text links

OPEN ACCESS TO FULL TEXT PLOS ONE Public Library of Science Free PMC article
Proceed to details
Cite
Share
565
Observational Study
High Blood Press Cardiovasc Prev
. 2022 Mar;29(2):163-167.
doi: 10.1007/s40292-021-00502-5. Epub 2022 Jan 3.

Humoral Immune Response to COVID-19 Vaccination in Hemodialysis Patients: A Retrospective, Observational Case-Control Pilot Study

Luca Piscitani [1], Rita Del Pinto [2], Andrea Basili [3], Marilena Tunno [3], Claudio Ferri [2]
Affiliations Expand

Affiliations

- [1] Nephrology and Dialysis Unit, Department of Medicine, S. Salvatore Hospital, Via Vetoio, 67100, L'Aquila, Italy. lucpis90@virgilio.it.
- [2] Internal Medicine and Nephrology Unit, Department of Life, Health, and Environmental Sciences, University of L'Aquila, S. Salvatore Hospital, Via Vetoio, 67100, L'Aquila, Italy.
- [3] Nephrology and Dialysis Unit, Department of Medicine, S. Salvatore Hospital, Via Vetoio, 67100, L'Aquila, Italy.

- PMID: **34978702**
- PMCID: PMC8721477
- DOI: 10.1007/s40292-021-00502-5

Free PMC article
Observational Study

Humoral Immune Response to COVID-19 Vaccination in Hemodialysis Patients: A Retrospective, Observational Case-Control Pilot Study

Luca Piscitani et al. High Blood Press Cardiovasc Prev. 2022 Mar.
Free PMC article

Show details

High Blood Press Cardiovasc Prev
. 2022 Mar;29(2):163-167.
doi: 10.1007/s40292-021-00502-5. Epub 2022 Jan 3.

Authors

Luca Piscitani [1], Rita Del Pinto [2], Andrea Basili [3], Marilena Tunno [3], Claudio Ferri [2]

Affiliations

- [1] Nephrology and Dialysis Unit, Department of Medicine, S. Salvatore Hospital, Via Vetoio, 67100, L'Aquila, Italy. lucpis90@virgilio.it.
- [2] Internal Medicine and Nephrology Unit, Department of Life, Health, and Environmental Sciences, University of L'Aquila, S. Salvatore Hospital, Via Vetoio, 67100, L'Aquila, Italy.
- [3] Nephrology and Dialysis Unit, Department of Medicine, S. Salvatore Hospital, Via Vetoio, 67100, L'Aquila, Italy.

- PMID: **34978702**
- PMCID: PMC8721477
- DOI: 10.1007/s40292-021-00502-5

Abstract

Introduction: Coronavirus 2 disease is associated with increased mortality and morbidity in chronic hemodialysis patients METHODS: A retrospective, observational case-control pilot study was conducted on consecutive hemodialysis outpatients (cases) and control group of individuals with preserved renal function. Complete SARS-CoV-2 vaccination with BNT162b2 mRNA vaccine, followed by determination of serum antibodies after the second dose, were required from participants in both groups. Previous COVID-19 was an exclusion criterium.

Results: 21 hemodialysis patients (M:F = 13:8, mean age 67.5 ± 13.4) and 16 controls without chronic kidney disease (M:F = 4:12, mean age 46.8 ± 12.7) were included. Hemodialysis patients had lower mean titers of serum antibodies to the SARS-CoV-2 spike antigen compared with controls (492.39 vs 1901.20 IU/mL, respectively; $p < 0.001$), a finding that was confirmed in the age-matched analysis on 18 participants (580.8 vs 1836.4 IU/mL, $p = 0.006$).

Conclusions: This study supports the finding of hyporesponsiveness to mRNA vaccination among hemodialysis patients.

Keywords: Antibody activity; COVID-19; Hemodialysis; mRNA vaccines.

© 2021. Italian Society of Hypertension.

Conflict of interest statement

All the authors declare no conflict of interest

- Cited by 1 article
- 11 references
- 2 figures

Supplementary info

Publication types, MeSH terms, Substances Expand

Publication types

- Observational Study

MeSH terms

- Adult
- Aged
- Aged, 80 and over
- Antibodies, Viral
- BNT162 Vaccine
- COVID-19 Vaccines* / adverse effects
- COVID-19* / prevention & control
- Case-Control Studies
- Humans
- Immunity, Humoral
- Middle Aged
- Pilot Projects
- Renal Dialysis / adverse effects
- Retrospective Studies
- SARS-CoV-2
- Vaccination
- Vaccines, Synthetic
- mRNA Vaccines

Substances

- Antibodies, Viral
- COVID-19 Vaccines
- Vaccines, Synthetic
- mRNA Vaccine
- mRNA Vaccines
- BNT162 Vaccine

Full text links

SpringerLink FULL-TEXT ARTICLE Springer Free PMC article
Proceed to details

Cite
Share
566
Observational Study
Clin Invest Med
. 2021 Jun 14;44(2):E48-54.
doi: 10.25011/cim.v44i2.36355.

Surgical Masks for Protection of Health Care Personnel Against Covid-19: Results from an Observational Study

Zhengqi Pan [1], Huijing Zhang [2], Jianming Yang [3], Shengli Tang [4], Zhenshun Cheng [5], Kaisong Wu [5], Bing Liu [5]
Affiliations Expand

Affiliations

- [1] Department of Joint Surgery and Sports Medicine, Zhongnan Hospital of Wuhan University, Wuhan, P. R. China; Hubei Key Laboratory of Big Data in Science and Technology (Wuhan Library of Chinese Academy of Science), Wuhan, P. R. China.
- [2] Wuhan Library of Chinese Academy of Science, Wuhan, P. R. China; Hubei Key Laboratory of Big Data in Science and Technology (Wuhan Library of Chinese Academy of Science), Wuhan, P. R. China.
- [3] Respiratory Department, Wu Xue NO.1 People's Hospital, Huanggang, P. R. China.
- [4] Department of Hepatopancreatobiliary Surgery, Zhongnan Hospital of Wuhan University, Wuhan, P. R. China.
- [5] Department of Respiratory and Critical Care Medicine, Zhongnan Hospital of Wuhan University, Wuhan, P. R. China; Wuhan Research Center for Infectious Diseases and Cancer, Chinese Academy of Medical Sciences, Wuhan, P. R. China.

- PMID: **34152707**
- DOI: 10.25011/cim.v44i2.36355

Observational Study

Surgical Masks for Protection of Health Care Personnel Against Covid-19: Results from an Observational Study

Zhengqi Pan et al. Clin Invest Med. 2021.
Show details
Clin Invest Med

. 2021 Jun 14;44(2):E48-54.
doi: 10.25011/cim.v44i2.36355.

Authors

Zhengqi Pan [1], Huijing Zhang [2], Jianming Yang [3], Shengli Tang [4], Zhenshun Cheng [5], Kaisong Wu [5], Bing Liu [5]

Affiliations

- [1] Department of Joint Surgery and Sports Medicine, Zhongnan Hospital of Wuhan University, Wuhan, P. R. China; Hubei Key Laboratory of Big Data in Science and Technology (Wuhan Library of Chinese Academy of Science), Wuhan, P. R. China.
- [2] Wuhan Library of Chinese Academy of Science, Wuhan, P. R. China; Hubei Key Laboratory of Big Data in Science and Technology (Wuhan Library of Chinese Academy of Science), Wuhan, P. R. China.
- [3] Respiratory Department, Wu Xue NO.1 People's Hospital, Huanggang, P. R. China.
- [4] Department of Hepatopancreatobiliary Surgery, Zhongnan Hospital of Wuhan University, Wuhan, P. R. China.
- [5] Department of Respiratory and Critical Care Medicine, Zhongnan Hospital of Wuhan University, Wuhan, P. R. China; Wuhan Research Center for Infectious Diseases and Cancer, Chinese Academy of Medical Sciences, Wuhan, P. R. China.

- PMID: **34152707**
- DOI: 10.25011/cim.v44i2.36355

Abstract

Purpose: The aim of the study was to describe the use of masks among health care personnel (HCP) exposed to index cases of coronavirus disease 2019 (COVID-19), and to evaluate any association with infection rate.

Methods: We did a retrospective, observational study of HCP at Zhongnan Hospital of Wuhan University for the management of COVID-19 (before person-to-person transmission was official confirmed, no additional protection was provided). A questionnaire was given to all staff listed on the roster in the clinical regions providing care for index patients with COVID-19. All participants were surveyed regarding hand-washing and use of surgical masks and gloves and were tested for severe acute respiratory syndrome coronavirus 2 (SARS-CoV-2). Data were analysed (Student's t test and Pearson $\chi 2$ test) for an association between infection and use of personal protective equipment.

Results: Exposure of a total of 299 non-infected and 30 infected staff was confirmed. None of the 149 staff who reported use of all three preventative measures (hand-washing and use of gloves and masks) became infect-ed. In contrast, all 30 of the staff who became infected had omitted at least one of the measures. Fewer staff who wore surgical masks (P=0.000003) became infected compared with those who did not. Infections rates were significantly lower in HCP from the internal medicine departments, as these personnel generally wore masks.

Conclusion: An association was found between SARS-CoV-2 infection of HCP and the non-use of masks when working with index cases in clinical settings. We recommend that all HCP follow

the strict instructions for prevention and treatment of nosocomial infection during intimate contact with COVID-19, especially staff from surgical departments.

Keywords: SARS-CoV-2; COVID-19; health care personnel; surgical masks; occupational protection.

Supplementary info

Publication types, MeSH terms Expand

Publication types

- Observational Study
- Research Support, Non-U.S. Gov't

MeSH terms

- Adult
- COVID-19 / prevention & control*
- COVID-19 / transmission
- China
- Delivery of Health Care
- Female
- Gloves, Surgical
- Hand Disinfection
- Health Personnel
- Humans
- Male
- Masks*
- Middle Aged
- Occupational Exposure / prevention & control*
- Personal Protective Equipment*
- Physicians*
- Retrospective Studies
- SARS-CoV-2*

Proceed to details
Cite
Share
567
Observational Study
J Am Heart Assoc
. 2021 Dec 21;10(24):e023535.
doi: 10.1161/JAHA.121.023535. Epub 2021 Dec 10.

Angiotensin-Converting Enzyme Inhibitors, Angiotensin II Receptor Blockers, and Outcomes in Patients Hospitalized for COVID-19

Michael Pan [1], Alexi Vasbinder [1, 2], Elizabeth Anderson [2], Toniemarie Catalan [2], Husam R Shadid [1], Hanna Berlin [1], Kishan Padalia [1], Patrick O'Hayer [1], Chelsea Meloche [1], Tariq U Azam [2], Ibrahim Khaleel [1], Erinleigh Michaud [1], Pennelope Blakely [2], Abbas Bitar [2], Yiyuan Huang [3], Lili Zhao [3], Rodica Pop-Busui [4], Sven H Loosen [5], Athanasios Chalkias [6, 7], Frank Tacke [8], Evangelos J Giamarellos-Bourboulis [9], Jochen Reiser [10], Jesper Eugen-Olsen [11], Salim S Hayek [2], ISIC Group
Affiliations Expand

Affiliations

- [1] Department of Internal Medicine University of Michigan Ann Arbor MI.
- [2] Division of Cardiology Department of Internal Medicine University of Michigan Ann Arbor MI.
- [3] Department of Biostatistics School of Public Health University of Michigan Ann Arbor MI.
- [4] Division of Metabolism, Endocrinology and Diabetes Department of Internal Medicine University of Michigan Ann Arbor MI.
- [5] Clinic for Gastroenterology, Hepatology and Infectious Diseases Medical Faculty University Hospital Düsseldorf Düsseldorf Germany.
- [6] Department of Anesthesiology School of Health Sciences Faculty of Medicine University of Thessaly Larisa Greece.
- [7] Outcomes Research Consortium Cleveland OH.
- [8] Department of Hepatology & Gastroenterology Campus Charité Mitte/Campus Virchow-KlinikumCharité University Medicine Berlin Berlin Germany.
- [9] 4th Department of Internal Medicine National and Kapodistrian University of Athens Greece.
- [10] Department of Medicine Rush University Medical Center Chicago IL.
- [11] Department of Clinical Research Copenhagen University Hospital Amager and Hvidovre Hvidovre Denmark.

- PMID: **34889102**
- DOI: 10.1161/JAHA.121.023535

Free article
Observational Study

Angiotensin-Converting Enzyme Inhibitors, Angiotensin II Receptor Blockers, and

Outcomes in Patients Hospitalized for COVID-19

Michael Pan et al. J Am Heart Assoc. 2021.
Free article
Show details
J Am Heart Assoc
. 2021 Dec 21;10(24):e023535.
doi: 10.1161/JAHA.121.023535. Epub 2021 Dec 10.

Authors

Michael Pan [1], Alexi Vasbinder [1 2], Elizabeth Anderson [2], Toniemarie Catalan [2], Husam R Shadid [1], Hanna Berlin [1], Kishan Padalia [1], Patrick O'Hayer [1], Chelsea Meloche [1], Tariq U Azam [2], Ibrahim Khaleel [1], Erinleigh Michaud [1], Pennelope Blakely [2], Abbas Bitar [2], Yiyuan Huang [3], Lili Zhao [3], Rodica Pop-Busui [4], Sven H Loosen [5], Athanasios Chalkias [6 7], Frank Tacke [8], Evangelos J Giamarellos-Bourboulis [9], Jochen Reiser [10], Jesper Eugen-Olsen [11], Salim S Hayek [2], ISIC Group

Affiliations

- [1] Department of Internal Medicine University of Michigan Ann Arbor MI.
- [2] Division of Cardiology Department of Internal Medicine University of Michigan Ann Arbor MI.
- [3] Department of Biostatistics School of Public Health University of Michigan Ann Arbor MI.
- [4] Division of Metabolism, Endocrinology and Diabetes Department of Internal Medicine University of Michigan Ann Arbor MI.
- [5] Clinic for Gastroenterology, Hepatology and Infectious Diseases Medical Faculty University Hospital Düsseldorf Düsseldorf Germany.
- [6] Department of Anesthesiology School of Health Sciences Faculty of Medicine University of Thessaly Larisa Greece.
- [7] Outcomes Research Consortium Cleveland OH.
- [8] Department of Hepatology & Gastroenterology Campus Charité Mitte/Campus Virchow-KlinikumCharité University Medicine Berlin Berlin Germany.
- [9] 4th Department of Internal Medicine National and Kapodistrian University of Athens Greece.
- [10] Department of Medicine Rush University Medical Center Chicago IL.
- [11] Department of Clinical Research Copenhagen University Hospital Amager and Hvidovre Hvidovre Denmark.

- PMID: **34889102**
- DOI: 10.1161/JAHA.121.023535

Abstract

Background Use of angiotensin-converting enzyme inhibitors and angiotensin receptor blockers (ACEi/ARB) is thought to affect COVID-19 through modulating levels of angiotensin-converting enzyme 2, the cell entry receptor for SARS-CoV2. We sought to assess the association between ACEi/ARB, biomarkers of inflammation, and outcomes in patients hospitalized for COVID-19. Methods and Results We leveraged the ISIC (International Study of Inflammation in COVID-19), identified patients admitted for symptomatic COVID-19 between February 1, 2020 and June 1, 2021 for COVID-19, and examined the association between in-hospital ACEi/ARB use and all-cause death, need for ventilation, and need for dialysis. We estimated the causal effect of ACEi/ARB on the composite outcomes using marginal structural models accounting for serial blood pressure and serum creatinine measures. Of 2044 patients in ISIC, 1686 patients met inclusion criteria, of whom 398 (23.6%) patients who were previously on ACEi/ARB received at least 1 dose during their hospitalization for COVID-19. There were 215 deaths, 407 patients requiring mechanical ventilation, and 124 patients who required dialysis during their hospitalization. Prior ACEi/ARB use was associated with lower levels of soluble urokinase plasminogen activator receptor and C-reactive protein. In multivariable analysis, in-hospital ACEi/ARB use was associated with a lower risk of the composite outcome of in-hospital death, mechanical ventilation, or dialysis (adjusted hazard ratio 0.49, 95% CI [0.36-0.65]). Conclusions In patients hospitalized for COVID-19, ACEi/ARB use was associated with lower levels of inflammation and lower risk of in-hospital outcomes. Clinical trials will define the role of ACEi/ARB in the treatment of COVID-19. Registration URL: https://www.clinicaltrials.gov; Unique identifier: NCT04818866.

Keywords: ACE inhibitors; COVID-19; angiotensin receptor blockers; mortality; outcomes.

Supplementary info

Publication types, MeSH terms, Substances, Associated data, Grant support Expand

Publication types

- Observational Study
- Research Support, N.I.H., Extramural
- Research Support, Non-U.S. Gov't

MeSH terms

- Angiotensin Receptor Antagonists / therapeutic use*
- Angiotensin-Converting Enzyme Inhibitors / therapeutic use*
- COVID-19* / drug therapy
- COVID-19* / mortality
- Hospital Mortality*
- Hospitalization
- Humans
- Inflammation
- RNA, Viral
- Retrospective Studies

Substances

- Angiotensin Receptor Antagonists
- Angiotensin-Converting Enzyme Inhibitors
- RNA, Viral

Associated data

- ClinicalTrials.gov/NCT04818866

Grant support

- T32 HL007853/HL/NHLBI NIH HHS/United States
- R01 HL153384/HL/NHLBI NIH HHS/United States
- U01 DK119083/DK/NIDDK NIH HHS/United States
- R01 DK107956/DK/NIDDK NIH HHS/United States
- P30 DK020572/DK/NIDDK NIH HHS/United States

- U2C DK110768/DK/NIDDK NIH HHS/United States

Show all 6 grants

Full text links

OPEN ACCESS Atypon
Proceed to details
Cite
Share
☐ 568
Observational Study
J Clin Neurosci
. 2021 Nov;93:241-246.
doi: 10.1016/j.jocn.2021.09.029. Epub 2021 Sep 20.

The significant impact of Coronavirus disease 2019 (COVID-19) on in-hospital mortality of elderly patients with moderate to severe traumatic brain injury: A retrospective observational study

Seyed Reza Bagheri [1], Alireza Abdi [2], Joseph Benson [3], Negin Naghdi [4], Sonia V Eden [5], Minoo Arjmand [6], Zahra Amini [7], Michael T Lawton [8], Ehsan Alimohammadi [9]
Affiliations Expand

Affiliations

- [1] Department of Neurosurgery, Kermanshah University of Medical Sciences, Kermanshah, Iran.
- [2] Nursing and Midwifery School, Kermanshah University of Medical Sciences, Imam Reza Hospital, Kermanshah, Iran.
- [3] M3 Student Meharry Medical College, USA. Electronic address: bjoseph19@email.mmc.edu.
- [4] Clinical Research Development Center, Taleghani and Imam Ali Hospital, Kermanshah University of Medical Sciences, Kermanshah, Iran.
- [5] Wayne State University School of Medicine, Detroit, MI, USA. Electronic address: seden@dmc.org.
- [6] Kermanshah University of Medical Sciences, Taleghani Hospital, Kermanshah, Iran.
- [7] Kermanshah University of Medical Sciences, Imam Reza Hospital, Kermanshah, Iran.
- [8] Department of Neurological Surgery, Barrow Neurological Institute, St. Joseph's Hospital and Medical Center, Phoenix, AZ, USA. Electronic address: michael.lawton@barrowbrainandspine.com.
- [9] Department of Neurosurgery, Kermanshah University of Medical Sciences, Kermanshah, Iran. Electronic address: ehsan.alimohammadi@kums.ac.ir.

- PMID: **34656255**
- PMCID: PMC8462266
- DOI: 10.1016/j.jocn.2021.09.029

Free PMC article
Observational Study

The significant impact of Coronavirus disease 2019 (COVID-19) on in-hospital mortality of elderly patients with moderate to severe traumatic brain injury: A retrospective observational study

Seyed Reza Bagheri et al. J Clin Neurosci. 2021 Nov.
Free PMC article
Show details
J Clin Neurosci
. 2021 Nov;93:241-246.
doi: 10.1016/j.jocn.2021.09.029. Epub 2021 Sep 20.

Authors

Seyed Reza Bagheri [1], Alireza Abdi [2], Joseph Benson [3], Negin Naghdi [4], Sonia V Eden [5], Minoo Arjmand [6], Zahra Amini [7], Michael T Lawton [8], Ehsan Alimohammadi [9]

Affiliations

- [1] Department of Neurosurgery, Kermanshah University of Medical Sciences, Kermanshah, Iran.
- [2] Nursing and Midwifery School, Kermanshah University of Medical Sciences, Imam Reza Hospital, Kermanshah, Iran.
- [3] M3 Student Meharry Medical College, USA. Electronic address: bjoseph19@email.mmc.edu.
- [4] Clinical Research Development Center, Taleghani and Imam Ali Hospital, Kermanshah University of Medical Sciences, Kermanshah, Iran.
- [5] Wayne State University School of Medicine, Detroit, MI, USA. Electronic address: seden@dmc.org.
- [6] Kermanshah University of Medical Sciences, Taleghani Hospital, Kermanshah, Iran.
- [7] Kermanshah University of Medical Sciences, Imam Reza Hospital, Kermanshah, Iran.
- [8] Department of Neurological Surgery, Barrow Neurological Institute, St. Joseph's Hospital and Medical Center, Phoenix, AZ, USA. Electronic address: michael.lawton@barrowbrainandspine.com.
- [9] Department of Neurosurgery, Kermanshah University of Medical Sciences, Kermanshah, Iran. Electronic address: ehsan.alimohammadi@kums.ac.ir.

- PMID: **34656255**
- PMCID: PMC8462266
- DOI: 10.1016/j.jocn.2021.09.029

Abstract

Background: Traumatic brain injury (TBI) is one of the main causes of death and disability among the elderly patient population. This study aimed to assess the predictors of in-hospital mortality of elderly patients with moderate to severe TBI who presented during the Coronavirus disease 2019 (COVID-19) pandemic.

Methods: In this retrospective analytical study, all elderly patients with moderate to severe TBI who were referred to our center between March 2nd, 2020 to August 1st, 2020 were investigated and compared against the TBI patients receiving treatment during the same time period within the year 2019. Patients were followed until discharge from the hospital or death. The demographic, clinical, radiological, and laboratory test data were evaluated. Data were analyzed using SPSS-21 software.

Findings: In this study, 359 elderly patients were evaluated (n = 162, Post-COVID-19). Fifty-four patients of the cohort had COVID-19 disease with a mortality rate was 33.3%. The patients with COVID-19 were 5.45 times more likely to expire before discharge ($P < 0.001$) than the TBI patients who were not COVID-19 positive. Other variables such as hypotension (OR, 4.57P < 0.001), hyperglycemia (OR, 2.39, $P = 0.002$), and use of anticoagulant drugs (OR, 2.41P = 0.001) were also associated with in-hospital death.According to the binary logistic regression analysis Age (OR, 1.72; 95% CI: 1.26-2.18; $P = 0.033$), Coronavirus infection (OR, 2.21; 95% CI: 1.83-2.92; $P = 0.011$) and Glasgow Coma Scale (GCS) (OR, 3.11; 95% CI: 2.12-4.53; $P < 0.001$) were independent risk factors correlated with increased risk of in-hospital mortality of elderly patients with moderate to severe TBI.

Conclusion: Our results showed that Coronavirus infection could increase the risk of in-hospital mortality of elderly patients with moderate to severe TBI significantly.

Keywords: COVID-19; Elderly patients; In-hospital mortality; Traumatic brain injury.

Copyright © 2021 Elsevier Ltd. All rights reserved.

Conflict of interest statement

Declaration of Competing Interest The authors declare that they have no known competing financial interests or personal relationships that could have appeared to influence the work reported in this paper.

- 44 references

Supplementary info

Publication types, MeSH terms Expand

Publication types

- Observational Study

MeSH terms

- Aged
- Brain Injuries, Traumatic*
- COVID-19*
- Glasgow Coma Scale
- Hospital Mortality
- Humans
- Retrospective Studies
- SARS-CoV-2

Full text links

ELSEVIER FULL-TEXT ARTICLE Elsevier Science Free PMC article

Proceed to details

Cite

Share

☐ 569

Observational Study

BMC Res Notes

. 2020 Dec 9;13(1):555.

doi: 10.1186/s13104-020-05402-w.

Biomarkers of acute respiratory distress syndrome in adults hospitalised for severe

SARS-CoV-2 infection in Tenerife Island, Spain

Juan Marco Figueira Gonçalves [1], José María Hernández Pérez [2], Marco Acosta Sorensen [2], Aurelio Luis Wangüemert Pérez [3], Elena Martín Ruiz de la Rosa [2], José Luis Trujillo Castilla [2], David Díaz Pérez [2], Yolanda Ramallo-Fariña [4] [5]
Affiliations Expand

Affiliations

- [1] Pneumology and Thoracic Surgery Service, University Hospital Nuestra Señora de Candelaria, Santa Cruz de Tenerife, Spain. juanmarcofigueira@gmail.com.
- [2] Pneumology and Thoracic Surgery Service, University Hospital Nuestra Señora de Candelaria, Santa Cruz de Tenerife, Spain.
- [3] Pneumology Service, San Juan de Dios Hospital, Tenerife, Spain.
- [4] Foundation of the Canary Islands Health Research Institute (FIISC), Santa Cruz de Tenerife, Spain.
- [5] Health Services Research On Chronic Patients Network (REDISSEC), Madrid, Spain.

- PMID: **33298124**
- PMCID: PMC7724618
- DOI: 10.1186/s13104-020-05402-w

Free PMC article
Observational Study

Biomarkers of acute respiratory distress syndrome in adults hospitalised for severe SARS-CoV-2 infection in Tenerife Island, Spain

Juan Marco Figueira Gonçalves et al. BMC Res Notes. 2020.
Free PMC article
Show details
BMC Res Notes
. 2020 Dec 9;13(1):555.
doi: 10.1186/s13104-020-05402-w.

Authors

Juan Marco Figueira Gonçalves [1], José María Hernández Pérez [2], Marco Acosta Sorensen [2], Aurelio Luis Wangüemert Pérez [3], Elena Martín Ruiz de la Rosa [2], José Luis Trujillo Castilla [2], David Díaz Pérez [2], Yolanda Ramallo-Fariña [4] [5]

Affiliations

- [1] Pneumology and Thoracic Surgery Service, University Hospital Nuestra Señora de Candelaria, Santa Cruz de Tenerife, Spain. juanmarcofigueira@gmail.com.
- [2] Pneumology and Thoracic Surgery Service, University Hospital Nuestra Señora de Candelaria, Santa Cruz de Tenerife, Spain.
- [3] Pneumology Service, San Juan de Dios Hospital, Tenerife, Spain.
- [4] Foundation of the Canary Islands Health Research Institute (FIISC), Santa Cruz de Tenerife, Spain.
- [5] Health Services Research On Chronic Patients Network (REDISSEC), Madrid, Spain.

- PMID: **33298124**
- PMCID: PMC7724618
- DOI: 10.1186/s13104-020-05402-w

Abstract

Objective: The dramatic spread of SARS-CoV-2 infections calls for reliable, inexpensive tools to quickly identify patients with a poor prognosis. In this study, acute respiratory distress syndrome (ARDS) was assessed within 72 h after admission of each of 153 consecutive, SARS-CoV-2 infected, adult patients to either of two hospitals in Tenerife, Spain, using suitable routine laboratory tests for lymphocyte counts, as well as ferritin, lactate dehydrogenase (LDH), and C-reactive protein levels. Results were correlated with the patients' respiratory function, defined through their pulse oximetric saturation/fraction of inspired oxygen (SpO2/FiO2) ratio.

Results: Within 72 h from admission, criteria matched ARDS (SpO2/FiO2 < 235) in 13.1% of cases. We found a significant, negative correlation between SpO2/FiO2 ratios and D-dimer, ferritin, and LDH levels (- 0.31, - 0.32, and - 0.41; p = 0.004, 0.004, and < 0.0001, respectively). In patients with ARDS, the mean LDH was 373 U/L ($CI_{95\%}$: 300.6-445.3), but only 298 U/L ($CI_{95\%}$: 274.7-323.1) when they did not develop the syndrome (p = 0.015). None of the additionally evaluated biomarkers correlated with the SpO2/FiO2 ratios. Serum LDH levels in patients hospitalised for COVID-19 correlate with ARDS, as defined by their SpO2/FiO2 ratio, and might help to predict said complication.

Keywords: Acute respiratory failure; Biomarkers; COVID-19; LDH.

Conflict of interest statement

The authors declare not to have any conflict of interest related to this article.

- Cited by 4 articles
- 42 references

Supplementary info

Publication types, MeSH terms, Substances Expand

Publication types

- Multicenter Study

- Observational Study

MeSH terms

- Aged
- Biomarkers / blood*
- COVID-19 / blood
- COVID-19 / complications*
- COVID-19 / diagnosis
- Cohort Studies
- Female
- Hospitalization
- Humans
- L-Lactate Dehydrogenase / blood
- Middle Aged
- Oximetry
- Oxygen / metabolism
- Patient Acuity
- Respiratory Distress Syndrome / blood
- Respiratory Distress Syndrome / diagnosis*
- Respiratory Distress Syndrome / virology
- Retrospective Studies
- Spain

Substances

- Biomarkers
- L-Lactate Dehydrogenase
- Oxygen

Full text links

Read free full text at BMC BioMed Central Free PMC article

Proceed to details

Cite

Share

☐ 570

Observational Study

Air Med J

. Jul-Aug 2021;40(4):220-224.

doi: 10.1016/j.amj.2021.04.001. Epub 2021 Apr 5.

Decision Making and Interventions During Interfacility Transport of High-Acuity Patients With Severe Acute Respiratory Syndrome Coronavirus 2 Infection

Ruben D Troncoso Jr [1], Eric M Garfinkel [2], David Leon [2], Sandra M Lopez [2], Andrew Lin [2], Dennis Jones [3], Shawn Trautman [3], Matthew J Levy [4], Asa M Margolis [4]
Affiliations Expand

Affiliations

- [1] Department of Emergency Medicine, Johns Hopkins University School of Medicine, Baltimore, MD; Johns Hopkins Lifeline Critical Care Transportation Program, Johns Hopkins University School of Medicine, Baltimore, MD. Electronic address: rtronco1@jhmi.edu.
- [2] Department of Emergency Medicine, Johns Hopkins University School of Medicine, Baltimore, MD.
- [3] Johns Hopkins Lifeline Critical Care Transportation Program, Johns Hopkins University School of Medicine, Baltimore, MD.
- [4] Department of Emergency Medicine, Johns Hopkins University School of Medicine, Baltimore, MD; Johns Hopkins Lifeline Critical Care Transportation Program, Johns Hopkins University School of Medicine, Baltimore, MD.

- PMID: **34172228**
- PMCID: PMC8020076
- DOI: 10.1016/j.amj.2021.04.001

Free PMC article
Observational Study

Decision Making and Interventions During Interfacility Transport of High-Acuity Patients With Severe Acute Respiratory Syndrome Coronavirus 2 Infection

Ruben D Troncoso Jr et al. Air Med J. Jul-Aug 2021.
Free PMC article
Show details
Air Med J
. Jul-Aug 2021;40(4):220-224.
doi: 10.1016/j.amj.2021.04.001. Epub 2021 Apr 5.

Authors

Ruben D Troncoso Jr [1], Eric M Garfinkel [2], David Leon [2], Sandra M Lopez [2], Andrew Lin [2], Dennis Jones [3], Shawn Trautman [3], Matthew J Levy [4], Asa M Margolis [4]

Affiliations

- [1] Department of Emergency Medicine, Johns Hopkins University School of Medicine, Baltimore, MD; Johns Hopkins Lifeline Critical Care Transportation Program, Johns Hopkins University School of Medicine, Baltimore, MD. Electronic address: rtronco1@jhmi.edu.
- [2] Department of Emergency Medicine, Johns Hopkins University School of Medicine, Baltimore, MD.
- [3] Johns Hopkins Lifeline Critical Care Transportation Program, Johns Hopkins University School of Medicine, Baltimore, MD.
- [4] Department of Emergency Medicine, Johns Hopkins University School of Medicine, Baltimore, MD; Johns Hopkins Lifeline Critical Care Transportation Program, Johns Hopkins University School of Medicine, Baltimore, MD.

- PMID: **34172228**
- PMCID: PMC8020076
- DOI: 10.1016/j.amj.2021.04.001

Abstract

Objective: There are limited data regarding the typical characteristics of coronavirus disease 2019 (COVID-19) patients requiring interfacility transport or the clinical capabilities of the out-of-hospital transport clinicians required to provide safe transport. The objective of this study is to provide epidemiologic data and highlight the clinical skill set and decision making needed to transport critically ill COVID-19 patients.

Methods: A retrospective chart review of persons under investigation for COVID-19 transported during the first 6 months of the pandemic by Johns Hopkins Lifeline was performed. Patients who required interfacility transport and tested positive for severe acute respiratory syndrome coronavirus 2 by polymerase chain reaction assay were included in the analysis.

Results: Sixty-eight patients (25.4%) required vasopressor support, 35 patients (13.1%) were pharmacologically paralyzed, 15 (5.60%) were prone, and 1 (0.75%) received an inhaled pulmonary vasodilator. At least 1 ventilator setting change occurred for 59 patients (22.0%), and ventilation mode was changed for 11 patients (4.10%) during transport.

Conclusion: The safe transport of critically ill patients with COVID-19 requires experience with vasopressors, paralytic medications, inhaled vasodilators, prone positioning, and ventilator management. The frequency of initiated critical interventions and ventilator adjustments underscores the tenuous nature of these patients and highlights the importance of transport clinician reassessment, critical thinking, and decision making.

Copyright © 2021 Air Medical Journal Associates. Published by Elsevier Inc. All rights reserved.

- Cited by 3 articles
- 21 references
- 3 figures

Supplementary info

Publication types, MeSH terms Expand

Publication types

- Observational Study

MeSH terms

- Adult
- Aged
- Aged, 80 and over
- COVID-19 / diagnosis
- COVID-19 / therapy*
- Clinical Competence*
- Clinical Decision-Making / methods*
- Combined Modality Therapy
- Critical Care / methods*
- Critical Care / standards
- Critical Care / statistics & numerical data
- Critical Illness
- Female
- Humans
- Male
- Maryland
- Middle Aged
- Patient Acuity
- Patient Transfer / methods
- Patient Transfer / standards
- Patient Transfer / statistics & numerical data
- Retrospective Studies
- Transportation of Patients / methods*
- Transportation of Patients / standards
- Transportation of Patients / statistics & numerical data

Full text links

ELSEVIER FULL-TEXT ARTICLE Elsevier Science Free PMC article
Proceed to details
Cite
Share
☐ 571

Observational Study

J Thromb Haemost
. 2021 Nov;19(11):2814-2824.
doi: 10.1111/jth.15517. Epub 2021 Sep 15.

Association of prehospital antiplatelet therapy with survival in patients hospitalized with COVID-19: A propensity score-matched analysis

Jonathan H Chow [1], Ying Yin [2], David P Yamane [1] [3], Danielle Davison [1], Ryan J Keneally [1], Katrina Hawkins [1], K Gage Parr [1], Mustafa Al-Mashat [1], Jeffery S Berger [1], Reamer L Bushardt [2], Michael A Mazzeffi [1], Stuart J Nelson [2]
Affiliations Expand

Affiliations

- [1] Department of Anesthesiology and Critical Care Medicine, George Washington University School of Medicine and Health Sciences, Washington, DC, USA.
- [2] George Washington University School of Medicine and Health Sciences, Washington, DC, USA.
- [3] Department of Emergency Medicine, George Washington University School of Medicine and Health Sciences, Washington, DC, USA.

- PMID: **34455688**
- PMCID: PMC8646433
- DOI: 10.1111/jth.15517

Free PMC article
Observational Study

Association of prehospital antiplatelet therapy with survival in patients hospitalized with COVID-19: A propensity score-matched analysis

Jonathan H Chow et al. J Thromb Haemost. 2021 Nov.
Free PMC article
Show details

J Thromb Haemost
. 2021 Nov;19(11):2814-2824.
doi: 10.1111/jth.15517. Epub 2021 Sep 15.

Authors

Jonathan H Chow [1], Ying Yin [2], David P Yamane [1 3], Danielle Davison [1], Ryan J Keneally [1], Katrina Hawkins [1], K Gage Parr [1], Mustafa Al-Mashat [1], Jeffery S Berger [1], Reamer L Bushardt [2], Michael A Mazzeffi [1], Stuart J Nelson [2]

Affiliations

- [1] Department of Anesthesiology and Critical Care Medicine, George Washington University School of Medicine and Health Sciences, Washington, DC, USA.
- [2] George Washington University School of Medicine and Health Sciences, Washington, DC, USA.
- [3] Department of Emergency Medicine, George Washington University School of Medicine and Health Sciences, Washington, DC, USA.

- PMID: **34455688**
- PMCID: PMC8646433
- DOI: 10.1111/jth.15517

Abstract

Purpose: Coronavirus disease 2019 (COVID-19) is associated with hypercoagulability and increased thrombotic risk. The impact of prehospital antiplatelet therapy on in-hospital mortality is uncertain.

Methods: This was an observational cohort study of 34 675 patients ≥50 years old from 90 health systems in the United States. Patients were hospitalized with laboratory-confirmed COVID-19 between February 2020 and September 2020. For all patients, the propensity to receive prehospital antiplatelet therapy was calculated using demographics and comorbidities. Patients were matched based on propensity scores, and in-hospital mortality was compared between the antiplatelet and non-antiplatelet groups.

Results: The propensity score-matched cohort of 17 347 patients comprised of 6781 and 10 566 patients in the antiplatelet and non-antiplatelet therapy groups, respectively. In-hospital mortality was significantly lower in patients receiving prehospital antiplatelet therapy (18.9% vs. 21.5%, $p < .001$), resulting in a 2.6% absolute reduction in mortality (HR: 0.81, 95% CI: 0.76-0.87, $p < .005$). On average, 39 patients needed to be treated to prevent one in-hospital death. In the antiplatelet therapy group, there was a significantly lower rate of pulmonary embolism (2.2% vs. 3.0%, $p = .002$) and higher rate of epistaxis (0.9% vs. 0.4%, $p < .001$). There was no difference in the rate of other hemorrhagic or thrombotic complications.

Conclusions: In the largest observational study to date of prehospital antiplatelet therapy in patients with COVID-19, there was an association with significantly lower in-hospital mortality. Randomized controlled trials in diverse patient populations with high rates of baseline comorbidities are needed to determine the ultimate utility of antiplatelet therapy in COVID-19.

Keywords: COVID-19; SARS-CoV-2; antiplatelet therapy; aspirin; clopidogrel; dipyridamole; prasugrel; ticagrelor.

© 2021 International Society on Thrombosis and Haemostasis.

Conflict of interest statement

Dr. Chow has served on the Speaker's Bureau for La Jolla Pharmaceutical Company, outside the scope of the submitted work. The other authors have no potential conflicts of interest to disclose.

- Cited by 2 articles
- 37 references
- 3 figures

Supplementary info

Publication types, MeSH terms, Substances Expand

Publication types

- Observational Study

MeSH terms

- COVID-19*
- Emergency Medical Services*
- Hospital Mortality
- Humans
- Middle Aged
- Platelet Aggregation Inhibitors / adverse effects
- Propensity Score
- Retrospective Studies
- SARS-CoV-2
- United States / epidemiology

Substances

- Platelet Aggregation Inhibitors

Full text links

WILEY Full Text Article Wiley Free PMC article
Proceed to details
Cite
Share
☐ 572
Observational Study
Gynecol Oncol
. 2022 Feb;164(2):304-310.
doi: 10.1016/j.ygyno.2021.12.004. Epub 2021 Dec 7.

COVID-19 outcomes of patients with gynecologic cancer in New York City: An updated analysis from the initial surge of the pandemic

Olivia D Lara [1], Maria Smith [1], Yuyan Wang [2], Roisin E O'Cearbhaill [3], Stephanie V Blank [4], Valentin Kolev [4], Caitlin Carr [4], Anne Knisely [5], Jennifer McEachron [6], Lisa Gabor [7], Eloise Chapman-Davis [8], Seth Cohen [3], Julia Fehniger [1], Yi-Chun Lee [6], Sara Isani [7], Mengling Liu [9], Jason D Wright [5], Bhavana Pothuri [10]

Affiliations Expand

Affiliations

- [1] Department of Obstetrics and Gynecology, Division of Gynecologic Oncology, Laura and Isaac Perlmutter Cancer Center, NYU Langone Health, New York, NY, United States of America.
- [2] Department of Population Health, NYU Langone Health, New York, NY, United States of America.
- [3] Department of Medical Oncology, Memorial Sloan Kettering Cancer Center, Weill Cornell Medical College, New York, NY, United States of America.
- [4] Department of Obstetrics, Gynecologic and Reproductive Science, Division of Gynecologic Oncology, Icahn School of Medicine at Mount Sinai, New York, NY, United States of America.
- [5] Department of Obstetrics and Gynecology, College of Physicians and Surgeons, Columbia University, New York, NY, United States of America.
- [6] Department of Obstetrics and Gynecology, State University of New York Downstate Medical Center, Brooklyn, NY, United States of America.
- [7] Department of Obstetrics & Gynecology and Women's Health, Montefiore Medical Center and Albert Einstein College of Medicine, Bronx, NY, United States of America.
- [8] Department of Obstetrics & Gynecology, Cornell University, New York, NY, United States of America.
- [9] Department of Population Health, NYU Langone Health, New York, NY, United States of America; Department of Environmental Medicine, NYU Langone Health, New York, NY, United States of America.
- [10] Department of Obstetrics and Gynecology, Division of Gynecologic Oncology, Laura and Isaac Perlmutter Cancer Center, NYU Langone Health, New York, NY, United States of America. Electronic address: bhavana.pothuri@nyulangone.org.

- PMID: **34922769**
- PMCID: PMC8648583
- DOI: 10.1016/j.ygyno.2021.12.004

Free PMC article
Observational Study

COVID-19 outcomes of patients with gynecologic cancer in New York City: An updated analysis from the initial surge of the pandemic

Olivia D Lara et al. Gynecol Oncol. 2022 Feb.
Free PMC article
Show details
Gynecol Oncol
. 2022 Feb;164(2):304-310.
doi: 10.1016/j.ygyno.2021.12.004. Epub 2021 Dec 7.

Authors

Olivia D Lara [1], Maria Smith [1], Yuyan Wang [2], Roisin E O'Cearbhaill [3], Stephanie V Blank [4], Valentin Kolev [4], Caitlin Carr [4], Anne Knisely [5], Jennifer McEachron [6], Lisa Gabor [7], Eloise Chapman-Davis [8], Seth Cohen [3], Julia Fehniger [1], Yi-Chun Lee [6], Sara Isani [7], Mengling Liu [9], Jason D Wright [5], Bhavana Pothuri [10]

Affiliations

- [1] Department of Obstetrics and Gynecology, Division of Gynecologic Oncology, Laura and Isaac Perlmutter Cancer Center, NYU Langone Health, New York, NY, United States of America.
- [2] Department of Population Health, NYU Langone Health, New York, NY, United States of America.
- [3] Department of Medical Oncology, Memorial Sloan Kettering Cancer Center, Weill Cornell Medical College, New York, NY, United States of America.
- [4] Department of Obstetrics, Gynecologic and Reproductive Science, Division of Gynecologic Oncology, Icahn School of Medicine at Mount Sinai, New York, NY, United States of America.
- [5] Department of Obstetrics and Gynecology, College of Physicians and Surgeons, Columbia University, New York, NY, United States of America.
- [6] Department of Obstetrics and Gynecology, State University of New York Downstate Medical Center, Brooklyn, NY, United States of America.
- [7] Department of Obstetrics & Gynecology and Women's Health, Montefiore Medical Center and Albert Einstein College of Medicine, Bronx, NY, United States of America.
- [8] Department of Obstetrics & Gynecology, Cornell University, New York, NY, United States of America.
- [9] Department of Population Health, NYU Langone Health, New York, NY, United States of America; Department of Environmental Medicine, NYU Langone Health, New York, NY, United States of America.
- [10] Department of Obstetrics and Gynecology, Division of Gynecologic Oncology, Laura and Isaac Perlmutter Cancer Center, NYU Langone Health, New York, NY, United States of America. Electronic address: bhavana.pothuri@nyulangone.org.

- PMID: **34922769**
- PMCID: PMC8648583
- DOI: 10.1016/j.ygyno.2021.12.004

Abstract

Background: Despite significant increase in COVID-19 publications, characterization of COVID-19 infection in patients with gynecologic cancer remains limited. Here we present an update of COVID-19 outcomes among people with gynecologic cancer in New York City (NYC) during the initial surge of severe acute respiratory syndrome coronavirus 2 (coronavirus disease 2019 [COVID-19]).

Methods: Data were abstracted from gynecologic oncology patients with COVID-19 infection among 8 NYC area hospital systems between March and June 2020. Multivariable logistic regression was utilized to estimate associations between factors and COVID-19 related hospitalization and mortality.

Results: Of 193 patients with gynecologic cancer and COVID-19, the median age at diagnosis was 65.0 years (interquartile range (IQR), 53.0-73.0 years). One hundred six of the 193 patients (54.9%) required hospitalization; among the hospitalized patients, 13 (12.3%) required invasive mechanical ventilation, 39 (36.8%) required ICU admission. Half of the cohort (49.2%) had not received anti-cancer treatment prior to COVID-19 diagnosis. No patients requiring mechanical ventilation survived. Thirty-four of 193 (17.6%) patients died of COVID-19 complications. In multivariable analysis, hospitalization was associated with an age ≥ 65 years (odds ratio [OR] 2.12, 95% confidence interval [CI] 1.11, 4.07), Black race (OR 2.53, CI 1.24, 5.32), performance status ≥2 (OR 3.67, CI 1.25, 13.55) and ≥ 3 comorbidities (OR 2.00, CI 1.05, 3.84). Only former or current history of smoking (OR 2.75, CI 1.21, 6.22) was associated with death due to COVID-19 in multivariable analysis. Administration of cytotoxic chemotherapy within 90 days of COVID-19 diagnosis was not predictive of COVID-19 hospitalization (OR 0.83, CI 0.41, 1.68) or mortality (OR 1.56, CI 0.67, 3.53).

Conclusions: The case fatality rate among patients with gynecologic malignancy with COVID-19 infection was 17.6%. Cancer-directed therapy was not associated with an increased risk of mortality related to COVID-19 infection.

Keywords: Coronavirus disease 2019 (COVID-19); Gynecologic cancer; Outcomes; Severe acute respiratory syndrome coronavirus 2 (SARS-CoV-2).

Copyright © 2021 Elsevier Inc. All rights reserved.

Conflict of interest statement

Declaration of Competing Interest B.P. reports grants, personal fees and non-financial support outside the submitted work; institutional PI for industry sponsored trials from Tesaro/GSK, AstraZeneca, Merck, Genentech/ Roche, Celison, Mersana and Clovis Oncology. Compensated advisory boards include Tesaro/GSK, AstraZeneca, Merck, Elevar, Arquer, Toray, and Eisai. J.J. reports a patent license from MDSeq Inc. R.OC reports personal fees from Tesaro, GlaxoSmithKline, Regeneron, Seagen, Fresenius Kabi, Genentech USA and Gynecologic Oncology Foundation, outside the submitted work and non-compensated steering committee member for the PRIMA, Moonstone (Tesaro/GSK) and DUO-O (AstraZeneca) studies. R.OC's institute receives funding for clinical research from Bayer/Celgene/Juno, Tesaro/GSK, Ludwig Cancer Institute, Abbvie/StemCentrx, Regeneron, TCR2 Therapeutics, Atara Biotherapeutics,

Marker Therapeutics, Syndax Pharmaceuticals, Genmab/Seagen Therapeutics, Sellas Therapeutics, Genentech, Kite Pharma, Gynecologic Oncology Foundation. S.V.B. has research collaborations with Roche/Genentech, Tesaro/GK, Seattle Genetics, Merck and Asta Zeneca, from which her institution receives funding.

- 29 references
- 1 figure

Supplementary info

Publication types, MeSH terms, Grant support Expand

Publication types

- Multicenter Study
- Observational Study
- Research Support, N.I.H., Extramural

MeSH terms

- Adult
- Aged
- Aged, 80 and over
- COVID-19 / complications*
- COVID-19 / mortality*
- COVID-19 / therapy
- Carcinoma / complications*
- Carcinoma / mortality*
- Carcinoma / therapy
- Female
- Genital Neoplasms, Female / complications*
- Genital Neoplasms, Female / mortality*
- Genital Neoplasms, Female / therapy
- Hospitalization / statistics & numerical data*
- Humans
- Logistic Models
- Middle Aged
- New York City / epidemiology
- Patient Acuity
- Retrospective Studies
- Risk Factors
- Treatment Outcome

Grant support

- P30 CA008748/CA/NCI NIH HHS/United States

Full text links

ELSEVIER FULL-TEXT ARTICLE Elsevier Science Free PMC article
Proceed to details
Cite
Share
573
Observational Study
Exp Oncol
. 2021 Mar;43(1):31-35.
doi: 10.32471/exp-oncology.2312-8852.vol-43-no-1.15964.

Emergency oncology admissions during COVID-19 pandemic: a major institution experience from Saudi Arabia

O Elemam [1], E Tashkandi [2], S Abdelkhalek [3], I Shalaby [4], R Baraka [5]
Affiliations Expand

Affiliations

- [1] Oncology Center, King Abdullah Medical City, Makkah 24246, Saudi Arabia.
- [2] College of Medicine, Umm AlQura University, Makkah 24246, Saudi Arabia.
- [3] Radiotherapy Department, Mansoura University, Mansoura 35516, Egypt.
- [4] Radiology Department, King Abdullah Medical City, Makkah 24246, Saudi Arabia.
- [5] Diagnostic Medicine Center, Colorado State University, Fort Collins 80523, USA.

- PMID: **33785710**
- DOI: 10.32471/exp-oncology.2312-8852.vol-43-no-1.15964

Observational Study

Emergency oncology admissions during COVID-19 pandemic: a major institution experience from Saudi Arabia

O Elemam et al. Exp Oncol. 2021 Mar.
Show details
Exp Oncol

. 2021 Mar;43(1):31-35.
doi: 10.32471/exp-oncology.2312-8852.vol-43-no-1.15964.

Authors

O Elemam [1], E Tashkandi [2], S Abdelkhalek [3], I Shalaby [4], R Baraka [5]

Affiliations

- [1] Oncology Center, King Abdullah Medical City, Makkah 24246, Saudi Arabia.
- [2] College of Medicine, Umm AlQura University, Makkah 24246, Saudi Arabia.
- [3] Radiotherapy Department, Mansoura University, Mansoura 35516, Egypt.
- [4] Radiology Department, King Abdullah Medical City, Makkah 24246, Saudi Arabia.
- [5] Diagnostic Medicine Center, Colorado State University, Fort Collins 80523, USA.

- PMID: **33785710**
- DOI: 10.32471/exp-oncology.2312-8852.vol-43-no-1.15964

Abstract

Background: The WHO has declared the coronavirus disease 2019 (COVID-19) pandemic in March 2020. Cancer patients are considered a highly susceptible group. The effect of this pandemic on cancer mortality is still unknown.

Aim: Our aim is to know whether or not we need to postpone cancer treatment during viral pandemics in the future.

Materials and methods: A retrospective observational study from March 1, 2020 to June 1, 2020, included cancer patients on active treatment, who have been admitted to our oncology center through the emergency unit, and patients who received oncology treatment in the outpatient treatment unit. COVID-19 positive cases were identified based on polymerase chain reaction testing of nasopharyngeal swab.

Results: A total of 1300 patients was included in the study, 1096 patients attended the outpatient clinics, 204 patients were admitted to our oncology floor for emergency care. The cancer diagnosis was mainly breast cancer, followed by colon cancer. The main cause of emergency room visit was mainly fever followed by pain. Admission diagnosis was mainly disease progression followed by symptom control, COVID-19 infection, and febrile neutropenia. 1288 cycles of anticancer therapy were provided to 513 patients in the outpatient treatment unit. Three out of the nineteen patients who had a confirmed COVID-19 infection (16%) died not only due to infection, but also disease progression.

Conclusion: Cancer treatment is not a risk factor for COVID-19 infection or its complications. Cancer treatment should not be interrupted during viral pandemics and every effort should be made to give cancer patients the standard of care.

Supplementary info

Publication types, MeSH terms Expand

Publication types

- Observational Study

MeSH terms

- Adolescent
- Adult
- Aged
- Aged, 80 and over
- COVID-19 / complications
- COVID-19 / epidemiology*
- COVID-19 / mortality
- COVID-19 Nucleic Acid Testing
- Comorbidity
- Emergencies
- Female
- Humans
- Immunotherapy
- Male
- Middle Aged
- Neoplasms / complications*
- Neoplasms / mortality
- Pandemics
- Patient Admission*
- Retrospective Studies
- Risk Factors
- Saudi Arabia / epidemiology
- Young Adult

Full text links

Full text article at www.exp-oncology.com.ua MORION LLC

Proceed to details

Cite

Share

☐ 574

Observational Study

Infection

. 2021 Apr;49(2):287-294.
doi: 10.1007/s15010-020-01550-0. Epub 2020 Nov 22.

Renin-angiotensin system inhibitors and mortality in patients with COVID-19

Luca Rossi [#, 1], Alessandro Malagoli [#, 2], Andrea Biagi [1], Alessia Zanni [1], Concetta Sticozzi [1], Greta Comastri [1], Luigi Pannone [3], Stefano Gandolfi [4], Pasquale Vergara [#, 3], Giovanni Quinto Villani [#, 1]

Affiliations Expand

Affiliations

- [1] Division of Cardiology, Cardiovascular and Emergency Department, Guglielmo da Saliceto Hospital, Piacenza, Italy.
- [2] Division of Cardiology, Nephro-Cardiovascular Department, "S. Agostino-Estense" Public Hospital, University of Modena and Reggio Emilia, 1355, Via Pietro Giardini - Baggiovara, 41126, Modena, Italy. ale.malagoli@gmail.com.
- [3] Arrhythmia Unit and Electrophysiology Laboratories, San Raffaele Hospital, Milano, Italy.
- [4] Health Management, Guglielmo da Saliceto Hospital, ASL Piacenza, Piacenza, Italy.

[#] Contributed equally.

- PMID: **33222020**
- PMCID: PMC7680554
- DOI: 10.1007/s15010-020-01550-0

Free PMC article
Observational Study

Renin-angiotensin system inhibitors and mortality in patients with COVID-19

Luca Rossi et al. Infection. 2021 Apr.
Free PMC article
Show details
Infection
. 2021 Apr;49(2):287-294.
doi: 10.1007/s15010-020-01550-0. Epub 2020 Nov 22.

Authors

Luca Rossi [#, 1], Alessandro Malagoli [#, 2], Andrea Biagi [1], Alessia Zanni [1], Concetta Sticozzi [1], Greta Comastri [1], Luigi Pannone [3], Stefano Gandolfi [4], Pasquale Vergara [#, 3], Giovanni Quinto Villani [#, 1]

Affiliations

- [1] Division of Cardiology, Cardiovascular and Emergency Department, Guglielmo da Saliceto Hospital, Piacenza, Italy.
- [2] Division of Cardiology, Nephro-Cardiovascular Department, "S. Agostino-Estense" Public Hospital, University of Modena and Reggio Emilia, 1355, Via Pietro Giardini - Baggiovara, 41126, Modena, Italy. ale.malagoli@gmail.com.
- [3] Arrhythmia Unit and Electrophysiology Laboratories, San Raffaele Hospital, Milano, Italy.
- [4] Health Management, Guglielmo da Saliceto Hospital, ASL Piacenza, Piacenza, Italy.

[#] Contributed equally.

- PMID: **33222020**
- PMCID: PMC7680554
- DOI: 10.1007/s15010-020-01550-0

Abstract

Association of renin-angiotensin system inhibitors with risk of death in patients with hypertension (HTN) and coronavirus disease 2019 (COVID-19) is not well characterized. The aim of this study was to evaluate the outcomes of patients with HTN and COVID-19 with respect to different chronic antihypertensive drug intake. We performed a retrospective, observational study from a large cohort of patients with HTN and with a laboratory-confirmed severe acute respiratory syndrome coronavirus 2 infection admitted to the Emergency Rooms (ER) of the Piacenza Hospital network from February 21, 2020 to March 20, 2020. There were 1050 patients admitted to the ERs of the Piacenza Hospital network with COVID-19. HTN was present in 590 patients [median age, 76.2 years (IQR 68.2-82.6)]; 399 (66.1%) patients were male. Of them, 248 patients were chronically treated with ACEi, 181 with ARBs, and 161 with other drugs (O-drugs) including beta blockers, diuretics and calcium-channel inhibitors. With respect to the antihypertensive use, there was no difference between comorbid conditions. During a follow-up of 38 days (IQR 7.0-46.0), 256 patients (43.4%) died, without any difference stratifying for antihypertensive drugs. Of them, 107 (43.1%) were in ACEi group vs 67 (37%) in ARBs group vs 82 (50.7%) in O-drugs group, (log-rank test: $p = 0.066$). In patients with HTN and COVID-19, neither ACEi nor ARBs were independently associated with mortality. After adjusting for potential confounders in risk prediction, the rate of death was similar. Our data confirm Specialty Societal recommendations, suggesting that treatment with ACEIs or ARBs should not be discontinued because of COVID-19.

Keywords: COVID-19; Hypertension; Mortality; Renin–angiotensin system (RAS) inhibitors; SARS-CoV-2.

Conflict of interest statement

The authors report no conflicts.

- Cited by 10 articles
- 23 references
- 2 figures

Supplementary info

Publication types, MeSH terms, Substances Expand

Publication types

- Observational Study

MeSH terms

- Adult
- Aged
- Aged, 80 and over
- Angiotensin Receptor Antagonists / therapeutic use
- Angiotensin-Converting Enzyme Inhibitors / therapeutic use
- Antihypertensive Agents / therapeutic use*
- COVID-19 / drug therapy*
- COVID-19 / mortality*
- Female
- Humans
- Hypertension / drug therapy
- Hypertension / mortality
- Male
- Middle Aged
- Retrospective Studies
- SARS-CoV-2
- Survival Rate

Substances

- Angiotensin Receptor Antagonists
- Angiotensin-Converting Enzyme Inhibitors
- Antihypertensive Agents

Full text links

SpringerLink FULL-TEXT ARTICLE Springer Free PMC article
Proceed to details
Cite
Share
☐ 575
Observational Study
Biomed Res Int
. 2020 Oct 5;2020:2138387.
doi: 10.1155/2020/2138387. eCollection 2020.

Clinical Features of COVID-19 Patients with Different Outcomes in Wuhan: A Retrospective Observational Study

Zhen Wang [1 2 3], Di Ye [1 2 3], Menglong Wang [1 2 3], Mengmeng Zhao [1 2 3], Dan Li [4], Jing Ye [1 2 3], Jianfang Liu [1 2 3], Yao Xu [1 2 3], Jishou Zhang [1 2 3], Wei Pan [1 2 3], Menglin Liu [5], Zhen Luo [1 2 3], Jun Wan [1 2 3]
Affiliations Expand

Affiliations

- [1] Department of Cardiology, Renmin Hospital of Wuhan University, Wuhan 430060, China.
- [2] Cardiovascular Research Institute, Wuhan University, Wuhan 430060, China.
- [3] Hubei Key Laboratory of Cardiology, Wuhan 430060, China.
- [4] Department of Pediatrics, Renmin Hospital of Wuhan University, Wuhan, China.
- [5] Department of Emergency, Renmin Hospital of Wuhan University, Wuhan, China.

- PMID: **33029494**
- PMCID: PMC7537706
- DOI: 10.1155/2020/2138387

Free PMC article
Observational Study

Clinical Features of COVID-19 Patients with Different Outcomes in Wuhan: A Retrospective Observational Study

Zhen Wang et al. Biomed Res Int. 2020.
Free PMC article
Show details
Biomed Res Int
. 2020 Oct 5;2020:2138387.
doi: 10.1155/2020/2138387. eCollection 2020.

Authors

Zhen Wang [1 2 3], Di Ye [1 2 3], Menglong Wang [1 2 3], Mengmeng Zhao [1 2 3], Dan Li [4], Jing Ye [1 2 3], Jianfang Liu [1 2 3], Yao Xu [1 2 3], Jishou Zhang [1 2 3], Wei Pan [1 2 3], Menglin Liu [5], Zhen Luo [1 2 3], Jun Wan [1 2 3]

Affiliations

- [1] Department of Cardiology, Renmin Hospital of Wuhan University, Wuhan 430060, China.
- [2] Cardiovascular Research Institute, Wuhan University, Wuhan 430060, China.
- [3] Hubei Key Laboratory of Cardiology, Wuhan 430060, China.
- [4] Department of Pediatrics, Renmin Hospital of Wuhan University, Wuhan, China.
- [5] Department of Emergency, Renmin Hospital of Wuhan University, Wuhan, China.

- PMID: **33029494**
- PMCID: PMC7537706
- DOI: 10.1155/2020/2138387

Abstract

Coronavirus disease 2019 (COVID-19) has caused considerable morbidity and mortality worldwide since December 2019. This retrospective study determined the characteristics and prognostic factors of COVID-19 patients, focusing on inpatients who died or were discharged between 30 December 2019 and 29 February 2020 at Renmin Hospital of Wuhan University. Patients' medical histories, comorbidities, symptoms, signs, laboratory findings, computed tomography (CT) findings, and clinical management were recorded. All 293 patients were divided into the nonsurviving (n = 116) and surviving (n = 177) groups. The median age was older in the nonsurviving group than in the surviving group; most patients were older than 65 years in the nonsurviving group. The incidence rates of lymphopenia, neutrophilia, and leukocytosis were significantly higher in the nonsurviving group than in the surviving group. More patients in the nonsurviving group had increased levels of nonspecific infection markers, abnormal liver and kidney function, cardiac injury, and blood coagulation abnormalities on admission. Immune and inflammatory responses were more severely disturbed in the nonsurviving group than in the surviving group. The incidence rates of complications during hospitalization were higher in the nonsurviving group than in the surviving group. Cox regression results also showed that older age, symptoms of dyspnea, comorbidities, and complications were all predictors of death. Close monitoring and timely treatment are needed for high-risk COVID-19 patients.

Copyright © 2020 Zhen Wang et al.

Conflict of interest statement

All the authors have no conflict of interest.

- Cited by 12 articles
- 27 references
- 1 figure

Supplementary info

Publication types, MeSH terms, Supplementary concepts Expand

Publication types

- Observational Study

MeSH terms

- Adult
- Aged
- Aged, 80 and over
- Betacoronavirus
- COVID-19
- COVID-19 Testing
- China / epidemiology
- Clinical Laboratory Techniques
- Coronavirus Infections / diagnosis
- Coronavirus Infections / drug therapy
- Coronavirus Infections / epidemiology
- Coronavirus Infections / etiology*
- Coronavirus Infections / mortality
- Female
- Hospital Mortality
- Humans
- Inpatients
- Male
- Middle Aged
- Pandemics
- Patient Discharge
- Pneumonia, Viral / epidemiology
- Pneumonia, Viral / etiology*
- Pneumonia, Viral / mortality
- Prognosis
- Retrospective Studies
- SARS-CoV-2

Supplementary concepts

- COVID-19 drug treatment

Full text links

Hindawi FREE Full Text Article Hindawi Limited Free PMC article
Proceed to details
Cite
Share
☐ 576
Observational Study

Med Sci Monit
. 2020 Jul 28;26:e925047.
doi: 10.12659/MSM.925047.

A Comparison of Clinical Characteristics and Outcomes in Elderly and Younger Patients with COVID-19

Jia Song [1], Weihang Hu [1], Yihua Yu [1], Xin Shen [1], Yueben Wang [1], Jin Yan [1], Xianghong Yang [2], Shijin Gong [1], Minjia Wang [1]
Affiliations Expand

Affiliations

- [1] Intensive Care Unit, Zhejiang Hospital, Hangzhou, Zhejiang, China (mainland).
- [2] Intensive Care Unit, Zhejiang Provincial People's Hospital, Hangzhou, Zhejiang, China (mainland).

- PMID: **32720649**
- PMCID: PMC7412913
- DOI: 10.12659/MSM.925047

Free PMC article
Observational Study

A Comparison of Clinical Characteristics and Outcomes in Elderly and Younger Patients with COVID-19

Jia Song et al. Med Sci Monit. 2020.
Free PMC article
Show details

Med Sci Monit
. 2020 Jul 28;26:e925047.
doi: 10.12659/MSM.925047.

Authors

Jia Song [1], Weihang Hu [1], Yihua Yu [1], Xin Shen [1], Yueben Wang [1], Jin Yan [1], Xianghong Yang [2], Shijin Gong [1], Minjia Wang [1]

Affiliations

- [1] Intensive Care Unit, Zhejiang Hospital, Hangzhou, Zhejiang, China (mainland).

- [2] Intensive Care Unit, Zhejiang Provincial People's Hospital, Hangzhou, Zhejiang, China (mainland).

- PMID: **32720649**
- PMCID: PMC7412913
- DOI: 10.12659/MSM.925047

Abstract

BACKGROUND The aim of this study was to describe the clinical characteristics and outcomes of patients with coronavirus disease 2019 (COVID-19) and compare these parameters in an elderly group with those in a younger group. MATERIAL AND METHODS This retrospective, single-center observational study included 69 hospitalized patients with laboratory-confirmed COVID-19 from a tertiary hospital in Wuhan, China, between January 14, 2020, and February 26, 2020. Epidemiological, demographic, clinical, and laboratory data, as well as treatments, complications, and outcomes were extracted from electronic medical records and compared between elderly patients (aged ≥60 years) and younger patients (aged <60 years). Patients were followed until March 19, 2020. RESULTS Elderly patients had more complications than younger patients, including acute respiratory distress syndrome (ARDS; 9/25, 36% vs. 5/44, 11.4%) and cardiac injury (7/25, 28% vs. 1/44, 2.3%), and they were more likely to be admitted to the intensive care unit (6/25, 24% vs. 2/44, 4.5%). As of March 19, 2020, 60/69 (87%) of the patients had been discharged, 6/69 (8.7%) had died, and 3/69 (4.3%) remained in the hospital. Of those who were discharged or died, the median duration of hospitalization was 13.5 days (interquartile range, 10-18 days). CONCLUSIONS Elderly patients with confirmed COVID-19 were more likely to develop ARDS and cardiac injury than younger patients and were more likely to be admitted to the intensive care unit. In addition to routine monitoring and respiratory support, cardiac monitoring and supportive care should be a focus in elderly patients with COVID-19.

Conflict of interest statement

Conflicts of interest

None.

- Cited by 8 articles
- 26 references

Supplementary info

Publication types, MeSH terms, Supplementary concepts Expand

Publication types

- Comparative Study
- Observational Study

MeSH terms

- Acute Kidney Injury / epidemiology

- Acute Kidney Injury / etiology
- Adult
- Age Factors*
- Aged
- Aged, 80 and over
- Betacoronavirus
- COVID-19
- China / epidemiology
- Combined Modality Therapy
- Coronavirus Infections / blood
- Coronavirus Infections / complications
- Coronavirus Infections / drug therapy
- Coronavirus Infections / epidemiology*
- Coronavirus Infections / therapy
- Heart Diseases / epidemiology*
- Heart Diseases / etiology
- Humans
- Inpatients
- Intensive Care Units / statistics & numerical data
- Length of Stay / statistics & numerical data
- Male
- Middle Aged
- Multiple Organ Failure / epidemiology
- Multiple Organ Failure / etiology
- Palliative Care / statistics & numerical data
- Pandemics*
- Pneumonia, Viral / blood
- Pneumonia, Viral / complications
- Pneumonia, Viral / epidemiology*
- Pneumonia, Viral / therapy
- Respiratory Distress Syndrome / epidemiology*
- Respiratory Distress Syndrome / etiology
- Retrospective Studies
- SARS-CoV-2
- Tertiary Care Centers
- Treatment Outcome
- Young Adult

Supplementary concepts

- COVID-19 drug treatment

Full text links

International Scientific Literature, Ltd. Free PMC article

Proceed to details

Cite

Share

☐ 577

Observational Study

Geriatr Gerontol Int

. 2021 Aug;21(8):629-635.

doi: 10.1111/ggi.14207. Epub 2021 Jun 9.

Characteristics and outcomes of older patients with coronavirus disease 2019 in Japan

Kenta Tanaka [1], Ling Zha [1], Tetsuhisa Kitamura [1], Yusuke Katayama [2], Taro Takeuchi [1], Sho Komukai [3], Atsushi Hirayama [4], Takeshi Shimazu [2], Tomotaka Sobue [1], COVID-19 Epidemiology Research Group of Osaka University

Affiliations Expand

Affiliations

- [1] Department of Social Medicine, Osaka University Graduate School of Medicine, Suita, Japan.
- [2] Department of Traumatology and Acute Critical Medicine, Osaka University Graduate School of Medicine, Suita, Japan.
- [3] Division of Biomedical Statistics, Department of Integrated Medicine, Graduate School of Medicine, Osaka University, Osaka, Japan.
- [4] Division of Public Health, Department of Social Medicine, Osaka University Graduate School of Medicine, Osaka, Japan.

- PMID: **34109721**
- DOI: 10.1111/ggi.14207

Observational Study

Characteristics and outcomes of older patients with coronavirus disease 2019 in Japan

Kenta Tanaka et al. Geriatr Gerontol Int. 2021 Aug.

Show details

Geriatr Gerontol Int
. 2021 Aug;21(8):629-635.
doi: 10.1111/ggi.14207. Epub 2021 Jun 9.

Authors

Kenta Tanaka [1], Ling Zha [1], Tetsuhisa Kitamura [1], Yusuke Katayama [2], Taro Takeuchi [1], Sho Komukai [3], Atsushi Hirayama [4], Takeshi Shimazu [2], Tomotaka Sobue [1], COVID-19 Epidemiology Research Group of Osaka University

Affiliations

- [1] Department of Social Medicine, Osaka University Graduate School of Medicine, Suita, Japan.
- [2] Department of Traumatology and Acute Critical Medicine, Osaka University Graduate School of Medicine, Suita, Japan.
- [3] Division of Biomedical Statistics, Department of Integrated Medicine, Graduate School of Medicine, Osaka University, Osaka, Japan.
- [4] Division of Public Health, Department of Social Medicine, Osaka University Graduate School of Medicine, Osaka, Japan.

- PMID: **34109721**
- DOI: 10.1111/ggi.14207

Abstract

Aim: The epidemiological characteristics, in-hospital treatments and outcomes of coronavirus disease 2019 among older patients have not been fully evaluated in Japan.

Methods: In this retrospective observational study carried out in Osaka Prefecture, Japan, we enrolled patients aged ≥60 years with laboratory-confirmed coronavirus disease 2019 from January to November 2020. The main outcome was mortality during the observation period, based on the Infectious Diseases Control Law. Cox regression analysis was used to evaluate the association between epidemiological factors and mortality among older patients with coronavirus disease 2019.

Results: Older patients accounted for 21.5% (3192/14 846) of the registered patients with coronavirus disease 2019. The number of patients according to age was as follows: 60-69 years, 1140 (35.7%); 70-79 years, 1058 (33.1%); 80-89 years, 749 (23.5%); and ≥90 years, 245 (7.7%). The proportion of deaths during the observation period was 8.5% (271/3192). The proportion of deaths increased with increasing age category (from 1.9% to 20.4%, P for trend <0.001). In multivariable Cox regression analysis, patients aged 70-79, 80-89 and ≥90 years had higher hazard ratios and 95% confidence intervals of death (2.62 [1.63-4.23], 5.99 [3.77-9.50] and 10.24 [6.03-17.40], respectively) than those aged 60-69 years. Factors such as male sex, presence of comorbidities, cluster cases in medical institutions and moderate/severe symptoms at diagnosis were also associated with mortality.

Conclusions: This study shows the epidemiological characteristics of older patients with coronavirus disease 2019 in Osaka Prefecture, Japan. The proportion of deaths was 8.5% in total and increased with increasing age. Geriatr Gerontol Int 2021; 21: 629-635.

Keywords: SARS-CoV-2; elderly; epidemiology; mortality; survival analysis.

© 2021 Japan Geriatrics Society.

- Cited by 1 article
- 29 references

Supplementary info

Publication types, MeSH terms Expand

Publication types

- Observational Study

MeSH terms

- Aged
- Aged, 80 and over
- COVID-19 / diagnosis
- COVID-19 / mortality*
- COVID-19 / therapy
- Comorbidity
- Female
- Humans
- Japan / epidemiology
- Male
- Middle Aged
- Pregnancy
- Retrospective Studies
- SARS-CoV-2
- Survival Analysis

Full text links

WILEY Full Text Article Wiley
Proceed to details
Cite
Share
578
Observational Study
Intensive Care Med
. 2021 Aug;47(8):887-895.
doi: 10.1007/s00134-021-06451-w. Epub 2021 Jun 22.

Implementation of new ECMO centers during the COVID-19 pandemic: experience and results from the Middle East and India

Ahmed A Rabie[1], Mohamed H Azzam[2], Abdulrahman A Al-Fares[3], Akram Abdelbary[4], Hani N Mufti[5 6], Ibrahim F Hassan[7], Arpan Chakraborty[8], Pranay Oza[9], Alyaa Elhazmi[10], Huda Alfoudri[11], Suneel Kumar Pooboni[12], Abdulrahman Alharthy[13], Daniel Brodie[14 15], Bishoy Zakhary[16], Kiran Shekar[17 18], Marta Velia Antonini[19], Nicholas A Barrett[20], Giles Peek[21], Alain Combes[22 23], Yaseen M Arabi[24]

Affiliations Expand

Affiliations

- [1] Critical Care Department, King Saud Medical City, 12746 Ulaishah discreet, Riyadh, Saudi Arabia. succenyl@gmail.com.
- [2] Critical Care Department, King Abdullah Medical Complex, Ministry of Health, Jeddah, Saudi Arabia.
- [3] Department of Anesthesia, Critical Care Medicine and Pain Medicine, Al-Amiri Hospital Center for Respiratory and Cardiac Failure, Kuwait Extracorporeal Life Support Program, Jaber Al-Ahmed Hospital Critical Care Unit, Ministry of Health, Kuwait City, Kuwait.
- [4] Critical Care Department, Cairo University, Cairo, Egypt.
- [5] Section of Cardiac Surgery, Department of Cardiac Sciences, King Faisal Cardiac Center, King Abdulaziz Medical City, MNGHA, Jeddah, Saudi Arabia.
- [6] College of Medicine, King Saud Bin Abdulaziz University for Health Sciences, King Abdullah International Medical Research Center, Jeddah, Saudi Arabia.
- [7] Medical Critical Care Division, Department of Medicine, Hamad Medical Corporation, Doha, Qatar.
- [8] Cardiac Anesthesia, Critical Care and ECMO Services, Medica Superspecialty Hospital, Kolkata, India.
- [9] Riddhi Vinayak Multispecialty Hospital, Mumbai, India.
- [10] Adult Critical Care Department, Dr. Sulaiman Alhabib Medical Group, Riyadh, Saudi Arabia.
- [11] Department of Anaesthesia, Critical Care, and Pain Management, Al-Adan Hospital Ministry of Health, Hadiya, Kuwait.
- [12] Department of Pediatric Critical Care, Mediclinic Airport Road Hospital, Abu Dhabi, United Arab Emirates.
- [13] Critical Care Department, King Saud Medical City, 12746 Ulaishah discreet, Riyadh, Saudi Arabia.
- [14] Division of Pulmonary, Allergy and Critical Care Medicine, Department of Medicine, Columbia College of Physicians and Surgeons, New York, NY, USA.
- [15] Center for Acute Respiratory Failure, New York-Presbyterian Hospital, New York, NY, USA.
- [16] Division of Pulmonary and Critical Care Medicine, Oregon Health and Science University, Portland, OR, USA.
- [17] Adult Intensive Care Services, The Prince Charles Hospital, Brisbane, QLD, Australia.

- [18] Faculty of Medicine, Institute of Health and Biomedical Innovation, Queensland University of Technology, Brisbane, Australia.
- [19] General Intensive Care Unit, University Hospital of Parma, Parma, Italy.
- [20] Faculty of Life Sciences and Medicine, Department of Critical Care, Centre of Human and Applied Physiological Sciences, Guy's and St Thomas' NHS Foundation Trust, King's College London, London, UK.
- [21] Department of Cardiothoracic Surgery, Congenital Heart Center, University of Florida, Gainesville, FL, USA.
- [22] Institute of Cardio-Metabolism and Nutrition, Sorbonne Université, INSERM, UMRS_1166-ICAN, 75013, Paris, France.
- [23] Service de Médecine Intensive-Réanimation, Institute de Cardiologie, APHP Hôpital Pitié-Salpêtrière, 75013, Paris, France.
- [24] Intensive Care Department, King Saud Bin Abdulaziz University for Health Sciences, King Abdullah International Medical Research Center, Ministry of National Guard Health Affairs, Riyadh, Saudi Arabia.

- PMID: **34156477**
- PMCID: PMC8217786
- DOI: 10.1007/s00134-021-06451-w

Free PMC article
Observational Study

Implementation of new ECMO centers during the COVID-19 pandemic: experience and results from the Middle East and India

Ahmed A Rabie et al. Intensive Care Med. 2021 Aug.
Free PMC article
Show details
Intensive Care Med
. 2021 Aug;47(8):887-895.
doi: 10.1007/s00134-021-06451-w. Epub 2021 Jun 22.

Authors

Ahmed A Rabie [1], Mohamed H Azzam [2], Abdulrahman A Al-Fares [3], Akram Abdelbary [4], Hani N Mufti [5] [6], Ibrahim F Hassan [7], Arpan Chakraborty [8], Pranay Oza [9], Alyaa Elhazmi [10], Huda Alfoudri [11], Suneel Kumar Pooboni [12], Abdulrahman Alharthy [13], Daniel Brodie [14] [15], Bishoy Zakhary [16], Kiran Shekar [17] [18], Marta Velia Antonini [19], Nicholas A Barrett [20], Giles Peek [21], Alain Combes [22] [23], Yaseen M Arabi [24]

Affiliations

- [1] Critical Care Department, King Saud Medical City, 12746 Ulaishah discreet, Riyadh, Saudi Arabia. succenyl@gmail.com.

- [2] Critical Care Department, King Abdullah Medical Complex, Ministry of Health, Jeddah, Saudi Arabia.
- [3] Department of Anesthesia, Critical Care Medicine and Pain Medicine, Al-Amiri Hospital Center for Respiratory and Cardiac Failure, Kuwait Extracorporeal Life Support Program, Jaber Al-Ahmed Hospital Critical Care Unit, Ministry of Health, Kuwait City, Kuwait.
- [4] Critical Care Department, Cairo University, Cairo, Egypt.
- [5] Section of Cardiac Surgery, Department of Cardiac Sciences, King Faisal Cardiac Center, King Abdulaziz Medical City, MNGHA, Jeddah, Saudi Arabia.
- [6] College of Medicine, King Saud Bin Abdulaziz University for Health Sciences, King Abdullah International Medical Research Center, Jeddah, Saudi Arabia.
- [7] Medical Critical Care Division, Department of Medicine, Hamad Medical Corporation, Doha, Qatar.
- [8] Cardiac Anesthesia, Critical Care and ECMO Services, Medica Superspecialty Hospital, Kolkata, India.
- [9] Riddhi Vinayak Multispecialty Hospital, Mumbai, India.
- [10] Adult Critical Care Department, Dr. Sulaiman Alhabib Medical Group, Riyadh, Saudi Arabia.
- [11] Department of Anaesthesia, Critical Care, and Pain Management, Al-Adan Hospital Ministry of Health, Hadiya, Kuwait.
- [12] Department of Pediatric Critical Care, Mediclinic Airport Road Hospital, Abu Dhabi, United Arab Emirates.
- [13] Critical Care Department, King Saud Medical City, 12746 Ulaishah discreet, Riyadh, Saudi Arabia.
- [14] Division of Pulmonary, Allergy and Critical Care Medicine, Department of Medicine, Columbia College of Physicians and Surgeons, New York, NY, USA.
- [15] Center for Acute Respiratory Failure, New York-Presbyterian Hospital, New York, NY, USA.
- [16] Division of Pulmonary and Critical Care Medicine, Oregon Health and Science University, Portland, OR, USA.
- [17] Adult Intensive Care Services, The Prince Charles Hospital, Brisbane, QLD, Australia.
- [18] Faculty of Medicine, Institute of Health and Biomedical Innovation, Queensland University of Technology, Brisbane, Australia.
- [19] General Intensive Care Unit, University Hospital of Parma, Parma, Italy.
- [20] Faculty of Life Sciences and Medicine, Department of Critical Care, Centre of Human and Applied Physiological Sciences, Guy's and St Thomas' NHS Foundation Trust, King's College London, London, UK.
- [21] Department of Cardiothoracic Surgery, Congenital Heart Center, University of Florida, Gainesville, FL, USA.
- [22] Institute of Cardio-Metabolism and Nutrition, Sorbonne Université, INSERM, UMRS_1166-ICAN, 75013, Paris, France.
- [23] Service de Médecine Intensive-Réanimation, Institute de Cardiologie, APHP Hôpital Pitié-Salpêtrière, 75013, Paris, France.
- [24] Intensive Care Department, King Saud Bin Abdulaziz University for Health Sciences, King Abdullah International Medical Research Center, Ministry of National Guard Health Affairs, Riyadh, Saudi Arabia.

- PMID: **34156477**
- PMCID: PMC8217786
- DOI: 10.1007/s00134-021-06451-w

Abstract

Purpose: Extracorporeal membrane oxygenation (ECMO) use for severe coronavirus disease 2019 (COVID-19) patients has increased during the course of the pandemic. As uncertainty existed regarding patient's outcomes, early guidelines recommended against establishing new ECMO centers. We aimed to explore the epidemiology and outcomes of ECMO for COVID-19 related cardiopulmonary failure in five countries in the Middle East and India and to evaluate the results of ECMO in 5 new centers.

Methods: This is a retrospective, multicenter international, observational study conducted in 19 ECMO centers in five countries in the Middle East and India from March 1, 2020, to September 30, 2020. We included patients with COVID-19 who received ECMO for refractory hypoxemia and severe respiratory acidosis with or without circulatory failure. Data collection included demographic data, ECMO-related specific data, pre-ECMO patient condition, 24 h post-ECMO initiation data, and outcome. The primary outcome was survival to home discharge. Secondary outcomes included mortality during ECMO, survival to decannulation, and outcomes stratified by center type.

Results: Three hundred and seven COVID-19 patients received ECMO support during the study period, of whom 78 (25%) were treated in the new ECMO centers. The median age was 45 years (interquartile range IQR 37-52), and 81% were men. New center patients were younger, were less frequently male, had received higher PEEP, more frequently inotropes and prone positioning before ECMO and were less frequently retrieved from a peripheral center on ECMO. Survival to home discharge was 45%. In patients treated in new and established centers, survival was 55 and 41% ($p = 0.03$), respectively. Multivariable analysis retained only a SOFA score < 12 at ECMO initiation as associated with survival (odds ratio, OR 1.93 (95% CI 1.05-3.58), $p = 0.034$), but not treatment in a new center (OR 1.65 (95% CI 0.75-3.67)).

Conclusions: During pandemics, ECMO may provide favorable outcomes in highly selected patients as resources allow. Newly formed ECMO centers with appropriate supervision of regional experts may have satisfactory results.

Keywords: COVID-19; ECMO; Pandemic; SARS-Cov2; SWAAC-ELSO.

© 2021. Springer-Verlag GmbH Germany, part of Springer Nature.

Conflict of interest statement

KS acknowledges research support from the Metro North Hospital and Health Service and the Prince Charles Hospital Foundation. DB receives research support from ALung Technologies. He has been on the medical advisory boards for Baxter, Abiomed, Xenios, and Hemovent and is the President-Elect of the Extracorporeal Life Support Organization (ELSO). AC reported receiving grants and personal fees from Maquet, Xenios, and Baxter and serving as the recent past president of the EuroELSO organization. Other authors have no conflict of interest.

- Cited by 8 articles
- 37 references
- 1 figure

Supplementary info

Publication types, MeSH terms Expand

Publication types

- Multicenter Study
- Observational Study

MeSH terms

- COVID-19*
- Extracorporeal Membrane Oxygenation*
- Humans
- India / epidemiology
- Male
- Middle Aged
- Middle East
- Pandemics
- Respiratory Distress Syndrome*
- Retrospective Studies
- SARS-CoV-2

Full text links

SpringerLink FULL-TEXT ARTICLE Springer Free PMC article
Proceed to details
Cite
Share
☐ 579
Observational Study
Vaccine
. 2022 Mar 15;40(12):1755-1760.
doi: 10.1016/j.vaccine.2022.02.013. Epub 2022 Feb 7.

Effect of the 2020/21 season influenza vaccine on SARS-CoV-2 infection in a cohort of Italian healthcare workers

Alexander Domnich [1], Andrea Orsi [2], Laura Sticchi [3], Donatella Panatto [4], Guglielmo Dini [5], Allegra Ferrari [6], Matilde Ogliastro [7], Simona Boccotti [8], Vanessa De Pace [9], Valentina Ricucci [10], Bianca Bruzzone [11], Paolo Durando [12], Giancarlo Icardi [13]
Affiliations Expand

Affiliations

- [1] Hygiene Unit, San Martino Policlinico Hospital - IRCCS for Oncology and Neurosciences, Genoa, Italy. Electronic address: alexander.domnich@hsanmartino.it.
- [2] Hygiene Unit, San Martino Policlinico Hospital - IRCCS for Oncology and Neurosciences, Genoa, Italy; Department of Health Sciences, University of Genoa, Genoa, Italy; Interuniversity Research Center on Influenza and Other Transmissible Infections (CIRI-IT), Genoa, Italy. Electronic address: andrea.orsi@unige.it.
- [3] Hygiene Unit, San Martino Policlinico Hospital - IRCCS for Oncology and Neurosciences, Genoa, Italy; Department of Health Sciences, University of Genoa, Genoa, Italy. Electronic address: sticchi@unige.it.
- [4] Department of Health Sciences, University of Genoa, Genoa, Italy; Interuniversity Research Center on Influenza and Other Transmissible Infections (CIRI-IT), Genoa, Italy. Electronic address: panatto@unige.it.
- [5] Department of Health Sciences, University of Genoa, Genoa, Italy; Occupational Medicine Unit, San Martino Policlinico Hospital - IRCCS for Oncology and Neurosciences, Genoa, Italy. Electronic address: guglielmo.dini@unige.it.
- [6] Department of Health Sciences, University of Genoa, Genoa, Italy. Electronic address: allegraferrari@virgilio.it.
- [7] Department of Health Sciences, University of Genoa, Genoa, Italy.
- [8] Department of Health Sciences, University of Genoa, Genoa, Italy. Electronic address: simona.boccotti@edu.unige.it.
- [9] Hygiene Unit, San Martino Policlinico Hospital - IRCCS for Oncology and Neurosciences, Genoa, Italy. Electronic address: vanessa.depace@hsanmartino.it.
- [10] Hygiene Unit, San Martino Policlinico Hospital - IRCCS for Oncology and Neurosciences, Genoa, Italy. Electronic address: valentina.ricucci@hsanmartino.it.
- [11] Hygiene Unit, San Martino Policlinico Hospital - IRCCS for Oncology and Neurosciences, Genoa, Italy. Electronic address: bianca.bruzzone@hsanmartino.it.
- [12] Department of Health Sciences, University of Genoa, Genoa, Italy; Interuniversity Research Center on Influenza and Other Transmissible Infections (CIRI-IT), Genoa, Italy; Occupational Medicine Unit, San Martino Policlinico Hospital - IRCCS for Oncology and Neurosciences, Genoa, Italy. Electronic address: durando@unige.it.
- [13] Hygiene Unit, San Martino Policlinico Hospital - IRCCS for Oncology and Neurosciences, Genoa, Italy; Department of Health Sciences, University of Genoa, Genoa, Italy; Interuniversity Research Center on Influenza and Other Transmissible Infections (CIRI-IT), Genoa, Italy. Electronic address: icardi@unige.it.

- PMID: **35153098**
- PMCID: PMC8829680
- DOI: 10.1016/j.vaccine.2022.02.013

Free PMC article
Observational Study

Effect of the 2020/21 season influenza vaccine on SARS-CoV-2 infection in a cohort of Italian healthcare workers

Alexander Domnich et al. Vaccine. 2022.
Free PMC article

Show details

Vaccine

. 2022 Mar 15;40(12):1755-1760.
doi: 10.1016/j.vaccine.2022.02.013. Epub 2022 Feb 7.

Authors

Alexander Domnich [1], Andrea Orsi [2], Laura Sticchi [3], Donatella Panatto [4], Guglielmo Dini [5], Allegra Ferrari [6], Matilde Ogliastro [7], Simona Boccotti [8], Vanessa De Pace [9], Valentina Ricucci [10], Bianca Bruzzone [11], Paolo Durando [12], Giancarlo Icardi [13]

Affiliations

- [1] Hygiene Unit, San Martino Policlinico Hospital - IRCCS for Oncology and Neurosciences, Genoa, Italy. Electronic address: alexander.domnich@hsanmartino.it.
- [2] Hygiene Unit, San Martino Policlinico Hospital - IRCCS for Oncology and Neurosciences, Genoa, Italy; Department of Health Sciences, University of Genoa, Genoa, Italy; Interuniversity Research Center on Influenza and Other Transmissible Infections (CIRI-IT), Genoa, Italy. Electronic address: andrea.orsi@unige.it.
- [3] Hygiene Unit, San Martino Policlinico Hospital - IRCCS for Oncology and Neurosciences, Genoa, Italy; Department of Health Sciences, University of Genoa, Genoa, Italy. Electronic address: sticchi@unige.it.
- [4] Department of Health Sciences, University of Genoa, Genoa, Italy; Interuniversity Research Center on Influenza and Other Transmissible Infections (CIRI-IT), Genoa, Italy. Electronic address: panatto@unige.it.
- [5] Department of Health Sciences, University of Genoa, Genoa, Italy; Occupational Medicine Unit, San Martino Policlinico Hospital - IRCCS for Oncology and Neurosciences, Genoa, Italy. Electronic address: guglielmo.dini@unige.it.
- [6] Department of Health Sciences, University of Genoa, Genoa, Italy. Electronic address: allegraferrari@virgilio.it.
- [7] Department of Health Sciences, University of Genoa, Genoa, Italy.
- [8] Department of Health Sciences, University of Genoa, Genoa, Italy. Electronic address: simona.boccotti@edu.unige.it.
- [9] Hygiene Unit, San Martino Policlinico Hospital - IRCCS for Oncology and Neurosciences, Genoa, Italy. Electronic address: vanessa.depace@hsanmartino.it.
- [10] Hygiene Unit, San Martino Policlinico Hospital - IRCCS for Oncology and Neurosciences, Genoa, Italy. Electronic address: valentina.ricucci@hsanmartino.it.
- [11] Hygiene Unit, San Martino Policlinico Hospital - IRCCS for Oncology and Neurosciences, Genoa, Italy. Electronic address: bianca.bruzzone@hsanmartino.it.
- [12] Department of Health Sciences, University of Genoa, Genoa, Italy; Interuniversity Research Center on Influenza and Other Transmissible Infections (CIRI-IT), Genoa, Italy; Occupational Medicine Unit, San Martino Policlinico Hospital - IRCCS for Oncology and Neurosciences, Genoa, Italy. Electronic address: durando@unige.it.
- [13] Hygiene Unit, San Martino Policlinico Hospital - IRCCS for Oncology and Neurosciences, Genoa, Italy; Department of Health Sciences, University of Genoa, Genoa, Italy; Interuniversity Research Center on Influenza and Other Transmissible Infections (CIRI-IT), Genoa, Italy. Electronic address: icardi@unige.it.

- PMID: **35153098**

- PMCID: PMC8829680
- DOI: 10.1016/j.vaccine.2022.02.013

Abstract

Objectives: Healthcare workers (HCWs) are a priority group for seasonal influenza vaccination (SIV). The 2020/21 SIV campaign was conducted during the second wave of the COVID-19 pandemic. Vaccines, including SIV, may exert non-specific protective effects on other infectious diseases which may be ascribable to the concept of trained immunity. The aim of this study was to explore the association between 2020/21 SIV and SARS-CoV-2 positivity in a cohort of Italian HCWs.

Methods: In this observational study, a cohort of HCWs employed by a large (ca 5000 employees) referral tertiary acute-care university hospital was followed up retrospectively until the start of the COVID-19 vaccination campaign. The independent variable of interest was the 2020/21 SIV uptake. Both egg-based and cell culture-derived quadrivalent SIVs were available. The study outcome was the incidence of new SARS-CoV-2 infections, as determined by RT-PCR. Multivariable Cox regression was applied in order to discern the association of interest.

Results: The final cohort consisted of 2561 HCWs who underwent ≥1 RT-PCR test and accounted for a total of 94,445 person-days of observation. SIV uptake was 35.6%. During the study period, a total of 290 new SARS-CoV-2 infections occurred. The incidence of new SARS-CoV-2 was 1.62 (95% CI: 1.22-2.10) and 3.91 (95% CI: 3.43-4.45) per 1000 person-days in vaccinated and non-vaccinated HCWs, respectively, with an adjusted non-proportional hazard ratio of 0.37 (95% CI: 0.22-0.62). E-values suggested that unmeasured confounding was unlikely to explain the association.

Conclusions: A lower risk of SARS-CoV-2 infection was observed among SIV recipients.

Keywords: COVID-19; Healthcare workers; Influenza; Influenza vaccines; SARS-CoV-2; Vaccination.

Copyright © 2022 Elsevier Ltd. All rights reserved.

Conflict of interest statement

Declaration of Competing Interest The authors declare that they have no known competing financial interests or personal relationships that could have appeared to influence the work reported in this paper.

- 47 references

Supplementary info

Publication types, MeSH terms, Substances Expand

Publication types

- Observational Study
- Research Support, Non-U.S. Gov't

MeSH terms

- COVID-19 Vaccines
- COVID-19* / epidemiology
- COVID-19* / prevention & control
- Health Personnel
- Humans
- Influenza Vaccines*
- Pandemics / prevention & control
- Retrospective Studies
- SARS-CoV-2
- Seasons

Substances

- COVID-19 Vaccines
- Influenza Vaccines

Full text links

ELSEVIER FULL-TEXT ARTICLE Elsevier Science Free PMC article

Proceed to details

Cite

Share

☐ 580

Observational Study

Ann Vasc Surg
. 2021 Apr;72:191-195.
doi: 10.1016/j.avsg.2020.12.001. Epub 2021 Jan 13.

Re-organization of the Vascular Surgery Department During the Acute Phase of the COVID19 Outbreak: Lessons Learned and Future Perspectives

Antonio Bozzani [1], Maura Pallini [2], Vittorio Arici [2], Guido Tavazzi [3], Giulia Ticozzelli [4], Mila Maria Franciscone [2], Vittorio Danesino [2], Francesco Mojoli [2], Franco Ragni [2], Antonio V Sterpetti [5]
Affiliations Expand

Affiliations

- [1] Vascular and Endovascular Surgery Unit, Fondazione IRCCS Policlinico San Matteo, Pavia, Italy. Electronic address: a.bozzani@smatteo.pv.it.
- [2] Vascular and Endovascular Surgery Unit, Fondazione IRCCS Policlinico San Matteo, Pavia, Italy.
- [3] Anesthesiology and Intensive Care Unit, Fondazione IRCCS Policlinico San Matteo, Pavia, Italy; Department of Medical, Surgical, Diagnostic and Pediatric Science, University of Pavia, Italy.
- [4] Anesthesiology and Intensive Care Unit, Fondazione IRCCS Policlinico San Matteo, Pavia, Italy.
- [5] The Sapienza University of Rome, Rome, Italy.

- PMID: **33333189**
- PMCID: PMC7832376
- DOI: 10.1016/j.avsg.2020.12.001

Free PMC article
Observational Study

Re-organization of the Vascular Surgery Department During the Acute Phase of the COVID19 Outbreak: Lessons Learned and Future Perspectives

Antonio Bozzani et al. Ann Vasc Surg. 2021 Apr.
Free PMC article
Show details
Ann Vasc Surg
. 2021 Apr;72:191-195.
doi: 10.1016/j.avsg.2020.12.001. Epub 2021 Jan 13.

Authors

Antonio Bozzani [1], Maura Pallini [2], Vittorio Arici [2], Guido Tavazzi [3], Giulia Ticozzelli [4], Mila Maria Franciscone [2], Vittorio Danesino [2], Francesco Mojoli [2], Franco Ragni [2], Antonio V Sterpetti [5]

Affiliations

- [1] Vascular and Endovascular Surgery Unit, Fondazione IRCCS Policlinico San Matteo, Pavia, Italy. Electronic address: a.bozzani@smatteo.pv.it.
- [2] Vascular and Endovascular Surgery Unit, Fondazione IRCCS Policlinico San Matteo, Pavia, Italy.

- [3] Anesthesiology and Intensive Care Unit, Fondazione IRCCS Policlinico San Matteo, Pavia, Italy; Department of Medical, Surgical, Diagnostic and Pediatric Science, University of Pavia, Italy.
- [4] Anesthesiology and Intensive Care Unit, Fondazione IRCCS Policlinico San Matteo, Pavia, Italy.
- [5] The Sapienza University of Rome, Rome, Italy.

- PMID: **33333189**
- PMCID: PMC7832376
- DOI: 10.1016/j.avsg.2020.12.001

Abstract

Introduction: Severe acute respiratory syndrome coronavirus 2 (SARS-CoV-2) infection represents a serious threat to public health because it leads to a wide spectrum of clinical manifestations. The region Lombardia (Italy) has suffered from severe problems during the acute phase of the outbreak in Italy (March-April 2020). The aim of our analysis is to report the experience of the Department of Vascular Surgery of Pavia, including the learned lessons and future perspectives, considering that the COVID-19 outbreak is in its acute phase in other continents.

Material and methods: Single-center, retrospective, observational study based on extracted data from the medical records of all consecutive COVID-19 patients observed in our Vascular Department between March 1st and April 30th, 2020. We reviewed the records for demographic information, comorbidities, laboratory tests, and anticoagulation treatment at the time of hospital admission.

Results: We observed an important reduction in elective and urgent interventions compared to the same period of the previous year; in parallel, we observed an increase in the diagnosis of deep vein thrombosis (DVT) in hospitalized patients, especially with severe infection. In our department, four infections were reported among health workers.

Conclusions: The impact of the COVID19 pandemic on health-care delivery has been massive. A wave of vascular-related complications is expected. Regular SARS-CoV-2 screening, adequate protection, and quick reorganization of health-care resources are still needed.

Copyright © 2020 Elsevier Inc. All rights reserved.

- Cited by 1 article
- 21 references

Supplementary info

Publication types, MeSH terms Expand

Publication types

- Observational Study

MeSH terms

- Adult
- Aged
- Aged, 80 and over
- COVID-19 / epidemiology*
- Female
- Humans
- Italy / epidemiology
- Male
- Middle Aged
- Pandemics
- Postoperative Complications / mortality
- Retrospective Studies
- SARS-CoV-2
- Surgery Department, Hospital / organization & administration*
- Vascular Surgical Procedures / statistics & numerical data*

Full text links

ELSEVIER FULL-TEXT ARTICLE Elsevier Science Free PMC article
Proceed to details
Cite
Share
581
Observational Study
Pulm Pharmacol Ther
. 2021 Aug;69:102038.
doi: 10.1016/j.pupt.2021.102038. Epub 2021 Jun 24.

The impact of SARS-COV2 pandemic on the management oF IPF patients: Our narrative experience

Alida Benfante [1], Riccardo Messina [2], Ilaria Piccionello [2], Rosangela Di Liberti [2], Stefania Principe [2], Nicola Scichilone [2]
Affiliations Expand

Affiliations

- [1] Dipartimento Universitario di Promozione Della Salute, Materno Infantile, Medicina Interna e Specialistica di Eccellenza "G. D'Alessandro" (PROMISE), Division of

Respiratory Medicine, "Paolo Giaccone" University Hospital, University of Palermo; Palermo; Italy. Electronic address: alida.benfante@policlinico.pa.it.
- [2] Dipartimento Universitario di Promozione Della Salute, Materno Infantile, Medicina Interna e Specialistica di Eccellenza "G. D'Alessandro" (PROMISE), Division of Respiratory Medicine, "Paolo Giaccone" University Hospital, University of Palermo; Palermo; Italy.

- PMID: **33965569**
- PMCID: PMC8221991
- DOI: 10.1016/j.pupt.2021.102038

Free PMC article
Observational Study

The impact of SARS-COV2 pandemic on the management oF IPF patients: Our narrative experience

Alida Benfante et al. Pulm Pharmacol Ther. 2021 Aug.
Free PMC article

Show details

Pulm Pharmacol Ther
. 2021 Aug;69:102038.
doi: 10.1016/j.pupt.2021.102038. Epub 2021 Jun 24.

Authors

Alida Benfante [1], Riccardo Messina [2], Ilaria Piccionello [2], Rosangela Di Liberti [2], Stefania Principe [2], Nicola Scichilone [2]

Affiliations

- [1] Dipartimento Universitario di Promozione Della Salute, Materno Infantile, Medicina Interna e Specialistica di Eccellenza "G. D'Alessandro" (PROMISE), Division of Respiratory Medicine, "Paolo Giaccone" University Hospital, University of Palermo; Palermo; Italy. Electronic address: alida.benfante@policlinico.pa.it.
- [2] Dipartimento Universitario di Promozione Della Salute, Materno Infantile, Medicina Interna e Specialistica di Eccellenza "G. D'Alessandro" (PROMISE), Division of Respiratory Medicine, "Paolo Giaccone" University Hospital, University of Palermo; Palermo; Italy.

- PMID: **33965569**
- PMCID: PMC8221991
- DOI: 10.1016/j.pupt.2021.102038

Abstract

Background: The SARS-CoV-2 pandemic has changed the health-care systems around the world in a remarkable way. We describe the strategies adopted to cope with the limitations imposed by the pandemic to the access to health care by patients diagnosed with idiopathic Pulmonary Fibrosis (IPF).

Material and methods: We conducted a retrospective observational analysis including IPF patients under antifibrotic drugs (nintedanib and pirfenidone) that accessed to the Outpatient clinic of the University of Palermo, Italy. Patients received a phone number and an email address in case of any urgency and a virtual meeting was settled up monthly.

Results: 40 patients (M/F: 30/10) were followed up, 33 under nintedanib treatment, 7 under pirfenidone. Among patients under nintedanib, 1 patient reported high fever (T max 39 °C) and purulent sputum with no sign of infections, 1 had hemoptysis that was spontaneously resolved. 2 patients accessed to the emergency department for the worsening of dyspnea; 5 patients had diarrhea that resolved with symptomatic drugs in few days. 3 patients had an increase of alkaline phosphatase levels, leading to the withdrawal of the antifibrotic drug for 15 days, and subsequent normalization of the plasmatic levels. Among patients under pirfenidone, one subject had an increase of ferritin serum levels with no symptoms. The remaining subjects were in stable clinical conditions. None of the patients reported hospitalization or exacerbations, and did not experience antifibrotic withdrawal.

Conclusions: We were able to demonstrate that by implementing alternative ways to monitor the disease, patients did not incur in increased rates of acute exacerbations or higher frequency of side effects and antifibrotic treatment withdrawal.

Keywords: COVID-19; IPF; Pulmonary fibrosis; SARS-CoV-2.

Copyright © 2021. Published by Elsevier Ltd.

Conflict of interest statement

The authors confirm that there are no known conflicts of interest associated with this publication.

- 14 references

Supplementary info

Publication types, MeSH terms, Substances Expand

Publication types

- Observational Study

MeSH terms

- COVID-19*
- Humans
- Idiopathic Pulmonary Fibrosis* / drug therapy
- Idiopathic Pulmonary Fibrosis* / epidemiology
- Pandemics

- Pyridones / therapeutic use
- RNA, Viral
- Retrospective Studies
- SARS-CoV-2

Substances

- Pyridones
- RNA, Viral

Full text links

Elsevier Science Free PMC article
Proceed to details
Cite
Share
582
Observational Study
Ginekol Pol
. 2020;91(12):755-763.
doi: 10.5603/GP.a2020.0130.

COVID-19 infection in symptomatic pregnant women at the midpoint of the pandemic in Spain: a retrospective analysis

Elias Ortiz Molina [1], Rafael Hernandez Pailos [2], Maria Pola Guillen [2], Ana Pascual Pedreno [2], Eduardo Rodriguez Rodriguez [2], Antonio Hernandez Martinez [3]
Affiliations Expand

Affiliations

- [1] Hospital Mancha Centro, Av Constitucion sn, Alcazar de San Juan, Spain, Spain. ortizmolina.e@hotmail.com.
- [2] Hospital Mancha Centro, Av Constitucion sn, Alcazar de San Juan, Spain, Spain.
- [3] University Castilla La Mancha, Ciudad Real, Spain, Spain.

- PMID: **33447995**
- DOI: 10.5603/GP.a2020.0130

Free article
Observational Study

COVID-19 infection in symptomatic pregnant women at the midpoint of the pandemic in Spain: a retrospective analysis

Elias Ortiz Molina et al. Ginekol Pol. 2020.
Free article
Show details
Ginekol Pol
. 2020;91(12):755-763.
doi: 10.5603/GP.a2020.0130.

Authors

Elias Ortiz Molina [1], Rafael Hernandez Pailos [2], Maria Pola Guillen [2], Ana Pascual Pedreno [2], Eduardo Rodriguez Rodriguez [2], Antonio Hernandez Martinez [3]

Affiliations

- [1] Hospital Mancha Centro, Av Constitucion sn, Alcazar de San Juan, Spain, Spain. ortizmolina.e@hotmail.com.
- [2] Hospital Mancha Centro, Av Constitucion sn, Alcazar de San Juan, Spain, Spain.
- [3] University Castilla La Mancha, Ciudad Real, Spain, Spain.

- PMID: **33447995**
- DOI: 10.5603/GP.a2020.0130

Abstract

Objectives: Determine the strengths and weakness of a symptomatic screening for COVID-19 in pregnant women. Analyze the clinical presentation, management, and outcomes.

Design: Descriptive retrospective observational study.

Setting: Mancha-Centro Hospital (Spain).

Material and methods: Population: Symptomatic pregnant women with confirmed diagnosis of COVID-19. Between the 12th of March and 17th of April 2020, all the symptomatic pregnancies were screened with diagnostic test for SARS-CoV-2. Data collection was done by reviewing the medical records and telephone interviews.

Main outcome measures: Clinical characteristics, management, treatment, and obstetric and neonatal outcomes.

Results: Twenty patients with positive COVID-19 diagnostic test out of thirty-four suspected. The most common symptoms were fever (70%), cough (65%) and myalgia (35%). A unique symptom of presentation in 20% of cases. COVID-19 pneumonia was diagnosed in 30% by chest X-ray and one case had pulmonary embolism associated diagnosed by CT-Scan. Thromboprophylaxis was indicated in 16 out of 20 patients. Eight women finished their pregnancy during the observation

period. Type of birth: 25% natural birth, 12.5% assisted vaginal delivery and 62.5% caesarean section. We had three severe cases, two of them with intensive care support. All neonates had negative test for COVID 19 infection.

Conclusions: We recommend universal screening of all pregnant woman for COVID-19 during the pandemic because of the limits of the symptomatic screening seen in this studio and the ratio of asymptomatic pregnancies with positive test for COVID-19 recently published.

Keywords: COVID-19; delivery; outcomes; pregnancy; screening; symptoms.

- Cited by 1 article

Supplementary info

Publication types, MeSH terms, Substances Expand

Publication types

- Observational Study

MeSH terms

- Adult
- Anosmia / physiopathology
- Anti-Bacterial Agents
- Anticoagulants / therapeutic use
- Antiviral Agents / therapeutic use
- C-Reactive Protein / metabolism
- COVID-19 / complications
- COVID-19 / metabolism
- COVID-19 / physiopathology*
- COVID-19 / therapy
- Cesarean Section*
- Cough / physiopathology*
- Critical Care
- Delivery, Obstetric
- Dysgeusia / physiopathology
- Dyspnea / physiopathology
- Enzyme Inhibitors / therapeutic use
- Extraction, Obstetrical
- Female
- Fever / physiopathology*
- Fibrin Fibrinogen Degradation Products / metabolism
- Gestational Age
- Heparin, Low-Molecular-Weight / therapeutic use

- Hospitalization
- Humans
- Hydroxychloroquine / therapeutic use
- Lung / diagnostic imaging*
- Lymphocyte Count
- Lymphopenia / physiopathology
- Middle Aged
- Myalgia / physiopathology*
- Obesity, Maternal / complications
- Oxygen Inhalation Therapy
- Pre-Eclampsia
- Pregnancy
- Pregnancy Complications, Cardiovascular / etiology
- Pregnancy Complications, Cardiovascular / physiopathology
- Pregnancy Complications, Cardiovascular / therapy
- Pregnancy Complications, Infectious / physiopathology*
- Pregnancy Complications, Infectious / therapy
- Premature Birth
- Pulmonary Embolism / etiology
- Pulmonary Embolism / physiopathology
- Pulmonary Embolism / therapy
- Respiration, Artificial
- Retrospective Studies
- SARS-CoV-2
- Spain

Substances

- Anti-Bacterial Agents
- Anticoagulants
- Antiviral Agents
- Enzyme Inhibitors
- Fibrin Fibrinogen Degradation Products
- Heparin, Low-Molecular-Weight
- fibrin fragment D
- Hydroxychloroquine
- C-Reactive Protein

Full text links

full text provider Via Medica Medical Publishers

Proceed to details

Cite

Share

☐ 583

Observational Study

J Med Virol

. 2022 Jan;94(1):372-379.

doi: 10.1002/jmv.27357. Epub 2021 Oct 8.

Do high-dose corticosteroids improve outcomes in hospitalized COVID-19 patients?

Gagan Kumar [1], Dhaval Patel [1], Martin Hererra [2], David Jefferies [1], Ankit Sakhuja [3], Mark Meersman [4], Drew Dalton [4], Rahul Nanchal [5], Achuta Kumar Guddati [6]

Affiliations Expand

Affiliations

- [1] Department of Pulmonary and Critical Care, Northeast Georgia Health System, Gainesville, Georgia, USA.
- [2] Department of Internal Medicine, Northeast Georgia Health System, Gainesville, Georgia, USA.
- [3] Division of Cardiovascular Critical Care, Department of Cardiovascular and Thoracic surgery, West Virginia University, West Virginia, USA.
- [4] IPC Global, Alpharetta, Georgia, USA.
- [5] Division of Pulmonary and Critical Care, Medical College of Wisconsin, Milwaukee, Wisconsin, USA.
- [6] Division of Hematology/Oncology, Georgia Cancer Center, Augusta University, Augusta, Georgia, USA.

- PMID: **34559436**
- PMCID: PMC8661573
- DOI: 10.1002/jmv.27357

Free PMC article

Observational Study

Do high-dose corticosteroids improve outcomes in hospitalized COVID-19 patients?

Gagan Kumar et al. J Med Virol. 2022 Jan.

Free PMC article

Show details

J Med Virol

. 2022 Jan;94(1):372-379.

doi: 10.1002/jmv.27357. Epub 2021 Oct 8.

Authors

Gagan Kumar [1], Dhaval Patel [1], Martin Hererra [2], David Jefferies [1], Ankit Sakhuja [3], Mark Meersman [4], Drew Dalton [4], Rahul Nanchal [5], Achuta Kumar Guddati [6]

Affiliations

- [1] Department of Pulmonary and Critical Care, Northeast Georgia Health System, Gainesville, Georgia, USA.
- [2] Department of Internal Medicine, Northeast Georgia Health System, Gainesville, Georgia, USA.
- [3] Division of Cardiovascular Critical Care, Department of Cardiovascular and Thoracic surgery, West Virginia University, West Virginia, USA.
- [4] IPC Global, Alpharetta, Georgia, USA.
- [5] Division of Pulmonary and Critical Care, Medical College of Wisconsin, Milwaukee, Wisconsin, USA.
- [6] Division of Hematology/Oncology, Georgia Cancer Center, Augusta University, Augusta, Georgia, USA.

- PMID: **34559436**
- PMCID: PMC8661573
- DOI: 10.1002/jmv.27357

Abstract

Coronavirus disease 2019 (COVID-19) is characterized by dysregulated hyperimmune response and steroids have been shown to decrease mortality. However, whether higher dosing of steroids results in better outcomes has been debated. This was a retrospective observation of COVID-19 admissions between March 1, 2020, and March 10, 2021. Adult patients (≥18 years) who received more than 10 mg daily methylprednisolone equivalent dosing (MED) within the first 14 days were included. We excluded patients who were discharged or died within 7 days of admission. We compared the standard dose of steroids (<40 mg MED) versus the high dose of steroids (>40 mg MED). Inverse probability weighted regression adjustment (IPWRA) was used to examine whether higher dose steroids resulted in improved outcomes. The outcomes studied were in-hospital mortality, rate of acute kidney injury (AKI) requiring hemodialysis, invasive mechanical ventilation (IMV), hospital-associated infections (HAI), and readmissions. Of the 1379 patients meeting study criteria, 506 received less than 40 mg of MED (median dose 30 mg MED) and 873 received more than or equal to 40 mg of MED (median dose 78 mg MED). Unadjusted in-hospital mortality was higher in patients who received high-dose corticosteroids (40.7% vs. 18.6%, $p < 0.001$). On IPWRA, the use of high-dose corticosteroids was associated with higher odds of death (odds ratio [OR] 2.14; 95% confidence interval [CI] 1.45-3.14, $p < 0.001$) but not with the development of HAI, readmissions, or requirement of IMV. High-dose corticosteroids were associated with lower rates of AKI requiring hemodialysis (OR 0.33; 95% CI 0.18-0.63). In COVID-19, corticosteroids more than or equal to 40 mg MED were associated with higher in-hospital mortality.

Keywords: COVID-19; corticosteroids; outcomes.

© 2021 Wiley Periodicals LLC.

Conflict of interest statement

The authors declare that there are no conflicts of interest.

- Cited by 2 articles
- 31 references
- 1 figure

Supplementary info

Publication types, MeSH terms, Substances, Grant support Expand

Publication types

- Observational Study
- Research Support, N.I.H., Extramural

MeSH terms

- Acute Kidney Injury / epidemiology*
- Adrenal Cortex Hormones / administration & dosage
- Adrenal Cortex Hormones / therapeutic use*
- Aged
- Aged, 80 and over
- COVID-19 / drug therapy*
- COVID-19 / mortality*
- Cross Infection / epidemiology
- Female
- Hospital Mortality
- Humans
- Male
- Methylprednisolone / administration & dosage
- Methylprednisolone / therapeutic use*
- Middle Aged
- Respiration, Artificial / statistics & numerical data
- Retrospective Studies
- SARS-CoV-2 / drug effects

Substances

- Adrenal Cortex Hormones
- Methylprednisolone

Grant support

- U54 GM104942/GM/NIGMS NIH HHS/United States

Full text links

WILEY Full Text Article Wiley Free PMC article
Proceed to details
Cite
Share
584
Clinical Trial
Nutrients
. 2021 May 19;13(5):1721.
doi: 10.3390/nu13051721.

Mediterranean Diet and SARS-COV-2 Infection: Is There Any Association? A Proof-of-Concept Study

Valentina Ponzo [1], Marianna Pellegrini [1], Chiara D'Eusebio [1], Fabio Bioletto [1], Ilaria Goitre [1], Silvio Buscemi [2] [3], Simone Frea [4], Ezio Ghigo [1], Simona Bo [1]
Affiliations Expand

Affiliations

- [1] Department of Medical Sciences, University of Torino, 10126 Torino, Italy.
- [2] Unit of Clinical Nutrition, AOU Policlinico "P. Giaccone", 90127 Palermo, Italy.
- [3] Dipartimento di Promozione della Salute, Materno-Infantile, Medicina Interna e Specialistica di Eccellenza (PROMISE), University of Palermo, 90127 Palermo, Italy.
- [4] Cardiology Unit, Città della Salute e della Scienza Hospital, University of Torino, 10126 Torino, Italy.

- PMID: **34069656**
- PMCID: PMC8160854
- DOI: 10.3390/nu13051721

Free PMC article
Clinical Trial

Mediterranean Diet and SARS-COV-2 Infection: Is There Any Association? A Proof-of-Concept Study

Valentina Ponzo et al. Nutrients. 2021.
Free PMC article
Show details
Nutrients
. 2021 May 19;13(5):1721.
doi: 10.3390/nu13051721.

Authors

Valentina Ponzo [1], Marianna Pellegrini [1], Chiara D'Eusebio [1], Fabio Bioletto [1], Ilaria Goitre [1], Silvio Buscemi [2] [3], Simone Frea [4], Ezio Ghigo [1], Simona Bo [1]

Affiliations

- [1] Department of Medical Sciences, University of Torino, 10126 Torino, Italy.
- [2] Unit of Clinical Nutrition, AOU Policlinico "P. Giaccone", 90127 Palermo, Italy.
- [3] Dipartimento di Promozione della Salute, Materno-Infantile, Medicina Interna e Specialistica di Eccellenza (PROMISE), University of Palermo, 90127 Palermo, Italy.
- [4] Cardiology Unit, Città della Salute e della Scienza Hospital, University of Torino, 10126 Torino, Italy.

- PMID: **34069656**
- PMCID: PMC8160854
- DOI: 10.3390/nu13051721

Abstract

The aim of this observational study was investigating the possible correlation between adherence to the Mediterranean diet (MeD) and SARS-COV-2 infection rates and severity among healthcare professionals (HCPs). An online self-administrated questionnaire (evaluating both MeD adherence and dietary habits) was filled out by HCPs working in Piedmont (Northern Italy) from 15 January to 28 February 2021. Out of the 1206 questionnaires collected, 900 were considered reliable and analyzed. Individuals who reported the SARS-COV-2 infection (n = 148) showed a significantly lower MeD score, with a lower adherence in fruit, vegetables, cereals, and olive oil consumption. In a logistic regression model, the risk of infection was inversely associated with the MeD score (OR = 0.88; 95% CI 0.81-0.97) and the consumption of cereals (OR = 0.64; 0.45-0.90). Asymptomatic individuals with SARS-COV-2 infection reported a lower intake of saturated fats than symptomatic; individuals requiring hospitalization were significantly older and reported worse dietary habits than both asymptomatic and symptomatic individuals. After combining all symptomatic individuals together, age (OR = 1.05; 1.01-1.09) and saturated fats intake (OR = 1.09; 1.01-1.17) were associated with the infection severity. HCPs who reported a SARS-COV-2 infection showed a significantly lower MeD score and cereal consumption. The infection severity was directly associated with higher age and saturated fat intake.

Keywords: Mediterranean diet; SARS-COV-2 infection; dietary habits; healthcare professionals.

Conflict of interest statement

The authors declare no conflict of interest.

- Cited by 5 articles
- 115 references

Supplementary info

Publication types, MeSH terms Expand

Publication types

- Clinical Trial
- Observational Study

MeSH terms

- Adult
- Age Factors
- Aged
- COVID-19 / epidemiology*
- Diet, Mediterranean*
- Female
- Humans
- Male
- Middle Aged
- Proof of Concept Study
- Retrospective Studies
- Risk Factors
- SARS-CoV-2*
- Surveys and Questionnaires*

Full text links

FULL TEXT OPEN ACCESS MDPI Multidisciplinary Digital Publishing Institute (MDPI) Free PMC article
Proceed to details
Cite
Share

☐ 585
Observational Study
Medicina (Kaunas)
. 2020 Oct 1;56(10):512.

doi: 10.3390/medicina56100512.

Non-COVID Diseases during the Pandemic: Where Have All Other Emergencies Gone?

Veronica Ojetti [1] [2], Marcello Covino [1], Mattia Brigida [2], Carmine Petruzziello [3], Angela Saviano [2], Alessio Migneco [1], Marcello Candelli [1], Francesco Franceschi [1] [2]
Affiliations Expand

Affiliations

- [1] Emergency Department-Fondazione Policlinico Universitario A. Gemelli, IRCCS-Largo A. Gemelli, 00168 1 Rome, Italy.
- [2] Università Cattolica del Sacro Cuore-Largo F. Vito, 00168 1 Rome, Italy.
- [3] Ospedale Cristo Re, Emergency Department-Via delle Calasanziane, 00168 25 Rome, Italy.

- PMID: **33019514**
- PMCID: PMC7599851
- DOI: 10.3390/medicina56100512

Free PMC article
Observational Study

Non-COVID Diseases during the Pandemic: Where Have All Other Emergencies Gone?

Veronica Ojetti et al. Medicina (Kaunas). 2020.
Free PMC article
Show details
Medicina (Kaunas)
. 2020 Oct 1;56(10):512.
doi: 10.3390/medicina56100512.

Authors

Veronica Ojetti [1] [2], Marcello Covino [1], Mattia Brigida [2], Carmine Petruzziello [3], Angela Saviano [2], Alessio Migneco [1], Marcello Candelli [1], Francesco Franceschi [1] [2]

Affiliations

- [1] Emergency Department-Fondazione Policlinico Universitario A. Gemelli, IRCCS-Largo A. Gemelli, 00168 1 Rome, Italy.
- [2] Università Cattolica del Sacro Cuore-Largo F. Vito, 00168 1 Rome, Italy.

- [3] Ospedale Cristo Re, Emergency Department-Via delle Calasanziane, 00168 25 Rome, Italy.

- PMID: **33019514**
- PMCID: PMC7599851
- DOI: 10.3390/medicina56100512

Abstract

Background and objectives: the emergency department (ED) is frequently identified by patients as a possible solution for all healthcare problems, leading to a high rate of misuse of the ED, possibly causing overcrowding. The coronavirus disease 2019 (COVID-19) pandemic started in China; it then spread throughout Italy, with the first cases confirmed in Lombardy, Italy, in February 2020. This has totally changed the type of patients referred to EDs. The aim of this study was to analyze the reduction of ED admissions at a Second level urban teaching (Fondazione Policlinico Universitario Agostino Gemelli IRCCS) during the COVID-19 pandemic. Materials and *Methods:* in this retrospective observational cross-sectional study, we reviewed and compared clinical records of all the patients consecutively admitted to our ED over a 40-day period (21 February -31 March) in the last three years (2018-2019-2020). Mean age, sex, triage urgency level, day/night admission, main presentation symptom, and final diagnosis, according to different medical specialties, hospitalization, and discharge rate, were analyzed. *Results:* we analyzed 16,281 patient clinical records. The overall reduction in ED admissions in 2020 was 37.6% compared to 2019. In 2020, we observed an increase in triage urgency levels for ED admissions (the main presentation symptom was a fever). We noticed a significant drop in admissions for cardio-thoracic, gastroenterological, urological, otolaryngologic/ophthalmologic, and traumatological diseases. Acute neurological conditions registered only a slight, but significant, reduction. Oncology admissions were stable. Admissions for infectious diseases were 30% in 2020, compared to 5% and 6% in 2018 and 2019, respectively. In 2020, the hospitalization rate increased to 42.9% compared to 27.7%, and 26.4% in previous years. *Conclusions:* the drastic reduction of ED admissions during the pandemic may be associated with fear of the virus, suggesting that patients with serious illnesses did not go to the emergency room. Moreover, there was possible misuse of the ED in the previous year. In particular, worrisome data emerged regarding a drop in cardiology and neurology admissions. Those patients postponed medical attention, possibly with fatal consequences, just for fear of exposure to COVID-19, leading to unnecessary morbidity and mortality.

Keywords: COVID-19; Emergency department access; fever; misuse.

Conflict of interest statement

The authors declare no conflict of interest.

- Cited by 16 articles
- 31 references
- 2 figures

Supplementary info

Publication types, MeSH terms Expand

Publication types

- Observational Study

MeSH terms

- Adolescent
- Adult
- Aged
- Aged, 80 and over
- Betacoronavirus*
- COVID-19
- Coronavirus Infections / epidemiology*
- Cross-Sectional Studies
- Emergency Service, Hospital / statistics & numerical data*
- Emergency Service, Hospital / trends
- Eye Diseases / epidemiology
- Female
- Gastrointestinal Diseases / epidemiology
- Humans
- Italy / epidemiology
- Male
- Middle Aged
- Neoplasms / epidemiology
- Nervous System Diseases / epidemiology
- Otorhinolaryngologic Diseases / epidemiology
- Pandemics*
- Patient Admission / statistics & numerical data*
- Patient Admission / trends
- Pneumonia, Viral / epidemiology*
- Retrospective Studies
- SARS-CoV-2
- Thoracic Diseases / epidemiology
- Urologic Diseases / epidemiology
- Wounds and Injuries / epidemiology
- Young Adult

Full text links

FULL TEXT OPEN ACCESS MDPI Multidisciplinary Digital Publishing Institute (MDPI) Free PMC article

Proceed to details

Cite

Share

586

Observational Study

Eur Heart J Cardiovasc Pharmacother

. 2021 Sep 21;7(5):426-434.

doi: 10.1093/ehjcvp/pvaa062.

Association between renin-angiotensin system inhibitors and COVID-19 complications

Sophie Liabeuf[1, 2], Julien Moragny[1], Youssef Bennis[1, 2], Benjamin Batteux[1, 2], Etienne Brochot[3, 4], Jean Luc Schmit[4, 5], Jean-Philippe Lanoix[4, 5], Claire Andrejak[4, 6], Olivier Ganry[7], Michel Slama[2, 8], Julien Maizel[2, 8], Yazine Mahjoub[9], Kamel Masmoudi[1], Valérie Gras-Champel[1, 2]

Affiliations Expand

Affiliations

- [1] Department of Clinical Pharmacology, Amiens University Hospital, Amiens, France.
- [2] MP3CV Laboratory, EA7517, Jules Verne University of Picardie, Amiens, France.
- [3] Department of Virology, Amiens University Medical Center, Amiens, France.
- [4] EA 4294, Jules Verne University of Picardie, Amiens, France.
- [5] Infectious Diseases Department, University Hospital, Amiens, France.
- [6] Department of Pneumology, University Hospital of Amiens-Picardie, Amiens, France.
- [7] Epidemiology and Public Health Service, Amiens University Hospital, Amiens, France.
- [8] Intensive Care Department and BoReal study group, Amiens University Hospital, Amiens, France.
- [9] Department of Anesthesiology and Critical Care Medicine, Amiens University Medical Center, Amiens, France.

- PMID: **32531040**
- PMCID: PMC7314068
- DOI: 10.1093/ehjcvp/pvaa062

Free PMC article

Observational Study

Association between renin-angiotensin system inhibitors and COVID-19 complications

Sophie Liabeuf et al. Eur Heart J Cardiovasc Pharmacother. 2021.

Free PMC article

Show details

Eur Heart J Cardiovasc Pharmacother

. 2021 Sep 21;7(5):426-434.

doi: 10.1093/ehjcvp/pvaa062.

Authors

Sophie Liabeuf [1, 2], Julien Moragny [1], Youssef Bennis [1, 2], Benjamin Batteux [1, 2], Etienne Brochot [3, 4], Jean Luc Schmit [4, 5], Jean-Philippe Lanoix [4, 5], Claire Andrejak [4, 6], Olivier Ganry [7], Michel Slama [2, 8], Julien Maizel [2, 8], Yazine Mahjoub [9], Kamel Masmoudi [1], Valérie Gras-Champel [1, 2]

Affiliations

- [1] Department of Clinical Pharmacology, Amiens University Hospital, Amiens, France.
- [2] MP3CV Laboratory, EA7517, Jules Verne University of Picardie, Amiens, France.
- [3] Department of Virology, Amiens University Medical Center, Amiens, France.
- [4] EA 4294, Jules Verne University of Picardie, Amiens, France.
- [5] Infectious Diseases Department, University Hospital, Amiens, France.
- [6] Department of Pneumology, University Hospital of Amiens-Picardie, Amiens, France.
- [7] Epidemiology and Public Health Service, Amiens University Hospital, Amiens, France.
- [8] Intensive Care Department and BoReal study group, Amiens University Hospital, Amiens, France.
- [9] Department of Anesthesiology and Critical Care Medicine, Amiens University Medical Center, Amiens, France.

- PMID: **32531040**
- PMCID: PMC7314068
- DOI: 10.1093/ehjcvp/pvaa062

Abstract

Aims: To describe the characteristics of patients hospitalized with COVID-19 (including their long-term at-home medication use), and compare them with regard to the course of the disease. To assess the association between renin-angiotensin system inhibitors (RASIs) and disease progression and critical outcomes.

Methods and results: All consecutive hospitalized patients with laboratory-confirmed COVID-19 in a university hospital in Amiens (France) were included in this study. The primary composite endpoint was admission to an intensive care unit (ICU) or death before ICU admission. Univariable and multivariable logistic regression models were used to identify factors associated with the composite endpoint. Between 28 February 2020 and 30 March 2020, a total of 499 local patients tested positive for SARS-CoV-2. Of these, 231 were not hospitalized {males 33%; median [interquartile range (IQR)] age: 44 (32-54)}, and 268 were hospitalized [males 58%; median (IQR) age: 73 (61-84)]. A total of 116 patients met the primary endpoint: 47 died before ICU admission, and 69 were admitted to the ICU. Patients meeting the primary endpoint were more likely than patients not meeting the primary endpoint to have coronary heart disease and to have been taking RASIs; however, the two subsets of patients did not differ with regard to median age. After adjustment for other associated variables, the risk of meeting the composite endpoint was 1.73 times higher (odds ratio 1.73, 95% confidence interval 1.02-2.93) in patients treated at baseline with a RASI than in patients not treated with this drug class. This association was confirmed when the analysis was restricted to patients treated with antihypertensive agents.

Conclusions: We highlighted a potential safety signal for RASIs, the long-term use of which was independently associated with a higher risk of severe COVID-19 and a poor outcome. Due to the widespread use of this important drug class, formal proof based on clinical trials is needed to better understand the association between RASIs and complications of COVID-19.

Keywords: Associated factors; COVID-19; Critical outcomes; Renin–angiotensin system inhibitors.

Published on behalf of the European Society of Cardiology. © The Author(s) 2020.

- Cited by 29 articles
- 31 references
- 2 figures

Supplementary info

Publication types, MeSH terms, Substances Expand

Publication types

- Observational Study

MeSH terms

- Adult
- Age Factors
- Aged
- Aged, 80 and over
- Antihypertensive Agents / adverse effects*
- COVID-19 / complications*
- Female
- Humans
- Hypertension / drug therapy
- Intensive Care Units
- Logistic Models
- Male
- Middle Aged
- Renin-Angiotensin System / drug effects*
- Retrospective Studies
- SARS-CoV-2*

Substances

- Antihypertensive Agents

Full text links

OXFORD ACADEMIC Silverchair Information Systems Free PMC article

Proceed to details

Cite

Share

587

Observational Study

PLoS One

. 2020 Dec 9;15(12):e0241956.

doi: 10.1371/journal.pone.0241956. eCollection 2020.

Comparison of COVID-19 infections among healthcare workers and non-healthcare workers

Rachel Kim [1], Sharon Nachman [2], Rafael Fernandes [1], Kristen Meyers [1], Maria Taylor [1], Debra LeBlanc [1], Adam J Singer [1]

Affiliations Expand

Affiliations

- [1] Department of Emergency Medicine, Renaissance School of Medicine at Stony Brook University, Stony Brook, New York, United States of America.
- [2] Department of Pediatrics, Renaissance School of Medicine at Stony Brook University, Stony Brook, New York, United States of America.

- PMID: **33296367**
- PMCID: PMC7725299
- DOI: 10.1371/journal.pone.0241956

Free PMC article

Observational Study

Comparison of COVID-19 infections among healthcare workers and non-healthcare workers

Rachel Kim et al. PLoS One. 2020.

Free PMC article

Show details

PLoS One

. 2020 Dec 9;15(12):e0241956.

doi: 10.1371/journal.pone.0241956. eCollection 2020.

Authors

Rachel Kim [1], Sharon Nachman [2], Rafael Fernandes [1], Kristen Meyers [1], Maria Taylor [1], Debra LeBlanc [1], Adam J Singer [1]

Affiliations

- [1] Department of Emergency Medicine, Renaissance School of Medicine at Stony Brook University, Stony Brook, New York, United States of America.
- [2] Department of Pediatrics, Renaissance School of Medicine at Stony Brook University, Stony Brook, New York, United States of America.

- PMID: **33296367**
- PMCID: PMC7725299
- DOI: 10.1371/journal.pone.0241956

Abstract

Objectives: Healthcare workers face distinct occupational challenges that affect their personal health, especially during a pandemic. In this study we compare the characteristics and outcomes of Covid-19 patients who are and who are not healthcare workers (HCW).

Methods: We retrospectively analyzed a cohort of 2,842 adult patients with known HCW status and a positive SARS-CoV-2 RT-PCR test presenting to a large academic medical center emergency department (ED) in New York State from March 21 2020 through June 2020. Early in the pandemic we instituted a policy to collect data on patient occupation and exposures to suspected Covid-19. The primary outcome was hospital admission. Secondary outcomes were ICU admission, need for invasive mechanical ventilation (IMV), and mortality. We compared baseline characteristics and outcomes of Covid-19 adult patients based on whether they were or were not HCW using univariable and multivariable analyses.

Results: Of 2,842 adult patients (mean age 53+/-19 years, 53% male) 193 (6.8%) were HCWs and 2,649 (93.2%) were not HCWs. Compared with non-HCW, HCWs were younger (43 vs 53 years, P<0.001), more likely female (118/193 [61%] vs 1211/2649 [46%], P<0.001), and more likely to have a known Covid-19 exposure (161/193 [83%] vs 946/2649 [36%], P<0.001), but had fewer comorbidities. On presentation to the ED, HCW also had lower frequencies of tachypnea (12/193 [6%] vs 426/2649 [16%], P<0.01), hypoxemia (15/193 [8%] vs 564/2649 [21%], P<0.01), bilateral opacities on imaging (38/193 [20%] vs 1189/2649 [45%], P<0.001), and lymphocytopenia (6/193 [3%] vs 532/2649 [20%], P<0.01) compared to non-HCWs. Direct discharges home from the ED were more frequent in HCW 154/193 (80%) vs 1275/2649 (48%) p<0.001). Hospital admissions (38/193 [20%] vs 1264/2694 [47%], P<0.001), ICU admissions (7/193 [3%] vs 321/2694 [12%], P<0.001), need for IMV (6/193 [3%] vs 321/2694 [12%], P<0.001) and mortality (2/193 [1%] vs 219/2694 [8%], P<0.01) were lower than among non-HCW. After controlling for age, sex, comorbidities, presenting vital signs and radiographic imaging, HCW were less likely to be admitted (OR 0.6, 95%CI 0.3-0.9) than non HCW.

Conclusions: Compared with non HCW, HCW with Covid-19 were younger, had less severe illness, and were less likely to be admitted.

Conflict of interest statement

The authors declare that no competing interest exits relevant to this study.

- Cited by 10 articles
- 19 references

Supplementary info

Publication types, MeSH terms, Grant support Expand

Publication types

- Comparative Study
- Observational Study

MeSH terms

- Adult
- Age Factors
- Aged
- COVID-19* / diagnostic imaging
- COVID-19* / mortality
- COVID-19* / therapy
- Cross-Sectional Studies
- Emergency Service, Hospital*
- Female
- Health Personnel*
- Humans
- Male
- Middle Aged
- Retrospective Studies
- SARS-CoV-2*
- Sex Factors

Grant support

No funding was received for this study.

Full text links

OPEN ACCESS TO FULL TEXT PLOS ONE Public Library of Science Free PMC article

Proceed to details

Cite

Share

☐ 588

Observational Study

J Clin Endocrinol Metab

. 2021 Sep 27;106(10):e4007-e4016.

doi: 10.1210/clinem/dgab409.

Use of Continuous Glucose Monitor in Critically Ill COVID-19 Patients Requiring Insulin Infusion: An Observational Study

Eileen R Faulds[1], Andrew Boutsicaris[2], Lyndsey Sumner[2], Laureen Jones[3], Molly McNett[4], Keaton S Smetana[3], Casey C May[3], Elizabeth Buschur[5], Matthew C Exline[6], Matthew D Ringel[5], Kathleen Dungan[5]

Affiliations Expand

Affiliations

- [1] The Ohio State University College of Nursing, The Ohio State University Medical Center, Columbus, OH, USA.
- [2] The Ohio State University College of Medicine, Columbus, OH, USA.
- [3] The Ohio State University Medical Center, Columbus, OH, USA.
- [4] Implementation/Translation Science Core, Helene Fuld Health Trust National Institute for EBP, Columbus, OH, USA.
- [5] Division of Endocrinology, Diabetes, and Metabolism, The Ohio State University Medical Center, Columbus, OH, USA.
- [6] Division of Critical Care Medicine, The Ohio State University Medical Center, Columbus, OH, USA.

- PMID: **34100545**
- DOI: 10.1210/clinem/dgab409

Observational Study

Use of Continuous Glucose Monitor in Critically Ill COVID-19 Patients Requiring Insulin Infusion: An Observational Study

Eileen R Faulds et al. J Clin Endocrinol Metab. 2021.

Show details

J Clin Endocrinol Metab

. 2021 Sep 27;106(10):e4007-e4016.

doi: 10.1210/clinem/dgab409.

Authors

Eileen R Faulds [1], Andrew Boutsicaris [2], Lyndsey Sumner [2], Laureen Jones [3], Molly McNett [4], Keaton S Smetana [3], Casey C May [3], Elizabeth Buschur [5], Matthew C Exline [6], Matthew D Ringel [5], Kathleen Dungan [5]

Affiliations

- [1] The Ohio State University College of Nursing, The Ohio State University Medical Center, Columbus, OH, USA.
- [2] The Ohio State University College of Medicine, Columbus, OH, USA.
- [3] The Ohio State University Medical Center, Columbus, OH, USA.
- [4] Implementation/Translation Science Core, Helene Fuld Health Trust National Institute for EBP, Columbus, OH, USA.
- [5] Division of Endocrinology, Diabetes, and Metabolism, The Ohio State University Medical Center, Columbus, OH, USA.
- [6] Division of Critical Care Medicine, The Ohio State University Medical Center, Columbus, OH, USA.

- PMID: **34100545**
- DOI: 10.1210/clinem/dgab409

Abstract

Context: The coronavirus disease 2019 (COVID-19) pandemic has created a need for remote blood glucose (BG) monitoring in the intensive care unit (ICU).

Objective: To evaluate feasibility and patient safety of a hybrid monitoring strategy of point-of-care (POC) BG plus continuous glucose monitor (CGM) in the ICU.

Design: Retrospective analysis.

Setting: ICU of an academic medical center.

Patients: Patients with COVID-19 on IV insulin.

Intervention: After meeting initial validation criteria, CGM was used for IV insulin titration and POC BG was performed every 6 hours or as needed.

Main outcome measures: Outcomes included frequency of POC BG, workflow, safety, and accuracy measures.

Results: The study included 19 patients, 18 with CGM data, mean age 58 years, 89% on mechanical ventilation, 37% on vasopressors, and 42% on dialysis. The median time to CGM validation was 137 minutes (interquartile range [IQR] 114-206). During IV insulin, the median number of POC values was 7 (IQR 6-16) on day 1, and declined slightly thereafter (71% reduction compared with standard of 24/day). The median number of CGM values used nonadjunctively to titrate IV insulin was 11.5 (IQR 0, 15) on day 1 and increased thereafter. Time in range 70 to 180 mg/dL was 64 ± 23% on day 1 and 72 ± 16% on days 2 through 7, whereas time <70 mg/dL was 1.5 ± 4.1% on day 1 and <1% on days 2 through 7.

Conclusions: This study provides data to support that CGM using a hybrid protocol is feasible, accurate, safe, and has potential to reduce nursing and staff workload.

Keywords: COVID-19; continuous glucose monitoring; glucose monitoring; hospitalized; inpatient; intensive care unit.

© The Author(s) 2021. Published by Oxford University Press on behalf of the Endocrine Society. All rights reserved. For permissions, please e-mail: journals.permissions@oup.com.

- Cited by 1 article

Supplementary info

Publication types, MeSH terms, Substances Expand

Publication types

- Observational Study

MeSH terms

- Adult
- Aged
- Blood Glucose / analysis
- Blood Glucose Self-Monitoring / methods*
- COVID-19 / epidemiology*
- COVID-19 / therapy
- Comorbidity
- Critical Illness / therapy
- Diabetes Complications / epidemiology
- Diabetes Complications / therapy
- Diabetes Complications / virology
- Diabetes Mellitus / epidemiology*
- Diabetes Mellitus / therapy*
- Female
- Glycemic Control / methods
- Humans
- Infusions, Intravenous
- Insulin / administration & dosage*
- Intensive Care Units
- Male
- Middle Aged
- Point-of-Care Systems
- Retrospective Studies
- SARS-CoV-2*

- Treatment Outcome

Substances

- Blood Glucose
- Insulin

Full text links

ENDOCRINE SOCIETY Silverchair Information Systems
Proceed to details
Cite
Share
589
Observational Study
An Pediatr (Engl Ed)
. 2020 Nov;93(5):313-322.
doi: 10.1016/j.anpedi.2020.06.021. Epub 2020 Jul 21.

[Impact of the COVID-19 pandemic on emergency department: Early findings from a hospital in Madrid]

[Article in Spanish]
Miguel Ángel Molina Gutiérrez [1], José Antonio Ruiz Domínguez [2], Marta Bueno Barriocanal [2], Begoña de Miguel Lavisier [2], Rosario López López [2], Julia Martín Sánchez [2], María de Ceano-Vivas la Calle [2]
Affiliations Expand

Affiliations

- [1] Servicio de Urgencias Pediátricas, Hospital Universitario La Paz, Madrid, España. Electronic address: malacatin@hotmail.com.
- [2] Servicio de Urgencias Pediátricas, Hospital Universitario La Paz, Madrid, España.

- PMID: **32800720**
- PMCID: PMC7373010
- DOI: 10.1016/j.anpedi.2020.06.021

Free PMC article
Observational Study

[Impact of the COVID-19 pandemic on emergency department: Early findings from a hospital in Madrid]

[Article in Spanish]
Miguel Ángel Molina Gutiérrez et al. An Pediatr (Engl Ed). 2020 Nov.
Free PMC article
Show details
An Pediatr (Engl Ed)
. 2020 Nov;93(5):313-322.
doi: 10.1016/j.anpedi.2020.06.021. Epub 2020 Jul 21.

Authors

Miguel Ángel Molina Gutiérrez [1], José Antonio Ruiz Domínguez [2], Marta Bueno Barriocanal [2], Begoña de Miguel Lavisier [2], Rosario López López [2], Julia Martín Sánchez [2], María de Ceano-Vivas la Calle [2]

Affiliations

- [1] Servicio de Urgencias Pediátricas, Hospital Universitario La Paz, Madrid, España. Electronic address: malacatin@hotmail.com.
- [2] Servicio de Urgencias Pediátricas, Hospital Universitario La Paz, Madrid, España.

- PMID: **32800720**
- PMCID: PMC7373010
- DOI: 10.1016/j.anpedi.2020.06.021

Abstract
in English, Spanish

Introduction: SARS-CoV-2, coronavirus that causes coronavirus disease 2019 (COVID-19), was first detected in Spain on 31 January 2020. On 14 March 2020, a state of emergency was declared in Spain in a bid to control the spread of the COVID-19 pandemic in the country. The aim of our study is to analyse the impact on emergency medicine attendance after the national lockdown, as well as the clinical presentation and the management of patients with suspected COVID-19 in the Paediatric Emergency Department.

Patients and methods: This retrospective observational study included children and adolescents under the age of 18, attended in our Paediatric Emergency Department during the period March 14 to April 17, 2020.

Results: A total of 1,666 patients were attended during the study period, 65.4% less than in the same period of 2019. Just over half (51.2%) were males, and mean age was 5.4 years. In triage, 39.9% were high priority levels, 6.5% more than 2019. Most frequent reasons for consultation at the Paediatric Emergency Department were fever (26.5%), respiratory symptoms (16.1%), and trauma (15.2%). A total of 218 patients (13%) received a diagnosis of possible COVID-19, with

SARS-CoV-2 infection confirmed in 18.4%, and 23.8% (52/218) were hospitalised. At discharge, 44% (96/218) were diagnosed with lower, and 33.9% (74/218) with upper respiratory infection.

Conclusions: During the SARS-CoV-2 outbreak, the demand for urgent paediatric care decreased, with the proportion of cases with high priority triage levels increasing. Most of the patients with suspected or microbiological confirmation of COVID-19 had mild respiratory symptoms.

Introducción: SARS-CoV-2, responsable de la enfermedad por coronavirus 2019 (COVID-19), fue detectado por primera vez en España el 31 de enero de 2020. El 14 de marzo fue declarado el estado de alarma con el objetivo de controlar la pandemia. El objetivo de este estudio es analizar las consecuencias de esta crisis sanitaria sobre el patrón de demanda asistencial, así como el manejo y las características de los pacientes con sospecha de COVID-19 en el Servicio de Urgencias Pediátricas.

Pacientes y métodos: Estudio retrospectivo observacional en niños y adolescentes menores de 18 años, atendidos en nuestro Servicio de Urgencias Pediátricas durante el periodo comprendido desde el 14 de marzo hasta el 17 de abril de 2020.

Resultados: Durante el periodo de estudio se atendieron 1.666 pacientes, un 65,4% menos que en el mismo periodo de 2019. La edad media fue de 5,4 años y el 51,2% eran varones. El 39,9% fueron clasificados con niveles de alta prioridad, un 6,5% más que en 2019. Los principales motivos de consulta fueron fiebre (26,5%), síntomas respiratorios (16,1%) y traumatismos (15,2%). Un total de 218 pacientes (13%) fueron diagnosticados de posible COVID-19, confirmándose la infección en el 18,4%. El 44% (96/218) fueron diagnosticados de infección respiratoria inferior y el 33,9% (74/218), superior. El 23,8% (52/218) fueron hospitalizados.

Conclusiones: Durante el brote epidémico SARS-CoV-2 disminuyó la demanda de asistencia pediátrica urgente, aumentando la proporción de casos con niveles de triaje de alta prioridad. La mayoría de los pacientes con sospecha o confirmación microbiológica de COVID-19 cursaron con clínica respiratoria leve.

Keywords: COVID-19; Medicina pediátrica de urgencias; Paediatric emergency medicine; Pandemia; Pandemics.

Copyright © 2020 Asociación Española de Pediatría. Publicado por Elsevier España, S.L.U. All rights reserved.

Comment in

- [Impact of the SARS-CoV-2 pandemic on the use of the emergency department and admissions in a tertiary hospital].
  Díaz Pérez D, Lorente Sorolla M, González Lago S, Osona B. Díaz Pérez D, et al. An Pediatr (Engl Ed). 2021 Feb;94(2):125-126. doi: 10.1016/j.anpedi.2020.10.014. Epub 2020 Oct 26. An Pediatr (Engl Ed). 2021. PMID: 33183961 Free PMC article. Spanish. No abstract available.
- [In response to the article «Impact of the COVID-19 pandemic in the emergency room: First findings in a hospital in Madrid»].
  Alonso Cadenas JA, Andina Martínez D, Martín Díaz MJ, Molina Cabañero JC. Alonso Cadenas JA, et al. An Pediatr (Engl Ed). 2021 Apr;94(4):270-272. doi: 10.1016/j.anpedi.2020.11.017. Epub 2020 Nov 25. An Pediatr (Engl Ed). 2021. PMID: 33342687 Free PMC article. Spanish. No abstract available.

- Cited by 9 articles
- 22 references

- 3 figures

Supplementary info

Publication types, MeSH terms Expand

Publication types

- Observational Study

MeSH terms

- Adolescent
- Betacoronavirus
- COVID-19
- Child
- Child, Preschool
- Coronavirus Infections* / diagnosis
- Coronavirus Infections* / epidemiology
- Coronavirus Infections* / therapy
- Emergency Service, Hospital / trends*
- Facilities and Services Utilization / trends*
- Female
- Health Care Rationing
- Health Policy
- Hospitals, Pediatric / trends*
- Humans
- Infant
- Infant, Newborn
- Male
- Pandemics*
- Pneumonia, Viral* / diagnosis
- Pneumonia, Viral* / epidemiology
- Pneumonia, Viral* / therapy
- Retrospective Studies
- SARS-CoV-2
- Spain / epidemiology
- Triage

Full text links

ELSEVIER OPEN ACCESS Elsevier Science Free PMC article
Proceed to details

Cite
Share
590
Observational Study
Artif Organs
. 2021 Jun;45(6):E158-E170.
doi: 10.1111/aor.13873. Epub 2020 Dec 28.

Role of extracorporeal membrane oxygenation in critically Ill COVID-19 patients and predictors of mortality

Rashad Zayat [1], Sebastian Kalverkamp [1], Oliver Grottke [2], Koray Durak [1], Michael Dreher [3], Rüdiger Autschbach [1], Gernot Marx [4], Nikolaus Marx [5], Jan Spillner [1], Alex Kersten [5]
Affiliations Expand

Affiliations

- [1] Department of Thoracic and Cardiovascular Surgery, RWTH University Hospital Aachen, Aachen, Germany.
- [2] Department of Anesthesiology, RWTH University Hospital Aachen, Aachen, Germany.
- [3] Department of Pneumology and Intensive Care Medicine, RWTH University Hospital Aachen, Aachen, Germany.
- [4] Department of Intensive Care and Intermediate Care Medicine, RWTH University Hospital Aachen, Aachen, Germany.
- [5] Department of Cardiology, Angiology and Intensive Care, RWTH University Hospital Aachen, Aachen, Germany.

- PMID: **33236373**
- PMCID: PMC7753822
- DOI: 10.1111/aor.13873

Free PMC article
Observational Study

Role of extracorporeal membrane oxygenation in critically Ill COVID-19 patients and predictors of mortality

Rashad Zayat et al. Artif Organs. 2021 Jun.
Free PMC article
Show details
Artif Organs

. 2021 Jun;45(6):E158-E170.
doi: 10.1111/aor.13873. Epub 2020 Dec 28.

Authors

Rashad Zayat [1], Sebastian Kalverkamp [1], Oliver Grottke [2], Koray Durak [1], Michael Dreher [3], Rüdiger Autschbach [1], Gernot Marx [4], Nikolaus Marx [5], Jan Spillner [1], Alex Kersten [5]

Affiliations

- [1] Department of Thoracic and Cardiovascular Surgery, RWTH University Hospital Aachen, Aachen, Germany.
- [2] Department of Anesthesiology, RWTH University Hospital Aachen, Aachen, Germany.
- [3] Department of Pneumology and Intensive Care Medicine, RWTH University Hospital Aachen, Aachen, Germany.
- [4] Department of Intensive Care and Intermediate Care Medicine, RWTH University Hospital Aachen, Aachen, Germany.
- [5] Department of Cardiology, Angiology and Intensive Care, RWTH University Hospital Aachen, Aachen, Germany.

- PMID: **33236373**
- PMCID: PMC7753822
- DOI: 10.1111/aor.13873

Abstract

The role of extracorporeal membrane oxygenation (ECMO) in the management of critically ill COVID-19 patients remains unclear. Our study aims to analyze the outcomes and risk factors from patients treated with ECMO. This retrospective, single-center study includes 17 COVID-19 patients treated with ECMO. Univariate and multivariate parametric survival regression identified predictors of survival. Nine patients (53%) were successfully weaned from ECMO and discharged. The incidence of in-hospital mortality was 47%. In a univariate analysis, only four out of 83 pre-ECMO variables were significantly different; IL-6, PCT, and NT-proBNP were significantly higher in non-survivors than in survivors. The Respiratory Extracorporeal Membrane Oxygenation Survival Prediction (RESP) score was significantly higher in survivors. After a multivariate parametric survival regression, IL-6, NT-proBNP and RESP scores remained significant independent predictors, with hazard ratios (HR) of 1.069 [95%-CI: 0.986-1.160], P = .016 1.001 [95%-CI: 1.000-1.001], P = .012; and .843 [95%-CI: 0.564-1.260], P = .040, respectively. A prediction model comprising IL-6, NT-proBNP, and RESP score showed an area under the curve (AUC) of 0.87, with a sensitivity of 87.5% and 77.8% specificity compared to an AUC of 0.79 for the RESP score alone. The present study suggests that ECMO is a potentially lifesaving treatment for selected critically ill COVID-19 patients. Considering IL-6 and NT-pro-BNP, in addition to the RESP score, may enhance outcome predictions.

Keywords: Acute respiratory distress syndrome; COVID-19; SARS-CoV-2; critical care; extra corporeal membrane oxygenation; risk factors.

© 2020 International Center for Artificial Organs and Transplantation and Wiley Periodicals, LLC.

- Cited by 10 articles

- 39 references

Supplementary info

Publication types, MeSH terms, Substances Expand

Publication types

- Observational Study

MeSH terms

- Biomarkers / blood
- COVID-19 / mortality*
- COVID-19 / therapy*
- Critical Illness*
- Extracorporeal Membrane Oxygenation*
- Female
- Humans
- Male
- Middle Aged
- Pneumonia, Viral / mortality*
- Pneumonia, Viral / therapy*
- Pneumonia, Viral / virology
- Predictive Value of Tests
- Retrospective Studies
- Risk Factors
- SARS-CoV-2
- Survival Rate

Substances

- Biomarkers

Full text links

WILEY Full Text Article Wiley Free PMC article

Proceed to details

Cite

Share

☐ 591

Observational Study

Clin Neurophysiol

. 2021 Dec;132(12):3019-3024.
doi: 10.1016/j.clinph.2021.10.001. Epub 2021 Oct 13.

Electrodiagnostic findings in COVID-19 patients: A single center experience

Sajid Hameed [1], Ayisha Farooq Khan [2], Sara Khan [3]
Affiliations Expand

Affiliations

- [1] Department of Neurology, Aga Khan University, Pakistan. Electronic address: sajid.hameed@aku.edu.
- [2] Department of Neurology, Aga Khan University, Pakistan. Electronic address: ayisha.farooq@aku.edu.
- [3] Department of Neurology, Aga Khan University, Pakistan. Electronic address: sara.khan@aku.edu.

- PMID: **34717222**
- PMCID: PMC8513511
- DOI: 10.1016/j.clinph.2021.10.001

Free PMC article
Observational Study

Electrodiagnostic findings in COVID-19 patients: A single center experience

Sajid Hameed et al. Clin Neurophysiol. 2021 Dec.
Free PMC article
Show details
Clin Neurophysiol
. 2021 Dec;132(12):3019-3024.
doi: 10.1016/j.clinph.2021.10.001. Epub 2021 Oct 13.

Authors

Sajid Hameed [1], Ayisha Farooq Khan [2], Sara Khan [3]

Affiliations

- [1] Department of Neurology, Aga Khan University, Pakistan. Electronic address: sajid.hameed@aku.edu.
- [2] Department of Neurology, Aga Khan University, Pakistan. Electronic address: ayisha.farooq@aku.edu.

- [3] Department of Neurology, Aga Khan University, Pakistan. Electronic address: sara.khan@aku.edu.

- PMID: **34717222**
- PMCID: PMC8513511
- DOI: 10.1016/j.clinph.2021.10.001

Abstract

Objective: Neurological manifestations in patients with coronavirus disease 2019 (COVID-19) have been reported from early features of anosmia and dysgeusia to widespread involvement of the central nervous system, peripheral nervous system, as well as the neuromuscular junction and muscle. Our study objective is to evaluate the electromyography and nerve conduction study (EMG/NCS) findings among COVID-19 patients and look for possible correlations.

Methods: This is a hospital-based retrospective observational study. All COVID-19 patients between the period of 1st January 2020 to 31st December 2020 undergoing an EMG/NCS were included.

Results: Eighteen patients (12 male and 6 female) were included. Mean age was 55 ± 12 years. 11 patients required intubation for a mean period of 18.6 days (range: 3-37 days). Electrodiagnostic findings were consistent with a myopathy in a majority of these patients (82%). Five of them also had a concurrent axonal neuropathy. In the remaining patients who did not require intubation (n = 7), three patients had myopathic EMG changes and one had Guillain Barre syndrome.

Conclusion: At this time, there are no neuromuscular-specific recommendations for patients who contract COVID-19. Only time and additional data will unveil the varying nature and potential neurological sequelae of COVID-19.

Significance: Myopathic EMG changes are commonly seen in critically ill COVID-19 patients, especially with a prolonged hospital stay.

Keywords: COVID-19; Coronavirus; EMG/NCS; Electromyography; Myopathy.

Copyright © 2021 International Federation of Clinical Neurophysiology. Published by Elsevier B.V. All rights reserved.

Conflict of interest statement

Declaration of Competing Interest The authors declare that they have no known competing financial interests or personal relationships that could have appeared to influence the work reported in this paper.

Comment in

- Digging deeper on the neurophysiological assessment in COVID-19 patients.
  Bocci T, Gentile F, Priori A. Bocci T, et al. Clin Neurophysiol. 2022 Feb;134:137-138. doi: 10.1016/j.clinph.2021.10.015. Epub 2021 Nov 27. Clin Neurophysiol. 2022. PMID: 34895820 Free PMC article. No abstract available.
- Myopathy in acute and long-term COVID-19.

Tankisi H, Ochala J. Tankisi H, et al. Clin Neurophysiol. 2022 Feb;134:141-142. doi: 10.1016/j.clinph.2021.11.006. Epub 2021 Dec 9. Clin Neurophysiol. 2022. PMID: 34930658 Free PMC article. No abstract available.

- Cited by 4 articles
- 18 references

Supplementary info

Publication types, MeSH terms Expand

Publication types

- Observational Study

MeSH terms

- Adult
- Aged
- COVID-19 / complications*
- Comorbidity
- Electromyography*
- Female
- Guillain-Barre Syndrome / diagnosis
- Humans
- Intubation, Intratracheal / statistics & numerical data
- Length of Stay
- Male
- Middle Aged
- Muscle, Skeletal
- Muscular Diseases / diagnosis*
- Nervous System Diseases / diagnosis*
- Neural Conduction*
- Retrospective Studies

Full text links

ELSEVIER FULL-TEXT ARTICLE Elsevier Science Free PMC article
Proceed to details
Cite
Share
☐ 592
Observational Study
Neuroepidemiology

. 2021;55(2):154-161.
doi: 10.1159/000514888. Epub 2021 Apr 1.

Risk Factors for Olfactory and Gustatory Dysfunctions in Patients with SARS-CoV-2 Infection

Francesca Galluzzi [1], Veronica Rossi [2], Cristina Bosetti [3], Werner Garavello [1 2]
Affiliations Expand

Affiliations

- [1] Department of Otorhinolaryngology, San Gerardo Hospital, Monza, Italy.
- [2] Department of Otorhinolaryngology, School of Medicine and Surgery, University of Milano-Bicocca, Monza, Italy.
- [3] Department of Oncology, Istituto di Ricerche Farmacologiche Mario Negri IRCCS, Milan, Italy.

- PMID: **33794531**
- DOI: 10.1159/000514888

Observational Study

Risk Factors for Olfactory and Gustatory Dysfunctions in Patients with SARS-CoV-2 Infection

Francesca Galluzzi et al. Neuroepidemiology. 2021.
Show details
Neuroepidemiology
. 2021;55(2):154-161.
doi: 10.1159/000514888. Epub 2021 Apr 1.

Authors

Francesca Galluzzi [1], Veronica Rossi [2], Cristina Bosetti [3], Werner Garavello [1 2]

Affiliations

- [1] Department of Otorhinolaryngology, San Gerardo Hospital, Monza, Italy.
- [2] Department of Otorhinolaryngology, School of Medicine and Surgery, University of Milano-Bicocca, Monza, Italy.
- [3] Department of Oncology, Istituto di Ricerche Farmacologiche Mario Negri IRCCS, Milan, Italy.

- PMID: **33794531**
- DOI: 10.1159/000514888

Abstract

Introduction: Smell and taste loss are characteristic symptoms of SARS-CoV-2 infection. The aim of this study is to investigate the prevalence and risk factors associated with olfactory and gustatory dysfunctions in coronavirus disease (COVID-19) patients.

Methods: We conducted an observational, retrospective study on 376 patients with documented SARS-CoV-2 infection admitted to the San Gerardo Hospital in Monza, Italy, from March to July 2020. All patients answered a phone questionnaire providing information on age, sex, smoking status, and clinical characteristics. Adjusted odds ratios (ORs) and corresponding 95% confidence intervals (CIs) were estimated through logistic regression models including relevant covariates.

Results: The prevalence of olfactory and gustatory dysfunctions in COVID-19 patients was 33.5 and 35.6%, respectively. Olfactory dysfunctions were significantly directly associated with current smoking and history of allergy, the multivariable ORs being 6.53 (95% CI 1.16-36.86) for current smokers versus never smokers, and 1.89 (95% CI 1.05-3.39) for those with an allergy compared to those without any allergy. Respiratory allergy in particular was significantly associated with olfactory dysfunctions (multivariable OR 2.30, 95% CI 1.02-5.17). Significant inverse associations were observed for patients aged 60 years or more (multivariable OR 0.33, 95% CI 0.19-0.57) and hospitalization (multivariable OR 0.22, 95% CI 0.06-0.89). Considering gustatory dysfunctions, after allowance of other variables a significant direct association was found for respiratory allergies (OR 2.24, 95% CI 1.03-4.86), and an inverse association was found only for hospitalization (OR 0.21, 95% CI 0.06-0.76).

Conclusion: Our study indicates that current smoking and history of allergy (particularly respiratory) significantly increase the risk for smell loss in COVID-19 patients; the latter is also significantly associated to taste loss. Hospitalization has an inverse association with the risk of olfactory and gustatory dysfunctions, suggesting that these may be symptoms characteristics of less severe SARS-CoV-2 infection.

Trial registration: ClinicalTrials.gov NCT04427332.

Keywords: COVID-19; Gustatory dysfunction; Olfactory dysfunction; Risk factors; SARS-CoV-2.

© 2021 S. Karger AG, Basel.

- Cited by 3 articles

Supplementary info

Publication types, MeSH terms, Associated data Expand

Publication types

- Observational Study

MeSH terms

- Age Factors
- Aged
- Anosmia / epidemiology*
- Anosmia / physiopathology
- COVID-19 / physiopathology*
- Dysgeusia / epidemiology*
- Dysgeusia / physiopathology
- Emergency Service, Hospital
- Female
- Hospitalization
- Humans
- Hypersensitivity / epidemiology
- Logistic Models
- Male
- Middle Aged
- Multivariate Analysis
- Olfaction Disorders / epidemiology
- Olfaction Disorders / physiopathology
- Prevalence
- Respiratory Hypersensitivity / epidemiology*
- Retrospective Studies
- Risk Factors
- SARS-CoV-2
- Smoking / epidemiology*
- Taste Disorders / epidemiology
- Taste Disorders / physiopathology

Associated data

- ClinicalTrials.gov/NCT04427332

Full text links

Karger Final Version S. Karger AG, Basel, Switzerland
Proceed to details
Cite
Share
593
Observational Study
JAMA Intern Med
. 2020 Oct 1;180(10):1328-1333.

doi: 10.1001/jamainternmed.2020.3288.

Trends in Emergency Department Visits and Hospital Admissions in Health Care Systems in 5 States in the First Months of the COVID-19 Pandemic in the US

Molly M Jeffery [1, 2], Gail D'Onofrio [3], Hyung Paek [4], Timothy F Platts-Mills [5], William E Soares 3rd [6], Jason A Hoppe [7], Nicholas Genes [8], Bidisha Nath [3], Edward R Melnick [3]
Affiliations Expand

Affiliations

- [1] Department of Emergency Medicine, Mayo Clinic, Rochester, Minnesota.
- [2] Department of Health Care Policy Research, Mayo Clinic, Rochester, Minnesota.
- [3] Department of Emergency Medicine, Yale University School of Medicine, New Haven, Connecticut.
- [4] Information Technology Services, Yale New Haven Health System, New Haven, Connecticut.
- [5] Department of Emergency Medicine, University of North Carolina School of Medicine, Chapel Hill.
- [6] Department of Emergency Medicine, University of Massachusetts Medical School-Baystate, Springfield.
- [7] Department of Emergency Medicine, University of Colorado, School of Medicine, Aurora.
- [8] Department of Emergency Medicine, Icahn School of Medicine at Mount Sinai, New York, New York.

- PMID: **32744612**
- PMCID: PMC7400214
- DOI: 10.1001/jamainternmed.2020.3288

Free PMC article
Observational Study

Trends in Emergency Department Visits and Hospital Admissions in Health Care Systems in 5 States in the First Months of the COVID-19 Pandemic in the US

Molly M Jeffery et al. JAMA Intern Med. 2020.
Free PMC article
Show details

JAMA Intern Med
. 2020 Oct 1;180(10):1328-1333.
doi: 10.1001/jamainternmed.2020.3288.

Authors

Molly M Jeffery [1, 2], Gail D'Onofrio [3], Hyung Paek [4], Timothy F Platts-Mills [5], William E Soares 3rd [6], Jason A Hoppe [7], Nicholas Genes [8], Bidisha Nath [3], Edward R Melnick [3]

Affiliations

- [1] Department of Emergency Medicine, Mayo Clinic, Rochester, Minnesota.
- [2] Department of Health Care Policy Research, Mayo Clinic, Rochester, Minnesota.
- [3] Department of Emergency Medicine, Yale University School of Medicine, New Haven, Connecticut.
- [4] Information Technology Services, Yale New Haven Health System, New Haven, Connecticut.
- [5] Department of Emergency Medicine, University of North Carolina School of Medicine, Chapel Hill.
- [6] Department of Emergency Medicine, University of Massachusetts Medical School-Baystate, Springfield.
- [7] Department of Emergency Medicine, University of Colorado, School of Medicine, Aurora.
- [8] Department of Emergency Medicine, Icahn School of Medicine at Mount Sinai, New York, New York.

- PMID: **32744612**
- PMCID: PMC7400214
- DOI: 10.1001/jamainternmed.2020.3288

Abstract

Importance: As coronavirus disease 2019 (COVID-19) spread throughout the US in the early months of 2020, acute care delivery changed to accommodate an influx of patients with a highly contagious infection about which little was known.

Objective: To examine trends in emergency department (ED) visits and visits that led to hospitalizations covering a 4-month period leading up to and during the COVID-19 outbreak in the US.

Design, setting, and participants: This retrospective, observational, cross-sectional study of 24 EDs in 5 large health care systems in Colorado (n = 4), Connecticut (n = 5), Massachusetts (n = 5), New York (n = 5), and North Carolina (n = 5) examined daily ED visit and hospital admission rates from January 1 to April 30, 2020, in relation to national and the 5 states' COVID-19 case counts.

Exposures: Time (day) as a continuous variable.

Main outcomes and measures: Daily counts of ED visits, hospital admissions, and COVID-19 cases.

Results: A total of 24 EDs were studied. The annual ED volume before the COVID-19 pandemic ranged from 13 000 to 115 000 visits per year; the decrease in ED visits ranged from 41.5% in Colorado to 63.5% in New York. The weeks with the most rapid rates of decrease in visits were in March 2020, which corresponded with national public health messaging about COVID-19. Hospital admission rates from the ED were stable until new COVID-19 case rates began to increase locally; the largest relative increase in admission rates was 149.0% in New York, followed by 51.7% in Massachusetts, 36.2% in Connecticut, 29.4% in Colorado, and 22.0% in North Carolina.

Conclusions and relevance: From January through April 2020, as the COVID-19 pandemic intensified in the US, temporal associations were observed with a decrease in ED visits and an increase in hospital admission rates in 5 health care systems in 5 states. These findings suggest that practitioners and public health officials should emphasize the importance of visiting the ED during the COVID-19 pandemic for serious symptoms, illnesses, and injuries that cannot be managed in other settings.

Conflict of interest statement

Conflict of Interest Disclosures: Drs. Jeffery, D'Onofrio, Platts-Mills, Soares, Hoppe, Nath, and Melnick reported receiving grants or contracts from the National Institutes of Health (NIH) during the conduct of the study. No other disclosures were reported.

Comment in

- Learning From the Decrease in US Emergency Department Visits in Response to the Coronavirus Disease 2019 Pandemic.
  Schriger DL. Schriger DL. JAMA Intern Med. 2020 Oct 1;180(10):1334-1335. doi: 10.1001/jamainternmed.2020.3265. JAMA Intern Med. 2020. PMID: 32744611 No abstract available.

- Cited by 139 articles
- 10 references
- 3 figures

Supplementary info

Publication types, MeSH terms, Grant support Expand

Publication types

- Observational Study
- Research Support, N.I.H., Extramural

MeSH terms

- Adult
- Betacoronavirus
- COVID-19
- Coronavirus Infections* / epidemiology

- Coronavirus Infections* / therapy
- Cross-Sectional Studies
- Delivery of Health Care / trends*
- Emergency Service, Hospital* / organization & administration
- Emergency Service, Hospital* / trends
- Female
- Hospitalization / statistics & numerical data*
- Humans
- Infection Control* / methods
- Infection Control* / organization & administration
- Male
- Organizational Innovation
- Pandemics* / prevention & control
- Pandemics* / statistics & numerical data
- Pneumonia, Viral* / epidemiology
- Pneumonia, Viral* / therapy
- Retrospective Studies
- SARS-CoV-2
- United States / epidemiology

Grant support

- K08 DA045933/DA/NIDA NIH HHS/United States
- UH3 DA047003/DA/NIDA NIH HHS/United States
- UL1 TR001863/TR/NCATS NIH HHS/United States
- UL1 TR002489/TR/NCATS NIH HHS/United States

Full text links

FULL TEXT JAMA Internal Medicine Silverchair Information Systems Free PMC article

Proceed to details

Cite

Share

☐ 594

Observational Study

Pediatr Emerg Care

. 2021 Sep 1;37(9):462-465.

doi: 10.1097/PEC.0000000000002468.

Pediatric Ocular Injury Due to Hand Sanitizer Exposure: An Emerging Hazard

Lauren M Wasser [1], Jordanna H Koppel [2], David Zadok [1], Liron Berkowitz [1], Adi Abulafia [1], Eyal Heiman [3], Ahmad Aryan [1], Eduardo Roditi [1], Yishay Weill [1]
Affiliations Expand

Affiliations

- [1] From the Department of Ophthalmology, Shaare Zedek Medical Center, Jerusalem.
- [2] Edmond and Lily Safra Children's Hospital, Chaim Sheba Medical Center, Tel Hashomer.
- [3] Pediatric Emergency Medicine, Shaare Zedek Medical Center, Jerusalem, Israel.

- PMID: **34116551**
- DOI: 10.1097/PEC.0000000000002468

Observational Study

Pediatric Ocular Injury Due to Hand Sanitizer Exposure: An Emerging Hazard

Lauren M Wasser et al. Pediatr Emerg Care. 2021.
Show details
Pediatr Emerg Care
. 2021 Sep 1;37(9):462-465.
doi: 10.1097/PEC.0000000000002468.

Authors

Lauren M Wasser [1], Jordanna H Koppel [2], David Zadok [1], Liron Berkowitz [1], Adi Abulafia [1], Eyal Heiman [3], Ahmad Aryan [1], Eduardo Roditi [1], Yishay Weill [1]

Affiliations

- [1] From the Department of Ophthalmology, Shaare Zedek Medical Center, Jerusalem.
- [2] Edmond and Lily Safra Children's Hospital, Chaim Sheba Medical Center, Tel Hashomer.
- [3] Pediatric Emergency Medicine, Shaare Zedek Medical Center, Jerusalem, Israel.

- PMID: **34116551**
- DOI: 10.1097/PEC.0000000000002468

Abstract

Objectives: The objective of this study was to describe the incidence and severity of ocular exposure to alcohol-based hand rub (ABHR) in children presenting to a tertiary medical center during the severe acute respiratory syndrome coronavirus 2 pandemic.

Methods: A retrospective single-center observational study conducted from February 21, 2020, to October 11, 2020. Subjects 10 years or younger who presented with ABHR-induced ocular injury were included. The same period from 2019 was studied and a comparison was performed between

the 2 years. Outcome measures included the number of subjects with ocular injury due to ABHR, extent of ocular epithelial defects, length of hospitalization and time to resolution.

Results: A total of 9 patients presented to the Pediatric Emergency Department after sustaining ocular chemical injuries from ABHR during this period. Treatment included immediate irrigation followed by topical antibiotics, steroids, and lubrication. Six children were discharged and followed as outpatients with no reported adverse ocular sequelae. Three patients exhibited epithelial defects involving 85% to 100% of the cornea, 30% to 75% of the conjunctiva and required inpatient treatment ranging from 4 to 11 days. All patients experienced a complete resolution of the ocular epithelial defects after 12 to 19 days. No long-term irreversible damage was observed and visual acuity returned to normal in all patients.

Conclusions: The utilization of ABHR during the severe acute respiratory syndrome coronavirus 2 pandemic resulted in childhood ocular injury. Prompt treatment led to good visual outcomes. These products should be regarded as potentially toxic and stored out of the reach of young children.

Copyright © 2021 Wolters Kluwer Health, Inc. All rights reserved.

Conflict of interest statement

Disclosure: The authors declare no conflict of interest.

- Cited by 1 article
- 21 references

Supplementary info

Publication types, MeSH terms, Substances Expand

Publication types

- Observational Study

MeSH terms

- COVID-19*
- Child
- Child, Preschool
- Eye Injuries*
- Hand Sanitizers*
- Humans
- Retrospective Studies
- SARS-CoV-2

Substances

- Hand Sanitizers

Full text links

Wolters Kluwer Wolters Kluwer

Proceed to details

Cite

Share

595

Observational Study

Rev Cardiovasc Med

. 2021 Sep 24;22(3):1063-1072.

doi: 10.31083/j.rcm2203116.

Early combination therapy with hydroxychloroquine and azithromycin reduces mortality in 10,429 COVID-19 outpatients

Matthieu Million [1], Jean-Christophe Lagier [1], Hervé Tissot-Dupont [1], Isabelle Ravaux [1], Catherine Dhiver [1], Christelle Tomei [1], Nadim Cassir [1], Léa Delorme [2], Sébastien Cortaredona [2], Sophie Amrane [2], Camille Aubry [2], Karim Bendamardji [1], Cyril Berenger [2], Barbara Doudier [1], Sophie Edouard [1], Marie Hocquart [2], Morgane Mailhe [1], Coralie Porcheto [1], Piseth Seng [1], Catherine Triquet [1], Stéphanie Gentile [3], Elisabeth Jouve [3], Audrey Giraud-Gatineau [2], Herve Chaudet [2], Laurence Camoin-Jau [4], Philippe Colson [1], Philippe Gautret [2], Pierre-Edouard Fournier [2], Baptiste Maille [5], Jean-Claude Deharo [5], Paul Habert [6], Jean-Yves Gaubert [6], Alexis Jacquier [6], Stéphane Honore [7], Katell Guillon-Lorvellec [1], Yolande Obadia [2], Philippe Parola [2], Philippe Brouqui [1], Didier Raoult [1]

Affiliations Expand

Affiliations

- [1] IHU-Méditerranée Infection, Aix Marseille Univ, Assistance Publique Hôpitaux de Marseille (AP-HM), Institut de recherche pour le développement (IRD), Unité Microbes Evolution Phylogénie et Infections (MEPHI),13005 Marseille, France.
- [2] IHU-Méditerranée Infection, Aix Marseille Univ, Assistance Publique Hôpitaux de Marseille (AP-HM), Institut de recherche pour le développement (IRD), Unité Vecteurs - Infections Tropicales et Méditerranéennes (VITROME), Service de santé des armées (SSA), 13005 Marseille, France.
- [3] EA 3279: CEReSS - Health Service Research and Quality of Life Center, Service d'Evaluation Médicale, Aix Marseille Univ, Assistance Publique Hôpitaux de Marseille, 13005 Marseille, France.
- [4] Laboratoire D'Hématologie, Hôpital de La Timone, Assistance Publique Hôpitaux de Marseille, 13005 Marseille, France.
- [5] Service de Cardiologie, Centre Hospitalier Universitaire La Timone, Assistance Publique Hôpitaux de Marseille, Aix Marseille Univ, C2VN, 13005 Marseille, France.

- [6] Radiology Department, La Timone Hospital, Assistance Publique Des Hôpitaux de Marseille, Aix Marseille Univ, LIIE, CERIMED, 13005 Marseille, France.
- [7] Service de Pharmacie, Hôpital Timone, Laboratoire de Pharmacie Clinique, Aix Marseille Université AP-HM, 13005 Marseille, France.

- PMID: **34565108**
- DOI: 10.31083/j.rcm2203116

Free article
Observational Study

Early combination therapy with hydroxychloroquine and azithromycin reduces mortality in 10,429 COVID-19 outpatients

Matthieu Million et al. Rev Cardiovasc Med. 2021.
Free article
Show details
Rev Cardiovasc Med
. 2021 Sep 24;22(3):1063-1072.
doi: 10.31083/j.rcm2203116.

Authors

Matthieu Million [1], Jean-Christophe Lagier [1], Hervé Tissot-Dupont [1], Isabelle Ravaux [1], Catherine Dhiver [1], Christelle Tomei [1], Nadim Cassir [1], Léa Delorme [2], Sébastien Cortaredona [2], Sophie Amrane [2], Camille Aubry [2], Karim Bendamardji [1], Cyril Berenger [2], Barbara Doudier [1], Sophie Edouard [1], Marie Hocquart [2], Morgane Mailhe [1], Coralie Porcheto [1], Piseth Seng [1], Catherine Triquet [1], Stéphanie Gentile [3], Elisabeth Jouve [3], Audrey Giraud-Gatineau [2], Herve Chaudet [2], Laurence Camoin-Jau [4], Philippe Colson [1], Philippe Gautret [2], Pierre-Edouard Fournier [2], Baptiste Maille [5], Jean-Claude Deharo [5], Paul Habert [6], Jean-Yves Gaubert [6], Alexis Jacquier [6], Stéphane Honore [7], Katell Guillon-Lorvellec [1], Yolande Obadia [2], Philippe Parola [2], Philippe Brouqui [1], Didier Raoult [1]

Affiliations

- [1] IHU-Méditerranée Infection, Aix Marseille Univ, Assistance Publique Hôpitaux de Marseille (AP-HM), Institut de recherche pour le développement (IRD), Unité Microbes Evolution Phylogénie et Infections (MEPHI),13005 Marseille, France.
- [2] IHU-Méditerranée Infection, Aix Marseille Univ, Assistance Publique Hôpitaux de Marseille (AP-HM), Institut de recherche pour le développement (IRD), Unité Vecteurs - Infections Tropicales et Méditerranéennes (VITROME), Service de santé des armées (SSA), 13005 Marseille, France.

- [3] EA 3279: CEReSS - Health Service Research and Quality of Life Center, Service d'Evaluation Médicale, Aix Marseille Univ, Assistance Publique Hôpitaux de Marseille, 13005 Marseille, France.
- [4] Laboratoire D'Hématologie, Hôpital de La Timone, Assistance Publique Hôpitaux de Marseille, 13005 Marseille, France.
- [5] Service de Cardiologie, Centre Hospitalier Universitaire La Timone, Assistance Publique Hôpitaux de Marseille, Aix Marseille Univ, C2VN, 13005 Marseille, France.
- [6] Radiology Department, La Timone Hospital, Assistance Publique Des Hôpitaux de Marseille, Aix Marseille Univ, LIIE, CERIMED, 13005 Marseille, France.
- [7] Service de Pharmacie, Hôpital Timone, Laboratoire de Pharmacie Clinique, Aix Marseille Université AP-HM, 13005 Marseille, France.

- PMID: **34565108**
- DOI: 10.31083/j.rcm2203116

Abstract

We evaluated the age-specific mortality of unselected adult outpatients infected with SARS-CoV-2 treated early in a dedicated COVID-19 day hospital and we assessed whether the use of hydroxychloroquine (HCQ) + azithromycin (AZ) was associated with improved survival in this cohort. A retrospective monocentric cohort study was conducted in the day hospital of our center from March to December 2020 in adults with PCR-proven infection who were treated as outpatients with a standardized protocol. The primary endpoint was 6-week mortality, and secondary endpoints were transfer to the intensive care unit and hospitalization rate. Among 10,429 patients (median age, 45 [IQR 32-57] years; 5597 [53.7%] women), 16 died (0.15%). The infection fatality rate was 0.06% among the 8315 patients treated with HCQ+AZ. No deaths occurred among the 8414 patients younger than 60 years. Older age and male sex were associated with a higher risk of death, ICU transfer, and hospitalization. Treatment with HCQ+AZ (0.17 [0.06-0.48]) was associated with a lower risk of death, independently of age, sex and epidemic period. Meta-analysis evidenced consistency with 4 previous outpatient studies (32,124 patients-Odds ratio 0.31 [0.20-0.47], $I^2 = 0\%$). Early ambulatory treatment of COVID-19 with HCQ+AZ as a standard of care is associated with very low mortality, and HCQ+AZ improve COVID-19 survival compared to other regimens.

Keywords: Ambulatory; Azithromycin; COVID-19; Hydroxychloroquine; Outpatients; SARS-CoV-2; Treatment.

© 2021 The Author(s). Published by IMR Press.

Conflict of interest statement

The authors declare no conflict of interest. The funders/sponsors had no role in the design and conduct of the study; collection, management, analysis, and interpretation of the data; preparation, review, or approval of the manuscript; or decision to submit the manuscript for publication. Funding sources had no role in the design and conduct of the study; collection, management, analysis, and interpretation of the data; and preparation, review, or approval of the manuscript. Our group used widely available generic drugs distributed by many pharmaceutical companies.

Comment in

- Role of hydroxychloroquine in multidrug treatment of COVID-19.

McCullough PA, Stricker RB, Risch HA. McCullough PA, et al. Rev Cardiovasc Med. 2021 Sep 24;22(3):545-546. doi: 10.31083/j.rcm2203063. Rev Cardiovasc Med. 2021. PMID: 34565055

- Cited by 3 articles

Supplementary info

Publication types, MeSH terms, Substances, Supplementary concepts Expand

Publication types

- Observational Study

MeSH terms

- Adolescent
- Adult
- Age Factors
- Aged
- Aged, 80 and over
- Ambulatory Care*
- Antiviral Agents / adverse effects
- Antiviral Agents / therapeutic use*
- Azithromycin / adverse effects
- Azithromycin / therapeutic use*
- COVID-19 / diagnosis
- COVID-19 / drug therapy*
- COVID-19 / mortality
- Drug Therapy, Combination
- Early Medical Intervention*
- Female
- France
- Hospitalization
- Humans
- Hydroxychloroquine / adverse effects
- Hydroxychloroquine / therapeutic use*
- Male
- Middle Aged
- Outpatients
- Retrospective Studies
- Risk Assessment
- Risk Factors

- Sex Factors
- Time Factors
- Treatment Outcome
- Young Adult

Substances

- Antiviral Agents
- Hydroxychloroquine
- Azithromycin

Supplementary concepts

- COVID-19 drug treatment

Full text links

IMR Press Free Full Text Online IMR Press

Proceed to details

Cite

Share

596

Observational Study

Am J Hypertens

. 2020 Dec 31;33(12):1102-1111.

doi: 10.1093/ajh/hpaa149.

Effects of Angiotensin Receptor Blockers (ARBs) on In-Hospital Outcomes of Patients With Hypertension and Confirmed or Clinically Suspected COVID-19

Abbas Soleimani [1], Sina Kazemian [2], Shahrokh Karbalai Saleh [1], Arya Aminorroaya [3] [4], Zahra Shajari [1], Azar Hadadi [5], Mohammad Talebpour [6], Hakimeh Sadeghian [7], Pooya Payandemehr [8], Mehran Sotoodehnia [8], Maryam Bahreini [8], Farhad Najmeddin [9], Ali Heidarzadeh [2], Ensieh Zivari [10], Haleh Ashraf [10] [11]

Affiliations Expand

Affiliations

- [1] Department of Cardiology, Sina Hospital, Tehran University of Medical Sciences, Tehran, Iran.

- [2] Students' Scientific Research Center (SSRC), Tehran University of Medical Sciences, Tehran, Iran.
- [3] Non-Communicable Diseases Research Center, Endocrinology and Metabolism Population Sciences Institute, Tehran University of Medical Sciences, Tehran, Iran.
- [4] Department of cardiology, Tehran Heart Center, Tehran University of Medical Sciences, Tehran, Iran.
- [5] Department of Infectious Diseases, Sina Hospital, Tehran University of Medical Sciences, Tehran, Iran.
- [6] Department of Surgery, Sina Hospital, Tehran University of Medical Sciences, Tehran, Iran.
- [7] Department of Cardiology, Shariati Hospital, Tehran University of Medical Sciences, Tehran, Iran.
- [8] Department of Emergency Medicine, Sina Hospital, Tehran University of Medical Sciences, Tehran, Iran.
- [9] Department of Clinical Pharmacy, Faculty of Pharmacy, Tehran University of Medical Sciences, Tehran, Iran.
- [10] Research Development Center, Sina Hospital, Tehran University of Medical Sciences, Tehran, Iran.
- [11] Cardiac Primary Prevention Research Center (CPPRC), Cardiovascular Diseases Research Institute, Tehran University of Medical Sciences, Tehran, Iran.

- PMID: **32920644**
- PMCID: PMC7543264
- DOI: 10.1093/ajh/hpaa149

Free PMC article
Observational Study

Effects of Angiotensin Receptor Blockers (ARBs) on In-Hospital Outcomes of Patients With Hypertension and Confirmed or Clinically Suspected COVID-19

Abbas Soleimani et al. Am J Hypertens. 2020.
Free PMC article
Show details
Am J Hypertens
. 2020 Dec 31;33(12):1102-1111.
doi: 10.1093/ajh/hpaa149.

Authors

Abbas Soleimani [1], Sina Kazemian [2], Shahrokh Karbalai Saleh [1], Arya Aminorroaya [3] [4], Zahra Shajari [1], Azar Hadadi [5], Mohammad Talebpour [6], Hakimeh Sadeghian [7], Pooya Payandemehr [8], Mehran Sotoodehnia [8], Maryam Bahreini [8], Farhad Najmeddin [9], Ali Heidarzadeh [2], Ensieh Zivari [10], Haleh Ashraf [10] [11]

Affiliations

- [1] Department of Cardiology, Sina Hospital, Tehran University of Medical Sciences, Tehran, Iran.
- [2] Students' Scientific Research Center (SSRC), Tehran University of Medical Sciences, Tehran, Iran.
- [3] Non-Communicable Diseases Research Center, Endocrinology and Metabolism Population Sciences Institute, Tehran University of Medical Sciences, Tehran, Iran.
- [4] Department of cardiology, Tehran Heart Center, Tehran University of Medical Sciences, Tehran, Iran.
- [5] Department of Infectious Diseases, Sina Hospital, Tehran University of Medical Sciences, Tehran, Iran.
- [6] Department of Surgery, Sina Hospital, Tehran University of Medical Sciences, Tehran, Iran.
- [7] Department of Cardiology, Shariati Hospital, Tehran University of Medical Sciences, Tehran, Iran.
- [8] Department of Emergency Medicine, Sina Hospital, Tehran University of Medical Sciences, Tehran, Iran.
- [9] Department of Clinical Pharmacy, Faculty of Pharmacy, Tehran University of Medical Sciences, Tehran, Iran.
- [10] Research Development Center, Sina Hospital, Tehran University of Medical Sciences, Tehran, Iran.
- [11] Cardiac Primary Prevention Research Center (CPPRC), Cardiovascular Diseases Research Institute, Tehran University of Medical Sciences, Tehran, Iran.

- PMID: **32920644**
- PMCID: PMC7543264
- DOI: 10.1093/ajh/hpaa149

Abstract

Background: There is an ongoing controversy about harms and benefits of angiotensin-converting enzyme inhibitors (ACEIs) and angiotensin II receptor blockers (ARBs) in hypertensive patients with coronavirus disease 2019 (COVID-19). Given the unresolved debate, we investigated the association of ARBs with in-hospital outcomes of these patients.

Methods: In this retrospective observational study, we studied patients with COVID-19 who referred to Sina Hospital in Tehran, Iran, from 20 February to 29 May 2020. Patients with either positive real-time reverse-transcriptase polymerase-chain-reaction test of swab specimens, or high clinical suspicion according to the World Health Organization's interim guidance were included. We followed-up patients for incurring death, severe COVID-19, and in-hospital complications.

Results: We evaluated 681 patients with COVID-19 of whom 37 patients were excluded due to incomplete medical records and 8 patients who used ACEIs which left 636 patients in the analysis. In this cohort, 108 (17.0%) patients expired and 407 (64.0%) patients incurred severe COVID-19. Of 254 (39.9%) patients with hypertension, 122 (48.0%) patients were receiving an ARB. After adjustment for possible confounders, we found no independent association between taking ARBs and in-hospital outcomes except for acute kidney injury (AKI), in patients with confirmed or clinically suspected COVID-19, either hypertensive or not-hypertensive. We found that discontinuation of ARBs during hospitalization was associated with a greater risk of mortality, invasive ventilation, and AKI (all $P < 0.002$).

Conclusions: We found that taking ARBs by patients with hypertension and confirmed or clinically suspected COVID-19 is not associated with poorer in-hospital outcomes after adjustment for possible confounders.

Keywords: COVID-19; SARS-CoV-2; angiotensin receptor antagonists; angiotensin-converting enzyme inhibitors; blood pressure; hypertension; renin–angiotensin system.

© American Journal of Hypertension, Ltd 2020. All rights reserved. For Permissions, please email: journals.permissions@oup.com.

- Cited by 20 articles

Supplementary info

Publication types, MeSH terms, Substances Expand

Publication types

- Observational Study
- Research Support, Non-U.S. Gov't

MeSH terms

- Acute Kidney Injury / mortality
- Aged
- Angiotensin Receptor Antagonists / adverse effects
- Angiotensin Receptor Antagonists / therapeutic use*
- Antihypertensive Agents / adverse effects
- Antihypertensive Agents / therapeutic use*
- COVID-19 / diagnosis
- COVID-19 / mortality
- COVID-19 / therapy*
- Female
- Hospital Mortality
- Hospitalization
- Humans
- Hypertension / diagnosis
- Hypertension / drug therapy*
- Hypertension / mortality
- Iran
- Male
- Middle Aged
- Retrospective Studies
- Risk Assessment
- Risk Factors

- Time Factors
- Treatment Outcome

Substances

- Angiotensin Receptor Antagonists
- Antihypertensive Agents

Full text links

OXFORD ACADEMIC Silverchair Information Systems Free PMC article
Proceed to details
Cite
Share
597
Observational Study
BMJ Open
. 2020 Oct 26;10(10):e040441.
doi: 10.1136/bmjopen-2020-040441.

Hospitalised COVID-19 patients of the Mount Sinai Health System: a retrospective observational study using the electronic medical records

Zichen Wang [1], Amanda Zheutlin [1], Yu-Han Kao [1], Kristin Ayers [1], Susan Gross [1, 2], Patricia Kovatch [3], Sharon Nirenberg [3], Alexander Charney [2, 4, 5], Girish Nadkarni [6, 7, 8], Jessica K De Freitas [2, 6], Paul O'Reilly [2, 4, 5], Allan Just [9, 10], Carol Horowitz [7, 10], Glenn Martin [5], Andrea Branch [7], Benjamin S Glicksberg [2, 4, 6], Dennis Charney [11], David Reich [12], William K Oh [13], Eric Schadt [1, 2], Rong Chen [1, 2], Li Li [14, 2]
Affiliations Expand

Affiliations

- [1] Sema4, Stamford, Connecticut, USA.
- [2] Department of Genetics and Genomic Sciences, The Icahn Institute for Genomics and Multiscale Biology, Icahn School of Medicine at Mount Sinai, New York, New York, USA.
- [3] Mount Sinai Data Warehouse, Icahn School of Medicine at Mount Sinai, New York, New York, USA.
- [4] The Pamela Sklar Division of Psychiatric Genomics, Icahn School of Medicine at Mount Sinai, New York, New York, USA.
- [5] Department of Psychiatry, Icahn School of Medicine at Mount Sinai, New York, New York, USA.

- [6] The Hasso Plattner Institute for Digital Health at the Mount Sinai, Icahn School of Medicine at Mount Sinai, New York, New York, USA.
- [7] Department of Medicine, Icahn School of Medicine at Mount Sinai, New York, New York, USA.
- [8] The Charles Bronfman Institute for Personalized Medicine, Icahn School of Medicine at Mount Sinai, New York, New York, USA.
- [9] Institute for Exposomic Research, Icahn School of Medicine at Mount Sinai, New York, New York, USA.
- [10] Department of Environmental Medicine and Public Health, Icahn School of Medicine at Mount Sinai, New York, New York, USA.
- [11] The Office of the Dean, Icahn School of Medicine at Mount Sinai, New York, New York, USA.
- [12] Department of Anesthesiology, Perioperative and Pain Medicine, Icahn School of Medicine at Mount Sinai, New York, New York, USA.
- [13] Tisch Cancer Institute and Division of Hematology and Medical Oncology, Icahn School of Medicine at Mount Sinai, New York, New York, USA.
- [14] Sema4, Stamford, Connecticut, USA li.li@mssm.edu.

- PMID: **33109676**
- PMCID: PMC7592304
- DOI: 10.1136/bmjopen-2020-040441

Free PMC article
Observational Study

Hospitalised COVID-19 patients of the Mount Sinai Health System: a retrospective observational study using the electronic medical records

Zichen Wang et al. BMJ Open. 2020.
Free PMC article
Show details
BMJ Open
. 2020 Oct 26;10(10):e040441.
doi: 10.1136/bmjopen-2020-040441.

Authors

Zichen Wang [1], Amanda Zheutlin [1], Yu-Han Kao [1], Kristin Ayers [1], Susan Gross [1 2], Patricia Kovatch [3], Sharon Nirenberg [3], Alexander Charney [2 4 5], Girish Nadkarni [6 7 8], Jessica K De Freitas [2 6], Paul O'Reilly [2 4 5], Allan Just [9 10], Carol Horowitz [7 10], Glenn Martin [5], Andrea Branch [7], Benjamin S Glicksberg [2 4 6], Dennis Charney [11], David Reich [12], William K Oh [13], Eric Schadt [1 2], Rong Chen [1 2], Li Li [14 2]

Affiliations

- [1] Sema4, Stamford, Connecticut, USA.
- [2] Department of Genetics and Genomic Sciences, The Icahn Institute for Genomics and Multiscale Biology, Icahn School of Medicine at Mount Sinai, New York, New York, USA.
- [3] Mount Sinai Data Warehouse, Icahn School of Medicine at Mount Sinai, New York, New York, USA.
- [4] The Pamela Sklar Division of Psychiatric Genomics, Icahn School of Medicine at Mount Sinai, New York, New York, USA.
- [5] Department of Psychiatry, Icahn School of Medicine at Mount Sinai, New York, New York, USA.
- [6] The Hasso Plattner Institute for Digital Health at the Mount Sinai, Icahn School of Medicine at Mount Sinai, New York, New York, USA.
- [7] Department of Medicine, Icahn School of Medicine at Mount Sinai, New York, New York, USA.
- [8] The Charles Bronfman Institute for Personalized Medicine, Icahn School of Medicine at Mount Sinai, New York, New York, USA.
- [9] Institute for Exposomic Research, Icahn School of Medicine at Mount Sinai, New York, New York, USA.
- [10] Department of Environmental Medicine and Public Health, Icahn School of Medicine at Mount Sinai, New York, New York, USA.
- [11] The Office of the Dean, Icahn School of Medicine at Mount Sinai, New York, New York, USA.
- [12] Department of Anesthesiology, Perioperative and Pain Medicine, Icahn School of Medicine at Mount Sinai, New York, New York, USA.
- [13] Tisch Cancer Institute and Division of Hematology and Medical Oncology, Icahn School of Medicine at Mount Sinai, New York, New York, USA.
- [14] Sema4, Stamford, Connecticut, USA li.li@mssm.edu.

- PMID: **33109676**
- PMCID: PMC7592304
- DOI: 10.1136/bmjopen-2020-040441

Abstract

Objective: To assess association of clinical features on COVID-19 patient outcomes.

Design: Retrospective observational study using electronic medical record data.

Setting: Five member hospitals from the Mount Sinai Health System in New York City (NYC).

Participants: 28 336 patients tested for SARS-CoV-2 from 24 February 2020 to 15 April 2020, including 6158 laboratory-confirmed COVID-19 cases.

Main outcomes and measures: Positive test rates and in-hospital mortality were assessed for different racial groups. Among positive cases admitted to the hospital (N=3273), we estimated HR for both discharge and death across various explanatory variables, including patient demographics, hospital site and unit, smoking status, vital signs, lab results and comorbidities.

Results: Hispanics (29%) and African Americans (25%) had disproportionately high positive case rates relative to their representation in the overall NYC population ($p<0.05$); however, no

differences in mortality rates were observed in hospitalised patients based on race. Outcomes differed significantly between hospitals (Gray's T=248.9; p<0.05), reflecting differences in average baseline age and underlying comorbidities. Significant risk factors for mortality included age (HR 1.05, 95% CI 1.04 to 1.06; p=1.15e-32), oxygen saturation (HR 0.985, 95% CI 0.982 to 0.988; p=1.57e-17), care in intensive care unit areas (HR 1.58, 95% CI 1.29 to 1.92; p=7.81e-6) and elevated creatinine (HR 1.75, 95% CI 1.47 to 2.10; p=7.48e-10), white cell count (HR 1.02, 95% CI 1.01 to 1.04; p=8.4e-3) and body mass index (BMI) (HR 1.02, 95% CI 1.00 to 1.03; p=1.09e-2). Deceased patients were more likely to have elevated markers of inflammation.

Conclusions: While race was associated with higher risk of infection, we did not find racial disparities in inpatient mortality suggesting that outcomes in a single tertiary care health system are comparable across races. In addition, we identified key clinical features associated with reduced mortality and discharge. These findings could help to identify which COVID-19 patients are at greatest risk of a severe infection response and predict survival.

Keywords: COVID-19; epidemiology; health informatics; infectious diseases.

© Author(s) (or their employer(s)) 2020. Re-use permitted under CC BY-NC. No commercial re-use. See rights and permissions. Published by BMJ.

Conflict of interest statement

Competing interests: WKO is a paid consultant to Astellas, Astra Zeneca, Bayer, Janssen, Sanofi, Sema4, and TeneoBio.

- Cited by 16 articles
- 26 references
- 5 figures

Supplementary info

Publication types, MeSH terms Expand

Publication types

- Observational Study

MeSH terms

- Age Factors
- Betacoronavirus / isolation & purification*
- COVID-19
- COVID-19 Testing
- Clinical Laboratory Techniques / statistics & numerical data
- Comorbidity
- Coronavirus Infections* / diagnosis
- Coronavirus Infections* / epidemiology
- Coronavirus Infections* / therapy

- Electronic Health Records / statistics & numerical data
- Ethnicity
- Female
- Hospital Mortality
- Hospitalization / statistics & numerical data*
- Humans
- Intensive Care Units / statistics & numerical data*
- Male
- Middle Aged
- Mortality
- New York City / epidemiology
- Pandemics*
- Pneumonia, Viral* / epidemiology
- Pneumonia, Viral* / therapy
- Retrospective Studies
- Risk Factors
- SARS-CoV-2

Full text links

BMJ Free Full Text HighWire Free PMC article
Proceed to details
Cite
Share
598
Observational Study
J Diabetes Complications
. 2020 Oct;34(10):107666.
doi: 10.1016/j.jdiacomp.2020.107666. Epub 2020 Jun 29.

Clinical analysis of risk factors for severe COVID-19 patients with type 2 diabetes

Qianhui Zhang [1], Yanhong Wei [1], Min Chen [1], Qianqian Wan [1], Xiaoqi Chen [2]
Affiliations Expand

Affiliations

- [1] Department of Rheumatology and Immunology, Zhongnan Hospital of Wuhan University, Wuhan, China.
- [2] Department of Rheumatology and Immunology, Zhongnan Hospital of Wuhan University, Wuhan, China. Electronic address: cxqznhospital@163.com.

- PMID: **32636061**
- PMCID: PMC7323648
- DOI: 10.1016/j.jdiacomp.2020.107666

Free PMC article
Observational Study

Clinical analysis of risk factors for severe COVID-19 patients with type 2 diabetes

Qianhui Zhang et al. J Diabetes Complications. 2020 Oct.
Free PMC article
Show details
J Diabetes Complications
. 2020 Oct;34(10):107666.
doi: 10.1016/j.jdiacomp.2020.107666. Epub 2020 Jun 29.

Authors

Qianhui Zhang [1], Yanhong Wei [1], Min Chen [1], Qianqian Wan [1], Xiaoqi Chen [2]

Affiliations

- [1] Department of Rheumatology and Immunology, Zhongnan Hospital of Wuhan University, Wuhan, China.
- [2] Department of Rheumatology and Immunology, Zhongnan Hospital of Wuhan University, Wuhan, China. Electronic address: cxqznhospital@163.com.

- PMID: **32636061**
- PMCID: PMC7323648
- DOI: 10.1016/j.jdiacomp.2020.107666

Abstract

Aims: To describe characteristics of COVID-19 patients with type 2 diabetes and to analyze risk factors for severity.

Methods: Demographics, comorbidities, symptoms, laboratory findings, treatments and outcomes of COVID-19 patients with diabetes were collected and analyzed.

Results: Seventy-fourCOVID-19 patients with diabetes were included. Twenty-seven patients (36.5%) were severe and 10 patients (13.5%) died. Higher levels of blood glucose, serum amyloid A (SAA), C reactive protein and interleukin 6 were associated with severe patients compared to non-severe ones ($P<0.05$). Levels of albumin, cholesterol, high density lipoprotein, small and dense low density lipoprotein and $CD4^{+}$T lymphocyte counts in severe patients were lower than those in non-severe patients ($P<0.05$). Logistic regression analysis identified decreased $CD4^{+}$T lymphocyte counts (odds ratio [OR]=0.988, 95%Confidence interval [95%CI] 0.979-0.997) and increased SAA levels (OR=1.029, 95%CI 1.002-1.058) as risk factors for severity of COVID-19 with diabetes ($P<0.05$).

Conclusions: Type 2 diabetic patients were more susceptible to COVID-19 than overall population, which might be associated with hyperglycemia and dyslipidemia. Aggressive treatment should be suggested, especially when these patients had low $CD4^{+}T$ lymphocyte counts and high SAA levels.

Keywords: CD4(+)T lymphocyte; COVID-19; Diabetes; Dislipidemia; Hyperglycemia.

Copyright © 2020 Elsevier Inc. All rights reserved.

- Cited by 30 articles
- 19 references

Supplementary info

Publication types, MeSH terms Expand

Publication types

- Observational Study

MeSH terms

- Aged
- Aged, 80 and over
- Betacoronavirus*
- COVID-19
- China
- Coronavirus Infections / complications*
- Coronavirus Infections / diagnosis
- Coronavirus Infections / epidemiology
- Diabetes Mellitus, Type 2 / complications*
- Female
- Hospitalization
- Humans
- Male
- Middle Aged
- Pandemics
- Pneumonia, Viral / complications*
- Pneumonia, Viral / diagnosis
- Pneumonia, Viral / epidemiology
- Prognosis
- Retrospective Studies
- Risk Factors
- SARS-CoV-2
- Severity of Illness Index

- Survival Rate
- Symptom Assessment

Full text links

ELSEVIER FULL-TEXT ARTICLE Elsevier Science Free PMC article
Proceed to details
Cite
Share
599
Observational Study
J Korean Med Sci
. 2020 Jun 15;35(23):e209.
doi: 10.3346/jkms.2020.35.e209.

Prognostic Factors for Severe Coronavirus Disease 2019 in Daegu, Korea

Jong Geol Jang [1], Jian Hur [2], Eun Young Choi [1], Kyung Soo Hong [1], Wonhwa Lee [3], June Hong Ahn [4]
Affiliations Expand

Affiliations

- [1] Division of Pulmonology and Allergy, Department of Internal Medicine, Regional Center for Respiratory Diseases, Yeungnam University Medical Center, College of Medicine, Yeungnam University, Daegu, Korea.
- [2] Division of Infection, Department of Internal Medicine, Yeungnam University Medical Center, College of Medicine, Yeungnam University, Daegu, Korea.
- [3] Aging Research Center, Korea Research Institute of Bioscience and Biotechnology, Daejeon, Korea. wonhwalee@kribb.re.kr.
- [4] Division of Pulmonology and Allergy, Department of Internal Medicine, Regional Center for Respiratory Diseases, Yeungnam University Medical Center, College of Medicine, Yeungnam University, Daegu, Korea. fireajh@gmail.com.

- PMID: **32537954**
- PMCID: PMC7295599
- DOI: 10.3346/jkms.2020.35.e209

Free PMC article
Observational Study

Prognostic Factors for Severe Coronavirus Disease 2019 in Daegu, Korea

Jong Geol Jang et al. J Korean Med Sci. 2020.
Free PMC article
Show details
J Korean Med Sci
. 2020 Jun 15;35(23):e209.
doi: 10.3346/jkms.2020.35.e209.

Authors

Jong Geol Jang [1], Jian Hur [2], Eun Young Choi [1], Kyung Soo Hong [1], Wonhwa Lee [3], June Hong Ahn [4]

Affiliations

- [1] Division of Pulmonology and Allergy, Department of Internal Medicine, Regional Center for Respiratory Diseases, Yeungnam University Medical Center, College of Medicine, Yeungnam University, Daegu, Korea.
- [2] Division of Infection, Department of Internal Medicine, Yeungnam University Medical Center, College of Medicine, Yeungnam University, Daegu, Korea.
- [3] Aging Research Center, Korea Research Institute of Bioscience and Biotechnology, Daejeon, Korea. wonhwalee@kribb.re.kr.
- [4] Division of Pulmonology and Allergy, Department of Internal Medicine, Regional Center for Respiratory Diseases, Yeungnam University Medical Center, College of Medicine, Yeungnam University, Daegu, Korea. fireajh@gmail.com.

- PMID: **32537954**
- PMCID: PMC7295599
- DOI: 10.3346/jkms.2020.35.e209

Abstract

Background: Since its first detection in December 2019, coronavirus disease 2019 (COVID-19) caused by severe acute respiratory syndrome coronavirus 2 infection has spread rapidly around the world. Although there have been several studies investigating prognostic factors for severe COVID-19, there have been no such studies in Korea.

Methods: We performed a retrospective observational study of 110 patients with confirmed COVID-19 hospitalized at a tertiary hospital in Daegu, Korea. Demographic, clinical, laboratory, and outcome data were collected and analyzed. Severe disease was defined as a composite outcome of acute respiratory distress syndrome, intensive care unit care, or death.

Results: Diabetes mellitus (odds ratio [OR], 19.15; 95% confidence interval [CI], 1.90-193.42; $P = 0.012$), body temperature ≥ 37.8°C (OR, 10.91; 95% CI, 1.35-88.36; $P = 0.025$), peripheral oxygen saturation < 92% (OR, 33.31; 95% CI, 2.45-452.22; $P = 0.008$), and creatine kinase-MB (CK-MB) > 6.3 (OR, 56.84; 95% CI, 2.64-1,223.78, $P = 0.010$) at admission were associated with higher risk of severe COVID-19. The likelihood of development of severe COVID-19 increased with an increasing number of prognostic factors.

Conclusion: In conclusion, we found that diabetes mellitus, body temperature ≥ 37.8°C, peripheral oxygen saturation < 92%, and CK-MB > 6.3 are independent predictors of severe disease in hospitalized COVID-19 patients. Appropriate assessment of prognostic factors and

close monitoring to provide the necessary interventions at the appropriate time in high-risk patients may reduce the case fatality rate of COVID-19.

Keywords: COVID-19; Korea; Prognostic Factor; Severe Disease.

© 2020 The Korean Academy of Medical Sciences.

Conflict of interest statement

The authors have no potential conflicts of interest to disclose.

- Cited by 36 articles
- 23 references
- 3 figures

Supplementary info

Publication types, MeSH terms, Grant support Expand

Publication types

- Observational Study

MeSH terms

- Adolescent
- Adult
- Aged
- Betacoronavirus
- COVID-19
- Child
- Child, Preschool
- Coronavirus Infections / pathology*
- Diabetes Complications / virology*
- Diabetes Mellitus / pathology*
- Female
- Fever / pathology*
- Hospitalization
- Humans
- Hypoxia / pathology*
- Infant
- Infant, Newborn
- Intensive Care Units
- Male
- Middle Aged

- Pandemics
- Pneumonia, Viral / pathology*
- Prognosis
- Republic of Korea
- Respiratory Distress Syndrome / etiology
- Retrospective Studies
- Risk Factors
- SARS-CoV-2
- Young Adult

Grant support

- Daegu Medical Association COVID-19 Scientific Committee/Korea

Full text links

Korean Academy of Medical Sciences Free PMC article
Proceed to details
Cite
Share
600
Observational Study
Acta Reumatol Port
. Jul-Sep 2021;46(3):252-256.

What happened to hip fragility fractures during COVID-19 pandemic?

Carolina Mazeda [1], Pedro Bernardo Santos [1], Paulo Vilas-Boas [1], Joana Antão [2], Anabela Barcelos [1]
Affiliations Expand

Affiliations

- [1] Serviço de Reumatologia - Centro Hospitalar Baixo Vouga.
- [2] Universidade de Aveiro.

- PMID: **34628458**

Free article
Observational Study

What happened to hip fragility fractures during COVID-19 pandemic?

Carolina Mazeda et al. Acta Reumatol Port. Jul-Sep 2021.
Free article
Show details
Acta Reumatol Port
. Jul-Sep 2021;46(3):252-256.

Authors

Carolina Mazeda [1], Pedro Bernardo Santos [1], Paulo Vilas-Boas [1], Joana Antão [2], Anabela Barcelos [1]

Affiliations

- [1] Serviço de Reumatologia - Centro Hospitalar Baixo Vouga.
- [2] Universidade de Aveiro.

- PMID: **34628458**

Abstract

Purpose: COVID-19 changed the dynamics of all healthcare system, leading to the restructuring of inpatient teams as well as the emergency department. Scheduled surgeries were suspended, operating rooms were closed, and anesthesiologists redistributed among the various intensive care units. At the Centro Hospitalar do Baixo Vouga the number of patients admitted to the emergency department decreased to approximately 8.000 during the period of lockdown which ranged from 18th March to 1st June 2020. The aim of this study was to compare the number of patients presenting with hip fractures during the first wave of the COVID-19 pandemic with the equivalent period in 2019 and to analyze postoperative outcomes.

Methods: An observational retrospective study was conducted in two different periods. Patients over the age of 50 years admitted with hip fracture were included for analysis. The data was collected from the hospital database. A general descriptive analysis was performed.

Results: There was an overall reduction in the number of admissions due to hip fractures in Period 2020 compared with homologous Period in 2019 (68 patients and 94 patients, respectively). No statistically significant differences could be found regarding age, gender, ASA grade and pre-admission residence among patients admitted during these both periods. Nursing home patients in Period 2020 had a longer hospital stay (p=0.03), independently of the functional status (p=0.07). There were no statistically significant differences in the time it took the patient to go to the emergency department after the fall, place where the fracture had occurred, waiting time to perform the surgery, type of treatment performed, post-surgical complications and mortality. There was no relationship between mortality and the time it took the patient to access the emergency department (p=0,487), or mortality and the mean length of stay in the hospital (p=0,151). All the patients admitted to the emergency department in Period 2020 were negative to PCR test for SARS-CoV-2.

Conclusion: The measures taken by the hospital during the pandemic had no impact in the healthcare provided to the admitted patients. This should be taken into account in order to optimize the efficiency of the health care system in future outbreaks.

Supplementary info

Publication types, MeSH terms Expand

Publication types

- Observational Study

MeSH terms

- COVID-19*
- Communicable Disease Control
- Hip Fractures* / epidemiology
- Hip Fractures* / surgery
- Humans
- Middle Aged
- Pandemics
- Retrospective Studies
- SARS-CoV-2

Full text links

Sociedade Portuguesa de Reumatologia
Proceed to details
Cite
Share
1,388 results
Show more results
[x]
Cite
Copy
Download .nbib
Format: NLM
[x]
Share

-
-

Permalink
Copy

first first First | previous previous Prev

Page

3

of 7

Next next next | Last last last

Send To

- Clipboard
- Email
- Save
- My Bibliography
- Collections
- Citation Manager

[x]

- Article type
- Species
- Language
- Sex
- Journal
- Age

- ☐ Address
- ☐ Autobiography
- ☐ Bibliography
- ☐ Biography
- ☐ Case Reports
- ☐ Classical Article
- ☐ Clinical Conference
- ☐ Clinical Study
- ☐ Clinical Trial Protocol
- ☐ Clinical Trial, Phase I
- ☐ Clinical Trial, Phase II
- ☐ Clinical Trial, Phase III
- ☐ Clinical Trial, Phase IV
- ☐ Clinical Trial, Veterinary
- ☐ Comment
- ☐ Comparative Study
- ☐ Congress
- ☐ Consensus Development Conference
- ☐ Consensus Development Conference, NIH
- ☐ Controlled Clinical Trial
- ☐ Corrected and Republished Article
- ☐ Dataset
- ☐ Dictionary
- ☐ Directory
- ☐ Duplicate Publication

- ☐ Editorial
- ☐ Electronic Supplementary Materials
- ☐ English Abstract
- ☐ Evaluation Study
- ☐ Festschrift
- ☐ Government Publication
- ☐ Guideline
- ☐ Historical Article
- ☐ Interactive Tutorial
- ☐ Interview
- ☐ Introductory Journal Article
- ☐ Lecture
- ☐ Legal Case
- ☐ Legislation
- ☐ Letter
- ☐ Multicenter Study
- ☐ News
- ☐ Newspaper Article
- ☐ Observational Study
- ☐ Observational Study, Veterinary
- ☐ Overall
- ☐ Patient Education Handout
- ☐ Periodical Index
- ☐ Personal Narrative
- ☐ Portrait
- ☐ Practice Guideline
- ☐ Pragmatic Clinical Trial
- ☐ Preprint
- ☐ Published Erratum
- ☐ Research Support, American Recovery and Reinvestment Act
- ☐ Research Support, N.I.H., Extramural
- ☐ Research Support, N.I.H., Intramural
- ☐ Research Support, Non-U.S. Gov't
- ☐ Research Support, U.S. Gov't, Non-P.H.S.
- ☐ Research Support, U.S. Gov't, P.H.S.
- ☐ Research Support, U.S. Gov't
- ☐ Retracted Publication
- ☐ Retraction of Publication
- ☐ Scientific Integrity Review
- ☐ Technical Report
- ☐ Twin Study
- ☐ Validation Study
- ☐ Video-Audio Media
- ☐ Webcast

- ☐ Humans
- ☐ Other Animals

- ☐ Afrikaans

- ☐ Albanian
- ☐ Arabic
- ☐ Armenian
- ☐ Azerbaijani
- ☐ Bosnian
- ☐ Bulgarian
- ☐ Catalan
- ☐ Chinese
- ☐ Croatian
- ☐ Czech
- ☐ Danish
- ☐ Dutch
- ☐ English
- ☐ Esperanto
- ☐ Estonian
- ☐ Finnish
- ☐ French
- ☐ Georgian
- ☐ German
- ☐ Greek, Modern
- ☐ Hebrew
- ☐ Hindi
- ☐ Hungarian
- ☐ Icelandic
- ☐ Indonesian
- ☐ Italian
- ☐ Japanese
- ☐ Kinyarwanda
- ☐ Korean
- ☐ Latin
- ☐ Latvian
- ☐ Lithuanian
- ☐ Macedonian
- ☐ Malay
- ☐ Malayalam
- ☐ Maori
- ☐ Multiple Languages
- ☐ Norwegian
- ☐ Persian
- ☐ Polish
- ☐ Portuguese
- ☐ Pushto
- ☐ Romanian
- ☐ Russian
- ☐ Sanskrit
- ☐ Scottish gaelic
- ☐ Serbian
- ☐ Slovak

- ☐ Slovenian
- ☐ Spanish
- ☐ Swedish
- ☐ Thai
- ☐ Turkish
- ☐ Ukrainian
- ☐ Undetermined
- ☐ Vietnamese
- ☐ Welsh

- ☐ Female
- ☐ Male

- ☐ MEDLINE

- ☐ Child: birth-18 years
- ☐ Newborn: birth-1 month
- ☐ Infant: birth-23 months
- ☐ Infant: 1-23 months
- ☐ Preschool Child: 2-5 years
- ☐ Child: 6-12 years
- ☐ Adolescent: 13-18 years
- ☐ Adult: 19+ years
- ☐ Young Adult: 19-24 years
- ☐ Adult: 19-44 years
- ☐ Middle Aged + Aged: 45+ years
- ☐ Middle Aged: 45-64 years
- ☐ Aged: 65+ years
- ☐ 80 and over: 80+ years

Cancel Show
Close dialog
Back to Top
Jump to page Close dialog
4 of 7
Jump

NCBI Literature Resources

MeSH PMC Bookshelf Disclaimer

Follow NCBI

Connect with NLM

•

-
-

National Library of Medicine
8600 Rockville Pike
Bethesda, MD 20894

Web Policies
FOIA
HHS Vulnerability Disclosure

Help
Accessibility
Careers

- NLM
- NIH
- HHS
- USA.gov

ERREUR p
du site :
Domaine
