# Supplementary material for: COVID-19 treatment of hospital patients worldwide at the onset of the pandemic in 2020: a systematic review
Source: BMC Infect Dis. 2025 Dec 17;26:107. doi: 10.1186/s12879-025-12368-2 (PMC12822144; doi:10.1186/s12879-025-12368-2)
Supplement: Supplementary file 5 — Supplementary Material 5 [file 12879_2025_12368_MOESM5_ESM.docx]

**Supplementary Material 5. Risk of bias assessment in included studies (overview, n=178)**

|  |  | SELECTION BIAS | | | | | | INFORMATION BIAS |  |
| --- | --- | --- | --- | --- | --- | --- | --- | --- | --- |
| Study number (PubMed search) | Study (first author)* | DUPLICATION BIAS  0= No, 1= Yes | Subpopulation bias  0= No, 1 = Yes | Unclear sampling  0= No, 1= Yes | Partial inclusion of ICU patients  0= No, 1= Yes | Partial inclusion of minors  0= No, 1= Yes | BIAS SELECTION* | About Traitement  0= No, 1= Yes | Web link to the article/DOI |
| 1 | GARCIA-VIDAL C | 1 | 0 | 0 | 1 | 0 | 1 | 0 | <https://www.ncbi.nlm.nih.gov/pmc/articles/PMC7836762/> |
| 6 | DE SMET R | 0 | 1 | 0 | 1 | 0 | 1 | 0 | <https://dx.doi.org/10.1016%2Fj.jamda.2020.06.008> |
| 7 | XIONG S | 0 | 0 | 0 | 0 | 0 | 0 | 0 | <https://doi.org/10.1186/s12879-020-05452-2> |
| 8 | BARTOLETTI M | 0 | 0 | 0 | 1 | 0 | 1 | 0 | <https://doi.org/10.1016/j.cmi.2020.09.014> |
| 14 | YAO JS | 0 | 0 | 0 | 1 | 0 | 1 | 1 | <https://doi.org/10.1016/j.chest.2020.06.082> |
| 19 | ARSHAD S | 0 | 0 | 0 | 1 | 0 | 1 | 0 | <https://doi.org/10.1016/j.ijid.2020.06.099> |
| 26 | GUGLIELMETTI L | 0 | 0 | 0 | 1 | 0 | 1 | 0 | <https://doi.org/10.1038/s41598-021-00243-4> |
| 31 | DUBERNET A | 0 | 0 | 0 | 1 | 0 | 1 | 1 | <https://doi.org/10.1016/j.jgar.2020.08.001> |
| 40 | OZTURK S | 1 | 0 | 0 | 1 | 0 | 1 | 0 | <https://doi.org/10.1093/ndt/gfaa271> |
| 42 | CHOPRA V | 1 | 0 | 1 | 1 | 0 | 1 | 0 | <http://dx.doi.org/10.1136/bmjopen-2020-044921> |
| 46 | KIM J | 0 | 0 | 0 | 1 | 0 | 1 | 0 | <https://dx.doi.org/10.1186%2Fs12879-021-06588-5> |
| 47 | ZHAO X | 1 | 0 | 0 | 0 | 0 | 0 | 0 | <https://doi.org/10.1002/jpen.1953> |
| 57 | RUIZ-QUINONEZ JA | 0 | 0 | 0 | 0 | 0 | 0 | 0 | <https://doi.org/10.1371/journal.pone.0245394> |
| 62 | ANGELIDI AM | 1 | 1 | 0 | 1 | 0 | 1 | 0 | <https://dx.doi.org/10.1016%2Fj.mayocp.2021.01.001> |
| 76 | CHEN K | 1 | 0 | 0 | 1 | 0 | 1 | 0 | <https://doi.org/10.1038/s41598-021-94570-1> |
| 77 | XU K | 1 | 0 | 0 | 0 | 0 | 0 | 0 | <https://doi.org/10.1093/cid/ciaa351> |
| 91 | BETTI M | 0 | 1 | 0 | 1 | 0 | 1 | 0 | https://doi.org/10.1371/journal.pone.0248829 |
| 92 | Wen XS | 0 | 0 | 0 | 0 | 0 | 0 | 0 | <https://doi.org/10.1186/s12879-020-05741-w> |
| 93 | Liu Q | 0 | 0 | 1 | 0 | 0 | 1 | 1 | <https://doi.org/10.1097/md.0000000000024544> |
| 96 | WANG Y | 1 | 0 | 1 | 0 | 0 | 1 | 1 | <https://doi.org/10.12659/msm.926751> |
| 101 | FRIED MW | 0 | 0 | 0 | 1 | 0 | 1 | 0 | <https://doi.org/10.1093/cid/ciaa1268> |
| 108 | HE XL | 1 | 0 | 1 | 0 | 0 | 1 | 0 | <https://doi.org/10.1007/s11596-021-2434-y> |
| 114 | TEJPAL A | 0 | 0 | 0 | 1 | 0 | 1 | 0 | <https://pubmed.ncbi.nlm.nih.gov/33885345/d> |
| 118 | XIA G | 1 | 0 | 1 | 0 | 0 | 1 | 0 | <https://doi.org/10.18632/aging.203503> |
| 121 | SAIB A | 0 | 0 | 0 | 0 | 0 | 0 | 0 | <https://doi.org/10.1371/journal.pone.0252388> |
| 129 | REGINA J | 0 | 0 | 0 | 1 | 0 | 1 | 0 | <https://doi.org/10.1371/journal.pone.0240781> |
| 158 | LOHIA P | 0 | 0 | 0 | 1 | 0 | 1 | 0 | <https://doi.org/10.1186/s12933-021-01336-0> |
| 164 | BEST JH | 0 | 0 | 1 | 0 | 1 | 1 | 0 | <https://doi.org/10.1002/jmv.27049> |
| 167 | SUARDI LR | 0 | 0 | 0 | 1 | 0 | 1 | 0 | <https://doi.org/10.1016/j.ijid.2020.09.012> |
| 184 | OKOH | 0 | 0 | 0 | 0 | 0 | 0 | 0 | <https://doi.org/10.1002/jmv.26471> |
| 200 | FRONTERA JA | 0 | 0 | 0 | 1 | 0 | 1 | 0 | <https://dx.doi.org/10.1007%2Fs12028-021-01220-5> |
| 202 | ZHENG Y | 0 | 0 | 0 | 1 | 0 | 1 | 0 | <https://doi.org/10.1097/md.0000000000024771> |
| 221 | VERNAZ N | 0 | 0 | 0 | 0 | 0 | 0 | 1 | <https://doi.org/10.4414/smw.2020.20446> |
| 222 | KUNAL S | 0 | 0 | 0 | 0 | 0 | 0 | 1 | <https://doi.org/10.1016/j.ihj.2020.10.005> |
| 231 | DU HW | 0 | 0 | 0 | 0 | 0 | 0 | 0 | <https://doi.org/10.1186/s12879-021-06970-3> |
| 242 | LOZANO-MONTOYA I | 0 | 0 | 0 | 0 | 0 | 0 | 0 | <https://doi.org/10.1007/s41999-021-00541-0> |
| 249 | YANG JY | 0 | 0 | 0 | 1 | 0 | 1 | 0 | <https://doi.org/10.1001/jamanetworkopen.2020.35699> |
| 253 | ZIELINSKA-TUREK J | 0 | 1 | 1 | 1 | 0 | 1 | 0 | <https://journals.viamedica.pl/neurologia_neurochirurgia_polska/article/view/70248> |
| 254 | YAN Q | 1 | 1 | 0 | 1 | 0 | 1 | 1 | <https://doi.org/10.1093/gerona/glaa181> |
| 262 | PATEL AJ | 0 | 1 | 0 | 1 | 0 | 1 | 0 | <https://doi.org/10.1016/j.eprac.2021.07.008> |
| 270 | FOX T | 0 | 0 | 0 | 0 | 0 | 0 | 0 | <https://doi.org/10.1007/s00592-020-01592-8> |
| 271 | MYLONA E | 0 | 0 | 1 | 1 | 0 | 1 | 0 | <https://doi.org/10.1080/20477724.2021.1893485> |
| 273 | TURGUTALP K | 1 | 1 | 1 | 1 | 0 | 1 | 0 | <https://doi.org/10.1186/s12882-021-02233-0> |
| 275 | PACCOUD O | 1 | 0 | 0 | 1 | 0 | 1 | 1 | <https://doi.org/10.1093/cid/ciaa791> |
| 278 | BARDAJI A | 1 | 0 | 0 | 1 | 0 | 1 | 0 | <https://doi.org/10.1016/j.rec.2020.08.027> |
| 284 | AWAD N | 0 | 0 | 0 | 1 | 0 | 1 | 0 | <https://pmc.ncbi.nlm.nih.gov/articles/PMC7929454/> |
| 297 | CROSSETTE-THAMBIAH C | 0 | 1 | 1 | 1 | 0 | 1 | 1 | <https://doi.org/10.1111/bjh.17579> |
| 300 | LEE HW | 0 | 1 | 0 | 0 | 0 | 1 | 0 | <https://doi.org/10.3904/kjim.2020.329> |
| 319 | ARIKAN H | 0 | 0 | 0 | 1 | 0 | 1 | 0 | <https://doi.org/10.1371/journal.pone.0256023> |
| 320 | PORTACCI A | 0 | 1 | 0 | 1 | 0 | 1 | 0 | <https://doi.org/10.1080/17476348.2021.1960824> |
| 331 | YEO I | 0 | 0 | 0 | 0 | 0 | 0 | 0 | <https://dx.doi.org/10.1111%2Fjoim.13241> |
| 334 | MEKOLO D | 0 | 0 | 0 | 1 | 0 | 1 | 0 | <https://doi.org/10.11604/pamj.2021.38.246.28169> |
| 335 | GONG X | 1 | 0 | 0 | 0 | 0 | 0 | 0 | <https://doi.org/10.1186/s12879-021-06282-6> |
| 337 | SNIPELISKY D | 0 | 0 | 0 | 1 | 0 | 1 | 0 | <https://doi.org/10.14423/smj.0000000000001182> |
| 342 | LAURIOLA M | 1 | 1 | 0 | 1 | 0 | 1 | 0 | <https://doi.org/10.1111/cts.12860> |
| 355 | MUSSINI C | 1 | 0 | 0 | 1 | 0 | 1 | 1 | <https://doi.org/10.1016/j.cmi.2020.12.010> |
| 357 | SHU Z | 1 | 0 | 0 | 0 | 0 | 0 | 0 | <https://doi.org/10.1007/s11684-020-0803-8> |
| 370 | PIETRI L | 0 | 0 | 1 | 1 | 0 | 1 | 0 | <https://doi.org/10.1016/j.metabol.2021.154703> |
| 376 | CHANG MC | 0 | 0 | 0 | 1 | 0 | 1 | 0 | <https://doi.org/10.1097/md.0000000000025917> |
| 382 | MONREAL E | 1 | 0 | 0 | 1 | 0 | 1 | 0 | <https://doi.org/10.1002/jmv.26656> |
| 395 | RAMIREZ GA | 1 | 1 | 0 | 0 | 0 | 1 | 1 | <https://doi.org/10.1016/j.cmi.2021.05.023> |
| 396 | LANZA E | 1 | 1 | 1 | 1 | 0 | 1 | 0 | <https://doi.org/10.1007/s00330-020-07013-2> |
| 421 | CATTELAN AM | 1 | 0 | 0 | 1 | 0 | 1 | 0 | <https://doi.org/10.1186/s12879-020-05647-7> |
| 428 | STEFAN G | 0 | 1 | 0 | 1 | 0 | 1 | 1 | <https://doi.org/10.1080/0886022X.2020.1853571> |
| 434 | SOH TV | 0 | 0 | 0 | 1 | 1 | 1 | 0 | <http://www.e-mjm.org/2020/v75n5/SARS-CoV2.pdf> |
| 453 | KUNO T | 0 | 0 | 0 | 1 | 0 | 1 | 0 | <https://doi.org/10.1016/j.jjcc.2021.12.012> |
| 462 | LEI C | 1 | 0 | 0 | 1 | 0 | 1 | 0 | <https://doi.org/10.1016/j.jcv.2020.104661> |
| 471 | FREEMAN A | 0 | 0 | 0 | 1 | 0 | 1 | 0 | <https://doi.org/10.1016/j.jcv.2021.105031> |
| 491 | LLANERA DK | 0 | 1 | 0 | 1 | 0 | 1 | 0 | <https://doi.org/10.3389/fendo.2021.777130> |
| 498 | DUARTE-MILLAN MA | 0 | 1 | 0 | 0 | 0 | 1 | 0 | <https://doi.org/10.1002/jmv.27488> |
| 503 | BAHL A | 1 | 0 | 0 | 1 | 0 | 1 | 0 | <https://doi.org/10.1007/s11739-021-02655-6> |
| 510 | HUANG J | 0 | 0 | 0 | 0 | 0 | 0 | 0 | <https://doi.org/10.1186/s12890-021-01487-6> |
| 514 | CARDINAL-FERNANDEZ P | 1 | 0 | 0 | 1 | 0 | 1 | 0 | <https://doi.org/10.37201/req/050.2021> |
| 526 | AL MUTAIR A | 0 | 0 | 1 | 0 | 0 | 1 | 0 | <https://doi.org/10.1186/s40001-020-00462-x> |
| 527 | CAPDEVILA-RENIU A | 1 | 1 | 0 | 0 | 0 | 1 | 0 | <https://doi.org/10.1097/md.0000000000024750> |
| 529 | PEREZ-NIETO OR | 0 | 0 | 0 | 1 | 0 | 1 | 0 | <https://doi.org/10.1183/13993003.00265-2021> |
| 539 | PEREZ-DE-LLANO L | 1 | 0 | 0 | 1 | 0 | 1 | 0 | <https://doi.org/10.1371/journal.pone.0253465> |
| 542 | PEPE M | 0 | 0 | 0 | 1 | 0 | 1 | 0 | <https://doi.org/10.1007/s10238-021-00684-1> |
| 548 | FUSINA F | 0 | 0 | 0 | 1 | 0 | 1 | 0 | <https://doi.org/10.1002/cpt.2245> |
| 554 | AKHTAR H | 0 | 0 | 1 | 0 | 0 | 1 | 1 | <https://doi.org/10.2196/28594> |
| 555 | LI Y | 0 | 1 | 1 | 0 | 0 | 1 | 1 | <https://doi.org/10.1097/md.0000000000023547> |
| 572 | LARA OD | 0 | 1 | 0 | 1 | 0 | 1 | 0 | <https://doi.org/10.1016/j.ygyno.2021.12.004> |
| 575 | WANG Z | 1 | 0 | 1 | 1 | 0 | 1 | 1 | <https://doi.org/10.1155/2020/2138387> |
| 576 | SONG J | 0 | 0 | 1 | 1 | 0 | 1 | 0 | <https://doi.org/10.12659/msm.925047> |
| 583 | KUMAR G | 0 | 0 | 0 | 1 | 0 | 1 | 0 | <https://doi.org/10.1002/jmv.27357> |
| 598 | ZHANG Q | 1 | 1 | 1 | 1 | 0 | 1 | 0 | <https://doi.org/10.1016/j.jdiacomp.2020.107666> |
| 628 | HUR K | 0 | 0 | 0 | 1 | 0 | 1 | 0 | <https://doi.org/10.1177/0194599820929640> |
| 630 | ZHANG Q | 0 | 0 | 0 | 0 | 0 | 0 | 1 | <https://doi.org/10.1097/md.0000000000025913> |
| 647 | BOTTIO T | 0 | 1 | 1 | 1 | 0 | 1 | 0 | <https://doi.org/10.1016/j.jchf.2020.10.009> |
| 649 | MILIC J | 1 | 0 | 0 | 1 | 0 | 1 | 0 | <https://doi.org/10.1089/aid.2020.0305> |
| 650 | VILLA L | 0 | 1 | 1 | 1 | 0 | 1 | 1 | <https://doi.org/10.1097/md.0000000000024893> |
| 654 | YANG D | 0 | 0 | 0 | 0 | 0 | 0 | 1 | <https://doi.org/10.1002/clc.23628> |
| 661 | SHEN L | 1 | 0 | 0 | 0 | 0 | 0 | 1 | <https://doi.org/10.1007/s10557-020-07133-3> |
| 673 | RHODES NJ | 0 | 0 | 1 | 1 | 0 | 1 | 0 | <https://doi.org/10.1093/ajhp/zxaa426> |
| 674 | CHEN Q | 0 | 0 | 1 | 1 | 0 | 1 | 1 | <https://doi.org/10.1007/s15010-020-01432-5> |
| 678 | SUN L | 0 | 0 | 1 | 0 | 1 | 1 | 0 | <https://doi.org/10.1002/jmv.25966> |
| 684 | KEVORKIAN JP | 1 | 0 | 0 | 1 | 0 | 1 | 1 | <https://doi.org/10.1016/j.jinf.2020.08.045> |
| 702 | CHEN Q | 1 | 0 | 0 | 1 | 0 | 1 | 0 | <https://doi.org/10.1161/jaha.120.018451> |
| 704 | PAFUNDI PC | 0 | 0 | 0 | 0 | 0 | 0 | 0 | <https://doi.org/10.1371/journal.pone.0256903> |
| 713 | RIVA G | 1 | 0 | 0 | 1 | 0 | 1 | 1 | <https://doi.org/10.1038/s41598-021-92236-6> |
| 724 | KHANUM I | 0 | 0 | 0 | 1 | 0 | 1 | 1 | <https://doi.org/10.4081/monaldi.2021.1561> |
| 728 | VAHEDI E | 0 | 1 | 1 | 0 | 0 | 1 | 1 | <https://doi.org/10.1007/s40199-020-00353-w> |
| 741 | CUI J | 1 | 0 | 1 | 0 | 0 | 1 | 0 | <https://doi.org/10.1097/md.0000000000027400> |
| 769 | ROSENBERG ES | 0 | 0 | 1 | 1 | 1 | 1 | 0 | <https://doi.org/10.1001/jama.2020.8630> |
| 775 | MATHER JF | 0 | 0 | 0 | 1 | 0 | 1 | 0 | <https://doi.org/10.14309/ajg.0000000000000832> |
| 794 | CHANGAL K | 0 | 0 | 0 | 0 | 0 | 0 | 0 | <https://doi.org/10.1186/s12872-021-01963-1> |
| 797 | DOUVILLE NJ | 0 | 0 | 0 | 1 | 0 | 1 | 0 | <https://doi.org/10.1016/j.bja.2020.11.034> |
| 801 | PONGPIRUL WA | 0 | 0 | 0 | 1 | 0 | 1 | 0 | <https://doi.org/10.1371/journal.pntd.0008806> |
| 804 | IP A | 0 | 0 | 0 | 1 | 0 | 1 | 0 | <https://doi.org/10.1371/journal.pone.0237693> |
| 833 | PELTZER B | 0 | 0 | 0 | 1 | 0 | 1 | 0 | <https://doi.org/10.1111/jce.14770> |
| 836 | ZHANG L | 0 | 0 | 1 | 1 | 0 | 1 | 0 | <https://doi.org/10.1016/j.phymed.2021.153531> |
| 840 | Favà A | 1 | 1 | 0 | 0 | 0 | 1 | 0 | <https://doi.org/10.1186/s13054-020-03340-4> |
| 841 | WU C | 1 | 1 | 0 | 0 | 0 | 1 | 0 | <https://doi.org/10.1186/s13054-020-03340-4> |
| 848 | BROSETA JJ | 1 | 1 | 0 | 1 | 0 | 1 | 0 | <https://doi.org/10.1159/000510557> |
| 864 | LI M | 0 | 0 | 0 | 1 | 0 | 1 | 0 | <https://doi.org/10.1016/j.amjms.2020.11.005> |
| 871 | RUSSO E | 0 | 0 | 0 | 0 | 0 | 0 | 0 | <https://doi.org/10.1007/s40620-020-00875-1> |
| 873 | DI CASTELNUOVO A | 1 | 0 | 1 | 0 | 0 | 1 | 0 | <https://doi.org/10.1155/2021/5556207> |
| 887 | THOREAU B | 0 | 1 | 0 | 1 | 0 | 1 | 1 | <https://doi.org/10.3390/v13050758> |
| 895 | GUNER R | 0 | 0 | 0 | 1 | 0 | 1 | 1 | <https://doi.org/10.1016/j.jiph.2020.12.017> |
| 905 | ZHANG Y | 1 | 0 | 0 | 1 | 0 | 1 | 0 | <https://doi.org/10.1111/dom.14086> |
| 908 | FERGUSON J | 0 | 0 | 0 | 0 | 0 | 0 | 0 | <https://doi.org/10.3201/eid2608.201776> |
| 922 | GARCIA-CABRERA L | 1 | 1 | 0 | 0 | 0 | 1 | 0 | <https://doi.org/10.1186/s12877-021-02565-4> |
| 923 | MOUSSEAUX E | 1 | 1 | 0 | 1 | 0 | 1 | 0 | <https://doi.org/10.1016/j.diii.2021.06.007> |
| 924 | YAN Y | 1 | 0 | 0 | 1 | 0 | 1 | 0 | <https://doi.org/10.1136/bmjdrc-2020-001343> |
| 928 | CAILLARD S | 0 | 1 | 0 | 1 | 0 | 1 | 1 | <https://doi.org/10.1016/j.kint.2020.08.005> |
| 952 | SISO-ALMIRALL A | 0 | 0 | 0 | 1 | 0 | 1 | 0 | <https://doi.org/10.1371/journal.pone.0237960> |
| 955 | FADINI GP | 1 | 0 | 0 | 0 | 0 | 0 | 0 | <https://doi.org/10.1016/j.diabres.2020.108374> |
| 957 | BERENGUER J | 1 | 0 | 0 | 1 | 0 | 1 | 0 | https://www.ncbi.nlm.nih.gov/pmc/articles/PMC7399713/bin/mmc1.pdf |
| 960 | XU B | 1 | 0 | 1 | 1 | 0 | 1 | 0 | <https://doi.org/10.1016/j.jinf.2020.04.012> |
| 961 | LIU J | 0 | 1 | 0 | 1 | 0 | 1 | 0 | <https://doi.org/10.1172/jci140617> |
| 964 | WU MA | 0 | 1 | 0 | 1 | 0 | 1 | 0 | <https://doi.org/10.1186/s13054-021-03846-5> |
| 967 | MONTEIRO AC | 0 | 0 | 0 | 1 | 0 | 1 | 0 | <https://doi.org/10.1371/journal.pone.0238552> |
| 986 | LORE NI | 1 | 0 | 0 | 1 | 0 | 1 | 1 | <https://doi.org/10.1186/s10020-021-00390-4> |
| 990 | ADAMI G | 0 | 0 | 0 | 1 | 0 | 1 | 0 | <https://doi.org/10.4081/reumatismo.2020.1333> |
| 999 | WANG N | 0 | 0 | 1 | 0 | 1 | 1 | 0 | <https://doi.org/10.1016/j.chom.2020.07.005> |
| 1001 | LI HY | 0 | 0 | 1 | 0 | 0 | 1 | 1 | <https://doi.org/10.1097/md.0000000000022847> |
| 1002 | CATTEAU L | 0 | 0 | 1 | 1 | 0 | 1 | 0 | <https://doi.org/10.1016/j.ijantimicag.2020.106144> |
| 1005 | SCUDIERO F | 0 | 1 | 0 | 1 | 0 | 1 | 0 | <https://doi.org/10.1016/j.thromres.2020.11.017> |
| 1007 | AOMAR-MILLAN IF | 0 | 0 | 0 | 1 | 0 | 1 | 1 | <https://doi.org/10.1007/s11739-020-02600-z> |
| 1010 | GALVAN-ROMAN JM | 1 | 1 | 0 | 1 | 0 | 1 | 0 | <https://doi.org/10.1016/j.jaci.2020.09.018> |
| 1012 | PIAZZA G | 0 | 0 | 0 | 0 | 0 | 0 | 0 | <https://doi.org/10.1016/j.jacc.2020.08.070> |
| 1027 | LOARCE-MARTOS J | 1 | 1 | 0 | 0 | 0 | 1 | 0 | <https://doi.org/10.1007/s00296-020-04699-x> |
| 1030 | MO Y | 0 | 0 | 0 | 1 | 0 | 1 | 0 | <https://doi.org/10.1002/jcph.1787> |
| 1032 | TZOUVELEKIS A | 0 | 0 | 1 | 1 | 0 | 1 | 1 | <https://doi.org/10.5603/arm.a2021.0087> |
| 1034 | SAEED O | 1 | 0 | 0 | 0 | 0 | 0 | 0 | <https://doi.org/10.1161/jaha.120.018475> |
| 1053 | LIU Z | 1 | 0 | 0 | 0 | 0 | 0 | 0 | <https://doi.org/10.3389/fendo.2020.00478> |
| 1057 | DU Y | 1 | 1 | 0 | 0 | 0 | 1 | 0 | <https://doi.org/10.1164/rccm.202003-0543oc> |
| 1080 | WEIZMAN O | 0 | 0 | 0 | 1 | 0 | 1 | 1 | <https://doi.org/10.1016/j.acvd.2021.04.002> |
| 1085 | ESCALERA-ANTEZANAN JP | 0 | 0 | 0 | 0 | 0 | 0 | 1 | <https://doi.org/10.1016/j.tmaid.2020.101653> |
| 1100 | FAN L | 0 | 0 | 0 | 0 | 0 | 0 | 1 | <https://doi.org/10.1097/md.0000000000023923> |
| 1107 | BRUNO PF | 0 | 1 | 0 | 1 | 0 | 1 | 1 | <https://doi.org/10.1159/000515128> |
| 1109 | MOEY MYY | 0 | 0 | 0 | 1 | 0 | 1 | 1 | <https://doi.org/10.1161/circep.120.009023> |
| 1118 | RAO X | 1 | 1 | 0 | 0 | 0 | 1 | 0 | <https://doi.org/10.1097/md.0000000000022766> |
| 1129 | MICALLEF S | 0 | 0 | 0 | 1 | 0 | 1 | 0 | <https://doi.org/10.1371/journal.pone.0239389> |
| 1136 | PASSAMONTI F | 1 | 1 | 0 | 1 | 0 | 1 | 0 | <https://doi.org/10.1016/s2352-3026(20)30251-9> |
| 1137 | DU H | 1 | 0 | 0 | 0 | 0 | 0 | 0 | <https://doi.org/10.1186/s12931-020-01510-0> |
| 1140 | GUO T | 1 | 0 | 0 | 1 | 0 | 1 | 0 | <https://doi.org/10.1001/jamacardio.2020.1017> |
| 1152 | CHENG Y | 1 | 0 | 0 | 1 | 0 | 1 | 0 | <https://doi.org/10.2215/cjn.04650420> |
| 1164 | ZHANG H | 1 | 1 | 0 | 0 | 0 | 1 | 0 | <https://doi.org/10.1002/cncr.33042> |
| 1167 | MCPADDEN J | 0 | 0 | 0 | 1 | 0 | 1 | 0 | <https://doi.org/10.1371/journal.pone.0243291> |
| 1181 | ALVISET S | 0 | 0 | 0 | 1 | 0 | 1 | 0 | <https://doi.org/10.1371/journal.pone.0240645> |
| 1196 | HUANG L | 1 | 0 | 0 | 0 | 0 | 0 | 1 | <https://doi.org/10.1016/j.jcmg.2020.05.004> |
| 1207 | ZHAO Y | 1 | 1 | 1 | 0 | 1 | 1 | 0 | <https://doi.org/10.1186/s40249-020-00723-1> |
| 1217 | RUIZ-IRASTORZA G | 1 | 1 | 0 | 0 | 0 | 1 | 0 | <https://doi.org/10.1371/journal.pone.0239401> |
| 1219 | XIONG F | 0 | 1 | 0 | 1 | 0 | 1 | 0 | <https://doi.org/10.1681/asn.2020030354> |
| 1228 | ZHENG J | 1 | 0 | 0 | 1 | 0 | 1 | 0 | <https://doi.org/10.1186/s12902-021-00896-2> |
| 1239 | PASCUAL PAREJA JF | 1 | 0 | 0 | 1 | 0 | 1 | 1 | <https://doi.org/10.1016/j.medcle.2020.11.006> |
| 1240 | CHEN SL | 1 | 0 | 0 | 0 | 0 | 0 | 0 | <https://doi.org/10.1093/ofid/ofaa432> |
| 1250 | LEO M | 0 | 1 | 0 | 1 | 0 | 1 | 0 | <https://doi.org/10.1016/j.dld.2021.12.014> |
| 1252 | BERTUZZI AF | 1 | 1 | 0 | 0 | 0 | 1 | 0 | <https://doi.org/10.3390/cancers12092352> |
| 1264 | CARDONA-PASCUAL I | 1 | 1 | 0 | 1 | 0 | 1 | 0 | <https://doi.org/10.1016/j.medcli.2021.03.005> |
| 1268 | SANCHEZ-RICO M | 1 | 0 | 0 | 1 | 0 | 1 | 0 | <https://doi.org/10.3390/jcm10245891> |
| 1296 | LOTFY SM | 0 | 0 | 1 | 1 | 0 | 1 | 1 | <https://doi.org/10.5152/turkthoracj.2021.20180> |
| 1306 | POLI D | 0 | 0 | 1 | 1 | 0 | 1 | 0 | <https://doi.org/10.1007/s11739-021-02891-w> |
| 1309 | JIANG S | 0 | 0 | 0 | 1 | 0 | 1 | 0 | <https://doi.org/10.3389/fmed.2020.00347> |
| 1310 | JOSA-LAORDEN C | 1 | 0 | 0 | 1 | 0 | 1 | 0 | <https://doi.org/10.3390/jcm10050899> |
| 1312 | HUANG F | 1 | 0 | 0 | 0 | 0 | 0 | 0 | <https://doi.org/10.21037/atm-21-1561> |
| 1354 | ZHAO X | 1 | 1 | 1 | 1 | 0 | 1 | 0 | <https://doi.org/10.2147/idr.s335868> |

* Selection bias was considered present if at least one of the following was identified: subpopulation bias, unclear sampling, partial inclusion of ICU patients, or partial inclusion of minors.
