# Supplementary material for: COVID-19 treatment of hospital patients worldwide at the onset of the pandemic in 2020: a systematic review
Source: BMC Infect Dis. 2025 Dec 17;26:107. doi: 10.1186/s12879-025-12368-2 (PMC12822144; doi:10.1186/s12879-025-12368-2)
Supplement: Supplementary file 6 — Supplementary Material 6 [file 12879_2025_12368_MOESM6_ESM.docx]

**Supplementary Material** **6. Studies included in the systematic review (overview, n=178)**

| Study number (PubMed search) | First author | Year of publication | Country | Continent | Inclusion period | Hospitals (n) | Patients (n) | web link to the article/DOI |
| --- | --- | --- | --- | --- | --- | --- | --- | --- |
| 334 | MEKOLO D | 2021 | Cameroon | Africa | 8/3-31/5/2020 | 1 | 282 | <https://doi.org/10.11604/pamj.2021.38.246.28169> |
| 1296 | LOFTY SM | 2021 | Egypt | Africa | 21/3-8/6/2020 | 1 | 202 | <https://doi.org/10.5152/turkthoracj.2021.20180> |
| 7 | XIONG S | 2020 | China | Asia | 20/1-8/3/2020 | 1 | 116 | <https://doi.org/10.1186/s12879-020-05452-2> |
| 47 | ZHAO X | 2021 | China | Asia | 29/01-19/2/2020 | 1 | 413 | <https://doi.org/10.1002/jpen.1953> |
| 76 | CHEN K | 2021 | China | Asia | 3/2-10/4/2020 | 3 | 1851 | <https://doi.org/10.1038/s41598-021-94570-1> |
| 77 | XU K | 2020 | China | Asia | 19/1-19/2/2020 | 2 | 113 | https://doi.org/10.1093/cid/ciaa351 |
| 92 | Wen XS | 2021 | China | Asia | until March 3, 2020 | 2 | 395 | <https://doi.org/10.1186/s12879-020-05741-w> |
| 93 | Liu Q | 2021 | China | Asia | 30/1-20/5/2020 | 1 | 232 | <https://doi.org/10.1097/md.0000000000024544> |
| 96 | WANG Y | 2021 | China | Asia | 20/2-20/3/2020 | 1 | 97 | <https://doi.org/10.12659/msm.926751> |
| 108 | HE XL | 2021 | China | Asia | January - May 2020 | 1 | 238 | <https://doi.org/10.1007/s11596-021-2434-y> |
| 118 | XIA G | 2021 | China | Asia | 1/2-10/3/2020 | 1 | 113 | https://doi.org/10.18632/aging.203503 |
| 202 | ZHENG Y | 2021 | China | Asia | January –March 2020 | 1 | 70 | <https://doi.org/10.1097/md.0000000000024771> |
| 231 | DU HW | 2021 | China | Asia | 15/2-14/3/2020 | 1 | 95 | https://doi.org/10.1186/s12879-021-06970-3 |
| 254 | YAN Q | 2021 | China | Asia | 27/1-17/2/2020 | 1 | 882 | <https://doi.org/10.1093/gerona/glaa181> |
| 335 | GONG X | 2021 | China | Asia | 11/1-13/3/2020 | 1 | 550 | <https://doi.org/10.1186/s12879-021-06282-6> |
| 357 | SHU Z | 2020 | China | Asia | 15/1-2/3/2020 | 1 | 293 | <https://doi.org/10.1007/s11684-020-0803-8> |
| 462 | LEI C | 2020 | China | Asia | 20/1-20/2/2020 | 1 | 297 | https://doi.org/10.1016/j.jcv.2020.104661 |
| 510 | HUANG J | 2021 | China | Asia | Janvier avril 2020 | 1 | 2425 | <https://doi.org/10.1186/s12890-021-01487-6> |
| 555 | LI Y | 2020 | China | Asia | March, April, 2020 | 1 | 34 | <https://doi.org/10.1097/md.0000000000023547> |
| 575 | WANG Z | 2020 | China | Asia | 30/12-29/2/2020 | 1 | 293 | <https://doi.org/10.1155/2020/2138387> |
| 576 | SONG J | 2020 | China | Asia | 14/1-26/2/2020 | 1 | 69 | <https://doi.org/10.12659/msm.925047> |
| 598 | ZHANG Q | 2021 | China | Asia | 3/1-14/4/2020 | 1 | 74 | <https://doi.org/10.1016/j.jdiacomp.2020.107666> |
| 630 | ZHANG Q | 2021 | China | Asia | Until March 27, 2020 | 1 | 157 | <https://doi.org/10.1097/md.0000000000025913> |
| 654 | YANG D | 2021 | China | Asia | 25/12/2019-10/3/2020 | 1 | 306 | <https://doi.org/10.1002/clc.23628> |
| 661 | SHEN L | 2021 | China | Asia | 26/1-26/3/2020 | 2 | 525 | https://doi.org/10.1007/s10557-020-07133-3 |
| 674 | CHEN Q | 2020 | China | Asia | 1/1-11/3/2020 | 1 | 145 | <https://doi.org/10.1007/s15010-020-01432-5> |
| 678 | SUN L | 2020 | China | Asia | 20/1-15/2/2020 | 1 | 55 | <https://doi.org/10.1002/jmv.25966> |
| 702 | CHEN Q | 2021 | China | Asia | 22/1-25/3/2020 | 6 | 1303 | <https://doi.org/10.1161/jaha.120.018451> |
| 741 | CUI J | 2021 | China | Asia | 11/1-13/3/2020 | 1 | 526 | <https://doi.org/10.1097/md.0000000000027400> |
| 836 | ZHANG L | 2021 | China | Asia | January – may 2020 | 15 | 8939 | <https://doi.org/10.1016/j.phymed.2021.153531> |
| 841 | WU C | 2020 | China | Asia | 20/1-24/2/2020 | 1 | 382 | <https://doi.org/10.1186/s13054-020-03340-4> |
| 905 | ZHANG Y | 2020 | China | Asia | 8/2-21/3/2020 | 1 | 166 | <https://doi.org/10.1111/dom.14086> |
| 924 | YAN Y | 2020 | China | Asia | 10/1-24/2/2020 | 1 | 193 | <https://doi.org/10.1136/bmjdrc-2020-001343> |
| 960 | XU B | 2020 | China | Asia | 26/12/2019-1/3/2020 | 1 | 187 | <https://doi.org/10.1016/j.jinf.2020.04.012> |
| 961 | LIU J | 2020 | China | Asia | 29/12/2019-16/3/2020 | 5 | 774 | <https://doi.org/10.1172/jci140617> |
| 999 | WANG N | 2020 | China | Asia | 15/1-31/3/2020 | 2 | 504 | <https://doi.org/10.1016/j.chom.2020.07.005> |
| 1001 | LI HY | 2020 | China | Asia | 1-29/2/2020 | 1 | 132 | <https://doi.org/10.1097/md.0000000000022847> |
| 1053 | LIU Z | 2020 | China | Asia | 8/2-15/4/2020 | 1 | 934 | <https://doi.org/10.3389/fendo.2020.00478> |
| 1057 | DU Y | 2020 | China | Asia | 9/1-15/2/2020 | 2 | 85 | <https://doi.org/10.1164/rccm.202003-0543oc> |
| 1100 | FAN L | 2021 | China | Asia | 20/1-15/3/2020 | 1 | 55 | <https://doi.org/10.1097/md.0000000000023923> |
| 1118 | RAO X | 2020 | China | Asia | 24/12/2019-25/3/2020 | 1 | 240 | <https://doi.org/10.1097/md.0000000000022766> |
| 1137 | DU H | 2020 | China | Asia | 15/2-14/3/2020 | 1 | 164 | https://doi.org/10.1186/s12931-020-01510-0 |
| 1140 | GUO T | 2020 | China | Asia | 23/1-23/2/2020 | 1 | 187 | <https://doi.org/10.1001/jamacardio.2020.1017> |
| 1152 | CHENG Y | 2020 | China | Asia | 18/1-28/2/2020 | 1 | 1392 | <https://doi.org/10.2215/cjn.04650420> |
| 1164 | ZHANG H | 2020 | China | Asia | 5/1-18/3/2020 | 5 | 107 | https://doi.org/10.1002/cncr.33042 |
| 1196 | HUANG L | 2020 | China | Asia | Until March 2020 | 1 | 26 | https://doi.org/10.1016/j.jcmg.2020.05.004 |
| 1207 | ZHAO Y | 2020 | China | Asia | 13/1-4/3/2020 | 1 | 539 | https://doi.org/10.1186/s40249-020-00723-1 |
| 1219 | XIONG F | 2020 | China | Asia | 1/1-10/3/2020 | 65 | 131 | https://doi.org/10.1681/asn.2020030354 |
| 1228 | ZHENG J | 2021 | China | Asia | 22/1-17/3/2020 | 1 | 235 | <https://doi.org/10.1186/s12902-021-00896-2> |
| 1240 | CHEN SL | 2020 | China | Asia | 14/1-10/3/2020 | 32 | 1282 | <https://doi.org/10.1093/ofid/ofaa432> |
| 1309 | JIANG S | 2020 | China | Asia | 19/1-20/2/2020 | 3 | 131 | <https://doi.org/10.3389/fmed.2020.00347> |
| 1312 | HUANG F | 2021 | China | Asia | 25/1-25/2/2020 | 1 | 349 | <https://doi.org/10.21037/atm-21-1561> |
| 1354 | ZHAO X | 2021 | China | Asia | 1/2-6/3/2020 | 3 | 368 | https://doi.org/10.2147/idr.s335868 |
| 222 | KUNAL S | 2020 | India | Asia | Article received 7/7/20* | 1 | 108 | https://doi.org/10.1016/j.ihj.2020.10.005 |
| 728 | VAHEDI E | 2020 | Iran | Asia | 29/2-23/3/2020 | 1 | 60 | https://doi.org/10.1007/s40199-020-00353-w |
| 434 | SOH TV | 2020 | Malaysia | Asia | 09/03-15/4/2020 | 1 | 247 | <http://www.e-mjm.org/2020/v75n5/SARS-CoV2.pdf> |
| 554 | AKHTAR H | 2021 | Pakistan | Asia | May-June 2020 | 4 | 1438 | <https://doi.org/10.2196/28594> |
| 724 | KHANUM I | 2021 | Pakistan | Asia | 1-31/5/2020 | 1 | 23 | <https://doi.org/10.4081/monaldi.2021.1561> |
| 526 | AL MUTAIR A | 2020 | Saudi Arabia | Asia | 22/4-22/5/2020 | 2 | 401 | <https://doi.org/10.1186/s40001-020-00462-x> |
| 46 | KIM J | 2021 | South Korea | Asia | 29/1-15/4/2020 | 1 | 140 | <https://dx.doi.org/10.1186%2Fs12879-021-06588-5> |
| 300 | LEE HW | 2021 | South Korea | Asia | 25/2-11/4/2020 | 1 | 127 | <https://doi.org/10.3904/kjim.2020.329> |
| 376 | CHANG MC | 2021 | South Korea | Asia | 22/1-29/2/2020 | 1 | 138 | <https://doi.org/10.1097/md.0000000000025917> |
| 801 | PONGPIRUL WA | 2020 | Thailand | Asia | 8/1-16/4/2020 | 1 | 193 | <https://doi.org/10.1371/journal.pntd.0008806> |
| 40 | OZTURK S | 2020 | Turkey | Asia | 17/4-6/5/2020 | 47 | 1210 | <https://doi.org/10.1093/ndt/gfaa271> |
| 273 | TURGUTALP K | 2021 | Turkey | Asia | 17/4-1/6/2020 | 47 | 567 | <https://doi.org/10.1186/s12882-021-02233-0> |
| 319 | ARIKAN H | 2021 | Turkey | Asia | 1/3-30/6/2020 | 34 | 578 | <https://doi.org/10.1371/journal.pone.0256023> |
| 895 | GUNER R | 2021 | Turkey | Asia | 15/3-1/6/2020 | 1 | 2441 | https://doi.org/10.1016/j.jiph.2020.12.017 |
| 6 | DE SMET R | 2020 | Belgium | Europa | 12/3-30/4/2020 | 1 | 81 | <https://dx.doi.org/10.1016%2Fj.jamda.2020.06.008> |
| 1002 | CATTEAU L | 2020 | Belgium | Europa | Until May 24, 2020 | 109 | 8910 | <https://doi.org/10.1016/j.ijantimicag.2020.106144> |
| 31 | DUBERNET A | 2020 | France | Europa | 11/3-17/4/2020 | 1 | 164 | <https://doi.org/10.1016/j.jgar.2020.08.001> |
| 121 | SAIB A | 2021 | France | Europa | 15/3-2/4/2020 | 1 | 203 | https://doi.org/10.1371/journal.pone.0252388 |
| 275 | PACCOUD O | 2021 | France | Europa | 28/1-19/3/2020 | 1 | 116 | https://doi.org/10.1093/cid/ciaa791 |
| 370 | PIETRI L | 2021 | France | Europa | 19/3-27/4/2020 | 1 | 113 | <https://doi.org/10.1016/j.metabol.2021.154703> |
| 684 | KEVORKIAN JP | 2021 | France | Europa | 11/3-27/4/2020 | 1 | 119 | <https://doi.org/10.1016/j.jinf.2020.08.045> |
| 887 | THOREAU B | 2021 | France | Europa | 6-28/4/2020 | 18 | 86 | <https://doi.org/10.3390/v13050758> |
| 923 | MOUSSEAUX E | 2021 | France | Europa | 13/3-1/4/2020 | 1 | 169 | https://doi.org/10.1016/j.diii.2021.06.007 |
| 928 | CAILLARD S | 2020 | France | Europa | 1/3-21/4/2020 | MD^‡^ | 243 | <https://doi.org/10.1016/j.kint.2020.08.005> |
| 1080 | WEIZMAN O | 2021 | France | Europa | 26/2-20/4/2020 | 24 | 2878 | <https://doi.org/10.1016/j.acvd.2021.04.002> |
| 1181 | ALVISET S | 2020 | France | Europa | 27/3-23/4/2022 | 1 | 49 | https://doi.org/10.1371/journal.pone.0240645 |
| 1268 | SANCHEZ-RICO M | 2021 | France | Europa | 24/1-1/5/2020 | 36 | 15103 | <https://doi.org/10.3390/jcm10245891> |
| 650 | VILLA L | 2021 | Germany | Europa | 24/2-17/6/2020 | 3 | 10 | <https://doi.org/10.1097/md.0000000000024893> |
| 271 | MYLONA E | 2021 | Greece | Europa | 1/3-18/5/2020 | 1 | 85 | https://doi.org/10.1080/20477724.2021.1893485 |
| 1032 | TZOUVELEKIS A | 2021 | Greece | Europa | 26/2-15/5/2020 | 6 | 187 | <https://doi.org/10.5603/arm.a2021.0087> |
| 8 | BARTOLETTI M | 2021 | Italy | Europa | 22/2-30/6/2020 | 9 | 1717 | <https://doi.org/10.1016/j.cmi.2020.09.014> |
| 26 | GUGLIELMETTI L | 2021 | Italy | Europa | 21/2-15/5/2020 | 2 | 600 | <https://doi.org/10.1038/s41598-021-00243-4> |
| 91 | BETTI M | 2021 | Italy | Europa | 1/3-30/4/2020 | 1 | 171 |  |
| 167 | SUARDI LR | 2020 | Italy | Europa | 25/2-25/4/2020 | 1 | 97 | https://doi.org/10.1016/j.ijid.2020.09.012 |
| 320 | PORTACCI A | 2021 | Italy | Europa | 11/3-31/5/2020 | 1 | 97 | <https://doi.org/10.1080/17476348.2021.1960824> |
| 342 | LAURIOLA M | 2020 | Italy | Europa | 27/2-20/4/2020 | 1 | 377 | <https://doi.org/10.1111/cts.12860> |
| 355 | MUSSINI C | 2021 | Italy | Europa | 21/2-25/5/2020 | 1 | 415 | <https://doi.org/10.1016/j.cmi.2020.12.010> |
| 395 | RAMIREZ GA | 2021 | Italy | Europa | February - March, 2020 | 1 | 5 | https://doi.org/10.1016/j.cmi.2021.05.023 |
| 396 | LANZA E | 2020 | Italy | Europa | 25/1-28/4/2020 | 1 | 222 | <https://doi.org/10.1007/s00330-020-07013-2> |
| 421 | CATTELAN AM | 2020 | Italy | Europa | 22/2-20/5/2020 | 1 | 303 | <https://doi.org/10.1186/s12879-020-05647-7> |
| 548 | FUSINA F | 2021 | Italy | Europa | 20/2-10/5/2020 | 1 | 1311 | <https://doi.org/10.1002/cpt.2245> |
| 647 | BOTTIO T | 2021 | Italy | Europa | Until July 01, 2020 | 7 | 38 | <https://doi.org/10.1016/j.jchf.2020.10.009> |
| 649 | MILIC J | 2021 | Italy | Europa | 21/2-9/4/2020 | 1 | 273 | <https://doi.org/10.1089/aid.2020.0305> |
| 704 | PAFUNDI PC | 2021 | Italy | Europa | 13/3-30/6/2020 | 18 | 618 | <https://doi.org/10.1371/journal.pone.0256903> |
| 713 | RIVA G | 2021 | Italy | Europa | April-June 2020 | 1 | 87 | <https://doi.org/10.1038/s41598-021-92236-6> |
| 871 | RUSSO E | 2021 | Italy | Europa | 25/2-13/4/2020 | 1 | 777 | https://doi.org/10.1007/s40620-020-00875-1 |
| 873 | DI CASTELNUOVO A | 2021 | Italy | Europa | February – May 2020 | 35 | 4396 | <https://doi.org/10.1155/2021/5556207> |
| 955 | FADINI GP | 2020 | Italy | Europa | 21/2-20/4/2020 | 1 | 413 | <https://doi.org/10.1016/j.diabres.2020.108374> |
| 964 | WU MA | 2021 | Italy | Europa | 29/2-30/6/2020 | MD^‡^ | 48 | https://doi.org/10.1186/s13054-021-03846-5 |
| 986 | LORE NI | 2021 | Italy | Europa | 18/3-5/5/2020 | 1 | 111 | <https://doi.org/10.1186/s10020-021-00390-4> |
| 990 | ADAMI G | 2021 | Italy | Europa | 8/3-8/5/2020 | 1 | 61 | <https://doi.org/10.4081/reumatismo.2020.1333> |
| 1005 | SCUDIERO F | 2021 | Italy | Europa | 28/2-20/4/2020 | 7 | 224 | <https://doi.org/10.1016/j.thromres.2020.11.017> |
| 1107 | BRUNO PF | 2021 | Italy | Europa | February - April, 2020 | 2 | 49 | https://doi.org/10.1159/000515128 |
| 1136 | PASSAMONTI F | 2020 | Italy | Europa | 25/2-18/5/2020 | 66 | 451 | <https://doi.org/10.1016/s2352-3026(20)30251-9> |
| 1252 | BERTUZZI AF | 2020 | Italy | Europa | 21/2-30/4/2020 | 1 | 14 | https://doi.org/10.3390/cancers12092352 |
| 1306 | POLI D | 2022 | Italy | Europa | 1/3-30/6/2020 | 22 | 1091 | <https://doi.org/10.1007/s11739-021-02891-w> |
| 1129 | MICALLEF S | 2021 | Malta | Europa | 7-24/3/2020 | 1 | 93 | <https://doi.org/10.1371/journal.pone.0239389> |
| 253 | ZIELINSKA-TUREK J | 2021 | Poland | Europa | 16/3-15/6/2020 | 1 | 70 | <https://journals.viamedica.pl/neurologia_neurochirurgia_polska/article/view/70248> |
| 428 | STEFAN G | 2021 | Romania | Europa | 24/3-22/5/2020 | 1 | 37 | <https://doi.org/10.1080/0886022x.2020.1853571> |
| 1 | GARCIA-VIDAL C | 2021 | Spain | Europa | 28/2-22/4/2020 | 1 | 917 | <https://www.ncbi.nlm.nih.gov/pmc/articles/PMC7836762/> |
| 242 | LOZANO-MONTOYA I | 2021 | Spain | Europa | March – May 2020 | 1 | 300 | <https://doi.org/10.1007/s41999-021-00541-0> |
| 278 | BARDAJI A | 2021 | Spain | Europa | 1/3-16/4/2020 | 1 | 186 | https://doi.org/10.1016/j.rec.2020.08.027 |
| 382 | MONREAL E | 2021 | Spain | Europa | 18/3-4/4/2020 | 1 | 138 | <https://doi.org/10.1002/jmv.26656> |
| 498 | DUARTE-MILLAN MA | 2022 | Spain | Europa | 1/3-30/4/2020 | 1 | 163 | <https://doi.org/10.1002/jmv.27488> |
| 514 | CARDINAL-FERNANDEZ P | 2021 | Spain | Europa | 1/3-5/4/2020 | 8 | 1331 | <https://doi.org/10.37201/req/050.2021> |
| 527 | CAPDEVILA-RENIU A | 2021 | Spain | Europa | 20/3-30/4/2020 | 1 | 159 | <https://doi.org/10.1097/md.0000000000024750> |
| 539 | PEREZ-DE-LLANO L | 2021 | Spain | Europa | 1/3-24/4/2020 | 8 | 1292 | <https://doi.org/10.1371/journal.pone.0253465> |
| 840 | FAVA A | 2020 | Spain | Europa | 4/3-17/4/2020 | 5 | 104 | https://doi.org/10.1186/s13054-020-03340-4 |
| 848 | BROSETA JJ | 2021 | Spain | Europa | Until May 31, 2020 | 1 | 29 | <https://doi.org/10.1159/000510557> |
| 922 | GARCIA-CABRERA L | 2021 | Spain | Europa | 30/3-30/4/2020 | 1 | 130 | https://doi.org/10.1186/s12877-021-02565-4 |
| 952 | SISO-ALMIRALL A | 2020 | Spain | Europa | 29/2-4/4/2020 | 3 | 158 | <https://doi.org/10.1371/journal.pone.0237960> |
| 957 | BERENGUER J | 2020 | Spain | Europa | Until March 17, 2020 | 127 | 4035 | [https://doi.org/10.1016/j.cmi.2020.07.024 https://www.ncbi.nlm.nih.gov/pmc/articles/PMC7399713/bin/mmc1.pdf](https://doi.org/10.1016/j.cmi.2020.07.024) |
| 1007 | AOMAR-MILLAN IF | 2021 | Spain | Europa | 15/3-15/5/2020 | 1 | 143 | https://doi.org/10.1007/s11739-020-02600-z |
| 1010 | GALVAN-ROMAN JM | 2021 | Spain | Europa | 24/2-23/3/2020 | 1 | 146 | https://doi.org/10.1016/j.jaci.2020.09.018 |
| 1027 | LOARCE-MARTOS J | 2020 | Spain | Europa | 2/1-26/5/2020 | 1 | 8 | https://doi.org/10.1007/s00296-020-04699-x |
| 1217 | RUIZ-IRASTORZA G | 2020 | Spain | Europa | 1/3-30/4/2020 | 1 | 242 | <https://doi.org/10.1371/journal.pone.0239401> |
| 1239 | PASCUAL PAREJA JF | 2020 | Spain | Europa | 20/2-9/4/2020 | 1 | 259 | <https://doi.org/10.1016/j.medcle.2020.11.006> |
| 1264 | CARDONA-PASCUAL I | 2021 | Spain | Europa | March - April, 2020 | 1 | 1480 | <https://doi.org/10.1016/j.medcli.2021.03.005> |
| 1310 | JOSA-LAORDEN C | 2021 | Spain | Europa | 1/3-2/6/2020 | 150 | 12063 | <https://doi.org/10.3390/jcm10050899> |
| 129 | REGINA J | 2020 | Switzerland | Europa | 1-25/3/2020 | 1 | 145 | <https://doi.org/10.1371/journal.pone.0240781> |
| 221 | VERNAZ N | 2020 | Switzerland | Europa | 26/2-31/5/2020 | 1 | 930 | <https://doi.org/10.4414/smw.2020.20446> |
| 1250 | LEO M | 2021 | Switzerland | Europa | 25/2-11/5/2020 | 7 | 292 | https://doi.org/10.1016/j.dld.2021.12.014 |
| 297 | CROSSETTE-THAMBIAH C | 2021 | UK | Europa | 1/3-31/5/2020 | MD^‡^ | 72 | <https://doi.org/10.1111/bjh.17579> |
| 471 | FREEMAN A | 2022 | UK | Europa | 16/06/2020 | 1 | 680 | <https://doi.org/10.1016/j.jcv.2021.105031> |
| 491 | LLANERA DK | 2022 | UK | Europa | 1/1-30/6/2020 | 7 | 1004 | https://doi.org/10.3389/fendo.2021.777130 |
| 529 | PEREZ-NIETO OR | 2022 | International | Multicontinental | 1/5-12/6/2020 | 27 | 827 | <https://doi.org/10.1183/13993003.00265-2021> |
| 542 | PEPE M | 2021 | International | Multicontinental | Until May 5, 2020 | 39 | 5746 | https://doi.org/10.1007/s10238-021-00684-1 |
| 249 | YANG JY | 2021 | International | North America | 15/4-5/6-2020 | 36 | 1790 | <https://doi.org/10.1001/jamanetworkopen.2020.35699> |
| 57 | RUIZ-QUINONEZ JA | 2021 | Mexico | North America | 15/4-12/5/2020 | 1 | 185 | https://doi.org/10.1371/journal.pone.0245394 |
| 14 | YAO JS | 2021 | USA | North America | Until April 11, 2020 | 1 | 242 | <https://doi.org/10.1016/j.chest.2020.06.082> |
| 19 | ARSHAD S | 2020 | USA | North America | 10/3-2/05/2020 | 6 | 2541 | <https://doi.org/10.1016/j.ijid.2020.06.099> |
| 42 | CHOPRA V | 2021 | USA | North America | 16/3-11/5/2020 | 32 | 792 | <http://dx.doi.org/10.1136/bmjopen-2020-044921> |
| 62 | ANGELIDI AM | 2021 | USA | North America | 1/2-15/5/2020 | 2 | 144 | <https://dx.doi.org/10.1016%2Fj.mayocp.2021.01.001> |
| 101 | FRIED MW | 2021 | USA | North America | 15/2-20/4/2020 | 245 | 11721 | <https://doi.org/10.1093/cid/ciaa1268> |
| 114 | TEJPAL A | 2021 | USA | North America | 1/3-27/4/2020 | 13 | 10630 | [https://pubmed.ncbi.nlm.nih.gov/33885345/d](http://antiphishing.aphp.fr/v4?f=anVKRFdQdUFFUmYyczNxNs-N4Coj9PmsS1LMqPp3ATjwx4yfdkzo_Fa3Xda1nJ-t&i=a0FMN2hiQk1KRmZRT3Vjd0Nh4GTa7P8Hq-artTbFwtU&k=2tdk&r=elhOU0JBS3RqT1RUejJhSxNuDsIOcOg3De767PrbOlan_oh44m9loLU8F82y-iH8&s=04a6f9a97f69e254421850cdb131b2a19d45f749c32ff1b211048c6ce7e962c4&u=https%3A%2F%2Fpubmed.ncbi.nlm.nih.gov%2F33885345%2Fd) |
| 158 | LOHIA P | 2021 | USA | North America | 10/3-30/6/2020 | 2 | 922 | https://doi.org/10.1186/s12933-021-01336-0 |
| 164 | BEST JH | 2021 | USA | North America | 1/12/2019-27/08/2020^†^ | 400 | 4817 | <https://doi.org/10.1002/jmv.27049> |
| 184 | OKOH | 2020 | USA | North America | 10/3-10/4/2020 | 1 | 343 | [Tocilizumab use in COVID‐19‐associated pneumonia (wiley.com)](https://onlinelibrary.wiley.com/doi/epdf/10.1002/jmv.26471) |
| 200 | FRONTERA JA | 2021 | USA | North America | 1/3-20/5/2020 | 4 | 4491 | <https://dx.doi.org/10.1007%2Fs12028-021-01220-5> |
| 262 | PATEL AJ | 2021 | USA | North America | 3/3-5/5/2020 | 1 | 506 | <https://doi.org/10.1016/j.eprac.2021.07.008> |
| 270 | FOX T | 2021 | USA | North America | 1/3-24/4/2020 | 1 | 355 | <https://doi.org/10.1007/s00592-020-01592-8> |
| 284 | AWAD N | 2021 | USA | North America | 20/3-20/4/2020 | 1 | 336 | <https://doi.org/10.1371/journal.pone.0256023*> |
| 331 | YEO I | 2021 | USA | North America | 13/3-9/4/2020 | 1 | 1062 | <https://dx.doi.org/10.1111%2Fjoim.13241> |
| 337 | SNIPELISKY D | 2020 | USA | North America | 6-31/3/2020 | 1 | 114 | https://doi.org/10.14423/smj.0000000000001182 |
| 453 | KUNO T | 2022 | USA | North America | 1/3-7/5/2020 | 1 | 6095 | <https://doi.org/10.1016/j.jjcc.2021.12.012> |
| 503 | BAHL A | 2021 | USA | North America | 1-31/3/2020 | 8 | 1461 | <https://doi.org/10.1007/s11739-021-02655-6> |
| 572 | LARA OD | 2022 | USA | North America | 1/3-1/6/2020 | 8 | 106 | <https://doi.org/10.1016/j.ygyno.2021.12.004> |
| 583 | KUMAR G | 2022 | USA | North America | 1-10/3/2020 | 1 | 3121 | https://doi.org/10.1002/jmv.27357 |
| 628 | HUR K | 2020 | USA | North America | 1/3-8/4/2020 | 10 | 486 | <https://doi.org/10.1177/0194599820929640> |
| 673 | RHODES NJ | 2021 | USA | North America | 18/4-8/5/2020 | 15 | 352 | <https://doi.org/10.1093/ajhp/zxaa426> |
| 769 | ROSENBERG ES | 2020 | USA | North America | 15/2-8/3/2020 | 25 | 1438 | <https://doi.org/10.1001/jama.2020.8630> |
| 775 | MATHER JF | 2020 | USA | North America | 24/2-13/5/2020 | 1 | 772 | https://doi.org/10.14309/ajg.0000000000000832 |
| 794 | CHANGAL K | 2021 | USA | North America | 1/1-1/5/2020 | 2 | 279 | https://doi.org/10.1186/s12872-021-01963-1 |
| 797 | DOUVILLE NJ | 2021 | USA | North America | 1/3-5/5/2020 | 1 | 398 | <https://doi.org/10.1016/j.bja.2020.11.034> |
| 804 | IP A | 2020 | USA | North America | 1/3-22/4/2020 | 13 | 2512 | <https://doi.org/10.1371/journal.pone.0237693> |
| 833 | PELTZER B | 2020 | USA | North America | 3/3-6/4/2020 | 2 | 1053 | <https://doi.org/10.1111/jce.14770> |
| 864 | LI M | 2021 | USA | North America | 25/3-17/6/2020 | 7 | 1938 | <https://doi.org/10.1016/j.amjms.2020.11.005> |
| 908 | FERGUSON J | 2020 | USA | North America | 13/3-11/4/2020 | 2 | 72 | <https://doi.org/10.3201/eid2608.201776> |
| 967 | MONTEIRO AC | 2020 | USA | North America | 12/3-16/4/2020 | 2 | 112 | <https://doi.org/10.1371/journal.pone.0238552> |
| 1012 | PIAZZA G | 2020 | USA | North America | 13/3-3/4/2020 | 12 | 229 | <https://doi.org/10.1016/j.jacc.2020.08.070> |
| 1030 | MO Y | 2021 | USA | North America | 1/2-22/5/2020 | 1 | 617 | https://doi.org/10.1002/jcph.1787 |
| 1034 | SAEED O | 2020 | USA | North America | 1/3-2/5/2020 | 1 | 4252 | <https://doi.org/10.1161/jaha.120.018475> |
| 1109 | MOEY MYY | 2020 | USA | North America | 1/3-26/4/2020 | 1 | 107 | https://doi.org/10.1161/circep.120.009023 |
| 1167 | MCPADDEN J | 2021 | USA | North America | 1/3-30/4/2020 | 5 | 2154 | <https://doi.org/10.1371/journal.pone.0243291> |
| 1085 | ESCALERA-ANTEZANAN JP | 2020 | Bolivia | South America | 2-15/3/2020 | 1 | 1 | https://doi.org/10.1016/j.tmaid.2020.101653 |

* The enrollment period was not explicitly reported in the article. However, given that the manuscript was received on July 7, 2020, patient inclusion must have occurred before June 30, 2020.

^‡^ Missing Data

^†^ Treatment information was provided separately for each month, which allowed us to restrict the analysis strictly to treatments administered during the inclusion period of our study (up to June 30, 2020 only).
