# Supplementary material for: COVID-19 treatment of hospital patients worldwide at the onset of the pandemic in 2020: a systematic review
Source: BMC Infect Dis. 2025 Dec 17;26:107. doi: 10.1186/s12879-025-12368-2 (PMC12822144; doi:10.1186/s12879-025-12368-2)
Supplement: Supplementary file 7 — Supplementary Material 7 [file 12879_2025_12368_MOESM7_ESM.docx]

**Supplementary Material** **7. Scale of included studies (department, hospital, city, region, country, multinational, continent)**

|  | **Studies Total** | | | **Departement wide studies*** | | | **Hospital/center wide studies*** | | | **City wide studies*** | | | **Region or state wide studies*** | | | **Country wide studies*** | | | **International studies** | | |
| --- | --- | --- | --- | --- | --- | --- | --- | --- | --- | --- | --- | --- | --- | --- | --- | --- | --- | --- | --- | --- | --- |
| **Location (continent/country)** | **n** | **hospitals/centers (n)** | **patients (n)** | **n** | **hospitals/centers (n)** | **patients (n)** | **n** | **hospitals/centers (n)** | **patients (n)** | **n** | **hospitals/centers (n)** | **patients (n)** | **n** | **hospitals/centers (n)** | **patients (n)** | **n** | **hospitals/centers (n)** | **patients (n)** | **n** | **hospitals/centers (n)** | **patients (n)** |
| **Africa** | **2** | **2** | **484** | **0** | **0** | **0** | **2** | **2** | **484** | **0** | **0** | **0** | **0** | **0** | **0** | **0** | **0** | **0** | **0** | **0** | **0** |
| Cameroon | 1 | 1 | 282 | 0 | 0 | 0 | 1 | 1 | 282 | 0 | 0 | 0 | 0 | 0 | 0 | 0 | 0 | 0 | 0 | 0 | 0 |
| Egypt | 1 | 1 | 202 | 0 | 0 | 0 | 1 | 1 | 202 | 0 | 0 | 0 | 0 | 0 | 0 | 0 | 0 | 0 | 0 | 0 | 0 |
| **Asia** | **65** | **327†** | **36840** | **3** | **3** | **768** | **43** | **43** | **15370** | **8** | **85** | **3599** | **7** | **64** | **13310** | **4** | **132†** | **3793** | **0** | **0** | **0** |
| China | 51 | 184 | 29169 | 3 | 3 | 768 | 34 | 34 | 11893 | 7 | 83 | 3198 | 7 | 64 | 13310 | 0 | 0 | 0 | 0 | 0 | 0 |
| India | 1 | 1 | 108 | 0 | 0 | 0 | 1 | 1 | 108 | 0 | 0 | 0 | 0 | 0 | 0 | 0 | 0 | 0 | 0 | 0 | 0 |
| Iran | 1 | 1 | 60 | 0 | 0 | 0 | 1 | 1 | 60 | 0 | 0 | 0 | 0 | 0 | 0 | 0 | 0 | 0 | 0 | 0 | 0 |
| Malaysia | 1 | 1 | 247 | 0 | 0 | 0 | 1 | 1 | 247 | 0 | 0 | 0 | 0 | 0 | 0 | 0 | 0 | 0 | 0 | 0 | 0 |
| Pakistan | 2 | 5 | 1461 | 0 | 0 | 0 | 1 | 1 | 23 | 0 | 0 | 0 | 0 | 0 | 0 | 1 | 4 | 1438 | 0 | 0 | 0 |
| Saudi Arabia | 1 | 2 | 401 | 0 | 0 | 0 | 0 | 0 | 0 | 1 | 1 | 401 | 0 | 0 | 0 | 0 | 0 | 0 | 0 | 0 | 0 |
| South Korea | 3 | 3 | 405 | 0 | 0 | 0 | 3 | 3 | 405 | 0 | 0 | 0 | 0 | 0 | 0 | 0 | 0 | 0 | 0 | 0 | 0 |
| Thailand | 1 | 1 | 193 | 0 | 0 | 0 | 1 | 1 | 193 | 0 | 0 | 0 | 0 | 0 | 0 | 0 | 0 | 0 | 0 | 0 | 0 |
| Turkey | 4 | 129**†** | 4796 | 0 | 0 | 0 | 1 | 1 | 2441 | 0 | 0 | 0 | 0 | 0 | 0 | 3 | 128**†** | 2355 | 0 | 0 | 0 |
| **Europa** | **71** | **724**‡ | **69088** | **9** | **9** | **1102** | **36** | **36** | **10986** | **1** | **3** | **158** | **12** | **105** | **20609** | **13** | **571**‡ | **36233** | **0** | **0** | **0** |
| Belgium | 2 | 110 | 8991 | 1 | 1 | 81 | 0 | 0 | 0 | 0 | 0 | 0 | 0 | 0 | 0 | 1 | 109 | 8910 | 0 | 0 | 0 |
| France | 11 | 85‡ | 19243 | 2 | 2 | 229 | 5 | 5 | 704 | 0 | 0 | 0 | 1 | 1 | 151030 | 3 | 42‡ | 3207 | 0 | 0 | 0 |
| Germany | 1 | 3 | 10 | 0 | 0 | 0 | 0 | 0 | 0 | 0 | 0 | 0 | 1 | 3 | 10 | 0 | 0 | 0 | 0 | 0 | 0 |
| Greece | 2 | 7 | 272 | 0 | 0 | 0 | 1 | 1 | 85 | 0 | 0 | 0 | 0 | 0 | 0 | 1 | 6 | 187 | 0 | 0 | 0 |
| Italy | 26 | 184‡ | 13966 | 3 | 4 | 414 | 13 | 13 | 4320 | 0 | 0 | 0 | 6 | 36‡ | 1577 | 4 | 132 | 7655 | 0 | 0 | 0 |
| Malta | 1 | 1 | 93 | 0 | 0 | 0 | 1 | 1 | 93 | 0 | 0 | 0 | 0 | 0 | 0 | 0 | 0 | 0 | 0 | 0 | 0 |
| Poland | 1 | 1 | 70 | 1 | 1 | 70 | 0 | 0 | 0 | 0 | 0 | 0 | 0 | 0 | 0 | 0 | 0 | 0 | 0 | 0 | 0 |
| Romania | 1 | 1 | 37 | 0 | 0 | 0 | 1 | 1 | 37 | 0 | 0 | 0 | 0 | 0 | 0 | 0 | 0 | 0 | 0 | 0 | 0 |
| Spain | 20 | 315 | 23283 | 2 | 2 | 308 | 12 | 12 | 3992 | 1 | 3 | 158 | 2 | 16 | 2623 | 3 | 282 | 16202 | 0 | 0 | 0 |
| Swiss | 3 | 9 | 1367 | 0 | 0 | 0 | 2 | 2 | 1075 | 0 | 0 | 0 | 1 | 7 | 292 | 0 | 0 | 0 | 0 | 0 | 0 |
| UK | 3 | 8‡ | 1756 | 0 | 0 | 0 | 1 | 1 | 680 | 0 | 0 | 0 | 1 | 7 | 1004 | 1 | MD‡ | 72 | 0 | 0 | 0 |
| **North America** | **37** | **866** | **68524** | **0** | **0** | **0** | **15** | **15** | **18505** | **5** | **79** | **23020** | **7** | **74** | **8196** | **4** | **662** | **17034** | **1** | **36** | **1790** |
| international | 1 | 36 | 1790 | 0 | 0 | 0 | 0 | 0 | 0 | 0 | 0 | 0 | 0 | 0 | 0 | 0 | 0 | 0 | 1 | 36 | 1790 |
| Mexico | 1 | 1 | 185 | 0 | 0 | 0 | 1 | 1 | 185 | 0 | 0 | 0 | 0 | 0 | 0 | 0 | 0 | 0 | 0 | 0 | 0 |
| USA | 35 | 829 | 66549 | 0 | 0 | 0 | 14 | 14 | 18320 | 10 | 79 | 23020 | 7 | 74 | 8196 | 4 | 662 | 17034 | 0 | 0 | 0 |
| **South America** | **1** | **1** | **1** | **0** | **0** | **0** | **0** | **0** | **0** | **0** | **0** | **0** | **0** | **0** | **0** | **1** | **1** | **1** | **0** | **0** | **0** |
| Bolivia | 1 | 1 | 1 | 0 | 0 | 0 | 0 | 0 | 0 | 0 | 0 | 0 | 0 | 0 | 0 | 1 | 1 | 1 | 0 | 0 | 0 |
| **Multicontinental** | **2** | **66** | **6573** | **0** | **0** | **0** | **0** | **0** | **0** | **0** | **0** | **0** | **0** | **0** | **0** | **0** | **0** | **0** | **2** | **66** | **6573** |
| international | 2 | 66 | 6573 | 0 | 0 | 0 | 0 | 0 | 0 | 0 | 0 | 0 | 0 | 0 | 0 | 0 | 0 | 0 | 2 | 66 | 6573 |
| **Total (all studies)**  **n (%)** | **178** | **1986*†** | **181510**  **(100.0)** | **12** | **12** | **1870**  **(1.0)** | **96** | **96** | **45345**  **(25.0)** | **19** | **167** | **26777**  **(14.7)** | **26** | **243** | **42116**  **(23.2)** | **22** | **1366** | **57060**  **(31.4)** | **3** | **102** | **8363**  **(4.6)** |

* A ward-wide study is a study that includes patients from only one ward. A hospital-wide study (or center-wide study) is a study that included patients from more than one ward of the hospital (or center) concerned. A city-wide study is a study that included patients treated in more than one hospital but in a single city. A region-wide (or state-wide) study is a study that included patients treated in more than one city but in a single region (or state in the case of a country comprising several states). A country-wide study is a study that included patients treated in hospitals belonging to more than one region (or state in the case of countries comprising several states).

**†** Of the 4 Turkish studies, 2 were organized under the aegis of the Turkish Nephrology Society and have identical characteristics: country wide study, each comprising 47 centres. These are studies number 567 and 1210.

‡ For 3 studies, the number of centres or hospitals is not known. These are studies 297 from Crossette-Thambiah C. et al. (UK, country wide study, 72 patients), 928 from Caillard S. et al. (France, country wide study, 243 patients) and 964 from Wu MA. et al. (Italy, region wide study, 48 patients).
