# Supplementary material for: COVID-19 treatment of hospital patients worldwide at the onset of the pandemic in 2020: a systematic review
Source: BMC Infect Dis. 2025 Dec 17;26:107. doi: 10.1186/s12879-025-12368-2 (PMC12822144; doi:10.1186/s12879-025-12368-2)
Supplement: Supplementary file 9 — Supplementary Material 9 [file 12879_2025_12368_MOESM9_ESM.docx]

**Supplementary Material** **9. Treatments for Covid-19 inpatients in different countries.**

| **Country** | **Patients** | **Number of different treatments** | **HCQ**‡ | **CS**‡ | **L-R**‡ | **AZM**‡ | **IL-6i** | **Umifénovir (Arbidol)** | **IFN**‡ | **TCM**‡ | **Oseltamivir** | **Ribavirin** | **IVIg**‡ | **IL-1i**‡ | **Darunavir** | **Remdesivir** | **Favipavir** | **Ganciclovir** | **Zinc** | **Colchicine** | **Atazanavir** | **thymosin** | **CP**‡ | **Infliximab** | **Neuraminidase inhibitor** | **Ivermectine** | **UCMSC**‡ | **leronlumab** | **Jak2i**‡ | **Thymalfasin** | **Aciclovir** | **A/IA**‡ | **Vidarabine** | **Rituximab** | **Any Antiviral** | **Antivirals (others)** | **Standard Of Care** |
| --- | --- | --- | --- | --- | --- | --- | --- | --- | --- | --- | --- | --- | --- | --- | --- | --- | --- | --- | --- | --- | --- | --- | --- | --- | --- | --- | --- | --- | --- | --- | --- | --- | --- | --- | --- | --- | --- |
| **Africa** | **484** | **3** | **358/484 (74.0)** | **62/282 (22.0)** | **6/282 (2.1)** |  |  |  |  |  |  |  |  |  |  |  |  |  |  |  |  |  |  |  |  |  |  |  |  |  |  |  |  |  |  |  |  |
| Cameroon | 282 | 3 | 259/282 (91.8) | 62/282 (22.0) | 6/282 (2.1) |  |  |  |  |  |  |  |  |  |  |  |  |  |  |  |  |  |  |  |  |  |  |  |  |  |  |  |  |  |  |  |  |
| Egypt | 202 | 1 | 99/202 (49.0) |  |  |  |  |  |  |  |  |  |  |  |  |  |  |  |  |  |  |  |  |  |  |  |  |  |  |  |  |  |  |  |  |  |  |
| **Asia** | **36840** | **23** | **3834/8490 (45.2)** | **9233/31606 (28.9)** | **2751/16069 (17.1)** | **1619/2223 (72.8)** | **153/4176 (3.7)** | **7382/13124 (56.2)** | **4364/12988 (33.6)** | **7192/14589 (49.3)** | **4398/16473 (26.7)** | **2347/11586 (20.3)** | **1778/6376 (27.9)** | **11/2063 (0.5)** | **36/314 (11.5)** | **16/571 (2.8)** | **929/4557 (20.4)** | **738/10977 (6.7)** |  |  |  | **134/1908 (7.0)** | **48/4517 (1.1)** |  | **0/348 (0.0)** |  | **44/1728 (2.5)** |  | **1/578 (0.2)** | **9/55 (16.4)** | **9/2485 (0.4)** | **9/578 (1.6)** |  |  | **13197/17865 (73.9)** | **783/2325 (33.7)** | **371/1706 (21.7)** |
| China | 29169 | 19 | 95/830 (11.4) | 8165/27822 (29.3) | 2480/13425 (18.5) |  | 28/1894 (1.5) | 7382/13124 (56.2) | 4353/12741 (34.2) | 7192/14589 (49.3) | 2767/12060 (22.9) | 2343/11339 (20.7) | 1778/6376 (27.9) |  | 36/314 (11.5) | 0/238 (0.0) | 4/778 (0.5) | 738/10977 (6.7) |  |  |  | 134/1908 (7.0) | 18/2240 (0.8) |  | 0/348 (0.0) |  | 44/1728 (2.5) |  |  | 9/55 (16.4) | 1/1047 (0.1) |  |  |  | 13137/17805 (73.8) | 198/718 (27.6) | 244/1175 (20.8) |
| India | 108 | 6 | 99/108 (91.7) |  |  | 79/108 (73.9) | 14/30 (46.7) |  |  |  |  |  |  |  |  |  |  |  |  |  |  |  |  |  |  |  |  |  |  |  |  |  |  |  |  |  |  |
| Iran | 60 | 5 | 30/60 (50.0) | 30/60 (50.0) | 30/60 (50.0) | 30/60 (50.0) |  |  |  |  | 30/60 (50.0) |  |  |  |  |  |  |  |  |  |  |  |  |  |  |  |  |  |  |  |  |  |  |  | 60/60 (100.0) |  | 0/60 (0.0) |
| Malaysia | 247 | 5 | 68/247 (27.5) |  | 37/247 (15.0) |  |  |  | 11/247 (4.5) |  | 11/247 (4.5) | 4/247 (1.6) |  |  |  |  |  |  |  |  |  |  |  |  |  |  |  |  |  |  |  |  |  |  |  |  |  |
| Pakistan | 1461 | 7 | 211/1461 (14.4) | 710/1461 (48.6) |  | 1283/1461 (87.8) | 13/23 (56.5) |  |  |  | 12/1438 (0.8) |  |  |  |  |  |  |  |  |  |  |  | 2/23 (8.7) |  |  |  |  |  |  |  | 8/1438 (0.6) |  |  |  |  |  |  |
| Saudi Arabia | 401 | 3 | 66/401 (16.5) |  |  | 193/401 (48.1) |  |  |  |  | 134/401 (33.4) |  |  |  |  |  |  |  |  |  |  |  |  |  |  |  |  |  |  |  |  |  |  |  |  | 134/401 (33.4) |  |
| South Korea | 405 | 4 | 151/405 (37.3) |  | 106/267 (39.7) |  |  |  |  |  |  |  |  |  |  | 9/140 (6.4) |  |  |  |  |  |  |  |  |  |  |  |  |  |  |  |  |  |  |  |  | 53/278 (19.1) |
| Thailand | 193 | 7 | 109/193 (56.5) | 2/193 (1.0) |  | 34/193 (17.6) | 3/193 (1.6) |  |  |  |  |  |  |  |  | 7/193 (3.6) | 50/193 (25.9) |  |  |  |  |  | 2/193 (1.0) |  |  |  |  |  |  |  |  |  |  |  |  | 91/193 (47.2) | 74/193 (38.3) |
| Turkey | 4796 | 9 | 3005/4785 (62.8) | 226/2070 (10.9) | 98/2070 (4.7) |  | 109/2066 (5.3) |  |  |  | 1444/2267 (63.7) |  |  | 11/2063 (0.5) |  | 0 | 875/3586 (24.4) |  |  |  |  |  | 26/2061 (1.3) |  |  |  |  |  | 1/578 (0.2) |  |  | 9/578 (1.6) |  |  |  | 360/1013 (35.5) |  |
| **Europa** | **69088** | **18** | **33683/47015** (**71.6)** | **12296/37575 (32.7)** | **18430/41523 (44.4)** | **5369/10714 (50.1)** | **4389/43057 (10.2)** |  | **2639/20061 (13.2)** |  | **109/17077 (0.6)** | **1/16117 (0.0)** | **74/17060 (0.4)** | **162/15016 (1.1)** | **1181/8373 (14.1)** | **382/33672 (1.1)** |  |  |  | **162/14064 (1.2)** |  |  | **11/975 (1.1)** | **104/618 (16.8)** | **103/3960 (2.6)** |  |  |  | **12/12772 (0.1)** |  |  |  |  | **4/158 (2.5)** | **2754/6840 (40.3)** | **37/757 (4.9)** | **17828/27222 (65.5)** |
| Belgium | 8991 | 5 | 5368/8991 (59.7) | 25/81 (30.9) | 30/8910 (0.3) |  | 24/8910 (0.3) |  |  |  |  |  |  |  |  | 4/8910 (0.0) |  |  |  |  |  |  |  |  |  |  |  |  |  |  |  |  |  |  |  | 27/81 (33.3) | 3533/8910 (39.7) |
| France | 19243 | 10 | 849/3971 (21.4) | 297/3296 (9.0) | 54/777 (6.9) | 234/559 (41.9) | 22/612 (3.6) |  | 13/2878 (0.5) |  | 6/3207 (0.2) |  | 2/2878 (0.1) | 7/213 (3.3) |  | 2/407 (0.5) |  |  |  |  |  |  | 0/164 (0.0) |  |  |  |  |  |  |  |  |  |  |  | 423/3133 (13.5) | 0/164 (0.0) | 13232/15152 (87.3) |
| Germany | 10 | 0 | 0/10 (0.0) | 0/10 (0.0) | 0/10 (0.0) |  | 0/10 (0.0) |  |  |  |  |  |  |  |  | 0/10 (0.0) |  |  |  |  |  |  | 0/10 (0.0) |  |  |  |  |  |  |  |  |  |  |  | 0/10 (0.0) | 0/10 (0.0) | 10/10 (100.0) |
| Greece | 272 | 9 | 246/272 (90.4) | 5/85 (5.9) | 33/187 (17.6) | 169/187 (90.4) | 5/187 (2.7) |  |  |  |  |  |  | 2/187 (1.1) |  | 6/272 (2.2) |  |  |  | 16/272 (5.9) |  |  | 1/85 (1.2) |  |  |  |  |  |  |  |  |  |  |  |  |  |  |
| Italy | 13966 | 12 | 8123/10441 (77.8) | 4149/10601 (39.1) | 4040/8444 (47.8) | 1197/1905 (62.8) | 1725/9686 (17.8) |  | 0/5 (0.0) |  | 1/1714 (0.1) | 0/5 (0.0) | 9/623 (1.4) | 66/1722 (3.8) | 877/3683 (23.8) | 176/5664 (3.1) |  |  |  | 0/5 (0.0) |  |  | 10/623 (1.6) | 104/618 (16.8) |  |  |  |  |  |  |  |  |  |  | 1952/3100 (63.0) | 0/5 (0.0) | 391/1582 (24.7) |
| Malta | 93 | 2 | 19/93 (20.4) | 0/93 (0.0) | 0/93 (0.0) | 19/93 (20.4) | 0/93 (0.0) |  | 0/93 (0.0) |  | 0/93 (0.0) | 0/93 (0.0) | 0/93 (0.0) | 0/93 (0.0) | 0/93 (0.0) | 0/93 (0.0) |  |  |  | 0/93 (0.0) |  |  | 0/93 (0.0) |  |  |  |  |  |  |  |  |  |  |  | 0/93 (0.0) | 0/93 (0.0) | 70/93 (75.3) |
| Poland | 70 | 3 | 52/70 (74.3) |  | 9/70 (12.9) | 30/70 (42.9) |  |  |  |  |  |  |  |  |  |  |  |  |  |  |  |  |  |  |  |  |  |  |  |  |  |  |  |  |  |  |  |
| Romania | 37 | 4 | 22/37 (59.5) | 9/37 (24.3) | 8/37 (21.6) |  | 4/37 (10.8) |  |  |  |  |  |  |  |  |  |  |  |  |  |  |  |  |  |  |  |  |  |  |  |  |  |  |  |  |  |  |
| Spain | 23283 | 16 | 18513/21763 (85.1) | 7590/21629 (35.1) | 13898/21773 (63.8) | 3667/6980 (52.5) | 2584/22333 (11.6) |  | 2626/17085 (15.4) |  | 102/12063 (0.8) | 1/16019 (0.0) | 63/13394 (0.5) | 87/12791 (0.7) | 304/4597 (6.6) | 151/17199 (0.9) |  |  |  | 146/13694 (1.1) |  |  |  |  | 103/3960 (2.6) |  |  |  | 12/12772 (0.1) |  |  |  |  | 4/158 (2.5) | 379/504 (75.2) | 10/404 (2.5) | 38/400 (9.5) |
| Switzerland | 1367 | 5 | 491/1367 (35.9) |  | 358/1222 (29.3) | 53/930 (5.7) | 23/437 (5.3) |  |  |  |  |  |  |  |  | 33/437 (7.6) |  |  |  |  |  |  |  |  |  |  |  |  |  |  |  |  |  |  |  |  | 554/1075 (51.5) |
| UK | 1756 | 3 |  | 221/1743 (12.7) |  |  | 2/752 (0.3) |  |  |  |  |  | 0/72 (0.0) |  |  | 10/680 (1.5) |  |  |  |  |  |  |  |  |  |  |  |  | 0/680 (0.0) |  |  |  |  |  |  |  |  |
| **North America** | **68524** | **13** | **33085/55831 (59.3)** | **15014/47148 (31.8)** | **560/12262 (4.6)** | **8384/15187 (55.2)** | **3910/37762 (10.4)** |  | **1/4817 (0.0)** |  | **34/1975 (1.7)** | **0/4817 (0.0)** | **10/5870 (0.2)** | **1142/10630 (10.7)** |  | **714/40328 (1.8)** |  |  | **411/1647 (25.5)** |  | **140/2154 (6.5)** |  | **41/1896 (2.2)** | **1/4817 (0.0)** |  |  |  | **14/184 (7.6)** |  |  |  |  |  |  | **281/2154 (13.0)** | **28/936 (3.0)** | **641/3410 (18.8)** |
| International | 1790 | 5 | 925/1790 (51.7) | 218/1790 (12.2) |  |  | 92/1790 (5.1) |  |  |  |  |  |  |  |  | 99/1790 (5.5) |  |  |  |  |  |  | 36/1790 (2.0) |  |  |  |  |  |  |  |  |  |  |  |  |  |  |
| Mexico | 185 | 5 | 54/185 (29.2) |  | 23/185 (12.4) | 61/185 (33.3) | 20/185 (10.8) |  |  |  | 34/185 (18.4) |  |  |  |  |  |  |  |  |  |  |  |  |  |  |  |  |  |  |  |  |  |  |  |  |  |  |
| USA | 66549 | 14 | 32106/53856 (59.6) | 14796/45358 (32.6) | 537/12077 (4.4) | 8323/15002 (55.5) | **3798/35787 (10.6)** |  | 1/4817 (0.0 |  | 0/1790 (0.0) | 0/4817 (0.0) | 10/5870 (0.2) | 1142/10630 (10.7) |  | 615/38538 (1.6) |  |  | 411/1647 (25.5) |  | 140/2154 (6.5) |  | 5/106 (4.7) | 1/4817 (0.0) |  |  |  | 14/184 (7.6) |  |  |  |  |  |  | 281/2154 (13.0) | 28/936 (3.0) | 641/3410 (18.8) |
| **South America** | **1** | **0** | **0/1** (**0.0)** |  | **0/1 (0.0)** |  |  | **0/1 (0.0)** | **0/1 (0.0** |  | **0/1 (0.0)** | **0/1 (0.0)** |  |  | **0/1 (0.0)** | **0/1 (0.0)** | **0/1 (0.0)** | **0/1 (0.0)** |  |  |  |  | **0/1 (0.0)** |  |  |  |  |  |  |  | **0** |  | **0/1 (0.0)** |  | **0/1 (0.0)** | **0/1 (0.0)** |  |
| Bolivia | 1 |  | 0/1 (0.0) |  | 0/1 (0.0) |  |  | 0/1 (0.0) | 0/1 (0.0 |  | 0/1 (0.0) | 0/1 (0.0) |  |  | 0/1 (0.0) | 0/1 (0.0) | 0/1 (0.0) | 0/1 (0.0) |  |  |  |  | 0/1 (0.0) |  |  |  |  |  |  |  |  |  | 0/1 (0.0) |  | 0/1 (0.0) | 0/1 (0.0) |  |
| **Multicontinental Studies** | **6573** | **8** | **5132/6467 (79.4)** | **1669/6391 (26.1)** | **81/827 (9.8)** | **549/827 (66.4)** | **514/6382 (8.1)** |  | **735/5539 (14.3)** |  | **130/827 (15.7)** |  |  |  |  |  |  |  |  |  |  |  |  |  |  | **57/827 (6.9)** |  |  |  |  |  |  |  |  | **3400/5626 (60.4)** |  |  |
| **World** | **181510** | **32** | **76092/118288 (64.3)** | **38174/123002 (31.0)** | **21828/70964 (30.8)** | **15921/28961 (55.0)** | **8966/91377 (9.8)** | **7382/13124 (56.2)** | **7739/43406 (17.8)** | **7192/14589 (49.3)** | **4671/36353 (12.8)** | **2348/32521 (7.2)** | **1862/29306 (6.4)** | **1315/27709 (4.8)** | **1217/8688 (14.0)** | **1112/74572 (1.5)** | **929/4558 (20.4)** | **738/10978 (6.7)** | **411/1647 (25.0)** | **162/14064 (1.2)** | **140/2154 (6.5)** | **134/1908 (7.0)** | **100/7388 (1.4)** | **105/5435 (1.9)** | **103/4308 (2.4)** | **57/827 (6.9)** | **44/1728 (2.5)** | **14/184 (7.6)** | **13/13350 (0.1)** | **9/55 (16.4)** | **9/2486 (0.4)** | **9/578 (1.6)** | **0/1 (0.0)** | **4/158 (2.5)** | **19632/32486 (60.4)** | **848/4019 (21.1)** | **18840/32338 (58.3)** |

NB: In case of missing data, the denominator used to calculate percentages does not correspond to the total number of patients in the country or continent. To avoid overloading the table, only percentages based on available data are shown.

^*^ For these treatments (IL-6 and IL-1 inhibitors, JAK2 inhibitors, Traditional Chinese Medicine), there may be several different molecules.

^‡^ HCQ: hydroxychloroquine, CS: corticosteroids, L-R: lopinavir-ritonavir, AZM: azithromycin, IL-6i: IL-6 inhibitors, IFN: interferons, TCM: traditional Chinese medicine, IVIg: intravenous immunoglobulins, IL-1i: IL-1 inhibitors, CP: convalescent plasma, UCMSC: Umbilical cord mesenchymal stem cells, JAK2i: Jak2 inhibitors, A/IA: apheresis/immunoabsorption.
