# Supplementary material for: COVID-19 treatment of hospital patients worldwide at the onset of the pandemic in 2020: a systematic review
Source: BMC Infect Dis. 2025 Dec 17;26:107. doi: 10.1186/s12879-025-12368-2 (PMC12822144; doi:10.1186/s12879-025-12368-2)
Supplement: Supplementary file 10 — Supplementary Material 10 [file 12879_2025_12368_MOESM10_ESM.docx]

**Supplementary Material 10. Top ten most commonly prescribed hospital treatments for Covid-19 during the first wave of the pandemic in countries with analysis of over 1000 patients.**

| **Country** | **Rank** | **Treatment** | **Treated patients**  **(n)** | **Patients without MD**‡ **(n)** | **Treated patients**  **(%)** | **Treated patients (overall^*^)**  **(%)** |
| --- | --- | --- | --- | --- | --- | --- |
| **China** | **1** | Corticosteroids | 8165 | 27822 | 29.3 | 27.9 |
| n=29169 | **2** | Umifénovir | 7382 | 13124 | 56.2 | 25.2 |
| 19 drugs^†^ | **3** | TCM‡ | 7192 | 14589 | 49.3 | 24.6 |
|  | **4** | Interferons | 4353 | 12741 | 34.2 | 14.9 |
|  | **5** | Oseltamivir | 2767 | 12060 | 22.9 | 9.4 |
|  | **6** | Lopinavir-ritonavir | 2480 | 13425 | 18.5 | 8.5 |
|  | **7** | Ribavirin | 2343 | 11339 | 20.7 | 8.0 |
|  | **8** | IVIg‡ | 1778 | 6376 | 27.9 | 6.1 |
|  | **9** | Ganciclovir | 738 | 10977 | 6.7 | 2.5 |
|  | 10 | Thymosin | 134 | 1908 | 7.0 | 0.5 |
| **Turkey** | 1 | Hydroxychloroquine | 3005 | 4785 | 62.8 | 62.7 |
| n=4796 | 2 | Oseltamivir | 1444 | 2267 | 63.7 | 30.1 |
| 10 drugs^†^ | 3 | Favipavir | 875 | 3586 | 24.4 | 18.2 |
|  | 4 | Corticosteroids | 226 | 2070 | 10.9 | 4.7 |
|  | 5 | IL-6 inhibitors | 109 | 2066 | 5.3 | 2.3 |
|  | 6 | Lopinavi-ritonavir | 98 | 2070 | 4.7 | 2.0 |
|  | 7 | Convalescent Plasma | 26 | 2061 | 1.3 | 0.5 |
|  | 8 | IL-1 inhibitors | 11 | 2063 | 0.5 | 0.2 |
|  | 9 | A/IA‡ | 9 | 578 | 1.6 | 0,2 |
|  | 10 | Jak2 inhibitors | 1 | 578 | 0.2 | 0,0 |
| **Pakistan** | 1 | Azithromycin | 1283 | 1461 | 87.8 | 87.8 |
| n=1461 | 2 | Corticosteroids | 710 | 1461 | 48.6 | 48.6 |
| 7 drugs^†^ | 3 | Hydroxychloroquine | 211 | 1461 | 14.4 | 14.4 |
|  | 4 | IL-6 inhibitors | 13 | 23 | 56.5 | 0.9 |
|  | 5 | Oseltamivir | 12 | 1438 | 0.8 | 0.8 |
|  | 6 | Aciclovir | 8 | 1438 | 0.6 | 0.5 |
|  | 7 | Convalescent Plasma | 2 | 23 | 8.7 | 0.1 |
| **Belgium** | 1 | Hydroxychloroquine | 5368 | 8991 | 59.7 | 59.7 |
| n=8991 | 2 | Lopinavir-ritonavir | 30 | 8910 | 0.3 | 0.3 |
| 5 drugs^†^ | 3 | Corticosteroids | 25 | 81 | 30.9 | 0.3 |
|  | 4 | IL-6 inhibitors | 24 | 8910 | 0.3 | 0.3 |
|  | 5 | remdesivir | 4 | 8910 | 0.0 | 0.0 |
| **France** | 1 | Hydroxychloroquine | 849 | 3971 | 21.4 | 4.4 |
| n=19243 | 2 | Corticosteroids | 297 | 3296 | 9.0 | 1.5 |
| 10 drugs^†^ | 3 | Azithromycin | 234 | 559 | 41.9 | 1.2 |
|  | 4 | Lopinavir-ritonavir | 54 | 777 | 6.9 | 0.3 |
|  | 5 | IL-6 inhibitors | 22 | 612 | 3.6 | 0.1 |
|  | 6 | Interferons | 13 | 2878 | 0.5 | 0.1 |
|  | 7 | IL-1 inhibitors | 7 | 213 | 3.3 | 0.0 |
|  | 8 | Oseltamivir | 6 | 3207 | 0.2 | 0.0 |
|  | 9 | Remdesivir | 2 | 407 | 0.5 | 0.0 |
|  | 10 | IVIg‡ | 2 | 2878 | 0.1 | 0.0 |
| **Italy** | 1 | Hydroxychloroquine | 8123 | 10441 | 77.8 | 58.2 |
| n=13966 | 2 | Corticosteroids | 4149 | 10601 | 39.1 | 29,7 |
| 12 drugs^†^ | 3 | Lopinavir-ritonavir | 4040 | 8444 | 47.8 | 28.9 |
|  | 4 | IL-6 inhibitors | 1725 | 9686 | 17.8 | 12.4 |
|  | 5 | Azithromycin | 1197 | 1905 | 62.8 | 8.6 |
|  | 6 | Darunavir | 877 | 3683 | 23.8 | 6.3 |
|  | 7 | Remdesivir | 176 | 5664 | 3.1 | 1.3 |
|  | 8 | Infliximab | 104 | 618 | 16.8 | 0.7 |
|  | 9 | IL-1 inhibitors | 66 | 1722 | 3.8 | 0.5 |
|  | 10 | Convalescent Plasma | 10 | 623 | 1.6 | 0.1 |
| **Spain** | 1 | Hydroxychloroquine | 18513 | 21763 | 85.1 | 79.5 |
| n=23283 | 2 | Lopinavir-ritonavir | 13898 | 21773 | 63.8 | 59.7 |
| 16 drugs^†^ | 3 | Corticosteroids | 7590 | 21629 | 35.1 | 32.6 |
|  | 4 | Azithromycin | 3667 | 6980 | 52.5 | 15.7 |
|  | 5 | Interferons | 2626 | 17085 | 15.4 | 11.3 |
|  | 6 | IL-6 inhibitors | 2584 | 22333 | 11.6 | 11.1 |
|  | 7 | Darunavir | 304 | 4597 | 6.6 | 1.3 |
|  | 8 | Remdesivir | 151 | 17199 | 0.9 | 0.6 |
|  | 9 | Colchicine | 146 | 13694 | 1.1 | 0.6 |
|  | 10 | Neuraminidase Inh‡ | 103 | 3960 | 2.6 | 0.4 |
| **Swizterland** | 1 | Hydroxychloroquine | 491 | 1367 | 35.9 | 35.9 |
| n=1367 | 2 | Lopinavir-ritonavir | 358 | 1222 | 29.3 | 26.2 |
| 5 drugs^†^ | 3 | Azithromycin | 53 | 930 | 5.7 | 3.9 |
|  | 4 | Remdesivir | 33 | 437 | 7.6 | 2.4 |
|  | 5 | IL-6 inhibitors | 23 | 437 | 5.3 | 1.7 |
| **UK** | 1 | Corticosteroids | 221 | 1743 | 12.7 | 12.6 |
| n=1756 | 2 | remdesivir | 10 | 680 | 1.5 | 0.6 |
| 3 drugs^†^ | 3 | IL-6 inhibitors | 2 | 752 | 0.3 | 0.1 |
| **USA** | 1 | Hydroxychloroquine | 32106 | 53856 | 59.6 | 47.7 |
| n=66549 | 2 | Corticosteroids | 14796 | 45358 | 32.6 | 22.0 |
| 14 drugs^†^ | 3 | Azithromycin | 8323 | 15002 | 55.5 | 12.4 |
|  | 4 | IL-6 inhibitors | 3798 | 35787 | 10.6 | 5.6 |
|  | 5 | IL-1 inhibitors | 1142 | 10630 | 10.7 | 1.7 |
|  | 6 | Remdesivir | 615 | 38538 | 1.6 | 0.9 |
|  | 7 | Lopinavir-ritonavir | 537 | 12077 | 4.4 | 0.8 |
|  | 8 | Zinc | 411 | 1647 | 25.5 | 0.6 |
|  | 9 | Atanazavir | 140 | 2154 | 6.5 | 0.2 |
|  | 10 | Leronumab | 14 | 184 | 7.6 | 0.0 |

^*^ Percentage of the number of patients treated out of the total number of patients in the country including patients whose information on drug use is not known.

^†^ The number indicated corresponds to the total number of different treatments in a country.

^‡^ TCM: Traditional Chinese Medicine, IVIg: intravenous immunoglobulins, A/IA: apheresis/ immunoadsorption, Neuraminidase Inh: neuraminidase inhibitor, MD: Missing Data.
